# Structure-property relationships of photofunctional diiridium(II) complexes with tetracationic charge and an unsupported Ir–Ir bond

**DOI:** 10.1038/s42004-022-00775-4

**Published:** 2022-11-23

**Authors:** Fangrui Zheng, Yuhong Yang, Siye Wu, Shunan Zhao, Yifan Zhu, Huimin Su, Jun-Feng Dai, Zeyin Yan, Lung Wa Chung, Keith Man-Chung Wong

**Affiliations:** 1 Department of Chemistry, Southern University of Science and Technology, 1088 Xueyuan Blvd., Shenzhen, 518055 P.R. China; 2 Analysis and Testing Center, Shenzhen Technology University, 3002 Lantian Road, Shenzhen, 518118 P. R. China; 3 Shenzhen Institute for Quantum Science and Engineering, Southern University of Science and Technology, International Quantum Academy (SIQA), and Shenzhen Branch, Hefei National Laboratory, Shenzhen Key Laboratory of Quantum Science and Engineering, Futian District, Shenzhen, 518055 China

**Keywords:** Coordination chemistry, Photochemistry, Chemical bonding

## Abstract

In contrast to the extensively studied dirhodium(II) complexes and iridium(III) complexes, neutral or dicationic dinuclear iridium(II) complexes with an unsupported ligand are underdeveloped. Here, a series of tetracationic dinuclear iridium(II) complexes, featuring the unsupported Ir(II)–Ir(II) single bond with long bond distances (2.8942(4)–2.9731(4) Å), are synthesized and structurally characterized. Interestingly, compared to the previous unsupported neutral or dicationic diiridium(II) complexes, our DFT and high-level DLPNO-CCSD(T) results found the largest binding energy in these tetracationic complexes even with the long Ir(II)–Ir(II) bond. Our study further reveals that London dispersion interactions enhance the stability cooperatively and significantly to overcome the strong electrostatic repulsion between two half dicationic metal fragments. This class of complexes also exhibit photoluminescence in solution and solid states, which, to our knowledge, represents the first example of this unsupported dinuclear iridium(II) system. In addition, their photoreactivity involving the generation of iridium(II) radical monomer from homolytic cleavage was also explored. The experimental results of photophysical and photochemical behaviours were also correlated with computational studies.

## Abstract (web summary)

Luminescent iridium(III) complexes have several appealing properties such as wide color-tunability and high photo- and chemical stabilities, whereas the properties of related diiridium(II) systems are largely unexplored. Here, the authors synthesize and characterize a series of tetracationic dinuclear iridium(II) complexes, which feature unsupported Ir(II)–Ir(II) single bonds and exhibit photoluminescence in various media.

## Introduction

**Fig. 1:**
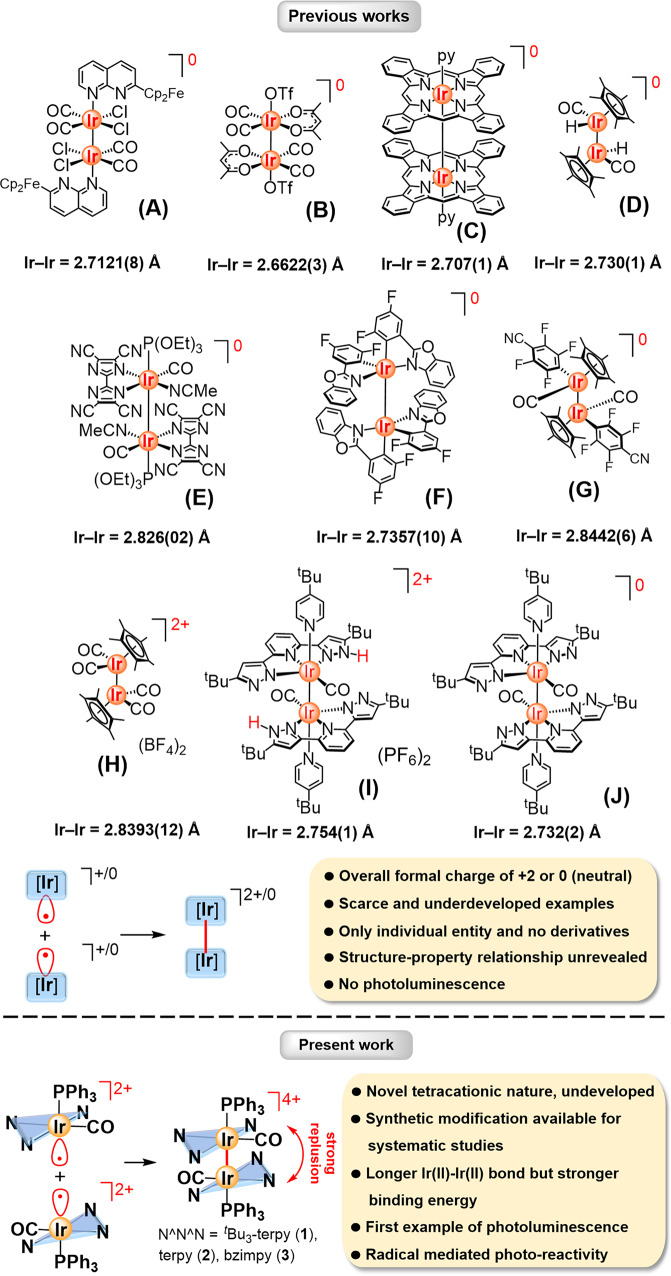
Previous examples with neutral or dicationic charge and this work with tetracationic charge. Previous works (**A**–**J**). Diiridium(II) complexes with unsupported Ir(II)–Ir(II) bond, formed from two d^7^ Ir(II) fragments and the uniqueness of tetracationic diiridium(II) complexes (**1**–**3**) in this study.

The most common oxidation state of iridium complexes is +3; while those of iridium(II) complexes of d^7^ electronic configuration with radical character are rare probably because of their low air- and moisture-stabilities^1–3^. One way to stabilize the iridium(II) complexes is through the formation of a Ir(II)–Ir(II) bond, leading to the pairing up of two individual radicals in the bonding orbital. The Ir(II)–Ir(II) bonds in such dinuclear iridium(II) complexes are usually supported and stabilized by bridging ligands^4–11^. Whereas, diiridium(II) complexes with an unsupported Ir(II)–Ir(II) bond are much less explored and the examples with structurally characterized are even more scarce^12–23^. In all reported complexes, the unsupported iridium(II) metal centres are found to coordinate with anionic ligands leading to the overall formal charge of +2 or 0 (neutral) (Fig. 1). The nature of low formal charge should facilitate the formation of these diiridium(II) complexes with reduced electrostatic repulsion between two half metal fragments. On the other hand, all reported examples with structural characterization were found to exist as only one entity without any derivatives, which precludes systematic study for the understanding of their structure-property relationships, presumably due to challenging synthesis of stable diiridium(II) complexes. In contrast, the isoelectronic dirhodium(II) compounds have been well known and extensively studied for the wide range of applications, such as catalysis^24–33^, antitumor metallopharmaceuticals^34–37^, phototherapeutic agents^38–40^, photochemistry^41–45^ and design of supramolecular arrays^46–48^.

The photophysical properties of polypyridine or cyclometalated iridium(III) system have also received tremendous attentions. In the past two decades, diversified potential applications^49–52^ of the luminescent iridium(III) complexes have been exploited because of their various advantages, such as wide colour-tunability, less thermally accessible ^3^d–d state, synthetic versatility and, photo- and chemical-stabilities. In sharp contrast, the photophysical and photochemical studies of unsupported diiridium(II) system are unknown from the limited examples. The exploration and exploitation of such photophysical and photochemical behaviours for the underdeveloped diiridium(II) complexes could open up an avenue for the development of new photofunctional materials. Therefore, synthesis and modification of series of diiridium(II) complexes are crucial and urged for the systematic study.

Herein, we report the synthesis and structural characterization of a series of tetracationic dinuclear iridium(II) complexes, [Ir(II)(N^N^N)(CO)(PPh_3_)]_2_[X]_4_ (**1**–**3**) [N^N^N = 4,4′,4′′-tri-*tert*-butyl-2,2′,6′,2′′-terpyridine (^*t*^Bu_3_-terpy), 2,2′,6′,2′′-terpyridine (terpy) and 2,6-bis(*N*-^*n*^butylbenzimidazol-2'-yl)pyridine (bzimpy); X = OTf^–^ or BF_4_^–^], featuring an unsupported and long Ir(II)–Ir(II) single bond for the first time (Fig. 1). Remarkably, state-of-the-art DFT and DLPNO-CCSD(T) studies revealed that two dicationic Ir(II) fragments are significantly stabilized by considerable London dispersion interactions in these tetracationic diiridium(II) complexes **1**–**3**. Such non-covalent interactions are responsible for the computed exceptionally largest Ir(II)–Ir(II) binding energy in **1**–**3**, even though they have a relatively long Ir(II)–Ir(II) bond. It is noteworthy that all of them are found to exhibit photoluminescence in various media, as the first example of the unsupported Ir(II) system. Their electrochemical and photophysical behaviours with different pincer ligands were determined and correlated with the electronic structures obtained from computational studies.

## Results and discussion

### Design and synthesis

**Fig. 2:**
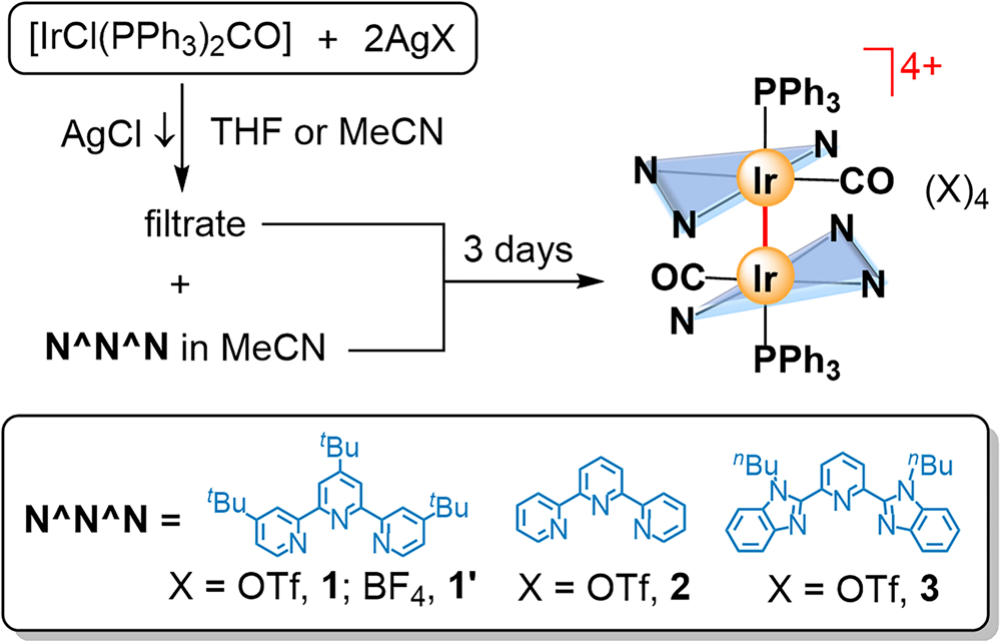
Synthetic route of tetracationic diiridium(II) complexes with an unsupported Ir–Ir bond tetracationic diiridium(II) complexes. Synthesis of **1**–**3** with different pincer ligands.

Reaction of [IrCl(PPh_3_)_2_(CO)] with AgX (X = OTf^–^ or BF_4_^–^), followed by treatment with the N^N^N pincer ligand in THF or MeCN at room temperature for 3 days afforded complexes **1**–**3** in 52–62% yield (Fig. 2). It is noteworthy that excess Ag(I) ion was added to serve as halide abstraction agent to remove the chloro group in the iridium(I) starting material, and as the oxidizing agent to generate the desired complexes. After addition of the pincer ligand into the pale yellow filtrate from the reaction mixture of [IrCl(PPh_3_)_2_(CO)] with AgX, greenish black solution was immediately formed. Such dark colour species was identified as [Ir(N^N^N)(CO)]^+^, based on the observation of [Ir(^*t*^Bu_3_-terpy)(CO)]^+^ at m/z = 622.23950 (calc. for [C_28_H_35_IrN_3_O]^+^ as 622.24039) in high-resolution mass spectrum (HRMS) during the formation of **1** (Supplementary Fig. S1). Interestingly, upon prolonged stirring, the iridium(I) species was further oxidized to form the desired diiridium(II) complex and red solution with dark red suspension was obtained. Non-covalent Ir(I)–Ir(I)^53,54^ and π–π interactions are suggested to facilitate the dimerization by holding the molecules into close proximity in the solution. Complexes **1**–**3** are stable toward air and moisture in the solid state. In dry and degassed CD_3_CN or (CD_3_)_2_SO solution of **1**–**3**, no observable change from their ^1^H NMR spectra was found for at least 24 h. Because of the highly charged nature, their solubilities are good in polar CH_3_CN and DMSO solvents, whereas only slightly to moderately soluble in CHCl_3_ and insoluble in toluene. Complexes **1**–**3** were fully characterized by ^1^H, ^13^C{1H} and ^31^P{1H} NMR spectrometry, HRMS, IR spectroscopy and satisfactory elemental analysis (Supplementary Figs. S2–S24). ^19^F{1H} NMR spectrum of **1'** was also record to show the signal at *δ* = –151.16 ppm for the BF_4_^–^ anon (Supplementary Fig. S12). The ^1^H NMR spectra of **1** and **1'** (Fig. 2) in CD_3_CN at room temperature show broad peaks for the pyridyl signals (Supplementary Figs. S2 and S9), whereas only sharp peaks were observed for those of **2** and **3** under the same conditions (Supplementary Figs. S15 and S20). It is attributable to the restricted rotation between two half units in **1** resulting from the presence of bulky *tert*-butyl groups. These signals can be restored into sharp peaks in other solvents, such as CDCl_3_ or (CD_3_)_2_SO (Supplementary Figs. S5 and S6), indicating the freely rotation about the Ir–Ir bond in such media. Their IR spectra show an absorption peak at 2035-2060 cm^-1^, assignable to the ν(C ≡ O) stretching frequency.

### Structure and Bonding

**Fig. 3:**
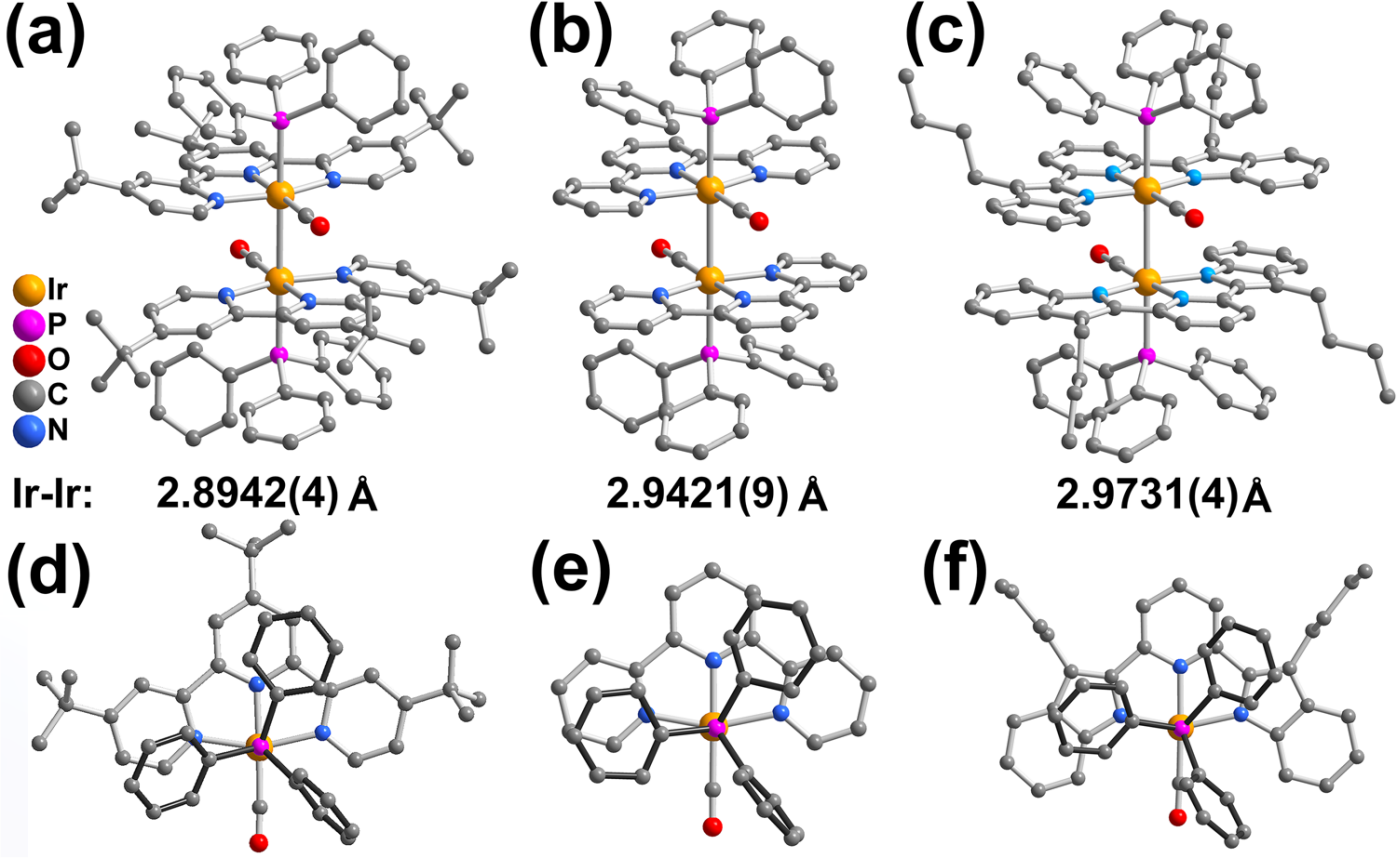
X-ray crystal structures of 1–3. Complex cations of **1**–**3** (**a**–**c**) and their corresponding half fragments from top view (**d**–**f**). Hydrogen atoms and solvent molecules are omitted for clarity.

The molecular structures of **1**–**3** were determined by X-ray crystallography. Their structural data, selected bond distances and angles are summarized in Supplementary Tables S1–S12. This class of complexes represent the first structural characterized example of tetracationic diiridium(II) system without any bridging ligands. As depicted in Fig. 3a–c, all the complex cations consist of two [Ir(II)(N^N^N)(CO)(PPh_3_)]^2+^ fragments in a head-to-tail arrangement and connected by an unsupported Ir(II)–Ir(II) single bond. Each iridium(II) metal centre is coordinated with one N^N^N pincer and one CO ligands on the equatorial plane, while the PPh_3_ ligand and another iridium(II) metal centre are bound in the axial position to exhibit a distorted octahedral geometry. In all cases, four counter-anions of OTf^–^ (or BF_4_^–^) are positioned around the corresponding complex cation (Supplementary Fig. S25). It is noteworthy that an interesting structural feature of these complexes is generally the longer Ir(II)–Ir(II) bond distance (**1**, 2.8942(4) Å; **2**, 2.9421(9) Å; **3**, 2.9731(4) Å), compared to the previously reported unsupported neutral or dicationic diiridium(II) complexes (2.66–2.84 Å)^12–23^.

The shortest Ir–Ir bond was observed in **1** even with bulky *tert*-butyl groups, whereas the longest one was found in **3** with the larger π-conjugated pincer ligand of bzimpy. By changing the counter-anion from OTf^–^ to BF_4_^–^ in **1'** as shown in Supplementary Fig. S25, the complex cation exhibited similar structural features with elongation of the Ir–Ir bond (2.9135(5) Å). The change in this Ir–Ir bond length is ascribed to the different non-covalent interaction between the complex cation and counter anions in crystal packing. The C ≡ O bond distances of 1.121(8)–1.135(4) Å are in the typical range of transition metal complexes. In **1**, the peripheral pyridine rings of ^*t*^Bu_3_-terpy are found to tile from the central pyridine group with the interplanar angles of 10.674(67)–13.455(59)°, attributable to the mutual repulsion from bulky *tert*-butyl groups. On the other hand, such deviation from coplanarity in the pincer ligand is diminished in **2** and **3** with the interplanar angles of 4.496(252)–5.383(258)° and 4.430(94)–4.507(102)°, respectively. The interplanar distances between the peripheral rings of pincer ligands on two half units are 3.2050(20)–3.2738(21) Å (**1**), 3.0761(71)–3.1702(77) Å (**2**) and 3.1890(36)–3.1981(37) Å (**3**), indicating the presence of π–π interactions. In addition, two phenyl rings on the axial PPh_3_ ligand are arranged in a parallel way to the pincer ligand with small tilted angles and short distances for the better π–π stacking (Fig. 3d–f).

**Fig. 4:**
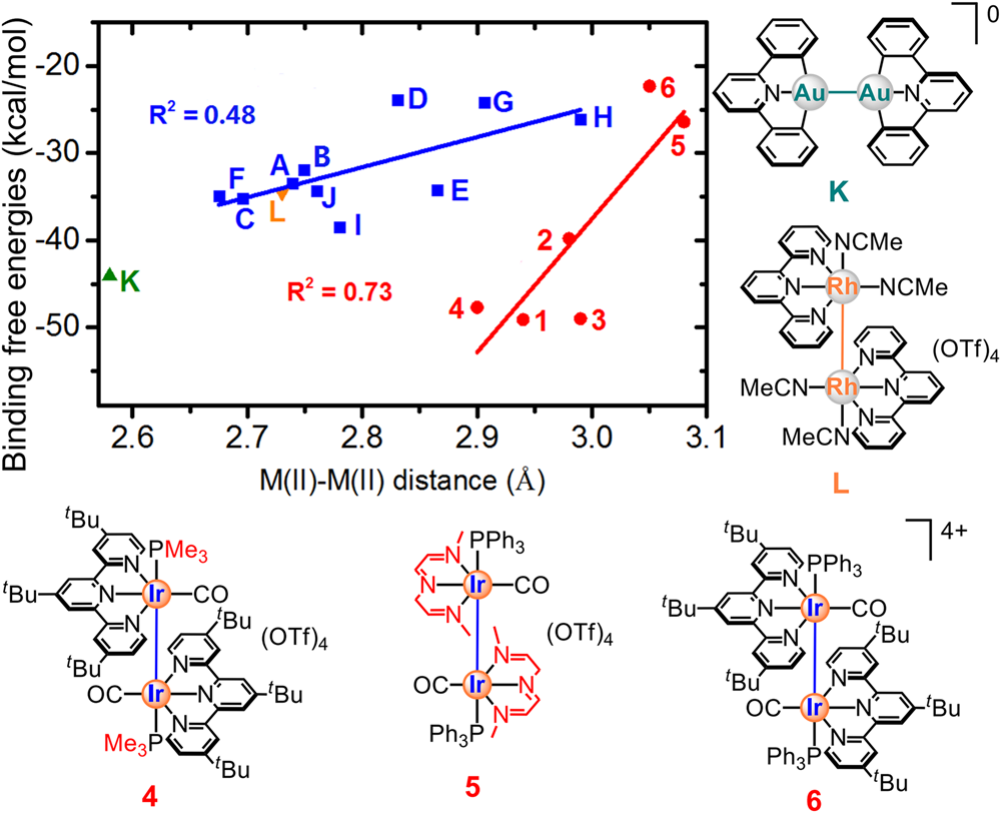
Correlation of the computed M(II)-M(II) bond distances and binding free energies. The plot of metal(II)–metal(II) distances (Å) and their relative binding free energies (in kcal/mol) for some unsupported metal(II)–metal(II) complexes (M: Ir, Rh or Au) in acetonitrile by the SMD M06-L//M06-L method.

DFT (including M06-L, M06, B3LYP-D3, PBE0-D3 and MN15 methods) and high-level DLPNO-CCSD(T) calculations^55–62^ were performed to examine the unusual tetracationic Ir(II) complexes **1**–**3** with unsupported long Ir(II)–Ir(II) bond. In addition, three model complexes (**4–6**), other previously reported complexes with unsupported metal–metal bond, including ten Ir(II)–Ir(II), one Rh(II)–Rh(II) and one Au(II)–Au(II) complexes (**A–L**), were also examined for comparison (Fig. 4)^12–23,63,64^. Despite the computed Ir–Ir bonds of **1**–**3** (2.94–2.99 Å) are generally longer, the present system was surprisingly computed to have a larger binding free energy (ΔG_soln_: *ca*. −49 kcal/mol by the SMD M06-L//M06-L method), compared to **A**–**J** (ΔG_soln_: *ca*. −24 to −39 kcal/mol), **K** (ΔG_soln_: −44 kcal/mol) and **L** (ΔG_soln_: −35 kcal/mol), as shown in Fig. 4. Such energetic trend was qualitatively supported by different DFT and high-level DLPNO-CCSD(T) methods (Supplementary Fig. S26 and Supplementary Table S13). It is noteworthy that the diiridium(II) complexes **1**–**3** were found to have such large binding energies, even though they have smaller electron density (ρ) and positive electron density (∇^2^ρ) values^65–69^ (Supplementary Table S13). Our computational study clearly manifests that the bonding features (a longer bond distance with a larger binding energy) for the tetracationic complexes **1**–**3** are unprecedented.

**Fig. 5:**
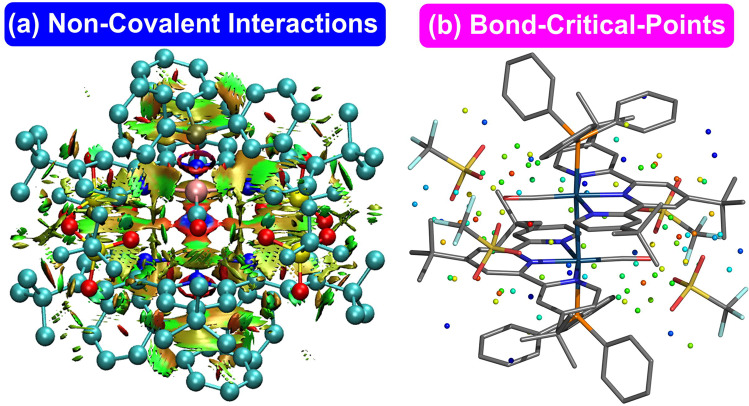
Non-covalent interactions of 1. **a** Non-covalent interactions (NCIs) analysis (red: strong repulsion; green: weak attraction; blue: strong attraction) and **b** the QTAIM analysis (Bond-Critical-Points (BCPs) in a sphere form; red: highest ρ; blue lowest ρ) in **1** based on the SMD M06-L//M06-L methods.

In order to further unravel the bonding features of **1**–**3**, distortion/interaction analysis^70^ was employed and these results showed that the large binding energy is mainly attributed to the considerable interaction energy (Supplementary Fig. S27a). Interestingly, a much larger interaction energy plays the key role of the largest binding energy determined in **1** (ΔE_int,soln_: *ca*. −91 kcal/mol), relative to **2** and **3** (*ca*. −65 to −68 kcal/mol). While, entropy effect favors **3** >**1**, which reduces their binding free-energy difference. Empirical dispersion (e.g. D3 contribution for B3LYP method) correction^61^ (Supplementary Fig. S27b and Supplementary Table S14) and non-covalent interactions (NCIs) analysis^71^ further demonstrated that London dispersion (Fig. 5a for **1** and Supplementary Fig. S28a for **2** and **3**), including π–π interactions among the two pincer ligands and the four OTf^–^ counterions, play one of the key roles in their high binding energies. These interactions can also be visualized by the bond-critical-points (BCPs) from the results of the quantum theory of atoms in molecules (QTAIM) method^72,73^. (Fig. 5b for **1** and Supplementary Fig. S28b for **2** and **3**). In this connection, a longer Ir–Ir bond distance (3.08 Å) and much smaller binding energy (ΔG_soln_: *ca*. −26.4 kcal/mol) were also computed for model complex **5**, in which a smaller and less conjugated tridentate pincer ligand with smaller dispersion stabilization was adopted. This result is in agreement with other previous computational studies suggesting the importance of London dispersion in some metal complexes^74–81^. As shown in Supplementary Table S14, both metal–metal^82^ and non-covalent interactions among the tridentate pincer ligands and counterions should be generally the critical factors in stabilizing the rare Ir(II)–Ir(II) bond and rendering the unusually large binding energies for tetracationic complexes **1**–**3** by conquering unfavorable and strong electrostatic repulsions.

### Photophysical and electrochemical behaviours

**Fig. 6:**
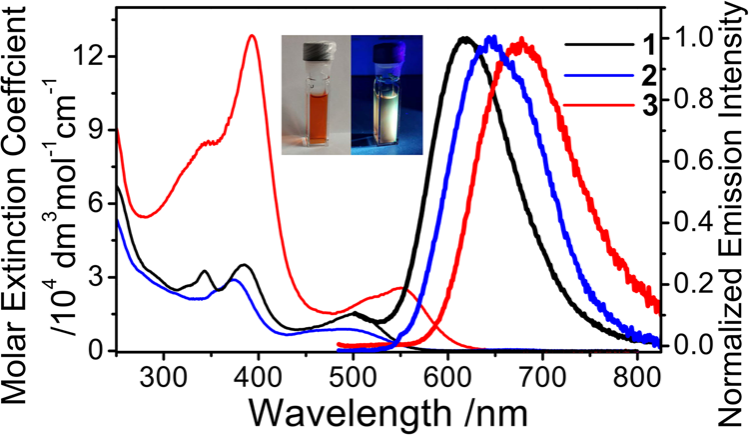
Photophysical studies of 1–3 in solution state. UV-vis absorption (thin line) and emission (thick line) spectra of complexes **1**–**3** in CH_3_CN solution at 298 K. Excitation at *λ*_ex_ = 470 nm. Inset shows the photographs of **1** in degassed CH_3_CN solution under ambient light and UV light.

The photophysical and electrochemical behaviours of **1**–**3** have been investigated and the data are summarized in Table 1. Their UV-vis absorption spectra of **1**–**3** in CH_3_CN are also depicted in Fig. 6. In addition to the intraligand (IL) π–π* absorptions of N^N^N pincer ligand at 343 and 385 nm, the UV-Vis absorption spectra exhibit a low-energy absorption band at 493–553 nm. Our TD-DFT (CPCM TD-B3LYP-D3//M06-L) calculations^18^ suggested that the low-energy absorption is mainly ascribed to metal–metal bond-to-ligand charge transfer (MMLCT) dσ(Ir–Ir) → π*(N^N^N ligand) transition with some mixing of dσ(Ir–Ir) → dσ*(Ir–Ir) character (Supplementary Figs. S29–S31). Such an assignment is also consistent with the computed composition of the donor/highest occupied molecular orbital (Ir–Ir, 31–37%; P, 18–23%; N^N^N ligand, 15–18%) and the acceptor/lowest unoccupied orbital (Ir–Ir, 13–18%; P, 1–2%; N^N^N ligand, 62–80%) for **1**–**3** by the M06-L method (Table 2). Figure 5a also shows the schematic frontier molecular orbital (FMO) diagram of the ground-state metal complexes, illustrating the key MOs involved in the lowest-lying electronic transition of the MMLCT character. Interestingly, this absorption band in **1** with electron-rich *tert*-buty groups exhibited a slight red-shift, relative to **2**. The change in the pincer ligand was found to vary the energy levels of HOMO and LUMO at the same time, as revealed from the potentials for the first oxidation and reduction (vide infra). In view of this, the MMLCT absorption energy could not simply correlate to the π* orbital energy level by subtle modification in the pincer ligand. For complex **3** with substantial lower-lying π* orbital in bzimpy ligand, this absorption band was found to shift to lower energy significantly. Despite of this, our TD-DFT calculations also suggested the red-shift absorption trend of the two key low-lying transitions from **1** to **3** (**1**: 377 and 496 nm; **3**: 398 and 540 nm; Supplementary Tables S15–S17). This result further supports the assignment of such low-energy absorption band as MMLCT transition based on the substantial lower-lying π* orbital energy level of bzimpy ligand in **3** (540 nm), relative to **1** (496 nm) with terpyridine ligand.

**Table 1:** Photophysical and electrochemical data of **1**–**3**.

	Absorption^a^ λ_abs_, nm (ε, ﻿10^4^ dm^3^ mol^–1^ cm^–1^)	Medium	Emission λ_em_, nm (τ, ps)	Emission quantum yield Φ_lum_	Ox.^b^ E_pa_^c^, V vs. SCE	Red.^b^ E_pc_^d^, V vs. SCE
**1**	343 (3.29), 385 (3.52), 502 (1.52)	CH_3_CN^e^ (298 K) solid (298 K) solid (77 K)	620 (63; 294) 653 (90; 360) 624	4.99 × 10^–4^ _^f^ _^f^	+1.80	–0.4
**2**	352 (2.37), 375 (2.89), 494 (0.89)	CH_3_CN^e^ (298 K) solid (298 K) solid (77 K)	650 (55; 359) 632 (135) 610	2.62 × 10^–4^ _^f^ _^f^	+1.90	–0.30
**3**	343 (8.47), 394 (12.90), 513 (2.07), 551 (2.89)	CH_3_CN^e^ (298 K) solid (298 K) solid (77 K)	678 (55; 292) 673 (68; 396) 647	0.85 × 10^–4^ _^f^ _^f^	+1.73	–0.26

^a^ In acetonitrile. ^b^ In acetonitrile solution with ^*n*^Bu_4_NPF_6_ (0.1 M) as the supporting electrolyte at room temperature; scan rate 100 mV s^-1^. ^c^ E_pa_ refers to the anodic peak potential for the irreversible oxidation waves. ^d^ E_pc_ refers to the cathodic peak potentials for irreversible reduction waves. ^e^ In degassed solution at 298 K. ^f^ Not determined.

**Table 2:** Composition of donor and acceptor molecular orbitals of **1**–**3** in gas phase by the M06-L method.

	MO	Ir, %	P, %	Pincer, %
**1**	Acceptor (LUMO)	18	2	62
	Donor (HOMO)	37	23	17
**2**	Acceptor (LUMO)	15	1	76
	Donor (HOMO)	31	19	15
**3**	Acceptor (LUMO)	13	1	80
	Donor (HOMO-2)	31	18	18

**Fig. 7:**
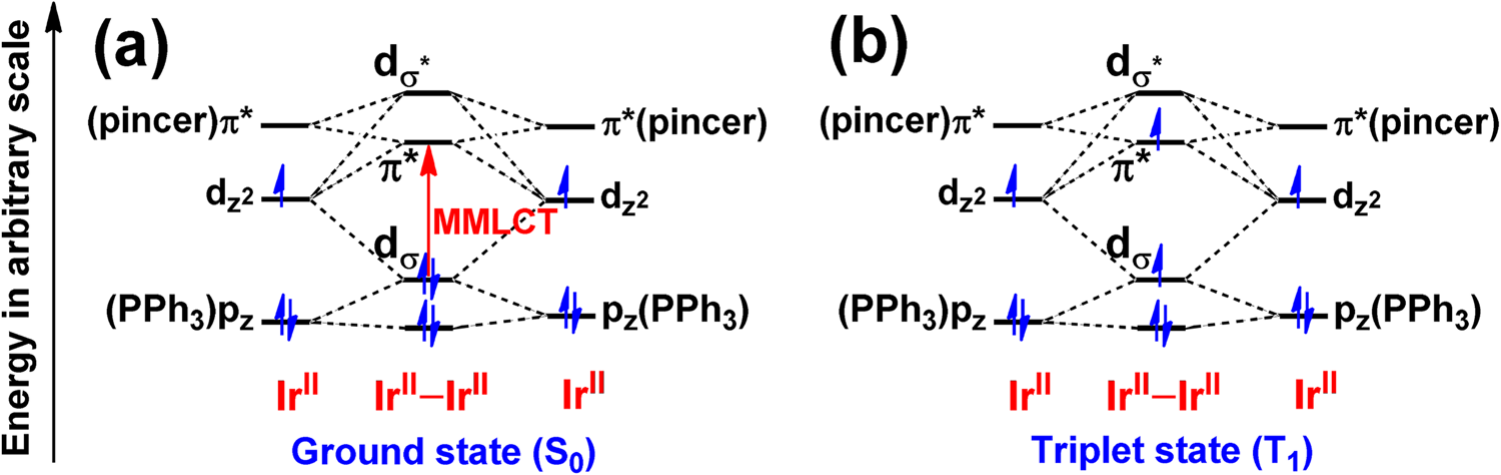
Illustration of the origin and Ir–Ir bond wenkening in triplet excited state. Schematic frontier molecular orbital (FMO) diagrams of **1**–**3** in the ground (**a**) and the lowest-lying triplet (**b**) states. Note that the relative energy is an arbitrary value. Only the key orbitals with main contribution are shown.

Upon excitation at λ_ex_ > 450 nm on their MMLCT absorption bands, **1**–**3** were found to exhibit orange-red luminescence at 620–678 nm in degassed CH_3_CN solution at 298 K (Fig. 6). To the best of our knowledge, this is the first example of unsupported diiridium(II) systems showing luminescence. Their excitation bands from the excitation spectra in degassed CH_3_CN solution were found to resemble to the corresponding low-energy absorption bands (Supplementary Fig. S32). In addition, the luminescence intensity of **1** diminishes to around 50% as in aerated CH_3_CN solution. Collectively, together with the large Stokes shift, such luminescence of **1**–**3** is assigned to be originated from the triplet metal–metal bond-to-ligand charge transfer (^3^MMLCT), with some mixing of dσ(Ir–Ir) → dσ*(Ir–Ir) character, as shown in Fig. 7b. This assignment is also qualitatively consistent with the spin density of the optimized triplet structure and emission transition for ^**3**^**1** by the M06-L and TD-B3LYP-D3//M06-L methods, respectively (Supplementary Figs. S33–S36). Similar to the low-energy absorption bands, the luminescence energy of **3** (678 nm) is lower than those of **1** (620 nm) and **2** (650 nm), which further supports the nature of ^3^MMLCT origin. Qualitatively, our CPCM TD-B3LYP-D3//M06-L (including effect of spin-orbital coupling) calculations^83–85^ also supported the observed red-shift luminescence of **3** for the assignment of ^3^MMLCT excited state (**1**: ~611 nm vs. **3**: ~644 nm; Supplementary Tables S18–S20). It is noteworthy that the UV-vis absorption and luminescence spectra of **1'** (Supplementary Fig. S37) with the counter anion of BF_4_^–^ showed essentially the same spectra as in **1**, indicative of insignificant influence from its counter anion in the solution state.

The luminescence quantum yield (Φ_lum_) of **1**–**3** (**1**, 4.99 × 10^–4^; **1'**, 4.86 × 10^–4^; **2**, 2.62 × 10^–4^; **3**, 0.85 × 10^–4^) were obtained in degassed CH_3_CN. The decrease in Φ_lum_ from **1** to **3** with lower luminescence energy is probably due to the effect arising from the energy gap law. It is noteworthy that very short luminescence lifetimes (τ_1_ = 55-63 ps; τ_2_ = 291–389 ps) were revealed in degassed CH_3_CN or in solid state 298 K (Table 2 and Supplementary Figs. S38–S39). Such short luminescence lifetime and low Φ_lum_ could be realized by intersystem crossing back to the ground state from the triplet-state minimum, which could facilitate non-radiative decay pathway with a low-energy barrier at their minimum crossing point (MECP)^86^ as suggested from our DFT calculations for **1** (Supplementary Fig. S40). In addition, photogeneration of radical monomer as a competing process is also responsible for the rapid deactivation process for the ^3^MMLCT excited state (vide infra). On the other hand, **1**–**3** were also found to exhibit photoluminescence at 632–673 nm in the solid state at 298 K (Supplementary Fig. S41a), while the corresponding luminescence at 77 K showed a blue-shift energy at 610–648 nm with narrower band shape (Supplementary Fig. S41b). It is interesting to note that the emission energies of **1'** in solid state at room temperature and 77 K are found to be higher than those of **1** (Supplementary Fig. S42). This could be rationalized by the lower-lying HOMO in **1'** resulting from the longer Ir–Ir bond, which was observed from their crystal structures. Since the LUMOs of **1** and **1'** are predominantly of π* orbital of the same terpyridine ligand, larger HOMO-LUMO energy gap in **1'** could give rise to higher emission energy in solid state.

The electrochemical behaviours of **1–3** were also studied by cyclic voltammetry in CH_3_CN (0.1 M ^*n*^Bu_4_NPF_6_) at 298 K (Table 1 and Supplementary Fig. S43). The oxidative scan showed an irreversible anodic wave (**1**, +1.80 V; **2**, +1.90 V; **3**, +1.73 V) (*vs*. SCE), attributed to the Ir(II) metal centre oxidation with some mixing of PPh_3_ ligand. The less positive potential for this oxidation in **1**, relative to **2**, is ascribed to the more electron-rich Ir(II) metal centre, through the incorporation of terpyridine ligand with electron-donating *tert-*butyl groups. Upon reductive scan, an irreversible cathodic wave at –0.26 V to –0.40 V (*vs*. SCE) was observed, which is reasonably assigned as the reduction on the terpyridine ligand with some σ*(Ir(II)–Ir(II)) character. The reduction potentials of these cathodic waves (**1**, –0.26 V; **2**, –0.30 V; 3, –0.40 V) are in agreement with the π* orbital energy level of the pincer ligand. Compared to **1** and **2**, the smallest potential difference between the potentials for oxidation and reduction in **3** is well correlated with the observation of the smallest MMLCT absorption energy. The essentially irreversible nature of this reduction process is probably due to dissociation of the diiridium(II) framework, resulting from the population of σ*(Ir(II)–Ir(II)) orbital. In **1**, an additional anodic peak at –0.16 V is only emerged after the first reduction scan beyond –0.40 V (Supplementary Fig. S43a), indicative of the oxidation of the decomposed product. Similar to the photophysical properties, the observation of almost the same reduction potentials for the reduction and oxidation of **1'** (Supplementary Fig. S44) and **1**, indicated the insignificant effect from the change of counter anion in solution state.

### Photoreactivity

**Fig. 8:**
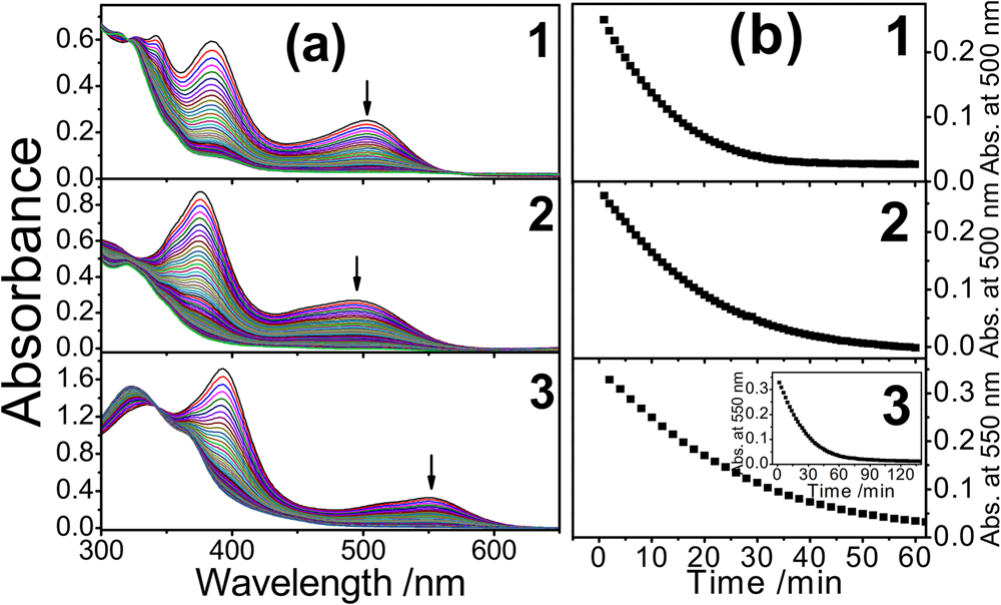
Photostability studies of 1–3. **a** UV-vis spectral changes of **1**–**3** in CH_3_CN solution at 298 K upon irradiation at 500 nm. **b** The plot of absorbance versus time for **1** and **2** (1-min time interval), and **3** (2-min time interval).

In connection with the unprecedentedly high binding energy by our DFT calculations, **1**–**3** were found to be inert towards O_2_, H_2_O, as well as Br_2_ and I_2_ in CH_3_CN solution. In view of the corresponding bonding(Ir–Ir) and anti-bonding(Ir–Ir) characters in their donor and acceptor orbitals (Fig. 7), the Ir–Ir bond cleavage resulting from photoexcitation would be anticipated to generate the respective radical monomer [Ir(N^N^N)(CO)(PPh_3_)]^2+•^^20^. As shown in Fig. 8, such photoreactivity behaviour was realized by the UV-vis spectral changes of **1**–**3** in CH_3_CN solution upon photoirradiation at the region of the MMLCT absorption band (Fig. 8a). By keeping the absorbance of **1**–**3** the same at 500 nm for the photoirradiation, the absorbance changes with time were monitored and their relative photostabilities could be qualified as **1** < **2** < **3** (Fig. 8b). The higher photostability would be envisioned for the complex with the computed smaller contribution from Ir(II) metal centre for the donor and acceptor orbitals (Table 2), which is in line with the experimental results of their relative photostabilities. The vanishment of the MMLCT absorption bands suggests that the photogenerated radical monomers would repel from each other to avoid the radical-radical coupling for the backward formation of the diiridium(II) complex. The photostability of **1'** was also found to be similar to that of **1**, suggesting that the influence of counter anion is insignificant in the solution state.

**Fig. 9:**
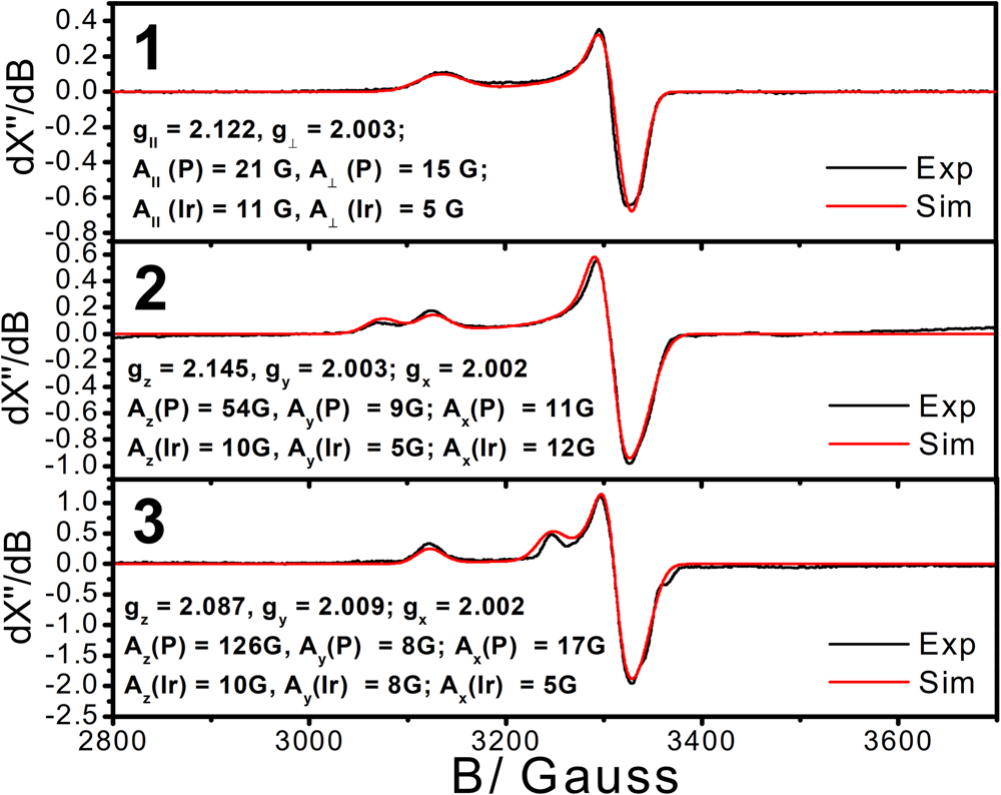
X-Band EPR spectra of 1–3. The spectra were recorded for the samples in CH_3_CN at 100 K after white light photoirradiation for 5 min at 298 K (black) and the simulated spectra (red). Frequency: 9.299527 GHz; modulation amplitude: 8.0 G; power: 2.0 mW.

Electron paramagnetic resonance (EPR) spectroscopy is a suitable technique to probe the formation of radical monomer upon photoirradiation. The EPR spectra of **1**–**3** in CH_3_CN at 100 K after photoirradiation for 5 min in solution state are depicted in Fig. 9. The EPR spectra clearly indicate that the photogenerated species are of S = 1/2 paramagnetic nature with typical axial symmetry^87,88^. On the basis of the *g*-tensor values (g_z_ > g_x_,g_y_ > 2) suggestive of a compressed octahedral structure^88^, [Ir(N^N^N)(CO)(PPh_3_)(CH_3_CN)]^2+•^ species is likely generated arising from readily occupation of the vacant site in 5-coordinated [Ir(N^N^N)(CO)(PPh_3_)]^2+•^ by a CH_3_CN solvent molecule. It is noteworthy that no corresponding EPR signal would be observed for**1**–**3** in the absence of photoirradiation, which further supports the formation of radical being orginated from the photocleavage process.

**Fig. 10:**
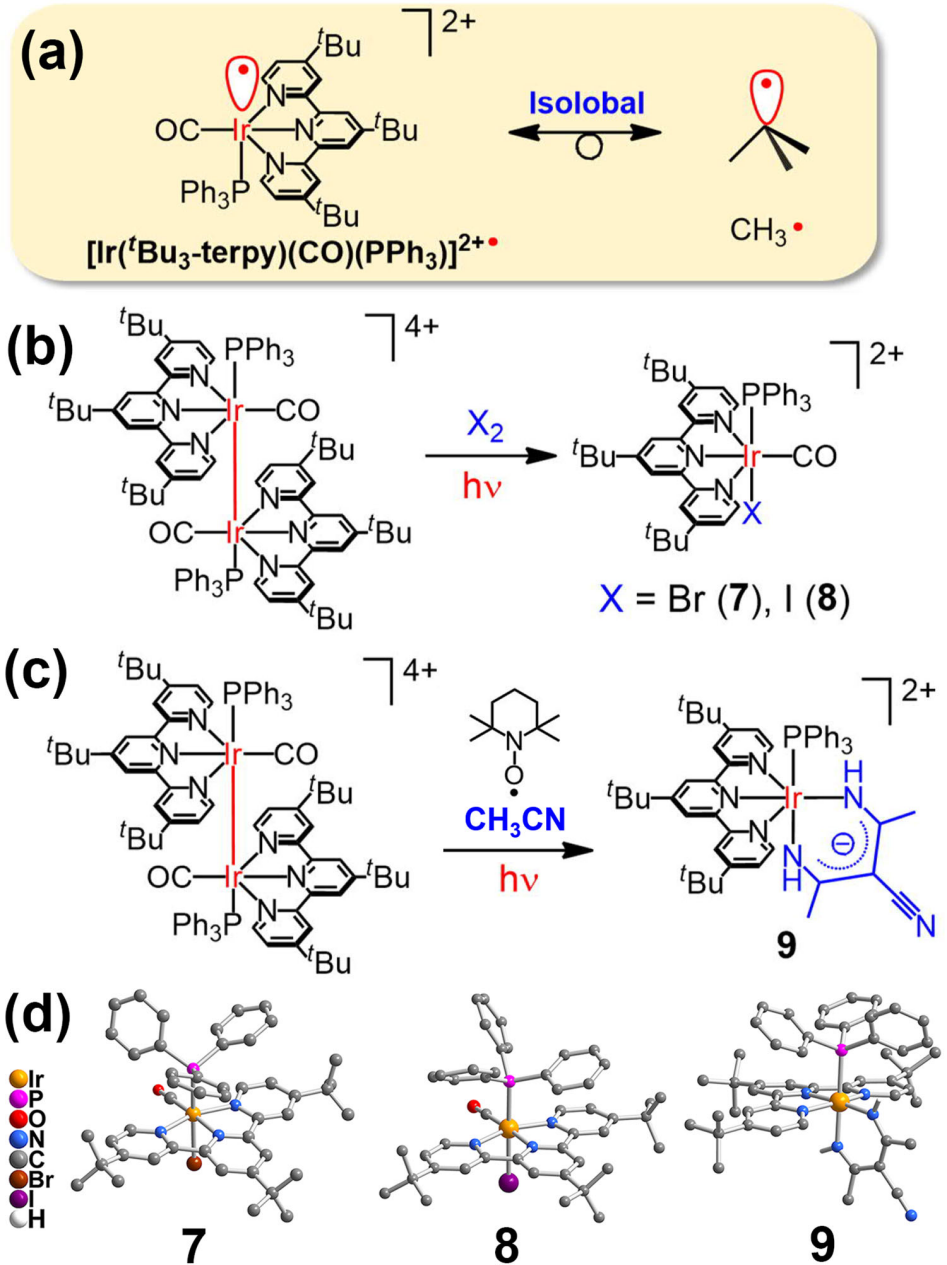
Photoreactivity of 1. **a** Isolobal analogy of [**1**_1/2_]^2+•^ radical. Photoreaction of **1** with Br_2_ or I_2_ (**b**); and with TEMPO (**c**) in CH_3_CN solution at 298 K. The perspective drawing of molecular cations of **7**–**9** (**d**). Hydrogen atoms are omitted for clarity, except for the imine groups in **9**.

According to the isolobal analogy, the d^7^ five-coordinate [**1**_1/2_]^2+•^ species upon photocleavage is isolobal to CH_3_^•^ radical with similar reactivity towards Br_2_ or I_2_ (Fig. 10a). Interestingly, mononuclear iridium(III) complexes, [Ir(^*t*^Bu_3_-terpy)(CO)(PPh_3_)X]^2+^ [X = Br, **7** (yield = 83%); I, **8** (yield = 78%)] were afforded from the reactions of **1** with X_2_ under photoirradiation at room temperature (Fig. 10b). Such photoreactions are possibly triggered from the photo-induced cleavage of the Ir–Ir bond in **1** because the related reactions were not observed in the dark. Based on the photoreactivity and EPR studies, generation of a reactive radical monomer is suggested for these reactions. Although the excited-state potential for the oxidation process of **1**^III/II^* (–0.45 V *vs*. SCE) is comparable or sufficient for the reduction of Br_2_ (+0.47 V) or I_2_ (+0.26 V), the very short excited-state lifetime should unfavour this bimolecular photo-induced electron-transfer process from the ^3^MMLCT excited state.

Attempts have been made to trap the radical species by a common spin trapping agent, TEMPO. Surprisingly, an unprecedented complex **9**, [Ir(^*t*^Bu_3_-terpy)(PPh_3_){HNC(CH_3_)}_2_C(CN)]^2+^ (86%), was isolated from the reaction of **1** with TEMPO upon photoirradiation (Fig. 10c). Uncommon trimerization of acetonitrile is ascribed to the formation of the chelating ligand, {HNC(CH_3_)}_2_C(CN), and the proposed mechanism is suggested in Supplementary Fig. S45. It is noteworthy that this reaction cannot occur in the dark or by replacing TEMPO with potassium *tert*-butoxide. These results indicate that this reaction should involve the photogeneration of radical monomer and could not be initiated by simple deprotonation of acetonitrile. One related example about trimethylgallium-induced trimerization of acetonitrile was also reported in the presence of halide ions at 60 °C^89^. No related trapped species could be isolated from the reaction of **1** with other radical trapping reagents, butylated hydroxytoluene (BHT) and diphenylethylene (DPE). All the products **7**–**9** from these photoreactions were fully characterized by ^1^H, ^13^C{^1^H} and ^31^P{^1^H} NMR spectroscopy, HRMS, IR spectroscopy and satisfactory elemental analysis (Supplementary Figs. S46–S60). Their molecular structures were also confirmed by X-ray crystallography, as shown in Fig. 10d. Their structural data, selected bond distances and angles are summarized in Supplementary Tables S21–S29.

## Conclusions

We report a series of tetracationic diiridium(II) complexes with an unsupported and long Ir(II)–Ir(II) bond for the first time. Our systematic computational study unveils that they possess the largest binding (dimerization) energy than the other related diiridium(II) complexes with the shorter metal–metal bond. Significant non-covalent London dispersion interactions were realized to overcome the considerable electrostatic repulsion between the two corresponding dicationic metal fragments. Moreover, these complexes were found to exhibit photo-luminescence in both solution and solid states, as the first example of luminescent unsupported diiridium(II) system. Photoreactions, including an interesting trimerization of acetonitrile, initiated from the generation of radical monomer were also explored. Experimental and computational studies on a series of unsupported diiridium(II) complexes were investigated for the understanding of their electronic structures. Tuning of photophysical and photoreactivity properties of these dirridium(II) complexes was achieved by changing the pincer ligands in this study. Further research for the improvement of photoluminescence efficiency or exploration of other possible photoactivated radical mediated reactions by variation of different ligands is ongoing. This work provides the first systematic study of a series of unsupported diiridium(II) system to open up an avenue for the fundamental understanding about the structural, bonding and photofunctional properties of these rare complexes.

## Method

### Experimental and computational details

See Supplementary Methods in the Supplementary Information.

### Analytical data including ^1^H, ^13^C{1H} and ^31^P{1H} NMR spectroscopy, HRMS, IR spectrometry

See Figs. S1–S24 and Figs. 46–60 in the Supplementary Information.

### Computaional results

See Tables S13–S20 and Tables S30–S48 in the Supplementary Information.

### General procedures

Unless otherwise noted, all experiments were performed under an atmosphere of nitrogen with the rigid exclusion of air and moisture using standard Schlenk or cannula techniques, or in a glovebox.

### Synthesis. Preparation of [Ir(II)(^*t*^Bu_3_-terpy)(CO)(PPh_3_)]_2_[OTf]_4_ (1)

AgOTf (56 mg, 218 μmol) was added to [IrCl(CO)(PPh_3_)_2_] (80 mg, 102 μmol) in dry and degassed THF (15 mL). The mixture was allowed to stir for 1 h at room temperature, and then the filtrate was transferred in a dropwise fashion to a solution of 4,4′,4″-tri-*tert*-butyl-2,2′:6′,2″-terpyridine (42 mg, 103 μmol) in THF (15 mL). The resulting solution was allowed to stir for 72 h at room temperature in the absence of light, after which, the red suspension was filtered and washed with multiple portions of tetrahydrofuran (20 mL) to give a red solid mixed with metallic Ag. The mixture was dissolved in a minimum amount of CH_3_CN to give a deep red solution. Recrystallization by the slow diffusion of diethyl ether to the filtrate gave 1 as red crystals (77 mg, 62%). ^1^H NMR (400 MHz, CD_3_CN, 298 K): *δ* 8.74 (br, 4H, ^*t*^Bu_3_-terpy C*H*), 8.31 (br, 4H, ^*t*^Bu_3_-terpy C*H*), 7.65 (br, 4H, ^*t*^Bu_3_-terpy C*H*), 7.45 (t, *J* = 7.5 Hz, 6H, PPh_3_ C*H*), 7.16 (t, *J* = 7.8 Hz, 12H, PPh_3_ C*H*), 6.69 (m, 16H, ^*t*^Bu_3_-terpy and PPh_3_ C*H*), 1.74 (s, 18H, ^*t*^Bu_3_-terpy CH_3_), 1.35 ppm (s, 36H, ^*t*^Bu_3_-terpy CH_3_). ^13^C{^1^H} NMR (100 MHz, CD_3_CN, 298 K): *δ* 171.6 (*C*O), 169.2, 166.7, 155.8, 155.2, 152.3, 133.5, 133.4, 131.0, 130.9, 128.2, 127.7, 127.2, 124.3, 124.0, 123.8, 123.7, 120.6 (aromatic *C* and *C*H), 38.3, 36.9 (*C*Me_3_), 31.0, 30.5 ppm (*C*H_3_). ^31^P{^1^H} NMR (162 MHz, CD_3_CN, 298 K): *δ* −14.2 ppm (*P*Ph_3_). IR (KBr disk): *v* = 2060 cm^−1^ (*v*(C ≡ O)), 1155 cm^−1^ (*v*(S = O)). HRMS (ESI). Calcd for C_95_H_100_F_9_Ir_2_N_6_O_11_P_2_S_3_ ([M − OTf]^+^): m/z 2215.5197. Found: m/z 2215.5212. Elemental analyses calcd for C_100_H_106_F_12_Ir_2_N_8_O_14_P_2_S_4_ (1·2CH_3_CN), found (calcd): C, 49.17 (49.09); H, 4.47 (4.37); N, 4.65 (4.58).

### Preparation of [Ir(II)(^*t*^Bu_3_-terpy)(CO)(PPh_3_)]_2_[BF_4_]_4_ (1')

This complex was prepared as red crystals from AgBF_4_ (42 mg, 218 μmol), [IrCl(CO)(PPh_3_)_2_] (80 mg, 102 μmol) and 4,4′,4″-tri-*tert*-butyl-2,2′:6′,2″-terpyridine (42 mg, 103 μmol) in THF using the same procedure reported for 1: yield 65 mg (60%). X-ray-quality crystals were obtained by the slow diffusion of diethyl ether to the filtrate at room temperature. ^1^H NMR (400 MHz, CD_3_CN, 298 K): *δ* 8.71 (br, 4H, ^*t*^Bu_3_-terpy CH), 8.30 (br, 4H, ^*t*^Bu_3_-terpy CH), 7.60 (br, 4H, ^*t*^Bu_3_-terpy C*H*), 7.44 (t, *J* = 7.6 Hz, 6H, PPh_3_ CH), 7.17 (t, *J* = 7.6 Hz, 12H, PPh_3_ CH), 6.71 (dd, *J1* = 12.8, *J2* = 6.4 Hz, 12H, PPh_3_ C*H*), 6.63 (br, 4H, ^*t*^Bu_3_-terpy CH), 1.73 (s, 18H, ^*t*^Bu_3_-terpy CH_3_), 1.35 ppm (s, 36H, ^*t*^Bu_3_-terpy CH_3_). ^13^C{^1^H} NMR (100 MHz, CD_3_CN, 298 K): *δ* 171.8 (*C*O), 169.5, 166.9, 155.5, 155.2, 152.2, 133.5, 133.4, 133.3, 131.0, 130.9, 128.0, 124.4, 124.1, 123.9 (aromatic *C* and CH), 38.2, 36.8 (*C*Me_3_), 31.0, 30.5 ppm (CH_3_). ^31^P{^1^H} NMR (162 MHz, CD_3_CN, 298 K): *δ* −13.7 ppm (*P*Ph_3_). ^19^F{^1^H} NMR (376 MHz, CD_3_CN, 298 K): *δ* −151.16 ppm (B*F*_4_^–^). IR (KBr disk): *v* = 2056 cm^−1^ (*v*(C ≡ O)). HRMS (ESI). Calcd for C_92_H_100_B_3_F_12_Ir_2_N_6_O_2_P_2_ ([M – BF_4_]^+^): m/z 2029.6724. Found: m/z 2029.6755. Elemental analyses calcd for C_92_H_100_B_4_F_16_Ir_2_N_6_O_2_P_2_ (1'), found (calcd): C, 52.01 (52.23); H, 4.88 (4.76); N, 4.04 (3.97).

### Preparation of [Ir(II)(terpyridine)(CO)(PPh_3_)]_2_[OTf]_4_ (2)

AgOTf (56 mg, 218 μmol) was added to [IrCl(CO)(PPh_3_)_2_] (80 mg, 102 μmol) in dry and degassed CH_3_CN (15 mL). The mixture was allowed to stir for 1 h at room temperature, and then the filtrate was transferred in a dropwise fashion to a solution of 2, 2':6',2''-terpyridine (24 mg, 103 μmol) in CH_3_CN (15 mL). The resulting solution was allowed to stir for 7 days at room temperature in the absence of light, after which, the deep brown suspension was filtered and the filtrate was concentrated to 2 mL. Recrystallization by the slow diffusion of diethyl ether to the filtrate gave 2 as leaf-shaped brown crystals (59 mg, 55%). ^1^H NMR (400 MHz, CD_3_CN, 298 K): *δ* 8.35 (d, *J* = 5.6 Hz, 4H, terpyridine CH), 8.31 (t, *J* = 8.2 Hz, 2H, terpyridine CH), 8.02 (t, *J* = 8.0 Hz, 4H, terpyridine CH), 7.90 (d, *J* = 8.2 Hz, 4H, terpyridine CH), 7.79 (d, *J* = 8.0 Hz, 4H, terpyridine CH), 7.46 (t, *J* = 7.5 Hz, 6H, PPh_3_ CH), 7.38 (t, *J* = 6.1 Hz, 4H, terpyridine CH), 7.18 (t, *J* = 7.9 Hz, 12H, PPh_3_ CH), 6.72 ppm (m, 12H, PPh_3_ CH). ^13^C{^1^H} NMR (100 MHz, CD_3_CN, 298 K): *δ* 173.1 (CO), 156.9, 154.1, 150.6, 143.2, 143.0, 133.9, 133.3, 133.2, 133.1, 131.2, 131.1, 128.5, 127.6, 123.8, 123.5, 123.2, 123.0, 120.6 ppm (aromatic *C* and CH). ^31^P{^1^H} NMR (162 MHz, CD_3_CN, 298 K): *δ* −12.3 ppm (PPh_3_). IR (KBr disk): *v* = 2060 cm^−1^ (*v*(C ≡ O)), 1157 cm^−1^ (*v*(S = O)). HRMS (ESI). Calcd for C_71_H_52_F_9_Ir_2_N_6_O_11_P_2_S_3_ ([M–OTf]^+^): m/z 1879.1441. Found: m/z 1879.1433. Elemental analyses calcd for C_72_H_56_F_12_Ir_2_N_6_O_16_P_2_S_4_ (2·2H_2_O), found (calcd): C, 41.38 (41.90); H, 2.83 (2.73); N, 4.19 (4.07).

### Preparation of [Ir(II)(*n*-Bu_2_bzimb)(CO)(PPh_3_)]_2_[OTf]_4_ (3)

This complex was prepared as deep red crystals from AgOTf (56 mg, 218 μmol), [IrCl(CO)(PPh_3_)_2_] (80 mg, 102 μmol) and 2,2′-(1,3-phenylene)bis[1-butyl-1*H*-benzimidazole] (44 mg, 103 μmol) (*n*-Bu_2_bzimb) in THF using the same procedure reported for 1: yield 71 mg (58%). X-ray-quality crystals were obtained by the slow diffusion of diethyl ether to the filtrate at room temperature. ^1^H NMR (400 MHz, CD_3_CN, 298 K): *δ* 8.92 (t, *J* = 8.2 Hz, 2H, *n*-Bu_2_bzimb CH), 8.55 (d, *J* = 8.3 Hz, 4H, *n*-Bu_2_bzimb CH), 7.53 (d, *J* = 7.3 Hz, 4H, *n*-Bu_2_bzimb CH), 7.46 (d, *J* = 7.3 Hz, 4H, *n*-Bu_2_bzimb CH), 7.27 (m, 10H, *n*-Bu_2_bzimb and PPh_3_ CH), 6.90 (m, 16H, *n*-Bu_2_bzimb and PPh_3_ CH), 6.36 (dd, *J1* = 12.8, *J2* = 6.4 Hz, 12H, PPh_3_ CH), 6.63 (s, 18H, ^*t*^Bu_3_-terpy CH_3_), 4.41 (m, 4H, NCH_2_CH_2_CH_2_CH_3_), 4.16 (m, 4H, NC*H*_2_CH_2_CH_2_CH_3_), 1.57 (m, 16H, NCH_2_C*H*_2_C*H*_2_CH_3_), 1.12 ppm (t, *J* = 6.8 Hz, 12H, NCH_2_CH_2_CH_2_CH_3_). ^13^C{^1^H} NMR (100 MHz, CD_3_CN, 298 K): *δ* 173.0 (*C*O), 147.8, 145.6, 144.7, 137.9, 134.9, 133.9, 133.4, 133.3, 130.3, 130.2, 129.3, 128.5, 123.8, 123.0, 122.8, 122.5, 120.6, 116.4, 114.7 (aromatic *C* and CH), 47.8 (N*C*H_2_CH_2_CH_2_CH_3_), 33.4 (NCH_2_*C*H_2_CH_2_CH_3_), 20.5 (NCH_2_CH_2_*C*H_2_CH_3_), 13.9 ppm (NCH_2_CH_2_CH_2_*C*H_3_). ^31^P{^1^H} NMR (162 MHz, CD_3_CN, 298 K): *δ* −11.0 ppm (*P*Ph_3_). IR (KBr disk): *v* = 2035 cm^−1^ (*v*(C ≡ O)), 1157 cm^−1^ (*v*(S = O)). HRMS (ESI). Calcd for C_95_H_88_F_9_Ir_2_N_10_O_11_P_2_S_3_ ([M–OTf]^+^): m/z 2259.4381. Found: m/z 2259.4390. Elemental analyses calcd for C_96_H_88_F_12_Ir_2_N_10_O_14_P_2_S_4_ (3), found (calcd): C, 47.68 (47.88); H, 3.86 (3.68); N, 5.80 (5.82).

### Photochemical reaction of 1. Preparation of [Ir(III)(^*t*^Bu_3_-terpy)(CO)(PPh_3_)Br][OTf]_2_ (7)

Bromine (7 mg, 42 μmol) was added to 1 (66 mg, 28 μmol) in dry and degassed CH_3_CN (15 mL). The mixture was allowed to stir for 12 h at room temperature upon irradiation of light, giving a brown solution which was then concentrated to 2 mL. Recrystallization by the slow diffusion of diethyl ether to the concentrated solution gave 7 as yellow crystals (55 mg, 78%). ^1^H NMR (400 MHz, CD_3_CN, 298 K): *δ* 8.89 (d, *J* = 6.2 Hz, 2H, ^*t*^Bu_3_-terpy CH), 8.39 (s, 2H, ^*t*^Bu_3_-terpy CH), 8.21 (d, *J* = 2.1 Hz, 2H, ^*t*^Bu_3_-terpy CH), 7.62 (m, 5H, ^*t*^Bu_3_-terpy and PPh_3_ CH), 7.37 (m, 6H, PPh_3_ CH), 7.14 (m, 6H, PPh_3_ CH), 1.61 (s, 9H, ^*t*^Bu_3_-terpy CH_3_), 1.41 ppm (s, 18H, ^*t*^Bu_3_-terpy CH_3_). ^13^C{^1^H} NMR (100 MHz, CD_3_CN, 298 K): *δ* 171.4 (*C*O), 169.5, 161.9, 161.8, 157.4, 156.8, 153.7, 134.6, 134.5, 134.2, 134.1, 131.2, 131.0, 129.0, 126.6, 125.6, 123.8, 123.5, 122.9, 120.6 (aromatic *C* and CH), 38.4, 37.2 (*C*Me_3_), 30.7, 30.2 ppm (CH_3_). ^31^P{^1^H} NMR (162 MHz, CD_3_CN, 298 K): *δ* −8.0 ppm (*P*Ph_3_). IR (KBr disk): *v* = 2102 cm^−1^ (*v*(C ≡ O)), 1157 cm^−1^ (*v*(S = O)). HRMS (ESI). Calcd for C_46_H_50_BrIrN_3_OP ([M − 2OTf]^2+^): m/z 481.6247. Found: m/z 481.6233. Elemental analyses calcd for C_50_H_53_BrF_6_IrN_4_O_7_PS_2_ (7·CH_3_CN), found (calcd): C, 45.97 (46.08); H, 4.22 (4.10); N, 4.09 (4.30).

### Preparation of [Ir(III)(^*t*^Bu_3_-terpy)(CO)(PPh_3_)I][OTf]_2_ (8)

This complex was prepared as yellow crystals from iodine (11 mg, 42 μmol) and 1 (66 mg, 28 μmol) in CH_3_CN using the same procedure reported for 7: yield 61 mg (83%). X-ray-quality crystals were obtained by the slow diffusion of diethyl ether to the concentrated solution at room temperature. ^1^H NMR (400 MHz, CD_3_CN, 298 K): *δ* 8.90 (d, *J* = 6.2 Hz, 2H, ^*t*^Bu_3_-terpy CH), 8.39 (s, 2H, ^*t*^Bu_3_-terpy CH), 8.21 (d, *J* = 2.1 Hz, 2H, ^*t*^Bu_3_-terpy CH), 7.62 (m, 5H, ^*t*^Bu_3_-terpy and PPh_3_ CH), 7.36 (m, 6H, PPh_3_ CH), 7.13 (m, 6H, PPh_3_ CH), 1.62 (s, 9H, ^*t*^Bu_3_-terpy CH_3_), 1.41 ppm (s, 18H, ^*t*^Bu_3_-terpy CH_3_). ^13^C{^1^H} NMR (100 MHz, CD_3_CN, 298 K): *δ* 171.1 (*C*O), 169.4, 162.3, 157.8, 157.0, 153.7, 134.5, 134.2, 134.1, 131.2, 131.1, 129.0, 126.6, 125.7, 123.8, 123.2, 122.6, 120.6 (aromatic *C* and CH), 38.4, 37.2 (*C*Me_3_), 30.7, 30.2 ppm (CH_3_). ^31^P{^1^H} NMR (162 MHz, CD_3_CN, 298 K): *δ* −9.4 ppm (*P*Ph_3_). IR (KBr disk): *v* = 2112 cm^−1^ (*v*(C ≡ O)), 1155 cm^−1^ (*v*(S = O)). HRMS (ESI). Calcd for C_47_H_50_F_3_IrIN_3_O_4_PS ([M–OTf]^+^): m/z 1160.1880. Found: m/z 1160.1893. Elemental analyses calcd for C_48_H_50_F_6_IrIN_3_O_7_PS_2_ (8), found (calcd): C, 44.00 (44.04); H, 4.01 (3.85); N, 3.12 (3.21).

### Preparation of [Ir(III)(^*t*^Bu_3_-terpy)(HNC(CH_3_)C(CN)C(CH_3_)NH)(PPh_3_)] [OTf]_2_ (9)

TEMPO (13 mg, 83 μmol) was added to 1 (24 mg, 10 μmol) in dry and degassed CH_3_CN (3 mL). The mixture was allowed to stir for 72 h at room temperature upon irradiation of 355 nm Xe lamp, giving a yellow solution which was then concentrated to 1 mL. Recrystallization by the slow diffusion of diethyl ether to the concentrated solution gave 9 as yellow crystals (22 mg, 86%). ^1^H NMR (400 MHz, CD_3_CN, 298 K): *δ* 8.42 (d, *J* = 6.1 Hz, 2H, ^*t*^Bu_3_-terpy CH), 8.27 (s, 2H, ^*t*^Bu_3_-terpy CH), 8.00 (d, *J* = 2.0 Hz, 2H, ^*t*^Bu_3_-terpy CH), 7.77 (br, 1H, N*H*), 7.64 (dd, *J1* = 6.1, *J2* = 2.1 Hz, 22H, ^*t*^Bu_3_-terpy CH), 7.50 (m, 3H, PPh_3_ CH), 7.27 (m, 6H, PPh_3_ CH), 6.89 (m, 6H, PPh_3_ C*H*), 6.77 (br, 1H, N*H*), 2.57, (s, 3H, HNC(C*H*_3_)), 1.89 (s, 3H, HNC(CH_3_)), 1.61 (s, 9H, ^*t*^Bu_3_-terpy CH_3_), 1.39 ppm (s, 18H, ^*t*^Bu_3_-terpy CH_3_). ^13^C{^1^H} NMR (100 MHz, CD_3_CN, 298 K): *δ* 168.8, 167.9, 166.1, 165.8, 159.0, 156.6, 153.6, 134.0, 133.9, 133.7, 133.0, 130.5, 130.4, 126.9, 124.7, 124.2, 124.0, 123.7, 123.6, 120.6 (aromatic *C* and CH, HN*C*(CH_3_)C(CN)*C*(CH_3_)NH), 122.1 (*C*N), 80.4 (*C*CN), 37.9, 36.8 (*C*Me_3_), 31.0, 30.4 (C(CH_3_)_3_), 28.3, 25.9 ppm (HNC(CH_3_)C(CN)C(CH_3_)NH). ^31^P{^1^H} NMR (162 MHz, CD_3_CN, 298 K): *δ* −16.8 ppm (*P*Ph_3_). IR (KBr disk): *v* = 2191 cm^−1^ (*v*(C ≡ N)), 1157 cm^−1^ (*v*(S = O)). HRMS (ESI). Calcd for C_53_H_57_F_6_IrN_6_O_6_PS_2_ ([M − H]^-^): m/z 1275.3058. Found: m/z 1275.3064. Elemental analyses calcd for C_53_H_58_F_6_IrN_6_O_6_PS_2_ (9), found (calcd): C, 49.46 (49.87); H, 4.73 (4.58); N, 6.54 (6.58).

## Supplementary information

Wong_PR File

Supplementary Information

Description of Additional Supplementary Files

Supplementary Data 1

Supplementary Data 2

Supplementary Data 3

Supplementary Data 4

Supplementary Data 5

Supplementary Data 6

Supplementary Data 7

Supplementary Data 8

## Data Availability

Experimental and computational details can be accessed from Supplementary Methods in the Supplementary Information. Analytical data including ^1^H, ^13^C{1H} and ^31^P{1H} NMR spectroscopy, HRMS, IR spectrometry can be obtained from Figs. S1–S24 and Figs. 46–60 in the Supplementary Information. Computaional results can be found from Tables S13–S20 and Tables S30–S48 in the Supplementary Information. Cartesian coordinates from computational studies can be accessed from Supplementary Data 1 from this article. The X-ray crystallographic coordinates for structures reported in this Article have been deposited at the Cambridge Crystallographic Data Centre (CCDC), under deposition number CCDC-2167632 (**1**), CCDC-2167633 (**1'**), CCDC-2167634 (**2**), CCDC-2167635 (**3**), CCDC-2167638 (**7**), CCDC-2167636 (**8**), CCDC-2167637 (**9**). These data can be obtained free of charge from The Cambridge Crystallographic Data Centre via www.ccdc.cam.ac.uk/data_request/cif, or accessed from Supplementary Data 2–8 from this article.
