# Author Correction: Structure-property relationships of photofunctional diiridium(II) complexes with tetracationic charge and an unsupported Ir–Ir bond

**DOI:** 10.1038/s42004-025-01538-7

**Published:** 2025-05-12

**Authors:** Fangrui Zheng, Yuhong Yang, Siye Wu, Shunan Zhao, Yifan Zhu, Huimin Su, Jun-Feng Dai, Zeyin Yan, Lung Wa Chung, Keith Man-Chung Wong

**Affiliations:** 1 Department of Chemistry, Southern University of Science and Technology, 1088 Xueyuan Blvd., Shenzhen, 518055 P.R. China; 2 Analysis and Testing Center, Shenzhen Technology University, 3002 Lantian Road, Shenzhen, 518118 P. R. China; 3 Shenzhen Institute for Quantum Science and Engineering, Southern University of Science and Technology, International Quantum Academy (SIQA), and Shenzhen Branch, Hefei National Laboratory, Shenzhen Key Laboratory of Quantum Science and Engineering, Futian District, Shenzhen, 518055 China

**Keywords:** Coordination chemistry, Photochemistry, Chemical bonding

Correction to: *Communications Chemistry* 10.1038/s42004-022-00775-4, published online 23 November 2022

In the version of this article initially published, there were typographical errors in the first paragraph of the “Photophysical and electrochemical behaviours” section, where in the text now reading “Our TD-DFT (CPCM TD-B3LYP-D3//M06-L) calculations18 suggested that the low-energy absorption is mainly ascribed to metal–metal bond-to-ligand charge transfer (MMLCT) dσ(Ir–Ir) → π*(N^N^N ligand) transition with some mixing of dσ(Ir–Ir) → dσ*(Ir–Ir) character…,” dσ(Ir–Ir) instead appeared as “dπ(Ir–Ir)” in two instances and dσ*(Ir–Ir) instead appeared as “dπ*(Ir–Ir).” In the second paragraph of that section, in the text now reading “Collectively, together with the large Stokes shift, such luminescence of 1–3 is assigned to be originated from the triplet metal–metal bond-to-ligand charge transfer (3MMLCT), with some mixing of dσ(Ir–Ir) → dσ*(Ir–Ir) character…,” dσ(Ir–Ir) → dσ*(Ir–Ir) instead appeared as “dπ(Ir–Ir) → dπ*(Ir–Ir).” In the fourth paragraph of that section, in the text now reading “Upon reductive scan … with some σ*(Ir(II)–Ir(II)) character,” σ*(Ir(II)–Ir(II)) instead appeared as “π*(Ir(II)–Ir(II)),” and later in that paragraph, in the sentence now reading “The essentially irreversible nature of this reduction process is probably due to dissociation of the diiridium(II) framework, resulting from the population of σ*(Ir(II)–Ir(II)) orbital,” σ*(Ir(II)–Ir(II)) instead appeared as “π*(Ir(II)–Ir(II)).” The orbitals have been amended in the HTML and PDF versions of the article.

