# Supplementary material for: Structure-property relationships of photofunctional diiridium(II) complexes with tetracationic charge and an unsupported Ir–Ir bond
Source: Commun Chem. 2022 Nov 23;5:159. doi: 10.1038/s42004-022-00775-4 (PMC9814866; doi:10.1038/s42004-022-00775-4)
Supplement: Supplementary file 2 — Supplementary Information [file 42004_2022_775_MOESM2_ESM.pdf]

Structure-property relationships of photofunctional diiridium(II) complexes with tetracationic charge and an unsupported Ir–Ir bond

Fangrui Zheng,[1,3,4] Yuhong Yang,[1,4] Siye Wu,[1] Shunan Zhao,[1] Yifan Zhu,[1] Huimin Su,[2] Jun-Feng Dai,[2] Zeyin Yan,[1] Lung Wa Chung*[1] and Keith Man-Chung Wong*[1]

[1] Department of Chemistry, Southern University of Science and Technology, 1088 Xueyuan Blvd., Shenzhen 518055, P.R. China

[2] Shenzhen Institute for Quantum Science and Engineering, Southern University of Science and Technology; International Quantum Academy (SIQA), and Shenzhen Branch, Hefei National Laboratory, Futian District; Shenzhen Key Laboratory of Quantum Science and Engineering, Shenzhen 518055, China

[3] Analysis and Testing Center, Shenzhen Technology University, 3002 Lantian Road, Shenzhen 518118, P. R. China

[4] These authors contributed equally: Fangrui Zheng, Yuhong Yang.

*Lung Wa Chung and *Keith Man-Chung Wong

Email: oscarchung@sustech.edu.cn and keithwongmc@sustech.edu.cn

Supporting Information

Supplementary Methods

Materials. All solvents were purified and distilled by using standard procedures before use. 2,2′-(1,3-phenylene)bis[1-butyl-1H-benzimidazole] was prepared according to literature methods.[1] All other chemicals were purchased from either Aldrich or Acros Chemical Company and used as received unless otherwise noted.

Physical Measurements and Instrumentation. NMR spectra were recorded on Bruker, AV 400 spectrometers. ^{1}H and ^{13}C chemical shifts are determined by reference to residual ^{1}H and ^{13}C solvent signals. Elemental analyses were performed with an elementary VARIO EL III elemental analyzer, Shanghai Institute of Organic Chemistry, CAS. EPR spectra were recorded on Bruker, EMXplus-10/12 spectrometers. The UV−vis absorption spectra were taken on an Agilent Technologies Cary 60 UV−vis spectrophotometer. Steady-state emission spectra at room temperature were recorded on an Edinburgh Instruments FLS980 or FS5 fluorescence spectrometer. Quartz cuvettes (path length = 1 cm) were used in all solution state spectrophotometric and fluorometric measurements. Luminescence quantum yields were measured by the optical dilute method reported by Demas and Crosby.[2,3] A degassed solution of $[Ru(bpy)_3]Cl_2$ in aqueous state (Φ_{lum} = 0.042, excitation wavelength at 436 nm) was used as the reference.[2,3] The time-resolved photoluminescence decay was measured by PicoQuant PicoHarp300 Time Correlated Single Photon Counting (TCSPC) system with excitation wavelength at 475 nm (150fs, 80MHz). The excitation fluence on the sample was in the range from 6 to 15 μJ cm^{-2} per pulse. High resolution mass spectra (HRMS) were performed on an Orbitrap Fusion Tribrid mass spectrometer. Cyclic voltammetric measurements were performed by using a CH Instruments, Inc., model CHI 600A electrochemical analyzer. Samples were in acetonitrile solution with $^{n}Bu_4NPF_6$ (0.1 M) as the supporting electrolyte at room temperature; scan rate 100 mV s^{-1}.

Computational Details. Density functional theory (DFT) calculations by Gaussian 09 and 16 (for MN15 only) packages[4, 5] were mainly carried out to understand the binding energies of Ir(II)-Ir(II) complexes

reported in this work (Figure S1). Several other unsupported Ir(II)-Ir(II) and Au(II)-Au(II), Rh(II)-Rh(II) complexes from the previous works (Figure S2) [6-14] were also investigated to compare with our Ir(II)-Ir(II) complexes in this work. M06-L [15] method combined with mixed basis sets of SDD[16, 17] for the metal (plus SDD's effective core potentials) and 6-31G*[18-20] for the other atoms were mainly used to optimize all the structures in gas phase. Then, vibrational frequency calculations on these optimized structures were carried out at the same level of theory to ensure no imaginary frequency for each local minimum. However, one very small imaginary frequency was found in few structures and cannot be eliminated by several attempts (these additional imaginary frequencies are given in the below corresponding tables). In addition, the key metal complexes were also optimized by the B3LYP-D3BJ and B3LYP methods with the same basis sets.[21-23] The effect of the solvent (acetonitrile) was then included by single-point calculations on the gas-optimized structures with SMD solvent model.[24] Moreover, to examine the effect of the DFT functional, M06-D3, M06, PBE0-D3BJ, PBE0, B3LYP-D3BJ, B3LYP and MN15 methods[21-23,25-27] were used for the single-point energy calculations in acetonitrile solvent. To examine the spin crossing of the excited-state 1, its minimum energy crossing point (MECP) was located by the program developed by Harvey.[28] The absorption and emission (including SOC for phosphorescence) calculations of the key structures were used by CPCM TD ZORA B3LYP-D3//M06-L method combined with mixed basis sets of SARC-ZORA-TZVP for the metal and ZORA def2-TZVP for the other atoms (additionally using the RI-SOMF(1X) for the emission calculations) based on the M06-L optimized geometries by ORCA 5.01.[29-37] We have also performed additional energy calculations of the key complexes by the ZORA RI-M06-L/def2-TZVP(-f) and high-level DLPNO-CCSD(T)/def2-TZVP(-f) with RIJCOSX methods using ORCA 5.01 (for the ZORA RI-M06-L method) and 4.01,[29-42] respectively. Decomposition of the dispersion correction energies were conducted by dftd3 code by Grimme group.[23, 43]. Distortion-interaction energy analysis was also carried out to understand the binding on the key Ir_2 complex.[44, 45] Non-covalent Interactions (NCIs) and topological analysis of the metal-metal bonds (based on the SMD M06-L//M06 method) were further analyzed based on the Quantum Theory of

Atoms-In-Molecules (QTAIM) method by using MultiWFN and AIMALL programs, respectively.[46-51]. All 3D images of the optimized structures and the key bond critical points (BCP) were illustrated by CYLview and Chimera,[52, 53] respectively.

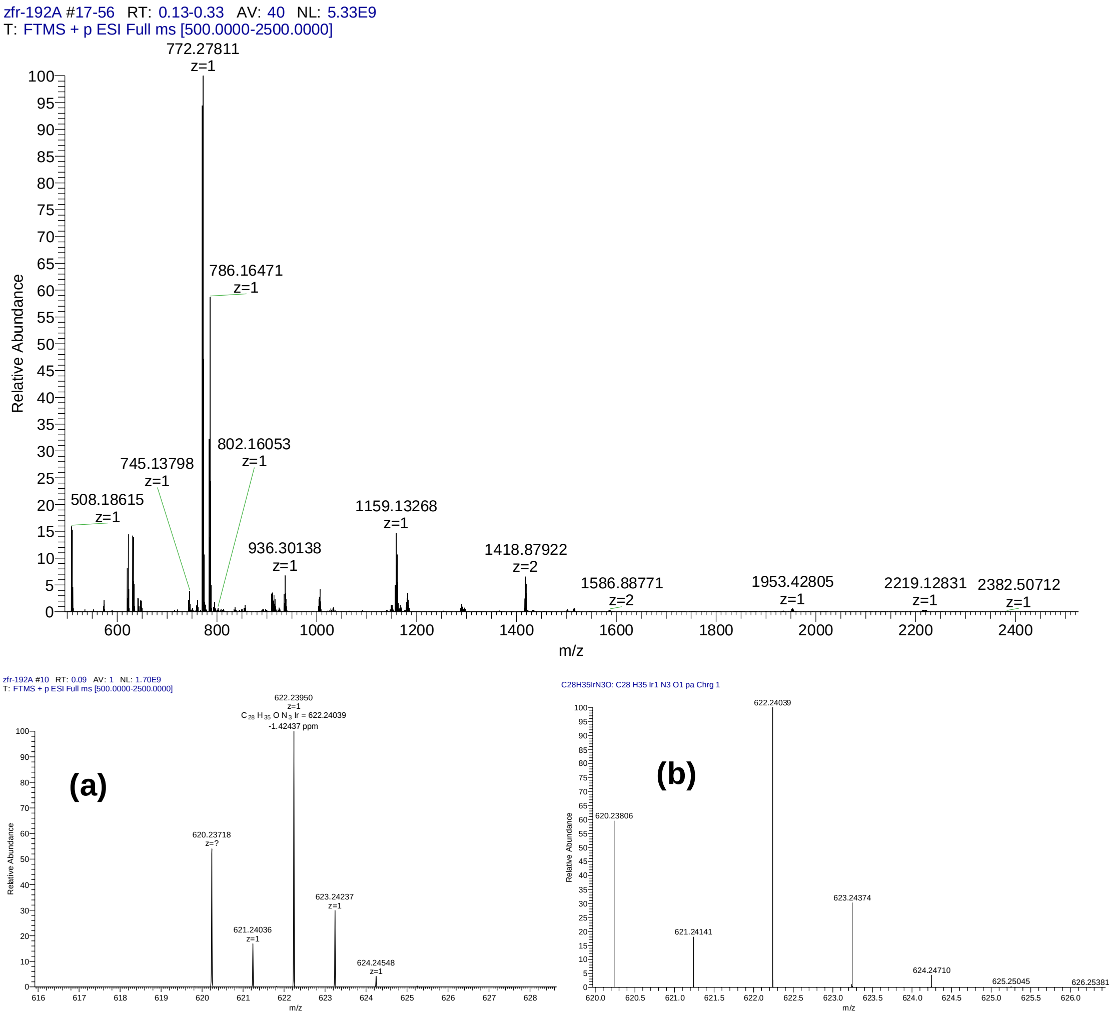

Fig. S1. (Top) HRMS (ESI) showing the formation of [Ir(^{t}Bu$_3$-terpy)(CO)]$^+$ upon addition of ^{t}Bu$_3$-terpy ligand into the pale yellow filtrate, from the reaction mixture of [Ir(PPh$_3$)$_2$(CO)Cl] with AgX, at 298 K. (Bottom) Expanded peak at m/z = 622 (a) and theoretical isotope pattern of [Ir(^{t}Bu$_3$-terpy)(CO)]$^+$ (b).

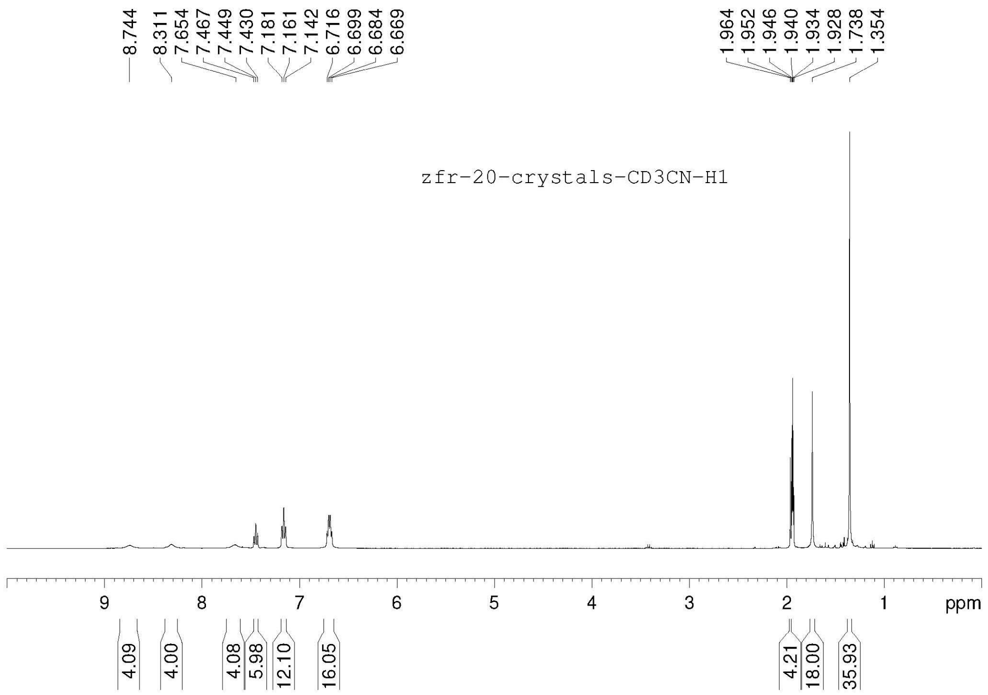

Fig. S2. ^{1}H NMR spectrum of complex **1** in CD_3CN.

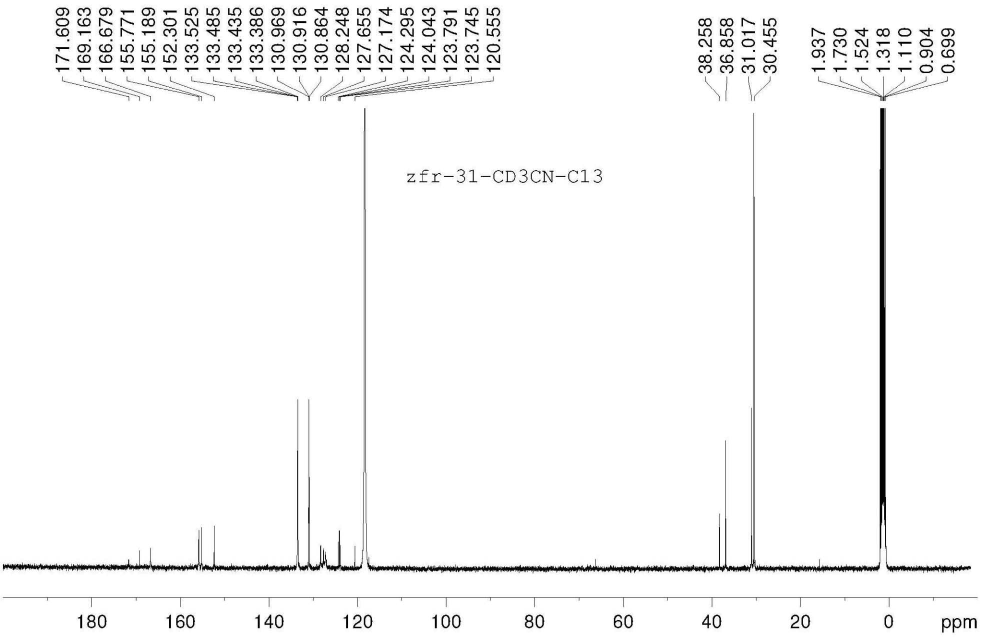

Fig. S3. $^{13}C\{^{1}H\}$ NMR spectrum of complex **1** in CD_3CN.

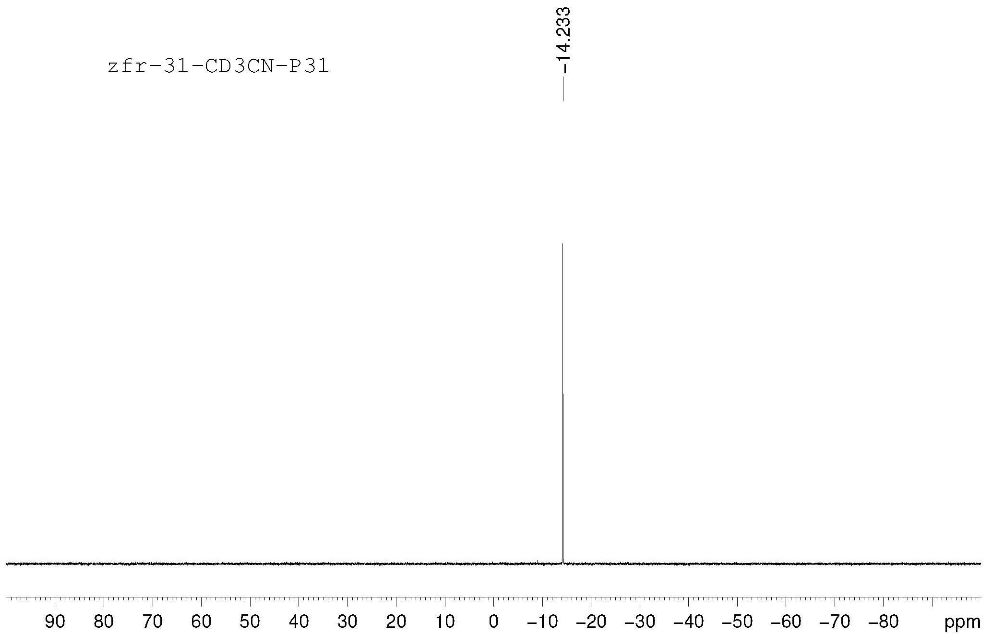

Fig. S4. $^{31}P\{^{1}H\}$ NMR spectrum of complex **1** in CD_3CN.

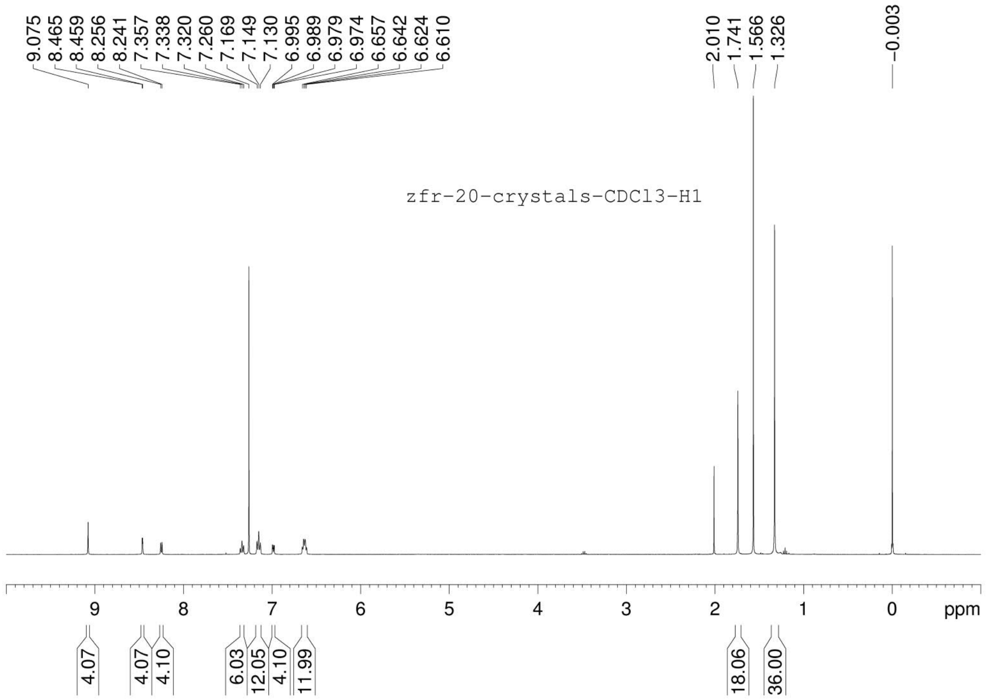

Fig. S5. ^{1}H NMR spectrum of complex **1** in $CDCl_3$.

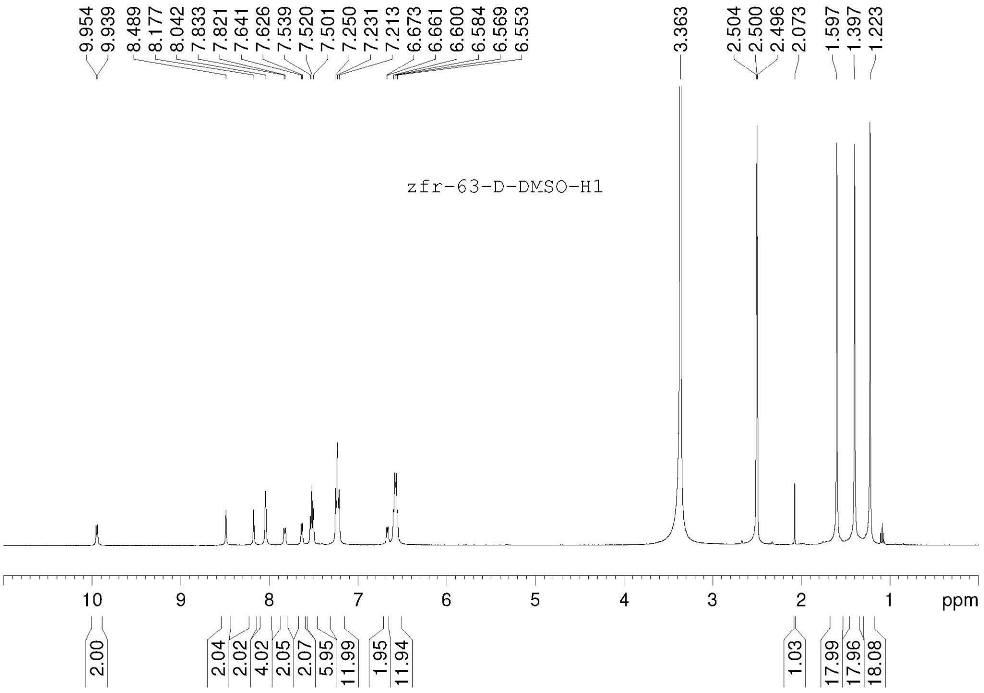

Fig. S6. ^{1}H NMR spectrum of complex **1** in $(CD_3)_2SO$.

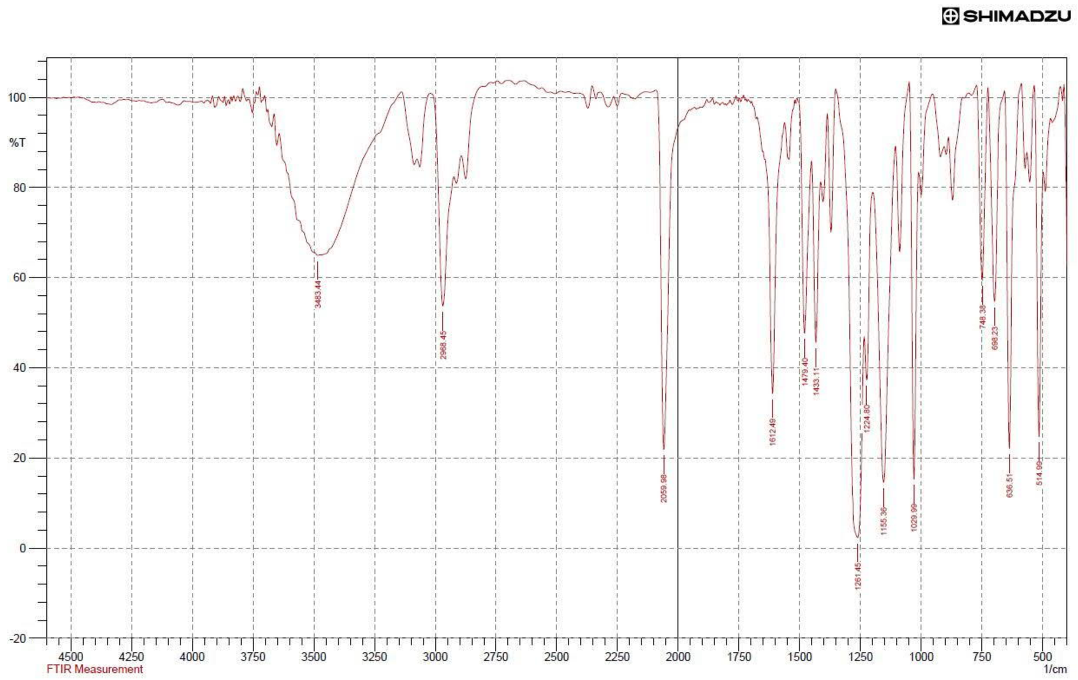

Fig. S7. IR spectrum of complex **1** in KBr disk.

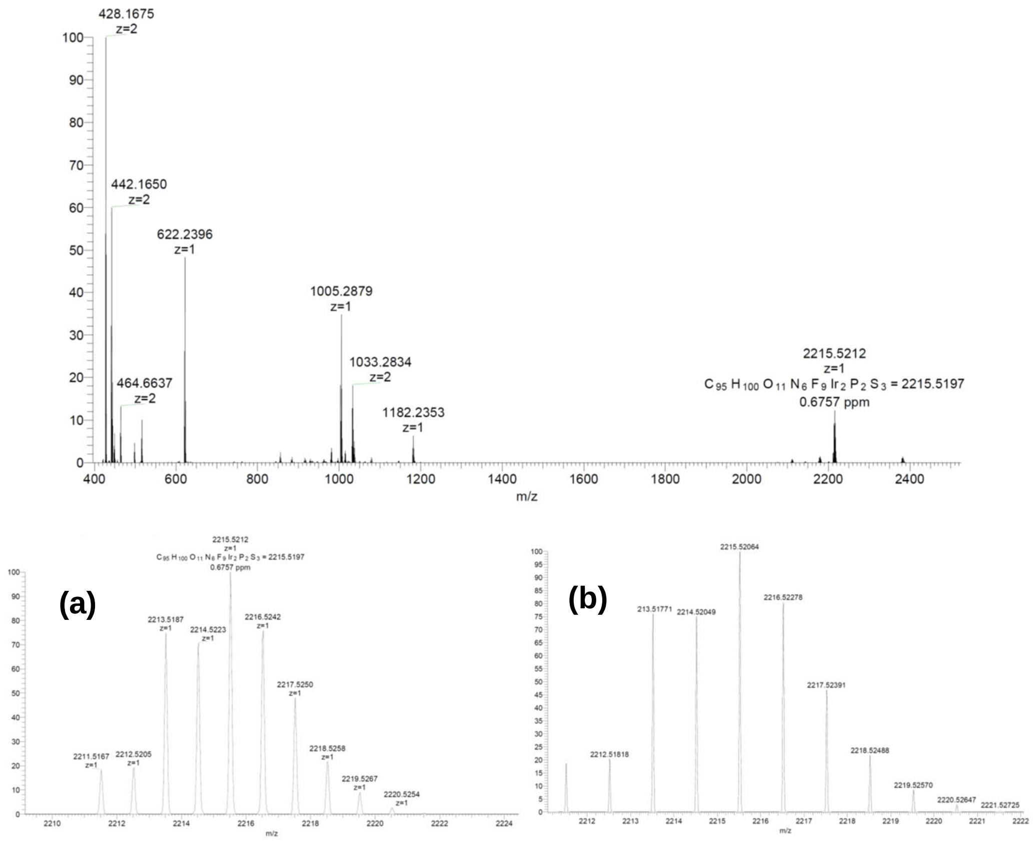

Fig. S8. (Top) HRMS (ESI) spectrum of complex **1**. (Bottom) Expanded peak at m/z = 2215 (a) and theoretical isotope pattern of $[M - OTf^{-}]^{+}$ (b).

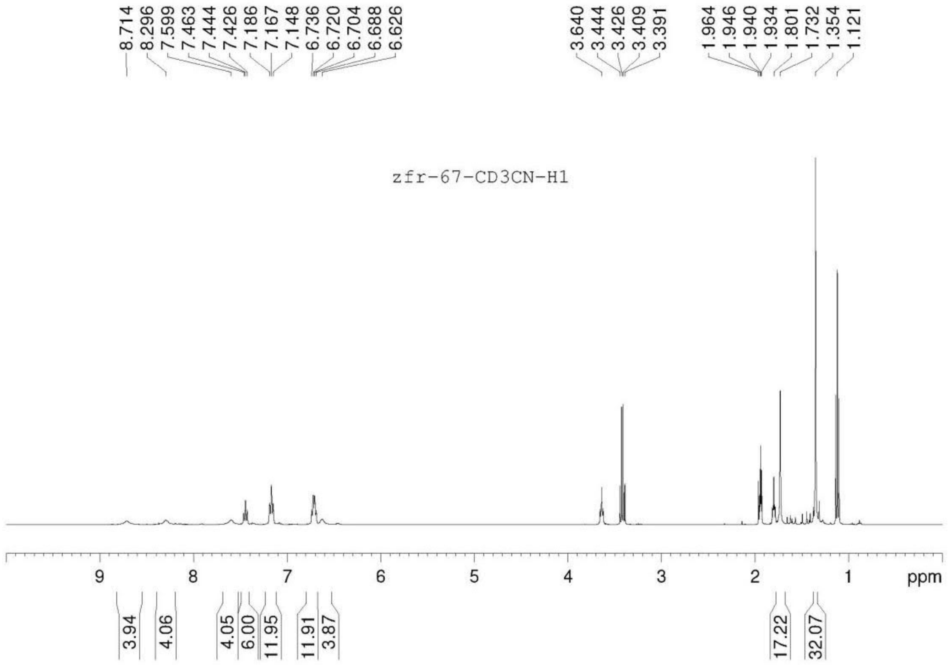

Fig. S9. ^{1}H NMR spectrum of complex **1'** in CD_3CN.

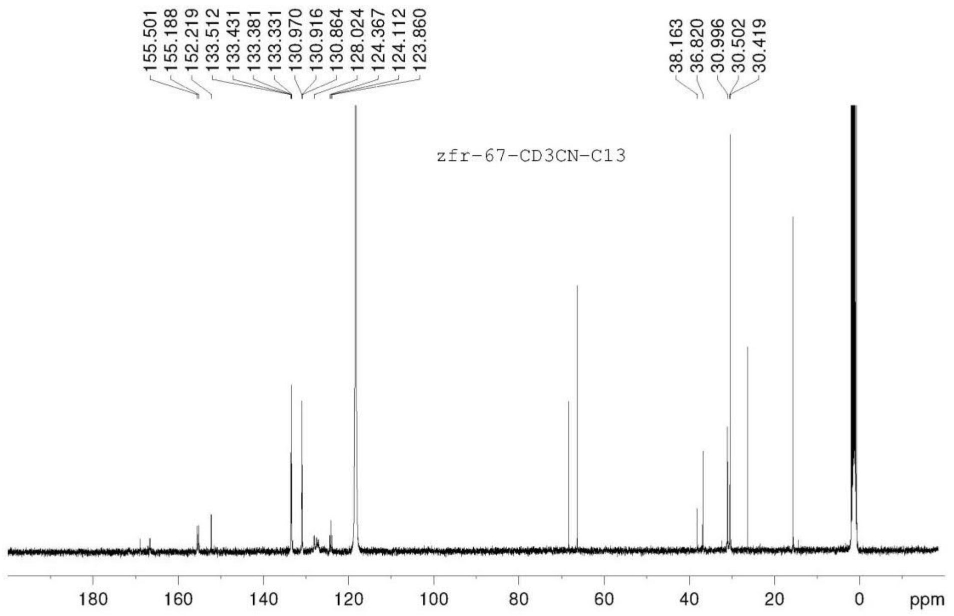

Fig. S10. $^{13}C\{^{1}H\}$ NMR spectrum of complex **1'** in CD_3CN.

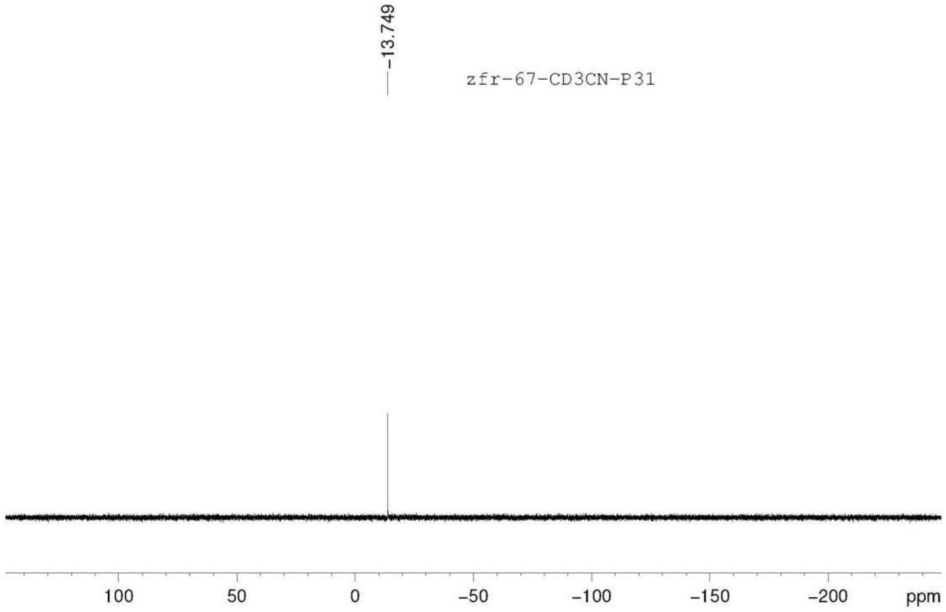

Fig. S11. $^{31}P\{^1H\}$ NMR spectrum of complex **1'** in CD_3CN.

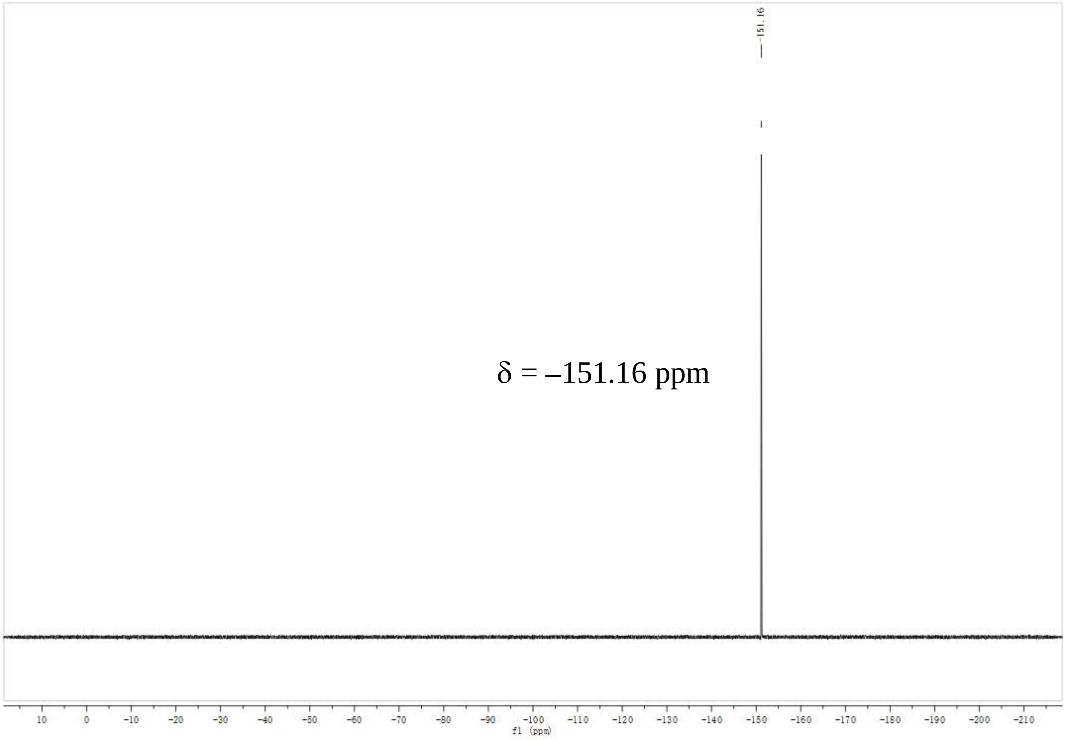

Fig. S12. $^{19}F\{^{1}H\}$ NMR spectrum of complex **1'** in CD_3CN.

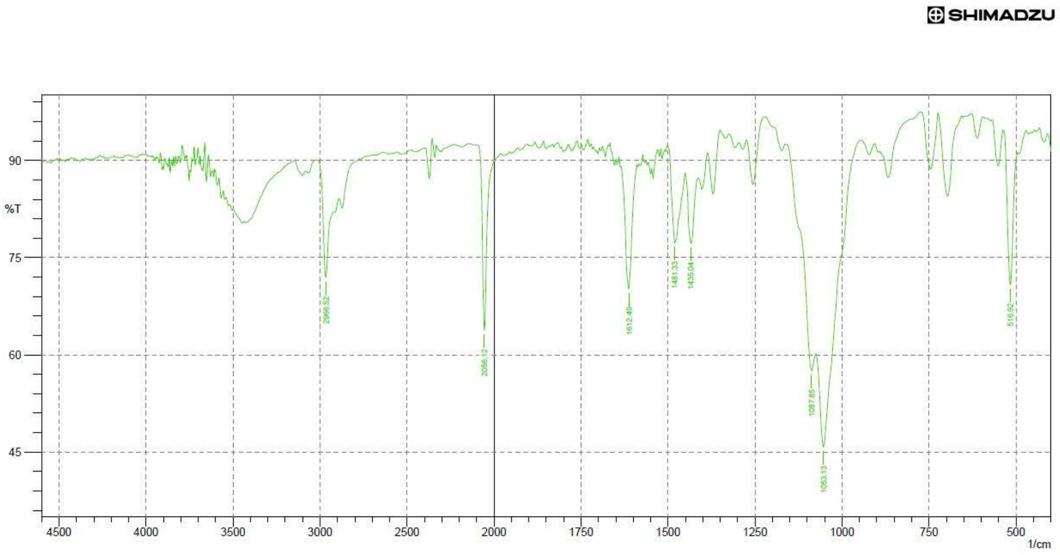

Fig. S13. IR spectrum of complex **1'** in KBr disk.

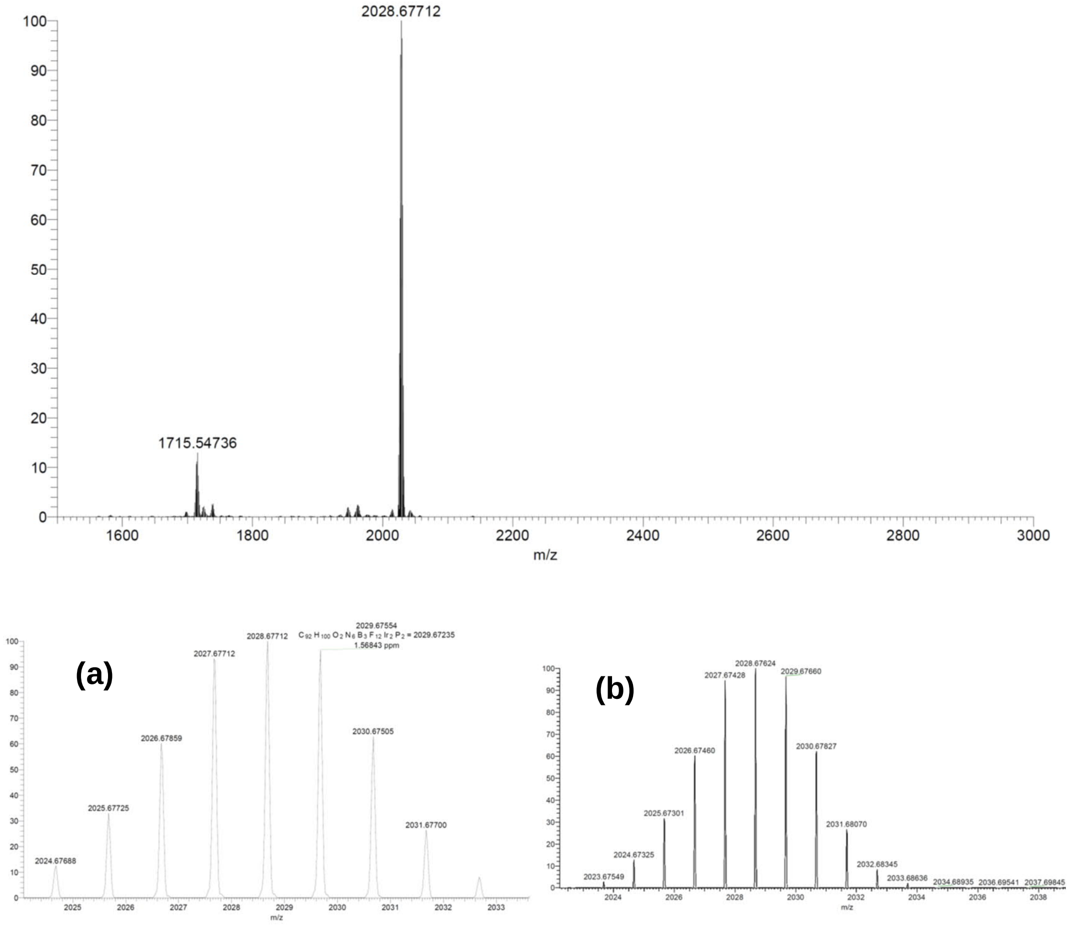

Fig. S14. (Top) HRMS (ESI) spectrum of complex **1'**. (Bottom) Expanded peak at m/z = 2029 (a) and theoretical isotope pattern of $[M – BF_4^{–}]^+$ (b).

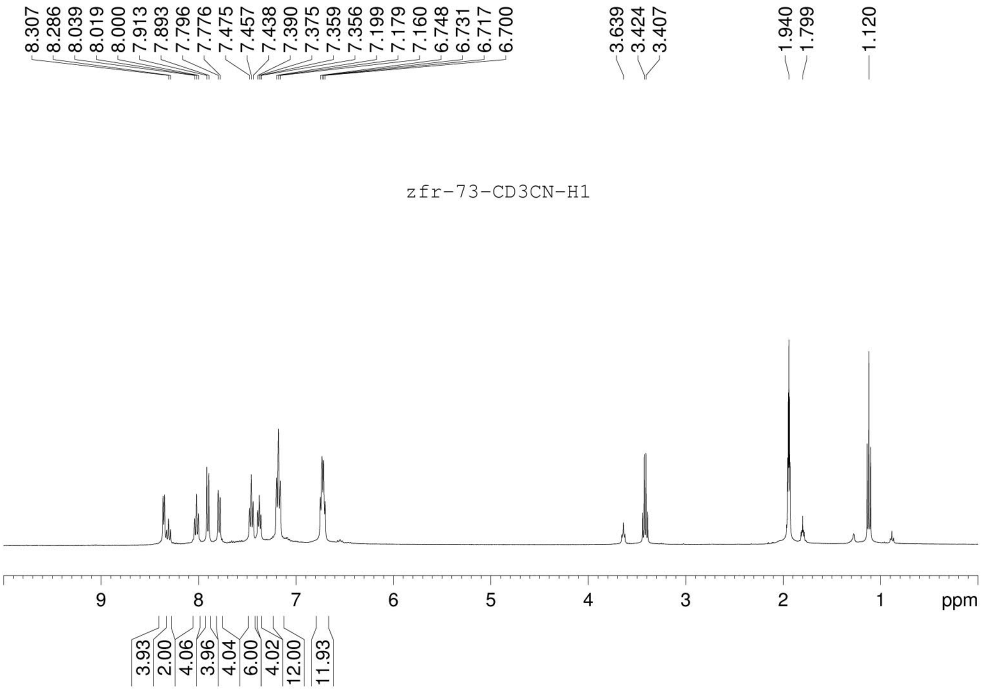

Fig. S15. ^1H NMR spectrum of complex **2** in CD_3CN.

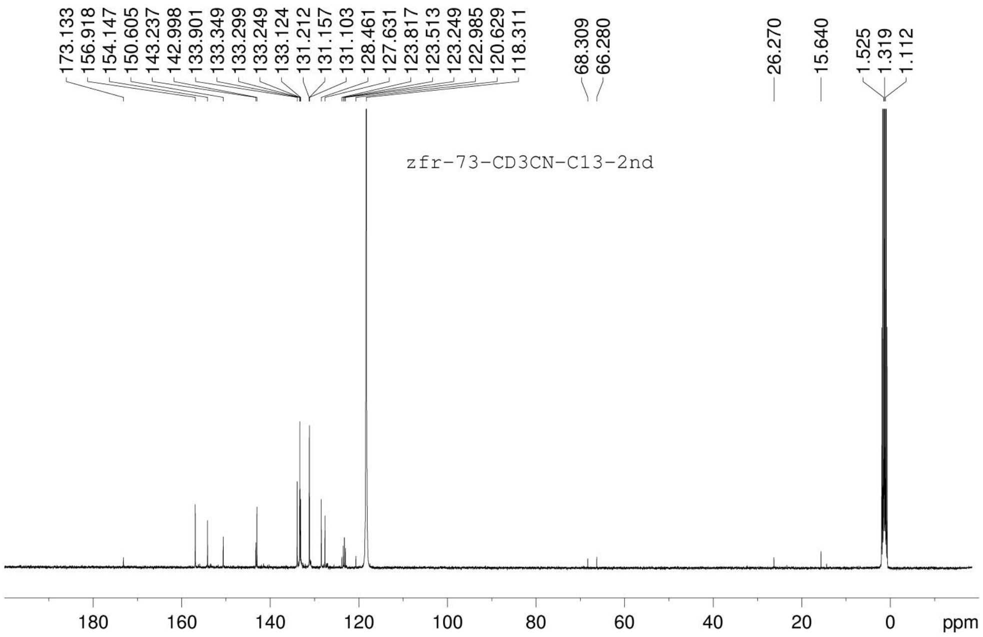

Fig. S16. $^{13}C\{^{1}H\}$ NMR spectrum of complex **2** in CD_3CN.

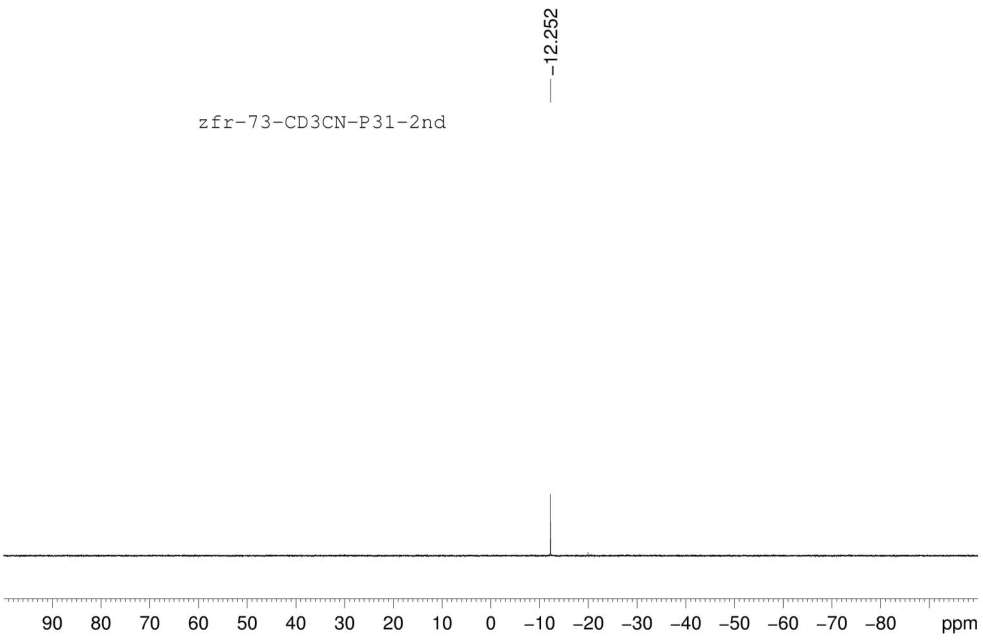

Fig. S17. $^{31}P\{^1H\}$ NMR spectrum of complex **2** in CD_3CN.

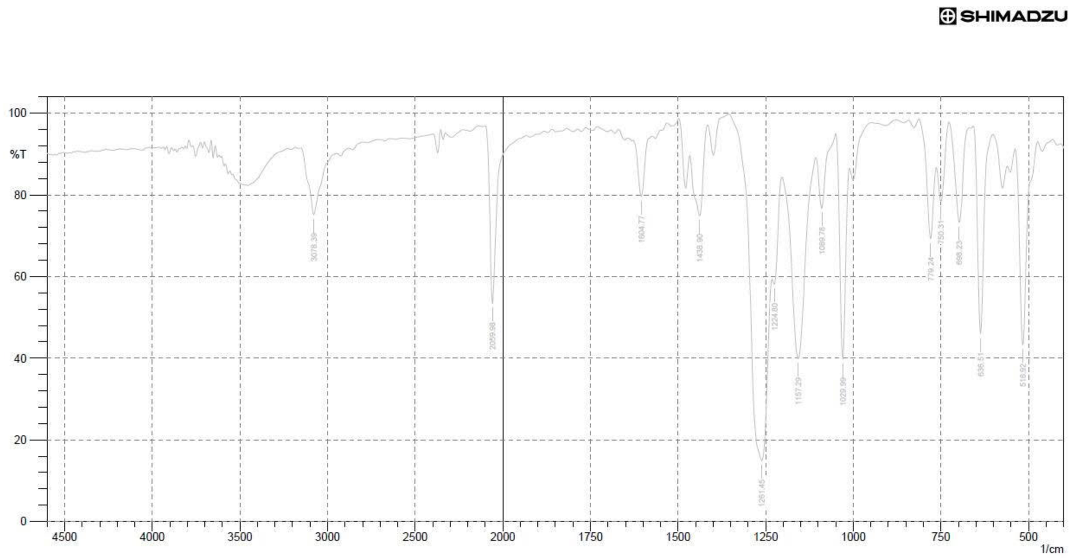

Fig. S18. IR spectrum of complex **2** in KBr disk.

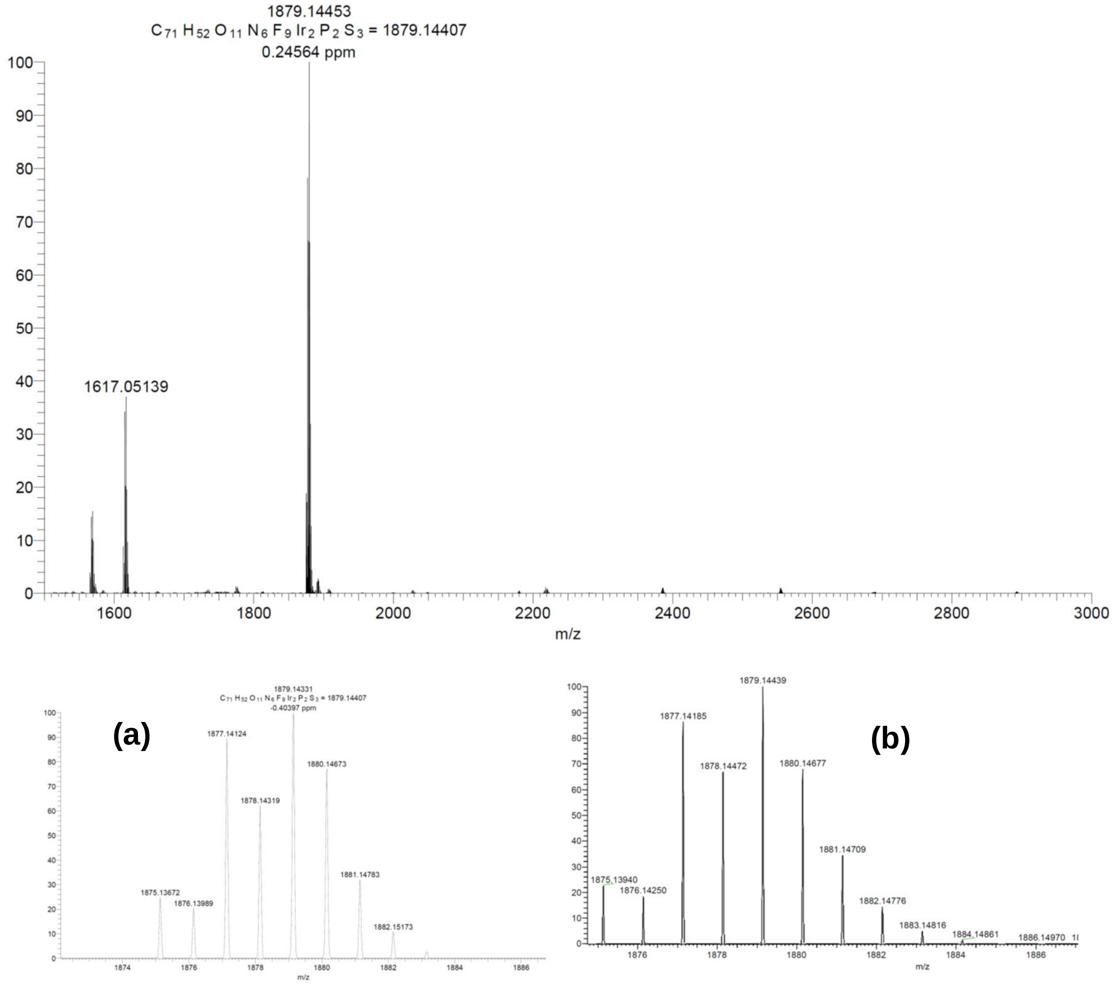

Fig. S19. (Top) HRMS (ESI) spectrum of complex **2**. (Bottom) Expanded peak at m/z = 1879 (a) and theoretical isotope pattern of [M – OTf⁻]$^+$ (b).

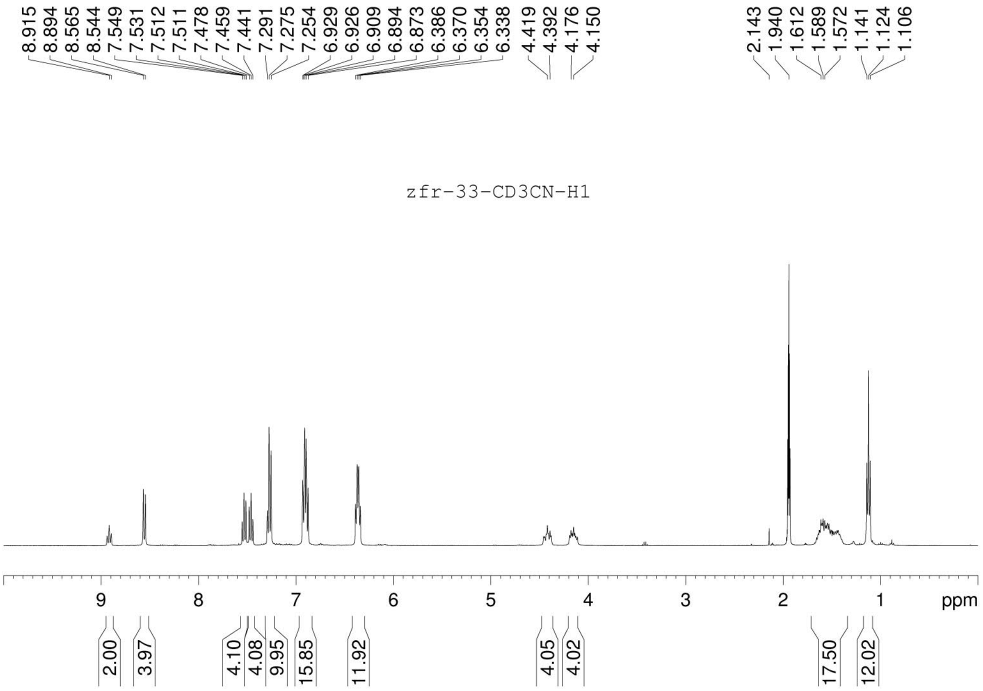

Fig. S20. ^{1}H NMR spectrum of complex **3** in CD_3CN.

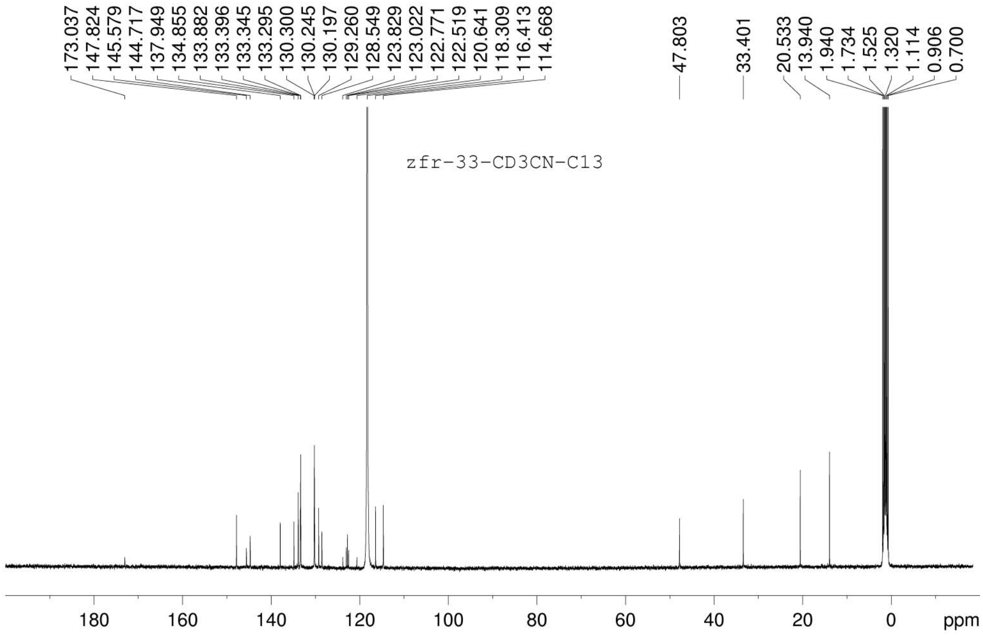

Fig. S21. $^{13}C\{^1H\}$ NMR spectrum of complex **3** in CD_3CN.

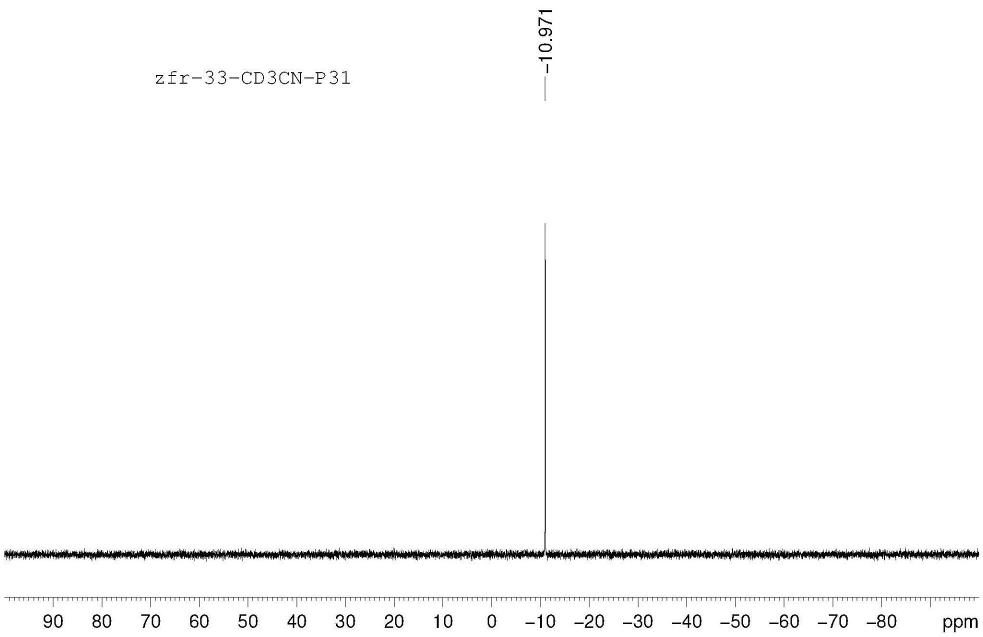

Fig. S22. $^{31}P\{^{1}H\}$ NMR spectrum of complex **3** in CD_3CN.

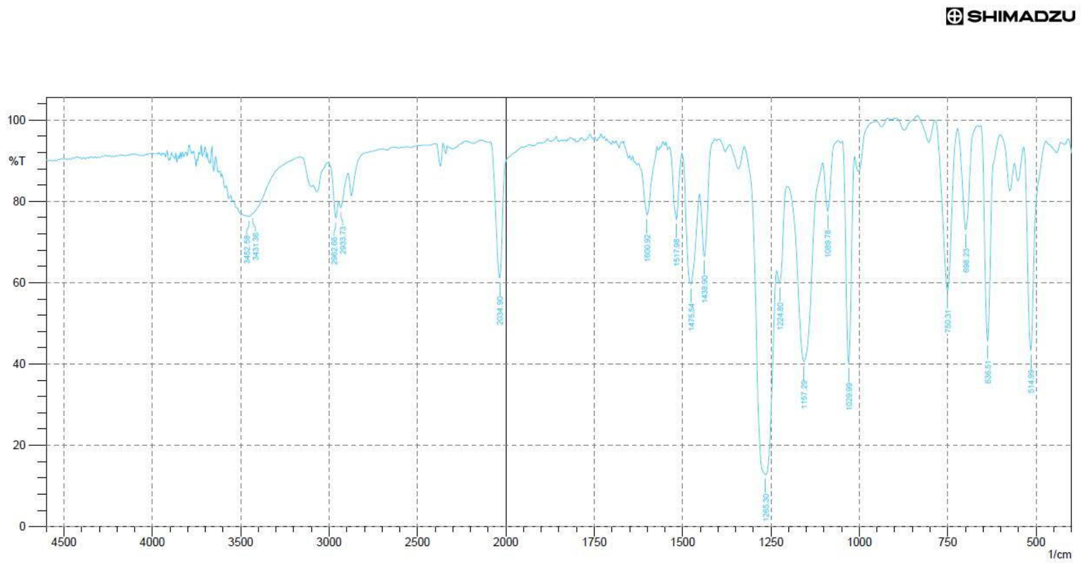

Fig. S23. IR spectrum of complex **3** in KBr disk.

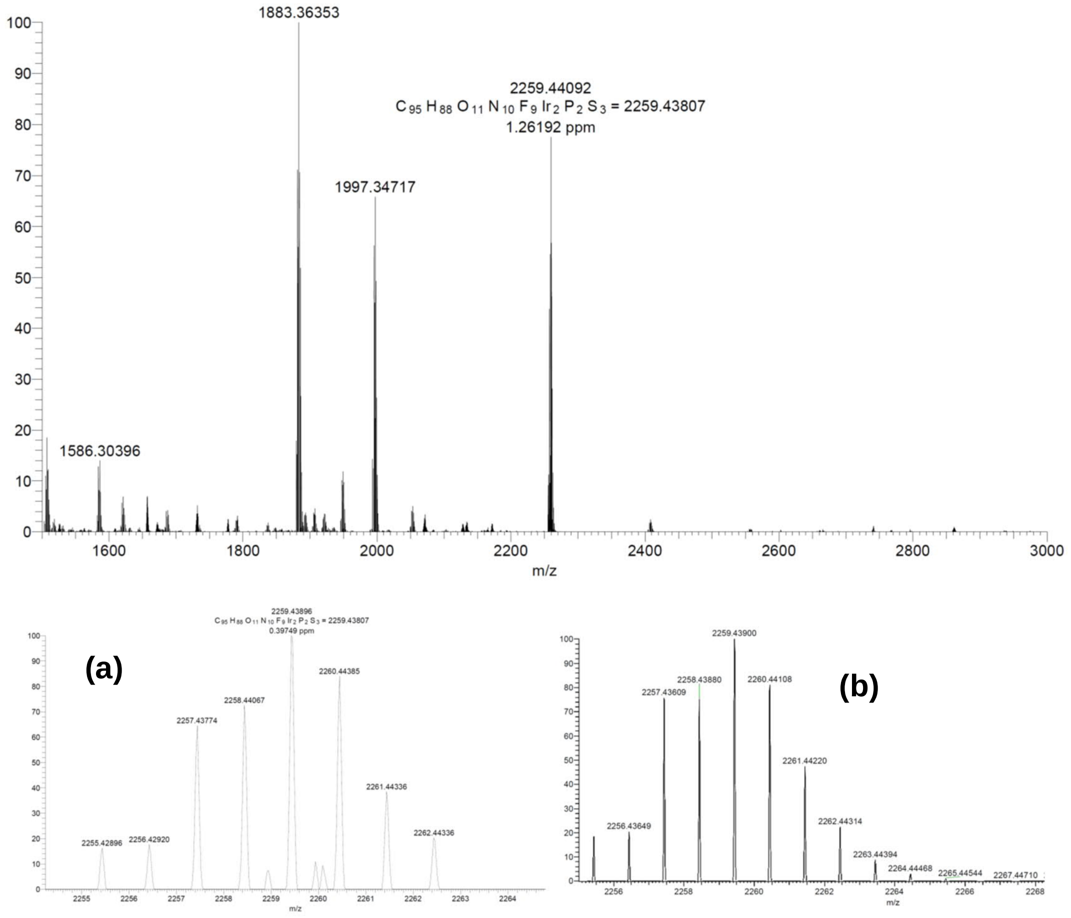

Fig. S24. (Top) HRMS (ESI) spectrum of complex **3**. (Bottom) Expanded peak at m/z = 2259 (a) and theoretical isotope pattern of $[M – OTf^{–}]^{+}$ (b).

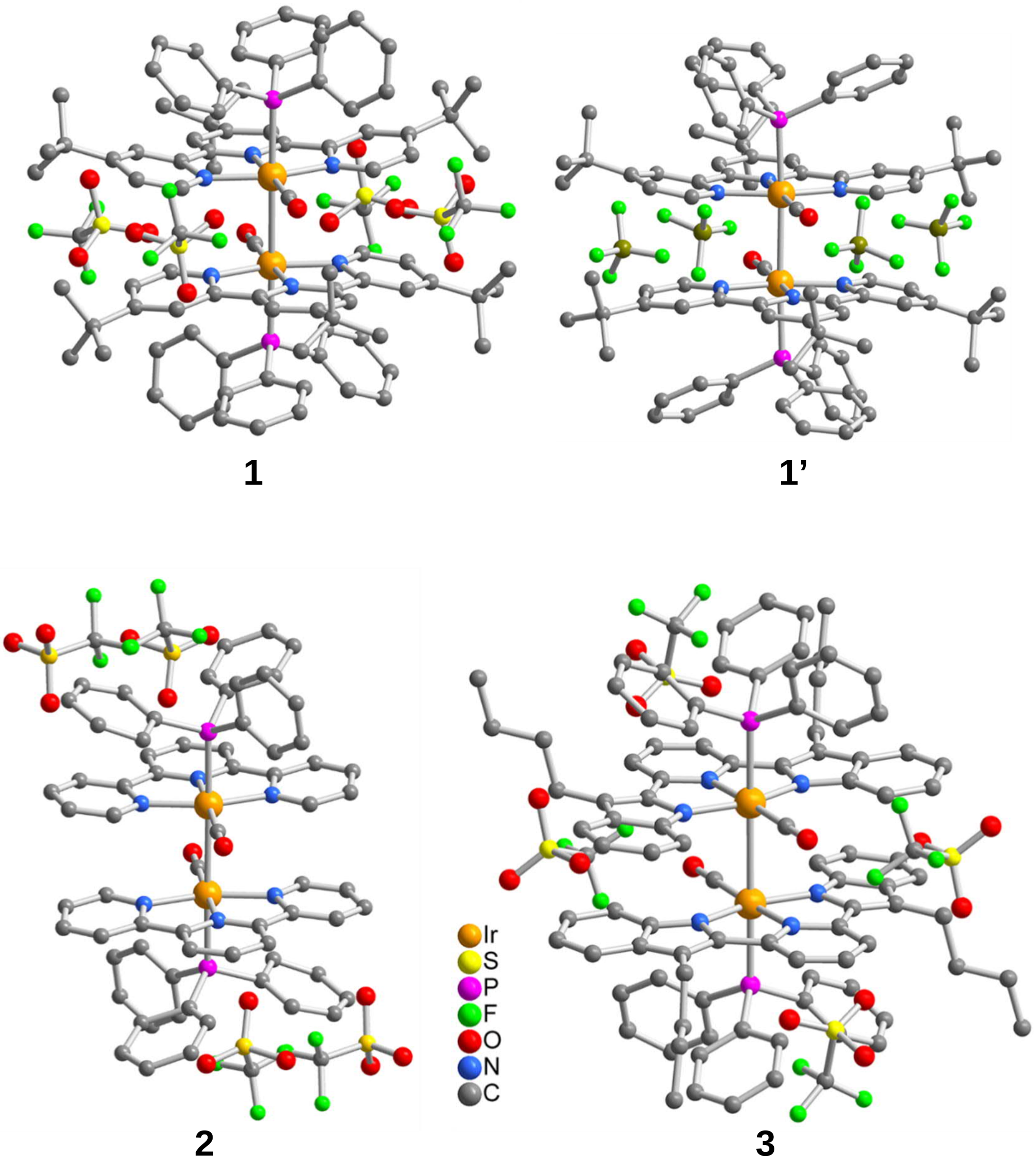

Fig. S25. Perspective drawing of **1–3** showing the position of their counter anions.

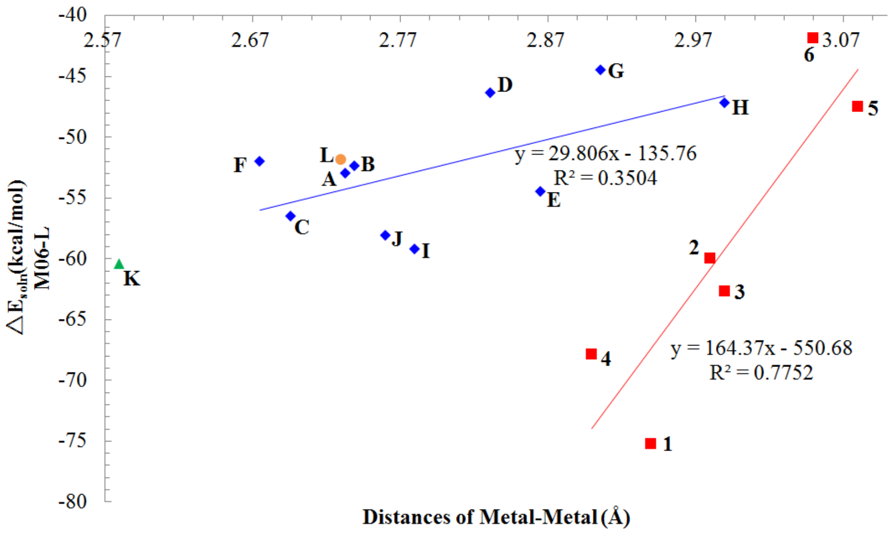

Fig. S26(a). Correlation of the computed metal(II)-metal(II) distances (Å) and relative binding (electronic) energies (in kcal/mol) in acetonitrile solvent by the SMD M06-L//M06-L method. The current Ir-Ir dimer systems are in red color and the previous metal-metal complexes are in blue (Ir-Ir), yellow (Rh-Rh) and green (Au-Au) color.

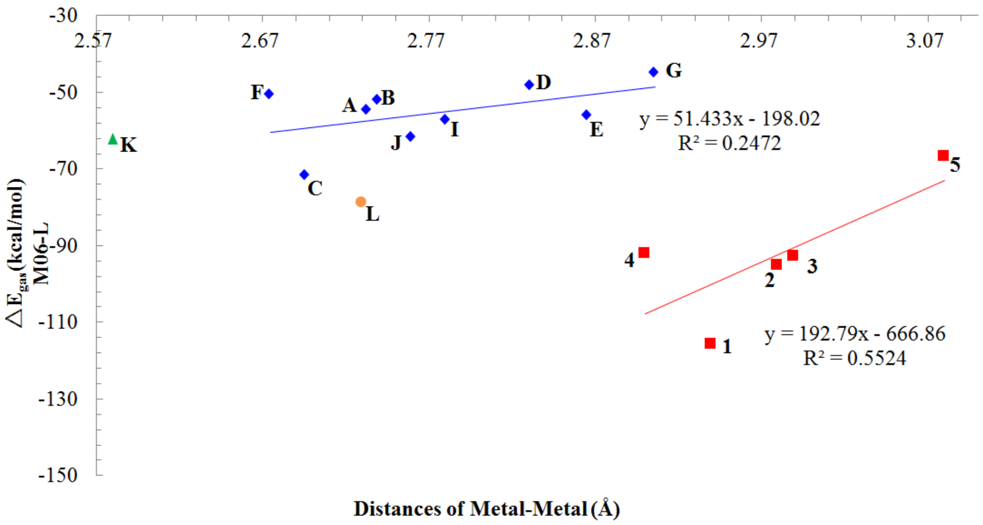

Fig. S26(b). Correlation of the computed metal(II)-metal(II) distances (Å) and relative binding (electronic) energies (in kcal/mol) in gas phase by the M06-L method. The current Ir-Ir dimer systems are in red color and the previous metal-metal complexes are in blue (Ir-Ir), yellow (Rh-Rh) and green (Au-Au) color.

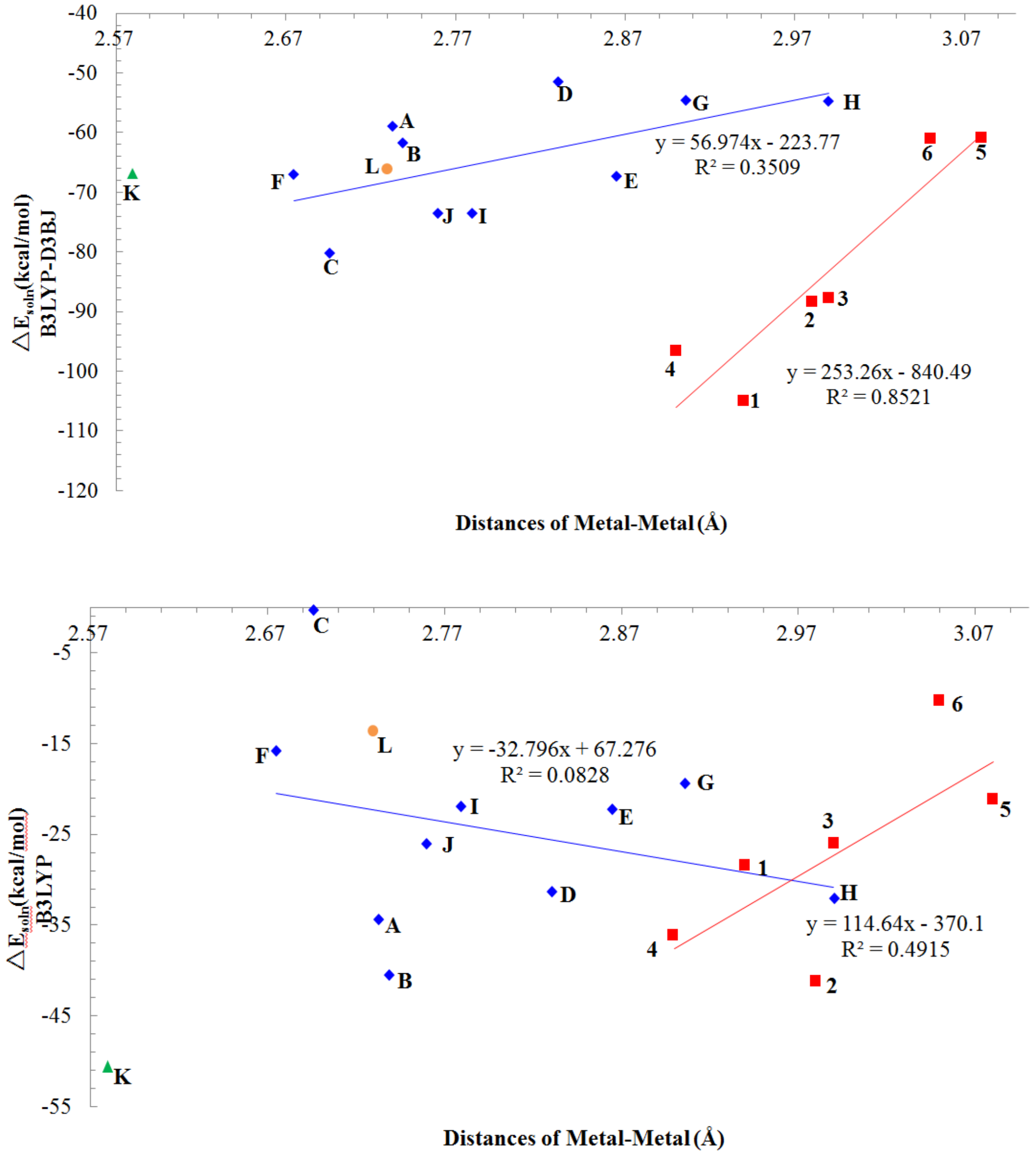

Fig. S26(c). Correlation of the computed metal(II)-metal(II) distances (Å) and relative binding (electronic) energies (in kcal/mol) in acetonitrile solvent by the (top) SMD B3LYP-D3BJ//M06-L and (bottom) SMD B3LYP//M06-L methods based on the M06-L-optimized geometries. The current Ir-Ir dimer systems are in red color and the previous metal-metal complexes are in blue (Ir-Ir), yellow (Rh-Rh) and green (Au-Au) color.

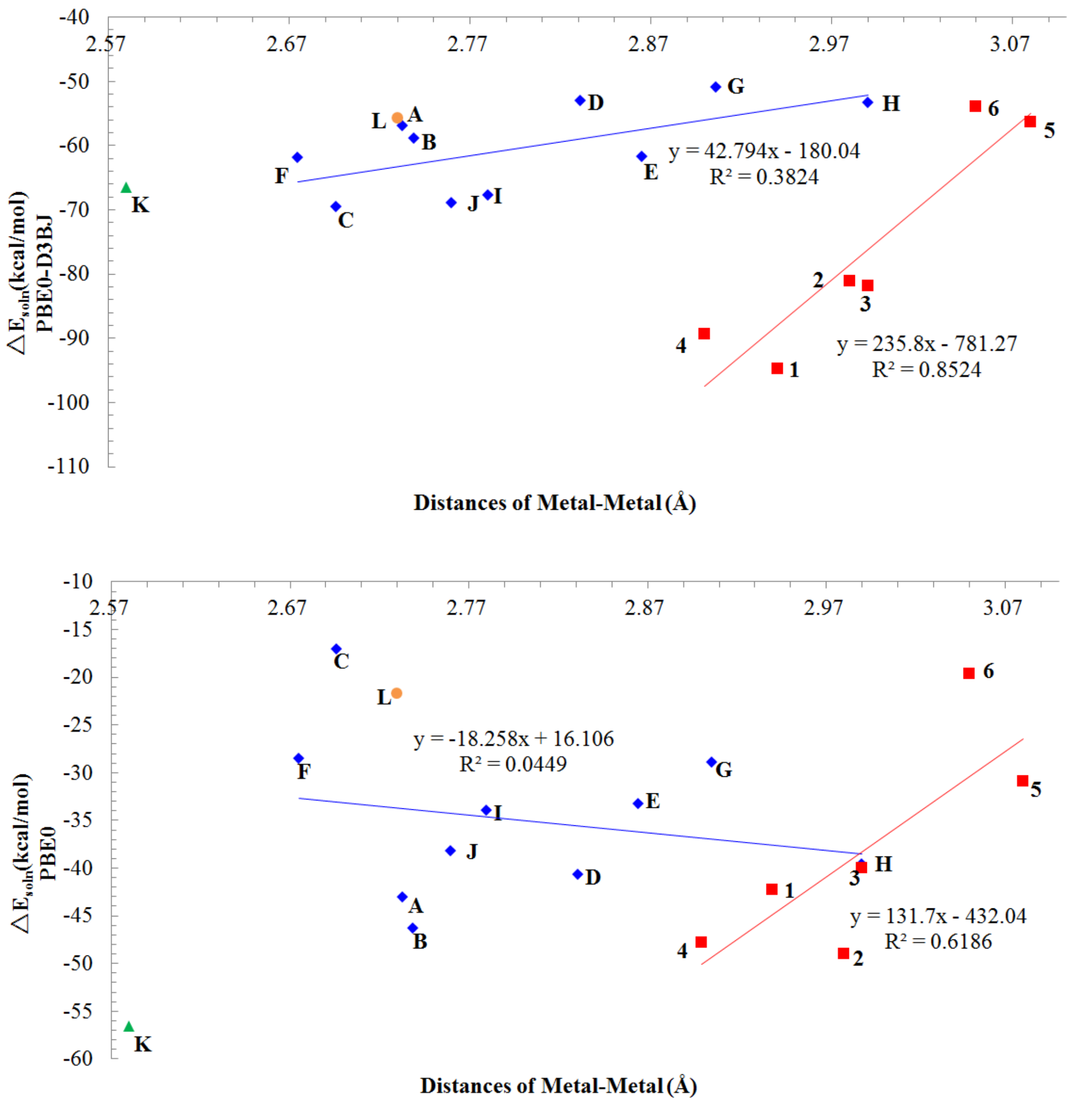

Fig. S26(d). Correlation of the computed metal(II)-metal(II) distances (Å) and relative binding (electronic) energies (in kcal/mol) in acetonitrile solvent by the (top) SMD PBE0-D3BJ//M06-L and (bottom) SMD PBE0//M06-L methods based on the M06-L-optimized geometries. The current Ir-Ir dimer systems are in red color and the previous metal-metal complexes are in blue (Ir-Ir), yellow (Rh-Rh) and green (Au-Au) color.

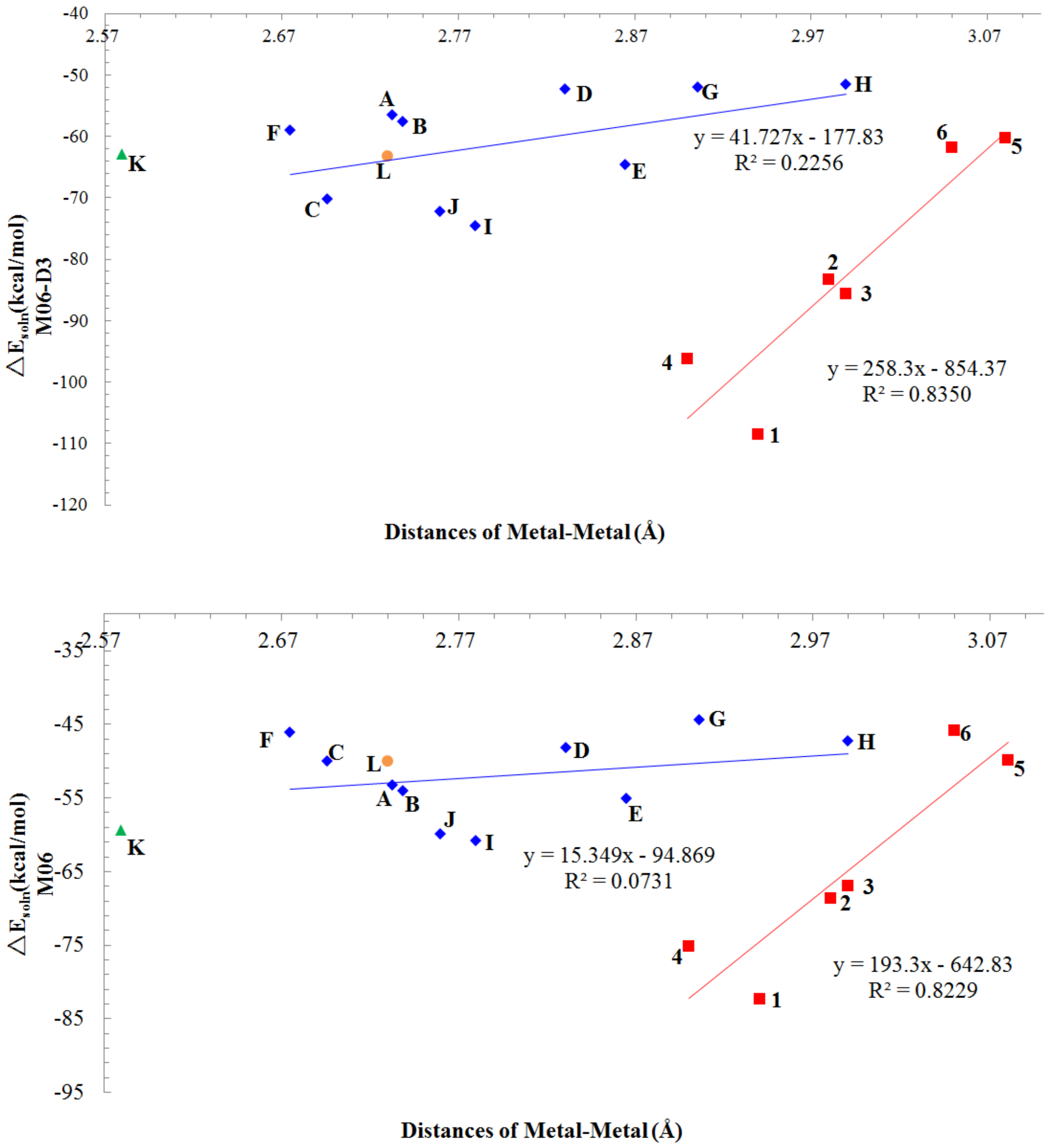

Fig. S26(e). Correlation of the computed metal(II)-metal(II) distances (Å) and relative binding (electronic) energies (in kcal/mol) in acetonitrile solvent by the (top) SMD M06-D3//M06-L and (bottom) SMD M06//M06-L methods based on the M06-L-optimized geometries. The current Ir-Ir dimer systems are in red color and the previous metal-metal complexes are in blue (Ir-Ir), yellow (Rh-Rh) and green (Au-Au) color.

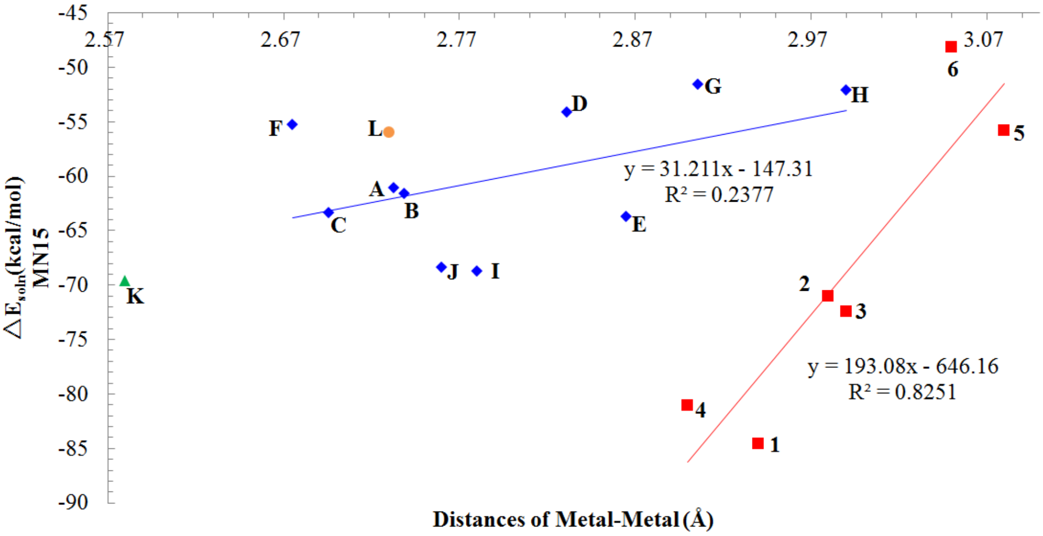

Fig. S26(f). Correlation of the computed metal(II)-metal(II) distances (Å) and relative binding (electronic) energies (in kcal/mol) in acetonitrile solvent by the SMD MN15//M06-L method based on the M06-L-optimized geometries. The current Ir-Ir dimer systems are in red color and the previous metal-metal complexes are in blue (Ir-Ir), yellow (Rh-Rh) and green (Au-Au) color.

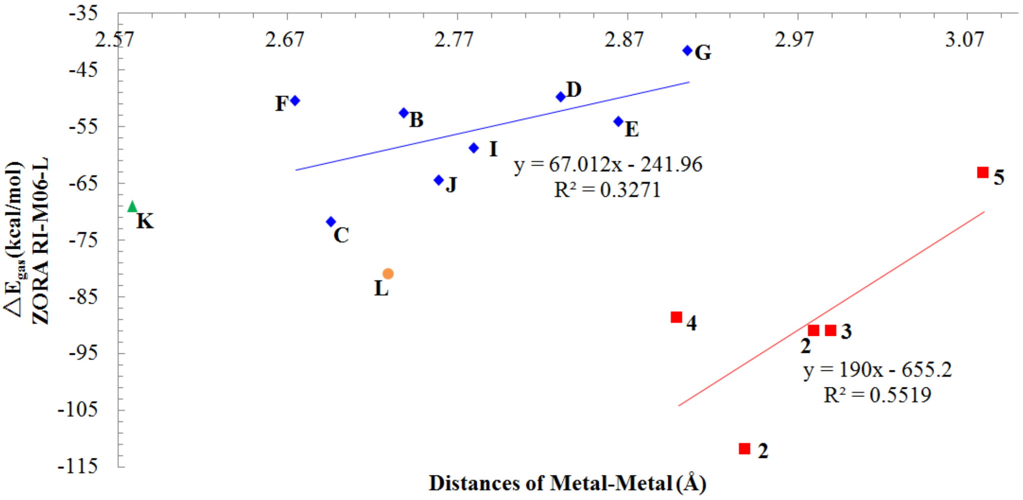

Fig. S26(g). Correlation of the computed metal(II)-metal(II) distances (Å) and relative binding (electronic) energies (in kcal/mol) in gas phase by the ZORA RI-M06-L//M06-L method based on the M06-L-optimized geometries. The current Ir-Ir dimer systems are in red color and the previous metal-metal complexes are in blue (Ir-Ir), yellow (Rh-Rh) and green (Au-Au) color.

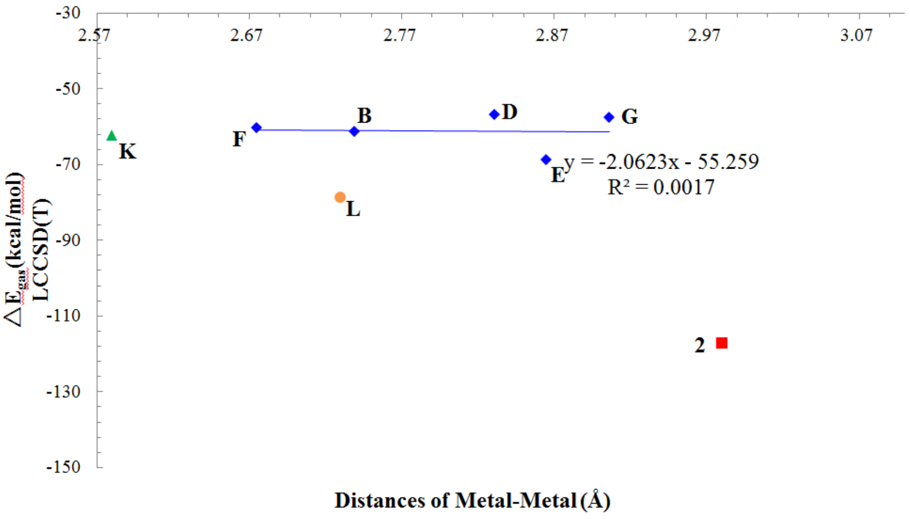

Fig. S26(h). Correlation of the computed metal(II)-metal(II) distances (Å) and relative binding (electronic) energies (in kcal/mol) in gas phase by the DLPNO-CCSD(T)//M06-L method based on the M06-L-optimized geometries. The current Ir-Ir dimer systems are in red color and the previous metal-metal complexes are in blue (Ir-Ir), yellow (Rh-Rh) and green (Au-Au) color.

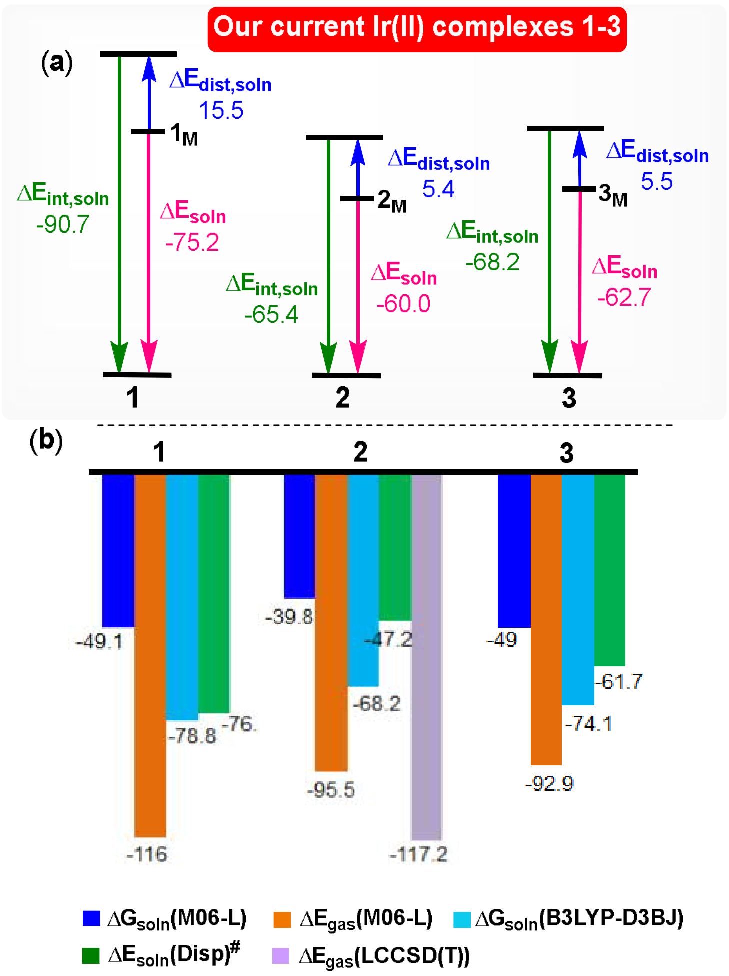

Fig. S27. (a) Distortion/interaction analysis (in kcal/mol) to form the key diiridium(II) complexes **1-3** from its corresponding monomeric forms ($\mathbf{1_M}$–$\mathbf{3_M}$) in acetonitrile by the SMD M06-L//M06-L method; (b) The binding energies (in kcal/mol) in solution (soln) and gas phase for **1-3** by the SMD M06-L//M06-L and other computational methods based on the M06-L-optimized structures. $\triangle E_{soln}$(Disp) = $\triangle$E(SMD B3LYP-D3BJ//M06-L) - $\triangle$E(SMD B3LYP//M06-L).

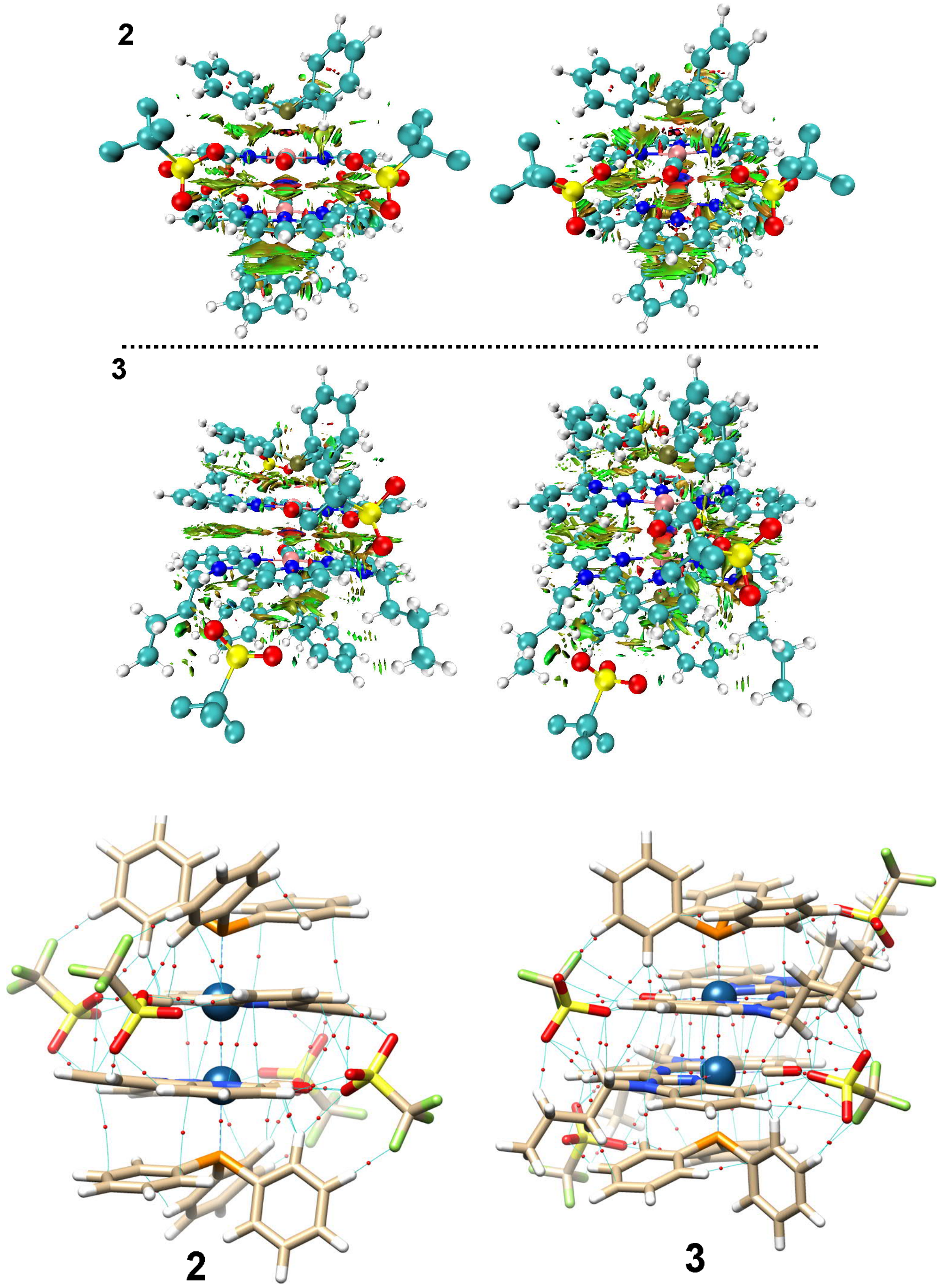

Fig. S28. (Top) Non-covalent interactions (NCIs) analysis (red: strong repulsion; green: weak attraction; blue: strong attraction) of **2** and **3**. (Bottom) The QTAIM analysis (Bond-Critical-Points, BCPs) on the key Ir_2 complexes in this work calculated on the SMD M06-L//M06-L optimized structures.

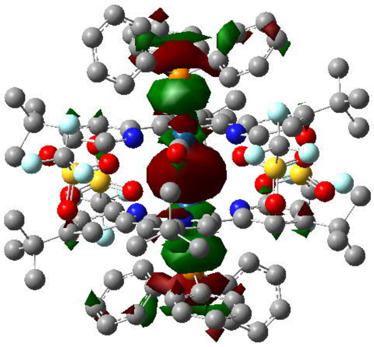

MO: 592

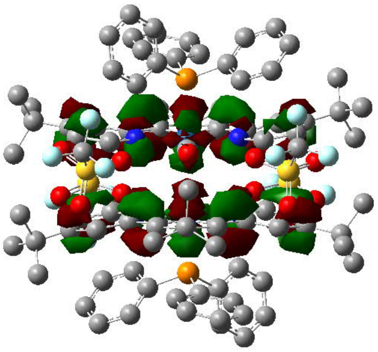

MO: 593

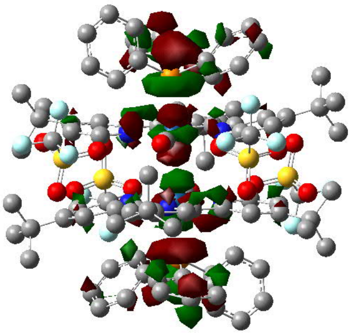

MO: 597

Fig. S29. The corresponding absorption orbitals of complex **1** in acetonitrile solvent by the CPCM TD-B3LYP-D3//M06-L method based on the M06-L optimized geometries using ORCA 5.01. Unimportant hydrogen atoms are not shown for clarity.

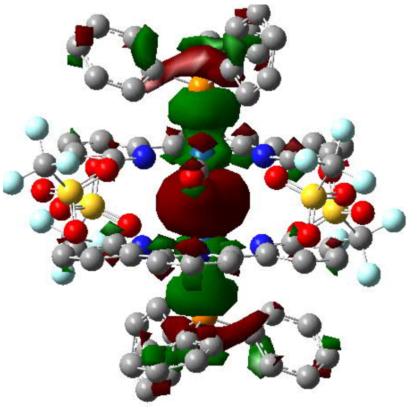

MO: 496

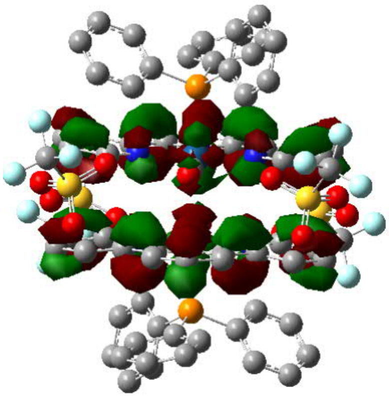

MO: 497

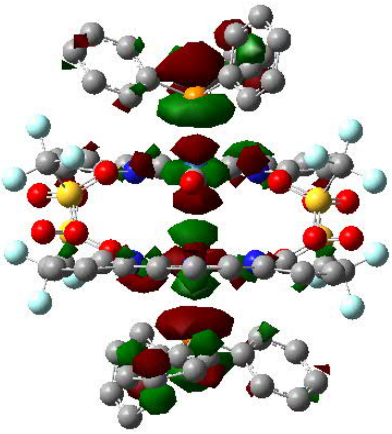

MO: 501

Fig. S30. The corresponding absorption orbitals of complex **2** in acetonitrile solvent by the CPCM TD-B3LYP-D3//M06-L method based on the M06-L optimized geometries using ORCA 5.01. Unimportant hydrogen atoms are not shown for clarity.

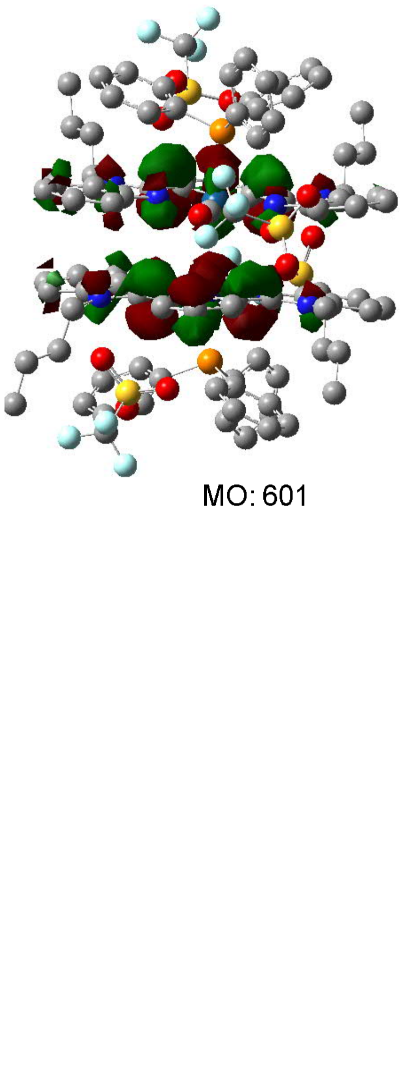

Fig. S31. The corresponding absorption orbitals of complex **3** in acetonitrile solvent by the CPCM TD-B3LYP-D3//M06-L method based on the M06-L optimized geometries using ORCA 5.01. Unimportant hydrogen atoms are not shown for clarity.

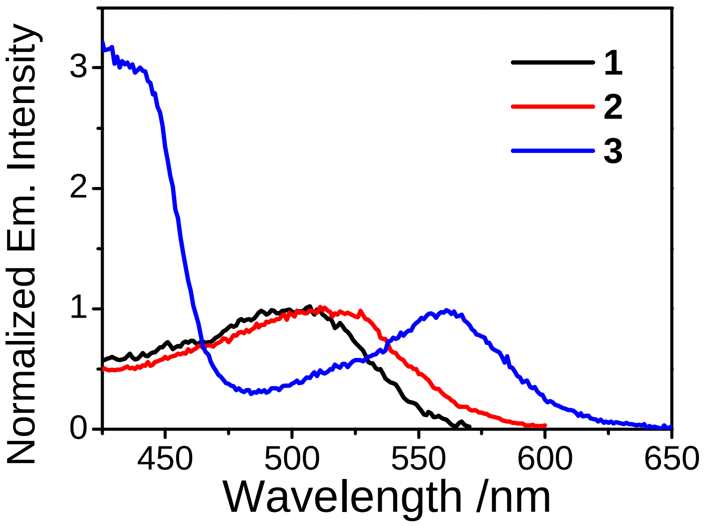

Fig. S32. Normalized excitation spectra of **1–3** in CH_3CN solution at 298 K. Emission intensity monitored at 620 nm for **1**, 650 nm for **2** and 678 nm for **3**.

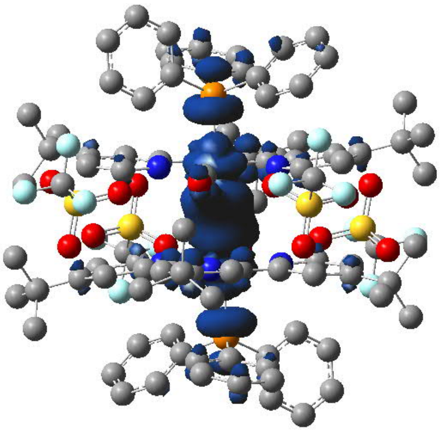

1

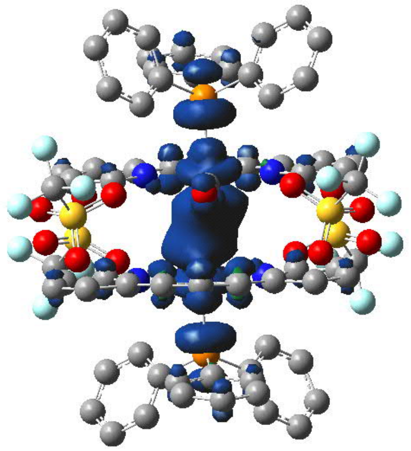

2

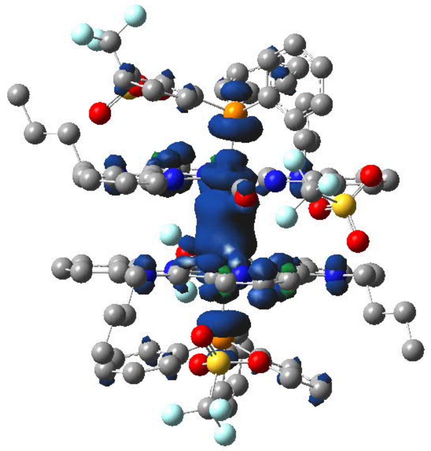

3

Fig. S33. The computed spin density of triplet **1**-**3** by the M06-L method.

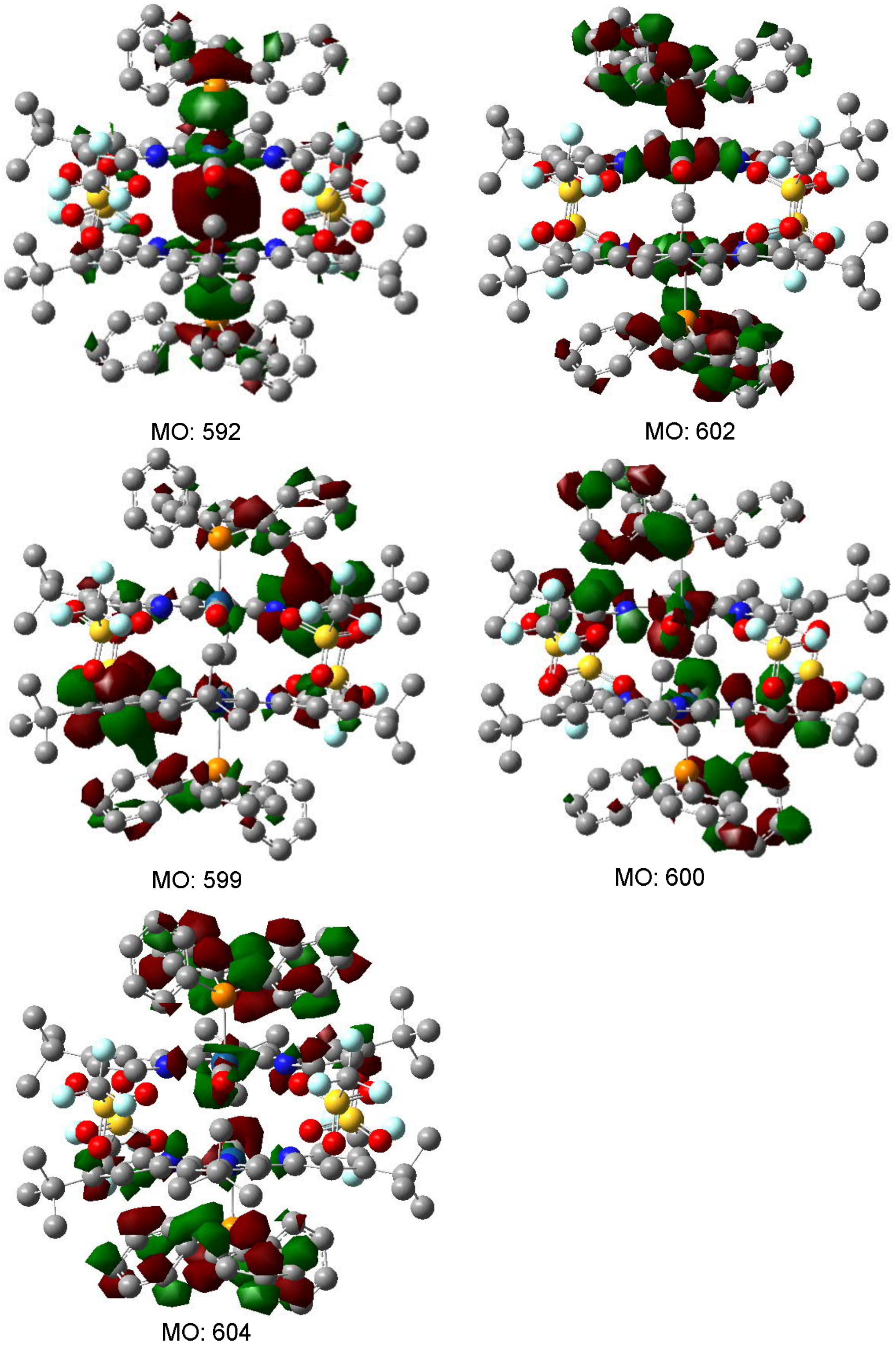

Fig. S34. The corresponding emission orbitals of triplet **1** in acetonitrile solvent by the CPCM TD-B3LYP-D3//M06-L method based on the M06-L optimized geometries using ORCA 5.01. Unimportant hydrogen atoms are not shown for clarity.

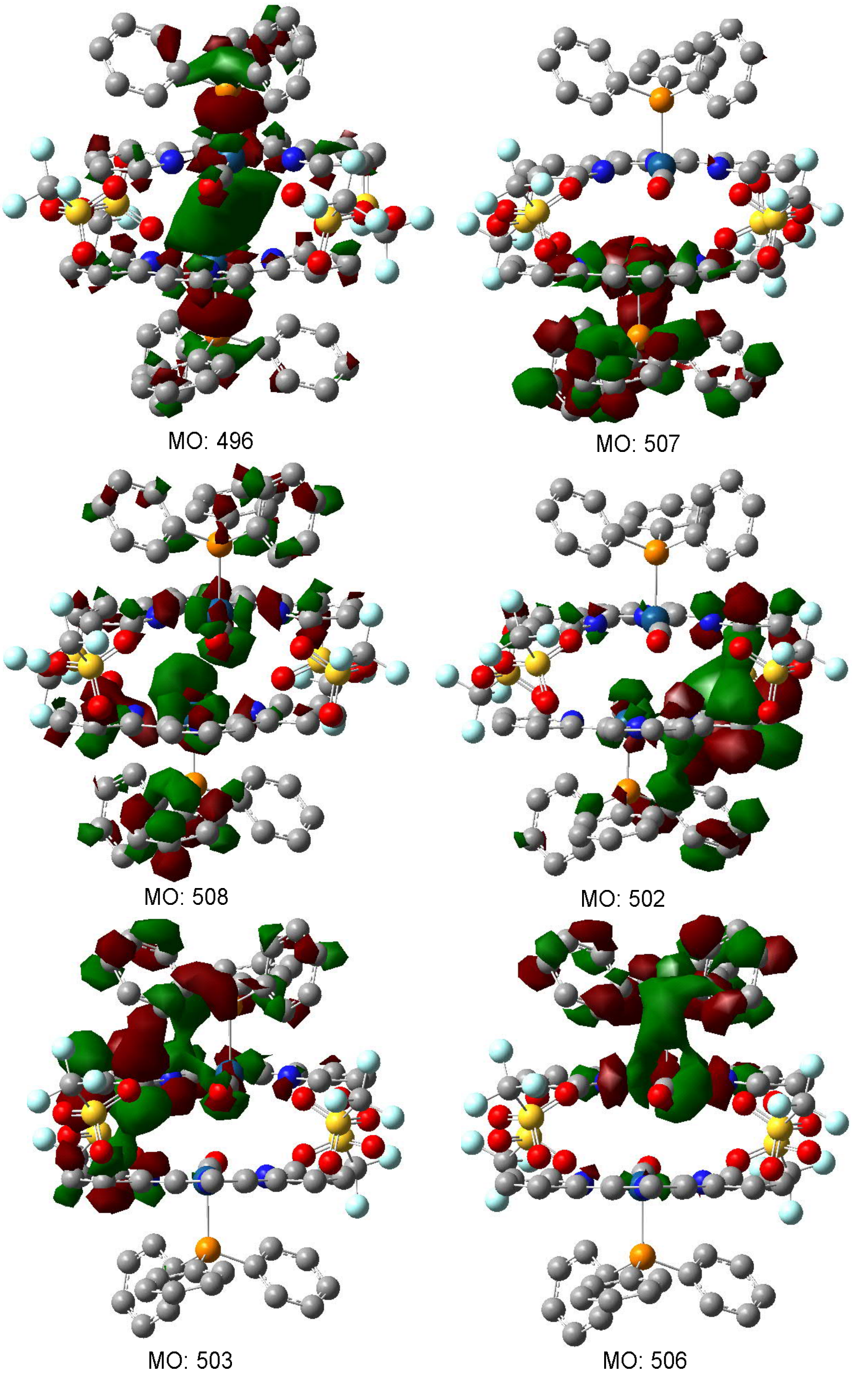

Fig. S35. The corresponding emission orbitals of triplet **2** in acetonitrile solvent by the CPCM TD-B3LYP-D3//M06-L method based on the M06-L optimized geometries using ORCA 5.01. Unimportant hydrogen atoms are not shown for clarity.

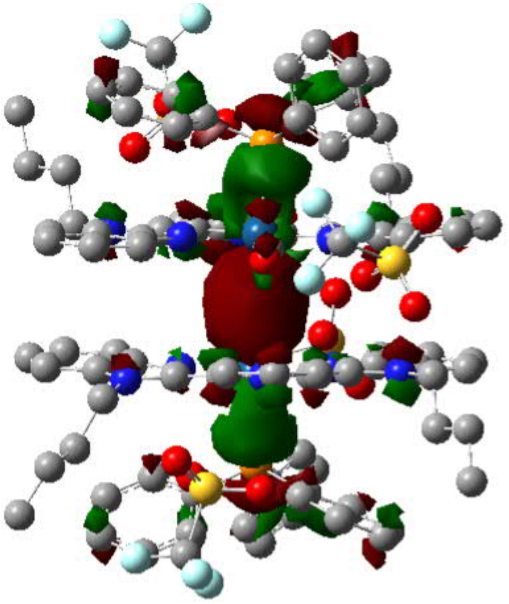

MO: 600

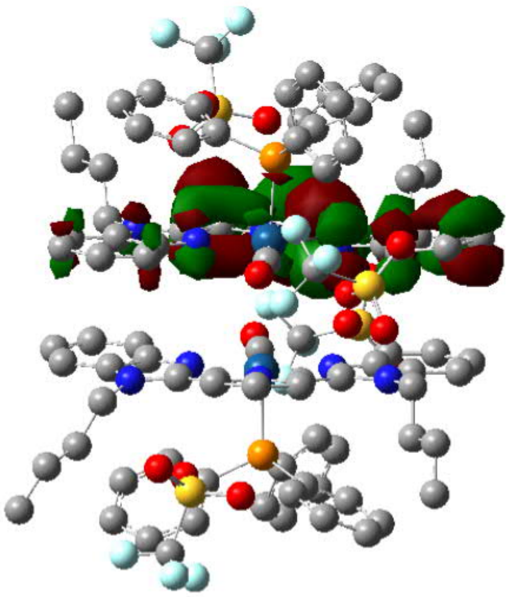

MO: 605

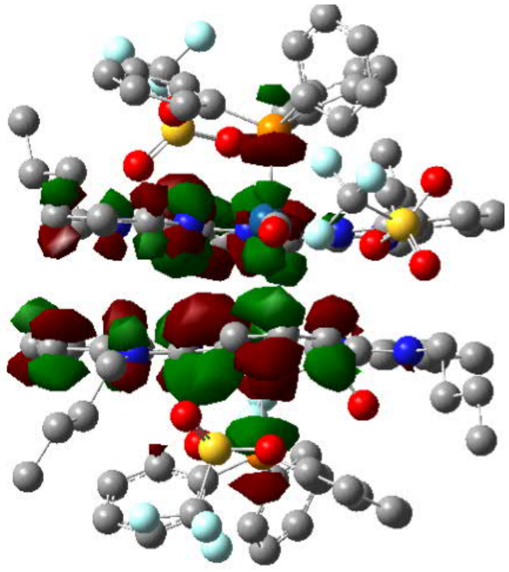

MO: 603

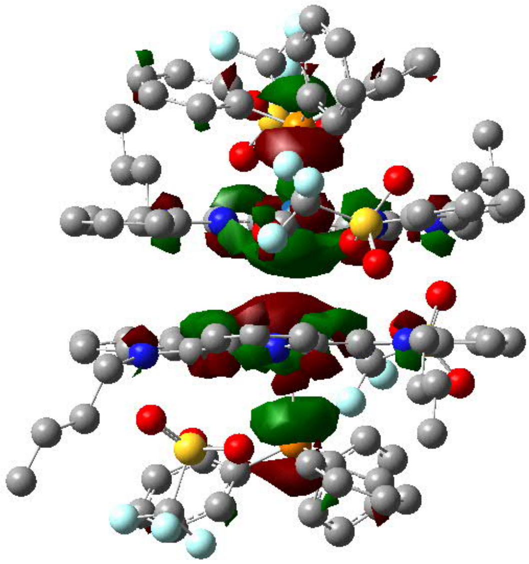

MO: 601

Fig. S36. The corresponding emission orbitals of triplet **3** in acetonitrile solvent by the CPCM TD-B3LYP-D3//M06-L method based on the M06-L optimized geometries using ORCA 5.01. Unimportant hydrogen atoms are not shown for clarity.

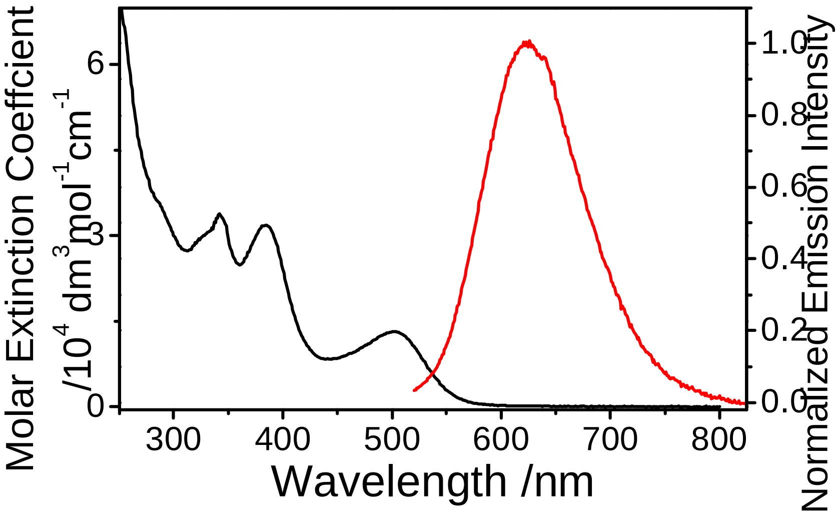

Fig. S37. UV-Vis absorption and emission spectra of complex **1'** in CH_3CN solution at 298 K. Excitation at λ_{ex} = 470 nm.

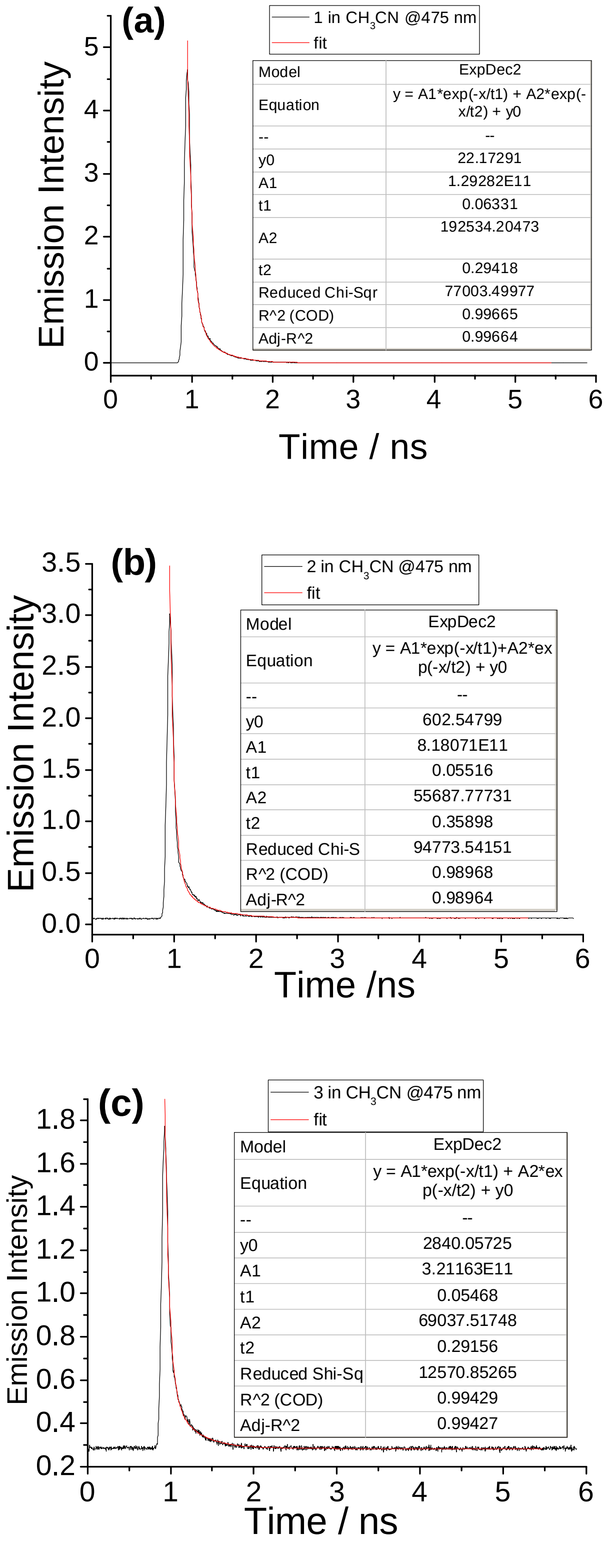

Fig. S38. Luminescence life-time measurement and fitting of **1** (a), **2** (b) and **3** (c) in degassed CH_3CN at 298 K. Excitation at λ_{ex} = 475 nm.

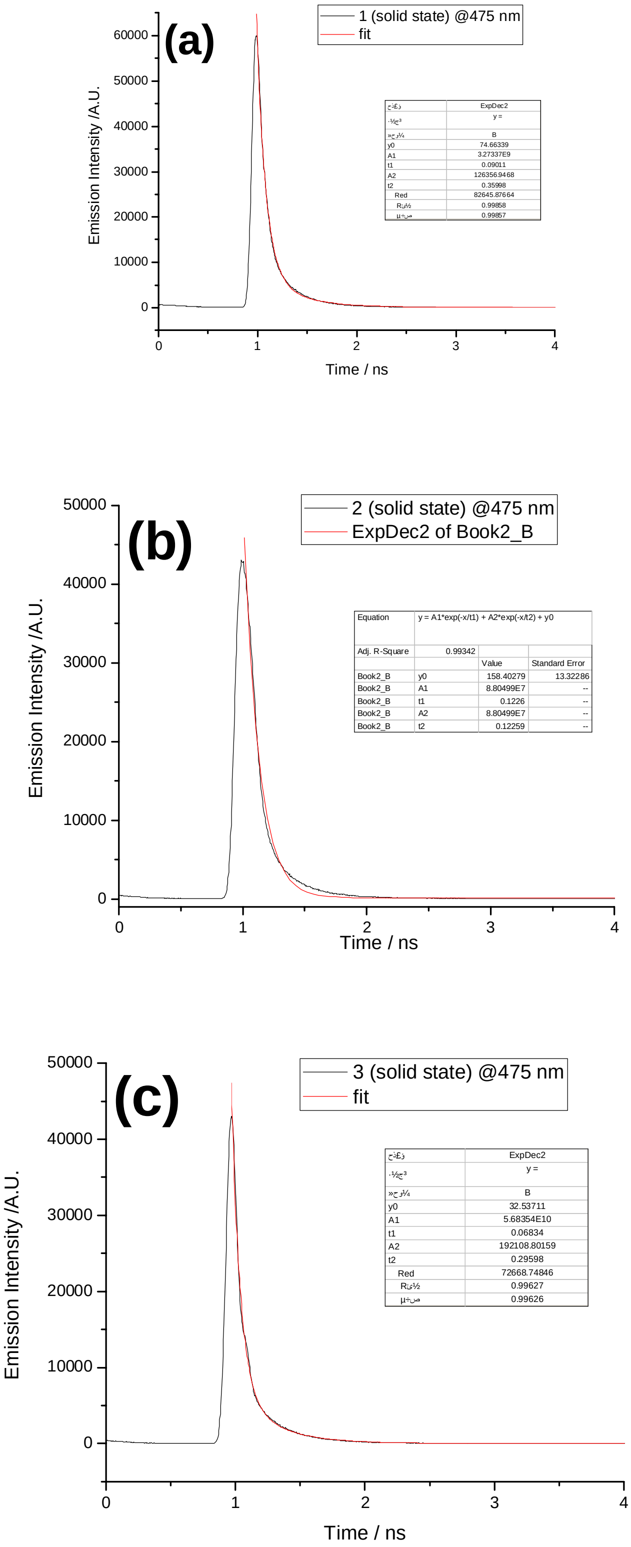

Fig. S39. Luminescence life-time measurement and fitting of **1** (a), **2** (b) and **3** (c) in solid state at 298 K. Excitation at λ_{ex} = 475 nm.

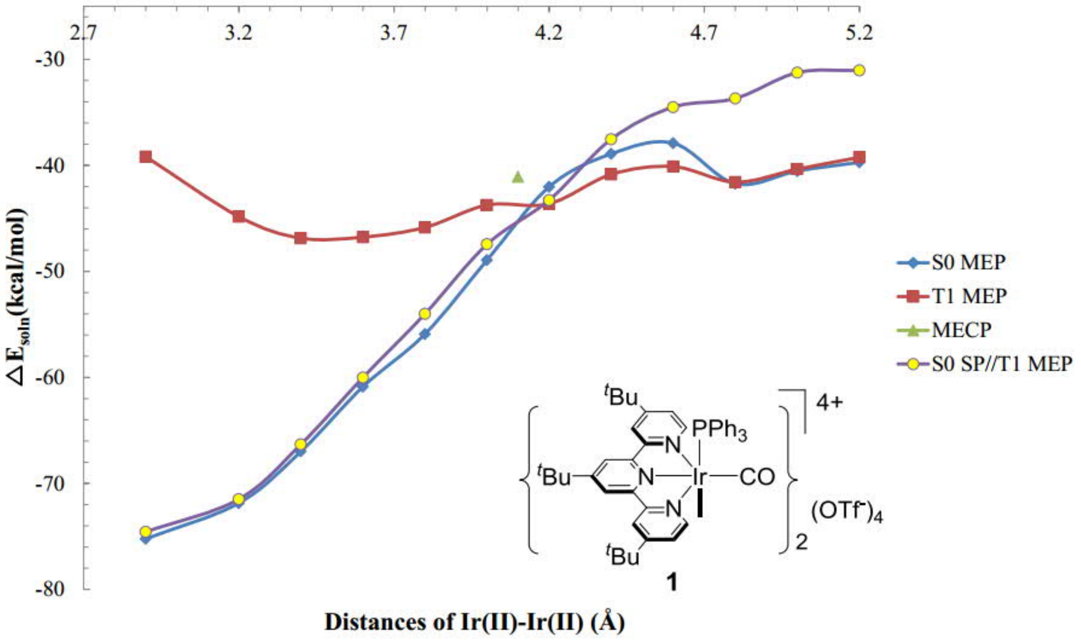

Fig. S40. Potential energy scan along various Ir-Ir bond distances for **1** in acetonitrile solvent by the SMD M06-L//M06-L method.

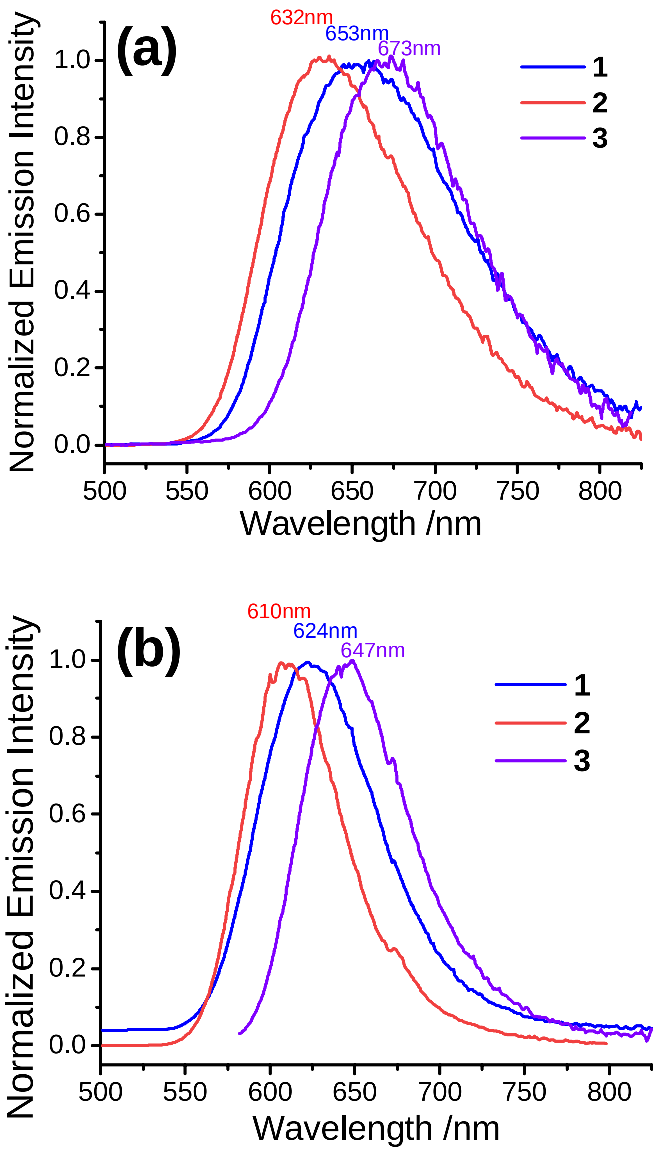

Fig. S41. Luminescence spectra of **1**–**3** in the solid state at 298 K (a) and 77 K (b).

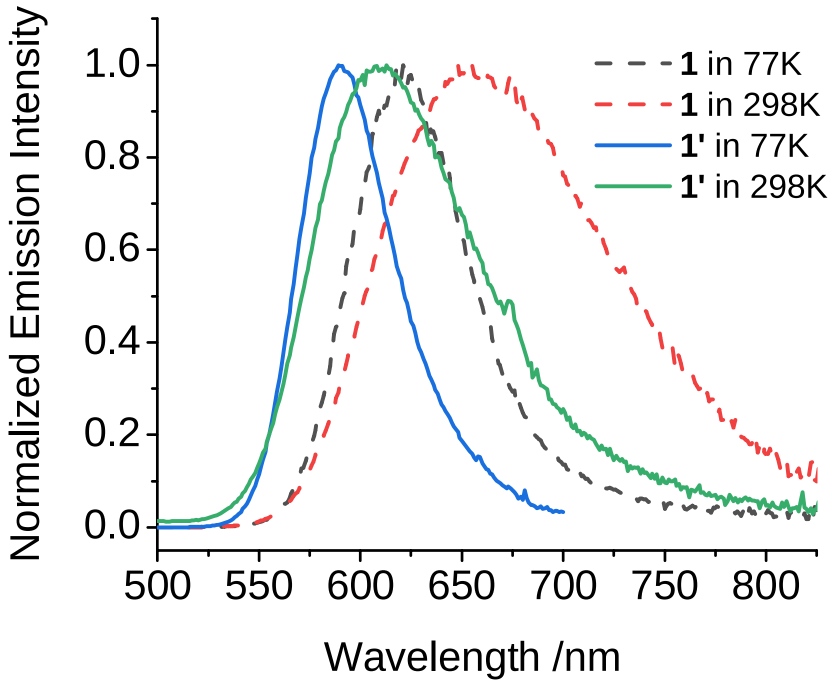

Fig. S42. Emission spectra of 1 and 1’ in solid state 77 and 298 K.

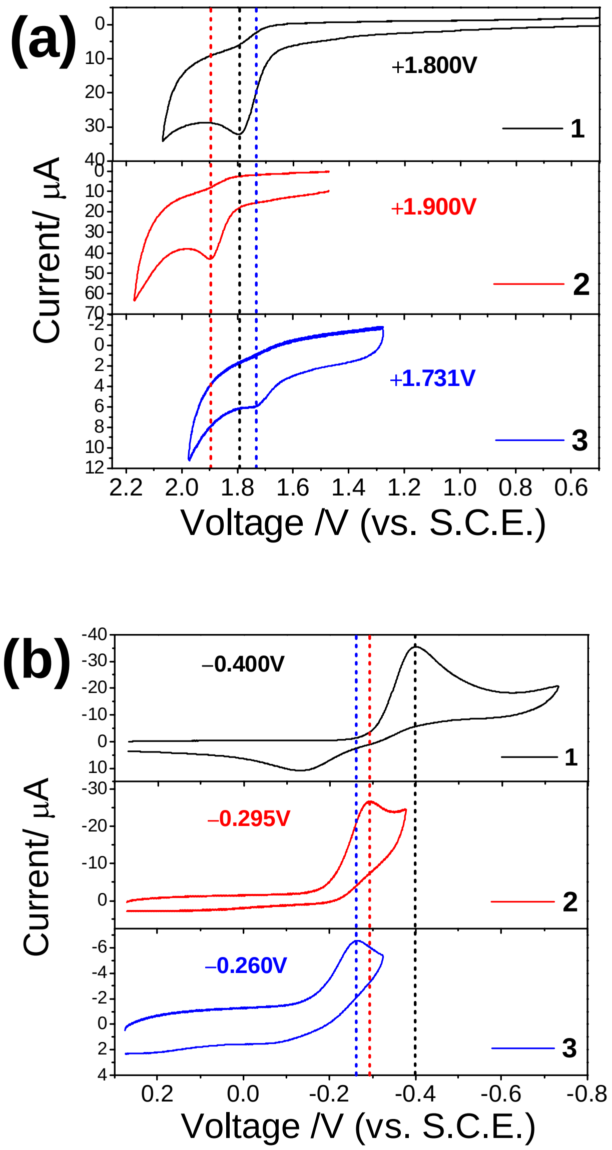

Fig. S43. Cyclic voltammograms for oxidative (a) and reductive (b) scans of **1–3** in CH_3CN solution (0.1 M $^nBu_4NPF_6$) at 298 K. Scan rate = 100 mV/s.

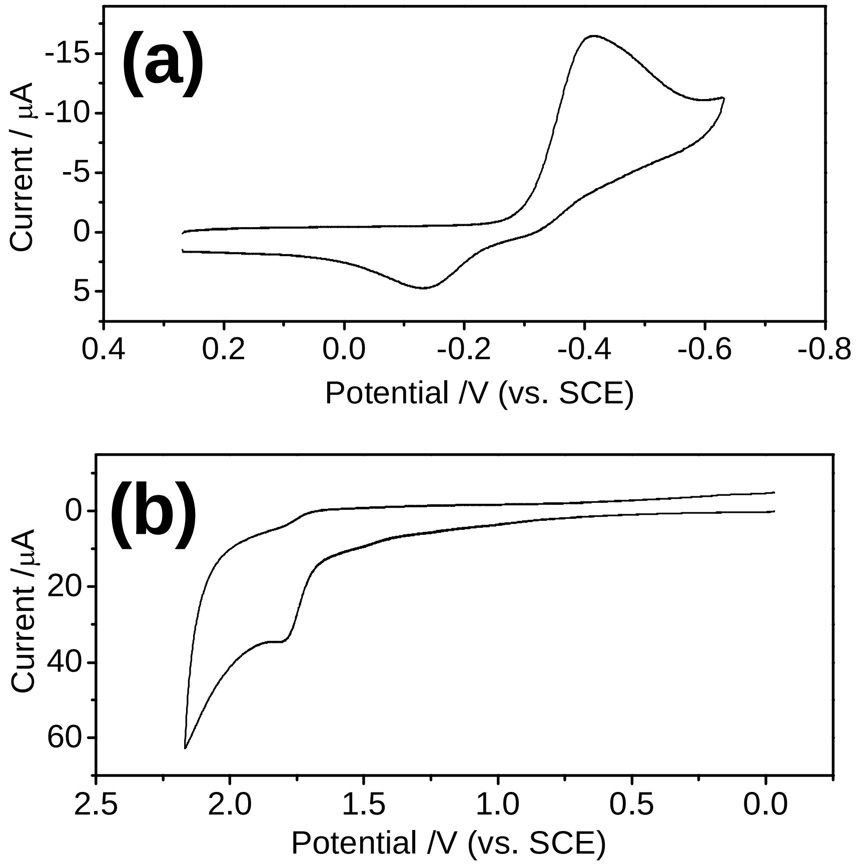

Fig. S44. Cyclic voltammograms for oxidative (a) and reductive (b) scans of **1'** in CH_3CN solution (0.1 M $^nBu_4NPF_6$) at 298 K. Scan rate = 100 mV/s.

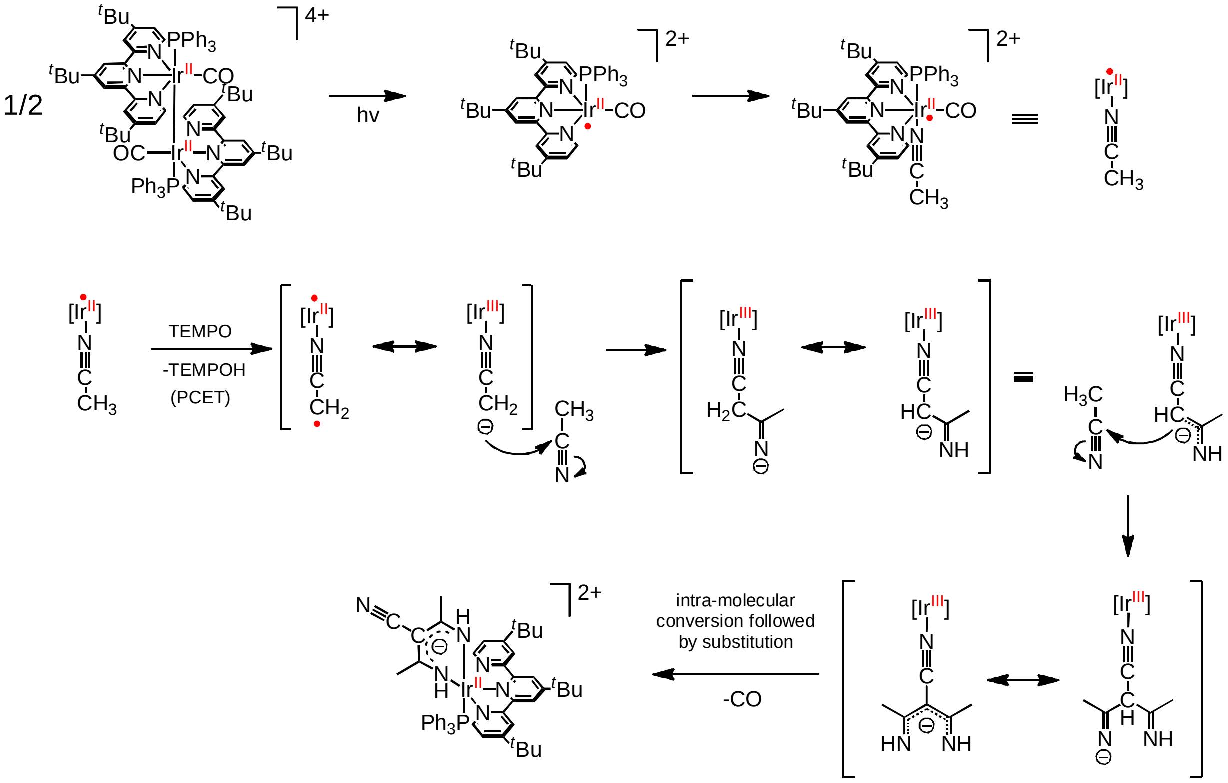

Fig. S45. Proposed mechanism for the formation of complex **9**.

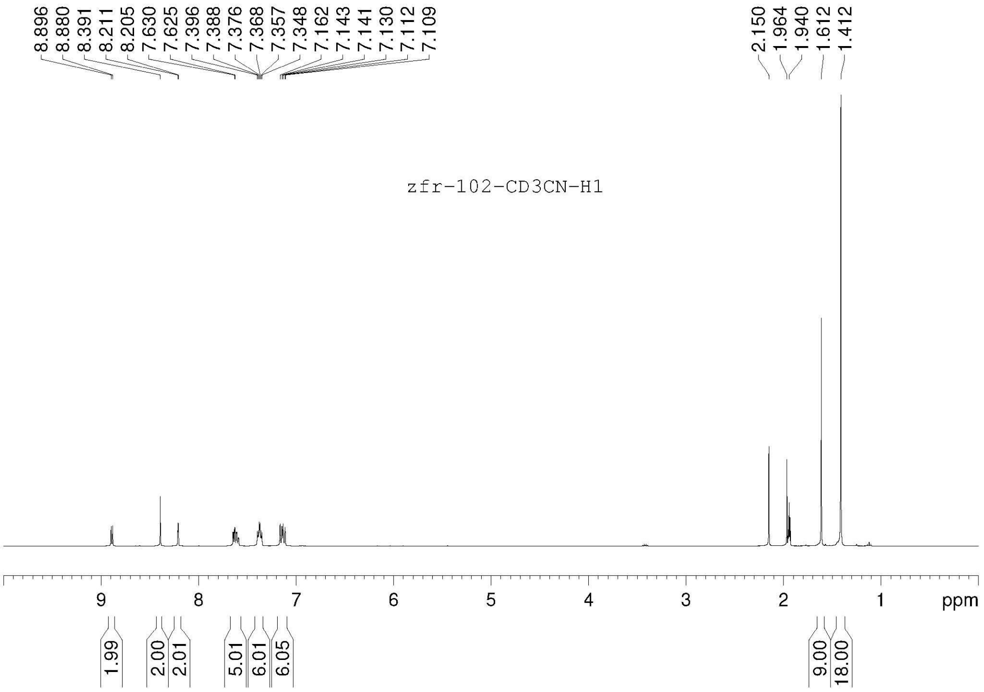

Fig. S46. ^{1}H NMR spectrum of complex **7** in CD_3CN.

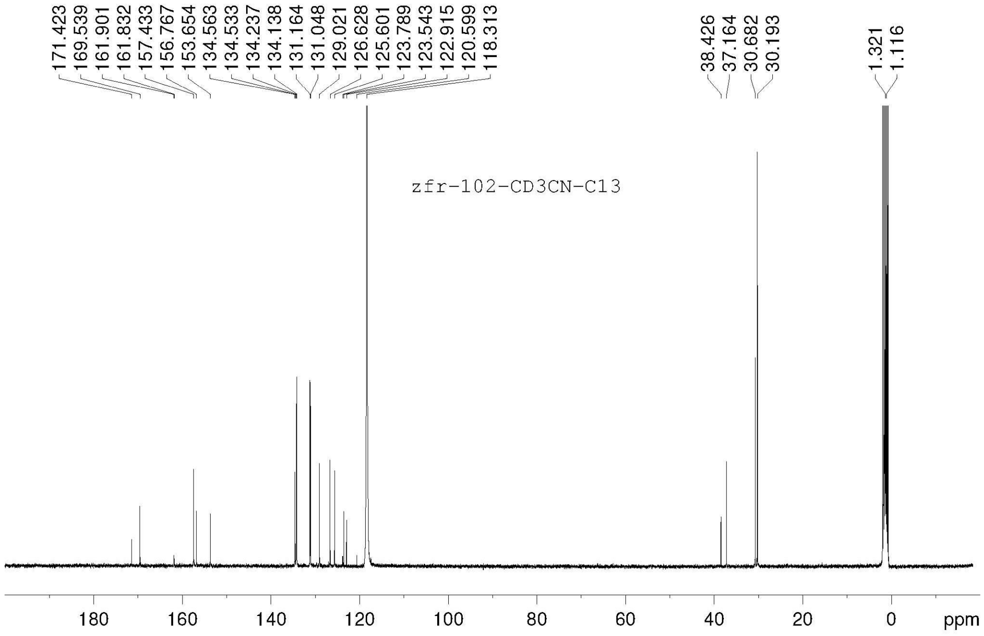

Fig. S47. $^{13}C\{^1H\}$ NMR spectrum of complex **7** in CD_3CN.

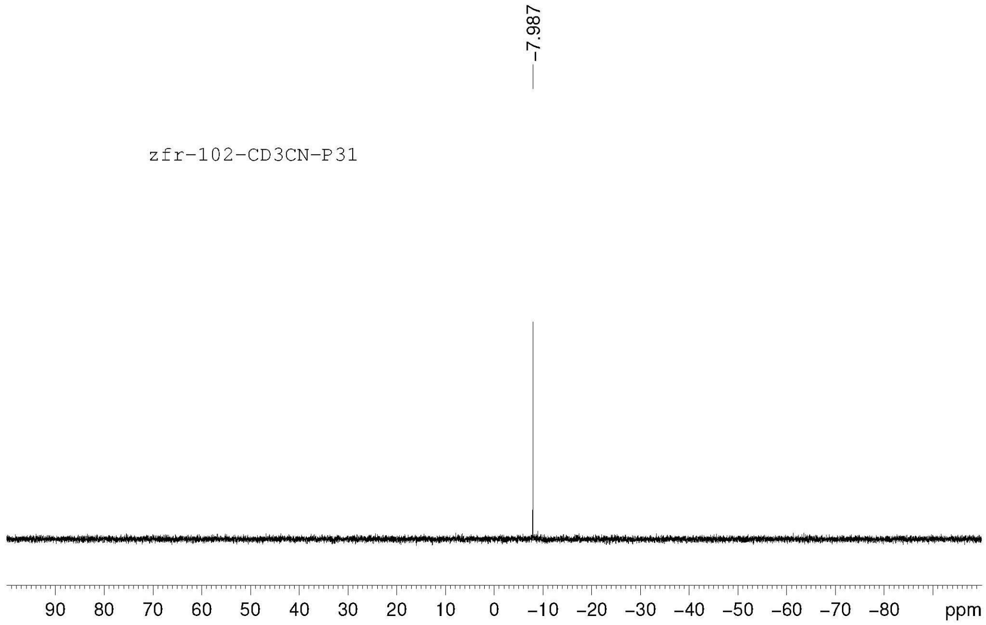

Fig. S48. $^{31}P\{^{1}H\}$ NMR spectrum of complex **7** in CD_3CN.

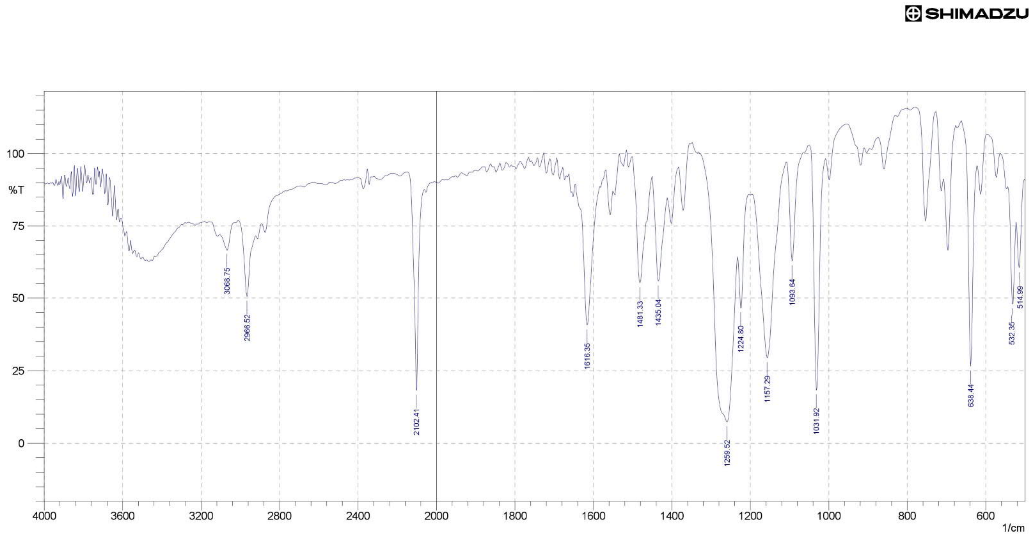

Fig. S49. IR spectrum of complex **7** in KBr disk.

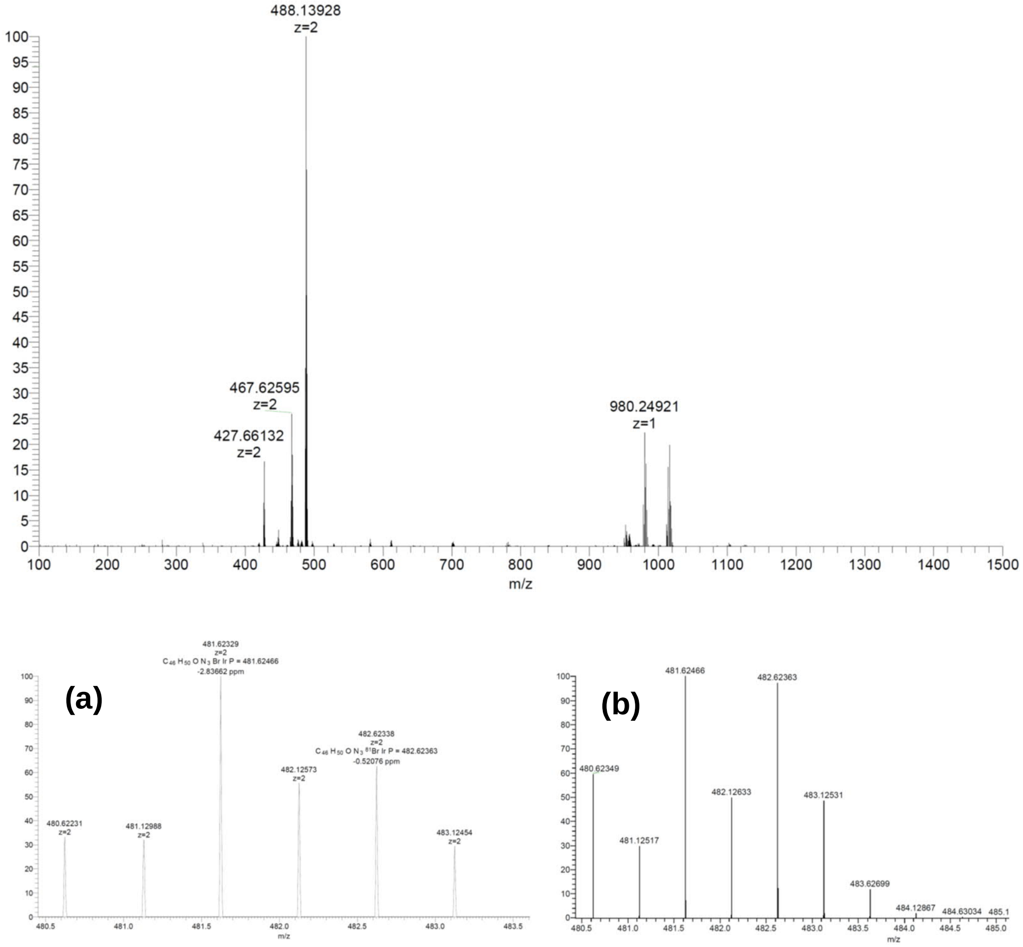

Fig. S50. (Top) HRMS (ESI) spectrum of complex **7**. (Bottom) Expanded peak at m/z = 481 (a) and theoretical isotope pattern of [M – 2OTf⁻]$^{2+}$ (b).

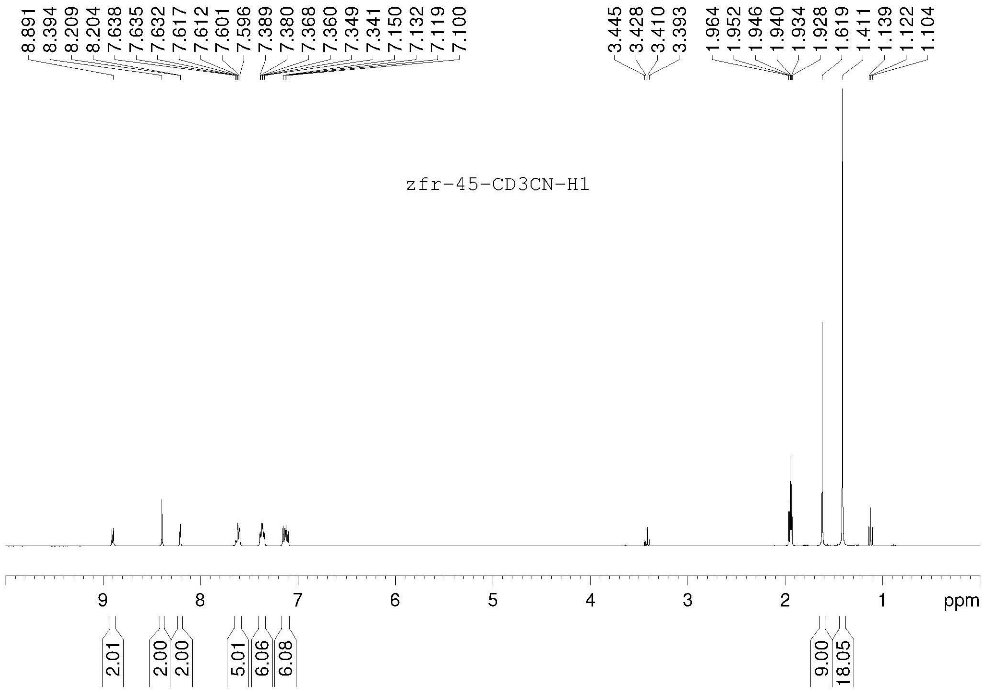

Fig. S51. ^{1}H NMR spectrum of complex **8** in CD_3CN.

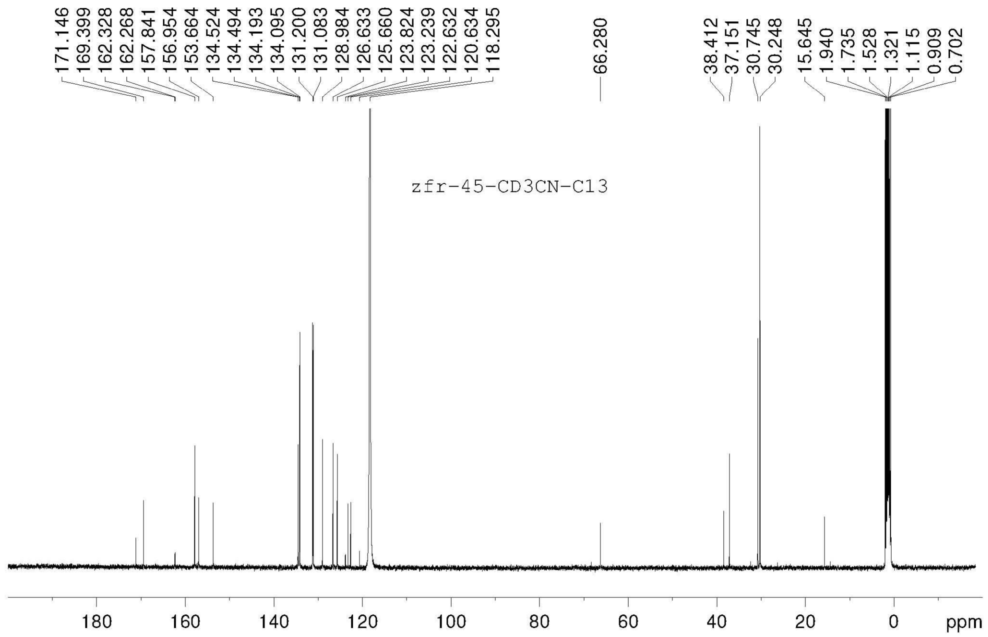

Fig. S52. ^{13}C{1H} NMR spectrum of complex **8** in CD_3CN.

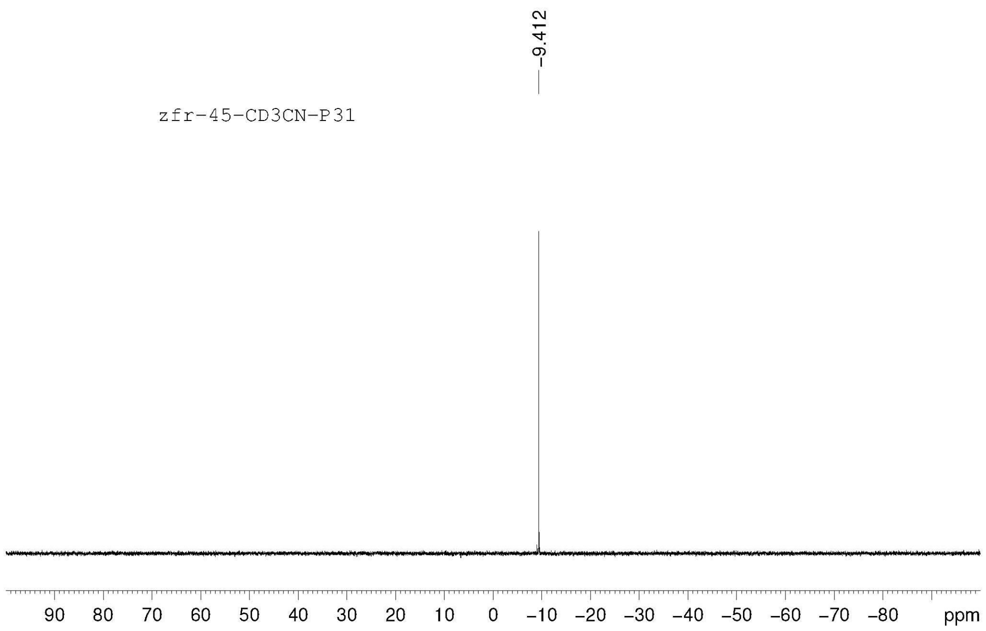

Fig. S53. $^{31}P\{^1H\}$ NMR spectrum of complex **8** in CD_3CN.

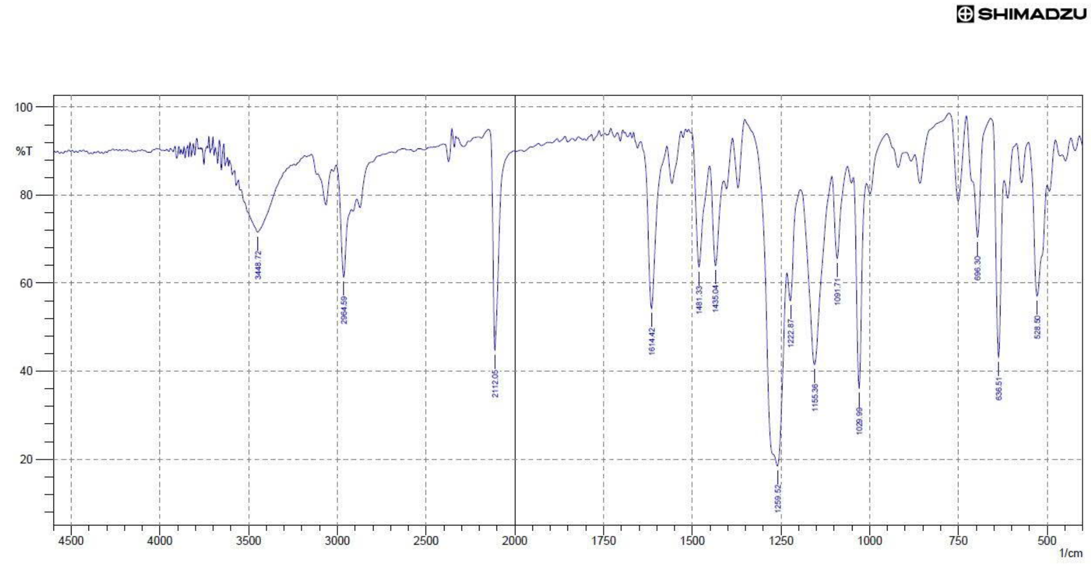

Fig. S54. IR spectrum of complex **8** in KBr disk.

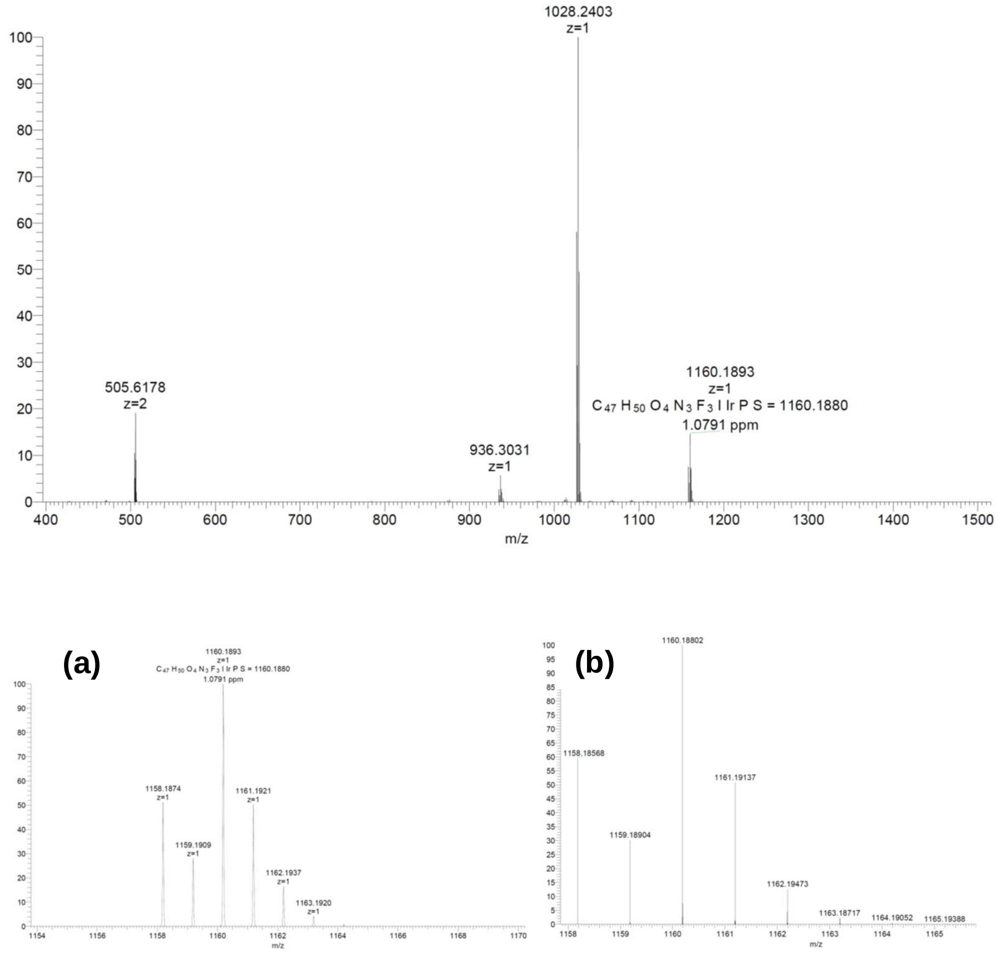

Fig. S55. (Top) HRMS (ESI) spectrum of complex **8**. (Bottom) Expanded peak at m/z = 1160 (a) and theoretical isotope pattern of $[M - OTf^{-}]^{+}$ (b).

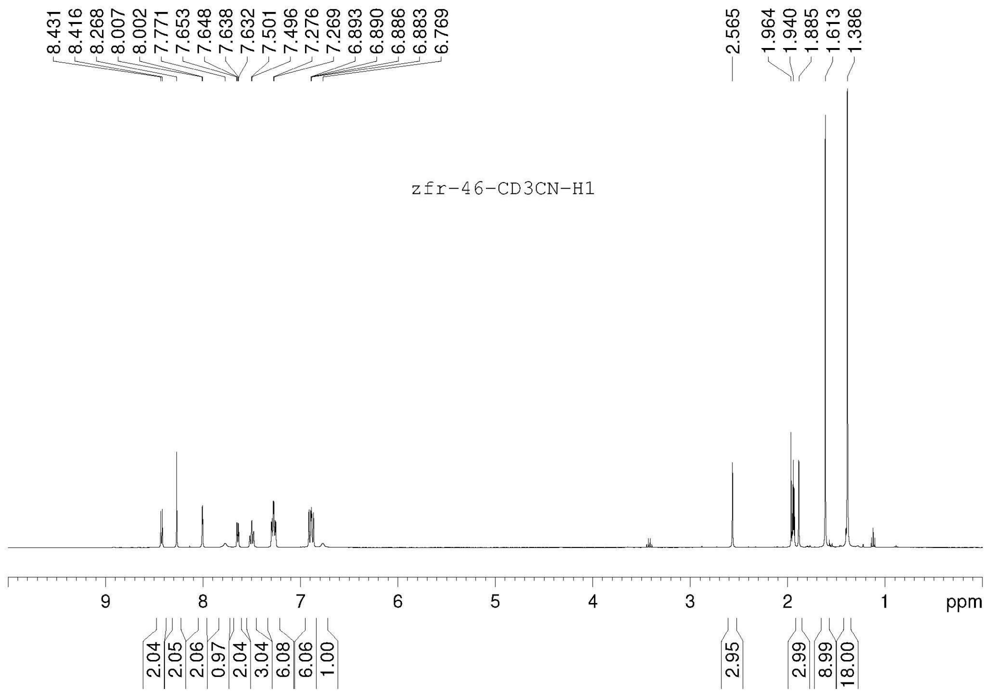

Fig. S56. ^{1}H NMR spectrum of complex **9** in CD_3CN.

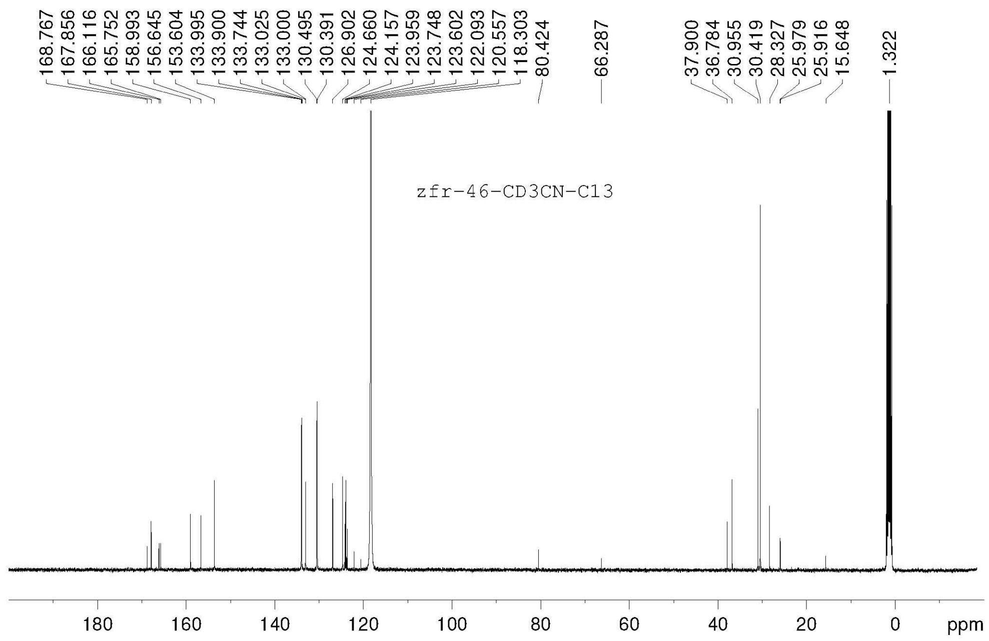

Fig. S57. $^{13}C\{^{1}H\}$ NMR spectrum of complex **9** in CD_3CN.

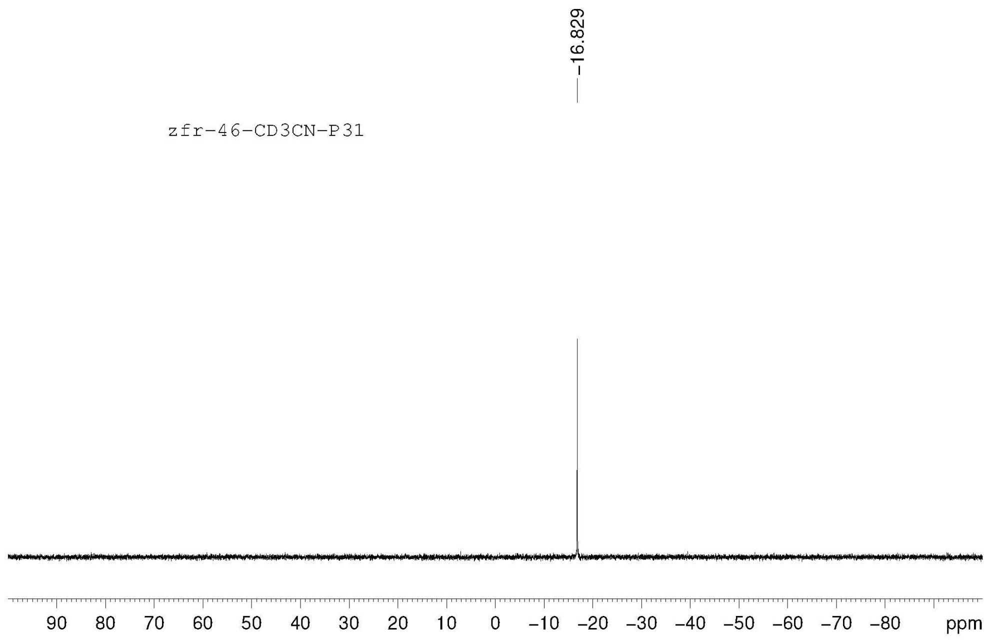

Fig. S58. $^{31}P\{^1H\}$ NMR spectrum of complex **9** in CD_3CN.

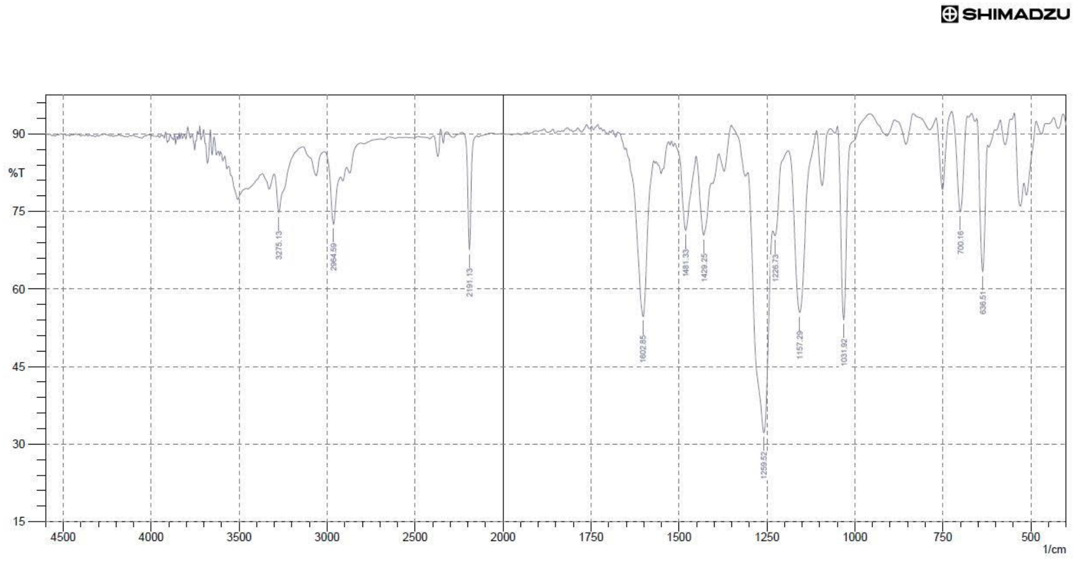

Fig. S59. IR spectrum of complex **9** in KBr disk.

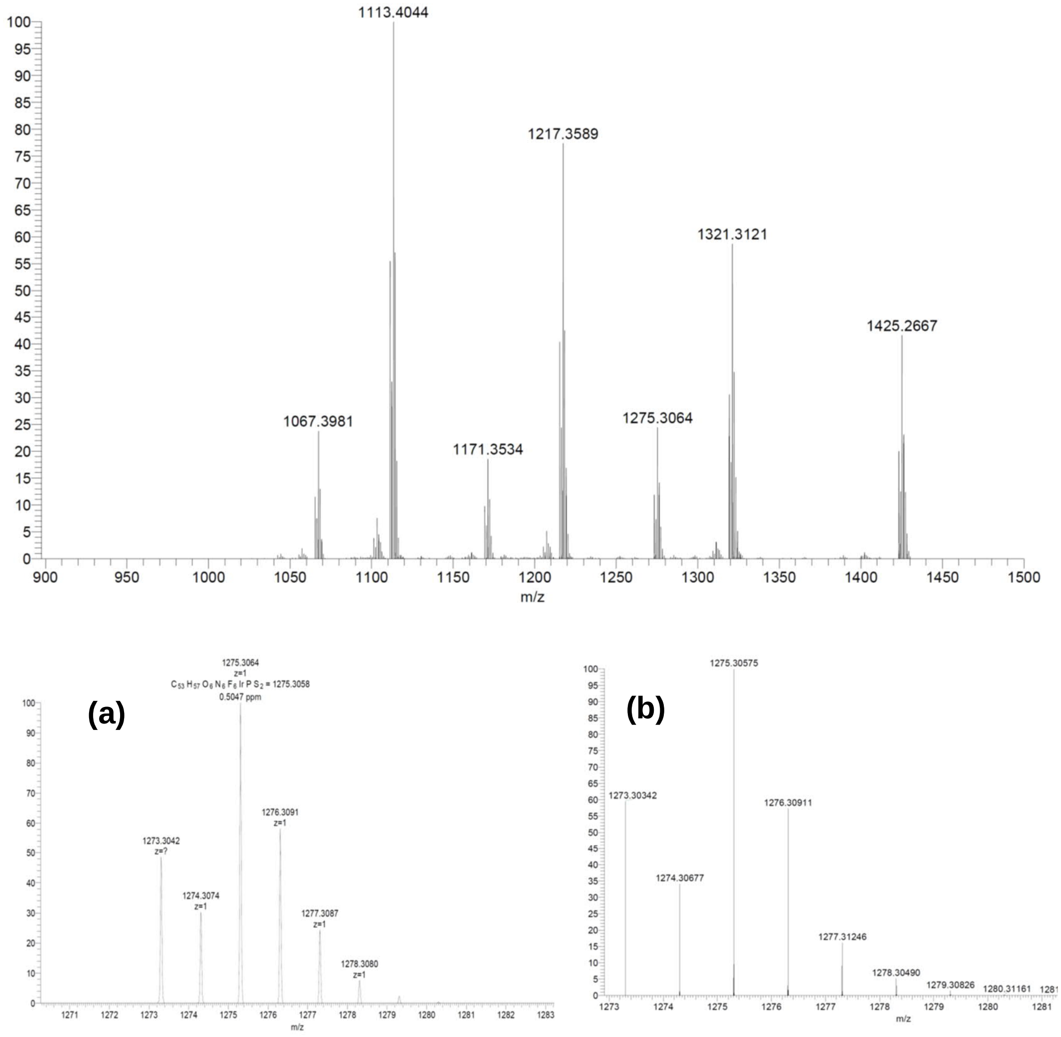

Fig. S60. (Top) HRMS (ESI) spectrum of complex **9**. (Bottom) Expanded peak at m/z = 1275 (a) and theoretical isotope pattern of $[M – H]^+$ (b).

Table S1. Crystallographic Data for **1**.

Empirical formula	$C_{50}H_{53}F_6IrN_4O_7PS_2$
Formula weight	1223.25
Temperature	150(2) K
Wavelength	0.710 Å
Crystal system, space group	Monoclinic, P 21/n
	a = 16.4301(19) Å alpha = 90 deg.
Unit cell dimensions	b = 17.620(2) Å beta = 95.138(4) deg.
	c = 17.470(2) Å gamma = 90 deg.
Volume	5037.4(10) $Å^3$
Z, Calculated density	4, 1.613 Mg/m^3
Absorption coefficient	2.842 mm^{-1}
F(000)	2460
Crystal size	0.210 x 0.190 x 0.170 mm
Theta range for data collection	2.123 to 27.505 deg.
Limiting indices	-21<=h<=21, -22<=k<=22, -22<=l<=22
Reflections collected / unique	108375 / 11594 [R_{int} = 0.0494]
Completeness to theta = 25.214	99.90%
Absorption correction	Semi-empirical from equivalents
Max. and min. transmission	0.7456 and 0.5830
Refinement method	Full-matrix least-squares on F^2
Data / restraints / parameters	11594 / 0 / 641
Goodness-of-fit on F^2	1.051
Final R indices [I>2sigma(I)]	R_1 = 0.0206, wR_2 = 0.0485
R indices (all data)	R_1 = 0.0291, wR_2 = 0.0513
Largest diff. peak and hole	0.405 and -0.700 e. $Å^{-3}$

Table S2. Selected bond lengths [Å] for **1**.

Ir(1)-C(50)	1.897(2)
Ir(1)-N(2)	1.9888(17)
Ir(1)-N(1)	2.0521(17)
Ir(1)-N(3)	2.0544(17)
Ir(1)-P(1)	2.4460(6)
Ir(1)-Ir(1)#1	2.8942(4)
O(1)-C(50)	1.131(3)

Table S3. Selected angles [deg] for **1**.

C(50)-Ir(1)-N(2)	178.12(8)
C(50)-Ir(1)-N(1)	102.31(8)
N(2)-Ir(1)-N(1)	79.35(7)
C(50)-Ir(1)-N(3)	98.95(8)
N(2)-Ir(1)-N(3)	79.42(7)
N(1)-Ir(1)-N(3)	158.73(7)
P(1)-Ir(1)-Ir(1)#1	177.004(14)

Table S4. Crystallographic Data for **1'**.

Empirical formula	$C_{46}H_{50}B_2F_8IrN_3OP$
Formula weight	1057.68
Temperature	99.99 K
Wavelength	1.54178 Å
Crystal system, space group	Monoclinic, P 1 21/n 1
Unit cell dimensions	a = 14.7269(10) Å alpha = 90 deg.<br>b = 17.4747(11) Å beta = 101.355(3) deg.<br>c = 17.3176(11) Å gamma = 90 deg.
Volume	4369.4(5) $Å^3$
Z, Calculated density	4, 1.608 Mg/m^3
Absorption coefficient	6.897 mm^{-1}
F(000)	2116
Crystal size	0.21 x 0.19 x 0.10 mm
Theta range for data collection	4.407 to 65.271 deg.
Limiting indices	-17<=h<=17, -20<=k<=20, -19<=l<=20
Reflections collected / unique	26298 / 7408 [R_{int}= 0.0435]
Completeness to theta = 67.679	93.50%
Absorption correction	Semi-empirical from equivalents
Max. and min. transmission	0.1621 and 0.0466
Refinement method	Full-matrix least-squares on F^2
Data / restraints / parameters	7408 / 30 / 599
Goodness-of-fit on F^2	1.064
Final R indices [I>2sigma(I)]	R_1 = 0.0423, wR_2 = 0.1100
R indices (all data)	R1 = 0.0474, wR_2 = 0.1150
Largest diff. peak and hole	2.659 and -0.826 e. $Å^{-3}$

Table S5. Selected bond lengths [Å] for **1'**.

Ir(1)-Ir(1)#1	2.9135(5)
Ir(1)-P(1)	2.4471(14)
Ir(1)-N(1)	2.058(5)
Ir(1)-N(2)	2.000(4)
Ir(1)-N(3)	2.046(4)
Ir(1)-C(46)	1.896(5)
O(1)-C(46)	1.132(6)

Table S6. Selected angles [deg] for **1'**.

P(1)-Ir(1)-Ir(1)#1	177.27(4)
N(2)-Ir(1)-N(1)	78.94(16)
N(2)-Ir(1)-N(3)	79.38(17)
N(3)-Ir(1)-N(1)	158.30(16)
C(46)-Ir(1)-N(1)	104.36(19)
C(46)-Ir(1)-N(2)	176.05(18)
C(46)-Ir(1)-N(3)	97.34(19)

Table S7. Crystallographic Data for **2**.

Empirical formula	$C_{38}H_{16}F_6IrN_3O_7PS_2$
Formula weight	1041.84
Temperature	141(2) K
Wavelength	0.710 Å
Crystal system, space group	Triclinic, P -1
Unit cell dimensions	a = 12.732(3) Å alpha = 72.951(7) deg.<br>b = 13.152(3) Å beta = 81.397(8) deg.<br>c = 14.553(4) Å gamma = 66.186(8) deg.
Volume	2130.1(9) Å^3
Z, Calculated density	2, 1.624 Mg/m^3
Absorption coefficient	3.346 mm^{-1}
F(000)	1012
Crystal size	0.210 x 0.170 x 0.150 mm
Theta range for data collection	2.239 to 26.702 deg.
Limiting indices	-16<=h<=15, -16<=k<=16, -18<=l<=18
Reflections collected / unique	41064 / 8923 [R_{int} = 0.0689]
Completeness to theta = 25.214	98.90%
Absorption correction	Semi-empirical from equivalents
Max. and min. transmission	0.7455 and 0.4376
Refinement method	Full-matrix least-squares on F^2
Data / restraints / parameters	8923 / 258 / 767
Goodness-of-fit on F^2	1.042
Final R indices [I>2sigma(I)]	R_1 = 0.0496, wR_2 = 0.1197
R indices (all data)	R_1 = 0.0680, wR_2 = 0.1354
Largest diff. peak and hole	2.151 and -2.974 e. Å^{-3}

Table S8. Selected bond lengths [Å] for **2**.

Ir(1)-C(1)	1.902(7)
Ir(1)-N(2)	1.997(5)
Ir(1)-N(1)	2.055(6)
Ir(1)-N(3)	2.069(6)
Ir(1)-P(1)	2.455(2)
Ir(1)-Ir(1)#1	2.9421(9)
C(1)-O(1)	1.121(8)

Table S9. Selected angles [deg] for **2**.

C(1)-Ir(1)-N(2)	177.7(2)
C(1)-Ir(1)-N(1)	99.7(3)
N(2)-Ir(1)-N(1)	79.7(2)
C(1)-Ir(1)-N(3)	102.1(3)
N(2)-Ir(1)-N(3)	78.5(2)
N(1)-Ir(1)-N(3)	158.2(2)
P(1)-Ir(1)-Ir(1)#1	176.64(5)

Table S10. Crystallographic Data for **3**.

Empirical formula	$C_{54}H_{50}F_6IrN_5O_7PS_2$
Formula weight	1282.28
Temperature	150(2) K
Wavelength	0.70999 A
Crystal system, space group	Triclinic, P -1
Unit cell dimensions	a = 14.0846(12) Å alpha = 99.697(3) deg.<br>b = 14.6810(12) Å beta = 111.760(3) deg.<br>c = 15.1434(13) Å gamma = 108.904(3) deg.
Volume	2598.8(4) $Å^3$
Z, Calculated density	2, 1.639 Mg/m^3
Absorption coefficient	2.760 mm^{-1}
F(000)	1286
Crystal size	0.250 x 0.190 x 0.150 mm
Theta range for data collection	2.446 to 27.497 deg.
Limiting indices	-18<=h<=18, -19<=k<=19, -19<=l<=19
Reflections collected / unique	55676 / 11940 [R_{int} = 0.0460]
Completeness to theta = 25.214	99.90%
Absorption correction	Semi-empirical from equivalents
Max. and min. transmission	0.7455 and 0.5501
Refinement method	Full-matrix least-squares on F^2
Data / restraints / parameters	11940 / 7 / 708
Goodness-of-fit on F^2	1.059
Final R indices [I>2sigma(I)]	R_1 = 0.0321, wR_2 = 0.0717
R indices (all data)	R_1 = 0.0411, wR_2 = 0.0747
Largest diff. peak and hole	1.361 and -0.797 e. $Å^{-3}$

Table S11. Selected bond lengths [Å] for **3**.

Ir(1)-C(48)	1.874(4)
Ir(1)-N(2)	2.029(3)
Ir(1)-N(3)	2.037(3)
Ir(1)-N(1)	2.057(3)
Ir(1)-P(1)	2.4467(9)
Ir(1)-Ir(1)#1	2.9731(4)
O(1)-C(48)	1.135(4)

Table S12. Selected angles [deg] for **3**.

C(48)-Ir(1)-N(2)	177.23(13)
C(48)-Ir(1)-N(3)	100.08(13)
N(2)-Ir(1)-N(3)	78.44(11)
C(48)-Ir(1)-N(1)	103.36(13)
N(2)-Ir(1)-N(1)	78.06(11)
N(3)-Ir(1)-N(1)	156.50(11)
P(1)-Ir(1)-Ir(1)#1	177.15(2)

Table S13. The binding energies (in kcal/mol) in gas (gas) and solution (soln) for various unsupported metal(II)-metal(II) complexes (M: Ir, Rh or Au) by the SMD M06-L and other computational (SMD B3LYP-D3BJ, SMD MN15, SMD M06, SMD PBE0-D3 and DLPNO-CCSD(T)) methods based on the M06-L-optimized structures. The calculated key topological properties (bond-critical-point (BCP), its electron density (ρ, in e/Å^3) and Laplacian of the electron density ($\nabla^2\rho$, in e/Å^5)) associated with the metal-metal bonds as well as their metal-metal distance (d, in Å) by the SMD M06-L//M06-L method are also given. Thermal corrections to free energy for the other DFT methods were taken from the M06-L method. The dispersion correction contribution for the B3LYP method was given in a square bracket.[a]

	**d**	**ΔG_{soln}** M06-L	**$\Delta G_{soln}[\Delta E_{Disp}]^a$** B3LYP-D3BJ	**ΔG_{soln}** MN15	**ΔG_{soln}** M06	**$\Delta G_{soln}[\Delta E_{Disp}]^b$** PBE0-D3BJ	**ΔE_{gas}** LCCSD(T)	ρ M06-L	$\nabla^2\rho$ M06-L
				The new Ir complexes in this work					
**1**	2.94	-49.1	-78.8[-76.3]	-58.5	-56.3	-68.6[-52.4]	-	0.27	1.17
**2**	2.98	-39.8	-68.2[-47.2]	-50.8	-48.6	-60.9[-32.1]	-117.2	0.25	1.14
**3**	2.99	-49.0	-74.1[-61.7]	-58.8	-53.3	-68.1[-41.8]	-	0.24	1.17
**4**	2.90	-47.7	-76.4[-60.3]	-61.0	-55.0	-69.2[-41.6]	-	0.28	1.32
**5**	3.08	-26.4	-39.7[-39.5]	-34.6	-28.7	-35.2[-25.5]	-	0.19	1.14
**6**	3.05	-22.3	-41.4[-50.6]	-28.6	-26.3	-34.3[-34.3]	-	-	-
				The other complexes in previous work					
**A**	2.73	-34.0	-40.1[-24.5]	-42.2	-34.3	-38.1[-14.0]	-	-	-
**B**	2.74	-33.4	-42.8[-21.3]	-42.6	-35.0	-40.0[-12.7]	-61.3	0.40	1.27
**C**	2.70	-35.2	-59.1[-80.0]	-42.1	-28.8	-48.4[-52.6]	-	0.44	1.25
**D**	2.83	-23.9	-29.1[-20.3]	-31.5	-25.7	-30.5[-12.3]	-56.9	0.33	1.38
**E**	2.87	-34.2	-47.2[-45.1]	-43.4	-34.9	-41.6[-28.5]	-68.8	0.30	1.33
**F**	2.68	-34.9	-50.1[-51.2]	-38.3	-29.1	-44.9[-33.4]	-60.5	0.44	2.12
**G**	2.91	-24.2	-34.4[-35.2]	-31.3	-24.3	-30.6[-21.9]	-57.7	0.28	1.30
**H**	2.99	-26.1	-33.6[-22.6]	-30.9	-26.2	-32.1[-13.6]	-4.3	0.23	1.26
**I**	2.78	-38.5	-52.9[-51.7]	-48.0	-40.0	-47.0[-33.8]	-	0.36	1.23
**J**	2.76	-34.3	-49.8[-47.5]	-44.6	-36.1	-45.2[-30.8]	-	-	-
**K**	2.58	-44.1	-50.6[-16.3]	-53.3	-43.1	-50.3[-10.0]	-72.0	0.51	2.64
**L**	2.73	-34.5	-48.6[-52.3]	-38.6	-32.6	-38.3[-34.0]	-96.2	0.31	1.22

a. ΔE(SMD B3LYP-D3BJ//M06-L) - ΔE(SMD B3LYP//M06-L) in a square bracket; b. ΔE(SMD PBE0-D3BJ//M06-L) - ΔE(SMD PBE0//M06-L) in a square bracket.

Table S14. The rough decomposition of the binding (electronic) energies (in kcal/mol) contribution from different key parts[a-c] taken from the optimized complete Ir_2(II/II) complexes **1-3** in acetonitrile solvent by the SMD M06-L//M06-L method. The dispersion (Disp) correction contribution for the B3LYP method based on the M06-L optimized structure was given in square bracket.[d]

	$\Delta E_{SMD\ M06-L//M06-L}$ [ΔE_{Disp}^{d}]		
	**(L_{pin}+CI)[a]**	**(L_{pin}+CI+Ir2)[b]**	**(All)[c]**
**1**	-23.5 [-55.5]	-67.1 [-67.3]	-75.2 [-76.3]
**2**	-16.6 [-36.4]	-53.5 [-46.5]	-60.0 [-47.2]
**3**	-7.4 [-35.2]	-32.4 [-43.9]	-62.7 [-61.7]

a. The fragments containing the whole tridentate pincer ligands (L_{pin}) and counterions (CI) only; b. The fragments containing the whole tridentate ligands, counteranions and metals only; c. The structures of Ir_2(II/II) complexes; d. ΔE(SMD B3LYP-D3BJ//M06-L) - ΔE(SMD B3LYP//M06-L)

Table S15. The key absorption calculations from **1** in acetonitrile solvent by the CPCM TD-B3LYP-D3//M06-L method based on the M06-L optimized geometries using ORCA 5.01. States with fosc (oscillation strength) > 0.1 are shown only.

State	Wavelength (nm)	fosc (au**2)	orbitals
1	**496**	**0.22**	592 -> 593
6	**377**	**1.04**	592 -> 597
exp	**343/385/502**		

Table S16. The key absorption calculations from **2** in acetonitrile solvent by the CPCM TD-B3LYP-D3//M06-L method based on the M06-L optimized geometries using ORCA 5.01. States with fosc > 0.1 are shown only.

State	Wavelength (nm)	fosc (au**2)	orbitals
1	**518**	**0.23**	496a -> 497
6	**381**	**0.97**	496a -> 501
exp	**373/490**		

Table S17. The key absorption calculations from **3** in acetonitrile solvent by the CPCM TD-B3LYP-D3//M06-L method based on the M06-L optimized geometries using ORCA 5.01. States with fosc > 0.1 are shown only.

State	Wavelength (nm)	fosc (au**2)	orbitals
1	**540**	**0.25**	600 -> 601
9	**398**	**1.07**	600 -> 605
exp	**341/393/550**		

Table S18. The key emission calculations including SOC from triplet 1 (TDA:TRUE) and composition of each state in acetonitrile solvent by the CPCM TD ZORA B3LYP-D3//M06-L method based on the M06-L optimized geometries using ORCA 5.01.

State	Wavelength (nm)	Weight	Root	Spin	Ms	fosc (au**2)	orbitals
5	623	0.37	2	1	0	0.00000	592 -> 597
		0.31	2	1	-1		
		0.31	2	1	1		
6	623	0.08	2	1	0	0.00000	590 -> 593
		0.46	2	1	-1		
		0.46	2	1	1		
7	623	0.55	2	1	0	0.00000	591 -> 593
		0.22	2	1	-1		
		0.22	2	1	1		
8	611	0.64	3	1	0	0.00001	592 -> 598
		0.17	3	1	-1		592 -> 603
		0.17	3	1	1		592 -> 606<br>592 -> 601
9	611	0.03	3	1	0	0.00006	583 -> 593
		0.48	3	1	-1		585 -> 593
		0.48	3	1	1		590 -> 593
10	611	0.31	3	1	0	0.00060	592 -> 602
		0.34	3	1	-1		592 -> 599
		0.34	3	1	1		592 -> 600<br>592 -> 604
exp	**620/655/626/595**						

Table S19. The key emission calculations including SOC from triplet **2** (TDA:TRUE) and composition of each state in acetonitrile solvent by the CPCM TD ZORA B3LYP-D3//M06-L method based on the M06-L optimized geometries using ORCA 5.01.

State	Wavelength (nm)	Weight	Root	Spin	Ms	fosc (au**2)	orbitals
9	698	0.25	3	1	0	0.00002	492 -> 497
		0.37	3	1	-1		493 -> 497
		0.37	3	1	1		
10	698	0.56	3	1	0	0.00004	491 -> 497
		0.22	3	1	-1		492 -> 497
		0.22	3	1	1		493 -> 497
11	637	0.05	2	0	0	0.00006	496 -> 499
		0.67	4	1	0		496 -> 497
		0.14	4	1	-1		
		0.14	4	1	1		
12	636	0.29	4	1	0	0.00000	483 -> 497
		0.35	4	1	-1		486 -> 497
		0.35	4	1	1		
13	636	0.50	4	1	-1	0.00000	487 -> 497
		0.50	4	1	1		482 -> 497
							486 -> 497
15	606	0.02	5	0	0	0.00871	496 -> 507
		0.69	5	1	0		496 -> 508
		0.14	5	1	-1		496 -> 502
		0.14	5	1	1		496 -> 503
							496 -> 506
exp	**642/632/611/605**						

Table S20. The key emission calculations including SOC from triplet **3** (TDA:TRUE) and composition of each state in acetonitrile solvent by the CPCM TD ZORA B3LYP-D3//M06-L method based on the M06-L optimized geometries using ORCA 5.01.

State	Wavelength (nm)	Weight	Root	Spin	Ms	fosc (au**2)	orbitals
5	644	0.12	2	1	0	0.00001	598 -> 601
		0.44	2	1	-1		595 -> 601
		0.44	2	1	1		596 -> 601
6	644	0.03	2	1	0	0.00008	600 -> 604
		0.48	2	1	-1		600 -> 605
		0.48	2	1	1		600 -> 603
7	644	0.85	2	1	0	0.00108	600 -> 605
		0.08	2	1	-1		600 -> 603
		0.08	2	1	1		600 -> 604<br>600 -> 601
exp	**678/672/647/635**						

Table S21. Crystallographic Data for **8**.

Empirical formula	$C_{48}H_{50}F_6IIrN_3O_7PS_2$
Formula weight	1309.1
Temperature	100.0 K
Wavelength	1.54178 Å
Crystal system, space group	Monoclinic, P 1 21 1
	a = 17.4944(5) Å alpha = 90 deg.
Unit cell dimensions	b = 14.4498(4) Å beta = 91.476(2) deg.
	c = 20.3284(6) Å gamma = 90 deg.
Volume	5137.1(3) Å^3
Z, Calculated density	4, 1.693 Mg/m^3
Absorption coefficient	11.439 mm^{-1}
F(000)	2584
Crystal size	0.21 x 0.19 x 0.1 mm
Theta range for data collection	2.174 to 65.344 deg.
Limiting indices	-20<=h<=20, -17<=k<=17, -23<=l<=23
Reflections collected / unique	72563 / 17015 [R_{int} = 0.0753]
Completeness to theta = 67.679	94.50%
Absorption correction	Semi-empirical from equivalents
Max. and min. transmission	0.7526 and 0.4913
Refinement method	Full-matrix least-squares on F^2
Data / restraints / parameters	17015 / 13 / 1262
Goodness-of-fit on F^2	1.059
Final R indices [I>2sigma(I)]	R_1 = 0.0441, wR_2 = 0.0957
R indices (all data)	R_1 = 0.0533, wR_2 = 0.0997
Absolute structure parameter	0.077(8)
Largest diff. peak and hole	3.485 and -1.355 e. Å^{-3}

Table S22. Crystallographic Data for 7.

Empirical formula	$C_{33}H_{45}BrF_3IrN_3O_5S$
Formula weight	924.89
Temperature	100.01 K
Wavelength	1.54178 Å
Crystal system, space group	Monoclinic, P 1 21/n 1
Unit cell dimensions	a = 21.0482(6) Å alpha = 90 deg.<br>b = 13.2791(4) Å beta = 96.5460(10) deg.<br>c = 26.4452(8) Å gamma = 90 deg.
Volume	7343.3(4) $Å^3$
Z, Calculated density	8, 1.673 Mg/m^3
Absorption coefficient	9.336 mm^{-1}
F(000)	3672
Crystal size	0.21 x 0.19 x 0.13 mm
Theta range for data collection	2.546 to 65.196 deg.
Limiting indices	-24<=h<=24, -15<=k<=14, -31<=l<=31
Reflections collected / unique	150824 / 12534 [R_{int} = 0.0519]
Completeness to theta = 65.196	99.80%
Absorption correction	Semi-empirical from equivalents
Max. and min. transmission	0.3165 and 0.1508
Refinement method	Full-matrix least-squares on F^2
Data / restraints / parameters	12534 / 42 / 869
Goodness-of-fit on F^2	1.108
Final R indices [I>2sigma(I)]	R_1 = 0.0607, wR_2 = 0.1675
R indices (all data)	R_1 = 0.0652, wR_2 = 0.1712
Largest diff. peak and hole	5.892 and -2.300 e.$Å^3$

Table S23. Crystallographic Data for **9**.

Empirical formula	$C_{55}H_{61}F_6IrN_7O_6PS_2$
Formula weight	1317.39
Temperature	100.01 K
Wavelength	0.71073 Å
Crystal system, space group	Monoclinic, P 1 21/c 1
Unit cell dimensions	a = 12.5751(6) Å alpha = 90 deg.<br>b = 21.4466(10) Å beta = 90.2280(10) deg.<br>c = 20.6816(9) Å gamma = 90 deg.
Volume	5577.6(4) Å ^{3}
Z, Calculated density	4, 1.569 Mg/m^3
Absorption coefficient	2.574 mm^{-1}
F(000)	2664
Crystal size	0.21 x 0.18 x 0.14 mm
Theta range for data collection	2.123 to 27.549 deg.
Limiting indices	-16<=h<=13, -23<=k<=27, -26<=l<=26
Reflections collected / unique	57582 / 12822 [R_{int} = 0.0420]
Completeness to theta = 25.242	99.80%
Absorption correction	Semi-empirical from equivalents
Max. and min. transmission	0.7456 and 0.4113
Refinement method	Full-matrix least-squares on F^2
Data / restraints / parameters	12822 / 36 / 715
Goodness-of-fit on F^2	1.145
Final R indices [I>2sigma(I)]	R_1 = 0.0460, wR_2 = 0.1170
R indices (all data)	R_1 = 0.0557, wR_2 = 0.1280
Largest diff. peak and hole	2.627 and -1.990 e.Å^{-3}

Table S24. Selected bond lengths [Å] for **8**.

Ir(2)-I(2)	2.7077(10)
Ir(2)-P(2)	2.362(3)
Ir(2)-N(4)	2.069(9)
Ir(2)-N(6)	2.085(9)
Ir(2)-N(5)	2.007(8)
Ir(2)-C(62)	1.911(13)
O(2)-C(62)	1.120(15)
Ir(1)-I(1)	2.7057(10)
Ir(1)-P(1)	2.371(3)
Ir(1)-N(1)	2.074(8)
Ir(1)-N(3)	2.062(9)
Ir(1)-N(2)	2.009(9)
Ir(1)-C(16)	1.908(12)
O(1)-C(16)	1.115(15)

Table S25. Selected angles [deg] for **8**.

P(2)-Ir(2)-I(2)	178.60(7)
N(4)-Ir(2)-N(6)	158.4(3)
N(5)-Ir(2)-N(4)	79.1(3)
N(5)-Ir(2)-N(6)	79.4(3)
C(62)-Ir(2)-N(4)	97.9(4)
C(62)-Ir(2)-N(6)	103.6(4)
C(62)-Ir(2)-N(5)	171.2(5)
P(1)-Ir(1)-I(1)	178.63(8)
N(3)-Ir(1)-N(1)	158.1(3)
N(2)-Ir(1)-N(1)	78.5(3)
N(2)-Ir(1)-N(3)	79.6(4)
C(16)-Ir(1)-N(1)	102.8(4)
C(16)-Ir(1)-N(3)	99.0(5)
C(16)-Ir(1)-N(2)	173.4(5)

Table S26. Selected bond lengths [Å] for **7**.

Ir(1)-Ir(2)	2.7634(5)
Ir(1)-Br(1)	2.5764(9)
Ir(1)-N(3)	2.060(7)
Ir(1)-N(1)	2.056(7)
Ir(1)-N(2)	1.990(6)
Ir(1)-C(28)	1.883(9)
Ir(2)-Br(2)	2.5723(9)
Ir(2)-N(6)	2.046(7)
Ir(2)-N(4)	2.066(7)
Ir(2)-N(5)	1.986(7)
Ir(2)-C(56)	1.874(9)
O(1)-C(28)	1.138(10)
O(2)-C(56)	1.142(10)

Table S27. Selected angles [deg] for **7**.

Br(1)-Ir(1)-Ir(2)	177.78(2)
N(1)-Ir(1)-N(3)	157.9(3)
N(2)-Ir(1)-N(3)	79.4(3)
N(2)-Ir(1)-N(1)	79.1(3)
C(28)-Ir(1)-N(3)	101.4(3)
C(28)-Ir(1)-N(1)	99.9(3)
C(28)-Ir(1)-N(2)	177.8(3)
Br(2)-Ir(2)-Ir(1)	177.32(2)
N(6)-Ir(2)-N(4)	158.6(3)
N(5)-Ir(2)-N(6)	79.6(3)
N(5)-Ir(2)-N(4)	79.3(3)
C(56)-Ir(2)-N(6)	99.7(3)
C(56)-Ir(2)-N(4)	101.2(3)
C(56)-Ir(2)-N(5)	176.8(3)

Table S28. Selected bond lengths [Å] for **9**.

Ir(1)-P(1)	2.3517(13)
Ir(1)-N(1)	2.045(4)
Ir(1)-N(2)	1.977(4)
Ir(1)-N(3)	2.058(4)
Ir(1)-N(4)	2.042(4)
Ir(1)-N(5)	2.051(4)

Table S29. Selected angles [deg] for **9**.

N(1)-Ir(1)-N(3)	159.14(16)
N(2)-Ir(1)-N(1)	80.11(16)
N(2)-Ir(1)-N(3)	80.00(16)
N(2)-Ir(1)-N(4)	175.25(17)
N(4)-Ir(1)-N(1)	98.13(16)
N(4)-Ir(1)-N(3)	101.22(16)
N(5)-Ir(1)-P(1)	178.01(11)

Table S30. The absolute (in Hartree) and relative (in kcal/mol) energies in gas phase for the monomer and dimer forms of the Ir complexes in this work by the M06-L method.

	E	E_{+ZPE}	G	△E	△E_{+ZPE}	△G
**1_M**	-4390.93193	-4390.015665	-4390.119065	0.0	0.0	0.0
**1**	-8782.048663	-8780.211865	-8780.381317	-116.0	-113.3	-89.9
**^{3}1**	-8781.998866	-8780.160488	-8780.331162	-84.7	-81.0	-58.4
**2_M**	-3919.220685	-3918.644989	-3918.731993	0.0	0.0	0.0
**2**	-7838.593602	-7837.438981	-7837.584062	-95.5	-93.5	-75.3
**^{3}2**	-7838.546044	-7837.390462	-7837.536751	-65.7	-63.1	-45.7
**3_M**	-4496.827111	-4495.959968	-4496.056946	0.0	0.0	0.0
**3**	-8993.802235	-8992.067652	-8992.240114	-92.9	-92.7	-79.2
**6_M**	-2467.701381	-2466.844741	-2466.927685	0.0	0.0	0.0
**6**	-4935.263689	-4933.548426	-4933.685040	87.3	88.5	106.9
**4_M**	-3815.80188	-3815.0458	-3815.136867	0.0	0.0	0.0
**4**	-7631.751138	-7630.237852	-7630.388972	-92.4	-91.7	-72.3
**5_M**	-3576.217176	-3575.697917	-3575.781308	0.0	0.0	0.0
**5**	-7152.540962	-7151.498636	-7151.635485	-66.9	-64.5	-45.7

Table S31. The absolute (in Hartree) and relative (in kcal/mol) energies in acetonitrile solvent for the monomer and dimer forms of the Ir complexes in this work by the SMD M06-L//M06-L method. The corrections to the free energies were estimated and taken from the M06-L method in gas phase.

	E	△E	△G
**1$_M$**	-4391.012123	0.0	0.0
**1**	-8782.144135	-75.2	-49.1
**^{3}1**	-8782.100106	-47.6	-21.3
**2$_M$**	-3919.301293	0.0	0.0
**2**	-7838.698229	-60.0	-39.8
**^{3}2**	-7838.656504	-33.8	-13.8
**3$_M$**	-4496.914884	0.0	0.0
**3**	-8993.929637	-62.7	-49.0
**6$_M$**	-2467.931410	0.0	0.0
**6**	-4935.929657	-41.9	-22.3
**4$_M$**	-3815.866392	0.0	0.0
**4**	-7631.840970	-67.9	-47.7
**5$_M$**	-3576.291738	0.0	0.0
**5**	-7152.659228	-47.5	-26.4

Table S32. The absolute (in Hartree) and relative (in kcal/mol) single-point energies of the minimum energy path, MEP, for the Ir-Ir bond elongation in 1 in S_0 (close-shell) and T_1 states in acetonitrile solvent by the SMD M06-L//M06-L method.

(Å)	**S0 MEP**		**T1 MEP**		**S0 SP//T1 MEP**	
	**E**	**△E**	**E**	**△E**	**E**	**△E**
2.9	-8782.144135	-75.2	-8782.086678	-39.2	-8782.143078	-74.6
3.2	-8782.138756	-71.9	-8782.095701	-44.8	-8782.138172	-71.5
3.4	-8782.130988	-67.0	-8782.098934	-46.9	-8782.129918	-66.3
3.6	-8782.121261	-60.9	-8782.098775	-46.8	-8782.11987	-60.0
3.8	-8782.113337	-55.9	-8782.097311	-45.8	-8782.110297	-54.0
4.0	-8782.10222	-48.9	-8782.093952	-43.7	-8782.099844	-47.4
4.2	-8782.09121	-42.0	-8782.093758	-43.6	-8782.093211	-43.3
4.4	-8782.086265	-38.9	-8782.089345	-40.9	-8782.084049	-37.5
4.6	-8782.084653	-37.9	-8782.08817	-40.1	-8782.079226	-34.5
4.8	-8782.090745	-41.7	-8782.090505	-41.6	-8782.077932	-33.7
5.0	-8782.088836	-40.5	-8782.08855	-40.4	-8782.074046	-31.2
5.2	-8782.087526	-39.7	-8782.086778	-39.2	-8782.073716	-31.0

Table S33. The absolute (in Hartree) and relative (in kcal/mol) single-point energies in acetonitrile solvent for the complexes in this work by the SMD B3LYP//M06-L and SMD B3LYP-D3BJ//M06-L methods based on the M06-L-optimized geometries.

	SMD B3LYP//M06-L			SMD B3LYP-D3BJ//M06-L		
	E	△E	△G	E	△E	△G
**1_M**	-4391.306774	0.0	0.0	-4391.685279	0.0	0.0
**1**	-8782.659203	-28.6	-2.5	-8783.537784	-104.9	-78.8
**2_M**	-3919.525288	0.0	0.0	-3919.818689	0.0	0.0
**2**	-7839.116162	-41.2	-21.0	-7839.778247	-88.4	-68.2
**3_M**	-4497.201664	0.0	0.0	-4497.582425	0.0	0.0
**3**	-8994.444887	-26.1	-12.4	-8995.304765	-87.8	-74.1
**6_M**	-2468.156415	0.0	0.0	-2468.464129	0.0	0.0
**6**	-4936.329395	-10.4	9.2	-4937.025497	-61.0	-41.4
**4_M**	-3816.107304	0.0	0.0	-3816.39099	0.0	0.0
**4**	-7632.272396	-36.3	-16.1	-7632.935824	-96.5	-76.4
**5_M**	-3576.500031	0.0	0.0	-3576.73733	0.0	0.0
**5**	-7153.034065	-21.3	-0.2	-7153.571732	-60.9	-39.7

Table S34. The absolute (in Hartree) and relative (in kcal/mol) single-point energies in acetonitrile solvent for the complexes in this work by the SMD PBE0//M06-L and SMD PBE0-D3BJ//M06-L methods based on the M06-L-optimized geometries.

	**SMD PBE0//M06-L**			**SMD PBE0-D3BJ//M06-L**		
	**E**	**△E**	**△G**	**E**	**△E**	**△G**
**1_M**	-4387.395451	0.0	0.0	-4387.606491	0.0	0.0
**1**	-8774.858372	-42.3	-16.2	-8775.363964	-94.7	-68.6
**2_M**	-3916.190317	0.0	0.0	-3916.352783	0.0	0.0
**2**	-7832.4587	-49.0	-28.8	-7832.834799	-81.1	-60.9
**3_M**	-4493.197721	0.0	0.0	-4493.412199	0.0	0.0
**3**	-8986.459185	-40.0	-26.3	-8986.954764	-81.8	-68.1
**6_M**	-2465.607756	0.0	0.0	-2465.777467	0.0	0.0
**6**	-4931.246727	-19.6	0.0	-4931.640775	-53.9	-34.3
**4_M**	-3812.859185	0.0	0.0	-3813.015018	0.0	0.0
**4**	-7625.794543	-47.8	-27.6	-7626.172496	-89.4	-69.2
**5_M**	-3573.545248	0.0	0.0	-3573.675893	0.0	0.0
**5**	-7147.139704	-30.9	-9.7	-7147.441649	-56.4	-35.2

Table S35. The absolute (in Hartree) and relative (in kcal/mol) single-point energies in acetonitrile solvent for the complexes in this work by the SMD M06//M06-L and M06-D3//M06-L methods based on the M06-L-optimized geometries.

	**SMD M06//M06-L**			**SMD M06-D3//M06-L**		
	**E**	**△E**	**△G**	**E**	**△E**	**△G**
**1_M**	-4389.354554	0.0	0.0	-4389.402472	0.0	0.0
**1**	-8778.840405	-82.4	-56.3	-8778.977932	-108.6	-82.4
**2_M**	-3917.944272	0.0	0.0	-3917.978614	0.0	0.0
**2**	-7835.998087	-68.7	-48.6	-7836.089942	-83.3	-63.1
**3_M**	-4495.208517	0.0	0.0	-4495.260075	0.0	0.0
**3**	-8990.523746	-67.0	-53.3	-8990.656574	-85.6	-71.9
**6_M**	-2466.588859	0.0	0.0	-2466.624305	0.0	0.0
**6**	-4933.250935	-45.9	-26.3	-4933.347037	-61.8	-42.1
**4_M**	-3814.57907	0.0	0.0	-3814.612214	0.0	0.0
**4**	-7629.277957	-75.2	-55.0	-7629.377800	-96.2	-76.1
**5_M**	-3575.158528	0.0	0.0	-3575.186000	0.0	0.0
**5**	-7150.396555	-49.9	-28.7	-7150.468153	-60.3	-39.2

Table S36. The absolute (in Hartree) and relative (in kcal/mol) single-point energies in acetonitrile solvent for the complexes in this work by the SMD MN15//M06-L method based on the M06-L-optimized geometries.

	**SMD MN15//M06-L**		
	**E**	**△E**	**△G**
**1_M**	-4387.335783	0.0	0.0
**1**	-8774.806334	-84.6	-58.5
**2_M**	-3916.170589	0.0	0.0
**2**	-7832.454359	-71.0	-50.8
**3_M**	-4493.147045	0.0	0.0
**3**	-8986.409538	-72.4	-58.8
**6_M**	-2465.399113	0.0	0.0
**6**	-4930.875072	-48.2	-28.6
**4_M**	-3812.802733	0.0	0.0
**4**	-7625.734726	-81.1	-61.0
**5_M**	-3573.52492	0.0	0.0
**5**	-7147.13876	-55.8	-34.6

Table S37. The absolute (in Hartree) and relative (in kcal/mol) single-point energies in gas phase for the complexes in this work by the ZORA RI-M06-L//M06-L and DLPNO-CCSD(T)//M06-L methods based on the M06-L-optimized geometries.

	RI-M06-L		DLPNO-CCSD(T)	
	E	△E	E	△E
$\mathbf{1_M}$	-22711.354191	0.0	-	-
**1**	-45422.886936	-112.0	-	-
$\mathbf{2_M}$	-22239.169563	0.0	-3912.707385	0.0
**2**	-44478.484527	-91.2	-7825.601542	-117.2
$\mathbf{3_M}$	-22817.369945	0.0		
**3**	-45634.885067	-91.1		
$\mathbf{6_M}$	-20782.659264	0.0	-	-
**6**	-41565.175469	89.8	-	-
$\mathbf{4_M}$	-22135.672677	0.0	-	-
**4**	-44271.487194	-89.0	-	-
$\mathbf{5_M}$	-21895.843041	0.0	-	-
**5**	-43791.787386	-63.6	-	-

Table S38. The absolute energies (in Hartree) for the distortion to form the key dimer structures from the monomer parts (M-A & M-B) in acetonitrile solvent by the SMD M06-L//M06-L method.

	**E**	**$E_{dist,M-A}$**	**$E_{dist,M-B}$**
**1_M**	-4391.012123		
**1**	-8782.144135	-4390.999941	-4390.999584
**2_M**	-3919.301293		
**2**	-7838.698229	-3919.297042	-3919.297039
**3_M**	-4496.914884		
**3**	-8993.929637	-4496.91176	-4496.909304

Table S39. The distortion and interaction energies (in kcal/mol) to form the key dimer structures from the monomer parts (M-A & M-B) in acetonitrile solvent by the SMD M06-L//M06-L method.

	$\triangle E$	$\triangle E_{dist,M-A}$	$\triangle E_{dist,M-B}$	ΔE_{dist}	ΔE_{int}
**1**	-75.2	7.6	7.9	15.5	-90.7
**2**	-60.0	2.7	2.7	5.3	-65.4
**3**	-62.7	2.0	3.5	5.5	-68.1

Table S40. The absolute single point electronic energies (in Hartree) between the key fragments of the key structures (see Table S14) in acetonitrile solvent by the SMD B3LYP-D3BJ//M06-L method.

	E(2)[a]	E(4)[b]
**1_M**	-3137.582887	-3241.803668
**1**	-6275.232768	-6483.785089
**2_M**	-2665.724128	-2769.955984
**2**	-5331.498108	-5540.030112
**3_M**	-3243.490889	-3347.711372
**3**	-6487.015594	-6695.528909

a. The fragments containing the whole tridentate ligands and counterions; b. The fragments containing the whole tridentate ligands, counteranions and metals.

Table S41. The absolute single point electronic energies (in Hartree) between the key fragments of the key structures (see Table S14) in acetonitrile solvent by the SMD B3LYP//M06-L method.

	**E(2)[a]**	**E(4)[b]**
**1_M**	-3137.39684	-3241.600124
**1**	-6274.772162	-6483.270644
**2_M**	-2665.62006	-2769.834122
**2**	-5331.231953	-5539.712228
**3_M**	-3243.304775	-3347.506513
**3**	-6486.587307	-6695.049164

a. The fragments containing the whole tridentate ligands and counterions; b. The fragments containing the whole tridentate ligands, counteranions and metals.

Table S42. The absolute (in Hartree) and relative (in kcal/mol) energies in gas phase for the metal(II)-metal(II) complexes in the previous works by the M06-L method.

	E	E_{+ZPE}	G	△E	$△E_{+ZPE}$	△G
The Previous Ir(II)-Ir(II) Complexes						
A_M	-3318.948101	-3318.653147	-3318.712299	0.0	0.0	0.0
A	-6637.983059	-6637.390936	-6637.481235	-54.5	-53.1	-35.5
B_M	-1637.694275	-1637.533439	-1637.586327	0.0	0.0	0.0
B	-3275.471763	-3275.146986	-3275.225579	-52.2	-50.2	-33.2
C_M	-2019.777302	-2019.270158	-2019.335552	0.0	0.0	0.0
C	-4039.6689	-4038.652327	-4038.751497	-71.7	-70.3	-50.4
D_M	-608.433205	-608.194772	-608.239844	0.0	0.0	0.0
D	-1216.943131	-1216.458942	-1216.520458	-48.1	-43.5	-25.6
E_M	-1974.217658	-1973.849228	-1973.920496	0.0	0.0	0.0
E	-3948.524953	-3947.784112	-3947.89837	-56.2	-53.7	-36.0
F_M	-1761.584922	-1761.264675	-1761.323027	0.0	0.0	0.0
F	-3523.25027	-3522.608194	-3522.699335	-50.5	-49.5	-33.4
G_M	-1328.601309	-1328.312869	-1328.369873	0.0	0.0	0.0
G	-2657.273892	-2656.693883	-2656.778742	-44.7	-42.8	-24.5
H_M	-720.973407	-720.734014	-720.783074	0.0	0.0	0.0
H	-1441.935916	-1441.452868	-1441.521537	6.8	9.5	28.0
I_M	-2575.975937	-2575.332685	-2575.409311	0.0	0.0	0.0
I	-5152.043572	-5150.753641	-5150.877209	-57.5	-55.4	-36.8
J_M	-1634.846331	-1634.238662	-1634.310598	0.0	0.0	0.0
J	-3269.790941	-3268.570952	-3268.68155	-61.7	-58.8	-37.9
The Previous Au(II)-Au(II) and Rh(II)-Rh(II) Complexes						
K_M	-844.881171	-844.653549	-844.696679	0.0	0.0	0.0
K	-1689.861571	-1689.403396	-1689.466586	-62.3	-60.4	-46.0
L_M	-3041.364351	-3040.978435	-3041.053801	0.0	0.0	0.0
L	-6082.854338	-6082.081446	-6082.205424	-78.8	-78.2	-61.4

Table S43. The absolute (in Hartree) and relative (in kcal/mol) single-point energies in acetonitrile solvent for the metal(II)-metal(II) complexes in the previous works by the SMD M06-L//M06-L method.

	E	△E	△G
The Previous Ir(II)-Ir(II) Complexes			
**A_M**	-3318.995191	0.0	0.0
**A**	-6638.074787	-53.0	-34.0
**B_M**	-1637.719545	0.0	0.0
**B**	-3275.522698	-52.4	-33.4
**C_M**	-2019.837324	0.0	0.0
**C**	-4039.764743	-56.5	-35.2
**D_M**	-608.453975	0.0	0.0
**D**	-1216.981959	-46.4	-23.9
**E_M**	-1974.272879	0.0	0.0
**E**	-3948.632594	-54.5	-34.2
**F_M**	-1761.622183	0.0	0.0
**F**	-3523.327251	-52.0	-34.9
**G_M**	-1328.627483	0.0	0.0
**G**	-2657.325809	-44.5	-24.2
**H_M**	-721.062651	0.0	0.0
**H**	-1442.200576	-47.2	-26.1
**I_M**	-2576.028389	0.0	0.0
**I**	-5152.151187	-59.2	-38.5
**J_M**	-1634.886446	0.0	0.0
**J**	-3269.865556	-58.1	-34.3
The Previous Au(II)-Au(II) and Rh(II)-Rh(II) Complexes			
**K_M**	-844.904377	0.0	0.0
**K**	1689.905002	-60.4	-44.1
**L_M**	-3041.428686	0.0	0.0
**L**	-6082.940140	-51.9	-34.5

Table S44. The absolute (in Hartree) and relative (in kcal/mol) single-point energies in acetonitrile solvent for the metal(II)-metal(II) complexes in the previous works by the SMD B3LYP//M06-L and SMD B3LYP-D3BJ//M06-L methods based on the M06-L-optimized geometries.

	SMD B3LYP//M06-L			SMD B3LYP-D3BJ//M06-L		
	E	△E	△G	E	△E	△G
The Previous Ir(II)-Ir(II) Complexes						
A_M	-3319.121696	0.0	0.0	-3319.248241	0.0	0.0
A	-6638.298446	-34.5	-15.6	-6638.590562	-59.0	-40.1
B_M	-1637.787762	0.0	0.0	-1637.852456	0.0	0.0
B	-3275.640113	-40.5	-21.5	-3275.803499	-61.8	-42.8
C_M	-2019.99423	0.0	0.0	-2020.203249	0.0	0.0
C	-4039.989133	-0.4	20.9	-4040.534586	-80.3	-59.1
D_M	-608.4851315	0.0	0.0	-608.547666	0.0	0.0
D	-1217.020345	-31.4	-8.8	-1217.177625	-51.6	-29.1
E_M	-1974.392779	0.0	0.0	-1974.537750	0.0	0.0
E	-3948.821209	-22.4	-2.1	-3949.182981	-67.4	-47.2
F_M	-1761.760392	0.0	0.0	-1761.890718	0.0	0.0
F	-3523.546128	-15.9	1.1	-3523.88846	-67.1	-50.1
G_M	-1328.710356	0.0	0.0	-1328.81263	0.0	0.0
G	-2657.451689	-19.4	0.8	-2657.712437	-54.7	-34.4
H_M	-721.0940337	0.0	0.0	-721.162735	0.0	0.0
H	-1442.239322	-32.2	-11.0	-1442.41272	-54.8	-33.6
I_M	-2576.242447	0.0	0.0	-2576.453238	0.0	0.0
I	-5152.519864	-21.9	-1.2	-5153.023815	-73.6	-52.9
J_M	-1635.036837	0.0	0.0	-1635.216739	0.0	0.0
J	-3270.115273	-26.1	-2.3	-3270.550699	-73.6	-49.8
The Previous Au(II)-Au(II) and Rh(II)-Rh(II) Complexes						
K_M	-844.9568409	0.0	0.0	-845.039022	0.0	0.0
K	-1689.994393	-50.6	-34.3	-1690.18467	-66.9	-50.6
L_M	-3041.600121	0.0	0.0	-3041.764653	0.0	0.0
L	-6083.222163	-13.8	3.7	-6083.634639	-66.1	-48.6

Table S45. The absolute (in Hartree) and relative (in kcal/mol) single-point energies in acetonitrile solvent for the metal(II)-metal(II) complexes in the previous works by the SMD PBE0//M06-L and SMD PBE0-D3BJ//M06-L methods based on the M06-L-optimized geometries.

	**SMD PBE0//M06-L**			**SMD PBE0-D3BJ//M06-L**		
	**E**	**△E**	**△G**	**E**	**△E**	**△G**
The Previous Ir(II)-Ir(II) Complexes						
**A_M**	-3317.34134	0.0	0.0	-3317.408601	0.0	0.0
**A**	-6634.751337	-43.1	-24.1	-6634.908084	-57.0	-38.1
**B_M**	-1636.423024	0.0	0.0	-1636.456946	0.0	0.0
**B**	-3272.919839	-46.3	-27.3	-3273.007854	-58.9	-39.9
**C_M**	-2017.796125	0.0	0.0	-2017.907411	0.0	0.0
**C**	-4035.619488	-17.1	4.2	-4035.925809	-69.6	-48.3
**D_M**	-607.8551695	0.0	0.0	-607.887973	0.0	0.0
**D**	-1215.775251	-40.7	-18.2	-1215.860535	-53.0	-30.5
**E_M**	-1972.488463	0.0	0.0	-1972.567986	0.0	0.0
**E**	-3945.030043	-33.3	-13.1	-3945.234497	-61.8	-41.5
**F_M**	-1759.944292	0.0	0.0	-1760.012581	0.0	0.0
**F**	-3519.934044	-28.5	-11.5	-3520.123865	-61.9	-44.9
**G_M**	-1327.358086	0.0	0.0	-1327.412525	0.0	0.0
**G**	-2654.762303	-28.9	-8.7	-2654.906117	-50.9	-30.6
**H_M**	-720.3451599	0.0	0.0	-720.381392	0.0	0.0
**H**	-1440.753489	-39.6	-18.5	-1440.84772	-53.3	-32.1
**I_M**	-2573.74476	0.0	0.0	-2573.859769	0.0	0.0
**I**	-5147.543677	-34.0	-13.2	-5147.827602	-67.8	-47.0
**J_M**	-1633.228885	0.0	0.0	-1633.325452	0.0	0.0
**J**	-3266.518567	-38.2	-14.4	-3266.760914	-69.0	-45.2
	The Previous Au(II)-Au(II) and Rh(II)-Rh(II) Complexes					
**K_M**	-844.0821882	0.0	0.0	-844.124344	0.0	0.0
**K**	-1688.254549	-56.6	-40.3	-1688.354769	-66.6	-50.3
**L_M**	-3039.009177	0.0	0.0	-3039.09832	0.0	0.0
**L**	-6078.052978	-21.7	-4.3	-6078.285549	-55.8	-38.3

Table S46. The absolute (in Hartree) and relative (in kcal/mol) single-point energies in acetonitrile solvent for the metal(II)-metal(II) complexes in the previous works by the SMD M06//M06-L and SMD M06-D3//M06-L methods based on the M06-L-optimized geometries.

	**SMD M06//M06-L**			**SMD M06-D3//M06-L**		
	**E**	**△E**	**△G**	**E**	**△E**	**△G**
The Previous Ir(II)-Ir(II) Complexes						
A_M	-3318.257286	0.0	0.0	-3318.268473	0.0	0.0
**A**	-6636.599532	-53.3	-34.3	-6636.627209	-56.6	-37.7
B_M	-1637.212856	0.0	0.0	-1637.218448	0.0	0.0
**B**	-3274.511851	-54.1	-35.0	-3274.528885	-57.7	-38.7
C_M	-2018.606463	0.0	0.0	-2018.624647	0.0	0.0
**C**	-4037.292676	-50.0	-28.7	-4037.361247	-70.2	-48.9
D_M	-608.0792422	0.0	0.0	-608.084300	0.0	0.0
**D**	-1216.235383	-48.2	-25.7	-1216.25204	-52.3	-29.8
E_M	-1973.392343	0.0	0.0	-1973.408296	0.0	0.0
**E**	-3946.872578	-55.1	-34.9	-3946.919744	-64.7	-44.4
F_M	-1760.754964	0.0	0.0	-1760.765237	0.0	0.0
**F**	-3521.583472	-46.1	-29.1	-3521.624646	-59.1	-42.0
G_M	-1327.993767	0.0	0.0	-1328.003247	0.0	0.0
**G**	-2656.058466	-44.5	-24.2	-2656.089581	-52.1	-31.9
H_M	-720.6376004	0.0	0.0	-720.643357	0.0	0.0
**H**	-1441.350600	-47.3	-26.2	-1441.368845	-51.5	-30.4
I_M	-2574.934283	0.0	0.0	-2574.957991	0.0	0.0
**I**	-5149.965457	-60.8	-40.0	-5150.034879	-74.6	-53.8
J_M	-1633.907949	0.0	0.0	-1633.925396	0.0	0.0
**J**	-3267.911329	-59.9	-36.1	-3267.96592	-72.2	-48.4
The Previous Au(II)-Au(II) and Rh(II)-Rh(II) Complexes						
K_M	-844.3886456	0.0	0.0	-844.393448	0.0	0.0
**K**	-1688.871928	-59.4	-43.1	-1688.887073	-62.9	-46.5
L_M	-3040.422576	0.0	0.0	-3040.439771	0.0	0.0
**L**	-6080.924934	-50.1	-32.6	-6080.980311	-63.2	-45.8

Table S47. The absolute (in Hartree) and relative (in kcal/mol) single-point energies in acetonitrile solvent for the metal(II)-metal(II) complexes in the previous works by the SMD MN15//M06-L method based on the M06-L-optimized geometries.

	**SMD MN15//M06-L**		
	**E**	**△E**	**△G**
The Previous Ir(II)-Ir(II) Complexes			
**A_M**	-3317.624990	0.0	0.0
**A**	-6635.347387	-61.1	-42.2
**B_M**	-1636.368506	0.0	0.0
**B**	-3272.835247	-61.6	-42.6
**C_M**	-2017.594535	0.0	0.0
**C**	-4035.290051	-63.4	-42.1
**D_M**	-607.705197	0.0	0.0
**D**	-1215.496596	-54.1	-31.5
**E_M**	-1972.381515	0.0	0.0
**E**	-3944.864503	-63.7	-43.4
**F_M**	-1759.843921	0.0	0.0
**F**	-3519.77603	-55.3	-38.3
**G_M**	-1327.263142	0.0	0.0
**G**	-2654.60849	-51.6	-31.3
**H_M**	-720.200516	0.0	0.0
**H**	-1440.484059	-52.1	-30.9
**I_M**	-2573.645998	0.0	0.0
**I**	-5147.40154	-68.7	-48.0
**J_M**	-1633.032622	0.0	0.0
**J**	-3266.174218	-68.4	-44.6
The Previous Au(II)-Au(II) and Rh(II)-Rh(II) Complexes			
**K_M**	-843.837957	0.0	0.0
**K**	-1687.786838	-69.6	-53.3
**L_M**	-3038.921631	0.0	0.0
**L**	-6077.93252	-56.0	-38.6

Table S48. The absolute (in Hartree) and relative (in kcal/mol) single-point energies in gas phase for the metal(II)-metal(II) complexes in the previous works by the ZORA RI-M06-L//M06-L and DLPNO-CCSD(T)//M06-L methods based on the M06-L-optimized geometries.

	ZORA RI-M06-L		DLPNO-CCSD(T)	
	E	△E	E	△E
The Previous Ir(II)-Ir(II) Complexes				
**A_M**	-19986.355451	0.0	-1666.763619	0.0
**A**	-39972.793814	-52.0	-3333.620034	-58.2
**B_M**	-19952.702423	0.0	-1634.962022	0.0
**B**	-39905.488829	-52.7	-3270.021706	-61.3
**C_M**	-20333.286965	0.0	-	-
**C**	-40666.688878	-72.1	-	-
**D_M**	-18920.532072	0.0	-606.894782	0.0
**D**	-37841.143685	-49.9	-1213.880266	-56.9
**E_M**	-20288.902182	0.0	-1970.443323	0.0
**E**	-40577.890513	-54.1	-3940.996312	-68.8
**F_M**	-20075.192960	0.0	-1758.061762	0.0
**F**	-40150.466292	-50.4	-3516.220016	-60.5
**G_M**	-19641.756050	0.0	-1325.93847	0.0
**G**	-39283.578513	-41.7	-2651.96888	-57.7
**H_M**	-19033.223468	0.0	-719.250137	0.0
**H**	-38066.437147	6.1	-1438.507052	-4.3
**I_M**	-20891.609877	0.0	-	-
**I**	-41783.313636	-58.9	-	-
**J_M**	-19948.017838	0.0	-	-
**J**	-39896.138755	-64.7	-	-
The Previous Au(II)-Au(II) and Rh(II)-Rh(II) Complexes				
**K_M**	-20385.654855	0.0	-842.771599	0.0
**K**	-40771.419782	-69.1	-1685.65794	-72.0
**L_M**	-7774.361116	0.0	-3036.533336	0.0
**L**	-15548.851981	-81.4	-6073.220017	-96.2

Supplementary References

1 Addison, A. W. & Burke, P. J. Synthesis of some imidazole- and pyrazole- derived chelating agents. *J. Heterocycl. Chem.* **18**, 803-805 (1981).

2 Crosby, G. A. & Demas, J. N. Measurement of photoluminescence quantum yields. Review. *J. Phys. Chem.* **75**, 991-1024 (1971).

3 Wallace, L. & Rillema, D. P. Photophysical properties of rhenium(I) tricarbonyl complexes containing alkyl- and aryl-substituted phenanthrolines as ligands. *Inorg. Chem.* **32**, 3836-3843 (1993).

4 Frisch, M. J., et al. Gaussian 09 (Revision D.01). (2009).

5 Frisch, M. J., et al. Gaussian 16 (Revision C.01). (2016).

6 Patra, S. K., Rahaman, S. M. W., Majumdar, M., Sinha, A. & Bera, J. K. A rare unsupported iridium(II) dimer, $[IrCl_2(CO)_2]_2$. *Chem. Commun.* **22**, 2511-2513 (2008).

7 Huang, H., Rheingold, A. L. & Hughes, R. P. Serendipitous discovery of a simple compound with an unsupported Ir-Ir bond. *Organometallics* **28**, 1575-1578 (2009).

8 Rasmussen, P. G., Anderson, J. E., Bailey, O. H., Tamres, M. & Bayon, J. C. A novel metal-metal bonded iridium(II) dimer. *J. Am. Chem. Soc.* **107**, 279-281 (1985).

9 Heinekey, D. M., Fine, D. A. & Barnhart, D. Protonation of metal-metal bonds in dinuclear iridium complexes: consequences for structure and reactivity. *Organometallics* **16**, 2530-2538 (1997).

10 Lee, H.-P., et al. A novel cyclometalated dimeric iridium complex, $[(dfpbo)_2Ir]_2$ [dfpbo = 2-(3,5-Difluorophenyl)benzoxazolato-N,C2′], containing an unsupported Ir^{II}-Ir^{II} bond. *Inorg. Chem.* **48**, 1263-1265 (2009).

11 Mak, K. H. G., Chan, P. K., Fan, W. Y., Ganguly, R. & Leong, W. K. Photochemical reaction of $Cp^*Ir(CO)_2$ with C_6F_5X (X = CN, F): Formation of diiridium(II) complexes. *Organometallics* **32**, 1053-1059 (2013).

12 Dann, T., Roşca, D.-A., Wright, J. A., Wildgoose, G. G. & Bochmann, M. Electrochemistry of Au^{II} and Au^{III} pincer complexes: determination of the Au^{II}-Au^{II} bond energy. *Chem. Commun.* **49**, 10169-10171 (2013).

13 Inoki, D., Matsumoto, T., Nakai, H. & Ogo, S. Experimental study of reductive elimination of H_2 from rhodium hydride species. *Organometallics* **31**, 2996-3001 (2012).

14 Kuo, J. L. & Goldberg, K. I. Metal/ligand proton tautomerism facilitates dinuclear H_2 reductive elimination. *J. Am. Chem. Soc.* **142**, 21439-21449 (2020).

15 Zhao, Y. & Truhlar, D. G. A new local density functional for main-group thermochemistry, transition metal bonding, thermochemical kinetics, and noncovalent interactions. *J. Chem. Phys.* **125**, 194101 (2006).

16 Dolg, M., Wedig, U., Stoll, H. & Preuss, H. Energy-adjusted ab initio pseudopotentials for the first row transition elements. *J. Chem. Phys.* **86**, 866-872 (1987).

17 Andrae, D., Häußermann, U., Dolg, M., Stoll, H. & Preuß, H. Energy-adjustedab initio pseudopotentials for the second and third row transition elements. *Theor. Chim. Acta* **77**, 123-141 (1990).

18 Ditchfield, R., Hehre, W. J. & Pople, J. A. Self-consistent molecular-orbital methods. IX. An extended gaussian-type basis for molecular-orbital studies of organic molecules. *J. Chem. Phys.* **54**, 724-728 (1971).

19 Hehre, W. J., Ditchfield, R. & Pople, J. A. Self-consistent molecular orbital methods. XII. Further extensions of Gaussian-type basis sets for use in molecular orbital studies of organic molecules. *J. Chem. Phys.* **56**, 2257-2261 (1972).

20 Hariharan, P. C. & Pople, J. A. The influence of polarization functions on molecular orbital hydrogenation energies. *Theor. Chim. Acta* **28**, 213-222 (1973).

21 Lee, C., Yang, W. & Parr, R. G. Development of the Colle-Salvetti correlation-energy formula into a functional of the electron density. *Phys. Rev. B: Condens. Matter* **37**, 785-789 (1988).

22 Becke, A. D. Density-functional thermochemistry. III. The role of exact exchange. *J. Chem. Phys.* **98**, 5648-5652 (1993).

23 Grimme, S., Antony, J., Ehrlich, S. & Krieg, H. A consistent and accurate ab initio parametrization of density functional dispersion correction (DFT-D) for the 94 elements H-Pu. *J. Chem. Phys.* **132**, 154104 (2010).

24 Marenich, A. V., Cramer, C. J. & Truhlar, D. G. Universal solvation model based on solute electron density and on a continuum model of the solvent defined by the bulk dielectric constant and atomic surface tensions. *J. Phys Chem B*. **113**, 6378-6396 (2009).

25 Zhao, Y. & Truhlar, D. G. The M06 suite of density functionals for main group thermochemistry, thermochemical kinetics, noncovalent interactions, excited states, and transition elements: two new functionals and systematic testing of four M06-class functionals and 12 other functionals. *Theor. Chem. Acc.* **120**, 215-241 (2008).

26 Adamo, C. & Barone, V. Toward reliable density functional methods without adjustable parameters: The PBE0 model. *J. Chem. Phys.* **110**, 6158-6170 (1999).

27 Yu, H. S., He, X., Li, S. L. & Truhlar, D. G. MN15: A Kohn-Sham global-hybrid exchange-correlation density functional with broad accuracy for multi-reference and single-reference systems and noncovalent interactions. *Chem. Sci.* **7**, 5032-5051 (2016).

28 Harvey, J. N., Aschi, M., Schwarz, H. & Koch, W. The singlet and triplet states of phenyl cation. A hybrid approach for locating minimum energy crossing points between non-interacting potential energy surfaces. *Theor. Chem. Acc.* **99**, 95-99 (1998).

29 Cossi, M., Rega, N., Scalmani, G. & Barone, V. Energies, structures, and electronic properties of molecules in solution with the C-PCM solvation model. *J. Comp. Chem.* **24**, 669-681 (2003).

30 Furche, F. & Ahlrichs, R. Adiabatic time-dependent density functional methods for excited state properties. *J. Chem. Phys.* **117**, 7433-7447 (2002).

31 de Souza, B., Farias, G., Neese, F. & Izsák, R. Predicting phosphorescence rates of light organic molecules using time-dependent density functional theory and the path integral approach to dynamics. *J. Chem. Theory Comput.* **15**, 1896-1904 (2019).

32 Neese, F. Efficient and accurate approximations to the molecular spin-orbit coupling operator and their use in molecular g-tensor calculations. *J. Chem. Phys.* **122**, 034107 (2005).

33 van Lenthe, E., Snijders, J. G. & Baerends, E. J. The zero-order regular approximation for relativistic effects: The effect of spin-orbit coupling in closed shell molecules. *J. Chem. Phys.* **105**, 6505-6505 (1996).

34 Weigend, F. & Ahlrichs, R. Balanced basis sets of split valence, triple zeta valence and quadruple zeta valence quality for H to Rn: Design and assessment of accuracy. *Phys. Chem. Chem. Phys.* **7**, 3297-305 (2005).

35 Schäfer, A., Huber, C. & Ahlrichs, R. Fully optimized contracted Gaussian basis sets of triple zeta valence quality for atoms Li to Kr. *J. Chem. Phys.* **100**, 5829-5835 (1994).

36 Riplinger, C., Sandhoefer, B., Hansen, A. & Neese, F. Natural triple excitations in local coupled cluster calculations with pair natural orbitals. *J. Chem. Phys.* **139**, 134101 (2013).

37 Neese, F. The ORCA program system. *Wiley Interdiscip. Rev. Comput. Mol. Sci.* **2**, 73–78 (2012).

38 Guo, Y., et al. Communication: An improved linear scaling perturbative triples correction for the domain based local pair-natural orbital based singles and doubles coupled cluster method [DLPNO-CCSD(T)]. *J. Chem. Phys.* **148**, 011101 (2018).

39 Neese, F. Software update: the ORCA program system, version 4.0. *Wiley Interdiscip. Rev. Comput. Mol. Sci*. **8**, e1327 (2018).

40 Weigend, F. Accurate Coulomb-fitting basis sets for H to Rn. *Phys. Chem. Chem. Phys.* **8**, 1057-65 (2006).

41 Izsák, R. & Neese, F. An overlap fitted chain of spheres exchange method. *J. Chem. Phys.* **135**, 144105 (2011).

42 Zhao, Y. & Truhlar, D. G. Design of density functionals that are broadly accurate for thermochemistry, thermochemical kinetics, and nonbonded interactions. *J. Phys. Chem. A.* **109**, 5656-5667 (2005).

43 Grimme, S., Ehrlich, S. & Goerigk, L. Effect of the damping function in dispersion corrected density functional theory. *J. Comp. Chem.* **32**, 1456-1465 (2011).

44 Ess, D. H. & Houk, K. N. Distortion/interaction energy control of 1,3-dipolar cycloaddition reactivity. *J. Am. Chem. Soc.* **129**, 10646-10647 (2007).

45 Bickelhaupt, F. M. & Houk, K. N. Analyzing reaction rates with the distortion/interaction-activation strain model. *Angew. Chem. Int. Ed.* **56**, 10070-10086 (2017).

46 Johnson, E. R., et al. Revealing noncovalent interactions. *J. Am. Chem. Soc.* **132**, 6498-6506 (2010).

47 Grabowski, S. J. QTAIM characteristics of halogen bond and related interactions. *J. Phys. Chem. A.* **116**, 1838-1845 (2012).

48 Wu, L.-C., et al. Bond characterization on a Cr–Cr quintuple bond: A combined experimental and theoretical study. *J. Phys. Chem. A.* **115**, 12602-12615 (2011).

49 Lu, T. & Chen, F. Multiwfn: A multifunctional wavefunction analyzer. *J. Comp. Chem.* **33**, 580-592 (2012).

50 Bader, R. F. W. *Atoms in Molecules: A Quantum Theory.* (Oxford Univ. Press, 1990).

51 T. A. Keith, AIMAll (Version 19.10.12), TK Gristmill Software (Overland Park KS, USA, 2019). Available at http://aim.tkgristmill.com.

52 C. Y. Legault, CYL View (version 1.0 b), Universite de Sherbrooke (Sherbrooke, Quebec, Canada, 2009). http://www.cylview.org.

53 Pettersen, E. F., et al. UCSF Chimera—A visualization system for exploratory research and analysis. *J. Comput. Chem.* **25**, 1605-1612 (2004).
