# Supplementary material for: Structure-property relationships of photofunctional diiridium(II) complexes with tetracationic charge and an unsupported Ir–Ir bond
Source: Commun Chem. 2022 Nov 23;5:159. doi: 10.1038/s42004-022-00775-4 (PMC9814866; doi:10.1038/s42004-022-00775-4)
Supplement: Supplementary file 3 — Description of Additional Supplementary Files [file 42004_2022_775_MOESM3_ESM.pdf]

Description of Additional Supplementary Files

File name: Supplementary Data 1

Description: Cartesian coordinates from computational studies

File name: Supplementary Data 2

Description: cif file of complex 1

File name: Supplementary Data 3

Description: cif file of complex 1'

File name: Supplementary Data 4

Description: cif file of complex 2

File name: Supplementary Data 5

Description: cif file of complex 3

File name: Supplementary Data 6

Description: cif file of complex 7

File name: Supplementary Data 7

Description: cif file of complex 8

File name: Supplementary Data 8

Description: cif file of complex 9
