# Supplementary material for: Structure-property relationships of photofunctional diiridium(II) complexes with tetracationic charge and an unsupported Ir–Ir bond
Source: Commun Chem. 2022 Nov 23;5:159. doi: 10.1038/s42004-022-00775-4 (PMC9814866; doi:10.1038/s42004-022-00775-4)
Supplement: Supplementary file 4 — Supplementary Data 1 [file 42004_2022_775_MOESM4_ESM.pdf]

Cartesian coordinates of all stationary points optimized by the M06-L method (**1-3**, **A-L**)

Cartesian coordinates of key structures optimized by the B3LYP-D3BJ method (**1-3**)

Cartesian coordinates of key structures optimized by the B3LYP method (**1-3**)

Cartesian coordinates of all stationary points optimized by the M06-L method

## The Ir(II)-Ir(II) Complexes

**1$_M$**

| | | | |
|---|---|---|---|
| Ir | -0.329401 | -0.287397 | -1.219529 |
| P | -0.106447 | 0.383733 | 1.115586 |
| N | -0.481822 | -2.248904 | -0.773119 |
| N | 1.677716 | -0.909397 | -1.321606 |
| N | -2.396144 | -0.597976 | -1.292596 |
| C | -0.200034 | 1.479238 | -1.903014 |
| C | 0.354739 | 2.124082 | 1.211641 |
| C | -1.584946 | 0.108299 | 2.135069 |
| C | 1.187310 | -0.661483 | 1.848241 |
| C | -1.713356 | -2.758265 | -0.515832 |
| C | 0.654696 | -2.984035 | -0.669929 |
| C | 1.868923 | -2.224761 | -0.963916 |
| C | -2.794379 | -1.809647 | -0.769907 |
| O | -0.079729 | 2.520387 | -2.381205 |
| C | -0.686369 | -4.887342 | 0.012367 |
| C | 4.280422 | -1.999374 | -1.184453 |
| C | -5.127509 | -1.138436 | -0.878368 |
| C | -0.751352 | -6.338806 | 0.474861 |
| C | 5.696973 | -2.526907 | -1.027326 |
| C | -6.589492 | -1.415579 | -0.561847 |
| O | 4.291354 | 2.402290 | -1.108570 |
| O | -2.505622 | 2.825380 | -0.492144 |
| S | -3.938581 | 3.205888 | -0.621761 |
| S | 5.332207 | 1.989797 | -0.138539 |
| O | 6.434812 | 1.195767 | -0.716700 |
| O | 4.804625 | 1.480295 | 1.144288 |

| | | | |
|---|---|---|---|
| C | 6.144166 | 3.581133 | 0.327269 |
| F | 6.979524 | 3.401295 | 1.356979 |
| F | 5.223415 | 4.491710 | 0.691777 |
| F | 6.841453 | 4.083547 | -0.697517 |
| O | -4.444382 | 3.192720 | -2.004707 |
| O | -4.828685 | 2.575746 | 0.376368 |
| C | -3.917319 | 4.980852 | -0.128210 |
| F | -3.470739 | 5.096250 | 1.134382 |
| F | -5.143212 | 5.514463 | -0.186351 |
| F | -3.107775 | 5.686995 | -0.922891 |
| C | -1.518926 | -0.490347 | 3.404644 |
| H | -0.567306 | -0.825545 | 3.809328 |
| C | -2.674309 | -0.632510 | 4.165449 |
| H | -2.610729 | -1.093108 | 5.149863 |
| C | -3.897306 | -0.164983 | 3.686587 |
| H | -4.794684 | -0.269196 | 4.295141 |
| C | -3.961246 | 0.465502 | 2.446980 |
| H | -4.883056 | 0.905912 | 2.071595 |
| C | -2.814655 | 0.591094 | 1.670269 |
| H | -2.869367 | 1.135113 | 0.730734 |
| C | -0.494964 | 3.060983 | 1.815883 |
| H | -1.432802 | 2.749640 | 2.270409 |
| C | -0.153730 | 4.408385 | 1.797550 |
| H | -0.825582 | 5.132669 | 2.251947 |
| C | 1.023135 | 4.829015 | 1.183852 |
| H | 1.278852 | 5.886773 | 1.170198 |
| C | 1.867127 | 3.903429 | 0.571672 |
| H | 2.788518 | 4.206095 | 0.081028 |
| C | 1.528093 | 2.558033 | 0.571301 |
| H | 2.203277 | 1.865452 | 0.069150 |

| | | | |
|---|---|---|---|
| C | 2.498285 | -0.203074 | 2.027032 |
| H | 2.788138 | 0.816832 | 1.792878 |
| C | 3.482395 | -1.061186 | 2.510086 |
| H | 4.491856 | -0.670986 | 2.619499 |
| C | 3.171294 | -2.379705 | 2.833929 |
| H | 3.941643 | -3.045074 | 3.222057 |
| C | 1.868983 | -2.846534 | 2.660001 |
| H | 1.613912 | -3.875116 | 2.913031 |
| C | 0.887170 | -2.001510 | 2.156186 |
| H | -0.125776 | -2.383550 | 2.014661 |
| C | 4.046545 | -0.678438 | -1.582860 |
| H | 4.865237 | -0.007329 | -1.827207 |
| C | 2.761271 | -0.169883 | -1.624323 |
| H | 2.595338 | 0.871288 | -1.888701 |
| C | 3.148904 | -2.761725 | -0.884790 |
| H | 3.254144 | -3.796725 | -0.567823 |
| C | 6.608059 | -1.952755 | -2.119322 |
| H | 6.740811 | -0.871078 | -2.010221 |
| H | 7.601620 | -2.409473 | -2.039937 |
| H | 6.221951 | -2.165213 | -3.124691 |
| C | 5.738808 | -4.054730 | -1.086359 |
| H | 5.203280 | -4.520706 | -0.248500 |
| H | 5.316950 | -4.442846 | -2.023088 |
| H | 6.778187 | -4.396345 | -1.024440 |
| C | 6.213261 | -2.056170 | 0.344143 |
| H | 6.180364 | -0.963122 | 0.432858 |
| H | 5.621183 | -2.495861 | 1.157406 |
| H | 7.255024 | -2.377201 | 0.471636 |
| C | -3.341181 | 0.264858 | -1.699752 |
| H | -2.997382 | 1.193171 | -2.143414 |

| | | | |
|---|---|---|---|
| C | -4.694371 | 0.031674 | -1.505679 |
| H | -5.372626 | 0.823294 | -1.810422 |
| C | -4.139122 | -2.076940 | -0.559111 |
| H | -4.417506 | -3.032267 | -0.116330 |
| C | -7.459544 | -0.189686 | -0.836549 |
| H | -7.477118 | 0.071824 | -1.901187 |
| H | -8.493340 | -0.394845 | -0.535458 |
| H | -7.111536 | 0.689509 | -0.279839 |
| C | -6.728594 | -1.793487 | 0.919512 |
| H | -6.140267 | -2.681537 | 1.182820 |
| H | -6.407063 | -0.973262 | 1.571574 |
| H | -7.777667 | -2.016265 | 1.148662 |
| C | -7.067542 | -2.589065 | -1.429153 |
| H | -8.121275 | -2.808712 | -1.217842 |
| H | -6.979547 | -2.359327 | -2.497918 |
| H | -6.492522 | -3.503451 | -1.232653 |
| C | -1.826862 | -4.082947 | -0.106554 |
| H | -2.810728 | -4.490307 | 0.106627 |
| C | 0.558226 | -4.316725 | -0.292498 |
| H | 1.463289 | -4.915206 | -0.213348 |
| C | -2.183746 | -6.793202 | 0.748516 |
| H | -2.659428 | -6.203483 | 1.542122 |
| H | -2.812475 | -6.735416 | -0.148723 |
| H | -2.182560 | -7.838147 | 1.077731 |
| C | -0.145987 | -7.244925 | -0.605821 |
| H | 0.901189 | -6.996514 | -0.814410 |
| H | -0.175295 | -8.291416 | -0.279308 |
| H | -0.701954 | -7.170804 | -1.548056 |
| C | 0.062581 | -6.475931 | 1.770145 |
| H | 1.116995 | -6.212122 | 1.621504 |

| | | | |
|---|---|---|---|
| H | -0.337915 | -5.830226 | 2.562278 |
| H | 0.029946 | -7.511117 | 2.131060 |

**1**

| | | | |
|---|---|---|---|
| Ir | 0.241300 | -0.410086 | -1.376408 |
| P | 0.608676 | -0.995593 | -3.763199 |
| N | -0.666144 | 1.318252 | -1.915864 |
| N | -1.773589 | -1.010100 | -1.525032 |
| N | 1.868556 | 0.907233 | -1.460404 |
| C | 1.102823 | -2.001458 | -0.824257 |
| C | 0.189624 | -2.681572 | -4.302422 |
| C | 2.342227 | -0.761839 | -4.268293 |
| C | -0.460653 | 0.064017 | -4.778834 |
| C | 0.093215 | 2.423147 | -2.062911 |
| C | -2.002302 | 1.314017 | -2.114411 |
| C | -2.610850 | -0.013025 | -1.961018 |
| C | 1.528823 | 2.169279 | -1.892996 |
| O | 1.650212 | -2.981141 | -0.566173 |
| C | -1.913818 | 3.703894 | -2.475238 |
| C | -4.460880 | -1.575329 | -2.139450 |
| C | 3.876174 | 2.765417 | -2.113838 |
| C | -2.629763 | 5.040643 | -2.587834 |
| C | -5.878779 | -1.939402 | -2.547268 |
| C | 4.936515 | 3.753555 | -2.574057 |
| S | -1.460172 | -5.745519 | 0.089249 |
| O | -1.647332 | -5.934232 | 1.546842 |
| O | -0.388992 | -4.781692 | -0.265360 |
| O | -2.687226 | -5.600842 | -0.710705 |
| C | -0.764894 | -7.382355 | -0.412439 |
| F | -1.660315 | -8.351994 | -0.209517 |
| F | -0.422167 | -7.393776 | -1.711717 |

| | | | |
|---|---|---|---|
| F | 0.332590 | -7.661906 | 0.305343 |
| S | 5.627396 | -1.979205 | 0.213698 |
| O | 5.819060 | -2.176492 | 1.667419 |
| O | 6.235646 | -0.761209 | -0.352745 |
| O | 4.251903 | -2.251398 | -0.266760 |
| C | 6.607793 | -3.369659 | -0.499453 |
| F | 6.519050 | -3.383462 | -1.841435 |
| F | 7.896745 | -3.258576 | -0.169830 |
| F | 6.151635 | -4.543202 | -0.041929 |
| Ir | -0.231052 | 0.447925 | 1.396143 |
| P | -0.581156 | 1.009566 | 3.791162 |
| N | 0.677027 | -1.284327 | 1.921770 |
| N | 1.785455 | 1.044754 | 1.542604 |
| N | -1.859884 | -0.865670 | 1.478870 |
| C | -1.106446 | 2.032727 | 0.843765 |
| C | -0.146926 | 2.687978 | 4.341107 |
| C | -2.312841 | 0.781936 | 4.304831 |
| C | 0.483653 | -0.069604 | 4.791463 |
| C | -0.084649 | -2.388830 | 2.066452 |
| C | 2.013660 | -1.283296 | 2.115207 |
| C | 2.624880 | 0.042577 | 1.961890 |
| C | -1.520927 | -2.131197 | 1.903209 |
| O | -1.678323 | 2.996677 | 0.577668 |
| C | 1.923085 | -3.674129 | 2.466827 |
| C | 4.488938 | 1.593672 | 2.105692 |
| C | -3.868815 | -2.724542 | 2.124049 |
| C | 2.629975 | -5.016337 | 2.570967 |
| C | 5.930163 | 1.939231 | 2.444278 |
| C | -4.932550 | -3.711436 | 2.578140 |
| O | 0.395774 | 4.761968 | 0.267602 |

| | | | |
|---|---|---|---|
| O | -4.319369 | 2.270525 | 0.290301 |
| S | -5.643723 | 1.927453 | -0.279180 |
| S | 1.454779 | 5.747273 | -0.064023 |
| O | 1.653248 | 5.958584 | -1.516419 |
| O | 2.676045 | 5.613209 | 0.746793 |
| C | 0.722371 | 7.362454 | 0.451183 |
| F | 1.593869 | 8.355088 | 0.258389 |
| F | 0.376293 | 7.354730 | 1.749816 |
| F | -0.381230 | 7.620142 | -0.266265 |
| O | -5.789145 | 2.221078 | -1.721757 |
| O | -6.170537 | 0.620535 | 0.158597 |
| C | -6.774918 | 3.172762 | 0.477205 |
| F | -6.733264 | 3.121991 | 1.821138 |
| F | -8.037595 | 2.959357 | 0.096318 |
| F | -6.417569 | 4.404974 | 0.098877 |
| C | -2.713177 | 0.013270 | 5.404391 |
| H | -1.972631 | -0.476132 | 6.033768 |
| C | -4.065566 | -0.108081 | 5.714418 |
| H | -4.365495 | -0.708113 | 6.571823 |
| C | -5.025721 | 0.538287 | 4.939996 |
| H | -6.081857 | 0.434028 | 5.182371 |
| C | -4.634299 | 1.326620 | 3.860134 |
| H | -5.372280 | 1.835526 | 3.245549 |
| C | -3.286389 | 1.448820 | 3.544735 |
| H | -3.004549 | 2.064975 | 2.689959 |
| C | -0.177444 | 2.959621 | 5.718770 |
| H | -0.488407 | 2.184650 | 6.420191 |
| C | 0.199943 | 4.209785 | 6.189163 |
| H | 0.174029 | 4.413843 | 7.258011 |
| C | 0.618128 | 5.196431 | 5.294798 |

| | | | |
|---|---|---|---|
| H | 0.928660 | 6.170531 | 5.668174 |
| C | 0.633999 | 4.939894 | 3.928674 |
| H | 0.956144 | 5.697188 | 3.219802 |
| C | 0.239245 | 3.691686 | 3.451306 |
| H | 0.236947 | 3.531206 | 2.377304 |
| C | 1.760607 | 0.383918 | 5.157539 |
| H | 2.045308 | 1.418702 | 4.965779 |
| C | 2.671356 | -0.478362 | 5.760019 |
| H | 3.658523 | -0.109052 | 6.033375 |
| C | 2.325082 | -1.804612 | 6.005530 |
| H | 3.039165 | -2.478420 | 6.474879 |
| C | 1.064326 | -2.269334 | 5.636884 |
| H | 0.790252 | -3.308574 | 5.812686 |
| C | 0.152676 | -1.414114 | 5.027341 |
| H | -0.820188 | -1.799552 | 4.721590 |
| C | 3.619374 | 2.577753 | 1.614463 |
| H | 3.931837 | 3.607147 | 1.442208 |
| C | 2.296726 | 2.276713 | 1.349266 |
| H | 1.620428 | 3.042955 | 0.967967 |
| C | 3.961567 | 0.308883 | 2.249169 |
| H | 4.590629 | -0.513652 | 2.579821 |
| C | 6.657113 | 2.298833 | 1.140488 |
| H | 6.160532 | 3.127914 | 0.618705 |
| H | 7.685349 | 2.613011 | 1.360010 |
| H | 6.698730 | 1.431244 | 0.470767 |
| C | 6.659934 | 0.774503 | 3.108990 |
| H | 6.168292 | 0.469471 | 4.043239 |
| H | 6.724328 | -0.102685 | 2.455507 |
| H | 7.681938 | 1.079746 | 3.362537 |
| C | 5.941846 | 3.148479 | 3.390205 |

| | | | |
|---|---|---|---|
| H | 5.487170 | 4.038437 | 2.939035 |
| H | 5.404631 | 2.931599 | 4.323185 |
| H | 6.975949 | 3.403677 | 3.651392 |
| C | -3.909522 | 4.917392 | -3.421620 |
| H | -3.703153 | 4.518846 | -4.424799 |
| H | -4.664304 | 4.281026 | -2.944777 |
| H | -4.359514 | 5.909376 | -3.545825 |
| C | -1.729961 | 6.114886 | -3.204103 |
| H | -0.848364 | 6.337516 | -2.592014 |
| H | -1.387420 | 5.831324 | -4.209272 |
| H | -2.294681 | 7.049530 | -3.300986 |
| C | -3.002254 | 5.451118 | -1.150335 |
| H | -3.537003 | 6.409897 | -1.170087 |
| H | -3.649387 | 4.701528 | -0.678454 |
| H | -2.107700 | 5.566613 | -0.528037 |
| C | -2.650803 | 2.511439 | -2.390059 |
| H | -3.736672 | 2.521230 | -2.462446 |
| C | -0.519257 | 3.638447 | -2.346369 |
| H | 0.092188 | 4.536446 | -2.390495 |
| C | -3.156688 | -0.569266 | 1.297940 |
| H | -3.398556 | 0.426692 | 0.919568 |
| C | -4.171330 | -1.467055 | 1.600986 |
| H | -5.190035 | -1.119106 | 1.444715 |
| C | -2.508278 | -3.051612 | 2.223498 |
| H | -2.203022 | -4.045497 | 2.541906 |
| C | -6.316590 | -3.064169 | 2.606667 |
| H | -6.652766 | -2.760936 | 1.607521 |
| H | -7.050309 | -3.781245 | 2.992393 |
| H | -6.342257 | -2.177172 | 3.253495 |
| C | -4.587549 | -4.203910 | 3.991474 |

| | | | |
|---|---|---|---|
| H | -3.643520 | -4.761195 | 4.012808 |
| H | -4.513803 | -3.370150 | 4.702424 |
| H | -5.371421 | -4.883073 | 4.348132 |
| C | -4.944713 | -4.912863 | 1.623906 |
| H | -5.717601 | -5.625159 | 1.939774 |
| H | -5.169016 | -4.608481 | 0.594011 |
| H | -3.981584 | -5.433184 | 1.604138 |
| C | 0.527267 | -3.605862 | 2.341664 |
| H | -0.085149 | -4.503662 | 2.382520 |
| C | 2.661426 | -2.483026 | 2.384883 |
| H | 3.747027 | -2.493497 | 2.458046 |
| C | 1.757791 | -6.055762 | 3.281223 |
| H | 1.491086 | -5.734732 | 4.298141 |
| H | 0.832514 | -6.280666 | 2.738139 |
| H | 2.312621 | -6.997335 | 3.367714 |
| C | 2.895544 | -5.475163 | 1.124465 |
| H | 3.531304 | -4.756737 | 0.593118 |
| H | 3.405695 | -6.447593 | 1.137332 |
| H | 1.959770 | -5.582098 | 0.564384 |
| C | 3.966158 | -4.892103 | 3.309748 |
| H | 4.698337 | -4.287662 | 2.760743 |
| H | 3.839469 | -4.456675 | 4.311066 |
| H | 4.405114 | -5.888692 | 3.436089 |
| C | 3.165414 | 0.613677 | -1.276759 |
| H | 3.403268 | -0.382332 | -0.896930 |
| C | 4.179734 | 1.511147 | -1.582988 |
| H | 5.199198 | 1.165481 | -1.427106 |
| C | 2.515249 | 3.088651 | -2.217827 |
| H | 2.208940 | 4.079401 | -2.543728 |
| C | 6.318762 | 3.103967 | -2.629325 |

| | | | |
|---|---|---|---|
| H | 7.048355 | 3.824313 | -3.016828 |
| H | 6.669981 | 2.786364 | -1.640374 |
| H | 6.333272 | 2.225548 | -3.287875 |
| C | 4.963086 | 4.948216 | -1.610589 |
| H | 5.212650 | 4.640042 | -0.587175 |
| H | 5.724453 | 5.667477 | -1.938399 |
| H | 3.997287 | 5.462452 | -1.566100 |
| C | 4.574613 | 4.258186 | -3.979104 |
| H | 4.481355 | 3.428759 | -4.693117 |
| H | 3.636796 | 4.826088 | -3.983241 |
| H | 5.360329 | 4.930786 | -4.343977 |
| C | -2.283838 | -2.242097 | -1.329104 |
| H | -1.610479 | -3.002426 | -0.930895 |
| C | -3.599210 | -2.551790 | -1.620461 |
| H | -3.911551 | -3.582040 | -1.451748 |
| C | -3.940282 | -0.286908 | -2.271144 |
| H | -4.568717 | 0.531521 | -2.613197 |
| C | -5.800822 | -3.061380 | -3.593552 |
| H | -5.248036 | -2.734764 | -4.485045 |
| H | -5.309115 | -3.961200 | -3.204474 |
| H | -6.811283 | -3.345484 | -3.911983 |
| C | -6.639105 | -2.436559 | -1.310458 |
| H | -7.655813 | -2.736390 | -1.593728 |
| H | -6.152858 | -3.310452 | -0.856693 |
| H | -6.713055 | -1.639202 | -0.561464 |
| C | -6.630453 | -0.750887 | -3.140268 |
| H | -6.727664 | 0.072774 | -2.424897 |
| H | -6.134158 | -0.363641 | -4.040966 |
| H | -7.638633 | -1.065709 | -3.434336 |
| C | 2.744515 | -0.000521 | -5.372368 |

| | | | |
|---|---|---|---|
| H | 2.004789 | 0.477574 | -6.011488 |
| C | 4.098367 | 0.127677 | -5.673572 |
| H | 4.400377 | 0.720711 | -6.535137 |
| C | 5.057640 | -0.500723 | -4.883179 |
| H | 6.114806 | -0.387939 | -5.117197 |
| C | 4.665388 | -1.279643 | -3.796738 |
| H | 5.403643 | -1.766254 | -3.163876 |
| C | 3.315403 | -1.413202 | -3.493862 |
| H | 3.032683 | -2.023380 | -2.634936 |
| C | -0.141086 | 1.408677 | -5.029049 |
| H | 0.825002 | 1.808141 | -4.719563 |
| C | -1.055096 | 2.245462 | -5.660264 |
| H | -0.789499 | 3.284390 | -5.849805 |
| C | -2.308159 | 1.763184 | -6.032733 |
| H | -3.024447 | 2.422788 | -6.518656 |
| C | -2.644273 | 0.437590 | -5.769930 |
| H | -3.625820 | 0.054881 | -6.045524 |
| C | -1.729951 | -0.407370 | -5.148783 |
| H | -2.006043 | -1.442365 | -4.945353 |
| C | 0.251956 | -2.968282 | -5.676061 |
| H | 0.582927 | -2.202434 | -6.378363 |
| C | -0.118451 | -4.222336 | -6.141648 |
| H | -0.067496 | -4.438163 | -7.207281 |
| C | -0.562100 | -5.197849 | -5.247165 |
| H | -0.866979 | -6.174923 | -5.617449 |
| C | -0.609422 | -4.926369 | -3.884710 |
| H | -0.950715 | -5.674422 | -3.174948 |
| C | -0.221019 | -3.674592 | -3.411878 |
| H | -0.241522 | -3.504442 | -2.340160 |

**$^{3}$1**

| | | | |
|---|---|---|---|
| Ir | 0.552062 | -0.769086 | -1.537844 |
| P | 1.124267 | -1.481515 | -3.748341 |
| N | -0.635448 | 0.773481 | -2.102981 |
| N | -1.378442 | -1.664573 | -1.563372 |
| N | 1.910997 | 0.850693 | -1.571921 |
| C | 1.639979 | -2.089300 | -0.718614 |
| C | 1.035240 | -3.261689 | -4.100526 |
| C | 2.808914 | -0.965717 | -4.191510 |
| C | -0.086427 | -0.724401 | -4.864057 |
| C | -0.078999 | 1.991508 | -2.295726 |
| C | -1.958313 | 0.550859 | -2.296014 |
| C | -2.356800 | -0.832678 | -2.052348 |
| C | 1.365578 | 2.012470 | -2.073841 |
| O | 2.346481 | -2.859272 | -0.233257 |
| C | -2.248071 | 2.880752 | -2.890352 |
| C | -3.967706 | -2.646221 | -2.104298 |
| C | 3.562646 | 3.033047 | -2.238109 |
| C | -3.158440 | 4.040488 | -3.265048 |
| C | -5.313115 | -3.229929 | -2.503443 |
| C | 4.434584 | 4.174370 | -2.736546 |
| S | -0.425518 | -5.882439 | 0.666926 |
| O | -0.712061 | -5.878438 | 2.120074 |
| O | 0.483569 | -4.795748 | 0.224376 |
| O | -1.578709 | -6.103704 | -0.219620 |
| C | 0.615268 | -7.396453 | 0.475624 |
| F | -0.049494 | -8.490801 | 0.853485 |
| F | 0.996788 | -7.558113 | -0.802822 |
| F | 1.723011 | -7.294149 | 1.224235 |
| S | 5.920070 | -0.929500 | 0.687796 |
| O | 5.886336 | -1.043782 | 2.162736 |

| | | | |
|---|---|---|---|
| O | 6.246288 | 0.414237 | 0.173069 |
| O | 4.802267 | -1.600529 | -0.014179 |
| C | 7.371017 | -1.968843 | 0.232775 |
| F | 7.558818 | -1.966728 | -1.099679 |
| F | 8.485906 | -1.513005 | 0.810635 |
| F | 7.175820 | -3.233923 | 0.621402 |
| Ir | -0.551981 | 0.769268 | 1.537902 |
| P | -1.124451 | 1.481493 | 3.748494 |
| N | 0.635495 | -0.773303 | 2.103053 |
| N | 1.378576 | 1.664775 | 1.563465 |
| N | -1.910925 | -0.850574 | 1.571698 |
| C | -1.640014 | 2.089633 | 0.718922 |
| C | -1.035640 | 3.261661 | 4.100764 |
| C | -2.809067 | 0.965538 | 4.191604 |
| C | 0.086302 | 0.724432 | 4.864187 |
| C | 0.079029 | -1.991351 | 2.295754 |
| C | 1.958353 | -0.550691 | 2.296206 |
| C | 2.356902 | 0.832840 | 2.052466 |
| C | -1.365541 | -2.012335 | 2.073708 |
| O | -2.346664 | 2.859664 | 0.233889 |
| C | 2.248047 | -2.880591 | 2.890611 |
| C | 3.968006 | 2.646250 | 2.104068 |
| C | -3.562607 | -3.032946 | 2.237728 |
| C | 3.158350 | -4.040362 | 3.265316 |
| C | 5.313528 | 3.229876 | 2.502962 |
| C | -4.434572 | -4.174270 | 2.736098 |
| O | -0.483324 | 4.795530 | -0.224209 |
| O | -4.802442 | 1.600356 | 0.013736 |
| S | -5.920264 | 0.929283 | -0.688167 |
| S | 0.425666 | 5.882301 | -0.666726 |

| | | | |
|---|---|---|---|
| O | 0.712088 | 5.878499 | -2.119894 |
| O | 1.578931 | 6.103499 | 0.219751 |
| C | -0.615145 | 7.396247 | -0.475129 |
| F | 0.049593 | 8.490699 | -0.852747 |
| F | -0.996662 | 7.557616 | 0.803349 |
| F | -1.722868 | 7.294065 | -1.223774 |
| O | -5.886568 | 1.043497 | -2.163099 |
| O | -6.246457 | -0.414419 | -0.173379 |
| C | -7.371175 | 1.968631 | -0.233110 |
| F | -7.558874 | 1.966562 | 1.099356 |
| F | -8.486105 | 1.512789 | -0.810872 |
| F | -7.175990 | 3.233694 | -0.621791 |
| C | -3.122682 | 0.201530 | 5.323254 |
| H | -2.346998 | -0.072888 | 6.035699 |
| C | -4.438701 | -0.191473 | 5.554083 |
| H | -4.672875 | -0.784188 | 6.436708 |
| C | -5.449703 | 0.174013 | 4.668341 |
| H | -6.474299 | -0.145737 | 4.849703 |
| C | -5.150419 | 0.953949 | 3.552760 |
| H | -5.924986 | 1.240962 | 2.844487 |
| C | -3.838945 | 1.347640 | 3.316378 |
| H | -3.627588 | 1.944074 | 2.427984 |
| C | -1.224996 | 3.668058 | 5.431579 |
| H | -1.443864 | 2.927611 | 6.201734 |
| C | -1.121547 | 5.010415 | 5.769282 |
| H | -1.269610 | 5.319362 | 6.802364 |
| C | -0.826005 | 5.956871 | 4.787263 |
| H | -0.738570 | 7.007774 | 5.056615 |
| C | -0.649744 | 5.562662 | 3.465646 |
| H | -0.422369 | 6.290526 | 2.691497 |

| | | | |
|---|---|---|---|
| C | -0.762033 | 4.217321 | 3.119307 |
| H | -0.638041 | 3.942629 | 2.074961 |
| C | 1.238222 | 1.452446 | 5.202657 |
| H | 1.323788 | 2.501115 | 4.916450 |
| C | 2.277805 | 0.841887 | 5.897066 |
| H | 3.165403 | 1.419994 | 6.148977 |
| C | 2.185647 | -0.498313 | 6.263125 |
| H | 2.999776 | -0.973516 | 6.806439 |
| C | 1.053356 | -1.234989 | 5.917373 |
| H | 0.984256 | -2.288941 | 6.183170 |
| C | 0.017182 | -0.636232 | 5.209605 |
| H | -0.843675 | -1.233555 | 4.908228 |
| C | 2.977971 | 3.447633 | 1.515743 |
| H | 3.142635 | 4.493631 | 1.260157 |
| C | 1.717942 | 2.936287 | 1.262613 |
| H | 0.945619 | 3.557794 | 0.809994 |
| C | 3.636332 | 1.308762 | 2.322127 |
| H | 4.376870 | 0.614866 | 2.713509 |
| C | 6.068352 | 3.664553 | 1.240261 |
| H | 5.486196 | 4.382696 | 0.648354 |
| H | 7.010664 | 4.152967 | 1.519241 |
| H | 6.306080 | 2.794693 | 0.616152 |
| C | 6.171749 | 2.221916 | 3.264271 |
| H | 5.674333 | 1.875458 | 4.180724 |
| H | 6.417234 | 1.344233 | 2.656460 |
| H | 7.113213 | 2.697662 | 3.563335 |
| C | 5.059016 | 4.451123 | 3.399420 |
| H | 4.505389 | 5.242543 | 2.880637 |
| H | 4.488706 | 4.174165 | 4.296680 |
| H | 6.014645 | 4.876913 | 3.728845 |

| | | | |
|---|---|---|---|
| C | -4.271729 | 3.573106 | -4.210859 |
| H | -3.862686 | 3.116544 | -5.123158 |
| H | -4.953695 | 2.857142 | -3.737924 |
| H | -4.873506 | 4.437524 | -4.515608 |
| C | -2.373495 | 5.167236 | -3.942134 |
| H | -1.622808 | 5.620282 | -3.285445 |
| H | -1.864390 | 4.816635 | -4.851309 |
| H | -3.064725 | 5.964769 | -4.239040 |
| C | -3.792965 | 4.571418 | -1.967071 |
| H | -4.455499 | 5.412993 | -2.208168 |
| H | -4.386001 | 3.798085 | -1.462545 |
| H | -3.025492 | 4.926086 | -1.270527 |
| C | -2.782944 | 1.599569 | -2.680780 |
| H | -3.853631 | 1.429057 | -2.774281 |
| C | -0.872373 | 3.061377 | -2.693278 |
| H | -0.416874 | 4.042241 | -2.802656 |
| C | -3.228384 | -0.827920 | 1.306255 |
| H | -3.629962 | 0.079901 | 0.852941 |
| C | -4.069761 | -1.885302 | 1.626415 |
| H | -5.125360 | -1.751311 | 1.402241 |
| C | -2.171412 | -3.093509 | 2.396486 |
| H | -1.700025 | -3.998983 | 2.774073 |
| C | -5.918356 | -3.816512 | 2.667396 |
| H | -6.257027 | -3.643780 | 1.638495 |
| H | -6.514737 | -4.642715 | 3.071793 |
| H | -6.151148 | -2.918028 | 3.255701 |
| C | -4.065558 | -4.469348 | 4.198460 |
| H | -3.035798 | -4.833903 | 4.294373 |
| H | -4.178038 | -3.577368 | 4.828819 |
| H | -4.724747 | -5.250205 | 4.596882 |

| | | | |
|---|---|---|---|
| C | -4.161014 | -5.430749 | 1.898724 |
| H | -4.786426 | -6.257146 | 2.259960 |
| H | -4.399266 | -5.270182 | 0.840016 |
| H | -3.113629 | -5.746188 | 1.954035 |
| C | 0.872372 | -3.061222 | 2.693387 |
| H | 0.416895 | -4.042116 | 2.802676 |
| C | 2.782948 | -1.599411 | 2.681089 |
| H | 3.853641 | -1.428904 | 2.774623 |
| C | 2.373349 | -5.167087 | 3.942361 |
| H | 1.864337 | -4.816520 | 4.851600 |
| H | 1.622570 | -5.619995 | 3.285681 |
| H | 3.064520 | -5.964720 | 4.239144 |
| C | 3.792833 | -4.571283 | 1.967309 |
| H | 4.385956 | -3.797963 | 1.462840 |
| H | 4.455278 | -5.412949 | 2.208346 |
| H | 3.025316 | -4.925817 | 1.270730 |
| C | 4.271682 | -3.573080 | 4.211118 |
| H | 4.953734 | -2.857203 | 3.738164 |
| H | 3.862695 | -3.116448 | 5.123412 |
| H | 4.873366 | -4.437559 | 4.515887 |
| C | 3.228488 | 0.827991 | -1.306628 |
| H | 3.630128 | -0.079831 | -0.853343 |
| C | 4.069858 | 1.885364 | -1.626887 |
| H | 5.125494 | 1.751351 | -1.402840 |
| C | 2.171437 | 3.093622 | -2.396701 |
| H | 1.700055 | 3.999116 | -2.774231 |
| C | 5.918361 | 3.816541 | -2.668050 |
| H | 6.514735 | 4.642725 | -3.072503 |
| H | 6.257162 | 3.643755 | -1.639193 |
| H | 6.151027 | 2.918052 | -3.256420 |

| | | | |
|---|---|---|---|
| C | 4.161183 | 5.430821 | -1.899077 |
| H | 4.399711 | 5.270230 | -0.840432 |
| H | 4.786488 | 6.257247 | -2.260440 |
| H | 3.113773 | 5.746236 | -1.954116 |
| C | 4.065412 | 4.469510 | -4.198852 |
| H | 4.177850 | 3.577556 | -4.829261 |
| H | 3.035639 | 4.834057 | -4.294642 |
| H | 4.724555 | 5.250392 | -4.597305 |
| C | -1.717724 | -2.936136 | -1.262627 |
| H | -0.945376 | -3.557603 | -0.810001 |
| C | -2.977685 | -3.447570 | -1.515910 |
| H | -3.142300 | -4.493601 | -1.260441 |
| C | -3.636139 | -1.308694 | -2.322178 |
| H | -4.376675 | -0.614859 | -2.713623 |
| C | -5.058388 | -4.451221 | -3.399785 |
| H | -4.487867 | -4.174307 | -4.296922 |
| H | -4.504877 | -5.242610 | -2.880839 |
| H | -6.013934 | -4.877035 | -3.729409 |
| C | -6.068168 | -3.664556 | -1.240859 |
| H | -7.010469 | -4.152903 | -1.519982 |
| H | -5.486157 | -4.382736 | -0.648860 |
| H | -6.305914 | -2.794682 | -0.616794 |
| C | -6.171198 | -2.222036 | -3.265004 |
| H | -6.416819 | -1.344298 | -2.657342 |
| H | -5.673593 | -1.875662 | -4.181387 |
| H | -7.112590 | -2.697818 | -3.564229 |
| C | 3.122561 | -0.201620 | -5.323089 |
| H | 2.346896 | 0.072926 | -6.035502 |
| C | 4.438602 | 0.191335 | -5.553894 |
| H | 4.672808 | 0.784145 | -6.436450 |

| | | | |
|---|---|---|---|
| C | 5.449601 | -0.174343 | -4.668217 |
| H | 6.474223 | 0.145366 | -4.849544 |
| C | 5.150283 | -0.954430 | -3.552736 |
| H | 5.924899 | -1.241622 | -2.844577 |
| C | 3.838774 | -1.348029 | -3.316360 |
| H | 3.627377 | -1.944582 | -2.428048 |
| C | -0.017198 | 0.636254 | -5.209484 |
| H | 0.843647 | 1.233539 | -4.908017 |
| C | -1.053257 | 1.235060 | -5.917366 |
| H | -0.984067 | 2.288994 | -6.183198 |
| C | -2.185573 | 0.498463 | -6.263184 |
| H | -2.999616 | 0.973703 | -6.806591 |
| C | -2.277866 | -0.841714 | -5.897080 |
| H | -3.165475 | -1.419764 | -6.149061 |
| C | -1.238377 | -1.452332 | -5.202585 |
| H | -1.324034 | -2.500989 | -4.916380 |
| C | 1.223781 | -3.668101 | -5.431457 |
| H | 1.441999 | -2.927635 | -6.201779 |
| C | 1.120315 | -5.010484 | -5.769064 |
| H | 1.267731 | -5.319428 | -6.802239 |
| C | 0.825596 | -5.956964 | -4.786820 |
| H | 0.738163 | -7.007888 | -5.056082 |
| C | 0.650154 | -5.562743 | -3.465104 |
| H | 0.423430 | -6.290610 | -2.690773 |
| C | 0.762440 | -4.217374 | -3.118862 |
| H | 0.639078 | -3.942726 | -2.074419 |

**$2_M$**

| | | | |
|---|---|---|---|
| Ir | -0.562940 | -1.021021 | -1.246841 |
| P | -0.290939 | -0.109814 | 1.003910 |
| N | -0.833820 | -2.921187 | -0.618123 |

| | | | |
|---|---|---|---|
| N | 1.402330 | -1.766175 | -1.288957 |
| N | -2.642039 | -1.230550 | -1.368160 |
| C | -0.337105 | 0.661942 | -2.097889 |
| C | 0.254021 | 1.606941 | 1.007901 |
| C | -1.707322 | -0.278971 | 2.131405 |
| C | 1.009895 | -1.135315 | 1.763236 |
| C | -2.105071 | -3.348256 | -0.384745 |
| C | 0.263511 | -3.697312 | -0.423096 |
| C | 1.515750 | -3.044158 | -0.781791 |
| C | -3.122094 | -2.385079 | -0.788432 |
| O | -0.167504 | 1.637202 | -2.686971 |
| C | -1.193198 | -5.444986 | 0.345828 |
| C | 3.907449 | -2.959917 | -1.049705 |
| C | -5.383617 | -1.657035 | -1.179984 |
| O | 4.111137 | 1.208799 | -1.108506 |
| O | -2.384096 | 1.878678 | -0.503395 |
| S | -3.733608 | 2.478387 | -0.705687 |
| S | 5.063297 | 1.040369 | 0.020383 |
| O | 5.807009 | -0.235358 | 0.002139 |
| O | 4.537393 | 1.449965 | 1.333397 |
| C | 6.334274 | 2.317981 | -0.366284 |
| F | 7.314141 | 2.325762 | 0.545639 |
| F | 5.771049 | 3.536853 | -0.389807 |
| F | 6.881074 | 2.087915 | -1.568475 |
| O | -4.078013 | 2.700542 | -2.119241 |
| O | -4.794753 | 1.864425 | 0.116782 |
| C | -3.529396 | 4.160299 | 0.018467 |
| F | -3.283870 | 4.066111 | 1.338876 |
| F | -4.637609 | 4.888658 | -0.145139 |
| F | -2.506563 | 4.807235 | -0.549402 |

| | | | |
|---|---|---|---|
| C | -1.476327 | -0.481234 | 3.504354 |
| H | -0.461600 | -0.593625 | 3.881058 |
| C | -2.545317 | -0.516073 | 4.391425 |
| H | -2.354186 | -0.665093 | 5.452616 |
| C | -3.849798 | -0.340934 | 3.929990 |
| H | -4.681105 | -0.356898 | 4.632964 |
| C | -4.081826 | -0.118480 | 2.576872 |
| H | -5.083312 | 0.075519 | 2.198496 |
| C | -3.017857 | -0.091995 | 1.680715 |
| H | -3.211255 | 0.154643 | 0.644547 |
| C | -0.459562 | 2.581247 | 1.719746 |
| H | -1.378134 | 2.323960 | 2.242627 |
| C | -0.005521 | 3.893409 | 1.718878 |
| H | -0.574685 | 4.652778 | 2.250986 |
| C | 1.154805 | 4.239296 | 1.027273 |
| H | 1.506172 | 5.269278 | 1.041783 |
| C | 1.859099 | 3.275805 | 0.309375 |
| H | 2.776883 | 3.517563 | -0.222878 |
| C | 1.394835 | 1.967675 | 0.278854 |
| H | 1.963255 | 1.239338 | -0.298580 |
| C | 2.357429 | -0.749995 | 1.735078 |
| H | 2.669767 | 0.232086 | 1.387440 |
| C | 3.348139 | -1.613925 | 2.192481 |
| H | 4.384368 | -1.283829 | 2.133590 |
| C | 3.005845 | -2.863298 | 2.705649 |
| H | 3.780326 | -3.535558 | 3.072237 |
| C | 1.665856 | -3.245544 | 2.765250 |
| H | 1.386523 | -4.212345 | 3.183656 |
| C | 0.674265 | -2.396695 | 2.285150 |
| H | -0.371214 | -2.705518 | 2.333509 |

| | | | |
|---|---|---|---|
| C | 3.778187 | -1.687026 | -1.591004 |
| H | 4.637577 | -1.092010 | -1.877060 |
| C | 2.514204 | -1.119595 | -1.680618 |
| H | 2.392079 | -0.105999 | -2.049387 |
| C | 2.765831 | -3.640295 | -0.647640 |
| H | 2.837340 | -4.634983 | -0.213876 |
| C | -3.509891 | -0.357183 | -1.909824 |
| H | -3.087252 | 0.525774 | -2.376514 |
| C | -4.884021 | -0.530012 | -1.822830 |
| H | -5.524556 | 0.248449 | -2.226669 |
| C | -4.492627 | -2.599639 | -0.680754 |
| H | -4.855264 | -3.505434 | -0.200389 |
| C | -2.302101 | -4.628802 | 0.121259 |
| H | -3.305872 | -4.993826 | 0.321090 |
| C | 0.092269 | -4.991840 | 0.062836 |
| H | 0.952535 | -5.635447 | 0.225335 |
| H | 4.891225 | -3.404122 | -0.921746 |
| H | -1.335935 | -6.449964 | 0.734878 |
| H | -6.455022 | -1.806433 | -1.073509 |

**2**

| | | | |
|---|---|---|---|
| Ir | -1.047795 | 0.170466 | -1.045195 |
| P | -2.735311 | 0.420201 | -2.847542 |
| N | 0.449997 | 0.071084 | -2.415948 |
| N | -0.537545 | 2.211304 | -1.269478 |
| N | -0.949474 | -1.892170 | -1.407862 |
| C | -2.374619 | 0.202589 | 0.300806 |
| C | -4.008950 | 1.702506 | -2.637145 |
| C | -3.687389 | -1.105354 | -3.117632 |
| C | -1.869698 | 0.861698 | -4.379045 |
| C | 0.801713 | -1.145192 | -2.889037 |

| | | | |
|---|---|---|---|
| C | 1.043765 | 1.221878 | -2.803720 |
| C | 0.471767 | 2.418487 | -2.184599 |
| C | -0.031316 | -2.235055 | -2.377691 |
| O | -3.218802 | 0.191611 | 1.086542 |
| C | 2.526208 | -0.079500 | -4.157555 |
| C | 0.294700 | 4.796320 | -1.875640 |
| C | -0.796105 | -4.515242 | -2.311343 |
| S | -3.164331 | 4.417191 | 2.299755 |
| O | -3.014863 | 4.227260 | 3.757054 |
| O | -3.415993 | 3.165452 | 1.543503 |
| O | -2.173687 | 5.315119 | 1.675355 |
| C | -4.742371 | 5.361241 | 2.147546 |
| F | -4.707486 | 6.456799 | 2.907403 |
| F | -4.931998 | 5.744049 | 0.870781 |
| F | -5.792585 | 4.615953 | 2.513031 |
| S | -4.173735 | -3.882244 | 1.845127 |
| O | -3.913985 | -3.741449 | 3.293422 |
| O | -3.426281 | -4.966035 | 1.176433 |
| O | -4.202649 | -2.606043 | 1.092594 |
| C | -5.926708 | -4.448601 | 1.795074 |
| F | -6.311518 | -4.675113 | 0.523551 |
| F | -6.080997 | -5.584944 | 2.479132 |
| F | -6.738302 | -3.524294 | 2.316355 |
| Ir | 1.047789 | -0.170382 | 1.045377 |
| P | 2.735423 | -0.420436 | 2.847579 |
| N | -0.450080 | -0.071399 | 2.416035 |
| N | 0.537712 | -2.211267 | 1.269425 |
| N | 0.949247 | 1.892237 | 1.408289 |
| C | 2.374874 | -0.202491 | -0.300492 |
| C | 4.009698 | -1.702124 | 2.636927 |

| | | | |
|---|---|---|---|
| C | 3.687000 | 1.105295 | 3.118404 |
| C | 1.869871 | -0.862932 | 4.378823 |
| C | -0.801802 | 1.144741 | 2.889417 |
| C | -1.043697 | -1.222331 | 2.803769 |
| C | -0.471610 | -2.418787 | 2.184448 |
| C | 0.030895 | 2.234833 | 2.378038 |
| O | 3.219305 | -0.191708 | -1.085948 |
| C | -2.525917 | 0.078717 | 4.158207 |
| C | -0.294383 | -4.796516 | 1.874537 |
| C | 0.794840 | 4.515273 | 2.311790 |
| O | 3.410953 | -3.165452 | -1.544865 |
| O | 4.203148 | 2.604514 | -1.092228 |
| S | 4.174022 | 3.880922 | -1.844411 |
| S | 3.162504 | -4.417562 | -2.301640 |
| O | 3.013431 | -4.227209 | -3.758899 |
| O | 2.173536 | -5.317893 | -1.678124 |
| C | 4.743060 | -5.357341 | -2.149079 |
| F | 4.713027 | -6.451736 | -2.910676 |
| F | 4.932422 | -5.741481 | -0.872678 |
| F | 5.791201 | -4.607671 | -2.512007 |
| O | 3.914378 | 3.740540 | -3.292750 |
| O | 3.426371 | 4.964396 | -1.175413 |
| C | 5.926917 | 4.447557 | -1.794235 |
| F | 6.311669 | 4.674172 | -0.522709 |
| F | 6.081079 | 5.583892 | -2.478317 |
| F | 6.738669 | 3.523338 | -2.315458 |
| C | 3.872348 | 1.698660 | 4.373544 |
| H | 3.436350 | 1.253498 | 5.265860 |
| C | 4.639977 | 2.854664 | 4.487683 |
| H | 4.777652 | 3.309934 | 5.466651 |

| | | | |
|---|---|---|---|
| C | 5.232438 | 3.420563 | 3.361284 |
| H | 5.824545 | 4.328751 | 3.455881 |
| C | 5.074660 | 2.821190 | 2.113664 |
| H | 5.525497 | 3.249780 | 1.221901 |
| C | 4.307862 | 1.668238 | 1.993812 |
| H | 4.202244 | 1.218399 | 1.006180 |
| C | 4.816784 | -2.026198 | 3.740728 |
| H | 4.638120 | -1.552927 | 4.706706 |
| C | 5.842450 | -2.951664 | 3.603105 |
| H | 6.460530 | -3.200688 | 4.463539 |
| C | 6.082453 | -3.554072 | 2.367215 |
| H | 6.888859 | -4.277435 | 2.263307 |
| C | 5.296222 | -3.227981 | 1.268262 |
| H | 5.469852 | -3.682723 | 0.297228 |
| C | 4.261376 | -2.305625 | 1.403861 |
| H | 3.670758 | -2.075918 | 0.524254 |
| C | 1.726647 | -2.217025 | 4.718445 |
| H | 2.250274 | -2.979685 | 4.141681 |
| C | 0.921701 | -2.593776 | 5.789095 |
| H | 0.822991 | -3.647963 | 6.040979 |
| C | 0.244736 | -1.629224 | 6.530848 |
| H | -0.384658 | -1.925720 | 7.367395 |
| C | 0.368625 | -0.282872 | 6.193471 |
| H | -0.168183 | 0.476259 | 6.759529 |
| C | 1.165796 | 0.099542 | 5.120668 |
| H | 1.235342 | 1.154001 | 4.853066 |
| C | 0.725684 | -4.577829 | 0.958058 |
| H | 1.220857 | -5.376175 | 0.410841 |
| C | 1.111289 | -3.272615 | 0.673239 |
| H | 1.885061 | -3.077535 | -0.071040 |

| | | | |
|---|---|---|---|
| C | -0.893594 | -3.707036 | 2.494377 |
| H | -1.721440 | -3.849440 | 3.186120 |
| C | 2.115388 | 1.168742 | -3.693660 |
| H | 2.663520 | 2.075003 | -3.949324 |
| C | 1.875029 | -1.249131 | -3.772778 |
| H | 2.231467 | -2.228208 | -4.094226 |
| C | 1.742988 | 2.841549 | 0.886689 |
| H | 2.445558 | 2.542554 | 0.106158 |
| C | 1.692328 | 4.164497 | 1.315143 |
| H | 2.359973 | 4.871948 | 0.826443 |
| C | -0.042562 | 3.540313 | 2.846625 |
| H | -0.786075 | 3.801029 | 3.596846 |
| C | -1.874807 | 1.248404 | 3.773571 |
| H | -2.230784 | 2.227370 | 4.095711 |
| C | -2.115199 | -1.169408 | 3.693857 |
| H | -2.663365 | -2.075718 | 3.949333 |
| C | -1.743384 | -2.841231 | -0.886062 |
| H | -2.445789 | -2.541866 | -0.105536 |
| C | -1.693203 | -4.164215 | -1.314426 |
| H | -2.360913 | -4.871473 | -0.825509 |
| C | 0.041493 | -3.540553 | -2.846389 |
| H | 0.784433 | -3.801501 | -3.597109 |
| C | -1.110962 | 3.272957 | -0.673601 |
| H | -1.884617 | 3.078345 | 0.070918 |
| C | -0.725355 | 4.578011 | -0.959071 |
| H | -1.220549 | 5.376576 | -0.412245 |
| C | 0.893908 | 3.706578 | -2.494970 |
| H | 1.721831 | 3.848602 | -3.186681 |
| C | -3.873196 | -1.698975 | -4.372586 |
| H | -3.437265 | -1.254162 | -5.265104 |

| | | | |
|---|---|---|---|
| C | -4.641209 | -2.854768 | -4.486294 |
| H | -4.779210 | -3.310228 | -5.465129 |
| C | -5.233595 | -3.420219 | -3.359634 |
| H | -5.825989 | -4.328256 | -3.453870 |
| C | -5.075383 | -2.820578 | -2.112197 |
| H | -5.526147 | -3.248854 | -1.220251 |
| C | -4.308211 | -1.667832 | -1.992786 |
| H | -4.202292 | -1.217745 | -1.005309 |
| C | -1.166230 | -0.101276 | -5.120796 |
| H | -1.236115 | -1.155636 | -4.852873 |
| C | -0.369209 | 0.280487 | -6.193958 |
| H | 0.167096 | -0.479061 | -6.759936 |
| C | -0.244786 | 1.626683 | -6.531724 |
| H | 0.384496 | 1.922668 | -7.368533 |
| C | -0.921069 | 2.591740 | -5.790008 |
| H | -0.821906 | 3.645818 | -6.042163 |
| C | -1.725899 | 2.215641 | -4.719054 |
| H | -2.248992 | 2.978704 | -4.142356 |
| C | -4.815769 | 2.027566 | -3.740826 |
| H | -4.637359 | 1.554700 | -4.707048 |
| C | -5.840895 | 2.953573 | -3.602751 |
| H | -6.458787 | 3.203341 | -4.463104 |
| C | -6.080573 | 3.555617 | -2.366611 |
| H | -6.886520 | 4.279454 | -2.262465 |
| C | -5.294588 | 3.228576 | -1.267767 |
| H | -5.467667 | 3.682982 | -0.296471 |
| C | -4.260385 | 2.305582 | -1.403827 |
| H | -3.669941 | 2.074907 | -0.524386 |
| H | -0.656140 | -5.798866 | 2.089139 |
| H | -3.389953 | 0.142119 | 4.814921 |

| | | | |
|---|---|---|---|
| H | 0.707935 | 5.541911 | 2.657745 |
| H | 0.656383 | 5.798606 | -2.090675 |
| H | 3.390433 | -0.143113 | -4.814003 |
| H | -0.709680 | -5.541879 | -2.657428 |

**$^3$2**

| | | | |
|---|---|---|---|
| Ir | -1.581073 | 0.515001 | -0.986226 |
| P | -3.290259 | 0.765299 | -2.641908 |
| N | -0.012686 | 0.934476 | -2.208239 |
| N | -1.330902 | 2.604804 | -0.693360 |
| N | -0.990825 | -1.406262 | -1.610310 |
| C | -2.790860 | -0.058316 | 0.360929 |
| C | -4.726624 | 1.765148 | -2.159064 |
| C | -3.950853 | -0.824806 | -3.220980 |
| C | -2.522941 | 1.631310 | -4.032359 |
| C | 0.594663 | -0.093646 | -2.852483 |
| C | 0.399951 | 2.219879 | -2.325904 |
| C | -0.358803 | 3.157175 | -1.503081 |
| C | 0.010863 | -1.397521 | -2.556632 |
| O | -3.502857 | -0.557196 | 1.120714 |
| C | 2.137416 | 1.472178 | -3.794574 |
| C | -0.833614 | 5.343284 | -0.622544 |
| C | -0.125141 | -3.790192 | -2.751228 |
| S | -3.793617 | 3.078129 | 3.355510 |
| O | -3.281682 | 2.509954 | 4.620221 |
| O | -3.982161 | 2.081180 | 2.271430 |
| O | -3.146894 | 4.330508 | 2.922186 |
| C | -5.514118 | 3.575715 | 3.789671 |
| F | -5.513274 | 4.500026 | 4.753581 |
| F | -6.140311 | 4.094301 | 2.716082 |
| F | -6.216436 | 2.519493 | 4.208470 |

| | | | |
|---|---|---|---|
| S | -3.294437 | -5.090357 | 1.080571 |
| O | -2.985837 | -5.168723 | 2.523942 |
| O | -2.364295 | -5.844237 | 0.214142 |
| O | -3.642427 | -3.735763 | 0.590422 |
| C | -4.876199 | -6.021021 | 0.910158 |
| F | -5.270160 | -6.015704 | -0.376557 |
| F | -4.719867 | -7.291241 | 1.291772 |
| F | -5.848934 | -5.472690 | 1.644783 |
| Ir | 1.573873 | -0.507174 | 1.012552 |
| P | 3.274162 | -0.731598 | 2.676142 |
| N | 0.003427 | -0.910180 | 2.235531 |
| N | 1.320045 | -2.597664 | 0.736401 |
| N | 0.999609 | 1.419355 | 1.630946 |
| C | 2.783018 | 0.052389 | -0.341142 |
| C | 4.733439 | -1.702896 | 2.207113 |
| C | 3.901544 | 0.887459 | 3.212578 |
| C | 2.513807 | -1.605226 | 4.066316 |
| C | -0.597269 | 0.124529 | 2.876038 |
| C | -0.415916 | -2.192702 | 2.359463 |
| C | 0.342575 | -3.139752 | 1.547743 |
| C | -0.002164 | 1.422911 | 2.577124 |
| O | 3.494521 | 0.543597 | -1.106127 |
| C | -2.152078 | -1.427121 | 3.820644 |
| C | 0.805347 | -5.333686 | 0.681210 |
| C | 0.164973 | 3.813884 | 2.769455 |
| O | 3.984717 | -2.083749 | -2.208171 |
| O | 3.455042 | 3.648116 | -0.848643 |
| S | 3.272904 | 5.083365 | -1.171884 |
| S | 3.799487 | -3.075447 | -3.297579 |
| O | 3.302001 | -2.499082 | -4.564132 |

| | | | |
|---|---|---|---|
| O | 3.142989 | -4.325772 | -2.872600 |
| C | 5.521061 | -3.581124 | -3.718825 |
| F | 5.523224 | -4.507902 | -4.680379 |
| F | 6.138083 | -4.099495 | -2.639498 |
| F | 6.230627 | -2.528823 | -4.135448 |
| O | 2.825525 | 5.344291 | -2.557501 |
| O | 2.575858 | 5.869295 | -0.135612 |
| C | 4.998425 | 5.729656 | -1.151246 |
| F | 5.556734 | 5.543413 | 0.060566 |
| F | 5.026838 | 7.037105 | -1.422134 |
| F | 5.747504 | 5.089564 | -2.054927 |
| C | 3.954361 | 1.296484 | 4.551141 |
| H | 3.650226 | 0.620096 | 5.347666 |
| C | 4.420681 | 2.568926 | 4.870551 |
| H | 4.455866 | 2.878146 | 5.913310 |
| C | 4.840924 | 3.438741 | 3.867386 |
| H | 5.189876 | 4.436971 | 4.123793 |
| C | 4.818607 | 3.030799 | 2.534874 |
| H | 5.135233 | 3.701190 | 1.738141 |
| C | 4.354235 | 1.761774 | 2.211195 |
| H | 4.342851 | 1.462908 | 1.163322 |
| C | 5.638769 | -2.070271 | 3.216175 |
| H | 5.439883 | -1.801512 | 4.254156 |
| C | 6.787239 | -2.780140 | 2.891805 |
| H | 7.483989 | -3.064373 | 3.678101 |
| C | 7.047727 | -3.123348 | 1.564249 |
| H | 7.949543 | -3.679305 | 1.315237 |
| C | 6.159107 | -2.754946 | 0.560258 |
| H | 6.344374 | -3.011752 | -0.479687 |
| C | 5.003589 | -2.045895 | 0.880400 |

| | | | |
|---|---|---|---|
| H | 4.331442 | -1.773073 | 0.071721 |
| C | 2.603706 | -3.005267 | 4.121922 |
| H | 3.256595 | -3.535738 | 3.428249 |
| C | 1.862849 | -3.720301 | 5.058255 |
| H | 1.943294 | -4.805010 | 5.090482 |
| C | 1.020700 | -3.053755 | 5.944273 |
| H | 0.441208 | -3.615906 | 6.673620 |
| C | 0.911964 | -1.665000 | 5.886213 |
| H | 0.242451 | -1.138410 | 6.563770 |
| C | 1.640627 | -0.945005 | 4.947106 |
| H | 1.518093 | 0.136218 | 4.888821 |
| C | 1.776595 | -4.777577 | -0.140606 |
| H | 2.346912 | -5.354768 | -0.863237 |
| C | 2.005626 | -3.406875 | -0.090851 |
| H | 2.727036 | -2.932545 | -0.756435 |
| C | 0.081900 | -4.505055 | 1.532019 |
| H | -0.718868 | -4.911623 | 2.148035 |
| C | 1.496739 | 2.518021 | -3.133055 |
| H | 1.884877 | 3.535267 | -3.190754 |
| C | 1.698805 | 0.156483 | -3.665348 |
| H | 2.241537 | -0.668002 | -4.131646 |
| C | 1.541959 | 2.587642 | 1.243717 |
| H | 2.306367 | 2.561827 | 0.467985 |
| C | 1.144912 | 3.805510 | 1.783603 |
| H | 1.614120 | 4.709626 | 1.391790 |
| C | -0.409793 | 2.612123 | 3.170738 |
| H | -1.199814 | 2.601067 | 3.920190 |
| C | -1.701164 | -0.115472 | 3.691866 |
| H | -2.234893 | 0.712876 | 4.161713 |
| C | -1.519054 | -2.479423 | 3.161962 |

| | | | |
|---|---|---|---|
| H | -1.921576 | -3.491575 | 3.214175 |
| C | -1.513636 | -2.582928 | -1.220964 |
| H | -2.280553 | -2.568980 | -0.447071 |
| C | -1.104175 | -3.794487 | -1.764950 |
| H | -1.560613 | -4.704629 | -1.372973 |
| C | 0.433436 | -2.580885 | -3.151475 |
| H | 1.223509 | -2.558644 | -3.900550 |
| C | -2.017882 | 3.404758 | 0.142758 |
| H | -2.735462 | 2.922052 | 0.806234 |
| C | -1.797110 | 4.775940 | 0.201307 |
| H | -2.369323 | 5.345245 | 0.928460 |
| C | -0.107317 | 4.524864 | -1.480309 |
| H | 0.683869 | 4.940962 | -2.103186 |
| C | -4.158391 | -1.110664 | -4.577598 |
| H | -3.926794 | -0.366697 | -5.338289 |
| C | -4.672794 | -2.347468 | -4.955424 |
| H | -4.829223 | -2.561703 | -6.011011 |
| C | -4.984524 | -3.305163 | -3.992632 |
| H | -5.371263 | -4.275449 | -4.297688 |
| C | -4.805201 | -3.021191 | -2.640800 |
| H | -5.034316 | -3.758390 | -1.873976 |
| C | -4.298652 | -1.784397 | -2.258355 |
| H | -4.177563 | -1.581689 | -1.195316 |
| C | -1.696081 | 0.945065 | -4.937497 |
| H | -1.632079 | -0.143079 | -4.900952 |
| C | -0.951585 | 1.647689 | -5.877529 |
| H | -0.316339 | 1.103951 | -6.574357 |
| C | -1.004044 | 3.040711 | -5.915823 |
| H | -0.412704 | 3.588122 | -6.646939 |
| C | -1.806730 | 3.730482 | -5.010878 |

| | | | |
|---|---|---|---|
| H | -1.844253 | 4.817912 | -5.030336 |
| C | -2.559408 | 3.033854 | -4.070234 |
| H | -3.180000 | 3.579590 | -3.358558 |
| C | -5.613261 | 2.183045 | -3.164703 |
| H | -5.410840 | 1.940406 | -4.208310 |
| C | -6.748933 | 2.908910 | -2.830541 |
| H | -7.431067 | 3.232034 | -3.614571 |
| C | -7.014445 | 3.219871 | -1.496206 |
| H | -7.905691 | 3.788995 | -1.239206 |
| C | -6.143853 | 2.802560 | -0.495442 |
| H | -6.332905 | 3.034486 | 0.549573 |
| C | -5.002246 | 2.075632 | -0.825402 |
| H | -4.344622 | 1.763427 | -0.019015 |
| H | 0.568020 | -6.393838 | 0.642664 |
| H | -3.036943 | -1.631267 | 4.418225 |
| H | -0.188651 | 4.743987 | 3.208872 |
| H | -0.610703 | 6.407013 | -0.583418 |
| H | 3.019128 | 1.684939 | -4.393746 |
| H | 0.238624 | -4.715384 | -3.192456 |

**$3_M$**

| | | | |
|---|---|---|---|
| Ir | -0.808553 | 0.428794 | -0.901566 |
| P | -0.830866 | 0.604279 | 1.498638 |
| O | -3.426376 | 1.842162 | -1.386932 |
| N | -0.895703 | -1.683555 | -0.870045 |
| N | 1.036359 | -0.116324 | -1.617079 |
| N | 0.112413 | 2.270847 | -1.240752 |
| C | -1.652471 | -2.696370 | -0.313310 |
| C | -2.855073 | -2.672498 | 0.392137 |
| H | -3.406602 | -1.755660 | 0.555488 |
| C | -3.355826 | -3.881536 | 0.841388 |

| | | | |
|---|---|---|---|
| H | -4.311737 | -3.881841 | 1.359475 |
| C | -2.673490 | -5.093178 | 0.624518 |
| H | -3.103704 | -6.021251 | 0.994521 |
| C | -1.461495 | -5.132278 | -0.046136 |
| H | -0.924945 | -6.066496 | -0.197557 |
| C | -0.967217 | -3.913703 | -0.509338 |
| C | 0.230050 | -2.261869 | -1.350931 |
| C | 1.294074 | -1.431643 | -1.863500 |
| C | 2.460772 | -1.790100 | -2.532956 |
| H | 2.691507 | -2.827598 | -2.743797 |
| C | 3.352940 | -0.797421 | -2.921703 |
| H | 4.275297 | -1.070627 | -3.426041 |
| C | 3.095434 | 0.535038 | -2.631174 |
| H | 3.812501 | 1.297385 | -2.914526 |
| C | 1.918044 | 0.861946 | -1.966745 |
| C | 1.406548 | 2.161938 | -1.614790 |
| C | 1.076057 | 4.310840 | -1.232492 |
| C | 1.174308 | 5.686130 | -1.033212 |
| H | 2.106638 | 6.224712 | -1.187979 |
| C | 0.022541 | 6.343896 | -0.627465 |
| H | 0.055334 | 7.419161 | -0.467117 |
| C | -1.186941 | 5.655275 | -0.425245 |
| H | -2.068864 | 6.211380 | -0.116623 |
| C | -1.282873 | 4.284947 | -0.610179 |
| H | -2.218533 | 3.757071 | -0.444807 |
| C | -0.127589 | 3.609708 | -1.011034 |
| C | 1.271430 | -4.567289 | -1.454750 |
| H | 1.615973 | -4.418727 | -2.485194 |
| H | 0.806779 | -5.559477 | -1.444926 |
| C | 2.408317 | -4.488513 | -0.446173 |

| | | | |
|---|---|---|---|
| H | 2.743263 | -3.447910 | -0.315025 |
| H | 2.017616 | -4.795910 | 0.534788 |
| C | 3.588962 | -5.363567 | -0.841167 |
| H | 4.014088 | -4.989784 | -1.787033 |
| H | 3.235958 | -6.384370 | -1.058323 |
| C | 4.666310 | -5.400504 | 0.228566 |
| H | 4.273974 | -5.812435 | 1.166795 |
| H | 5.039024 | -4.395564 | 0.451964 |
| H | 5.513464 | -6.023383 | -0.078811 |
| C | 3.455676 | 3.631136 | -1.744328 |
| H | 3.572199 | 4.718592 | -1.808767 |
| H | 3.805649 | 3.242071 | -2.708329 |
| C | 4.245351 | 3.070779 | -0.570748 |
| H | 3.909087 | 3.582739 | 0.343149 |
| H | 4.010337 | 2.005332 | -0.411092 |
| C | 5.745551 | 3.246348 | -0.757014 |
| H | 5.965245 | 4.288875 | -1.038493 |
| H | 6.078208 | 2.629264 | -1.606781 |
| C | 6.522146 | 2.868040 | 0.491841 |
| H | 6.244405 | 3.513954 | 1.335519 |
| H | 7.602092 | 2.970002 | 0.339516 |
| H | 6.312107 | 1.835951 | 0.792971 |
| C | 0.400725 | 1.836817 | 2.026203 |
| C | 1.746774 | 1.493099 | 2.210948 |
| H | 2.080845 | 0.465288 | 2.127740 |
| C | 2.692216 | 2.471712 | 2.499713 |
| H | 3.731124 | 2.166210 | 2.617445 |
| C | 2.306149 | 3.805152 | 2.612244 |
| H | 3.047403 | 4.570766 | 2.839663 |
| C | 0.969096 | 4.158935 | 2.436189 |

| | | | |
|---|---|---|---|
| H | 0.656564 | 5.198462 | 2.524077 |
| C | 0.022772 | 3.185244 | 2.136213 |
| H | -1.019355 | 3.470861 | 2.002492 |
| C | -2.399895 | 1.102657 | 2.271253 |
| C | -2.377923 | 1.713137 | 3.537672 |
| H | -1.429024 | 1.983553 | 3.998140 |
| C | -3.565901 | 1.955090 | 4.216228 |
| H | -3.535974 | 2.430415 | 5.195209 |
| C | -4.784733 | 1.576238 | 3.654460 |
| H | -5.712072 | 1.760389 | 4.194234 |
| C | -4.815369 | 0.952091 | 2.411791 |
| H | -5.746082 | 0.618573 | 1.961111 |
| C | -3.628874 | 0.718734 | 1.720556 |
| H | -3.699360 | 0.209527 | 0.759736 |
| C | -0.420621 | -0.988318 | 2.277769 |
| C | -1.305491 | -1.564736 | 3.200932 |
| H | -2.243451 | -1.072934 | 3.448995 |
| C | -0.993588 | -2.779856 | 3.800620 |
| H | -1.694416 | -3.220753 | 4.506888 |
| C | 0.204994 | -3.422078 | 3.504380 |
| H | 0.453372 | -4.363624 | 3.991551 |
| C | 1.085452 | -2.860375 | 2.581179 |
| H | 2.044401 | -3.323732 | 2.356953 |
| C | 0.763810 | -1.666058 | 1.944994 |
| H | 1.460208 | -1.263413 | 1.206947 |
| C | -2.548470 | 1.138812 | -1.120994 |
| S | -5.500253 | -1.221869 | -1.096819 |
| F | -6.134378 | 0.793433 | -2.696207 |
| F | -6.123900 | 1.302277 | -0.584372 |
| F | -7.752351 | 0.131393 | -1.408513 |

| | | | |
|---|---|---|---|
| O | -4.112452 | -0.883345 | -1.517684 |
| O | -5.656132 | -1.334903 | 0.371715 |
| O | -6.163539 | -2.241136 | -1.908754 |
| C | -6.430862 | 0.331705 | -1.473865 |
| S | 4.342169 | -0.930230 | 1.017252 |
| F | 5.250506 | -2.227701 | -1.101752 |
| F | 6.800554 | -1.592540 | 0.277433 |
| F | 5.836840 | -0.135354 | -1.018053 |
| O | 4.128074 | -2.261894 | 1.604063 |
| O | 4.941363 | 0.095788 | 1.881673 |
| O | 3.215108 | -0.441736 | 0.183164 |
| C | 5.634896 | -1.238519 | -0.262668 |
| N | 0.202890 | -3.611765 | -1.179478 |
| N | 2.019239 | 3.378507 | -1.627512 |

**3**

| | | | |
|---|---|---|---|
| Ir | 1.009244 | 0.385522 | -1.050933 |
| P | 2.653564 | 1.009708 | -2.787890 |
| O | -1.339285 | 0.829481 | -2.905811 |
| N | 1.517303 | 2.126532 | 0.014895 |
| N | 2.574852 | -0.234515 | 0.157789 |
| N | 1.184427 | -1.632875 | -1.532152 |
| C | 1.246802 | 3.473857 | -0.038172 |
| C | 0.289249 | 4.184071 | -0.760127 |
| H | -0.447074 | 3.694033 | -1.393854 |
| C | 0.284206 | 5.562104 | -0.621354 |
| H | -0.441613 | 6.138534 | -1.193714 |
| C | 1.191250 | 6.218360 | 0.234145 |
| H | 1.157794 | 7.302835 | 0.314014 |
| C | 2.124434 | 5.517025 | 0.983714 |
| H | 2.815412 | 6.026286 | 1.652253 |

| | | | |
|---|---|---|---|
| C | 2.139124 | 4.129980 | 0.832875 |
| C | 2.543693 | 1.962559 | 0.883859 |
| C | 3.150941 | 0.649158 | 1.010597 |
| C | 4.209328 | 0.237467 | 1.821808 |
| H | 4.637575 | 0.890177 | 2.575769 |
| C | 4.699438 | -1.053152 | 1.674862 |
| H | 5.552414 | -1.376602 | 2.261325 |
| C | 4.128007 | -1.935977 | 0.761568 |
| H | 4.587729 | -2.908126 | 0.620206 |
| C | 3.028506 | -1.512143 | 0.021591 |
| C | 2.226002 | -2.251425 | -0.932545 |
| C | 1.335976 | -3.745949 | -2.288650 |
| C | 1.019183 | -4.865308 | -3.055082 |
| H | 1.596348 | -5.785277 | -2.987809 |
| C | -0.065241 | -4.750545 | -3.912772 |
| H | -0.344710 | -5.600204 | -4.531651 |
| C | -0.808231 | -3.558656 | -4.005235 |
| H | -1.649669 | -3.509392 | -4.693710 |
| C | -0.485803 | -2.440019 | -3.251257 |
| H | -1.056308 | -1.518383 | -3.337798 |
| C | 0.607855 | -2.542900 | -2.387500 |
| C | 3.933639 | 3.406466 | 2.448774 |
| H | 3.823252 | 2.638418 | 3.220977 |
| H | 3.652782 | 4.356790 | 2.918323 |
| C | 5.346266 | 3.458745 | 1.897713 |
| H | 5.548529 | 2.537417 | 1.332226 |
| H | 5.429371 | 4.273564 | 1.160887 |
| C | 6.384400 | 3.606804 | 3.000431 |
| H | 6.211212 | 2.832992 | 3.761193 |
| H | 6.242718 | 4.569646 | 3.514630 |

| | | | |
|---|---|---|---|
| C | 7.800020 | 3.503087 | 2.460504 |
| H | 8.004896 | 4.281714 | 1.713286 |
| H | 7.966283 | 2.530052 | 1.979154 |
| H | 8.544181 | 3.607010 | 3.257126 |
| C | 3.255871 | -4.576666 | -0.894698 |
| H | 2.726465 | -5.529492 | -1.027491 |
| H | 3.385511 | -4.454694 | 0.187377 |
| C | 4.578371 | -4.566025 | -1.636770 |
| H | 4.374962 | -4.733335 | -2.706345 |
| H | 5.051542 | -3.574232 | -1.565071 |
| C | 5.545320 | -5.616201 | -1.113311 |
| H | 5.067463 | -6.609462 | -1.125586 |
| H | 5.785710 | -5.389917 | -0.065337 |
| C | 6.825007 | -5.631574 | -1.928029 |
| H | 6.627667 | -5.895457 | -2.976018 |
| H | 7.548979 | -6.352060 | -1.533364 |
| H | 7.296495 | -4.643635 | -1.911649 |
| C | 3.155972 | -0.470948 | -3.724035 |
| C | 4.367123 | -1.144824 | -3.545843 |
| H | 5.128621 | -0.793301 | -2.859169 |
| C | 4.635751 | -2.302815 | -4.274022 |
| H | 5.586364 | -2.808632 | -4.110393 |
| C | 3.706583 | -2.793718 | -5.185519 |
| H | 3.922031 | -3.696454 | -5.755426 |
| C | 2.500550 | -2.119183 | -5.377314 |
| H | 1.770847 | -2.489376 | -6.095217 |
| C | 2.220343 | -0.971548 | -4.646529 |
| H | 1.273147 | -0.450572 | -4.800758 |
| C | 2.067607 | 2.131611 | -4.095963 |
| C | 2.837738 | 2.210599 | -5.268511 |

| | | | |
|---|---|---|---|
| H | 3.680747 | 1.535777 | -5.414275 |
| C | 2.532061 | 3.157624 | -6.236270 |
| H | 3.132379 | 3.210779 | -7.142575 |
| C | 1.468156 | 4.039998 | -6.044593 |
| H | 1.237566 | 4.785093 | -6.803752 |
| C | 0.696583 | 3.966418 | -4.889646 |
| H | -0.150103 | 4.633700 | -4.728964 |
| C | 0.995328 | 3.007775 | -3.922543 |
| H | 0.376280 | 2.961490 | -3.032398 |
| C | 4.124517 | 1.862294 | -2.130662 |
| C | 4.247958 | 3.246045 | -2.352817 |
| H | 3.460028 | 3.791595 | -2.869146 |
| C | 5.394904 | 3.922000 | -1.952359 |
| H | 5.476806 | 4.990925 | -2.142063 |
| C | 6.439396 | 3.230621 | -1.343572 |
| H | 7.350508 | 3.756112 | -1.061040 |
| C | 6.309552 | 1.871388 | -1.073687 |
| H | 7.093984 | 1.307334 | -0.571232 |
| C | 5.145937 | 1.195649 | -1.432315 |
| H | 5.085098 | 0.137959 | -1.177179 |
| C | -0.467887 | 0.753833 | -2.147218 |
| S | -2.944927 | 4.265276 | -3.383585 |
| F | -3.864018 | 1.990718 | -4.377186 |
| F | -2.042333 | 2.701013 | -5.313195 |
| F | -3.936648 | 3.667096 | -5.753771 |
| O | -2.127544 | 3.455064 | -2.438643 |
| O | -2.229401 | 5.392990 | -3.992944 |
| O | -4.309370 | 4.523567 | -2.886446 |
| C | -3.214540 | 3.094874 | -4.788273 |
| S | 7.287269 | -1.869069 | -0.255149 |

| | | | |
|---|---|---|---|
| F | 9.828545 | -2.239008 | 0.330953 |
| F | 9.478803 | -1.032901 | -1.440381 |
| F | 9.202655 | -3.183854 | -1.518266 |
| O | 7.321321 | -0.614715 | 0.523770 |
| O | 6.579073 | -1.769148 | -1.555564 |
| O | 6.959840 | -3.085767 | 0.514307 |
| C | 9.051122 | -2.096701 | -0.746481 |
| Ir | -1.037451 | -0.244002 | 1.040183 |
| P | -2.700344 | -0.701144 | 2.836785 |
| O | 1.293800 | -1.284801 | 2.665095 |
| N | -1.321568 | 1.821223 | 1.347438 |
| N | -2.611821 | 0.201864 | -0.227412 |
| N | -1.435710 | -2.074631 | 0.136086 |
| C | -0.899742 | 2.808681 | 2.209273 |
| C | 0.110564 | 2.820586 | 3.170123 |
| H | 0.764914 | 1.969601 | 3.343480 |
| C | 0.279889 | 3.984866 | 3.900502 |
| H | 1.049558 | 4.006084 | 4.670962 |
| C | -0.515655 | 5.124122 | 3.666932 |
| H | -0.352311 | 6.019170 | 4.263509 |
| C | -1.497758 | 5.135703 | 2.688068 |
| H | -2.102014 | 6.020152 | 2.497633 |
| C | -1.678389 | 3.956684 | 1.964161 |
| C | -2.323964 | 2.346740 | 0.602619 |
| C | -3.064382 | 1.477820 | -0.294588 |
| C | -4.144403 | 1.789880 | -1.123165 |
| H | -4.487952 | 2.811756 | -1.250710 |
| C | -4.758081 | 0.763753 | -1.824287 |
| H | -5.622024 | 0.974257 | -2.445434 |
| C | -4.297652 | -0.548281 | -1.728222 |

| | | | |
|---|---|---|---|
| H | -4.850741 | -1.330586 | -2.236637 |
| C | -3.201242 | -0.817342 | -0.917525 |
| C | -2.532887 | -2.083495 | -0.659400 |
| C | -1.835312 | -4.173858 | -0.548750 |
| C | -1.659648 | -5.553791 | -0.649166 |
| H | -2.330430 | -6.171966 | -1.241884 |
| C | -0.590504 | -6.101387 | 0.043689 |
| H | -0.419582 | -7.174661 | -0.004572 |
| C | 0.273947 | -5.300740 | 0.813761 |
| H | 1.093596 | -5.771993 | 1.352870 |
| C | 0.097946 | -3.929208 | 0.915718 |
| H | 0.762238 | -3.321306 | 1.525317 |
| C | -0.978918 | -3.367325 | 0.222827 |
| C | -3.451147 | 4.615491 | 0.313892 |
| H | -3.438835 | 4.449829 | -0.767126 |
| H | -2.993373 | 5.599559 | 0.474954 |
| C | -4.857471 | 4.561415 | 0.880269 |
| H | -5.246571 | 3.536871 | 0.779733 |
| H | -4.834362 | 4.761526 | 1.963633 |
| C | -5.786668 | 5.536807 | 0.172006 |
| H | -5.730077 | 5.364483 | -0.911756 |
| H | -5.425919 | 6.564567 | 0.329933 |
| C | -7.221310 | 5.410499 | 0.652476 |
| H | -7.300879 | 5.584624 | 1.734217 |
| H | -7.617670 | 4.406674 | 0.451402 |
| H | -7.879754 | 6.128825 | 0.152789 |
| C | -3.916066 | -3.902707 | -1.873440 |
| H | -3.461667 | -4.595363 | -2.597501 |
| H | -4.401272 | -3.111844 | -2.447269 |
| C | -4.908390 | -4.597076 | -0.958820 |

| | | | |
|---|---|---|---|
| H | -4.358626 | -5.274616 | -0.286057 |
| H | -5.385066 | -3.835354 | -0.327571 |
| C | -5.985699 | -5.371472 | -1.700378 |
| H | -5.527003 | -6.129879 | -2.356199 |
| H | -6.542119 | -4.679703 | -2.345636 |
| C | -6.936631 | -6.031778 | -0.716522 |
| H | -6.412084 | -6.753105 | -0.073626 |
| H | -7.745349 | -6.566546 | -1.226548 |
| H | -7.396921 | -5.277980 | -0.066874 |
| C | -3.298134 | -2.421270 | 2.761653 |
| C | -4.611684 | -2.801025 | 2.468792 |
| H | -5.386266 | -2.071751 | 2.255998 |
| C | -4.957506 | -4.151145 | 2.450219 |
| H | -5.982364 | -4.421876 | 2.204130 |
| C | -4.006322 | -5.128180 | 2.723339 |
| H | -4.283795 | -6.181148 | 2.701132 |
| C | -2.697434 | -4.755637 | 3.031488 |
| H | -1.946085 | -5.511624 | 3.253046 |
| C | -2.344541 | -3.412778 | 3.048081 |
| H | -1.321648 | -3.126672 | 3.301937 |
| C | -2.097055 | -0.563736 | 4.547691 |
| C | -2.924736 | -1.055158 | 5.571351 |
| H | -3.858744 | -1.560174 | 5.324126 |
| C | -2.554475 | -0.889648 | 6.898846 |
| H | -3.199237 | -1.273997 | 7.687202 |
| C | -1.363873 | -0.235318 | 7.220262 |
| H | -1.078787 | -0.109208 | 8.263163 |
| C | -0.535111 | 0.247111 | 6.213793 |
| H | 0.407679 | 0.742686 | 6.446103 |
| C | -0.903643 | 0.076234 | 4.880271 |

| | | | |
|---|---|---|---|
| H | -0.241622 | 0.451790 | 4.107385 |
| C | -4.135946 | 0.415348 | 2.775131 |
| C | -4.272300 | 1.425435 | 3.742571 |
| H | -3.516205 | 1.548482 | 4.516749 |
| C | -5.392710 | 2.249374 | 3.737401 |
| H | -5.493566 | 3.018428 | 4.501327 |
| C | -6.386783 | 2.079829 | 2.775428 |
| H | -7.271252 | 2.714800 | 2.790382 |
| C | -6.244119 | 1.109185 | 1.786830 |
| H | -6.996201 | 0.956972 | 1.013133 |
| C | -5.116137 | 0.293881 | 1.777017 |
| H | -5.041176 | -0.461160 | 0.994899 |
| C | 0.435583 | -0.792420 | 2.062802 |
| S | 3.193296 | 0.914270 | 5.178684 |
| F | 3.816046 | -1.577534 | 4.545730 |
| F | 2.017831 | -1.378968 | 5.746627 |
| F | 3.970104 | -1.124535 | 6.663085 |
| O | 2.336287 | 0.948232 | 3.961269 |
| O | 2.578745 | 1.510456 | 6.371261 |
| O | 4.599289 | 1.261611 | 4.903331 |
| C | 3.256895 | -0.891965 | 5.559459 |
| S | -7.321668 | -1.684849 | -0.872738 |
| F | -9.709673 | -1.740640 | -1.986337 |
| F | -9.724358 | -1.823616 | 0.182268 |
| F | -9.153029 | -3.574980 | -0.970993 |
| O | -7.406742 | -0.212896 | -0.878525 |
| O | -6.806274 | -2.270281 | 0.386302 |
| O | -6.752824 | -2.281373 | -2.103857 |
| C | -9.079959 | -2.234554 | -0.916366 |
| N | -2.797244 | -3.346827 | -1.094869 |

| | | | |
|---|---|---|---|
| N | -2.543781 | 3.647356 | 0.935423 |
| N | 2.332060 | -3.538527 | -1.356186 |
| N | 2.925484 | 3.155546 | 1.413407 |

**6$_M$**

| | | | |
|---|---|---|---|
| Ir | 0.045122 | -0.619830 | -1.371585 |
| P | 0.017230 | -1.674701 | 0.813615 |
| N | -0.000712 | 1.286827 | -0.703094 |
| N | -2.025150 | -0.231408 | -1.302766 |
| N | 2.085410 | -0.139486 | -1.289928 |
| C | 0.147731 | -2.191223 | -2.409728 |
| C | -1.119798 | -3.069565 | 1.049571 |
| C | 1.659921 | -2.323797 | 1.228976 |
| C | -0.510484 | -0.385175 | 1.967157 |
| C | 1.168892 | 1.930645 | -0.481851 |
| C | -1.204920 | 1.870222 | -0.476351 |
| C | -2.340718 | 0.997085 | -0.777032 |
| C | 2.346202 | 1.104785 | -0.757028 |
| O | 0.259645 | -3.121693 | -3.089602 |
| C | -0.063301 | 3.904521 | 0.196411 |
| C | -4.719457 | 0.491243 | -0.843853 |
| C | 4.740785 | 0.655703 | -0.748062 |
| C | -0.144233 | 5.353780 | 0.654777 |
| C | -6.174100 | 0.825769 | -0.565745 |
| C | 6.150570 | 1.094182 | -0.394020 |
| C | 2.406189 | -1.953900 | 2.354612 |
| H | 2.006754 | -1.248411 | 3.078832 |
| C | 3.652670 | -2.534335 | 2.582137 |
| H | 4.220589 | -2.250133 | 3.465320 |
| C | 4.156215 | -3.492811 | 1.706832 |
| H | 5.124203 | -3.950380 | 1.898250 |

| | | | |
|---|---|---|---|
| C | 3.405187 | -3.886395 | 0.599197 |
| H | 3.779450 | -4.656422 | -0.072088 |
| C | 2.168737 | -3.302120 | 0.358206 |
| H | 1.579929 | -3.628007 | -0.501886 |
| C | -1.443746 | -3.445211 | 2.364413 |
| H | -1.079175 | -2.860955 | 3.208938 |
| C | -2.226249 | -4.570136 | 2.592740 |
| H | -2.472824 | -4.854021 | 3.613105 |
| C | -2.684072 | -5.335841 | 1.521112 |
| H | -3.290964 | -6.219071 | 1.706001 |
| C | -2.356418 | -4.976687 | 0.216983 |
| H | -2.700098 | -5.579508 | -0.620421 |
| C | -1.577361 | -3.847032 | -0.018986 |
| H | -1.312690 | -3.586772 | -1.041257 |
| C | -1.879882 | -0.264876 | 2.259775 |
| H | -2.583070 | -1.014760 | 1.896445 |
| C | -2.346522 | 0.801045 | 3.022390 |
| H | -3.408665 | 0.872212 | 3.250513 |
| C | -1.459821 | 1.762106 | 3.502486 |
| H | -1.823234 | 2.584107 | 4.115752 |
| C | -0.102755 | 1.667523 | 3.194009 |
| H | 0.595666 | 2.417426 | 3.561605 |
| C | 0.368972 | 0.614751 | 2.418449 |
| H | 1.427233 | 0.573124 | 2.162473 |
| C | -4.362649 | -0.743888 | -1.409060 |
| H | -5.121844 | -1.470109 | -1.690317 |
| C | -3.037612 | -1.067222 | -1.616741 |
| H | -2.748787 | -2.021320 | -2.048127 |
| C | -3.665152 | 1.355370 | -0.546930 |
| H | -3.868316 | 2.328828 | -0.109054 |

| | | | |
|---|---|---|---|
| C | -6.964878 | 0.748002 | -1.880868 |
| H | -6.917842 | -0.248223 | -2.335236 |
| H | -8.020892 | 0.968473 | -1.690340 |
| H | -6.599257 | 1.473646 | -2.616433 |
| C | -6.335505 | 2.219904 | 0.035809 |
| H | -5.821122 | 2.315105 | 1.001496 |
| H | -5.967822 | 3.005997 | -0.635676 |
| H | -7.395508 | 2.423604 | 0.217293 |
| C | -6.722442 | -0.214429 | 0.424771 |
| H | -6.677366 | -1.233206 | 0.022279 |
| H | -6.169370 | -0.198858 | 1.372753 |
| H | -7.772819 | 0.002626 | 0.647612 |
| C | 3.126579 | -0.938895 | -1.577754 |
| H | 2.881932 | -1.901049 | -2.018224 |
| C | 4.439923 | -0.581263 | -1.324996 |
| H | 5.223113 | -1.289960 | -1.578382 |
| C | 3.648584 | 1.495518 | -0.487155 |
| H | 3.819062 | 2.477940 | -0.051345 |
| C | 7.179899 | 0.016934 | -0.728608 |
| H | 7.206918 | -0.213002 | -1.800582 |
| H | 8.180527 | 0.361926 | -0.449579 |
| H | 6.994106 | -0.914589 | -0.178324 |
| C | 6.201641 | 1.382895 | 1.115105 |
| H | 5.527334 | 2.198068 | 1.405678 |
| H | 5.936614 | 0.495132 | 1.703004 |
| H | 7.215619 | 1.683643 | 1.400542 |
| C | 6.485120 | 2.375009 | -1.173672 |
| H | 7.495529 | 2.710790 | -0.915872 |
| H | 6.454227 | 2.209330 | -2.256674 |
| H | 5.798235 | 3.196505 | -0.935835 |

| | | | |
|---|---|---|---|
| C | 1.151827 | 3.248128 | -0.035356 |
| H | 2.090548 | 3.772056 | 0.120181 |
| C | -1.247597 | 3.180703 | -0.026508 |
| H | -2.208819 | 3.662043 | 0.139593 |
| C | 1.237862 | 5.971500 | 0.853684 |
| H | 1.819846 | 5.444206 | 1.620697 |
| H | 1.820964 | 5.991840 | -0.075326 |
| H | 1.134294 | 7.008655 | 1.188158 |
| C | -0.905044 | 6.159032 | -0.411381 |
| H | -1.928932 | 5.793122 | -0.554866 |
| H | -0.974024 | 7.207955 | -0.103166 |
| H | -0.395685 | 6.128625 | -1.381561 |
| C | -0.911871 | 5.416281 | 1.984215 |
| H | -1.919825 | 4.990436 | 1.905745 |
| H | -0.378989 | 4.884073 | 2.781534 |
| H | -1.023110 | 6.459688 | 2.299086 |

**6**

| | | | |
|---|---|---|---|
| Ir | 1.393884 | -0.005265 | 0.630833 |
| P | 3.673237 | -0.058481 | 1.684188 |
| N | 2.244061 | -0.123890 | -1.213107 |
| N | 1.518923 | -2.093360 | 0.330432 |
| N | 1.677591 | 2.019037 | 0.146244 |
| C | 0.496512 | 0.191730 | 2.266178 |
| C | 3.867953 | -1.139576 | 3.128972 |
| C | 4.134604 | 1.587237 | 2.304329 |
| C | 4.866248 | -0.654829 | 0.461459 |
| C | 2.570025 | 1.017622 | -1.855954 |
| C | 2.528546 | -1.344726 | -1.724399 |
| C | 2.145212 | -2.448357 | -0.839417 |
| C | 2.301912 | 2.223464 | -1.066084 |

| | | | |
|---|---|---|---|
| O | -0.039653 | 0.383017 | 3.278339 |
| C | 3.413866 | -0.283937 | -3.721084 |
| C | 2.089593 | -4.808111 | -0.242000 |
| C | 2.563759 | 4.603036 | -0.594348 |
| C | 3.962246 | -0.415293 | -5.133884 |
| C | 2.424829 | -6.267728 | -0.490089 |
| C | 3.126159 | 5.957766 | -0.988841 |
| Ir | -1.387121 | 0.024448 | -0.625390 |
| P | -3.664408 | 0.067164 | -1.685347 |
| N | -2.251560 | 0.115383 | 1.213788 |
| N | -1.522965 | 2.107788 | -0.296194 |
| N | -1.649325 | -2.008222 | -0.162609 |
| C | -0.496239 | -0.141940 | -2.267616 |
| C | -3.861636 | 1.147686 | -3.130057 |
| C | -4.120848 | -1.579417 | -2.306080 |
| C | -4.864117 | 0.657371 | -0.466497 |
| C | -2.578053 | -1.036355 | 1.837969 |
| C | -2.557759 | 1.327691 | 1.733329 |
| C | -2.164372 | 2.445270 | 0.870654 |
| C | -2.286884 | -2.231145 | 1.039244 |
| O | 0.030568 | -0.310974 | -3.288571 |
| C | -3.477891 | 0.235780 | 3.697193 |
| C | -2.093039 | 4.814431 | 0.313150 |
| C | -2.512656 | -4.609720 | 0.544569 |
| C | -4.061452 | 0.344893 | 5.098011 |
| C | -2.417793 | 6.272195 | 0.583890 |
| C | -3.070145 | -5.972354 | 0.919340 |
| C | -5.275459 | -2.285549 | -1.945332 |
| H | -5.984043 | -1.870560 | -1.232895 |
| C | -5.556813 | -3.510920 | -2.549190 |

| | | | |
|---|---|---|---|
| H | -6.466904 | -4.042233 | -2.278893 |
| C | -4.706135 | -4.033825 | -3.519858 |
| H | -4.946092 | -4.979102 | -4.001722 |
| C | -3.568592 | -3.322426 | -3.902612 |
| H | -2.922611 | -3.705675 | -4.689849 |
| C | -3.275325 | -2.107942 | -3.296705 |
| H | -2.403655 | -1.540982 | -3.630174 |
| C | -5.160833 | 1.293467 | -3.647054 |
| H | -6.006044 | 0.814077 | -3.153291 |
| C | -5.371456 | 2.045046 | -4.795874 |
| H | -6.378537 | 2.151182 | -5.191888 |
| C | -4.295454 | 2.652133 | -5.443900 |
| H | -4.465244 | 3.233898 | -6.346755 |
| C | -3.005909 | 2.505653 | -4.941375 |
| H | -2.166123 | 2.969952 | -5.453566 |
| C | -2.789482 | 1.755050 | -3.788348 |
| H | -1.777460 | 1.630549 | -3.411048 |
| C | -5.214508 | 2.018472 | -0.460677 |
| H | -4.848517 | 2.680207 | -1.246567 |
| C | -6.055672 | 2.524257 | 0.525890 |
| H | -6.342609 | 3.573803 | 0.501921 |
| C | -6.555110 | 1.684900 | 1.519591 |
| H | -7.235169 | 2.075965 | 2.273532 |
| C | -6.193219 | 0.337439 | 1.536061 |
| H | -6.590007 | -0.326407 | 2.302988 |
| C | -5.346994 | -0.173156 | 0.558900 |
| H | -5.069670 | -1.226395 | 0.590937 |
| C | -1.421907 | 4.435190 | -0.861476 |
| H | -1.109658 | 5.179641 | -1.591126 |
| C | -1.157648 | 3.106970 | -1.126043 |

| | | | |
|---|---|---|---|
| H | -0.635436 | 2.806939 | -2.030640 |
| C | -2.436501 | 3.775918 | 1.178833 |
| H | -2.958914 | 3.996208 | 2.106097 |
| C | -1.101655 | 7.064874 | 0.624716 |
| H | -0.532403 | 6.966135 | -0.309052 |
| H | -1.309719 | 8.130506 | 0.768817 |
| H | -0.463197 | 6.737884 | 1.456152 |
| C | -3.159832 | 6.460010 | 1.905136 |
| H | -4.123483 | 5.934531 | 1.914836 |
| H | -2.569573 | 6.123967 | 2.767204 |
| H | -3.376270 | 7.521108 | 2.062323 |
| C | -3.296981 | 6.789583 | -0.566754 |
| H | -2.790744 | 6.733384 | -1.537512 |
| H | -4.234787 | 6.224742 | -0.638676 |
| H | -3.554805 | 7.839694 | -0.393445 |
| C | 5.214906 | -1.303541 | -5.135444 |
| H | 6.038073 | -0.842161 | -4.577522 |
| H | 5.031282 | -2.299001 | -4.713080 |
| H | 5.558666 | -1.450801 | -6.164632 |
| C | 4.318257 | 0.940520 | -5.740035 |
| H | 3.443512 | 1.594820 | -5.842870 |
| H | 5.084191 | 1.466616 | -5.155029 |
| H | 4.726768 | 0.800582 | -6.745462 |
| C | 2.864673 | -1.078068 | -5.986678 |
| H | 3.207708 | -1.172579 | -7.022638 |
| H | 2.618562 | -2.085972 | -5.629734 |
| H | 1.942700 | -0.483727 | -5.990773 |
| C | 3.117010 | -1.438483 | -2.977439 |
| H | 3.335881 | -2.416777 | -3.398874 |
| C | 3.142798 | 0.953858 | -3.123749 |

| | | | |
|---|---|---|---|
| H | 3.384142 | 1.871518 | -3.653159 |
| C | -1.416841 | -3.064749 | -0.960026 |
| H | -0.907206 | -2.851490 | -1.896879 |
| C | -1.822054 | -4.352379 | -0.643820 |
| H | -1.624850 | -5.145552 | -1.360673 |
| C | -2.698888 | -3.507972 | 1.393106 |
| H | -3.222392 | -3.642316 | 2.337621 |
| C | -2.863713 | -6.995273 | -0.195636 |
| H | -1.801921 | -7.183572 | -0.398896 |
| H | -3.306473 | -7.953312 | 0.093585 |
| H | -3.347087 | -6.685181 | -1.131265 |
| C | -4.578181 | -5.826220 | 1.185274 |
| H | -4.793861 | -5.162582 | 2.031089 |
| H | -5.106337 | -5.442498 | 0.304147 |
| H | -5.005537 | -6.804539 | 1.429271 |
| C | -2.373229 | -6.458993 | 2.199751 |
| H | -2.786418 | -7.426724 | 2.503728 |
| H | -1.295005 | -6.596090 | 2.047121 |
| H | -2.515145 | -5.767158 | 3.039536 |
| C | -3.176853 | -0.992393 | 3.094546 |
| H | -3.419190 | -1.918205 | 3.609198 |
| C | -3.175984 | 1.401249 | 2.973038 |
| H | -3.414583 | 2.372599 | 3.399700 |
| C | -4.436432 | -1.020166 | 5.671334 |
| H | -5.184672 | -1.535914 | 5.054895 |
| H | -3.565716 | -1.677187 | 5.789502 |
| H | -4.874166 | -0.896269 | 6.666440 |
| C | -2.982964 | 0.988891 | 5.988746 |
| H | -2.719380 | 1.998796 | 5.650721 |
| H | -3.353502 | 1.073348 | 7.016076 |

| | | | |
|---|---|---|---|
| H | -2.066100 | 0.387171 | 6.011399 |
| C | -5.310885 | 1.237548 | 5.085897 |
| H | -5.112786 | 2.242886 | 4.694735 |
| H | -6.119741 | 0.794002 | 4.493437 |
| H | -5.683157 | 1.360568 | 6.108296 |
| C | 1.472433 | 3.084178 | 0.939636 |
| H | 0.976015 | 2.884183 | 1.886178 |
| C | 1.891651 | 4.363285 | 0.608156 |
| H | 1.718964 | 5.163748 | 1.322852 |
| C | 2.723491 | 3.492519 | -1.437008 |
| H | 3.234505 | 3.613237 | -2.390254 |
| C | 2.951707 | 6.989969 | 0.123264 |
| H | 3.396047 | 7.942145 | -0.182280 |
| H | 1.896307 | 7.189230 | 0.348235 |
| H | 3.452293 | 6.682968 | 1.050713 |
| C | 2.404758 | 6.442265 | -2.256648 |
| H | 1.331173 | 6.588432 | -2.080500 |
| H | 2.818241 | 7.405023 | -2.575702 |
| H | 2.522991 | 5.744310 | -3.095079 |
| C | 4.626955 | 5.796531 | -1.284789 |
| H | 5.169580 | 5.412498 | -0.412411 |
| H | 4.819044 | 5.126333 | -2.131124 |
| H | 5.058482 | 6.769289 | -1.543240 |
| C | 1.166673 | -3.079916 | 1.180932 |
| H | 0.660664 | -2.765519 | 2.089770 |
| C | 1.430177 | -4.411929 | 0.933915 |
| H | 1.131383 | -5.145077 | 1.680482 |
| C | 2.418072 | -3.782880 | -1.128899 |
| H | 2.934568 | -4.015362 | -2.056393 |
| C | 3.317157 | -6.757815 | 0.662546 |

| | | | |
|---|---|---|---|
| H | 4.250971 | -6.184461 | 0.717162 |
| H | 2.818341 | -6.688679 | 1.636275 |
| H | 3.581966 | -7.808767 | 0.505438 |
| C | 1.115948 | -7.072454 | -0.507328 |
| H | 1.331752 | -8.138758 | -0.634356 |
| H | 0.552635 | -6.962089 | 0.428697 |
| H | 0.468601 | -6.765330 | -1.339507 |
| C | 3.159050 | -6.472283 | -1.813261 |
| H | 2.559225 | -6.158077 | -2.676952 |
| H | 4.117180 | -5.937325 | -1.839357 |
| H | 3.384862 | -7.533934 | -1.952569 |
| C | 5.281855 | 2.299390 | 1.932245 |
| H | 5.986733 | 1.887772 | 1.214256 |
| C | 5.561196 | 3.527645 | 2.531430 |
| H | 6.465715 | 4.063846 | 2.252179 |
| C | 4.716861 | 4.046526 | 3.509751 |
| H | 4.955662 | 4.993944 | 3.987965 |
| C | 3.587617 | 3.328655 | 3.904602 |
| H | 2.947307 | 3.708546 | 4.698046 |
| C | 3.295793 | 2.111744 | 3.302879 |
| H | 2.430585 | 1.540177 | 3.645376 |
| C | 5.353003 | 0.174040 | -0.563633 |
| H | 5.079510 | 1.228283 | -0.595943 |
| C | 6.198976 | -0.339121 | -1.540104 |
| H | 6.599722 | 0.323367 | -2.305970 |
| C | 6.552117 | -1.688787 | -1.526569 |
| H | 7.230378 | -2.082415 | -2.280722 |
| C | 6.046221 | -2.527183 | -0.535274 |
| H | 6.326718 | -3.578592 | -0.513473 |
| C | 5.209178 | -2.017973 | 0.453018 |

| | | | |
|---|---|---|---|
| H | 4.842344 | -2.678339 | 1.239574 |
| C | 5.172037 | -1.327615 | 3.619730 |
| H | 6.023507 | -0.886180 | 3.101890 |
| C | 5.380068 | -2.071760 | 4.773911 |
| H | 6.391143 | -2.209946 | 5.149472 |
| C | 4.297058 | -2.629468 | 5.453706 |
| H | 4.465195 | -3.205786 | 6.360370 |
| C | 3.002769 | -2.438582 | 4.979124 |
| H | 2.157541 | -2.862247 | 5.517040 |
| C | 2.788627 | -1.694739 | 3.821449 |
| H | 1.772991 | -1.533043 | 3.468299 |

**The ligand effects on the Ir(II)-Ir(II) Complexes**

**$4_M$**

| | | | |
|---|---|---|---|
| Ir | 0.021505 | 0.046065 | -1.175164 |
| P | 0.147466 | -1.027692 | 0.967760 |
| N | -0.082471 | 1.865653 | -0.306376 |
| N | -2.039842 | 0.443473 | -1.215341 |
| N | 2.024036 | 0.676963 | -1.215521 |
| C | 0.114485 | -1.598148 | -2.099943 |
| C | 1.078097 | 2.491859 | 0.029624 |
| C | -1.305352 | 2.373295 | -0.004242 |
| C | -2.408791 | 1.577762 | -0.531309 |
| C | 2.269405 | 1.805623 | -0.465470 |
| O | 0.160746 | -2.606717 | -2.660364 |
| C | -0.220809 | 4.236954 | 1.104319 |
| C | -4.750169 | 1.205275 | -1.051491 |
| C | 4.662277 | 1.557506 | -0.792941 |
| C | -0.250590 | 5.540143 | 1.894124 |
| C | -6.229796 | 1.517275 | -0.915800 |
| C | 6.082659 | 1.933387 | -0.406748 |

| | | | |
|---|---|---|---|
| O | -2.668149 | -2.498451 | -0.270260 |
| O | 2.728014 | -2.255404 | -0.440898 |
| S | 4.162344 | -2.283173 | -0.041900 |
| S | -4.024366 | -2.328235 | 0.313894 |
| O | -5.137716 | -2.655390 | -0.586416 |
| O | -4.178856 | -1.077680 | 1.092266 |
| C | -4.044133 | -3.633015 | 1.618743 |
| F | -5.147610 | -3.557515 | 2.369801 |
| F | -2.977449 | -3.470585 | 2.428587 |
| F | -3.977695 | -4.855571 | 1.083550 |
| O | 5.103212 | -2.550238 | -1.135644 |
| O | 4.541108 | -1.182252 | 0.872151 |
| C | 4.217280 | -3.769378 | 1.045130 |
| F | 3.398056 | -3.586137 | 2.100040 |
| F | 5.450594 | -3.988659 | 1.508776 |
| F | 3.805126 | -4.860508 | 0.391281 |
| C | -4.332736 | 0.096199 | -1.795823 |
| H | -5.050155 | -0.552102 | -2.290722 |
| C | -3.000785 | -0.265995 | -1.836663 |
| H | -2.676739 | -1.161423 | -2.352671 |
| C | -3.746409 | 1.949476 | -0.434304 |
| H | -3.997529 | 2.838016 | 0.138789 |
| C | -6.851341 | 1.660348 | -2.311829 |
| H | -6.752736 | 0.741944 | -2.901290 |
| H | -7.922958 | 1.875645 | -2.221774 |
| H | -6.389759 | 2.478622 | -2.879251 |
| C | -6.475034 | 2.805627 | -0.133190 |
| H | -6.092195 | 2.737940 | 0.892910 |
| H | -6.018261 | 3.679386 | -0.617987 |
| H | -7.552037 | 2.996146 | -0.064009 |

| | | | |
|---|---|---|---|
| C | -6.890576 | 0.344186 | -0.173920 |
| H | -6.726473 | -0.612614 | -0.682501 |
| H | -6.477921 | 0.229196 | 0.833700 |
| H | -7.971516 | 0.519736 | -0.099255 |
| C | 3.060277 | 0.079857 | -1.826496 |
| H | 2.829298 | -0.800107 | -2.415287 |
| C | 4.367009 | 0.505727 | -1.660200 |
| H | 5.148913 | -0.072525 | -2.140641 |
| C | 3.569728 | 2.233352 | -0.240301 |
| H | 3.733289 | 3.094059 | 0.406658 |
| C | 7.099091 | 0.975342 | -1.025509 |
| H | 7.102656 | 1.030238 | -2.121523 |
| H | 8.107322 | 1.237395 | -0.684412 |
| H | 6.902561 | -0.063307 | -0.731919 |
| C | 6.199806 | 1.846130 | 1.122905 |
| H | 5.534112 | 2.559501 | 1.625893 |
| H | 5.952789 | 0.838344 | 1.474296 |
| H | 7.225986 | 2.083525 | 1.429928 |
| C | 6.377738 | 3.366616 | -0.867897 |
| H | 7.393130 | 3.655992 | -0.569964 |
| H | 6.308079 | 3.461764 | -1.958675 |
| H | 5.685200 | 4.092818 | -0.422352 |
| C | 1.017542 | 3.680501 | 0.741181 |
| H | 1.940098 | 4.190202 | 1.011084 |
| C | -1.383578 | 3.566049 | 0.710678 |
| H | -2.360182 | 3.971924 | 0.957279 |
| C | 0.493803 | 5.334138 | 3.221255 |
| H | 0.017867 | 4.556113 | 3.830183 |
| H | 1.539948 | 5.044273 | 3.065126 |
| H | 0.493506 | 6.263966 | 3.802734 |

| | | | |
|---|---|---|---|
| C | 0.454282 | 6.635248 | 1.080711 |
| H | -0.046575 | 6.806401 | 0.120135 |
| H | 0.447652 | 7.580122 | 1.637479 |
| H | 1.501392 | 6.384792 | 0.872507 |
| C | -1.676042 | 5.997576 | 2.197765 |
| H | -2.250228 | 6.194264 | 1.283640 |
| H | -2.226035 | 5.262514 | 2.798483 |
| H | -1.650899 | 6.931289 | 2.770656 |
| C | 1.590607 | -0.452832 | 1.910548 |
| H | 2.541996 | -0.644266 | 1.402859 |
| H | 1.488354 | 0.619400 | 2.118544 |
| H | 1.603839 | -0.983192 | 2.871531 |
| C | 0.142862 | -2.833877 | 1.019630 |
| H | -0.795696 | -3.201766 | 0.595469 |
| H | 1.010387 | -3.214567 | 0.474349 |
| H | 0.211253 | -3.143317 | 2.070551 |
| C | -1.228233 | -0.467182 | 2.010474 |
| H | -1.141232 | 0.612856 | 2.183043 |
| H | -2.212299 | -0.690001 | 1.580442 |
| H | -1.157307 | -0.971230 | 2.982900 |

**4**

| | | | |
|---|---|---|---|
| Ir | -0.061848 | 0.305070 | -1.419485 |
| P | -0.157547 | 0.948522 | -3.742298 |
| N | 0.319684 | -1.650389 | -1.820834 |
| N | 2.044652 | 0.283440 | -1.526495 |
| N | -2.003266 | -0.505743 | -1.528420 |
| C | -0.456175 | 2.112842 | -1.010984 |
| C | -0.719521 | -2.509259 | -1.904720 |
| C | 1.608139 | -2.050795 | -1.921092 |
| C | 2.570368 | -0.948878 | -1.832104 |

| | | | |
|---|---|---|---|
| C | -2.027301 | -1.853942 | -1.810003 |
| O | -0.740423 | 3.221815 | -0.896724 |
| C | 0.847738 | -4.342949 | -2.072134 |
| C | 4.801492 | -0.006175 | -2.023020 |
| C | -4.448572 | -1.805896 | -2.050189 |
| C | 1.164373 | -5.828998 | -2.006777 |
| C | 6.281925 | -0.097417 | -2.353977 |
| C | -5.742097 | -2.510940 | -2.430787 |
| S | 2.991699 | 4.939780 | -0.471165 |
| O | 3.186116 | 5.467207 | 0.897476 |
| O | 1.740716 | 4.158279 | -0.640704 |
| O | 4.171912 | 4.333479 | -1.107564 |
| C | 2.662031 | 6.446268 | -1.480808 |
| F | 3.721745 | 7.257933 | -1.487574 |
| F | 2.384552 | 6.101509 | -2.747834 |
| F | 1.611028 | 7.124120 | -0.993800 |
| S | -4.810170 | 3.375459 | -0.228834 |
| O | -5.011233 | 4.066926 | 1.060085 |
| O | -5.701041 | 2.228326 | -0.489535 |
| O | -3.386603 | 3.124248 | -0.561950 |
| C | -5.297395 | 4.620304 | -1.498862 |
| F | -5.086399 | 4.119465 | -2.727139 |
| F | -6.588459 | 4.946735 | -1.393205 |
| F | -4.564331 | 5.733320 | -1.372158 |
| Ir | 0.053415 | -0.305777 | 1.416340 |
| P | 0.137678 | -0.975813 | 3.734185 |
| N | -0.324092 | 1.650354 | 1.819514 |
| N | -2.054032 | -0.278558 | 1.523436 |
| N | 1.995446 | 0.500362 | 1.526693 |
| C | 0.453270 | -2.111026 | 1.003795 |

| | | | |
|---|---|---|---|
| C | 0.717134 | 2.507062 | 1.903120 |
| C | -1.611448 | 2.054485 | 1.916778 |
| C | -2.576582 | 0.955266 | 1.827769 |
| C | 2.023243 | 1.848075 | 1.810831 |
| O | 0.749613 | -3.216837 | 0.888373 |
| C | -0.845731 | 4.344988 | 2.066104 |
| C | -4.810386 | 0.018832 | 2.017798 |
| C | 4.443907 | 1.791304 | 2.055680 |
| C | -1.158797 | 5.831894 | 2.000721 |
| C | -6.291272 | 0.114781 | 2.345590 |
| C | 5.738719 | 2.490638 | 2.442258 |
| O | -1.734365 | -4.138300 | 0.628877 |
| O | 3.395180 | -3.124819 | 0.550124 |
| S | 4.818558 | -3.374212 | 0.214838 |
| S | -2.981473 | -4.931908 | 0.487247 |
| O | -3.186255 | -5.484468 | -0.869416 |
| O | -4.158922 | -4.323611 | 1.126935 |
| C | -2.625298 | -6.413324 | 1.523282 |
| F | -3.679566 | -7.230374 | 1.571394 |
| F | -2.326017 | -6.035655 | 2.776773 |
| F | -1.579859 | -7.096971 | 1.033101 |
| O | 5.017941 | -4.061650 | -1.076560 |
| O | 5.709737 | -2.227996 | 0.478028 |
| C | 5.307564 | -4.621212 | 1.482163 |
| F | 5.096125 | -4.122168 | 2.711118 |
| F | 6.598987 | -4.946008 | 1.375776 |
| F | 4.575939 | -5.735081 | 1.354393 |
| C | -4.246986 | -1.213290 | 1.661483 |
| H | -4.836807 | -2.124323 | 1.574792 |
| C | -2.888852 | -1.331767 | 1.429508 |

| | | | |
|---|---|---|---|
| H | -2.442704 | -2.293341 | 1.167922 |
| C | -3.938572 | 1.108882 | 2.074212 |
| H | -4.313987 | 2.104827 | 2.299951 |
| C | -7.101590 | -0.303675 | 1.110785 |
| H | -6.841163 | -1.317429 | 0.776511 |
| H | -8.171813 | -0.301566 | 1.352150 |
| H | -6.933136 | 0.398589 | 0.287365 |
| C | -6.701597 | 1.526521 | 2.756214 |
| H | -6.169070 | 1.857612 | 3.657940 |
| H | -6.519392 | 2.257450 | 1.960824 |
| H | -7.772634 | 1.541864 | 2.989393 |
| C | -6.590907 | -0.855395 | 3.499285 |
| H | -6.357486 | -1.894724 | 3.238630 |
| H | -6.020885 | -0.595276 | 4.400620 |
| H | -7.656713 | -0.809019 | 3.754136 |
| C | 2.353522 | -6.174117 | -2.911473 |
| H | 2.159321 | -5.906342 | -3.958543 |
| H | 3.280231 | -5.683760 | -2.590763 |
| H | 2.536049 | -7.254552 | -2.874620 |
| C | -0.039668 | -6.686145 | -2.399790 |
| H | -0.886561 | -6.568093 | -1.712452 |
| H | -0.389821 | -6.459069 | -3.416061 |
| H | 0.246034 | -7.744039 | -2.379618 |
| C | 1.540575 | -6.128782 | -0.543615 |
| H | 1.804311 | -7.189783 | -0.445489 |
| H | 2.399105 | -5.529759 | -0.217179 |
| H | 0.700748 | -5.920148 | 0.129183 |
| C | 1.892306 | -3.404508 | -2.046563 |
| H | 2.930081 | -3.733147 | -2.026369 |
| C | -0.471352 | -3.871898 | -2.038573 |

| | | | |
|---|---|---|---|
| H | -1.310591 | -4.563739 | -2.021759 |
| C | 3.159788 | -0.167717 | 1.447669 |
| H | 3.113633 | -1.226366 | 1.177647 |
| C | 4.380230 | 0.438869 | 1.708385 |
| H | 5.261532 | -0.193970 | 1.633093 |
| C | 3.229957 | 2.493076 | 2.054880 |
| H | 3.214165 | 3.565081 | 2.243850 |
| C | 6.898202 | 1.502912 | 2.563746 |
| H | 7.129049 | 1.016516 | 1.608264 |
| H | 7.800802 | 2.036716 | 2.882447 |
| H | 6.700122 | 0.716765 | 3.303814 |
| C | 5.539559 | 3.185986 | 3.797581 |
| H | 4.762571 | 3.957850 | 3.751469 |
| H | 5.268827 | 2.469899 | 4.584534 |
| H | 6.470659 | 3.679877 | 4.100505 |
| C | 6.082047 | 3.550944 | 1.388052 |
| H | 7.015338 | 4.055020 | 1.669835 |
| H | 6.226999 | 3.103483 | 0.397078 |
| H | 5.298205 | 4.309138 | 1.287047 |
| C | 0.472426 | 3.870622 | 2.034206 |
| H | 1.313752 | 4.560163 | 2.017568 |
| C | -1.892362 | 3.409075 | 2.039982 |
| H | -2.929296 | 3.740001 | 2.018162 |
| C | 0.044226 | 6.685457 | 2.404753 |
| H | 0.384905 | 6.456062 | 3.423703 |
| H | 0.896807 | 6.566003 | 1.724910 |
| H | -0.238462 | 7.744152 | 2.383428 |
| C | -1.524288 | 6.135509 | 0.535543 |
| H | -2.381442 | 5.538192 | 0.201937 |
| H | -1.786275 | 7.197029 | 0.438312 |

| | | | |
|---|---|---|---|
| H | -0.680196 | 5.927814 | -0.132183 |
| C | -2.353529 | 6.178438 | 2.897518 |
| H | -3.279716 | 5.692691 | 2.568443 |
| H | -2.168183 | 5.906735 | 3.945193 |
| H | -2.531686 | 7.259665 | 2.862665 |
| C | -3.169506 | 0.159221 | -1.450276 |
| H | -3.125641 | 1.218944 | -1.183767 |
| C | -4.388341 | -0.451980 | -1.707729 |
| H | -5.271437 | 0.178334 | -1.633199 |
| C | -3.232406 | -2.503605 | -2.049616 |
| H | -3.213580 | -3.575925 | -2.236495 |
| C | -6.904050 | -1.526864 | -2.558046 |
| H | -7.805592 | -2.064949 | -2.872462 |
| H | -7.135456 | -1.034688 | -1.605682 |
| H | -6.708404 | -0.745248 | -3.303535 |
| C | -6.081621 | -3.564447 | -1.368495 |
| H | -6.228722 | -3.109995 | -0.380963 |
| H | -7.012790 | -4.074480 | -1.646566 |
| H | -5.294787 | -4.318762 | -1.261486 |
| C | -5.543004 | -3.215603 | -3.781282 |
| H | -5.272852 | -2.504892 | -4.573281 |
| H | -4.765822 | -3.986942 | -3.730227 |
| H | -6.473978 | -3.712019 | -4.080426 |
| C | 2.877025 | 1.338570 | -1.430738 |
| H | 2.429574 | 2.299628 | -1.168973 |
| C | 4.235424 | 1.223659 | -1.662884 |
| H | 4.822802 | 2.136098 | -1.573864 |
| C | 3.932517 | -1.098554 | -2.080157 |
| H | 4.310348 | -2.093216 | -2.307897 |
| C | 6.577330 | 0.878811 | -3.503671 |

| | | | |
|---|---|---|---|
| H | 6.005231 | 0.622342 | -4.404778 |
| H | 6.343430 | 1.916598 | -3.237437 |
| H | 7.642494 | 0.834954 | -3.761579 |
| C | 7.093562 | 0.316762 | -1.118626 |
| H | 8.163393 | 0.317249 | -1.361684 |
| H | 6.832101 | 1.328634 | -0.779459 |
| H | 6.927283 | -0.389427 | -0.298163 |
| C | 6.694039 | -1.506339 | -2.772338 |
| H | 6.515376 | -2.241490 | -1.980081 |
| H | 6.159882 | -1.834103 | -3.674328 |
| H | 7.764517 | -1.518563 | -3.008188 |
| C | 1.763879 | -1.630796 | 4.221840 |
| H | 2.050616 | -2.466177 | 3.571824 |
| H | 2.527747 | -0.851953 | 4.120706 |
| H | 1.753515 | -1.980164 | 5.260725 |
| C | -1.047408 | -2.300863 | 4.122462 |
| H | -2.072494 | -1.927787 | 4.020879 |
| H | -0.932285 | -3.141995 | 3.427698 |
| H | -0.907811 | -2.665167 | 5.146948 |
| C | -0.217524 | 0.343330 | 4.939314 |
| H | 0.508268 | 1.157063 | 4.832639 |
| H | -1.217228 | 0.756201 | 4.764037 |
| H | -0.171629 | -0.034943 | 5.967278 |
| C | 0.149377 | -0.402969 | -4.925040 |
| H | 1.149847 | -0.821476 | -4.768685 |
| H | -0.579888 | -1.206945 | -4.775305 |
| H | 0.076514 | -0.050479 | -5.960520 |
| C | 1.053707 | 2.237013 | -4.168859 |
| H | 0.966547 | 3.089181 | -3.483720 |
| H | 2.070752 | 1.840742 | -4.075767 |

| | | | |
|---|---|---|---|
| H | 0.908255 | 2.591031 | -5.196082 |
| C | -1.773757 | 1.633059 | -4.221992 |
| H | -2.552915 | 0.871670 | -4.106264 |
| H | -2.037129 | 2.480561 | -3.578083 |
| H | -1.765395 | 1.971259 | -5.264532 |

**$5_M$**

| | | | |
|---|---|---|---|
| Ir | -0.342313 | 0.081950 | -1.231907 |
| P | 0.093748 | 0.866697 | 0.943720 |
| N | 0.952423 | 1.185628 | -2.320035 |
| N | -1.527008 | 1.741928 | -1.802002 |
| N | 1.316007 | -1.207820 | -1.322229 |
| C | -1.497450 | -1.360726 | -0.709342 |
| C | -1.236972 | 1.757998 | 1.785962 |
| C | 0.494102 | -0.479020 | 2.090563 |
| C | 1.457855 | 2.027485 | 0.717031 |
| O | -1.866461 | -2.429696 | -0.492758 |
| O | -4.086524 | -1.313447 | 0.958789 |
| O | 4.887392 | -2.345783 | -1.548305 |
| S | 5.536198 | -1.197770 | -0.876572 |
| S | -4.769420 | -0.717319 | -0.206259 |
| O | -4.015150 | -0.851361 | -1.482924 |
| O | -5.334580 | 0.625505 | 0.019495 |
| C | -6.234699 | -1.806728 | -0.463512 |
| F | -6.955422 | -1.394339 | -1.513122 |
| F | -7.018291 | -1.789836 | 0.620329 |
| F | -5.843605 | -3.066496 | -0.685464 |
| O | 6.992281 | -1.150042 | -0.883865 |
| O | 4.838743 | 0.090977 | -1.141884 |
| C | 5.084856 | -1.477556 | 0.894778 |
| F | 3.762676 | -1.785148 | 0.979959 |

| | | | |
|---|---|---|---|
| F | 5.265096 | -0.368024 | 1.637511 |
| F | 5.767862 | -2.472868 | 1.455022 |
| C | 1.721258 | -0.606058 | 2.750079 |
| H | 2.523116 | 0.108489 | 2.587960 |
| C | 1.926524 | -1.666202 | 3.628506 |
| H | 2.891393 | -1.766923 | 4.121395 |
| C | 0.914885 | -2.591295 | 3.862877 |
| H | 1.084884 | -3.421907 | 4.544974 |
| C | -0.322848 | -2.447766 | 3.234359 |
| H | -1.127182 | -3.153369 | 3.429318 |
| C | -0.539295 | -1.397577 | 2.353300 |
| H | -1.521984 | -1.286597 | 1.891228 |
| C | -0.935087 | 2.731009 | 2.754107 |
| H | 0.095135 | 3.037856 | 2.929362 |
| C | -1.962795 | 3.296560 | 3.498455 |
| H | -1.728286 | 4.053528 | 4.244490 |
| C | -3.284386 | 2.894539 | 3.298072 |
| H | -4.081445 | 3.347113 | 3.885038 |
| C | -3.583161 | 1.910850 | 2.360955 |
| H | -4.600365 | 1.569812 | 2.180958 |
| C | -2.557119 | 1.343699 | 1.610398 |
| H | -2.799347 | 0.551141 | 0.907813 |
| C | 1.150451 | 3.382269 | 0.496047 |
| H | 0.121844 | 3.730642 | 0.578834 |
| C | 2.161299 | 4.283501 | 0.183150 |
| H | 1.914937 | 5.331994 | 0.025120 |
| C | 3.481825 | 3.848482 | 0.077182 |
| H | 4.271278 | 4.558940 | -0.160553 |
| C | 3.789565 | 2.501648 | 0.256213 |
| H | 4.801024 | 2.124239 | 0.129536 |

| | | | |
|---|---|---|---|
| C | 2.780451 | 1.589627 | 0.553314 |
| H | 3.041081 | 0.535313 | 0.621858 |
| C | 1.400118 | -2.550776 | -0.754226 |
| H | 0.651887 | -3.190250 | -1.231896 |
| H | 2.405299 | -2.959013 | -0.897382 |
| H | 1.174260 | -2.497793 | 0.316843 |
| C | -2.946843 | 1.972410 | -1.637223 |
| H | -3.198143 | 2.107973 | -0.579819 |
| H | -3.254091 | 2.861900 | -2.198761 |
| H | -3.500096 | 1.083981 | -1.964399 |
| C | 2.324184 | -0.745492 | -1.988112 |
| H | 3.221016 | -1.363223 | -2.119604 |
| C | 2.270459 | 0.620844 | -2.561222 |
| H | 3.071805 | 1.217350 | -2.100556 |
| H | 2.489303 | 0.585255 | -3.638728 |
| C | 0.542572 | 2.372463 | -2.724420 |
| H | 1.189624 | 3.045671 | -3.286199 |
| C | -0.813283 | 2.659484 | -2.432276 |
| H | -1.292674 | 3.579683 | -2.767183 |

**5**

| | | | |
|---|---|---|---|
| Ir | -1.002704 | 0.074646 | -1.137923 |
| P | -2.920137 | 0.018914 | -2.596524 |
| N | 0.355605 | 0.460444 | -2.612009 |
| N | -0.882870 | 2.168433 | -1.145158 |
| N | -0.483158 | -1.890131 | -1.781964 |
| C | -2.119870 | -0.196907 | 0.371538 |
| C | -3.954731 | 1.503145 | -2.434596 |
| C | -4.095765 | -1.350708 | -2.367754 |
| C | -2.291533 | -0.113903 | -4.289817 |
| O | -2.852264 | -0.299451 | 1.256295 |

| | | | |
|---|---|---|---|
| S | -3.525646 | 3.672448 | 2.468197 |
| O | -2.434429 | 3.876647 | 3.444928 |
| O | -3.680758 | 2.247037 | 2.054734 |
| O | -3.595415 | 4.633389 | 1.358611 |
| C | -5.063056 | 3.984269 | 3.435640 |
| F | -5.097881 | 5.244694 | 3.875249 |
| F | -6.142079 | 3.776403 | 2.662995 |
| F | -5.137030 | 3.163253 | 4.489675 |
| S | -2.495039 | -4.477631 | 1.565213 |
| O | -1.237516 | -5.038330 | 2.101401 |
| O | -2.950789 | -5.000885 | 0.272095 |
| O | -2.570530 | -2.989461 | 1.680480 |
| C | -3.781935 | -5.046230 | 2.756025 |
| F | -4.987616 | -4.580861 | 2.393564 |
| F | -3.835226 | -6.378275 | 2.788590 |
| F | -3.516797 | -4.601683 | 3.993616 |
| Ir | 1.053573 | 0.017566 | 1.154346 |
| P | 2.973378 | -0.031036 | 2.613270 |
| N | -0.355570 | 0.001866 | 2.646046 |
| N | 0.737709 | -2.039420 | 1.500964 |
| N | 0.757955 | 2.099392 | 1.511064 |
| C | 2.166488 | 0.065840 | -0.377826 |
| C | 4.179688 | -1.361843 | 2.328316 |
| C | 3.982661 | 1.480465 | 2.579134 |
| C | 2.327053 | -0.262632 | 4.287626 |
| O | 2.893901 | 0.158710 | -1.267878 |
| O | 2.888166 | -2.495660 | -1.961507 |
| O | 3.216770 | 2.942757 | -1.802729 |
| S | 2.757541 | 4.245263 | -2.356338 |
| S | 2.992962 | -3.971139 | -2.175513 |

| | | | |
|---|---|---|---|
| O | 1.865519 | -4.522904 | -2.955551 |
| O | 3.404459 | -4.723101 | -0.985040 |
| C | 4.432938 | -4.100760 | -3.318863 |
| F | 4.613307 | -5.360315 | -3.719099 |
| F | 5.552586 | -3.686593 | -2.703767 |
| F | 4.246441 | -3.333898 | -4.402608 |
| O | 2.159849 | 4.144104 | -3.701740 |
| O | 2.005404 | 5.079708 | -1.399780 |
| C | 4.326778 | 5.174083 | -2.629756 |
| F | 5.013731 | 5.294130 | -1.477381 |
| F | 4.070942 | 6.399738 | -3.090152 |
| F | 5.104413 | 4.538503 | -3.511201 |
| C | 4.490212 | 2.061535 | 3.749552 |
| H | 4.244928 | 1.643622 | 4.724678 |
| C | 5.322673 | 3.173881 | 3.667475 |
| H | 5.707346 | 3.621940 | 4.581570 |
| C | 5.660443 | 3.710333 | 2.426990 |
| H | 6.299807 | 4.588826 | 2.371273 |
| C | 5.178625 | 3.123468 | 1.259188 |
| H | 5.412657 | 3.536618 | 0.281400 |
| C | 4.351530 | 2.009396 | 1.334362 |
| H | 3.979476 | 1.588891 | 0.402772 |
| C | 5.053718 | -1.682493 | 3.381358 |
| H | 4.944825 | -1.199013 | 4.352198 |
| C | 6.059213 | -2.620478 | 3.189291 |
| H | 6.726084 | -2.868449 | 4.012802 |
| C | 6.209831 | -3.240869 | 1.949259 |
| H | 6.992791 | -3.982415 | 1.804881 |
| C | 5.358316 | -2.918213 | 0.898574 |
| H | 5.446005 | -3.409942 | -0.066266 |

| | | | |
|---|---|---|---|
| C | 4.348206 | -1.975869 | 1.082474 |
| H | 3.694460 | -1.766046 | 0.237783 |
| C | 2.160368 | -1.559755 | 4.794204 |
| H | 2.567787 | -2.412025 | 4.250245 |
| C | 1.484056 | -1.763461 | 5.994924 |
| H | 1.366125 | -2.774365 | 6.379559 |
| C | 0.960108 | -0.680557 | 6.695931 |
| H | 0.433736 | -0.841401 | 7.634401 |
| C | 1.109295 | 0.611496 | 6.192377 |
| H | 0.699935 | 1.461058 | 6.735977 |
| C | 1.779822 | 0.821382 | 4.992598 |
| H | 1.879528 | 1.834187 | 4.598990 |
| C | -5.093813 | -1.492457 | -3.348608 |
| H | -5.091033 | -0.848712 | -4.228968 |
| C | -6.085574 | -2.451334 | -3.196480 |
| H | -6.850525 | -2.559082 | -3.962871 |
| C | -6.102202 | -3.267647 | -2.064837 |
| H | -6.882122 | -4.017355 | -1.948485 |
| C | -5.123469 | -3.128387 | -1.088163 |
| H | -5.109308 | -3.769345 | -0.210754 |
| C | -4.120186 | -2.172101 | -1.237079 |
| H | -3.367354 | -2.110082 | -0.455287 |
| C | -2.194868 | -1.376932 | -4.893672 |
| H | -2.638644 | -2.246478 | -4.409101 |
| C | -1.539954 | -1.522865 | -6.113644 |
| H | -1.473086 | -2.506908 | -6.572568 |
| C | -0.967496 | -0.416860 | -6.738363 |
| H | -0.453268 | -0.533926 | -7.689938 |
| C | -1.052120 | 0.841898 | -6.142172 |
| H | -0.603099 | 1.708458 | -6.623530 |

| | | | |
|---|---|---|---|
| C | -1.704936 | 0.994647 | -4.923472 |
| H | -1.751957 | 1.978699 | -4.457456 |
| C | -4.335231 | 2.300907 | -3.521374 |
| H | -4.005469 | 2.059150 | -4.529935 |
| C | -5.163066 | 3.401181 | -3.313227 |
| H | -5.450162 | 4.020482 | -4.160689 |
| C | -5.620476 | 3.708063 | -2.034041 |
| H | -6.252813 | 4.579276 | -1.877200 |
| C | -5.267266 | 2.903114 | -0.952800 |
| H | -5.600686 | 3.139935 | 0.054622 |
| C | -4.441587 | 1.803998 | -1.152823 |
| H | -4.166103 | 1.205516 | -0.285800 |
| C | 1.472367 | 3.219978 | 0.898856 |
| H | 1.865451 | 2.941239 | -0.079865 |
| H | 0.820789 | 4.092448 | 0.799750 |
| H | 2.322305 | 3.501007 | 1.527923 |
| C | 1.379483 | -3.182233 | 0.864701 |
| H | 2.331968 | -3.402020 | 1.360342 |
| H | 0.735166 | -4.066389 | 0.936606 |
| H | 1.611942 | -2.935945 | -0.172203 |
| C | -1.678804 | 3.112095 | -0.371769 |
| H | -2.153510 | 2.608711 | 0.471298 |
| H | -1.066334 | 3.954109 | -0.031980 |
| H | -2.482233 | 3.523465 | -0.992668 |
| C | -0.989983 | -3.161196 | -1.270143 |
| H | -0.287726 | -3.971308 | -1.498471 |
| H | -1.148853 | -3.070132 | -0.195033 |
| H | -1.959949 | -3.387966 | -1.723297 |
| C | -0.142968 | 2.360066 | 2.396832 |
| H | -0.402318 | 3.392936 | 2.648221 |

| | | | |
|---|---|---|---|
| C | -0.911817 | 1.282524 | 3.054863 |
| H | -0.913144 | 1.403940 | 4.146767 |
| H | -1.978745 | 1.375759 | 2.754390 |
| C | -0.724418 | -1.155441 | 3.109121 |
| H | -1.487452 | -1.263139 | 3.879219 |
| C | -0.096562 | -2.281981 | 2.480174 |
| H | -0.318990 | -3.313986 | 2.761380 |
| C | -0.043071 | 2.647067 | -2.032801 |
| H | 0.114744 | 3.723450 | -2.130507 |
| C | 0.655961 | 1.705103 | -2.856529 |
| H | 1.374319 | 2.024890 | -3.611992 |
| C | 0.890064 | -0.673084 | -3.352585 |
| H | 1.987918 | -0.706487 | -3.297273 |
| H | 0.617095 | -0.589555 | -4.417817 |
| C | 0.346195 | -1.921237 | -2.770330 |
| H | 0.679323 | -2.887106 | -3.169440 |

**The Previous Ir(II)-Ir(II) Complexes**

**$A_M$**

| | | | |
|---|---|---|---|
| Ir | -2.296739 | -0.915871 | 0.060180 |
| Fe | 3.829474 | -0.409109 | -0.037933 |
| Cl | -3.420947 | -0.843531 | -2.060764 |
| Cl | -4.347355 | -0.317378 | 1.151857 |
| C | 3.949599 | 0.811094 | 1.536265 |
| H | 4.421746 | 1.787836 | 1.556829 |
| C | 4.260313 | 0.652206 | -1.685496 |
| H | 4.275416 | 1.735104 | -1.757205 |
| C | 4.597366 | -0.422939 | 1.822007 |
| H | 5.643720 | -0.543179 | 2.080917 |
| C | 4.905977 | -1.532237 | -1.319381 |

| | | | |
|---|---|---|---|
| H | 5.496052 | -2.402055 | -1.051609 |
| C | 2.404765 | -0.894655 | 1.282583 |
| H | 1.487504 | -1.424658 | 1.056563 |
| C | 3.128455 | -0.189453 | -1.911636 |
| H | 2.125468 | 0.139984 | -2.164359 |
| C | 0.923609 | 3.735197 | 0.205810 |
| H | 1.139203 | 4.796927 | 0.090472 |
| C | 3.645613 | -1.472421 | 1.664008 |
| H | 3.843083 | -2.532670 | 1.777450 |
| C | 2.573910 | 0.530068 | 1.215096 |
| C | 5.360297 | -0.180473 | -1.321648 |
| H | 6.357177 | 0.156752 | -1.058373 |
| C | -1.439425 | 4.067947 | -0.523554 |
| H | -1.276296 | 5.137389 | -0.655065 |
| C | -2.665328 | 3.497922 | -0.778131 |
| H | -3.512177 | 4.087014 | -1.117313 |
| C | 1.885038 | 2.860671 | 0.628685 |
| H | 2.889044 | 3.208277 | 0.859425 |
| C | 3.528387 | -1.536858 | -1.686169 |
| H | 2.882729 | -2.406106 | -1.739334 |
| C | -2.820305 | 2.114687 | -0.592382 |
| H | -3.768699 | 1.613531 | -0.780435 |
| N | -1.831853 | 1.332660 | -0.179585 |
| C | -0.599591 | 1.857496 | 0.085286 |
| C | -0.376116 | 3.257251 | -0.085207 |
| N | 0.349386 | 1.001380 | 0.504367 |
| C | 1.565399 | 1.479711 | 0.772091 |
| O | 0.149152 | -1.907554 | -1.437152 |
| O | -1.071397 | -1.200576 | 2.825566 |
| C | -1.532849 | -1.093224 | 1.774065 |

| | | | |
|---|---|---|---|
| C | -0.779289 | -1.530823 | -0.861454 |
| A | | | |
| Ir | 0.899150 | 1.028741 | -0.238096 |
| Fe | 6.454470 | -1.385091 | 0.348419 |
| Cl | 0.175208 | 1.783691 | -2.417920 |
| Cl | -0.782926 | 2.498755 | 0.758073 |
| C | 6.913027 | -0.332710 | 1.978554 |
| H | 7.779284 | 0.307368 | 2.111853 |
| C | 7.629968 | -0.742694 | -1.145096 |
| H | 8.228347 | 0.162882 | -1.138867 |
| C | 6.854105 | -1.719950 | 2.290745 |
| H | 7.671998 | -2.315490 | 2.681874 |
| C | 6.949822 | -2.927401 | -0.846098 |
| H | 6.936522 | -3.973564 | -0.559965 |
| C | 4.796286 | -1.110341 | 1.441389 |
| H | 3.777788 | -1.159901 | 1.071917 |
| C | 6.275463 | -0.852118 | -1.585579 |
| H | 5.653514 | -0.040526 | -1.949852 |
| C | 5.800374 | 3.663364 | 0.438957 |
| H | 6.500231 | 4.496924 | 0.387542 |
| C | 5.551813 | -2.196048 | 1.958236 |
| H | 5.204507 | -3.219633 | 2.044362 |
| C | 5.626342 | 0.063022 | 1.465086 |
| C | 8.047712 | -2.029654 | -0.690394 |
| H | 9.016945 | -2.275173 | -0.269265 |
| C | 3.938970 | 5.082656 | -0.435448 |
| H | 4.592201 | 5.952718 | -0.500099 |
| C | 2.613841 | 5.157628 | -0.799043 |
| H | 2.171344 | 6.081586 | -1.159549 |
| C | 6.196650 | 2.436736 | 0.895153 |

| | | | |
|---|---|---|---|
| H | 7.222138 | 2.266917 | 1.214904 |
| C | 5.856163 | -2.199694 | -1.400719 |
| H | 4.862806 | -2.586733 | -1.597474 |
| C | 1.817068 | 4.004923 | -0.706163 |
| H | 0.765983 | 4.008200 | -0.990801 |
| N | 2.284179 | 2.840947 | -0.275894 |
| C | 3.591813 | 2.731523 | 0.110689 |
| C | 4.458946 | 3.861502 | 0.031148 |
| N | 3.987738 | 1.530876 | 0.564810 |
| C | 5.251019 | 1.372581 | 0.958991 |
| O | 2.944861 | -0.730987 | -1.616718 |
| O | 1.718177 | 0.321858 | 2.598317 |
| C | 1.420877 | 0.548397 | 1.509428 |
| C | 2.139230 | -0.099675 | -1.091613 |
| Ir | -0.899248 | -1.028780 | -0.238093 |
| Fe | -6.454271 | 1.385126 | 0.348546 |
| Cl | -0.175386 | -1.783653 | -2.417968 |
| Cl | 0.782825 | -2.498856 | 0.757965 |
| C | -6.912995 | 0.332904 | 1.978610 |
| H | -7.779293 | -0.307114 | 2.111928 |
| C | -7.629597 | 0.742711 | -1.145248 |
| H | -8.227960 | -0.162876 | -1.139063 |
| C | -6.853946 | 1.720195 | 2.290738 |
| H | -7.671807 | 2.315815 | 2.681812 |
| C | -6.949527 | 2.927377 | -0.846265 |
| H | -6.936290 | 3.973563 | -0.560213 |
| C | -4.796137 | 1.110356 | 1.441384 |
| H | -3.777659 | 1.159840 | 1.071843 |
| C | -6.275062 | 0.852103 | -1.585529 |
| H | -5.653041 | 0.040509 | -1.949663 |

| | | | |
|---|---|---|---|
| C | -5.800511 | -3.663298 | 0.438999 |
| H | -6.500401 | -4.496829 | 0.387569 |
| C | -5.551587 | 2.196194 | 1.958163 |
| H | -5.204234 | 3.219770 | 2.044201 |
| C | -5.626313 | -0.062963 | 1.465151 |
| C | -8.047406 | 2.029649 | -0.690662 |
| H | -9.016718 | 2.275187 | -0.269725 |
| C | -3.939150 | -5.082647 | -0.435427 |
| H | -4.592403 | -5.952691 | -0.500084 |
| C | -2.614025 | -5.157651 | -0.799035 |
| H | -2.171558 | -6.081619 | -1.159553 |
| C | -6.196746 | -2.436659 | 0.895207 |
| H | -7.222231 | -2.266794 | 1.214945 |
| C | -5.855817 | 2.199661 | -1.400698 |
| H | -4.862433 | 2.586707 | -1.597340 |
| C | -1.817218 | -4.004966 | -0.706166 |
| H | -0.766138 | -4.008263 | -0.990826 |
| N | -2.284298 | -2.840978 | -0.275892 |
| C | -3.591920 | -2.731535 | 0.110721 |
| C | -4.459089 | -3.861483 | 0.031182 |
| N | -3.987802 | -1.530887 | 0.564870 |
| C | -5.251072 | -1.372545 | 0.959061 |
| O | -2.944960 | 0.731025 | -1.616623 |
| O | -1.718077 | -0.322036 | 2.598413 |
| C | -1.420872 | -0.548510 | 1.509484 |
| C | -2.139336 | 0.099690 | -1.091533 |

**$B_M$**

| | | | |
|---|---|---|---|
| Ir | -0.956683 | -0.666587 | -0.380121 |
| S | 1.953947 | -0.411544 | 1.210855 |
| F | 2.402072 | 0.473365 | -1.231180 |

| | | | |
|---|---|---|---|
| F | 2.465645 | 2.007846 | 0.303280 |
| F | 4.114380 | 0.609394 | 0.097589 |
| O | -1.717708 | -3.200493 | 1.100695 |
| O | 0.820336 | -2.153331 | -2.345075 |
| O | -2.273394 | 0.398380 | 0.822033 |
| O | -0.549723 | 1.085100 | -1.409810 |
| O | 0.514864 | 0.125502 | 1.147149 |
| O | 2.077502 | -1.736438 | 0.608084 |
| O | 2.546341 | -0.129196 | 2.507754 |
| C | -1.416080 | -2.251240 | 0.523929 |
| C | 0.157599 | -1.611048 | -1.578773 |
| C | -2.270162 | 1.677448 | 0.947844 |
| C | -1.542187 | 2.581542 | 0.168072 |
| H | -1.631822 | 3.633084 | 0.423416 |
| C | -0.774080 | 2.266849 | -0.955508 |
| C | -3.156841 | 2.182165 | 2.041072 |
| H | -4.160229 | 1.756270 | 1.937365 |
| H | -3.225555 | 3.272535 | 2.053798 |
| H | -2.768054 | 1.840641 | 3.007440 |
| C | -0.141528 | 3.353242 | -1.764245 |
| H | 0.946394 | 3.216645 | -1.758641 |
| H | -0.379027 | 4.350023 | -1.384968 |
| H | -0.462107 | 3.278689 | -2.809037 |
| C | 2.792815 | 0.739776 | 0.026354 |

**B**

| | | | |
|---|---|---|---|
| Ir | -1.318146 | 0.062101 | 0.381266 |
| Ir | 1.310711 | -0.062269 | -0.377420 |
| S | -4.493492 | 0.749463 | 0.898973 |
| S | 4.488027 | -0.749596 | -0.890336 |
| F | -4.211586 | 0.668793 | -1.722601 |

| | | | |
|---|---|---|---|
| F | -5.496452 | -0.881596 | -0.916213 |
| F | -6.214001 | 1.167174 | -1.044963 |
| F | 4.204235 | -0.653914 | 1.730641 |
| F | 5.491152 | 0.890575 | 0.916329 |
| F | 6.206724 | -1.158190 | 1.057397 |
| O | -0.686261 | 1.756709 | 2.814807 |
| O | -1.747668 | 2.501707 | -1.382312 |
| O | -1.078211 | -1.641213 | 1.557055 |
| O | -1.869590 | -1.125872 | -1.233190 |
| O | 0.684797 | -1.771566 | -2.802046 |
| O | 1.741074 | -2.490161 | 1.401640 |
| O | 1.857637 | 1.138318 | 1.229673 |
| O | 1.076261 | 1.633066 | -1.567025 |
| O | -3.405959 | -0.332220 | 0.996945 |
| O | -3.917954 | 2.092589 | 0.817255 |
| O | -5.593645 | 0.469166 | 1.803452 |
| O | 3.399970 | 0.330837 | -0.993254 |
| O | 3.913482 | -2.092761 | -0.802680 |
| O | 5.588222 | -0.472684 | -1.795888 |
| C | -0.891260 | 1.127027 | 1.875939 |
| C | -1.572228 | 1.592375 | -0.705507 |
| C | -1.506466 | -2.808627 | 1.226937 |
| C | -2.043798 | -3.178324 | -0.010022 |
| H | -2.367640 | -4.209946 | -0.107916 |
| C | -2.198273 | -2.366249 | -1.136496 |
| C | -1.372556 | -3.824125 | 2.315072 |
| H | -0.336061 | -3.858055 | 2.668313 |
| H | -1.678389 | -4.822358 | 1.993726 |
| H | -1.987551 | -3.520237 | 3.169953 |
| C | -2.775393 | -2.927556 | -2.395097 |

| | | | |
|---|---|---|---|
| H | -2.072227 | -2.789285 | -3.224172 |
| H | -3.681150 | -2.366174 | -2.653126 |
| H | -3.023512 | -3.987599 | -2.305060 |
| C | 0.887374 | -1.136547 | -1.866263 |
| C | 1.564943 | -1.584715 | 0.719484 |
| C | 2.201501 | 2.373740 | 1.119801 |
| C | 2.072136 | 3.171071 | -0.019961 |
| H | 2.416912 | 4.197080 | 0.064457 |
| C | 1.537271 | 2.793133 | -1.255546 |
| C | 2.773587 | 2.944638 | 2.376448 |
| H | 3.676702 | 2.383000 | 2.643648 |
| H | 2.066099 | 2.815629 | 3.203185 |
| H | 3.025702 | 4.003024 | 2.278080 |
| C | 1.459066 | 3.787928 | -2.367648 |
| H | 2.156244 | 3.489164 | -3.159897 |
| H | 1.711134 | 4.799593 | -2.041456 |
| H | 0.458067 | 3.784304 | -2.810786 |
| C | -5.152694 | 0.405388 | -0.798203 |
| C | 5.146419 | -0.396819 | 0.805391 |

**$C_M$**

| | | | |
|---|---|---|---|
| Ir | 0.008228 | -0.000104 | -0.383977 |
| N | -1.411621 | -1.419323 | -0.432401 |
| N | -3.379286 | -0.000350 | -0.437559 |
| N | -1.411801 | 1.418903 | -0.432612 |
| N | 0.008258 | 3.384621 | -0.414971 |
| N | 1.428556 | 1.418727 | -0.413862 |
| N | 3.396300 | 0.000094 | -0.402631 |
| N | 1.428711 | -1.418786 | -0.413654 |
| N | 0.008678 | -3.384864 | -0.414524 |
| C | -2.765784 | -1.178714 | -0.441277 |

| | | | |
|---|---|---|---|
| C | -2.765940 | 1.178097 | -0.441492 |
| C | -3.448866 | 2.460403 | -0.445292 |
| C | -4.798132 | 2.798720 | -0.454337 |
| H | -5.562310 | 2.023646 | -0.464366 |
| C | -5.131396 | 4.150580 | -0.452516 |
| H | -6.179016 | 4.445973 | -0.459525 |
| C | -4.139006 | 5.141350 | -0.443386 |
| H | -4.432853 | 6.189413 | -0.443170 |
| C | -2.787646 | 4.806132 | -0.436274 |
| H | -2.011365 | 5.568993 | -0.430706 |
| C | -2.451031 | 3.456558 | -0.436460 |
| C | -1.169127 | 2.772437 | -0.427543 |
| C | 1.185643 | 2.772412 | -0.416006 |
| C | 2.467488 | 3.456705 | -0.417675 |
| C | 2.803831 | 4.806344 | -0.421980 |
| H | 2.027410 | 5.569082 | -0.426014 |
| C | 4.155172 | 5.141754 | -0.422173 |
| H | 4.448833 | 6.189860 | -0.425613 |
| C | 5.147731 | 4.151122 | -0.419849 |
| H | 6.195311 | 4.446687 | -0.421951 |
| C | 4.814702 | 2.799201 | -0.417097 |
| H | 5.578997 | 2.024202 | -0.418442 |
| C | 3.465470 | 2.460709 | -0.415150 |
| C | 2.782803 | 1.178225 | -0.411900 |
| C | 2.782930 | -1.178114 | -0.411828 |
| C | 3.465773 | -2.460490 | -0.415070 |
| C | 4.815052 | -2.798802 | -0.417130 |
| H | 5.579275 | -2.023727 | -0.418554 |
| C | 5.148265 | -4.150685 | -0.419894 |
| H | 6.195890 | -4.446107 | -0.422057 |

| | | | |
|---|---|---|---|
| C | 4.155838 | -5.141454 | -0.422134 |
| H | 4.449650 | -6.189522 | -0.425584 |
| C | 2.804450 | -4.806215 | -0.421851 |
| H | 2.028122 | -5.569057 | -0.425782 |
| C | 2.467917 | -3.456619 | -0.417498 |
| C | 1.185983 | -2.772502 | -0.415689 |
| C | -1.168784 | -2.772809 | -0.427117 |
| C | -2.450572 | -3.457088 | -0.435821 |
| C | -2.787001 | -4.806713 | -0.435355 |
| H | -2.010603 | -5.569475 | -0.429712 |
| C | -4.138312 | -5.142115 | -0.442253 |
| H | -4.431999 | -6.190220 | -0.441797 |
| C | -5.130837 | -4.151472 | -0.451506 |
| H | -6.178411 | -4.447001 | -0.458372 |
| C | -4.797739 | -2.799591 | -0.453612 |
| H | -5.561994 | -2.024599 | -0.463747 |
| C | -3.448544 | -2.461078 | -0.444774 |
| N | -0.033468 | 0.000107 | 1.995347 |
| C | -1.203701 | 0.000378 | 2.646270 |
| H | -2.097043 | -0.000021 | 2.020471 |
| C | -1.282634 | 0.001191 | 4.032925 |
| H | -2.254672 | 0.001417 | 4.520043 |
| C | -0.102346 | 0.001725 | 4.769829 |
| H | -0.129699 | 0.002377 | 5.857605 |
| C | 1.113207 | 0.001448 | 4.092756 |
| H | 2.059611 | 0.001830 | 4.627952 |
| C | 1.102933 | 0.000637 | 2.703573 |
| H | 2.026247 | 0.000374 | 2.123149 |

**C**

| | | | |
|---|---|---|---|
| Ir | -0.000048 | -0.000091 | 1.347841 |

| | | | |
|---|---|---|---|
| N | 1.837136 | 0.810253 | 1.434310 |
| N | 3.162448 | -1.221627 | 1.521625 |
| N | 0.815209 | -1.834197 | 1.431007 |
| N | -1.217586 | -3.153000 | 1.566966 |
| N | -1.837227 | -0.810434 | 1.434277 |
| N | -3.162541 | 1.221411 | 1.521669 |
| N | -0.815307 | 1.833995 | 1.431100 |
| N | 1.217490 | 3.152818 | 1.567178 |
| C | 3.012112 | 0.098028 | 1.506829 |
| C | 2.163891 | -2.096853 | 1.495715 |
| C | 2.336525 | -3.536655 | 1.566740 |
| C | 3.473763 | -4.336794 | 1.590280 |
| H | 4.465595 | -3.888279 | 1.565112 |
| C | 3.296487 | -5.717324 | 1.623219 |
| H | 4.167206 | -6.371027 | 1.633873 |
| C | 2.013030 | -6.283447 | 1.638718 |
| H | 1.909468 | -7.367156 | 1.662117 |
| C | 0.872857 | -5.483845 | 1.622768 |
| H | -0.127767 | -5.913145 | 1.624396 |
| C | 1.047287 | -4.104618 | 1.583876 |
| C | 0.100217 | -3.006070 | 1.522725 |
| C | -2.094195 | -2.157693 | 1.534954 |
| C | -3.533147 | -2.333472 | 1.614138 |
| C | -4.328355 | -3.473083 | 1.667815 |
| H | -3.874633 | -4.462858 | 1.668516 |
| C | -5.709634 | -3.301325 | 1.696834 |
| H | -6.359454 | -4.174345 | 1.730826 |
| C | -6.281704 | -2.020459 | 1.677702 |
| H | -7.365869 | -1.921166 | 1.698748 |
| C | -5.487305 | -0.877555 | 1.630329 |

| | | | |
|---|---|---|---|
| H | -5.921693 | 0.120548 | 1.604577 |
| C | -4.107248 | -1.046916 | 1.594872 |
| C | -3.012191 | -0.098218 | 1.506863 |
| C | -2.163966 | 2.096650 | 1.495760 |
| C | -2.336628 | 3.536438 | 1.566786 |
| C | -3.473875 | 4.336546 | 1.590312 |
| H | -4.465691 | 3.887999 | 1.565073 |
| C | -3.296637 | 5.717066 | 1.623345 |
| H | -4.167371 | 6.370745 | 1.633986 |
| C | -2.013195 | 6.283218 | 1.638974 |
| H | -1.909655 | 7.366928 | 1.662478 |
| C | -0.873005 | 5.483648 | 1.623034 |
| H | 0.127618 | 5.912978 | 1.624782 |
| C | -1.047394 | 4.104412 | 1.584031 |
| C | -0.100303 | 3.005889 | 1.522860 |
| C | 2.094117 | 2.157511 | 1.535084 |
| C | 3.533065 | 2.333273 | 1.614329 |
| C | 4.328310 | 3.472862 | 1.668190 |
| H | 3.874613 | 4.462663 | 1.669056 |
| C | 5.709587 | 3.301078 | 1.697160 |
| H | 6.359422 | 4.174088 | 1.731289 |
| C | 6.281647 | 2.020201 | 1.677795 |
| H | 7.365815 | 1.920901 | 1.698800 |
| C | 5.487223 | 0.877323 | 1.630263 |
| H | 5.921593 | -0.120790 | 1.604334 |
| C | 4.107166 | 1.046705 | 1.594864 |
| N | -0.000026 | -0.000174 | 3.717282 |
| C | 1.074412 | -0.416903 | 4.401725 |
| H | 1.923821 | -0.745939 | 3.803456 |
| C | 1.116401 | -0.432763 | 5.789860 |

| | | | |
|---|---|---|---|
| H | 2.012006 | -0.779194 | 6.299570 |
| C | 0.000043 | 0.000023 | 6.498116 |
| H | 0.000072 | 0.000097 | 7.586342 |
| C | -1.116355 | 0.432703 | 5.789862 |
| H | -2.011933 | 0.779215 | 6.299566 |
| C | -1.074431 | 0.416649 | 4.401727 |
| H | -1.923868 | 0.745602 | 3.803451 |
| Ir | 0.000030 | 0.000105 | -1.347725 |
| N | -0.815884 | -1.833696 | -1.430979 |
| N | 1.216425 | -3.153236 | -1.566994 |
| N | 1.836896 | -0.810897 | -1.434202 |
| N | 3.162961 | 1.220499 | -1.521513 |
| N | 0.815919 | 1.833890 | -1.430930 |
| N | -1.216384 | 3.153458 | -1.566912 |
| N | -1.836853 | 0.811100 | -1.434326 |
| N | -3.162885 | -1.220281 | -1.521697 |
| C | -0.101320 | -3.005840 | -1.522685 |
| C | 2.093397 | -2.158245 | -1.535018 |
| C | 3.532276 | -2.334532 | -1.614336 |
| C | 4.327100 | -3.474414 | -1.668210 |
| H | 3.873043 | -4.464037 | -1.669008 |
| C | 5.708441 | -3.303132 | -1.697317 |
| H | 6.357973 | -4.176363 | -1.731465 |
| C | 6.280951 | -2.022460 | -1.678067 |
| H | 7.365151 | -1.923536 | -1.699197 |
| C | 5.486944 | -0.879295 | -1.630494 |
| H | 5.921672 | 0.118665 | -1.604602 |
| C | 4.106825 | -1.048170 | -1.594960 |
| C | 3.012118 | -0.099102 | -1.506802 |
| C | 2.164697 | 2.096076 | -1.495531 |

| | | | |
|---|---|---|---|
| C | 2.337866 | 3.535814 | -1.566509 |
| C | 3.475404 | 4.335522 | -1.590030 |
| H | 4.467064 | 3.886609 | -1.564966 |
| C | 3.298662 | 5.716132 | -1.622847 |
| H | 4.169639 | 6.369503 | -1.633472 |
| C | 2.015414 | 6.282728 | -1.638255 |
| H | 1.912237 | 7.366482 | -1.661553 |
| C | 0.874931 | 5.483549 | -1.622382 |
| H | -0.125533 | 5.913239 | -1.623981 |
| C | 1.048831 | 4.104247 | -1.583617 |
| C | 0.101348 | 3.006056 | -1.522556 |
| C | -2.093342 | 2.158426 | -1.535017 |
| C | -3.532223 | 2.334732 | -1.614371 |
| C | -4.327038 | 3.474619 | -1.668242 |
| H | -3.872965 | 4.464239 | -1.668990 |
| C | -5.708375 | 3.303350 | -1.697366 |
| H | -6.357894 | 4.176589 | -1.731487 |
| C | -6.280889 | 2.022678 | -1.678161 |
| H | -7.365086 | 1.923766 | -1.699312 |
| C | -5.486891 | 0.879515 | -1.630595 |
| H | -5.921644 | -0.118427 | -1.604761 |
| C | -4.106777 | 1.048372 | -1.595038 |
| C | -3.012082 | 0.099294 | -1.506929 |
| C | -2.164649 | -2.095867 | -1.495706 |
| C | -2.337818 | -3.535600 | -1.566701 |
| C | -3.475351 | -4.335313 | -1.590309 |
| H | -4.467009 | -3.886418 | -1.565237 |
| C | -3.298605 | -5.715909 | -1.623214 |
| H | -4.169563 | -6.369293 | -1.633902 |
| C | -2.015349 | -6.282501 | -1.638630 |

| | | | |
|---|---|---|---|
| H | -1.912191 | -7.366247 | -1.662010 |
| C | -0.874886 | -5.483326 | -1.622662 |
| H | 0.125576 | -5.913010 | -1.624261 |
| C | -1.048789 | -4.104023 | -1.583800 |
| N | 0.000130 | 0.000188 | -3.717717 |
| C | -1.074395 | -0.416309 | -4.402121 |
| H | -1.923943 | -0.744952 | -3.803858 |
| C | -1.116330 | -0.432392 | -5.790254 |
| H | -2.012001 | -0.778625 | -6.299965 |
| C | 0.000186 | -0.000035 | -6.498535 |
| H | 0.000182 | -0.000086 | -7.586757 |
| C | 1.116674 | 0.432381 | -5.790298 |
| H | 2.012386 | 0.778522 | -6.300003 |
| C | 1.074690 | 0.416526 | -4.402154 |
| H | 1.924229 | 0.745238 | -3.803905 |

**$D_M$**

| | | | |
|---|---|---|---|
| Ir | -0.465535 | 0.158695 | -0.543584 |
| C | -2.315204 | 0.073515 | -0.556784 |
| O | -3.482498 | 0.039900 | -0.522141 |
| C | 0.538604 | -1.271294 | 0.961537 |
| C | 0.041567 | -2.609011 | 1.389548 |
| C | 1.476896 | -1.022209 | -0.097649 |
| C | 2.064879 | -2.054503 | -0.991611 |
| C | 1.737772 | 0.389312 | -0.146502 |
| C | 2.718918 | 1.070654 | -1.039316 |
| C | 1.006158 | 1.015154 | 0.929499 |
| C | 1.090348 | 2.442955 | 1.334637 |
| C | 0.253046 | -0.017338 | 1.597822 |
| C | -0.605137 | 0.181497 | 2.797980 |
| H | 0.732324 | -3.083049 | 2.101742 |

| | | | |
|---|---|---|---|
| H | -0.934781 | -2.539659 | 1.881324 |
| H | -0.069292 | -3.292343 | 0.540427 |
| H | 2.956131 | -2.510466 | -0.535229 |
| H | 1.355849 | -2.864651 | -1.195283 |
| H | 2.373130 | -1.631785 | -1.953621 |
| H | 3.720137 | 1.094151 | -0.586239 |
| H | 2.807789 | 0.563631 | -2.006040 |
| H | 2.425451 | 2.105611 | -1.243502 |
| H | 1.886601 | 2.598114 | 2.078075 |
| H | 1.309816 | 3.091283 | 0.479917 |
| H | 0.153309 | 2.793019 | 1.780592 |
| H | -0.013248 | 0.110134 | 3.721467 |
| H | -1.085772 | 1.165889 | 2.792881 |
| H | -1.397801 | -0.571960 | 2.859990 |
| H | -0.560541 | 1.240995 | -1.758342 |

**D**

| | | | |
|---|---|---|---|
| Ir | 1.359432 | 0.232094 | -0.827670 |
| N | 1.219971 | 1.370689 | 0.877240 |
| C | 1.638310 | 0.813089 | 2.052803 |
| N | 1.619484 | 1.652561 | 3.083162 |
| C | 1.163840 | 2.819168 | 2.552727 |
| C | 0.916277 | 2.672481 | 1.177104 |
| C | 0.998219 | 4.004618 | 3.311245 |
| C | 0.472862 | 3.578523 | 0.198774 |
| N | 0.881651 | 5.004881 | 3.902983 |
| N | 0.143166 | 4.303985 | -0.660488 |
| N | 2.060927 | -1.092221 | 0.704223 |
| C | 2.118034 | -0.541349 | 1.953486 |
| N | 2.728043 | -1.290272 | 2.867742 |
| C | 3.119971 | -2.386401 | 2.171564 |

| | | | |
|---|---|---|---|
| C | 2.733636 | -2.281507 | 0.824649 |
| C | 3.828684 | -3.468403 | 2.751054 |
| C | 3.009520 | -3.156743 | -0.238604 |
| N | 4.433815 | -4.367631 | 3.186456 |
| N | 3.284486 | -3.884370 | -1.114847 |
| P | 3.713470 | 0.813421 | -0.952435 |
| O | 4.403563 | -0.365471 | -1.784330 |
| O | 4.242940 | 2.187352 | -1.621643 |
| O | 4.516774 | 0.724361 | 0.443096 |
| C | 5.820332 | -0.688772 | -1.685533 |
| C | 6.076584 | -1.818735 | -2.642745 |
| C | 3.414859 | 3.348244 | -1.894817 |
| C | 3.656473 | 4.442053 | -0.891277 |
| C | 4.632831 | 1.876525 | 1.327924 |
| C | 5.135407 | 1.392095 | 2.657209 |
| C | 0.565272 | 1.408008 | -2.053896 |
| O | 0.085182 | 2.036905 | -2.898215 |
| N | 1.580919 | -1.176465 | -2.299511 |
| C | 1.822695 | -2.028502 | -3.046447 |
| C | 2.142944 | -3.123107 | -3.923759 |
| Ir | -1.324713 | -0.701795 | -0.468813 |
| N | -1.148886 | -0.418052 | 1.557087 |
| C | -1.580539 | 0.771178 | 2.071986 |
| N | -1.572686 | 0.820255 | 3.398083 |
| C | -1.125543 | -0.408143 | 3.771715 |
| C | -0.867926 | -1.203731 | 2.644090 |
| C | -0.968563 | -0.797095 | 5.125093 |
| C | -0.447640 | -2.538382 | 2.517470 |
| N | -0.858149 | -1.153336 | 6.232028 |
| N | -0.147172 | -3.660773 | 2.362369 |

| | | | |
|---|---|---|---|
| N | -2.049448 | 1.293991 | -0.193601 |
| C | -2.089515 | 1.711785 | 1.107555 |
| N | -2.715394 | 2.869444 | 1.300594 |
| C | -3.126742 | 3.222951 | 0.055263 |
| C | -2.739042 | 2.256694 | -0.887377 |
| C | -3.866507 | 4.400247 | -0.218229 |
| C | -3.024404 | 2.200644 | -2.261810 |
| N | -4.502699 | 5.345335 | -0.476503 |
| N | -3.301911 | 2.152236 | -3.398772 |
| P | -3.620531 | -1.257834 | -0.133160 |
| O | -4.500876 | -0.603178 | -1.295455 |
| O | -4.131195 | -2.795079 | -0.147382 |
| O | -4.045628 | -0.643243 | 1.285782 |
| C | -5.887209 | -0.945292 | -1.583272 |
| C | -6.503230 | 0.242468 | -2.268177 |
| C | -3.635244 | -3.658591 | 0.921676 |
| C | -3.428572 | -5.042443 | 0.378279 |
| C | -5.365968 | -0.151728 | 1.639259 |
| C | -5.250767 | 0.538362 | 2.969466 |
| C | -0.558158 | -2.412714 | -0.588700 |
| O | -0.113576 | -3.462374 | -0.776604 |
| N | -1.552860 | -0.623316 | -2.509061 |
| C | -1.727710 | -0.445869 | -3.640691 |
| C | -1.927712 | -0.143721 | -5.033120 |
| H | 6.401190 | 0.209206 | -1.931774 |
| H | 6.029429 | -0.968899 | -0.647493 |
| H | 7.129490 | -2.116082 | -2.598702 |
| H | 5.850282 | -1.520113 | -3.672543 |
| H | 5.464497 | -2.689414 | -2.382665 |
| H | 3.687342 | 3.655865 | -2.909155 |

| | | | |
|---|---|---|---|
| H | 2.358785 | 3.053435 | -1.904569 |
| H | 3.076850 | 5.327635 | -1.171851 |
| H | 4.716004 | 4.715665 | -0.848306 |
| H | 3.330620 | 4.146466 | 0.113224 |
| H | 5.316536 | 2.589844 | 0.849676 |
| H | 3.650782 | 2.361019 | 1.426613 |
| H | 6.109829 | 0.903543 | 2.552709 |
| H | 5.246991 | 2.244373 | 3.335576 |
| H | 4.434546 | 0.686861 | 3.115232 |
| H | 2.820309 | -2.793752 | -4.718532 |
| H | 1.236609 | -3.544514 | -4.370618 |
| H | 2.642399 | -3.891456 | -3.317659 |
| H | -5.876214 | -1.840631 | -2.215160 |
| H | -6.404278 | -1.201907 | -0.650074 |
| H | -7.541451 | 0.024452 | -2.539571 |
| H | -5.950383 | 0.499935 | -3.177190 |
| H | -6.494584 | 1.122736 | -1.615534 |
| H | -2.695194 | -3.249959 | 1.319985 |
| H | -4.377404 | -3.627013 | 1.728260 |
| H | -4.360535 | -5.457613 | -0.019113 |
| H | -2.677893 | -5.042955 | -0.418634 |
| H | -3.067526 | -5.697820 | 1.177668 |
| H | -5.693290 | 0.538401 | 0.848773 |
| H | -6.055978 | -1.008193 | 1.669528 |
| H | -4.568574 | 1.392708 | 2.911497 |
| H | -6.234646 | 0.902493 | 3.283853 |
| H | -4.875954 | -0.148136 | 3.735596 |
| H | -2.530559 | -0.915548 | -5.521451 |
| H | -0.965022 | -0.056041 | -5.548060 |
| H | -2.455316 | 0.818371 | -5.084332 |

**$E_M$**

| | | | |
|---|---|---|---|
| Ir | 0.121513 | -0.782497 | -1.027526 |
| N | 1.607797 | 0.639399 | -0.955805 |
| C | 1.221559 | 1.951070 | -0.916581 |
| N | 2.217012 | 2.805977 | -0.719169 |
| C | 3.313654 | 2.009717 | -0.600089 |
| C | 2.959813 | 0.658247 | -0.730902 |
| C | 4.616318 | 2.515333 | -0.366933 |
| C | 3.728448 | -0.510848 | -0.601466 |
| N | 5.699620 | 2.902080 | -0.161968 |
| N | 4.335893 | -1.500484 | -0.443760 |
| N | -0.963646 | 1.042539 | -1.056519 |
| C | -0.202551 | 2.173173 | -0.947942 |
| N | -0.892035 | 3.290206 | -0.742289 |
| C | -2.183034 | 2.865197 | -0.695435 |
| C | -2.253170 | 1.473879 | -0.873499 |
| C | -3.280954 | 3.735216 | -0.482421 |
| C | -3.377688 | 0.631067 | -0.845779 |
| N | -4.208178 | 4.421057 | -0.294630 |
| N | -4.321908 | -0.060317 | -0.796398 |
| P | -0.206392 | -0.687967 | 1.321468 |
| O | -1.761321 | -0.803323 | 1.679103 |
| O | 0.451929 | -1.751423 | 2.352929 |
| O | 0.337658 | 0.765845 | 1.748469 |
| C | -2.294631 | -1.057926 | 3.010907 |
| C | -3.673357 | -0.461044 | 3.058825 |
| C | 1.909736 | -1.829004 | 2.374645 |
| C | 2.322816 | -3.259942 | 2.562261 |
| C | -0.072257 | 1.486213 | 2.938665 |
| C | 0.652582 | 2.803213 | 2.940615 |

| | | | |
|---|---|---|---|
| C | 1.206815 | -2.316175 | -1.198698 |
| O | 1.791214 | -3.309849 | -1.279945 |
| N | -1.560960 | -1.891281 | -1.412758 |
| C | -2.574211 | -2.417984 | -1.611237 |
| C | -3.858087 | -3.021790 | -1.851785 |
| H | -2.298171 | -2.144143 | 3.155185 |
| H | -1.626155 | -0.623951 | 3.764817 |
| H | -4.137227 | -0.658989 | 4.031065 |
| H | -4.309567 | -0.884745 | 2.275499 |
| H | -3.639383 | 0.623800 | 2.908130 |
| H | 2.313952 | -1.426484 | 1.434692 |
| H | 2.252656 | -1.179455 | 3.189312 |
| H | 1.922867 | -3.671369 | 3.494810 |
| H | 1.975748 | -3.879841 | 1.729290 |
| H | 3.415431 | -3.322572 | 2.594026 |
| H | -1.162650 | 1.618765 | 2.893570 |
| H | 0.173932 | 0.876134 | 3.820721 |
| H | 0.393007 | 3.395866 | 2.057751 |
| H | 0.382233 | 3.377499 | 3.833020 |
| H | 1.736893 | 2.650767 | 2.943622 |
| H | -3.988634 | -3.910787 | -1.226857 |
| H | -3.959840 | -3.307100 | -2.903706 |
| H | -4.627477 | -2.281486 | -1.597761 |

**E**

| | | | |
|---|---|---|---|
| Ir | -1.391886 | -0.257258 | -0.453295 |
| Ir | 1.391947 | 0.257245 | -0.453623 |
| C | -0.838168 | -1.914064 | -1.025804 |
| O | -0.562838 | -2.977760 | -1.430988 |
| C | 0.838255 | 1.914059 | -1.026034 |
| O | 0.562632 | 2.978191 | -1.429891 |

| | | | |
|---|---|---|---|
| C | -2.568825 | -0.337009 | 1.578481 |
| C | -2.443451 | -1.288158 | 2.719011 |
| C | -2.000236 | 0.963469 | 1.466258 |
| C | -1.090156 | 1.625780 | 2.438627 |
| C | -2.505422 | 1.583177 | 0.268868 |
| C | -2.315662 | 3.009403 | -0.121337 |
| C | -3.435307 | 0.663943 | -0.345216 |
| C | -4.301177 | 0.957145 | -1.521175 |
| C | -3.447540 | -0.530614 | 0.444319 |
| C | -4.314690 | -1.722917 | 0.227193 |
| C | 2.503421 | -1.583466 | 0.271293 |
| C | 2.311526 | -3.010325 | -0.115571 |
| C | 2.000151 | -0.960780 | 1.467980 |
| C | 1.090669 | -1.620358 | 2.442836 |
| C | 2.570591 | 0.339052 | 1.577190 |
| C | 2.446751 | 1.292864 | 2.715691 |
| C | 3.448822 | 0.529268 | 0.442039 |
| C | 4.317257 | 1.720128 | 0.222111 |
| C | 3.434274 | -0.666832 | -0.345128 |
| C | 4.298528 | -0.963791 | -1.521368 |
| H | -3.337697 | -1.251171 | 3.358593 |
| H | -2.336897 | -2.327075 | 2.383747 |
| H | -1.582527 | -1.058411 | 3.354409 |
| H | -1.635933 | 2.354532 | 3.055491 |
| H | -0.631337 | 0.900604 | 3.120000 |
| H | -0.280522 | 2.163660 | 1.930819 |
| H | -3.139876 | 3.629714 | 0.261520 |
| H | -1.382884 | 3.421291 | 0.274609 |
| H | -2.281645 | 3.139848 | -1.207996 |
| H | -5.241201 | 1.434108 | -1.207354 |

| | | | |
|---|---|---|---|
| H | -3.808389 | 1.635994 | -2.224826 |
| H | -4.561619 | 0.046583 | -2.071245 |
| H | -5.243076 | -1.647654 | 0.811708 |
| H | -4.596753 | -1.831337 | -0.825467 |
| H | -3.815431 | -2.650379 | 0.530623 |
| H | 3.135116 | -3.630892 | 0.268222 |
| H | 1.378438 | -3.419958 | 0.282044 |
| H | 2.276542 | -3.143322 | -1.201891 |
| H | 1.636167 | -2.350403 | 3.058430 |
| H | 0.635743 | -0.893804 | 3.125390 |
| H | 0.278255 | -2.156351 | 1.937369 |
| H | 3.340907 | 1.255863 | 3.355417 |
| H | 2.341816 | 2.331224 | 2.378250 |
| H | 1.585401 | 1.065778 | 3.351489 |
| H | 5.245231 | 1.645683 | 0.807369 |
| H | 4.599971 | 1.825358 | -0.830681 |
| H | 3.818632 | 2.648869 | 0.522675 |
| H | 5.237298 | -1.443287 | -1.207643 |
| H | 3.803242 | -1.641894 | -2.223998 |
| H | 4.561361 | -0.054600 | -2.072555 |
| H | -1.141998 | 0.206302 | -1.968254 |
| H | 1.142971 | -0.207154 | -1.968082 |

**$F_M$**

| | | | |
|---|---|---|---|
| Ir | 0.000047 | 0.000036 | -0.373439 |
| F | -1.485341 | 5.447431 | 2.248419 |
| F | 1.898837 | 2.441311 | 1.062848 |
| F | 1.484765 | -5.447427 | 2.248441 |
| F | -1.899010 | -2.440982 | 1.062834 |
| O | -3.900464 | 1.380758 | -0.105863 |
| O | 3.900457 | -1.380796 | -0.105390 |

| | | | |
|---|---|---|---|
| N | -2.063596 | 0.132080 | -0.424586 |
| N | 2.063640 | -0.132016 | -0.424493 |
| C | -0.311685 | 1.867610 | 0.433985 |
| C | 0.584157 | 2.747951 | 1.043109 |
| C | 0.220391 | 3.948585 | 1.638058 |
| H | 0.967683 | 4.610327 | 2.066074 |
| C | -1.129523 | 4.286892 | 1.671441 |
| C | -2.105039 | 3.461213 | 1.144186 |
| H | -3.155901 | 3.731296 | 1.208701 |
| C | -1.678433 | 2.268883 | 0.548861 |
| C | -2.557828 | 1.277890 | 0.015645 |
| C | -4.285731 | 0.198339 | -0.704441 |
| C | -5.559502 | -0.191087 | -1.069916 |
| H | -6.426664 | 0.438803 | -0.892709 |
| C | -5.654507 | -1.439537 | -1.684221 |
| H | -6.631473 | -1.800436 | -1.997616 |
| C | -4.522748 | -2.236716 | -1.911003 |
| H | -4.647084 | -3.202716 | -2.395380 |
| C | -3.249677 | -1.827767 | -1.529787 |
| H | -2.369108 | -2.443391 | -1.696110 |
| C | -3.145996 | -0.584147 | -0.913373 |
| C | 0.311547 | -1.867371 | 0.434139 |
| C | -0.584380 | -2.747680 | 1.043152 |
| C | -0.220761 | -3.948379 | 1.637988 |
| H | -0.968137 | -4.610097 | 2.065872 |
| C | 1.129118 | -4.286805 | 1.671526 |
| C | 2.104766 | -3.461152 | 1.144467 |
| H | 3.155620 | -3.731294 | 1.209051 |
| C | 1.678273 | -2.268753 | 0.549133 |
| C | 2.557821 | -1.277849 | 0.015940 |

| | | | |
|---|---|---|---|
| C | 4.285865 | -0.198513 | -0.704153 |
| C | 5.559719 | 0.190747 | -1.069523 |
| H | 6.426784 | -0.439191 | -0.892045 |
| C | 5.654916 | 1.439043 | -1.684087 |
| H | 6.631946 | 1.799790 | -1.997431 |
| C | 4.523264 | 2.236269 | -1.911186 |
| H | 4.647715 | 3.202162 | -2.395745 |
| C | 3.250095 | 1.827546 | -1.530041 |
| H | 2.369607 | 2.443243 | -1.696591 |
| C | 3.146189 | 0.584047 | -0.913329 |

**F**

| | | | |
|---|---|---|---|
| Ir | -1.331904 | 0.005934 | -0.012672 |
| F | -4.807138 | 3.822150 | 3.369679 |
| F | -3.488924 | -0.585623 | 2.451843 |
| F | -5.864915 | -3.147816 | -2.847347 |
| F | -3.738484 | 0.975388 | -2.147520 |
| O | -1.655511 | 4.039642 | -0.930335 |
| O | -2.121702 | -3.993500 | 0.816181 |
| N | -1.163630 | 1.847726 | -0.931013 |
| N | -1.346482 | -1.886125 | 0.862717 |
| C | -2.661516 | 1.119863 | 1.030197 |
| C | -3.437138 | 0.724421 | 2.120066 |
| C | -4.165423 | 1.606096 | 2.902346 |
| H | -4.775911 | 1.246964 | 3.726089 |
| C | -4.113815 | 2.966978 | 2.594381 |
| C | -3.378468 | 3.452377 | 1.534766 |
| H | -3.342134 | 4.517946 | 1.323895 |
| C | -2.669031 | 2.520943 | 0.760577 |
| C | -1.841047 | 2.828728 | -0.349229 |
| C | -0.809728 | 3.786627 | -1.991614 |

| | | | |
|---|---|---|---|
| C | -0.305807 | 4.687230 | -2.910115 |
| H | -0.549815 | 5.745113 | -2.870806 |
| C | 0.525855 | 4.149308 | -3.893160 |
| H | 0.951912 | 4.812055 | -4.642925 |
| C | 0.825522 | 2.780232 | -3.937738 |
| H | 1.486521 | 2.406387 | -4.716871 |
| C | 0.315013 | 1.890614 | -2.996992 |
| H | 0.568229 | 0.830978 | -3.002248 |
| C | -0.510436 | 2.420386 | -2.010208 |
| C | -2.992473 | -0.866128 | -0.862863 |
| C | -3.844642 | -0.334586 | -1.827623 |
| C | -4.814177 | -1.071494 | -2.492016 |
| H | -5.480147 | -0.602253 | -3.210581 |
| C | -4.931731 | -2.429818 | -2.194838 |
| C | -4.122938 | -3.050505 | -1.266230 |
| H | -4.214779 | -4.115828 | -1.071309 |
| C | -3.172367 | -2.254880 | -0.608552 |
| C | -2.234232 | -2.727289 | 0.349498 |
| C | -1.084797 | -3.937247 | 1.726200 |
| C | -0.550261 | -4.967790 | 2.473630 |
| H | -0.933570 | -5.982394 | 2.410460 |
| C | 0.509558 | -4.621138 | 3.314638 |
| H | 0.971755 | -5.393397 | 3.925359 |
| C | 0.988588 | -3.306454 | 3.392290 |
| H | 1.820842 | -3.080224 | 4.055885 |
| C | 0.437194 | -2.283067 | 2.626024 |
| H | 0.818156 | -1.262737 | 2.662784 |
| C | -0.608719 | -2.622972 | 1.774887 |
| Ir | 1.341986 | -0.044771 | 0.014342 |
| F | 4.935831 | 3.679595 | -3.344658 |

| | | | |
|---|---|---|---|
| F | 3.464901 | -0.687958 | -2.464883 |
| F | 5.746937 | -3.421293 | 2.792898 |
| F | 3.820975 | 0.803380 | 2.125172 |
| O | 1.809123 | 3.965522 | 0.969210 |
| O | 1.930979 | -4.072253 | -0.832040 |
| N | 1.252186 | 1.789300 | 0.959189 |
| N | 1.271080 | -1.925905 | -0.882716 |
| C | 2.710650 | 1.030035 | -1.019380 |
| C | 3.464837 | 0.619028 | -2.118444 |
| C | 4.218857 | 1.482668 | -2.896496 |
| H | 4.810197 | 1.111095 | -3.728613 |
| C | 4.217926 | 2.841113 | -2.573625 |
| C | 3.507453 | 3.340797 | -1.503593 |
| H | 3.509925 | 4.404576 | -1.281286 |
| C | 2.770606 | 2.426799 | -0.734357 |
| C | 1.958213 | 2.752531 | 0.381984 |
| C | 0.953706 | 3.733223 | 2.027938 |
| C | 0.471190 | 4.644352 | 2.947697 |
| H | 0.744864 | 5.695084 | 2.913534 |
| C | -0.381398 | 4.126891 | 3.923959 |
| H | -0.792903 | 4.799157 | 4.673391 |
| C | -0.720737 | 2.766812 | 3.961711 |
| H | -1.397056 | 2.409224 | 4.735244 |
| C | -0.230623 | 1.866500 | 3.020280 |
| H | -0.513925 | 0.814513 | 3.019669 |
| C | 0.614697 | 2.376215 | 2.039950 |
| C | 2.966815 | -0.995605 | 0.847918 |
| C | 3.856199 | -0.509561 | 1.803625 |
| C | 4.795940 | -1.295688 | 2.453668 |
| H | 5.493628 | -0.862060 | 3.164337 |

| | | | |
|---|---|---|---|
| C | 4.842555 | -2.657336 | 2.152281 |
| C | 3.993393 | -3.234539 | 1.231382 |
| H | 4.030839 | -4.302233 | 1.030996 |
| C | 3.076424 | -2.390387 | 0.586896 |
| C | 2.113618 | -2.812602 | -0.369655 |
| C | 0.896336 | -3.961973 | -1.739795 |
| C | 0.304169 | -4.964620 | -2.481654 |
| H | 0.630699 | -5.998641 | -2.414994 |
| C | -0.735755 | -4.563360 | -3.323008 |
| H | -1.241524 | -5.311783 | -3.928902 |
| C | -1.140616 | -3.224350 | -3.406766 |
| H | -1.960975 | -2.955947 | -4.069389 |
| C | -0.532817 | -2.229702 | -2.645123 |
| H | -0.857040 | -1.190151 | -2.686549 |
| C | 0.493395 | -2.623621 | -1.793011 |

**$G_M$**

| | | | |
|---|---|---|---|
| Ir | 0.988259 | -0.180493 | -0.391433 |
| C | 1.096471 | -1.457327 | -1.741757 |
| C | 2.822427 | -0.488613 | 0.854849 |
| C | 3.035639 | 0.682520 | 0.048451 |
| C | 2.079720 | 1.683308 | 0.452514 |
| C | 1.228397 | 1.099915 | 1.448211 |
| C | 1.710314 | -0.232352 | 1.737623 |
| C | 3.648317 | -1.727711 | 0.841407 |
| H | 4.455511 | -1.666558 | 1.583922 |
| H | 4.114014 | -1.894338 | -0.135574 |
| H | 3.050393 | -2.614451 | 1.077379 |
| C | 4.137485 | 0.887034 | -0.933147 |
| H | 3.827407 | 1.532718 | -1.761766 |
| H | 4.476811 | -0.060267 | -1.364890 |

| | | | |
|---|---|---|---|
| H | 5.007790 | 1.359830 | -0.456762 |
| C | 1.993207 | 3.064855 | -0.085833 |
| H | 2.719735 | 3.721670 | 0.414616 |
| H | 0.999956 | 3.498100 | 0.061387 |
| H | 2.213603 | 3.098587 | -1.158565 |
| C | 0.113593 | 1.781918 | 2.166980 |
| H | 0.465338 | 2.226126 | 3.107619 |
| H | -0.691550 | 1.081040 | 2.416693 |
| H | -0.326028 | 2.583237 | 1.564905 |
| C | 1.175569 | -1.146781 | 2.779697 |
| H | 1.389488 | -2.194986 | 2.546622 |
| H | 0.089133 | -1.048382 | 2.882755 |
| H | 1.618738 | -0.929618 | 3.762631 |
| C | -1.081108 | -0.154293 | -0.347671 |
| C | -1.858151 | -1.245105 | 0.037253 |
| C | -3.232268 | -1.163972 | 0.207867 |
| C | -3.909092 | 0.043767 | -0.016381 |
| C | -3.146079 | 1.152355 | -0.409302 |
| C | -1.774526 | 1.034760 | -0.562753 |
| C | -5.312430 | 0.141692 | 0.157642 |
| F | -1.273724 | -2.433503 | 0.291450 |
| F | -3.921755 | -2.235720 | 0.602138 |
| F | -3.749025 | 2.322783 | -0.629900 |
| F | -1.094344 | 2.148183 | -0.926055 |
| N | -6.468317 | 0.223510 | 0.304759 |
| O | 1.219584 | -2.288857 | -2.548314 |

**G**

| | | | |
|---|---|---|---|
| Ir | 0.662109 | -1.290896 | 0.082044 |
| C | 0.433794 | -0.929450 | 1.880522 |
| C | 0.519191 | -3.534073 | 0.147615 |

| | | | |
|---|---|---|---|
| C | -0.742636 | -3.057428 | -0.375894 |
| C | -0.486989 | -2.447592 | -1.648116 |
| C | 0.923944 | -2.452911 | -1.878721 |
| C | 1.549859 | -3.163181 | -0.774004 |
| C | 0.687642 | -4.327928 | 1.396210 |
| H | 0.586710 | -5.402206 | 1.188901 |
| H | -0.070297 | -4.068641 | 2.143661 |
| H | 1.670853 | -4.170129 | 1.852027 |
| C | -2.085346 | -3.391454 | 0.178709 |
| H | -2.873785 | -2.810565 | -0.310998 |
| H | -2.156480 | -3.205897 | 1.256680 |
| H | -2.315206 | -4.454014 | 0.016095 |
| C | -1.536192 | -2.017990 | -2.608923 |
| H | -1.996613 | -2.905928 | -3.065107 |
| H | -1.135249 | -1.400863 | -3.415974 |
| H | -2.341009 | -1.457379 | -2.120418 |
| C | 1.615113 | -2.055053 | -3.137709 |
| H | 1.635573 | -2.896202 | -3.845459 |
| H | 2.654366 | -1.763570 | -2.953557 |
| H | 1.122050 | -1.213264 | -3.632034 |
| C | 2.991552 | -3.523760 | -0.684439 |
| H | 3.297540 | -3.722218 | 0.348447 |
| H | 3.635840 | -2.725553 | -1.072226 |
| H | 3.202469 | -4.427197 | -1.273103 |
| C | 2.519787 | -0.353320 | 0.047598 |
| C | 3.466814 | -0.621987 | 1.044963 |
| C | 4.776335 | -0.170018 | 1.010368 |
| C | 5.241087 | 0.590921 | -0.071656 |
| C | 4.325485 | 0.854332 | -1.096502 |
| C | 3.021317 | 0.386701 | -1.024456 |

| | | | |
|---|---|---|---|
| C | 6.573473 | 1.070914 | -0.123701 |
| F | 3.129105 | -1.382475 | 2.110388 |
| F | 5.611694 | -0.470076 | 2.006301 |
| F | 4.703514 | 1.588687 | -2.145837 |
| F | 2.229801 | 0.710538 | -2.067508 |
| N | 7.670483 | 1.470073 | -0.164969 |
| O | 0.330725 | -0.856802 | 3.039307 |
| Ir | -0.662097 | 1.291020 | -0.081774 |
| C | -0.434162 | 0.928652 | -1.880153 |
| C | -0.517657 | 3.533919 | -0.147860 |
| C | 0.743432 | 3.056645 | 0.376792 |
| C | 0.486296 | 2.447161 | 1.648969 |
| C | -0.924824 | 2.453503 | 1.878432 |
| C | -1.549359 | 3.163923 | 0.772982 |
| C | -0.684656 | 4.327547 | -1.396803 |
| H | -0.583709 | 5.401851 | -1.189602 |
| H | 0.074011 | 4.067890 | -2.143390 |
| H | -1.667424 | 4.169824 | -1.853597 |
| C | 2.086999 | 3.389449 | -0.176486 |
| H | 2.874061 | 2.805738 | 0.312185 |
| H | 2.158137 | 3.206088 | -1.254846 |
| H | 2.318866 | 4.451189 | -0.011291 |
| C | 1.534408 | 2.017125 | 2.610879 |
| H | 1.994093 | 2.904923 | 3.068144 |
| H | 1.132484 | 1.399432 | 3.417069 |
| H | 2.339962 | 1.456894 | 2.123042 |
| C | -1.617170 | 2.055760 | 3.136862 |
| H | -1.637149 | 2.896581 | 3.845041 |
| H | -2.656613 | 1.765384 | 2.951918 |
| H | -1.125156 | 1.213156 | 3.630957 |

| | | | |
|---|---|---|---|
| C | -2.990727 | 3.525666 | 0.682446 |
| H | -3.295607 | 3.725460 | -0.350495 |
| H | -3.635963 | 2.727539 | 1.068838 |
| H | -3.201441 | 4.428621 | 1.271947 |
| C | -2.520132 | 0.353799 | -0.047442 |
| C | -3.467102 | 0.623120 | -1.044639 |
| C | -4.776528 | 0.170909 | -1.010453 |
| C | -5.241152 | -0.590964 | 0.070916 |
| C | -4.325656 | -0.854859 | 1.095690 |
| C | -3.021607 | -0.386829 | 1.024171 |
| C | -6.573304 | -1.071440 | 0.122419 |
| F | -3.129371 | 1.384291 | -2.109540 |
| F | -5.611845 | 0.471473 | -2.006225 |
| F | -4.703679 | -1.590022 | 2.144450 |
| F | -2.230236 | -0.710848 | 2.067279 |
| N | -7.670167 | -1.471062 | 0.163194 |
| O | -0.331629 | 0.855233 | -3.038874 |

**$H_M$**

| | | | |
|---|---|---|---|
| Ir | 0.537311 | -0.000104 | -0.145508 |
| C | 1.825090 | -1.420722 | -0.153853 |
| C | -1.162226 | -0.722022 | 1.171474 |
| C | -1.374294 | -1.166057 | -0.186537 |
| C | -1.582050 | 0.000264 | -1.013568 |
| C | -1.373827 | 1.166438 | -0.186418 |
| C | -1.161984 | 0.722180 | 1.171565 |
| C | -1.027197 | -1.590287 | 2.365703 |
| H | -2.016536 | -1.765340 | 2.810713 |
| H | -0.608799 | -2.570518 | 2.118021 |
| H | -0.400233 | -1.136020 | 3.138836 |
| C | -1.535802 | -2.580563 | -0.624782 |

| | | | |
|---|---|---|---|
| H | -1.220894 | -2.728045 | -1.662165 |
| H | -0.961379 | -3.271855 | -0.000468 |
| H | -2.589386 | -2.879821 | -0.556809 |
| C | -1.930780 | 0.000547 | -2.451818 |
| H | -3.024597 | 0.002398 | -2.567334 |
| H | -1.553959 | 0.887707 | -2.969584 |
| H | -1.556907 | -0.888026 | -2.969250 |
| C | -1.534868 | 2.581017 | -0.624629 |
| H | -2.588340 | 2.880649 | -0.556523 |
| H | -0.960087 | 3.272088 | -0.000407 |
| H | -1.220057 | 2.728330 | -1.662077 |
| C | -1.026674 | 1.590273 | 2.365878 |
| H | -0.399630 | 1.135873 | 3.138873 |
| H | -0.608281 | 2.570520 | 2.118249 |
| H | -2.015929 | 1.765330 | 2.811076 |
| O | 2.544153 | -2.318644 | -0.129125 |
| C | 1.825296 | 1.420275 | -0.153628 |
| O | 2.544338 | 2.318229 | -0.128797 |

**H**

| | | | |
|---|---|---|---|
| Ir | 1.444769 | 0.000234 | 0.392997 |
| C | 0.797255 | -1.437960 | 1.465606 |
| C | 3.578821 | -0.719039 | 0.232335 |
| C | 2.853872 | -1.163609 | -0.950002 |
| C | 2.482630 | 0.003556 | -1.705520 |
| C | 2.856658 | 1.166230 | -0.944613 |
| C | 3.580552 | 0.714503 | 0.235847 |
| C | 4.283843 | -1.603034 | 1.193839 |
| H | 5.303917 | -1.788923 | 0.830826 |
| H | 3.800478 | -2.579285 | 1.296834 |
| H | 4.372547 | -1.154329 | 2.187301 |

| | | | |
|---|---|---|---|
| C | 2.771285 | -2.577061 | -1.411253 |
| H | 2.016002 | -2.720200 | -2.187425 |
| H | 2.554730 | -3.273453 | -0.594638 |
| H | 3.736284 | -2.879180 | -1.839017 |
| C | 1.913050 | 0.007346 | -3.074220 |
| H | 2.742372 | 0.006612 | -3.796050 |
| H | 1.319928 | 0.900666 | -3.287714 |
| H | 1.315590 | -0.882326 | -3.290897 |
| C | 2.776516 | 2.581958 | -1.399174 |
| H | 3.738513 | 2.880943 | -1.835910 |
| H | 2.572337 | 3.275722 | -0.577193 |
| H | 2.013903 | 2.732390 | -2.166863 |
| C | 4.288336 | 1.591968 | 1.201322 |
| H | 4.377146 | 1.137765 | 2.192268 |
| H | 3.807086 | 2.568630 | 1.310251 |
| H | 5.308398 | 1.777802 | 0.838236 |
| O | 0.513399 | -2.355329 | 2.104170 |
| Ir | -1.444914 | -0.000300 | -0.393057 |
| C | -0.797165 | 1.438162 | -1.465118 |
| C | -3.579196 | 0.718346 | -0.232338 |
| C | -2.854251 | 1.163531 | 0.949776 |
| C | -2.482514 | -0.003188 | 1.705660 |
| C | -2.856252 | -1.166255 | 0.945227 |
| C | -3.580454 | -0.715209 | -0.235286 |
| C | -4.284611 | 1.601719 | -1.194097 |
| H | -5.304570 | 1.787770 | -0.830895 |
| H | -3.801356 | 2.577952 | -1.297868 |
| H | -4.373586 | 1.152444 | -2.187273 |
| C | -2.772006 | 2.577086 | 1.410645 |
| H | -2.015298 | 2.721092 | 2.185270 |

| | | | |
|---|---|---|---|
| H | -2.557822 | 3.273558 | 0.593468 |
| H | -3.736396 | 2.878361 | 1.840365 |
| C | -1.912489 | -0.006114 | 3.074122 |
| H | -2.741501 | -0.003865 | 3.796271 |
| H | -1.320083 | -0.899700 | 3.288315 |
| H | -1.314232 | 0.883261 | 3.289768 |
| C | -2.775616 | -2.581767 | 1.400268 |
| H | -3.737170 | -2.880684 | 1.837979 |
| H | -2.572140 | -3.275797 | 0.578376 |
| H | -2.012367 | -2.731943 | 2.167319 |
| C | -4.287891 | -1.593206 | -1.200489 |
| H | -4.376872 | -1.139291 | -2.191545 |
| H | -3.806240 | -2.569673 | -1.309216 |
| H | -5.307865 | -1.779401 | -0.837419 |
| O | -0.512880 | 2.355936 | -2.103061 |
| C | 0.795521 | 1.438287 | 1.464762 |
| C | -0.795589 | -1.437648 | -1.465821 |
| O | -0.509682 | -2.354097 | -2.104767 |
| O | 0.510122 | 2.355561 | 2.102764 |

**$I_M$**

| | | | |
|---|---|---|---|
| Ir | -0.838463 | -0.989832 | -0.637141 |
| O | -0.329518 | 0.572725 | -3.189053 |
| N | 0.966343 | -1.952166 | -0.134276 |
| N | 2.246745 | -1.911694 | -0.519553 |
| H | 2.528051 | -1.167991 | -1.178816 |
| N | -1.353811 | -2.241014 | 0.934118 |
| N | -3.682105 | 0.170314 | -1.084632 |
| N | -2.844194 | -0.663117 | -0.477940 |
| N | -0.503779 | 0.788580 | 0.751025 |
| C | 3.030057 | -2.709562 | 0.239631 |

| | | | |
|---|---|---|---|
| C | 2.187157 | -3.331198 | 1.156713 |
| H | 2.474911 | -4.051338 | 1.911721 |
| C | 0.904820 | -2.831223 | 0.902043 |
| C | -0.404799 | -3.017693 | 1.506897 |
| C | -0.747988 | -3.855492 | 2.562835 |
| H | 0.002254 | -4.485836 | 3.031996 |
| C | -2.076661 | -3.861474 | 2.995959 |
| H | -2.364691 | -4.508124 | 3.822249 |
| C | -3.035339 | -3.059762 | 2.391068 |
| H | -4.069132 | -3.068799 | 2.725068 |
| C | -2.654629 | -2.230796 | 1.327433 |
| C | -3.466104 | -1.329803 | 0.555632 |
| C | -4.792125 | -0.893305 | 0.582858 |
| H | -5.576309 | -1.206576 | 1.262090 |
| C | -4.876952 | 0.040937 | -0.452966 |
| C | -6.050506 | 0.872168 | -0.908879 |
| C | -5.683824 | 2.357350 | -0.796709 |
| H | -6.520939 | 2.987902 | -1.124077 |
| H | -4.813877 | 2.591978 | -1.420557 |
| H | -5.440960 | 2.628501 | 0.239087 |
| C | -7.270368 | 0.579247 | -0.038682 |
| H | -7.563304 | -0.477140 | -0.097665 |
| H | -8.127404 | 1.179321 | -0.367968 |
| H | -7.081574 | 0.819926 | 1.015706 |
| C | -6.370312 | 0.542051 | -2.371321 |
| H | -7.207447 | 1.155133 | -2.730643 |
| H | -6.649065 | -0.512733 | -2.488294 |
| H | -5.502996 | 0.733292 | -3.012565 |
| C | 0.591373 | 0.860129 | 1.513863 |
| H | 1.118424 | -0.074266 | 1.694397 |

| | | | |
|---|---|---|---|
| C | 1.085213 | 2.061393 | 1.997769 |
| H | 2.018158 | 2.047690 | 2.553261 |
| C | 0.441356 | 3.255977 | 1.683242 |
| C | -0.749153 | 3.150267 | 0.949463 |
| H | -1.319221 | 4.037813 | 0.681315 |
| C | -1.180852 | 1.922285 | 0.489026 |
| H | -2.069789 | 1.814532 | -0.133560 |
| C | 1.002629 | 4.623725 | 2.038773 |
| C | 1.230754 | 5.396333 | 0.729954 |
| H | 1.669234 | 6.378472 | 0.947809 |
| H | 0.293072 | 5.565648 | 0.187379 |
| H | 1.912229 | 4.852528 | 0.066568 |
| C | 2.335551 | 4.515499 | 2.775941 |
| H | 2.236038 | 3.980775 | 3.729753 |
| H | 2.715981 | 5.518689 | 3.001353 |
| H | 3.088610 | 4.003575 | 2.165388 |
| C | -0.003955 | 5.378032 | 2.916424 |
| H | 0.375761 | 6.381224 | 3.147508 |
| H | -0.178792 | 4.857370 | 3.866532 |
| H | -0.973199 | 5.499554 | 2.417034 |
| C | -0.483769 | -0.008213 | -2.204606 |
| F | 1.555970 | 1.911888 | -1.078620 |
| P | 3.150817 | 1.580202 | -1.215527 |
| F | 3.523042 | 2.858433 | -0.291211 |
| F | 2.727642 | 0.210147 | -2.114925 |
| F | 4.709106 | 1.142465 | -1.339966 |
| F | 3.039692 | 0.629491 | 0.137031 |
| F | 3.239182 | 2.431735 | -2.572039 |
| C | 4.524575 | -2.774696 | 0.062361 |
| C | 4.917808 | -2.373732 | -1.361861 |

| | | | |
|---|---|---|---|
| H | 5.999705 | -2.494365 | -1.487789 |
| H | 4.421939 | -3.001999 | -2.112451 |
| H | 4.688625 | -1.323226 | -1.574850 |
| C | 5.177087 | -1.800994 | 1.056792 |
| H | 4.917935 | -2.055068 | 2.092487 |
| H | 6.268550 | -1.851953 | 0.959525 |
| H | 4.864896 | -0.770279 | 0.860828 |
| C | 4.995744 | -4.201640 | 0.354098 |
| H | 6.084408 | -4.264864 | 0.241485 |
| H | 4.756005 | -4.508580 | 1.380147 |
| H | 4.542600 | -4.927178 | -0.332432 |

**I**

| | | | |
|---|---|---|---|
| Ir | 0.998335 | 0.125754 | 0.703922 |
| Ir | -1.583138 | 0.123472 | -0.339402 |
| O | -2.214980 | 1.347795 | 2.351681 |
| O | -0.024431 | -1.591012 | 2.975619 |
| N | -1.646646 | -1.964201 | -0.086019 |
| N | -1.951531 | -2.830444 | 0.886665 |
| H | -2.435842 | -2.467765 | 1.722334 |
| N | -0.963276 | -0.626586 | -2.173096 |
| N | -1.495163 | 3.127466 | -1.174653 |
| N | -1.262195 | 1.836788 | -1.416658 |
| N | -3.692527 | 0.165951 | -1.171675 |
| N | 1.594854 | -1.351582 | -0.553929 |
| N | 1.738085 | -2.665296 | -0.388617 |
| N | 1.738235 | 1.209256 | -0.902141 |
| N | 0.309724 | 2.841426 | 2.347371 |
| H | -0.237733 | 2.354800 | 3.046347 |
| N | 0.724737 | 2.154666 | 1.268558 |
| N | 3.109628 | 0.144968 | 1.520631 |

| | | | |
|---|---|---|---|
| C | -1.824037 | -4.113977 | 0.470652 |
| C | -1.396234 | -4.061989 | -0.848692 |
| H | -1.166008 | -4.904898 | -1.485500 |
| C | -1.280190 | -2.704091 | -1.162431 |
| C | -0.884840 | -1.964677 | -2.346366 |
| C | -0.475169 | -2.474073 | -3.574192 |
| H | -0.387483 | -3.547338 | -3.720556 |
| C | -0.178079 | -1.573287 | -4.598612 |
| H | 0.153178 | -1.952107 | -5.563104 |
| C | -0.293780 | -0.202336 | -4.404851 |
| H | -0.066076 | 0.501825 | -5.201105 |
| C | -0.701992 | 0.270836 | -3.152022 |
| C | -0.907079 | 1.633564 | -2.729140 |
| C | -0.902863 | 2.880823 | -3.352139 |
| H | -0.659494 | 3.097026 | -4.385582 |
| C | -1.281578 | 3.777445 | -2.349287 |
| C | -1.545865 | 5.259748 | -2.456313 |
| C | -3.055158 | 5.500201 | -2.309645 |
| H | -3.291642 | 6.568746 | -2.401717 |
| H | -3.411621 | 5.154467 | -1.331722 |
| H | -3.617292 | 4.961099 | -3.083109 |
| C | -1.075007 | 5.777298 | -3.814021 |
| H | -0.003319 | 5.587061 | -3.963690 |
| H | -1.237796 | 6.859283 | -3.891145 |
| H | -1.619412 | 5.301818 | -4.639635 |
| C | -0.812314 | 6.010481 | -1.343665 |
| H | -1.054970 | 7.081209 | -1.370260 |
| H | 0.275906 | 5.910196 | -1.454883 |
| H | -1.089108 | 5.614204 | -0.360468 |
| C | -4.420443 | -0.953453 | -1.262751 |

| | | | |
|---|---|---|---|
| H | -3.871835 | -1.891529 | -1.239942 |
| C | -5.805511 | -0.945218 | -1.305438 |
| H | -6.324962 | -1.898728 | -1.306354 |
| C | -6.499831 | 0.261413 | -1.244957 |
| C | -5.719149 | 1.425418 | -1.234708 |
| H | -6.183954 | 2.409719 | -1.212881 |
| C | -4.341923 | 1.345374 | -1.182510 |
| H | -3.718289 | 2.235584 | -1.108513 |
| C | -8.015742 | 0.347792 | -1.156100 |
| C | -8.386822 | 1.178358 | 0.081611 |
| H | -9.478150 | 1.227142 | 0.183592 |
| H | -8.016997 | 2.208413 | 0.010196 |
| H | -7.974320 | 0.732313 | 0.993173 |
| C | -8.655061 | -1.032918 | -1.021316 |
| H | -8.461447 | -1.664579 | -1.897758 |
| H | -9.742593 | -0.928973 | -0.930003 |
| H | -8.291837 | -1.554757 | -0.127923 |
| C | -8.551927 | 1.037094 | -2.417874 |
| H | -9.643190 | 1.135560 | -2.359638 |
| H | -8.313719 | 0.461761 | -3.321402 |
| H | -8.133868 | 2.044041 | -2.541227 |
| C | -2.031725 | 0.850988 | 1.319850 |
| C | 2.431446 | -3.102709 | -1.473102 |
| C | 2.711946 | -2.050332 | -2.346264 |
| H | 3.320455 | -2.069949 | -3.239564 |
| C | 2.193959 | -0.922183 | -1.719252 |
| C | 2.262101 | 0.498424 | -1.928658 |
| C | 2.813085 | 1.197006 | -3.013200 |
| H | 3.262701 | 0.644192 | -3.829945 |
| C | 2.842187 | 2.580071 | -2.974722 |

| | | | |
|---|---|---|---|
| H | 3.289802 | 3.128023 | -3.800809 |
| C | 2.335689 | 3.285297 | -1.876465 |
| H | 2.377719 | 4.370809 | -1.832298 |
| C | 1.785449 | 2.559228 | -0.831960 |
| C | 1.228597 | 3.073409 | 0.410853 |
| C | 1.115002 | 4.349727 | 0.976251 |
| H | 1.430187 | 5.289986 | 0.543756 |
| C | 0.496686 | 4.177957 | 2.205402 |
| C | 2.911833 | -4.532122 | -1.538420 |
| C | 1.725115 | -5.497062 | -1.589993 |
| H | 2.069273 | -6.539698 | -1.612332 |
| H | 1.112373 | -5.327867 | -2.487139 |
| H | 1.084560 | -5.364998 | -0.708873 |
| C | 3.794045 | -4.713788 | -2.772084 |
| H | 4.172045 | -5.742362 | -2.825055 |
| H | 4.656488 | -4.035541 | -2.741515 |
| H | 3.236676 | -4.515356 | -3.698095 |
| C | 3.739172 | -4.827449 | -0.279550 |
| H | 3.131673 | -4.701023 | 0.625276 |
| H | 4.599545 | -4.151617 | -0.214593 |
| H | 4.114326 | -5.859554 | -0.299478 |
| C | 0.088705 | 5.178283 | 3.255137 |
| C | -0.736249 | 4.509677 | 4.355893 |
| H | -1.653275 | 4.050771 | 3.964284 |
| H | -1.039409 | 5.256605 | 5.097291 |
| H | -0.162882 | 3.743536 | 4.894744 |
| C | 1.356095 | 5.793266 | 3.864519 |
| H | 1.979908 | 5.030049 | 4.345370 |
| H | 1.085900 | 6.537580 | 4.623398 |
| H | 1.963648 | 6.294673 | 3.101867 |

| | | | |
|---|---|---|---|
| C | -0.745624 | 6.277930 | 2.588418 |
| H | -0.183698 | 6.788264 | 1.796398 |
| H | -1.034615 | 7.031380 | 3.330990 |
| H | -1.660884 | 5.870963 | 2.142132 |
| C | 3.677444 | 1.266060 | 2.000794 |
| H | 3.063691 | 2.163935 | 1.994356 |
| C | 4.972170 | 1.295719 | 2.476639 |
| H | 5.368553 | 2.238672 | 2.848232 |
| C | 5.764566 | 0.140935 | 2.458982 |
| C | 5.158357 | -1.008063 | 1.954727 |
| H | 5.707655 | -1.938865 | 1.853273 |
| C | 3.850396 | -0.973241 | 1.498171 |
| H | 3.377024 | -1.859153 | 1.079541 |
| C | 7.191568 | 0.178157 | 2.984382 |
| C | 7.139908 | 0.482605 | 4.489591 |
| H | 8.157063 | 0.523227 | 4.899573 |
| H | 6.586654 | -0.290515 | 5.037936 |
| H | 6.661476 | 1.448847 | 4.695717 |
| C | 7.911553 | -1.151805 | 2.770423 |
| H | 7.955678 | -1.413982 | 1.708464 |
| H | 7.426444 | -1.971787 | 3.315798 |
| H | 8.939641 | -1.077427 | 3.144353 |
| C | 7.976154 | 1.287652 | 2.269964 |
| H | 9.005340 | 1.313011 | 2.650028 |
| H | 7.539651 | 2.278414 | 2.447874 |
| H | 8.006230 | 1.113859 | 1.190353 |
| C | 0.351474 | -0.926487 | 2.104368 |
| P | 6.106095 | -0.438245 | -1.588065 |
| F | 6.847061 | 0.886675 | -2.151586 |
| F | 4.999406 | 0.524310 | -0.824376 |

| | | | |
|---|---|---|---|
| F | 5.134150 | -0.473755 | -2.913810 |
| F | 7.044015 | -0.406557 | -0.241256 |
| F | 5.323280 | -1.755098 | -0.987806 |
| F | 7.167432 | -1.407214 | -2.329140 |
| F | -4.526134 | -0.200138 | 1.478484 |
| P | -5.006495 | -1.451138 | 2.430732 |
| F | -6.534758 | -1.263095 | 1.922849 |
| F | -3.383598 | -1.647483 | 2.878247 |
| F | -5.396927 | -2.713954 | 3.357549 |
| F | -4.709543 | -2.479476 | 1.174303 |
| F | -5.207728 | -0.430584 | 3.658339 |
| C | -2.029924 | -5.274053 | 1.405224 |
| C | -3.392662 | -5.178659 | 2.099424 |
| H | -3.521035 | -6.037039 | 2.770030 |
| H | -3.496931 | -4.270233 | 2.701138 |
| H | -4.216750 | -5.180619 | 1.377740 |
| C | -1.940486 | -6.575437 | 0.609173 |
| H | -0.957974 | -6.696799 | 0.134372 |
| H | -2.092290 | -7.431045 | 1.277015 |
| H | -2.708093 | -6.623069 | -0.173411 |
| C | -0.911882 | -5.238434 | 2.460037 |
| H | -0.984860 | -6.117188 | 3.112714 |
| H | 0.082145 | -5.237246 | 1.993467 |
| H | -0.987623 | -4.345288 | 3.091691 |

**$J_M$**

| | | | |
|---|---|---|---|
| Ir | 0.069249 | -1.133799 | -0.683148 |
| O | 0.064766 | -0.905691 | -3.701981 |
| N | 2.094398 | -1.135169 | -0.232846 |
| N | 3.196001 | -0.832293 | -0.921080 |
| N | 0.082063 | -1.537831 | 1.353823 |

| | | | |
|---|---|---|---|
| N | -3.068131 | -1.083917 | -0.929069 |
| N | -1.949767 | -1.313971 | -0.239043 |
| N | -0.051604 | 1.151192 | -0.268921 |
| C | 4.224093 | -0.862933 | -0.035793 |
| C | 3.768044 | -1.184080 | 1.245897 |
| H | 4.347985 | -1.279934 | 2.156520 |
| C | 2.390201 | -1.348443 | 1.094627 |
| C | 1.282989 | -1.599589 | 1.981793 |
| C | 1.309498 | -1.859520 | 3.355396 |
| H | 2.259351 | -1.917914 | 3.879778 |
| C | 0.100499 | -2.039234 | 4.022851 |
| H | 0.108088 | -2.244314 | 5.092025 |
| C | -1.118141 | -1.959880 | 3.353337 |
| H | -2.061253 | -2.095470 | 3.875659 |
| C | -1.110570 | -1.698449 | 1.979801 |
| C | -2.232366 | -1.536424 | 1.089433 |
| C | -3.618125 | -1.459479 | 1.239330 |
| H | -4.191300 | -1.586875 | 2.150427 |
| C | -4.092760 | -1.173580 | -0.043811 |
| C | -5.506325 | -0.946639 | -0.520822 |
| C | -5.605440 | 0.455366 | -1.135088 |
| H | -6.619983 | 0.646030 | -1.510487 |
| H | -4.903113 | 0.563861 | -1.969337 |
| H | -5.370200 | 1.229595 | -0.391752 |
| C | -6.482976 | -1.060600 | 0.647445 |
| H | -6.446051 | -2.057019 | 1.106610 |
| H | -7.511416 | -0.890041 | 0.305087 |
| H | -6.265281 | -0.320778 | 1.429182 |
| C | -5.858575 | -1.988539 | -1.588295 |
| H | -6.874173 | -1.822687 | -1.972747 |

| | | | |
|---|---|---|---|
| H | -5.813466 | -3.005003 | -1.177774 |
| H | -5.157559 | -1.935380 | -2.428367 |
| C | 1.058453 | 1.885459 | -0.120695 |
| H | 2.002477 | 1.348423 | -0.213917 |
| C | 1.016635 | 3.253852 | 0.116143 |
| H | 1.956275 | 3.789236 | 0.224207 |
| C | -0.209780 | 3.915646 | 0.204932 |
| C | -1.354930 | 3.125429 | 0.040682 |
| H | -2.349493 | 3.566565 | 0.088478 |
| C | -1.243939 | 1.765624 | -0.192381 |
| H | -2.119566 | 1.133602 | -0.341235 |
| C | -0.338910 | 5.412836 | 0.452476 |
| C | -1.064339 | 6.051297 | -0.740475 |
| H | -1.176357 | 7.130961 | -0.580885 |
| H | -2.067060 | 5.631000 | -0.881497 |
| H | -0.505954 | 5.903925 | -1.672868 |
| C | 1.021052 | 6.087363 | 0.619980 |
| H | 1.579689 | 5.678705 | 1.471253 |
| H | 0.883713 | 7.159518 | 0.802145 |
| H | 1.643382 | 5.984879 | -0.277542 |
| C | -1.163666 | 5.639341 | 1.727109 |
| H | -1.277654 | 6.713889 | 1.917128 |
| H | -0.677779 | 5.191362 | 2.602509 |
| H | -2.168964 | 5.209581 | 1.645546 |
| C | 0.066432 | -0.982641 | -2.545015 |
| C | 5.622760 | -0.557577 | -0.512865 |
| C | 6.032237 | -1.581671 | -1.577572 |
| H | 7.039191 | -1.363515 | -1.958699 |
| H | 6.039213 | -2.598599 | -1.165612 |
| H | 5.332602 | -1.566602 | -2.420394 |

| | | | |
|---|---|---|---|
| C | 5.645536 | 0.845480 | -1.131657 |
| H | 5.367593 | 1.608452 | -0.391797 |
| H | 6.648687 | 1.089502 | -1.506819 |
| H | 4.939320 | 0.912706 | -1.966903 |
| C | 6.603873 | -0.615954 | 0.655631 |
| H | 7.621412 | -0.388951 | 0.313586 |
| H | 6.345072 | 0.111166 | 1.436762 |
| H | 6.622763 | -1.612646 | 1.115508 |

**J**

| | | | |
|---|---|---|---|
| Ir | 1.389406 | -0.017612 | 0.418738 |
| Ir | -1.365555 | 0.039408 | 0.415284 |
| O | -1.264964 | 1.389899 | 3.119886 |
| O | 1.300370 | -1.324082 | 3.144428 |
| N | -1.396051 | -1.976122 | 0.891263 |
| N | -1.464348 | -2.651056 | 2.037626 |
| N | -1.473486 | -0.869293 | -1.451995 |
| N | -1.626113 | 2.972652 | -0.700570 |
| N | -1.471699 | 1.659886 | -0.879812 |
| N | -3.668034 | 0.016340 | 0.406303 |
| N | 1.485301 | -1.632907 | -0.880459 |
| N | 1.628813 | -2.946765 | -0.704816 |
| N | 1.498112 | 0.900720 | -1.441115 |
| N | 1.467845 | 2.651620 | 2.061435 |
| N | 1.423011 | 1.988464 | 0.906935 |
| N | 3.687613 | -0.030778 | 0.397271 |
| C | -1.623688 | -3.957827 | 1.703133 |
| C | -1.677420 | -4.113428 | 0.316507 |
| H | -1.790850 | -5.031485 | -0.247854 |
| C | -1.524142 | -2.821456 | -0.185795 |
| C | -1.535729 | -2.223196 | -1.494092 |

| | | | |
|---|---|---|---|
| C | -1.635532 | -2.851330 | -2.739821 |
| H | -1.670769 | -3.936288 | -2.800023 |
| C | -1.692188 | -2.059757 | -3.883749 |
| H | -1.763476 | -2.538031 | -4.859578 |
| C | -1.668702 | -0.669636 | -3.804690 |
| H | -1.727042 | -0.047784 | -4.694361 |
| C | -1.568467 | -0.074383 | -2.543098 |
| C | -1.592220 | 1.329272 | -2.209239 |
| C | -1.812994 | 2.512840 | -2.914692 |
| H | -1.937508 | 2.627345 | -3.985628 |
| C | -1.827753 | 3.506955 | -1.932552 |
| C | -2.095170 | 4.985395 | -2.072298 |
| C | -3.466407 | 5.297635 | -1.456914 |
| H | -3.701204 | 6.367611 | -1.541145 |
| H | -3.483698 | 5.025899 | -0.394853 |
| H | -4.262602 | 4.736094 | -1.964443 |
| C | -2.095773 | 5.381681 | -3.546974 |
| H | -1.137377 | 5.136373 | -4.025035 |
| H | -2.260005 | 6.461191 | -3.656499 |
| H | -2.889578 | 4.867318 | -4.104283 |
| C | -1.021605 | 5.780867 | -1.325792 |
| H | -1.245549 | 6.856157 | -1.349804 |
| H | -0.034251 | 5.631443 | -1.782559 |
| H | -0.954763 | 5.454935 | -0.282034 |
| C | -4.351828 | -1.055710 | 0.844925 |
| H | -3.754915 | -1.887002 | 1.215316 |
| C | -5.735216 | -1.099301 | 0.851094 |
| H | -6.224025 | -1.999385 | 1.220824 |
| C | -6.485812 | -0.006853 | 0.398920 |
| C | -5.756376 | 1.101538 | -0.035592 |

| | | | |
|---|---|---|---|
| H | -6.252048 | 2.001398 | -0.391096 |
| C | -4.367974 | 1.079338 | -0.016947 |
| H | -3.783753 | 1.942696 | -0.332665 |
| C | -8.007664 | -0.060405 | 0.409279 |
| C | -8.488603 | -0.267119 | 1.852351 |
| H | -9.584039 | -0.322287 | 1.881874 |
| H | -8.174924 | 0.560597 | 2.499867 |
| H | -8.098320 | -1.196000 | 2.284405 |
| C | -8.471780 | -1.242206 | -0.453315 |
| H | -8.139270 | -1.133224 | -1.492880 |
| H | -9.567265 | -1.302626 | -0.453657 |
| H | -8.087954 | -2.198077 | -0.077840 |
| C | -8.631121 | 1.221914 | -0.137738 |
| H | -9.724209 | 1.141437 | -0.117621 |
| H | -8.334808 | 1.410949 | -1.177140 |
| H | -8.356269 | 2.099545 | 0.460147 |
| C | -1.269387 | 0.874740 | 2.083396 |
| C | 1.831296 | -3.478924 | -1.937088 |
| C | 1.827878 | -2.481365 | -2.916337 |
| H | 1.955172 | -2.593973 | -3.987201 |
| C | 1.613864 | -1.297489 | -2.207709 |
| C | 1.594261 | 0.109107 | -2.535172 |
| C | 1.695299 | 0.711334 | -3.793539 |
| H | 1.754355 | 0.094522 | -4.686713 |
| C | 1.716673 | 2.101782 | -3.865476 |
| H | 1.788099 | 2.584635 | -4.839031 |
| C | 1.657226 | 2.888905 | -2.718011 |
| H | 1.689927 | 3.974182 | -2.774489 |
| C | 1.557512 | 2.255811 | -1.475161 |
| C | 1.537915 | 2.847738 | -0.161443 |

| | | | |
|---|---|---|---|
| C | 1.660699 | 4.135795 | 0.357056 |
| H | 1.751875 | 5.064116 | -0.196067 |
| C | 1.599466 | 3.962439 | 1.745192 |
| C | 2.093245 | -4.958676 | -2.074518 |
| C | 1.018324 | -5.752119 | -1.326596 |
| H | 1.237845 | -6.828224 | -1.356397 |
| H | 0.029857 | -5.597137 | -1.779066 |
| H | 0.953912 | -5.432554 | -0.280239 |
| C | 2.093962 | -5.357036 | -3.548627 |
| H | 2.254912 | -6.437167 | -3.656888 |
| H | 2.890157 | -4.845788 | -4.105367 |
| H | 1.137227 | -5.109117 | -4.028491 |
| C | 3.464075 | -5.272881 | -1.458508 |
| H | 3.478836 | -5.007388 | -0.394742 |
| H | 4.260565 | -4.706902 | -1.960810 |
| H | 3.700468 | -6.342103 | -1.548059 |
| C | 1.576535 | 5.021218 | 2.819856 |
| C | 1.751202 | 4.391057 | 4.200586 |
| H | 0.963687 | 3.657669 | 4.404106 |
| H | 1.716067 | 5.167670 | 4.976081 |
| H | 2.711129 | 3.867312 | 4.283289 |
| C | 2.691842 | 6.041403 | 2.574236 |
| H | 3.681105 | 5.567952 | 2.617343 |
| H | 2.664082 | 6.834233 | 3.333700 |
| H | 2.592519 | 6.521245 | 1.591922 |
| C | 0.219196 | 5.736974 | 2.762786 |
| H | 0.076914 | 6.241178 | 1.797936 |
| H | 0.143543 | 6.495117 | 3.554700 |
| H | -0.603080 | 5.021398 | 2.891299 |
| C | 4.400259 | 1.059230 | 0.731212 |

| | | | |
|---|---|---|---|
| H | 3.824656 | 1.941645 | 1.002921 |
| C | 5.784233 | 1.061373 | 0.747265 |
| H | 6.300968 | 1.977513 | 1.028576 |
| C | 6.503754 | -0.093510 | 0.416132 |
| C | 5.744113 | -1.215623 | 0.080358 |
| H | 6.213078 | -2.160618 | -0.182527 |
| C | 4.357054 | -1.149390 | 0.082390 |
| H | 3.747948 | -2.019338 | -0.161046 |
| C | 8.026065 | -0.083791 | 0.442444 |
| C | 8.497991 | 0.271128 | 1.859319 |
| H | 9.594238 | 0.294461 | 1.897628 |
| H | 8.150132 | -0.466446 | 2.592581 |
| H | 8.134055 | 1.255179 | 2.178200 |
| C | 8.615932 | -1.437849 | 0.053363 |
| H | 8.329370 | -1.734223 | -0.963443 |
| H | 8.304170 | -2.233668 | 0.741151 |
| H | 9.710658 | -1.388664 | 0.083997 |
| C | 8.535096 | 0.978130 | -0.542200 |
| H | 9.631732 | 1.011465 | -0.528113 |
| H | 8.169565 | 1.980309 | -0.288169 |
| H | 8.217189 | 0.756292 | -1.568313 |
| C | 1.298079 | -0.836342 | 2.095067 |
| C | -1.690492 | -5.002621 | 2.788244 |
| C | -2.878246 | -4.706431 | 3.711165 |
| H | -2.932364 | -5.440332 | 4.527057 |
| H | -2.785774 | -3.707667 | 4.152231 |
| H | -3.827431 | -4.746085 | 3.159346 |
| C | -1.854693 | -6.390180 | 2.171525 |
| H | -1.013072 | -6.639344 | 1.511300 |
| H | -1.900107 | -7.155666 | 2.956616 |

| | | | |
|---|---|---|---|
| H | -2.777990 | -6.463485 | 1.581336 |
| C | -0.393693 | -4.953290 | 3.604468 |
| H | -0.402973 | -5.714984 | 4.396387 |
| H | 0.480508 | -5.133470 | 2.965134 |
| H | -0.268067 | -3.969564 | 4.068739 |

**The Previous Au(II)-Au(II) and Rh(II)-Rh(II) Complexes**

**$K_M$**

| | | | |
|---|---|---|---|
| C | 4.485171 | 1.047414 | 0.000125 |
| C | 3.174053 | 1.528129 | 0.000159 |
| C | 4.729986 | -0.323902 | -0.000041 |
| C | 2.073373 | 0.671655 | 0.000035 |
| C | 3.660395 | -1.209698 | -0.000130 |
| C | 2.343903 | -0.732506 | -0.000086 |
| C | 1.204875 | -1.663941 | -0.000078 |
| C | -2.073373 | 0.671655 | -0.000040 |
| C | -3.174054 | 1.528129 | -0.000167 |
| C | 1.216702 | -3.064441 | -0.000096 |
| C | -1.204874 | -1.663941 | 0.000085 |
| C | -4.485172 | 1.047413 | -0.000122 |
| C | -2.343903 | -0.732506 | 0.000095 |
| C | 0.000001 | -3.742217 | -0.000012 |
| C | -1.216700 | -3.064441 | 0.000081 |
| C | -3.660395 | -1.209698 | 0.000149 |
| C | -4.729987 | -0.323903 | 0.000058 |
| H | 5.750859 | -0.701725 | -0.000095 |
| H | 3.856453 | -2.282583 | -0.000228 |
| H | -3.013365 | 2.606569 | -0.000302 |
| H | 2.155222 | -3.612763 | -0.000153 |
| H | 0.000001 | -4.830896 | -0.000020 |
| H | -3.856452 | -2.282584 | 0.000259 |

| | | | |
|---|---|---|---|
| H | -2.155221 | -3.612763 | 0.000126 |
| H | -5.750859 | -0.701727 | 0.000121 |
| Au | 0.000000 | 1.066549 | -0.000002 |
| N | 0.000001 | -1.074021 | 0.000011 |
| H | 3.013363 | 2.606569 | 0.000284 |
| H | -5.321255 | 1.746749 | -0.000195 |
| H | 5.321254 | 1.746750 | 0.000196 |

**K**

| | | | |
|---|---|---|---|
| C | -3.546997 | -3.103641 | 1.939132 |
| C | -2.657590 | -4.013897 | 2.495429 |
| C | -5.399700 | -1.028762 | 0.645155 |
| C | -6.079363 | 0.000794 | -0.000723 |
| C | -3.073818 | -1.987227 | 1.237925 |
| C | -1.286784 | -3.814405 | 2.347259 |
| C | -4.000679 | -1.017072 | 0.636816 |
| C | -5.399184 | 1.030198 | -0.646296 |
| C | -0.806738 | -2.705114 | 1.648763 |
| C | -1.670000 | -1.765554 | 1.084480 |
| C | 1.284173 | -3.814425 | -2.347105 |
| C | -4.000169 | 1.018207 | -0.637292 |
| C | 0.804921 | -2.705014 | -1.648237 |
| C | 2.654834 | -4.014404 | -2.495985 |
| C | -3.072830 | 1.988121 | -1.238050 |
| C | 1.668795 | -1.765925 | -1.084261 |
| C | -3.545457 | 3.104651 | -1.939438 |
| C | 3.544838 | -3.104544 | -1.939982 |
| C | -1.669141 | 1.766104 | -1.084097 |
| C | 3.072418 | -1.988096 | -1.238355 |
| C | -2.655602 | 4.014731 | -2.495304 |
| C | -0.805419 | 2.705447 | -1.647961 |

| | | | |
|---|---|---|---|
| C | 3.999874 | -1.018437 | -0.637545 |
| C | -1.284912 | 3.814925 | -2.346537 |
| C | 1.670471 | 1.765547 | 1.084317 |
| C | 0.807347 | 2.705260 | 1.648443 |
| C | 5.398864 | -1.030845 | -0.646454 |
| C | 4.000832 | 1.016665 | 0.636598 |
| C | 1.287569 | 3.814523 | 2.346907 |
| C | 3.074268 | 1.986950 | 1.237741 |
| C | 6.079301 | -0.001711 | -0.000735 |
| C | 5.399824 | 1.027945 | 0.645175 |
| C | 3.547657 | 3.103215 | 1.938983 |
| C | 2.658421 | 4.013693 | 2.495206 |
| H | -4.619609 | -3.265540 | 2.053313 |
| H | -3.032930 | -4.878047 | 3.040837 |
| H | -5.946640 | -1.824097 | 1.144372 |
| H | -7.168190 | 0.000894 | -0.000998 |
| H | -5.945715 | 1.825627 | -1.145803 |
| H | 0.269751 | -2.573653 | 1.537049 |
| H | 3.029586 | -4.878607 | -3.041729 |
| H | -4.617981 | 3.266745 | -2.054164 |
| H | 4.617316 | -3.266819 | -2.054817 |
| H | -0.269180 | 2.574032 | 1.536733 |
| H | -3.030508 | 4.878973 | -3.040869 |
| H | 0.270996 | 2.573701 | -1.535919 |
| H | 5.945225 | -1.826397 | -1.145935 |
| H | 7.168126 | -0.002138 | -0.000855 |
| H | 4.620277 | 3.264893 | 2.053279 |
| H | 5.946928 | 1.823019 | 1.144599 |
| H | 3.033902 | 4.877766 | 3.040646 |
| Au | -1.291227 | 0.000298 | 0.000388 |

| | | | |
|---|---|---|---|
| Au | 1.291228 | -0.000107 | 0.000185 |
| N | -3.392237 | 0.000519 | -0.000086 |
| N | 3.392218 | -0.000614 | -0.000458 |
| H | -0.271505 | -2.573174 | -1.536042 |
| H | 0.586227 | 4.528927 | 2.777503 |
| H | -0.585317 | -4.528634 | 2.777912 |
| H | -0.583109 | 4.529018 | -2.776873 |
| H | 0.582210 | -4.528325 | -2.777533 |

**$L_M$**

| | | | |
|---|---|---|---|
| N | 3.555622 | -0.006788 | -0.027529 |
| N | 2.054007 | 2.050438 | -0.546781 |
| N | 2.043079 | -2.050938 | -0.566543 |
| C | 4.145227 | -1.201925 | 0.213036 |
| C | 4.151550 | 1.182791 | 0.224505 |
| C | 3.301400 | 2.342557 | -0.058375 |
| C | 3.289112 | -2.354480 | -0.081383 |
| C | 6.096106 | -0.018227 | 0.957213 |
| C | 2.781998 | 4.689056 | -0.114075 |
| C | 2.757015 | -4.697384 | -0.160711 |
| O | -1.853740 | 2.968547 | -1.394018 |
| O | -2.029745 | -1.940572 | 0.857701 |
| S | -2.045325 | -3.189214 | 0.064417 |
| S | -2.034151 | 3.219221 | 0.056256 |
| O | -1.286145 | 4.374347 | 0.579671 |
| O | -1.980436 | 1.996504 | 0.888078 |
| C | -3.804377 | 3.720766 | 0.153559 |
| F | -4.172148 | 3.965074 | 1.417616 |
| F | -4.586076 | 2.737488 | -0.327116 |
| F | -4.028949 | 4.822619 | -0.570901 |
| O | -1.873195 | -2.976948 | -1.393420 |

| | | | |
|---|---|---|---|
| O | -1.262787 | -4.305658 | 0.620866 |
| C | -3.798826 | -3.741262 | 0.179801 |
| F | -4.154202 | -3.971791 | 1.449988 |
| F | -3.995151 | -4.863054 | -0.522242 |
| F | -4.609551 | -2.790655 | -0.316286 |
| C | 1.520373 | 4.375604 | -0.596316 |
| H | 0.757834 | 5.126139 | -0.778575 |
| C | 1.192727 | 3.037671 | -0.810566 |
| H | 0.211600 | 2.749300 | -1.191446 |
| C | 3.683524 | 3.660882 | 0.154315 |
| H | 4.675111 | 3.882836 | 0.542331 |
| C | 1.176090 | -3.030592 | -0.839036 |
| H | 0.195921 | -2.733388 | -1.215085 |
| C | 1.496764 | -4.372307 | -0.638330 |
| H | 0.730021 | -5.116945 | -0.826744 |
| C | 3.664219 | -3.676739 | 0.117535 |
| H | 4.654835 | -3.908169 | 0.502456 |
| C | 5.446624 | -1.224487 | 0.710576 |
| H | 5.944044 | -2.170292 | 0.907755 |
| C | 5.453043 | 1.193769 | 0.722046 |
| H | 5.955554 | 2.134931 | 0.928444 |
| H | 3.066444 | 5.722792 | 0.067586 |
| H | 7.111997 | -0.022774 | 1.344225 |
| H | 3.036104 | -5.734362 | 0.010430 |
| Rh | 1.680850 | 0.000552 | -0.626171 |
| N | 0.879123 | -0.006936 | 1.489689 |
| N | -0.262104 | 0.006612 | -1.256134 |
| C | -0.076931 | -0.003206 | 2.154744 |
| C | -1.421749 | 0.013900 | -1.310487 |
| C | -1.232165 | 0.001656 | 3.011070 |

| | | | |
|---|---|---|---|
| H | -1.817669 | 0.899818 | 2.784646 |
| H | -1.830856 | -0.885617 | 2.776457 |
| H | -0.931074 | -0.005042 | 4.063220 |
| C | -2.853953 | 0.019524 | -1.410126 |
| H | -3.171565 | 0.851595 | -2.045076 |
| H | -3.189603 | -0.949103 | -1.793112 |
| H | -3.256973 | 0.174290 | -0.404039 |

**L**

| | | | |
|---|---|---|---|
| N | 1.370076 | 0.018403 | -1.945337 |
| N | -0.189189 | 2.048693 | -1.465622 |
| N | -0.129453 | -2.058326 | -1.474154 |
| C | 1.985708 | -1.162980 | -2.166647 |
| C | 1.947412 | 1.218038 | -2.171390 |
| C | 1.061375 | 2.358617 | -1.930957 |
| C | 1.133577 | -2.329716 | -1.931105 |
| C | 3.944357 | 0.057718 | -2.800505 |
| C | 0.490097 | 4.690167 | -1.945161 |
| C | 0.632198 | -4.677539 | -1.956049 |
| S | -4.342350 | 3.965985 | 0.483992 |
| O | -4.764009 | 3.996134 | 1.897825 |
| O | -3.924626 | 2.619156 | 0.010397 |
| O | -3.454550 | 5.058221 | 0.054162 |
| C | -5.903819 | 4.230531 | -0.459114 |
| F | -6.474773 | 5.396074 | -0.151539 |
| F | -5.660713 | 4.210497 | -1.778845 |
| F | -6.777626 | 3.244008 | -0.187697 |
| S | -4.235114 | -4.071330 | 0.461740 |
| O | -4.661176 | -4.109313 | 1.874247 |
| O | -3.351651 | -5.163042 | 0.023754 |
| O | -3.809652 | -2.722337 | 0.001838 |

| | | | |
|---|---|---|---|
| C | -5.797317 | -4.309611 | -0.487309 |
| F | -5.549942 | -4.280102 | -1.806709 |
| F | -6.383882 | -5.470716 | -0.192342 |
| F | -6.657997 | -3.314272 | -0.210089 |
| N | -1.369536 | -0.016411 | 1.950707 |
| N | 0.186133 | -2.048261 | 1.468660 |
| N | 0.132209 | 2.058811 | 1.477673 |
| C | -1.982074 | 1.165725 | 2.176014 |
| C | -1.948972 | -1.215362 | 2.175129 |
| C | -1.065709 | -2.357149 | 1.931020 |
| C | -1.129636 | 2.331605 | 1.937255 |
| C | -3.940009 | -0.052017 | 2.816803 |
| C | -0.499409 | -4.690029 | 1.938322 |
| C | -0.627810 | 4.679407 | 1.953905 |
| O | 3.910982 | -2.639570 | 0.000339 |
| O | 3.807378 | 2.740532 | -0.013990 |
| S | 4.231198 | 4.090101 | -0.474907 |
| S | 4.343432 | -3.981506 | -0.474379 |
| O | 4.765420 | -4.003855 | -1.888429 |
| O | 3.468080 | -5.083698 | -0.045490 |
| C | 5.909107 | -4.224507 | 0.467876 |
| F | 6.501116 | -5.378306 | 0.155251 |
| F | 5.665454 | -4.215236 | 1.787456 |
| F | 6.764487 | -3.221030 | 0.200905 |
| O | 4.646100 | 4.129608 | -1.890738 |
| O | 3.353994 | 5.182652 | -0.027375 |
| C | 5.802194 | 4.320415 | 0.461271 |
| F | 5.564673 | 4.302129 | 1.781955 |
| F | 6.399241 | 5.473026 | 0.154477 |
| F | 6.650720 | 3.313765 | 0.183266 |

| | | | |
|---|---|---|---|
| C | 0.776113 | -4.360597 | 1.497066 |
| H | 1.544257 | -5.102179 | 1.288641 |
| C | 1.084931 | -3.021812 | 1.268448 |
| H | 2.066061 | -2.722809 | 0.891340 |
| C | -1.426397 | -3.678850 | 2.170306 |
| H | -2.435532 | -3.915935 | 2.503134 |
| C | 3.272367 | 1.262481 | -2.603361 |
| H | 3.777451 | 2.220185 | -2.726216 |
| C | 3.312552 | -1.167086 | -2.594976 |
| H | 3.848577 | -2.108647 | -2.711592 |
| C | 1.004146 | 3.057544 | 1.284714 |
| H | 1.995036 | 2.788797 | 0.910471 |
| C | 0.657345 | 4.386818 | 1.515659 |
| H | 1.405483 | 5.149997 | 1.312267 |
| C | -1.526618 | 3.641835 | 2.181065 |
| H | -2.541529 | 3.850320 | 2.515007 |
| C | -3.306662 | 1.171684 | 2.610888 |
| H | -3.840512 | 2.113759 | 2.731976 |
| C | -3.272495 | -1.257979 | 2.611464 |
| H | -3.779820 | -2.214854 | 2.732134 |
| C | -1.001771 | -3.057804 | -1.286267 |
| H | -1.993595 | -2.789609 | -0.913897 |
| C | -0.654296 | -4.386275 | -1.520606 |
| H | -1.402863 | -5.150154 | -1.321816 |
| C | 1.531586 | -3.639270 | -2.177203 |
| H | 2.547494 | -3.846425 | -2.509004 |
| C | -1.089421 | 3.021079 | -1.265618 |
| H | -2.069169 | 2.720782 | -0.885598 |
| C | -0.783428 | 4.359712 | -1.498942 |
| H | -1.552296 | 5.100295 | -1.290427 |

| | | | |
|---|---|---|---|
| C | 1.418744 | 3.680226 | -2.175733 |
| H | 2.426469 | 3.918106 | -2.512293 |
| H | -0.791574 | -5.726870 | 2.088796 |
| H | -4.980985 | -0.065666 | 3.132216 |
| H | -0.949926 | 5.707047 | 2.106242 |
| H | 0.779194 | 5.727115 | -2.100718 |
| H | 4.987031 | 0.073494 | -3.110000 |
| H | 0.954675 | -5.704701 | -2.110895 |
| Rh | -0.476122 | -0.007822 | -1.277590 |
| Rh | 0.476237 | 0.008704 | 1.282093 |
| N | 2.462707 | 0.037911 | 0.715794 |
| N | -2.463006 | -0.038697 | -0.711306 |
| C | 3.616752 | 0.068192 | 0.599882 |
| C | -3.616683 | -0.082140 | -0.596675 |
| C | 5.051270 | 0.103520 | 0.471660 |
| H | 5.330731 | 0.943898 | -0.169979 |
| H | 5.503699 | 0.266809 | 1.455740 |
| H | 5.394052 | -0.855187 | 0.070702 |
| C | -5.052167 | -0.136079 | -0.480894 |
| H | -5.475018 | -0.583005 | -1.386983 |
| H | -5.323029 | -0.787435 | 0.354823 |
| H | -5.436685 | 0.878303 | -0.336131 |
| N | 1.272539 | 0.006832 | 3.400533 |
| N | -1.274869 | -0.011855 | -3.394882 |
| C | 1.781435 | -0.020011 | 4.443678 |
| C | -1.790777 | -0.000617 | -4.434878 |
| C | 2.418855 | -0.054910 | 5.739267 |
| H | 2.920957 | -1.016165 | 5.887034 |
| H | 3.162250 | 0.744515 | 5.816937 |
| H | 1.676910 | 0.078757 | 6.532528 |

| | | | |
|---|---|---|---|
| C | -2.437450 | 0.012587 | -5.726303 |
| H | -2.961157 | -0.933333 | -5.896355 |
| H | -3.163874 | 0.829708 | -5.776302 |
| H | -1.698240 | 0.151937 | -6.521145 |

**Cartesian coordinates of key structures optimized by the B3LYP-D3BJ method**

**$1_M$**

| | | | |
|---|---|---|---|
| Ir | -0.281101 | -0.317508 | -1.249848 |
| P | -0.097202 | 0.378034 | 1.065450 |
| N | -0.469191 | -2.256458 | -0.762922 |
| N | 1.707476 | -0.949181 | -1.303342 |
| N | -2.336893 | -0.589056 | -1.379467 |
| C | -0.130420 | 1.453356 | -1.946829 |
| C | 0.300633 | 2.140415 | 1.184757 |
| C | -1.569316 | 0.065481 | 2.097490 |
| C | 1.226112 | -0.628828 | 1.816912 |
| C | -1.712129 | -2.735209 | -0.510123 |
| C | 0.655169 | -2.998438 | -0.614721 |
| C | 1.885533 | -2.259631 | -0.925066 |
| C | -2.775932 | -1.778297 | -0.839928 |
| O | -0.018905 | 2.494397 | -2.412701 |
| C | -0.720189 | -4.848211 | 0.143122 |
| C | 4.298061 | -2.058803 | -1.146615 |
| C | -5.090083 | -1.077320 | -1.080774 |
| C | -0.811243 | -6.276232 | 0.688297 |
| C | 5.717367 | -2.601241 | -1.001236 |
| C | -6.576134 | -1.302006 | -0.807956 |
| O | 4.158822 | 2.359516 | -1.008065 |
| O | -2.580655 | 2.666892 | -0.485539 |
| S | -3.997551 | 3.128203 | -0.577979 |
| S | 5.306141 | 2.037532 | -0.115176 |

| | | | |
|---|---|---|---|
| O | 6.376555 | 1.239421 | -0.761971 |
| O | 4.919975 | 1.578661 | 1.240396 |
| C | 6.087444 | 3.696238 | 0.176261 |
| F | 7.124623 | 3.592158 | 1.017747 |
| F | 5.190731 | 4.543407 | 0.716719 |
| F | 6.522570 | 4.224595 | -0.974871 |
| O | -4.478974 | 3.315848 | -1.962012 |
| O | -4.932285 | 2.398680 | 0.316724 |
| C | -3.909152 | 4.826608 | 0.168429 |
| F | -3.539965 | 4.737229 | 1.463486 |
| F | -5.100391 | 5.435258 | 0.115502 |
| F | -3.009367 | 5.584017 | -0.470003 |
| C | -1.457905 | -0.459061 | 3.398224 |
| H | -0.490103 | -0.727466 | 3.803014 |
| C | -2.596357 | -0.613775 | 4.185949 |
| H | -2.499365 | -1.019404 | 5.189078 |
| C | -3.847690 | -0.221728 | 3.703501 |
| H | -4.728295 | -0.326975 | 4.331211 |
| C | -3.956000 | 0.336852 | 2.430749 |
| H | -4.895185 | 0.723337 | 2.052013 |
| C | -2.825931 | 0.466174 | 1.625603 |
| H | -2.917179 | 0.960044 | 0.669934 |
| C | -0.606810 | 3.032369 | 1.776285 |
| H | -1.535189 | 2.676451 | 2.202603 |
| C | -0.329346 | 4.397125 | 1.776784 |
| H | -1.054096 | 5.082397 | 2.202506 |
| C | 0.849454 | 4.877208 | 1.205725 |
| H | 1.058684 | 5.943028 | 1.211348 |
| C | 1.752602 | 3.993394 | 0.611077 |
| H | 2.677776 | 4.340538 | 0.165585 |

| | | | |
|---|---|---|---|
| C | 1.471377 | 2.631744 | 0.581037 |
| H | 2.186895 | 1.988327 | 0.085234 |
| C | 2.531958 | -0.142621 | 1.965724 |
| H | 2.800645 | 0.874638 | 1.717944 |
| C | 3.541350 | -0.977244 | 2.444459 |
| H | 4.542176 | -0.568000 | 2.521319 |
| C | 3.258170 | -2.297181 | 2.795426 |
| H | 4.045346 | -2.941446 | 3.177348 |
| C | 1.957466 | -2.788276 | 2.658091 |
| H | 1.723728 | -3.812528 | 2.935510 |
| C | 0.950762 | -1.967061 | 2.158014 |
| H | -0.054593 | -2.360532 | 2.050485 |
| C | 4.077968 | -0.735050 | -1.544158 |
| H | 4.900267 | -0.066318 | -1.759549 |
| C | 2.794980 | -0.215729 | -1.599922 |
| H | 2.644565 | 0.823916 | -1.854363 |
| C | 3.158304 | -2.812139 | -0.841012 |
| H | 3.253004 | -3.841122 | -0.521497 |
| C | 6.601834 | -2.095413 | -2.161113 |
| H | 6.738896 | -1.012055 | -2.118812 |
| H | 7.593139 | -2.554541 | -2.085353 |
| H | 6.176744 | -2.363949 | -3.135214 |
| C | 5.740026 | -4.139056 | -0.983865 |
| H | 5.222345 | -4.549765 | -0.109126 |
| H | 5.287035 | -4.563381 | -1.888036 |
| H | 6.776625 | -4.486184 | -0.933811 |
| C | 6.290317 | -2.063746 | 0.332594 |
| H | 6.291486 | -0.970460 | 0.348877 |
| H | 5.704980 | -2.430925 | 1.181623 |
| H | 7.322695 | -2.412771 | 0.450191 |

| | | | |
|---|---|---|---|
| C | -3.244433 | 0.272487 | -1.865251 |
| H | -2.870320 | 1.185439 | -2.301450 |
| C | -4.610386 | 0.058190 | -1.736692 |
| H | -5.262690 | 0.841484 | -2.095098 |
| C | -4.132086 | -2.022001 | -0.680843 |
| H | -4.443403 | -2.950525 | -0.217383 |
| C | -7.393117 | -0.030528 | -1.097020 |
| H | -7.385445 | 0.223745 | -2.162234 |
| H | -8.436262 | -0.195478 | -0.808566 |
| H | -7.007982 | 0.828183 | -0.537266 |
| C | -6.774242 | -1.680724 | 0.675899 |
| H | -6.263886 | -2.612971 | 0.940670 |
| H | -6.399945 | -0.891054 | 1.333188 |
| H | -7.841176 | -1.821195 | 0.878891 |
| C | -7.075102 | -2.454751 | -1.706633 |
| H | -8.140462 | -2.634909 | -1.525512 |
| H | -6.945954 | -2.210307 | -2.766413 |
| H | -6.536490 | -3.388010 | -1.505324 |
| C | -1.851124 | -4.036580 | -0.036060 |
| H | -2.839531 | -4.414945 | 0.183395 |
| C | 0.537669 | -4.312274 | -0.178968 |
| H | 1.430740 | -4.910724 | -0.055996 |
| C | -2.260245 | -6.695500 | 0.983487 |
| H | -2.725882 | -6.050707 | 1.737012 |
| H | -2.880491 | -6.682144 | 0.080463 |
| H | -2.270643 | -7.717473 | 1.374423 |
| C | -0.217103 | -7.255272 | -0.348101 |
| H | 0.833875 | -7.037749 | -0.562677 |
| H | -0.273319 | -8.280466 | 0.033181 |
| H | -0.771186 | -7.210835 | -1.291739 |

| | | | |
|---|---|---|---|
| C | -0.001526 | -6.354593 | 2.002615 |
| H | 1.054345 | -6.111454 | 1.846567 |
| H | -0.401946 | -5.661804 | 2.750961 |
| H | -0.052768 | -7.368257 | 2.414369 |

**1**

| | | | |
|---|---|---|---|
| Ir | 0.115517 | -0.510765 | -1.332274 |
| P | 0.327717 | -1.240611 | -3.677397 |
| N | 0.172779 | 1.414343 | -1.928787 |
| N | -1.910227 | -0.121469 | -1.644494 |
| N | 2.163600 | -0.138089 | -1.304603 |
| C | 0.060048 | -2.316441 | -0.744114 |
| C | -0.770410 | -2.563539 | -4.291263 |
| C | 2.001439 | -1.856583 | -4.085355 |
| C | -0.061473 | 0.189055 | -4.740703 |
| C | 1.371561 | 2.026023 | -2.012039 |
| C | -0.990733 | 2.029413 | -2.223246 |
| C | -2.159705 | 1.139130 | -2.125014 |
| C | 2.502326 | 1.112465 | -1.770326 |
| O | 0.016543 | -3.430434 | -0.484663 |
| C | 0.231087 | 4.093520 | -2.528211 |
| C | -4.512326 | 0.613674 | -2.416051 |
| C | 4.838572 | 0.458301 | -1.921490 |
| C | 0.252360 | 5.614547 | -2.664790 |
| C | -5.927915 | 0.968525 | -2.864148 |
| C | 6.254434 | 0.755071 | -2.408832 |
| S | -4.139865 | -4.281035 | -0.023361 |
| O | -4.307462 | -4.381610 | 1.451514 |
| O | -2.745434 | -3.986489 | -0.455402 |
| O | -5.173177 | -3.499330 | -0.732284 |
| C | -4.400644 | -6.037199 | -0.569866 |

| | | | |
|---|---|---|---|
| F | -5.598387 | -6.481084 | -0.176027 |
| F | -4.337435 | -6.140217 | -1.911599 |
| F | -3.453910 | -6.829210 | -0.043359 |
| S | 3.952862 | -4.357256 | 0.509983 |
| O | 3.996388 | -4.547409 | 1.982285 |
| O | 5.108802 | -3.643816 | -0.078241 |
| O | 2.631239 | -3.906862 | -0.004577 |
| C | 4.085331 | -6.088028 | -0.148919 |
| F | 3.954190 | -6.091902 | -1.489575 |
| F | 5.267784 | -6.628082 | 0.160745 |
| F | 3.113179 | -6.856435 | 0.366476 |
| Ir | -0.115721 | 0.510537 | 1.332040 |
| P | -0.328349 | 1.240781 | 3.677192 |
| N | -0.172822 | -1.414493 | 1.928716 |
| N | 1.910061 | 0.121461 | 1.644255 |
| N | -2.163803 | 0.137670 | 1.304356 |
| C | -0.060070 | 2.316368 | 0.744036 |
| C | 0.768973 | 2.564507 | 4.290949 |
| C | -2.002412 | 1.856023 | 4.085006 |
| C | 0.061577 | -0.188526 | 4.740796 |
| C | -1.371547 | -2.026304 | 2.012018 |
| C | 0.990742 | -2.029387 | 2.223430 |
| C | 2.159636 | -1.138986 | 2.125126 |
| C | -2.502408 | -1.112884 | 1.770175 |
| O | -0.016107 | 3.430398 | 0.484889 |
| C | -0.230895 | -4.093592 | 2.528585 |
| C | 4.512231 | -0.613264 | 2.416027 |
| C | -4.838719 | -0.458956 | 1.921317 |
| C | -0.251954 | -5.614587 | 2.665421 |
| C | 5.927832 | -0.967799 | 2.864342 |

| | | | |
|---|---|---|---|
| C | -6.254588 | -0.755949 | 2.408489 |
| O | 2.745950 | 3.986372 | 0.455109 |
| O | -2.630664 | 3.906072 | 0.004365 |
| S | -3.952420 | 4.357349 | -0.509101 |
| S | 4.140317 | 4.280866 | 0.022833 |
| O | 4.307691 | 4.381233 | -1.452093 |
| O | 5.173762 | 3.499247 | 0.731647 |
| C | 4.401051 | 6.037074 | 0.569206 |
| F | 5.599033 | 6.480799 | 0.175901 |
| F | 4.337163 | 6.140025 | 1.910903 |
| F | 3.454676 | 6.829172 | 0.042168 |
| O | -3.996920 | 4.547676 | -1.981333 |
| O | -5.108322 | 3.644556 | 0.079977 |
| C | -4.083302 | 6.088072 | 0.150236 |
| F | -3.950118 | 6.091626 | 1.490688 |
| F | -5.266032 | 6.628584 | -0.157535 |
| F | -3.111662 | 6.856259 | -0.366472 |
| C | -2.770461 | 1.365880 | 5.149878 |
| H | -2.377172 | 0.597271 | 5.804068 |
| C | -4.046259 | 1.879886 | 5.385843 |
| H | -4.633657 | 1.492016 | 6.213198 |
| C | -4.560181 | 2.890253 | 4.571710 |
| H | -5.556891 | 3.281287 | 4.753857 |
| C | -3.788699 | 3.405354 | 3.529282 |
| H | -4.177166 | 4.185468 | 2.885697 |
| C | -2.516050 | 2.892784 | 3.287289 |
| H | -1.943478 | 3.306575 | 2.464233 |
| C | 0.742393 | 2.852254 | 5.667602 |
| H | 0.098193 | 2.281549 | 6.329820 |
| C | 1.541638 | 3.866425 | 6.183146 |

| | | | |
|---|---|---|---|
| H | 1.517740 | 4.081717 | 7.247496 |
| C | 2.367827 | 4.608743 | 5.332479 |
| H | 2.989922 | 5.401546 | 5.737623 |
| C | 2.387647 | 4.336684 | 3.966997 |
| H | 3.012733 | 4.907139 | 3.291309 |
| C | 1.589806 | 3.315964 | 3.446363 |
| H | 1.626045 | 3.144764 | 2.382797 |
| C | 1.383289 | -0.351952 | 5.186850 |
| H | 2.110920 | 0.435819 | 5.023785 |
| C | 1.771249 | -1.526776 | 5.828824 |
| H | 2.799238 | -1.641027 | 6.159694 |
| C | 0.849314 | -2.553810 | 6.031221 |
| H | 1.154726 | -3.472570 | 6.522695 |
| C | -0.465741 | -2.401187 | 5.588241 |
| H | -1.185516 | -3.201921 | 5.727365 |
| C | -0.857812 | -1.231634 | 4.941762 |
| H | -1.873847 | -1.141320 | 4.576423 |
| C | 4.233034 | 0.641396 | 1.854636 |
| H | 4.991138 | 1.398864 | 1.693812 |
| C | 2.939887 | 0.974785 | 1.491490 |
| H | 2.726144 | 1.946370 | 1.063733 |
| C | 3.442157 | -1.508367 | 2.517487 |
| H | 3.597262 | -2.512882 | 2.882677 |
| C | 6.807399 | -1.118221 | 1.602636 |
| H | 6.810806 | -0.196104 | 1.011646 |
| H | 7.840880 | -1.332885 | 1.897552 |
| H | 6.445666 | -1.940835 | 0.979071 |
| C | 5.968345 | -2.283043 | 3.659136 |
| H | 5.338812 | -2.225881 | 4.556316 |
| H | 5.642088 | -3.137711 | 3.061006 |

| | | | |
|---|---|---|---|
| H | 6.995245 | -2.473348 | 3.987833 |
| C | 6.479659 | 0.169435 | 3.751114 |
| H | 6.541478 | 1.122017 | 3.216674 |
| H | 5.852268 | 0.316894 | 4.638024 |
| H | 7.489103 | -0.088225 | 4.089201 |
| C | -0.964637 | 6.125336 | -3.459443 |
| H | -1.019633 | 5.658681 | -4.451031 |
| H | -1.909165 | 5.944958 | -2.938181 |
| H | -0.873110 | 7.206923 | -3.602138 |
| C | 1.546164 | 6.092570 | -3.353439 |
| H | 2.437746 | 5.886503 | -2.754518 |
| H | 1.671824 | 5.626255 | -4.338935 |
| H | 1.496576 | 7.176318 | -3.500597 |
| C | 0.200201 | 6.192783 | -1.227607 |
| H | 0.218196 | 7.287687 | -1.280258 |
| H | -0.715232 | 5.884824 | -0.716487 |
| H | 1.056919 | 5.858017 | -0.636938 |
| C | -0.982127 | 3.385576 | -2.529656 |
| H | -1.922314 | 3.894679 | -2.687535 |
| C | 1.422013 | 3.382709 | -2.305873 |
| H | 2.380187 | 3.884285 | -2.287774 |
| C | -3.138038 | 1.022861 | 1.058563 |
| H | -2.848073 | 1.982164 | 0.647958 |
| C | -4.470365 | 0.758744 | 1.351412 |
| H | -5.171723 | 1.558974 | 1.159608 |
| C | -3.820689 | -1.417786 | 2.071559 |
| H | -4.053022 | -2.415274 | 2.418902 |
| C | -7.155708 | 0.485443 | 2.309541 |
| H | -7.283605 | 0.818160 | 1.275447 |
| H | -8.147839 | 0.244380 | 2.704600 |

| | | | |
|---|---|---|---|
| H | -6.754509 | 1.322986 | 2.891237 |
| C | -6.176776 | -1.184353 | 3.892055 |
| H | -5.603526 | -2.107872 | 4.017947 |
| H | -5.711257 | -0.400961 | 4.500588 |
| H | -7.186369 | -1.366268 | 4.276365 |
| C | -6.859758 | -1.904229 | 1.574115 |
| H | -7.877640 | -2.110230 | 1.924924 |
| H | -6.908203 | -1.642090 | 0.513259 |
| H | -6.272213 | -2.820866 | 1.652600 |
| C | -1.421872 | -3.382947 | 2.306047 |
| H | -2.380008 | -3.884586 | 2.287906 |
| C | 0.982242 | -3.385501 | 2.530050 |
| H | 1.922479 | -3.894457 | 2.688135 |
| C | -1.545877 | -6.092742 | 3.353749 |
| H | -1.671894 | -5.626362 | 4.339169 |
| H | -2.437325 | -5.886873 | 2.754569 |
| H | -1.496160 | -7.176468 | 3.501028 |
| C | -0.199237 | -6.193079 | 1.228359 |
| H | 0.716235 | -5.884958 | 0.717400 |
| H | -0.216925 | -7.287977 | 1.281222 |
| H | -1.055909 | -5.858685 | 0.637412 |
| C | 0.964899 | -6.124998 | 3.460549 |
| H | 1.909540 | -5.944624 | 2.939487 |
| H | 1.019558 | -5.658050 | 4.452020 |
| H | 0.873480 | -7.206558 | 3.603521 |
| C | 3.137745 | -1.023333 | -1.058709 |
| H | 2.847745 | -1.982608 | -0.648063 |
| C | 4.470107 | -0.759356 | -1.351541 |
| H | 5.171400 | -1.559638 | -1.159657 |
| C | 3.820641 | 1.417255 | -2.071687 |

| | | | |
|---|---|---|---|
| H | 4.053117 | 2.414744 | -2.418960 |
| C | 7.155543 | -0.486290 | -2.309384 |
| H | 8.147684 | -0.245385 | -2.704516 |
| H | 7.283396 | -0.818604 | -1.275150 |
| H | 6.754359 | -1.324061 | -2.890770 |
| C | 6.859669 | 1.903712 | -1.575010 |
| H | 6.908173 | 1.642050 | -0.514034 |
| H | 7.877534 | 2.109550 | -1.925965 |
| H | 6.272140 | 2.820332 | -1.653856 |
| C | 6.176469 | 1.182782 | -3.892594 |
| H | 5.710932 | 0.399071 | -4.500709 |
| H | 5.603138 | 2.106205 | -4.018845 |
| H | 7.186016 | 1.364576 | -4.277086 |
| C | -2.940103 | -0.974776 | -1.492018 |
| H | -2.726442 | -1.946504 | -1.064548 |
| C | -4.233208 | -0.641191 | -1.855105 |
| H | -4.991332 | -1.398682 | -1.694553 |
| C | -3.442197 | 1.508737 | -2.517210 |
| H | -3.597233 | 2.513380 | -2.882066 |
| C | -6.480267 | -0.168584 | -3.750749 |
| H | -5.853114 | -0.316297 | -4.637776 |
| H | -6.542309 | -1.121117 | -3.216259 |
| H | -7.489694 | 0.089385 | -4.088649 |
| C | -6.807189 | 1.119270 | -1.602275 |
| H | -7.840646 | 1.334315 | -1.896993 |
| H | -6.810823 | 0.197156 | -1.011289 |
| H | -6.445054 | 1.941743 | -0.978761 |
| C | -5.968246 | 2.283737 | -3.659009 |
| H | -5.641878 | 3.138391 | -3.060930 |
| H | -5.338743 | 2.226441 | -4.556195 |

| | | | |
|---|---|---|---|
| H | -6.995125 | 2.474174 | -3.987693 |
| C | 2.769340 | -1.367108 | -5.150628 |
| H | 2.376139 | -0.598618 | -5.804997 |
| C | 4.044908 | -1.881594 | -5.386764 |
| H | 4.632208 | -1.494201 | -6.214412 |
| C | 4.558730 | -2.891839 | -4.572409 |
| H | 5.555262 | -3.283269 | -4.754688 |
| C | 3.787339 | -3.406322 | -3.529611 |
| H | 4.175664 | -4.186427 | -2.885925 |
| C | 2.514947 | -2.893222 | -3.287448 |
| H | 1.942504 | -3.306585 | -2.464119 |
| C | 0.858611 | 1.231469 | -4.941872 |
| H | 1.874662 | 1.140427 | -4.576788 |
| C | 0.467266 | 2.401294 | -5.588286 |
| H | 1.187606 | 3.201482 | -5.727624 |
| C | -0.847779 | 2.554892 | -6.030951 |
| H | -1.152618 | 3.473863 | -6.522385 |
| C | -1.770423 | 1.528556 | -5.828274 |
| H | -2.798409 | 1.643578 | -6.158869 |
| C | -1.383157 | 0.353473 | -5.186392 |
| H | -2.111335 | -0.433735 | -5.023174 |
| C | -0.745484 | -2.849829 | -5.668235 |
| H | -0.102246 | -2.278319 | -6.330673 |
| C | -1.545224 | -3.863533 | -6.183892 |
| H | -1.522651 | -4.077648 | -7.248507 |
| C | -2.370241 | -4.606878 | -5.333000 |
| H | -2.992756 | -5.399312 | -5.738224 |
| C | -2.388388 | -4.336292 | -3.967201 |
| H | -3.012649 | -4.907577 | -3.291463 |
| C | -1.590076 | -3.316007 | -3.446462 |

| | | | |
|---|---|---|---|
| H | -1.625142 | -3.145925 | -2.382678 |

**$2_M$**

| | | | |
|---|---|---|---|
| Ir | -0.466392 | -1.023683 | -1.220545 |
| P | -0.255445 | -0.425433 | 1.111481 |
| N | -0.825283 | -2.955731 | -0.807542 |
| N | 1.460267 | -1.820540 | -1.329445 |
| N | -2.546054 | -1.092689 | -1.314505 |
| C | -0.137948 | 0.738818 | -1.898033 |
| C | 0.212164 | 1.301324 | 1.366479 |
| C | -1.796639 | -0.724214 | 2.033450 |
| C | 1.034300 | -1.515483 | 1.794419 |
| C | -2.110990 | -3.331351 | -0.568133 |
| C | 0.234263 | -3.799363 | -0.717033 |
| C | 1.517996 | -3.160563 | -1.011387 |
| C | -3.083102 | -2.270190 | -0.842078 |
| O | 0.101361 | 1.747371 | -2.382433 |
| C | -1.295798 | -5.536942 | -0.072104 |
| C | 3.913018 | -3.137269 | -1.257147 |
| C | -5.304569 | -1.375682 | -1.095959 |
| O | 3.847579 | 1.451135 | -1.099391 |
| O | -2.222118 | 2.215271 | -0.447534 |
| S | -3.560467 | 2.870510 | -0.570048 |
| S | 4.983815 | 1.161543 | -0.177271 |
| O | 5.991253 | 0.227574 | -0.729679 |
| O | 4.577935 | 0.902559 | 1.224646 |
| C | 5.868622 | 2.791379 | -0.091935 |
| F | 6.921807 | 2.718088 | 0.732515 |
| F | 5.041928 | 3.750440 | 0.368805 |
| F | 6.300694 | 3.159969 | -1.304801 |
| O | -4.077616 | 2.901221 | -1.957324 |

| | | | |
|---|---|---|---|
| O | -4.535671 | 2.436775 | 0.456989 |
| C | -3.191348 | 4.640932 | -0.149634 |
| F | -2.776370 | 4.739488 | 1.125824 |
| F | -4.287110 | 5.397730 | -0.294620 |
| F | -2.228367 | 5.121378 | -0.945512 |
| C | -1.875192 | -1.536621 | 3.176764 |
| H | -0.992418 | -2.019456 | 3.575472 |
| C | -3.095729 | -1.692302 | 3.833164 |
| H | -3.148034 | -2.320390 | 4.718005 |
| C | -4.235022 | -1.024569 | 3.377486 |
| H | -5.178353 | -1.142966 | 3.903478 |
| C | -4.148557 | -0.176462 | 2.272272 |
| H | -4.995806 | 0.407651 | 1.929252 |
| C | -2.938509 | -0.032935 | 1.600103 |
| H | -2.867856 | 0.669168 | 0.780408 |
| C | -0.544724 | 2.132234 | 2.206753 |
| H | -1.418097 | 1.752207 | 2.720967 |
| C | -0.185791 | 3.468235 | 2.355048 |
| H | -0.787978 | 4.113094 | 2.986319 |
| C | 0.924021 | 3.979414 | 1.680384 |
| H | 1.194889 | 5.024411 | 1.798822 |
| C | 1.682209 | 3.153459 | 0.849629 |
| H | 2.557681 | 3.523623 | 0.329562 |
| C | 1.320358 | 1.821283 | 0.681290 |
| H | 1.934053 | 1.217170 | 0.025419 |
| C | 2.353546 | -1.050840 | 1.906876 |
| H | 2.622500 | -0.022877 | 1.701840 |
| C | 3.376289 | -1.922431 | 2.277826 |
| H | 4.386120 | -1.528414 | 2.324449 |
| C | 3.093768 | -3.257971 | 2.567303 |

| | | | |
|---|---|---|---|
| H | 3.890846 | -3.932315 | 2.867526 |
| C | 1.781276 | -3.726149 | 2.469962 |
| H | 1.550038 | -4.763872 | 2.695271 |
| C | 0.760257 | -2.869588 | 2.065253 |
| H | -0.246956 | -3.256823 | 1.963571 |
| C | 3.844396 | -1.785762 | -1.579736 |
| H | 4.732392 | -1.183385 | -1.744409 |
| C | 2.601036 | -1.160984 | -1.602327 |
| H | 2.539005 | -0.099152 | -1.799245 |
| C | 2.738652 | -3.826931 | -0.971138 |
| H | 2.766424 | -4.875322 | -0.699109 |
| C | -3.367261 | -0.115007 | -1.734438 |
| H | -2.907273 | 0.784575 | -2.110256 |
| C | -4.750789 | -0.215620 | -1.628997 |
| H | -5.343054 | 0.638107 | -1.934833 |
| C | -4.460912 | -2.418609 | -0.718603 |
| H | -4.868095 | -3.342935 | -0.325340 |
| C | -2.365263 | -4.641574 | -0.173875 |
| H | -3.376883 | -4.967405 | 0.035485 |
| C | 0.006766 | -5.126952 | -0.356996 |
| H | 0.831416 | -5.824778 | -0.282165 |
| H | 4.871062 | -3.643931 | -1.209192 |
| H | -1.482980 | -6.563359 | 0.224746 |
| H | -6.378662 | -1.475038 | -0.980314 |

**2**

| | | | |
|---|---|---|---|
| Ir | 0.987769 | 0.067507 | 1.059549 |
| P | 2.591693 | 0.157878 | 2.921533 |
| N | -0.552011 | 0.034883 | 2.369689 |
| N | 0.617691 | 2.113788 | 1.300622 |
| N | 0.767403 | -1.983916 | 1.369016 |

| | | | |
|---|---|---|---|
| C | 2.405342 | 0.070562 | -0.207757 |
| C | 3.972012 | 1.349508 | 2.846845 |
| C | 3.434038 | -1.436606 | 3.204467 |
| C | 1.675428 | 0.627658 | 4.423583 |
| C | -0.999965 | -1.165846 | 2.795613 |
| C | -1.078359 | 1.214261 | 2.764479 |
| C | -0.386920 | 2.382481 | 2.202486 |
| C | -0.206712 | -2.296458 | 2.291968 |
| O | 3.307988 | 0.061298 | -0.912551 |
| C | -2.712910 | -0.015540 | 4.007228 |
| C | -0.001518 | 4.744476 | 1.956410 |
| C | 0.425619 | -4.616347 | 2.189619 |
| S | 3.573245 | 4.254428 | -2.067456 |
| O | 3.353299 | 4.095372 | -3.527297 |
| O | 3.651939 | 2.965613 | -1.322803 |
| O | 2.732332 | 5.282715 | -1.414550 |
| C | 5.298036 | 4.935952 | -1.966355 |
| F | 5.406040 | 6.054536 | -2.688939 |
| F | 5.607589 | 5.231707 | -0.686472 |
| F | 6.186988 | 4.041258 | -2.413512 |
| S | 3.951995 | -4.038679 | -1.838382 |
| O | 3.708131 | -3.943259 | -3.300352 |
| O | 3.218234 | -5.126498 | -1.151825 |
| O | 3.913481 | -2.730963 | -1.126217 |
| C | 5.736966 | -4.542736 | -1.736599 |
| F | 6.099628 | -4.739693 | -0.451446 |
| F | 5.943245 | -5.683095 | -2.403007 |
| F | 6.523999 | -3.591488 | -2.251153 |
| Ir | -0.987750 | -0.067462 | -1.059602 |
| P | -2.591712 | -0.157723 | -2.921558 |

| | | | |
|---|---|---|---|
| N | 0.552022 | -0.034770 | -2.369752 |
| N | -0.617624 | -2.113727 | -1.300724 |
| N | -0.767413 | 1.983981 | -1.369007 |
| C | -2.405336 | -0.070621 | 0.207693 |
| C | -3.972034 | -1.349353 | -2.846969 |
| C | -3.434081 | 1.436775 | -3.204352 |
| C | -1.675461 | -0.627385 | -4.423649 |
| C | 0.999956 | 1.165978 | -2.795644 |
| C | 1.078390 | -1.214127 | -2.764578 |
| C | 0.386990 | -2.382375 | -2.202601 |
| C | 0.206698 | 2.296565 | -2.291947 |
| O | -3.308005 | -0.061450 | 0.912457 |
| C | 2.712907 | 0.015733 | -4.007311 |
| C | 0.001691 | -4.744387 | -1.956532 |
| C | -0.425648 | 4.616448 | -2.189500 |
| O | -3.651934 | -2.965634 | 1.322549 |
| O | -3.913000 | 2.730688 | 1.126860 |
| S | -3.951971 | 4.038594 | 1.838660 |
| S | -3.573176 | -4.254398 | 2.067282 |
| O | -3.353371 | -4.095240 | 3.527132 |
| O | -2.732111 | -5.282640 | 1.414499 |
| C | -5.297886 | -4.936106 | 1.966051 |
| F | -5.405837 | -6.054678 | 2.688660 |
| F | -5.607285 | -5.231938 | 0.686146 |
| F | -6.186980 | -4.041496 | 2.413092 |
| O | -3.707769 | 3.943679 | 3.300610 |
| O | -3.218855 | 5.126551 | 1.151648 |
| C | -5.737216 | 4.541744 | 1.737172 |
| F | -6.100315 | 4.738211 | 0.452068 |
| F | -5.943911 | 5.682154 | 2.403362 |

| | | | |
|---|---|---|---|
| F | -6.523632 | 3.590214 | 2.252152 |
| C | -3.494084 | 2.076033 | -4.449986 |
| H | -3.027870 | 1.631356 | -5.320951 |
| C | -4.176758 | 3.285987 | -4.579897 |
| H | -4.214655 | 3.776061 | -5.548508 |
| C | -4.811338 | 3.858816 | -3.477152 |
| H | -5.334863 | 4.804548 | -3.581321 |
| C | -4.780046 | 3.211939 | -2.240934 |
| H | -5.264880 | 3.642003 | -1.373160 |
| C | -4.097100 | 2.006596 | -2.105297 |
| H | -4.091306 | 1.529169 | -1.132233 |
| C | -4.763577 | -1.495050 | -4.001152 |
| H | -4.527568 | -0.930620 | -4.898239 |
| C | -5.850434 | -2.361721 | -3.995962 |
| H | -6.455856 | -2.470408 | -4.891085 |
| C | -6.162968 | -3.086087 | -2.840803 |
| H | -7.011837 | -3.763554 | -2.839046 |
| C | -5.391090 | -2.934495 | -1.692004 |
| H | -5.618650 | -3.488008 | -0.789939 |
| C | -4.297368 | -2.066266 | -1.693187 |
| H | -3.734021 | -1.970910 | -0.778730 |
| C | -1.605985 | -1.984979 | -4.775913 |
| H | -2.202141 | -2.714523 | -4.238323 |
| C | -0.773094 | -2.401498 | -5.813005 |
| H | -0.726680 | -3.454832 | -6.072204 |
| C | 0.001874 | -1.472450 | -6.507438 |
| H | 0.653326 | -1.799009 | -7.312248 |
| C | -0.056561 | -0.121916 | -6.159066 |
| H | 0.552938 | 0.605557 | -6.686200 |
| C | -0.881653 | 0.299331 | -5.119330 |

| | | | |
|---|---|---|---|
| H | -0.896385 | 1.347064 | -4.842650 |
| C | -1.001210 | -4.461755 | -1.035484 |
| H | -1.565569 | -5.223044 | -0.509163 |
| C | -1.283470 | -3.133756 | -0.731342 |
| H | -2.042578 | -2.894892 | 0.003480 |
| C | 0.697287 | -3.693233 | -2.547848 |
| H | 1.511832 | -3.889230 | -3.233684 |
| C | -2.195110 | 1.214567 | 3.599160 |
| H | -2.691263 | 2.144843 | 3.853860 |
| C | -2.118798 | -1.218966 | 3.626334 |
| H | -2.549977 | -2.174226 | 3.908046 |
| C | -1.532136 | 2.960251 | -0.856985 |
| H | -2.276522 | 2.687273 | -0.118306 |
| C | -1.384628 | 4.291504 | -1.240143 |
| H | -2.027961 | 5.020226 | -0.760125 |
| C | 0.374080 | 3.606283 | -2.724237 |
| H | 1.158422 | 3.843189 | -3.432346 |
| C | 2.118771 | 1.219137 | -3.626385 |
| H | 2.549922 | 2.174410 | -3.908096 |
| C | 2.195138 | -1.214393 | -3.599263 |
| H | 2.691330 | -2.144649 | -3.853959 |
| C | 1.532112 | -2.960214 | 0.857029 |
| H | 2.276508 | -2.687275 | 0.118347 |
| C | 1.384593 | -4.291450 | 1.240241 |
| H | 2.027901 | -5.020197 | 0.760231 |
| C | -0.374099 | -3.606153 | 2.724316 |
| H | -1.158445 | -3.843024 | 3.432432 |
| C | 1.283590 | 3.133787 | 0.731242 |
| H | 2.042704 | 2.894885 | -0.003561 |
| C | 1.001377 | 4.461799 | 1.035367 |

| | | | |
|---|---|---|---|
| H | 1.565776 | 5.223062 | 0.509050 |
| C | -0.697158 | 3.693357 | 2.547732 |
| H | -1.511674 | 3.889391 | 3.233592 |
| C | 3.494033 | -2.075748 | 4.450161 |
| H | 3.027838 | -1.630977 | 5.321088 |
| C | 4.176672 | -3.285710 | 4.580177 |
| H | 4.214565 | -3.775695 | 5.548833 |
| C | 4.811225 | -3.858660 | 3.477478 |
| H | 5.334720 | -4.804398 | 3.581731 |
| C | 4.779945 | -3.211896 | 2.241201 |
| H | 5.264749 | -3.642055 | 1.373456 |
| C | 4.097034 | -2.006544 | 2.105461 |
| H | 4.091251 | -1.529213 | 1.132351 |
| C | 0.881605 | -0.299012 | 5.119311 |
| H | 0.896332 | -1.346761 | 4.842692 |
| C | 0.056509 | 0.122299 | 6.159018 |
| H | -0.553002 | -0.605140 | 6.686186 |
| C | -0.001914 | 1.472852 | 6.507318 |
| H | -0.653370 | 1.799462 | 7.312105 |
| C | 0.773068 | 2.401854 | 5.812840 |
| H | 0.726662 | 3.455203 | 6.071981 |
| C | 1.605959 | 1.985271 | 4.775773 |
| H | 2.202120 | 2.714783 | 4.238146 |
| C | 4.763459 | 1.495443 | 4.001065 |
| H | 4.527360 | 0.931216 | 4.898255 |
| C | 5.850325 | 2.362104 | 3.995786 |
| H | 6.455666 | 2.470978 | 4.890941 |
| C | 6.162970 | 3.086217 | 2.840500 |
| H | 7.011845 | 3.763676 | 2.838672 |
| C | 5.391189 | 2.934390 | 1.691667 |

| | | | |
|---|---|---|---|
| H | 5.618826 | 3.487717 | 0.789507 |
| C | 4.297455 | 2.066177 | 1.692940 |
| H | 3.734173 | 1.970653 | 0.778460 |
| H | 0.274489 | -5.766141 | -2.195708 |
| H | 3.609546 | 0.036914 | -4.617266 |
| H | -0.265490 | 5.642890 | -2.499739 |
| H | -0.274255 | 5.766246 | 2.195596 |
| H | -3.609555 | -0.036687 | 4.617175 |
| H | 0.265452 | -5.642775 | 2.499898 |

**$3_M$**

| | | | |
|---|---|---|---|
| Ir | -0.822987 | 0.371977 | -0.947500 |
| P | -0.716299 | 0.596287 | 1.441714 |
| O | -3.482908 | 1.729762 | -1.399763 |
| N | -0.894526 | -1.721791 | -0.851031 |
| N | 1.032611 | -0.158961 | -1.615702 |
| N | 0.040205 | 2.218522 | -1.350302 |
| C | -1.668174 | -2.721090 | -0.280444 |
| C | -2.892420 | -2.678995 | 0.387607 |
| H | -3.448342 | -1.766957 | 0.505164 |
| C | -3.407333 | -3.874641 | 0.859482 |
| H | -4.376704 | -3.856327 | 1.345222 |
| C | -2.713653 | -5.093355 | 0.701355 |
| H | -3.150859 | -6.008269 | 1.089112 |
| C | -1.483312 | -5.151576 | 0.060213 |
| H | -0.944125 | -6.086496 | -0.046361 |
| C | -0.973886 | -3.944977 | -0.427879 |
| C | 0.242151 | -2.309203 | -1.296646 |
| C | 1.314479 | -1.476848 | -1.806976 |
| C | 2.503722 | -1.839815 | -2.435791 |
| H | 2.757423 | -2.875586 | -2.598809 |

| | | | |
|---|---|---|---|
| C | 3.380498 | -0.841852 | -2.854380 |
| H | 4.314147 | -1.115058 | -3.329504 |
| C | 3.088880 | 0.498533 | -2.626087 |
| H | 3.789802 | 1.262247 | -2.926893 |
| C | 1.895360 | 0.824361 | -1.989281 |
| C | 1.344312 | 2.132007 | -1.696546 |
| C | 0.951114 | 4.286800 | -1.364539 |
| C | 1.013654 | 5.669550 | -1.178353 |
| H | 1.933276 | 6.225905 | -1.321115 |
| C | -0.159336 | 6.306211 | -0.792587 |
| H | -0.152675 | 7.380681 | -0.639799 |
| C | -1.360341 | 5.590414 | -0.603704 |
| H | -2.256906 | 6.127408 | -0.311722 |
| C | -1.423973 | 4.215136 | -0.782002 |
| H | -2.347437 | 3.672503 | -0.629815 |
| C | -0.243993 | 3.560524 | -1.155307 |
| C | 1.251245 | -4.632905 | -1.422863 |
| H | 1.564025 | -4.451912 | -2.455290 |
| H | 0.767043 | -5.611692 | -1.424738 |
| C | 2.437802 | -4.607622 | -0.456057 |
| H | 2.757537 | -3.579551 | -0.260578 |
| H | 2.109291 | -5.007383 | 0.509954 |
| C | 3.627609 | -5.413875 | -0.984428 |
| H | 3.944411 | -4.994978 | -1.951817 |
| H | 3.318440 | -6.449051 | -1.188499 |
| C | 4.810567 | -5.395758 | -0.013367 |
| H | 4.534227 | -5.845638 | 0.947336 |
| H | 5.131305 | -4.369578 | 0.184632 |
| H | 5.658740 | -5.959399 | -0.416744 |
| C | 3.369697 | 3.644883 | -1.771867 |

| | | | |
|---|---|---|---|
| H | 3.471551 | 4.725717 | -1.890354 |
| H | 3.783243 | 3.200210 | -2.680280 |
| C | 4.080346 | 3.159057 | -0.507265 |
| H | 3.665620 | 3.710334 | 0.343320 |
| H | 3.852553 | 2.102936 | -0.325520 |
| C | 5.597394 | 3.349898 | -0.576493 |
| H | 5.833177 | 4.384790 | -0.865331 |
| H | 6.006252 | 2.705128 | -1.365698 |
| C | 6.270514 | 3.014670 | 0.757275 |
| H | 5.931166 | 3.698325 | 1.545163 |
| H | 7.359333 | 3.106278 | 0.682212 |
| H | 6.031176 | 1.996405 | 1.077657 |
| C | 0.563551 | 1.784426 | 1.978729 |
| C | 1.826697 | 1.379908 | 2.430504 |
| H | 2.078487 | 0.334611 | 2.510967 |
| C | 2.783146 | 2.327327 | 2.793574 |
| H | 3.761481 | 1.979551 | 3.106777 |
| C | 2.484033 | 3.687187 | 2.721290 |
| H | 3.229086 | 4.423876 | 3.009405 |
| C | 1.224056 | 4.102037 | 2.279443 |
| H | 0.977801 | 5.158141 | 2.223182 |
| C | 0.270820 | 3.158636 | 1.907781 |
| H | -0.711426 | 3.490179 | 1.595782 |
| C | -2.251606 | 1.149434 | 2.257663 |
| C | -2.185763 | 1.858581 | 3.470556 |
| H | -1.226723 | 2.155849 | 3.878915 |
| C | -3.356241 | 2.174819 | 4.155603 |
| H | -3.295958 | 2.727663 | 5.088760 |
| C | -4.597665 | 1.775976 | 3.652575 |
| H | -5.506435 | 2.021745 | 4.194503 |

| | | | |
|---|---|---|---|
| C | -4.669177 | 1.054250 | 2.461922 |
| H | -5.611622 | 0.708936 | 2.054087 |
| C | -3.500361 | 0.745839 | 1.767083 |
| H | -3.596236 | 0.170497 | 0.857389 |
| C | -0.334559 | -1.020250 | 2.183090 |
| C | -1.257433 | -1.644521 | 3.034347 |
| H | -2.193639 | -1.158016 | 3.279750 |
| C | -0.978101 | -2.907386 | 3.554426 |
| H | -1.706513 | -3.391528 | 4.197715 |
| C | 0.226167 | -3.541227 | 3.251356 |
| H | 0.446246 | -4.518037 | 3.672740 |
| C | 1.152101 | -2.917826 | 2.411652 |
| H | 2.112897 | -3.368691 | 2.194031 |
| C | 0.862965 | -1.676295 | 1.852487 |
| H | 1.584206 | -1.222455 | 1.181413 |
| C | -2.594675 | 1.046279 | -1.147042 |
| S | -5.642997 | -1.211794 | -0.938728 |
| F | -6.426611 | 0.789343 | -2.509458 |
| F | -6.083423 | 1.360927 | -0.437453 |
| F | -7.868406 | 0.243243 | -0.974394 |
| O | -4.291032 | -0.931447 | -1.506358 |
| O | -5.644504 | -1.299443 | 0.547951 |
| O | -6.427433 | -2.227708 | -1.652512 |
| C | -6.561667 | 0.380072 | -1.239499 |
| S | 4.397090 | -0.877950 | 0.997796 |
| F | 5.467022 | -2.017569 | -1.159511 |
| F | 6.928489 | -1.282654 | 0.270845 |
| F | 5.853574 | 0.118826 | -1.002181 |
| O | 4.111440 | -2.289211 | 1.337252 |
| O | 5.004438 | -0.051327 | 2.057522 |

| | | | |
|---|---|---|---|
| O | 3.303524 | -0.205797 | 0.242819 |
| C | 5.744268 | -1.022989 | -0.284311 |
| N | 0.212242 | -3.655315 | -1.087203 |
| N | 1.927429 | 3.364050 | -1.721945 |

**3**

| | | | |
|---|---|---|---|
| Ir | -0.947364 | -0.091355 | -1.117739 |
| P | -2.485278 | -0.169934 | -3.015341 |
| O | 1.438438 | 0.306062 | -2.928914 |
| N | -1.342619 | -2.115667 | -0.772364 |
| N | -2.540557 | -0.020200 | 0.182037 |
| N | -1.254422 | 1.947130 | -0.911132 |
| C | -0.951065 | -3.348206 | -1.263369 |
| C | 0.052477 | -3.711157 | -2.162532 |
| H | 0.735567 | -2.995383 | -2.596561 |
| C | 0.180071 | -5.059001 | -2.466771 |
| H | 0.938997 | -5.355567 | -3.184516 |
| C | -0.651906 | -6.032879 | -1.872987 |
| H | -0.520883 | -7.077417 | -2.138001 |
| C | -1.632518 | -5.686493 | -0.951628 |
| H | -2.265035 | -6.434828 | -0.487877 |
| C | -1.772909 | -4.326987 | -0.660374 |
| C | -2.371090 | -2.329682 | 0.083823 |
| C | -3.080494 | -1.179782 | 0.627319 |
| C | -4.197606 | -1.147957 | 1.463198 |
| H | -4.624375 | -2.047409 | 1.878091 |
| C | -4.753996 | 0.083168 | 1.785830 |
| H | -5.644207 | 0.128455 | 2.397398 |
| C | -4.198637 | 1.268006 | 1.300988 |
| H | -4.704987 | 2.199476 | 1.505926 |
| C | -3.067487 | 1.196018 | 0.495866 |

| | | | |
|---|---|---|---|
| C | -2.310834 | 2.271702 | -0.129016 |
| C | -1.521758 | 4.182563 | -0.924478 |
| C | -1.269067 | 5.513441 | -1.262028 |
| H | -1.874399 | 6.322665 | -0.870836 |
| C | -0.213179 | 5.750651 | -2.132553 |
| H | 0.011170 | 6.770151 | -2.429682 |
| C | 0.568471 | 4.694595 | -2.646748 |
| H | 1.376201 | 4.920237 | -3.335853 |
| C | 0.322045 | 3.371475 | -2.305827 |
| H | 0.918175 | 2.570695 | -2.719280 |
| C | -0.742620 | 3.119408 | -1.433082 |
| C | -3.535835 | -4.339021 | 1.139654 |
| H | -3.532189 | -3.773269 | 2.067611 |
| H | -3.065519 | -5.300680 | 1.361062 |
| C | -4.948742 | -4.529311 | 0.598775 |
| H | -5.374937 | -3.551498 | 0.352382 |
| H | -4.913097 | -5.090083 | -0.344431 |
| C | -5.844721 | -5.239189 | 1.618213 |
| H | -5.810082 | -4.691052 | 2.566853 |
| H | -5.442077 | -6.241331 | 1.823008 |
| C | -7.289317 | -5.352135 | 1.126414 |
| H | -7.347611 | -5.909637 | 0.182918 |
| H | -7.722637 | -4.359415 | 0.955537 |
| H | -7.918894 | -5.867457 | 1.859109 |
| C | -3.413922 | 4.432670 | 0.700707 |
| H | -2.822134 | 5.271861 | 1.078834 |
| H | -3.712591 | 3.850247 | 1.570956 |
| C | -4.626065 | 4.921474 | -0.087099 |
| H | -4.277209 | 5.480547 | -0.965731 |
| H | -5.195633 | 4.061893 | -0.450338 |

| | | | |
|---|---|---|---|
| C | -5.515750 | 5.807527 | 0.788925 |
| H | -4.919472 | 6.633105 | 1.206733 |
| H | -5.883062 | 5.209226 | 1.630070 |
| C | -6.710426 | 6.367436 | 0.016096 |
| H | -6.383907 | 6.954114 | -0.852162 |
| H | -7.318471 | 7.020373 | 0.651430 |
| H | -7.349565 | 5.555709 | -0.339314 |
| C | -2.930088 | 1.532081 | -3.512904 |
| C | -4.074565 | 2.208391 | -3.077483 |
| H | -4.838562 | 1.732331 | -2.483157 |
| C | -4.266835 | 3.547797 | -3.421917 |
| H | -5.164153 | 4.046828 | -3.071331 |
| C | -3.325513 | 4.221416 | -4.198109 |
| H | -3.477619 | 5.264245 | -4.460877 |
| C | -2.188190 | 3.545988 | -4.646794 |
| H | -1.450036 | 4.059137 | -5.255347 |
| C | -1.986972 | 2.212328 | -4.305062 |
| H | -1.100157 | 1.696281 | -4.660885 |
| C | -1.885576 | -0.885194 | -4.585620 |
| C | -2.733616 | -0.751921 | -5.701947 |
| H | -3.661769 | -0.195370 | -5.616467 |
| C | -2.385452 | -1.338940 | -6.912659 |
| H | -3.043797 | -1.231674 | -7.769875 |
| C | -1.195384 | -2.066767 | -7.023783 |
| H | -0.928711 | -2.527637 | -7.970480 |
| C | -0.348897 | -2.198139 | -5.926369 |
| H | 0.580281 | -2.754534 | -5.996781 |
| C | -0.694493 | -1.601638 | -4.710056 |
| H | -0.014403 | -1.709644 | -3.881163 |
| C | -3.959369 | -1.179910 | -2.641343 |

| | | | |
|---|---|---|---|
| C | -4.006639 | -2.489769 | -3.156573 |
| H | -3.200389 | -2.861925 | -3.777997 |
| C | -5.101370 | -3.306680 | -2.888480 |
| H | -5.128877 | -4.311870 | -3.299161 |
| C | -6.160516 | -2.832970 | -2.110307 |
| H | -7.018102 | -3.470095 | -1.914433 |
| C | -6.104406 | -1.551457 | -1.564174 |
| H | -6.897825 | -1.159371 | -0.935146 |
| C | -5.000716 | -0.734862 | -1.813226 |
| H | -4.997443 | 0.244991 | -1.356988 |
| C | 0.567334 | 0.042630 | -2.224394 |
| S | 3.263849 | -2.666643 | -4.501457 |
| F | 4.065074 | -0.141871 | -4.655879 |
| F | 2.269956 | -0.580890 | -5.800156 |
| F | 4.212846 | -1.241137 | -6.527162 |
| O | 2.353431 | -2.264826 | -3.386532 |
| O | 2.655315 | -3.589096 | -5.477615 |
| O | 4.629868 | -2.992427 | -4.034087 |
| C | 3.469687 | -1.067930 | -5.430984 |
| S | -7.175643 | 1.941172 | 0.149694 |
| F | -9.730638 | 2.562390 | 0.446371 |
| F | -9.230420 | 1.697672 | -1.487094 |
| F | -8.694002 | 3.757366 | -1.049240 |
| O | -7.424798 | 0.527214 | 0.508084 |
| O | -6.246513 | 2.130002 | -1.001219 |
| O | -6.888386 | 2.845864 | 1.289129 |
| C | -8.808030 | 2.523955 | -0.520204 |
| Ir | 0.947771 | -0.090626 | 1.117950 |
| P | 2.485486 | -0.167950 | 3.015562 |
| O | -1.438111 | 0.308152 | 2.928687 |

| | | | |
|---|---|---|---|
| N | 1.342764 | -2.115211 | 0.773827 |
| N | 2.540988 | -0.020552 | -0.181946 |
| N | 1.255026 | 1.947647 | 0.909791 |
| C | 0.950899 | -3.347392 | 1.265458 |
| C | -0.052791 | -3.709642 | 2.164737 |
| H | -0.735751 | -2.993490 | 2.598363 |
| C | -0.180756 | -5.057306 | 2.469644 |
| H | -0.939801 | -5.353333 | 3.187488 |
| C | 0.650976 | -6.031701 | 1.876360 |
| H | 0.519668 | -7.076082 | 2.141862 |
| C | 1.631710 | -5.686023 | 0.954859 |
| H | 2.264032 | -6.434751 | 0.491479 |
| C | 1.772485 | -4.326692 | 0.662965 |
| C | 2.371159 | -2.329911 | -0.082276 |
| C | 3.080717 | -1.180487 | -0.626522 |
| C | 4.197726 | -1.149367 | -1.462549 |
| H | 4.624310 | -2.049138 | -1.876912 |
| C | 4.754227 | 0.081468 | -1.786063 |
| H | 5.644389 | 0.126230 | -2.397735 |
| C | 4.199046 | 1.266704 | -1.302003 |
| H | 4.705605 | 2.197930 | -1.507546 |
| C | 3.067985 | 1.195380 | -0.496699 |
| C | 2.311456 | 2.271569 | 0.127428 |
| C | 1.522247 | 4.183087 | 0.921198 |
| C | 1.269552 | 5.514230 | 1.257697 |
| H | 1.874894 | 6.323149 | 0.865898 |
| C | 0.213646 | 5.752131 | 2.128016 |
| H | -0.010698 | 6.771867 | 2.424343 |
| C | -0.568040 | 4.696486 | 2.643015 |
| H | -1.375798 | 4.922680 | 3.331912 |

| | | | |
|---|---|---|---|
| C | -0.321608 | 3.373090 | 2.303139 |
| H | -0.917785 | 2.572642 | 2.717193 |
| C | 0.743100 | 3.120346 | 1.430630 |
| C | 3.535542 | -4.340125 | -1.136882 |
| H | 3.531988 | -3.775069 | -2.065256 |
| H | 3.065159 | -5.301903 | -1.357617 |
| C | 4.948405 | -4.530164 | -0.595789 |
| H | 5.374680 | -3.552233 | -0.350023 |
| H | 4.912655 | -5.090306 | 0.347786 |
| C | 5.844361 | -5.240805 | -1.614719 |
| H | 5.809807 | -4.693298 | -2.563723 |
| H | 5.441615 | -6.243038 | -1.818872 |
| C | 7.288926 | -5.353584 | -1.122797 |
| H | 7.347149 | -5.910626 | -0.179029 |
| H | 7.722284 | -4.360802 | -0.952386 |
| H | 7.918510 | -5.869289 | -1.855215 |
| C | 3.414574 | 4.431953 | -0.703923 |
| H | 2.822707 | 5.270710 | -1.082884 |
| H | 3.713547 | 3.848843 | -1.573607 |
| C | 4.626467 | 4.921665 | 0.083707 |
| H | 4.277331 | 5.481364 | 0.961835 |
| H | 5.196203 | 4.062510 | 0.447706 |
| C | 5.516015 | 5.807235 | -0.792953 |
| H | 4.919527 | 6.632219 | -1.211634 |
| H | 5.883679 | 5.208293 | -1.633488 |
| C | 6.710349 | 6.368335 | -0.020455 |
| H | 6.383458 | 6.955920 | 0.847048 |
| H | 7.318368 | 7.020712 | -0.656389 |
| H | 7.349638 | 5.557209 | 0.336051 |
| C | 2.930591 | 1.534270 | 3.512006 |

| | | | |
|---|---|---|---|
| C | 4.075719 | 2.209665 | 3.076913 |
| H | 4.839969 | 1.732589 | 2.483754 |
| C | 4.268249 | 3.549350 | 3.420091 |
| H | 5.165969 | 4.047726 | 3.069597 |
| C | 3.326607 | 4.224082 | 4.194939 |
| H | 3.478898 | 5.267122 | 4.456765 |
| C | 2.188728 | 3.549506 | 4.643526 |
| H | 1.450369 | 4.063536 | 5.251089 |
| C | 1.987197 | 2.215609 | 4.302880 |
| H | 1.099900 | 1.700276 | 4.658548 |
| C | 1.885477 | -0.882057 | 4.586225 |
| C | 2.733138 | -0.747784 | 5.702713 |
| H | 3.661181 | -0.191068 | 5.617147 |
| C | 2.384747 | -1.334016 | 6.913749 |
| H | 3.042799 | -1.225969 | 7.771093 |
| C | 1.194810 | -2.062038 | 7.025053 |
| H | 0.927962 | -2.522272 | 7.972012 |
| C | 0.348671 | -2.194382 | 5.927474 |
| H | -0.580457 | -2.750851 | 5.998052 |
| C | 0.694521 | -1.598686 | 4.710841 |
| H | 0.014695 | -1.707438 | 3.881837 |
| C | 3.959435 | -1.178337 | 2.642292 |
| C | 4.006603 | -2.487837 | 3.158435 |
| H | 3.200354 | -2.859497 | 3.780163 |
| C | 5.101227 | -3.305058 | 2.890817 |
| H | 5.128660 | -4.309984 | 3.302156 |
| C | 6.160382 | -2.831971 | 2.112263 |
| H | 7.017893 | -3.469312 | 1.916772 |
| C | 6.104360 | -1.550822 | 1.565272 |
| H | 6.897799 | -1.159251 | 0.935961 |

| | | | |
|---|---|---|---|
| C | 5.000767 | -0.733963 | 1.813818 |
| H | 4.997558 | 0.245552 | 1.356861 |
| C | -0.566933 | 0.044243 | 2.224432 |
| S | -3.264206 | -2.663129 | 4.503216 |
| F | -4.063692 | -0.137667 | 4.655050 |
| F | -2.270021 | -0.577187 | 5.801406 |
| F | -4.214107 | -1.235486 | 6.526964 |
| O | -2.353401 | -2.262539 | 3.388162 |
| O | -2.656223 | -3.585003 | 5.480243 |
| O | -4.630245 | -2.988786 | 4.035806 |
| C | -3.469734 | -1.063613 | 5.431406 |
| S | 7.175210 | 1.940900 | -0.151064 |
| F | 9.730349 | 2.562566 | -0.445761 |
| F | 9.228588 | 1.698654 | 1.487665 |
| F | 8.692059 | 3.758010 | 1.048351 |
| O | 7.424751 | 0.526618 | -0.507887 |
| O | 6.244867 | 2.130828 | 0.998745 |
| O | 6.888935 | 2.844544 | -1.291576 |
| C | 8.806885 | 2.524368 | 0.519998 |
| N | 2.484134 | 3.621643 | 0.095133 |
| N | 2.633585 | -3.660813 | -0.195263 |
| N | -2.483563 | 3.621805 | -0.097868 |
| N | -2.633860 | -3.660457 | 0.197491 |

**Cartesian coordinates of key structures optimized by the B3LYP method**

**$1_M$**

| | | | |
|---|---|---|---|
| Ir | -0.129116 | -0.436262 | -1.022793 |
| P | 0.012600 | 0.550878 | 1.264194 |
| N | -0.202355 | -2.397968 | -0.546272 |
| N | 1.902141 | -0.974075 | -1.108409 |
| N | -2.188555 | -0.820157 | -1.110640 |

| | | | |
|---|---|---|---|
| C | -0.085728 | 1.258913 | -1.895538 |
| C | 0.250364 | 2.362921 | 1.248892 |
| C | -1.465643 | 0.219742 | 2.298934 |
| C | 1.450623 | -0.214299 | 2.112948 |
| C | -1.414049 | -2.984911 | -0.393554 |
| C | 0.960923 | -3.092495 | -0.459858 |
| C | 2.149442 | -2.283505 | -0.773544 |
| C | -2.539946 | -2.087082 | -0.695368 |
| O | -0.066609 | 2.235996 | -2.497414 |
| C | -0.308142 | -5.094337 | 0.086152 |
| C | 4.546873 | -1.987869 | -1.092889 |
| C | -4.905172 | -1.611188 | -1.040635 |
| C | -0.326453 | -6.587343 | 0.450043 |
| C | 5.994638 | -2.484923 | -1.048241 |
| C | -6.375200 | -2.023018 | -0.908699 |
| O | 4.041831 | 2.586151 | -1.076024 |
| O | -3.353233 | 2.881063 | -0.390557 |
| S | -4.828572 | 2.888296 | -0.609199 |
| S | 5.254822 | 2.533255 | -0.208621 |
| O | 6.319382 | 1.632847 | -0.720182 |
| O | 4.971421 | 2.424632 | 1.240838 |
| C | 5.974340 | 4.237822 | -0.406129 |
| F | 7.076926 | 4.380716 | 0.342112 |
| F | 5.081838 | 5.170479 | -0.021096 |
| F | 6.296307 | 4.472621 | -1.685808 |
| O | -5.235088 | 2.622512 | -2.009655 |
| O | -5.598655 | 2.130241 | 0.411876 |
| C | -5.305874 | 4.662731 | -0.318759 |
| F | -4.983195 | 5.038364 | 0.930297 |
| F | -6.627379 | 4.826403 | -0.478284 |

| | | | |
|---|---|---|---|
| F | -4.671329 | 5.470704 | -1.179190 |
| C | -1.387771 | -0.401999 | 3.558530 |
| H | -0.433344 | -0.718000 | 3.961818 |
| C | -2.542042 | -0.586678 | 4.319908 |
| H | -2.466175 | -1.061957 | 5.294430 |
| C | -3.778774 | -0.142440 | 3.847722 |
| H | -4.671559 | -0.278816 | 4.452493 |
| C | -3.861456 | 0.501643 | 2.611632 |
| H | -4.796817 | 0.902903 | 2.231596 |
| C | -2.713219 | 0.677121 | 1.837447 |
| H | -2.804735 | 1.237738 | 0.911187 |
| C | -0.623633 | 3.220623 | 1.938528 |
| H | -1.471394 | 2.828210 | 2.485477 |
| C | -0.409223 | 4.597786 | 1.912146 |
| H | -1.099930 | 5.251034 | 2.437079 |
| C | 0.675113 | 5.132322 | 1.215341 |
| H | 0.837640 | 6.206460 | 1.204382 |
| C | 1.549307 | 4.286036 | 0.530947 |
| H | 2.413931 | 4.671856 | 0.001328 |
| C | 1.330197 | 2.910547 | 0.533701 |
| H | 2.042222 | 2.294871 | -0.004127 |
| C | 2.642753 | 0.494215 | 2.328079 |
| H | 2.751369 | 1.531539 | 2.038248 |
| C | 3.744093 | -0.137015 | 2.911508 |
| H | 4.658078 | 0.435566 | 3.030159 |
| C | 3.664003 | -1.471304 | 3.307408 |
| H | 4.517117 | -1.954905 | 3.775841 |
| C | 2.481930 | -2.187918 | 3.095423 |
| H | 2.409475 | -3.228499 | 3.401947 |
| C | 1.390583 | -1.572818 | 2.485676 |

| | | | |
|---|---|---|---|
| H | 0.482128 | -2.145160 | 2.322659 |
| C | 4.260126 | -0.657705 | -1.429149 |
| H | 5.043205 | 0.063573 | -1.638416 |
| C | 2.952829 | -0.193047 | -1.428083 |
| H | 2.761314 | 0.847011 | -1.656516 |
| C | 3.446389 | -2.791397 | -0.768831 |
| H | 3.592796 | -3.828815 | -0.497352 |
| C | 6.704203 | -2.129355 | -2.376897 |
| H | 6.743137 | -1.048900 | -2.540827 |
| H | 7.736652 | -2.494789 | -2.348144 |
| H | 6.204017 | -2.593705 | -3.235091 |
| C | 6.079639 | -4.007589 | -0.834717 |
| H | 5.662596 | -4.312537 | 0.132711 |
| H | 5.564882 | -4.564645 | -1.627658 |
| H | 7.129412 | -4.317917 | -0.843977 |
| C | 6.713445 | -1.765095 | 0.122449 |
| H | 6.711580 | -0.678014 | -0.005182 |
| H | 6.232413 | -1.999729 | 1.079411 |
| H | 7.754942 | -2.104152 | 0.173462 |
| C | -3.165341 | 0.000531 | -1.534078 |
| H | -2.882534 | 1.001758 | -1.832421 |
| C | -4.508460 | -0.366301 | -1.532776 |
| H | -5.210626 | 0.384495 | -1.873829 |
| C | -3.872311 | -2.476542 | -0.643145 |
| H | -4.107637 | -3.471348 | -0.282141 |
| C | -7.321512 | -0.888250 | -1.345144 |
| H | -7.201309 | -0.642761 | -2.406097 |
| H | -8.359332 | -1.205558 | -1.196820 |
| H | -7.157235 | 0.024854 | -0.763472 |
| C | -6.662122 | -2.356550 | 0.576012 |

| | | | |
|---|---|---|---|
| H | -6.054930 | -3.193954 | 0.939001 |
| H | -6.465190 | -1.490028 | 1.215667 |
| H | -7.714977 | -2.635958 | 0.694759 |
| C | -6.639561 | -3.271260 | -1.783662 |
| H | -7.688091 | -3.575165 | -1.688918 |
| H | -6.443462 | -3.061653 | -2.841119 |
| H | -6.019748 | -4.125436 | -1.486321 |
| C | -1.480473 | -4.337891 | -0.059912 |
| H | -2.448716 | -4.802250 | 0.066302 |
| C | 0.919818 | -4.445221 | -0.138548 |
| H | 1.846545 | -5.001050 | -0.069750 |
| C | -1.755640 | -7.116907 | 0.672525 |
| H | -2.265847 | -6.592033 | 1.488263 |
| H | -2.369125 | -7.036011 | -0.231973 |
| H | -1.712723 | -8.176859 | 0.942538 |
| C | 0.321920 | -7.396379 | -0.699912 |
| H | 1.362340 | -7.103370 | -0.875218 |
| H | 0.316105 | -8.463943 | -0.453571 |
| H | -0.228667 | -7.261660 | -1.637520 |
| C | 0.480540 | -6.800863 | 1.754012 |
| H | 1.525284 | -6.490014 | 1.649189 |
| H | 0.042483 | -6.239680 | 2.587064 |
| H | 0.477726 | -7.862867 | 2.023229 |

**1**

| | | | |
|---|---|---|---|
| Ir | 0.354502 | -0.466312 | -1.409314 |
| P | 0.946103 | -1.230826 | -3.790432 |
| N | -0.476897 | 1.233184 | -2.131947 |
| N | -1.636039 | -1.074955 | -1.725932 |
| N | 2.010928 | 0.823914 | -1.446973 |
| C | 1.133784 | -2.030457 | -0.660930 |

| | | | |
|---|---|---|---|
| C | 0.555194 | -2.960708 | -4.296312 |
| C | 2.740932 | -1.135917 | -4.205864 |
| C | 0.042942 | -0.190694 | -5.008928 |
| C | 0.301762 | 2.327898 | -2.265021 |
| C | -1.794034 | 1.231098 | -2.430426 |
| C | -2.426328 | -0.098154 | -2.281460 |
| C | 1.730842 | 2.061901 | -1.985445 |
| O | 1.628292 | -2.987444 | -0.270059 |
| C | -1.661774 | 3.611008 | -2.880422 |
| C | -4.258469 | -1.676216 | -2.590204 |
| C | 4.108821 | 2.592986 | -2.116478 |
| C | -2.353636 | 4.956513 | -3.138446 |
| C | -5.650900 | -2.051893 | -3.110946 |
| C | 5.233068 | 3.504085 | -2.623151 |
| S | -1.681412 | -5.813685 | 0.197799 |
| O | -1.795834 | -5.856492 | 1.681318 |
| O | -0.619063 | -4.891677 | -0.296510 |
| O | -2.957568 | -5.727909 | -0.542187 |
| C | -1.010408 | -7.504920 | -0.199082 |
| F | -1.834812 | -8.459684 | 0.245222 |
| F | -0.864436 | -7.667824 | -1.529171 |
| F | 0.191244 | -7.676829 | 0.375732 |
| S | 5.684130 | -1.954918 | 0.692494 |
| O | 5.605957 | -2.143658 | 2.165602 |
| O | 6.325529 | -0.694659 | 0.251597 |
| O | 4.429410 | -2.290746 | -0.035792 |
| C | 6.861417 | -3.293053 | 0.156534 |
| F | 7.061152 | -3.245224 | -1.176080 |
| F | 8.048342 | -3.152408 | 0.757743 |
| F | 6.366789 | -4.501351 | 0.460974 |

| | | | |
|---|---|---|---|
| Ir | -0.354805 | 0.465966 | 1.409324 |
| P | -0.946387 | 1.230479 | 3.790425 |
| N | 0.476578 | -1.233524 | 2.131983 |
| N | 1.635726 | 1.074633 | 1.725958 |
| N | -2.011217 | -0.824292 | 1.446984 |
| C | -1.133979 | 2.030177 | 0.660947 |
| C | -0.555523 | 2.960374 | 4.296305 |
| C | -2.741200 | 1.135510 | 4.205920 |
| C | -0.043141 | 0.190409 | 5.008909 |
| C | -0.302067 | -2.328241 | 2.265089 |
| C | 1.793719 | -1.231421 | 2.430452 |
| C | 2.426007 | 0.097839 | 2.281517 |
| C | -1.731146 | -2.062255 | 1.985508 |
| O | -1.628282 | 2.987286 | 0.270123 |
| C | 1.661498 | -3.611334 | 2.880410 |
| C | 4.258058 | 1.675955 | 2.590494 |
| C | -4.109139 | -2.593303 | 2.116562 |
| C | 2.353462 | -4.956810 | 3.138251 |
| C | 5.650346 | 2.051721 | 3.111537 |
| C | -5.233387 | -3.504385 | 2.623268 |
| O | 0.618650 | 4.891565 | 0.296548 |
| O | -4.428002 | 2.288310 | 0.034126 |
| S | -5.684210 | 1.955103 | -0.692845 |
| S | 1.681103 | 5.813454 | -0.197763 |
| O | 1.795574 | 5.856196 | -1.681281 |
| O | 2.957230 | 5.727582 | 0.542266 |
| C | 1.010245 | 7.504762 | 0.199044 |
| F | 1.834872 | 8.459429 | -0.245057 |
| F | 0.864025 | 7.667608 | 1.529112 |
| F | -0.191265 | 7.676866 | -0.376003 |

| | | | |
|---|---|---|---|
| O | -5.606741 | 2.142595 | -2.166155 |
| O | -6.328462 | 0.696874 | -0.250398 |
| C | -6.857302 | 3.296965 | -0.157039 |
| F | -7.056143 | 3.250919 | 1.175761 |
| F | -8.045086 | 3.159334 | -0.757223 |
| F | -6.359324 | 4.503564 | -0.462925 |
| C | -3.231247 | 0.578878 | 5.398247 |
| H | -2.557718 | 0.117475 | 6.111206 |
| C | -4.595471 | 0.632947 | 5.689919 |
| H | -4.960025 | 0.199421 | 6.617401 |
| C | -5.481937 | 1.250179 | 4.805785 |
| H | -6.542602 | 1.291341 | 5.037595 |
| C | -5.001152 | 1.822688 | 3.627400 |
| H | -5.674010 | 2.305498 | 2.927271 |
| C | -3.640195 | 1.765070 | 3.328782 |
| H | -3.300808 | 2.222442 | 2.405632 |
| C | -0.699519 | 3.303156 | 5.655217 |
| H | -0.975595 | 2.547793 | 6.384629 |
| C | -0.495193 | 4.613795 | 6.074862 |
| H | -0.607100 | 4.863915 | 7.126372 |
| C | -0.157102 | 5.603501 | 5.146419 |
| H | -0.001842 | 6.627248 | 5.475542 |
| C | -0.027990 | 5.276475 | 3.799223 |
| H | 0.224735 | 6.030725 | 3.063373 |
| C | -0.226411 | 3.959444 | 3.374107 |
| H | -0.126410 | 3.754655 | 2.317794 |
| C | 1.168210 | 0.647665 | 5.556140 |
| H | 1.530265 | 1.642391 | 5.318761 |
| C | 1.906487 | -0.161395 | 6.420340 |
| H | 2.836741 | 0.212214 | 6.838934 |

| | | | |
|---|---|---|---|
| C | 1.452568 | -1.439501 | 6.747996 |
| H | 2.025138 | -2.065608 | 7.426358 |
| C | 0.259168 | -1.910596 | 6.197665 |
| H | -0.100026 | -2.906228 | 6.442195 |
| C | -0.479476 | -1.108028 | 5.328157 |
| H | -1.401755 | -1.495321 | 4.907631 |
| C | 3.445647 | 2.629560 | 1.958465 |
| H | 3.768484 | 3.649475 | 1.776444 |
| C | 2.161734 | 2.302539 | 1.546791 |
| H | 1.539014 | 3.051583 | 1.069146 |
| C | 3.720436 | 0.386208 | 2.711343 |
| H | 4.314436 | -0.417097 | 3.124312 |
| C | 6.558125 | 2.399067 | 1.905846 |
| H | 6.144524 | 3.225975 | 1.317244 |
| H | 7.544545 | 2.709872 | 2.269591 |
| H | 6.687479 | 1.527353 | 1.257148 |
| C | 6.302942 | 0.906021 | 3.908115 |
| H | 5.703536 | 0.631527 | 4.785617 |
| H | 6.454805 | 0.010434 | 3.298755 |
| H | 7.282642 | 1.234385 | 4.271880 |
| C | 5.521853 | 3.287801 | 4.035184 |
| H | 5.119491 | 4.160412 | 3.510930 |
| H | 4.871687 | 3.077940 | 4.893165 |
| H | 6.510388 | 3.560171 | 4.421671 |
| C | -3.582435 | 4.784603 | -4.059384 |
| H | -3.308689 | 4.314836 | -5.012594 |
| H | -4.375357 | 4.192172 | -3.593035 |
| H | -4.004794 | 5.770392 | -4.282271 |
| C | -1.388757 | 5.976436 | -3.780928 |
| H | -0.562726 | 6.250050 | -3.117624 |

| | | | |
|---|---|---|---|
| H | -0.969624 | 5.599407 | -4.722690 |
| H | -1.938755 | 6.895929 | -4.007989 |
| C | -2.824138 | 5.505158 | -1.763882 |
| H | -3.336739 | 6.462661 | -1.916372 |
| H | -3.521129 | 4.816973 | -1.275364 |
| H | -1.975278 | 5.673241 | -1.094064 |
| C | -2.411270 | 2.421792 | -2.804708 |
| H | -3.483625 | 2.431051 | -2.954065 |
| C | -0.278423 | 3.539826 | -2.633950 |
| H | 0.339002 | 4.429587 | -2.649294 |
| C | -3.289204 | -0.520749 | 1.168821 |
| H | -3.492469 | 0.441520 | 0.709864 |
| C | -4.345635 | -1.371761 | 1.481946 |
| H | -5.338415 | -1.015924 | 1.235028 |
| C | -2.757964 | -2.939422 | 2.315262 |
| H | -2.496519 | -3.909127 | 2.719062 |
| C | -6.622637 | -2.891737 | 2.364722 |
| H | -6.818652 | -2.756579 | 1.295833 |
| H | -7.392666 | -3.564176 | 2.757407 |
| H | -6.744651 | -1.921173 | 2.859233 |
| C | -5.057228 | -3.684414 | 4.152486 |
| H | -4.108652 | -4.172832 | 4.399531 |
| H | -5.094376 | -2.719917 | 4.673137 |
| H | -5.863921 | -4.314380 | 4.544170 |
| C | -5.154770 | -4.885754 | 1.932700 |
| H | -5.959314 | -5.525246 | 2.315094 |
| H | -5.274223 | -4.798834 | 0.848433 |
| H | -4.201633 | -5.390105 | 2.109482 |
| C | 0.278134 | -3.540163 | 2.634027 |
| H | -0.339279 | -4.429931 | 2.649398 |

| | | | |
|---|---|---|---|
| C | 2.410983 | -2.422107 | 2.804684 |
| H | 3.483358 | -2.431357 | 2.953931 |
| C | 1.388665 | -5.977014 | 3.780391 |
| H | 0.969495 | -5.600318 | 4.722271 |
| H | 0.562661 | -6.250485 | 3.116996 |
| H | 1.938736 | -6.896532 | 4.007168 |
| C | 2.824185 | -5.505013 | 1.763583 |
| H | 3.520581 | -4.816250 | 1.275023 |
| H | 3.337478 | -6.462172 | 1.915905 |
| H | 1.975347 | -5.673612 | 1.093864 |
| C | 3.582133 | -4.784924 | 4.059372 |
| H | 4.375043 | -4.192326 | 3.593222 |
| H | 3.308209 | -4.315349 | 5.012626 |
| H | 4.004568 | -5.770707 | 4.282143 |
| C | 3.288928 | 0.520384 | -1.168829 |
| H | 3.492219 | -0.441930 | -0.710001 |
| C | 4.345340 | 1.371439 | -1.481885 |
| H | 5.338093 | 1.015623 | -1.234866 |
| C | 2.757646 | 2.939089 | -2.315182 |
| H | 2.496200 | 3.908800 | -2.718963 |
| C | 6.622316 | 2.891445 | -2.364577 |
| H | 7.392352 | 3.563894 | -2.757232 |
| H | 6.818298 | 2.756272 | -1.295682 |
| H | 6.744346 | 1.920887 | -2.859097 |
| C | 5.154423 | 4.885449 | -1.932578 |
| H | 5.273846 | 4.798523 | -0.848309 |
| H | 5.958972 | 5.524950 | -2.314949 |
| H | 4.201286 | 5.389792 | -2.109384 |
| C | 5.056941 | 3.684118 | -4.152372 |
| H | 5.094112 | 2.719624 | -4.673025 |

| | | | |
|---|---|---|---|
| H | 4.108365 | 4.172526 | -4.399436 |
| H | 5.863635 | 4.314096 | -4.544037 |
| C | -2.162063 | -2.302842 | -1.546696 |
| H | -1.539324 | -3.051890 | -1.069086 |
| C | -3.446026 | -2.629832 | -1.958229 |
| H | -3.768884 | -3.649726 | -1.776130 |
| C | -3.720799 | -0.386502 | -2.711180 |
| H | -4.314825 | 0.416819 | -3.124079 |
| C | -5.522867 | -3.288439 | -4.034025 |
| H | -4.872750 | -3.079149 | -4.892182 |
| H | -5.120690 | -4.160931 | -3.509434 |
| H | -6.511528 | -3.560703 | -4.420262 |
| C | -6.558617 | -2.398418 | -1.904964 |
| H | -7.545164 | -2.709143 | -2.268430 |
| H | -6.145143 | -3.225152 | -1.316026 |
| H | -6.687718 | -1.526380 | -1.256633 |
| C | -6.303304 | -0.906403 | -3.907986 |
| H | -6.455006 | -0.010529 | -3.299008 |
| H | -5.703856 | -0.632393 | -4.785610 |
| H | -7.283069 | -1.234746 | -4.271598 |
| C | 3.231056 | -0.579206 | -5.398124 |
| H | 2.557575 | -0.117742 | -6.111088 |
| C | 4.595296 | -0.633278 | -5.689716 |
| H | 4.959911 | -0.199695 | -6.617146 |
| C | 5.481703 | -1.250569 | -4.805565 |
| H | 6.542385 | -1.291710 | -5.037307 |
| C | 5.000843 | -1.823156 | -3.627247 |
| H | 5.673648 | -2.305949 | -2.927055 |
| C | 3.639864 | -1.765560 | -3.328723 |
| H | 3.300441 | -2.223013 | -2.405628 |

| | | | |
|---|---|---|---|
| C | 0.479332 | 1.107751 | -5.328072 |
| H | 1.401580 | 1.495004 | -4.907438 |
| C | -0.259212 | 1.910375 | -6.197612 |
| H | 0.100022 | 2.906013 | -6.442057 |
| C | -1.452566 | 1.439327 | -6.748081 |
| H | -2.025060 | 2.065474 | -7.426471 |
| C | -1.906545 | 0.161216 | -6.420526 |
| H | -2.836768 | -0.212353 | -6.839223 |
| C | -1.168370 | -0.647900 | -5.556289 |
| H | -1.530473 | -1.642623 | -5.318978 |
| C | 0.699124 | -3.303487 | -5.655230 |
| H | 0.975150 | -2.548121 | -6.384657 |
| C | 0.494794 | -4.614130 | -6.074862 |
| H | 0.606647 | -4.864252 | -7.126378 |
| C | 0.156770 | -5.603838 | -5.146395 |
| H | 0.001509 | -6.627588 | -5.475507 |
| C | 0.027724 | -5.276811 | -3.799192 |
| H | -0.224958 | -6.031075 | -3.063344 |
| C | 0.226148 | -3.959780 | -3.374092 |
| H | 0.126191 | -3.754975 | -2.317780 |

**$2_M$**

| | | | |
|---|---|---|---|
| Ir | -0.359547 | -1.173872 | -1.118835 |
| P | -0.136040 | -0.327615 | 1.212003 |
| N | -0.495094 | -3.159134 | -0.779589 |
| N | 1.655000 | -1.764921 | -1.288069 |
| N | -2.434849 | -1.478105 | -1.205066 |
| C | -0.264518 | 0.580271 | -1.878032 |
| C | 0.159689 | 1.471153 | 1.298818 |
| C | -1.614779 | -0.680672 | 2.235307 |
| C | 1.285262 | -1.201280 | 1.975579 |

| | | | |
|---|---|---|---|
| C | -1.731509 | -3.711702 | -0.647557 |
| C | 0.650509 | -3.890356 | -0.752495 |
| C | 1.855901 | -3.106449 | -1.042847 |
| C | -2.823785 | -2.762517 | -0.890759 |
| O | -0.195290 | 1.583298 | -2.426796 |
| C | -0.677208 | -5.845570 | -0.304262 |
| C | 4.223584 | -2.853775 | -1.422181 |
| C | -5.137195 | -2.178819 | -1.247472 |
| O | 3.880194 | 1.702259 | -1.086226 |
| O | -2.851540 | 2.158632 | -0.538639 |
| S | -4.291234 | 2.560256 | -0.453890 |
| S | 5.102408 | 1.553540 | -0.239615 |
| O | 6.117066 | 0.636909 | -0.810207 |
| O | 4.825151 | 1.385087 | 1.206127 |
| C | 5.884282 | 3.237520 | -0.356998 |
| F | 7.002109 | 3.298439 | 0.379801 |
| F | 5.034534 | 4.182140 | 0.091496 |
| F | 6.197055 | 3.528803 | -1.627353 |
| O | -5.074330 | 2.204774 | -1.661891 |
| O | -4.930956 | 2.235096 | 0.840221 |
| C | -4.204273 | 4.418275 | -0.475917 |
| F | -3.484633 | 4.863462 | 0.568444 |
| F | -5.432283 | 4.949891 | -0.398277 |
| F | -3.623374 | 4.851822 | -1.603012 |
| C | -1.559445 | -1.415744 | 3.433419 |
| H | -0.620498 | -1.816768 | 3.795259 |
| C | -2.716349 | -1.603978 | 4.190153 |
| H | -2.659853 | -2.168985 | 5.116777 |
| C | -3.929688 | -1.049047 | 3.777541 |
| H | -4.822859 | -1.187022 | 4.381173 |

| | | | |
|---|---|---|---|
| C | -3.985306 | -0.290230 | 2.606907 |
| H | -4.896808 | 0.206342 | 2.289633 |
| C | -2.837956 | -0.114028 | 1.833839 |
| H | -2.893415 | 0.528396 | 0.960464 |
| C | -0.695882 | 2.318867 | 2.023781 |
| H | -1.554865 | 1.926474 | 2.552070 |
| C | -0.452397 | 3.690447 | 2.053471 |
| H | -1.134757 | 4.336658 | 2.597117 |
| C | 0.644670 | 4.228683 | 1.379997 |
| H | 0.828490 | 5.299008 | 1.409809 |
| C | 1.503053 | 3.392049 | 0.664378 |
| H | 2.375588 | 3.780493 | 0.150169 |
| C | 1.254139 | 2.023057 | 0.609506 |
| H | 1.953662 | 1.417165 | 0.044921 |
| C | 2.504995 | -0.549174 | 2.215920 |
| H | 2.644797 | 0.502284 | 1.999910 |
| C | 3.595256 | -1.258443 | 2.725707 |
| H | 4.531353 | -0.727255 | 2.862970 |
| C | 3.476988 | -2.614911 | 3.024225 |
| H | 4.323456 | -3.160537 | 3.432365 |
| C | 2.266224 | -3.274398 | 2.789842 |
| H | 2.163908 | -4.331457 | 3.022497 |
| C | 1.184892 | -2.580841 | 2.250382 |
| H | 0.254679 | -3.109801 | 2.065518 |
| C | 4.015647 | -1.499031 | -1.660243 |
| H | 4.835193 | -0.808136 | -1.838474 |
| C | 2.718855 | -0.993316 | -1.585686 |
| H | 2.554293 | 0.065798 | -1.734941 |
| C | 3.132248 | -3.660525 | -1.111174 |
| H | 3.270161 | -4.717613 | -0.913537 |

| | | | |
|---|---|---|---|
| C | -3.374849 | -0.578177 | -1.553032 |
| H | -3.042405 | 0.434235 | -1.745919 |
| C | -4.732420 | -0.892525 | -1.588686 |
| H | -5.420685 | -0.093244 | -1.844257 |
| C | -4.170342 | -3.120034 | -0.897670 |
| H | -4.458818 | -4.132573 | -0.638364 |
| C | -1.840507 | -5.077012 | -0.387100 |
| H | -2.812352 | -5.540808 | -0.266976 |
| C | 0.574182 | -5.261569 | -0.505336 |
| H | 1.471846 | -5.867344 | -0.475327 |
| H | 5.223295 | -3.274314 | -1.462076 |
| H | -0.748807 | -6.909621 | -0.103519 |
| H | -6.187900 | -2.451270 | -1.250405 |

**2**

| | | | |
|---|---|---|---|
| Ir | 1.149723 | 0.020087 | 1.051485 |
| P | 3.050801 | 0.035495 | 2.754516 |
| N | -0.245355 | -0.049105 | 2.525591 |
| N | 0.813832 | 2.073061 | 1.402590 |
| N | 0.940984 | -2.053314 | 1.320120 |
| C | 2.372496 | 0.066521 | -0.406122 |
| C | 4.416938 | 1.254600 | 2.545929 |
| C | 3.983542 | -1.548701 | 2.873859 |
| C | 2.367048 | 0.397528 | 4.420822 |
| C | -0.665345 | -1.262666 | 2.949371 |
| C | -0.726972 | 1.118757 | 3.007161 |
| C | -0.090039 | 2.310146 | 2.415732 |
| C | 0.053036 | -2.387342 | 2.321043 |
| O | 3.146484 | 0.086306 | -1.251219 |
| C | -2.239533 | -0.146848 | 4.368848 |
| C | 0.274788 | 4.684503 | 2.215907 |

| | | | |
|---|---|---|---|
| C | 0.610425 | -4.718483 | 2.062374 |
| S | 3.444627 | 4.409682 | -2.272730 |
| O | 3.033069 | 4.254340 | -3.692556 |
| O | 3.630597 | 3.118830 | -1.549654 |
| O | 2.690056 | 5.428719 | -1.506994 |
| C | 5.167425 | 5.102310 | -2.401651 |
| F | 5.159141 | 6.276902 | -3.040340 |
| F | 5.686971 | 5.299246 | -1.171373 |
| F | 5.969972 | 4.257909 | -3.061079 |
| S | 3.766016 | -4.068564 | -2.347948 |
| O | 3.364356 | -3.863011 | -3.763619 |
| O | 3.062981 | -5.171602 | -1.651387 |
| O | 3.868301 | -2.812699 | -1.551637 |
| C | 5.529014 | -4.650667 | -2.478763 |
| F | 6.033814 | -4.901466 | -1.251921 |
| F | 5.604291 | -5.778639 | -3.193989 |
| F | 6.294122 | -3.719732 | -3.061014 |
| Ir | -1.149724 | -0.020087 | -1.051484 |
| P | -3.050800 | -0.035495 | -2.754515 |
| N | 0.245354 | 0.049105 | -2.525591 |
| N | -0.813833 | -2.073061 | -1.402590 |
| N | -0.940984 | 2.053314 | -1.320120 |
| C | -2.372496 | -0.066521 | 0.406123 |
| C | -4.416937 | -1.254601 | -2.545928 |
| C | -3.983541 | 1.548700 | -2.873860 |
| C | -2.367047 | -0.397530 | -4.420822 |
| C | 0.665345 | 1.262665 | -2.949371 |
| C | 0.726972 | -1.118758 | -3.007160 |
| C | 0.090038 | -2.310147 | -2.415731 |
| C | -0.053036 | 2.387341 | -2.321042 |

| | | | |
|---|---|---|---|
| O | -3.146483 | -0.086305 | 1.251220 |
| C | 2.239535 | 0.146846 | -4.368846 |
| C | -0.274789 | -4.684504 | -2.215906 |
| C | -0.610424 | 4.718483 | -2.062374 |
| O | -3.630597 | -3.118828 | 1.549655 |
| O | -3.868306 | 2.812699 | 1.551632 |
| S | -3.766019 | 4.068562 | 2.347945 |
| S | -3.444628 | -4.409680 | 2.272731 |
| O | -3.033069 | -4.254339 | 3.692557 |
| O | -2.690058 | -5.428718 | 1.506994 |
| C | -5.167426 | -5.102306 | 2.401653 |
| F | -5.159143 | -6.276898 | 3.040342 |
| F | -5.686972 | -5.299242 | 1.171374 |
| F | -5.969972 | -4.257904 | 3.061080 |
| O | -3.364360 | 3.863006 | 3.763616 |
| O | -3.062981 | 5.171599 | 1.651386 |
| C | -5.529015 | 4.650669 | 2.478759 |
| F | -6.033813 | 4.901471 | 1.251917 |
| F | -5.604289 | 5.778641 | 3.193986 |
| F | -6.294126 | 3.719736 | 3.061008 |
| C | -4.307646 | 2.159712 | -4.096156 |
| H | -3.958275 | 1.742706 | -5.033668 |
| C | -5.104270 | 3.305508 | -4.117834 |
| H | -5.346912 | 3.767913 | -5.070770 |
| C | -5.591073 | 3.849105 | -2.928268 |
| H | -6.207865 | 4.743153 | -2.950178 |
| C | -5.287409 | 3.239341 | -1.710210 |
| H | -5.649333 | 3.650253 | -0.774226 |
| C | -4.491090 | 2.095109 | -1.682997 |
| H | -4.283788 | 1.643715 | -0.718702 |

| | | | |
|---|---|---|---|
| C | -5.368603 | -1.358650 | -3.579934 |
| H | -5.263528 | -0.766960 | -4.484314 |
| C | -6.456019 | -2.216549 | -3.449484 |
| H | -7.182024 | -2.287804 | -4.254720 |
| C | -6.615512 | -2.976717 | -2.286284 |
| H | -7.465435 | -3.646098 | -2.186900 |
| C | -5.687711 | -2.869156 | -1.253747 |
| H | -5.790942 | -3.451302 | -0.345470 |
| C | -4.593624 | -2.008198 | -1.381127 |
| H | -3.908388 | -1.945617 | -0.548813 |
| C | -2.417387 | -1.704988 | -4.934198 |
| H | -2.929358 | -2.486719 | -4.382963 |
| C | -1.829844 | -2.005959 | -6.163743 |
| H | -1.884991 | -3.020262 | -6.548654 |
| C | -1.179693 | -1.011704 | -6.895636 |
| H | -0.727479 | -1.247237 | -7.854724 |
| C | -1.112707 | 0.287844 | -6.389358 |
| H | -0.606840 | 1.068181 | -6.950752 |
| C | -1.693779 | 0.592830 | -5.158775 |
| H | -1.631231 | 1.608671 | -4.781914 |
| C | -1.174558 | -4.436337 | -1.185047 |
| H | -1.685534 | -5.218863 | -0.633143 |
| C | -1.416415 | -3.118019 | -0.804323 |
| H | -2.100510 | -2.911147 | 0.011469 |
| C | 0.362618 | -3.610086 | -2.835318 |
| H | 1.112414 | -3.785089 | -3.597844 |
| C | -1.757351 | 1.094781 | 3.949783 |
| H | -2.226492 | 2.016412 | 4.279738 |
| C | -1.698710 | -1.339908 | 3.885757 |
| H | -2.118107 | -2.301537 | 4.167577 |

| | | | |
|---|---|---|---|
| C | -1.631006 | 3.027912 | -0.701605 |
| H | -2.314579 | 2.743742 | 0.092219 |
| C | -1.488624 | 4.372705 | -1.043480 |
| H | -2.064598 | 5.096234 | -0.475557 |
| C | 0.110746 | 3.713855 | -2.709524 |
| H | 0.840512 | 3.968437 | -3.469350 |
| C | 1.698711 | 1.339907 | -3.885756 |
| H | 2.118109 | 2.301536 | -4.167575 |
| C | 1.757352 | -1.094782 | -3.949781 |
| H | 2.226493 | -2.016414 | -4.279735 |
| C | 1.631006 | -3.027912 | 0.701605 |
| H | 2.314579 | -2.743741 | -0.092220 |
| C | 1.488624 | -4.372705 | 1.043479 |
| H | 2.064599 | -5.096234 | 0.475556 |
| C | -0.110745 | -3.713856 | 2.709524 |
| H | -0.840511 | -3.968438 | 3.469350 |
| C | 1.416414 | 3.118019 | 0.804324 |
| H | 2.100509 | 2.911146 | -0.011469 |
| C | 1.174557 | 4.436337 | 1.185048 |
| H | 1.685532 | 5.218864 | 0.633144 |
| C | -0.362619 | 3.610085 | 2.835319 |
| H | -1.112415 | 3.785088 | 3.597845 |
| C | 4.307648 | -2.159712 | 4.096156 |
| H | 3.958277 | -1.742706 | 5.033668 |
| C | 5.104273 | -3.305509 | 4.117833 |
| H | 5.346915 | -3.767913 | 5.070769 |
| C | 5.591075 | -3.849105 | 2.928267 |
| H | 6.207868 | -4.743153 | 2.950176 |
| C | 5.287411 | -3.239341 | 1.710209 |
| H | 5.649335 | -3.650253 | 0.774225 |

| | | | |
|---|---|---|---|
| C | 4.491091 | -2.095110 | 1.682996 |
| H | 4.283788 | -1.643715 | 0.718702 |
| C | 1.693780 | -0.592832 | 5.158776 |
| H | 1.631232 | -1.608672 | 4.781914 |
| C | 1.112709 | -0.287846 | 6.389359 |
| H | 0.606842 | -1.068183 | 6.950752 |
| C | 1.179696 | 1.011701 | 6.895638 |
| H | 0.727483 | 1.247233 | 7.854726 |
| C | 1.829847 | 2.005956 | 6.163745 |
| H | 1.884995 | 3.020259 | 6.548657 |
| C | 2.417389 | 1.704987 | 4.934200 |
| H | 2.929361 | 2.486717 | 4.382965 |
| C | 5.368605 | 1.358647 | 3.579934 |
| H | 5.263530 | 0.766956 | 4.484313 |
| C | 6.456021 | 2.216547 | 3.449485 |
| H | 7.182027 | 2.287800 | 4.254721 |
| C | 6.615513 | 2.976716 | 2.286286 |
| H | 7.465437 | 3.646097 | 2.186902 |
| C | 5.687712 | 2.869157 | 1.253749 |
| H | 5.790942 | 3.451305 | 0.345473 |
| C | 4.593625 | 2.008200 | 1.381129 |
| H | 3.908388 | 1.945620 | 0.548815 |
| H | -0.035098 | -5.697206 | -2.522182 |
| H | 3.071652 | 0.184802 | -5.064439 |
| H | -0.453753 | 5.754272 | -2.344152 |
| H | 0.035097 | 5.697205 | 2.522184 |
| H | -3.071650 | -0.184804 | 5.064443 |
| H | 0.453754 | -5.754272 | 2.344152 |

**$3_M$**

| | | | |
|---|---|---|---|
| Ir | -0.728941 | 0.375468 | -0.973286 |

| | | | |
|---|---|---|---|
| P | -0.639062 | 0.524491 | 1.515353 |
| O | -3.614931 | 1.240859 | -1.386877 |
| N | -0.762472 | -1.708565 | -1.111472 |
| N | 1.198009 | -0.058057 | -1.535564 |
| N | 0.077639 | 2.269948 | -1.308257 |
| C | -1.647469 | -2.754369 | -0.912559 |
| C | -3.001608 | -2.755934 | -0.567058 |
| H | -3.574918 | -1.851495 | -0.396396 |
| C | -3.626439 | -3.989695 | -0.461370 |
| H | -4.682198 | -4.011045 | -0.209146 |
| C | -2.924938 | -5.194113 | -0.688461 |
| H | -3.452179 | -6.139027 | -0.596346 |
| C | -1.578717 | -5.204997 | -1.033008 |
| H | -1.050966 | -6.137449 | -1.203768 |
| C | -0.949433 | -3.960543 | -1.147128 |
| C | 0.426666 | -2.249680 | -1.456204 |
| C | 1.537036 | -1.356540 | -1.759236 |
| C | 2.802523 | -1.658441 | -2.266497 |
| H | 3.109606 | -2.679265 | -2.434541 |
| C | 3.694228 | -0.623612 | -2.519366 |
| H | 4.698849 | -0.848736 | -2.856424 |
| C | 3.327074 | 0.701155 | -2.293628 |
| H | 4.044879 | 1.486760 | -2.465668 |
| C | 2.048729 | 0.971381 | -1.811706 |
| C | 1.392536 | 2.261167 | -1.617011 |
| C | 0.800545 | 4.395135 | -1.555221 |
| C | 0.717587 | 5.791821 | -1.577953 |
| H | 1.579113 | 6.416448 | -1.788114 |
| C | -0.525114 | 6.353740 | -1.314652 |
| H | -0.629890 | 7.434260 | -1.326076 |

| | | | |
|---|---|---|---|
| C | -1.655015 | 5.554993 | -1.036228 |
| H | -2.609017 | 6.034844 | -0.841919 |
| C | -1.574758 | 4.169674 | -1.008336 |
| H | -2.445925 | 3.560505 | -0.797632 |
| C | -0.324931 | 3.591919 | -1.269778 |
| C | 1.421075 | -4.579500 | -1.777661 |
| H | 1.924187 | -4.279847 | -2.701937 |
| H | 0.923276 | -5.525716 | -2.001409 |
| C | 2.414529 | -4.760739 | -0.622370 |
| H | 2.874107 | -3.799565 | -0.359706 |
| H | 1.858458 | -5.090875 | 0.264039 |
| C | 3.508656 | -5.780453 | -0.969998 |
| H | 4.039384 | -5.451128 | -1.875285 |
| H | 3.045273 | -6.744992 | -1.221834 |
| C | 4.517472 | -5.970323 | 0.167219 |
| H | 4.022996 | -6.330576 | 1.077423 |
| H | 5.022226 | -5.029763 | 0.409162 |
| H | 5.282835 | -6.704174 | -0.107333 |
| C | 3.248419 | 3.962906 | -2.049567 |
| H | 3.183343 | 5.005612 | -2.369444 |
| H | 3.616090 | 3.404811 | -2.915561 |
| C | 4.181408 | 3.821159 | -0.841285 |
| H | 3.783623 | 4.429370 | -0.019237 |
| H | 4.195585 | 2.784170 | -0.486764 |
| C | 5.616788 | 4.251975 | -1.174524 |
| H | 5.627207 | 5.304192 | -1.494967 |
| H | 5.980975 | 3.665903 | -2.030823 |
| C | 6.566259 | 4.049069 | 0.010826 |
| H | 6.252446 | 4.647123 | 0.875169 |
| H | 7.587314 | 4.349460 | -0.248458 |

| | | | |
|---|---|---|---|
| H | 6.580978 | 2.996683 | 0.312553 |
| C | 0.702165 | 1.675462 | 2.031162 |
| C | 1.955312 | 1.241984 | 2.491135 |
| H | 2.194373 | 0.190739 | 2.574179 |
| C | 2.937553 | 2.168638 | 2.850972 |
| H | 3.903713 | 1.795912 | 3.174902 |
| C | 2.678765 | 3.535799 | 2.766148 |
| H | 3.440502 | 4.253606 | 3.058722 |
| C | 1.436808 | 3.980089 | 2.302761 |
| H | 1.221930 | 5.043404 | 2.236460 |
| C | 0.460054 | 3.060273 | 1.927995 |
| H | -0.503857 | 3.423647 | 1.588017 |
| C | -2.149250 | 1.160546 | 2.348814 |
| C | -2.055977 | 1.974745 | 3.494391 |
| H | -1.089720 | 2.285531 | 3.874195 |
| C | -3.211703 | 2.383763 | 4.157421 |
| H | -3.125013 | 3.014823 | 5.037914 |
| C | -4.469406 | 1.982333 | 3.699491 |
| H | -5.366382 | 2.308152 | 4.219183 |
| C | -4.570444 | 1.151887 | 2.583968 |
| H | -5.529081 | 0.804021 | 2.214486 |
| C | -3.415482 | 0.740574 | 1.915047 |
| H | -3.550657 | 0.064449 | 1.079762 |
| C | -0.323782 | -1.112256 | 2.286175 |
| C | -1.263473 | -1.674244 | 3.167421 |
| H | -2.184510 | -1.151539 | 3.396383 |
| C | -1.017442 | -2.913467 | 3.758699 |
| H | -1.754266 | -3.335220 | 4.436272 |
| C | 0.167300 | -3.599225 | 3.490142 |
| H | 0.362902 | -4.556349 | 3.966041 |

| | | | |
|---|---|---|---|
| C | 1.101382 | -3.048181 | 2.610832 |
| H | 2.038307 | -3.557943 | 2.407238 |
| C | 0.853842 | -1.822403 | 1.992988 |
| H | 1.609810 | -1.425065 | 1.323905 |
| C | -2.562742 | 0.851401 | -1.148952 |
| S | -6.282640 | -1.169489 | -0.700155 |
| F | -8.286664 | 0.469641 | -1.358603 |
| F | -6.548658 | 1.470799 | -0.514339 |
| F | -7.888307 | 0.352572 | 0.778646 |
| O | -5.635319 | -0.947561 | -2.011411 |
| O | -5.337204 | -1.081153 | 0.457522 |
| O | -7.242805 | -2.284021 | -0.617361 |
| C | -7.314570 | 0.360421 | -0.442727 |
| S | 4.928888 | -0.475665 | 0.884120 |
| F | 5.906961 | -2.296074 | -0.787961 |
| F | 5.959027 | -2.889222 | 1.305042 |
| F | 7.392849 | -1.437461 | 0.552420 |
| O | 3.614437 | -1.129486 | 0.618976 |
| O | 5.198752 | -0.162252 | 2.297848 |
| O | 5.283647 | 0.584159 | -0.097179 |
| C | 6.122677 | -1.845708 | 0.471785 |
| N | 0.351986 | -3.612545 | -1.494331 |
| N | 1.866414 | 3.531398 | -1.778134 |

**3**

| | | | |
|---|---|---|---|
| Ir | -1.154369 | -0.147015 | -1.109331 |
| P | -2.793252 | -0.363022 | -3.036356 |
| O | 1.252453 | 0.072447 | -2.937197 |
| N | -1.516932 | -2.158907 | -0.606701 |
| N | -2.688458 | 0.002297 | 0.260345 |
| N | -1.476372 | 1.911924 | -1.022041 |

| | | | |
|---|---|---|---|
| C | -1.146020 | -3.425058 | -1.027804 |
| C | -0.178701 | -3.857744 | -1.942916 |
| H | 0.495060 | -3.181655 | -2.455204 |
| C | -0.078408 | -5.223540 | -2.170151 |
| H | 0.647903 | -5.573871 | -2.897882 |
| C | -0.898362 | -6.149797 | -1.489798 |
| H | -0.792443 | -7.209879 | -1.700610 |
| C | -1.835814 | -5.735568 | -0.553075 |
| H | -2.460651 | -6.448524 | -0.026364 |
| C | -1.948630 | -4.357317 | -0.335496 |
| C | -2.506489 | -2.318652 | 0.304180 |
| C | -3.188202 | -1.129179 | 0.816188 |
| C | -4.250798 | -1.046661 | 1.720588 |
| H | -4.635565 | -1.918637 | 2.228553 |
| C | -4.813326 | 0.199036 | 1.975524 |
| H | -5.674867 | 0.280046 | 2.625945 |
| C | -4.302214 | 1.352040 | 1.377560 |
| H | -4.820170 | 2.287578 | 1.535964 |
| C | -3.203559 | 1.235261 | 0.529294 |
| C | -2.484454 | 2.281991 | -0.199892 |
| C | -1.750429 | 4.145626 | -1.136903 |
| C | -1.515533 | 5.458629 | -1.558401 |
| H | -2.093822 | 6.291634 | -1.175174 |
| C | -0.518366 | 5.648032 | -2.505843 |
| H | -0.315102 | 6.650790 | -2.869422 |
| C | 0.228340 | 4.564113 | -3.014802 |
| H | 0.988596 | 4.751021 | -3.767541 |
| C | 0.002703 | 3.261443 | -2.589423 |
| H | 0.573542 | 2.436697 | -2.996243 |
| C | -1.004539 | 3.056988 | -1.635642 |

| | | | |
|---|---|---|---|
| C | -3.651102 | -4.272422 | 1.517295 |
| H | -3.638179 | -3.663890 | 2.420199 |
| H | -3.156719 | -5.211935 | 1.780986 |
| C | -5.076060 | -4.540146 | 1.025225 |
| H | -5.564059 | -3.591733 | 0.769366 |
| H | -5.043660 | -5.127692 | 0.097611 |
| C | -5.895997 | -5.275192 | 2.096383 |
| H | -5.864328 | -4.700054 | 3.029624 |
| H | -5.419632 | -6.241664 | 2.315532 |
| C | -7.348785 | -5.506606 | 1.670096 |
| H | -7.407309 | -6.101806 | 0.749946 |
| H | -7.863881 | -4.556057 | 1.486947 |
| H | -7.905769 | -6.039980 | 2.447824 |
| C | -3.540754 | 4.498003 | 0.587911 |
| H | -2.909429 | 5.331228 | 0.915430 |
| H | -3.817289 | 3.949636 | 1.487274 |
| C | -4.779584 | 5.008613 | -0.154230 |
| H | -4.461597 | 5.540121 | -1.061658 |
| H | -5.394262 | 4.159968 | -0.470555 |
| C | -5.600349 | 5.945531 | 0.743250 |
| H | -4.956534 | 6.754656 | 1.121617 |
| H | -5.954018 | 5.378182 | 1.612074 |
| C | -6.807061 | 6.545165 | 0.015549 |
| H | -6.499038 | 7.110527 | -0.873447 |
| H | -7.357232 | 7.229095 | 0.671203 |
| H | -7.497158 | 5.758015 | -0.300716 |
| C | -3.320068 | 1.288405 | -3.673212 |
| C | -4.507201 | 1.937478 | -3.304368 |
| H | -5.218473 | 1.504213 | -2.615827 |
| C | -4.819033 | 3.189882 | -3.841015 |

| | | | |
|---|---|---|---|
| H | -5.747007 | 3.664629 | -3.537068 |
| C | -3.959886 | 3.806644 | -4.748625 |
| H | -4.212649 | 4.775065 | -5.171844 |
| C | -2.773636 | 3.167786 | -5.118629 |
| H | -2.097692 | 3.634418 | -5.829757 |
| C | -2.451243 | 1.923742 | -4.581982 |
| H | -1.534575 | 1.434164 | -4.896919 |
| C | -2.201709 | -1.169266 | -4.585770 |
| C | -3.053892 | -1.099892 | -5.707488 |
| H | -3.989649 | -0.552515 | -5.651201 |
| C | -2.704386 | -1.737470 | -6.893430 |
| H | -3.370389 | -1.676023 | -7.749798 |
| C | -1.505927 | -2.454044 | -6.981063 |
| H | -1.237582 | -2.953114 | -7.908096 |
| C | -0.654567 | -2.526128 | -5.881553 |
| H | 0.278940 | -3.078780 | -5.931288 |
| C | -1.003040 | -1.880665 | -4.689405 |
| H | -0.317962 | -1.949479 | -3.858148 |
| C | -4.284740 | -1.352789 | -2.595984 |
| C | -4.429896 | -2.646229 | -3.135409 |
| H | -3.676014 | -3.053493 | -3.798870 |
| C | -5.561225 | -3.407726 | -2.845267 |
| H | -5.661509 | -4.397629 | -3.282181 |
| C | -6.563335 | -2.896973 | -2.017645 |
| H | -7.452108 | -3.486670 | -1.810216 |
| C | -6.417738 | -1.629532 | -1.452820 |
| H | -7.173888 | -1.202941 | -0.799558 |
| C | -5.278791 | -0.869411 | -1.727076 |
| H | -5.216887 | 0.109058 | -1.267631 |
| C | 0.361761 | -0.105542 | -2.228914 |

| | | | |
|---|---|---|---|
| S | 3.089146 | -3.168219 | -4.498243 |
| F | 4.077307 | -0.734258 | -4.917344 |
| F | 2.237202 | -1.148707 | -5.999580 |
| F | 4.113933 | -2.026182 | -6.666565 |
| O | 2.227838 | -2.600687 | -3.414781 |
| O | 2.401272 | -4.130704 | -5.379960 |
| O | 4.440481 | -3.545569 | -4.020519 |
| C | 3.401079 | -1.687636 | -5.587881 |
| S | -7.407827 | 1.994825 | 0.246463 |
| F | -9.943823 | 2.632251 | 0.683229 |
| F | -9.552230 | 1.810120 | -1.292574 |
| F | -8.971040 | 3.853542 | -0.833157 |
| O | -7.662369 | 0.592485 | 0.647277 |
| O | -6.543418 | 2.143448 | -0.961088 |
| O | -7.037990 | 2.913906 | 1.352436 |
| C | -9.069535 | 2.607839 | -0.328808 |
| Ir | 1.145241 | 0.031702 | 1.116191 |
| P | 2.785061 | 0.099166 | 3.050036 |
| O | -1.266152 | 0.497353 | 2.892709 |
| N | 1.505411 | -2.028488 | 0.894781 |
| N | 2.674188 | -0.011577 | -0.267147 |
| N | 1.464264 | 2.059586 | 0.739870 |
| C | 1.140770 | -3.220600 | 1.497971 |
| C | 0.177876 | -3.517766 | 2.469762 |
| H | -0.497012 | -2.775629 | 2.878791 |
| C | 0.083032 | -4.836138 | 2.894175 |
| H | -0.639938 | -5.078831 | 3.667648 |
| C | 0.904140 | -5.849307 | 2.352916 |
| H | 0.802350 | -6.867650 | 2.716312 |
| C | 1.837249 | -5.573363 | 1.362577 |

| | | | |
|---|---|---|---|
| H | 2.463128 | -6.353473 | 0.943278 |
| C | 1.944977 | -4.241105 | 0.946530 |
| C | 2.494550 | -2.315605 | 0.015166 |
| C | 3.172353 | -1.209695 | -0.662639 |
| C | 4.229991 | -1.253692 | -1.575467 |
| H | 4.612364 | -2.186952 | -1.962023 |
| C | 4.792524 | -0.055425 | -2.001254 |
| H | 5.651253 | -0.065822 | -2.660143 |
| C | 4.285700 | 1.169516 | -1.564123 |
| H | 4.807192 | 2.073180 | -1.847062 |
| C | 3.189822 | 1.171966 | -0.704286 |
| C | 2.473151 | 2.310642 | -0.125312 |
| C | 1.741466 | 4.287095 | 0.544333 |
| C | 1.512500 | 5.646163 | 0.784029 |
| H | 2.094253 | 6.417151 | 0.291697 |
| C | 0.517641 | 5.966833 | 1.697874 |
| H | 0.318437 | 7.010492 | 1.921658 |
| C | -0.231651 | 4.964955 | 2.350817 |
| H | -0.989983 | 5.255294 | 3.072057 |
| C | -0.011379 | 3.615603 | 2.106362 |
| H | -0.583646 | 2.856576 | 2.624252 |
| C | 0.993302 | 3.279061 | 1.187925 |
| C | 3.648304 | -4.419958 | -0.899397 |
| H | 3.640761 | -3.944184 | -1.878254 |
| H | 3.155997 | -5.387905 | -1.030921 |
| C | 5.071054 | -4.612636 | -0.367830 |
| H | 5.557029 | -3.636511 | -0.250185 |
| H | 5.034836 | -5.059605 | 0.634905 |
| C | 5.896161 | -5.494333 | -1.317448 |
| H | 5.869145 | -5.060810 | -2.324431 |

| | | | |
|---|---|---|---|
| H | 5.421421 | -6.482994 | -1.396260 |
| C | 7.346915 | -5.659665 | -0.854750 |
| H | 7.400646 | -6.115919 | 0.141909 |
| H | 7.860093 | -4.691781 | -0.807837 |
| H | 7.908567 | -6.298779 | -1.544454 |
| C | 3.554725 | 4.397526 | -1.188096 |
| H | 2.942559 | 5.197575 | -1.618128 |
| H | 3.829575 | 3.741656 | -2.013185 |
| C | 4.794384 | 4.968623 | -0.492950 |
| H | 4.474284 | 5.603002 | 0.345038 |
| H | 5.391113 | 4.151709 | -0.074980 |
| C | 5.644885 | 5.785284 | -1.475984 |
| H | 5.022890 | 6.558617 | -1.952727 |
| H | 6.000977 | 5.118475 | -2.270036 |
| C | 6.852197 | 6.439632 | -0.798229 |
| H | 6.542173 | 7.111692 | 0.012385 |
| H | 7.430297 | 7.030232 | -1.517476 |
| H | 7.517510 | 5.679684 | -0.379052 |
| C | 3.335302 | 1.819789 | 3.438613 |
| C | 4.542098 | 2.381958 | 2.999086 |
| H | 5.250743 | 1.836949 | 2.392511 |
| C | 4.877262 | 3.692073 | 3.352945 |
| H | 5.820901 | 4.096182 | 2.999416 |
| C | 4.020598 | 4.453914 | 4.145148 |
| H | 4.290915 | 5.468094 | 4.426623 |
| C | 2.813887 | 3.902432 | 4.583653 |
| H | 2.139410 | 4.482792 | 5.206991 |
| C | 2.469664 | 2.600133 | 4.230185 |
| H | 1.538344 | 2.181952 | 4.600698 |
| C | 2.190319 | -0.461932 | 4.703597 |

| | | | |
|---|---|---|---|
| C | 3.039909 | -0.221285 | 5.803458 |
| H | 3.975091 | 0.312469 | 5.666417 |
| C | 2.688362 | -0.671341 | 7.071948 |
| H | 3.352159 | -0.479222 | 7.910499 |
| C | 1.491109 | -1.368781 | 7.264801 |
| H | 1.221273 | -1.721600 | 8.256371 |
| C | 0.642733 | -1.609826 | 6.187169 |
| H | -0.289804 | -2.150766 | 6.318419 |
| C | 0.992895 | -1.152549 | 4.911503 |
| H | 0.310406 | -1.348495 | 4.098657 |
| C | 4.265446 | -0.959022 | 2.760033 |
| C | 4.397599 | -2.159033 | 3.486016 |
| H | 3.636650 | -2.457036 | 4.197768 |
| C | 5.523801 | -2.963778 | 3.318620 |
| H | 5.614121 | -3.878286 | 3.898601 |
| C | 6.533520 | -2.589125 | 2.429751 |
| H | 7.418689 | -3.209198 | 2.317347 |
| C | 6.399711 | -1.419653 | 1.680549 |
| H | 7.160796 | -1.100831 | 0.973774 |
| C | 5.265839 | -0.618732 | 1.831911 |
| H | 5.213656 | 0.279425 | 1.229170 |
| C | -0.372716 | 0.225806 | 2.218493 |
| S | -3.088212 | -2.487958 | 4.897351 |
| F | -4.129323 | -0.040952 | 4.947594 |
| F | -2.271243 | -0.243531 | 6.059106 |
| F | -4.121709 | -1.047205 | 6.875502 |
| O | -2.238163 | -2.076954 | 3.736985 |
| O | -2.382022 | -3.291706 | 5.913678 |
| O | -4.432681 | -2.958241 | 4.488065 |
| C | -3.425779 | -0.864395 | 5.749214 |

| | | | |
|---|---|---|---|
| S | 7.418823 | 1.908956 | -0.525739 |
| F | 9.961048 | 2.358527 | -1.122805 |
| F | 9.599934 | 1.897142 | 0.972493 |
| F | 9.081863 | 3.856869 | 0.187607 |
| O | 7.620479 | 0.456334 | -0.731634 |
| O | 6.604326 | 2.253623 | 0.675838 |
| O | 7.035040 | 2.672493 | -1.740370 |
| C | 9.117905 | 2.541419 | -0.100411 |
| N | 2.652860 | 3.652501 | -0.286040 |
| N | 2.766763 | -3.649502 | 0.000595 |
| N | -2.661810 | 3.633514 | -0.225976 |
| N | -2.774390 | -3.636952 | 0.512835 |
