# Supplementary material for: Structure-property relationships of photofunctional diiridium(II) complexes with tetracationic charge and an unsupported Ir–Ir bond
Source: Commun Chem. 2022 Nov 23;5:159. doi: 10.1038/s42004-022-00775-4 (PMC9814866; doi:10.1038/s42004-022-00775-4)
Supplement: Supplementary file 6 — Supplementary Data 3 [file 42004_2022_775_MOESM6_ESM.pdf]

```
data_[Ir(II)(t-Bu-terpyr)(PPh3)(CO)]2[BF4]4
_audit_creation_date              2017-12-05
_audit_creation_method
;
Olex2 1.2
(compiled 2017.08.10 svn.r3458 for OlexSys, GUI svn.r5381)
;
_shelxl_version_number            2013-2
_publ_contact_author_address      ?
_publ_contact_author_email        ?
_publ_contact_author_id_orcid     ?
_publ_contact_author_name         ''
_publ_contact_author_phone        ?
_publ_section_references
;
Dolomanov, O.V., Bourhis, L.J., Gildea, R.J, Howard, J.A.K. & Puschmann,
H.
 (2009), J. Appl. Cryst. 42, 339-341.

Sheldrick, G.M. (2015). Acta Cryst. A71, 3-8.

Sheldrick, G.M. (2015). Acta Cryst. C71, 3-8.
;
_chemical_name_common             ?
_chemical_name_systematic
;
 ?
;
_chemical_formula_moiety          'C46 H50 Ir N3 O P, 2(B F4)'
_chemical_formula_sum             'C46 H50 B2 F8 Ir N3 O P'
_chemical_formula_weight          1057.68
_chemical_melting_point           ?
loop_
  _atom_type_symbol
  _atom_type_description
  _atom_type_scat_dispersion_real
  _atom_type_scat_dispersion_imag
  _atom_type_scat_source
 'C' 'C' 0.0181 0.0091 'International Tables Vol C Tables 4.2.6.8 and
6.1.1.4'
 'H' 'H' 0.0000 0.0000 'International Tables Vol C Tables 4.2.6.8 and
6.1.1.4'
 'B' 'B' 0.0090 0.0039 'International Tables Vol C Tables 4.2.6.8 and
6.1.1.4'
```

```
 'F' 'F' 0.0727 0.0534 'International Tables Vol C Tables 4.2.6.8 and
6.1.1.4'
 'Ir' 'Ir' -4.7710 6.5667
 'International Tables Vol C Tables 4.2.6.8 and 6.1.1.4'
 'N' 'N' 0.0311 0.0180 'International Tables Vol C Tables 4.2.6.8 and
6.1.1.4'
 'O' 'O' 0.0492 0.0322 'International Tables Vol C Tables 4.2.6.8 and
6.1.1.4'
 'P' 'P' 0.2955 0.4335 'International Tables Vol C Tables 4.2.6.8 and
6.1.1.4'

_shelx_space_group_comment
;
The symmetry employed for this shelxl refinement is uniquely defined
by the following loop, which should always be used as a source of
symmetry information in preference to the above space-group names.
They are only intended as comments.
;
_space_group_crystal_system       'monoclinic'
_space_group_IT_number            14
_space_group_name_H-M_alt         'P 1 21/n 1'
_space_group_name_Hall            '-P 2yn'
loop_
  _space_group_symop_operation_xyz
 'x, y, z'
 '-x+1/2, y+1/2, -z+1/2'
 '-x, -y, -z'
 'x-1/2, -y-1/2, z-1/2'

_cell_length_a                    14.7269(10)
_cell_length_b                    17.4747(11)
_cell_length_c                    17.3176(11)
_cell_angle_alpha                 90
_cell_angle_beta                  101.355(3)
_cell_angle_gamma                 90
_cell_volume                      4369.4(5)
_cell_formula_units_Z             4
_cell_measurement_reflns_used     9062
_cell_measurement_temperature     100(2)
_cell_measurement_theta_max       65.25
_cell_measurement_theta_min       4.41
_shelx_estimated_absorpt_T_max    0.387
_shelx_estimated_absorpt_T_min    0.325
_exptl_absorpt_coefficient_mu     6.897
```

```
_exptl_absorpt_correction_T_max   0.1621
_exptl_absorpt_correction_T_min   0.0466
_exptl_absorpt_correction_type    multi-scan
_exptl_absorpt_process_details
'SADABS-2014/5 (Bruker,2014/5) was used for absorption correction.
wR2(int) was 0.1284 before and 0.0577 after correction. The Ratio of
minimum to maximum transmission is 0.2875. The \l/2 correction factor
is Not present.'
_exptl_crystal_colour             brown
_exptl_crystal_colour_primary     brown
_exptl_crystal_density_diffrn     1.608
_exptl_crystal_density_meas       ?
_exptl_crystal_density_method     ?
_exptl_crystal_description        block
_exptl_crystal_F_000              2116
_exptl_crystal_size_max           0.21
_exptl_crystal_size_mid           0.19
_exptl_crystal_size_min           0.17
_exptl_special_details
;
 ?
;
_exptl_transmission_factor_max    ?
_exptl_transmission_factor_min    ?
_diffrn_reflns_av_R_equivalents   0.0435
_diffrn_reflns_av_unetI/netI      0.0412
_diffrn_reflns_Laue_measured_fraction_full 0.935
_diffrn_reflns_Laue_measured_fraction_max 0.992
_diffrn_reflns_limit_h_max        17
_diffrn_reflns_limit_h_min        -17
_diffrn_reflns_limit_k_max        20
_diffrn_reflns_limit_k_min        -20
_diffrn_reflns_limit_l_max        20
_diffrn_reflns_limit_l_min        -19
_diffrn_reflns_number             26298
_diffrn_reflns_point_group_measured_fraction_full 0.935
_diffrn_reflns_point_group_measured_fraction_max 0.992
_diffrn_reflns_theta_full         67.679
_diffrn_reflns_theta_max          65.271
_diffrn_reflns_theta_min          4.407
_diffrn_ambient_temperature       99.99
_diffrn_detector                  'Bruker PHOTO 100 area detector'
_diffrn_detector_area_resol_mean  7.9
_diffrn_detector_type             'CCD area detector'
```

```
_diffrn_measured_fraction_theta_full 0.935
_diffrn_measured_fraction_theta_max 0.992
_diffrn_measurement_device        'three-circle diffractometer'
_diffrn_measurement_device_type   'Bruker APEX-II CCD'
_diffrn_measurement_method        '\f and \w scans'
_diffrn_radiation_monochromator   'mirror optics'
_diffrn_radiation_probe           x-ray
_diffrn_radiation_type            CuK\a
_diffrn_radiation_wavelength      1.54178
_diffrn_source                    'microfocus sealed X-ray tube'
_diffrn_source_type               'Incoatec I\ms'
_diffrn_standards_number          0
_reflns_Friedel_coverage          0.000
_reflns_Friedel_fraction_full     .
_reflns_Friedel_fraction_max      .
_reflns_number_gt                 6537
_reflns_number_total              7408
_reflns_special_details
;
 Reflections were merged by SHELXL according to the crystal
 class for the calculation of statistics and refinement.

 _reflns_Friedel_fraction is defined as the number of unique
 Friedel pairs measured divided by the number that would be
 possible theoretically, ignoring centric projections and
 systematic absences.
;
_reflns_threshold_expression      'I > 2\s(I)'
_computing_cell_refinement        'SAINT v8.37A (Bruker, 2015)'
_computing_data_collection        ?
_computing_data_reduction         'SAINT v8.37A (Bruker, 2015)'
_computing_molecular_graphics     'Olex2 (Dolomanov et al., 2009)'
_computing_publication_material   'Olex2 (Dolomanov et al., 2009)'
_computing_structure_refinement   'ShelXL (Sheldrick, 2015)'
_computing_structure_solution     'ShelXT (Sheldrick, 2015)'
_refine_diff_density_max          2.659
_refine_diff_density_min          -0.826
_refine_diff_density_rms          0.117
_refine_ls_extinction_coef        .
_refine_ls_extinction_method      none
_refine_ls_goodness_of_fit_ref    1.064
_refine_ls_hydrogen_treatment     constr
_refine_ls_matrix_type            full
_refine_ls_number_parameters      599
```

```
_refine_ls_number_reflns          7408
_refine_ls_number_restraints      30
_refine_ls_R_factor_all           0.0474
_refine_ls_R_factor_gt            0.0423
_refine_ls_restrained_S_all       1.086
_refine_ls_shift/su_max           0.002
_refine_ls_shift/su_mean          0.000
_refine_ls_structure_factor_coef  Fsqd
_refine_ls_weighting_details
;
 w=1/[\s^2^(Fo^2^)+(0.0470P)^2^+15.7140P]
 where P=(Fo^2^+2Fc^2^)/3
;
_refine_ls_weighting_scheme       calc
_refine_ls_wR_factor_gt           0.1100
_refine_ls_wR_factor_ref          0.1150
_refine_special_details
;
 ?
;
_olex2_refinement_description
;
1. Fixed Uiso
 At 1.2 times of:
  All C(H) groups
 At 1.5 times of:
  All C(H,H,H) groups
2. Uiso/Uaniso restraints and constraints
Uanis(C25) \\sim Ueq, Uanis(C26) \\sim Ueq, Uanis(C27) \\sim Ueq: with
sigma of
0.01 and sigma for terminal atoms of 0.02
Uanis(F7) \\sim Ueq, Uanis(F8) \\sim Ueq: with sigma of 0.002 and sigma
for
terminal atoms of 0.004
3. Others

Sof(C25')=Sof(H25D)=Sof(H25E)=Sof(H25F)=Sof(C26')=Sof(H26D)=Sof(H26E)=
 Sof(H26F)=Sof(C27')=Sof(H27D)=Sof(H27E)=Sof(H27F)=1-FVAR(1)

Sof(C25)=Sof(H25A)=Sof(H25B)=Sof(H25C)=Sof(C26)=Sof(H26A)=Sof(H26B)=So
f(H26C)=
 Sof(C27)=Sof(H27A)=Sof(H27B)=Sof(H27C)=FVAR(1)
4.a Aromatic/amide H refined with riding coordinates:
 C1(H1), C2(H2), C4(H4), C7(H7), C9(H9), C12(H12), C14(H14), C15(H15),
```

```
 C29(H29), C30(H30), C31(H31), C32(H32), C33(H33), C35(H35), C36(H36),
C37(H37),
  C38(H38), C39(H39), C41(H41), C42(H42), C43(H43), C44(H44), C45(H45)
4.b Idealised Me refined as rotating group:
 C17(H17A,H17B,H17C),     C18(H18A,H18B,H18C),     C19(H19A,H19B,H19C),
C21(H21A,H21B,
 H21C), C22(H22A,H22B,H22C), C23(H23A,H23B,H23C), C25(H25A,H25B,H25C),
C26(H26A,
 H26B,H26C),         C27(H27A,H27B,H27C),         C25'(H25D,H25E,H25F),
C26'(H26D,H26E,H26F),
 C27'(H27D,H27E,H27F)
;
_atom_sites_solution_hydrogens    geom
_atom_sites_solution_primary      dual
_atom_sites_solution_secondary    ?
loop_
  _atom_site_label
  _atom_site_type_symbol
  _atom_site_fract_x
  _atom_site_fract_y
  _atom_site_fract_z
  _atom_site_U_iso_or_equiv
  _atom_site_adp_type
  _atom_site_occupancy
  _atom_site_site_symmetry_order
  _atom_site_calc_flag
  _atom_site_refinement_flags_posn
  _atom_site_refinement_flags_adp
  _atom_site_refinement_flags_occupancy
  _atom_site_disorder_assembly
  _atom_site_disorder_group
 Ir1 Ir 0.60015(2) 0.49506(2) 0.50788(2) 0.03261(10) Uani 1 1 d . . . . .
 P1 P 0.76757(10) 0.48924(8) 0.51477(9) 0.0403(3) Uani 1 1 d . . . . .
 O1 O 0.6073(3) 0.6625(2) 0.5530(2) 0.0428(8) Uani 1 1 d . . . . .
 N1 N 0.5866(3) 0.4921(2) 0.3874(3) 0.0367(9) Uani 1 1 d . . . . .
 N2 N 0.5925(3) 0.3823(2) 0.4879(2) 0.0346(9) Uani 1 1 d . . . . .
 N3 N 0.6130(3) 0.4546(2) 0.6204(2) 0.0364(9) Uani 1 1 d . . . . .
 C1 C 0.6272(4) 0.4953(3) 0.6881(3) 0.0374(11) Uani 1 1 d . . . . .
 H1 H 0.6268 0.5496 0.6849 0.045 Uiso 1 1 calc R . . . .
 C2 C 0.6422(4) 0.4617(3) 0.7611(3) 0.0412(12) Uani 1 1 d . . . . .
 H2 H 0.6506 0.4928 0.8069 0.049 Uiso 1 1 calc R . . . .
 C3 C 0.6453(4) 0.3821(3) 0.7689(3) 0.0435(12) Uani 1 1 d . . . . .
 C4 C 0.6286(4) 0.3400(3) 0.6988(3) 0.0399(11) Uani 1 1 d . . . . .
 H4 H 0.6276 0.2857 0.7011 0.048 Uiso 1 1 calc R . . . .
```

C5 C 0.6136(3) 0.3758(3) 0.6262(3) 0.0361(10) Uani 1 1 d . . . . .
C6 C 0.6022(3) 0.3351(3) 0.5511(3) 0.0360(10) Uani 1 1 d . . . . .
C7 C 0.6023(4) 0.2571(3) 0.5385(3) 0.0410(12) Uani 1 1 d . . . . .
H7 H 0.6086 0.2230 0.5820 0.049 Uiso 1 1 calc R . . . .
C8 C 0.5932(4) 0.2277(3) 0.4623(3) 0.0419(12) Uani 1 1 d . . . . .
C9 C 0.5843(4) 0.2788(3) 0.3995(3) 0.0416(12) Uani 1 1 d . . . . .
H9 H 0.5772 0.2603 0.3470 0.050 Uiso 1 1 calc R . . . .
C10 C 0.5858(3) 0.3569(3) 0.4140(3) 0.0368(11) Uani 1 1 d . . . . .
C11 C 0.5835(3) 0.4193(3) 0.3562(3) 0.0374(11) Uani 1 1 d . . . . .
C12 C 0.5880(4) 0.4071(3) 0.2783(3) 0.0414(12) Uani 1 1 d . . . . .
H12 H 0.5838 0.3564 0.2581 0.050 Uiso 1 1 calc R . . . .
C13 C 0.5988(4) 0.4684(3) 0.2286(3) 0.0449(12) Uani 1 1 d . . . . .
C14 C 0.5966(4) 0.5409(3) 0.2607(3) 0.0454(12) Uani 1 1 d . . . . .
H14 H 0.6002 0.5844 0.2286 0.054 Uiso 1 1 calc R . . . .
C15 C 0.5893(4) 0.5514(3) 0.3378(3) 0.0394(11) Uani 1 1 d . . . . .
H15 H 0.5861 0.6021 0.3569 0.047 Uiso 1 1 calc R . . . .
C16 C 0.6650(5) 0.3417(4) 0.8491(3) 0.0519(14) Uani 1 1 d . . . . .
C17 C 0.7045(6) 0.3973(5) 0.9147(4) 0.079(2) Uani 1 1 d . . . . .
H17A H 0.7601 0.4216 0.9029 0.119 Uiso 1 1 calc GR . . . .
H17B H 0.7205 0.3694 0.9647 0.119 Uiso 1 1 calc GR . . . .
H17C H 0.6582 0.4366 0.9188 0.119 Uiso 1 1 calc GR . . . .
C18 C 0.7350(7) 0.2786(5) 0.8480(4) 0.089(3) Uani 1 1 d . . . . .
H18A H 0.7069 0.2379 0.8122 0.133 Uiso 1 1 calc GR . . . .
H18B H 0.7545 0.2577 0.9012 0.133 Uiso 1 1 calc GR . . . .
H18C H 0.7891 0.2993 0.8299 0.133 Uiso 1 1 calc GR . . . .
C19 C 0.5760(6) 0.3083(6) 0.8645(5) 0.097(3) Uani 1 1 d . . . . .
H19A H 0.5352 0.3497 0.8750 0.146 Uiso 1 1 calc GR . . . .
H19B H 0.5896 0.2742 0.9103 0.146 Uiso 1 1 calc GR . . . .
H19C H 0.5453 0.2792 0.8183 0.146 Uiso 1 1 calc GR . . . .
C20 C 0.5899(4) 0.1407(3) 0.4497(4) 0.0506(14) Uani 1 1 d . . . . .
C21 C 0.4939(5) 0.1138(4) 0.4590(5) 0.0696(19) Uani 1 1 d . . . . .
H21A H 0.4829 0.1296 0.5106 0.104 Uiso 1 1 calc GR . . . .
H21B H 0.4905 0.0579 0.4548 0.104 Uiso 1 1 calc GR . . . .
H21C H 0.4469 0.1367 0.4175 0.104 Uiso 1 1 calc GR . . . .
C22 C 0.6657(6) 0.1011(4) 0.5085(5) 0.072(2) Uani 1 1 d . . . . .
H22A H 0.7259 0.1233 0.5050 0.108 Uiso 1 1 calc GR . . . .
H22B H 0.6658 0.0463 0.4964 0.108 Uiso 1 1 calc GR . . . .
H22C H 0.6543 0.1083 0.5619 0.108 Uiso 1 1 calc GR . . . .
C23 C 0.6034(6) 0.1209(4) 0.3652(4) 0.0652(18) Uani 1 1 d . . . . .
H23A H 0.5531 0.1436 0.3266 0.098 Uiso 1 1 calc GR . . . .
H23B H 0.6028 0.0652 0.3585 0.098 Uiso 1 1 calc GR . . . .
H23C H 0.6629 0.1414 0.3574 0.098 Uiso 1 1 calc GR . . . .
C24 C 0.6171(5) 0.4542(4) 0.1468(3) 0.0528(14) Uani 1 1 d . . . . .
C28 C 0.8376(4) 0.5032(3) 0.6122(4) 0.0492(14) Uani 1 1 d . . . . .

C29 C 0.8355(4) 0.4536(4) 0.6736(4) 0.0556(15) Uani 1 1 d . . . . .
H29 H 0.7977 0.4092 0.6649 0.067 Uiso 1 1 calc R . . . .
C30 C 0.8877(5) 0.4676(5) 0.7479(4) 0.0639(17) Uani 1 1 d . . . . .
H30 H 0.8844 0.4332 0.7898 0.077 Uiso 1 1 calc R . . . .
C31 C 0.9440(6) 0.5306(4) 0.7616(4) 0.0677(18) Uani 1 1 d . . . . .
H31 H 0.9779 0.5408 0.8131 0.081 Uiso 1 1 calc R . . . .
C32 C 0.9516(6) 0.5803(4) 0.6993(4) 0.071(2) Uani 1 1 d . . . . .
H32 H 0.9925 0.6228 0.7082 0.086 Uiso 1 1 calc R . . . .
C33 C 0.8990(5) 0.5667(4) 0.6243(4) 0.0626(17) Uani 1 1 d . . . . .
H33 H 0.9041 0.5996 0.5817 0.075 Uiso 1 1 calc R . . . .
C34 C 0.8149(4) 0.5601(4) 0.4558(4) 0.0545(15) Uani 1 1 d . . . . .
C35 C 0.7749(4) 0.6310(3) 0.4392(4) 0.0504(14) Uani 1 1 d . . . . .
H35 H 0.7186 0.6426 0.4557 0.060 Uiso 1 1 calc R . . . .
C36 C 0.8148(5) 0.6854(4) 0.3993(5) 0.0656(18) Uani 1 1 d . . . . .
H36 H 0.7878 0.7348 0.3908 0.079 Uiso 1 1 calc R . . . .
C37 C 0.8944(5) 0.6686(5) 0.3713(6) 0.083(2) Uani 1 1 d . . . . .
H37 H 0.9207 0.7059 0.3424 0.100 Uiso 1 1 calc R . . . .
C38 C 0.9356(5) 0.5972(5) 0.3854(5) 0.076(2) Uani 1 1 d . . . . .
H38 H 0.9902 0.5859 0.3661 0.092 Uiso 1 1 calc R . . . .
C39 C 0.8979(5) 0.5426(4) 0.4273(4) 0.0619(16) Uani 1 1 d . . . . .
H39 H 0.9264 0.4939 0.4372 0.074 Uiso 1 1 calc R . . . .
C40 C 0.8016(4) 0.3989(3) 0.4759(3) 0.0465(13) Uani 1 1 d . . . . .
C41 C 0.7982(4) 0.3920(4) 0.3949(4) 0.0592(16) Uani 1 1 d . . . . .
H41 H 0.7802 0.4345 0.3611 0.071 Uiso 1 1 calc R . . . .
C42 C 0.8214(5) 0.3222(5) 0.3638(5) 0.073(2) Uani 1 1 d . . . . .
H42 H 0.8178 0.3177 0.3086 0.088 Uiso 1 1 calc R . . . .
C43 C 0.8488(5) 0.2612(5) 0.4104(6) 0.080(2) Uani 1 1 d . . . . .
H43 H 0.8666 0.2151 0.3884 0.096 Uiso 1 1 calc R . . . .
C44 C 0.8507(6) 0.2662(4) 0.4898(5) 0.075(2) Uani 1 1 d . . . . .
H44 H 0.8680 0.2229 0.5225 0.090 Uiso 1 1 calc R . . . .
C45 C 0.8271(5) 0.3350(3) 0.5230(4) 0.0588(16) Uani 1 1 d . . . . .
H45 H 0.8286 0.3379 0.5781 0.071 Uiso 1 1 calc R . . . .
C46 C 0.6031(3) 0.6008(3) 0.5327(3) 0.0366(11) Uani 1 1 d . . . . .
C25 C 0.5438(13) 0.4080(9) 0.1000(8) 0.076(5) Uani 0.520(12) 1 d . U P
A 1
H25A H 0.5398 0.3588 0.1263 0.114 Uiso 0.520(12) 1 calc GR . P A 1
H25B H 0.5578 0.3991 0.0477 0.114 Uiso 0.520(12) 1 calc GR . P A 1
H25C H 0.4845 0.4350 0.0947 0.114 Uiso 0.520(12) 1 calc GR . P A 1
C26 C 0.6292(11) 0.5318(8) 0.1029(7) 0.061(4) Uani 0.520(12) 1 d . U P
A 1
H26A H 0.5702 0.5594 0.0920 0.092 Uiso 0.520(12) 1 calc GR . P A 1
H26B H 0.6488 0.5207 0.0531 0.092 Uiso 0.520(12) 1 calc GR . P A 1
H26C H 0.6763 0.5634 0.1362 0.092 Uiso 0.520(12) 1 calc GR . P A 1
C27 C 0.7128(12) 0.4156(10) 0.1601(8) 0.081(5) Uani 0.520(12) 1 d . U

```
P A 1
 H27A H 0.7593 0.4502 0.1900 0.122 Uiso 0.520(12) 1 calc GR . P A 1
 H27B H 0.7290 0.4042 0.1091 0.122 Uiso 0.520(12) 1 calc GR . P A 1
 H27C H 0.7112 0.3680 0.1897 0.122 Uiso 0.520(12) 1 calc GR . P A 1
 C25' C 0.7070(12) 0.4875(9) 0.1387(10) 0.065(5) Uani 0.480(12) 1 d . .
P A 2
 H25D H 0.7040 0.5434 0.1422 0.098 Uiso 0.480(12) 1 calc GR . P A 2
 H25E H 0.7207 0.4730 0.0875 0.098 Uiso 0.480(12) 1 calc GR . P A 2
 H25F H 0.7559 0.4681 0.1809 0.098 Uiso 0.480(12) 1 calc GR . P A 2
 C26' C 0.6163(10) 0.3670(8) 0.1260(7) 0.055(4) Uani 0.480(12) 1 d . .
P A 2
 H26D H 0.6700 0.3420 0.1587 0.082 Uiso 0.480(12) 1 calc GR . P A 2
 H26E H 0.6192 0.3609 0.0703 0.082 Uiso 0.480(12) 1 calc GR . P A 2
 H26F H 0.5593 0.3436 0.1360 0.082 Uiso 0.480(12) 1 calc GR . P A 2
 C27' C 0.5354(13) 0.4898(9) 0.0856(9) 0.073(5) Uani 0.480(12) 1 d . .
P A 2
 H27D H 0.4762 0.4730 0.0977 0.109 Uiso 0.480(12) 1 calc GR . P A 2
 H27E H 0.5396 0.4730 0.0325 0.109 Uiso 0.480(12) 1 calc GR . P A 2
 H27F H 0.5392 0.5458 0.0884 0.109 Uiso 0.480(12) 1 calc GR . P A 2
 F1 F 0.4526(3) 0.2578(2) 0.2045(3) 0.0784(12) Uani 1 1 d . . . . .
 F2 F 0.3768(3) 0.3291(2) 0.2780(3) 0.0744(11) Uani 1 1 d . . . . .
 F3 F 0.3736(4) 0.3635(3) 0.1538(3) 0.1041(17) Uani 1 1 d . . . . .
 F4 F 0.2951(3) 0.2583(3) 0.1773(3) 0.0845(13) Uani 1 1 d . . . . .
 B1 B 0.3742(5) 0.3010(5) 0.2049(5) 0.0590(18) Uani 1 1 d . . . . .
 F5 F 0.4169(3) 0.2776(3) 0.5991(3) 0.0790(12) Uani 1 1 d . . . . .
 F6 F 0.5021(3) 0.1870(3) 0.6661(3) 0.0897(15) Uani 1 1 d . . . . .
 F7 F 0.4006(5) 0.2624(4) 0.7198(4) 0.1220(19) Uani 1 1 d . U . . .
 F8 F 0.3520(5) 0.1705(4) 0.6453(5) 0.151(3) Uani 1 1 d . U . . .
 B2 B 0.4156(6) 0.2214(4) 0.6512(6) 0.068(2) Uani 1 1 d . . . . .

loop_
  _atom_site_aniso_label
  _atom_site_aniso_U_11
  _atom_site_aniso_U_22
  _atom_site_aniso_U_33
  _atom_site_aniso_U_23
  _atom_site_aniso_U_13
  _atom_site_aniso_U_12
 Ir1  0.03647(14)  0.03404(14)  0.02789(15)  -0.00027(8)  0.00770(9)
0.00024(8)
 P1 0.0377(7) 0.0421(7) 0.0404(7) 0.0005(5) 0.0057(6) 0.0011(5)
 O1 0.048(2) 0.038(2) 0.043(2) -0.0029(16) 0.0111(16) -0.0029(16)
 N1 0.038(2) 0.040(2) 0.035(2) -0.0028(17) 0.0121(19) 0.0001(17)
 N2 0.036(2) 0.034(2) 0.035(2) -0.0026(17) 0.0112(17) 0.0011(16)
```

N3 0.041(2) 0.038(2) 0.032(2) 0.0008(17) 0.0092(18) 0.0004(17)
C1 0.040(3) 0.038(3) 0.035(3) -0.004(2) 0.008(2) 0.001(2)
C2 0.043(3) 0.049(3) 0.032(3) -0.004(2) 0.008(2) 0.004(2)
C3 0.042(3) 0.050(3) 0.040(3) 0.005(2) 0.013(2) 0.005(2)
C4 0.041(3) 0.040(3) 0.039(3) 0.002(2) 0.008(2) 0.004(2)
C5 0.036(3) 0.039(3) 0.034(3) 0.001(2) 0.008(2) 0.004(2)
C6 0.035(2) 0.040(3) 0.035(3) 0.002(2) 0.011(2) 0.002(2)
C7 0.046(3) 0.035(3) 0.043(3) 0.002(2) 0.011(2) -0.001(2)
C8 0.042(3) 0.038(3) 0.047(3) -0.003(2) 0.012(2) 0.000(2)
C9 0.044(3) 0.043(3) 0.039(3) -0.008(2) 0.011(2) -0.002(2)
C10 0.036(3) 0.042(3) 0.034(3) -0.004(2) 0.010(2) 0.002(2)
C11 0.035(2) 0.043(3) 0.035(3) -0.002(2) 0.009(2) 0.000(2)
C12 0.046(3) 0.047(3) 0.034(3) -0.003(2) 0.013(2) 0.001(2)
C13 0.045(3) 0.057(3) 0.035(3) -0.001(2) 0.012(2) 0.001(3)
C14 0.043(3) 0.053(3) 0.041(3) 0.007(2) 0.011(2) -0.001(2)
C15 0.043(3) 0.044(3) 0.032(3) 0.004(2) 0.010(2) 0.000(2)
C16 0.062(4) 0.057(3) 0.037(3) 0.010(3) 0.011(3) 0.010(3)
C17 0.098(6) 0.090(5) 0.045(4) 0.007(4) 0.005(4) 0.013(5)
C18 0.124(7) 0.094(6) 0.048(4) 0.016(4) 0.015(4) 0.054(5)
C19 0.090(6) 0.127(8) 0.081(6) 0.055(6) 0.030(5) 0.006(5)
C20 0.059(4) 0.037(3) 0.057(4) -0.007(2) 0.015(3) -0.004(2)
C21 0.077(5) 0.050(4) 0.087(5) -0.012(4) 0.028(4) -0.015(3)
C22 0.098(6) 0.041(3) 0.075(5) -0.004(3) 0.008(4) 0.015(3)
C23 0.087(5) 0.043(3) 0.068(4) -0.015(3) 0.022(4) -0.003(3)
C24 0.069(4) 0.058(3) 0.035(3) -0.001(3) 0.020(3) 0.004(3)
C28 0.050(3) 0.049(3) 0.044(3) -0.010(2) -0.001(3) 0.006(2)
C29 0.049(3) 0.069(4) 0.049(3) -0.001(3) 0.012(3) -0.010(3)
C30 0.055(4) 0.084(5) 0.052(4) 0.008(3) 0.008(3) 0.002(3)
C31 0.079(5) 0.066(4) 0.051(4) -0.004(3) -0.004(3) 0.007(4)
C32 0.078(5) 0.060(4) 0.068(5) -0.004(3) -0.007(4) -0.007(3)
C33 0.071(4) 0.057(4) 0.057(4) 0.000(3) 0.005(3) -0.001(3)
C34 0.039(3) 0.055(3) 0.070(4) 0.017(3) 0.012(3) -0.002(3)
C35 0.043(3) 0.054(3) 0.056(4) 0.007(3) 0.016(3) -0.001(3)
C36 0.052(4) 0.064(4) 0.086(5) 0.020(4) 0.025(3) 0.001(3)
C37 0.065(4) 0.077(5) 0.116(7) 0.034(5) 0.039(5) -0.003(4)
C38 0.053(4) 0.090(5) 0.092(6) 0.021(4) 0.029(4) 0.003(4)
C39 0.050(3) 0.071(4) 0.067(4) 0.010(3) 0.017(3) 0.005(3)
C40 0.036(3) 0.055(3) 0.049(3) -0.009(3) 0.010(2) -0.004(2)
C41 0.049(3) 0.084(5) 0.048(3) -0.005(3) 0.015(3) 0.007(3)
C42 0.050(4) 0.110(6) 0.062(4) -0.033(4) 0.017(3) 0.005(4)
C43 0.058(4) 0.082(5) 0.100(6) -0.044(5) 0.015(4) 0.007(4)
C44 0.078(5) 0.051(4) 0.101(6) -0.012(4) 0.029(4) 0.003(3)
C45 0.066(4) 0.046(3) 0.070(4) -0.001(3) 0.026(3) 0.004(3)
C46 0.039(3) 0.039(3) 0.033(3) 0.002(2) 0.010(2) 0.001(2)

```
 C25 0.118(12) 0.073(9) 0.041(7) -0.004(6) 0.026(7) -0.013(8)
 C26 0.086(9) 0.066(8) 0.038(6) -0.003(5) 0.028(6) -0.011(7)
 C27 0.099(10) 0.100(11) 0.053(7) 0.011(7) 0.036(7) 0.028(8)
 C25' 0.071(10) 0.073(10) 0.060(9) -0.015(7) 0.034(8) -0.017(8)
 C26' 0.075(9) 0.067(8) 0.028(6) -0.004(5) 0.026(6) 0.002(7)
 C27' 0.096(12) 0.085(11) 0.037(7) -0.005(6) 0.010(7) 0.035(9)
 F1 0.066(2) 0.072(3) 0.102(3) -0.015(2) 0.027(2) 0.006(2)
 F2 0.077(3) 0.063(2) 0.088(3) -0.028(2) 0.028(2) -0.0158(19)
 F3 0.118(4) 0.085(3) 0.098(4) 0.012(3) -0.006(3) -0.015(3)
 F4 0.064(2) 0.106(3) 0.088(3) -0.036(3) 0.026(2) -0.034(2)
 B1 0.048(4) 0.063(4) 0.068(5) -0.010(4) 0.017(3) -0.011(3)
 F5 0.096(3) 0.078(3) 0.073(3) 0.023(2) 0.042(2) 0.031(2)
 F6 0.071(3) 0.087(3) 0.118(4) 0.050(3) 0.038(3) 0.022(2)
 F7 0.131(3) 0.148(4) 0.100(3) 0.025(3) 0.052(3) 0.024(3)
 F8 0.136(4) 0.130(4) 0.185(4) 0.005(3) 0.026(3) -0.011(3)
 B2 0.060(5) 0.046(4) 0.098(7) 0.013(4) 0.017(4) -0.006(3)

_geom_special_details
;
 All esds (except the esd in the dihedral angle between two l.s. planes)
 are estimated using the full covariance matrix.  The cell esds are
taken
 into account individually in the estimation of esds in distances,
angles
 and torsion angles; correlations between esds in cell parameters are
only
 used when they are defined by crystal symmetry.  An approximate
(isotropic)
 treatment of cell esds is used for estimating esds involving l.s.
planes.
;
loop_
  _geom_bond_atom_site_label_1
  _geom_bond_atom_site_label_2
  _geom_bond_distance
  _geom_bond_site_symmetry_2
  _geom_bond_publ_flag
 Ir1 Ir1 2.9135(5) 3_666 ?
 Ir1 P1 2.4471(14) . ?
 Ir1 N1 2.058(5) . ?
 Ir1 N2 2.000(4) . ?
 Ir1 N3 2.046(4) . ?
 Ir1 C46 1.896(5) . ?
 P1 C28 1.813(6) . ?
```

P1 C34 1.828(6) . ?
P1 C40 1.824(6) . ?
O1 C46 1.132(6) . ?
N1 C11 1.379(6) . ?
N1 C15 1.353(7) . ?
N2 C6 1.356(7) . ?
N2 C10 1.340(6) . ?
N3 C1 1.353(7) . ?
N3 C5 1.381(6) . ?
C1 H1 0.9500 . ?
C1 C2 1.373(8) . ?
C2 H2 0.9500 . ?
C2 C3 1.398(8) . ?
C3 C4 1.398(8) . ?
C3 C16 1.534(8) . ?
C4 H4 0.9500 . ?
C4 C5 1.382(7) . ?
C5 C6 1.463(7) . ?
C6 C7 1.381(7) . ?
C7 H7 0.9500 . ?
C7 C8 1.397(8) . ?
C8 C9 1.394(8) . ?
C8 C20 1.535(7) . ?
C9 H9 0.9500 . ?
C9 C10 1.387(7) . ?
C10 C11 1.475(7) . ?
C11 C12 1.380(7) . ?
C12 H12 0.9500 . ?
C12 C13 1.403(8) . ?
C13 C14 1.387(9) . ?
C13 C24 1.512(8) . ?
C14 H14 0.9500 . ?
C14 C15 1.372(8) . ?
C15 H15 0.9500 . ?
C16 C17 1.520(10) . ?
C16 C18 1.513(10) . ?
C16 C19 1.507(11) . ?
C17 H17A 0.9800 . ?
C17 H17B 0.9800 . ?
C17 H17C 0.9800 . ?
C18 H18A 0.9800 . ?
C18 H18B 0.9800 . ?
C18 H18C 0.9800 . ?
C19 H19A 0.9800 . ?

C19 H19B 0.9800 . ?
C19 H19C 0.9800 . ?
C20 C21 1.527(9) . ?
C20 C22 1.521(9) . ?
C20 C23 1.553(9) . ?
C21 H21A 0.9800 . ?
C21 H21B 0.9800 . ?
C21 H21C 0.9800 . ?
C22 H22A 0.9800 . ?
C22 H22B 0.9800 . ?
C22 H22C 0.9800 . ?
C23 H23A 0.9800 . ?
C23 H23B 0.9800 . ?
C23 H23C 0.9800 . ?
C24 C25 1.460(17) . ?
C24 C26 1.583(14) . ?
C24 C27 1.539(16) . ?
C24 C25' 1.479(16) . ?
C24 C26' 1.565(15) . ?
C24 C27' 1.568(17) . ?
C28 C29 1.376(9) . ?
C28 C33 1.421(9) . ?
C29 H29 0.9500 . ?
C29 C30 1.384(9) . ?
C30 H30 0.9500 . ?
C30 C31 1.372(11) . ?
C31 H31 0.9500 . ?
C31 C32 1.406(11) . ?
C32 H32 0.9500 . ?
C32 C33 1.395(9) . ?
C33 H33 0.9500 . ?
C34 C35 1.378(8) . ?
C34 C39 1.438(9) . ?
C35 H35 0.9500 . ?
C35 C36 1.374(9) . ?
C36 H36 0.9500 . ?
C36 C37 1.385(10) . ?
C37 H37 0.9500 . ?
C37 C38 1.387(11) . ?
C38 H38 0.9500 . ?
C38 C39 1.381(10) . ?
C39 H39 0.9500 . ?
C40 C41 1.399(9) . ?
C40 C45 1.390(9) . ?

```
 C41 H41 0.9500 . ?
 C41 C42 1.404(10) . ?
 C42 H42 0.9500 . ?
 C42 C43 1.349(12) . ?
 C43 H43 0.9500 . ?
 C43 C44 1.372(12) . ?
 C44 H44 0.9500 . ?
 C44 C45 1.406(9) . ?
 C45 H45 0.9500 . ?
 C25 H25A 0.9800 . ?
 C25 H25B 0.9800 . ?
 C25 H25C 0.9800 . ?
 C26 H26A 0.9800 . ?
 C26 H26B 0.9800 . ?
 C26 H26C 0.9800 . ?
 C27 H27A 0.9800 . ?
 C27 H27B 0.9800 . ?
 C27 H27C 0.9800 . ?
 C25' H25D 0.9800 . ?
 C25' H25E 0.9800 . ?
 C25' H25F 0.9800 . ?
 C26' H26D 0.9800 . ?
 C26' H26E 0.9800 . ?
 C26' H26F 0.9800 . ?
 C27' H27D 0.9800 . ?
 C27' H27E 0.9800 . ?
 C27' H27F 0.9800 . ?
 F1 B1 1.381(9) . ?
 F2 B1 1.351(9) . ?
 F3 B1 1.404(10) . ?
 F4 B1 1.388(8) . ?
 F5 B2 1.337(9) . ?
 F6 B2 1.386(9) . ?
 F7 B2 1.441(11) . ?
 F8 B2 1.281(10) . ?

loop_
  _geom_angle_atom_site_label_1
  _geom_angle_atom_site_label_2
  _geom_angle_atom_site_label_3
  _geom_angle
  _geom_angle_site_symmetry_1
  _geom_angle_site_symmetry_3
  _geom_angle_publ_flag
```

P1 Ir1 Ir1 177.27(4) . 3_666 ?
N1 Ir1 Ir1 90.70(12) . 3_666 ?
N1 Ir1 P1 86.79(13) . . ?
N2 Ir1 Ir1 91.20(11) . 3_666 ?
N2 Ir1 P1 89.37(12) . . ?
N2 Ir1 N1 78.94(16) . . ?
N2 Ir1 N3 79.38(17) . . ?
N3 Ir1 Ir1 90.70(12) . 3_666 ?
N3 Ir1 P1 92.03(12) . . ?
N3 Ir1 N1 158.30(16) . . ?
C46 Ir1 Ir1 86.63(15) . 3_666 ?
C46 Ir1 P1 92.95(15) . . ?
C46 Ir1 N1 104.36(19) . . ?
C46 Ir1 N2 176.05(18) . . ?
C46 Ir1 N3 97.34(19) . . ?
C28 P1 Ir1 115.0(2) . . ?
C28 P1 C34 102.7(3) . . ?
C28 P1 C40 108.2(3) . . ?
C34 P1 Ir1 115.83(19) . . ?
C40 P1 Ir1 111.45(18) . . ?
C40 P1 C34 102.6(3) . . ?
C11 N1 Ir1 114.2(3) . . ?
C15 N1 Ir1 128.0(3) . . ?
C15 N1 C11 117.5(4) . . ?
C6 N2 Ir1 117.8(3) . . ?
C10 N2 Ir1 118.9(3) . . ?
C10 N2 C6 123.1(4) . . ?
C1 N3 Ir1 127.8(3) . . ?
C1 N3 C5 117.6(4) . . ?
C5 N3 Ir1 114.4(3) . . ?
N3 C1 H1 118.6 . . ?
N3 C1 C2 122.9(5) . . ?
C2 C1 H1 118.6 . . ?
C1 C2 H2 119.6 . . ?
C1 C2 C3 120.7(5) . . ?
C3 C2 H2 119.6 . . ?
C2 C3 C4 116.3(5) . . ?
C2 C3 C16 122.7(5) . . ?
C4 C3 C16 121.0(5) . . ?
C3 C4 H4 119.3 . . ?
C5 C4 C3 121.4(5) . . ?
C5 C4 H4 119.3 . . ?
N3 C5 C4 121.0(5) . . ?
N3 C5 C6 114.9(4) . . ?

C4 C5 C6 124.0(5) . . ?
N2 C6 C5 113.4(4) . . ?
N2 C6 C7 118.5(5) . . ?
C7 C6 C5 128.1(5) . . ?
C6 C7 H7 119.7 . . ?
C6 C7 C8 120.6(5) . . ?
C8 C7 H7 119.7 . . ?
C7 C8 C20 119.5(5) . . ?
C9 C8 C7 118.6(5) . . ?
C9 C8 C20 121.9(5) . . ?
C8 C9 H9 120.2 . . ?
C10 C9 C8 119.6(5) . . ?
C10 C9 H9 120.2 . . ?
N2 C10 C9 119.6(5) . . ?
N2 C10 C11 113.0(4) . . ?
C9 C10 C11 127.3(5) . . ?
N1 C11 C10 114.9(4) . . ?
N1 C11 C12 121.4(5) . . ?
C12 C11 C10 123.3(5) . . ?
C11 C12 H12 119.5 . . ?
C11 C12 C13 121.0(5) . . ?
C13 C12 H12 119.5 . . ?
C12 C13 C24 120.8(5) . . ?
C14 C13 C12 115.9(5) . . ?
C14 C13 C24 123.2(5) . . ?
C13 C14 H14 119.1 . . ?
C15 C14 C13 121.7(5) . . ?
C15 C14 H14 119.1 . . ?
N1 C15 C14 122.2(5) . . ?
N1 C15 H15 118.9 . . ?
C14 C15 H15 118.9 . . ?
C17 C16 C3 111.1(5) . . ?
C18 C16 C3 109.4(5) . . ?
C18 C16 C17 108.3(6) . . ?
C19 C16 C3 108.8(5) . . ?
C19 C16 C17 109.5(7) . . ?
C19 C16 C18 109.8(7) . . ?
C16 C17 H17A 109.5 . . ?
C16 C17 H17B 109.5 . . ?
C16 C17 H17C 109.5 . . ?
H17A C17 H17B 109.5 . . ?
H17A C17 H17C 109.5 . . ?
H17B C17 H17C 109.5 . . ?
C16 C18 H18A 109.5 . . ?

C16 C18 H18B 109.5 . . ?
C16 C18 H18C 109.5 . . ?
H18A C18 H18B 109.5 . . ?
H18A C18 H18C 109.5 . . ?
H18B C18 H18C 109.5 . . ?
C16 C19 H19A 109.5 . . ?
C16 C19 H19B 109.5 . . ?
C16 C19 H19C 109.5 . . ?
H19A C19 H19B 109.5 . . ?
H19A C19 H19C 109.5 . . ?
H19B C19 H19C 109.5 . . ?
C8 C20 C23 110.4(5) . . ?
C21 C20 C8 107.1(5) . . ?
C21 C20 C23 108.9(6) . . ?
C22 C20 C8 110.9(5) . . ?
C22 C20 C21 111.2(6) . . ?
C22 C20 C23 108.4(6) . . ?
C20 C21 H21A 109.5 . . ?
C20 C21 H21B 109.5 . . ?
C20 C21 H21C 109.5 . . ?
H21A C21 H21B 109.5 . . ?
H21A C21 H21C 109.5 . . ?
H21B C21 H21C 109.5 . . ?
C20 C22 H22A 109.5 . . ?
C20 C22 H22B 109.5 . . ?
C20 C22 H22C 109.5 . . ?
H22A C22 H22B 109.5 . . ?
H22A C22 H22C 109.5 . . ?
H22B C22 H22C 109.5 . . ?
C20 C23 H23A 109.5 . . ?
C20 C23 H23B 109.5 . . ?
C20 C23 H23C 109.5 . . ?
H23A C23 H23B 109.5 . . ?
H23A C23 H23C 109.5 . . ?
H23B C23 H23C 109.5 . . ?
C13 C24 C26 111.6(6) . . ?
C13 C24 C27 105.0(7) . . ?
C13 C24 C26' 112.4(6) . . ?
C13 C24 C27' 108.3(7) . . ?
C25 C24 C13 111.2(7) . . ?
C25 C24 C26 110.4(9) . . ?
C25 C24 C27 113.4(11) . . ?
C27 C24 C26 105.1(9) . . ?
C25' C24 C13 110.7(8) . . ?

C25' C24 C26' 109.1(9) . . ?
C25' C24 C27' 111.2(11) . . ?
C26' C24 C27' 105.1(9) . . ?
C29 C28 P1 122.7(5) . . ?
C29 C28 C33 119.2(6) . . ?
C33 C28 P1 118.1(5) . . ?
C28 C29 H29 119.5 . . ?
C28 C29 C30 121.0(6) . . ?
C30 C29 H29 119.5 . . ?
C29 C30 H30 119.8 . . ?
C31 C30 C29 120.5(7) . . ?
C31 C30 H30 119.8 . . ?
C30 C31 H31 120.0 . . ?
C30 C31 C32 120.1(6) . . ?
C32 C31 H31 120.0 . . ?
C31 C32 H32 120.2 . . ?
C33 C32 C31 119.7(7) . . ?
C33 C32 H32 120.2 . . ?
C28 C33 H33 120.3 . . ?
C32 C33 C28 119.4(7) . . ?
C32 C33 H33 120.3 . . ?
C35 C34 P1 122.1(5) . . ?
C35 C34 C39 118.5(6) . . ?
C39 C34 P1 119.4(5) . . ?
C34 C35 H35 119.4 . . ?
C36 C35 C34 121.3(6) . . ?
C36 C35 H35 119.4 . . ?
C35 C36 H36 119.8 . . ?
C35 C36 C37 120.3(7) . . ?
C37 C36 H36 119.8 . . ?
C36 C37 H37 120.0 . . ?
C36 C37 C38 120.1(7) . . ?
C38 C37 H37 120.0 . . ?
C37 C38 H38 119.8 . . ?
C39 C38 C37 120.4(7) . . ?
C39 C38 H38 119.8 . . ?
C34 C39 H39 120.3 . . ?
C38 C39 C34 119.4(7) . . ?
C38 C39 H39 120.3 . . ?
C41 C40 P1 119.1(5) . . ?
C45 C40 P1 122.7(5) . . ?
C45 C40 C41 118.2(6) . . ?
C40 C41 H41 120.1 . . ?
C40 C41 C42 119.7(7) . . ?

C42 C41 H41 120.1 . . ?
C41 C42 H42 119.2 . . ?
C43 C42 C41 121.6(7) . . ?
C43 C42 H42 119.2 . . ?
C42 C43 H43 120.2 . . ?
C42 C43 C44 119.5(7) . . ?
C44 C43 H43 120.2 . . ?
C43 C44 H44 119.8 . . ?
C43 C44 C45 120.5(8) . . ?
C45 C44 H44 119.8 . . ?
C40 C45 C44 120.4(7) . . ?
C40 C45 H45 119.8 . . ?
C44 C45 H45 119.8 . . ?
O1 C46 Ir1 175.0(5) . . ?
C24 C25 H25A 109.5 . . ?
C24 C25 H25B 109.5 . . ?
C24 C25 H25C 109.5 . . ?
H25A C25 H25B 109.5 . . ?
H25A C25 H25C 109.5 . . ?
H25B C25 H25C 109.5 . . ?
C24 C26 H26A 109.5 . . ?
C24 C26 H26B 109.5 . . ?
C24 C26 H26C 109.5 . . ?
H26A C26 H26B 109.5 . . ?
H26A C26 H26C 109.5 . . ?
H26B C26 H26C 109.5 . . ?
C24 C27 H27A 109.5 . . ?
C24 C27 H27B 109.5 . . ?
C24 C27 H27C 109.5 . . ?
H27A C27 H27B 109.5 . . ?
H27A C27 H27C 109.5 . . ?
H27B C27 H27C 109.5 . . ?
C24 C25' H25D 109.5 . . ?
C24 C25' H25E 109.5 . . ?
C24 C25' H25F 109.5 . . ?
H25D C25' H25E 109.5 . . ?
H25D C25' H25F 109.5 . . ?
H25E C25' H25F 109.5 . . ?
C24 C26' H26D 109.5 . . ?
C24 C26' H26E 109.5 . . ?
C24 C26' H26F 109.5 . . ?
H26D C26' H26E 109.5 . . ?
H26D C26' H26F 109.5 . . ?
H26E C26' H26F 109.5 . . ?

```
 C24 C27' H27D 109.5 . . ?
 C24 C27' H27E 109.5 . . ?
 C24 C27' H27F 109.5 . . ?
 H27D C27' H27E 109.5 . . ?
 H27D C27' H27F 109.5 . . ?
 H27E C27' H27F 109.5 . . ?
 F1 B1 F3 108.9(6) . . ?
 F1 B1 F4 110.5(6) . . ?
 F2 B1 F1 109.4(6) . . ?
 F2 B1 F3 107.7(6) . . ?
 F2 B1 F4 112.7(6) . . ?
 F4 B1 F3 107.5(6) . . ?
 F5 B2 F6 108.0(6) . . ?
 F5 B2 F7 102.5(6) . . ?
 F6 B2 F7 110.3(7) . . ?
 F8 B2 F5 124.3(9) . . ?
 F8 B2 F6 110.2(7) . . ?
 F8 B2 F7 100.5(8) . . ?

_shelx_res_file
;
TITL z1_a.res in P2(1)/n
REM Old TITL z1 in P2(1)/n
REM SHELXT solution in P2(1)/n
REM R1 0.087, Rweak 0.002, Alpha 0.040, Orientation as input
REM Formula found by SHELXT: B1 N5 C26' F6 S Ir
CELL 1.54178 14.7269 17.4747 17.3176 90 101.355 90
ZERR 4 0.001 0.0011 0.0011 0 0.003 0
LATT 1
SYMM 0.5-X,0.5+Y,0.5-Z
SFAC C H B F Ir N O P
UNIT 184 200 8 32 4 12 4 4
ISOR 0.01 0.02 C25 C26 C27
ISOR 0.002 0.004 F7 F8

L.S. 20
PLAN  1
SIZE 0.21 0.19 0.17
TEMP -173.16
BOND $H
list 4
fmap 2
acta
REM <olex2.extras>
```

```
REM <HklSrc "%.\\z1.hkl">
REM </olex2.extras>

WGHT    0.047000   15.714000
FVAR       0.32700   0.51989
IR1   5    0.600149    0.495063    0.507881    11.00000    0.03647
0.03404 =
         0.02789   -0.00027    0.00770    0.00024
P1    8    0.767574    0.489236    0.514774    11.00000    0.03770
0.04213 =
         0.04041    0.00054    0.00575    0.00106
O1    7    0.607350    0.662484    0.552992    11.00000    0.04794
0.03832 =
         0.04296   -0.00286    0.01113   -0.00290
N1    6    0.586598    0.492056    0.387366    11.00000    0.03754
0.03975 =
         0.03486   -0.00276    0.01211    0.00014
N2    6    0.592537    0.382310    0.487879    11.00000    0.03638
0.03415 =
         0.03492   -0.00263    0.01117    0.00107
N3    6    0.613042    0.454576    0.620350    11.00000    0.04054
0.03778 =
         0.03174    0.00080    0.00922    0.00040
C1    1    0.627214    0.495332    0.688062    11.00000    0.03984
0.03828 =
         0.03466   -0.00394    0.00846    0.00147
AFIX  43
H1    2    0.626751    0.549611    0.684936    11.00000   -1.20000
AFIX   0
C2    1    0.642244    0.461670    0.761143    11.00000    0.04257
0.04885 =
         0.03236   -0.00411    0.00752    0.00353
AFIX  43
H2    2    0.650621    0.492845    0.806942    11.00000   -1.20000
AFIX   0
C3    1    0.645349    0.382057    0.768864    11.00000    0.04209
0.05005 =
         0.04014    0.00508    0.01261    0.00491
C4    1    0.628630    0.340041    0.698790    11.00000    0.04059
0.04021 =
         0.03913    0.00190    0.00827    0.00392
AFIX  43
H4    2    0.627565    0.285741    0.701129    11.00000   -1.20000
AFIX   0
```

```
C5     1     0.613623     0.375766     0.626218     11.00000     0.03592
0.03950 =
         0.03353    0.00121    0.00843    0.00411
C6     1     0.602150     0.335068     0.551090     11.00000     0.03469
0.04019 =
         0.03465    0.00161    0.01064    0.00238
C7     1     0.602285     0.257063     0.538465     11.00000     0.04606
0.03476 =
         0.04299    0.00175    0.01077   -0.00080
AFIX  43
H7    2    0.608624    0.223038    0.581983    11.00000   -1.20000
AFIX   0
C8     1     0.593188     0.227696     0.462297     11.00000     0.04231
0.03799 =
         0.04670   -0.00315    0.01229    0.00014
C9     1     0.584286     0.278838     0.399460     11.00000     0.04371
0.04287 =
         0.03945   -0.00783    0.01088   -0.00159
AFIX  43
H9    2    0.577245    0.260317    0.347041    11.00000   -1.20000
AFIX   0
C10    1     0.585776     0.356930     0.413975     11.00000     0.03616
0.04158 =
         0.03404   -0.00439    0.01019    0.00223
C11    1     0.583539     0.419283     0.356223     11.00000     0.03467
0.04316 =
         0.03515   -0.00226    0.00893    0.00037
C12    1     0.588022     0.407083     0.278339     11.00000     0.04618
0.04652 =
         0.03351   -0.00327    0.01252    0.00145
AFIX  43
H12   2    0.583778    0.356399    0.258104    11.00000   -1.20000
AFIX   0
C13    1     0.598755     0.468388     0.228555     11.00000     0.04456
0.05681 =
         0.03495   -0.00126    0.01182    0.00128
C14    1     0.596575     0.540891     0.260721     11.00000     0.04286
0.05343 =
         0.04096    0.00672    0.01088   -0.00123
AFIX  43
H14   2    0.600190    0.584383    0.228551    11.00000   -1.20000
AFIX   0
C15    1     0.589335     0.551447     0.337763     11.00000     0.04276
0.04403 =
```

```
         0.03244    0.00426    0.00986   -0.00016
AFIX  43
H15   2    0.586139    0.602121    0.356917    11.00000   -1.20000
AFIX   0
C16    1     0.665036     0.341718     0.849112     11.00000     0.06249
0.05728 =
         0.03652    0.01049    0.01143    0.00955
C17    1     0.704483     0.397250     0.914693     11.00000     0.09849
0.08955 =
         0.04537    0.00684    0.00488    0.01320
AFIX 137
H17A  2    0.760130    0.421585    0.902872    11.00000   -1.50000
H17B  2    0.720542    0.369428    0.964657    11.00000   -1.50000
H17C  2    0.658215    0.436552    0.918844    11.00000   -1.50000
AFIX   0
C18    1     0.735039     0.278579     0.848014     11.00000     0.12386
0.09388 =
         0.04769    0.01605    0.01484    0.05430
AFIX 137
H18A  2    0.706933    0.237873    0.812226    11.00000   -1.50000
H18B  2    0.754480    0.257703    0.901229    11.00000   -1.50000
H18C  2    0.789055    0.299255    0.829877    11.00000   -1.50000
AFIX   0
C19    1     0.575994     0.308262     0.864537     11.00000     0.08975
0.12651 =
         0.08094    0.05531    0.03011    0.00621
AFIX 137
H19A  2    0.535194    0.349654    0.875046    11.00000   -1.50000
H19B  2    0.589551    0.274167    0.910292    11.00000   -1.50000
H19C  2    0.545327    0.279206    0.818304    11.00000   -1.50000
AFIX   0
C20    1     0.589888     0.140697     0.449674     11.00000     0.05922
0.03710 =
         0.05681   -0.00729    0.01467   -0.00421
C21    1     0.493950     0.113836     0.458976     11.00000     0.07682
0.04967 =
         0.08712   -0.01165    0.02809   -0.01513
AFIX 137
H21A  2    0.482883    0.129628    0.510633    11.00000   -1.50000
H21B  2    0.490545    0.057942    0.454764    11.00000   -1.50000
H21C  2    0.446855    0.136701    0.417500    11.00000   -1.50000
AFIX   0
C22    1     0.665718     0.101126     0.508475     11.00000     0.09754
0.04078 =
```

```
         0.07464   -0.00366    0.00778    0.01467
AFIX 137
H22A  2    0.725925    0.123266    0.505011    11.00000   -1.50000
H22B  2    0.665822    0.046334    0.496431    11.00000   -1.50000
H22C  2    0.654255    0.108288    0.561863    11.00000   -1.50000
AFIX   0
C23    1     0.603398     0.120884     0.365238     11.00000     0.08734
0.04268 =
         0.06836   -0.01464    0.02208   -0.00272
AFIX 137
H23A  2    0.553054    0.143599    0.326557    11.00000   -1.50000
H23B  2    0.602811    0.065189    0.358523    11.00000   -1.50000
H23C  2    0.662853    0.141361    0.357355    11.00000   -1.50000
AFIX   0
C24    1     0.617073     0.454214     0.146845     11.00000     0.06857
0.05845 =
         0.03540   -0.00128    0.01986    0.00433
C28    1     0.837582     0.503169     0.612235     11.00000     0.05034
0.04873 =
         0.04399   -0.01024   -0.00143    0.00603
C29    1     0.835519     0.453631     0.673640     11.00000     0.04902
0.06944 =
         0.04943   -0.00050    0.01216   -0.01017
AFIX  43
H29   2    0.797729    0.409193    0.664892    11.00000   -1.20000
AFIX   0
C30    1     0.887650     0.467555     0.747938     11.00000     0.05468
0.08381 =
         0.05242    0.00821    0.00837    0.00212
AFIX  43
H30   2    0.884396    0.433183    0.789808    11.00000   -1.20000
AFIX   0
C31    1     0.944001     0.530638     0.761570     11.00000     0.07911
0.06613 =
         0.05117   -0.00407   -0.00376    0.00748
AFIX  43
H31   2    0.977877    0.540783    0.813142    11.00000   -1.20000
AFIX   0
C32    1     0.951650     0.580270     0.699293     11.00000     0.07815
0.05988 =
         0.06790   -0.00395   -0.00653   -0.00728
AFIX  43
H32   2    0.992493    0.622843    0.708198    11.00000   -1.20000
AFIX   0
```

```
C33   1    0.898953    0.566699    0.624322    11.00000    0.07100
0.05651 =
         0.05729    0.00011    0.00546   -0.00052
AFIX  43
H33   2    0.904069    0.599643    0.581749    11.00000   -1.20000
AFIX   0
C34   1    0.814892    0.560118    0.455847    11.00000    0.03900
0.05488 =
         0.07013    0.01746    0.01216   -0.00167
C35   1    0.774895    0.631048    0.439230    11.00000    0.04334
0.05414 =
         0.05610    0.00707    0.01558   -0.00093
AFIX  43
H35   2    0.718616    0.642580    0.455732    11.00000   -1.20000
AFIX   0
C36   1    0.814844    0.685418    0.399252    11.00000    0.05177
0.06357 =
         0.08603    0.01971    0.02491    0.00067
AFIX  43
H36   2    0.787756    0.734807    0.390769    11.00000   -1.20000
AFIX   0
C37   1    0.894379    0.668560    0.371280    11.00000    0.06465
0.07730 =
         0.11577    0.03377    0.03884   -0.00272
AFIX  43
H37   2    0.920727    0.705883    0.342400    11.00000   -1.20000
AFIX   0
C38   1    0.935642    0.597212    0.385385    11.00000    0.05347
0.08969 =
         0.09235    0.02064    0.02913    0.00343
AFIX  43
H38   2    0.990203    0.585865    0.366080    11.00000   -1.20000
AFIX   0
C39   1    0.897871    0.542603    0.427316    11.00000    0.05035
0.07112 =
         0.06672    0.01026    0.01742    0.00511
AFIX  43
H39   2    0.926434    0.493894    0.437235    11.00000   -1.20000
AFIX   0
C40   1    0.801645    0.398926    0.475914    11.00000    0.03616
0.05517 =
         0.04876   -0.00899    0.01009   -0.00422
C41   1    0.798206    0.392033    0.394900    11.00000    0.04856
0.08381 =
```

```
         0.04759   -0.00535    0.01495    0.00667
AFIX  43
H41   2    0.780216    0.434472    0.361059    11.00000   -1.20000
AFIX   0
C42   1     0.821445     0.322158     0.363827     11.00000     0.05005
0.11043 =
         0.06179   -0.03328    0.01718    0.00477
AFIX  43
H42   2    0.817847    0.317696    0.308635    11.00000   -1.20000
AFIX   0
C43   1     0.848769     0.261167     0.410402     11.00000     0.05759
0.08203 =
         0.10034   -0.04350    0.01474    0.00733
AFIX  43
H43   2    0.866566    0.215061    0.388388    11.00000   -1.20000
AFIX   0
C44   1     0.850677     0.266190     0.489794     11.00000     0.07759
0.05089 =
         0.10108   -0.01201    0.02949    0.00320
AFIX  43
H44   2    0.868042    0.222869    0.522513    11.00000   -1.20000
AFIX   0
C45   1     0.827099     0.335033     0.523049     11.00000     0.06554
0.04579 =
         0.07010   -0.00094    0.02565    0.00381
AFIX  43
H45   2    0.828576    0.337891    0.578064    11.00000   -1.20000
AFIX   0
C46   1     0.603116     0.600802     0.532683     11.00000     0.03912
0.03906 =
         0.03259    0.00183    0.00969    0.00128
PART 1
C25   1     0.543767     0.407978     0.100037     21.00000     0.11800
0.07278 =
         0.04119   -0.00373    0.02644   -0.01258
AFIX 137
H25A  2    0.539787    0.358762    0.126296    21.00000   -1.50000
H25B  2    0.557765    0.399142    0.047730    21.00000   -1.50000
H25C  2    0.484534    0.434988    0.094659    21.00000   -1.50000
AFIX   0
C26   1     0.629244     0.531810     0.102862     21.00000     0.08626
0.06640 =
         0.03794   -0.00298    0.02755   -0.01054
AFIX 137
```

```
H26A  2    0.570162    0.559398    0.092014    21.00000   -1.50000
H26B  2    0.648842    0.520660    0.053145    21.00000   -1.50000
H26C  2    0.676264    0.563421    0.136192    21.00000   -1.50000
AFIX   0
C27    1     0.712834     0.415646     0.160144     21.00000     0.09886
0.09957 =
         0.05329    0.01117    0.03627    0.02784
AFIX 137
H27A  2    0.759287    0.450164    0.190039    21.00000   -1.50000
H27B  2    0.729046    0.404184    0.109138    21.00000   -1.50000
H27C  2    0.711151    0.368029    0.189724    21.00000   -1.50000
AFIX   0
PART 0
PART 2
C25'  1     0.707015     0.487477     0.138668    -21.00000     0.07148
0.07333 =
         0.06006   -0.01507    0.03396   -0.01711
AFIX 137
H25D  2    0.704003    0.543359    0.142200   -21.00000   -1.50000
H25E  2    0.720738    0.473014    0.087500   -21.00000   -1.50000
H25F  2    0.755916    0.468102    0.180883   -21.00000   -1.50000
AFIX   0
C26'  1     0.616308     0.367024     0.126007    -21.00000     0.07469
0.06738 =
         0.02835   -0.00372    0.02596    0.00241
AFIX 137
H26D  2    0.669964    0.341968    0.158694   -21.00000   -1.50000
H26E  2    0.619223    0.360912    0.070294   -21.00000   -1.50000
H26F  2    0.559258    0.343604    0.135985   -21.00000   -1.50000
AFIX   0
C27'  1     0.535358     0.489840     0.085611    -21.00000     0.09569
0.08490 =
         0.03691   -0.00469    0.00978    0.03457
AFIX 137
H27D  2    0.476193    0.473042    0.097654   -21.00000   -1.50000
H27E  2    0.539594    0.472959    0.032471   -21.00000   -1.50000
H27F  2    0.539204    0.545789    0.088429   -21.00000   -1.50000
AFIX   0

PART 0
F1     4     0.452610     0.257808     0.204545     11.00000     0.06566
0.07237 =
         0.10166   -0.01509    0.02716    0.00632
F2     4     0.376848     0.329069     0.278041     11.00000     0.07740
```

```
0.06304 =
         0.08790   -0.02750    0.02846   -0.01580
F3     4     0.373577     0.363544     0.153837     11.00000     0.11814
0.08515 =
         0.09778    0.01245   -0.00606   -0.01527
F4     4     0.295096     0.258279     0.177271     11.00000     0.06435
0.10559 =
         0.08829   -0.03562    0.02644   -0.03357
B1     3     0.374206     0.301049     0.204913     11.00000     0.04791
0.06322 =
         0.06802   -0.01034    0.01698   -0.01128

F5     4     0.416879     0.277589     0.599106     11.00000     0.09640
0.07769 =
         0.07325    0.02322    0.04167    0.03085
F6     4     0.502083     0.186970     0.666140     11.00000     0.07143
0.08712 =
         0.11842    0.05015    0.03769    0.02200
F7     4     0.400571     0.262370     0.719803     11.00000     0.13051
0.14792 =
         0.09969    0.02508    0.05241    0.02422
F8     4     0.351952     0.170504     0.645278     11.00000     0.13579
0.12968 =
         0.18469    0.00502    0.02645   -0.01077
B2     3     0.415558     0.221379     0.651221     11.00000     0.06047
0.04636 =
         0.09826    0.01308    0.01747   -0.00636
HKLF 4

REM  z1_a.res in P2(1)/n
REM R1 =  0.0423 for    6537 Fo > 4sig(Fo)  and  0.0474 for all    7408
data
REM    599 parameters refined using     30 restraints

END

WGHT      0.0468     15.7856

REM Highest difference peak  2.659,  deepest hole -0.826,  1-sigma
level  0.117
Q1    1   0.3511  0.1787  0.5784  11.00000  0.05    2.66
;
_shelx_res_checksum               85706
_shelx_hkl_file
```

;

| | | | | |
|---|---|---|---|---|
| 1 | 0 | 0 | 3.90 | 0.60 |
| -1 | 0 | 0 | 2.30 | 0.50 |
| -2 | 0 | 0 | 1397.06 | 66.79 |
| 2 | 0 | 0 | 1092.69 | 66.79 |
| 3 | 0 | 0 | 1.10 | 1.30 |
| -3 | 0 | 0 | 1.80 | 0.80 |
| 7 | 0 | 0 | -3.10 | 4.10 |
| 8 | 0 | 0 | 1309.07 | 72.29 |
| 9 | 0 | 0 | 16.90 | 4.80 |
| 10 | 0 | 0 | 6445.35 | 333.77 |
| 10 | 0 | 0 | 6031.40 | 333.27 |
| 10 | 0 | 0 | 6095.69 | 333.67 |
| 11 | 0 | 0 | 24.80 | 4.80 |
| 11 | 0 | 0 | 5.60 | 5.30 |
| 12 | 0 | 0 | 377.66 | 22.50 |
| 12 | 0 | 0 | 386.46 | 22.30 |
| 12 | 0 | 0 | 330.27 | 23.60 |
| 13 | 0 | 0 | 1.20 | 3.20 |
| 13 | 0 | 0 | 4.60 | 3.90 |
| 13 | 0 | 0 | 4.80 | 4.60 |
| 14 | 0 | 0 | 749.82 | 44.90 |
| 14 | 0 | 0 | 797.82 | 45.70 |
| 14 | 0 | 0 | 853.71 | 44.80 |
| 15 | 0 | 0 | -2.20 | 2.60 |
| 15 | 0 | 0 | 2.00 | 3.30 |
| 15 | 0 | 0 | -3.30 | 3.80 |
| 16 | 0 | 0 | 526.85 | 30.90 |
| 16 | 0 | 0 | 544.35 | 30.50 |
| 16 | 0 | 0 | 577.84 | 31.20 |
| 17 | 0 | 0 | -3.70 | 2.60 |
| 0 | -1 | 0 | 11.10 | 1.20 |
| 0 | 1 | 0 | 7.70 | 0.90 |
| 3 | 1 | 0 | 1423.96 | 72.19 |
| -3 | 1 | 0 | 1251.37 | 71.79 |
| 4 | 1 | 0 | 1245.68 | 67.29 |
| 7 | -1 | 0 | 197.58 | 13.30 |
| 7 | 1 | 0 | 176.78 | 13.30 |
| 8 | -1 | 0 | 466.05 | 27.20 |
| 8 | 1 | 0 | 452.35 | 27.30 |
| 9 | -1 | 0 | 3514.85 | 180.28 |
| 9 | -1 | 0 | 3214.78 | 179.68 |
| 9 | -1 | 0 | 3082.19 | 179.48 |
| 9 | 1 | 0 | 3323.87 | 179.98 |

| | | | | |
|---|---|---|---|---|
| 9 | 1 | 0 | 3515.45 | 179.98 |
| 9 | 1 | 0 | 3254.17 | 179.38 |
| 10 | 1 | 0 | 67.29 | 8.60 |
| 10 | 1 | 0 | 80.49 | 8.90 |
| 10 | 1 | 0 | 44.30 | 10.30 |
| 11 | -1 | 0 | 1527.45 | 87.89 |
| 11 | -1 | 0 | 1636.74 | 87.09 |
| 11 | -1 | 0 | 1578.14 | 87.19 |
| 11 | 1 | 0 | 1670.13 | 87.39 |
| 11 | 1 | 0 | 1505.35 | 87.69 |
| 11 | 1 | 0 | 1586.84 | 86.79 |
| 12 | -1 | 0 | 14.30 | 6.00 |
| 12 | -1 | 0 | 26.40 | 4.70 |
| 12 | -1 | 0 | 23.50 | 4.30 |
| 12 | 1 | 0 | 14.60 | 6.00 |
| 12 | 1 | 0 | 17.20 | 4.00 |
| 12 | 1 | 0 | 18.60 | 4.20 |
| 13 | -1 | 0 | 252.27 | 16.20 |
| 13 | -1 | 0 | 250.17 | 16.20 |
| 13 | -1 | 0 | 235.18 | 17.30 |
| 13 | 1 | 0 | 245.48 | 15.90 |
| 13 | 1 | 0 | 241.08 | 16.20 |
| 13 | 1 | 0 | 258.57 | 17.70 |
| 14 | -1 | 0 | 53.39 | 8.40 |
| 14 | -1 | 0 | 52.39 | 7.00 |
| 14 | -1 | 0 | 53.19 | 6.60 |
| 14 | 1 | 0 | 48.90 | 7.00 |
| 14 | 1 | 0 | 48.90 | 6.50 |
| 14 | 1 | 0 | 41.20 | 8.10 |
| 15 | -1 | 0 | 467.35 | 26.90 |
| 15 | -1 | 0 | 469.95 | 27.60 |
| 15 | -1 | 0 | 478.75 | 26.60 |
| 15 | 1 | 0 | 476.15 | 27.70 |
| 15 | 1 | 0 | 429.26 | 26.70 |
| 15 | 1 | 0 | 465.45 | 26.50 |
| 16 | -1 | 0 | -1.80 | 2.10 |
| 16 | -1 | 0 | -2.50 | 2.60 |
| 16 | -1 | 0 | 0.50 | 2.50 |
| 16 | 1 | 0 | 1.50 | 2.10 |
| 16 | 1 | 0 | 1.50 | 2.60 |
| 16 | 1 | 0 | -1.30 | 2.70 |
| -1 | 2 | 0 | 1326.37 | 72.29 |
| 1 | 2 | 0 | 1361.06 | 72.49 |
| -2 | 2 | 0 | 210.88 | 11.70 |

| | | | | |
|---|---|---|---|---|
| 2 | 2 | 0 | 207.88 | 12.00 |
| 3 | 2 | 0 | 85.59 | 7.30 |
| -3 | 2 | 0 | 78.19 | 4.90 |
| -4 | 2 | 0 | 3278.17 | 176.08 |
| 4 | 2 | 0 | 3291.77 | 176.68 |
| 5 | 2 | 0 | 152.78 | 9.80 |
| 6 | 2 | 0 | 4160.38 | 223.78 |
| 7 | -2 | 0 | 26.10 | 4.80 |
| 7 | 2 | 0 | 32.40 | 5.30 |
| 7 | 2 | 0 | 35.70 | 7.30 |
| 8 | -2 | 0 | 699.33 | 39.80 |
| 8 | 2 | 0 | 700.33 | 39.70 |
| 9 | -2 | 0 | 1.70 | 6.50 |
| 9 | -2 | 0 | 2.90 | 5.20 |
| 9 | -2 | 0 | 10.70 | 6.50 |
| 9 | 2 | 0 | 3.20 | 5.00 |
| 9 | 2 | 0 | 4.30 | 4.40 |
| 9 | 2 | 0 | 3.10 | 6.00 |
| 10 | -2 | 0 | 1924.51 | 103.09 |
| 10 | -2 | 0 | 1802.42 | 103.59 |
| 10 | -2 | 0 | 1824.72 | 102.89 |
| 10 | 2 | 0 | 1879.71 | 103.19 |
| 10 | 2 | 0 | 1931.01 | 103.49 |
| 10 | 2 | 0 | 1925.61 | 102.59 |
| 11 | -2 | 0 | 10.10 | 4.60 |
| 11 | -2 | 0 | 10.30 | 4.00 |
| 11 | -2 | 0 | 13.60 | 6.40 |
| 11 | 2 | 0 | 3.80 | 5.30 |
| 11 | 2 | 0 | 5.00 | 3.70 |
| 11 | 2 | 0 | 2.60 | 4.00 |
| 12 | -2 | 0 | 108.39 | 11.90 |
| 12 | -2 | 0 | 99.59 | 10.10 |
| 12 | -2 | 0 | 119.99 | 9.50 |
| 12 | 2 | 0 | 103.99 | 9.20 |
| 12 | 2 | 0 | 92.79 | 9.70 |
| 12 | 2 | 0 | 118.79 | 11.90 |
| 13 | -2 | 0 | -1.10 | 4.30 |
| 13 | -2 | 0 | 1.60 | 3.30 |
| 13 | -2 | 0 | 5.50 | 3.90 |
| 13 | 2 | 0 | 5.20 | 3.40 |
| 13 | 2 | 0 | 2.10 | 3.60 |
| 13 | 2 | 0 | 4.50 | 4.90 |
| 14 | -2 | 0 | 622.84 | 35.50 |
| 14 | -2 | 0 | 608.34 | 36.10 |

| | | | | |
|---|---|---|---|---|
| 14 | -2 | 0 | 650.53 | 35.30 |
| 14 | 2 | 0 | 611.24 | 36.10 |
| 14 | 2 | 0 | 642.14 | 35.00 |
| 14 | 2 | 0 | 590.14 | 35.30 |
| 15 | -2 | 0 | 19.00 | 3.60 |
| 15 | -2 | 0 | 15.50 | 4.10 |
| 15 | -2 | 0 | 23.10 | 4.90 |
| 15 | 2 | 0 | 24.50 | 4.70 |
| 15 | 2 | 0 | 26.60 | 4.80 |
| 15 | 2 | 0 | 19.60 | 4.00 |
| 16 | -2 | 0 | 335.57 | 19.00 |
| 16 | -2 | 0 | 325.17 | 18.30 |
| 16 | -2 | 0 | 317.87 | 18.70 |
| 16 | 2 | 0 | 331.57 | 18.90 |
| 16 | 2 | 0 | 273.87 | 19.60 |
| 16 | 2 | 0 | 326.97 | 18.30 |
| 0 | 3 | 0 | 1.50 | 1.20 |
| 1 | 3 | 0 | 4949.70 | 265.67 |
| 2 | 3 | 0 | 7594.14 | 407.26 |
| 3 | 3 | 0 | 259.57 | 15.10 |
| -4 | 3 | 0 | 451.35 | 25.60 |
| 4 | 3 | 0 | 484.35 | 26.40 |
| 5 | 3 | 0 | 1976.80 | 107.99 |
| -5 | 3 | 0 | 2007.70 | 107.09 |
| 6 | 3 | 0 | 21.80 | 5.10 |
| 7 | -3 | 0 | 77.39 | 8.10 |
| 7 | 3 | 0 | 71.19 | 7.50 |
| 8 | -3 | 0 | 23.10 | 4.90 |
| 8 | 3 | 0 | 20.50 | 4.30 |
| 9 | -3 | 0 | 954.90 | 56.89 |
| 9 | -3 | 0 | 980.00 | 56.69 |
| 9 | -3 | 0 | 1002.80 | 57.79 |
| 9 | 3 | 0 | 1129.39 | 57.59 |
| 9 | 3 | 0 | 1020.70 | 56.49 |
| 10 | -3 | 0 | 14.80 | 6.40 |
| 10 | -3 | 0 | 7.30 | 4.80 |
| 10 | -3 | 0 | 7.20 | 3.90 |
| 10 | 3 | 0 | 10.80 | 4.00 |
| 10 | 3 | 0 | -1.90 | 6.10 |
| 10 | 3 | 0 | 9.50 | 4.10 |
| 11 | -3 | 0 | 1572.34 | 96.49 |
| 11 | -3 | 0 | 1801.42 | 92.49 |
| 11 | 3 | 0 | 1737.83 | 92.79 |
| 11 | 3 | 0 | 1630.14 | 92.39 |

| | | | | |
|---|---|---|---|---|
| 11 | 3 | 0 | 1650.53 | 95.99 |
| 12 | -3 | 0 | -8.40 | 5.00 |
| 12 | -3 | 0 | -1.20 | 3.80 |
| 12 | -3 | 0 | -4.00 | 3.20 |
| 12 | 3 | 0 | -6.10 | 3.20 |
| 12 | 3 | 0 | 2.80 | 5.00 |
| 12 | 3 | 0 | 3.30 | 4.00 |
| 13 | -3 | 0 | 114.79 | 10.70 |
| 13 | -3 | 0 | 120.19 | 11.20 |
| 13 | 3 | 0 | 111.69 | 9.10 |
| 13 | 3 | 0 | 103.39 | 11.20 |
| 14 | -3 | 0 | 2.40 | 4.00 |
| 14 | -3 | 0 | -1.00 | 2.70 |
| 14 | 3 | 0 | -6.70 | 3.10 |
| 14 | 3 | 0 | 1.10 | 4.20 |
| 14 | 3 | 0 | -1.70 | 2.60 |
| 15 | -3 | 0 | 357.86 | 20.70 |
| 15 | -3 | 0 | 351.86 | 19.90 |
| 15 | -3 | 0 | 334.37 | 20.20 |
| 15 | 3 | 0 | 314.97 | 20.20 |
| 15 | 3 | 0 | 346.87 | 20.90 |
| 15 | 3 | 0 | 338.47 | 19.80 |
| 16 | -3 | 0 | -3.90 | 2.30 |
| 16 | -3 | 0 | -2.80 | 1.90 |
| 16 | 3 | 0 | -1.00 | 2.00 |
| 16 | 3 | 0 | 2.70 | 2.60 |
| 16 | 3 | 0 | -1.50 | 2.90 |
| 1 | 4 | 0 | 165.88 | 10.40 |
| -1 | 4 | 0 | 177.48 | 10.30 |
| -2 | 4 | 0 | 408.26 | 23.30 |
| 2 | 4 | 0 | 428.06 | 23.60 |
| -3 | 4 | 0 | 30.10 | 3.10 |
| 3 | 4 | 0 | 30.50 | 4.00 |
| 4 | 4 | 0 | 2875.21 | 159.88 |
| -4 | 4 | 0 | 3048.29 | 159.28 |
| -5 | 4 | 0 | 27.40 | 3.30 |
| 5 | 4 | 0 | 22.30 | 5.00 |
| 6 | 4 | 0 | 4438.26 | 247.98 |
| 6 | 4 | 0 | 4537.55 | 248.48 |
| -6 | 4 | 0 | 4858.91 | 247.28 |
| 7 | -4 | 0 | 722.63 | 40.30 |
| 7 | 4 | 0 | 675.43 | 39.70 |
| 7 | 4 | 0 | 726.33 | 39.60 |
| 8 | -4 | 0 | 263.17 | 17.40 |

| | | | | |
|---|---|---|---|---|
| 8 | 4 | 0 | 278.97 | 17.00 |
| 9 | -4 | 0 | 2.10 | 3.30 |
| 9 | -4 | 0 | 7.40 | 5.30 |
| 9 | -4 | 0 | 13.30 | 6.90 |
| 9 | 4 | 0 | 8.00 | 4.40 |
| 9 | 4 | 0 | 19.60 | 7.10 |
| 9 | 4 | 0 | 6.20 | 4.00 |
| 10 | -4 | 0 | 1158.18 | 65.39 |
| 10 | -4 | 0 | 1133.89 | 64.39 |
| 10 | -4 | 0 | 1152.08 | 64.29 |
| 10 | 4 | 0 | 1117.69 | 63.69 |
| 10 | 4 | 0 | 1151.18 | 64.29 |
| 10 | 4 | 0 | 1228.08 | 65.09 |
| 11 | -4 | 0 | 183.68 | 13.60 |
| 11 | -4 | 0 | 165.68 | 15.30 |
| 11 | -4 | 0 | 164.88 | 12.60 |
| 11 | 4 | 0 | 183.58 | 12.50 |
| 11 | 4 | 0 | 180.68 | 13.30 |
| 11 | 4 | 0 | 168.38 | 15.20 |
| 12 | -4 | 0 | 134.39 | 10.70 |
| 12 | -4 | 0 | 147.99 | 11.70 |
| 12 | -4 | 0 | 149.59 | 13.30 |
| 12 | 4 | 0 | 124.79 | 12.50 |
| 12 | 4 | 0 | 136.09 | 11.20 |
| 12 | 4 | 0 | 146.79 | 10.30 |
| 13 | -4 | 0 | 1.40 | 3.40 |
| 13 | -4 | 0 | 0.70 | 3.50 |
| 13 | -4 | 0 | 0.70 | 4.70 |
| 13 | 4 | 0 | -0.20 | 4.00 |
| 13 | 4 | 0 | 1.70 | 4.40 |
| 13 | 4 | 0 | 1.10 | 2.80 |
| 14 | -4 | 0 | 499.05 | 28.10 |
| 14 | -4 | 0 | 510.05 | 28.30 |
| 14 | -4 | 0 | 472.35 | 28.70 |
| 14 | 4 | 0 | 469.25 | 28.10 |
| 14 | 4 | 0 | 480.65 | 27.70 |
| 14 | 4 | 0 | 484.25 | 28.90 |
| 15 | -4 | 0 | -0.60 | 2.60 |
| 15 | -4 | 0 | -3.60 | 3.20 |
| 15 | -4 | 0 | -2.10 | 2.50 |
| 15 | 4 | 0 | 0.40 | 3.50 |
| 15 | 4 | 0 | -1.20 | 2.30 |
| 15 | 4 | 0 | 1.60 | 3.10 |
| 16 | -4 | 0 | 305.67 | 18.50 |

| | | | | |
|---|---|---|---|---|
| 16 | -4 | 0 | 305.07 | 17.70 |
| 16 | 4 | 0 | 310.07 | 17.60 |
| 16 | 4 | 0 | 308.97 | 18.20 |
| 16 | 4 | 0 | 311.37 | 18.10 |
| 0 | 5 | 0 | 4.60 | 1.80 |
| -1 | 5 | 0 | 5789.72 | 309.57 |
| 1 | 5 | 0 | 5739.33 | 309.67 |
| 2 | 5 | 0 | 34.20 | 4.10 |
| -2 | 5 | 0 | 32.60 | 3.60 |
| 3 | 5 | 0 | 226.58 | 14.30 |
| -3 | 5 | 0 | 240.48 | 13.80 |
| -4 | 5 | 0 | 6.10 | 2.10 |
| 4 | 5 | 0 | 8.40 | 2.80 |
| -5 | 5 | 0 | 6802.92 | 343.57 |
| 5 | 5 | 0 | 5999.80 | 344.37 |
| 6 | -5 | 0 | 285.27 | 19.60 |
| -6 | 5 | 0 | 320.87 | 17.40 |
| 6 | 5 | 0 | 295.47 | 18.70 |
| 6 | 5 | 0 | 302.17 | 17.90 |
| 7 | -5 | 0 | 599.24 | 35.00 |
| 7 | 5 | 0 | 612.14 | 33.90 |
| 7 | 5 | 0 | 598.64 | 34.60 |
| 8 | -5 | 0 | 0.80 | 4.80 |
| 8 | -5 | 0 | 5.40 | 4.10 |
| 8 | 5 | 0 | -2.10 | 2.90 |
| 9 | -5 | 0 | 1317.47 | 76.99 |
| 9 | -5 | 0 | 1411.96 | 77.09 |
| 9 | -5 | 0 | 1325.97 | 78.09 |
| 9 | 5 | 0 | 1331.87 | 76.39 |
| 9 | 5 | 0 | 1558.14 | 76.89 |
| 9 | 5 | 0 | 1429.36 | 77.59 |
| 10 | -5 | 0 | -0.40 | 6.20 |
| 10 | -5 | 0 | -0.50 | 4.60 |
| 10 | -5 | 0 | 0.60 | 3.30 |
| 10 | 5 | 0 | 5.40 | 4.00 |
| 10 | 5 | 0 | 5.20 | 6.40 |
| 10 | 5 | 0 | 7.40 | 3.90 |
| 11 | -5 | 0 | 842.52 | 47.90 |
| 11 | -5 | 0 | 900.01 | 49.30 |
| 11 | -5 | 0 | 855.71 | 48.10 |
| 11 | 5 | 0 | 854.11 | 48.10 |
| 11 | 5 | 0 | 809.32 | 48.70 |
| 11 | 5 | 0 | 838.92 | 47.20 |
| 12 | -5 | 0 | 78.99 | 11.20 |

| | | | | |
|---|---|---|---|---|
| 12 | -5 | 0 | 103.29 | 8.50 |
| 12 | -5 | 0 | 91.19 | 9.30 |
| 12 | 5 | 0 | 82.49 | 10.70 |
| 12 | 5 | 0 | 89.19 | 7.90 |
| 12 | 5 | 0 | 77.39 | 9.10 |
| 13 | -5 | 0 | 302.17 | 18.50 |
| 13 | -5 | 0 | 283.27 | 18.30 |
| 13 | -5 | 0 | 311.07 | 19.40 |
| 13 | 5 | 0 | 289.67 | 17.70 |
| 13 | 5 | 0 | 274.37 | 19.30 |
| 13 | 5 | 0 | 298.57 | 18.70 |
| 14 | -5 | 0 | 2.80 | 3.00 |
| 14 | -5 | 0 | 2.00 | 3.00 |
| 14 | -5 | 0 | 2.30 | 3.80 |
| 14 | 5 | 0 | 6.30 | 3.20 |
| 14 | 5 | 0 | 1.90 | 3.80 |
| 14 | 5 | 0 | 6.10 | 5.30 |
| 15 | -5 | 0 | 235.08 | 14.70 |
| 15 | -5 | 0 | 254.57 | 15.10 |
| 15 | 5 | 0 | 251.17 | 15.20 |
| 15 | 5 | 0 | 250.77 | 14.40 |
| 15 | 5 | 0 | 220.88 | 15.40 |
| 16 | -5 | 0 | 0.40 | 2.40 |
| 16 | -5 | 0 | 0.10 | 1.80 |
| 16 | 5 | 0 | 8.00 | 3.20 |
| 16 | 5 | 0 | 1.10 | 2.10 |
| 16 | 5 | 0 | 2.50 | 2.80 |
| 0 | 6 | 0 | 6379.96 | 342.77 |
| 1 | 6 | 0 | 180.48 | 11.70 |
| -1 | 6 | 0 | 189.98 | 11.50 |
| 2 | 6 | 0 | 70.39 | 5.90 |
| -2 | 6 | 0 | 66.79 | 5.50 |
| -3 | 6 | 0 | 4.00 | 2.10 |
| 3 | 6 | 0 | 2.60 | 2.40 |
| 4 | 6 | 0 | 4313.67 | 243.08 |
| -4 | 6 | 0 | 4695.53 | 242.38 |
| 5 | -6 | 0 | 49.40 | 7.50 |
| 5 | 6 | 0 | 66.49 | 6.50 |
| 5 | 6 | 0 | 63.89 | 7.20 |
| -5 | 6 | 0 | 69.19 | 5.80 |
| 6 | -6 | 0 | 1412.26 | 75.59 |
| -6 | 6 | 0 | 1481.65 | 73.99 |
| 6 | 6 | 0 | 1227.98 | 73.99 |
| 6 | 6 | 0 | 1314.47 | 75.09 |

| | | | | |
|---|---|---|---|---|
| 7 | -6 | 0 | 353.96 | 22.10 |
| 7 | 6 | 0 | 332.37 | 20.60 |
| 7 | 6 | 0 | 371.56 | 21.90 |
| 8 | -6 | 0 | 1623.24 | 81.89 |
| 8 | -6 | 0 | 1444.06 | 81.29 |
| 8 | 6 | 0 | 1488.45 | 81.49 |
| 8 | 6 | 0 | 1488.25 | 81.89 |
| 8 | 6 | 0 | 1356.06 | 80.79 |
| 9 | -6 | 0 | 54.69 | 9.20 |
| 9 | -6 | 0 | 50.69 | 8.90 |
| 9 | -6 | 0 | 51.89 | 7.40 |
| 9 | 6 | 0 | 49.80 | 6.80 |
| 9 | 6 | 0 | 67.39 | 8.00 |
| 9 | 6 | 0 | 55.89 | 12.70 |
| 10 | -6 | 0 | 1120.69 | 62.59 |
| 10 | -6 | 0 | 1150.08 | 63.59 |
| 10 | 6 | 0 | 1082.89 | 61.69 |
| 10 | 6 | 0 | 1064.19 | 63.09 |
| 10 | 6 | 0 | 1198.38 | 62.49 |
| 11 | -6 | 0 | 2.30 | 4.20 |
| 11 | -6 | 0 | 3.30 | 3.30 |
| 11 | -6 | 0 | -1.70 | 5.50 |
| 11 | 6 | 0 | 0.70 | 3.20 |
| 11 | 6 | 0 | 7.00 | 6.10 |
| 11 | 6 | 0 | 4.60 | 4.10 |
| 12 | -6 | 0 | 117.79 | 11.60 |
| 12 | -6 | 0 | 127.19 | 10.40 |
| 12 | -6 | 0 | 118.39 | 9.80 |
| 12 | 6 | 0 | 107.69 | 12.10 |
| 12 | 6 | 0 | 115.19 | 9.00 |
| 12 | 6 | 0 | 103.09 | 10.30 |
| 13 | -6 | 0 | 14.60 | 3.50 |
| 13 | -6 | 0 | 33.60 | 7.50 |
| 13 | -6 | 0 | 9.90 | 3.50 |
| 13 | 6 | 0 | 15.20 | 4.40 |
| 13 | 6 | 0 | 25.20 | 5.30 |
| 13 | 6 | 0 | 13.90 | 3.00 |
| 14 | -6 | 0 | 536.55 | 30.70 |
| 14 | -6 | 0 | 527.75 | 30.10 |
| 14 | 6 | 0 | 495.75 | 30.40 |
| 14 | 6 | 0 | 532.25 | 30.20 |
| 14 | 6 | 0 | 523.75 | 29.50 |
| 15 | -6 | 0 | -1.70 | 2.50 |
| 15 | -6 | 0 | -2.10 | 2.10 |

| | | | | |
|---|---|---|---|---|
| 15 | 6 | 0 | 2.00 | 3.30 |
| 15 | 6 | 0 | 2.90 | 3.00 |
| 15 | 6 | 0 | -0.10 | 2.10 |
| 16 | -6 | 0 | 158.48 | 10.30 |
| 16 | -6 | 0 | 169.18 | 9.90 |
| 16 | 6 | 0 | 181.58 | 11.20 |
| 16 | 6 | 0 | 144.99 | 10.60 |
| 0 | 7 | 0 | -1.80 | 1.90 |
| 1 | 7 | 0 | 4518.45 | 247.98 |
| -1 | 7 | 0 | 4685.13 | 247.88 |
| 2 | 7 | 0 | 46.80 | 5.60 |
| -2 | 7 | 0 | 53.69 | 5.10 |
| -3 | 7 | 0 | 1410.86 | 75.99 |
| 3 | 7 | 0 | 1371.06 | 76.59 |
| 4 | -7 | 0 | 103.39 | 10.40 |
| -4 | 7 | 0 | 93.79 | 7.30 |
| 4 | 7 | 0 | 97.49 | 8.60 |
| 4 | 7 | 0 | 92.59 | 7.80 |
| 5 | -7 | 0 | 4421.86 | 247.88 |
| -5 | 7 | 0 | 4730.53 | 246.88 |
| 5 | 7 | 0 | 4451.55 | 247.98 |
| 5 | 7 | 0 | 4742.23 | 246.78 |
| 6 | -7 | 0 | 1.50 | 5.10 |
| 6 | 7 | 0 | 7.20 | 4.60 |
| -6 | 7 | 0 | 3.40 | 3.30 |
| 6 | 7 | 0 | -0.30 | 2.90 |
| 7 | -7 | 0 | 26.60 | 5.40 |
| 7 | -7 | 0 | 14.90 | 5.60 |
| 7 | 7 | 0 | 20.30 | 5.60 |
| 7 | 7 | 0 | 24.80 | 3.60 |
| 8 | -7 | 0 | 42.30 | 5.90 |
| 8 | -7 | 0 | 15.00 | 8.60 |
| 8 | -7 | 0 | 36.20 | 7.70 |
| 8 | 7 | 0 | 32.90 | 5.30 |
| 8 | 7 | 0 | 31.60 | 6.50 |
| 8 | 7 | 0 | 25.50 | 5.20 |
| 9 | -7 | 0 | 992.80 | 55.49 |
| 9 | -7 | 0 | 979.60 | 54.39 |
| 9 | -7 | 0 | 965.80 | 54.59 |
| 9 | 7 | 0 | 896.51 | 53.59 |
| 9 | 7 | 0 | 1023.70 | 53.89 |
| 9 | 7 | 0 | 957.60 | 55.59 |
| 10 | -7 | 0 | -4.70 | 3.40 |
| 10 | -7 | 0 | -8.40 | 9.50 |

| | | | | |
|---|---|---|---|---|
| 10 | -7 | 0 | 10.30 | 4.60 |
| 10 | 7 | 0 | -1.20 | 3.70 |
| 10 | 7 | 0 | 0.10 | 3.40 |
| 10 | 7 | 0 | 7.20 | 6.90 |
| 11 | -7 | 0 | 1041.40 | 57.19 |
| 11 | -7 | 0 | 1025.60 | 56.59 |
| 11 | -7 | 0 | 1008.10 | 56.59 |
| 11 | 7 | 0 | 1050.59 | 55.69 |
| 11 | 7 | 0 | 1003.60 | 56.49 |
| 11 | 7 | 0 | 927.71 | 57.09 |
| 12 | -7 | 0 | 137.39 | 10.90 |
| 12 | -7 | 0 | 115.49 | 10.00 |
| 12 | -7 | 0 | 109.59 | 10.90 |
| 12 | 7 | 0 | 102.49 | 8.50 |
| 12 | 7 | 0 | 95.79 | 12.20 |
| 12 | 7 | 0 | 97.39 | 12.20 |
| 13 | -7 | 0 | 311.37 | 19.90 |
| 13 | -7 | 0 | 307.27 | 18.90 |
| 13 | -7 | 0 | 315.57 | 18.90 |
| 13 | 7 | 0 | 305.27 | 20.10 |
| 13 | 7 | 0 | 302.77 | 19.30 |
| 13 | 7 | 0 | 304.97 | 18.10 |
| 14 | -7 | 0 | 13.50 | 3.30 |
| 14 | -7 | 0 | 9.50 | 3.10 |
| 14 | 7 | 0 | 22.60 | 4.50 |
| 14 | 7 | 0 | 15.80 | 3.90 |
| 14 | 7 | 0 | 11.60 | 4.70 |
| 15 | -7 | 0 | 258.57 | 15.30 |
| 15 | -7 | 0 | 262.77 | 15.60 |
| 15 | 7 | 0 | 251.97 | 16.00 |
| 15 | 7 | 0 | 267.27 | 15.10 |
| 15 | 7 | 0 | 251.67 | 15.60 |
| 0 | 8 | 0 | 9078.09 | 487.75 |
| 1 | 8 | 0 | 417.26 | 24.70 |
| -1 | 8 | 0 | 420.66 | 24.40 |
| -2 | 8 | 0 | 96.09 | 7.30 |
| 2 | 8 | 0 | 90.79 | 8.20 |
| 2 | 8 | 0 | 79.69 | 7.70 |
| 3 | -8 | 0 | 451.85 | 26.00 |
| 3 | 8 | 0 | 432.76 | 26.10 |
| 3 | 8 | 0 | 407.46 | 24.90 |
| -3 | 8 | 0 | 444.86 | 25.20 |
| 4 | -8 | 0 | 1963.70 | 103.49 |
| -4 | 8 | 0 | 1946.91 | 102.79 |

| | | | | |
|---|---|---|---|---|
| 4 | 8 | 0 | 1838.22 | 103.79 |
| 4 | 8 | 0 | 1804.92 | 102.29 |
| 5 | -8 | 0 | 17.70 | 5.10 |
| -5 | 8 | 0 | 17.90 | 3.60 |
| 5 | 8 | 0 | 18.50 | 3.60 |
| 6 | -8 | 0 | 3092.09 | 177.88 |
| 6 | -8 | 0 | 3261.27 | 179.08 |
| 6 | 8 | 0 | 3368.76 | 177.68 |
| 6 | 8 | 0 | 3161.18 | 179.28 |
| -6 | 8 | 0 | 3582.34 | 178.08 |
| 7 | -8 | 0 | 119.69 | 11.00 |
| 7 | -8 | 0 | 90.39 | 9.90 |
| 7 | 8 | 0 | 95.89 | 10.90 |
| 7 | 8 | 0 | 91.09 | 11.00 |
| 7 | 8 | 0 | 98.39 | 8.20 |
| 8 | -8 | 0 | 229.08 | 21.50 |
| 8 | -8 | 0 | 273.77 | 17.60 |
| 8 | -8 | 0 | 247.78 | 17.10 |
| 8 | 8 | 0 | 244.28 | 15.70 |
| 8 | 8 | 0 | 260.07 | 16.70 |
| 8 | 8 | 0 | 264.37 | 18.60 |
| 9 | -8 | 0 | 8.90 | 7.30 |
| 9 | -8 | 0 | 1.40 | 4.20 |
| 9 | 8 | 0 | -4.70 | 3.00 |
| 9 | 8 | 0 | -1.00 | 3.30 |
| 10 | -8 | 0 | 1092.29 | 62.49 |
| 10 | -8 | 0 | 1106.19 | 62.49 |
| 10 | -8 | 0 | 1125.79 | 63.09 |
| 10 | 8 | 0 | 1202.08 | 62.19 |
| 10 | 8 | 0 | 1071.69 | 61.49 |
| 11 | -8 | 0 | -6.00 | 5.40 |
| 11 | -8 | 0 | 3.20 | 4.00 |
| 11 | -8 | 0 | 3.40 | 3.40 |
| 11 | 8 | 0 | 4.20 | 4.20 |
| 11 | 8 | 0 | 0.80 | 7.10 |
| 11 | 8 | 0 | 5.60 | 3.20 |
| 12 | -8 | 0 | 44.40 | 6.40 |
| 12 | -8 | 0 | 46.50 | 7.10 |
| 12 | 8 | 0 | 45.90 | 7.70 |
| 12 | 8 | 0 | 39.70 | 5.80 |
| 12 | 8 | 0 | 34.30 | 7.00 |
| 13 | -8 | 0 | 49.20 | 6.10 |
| 13 | -8 | 0 | 50.19 | 6.50 |
| 13 | 8 | 0 | 48.20 | 7.30 |

| | | | | |
|---|---|---|---|---|
| 13 | 8 | 0 | 39.10 | 7.20 |
| 13 | 8 | 0 | 48.20 | 7.40 |
| 14 | -8 | 0 | 230.38 | 14.10 |
| 14 | -8 | 0 | 220.58 | 14.20 |
| 14 | 8 | 0 | 229.18 | 14.50 |
| 14 | 8 | 0 | 228.08 | 13.60 |
| 14 | 8 | 0 | 224.48 | 18.10 |
| 15 | -8 | 0 | 3.60 | 2.10 |
| 15 | -8 | 0 | 6.90 | 3.10 |
| 15 | 8 | 0 | 2.00 | 3.40 |
| 15 | 8 | 0 | 7.80 | 2.40 |
| 0 | 9 | 0 | 0.10 | 2.80 |
| -1 | 9 | 0 | 2929.11 | 155.08 |
| 1 | 9 | 0 | 2827.32 | 155.28 |
| 1 | 9 | 0 | 2817.42 | 154.38 |
| -2 | 9 | 0 | 438.56 | 25.80 |
| 2 | 9 | 0 | 426.26 | 25.30 |
| 2 | 9 | 0 | 460.05 | 26.50 |
| 3 | -9 | 0 | 218.68 | 14.10 |
| -3 | 9 | 0 | 213.08 | 13.20 |
| 3 | 9 | 0 | 183.88 | 12.70 |
| 3 | 9 | 0 | 174.98 | 13.80 |
| 4 | -9 | 0 | 116.89 | 11.60 |
| 4 | 9 | 0 | 150.68 | 9.90 |
| 4 | 9 | 0 | 134.89 | 11.40 |
| -4 | 9 | 0 | 136.19 | 10.20 |
| 5 | -9 | 0 | 2779.62 | 148.69 |
| 5 | -9 | 0 | 2521.25 | 147.59 |
| -5 | 9 | 0 | 2896.91 | 147.99 |
| 5 | 9 | 0 | 2916.81 | 147.49 |
| 5 | 9 | 0 | 2522.15 | 149.09 |
| -6 | -9 | 0 | 430.76 | 28.40 |
| 6 | -9 | 0 | 535.85 | 29.70 |
| 6 | -9 | 0 | 471.25 | 28.10 |
| -6 | 9 | 0 | 521.25 | 28.10 |
| 6 | 9 | 0 | 436.96 | 29.50 |
| 7 | -9 | 0 | 276.47 | 19.10 |
| 7 | -9 | 0 | 299.07 | 18.00 |
| -7 | -9 | 0 | 252.47 | 18.60 |
| 7 | 9 | 0 | 274.57 | 18.60 |
| 7 | 9 | 0 | 277.07 | 16.80 |
| 7 | 9 | 0 | 284.37 | 19.80 |
| 8 | -9 | 0 | 254.27 | 17.70 |
| 8 | -9 | 0 | 243.08 | 16.80 |

| | | | | |
|---|---|---|---|---|
| 8 | 9 | 0 | 245.58 | 15.60 |
| 8 | 9 | 0 | 280.57 | 19.60 |
| 8 | 9 | 0 | 236.98 | 16.60 |
| 9 | -9 | 0 | 476.05 | 28.50 |
| 9 | -9 | 0 | 469.95 | 28.10 |
| 9 | 9 | 0 | 463.95 | 27.10 |
| 9 | 9 | 0 | 467.95 | 27.20 |
| 10 | -9 | 0 | -2.20 | 3.60 |
| 10 | -9 | 0 | 2.00 | 3.80 |
| 10 | 9 | 0 | -0.60 | 3.10 |
| 10 | 9 | 0 | 3.10 | 3.60 |
| 11 | -9 | 0 | 915.81 | 51.69 |
| 11 | 9 | 0 | 922.71 | 50.59 |
| 12 | -9 | 0 | 158.38 | 11.30 |
| 12 | -9 | 0 | 153.28 | 11.00 |
| 12 | 9 | 0 | 150.18 | 10.10 |
| 12 | 9 | 0 | 139.99 | 11.70 |
| 13 | -9 | 0 | 101.49 | 8.80 |
| 13 | -9 | 0 | 98.99 | 8.00 |
| 13 | 9 | 0 | 100.69 | 7.40 |
| 13 | 9 | 0 | 96.59 | 8.90 |
| 14 | -9 | 0 | 5.90 | 2.60 |
| 14 | -9 | 0 | 4.60 | 2.20 |
| 14 | 9 | 0 | 13.20 | 5.40 |
| 14 | 9 | 0 | 5.40 | 2.40 |
| 15 | -9 | 0 | 293.47 | 17.10 |
| 15 | -9 | 0 | 283.77 | 16.80 |
| 15 | 9 | 0 | 304.77 | 16.80 |
| 0 | 10 | 0 | 1840.62 | 103.29 |
| 0 | 10 | 0 | 1929.11 | 102.49 |
| 1 | 10 | 0 | 375.36 | 23.80 |
| 1 | 10 | 0 | 400.26 | 23.10 |
| -1 | 10 | 0 | 405.56 | 23.70 |
| -2 | 10 | 0 | 107.79 | 8.60 |
| 2 | 10 | 0 | 89.99 | 9.00 |
| 2 | 10 | 0 | 99.19 | 8.10 |
| -3 | -10 | 0 | 326.17 | 23.80 |
| 3 | 10 | 0 | 303.17 | 21.10 |
| -3 | 10 | 0 | 374.46 | 20.60 |
| 3 | 10 | 0 | 325.07 | 19.70 |
| -4 | -10 | 0 | 197.58 | 14.20 |
| 4 | -10 | 0 | 197.28 | 14.10 |
| 4 | -10 | 0 | 173.78 | 13.40 |
| 4 | 10 | 0 | 176.68 | 14.40 |

| | | | | |
|---|---|---|---|---|
| 4 | 10 | 0 | 197.68 | 12.40 |
| -4 | 10 | 0 | 194.88 | 13.20 |
| -5 | -10 | 0 | -2.20 | 4.10 |
| 5 | -10 | 0 | 9.00 | 5.00 |
| 5 | -10 | 0 | 9.60 | 4.00 |
| 5 | 10 | 0 | 2.60 | 3.20 |
| -5 | 10 | 0 | 1.30 | 3.60 |
| 5 | 10 | 0 | -1.10 | 4.80 |
| 6 | -10 | 0 | 1186.68 | 64.69 |
| -6 | -10 | 0 | 1024.70 | 63.19 |
| 6 | -10 | 0 | 1168.78 | 63.39 |
| 6 | 10 | 0 | 1081.69 | 65.19 |
| -6 | 10 | 0 | 1164.68 | 63.59 |
| 6 | 10 | 0 | 1246.98 | 62.89 |
| 7 | -10 | 0 | 507.75 | 28.30 |
| -7 | -10 | 0 | 458.65 | 28.30 |
| 7 | -10 | 0 | 499.85 | 29.70 |
| 7 | 10 | 0 | 460.15 | 28.40 |
| 7 | 10 | 0 | 474.85 | 30.50 |
| 7 | 10 | 0 | 481.35 | 27.40 |
| 8 | -10 | 0 | 59.39 | 9.20 |
| 8 | -10 | 0 | 55.29 | 8.50 |
| 8 | 10 | 0 | 85.49 | 14.10 |
| 8 | 10 | 0 | 57.29 | 6.50 |
| 9 | -10 | 0 | 4.60 | 4.00 |
| 9 | -10 | 0 | 7.30 | 4.50 |
| 9 | 10 | 0 | 11.00 | 3.40 |
| 9 | 10 | 0 | 11.10 | 3.40 |
| 10 | -10 | 0 | 795.22 | 45.40 |
| 10 | -10 | 0 | 839.22 | 45.80 |
| 10 | 10 | 0 | 826.62 | 45.00 |
| 10 | 10 | 0 | 742.63 | 44.30 |
| 11 | -10 | 0 | 72.69 | 8.60 |
| 11 | -10 | 0 | 75.09 | 8.10 |
| 11 | 10 | 0 | 88.89 | 7.30 |
| 11 | 10 | 0 | 95.99 | 8.80 |
| 12 | -10 | 0 | 101.89 | 8.80 |
| 12 | -10 | 0 | 103.69 | 8.40 |
| 12 | 10 | 0 | 87.59 | 9.20 |
| 13 | -10 | 0 | 66.59 | 6.40 |
| 13 | -10 | 0 | 65.49 | 6.70 |
| 13 | 10 | 0 | 58.99 | 7.60 |
| 13 | 10 | 0 | 79.59 | 5.90 |
| 14 | -10 | 0 | 123.19 | 8.00 |

| | | | | |
|---|---|---|---|---|
| 14 | -10 | 0 | 105.89 | 8.20 |
| 14 | 10 | 0 | 126.39 | 7.90 |
| 14 | 10 | 0 | 111.39 | 9.20 |
| 0 | -11 | 0 | -1.70 | 4.70 |
| 0 | 11 | 0 | -0.70 | 2.80 |
| 0 | 11 | 0 | -1.50 | 3.10 |
| -1 | -11 | 0 | 892.21 | 49.20 |
| 1 | -11 | 0 | 864.81 | 49.10 |
| 1 | -11 | 0 | 859.91 | 49.60 |
| -1 | -11 | 0 | 844.92 | 49.10 |
| -1 | 11 | 0 | 891.61 | 49.70 |
| 1 | 11 | 0 | 900.11 | 48.70 |
| 1 | 11 | 0 | 881.11 | 49.90 |
| 2 | -11 | 0 | 669.23 | 41.40 |
| 2 | -11 | 0 | 732.93 | 42.70 |
| -2 | -11 | 0 | 689.43 | 41.50 |
| -2 | -11 | 0 | 743.83 | 41.90 |
| -2 | 11 | 0 | 779.02 | 42.10 |
| 2 | 11 | 0 | 740.73 | 42.70 |
| 2 | 11 | 0 | 786.52 | 41.20 |
| -3 | -11 | 0 | 56.09 | 7.90 |
| 3 | -11 | 0 | 66.49 | 8.60 |
| 3 | -11 | 0 | 71.39 | 9.00 |
| -3 | -11 | 0 | 71.59 | 10.50 |
| 3 | 11 | 0 | 65.69 | 8.50 |
| -3 | 11 | 0 | 58.19 | 7.30 |
| -4 | -11 | 0 | 129.49 | 11.00 |
| 4 | -11 | 0 | 100.59 | 16.30 |
| 4 | -11 | 0 | 133.29 | 10.70 |
| 4 | 11 | 0 | 128.29 | 9.40 |
| 4 | 11 | 0 | 136.39 | 12.70 |
| -4 | 11 | 0 | 138.09 | 10.80 |
| 5 | -11 | 0 | 2278.07 | 126.29 |
| -5 | -11 | 0 | 2259.37 | 126.39 |
| 5 | -11 | 0 | 2537.45 | 127.59 |
| 5 | 11 | 0 | 2056.19 | 128.39 |
| -5 | 11 | 0 | 2414.76 | 127.09 |
| 5 | 11 | 0 | 2421.66 | 126.09 |
| 6 | -11 | 0 | 121.79 | 10.10 |
| 6 | -11 | 0 | 128.39 | 11.70 |
| 6 | 11 | 0 | 123.19 | 8.60 |
| 6 | 11 | 0 | 91.49 | 13.00 |
| 7 | -11 | 0 | 114.19 | 11.40 |
| 7 | -11 | 0 | 115.29 | 10.00 |

| | | | | |
|---|---|---|---|---|
| -7 | -11 | 0 | 122.29 | 10.60 |
| 7 | 11 | 0 | 106.69 | 14.50 |
| 7 | 11 | 0 | 112.29 | 8.50 |
| -8 | -11 | 0 | 29.00 | 5.10 |
| 8 | -11 | 0 | 26.00 | 5.80 |
| 8 | -11 | 0 | 26.30 | 4.70 |
| 8 | 11 | 0 | 25.20 | 5.30 |
| 8 | 11 | 0 | 24.20 | 4.30 |
| 9 | -11 | 0 | 569.84 | 32.60 |
| 9 | -11 | 0 | 570.84 | 33.30 |
| 9 | 11 | 0 | 569.24 | 31.90 |
| 9 | 11 | 0 | 528.05 | 31.60 |
| 10 | -11 | 0 | 3.30 | 4.20 |
| 10 | -11 | 0 | 4.50 | 3.30 |
| 10 | 11 | 0 | 6.60 | 3.70 |
| 10 | 11 | 0 | 1.70 | 2.90 |
| 11 | -11 | 0 | 466.85 | 29.30 |
| 11 | -11 | 0 | 520.85 | 29.60 |
| 11 | 11 | 0 | 512.15 | 29.30 |
| 11 | 11 | 0 | 518.05 | 28.40 |
| 12 | -11 | 0 | 51.69 | 6.90 |
| 12 | -11 | 0 | 59.19 | 6.40 |
| 12 | 11 | 0 | 59.19 | 7.70 |
| 12 | 11 | 0 | 56.29 | 5.80 |
| 13 | -11 | 0 | 63.19 | 6.00 |
| 13 | -11 | 0 | 56.09 | 5.90 |
| 13 | 11 | 0 | 76.99 | 5.70 |
| 13 | 11 | 0 | 62.29 | 7.40 |
| 14 | -11 | 0 | 4.80 | 1.90 |
| 14 | 11 | 0 | 5.20 | 2.80 |
| 0 | -12 | 0 | 2921.81 | 158.68 |
| 0 | -12 | 0 | 2935.71 | 158.98 |
| 0 | 12 | 0 | 3013.30 | 160.08 |
| 0 | 12 | 0 | 2860.21 | 158.58 |
| 1 | -12 | 0 | 475.65 | 28.10 |
| -1 | -12 | 0 | 489.95 | 28.40 |
| 1 | -12 | 0 | 476.35 | 28.90 |
| -1 | -12 | 0 | 473.15 | 28.20 |
| 1 | 12 | 0 | 531.55 | 27.80 |
| -1 | 12 | 0 | 465.75 | 28.80 |
| -1 | 12 | 0 | 458.35 | 27.60 |
| 1 | 12 | 0 | 467.45 | 29.10 |
| 2 | -12 | 0 | 25.70 | 4.90 |
| -2 | -12 | 0 | 29.70 | 5.30 |

| | | | | |
|---|---|---|---|---|
| 2 | -12 | 0 | 23.60 | 6.00 |
| -2 | -12 | 0 | 39.80 | 7.40 |
| 2 | 12 | 0 | 35.40 | 7.50 |
| 2 | 12 | 0 | 34.10 | 5.70 |
| -2 | 12 | 0 | 47.80 | 6.90 |
| 3 | -12 | 0 | 150.38 | 12.00 |
| 3 | -12 | 0 | 196.68 | 15.30 |
| -3 | -12 | 0 | 153.78 | 12.00 |
| -3 | -12 | 0 | 156.28 | 13.20 |
| -3 | 12 | 0 | 166.48 | 12.60 |
| 3 | 12 | 0 | 163.08 | 11.10 |
| 3 | 12 | 0 | 153.38 | 13.80 |
| -4 | -12 | 0 | 810.82 | 46.40 |
| 4 | -12 | 0 | 806.42 | 46.30 |
| 4 | -12 | 0 | 802.22 | 48.00 |
| -4 | 12 | 0 | 903.51 | 47.50 |
| 4 | 12 | 0 | 838.52 | 46.00 |
| 4 | 12 | 0 | 805.02 | 48.90 |
| 5 | -12 | 0 | 63.99 | 9.40 |
| -5 | -12 | 0 | 71.99 | 8.80 |
| 5 | -12 | 0 | 90.39 | 8.50 |
| 5 | 12 | 0 | 94.29 | 7.30 |
| 5 | 12 | 0 | 66.99 | 12.30 |
| -5 | 12 | 0 | 73.29 | 10.10 |
| -6 | -12 | 0 | 652.43 | 36.40 |
| 6 | -12 | 0 | 662.13 | 36.40 |
| 6 | 12 | 0 | 657.23 | 35.70 |
| 6 | 12 | 0 | 567.34 | 39.20 |
| 7 | -12 | 0 | 281.47 | 17.60 |
| 7 | -12 | 0 | 275.47 | 19.00 |
| -7 | -12 | 0 | 269.57 | 17.30 |
| 7 | 12 | 0 | 264.27 | 16.50 |
| 7 | 12 | 0 | 270.77 | 17.70 |
| 7 | 12 | 0 | 285.47 | 22.30 |
| 8 | -12 | 0 | 29.10 | 5.00 |
| -8 | -12 | 0 | 38.00 | 5.20 |
| 8 | -12 | 0 | 46.20 | 8.70 |
| 8 | 12 | 0 | 43.00 | 5.70 |
| 8 | 12 | 0 | 36.90 | 15.30 |
| 8 | 12 | 0 | 39.50 | 6.80 |
| 9 | -12 | 0 | 148.89 | 12.50 |
| 9 | -12 | 0 | 144.89 | 11.10 |
| 9 | 12 | 0 | 136.19 | 10.20 |
| 9 | 12 | 0 | 143.59 | 10.70 |

| | | | | |
|---|---|---|---|---|
| 10 | -12 | 0 | 542.35 | 29.70 |
| 10 | -12 | 0 | 484.15 | 29.10 |
| 10 | 12 | 0 | 466.05 | 28.60 |
| 10 | 12 | 0 | 507.05 | 28.20 |
| 11 | -12 | 0 | 72.99 | 7.40 |
| 11 | -12 | 0 | 71.39 | 8.80 |
| 11 | 12 | 0 | 82.39 | 6.50 |
| 11 | 12 | 0 | 62.99 | 8.00 |
| 12 | -12 | 0 | 48.60 | 5.60 |
| 12 | 12 | 0 | 45.10 | 5.00 |
| 12 | 12 | 0 | 41.30 | 7.40 |
| 13 | -12 | 0 | 73.09 | 5.60 |
| 13 | 12 | 0 | 59.79 | 6.70 |
| 0 | -13 | 0 | 1.60 | 4.00 |
| 0 | -13 | 0 | -1.90 | 3.70 |
| 0 | 13 | 0 | 0.70 | 2.80 |
| 0 | 13 | 0 | 2.40 | 4.10 |
| -1 | -13 | 0 | 1486.85 | 79.99 |
| 1 | -13 | 0 | 1462.75 | 79.89 |
| 1 | -13 | 0 | 1497.15 | 80.49 |
| -1 | -13 | 0 | 1505.95 | 80.19 |
| -1 | 13 | 0 | 1406.26 | 79.89 |
| 1 | 13 | 0 | 1402.76 | 79.79 |
| -1 | 13 | 0 | 1433.16 | 81.19 |
| 1 | 13 | 0 | 1473.15 | 81.59 |
| 2 | -13 | 0 | 79.59 | 9.20 |
| -2 | -13 | 0 | 87.69 | 10.00 |
| 2 | -13 | 0 | 98.89 | 11.00 |
| -2 | -13 | 0 | 102.99 | 9.10 |
| 2 | 13 | 0 | 90.99 | 8.00 |
| -2 | 13 | 0 | 98.09 | 9.90 |
| 2 | 13 | 0 | 91.19 | 10.90 |
| -2 | 13 | 0 | 88.49 | 8.10 |
| -3 | -13 | 0 | 36.20 | 7.70 |
| 3 | -13 | 0 | 62.79 | 10.90 |
| 3 | -13 | 0 | 31.70 | 5.00 |
| -3 | -13 | 0 | 36.10 | 5.60 |
| -3 | 13 | 0 | 38.60 | 7.90 |
| 3 | 13 | 0 | 24.70 | 6.50 |
| 3 | 13 | 0 | 40.30 | 5.50 |
| 4 | -13 | 0 | 253.77 | 19.20 |
| 4 | -13 | 0 | 277.97 | 16.70 |
| -4 | -13 | 0 | 256.97 | 16.70 |
| 4 | 13 | 0 | 245.78 | 20.10 |

| | | | | |
|---|---|---|---|---|
| -4 | 13 | 0 | 259.87 | 18.40 |
| 4 | 13 | 0 | 258.37 | 15.90 |
| 5 | -13 | 0 | 973.50 | 54.09 |
| 5 | -13 | 0 | 887.71 | 52.39 |
| -5 | -13 | 0 | 938.61 | 52.59 |
| 5 | 13 | 0 | 968.70 | 52.09 |
| -5 | 13 | 0 | 954.10 | 53.99 |
| -6 | -13 | 0 | 94.09 | 8.80 |
| 6 | -13 | 0 | 100.49 | 9.00 |
| 6 | 13 | 0 | 91.49 | 15.50 |
| 6 | 13 | 0 | 88.89 | 7.50 |
| -7 | -13 | 0 | 121.49 | 10.60 |
| 7 | -13 | 0 | 148.69 | 10.90 |
| 7 | 13 | 0 | 130.39 | 9.50 |
| 7 | 13 | 0 | 149.88 | 18.70 |
| 7 | 13 | 0 | 141.29 | 11.00 |
| -8 | -13 | 0 | 54.89 | 7.60 |
| 8 | -13 | 0 | 59.39 | 7.30 |
| 8 | 13 | 0 | 46.90 | 6.60 |
| 8 | 13 | 0 | 56.69 | 7.00 |
| 9 | -13 | 0 | 476.65 | 27.50 |
| 9 | 13 | 0 | 482.55 | 26.90 |
| 9 | 13 | 0 | 446.16 | 26.50 |
| 10 | -13 | 0 | 42.70 | 6.30 |
| 10 | 13 | 0 | 29.20 | 6.20 |
| 10 | 13 | 0 | 24.90 | 3.70 |
| 11 | -13 | 0 | 468.15 | 26.50 |
| 11 | 13 | 0 | 479.75 | 25.80 |
| 11 | 13 | 0 | 436.16 | 26.70 |
| 12 | -13 | 0 | 61.19 | 5.70 |
| 12 | 13 | 0 | 70.19 | 5.80 |
| 13 | -13 | 0 | 51.49 | 4.50 |
| 0 | -14 | 0 | 867.61 | 46.50 |
| 0 | -14 | 0 | 868.01 | 46.00 |
| 0 | 14 | 0 | 847.62 | 45.90 |
| 0 | 14 | 0 | 689.03 | 49.40 |
| -1 | -14 | 0 | 418.86 | 25.00 |
| 1 | -14 | 0 | 413.36 | 25.50 |
| 1 | -14 | 0 | 399.56 | 24.60 |
| -1 | -14 | 0 | 433.26 | 24.90 |
| 1 | 14 | 0 | 424.76 | 24.50 |
| -1 | 14 | 0 | 415.46 | 24.50 |
| -2 | -14 | 0 | 94.49 | 8.60 |
| -2 | -14 | 0 | 92.69 | 9.10 |

| | | | | |
|---|---|---|---|---|
| 2 | -14 | 0 | 81.79 | 8.50 |
| 2 | -14 | 0 | 78.29 | 10.30 |
| 2 | 14 | 0 | 84.69 | 7.40 |
| -2 | 14 | 0 | 71.89 | 7.60 |
| -2 | 14 | 0 | 75.79 | 14.10 |
| -3 | -14 | 0 | 63.29 | 7.50 |
| 3 | -14 | 0 | 84.29 | 10.50 |
| 3 | -14 | 0 | 76.39 | 7.90 |
| -3 | 14 | 0 | 78.89 | 10.50 |
| 3 | 14 | 0 | 67.59 | 11.70 |
| -3 | 14 | 0 | 65.19 | 7.20 |
| 3 | 14 | 0 | 63.49 | 6.80 |
| 4 | -14 | 0 | 467.05 | 26.50 |
| 4 | -14 | 0 | 409.36 | 24.50 |
| -4 | -14 | 0 | 400.96 | 24.60 |
| 4 | 14 | 0 | 343.27 | 28.30 |
| 4 | 14 | 0 | 434.16 | 24.10 |
| -4 | 14 | 0 | 439.66 | 26.90 |
| -5 | -14 | 0 | 1.80 | 3.00 |
| 5 | -14 | 0 | 3.50 | 3.20 |
| 5 | -14 | 0 | -9.10 | 5.80 |
| 5 | 14 | 0 | -3.20 | 7.80 |
| 5 | 14 | 0 | 5.50 | 2.70 |
| -6 | -14 | 0 | 1139.49 | 60.39 |
| 6 | -14 | 0 | 1098.69 | 60.49 |
| 6 | 14 | 0 | 1042.20 | 64.79 |
| 6 | 14 | 0 | 1089.49 | 59.99 |
| -7 | -14 | 0 | 191.88 | 12.80 |
| 7 | -14 | 0 | 190.28 | 13.10 |
| 7 | 14 | 0 | 192.08 | 12.20 |
| 7 | 14 | 0 | 200.98 | 22.90 |
| 7 | 14 | 0 | 178.28 | 13.00 |
| -8 | -14 | 0 | 54.59 | 7.10 |
| 8 | -14 | 0 | 55.59 | 7.00 |
| 8 | 14 | 0 | 54.99 | 6.30 |
| 8 | 14 | 0 | 44.30 | 6.00 |
| 9 | 14 | 0 | 56.49 | 6.00 |
| 9 | 14 | 0 | 44.00 | 6.40 |
| 10 | -14 | 0 | 291.57 | 18.30 |
| 10 | 14 | 0 | 298.97 | 18.40 |
| 10 | 14 | 0 | 320.37 | 17.60 |
| 11 | -14 | 0 | 9.60 | 2.60 |
| 11 | 14 | 0 | 12.20 | 2.70 |
| 12 | -14 | 0 | 73.59 | 5.60 |

| | | | | |
|---|---|---|---|---|
| 0 | -15 | 0 | -0.40 | 3.50 |
| 0 | -15 | 0 | 10.20 | 3.60 |
| 0 | 15 | 0 | -5.40 | 5.20 |
| 0 | 15 | 0 | 6.70 | 2.90 |
| -1 | -15 | 0 | 491.05 | 28.20 |
| 1 | -15 | 0 | 489.95 | 29.00 |
| -1 | -15 | 0 | 506.15 | 28.50 |
| 1 | -15 | 0 | 454.45 | 28.00 |
| 1 | 15 | 0 | 524.15 | 31.10 |
| -1 | 15 | 0 | 454.75 | 30.20 |
| 1 | 15 | 0 | 512.85 | 28.00 |
| -1 | 15 | 0 | 447.66 | 27.90 |
| 2 | -15 | 0 | 237.38 | 15.60 |
| 2 | -15 | 0 | 248.58 | 17.10 |
| -2 | -15 | 0 | 243.18 | 15.90 |
| -2 | -15 | 0 | 264.67 | 16.20 |
| -2 | 15 | 0 | 251.67 | 18.60 |
| 2 | 15 | 0 | 252.97 | 15.50 |
| -2 | 15 | 0 | 247.48 | 15.60 |
| 2 | 15 | 0 | 230.78 | 19.40 |
| 3 | -15 | 0 | 97.09 | 10.40 |
| -3 | -15 | 0 | 81.49 | 7.60 |
| 3 | -15 | 0 | 65.59 | 7.70 |
| -3 | 15 | 0 | 71.69 | 12.10 |
| -3 | 15 | 0 | 78.19 | 7.50 |
| 3 | 15 | 0 | 82.19 | 7.20 |
| 3 | 15 | 0 | 68.39 | 13.40 |
| 4 | -15 | 0 | 170.28 | 11.60 |
| 4 | -15 | 0 | 143.59 | 13.70 |
| -4 | -15 | 0 | 171.18 | 11.50 |
| 4 | 15 | 0 | 161.28 | 11.10 |
| -4 | 15 | 0 | 173.08 | 16.60 |
| 4 | 15 | 0 | 160.88 | 17.90 |
| -4 | 15 | 0 | 161.98 | 11.90 |
| -5 | -15 | 0 | 637.04 | 34.70 |
| 5 | -15 | 0 | 632.34 | 34.70 |
| 5 | -15 | 0 | 664.83 | 35.80 |
| 5 | 15 | 0 | 505.75 | 39.70 |
| 5 | 15 | 0 | 599.74 | 34.40 |
| -6 | -15 | 0 | 16.00 | 3.40 |
| 6 | -15 | 0 | 12.50 | 3.70 |
| 6 | 15 | 0 | 17.70 | 3.30 |
| 6 | 15 | 0 | 22.40 | 4.20 |
| 7 | -15 | 0 | 12.10 | 3.70 |

| | | | | |
|---|---|---|---|---|
| -7 | -15 | 0 | 5.20 | 3.10 |
| 7 | 15 | 0 | 10.90 | 3.40 |
| 7 | 15 | 0 | 6.30 | 3.10 |
| -8 | -15 | 0 | 57.99 | 7.10 |
| 8 | -15 | 0 | 66.89 | 8.60 |
| 8 | 15 | 0 | 54.59 | 6.50 |
| 8 | 15 | 0 | 60.49 | 6.30 |
| 9 | -15 | 0 | 105.99 | 8.50 |
| 9 | 15 | 0 | 113.19 | 7.80 |
| 9 | 15 | 0 | 102.89 | 8.90 |
| 10 | -15 | 0 | 46.90 | 5.00 |
| 10 | 15 | 0 | 33.50 | 5.00 |
| 11 | -15 | 0 | 215.58 | 12.90 |
| 0 | -16 | 0 | 748.03 | 41.30 |
| 0 | -16 | 0 | 761.02 | 40.80 |
| 0 | 16 | 0 | 727.63 | 43.40 |
| 0 | 16 | 0 | 663.53 | 43.30 |
| 1 | -16 | 0 | 133.89 | 10.40 |
| -1 | -16 | 0 | 107.39 | 9.30 |
| 1 | -16 | 0 | 121.89 | 9.20 |
| -1 | -16 | 0 | 106.39 | 9.10 |
| -1 | 16 | 0 | 84.29 | 12.70 |
| 1 | 16 | 0 | 89.39 | 13.40 |
| -2 | -16 | 0 | 17.20 | 3.70 |
| 2 | -16 | 0 | 15.50 | 3.30 |
| 2 | -16 | 0 | 19.10 | 5.10 |
| -2 | 16 | 0 | 10.20 | 3.50 |
| 2 | 16 | 0 | 24.80 | 4.30 |
| -2 | 16 | 0 | 18.40 | 7.60 |
| 2 | 16 | 0 | 6.50 | 6.70 |
| -3 | -16 | 0 | 182.28 | 12.30 |
| 3 | -16 | 0 | 187.18 | 12.20 |
| 3 | -16 | 0 | 163.08 | 13.80 |
| 3 | 16 | 0 | 147.09 | 18.90 |
| -3 | 16 | 0 | 181.18 | 17.30 |
| -3 | 16 | 0 | 178.68 | 12.50 |
| 3 | 16 | 0 | 210.38 | 12.20 |
| 4 | -16 | 0 | 245.48 | 16.70 |
| -4 | -16 | 0 | 225.38 | 14.80 |
| 4 | -16 | 0 | 230.98 | 14.90 |
| 4 | 16 | 0 | 238.98 | 14.60 |
| -4 | 16 | 0 | 241.68 | 15.40 |
| 5 | -16 | 0 | 105.39 | 8.20 |
| -5 | -16 | 0 | 74.59 | 7.60 |

| | | | | |
|---|---|---|---|---|
| 5 | -16 | 0 | 89.29 | 10.70 |
| 5 | 16 | 0 | 87.29 | 7.20 |
| -5 | 16 | 0 | 90.99 | 8.50 |
| 6 | -16 | 0 | 214.38 | 14.40 |
| -6 | -16 | 0 | 223.38 | 14.20 |
| -6 | 16 | 0 | 237.38 | 15.10 |
| 6 | 16 | 0 | 226.78 | 14.00 |
| 7 | -16 | 0 | 339.47 | 19.90 |
| -7 | -16 | 0 | 327.27 | 19.50 |
| -7 | 16 | 0 | 338.57 | 20.50 |
| 7 | 16 | 0 | 314.87 | 19.30 |
| 7 | 16 | 0 | 332.67 | 19.40 |
| -8 | -16 | 0 | 51.29 | 6.60 |
| 8 | -16 | 0 | 71.19 | 6.70 |
| 8 | 16 | 0 | 60.29 | 6.00 |
| 8 | 16 | 0 | 57.89 | 6.80 |
| 9 | -16 | 0 | 82.49 | 6.70 |
| 9 | 16 | 0 | 77.89 | 6.60 |
| 10 | -16 | 0 | 303.27 | 17.50 |
| -10 | 16 | 0 | 303.27 | 17.90 |
| 0 | -17 | 0 | 2.90 | 3.10 |
| 0 | -17 | 0 | -3.40 | 3.00 |
| 0 | 17 | 0 | 1.80 | 2.60 |
| 1 | -17 | 0 | 351.76 | 21.30 |
| 1 | -17 | 0 | 361.76 | 22.10 |
| -1 | -17 | 0 | 362.26 | 21.60 |
| 1 | 17 | 0 | 371.26 | 21.20 |
| -1 | 17 | 0 | 357.46 | 21.30 |
| 2 | -17 | 0 | 354.66 | 20.70 |
| 2 | -17 | 0 | 337.47 | 19.60 |
| -2 | -17 | 0 | 326.07 | 19.70 |
| -2 | 17 | 0 | 307.17 | 19.50 |
| 2 | 17 | 0 | 316.17 | 19.30 |
| 3 | -17 | 0 | 11.60 | 3.30 |
| 3 | -17 | 0 | 19.40 | 4.70 |
| -3 | -17 | 0 | 17.00 | 3.20 |
| -3 | 17 | 0 | 11.40 | 3.40 |
| 3 | 17 | 0 | 18.60 | 3.40 |
| 4 | -17 | 0 | 159.88 | 10.80 |
| -4 | -17 | 0 | 141.89 | 10.40 |
| 4 | -17 | 0 | 166.98 | 16.80 |
| -4 | 17 | 0 | 150.58 | 10.80 |
| 4 | 17 | 0 | 153.48 | 10.30 |
| -5 | -17 | 0 | 747.83 | 42.50 |

| | | | | |
|---|---|---|---|---|
| 5 | -17 | 0 | 765.32 | 42.70 |
| 5 | -17 | 0 | 784.62 | 43.10 |
| 5 | 17 | 0 | 721.13 | 42.20 |
| 5 | 17 | 0 | 750.62 | 42.50 |
| -5 | 17 | 0 | 820.92 | 43.10 |
| 6 | -17 | 0 | 78.29 | 7.10 |
| -6 | -17 | 0 | 70.09 | 6.70 |
| 6 | 17 | 0 | 75.79 | 7.00 |
| 6 | 17 | 0 | 78.99 | 6.90 |
| -6 | 17 | 0 | 76.09 | 7.70 |
| 7 | -17 | 0 | 48.30 | 5.70 |
| -7 | -17 | 0 | 42.40 | 5.50 |
| 7 | 17 | 0 | 51.59 | 7.10 |
| -7 | 17 | 0 | 37.50 | 6.60 |
| 7 | 17 | 0 | 40.50 | 5.40 |
| 8 | -17 | 0 | 160.68 | 10.50 |
| -8 | -17 | 0 | 161.58 | 10.50 |
| 8 | 17 | 0 | 163.08 | 10.30 |
| 9 | -17 | 0 | 228.18 | 13.30 |
| -9 | 17 | 0 | 215.48 | 13.80 |
| 0 | -18 | 0 | 1015.20 | 58.19 |
| 0 | 18 | 0 | 1003.40 | 55.39 |
| -1 | -18 | 0 | 110.69 | 11.60 |
| 1 | -18 | 0 | 94.09 | 8.60 |
| 1 | -18 | 0 | 100.49 | 9.70 |
| 1 | 18 | 0 | 101.79 | 7.80 |
| -1 | 18 | 0 | 105.79 | 8.00 |
| 2 | -18 | 0 | 7.90 | 3.10 |
| -2 | -18 | 0 | 5.30 | 2.70 |
| 2 | -18 | 0 | 15.70 | 4.10 |
| 2 | 18 | 0 | 10.60 | 3.20 |
| -2 | 18 | 0 | 17.70 | 3.30 |
| 3 | -18 | 0 | 141.09 | 11.20 |
| -3 | -18 | 0 | 139.79 | 9.70 |
| 3 | -18 | 0 | 152.48 | 10.20 |
| -3 | 18 | 0 | 139.79 | 10.00 |
| 3 | 18 | 0 | 136.19 | 9.60 |
| 4 | -18 | 0 | 492.95 | 26.60 |
| -4 | -18 | 0 | 424.46 | 25.80 |
| 4 | -18 | 0 | 457.35 | 26.00 |
| -4 | 18 | 0 | 452.55 | 26.20 |
| 4 | 18 | 0 | 449.26 | 25.70 |
| 5 | -18 | 0 | 8.30 | 4.50 |
| 5 | -18 | 0 | 7.10 | 2.70 |

| | | | | |
|---|---|---|---|---|
| -5 | -18 | 0 | 1.70 | 2.20 |
| -5 | 18 | 0 | 2.50 | 3.10 |
| 5 | 18 | 0 | 10.00 | 3.60 |
| 5 | 18 | 0 | 4.00 | 3.20 |
| -6 | -18 | 0 | 224.78 | 13.40 |
| 6 | -18 | 0 | 217.88 | 13.70 |
| -6 | 18 | 0 | 202.18 | 15.80 |
| 6 | 18 | 0 | 239.78 | 13.60 |
| 7 | -18 | 0 | 190.88 | 11.50 |
| -7 | -18 | 0 | 187.98 | 11.30 |
| -7 | 18 | 0 | 171.78 | 12.10 |
| 8 | -18 | 0 | 57.39 | 5.00 |
| -8 | -18 | 0 | 54.99 | 5.80 |
| -8 | 18 | 0 | 50.09 | 6.00 |
| 0 | -19 | 0 | -1.00 | 2.70 |
| 0 | 19 | 0 | -3.40 | 2.90 |
| 1 | -19 | 0 | 316.67 | 19.80 |
| -1 | -19 | 0 | 335.97 | 19.50 |
| -1 | 19 | 0 | 337.27 | 19.30 |
| 2 | -19 | 0 | 149.79 | 9.80 |
| 2 | -19 | 0 | 131.49 | 10.30 |
| -2 | -19 | 0 | 137.79 | 9.40 |
| 2 | 19 | 0 | 136.59 | 9.40 |
| -2 | 19 | 0 | 145.69 | 10.70 |
| -3 | -19 | 0 | 35.20 | 4.30 |
| 3 | -19 | 0 | 36.00 | 6.30 |
| 3 | -19 | 0 | 26.50 | 5.30 |
| -3 | 19 | 0 | 40.80 | 5.60 |
| 3 | 19 | 0 | 23.20 | 5.10 |
| 4 | -19 | 0 | 60.19 | 6.20 |
| 4 | -19 | 0 | 68.59 | 7.50 |
| -4 | -19 | 0 | 52.99 | 5.20 |
| -4 | 19 | 0 | 69.49 | 6.50 |
| 4 | 19 | 0 | 61.99 | 6.90 |
| -5 | -19 | 0 | 420.36 | 25.10 |
| 5 | -19 | 0 | 450.05 | 25.30 |
| -5 | 19 | 0 | 450.05 | 25.50 |
| 5 | 19 | 0 | 473.75 | 26.00 |
| 6 | -19 | 0 | 80.59 | 6.40 |
| -6 | -19 | 0 | 84.89 | 6.20 |
| -6 | 19 | 0 | 75.79 | 7.10 |
| 0 | -20 | 0 | 303.37 | 17.90 |
| 1 | -20 | 0 | 118.09 | 8.90 |
| -1 | -20 | 0 | 123.39 | 8.50 |

| | | | | |
|---|---|---|---|---|
| -1 | 20 | 0 | 132.19 | 8.90 |
| 2 | -20 | 0 | 7.40 | 2.80 |
| -2 | -20 | 0 | 5.00 | 2.00 |
| -2 | 20 | 0 | -2.40 | 3.10 |
| 3 | -20 | 0 | 60.39 | 7.40 |
| -3 | -20 | 0 | 65.29 | 5.10 |
| 3 | 20 | 0 | 56.59 | 7.30 |
| -3 | 20 | 0 | 58.89 | 6.60 |
| -4 | -20 | 0 | 145.49 | 9.00 |
| 4 | -20 | 0 | 139.49 | 10.20 |
| 4 | -20 | 0 | 151.48 | 10.00 |
| 17 | 0 | -1 | 162.78 | 10.30 |
| 17 | 0 | -1 | 155.58 | 9.50 |
| 16 | 0 | -1 | -3.60 | 1.90 |
| 16 | 0 | -1 | -0.60 | 2.60 |
| 16 | 0 | -1 | -3.70 | 2.80 |
| 15 | 0 | -1 | 1175.28 | 67.29 |
| 15 | 0 | -1 | 1279.37 | 67.79 |
| 14 | 0 | -1 | -2.90 | 3.00 |
| 14 | 0 | -1 | -7.00 | 3.00 |
| 14 | 0 | -1 | -1.50 | 4.00 |
| 13 | 0 | -1 | 491.45 | 28.80 |
| 13 | 0 | -1 | 486.15 | 29.90 |
| 13 | 0 | -1 | 493.55 | 28.40 |
| 12 | 0 | -1 | -2.30 | 3.20 |
| 12 | 0 | -1 | -4.50 | 4.80 |
| 12 | 0 | -1 | -5.00 | 3.70 |
| 11 | 0 | -1 | 3950.70 | 234.48 |
| 11 | 0 | -1 | 4797.62 | 234.48 |
| 11 | 0 | -1 | 4251.17 | 233.78 |
| 10 | 0 | -1 | 124.89 | 10.20 |
| 10 | 0 | -1 | 82.69 | 12.60 |
| 9 | 0 | -1 | 8969.80 | 483.15 |
| 9 | 0 | -1 | 8885.21 | 482.65 |
| 9 | 0 | -1 | 9101.89 | 483.05 |
| 8 | 0 | -1 | -1.30 | 4.20 |
| 7 | 0 | -1 | 3.70 | 4.50 |
| 2 | 0 | -1 | 0.50 | 1.00 |
| 0 | 0 | 1 | 9.70 | 1.60 |
| 2 | 0 | 1 | -0.50 | 1.50 |
| 3 | 0 | 1 | 1437.06 | 77.79 |
| 4 | 0 | 1 | 0.80 | 2.00 |
| 6 | 0 | 1 | 1.60 | 1.90 |
| 7 | 0 | 1 | 437.26 | 26.00 |

| | | | | |
|---|---|---|---|---|
| 8 | 0 | 1 | 2.20 | 3.80 |
| 9 | 0 | 1 | 615.04 | 37.40 |
| 9 | 0 | 1 | 678.13 | 37.80 |
| 10 | 0 | 1 | 2.10 | 5.00 |
| 10 | 0 | 1 | 14.20 | 5.20 |
| 11 | 0 | 1 | 2224.68 | 123.19 |
| 11 | 0 | 1 | 2301.27 | 123.59 |
| 12 | 0 | 1 | 1.80 | 4.20 |
| 12 | 0 | 1 | 1.60 | 3.50 |
| 12 | 0 | 1 | -1.00 | 4.90 |
| 13 | 0 | 1 | 10.90 | 4.80 |
| 13 | 0 | 1 | 5.20 | 4.40 |
| 13 | 0 | 1 | 8.10 | 3.70 |
| 14 | 0 | 1 | 4.00 | 3.30 |
| 14 | 0 | 1 | -2.00 | 3.90 |
| 14 | 0 | 1 | -0.50 | 2.80 |
| 15 | 0 | 1 | 288.07 | 17.00 |
| 15 | 0 | 1 | 284.57 | 18.10 |
| 15 | 0 | 1 | 282.77 | 17.30 |
| 16 | 0 | 1 | 1.70 | 3.40 |
| 16 | 0 | 1 | 2.90 | 2.10 |
| 16 | 0 | 1 | -1.40 | 2.40 |
| 17 | -1 | -1 | 1.10 | 1.60 |
| 17 | -1 | -1 | -1.00 | 2.60 |
| 17 | 1 | -1 | 3.40 | 3.00 |
| 17 | 1 | -1 | -0.30 | 1.70 |
| 16 | -1 | -1 | 381.36 | 22.00 |
| 16 | -1 | -1 | 364.86 | 21.20 |
| 16 | -1 | -1 | 373.16 | 21.70 |
| 16 | 1 | -1 | 398.86 | 22.10 |
| 16 | 1 | -1 | 331.47 | 21.60 |
| 16 | 1 | -1 | 395.96 | 21.30 |
| 15 | -1 | -1 | -0.50 | 3.00 |
| 15 | -1 | -1 | 8.00 | 4.10 |
| 15 | -1 | -1 | 3.30 | 5.50 |
| 15 | 1 | -1 | -0.20 | 5.10 |
| 15 | 1 | -1 | 5.60 | 3.20 |
| 15 | 1 | -1 | 6.40 | 3.50 |
| 14 | -1 | -1 | 1342.07 | 73.49 |
| 14 | -1 | -1 | 1330.97 | 72.89 |
| 14 | -1 | -1 | 1349.86 | 72.49 |
| 14 | 1 | -1 | 1289.57 | 73.39 |
| 14 | 1 | -1 | 1300.27 | 72.39 |
| 14 | 1 | -1 | 1318.47 | 72.79 |

| | | | | |
|---|---|---|---|---|
| 13 | -1 | -1 | 4.90 | 3.90 |
| 13 | -1 | -1 | 4.20 | 4.80 |
| 13 | -1 | -1 | 1.20 | 3.20 |
| 13 | 1 | -1 | 0.10 | 4.80 |
| 13 | 1 | -1 | -1.40 | 3.60 |
| 13 | 1 | -1 | 0.60 | 3.10 |
| 12 | -1 | -1 | 208.08 | 16.10 |
| 12 | -1 | -1 | 208.58 | 14.00 |
| 12 | 1 | -1 | 214.28 | 13.70 |
| 12 | 1 | -1 | 197.48 | 16.10 |
| 12 | 1 | -1 | 188.08 | 14.20 |
| 11 | -1 | -1 | 12.80 | 4.50 |
| 11 | 1 | -1 | -0.70 | 3.40 |
| 11 | 1 | -1 | 1.40 | 5.70 |
| 11 | 1 | -1 | 3.60 | 3.80 |
| 10 | -1 | -1 | 4842.82 | 274.27 |
| 10 | -1 | -1 | 4761.22 | 274.47 |
| 10 | -1 | -1 | 5275.27 | 275.27 |
| 10 | 1 | -1 | 5282.97 | 274.27 |
| 10 | 1 | -1 | 5071.19 | 275.07 |
| 10 | 1 | -1 | 5309.27 | 274.77 |
| 9 | 1 | -1 | 6.50 | 6.30 |
| 9 | 1 | -1 | 9.80 | 4.80 |
| 8 | -1 | -1 | 701.33 | 37.10 |
| 8 | 1 | -1 | 593.04 | 37.00 |
| 7 | -1 | -1 | 350.66 | 21.90 |
| 7 | 1 | -1 | 364.36 | 22.00 |
| 3 | 1 | -1 | 578.04 | 31.40 |
| 2 | -1 | -1 | 65.79 | 3.90 |
| -2 | 1 | 1 | 61.39 | 3.80 |
| -1 | 1 | 1 | 304.37 | 16.60 |
| 1 | -1 | 1 | 428.36 | 23.20 |
| 1 | 1 | 1 | 420.06 | 23.20 |
| 2 | -1 | 1 | 41.70 | 3.20 |
| 2 | 1 | 1 | 35.40 | 3.30 |
| 3 | -1 | 1 | 238.08 | 12.60 |
| 3 | 1 | 1 | 205.38 | 13.00 |
| 4 | -1 | 1 | 3172.08 | 167.18 |
| 4 | 1 | 1 | 3055.99 | 168.38 |
| 5 | -1 | 1 | 112.09 | 6.90 |
| 7 | -1 | 1 | 98.49 | 9.60 |
| 7 | 1 | 1 | 113.79 | 9.40 |
| 8 | -1 | 1 | 23.20 | 4.60 |
| 9 | -1 | 1 | 75.09 | 8.70 |

| | | | | |
|---|---|---|---|---|
| 9 | -1 | 1 | 67.09 | 10.70 |
| 9 | -1 | 1 | 74.89 | 9.20 |
| 9 | 1 | 1 | 99.39 | 10.60 |
| 9 | 1 | 1 | 61.59 | 11.70 |
| 9 | 1 | 1 | 80.89 | 10.80 |
| 10 | -1 | 1 | 2005.00 | 112.29 |
| 10 | -1 | 1 | 2165.98 | 112.79 |
| 10 | -1 | 1 | 1956.50 | 112.09 |
| 10 | 1 | 1 | 2142.19 | 112.59 |
| 10 | 1 | 1 | 2016.30 | 112.09 |
| 10 | 1 | 1 | 2044.10 | 112.59 |
| 11 | -1 | 1 | 10.60 | 4.80 |
| 11 | 1 | 1 | 25.70 | 4.90 |
| 12 | -1 | 1 | 282.87 | 19.20 |
| 12 | -1 | 1 | 305.17 | 19.10 |
| 12 | -1 | 1 | 285.97 | 20.00 |
| 12 | 1 | 1 | 282.27 | 20.10 |
| 12 | 1 | 1 | 325.87 | 19.30 |
| 12 | 1 | 1 | 315.07 | 19.00 |
| 13 | -1 | 1 | 63.39 | 8.20 |
| 13 | -1 | 1 | 48.70 | 9.20 |
| 13 | 1 | 1 | 84.09 | 8.20 |
| 13 | 1 | 1 | 65.79 | 10.70 |
| 13 | 1 | 1 | 59.89 | 9.10 |
| 14 | -1 | 1 | 393.66 | 26.00 |
| 14 | -1 | 1 | 425.96 | 25.30 |
| 14 | -1 | 1 | 452.85 | 25.60 |
| 14 | 1 | 1 | 414.46 | 26.20 |
| 14 | 1 | 1 | 469.35 | 25.30 |
| 14 | 1 | 1 | 441.26 | 25.50 |
| 15 | -1 | 1 | -3.00 | 3.20 |
| 15 | -1 | 1 | 2.80 | 2.70 |
| 15 | -1 | 1 | 4.80 | 2.80 |
| 15 | 1 | 1 | 1.50 | 2.80 |
| 15 | 1 | 1 | -0.60 | 3.30 |
| 15 | 1 | 1 | 7.00 | 3.00 |
| 16 | -1 | 1 | 378.46 | 20.90 |
| 16 | -1 | 1 | 319.97 | 20.90 |
| 16 | -1 | 1 | 372.06 | 20.30 |
| 16 | 1 | 1 | 314.37 | 21.10 |
| 16 | 1 | 1 | 384.46 | 20.40 |
| 16 | 1 | 1 | 372.46 | 20.90 |
| 17 | -2 | -1 | 62.79 | 5.80 |
| 17 | -2 | -1 | 66.59 | 4.80 |

| | | | | |
|---|---|---|---|---|
| 17 | 2 | -1 | 68.39 | 6.20 |
| 17 | 2 | -1 | 67.69 | 4.90 |
| 16 | -2 | -1 | -1.90 | 2.00 |
| 16 | -2 | -1 | -2.40 | 2.70 |
| 16 | 2 | -1 | -6.20 | 3.10 |
| 16 | 2 | -1 | 0.70 | 1.90 |
| 16 | 2 | -1 | 3.80 | 2.80 |
| 15 | -2 | -1 | 364.06 | 24.70 |
| 15 | -2 | -1 | 396.56 | 22.70 |
| 15 | -2 | -1 | 393.66 | 22.40 |
| 15 | 2 | -1 | 389.26 | 22.20 |
| 15 | 2 | -1 | 378.46 | 23.40 |
| 15 | 2 | -1 | 382.46 | 22.60 |
| 14 | -2 | -1 | 18.10 | 4.40 |
| 14 | -2 | -1 | 12.70 | 3.90 |
| 14 | -2 | -1 | 8.20 | 3.50 |
| 14 | 2 | -1 | 9.80 | 4.70 |
| 14 | 2 | -1 | 10.30 | 4.10 |
| 14 | 2 | -1 | 4.50 | 3.00 |
| 13 | -2 | -1 | 166.08 | 12.10 |
| 13 | -2 | -1 | 178.68 | 11.90 |
| 13 | -2 | -1 | 154.98 | 13.20 |
| 13 | 2 | -1 | 163.88 | 12.00 |
| 13 | 2 | -1 | 139.89 | 13.20 |
| 13 | 2 | -1 | 153.08 | 11.20 |
| 12 | -2 | -1 | 1.30 | 4.90 |
| 12 | -2 | -1 | -0.90 | 3.80 |
| 12 | -2 | -1 | 0.90 | 3.50 |
| 12 | 2 | -1 | 4.20 | 5.40 |
| 12 | 2 | -1 | 0.00 | 3.70 |
| 12 | 2 | -1 | -4.60 | 3.00 |
| 11 | -2 | -1 | 763.32 | 43.40 |
| 11 | -2 | -1 | 769.52 | 43.50 |
| 11 | -2 | -1 | 744.33 | 44.70 |
| 11 | 2 | -1 | 752.22 | 43.60 |
| 11 | 2 | -1 | 798.02 | 44.70 |
| 11 | 2 | -1 | 768.12 | 42.90 |
| 10 | -2 | -1 | 23.80 | 5.10 |
| 10 | -2 | -1 | 21.90 | 4.40 |
| 10 | -2 | -1 | 20.70 | 6.80 |
| 10 | 2 | -1 | 14.60 | 4.70 |
| 10 | 2 | -1 | 27.60 | 6.60 |
| 10 | 2 | -1 | 23.10 | 4.50 |
| 9 | -2 | -1 | 1122.79 | 59.89 |

| | | | | |
|---|---|---|---|---|
| 9 | -2 | -1 | 1070.19 | 61.19 |
| 9 | -2 | -1 | 1047.20 | 59.99 |
| 9 | 2 | -1 | 1025.50 | 60.69 |
| 9 | 2 | -1 | 1132.39 | 60.19 |
| 9 | 2 | -1 | 1066.29 | 59.49 |
| 8 | -2 | -1 | 37.80 | 7.00 |
| 8 | 2 | -1 | 33.20 | 6.10 |
| 7 | -2 | -1 | 13.50 | 4.40 |
| 7 | 2 | -1 | 24.30 | 5.10 |
| 6 | 2 | -1 | 133.29 | 8.70 |
| 5 | 2 | -1 | 8204.28 | 440.16 |
| 4 | 2 | -1 | 1577.44 | 85.69 |
| 3 | 2 | -1 | 322.97 | 18.50 |
| -3 | 2 | 1 | 339.07 | 18.20 |
| -2 | 2 | 1 | 223.08 | 12.20 |
| 2 | 2 | -1 | 212.08 | 12.40 |
| -1 | 2 | 1 | 2740.33 | 147.19 |
| 0 | 2 | 1 | 38.10 | 3.00 |
| 1 | 2 | 1 | 2372.46 | 127.69 |
| 2 | 2 | 1 | 169.98 | 10.10 |
| 3 | 2 | 1 | 1272.47 | 69.09 |
| 4 | 2 | 1 | 586.34 | 32.60 |
| 5 | 2 | 1 | 2017.20 | 109.19 |
| 6 | 2 | 1 | 200.78 | 12.90 |
| 7 | -2 | 1 | 45.90 | 7.70 |
| 7 | 2 | 1 | 59.09 | 6.90 |
| 7 | 2 | 1 | 48.50 | 6.90 |
| 8 | -2 | 1 | 54.49 | 7.50 |
| 8 | 2 | 1 | 53.09 | 7.40 |
| 9 | -2 | 1 | 539.75 | 32.10 |
| 9 | -2 | 1 | 514.45 | 30.80 |
| 9 | -2 | 1 | 486.75 | 31.20 |
| 9 | 2 | 1 | 552.14 | 31.70 |
| 9 | 2 | 1 | 481.35 | 31.20 |
| 9 | 2 | 1 | 593.94 | 31.20 |
| 10 | -2 | 1 | -5.80 | 5.10 |
| 10 | -2 | 1 | 2.00 | 3.70 |
| 10 | -2 | 1 | -0.10 | 4.90 |
| 10 | 2 | 1 | -9.90 | 5.10 |
| 10 | 2 | 1 | -2.90 | 4.40 |
| 10 | 2 | 1 | -0.80 | 4.20 |
| 11 | -2 | 1 | 1450.45 | 73.29 |
| 11 | -2 | 1 | 1185.98 | 73.79 |
| 11 | 2 | 1 | 1282.37 | 73.19 |

| | | | | |
|---|---|---|---|---|
| 11 | 2 | 1 | 1342.67 | 73.09 |
| 11 | 2 | 1 | 1333.67 | 74.59 |
| 12 | -2 | 1 | 3.00 | 3.30 |
| 12 | -2 | 1 | 6.00 | 5.40 |
| 12 | -2 | 1 | 9.50 | 4.40 |
| 12 | 2 | 1 | 9.00 | 4.10 |
| 12 | 2 | 1 | 8.30 | 4.10 |
| 12 | 2 | 1 | 4.20 | 4.90 |
| 13 | -2 | 1 | 216.18 | 16.20 |
| 13 | -2 | 1 | 216.38 | 15.30 |
| 13 | -2 | 1 | 263.57 | 20.00 |
| 13 | 2 | 1 | 223.28 | 16.70 |
| 13 | 2 | 1 | 231.38 | 14.90 |
| 13 | 2 | 1 | 228.48 | 16.50 |
| 14 | -2 | 1 | 0.50 | 3.10 |
| 14 | -2 | 1 | -0.10 | 3.30 |
| 14 | -2 | 1 | -1.70 | 4.00 |
| 14 | 2 | 1 | -0.80 | 3.10 |
| 14 | 2 | 1 | -1.70 | 3.80 |
| 14 | 2 | 1 | -0.60 | 3.40 |
| 15 | -2 | 1 | 296.87 | 18.20 |
| 15 | -2 | 1 | 278.67 | 17.60 |
| 15 | -2 | 1 | 297.97 | 17.50 |
| 15 | 2 | 1 | 292.97 | 18.30 |
| 15 | 2 | 1 | 304.47 | 17.40 |
| 15 | 2 | 1 | 287.57 | 17.70 |
| 16 | -2 | 1 | 0.40 | 3.10 |
| 16 | -2 | 1 | -1.20 | 2.50 |
| 16 | -2 | 1 | 1.80 | 2.10 |
| 16 | 2 | 1 | -1.90 | 3.30 |
| 16 | 2 | 1 | -0.30 | 2.30 |
| 16 | 2 | 1 | -1.60 | 2.40 |
| 16 | -3 | -1 | 215.38 | 13.30 |
| 16 | -3 | -1 | 214.38 | 12.80 |
| 16 | 3 | -1 | 218.48 | 13.40 |
| 16 | 3 | -1 | 214.38 | 12.70 |
| 16 | 3 | -1 | 202.78 | 13.40 |
| 15 | -3 | -1 | 1.80 | 3.50 |
| 15 | -3 | -1 | 2.40 | 2.90 |
| 15 | -3 | -1 | 0.70 | 3.00 |
| 15 | 3 | -1 | -1.40 | 3.60 |
| 15 | 3 | -1 | 1.60 | 3.20 |
| 15 | 3 | -1 | 2.10 | 2.40 |
| 14 | -3 | -1 | 386.46 | 23.40 |

| | | | | |
|---|---|---|---|---|
| 14 | -3 | -1 | 378.06 | 22.80 |
| 14 | -3 | -1 | 361.36 | 22.40 |
| 14 | 3 | -1 | 379.36 | 23.60 |
| 14 | 3 | -1 | 397.86 | 22.90 |
| 14 | 3 | -1 | 373.26 | 22.00 |
| 13 | -3 | -1 | 68.79 | 9.60 |
| 13 | -3 | -1 | 68.39 | 7.70 |
| 13 | -3 | -1 | 77.89 | 7.90 |
| 13 | 3 | -1 | 73.09 | 9.90 |
| 13 | 3 | -1 | 70.09 | 7.00 |
| 13 | 3 | -1 | 70.29 | 8.50 |
| 12 | -3 | -1 | 189.68 | 15.90 |
| 12 | -3 | -1 | 218.18 | 14.60 |
| 12 | -3 | -1 | 228.08 | 14.40 |
| 12 | 3 | -1 | 193.98 | 13.40 |
| 12 | 3 | -1 | 229.58 | 14.70 |
| 12 | 3 | -1 | 175.88 | 15.80 |
| 11 | -3 | -1 | 57.99 | 11.70 |
| 11 | -3 | -1 | 57.49 | 9.60 |
| 11 | -3 | -1 | 53.59 | 7.50 |
| 11 | 3 | -1 | 56.29 | 7.30 |
| 11 | 3 | -1 | 81.59 | 11.00 |
| 11 | 3 | -1 | 55.59 | 8.30 |
| 10 | -3 | -1 | 1009.30 | 57.99 |
| 10 | -3 | -1 | 1000.00 | 58.99 |
| 10 | -3 | -1 | 1105.89 | 57.99 |
| 10 | 3 | -1 | 1030.60 | 57.99 |
| 10 | 3 | -1 | 1070.29 | 57.29 |
| 10 | 3 | -1 | 1006.70 | 58.79 |
| 9 | -3 | -1 | -2.40 | 7.10 |
| 9 | -3 | -1 | -2.80 | 4.50 |
| 9 | 3 | -1 | -10.60 | 7.10 |
| 9 | 3 | -1 | 5.30 | 4.40 |
| 9 | 3 | -1 | -0.60 | 4.10 |
| 8 | -3 | -1 | 973.80 | 53.79 |
| 8 | 3 | -1 | 955.70 | 53.69 |
| 7 | -3 | -1 | 64.39 | 8.00 |
| 7 | 3 | -1 | 68.49 | 6.80 |
| 7 | 3 | -1 | 67.79 | 7.90 |
| 6 | 3 | -1 | 1572.44 | 85.39 |
| 5 | 3 | -1 | 438.46 | 25.70 |
| -5 | 3 | 1 | 470.45 | 24.70 |
| 4 | 3 | -1 | 1764.72 | 99.89 |
| -4 | 3 | 1 | 1927.11 | 99.39 |

| | | | | |
|---|---|---|---|---|
| 3 | 3 | -1 | 5.60 | 1.90 |
| -3 | 3 | 1 | 5.70 | 1.50 |
| 2 | 3 | -1 | 3841.32 | 209.78 |
| -2 | 3 | 1 | 3966.80 | 209.58 |
| 1 | 3 | -1 | 143.89 | 9.10 |
| -1 | 3 | 1 | 157.38 | 9.00 |
| 1 | 3 | 1 | 185.18 | 11.00 |
| 2 | 3 | 1 | 9259.57 | 496.45 |
| 3 | 3 | 1 | 13.80 | 2.50 |
| 4 | 3 | 1 | 2555.54 | 138.09 |
| -5 | 3 | -1 | 249.58 | 14.60 |
| 5 | 3 | 1 | 269.97 | 15.90 |
| 6 | 3 | 1 | 4379.56 | 225.88 |
| -6 | 3 | -1 | 4078.69 | 224.78 |
| 6 | 3 | 1 | 4116.49 | 225.58 |
| 7 | -3 | 1 | 83.69 | 9.10 |
| 7 | 3 | 1 | 97.99 | 8.20 |
| 7 | 3 | 1 | 104.49 | 9.10 |
| 8 | -3 | 1 | 613.84 | 36.20 |
| 8 | 3 | 1 | 650.33 | 36.00 |
| 9 | -3 | 1 | 109.29 | 9.40 |
| 9 | -3 | 1 | 87.89 | 11.10 |
| 9 | -3 | 1 | 99.19 | 12.20 |
| 9 | 3 | 1 | 120.89 | 9.70 |
| 9 | 3 | 1 | 93.29 | 11.70 |
| 9 | 3 | 1 | 104.19 | 11.40 |
| 10 | -3 | 1 | 1474.65 | 83.49 |
| 10 | -3 | 1 | 1573.04 | 83.59 |
| 10 | -3 | 1 | 1527.25 | 84.29 |
| 10 | 3 | 1 | 1560.74 | 83.89 |
| 10 | 3 | 1 | 1525.05 | 83.19 |
| 10 | 3 | 1 | 1440.56 | 83.59 |
| 11 | -3 | 1 | 67.89 | 9.10 |
| 11 | -3 | 1 | 47.10 | 7.80 |
| 11 | -3 | 1 | 49.20 | 7.20 |
| 11 | 3 | 1 | 45.60 | 9.50 |
| 11 | 3 | 1 | 47.80 | 7.90 |
| 12 | -3 | 1 | 276.27 | 17.70 |
| 12 | -3 | 1 | 295.77 | 17.40 |
| 12 | -3 | 1 | 266.17 | 18.80 |
| 12 | 3 | 1 | 247.08 | 17.40 |
| 12 | 3 | 1 | 256.37 | 18.60 |
| 12 | 3 | 1 | 262.37 | 17.10 |
| 13 | -3 | 1 | 0.80 | 4.80 |

| | | | | |
|---|---|---|---|---|
| 13 | -3 | 1 | 8.80 | 4.00 |
| 13 | -3 | 1 | 9.00 | 3.70 |
| 13 | 3 | 1 | 6.30 | 4.40 |
| 13 | 3 | 1 | 5.90 | 3.50 |
| 13 | 3 | 1 | 11.20 | 4.70 |
| 14 | -3 | 1 | 349.37 | 21.50 |
| 14 | -3 | 1 | 337.07 | 22.20 |
| 14 | -3 | 1 | 380.76 | 21.50 |
| 14 | 3 | 1 | 369.36 | 21.20 |
| 14 | 3 | 1 | 370.56 | 21.60 |
| 14 | 3 | 1 | 343.47 | 22.20 |
| 15 | -3 | 1 | 6.50 | 3.10 |
| 15 | -3 | 1 | 10.40 | 2.90 |
| 15 | -3 | 1 | 4.00 | 2.80 |
| 15 | 3 | 1 | 5.10 | 3.00 |
| 15 | 3 | 1 | 6.60 | 2.80 |
| 15 | 3 | 1 | 2.90 | 3.50 |
| 16 | -3 | 1 | 358.56 | 19.80 |
| 16 | -3 | 1 | 352.96 | 20.30 |
| 16 | 3 | 1 | 352.36 | 19.80 |
| 16 | 3 | 1 | 336.07 | 20.10 |
| 16 | 3 | 1 | 344.47 | 20.30 |
| 16 | -4 | -1 | 8.80 | 2.10 |
| 16 | -4 | -1 | 9.40 | 3.30 |
| 16 | 4 | -1 | 7.20 | 2.10 |
| 16 | 4 | -1 | 6.00 | 3.10 |
| 16 | 4 | -1 | 12.60 | 3.60 |
| 15 | -4 | -1 | 355.46 | 20.90 |
| 15 | -4 | -1 | 348.77 | 20.50 |
| 15 | -4 | -1 | 341.17 | 20.10 |
| 15 | 4 | -1 | 333.27 | 20.50 |
| 15 | 4 | -1 | 355.26 | 19.90 |
| 15 | 4 | -1 | 335.27 | 20.90 |
| 14 | -4 | -1 | 0.60 | 2.80 |
| 14 | -4 | -1 | 1.80 | 4.00 |
| 14 | -4 | -1 | 0.60 | 3.20 |
| 14 | 4 | -1 | -2.30 | 2.30 |
| 14 | 4 | -1 | 2.20 | 4.00 |
| 14 | 4 | -1 | 4.80 | 4.10 |
| 13 | -4 | -1 | 330.07 | 21.40 |
| 13 | -4 | -1 | 342.97 | 20.20 |
| 13 | 4 | -1 | 310.27 | 21.30 |
| 13 | 4 | -1 | 325.27 | 19.40 |
| 12 | -4 | -1 | 80.09 | 7.80 |

| | | | | |
|---|---|---|---|---|
| 12 | -4 | -1 | 67.39 | 10.50 |
| 12 | -4 | -1 | 75.39 | 8.50 |
| 12 | 4 | -1 | 50.69 | 10.70 |
| 12 | 4 | -1 | 74.29 | 8.60 |
| 12 | 4 | -1 | 64.79 | 7.20 |
| 11 | -4 | -1 | 1323.97 | 72.99 |
| 11 | -4 | -1 | 1399.26 | 73.19 |
| 11 | -4 | -1 | 1362.76 | 74.79 |
| 11 | 4 | -1 | 1241.08 | 72.99 |
| 11 | 4 | -1 | 1346.37 | 73.89 |
| 11 | 4 | -1 | 1241.88 | 76.39 |
| 10 | -4 | -1 | 49.00 | 7.20 |
| 10 | -4 | -1 | 48.90 | 11.30 |
| 10 | -4 | -1 | 45.40 | 9.20 |
| 10 | 4 | -1 | 56.39 | 7.30 |
| 10 | 4 | -1 | 61.89 | 8.20 |
| 10 | 4 | -1 | 55.89 | 8.40 |
| 9 | -4 | -1 | 879.41 | 53.29 |
| 9 | -4 | -1 | 1051.99 | 53.29 |
| 9 | -4 | -1 | 850.91 | 54.29 |
| 9 | 4 | -1 | 957.10 | 52.59 |
| 9 | 4 | -1 | 964.40 | 54.29 |
| 9 | 4 | -1 | 990.90 | 53.69 |
| 8 | -4 | -1 | 493.85 | 28.20 |
| 8 | 4 | -1 | 465.75 | 27.80 |
| 7 | -4 | -1 | 193.88 | 13.90 |
| 7 | 4 | -1 | 193.08 | 13.10 |
| 7 | 4 | -1 | 202.68 | 13.00 |
| 6 | -4 | -1 | 665.23 | 37.50 |
| 6 | 4 | -1 | 637.84 | 36.60 |
| 5 | 4 | -1 | 3298.07 | 194.28 |
| -5 | 4 | 1 | 3910.61 | 193.88 |
| 4 | 4 | -1 | 1513.85 | 82.39 |
| -4 | 4 | 1 | 1511.45 | 81.69 |
| -3 | 4 | 1 | 972.30 | 52.09 |
| 3 | 4 | -1 | 942.61 | 52.59 |
| 2 | 4 | -1 | 190.08 | 11.60 |
| -2 | 4 | 1 | 188.98 | 11.20 |
| 1 | 4 | -1 | 3827.12 | 206.78 |
| -1 | 4 | 1 | 3863.11 | 206.68 |
| 0 | 4 | 1 | 251.97 | 15.30 |
| 0 | 4 | -1 | 276.67 | 15.30 |
| 1 | 4 | 1 | 1737.43 | 96.19 |
| -1 | 4 | -1 | 1819.02 | 96.09 |

| | | | | |
|---|---|---|---|---|
| 2 | 4 | 1 | 222.28 | 13.60 |
| -2 | 4 | -1 | 235.48 | 13.30 |
| -3 | 4 | -1 | 30.00 | 3.30 |
| 4 | 4 | 1 | 12.80 | 2.90 |
| -4 | 4 | -1 | 16.30 | 2.90 |
| 5 | 4 | 1 | 6754.82 | 368.46 |
| -5 | 4 | -1 | 6950.80 | 367.56 |
| 6 | -4 | 1 | 36.30 | 6.30 |
| -6 | 4 | -1 | 46.00 | 4.60 |
| 6 | 4 | 1 | 51.39 | 6.70 |
| 6 | 4 | 1 | 38.20 | 5.60 |
| 7 | -4 | 1 | 1274.57 | 69.19 |
| 7 | 4 | 1 | 1312.07 | 68.79 |
| 7 | 4 | 1 | 1157.38 | 68.19 |
| 8 | -4 | 1 | 74.99 | 8.10 |
| 8 | -4 | 1 | 63.99 | 10.10 |
| 8 | 4 | 1 | 72.89 | 7.30 |
| 9 | -4 | 1 | 1075.49 | 60.69 |
| 9 | -4 | 1 | 1180.28 | 60.79 |
| 9 | -4 | 1 | 1103.69 | 61.69 |
| 9 | 4 | 1 | 1063.39 | 60.89 |
| 9 | 4 | 1 | 1031.50 | 60.29 |
| 9 | 4 | 1 | 1075.69 | 60.29 |
| 10 | -4 | 1 | 16.10 | 4.40 |
| 10 | -4 | 1 | 23.70 | 5.00 |
| 10 | -4 | 1 | 22.20 | 6.30 |
| 10 | 4 | 1 | 27.30 | 5.10 |
| 10 | 4 | 1 | 23.40 | 5.10 |
| 10 | 4 | 1 | 18.00 | 6.20 |
| 11 | -4 | 1 | 1162.98 | 64.79 |
| 11 | -4 | 1 | 1155.58 | 64.79 |
| 11 | -4 | 1 | 1142.09 | 65.59 |
| 11 | 4 | 1 | 1081.09 | 64.79 |
| 11 | 4 | 1 | 1168.58 | 64.19 |
| 11 | 4 | 1 | 1275.27 | 65.39 |
| 12 | -4 | 1 | 29.50 | 6.20 |
| 12 | -4 | 1 | 22.40 | 6.00 |
| 12 | -4 | 1 | 40.70 | 7.70 |
| 12 | 4 | 1 | 35.00 | 6.90 |
| 12 | 4 | 1 | 26.40 | 5.80 |
| 12 | 4 | 1 | 18.10 | 4.40 |
| 13 | -4 | 1 | 173.18 | 12.40 |
| 13 | -4 | 1 | 165.28 | 12.20 |
| 13 | -4 | 1 | 170.98 | 13.40 |

| | | | | |
|---|---|---|---|---|
| 13 | 4 | 1 | 162.08 | 12.60 |
| 13 | 4 | 1 | 163.58 | 13.20 |
| 13 | 4 | 1 | 185.08 | 11.90 |
| 14 | -4 | 1 | 2.40 | 3.80 |
| 14 | -4 | 1 | 2.60 | 3.10 |
| 14 | -4 | 1 | 1.90 | 3.00 |
| 14 | 4 | 1 | 1.00 | 3.10 |
| 14 | 4 | 1 | -1.30 | 3.90 |
| 15 | -4 | 1 | 234.58 | 13.90 |
| 15 | -4 | 1 | 220.68 | 14.40 |
| 15 | -4 | 1 | 233.98 | 14.10 |
| 15 | 4 | 1 | 221.28 | 14.20 |
| 15 | 4 | 1 | 211.98 | 14.50 |
| 15 | 4 | 1 | 227.98 | 13.70 |
| 16 | -4 | 1 | 2.60 | 1.90 |
| 16 | -4 | 1 | 1.40 | 2.40 |
| 16 | 4 | 1 | 1.40 | 2.70 |
| 16 | 4 | 1 | -3.20 | 2.50 |
| 16 | 4 | 1 | 1.50 | 2.40 |
| 16 | -5 | -1 | 263.07 | 15.80 |
| 16 | -5 | -1 | 272.67 | 15.50 |
| 16 | 5 | -1 | 253.47 | 16.00 |
| 16 | 5 | -1 | 276.57 | 15.30 |
| 15 | -5 | -1 | 0.10 | 2.80 |
| 15 | -5 | -1 | 9.10 | 2.60 |
| 15 | 5 | -1 | 1.20 | 2.00 |
| 15 | 5 | -1 | 6.90 | 3.50 |
| 15 | 5 | -1 | 1.50 | 3.50 |
| 14 | -5 | -1 | 577.44 | 31.80 |
| 14 | -5 | -1 | 559.44 | 32.50 |
| 14 | -5 | -1 | 516.85 | 31.90 |
| 14 | 5 | -1 | 563.84 | 32.50 |
| 14 | 5 | -1 | 565.54 | 31.30 |
| 14 | 5 | -1 | 561.54 | 32.00 |
| 13 | -5 | -1 | -6.70 | 4.30 |
| 13 | -5 | -1 | -1.50 | 3.20 |
| 13 | -5 | -1 | 0.20 | 3.30 |
| 13 | 5 | -1 | -5.40 | 4.90 |
| 13 | 5 | -1 | 1.30 | 2.40 |
| 13 | 5 | -1 | -6.80 | 4.00 |
| 12 | -5 | -1 | 252.07 | 17.60 |
| 12 | -5 | -1 | 249.58 | 16.30 |
| 12 | -5 | -1 | 255.87 | 16.00 |
| 12 | 5 | -1 | 211.98 | 17.80 |

| | | | | |
|---|---|---|---|---|
| 12 | 5 | -1 | 241.88 | 16.40 |
| 12 | 5 | -1 | 245.58 | 15.20 |
| 11 | -5 | -1 | -4.50 | 5.40 |
| 11 | -5 | -1 | 1.00 | 4.20 |
| 11 | -5 | -1 | 0.20 | 3.40 |
| 11 | 5 | -1 | 3.70 | 3.20 |
| 11 | 5 | -1 | -3.50 | 4.10 |
| 11 | 5 | -1 | 5.60 | 6.00 |
| 10 | -5 | -1 | 1519.15 | 83.59 |
| 10 | -5 | -1 | 1393.66 | 82.69 |
| 10 | -5 | -1 | 1530.95 | 82.69 |
| 10 | 5 | -1 | 1576.64 | 82.79 |
| 10 | 5 | -1 | 1489.35 | 81.89 |
| 10 | 5 | -1 | 1501.25 | 83.49 |
| 9 | -5 | -1 | -3.50 | 5.10 |
| 9 | -5 | -1 | -5.90 | 6.40 |
| 9 | -5 | -1 | -4.20 | 3.30 |
| 9 | 5 | -1 | -2.80 | 3.80 |
| 9 | 5 | -1 | 3.20 | 3.80 |
| 9 | 5 | -1 | 0.40 | 7.00 |
| 8 | -5 | -1 | 499.25 | 28.60 |
| 8 | -5 | -1 | 438.26 | 29.10 |
| 8 | 5 | -1 | 495.25 | 28.10 |
| 8 | 5 | -1 | 508.45 | 29.90 |
| 7 | -5 | -1 | 34.50 | 7.20 |
| 7 | 5 | -1 | 17.80 | 3.60 |
| 7 | 5 | -1 | 23.20 | 4.60 |
| 6 | -5 | -1 | 3410.66 | 176.18 |
| 6 | 5 | -1 | 2939.51 | 174.88 |
| -6 | 5 | 1 | 3423.06 | 174.58 |
| 6 | 5 | -1 | 3217.48 | 175.68 |
| -5 | 5 | 1 | 622.54 | 33.80 |
| 5 | 5 | -1 | 600.74 | 34.80 |
| 4 | 5 | -1 | 3144.49 | 174.98 |
| -4 | 5 | 1 | 3331.37 | 174.28 |
| 3 | 5 | -1 | 41.80 | 4.80 |
| -3 | 5 | 1 | 42.40 | 4.20 |
| 2 | 5 | -1 | 641.04 | 36.00 |
| -2 | 5 | 1 | 648.44 | 35.70 |
| -1 | 5 | 1 | 18.30 | 3.20 |
| 1 | 5 | -1 | 14.30 | 3.30 |
| -1 | 5 | -1 | 860.91 | 45.90 |
| 1 | 5 | 1 | 810.22 | 46.00 |
| -2 | 5 | -1 | 49.80 | 4.60 |

| | | | | |
|---|---|---|---|---|
| 2 | 5 | 1 | 63.89 | 5.20 |
| 3 | 5 | 1 | -0.50 | 2.10 |
| -3 | 5 | -1 | 4.00 | 1.80 |
| -4 | 5 | -1 | 3388.76 | 185.88 |
| 4 | 5 | 1 | 3520.45 | 186.78 |
| 5 | 5 | 1 | 716.23 | 41.30 |
| -5 | 5 | -1 | 820.42 | 40.30 |
| 5 | 5 | 1 | 652.73 | 40.30 |
| 6 | -5 | 1 | 1490.25 | 84.49 |
| 6 | 5 | 1 | 1466.75 | 83.29 |
| -6 | 5 | -1 | 1662.33 | 83.09 |
| 6 | 5 | 1 | 1505.05 | 84.19 |
| 7 | -5 | 1 | 76.09 | 9.00 |
| 7 | 5 | 1 | 72.69 | 6.70 |
| 7 | 5 | 1 | 60.49 | 8.40 |
| 8 | -5 | 1 | 379.46 | 27.20 |
| 8 | -5 | 1 | 429.56 | 25.20 |
| 8 | -5 | 1 | 385.16 | 28.00 |
| 8 | 5 | 1 | 423.46 | 27.70 |
| 8 | 5 | 1 | 440.56 | 24.30 |
| 9 | -5 | 1 | 141.79 | 11.20 |
| 9 | -5 | 1 | 143.79 | 15.10 |
| 9 | -5 | 1 | 112.49 | 12.10 |
| 9 | 5 | 1 | 145.19 | 10.60 |
| 9 | 5 | 1 | 141.69 | 13.60 |
| 9 | 5 | 1 | 150.28 | 12.10 |
| 10 | -5 | 1 | 1044.30 | 57.19 |
| 10 | -5 | 1 | 1007.30 | 57.19 |
| 10 | -5 | 1 | 944.31 | 57.99 |
| 10 | 5 | 1 | 941.41 | 56.79 |
| 10 | 5 | 1 | 1139.19 | 57.69 |
| 10 | 5 | 1 | 1048.00 | 56.59 |
| 11 | -5 | 1 | 11.50 | 6.20 |
| 11 | -5 | 1 | 0.90 | 3.20 |
| 11 | -5 | 1 | 10.90 | 4.50 |
| 11 | 5 | 1 | 6.70 | 4.20 |
| 11 | 5 | 1 | 4.10 | 5.20 |
| 11 | 5 | 1 | 3.80 | 3.70 |
| 12 | -5 | 1 | 135.69 | 12.90 |
| 12 | -5 | 1 | 145.09 | 11.40 |
| 12 | -5 | 1 | 154.18 | 10.70 |
| 12 | 5 | 1 | 132.39 | 12.30 |
| 12 | 5 | 1 | 130.59 | 11.20 |
| 12 | 5 | 1 | 125.69 | 10.40 |

| | | | | |
|---|---|---|---|---|
| 13 | -5 | 1 | 3.60 | 3.10 |
| 13 | -5 | 1 | -4.00 | 4.40 |
| 13 | -5 | 1 | 1.50 | 3.50 |
| 13 | 5 | 1 | -0.10 | 3.80 |
| 13 | 5 | 1 | 0.60 | 3.00 |
| 13 | 5 | 1 | -5.30 | 4.40 |
| 14 | -5 | 1 | 435.46 | 25.20 |
| 14 | 5 | 1 | 426.96 | 24.70 |
| 14 | 5 | 1 | 428.66 | 26.50 |
| 14 | 5 | 1 | 433.66 | 25.30 |
| 15 | -5 | 1 | -1.80 | 2.40 |
| 15 | -5 | 1 | 0.30 | 2.50 |
| 15 | -5 | 1 | -1.00 | 2.90 |
| 15 | 5 | 1 | 0.60 | 2.60 |
| 15 | 5 | 1 | 1.90 | 2.90 |
| 15 | 5 | 1 | 0.90 | 3.40 |
| 16 | -5 | 1 | 212.28 | 12.60 |
| 16 | -5 | 1 | 212.28 | 13.00 |
| 16 | 5 | 1 | 211.78 | 12.70 |
| 16 | 5 | 1 | 225.98 | 13.10 |
| 16 | 5 | 1 | 207.58 | 13.00 |
| 16 | -6 | -1 | -1.40 | 2.30 |
| 16 | -6 | -1 | 1.30 | 1.70 |
| 16 | 6 | -1 | -2.80 | 1.80 |
| 16 | 6 | -1 | 0.10 | 4.00 |
| 15 | -6 | -1 | 196.18 | 12.30 |
| 15 | -6 | -1 | 201.18 | 12.70 |
| 15 | 6 | -1 | 199.58 | 12.00 |
| 15 | 6 | -1 | 194.18 | 13.20 |
| 15 | 6 | -1 | 204.98 | 13.00 |
| 14 | -6 | -1 | -2.10 | 2.60 |
| 14 | -6 | -1 | 0.30 | 2.90 |
| 14 | 6 | -1 | -5.20 | 3.50 |
| 14 | 6 | -1 | 3.80 | 4.60 |
| 13 | -6 | -1 | 193.48 | 13.00 |
| 13 | -6 | -1 | 213.98 | 14.30 |
| 13 | -6 | -1 | 185.98 | 12.90 |
| 13 | 6 | -1 | 182.78 | 13.60 |
| 13 | 6 | -1 | 170.28 | 11.90 |
| 13 | 6 | -1 | 177.88 | 14.40 |
| 12 | -6 | -1 | 104.89 | 9.40 |
| 12 | -6 | -1 | 125.29 | 11.80 |
| 12 | -6 | -1 | 115.69 | 10.10 |
| 12 | 6 | -1 | 108.19 | 12.60 |

| | | | | |
|---|---|---|---|---|
| 12 | 6 | -1 | 109.49 | 10.30 |
| 12 | 6 | -1 | 114.09 | 8.60 |
| 11 | -6 | -1 | 769.22 | 46.00 |
| 11 | -6 | -1 | 874.11 | 46.90 |
| 11 | -6 | -1 | 858.41 | 46.00 |
| 11 | 6 | -1 | 810.32 | 45.10 |
| 11 | 6 | -1 | 754.42 | 46.90 |
| 11 | 6 | -1 | 799.82 | 46.00 |
| 10 | -6 | -1 | 26.60 | 6.90 |
| 10 | -6 | -1 | 39.50 | 6.30 |
| 10 | -6 | -1 | 28.50 | 5.10 |
| 10 | 6 | -1 | 35.20 | 6.30 |
| 10 | 6 | -1 | 34.70 | 7.40 |
| 10 | 6 | -1 | 36.30 | 8.30 |
| 9 | -6 | -1 | 1289.77 | 68.09 |
| 9 | -6 | -1 | 1217.98 | 68.79 |
| 9 | -6 | -1 | 1203.08 | 67.99 |
| 9 | 6 | -1 | 1162.38 | 69.19 |
| 9 | 6 | -1 | 1171.68 | 67.09 |
| 9 | 6 | -1 | 1298.97 | 67.59 |
| 8 | -6 | -1 | 38.50 | 7.30 |
| 8 | -6 | -1 | 23.90 | 8.30 |
| 8 | -6 | -1 | 41.70 | 6.60 |
| 8 | 6 | -1 | 42.80 | 8.70 |
| 8 | 6 | -1 | 32.40 | 5.00 |
| 8 | 6 | -1 | 53.59 | 6.10 |
| 7 | -6 | -1 | 992.90 | 56.39 |
| 7 | 6 | -1 | 994.90 | 55.29 |
| 7 | 6 | -1 | 1025.30 | 56.19 |
| 6 | -6 | -1 | 0.60 | 4.60 |
| 6 | 6 | -1 | 4.50 | 3.50 |
| -6 | 6 | 1 | 0.70 | 2.80 |
| 6 | 6 | -1 | 8.80 | 3.30 |
| -5 | 6 | 1 | 5735.63 | 295.37 |
| 5 | 6 | -1 | 5257.47 | 296.27 |
| 4 | 6 | -1 | 80.89 | 7.60 |
| -4 | 6 | 1 | 90.59 | 6.60 |
| -3 | 6 | 1 | 33.60 | 4.20 |
| 3 | 6 | -1 | 32.60 | 4.90 |
| 2 | 6 | -1 | 648.94 | 37.30 |
| -2 | 6 | 1 | 680.13 | 37.00 |
| 1 | 6 | -1 | 5648.94 | 300.07 |
| -1 | 6 | 1 | 5509.45 | 299.87 |
| 0 | 6 | -1 | 6.00 | 2.30 |

| | | | | |
|---|---|---|---|---|
| 0 | 6 | 1 | 5.30 | 2.00 |
| 1 | 6 | 1 | 2695.83 | 150.58 |
| -1 | 6 | -1 | 2876.21 | 150.48 |
| -2 | 6 | -1 | 173.38 | 10.60 |
| 2 | 6 | 1 | 162.78 | 10.90 |
| 3 | 6 | 1 | 2027.00 | 109.49 |
| -3 | 6 | -1 | 1995.20 | 108.89 |
| -4 | 6 | -1 | 348.77 | 19.60 |
| 4 | 6 | 1 | 326.47 | 20.50 |
| 5 | -6 | 1 | 2442.26 | 141.49 |
| -5 | 6 | -1 | 2740.33 | 140.39 |
| 5 | 6 | 1 | 2637.84 | 141.59 |
| 5 | 6 | 1 | 2576.14 | 140.39 |
| 6 | -6 | 1 | 180.18 | 14.20 |
| 6 | 6 | 1 | 184.58 | 11.90 |
| 6 | 6 | 1 | 177.88 | 13.50 |
| -6 | 6 | -1 | 194.48 | 11.90 |
| 7 | -6 | 1 | 41.30 | 6.10 |
| 7 | -6 | 1 | 56.89 | 8.10 |
| 7 | 6 | 1 | 37.40 | 5.30 |
| 7 | 6 | 1 | 35.80 | 6.20 |
| 8 | -6 | 1 | 71.39 | 8.40 |
| 8 | -6 | 1 | 79.49 | 9.30 |
| 8 | -6 | 1 | 71.99 | 10.90 |
| 8 | 6 | 1 | 57.59 | 10.00 |
| 8 | 6 | 1 | 81.09 | 12.40 |
| 8 | 6 | 1 | 57.79 | 11.20 |
| 8 | 6 | 1 | 62.89 | 6.70 |
| 9 | -6 | 1 | 1114.39 | 59.99 |
| 9 | -6 | 1 | 959.80 | 60.89 |
| 9 | -6 | 1 | 1076.09 | 59.69 |
| 9 | 6 | 1 | 1050.09 | 59.29 |
| 9 | 6 | 1 | 1154.58 | 60.19 |
| 9 | 6 | 1 | 1068.79 | 59.29 |
| 10 | -6 | 1 | 3.20 | 4.70 |
| 10 | -6 | 1 | 2.10 | 3.80 |
| 10 | -6 | 1 | -10.60 | 6.20 |
| 10 | 6 | 1 | 0.50 | 3.60 |
| 10 | 6 | 1 | -2.20 | 4.00 |
| 10 | 6 | 1 | -7.50 | 5.30 |
| 11 | -6 | 1 | 744.63 | 42.90 |
| 11 | -6 | 1 | 756.62 | 42.00 |
| 11 | -6 | 1 | 752.92 | 42.10 |
| 11 | 6 | 1 | 741.53 | 42.50 |

| | | | | |
|---|---|---|---|---|
| 11 | 6 | 1 | 728.33 | 41.20 |
| 11 | 6 | 1 | 689.43 | 41.80 |
| 12 | -6 | 1 | 246.28 | 15.10 |
| 12 | -6 | 1 | 202.98 | 14.70 |
| 12 | -6 | 1 | 229.88 | 16.00 |
| 12 | 6 | 1 | 199.68 | 15.70 |
| 12 | 6 | 1 | 180.88 | 15.50 |
| 12 | 6 | 1 | 217.68 | 13.90 |
| 13 | -6 | 1 | 246.88 | 15.90 |
| 13 | -6 | 1 | 245.58 | 15.60 |
| 13 | -6 | 1 | 249.97 | 16.80 |
| 13 | 6 | 1 | 218.18 | 16.30 |
| 13 | 6 | 1 | 242.18 | 15.80 |
| 13 | 6 | 1 | 250.67 | 15.20 |
| 14 | -6 | 1 | 16.80 | 4.30 |
| 14 | -6 | 1 | 16.70 | 3.80 |
| 14 | -6 | 1 | 13.30 | 3.30 |
| 14 | 6 | 1 | 13.30 | 3.10 |
| 14 | 6 | 1 | 21.90 | 4.60 |
| 14 | 6 | 1 | 18.40 | 3.90 |
| 15 | -6 | 1 | 221.88 | 13.50 |
| 15 | -6 | 1 | 229.38 | 13.80 |
| 15 | 6 | 1 | 210.88 | 13.80 |
| 15 | 6 | 1 | 216.28 | 14.00 |
| 15 | 6 | 1 | 226.98 | 13.40 |
| 16 | -6 | 1 | 1.10 | 2.20 |
| 16 | -6 | 1 | 2.50 | 1.80 |
| 16 | 6 | 1 | 4.60 | 2.80 |
| 16 | 6 | 1 | 6.90 | 4.10 |
| 16 | -7 | -1 | 285.37 | 15.80 |
| 16 | -7 | -1 | 267.57 | 16.20 |
| 15 | -7 | -1 | 8.00 | 2.40 |
| 15 | -7 | -1 | 9.70 | 2.90 |
| 15 | 7 | -1 | -1.60 | 3.70 |
| 15 | 7 | -1 | 7.70 | 2.10 |
| 14 | -7 | -1 | 351.06 | 21.70 |
| 14 | -7 | -1 | 363.46 | 21.60 |
| 14 | 7 | -1 | 371.46 | 21.00 |
| 14 | 7 | -1 | 370.76 | 22.30 |
| 14 | 7 | -1 | 374.36 | 21.90 |
| 13 | -7 | -1 | -0.20 | 2.80 |
| 13 | -7 | -1 | 2.70 | 3.00 |
| 13 | 7 | -1 | -2.80 | 2.40 |
| 13 | 7 | -1 | -1.90 | 3.90 |

| | | | | |
|---|---|---|---|---|
| 13 | 7 | -1 | -2.50 | 5.30 |
| 12 | -7 | -1 | 132.99 | 11.40 |
| 12 | -7 | -1 | 148.89 | 12.60 |
| 12 | -7 | -1 | 165.48 | 13.20 |
| 12 | 7 | -1 | 185.78 | 16.30 |
| 12 | 7 | -1 | 135.39 | 14.30 |
| 12 | 7 | -1 | 149.88 | 10.30 |
| 11 | -7 | -1 | -0.10 | 3.00 |
| 11 | -7 | -1 | 7.20 | 4.40 |
| 11 | -7 | -1 | -5.90 | 5.00 |
| 11 | 7 | -1 | -8.00 | 6.90 |
| 11 | 7 | -1 | -0.60 | 4.20 |
| 11 | 7 | -1 | 3.80 | 2.80 |
| 10 | -7 | -1 | 1396.66 | 78.49 |
| 10 | -7 | -1 | 1520.05 | 82.59 |
| 10 | -7 | -1 | 1360.06 | 78.39 |
| 10 | 7 | -1 | 1415.86 | 78.09 |
| 10 | 7 | -1 | 1490.55 | 78.19 |
| 10 | 7 | -1 | 1342.77 | 79.49 |
| 9 | -7 | -1 | 46.20 | 7.30 |
| 9 | -7 | -1 | 28.20 | 7.30 |
| 9 | -7 | -1 | 34.90 | 6.10 |
| 9 | 7 | -1 | 51.99 | 6.60 |
| 9 | 7 | -1 | 47.00 | 6.80 |
| 8 | -7 | -1 | 406.06 | 25.30 |
| 8 | -7 | -1 | 372.06 | 27.40 |
| 8 | -7 | -1 | 457.95 | 25.50 |
| 8 | 7 | -1 | 439.56 | 25.80 |
| 8 | 7 | -1 | 404.16 | 24.60 |
| 8 | 7 | -1 | 396.76 | 24.60 |
| 7 | -7 | -1 | 57.29 | 9.50 |
| 7 | -7 | -1 | 43.20 | 8.00 |
| 7 | 7 | -1 | 37.70 | 6.10 |
| 7 | 7 | -1 | 35.90 | 5.70 |
| 7 | 7 | -1 | 39.20 | 8.70 |
| 6 | -7 | -1 | 4831.92 | 268.97 |
| 6 | 7 | -1 | 4998.70 | 267.77 |
| 6 | 7 | -1 | 4755.02 | 268.97 |
| -6 | 7 | 1 | 5327.17 | 267.87 |
| 5 | -7 | -1 | 50.59 | 7.50 |
| 5 | 7 | -1 | 45.40 | 7.40 |
| -5 | 7 | 1 | 48.60 | 5.60 |
| 5 | 7 | -1 | 49.20 | 5.90 |
| 4 | 7 | -1 | 863.41 | 49.30 |

| | | | | |
|---|---|---|---|---|
| -4 | 7 | 1 | 884.71 | 48.30 |
| 3 | 7 | -1 | 421.66 | 25.70 |
| -3 | 7 | 1 | 449.36 | 25.00 |
| -2 | 7 | 1 | 1259.47 | 67.59 |
| 2 | 7 | -1 | 1204.18 | 67.89 |
| 1 | 7 | -1 | 616.44 | 34.80 |
| -1 | 7 | 1 | 613.54 | 34.60 |
| 0 | 7 | 1 | 5955.90 | 319.47 |
| 0 | 7 | -1 | 5921.41 | 319.47 |
| -1 | 7 | -1 | 38.60 | 4.50 |
| 1 | 7 | 1 | 33.20 | 4.70 |
| -2 | 7 | -1 | 553.14 | 31.20 |
| 2 | 7 | 1 | 551.44 | 31.60 |
| 3 | 7 | 1 | 65.29 | 7.40 |
| 3 | 7 | 1 | 82.29 | 7.30 |
| -3 | 7 | -1 | 79.59 | 6.40 |
| 4 | -7 | 1 | 2276.77 | 124.29 |
| 4 | 7 | 1 | 2184.88 | 124.49 |
| -4 | 7 | -1 | 2363.86 | 123.69 |
| 4 | 7 | 1 | 2300.27 | 123.39 |
| 5 | -7 | 1 | 373.66 | 24.30 |
| 5 | 7 | 1 | 383.86 | 24.40 |
| 5 | 7 | 1 | 409.16 | 22.60 |
| -5 | 7 | -1 | 396.46 | 22.70 |
| 6 | -7 | 1 | 1445.86 | 83.59 |
| -6 | 7 | -1 | 1629.24 | 82.49 |
| 6 | 7 | 1 | 1603.74 | 82.39 |
| 6 | 7 | 1 | 1363.66 | 83.69 |
| 7 | -7 | 1 | 190.08 | 14.10 |
| 7 | -7 | 1 | 162.98 | 16.70 |
| 7 | 7 | 1 | 206.68 | 15.00 |
| 7 | 7 | 1 | 201.08 | 12.50 |
| 8 | -7 | 1 | 647.14 | 42.70 |
| 8 | -7 | 1 | 753.62 | 41.10 |
| 8 | -7 | 1 | 701.03 | 40.30 |
| 8 | 7 | 1 | 662.53 | 40.10 |
| 8 | 7 | 1 | 730.23 | 41.40 |
| 8 | 7 | 1 | 766.32 | 39.80 |
| 8 | 7 | 1 | 685.53 | 41.70 |
| 9 | -7 | 1 | 9.80 | 4.50 |
| 9 | -7 | 1 | 4.60 | 4.90 |
| 9 | -7 | 1 | 11.40 | 7.60 |
| 9 | 7 | 1 | 12.50 | 7.00 |
| 9 | 7 | 1 | 15.50 | 4.80 |

| | | | | |
|---|---|---|---|---|
| 9 | 7 | 1 | 7.90 | 3.40 |
| 10 | -7 | 1 | 1123.49 | 62.09 |
| 10 | -7 | 1 | 1049.49 | 62.59 |
| 10 | -7 | 1 | 1189.38 | 61.99 |
| 10 | 7 | 1 | 1086.29 | 61.59 |
| 10 | 7 | 1 | 1110.99 | 62.49 |
| 10 | 7 | 1 | 1102.59 | 61.09 |
| 11 | -7 | 1 | 3.20 | 4.20 |
| 11 | -7 | 1 | -5.40 | 5.30 |
| 11 | -7 | 1 | 5.60 | 3.60 |
| 11 | 7 | 1 | 0.80 | 3.70 |
| 11 | 7 | 1 | -0.90 | 5.50 |
| 11 | 7 | 1 | 3.60 | 3.40 |
| 12 | -7 | 1 | 31.40 | 5.00 |
| 12 | -7 | 1 | 41.60 | 6.70 |
| 12 | -7 | 1 | 38.80 | 8.90 |
| 12 | 7 | 1 | 51.59 | 7.80 |
| 12 | 7 | 1 | 39.50 | 6.20 |
| 12 | 7 | 1 | 34.10 | 6.40 |
| 13 | -7 | 1 | 61.69 | 6.50 |
| 13 | -7 | 1 | 58.09 | 7.20 |
| 13 | -7 | 1 | 49.20 | 8.60 |
| 13 | 7 | 1 | 35.40 | 7.10 |
| 13 | 7 | 1 | 53.09 | 6.60 |
| 13 | 7 | 1 | 54.59 | 9.30 |
| 14 | -7 | 1 | 403.76 | 23.10 |
| 14 | -7 | 1 | 398.26 | 22.90 |
| 14 | 7 | 1 | 361.86 | 23.40 |
| 14 | 7 | 1 | 418.46 | 22.60 |
| 14 | 7 | 1 | 389.16 | 23.10 |
| 15 | -7 | 1 | 3.20 | 2.40 |
| 15 | -7 | 1 | -0.40 | 2.10 |
| 15 | 7 | 1 | -1.60 | 2.80 |
| 15 | 7 | 1 | -1.80 | 3.40 |
| 15 | 7 | 1 | -3.30 | 2.90 |
| 15 | -8 | -1 | 433.86 | 26.00 |
| 15 | -8 | -1 | 495.95 | 27.20 |
| 15 | 8 | -1 | 467.15 | 25.80 |
| 14 | -8 | -1 | 2.40 | 2.40 |
| 14 | -8 | -1 | 3.80 | 2.50 |
| 14 | 8 | -1 | 5.20 | 4.10 |
| 14 | 8 | -1 | 1.90 | 2.10 |
| 13 | -8 | -1 | 191.98 | 12.70 |
| 13 | -8 | -1 | 170.48 | 12.60 |

| | | | | |
|---|---|---|---|---|
| 13 | 8 | -1 | 192.68 | 13.40 |
| 13 | 8 | -1 | 201.98 | 11.90 |
| 12 | -8 | -1 | 105.99 | 9.40 |
| 12 | -8 | -1 | 102.39 | 9.00 |
| 12 | 8 | -1 | 99.69 | 10.20 |
| 12 | 8 | -1 | 105.39 | 8.00 |
| 11 | -8 | -1 | 1776.72 | 96.09 |
| 11 | -8 | -1 | 1732.83 | 95.99 |
| 11 | 8 | -1 | 1771.02 | 95.99 |
| 11 | 8 | -1 | 1722.83 | 94.99 |
| 10 | -8 | -1 | 1.80 | 3.70 |
| 10 | -8 | -1 | -6.70 | 4.00 |
| 10 | -8 | -1 | -3.10 | 6.00 |
| 10 | 8 | -1 | 2.70 | 3.30 |
| 10 | 8 | -1 | 12.20 | 4.00 |
| 9 | -8 | -1 | 996.30 | 55.09 |
| 9 | -8 | -1 | 965.70 | 55.79 |
| 9 | -8 | -1 | 965.90 | 54.89 |
| 9 | 8 | -1 | 937.01 | 53.89 |
| 9 | 8 | -1 | 1020.70 | 54.29 |
| 8 | -8 | -1 | 111.29 | 10.40 |
| 8 | -8 | -1 | 122.59 | 11.00 |
| 8 | -8 | -1 | 101.49 | 12.40 |
| 8 | 8 | -1 | 79.49 | 9.90 |
| 8 | 8 | -1 | 110.29 | 8.60 |
| 8 | 8 | -1 | 112.59 | 11.90 |
| 7 | -8 | -1 | 136.59 | 12.50 |
| 7 | -8 | -1 | 133.39 | 12.00 |
| 7 | 8 | -1 | 149.29 | 10.10 |
| 7 | 8 | -1 | 130.39 | 12.40 |
| 7 | 8 | -1 | 145.09 | 12.50 |
| 6 | -8 | -1 | 171.08 | 13.60 |
| 6 | -8 | -1 | 169.18 | 14.20 |
| -6 | 8 | 1 | 197.88 | 12.70 |
| 6 | 8 | -1 | 158.88 | 13.80 |
| 6 | 8 | -1 | 183.88 | 11.70 |
| 5 | -8 | -1 | 6463.85 | 347.67 |
| 5 | 8 | -1 | 6488.25 | 346.37 |
| 5 | 8 | -1 | 6135.99 | 347.97 |
| -5 | 8 | 1 | 6706.53 | 346.87 |
| 4 | -8 | -1 | 156.48 | 12.70 |
| 4 | 8 | -1 | 146.99 | 12.20 |
| -4 | 8 | 1 | 159.88 | 10.60 |
| 4 | 8 | -1 | 146.19 | 10.50 |

| | | | | |
|---|---|---|---|---|
| 3 | -8 | -1 | 420.46 | 26.70 |
| 3 | 8 | -1 | 454.95 | 27.00 |
| 3 | 8 | -1 | 439.86 | 26.10 |
| -3 | 8 | 1 | 483.75 | 26.10 |
| 2 | 8 | -1 | 720.43 | 42.00 |
| -2 | 8 | 1 | 762.52 | 41.60 |
| -1 | 8 | 1 | 5771.92 | 302.87 |
| 1 | 8 | -1 | 5474.65 | 302.97 |
| 0 | 8 | 1 | 122.89 | 9.20 |
| 0 | 8 | -1 | 124.49 | 9.20 |
| 1 | 8 | 1 | 2974.80 | 159.38 |
| -1 | 8 | -1 | 2866.71 | 159.78 |
| 1 | 8 | 1 | 3014.80 | 160.08 |
| 2 | 8 | 1 | 511.65 | 30.40 |
| -2 | 8 | -1 | 550.44 | 30.00 |
| 2 | 8 | 1 | 507.05 | 29.60 |
| 3 | -8 | 1 | 171.88 | 13.40 |
| 3 | 8 | 1 | 192.78 | 12.10 |
| -3 | 8 | -1 | 204.78 | 12.30 |
| 3 | 8 | 1 | 160.28 | 15.80 |
| 4 | -8 | 1 | -7.80 | 4.00 |
| 4 | 8 | 1 | 4.70 | 2.60 |
| -4 | 8 | -1 | 0.90 | 2.80 |
| 4 | 8 | 1 | 0.50 | 3.60 |
| 5 | -8 | 1 | 5295.57 | 276.37 |
| 5 | 8 | 1 | 5193.28 | 275.17 |
| -5 | 8 | -1 | 5093.19 | 275.57 |
| 5 | 8 | 1 | 4888.61 | 276.77 |
| 6 | -8 | 1 | 149.99 | 13.40 |
| 6 | -8 | 1 | 151.98 | 12.40 |
| -6 | 8 | -1 | 170.58 | 11.80 |
| 6 | 8 | 1 | 150.48 | 13.30 |
| 6 | 8 | 1 | 175.08 | 10.80 |
| 7 | -8 | 1 | 419.16 | 25.10 |
| 7 | -8 | 1 | 451.65 | 26.50 |
| 7 | 8 | 1 | 431.96 | 24.40 |
| 7 | 8 | 1 | 438.46 | 26.80 |
| 7 | 8 | 1 | 362.56 | 25.80 |
| 8 | -8 | 1 | 136.29 | 17.70 |
| 8 | -8 | 1 | 93.39 | 10.60 |
| 8 | -8 | 1 | 114.49 | 10.30 |
| 8 | 8 | 1 | 115.79 | 11.20 |
| 8 | 8 | 1 | 99.09 | 13.30 |
| 8 | 8 | 1 | 118.59 | 8.60 |

| | | | | |
|---|---|---|---|---|
| 9 | -8 | 1 | 902.21 | 54.69 |
| 9 | -8 | 1 | 948.41 | 53.69 |
| 9 | -8 | 1 | 973.30 | 53.29 |
| 9 | 8 | 1 | 937.41 | 52.59 |
| 9 | 8 | 1 | 947.81 | 52.59 |
| 9 | 8 | 1 | 980.50 | 54.89 |
| 10 | -8 | 1 | 9.30 | 4.30 |
| 10 | -8 | 1 | 20.50 | 4.70 |
| 10 | -8 | 1 | 9.90 | 6.50 |
| 10 | 8 | 1 | 15.40 | 4.00 |
| 10 | 8 | 1 | 12.50 | 6.90 |
| 10 | 8 | 1 | 13.90 | 3.90 |
| 11 | -8 | 1 | 1506.85 | 85.19 |
| 11 | -8 | 1 | 1578.34 | 84.99 |
| 11 | -8 | 1 | 1579.94 | 85.19 |
| 11 | 8 | 1 | 1555.54 | 84.09 |
| 11 | 8 | 1 | 1467.75 | 85.19 |
| 11 | 8 | 1 | 1571.64 | 84.89 |
| 12 | -8 | 1 | 85.79 | 8.90 |
| 12 | -8 | 1 | 110.39 | 10.80 |
| 12 | -8 | 1 | 99.89 | 8.70 |
| 12 | 8 | 1 | 84.39 | 11.50 |
| 12 | 8 | 1 | 92.79 | 9.40 |
| 12 | 8 | 1 | 104.99 | 8.30 |
| 13 | -8 | 1 | 199.98 | 14.30 |
| 13 | -8 | 1 | 206.18 | 13.60 |
| 13 | 8 | 1 | 197.08 | 13.90 |
| 13 | 8 | 1 | 231.18 | 13.30 |
| 13 | 8 | 1 | 213.38 | 15.90 |
| 14 | -8 | 1 | 2.60 | 2.50 |
| 14 | -8 | 1 | -2.30 | 2.30 |
| 14 | 8 | 1 | -2.60 | 2.80 |
| 14 | 8 | 1 | -1.20 | 3.20 |
| 14 | 8 | 1 | 9.10 | 4.70 |
| 15 | -8 | 1 | 333.77 | 19.10 |
| 15 | -8 | 1 | 326.97 | 18.80 |
| 15 | 8 | 1 | 310.17 | 20.20 |
| 15 | 8 | 1 | 345.17 | 19.20 |
| 15 | -9 | -1 | -1.10 | 1.70 |
| 15 | -9 | -1 | -0.30 | 2.30 |
| 15 | 9 | -1 | -2.40 | 1.80 |
| 14 | -9 | -1 | 155.48 | 10.60 |
| 14 | -9 | -1 | 157.08 | 10.30 |
| 14 | 9 | -1 | 150.78 | 11.60 |

| | | | | |
|---|---|---|---|---|
| 14 | 9 | -1 | 165.08 | 9.80 |
| 13 | -9 | -1 | 55.59 | 6.40 |
| 13 | 9 | -1 | 70.69 | 8.20 |
| 13 | 9 | -1 | 55.29 | 5.80 |
| 12 | -9 | -1 | 99.49 | 8.60 |
| 12 | -9 | -1 | 83.79 | 8.00 |
| 12 | 9 | -1 | 83.39 | 9.50 |
| 12 | 9 | -1 | 85.99 | 7.00 |
| 11 | -9 | -1 | 24.50 | 4.70 |
| 11 | -9 | -1 | 22.50 | 4.90 |
| 11 | 9 | -1 | 27.00 | 6.20 |
| 11 | 9 | -1 | 17.60 | 3.40 |
| 10 | -9 | -1 | 871.71 | 49.60 |
| 10 | -9 | -1 | 879.51 | 49.60 |
| 10 | 9 | -1 | 926.71 | 49.10 |
| 10 | 9 | -1 | 826.62 | 48.40 |
| 9 | -9 | -1 | 70.09 | 8.50 |
| 9 | -9 | -1 | 53.69 | 8.80 |
| 9 | 9 | -1 | 51.19 | 7.30 |
| 9 | 9 | -1 | 61.79 | 7.20 |
| 8 | -9 | -1 | 121.29 | 11.50 |
| 8 | -9 | -1 | 126.19 | 11.50 |
| 8 | 9 | -1 | 113.99 | 10.50 |
| 8 | 9 | -1 | 138.79 | 9.60 |
| 8 | 9 | -1 | 129.69 | 13.60 |
| 7 | -9 | -1 | 593.04 | 33.90 |
| 7 | -9 | -1 | 550.44 | 32.70 |
| -7 | -9 | 1 | 521.25 | 34.20 |
| 7 | 9 | -1 | 596.64 | 34.20 |
| 7 | 9 | -1 | 517.45 | 32.80 |
| 7 | 9 | -1 | 588.14 | 31.80 |
| -6 | -9 | 1 | 1724.53 | 94.29 |
| 6 | -9 | -1 | 1740.93 | 94.29 |
| 6 | -9 | -1 | 1625.04 | 92.89 |
| 6 | 9 | -1 | 1749.53 | 92.49 |
| 6 | 9 | -1 | 1666.63 | 94.49 |
| 5 | -9 | -1 | 99.49 | 10.90 |
| 5 | -9 | -1 | 96.29 | 11.60 |
| -5 | 9 | 1 | 130.29 | 9.70 |
| 5 | 9 | -1 | 102.89 | 11.00 |
| 5 | 9 | -1 | 132.19 | 9.00 |
| 4 | -9 | -1 | 1330.07 | 71.59 |
| 4 | 9 | -1 | 1251.77 | 70.29 |
| 4 | 9 | -1 | 1243.28 | 71.99 |

| | | | | |
|---|---|---|---|---|
| -4 | 9 | 1 | 1325.27 | 70.99 |
| 3 | -9 | -1 | 1390.86 | 73.39 |
| 3 | 9 | -1 | 1264.57 | 72.39 |
| 3 | 9 | -1 | 1304.97 | 73.89 |
| -3 | 9 | 1 | 1348.47 | 73.09 |
| 2 | 9 | -1 | 84.39 | 7.50 |
| 2 | 9 | -1 | 72.19 | 7.70 |
| -2 | 9 | 1 | 85.99 | 7.40 |
| 1 | 9 | -1 | 1122.29 | 60.69 |
| 1 | 9 | -1 | 1044.90 | 59.69 |
| -1 | 9 | 1 | 1095.69 | 60.39 |
| 0 | 9 | 1 | 3700.53 | 202.88 |
| 0 | 9 | -1 | 3929.61 | 203.68 |
| 0 | 9 | 1 | 3659.33 | 203.48 |
| -1 | 9 | -1 | 536.25 | 29.70 |
| 1 | 9 | 1 | 492.05 | 29.00 |
| 1 | 9 | 1 | 503.85 | 29.80 |
| 2 | 9 | 1 | 239.28 | 15.50 |
| 2 | 9 | 1 | 251.57 | 16.40 |
| -2 | 9 | -1 | 268.97 | 16.10 |
| 3 | -9 | 1 | 549.45 | 30.40 |
| -3 | 9 | -1 | 531.45 | 30.10 |
| 3 | 9 | 1 | 492.05 | 29.50 |
| 3 | 9 | 1 | 514.25 | 30.90 |
| 4 | -9 | 1 | 1536.15 | 82.39 |
| 4 | 9 | 1 | 1458.15 | 81.39 |
| -4 | 9 | -1 | 1541.95 | 82.09 |
| 4 | 9 | 1 | 1441.56 | 83.09 |
| 5 | -9 | 1 | 43.90 | 8.00 |
| 5 | 9 | 1 | 31.60 | 5.10 |
| -5 | 9 | -1 | 28.50 | 4.60 |
| 5 | 9 | 1 | 18.80 | 5.30 |
| -6 | -9 | -1 | 2326.87 | 127.79 |
| 6 | -9 | 1 | 2453.65 | 128.49 |
| 6 | -9 | 1 | 2275.87 | 127.19 |
| 6 | 9 | 1 | 2438.76 | 126.99 |
| 6 | 9 | 1 | 2126.69 | 128.79 |
| -6 | 9 | -1 | 2442.46 | 127.49 |
| -7 | -9 | -1 | 204.58 | 16.70 |
| 7 | -9 | 1 | 248.38 | 15.60 |
| 7 | -9 | 1 | 237.78 | 16.90 |
| 7 | 9 | 1 | 228.58 | 14.40 |
| 7 | 9 | 1 | 221.58 | 16.50 |
| 7 | 9 | 1 | 246.28 | 18.00 |

| | | | | |
|---|---|---|---|---|
| 8 | -9 | 1 | 279.27 | 18.60 |
| 8 | -9 | 1 | 311.77 | 19.80 |
| 8 | 9 | 1 | 272.67 | 17.60 |
| 8 | 9 | 1 | 290.77 | 21.60 |
| 8 | 9 | 1 | 305.27 | 18.70 |
| 9 | -9 | 1 | 79.29 | 9.00 |
| 9 | -9 | 1 | 81.09 | 9.20 |
| 9 | 9 | 1 | 78.69 | 8.70 |
| 9 | 9 | 1 | 71.89 | 7.20 |
| 10 | -9 | 1 | 547.45 | 34.40 |
| 10 | -9 | 1 | 590.44 | 34.20 |
| 10 | -9 | 1 | 598.14 | 35.60 |
| 10 | 9 | 1 | 622.44 | 33.70 |
| 10 | 9 | 1 | 584.04 | 33.20 |
| 11 | -9 | 1 | 2.30 | 3.80 |
| 11 | -9 | 1 | 7.50 | 4.20 |
| 11 | 9 | 1 | 7.70 | 3.40 |
| 11 | 9 | 1 | 2.10 | 4.20 |
| 12 | -9 | 1 | 84.09 | 8.30 |
| 12 | -9 | 1 | 76.59 | 7.80 |
| 12 | 9 | 1 | 75.69 | 8.40 |
| 12 | 9 | 1 | 93.69 | 7.60 |
| 13 | -9 | 1 | 86.29 | 8.00 |
| 13 | -9 | 1 | 78.79 | 7.20 |
| 13 | 9 | 1 | 79.99 | 8.20 |
| 13 | 9 | 1 | 96.59 | 7.30 |
| 14 | -9 | 1 | 129.99 | 8.80 |
| 14 | -9 | 1 | 121.69 | 8.90 |
| 14 | 9 | 1 | 135.29 | 9.10 |
| 14 | 9 | 1 | 126.09 | 9.70 |
| 15 | -9 | 1 | 2.60 | 2.20 |
| 15 | -9 | 1 | 0.60 | 1.70 |
| 15 | 9 | 1 | 2.20 | 3.80 |
| 15 | 10 | -1 | 273.87 | 15.50 |
| 14 | -10 | -1 | 6.60 | 2.50 |
| 14 | -10 | -1 | 4.40 | 2.00 |
| 14 | 10 | -1 | 12.20 | 2.10 |
| 13 | -10 | -1 | 13.80 | 3.10 |
| 13 | -10 | -1 | 8.50 | 2.80 |
| 13 | 10 | -1 | 14.80 | 4.80 |
| 13 | 10 | -1 | 21.20 | 4.00 |
| 12 | -10 | -1 | 88.29 | 7.90 |
| 12 | -10 | -1 | 85.59 | 7.90 |
| 12 | 10 | -1 | 94.49 | 9.40 |

| | | | | |
|---|---|---|---|---|
| 12 | 10 | -1 | 79.89 | 6.90 |
| 11 | -10 | -1 | 757.02 | 43.40 |
| 11 | -10 | -1 | 731.73 | 43.40 |
| 11 | 10 | -1 | 799.42 | 43.40 |
| 11 | 10 | -1 | 776.02 | 42.40 |
| 10 | -10 | -1 | 0.80 | 3.40 |
| 10 | -10 | -1 | 3.90 | 4.10 |
| 10 | 10 | -1 | 0.40 | 3.00 |
| 10 | 10 | -1 | 3.10 | 3.80 |
| 9 | -10 | -1 | 565.44 | 32.50 |
| 9 | -10 | -1 | 524.05 | 32.00 |
| 9 | 10 | -1 | 500.95 | 30.90 |
| 9 | 10 | -1 | 587.74 | 31.30 |
| 8 | -10 | -1 | 317.47 | 20.00 |
| 8 | -10 | -1 | 315.57 | 20.80 |
| 8 | 10 | -1 | 315.27 | 22.40 |
| 8 | 10 | -1 | 302.87 | 18.80 |
| -7 | -10 | 1 | 124.99 | 11.20 |
| 7 | -10 | -1 | 100.09 | 11.30 |
| 7 | -10 | -1 | 90.59 | 10.40 |
| 7 | 10 | -1 | 114.99 | 12.70 |
| 7 | 10 | -1 | 100.89 | 10.80 |
| 7 | 10 | -1 | 110.79 | 8.60 |
| 6 | -10 | -1 | 93.59 | 9.70 |
| 6 | -10 | -1 | 100.69 | 11.00 |
| -6 | -10 | 1 | 71.99 | 10.30 |
| -6 | 10 | 1 | 108.79 | 9.80 |
| 6 | 10 | -1 | 80.39 | 11.40 |
| 6 | 10 | -1 | 106.09 | 7.90 |
| 5 | -10 | -1 | 2465.05 | 123.49 |
| 5 | -10 | -1 | 2110.89 | 122.09 |
| -5 | -10 | 1 | 2111.49 | 122.19 |
| -5 | 10 | 1 | 2465.95 | 122.69 |
| 5 | 10 | -1 | 2237.88 | 121.79 |
| 5 | 10 | -1 | 2104.89 | 123.89 |
| 4 | -10 | -1 | 518.25 | 28.80 |
| 4 | -10 | -1 | 430.36 | 26.90 |
| -4 | -10 | 1 | 422.86 | 27.00 |
| -4 | 10 | 1 | 473.55 | 26.90 |
| 4 | 10 | -1 | 427.86 | 28.10 |
| 4 | 10 | -1 | 457.85 | 26.10 |
| -3 | -10 | 1 | 241.48 | 20.10 |
| 3 | 10 | -1 | 266.57 | 15.90 |
| 3 | 10 | -1 | 254.87 | 17.50 |

| | | | | |
|---|---|---|---|---|
| -3 | 10 | 1 | 262.37 | 16.50 |
| -2 | 10 | 1 | 135.69 | 9.90 |
| 2 | 10 | -1 | 120.09 | 9.40 |
| 2 | 10 | -1 | 126.49 | 10.50 |
| 1 | 10 | -1 | 1716.13 | 90.49 |
| 1 | 10 | -1 | 1629.14 | 89.19 |
| -1 | 10 | 1 | 1570.94 | 89.99 |
| 0 | 10 | -1 | 319.87 | 18.60 |
| 0 | 10 | 1 | 298.27 | 18.40 |
| 0 | 10 | -1 | 291.77 | 19.00 |
| 0 | 10 | 1 | 316.57 | 19.20 |
| -1 | 10 | -1 | 2339.07 | 129.79 |
| 1 | 10 | 1 | 2444.96 | 129.09 |
| 1 | 10 | 1 | 2362.76 | 130.09 |
| 2 | 10 | 1 | 172.38 | 12.10 |
| -2 | 10 | -1 | 196.68 | 12.80 |
| 2 | 10 | 1 | 190.48 | 13.40 |
| 3 | -10 | 1 | 192.78 | 15.40 |
| -3 | -10 | -1 | 226.38 | 15.80 |
| 3 | 10 | 1 | 255.27 | 16.40 |
| -3 | 10 | -1 | 227.28 | 15.00 |
| 3 | 10 | 1 | 247.28 | 14.50 |
| 4 | -10 | 1 | 21.60 | 4.70 |
| 4 | -10 | 1 | 29.20 | 4.80 |
| -4 | -10 | -1 | 25.10 | 5.30 |
| 4 | 10 | 1 | 29.50 | 6.00 |
| 4 | 10 | 1 | 23.40 | 5.10 |
| -4 | 10 | -1 | 23.20 | 5.80 |
| 5 | -10 | 1 | 2017.80 | 109.39 |
| -5 | -10 | -1 | 1906.61 | 109.49 |
| 5 | -10 | 1 | 2162.58 | 110.39 |
| -5 | 10 | -1 | 2198.88 | 109.99 |
| 5 | 10 | 1 | 2016.20 | 109.19 |
| 5 | 10 | 1 | 1765.72 | 111.09 |
| -6 | -10 | -1 | 108.29 | 11.40 |
| 6 | -10 | 1 | 136.09 | 10.60 |
| 6 | -10 | 1 | 151.68 | 12.20 |
| -6 | 10 | -1 | 147.89 | 11.30 |
| 6 | 10 | 1 | 113.69 | 13.40 |
| 6 | 10 | 1 | 138.99 | 9.40 |
| -7 | -10 | -1 | 47.00 | 9.00 |
| 7 | -10 | 1 | 47.10 | 7.40 |
| 7 | -10 | 1 | 26.40 | 5.70 |
| 7 | 10 | 1 | 33.60 | 5.40 |

| | | | | |
|---|---|---|---|---|
| 7 | 10 | 1 | 44.10 | 6.70 |
| 7 | 10 | 1 | 44.70 | 8.80 |
| 8 | -10 | 1 | 1.40 | 4.40 |
| 8 | -10 | 1 | 5.60 | 3.80 |
| 8 | 10 | 1 | 14.00 | 6.10 |
| 8 | 10 | 1 | 4.80 | 8.10 |
| 8 | 10 | 1 | 12.00 | 3.10 |
| 9 | -10 | 1 | 397.46 | 24.70 |
| 9 | -10 | 1 | 409.56 | 25.50 |
| 9 | 10 | 1 | 421.86 | 24.00 |
| 9 | 10 | 1 | 408.76 | 24.10 |
| 10 | -10 | 1 | -1.20 | 3.80 |
| 10 | -10 | 1 | 3.40 | 3.40 |
| 10 | 10 | 1 | -5.90 | 3.20 |
| 10 | 10 | 1 | -1.40 | 3.10 |
| 11 | -10 | 1 | 671.23 | 35.50 |
| 11 | -10 | 1 | 600.64 | 35.00 |
| 11 | 10 | 1 | 555.24 | 34.80 |
| 11 | 10 | 1 | 618.14 | 34.20 |
| 12 | -10 | 1 | 130.09 | 9.50 |
| 12 | -10 | 1 | 116.29 | 9.80 |
| 12 | 10 | 1 | 117.19 | 9.90 |
| 12 | 10 | 1 | 136.39 | 9.30 |
| 13 | -10 | 1 | 87.19 | 7.50 |
| 13 | -10 | 1 | 87.69 | 7.50 |
| 13 | 10 | 1 | 89.69 | 8.10 |
| 13 | 10 | 1 | 108.79 | 7.60 |
| 14 | -10 | 1 | 1.60 | 2.50 |
| 14 | -10 | 1 | 2.40 | 1.90 |
| 14 | 10 | 1 | 6.10 | 3.30 |
| 14 | -11 | -1 | 318.77 | 18.00 |
| 14 | 11 | -1 | 311.27 | 17.70 |
| 13 | -11 | -1 | 61.39 | 5.70 |
| 13 | -11 | -1 | 65.69 | 6.00 |
| 13 | 11 | -1 | 66.69 | 5.30 |
| 12 | -11 | -1 | 122.59 | 10.10 |
| 12 | -11 | -1 | 109.79 | 9.00 |
| 12 | 11 | -1 | 129.39 | 8.30 |
| 12 | 11 | -1 | 117.99 | 10.90 |
| 11 | -11 | -1 | 77.09 | 7.90 |
| 11 | -11 | -1 | 71.49 | 7.60 |
| 11 | 11 | -1 | 70.79 | 8.90 |
| 11 | 11 | -1 | 78.09 | 6.60 |
| 10 | -11 | -1 | 751.92 | 43.70 |

| | | | | |
|---|---|---|---|---|
| 10 | -11 | -1 | 746.23 | 43.20 |
| 10 | 11 | -1 | 777.72 | 43.00 |
| 10 | 11 | -1 | 771.72 | 42.20 |
| 9 | -11 | -1 | 15.60 | 4.50 |
| 9 | -11 | -1 | 7.90 | 3.90 |
| 9 | 11 | -1 | 7.40 | 3.00 |
| 9 | 11 | -1 | 13.80 | 3.90 |
| -8 | -11 | 1 | 65.89 | 9.90 |
| 8 | -11 | -1 | 80.79 | 9.70 |
| 8 | -11 | -1 | 77.59 | 9.00 |
| 8 | 11 | -1 | 70.09 | 7.30 |
| 8 | 11 | -1 | 73.49 | 8.10 |
| 8 | 11 | -1 | 87.99 | 16.50 |
| 7 | -11 | -1 | 269.67 | 18.60 |
| -7 | -11 | 1 | 251.07 | 17.30 |
| 7 | -11 | -1 | 267.07 | 17.40 |
| 7 | 11 | -1 | 272.37 | 20.20 |
| 7 | 11 | -1 | 261.57 | 16.20 |
| -6 | -11 | 1 | 1963.60 | 109.79 |
| 6 | -11 | -1 | 1950.50 | 109.79 |
| 6 | -11 | -1 | 2077.79 | 111.19 |
| 6 | 11 | -1 | 1941.41 | 111.99 |
| 6 | 11 | -1 | 2148.39 | 109.49 |
| 5 | -11 | -1 | 7.70 | 4.10 |
| -5 | -11 | 1 | 1.50 | 4.20 |
| 5 | 11 | -1 | 7.30 | 3.20 |
| 5 | 11 | -1 | -2.40 | 5.50 |
| -5 | 11 | 1 | -2.40 | 4.00 |
| 4 | -11 | -1 | 958.70 | 53.39 |
| 4 | -11 | -1 | 859.31 | 54.79 |
| -4 | -11 | 1 | 971.70 | 53.59 |
| -4 | 11 | 1 | 1044.90 | 54.19 |
| 4 | 11 | -1 | 982.00 | 53.09 |
| 4 | 11 | -1 | 940.91 | 55.49 |
| 3 | -11 | -1 | 118.49 | 15.30 |
| -3 | -11 | 1 | 150.78 | 12.60 |
| 3 | -11 | -1 | 149.99 | 12.00 |
| -3 | -11 | 1 | 152.78 | 11.80 |
| 3 | 11 | -1 | 141.59 | 12.40 |
| -3 | 11 | 1 | 165.68 | 11.50 |
| 3 | 11 | -1 | 141.89 | 10.40 |
| -2 | -11 | 1 | 200.48 | 15.00 |
| -2 | -11 | 1 | 218.38 | 15.20 |
| 2 | -11 | -1 | 188.28 | 15.30 |

| | | | | |
|---|---|---|---|---|
| 2 | -11 | -1 | 221.58 | 17.10 |
| 2 | 11 | -1 | 225.48 | 13.90 |
| -2 | 11 | 1 | 244.68 | 14.90 |
| 2 | 11 | -1 | 205.58 | 15.30 |
| -1 | -11 | 1 | 207.98 | 15.70 |
| 1 | -11 | -1 | 217.78 | 16.40 |
| 1 | -11 | -1 | 232.38 | 16.30 |
| -1 | -11 | 1 | 245.48 | 15.70 |
| 1 | 11 | -1 | 235.98 | 14.50 |
| -1 | 11 | 1 | 226.98 | 15.30 |
| 1 | 11 | -1 | 223.28 | 15.70 |
| 0 | -11 | 1 | 3258.07 | 182.18 |
| 0 | -11 | -1 | 3322.97 | 182.68 |
| 0 | -11 | 1 | 3268.57 | 182.48 |
| 0 | 11 | -1 | 3380.16 | 183.28 |
| 0 | 11 | 1 | 3435.86 | 182.28 |
| 0 | 11 | 1 | 3552.34 | 183.48 |
| 0 | 11 | -1 | 3405.56 | 182.18 |
| 1 | -11 | 1 | 497.05 | 28.10 |
| 1 | -11 | 1 | 486.15 | 28.90 |
| -1 | -11 | -1 | 414.96 | 32.70 |
| -1 | -11 | -1 | 453.15 | 28.30 |
| 1 | 11 | 1 | 510.15 | 27.60 |
| 1 | 11 | 1 | 466.35 | 28.70 |
| -1 | 11 | -1 | 503.45 | 28.60 |
| -2 | -11 | -1 | 20.20 | 5.80 |
| 2 | -11 | 1 | 10.40 | 5.90 |
| -2 | -11 | -1 | 16.70 | 4.70 |
| 2 | -11 | 1 | 14.80 | 4.40 |
| 2 | 11 | 1 | 18.40 | 4.70 |
| -2 | 11 | -1 | 8.60 | 3.30 |
| 3 | -11 | 1 | 199.48 | 14.00 |
| -3 | -11 | -1 | 203.38 | 16.00 |
| 3 | -11 | 1 | 191.18 | 17.30 |
| -3 | -11 | -1 | 180.28 | 14.00 |
| 3 | 11 | 1 | 203.28 | 13.00 |
| -3 | 11 | -1 | 223.78 | 14.20 |
| 3 | 11 | 1 | 208.78 | 15.30 |
| -4 | -11 | -1 | 1282.97 | 73.29 |
| 4 | -11 | 1 | 1326.67 | 73.09 |
| 4 | -11 | 1 | 1318.37 | 74.69 |
| 4 | 11 | 1 | 1299.87 | 72.89 |
| 4 | 11 | 1 | 1309.87 | 75.29 |
| -4 | 11 | -1 | 1457.55 | 74.09 |

| | | | | |
|---|---|---|---|---|
| 5 | -11 | 1 | 12.30 | 4.40 |
| -5 | -11 | -1 | 0.00 | 3.90 |
| 5 | -11 | 1 | -15.00 | 8.30 |
| 5 | -11 | 1 | 6.70 | 3.50 |
| 5 | 11 | 1 | 5.30 | 6.10 |
| 5 | 11 | 1 | 12.70 | 3.20 |
| -5 | 11 | -1 | 5.90 | 4.30 |
| 6 | -11 | 1 | 1132.09 | 62.39 |
| -6 | -11 | -1 | 1174.68 | 62.49 |
| 6 | -11 | 1 | 1123.99 | 63.49 |
| 6 | 11 | 1 | 1202.98 | 62.09 |
| 6 | 11 | 1 | 1006.90 | 64.69 |
| 7 | -11 | 1 | 382.26 | 23.80 |
| 7 | -11 | 1 | 403.66 | 25.20 |
| 7 | 11 | 1 | 402.56 | 27.80 |
| 7 | 11 | 1 | 404.16 | 23.20 |
| -8 | -11 | -1 | 176.18 | 14.10 |
| 8 | -11 | 1 | 207.18 | 13.80 |
| 8 | -11 | 1 | 194.68 | 15.10 |
| 8 | 11 | 1 | 206.68 | 20.20 |
| 8 | 11 | 1 | 198.38 | 12.70 |
| 8 | 11 | 1 | 202.28 | 13.70 |
| 9 | -11 | 1 | 51.99 | 8.90 |
| 9 | -11 | 1 | 57.69 | 7.60 |
| 9 | 11 | 1 | 52.79 | 7.30 |
| 9 | 11 | 1 | 60.09 | 6.40 |
| 10 | -11 | 1 | 999.80 | 55.39 |
| 10 | -11 | 1 | 1051.89 | 55.99 |
| 10 | 11 | 1 | 975.20 | 54.49 |
| 10 | 11 | 1 | 944.01 | 54.89 |
| 11 | -11 | 1 | 34.90 | 6.30 |
| 11 | -11 | 1 | 36.80 | 6.50 |
| 11 | 11 | 1 | 34.80 | 6.30 |
| 12 | -11 | 1 | 7.30 | 2.80 |
| 12 | -11 | 1 | 6.40 | 3.00 |
| 12 | 11 | 1 | 8.00 | 3.00 |
| 12 | 11 | 1 | 9.20 | 3.70 |
| 13 | -11 | 1 | 46.80 | 5.40 |
| 13 | -11 | 1 | 53.99 | 5.20 |
| 13 | 11 | 1 | 48.80 | 6.40 |
| 14 | -11 | 1 | 229.98 | 13.20 |
| 14 | 11 | 1 | 219.28 | 15.80 |
| 13 | -12 | -1 | 64.59 | 5.50 |
| 13 | 12 | -1 | 68.49 | 5.20 |

| | | | | |
|---|---|---|---|---|
| 12 | -12 | -1 | 109.29 | 8.40 |
| 12 | 12 | -1 | 110.19 | 7.50 |
| 11 | -12 | -1 | 548.95 | 31.20 |
| 11 | 12 | -1 | 517.65 | 30.70 |
| 11 | 12 | -1 | 556.84 | 31.40 |
| 10 | -12 | -1 | -2.00 | 3.10 |
| 10 | -12 | -1 | 1.10 | 3.40 |
| 10 | 12 | -1 | 5.40 | 2.50 |
| 10 | 12 | -1 | 5.00 | 4.10 |
| 9 | -12 | -1 | 588.04 | 35.30 |
| 9 | -12 | -1 | 528.45 | 31.00 |
| 9 | 12 | -1 | 511.15 | 31.70 |
| 9 | 12 | -1 | 505.75 | 30.00 |
| -8 | -12 | 1 | 36.50 | 8.00 |
| 8 | -12 | -1 | 30.40 | 5.40 |
| 8 | -12 | -1 | 46.90 | 8.70 |
| 8 | 12 | -1 | 42.80 | 7.20 |
| 8 | 12 | -1 | 43.30 | 5.90 |
| 7 | -12 | -1 | 96.19 | 9.50 |
| -7 | -12 | 1 | 88.89 | 9.40 |
| 7 | 12 | -1 | 80.09 | 9.50 |
| 7 | 12 | -1 | 91.59 | 14.70 |
| 7 | 12 | -1 | 93.39 | 7.70 |
| 6 | -12 | -1 | 260.87 | 16.00 |
| -6 | -12 | 1 | 235.48 | 17.40 |
| 6 | 12 | -1 | 207.88 | 19.50 |
| 6 | 12 | -1 | 245.98 | 14.80 |
| 5 | -12 | -1 | 2247.98 | 110.59 |
| 5 | -12 | -1 | 1941.41 | 109.49 |
| -5 | -12 | 1 | 1991.70 | 109.59 |
| 5 | 12 | -1 | 2049.49 | 109.19 |
| -5 | 12 | 1 | 2042.90 | 110.49 |
| 5 | 12 | -1 | 1799.72 | 111.89 |
| 4 | -12 | -1 | 271.87 | 17.20 |
| 4 | -12 | -1 | 227.38 | 20.20 |
| -4 | -12 | 1 | 250.57 | 17.00 |
| -4 | 12 | 1 | 290.87 | 18.00 |
| 4 | 12 | -1 | 247.58 | 19.60 |
| 4 | 12 | -1 | 280.57 | 16.20 |
| 3 | -12 | -1 | 49.40 | 8.50 |
| -3 | -12 | 1 | 72.89 | 8.30 |
| 3 | -12 | -1 | 62.39 | 12.20 |
| -3 | -12 | 1 | 78.69 | 9.10 |
| -3 | 12 | 1 | 63.99 | 8.40 |

| | | | | |
|---|---|---|---|---|
| 3 | 12 | -1 | 66.69 | 9.80 |
| 3 | 12 | -1 | 63.39 | 6.80 |
| -2 | -12 | 1 | 287.67 | 17.10 |
| 2 | -12 | -1 | 224.28 | 16.90 |
| -2 | -12 | 1 | 255.97 | 17.00 |
| 2 | -12 | -1 | 272.87 | 18.60 |
| 2 | 12 | -1 | 255.27 | 16.10 |
| 2 | 12 | -1 | 247.88 | 18.00 |
| -2 | 12 | 1 | 277.27 | 17.50 |
| -1 | -12 | 1 | 1485.25 | 80.09 |
| 1 | -12 | -1 | 1415.16 | 79.89 |
| 1 | -12 | -1 | 1433.66 | 80.29 |
| -1 | -12 | 1 | 1497.65 | 79.89 |
| 1 | 12 | -1 | 1469.45 | 81.29 |
| -1 | 12 | 1 | 1387.86 | 79.69 |
| -1 | 12 | 1 | 1436.86 | 80.89 |
| 1 | 12 | -1 | 1524.85 | 79.69 |
| 0 | -12 | -1 | 14.50 | 4.60 |
| 0 | -12 | 1 | 7.60 | 4.40 |
| 0 | -12 | -1 | -1.10 | 4.20 |
| 0 | -12 | 1 | 8.50 | 4.60 |
| 0 | 12 | -1 | 8.20 | 3.40 |
| 0 | 12 | 1 | 7.60 | 3.20 |
| 0 | 12 | 1 | 12.40 | 4.10 |
| 0 | 12 | -1 | 7.20 | 4.00 |
| 1 | -12 | 1 | 763.92 | 44.50 |
| -1 | -12 | -1 | 800.72 | 44.90 |
| 1 | -12 | 1 | 790.62 | 45.20 |
| -1 | -12 | -1 | 794.02 | 44.90 |
| -1 | 12 | -1 | 770.42 | 44.30 |
| 1 | 12 | 1 | 860.31 | 44.50 |
| -1 | 12 | -1 | 819.52 | 45.70 |
| 1 | 12 | 1 | 754.82 | 45.70 |
| 2 | -12 | 1 | 182.08 | 14.40 |
| -2 | -12 | -1 | 164.08 | 12.00 |
| -2 | -12 | -1 | 169.28 | 13.70 |
| 2 | -12 | 1 | 161.78 | 12.30 |
| 2 | 12 | 1 | 162.28 | 11.30 |
| -2 | 12 | -1 | 165.98 | 12.50 |
| 2 | 12 | 1 | 163.18 | 13.40 |
| 3 | -12 | 1 | 351.46 | 21.80 |
| 3 | -12 | 1 | 390.76 | 24.20 |
| -3 | -12 | -1 | 362.46 | 22.00 |
| 3 | 12 | 1 | 329.27 | 23.70 |

| | | | | |
|---|---|---|---|---|
| 3 | 12 | 1 | 360.06 | 21.30 |
| -3 | 12 | -1 | 380.36 | 22.80 |
| 4 | -12 | 1 | 178.08 | 16.90 |
| -4 | -12 | -1 | 185.38 | 13.10 |
| 4 | -12 | 1 | 191.48 | 12.80 |
| -4 | 12 | -1 | 179.38 | 14.00 |
| 4 | 12 | 1 | 184.78 | 12.00 |
| 4 | 12 | 1 | 178.08 | 15.90 |
| -5 | -12 | -1 | 2398.16 | 130.29 |
| 5 | -12 | 1 | 2420.06 | 131.29 |
| 5 | 12 | 1 | 2476.75 | 130.09 |
| -5 | 12 | -1 | 2489.55 | 131.29 |
| 5 | 12 | 1 | 2217.18 | 132.79 |
| -6 | -12 | -1 | 278.27 | 17.30 |
| 6 | -12 | 1 | 303.57 | 17.20 |
| 6 | 12 | 1 | 241.08 | 21.40 |
| 6 | 12 | 1 | 249.08 | 16.20 |
| 7 | -12 | 1 | -2.30 | 3.10 |
| -7 | -12 | -1 | -1.00 | 3.60 |
| 7 | -12 | 1 | 7.20 | 4.60 |
| 7 | 12 | 1 | 6.80 | 9.40 |
| 7 | 12 | 1 | 2.80 | 2.70 |
| 7 | 12 | 1 | -1.20 | 4.20 |
| 8 | -12 | 1 | 23.30 | 4.20 |
| -8 | -12 | -1 | 41.50 | 7.10 |
| 8 | -12 | 1 | 26.10 | 5.50 |
| 8 | 12 | 1 | 41.50 | 7.20 |
| 8 | 12 | 1 | 28.20 | 5.30 |
| 9 | -12 | 1 | 947.51 | 50.39 |
| 9 | -12 | 1 | 912.81 | 49.30 |
| 9 | 12 | 1 | 844.02 | 48.50 |
| 9 | 12 | 1 | 816.62 | 48.70 |
| 10 | -12 | 1 | 49.90 | 6.80 |
| 10 | -12 | 1 | 42.50 | 7.20 |
| 10 | 12 | 1 | 51.09 | 6.50 |
| 10 | 12 | 1 | 43.00 | 6.20 |
| 11 | -12 | 1 | 249.28 | 16.30 |
| 11 | -12 | 1 | 260.77 | 16.20 |
| 11 | 12 | 1 | 254.87 | 15.40 |
| 11 | 12 | 1 | 253.07 | 15.90 |
| 12 | -12 | 1 | 28.90 | 4.60 |
| 12 | 12 | 1 | 26.40 | 3.80 |
| 12 | 12 | 1 | 34.10 | 6.10 |
| 13 | -12 | 1 | 103.49 | 7.30 |

| | | | | |
|---|---|---|---|---|
| 13 | 12 | 1 | 106.69 | 8.50 |
| 13 | -13 | -1 | 46.30 | 4.50 |
| 13 | 13 | -1 | 55.59 | 4.60 |
| 12 | -13 | -1 | 105.89 | 7.70 |
| 12 | 13 | -1 | 107.99 | 7.10 |
| 11 | -13 | -1 | 19.80 | 3.50 |
| 11 | 13 | -1 | 24.20 | 4.00 |
| 10 | -13 | -1 | 602.54 | 35.30 |
| 10 | 13 | -1 | 646.54 | 35.30 |
| 10 | 13 | -1 | 608.44 | 36.40 |
| 9 | -13 | -1 | 37.90 | 6.80 |
| 9 | 13 | -1 | 23.80 | 4.30 |
| 9 | 13 | -1 | 20.00 | 3.40 |
| 8 | -13 | -1 | 181.78 | 13.20 |
| -8 | -13 | 1 | 198.58 | 12.80 |
| 8 | 13 | -1 | 167.48 | 13.80 |
| 8 | 13 | -1 | 175.18 | 12.40 |
| -7 | -13 | 1 | 302.57 | 19.60 |
| 7 | -13 | -1 | 327.07 | 19.90 |
| 7 | 13 | -1 | 341.77 | 25.60 |
| 7 | 13 | -1 | 296.37 | 19.80 |
| 7 | 13 | -1 | 338.67 | 19.10 |
| -6 | -13 | 1 | 828.42 | 45.50 |
| 6 | -13 | -1 | 841.82 | 45.60 |
| 6 | 13 | -1 | 795.22 | 44.90 |
| 6 | 13 | -1 | 775.92 | 49.10 |
| 5 | -13 | -1 | -7.90 | 8.80 |
| -5 | -13 | 1 | 4.00 | 3.50 |
| 5 | -13 | -1 | -5.90 | 3.50 |
| 5 | 13 | -1 | -5.00 | 7.10 |
| -5 | 13 | 1 | -5.20 | 5.60 |
| 5 | 13 | -1 | 0.80 | 2.70 |
| -4 | -13 | 1 | 885.81 | 49.70 |
| 4 | -13 | -1 | 856.81 | 49.99 |
| 4 | -13 | -1 | 910.91 | 50.79 |
| 4 | 13 | -1 | 831.22 | 52.59 |
| 4 | 13 | -1 | 905.11 | 49.10 |
| -4 | 13 | 1 | 935.31 | 50.99 |
| -3 | -13 | 1 | 72.39 | 8.50 |
| 3 | -13 | -1 | 75.29 | 8.70 |
| 3 | -13 | -1 | 70.79 | 11.30 |
| -3 | -13 | 1 | 61.09 | 8.70 |
| 3 | 13 | -1 | 65.49 | 11.00 |
| 3 | 13 | -1 | 67.79 | 7.00 |

| | | | | |
|---|---|---|---|---|
| -3 | 13 | 1 | 73.49 | 9.30 |
| 2 | -13 | -1 | 145.49 | 13.20 |
| -2 | -13 | 1 | 137.99 | 11.50 |
| 2 | -13 | -1 | 163.68 | 12.10 |
| -2 | -13 | 1 | 156.28 | 11.40 |
| -2 | 13 | 1 | 147.39 | 10.80 |
| 2 | 13 | -1 | 154.18 | 13.40 |
| -2 | 13 | 1 | 155.48 | 12.50 |
| 2 | 13 | -1 | 140.89 | 10.70 |
| 1 | -13 | -1 | 478.45 | 28.00 |
| -1 | -13 | 1 | 503.85 | 27.90 |
| -1 | -13 | 1 | 457.95 | 28.00 |
| 1 | -13 | -1 | 474.85 | 28.70 |
| -1 | 13 | 1 | 518.35 | 29.40 |
| 1 | 13 | -1 | 461.15 | 29.40 |
| 1 | 13 | -1 | 462.95 | 27.50 |
| -1 | 13 | 1 | 461.65 | 27.60 |
| 0 | -13 | 1 | 2820.72 | 150.98 |
| 0 | -13 | -1 | 2827.52 | 150.68 |
| 0 | -13 | 1 | 2819.42 | 150.58 |
| 0 | -13 | -1 | 2707.43 | 150.98 |
| 0 | 13 | 1 | 2719.33 | 152.08 |
| 0 | 13 | -1 | 2800.12 | 152.18 |
| 0 | 13 | 1 | 2664.23 | 150.58 |
| 0 | 13 | -1 | 2895.81 | 150.68 |
| -1 | -13 | -1 | 228.28 | 15.40 |
| 1 | -13 | 1 | 225.08 | 15.50 |
| 1 | -13 | 1 | 212.28 | 14.30 |
| -1 | -13 | -1 | 209.28 | 14.60 |
| -1 | 13 | -1 | 212.18 | 13.80 |
| 1 | 13 | 1 | 193.68 | 13.70 |
| 1 | 13 | 1 | 207.78 | 15.90 |
| -1 | 13 | -1 | 220.38 | 15.60 |
| 2 | -13 | 1 | 149.19 | 12.80 |
| -2 | -13 | -1 | 125.59 | 11.60 |
| 2 | -13 | 1 | 124.49 | 10.50 |
| -2 | 13 | -1 | 129.39 | 11.60 |
| 2 | 13 | 1 | 136.39 | 12.60 |
| 2 | 13 | 1 | 130.99 | 9.60 |
| 3 | -13 | 1 | 6.10 | 5.40 |
| 3 | 13 | 1 | 5.10 | 5.10 |
| -3 | 13 | -1 | 13.40 | 5.20 |
| 3 | 13 | 1 | 9.50 | 3.00 |
| 4 | -13 | 1 | 930.91 | 53.19 |

| | | | | |
|---|---|---|---|---|
| -4 | -13 | -1 | 970.10 | 53.49 |
| 4 | 13 | 1 | 953.80 | 53.09 |
| -4 | 13 | -1 | 1006.90 | 54.79 |
| 4 | 13 | 1 | 938.21 | 56.79 |
| 5 | -13 | 1 | 62.39 | 13.50 |
| 5 | -13 | 1 | 55.39 | 7.20 |
| -5 | -13 | -1 | 46.40 | 7.70 |
| 5 | 13 | 1 | 71.49 | 6.30 |
| -5 | 13 | -1 | 59.49 | 9.60 |
| -6 | -13 | -1 | 560.04 | 33.30 |
| 6 | -13 | 1 | 602.24 | 33.50 |
| 6 | 13 | 1 | 545.35 | 37.80 |
| 6 | 13 | 1 | 600.64 | 34.00 |
| 6 | 13 | 1 | 612.84 | 33.00 |
| 7 | -13 | 1 | 32.40 | 6.40 |
| -7 | -13 | -1 | 36.00 | 6.60 |
| 7 | 13 | 1 | 38.90 | 7.90 |
| 7 | 13 | 1 | 29.30 | 5.20 |
| 7 | 13 | 1 | 55.89 | 12.30 |
| -8 | -13 | -1 | 149.99 | 11.70 |
| 8 | -13 | 1 | 168.48 | 11.80 |
| 8 | 13 | 1 | 159.98 | 11.20 |
| 8 | 13 | 1 | 179.68 | 11.70 |
| 9 | -13 | 1 | 75.49 | 7.40 |
| 9 | 13 | 1 | 57.49 | 6.40 |
| 9 | 13 | 1 | 68.19 | 7.30 |
| 10 | -13 | 1 | 321.47 | 22.70 |
| 10 | 13 | 1 | 352.46 | 20.40 |
| 10 | 13 | 1 | 374.26 | 20.80 |
| 11 | -13 | 1 | -1.00 | 2.60 |
| 11 | 13 | 1 | 5.20 | 3.20 |
| 11 | 13 | 1 | 4.70 | 3.70 |
| 12 | -13 | 1 | 51.09 | 5.00 |
| 12 | 13 | 1 | 36.90 | 6.90 |
| 12 | -14 | -1 | 91.59 | 6.80 |
| 12 | 14 | -1 | 107.79 | 7.60 |
| 11 | -14 | -1 | 409.16 | 24.50 |
| 11 | 14 | -1 | 451.65 | 24.00 |
| 10 | -14 | -1 | 43.20 | 6.10 |
| 9 | -14 | -1 | 266.67 | 16.40 |
| 9 | 14 | -1 | 247.68 | 16.20 |
| 9 | 14 | -1 | 256.47 | 16.70 |
| -8 | -14 | 1 | 154.48 | 11.60 |
| 8 | -14 | -1 | 184.88 | 12.00 |

| | | | | |
|---|---|---|---|---|
| 8 | 14 | -1 | 162.38 | 11.20 |
| 8 | 14 | -1 | 153.98 | 11.10 |
| 7 | -14 | -1 | 220.58 | 14.40 |
| -7 | -14 | 1 | 214.88 | 13.90 |
| 7 | 14 | -1 | 194.58 | 22.50 |
| 7 | 14 | -1 | 219.38 | 13.80 |
| 7 | 14 | -1 | 207.48 | 14.10 |
| -6 | -14 | 1 | 182.88 | 12.80 |
| 6 | -14 | -1 | 192.48 | 13.30 |
| 6 | 14 | -1 | 192.88 | 12.20 |
| 6 | 14 | -1 | 170.88 | 20.70 |
| 5 | -14 | -1 | 1440.46 | 75.89 |
| -5 | -14 | 1 | 1335.17 | 75.09 |
| 5 | -14 | -1 | 1345.37 | 75.19 |
| 5 | 14 | -1 | 1354.46 | 74.69 |
| 4 | -14 | -1 | 192.28 | 12.90 |
| -4 | -14 | 1 | 178.08 | 12.60 |
| 4 | -14 | -1 | 174.48 | 14.60 |
| 4 | 14 | -1 | 179.78 | 11.80 |
| -4 | 14 | 1 | 185.18 | 15.20 |
| 4 | 14 | -1 | 147.19 | 17.30 |
| -3 | -14 | 1 | 184.58 | 12.30 |
| 3 | -14 | -1 | 158.88 | 12.30 |
| 3 | -14 | -1 | 168.38 | 14.30 |
| 3 | 14 | -1 | 170.78 | 16.20 |
| 3 | 14 | -1 | 181.08 | 11.80 |
| -3 | 14 | 1 | 172.78 | 14.40 |
| -3 | 14 | 1 | 158.58 | 12.00 |
| 2 | -14 | -1 | 99.49 | 10.10 |
| -2 | -14 | 1 | 125.49 | 9.80 |
| 2 | -14 | -1 | 115.79 | 11.60 |
| -2 | -14 | 1 | 122.99 | 10.10 |
| -2 | 14 | 1 | 115.29 | 9.30 |
| -2 | 14 | 1 | 146.99 | 18.50 |
| 2 | 14 | -1 | 116.19 | 9.00 |
| 2 | 14 | -1 | 100.79 | 12.40 |
| -1 | -14 | 1 | 911.21 | 51.59 |
| 1 | -14 | -1 | 909.31 | 52.09 |
| 1 | -14 | -1 | 903.41 | 51.39 |
| -1 | -14 | 1 | 917.81 | 51.39 |
| 1 | 14 | -1 | 928.41 | 51.29 |
| 1 | 14 | -1 | 1041.80 | 58.09 |
| -1 | 14 | 1 | 868.71 | 51.29 |
| 0 | -14 | -1 | 61.89 | 8.40 |

| | | | | |
|---|---|---|---|---|
| 0 | -14 | -1 | 68.89 | 8.90 |
| 0 | -14 | 1 | 71.69 | 8.70 |
| 0 | -14 | 1 | 92.59 | 8.10 |
| 0 | 14 | -1 | 70.09 | 7.20 |
| 0 | 14 | 1 | 69.89 | 7.00 |
| -1 | -14 | -1 | 789.02 | 45.90 |
| 1 | -14 | 1 | 861.71 | 46.60 |
| -1 | -14 | -1 | 822.52 | 46.10 |
| 1 | -14 | 1 | 812.92 | 45.70 |
| -1 | 14 | -1 | 818.02 | 45.70 |
| 1 | 14 | 1 | 830.22 | 45.70 |
| 1 | 14 | 1 | 789.52 | 49.90 |
| 2 | -14 | 1 | 44.20 | 7.10 |
| -2 | -14 | -1 | 47.40 | 7.20 |
| -2 | -14 | -1 | 46.30 | 8.80 |
| 2 | 14 | 1 | 50.19 | 6.00 |
| -2 | 14 | -1 | 49.80 | 9.20 |
| -2 | 14 | -1 | 44.00 | 6.20 |
| 2 | 14 | 1 | 48.70 | 10.00 |
| 3 | -14 | 1 | 56.49 | 10.10 |
| -3 | -14 | -1 | 61.59 | 7.20 |
| 3 | -14 | 1 | 41.60 | 7.30 |
| 3 | 14 | 1 | 55.89 | 11.20 |
| 3 | 14 | 1 | 70.79 | 6.50 |
| -3 | 14 | -1 | 80.79 | 10.20 |
| 4 | -14 | 1 | 336.97 | 21.00 |
| -4 | -14 | -1 | 349.47 | 21.20 |
| 4 | -14 | 1 | 332.57 | 23.10 |
| -4 | 14 | -1 | 363.66 | 23.80 |
| 4 | 14 | 1 | 377.06 | 20.80 |
| 4 | 14 | 1 | 342.37 | 25.60 |
| -5 | -14 | -1 | 961.30 | 53.29 |
| 5 | -14 | 1 | 944.61 | 53.19 |
| 5 | -14 | 1 | 1053.39 | 54.59 |
| 5 | 14 | 1 | 826.52 | 56.89 |
| 5 | 14 | 1 | 1004.90 | 52.99 |
| 6 | -14 | 1 | 38.80 | 6.40 |
| -6 | -14 | -1 | 47.10 | 6.50 |
| 6 | 14 | 1 | 43.10 | 6.00 |
| 6 | 14 | 1 | 43.80 | 5.60 |
| 6 | 14 | 1 | 39.30 | 11.30 |
| -7 | -14 | -1 | 135.29 | 10.00 |
| 7 | -14 | 1 | 123.69 | 10.00 |
| 7 | 14 | 1 | 137.49 | 9.40 |

| | | | | |
|---|---|---|---|---|
| 7 | 14 | 1 | 125.69 | 10.60 |
| 7 | 14 | 1 | 115.79 | 22.60 |
| 8 | -14 | 1 | 30.00 | 6.00 |
| -8 | -14 | -1 | 29.70 | 4.20 |
| 8 | 14 | 1 | 32.30 | 5.20 |
| 8 | 14 | 1 | 28.50 | 4.20 |
| 9 | -14 | 1 | 193.08 | 12.90 |
| 9 | 14 | 1 | 182.58 | 11.90 |
| 9 | 14 | 1 | 171.28 | 11.80 |
| 10 | -14 | 1 | 18.50 | 3.50 |
| 10 | 14 | 1 | 15.60 | 3.80 |
| 10 | 14 | 1 | 14.50 | 3.80 |
| 11 | -14 | 1 | 289.57 | 16.40 |
| 11 | 14 | 1 | 262.47 | 16.90 |
| 12 | -14 | 1 | 50.59 | 4.50 |
| 11 | -15 | -1 | 28.00 | 3.80 |
| 11 | 15 | -1 | 19.90 | 2.90 |
| 10 | -15 | -1 | 234.78 | 14.30 |
| 10 | 15 | -1 | 230.28 | 13.50 |
| 9 | -15 | -1 | 28.00 | 5.30 |
| 9 | 15 | -1 | 14.70 | 2.60 |
| -8 | -15 | 1 | 7.00 | 3.60 |
| 8 | -15 | -1 | 8.10 | 3.70 |
| 8 | 15 | -1 | 14.50 | 4.00 |
| 8 | 15 | -1 | 16.90 | 3.90 |
| 7 | -15 | -1 | 263.57 | 16.70 |
| -7 | -15 | 1 | 260.37 | 16.20 |
| 7 | 15 | -1 | 246.68 | 16.20 |
| 7 | 15 | -1 | 284.27 | 16.30 |
| 6 | -15 | -1 | 524.75 | 28.90 |
| -6 | -15 | 1 | 533.05 | 28.70 |
| 6 | 15 | -1 | 502.45 | 28.30 |
| 6 | 15 | -1 | 399.26 | 37.30 |
| -5 | -15 | 1 | 2.00 | 3.20 |
| 5 | -15 | -1 | -0.50 | 5.10 |
| 5 | -15 | -1 | 2.40 | 3.50 |
| 5 | 15 | -1 | 5.10 | 3.70 |
| 5 | 15 | -1 | 2.90 | 9.20 |
| 4 | -15 | -1 | 168.08 | 11.90 |
| 4 | -15 | -1 | 188.38 | 13.50 |
| -4 | -15 | 1 | 165.28 | 11.50 |
| 4 | 15 | -1 | 165.78 | 11.10 |
| -4 | 15 | 1 | 148.49 | 16.50 |
| 4 | 15 | -1 | 116.39 | 17.60 |

| | | | | |
|---|---|---|---|---|
| -4 | 15 | 1 | 162.88 | 12.00 |
| 3 | -15 | -1 | 134.09 | 10.50 |
| -3 | -15 | 1 | 132.39 | 10.20 |
| 3 | -15 | -1 | 141.69 | 12.10 |
| 3 | 15 | -1 | 135.29 | 9.70 |
| 3 | 15 | -1 | 133.19 | 15.90 |
| -3 | 15 | 1 | 138.69 | 10.30 |
| -3 | 15 | 1 | 123.29 | 14.00 |
| -2 | -15 | 1 | 11.60 | 3.90 |
| 2 | -15 | -1 | 13.30 | 4.80 |
| 2 | -15 | -1 | 25.00 | 4.20 |
| -2 | -15 | 1 | 22.00 | 4.10 |
| 2 | 15 | -1 | 9.60 | 6.20 |
| -2 | 15 | 1 | 16.40 | 3.50 |
| -2 | 15 | 1 | 17.60 | 5.80 |
| 2 | 15 | -1 | 28.50 | 5.60 |
| 1 | -15 | -1 | 204.28 | 14.30 |
| 1 | -15 | -1 | 193.78 | 13.50 |
| -1 | -15 | 1 | 186.68 | 13.70 |
| -1 | -15 | 1 | 198.98 | 13.20 |
| 1 | 15 | -1 | 203.48 | 13.10 |
| 1 | 15 | -1 | 191.88 | 16.80 |
| -1 | 15 | 1 | 193.58 | 12.90 |
| -1 | 15 | 1 | 199.68 | 15.90 |
| 0 | -15 | -1 | 1016.20 | 55.99 |
| 0 | -15 | 1 | 1032.00 | 55.99 |
| 0 | -15 | 1 | 987.10 | 55.29 |
| 0 | -15 | -1 | 975.40 | 55.49 |
| 0 | 15 | 1 | 1015.60 | 57.59 |
| 0 | 15 | -1 | 1013.60 | 57.69 |
| 0 | 15 | 1 | 959.10 | 55.39 |
| 0 | 15 | -1 | 1005.40 | 55.39 |
| 1 | -15 | 1 | 120.59 | 11.20 |
| -1 | -15 | -1 | 126.49 | 10.40 |
| -1 | -15 | -1 | 133.19 | 11.00 |
| 1 | -15 | 1 | 133.09 | 9.80 |
| -1 | 15 | -1 | 124.59 | 13.00 |
| 1 | 15 | 1 | 120.09 | 13.00 |
| 1 | 15 | 1 | 132.59 | 9.50 |
| -1 | 15 | -1 | 123.29 | 9.50 |
| 2 | -15 | 1 | 19.10 | 3.40 |
| 2 | -15 | 1 | 28.20 | 5.70 |
| 2 | 15 | 1 | 10.40 | 5.80 |
| -2 | 15 | -1 | 42.90 | 10.50 |

| | | | | |
|---|---|---|---|---|
| -2 | 15 | -1 | 15.90 | 3.40 |
| -3 | -15 | -1 | 123.39 | 9.40 |
| 3 | -15 | 1 | 94.89 | 11.80 |
| 3 | -15 | 1 | 114.79 | 9.20 |
| 3 | 15 | 1 | 102.99 | 14.80 |
| -3 | 15 | -1 | 126.09 | 14.10 |
| 3 | 15 | 1 | 125.89 | 8.90 |
| -3 | 15 | -1 | 113.29 | 9.30 |
| 4 | -15 | 1 | 374.86 | 23.30 |
| 4 | -15 | 1 | 442.16 | 25.00 |
| -4 | -15 | -1 | 363.56 | 23.40 |
| 4 | 15 | 1 | 415.96 | 23.20 |
| -4 | 15 | -1 | 393.06 | 27.00 |
| -4 | 15 | -1 | 403.16 | 23.70 |
| 5 | -15 | 1 | 24.70 | 6.10 |
| 5 | -15 | 1 | 16.40 | 3.50 |
| -5 | -15 | -1 | 9.10 | 3.00 |
| 5 | 15 | 1 | 24.40 | 5.20 |
| 5 | 15 | 1 | 15.30 | 9.70 |
| -6 | -15 | -1 | 332.37 | 19.90 |
| 6 | -15 | 1 | 336.37 | 20.00 |
| 6 | 15 | 1 | 333.77 | 19.60 |
| 6 | 15 | 1 | 332.27 | 20.40 |
| -7 | -15 | -1 | 186.18 | 12.40 |
| 7 | -15 | 1 | 199.78 | 12.60 |
| 7 | 15 | 1 | 171.28 | 14.50 |
| 7 | 15 | 1 | 197.08 | 12.20 |
| -8 | -15 | -1 | 80.89 | 7.50 |
| 8 | 15 | 1 | 87.09 | 6.90 |
| 8 | 15 | 1 | 66.09 | 7.50 |
| 9 | -15 | 1 | 70.99 | 6.70 |
| 9 | 15 | 1 | 67.19 | 6.70 |
| 9 | 15 | 1 | 66.09 | 8.90 |
| 10 | -15 | 1 | 269.57 | 15.70 |
| 10 | 15 | 1 | 256.47 | 16.10 |
| 11 | -15 | 1 | -0.10 | 1.90 |
| 10 | -16 | -1 | 3.60 | 2.20 |
| -10 | 16 | 1 | 4.70 | 2.60 |
| 10 | 16 | -1 | 5.10 | 2.60 |
| 9 | -16 | -1 | 162.68 | 10.90 |
| 9 | 16 | -1 | 160.08 | 10.10 |
| -9 | 16 | 1 | 175.28 | 11.40 |
| 8 | -16 | -1 | 68.39 | 7.30 |
| -8 | -16 | 1 | 69.59 | 7.00 |

| | | | | |
|---|---|---|---|---|
| 8 | 16 | -1 | 68.39 | 5.90 |
| -8 | 16 | 1 | 71.79 | 7.40 |
| -7 | -16 | 1 | 55.09 | 6.70 |
| 7 | -16 | -1 | 53.89 | 7.50 |
| -7 | 16 | 1 | 49.70 | 7.00 |
| 7 | 16 | -1 | 63.99 | 6.10 |
| 7 | 16 | -1 | 51.39 | 7.70 |
| 6 | -16 | -1 | 154.38 | 11.10 |
| -6 | -16 | 1 | 143.59 | 10.40 |
| 6 | 16 | -1 | 138.19 | 10.30 |
| 6 | 16 | -1 | 140.39 | 10.50 |
| -6 | 16 | 1 | 153.68 | 11.50 |
| 5 | -16 | -1 | 538.95 | 30.60 |
| 5 | -16 | -1 | 517.75 | 29.90 |
| -5 | -16 | 1 | 518.95 | 29.80 |
| -5 | 16 | 1 | 527.45 | 30.50 |
| 5 | 16 | -1 | 500.65 | 29.30 |
| -4 | -16 | 1 | 6.60 | 2.80 |
| 4 | -16 | -1 | 0.60 | 4.40 |
| 4 | -16 | -1 | 8.20 | 3.50 |
| 4 | 16 | -1 | 4.10 | 3.20 |
| -4 | 16 | 1 | 4.10 | 3.30 |
| 3 | -16 | -1 | 9.70 | 3.40 |
| 3 | -16 | -1 | 9.00 | 4.20 |
| -3 | -16 | 1 | 4.20 | 2.70 |
| -3 | 16 | 1 | 4.20 | 3.20 |
| 3 | 16 | -1 | 2.40 | 3.30 |
| -3 | 16 | 1 | -4.60 | 7.90 |
| 3 | 16 | -1 | 1.20 | 8.70 |
| 2 | -16 | -1 | 421.26 | 25.20 |
| -2 | -16 | 1 | 408.86 | 25.20 |
| 2 | -16 | -1 | 460.45 | 26.30 |
| 2 | 16 | -1 | 464.15 | 25.30 |
| -2 | 16 | 1 | 416.06 | 25.30 |
| -2 | 16 | 1 | 427.06 | 28.30 |
| 2 | 16 | -1 | 418.56 | 29.40 |
| 1 | -16 | -1 | 565.64 | 31.30 |
| 1 | -16 | -1 | 535.95 | 30.60 |
| -1 | -16 | 1 | 541.75 | 30.80 |
| -1 | -16 | 1 | 557.14 | 30.50 |
| -1 | 16 | 1 | 493.05 | 33.00 |
| -1 | 16 | 1 | 490.55 | 33.40 |
| 0 | -16 | 1 | 21.80 | 3.50 |
| 0 | -16 | 1 | 12.30 | 3.80 |

| | | | | |
|---|---|---|---|---|
| 0 | -16 | -1 | 15.30 | 4.10 |
| 0 | -16 | -1 | 20.20 | 4.00 |
| 0 | 16 | -1 | 17.80 | 6.40 |
| 0 | 16 | 1 | 11.30 | 7.20 |
| -1 | -16 | -1 | 338.07 | 20.10 |
| 1 | -16 | 1 | 334.57 | 20.60 |
| 1 | -16 | 1 | 298.17 | 19.40 |
| -1 | -16 | -1 | 336.17 | 20.10 |
| -1 | 16 | -1 | 291.97 | 22.70 |
| 1 | 16 | 1 | 307.37 | 23.20 |
| 1 | 16 | 1 | 339.77 | 21.30 |
| -1 | 16 | -1 | 368.96 | 21.10 |
| 2 | -16 | 1 | 250.97 | 15.30 |
| 2 | -16 | 1 | 263.07 | 17.00 |
| -2 | -16 | -1 | 243.98 | 15.60 |
| 2 | 16 | 1 | 272.27 | 15.60 |
| -2 | 16 | -1 | 245.38 | 15.70 |
| 2 | 16 | 1 | 213.08 | 20.10 |
| -2 | 16 | -1 | 215.58 | 19.40 |
| 3 | -16 | 1 | 114.99 | 9.20 |
| -3 | -16 | -1 | 124.39 | 9.30 |
| 3 | -16 | 1 | 126.79 | 11.70 |
| -3 | 16 | -1 | 106.89 | 15.80 |
| 3 | 16 | 1 | 122.29 | 17.30 |
| 3 | 16 | 1 | 132.59 | 9.10 |
| -3 | 16 | -1 | 121.39 | 9.60 |
| 4 | -16 | 1 | 123.69 | 11.70 |
| 4 | -16 | 1 | 126.49 | 9.40 |
| -4 | -16 | -1 | 132.79 | 9.50 |
| 4 | 16 | 1 | 127.89 | 9.30 |
| -4 | 16 | -1 | 132.59 | 10.10 |
| -5 | -16 | -1 | 605.44 | 35.90 |
| 5 | -16 | 1 | 669.93 | 37.10 |
| 5 | -16 | 1 | 633.94 | 35.90 |
| -5 | 16 | -1 | 659.23 | 36.50 |
| 5 | 16 | 1 | 644.04 | 35.70 |
| 5 | 16 | 1 | 622.64 | 36.30 |
| 6 | -16 | 1 | 69.29 | 6.80 |
| -6 | -16 | -1 | 59.49 | 6.90 |
| -6 | 16 | -1 | 65.39 | 7.60 |
| 6 | 16 | 1 | 66.59 | 8.30 |
| 6 | 16 | 1 | 62.99 | 6.00 |
| 7 | -16 | 1 | 3.30 | 2.80 |
| -7 | -16 | -1 | -2.90 | 2.60 |

| | | | | |
|---|---|---|---|---|
| -7 | 16 | -1 | 4.10 | 3.80 |
| 7 | 16 | 1 | 0.80 | 2.60 |
| 7 | 16 | 1 | 0.60 | 3.40 |
| 8 | -16 | 1 | 72.79 | 6.70 |
| -8 | -16 | -1 | 74.39 | 6.80 |
| 8 | 16 | 1 | 67.99 | 6.60 |
| 9 | -16 | 1 | 287.17 | 17.00 |
| 9 | 16 | 1 | 291.97 | 17.40 |
| 10 | -16 | 1 | 31.30 | 4.00 |
| -10 | 16 | -1 | 27.40 | 5.00 |
| 9 | -17 | -1 | 15.30 | 2.80 |
| -9 | 17 | 1 | 19.80 | 3.50 |
| 9 | 17 | -1 | 17.70 | 3.00 |
| 8 | -17 | -1 | 15.60 | 3.20 |
| -8 | -17 | 1 | 25.60 | 5.40 |
| 8 | 17 | -1 | 23.60 | 3.90 |
| -8 | 17 | 1 | 22.60 | 4.10 |
| -7 | -17 | 1 | 396.46 | 22.90 |
| 7 | -17 | -1 | 406.66 | 23.20 |
| -7 | 17 | 1 | 393.56 | 23.70 |
| 7 | 17 | -1 | 397.86 | 22.50 |
| -6 | -17 | 1 | 396.86 | 22.70 |
| 6 | -17 | -1 | 399.56 | 23.10 |
| 6 | 17 | -1 | 383.06 | 22.50 |
| -6 | 17 | 1 | 395.46 | 23.70 |
| -5 | -17 | 1 | 12.10 | 3.70 |
| 5 | -17 | -1 | 17.10 | 4.10 |
| 5 | -17 | -1 | 7.50 | 4.60 |
| 5 | 17 | -1 | 12.90 | 3.60 |
| -5 | 17 | 1 | 13.70 | 3.60 |
| -4 | -17 | 1 | 404.16 | 25.00 |
| 4 | -17 | -1 | 426.26 | 28.70 |
| 4 | -17 | -1 | 482.95 | 26.20 |
| -4 | 17 | 1 | 422.76 | 25.50 |
| 4 | 17 | -1 | 439.76 | 24.90 |
| 3 | -17 | -1 | 166.08 | 13.00 |
| -3 | -17 | 1 | 182.58 | 11.60 |
| 3 | -17 | -1 | 167.88 | 12.00 |
| 3 | 17 | -1 | 175.68 | 11.50 |
| -3 | 17 | 1 | 166.48 | 11.80 |
| 2 | -17 | -1 | 92.89 | 9.90 |
| 2 | -17 | -1 | 113.09 | 9.10 |
| -2 | -17 | 1 | 104.09 | 8.40 |
| 2 | 17 | -1 | 99.99 | 8.50 |

| | | | | |
|---|---|---|---|---|
| -2 | 17 | 1 | 109.79 | 8.30 |
| 1 | -17 | -1 | 210.98 | 13.20 |
| 1 | -17 | -1 | 191.28 | 13.50 |
| -1 | -17 | 1 | 195.68 | 13.10 |
| -1 | -17 | 1 | 186.88 | 12.70 |
| -1 | 17 | 1 | 178.98 | 12.60 |
| 1 | 17 | -1 | 198.88 | 12.70 |
| 0 | -17 | 1 | 1064.59 | 59.39 |
| 0 | -17 | -1 | 1100.49 | 59.39 |
| 0 | -17 | -1 | 1072.79 | 58.99 |
| 0 | -17 | 1 | 1082.09 | 58.79 |
| 0 | 17 | -1 | 1056.89 | 58.79 |
| 0 | 17 | 1 | 1041.40 | 58.79 |
| 1 | -17 | 1 | 111.69 | 9.80 |
| -1 | -17 | -1 | 113.49 | 8.90 |
| 1 | -17 | 1 | 99.59 | 8.30 |
| -1 | 17 | -1 | 108.79 | 8.40 |
| 1 | 17 | 1 | 106.19 | 8.40 |
| 2 | -17 | 1 | 7.90 | 4.00 |
| -2 | -17 | -1 | 10.80 | 2.90 |
| 2 | -17 | 1 | 7.70 | 2.70 |
| -2 | 17 | -1 | 7.80 | 3.10 |
| 2 | 17 | 1 | 6.90 | 2.90 |
| 3 | -17 | 1 | 321.37 | 19.60 |
| -3 | -17 | -1 | 329.27 | 19.70 |
| 3 | -17 | 1 | 387.46 | 21.20 |
| -3 | 17 | -1 | 309.67 | 19.70 |
| 3 | 17 | 1 | 323.07 | 19.50 |
| -4 | -17 | -1 | 580.94 | 34.10 |
| 4 | -17 | 1 | 595.04 | 34.30 |
| 4 | -17 | 1 | 645.74 | 35.50 |
| 4 | 17 | 1 | 605.14 | 34.00 |
| -4 | 17 | -1 | 616.74 | 34.40 |
| 5 | -17 | 1 | 15.20 | 4.80 |
| -5 | -17 | -1 | 11.10 | 2.90 |
| 5 | -17 | 1 | 5.60 | 2.80 |
| 5 | 17 | 1 | 18.50 | 4.10 |
| -5 | 17 | -1 | 13.60 | 3.60 |
| 5 | 17 | 1 | 14.50 | 3.00 |
| -6 | -17 | -1 | 286.07 | 17.40 |
| 6 | -17 | 1 | 306.17 | 17.70 |
| 6 | 17 | 1 | 296.07 | 17.40 |
| -6 | 17 | -1 | 301.77 | 18.20 |
| 6 | 17 | 1 | 294.77 | 17.90 |

| | | | | |
|---|---|---|---|---|
| -7 | -17 | -1 | 264.37 | 15.40 |
| 7 | -17 | 1 | 263.67 | 15.50 |
| 7 | 17 | 1 | 243.48 | 16.30 |
| -7 | 17 | -1 | 256.97 | 16.20 |
| -8 | -17 | -1 | 73.79 | 6.30 |
| 8 | -17 | 1 | 74.69 | 6.00 |
| -8 | 17 | -1 | 56.19 | 6.90 |
| 8 | 17 | 1 | 65.69 | 6.90 |
| 9 | -17 | 1 | 32.10 | 4.00 |
| -9 | 17 | -1 | 27.30 | 5.40 |
| -8 | -18 | 1 | 96.69 | 7.50 |
| 8 | -18 | -1 | 99.79 | 6.90 |
| -8 | 18 | 1 | 88.49 | 7.60 |
| 8 | 18 | -1 | 99.89 | 6.80 |
| -7 | -18 | 1 | 41.40 | 5.10 |
| 7 | -18 | -1 | 38.80 | 5.10 |
| 7 | 18 | -1 | 43.50 | 4.60 |
| -7 | 18 | 1 | 33.60 | 5.70 |
| 6 | -18 | -1 | 32.90 | 5.30 |
| -6 | -18 | 1 | 36.40 | 5.00 |
| -6 | 18 | 1 | 37.60 | 6.00 |
| 6 | 18 | -1 | 24.90 | 4.60 |
| 5 | -18 | -1 | 832.02 | 44.80 |
| 5 | -18 | -1 | 809.82 | 44.70 |
| -5 | -18 | 1 | 796.42 | 44.40 |
| -5 | 18 | 1 | 802.32 | 45.00 |
| 5 | 18 | -1 | 785.12 | 44.30 |
| 4 | -18 | -1 | 62.89 | 8.00 |
| 4 | -18 | -1 | 72.59 | 7.30 |
| -4 | -18 | 1 | 72.99 | 6.40 |
| -4 | 18 | 1 | 71.29 | 7.10 |
| 3 | -18 | -1 | 31.80 | 6.20 |
| -3 | -18 | 1 | 39.30 | 5.50 |
| 3 | -18 | -1 | 48.00 | 7.10 |
| 3 | 18 | -1 | 47.70 | 5.90 |
| -3 | 18 | 1 | 44.60 | 5.60 |
| -2 | -18 | 1 | 275.17 | 16.90 |
| 2 | -18 | -1 | 294.97 | 17.60 |
| 2 | -18 | -1 | 266.97 | 17.00 |
| -2 | 18 | 1 | 278.07 | 16.70 |
| 2 | 18 | -1 | 261.77 | 16.60 |
| -1 | -18 | 1 | 460.25 | 32.60 |
| 1 | -18 | -1 | 574.94 | 30.80 |
| -1 | 18 | 1 | 556.84 | 30.50 |

| | | | | |
|---|---|---|---|---|
| 1 | 18 | -1 | 557.64 | 30.40 |
| 0 | -18 | 1 | 12.60 | 3.40 |
| 0 | -18 | -1 | 2.30 | 4.30 |
| 0 | -18 | 1 | 8.30 | 6.00 |
| 0 | 18 | 1 | 5.20 | 2.80 |
| 0 | 18 | -1 | 7.90 | 3.20 |
| -1 | -18 | -1 | 390.76 | 25.20 |
| 1 | -18 | 1 | 428.46 | 24.80 |
| 1 | -18 | 1 | 420.46 | 23.80 |
| 1 | 18 | 1 | 407.96 | 23.60 |
| -1 | 18 | -1 | 411.76 | 23.70 |
| 2 | -18 | 1 | 270.77 | 16.90 |
| 2 | -18 | 1 | 253.67 | 15.70 |
| -2 | -18 | -1 | 270.57 | 16.10 |
| 2 | 18 | 1 | 245.78 | 15.50 |
| -2 | 18 | -1 | 258.67 | 15.80 |
| 3 | -18 | 1 | 27.30 | 4.90 |
| -3 | -18 | -1 | 22.80 | 4.50 |
| 3 | -18 | 1 | 32.70 | 6.90 |
| -3 | 18 | -1 | 16.80 | 3.50 |
| 3 | 18 | 1 | 22.90 | 4.50 |
| 4 | -18 | 1 | 81.79 | 8.50 |
| 4 | -18 | 1 | 83.59 | 6.70 |
| -4 | -18 | -1 | 62.29 | 6.20 |
| 4 | 18 | 1 | 71.99 | 8.50 |
| -4 | 18 | -1 | 75.09 | 7.10 |
| 4 | 18 | 1 | 70.49 | 6.10 |
| 5 | -18 | 1 | 453.75 | 27.80 |
| 5 | -18 | 1 | 483.05 | 27.10 |
| -5 | -18 | -1 | 461.45 | 27.00 |
| -5 | 18 | -1 | 484.95 | 27.40 |
| 5 | 18 | 1 | 484.65 | 27.80 |
| 5 | 18 | 1 | 519.25 | 29.10 |
| 6 | -18 | 1 | 89.39 | 6.90 |
| -6 | -18 | -1 | 84.99 | 6.80 |
| 6 | 18 | 1 | 99.69 | 7.30 |
| -6 | 18 | -1 | 89.69 | 7.80 |
| 7 | -18 | 1 | 2.20 | 2.10 |
| -7 | -18 | -1 | 8.20 | 2.30 |
| -7 | 18 | -1 | 2.50 | 3.20 |
| 8 | -18 | 1 | 72.09 | 5.20 |
| -8 | -18 | -1 | 62.49 | 6.00 |
| -8 | 18 | -1 | 50.89 | 6.40 |
| -6 | -19 | 1 | 313.77 | 17.60 |

| | | | | |
|---|---|---|---|---|
| 6 | -19 | -1 | 295.57 | 17.70 |
| 6 | 19 | -1 | 318.97 | 17.50 |
| -6 | 19 | 1 | 297.77 | 18.20 |
| -5 | -19 | 1 | 27.20 | 4.10 |
| 5 | -19 | -1 | 30.40 | 5.30 |
| -5 | 19 | 1 | 32.50 | 5.40 |
| 5 | 19 | -1 | 34.10 | 4.70 |
| 4 | -19 | -1 | 201.68 | 13.20 |
| 4 | -19 | -1 | 214.88 | 13.60 |
| -4 | -19 | 1 | 204.18 | 12.60 |
| -4 | 19 | 1 | 216.18 | 13.30 |
| 4 | 19 | -1 | 212.68 | 12.90 |
| 3 | -19 | -1 | 127.29 | 9.90 |
| 3 | -19 | -1 | 149.59 | 10.20 |
| -3 | -19 | 1 | 135.49 | 9.20 |
| -3 | 19 | 1 | 141.89 | 10.10 |
| 2 | -19 | -1 | 74.89 | 7.10 |
| 2 | -19 | -1 | 64.79 | 7.30 |
| -2 | -19 | 1 | 74.59 | 6.30 |
| -2 | 19 | 1 | 72.29 | 6.40 |
| -1 | -19 | 1 | 70.59 | 6.50 |
| 1 | -19 | -1 | 61.49 | 7.00 |
| -1 | 19 | 1 | 62.39 | 6.10 |
| 0 | -19 | -1 | 514.35 | 29.70 |
| 0 | -19 | 1 | 535.85 | 29.80 |
| 0 | 19 | -1 | 550.14 | 29.50 |
| 0 | 19 | 1 | 491.55 | 29.20 |
| -1 | -19 | -1 | 167.58 | 10.70 |
| 1 | -19 | 1 | 166.98 | 11.40 |
| -1 | 19 | -1 | 150.08 | 10.70 |
| 1 | 19 | 1 | 155.58 | 10.20 |
| -2 | -19 | -1 | 10.50 | 2.40 |
| 2 | -19 | 1 | 14.30 | 3.60 |
| 2 | -19 | 1 | 10.90 | 2.60 |
| -2 | 19 | -1 | 15.30 | 3.50 |
| 2 | 19 | 1 | 9.00 | 3.60 |
| 3 | -19 | 1 | 57.29 | 5.80 |
| 3 | -19 | 1 | 70.29 | 7.40 |
| -3 | -19 | -1 | 67.49 | 5.60 |
| -3 | 19 | -1 | 74.49 | 6.70 |
| 3 | 19 | 1 | 58.89 | 5.70 |
| 3 | 19 | 1 | 68.89 | 8.60 |
| 4 | -19 | 1 | 310.17 | 19.00 |
| 4 | -19 | 1 | 324.47 | 18.30 |

| | | | | |
|---|---|---|---|---|
| -4 | -19 | -1 | 318.77 | 18.20 |
| -4 | 19 | -1 | 316.77 | 18.70 |
| 4 | 19 | 1 | 314.37 | 18.20 |
| 5 | -19 | 1 | 4.10 | 2.30 |
| -5 | -19 | -1 | 3.50 | 1.90 |
| -5 | 19 | -1 | 7.80 | 3.50 |
| -6 | -19 | -1 | 172.88 | 10.60 |
| 6 | -19 | 1 | 179.08 | 10.70 |
| -6 | 19 | -1 | 172.28 | 11.60 |
| 4 | -20 | -1 | 71.09 | 7.30 |
| -4 | -20 | 1 | 75.19 | 5.70 |
| 4 | 20 | -1 | 88.79 | 6.70 |
| -4 | 20 | 1 | 72.19 | 6.60 |
| -3 | -20 | 1 | 18.60 | 3.40 |
| 3 | -20 | -1 | 16.10 | 4.20 |
| 3 | 20 | -1 | 15.50 | 4.90 |
| -3 | 20 | 1 | 14.40 | 3.30 |
| -2 | -20 | 1 | 106.39 | 7.40 |
| 2 | -20 | -1 | 108.39 | 7.90 |
| -2 | 20 | 1 | 101.19 | 7.70 |
| 1 | -20 | -1 | 216.38 | 12.90 |
| -1 | -20 | 1 | 207.08 | 12.70 |
| -1 | 20 | 1 | 194.68 | 12.50 |
| 0 | -20 | -1 | 1.60 | 2.40 |
| 0 | -20 | 1 | 5.20 | 2.50 |
| 0 | 20 | 1 | 9.00 | 2.80 |
| 1 | -20 | 1 | 102.29 | 8.00 |
| -1 | -20 | -1 | 100.39 | 7.40 |
| 1 | 20 | 1 | 105.59 | 7.30 |
| 2 | -20 | 1 | 163.18 | 10.10 |
| -2 | -20 | -1 | 154.98 | 9.50 |
| 2 | 20 | 1 | 123.69 | 9.50 |
| -3 | -20 | -1 | 33.10 | 3.80 |
| 3 | -20 | 1 | 21.60 | 5.20 |
| 17 | 0 | -2 | -0.10 | 1.50 |
| 17 | 0 | -2 | 1.50 | 3.20 |
| 16 | 0 | -2 | 891.11 | 50.09 |
| 16 | 0 | -2 | 925.71 | 49.70 |
| 15 | 0 | -2 | -1.70 | 2.70 |
| 15 | 0 | -2 | -2.50 | 3.60 |
| 14 | 0 | -2 | 1737.73 | 96.59 |
| 14 | 0 | -2 | 1732.43 | 96.09 |
| 14 | 0 | -2 | 1828.42 | 97.19 |
| 13 | 0 | -2 | 3.10 | 4.50 |

| | | | | |
|---|---|---|---|---|
| 13 | 0 | -2 | -0.50 | 2.70 |
| 13 | 0 | -2 | -4.90 | 3.40 |
| 12 | 0 | -2 | 1413.96 | 75.99 |
| 12 | 0 | -2 | 1387.86 | 76.39 |
| 12 | 0 | -2 | 1351.76 | 77.39 |
| 10 | 0 | -2 | 6766.42 | 341.57 |
| 10 | 0 | -2 | 5668.13 | 341.67 |
| 10 | 0 | -2 | 6581.94 | 341.07 |
| 9 | 0 | -2 | 22.20 | 7.20 |
| 9 | 0 | -2 | 23.80 | 5.00 |
| 8 | 0 | -2 | 135.09 | 11.10 |
| 4 | 0 | -2 | 3639.84 | 187.48 |
| 4 | 0 | -2 | 3359.46 | 187.58 |
| -3 | 0 | 2 | 2.30 | 1.50 |
| -2 | 0 | 2 | 567.54 | 31.10 |
| -1 | 0 | 2 | 0.50 | 1.50 |
| 0 | 0 | 2 | 7899.21 | 423.56 |
| 1 | 0 | 2 | 1.70 | 2.00 |
| 3 | 0 | 2 | 3.80 | 2.50 |
| 4 | 0 | 2 | 169.28 | 10.90 |
| 5 | 0 | 2 | 0.30 | 2.50 |
| 6 | 0 | 2 | 2826.52 | 152.18 |
| 7 | 0 | 2 | 5.00 | 4.10 |
| 8 | 0 | 2 | 108.09 | 9.90 |
| 9 | 0 | 2 | -0.60 | 5.30 |
| 9 | 0 | 2 | -7.30 | 3.90 |
| 9 | 0 | 2 | 4.80 | 5.60 |
| 10 | 0 | 2 | 1109.89 | 66.99 |
| 10 | 0 | 2 | 1226.38 | 67.29 |
| 10 | 0 | 2 | 1289.87 | 67.59 |
| 11 | 0 | 2 | 10.50 | 5.50 |
| 11 | 0 | 2 | 18.40 | 4.80 |
| 12 | 0 | 2 | 97.59 | 10.40 |
| 12 | 0 | 2 | 89.59 | 10.20 |
| 12 | 0 | 2 | 98.29 | 9.20 |
| 13 | 0 | 2 | 2.20 | 4.00 |
| 13 | 0 | 2 | 3.50 | 3.90 |
| 13 | 0 | 2 | 1.10 | 4.00 |
| 14 | 0 | 2 | 186.88 | 14.50 |
| 14 | 0 | 2 | 229.68 | 14.00 |
| 14 | 0 | 2 | 211.78 | 13.80 |
| 15 | 0 | 2 | -2.50 | 2.50 |
| 15 | 0 | 2 | -4.40 | 3.00 |
| 15 | 0 | 2 | -1.20 | 2.80 |

| | | | | |
|---|---|---|---|---|
| 16 | 0 | 2 | 194.18 | 11.50 |
| 16 | 0 | 2 | 178.48 | 11.60 |
| 16 | 0 | 2 | 190.48 | 11.90 |
| 17 | -1 | -2 | 127.99 | 7.90 |
| 17 | -1 | -2 | 125.39 | 8.80 |
| 17 | 1 | -2 | 121.19 | 9.10 |
| 17 | 1 | -2 | 130.29 | 7.90 |
| 16 | -1 | -2 | 21.50 | 3.80 |
| 16 | -1 | -2 | 26.30 | 5.30 |
| 16 | 1 | -2 | 32.90 | 4.00 |
| 16 | 1 | -2 | 35.00 | 5.40 |
| 16 | 1 | -2 | 21.80 | 4.00 |
| 15 | -1 | -2 | 870.71 | 51.19 |
| 15 | -1 | -2 | 1027.70 | 51.69 |
| 15 | -1 | -2 | 867.31 | 52.09 |
| 15 | 1 | -2 | 1011.80 | 52.89 |
| 15 | 1 | -2 | 894.51 | 53.09 |
| 15 | 1 | -2 | 879.21 | 51.29 |
| 14 | -1 | -2 | -4.30 | 2.60 |
| 14 | -1 | -2 | -1.70 | 3.80 |
| 14 | -1 | -2 | -1.00 | 3.60 |
| 14 | 1 | -2 | 1.30 | 2.40 |
| 14 | 1 | -2 | -2.00 | 3.60 |
| 14 | 1 | -2 | -2.80 | 3.80 |
| 13 | -1 | -2 | 275.67 | 19.00 |
| 13 | -1 | -2 | 269.47 | 17.70 |
| 13 | -1 | -2 | 288.77 | 17.30 |
| 13 | 1 | -2 | 293.37 | 17.90 |
| 13 | 1 | -2 | 257.57 | 19.00 |
| 13 | 1 | -2 | 277.37 | 17.10 |
| 12 | -1 | -2 | 230.78 | 16.20 |
| 12 | -1 | -2 | 215.78 | 14.40 |
| 12 | -1 | -2 | 219.28 | 14.10 |
| 12 | 1 | -2 | 179.48 | 16.00 |
| 12 | 1 | -2 | 203.98 | 13.80 |
| 12 | 1 | -2 | 199.38 | 14.50 |
| 11 | -1 | -2 | 2403.66 | 137.99 |
| 11 | -1 | -2 | 2723.43 | 137.39 |
| 11 | -1 | -2 | 2499.45 | 136.89 |
| 11 | 1 | -2 | 2614.24 | 137.39 |
| 11 | 1 | -2 | 2356.86 | 137.89 |
| 11 | 1 | -2 | 2534.95 | 136.69 |
| 10 | 1 | -2 | 9.90 | 6.30 |
| 10 | 1 | -2 | 8.70 | 3.80 |

| | | | | |
|---|---|---|---|---|
| 9 | -1 | -2 | 2290.17 | 126.39 |
| 9 | -1 | -2 | 2183.38 | 124.99 |
| 9 | -1 | -2 | 2373.66 | 125.39 |
| 9 | 1 | -2 | 2223.88 | 124.89 |
| 9 | 1 | -2 | 2251.77 | 126.29 |
| 9 | 1 | -2 | 2496.45 | 125.59 |
| 8 | -1 | -2 | 812.52 | 44.00 |
| 8 | 1 | -2 | 746.93 | 44.10 |
| 7 | -1 | -2 | 1139.29 | 62.89 |
| 4 | 1 | -2 | 1002.00 | 54.19 |
| -3 | 1 | 2 | 1173.78 | 63.29 |
| -2 | -1 | 2 | 303.97 | 18.30 |
| -2 | 1 | 2 | 350.36 | 18.20 |
| -1 | -1 | 2 | 2977.20 | 182.48 |
| -1 | 1 | 2 | 3813.92 | 182.38 |
| 0 | -1 | 2 | 650.63 | 37.60 |
| 0 | 1 | 2 | 717.63 | 37.40 |
| 1 | -1 | 2 | 1468.45 | 76.99 |
| 1 | 1 | 2 | 1368.66 | 77.89 |
| 2 | -1 | 2 | 1482.95 | 87.69 |
| 2 | 1 | 2 | 1738.83 | 87.39 |
| 3 | -1 | 2 | 22.50 | 3.40 |
| 3 | 1 | 2 | 21.80 | 4.10 |
| 4 | -1 | 2 | 233.48 | 13.20 |
| 4 | 1 | 2 | 212.68 | 13.80 |
| 5 | -1 | 2 | 3954.80 | 205.08 |
| 5 | 1 | 2 | 3676.33 | 205.58 |
| 6 | -1 | 2 | 9.50 | 2.60 |
| 6 | 1 | 2 | 11.70 | 3.60 |
| 7 | -1 | 2 | 18.20 | 4.90 |
| 7 | 1 | 2 | 13.00 | 3.60 |
| 7 | 1 | 2 | 9.90 | 4.00 |
| 8 | -1 | 2 | 72.19 | 8.60 |
| 8 | 1 | 2 | 91.69 | 9.00 |
| 9 | -1 | 2 | 342.57 | 21.70 |
| 9 | -1 | 2 | 347.07 | 20.90 |
| 9 | -1 | 2 | 329.47 | 21.60 |
| 9 | 1 | 2 | 314.57 | 20.80 |
| 9 | 1 | 2 | 302.57 | 21.80 |
| 9 | 1 | 2 | 359.26 | 21.60 |
| 10 | -1 | 2 | 0.10 | 5.30 |
| 10 | -1 | 2 | 4.30 | 5.20 |
| 10 | -1 | 2 | 13.60 | 4.20 |
| 10 | 1 | 2 | 7.10 | 4.20 |

| | | | | |
|---|---|---|---|---|
| 10 | 1 | 2 | 6.00 | 5.20 |
| 10 | 1 | 2 | 6.10 | 5.50 |
| 11 | -1 | 2 | 774.72 | 44.80 |
| 11 | -1 | 2 | 675.43 | 45.00 |
| 11 | -1 | 2 | 839.12 | 44.50 |
| 11 | 1 | 2 | 771.82 | 44.80 |
| 11 | 1 | 2 | 828.52 | 44.60 |
| 12 | -1 | 2 | -1.20 | 4.20 |
| 12 | -1 | 2 | -1.00 | 3.40 |
| 12 | -1 | 2 | -5.20 | 4.30 |
| 12 | 1 | 2 | -5.60 | 4.10 |
| 12 | 1 | 2 | -3.70 | 3.60 |
| 12 | 1 | 2 | -4.00 | 3.90 |
| 13 | -1 | 2 | 339.07 | 22.40 |
| 13 | -1 | 2 | 355.46 | 21.80 |
| 13 | -1 | 2 | 348.67 | 21.70 |
| 13 | 1 | 2 | 366.26 | 21.70 |
| 13 | 1 | 2 | 362.16 | 22.50 |
| 13 | 1 | 2 | 358.46 | 21.90 |
| 14 | -1 | 2 | -4.70 | 3.40 |
| 14 | -1 | 2 | -0.10 | 3.40 |
| 14 | -1 | 2 | 0.90 | 2.80 |
| 14 | 1 | 2 | -1.80 | 2.80 |
| 14 | 1 | 2 | -0.20 | 3.60 |
| 14 | 1 | 2 | -0.80 | 3.50 |
| 15 | -1 | 2 | 255.17 | 15.50 |
| 15 | -1 | 2 | 262.07 | 15.40 |
| 15 | -1 | 2 | 240.98 | 16.00 |
| 15 | 1 | 2 | 270.57 | 15.40 |
| 15 | 1 | 2 | 237.68 | 16.10 |
| 15 | 1 | 2 | 246.78 | 15.50 |
| 16 | -1 | 2 | -1.00 | 2.30 |
| 16 | -1 | 2 | 0.40 | 2.30 |
| 16 | -1 | 2 | -3.70 | 2.30 |
| 16 | 1 | 2 | 0.60 | 2.40 |
| 16 | 1 | 2 | 0.60 | 2.60 |
| 16 | 1 | 2 | 2.90 | 2.40 |
| 17 | -2 | -2 | 4.60 | 2.90 |
| 17 | -2 | -2 | 1.20 | 1.50 |
| 17 | 2 | -2 | -0.90 | 1.50 |
| 17 | 2 | -2 | -2.40 | 4.90 |
| 16 | -2 | -2 | 184.38 | 11.70 |
| 16 | -2 | -2 | 193.28 | 12.40 |
| 16 | 2 | -2 | 211.98 | 15.80 |

| | | | | |
|---|---|---|---|---|
| 16 | 2 | -2 | 190.58 | 11.70 |
| 15 | -2 | -2 | 1.20 | 3.20 |
| 15 | -2 | -2 | 0.40 | 4.20 |
| 15 | 2 | -2 | -2.40 | 3.30 |
| 15 | 2 | -2 | 0.20 | 3.40 |
| 15 | 2 | -2 | 4.80 | 5.10 |
| 14 | -2 | -2 | 700.53 | 39.70 |
| 14 | -2 | -2 | 708.03 | 40.60 |
| 14 | -2 | -2 | 743.43 | 40.20 |
| 14 | 2 | -2 | 650.23 | 39.90 |
| 14 | 2 | -2 | 730.43 | 40.70 |
| 14 | 2 | -2 | 705.23 | 39.40 |
| 13 | -2 | -2 | 112.19 | 9.20 |
| 13 | -2 | -2 | 104.99 | 9.60 |
| 13 | -2 | -2 | 107.69 | 10.90 |
| 13 | 2 | -2 | 106.49 | 11.40 |
| 13 | 2 | -2 | 104.49 | 8.60 |
| 13 | 2 | -2 | 111.69 | 10.00 |
| 12 | -2 | -2 | 95.29 | 11.10 |
| 12 | -2 | -2 | 83.69 | 8.70 |
| 12 | -2 | -2 | 93.09 | 8.80 |
| 12 | 2 | -2 | 83.29 | 7.90 |
| 12 | 2 | -2 | 83.79 | 11.50 |
| 12 | 2 | -2 | 108.99 | 9.40 |
| 11 | -2 | -2 | 80.09 | 8.70 |
| 11 | -2 | -2 | 82.39 | 8.70 |
| 11 | -2 | -2 | 98.89 | 11.50 |
| 11 | 2 | -2 | 73.99 | 11.80 |
| 11 | 2 | -2 | 66.69 | 8.10 |
| 11 | 2 | -2 | 98.29 | 9.30 |
| 10 | -2 | -2 | 2232.38 | 123.29 |
| 10 | -2 | -2 | 2224.58 | 122.19 |
| 10 | -2 | -2 | 2266.77 | 122.09 |
| 10 | 2 | -2 | 2237.88 | 121.69 |
| 10 | 2 | -2 | 2386.76 | 122.49 |
| 10 | 2 | -2 | 2111.39 | 122.99 |
| 9 | -2 | -2 | 120.79 | 15.00 |
| 9 | -2 | -2 | 166.98 | 11.70 |
| 9 | -2 | -2 | 153.78 | 12.60 |
| 9 | 2 | -2 | 160.68 | 15.40 |
| 9 | 2 | -2 | 154.18 | 11.60 |
| 9 | 2 | -2 | 150.98 | 12.80 |
| 8 | -2 | -2 | 47.90 | 7.40 |
| 8 | 2 | -2 | 40.30 | 6.40 |

| | | | | |
|---|---|---|---|---|
| 7 | -2 | -2 | 1.50 | 4.00 |
| 6 | 2 | -2 | 2729.13 | 146.79 |
| -4 | 2 | 2 | 59.19 | 4.10 |
| 4 | 2 | -2 | 55.29 | 4.70 |
| -3 | 2 | 2 | 13.50 | 2.40 |
| -2 | 2 | 2 | 1824.52 | 98.19 |
| -1 | -2 | 2 | 101.09 | 6.70 |
| 1 | -2 | 2 | 59.29 | 4.70 |
| 1 | 2 | 2 | 59.19 | 4.60 |
| 2 | -2 | 2 | 765.12 | 41.10 |
| 2 | 2 | 2 | 735.53 | 41.30 |
| 3 | -2 | 2 | 8.30 | 1.80 |
| 3 | 2 | 2 | 9.90 | 2.50 |
| 4 | -2 | 2 | 1749.92 | 85.19 |
| 4 | 2 | 2 | 1398.16 | 85.59 |
| 5 | -2 | 2 | 5.60 | 2.10 |
| 5 | 2 | 2 | 6.80 | 3.20 |
| 7 | -2 | 2 | 3.20 | 3.90 |
| 7 | 2 | 2 | 7.30 | 4.40 |
| 7 | 2 | 2 | 5.10 | 3.40 |
| 8 | -2 | 2 | 858.31 | 50.59 |
| 8 | 2 | 2 | 947.31 | 50.59 |
| 9 | -2 | 2 | 39.80 | 7.00 |
| 9 | -2 | 2 | 29.30 | 5.90 |
| 9 | -2 | 2 | 30.40 | 6.50 |
| 9 | 2 | 2 | 39.50 | 6.50 |
| 9 | 2 | 2 | 43.30 | 7.40 |
| 9 | 2 | 2 | 43.80 | 9.00 |
| 10 | -2 | 2 | 1154.18 | 65.89 |
| 10 | -2 | 2 | 1189.58 | 66.39 |
| 10 | -2 | 2 | 1094.99 | 65.79 |
| 10 | 2 | 2 | 1147.49 | 65.79 |
| 10 | 2 | 2 | 1178.28 | 66.09 |
| 10 | 2 | 2 | 1350.66 | 66.39 |
| 11 | -2 | 2 | 0.50 | 3.90 |
| 11 | -2 | 2 | -0.50 | 4.70 |
| 11 | -2 | 2 | 0.70 | 5.00 |
| 11 | 2 | 2 | 6.50 | 4.00 |
| 11 | 2 | 2 | -0.20 | 4.60 |
| 11 | 2 | 2 | 1.50 | 5.50 |
| 12 | -2 | 2 | 19.90 | 4.80 |
| 12 | -2 | 2 | 12.40 | 3.50 |
| 12 | -2 | 2 | 13.30 | 5.50 |
| 12 | 2 | 2 | 11.90 | 4.20 |

| | | | | |
|---|---|---|---|---|
| 12 | 2 | 2 | 20.10 | 4.70 |
| 12 | 2 | 2 | 11.40 | 4.70 |
| 13 | -2 | 2 | 63.79 | 8.10 |
| 13 | -2 | 2 | 47.30 | 8.80 |
| 13 | -2 | 2 | 58.09 | 7.50 |
| 13 | 2 | 2 | 81.39 | 8.20 |
| 13 | 2 | 2 | 62.19 | 7.90 |
| 13 | 2 | 2 | 61.39 | 8.80 |
| 14 | -2 | 2 | 305.97 | 18.80 |
| 14 | -2 | 2 | 299.07 | 18.50 |
| 14 | -2 | 2 | 285.07 | 19.20 |
| 14 | 2 | 2 | 266.17 | 19.10 |
| 14 | 2 | 2 | 313.47 | 18.60 |
| 14 | 2 | 2 | 345.67 | 19.80 |
| 15 | -2 | 2 | 1.30 | 2.60 |
| 15 | -2 | 2 | -1.30 | 2.40 |
| 15 | -2 | 2 | -2.10 | 3.00 |
| 15 | 2 | 2 | -1.50 | 3.20 |
| 15 | 2 | 2 | 1.50 | 2.60 |
| 15 | 2 | 2 | 1.10 | 3.00 |
| 16 | -2 | 2 | 162.18 | 9.90 |
| 16 | -2 | 2 | 163.68 | 10.20 |
| 16 | -2 | 2 | 155.98 | 10.30 |
| 16 | 2 | 2 | 152.48 | 10.40 |
| 16 | 2 | 2 | 160.18 | 10.20 |
| 16 | 2 | 2 | 153.58 | 10.30 |
| 17 | -3 | -2 | 48.80 | 4.00 |
| 17 | -3 | -2 | 45.90 | 5.30 |
| 17 | 3 | -2 | 53.89 | 4.10 |
| 16 | -3 | -2 | 5.80 | 2.10 |
| 16 | -3 | -2 | 2.30 | 3.00 |
| 16 | 3 | -2 | -3.70 | 3.40 |
| 16 | 3 | -2 | 6.20 | 2.10 |
| 15 | -3 | -2 | 348.37 | 21.60 |
| 15 | -3 | -2 | 347.17 | 20.60 |
| 15 | -3 | -2 | 332.77 | 20.40 |
| 15 | 3 | -2 | 350.36 | 20.60 |
| 15 | 3 | -2 | 353.16 | 21.30 |
| 15 | 3 | -2 | 333.47 | 19.90 |
| 14 | -3 | -2 | 36.80 | 6.60 |
| 14 | -3 | -2 | 37.20 | 7.70 |
| 14 | -3 | -2 | 42.40 | 6.00 |
| 14 | 3 | -2 | 54.99 | 7.20 |
| 14 | 3 | -2 | 43.30 | 8.20 |

| | | | | |
|---|---|---|---|---|
| 14 | 3 | -2 | 42.60 | 5.30 |
| 13 | -3 | -2 | 192.68 | 14.40 |
| 13 | -3 | -2 | 195.98 | 13.30 |
| 13 | -3 | -2 | 178.78 | 12.80 |
| 13 | 3 | -2 | 180.08 | 14.70 |
| 13 | 3 | -2 | 177.38 | 13.30 |
| 13 | 3 | -2 | 185.28 | 12.10 |
| 12 | -3 | -2 | 0.30 | 5.10 |
| 12 | -3 | -2 | 3.80 | 3.30 |
| 12 | -3 | -2 | 5.50 | 4.00 |
| 12 | 3 | -2 | 14.00 | 4.30 |
| 12 | 3 | -2 | 7.20 | 2.90 |
| 12 | 3 | -2 | -0.30 | 5.20 |
| 11 | -3 | -2 | 844.32 | 48.80 |
| 11 | -3 | -2 | 851.71 | 47.60 |
| 11 | -3 | -2 | 871.51 | 47.60 |
| 11 | 3 | -2 | 839.62 | 46.90 |
| 11 | 3 | -2 | 880.21 | 47.80 |
| 11 | 3 | -2 | 770.82 | 48.60 |
| 10 | -3 | -2 | 16.40 | 6.50 |
| 10 | -3 | -2 | 20.30 | 5.00 |
| 10 | -3 | -2 | 10.00 | 4.10 |
| 10 | 3 | -2 | 5.20 | 5.00 |
| 10 | 3 | -2 | 14.80 | 4.20 |
| 10 | 3 | -2 | 11.70 | 6.60 |
| 9 | -3 | -2 | 433.86 | 28.70 |
| 9 | -3 | -2 | 477.55 | 28.20 |
| 9 | -3 | -2 | 481.95 | 30.60 |
| 9 | 3 | -2 | 548.25 | 29.10 |
| 9 | 3 | -2 | 476.15 | 30.80 |
| 9 | 3 | -2 | 484.45 | 28.00 |
| 8 | -3 | -2 | 98.59 | 9.00 |
| 8 | 3 | -2 | 85.09 | 10.10 |
| 7 | -3 | -2 | 16.00 | 4.80 |
| 7 | 3 | -2 | 28.00 | 5.30 |
| 7 | 3 | -2 | 15.80 | 3.60 |
| 6 | 3 | -2 | 208.58 | 12.90 |
| -5 | 3 | 2 | 3756.52 | 194.58 |
| 5 | 3 | -2 | 3500.25 | 195.38 |
| -4 | 3 | 2 | 48.30 | 3.90 |
| 4 | 3 | -2 | 44.00 | 4.70 |
| 3 | 3 | -2 | 1710.23 | 91.29 |
| -3 | 3 | 2 | 1660.53 | 90.89 |
| -2 | 3 | 2 | 2.60 | 1.50 |

| | | | | |
|---|---|---|---|---|
| 0 | 3 | 2 | 188.78 | 11.30 |
| 1 | 3 | 2 | 1037.20 | 56.59 |
| 2 | 3 | 2 | 330.67 | 19.00 |
| 3 | 3 | 2 | 586.44 | 32.90 |
| 4 | 3 | 2 | 317.97 | 19.00 |
| 5 | 3 | 2 | 3941.01 | 212.68 |
| 6 | -3 | 2 | 48.70 | 6.90 |
| 6 | 3 | 2 | 51.59 | 5.90 |
| 6 | 3 | 2 | 40.30 | 7.00 |
| 7 | -3 | 2 | 124.99 | 10.90 |
| 7 | 3 | 2 | 143.59 | 11.20 |
| 7 | 3 | 2 | 134.39 | 9.90 |
| 8 | -3 | 2 | 83.79 | 9.20 |
| 8 | 3 | 2 | 108.99 | 8.70 |
| 8 | 3 | 2 | 106.59 | 11.70 |
| 9 | -3 | 2 | 778.32 | 43.50 |
| 9 | -3 | 2 | 751.12 | 43.60 |
| 9 | -3 | 2 | 810.92 | 44.40 |
| 9 | 3 | 2 | 713.53 | 43.30 |
| 9 | 3 | 2 | 816.92 | 43.80 |
| 9 | 3 | 2 | 724.83 | 43.70 |
| 10 | -3 | 2 | 157.08 | 12.60 |
| 10 | -3 | 2 | 137.89 | 11.20 |
| 10 | -3 | 2 | 133.39 | 13.30 |
| 10 | 3 | 2 | 149.88 | 12.70 |
| 10 | 3 | 2 | 150.78 | 12.50 |
| 10 | 3 | 2 | 141.29 | 11.60 |
| 11 | -3 | 2 | 723.23 | 41.80 |
| 11 | -3 | 2 | 788.12 | 42.00 |
| 11 | -3 | 2 | 686.33 | 42.50 |
| 11 | 3 | 2 | 688.73 | 42.00 |
| 11 | 3 | 2 | 784.92 | 42.40 |
| 11 | 3 | 2 | 729.23 | 41.70 |
| 12 | -3 | 2 | 2.00 | 5.30 |
| 12 | -3 | 2 | 13.50 | 4.70 |
| 12 | -3 | 2 | 5.20 | 3.20 |
| 12 | 3 | 2 | 5.40 | 4.00 |
| 12 | 3 | 2 | 8.10 | 4.80 |
| 12 | 3 | 2 | 12.00 | 4.20 |
| 13 | -3 | 2 | 239.88 | 15.10 |
| 13 | -3 | 2 | 230.38 | 15.50 |
| 13 | -3 | 2 | 215.78 | 16.20 |
| 13 | 3 | 2 | 219.68 | 15.20 |
| 13 | 3 | 2 | 237.48 | 15.20 |

| | | | | |
|---|---|---|---|---|
| 13 | 3 | 2 | 229.08 | 16.20 |
| 14 | -3 | 2 | -1.60 | 3.60 |
| 14 | -3 | 2 | 3.00 | 3.00 |
| 14 | -3 | 2 | -4.70 | 4.40 |
| 14 | 3 | 2 | 7.20 | 4.10 |
| 14 | 3 | 2 | 2.10 | 4.20 |
| 14 | 3 | 2 | 4.10 | 2.80 |
| 15 | -3 | 2 | 231.78 | 15.00 |
| 15 | -3 | 2 | 243.68 | 14.50 |
| 15 | -3 | 2 | 247.18 | 14.70 |
| 15 | 3 | 2 | 218.88 | 15.10 |
| 15 | 3 | 2 | 254.47 | 14.60 |
| 15 | 3 | 2 | 228.88 | 14.50 |
| 16 | -3 | 2 | -2.70 | 2.10 |
| 16 | -3 | 2 | -1.40 | 2.30 |
| 16 | -3 | 2 | 4.00 | 2.40 |
| 16 | 3 | 2 | 4.30 | 3.10 |
| 16 | 3 | 2 | -1.70 | 2.30 |
| 16 | 3 | 2 | -3.40 | 2.30 |
| 16 | -4 | -2 | 300.37 | 17.90 |
| 16 | -4 | -2 | 277.77 | 16.40 |
| 16 | 4 | -2 | 278.47 | 17.30 |
| 16 | 4 | -2 | 283.47 | 16.30 |
| 15 | -4 | -2 | 2.30 | 2.60 |
| 15 | -4 | -2 | -2.50 | 2.90 |
| 15 | 4 | -2 | -3.00 | 3.50 |
| 15 | 4 | -2 | 4.50 | 3.80 |
| 15 | 4 | -2 | 1.10 | 2.10 |
| 14 | -4 | -2 | 359.36 | 22.50 |
| 14 | -4 | -2 | 369.16 | 21.90 |
| 14 | -4 | -2 | 363.66 | 21.40 |
| 14 | 4 | -2 | 353.46 | 22.50 |
| 14 | 4 | -2 | 363.76 | 21.00 |
| 14 | 4 | -2 | 361.86 | 21.80 |
| 13 | -4 | -2 | 10.10 | 3.50 |
| 13 | -4 | -2 | 10.60 | 4.90 |
| 13 | -4 | -2 | 5.60 | 4.50 |
| 13 | 4 | -2 | 11.80 | 5.10 |
| 13 | 4 | -2 | 8.40 | 2.70 |
| 13 | 4 | -2 | 6.30 | 5.30 |
| 12 | -4 | -2 | 301.57 | 19.40 |
| 12 | -4 | -2 | 308.07 | 20.90 |
| 12 | -4 | -2 | 303.57 | 19.30 |
| 12 | 4 | -2 | 301.37 | 18.50 |

| | | | | |
|---|---|---|---|---|
| 12 | 4 | -2 | 296.47 | 21.00 |
| 12 | 4 | -2 | 339.87 | 19.70 |
| 11 | -4 | -2 | 1.10 | 4.90 |
| 11 | -4 | -2 | 2.60 | 3.40 |
| 11 | 4 | -2 | -13.00 | 6.20 |
| 11 | 4 | -2 | -7.70 | 4.10 |
| 10 | -4 | -2 | 1116.09 | 62.29 |
| 10 | -4 | -2 | 1152.88 | 63.29 |
| 10 | -4 | -2 | 1097.39 | 62.09 |
| 10 | 4 | -2 | 1124.79 | 61.49 |
| 10 | 4 | -2 | 1076.49 | 63.29 |
| 10 | 4 | -2 | 1144.59 | 62.39 |
| 9 | -4 | -2 | 255.67 | 15.90 |
| 9 | -4 | -2 | 234.18 | 16.90 |
| 9 | -4 | -2 | 268.87 | 18.90 |
| 9 | 4 | -2 | 216.68 | 20.50 |
| 9 | 4 | -2 | 235.28 | 15.50 |
| 9 | 4 | -2 | 234.98 | 16.50 |
| 8 | -4 | -2 | 1296.97 | 70.59 |
| 8 | 4 | -2 | 1262.37 | 70.39 |
| 7 | -4 | -2 | 92.99 | 9.00 |
| 7 | 4 | -2 | 90.49 | 9.10 |
| 7 | 4 | -2 | 93.99 | 8.20 |
| 6 | -4 | -2 | 5515.35 | 284.27 |
| 6 | 4 | -2 | 5034.20 | 283.67 |
| -5 | 4 | 2 | 703.83 | 37.40 |
| 5 | 4 | -2 | 662.03 | 38.20 |
| 4 | 4 | -2 | 3236.18 | 184.18 |
| -4 | 4 | 2 | 3594.84 | 183.68 |
| 3 | 4 | -2 | 31.80 | 4.20 |
| -3 | 4 | 2 | 34.90 | 3.70 |
| 2 | 4 | -2 | 46.50 | 5.00 |
| -2 | 4 | 2 | 66.29 | 4.70 |
| -1 | 4 | 2 | 1676.43 | 90.79 |
| 0 | 4 | 2 | 2214.08 | 119.59 |
| 1 | 4 | 2 | 325.37 | 18.80 |
| 2 | 4 | 2 | 127.79 | 8.60 |
| 3 | 4 | 2 | 18.30 | 4.10 |
| 4 | 4 | 2 | 2989.10 | 161.78 |
| 5 | 4 | 2 | 258.07 | 15.60 |
| 5 | 4 | 2 | 257.47 | 16.50 |
| 6 | -4 | 2 | 679.23 | 39.50 |
| 6 | 4 | 2 | 706.43 | 39.20 |
| 6 | 4 | 2 | 702.93 | 38.20 |

| | | | | |
|---|---|---|---|---|
| -6 | 4 | -2 | 654.53 | 37.60 |
| 7 | -4 | 2 | -1.80 | 4.10 |
| 7 | 4 | 2 | 0.70 | 2.80 |
| 7 | 4 | 2 | 2.40 | 4.40 |
| 8 | -4 | 2 | 347.37 | 22.40 |
| 8 | -4 | 2 | 341.37 | 24.10 |
| 8 | -4 | 2 | 344.37 | 23.50 |
| 8 | 4 | 2 | 391.56 | 22.10 |
| 8 | 4 | 2 | 359.26 | 22.80 |
| 9 | -4 | 2 | 6.50 | 6.30 |
| 9 | -4 | 2 | 9.50 | 4.50 |
| 9 | -4 | 2 | -3.60 | 3.90 |
| 9 | 4 | 2 | 3.90 | 5.40 |
| 9 | 4 | 2 | 5.20 | 3.40 |
| 9 | 4 | 2 | 12.70 | 5.70 |
| 10 | -4 | 2 | 695.03 | 41.30 |
| 10 | -4 | 2 | 729.03 | 42.40 |
| 10 | -4 | 2 | 711.03 | 41.30 |
| 10 | 4 | 2 | 712.23 | 41.30 |
| 10 | 4 | 2 | 765.72 | 41.40 |
| 10 | 4 | 2 | 727.83 | 41.60 |
| 11 | -4 | 2 | 19.50 | 4.80 |
| 11 | -4 | 2 | 14.20 | 4.20 |
| 11 | -4 | 2 | 14.80 | 5.70 |
| 11 | 4 | 2 | 17.20 | 5.00 |
| 11 | 4 | 2 | 20.70 | 5.40 |
| 11 | 4 | 2 | 14.90 | 4.60 |
| 12 | -4 | 2 | 97.59 | 11.00 |
| 12 | -4 | 2 | 101.49 | 8.80 |
| 12 | -4 | 2 | 79.69 | 9.70 |
| 12 | 4 | 2 | 85.79 | 9.30 |
| 12 | 4 | 2 | 92.29 | 10.10 |
| 12 | 4 | 2 | 78.09 | 8.80 |
| 13 | -4 | 2 | -0.70 | 3.50 |
| 13 | -4 | 2 | 3.10 | 4.40 |
| 13 | -4 | 2 | -3.50 | 2.80 |
| 13 | 4 | 2 | 4.90 | 3.50 |
| 13 | 4 | 2 | 0.80 | 4.10 |
| 13 | 4 | 2 | -4.40 | 3.50 |
| 14 | -4 | 2 | 336.47 | 20.30 |
| 14 | 4 | 2 | 356.46 | 20.10 |
| 14 | 4 | 2 | 368.06 | 20.40 |
| 14 | 4 | 2 | 283.57 | 22.80 |
| 15 | -4 | 2 | -2.20 | 2.50 |

| | | | | |
|---|---|---|---|---|
| 15 | -4 | 2 | -1.30 | 2.80 |
| 15 | -4 | 2 | 0.60 | 2.60 |
| 15 | 4 | 2 | 4.40 | 2.90 |
| 15 | 4 | 2 | 4.90 | 3.20 |
| 16 | -4 | 2 | 235.58 | 13.90 |
| 16 | -4 | 2 | 238.48 | 13.50 |
| 16 | 4 | 2 | 223.38 | 13.80 |
| 16 | 4 | 2 | 226.68 | 13.90 |
| 16 | -5 | -2 | -3.40 | 2.60 |
| 16 | -5 | -2 | 6.70 | 2.10 |
| 16 | 5 | -2 | 3.30 | 1.80 |
| 15 | -5 | -2 | 247.58 | 15.20 |
| 15 | -5 | -2 | 262.67 | 15.90 |
| 15 | 5 | -2 | 249.18 | 16.10 |
| 15 | 5 | -2 | 262.47 | 15.00 |
| 15 | 5 | -2 | 247.58 | 16.00 |
| 14 | -5 | -2 | 11.00 | 3.40 |
| 14 | -5 | -2 | 11.00 | 3.50 |
| 14 | 5 | -2 | 11.10 | 4.60 |
| 14 | 5 | -2 | 11.60 | 2.80 |
| 14 | 5 | -2 | 19.30 | 4.60 |
| 13 | -5 | -2 | 153.48 | 11.20 |
| 13 | -5 | -2 | 164.58 | 11.70 |
| 13 | -5 | -2 | 163.58 | 12.80 |
| 13 | 5 | -2 | 149.19 | 12.40 |
| 13 | 5 | -2 | 142.89 | 12.90 |
| 13 | 5 | -2 | 137.49 | 10.10 |
| 12 | -5 | -2 | 175.88 | 12.70 |
| 12 | -5 | -2 | 181.08 | 12.50 |
| 12 | -5 | -2 | 170.98 | 14.10 |
| 12 | 5 | -2 | 158.78 | 14.60 |
| 12 | 5 | -2 | 164.98 | 13.00 |
| 12 | 5 | -2 | 169.58 | 11.50 |
| 11 | -5 | -2 | 1768.12 | 94.79 |
| 11 | -5 | -2 | 1756.02 | 93.99 |
| 11 | -5 | -2 | 1764.22 | 93.99 |
| 11 | 5 | -2 | 1681.23 | 93.09 |
| 11 | 5 | -2 | 1594.34 | 93.89 |
| 10 | -5 | -2 | 183.88 | 15.10 |
| 10 | -5 | -2 | 170.48 | 13.50 |
| 10 | -5 | -2 | 181.28 | 12.50 |
| 10 | 5 | -2 | 140.79 | 16.90 |
| 10 | 5 | -2 | 168.28 | 13.30 |
| 10 | 5 | -2 | 179.08 | 12.00 |

| | | | | |
|---|---|---|---|---|
| 9 | -5 | -2 | 1157.08 | 66.29 |
| 9 | -5 | -2 | 1160.48 | 67.09 |
| 9 | -5 | -2 | 1203.58 | 66.09 |
| 9 | 5 | -2 | 1258.17 | 68.29 |
| 9 | 5 | -2 | 1193.88 | 65.39 |
| 9 | 5 | -2 | 1182.28 | 66.29 |
| 8 | -5 | -2 | 3.40 | 3.80 |
| 8 | -5 | -2 | -9.00 | 5.50 |
| 8 | 5 | -2 | 3.20 | 4.10 |
| 8 | 5 | -2 | -2.40 | 3.90 |
| 7 | -5 | -2 | 204.38 | 14.10 |
| 7 | 5 | -2 | 188.18 | 13.30 |
| 7 | 5 | -2 | 205.98 | 13.50 |
| 6 | -5 | -2 | 65.29 | 9.10 |
| -6 | 5 | 2 | 61.59 | 5.70 |
| 6 | 5 | -2 | 61.39 | 7.10 |
| -5 | 5 | 2 | 4707.13 | 244.48 |
| 5 | 5 | -2 | 4391.16 | 245.28 |
| -4 | 5 | 2 | 134.69 | 8.60 |
| 4 | 5 | -2 | 127.79 | 9.40 |
| 3 | 5 | -2 | 388.56 | 23.00 |
| -3 | 5 | 2 | 402.56 | 22.50 |
| 2 | 5 | -2 | 203.78 | 12.90 |
| -2 | 5 | 2 | 210.38 | 12.50 |
| 1 | 5 | -2 | 5743.33 | 302.87 |
| -1 | 5 | 2 | 5523.45 | 302.67 |
| 0 | 5 | -2 | 443.56 | 24.20 |
| 0 | 5 | 2 | 405.36 | 24.10 |
| 1 | 5 | 2 | 2973.90 | 160.58 |
| 2 | 5 | 2 | 839.92 | 46.60 |
| 3 | 5 | 2 | 51.19 | 5.50 |
| 4 | 5 | 2 | 32.40 | 5.50 |
| -4 | 5 | -2 | 29.70 | 4.10 |
| -5 | 5 | -2 | 1878.11 | 98.89 |
| 5 | 5 | 2 | 1713.63 | 98.89 |
| 5 | 5 | 2 | 1884.41 | 100.09 |
| 6 | -5 | 2 | 211.98 | 15.50 |
| 6 | 5 | 2 | 207.18 | 14.80 |
| 6 | 5 | 2 | 218.08 | 13.30 |
| -6 | 5 | -2 | 210.08 | 12.90 |
| 7 | -5 | 2 | 144.99 | 11.40 |
| 7 | 5 | 2 | 115.29 | 9.10 |
| 7 | 5 | 2 | 129.49 | 11.40 |
| 8 | -5 | 2 | 315.67 | 18.80 |

| | | | | |
|---|---|---|---|---|
| 8 | -5 | 2 | 247.88 | 18.50 |
| 8 | -5 | 2 | 257.97 | 21.40 |
| 8 | 5 | 2 | 304.57 | 17.60 |
| 8 | 5 | 2 | 290.57 | 19.20 |
| 8 | 5 | 2 | 287.17 | 20.00 |
| 9 | -5 | 2 | 691.73 | 38.60 |
| 9 | -5 | 2 | 640.24 | 40.10 |
| 9 | -5 | 2 | 670.53 | 38.60 |
| 9 | 5 | 2 | 699.13 | 38.20 |
| 9 | 5 | 2 | 710.83 | 38.90 |
| 9 | 5 | 2 | 612.74 | 38.50 |
| 10 | -5 | 2 | 22.30 | 7.50 |
| 10 | -5 | 2 | 19.80 | 4.40 |
| 10 | -5 | 2 | 20.50 | 6.60 |
| 10 | 5 | 2 | 16.60 | 5.20 |
| 10 | 5 | 2 | 37.60 | 8.40 |
| 10 | 5 | 2 | 16.40 | 4.60 |
| 11 | -5 | 2 | 1101.79 | 64.29 |
| 11 | -5 | 2 | 1162.98 | 65.19 |
| 11 | -5 | 2 | 1174.38 | 64.39 |
| 11 | 5 | 2 | 1209.88 | 64.59 |
| 11 | 5 | 2 | 1105.99 | 64.29 |
| 11 | 5 | 2 | 1181.28 | 63.89 |
| 12 | -5 | 2 | 173.88 | 14.50 |
| 12 | -5 | 2 | 181.08 | 13.40 |
| 12 | -5 | 2 | 179.68 | 12.70 |
| 12 | 5 | 2 | 189.28 | 12.90 |
| 12 | 5 | 2 | 173.28 | 13.20 |
| 12 | 5 | 2 | 178.98 | 13.80 |
| 13 | -5 | 2 | 85.29 | 9.10 |
| 13 | -5 | 2 | 106.29 | 8.60 |
| 13 | -5 | 2 | 103.89 | 10.10 |
| 13 | 5 | 2 | 102.09 | 9.00 |
| 13 | 5 | 2 | 82.59 | 9.40 |
| 13 | 5 | 2 | 103.79 | 9.10 |
| 14 | -5 | 2 | 7.70 | 2.90 |
| 14 | -5 | 2 | 10.40 | 3.60 |
| 14 | -5 | 2 | 2.90 | 3.80 |
| 14 | 5 | 2 | 12.90 | 4.20 |
| 14 | 5 | 2 | 3.10 | 3.60 |
| 14 | 5 | 2 | 17.10 | 3.80 |
| 15 | -5 | 2 | 320.07 | 18.70 |
| 15 | -5 | 2 | 312.57 | 18.40 |
| 15 | -5 | 2 | 325.77 | 19.00 |

| | | | | |
|---|---|---|---|---|
| 15 | 5 | 2 | 283.77 | 18.70 |
| 15 | 5 | 2 | 335.57 | 18.80 |
| 15 | 5 | 2 | 325.47 | 18.60 |
| 16 | -5 | 2 | 8.50 | 2.10 |
| 16 | -5 | 2 | 4.90 | 2.40 |
| 16 | 5 | 2 | 7.30 | 2.70 |
| 16 | 5 | 2 | 9.90 | 2.80 |
| 16 | -6 | -2 | 377.96 | 23.00 |
| 16 | -6 | -2 | 402.36 | 22.70 |
| 16 | 6 | -2 | 433.06 | 22.60 |
| 15 | -6 | -2 | 2.90 | 2.90 |
| 15 | -6 | -2 | 7.90 | 2.40 |
| 15 | 6 | -2 | 5.20 | 2.00 |
| 15 | 6 | -2 | 11.80 | 5.40 |
| 14 | -6 | -2 | 581.94 | 33.70 |
| 14 | -6 | -2 | 609.24 | 33.40 |
| 14 | 6 | -2 | 590.84 | 34.30 |
| 14 | 6 | -2 | 599.64 | 32.90 |
| 14 | 6 | -2 | 567.84 | 33.70 |
| 13 | -6 | -2 | 2.40 | 3.40 |
| 13 | -6 | -2 | 5.30 | 3.30 |
| 13 | 6 | -2 | 7.10 | 2.50 |
| 13 | 6 | -2 | 4.10 | 4.30 |
| 13 | 6 | -2 | 8.90 | 5.30 |
| 12 | -6 | -2 | 562.04 | 31.20 |
| 12 | -6 | -2 | 537.05 | 30.20 |
| 12 | -6 | -2 | 493.05 | 30.00 |
| 12 | 6 | -2 | 519.25 | 31.50 |
| 12 | 6 | -2 | 511.65 | 29.20 |
| 12 | 6 | -2 | 474.95 | 30.30 |
| 11 | -6 | -2 | 10.70 | 5.10 |
| 11 | -6 | -2 | 1.60 | 4.30 |
| 11 | -6 | -2 | 4.40 | 3.40 |
| 11 | 6 | -2 | 6.00 | 3.00 |
| 11 | 6 | -2 | 5.50 | 4.80 |
| 11 | 6 | -2 | -1.70 | 6.80 |
| 10 | -6 | -2 | 1589.34 | 85.79 |
| 10 | -6 | -2 | 1468.35 | 85.09 |
| 10 | -6 | -2 | 1577.24 | 85.19 |
| 10 | 6 | -2 | 1554.04 | 85.09 |
| 10 | 6 | -2 | 1510.65 | 86.39 |
| 10 | 6 | -2 | 1580.04 | 84.29 |
| 9 | -6 | -2 | 0.30 | 5.10 |
| 9 | -6 | -2 | -9.60 | 6.50 |

| | | | | |
|---|---|---|---|---|
| 9 | -6 | -2 | -3.00 | 3.60 |
| 9 | 6 | -2 | 8.80 | 10.10 |
| 9 | 6 | -2 | 0.50 | 4.30 |
| 9 | 6 | -2 | -2.10 | 3.10 |
| 8 | -6 | -2 | 139.09 | 15.00 |
| 8 | -6 | -2 | 99.39 | 9.90 |
| 8 | -6 | -2 | 91.89 | 11.40 |
| 8 | 6 | -2 | 110.59 | 10.40 |
| 8 | 6 | -2 | 101.99 | 9.90 |
| 8 | 6 | -2 | 100.39 | 9.10 |
| 7 | -6 | -2 | 9.20 | 4.30 |
| 7 | 6 | -2 | 1.40 | 3.30 |
| 7 | 6 | -2 | 7.70 | 4.20 |
| 6 | -6 | -2 | 5018.10 | 269.07 |
| 6 | 6 | -2 | 4859.01 | 267.87 |
| -6 | 6 | 2 | 5116.89 | 267.77 |
| 6 | 6 | -2 | 4937.81 | 268.87 |
| 5 | -6 | -2 | 214.68 | 16.70 |
| -5 | 6 | 2 | 248.88 | 14.20 |
| 5 | 6 | -2 | 232.78 | 15.30 |
| 4 | 6 | -2 | 206.58 | 14.00 |
| -4 | 6 | 2 | 219.28 | 13.10 |
| 3 | 6 | -2 | 41.60 | 5.50 |
| -3 | 6 | 2 | 46.80 | 4.70 |
| -2 | 6 | 2 | 1576.04 | 85.39 |
| 2 | 6 | -2 | 1561.74 | 85.69 |
| 1 | 6 | -2 | 266.07 | 16.80 |
| -1 | 6 | 2 | 297.37 | 16.80 |
| 0 | 6 | 2 | 5807.42 | 313.37 |
| 0 | 6 | -2 | 5847.22 | 313.27 |
| -1 | 6 | -2 | 3.50 | 2.00 |
| 1 | 6 | 2 | 3.00 | 1.90 |
| -2 | 6 | -2 | 20.00 | 3.70 |
| 2 | 6 | 2 | 20.40 | 4.20 |
| -3 | 6 | -2 | 81.39 | 6.40 |
| 3 | 6 | 2 | 81.29 | 7.20 |
| 4 | 6 | 2 | 2227.78 | 131.69 |
| -4 | 6 | -2 | 2672.83 | 132.09 |
| 4 | 6 | 2 | 2417.86 | 132.79 |
| 5 | -6 | 2 | 17.10 | 5.50 |
| 5 | 6 | 2 | 13.90 | 3.10 |
| 5 | 6 | 2 | 8.40 | 4.10 |
| -5 | 6 | -2 | 17.50 | 3.10 |
| 6 | -6 | 2 | 3431.06 | 193.28 |

| | | | | |
|---|---|---|---|---|
| 6 | 6 | 2 | 3912.11 | 192.38 |
| -6 | 6 | -2 | 3499.75 | 192.08 |
| 6 | 6 | 2 | 3422.66 | 193.48 |
| 7 | -6 | 2 | 273.87 | 18.60 |
| 7 | -6 | 2 | 297.37 | 19.10 |
| 7 | 6 | 2 | 291.37 | 17.30 |
| 8 | -6 | 2 | 603.44 | 35.60 |
| 8 | -6 | 2 | 682.83 | 36.40 |
| 8 | -6 | 2 | 587.14 | 38.00 |
| 8 | 6 | 2 | 573.44 | 36.00 |
| 8 | 6 | 2 | 625.44 | 36.80 |
| 8 | 6 | 2 | 691.13 | 35.20 |
| 8 | 6 | 2 | 568.14 | 36.10 |
| 9 | -6 | 2 | 8.30 | 7.80 |
| 9 | -6 | 2 | 6.80 | 4.20 |
| 9 | -6 | 2 | -2.20 | 4.10 |
| 9 | 6 | 2 | -1.70 | 5.60 |
| 9 | 6 | 2 | 0.70 | 3.20 |
| 9 | 6 | 2 | 13.10 | 5.50 |
| 10 | -6 | 2 | 763.92 | 51.19 |
| 10 | -6 | 2 | 964.80 | 49.90 |
| 10 | -6 | 2 | 926.81 | 49.99 |
| 10 | 6 | 2 | 838.72 | 49.50 |
| 10 | 6 | 2 | 865.11 | 50.69 |
| 10 | 6 | 2 | 934.01 | 50.19 |
| 11 | -6 | 2 | 3.80 | 4.20 |
| 11 | -6 | 2 | -11.00 | 5.50 |
| 11 | -6 | 2 | -1.30 | 3.10 |
| 11 | 6 | 2 | 8.10 | 4.20 |
| 11 | 6 | 2 | 7.30 | 3.80 |
| 11 | 6 | 2 | -5.50 | 4.50 |
| 12 | -6 | 2 | 5.00 | 3.80 |
| 12 | -6 | 2 | 0.00 | 4.80 |
| 12 | -6 | 2 | 6.60 | 3.50 |
| 12 | 6 | 2 | 6.90 | 4.00 |
| 12 | 6 | 2 | 13.60 | 5.10 |
| 12 | 6 | 2 | -2.00 | 4.40 |
| 13 | -6 | 2 | 1.80 | 4.20 |
| 13 | -6 | 2 | -0.50 | 3.50 |
| 13 | -6 | 2 | 3.50 | 3.10 |
| 13 | 6 | 2 | 14.40 | 3.90 |
| 13 | 6 | 2 | 7.50 | 4.20 |
| 13 | 6 | 2 | 1.10 | 3.40 |
| 14 | -6 | 2 | 314.97 | 19.00 |

| | | | | |
|---|---|---|---|---|
| 14 | -6 | 2 | 344.77 | 19.60 |
| 14 | -6 | 2 | 292.37 | 18.90 |
| 14 | 6 | 2 | 285.27 | 19.40 |
| 14 | 6 | 2 | 319.67 | 19.10 |
| 14 | 6 | 2 | 339.67 | 18.90 |
| 15 | -6 | 2 | -3.10 | 2.30 |
| 15 | -6 | 2 | -3.70 | 2.20 |
| 15 | 6 | 2 | 2.40 | 3.10 |
| 15 | 6 | 2 | 0.20 | 3.10 |
| 16 | -7 | -2 | 4.60 | 2.40 |
| 16 | -7 | -2 | 1.10 | 1.60 |
| 16 | 7 | -2 | 1.00 | 1.60 |
| 15 | -7 | -2 | 420.86 | 23.80 |
| 15 | -7 | -2 | 408.96 | 23.40 |
| 15 | 7 | -2 | 408.46 | 23.10 |
| 14 | -7 | -2 | 11.00 | 3.20 |
| 14 | -7 | -2 | 5.90 | 4.10 |
| 14 | 7 | -2 | 6.10 | 2.50 |
| 14 | 7 | -2 | 15.50 | 4.70 |
| 14 | 7 | -2 | 5.40 | 5.20 |
| 13 | -7 | -2 | 123.79 | 10.20 |
| 13 | -7 | -2 | 135.29 | 10.10 |
| 13 | 7 | -2 | 119.59 | 11.20 |
| 13 | 7 | -2 | 143.09 | 9.10 |
| 13 | 7 | -2 | 149.49 | 12.70 |
| 12 | -7 | -2 | 50.19 | 6.90 |
| 12 | -7 | -2 | 52.59 | 7.50 |
| 12 | 7 | -2 | 53.89 | 5.80 |
| 12 | 7 | -2 | 48.80 | 8.90 |
| 12 | 7 | -2 | 44.40 | 7.90 |
| 11 | -7 | -2 | 1612.54 | 87.89 |
| 11 | -7 | -2 | 1622.74 | 87.49 |
| 11 | -7 | -2 | 1554.54 | 87.49 |
| 11 | 7 | -2 | 1615.04 | 86.59 |
| 11 | 7 | -2 | 1606.14 | 88.39 |
| 11 | 7 | -2 | 1531.95 | 87.49 |
| 10 | -7 | -2 | 70.69 | 8.20 |
| 10 | -7 | -2 | 96.59 | 10.30 |
| 10 | -7 | -2 | 86.09 | 13.30 |
| 10 | 7 | -2 | 88.99 | 8.20 |
| 10 | 7 | -2 | 86.49 | 9.20 |
| 9 | -7 | -2 | 838.42 | 44.70 |
| 9 | -7 | -2 | 786.92 | 45.60 |
| 9 | -7 | -2 | 779.52 | 44.70 |

| | | | | |
|---|---|---|---|---|
| 9 | 7 | -2 | 771.22 | 43.60 |
| 9 | 7 | -2 | 739.33 | 43.90 |
| 8 | -7 | -2 | 217.98 | 16.30 |
| 8 | -7 | -2 | 229.98 | 15.40 |
| 8 | -7 | -2 | 176.98 | 18.90 |
| 8 | 7 | -2 | 210.78 | 14.80 |
| 8 | 7 | -2 | 235.88 | 14.40 |
| 8 | 7 | -2 | 235.38 | 16.10 |
| 7 | -7 | -2 | 620.34 | 38.60 |
| 7 | -7 | -2 | 760.12 | 39.20 |
| 7 | 7 | -2 | 595.84 | 38.30 |
| 7 | 7 | -2 | 699.53 | 37.50 |
| 6 | -7 | -2 | 494.55 | 29.00 |
| -6 | 7 | 2 | 499.75 | 27.30 |
| 6 | 7 | -2 | 437.46 | 28.50 |
| 6 | 7 | -2 | 470.95 | 27.20 |
| 5 | -7 | -2 | 4548.54 | 257.27 |
| 5 | 7 | -2 | 4448.75 | 257.17 |
| 5 | 7 | -2 | 5024.00 | 257.37 |
| -5 | 7 | 2 | 5022.40 | 256.27 |
| -4 | 7 | 2 | 107.59 | 8.00 |
| 4 | 7 | -2 | 98.19 | 9.00 |
| 3 | 7 | -2 | 32.50 | 5.60 |
| -3 | 7 | 2 | 28.50 | 4.50 |
| 2 | 7 | -2 | 98.69 | 8.20 |
| -2 | 7 | 2 | 105.49 | 7.70 |
| 1 | 7 | -2 | 6356.46 | 342.27 |
| -1 | 7 | 2 | 6365.46 | 342.07 |
| 0 | 7 | 2 | 728.63 | 41.10 |
| 0 | 7 | -2 | 740.63 | 41.10 |
| -1 | 7 | -2 | 1425.36 | 75.79 |
| 1 | 7 | 2 | 1344.57 | 75.99 |
| 2 | 7 | 2 | 102.29 | 8.30 |
| -2 | 7 | -2 | 106.19 | 7.80 |
| -3 | 7 | -2 | 959.40 | 51.09 |
| 3 | 7 | 2 | 864.61 | 50.79 |
| 3 | 7 | 2 | 945.81 | 51.79 |
| 4 | -7 | 2 | 14.50 | 4.80 |
| 4 | 7 | 2 | 19.50 | 3.50 |
| 4 | 7 | 2 | 14.30 | 4.40 |
| -4 | 7 | -2 | 18.70 | 4.60 |
| 5 | -7 | 2 | 4979.00 | 262.37 |
| 5 | 7 | 2 | 4852.31 | 261.37 |
| -5 | 7 | -2 | 5237.38 | 261.77 |

| | | | | |
|---|---|---|---|---|
| 5 | 7 | 2 | 4368.86 | 262.97 |
| 6 | -7 | 2 | 170.48 | 13.60 |
| 6 | -7 | 2 | 171.38 | 13.50 |
| -6 | 7 | -2 | 184.38 | 11.70 |
| 6 | 7 | 2 | 161.38 | 11.00 |
| 6 | 7 | 2 | 152.38 | 13.70 |
| 7 | -7 | 2 | 122.09 | 12.20 |
| 7 | -7 | 2 | 146.69 | 11.50 |
| 7 | 7 | 2 | 138.59 | 13.10 |
| 7 | 7 | 2 | 144.69 | 9.70 |
| 8 | -7 | 2 | -1.70 | 4.30 |
| 8 | -7 | 2 | -17.70 | 8.90 |
| 8 | -7 | 2 | -5.60 | 4.30 |
| 8 | 7 | 2 | 12.00 | 5.60 |
| 8 | 7 | 2 | -10.00 | 6.00 |
| 8 | 7 | 2 | 9.60 | 6.40 |
| 8 | 7 | 2 | 2.10 | 2.60 |
| 9 | -7 | 2 | 1038.10 | 57.39 |
| 9 | -7 | 2 | 996.80 | 56.89 |
| 9 | -7 | 2 | 962.80 | 58.39 |
| 9 | 7 | 2 | 1030.70 | 56.59 |
| 9 | 7 | 2 | 1041.30 | 56.69 |
| 9 | 7 | 2 | 1042.90 | 57.09 |
| 10 | -7 | 2 | 16.00 | 4.70 |
| 10 | -7 | 2 | 20.30 | 4.60 |
| 10 | -7 | 2 | 25.90 | 7.40 |
| 10 | 7 | 2 | 27.80 | 5.90 |
| 10 | 7 | 2 | 7.90 | 4.80 |
| 10 | 7 | 2 | 25.90 | 6.10 |
| 11 | -7 | 2 | 1120.99 | 65.89 |
| 11 | -7 | 2 | 1223.28 | 65.49 |
| 11 | -7 | 2 | 1228.58 | 65.39 |
| 11 | 7 | 2 | 1194.08 | 64.79 |
| 11 | 7 | 2 | 1081.19 | 65.29 |
| 11 | 7 | 2 | 1206.18 | 65.39 |
| 12 | -7 | 2 | 174.08 | 13.50 |
| 12 | -7 | 2 | 185.08 | 12.30 |
| 12 | -7 | 2 | 171.48 | 12.60 |
| 12 | 7 | 2 | 153.28 | 13.50 |
| 12 | 7 | 2 | 163.18 | 12.50 |
| 12 | 7 | 2 | 187.88 | 12.50 |
| 13 | -7 | 2 | 197.98 | 17.30 |
| 13 | -7 | 2 | 192.98 | 12.90 |
| 13 | -7 | 2 | 192.08 | 13.10 |

| | | | | |
|---|---|---|---|---|
| 13 | 7 | 2 | 179.28 | 13.00 |
| 13 | 7 | 2 | 172.08 | 18.10 |
| 13 | 7 | 2 | 210.58 | 13.20 |
| 14 | -7 | 2 | -0.90 | 2.60 |
| 14 | -7 | 2 | 0.80 | 2.70 |
| 14 | 7 | 2 | -0.50 | 3.70 |
| 14 | 7 | 2 | -2.40 | 3.60 |
| 14 | 7 | 2 | 0.60 | 2.90 |
| 15 | -7 | 2 | 307.97 | 18.60 |
| 15 | -7 | 2 | 335.97 | 18.40 |
| 15 | 7 | 2 | 316.87 | 18.60 |
| 15 | 7 | 2 | 314.77 | 18.70 |
| 15 | -8 | -2 | 1.70 | 2.50 |
| 15 | -8 | -2 | 1.40 | 1.80 |
| 15 | 8 | -2 | 0.40 | 1.70 |
| 14 | -8 | -2 | 312.77 | 18.80 |
| 14 | -8 | -2 | 297.67 | 18.40 |
| 14 | 8 | -2 | 313.67 | 17.90 |
| 14 | 8 | -2 | 323.87 | 19.50 |
| 13 | -8 | -2 | 108.79 | 8.70 |
| 13 | -8 | -2 | 113.09 | 8.70 |
| 13 | 8 | -2 | 100.09 | 10.10 |
| 13 | 8 | -2 | 101.59 | 7.70 |
| 12 | -8 | -2 | 145.49 | 11.00 |
| 12 | -8 | -2 | 137.79 | 10.90 |
| 12 | 8 | -2 | 145.29 | 9.70 |
| 12 | 8 | -2 | 128.19 | 12.40 |
| 11 | -8 | -2 | 4.60 | 3.30 |
| 11 | -8 | -2 | 0.30 | 3.80 |
| 11 | 8 | -2 | 6.30 | 3.00 |
| 11 | 8 | -2 | -1.90 | 4.70 |
| 10 | -8 | -2 | 1602.34 | 94.09 |
| 10 | -8 | -2 | 1716.23 | 94.29 |
| 10 | 8 | -2 | 1707.33 | 93.29 |
| 10 | 8 | -2 | 1832.82 | 93.99 |
| 9 | -8 | -2 | 108.09 | 11.90 |
| 9 | -8 | -2 | 126.29 | 10.80 |
| 9 | 8 | -2 | 138.79 | 10.40 |
| 9 | 8 | -2 | 127.79 | 9.80 |
| 8 | -8 | -2 | 625.74 | 37.90 |
| 8 | -8 | -2 | 667.13 | 37.90 |
| 8 | 8 | -2 | 671.43 | 36.80 |
| 8 | 8 | -2 | 626.94 | 37.00 |
| 8 | 8 | -2 | 669.53 | 38.20 |

| | | | | |
|---|---|---|---|---|
| 7 | -8 | -2 | 167.78 | 13.50 |
| 7 | -8 | -2 | 179.98 | 13.60 |
| 7 | 8 | -2 | 145.79 | 13.20 |
| 7 | 8 | -2 | 170.88 | 13.40 |
| 7 | 8 | -2 | 159.68 | 11.30 |
| 6 | -8 | -2 | 3436.16 | 197.18 |
| 6 | -8 | -2 | 4207.68 | 199.48 |
| 6 | 8 | -2 | 3537.75 | 195.68 |
| -6 | 8 | 2 | 3824.72 | 196.18 |
| 6 | 8 | -2 | 3180.78 | 196.98 |
| 5 | -8 | -2 | 311.17 | 21.10 |
| -5 | 8 | 2 | 344.17 | 19.40 |
| 5 | 8 | -2 | 289.67 | 20.70 |
| 5 | 8 | -2 | 330.57 | 19.00 |
| 4 | -8 | -2 | 1762.02 | 93.79 |
| -4 | 8 | 2 | 1815.72 | 93.09 |
| 4 | 8 | -2 | 1611.24 | 92.39 |
| 4 | 8 | -2 | 1625.54 | 93.89 |
| 3 | -8 | -2 | 72.99 | 8.60 |
| 3 | 8 | -2 | 69.79 | 11.40 |
| -3 | 8 | 2 | 78.19 | 6.70 |
| 2 | 8 | -2 | 102.69 | 8.90 |
| -2 | 8 | 2 | 97.59 | 8.00 |
| -1 | 8 | 2 | 117.89 | 8.70 |
| 1 | 8 | -2 | 103.29 | 8.80 |
| 0 | 8 | 2 | 8196.18 | 451.55 |
| 0 | 8 | -2 | 8497.05 | 451.55 |
| 0 | 8 | 2 | 8509.75 | 450.95 |
| 1 | 8 | 2 | 42.40 | 5.80 |
| 1 | 8 | 2 | 35.10 | 5.70 |
| -1 | 8 | -2 | 47.90 | 5.70 |
| -2 | 8 | -2 | 636.94 | 34.40 |
| 2 | 8 | 2 | 571.24 | 33.90 |
| 3 | -8 | 2 | 308.27 | 18.90 |
| 3 | 8 | 2 | 278.17 | 17.50 |
| 3 | 8 | 2 | 288.07 | 18.70 |
| -3 | 8 | -2 | 306.97 | 18.10 |
| 4 | -8 | 2 | 2317.77 | 124.09 |
| 4 | 8 | 2 | 2223.28 | 123.39 |
| 4 | 8 | 2 | 2120.19 | 124.69 |
| -4 | 8 | -2 | 2458.25 | 123.99 |
| 5 | -8 | 2 | -0.70 | 4.50 |
| -5 | 8 | -2 | 5.60 | 3.40 |
| 5 | 8 | 2 | 1.90 | 4.10 |

| | | | | |
|---|---|---|---|---|
| 5 | 8 | 2 | 1.30 | 2.50 |
| 6 | -8 | 2 | 2186.98 | 117.99 |
| -6 | 8 | -2 | 2423.76 | 118.39 |
| 6 | 8 | 2 | 2129.29 | 118.89 |
| 6 | 8 | 2 | 1947.61 | 119.39 |
| 7 | -8 | 2 | 307.37 | 20.30 |
| 7 | -8 | 2 | 321.57 | 19.20 |
| 7 | 8 | 2 | 302.57 | 18.20 |
| 7 | 8 | 2 | 271.07 | 20.50 |
| 7 | 8 | 2 | 317.07 | 21.30 |
| 8 | -8 | 2 | 408.46 | 25.60 |
| 8 | -8 | 2 | 441.96 | 26.70 |
| 8 | 8 | 2 | 442.06 | 28.30 |
| 8 | 8 | 2 | 430.66 | 27.50 |
| 8 | 8 | 2 | 422.36 | 26.00 |
| 8 | 8 | 2 | 438.56 | 25.00 |
| 9 | -8 | 2 | 14.30 | 5.00 |
| 9 | -8 | 2 | 15.50 | 8.30 |
| 9 | -8 | 2 | 16.40 | 4.90 |
| 9 | 8 | 2 | 27.80 | 6.80 |
| 9 | 8 | 2 | 13.50 | 5.20 |
| 9 | 8 | 2 | 11.40 | 3.30 |
| 10 | -8 | 2 | 1084.99 | 65.59 |
| 10 | -8 | 2 | 1240.78 | 64.99 |
| 10 | -8 | 2 | 1152.88 | 65.19 |
| 10 | 8 | 2 | 1146.19 | 64.99 |
| 10 | 8 | 2 | 1249.57 | 64.69 |
| 10 | 8 | 2 | 1129.59 | 64.29 |
| 11 | -8 | 2 | 3.40 | 5.60 |
| 11 | -8 | 2 | 0.50 | 3.50 |
| 11 | -8 | 2 | 4.00 | 4.80 |
| 11 | 8 | 2 | 8.70 | 4.10 |
| 11 | 8 | 2 | -0.90 | 5.40 |
| 12 | -8 | 2 | 199.08 | 14.20 |
| 12 | -8 | 2 | 191.88 | 13.10 |
| 12 | -8 | 2 | 177.28 | 12.60 |
| 12 | 8 | 2 | 192.68 | 12.70 |
| 12 | 8 | 2 | 174.28 | 12.80 |
| 12 | 8 | 2 | 162.78 | 14.30 |
| 13 | -8 | 2 | 72.69 | 7.90 |
| 13 | -8 | 2 | 68.99 | 6.90 |
| 13 | 8 | 2 | 81.59 | 7.80 |
| 13 | 8 | 2 | 76.99 | 7.90 |
| 13 | 8 | 2 | 74.59 | 9.60 |

| | | | | |
|---|---|---|---|---|
| 14 | -8 | 2 | 300.97 | 18.50 |
| 14 | -8 | 2 | 324.37 | 18.40 |
| 14 | 8 | 2 | 316.47 | 18.60 |
| 14 | 8 | 2 | 307.17 | 19.30 |
| 15 | -8 | 2 | 8.30 | 2.60 |
| 15 | -8 | 2 | 2.20 | 1.80 |
| 15 | 8 | 2 | -1.80 | 2.60 |
| 15 | -9 | -2 | 386.76 | 22.00 |
| 15 | -9 | -2 | 389.36 | 22.40 |
| 15 | 9 | -2 | 386.76 | 21.70 |
| -15 | 9 | 2 | 402.86 | 22.10 |
| 14 | -9 | -2 | 66.19 | 5.90 |
| 14 | -9 | -2 | 63.89 | 6.20 |
| 14 | 9 | -2 | 69.09 | 5.20 |
| 13 | -9 | -2 | 35.80 | 5.70 |
| 13 | -9 | -2 | 35.40 | 6.30 |
| 13 | 9 | -2 | 56.79 | 7.70 |
| 13 | 9 | -2 | 42.30 | 8.50 |
| 12 | -9 | -2 | 404.56 | 22.40 |
| 12 | -9 | -2 | 354.66 | 22.20 |
| 12 | 9 | -2 | 387.46 | 21.30 |
| 12 | 9 | -2 | 328.57 | 22.80 |
| 11 | -9 | -2 | 1068.39 | 60.29 |
| 11 | -9 | -2 | 1067.59 | 60.29 |
| 11 | 9 | -2 | 1106.39 | 60.39 |
| 11 | 9 | -2 | 1083.79 | 59.29 |
| 10 | -9 | -2 | 92.59 | 10.70 |
| 10 | -9 | -2 | 107.59 | 9.50 |
| 10 | 9 | -2 | 109.79 | 10.30 |
| 10 | 9 | -2 | 112.69 | 8.60 |
| 9 | -9 | -2 | 632.34 | 35.50 |
| 9 | -9 | -2 | 585.94 | 35.30 |
| 9 | 9 | -2 | 595.94 | 34.30 |
| 9 | 9 | -2 | 604.74 | 34.50 |
| 8 | -9 | -2 | 520.75 | 31.30 |
| 8 | -9 | -2 | 557.34 | 31.80 |
| 8 | 9 | -2 | 529.95 | 30.20 |
| 8 | 9 | -2 | 500.75 | 30.60 |
| 8 | 9 | -2 | 537.95 | 32.30 |
| -7 | -9 | 2 | 99.59 | 15.70 |
| 7 | -9 | -2 | 145.19 | 12.40 |
| 7 | -9 | -2 | 139.59 | 12.00 |
| 7 | 9 | -2 | 124.89 | 10.40 |
| 7 | 9 | -2 | 131.29 | 12.50 |

| | | | | |
|---|---|---|---|---|
| 6 | -9 | -2 | 55.29 | 9.70 |
| 6 | -9 | -2 | 58.19 | 9.60 |
| -6 | -9 | 2 | 54.29 | 11.30 |
| 6 | 9 | -2 | 61.69 | 6.60 |
| 6 | 9 | -2 | 39.20 | 8.80 |
| 5 | -9 | -2 | 4324.07 | 237.38 |
| -5 | 9 | 2 | 4843.32 | 236.88 |
| 5 | 9 | -2 | 4656.53 | 236.08 |
| 5 | 9 | -2 | 3712.73 | 237.48 |
| 4 | -9 | -2 | 1010.60 | 54.79 |
| -4 | 9 | 2 | 1047.80 | 54.09 |
| 4 | 9 | -2 | 914.81 | 54.99 |
| 4 | 9 | -2 | 902.81 | 53.29 |
| 3 | 9 | -2 | 459.35 | 28.20 |
| -3 | 9 | 2 | 486.15 | 27.40 |
| 3 | 9 | -2 | 457.15 | 26.80 |
| 2 | 9 | -2 | 642.84 | 36.90 |
| 2 | 9 | -2 | 645.64 | 38.00 |
| -2 | 9 | 2 | 685.43 | 37.50 |
| 1 | 9 | -2 | 1145.79 | 63.59 |
| -1 | 9 | 2 | 1149.69 | 63.29 |
| 1 | 9 | -2 | 1129.69 | 62.59 |
| 0 | 9 | -2 | 228.28 | 14.60 |
| 0 | 9 | 2 | 227.38 | 14.60 |
| 0 | 9 | 2 | 210.98 | 14.10 |
| 1 | 9 | 2 | 2945.11 | 158.08 |
| -1 | 9 | -2 | 2948.80 | 158.68 |
| 1 | 9 | 2 | 2883.31 | 158.88 |
| 2 | 9 | 2 | 303.37 | 17.90 |
| -2 | 9 | -2 | 309.67 | 18.30 |
| 2 | 9 | 2 | 278.87 | 18.70 |
| 3 | -9 | 2 | 158.58 | 12.00 |
| 3 | 9 | 2 | 155.98 | 10.60 |
| -3 | 9 | -2 | 160.68 | 11.10 |
| 3 | 9 | 2 | 153.58 | 12.00 |
| 4 | -9 | 2 | 198.98 | 14.00 |
| 4 | -9 | 2 | 215.08 | 14.20 |
| 4 | 9 | 2 | 188.78 | 14.50 |
| -4 | 9 | -2 | 195.18 | 13.30 |
| 4 | 9 | 2 | 199.68 | 12.70 |
| -5 | -9 | -2 | 3255.77 | 183.98 |
| 5 | -9 | 2 | 3672.43 | 184.78 |
| 5 | -9 | 2 | 3275.57 | 183.78 |
| -5 | 9 | -2 | 3579.84 | 184.28 |

| | | | | |
|---|---|---|---|---|
| 5 | 9 | 2 | 3241.38 | 185.58 |
| 6 | -9 | 2 | 223.68 | 14.80 |
| -6 | -9 | -2 | 222.08 | 15.70 |
| 6 | -9 | 2 | 242.38 | 16.00 |
| 6 | 9 | 2 | 200.68 | 16.90 |
| -6 | 9 | -2 | 228.38 | 14.70 |
| 6 | 9 | 2 | 218.68 | 13.70 |
| 6 | 9 | 2 | 176.08 | 18.00 |
| 7 | -9 | 2 | 134.19 | 10.50 |
| -7 | -9 | -2 | 124.19 | 12.70 |
| 7 | -9 | 2 | 124.79 | 11.60 |
| 7 | 9 | 2 | 128.79 | 14.00 |
| 7 | 9 | 2 | 100.29 | 13.20 |
| 7 | 9 | 2 | 116.29 | 8.90 |
| 8 | -9 | 2 | 221.38 | 16.00 |
| 8 | -9 | 2 | 225.08 | 15.80 |
| 8 | 9 | 2 | 212.58 | 13.90 |
| 8 | 9 | 2 | 220.48 | 15.90 |
| 8 | 9 | 2 | 215.48 | 19.00 |
| 9 | -9 | 2 | 761.42 | 42.70 |
| 9 | -9 | 2 | 761.22 | 41.90 |
| 9 | 9 | 2 | 705.73 | 41.30 |
| 9 | 9 | 2 | 721.43 | 41.60 |
| 10 | -9 | 2 | -0.70 | 3.40 |
| 10 | -9 | 2 | -2.60 | 3.60 |
| 10 | -9 | 2 | -6.30 | 8.10 |
| 10 | 9 | 2 | 3.20 | 2.90 |
| 10 | 9 | 2 | -1.90 | 4.30 |
| 11 | -9 | 2 | 862.41 | 46.80 |
| 11 | -9 | 2 | 874.31 | 47.20 |
| 11 | 9 | 2 | 849.72 | 46.30 |
| 11 | 9 | 2 | 769.92 | 47.20 |
| 11 | 9 | 2 | 798.02 | 46.60 |
| 12 | -9 | 2 | 76.49 | 7.30 |
| 12 | -9 | 2 | 76.29 | 7.90 |
| 12 | 9 | 2 | 62.49 | 8.50 |
| 12 | 9 | 2 | 75.69 | 7.90 |
| 12 | 9 | 2 | 62.19 | 11.60 |
| 13 | -9 | 2 | 267.17 | 15.80 |
| 13 | -9 | 2 | 237.08 | 15.60 |
| 13 | 9 | 2 | 254.27 | 15.80 |
| 14 | -9 | 2 | -3.80 | 2.40 |
| 14 | -9 | 2 | 4.70 | 2.30 |
| 14 | 9 | 2 | 0.80 | 3.00 |

| | | | | |
|---|---|---|---|---|
| 15 | -10 | -2 | 0.70 | 1.50 |
| 15 | 10 | -2 | -2.10 | 1.90 |
| 14 | -10 | -2 | 272.87 | 16.20 |
| 14 | -10 | -2 | 275.07 | 16.70 |
| 14 | 10 | -2 | 283.77 | 15.90 |
| -14 | 10 | 2 | 281.37 | 16.70 |
| 13 | -10 | -2 | 114.09 | 8.70 |
| 13 | -10 | -2 | 119.09 | 8.80 |
| 13 | 10 | -2 | 122.59 | 7.90 |
| 12 | -10 | -2 | 73.89 | 7.50 |
| 12 | -10 | -2 | 65.59 | 7.10 |
| 12 | 10 | -2 | 78.49 | 6.30 |
| 12 | 10 | -2 | 60.19 | 9.90 |
| 11 | -10 | -2 | 1.80 | 3.70 |
| 11 | -10 | -2 | 2.70 | 3.30 |
| 11 | 10 | -2 | 1.20 | 5.50 |
| 11 | 10 | -2 | 6.50 | 2.70 |
| 10 | -10 | -2 | 668.03 | 39.70 |
| 10 | -10 | -2 | 696.53 | 39.80 |
| 10 | 10 | -2 | 704.63 | 38.60 |
| 10 | 10 | -2 | 694.63 | 39.40 |
| 9 | -10 | -2 | 138.69 | 13.00 |
| 9 | -10 | -2 | 172.18 | 12.60 |
| 9 | 10 | -2 | 157.48 | 11.20 |
| 9 | 10 | -2 | 162.38 | 11.80 |
| 8 | -10 | -2 | 147.79 | 12.50 |
| 8 | -10 | -2 | 138.49 | 12.50 |
| 8 | 10 | -2 | 149.49 | 10.90 |
| 8 | 10 | -2 | 145.39 | 12.30 |
| 8 | 10 | -2 | 159.88 | 14.90 |
| -7 | -10 | 2 | 299.67 | 21.70 |
| 7 | -10 | -2 | 350.16 | 22.60 |
| 7 | -10 | -2 | 355.26 | 21.70 |
| 7 | 10 | -2 | 360.46 | 20.40 |
| 7 | 10 | -2 | 350.66 | 23.20 |
| -6 | -10 | 2 | 824.62 | 48.90 |
| 6 | -10 | -2 | 873.51 | 50.29 |
| 6 | -10 | -2 | 861.51 | 49.10 |
| 6 | 10 | -2 | 885.31 | 48.60 |
| 6 | 10 | -2 | 857.01 | 50.69 |
| -6 | 10 | 2 | 941.01 | 49.40 |
| -5 | -10 | 2 | 213.38 | 16.50 |
| 5 | -10 | -2 | 260.17 | 17.50 |
| 5 | -10 | -2 | 226.28 | 16.50 |

| | | | | |
|---|---|---|---|---|
| -5 | 10 | 2 | 265.37 | 16.10 |
| 5 | 10 | -2 | 222.38 | 17.80 |
| 5 | 10 | -2 | 258.47 | 15.20 |
| -4 | -10 | 2 | 1233.68 | 68.19 |
| -4 | -10 | 2 | 1337.27 | 68.29 |
| 4 | -10 | -2 | 1170.78 | 68.09 |
| 4 | 10 | -2 | 1172.58 | 67.39 |
| -4 | 10 | 2 | 1313.27 | 68.49 |
| 4 | 10 | -2 | 1177.38 | 69.59 |
| -3 | -10 | 2 | 377.86 | 24.50 |
| -3 | -10 | 2 | 381.16 | 24.40 |
| -3 | 10 | 2 | 445.76 | 24.40 |
| 3 | 10 | -2 | 415.86 | 23.50 |
| 3 | 10 | -2 | 390.56 | 25.10 |
| 2 | 10 | -2 | 140.69 | 11.60 |
| 2 | 10 | -2 | 135.79 | 10.70 |
| -2 | 10 | 2 | 170.58 | 11.30 |
| -1 | 10 | 2 | 44.20 | 6.40 |
| 1 | 10 | -2 | 53.99 | 6.70 |
| 1 | 10 | -2 | 55.69 | 6.90 |
| 0 | 10 | -2 | 4729.63 | 255.87 |
| 0 | 10 | 2 | 4665.03 | 254.97 |
| 0 | 10 | 2 | 4810.82 | 255.97 |
| 1 | 10 | 2 | 845.82 | 50.79 |
| 1 | 10 | 2 | 899.71 | 51.89 |
| -1 | 10 | -2 | 1012.20 | 51.89 |
| 2 | -10 | 2 | 23.90 | 4.60 |
| -2 | -10 | -2 | 21.60 | 4.90 |
| 2 | 10 | 2 | 29.20 | 6.00 |
| -2 | 10 | -2 | 25.90 | 5.20 |
| 2 | 10 | 2 | 18.30 | 3.30 |
| -3 | -10 | -2 | 20.40 | 5.00 |
| 3 | -10 | 2 | 19.20 | 4.40 |
| 3 | -10 | 2 | 17.30 | 4.30 |
| 3 | 10 | 2 | 8.60 | 4.00 |
| 3 | 10 | 2 | 10.90 | 3.40 |
| -3 | 10 | -2 | 13.10 | 3.50 |
| 4 | -10 | 2 | 865.41 | 51.99 |
| -4 | -10 | -2 | 860.61 | 50.19 |
| 4 | -10 | 2 | 913.21 | 49.99 |
| 4 | 10 | 2 | 904.01 | 49.70 |
| -4 | 10 | -2 | 993.90 | 50.69 |
| 4 | 10 | 2 | 846.02 | 51.59 |
| 5 | -10 | 2 | 3.40 | 4.30 |

| | | | | |
|---|---|---|---|---|
| 5 | -10 | 2 | 16.70 | 3.90 |
| -5 | -10 | -2 | 4.30 | 4.40 |
| 5 | 10 | 2 | 16.20 | 3.20 |
| 5 | 10 | 2 | 7.70 | 5.40 |
| -5 | 10 | -2 | 7.30 | 3.90 |
| 6 | -10 | 2 | 1682.13 | 92.29 |
| 6 | -10 | 2 | 1703.23 | 91.39 |
| -6 | -10 | -2 | 1741.43 | 91.59 |
| 6 | 10 | 2 | 1532.85 | 91.79 |
| 6 | 10 | 2 | 1787.42 | 91.19 |
| 6 | 10 | 2 | 1534.65 | 93.49 |
| -6 | 10 | -2 | 1723.23 | 91.89 |
| -7 | -10 | -2 | 57.19 | 8.50 |
| 7 | -10 | 2 | 20.00 | 5.20 |
| 7 | 10 | 2 | 32.90 | 5.10 |
| 7 | 10 | 2 | 30.90 | 8.50 |
| 7 | 10 | 2 | 36.70 | 6.50 |
| 8 | -10 | 2 | 65.89 | 8.20 |
| 8 | -10 | 2 | 91.79 | 10.20 |
| 8 | 10 | 2 | 75.89 | 15.60 |
| 8 | 10 | 2 | 91.69 | 10.60 |
| 8 | 10 | 2 | 81.49 | 7.20 |
| 9 | -10 | 2 | 13.10 | 4.70 |
| 9 | -10 | 2 | 11.60 | 3.60 |
| 9 | 10 | 2 | 17.90 | 3.60 |
| 9 | 10 | 2 | 20.80 | 4.80 |
| 10 | -10 | 2 | 901.01 | 51.69 |
| 10 | -10 | 2 | 885.31 | 50.09 |
| 10 | 10 | 2 | 873.51 | 49.50 |
| 10 | 10 | 2 | 913.91 | 50.09 |
| 11 | -10 | 2 | 43.10 | 6.90 |
| 11 | 10 | 2 | 56.39 | 6.60 |
| 11 | 10 | 2 | 44.00 | 7.60 |
| 12 | -10 | 2 | 6.50 | 3.30 |
| 12 | -10 | 2 | 8.20 | 3.90 |
| 12 | 10 | 2 | 12.00 | 6.00 |
| 12 | 10 | 2 | 3.90 | 3.50 |
| 13 | -10 | 2 | 31.90 | 4.90 |
| 13 | -10 | 2 | 29.50 | 4.90 |
| 13 | 10 | 2 | 32.40 | 5.90 |
| 14 | -10 | 2 | 216.58 | 13.00 |
| 14 | -10 | 2 | 216.88 | 12.70 |
| 14 | 10 | 2 | 205.78 | 13.10 |
| 14 | -11 | -2 | 10.80 | 2.10 |

| | | | | |
|---|---|---|---|---|
| 14 | 11 | -2 | 10.70 | 1.90 |
| 13 | -11 | -2 | 53.79 | 5.80 |
| 13 | 11 | -2 | 63.79 | 5.00 |
| 12 | -11 | -2 | 79.19 | 8.90 |
| 12 | -11 | -2 | 72.99 | 7.10 |
| 12 | 11 | -2 | 78.99 | 6.40 |
| 11 | -11 | -2 | 492.15 | 29.20 |
| 11 | -11 | -2 | 503.75 | 29.10 |
| 11 | 11 | -2 | 483.75 | 29.50 |
| 11 | 11 | -2 | 515.75 | 28.10 |
| 10 | -11 | -2 | 4.60 | 3.80 |
| 10 | -11 | -2 | 7.10 | 4.00 |
| 10 | 11 | -2 | -1.20 | 2.60 |
| 10 | 11 | -2 | 4.30 | 5.50 |
| 9 | -11 | -2 | 874.11 | 50.29 |
| 9 | -11 | -2 | 928.81 | 50.89 |
| 9 | 11 | -2 | 916.41 | 49.70 |
| 9 | 11 | -2 | 848.82 | 49.40 |
| -8 | -11 | 2 | 83.39 | 10.30 |
| 8 | -11 | -2 | 97.59 | 9.80 |
| 8 | -11 | -2 | 87.49 | 10.20 |
| 8 | 11 | -2 | 83.79 | 8.70 |
| 8 | 11 | -2 | 91.39 | 8.20 |
| 8 | 11 | -2 | 80.59 | 14.20 |
| 7 | -11 | -2 | 137.99 | 12.20 |
| -7 | -11 | 2 | 107.19 | 11.10 |
| 7 | -11 | -2 | 115.69 | 12.00 |
| 7 | 11 | -2 | 140.29 | 14.20 |
| 7 | 11 | -2 | 128.69 | 9.50 |
| 6 | -11 | -2 | 110.69 | 10.40 |
| -6 | -11 | 2 | 108.19 | 10.60 |
| 6 | 11 | -2 | 110.49 | 12.80 |
| 6 | 11 | -2 | 106.59 | 8.60 |
| 5 | -11 | -2 | 1906.11 | 106.89 |
| -5 | -11 | 2 | 2003.00 | 106.89 |
| 5 | 11 | -2 | 1740.83 | 108.79 |
| 5 | 11 | -2 | 2065.29 | 106.39 |
| -5 | 11 | 2 | 2087.39 | 107.59 |
| 4 | -11 | -2 | 79.09 | 10.00 |
| 4 | -11 | -2 | 89.89 | 14.40 |
| -4 | -11 | 2 | 100.79 | 9.70 |
| 4 | 11 | -2 | 84.69 | 11.00 |
| 4 | 11 | -2 | 105.99 | 8.10 |
| -4 | 11 | 2 | 96.69 | 9.20 |

| | | | | |
|---|---|---|---|---|
| -3 | -11 | 2 | 95.79 | 10.30 |
| 3 | -11 | -2 | 84.49 | 10.90 |
| 3 | -11 | -2 | 127.19 | 13.90 |
| -3 | -11 | 2 | 104.69 | 10.00 |
| 3 | 11 | -2 | 112.59 | 8.80 |
| 3 | 11 | -2 | 108.59 | 10.90 |
| -3 | 11 | 2 | 119.39 | 9.60 |
| -2 | -11 | 2 | 103.59 | 10.10 |
| -2 | -11 | 2 | 103.99 | 10.20 |
| 2 | -11 | -2 | 114.39 | 12.20 |
| 2 | -11 | -2 | 118.39 | 13.10 |
| 2 | 11 | -2 | 105.29 | 10.20 |
| -2 | 11 | 2 | 117.89 | 9.70 |
| 2 | 11 | -2 | 109.89 | 8.90 |
| -1 | -11 | 2 | 2773.52 | 151.98 |
| 1 | -11 | -2 | 2751.62 | 152.28 |
| -1 | -11 | 2 | 2789.62 | 151.68 |
| -1 | 11 | 2 | 2934.41 | 153.38 |
| 1 | 11 | -2 | 2775.02 | 151.58 |
| -1 | 11 | 2 | 2789.62 | 152.78 |
| 1 | 11 | -2 | 2815.52 | 153.08 |
| 0 | -11 | -2 | 7.90 | 5.00 |
| 0 | -11 | 2 | 0.30 | 4.50 |
| 0 | -11 | 2 | 1.70 | 4.00 |
| 0 | 11 | -2 | -1.00 | 2.90 |
| 0 | 11 | -2 | -0.20 | 3.40 |
| 0 | 11 | 2 | -1.80 | 3.30 |
| 1 | -11 | 2 | 1370.26 | 75.89 |
| 1 | -11 | 2 | 1350.26 | 76.49 |
| -1 | -11 | -2 | 1363.26 | 76.29 |
| 1 | 11 | 2 | 1439.26 | 76.19 |
| 1 | 11 | 2 | 1367.16 | 77.09 |
| -1 | 11 | -2 | 1425.36 | 76.89 |
| 2 | -11 | 2 | 214.58 | 14.70 |
| 2 | -11 | 2 | 231.58 | 16.80 |
| -2 | -11 | -2 | 212.78 | 14.80 |
| -2 | 11 | -2 | 226.88 | 15.00 |
| 2 | 11 | 2 | 205.58 | 15.40 |
| 2 | 11 | 2 | 221.48 | 13.90 |
| -3 | -11 | -2 | 116.19 | 10.10 |
| 3 | -11 | 2 | 93.39 | 9.50 |
| 3 | -11 | 2 | 120.49 | 13.80 |
| 3 | 11 | 2 | 106.99 | 8.30 |
| -3 | 11 | -2 | 109.09 | 9.50 |

| | | | | |
|---|---|---|---|---|
| 3 | 11 | 2 | 104.29 | 10.70 |
| 4 | -11 | 2 | 42.80 | 9.20 |
| -4 | -11 | -2 | 54.49 | 7.90 |
| 4 | -11 | 2 | 53.09 | 7.50 |
| 4 | 11 | 2 | 57.99 | 5.80 |
| -4 | 11 | -2 | 53.59 | 7.40 |
| 4 | 11 | 2 | 40.20 | 9.20 |
| 5 | -11 | 2 | 2313.77 | 128.09 |
| -5 | -11 | -2 | 2480.05 | 128.39 |
| 5 | -11 | 2 | 2436.36 | 128.79 |
| 5 | -11 | 2 | 2342.97 | 129.69 |
| 5 | 11 | 2 | 2219.58 | 130.49 |
| 5 | 11 | 2 | 2276.97 | 127.99 |
| -5 | 11 | -2 | 2462.95 | 129.09 |
| 6 | -11 | 2 | 255.67 | 16.20 |
| -6 | -11 | -2 | 220.88 | 15.20 |
| 6 | 11 | 2 | 230.98 | 14.00 |
| 6 | 11 | 2 | 186.98 | 18.60 |
| 6 | 11 | 2 | 223.98 | 17.60 |
| -7 | -11 | -2 | 60.09 | 8.30 |
| 7 | -11 | 2 | 42.70 | 9.00 |
| 7 | -11 | 2 | 53.59 | 7.10 |
| 7 | 11 | 2 | 49.20 | 5.80 |
| 7 | 11 | 2 | 65.29 | 14.80 |
| 8 | -11 | 2 | 29.80 | 6.30 |
| -8 | -11 | -2 | 29.20 | 5.00 |
| 8 | -11 | 2 | 39.80 | 6.80 |
| 8 | 11 | 2 | 31.20 | 5.40 |
| 8 | 11 | 2 | 45.80 | 11.00 |
| 8 | 11 | 2 | 40.00 | 5.30 |
| 9 | -11 | 2 | 489.85 | 29.20 |
| 9 | -11 | 2 | 477.05 | 27.80 |
| 9 | 11 | 2 | 466.05 | 27.50 |
| 9 | 11 | 2 | 468.35 | 27.60 |
| 10 | -11 | 2 | 11.80 | 3.80 |
| 10 | -11 | 2 | 12.80 | 4.20 |
| 10 | 11 | 2 | 12.90 | 4.30 |
| 10 | 11 | 2 | 13.90 | 3.70 |
| 11 | -11 | 2 | 584.24 | 31.80 |
| 11 | -11 | 2 | 526.65 | 31.30 |
| 11 | 11 | 2 | 525.35 | 31.10 |
| 12 | -11 | 2 | 102.59 | 8.30 |
| 12 | -11 | 2 | 99.19 | 8.30 |
| 12 | 11 | 2 | 99.99 | 8.40 |

| | | | | |
|---|---|---|---|---|
| 13 | -11 | 2 | 65.49 | 5.80 |
| 13 | -11 | 2 | 60.49 | 6.20 |
| 13 | 11 | 2 | 67.29 | 6.60 |
| 14 | -12 | -2 | 244.58 | 14.50 |
| 14 | 12 | -2 | 264.67 | 14.40 |
| 13 | -12 | -2 | 66.39 | 5.50 |
| 13 | 12 | -2 | 68.69 | 5.10 |
| 12 | -12 | -2 | 124.09 | 9.10 |
| 12 | 12 | -2 | 122.79 | 8.10 |
| 11 | -12 | -2 | 35.20 | 6.50 |
| 11 | 12 | -2 | 42.40 | 6.70 |
| 10 | -12 | -2 | 564.64 | 33.70 |
| 10 | 12 | -2 | 607.54 | 34.70 |
| 10 | 12 | -2 | 584.54 | 32.80 |
| 9 | -12 | -2 | 23.50 | 4.80 |
| 9 | 12 | -2 | 24.50 | 3.70 |
| -8 | -12 | 2 | 101.99 | 10.10 |
| 8 | -12 | -2 | 99.19 | 10.10 |
| 8 | 12 | -2 | 105.29 | 8.70 |
| 8 | 12 | -2 | 96.19 | 9.00 |
| -7 | -12 | 2 | 215.98 | 14.90 |
| 7 | -12 | -2 | 211.38 | 15.40 |
| 7 | 12 | -2 | 245.58 | 19.70 |
| 7 | 12 | -2 | 222.08 | 14.10 |
| -6 | -12 | 2 | 1343.07 | 74.89 |
| 6 | -12 | -2 | 1342.67 | 74.29 |
| 6 | 12 | -2 | 1348.47 | 76.99 |
| 6 | 12 | -2 | 1360.66 | 73.69 |
| -5 | -12 | 2 | 0.60 | 4.00 |
| 5 | -12 | -2 | 10.80 | 4.20 |
| 5 | -12 | -2 | -13.60 | 6.90 |
| 5 | 12 | -2 | 2.70 | 6.20 |
| 5 | 12 | -2 | 9.30 | 3.20 |
| -5 | 12 | 2 | 2.80 | 4.90 |
| 4 | -12 | -2 | 925.21 | 51.19 |
| -4 | -12 | 2 | 903.51 | 49.80 |
| 4 | -12 | -2 | 890.21 | 49.80 |
| -4 | 12 | 2 | 949.01 | 50.79 |
| 4 | 12 | -2 | 766.12 | 51.89 |
| 4 | 12 | -2 | 904.81 | 49.20 |
| -3 | -12 | 2 | 47.50 | 6.10 |
| 3 | -12 | -2 | 39.40 | 7.20 |
| -3 | -12 | 2 | 64.99 | 7.90 |
| 3 | -12 | -2 | 53.09 | 8.80 |

| | | | | |
|---|---|---|---|---|
| 3 | 12 | -2 | 47.70 | 9.20 |
| -3 | 12 | 2 | 58.89 | 7.90 |
| 3 | 12 | -2 | 29.90 | 6.40 |
| -2 | -12 | 2 | 104.29 | 9.60 |
| 2 | -12 | -2 | 94.29 | 11.50 |
| -2 | -12 | 2 | 84.59 | 9.70 |
| 2 | -12 | -2 | 121.89 | 12.40 |
| 2 | 12 | -2 | 120.79 | 8.90 |
| -2 | 12 | 2 | 113.19 | 10.10 |
| 2 | 12 | -2 | 115.39 | 11.10 |
| -1 | -12 | 2 | 518.75 | 30.10 |
| 1 | -12 | -2 | 531.35 | 30.80 |
| -1 | -12 | 2 | 530.05 | 29.90 |
| 1 | -12 | -2 | 493.75 | 30.60 |
| 1 | 12 | -2 | 537.35 | 29.60 |
| -1 | 12 | 2 | 483.25 | 29.60 |
| 1 | 12 | -2 | 511.95 | 31.20 |
| -1 | 12 | 2 | 516.65 | 30.90 |
| 0 | -12 | 2 | 2626.74 | 143.69 |
| 0 | -12 | -2 | 2626.64 | 143.89 |
| 0 | -12 | -2 | 2676.33 | 144.29 |
| 0 | -12 | 2 | 2618.24 | 144.09 |
| 0 | 12 | 2 | 2683.73 | 143.89 |
| 0 | 12 | 2 | 2663.73 | 145.19 |
| 0 | 12 | -2 | 2720.13 | 143.69 |
| 0 | 12 | -2 | 2618.64 | 145.09 |
| -1 | -12 | -2 | 22.50 | 6.10 |
| 1 | -12 | 2 | 26.30 | 5.80 |
| -1 | -12 | -2 | 22.10 | 4.60 |
| 1 | -12 | 2 | 21.60 | 4.50 |
| 1 | 12 | 2 | 29.50 | 5.80 |
| 1 | 12 | 2 | 13.60 | 3.30 |
| -1 | 12 | -2 | 23.10 | 3.90 |
| -1 | 12 | -2 | 19.30 | 4.80 |
| 2 | -12 | 2 | 4.60 | 5.50 |
| -2 | -12 | -2 | 10.40 | 5.80 |
| -2 | -12 | -2 | 15.60 | 4.40 |
| 2 | -12 | 2 | 11.30 | 4.10 |
| 2 | 12 | 2 | 7.50 | 3.00 |
| 2 | 12 | 2 | 12.50 | 4.90 |
| -2 | 12 | -2 | 3.90 | 3.50 |
| 3 | -12 | 2 | 68.19 | 7.90 |
| -3 | -12 | -2 | 79.39 | 8.50 |
| 3 | 12 | 2 | 66.39 | 9.80 |

| | | | | |
|---|---|---|---|---|
| -3 | 12 | -2 | 72.39 | 8.60 |
| 3 | 12 | 2 | 66.59 | 6.70 |
| 4 | -12 | 2 | 1792.42 | 96.29 |
| -4 | -12 | -2 | 1751.02 | 94.99 |
| 4 | -12 | 2 | 1634.34 | 94.69 |
| 4 | 12 | 2 | 1750.02 | 94.69 |
| 4 | 12 | 2 | 1695.93 | 97.29 |
| -4 | 12 | -2 | 1805.22 | 95.99 |
| 5 | -12 | 2 | 132.49 | 15.80 |
| 5 | -12 | 2 | 104.49 | 9.50 |
| 5 | 12 | 2 | 119.69 | 8.70 |
| -5 | 12 | -2 | 125.49 | 11.70 |
| 5 | 12 | 2 | 97.59 | 14.10 |
| 6 | -12 | 2 | 624.94 | 35.40 |
| -6 | -12 | -2 | 628.24 | 35.40 |
| 6 | -12 | 2 | 678.93 | 36.50 |
| 6 | 12 | 2 | 600.64 | 36.40 |
| 6 | 12 | 2 | 604.24 | 35.00 |
| 6 | 12 | 2 | 593.54 | 40.60 |
| 7 | -12 | 2 | 249.38 | 16.50 |
| 7 | -12 | 2 | 235.68 | 15.10 |
| -7 | -12 | -2 | 204.38 | 15.10 |
| 7 | 12 | 2 | 248.58 | 22.60 |
| 7 | 12 | 2 | 221.28 | 16.30 |
| 7 | 12 | 2 | 236.68 | 14.40 |
| -8 | -12 | -2 | 315.17 | 19.70 |
| 8 | -12 | 2 | 329.07 | 21.10 |
| 8 | -12 | 2 | 332.77 | 19.70 |
| 8 | 12 | 2 | 319.37 | 20.00 |
| 8 | 12 | 2 | 314.47 | 19.10 |
| 9 | -12 | 2 | 70.69 | 9.20 |
| 9 | -12 | 2 | 84.69 | 7.80 |
| 9 | 12 | 2 | 72.29 | 8.60 |
| 9 | 12 | 2 | 69.09 | 6.90 |
| 10 | -12 | 2 | 667.63 | 38.30 |
| 10 | -12 | 2 | 675.03 | 37.70 |
| 10 | 12 | 2 | 653.13 | 37.40 |
| 11 | -12 | 2 | 28.90 | 6.30 |
| 11 | -12 | 2 | 40.90 | 5.80 |
| 11 | 12 | 2 | 35.90 | 6.00 |
| 12 | -12 | 2 | 24.90 | 4.60 |
| 12 | 12 | 2 | 29.50 | 5.90 |
| 13 | -12 | 2 | 41.60 | 4.20 |
| 13 | 12 | 2 | 29.20 | 5.50 |

| | | | | |
|---|---|---|---|---|
| 13 | -13 | -2 | 55.19 | 4.80 |
| 13 | 13 | -2 | 63.19 | 4.50 |
| 12 | -13 | -2 | 80.39 | 6.50 |
| 12 | 13 | -2 | 80.99 | 5.80 |
| 11 | -13 | -2 | 561.14 | 31.70 |
| 11 | 13 | -2 | 555.34 | 30.90 |
| 10 | -13 | -2 | 2.40 | 3.20 |
| 10 | 13 | -2 | 5.00 | 2.40 |
| 9 | -13 | -2 | 463.05 | 27.10 |
| 9 | 13 | -2 | 454.05 | 26.20 |
| 8 | -13 | -2 | 13.40 | 4.40 |
| -8 | -13 | 2 | 17.30 | 4.20 |
| 8 | 13 | -2 | 9.80 | 6.30 |
| 8 | 13 | -2 | 13.60 | 3.70 |
| -7 | -13 | 2 | 31.60 | 6.70 |
| 7 | -13 | -2 | 44.10 | 7.70 |
| 7 | 13 | -2 | 36.70 | 6.20 |
| 7 | 13 | -2 | 32.60 | 5.30 |
| 7 | 13 | -2 | 32.30 | 9.30 |
| -6 | -13 | 2 | 58.39 | 7.80 |
| 6 | -13 | -2 | 79.19 | 8.90 |
| 6 | 13 | -2 | 73.59 | 7.10 |
| 6 | 13 | -2 | 64.19 | 13.90 |
| 5 | -13 | -2 | 1422.96 | 79.79 |
| 5 | -13 | -2 | 1501.75 | 81.49 |
| -5 | -13 | 2 | 1531.25 | 79.79 |
| 5 | 13 | -2 | 1464.15 | 79.19 |
| 5 | 13 | -2 | 1314.77 | 82.99 |
| -5 | 13 | 2 | 1481.85 | 80.99 |
| 4 | -13 | -2 | 31.90 | 6.80 |
| -4 | -13 | 2 | 33.10 | 7.00 |
| 4 | -13 | -2 | 17.90 | 6.60 |
| 4 | 13 | -2 | 20.10 | 6.90 |
| 4 | 13 | -2 | 34.80 | 6.00 |
| -4 | 13 | 2 | 40.90 | 8.80 |
| -3 | -13 | 2 | 108.59 | 13.10 |
| -3 | -13 | 2 | 143.89 | 11.60 |
| 3 | -13 | -2 | 128.99 | 11.50 |
| 3 | -13 | -2 | 127.59 | 12.90 |
| 3 | 13 | -2 | 128.49 | 13.60 |
| -3 | 13 | 2 | 143.89 | 11.80 |
| 3 | 13 | -2 | 124.69 | 9.80 |
| -2 | -13 | 2 | 43.90 | 7.40 |
| 2 | -13 | -2 | 43.90 | 9.40 |

| | | | | |
|---|---|---|---|---|
| 2 | -13 | -2 | 45.50 | 8.70 |
| -2 | -13 | 2 | 54.39 | 7.30 |
| -2 | 13 | 2 | 40.50 | 6.10 |
| 2 | 13 | -2 | 41.50 | 6.50 |
| -2 | 13 | 2 | 53.69 | 8.20 |
| 2 | 13 | -2 | 43.20 | 9.50 |
| 1 | -13 | -2 | 1154.28 | 66.39 |
| 1 | -13 | -2 | 1176.48 | 65.99 |
| -1 | -13 | 2 | 1233.48 | 66.19 |
| -1 | -13 | 2 | 1199.78 | 65.89 |
| -1 | 13 | 2 | 1172.98 | 67.29 |
| -1 | 13 | 2 | 1214.78 | 65.99 |
| 1 | 13 | -2 | 1197.08 | 67.69 |
| 1 | 13 | -2 | 1215.18 | 65.79 |
| 0 | -13 | -2 | 4.30 | 4.70 |
| 0 | -13 | 2 | -2.30 | 4.30 |
| 0 | -13 | 2 | 1.10 | 3.40 |
| 0 | -13 | -2 | -4.40 | 3.70 |
| 0 | 13 | 2 | -0.60 | 4.00 |
| 0 | 13 | -2 | 0.60 | 3.30 |
| 0 | 13 | 2 | 4.10 | 2.60 |
| 0 | 13 | -2 | -2.90 | 4.50 |
| 1 | -13 | 2 | 861.31 | 47.10 |
| -1 | -13 | -2 | 787.52 | 46.50 |
| -1 | -13 | -2 | 821.42 | 46.80 |
| 1 | -13 | 2 | 792.22 | 46.20 |
| 1 | 13 | 2 | 848.42 | 48.10 |
| 1 | 13 | 2 | 873.41 | 46.40 |
| -1 | 13 | -2 | 850.91 | 46.30 |
| -1 | 13 | -2 | 793.32 | 47.60 |
| 2 | -13 | 2 | 109.69 | 9.80 |
| -2 | -13 | -2 | 117.19 | 10.10 |
| 2 | -13 | 2 | 129.29 | 12.20 |
| -2 | -13 | -2 | 116.59 | 12.10 |
| 2 | 13 | 2 | 118.59 | 9.00 |
| 2 | 13 | 2 | 123.59 | 11.80 |
| -2 | 13 | -2 | 114.59 | 10.90 |
| -3 | -13 | -2 | 88.39 | 8.70 |
| 3 | -13 | 2 | 85.99 | 8.60 |
| 3 | -13 | 2 | 107.59 | 13.20 |
| 3 | 13 | 2 | 94.69 | 11.90 |
| 3 | 13 | 2 | 102.79 | 7.90 |
| -3 | 13 | -2 | 104.49 | 10.30 |
| 4 | -13 | 2 | 234.48 | 18.40 |

| | | | | |
|---|---|---|---|---|
| -4 | -13 | -2 | 218.38 | 15.10 |
| 4 | -13 | 2 | 235.68 | 14.80 |
| -4 | 13 | -2 | 240.58 | 17.10 |
| 4 | 13 | 2 | 203.18 | 23.60 |
| 4 | 13 | 2 | 232.38 | 14.40 |
| 5 | -13 | 2 | 1296.97 | 69.79 |
| -5 | -13 | -2 | 1276.37 | 69.89 |
| 5 | -13 | 2 | 1286.27 | 71.09 |
| -5 | 13 | -2 | 1333.67 | 71.69 |
| 5 | 13 | 2 | 1116.99 | 72.89 |
| 5 | 13 | 2 | 1302.47 | 69.59 |
| -6 | -13 | -2 | 92.29 | 8.80 |
| 6 | -13 | 2 | 100.49 | 8.40 |
| 6 | 13 | 2 | 87.39 | 17.50 |
| 6 | 13 | 2 | 91.99 | 11.40 |
| 6 | 13 | 2 | 98.29 | 7.50 |
| 7 | -13 | 2 | 51.49 | 6.60 |
| -7 | -13 | -2 | 43.90 | 6.70 |
| 7 | 13 | 2 | 46.30 | 5.70 |
| 7 | 13 | 2 | 56.59 | 12.80 |
| 7 | 13 | 2 | 45.70 | 6.10 |
| -8 | -13 | -2 | 25.80 | 4.10 |
| 8 | -13 | 2 | 16.00 | 3.70 |
| 8 | 13 | 2 | 20.60 | 4.50 |
| 8 | 13 | 2 | 22.50 | 4.80 |
| 9 | -13 | 2 | 402.86 | 23.30 |
| 9 | 13 | 2 | 385.56 | 22.90 |
| 10 | -13 | 2 | 0.70 | 3.20 |
| 10 | 13 | 2 | 2.00 | 3.00 |
| 11 | -13 | 2 | 254.87 | 15.70 |
| 11 | 13 | 2 | 263.67 | 15.90 |
| 12 | -13 | 2 | 99.89 | 6.90 |
| 12 | 13 | 2 | 88.29 | 7.80 |
| 12 | -14 | -2 | 25.20 | 3.70 |
| 12 | 14 | -2 | 23.00 | 3.30 |
| 11 | -14 | -2 | 11.10 | 2.70 |
| 11 | 14 | -2 | 12.50 | 2.20 |
| 10 | -14 | -2 | 540.35 | 30.20 |
| 10 | 14 | -2 | 517.65 | 29.40 |
| 9 | -14 | -2 | 15.00 | 3.90 |
| 9 | 14 | -2 | 2.20 | 2.40 |
| 8 | -14 | -2 | 191.88 | 13.20 |
| -8 | -14 | 2 | 178.78 | 12.80 |
| 8 | 14 | -2 | 189.18 | 12.30 |

| | | | | |
|---|---|---|---|---|
| -7 | -14 | 2 | 86.59 | 7.90 |
| 7 | -14 | -2 | 85.19 | 8.90 |
| 7 | 14 | -2 | 83.69 | 8.30 |
| 7 | 14 | -2 | 54.99 | 12.50 |
| 7 | 14 | -2 | 76.29 | 7.90 |
| 6 | -14 | -2 | 743.53 | 41.70 |
| -6 | -14 | 2 | 748.23 | 41.40 |
| 6 | 14 | -2 | 694.63 | 46.00 |
| 6 | 14 | -2 | 750.82 | 40.90 |
| 5 | -14 | -2 | 18.50 | 4.30 |
| 5 | -14 | -2 | 6.40 | 5.70 |
| -5 | -14 | 2 | 12.60 | 3.60 |
| 5 | 14 | -2 | 11.60 | 3.30 |
| 5 | 14 | -2 | 5.90 | 8.00 |
| 4 | -14 | -2 | 875.01 | 50.69 |
| -4 | -14 | 2 | 892.31 | 49.90 |
| 4 | -14 | -2 | 907.21 | 49.99 |
| -4 | 14 | 2 | 928.81 | 51.59 |
| 4 | 14 | -2 | 864.31 | 49.30 |
| 3 | -14 | -2 | 14.00 | 4.40 |
| -3 | -14 | 2 | 9.40 | 3.60 |
| -3 | -14 | 2 | 5.90 | 3.60 |
| 3 | -14 | -2 | 7.70 | 5.00 |
| -3 | 14 | 2 | 6.20 | 3.20 |
| -3 | 14 | 2 | 11.80 | 5.80 |
| 3 | 14 | -2 | 6.20 | 3.40 |
| 3 | 14 | -2 | 12.90 | 7.30 |
| -2 | -14 | 2 | 4.30 | 3.60 |
| -2 | -14 | 2 | 5.70 | 3.60 |
| 2 | -14 | -2 | 2.60 | 4.60 |
| 2 | -14 | -2 | 7.20 | 4.20 |
| 2 | 14 | -2 | 2.10 | 5.00 |
| 2 | 14 | -2 | 4.40 | 3.30 |
| -2 | 14 | 2 | 0.50 | 4.60 |
| -2 | 14 | 2 | 6.60 | 3.00 |
| 1 | -14 | -2 | 422.16 | 25.70 |
| 1 | -14 | -2 | 402.86 | 25.10 |
| -1 | -14 | 2 | 410.16 | 25.10 |
| -1 | -14 | 2 | 419.66 | 24.70 |
| -1 | 14 | 2 | 388.06 | 24.60 |
| 1 | 14 | -2 | 443.96 | 24.60 |
| -1 | 14 | 2 | 465.35 | 30.10 |
| 1 | 14 | -2 | 418.26 | 27.20 |
| 0 | -14 | -2 | 1769.92 | 93.59 |

| | | | | |
|---|---|---|---|---|
| 0 | -14 | 2 | 1694.23 | 92.99 |
| 0 | -14 | 2 | 1726.03 | 93.49 |
| 0 | -14 | -2 | 1642.74 | 93.29 |
| 0 | 14 | -2 | 1692.93 | 92.99 |
| 0 | 14 | 2 | 1740.23 | 95.39 |
| 0 | 14 | 2 | 1603.74 | 93.09 |
| 0 | 14 | -2 | 1774.92 | 95.49 |
| 1 | -14 | 2 | 127.09 | 9.90 |
| 1 | -14 | 2 | 131.69 | 11.70 |
| -1 | -14 | -2 | 135.29 | 10.70 |
| -1 | -14 | -2 | 115.39 | 11.70 |
| 1 | 14 | 2 | 141.09 | 12.60 |
| -1 | 14 | -2 | 135.89 | 10.00 |
| 1 | 14 | 2 | 121.19 | 9.50 |
| -1 | 14 | -2 | 121.69 | 12.20 |
| -2 | -14 | -2 | 378.76 | 22.90 |
| 2 | -14 | 2 | 395.26 | 24.10 |
| 2 | -14 | 2 | 384.86 | 22.70 |
| 2 | 14 | 2 | 389.16 | 22.60 |
| 2 | 14 | 2 | 375.36 | 25.40 |
| -2 | 14 | -2 | 370.26 | 22.60 |
| -2 | 14 | -2 | 385.36 | 24.60 |
| 3 | -14 | 2 | 66.99 | 10.70 |
| -3 | -14 | -2 | 81.39 | 7.80 |
| 3 | -14 | 2 | 78.69 | 7.60 |
| 3 | 14 | 2 | 69.29 | 6.80 |
| 3 | 14 | 2 | 72.39 | 11.90 |
| -3 | 14 | -2 | 71.49 | 10.40 |
| 4 | -14 | 2 | 333.97 | 22.80 |
| 4 | -14 | 2 | 348.17 | 20.30 |
| -4 | -14 | -2 | 340.27 | 20.50 |
| -4 | 14 | -2 | 379.86 | 23.30 |
| 4 | 14 | 2 | 323.07 | 20.00 |
| 4 | 14 | 2 | 300.97 | 24.70 |
| -5 | -14 | -2 | 114.09 | 9.50 |
| 5 | -14 | 2 | 127.69 | 9.30 |
| 5 | -14 | 2 | 110.69 | 13.90 |
| 5 | 14 | 2 | 105.29 | 17.80 |
| 5 | 14 | 2 | 120.79 | 8.70 |
| -6 | -14 | -2 | 881.01 | 46.00 |
| 6 | -14 | 2 | 846.52 | 45.90 |
| 6 | 14 | 2 | 695.63 | 51.09 |
| 6 | 14 | 2 | 817.72 | 47.40 |
| 6 | 14 | 2 | 854.91 | 46.30 |

| | | | | |
|---|---|---|---|---|
| 7 | -14 | 2 | 80.29 | 7.30 |
| -7 | -14 | -2 | 63.79 | 7.50 |
| 7 | 14 | 2 | 78.39 | 6.70 |
| 7 | 14 | 2 | 90.19 | 9.00 |
| 8 | -14 | 2 | 12.30 | 3.30 |
| 8 | 14 | 2 | 16.60 | 3.10 |
| 9 | -14 | 2 | 37.00 | 6.10 |
| 9 | 14 | 2 | 25.20 | 5.20 |
| 10 | -14 | 2 | 254.27 | 15.50 |
| 10 | 14 | 2 | 255.17 | 15.60 |
| 11 | -14 | 2 | 7.20 | 2.30 |
| 11 | 14 | 2 | 4.30 | 3.30 |
| 12 | -14 | 2 | 45.70 | 4.30 |
| 11 | -15 | -2 | 288.67 | 16.80 |
| 11 | 15 | -2 | 289.97 | 16.30 |
| 10 | -15 | -2 | 3.20 | 2.70 |
| 10 | 15 | -2 | 1.60 | 1.80 |
| 9 | -15 | -2 | 127.79 | 9.90 |
| 9 | 15 | -2 | 136.09 | 8.80 |
| 8 | -15 | -2 | 57.49 | 11.20 |
| -8 | -15 | 2 | 55.79 | 7.60 |
| -7 | -15 | 2 | 14.10 | 3.40 |
| 7 | -15 | -2 | 14.40 | 4.00 |
| 7 | 15 | -2 | 12.70 | 3.30 |
| -6 | -15 | 2 | 151.28 | 10.60 |
| 6 | -15 | -2 | 146.89 | 11.60 |
| 6 | 15 | -2 | 139.39 | 12.70 |
| 6 | 15 | -2 | 146.49 | 32.70 |
| -5 | -15 | 2 | 665.63 | 37.40 |
| 5 | -15 | -2 | 661.73 | 37.70 |
| 5 | -15 | -2 | 695.13 | 38.10 |
| 5 | 15 | -2 | 554.34 | 42.40 |
| 5 | 15 | -2 | 678.13 | 37.40 |
| -5 | 15 | 2 | 699.93 | 39.20 |
| -4 | -15 | 2 | 19.00 | 4.00 |
| 4 | -15 | -2 | 31.70 | 5.70 |
| 4 | -15 | -2 | 44.50 | 7.70 |
| -4 | 15 | 2 | 27.60 | 8.00 |
| -4 | 15 | 2 | 25.70 | 4.70 |
| 4 | 15 | -2 | 30.70 | 6.00 |
| 4 | 15 | -2 | 21.00 | 9.00 |
| -3 | -15 | 2 | 119.49 | 9.30 |
| 3 | -15 | -2 | 97.39 | 10.90 |
| 3 | -15 | -2 | 107.39 | 10.20 |

| | | | | |
|---|---|---|---|---|
| 3 | 15 | -2 | 107.89 | 15.80 |
| -3 | 15 | 2 | 109.19 | 9.40 |
| 3 | 15 | -2 | 116.69 | 9.00 |
| -3 | 15 | 2 | 123.19 | 13.80 |
| 2 | -15 | -2 | 112.99 | 9.80 |
| 2 | -15 | -2 | 114.29 | 10.90 |
| -2 | -15 | 2 | 119.39 | 9.50 |
| -2 | -15 | 2 | 103.09 | 9.00 |
| -2 | 15 | 2 | 105.79 | 9.00 |
| 2 | 15 | -2 | 108.69 | 9.00 |
| -2 | 15 | 2 | 119.89 | 12.60 |
| 2 | 15 | -2 | 97.89 | 13.70 |
| 1 | -15 | -2 | 901.41 | 49.90 |
| 1 | -15 | -2 | 907.31 | 50.49 |
| -1 | -15 | 2 | 902.81 | 49.60 |
| -1 | -15 | 2 | 897.51 | 50.09 |
| -1 | 15 | 2 | 908.21 | 51.89 |
| 1 | 15 | -2 | 908.51 | 49.60 |
| -1 | 15 | 2 | 863.71 | 49.80 |
| 1 | 15 | -2 | 853.91 | 52.29 |
| 0 | -15 | 2 | 40.50 | 6.20 |
| 0 | -15 | 2 | 28.40 | 5.00 |
| 0 | -15 | -2 | 42.30 | 8.50 |
| 0 | -15 | -2 | 45.50 | 7.60 |
| 0 | 15 | 2 | 32.90 | 5.50 |
| 0 | 15 | 2 | 34.20 | 7.10 |
| 0 | 15 | -2 | 24.90 | 6.20 |
| 0 | 15 | -2 | 40.80 | 6.30 |
| 1 | -15 | 2 | 668.13 | 36.50 |
| -1 | -15 | -2 | 610.54 | 35.90 |
| -1 | -15 | -2 | 634.34 | 36.00 |
| 1 | -15 | 2 | 608.44 | 35.40 |
| -1 | 15 | -2 | 611.44 | 35.50 |
| -1 | 15 | -2 | 599.94 | 37.70 |
| 1 | 15 | 2 | 650.83 | 38.30 |
| 1 | 15 | 2 | 638.24 | 35.50 |
| 2 | -15 | 2 | 46.30 | 9.20 |
| 2 | -15 | 2 | 40.70 | 6.00 |
| -2 | -15 | -2 | 61.09 | 6.80 |
| 2 | 15 | 2 | 46.40 | 5.70 |
| -2 | 15 | -2 | 53.49 | 10.50 |
| 2 | 15 | 2 | 46.10 | 8.50 |
| -2 | 15 | -2 | 45.30 | 6.40 |
| 3 | -15 | 2 | 96.19 | 8.50 |

| | | | | |
|---|---|---|---|---|
| 3 | -15 | 2 | 126.39 | 11.60 |
| -3 | -15 | -2 | 110.09 | 8.90 |
| -3 | 15 | -2 | 101.89 | 8.80 |
| 3 | 15 | 2 | 92.49 | 14.40 |
| 3 | 15 | 2 | 110.59 | 8.20 |
| -3 | 15 | -2 | 93.89 | 12.80 |
| -4 | -15 | -2 | 138.09 | 10.50 |
| 4 | -15 | 2 | 157.68 | 13.70 |
| 4 | -15 | 2 | 147.59 | 10.30 |
| -4 | 15 | -2 | 142.09 | 16.60 |
| 4 | 15 | 2 | 145.39 | 18.50 |
| 5 | -15 | 2 | 741.63 | 41.90 |
| 5 | -15 | 2 | 817.42 | 43.40 |
| -5 | -15 | -2 | 749.42 | 42.00 |
| 5 | 15 | 2 | 737.23 | 41.80 |
| 5 | 15 | 2 | 711.43 | 42.50 |
| -6 | -15 | -2 | 88.59 | 7.50 |
| 6 | -15 | 2 | 73.99 | 7.40 |
| 6 | 15 | 2 | 83.89 | 7.10 |
| 6 | 15 | 2 | 96.49 | 10.50 |
| 7 | -15 | 2 | 17.40 | 3.30 |
| -7 | -15 | -2 | 16.00 | 3.30 |
| 7 | 15 | 2 | 17.80 | 3.20 |
| -8 | -15 | -2 | 21.20 | 3.60 |
| 8 | -15 | 2 | 27.80 | 5.50 |
| 8 | 15 | 2 | 23.30 | 4.80 |
| 9 | -15 | 2 | 226.58 | 13.90 |
| 9 | 15 | 2 | 220.08 | 13.80 |
| 10 | -15 | 2 | 7.70 | 2.40 |
| 10 | 15 | 2 | 11.70 | 3.50 |
| 11 | -15 | 2 | 207.78 | 11.20 |
| 11 | 15 | 2 | 157.28 | 12.60 |
| 10 | -16 | -2 | 256.97 | 15.30 |
| -10 | 16 | 2 | 255.07 | 15.50 |
| 10 | 16 | -2 | 265.07 | 14.70 |
| 9 | -16 | -2 | 6.10 | 2.80 |
| 9 | 16 | -2 | 2.00 | 1.70 |
| -9 | 16 | 2 | 1.80 | 2.70 |
| -8 | -16 | 2 | 31.80 | 6.00 |
| 8 | -16 | -2 | 30.40 | 6.10 |
| -8 | 16 | 2 | 21.60 | 4.20 |
| 8 | 16 | -2 | 32.60 | 4.30 |
| 7 | -16 | -2 | 301.07 | 17.90 |
| -7 | -16 | 2 | 284.77 | 17.00 |

| | | | | |
|---|---|---|---|---|
| -7 | 16 | 2 | 260.67 | 17.80 |
| 7 | 16 | -2 | 273.27 | 16.80 |
| 6 | -16 | -2 | 463.95 | 27.90 |
| -6 | -16 | 2 | 475.65 | 27.40 |
| -6 | 16 | 2 | 494.15 | 28.50 |
| -5 | -16 | 2 | -1.40 | 2.90 |
| 5 | -16 | -2 | -4.20 | 4.30 |
| 5 | -16 | -2 | -4.40 | 3.60 |
| -5 | 16 | 2 | -1.60 | 3.20 |
| 4 | -16 | -2 | 383.46 | 23.10 |
| 4 | -16 | -2 | 375.26 | 22.70 |
| -4 | -16 | 2 | 353.96 | 22.30 |
| -4 | 16 | 2 | 402.46 | 23.10 |
| 4 | 16 | -2 | 380.06 | 22.20 |
| 3 | -16 | -2 | 148.19 | 11.10 |
| -3 | -16 | 2 | 143.49 | 10.40 |
| 3 | -16 | -2 | 146.19 | 11.80 |
| -3 | 16 | 2 | 137.89 | 16.20 |
| -3 | 16 | 2 | 136.79 | 10.50 |
| 3 | 16 | -2 | 105.49 | 18.10 |
| 3 | 16 | -2 | 151.28 | 10.60 |
| -2 | -16 | 2 | 72.59 | 7.20 |
| 2 | -16 | -2 | 68.19 | 8.30 |
| -2 | -16 | 2 | 64.29 | 7.30 |
| 2 | -16 | -2 | 74.99 | 9.00 |
| -2 | 16 | 2 | 79.79 | 12.60 |
| 2 | 16 | -2 | 53.59 | 10.10 |
| -2 | 16 | 2 | 61.09 | 7.40 |
| 2 | 16 | -2 | 78.69 | 8.10 |
| -1 | -16 | 2 | 107.59 | 9.50 |
| -1 | -16 | 2 | 125.49 | 10.10 |
| 1 | -16 | -2 | 144.89 | 11.00 |
| 1 | -16 | -2 | 129.09 | 10.70 |
| 1 | 16 | -2 | 116.59 | 14.70 |
| 1 | 16 | -2 | 138.29 | 10.90 |
| -1 | 16 | 2 | 116.59 | 10.00 |
| -1 | 16 | 2 | 132.89 | 14.00 |
| 0 | -16 | 2 | 996.70 | 54.29 |
| 0 | -16 | 2 | 1007.10 | 54.99 |
| 0 | -16 | -2 | 999.20 | 54.59 |
| 0 | -16 | -2 | 982.20 | 54.89 |
| 0 | 16 | -2 | 903.31 | 56.89 |
| 0 | 16 | -2 | 1017.10 | 54.89 |
| 0 | 16 | 2 | 1003.80 | 55.19 |

| | | | | |
|---|---|---|---|---|
| 0 | 16 | 2 | 950.30 | 56.79 |
| 1 | -16 | 2 | 135.79 | 11.10 |
| 1 | -16 | 2 | 125.99 | 9.30 |
| -1 | -16 | -2 | 122.39 | 9.90 |
| -1 | 16 | -2 | 118.99 | 10.00 |
| 1 | 16 | 2 | 114.79 | 9.30 |
| -1 | 16 | -2 | 110.79 | 14.40 |
| 1 | 16 | 2 | 123.59 | 14.20 |
| -2 | -16 | -2 | 30.20 | 5.60 |
| 2 | -16 | 2 | 33.10 | 5.20 |
| 2 | -16 | 2 | 23.10 | 5.10 |
| -2 | 16 | -2 | 19.50 | 7.60 |
| 2 | 16 | 2 | 27.80 | 5.20 |
| -2 | 16 | -2 | 31.10 | 5.80 |
| 2 | 16 | 2 | 10.70 | 7.10 |
| 3 | -16 | 2 | 63.59 | 9.90 |
| -3 | -16 | -2 | 49.50 | 6.50 |
| 3 | -16 | 2 | 69.09 | 6.30 |
| 3 | 16 | 2 | 68.19 | 16.00 |
| -3 | 16 | -2 | 58.99 | 6.80 |
| 3 | 16 | 2 | 64.69 | 6.20 |
| 4 | -16 | 2 | 419.46 | 23.90 |
| -4 | -16 | -2 | 356.86 | 22.10 |
| 4 | -16 | 2 | 372.56 | 22.00 |
| 4 | 16 | 2 | 378.06 | 21.90 |
| -4 | 16 | -2 | 367.96 | 22.30 |
| 5 | -16 | 2 | 11.00 | 2.90 |
| 5 | -16 | 2 | 0.80 | 8.10 |
| -5 | -16 | -2 | 4.00 | 2.60 |
| 5 | 16 | 2 | 9.70 | 2.70 |
| -5 | 16 | -2 | 10.80 | 3.60 |
| -6 | -16 | -2 | 481.75 | 29.50 |
| 6 | -16 | 2 | 508.35 | 28.80 |
| 6 | 16 | 2 | 488.15 | 28.20 |
| -6 | 16 | -2 | 524.75 | 29.10 |
| -7 | -16 | -2 | 77.69 | 7.00 |
| 7 | -16 | 2 | 79.39 | 7.00 |
| 7 | 16 | 2 | 78.19 | 6.60 |
| -7 | 16 | -2 | 84.39 | 8.20 |
| 8 | -16 | 2 | 81.39 | 6.50 |
| -8 | -16 | -2 | 66.29 | 6.80 |
| 8 | 16 | 2 | 76.89 | 6.50 |
| -8 | 16 | -2 | 85.39 | 7.70 |
| 9 | -16 | 2 | 10.30 | 2.40 |

| | | | | |
|---|---|---|---|---|
| -9 | 16 | -2 | 15.10 | 3.60 |
| 9 | 16 | 2 | 14.20 | 3.30 |
| 10 | -16 | 2 | 282.17 | 15.00 |
| 10 | 16 | 2 | 224.08 | 16.50 |
| -10 | 16 | -2 | 254.67 | 15.40 |
| 9 | -17 | -2 | 212.88 | 12.50 |
| -9 | 17 | 2 | 208.88 | 12.80 |
| 9 | 17 | -2 | 194.88 | 11.80 |
| 8 | -17 | -2 | 124.99 | 9.00 |
| -8 | -17 | 2 | 122.99 | 9.10 |
| 8 | 17 | -2 | 123.29 | 7.90 |
| -8 | 17 | 2 | 127.49 | 9.30 |
| -7 | -17 | 2 | 126.09 | 9.10 |
| 7 | -17 | -2 | 119.29 | 9.30 |
| 7 | 17 | -2 | 121.39 | 8.40 |
| -7 | 17 | 2 | 126.89 | 9.80 |
| 6 | -17 | -2 | 131.49 | 10.20 |
| -6 | -17 | 2 | 137.29 | 9.50 |
| 6 | 17 | -2 | 114.79 | 9.20 |
| -6 | 17 | 2 | 135.89 | 10.20 |
| -5 | -17 | 2 | 734.73 | 42.50 |
| 5 | -17 | -2 | 776.82 | 42.60 |
| 5 | -17 | -2 | 750.02 | 42.70 |
| -5 | 17 | 2 | 778.42 | 43.10 |
| 4 | -17 | -2 | 11.90 | 6.40 |
| -4 | -17 | 2 | 9.20 | 2.80 |
| -4 | 17 | 2 | 16.20 | 3.60 |
| 3 | -17 | -2 | 8.30 | 4.10 |
| 3 | -17 | -2 | 6.80 | 3.80 |
| -3 | -17 | 2 | 3.20 | 2.50 |
| -3 | 17 | 2 | 3.10 | 2.70 |
| 2 | -17 | -2 | 358.26 | 22.00 |
| 2 | -17 | -2 | 362.36 | 21.80 |
| -2 | -17 | 2 | 344.07 | 21.40 |
| 2 | 17 | -2 | 380.16 | 21.40 |
| -2 | 17 | 2 | 353.76 | 21.30 |
| -1 | -17 | 2 | 444.16 | 25.70 |
| -1 | -17 | 2 | 429.16 | 26.20 |
| 1 | -17 | -2 | 436.06 | 26.30 |
| 1 | -17 | -2 | 458.25 | 26.60 |
| -1 | 17 | 2 | 446.46 | 25.90 |
| 1 | 17 | -2 | 472.95 | 26.00 |
| 0 | -17 | 2 | 1.10 | 3.20 |
| 0 | -17 | 2 | -2.40 | 2.50 |

| | | | | |
|---|---|---|---|---|
| 0 | -17 | -2 | -3.40 | 3.00 |
| 0 | 17 | -2 | 2.80 | 3.30 |
| 0 | 17 | 2 | 0.50 | 2.70 |
| -1 | -17 | -2 | 218.78 | 14.30 |
| 1 | -17 | 2 | 233.68 | 15.10 |
| 1 | -17 | 2 | 213.48 | 13.60 |
| 1 | 17 | 2 | 219.88 | 13.70 |
| -1 | 17 | -2 | 210.28 | 14.00 |
| 2 | -17 | 2 | 102.69 | 8.20 |
| 2 | -17 | 2 | 111.99 | 10.60 |
| -2 | -17 | -2 | 110.49 | 8.60 |
| 2 | 17 | 2 | 103.69 | 8.00 |
| -2 | 17 | -2 | 107.99 | 8.70 |
| -3 | -17 | -2 | 129.09 | 9.30 |
| 3 | -17 | 2 | 144.79 | 11.50 |
| 3 | -17 | 2 | 123.59 | 9.00 |
| 3 | 17 | 2 | 121.89 | 8.90 |
| -3 | 17 | -2 | 116.99 | 9.40 |
| -4 | -17 | -2 | 142.29 | 13.90 |
| 4 | -17 | 2 | 182.38 | 13.00 |
| 4 | -17 | 2 | 148.69 | 13.10 |
| 4 | 17 | 2 | 153.18 | 10.30 |
| -4 | 17 | -2 | 162.98 | 11.10 |
| 5 | -17 | 2 | 595.74 | 32.90 |
| 5 | -17 | 2 | 543.65 | 33.60 |
| -5 | -17 | -2 | 579.24 | 32.80 |
| -5 | 17 | -2 | 595.14 | 33.20 |
| 5 | 17 | 2 | 613.34 | 32.70 |
| 6 | -17 | 2 | 44.20 | 5.20 |
| -6 | -17 | -2 | 43.50 | 5.30 |
| -6 | 17 | -2 | 45.70 | 6.40 |
| 6 | 17 | 2 | 44.60 | 4.90 |
| 7 | -17 | 2 | 5.60 | 2.30 |
| -7 | -17 | -2 | 1.90 | 2.20 |
| -7 | 17 | -2 | 1.70 | 5.40 |
| 7 | 17 | 2 | 9.00 | 3.90 |
| -8 | -17 | -2 | 49.50 | 5.60 |
| 8 | -17 | 2 | 46.30 | 4.80 |
| 8 | 17 | 2 | 53.09 | 5.70 |
| -8 | 17 | -2 | 46.30 | 6.50 |
| 9 | -17 | 2 | 201.68 | 11.00 |
| -9 | 17 | -2 | 156.78 | 11.40 |
| -8 | -18 | 2 | 4.70 | 3.00 |
| 8 | -18 | -2 | 5.40 | 2.40 |

| | | | | |
|---|---|---|---|---|
| 8 | 18 | -2 | 2.90 | 2.60 |
| -8 | 18 | 2 | 0.20 | 2.60 |
| 7 | -18 | -2 | 230.78 | 13.30 |
| -7 | -18 | 2 | 217.88 | 13.10 |
| 7 | 18 | -2 | 212.98 | 12.50 |
| -7 | 18 | 2 | 198.58 | 13.60 |
| 6 | -18 | -2 | 319.87 | 19.70 |
| -6 | -18 | 2 | 341.17 | 19.40 |
| -6 | 18 | 2 | 329.57 | 20.10 |
| 6 | 18 | -2 | 344.97 | 19.10 |
| 5 | -18 | -2 | 5.80 | 4.10 |
| 5 | -18 | -2 | 13.60 | 3.50 |
| -5 | -18 | 2 | 7.30 | 2.60 |
| -5 | 18 | 2 | 11.50 | 3.40 |
| 5 | 18 | -2 | 11.70 | 2.80 |
| 4 | -18 | -2 | 163.08 | 12.00 |
| 4 | -18 | -2 | 179.08 | 12.00 |
| -4 | -18 | 2 | 165.68 | 11.20 |
| -4 | 18 | 2 | 178.18 | 11.80 |
| 3 | -18 | -2 | 263.37 | 16.60 |
| 3 | -18 | -2 | 258.37 | 16.60 |
| -3 | -18 | 2 | 259.37 | 16.00 |
| -3 | 18 | 2 | 270.07 | 16.30 |
| 2 | -18 | -2 | 91.09 | 8.50 |
| 2 | -18 | -2 | 77.39 | 9.10 |
| -2 | -18 | 2 | 96.69 | 8.00 |
| -2 | 18 | 2 | 100.69 | 7.80 |
| -1 | -18 | 2 | 104.39 | 9.60 |
| 1 | -18 | -2 | 137.09 | 10.30 |
| -1 | 18 | 2 | 122.49 | 8.90 |
| 0 | -18 | 2 | 787.02 | 44.10 |
| 0 | -18 | -2 | 822.32 | 44.80 |
| 0 | -18 | 2 | 771.12 | 44.80 |
| 0 | 18 | 2 | 759.22 | 44.10 |
| 0 | 18 | -2 | 851.21 | 44.40 |
| 1 | -18 | 2 | 310.07 | 18.10 |
| 1 | -18 | 2 | 296.77 | 19.10 |
| -1 | -18 | -2 | 309.67 | 18.60 |
| -1 | 18 | -2 | 310.77 | 18.40 |
| 1 | 18 | 2 | 302.87 | 18.00 |
| 2 | -18 | 2 | 33.60 | 6.80 |
| 2 | -18 | 2 | 26.00 | 4.60 |
| -2 | -18 | -2 | 34.90 | 5.40 |
| 2 | 18 | 2 | 25.00 | 4.60 |

| | | | | |
|---|---|---|---|---|
| -2 | 18 | -2 | 22.20 | 4.10 |
| 3 | -18 | 2 | 142.59 | 9.60 |
| -3 | -18 | -2 | 143.59 | 9.70 |
| 3 | -18 | 2 | 156.38 | 11.50 |
| 3 | 18 | 2 | 140.09 | 9.40 |
| -3 | 18 | -2 | 140.29 | 10.20 |
| 4 | -18 | 2 | 322.57 | 18.70 |
| 4 | -18 | 2 | 304.57 | 17.50 |
| -4 | -18 | -2 | 295.87 | 17.50 |
| 4 | 18 | 2 | 290.47 | 17.30 |
| -4 | 18 | -2 | 282.37 | 17.90 |
| 5 | -18 | 2 | 0.90 | 4.20 |
| 5 | -18 | 2 | 1.50 | 2.00 |
| -5 | -18 | -2 | 5.20 | 2.30 |
| 5 | 18 | 2 | 10.00 | 3.70 |
| -5 | 18 | -2 | 6.50 | 3.50 |
| 6 | -18 | 2 | 184.88 | 11.10 |
| -6 | -18 | -2 | 176.98 | 11.00 |
| -6 | 18 | -2 | 180.58 | 11.90 |
| 6 | 18 | 2 | 173.08 | 11.10 |
| 7 | -18 | 2 | 178.38 | 10.50 |
| -7 | -18 | -2 | 168.08 | 10.40 |
| -7 | 18 | -2 | 149.09 | 11.40 |
| 7 | 18 | 2 | 180.88 | 10.70 |
| 6 | -19 | -2 | 48.30 | 6.10 |
| -6 | -19 | 2 | 37.50 | 4.60 |
| 6 | 19 | -2 | 49.40 | 4.80 |
| -6 | 19 | 2 | 44.90 | 5.70 |
| -5 | -19 | 2 | 433.86 | 25.40 |
| 5 | -19 | -2 | 446.56 | 25.70 |
| -5 | 19 | 2 | 468.85 | 26.10 |
| 5 | 19 | -2 | 467.15 | 25.30 |
| 4 | -19 | -2 | 69.69 | 7.60 |
| -4 | -19 | 2 | 81.29 | 6.20 |
| 4 | -19 | -2 | 81.29 | 7.60 |
| -3 | -19 | 2 | 5.50 | 2.10 |
| 3 | -19 | -2 | 9.10 | 3.10 |
| 3 | -19 | -2 | 5.80 | 3.80 |
| -3 | 19 | 2 | 7.70 | 2.90 |
| -2 | -19 | 2 | 155.18 | 10.40 |
| 2 | -19 | -2 | 162.68 | 10.90 |
| -2 | 19 | 2 | 157.98 | 10.60 |
| -1 | -19 | 2 | 321.97 | 19.00 |
| 1 | -19 | -2 | 324.97 | 19.20 |

| | | | | |
|---|---|---|---|---|
| -1 | 19 | 2 | 313.17 | 18.60 |
| 0 | -19 | 2 | -3.80 | 2.50 |
| 0 | -19 | -2 | 0.90 | 2.70 |
| 0 | 19 | 2 | 0.70 | 2.30 |
| 1 | -19 | 2 | 139.59 | 9.10 |
| -1 | -19 | -2 | 140.39 | 9.60 |
| 1 | -19 | 2 | 145.39 | 10.40 |
| 1 | 19 | 2 | 134.49 | 9.20 |
| -2 | -19 | -2 | 116.99 | 8.20 |
| 2 | -19 | 2 | 124.29 | 9.40 |
| 2 | -19 | 2 | 108.39 | 7.80 |
| 2 | 19 | 2 | 111.19 | 8.40 |
| 3 | -19 | 2 | 52.39 | 5.20 |
| -3 | -19 | -2 | 50.49 | 5.00 |
| 3 | -19 | 2 | 59.39 | 7.20 |
| 3 | 19 | 2 | 56.59 | 5.00 |
| -4 | -19 | -2 | 95.09 | 7.20 |
| 4 | -19 | 2 | 109.59 | 9.00 |
| 4 | -19 | 2 | 110.19 | 7.30 |
| 4 | 19 | 2 | 110.39 | 7.30 |
| 5 | -19 | 2 | 302.97 | 16.40 |
| -5 | -19 | -2 | 270.47 | 16.30 |
| 5 | 19 | 2 | 278.97 | 16.30 |
| -4 | 20 | 2 | 85.69 | 7.10 |
| -3 | -20 | 2 | 51.79 | 4.70 |
| 3 | 20 | -2 | 51.39 | 5.10 |
| 2 | -20 | -2 | 44.90 | 5.60 |
| -2 | -20 | 2 | 44.50 | 4.80 |
| -2 | 20 | 2 | 51.99 | 5.30 |
| -1 | -20 | 2 | 84.89 | 6.80 |
| 1 | -20 | -2 | 90.79 | 7.10 |
| -1 | 20 | 2 | 86.79 | 6.70 |
| 0 | -20 | 2 | 317.97 | 18.70 |
| 0 | -20 | -2 | 319.67 | 18.60 |
| 0 | 20 | 2 | 316.37 | 18.20 |
| 1 | -20 | 2 | 108.49 | 8.10 |
| -1 | -20 | -2 | 107.19 | 7.50 |
| 1 | 20 | 2 | 95.79 | 7.10 |
| 2 | -20 | 2 | 1.40 | 2.90 |
| -2 | -20 | -2 | -0.30 | 1.80 |
| 2 | 20 | 2 | 0.40 | 2.30 |
| 3 | -20 | 2 | 85.89 | 7.20 |
| -3 | -20 | -2 | 90.39 | 6.10 |
| 3 | -20 | 2 | 87.49 | 6.10 |

| | | | | |
|---|---|---|---|---|
| 3 | 20 | 2 | 68.19 | 7.30 |
| 17 | 0 | -3 | 234.58 | 15.00 |
| 17 | 0 | -3 | 249.48 | 13.90 |
| 16 | 0 | -3 | -0.60 | 3.40 |
| 16 | 0 | -3 | -0.40 | 1.90 |
| 15 | 0 | -3 | 998.10 | 52.79 |
| 15 | 0 | -3 | 928.21 | 51.79 |
| 15 | 0 | -3 | 898.51 | 52.19 |
| 14 | 0 | -3 | 0.60 | 4.20 |
| 14 | 0 | -3 | -5.80 | 3.60 |
| 14 | 0 | -3 | 1.30 | 2.70 |
| 13 | 0 | -3 | 30.80 | 5.10 |
| 13 | 0 | -3 | 32.60 | 5.80 |
| 13 | 0 | -3 | 27.20 | 5.90 |
| 12 | 0 | -3 | -1.20 | 5.30 |
| 12 | 0 | -3 | 4.10 | 3.90 |
| 12 | 0 | -3 | -2.40 | 3.20 |
| 11 | 0 | -3 | 2487.15 | 129.49 |
| 11 | 0 | -3 | 2282.47 | 127.89 |
| 11 | 0 | -3 | 2305.77 | 128.29 |
| 10 | 0 | -3 | 17.90 | 4.00 |
| 10 | 0 | -3 | 20.30 | 6.90 |
| 9 | 0 | -3 | 709.93 | 45.10 |
| 9 | 0 | -3 | 893.21 | 43.80 |
| 9 | 0 | -3 | 709.03 | 43.20 |
| 8 | 0 | -3 | -1.20 | 4.30 |
| -4 | 0 | 3 | 4.40 | 1.70 |
| -3 | 0 | 3 | 5838.12 | 313.27 |
| -1 | 0 | 3 | 2243.18 | 121.29 |
| 0 | 0 | 3 | 2.70 | 2.30 |
| 2 | 0 | 3 | -0.20 | 2.60 |
| 3 | 0 | 3 | 268.37 | 16.50 |
| 4 | 0 | 3 | 0.50 | 2.90 |
| 5 | 0 | 3 | 4532.25 | 244.08 |
| 6 | 0 | 3 | -3.90 | 3.80 |
| 6 | 0 | 3 | -3.70 | 2.60 |
| 7 | 0 | 3 | 32.90 | 7.20 |
| 7 | 0 | 3 | 33.60 | 5.70 |
| 8 | 0 | 3 | 2.10 | 3.90 |
| 8 | 0 | 3 | 7.10 | 5.40 |
| 9 | 0 | 3 | 1060.39 | 58.59 |
| 9 | 0 | 3 | 1060.39 | 58.69 |
| 9 | 0 | 3 | 1025.10 | 58.69 |
| 10 | 0 | 3 | 1.90 | 3.60 |

| | | | | |
|---|---|---|---|---|
| 10 | 0 | 3 | 1.90 | 5.20 |
| 10 | 0 | 3 | -0.10 | 4.90 |
| 11 | 0 | 3 | 783.22 | 43.40 |
| 11 | 0 | 3 | 688.03 | 42.90 |
| 11 | 0 | 3 | 793.92 | 43.20 |
| 12 | 0 | 3 | -0.50 | 3.50 |
| 12 | 0 | 3 | 3.10 | 4.50 |
| 12 | 0 | 3 | 5.50 | 4.70 |
| 13 | 0 | 3 | 787.72 | 46.50 |
| 13 | 0 | 3 | 825.52 | 46.00 |
| 13 | 0 | 3 | 834.92 | 45.90 |
| 14 | 0 | 3 | 2.80 | 4.00 |
| 14 | 0 | 3 | 1.30 | 3.00 |
| 14 | 0 | 3 | -3.30 | 3.70 |
| 15 | 0 | 3 | 419.16 | 24.50 |
| 15 | 0 | 3 | 388.56 | 24.70 |
| 15 | 0 | 3 | 469.65 | 24.50 |
| 16 | 0 | 3 | -6.30 | 3.10 |
| 16 | 0 | 3 | 0.30 | 2.30 |
| 16 | 0 | 3 | -1.20 | 2.20 |
| 17 | -1 | -3 | 8.00 | 3.60 |
| 17 | -1 | -3 | 5.80 | 1.70 |
| 17 | 1 | -3 | 5.70 | 1.70 |
| 16 | -1 | -3 | 615.84 | 33.90 |
| 16 | -1 | -3 | 617.24 | 34.40 |
| 16 | 1 | -3 | 576.94 | 34.90 |
| 16 | 1 | -3 | 638.14 | 33.90 |
| 15 | -1 | -3 | 1.60 | 2.60 |
| 15 | -1 | -3 | -2.50 | 3.30 |
| 15 | -1 | -3 | 1.60 | 3.70 |
| 15 | 1 | -3 | 0.20 | 3.50 |
| 15 | 1 | -3 | 4.30 | 4.40 |
| 15 | 1 | -3 | -1.30 | 2.20 |
| 14 | -1 | -3 | 652.13 | 36.20 |
| 14 | -1 | -3 | 672.33 | 36.70 |
| 14 | -1 | -3 | 645.54 | 37.30 |
| 14 | 1 | -3 | 629.84 | 36.70 |
| 14 | 1 | -3 | 620.94 | 36.00 |
| 14 | 1 | -3 | 634.94 | 37.30 |
| 13 | -1 | -3 | 78.69 | 9.80 |
| 13 | -1 | -3 | 73.99 | 8.50 |
| 13 | -1 | -3 | 80.09 | 7.50 |
| 13 | 1 | -3 | 80.99 | 10.10 |
| 13 | 1 | -3 | 81.89 | 7.10 |

| | | | | |
|---|---|---|---|---|
| 13 | 1 | -3 | 62.09 | 8.50 |
| 12 | -1 | -3 | 675.43 | 40.10 |
| 12 | -1 | -3 | 678.13 | 38.40 |
| 12 | -1 | -3 | 704.13 | 38.90 |
| 12 | 1 | -3 | 677.33 | 38.90 |
| 12 | 1 | -3 | 672.53 | 38.30 |
| 12 | 1 | -3 | 671.13 | 40.10 |
| 11 | -1 | -3 | 4.40 | 5.60 |
| 11 | -1 | -3 | 4.90 | 4.20 |
| 11 | 1 | -3 | 7.10 | 3.40 |
| 11 | 1 | -3 | -0.50 | 5.60 |
| 11 | 1 | -3 | -2.90 | 3.90 |
| 10 | -1 | -3 | 1890.21 | 104.19 |
| 10 | -1 | -3 | 1877.91 | 105.59 |
| 10 | -1 | -3 | 1925.11 | 104.49 |
| 10 | 1 | -3 | 2066.89 | 104.79 |
| 10 | 1 | -3 | 1871.51 | 103.99 |
| 10 | 1 | -3 | 1848.52 | 105.59 |
| 9 | -1 | -3 | 13.90 | 4.60 |
| 9 | -1 | -3 | 10.90 | 6.90 |
| 9 | 1 | -3 | 10.30 | 7.40 |
| 8 | -1 | -3 | 12.40 | 4.80 |
| 8 | 1 | -3 | 23.90 | 5.50 |
| 5 | 1 | -3 | 127.89 | 8.00 |
| -4 | 1 | 3 | 876.51 | 47.60 |
| -3 | -1 | 3 | 872.11 | 49.99 |
| -3 | 1 | 3 | 957.80 | 49.90 |
| -2 | -1 | 3 | 1046.10 | 61.59 |
| -2 | 1 | 3 | 1213.18 | 61.59 |
| -1 | -1 | 3 | 902.71 | 54.49 |
| -1 | 1 | 3 | 1089.39 | 54.39 |
| 0 | -1 | 3 | 8360.86 | 448.76 |
| 1 | -1 | 3 | 336.57 | 20.70 |
| 1 | 1 | 3 | 369.76 | 20.40 |
| 2 | -1 | 3 | 1273.17 | 74.99 |
| 2 | 1 | 3 | 1475.85 | 75.19 |
| 3 | -1 | 3 | 24.30 | 4.40 |
| 3 | 1 | 3 | 24.20 | 5.10 |
| 4 | -1 | 3 | 3370.16 | 172.98 |
| 4 | 1 | 3 | 3042.10 | 173.38 |
| 5 | -1 | 3 | 9.00 | 2.70 |
| 5 | 1 | 3 | 8.00 | 3.70 |
| 6 | -1 | 3 | 2431.06 | 148.09 |
| 6 | -1 | 3 | 3066.19 | 147.49 |

| | | | | |
|---|---|---|---|---|
| 6 | 1 | 3 | 2697.33 | 147.99 |
| 7 | -1 | 3 | 34.30 | 7.30 |
| 7 | -1 | 3 | 28.30 | 5.70 |
| 7 | 1 | 3 | 31.70 | 4.90 |
| 7 | 1 | 3 | 34.50 | 5.10 |
| 8 | -1 | 3 | 2299.37 | 121.39 |
| 8 | -1 | 3 | 2238.38 | 121.19 |
| 8 | 1 | 3 | 2102.99 | 120.99 |
| 8 | 1 | 3 | 2255.37 | 121.29 |
| 9 | -1 | 3 | 57.59 | 9.10 |
| 9 | -1 | 3 | 51.29 | 7.70 |
| 9 | -1 | 3 | 45.60 | 7.10 |
| 9 | 1 | 3 | 57.39 | 8.80 |
| 9 | 1 | 3 | 56.49 | 7.40 |
| 9 | 1 | 3 | 45.80 | 7.80 |
| 10 | -1 | 3 | 1862.61 | 95.69 |
| 10 | -1 | 3 | 1642.54 | 95.49 |
| 10 | -1 | 3 | 1725.73 | 95.39 |
| 10 | 1 | 3 | 1832.32 | 95.49 |
| 10 | 1 | 3 | 1722.73 | 95.49 |
| 10 | 1 | 3 | 1650.13 | 95.59 |
| 11 | -1 | 3 | 86.79 | 10.30 |
| 11 | -1 | 3 | 92.39 | 10.80 |
| 11 | -1 | 3 | 80.29 | 9.00 |
| 11 | 1 | 3 | 94.89 | 9.30 |
| 11 | 1 | 3 | 104.89 | 11.10 |
| 11 | 1 | 3 | 89.79 | 10.20 |
| 12 | -1 | 3 | 46.80 | 8.40 |
| 12 | -1 | 3 | 44.90 | 8.30 |
| 12 | -1 | 3 | 35.70 | 6.70 |
| 12 | 1 | 3 | 42.80 | 7.70 |
| 12 | 1 | 3 | 37.20 | 5.90 |
| 12 | 1 | 3 | 36.90 | 6.80 |
| 13 | -1 | 3 | 18.70 | 4.80 |
| 13 | -1 | 3 | 8.00 | 3.40 |
| 13 | -1 | 3 | 10.10 | 4.20 |
| 13 | 1 | 3 | 11.60 | 4.50 |
| 13 | 1 | 3 | 2.80 | 4.10 |
| 13 | 1 | 3 | 7.50 | 3.50 |
| 14 | -1 | 3 | 411.16 | 23.40 |
| 14 | -1 | 3 | 401.56 | 23.20 |
| 14 | -1 | 3 | 337.37 | 23.70 |
| 14 | 1 | 3 | 421.26 | 23.30 |
| 14 | 1 | 3 | 420.26 | 23.30 |

| | | | | |
|---|---|---|---|---|
| 14 | 1 | 3 | 358.76 | 23.60 |
| 15 | -1 | 3 | 13.10 | 3.20 |
| 15 | -1 | 3 | 11.10 | 3.00 |
| 15 | -1 | 3 | 6.80 | 3.10 |
| 15 | 1 | 3 | 5.30 | 2.60 |
| 15 | 1 | 3 | 7.20 | 3.50 |
| 15 | 1 | 3 | 11.00 | 3.40 |
| 16 | -1 | 3 | 110.79 | 7.80 |
| 16 | -1 | 3 | 96.59 | 7.40 |
| 16 | -1 | 3 | 103.79 | 7.50 |
| 16 | 1 | 3 | 107.79 | 7.70 |
| 16 | 1 | 3 | 110.39 | 7.40 |
| 17 | -2 | -3 | 95.79 | 8.10 |
| 17 | -2 | -3 | 116.19 | 7.10 |
| 17 | 2 | -3 | 115.69 | 7.00 |
| 16 | -2 | -3 | 1.20 | 2.00 |
| 16 | -2 | -3 | 4.00 | 3.40 |
| 16 | 2 | -3 | 4.50 | 4.30 |
| 16 | 2 | -3 | 2.60 | 2.10 |
| 15 | -2 | -3 | 335.07 | 20.60 |
| 15 | -2 | -3 | 382.46 | 20.80 |
| 15 | 2 | -3 | 288.77 | 23.30 |
| 15 | 2 | -3 | 345.97 | 21.60 |
| 15 | 2 | -3 | 344.87 | 20.60 |
| 14 | -2 | -3 | 44.30 | 6.90 |
| 14 | -2 | -3 | 38.70 | 5.70 |
| 14 | -2 | -3 | 43.00 | 7.70 |
| 14 | 2 | -3 | 24.60 | 5.10 |
| 14 | 2 | -3 | 36.30 | 7.30 |
| 14 | 2 | -3 | 42.10 | 5.20 |
| 13 | -2 | -3 | 2.00 | 2.90 |
| 13 | -2 | -3 | 14.00 | 4.10 |
| 13 | -2 | -3 | 9.60 | 5.10 |
| 13 | 2 | -3 | 6.30 | 2.70 |
| 13 | 2 | -3 | 9.90 | 4.10 |
| 13 | 2 | -3 | 17.70 | 5.50 |
| 12 | -2 | -3 | 2.70 | 4.90 |
| 12 | -2 | -3 | 7.10 | 3.70 |
| 12 | -2 | -3 | 0.70 | 3.70 |
| 12 | 2 | -3 | 2.00 | 2.80 |
| 12 | 2 | -3 | 0.60 | 3.80 |
| 12 | 2 | -3 | 4.80 | 5.50 |
| 11 | -2 | -3 | 1412.96 | 79.99 |
| 11 | -2 | -3 | 1423.96 | 80.99 |

| | | | | |
|---|---|---|---|---|
| 11 | -2 | -3 | 1469.35 | 79.79 |
| 11 | 2 | -3 | 1430.06 | 79.39 |
| 11 | 2 | -3 | 1551.64 | 80.19 |
| 11 | 2 | -3 | 1429.26 | 80.99 |
| 10 | -2 | -3 | 67.79 | 8.30 |
| 10 | -2 | -3 | 60.59 | 7.90 |
| 10 | -2 | -3 | 51.09 | 11.70 |
| 10 | 2 | -3 | 61.89 | 12.30 |
| 10 | 2 | -3 | 58.69 | 7.50 |
| 10 | 2 | -3 | 69.59 | 9.00 |
| 9 | -2 | -3 | 607.64 | 33.40 |
| 9 | -2 | -3 | 585.34 | 33.50 |
| 9 | -2 | -3 | 561.84 | 35.50 |
| 9 | 2 | -3 | 592.54 | 34.00 |
| 9 | 2 | -3 | 565.84 | 36.00 |
| 9 | 2 | -3 | 566.54 | 33.00 |
| 8 | -2 | -3 | 7.70 | 3.90 |
| 8 | 2 | -3 | 9.60 | 5.60 |
| 7 | -2 | -3 | 13.20 | 4.40 |
| 6 | 2 | -3 | 311.07 | 18.50 |
| 5 | 2 | -3 | 4705.33 | 276.77 |
| -5 | 2 | 3 | 5605.34 | 276.37 |
| -4 | 2 | 3 | 151.08 | 9.20 |
| -3 | 2 | 3 | 88.19 | 6.00 |
| -2 | -2 | 3 | 276.47 | 18.50 |
| -2 | 2 | 3 | 360.76 | 18.20 |
| -1 | -2 | 3 | 5460.15 | 293.47 |
| 0 | -2 | 3 | 544.85 | 30.90 |
| 0 | 2 | 3 | 556.94 | 30.60 |
| 1 | -2 | 3 | 1774.42 | 94.19 |
| 1 | 2 | 3 | 1694.63 | 93.99 |
| 2 | -2 | 3 | 113.59 | 7.90 |
| 2 | 2 | 3 | 112.69 | 8.00 |
| 3 | -2 | 3 | 795.02 | 42.10 |
| 3 | 2 | 3 | 730.83 | 42.50 |
| 4 | -2 | 3 | 164.68 | 9.50 |
| 4 | 2 | 3 | 124.89 | 10.40 |
| 5 | -2 | 3 | 4371.66 | 224.78 |
| 5 | 2 | 3 | 3986.80 | 225.68 |
| 6 | -2 | 3 | 2.30 | 4.60 |
| 6 | -2 | 3 | 1.30 | 2.80 |
| 6 | 2 | 3 | 4.40 | 4.10 |
| 6 | 2 | 3 | -1.20 | 2.90 |
| 7 | -2 | 3 | 214.58 | 15.20 |

| | | | | |
|---|---|---|---|---|
| 7 | 2 | 3 | 232.98 | 14.50 |
| 7 | 2 | 3 | 221.58 | 15.50 |
| 8 | -2 | 3 | 5.30 | 6.00 |
| 8 | -2 | 3 | 3.40 | 4.00 |
| 8 | 2 | 3 | 4.00 | 3.90 |
| 8 | 2 | 3 | 18.10 | 5.30 |
| 9 | -2 | 3 | 1121.69 | 62.99 |
| 9 | -2 | 3 | 1174.28 | 63.39 |
| 9 | -2 | 3 | 1094.29 | 63.09 |
| 9 | 2 | 3 | 1117.39 | 63.29 |
| 9 | 2 | 3 | 1145.79 | 63.19 |
| 9 | 2 | 3 | 1148.59 | 63.09 |
| 10 | -2 | 3 | 1.40 | 5.00 |
| 10 | -2 | 3 | 5.50 | 3.70 |
| 10 | -2 | 3 | 7.00 | 5.50 |
| 10 | 2 | 3 | -2.20 | 5.50 |
| 10 | 2 | 3 | 7.80 | 4.90 |
| 10 | 2 | 3 | 2.80 | 4.40 |
| 11 | -2 | 3 | 1126.59 | 58.29 |
| 11 | -2 | 3 | 952.60 | 58.49 |
| 11 | -2 | 3 | 981.20 | 58.19 |
| 11 | 2 | 3 | 1091.49 | 58.69 |
| 11 | 2 | 3 | 993.10 | 58.09 |
| 11 | 2 | 3 | 1110.39 | 58.69 |
| 12 | -2 | 3 | -0.90 | 4.30 |
| 12 | -2 | 3 | 0.50 | 4.40 |
| 12 | -2 | 3 | 7.00 | 3.60 |
| 12 | 2 | 3 | 14.10 | 4.60 |
| 12 | 2 | 3 | 3.10 | 3.70 |
| 12 | 2 | 3 | 6.80 | 4.70 |
| 13 | -2 | 3 | 243.48 | 17.80 |
| 13 | -2 | 3 | 261.67 | 17.10 |
| 13 | -2 | 3 | 278.97 | 16.90 |
| 13 | 2 | 3 | 266.57 | 17.10 |
| 13 | 2 | 3 | 283.27 | 17.30 |
| 13 | 2 | 3 | 255.67 | 17.50 |
| 14 | -2 | 3 | 6.20 | 6.30 |
| 14 | -2 | 3 | 2.60 | 2.80 |
| 14 | -2 | 3 | -3.00 | 3.80 |
| 14 | 2 | 3 | 0.50 | 3.80 |
| 14 | 2 | 3 | 2.20 | 2.80 |
| 14 | 2 | 3 | 8.70 | 5.40 |
| 15 | -2 | 3 | 365.56 | 21.80 |
| 15 | -2 | 3 | 380.26 | 22.20 |

| | | | | |
|---|---|---|---|---|
| 15 | -2 | 3 | 394.46 | 21.80 |
| 15 | 2 | 3 | 353.56 | 22.20 |
| 15 | 2 | 3 | 398.66 | 22.00 |
| 15 | 2 | 3 | 366.96 | 21.80 |
| 16 | -2 | 3 | 8.40 | 3.00 |
| 16 | -2 | 3 | -0.80 | 2.10 |
| 16 | -2 | 3 | -0.40 | 2.20 |
| 16 | 2 | 3 | -0.40 | 2.40 |
| 16 | 2 | 3 | 0.80 | 2.20 |
| 17 | -3 | -3 | -1.00 | 2.90 |
| 17 | -3 | -3 | 0.60 | 1.50 |
| 17 | 3 | -3 | -0.30 | 1.40 |
| 16 | -3 | -3 | 255.47 | 14.70 |
| 16 | -3 | -3 | 250.77 | 15.30 |
| 16 | 3 | -3 | 255.87 | 14.60 |
| 16 | 3 | -3 | 232.98 | 15.80 |
| 15 | -3 | -3 | 2.50 | 3.40 |
| 15 | -3 | -3 | 0.00 | 4.80 |
| 15 | 3 | -3 | 1.10 | 2.60 |
| 15 | 3 | -3 | -1.70 | 4.00 |
| 15 | 3 | -3 | 3.50 | 4.00 |
| 14 | -3 | -3 | 698.43 | 38.20 |
| 14 | -3 | -3 | 629.54 | 37.00 |
| 14 | -3 | -3 | 653.23 | 37.50 |
| 14 | 3 | -3 | 636.64 | 36.80 |
| 14 | 3 | -3 | 651.93 | 37.50 |
| 14 | 3 | -3 | 685.63 | 38.10 |
| 13 | -3 | -3 | -0.30 | 2.90 |
| 13 | -3 | -3 | 3.70 | 3.70 |
| 13 | -3 | -3 | 0.60 | 4.20 |
| 13 | 3 | -3 | 8.70 | 4.90 |
| 13 | 3 | -3 | 1.90 | 2.60 |
| 13 | 3 | -3 | -0.70 | 4.10 |
| 12 | -3 | -3 | 164.68 | 14.10 |
| 12 | -3 | -3 | 161.68 | 12.40 |
| 12 | -3 | -3 | 183.58 | 12.50 |
| 12 | 3 | -3 | 151.68 | 11.50 |
| 12 | 3 | -3 | 191.98 | 13.00 |
| 12 | 3 | -3 | 169.98 | 14.50 |
| 11 | -3 | -3 | 10.20 | 4.20 |
| 11 | -3 | -3 | 10.30 | 6.10 |
| 11 | -3 | -3 | 7.40 | 3.70 |
| 11 | 3 | -3 | 7.90 | 6.10 |
| 11 | 3 | -3 | 6.20 | 3.50 |

| | | | | |
|---|---|---|---|---|
| 11 | 3 | -3 | 9.50 | 4.70 |
| 10 | -3 | -3 | 1961.00 | 102.29 |
| 10 | -3 | -3 | 1879.41 | 101.09 |
| 10 | -3 | -3 | 1788.02 | 101.09 |
| 10 | 3 | -3 | 1767.02 | 100.49 |
| 10 | 3 | -3 | 1833.62 | 102.19 |
| 10 | 3 | -3 | 1871.51 | 101.39 |
| 9 | -3 | -3 | 77.29 | 12.90 |
| 9 | -3 | -3 | 96.99 | 8.80 |
| 9 | -3 | -3 | 72.29 | 9.90 |
| 9 | 3 | -3 | 107.99 | 15.20 |
| 9 | 3 | -3 | 112.79 | 10.80 |
| 9 | 3 | -3 | 87.39 | 8.70 |
| 8 | -3 | -3 | 175.58 | 12.10 |
| 8 | 3 | -3 | 155.18 | 13.80 |
| 7 | -3 | -3 | 190.38 | 13.70 |
| 7 | 3 | -3 | 199.78 | 12.30 |
| 6 | 3 | -3 | 6268.17 | 336.67 |
| -5 | 3 | 3 | 62.89 | 5.30 |
| 5 | 3 | -3 | 65.69 | 5.90 |
| -4 | 3 | 3 | 264.57 | 14.20 |
| 4 | 3 | -3 | 221.68 | 14.60 |
| -3 | 3 | 3 | 54.09 | 4.60 |
| -2 | 3 | 3 | 18.90 | 3.20 |
| -1 | -3 | 3 | 1192.58 | 67.79 |
| -1 | 3 | 3 | 1290.27 | 67.49 |
| 0 | -3 | 3 | 2736.03 | 149.29 |
| 0 | 3 | 3 | 2792.12 | 149.09 |
| 1 | -3 | 3 | 1039.00 | 56.59 |
| 1 | 3 | 3 | 1028.00 | 56.59 |
| 2 | -3 | 3 | 159.88 | 9.80 |
| 2 | 3 | 3 | 146.09 | 10.00 |
| 3 | -3 | 3 | 306.07 | 17.20 |
| 3 | 3 | 3 | 285.67 | 17.80 |
| 4 | -3 | 3 | 3621.64 | 176.78 |
| 4 | 3 | 3 | 2938.31 | 177.28 |
| 5 | 3 | 3 | 128.09 | 10.30 |
| 6 | -3 | 3 | 2351.76 | 129.99 |
| 6 | 3 | 3 | 2385.66 | 129.19 |
| 6 | 3 | 3 | 2421.86 | 130.09 |
| 7 | -3 | 3 | 43.00 | 7.90 |
| 7 | 3 | 3 | 28.10 | 5.60 |
| 7 | 3 | 3 | 42.30 | 8.50 |
| 8 | -3 | 3 | 322.17 | 24.00 |

| | | | | |
|---|---|---|---|---|
| 8 | -3 | 3 | 371.36 | 23.40 |
| 8 | 3 | 3 | 418.96 | 23.50 |
| 8 | 3 | 3 | 389.66 | 24.30 |
| 9 | -3 | 3 | 16.90 | 5.00 |
| 9 | -3 | 3 | 17.00 | 4.30 |
| 9 | -3 | 3 | 21.10 | 6.30 |
| 9 | 3 | 3 | 10.50 | 5.90 |
| 9 | 3 | 3 | 11.90 | 3.90 |
| 10 | -3 | 3 | 1351.06 | 75.09 |
| 10 | -3 | 3 | 1423.06 | 74.69 |
| 10 | -3 | 3 | 1418.06 | 74.89 |
| 10 | 3 | 3 | 1297.07 | 74.69 |
| 10 | 3 | 3 | 1209.68 | 74.69 |
| 10 | 3 | 3 | 1406.66 | 74.79 |
| 11 | -3 | 3 | 34.30 | 6.80 |
| 11 | -3 | 3 | 43.10 | 7.10 |
| 11 | -3 | 3 | 48.90 | 8.60 |
| 11 | 3 | 3 | 41.00 | 7.50 |
| 11 | 3 | 3 | 41.10 | 8.30 |
| 11 | 3 | 3 | 66.39 | 10.00 |
| 12 | -3 | 3 | 2.30 | 4.40 |
| 12 | -3 | 3 | 2.60 | 3.50 |
| 12 | -3 | 3 | 0.00 | 4.20 |
| 12 | 3 | 3 | 6.70 | 4.60 |
| 12 | 3 | 3 | 4.20 | 4.30 |
| 12 | 3 | 3 | 3.70 | 3.60 |
| 13 | -3 | 3 | 28.10 | 5.70 |
| 13 | -3 | 3 | 24.70 | 4.50 |
| 13 | -3 | 3 | 19.70 | 4.80 |
| 13 | 3 | 3 | 20.00 | 4.20 |
| 13 | 3 | 3 | 25.00 | 5.00 |
| 13 | 3 | 3 | 20.10 | 4.90 |
| 14 | -3 | 3 | 366.66 | 22.90 |
| 14 | -3 | 3 | 387.26 | 22.00 |
| 14 | -3 | 3 | 370.46 | 21.80 |
| 14 | 3 | 3 | 368.66 | 21.90 |
| 14 | 3 | 3 | 351.76 | 21.80 |
| 15 | -3 | 3 | -3.90 | 2.30 |
| 15 | -3 | 3 | -0.50 | 2.60 |
| 15 | -3 | 3 | -1.40 | 2.80 |
| 15 | 3 | 3 | 0.20 | 2.60 |
| 15 | 3 | 3 | 0.90 | 2.70 |
| 15 | 3 | 3 | 3.30 | 3.70 |
| 16 | -3 | 3 | 139.69 | 9.20 |

| | | | | |
|---|---|---|---|---|
| 16 | -3 | 3 | 156.48 | 9.50 |
| 16 | -3 | 3 | 157.38 | 9.40 |
| 16 | 3 | 3 | 139.39 | 9.30 |
| 16 | 3 | 3 | 132.59 | 9.30 |
| 17 | -4 | -3 | 87.79 | 6.90 |
| 16 | -4 | -3 | 2.20 | 1.90 |
| 16 | -4 | -3 | 1.10 | 3.80 |
| 16 | 4 | -3 | -2.00 | 1.60 |
| 15 | -4 | -3 | 349.47 | 20.50 |
| 15 | -4 | -3 | 346.67 | 20.90 |
| 15 | 4 | -3 | 353.86 | 20.20 |
| 15 | 4 | -3 | 352.76 | 21.20 |
| 15 | 4 | -3 | 349.67 | 21.20 |
| 14 | -4 | -3 | -6.00 | 3.20 |
| 14 | -4 | -3 | 1.70 | 2.70 |
| 14 | 4 | -3 | 0.10 | 2.30 |
| 14 | 4 | -3 | -1.20 | 4.00 |
| 14 | 4 | -3 | 13.10 | 4.50 |
| 13 | -4 | -3 | 188.58 | 13.20 |
| 13 | -4 | -3 | 207.78 | 14.00 |
| 13 | -4 | -3 | 214.88 | 14.90 |
| 13 | 4 | -3 | 193.48 | 12.50 |
| 13 | 4 | -3 | 193.18 | 15.00 |
| 13 | 4 | -3 | 194.68 | 15.20 |
| 12 | -4 | -3 | 18.00 | 5.70 |
| 12 | -4 | -3 | 16.50 | 4.30 |
| 12 | -4 | -3 | 16.30 | 4.20 |
| 12 | 4 | -3 | 15.00 | 3.40 |
| 12 | 4 | -3 | 25.30 | 6.50 |
| 12 | 4 | -3 | 22.10 | 5.30 |
| 11 | -4 | -3 | 1218.78 | 66.79 |
| 11 | -4 | -3 | 1228.08 | 66.09 |
| 11 | -4 | -3 | 1018.10 | 69.39 |
| 11 | 4 | -3 | 1267.37 | 66.29 |
| 11 | 4 | -3 | 1206.68 | 67.09 |
| 10 | -4 | -3 | 9.40 | 3.60 |
| 10 | -4 | -3 | 4.60 | 4.40 |
| 10 | -4 | -3 | 10.70 | 6.30 |
| 10 | 4 | -3 | 20.10 | 7.80 |
| 10 | 4 | -3 | 1.20 | 4.80 |
| 10 | 4 | -3 | 4.10 | 3.30 |
| 9 | -4 | -3 | 752.52 | 44.00 |
| 9 | -4 | -3 | 835.62 | 45.40 |
| 9 | -4 | -3 | 758.82 | 44.20 |

| | | | | |
|---|---|---|---|---|
| 9 | 4 | -3 | 736.33 | 43.30 |
| 9 | 4 | -3 | 829.12 | 44.40 |
| 9 | 4 | -3 | 751.22 | 46.40 |
| 8 | -4 | -3 | 19.70 | 4.40 |
| 8 | 4 | -3 | 18.50 | 5.70 |
| 7 | -4 | -3 | 1084.19 | 61.79 |
| 7 | 4 | -3 | 1145.79 | 61.29 |
| 6 | -4 | -3 | 263.57 | 17.90 |
| 6 | 4 | -3 | 264.27 | 16.50 |
| -6 | 4 | 3 | 280.57 | 15.70 |
| 5 | 4 | -3 | 2544.65 | 149.09 |
| -5 | 4 | 3 | 2969.30 | 148.49 |
| 4 | 4 | -3 | 80.69 | 6.90 |
| -4 | 4 | 3 | 90.19 | 6.30 |
| -3 | 4 | 3 | 1055.39 | 57.59 |
| -2 | 4 | 3 | 412.16 | 23.30 |
| -1 | 4 | 3 | 2820.32 | 152.18 |
| 0 | 4 | 3 | 116.59 | 8.00 |
| 1 | 4 | 3 | 1581.44 | 86.09 |
| 2 | 4 | 3 | 711.13 | 39.70 |
| 3 | 4 | 3 | 181.88 | 12.00 |
| 4 | 4 | 3 | 12.60 | 3.60 |
| 6 | -4 | 3 | 157.18 | 13.00 |
| 6 | 4 | 3 | 143.69 | 12.00 |
| 6 | 4 | 3 | 152.48 | 10.30 |
| 7 | -4 | 3 | 56.59 | 8.60 |
| 7 | 4 | 3 | 55.89 | 6.10 |
| 7 | 4 | 3 | 49.30 | 9.20 |
| 8 | -4 | 3 | 47.20 | 8.50 |
| 8 | -4 | 3 | 61.09 | 9.40 |
| 8 | -4 | 3 | 38.30 | 7.70 |
| 8 | 4 | 3 | 39.40 | 5.90 |
| 8 | 4 | 3 | 48.20 | 9.00 |
| 9 | -4 | 3 | 967.10 | 53.19 |
| 9 | -4 | 3 | 946.91 | 53.69 |
| 9 | -4 | 3 | 922.71 | 52.79 |
| 9 | 4 | 3 | 880.31 | 53.19 |
| 9 | 4 | 3 | 940.71 | 52.79 |
| 9 | 4 | 3 | 1009.70 | 53.09 |
| 10 | -4 | 3 | -3.30 | 4.70 |
| 10 | -4 | 3 | -2.00 | 3.80 |
| 10 | -4 | 3 | -6.80 | 6.80 |
| 10 | 4 | 3 | -4.00 | 3.60 |
| 10 | 4 | 3 | -6.30 | 5.50 |

| | | | | |
|---|---|---|---|---|
| 11 | -4 | 3 | 774.82 | 42.30 |
| 11 | -4 | 3 | 731.73 | 42.40 |
| 11 | -4 | 3 | 732.73 | 43.10 |
| 11 | 4 | 3 | 747.73 | 42.30 |
| 11 | 4 | 3 | 707.13 | 42.40 |
| 11 | 4 | 3 | 763.32 | 42.50 |
| 12 | -4 | 3 | 49.40 | 8.70 |
| 12 | -4 | 3 | 45.80 | 9.10 |
| 12 | -4 | 3 | 39.30 | 5.60 |
| 12 | 4 | 3 | 47.30 | 8.70 |
| 12 | 4 | 3 | 26.10 | 5.10 |
| 12 | 4 | 3 | 32.50 | 5.50 |
| 13 | -4 | 3 | 300.67 | 18.80 |
| 13 | -4 | 3 | 318.97 | 19.80 |
| 13 | -4 | 3 | 308.57 | 19.20 |
| 13 | 4 | 3 | 297.07 | 19.50 |
| 13 | 4 | 3 | 297.97 | 19.00 |
| 13 | 4 | 3 | 314.67 | 19.30 |
| 14 | -4 | 3 | 1.00 | 2.70 |
| 14 | -4 | 3 | 3.40 | 3.10 |
| 14 | -4 | 3 | -3.30 | 3.50 |
| 14 | 4 | 3 | 3.40 | 3.60 |
| 14 | 4 | 3 | -3.10 | 2.90 |
| 14 | 4 | 3 | 4.70 | 4.10 |
| 15 | -4 | 3 | 337.87 | 19.50 |
| 15 | -4 | 3 | 330.37 | 19.20 |
| 15 | -4 | 3 | 324.97 | 19.30 |
| 15 | 4 | 3 | 315.17 | 19.60 |
| 15 | 4 | 3 | 341.57 | 19.40 |
| 16 | -5 | -3 | 354.16 | 21.20 |
| 16 | -5 | -3 | 375.56 | 20.70 |
| 16 | 5 | -3 | 368.86 | 20.60 |
| 15 | -5 | -3 | 12.50 | 3.30 |
| 15 | -5 | -3 | 4.40 | 2.40 |
| 15 | 5 | -3 | -3.00 | 5.10 |
| 15 | 5 | -3 | 7.30 | 2.20 |
| 14 | -5 | -3 | 215.48 | 13.50 |
| 14 | -5 | -3 | 202.88 | 14.10 |
| 14 | 5 | -3 | 230.78 | 15.10 |
| 14 | 5 | -3 | 214.68 | 13.00 |
| 14 | 5 | -3 | 192.98 | 14.50 |
| 13 | -5 | -3 | 26.20 | 4.20 |
| 13 | -5 | -3 | 22.50 | 4.70 |
| 13 | 5 | -3 | 20.20 | 3.30 |

| | | | | |
|---|---|---|---|---|
| 13 | 5 | -3 | 19.50 | 5.70 |
| 13 | 5 | -3 | 26.00 | 6.00 |
| 12 | -5 | -3 | 499.25 | 30.40 |
| 12 | -5 | -3 | 538.65 | 29.60 |
| 12 | -5 | -3 | 504.65 | 29.40 |
| 12 | 5 | -3 | 490.75 | 29.70 |
| 12 | 5 | -3 | 494.75 | 31.00 |
| 12 | 5 | -3 | 492.25 | 28.60 |
| 11 | -5 | -3 | 14.30 | 3.90 |
| 11 | -5 | -3 | 5.20 | 4.00 |
| 11 | -5 | -3 | 14.60 | 5.70 |
| 11 | 5 | -3 | 19.70 | 3.80 |
| 11 | 5 | -3 | 19.90 | 5.20 |
| 10 | -5 | -3 | 1050.19 | 58.09 |
| 10 | -5 | -3 | 1060.29 | 57.89 |
| 10 | -5 | -3 | 1063.09 | 58.89 |
| 10 | 5 | -3 | 967.70 | 57.99 |
| 10 | 5 | -3 | 1041.20 | 59.69 |
| 10 | 5 | -3 | 1050.49 | 57.19 |
| 9 | -5 | -3 | 30.20 | 6.70 |
| 9 | -5 | -3 | 7.00 | 5.00 |
| 9 | -5 | -3 | 13.10 | 4.30 |
| 9 | 5 | -3 | 6.00 | 10.20 |
| 9 | 5 | -3 | 9.40 | 3.40 |
| 9 | 5 | -3 | 14.10 | 5.60 |
| 8 | -5 | -3 | 449.76 | 30.30 |
| 8 | -5 | -3 | 545.55 | 32.30 |
| 8 | -5 | -3 | 518.05 | 29.60 |
| 8 | 5 | -3 | 551.44 | 29.80 |
| 8 | 5 | -3 | 464.85 | 29.30 |
| 7 | -5 | -3 | 854.11 | 46.30 |
| 7 | 5 | -3 | 830.92 | 45.80 |
| 7 | 5 | -3 | 769.02 | 45.70 |
| 6 | -5 | -3 | 4065.29 | 221.08 |
| 6 | 5 | -3 | 4077.49 | 220.68 |
| -6 | 5 | 3 | 4124.59 | 219.78 |
| 5 | -5 | -3 | 224.58 | 20.40 |
| 5 | 5 | -3 | 207.68 | 14.40 |
| -5 | 5 | 3 | 236.28 | 13.50 |
| -4 | 5 | 3 | 417.56 | 22.30 |
| 4 | 5 | -3 | 360.86 | 22.90 |
| 3 | 5 | -3 | 645.54 | 37.70 |
| -3 | 5 | 3 | 691.83 | 37.20 |
| -2 | 5 | 3 | 481.95 | 27.20 |

| | | | | |
|---|---|---|---|---|
| -1 | 5 | 3 | 8.10 | 2.20 |
| 0 | 5 | 3 | 9701.23 | 520.65 |
| 1 | 5 | 3 | 660.83 | 37.00 |
| 2 | 5 | 3 | 591.24 | 33.50 |
| 3 | 5 | 3 | 80.79 | 7.20 |
| 4 | 5 | 3 | 1275.47 | 70.59 |
| 5 | -5 | 3 | 54.69 | 9.70 |
| 5 | 5 | 3 | 39.20 | 7.60 |
| 5 | 5 | 3 | 45.00 | 5.30 |
| 6 | -5 | 3 | 1267.67 | 73.19 |
| 6 | 5 | 3 | 1281.67 | 73.39 |
| 6 | 5 | 3 | 1399.76 | 72.09 |
| -6 | 5 | -3 | 1323.97 | 71.89 |
| 7 | -5 | 3 | 792.02 | 46.00 |
| 7 | -5 | 3 | 783.72 | 46.80 |
| 7 | 5 | 3 | 842.12 | 44.90 |
| 7 | 5 | 3 | 803.62 | 46.50 |
| 8 | -5 | 3 | 444.56 | 28.70 |
| 8 | -5 | 3 | 503.75 | 27.40 |
| 8 | -5 | 3 | 411.06 | 26.70 |
| 8 | 5 | 3 | 392.66 | 30.10 |
| 8 | 5 | 3 | 489.65 | 27.20 |
| 8 | 5 | 3 | 447.76 | 26.10 |
| 9 | -5 | 3 | 24.40 | 5.40 |
| 9 | -5 | 3 | 30.50 | 7.70 |
| 9 | -5 | 3 | 30.90 | 5.30 |
| 9 | 5 | 3 | 26.10 | 5.50 |
| 9 | 5 | 3 | 32.40 | 6.90 |
| 9 | 5 | 3 | 31.40 | 5.80 |
| 10 | -5 | 3 | 1250.87 | 71.09 |
| 10 | -5 | 3 | 1317.67 | 70.79 |
| 10 | -5 | 3 | 1224.58 | 71.79 |
| 10 | 5 | 3 | 1329.97 | 70.99 |
| 10 | 5 | 3 | 1267.87 | 70.79 |
| 10 | 5 | 3 | 1292.27 | 70.89 |
| 11 | -5 | 3 | 39.60 | 7.30 |
| 11 | -5 | 3 | 48.90 | 8.20 |
| 11 | -5 | 3 | 40.00 | 7.10 |
| 11 | 5 | 3 | 40.90 | 6.30 |
| 11 | 5 | 3 | 46.00 | 7.30 |
| 11 | 5 | 3 | 59.79 | 8.20 |
| 12 | -5 | 3 | 30.40 | 6.60 |
| 12 | -5 | 3 | 23.40 | 5.50 |
| 12 | -5 | 3 | 27.80 | 4.90 |

| | | | | |
|---|---|---|---|---|
| 12 | 5 | 3 | 16.30 | 4.50 |
| 12 | 5 | 3 | 7.30 | 5.00 |
| 12 | 5 | 3 | 14.10 | 5.00 |
| 13 | -5 | 3 | 12.10 | 4.90 |
| 13 | -5 | 3 | 11.90 | 3.90 |
| 13 | -5 | 3 | 4.70 | 3.10 |
| 13 | 5 | 3 | 9.20 | 4.00 |
| 13 | 5 | 3 | 7.60 | 3.40 |
| 14 | -5 | 3 | 409.76 | 22.30 |
| 14 | -5 | 3 | 372.26 | 21.70 |
| 14 | -5 | 3 | 373.86 | 21.70 |
| 14 | 5 | 3 | 332.87 | 21.90 |
| 14 | 5 | 3 | 341.37 | 22.20 |
| 14 | 5 | 3 | 374.76 | 21.70 |
| 15 | -5 | 3 | 4.90 | 2.50 |
| 15 | -5 | 3 | 0.50 | 2.40 |
| 15 | -5 | 3 | 2.00 | 2.80 |
| 15 | 5 | 3 | 3.70 | 2.90 |
| 15 | 5 | 3 | 1.70 | 3.00 |
| 16 | -6 | -3 | 8.50 | 2.00 |
| 16 | -6 | -3 | 9.90 | 2.90 |
| 16 | 6 | -3 | 6.10 | 1.70 |
| 15 | -6 | -3 | 456.65 | 26.40 |
| 15 | -6 | -3 | 449.16 | 26.00 |
| 15 | 6 | -3 | 472.15 | 25.80 |
| 14 | -6 | -3 | 14.80 | 3.70 |
| 14 | -6 | -3 | 4.70 | 2.70 |
| 14 | 6 | -3 | 9.90 | 2.30 |
| 14 | 6 | -3 | 10.20 | 5.60 |
| 14 | 6 | -3 | 6.40 | 5.00 |
| 13 | -6 | -3 | 78.19 | 7.70 |
| 13 | -6 | -3 | 90.39 | 8.30 |
| 13 | 6 | -3 | 75.29 | 10.20 |
| 13 | 6 | -3 | 83.19 | 6.80 |
| 13 | 6 | -3 | 86.59 | 10.60 |
| 12 | -6 | -3 | 147.39 | 11.10 |
| 12 | -6 | -3 | 152.18 | 11.30 |
| 12 | 6 | -3 | 134.29 | 14.40 |
| 12 | 6 | -3 | 136.29 | 10.00 |
| 12 | 6 | -3 | 156.48 | 12.40 |
| 11 | -6 | -3 | 1663.63 | 88.09 |
| 11 | -6 | -3 | 1612.04 | 87.49 |
| 11 | -6 | -3 | 1590.84 | 87.49 |
| 11 | 6 | -3 | 1562.54 | 88.39 |

| | | | | |
|---|---|---|---|---|
| 11 | 6 | -3 | 1479.05 | 87.39 |
| 11 | 6 | -3 | 1640.54 | 86.69 |
| 10 | -6 | -3 | 54.49 | 9.10 |
| 10 | -6 | -3 | 50.89 | 7.30 |
| 10 | -6 | -3 | 57.19 | 10.60 |
| 10 | 6 | -3 | 74.49 | 15.60 |
| 10 | 6 | -3 | 63.09 | 6.80 |
| 10 | 6 | -3 | 54.69 | 9.70 |
| 9 | -6 | -3 | 528.45 | 30.90 |
| 9 | -6 | -3 | 563.44 | 32.50 |
| 9 | -6 | -3 | 505.45 | 31.50 |
| 9 | 6 | -3 | 520.95 | 30.20 |
| 8 | -6 | -3 | 10.90 | 5.80 |
| 8 | -6 | -3 | 10.80 | 7.30 |
| 8 | -6 | -3 | 11.00 | 4.40 |
| 8 | 6 | -3 | 11.50 | 4.10 |
| 8 | 6 | -3 | 9.50 | 4.50 |
| 8 | 6 | -3 | 23.30 | 4.90 |
| 7 | -6 | -3 | 147.39 | 11.60 |
| 7 | 6 | -3 | 114.49 | 10.80 |
| 7 | 6 | -3 | 141.79 | 10.90 |
| 6 | -6 | -3 | 41.70 | 8.90 |
| -6 | 6 | 3 | 40.10 | 6.10 |
| 6 | 6 | -3 | 44.10 | 6.80 |
| 5 | -6 | -3 | 4577.44 | 253.87 |
| 5 | 6 | -3 | 4451.75 | 253.67 |
| -5 | 6 | 3 | 5067.39 | 252.87 |
| 4 | -6 | -3 | 130.69 | 13.00 |
| 4 | 6 | -3 | 161.98 | 11.80 |
| -4 | 6 | 3 | 189.48 | 10.90 |
| -3 | 6 | 3 | 570.14 | 31.70 |
| 3 | 6 | -3 | 554.44 | 32.30 |
| 2 | 6 | -3 | 337.87 | 21.10 |
| -2 | 6 | 3 | 369.66 | 20.70 |
| -1 | 6 | 3 | 2665.33 | 145.69 |
| 1 | 6 | -3 | 2719.63 | 145.89 |
| 0 | 6 | 3 | 81.19 | 6.70 |
| 1 | 6 | 3 | 1486.65 | 81.39 |
| 2 | 6 | 3 | 400.36 | 23.60 |
| 3 | 6 | 3 | 567.34 | 32.80 |
| 4 | -6 | 3 | -0.10 | 4.50 |
| 4 | 6 | 3 | 4.80 | 3.50 |
| -4 | 6 | -3 | 0.00 | 2.30 |
| 4 | 6 | 3 | 2.70 | 2.70 |

| | | | | |
|---|---|---|---|---|
| 5 | -6 | 3 | 5963.80 | 335.27 |
| 5 | 6 | 3 | 6180.98 | 334.47 |
| 5 | 6 | 3 | 6168.18 | 336.07 |
| -5 | 6 | -3 | 6590.44 | 334.77 |
| 6 | -6 | 3 | 188.18 | 15.20 |
| -6 | 6 | -3 | 213.18 | 13.10 |
| 6 | 6 | 3 | 211.48 | 15.70 |
| 6 | 6 | 3 | 196.08 | 12.80 |
| 7 | -6 | 3 | 237.48 | 16.60 |
| 7 | 6 | 3 | 231.58 | 17.50 |
| 7 | 6 | 3 | 260.97 | 18.20 |
| 7 | 6 | 3 | 256.97 | 15.30 |
| 8 | -6 | 3 | 38.00 | 6.10 |
| 8 | -6 | 3 | 42.40 | 5.90 |
| 8 | -6 | 3 | 44.60 | 9.40 |
| 8 | 6 | 3 | 32.00 | 5.50 |
| 8 | 6 | 3 | 38.30 | 7.90 |
| 8 | 6 | 3 | 54.49 | 9.00 |
| 9 | -6 | 3 | 916.71 | 49.70 |
| 9 | -6 | 3 | 829.02 | 48.90 |
| 9 | -6 | 3 | 827.02 | 50.49 |
| 9 | 6 | 3 | 890.51 | 49.20 |
| 9 | 6 | 3 | 947.21 | 49.00 |
| 9 | 6 | 3 | 819.72 | 49.30 |
| 10 | -6 | 3 | -0.30 | 3.60 |
| 10 | -6 | 3 | 5.70 | 6.50 |
| 10 | -6 | 3 | -2.10 | 3.90 |
| 10 | 6 | 3 | -4.40 | 5.60 |
| 10 | 6 | 3 | -2.40 | 3.20 |
| 10 | 6 | 3 | 4.90 | 4.80 |
| 11 | -6 | 3 | 1500.55 | 78.29 |
| 11 | -6 | 3 | 1354.76 | 78.89 |
| 11 | -6 | 3 | 1470.05 | 78.49 |
| 11 | 6 | 3 | 1413.96 | 77.99 |
| 11 | 6 | 3 | 1292.47 | 78.19 |
| 11 | 6 | 3 | 1485.05 | 78.29 |
| 12 | -6 | 3 | 38.70 | 7.50 |
| 12 | -6 | 3 | 43.90 | 6.10 |
| 12 | -6 | 3 | 30.60 | 5.90 |
| 12 | 6 | 3 | 31.40 | 7.10 |
| 12 | 6 | 3 | 46.20 | 8.80 |
| 12 | 6 | 3 | 29.50 | 6.60 |
| 13 | -6 | 3 | 220.88 | 14.30 |
| 13 | -6 | 3 | 255.67 | 18.20 |

| | | | | |
|---|---|---|---|---|
| 13 | -6 | 3 | 219.78 | 14.80 |
| 13 | 6 | 3 | 224.18 | 16.00 |
| 13 | 6 | 3 | 206.38 | 14.60 |
| 13 | 6 | 3 | 216.68 | 14.90 |
| 14 | -6 | 3 | -1.20 | 2.60 |
| 14 | -6 | 3 | 2.90 | 2.90 |
| 14 | -6 | 3 | 0.90 | 3.30 |
| 14 | 6 | 3 | 4.40 | 3.40 |
| 14 | 6 | 3 | 4.20 | 2.70 |
| 15 | -6 | 3 | 329.47 | 17.80 |
| 15 | -6 | 3 | 283.47 | 17.90 |
| 15 | 6 | 3 | 295.47 | 18.00 |
| 15 | 6 | 3 | 318.07 | 18.00 |
| 16 | -7 | -3 | 453.75 | 25.70 |
| 16 | -7 | -3 | 448.56 | 25.30 |
| 16 | 7 | -3 | 463.85 | 25.20 |
| 15 | -7 | -3 | 1.50 | 2.80 |
| 15 | -7 | -3 | 1.90 | 2.00 |
| 15 | 7 | -3 | 2.90 | 1.80 |
| 14 | -7 | -3 | 412.96 | 24.40 |
| 14 | 7 | -3 | 421.56 | 24.00 |
| 13 | -7 | -3 | 12.40 | 3.20 |
| 13 | -7 | -3 | 3.80 | 3.20 |
| 13 | 7 | -3 | 7.90 | 2.40 |
| 13 | 7 | -3 | 1.50 | 5.10 |
| 13 | 7 | -3 | 8.70 | 6.00 |
| 12 | -7 | -3 | 386.96 | 25.10 |
| 12 | -7 | -3 | 425.86 | 25.20 |
| 12 | 7 | -3 | 466.95 | 26.10 |
| 12 | 7 | -3 | 417.36 | 24.30 |
| 12 | 7 | -3 | 427.36 | 27.20 |
| 11 | -7 | -3 | 33.20 | 5.60 |
| 11 | -7 | -3 | 35.90 | 6.30 |
| 11 | 7 | -3 | 30.00 | 6.40 |
| 11 | 7 | -3 | 34.70 | 5.60 |
| 10 | -7 | -3 | 1439.66 | 84.39 |
| 10 | -7 | -3 | 1503.55 | 84.59 |
| 10 | 7 | -3 | 1653.23 | 84.59 |
| 10 | 7 | -3 | 1543.65 | 83.69 |
| 9 | -7 | -3 | 55.29 | 7.70 |
| 9 | -7 | -3 | 54.29 | 10.30 |
| 9 | 7 | -3 | 68.59 | 7.30 |
| 9 | 7 | -3 | 76.69 | 9.40 |
| 8 | -7 | -3 | 14.40 | 5.70 |

| | | | | |
|---|---|---|---|---|
| 8 | -7 | -3 | 2.00 | 3.90 |
| 8 | 7 | -3 | 7.90 | 5.20 |
| 8 | 7 | -3 | 8.80 | 4.10 |
| 8 | 7 | -3 | 0.90 | 4.20 |
| 7 | -7 | -3 | 676.93 | 34.90 |
| 7 | -7 | -3 | 538.45 | 34.60 |
| 7 | 7 | -3 | 585.34 | 33.40 |
| 7 | 7 | -3 | 545.95 | 34.00 |
| 7 | 7 | -3 | 593.44 | 34.60 |
| 6 | -7 | -3 | 4480.05 | 233.78 |
| -6 | 7 | 3 | 4403.16 | 232.48 |
| 6 | 7 | -3 | 4243.78 | 233.68 |
| 6 | 7 | -3 | 4143.49 | 232.28 |
| 5 | -7 | -3 | 375.66 | 26.00 |
| -5 | 7 | 3 | 466.05 | 24.50 |
| 5 | 7 | -3 | 412.46 | 25.70 |
| 4 | -7 | -3 | 1265.17 | 74.29 |
| -4 | 7 | 3 | 1444.06 | 73.59 |
| 4 | 7 | -3 | 1314.87 | 74.39 |
| 3 | -7 | -3 | 73.89 | 8.70 |
| -3 | 7 | 3 | 66.99 | 6.40 |
| 3 | 7 | -3 | 69.79 | 7.30 |
| 2 | 7 | -3 | 294.37 | 18.40 |
| -2 | 7 | 3 | 295.67 | 17.80 |
| -1 | 7 | 3 | 192.98 | 12.50 |
| 1 | 7 | -3 | 187.88 | 12.70 |
| 0 | 7 | 3 | 7171.58 | 393.66 |
| 0 | 7 | -3 | 7471.65 | 393.66 |
| 1 | 7 | 3 | 341.07 | 20.80 |
| -1 | 7 | -3 | 355.16 | 20.60 |
| -2 | 7 | -3 | 200.38 | 12.60 |
| 2 | 7 | 3 | 192.58 | 13.10 |
| -3 | 7 | -3 | 288.67 | 16.80 |
| 3 | 7 | 3 | 256.57 | 16.50 |
| 3 | 7 | 3 | 284.57 | 17.90 |
| 4 | -7 | 3 | 4224.88 | 236.08 |
| 4 | 7 | 3 | 4530.25 | 235.58 |
| -4 | 7 | -3 | 4508.05 | 236.08 |
| 4 | 7 | 3 | 4246.77 | 236.98 |
| 5 | -7 | 3 | 39.90 | 8.30 |
| 5 | 7 | 3 | 25.80 | 4.50 |
| 5 | 7 | 3 | 21.10 | 5.30 |
| -5 | 7 | -3 | 31.20 | 5.40 |
| 6 | -7 | 3 | 1871.81 | 100.59 |

| | | | | |
|---|---|---|---|---|
| 6 | -7 | 3 | 1830.42 | 101.49 |
| -6 | 7 | -3 | 1882.61 | 100.59 |
| 6 | 7 | 3 | 1939.21 | 100.39 |
| 6 | 7 | 3 | 1707.93 | 101.99 |
| 7 | -7 | 3 | 41.60 | 8.70 |
| 7 | -7 | 3 | 52.79 | 8.20 |
| 7 | 7 | 3 | 45.40 | 5.40 |
| 7 | 7 | 3 | 56.39 | 12.50 |
| 7 | 7 | 3 | 38.80 | 7.70 |
| 8 | -7 | 3 | 443.96 | 33.60 |
| 8 | -7 | 3 | 535.65 | 30.40 |
| 8 | -7 | 3 | 534.85 | 31.30 |
| 8 | 7 | 3 | 530.65 | 30.90 |
| 8 | 7 | 3 | 527.05 | 29.80 |
| 8 | 7 | 3 | 547.05 | 32.80 |
| 9 | -7 | 3 | 3.30 | 7.90 |
| 9 | -7 | 3 | 1.90 | 4.50 |
| 9 | -7 | 3 | -6.10 | 4.10 |
| 9 | 7 | 3 | 0.30 | 2.80 |
| 9 | 7 | 3 | -11.10 | 5.80 |
| 9 | 7 | 3 | -5.40 | 4.90 |
| 10 | -7 | 3 | 854.01 | 53.99 |
| 10 | -7 | 3 | 980.60 | 53.69 |
| 10 | -7 | 3 | 1009.30 | 53.29 |
| 10 | 7 | 3 | 965.90 | 53.19 |
| 10 | 7 | 3 | 951.80 | 53.39 |
| 10 | 7 | 3 | 944.21 | 53.19 |
| 11 | -7 | 3 | 27.50 | 4.90 |
| 11 | -7 | 3 | 22.50 | 5.00 |
| 11 | -7 | 3 | 25.10 | 6.40 |
| 11 | 7 | 3 | 19.70 | 5.00 |
| 11 | 7 | 3 | 38.10 | 6.60 |
| 11 | 7 | 3 | 21.20 | 5.30 |
| 12 | -7 | 3 | 56.89 | 6.90 |
| 12 | -7 | 3 | 67.79 | 8.90 |
| 12 | -7 | 3 | 57.69 | 7.60 |
| 12 | 7 | 3 | 52.69 | 7.60 |
| 12 | 7 | 3 | 53.09 | 8.20 |
| 12 | 7 | 3 | 51.59 | 9.10 |
| 13 | -7 | 3 | 44.90 | 7.30 |
| 13 | -7 | 3 | 51.09 | 5.90 |
| 13 | -7 | 3 | 45.80 | 7.80 |
| 13 | 7 | 3 | 38.40 | 6.10 |
| 13 | 7 | 3 | 42.40 | 7.90 |

| | | | | |
|---|---|---|---|---|
| 14 | -7 | 3 | 348.07 | 20.20 |
| 14 | -7 | 3 | 358.76 | 20.10 |
| 14 | 7 | 3 | 319.17 | 20.30 |
| 14 | 7 | 3 | 348.47 | 20.30 |
| 15 | -7 | 3 | 1.60 | 2.20 |
| 15 | -7 | 3 | -1.50 | 2.40 |
| 15 | 7 | 3 | -0.70 | 3.80 |
| 15 | 7 | 3 | -0.20 | 2.50 |
| 15 | -8 | -3 | 418.36 | 25.70 |
| 15 | -8 | -3 | 458.65 | 25.30 |
| 15 | 8 | -3 | 474.15 | 25.10 |
| 14 | -8 | -3 | 29.30 | 4.80 |
| 14 | -8 | -3 | 38.00 | 5.70 |
| 14 | 8 | -3 | 33.60 | 4.10 |
| 13 | -8 | -3 | 25.40 | 5.60 |
| 13 | -8 | -3 | 18.30 | 3.60 |
| 13 | 8 | -3 | 31.60 | 4.20 |
| 12 | -8 | -3 | 85.89 | 8.30 |
| 12 | -8 | -3 | 75.29 | 8.30 |
| 12 | 8 | -3 | 95.99 | 11.30 |
| 12 | 8 | -3 | 78.99 | 6.80 |
| 11 | -8 | -3 | 1462.05 | 78.29 |
| 11 | -8 | -3 | 1416.06 | 78.29 |
| 11 | 8 | -3 | 1412.16 | 77.29 |
| 11 | 8 | -3 | 1384.26 | 78.29 |
| 10 | -8 | -3 | 118.39 | 10.90 |
| 10 | -8 | -3 | 109.39 | 9.50 |
| 10 | 8 | -3 | 91.19 | 11.00 |
| 10 | 8 | -3 | 95.99 | 8.30 |
| 9 | -8 | -3 | 892.61 | 55.29 |
| 9 | -8 | -3 | 1003.00 | 55.39 |
| 9 | 8 | -3 | 990.10 | 54.49 |
| 9 | 8 | -3 | 1049.49 | 54.99 |
| 8 | -8 | -3 | 62.79 | 8.80 |
| 8 | -8 | -3 | 91.79 | 10.80 |
| 8 | 8 | -3 | 73.49 | 10.30 |
| 8 | 8 | -3 | 79.99 | 8.60 |
| 7 | -8 | -3 | 171.88 | 13.20 |
| 7 | -8 | -3 | 175.18 | 14.10 |
| 7 | 8 | -3 | 167.28 | 13.00 |
| 7 | 8 | -3 | 139.09 | 11.70 |
| 6 | -8 | -3 | 16.80 | 5.70 |
| 6 | 8 | -3 | 15.20 | 4.20 |
| -6 | 8 | 3 | 19.00 | 4.70 |

| | | | | |
|---|---|---|---|---|
| 6 | 8 | -3 | 18.10 | 5.00 |
| 5 | -8 | -3 | 3521.55 | 199.18 |
| 5 | 8 | -3 | 3697.63 | 197.78 |
| 5 | 8 | -3 | 3366.76 | 199.28 |
| -5 | 8 | 3 | 4100.39 | 198.48 |
| 4 | -8 | -3 | 240.08 | 16.50 |
| -4 | 8 | 3 | 251.47 | 15.30 |
| 4 | 8 | -3 | 225.58 | 16.40 |
| 3 | 8 | -3 | 249.28 | 17.90 |
| -3 | 8 | 3 | 303.07 | 17.30 |
| -2 | 8 | 3 | 1147.49 | 63.49 |
| 1 | 8 | -3 | 4712.03 | 260.07 |
| -1 | 8 | 3 | 4886.51 | 258.97 |
| 0 | 8 | 3 | 12.80 | 3.90 |
| 0 | 8 | -3 | 18.80 | 4.20 |
| -1 | 8 | -3 | 3138.39 | 173.28 |
| 1 | 8 | 3 | 3199.88 | 172.38 |
| 1 | 8 | 3 | 3241.28 | 173.38 |
| 2 | 8 | 3 | 342.87 | 20.00 |
| 2 | 8 | 3 | 336.37 | 20.80 |
| 3 | -8 | 3 | 157.68 | 11.90 |
| 3 | 8 | 3 | 140.89 | 11.30 |
| -3 | 8 | -3 | 151.88 | 11.10 |
| 3 | 8 | 3 | 145.69 | 10.10 |
| 4 | -8 | 3 | 105.69 | 10.20 |
| 4 | 8 | 3 | 110.69 | 8.10 |
| -4 | 8 | -3 | 110.69 | 8.80 |
| 4 | 8 | 3 | 103.89 | 10.00 |
| 5 | -8 | 3 | 1563.94 | 90.79 |
| 5 | -8 | 3 | 1705.13 | 91.39 |
| 5 | 8 | 3 | 1556.64 | 92.29 |
| 5 | 8 | 3 | 1794.92 | 90.69 |
| -5 | 8 | -3 | 1696.73 | 90.99 |
| 6 | -8 | 3 | 26.60 | 5.70 |
| 6 | -8 | 3 | 35.00 | 5.40 |
| -6 | -8 | -3 | 24.70 | 6.30 |
| 6 | 8 | 3 | 24.00 | 6.30 |
| -6 | 8 | -3 | 35.80 | 5.00 |
| 6 | 8 | 3 | 32.70 | 4.90 |
| -7 | -8 | -3 | 63.69 | 11.30 |
| 7 | -8 | 3 | 56.59 | 7.80 |
| 7 | -8 | 3 | 60.79 | 8.80 |
| 7 | 8 | 3 | 43.90 | 5.80 |
| 7 | 8 | 3 | 60.19 | 11.50 |

| | | | | |
|---|---|---|---|---|
| 7 | 8 | 3 | 49.20 | 7.60 |
| 8 | -8 | 3 | 90.49 | 10.40 |
| 8 | -8 | 3 | 94.49 | 9.40 |
| 8 | 8 | 3 | 86.59 | 11.60 |
| 8 | 8 | 3 | 75.59 | 14.30 |
| 8 | 8 | 3 | 106.89 | 7.80 |
| 9 | -8 | 3 | 714.33 | 40.50 |
| 9 | -8 | 3 | 717.13 | 39.70 |
| 9 | 8 | 3 | 657.43 | 40.00 |
| 9 | 8 | 3 | 699.33 | 39.30 |
| 10 | -8 | 3 | 18.90 | 4.90 |
| 10 | -8 | 3 | 21.10 | 7.30 |
| 10 | -8 | 3 | 14.00 | 4.00 |
| 10 | 8 | 3 | 17.80 | 3.80 |
| 10 | 8 | 3 | 24.20 | 5.60 |
| 10 | 8 | 3 | 23.00 | 6.50 |
| 11 | -8 | 3 | 776.52 | 46.30 |
| 11 | -8 | 3 | 847.22 | 45.80 |
| 11 | -8 | 3 | 819.12 | 46.10 |
| 11 | 8 | 3 | 806.22 | 45.90 |
| 11 | 8 | 3 | 808.42 | 45.80 |
| 12 | -8 | 3 | 99.59 | 8.40 |
| 12 | -8 | 3 | 87.29 | 8.60 |
| 12 | -8 | 3 | 95.79 | 11.50 |
| 12 | 8 | 3 | 98.39 | 8.60 |
| 12 | 8 | 3 | 83.99 | 10.10 |
| 13 | -8 | 3 | 169.18 | 11.10 |
| 13 | -8 | 3 | 157.88 | 10.80 |
| 13 | 8 | 3 | 148.09 | 12.00 |
| 13 | 8 | 3 | 144.59 | 10.90 |
| 14 | -8 | 3 | -2.50 | 2.30 |
| 14 | -8 | 3 | -3.40 | 2.40 |
| 14 | 8 | 3 | -2.60 | 3.80 |
| 14 | 8 | 3 | -0.20 | 3.20 |
| 15 | -9 | -3 | 9.80 | 2.80 |
| 15 | -9 | -3 | 6.70 | 1.90 |
| -15 | 9 | 3 | 11.10 | 2.70 |
| 15 | 9 | -3 | 15.20 | 2.70 |
| 14 | -9 | -3 | 444.56 | 25.10 |
| 14 | -9 | -3 | 426.96 | 24.60 |
| 14 | 9 | -3 | 430.56 | 24.20 |
| 13 | -9 | -3 | 314.87 | 18.90 |
| 13 | -9 | -3 | 298.17 | 18.40 |
| 13 | 9 | -3 | 297.87 | 19.20 |

| | | | | |
|---|---|---|---|---|
| 12 | -9 | -3 | 46.20 | 6.60 |
| 12 | -9 | -3 | 57.69 | 7.10 |
| 12 | 9 | -3 | 48.30 | 5.30 |
| 11 | -9 | -3 | 7.10 | 3.90 |
| 11 | -9 | -3 | 3.40 | 3.40 |
| 11 | 9 | -3 | 1.40 | 2.40 |
| 11 | 9 | -3 | 0.10 | 6.50 |
| 10 | -9 | -3 | 1340.47 | 72.79 |
| 10 | -9 | -3 | 1283.47 | 72.79 |
| 10 | 9 | -3 | 1288.97 | 71.79 |
| 10 | 9 | -3 | 1340.27 | 72.59 |
| 9 | -9 | -3 | 19.90 | 4.80 |
| 9 | -9 | -3 | 13.20 | 4.90 |
| 9 | 9 | -3 | 15.60 | 3.60 |
| 9 | 9 | -3 | 26.20 | 5.30 |
| 8 | -9 | -3 | 144.29 | 11.90 |
| 8 | -9 | -3 | 134.39 | 12.80 |
| 8 | 9 | -3 | 156.88 | 11.50 |
| 8 | 9 | -3 | 149.39 | 13.60 |
| 8 | 9 | -3 | 132.99 | 11.20 |
| 7 | -9 | -3 | 595.64 | 34.80 |
| 7 | -9 | -3 | 630.44 | 35.50 |
| -7 | -9 | 3 | 513.75 | 34.60 |
| 7 | 9 | -3 | 619.84 | 35.50 |
| 7 | 9 | -3 | 607.84 | 33.70 |
| 6 | -9 | -3 | 2698.33 | 152.18 |
| -6 | -9 | 3 | 2364.26 | 151.78 |
| 6 | -9 | -3 | 2842.82 | 153.28 |
| 6 | 9 | -3 | 2809.52 | 153.48 |
| 6 | 9 | -3 | 2946.21 | 151.58 |
| -6 | 9 | 3 | 3176.18 | 152.48 |
| 5 | -9 | -3 | 1.90 | 4.90 |
| 5 | 9 | -3 | 12.70 | 4.00 |
| -5 | 9 | 3 | 2.80 | 3.50 |
| 5 | 9 | -3 | -2.50 | 3.90 |
| 4 | 9 | -3 | 1449.46 | 87.99 |
| 4 | 9 | -3 | 1573.34 | 86.29 |
| -4 | 9 | 3 | 1732.93 | 87.09 |
| 3 | 9 | -3 | 100.09 | 9.10 |
| -3 | 9 | 3 | 79.39 | 7.70 |
| 3 | 9 | -3 | 64.69 | 8.70 |
| 2 | 9 | -3 | 9.70 | 3.90 |
| -2 | 9 | 3 | 8.60 | 3.00 |
| 1 | 9 | -3 | 205.58 | 14.20 |

| | | | | |
|---|---|---|---|---|
| -1 | 9 | 3 | 212.28 | 14.00 |
| 0 | 9 | 3 | 4301.57 | 231.38 |
| 0 | 9 | -3 | 4361.06 | 232.18 |
| 0 | 9 | 3 | 4218.98 | 232.08 |
| 1 | 9 | 3 | 31.60 | 5.40 |
| -1 | 9 | -3 | 25.60 | 5.20 |
| 1 | 9 | 3 | 20.80 | 3.60 |
| 2 | -9 | 3 | 88.69 | 10.20 |
| -2 | 9 | -3 | 111.89 | 8.80 |
| 2 | 9 | 3 | 99.09 | 9.00 |
| 2 | 9 | 3 | 125.29 | 8.50 |
| 3 | -9 | 3 | 226.68 | 15.20 |
| -3 | 9 | -3 | 226.48 | 14.50 |
| 3 | 9 | 3 | 216.28 | 15.30 |
| 3 | 9 | 3 | 217.78 | 13.90 |
| 4 | -9 | 3 | 1170.48 | 63.99 |
| 4 | -9 | 3 | 1204.08 | 64.29 |
| -4 | -9 | -3 | 1100.29 | 64.19 |
| 4 | 9 | 3 | 1157.68 | 63.69 |
| 4 | 9 | 3 | 1124.09 | 65.39 |
| -4 | 9 | -3 | 1203.98 | 64.39 |
| -5 | -9 | -3 | 11.70 | 4.80 |
| 5 | -9 | 3 | 11.70 | 3.90 |
| 5 | 9 | 3 | 13.10 | 3.00 |
| 5 | 9 | 3 | 7.20 | 5.50 |
| -5 | 9 | -3 | 10.60 | 3.80 |
| -6 | -9 | -3 | 2076.69 | 115.79 |
| 6 | -9 | 3 | 2119.89 | 115.59 |
| 6 | -9 | 3 | 2100.59 | 116.49 |
| 6 | 9 | 3 | 2275.97 | 115.59 |
| 6 | 9 | 3 | 1944.31 | 117.69 |
| -6 | 9 | -3 | 2249.57 | 116.09 |
| 7 | -9 | 3 | 246.98 | 17.40 |
| -7 | -9 | -3 | 232.98 | 17.20 |
| 7 | -9 | 3 | 265.97 | 16.30 |
| 7 | 9 | 3 | 257.67 | 20.10 |
| 7 | 9 | 3 | 253.27 | 15.50 |
| 8 | -9 | 3 | 396.36 | 26.00 |
| 8 | -9 | 3 | 405.06 | 24.70 |
| 8 | 9 | 3 | 419.66 | 24.30 |
| 8 | 9 | 3 | 451.25 | 29.30 |
| 9 | -9 | 3 | 67.19 | 8.30 |
| 9 | -9 | 3 | 69.89 | 9.50 |
| 9 | 9 | 3 | 77.19 | 6.90 |

| | | | | |
|---|---|---|---|---|
| 10 | -9 | 3 | 821.72 | 44.80 |
| 10 | -9 | 3 | 794.32 | 45.50 |
| 10 | 9 | 3 | 773.02 | 45.10 |
| 10 | 9 | 3 | 787.32 | 44.60 |
| 11 | -9 | 3 | 10.80 | 3.40 |
| 11 | -9 | 3 | 15.90 | 4.10 |
| 11 | 9 | 3 | 14.40 | 5.60 |
| 11 | 9 | 3 | 9.50 | 3.50 |
| 12 | -9 | 3 | 9.90 | 3.60 |
| 12 | -9 | 3 | 9.40 | 4.60 |
| 12 | 9 | 3 | 16.90 | 5.80 |
| 12 | 9 | 3 | 2.10 | 3.20 |
| 13 | -9 | 3 | 36.50 | 5.30 |
| 13 | -9 | 3 | 29.90 | 5.30 |
| 13 | 9 | 3 | 31.60 | 5.40 |
| 13 | 9 | 3 | 26.00 | 5.60 |
| 14 | -9 | 3 | 214.08 | 13.40 |
| 14 | -9 | 3 | 227.08 | 13.70 |
| 14 | 9 | 3 | 231.38 | 13.70 |
| 15 | -10 | -3 | 201.28 | 12.10 |
| -15 | 10 | 3 | 208.08 | 12.70 |
| 15 | 10 | -3 | 215.88 | 12.00 |
| 14 | -10 | -3 | 48.60 | 4.60 |
| -14 | 10 | 3 | 41.50 | 5.70 |
| 14 | 10 | -3 | 46.00 | 4.00 |
| 13 | -10 | -3 | 44.50 | 5.50 |
| 13 | -10 | -3 | 49.30 | 5.60 |
| 13 | 10 | -3 | 36.60 | 4.40 |
| -13 | 10 | 3 | 38.50 | 6.10 |
| 12 | -10 | -3 | 122.49 | 9.60 |
| 12 | -10 | -3 | 125.89 | 9.80 |
| 12 | 10 | -3 | 135.09 | 8.60 |
| 11 | -10 | -3 | 612.24 | 34.40 |
| 11 | -10 | -3 | 581.94 | 34.30 |
| 11 | 10 | -3 | 593.54 | 33.30 |
| 10 | -10 | -3 | 42.40 | 6.80 |
| 10 | -10 | -3 | 42.40 | 8.30 |
| 10 | 10 | -3 | 42.00 | 5.60 |
| 9 | -10 | -3 | 546.75 | 31.00 |
| 9 | -10 | -3 | 486.45 | 30.90 |
| 9 | 10 | -3 | 516.25 | 29.90 |
| 9 | 10 | -3 | 529.85 | 30.70 |
| 8 | -10 | -3 | 162.58 | 13.20 |
| 8 | -10 | -3 | 161.38 | 14.10 |

| | | | | |
|---|---|---|---|---|
| 8 | 10 | -3 | 183.78 | 15.70 |
| 8 | 10 | -3 | 153.18 | 12.20 |
| 8 | 10 | -3 | 179.88 | 12.50 |
| 7 | -10 | -3 | 113.69 | 11.30 |
| 7 | -10 | -3 | 124.79 | 11.60 |
| -7 | -10 | 3 | 116.09 | 11.50 |
| 7 | 10 | -3 | 105.99 | 9.60 |
| 7 | 10 | -3 | 107.49 | 12.50 |
| 6 | -10 | -3 | -2.10 | 4.60 |
| 6 | -10 | -3 | 5.50 | 5.20 |
| -6 | -10 | 3 | -4.00 | 4.50 |
| 6 | 10 | -3 | 3.80 | 5.10 |
| 6 | 10 | -3 | 2.50 | 3.80 |
| -6 | 10 | 3 | -0.70 | 4.70 |
| -5 | -10 | 3 | 2243.58 | 125.09 |
| 5 | -10 | -3 | 2202.88 | 125.19 |
| 5 | 10 | -3 | 2082.09 | 126.69 |
| -5 | 10 | 3 | 2561.54 | 125.69 |
| 5 | 10 | -3 | 2417.96 | 124.69 |
| 4 | -10 | -3 | 100.19 | 11.50 |
| -4 | -10 | 3 | 69.39 | 9.30 |
| -4 | 10 | 3 | 82.29 | 8.10 |
| 4 | 10 | -3 | 76.49 | 9.80 |
| 4 | 10 | -3 | 75.09 | 8.20 |
| -3 | -10 | 3 | 172.98 | 13.00 |
| -3 | -10 | 3 | 178.88 | 13.20 |
| 3 | 10 | -3 | 160.58 | 13.30 |
| 3 | 10 | -3 | 158.18 | 12.00 |
| -3 | 10 | 3 | 169.58 | 12.00 |
| -2 | -10 | 3 | 177.48 | 13.50 |
| -2 | -10 | 3 | 199.48 | 14.20 |
| 2 | 10 | -3 | 195.98 | 14.20 |
| 2 | 10 | -3 | 179.48 | 13.00 |
| -2 | 10 | 3 | 209.08 | 13.50 |
| 1 | 10 | -3 | 1907.41 | 104.69 |
| -1 | 10 | 3 | 1966.10 | 105.09 |
| 1 | 10 | -3 | 1900.01 | 106.09 |
| -1 | 10 | 3 | 1942.91 | 105.79 |
| 0 | 10 | 3 | -1.50 | 2.70 |
| 0 | 10 | 3 | 0.80 | 3.00 |
| 0 | 10 | -3 | 3.60 | 3.00 |
| 1 | -10 | 3 | 776.72 | 45.00 |
| -1 | -10 | -3 | 770.22 | 45.60 |
| -1 | 10 | -3 | 823.02 | 45.70 |

| | | | | |
|---|---|---|---|---|
| 1 | 10 | 3 | 828.52 | 46.00 |
| 1 | 10 | 3 | 827.02 | 44.90 |
| 2 | -10 | 3 | 373.96 | 23.20 |
| -2 | -10 | -3 | 369.46 | 23.80 |
| 2 | -10 | 3 | 410.66 | 25.80 |
| 2 | 10 | 3 | 370.76 | 23.90 |
| 2 | 10 | 3 | 400.46 | 22.80 |
| -2 | 10 | -3 | 409.96 | 23.60 |
| 3 | -10 | 3 | 187.68 | 18.70 |
| 3 | -10 | 3 | 240.18 | 15.10 |
| 3 | -10 | 3 | 209.88 | 14.90 |
| -3 | -10 | -3 | 205.58 | 15.40 |
| 3 | 10 | 3 | 231.08 | 16.00 |
| 3 | 10 | 3 | 244.28 | 14.20 |
| -3 | 10 | -3 | 226.08 | 14.80 |
| -4 | -10 | -3 | 399.86 | 24.20 |
| 4 | -10 | 3 | 351.76 | 27.90 |
| 4 | -10 | 3 | 384.26 | 23.70 |
| -4 | 10 | -3 | 430.16 | 24.30 |
| 4 | 10 | 3 | 403.36 | 23.20 |
| 4 | 10 | 3 | 417.76 | 25.70 |
| 5 | -10 | 3 | 2121.49 | 110.99 |
| 5 | -10 | 3 | 1975.90 | 112.79 |
| 5 | -10 | 3 | 2085.99 | 110.39 |
| -5 | -10 | -3 | 1964.70 | 110.49 |
| 5 | 10 | 3 | 1954.80 | 112.59 |
| -5 | 10 | -3 | 2137.79 | 111.09 |
| 5 | 10 | 3 | 1963.60 | 110.19 |
| -6 | -10 | -3 | 299.87 | 18.60 |
| 6 | -10 | 3 | 298.27 | 19.00 |
| 6 | -10 | 3 | 273.77 | 17.90 |
| 6 | 10 | 3 | 271.07 | 21.50 |
| -6 | 10 | -3 | 290.87 | 18.70 |
| 6 | 10 | 3 | 282.97 | 17.20 |
| 7 | -10 | 3 | 10.10 | 4.90 |
| 7 | -10 | 3 | 4.40 | 3.40 |
| -7 | -10 | -3 | 7.30 | 4.40 |
| 7 | 10 | 3 | 4.70 | 7.80 |
| 7 | 10 | 3 | 1.00 | 2.60 |
| 8 | -10 | 3 | 32.00 | 6.30 |
| 8 | -10 | 3 | 28.10 | 4.40 |
| -8 | -10 | -3 | 24.00 | 5.50 |
| 8 | 10 | 3 | 31.80 | 5.00 |
| 9 | -10 | 3 | 332.57 | 22.50 |

| | | | | |
|---|---|---|---|---|
| 9 | -10 | 3 | 359.16 | 21.50 |
| 9 | 10 | 3 | 366.16 | 21.10 |
| 10 | -10 | 3 | 0.70 | 4.20 |
| 10 | -10 | 3 | 0.10 | 3.00 |
| 10 | 10 | 3 | 1.30 | 3.10 |
| 11 | -10 | 3 | 544.25 | 30.10 |
| 11 | -10 | 3 | 513.75 | 30.40 |
| 11 | 10 | 3 | 504.05 | 29.90 |
| 12 | -10 | 3 | 98.09 | 7.90 |
| 12 | -10 | 3 | 87.79 | 7.90 |
| 12 | 10 | 3 | 78.89 | 7.90 |
| 13 | -10 | 3 | 86.19 | 7.20 |
| 13 | -10 | 3 | 100.89 | 7.50 |
| 13 | 10 | 3 | 96.29 | 7.90 |
| 14 | -10 | 3 | 1.00 | 2.50 |
| 14 | -10 | 3 | -0.60 | 2.00 |
| 14 | 10 | 3 | 3.10 | 2.70 |
| 14 | -11 | -3 | 256.97 | 15.50 |
| 14 | 11 | -3 | 279.47 | 15.20 |
| 13 | -11 | -3 | 77.39 | 6.30 |
| 13 | 11 | -3 | 70.99 | 5.30 |
| 12 | -11 | -3 | 162.78 | 13.20 |
| -12 | 11 | 3 | 161.98 | 11.90 |
| 12 | 11 | -3 | 155.78 | 10.20 |
| 11 | -11 | -3 | 3.40 | 3.40 |
| 11 | -11 | -3 | 3.60 | 3.40 |
| 11 | 11 | -3 | -2.00 | 2.20 |
| -11 | 11 | 3 | 5.10 | 5.40 |
| 10 | -11 | -3 | 824.72 | 44.80 |
| 10 | -11 | -3 | 758.62 | 44.50 |
| 10 | 11 | -3 | 774.02 | 43.50 |
| 9 | -11 | -3 | 100.29 | 10.50 |
| 9 | -11 | -3 | 105.09 | 10.00 |
| 9 | 11 | -3 | 101.69 | 8.50 |
| 8 | -11 | -3 | 128.39 | 11.80 |
| 8 | -11 | -3 | 134.29 | 11.90 |
| -8 | -11 | 3 | 155.28 | 12.50 |
| 8 | 11 | -3 | 115.79 | 10.30 |
| 8 | 11 | -3 | 134.99 | 17.80 |
| 7 | -11 | -3 | 63.09 | 10.90 |
| -7 | -11 | 3 | 84.39 | 9.30 |
| 7 | 11 | -3 | 85.99 | 8.90 |
| 7 | 11 | -3 | 86.29 | 12.30 |
| -6 | -11 | 3 | 1079.89 | 59.69 |

| | | | | |
|---|---|---|---|---|
| 6 | -11 | -3 | 1048.90 | 59.99 |
| 6 | 11 | -3 | 1071.39 | 59.19 |
| 6 | 11 | -3 | 1107.79 | 61.89 |
| 5 | -11 | -3 | 157.78 | 11.90 |
| 5 | -11 | -3 | 117.69 | 15.80 |
| -5 | -11 | 3 | 126.19 | 11.30 |
| -5 | 11 | 3 | 136.69 | 11.30 |
| 5 | 11 | -3 | 100.79 | 12.90 |
| -4 | -11 | 3 | 517.15 | 31.00 |
| -4 | -11 | 3 | 585.54 | 31.20 |
| 4 | -11 | -3 | 524.15 | 33.20 |
| 4 | -11 | -3 | 492.65 | 31.30 |
| 4 | 11 | -3 | 543.85 | 30.40 |
| -4 | 11 | 3 | 582.24 | 31.60 |
| 4 | 11 | -3 | 488.95 | 32.90 |
| -3 | -11 | 3 | 101.49 | 9.70 |
| 3 | -11 | -3 | 96.49 | 12.00 |
| 3 | -11 | -3 | 122.89 | 14.10 |
| -3 | -11 | 3 | 103.59 | 9.60 |
| 3 | 11 | -3 | 94.59 | 8.90 |
| -3 | 11 | 3 | 96.29 | 9.20 |
| 3 | 11 | -3 | 89.69 | 10.60 |
| -2 | -11 | 3 | 194.18 | 13.30 |
| -2 | -11 | 3 | 184.78 | 13.80 |
| 2 | -11 | -3 | 202.18 | 16.00 |
| 2 | -11 | -3 | 162.58 | 15.90 |
| -2 | 11 | 3 | 198.38 | 13.70 |
| 2 | 11 | -3 | 185.78 | 14.50 |
| 2 | 11 | -3 | 199.08 | 13.00 |
| -1 | -11 | 3 | 280.17 | 18.00 |
| -1 | -11 | 3 | 294.37 | 18.80 |
| 1 | -11 | -3 | 277.07 | 19.50 |
| -1 | 11 | 3 | 305.97 | 19.00 |
| 1 | 11 | -3 | 276.67 | 19.20 |
| 1 | 11 | -3 | 310.17 | 18.00 |
| -1 | 11 | 3 | 287.37 | 17.80 |
| 0 | -11 | -3 | 2013.70 | 112.79 |
| 0 | -11 | 3 | 2074.59 | 112.69 |
| 0 | -11 | 3 | 2072.69 | 112.29 |
| 0 | 11 | 3 | 2028.20 | 113.49 |
| 0 | 11 | -3 | 2124.69 | 112.19 |
| 0 | 11 | -3 | 2158.58 | 113.69 |
| 0 | 11 | 3 | 1996.30 | 112.39 |
| -1 | -11 | -3 | 96.89 | 10.30 |

| | | | | |
|---|---|---|---|---|
| 1 | -11 | 3 | 110.09 | 9.60 |
| 1 | -11 | 3 | 110.99 | 11.50 |
| 1 | 11 | 3 | 112.99 | 8.40 |
| 1 | 11 | 3 | 100.09 | 9.60 |
| -1 | 11 | -3 | 106.69 | 9.40 |
| 2 | -11 | 3 | 135.19 | 10.70 |
| 2 | -11 | 3 | 116.49 | 13.20 |
| -2 | -11 | -3 | 135.39 | 11.20 |
| 2 | 11 | 3 | 128.69 | 11.30 |
| -2 | 11 | -3 | 138.19 | 10.60 |
| 2 | 11 | 3 | 130.59 | 9.50 |
| -3 | -11 | -3 | 17.30 | 4.60 |
| 3 | -11 | 3 | 15.00 | 7.40 |
| 3 | -11 | 3 | 15.00 | 4.00 |
| 3 | 11 | 3 | 13.20 | 5.20 |
| 3 | 11 | 3 | 15.00 | 3.50 |
| -3 | 11 | -3 | 13.30 | 4.10 |
| 4 | -11 | 3 | 886.81 | 52.39 |
| -4 | -11 | -3 | 872.61 | 50.19 |
| 4 | -11 | 3 | 877.31 | 49.99 |
| 4 | 11 | 3 | 933.81 | 49.90 |
| -4 | 11 | -3 | 944.11 | 50.99 |
| 4 | 11 | 3 | 873.61 | 52.19 |
| 5 | -11 | 3 | 44.60 | 7.40 |
| 5 | -11 | 3 | 16.70 | 9.30 |
| 5 | -11 | 3 | 29.50 | 4.40 |
| -5 | -11 | -3 | 25.50 | 4.70 |
| 5 | 11 | 3 | 15.50 | 6.60 |
| -5 | 11 | -3 | 32.10 | 8.00 |
| 5 | 11 | 3 | 40.20 | 5.10 |
| 6 | -11 | 3 | 1095.99 | 59.39 |
| 6 | -11 | 3 | 1119.69 | 60.19 |
| 6 | 11 | 3 | 1091.09 | 59.09 |
| 6 | 11 | 3 | 980.10 | 62.29 |
| 7 | -11 | 3 | 196.58 | 13.60 |
| -7 | -11 | -3 | 213.18 | 14.00 |
| 7 | -11 | 3 | 201.18 | 15.00 |
| 7 | 11 | 3 | 203.58 | 13.00 |
| 7 | 11 | 3 | 201.58 | 20.80 |
| -8 | -11 | -3 | 181.88 | 13.20 |
| 8 | -11 | 3 | 165.68 | 14.10 |
| 8 | -11 | 3 | 197.68 | 12.60 |
| 8 | 11 | 3 | 180.18 | 12.00 |
| 9 | -11 | 3 | 49.40 | 8.90 |

| | | | | |
|---|---|---|---|---|
| 9 | -11 | 3 | 42.80 | 6.50 |
| 9 | 11 | 3 | 40.40 | 6.00 |
| 10 | -11 | 3 | 1127.09 | 58.79 |
| 10 | -11 | 3 | 1032.80 | 59.29 |
| 10 | 11 | 3 | 1020.00 | 58.49 |
| 11 | -11 | 3 | 35.50 | 6.50 |
| 11 | -11 | 3 | 26.30 | 6.40 |
| 11 | 11 | 3 | 37.70 | 5.90 |
| 12 | -11 | 3 | 52.49 | 5.90 |
| 12 | -11 | 3 | 65.59 | 6.50 |
| 12 | 11 | 3 | 62.19 | 6.50 |
| 13 | -11 | 3 | 0.60 | 2.20 |
| 13 | -11 | 3 | 5.40 | 2.80 |
| 13 | 11 | 3 | 7.80 | 3.10 |
| 14 | -12 | -3 | 3.40 | 1.80 |
| 14 | 12 | -3 | 5.50 | 1.50 |
| 13 | -12 | -3 | 45.40 | 4.70 |
| 13 | 12 | -3 | 51.69 | 4.10 |
| 12 | -12 | -3 | 80.99 | 7.10 |
| 12 | 12 | -3 | 89.29 | 6.20 |
| 11 | -12 | -3 | 581.34 | 33.20 |
| 11 | 12 | -3 | 576.14 | 32.40 |
| 10 | -12 | -3 | 6.00 | 3.80 |
| 10 | 12 | -3 | 7.20 | 2.30 |
| 9 | -12 | -3 | 310.17 | 20.20 |
| 9 | 12 | -3 | 324.97 | 19.10 |
| 8 | -12 | -3 | 164.28 | 12.90 |
| -8 | -12 | 3 | 146.79 | 12.60 |
| 8 | 12 | -3 | 163.98 | 11.50 |
| 7 | -12 | -3 | 533.05 | 32.00 |
| -7 | -12 | 3 | 539.75 | 31.40 |
| 7 | 12 | -3 | 560.74 | 34.70 |
| 6 | -12 | -3 | 39.70 | 5.80 |
| -6 | -12 | 3 | 43.50 | 7.70 |
| -5 | -12 | 3 | 1683.33 | 87.99 |
| 5 | -12 | -3 | 1618.04 | 88.69 |
| 5 | -12 | -3 | 1612.84 | 88.09 |
| -5 | 12 | 3 | 1644.74 | 88.89 |
| 5 | 12 | -3 | 1422.66 | 90.19 |
| 5 | 12 | -3 | 1650.73 | 87.39 |
| 4 | -12 | -3 | 313.57 | 21.50 |
| 4 | -12 | -3 | 381.46 | 23.30 |
| -4 | -12 | 3 | 333.07 | 20.70 |
| 4 | 12 | -3 | 336.17 | 20.10 |

| | | | | |
|---|---|---|---|---|
| -4 | 12 | 3 | 363.06 | 21.80 |
| 4 | 12 | -3 | 293.07 | 23.30 |
| 3 | -12 | -3 | 7.50 | 6.00 |
| -3 | -12 | 3 | 8.90 | 3.70 |
| -3 | -12 | 3 | 13.60 | 4.30 |
| 3 | -12 | -3 | 9.30 | 5.60 |
| -3 | 12 | 3 | 9.70 | 4.20 |
| 3 | 12 | -3 | 10.30 | 3.80 |
| 3 | 12 | -3 | 5.10 | 4.70 |
| -2 | -12 | 3 | 201.48 | 14.40 |
| -2 | -12 | 3 | 209.68 | 13.90 |
| 2 | -12 | -3 | 203.08 | 16.00 |
| 2 | -12 | -3 | 227.58 | 16.10 |
| 2 | 12 | -3 | 176.48 | 15.50 |
| -2 | 12 | 3 | 202.88 | 13.70 |
| -2 | 12 | 3 | 225.78 | 15.00 |
| 2 | 12 | -3 | 200.38 | 14.80 |
| 1 | -12 | -3 | 2019.30 | 107.29 |
| -1 | -12 | 3 | 1970.60 | 106.59 |
| 1 | -12 | -3 | 1873.21 | 106.79 |
| -1 | -12 | 3 | 2007.60 | 106.89 |
| 1 | 12 | -3 | 1882.41 | 108.19 |
| -1 | 12 | 3 | 2017.60 | 107.99 |
| -1 | 12 | 3 | 1937.91 | 106.79 |
| 0 | -12 | -3 | -1.40 | 4.30 |
| 0 | -12 | 3 | -1.40 | 4.60 |
| 0 | -12 | 3 | 6.90 | 3.20 |
| 0 | -12 | -3 | -1.90 | 6.00 |
| 0 | 12 | -3 | 4.10 | 3.70 |
| 0 | 12 | -3 | 4.90 | 4.00 |
| 0 | 12 | 3 | 0.40 | 2.50 |
| 0 | 12 | 3 | -0.40 | 3.40 |
| 1 | -12 | 3 | 663.43 | 38.20 |
| -1 | -12 | -3 | 685.53 | 37.90 |
| -1 | -12 | -3 | 630.44 | 40.70 |
| 1 | -12 | 3 | 660.43 | 37.40 |
| -1 | 12 | -3 | 654.23 | 38.50 |
| 1 | 12 | 3 | 673.53 | 37.40 |
| -1 | 12 | -3 | 650.33 | 37.30 |
| 1 | 12 | 3 | 662.23 | 38.90 |
| 2 | -12 | 3 | 418.96 | 25.20 |
| -2 | -12 | -3 | 411.46 | 24.00 |
| 2 | -12 | 3 | 399.06 | 23.70 |
| -2 | 12 | -3 | 399.76 | 24.70 |

| | | | | |
|---|---|---|---|---|
| 2 | 12 | 3 | 381.96 | 25.30 |
| 2 | 12 | 3 | 389.96 | 23.40 |
| -3 | -12 | -3 | 181.98 | 13.20 |
| 3 | -12 | 3 | 201.08 | 16.40 |
| 3 | -12 | 3 | 175.28 | 12.70 |
| -3 | 12 | -3 | 195.98 | 13.80 |
| 3 | 12 | 3 | 176.18 | 15.00 |
| 3 | 12 | 3 | 179.08 | 12.10 |
| -4 | -12 | -3 | 159.38 | 12.60 |
| 4 | -12 | 3 | 183.38 | 17.50 |
| -4 | 12 | -3 | 175.88 | 13.90 |
| 4 | 12 | 3 | 186.78 | 11.80 |
| 4 | 12 | 3 | 171.38 | 15.50 |
| 5 | -12 | 3 | 1339.77 | 75.29 |
| -5 | -12 | -3 | 1340.07 | 73.79 |
| 5 | -12 | 3 | 1335.07 | 73.49 |
| 5 | 12 | 3 | 1193.78 | 76.99 |
| 5 | 12 | 3 | 1401.16 | 73.49 |
| -5 | 12 | -3 | 1429.96 | 74.99 |
| -6 | -12 | -3 | 232.18 | 15.30 |
| 6 | -12 | 3 | 240.48 | 15.10 |
| 6 | -12 | 3 | 251.07 | 16.10 |
| 6 | 12 | 3 | 232.98 | 14.50 |
| 6 | 12 | 3 | 202.88 | 20.50 |
| 7 | -12 | 3 | 29.30 | 6.00 |
| -7 | -12 | -3 | 32.90 | 7.00 |
| 7 | 12 | 3 | 29.00 | 4.90 |
| 7 | 12 | 3 | 13.50 | 11.00 |
| 8 | -12 | 3 | 86.69 | 12.10 |
| -8 | -12 | -3 | 51.39 | 7.90 |
| 8 | -12 | 3 | 59.29 | 7.00 |
| 8 | 12 | 3 | 68.39 | 7.60 |
| 9 | -12 | 3 | 573.84 | 31.60 |
| 9 | -12 | 3 | 556.54 | 32.60 |
| 9 | 12 | 3 | 526.55 | 31.30 |
| 10 | -12 | 3 | 3.40 | 3.00 |
| 10 | -12 | 3 | -2.00 | 3.60 |
| 10 | 12 | 3 | 4.40 | 3.00 |
| 11 | -12 | 3 | 433.76 | 24.90 |
| 11 | -12 | 3 | 410.16 | 24.20 |
| 11 | 12 | 3 | 415.06 | 24.30 |
| 12 | -12 | 3 | 94.19 | 7.40 |
| 12 | 12 | 3 | 108.09 | 9.50 |
| 13 | -12 | 3 | 199.18 | 11.30 |

| | | | | |
|---|---|---|---|---|
| 13 | 12 | 3 | 174.98 | 11.70 |
| 13 | -13 | -3 | 48.90 | 4.60 |
| 13 | 13 | -3 | 54.59 | 4.10 |
| 12 | -13 | -3 | 38.60 | 4.80 |
| 12 | 13 | -3 | 37.10 | 3.90 |
| 11 | -13 | -3 | 1.40 | 2.70 |
| 11 | 13 | -3 | 7.50 | 2.10 |
| 10 | -13 | -3 | 524.55 | 30.20 |
| 10 | 13 | -3 | 520.05 | 29.30 |
| 9 | -13 | -3 | 14.80 | 4.20 |
| 9 | 13 | -3 | 14.20 | 3.00 |
| 8 | -13 | -3 | 113.89 | 10.40 |
| -8 | -13 | 3 | 95.29 | 9.90 |
| 8 | 13 | -3 | 106.69 | 8.90 |
| 7 | -13 | -3 | 115.99 | 11.20 |
| -7 | -13 | 3 | 122.49 | 10.30 |
| 7 | 13 | -3 | 124.09 | 17.20 |
| 6 | -13 | -3 | 810.82 | 45.00 |
| -6 | -13 | 3 | 761.22 | 44.40 |
| 6 | 13 | -3 | 806.02 | 48.10 |
| 5 | -13 | -3 | -5.40 | 5.80 |
| 5 | -13 | -3 | 9.10 | 4.80 |
| 5 | 13 | -3 | -2.90 | 7.10 |
| -4 | -13 | 3 | 412.06 | 24.50 |
| -4 | 13 | 3 | 449.56 | 26.00 |
| 4 | 13 | -3 | 424.36 | 23.80 |
| 4 | 13 | -3 | 339.97 | 28.10 |
| -3 | -13 | 3 | 185.98 | 13.20 |
| 3 | -13 | -3 | 160.38 | 14.70 |
| -3 | -13 | 3 | 182.68 | 13.50 |
| 3 | -13 | -3 | 172.58 | 13.90 |
| -3 | 13 | 3 | 197.28 | 14.20 |
| 3 | 13 | -3 | 178.48 | 12.30 |
| 3 | 13 | -3 | 170.68 | 16.20 |
| -3 | 13 | 3 | 168.08 | 12.60 |
| -2 | -13 | 3 | 4.90 | 4.50 |
| 2 | -13 | -3 | 15.60 | 5.40 |
| 2 | -13 | -3 | 0.00 | 4.60 |
| -2 | -13 | 3 | 0.30 | 3.50 |
| -2 | 13 | 3 | 4.80 | 4.50 |
| -2 | 13 | 3 | 8.90 | 3.40 |
| 2 | 13 | -3 | 1.90 | 3.60 |
| 2 | 13 | -3 | 6.20 | 5.10 |
| 1 | -13 | -3 | 154.98 | 12.80 |

| | | | | |
|---|---|---|---|---|
| -1 | -13 | 3 | 156.68 | 11.90 |
| -1 | -13 | 3 | 151.08 | 11.20 |
| 1 | -13 | -3 | 136.49 | 13.40 |
| -1 | 13 | 3 | 161.18 | 13.10 |
| -1 | 13 | 3 | 148.39 | 11.00 |
| 1 | 13 | -3 | 146.49 | 13.40 |
| 1 | 13 | -3 | 169.38 | 11.40 |
| 0 | -13 | -3 | 1561.24 | 89.19 |
| 0 | -13 | 3 | 1594.04 | 88.79 |
| 0 | -13 | 3 | 1639.94 | 89.39 |
| 0 | -13 | -3 | 1678.33 | 89.59 |
| 0 | 13 | 3 | 1683.03 | 90.69 |
| 0 | 13 | -3 | 1620.14 | 90.59 |
| 0 | 13 | -3 | 1626.34 | 88.79 |
| 0 | 13 | 3 | 1620.74 | 89.09 |
| -1 | -13 | -3 | 506.55 | 34.10 |
| 1 | -13 | 3 | 626.74 | 34.30 |
| -1 | -13 | -3 | 523.05 | 34.40 |
| 1 | -13 | 3 | 583.24 | 33.60 |
| -1 | 13 | -3 | 610.54 | 33.30 |
| 1 | 13 | 3 | 603.34 | 35.20 |
| -1 | 13 | -3 | 595.64 | 34.80 |
| 1 | 13 | 3 | 604.34 | 33.30 |
| 2 | -13 | 3 | 3.70 | 3.60 |
| -2 | -13 | -3 | 2.50 | 3.40 |
| 2 | 13 | 3 | 9.20 | 5.50 |
| 2 | 13 | 3 | 4.80 | 2.80 |
| -2 | 13 | -3 | 8.80 | 4.10 |
| 3 | -13 | 3 | 5.20 | 3.20 |
| 3 | -13 | 3 | 7.30 | 5.80 |
| -3 | -13 | -3 | 6.80 | 3.70 |
| 3 | 13 | 3 | 10.50 | 7.40 |
| 3 | 13 | 3 | 7.10 | 3.00 |
| -3 | 13 | -3 | 4.10 | 4.80 |
| 4 | -13 | 3 | 525.55 | 30.60 |
| -4 | -13 | -3 | 441.86 | 28.10 |
| 4 | -13 | 3 | 460.95 | 27.90 |
| 4 | 13 | 3 | 477.85 | 31.70 |
| -4 | 13 | -3 | 544.65 | 33.90 |
| 4 | 13 | 3 | 471.55 | 27.70 |
| -5 | -13 | -3 | 29.10 | 6.30 |
| 5 | -13 | 3 | 35.50 | 7.70 |
| 5 | -13 | 3 | 33.70 | 6.10 |
| 5 | 13 | 3 | 33.90 | 8.90 |

| | | | | |
|---|---|---|---|---|
| 5 | 13 | 3 | 31.90 | 4.80 |
| -5 | 13 | -3 | 39.60 | 7.80 |
| -6 | -13 | -3 | 275.77 | 16.60 |
| 6 | -13 | 3 | 251.57 | 16.20 |
| 6 | 13 | 3 | 201.08 | 24.10 |
| 6 | 13 | 3 | 286.47 | 16.00 |
| -7 | -13 | -3 | 109.19 | 9.30 |
| 7 | -13 | 3 | 110.99 | 8.90 |
| 7 | 13 | 3 | 98.29 | 15.70 |
| 7 | 13 | 3 | 117.29 | 8.80 |
| 8 | -13 | 3 | 55.19 | 6.60 |
| -8 | -13 | -3 | 51.99 | 7.10 |
| 8 | 13 | 3 | 58.09 | 5.80 |
| 9 | -13 | 3 | -0.60 | 2.80 |
| 9 | 13 | 3 | 1.40 | 2.70 |
| 10 | -13 | 3 | 343.27 | 19.70 |
| 10 | 13 | 3 | 322.47 | 19.60 |
| 11 | -13 | 3 | 39.30 | 5.10 |
| 11 | 13 | 3 | 51.89 | 5.70 |
| 12 | -13 | 3 | 20.90 | 3.70 |
| 12 | 13 | 3 | 15.20 | 3.50 |
| 12 | -14 | -3 | 75.19 | 5.90 |
| 12 | 14 | -3 | 81.09 | 5.40 |
| 11 | -14 | -3 | 295.07 | 17.60 |
| 11 | 14 | -3 | 302.47 | 17.00 |
| 10 | -14 | -3 | 2.50 | 3.10 |
| 10 | 14 | -3 | 2.90 | 1.90 |
| 9 | -14 | -3 | 500.55 | 29.90 |
| 9 | 14 | -3 | 528.55 | 29.00 |
| 8 | -14 | -3 | 40.30 | 8.00 |
| -8 | -14 | 3 | 49.90 | 7.60 |
| 8 | 14 | -3 | 49.10 | 6.00 |
| 7 | -14 | -3 | 12.90 | 4.40 |
| -7 | -14 | 3 | 6.00 | 3.40 |
| 7 | 14 | -3 | 14.70 | 10.10 |
| 7 | 14 | -3 | 9.10 | 3.60 |
| -6 | -14 | 3 | 34.20 | 7.00 |
| 6 | -14 | -3 | 51.99 | 8.40 |
| 6 | 14 | -3 | 34.80 | 10.00 |
| -5 | -14 | 3 | 1204.18 | 65.49 |
| 5 | -14 | -3 | 1207.78 | 65.79 |
| 5 | -14 | -3 | 1258.47 | 65.99 |
| 5 | 14 | -3 | 1070.49 | 68.89 |
| -4 | -14 | 3 | 32.70 | 5.00 |

| | | | | |
|---|---|---|---|---|
| 4 | -14 | -3 | 38.40 | 5.80 |
| 4 | -14 | -3 | 26.00 | 5.70 |
| -4 | 14 | 3 | 39.10 | 7.30 |
| 4 | 14 | -3 | 27.30 | 8.70 |
| -4 | 14 | 3 | 44.60 | 7.00 |
| -3 | -14 | 3 | 71.09 | 7.90 |
| 3 | -14 | -3 | 79.09 | 10.60 |
| 3 | -14 | -3 | 91.19 | 10.30 |
| -3 | -14 | 3 | 65.59 | 8.00 |
| 3 | 14 | -3 | 77.79 | 7.70 |
| -3 | 14 | 3 | 91.09 | 10.90 |
| -3 | 14 | 3 | 73.99 | 8.00 |
| 3 | 14 | -3 | 85.59 | 13.20 |
| 2 | -14 | -3 | 6.50 | 4.60 |
| -2 | -14 | 3 | 2.10 | 3.40 |
| -2 | -14 | 3 | 6.80 | 3.50 |
| 2 | -14 | -3 | 13.30 | 4.70 |
| 2 | 14 | -3 | 8.00 | 6.40 |
| 2 | 14 | -3 | 11.30 | 3.80 |
| -2 | 14 | 3 | 14.80 | 5.60 |
| -2 | 14 | 3 | 7.40 | 3.10 |
| 1 | -14 | -3 | 801.22 | 45.90 |
| 1 | -14 | -3 | 795.92 | 45.50 |
| -1 | -14 | 3 | 829.02 | 45.50 |
| -1 | -14 | 3 | 837.92 | 45.10 |
| -1 | 14 | 3 | 777.52 | 45.20 |
| 1 | 14 | -3 | 825.72 | 45.00 |
| -1 | 14 | 3 | 765.72 | 46.80 |
| 1 | 14 | -3 | 812.92 | 47.60 |
| 0 | -14 | 3 | 1.10 | 3.90 |
| 0 | -14 | -3 | 7.30 | 5.30 |
| 0 | -14 | -3 | -3.70 | 4.00 |
| 0 | -14 | 3 | -1.80 | 2.90 |
| 0 | 14 | -3 | 6.30 | 4.80 |
| 0 | 14 | 3 | 1.00 | 2.40 |
| 0 | 14 | -3 | -1.40 | 3.40 |
| 1 | -14 | 3 | 362.16 | 21.50 |
| -1 | -14 | -3 | 342.27 | 22.10 |
| 1 | -14 | 3 | 391.96 | 22.90 |
| -1 | -14 | -3 | 347.07 | 22.80 |
| 1 | 14 | 3 | 358.66 | 21.50 |
| -1 | 14 | -3 | 378.96 | 21.80 |
| 1 | 14 | 3 | 349.57 | 24.00 |
| -1 | 14 | -3 | 365.96 | 23.70 |

| | | | | |
|---|---|---|---|---|
| -2 | -14 | -3 | 9.00 | 3.50 |
| 2 | -14 | 3 | 6.90 | 4.90 |
| 2 | -14 | 3 | 6.40 | 3.20 |
| 2 | 14 | 3 | 13.40 | 5.40 |
| -2 | 14 | -3 | 5.10 | 3.50 |
| 2 | 14 | 3 | 5.30 | 3.00 |
| -2 | 14 | -3 | 14.00 | 5.70 |
| 3 | -14 | 3 | 30.00 | 6.60 |
| -3 | -14 | -3 | 29.50 | 6.20 |
| 3 | -14 | 3 | 32.40 | 6.00 |
| -3 | 14 | -3 | 44.20 | 10.00 |
| 3 | 14 | 3 | 44.50 | 11.20 |
| 3 | 14 | 3 | 34.40 | 5.00 |
| -4 | -14 | -3 | 37.30 | 6.20 |
| 4 | -14 | 3 | 33.10 | 5.50 |
| 4 | -14 | 3 | 23.50 | 6.30 |
| -4 | 14 | -3 | 26.60 | 7.20 |
| 4 | 14 | 3 | 29.80 | 5.10 |
| 4 | 14 | 3 | 18.90 | 8.30 |
| 5 | -14 | 3 | 630.84 | 34.50 |
| 5 | -14 | 3 | 566.74 | 32.20 |
| -5 | -14 | -3 | 566.04 | 32.40 |
| 5 | 14 | 3 | 575.84 | 32.10 |
| 5 | 14 | 3 | 490.05 | 37.80 |
| 6 | -14 | 3 | 164.08 | 10.90 |
| -6 | -14 | -3 | 156.98 | 11.00 |
| 6 | 14 | 3 | 152.28 | 10.40 |
| 6 | 14 | 3 | 103.69 | 22.50 |
| 7 | -14 | 3 | 31.40 | 5.10 |
| -7 | -14 | -3 | 21.10 | 3.60 |
| 7 | 14 | 3 | 24.60 | 4.70 |
| 8 | -14 | 3 | 45.20 | 6.00 |
| -8 | -14 | -3 | 57.59 | 8.00 |
| 8 | 14 | 3 | 49.30 | 5.60 |
| 9 | -14 | 3 | 158.88 | 10.40 |
| 9 | 14 | 3 | 141.39 | 10.00 |
| 10 | -14 | 3 | 0.60 | 2.30 |
| 10 | 14 | 3 | 0.10 | 2.80 |
| 11 | -14 | 3 | 179.38 | 11.20 |
| 11 | 14 | 3 | 182.78 | 11.70 |
| 11 | -15 | -3 | 4.00 | 2.40 |
| 11 | 15 | -3 | 8.60 | 1.80 |
| 10 | -15 | -3 | 390.16 | 22.00 |
| 10 | 15 | -3 | 371.06 | 21.30 |

| | | | | |
|---|---|---|---|---|
| 9 | -15 | -3 | 23.60 | 4.10 |
| -8 | -15 | 3 | 151.78 | 11.30 |
| 8 | -15 | -3 | 133.59 | 14.60 |
| 8 | 15 | -3 | 151.78 | 12.30 |
| -7 | -15 | 3 | 171.08 | 12.00 |
| 7 | -15 | -3 | 177.88 | 13.10 |
| 7 | 15 | -3 | 174.38 | 11.80 |
| -6 | -15 | 3 | 489.65 | 28.70 |
| 6 | -15 | -3 | 491.25 | 29.20 |
| -6 | 15 | 3 | 514.95 | 30.00 |
| 5 | -15 | -3 | 10.70 | 5.00 |
| -5 | -15 | 3 | 13.30 | 3.70 |
| 5 | -15 | -3 | 9.50 | 4.60 |
| 5 | 15 | -3 | -12.50 | 9.20 |
| -5 | 15 | 3 | 12.70 | 4.50 |
| 4 | -15 | -3 | 465.85 | 28.20 |
| 4 | -15 | -3 | 468.05 | 28.00 |
| -4 | -15 | 3 | 460.45 | 27.40 |
| -4 | 15 | 3 | 467.55 | 28.30 |
| -4 | 15 | 3 | 494.95 | 31.20 |
| 3 | -15 | -3 | 68.49 | 9.50 |
| 3 | -15 | -3 | 63.49 | 8.60 |
| -3 | -15 | 3 | 67.09 | 7.10 |
| 3 | 15 | -3 | 61.59 | 14.00 |
| -3 | 15 | 3 | 46.10 | 7.20 |
| -3 | 15 | 3 | 62.39 | 12.40 |
| 2 | -15 | -3 | 19.40 | 5.10 |
| 2 | -15 | -3 | 8.50 | 4.40 |
| -2 | -15 | 3 | 12.50 | 3.50 |
| -2 | -15 | 3 | 8.00 | 3.10 |
| -2 | 15 | 3 | 10.50 | 3.20 |
| -2 | 15 | 3 | 6.00 | 5.60 |
| 2 | 15 | -3 | 11.40 | 4.20 |
| 2 | 15 | -3 | 15.10 | 7.50 |
| -1 | -15 | 3 | 265.67 | 16.70 |
| -1 | -15 | 3 | 260.17 | 16.10 |
| 1 | -15 | -3 | 255.07 | 17.30 |
| 1 | -15 | -3 | 247.58 | 17.50 |
| 1 | 15 | -3 | 268.57 | 16.30 |
| -1 | 15 | 3 | 260.17 | 16.30 |
| 1 | 15 | -3 | 254.27 | 20.00 |
| -1 | 15 | 3 | 236.68 | 18.90 |
| 0 | -15 | -3 | 819.92 | 49.10 |
| 0 | -15 | 3 | 891.81 | 48.60 |

| | | | | |
|---|---|---|---|---|
| 0 | -15 | 3 | 899.31 | 49.30 |
| 0 | -15 | -3 | 917.21 | 49.40 |
| 0 | 15 | 3 | 896.61 | 51.09 |
| 0 | 15 | 3 | 819.82 | 48.70 |
| 0 | 15 | -3 | 891.91 | 48.70 |
| 0 | 15 | -3 | 859.91 | 51.19 |
| 1 | -15 | 3 | 154.58 | 10.70 |
| -1 | -15 | -3 | 167.38 | 11.70 |
| 1 | -15 | 3 | 156.08 | 12.50 |
| 1 | 15 | 3 | 157.58 | 10.70 |
| -1 | 15 | -3 | 149.59 | 11.10 |
| 1 | 15 | 3 | 134.39 | 14.30 |
| -1 | 15 | -3 | 145.19 | 14.40 |
| 2 | -15 | 3 | 126.69 | 11.80 |
| 2 | -15 | 3 | 116.29 | 9.20 |
| -2 | -15 | -3 | 135.39 | 10.00 |
| -2 | 15 | -3 | 124.49 | 9.80 |
| -2 | 15 | -3 | 109.19 | 13.20 |
| 2 | 15 | 3 | 125.69 | 9.20 |
| 2 | 15 | 3 | 121.69 | 14.30 |
| -3 | -15 | -3 | 52.39 | 6.40 |
| 3 | -15 | 3 | 34.30 | 5.70 |
| 3 | -15 | 3 | 31.70 | 6.20 |
| 3 | 15 | 3 | 40.80 | 5.30 |
| -3 | 15 | -3 | 49.30 | 11.70 |
| 3 | 15 | 3 | 43.30 | 9.30 |
| -3 | 15 | -3 | 33.30 | 6.70 |
| 4 | -15 | 3 | 271.77 | 19.40 |
| -4 | -15 | -3 | 278.27 | 16.90 |
| 4 | -15 | 3 | 277.67 | 16.60 |
| 4 | 15 | 3 | 280.77 | 16.70 |
| -4 | 15 | -3 | 271.07 | 17.20 |
| -4 | 15 | -3 | 262.97 | 22.00 |
| 4 | 15 | 3 | 246.08 | 23.30 |
| 5 | -15 | 3 | 17.30 | 5.90 |
| 5 | -15 | 3 | 26.30 | 5.10 |
| -5 | -15 | -3 | 16.10 | 3.40 |
| 5 | 15 | 3 | 29.80 | 4.60 |
| -6 | -15 | -3 | 458.25 | 25.10 |
| 6 | -15 | 3 | 426.46 | 24.90 |
| 6 | 15 | 3 | 417.16 | 24.80 |
| 7 | -15 | 3 | 52.69 | 5.90 |
| -7 | -15 | -3 | 52.19 | 7.60 |
| 7 | 15 | 3 | 53.09 | 5.40 |

| | | | | |
|---|---|---|---|---|
| -8 | -15 | -3 | 41.00 | 5.70 |
| 8 | -15 | 3 | 25.90 | 4.90 |
| 8 | 15 | 3 | 34.10 | 4.60 |
| 9 | -15 | 3 | 6.00 | 2.50 |
| 9 | 15 | 3 | 5.50 | 2.70 |
| 10 | -15 | 3 | 248.48 | 14.70 |
| 10 | 15 | 3 | 247.48 | 14.90 |
| 10 | -16 | -3 | 3.10 | 2.40 |
| -10 | 16 | 3 | 3.20 | 2.20 |
| 10 | 16 | -3 | -0.50 | 1.50 |
| 9 | -16 | -3 | 166.48 | 10.90 |
| 9 | 16 | -3 | 159.08 | 10.00 |
| -9 | 16 | 3 | 172.08 | 11.10 |
| -8 | -16 | 3 | 48.80 | 6.70 |
| 8 | -16 | -3 | 57.59 | 7.10 |
| 8 | 16 | -3 | 42.00 | 5.00 |
| -8 | 16 | 3 | 55.19 | 6.20 |
| -7 | -16 | 3 | 106.99 | 8.70 |
| 7 | -16 | -3 | 112.39 | 9.80 |
| -7 | 16 | 3 | 105.79 | 9.20 |
| 7 | 16 | -3 | 102.59 | 8.40 |
| -6 | -16 | 3 | 34.40 | 6.30 |
| 6 | -16 | -3 | 54.59 | 10.00 |
| -6 | 16 | 3 | 33.50 | 6.80 |
| 5 | -16 | -3 | 571.84 | 32.60 |
| -5 | -16 | 3 | 558.74 | 32.10 |
| 5 | -16 | -3 | 554.94 | 32.70 |
| -5 | 16 | 3 | 573.14 | 33.20 |
| 4 | -16 | -3 | 20.90 | 4.60 |
| 4 | -16 | -3 | 26.20 | 4.70 |
| -4 | -16 | 3 | 17.80 | 3.80 |
| -4 | 16 | 3 | 20.40 | 4.20 |
| 3 | -16 | -3 | 12.10 | 4.60 |
| 3 | -16 | -3 | 2.50 | 4.20 |
| -3 | -16 | 3 | 12.20 | 3.30 |
| -3 | 16 | 3 | 10.10 | 11.80 |
| 3 | 16 | -3 | 13.10 | 9.50 |
| -3 | 16 | 3 | 9.90 | 3.10 |
| 2 | -16 | -3 | 173.78 | 13.30 |
| -2 | -16 | 3 | 178.88 | 12.20 |
| -2 | -16 | 3 | 170.58 | 11.60 |
| 2 | -16 | -3 | 188.48 | 13.20 |
| -2 | 16 | 3 | 173.68 | 12.20 |
| 2 | 16 | -3 | 133.19 | 17.90 |

| | | | | |
|---|---|---|---|---|
| -2 | 16 | 3 | 185.28 | 17.50 |
| -1 | -16 | 3 | 513.05 | 29.70 |
| 1 | -16 | -3 | 478.45 | 30.40 |
| -1 | -16 | 3 | 539.25 | 30.30 |
| 1 | -16 | -3 | 531.75 | 30.70 |
| -1 | 16 | 3 | 470.05 | 32.80 |
| 1 | 16 | -3 | 593.54 | 32.30 |
| -1 | 16 | 3 | 522.05 | 30.10 |
| 0 | -16 | 3 | 0.80 | 2.80 |
| 0 | -16 | -3 | 1.00 | 3.40 |
| 0 | -16 | 3 | -1.00 | 3.60 |
| 0 | -16 | -3 | 11.60 | 5.20 |
| 0 | 16 | 3 | 2.30 | 3.00 |
| 0 | 16 | -3 | -2.40 | 4.20 |
| 0 | 16 | 3 | 0.00 | 6.70 |
| 0 | 16 | -3 | -8.10 | 6.60 |
| 1 | -16 | 3 | 451.25 | 27.20 |
| 1 | -16 | 3 | 467.65 | 26.00 |
| -1 | -16 | -3 | 451.05 | 26.60 |
| 1 | 16 | 3 | 443.06 | 26.20 |
| 1 | 16 | 3 | 426.86 | 29.80 |
| -1 | 16 | -3 | 434.76 | 29.50 |
| -1 | 16 | -3 | 487.45 | 26.50 |
| 2 | -16 | 3 | 54.99 | 8.90 |
| -2 | -16 | -3 | 53.29 | 6.60 |
| 2 | -16 | 3 | 55.59 | 6.10 |
| -2 | 16 | -3 | 63.69 | 7.20 |
| 2 | 16 | 3 | 56.89 | 5.80 |
| 2 | 16 | 3 | 36.90 | 9.20 |
| -2 | 16 | -3 | 46.20 | 10.20 |
| 3 | -16 | 3 | 143.59 | 10.30 |
| 3 | -16 | 3 | 181.58 | 13.30 |
| -3 | -16 | -3 | 141.59 | 10.60 |
| -3 | 16 | -3 | 155.88 | 11.00 |
| 3 | 16 | 3 | 156.68 | 10.50 |
| -4 | -16 | -3 | 34.10 | 5.50 |
| 4 | -16 | 3 | 41.20 | 5.30 |
| 4 | -16 | 3 | 52.19 | 9.20 |
| -4 | 16 | -3 | 36.60 | 6.50 |
| 4 | 16 | 3 | 42.10 | 5.00 |
| 5 | -16 | 3 | 673.73 | 39.10 |
| 5 | -16 | 3 | 683.03 | 37.50 |
| -5 | -16 | -3 | 641.24 | 37.50 |
| 5 | 16 | 3 | 707.03 | 37.40 |

| | | | | |
|---|---|---|---|---|
| -5 | 16 | -3 | 650.63 | 37.80 |
| -6 | -16 | -3 | 41.70 | 5.70 |
| 6 | -16 | 3 | 41.70 | 5.80 |
| -6 | 16 | -3 | 42.80 | 6.70 |
| 6 | 16 | 3 | 48.80 | 5.20 |
| 7 | -16 | 3 | 11.80 | 2.80 |
| -7 | -16 | -3 | 14.70 | 2.90 |
| -7 | 16 | -3 | 18.20 | 3.90 |
| 7 | 16 | 3 | 12.90 | 2.80 |
| -8 | -16 | -3 | 25.50 | 5.10 |
| 8 | -16 | 3 | 27.90 | 4.30 |
| -8 | 16 | -3 | 31.50 | 6.60 |
| 8 | 16 | 3 | 26.70 | 4.50 |
| 9 | -16 | 3 | 150.28 | 8.80 |
| 9 | 16 | 3 | 134.59 | 9.00 |
| -9 | 16 | -3 | 111.09 | 9.50 |
| 9 | -17 | -3 | 21.50 | 4.20 |
| -9 | 17 | 3 | 21.30 | 4.10 |
| 9 | 17 | -3 | 15.00 | 2.80 |
| 8 | -17 | -3 | 7.40 | 2.90 |
| -8 | -17 | 3 | 6.10 | 3.00 |
| -8 | 17 | 3 | 3.80 | 2.60 |
| 8 | 17 | -3 | 7.20 | 1.90 |
| -7 | -17 | 3 | 247.08 | 15.10 |
| 7 | -17 | -3 | 256.17 | 15.60 |
| 7 | 17 | -3 | 255.37 | 14.80 |
| -7 | 17 | 3 | 239.98 | 15.90 |
| 6 | -17 | -3 | 396.66 | 24.00 |
| -6 | -17 | 3 | 426.36 | 23.60 |
| 6 | 17 | -3 | 417.26 | 23.40 |
| -6 | 17 | 3 | 390.66 | 24.40 |
| 5 | -17 | -3 | 90.09 | 8.70 |
| -5 | -17 | 3 | 68.39 | 7.40 |
| 5 | -17 | -3 | 69.29 | 8.80 |
| -5 | 17 | 3 | 82.59 | 8.00 |
| 4 | -17 | -3 | 297.57 | 19.70 |
| 4 | -17 | -3 | 345.97 | 19.50 |
| -4 | -17 | 3 | 292.17 | 18.60 |
| -4 | 17 | 3 | 306.47 | 19.20 |
| 3 | -17 | -3 | 66.89 | 8.30 |
| -3 | -17 | 3 | 60.29 | 6.50 |
| -3 | 17 | 3 | 60.59 | 6.90 |
| 2 | -17 | -3 | 67.79 | 12.30 |
| -2 | -17 | 3 | 73.99 | 7.40 |

| | | | | |
|---|---|---|---|---|
| 2 | -17 | -3 | 82.99 | 8.80 |
| -2 | 17 | 3 | 78.89 | 7.20 |
| -1 | -17 | 3 | 24.90 | 4.90 |
| -1 | -17 | 3 | 16.60 | 3.90 |
| 1 | -17 | -3 | 23.00 | 4.10 |
| 1 | -17 | -3 | 26.70 | 6.20 |
| -1 | 17 | 3 | 19.50 | 3.50 |
| 0 | -17 | -3 | 685.73 | 38.40 |
| 0 | -17 | 3 | 670.43 | 37.70 |
| 0 | -17 | 3 | 670.73 | 38.50 |
| 0 | 17 | 3 | 678.63 | 38.00 |
| -1 | -17 | -3 | 99.09 | 8.30 |
| 1 | -17 | 3 | 100.39 | 7.50 |
| 1 | -17 | 3 | 93.39 | 9.40 |
| -1 | 17 | -3 | 82.99 | 11.70 |
| 1 | 17 | 3 | 90.69 | 7.40 |
| 2 | -17 | 3 | 21.30 | 4.30 |
| -2 | -17 | -3 | 12.50 | 2.90 |
| 2 | -17 | 3 | 16.10 | 4.50 |
| 2 | 17 | 3 | 15.50 | 3.00 |
| -2 | 17 | -3 | 16.50 | 4.10 |
| -3 | -17 | -3 | 64.89 | 6.40 |
| 3 | -17 | 3 | 51.69 | 5.90 |
| -3 | 17 | -3 | 63.09 | 7.30 |
| 3 | 17 | 3 | 72.19 | 5.90 |
| 4 | -17 | 3 | 229.98 | 16.00 |
| -4 | -17 | -3 | 226.18 | 14.30 |
| 4 | -17 | 3 | 221.98 | 14.20 |
| -4 | 17 | -3 | 234.88 | 14.70 |
| 4 | 17 | 3 | 239.78 | 14.00 |
| 5 | -17 | 3 | 3.90 | 2.30 |
| -5 | -17 | -3 | -1.10 | 2.20 |
| 5 | -17 | 3 | 7.30 | 4.50 |
| 5 | 17 | 3 | 2.70 | 2.20 |
| -5 | 17 | -3 | -5.60 | 3.20 |
| 6 | -17 | 3 | 252.17 | 14.70 |
| -6 | -17 | -3 | 234.58 | 14.60 |
| 6 | 17 | 3 | 248.98 | 14.60 |
| -7 | -17 | -3 | 127.79 | 8.70 |
| 7 | -17 | 3 | 133.49 | 8.50 |
| 7 | 17 | 3 | 125.29 | 8.70 |
| -7 | 17 | -3 | 130.79 | 9.90 |
| -8 | -17 | -3 | 24.90 | 3.70 |
| -8 | 17 | -3 | 29.80 | 6.40 |

| | | | | |
|---|---|---|---|---|
| 8 | 17 | 3 | 31.80 | 4.60 |
| 8 | -18 | -3 | 137.09 | 9.10 |
| -8 | -18 | 3 | 137.69 | 9.50 |
| -8 | 18 | 3 | 151.08 | 9.50 |
| 8 | 18 | -3 | 135.39 | 8.40 |
| 7 | -18 | -3 | 34.40 | 5.50 |
| -7 | -18 | 3 | 35.50 | 5.20 |
| 7 | 18 | -3 | 45.10 | 3.90 |
| -7 | 18 | 3 | 36.60 | 5.40 |
| 6 | -18 | -3 | 35.40 | 6.40 |
| -6 | -18 | 3 | 42.10 | 5.10 |
| 6 | 18 | -3 | 48.80 | 4.70 |
| -6 | 18 | 3 | 36.90 | 5.80 |
| 5 | -18 | -3 | 261.57 | 16.90 |
| -5 | -18 | 3 | 272.07 | 16.40 |
| 5 | -18 | -3 | 272.67 | 16.90 |
| -5 | 18 | 3 | 287.77 | 17.10 |
| 4 | -18 | -3 | 96.39 | 8.20 |
| -4 | -18 | 3 | 86.09 | 7.20 |
| 4 | -18 | -3 | 90.19 | 9.30 |
| -4 | 18 | 3 | 84.89 | 8.00 |
| 3 | -18 | -3 | 7.10 | 4.70 |
| 3 | -18 | -3 | 4.90 | 3.10 |
| -3 | -18 | 3 | 2.90 | 2.20 |
| -3 | 18 | 3 | 1.50 | 2.80 |
| 2 | -18 | -3 | 142.39 | 13.30 |
| 2 | -18 | -3 | 190.58 | 12.40 |
| -2 | -18 | 3 | 172.98 | 11.70 |
| -2 | 18 | 3 | 191.28 | 11.80 |
| -1 | -18 | 3 | 309.17 | 19.50 |
| 1 | -18 | -3 | 331.87 | 19.80 |
| -1 | 18 | 3 | 325.37 | 19.10 |
| 0 | -18 | -3 | 7.60 | 3.20 |
| 0 | -18 | 3 | 17.00 | 3.90 |
| 0 | -18 | 3 | 13.70 | 2.80 |
| 0 | 18 | 3 | 7.20 | 2.90 |
| 1 | -18 | 3 | 130.49 | 8.80 |
| -1 | -18 | -3 | 130.79 | 9.70 |
| 1 | -18 | 3 | 131.99 | 10.50 |
| 1 | 18 | 3 | 115.99 | 8.60 |
| 2 | -18 | 3 | 134.49 | 10.80 |
| -2 | -18 | -3 | 133.69 | 9.40 |
| 2 | -18 | 3 | 133.79 | 8.90 |
| 2 | 18 | 3 | 126.49 | 8.80 |

| | | | | |
|---|---|---|---|---|
| -3 | -18 | -3 | 30.00 | 4.80 |
| 3 | -18 | 3 | 33.10 | 4.40 |
| 3 | -18 | 3 | 42.30 | 7.10 |
| 3 | 18 | 3 | 29.90 | 4.40 |
| 4 | -18 | 3 | 57.59 | 5.10 |
| 4 | -18 | 3 | 61.59 | 7.90 |
| -4 | -18 | -3 | 53.49 | 5.30 |
| 4 | 18 | 3 | 57.89 | 5.20 |
| 5 | -18 | 3 | 313.17 | 16.90 |
| -5 | -18 | -3 | 294.47 | 16.90 |
| 5 | -18 | 3 | 257.27 | 19.20 |
| -6 | -18 | -3 | 117.99 | 7.90 |
| 6 | -18 | 3 | 126.59 | 7.90 |
| 6 | 18 | 3 | 112.09 | 7.90 |
| -7 | -18 | -3 | 9.70 | 4.30 |
| 7 | 18 | 3 | 12.40 | 2.90 |
| -6 | -19 | 3 | 235.78 | 14.40 |
| -6 | 19 | 3 | 244.18 | 15.00 |
| 6 | 19 | -3 | 258.57 | 14.10 |
| -5 | -19 | 3 | 13.80 | 2.60 |
| 5 | -19 | -3 | 14.40 | 4.90 |
| 5 | 19 | -3 | 21.20 | 3.50 |
| -5 | 19 | 3 | 21.00 | 3.70 |
| -4 | -19 | 3 | 119.79 | 8.40 |
| 4 | -19 | -3 | 127.59 | 9.20 |
| -3 | -19 | 3 | 95.69 | 7.20 |
| 3 | -19 | -3 | 107.19 | 7.90 |
| -3 | 19 | 3 | 86.69 | 7.70 |
| 2 | -19 | -3 | 28.70 | 5.10 |
| -2 | -19 | 3 | 20.50 | 4.40 |
| -2 | 19 | 3 | 22.60 | 4.80 |
| 1 | -19 | -3 | 137.39 | 9.70 |
| -1 | -19 | 3 | 130.89 | 9.60 |
| -1 | 19 | 3 | 136.19 | 9.20 |
| 0 | -19 | -3 | 311.17 | 18.50 |
| 0 | -19 | 3 | 308.17 | 18.60 |
| 0 | 19 | 3 | 310.17 | 18.10 |
| -1 | -19 | -3 | 169.18 | 10.90 |
| 1 | -19 | 3 | 175.28 | 11.50 |
| 1 | -19 | 3 | 155.18 | 10.20 |
| 1 | 19 | 3 | 164.38 | 10.40 |
| -2 | -19 | -3 | 8.00 | 2.20 |
| 2 | -19 | 3 | 9.20 | 2.10 |
| 2 | -19 | 3 | 7.10 | 3.30 |

| | | | | |
|---|---|---|---|---|
| 3 | -19 | 3 | 114.49 | 8.70 |
| -3 | -19 | -3 | 93.99 | 7.10 |
| 3 | -19 | 3 | 99.09 | 6.80 |
| 3 | 19 | 3 | 91.59 | 6.80 |
| -4 | -19 | -3 | 196.08 | 12.20 |
| 4 | -19 | 3 | 210.78 | 12.20 |
| 4 | -19 | 3 | 193.28 | 13.90 |
| 4 | 19 | 3 | 214.48 | 12.20 |
| 5 | -19 | 3 | 1.50 | 1.70 |
| -5 | -19 | -3 | -2.40 | 1.60 |
| 5 | 19 | 3 | 1.80 | 2.60 |
| 3 | -20 | -3 | 9.10 | 3.00 |
| -3 | -20 | 3 | 4.00 | 1.90 |
| -3 | 20 | 3 | 5.30 | 2.90 |
| 2 | -20 | -3 | 149.59 | 9.40 |
| -2 | -20 | 3 | 133.69 | 9.00 |
| -2 | 20 | 3 | 134.19 | 9.20 |
| 1 | -20 | -3 | 294.27 | 17.60 |
| -1 | -20 | 3 | 302.77 | 17.60 |
| -1 | 20 | 3 | 306.17 | 17.50 |
| 0 | -20 | -3 | -1.20 | 2.20 |
| 0 | -20 | 3 | 0.60 | 2.50 |
| 0 | 20 | 3 | -1.50 | 2.50 |
| -1 | -20 | -3 | 102.09 | 7.40 |
| 1 | -20 | 3 | 105.19 | 8.00 |
| 1 | 20 | 3 | 107.29 | 7.20 |
| 2 | -20 | 3 | 107.99 | 7.70 |
| -2 | -20 | -3 | 98.09 | 6.80 |
| 2 | 20 | 3 | 85.89 | 6.60 |
| 17 | 0 | -4 | 0.10 | 1.60 |
| 16 | 0 | -4 | 627.14 | 36.30 |
| 16 | 0 | -4 | 618.64 | 34.50 |
| 15 | 0 | -4 | -3.00 | 3.90 |
| 15 | 0 | -4 | -3.30 | 2.20 |
| 14 | 0 | -4 | 599.04 | 35.60 |
| 14 | 0 | -4 | 648.84 | 35.20 |
| 14 | 0 | -4 | 589.54 | 34.50 |
| 13 | 0 | -4 | 2.20 | 4.00 |
| 13 | 0 | -4 | -2.30 | 2.70 |
| 13 | 0 | -4 | -5.70 | 4.30 |
| 12 | 0 | -4 | 372.76 | 22.50 |
| 12 | 0 | -4 | 374.96 | 23.90 |
| 12 | 0 | -4 | 353.96 | 21.70 |
| 11 | 0 | -4 | -0.90 | 5.50 |

| | | | | |
|---|---|---|---|---|
| 11 | 0 | -4 | -0.50 | 4.30 |
| 11 | 0 | -4 | 3.20 | 2.90 |
| 10 | 0 | -4 | 1299.97 | 72.59 |
| 10 | 0 | -4 | 1321.67 | 74.39 |
| 10 | 0 | -4 | 1255.17 | 70.79 |
| 9 | 0 | -4 | 38.80 | 7.50 |
| 8 | 0 | -4 | 497.25 | 29.00 |
| 6 | 0 | -4 | 2609.14 | 140.09 |
| -4 | 0 | 4 | 2116.89 | 114.29 |
| -3 | 0 | 4 | 5.30 | 2.10 |
| -2 | 0 | 4 | 6879.61 | 369.46 |
| -1 | 0 | 4 | 0.40 | 2.60 |
| 0 | 0 | 4 | 2317.37 | 125.79 |
| 1 | 0 | 4 | 1.50 | 3.10 |
| 2 | 0 | 4 | 1273.57 | 70.29 |
| 3 | 0 | 4 | -3.30 | 3.00 |
| 4 | 0 | 4 | 2758.42 | 149.79 |
| 5 | 0 | 4 | 0.10 | 3.40 |
| 6 | 0 | 4 | 2580.24 | 126.79 |
| 6 | 0 | 4 | 2089.69 | 126.69 |
| 7 | 0 | 4 | 0.70 | 3.50 |
| 7 | 0 | 4 | -3.40 | 4.00 |
| 8 | 0 | 4 | 1146.69 | 62.79 |
| 8 | 0 | 4 | 1114.89 | 63.09 |
| 9 | 0 | 4 | -3.00 | 5.80 |
| 9 | 0 | 4 | -4.60 | 4.70 |
| 9 | 0 | 4 | -3.40 | 3.60 |
| 10 | 0 | 4 | 1173.08 | 61.79 |
| 10 | 0 | 4 | 1038.10 | 61.69 |
| 11 | 0 | 4 | -3.10 | 4.20 |
| 11 | 0 | 4 | 4.70 | 5.30 |
| 11 | 0 | 4 | 13.00 | 4.50 |
| 12 | 0 | 4 | 10.50 | 4.80 |
| 12 | 0 | 4 | 16.50 | 4.10 |
| 12 | 0 | 4 | 18.40 | 5.10 |
| 13 | 0 | 4 | 10.30 | 3.60 |
| 13 | 0 | 4 | 10.40 | 4.60 |
| 13 | 0 | 4 | 0.90 | 3.50 |
| 14 | 0 | 4 | 446.66 | 26.20 |
| 14 | 0 | 4 | 430.16 | 26.80 |
| 14 | 0 | 4 | 471.95 | 26.20 |
| 15 | 0 | 4 | 2.30 | 2.90 |
| 15 | 0 | 4 | 2.60 | 4.00 |
| 15 | 0 | 4 | -1.60 | 2.40 |

| | | | | |
|---|---|---|---|---|
| 16 | 0 | 4 | 123.39 | 8.20 |
| 17 | -1 | -4 | 253.87 | 14.50 |
| 17 | 1 | -4 | 250.27 | 14.40 |
| 16 | -1 | -4 | 7.40 | 2.20 |
| 16 | -1 | -4 | 2.90 | 4.80 |
| 16 | 1 | -4 | 5.60 | 2.20 |
| 16 | 1 | -4 | 4.40 | 4.40 |
| 15 | -1 | -4 | 510.45 | 28.00 |
| 15 | -1 | -4 | 474.75 | 27.40 |
| 15 | 1 | -4 | 457.05 | 27.90 |
| 15 | 1 | -4 | 488.35 | 27.40 |
| 14 | -1 | -4 | 38.10 | 5.50 |
| 14 | -1 | -4 | 34.40 | 5.50 |
| 14 | -1 | -4 | 35.70 | 5.80 |
| 14 | 1 | -4 | 43.00 | 7.70 |
| 14 | 1 | -4 | 33.40 | 5.40 |
| 14 | 1 | -4 | 40.90 | 5.30 |
| 13 | -1 | -4 | 9.50 | 4.10 |
| 13 | -1 | -4 | 5.60 | 2.80 |
| 13 | -1 | -4 | 3.40 | 4.80 |
| 13 | 1 | -4 | 2.90 | 4.20 |
| 13 | 1 | -4 | 3.10 | 2.60 |
| 13 | 1 | -4 | 0.90 | 4.80 |
| 12 | -1 | -4 | 16.50 | 3.60 |
| 12 | -1 | -4 | 27.00 | 5.00 |
| 12 | -1 | -4 | 15.40 | 5.30 |
| 12 | 1 | -4 | 33.60 | 5.40 |
| 12 | 1 | -4 | 21.90 | 5.80 |
| 12 | 1 | -4 | 29.20 | 5.50 |
| 11 | -1 | -4 | 1662.23 | 95.69 |
| 11 | -1 | -4 | 1851.11 | 96.89 |
| 11 | -1 | -4 | 1753.62 | 95.29 |
| 11 | 1 | -4 | 1772.32 | 96.69 |
| 11 | 1 | -4 | 1645.94 | 95.09 |
| 11 | 1 | -4 | 1805.72 | 95.79 |
| 10 | -1 | -4 | 13.00 | 3.70 |
| 10 | -1 | -4 | 21.10 | 4.90 |
| 10 | -1 | -4 | 6.10 | 6.60 |
| 10 | 1 | -4 | 8.80 | 6.60 |
| 10 | 1 | -4 | 16.60 | 3.70 |
| 9 | -1 | -4 | 992.40 | 55.49 |
| 9 | -1 | -4 | 999.20 | 57.19 |
| 9 | -1 | -4 | 960.40 | 55.29 |
| 9 | 1 | -4 | 922.21 | 54.99 |

| | | | | |
|---|---|---|---|---|
| 9 | 1 | -4 | 965.20 | 57.19 |
| 9 | 1 | -4 | 1150.38 | 55.89 |
| 8 | -1 | -4 | 126.69 | 10.70 |
| 8 | 1 | -4 | 118.39 | 12.20 |
| 6 | 1 | -4 | 7.50 | 3.00 |
| -5 | 1 | 4 | 7250.17 | 388.76 |
| -4 | -1 | 4 | 365.16 | 20.70 |
| -4 | 1 | 4 | 358.56 | 20.60 |
| -3 | -1 | 4 | 4.40 | 2.30 |
| -2 | -1 | 4 | 2.20 | 2.40 |
| -2 | 1 | 4 | 2.50 | 2.10 |
| -1 | -1 | 4 | 2614.34 | 140.59 |
| -1 | 1 | 4 | 2580.94 | 140.39 |
| 0 | -1 | 4 | 1426.46 | 81.59 |
| 0 | 1 | 4 | 1559.04 | 81.49 |
| 1 | -1 | 4 | 2596.24 | 153.38 |
| 1 | 1 | 4 | 3069.09 | 153.38 |
| 2 | -1 | 4 | 765.02 | 44.80 |
| 2 | 1 | 4 | 832.42 | 44.90 |
| 3 | -1 | 4 | 2477.15 | 126.99 |
| 3 | 1 | 4 | 2194.18 | 127.09 |
| 4 | -1 | 4 | 3.40 | 2.80 |
| 4 | 1 | 4 | 2.00 | 3.60 |
| 5 | -1 | 4 | 5912.71 | 306.67 |
| 5 | 1 | 4 | 5490.15 | 307.37 |
| 6 | -1 | 4 | -5.60 | 4.60 |
| 6 | -1 | 4 | 4.10 | 2.90 |
| 6 | 1 | 4 | 5.80 | 3.80 |
| 6 | 1 | 4 | 3.60 | 4.00 |
| 7 | -1 | 4 | 160.38 | 13.90 |
| 7 | -1 | 4 | 213.68 | 13.00 |
| 7 | 1 | 4 | 192.18 | 13.70 |
| 7 | 1 | 4 | 205.68 | 13.50 |
| 8 | -1 | 4 | -5.80 | 4.70 |
| 8 | -1 | 4 | 4.20 | 4.50 |
| 8 | 1 | 4 | -3.10 | 4.40 |
| 8 | 1 | 4 | 1.00 | 4.00 |
| 9 | -1 | 4 | 1148.19 | 58.89 |
| 9 | -1 | 4 | 1076.49 | 58.79 |
| 9 | -1 | 4 | 1119.59 | 59.09 |
| 9 | 1 | 4 | 944.01 | 58.79 |
| 9 | 1 | 4 | 1029.00 | 58.59 |
| 9 | 1 | 4 | 998.10 | 58.79 |
| 10 | -1 | 4 | 93.19 | 11.70 |

| | | | | |
|---|---|---|---|---|
| 10 | -1 | 4 | 104.39 | 9.60 |
| 10 | 1 | 4 | 96.59 | 12.80 |
| 10 | 1 | 4 | 101.79 | 9.70 |
| 11 | -1 | 4 | 1190.88 | 66.09 |
| 11 | -1 | 4 | 1177.88 | 66.29 |
| 11 | -1 | 4 | 1147.89 | 66.29 |
| 11 | 1 | 4 | 1259.87 | 66.49 |
| 11 | 1 | 4 | 1184.38 | 66.19 |
| 11 | 1 | 4 | 1189.68 | 66.49 |
| 12 | -1 | 4 | -5.10 | 4.30 |
| 12 | -1 | 4 | 1.40 | 3.70 |
| 12 | -1 | 4 | 0.70 | 4.40 |
| 12 | 1 | 4 | 1.20 | 4.00 |
| 12 | 1 | 4 | 0.10 | 3.60 |
| 12 | 1 | 4 | 3.40 | 4.90 |
| 13 | -1 | 4 | 349.07 | 20.90 |
| 13 | -1 | 4 | 327.97 | 20.40 |
| 13 | -1 | 4 | 321.17 | 21.10 |
| 13 | 1 | 4 | 343.77 | 20.70 |
| 13 | 1 | 4 | 332.67 | 21.00 |
| 13 | 1 | 4 | 345.07 | 21.10 |
| 14 | -1 | 4 | 2.30 | 3.70 |
| 14 | -1 | 4 | 4.70 | 4.00 |
| 14 | 1 | 4 | 7.10 | 4.00 |
| 14 | 1 | 4 | 7.80 | 2.90 |
| 14 | 1 | 4 | 10.60 | 4.30 |
| 15 | -1 | 4 | 425.26 | 24.90 |
| 15 | -1 | 4 | 449.36 | 24.70 |
| 15 | -1 | 4 | 415.36 | 24.50 |
| 15 | 1 | 4 | 416.36 | 24.80 |
| 15 | 1 | 4 | 442.36 | 24.60 |
| 17 | -2 | -4 | 2.50 | 1.50 |
| 17 | 2 | -4 | 0.20 | 1.40 |
| 16 | -2 | -4 | 304.07 | 16.70 |
| 16 | -2 | -4 | 248.08 | 18.30 |
| 16 | 2 | -4 | 302.87 | 16.60 |
| 15 | -2 | -4 | -6.90 | 3.10 |
| 15 | -2 | -4 | -0.20 | 3.70 |
| 15 | 2 | -4 | -1.20 | 2.90 |
| 15 | 2 | -4 | 1.40 | 4.30 |
| 14 | -2 | -4 | 240.38 | 15.00 |
| 14 | -2 | -4 | 224.78 | 15.70 |
| 14 | 2 | -4 | 255.87 | 16.20 |
| 14 | 2 | -4 | 248.38 | 14.70 |

| | | | | |
|---|---|---|---|---|
| 14 | 2 | -4 | 225.98 | 16.40 |
| 13 | -2 | -4 | 5.30 | 3.00 |
| 13 | -2 | -4 | 7.10 | 4.00 |
| 13 | -2 | -4 | 6.30 | 4.40 |
| 13 | 2 | -4 | 8.20 | 4.40 |
| 13 | 2 | -4 | 6.30 | 2.70 |
| 13 | 2 | -4 | 5.20 | 4.90 |
| 12 | -2 | -4 | 798.12 | 44.20 |
| 12 | -2 | -4 | 808.22 | 45.30 |
| 12 | -2 | -4 | 750.82 | 43.70 |
| 12 | 2 | -4 | 798.02 | 44.20 |
| 12 | 2 | -4 | 762.52 | 45.20 |
| 12 | 2 | -4 | 764.92 | 43.50 |
| 11 | -2 | -4 | 7.80 | 6.00 |
| 11 | -2 | -4 | 4.00 | 3.90 |
| 11 | -2 | -4 | 4.50 | 3.40 |
| 11 | 2 | -4 | 13.50 | 4.70 |
| 11 | 2 | -4 | 9.40 | 3.20 |
| 11 | 2 | -4 | 9.30 | 5.90 |
| 10 | -2 | -4 | 1010.40 | 54.99 |
| 10 | -2 | -4 | 935.21 | 53.19 |
| 10 | -2 | -4 | 1004.50 | 53.59 |
| 10 | 2 | -4 | 941.11 | 54.89 |
| 10 | 2 | -4 | 899.71 | 52.79 |
| 9 | -2 | -4 | 0.10 | 4.00 |
| 9 | -2 | -4 | -4.20 | 6.50 |
| 9 | -2 | -4 | -1.40 | 4.40 |
| 9 | 2 | -4 | -0.40 | 3.40 |
| 9 | 2 | -4 | -2.40 | 7.70 |
| 9 | 2 | -4 | 4.50 | 5.10 |
| 8 | -2 | -4 | 94.39 | 9.30 |
| 8 | 2 | -4 | 93.19 | 11.20 |
| 7 | -2 | -4 | 291.67 | 18.50 |
| 6 | 2 | -4 | 5139.29 | 276.07 |
| -4 | 2 | 4 | 651.63 | 36.10 |
| -3 | -2 | 4 | 337.07 | 21.00 |
| -3 | 2 | 4 | 384.06 | 20.70 |
| -2 | -2 | 4 | 164.98 | 11.50 |
| -2 | 2 | 4 | 189.58 | 11.00 |
| -1 | -2 | 4 | 62.39 | 6.10 |
| -1 | 2 | 4 | 66.29 | 5.40 |
| 0 | -2 | 4 | 8508.95 | 481.15 |
| 0 | 2 | 4 | 9414.46 | 480.95 |
| 1 | -2 | 4 | 309.07 | 19.60 |

| | | | | |
|---|---|---|---|---|
| 1 | 2 | 4 | 351.16 | 19.50 |
| 2 | -2 | 4 | 41.00 | 5.20 |
| 2 | 2 | 4 | 38.30 | 5.10 |
| 3 | -2 | 4 | 688.83 | 36.90 |
| 3 | 2 | 4 | 622.44 | 37.30 |
| 4 | -2 | 4 | 3047.60 | 148.49 |
| 4 | 2 | 4 | 2437.86 | 148.99 |
| 5 | -2 | 4 | 63.39 | 6.00 |
| 5 | 2 | 4 | 54.79 | 8.30 |
| 6 | -2 | 4 | 2075.39 | 100.99 |
| 6 | -2 | 4 | 1730.53 | 101.69 |
| 6 | 2 | 4 | 1801.32 | 101.99 |
| 6 | 2 | 4 | 1823.52 | 100.99 |
| 7 | -2 | 4 | -0.30 | 4.20 |
| 7 | 2 | 4 | 2.90 | 4.80 |
| 7 | 2 | 4 | 1.50 | 3.40 |
| 8 | -2 | 4 | 472.65 | 28.60 |
| 8 | -2 | 4 | 471.35 | 28.50 |
| 8 | -2 | 4 | 450.05 | 28.50 |
| 8 | 2 | 4 | 501.95 | 28.20 |
| 8 | 2 | 4 | 493.65 | 28.30 |
| 9 | -2 | 4 | 22.30 | 5.30 |
| 9 | -2 | 4 | 17.50 | 4.30 |
| 9 | -2 | 4 | 15.30 | 5.70 |
| 9 | 2 | 4 | 29.90 | 6.80 |
| 9 | 2 | 4 | 15.90 | 4.80 |
| 9 | 2 | 4 | 11.90 | 4.60 |
| 10 | -2 | 4 | 1755.12 | 105.59 |
| 10 | -2 | 4 | 1827.72 | 102.99 |
| 10 | -2 | 4 | 1975.80 | 103.59 |
| 10 | 2 | 4 | 1840.22 | 103.49 |
| 10 | 2 | 4 | 1948.71 | 103.59 |
| 10 | 2 | 4 | 1962.40 | 103.29 |
| 11 | -2 | 4 | 9.00 | 4.00 |
| 11 | -2 | 4 | 7.70 | 4.70 |
| 11 | -2 | 4 | 18.00 | 5.10 |
| 11 | 2 | 4 | 17.00 | 5.90 |
| 11 | 2 | 4 | 20.90 | 5.00 |
| 11 | 2 | 4 | 12.20 | 4.20 |
| 12 | -2 | 4 | 18.40 | 4.80 |
| 12 | -2 | 4 | 13.70 | 5.70 |
| 12 | -2 | 4 | 7.00 | 4.30 |
| 12 | 2 | 4 | 20.60 | 5.60 |
| 12 | 2 | 4 | 12.50 | 5.40 |

| | | | | |
|---|---|---|---|---|
| 12 | 2 | 4 | 9.80 | 4.10 |
| 13 | -2 | 4 | 18.10 | 4.60 |
| 13 | -2 | 4 | 7.40 | 4.20 |
| 13 | -2 | 4 | 8.80 | 3.00 |
| 13 | 2 | 4 | 11.40 | 3.70 |
| 13 | 2 | 4 | 14.00 | 3.70 |
| 13 | 2 | 4 | 20.80 | 5.20 |
| 14 | -2 | 4 | 443.86 | 26.60 |
| 14 | -2 | 4 | 486.85 | 26.80 |
| 14 | -2 | 4 | 471.15 | 27.30 |
| 14 | 2 | 4 | 470.95 | 26.70 |
| 14 | 2 | 4 | 430.76 | 27.10 |
| 14 | 2 | 4 | 459.55 | 26.90 |
| 15 | -2 | 4 | 6.50 | 2.40 |
| 15 | -2 | 4 | 9.40 | 3.10 |
| 15 | -2 | 4 | 12.00 | 3.60 |
| 15 | 2 | 4 | 8.70 | 2.80 |
| 15 | 2 | 4 | 3.60 | 2.50 |
| 17 | -3 | -4 | 77.09 | 10.00 |
| 17 | -3 | -4 | 101.99 | 6.30 |
| 17 | 3 | -4 | 94.19 | 6.10 |
| 16 | -3 | -4 | 0.30 | 1.90 |
| 16 | -3 | -4 | -0.60 | 5.40 |
| 16 | 3 | -4 | -1.20 | 1.70 |
| 15 | -3 | -4 | 443.66 | 25.90 |
| 15 | -3 | -4 | 375.46 | 23.60 |
| 15 | 3 | -4 | 422.66 | 23.80 |
| 15 | 3 | -4 | 377.46 | 24.30 |
| 14 | -3 | -4 | 0.80 | 3.60 |
| 14 | -3 | -4 | -3.40 | 2.70 |
| 14 | 3 | -4 | -3.30 | 2.20 |
| 14 | 3 | -4 | -4.50 | 4.50 |
| 14 | 3 | -4 | 4.80 | 4.50 |
| 13 | -3 | -4 | 83.49 | 9.60 |
| 13 | -3 | -4 | 74.29 | 8.60 |
| 13 | -3 | -4 | 73.99 | 7.60 |
| 13 | 3 | -4 | 73.19 | 9.20 |
| 13 | 3 | -4 | 75.69 | 6.80 |
| 13 | 3 | -4 | 84.79 | 10.20 |
| 12 | -3 | -4 | -2.50 | 3.30 |
| 12 | -3 | -4 | -2.80 | 3.80 |
| 12 | -3 | -4 | 0.30 | 5.00 |
| 12 | 3 | -4 | -1.50 | 4.50 |
| 12 | 3 | -4 | -2.00 | 2.70 |

| | | | | |
|---|---|---|---|---|
| 12 | 3 | -4 | -4.30 | 5.70 |
| 11 | -3 | -4 | 1482.25 | 84.59 |
| 11 | -3 | -4 | 1378.26 | 84.19 |
| 11 | -3 | -4 | 1598.14 | 86.09 |
| 11 | 3 | -4 | 1556.84 | 84.39 |
| 11 | 3 | -4 | 1612.64 | 84.79 |
| 11 | 3 | -4 | 1589.14 | 85.39 |
| 10 | -3 | -4 | 7.20 | 5.50 |
| 10 | -3 | -4 | 7.80 | 4.30 |
| 10 | -3 | -4 | 7.00 | 3.70 |
| 10 | 3 | -4 | 11.70 | 6.80 |
| 10 | 3 | -4 | 7.10 | 3.60 |
| 10 | 3 | -4 | 0.90 | 7.10 |
| 9 | -3 | -4 | 497.35 | 30.40 |
| 9 | -3 | -4 | 545.75 | 30.20 |
| 9 | -3 | -4 | 545.05 | 32.10 |
| 9 | 3 | -4 | 481.25 | 33.10 |
| 9 | 3 | -4 | 536.65 | 31.10 |
| 9 | 3 | -4 | 513.05 | 29.80 |
| 8 | -3 | -4 | 326.07 | 19.70 |
| 8 | 3 | -4 | 311.57 | 21.20 |
| 7 | -3 | -4 | 414.46 | 26.30 |
| 7 | 3 | -4 | 477.45 | 25.50 |
| 6 | 3 | -4 | 1.10 | 2.70 |
| -6 | 3 | 4 | 4.90 | 2.10 |
| 5 | 3 | -4 | 2540.95 | 146.09 |
| -5 | 3 | 4 | 2867.51 | 145.89 |
| -4 | 3 | 4 | 200.28 | 12.30 |
| -3 | 3 | 4 | 337.97 | 19.40 |
| -2 | 3 | 4 | 84.19 | 6.30 |
| -1 | -3 | 4 | 2312.27 | 138.89 |
| -1 | 3 | 4 | 2816.92 | 138.69 |
| 0 | -3 | 4 | 159.28 | 10.70 |
| 0 | 3 | 4 | 153.88 | 10.10 |
| 1 | -3 | 4 | 1758.12 | 93.79 |
| 1 | 3 | 4 | 1683.93 | 93.59 |
| 2 | -3 | 4 | 83.19 | 6.70 |
| 2 | 3 | 4 | 79.89 | 7.00 |
| 3 | -3 | 4 | 174.98 | 10.70 |
| 3 | 3 | 4 | 148.99 | 11.30 |
| 4 | -3 | 4 | 14.70 | 2.90 |
| 4 | 3 | 4 | 12.90 | 4.00 |
| 5 | -3 | 4 | 5634.54 | 267.07 |
| 5 | 3 | 4 | 4750.92 | 268.07 |

| | | | | |
|---|---|---|---|---|
| 5 | 3 | 4 | 4499.15 | 266.67 |
| 6 | -3 | 4 | 16.20 | 5.80 |
| 6 | 3 | 4 | 13.10 | 3.50 |
| 6 | 3 | 4 | 14.70 | 5.20 |
| 7 | -3 | 4 | 26.00 | 5.80 |
| 7 | 3 | 4 | 20.50 | 6.10 |
| 7 | 3 | 4 | 14.20 | 3.40 |
| 8 | -3 | 4 | 39.50 | 7.00 |
| 8 | -3 | 4 | 34.00 | 6.10 |
| 8 | -3 | 4 | 37.00 | 7.70 |
| 8 | 3 | 4 | 36.00 | 8.20 |
| 9 | -3 | 4 | 598.44 | 35.70 |
| 9 | -3 | 4 | 610.14 | 35.90 |
| 9 | -3 | 4 | 623.54 | 36.30 |
| 9 | 3 | 4 | 609.74 | 35.70 |
| 9 | 3 | 4 | 653.73 | 35.80 |
| 10 | -3 | 4 | 60.29 | 8.70 |
| 10 | -3 | 4 | 54.79 | 9.50 |
| 10 | -3 | 4 | 62.29 | 8.00 |
| 10 | 3 | 4 | 66.69 | 8.70 |
| 10 | 3 | 4 | 47.70 | 7.60 |
| 10 | 3 | 4 | 51.19 | 7.90 |
| 11 | -3 | 4 | 995.30 | 55.49 |
| 11 | -3 | 4 | 988.50 | 55.79 |
| 11 | -3 | 4 | 1008.70 | 55.29 |
| 11 | 3 | 4 | 1000.80 | 55.49 |
| 11 | 3 | 4 | 991.40 | 55.59 |
| 11 | 3 | 4 | 948.61 | 55.59 |
| 12 | -3 | 4 | 20.40 | 4.80 |
| 12 | -3 | 4 | 8.00 | 3.90 |
| 12 | -3 | 4 | 9.30 | 4.70 |
| 12 | 3 | 4 | 17.90 | 4.60 |
| 12 | 3 | 4 | 11.70 | 5.30 |
| 12 | 3 | 4 | 30.80 | 6.00 |
| 13 | -3 | 4 | 91.79 | 9.70 |
| 13 | -3 | 4 | 105.99 | 9.70 |
| 13 | -3 | 4 | 105.59 | 8.60 |
| 13 | 3 | 4 | 99.29 | 8.60 |
| 13 | 3 | 4 | 98.09 | 9.20 |
| 14 | -3 | 4 | 4.80 | 2.70 |
| 14 | -3 | 4 | 3.20 | 3.40 |
| 14 | -3 | 4 | 4.90 | 3.40 |
| 14 | 3 | 4 | 2.80 | 3.10 |
| 14 | 3 | 4 | 5.70 | 2.80 |

| | | | | |
|---|---|---|---|---|
| 15 | -3 | 4 | 426.46 | 23.50 |
| 15 | -3 | 4 | 404.16 | 23.20 |
| 15 | -3 | 4 | 412.06 | 23.10 |
| 15 | 3 | 4 | 388.26 | 23.40 |
| 15 | 3 | 4 | 391.46 | 23.10 |
| 17 | -4 | -4 | -1.10 | 1.40 |
| 17 | 4 | -4 | 1.60 | 1.40 |
| 16 | -4 | -4 | 266.47 | 14.90 |
| 16 | -4 | -4 | 236.28 | 15.50 |
| 16 | 4 | -4 | 258.57 | 14.70 |
| 15 | -4 | -4 | 1.10 | 2.50 |
| 15 | -4 | -4 | -2.00 | 3.30 |
| 15 | 4 | -4 | 1.70 | 2.20 |
| 14 | -4 | -4 | 657.63 | 36.80 |
| 14 | -4 | -4 | 647.14 | 36.20 |
| 14 | 4 | -4 | 635.54 | 36.90 |
| 14 | 4 | -4 | 635.64 | 37.10 |
| 14 | 4 | -4 | 646.94 | 35.90 |
| 13 | -4 | -4 | 23.70 | 5.40 |
| 13 | 4 | -4 | 6.00 | 6.60 |
| 13 | 4 | -4 | 20.20 | 5.50 |
| 13 | 4 | -4 | 24.70 | 4.70 |
| 12 | -4 | -4 | 182.58 | 14.00 |
| 12 | -4 | -4 | 179.68 | 12.30 |
| 12 | -4 | -4 | 156.28 | 12.00 |
| 12 | 4 | -4 | 161.28 | 13.00 |
| 12 | 4 | -4 | 164.18 | 11.30 |
| 12 | 4 | -4 | 165.78 | 14.50 |
| 11 | -4 | -4 | 8.30 | 3.80 |
| 11 | -4 | -4 | 4.70 | 3.60 |
| 11 | -4 | -4 | 8.00 | 5.30 |
| 11 | 4 | -4 | 3.20 | 6.70 |
| 11 | 4 | -4 | 2.80 | 3.00 |
| 11 | 4 | -4 | -5.70 | 4.90 |
| 10 | -4 | -4 | 1302.27 | 68.99 |
| 10 | -4 | -4 | 1204.18 | 67.79 |
| 10 | -4 | -4 | 1203.28 | 67.79 |
| 10 | 4 | -4 | 1207.78 | 67.29 |
| 10 | 4 | -4 | 1215.68 | 69.39 |
| 10 | 4 | -4 | 1232.88 | 68.09 |
| 9 | -4 | -4 | 52.59 | 8.10 |
| 9 | -4 | -4 | 37.30 | 6.50 |
| 9 | -4 | -4 | 52.69 | 9.30 |
| 9 | 4 | -4 | 40.90 | 9.50 |

| | | | | |
|---|---|---|---|---|
| 9 | 4 | -4 | 51.29 | 10.50 |
| 9 | 4 | -4 | 51.99 | 6.90 |
| 8 | -4 | -4 | 424.46 | 25.10 |
| 8 | 4 | -4 | 438.76 | 25.40 |
| 8 | 4 | -4 | 404.06 | 26.40 |
| 7 | -4 | -4 | 268.97 | 18.20 |
| 7 | 4 | -4 | 291.77 | 17.20 |
| 6 | -4 | -4 | 3670.63 | 209.18 |
| -6 | 4 | 4 | 3886.71 | 208.18 |
| 6 | 4 | -4 | 4053.79 | 208.78 |
| 5 | 4 | -4 | 35.10 | 5.50 |
| -5 | 4 | 4 | 53.49 | 5.40 |
| -4 | 4 | 4 | 2591.14 | 139.89 |
| -3 | 4 | 4 | 667.03 | 37.10 |
| -2 | 4 | 4 | 1288.07 | 70.29 |
| -1 | 4 | 4 | 498.95 | 28.20 |
| 0 | -4 | 4 | 7828.72 | 416.36 |
| 0 | 4 | 4 | 7664.33 | 415.96 |
| 1 | -4 | 4 | 6.70 | 2.50 |
| 1 | 4 | 4 | 5.90 | 2.40 |
| 2 | -4 | 4 | 31.10 | 4.50 |
| 2 | 4 | 4 | 37.80 | 5.00 |
| 3 | -4 | 4 | 96.39 | 7.30 |
| 3 | 4 | 4 | 93.69 | 8.20 |
| 5 | -4 | 4 | 33.90 | 6.40 |
| 5 | 4 | 4 | 24.00 | 5.20 |
| 5 | 4 | 4 | 25.10 | 3.80 |
| 6 | -4 | 4 | 2727.63 | 147.89 |
| 6 | 4 | 4 | 2630.24 | 146.89 |
| 6 | 4 | 4 | 2796.42 | 148.49 |
| 7 | -4 | 4 | 101.39 | 10.50 |
| 7 | 4 | 4 | 108.99 | 8.30 |
| 7 | 4 | 4 | 112.29 | 12.90 |
| 7 | 4 | 4 | 96.39 | 10.50 |
| 8 | -4 | 4 | 747.83 | 44.20 |
| 8 | -4 | 4 | 793.42 | 44.70 |
| 8 | -4 | 4 | 762.02 | 45.10 |
| 8 | 4 | 4 | 771.52 | 44.20 |
| 8 | 4 | 4 | 832.02 | 44.00 |
| 9 | -4 | 4 | 30.40 | 5.30 |
| 9 | -4 | 4 | 24.90 | 6.80 |
| 9 | -4 | 4 | 24.60 | 5.20 |
| 9 | 4 | 4 | 28.90 | 5.40 |
| 9 | 4 | 4 | 25.50 | 4.80 |

| | | | | |
|---|---|---|---|---|
| 10 | -4 | 4 | 1588.84 | 82.59 |
| 10 | -4 | 4 | 1428.06 | 81.99 |
| 10 | -4 | 4 | 1489.75 | 81.69 |
| 10 | 4 | 4 | 1565.84 | 81.89 |
| 10 | 4 | 4 | 1361.16 | 81.89 |
| 11 | -4 | 4 | 16.20 | 4.80 |
| 11 | -4 | 4 | 9.60 | 3.70 |
| 11 | -4 | 4 | 0.70 | 4.90 |
| 11 | 4 | 4 | 13.90 | 4.00 |
| 11 | 4 | 4 | 0.80 | 5.60 |
| 11 | 4 | 4 | 8.90 | 4.20 |
| 12 | -4 | 4 | 4.30 | 3.40 |
| 12 | -4 | 4 | -4.30 | 3.80 |
| 12 | -4 | 4 | 9.60 | 5.00 |
| 12 | 4 | 4 | 6.80 | 3.90 |
| 12 | 4 | 4 | 15.00 | 4.50 |
| 12 | 4 | 4 | 16.50 | 6.20 |
| 13 | -4 | 4 | 6.80 | 4.20 |
| 13 | -4 | 4 | 14.10 | 4.30 |
| 13 | -4 | 4 | 11.00 | 3.50 |
| 13 | 4 | 4 | 7.60 | 3.60 |
| 13 | 4 | 4 | 10.50 | 3.70 |
| 14 | -4 | 4 | 437.56 | 24.70 |
| 14 | -4 | 4 | 416.66 | 24.20 |
| 14 | -4 | 4 | 442.76 | 24.20 |
| 14 | 4 | 4 | 387.86 | 24.10 |
| 14 | 4 | 4 | 394.96 | 24.40 |
| 15 | -4 | 4 | 10.40 | 2.80 |
| 15 | -4 | 4 | 3.20 | 2.60 |
| 15 | -4 | 4 | 12.00 | 3.20 |
| 15 | 4 | 4 | 11.50 | 3.30 |
| 15 | 4 | 4 | 11.10 | 2.90 |
| 16 | -5 | -4 | 0.80 | 1.80 |
| 16 | -5 | -4 | 5.00 | 3.20 |
| 16 | 5 | -4 | 2.00 | 1.60 |
| 15 | -5 | -4 | 414.46 | 23.50 |
| 15 | -5 | -4 | 399.06 | 24.00 |
| 15 | 5 | -4 | 419.06 | 23.20 |
| 14 | -5 | -4 | 10.00 | 2.90 |
| 14 | -5 | -4 | 10.90 | 3.90 |
| 14 | 5 | -4 | 7.40 | 2.20 |
| 13 | -5 | -4 | 156.98 | 12.50 |
| 13 | -5 | -4 | 162.48 | 11.40 |
| 13 | 5 | -4 | 155.88 | 13.60 |

| | | | | |
|---|---|---|---|---|
| 13 | 5 | -4 | 163.08 | 10.90 |
| 13 | 5 | -4 | 187.98 | 14.00 |
| 12 | -5 | -4 | 47.60 | 6.90 |
| 12 | -5 | -4 | 43.30 | 7.10 |
| 12 | 5 | -4 | 37.60 | 5.70 |
| 12 | 5 | -4 | 47.30 | 10.70 |
| 12 | 5 | -4 | 36.10 | 6.10 |
| 11 | -5 | -4 | 1569.44 | 92.19 |
| 11 | -5 | -4 | 1815.02 | 93.29 |
| 11 | -5 | -4 | 1655.33 | 92.19 |
| 11 | 5 | -4 | 1651.33 | 91.59 |
| 11 | 5 | -4 | 1733.93 | 92.49 |
| 10 | -5 | -4 | 165.78 | 13.50 |
| 10 | -5 | -4 | 202.08 | 15.20 |
| 10 | -5 | -4 | 169.68 | 12.60 |
| 10 | 5 | -4 | 178.08 | 18.00 |
| 10 | 5 | -4 | 187.38 | 12.10 |
| 10 | 5 | -4 | 171.98 | 14.50 |
| 9 | -5 | -4 | 767.52 | 43.40 |
| 9 | -5 | -4 | 766.02 | 42.10 |
| 9 | -5 | -4 | 712.23 | 42.40 |
| 9 | 5 | -4 | 741.83 | 41.50 |
| 9 | 5 | -4 | 752.72 | 42.80 |
| 9 | 5 | -4 | 710.23 | 45.30 |
| 8 | -5 | -4 | 128.89 | 13.20 |
| 8 | -5 | -4 | 127.29 | 14.60 |
| 8 | -5 | -4 | 119.49 | 10.30 |
| 8 | 5 | -4 | 117.19 | 10.40 |
| 8 | 5 | -4 | 135.89 | 16.20 |
| 7 | -5 | -4 | 39.40 | 8.00 |
| 7 | 5 | -4 | 40.90 | 6.50 |
| 6 | -5 | -4 | 36.00 | 6.00 |
| -6 | 5 | 4 | 32.40 | 6.10 |
| 6 | 5 | -4 | 34.40 | 5.90 |
| 5 | -5 | -4 | 1169.78 | 67.89 |
| 5 | 5 | -4 | 1166.28 | 67.39 |
| -5 | 5 | 4 | 1326.57 | 66.79 |
| -4 | 5 | 4 | 34.00 | 4.80 |
| 4 | 5 | -4 | 34.60 | 5.70 |
| -3 | 5 | 4 | 1711.73 | 93.09 |
| -2 | 5 | 4 | 6.80 | 2.50 |
| -1 | 5 | 4 | 6335.37 | 340.57 |
| 0 | 5 | 4 | 85.29 | 6.90 |
| 1 | 5 | 4 | 1007.70 | 55.79 |

| | | | | |
|---|---|---|---|---|
| 2 | 5 | 4 | 365.16 | 21.80 |
| 3 | 5 | 4 | 404.56 | 24.20 |
| 4 | 5 | 4 | 2.40 | 2.90 |
| 4 | 5 | 4 | 9.10 | 4.20 |
| 5 | -5 | 4 | 3107.09 | 173.58 |
| 5 | 5 | 4 | 3042.50 | 174.08 |
| 5 | 5 | 4 | 3448.25 | 172.68 |
| 6 | -5 | 4 | 86.99 | 10.70 |
| 6 | 5 | 4 | 88.59 | 7.20 |
| 6 | 5 | 4 | 66.79 | 12.20 |
| 7 | -5 | 4 | 33.70 | 5.80 |
| 7 | -5 | 4 | 44.60 | 9.60 |
| 7 | 5 | 4 | 28.30 | 5.60 |
| 7 | 5 | 4 | 32.90 | 5.20 |
| 7 | 5 | 4 | 22.10 | 6.90 |
| 8 | -5 | 4 | 22.40 | 5.10 |
| 8 | -5 | 4 | 35.20 | 5.60 |
| 8 | -5 | 4 | 25.50 | 8.30 |
| 8 | 5 | 4 | 18.90 | 3.70 |
| 8 | 5 | 4 | 35.20 | 7.90 |
| 8 | 5 | 4 | 11.90 | 7.40 |
| 9 | -5 | 4 | 1001.60 | 54.79 |
| 9 | -5 | 4 | 964.40 | 53.59 |
| 9 | -5 | 4 | 942.31 | 54.19 |
| 9 | 5 | 4 | 929.31 | 53.59 |
| 9 | 5 | 4 | 968.80 | 53.69 |
| 10 | -5 | 4 | -3.10 | 5.90 |
| 10 | -5 | 4 | -2.90 | 3.80 |
| 10 | -5 | 4 | -0.70 | 4.20 |
| 10 | 5 | 4 | -3.20 | 3.20 |
| 10 | 5 | 4 | 6.30 | 4.50 |
| 11 | -5 | 4 | 552.14 | 36.00 |
| 11 | -5 | 4 | 601.34 | 35.40 |
| 11 | -5 | 4 | 685.43 | 35.40 |
| 11 | 5 | 4 | 573.64 | 35.30 |
| 11 | 5 | 4 | 644.34 | 35.30 |
| 12 | -5 | 4 | 35.60 | 6.00 |
| 12 | -5 | 4 | 25.40 | 5.60 |
| 12 | -5 | 4 | 36.40 | 7.10 |
| 12 | 5 | 4 | 31.20 | 6.60 |
| 12 | 5 | 4 | 35.20 | 6.80 |
| 13 | -5 | 4 | 333.47 | 20.30 |
| 13 | -5 | 4 | 316.47 | 23.70 |
| 13 | 5 | 4 | 338.87 | 21.40 |

| | | | | |
|---|---|---|---|---|
| 13 | 5 | 4 | 352.26 | 20.50 |
| 14 | -5 | 4 | 0.20 | 3.20 |
| 14 | -5 | 4 | -2.20 | 3.00 |
| 14 | -5 | 4 | -3.10 | 2.40 |
| 14 | 5 | 4 | -2.00 | 2.60 |
| 14 | 5 | 4 | -3.20 | 3.10 |
| 15 | -5 | 4 | 272.57 | 15.00 |
| 15 | -5 | 4 | 238.58 | 14.60 |
| 15 | -5 | 4 | 242.68 | 14.70 |
| 15 | 5 | 4 | 243.88 | 14.70 |
| 15 | 5 | 4 | 230.28 | 14.80 |
| 16 | -6 | -4 | 305.97 | 18.20 |
| 16 | -6 | -4 | 308.27 | 17.70 |
| 16 | 6 | -4 | 317.47 | 17.50 |
| 15 | -6 | -4 | -2.40 | 2.10 |
| 15 | -6 | -4 | -2.10 | 2.90 |
| 15 | 6 | -4 | -2.50 | 1.60 |
| 14 | -6 | -4 | 229.08 | 13.80 |
| 14 | -6 | -4 | 212.68 | 14.40 |
| 14 | 6 | -4 | 217.28 | 13.30 |
| 13 | -6 | -4 | 8.00 | 3.80 |
| 13 | -6 | -4 | 9.20 | 3.40 |
| 12 | -6 | -4 | 196.88 | 13.70 |
| 12 | -6 | -4 | 190.68 | 13.80 |
| 12 | 6 | -4 | 206.48 | 12.70 |
| 12 | 6 | -4 | 205.78 | 16.60 |
| 11 | -6 | -4 | 40.40 | 6.70 |
| 11 | -6 | -4 | 39.50 | 5.60 |
| 11 | 6 | -4 | 29.90 | 5.60 |
| 11 | 6 | -4 | 35.90 | 6.60 |
| 11 | 6 | -4 | 20.70 | 8.10 |
| 10 | -6 | -4 | 1312.97 | 71.99 |
| 10 | -6 | -4 | 1316.57 | 72.09 |
| 10 | 6 | -4 | 1224.48 | 72.09 |
| 10 | 6 | -4 | 1357.96 | 71.49 |
| 9 | -6 | -4 | 3.50 | 5.40 |
| 9 | -6 | -4 | -2.20 | 3.50 |
| 9 | 6 | -4 | 9.00 | 3.60 |
| 9 | 6 | -4 | 9.50 | 8.30 |
| 8 | -6 | -4 | 220.18 | 15.20 |
| 8 | -6 | -4 | 190.78 | 16.50 |
| 8 | 6 | -4 | 210.38 | 14.60 |
| 8 | 6 | -4 | 235.18 | 15.00 |
| 7 | -6 | -4 | 142.09 | 12.10 |

| | | | | |
|---|---|---|---|---|
| 7 | 6 | -4 | 152.68 | 11.30 |
| 7 | 6 | -4 | 141.69 | 12.70 |
| 6 | -6 | -4 | 6769.22 | 359.46 |
| -6 | 6 | 4 | 6580.74 | 358.46 |
| 6 | 6 | -4 | 6668.83 | 359.26 |
| 5 | -6 | -4 | 7.50 | 4.90 |
| -5 | 6 | 4 | 1.70 | 2.70 |
| 5 | 6 | -4 | -1.80 | 3.20 |
| 4 | -6 | -4 | 651.83 | 40.10 |
| 4 | 6 | -4 | 745.23 | 40.10 |
| -3 | 6 | 4 | 65.19 | 6.00 |
| 3 | 6 | -4 | 52.49 | 6.90 |
| -2 | 6 | 4 | 83.19 | 6.90 |
| -1 | 6 | 4 | 3.90 | 2.50 |
| 0 | 6 | 4 | 3735.83 | 201.78 |
| 1 | 6 | 4 | 117.09 | 8.90 |
| 2 | 6 | 4 | 640.34 | 36.60 |
| 3 | 6 | 4 | 1.00 | 3.40 |
| 3 | 6 | 4 | -1.40 | 2.60 |
| 4 | -6 | 4 | 1916.61 | 102.49 |
| 4 | 6 | 4 | 1870.01 | 104.39 |
| 4 | 6 | 4 | 1843.42 | 101.79 |
| 5 | -6 | 4 | 13.60 | 5.30 |
| 5 | 6 | 4 | 13.50 | 5.20 |
| 5 | 6 | 4 | 16.20 | 3.10 |
| 6 | -6 | 4 | 2063.19 | 115.09 |
| 6 | 6 | 4 | 2284.57 | 114.19 |
| 6 | 6 | 4 | 1908.01 | 115.79 |
| -6 | 6 | -4 | 2157.88 | 114.29 |
| 7 | -6 | 4 | 22.70 | 5.20 |
| 7 | -6 | 4 | 33.00 | 9.50 |
| 7 | -6 | 4 | 19.30 | 5.30 |
| 7 | 6 | 4 | 17.40 | 6.80 |
| 7 | 6 | 4 | 23.20 | 5.50 |
| 8 | -6 | 4 | 647.74 | 41.10 |
| 8 | -6 | 4 | 669.93 | 39.10 |
| 8 | -6 | 4 | 736.53 | 40.10 |
| 8 | 6 | 4 | 666.83 | 38.70 |
| 8 | 6 | 4 | 700.33 | 39.20 |
| 8 | 6 | 4 | 706.23 | 41.50 |
| 9 | -6 | 4 | -2.60 | 3.90 |
| 9 | -6 | 4 | -0.30 | 7.20 |
| 9 | 6 | 4 | -1.50 | 5.90 |
| 9 | 6 | 4 | 1.40 | 2.90 |

| | | | | |
|---|---|---|---|---|
| 10 | -6 | 4 | 1104.19 | 60.49 |
| 10 | -6 | 4 | 1004.30 | 61.39 |
| 10 | -6 | 4 | 1095.69 | 60.99 |
| 10 | 6 | 4 | 1182.08 | 60.79 |
| 10 | 6 | 4 | 1056.09 | 60.49 |
| 11 | -6 | 4 | 16.00 | 4.40 |
| 11 | -6 | 4 | 15.50 | 4.10 |
| 11 | -6 | 4 | 14.70 | 5.70 |
| 11 | 6 | 4 | 22.50 | 4.60 |
| 11 | 6 | 4 | 15.90 | 4.40 |
| 12 | -6 | 4 | 5.90 | 3.40 |
| 12 | -6 | 4 | 2.70 | 3.70 |
| 12 | -6 | 4 | 3.20 | 4.70 |
| 12 | 6 | 4 | -0.30 | 3.60 |
| 12 | 6 | 4 | -1.90 | 3.30 |
| 13 | -6 | 4 | 36.80 | 7.00 |
| 13 | -6 | 4 | 21.30 | 4.90 |
| 13 | -6 | 4 | 27.80 | 5.40 |
| 13 | 6 | 4 | 31.10 | 5.50 |
| 13 | 6 | 4 | 31.30 | 6.90 |
| 14 | -6 | 4 | 378.66 | 21.20 |
| 14 | -6 | 4 | 395.16 | 21.50 |
| 14 | -6 | 4 | 358.26 | 21.00 |
| 14 | 6 | 4 | 358.76 | 21.20 |
| 14 | 6 | 4 | 322.57 | 21.30 |
| 15 | -6 | 4 | 12.20 | 4.60 |
| 15 | -6 | 4 | 4.90 | 2.50 |
| 15 | 6 | 4 | 0.60 | 2.20 |
| 15 | 6 | 4 | 0.90 | 2.60 |
| 16 | -7 | -4 | -1.90 | 1.60 |
| 16 | -7 | -4 | -4.10 | 3.10 |
| 16 | 7 | -4 | -0.90 | 1.40 |
| 15 | -7 | -4 | 406.86 | 23.20 |
| 15 | -7 | -4 | 390.56 | 23.60 |
| 15 | 7 | -4 | 432.46 | 23.00 |
| 14 | -7 | -4 | -1.20 | 3.00 |
| 14 | -7 | -4 | 3.30 | 3.00 |
| 14 | 7 | -4 | -1.20 | 2.10 |
| 13 | -7 | -4 | 0.10 | 3.40 |
| 13 | -7 | -4 | 10.30 | 3.10 |
| 13 | 7 | -4 | 6.00 | 2.20 |
| 12 | -7 | -4 | 30.30 | 4.70 |
| 12 | -7 | -4 | 33.20 | 6.70 |
| 12 | 7 | -4 | 40.10 | 5.10 |

| | | | | |
|---|---|---|---|---|
| 11 | -7 | -4 | 1058.19 | 58.39 |
| 11 | -7 | -4 | 1029.70 | 58.39 |
| 11 | 7 | -4 | 1056.19 | 57.59 |
| 10 | -7 | -4 | 57.59 | 9.60 |
| 10 | -7 | -4 | 61.39 | 7.60 |
| 10 | 7 | -4 | 71.79 | 6.90 |
| 9 | -7 | -4 | 475.55 | 28.40 |
| 9 | -7 | -4 | 462.25 | 29.20 |
| 9 | 7 | -4 | 504.65 | 27.90 |
| 8 | -7 | -4 | 91.59 | 9.20 |
| 8 | -7 | -4 | 73.99 | 12.00 |
| 8 | 7 | -4 | 72.19 | 8.90 |
| 8 | 7 | -4 | 95.69 | 10.20 |
| 7 | -7 | -4 | 225.98 | 18.50 |
| 7 | -7 | -4 | 253.57 | 17.60 |
| 7 | 7 | -4 | 254.67 | 16.90 |
| 6 | -7 | -4 | 2.10 | 4.70 |
| -6 | 7 | 4 | -0.30 | 3.80 |
| 6 | 7 | -4 | 5.30 | 4.00 |
| 5 | -7 | -4 | 4471.05 | 259.27 |
| -5 | 7 | 4 | 5064.39 | 258.67 |
| 5 | 7 | -4 | 4867.11 | 262.97 |
| 4 | -7 | -4 | 55.19 | 9.00 |
| 4 | 7 | -4 | 53.69 | 8.20 |
| -4 | 7 | 4 | 70.49 | 6.70 |
| 3 | 7 | -4 | 178.48 | 13.50 |
| -3 | 7 | 4 | 198.78 | 12.60 |
| -2 | 7 | 4 | 232.48 | 14.20 |
| 2 | 7 | -4 | 206.48 | 14.90 |
| -1 | 7 | 4 | 4956.50 | 268.47 |
| 1 | 7 | -4 | 5004.10 | 268.77 |
| 0 | 7 | -4 | 185.88 | 12.20 |
| 0 | 7 | 4 | 163.98 | 11.80 |
| 2 | 7 | 4 | 83.59 | 7.20 |
| -3 | 7 | -4 | 991.70 | 53.49 |
| 3 | 7 | 4 | 913.61 | 54.19 |
| 3 | 7 | 4 | 984.00 | 52.99 |
| 4 | -7 | 4 | 149.59 | 11.50 |
| 4 | 7 | 4 | 129.99 | 9.40 |
| -4 | 7 | -4 | 121.49 | 11.90 |
| 4 | 7 | 4 | 137.29 | 11.40 |
| 5 | -7 | 4 | 2501.65 | 130.49 |
| 5 | 7 | 4 | 2308.47 | 129.59 |
| 5 | 7 | 4 | 2273.37 | 131.49 |

| | | | | |
|---|---|---|---|---|
| -5 | 7 | -4 | 2500.55 | 130.19 |
| 6 | -7 | 4 | -1.20 | 4.70 |
| 6 | -7 | 4 | 3.30 | 4.20 |
| -6 | 7 | -4 | 0.10 | 3.60 |
| 6 | 7 | 4 | 5.70 | 2.90 |
| 6 | 7 | 4 | 12.50 | 5.80 |
| 6 | 7 | 4 | 4.10 | 5.90 |
| 7 | -7 | 4 | 45.00 | 8.00 |
| 7 | -7 | 4 | 37.50 | 6.40 |
| 7 | 7 | 4 | 29.30 | 5.10 |
| 7 | 7 | 4 | 41.20 | 8.00 |
| 7 | 7 | 4 | 35.80 | 6.00 |
| 8 | -7 | 4 | 36.40 | 9.80 |
| 8 | -7 | 4 | 36.00 | 7.30 |
| 8 | -7 | 4 | 38.90 | 6.40 |
| 8 | 7 | 4 | 36.50 | 5.90 |
| 8 | 7 | 4 | 26.30 | 8.60 |
| 8 | 7 | 4 | 35.20 | 5.30 |
| 9 | -7 | 4 | 848.42 | 47.60 |
| 9 | -7 | 4 | 857.41 | 48.50 |
| 9 | 7 | 4 | 842.62 | 47.70 |
| 9 | 7 | 4 | 846.12 | 47.50 |
| 10 | -7 | 4 | 5.60 | 4.30 |
| 10 | -7 | 4 | 5.20 | 6.40 |
| 10 | -7 | 4 | 4.50 | 3.70 |
| 10 | 7 | 4 | 2.30 | 4.60 |
| 10 | 7 | 4 | 4.60 | 3.60 |
| 11 | -7 | 4 | 637.74 | 36.10 |
| 11 | -7 | 4 | 665.13 | 35.60 |
| 11 | -7 | 4 | 613.24 | 36.50 |
| 11 | 7 | 4 | 605.44 | 35.60 |
| 11 | 7 | 4 | 574.74 | 35.60 |
| 12 | -7 | 4 | 4.60 | 3.50 |
| 12 | -7 | 4 | 4.70 | 5.10 |
| 12 | -7 | 4 | 3.00 | 3.10 |
| 12 | 7 | 4 | 6.90 | 3.90 |
| 12 | 7 | 4 | 2.30 | 3.70 |
| 13 | -7 | 4 | 213.88 | 14.10 |
| 13 | -7 | 4 | 220.08 | 14.10 |
| 13 | -7 | 4 | 223.38 | 14.60 |
| 13 | 7 | 4 | 208.58 | 14.50 |
| 13 | 7 | 4 | 221.18 | 14.10 |
| 14 | -7 | 4 | 1.80 | 2.70 |
| 14 | -7 | 4 | 3.20 | 2.50 |

| | | | | |
|---|---|---|---|---|
| 14 | 7 | 4 | 2.10 | 3.30 |
| 14 | 7 | 4 | 4.70 | 2.70 |
| 15 | -8 | -4 | 3.00 | 1.90 |
| 15 | -8 | -4 | -3.00 | 3.90 |
| 15 | 8 | -4 | 1.80 | 1.50 |
| 14 | -8 | -4 | 403.86 | 23.70 |
| 14 | -8 | -4 | 400.76 | 23.20 |
| 14 | 8 | -4 | 409.86 | 22.80 |
| 13 | -8 | -4 | 101.79 | 8.60 |
| 13 | -8 | -4 | 101.29 | 8.30 |
| 13 | 8 | -4 | 103.49 | 7.40 |
| 12 | -8 | -4 | 265.77 | 16.70 |
| 12 | -8 | -4 | 268.07 | 17.10 |
| 12 | 8 | -4 | 255.87 | 15.80 |
| 11 | -8 | -4 | 55.69 | 7.00 |
| 11 | -8 | -4 | 59.29 | 8.00 |
| 11 | 8 | -4 | 51.19 | 5.90 |
| 10 | -8 | -4 | 1654.53 | 88.59 |
| 10 | -8 | -4 | 1529.55 | 88.39 |
| 10 | 8 | -4 | 1644.44 | 87.69 |
| 9 | -8 | -4 | -1.50 | 3.30 |
| 9 | -8 | -4 | -1.90 | 4.80 |
| 9 | 8 | -4 | -2.20 | 3.20 |
| 8 | -8 | -4 | 245.18 | 17.90 |
| 8 | -8 | -4 | 250.87 | 16.50 |
| 8 | 8 | -4 | 237.98 | 17.20 |
| 8 | 8 | -4 | 228.78 | 15.90 |
| 7 | -8 | -4 | 295.77 | 21.40 |
| 7 | -8 | -4 | 327.47 | 20.60 |
| 7 | 8 | -4 | 314.77 | 20.50 |
| 6 | -8 | -4 | 2115.49 | 117.89 |
| -6 | 8 | 4 | 2274.47 | 116.99 |
| 6 | 8 | -4 | 2048.70 | 117.69 |
| 5 | -8 | -4 | 17.40 | 5.60 |
| -5 | 8 | 4 | 14.10 | 4.30 |
| 5 | 8 | -4 | 9.30 | 4.50 |
| -4 | 8 | 4 | 1261.97 | 63.29 |
| 4 | 8 | -4 | 1018.60 | 63.89 |
| 3 | 8 | -4 | 8.40 | 4.30 |
| -3 | 8 | 4 | 3.90 | 3.00 |
| 2 | 8 | -4 | 718.63 | 41.40 |
| -1 | 8 | 4 | 452.05 | 26.40 |
| 1 | 8 | -4 | 441.16 | 26.70 |
| 0 | 8 | 4 | 5726.73 | 306.07 |

| | | | | |
|---|---|---|---|---|
| 0 | 8 | 4 | 5408.06 | 305.17 |
| 0 | 8 | -4 | 5895.31 | 306.17 |
| 1 | 8 | 4 | 32.10 | 5.60 |
| 1 | 8 | 4 | 37.10 | 5.40 |
| -1 | 8 | -4 | 41.30 | 5.70 |
| 2 | 8 | 4 | 63.39 | 7.10 |
| -2 | 8 | -4 | 76.49 | 6.90 |
| 2 | 8 | 4 | 60.09 | 6.40 |
| 3 | -8 | 4 | 83.19 | 9.50 |
| -3 | 8 | -4 | 80.59 | 7.40 |
| 3 | 8 | 4 | 79.99 | 7.00 |
| 3 | 8 | 4 | 87.09 | 8.60 |
| 4 | -8 | 4 | 1231.88 | 67.69 |
| -4 | 8 | -4 | 1259.57 | 67.69 |
| 4 | 8 | 4 | 1170.58 | 67.09 |
| 4 | 8 | 4 | 1243.68 | 68.99 |
| -5 | -8 | -4 | 53.69 | 10.30 |
| 5 | -8 | 4 | 63.29 | 8.50 |
| 5 | -8 | 4 | 79.69 | 8.90 |
| -5 | 8 | -4 | 72.99 | 7.40 |
| 5 | 8 | 4 | 62.09 | 10.10 |
| 5 | 8 | 4 | 68.09 | 6.30 |
| 6 | -8 | 4 | 1637.94 | 87.09 |
| 6 | -8 | 4 | 1603.94 | 87.79 |
| -6 | -8 | -4 | 1546.25 | 87.39 |
| 6 | 8 | 4 | 1515.45 | 89.19 |
| -6 | 8 | -4 | 1749.53 | 87.49 |
| 6 | 8 | 4 | 1489.55 | 87.19 |
| 6 | 8 | 4 | 1605.84 | 86.89 |
| 7 | -8 | 4 | 173.98 | 13.70 |
| 7 | -8 | 4 | 181.08 | 12.40 |
| -7 | -8 | -4 | 154.18 | 14.00 |
| 7 | 8 | 4 | 167.18 | 16.40 |
| 7 | 8 | 4 | 147.59 | 13.80 |
| 7 | 8 | 4 | 183.68 | 11.40 |
| 8 | -8 | 4 | 248.78 | 18.60 |
| 8 | -8 | 4 | 292.27 | 18.40 |
| 8 | 8 | 4 | 263.67 | 18.30 |
| 8 | 8 | 4 | 282.47 | 22.80 |
| 8 | 8 | 4 | 294.97 | 17.10 |
| 9 | -8 | 4 | 55.99 | 8.70 |
| 9 | -8 | 4 | 52.29 | 8.00 |
| 9 | 8 | 4 | 68.99 | 9.20 |
| 9 | 8 | 4 | 66.99 | 6.70 |

| | | | | |
|---|---|---|---|---|
| 10 | -8 | 4 | 703.93 | 37.70 |
| 10 | -8 | 4 | 704.63 | 38.60 |
| 10 | -8 | 4 | 622.34 | 39.10 |
| 10 | 8 | 4 | 664.33 | 37.70 |
| 10 | 8 | 4 | 610.04 | 37.50 |
| 11 | -8 | 4 | -4.80 | 3.40 |
| 11 | -8 | 4 | 0.40 | 5.90 |
| 11 | -8 | 4 | -1.70 | 3.20 |
| 11 | 8 | 4 | 9.90 | 3.90 |
| 11 | 8 | 4 | -0.60 | 4.20 |
| 12 | -8 | 4 | 61.39 | 11.90 |
| 12 | -8 | 4 | 66.69 | 7.20 |
| 12 | -8 | 4 | 60.39 | 9.70 |
| 12 | 8 | 4 | 53.19 | 6.80 |
| 12 | 8 | 4 | 75.19 | 9.00 |
| 13 | -8 | 4 | 25.80 | 5.20 |
| 13 | 8 | 4 | 25.60 | 5.20 |
| 13 | 8 | 4 | 17.40 | 4.30 |
| 14 | -8 | 4 | 240.08 | 14.70 |
| 14 | -8 | 4 | 260.07 | 14.60 |
| 14 | 8 | 4 | 236.78 | 14.60 |
| 14 | 8 | 4 | 235.88 | 15.10 |
| 15 | -9 | -4 | 386.06 | 22.20 |
| -15 | 9 | 4 | 402.16 | 22.40 |
| 15 | 9 | -4 | 399.46 | 22.00 |
| 14 | -9 | -4 | 3.20 | 2.90 |
| 14 | -9 | -4 | -0.20 | 2.20 |
| -14 | 9 | 4 | -1.40 | 2.40 |
| 14 | 9 | -4 | 1.20 | 1.60 |
| 13 | -9 | -4 | 57.49 | 6.20 |
| 13 | 9 | -4 | 48.50 | 6.30 |
| 12 | -9 | -4 | 73.29 | 7.70 |
| 12 | -9 | -4 | 65.49 | 7.50 |
| 12 | 9 | -4 | 80.49 | 6.40 |
| 11 | -9 | -4 | 895.31 | 49.70 |
| 11 | -9 | -4 | 881.91 | 49.90 |
| 11 | 9 | -4 | 878.21 | 48.80 |
| 10 | -9 | -4 | 19.30 | 4.80 |
| 10 | -9 | -4 | 20.00 | 4.10 |
| 10 | 9 | -4 | 35.70 | 5.40 |
| 9 | -9 | -4 | 590.84 | 33.40 |
| 9 | -9 | -4 | 546.55 | 33.70 |
| 9 | 9 | -4 | 570.04 | 32.50 |
| 8 | -9 | -4 | 398.96 | 23.00 |

| | | | | |
|---|---|---|---|---|
| 8 | -9 | -4 | 352.16 | 23.40 |
| 8 | 9 | -4 | 347.37 | 23.50 |
| 8 | 9 | -4 | 346.77 | 21.80 |
| 7 | -9 | -4 | 329.97 | 20.10 |
| -7 | -9 | 4 | 277.77 | 20.40 |
| 7 | -9 | -4 | 264.37 | 20.50 |
| 7 | 9 | -4 | 311.17 | 20.20 |
| 6 | -9 | -4 | 160.38 | 15.00 |
| -6 | -9 | 4 | 138.89 | 16.20 |
| 6 | -9 | -4 | 159.08 | 13.20 |
| 6 | 9 | -4 | 146.09 | 12.80 |
| -6 | 9 | 4 | 122.09 | 20.00 |
| -6 | 9 | 4 | 170.28 | 12.60 |
| -5 | -9 | 4 | 2551.44 | 142.89 |
| -5 | 9 | 4 | 2887.81 | 143.19 |
| 5 | 9 | -4 | 2455.85 | 144.09 |
| -4 | -9 | 4 | 6.50 | 4.40 |
| -4 | 9 | 4 | 0.10 | 2.90 |
| 4 | 9 | -4 | 3.60 | 3.80 |
| -3 | 9 | 4 | 78.19 | 7.30 |
| 3 | 9 | -4 | 56.99 | 8.30 |
| -2 | 9 | 4 | 538.15 | 30.50 |
| 2 | 9 | -4 | 505.95 | 31.20 |
| 1 | 9 | -4 | 2041.70 | 109.89 |
| -1 | 9 | 4 | 1957.60 | 109.39 |
| -1 | 9 | 4 | 2000.50 | 108.79 |
| 0 | 9 | 4 | 227.48 | 14.10 |
| 0 | 9 | -4 | 212.38 | 14.80 |
| 0 | 9 | 4 | 221.68 | 14.70 |
| 1 | 9 | 4 | 793.32 | 47.50 |
| -1 | 9 | -4 | 863.01 | 47.40 |
| 1 | 9 | 4 | 861.51 | 46.70 |
| 2 | -9 | 4 | 79.49 | 9.80 |
| 2 | 9 | 4 | 77.99 | 7.20 |
| -2 | 9 | -4 | 84.39 | 7.60 |
| 2 | 9 | 4 | 88.69 | 8.30 |
| -3 | -9 | -4 | 268.47 | 17.30 |
| 3 | -9 | 4 | 252.87 | 17.30 |
| 3 | -9 | 4 | 267.67 | 17.50 |
| 3 | 9 | 4 | 266.57 | 16.30 |
| -3 | 9 | -4 | 282.87 | 17.00 |
| 3 | 9 | 4 | 257.17 | 17.80 |
| 4 | -9 | 4 | 461.15 | 26.70 |
| -4 | -9 | -4 | 427.36 | 27.10 |

| | | | | |
|---|---|---|---|---|
| 4 | -9 | 4 | 463.75 | 27.00 |
| 4 | 9 | 4 | 437.26 | 28.30 |
| 4 | 9 | 4 | 467.15 | 26.20 |
| -4 | 9 | -4 | 474.95 | 27.00 |
| 5 | -9 | 4 | 2594.24 | 135.49 |
| -5 | -9 | -4 | 2514.65 | 135.29 |
| 5 | -9 | 4 | 2614.74 | 135.09 |
| -5 | 9 | -4 | 2539.05 | 135.59 |
| 5 | 9 | 4 | 2402.06 | 137.19 |
| 5 | 9 | 4 | 2282.37 | 134.79 |
| 6 | -9 | 4 | 26.30 | 4.50 |
| 6 | -9 | 4 | 23.80 | 5.10 |
| -6 | -9 | -4 | 41.10 | 5.90 |
| -6 | 9 | -4 | 39.60 | 7.70 |
| 6 | 9 | 4 | 36.20 | 7.70 |
| 6 | 9 | 4 | 43.40 | 5.10 |
| 7 | -9 | 4 | 10.80 | 5.10 |
| 7 | -9 | 4 | 6.00 | 3.80 |
| -7 | -9 | -4 | 14.90 | 4.90 |
| 7 | 9 | 4 | 6.30 | 2.90 |
| 7 | 9 | 4 | 18.40 | 6.00 |
| 7 | 9 | 4 | 7.90 | 7.70 |
| 8 | -9 | 4 | 0.00 | 3.40 |
| 8 | -9 | 4 | 4.50 | 4.60 |
| 8 | 9 | 4 | 2.60 | 5.40 |
| 8 | 9 | 4 | -4.70 | 9.60 |
| 8 | 9 | 4 | 3.70 | 2.70 |
| 9 | -9 | 4 | 298.97 | 19.60 |
| 9 | -9 | 4 | 297.47 | 18.30 |
| 9 | 9 | 4 | 279.07 | 19.30 |
| 9 | 9 | 4 | 296.47 | 18.00 |
| 10 | -9 | 4 | -0.70 | 3.30 |
| 10 | -9 | 4 | -4.80 | 4.80 |
| 10 | 9 | 4 | -0.40 | 2.90 |
| 10 | 9 | 4 | -6.10 | 5.00 |
| 11 | -9 | 4 | 496.95 | 27.60 |
| 11 | -9 | 4 | 490.65 | 28.10 |
| 11 | 9 | 4 | 441.06 | 28.00 |
| 11 | 9 | 4 | 463.05 | 27.50 |
| 12 | -9 | 4 | 46.10 | 6.60 |
| 12 | -9 | 4 | 45.30 | 6.30 |
| 12 | 9 | 4 | 45.30 | 8.50 |
| 12 | 9 | 4 | 47.90 | 6.20 |
| 13 | -9 | 4 | 139.09 | 9.50 |

| | | | | |
|---|---|---|---|---|
| 13 | -9 | 4 | 144.69 | 9.80 |
| 13 | 9 | 4 | 130.69 | 11.10 |
| 13 | 9 | 4 | 135.49 | 9.70 |
| 14 | -9 | 4 | 1.40 | 2.90 |
| 14 | -9 | 4 | 2.00 | 2.30 |
| 14 | 9 | 4 | 1.00 | 2.50 |
| 15 | -10 | -4 | 2.00 | 1.60 |
| -15 | 10 | 4 | 2.10 | 2.60 |
| 15 | 10 | -4 | -0.40 | 1.30 |
| 14 | -10 | -4 | 341.37 | 19.90 |
| 14 | 10 | -4 | 347.97 | 19.60 |
| -14 | 10 | 4 | 356.76 | 20.10 |
| 13 | -10 | -4 | 53.99 | 6.20 |
| 13 | -10 | -4 | 56.09 | 6.00 |
| -13 | 10 | 4 | 59.99 | 6.50 |
| 13 | 10 | -4 | 59.89 | 5.00 |
| 12 | -10 | -4 | 127.19 | 10.00 |
| 12 | -10 | -4 | 136.89 | 10.00 |
| 12 | 10 | -4 | 144.69 | 9.00 |
| -12 | 10 | 4 | 131.49 | 10.60 |
| 11 | -10 | -4 | 0.80 | 3.00 |
| 11 | -10 | -4 | 3.10 | 3.60 |
| 11 | 10 | -4 | -0.10 | 2.20 |
| 10 | -10 | -4 | 878.01 | 50.39 |
| 10 | -10 | -4 | 911.41 | 50.29 |
| 10 | 10 | -4 | 889.11 | 49.40 |
| 9 | -10 | -4 | 21.10 | 5.30 |
| 9 | -10 | -4 | 28.80 | 4.90 |
| 9 | 10 | -4 | 15.60 | 3.60 |
| 8 | -10 | -4 | 236.28 | 18.10 |
| 8 | -10 | -4 | 276.27 | 17.40 |
| 8 | 10 | -4 | 247.88 | 19.20 |
| 8 | 10 | -4 | 240.58 | 16.30 |
| -8 | 10 | 4 | 251.77 | 20.00 |
| -7 | -10 | 4 | 223.88 | 16.80 |
| 7 | -10 | -4 | 234.48 | 17.70 |
| 7 | -10 | -4 | 236.48 | 17.10 |
| -7 | 10 | 4 | 239.08 | 23.10 |
| 7 | 10 | -4 | 249.68 | 17.90 |
| -6 | -10 | 4 | 1653.53 | 99.59 |
| 6 | -10 | -4 | 1772.82 | 100.19 |
| 6 | 10 | -4 | 1864.91 | 101.59 |
| -6 | 10 | 4 | 2021.70 | 100.69 |
| -5 | -10 | 4 | 18.70 | 4.80 |

| | | | | |
|---|---|---|---|---|
| 5 | -10 | -4 | 18.10 | 5.80 |
| 5 | 10 | -4 | 16.40 | 5.20 |
| -5 | 10 | 4 | 21.80 | 5.00 |
| -4 | -10 | 4 | 840.02 | 43.80 |
| -4 | -10 | 4 | 772.52 | 43.80 |
| -4 | 10 | 4 | 814.02 | 44.10 |
| 4 | 10 | -4 | 668.53 | 45.10 |
| -3 | -10 | 4 | 252.27 | 15.50 |
| -3 | -10 | 4 | 211.18 | 15.70 |
| 3 | 10 | -4 | 222.38 | 16.70 |
| -3 | 10 | 4 | 233.38 | 15.40 |
| -2 | -10 | 4 | 52.69 | 7.80 |
| 2 | -10 | -4 | 40.80 | 8.00 |
| -2 | -10 | 4 | 47.30 | 8.40 |
| -2 | 10 | 4 | 59.69 | 6.80 |
| 2 | 10 | -4 | 42.20 | 7.50 |
| 1 | -10 | -4 | 290.47 | 19.80 |
| -1 | -10 | 4 | 269.97 | 18.90 |
| -1 | -10 | 4 | 293.77 | 18.20 |
| 1 | 10 | -4 | 282.57 | 19.10 |
| -1 | 10 | 4 | 300.27 | 18.70 |
| -1 | 10 | 4 | 303.97 | 18.00 |
| 0 | -10 | 4 | 2646.84 | 148.19 |
| 0 | -10 | -4 | 2681.23 | 148.89 |
| 0 | -10 | 4 | 2712.43 | 148.69 |
| 0 | 10 | 4 | 2691.13 | 149.29 |
| 0 | 10 | -4 | 2893.41 | 149.59 |
| 0 | 10 | -4 | 2672.53 | 147.99 |
| 0 | 10 | 4 | 2879.81 | 148.49 |
| 1 | -10 | 4 | 355.16 | 22.50 |
| 1 | -10 | 4 | 375.86 | 23.90 |
| -1 | -10 | -4 | 377.66 | 23.40 |
| 1 | 10 | 4 | 389.26 | 23.40 |
| -1 | 10 | -4 | 397.36 | 23.20 |
| 1 | 10 | 4 | 358.86 | 22.60 |
| 2 | -10 | 4 | 60.69 | 7.80 |
| 2 | -10 | 4 | 44.10 | 7.50 |
| -2 | -10 | -4 | 53.49 | 8.70 |
| 2 | 10 | 4 | 54.49 | 7.30 |
| 2 | 10 | 4 | 46.80 | 5.80 |
| -2 | 10 | -4 | 46.30 | 6.60 |
| 3 | -10 | 4 | 96.59 | 8.90 |
| 3 | -10 | 4 | 78.49 | 14.70 |
| -3 | -10 | -4 | 92.89 | 9.50 |

| | | | | |
|---|---|---|---|---|
| 3 | -10 | 4 | 75.29 | 9.20 |
| -3 | 10 | -4 | 91.79 | 8.30 |
| 3 | 10 | 4 | 82.49 | 7.30 |
| 3 | 10 | 4 | 85.59 | 9.50 |
| -4 | -10 | -4 | 1989.80 | 111.99 |
| 4 | -10 | 4 | 2113.19 | 111.79 |
| 4 | -10 | 4 | 2071.59 | 111.99 |
| 4 | -10 | 4 | 1969.90 | 113.59 |
| 4 | 10 | 4 | 2070.79 | 111.69 |
| 4 | 10 | 4 | 1996.70 | 113.79 |
| -4 | 10 | -4 | 2181.28 | 112.69 |
| 5 | -10 | 4 | 17.90 | 10.00 |
| -5 | -10 | -4 | 37.30 | 7.80 |
| 5 | -10 | 4 | 46.40 | 7.30 |
| 5 | -10 | 4 | 27.80 | 4.50 |
| 5 | 10 | 4 | 28.80 | 7.60 |
| -5 | 10 | -4 | 22.50 | 5.10 |
| 5 | 10 | 4 | 31.70 | 4.90 |
| 6 | -10 | 4 | 721.33 | 41.10 |
| -6 | -10 | -4 | 720.33 | 41.30 |
| 6 | -10 | 4 | 750.32 | 41.70 |
| -6 | 10 | -4 | 807.12 | 42.90 |
| 6 | 10 | 4 | 657.03 | 44.10 |
| 6 | 10 | 4 | 711.73 | 40.70 |
| 7 | -10 | 4 | 110.19 | 9.50 |
| 7 | -10 | 4 | 131.89 | 11.20 |
| -7 | -10 | -4 | 107.79 | 10.60 |
| 7 | 10 | 4 | 109.19 | 17.30 |
| 7 | 10 | 4 | 119.99 | 8.70 |
| 8 | -10 | 4 | 546.65 | 31.00 |
| -8 | -10 | -4 | 552.94 | 31.30 |
| 8 | -10 | 4 | 506.15 | 32.10 |
| 8 | 10 | 4 | 542.55 | 30.80 |
| 8 | 10 | 4 | 553.94 | 46.60 |
| 9 | -10 | 4 | 10.80 | 5.20 |
| 9 | -10 | 4 | 9.60 | 3.50 |
| 9 | 10 | 4 | 12.50 | 3.50 |
| 10 | -10 | 4 | 911.31 | 49.60 |
| 10 | -10 | 4 | 840.72 | 50.29 |
| 10 | 10 | 4 | 916.41 | 49.60 |
| 11 | -10 | 4 | 17.30 | 4.10 |
| 11 | -10 | 4 | 18.00 | 4.20 |
| 11 | 10 | 4 | 26.30 | 5.20 |
| 12 | -10 | 4 | 6.30 | 2.90 |

| | | | | |
|---|---|---|---|---|
| 12 | -10 | 4 | 7.50 | 3.40 |
| 12 | 10 | 4 | 9.30 | 3.40 |
| 13 | -10 | 4 | 5.90 | 2.90 |
| 13 | -10 | 4 | 4.10 | 2.50 |
| 13 | 10 | 4 | 7.80 | 3.30 |
| 14 | -11 | -4 | 6.90 | 2.10 |
| 14 | 11 | -4 | 8.80 | 1.70 |
| 13 | -11 | -4 | 10.80 | 2.80 |
| 13 | 11 | -4 | 16.80 | 3.00 |
| -13 | 11 | 4 | 15.20 | 3.60 |
| 12 | -11 | -4 | 69.69 | 7.50 |
| 12 | 11 | -4 | 72.69 | 6.10 |
| -12 | 11 | 4 | 74.89 | 8.20 |
| 11 | -11 | -4 | 612.84 | 35.10 |
| -11 | 11 | 4 | 617.04 | 35.20 |
| 11 | 11 | -4 | 618.84 | 34.30 |
| 10 | -11 | -4 | 1.90 | 3.90 |
| -10 | 11 | 4 | 9.30 | 5.20 |
| 10 | 11 | -4 | 2.10 | 2.50 |
| 9 | -11 | -4 | 476.65 | 29.70 |
| 9 | 11 | -4 | 488.15 | 28.50 |
| -9 | 11 | 4 | 524.15 | 31.50 |
| -8 | -11 | 4 | 48.30 | 6.60 |
| 8 | -11 | -4 | 31.80 | 5.90 |
| 8 | 11 | -4 | 46.60 | 7.10 |
| 8 | 11 | -4 | 33.80 | 11.90 |
| 7 | -11 | -4 | 175.78 | 15.20 |
| -7 | -11 | 4 | 137.79 | 14.30 |
| 7 | 11 | -4 | 197.78 | 15.90 |
| -6 | -11 | 4 | 13.70 | 4.60 |
| 6 | 11 | -4 | 17.30 | 6.30 |
| 5 | -11 | -4 | 2114.59 | 122.59 |
| -5 | -11 | 4 | 2266.87 | 121.99 |
| 5 | -11 | -4 | 2289.67 | 122.29 |
| -5 | 11 | 4 | 2448.66 | 122.89 |
| 5 | 11 | -4 | 2093.69 | 123.99 |
| -4 | -11 | 4 | 132.09 | 10.40 |
| 4 | -11 | -4 | 130.89 | 13.60 |
| 4 | -11 | -4 | 110.79 | 15.50 |
| -4 | -11 | 4 | 111.99 | 10.70 |
| -4 | 11 | 4 | 118.49 | 10.60 |
| 4 | 11 | -4 | 112.79 | 12.60 |
| 3 | -11 | -4 | 107.69 | 13.90 |
| -3 | -11 | 4 | 110.69 | 10.10 |

| | | | | |
|---|---|---|---|---|
| -3 | -11 | 4 | 117.39 | 9.70 |
| 3 | -11 | -4 | 65.79 | 14.10 |
| -3 | 11 | 4 | 118.09 | 10.00 |
| 3 | 11 | -4 | 101.89 | 11.50 |
| -2 | -11 | 4 | 417.86 | 26.60 |
| -2 | -11 | 4 | 441.16 | 26.20 |
| 2 | -11 | -4 | 472.45 | 28.30 |
| -2 | 11 | 4 | 451.15 | 26.50 |
| 2 | 11 | -4 | 427.76 | 28.00 |
| -2 | 11 | 4 | 484.05 | 27.30 |
| -1 | -11 | 4 | 3022.30 | 164.98 |
| 1 | -11 | -4 | 3025.20 | 165.78 |
| -1 | -11 | 4 | 2952.70 | 165.28 |
| -1 | 11 | 4 | 3137.89 | 165.38 |
| -1 | 11 | 4 | 3147.39 | 166.38 |
| 1 | 11 | -4 | 3029.80 | 166.68 |
| 0 | -11 | 4 | 9.40 | 4.50 |
| 0 | -11 | 4 | -0.10 | 3.30 |
| 0 | -11 | -4 | -1.20 | 4.30 |
| 0 | 11 | 4 | 4.00 | 3.60 |
| 0 | 11 | 4 | 0.70 | 2.60 |
| 0 | 11 | -4 | 5.30 | 4.30 |
| 0 | 11 | -4 | 2.20 | 3.70 |
| -1 | -11 | -4 | 1455.35 | 81.59 |
| 1 | -11 | 4 | 1457.05 | 80.99 |
| 1 | -11 | 4 | 1527.45 | 81.89 |
| 1 | 11 | 4 | 1524.85 | 82.59 |
| 1 | 11 | 4 | 1473.25 | 81.19 |
| -1 | 11 | -4 | 1460.45 | 82.19 |
| -2 | -11 | -4 | 258.27 | 17.30 |
| 2 | -11 | 4 | 279.97 | 19.20 |
| 2 | -11 | 4 | 261.67 | 16.90 |
| 2 | 11 | 4 | 265.47 | 16.40 |
| 2 | 11 | 4 | 260.47 | 18.10 |
| -2 | 11 | -4 | 267.87 | 17.40 |
| 3 | -11 | 4 | 633.34 | 35.40 |
| -3 | -11 | -4 | 585.14 | 35.70 |
| 3 | -11 | 4 | 626.74 | 37.70 |
| 3 | 11 | 4 | 627.24 | 37.40 |
| -3 | 11 | -4 | 639.94 | 36.30 |
| 3 | 11 | 4 | 625.64 | 35.20 |
| 4 | -11 | 4 | 84.99 | 16.40 |
| -4 | -11 | -4 | 92.09 | 9.40 |
| 4 | -11 | 4 | 110.39 | 9.00 |

| | | | | |
|---|---|---|---|---|
| 4 | 11 | 4 | 101.49 | 7.90 |
| 4 | 11 | 4 | 93.49 | 12.20 |
| -4 | 11 | -4 | 102.69 | 9.90 |
| -5 | -11 | -4 | 2196.78 | 114.39 |
| 5 | -11 | 4 | 2130.39 | 115.89 |
| 5 | -11 | 4 | 2094.79 | 114.39 |
| 5 | 11 | 4 | 1959.50 | 113.89 |
| 5 | 11 | 4 | 1984.70 | 116.79 |
| -5 | 11 | -4 | 2225.28 | 115.19 |
| -6 | -11 | -4 | 193.28 | 14.40 |
| 6 | -11 | 4 | 210.08 | 13.90 |
| 6 | -11 | 4 | 221.38 | 14.90 |
| 6 | 11 | 4 | 212.08 | 13.30 |
| 6 | 11 | 4 | 214.68 | 25.20 |
| -7 | -11 | -4 | 7.50 | 4.10 |
| 7 | -11 | 4 | 8.00 | 4.30 |
| 7 | -11 | 4 | 4.80 | 3.10 |
| 7 | 11 | 4 | 13.90 | 10.60 |
| 7 | 11 | 4 | 1.60 | 2.40 |
| 8 | -11 | 4 | 18.20 | 5.10 |
| 8 | -11 | 4 | 15.40 | 3.60 |
| -8 | -11 | -4 | 25.90 | 4.40 |
| 8 | 11 | 4 | 23.70 | 4.80 |
| 9 | -11 | 4 | 773.72 | 46.20 |
| 9 | -11 | 4 | 796.22 | 45.30 |
| 9 | 11 | 4 | 862.61 | 45.30 |
| 10 | -11 | 4 | 47.40 | 6.60 |
| 10 | -11 | 4 | 53.09 | 7.90 |
| 10 | 11 | 4 | 56.99 | 6.00 |
| 11 | -11 | 4 | 438.86 | 26.70 |
| 11 | -11 | 4 | 484.55 | 26.20 |
| 11 | 11 | 4 | 439.36 | 26.20 |
| 12 | -11 | 4 | 50.89 | 5.30 |
| 12 | -11 | 4 | 40.70 | 6.10 |
| 12 | 11 | 4 | 38.20 | 5.50 |
| 13 | -11 | 4 | 267.57 | 15.50 |
| 13 | 11 | 4 | 267.27 | 15.90 |
| 14 | -12 | -4 | 208.08 | 12.30 |
| 14 | 12 | -4 | 214.68 | 12.00 |
| 13 | -12 | -4 | 108.79 | 7.70 |
| 13 | 12 | -4 | 118.59 | 7.20 |
| 12 | -12 | -4 | 108.99 | 8.30 |
| 12 | 12 | -4 | 112.09 | 7.40 |
| 11 | -12 | -4 | -2.80 | 5.40 |

| | | | | |
|---|---|---|---|---|
| 11 | 12 | -4 | 2.70 | 2.20 |
| 10 | -12 | -4 | 515.65 | 29.50 |
| 10 | 12 | -4 | 494.85 | 28.50 |
| 9 | -12 | -4 | 59.19 | 8.90 |
| 9 | 12 | -4 | 59.39 | 6.50 |
| 8 | -12 | -4 | 57.49 | 9.70 |
| -8 | -12 | 4 | 60.69 | 9.60 |
| 8 | 12 | -4 | 63.89 | 7.30 |
| 7 | -12 | -4 | 269.17 | 18.80 |
| -7 | -12 | 4 | 268.47 | 17.90 |
| 7 | 12 | -4 | 300.07 | 21.50 |
| -6 | -12 | 4 | 1430.56 | 73.59 |
| 6 | -12 | -4 | 1284.07 | 73.89 |
| 6 | 12 | -4 | 1288.07 | 77.29 |
| 5 | -12 | -4 | 7.70 | 6.60 |
| 5 | -12 | -4 | 3.60 | 5.50 |
| 5 | 12 | -4 | 2.00 | 6.40 |
| -5 | 12 | 4 | 5.90 | 5.40 |
| -4 | -12 | 4 | 361.06 | 20.40 |
| 4 | -12 | -4 | 327.47 | 22.50 |
| 4 | -12 | -4 | 309.97 | 21.60 |
| 4 | 12 | -4 | 264.37 | 22.60 |
| -4 | 12 | 4 | 358.46 | 21.50 |
| -3 | -12 | 4 | 89.89 | 9.10 |
| -3 | -12 | 4 | 92.89 | 8.60 |
| 3 | -12 | -4 | 97.69 | 13.00 |
| 3 | -12 | -4 | 84.49 | 12.70 |
| 3 | 12 | -4 | 83.69 | 11.60 |
| -3 | 12 | 4 | 102.99 | 9.90 |
| 2 | -12 | -4 | 295.97 | 23.10 |
| 2 | -12 | -4 | 329.37 | 21.10 |
| -2 | -12 | 4 | 317.47 | 19.50 |
| -2 | -12 | 4 | 297.77 | 19.00 |
| 2 | 12 | -4 | 299.67 | 21.60 |
| -2 | 12 | 4 | 318.97 | 20.50 |
| -2 | 12 | 4 | 326.47 | 19.50 |
| 1 | -12 | -4 | 110.99 | 11.20 |
| -1 | -12 | 4 | 92.59 | 8.60 |
| -1 | -12 | 4 | 74.69 | 9.30 |
| -1 | 12 | 4 | 84.49 | 8.20 |
| 1 | 12 | -4 | 98.69 | 10.60 |
| -1 | 12 | 4 | 101.69 | 9.90 |
| 0 | -12 | 4 | 2120.89 | 117.89 |
| 0 | -12 | -4 | 2240.88 | 118.69 |

| | | | | |
|---|---|---|---|---|
| 0 | -12 | 4 | 2283.17 | 118.59 |
| 0 | 12 | -4 | 2167.18 | 119.69 |
| 0 | 12 | 4 | 2013.50 | 118.09 |
| 0 | 12 | -4 | 2313.67 | 118.89 |
| 0 | 12 | 4 | 2072.99 | 119.39 |
| 1 | -12 | 4 | 422.16 | 24.80 |
| -1 | -12 | -4 | 419.36 | 25.60 |
| 1 | -12 | 4 | 450.65 | 26.10 |
| -1 | 12 | -4 | 434.36 | 24.90 |
| 1 | 12 | 4 | 398.66 | 26.40 |
| 1 | 12 | 4 | 423.86 | 24.80 |
| -1 | 12 | -4 | 412.06 | 26.10 |
| -2 | -12 | -4 | 3.90 | 3.60 |
| 2 | -12 | 4 | -5.10 | 5.00 |
| 2 | -12 | 4 | -3.20 | 3.20 |
| 2 | 12 | 4 | 3.40 | 4.00 |
| 2 | 12 | 4 | 3.00 | 2.70 |
| -2 | 12 | -4 | 6.50 | 4.20 |
| 3 | -12 | 4 | 303.17 | 18.20 |
| 3 | -12 | 4 | 305.77 | 21.10 |
| -3 | -12 | -4 | 296.97 | 18.50 |
| -3 | 12 | -4 | 292.57 | 19.30 |
| 3 | 12 | 4 | 269.87 | 17.60 |
| 3 | 12 | 4 | 291.37 | 20.70 |
| 4 | -12 | 4 | 1928.41 | 105.29 |
| -4 | -12 | -4 | 1907.21 | 103.49 |
| 4 | -12 | 4 | 1878.51 | 103.19 |
| 4 | 12 | 4 | 1921.61 | 103.19 |
| 4 | 12 | 4 | 1841.42 | 105.99 |
| -4 | 12 | -4 | 1900.81 | 104.49 |
| -5 | -12 | -4 | 33.50 | 7.10 |
| 5 | -12 | 4 | 36.70 | 6.30 |
| 5 | -12 | 4 | 41.80 | 8.60 |
| 5 | 12 | 4 | 32.40 | 9.40 |
| 5 | 12 | 4 | 35.70 | 5.00 |
| -5 | 12 | -4 | 24.20 | 6.60 |
| -6 | -12 | -4 | 1099.09 | 59.49 |
| 6 | -12 | 4 | 1075.69 | 59.19 |
| 6 | 12 | 4 | 941.61 | 63.39 |
| 6 | 12 | 4 | 1167.08 | 59.29 |
| 7 | -12 | 4 | 226.48 | 14.60 |
| 7 | -12 | 4 | 226.98 | 16.00 |
| -7 | -12 | -4 | 227.48 | 14.90 |
| 7 | 12 | 4 | 236.48 | 14.30 |

| | | | | |
|---|---|---|---|---|
| 7 | 12 | 4 | 202.38 | 24.90 |
| -8 | -12 | -4 | 423.36 | 25.90 |
| 8 | -12 | 4 | 444.76 | 27.10 |
| 8 | -12 | 4 | 456.65 | 26.00 |
| 8 | 12 | 4 | 462.95 | 25.80 |
| 9 | -12 | 4 | 16.00 | 4.70 |
| 9 | -12 | 4 | 18.50 | 3.70 |
| 9 | 12 | 4 | 21.80 | 4.70 |
| 10 | -12 | 4 | 776.22 | 44.40 |
| 10 | -12 | 4 | 774.22 | 45.00 |
| 10 | 12 | 4 | 837.12 | 44.40 |
| 11 | -12 | 4 | 23.30 | 4.50 |
| 11 | 12 | 4 | 15.10 | 3.40 |
| 12 | -12 | 4 | 9.00 | 2.40 |
| 12 | 12 | 4 | 8.10 | 3.10 |
| 13 | -13 | -4 | 10.60 | 2.30 |
| 13 | 13 | -4 | 17.40 | 2.60 |
| 12 | -13 | -4 | 46.10 | 5.00 |
| 12 | 13 | -4 | 50.09 | 4.20 |
| 11 | -13 | -4 | 394.06 | 23.40 |
| 11 | 13 | -4 | 416.36 | 22.80 |
| 10 | -13 | -4 | 8.70 | 5.30 |
| 10 | 13 | -4 | 7.10 | 2.30 |
| 9 | -13 | -4 | 186.48 | 13.30 |
| 9 | 13 | -4 | 174.58 | 11.60 |
| -8 | -13 | 4 | 107.09 | 10.50 |
| 8 | -13 | -4 | 118.99 | 11.10 |
| 8 | 13 | -4 | 120.69 | 9.20 |
| -7 | -13 | 4 | 93.59 | 9.00 |
| 7 | -13 | -4 | 86.29 | 10.30 |
| 7 | 13 | -4 | 85.79 | 15.70 |
| -6 | -13 | 4 | 42.20 | 7.50 |
| 6 | -13 | -4 | 50.39 | 9.70 |
| 6 | 13 | -4 | 25.80 | 8.40 |
| -5 | -13 | 4 | 987.20 | 53.39 |
| 5 | -13 | -4 | 967.50 | 53.89 |
| 5 | -13 | -4 | 995.60 | 54.09 |
| 5 | 13 | -4 | 874.81 | 56.39 |
| 4 | -13 | -4 | 312.07 | 22.30 |
| -4 | -13 | 4 | 331.57 | 20.90 |
| 4 | -13 | -4 | 360.26 | 24.70 |
| -4 | 13 | 4 | 359.26 | 22.70 |
| 3 | -13 | -4 | 6.30 | 6.20 |
| -3 | -13 | 4 | 5.70 | 3.40 |

| | | | | |
|---|---|---|---|---|
| -3 | -13 | 4 | 10.60 | 3.70 |
| -3 | 13 | 4 | 0.10 | 3.40 |
| -3 | 13 | 4 | 7.40 | 4.80 |
| 3 | 13 | -4 | 4.60 | 5.70 |
| -2 | -13 | 4 | 77.79 | 7.30 |
| -2 | -13 | 4 | 66.69 | 8.30 |
| 2 | -13 | -4 | 81.09 | 12.10 |
| 2 | 13 | -4 | 73.09 | 11.20 |
| -2 | 13 | 4 | 58.29 | 7.40 |
| -2 | 13 | 4 | 59.89 | 9.30 |
| -1 | -13 | 4 | 1235.68 | 70.59 |
| -1 | 13 | 4 | 1341.37 | 71.19 |
| -1 | 13 | 4 | 1158.18 | 69.59 |
| 1 | 13 | -4 | 1315.77 | 71.89 |
| 0 | -13 | 4 | 7.20 | 4.00 |
| 0 | -13 | -4 | 20.60 | 8.10 |
| 0 | 13 | -4 | 25.20 | 6.40 |
| 0 | 13 | 4 | 10.90 | 3.00 |
| 0 | 13 | 4 | 14.30 | 4.90 |
| -1 | -13 | -4 | 308.97 | 19.20 |
| 1 | -13 | 4 | 297.17 | 18.40 |
| 1 | -13 | 4 | 300.37 | 20.20 |
| 1 | 13 | 4 | 285.17 | 20.40 |
| -1 | 13 | -4 | 297.57 | 20.30 |
| -1 | 13 | -4 | 310.87 | 18.80 |
| 1 | 13 | 4 | 301.77 | 18.30 |
| -2 | -13 | -4 | 271.57 | 17.00 |
| 2 | -13 | 4 | 303.67 | 18.80 |
| 2 | -13 | 4 | 247.58 | 16.40 |
| -2 | 13 | -4 | 260.67 | 18.10 |
| 2 | 13 | 4 | 231.78 | 23.30 |
| -2 | 13 | -4 | 263.87 | 16.70 |
| 2 | 13 | 4 | 251.77 | 16.20 |
| -3 | -13 | -4 | 130.09 | 10.60 |
| 3 | -13 | 4 | 156.08 | 14.00 |
| 3 | -13 | 4 | 135.59 | 10.10 |
| 3 | 13 | 4 | 129.39 | 9.60 |
| -3 | 13 | -4 | 130.79 | 14.20 |
| 3 | 13 | 4 | 138.09 | 14.10 |
| 4 | -13 | 4 | 2.70 | 6.60 |
| 4 | -13 | 4 | 10.70 | 3.30 |
| -4 | -13 | -4 | 9.70 | 3.80 |
| 4 | 13 | 4 | 11.00 | 2.90 |
| 4 | 13 | 4 | 12.60 | 7.40 |

| | | | | |
|---|---|---|---|---|
| -4 | 13 | -4 | 9.40 | 5.90 |
| -5 | -13 | -4 | 1251.37 | 70.59 |
| 5 | -13 | 4 | 1351.46 | 72.09 |
| 5 | -13 | 4 | 1272.47 | 70.49 |
| 5 | 13 | 4 | 1156.68 | 74.29 |
| -5 | 13 | -4 | 1374.46 | 72.79 |
| 5 | 13 | 4 | 1302.37 | 70.49 |
| 6 | -13 | 4 | 346.07 | 21.00 |
| -6 | -13 | -4 | 366.16 | 21.30 |
| 6 | 13 | 4 | 375.76 | 21.00 |
| 6 | 13 | 4 | 316.47 | 29.30 |
| 7 | -13 | 4 | 7.50 | 3.10 |
| -7 | -13 | -4 | 10.70 | 3.50 |
| 7 | 13 | 4 | 16.60 | 3.30 |
| 7 | 13 | 4 | 0.70 | 13.70 |
| -8 | -13 | -4 | 38.00 | 6.50 |
| 8 | -13 | 4 | 39.30 | 5.90 |
| 8 | 13 | 4 | 39.70 | 4.90 |
| 9 | 13 | 4 | 659.73 | 36.90 |
| 10 | -13 | 4 | 0.40 | 2.60 |
| 10 | 13 | 4 | -3.70 | 2.60 |
| 11 | -13 | 4 | 103.99 | 7.20 |
| 11 | 13 | 4 | 89.29 | 8.20 |
| 12 | -13 | 4 | 196.08 | 11.10 |
| 12 | 13 | 4 | 171.98 | 11.60 |
| 12 | -14 | -4 | 16.10 | 2.60 |
| 12 | 14 | -4 | 19.70 | 2.80 |
| 11 | -14 | -4 | 0.70 | 2.50 |
| 11 | 14 | -4 | -0.40 | 1.50 |
| 10 | -14 | -4 | 448.06 | 25.50 |
| 10 | 14 | -4 | 432.86 | 24.70 |
| 9 | 14 | -4 | 37.20 | 5.00 |
| -8 | -14 | 4 | 79.59 | 8.60 |
| 8 | -14 | -4 | 74.89 | 9.00 |
| 8 | 14 | -4 | 76.59 | 6.90 |
| 7 | -14 | -4 | 145.79 | 12.60 |
| -7 | -14 | 4 | 159.48 | 11.40 |
| 7 | 14 | -4 | 134.19 | 19.10 |
| -6 | -14 | 4 | 439.66 | 25.40 |
| 6 | -14 | -4 | 408.76 | 26.20 |
| 6 | 14 | -4 | 444.96 | 30.60 |
| -5 | -14 | 4 | 71.79 | 8.40 |
| 5 | -14 | -4 | 84.99 | 10.80 |
| 5 | -14 | -4 | 75.99 | 10.70 |

| | | | | |
|---|---|---|---|---|
| -5 | 14 | 4 | 85.89 | 9.10 |
| 5 | 14 | -4 | 62.39 | 14.80 |
| 4 | -14 | -4 | 214.58 | 15.20 |
| -4 | -14 | 4 | 216.88 | 14.10 |
| 4 | -14 | -4 | 185.48 | 15.70 |
| -4 | 14 | 4 | 210.48 | 17.30 |
| -4 | 14 | 4 | 200.88 | 14.50 |
| 4 | 14 | -4 | 182.48 | 19.30 |
| 3 | -14 | -4 | 165.08 | 13.10 |
| 3 | -14 | -4 | 161.98 | 13.70 |
| -3 | -14 | 4 | 146.59 | 11.50 |
| -3 | -14 | 4 | 157.18 | 11.00 |
| -3 | 14 | 4 | 153.88 | 14.30 |
| -3 | 14 | 4 | 156.28 | 11.70 |
| 3 | 14 | -4 | 118.89 | 16.30 |
| -2 | -14 | 4 | 6.60 | 3.60 |
| 2 | -14 | -4 | 3.20 | 4.40 |
| -2 | -14 | 4 | 9.40 | 3.40 |
| -2 | 14 | 4 | 6.60 | 3.10 |
| -2 | 14 | 4 | 8.80 | 5.70 |
| 2 | 14 | -4 | 20.50 | 7.80 |
| -1 | -14 | 4 | 59.79 | 6.90 |
| -1 | -14 | 4 | 51.89 | 7.80 |
| 1 | -14 | -4 | 67.69 | 8.80 |
| -1 | 14 | 4 | 49.10 | 10.20 |
| -1 | 14 | 4 | 59.69 | 6.60 |
| 1 | 14 | -4 | 54.29 | 10.90 |
| 0 | -14 | -4 | 625.54 | 35.20 |
| 0 | -14 | 4 | 601.54 | 35.10 |
| 0 | -14 | 4 | 613.54 | 34.30 |
| 0 | 14 | 4 | 616.34 | 34.50 |
| 0 | 14 | -4 | 563.14 | 36.50 |
| 0 | 14 | 4 | 601.34 | 36.60 |
| -1 | -14 | -4 | 234.88 | 15.60 |
| 1 | -14 | 4 | 223.58 | 16.20 |
| 1 | -14 | 4 | 236.28 | 14.70 |
| 1 | 14 | 4 | 229.78 | 14.60 |
| -1 | 14 | -4 | 220.88 | 17.10 |
| 1 | 14 | 4 | 219.48 | 17.70 |
| -1 | 14 | -4 | 227.88 | 14.90 |
| 2 | -14 | 4 | 20.10 | 5.50 |
| -2 | -14 | -4 | 34.30 | 6.70 |
| 2 | -14 | 4 | 34.30 | 5.70 |
| 2 | 14 | 4 | 47.60 | 10.80 |

| | | | | |
|---|---|---|---|---|
| -2 | 14 | -4 | 24.40 | 4.60 |
| -2 | 14 | -4 | 24.20 | 6.20 |
| 2 | 14 | 4 | 34.70 | 5.10 |
| -3 | -14 | -4 | 14.40 | 3.60 |
| 3 | -14 | 4 | 19.70 | 6.00 |
| 3 | -14 | 4 | 9.80 | 3.40 |
| 3 | 14 | 4 | 11.40 | 3.10 |
| -3 | 14 | -4 | 10.30 | 3.80 |
| -3 | 14 | -4 | 5.00 | 5.90 |
| 3 | 14 | 4 | 27.50 | 8.10 |
| -4 | -14 | -4 | 311.27 | 19.00 |
| 4 | -14 | 4 | 298.67 | 18.60 |
| 4 | -14 | 4 | 333.47 | 21.70 |
| 4 | 14 | 4 | 295.77 | 18.50 |
| 4 | 14 | 4 | 296.67 | 24.10 |
| -4 | 14 | -4 | 328.57 | 22.40 |
| 5 | -14 | 4 | 29.80 | 5.40 |
| 5 | -14 | 4 | 32.80 | 7.20 |
| -5 | -14 | -4 | 31.10 | 5.80 |
| 5 | 14 | 4 | 16.00 | 10.50 |
| 5 | 14 | 4 | 23.80 | 4.80 |
| -6 | -14 | -4 | 261.87 | 15.70 |
| 6 | -14 | 4 | 235.18 | 15.30 |
| 6 | 14 | 4 | 249.58 | 15.20 |
| -7 | -14 | -4 | 279.97 | 17.00 |
| 7 | -14 | 4 | 280.57 | 16.90 |
| 7 | 14 | 4 | 276.17 | 16.60 |
| 8 | -14 | 4 | 74.69 | 9.50 |
| -8 | -14 | -4 | 96.69 | 8.10 |
| 8 | 14 | 4 | 96.69 | 7.20 |
| 9 | -14 | 4 | 6.40 | 2.70 |
| 9 | 14 | 4 | 7.00 | 2.60 |
| 10 | -14 | 4 | 277.27 | 15.40 |
| 10 | 14 | 4 | 240.68 | 15.80 |
| 11 | -14 | 4 | 107.99 | 7.10 |
| 11 | 14 | 4 | 95.79 | 7.60 |
| 11 | -15 | -4 | 217.98 | 13.10 |
| -11 | 15 | 4 | 221.88 | 13.30 |
| 11 | 15 | -4 | 226.08 | 12.70 |
| 10 | -15 | -4 | 1.30 | 2.80 |
| 10 | 15 | -4 | 4.60 | 1.70 |
| 9 | -15 | -4 | 177.38 | 12.10 |
| 9 | 15 | -4 | 171.68 | 10.80 |
| -8 | -15 | 4 | 77.99 | 8.50 |

| | | | | |
|---|---|---|---|---|
| 8 | -15 | -4 | 108.79 | 9.90 |
| -7 | -15 | 4 | 58.89 | 7.30 |
| 7 | -15 | -4 | 60.29 | 9.10 |
| 7 | 15 | -4 | 56.19 | 6.50 |
| -7 | 15 | 4 | 51.09 | 7.40 |
| -6 | -15 | 4 | 131.79 | 9.90 |
| 6 | -15 | -4 | 128.69 | 11.80 |
| -6 | 15 | 4 | 119.19 | 10.90 |
| 5 | -15 | -4 | 570.24 | 32.20 |
| 5 | -15 | -4 | 545.15 | 32.50 |
| -5 | -15 | 4 | 572.64 | 31.90 |
| -5 | 15 | 4 | 576.24 | 33.10 |
| 5 | 15 | -4 | 490.85 | 37.60 |
| -4 | -15 | 4 | 52.99 | 7.00 |
| 4 | -15 | -4 | 44.00 | 8.70 |
| 4 | -15 | -4 | 60.19 | 10.30 |
| 4 | 15 | -4 | 31.00 | 9.70 |
| -4 | 15 | 4 | 59.89 | 10.60 |
| 3 | -15 | -4 | 40.40 | 8.70 |
| -3 | -15 | 4 | 49.30 | 6.90 |
| 3 | -15 | -4 | 51.79 | 9.90 |
| 3 | 15 | -4 | 29.70 | 9.00 |
| -3 | 15 | 4 | 52.09 | 9.40 |
| -3 | 15 | 4 | 44.40 | 7.00 |
| -2 | -15 | 4 | 119.79 | 9.90 |
| 2 | -15 | -4 | 93.79 | 12.40 |
| -2 | -15 | 4 | 113.29 | 9.10 |
| 2 | -15 | -4 | 126.89 | 11.00 |
| -2 | 15 | 4 | 120.49 | 14.00 |
| 2 | 15 | -4 | 105.39 | 15.10 |
| -2 | 15 | 4 | 123.09 | 9.50 |
| 1 | -15 | -4 | 496.35 | 29.40 |
| -1 | -15 | 4 | 488.85 | 28.20 |
| -1 | -15 | 4 | 472.35 | 28.90 |
| -1 | 15 | 4 | 524.25 | 28.80 |
| -1 | 15 | 4 | 479.85 | 31.30 |
| 1 | 15 | -4 | 492.85 | 32.20 |
| 0 | -15 | 4 | 10.10 | 2.90 |
| 0 | -15 | 4 | 8.50 | 4.40 |
| 0 | -15 | -4 | 4.80 | 3.60 |
| 0 | 15 | 4 | 5.10 | 6.10 |
| 0 | 15 | -4 | 17.90 | 7.20 |
| 0 | 15 | 4 | 11.80 | 3.10 |
| -1 | -15 | -4 | 288.27 | 18.30 |

| | | | | |
|---|---|---|---|---|
| 1 | -15 | 4 | 296.27 | 17.60 |
| 1 | -15 | 4 | 302.77 | 19.30 |
| 1 | 15 | 4 | 272.57 | 21.30 |
| -1 | 15 | -4 | 304.77 | 21.10 |
| 1 | 15 | 4 | 276.77 | 17.60 |
| 2 | -15 | 4 | 213.98 | 15.60 |
| 2 | -15 | 4 | 199.48 | 13.20 |
| -2 | -15 | -4 | 196.18 | 13.80 |
| 2 | 15 | 4 | 221.48 | 13.40 |
| -2 | 15 | -4 | 206.88 | 17.00 |
| 2 | 15 | 4 | 212.58 | 18.10 |
| -2 | 15 | -4 | 196.78 | 13.80 |
| 3 | -15 | 4 | 128.69 | 13.20 |
| -3 | -15 | -4 | 122.19 | 10.00 |
| 3 | -15 | 4 | 144.29 | 9.90 |
| -3 | 15 | -4 | 138.99 | 11.70 |
| 3 | 15 | 4 | 142.49 | 10.00 |
| -3 | 15 | -4 | 141.39 | 15.20 |
| 3 | 15 | 4 | 133.59 | 17.10 |
| -4 | -15 | -4 | 290.87 | 17.90 |
| 4 | -15 | 4 | 291.37 | 17.60 |
| 4 | -15 | 4 | 299.47 | 20.40 |
| 4 | 15 | 4 | 263.47 | 25.20 |
| 4 | 15 | 4 | 305.67 | 17.60 |
| -4 | 15 | -4 | 298.37 | 18.20 |
| 5 | -15 | 4 | 306.17 | 20.50 |
| 5 | -15 | 4 | 297.47 | 17.90 |
| -5 | -15 | -4 | 288.57 | 18.10 |
| 5 | 15 | 4 | 301.87 | 17.90 |
| -5 | 15 | -4 | 314.37 | 18.60 |
| 6 | -15 | 4 | 132.49 | 9.40 |
| -6 | -15 | -4 | 136.59 | 9.70 |
| 6 | 15 | 4 | 135.19 | 9.20 |
| 7 | -15 | 4 | -1.40 | 2.60 |
| -7 | -15 | -4 | 4.60 | 2.70 |
| 7 | 15 | 4 | 3.10 | 2.30 |
| 8 | -15 | 4 | 82.49 | 6.60 |
| -8 | -15 | -4 | 75.89 | 7.10 |
| 8 | 15 | 4 | 79.49 | 6.40 |
| 9 | -15 | 4 | 141.39 | 8.70 |
| 9 | 15 | 4 | 118.09 | 9.00 |
| 10 | -15 | 4 | 0.80 | 2.00 |
| 10 | 15 | 4 | -4.60 | 2.70 |
| 10 | -16 | -4 | 188.08 | 11.80 |

| | | | | |
|---|---|---|---|---|
| 10 | 16 | -4 | 190.48 | 11.20 |
| -10 | 16 | 4 | 204.28 | 11.90 |
| 9 | -16 | -4 | 6.20 | 3.00 |
| -9 | 16 | 4 | 6.50 | 2.50 |
| 9 | 16 | -4 | 5.00 | 1.80 |
| 8 | -16 | -4 | 9.50 | 3.80 |
| -8 | -16 | 4 | 11.10 | 3.40 |
| 8 | 16 | -4 | 9.30 | 2.40 |
| -8 | 16 | 4 | 11.10 | 3.10 |
| -7 | -16 | 4 | 110.29 | 9.30 |
| 7 | -16 | -4 | 128.19 | 10.70 |
| 7 | 16 | -4 | 109.49 | 8.70 |
| -7 | 16 | 4 | 130.79 | 9.80 |
| 6 | -16 | -4 | 387.66 | 24.90 |
| -6 | -16 | 4 | 407.36 | 24.50 |
| -6 | 16 | 4 | 424.76 | 24.80 |
| 5 | -16 | -4 | 20.50 | 5.30 |
| 5 | -16 | -4 | 8.70 | 4.60 |
| -5 | -16 | 4 | 14.30 | 3.60 |
| -5 | 16 | 4 | 9.40 | 3.80 |
| 4 | -16 | -4 | 136.99 | 10.50 |
| -4 | -16 | 4 | 133.99 | 9.90 |
| 4 | -16 | -4 | 112.89 | 11.90 |
| -4 | 16 | 4 | 122.79 | 10.40 |
| -3 | -16 | 4 | 48.40 | 6.70 |
| 3 | -16 | -4 | 71.79 | 8.10 |
| 3 | -16 | -4 | 37.30 | 6.50 |
| -3 | 16 | 4 | 63.49 | 7.00 |
| 2 | -16 | -4 | 152.68 | 11.60 |
| -2 | -16 | 4 | 145.29 | 10.00 |
| -2 | -16 | 4 | 130.99 | 10.70 |
| -2 | 16 | 4 | 118.99 | 18.80 |
| -2 | 16 | 4 | 139.79 | 10.50 |
| -1 | -16 | 4 | 99.39 | 7.90 |
| 1 | -16 | -4 | 84.39 | 9.50 |
| -1 | -16 | 4 | 81.99 | 8.90 |
| 1 | 16 | -4 | 123.19 | 15.40 |
| -1 | 16 | 4 | 89.89 | 14.50 |
| -1 | 16 | 4 | 104.49 | 8.30 |
| 0 | -16 | 4 | 506.35 | 29.60 |
| 0 | -16 | -4 | 515.65 | 29.50 |
| 0 | -16 | 4 | 501.35 | 28.60 |
| 0 | 16 | 4 | 549.74 | 29.00 |
| 0 | 16 | 4 | 484.65 | 32.40 |

| | | | | |
|---|---|---|---|---|
| 0 | 16 | -4 | 444.56 | 32.30 |
| -1 | -16 | -4 | 175.08 | 12.10 |
| 1 | -16 | 4 | 169.28 | 11.10 |
| 1 | -16 | 4 | 174.78 | 13.00 |
| 1 | 16 | 4 | 150.08 | 19.00 |
| 1 | 16 | 4 | 168.98 | 11.20 |
| -1 | 16 | -4 | 132.69 | 25.00 |
| 2 | -16 | 4 | 5.40 | 2.40 |
| 2 | -16 | 4 | 5.60 | 4.50 |
| -2 | -16 | -4 | 1.90 | 3.00 |
| 2 | 16 | 4 | 2.30 | 2.60 |
| 3 | -16 | 4 | 106.99 | 8.00 |
| 3 | -16 | 4 | 114.59 | 11.20 |
| -3 | -16 | -4 | 111.69 | 8.60 |
| 3 | 16 | 4 | 107.69 | 8.10 |
| -3 | 16 | -4 | 104.69 | 11.60 |
| 4 | -16 | 4 | 211.98 | 13.30 |
| 4 | -16 | 4 | 210.18 | 16.10 |
| -4 | -16 | -4 | 205.98 | 13.60 |
| -4 | 16 | -4 | 216.78 | 14.30 |
| 4 | 16 | 4 | 226.48 | 13.30 |
| 5 | -16 | 4 | 9.80 | 5.10 |
| 5 | -16 | 4 | 6.40 | 3.10 |
| 5 | 16 | 4 | 9.60 | 2.50 |
| -5 | 16 | -4 | 5.90 | 3.90 |
| -6 | -16 | -4 | 347.27 | 20.70 |
| 6 | -16 | 4 | 361.96 | 20.80 |
| -6 | 16 | -4 | 362.36 | 21.40 |
| 6 | 16 | 4 | 361.96 | 20.60 |
| 7 | -16 | 4 | 216.28 | 12.80 |
| -7 | -16 | -4 | 218.38 | 12.80 |
| 7 | 16 | 4 | 210.38 | 12.70 |
| -7 | 16 | -4 | 180.58 | 15.10 |
| -8 | -16 | -4 | 69.99 | 6.20 |
| 8 | -16 | 4 | 75.79 | 5.80 |
| 8 | 16 | 4 | 68.79 | 6.50 |
| -8 | 16 | -4 | 61.29 | 7.60 |
| 9 | -16 | 4 | 5.60 | 2.00 |
| 9 | 16 | 4 | 6.30 | 2.70 |
| 9 | -17 | -4 | 107.59 | 8.50 |
| 9 | 17 | -4 | 99.99 | 6.30 |
| -9 | 17 | 4 | 95.29 | 7.20 |
| -8 | -17 | 4 | 86.59 | 7.90 |
| 8 | -17 | -4 | 107.39 | 7.90 |

| | | | | |
|---|---|---|---|---|
| 8 | 17 | -4 | 99.09 | 6.60 |
| -8 | 17 | 4 | 103.09 | 7.60 |
| -7 | -17 | 4 | 64.19 | 6.80 |
| 7 | -17 | -4 | 87.79 | 7.90 |
| -7 | 17 | 4 | 79.99 | 7.30 |
| 7 | 17 | -4 | 70.69 | 5.90 |
| 6 | -17 | -4 | 18.10 | 4.20 |
| -6 | -17 | 4 | 15.60 | 3.20 |
| 6 | 17 | -4 | 23.90 | 4.70 |
| -6 | 17 | 4 | 26.10 | 5.40 |
| 5 | -17 | -4 | 373.66 | 24.50 |
| 5 | -17 | -4 | 459.35 | 24.20 |
| -5 | -17 | 4 | 408.76 | 23.90 |
| -5 | 17 | 4 | 397.76 | 24.60 |
| 4 | -17 | -4 | 51.19 | 10.80 |
| 4 | -17 | -4 | 88.99 | 8.00 |
| -4 | 17 | 4 | 86.09 | 8.00 |
| -3 | -17 | 4 | 30.30 | 5.60 |
| 3 | -17 | -4 | 29.10 | 8.50 |
| -3 | 17 | 4 | 28.80 | 5.60 |
| 2 | -17 | -4 | 186.68 | 12.30 |
| -2 | -17 | 4 | 151.28 | 11.40 |
| -2 | 17 | 4 | 161.28 | 11.50 |
| -1 | -17 | 4 | 593.24 | 33.80 |
| -1 | -17 | 4 | 580.54 | 33.10 |
| 1 | -17 | -4 | 570.34 | 34.00 |
| -1 | 17 | 4 | 617.94 | 33.70 |
| 0 | -17 | -4 | 41.30 | 6.60 |
| 0 | -17 | 4 | 43.50 | 5.50 |
| 0 | -17 | 4 | 38.70 | 7.00 |
| 0 | 17 | 4 | 49.50 | 5.60 |
| 1 | -17 | 4 | 387.26 | 22.00 |
| 1 | -17 | 4 | 391.16 | 23.50 |
| -1 | -17 | -4 | 376.16 | 22.70 |
| 1 | 17 | 4 | 371.76 | 22.10 |
| -2 | -17 | -4 | 117.69 | 9.10 |
| 2 | -17 | 4 | 116.79 | 8.50 |
| 2 | -17 | 4 | 146.59 | 13.80 |
| 2 | 17 | 4 | 117.99 | 8.40 |
| 3 | -17 | 4 | 122.29 | 9.10 |
| 3 | -17 | 4 | 133.89 | 11.80 |
| -3 | -17 | -4 | 119.19 | 10.60 |
| 3 | 17 | 4 | 107.69 | 8.20 |
| 4 | -17 | 4 | 26.50 | 4.30 |

| | | | | |
|---|---|---|---|---|
| 4 | -17 | 4 | 19.70 | 4.80 |
| -4 | -17 | -4 | 30.00 | 4.60 |
| 4 | 17 | 4 | 27.70 | 4.00 |
| 5 | -17 | 4 | 571.64 | 31.00 |
| -5 | -17 | -4 | 538.95 | 31.00 |
| 5 | -17 | 4 | 527.45 | 31.70 |
| 5 | 17 | 4 | 580.44 | 31.00 |
| -6 | -17 | -4 | 194.48 | 12.30 |
| 6 | -17 | 4 | 212.08 | 12.40 |
| 6 | 17 | 4 | 202.68 | 12.30 |
| 7 | -17 | 4 | 1.80 | 1.80 |
| -7 | -17 | -4 | -0.10 | 2.10 |
| 7 | 17 | 4 | -1.70 | 2.20 |
| 8 | -17 | 4 | 42.50 | 4.00 |
| -8 | -17 | -4 | 39.80 | 5.10 |
| 8 | 17 | 4 | 42.90 | 4.70 |
| -8 | -18 | 4 | 6.60 | 3.00 |
| 8 | 18 | -4 | 2.60 | 1.40 |
| -8 | 18 | 4 | 0.20 | 2.40 |
| -7 | -18 | 4 | 230.38 | 13.80 |
| -7 | 18 | 4 | 224.08 | 14.20 |
| 7 | 18 | -4 | 232.08 | 13.30 |
| -6 | -18 | 4 | 199.78 | 13.00 |
| -6 | 18 | 4 | 217.18 | 13.60 |
| 6 | 18 | -4 | 216.68 | 12.60 |
| -5 | -18 | 4 | 42.80 | 5.50 |
| 5 | -18 | -4 | 45.50 | 6.80 |
| -5 | 18 | 4 | 44.40 | 6.00 |
| -4 | -18 | 4 | 117.29 | 9.20 |
| 4 | -18 | -4 | 141.09 | 9.60 |
| -4 | 18 | 4 | 132.29 | 9.90 |
| -3 | -18 | 4 | 115.89 | 9.10 |
| 3 | -18 | -4 | 135.29 | 9.60 |
| -3 | 18 | 4 | 127.89 | 9.50 |
| -2 | -18 | 4 | 42.80 | 6.00 |
| 2 | -18 | -4 | 52.29 | 6.60 |
| -2 | 18 | 4 | 50.39 | 5.60 |
| -1 | -18 | 4 | 73.79 | 7.60 |
| 1 | -18 | -4 | 86.09 | 8.00 |
| -1 | 18 | 4 | 80.49 | 7.00 |
| 0 | -18 | 4 | 406.16 | 24.10 |
| 0 | -18 | -4 | 409.36 | 23.90 |
| 0 | -18 | 4 | 395.96 | 23.10 |
| 0 | 18 | 4 | 417.46 | 23.50 |

| | | | | |
|---|---|---|---|---|
| 1 | -18 | 4 | 253.67 | 15.50 |
| -1 | -18 | -4 | 268.27 | 16.20 |
| 1 | -18 | 4 | 271.17 | 16.80 |
| 1 | 18 | 4 | 256.97 | 15.60 |
| -2 | -18 | -4 | 4.40 | 2.50 |
| 2 | -18 | 4 | 3.60 | 2.00 |
| 2 | -18 | 4 | -0.10 | 3.50 |
| 2 | 18 | 4 | 4.40 | 2.20 |
| 3 | -18 | 4 | 99.09 | 9.00 |
| -3 | -18 | -4 | 82.79 | 6.70 |
| 3 | -18 | 4 | 86.59 | 6.40 |
| 3 | 18 | 4 | 84.29 | 6.40 |
| 4 | -18 | 4 | 262.77 | 15.30 |
| -4 | -18 | -4 | 258.37 | 15.50 |
| 4 | -18 | 4 | 265.47 | 16.50 |
| 4 | 18 | 4 | 260.87 | 15.30 |
| -5 | -18 | -4 | 26.00 | 4.10 |
| 5 | -18 | 4 | 27.90 | 3.70 |
| 5 | 18 | 4 | 34.30 | 3.90 |
| 6 | -18 | 4 | 115.19 | 7.50 |
| -6 | -18 | -4 | 107.19 | 7.50 |
| 6 | 18 | 4 | 118.69 | 7.50 |
| -6 | -19 | 4 | 24.10 | 4.30 |
| -6 | 19 | 4 | 28.20 | 4.80 |
| 6 | 19 | -4 | 22.80 | 3.20 |
| -5 | -19 | 4 | 319.17 | 18.20 |
| 5 | 19 | -4 | 320.97 | 17.90 |
| -5 | 19 | 4 | 305.37 | 18.70 |
| 4 | -19 | -4 | 85.19 | 7.20 |
| -4 | -19 | 4 | 77.09 | 6.20 |
| -4 | 19 | 4 | 74.39 | 7.00 |
| 3 | -19 | -4 | 3.00 | 2.60 |
| -3 | -19 | 4 | 2.50 | 2.00 |
| -3 | 19 | 4 | 7.70 | 4.00 |
| 2 | -19 | -4 | 142.49 | 9.90 |
| -2 | -19 | 4 | 150.48 | 9.90 |
| -2 | 19 | 4 | 146.59 | 9.90 |
| 1 | -19 | -4 | 271.17 | 17.00 |
| -1 | -19 | 4 | 287.37 | 17.00 |
| -1 | 19 | 4 | 292.57 | 16.80 |
| 0 | -19 | -4 | 22.00 | 4.70 |
| 0 | -19 | 4 | 18.20 | 3.50 |
| 0 | 19 | 4 | 25.40 | 4.10 |
| -1 | -19 | -4 | 152.58 | 10.60 |

| | | | | |
|---|---|---|---|---|
| 1 | -19 | 4 | 180.68 | 11.30 |
| 1 | 19 | 4 | 154.38 | 10.70 |
| 2 | -19 | 4 | 87.99 | 7.50 |
| 2 | -19 | 4 | 65.09 | 5.50 |
| -2 | -19 | -4 | 85.09 | 6.30 |
| 2 | 19 | 4 | 70.99 | 5.90 |
| 3 | -19 | 4 | 59.09 | 4.80 |
| 3 | -19 | 4 | 54.39 | 6.70 |
| -3 | -19 | -4 | 66.49 | 5.20 |
| 3 | 19 | 4 | 60.09 | 5.00 |
| 4 | -19 | 4 | 45.00 | 6.20 |
| 4 | -19 | 4 | 53.79 | 4.30 |
| -4 | -19 | -4 | 49.50 | 4.60 |
| 4 | 19 | 4 | 48.00 | 4.50 |
| 2 | -20 | -4 | 75.69 | 6.00 |
| -2 | -20 | 4 | 73.29 | 5.80 |
| -2 | 20 | 4 | 69.69 | 6.20 |
| 1 | -20 | -4 | 64.99 | 5.80 |
| -1 | -20 | 4 | 63.09 | 5.70 |
| -1 | 20 | 4 | 70.89 | 5.70 |
| 0 | -20 | 4 | 258.27 | 15.30 |
| 0 | -20 | -4 | 253.37 | 15.10 |
| 0 | 20 | 4 | 256.87 | 15.00 |
| -1 | -20 | -4 | 103.59 | 7.30 |
| 1 | -20 | 4 | 107.09 | 7.90 |
| 1 | 20 | 4 | 104.69 | 7.10 |
| 17 | 0 | -5 | 115.29 | 7.30 |
| 16 | 0 | -5 | 0.90 | 1.90 |
| 15 | 0 | -5 | 510.05 | 28.70 |
| 15 | 0 | -5 | 499.25 | 29.30 |
| 14 | 0 | -5 | -4.30 | 2.10 |
| 14 | 0 | -5 | -2.40 | 4.40 |
| 13 | 0 | -5 | 126.99 | 11.60 |
| 13 | 0 | -5 | 117.99 | 9.10 |
| 13 | 0 | -5 | 124.19 | 10.60 |
| 12 | 0 | -5 | -5.90 | 4.30 |
| 12 | 0 | -5 | 1.40 | 5.20 |
| 12 | 0 | -5 | -5.60 | 2.70 |
| 11 | 0 | -5 | 2443.36 | 125.89 |
| 11 | 0 | -5 | 2257.67 | 124.89 |
| 11 | 0 | -5 | 2187.88 | 124.39 |
| 10 | 0 | -5 | 11.30 | 6.50 |
| 10 | 0 | -5 | 7.90 | 3.30 |
| 10 | 0 | -5 | -1.60 | 5.60 |

| | | | | |
|---|---|---|---|---|
| 9 | 0 | -5 | 2267.17 | 124.49 |
| 9 | 0 | -5 | 2200.18 | 122.89 |
| 9 | 0 | -5 | 2337.57 | 123.29 |
| 8 | 0 | -5 | 4.90 | 5.20 |
| -5 | 0 | 5 | 7611.54 | 408.36 |
| -4 | 0 | 5 | -0.30 | 2.20 |
| -3 | 0 | 5 | 36.70 | 4.60 |
| -2 | 0 | 5 | 3.70 | 2.40 |
| -1 | 0 | 5 | 9292.47 | 499.05 |
| 0 | 0 | 5 | 1.10 | 2.80 |
| 1 | 0 | 5 | 2972.10 | 161.38 |
| 2 | 0 | 5 | 0.10 | 3.50 |
| 3 | 0 | 5 | 2521.95 | 137.59 |
| 4 | 0 | 5 | 3.80 | 3.80 |
| 5 | 0 | 5 | 4300.67 | 209.78 |
| 5 | 0 | 5 | 3445.46 | 208.78 |
| 6 | 0 | 5 | -2.00 | 3.40 |
| 6 | 0 | 5 | -1.30 | 4.20 |
| 7 | 0 | 5 | 323.27 | 20.10 |
| 7 | 0 | 5 | 321.07 | 20.60 |
| 7 | 0 | 5 | 336.77 | 20.80 |
| 8 | 0 | 5 | 0.20 | 4.30 |
| 8 | 0 | 5 | -2.40 | 3.90 |
| 9 | 0 | 5 | 1399.76 | 76.29 |
| 9 | 0 | 5 | 1185.58 | 76.39 |
| 9 | 0 | 5 | 1559.94 | 76.39 |
| 10 | 0 | 5 | 0.40 | 4.40 |
| 10 | 0 | 5 | 1.30 | 3.70 |
| 11 | 0 | 5 | 825.42 | 46.00 |
| 11 | 0 | 5 | 813.52 | 46.10 |
| 11 | 0 | 5 | 794.82 | 46.00 |
| 12 | 0 | 5 | -1.30 | 3.50 |
| 12 | 0 | 5 | -2.20 | 3.70 |
| 12 | 0 | 5 | -11.80 | 5.00 |
| 13 | 0 | 5 | 359.76 | 21.00 |
| 13 | 0 | 5 | 297.97 | 19.90 |
| 13 | 0 | 5 | 309.57 | 19.50 |
| 14 | 0 | 5 | 2.40 | 2.50 |
| 14 | 0 | 5 | 1.50 | 3.00 |
| 15 | 0 | 5 | 252.77 | 15.20 |
| 15 | 0 | 5 | 242.68 | 15.80 |
| 17 | -1 | -5 | 1.80 | 1.50 |
| 17 | 1 | -5 | 3.90 | 1.70 |
| 16 | -1 | -5 | 283.57 | 16.30 |

| | | | | |
|---|---|---|---|---|
| 16 | 1 | -5 | 275.87 | 16.20 |
| 15 | -1 | -5 | 10.60 | 4.40 |
| 15 | -1 | -5 | 8.20 | 2.80 |
| 15 | 1 | -5 | 17.70 | 3.30 |
| 14 | -1 | -5 | 448.96 | 26.20 |
| 14 | -1 | -5 | 449.36 | 25.40 |
| 14 | 1 | -5 | 420.46 | 25.20 |
| 14 | 1 | -5 | 444.66 | 26.30 |
| 13 | -1 | -5 | 10.80 | 5.00 |
| 13 | -1 | -5 | 5.70 | 4.30 |
| 13 | -1 | -5 | 4.80 | 2.60 |
| 13 | 1 | -5 | 1.80 | 2.50 |
| 13 | 1 | -5 | 11.00 | 4.50 |
| 13 | 1 | -5 | 6.50 | 4.60 |
| 12 | -1 | -5 | 327.67 | 20.50 |
| 12 | -1 | -5 | 379.06 | 22.60 |
| 12 | -1 | -5 | 362.66 | 21.30 |
| 12 | 1 | -5 | 346.27 | 21.40 |
| 12 | 1 | -5 | 319.27 | 20.20 |
| 12 | 1 | -5 | 323.17 | 22.30 |
| 11 | -1 | -5 | 48.40 | 8.10 |
| 11 | -1 | -5 | 48.00 | 7.80 |
| 11 | -1 | -5 | 47.50 | 6.90 |
| 11 | 1 | -5 | 60.59 | 8.90 |
| 11 | 1 | -5 | 44.00 | 6.60 |
| 11 | 1 | -5 | 44.70 | 7.70 |
| 10 | -1 | -5 | 1238.98 | 72.09 |
| 10 | -1 | -5 | 1389.16 | 73.89 |
| 10 | -1 | -5 | 1335.27 | 72.69 |
| 10 | 1 | -5 | 1268.27 | 71.99 |
| 10 | 1 | -5 | 1369.36 | 72.69 |
| 10 | 1 | -5 | 1303.47 | 73.79 |
| 9 | -1 | -5 | 14.50 | 4.70 |
| 9 | -1 | -5 | 8.60 | 4.00 |
| 9 | 1 | -5 | 6.60 | 6.90 |
| 9 | 1 | -5 | 6.00 | 3.70 |
| 9 | 1 | -5 | 8.10 | 5.50 |
| 8 | -1 | -5 | 47.00 | 8.90 |
| 8 | 1 | -5 | 51.09 | 7.40 |
| -6 | 1 | 5 | 5077.49 | 272.67 |
| -5 | -1 | 5 | 117.79 | 8.00 |
| -5 | 1 | 5 | 99.19 | 7.70 |
| -4 | -1 | 5 | 2509.75 | 130.79 |
| -4 | 1 | 5 | 2325.77 | 130.69 |

| | | | | |
|---|---|---|---|---|
| -3 | -1 | 5 | 205.28 | 11.80 |
| -3 | 1 | 5 | 159.48 | 11.60 |
| -2 | -1 | 5 | 1580.84 | 80.39 |
| -2 | 1 | 5 | 1357.46 | 80.19 |
| -1 | -1 | 5 | 2390.76 | 129.29 |
| -1 | 1 | 5 | 2372.46 | 129.19 |
| 0 | -1 | 5 | 7565.64 | 404.86 |
| 0 | 1 | 5 | 7487.05 | 404.66 |
| 1 | -1 | 5 | 19.90 | 4.20 |
| 1 | 1 | 5 | 16.40 | 4.00 |
| 2 | -1 | 5 | 102.09 | 8.70 |
| 2 | 1 | 5 | 87.19 | 8.50 |
| 3 | -1 | 5 | 17.20 | 4.10 |
| 3 | 1 | 5 | 15.30 | 4.50 |
| 4 | -1 | 5 | 1676.63 | 85.39 |
| 4 | 1 | 5 | 1429.56 | 85.89 |
| 5 | -1 | 5 | 27.50 | 6.30 |
| 5 | -1 | 5 | 41.40 | 6.40 |
| 5 | 1 | 5 | 38.50 | 5.20 |
| 5 | 1 | 5 | 48.70 | 6.90 |
| 6 | -1 | 5 | 1803.12 | 93.09 |
| 6 | -1 | 5 | 1712.73 | 93.59 |
| 6 | 1 | 5 | 1781.92 | 93.89 |
| 6 | 1 | 5 | 1516.45 | 92.79 |
| 7 | -1 | 5 | 23.50 | 5.00 |
| 7 | -1 | 5 | 25.30 | 5.70 |
| 7 | -1 | 5 | 35.00 | 6.80 |
| 7 | 1 | 5 | 32.00 | 5.20 |
| 7 | 1 | 5 | 23.60 | 5.30 |
| 7 | 1 | 5 | 29.20 | 5.30 |
| 8 | -1 | 5 | 1059.29 | 56.99 |
| 8 | -1 | 5 | 983.30 | 57.39 |
| 8 | -1 | 5 | 1010.60 | 57.19 |
| 8 | 1 | 5 | 1046.60 | 56.89 |
| 8 | 1 | 5 | 1010.80 | 57.29 |
| 9 | -1 | 5 | 17.80 | 5.20 |
| 9 | -1 | 5 | 26.10 | 6.40 |
| 9 | -1 | 5 | 20.30 | 5.30 |
| 9 | 1 | 5 | 15.20 | 5.10 |
| 9 | 1 | 5 | 11.40 | 4.40 |
| 10 | -1 | 5 | 1346.37 | 75.19 |
| 10 | -1 | 5 | 1485.75 | 75.09 |
| 10 | -1 | 5 | 1242.38 | 75.19 |
| 10 | 1 | 5 | 1288.97 | 75.09 |

| | | | | |
|---|---|---|---|---|
| 10 | 1 | 5 | 1390.96 | 74.89 |
| 10 | 1 | 5 | 1369.76 | 77.39 |
| 11 | -1 | 5 | 23.10 | 5.20 |
| 11 | -1 | 5 | 9.00 | 3.70 |
| 11 | -1 | 5 | 17.40 | 5.50 |
| 11 | 1 | 5 | 8.50 | 5.90 |
| 11 | 1 | 5 | 16.70 | 4.40 |
| 11 | 1 | 5 | 9.70 | 4.40 |
| 12 | -1 | 5 | 7.60 | 3.80 |
| 12 | -1 | 5 | 2.50 | 4.80 |
| 12 | -1 | 5 | 8.80 | 3.90 |
| 12 | 1 | 5 | 18.90 | 5.70 |
| 12 | 1 | 5 | 6.00 | 4.10 |
| 12 | 1 | 5 | -1.80 | 3.90 |
| 13 | -1 | 5 | 5.80 | 3.80 |
| 13 | -1 | 5 | 11.40 | 5.90 |
| 13 | -1 | 5 | 5.70 | 3.00 |
| 13 | 1 | 5 | 6.10 | 3.10 |
| 13 | 1 | 5 | 8.50 | 3.80 |
| 14 | -1 | 5 | 501.05 | 26.70 |
| 14 | -1 | 5 | 424.06 | 26.90 |
| 14 | -1 | 5 | 445.06 | 27.20 |
| 14 | 1 | 5 | 479.65 | 26.60 |
| 14 | 1 | 5 | 451.45 | 26.90 |
| 15 | -1 | 5 | 1.50 | 2.60 |
| 15 | -1 | 5 | 0.30 | 3.50 |
| 15 | 1 | 5 | 3.10 | 2.70 |
| 15 | 1 | 5 | 2.80 | 2.50 |
| 17 | -2 | -5 | 144.29 | 8.70 |
| 17 | 2 | -5 | 141.69 | 8.70 |
| 16 | -2 | -5 | 4.00 | 2.10 |
| 16 | -2 | -5 | -2.60 | 4.10 |
| 16 | 2 | -5 | 4.00 | 1.90 |
| 15 | -2 | -5 | 240.68 | 15.50 |
| 14 | -2 | -5 | 38.00 | 7.60 |
| 14 | -2 | -5 | 36.60 | 5.60 |
| 14 | 2 | -5 | 35.20 | 5.00 |
| 14 | 2 | -5 | 25.60 | 5.80 |
| 13 | -2 | -5 | 151.48 | 10.60 |
| 13 | -2 | -5 | 155.88 | 11.80 |
| 13 | 2 | -5 | 136.79 | 10.10 |
| 13 | 2 | -5 | 130.19 | 12.20 |
| 13 | 2 | -5 | 158.98 | 12.90 |
| 12 | -2 | -5 | -2.90 | 3.30 |

| | | | | |
|---|---|---|---|---|
| 12 | -2 | -5 | 3.20 | 5.10 |
| 12 | -2 | -5 | -5.20 | 4.00 |
| 12 | 2 | -5 | -2.00 | 5.00 |
| 12 | 2 | -5 | -0.60 | 4.80 |
| 12 | 2 | -5 | -5.30 | 2.60 |
| 11 | -2 | -5 | 1293.27 | 68.19 |
| 11 | -2 | -5 | 1201.98 | 66.49 |
| 11 | -2 | -5 | 1163.88 | 65.99 |
| 11 | 2 | -5 | 1227.68 | 67.49 |
| 11 | 2 | -5 | 1104.09 | 66.29 |
| 11 | 2 | -5 | 1211.98 | 66.09 |
| 10 | -2 | -5 | -10.80 | 5.40 |
| 10 | -2 | -5 | -2.80 | 3.80 |
| 10 | -2 | -5 | 0.00 | 4.00 |
| 10 | 2 | -5 | -2.50 | 5.30 |
| 10 | 2 | -5 | 0.30 | 3.40 |
| 10 | 2 | -5 | -3.90 | 6.60 |
| 9 | -2 | -5 | 521.25 | 32.10 |
| 9 | -2 | -5 | 560.34 | 33.90 |
| 9 | -2 | -5 | 573.34 | 32.30 |
| 9 | 2 | -5 | 574.84 | 32.90 |
| 9 | 2 | -5 | 537.45 | 31.70 |
| 9 | 2 | -5 | 590.04 | 34.80 |
| 8 | -2 | -5 | 47.70 | 8.20 |
| 7 | -2 | -5 | 1200.58 | 73.49 |
| 7 | 2 | -5 | 1466.45 | 72.69 |
| -6 | 2 | 5 | 81.39 | 7.30 |
| -5 | 2 | 5 | 3030.40 | 163.38 |
| -4 | -2 | 5 | 14.50 | 3.10 |
| -4 | 2 | 5 | 14.20 | 3.10 |
| -3 | -2 | 5 | 275.87 | 16.60 |
| -3 | 2 | 5 | 266.97 | 16.30 |
| -2 | -2 | 5 | 15.00 | 3.20 |
| -2 | 2 | 5 | 9.80 | 2.80 |
| -1 | -2 | 5 | 9685.83 | 525.65 |
| -1 | 2 | 5 | 9887.21 | 525.25 |
| 0 | -2 | 5 | 306.17 | 19.60 |
| 0 | 2 | 5 | 339.27 | 19.30 |
| 1 | -2 | 5 | 1836.02 | 113.29 |
| 1 | 2 | 5 | 2329.27 | 113.59 |
| 2 | -2 | 5 | 246.58 | 15.50 |
| 2 | 2 | 5 | 235.48 | 15.60 |
| 3 | -2 | 5 | 503.25 | 28.70 |
| 3 | 2 | 5 | 483.95 | 29.30 |

| | | | | |
|---|---|---|---|---|
| 4 | -2 | 5 | 338.07 | 19.20 |
| 4 | 2 | 5 | 290.67 | 20.30 |
| 5 | -2 | 5 | 4844.12 | 282.77 |
| 5 | -2 | 5 | 5576.74 | 282.47 |
| 5 | 2 | 5 | 5551.34 | 282.07 |
| 5 | 2 | 5 | 5001.90 | 283.57 |
| 6 | -2 | 5 | 171.68 | 13.70 |
| 6 | -2 | 5 | 169.48 | 11.60 |
| 6 | 2 | 5 | 165.68 | 14.00 |
| 6 | 2 | 5 | 164.58 | 11.60 |
| 7 | -2 | 5 | 14.70 | 4.60 |
| 7 | -2 | 5 | 6.30 | 4.20 |
| 7 | -2 | 5 | 4.80 | 5.00 |
| 7 | 2 | 5 | 6.70 | 4.70 |
| 7 | 2 | 5 | 10.30 | 3.90 |
| 7 | 2 | 5 | 0.80 | 5.50 |
| 8 | -2 | 5 | 3.60 | 4.60 |
| 8 | -2 | 5 | 1.30 | 4.90 |
| 8 | -2 | 5 | 9.80 | 6.20 |
| 8 | 2 | 5 | 8.40 | 4.60 |
| 8 | 2 | 5 | 2.30 | 3.80 |
| 9 | -2 | 5 | 790.62 | 46.70 |
| 9 | -2 | 5 | 819.32 | 46.90 |
| 9 | -2 | 5 | 749.23 | 46.50 |
| 9 | 2 | 5 | 934.41 | 46.80 |
| 9 | 2 | 5 | 833.22 | 46.90 |
| 10 | -2 | 5 | 2.30 | 4.20 |
| 10 | -2 | 5 | -1.80 | 4.50 |
| 10 | -2 | 5 | 2.60 | 7.00 |
| 10 | 2 | 5 | -1.50 | 4.10 |
| 10 | 2 | 5 | 4.50 | 4.30 |
| 11 | -2 | 5 | 842.82 | 46.30 |
| 11 | -2 | 5 | 848.72 | 45.90 |
| 11 | -2 | 5 | 789.32 | 46.10 |
| 11 | 2 | 5 | 740.53 | 46.00 |
| 11 | 2 | 5 | 837.72 | 46.00 |
| 12 | -2 | 5 | 8.30 | 4.70 |
| 12 | -2 | 5 | -3.40 | 3.10 |
| 12 | -2 | 5 | 2.80 | 4.30 |
| 12 | 2 | 5 | 6.20 | 4.10 |
| 12 | 2 | 5 | 1.10 | 3.20 |
| 13 | -2 | 5 | 180.48 | 12.40 |
| 13 | -2 | 5 | 153.98 | 12.90 |
| 13 | -2 | 5 | 194.98 | 14.30 |

| | | | | |
|---|---|---|---|---|
| 13 | 2 | 5 | 193.58 | 12.80 |
| 13 | 2 | 5 | 187.88 | 12.50 |
| 14 | -2 | 5 | -2.60 | 2.50 |
| 14 | -2 | 5 | 4.30 | 3.30 |
| 14 | -2 | 5 | 0.60 | 4.30 |
| 14 | 2 | 5 | 2.30 | 2.60 |
| 14 | 2 | 5 | 2.10 | 2.90 |
| 15 | -2 | 5 | 360.46 | 19.70 |
| 15 | -2 | 5 | 340.77 | 19.80 |
| 15 | 2 | 5 | 320.37 | 19.70 |
| 15 | 2 | 5 | 334.37 | 19.60 |
| 17 | -3 | -5 | 0.00 | 1.50 |
| 17 | 3 | -5 | 0.00 | 1.40 |
| 16 | -3 | -5 | 244.28 | 14.20 |
| 16 | -3 | -5 | 227.98 | 15.20 |
| 16 | 3 | -5 | 248.18 | 14.10 |
| 15 | -3 | -5 | 3.50 | 2.70 |
| 15 | -3 | -5 | 2.30 | 3.80 |
| 15 | 3 | -5 | -2.10 | 2.20 |
| 14 | -3 | -5 | 263.07 | 16.30 |
| 14 | -3 | -5 | 266.77 | 17.20 |
| 14 | 3 | -5 | 271.97 | 16.00 |
| 13 | -3 | -5 | 6.40 | 2.90 |
| 13 | -3 | -5 | 7.70 | 4.10 |
| 13 | 3 | -5 | 5.60 | 4.90 |
| 13 | 3 | -5 | 3.60 | 2.50 |
| 12 | -3 | -5 | 384.06 | 22.60 |
| 12 | -3 | -5 | 343.37 | 22.00 |
| 12 | 3 | -5 | 390.06 | 23.80 |
| 12 | 3 | -5 | 369.66 | 22.80 |
| 12 | 3 | -5 | 358.66 | 21.60 |
| 11 | -3 | -5 | 0.60 | 8.60 |
| 11 | -3 | -5 | 1.10 | 4.10 |
| 11 | 3 | -5 | 4.80 | 4.30 |
| 11 | 3 | -5 | -1.40 | 5.50 |
| 11 | 3 | -5 | -2.50 | 5.60 |
| 10 | -3 | -5 | 872.51 | 48.10 |
| 10 | -3 | -5 | 785.12 | 46.60 |
| 10 | -3 | -5 | 836.72 | 46.60 |
| 10 | 3 | -5 | 838.22 | 47.90 |
| 10 | 3 | -5 | 824.72 | 48.20 |
| 10 | 3 | -5 | 828.42 | 46.10 |
| 9 | -3 | -5 | 40.30 | 7.20 |
| 9 | -3 | -5 | 39.10 | 7.30 |

| | | | | |
|---|---|---|---|---|
| 9 | -3 | -5 | 41.60 | 5.80 |
| 9 | 3 | -5 | 52.59 | 9.50 |
| 9 | 3 | -5 | 36.40 | 6.70 |
| 8 | -3 | -5 | 363.66 | 22.20 |
| 7 | -3 | -5 | 148.99 | 13.60 |
| 7 | 3 | -5 | 191.28 | 11.80 |
| -6 | 3 | 5 | 3914.11 | 203.48 |
| 6 | 3 | -5 | 3646.14 | 203.58 |
| -5 | 3 | 5 | 62.89 | 6.50 |
| -4 | 3 | 5 | 1508.65 | 82.19 |
| -3 | -3 | 5 | 2.50 | 2.70 |
| -3 | 3 | 5 | 3.30 | 2.40 |
| -2 | -3 | 5 | 1497.45 | 87.99 |
| -2 | 3 | 5 | 1719.33 | 87.69 |
| -1 | -3 | 5 | 6.30 | 3.00 |
| -1 | 3 | 5 | 6.60 | 2.40 |
| 0 | -3 | 5 | 5940.41 | 345.27 |
| 0 | 3 | 5 | 6890.21 | 345.07 |
| 1 | -3 | 5 | 4.30 | 3.00 |
| 1 | 3 | 5 | 2.20 | 2.30 |
| 2 | -3 | 5 | 166.98 | 11.60 |
| 2 | 3 | 5 | 171.48 | 11.80 |
| 3 | -3 | 5 | 22.90 | 4.80 |
| 3 | 3 | 5 | 23.10 | 5.80 |
| 4 | -3 | 5 | 841.42 | 44.10 |
| 4 | 3 | 5 | 730.43 | 45.10 |
| 5 | -3 | 5 | 2.70 | 6.00 |
| 5 | -3 | 5 | 4.00 | 3.00 |
| 5 | 3 | 5 | 3.40 | 4.90 |
| 5 | 3 | 5 | 6.70 | 3.20 |
| 6 | -3 | 5 | 1669.43 | 94.39 |
| 6 | -3 | 5 | 1850.71 | 93.69 |
| 6 | 3 | 5 | 1665.63 | 93.39 |
| 6 | 3 | 5 | 1674.33 | 95.19 |
| 7 | -3 | 5 | 3.30 | 6.20 |
| 7 | -3 | 5 | 5.90 | 4.60 |
| 7 | 3 | 5 | 4.40 | 4.70 |
| 7 | 3 | 5 | 2.60 | 7.10 |
| 8 | -3 | 5 | 291.67 | 18.10 |
| 8 | -3 | 5 | 260.07 | 18.50 |
| 8 | -3 | 5 | 252.87 | 18.20 |
| 8 | 3 | 5 | 293.07 | 17.70 |
| 8 | 3 | 5 | 250.67 | 17.10 |
| 9 | -3 | 5 | 130.09 | 10.80 |

| | | | | |
|---|---|---|---|---|
| 9 | -3 | 5 | 95.79 | 11.90 |
| 9 | -3 | 5 | 121.99 | 11.40 |
| 9 | 3 | 5 | 126.39 | 10.40 |
| 9 | 3 | 5 | 104.49 | 10.00 |
| 10 | -3 | 5 | 1959.50 | 106.89 |
| 10 | -3 | 5 | 2215.68 | 107.69 |
| 10 | -3 | 5 | 1883.31 | 107.49 |
| 10 | 3 | 5 | 1693.73 | 107.29 |
| 10 | 3 | 5 | 2057.19 | 107.19 |
| 11 | -3 | 5 | 35.80 | 6.80 |
| 11 | -3 | 5 | 53.99 | 8.50 |
| 11 | -3 | 5 | 33.30 | 5.50 |
| 11 | 3 | 5 | 26.20 | 5.00 |
| 11 | 3 | 5 | 33.70 | 7.10 |
| 12 | -3 | 5 | -2.60 | 4.20 |
| 12 | -3 | 5 | 0.70 | 4.40 |
| 12 | -3 | 5 | 3.50 | 3.00 |
| 12 | 3 | 5 | 0.60 | 3.40 |
| 13 | -3 | 5 | 69.09 | 8.40 |
| 13 | -3 | 5 | 46.20 | 6.40 |
| 13 | -3 | 5 | 43.10 | 7.50 |
| 13 | 3 | 5 | 52.49 | 6.10 |
| 13 | 3 | 5 | 38.70 | 6.50 |
| 14 | -3 | 5 | 383.66 | 23.10 |
| 14 | -3 | 5 | 425.06 | 23.40 |
| 14 | -3 | 5 | 419.76 | 23.60 |
| 14 | 3 | 5 | 372.56 | 23.30 |
| 14 | 3 | 5 | 391.16 | 23.10 |
| 15 | -3 | 5 | 21.30 | 4.40 |
| 15 | -3 | 5 | 10.60 | 2.70 |
| 15 | 3 | 5 | 5.80 | 2.40 |
| 15 | 3 | 5 | 10.40 | 2.70 |
| 16 | -4 | -5 | 8.60 | 3.80 |
| 16 | -4 | -5 | 0.10 | 1.70 |
| 15 | -4 | -5 | 441.76 | 25.40 |
| 15 | -4 | -5 | 440.66 | 25.90 |
| 15 | 4 | -5 | 455.05 | 25.20 |
| 14 | -4 | -5 | 8.30 | 4.00 |
| 14 | -4 | -5 | 2.80 | 2.50 |
| 14 | 4 | -5 | 3.50 | 2.30 |
| 13 | -4 | -5 | 102.09 | 8.60 |
| 13 | 4 | -5 | 104.69 | 7.90 |
| 12 | -4 | -5 | 123.79 | 10.10 |
| 12 | -4 | -5 | 112.69 | 10.30 |

| | | | | |
|---|---|---|---|---|
| 12 | 4 | -5 | 131.69 | 13.20 |
| 12 | 4 | -5 | 130.39 | 9.10 |
| 11 | -4 | -5 | 1236.98 | 72.39 |
| 11 | -4 | -5 | 1332.97 | 72.29 |
| 11 | 4 | -5 | 1323.27 | 71.79 |
| 11 | 4 | -5 | 1363.96 | 74.19 |
| 10 | -4 | -5 | 12.90 | 4.30 |
| 10 | -4 | -5 | 8.70 | 3.80 |
| 10 | 4 | -5 | 9.00 | 7.70 |
| 10 | 4 | -5 | 16.70 | 3.80 |
| 9 | -4 | -5 | 576.34 | 38.90 |
| 9 | -4 | -5 | 712.03 | 39.50 |
| 9 | -4 | -5 | 677.03 | 38.30 |
| 9 | 4 | -5 | 669.03 | 37.50 |
| 9 | 4 | -5 | 698.43 | 40.60 |
| 8 | -4 | -5 | 79.09 | 9.00 |
| 8 | 4 | -5 | 87.99 | 8.80 |
| 7 | -4 | -5 | 132.59 | 12.10 |
| 7 | 4 | -5 | 154.78 | 10.60 |
| 6 | -4 | -5 | 155.38 | 14.30 |
| 6 | 4 | -5 | 167.88 | 11.40 |
| -6 | 4 | 5 | 177.88 | 11.80 |
| -5 | 4 | 5 | 3047.60 | 164.58 |
| -4 | 4 | 5 | 49.60 | 5.50 |
| -3 | 4 | 5 | 187.68 | 12.00 |
| -2 | 4 | 5 | 25.40 | 4.20 |
| -1 | -4 | 5 | 1678.73 | 105.59 |
| -1 | 4 | 5 | 2193.58 | 105.49 |
| 0 | -4 | 5 | 21.50 | 5.10 |
| 0 | 4 | 5 | 21.40 | 3.70 |
| 1 | -4 | 5 | 1417.26 | 79.49 |
| 1 | 4 | 5 | 1476.55 | 79.39 |
| 2 | -4 | 5 | 203.78 | 13.00 |
| 2 | 4 | 5 | 194.18 | 13.30 |
| 3 | -4 | 5 | 855.21 | 46.80 |
| 3 | 4 | 5 | 821.32 | 47.50 |
| 4 | -4 | 5 | 52.99 | 6.10 |
| 4 | 4 | 5 | 47.00 | 7.80 |
| 4 | 4 | 5 | 59.99 | 6.00 |
| 5 | -4 | 5 | 4651.53 | 251.77 |
| 5 | 4 | 5 | 4506.45 | 252.57 |
| 5 | 4 | 5 | 4822.22 | 250.77 |
| 6 | -4 | 5 | -1.40 | 5.40 |
| 6 | 4 | 5 | -1.10 | 2.90 |

| | | | | |
|---|---|---|---|---|
| 6 | 4 | 5 | 5.40 | 6.00 |
| 7 | -4 | 5 | 316.27 | 21.80 |
| 7 | -4 | 5 | 334.77 | 22.50 |
| 7 | 4 | 5 | 375.16 | 21.30 |
| 7 | 4 | 5 | 335.77 | 20.30 |
| 7 | 4 | 5 | 337.07 | 23.30 |
| 8 | -4 | 5 | 13.50 | 6.30 |
| 8 | -4 | 5 | 3.70 | 5.20 |
| 8 | -4 | 5 | 9.10 | 4.80 |
| 8 | 4 | 5 | 9.80 | 4.70 |
| 8 | 4 | 5 | 3.50 | 3.10 |
| 9 | -4 | 5 | 945.61 | 54.29 |
| 9 | -4 | 5 | 913.31 | 53.69 |
| 9 | -4 | 5 | 1019.20 | 54.59 |
| 9 | 4 | 5 | 1005.60 | 54.09 |
| 9 | 4 | 5 | 934.51 | 54.59 |
| 10 | -4 | 5 | 22.30 | 6.20 |
| 10 | -4 | 5 | 14.60 | 4.60 |
| 10 | -4 | 5 | 11.30 | 4.30 |
| 10 | 4 | 5 | 16.00 | 4.60 |
| 10 | 4 | 5 | 10.90 | 4.10 |
| 11 | -4 | 5 | 545.55 | 34.00 |
| 11 | -4 | 5 | 648.84 | 33.40 |
| 11 | -4 | 5 | 604.74 | 33.80 |
| 11 | 4 | 5 | 523.55 | 33.60 |
| 11 | 4 | 5 | 565.84 | 33.40 |
| 12 | -4 | 5 | 6.20 | 4.80 |
| 12 | -4 | 5 | 8.20 | 4.20 |
| 12 | -4 | 5 | 4.30 | 3.40 |
| 12 | 4 | 5 | -2.60 | 3.20 |
| 12 | 4 | 5 | -1.30 | 3.30 |
| 13 | -4 | 5 | 224.88 | 17.30 |
| 13 | -4 | 5 | 284.07 | 16.40 |
| 13 | -4 | 5 | 261.07 | 16.10 |
| 13 | 4 | 5 | 254.77 | 19.40 |
| 13 | 4 | 5 | 246.98 | 16.00 |
| 14 | -4 | 5 | -2.80 | 3.60 |
| 14 | -4 | 5 | -1.80 | 2.60 |
| 14 | -4 | 5 | -5.00 | 3.10 |
| 14 | 4 | 5 | -2.20 | 2.30 |
| 14 | 4 | 5 | 4.50 | 2.90 |
| 15 | -4 | 5 | 274.87 | 15.70 |
| 15 | -4 | 5 | 276.87 | 15.70 |
| 15 | 4 | 5 | 261.67 | 15.50 |

| | | | | |
|---|---|---|---|---|
| 15 | 4 | 5 | 245.58 | 15.60 |
| 16 | -5 | -5 | 233.28 | 14.10 |
| 16 | -5 | -5 | 232.98 | 14.80 |
| 16 | 5 | -5 | 253.97 | 13.90 |
| 15 | -5 | -5 | 0.40 | 2.10 |
| 15 | -5 | -5 | 1.90 | 3.30 |
| 15 | 5 | -5 | -1.00 | 1.80 |
| 14 | -5 | -5 | 279.47 | 17.90 |
| 14 | -5 | -5 | 292.87 | 17.30 |
| 14 | 5 | -5 | 284.97 | 16.80 |
| 13 | -5 | -5 | -0.20 | 4.00 |
| 13 | -5 | -5 | 1.00 | 2.90 |
| 13 | 5 | -5 | -1.30 | 2.30 |
| 12 | -5 | -5 | 277.07 | 17.70 |
| 12 | -5 | -5 | 268.17 | 17.30 |
| 12 | 5 | -5 | 280.17 | 19.70 |
| 12 | 5 | -5 | 276.17 | 16.60 |
| 11 | -5 | -5 | 0.70 | 3.50 |
| 11 | -5 | -5 | -1.90 | 3.50 |
| 11 | 5 | -5 | -10.50 | 9.30 |
| 11 | 5 | -5 | 5.70 | 2.80 |
| 10 | -5 | -5 | 878.41 | 49.60 |
| 10 | -5 | -5 | 832.22 | 49.50 |
| 10 | 5 | -5 | 879.31 | 49.00 |
| 10 | 5 | -5 | 949.90 | 51.69 |
| 9 | -5 | -5 | 0.10 | 3.70 |
| 9 | -5 | -5 | 0.90 | 5.00 |
| 9 | 5 | -5 | 5.10 | 11.30 |
| 9 | 5 | -5 | 1.60 | 3.20 |
| 8 | -5 | -5 | 463.15 | 26.40 |
| 8 | -5 | -5 | 409.26 | 27.40 |
| 8 | 5 | -5 | 446.16 | 25.90 |
| 7 | -5 | -5 | 38.50 | 8.30 |
| 7 | 5 | -5 | 32.30 | 6.50 |
| 6 | -5 | -5 | 3784.22 | 220.68 |
| -6 | 5 | 5 | 4274.57 | 220.18 |
| 6 | 5 | -5 | 4190.28 | 220.58 |
| 5 | -5 | -5 | 57.39 | 7.60 |
| -5 | 5 | 5 | 65.59 | 6.50 |
| 5 | 5 | -5 | 60.09 | 7.00 |
| -4 | 5 | 5 | 655.83 | 36.80 |
| -3 | 5 | 5 | 198.68 | 12.70 |
| -2 | 5 | 5 | 182.88 | 11.90 |
| -1 | 5 | 5 | 164.98 | 11.10 |

| | | | | |
|---|---|---|---|---|
| 0 | 5 | 5 | 3168.78 | 171.48 |
| 1 | 5 | 5 | 575.24 | 33.00 |
| 2 | 5 | 5 | 113.69 | 9.20 |
| 3 | 5 | 5 | 49.30 | 7.00 |
| 3 | 5 | 5 | 31.20 | 5.50 |
| 4 | 5 | 5 | 3128.99 | 171.48 |
| 4 | 5 | 5 | 3121.79 | 168.58 |
| 5 | -5 | 5 | 97.19 | 11.30 |
| 5 | 5 | 5 | 94.99 | 7.60 |
| 5 | 5 | 5 | 86.69 | 10.80 |
| 6 | -5 | 5 | 375.56 | 24.00 |
| 6 | 5 | 5 | 352.26 | 23.00 |
| 6 | 5 | 5 | 396.06 | 22.10 |
| 6 | 5 | 5 | 388.86 | 25.20 |
| 7 | -5 | 5 | 7.80 | 5.20 |
| 7 | -5 | 5 | -7.80 | 7.50 |
| 7 | -5 | 5 | 4.40 | 5.10 |
| 7 | 5 | 5 | -7.90 | 4.40 |
| 7 | 5 | 5 | 0.00 | 2.60 |
| 7 | 5 | 5 | 2.00 | 6.40 |
| 8 | -5 | 5 | 849.91 | 47.20 |
| 8 | -5 | 5 | 887.91 | 48.50 |
| 8 | -5 | 5 | 894.61 | 48.10 |
| 8 | 5 | 5 | 699.83 | 50.19 |
| 8 | 5 | 5 | 782.12 | 46.90 |
| 8 | 5 | 5 | 924.11 | 47.40 |
| 9 | -5 | 5 | 36.40 | 5.90 |
| 9 | -5 | 5 | 27.00 | 9.30 |
| 9 | -5 | 5 | 23.30 | 6.60 |
| 9 | 5 | 5 | 34.50 | 6.10 |
| 9 | 5 | 5 | 36.90 | 7.20 |
| 10 | -5 | 5 | 786.22 | 43.00 |
| 10 | -5 | 5 | 752.52 | 43.30 |
| 10 | -5 | 5 | 769.82 | 42.30 |
| 10 | 5 | 5 | 696.13 | 42.50 |
| 10 | 5 | 5 | 738.93 | 42.30 |
| 11 | -5 | 5 | 11.70 | 6.40 |
| 11 | -5 | 5 | 9.30 | 3.90 |
| 11 | -5 | 5 | 9.80 | 5.00 |
| 11 | 5 | 5 | 8.60 | 4.20 |
| 12 | -5 | 5 | 21.50 | 4.30 |
| 12 | -5 | 5 | 8.20 | 4.80 |
| 12 | -5 | 5 | 23.30 | 4.50 |
| 12 | 5 | 5 | 19.10 | 4.00 |

| | | | | |
|---|---|---|---|---|
| 12 | 5 | 5 | 13.90 | 3.80 |
| 13 | -5 | 5 | 30.80 | 6.90 |
| 13 | -5 | 5 | 43.00 | 7.00 |
| 13 | 5 | 5 | 31.30 | 6.30 |
| 13 | 5 | 5 | 36.90 | 5.60 |
| 14 | -5 | 5 | 323.17 | 18.30 |
| 14 | -5 | 5 | 296.47 | 17.90 |
| 14 | -5 | 5 | 282.87 | 18.00 |
| 14 | 5 | 5 | 282.27 | 18.00 |
| 14 | 5 | 5 | 321.17 | 18.00 |
| 15 | -5 | 5 | 5.70 | 4.30 |
| 15 | -5 | 5 | -1.00 | 2.40 |
| 15 | -5 | 5 | 2.80 | 3.30 |
| 15 | 5 | 5 | -4.60 | 2.30 |
| 15 | 5 | 5 | -2.20 | 2.50 |
| 16 | -6 | -5 | -1.50 | 3.30 |
| 16 | -6 | -5 | -2.30 | 1.60 |
| 16 | 6 | -5 | -1.20 | 1.40 |
| 15 | -6 | -5 | 348.67 | 21.30 |
| 15 | -6 | -5 | 369.76 | 20.80 |
| 15 | 6 | -5 | 367.16 | 20.50 |
| 14 | -6 | -5 | 2.70 | 2.60 |
| 14 | -6 | -5 | 5.60 | 3.50 |
| 14 | 6 | -5 | 2.50 | 2.00 |
| 13 | -6 | -5 | 111.69 | 9.50 |
| 13 | -6 | -5 | 98.19 | 8.70 |
| 13 | 6 | -5 | 109.99 | 7.80 |
| 12 | -6 | -5 | -2.30 | 3.10 |
| 12 | -6 | -5 | 1.40 | 3.60 |
| 12 | 6 | -5 | 3.10 | 2.50 |
| 11 | -6 | -5 | 1246.88 | 68.99 |
| 11 | -6 | -5 | 1229.08 | 69.19 |
| 11 | 6 | -5 | 1277.17 | 68.49 |
| 10 | -6 | -5 | 168.68 | 13.40 |
| 10 | -6 | -5 | 179.08 | 12.70 |
| 9 | -6 | -5 | 451.25 | 28.40 |
| 9 | -6 | -5 | 472.25 | 27.70 |
| 9 | 6 | -5 | 485.55 | 27.20 |
| 8 | -6 | -5 | 6.00 | 4.40 |
| 8 | -6 | -5 | 22.80 | 6.70 |
| 8 | 6 | -5 | 8.40 | 5.00 |
| 8 | 6 | -5 | -0.10 | 3.60 |
| 7 | -6 | -5 | 538.45 | 30.50 |
| 7 | 6 | -5 | 451.65 | 30.00 |

| | | | | |
|---|---|---|---|---|
| 7 | 6 | -5 | 543.45 | 30.30 |
| 6 | -6 | -5 | 47.10 | 9.30 |
| -6 | 6 | 5 | 57.39 | 7.70 |
| 6 | 6 | -5 | 62.19 | 8.00 |
| 5 | -6 | -5 | 3090.09 | 182.78 |
| -5 | 6 | 5 | 3589.44 | 182.38 |
| 5 | 6 | -5 | 3442.86 | 182.98 |
| 4 | -6 | -5 | 220.38 | 17.50 |
| 4 | 6 | -5 | 260.27 | 17.20 |
| -4 | 6 | 5 | 305.67 | 16.70 |
| -3 | 6 | 5 | 118.79 | 9.00 |
| -2 | 6 | 5 | 107.99 | 8.60 |
| -1 | 6 | 5 | 7581.54 | 407.76 |
| 0 | 6 | 5 | 272.77 | 17.00 |
| 2 | 6 | 5 | -0.70 | 2.80 |
| 2 | 6 | 5 | 5.30 | 3.70 |
| 3 | 6 | 5 | 913.51 | 50.69 |
| 3 | 6 | 5 | 872.41 | 49.20 |
| 4 | -6 | 5 | 51.79 | 9.50 |
| 4 | 6 | 5 | 70.79 | 8.60 |
| 4 | 6 | 5 | 61.79 | 6.20 |
| 5 | -6 | 5 | 2140.09 | 111.99 |
| 5 | 6 | 5 | 2127.19 | 111.19 |
| 5 | 6 | 5 | 1877.21 | 112.99 |
| 6 | -6 | 5 | 67.29 | 9.40 |
| 6 | -6 | 5 | 83.19 | 9.80 |
| 6 | 6 | 5 | 45.10 | 8.50 |
| 6 | 6 | 5 | 72.49 | 6.40 |
| 6 | 6 | 5 | 52.29 | 8.80 |
| 7 | -6 | 5 | 65.69 | 9.70 |
| 7 | -6 | 5 | 78.09 | 10.10 |
| 7 | -6 | 5 | 71.39 | 15.30 |
| 7 | 6 | 5 | 80.59 | 8.90 |
| 7 | 6 | 5 | 76.89 | 12.90 |
| 7 | 6 | 5 | 75.29 | 6.90 |
| 8 | -6 | 5 | -0.30 | 7.90 |
| 8 | -6 | 5 | -5.10 | 4.10 |
| 8 | -6 | 5 | 2.50 | 4.20 |
| 8 | 6 | 5 | 2.30 | 2.70 |
| 8 | 6 | 5 | -3.80 | 4.00 |
| 8 | 6 | 5 | -8.00 | 8.30 |
| 9 | -6 | 5 | 876.01 | 50.79 |
| 9 | -6 | 5 | 890.31 | 49.40 |
| 9 | -6 | 5 | 913.41 | 50.29 |

| | | | | |
|---|---|---|---|---|
| 9 | 6 | 5 | 844.82 | 49.30 |
| 9 | 6 | 5 | 889.41 | 49.50 |
| 10 | -6 | 5 | 82.89 | 9.20 |
| 10 | -6 | 5 | 89.79 | 12.10 |
| 10 | -6 | 5 | 89.49 | 9.50 |
| 10 | 6 | 5 | 95.59 | 8.80 |
| 10 | 6 | 5 | 84.59 | 8.40 |
| 11 | -6 | 5 | 165.08 | 14.10 |
| 11 | -6 | 5 | 187.68 | 12.60 |
| 11 | -6 | 5 | 182.98 | 13.20 |
| 11 | 6 | 5 | 157.98 | 12.90 |
| 11 | 6 | 5 | 178.68 | 12.60 |
| 12 | -6 | 5 | 7.10 | 3.40 |
| 12 | -6 | 5 | 5.20 | 3.80 |
| 12 | -6 | 5 | 3.60 | 4.90 |
| 12 | 6 | 5 | 9.80 | 3.50 |
| 12 | 6 | 5 | 10.00 | 3.70 |
| 13 | -6 | 5 | 471.55 | 25.80 |
| 13 | -6 | 5 | 446.56 | 26.20 |
| 13 | -6 | 5 | 443.36 | 25.80 |
| 13 | 6 | 5 | 417.36 | 25.90 |
| 13 | 6 | 5 | 444.56 | 25.90 |
| 14 | -6 | 5 | 27.50 | 5.40 |
| 14 | -6 | 5 | 15.60 | 4.00 |
| 14 | -6 | 5 | 16.70 | 3.50 |
| 14 | 6 | 5 | 23.10 | 5.10 |
| 14 | 6 | 5 | 11.60 | 2.80 |
| 16 | -7 | -5 | 183.58 | 11.30 |
| -16 | 7 | 5 | 201.78 | 11.50 |
| 16 | 7 | -5 | 190.78 | 11.10 |
| 15 | -7 | -5 | 2.20 | 2.10 |
| 15 | -7 | -5 | 4.00 | 3.20 |
| 15 | 7 | -5 | 0.80 | 1.70 |
| 14 | -7 | -5 | 295.97 | 17.80 |
| 14 | -7 | -5 | 300.97 | 18.40 |
| 14 | 7 | -5 | 299.47 | 17.50 |
| 13 | -7 | -5 | 41.40 | 6.50 |
| 13 | -7 | -5 | 30.40 | 5.70 |
| 13 | 7 | -5 | 32.40 | 4.40 |
| 12 | -7 | -5 | 105.49 | 9.20 |
| 12 | 7 | -5 | 116.29 | 8.20 |
| 11 | -7 | -5 | 13.80 | 3.90 |
| 11 | -7 | -5 | 7.00 | 4.00 |
| 11 | 7 | -5 | 10.10 | 2.80 |

| | | | | |
|---|---|---|---|---|
| 10 | -7 | -5 | 1300.97 | 71.19 |
| 10 | -7 | -5 | 1288.57 | 71.19 |
| 10 | 7 | -5 | 1271.27 | 70.49 |
| 9 | -7 | -5 | 11.70 | 5.60 |
| 9 | -7 | -5 | 1.10 | 3.80 |
| 9 | 7 | -5 | 6.30 | 3.20 |
| 8 | -7 | -5 | 105.29 | 10.00 |
| 8 | -7 | -5 | 94.49 | 12.90 |
| 8 | 7 | -5 | 112.89 | 10.90 |
| 8 | 7 | -5 | 104.99 | 9.60 |
| 7 | -7 | -5 | 46.50 | 8.00 |
| 7 | 7 | -5 | 36.70 | 7.60 |
| 6 | -7 | -5 | 2969.80 | 162.18 |
| -6 | 7 | 5 | 3021.00 | 161.48 |
| 6 | 7 | -5 | 2955.40 | 162.18 |
| 5 | -7 | -5 | -4.80 | 4.90 |
| -5 | 7 | 5 | 0.50 | 3.30 |
| 5 | 7 | -5 | 3.50 | 4.10 |
| -4 | 7 | 5 | 973.80 | 48.50 |
| 4 | 7 | -5 | 752.22 | 48.90 |
| 3 | 7 | -5 | 205.58 | 16.90 |
| -3 | 7 | 5 | 279.07 | 15.90 |
| -2 | 7 | 5 | 324.07 | 19.90 |
| -1 | 7 | 5 | 286.87 | 16.70 |
| -1 | 7 | 5 | 227.78 | 16.10 |
| 0 | 7 | 5 | 6052.19 | 325.47 |
| 1 | 7 | 5 | 63.79 | 6.60 |
| 1 | 7 | 5 | 60.99 | 7.10 |
| 2 | 7 | 5 | 133.99 | 10.60 |
| 2 | 7 | 5 | 134.49 | 9.60 |
| 3 | 7 | 5 | 218.68 | 14.90 |
| 3 | 7 | 5 | 203.28 | 13.20 |
| 4 | -7 | 5 | 2715.93 | 148.99 |
| 4 | 7 | 5 | 2665.23 | 150.38 |
| 4 | 7 | 5 | 2867.71 | 148.69 |
| 5 | -7 | 5 | 103.09 | 10.70 |
| 5 | -7 | 5 | 107.79 | 10.70 |
| -5 | 7 | -5 | 103.19 | 8.80 |
| 5 | 7 | 5 | 107.29 | 8.40 |
| 5 | 7 | 5 | 88.59 | 11.60 |
| 6 | -7 | 5 | 1388.56 | 75.59 |
| 6 | -7 | 5 | 1358.86 | 76.19 |
| 6 | 7 | 5 | 1360.16 | 75.69 |
| 6 | 7 | 5 | 1447.66 | 75.39 |

| | | | | |
|---|---|---|---|---|
| -6 | 7 | -5 | 1493.65 | 75.89 |
| 6 | 7 | 5 | 1205.28 | 77.59 |
| -7 | -7 | -5 | 134.29 | 12.90 |
| 7 | -7 | 5 | 110.59 | 11.50 |
| 7 | -7 | 5 | 125.09 | 10.90 |
| 7 | 7 | 5 | 135.79 | 15.30 |
| 7 | 7 | 5 | 114.09 | 10.80 |
| 7 | 7 | 5 | 125.89 | 9.10 |
| 8 | -7 | 5 | 841.82 | 51.49 |
| 8 | -7 | 5 | 844.12 | 51.19 |
| 8 | -7 | 5 | 904.01 | 50.59 |
| 8 | 7 | 5 | 862.31 | 49.40 |
| 8 | 7 | 5 | 962.50 | 49.99 |
| 8 | 7 | 5 | 905.91 | 53.19 |
| 9 | -7 | 5 | -2.00 | 4.40 |
| 9 | -7 | 5 | 3.40 | 3.80 |
| 9 | -7 | 5 | 3.90 | 7.70 |
| 9 | 7 | 5 | 0.70 | 3.10 |
| 9 | 7 | 5 | 0.60 | 3.90 |
| 10 | -7 | 5 | 927.21 | 49.40 |
| 10 | -7 | 5 | 936.01 | 50.39 |
| 10 | -7 | 5 | 814.42 | 50.49 |
| 10 | 7 | 5 | 804.62 | 49.50 |
| 10 | 7 | 5 | 938.71 | 49.50 |
| 11 | -7 | 5 | -5.20 | 5.20 |
| 11 | -7 | 5 | -4.30 | 3.70 |
| 11 | -7 | 5 | -4.10 | 3.30 |
| 11 | 7 | 5 | 5.80 | 3.50 |
| 11 | 7 | 5 | 3.80 | 3.70 |
| 12 | -7 | 5 | 2.50 | 3.30 |
| 12 | -7 | 5 | 12.70 | 5.20 |
| 12 | -7 | 5 | 2.10 | 4.30 |
| 12 | 7 | 5 | 3.00 | 5.60 |
| 12 | 7 | 5 | -3.20 | 3.10 |
| 13 | -7 | 5 | 13.80 | 3.40 |
| 13 | -7 | 5 | 24.00 | 4.10 |
| 13 | -7 | 5 | 17.40 | 4.40 |
| 13 | 7 | 5 | 18.60 | 4.10 |
| 13 | 7 | 5 | 32.10 | 5.00 |
| 14 | -7 | 5 | 293.87 | 16.80 |
| 14 | -7 | 5 | 281.87 | 16.50 |
| 14 | 7 | 5 | 279.17 | 16.90 |
| 14 | 7 | 5 | 269.97 | 16.60 |
| 15 | -8 | -5 | 352.26 | 20.90 |

| | | | | |
|---|---|---|---|---|
| -15 | 8 | 5 | 371.06 | 21.10 |
| 15 | 8 | -5 | 385.96 | 20.70 |
| 14 | -8 | -5 | 3.80 | 3.30 |
| 14 | -8 | -5 | 6.10 | 2.50 |
| 14 | 8 | -5 | 3.00 | 1.80 |
| 13 | -8 | -5 | 3.70 | 2.80 |
| 13 | -8 | -5 | 1.10 | 3.10 |
| 13 | 8 | -5 | 8.10 | 2.40 |
| 12 | -8 | -5 | 49.60 | 7.00 |
| 12 | -8 | -5 | 49.90 | 6.70 |
| 12 | 8 | -5 | 47.20 | 5.40 |
| 11 | -8 | -5 | 881.61 | 49.90 |
| 11 | -8 | -5 | 891.51 | 49.90 |
| 11 | 8 | -5 | 896.71 | 49.10 |
| 10 | -8 | -5 | -1.20 | 3.30 |
| 10 | -8 | -5 | 9.10 | 4.90 |
| 10 | 8 | -5 | 0.70 | 2.60 |
| 9 | -8 | -5 | 456.25 | 27.00 |
| 9 | -8 | -5 | 431.26 | 26.20 |
| 9 | 8 | -5 | 424.46 | 25.90 |
| 8 | -8 | -5 | 53.79 | 7.90 |
| 8 | -8 | -5 | 46.70 | 8.00 |
| 8 | 8 | -5 | 52.59 | 7.60 |
| 8 | 8 | -5 | 55.19 | 9.70 |
| 7 | -8 | -5 | 218.88 | 15.30 |
| 7 | -8 | -5 | 200.98 | 17.90 |
| 7 | 8 | -5 | 211.18 | 15.20 |
| 6 | -8 | -5 | 50.89 | 9.50 |
| -6 | 8 | 5 | 57.49 | 9.50 |
| 6 | 8 | -5 | 44.00 | 8.10 |
| 5 | 8 | -5 | 2047.30 | 128.09 |
| -5 | 8 | 5 | 2638.14 | 128.49 |
| 4 | 8 | -5 | 16.40 | 4.90 |
| -4 | 8 | 5 | 24.90 | 6.00 |
| -3 | 8 | 5 | 192.48 | 12.70 |
| 3 | 8 | -5 | 163.18 | 13.40 |
| 2 | 8 | -5 | 6.30 | 4.00 |
| -2 | 8 | 5 | 3.70 | 3.00 |
| 1 | 8 | -5 | 1692.63 | 93.29 |
| -1 | 8 | 5 | 1740.23 | 92.89 |
| -1 | 8 | 5 | 1635.94 | 92.19 |
| 0 | 8 | -5 | 70.69 | 7.50 |
| 0 | 8 | 5 | 72.29 | 6.90 |
| 0 | 8 | 5 | 66.89 | 7.30 |

| | | | | |
|---|---|---|---|---|
| 1 | 8 | 5 | 1453.55 | 76.79 |
| 1 | 8 | 5 | 1351.66 | 77.59 |
| -1 | 8 | -5 | 1402.46 | 77.49 |
| 2 | 8 | 5 | 145.49 | 10.30 |
| 2 | 8 | 5 | 154.08 | 11.50 |
| -2 | 8 | -5 | 148.59 | 10.80 |
| 3 | -8 | 5 | 575.64 | 34.30 |
| 3 | 8 | 5 | 614.54 | 33.70 |
| -3 | 8 | -5 | 589.74 | 34.20 |
| 3 | 8 | 5 | 604.84 | 35.30 |
| 4 | -8 | 5 | 10.80 | 4.60 |
| 4 | -8 | 5 | -3.60 | 4.50 |
| 4 | 8 | 5 | 1.30 | 4.60 |
| -4 | 8 | -5 | 1.10 | 3.00 |
| 4 | 8 | 5 | -2.10 | 3.60 |
| 5 | -8 | 5 | 2756.62 | 143.49 |
| 5 | -8 | 5 | 2735.13 | 143.89 |
| -5 | 8 | -5 | 2802.72 | 144.09 |
| 5 | 8 | 5 | 2565.84 | 143.29 |
| 5 | 8 | 5 | 2514.05 | 143.49 |
| 5 | 8 | 5 | 2520.45 | 145.69 |
| 6 | -8 | 5 | 64.39 | 9.10 |
| -6 | -8 | -5 | 62.39 | 9.90 |
| 6 | -8 | 5 | 79.59 | 8.70 |
| -6 | 8 | -5 | 71.19 | 8.50 |
| 6 | 8 | 5 | 59.49 | 12.10 |
| 6 | 8 | 5 | 86.19 | 6.70 |
| 6 | 8 | 5 | 66.39 | 9.60 |
| -7 | -8 | -5 | 8.50 | 4.90 |
| 7 | -8 | 5 | 5.60 | 3.80 |
| 7 | -8 | 5 | 5.10 | 4.60 |
| 7 | 8 | 5 | -0.50 | 8.10 |
| 7 | 8 | 5 | -2.10 | 4.60 |
| 7 | 8 | 5 | 1.20 | 2.80 |
| 8 | -8 | 5 | -2.80 | 4.70 |
| 8 | -8 | 5 | 6.30 | 3.80 |
| 8 | 8 | 5 | 13.20 | 11.00 |
| 8 | 8 | 5 | 5.20 | 2.70 |
| 8 | 8 | 5 | 2.10 | 4.40 |
| 9 | -8 | 5 | 615.54 | 34.50 |
| 9 | -8 | 5 | 623.34 | 35.70 |
| 9 | 8 | 5 | 584.14 | 34.40 |
| 9 | 8 | 5 | 594.44 | 34.60 |
| 10 | -8 | 5 | 6.90 | 3.30 |

| | | | | |
|---|---|---|---|---|
| 10 | -8 | 5 | 2.00 | 7.30 |
| 10 | -8 | 5 | 0.10 | 4.90 |
| 10 | 8 | 5 | 6.80 | 3.60 |
| 10 | 8 | 5 | 3.40 | 4.30 |
| 11 | -8 | 5 | 378.16 | 24.00 |
| 11 | -8 | 5 | 389.66 | 22.30 |
| 11 | -8 | 5 | 376.26 | 22.80 |
| 11 | 8 | 5 | 377.96 | 22.30 |
| 11 | 8 | 5 | 335.77 | 22.10 |
| 12 | -8 | 5 | 32.70 | 6.20 |
| 12 | -8 | 5 | 41.10 | 6.20 |
| 12 | -8 | 5 | 33.00 | 5.80 |
| 12 | 8 | 5 | 37.50 | 6.80 |
| 12 | 8 | 5 | 33.70 | 5.80 |
| 13 | -8 | 5 | 205.38 | 12.60 |
| 13 | -8 | 5 | 209.28 | 13.00 |
| 13 | 8 | 5 | 185.08 | 12.70 |
| 13 | 8 | 5 | 196.68 | 13.30 |
| 14 | -8 | 5 | -3.40 | 2.70 |
| 14 | -8 | 5 | 0.90 | 3.90 |
| 14 | 8 | 5 | -3.90 | 2.50 |
| 14 | 8 | 5 | 1.70 | 3.50 |
| 15 | -9 | -5 | 4.90 | 1.90 |
| 15 | 9 | -5 | 7.90 | 1.60 |
| -15 | 9 | 5 | 7.60 | 2.20 |
| 14 | -9 | -5 | 360.26 | 20.80 |
| 14 | 9 | -5 | 357.76 | 20.40 |
| -14 | 9 | 5 | 368.96 | 21.00 |
| 13 | -9 | -5 | 57.49 | 7.50 |
| 13 | -9 | -5 | 51.19 | 6.50 |
| -13 | 9 | 5 | 55.59 | 6.50 |
| 12 | -9 | -5 | 139.19 | 10.80 |
| 12 | -9 | -5 | 149.49 | 10.70 |
| 12 | 9 | -5 | 145.89 | 9.60 |
| 11 | -9 | -5 | 1.60 | 4.00 |
| 11 | 9 | -5 | 6.20 | 2.40 |
| 10 | -9 | -5 | 994.10 | 55.09 |
| 10 | -9 | -5 | 922.51 | 54.69 |
| 10 | 9 | -5 | 1024.30 | 54.09 |
| 9 | -9 | -5 | -3.50 | 3.70 |
| 9 | -9 | -5 | 6.30 | 5.30 |
| 9 | 9 | -5 | 1.80 | 3.00 |
| 8 | -9 | -5 | 317.77 | 20.50 |
| 8 | -9 | -5 | 306.17 | 21.70 |

| | | | | |
|---|---|---|---|---|
| 8 | 9 | -5 | 332.57 | 21.60 |
| 8 | 9 | -5 | 315.87 | 21.30 |
| -7 | -9 | 5 | 206.18 | 16.60 |
| 7 | -9 | -5 | 207.48 | 17.90 |
| 7 | -9 | -5 | 214.38 | 15.80 |
| 7 | 9 | -5 | 232.18 | 16.20 |
| -7 | 9 | 5 | 218.88 | 18.70 |
| 6 | -9 | -5 | 1915.11 | 106.39 |
| -6 | -9 | 5 | 1767.52 | 105.59 |
| -6 | 9 | 5 | 1650.93 | 106.99 |
| -6 | 9 | 5 | 2190.78 | 106.39 |
| 6 | 9 | -5 | 2160.18 | 107.29 |
| -5 | -9 | 5 | 113.09 | 11.50 |
| 5 | 9 | -5 | 103.39 | 11.10 |
| -5 | 9 | 5 | 112.39 | 10.00 |
| -4 | -9 | 5 | 1135.99 | 61.09 |
| -4 | -9 | 5 | 1026.10 | 60.99 |
| -4 | 9 | 5 | 1136.79 | 61.29 |
| -3 | -9 | 5 | 45.70 | 8.60 |
| -3 | -9 | 5 | 49.80 | 8.40 |
| 3 | 9 | -5 | 63.39 | 8.80 |
| -3 | 9 | 5 | 63.99 | 7.20 |
| -2 | 9 | 5 | 227.88 | 14.80 |
| 2 | 9 | -5 | 205.98 | 15.70 |
| 1 | 9 | -5 | 3.90 | 3.90 |
| -1 | 9 | 5 | 10.70 | 3.40 |
| -1 | 9 | 5 | 11.60 | 3.90 |
| 0 | 9 | 5 | 2107.79 | 108.99 |
| 0 | 9 | -5 | 2002.40 | 108.89 |
| 0 | 9 | 5 | 1839.02 | 107.79 |
| 1 | 9 | 5 | 377.86 | 21.00 |
| -1 | 9 | -5 | 356.96 | 21.70 |
| 1 | 9 | 5 | 318.87 | 21.70 |
| 2 | -9 | 5 | 18.10 | 4.80 |
| 2 | -9 | 5 | 27.40 | 7.70 |
| 2 | 9 | 5 | 18.60 | 4.50 |
| -2 | 9 | -5 | 22.10 | 4.30 |
| 3 | -9 | 5 | 13.70 | 4.90 |
| 3 | -9 | 5 | 3.30 | 8.70 |
| 3 | -9 | 5 | 8.00 | 4.40 |
| -3 | 9 | -5 | 6.60 | 3.10 |
| 3 | 9 | 5 | 8.50 | 4.70 |
| 3 | 9 | 5 | 6.50 | 3.20 |
| -4 | -9 | -5 | 803.12 | 47.60 |

| | | | | |
|---|---|---|---|---|
| 4 | -9 | 5 | 867.81 | 47.30 |
| 4 | -9 | 5 | 837.82 | 50.89 |
| 4 | -9 | 5 | 872.21 | 47.60 |
| 4 | 9 | 5 | 870.91 | 49.40 |
| 4 | 9 | 5 | 803.52 | 47.00 |
| -4 | 9 | -5 | 872.41 | 47.90 |
| -5 | -9 | -5 | -0.70 | 4.60 |
| 5 | -9 | 5 | 11.60 | 4.60 |
| 5 | -9 | 5 | 9.10 | 4.00 |
| 5 | -9 | 5 | 33.00 | 10.00 |
| 5 | 9 | 5 | 7.50 | 5.70 |
| 5 | 9 | 5 | 1.30 | 5.60 |
| -5 | 9 | -5 | 7.30 | 4.20 |
| 5 | 9 | 5 | 12.60 | 2.90 |
| -6 | -9 | -5 | 1211.88 | 65.99 |
| 6 | -9 | 5 | 1180.18 | 65.59 |
| 6 | -9 | 5 | 1246.88 | 66.29 |
| 6 | 9 | 5 | 1210.28 | 65.49 |
| -6 | 9 | -5 | 1244.28 | 66.49 |
| 6 | 9 | 5 | 1053.79 | 68.39 |
| 6 | 9 | 5 | 1185.88 | 65.89 |
| 7 | -9 | 5 | 11.90 | 4.70 |
| -7 | -9 | -5 | 22.70 | 5.30 |
| 7 | -9 | 5 | 15.00 | 4.00 |
| 7 | 9 | 5 | 10.90 | 3.10 |
| 7 | 9 | 5 | 10.60 | 5.50 |
| 7 | 9 | 5 | 28.20 | 9.70 |
| -8 | -9 | -5 | 232.28 | 20.10 |
| 8 | -9 | 5 | 233.08 | 15.60 |
| 8 | -9 | 5 | 263.47 | 17.30 |
| 8 | 9 | 5 | 249.38 | 15.30 |
| 8 | 9 | 5 | 241.08 | 16.50 |
| 9 | -9 | 5 | -2.30 | 3.10 |
| 9 | -9 | 5 | 4.80 | 4.70 |
| 9 | 9 | 5 | 5.40 | 4.70 |
| 9 | 9 | 5 | 2.50 | 2.90 |
| 10 | -9 | 5 | 1127.39 | 63.69 |
| 10 | -9 | 5 | 1126.99 | 62.79 |
| 10 | 9 | 5 | 1129.39 | 62.99 |
| 10 | 9 | 5 | 1172.88 | 62.99 |
| 11 | -9 | 5 | 36.50 | 7.20 |
| 11 | -9 | 5 | 36.90 | 6.60 |
| 11 | 9 | 5 | 47.60 | 9.10 |
| 11 | 9 | 5 | 32.30 | 5.90 |

| | | | | |
|---|---|---|---|---|
| 12 | -9 | 5 | -0.30 | 3.30 |
| 12 | -9 | 5 | 1.50 | 2.80 |
| 12 | 9 | 5 | -1.60 | 2.90 |
| 12 | 9 | 5 | -1.70 | 4.20 |
| 13 | -9 | 5 | 3.60 | 2.60 |
| 13 | -9 | 5 | 4.80 | 3.00 |
| 13 | 9 | 5 | 6.30 | 4.20 |
| 13 | 9 | 5 | 3.00 | 3.00 |
| 15 | -10 | -5 | 222.48 | 12.80 |
| 15 | 10 | -5 | 221.08 | 12.60 |
| -15 | 10 | 5 | 220.48 | 13.10 |
| 14 | -10 | -5 | -2.50 | 1.90 |
| 14 | 10 | -5 | -1.30 | 1.40 |
| -14 | 10 | 5 | 3.20 | 2.50 |
| 13 | -10 | -5 | 14.00 | 3.10 |
| -13 | 10 | 5 | 5.90 | 2.80 |
| 13 | 10 | -5 | 11.00 | 2.10 |
| 12 | -10 | -5 | 173.18 | 11.70 |
| 12 | 10 | -5 | 167.28 | 10.50 |
| -12 | 10 | 5 | 163.78 | 12.80 |
| 11 | -10 | -5 | 840.62 | 48.10 |
| 11 | -10 | -5 | 851.01 | 48.10 |
| 11 | 10 | -5 | 872.11 | 47.40 |
| -11 | 10 | 5 | 874.31 | 48.10 |
| 10 | -10 | -5 | 14.00 | 4.80 |
| 10 | -10 | -5 | 10.80 | 3.90 |
| -10 | 10 | 5 | 22.30 | 4.90 |
| 10 | 10 | -5 | 13.60 | 2.70 |
| 9 | -10 | -5 | 160.28 | 11.60 |
| 9 | -10 | -5 | 146.49 | 13.40 |
| -9 | 10 | 5 | 144.79 | 13.30 |
| 9 | 10 | -5 | 145.39 | 10.80 |
| 8 | -10 | -5 | 240.88 | 18.00 |
| 8 | 10 | -5 | 237.88 | 15.80 |
| 8 | 10 | -5 | 245.58 | 18.50 |
| -7 | -10 | 5 | 352.06 | 22.80 |
| 7 | -10 | -5 | 338.07 | 23.90 |
| -7 | 10 | 5 | 343.07 | 25.40 |
| 7 | 10 | -5 | 398.06 | 23.90 |
| -6 | -10 | 5 | 1.10 | 4.60 |
| 6 | -10 | -5 | 5.90 | 6.70 |
| -6 | 10 | 5 | 7.10 | 9.00 |
| 6 | 10 | -5 | 8.50 | 4.90 |
| -6 | 10 | 5 | -12.60 | 6.90 |

| | | | | |
|---|---|---|---|---|
| -5 | -10 | 5 | 1292.07 | 72.39 |
| 5 | -10 | -5 | 1285.77 | 73.19 |
| 5 | 10 | -5 | 1294.07 | 74.19 |
| -5 | 10 | 5 | 1377.06 | 72.99 |
| -4 | -10 | 5 | 276.47 | 16.90 |
| 4 | -10 | -5 | 296.37 | 21.40 |
| -4 | -10 | 5 | 249.28 | 17.00 |
| 4 | 10 | -5 | 211.28 | 18.20 |
| -4 | 10 | 5 | 265.77 | 17.20 |
| 3 | -10 | -5 | 104.79 | 15.10 |
| -3 | -10 | 5 | 117.29 | 10.90 |
| -3 | -10 | 5 | 135.29 | 10.40 |
| -3 | 10 | 5 | 122.79 | 10.30 |
| 3 | 10 | -5 | 108.59 | 11.70 |
| -2 | -10 | 5 | 226.58 | 16.00 |
| -2 | -10 | 5 | 237.18 | 15.50 |
| 2 | -10 | -5 | 239.78 | 18.50 |
| -2 | 10 | 5 | 255.47 | 16.10 |
| 2 | 10 | -5 | 207.28 | 16.90 |
| -2 | 10 | 5 | 238.08 | 15.50 |
| -1 | -10 | 5 | 3670.53 | 207.48 |
| 1 | -10 | -5 | 3798.42 | 207.98 |
| -1 | -10 | 5 | 3820.92 | 207.18 |
| -1 | 10 | 5 | 3658.03 | 207.78 |
| 1 | 10 | -5 | 3982.20 | 209.08 |
| -1 | 10 | 5 | 4123.29 | 208.68 |
| 0 | -10 | -5 | 115.59 | 12.50 |
| 0 | -10 | 5 | 141.19 | 12.10 |
| 0 | -10 | 5 | 120.19 | 10.40 |
| 0 | 10 | 5 | 123.69 | 9.60 |
| 0 | 10 | -5 | 141.99 | 11.20 |
| 0 | 10 | 5 | 123.29 | 10.70 |
| -1 | -10 | -5 | 1063.29 | 61.39 |
| 1 | -10 | 5 | 1153.28 | 61.89 |
| 1 | -10 | 5 | 1063.09 | 60.69 |
| -1 | 10 | -5 | 1154.48 | 62.09 |
| 1 | 10 | 5 | 1052.49 | 62.09 |
| 1 | 10 | 5 | 1134.79 | 60.99 |
| -2 | -10 | -5 | 138.79 | 11.70 |
| 2 | -10 | 5 | 125.49 | 10.70 |
| 2 | 10 | 5 | 139.99 | 11.60 |
| -2 | 10 | -5 | 141.09 | 10.90 |
| 2 | 10 | 5 | 134.09 | 9.80 |
| 3 | -10 | 5 | 484.25 | 29.50 |

| | | | | |
|---|---|---|---|---|
| -3 | -10 | -5 | 493.85 | 30.00 |
| 3 | -10 | 5 | 494.85 | 32.90 |
| 3 | 10 | 5 | 493.85 | 31.30 |
| 3 | 10 | 5 | 538.45 | 29.40 |
| -3 | 10 | -5 | 559.74 | 30.50 |
| 4 | -10 | 5 | 1.20 | 4.20 |
| -4 | -10 | -5 | 1.70 | 4.10 |
| 4 | -10 | 5 | 5.30 | 8.70 |
| 4 | -10 | 5 | 8.90 | 3.90 |
| 4 | 10 | 5 | 0.10 | 2.40 |
| 4 | 10 | 5 | 9.70 | 5.50 |
| -4 | 10 | -5 | 2.80 | 3.90 |
| 5 | -10 | 5 | 1330.87 | 76.69 |
| 5 | -10 | 5 | 1404.16 | 74.39 |
| -5 | -10 | -5 | 1396.96 | 74.69 |
| 5 | -10 | 5 | 1364.26 | 74.69 |
| 5 | 10 | 5 | 1285.87 | 74.19 |
| -5 | 10 | -5 | 1396.76 | 75.39 |
| 5 | 10 | 5 | 1309.17 | 77.19 |
| 6 | -10 | 5 | 232.18 | 14.50 |
| 6 | -10 | 5 | 229.28 | 15.20 |
| 6 | 10 | 5 | 210.18 | 13.70 |
| 6 | 10 | 5 | 188.18 | 19.80 |
| 7 | -10 | 5 | 3.50 | 4.50 |
| -7 | -10 | -5 | 2.10 | 4.00 |
| 7 | 10 | 5 | 8.40 | 5.70 |
| 7 | 10 | 5 | -1.00 | 2.60 |
| 7 | 10 | 5 | 13.70 | 10.40 |
| 8 | -10 | 5 | 9.10 | 4.70 |
| 8 | -10 | 5 | 9.50 | 3.20 |
| -8 | -10 | -5 | 7.70 | 4.40 |
| 8 | 10 | 5 | 6.40 | 2.90 |
| 8 | 10 | 5 | 8.80 | 5.40 |
| 9 | -10 | 5 | 644.24 | 37.90 |
| 9 | -10 | 5 | 650.43 | 36.70 |
| 9 | 10 | 5 | 653.43 | 36.80 |
| 9 | 10 | 5 | 650.73 | 37.30 |
| 10 | -10 | 5 | -0.90 | 2.90 |
| 10 | -10 | 5 | -8.50 | 3.90 |
| 10 | 10 | 5 | 4.40 | 5.20 |
| 10 | 10 | 5 | -1.00 | 2.60 |
| 11 | -10 | 5 | 409.36 | 24.80 |
| 11 | -10 | 5 | 428.76 | 24.10 |
| 11 | 10 | 5 | 398.56 | 25.10 |

| | | | | |
|---|---|---|---|---|
| 11 | 10 | 5 | 423.36 | 24.30 |
| 12 | -10 | 5 | 73.99 | 7.50 |
| 12 | -10 | 5 | 83.29 | 6.60 |
| 12 | 10 | 5 | 62.59 | 6.80 |
| 13 | -10 | 5 | 195.78 | 12.40 |
| 13 | -10 | 5 | 189.58 | 11.60 |
| 13 | 10 | 5 | 188.98 | 12.00 |
| 14 | -11 | -5 | 201.48 | 12.50 |
| -14 | 11 | 5 | 218.48 | 13.10 |
| 14 | 11 | -5 | 218.08 | 12.20 |
| 13 | -11 | -5 | 87.59 | 6.70 |
| 13 | 11 | -5 | 84.59 | 5.90 |
| -13 | 11 | 5 | 84.59 | 7.40 |
| 12 | -11 | -5 | 161.68 | 10.90 |
| 12 | 11 | -5 | 150.38 | 9.80 |
| -12 | 11 | 5 | 148.69 | 11.10 |
| 11 | -11 | -5 | 1.80 | 3.40 |
| 11 | 11 | -5 | -0.70 | 2.00 |
| -11 | 11 | 5 | 0.20 | 3.90 |
| 10 | -11 | -5 | 873.41 | 49.30 |
| 10 | 11 | -5 | 860.71 | 48.40 |
| -10 | 11 | 5 | 897.51 | 49.40 |
| 9 | -11 | -5 | -3.50 | 4.50 |
| -9 | 11 | 5 | -1.20 | 5.70 |
| 9 | 11 | -5 | 0.80 | 2.70 |
| -8 | -11 | 5 | 4.80 | 5.40 |
| 8 | -11 | -5 | 9.50 | 5.70 |
| 8 | 11 | -5 | 6.40 | 8.30 |
| 8 | 11 | -5 | 9.30 | 4.00 |
| -8 | 11 | 5 | 19.90 | 7.60 |
| 7 | -11 | -5 | 221.38 | 18.00 |
| -7 | -11 | 5 | 236.28 | 16.50 |
| 7 | 11 | -5 | 247.48 | 18.60 |
| -6 | -11 | 5 | 2335.57 | 125.89 |
| 6 | -11 | -5 | 2125.99 | 126.19 |
| 6 | 11 | -5 | 2483.75 | 128.09 |
| -5 | -11 | 5 | 25.00 | 5.20 |
| 5 | -11 | -5 | 27.50 | 8.00 |
| 5 | -11 | -5 | 16.70 | 7.40 |
| 5 | 11 | -5 | 31.40 | 7.40 |
| -5 | 11 | 5 | 34.60 | 6.30 |
| -4 | -11 | 5 | 458.15 | 26.30 |
| 4 | -11 | -5 | 464.95 | 28.80 |
| -4 | -11 | 5 | 449.86 | 26.80 |

| | | | | |
|---|---|---|---|---|
| -4 | 11 | 5 | 479.65 | 27.40 |
| 4 | 11 | -5 | 382.26 | 28.60 |
| -3 | -11 | 5 | 314.77 | 19.80 |
| -3 | -11 | 5 | 339.17 | 19.50 |
| 3 | -11 | -5 | 336.07 | 22.20 |
| 3 | 11 | -5 | 266.17 | 21.60 |
| -3 | 11 | 5 | 322.37 | 20.50 |
| 2 | -11 | -5 | 655.93 | 37.70 |
| -2 | -11 | 5 | 640.04 | 36.00 |
| -2 | -11 | 5 | 612.54 | 36.50 |
| 2 | 11 | -5 | 579.14 | 38.20 |
| -2 | 11 | 5 | 648.14 | 37.30 |
| -2 | 11 | 5 | 675.33 | 36.60 |
| 1 | -11 | -5 | 64.59 | 10.50 |
| -1 | -11 | 5 | 47.80 | 8.90 |
| -1 | -11 | 5 | 67.39 | 7.60 |
| -1 | 11 | 5 | 61.89 | 7.10 |
| 1 | 11 | -5 | 54.79 | 9.00 |
| -1 | 11 | 5 | 69.19 | 8.60 |
| 0 | -11 | 5 | 3307.87 | 179.38 |
| 0 | -11 | 5 | 3239.18 | 179.98 |
| 0 | -11 | -5 | 3300.37 | 180.18 |
| 0 | 11 | 5 | 3382.96 | 181.08 |
| 0 | 11 | 5 | 3418.56 | 179.88 |
| 0 | 11 | -5 | 3294.77 | 181.18 |
| 1 | -11 | 5 | 219.78 | 16.20 |
| -1 | -11 | -5 | 212.48 | 15.40 |
| 1 | -11 | 5 | 217.88 | 14.60 |
| -1 | 11 | -5 | 233.68 | 15.70 |
| 1 | 11 | 5 | 222.68 | 14.30 |
| 1 | 11 | 5 | 224.38 | 16.10 |
| 2 | -11 | 5 | 1.20 | 3.40 |
| -2 | -11 | -5 | -2.30 | 3.90 |
| 2 | -11 | 5 | -1.00 | 5.60 |
| -2 | 11 | -5 | 4.50 | 4.10 |
| 2 | 11 | 5 | 1.10 | 4.50 |
| 2 | 11 | 5 | -0.90 | 2.50 |
| 3 | -11 | 5 | 1.20 | 3.60 |
| -3 | -11 | -5 | -0.90 | 3.80 |
| 3 | -11 | 5 | 6.50 | 6.90 |
| 3 | 11 | 5 | 5.70 | 5.40 |
| -3 | 11 | -5 | 0.40 | 3.50 |
| 3 | 11 | 5 | 5.60 | 3.00 |
| 4 | -11 | 5 | 1676.43 | 95.29 |

| | | | | |
|---|---|---|---|---|
| -4 | -11 | -5 | 1717.13 | 93.59 |
| 4 | -11 | 5 | 1709.53 | 93.19 |
| -4 | 11 | -5 | 1730.03 | 94.39 |
| 4 | 11 | 5 | 1807.12 | 93.29 |
| 4 | 11 | 5 | 1614.44 | 95.79 |
| 5 | -11 | 5 | 18.50 | 4.00 |
| -5 | -11 | -5 | 18.00 | 4.40 |
| 5 | -11 | 5 | 21.90 | 8.90 |
| 5 | -11 | 5 | 23.80 | 4.80 |
| 5 | 11 | 5 | 23.80 | 4.60 |
| 5 | 11 | 5 | 12.60 | 7.80 |
| -5 | 11 | -5 | 15.70 | 5.60 |
| -6 | -11 | -5 | 988.60 | 53.39 |
| 6 | -11 | 5 | 1012.00 | 53.19 |
| 6 | -11 | 5 | 979.40 | 53.69 |
| 6 | 11 | 5 | 957.80 | 53.09 |
| 6 | 11 | 5 | 849.91 | 57.19 |
| -7 | -11 | -5 | 62.19 | 8.30 |
| 7 | -11 | 5 | 83.29 | 9.20 |
| 7 | -11 | 5 | 72.69 | 7.50 |
| 7 | 11 | 5 | 80.29 | 6.70 |
| -8 | -11 | -5 | 551.44 | 33.00 |
| 8 | -11 | 5 | 653.13 | 35.10 |
| 8 | -11 | 5 | 559.84 | 33.00 |
| 8 | 11 | 5 | 566.24 | 33.10 |
| 9 | -11 | 5 | 97.69 | 8.00 |
| 9 | -11 | 5 | 72.69 | 9.10 |
| 9 | 11 | 5 | 93.89 | 7.60 |
| 10 | -11 | 5 | 539.75 | 30.90 |
| 10 | 11 | 5 | 514.05 | 30.00 |
| 11 | -11 | 5 | 11.10 | 2.90 |
| 11 | -11 | 5 | 3.40 | 3.70 |
| 11 | 11 | 5 | 6.60 | 2.90 |
| 12 | -11 | 5 | 18.00 | 3.90 |
| 12 | -11 | 5 | 8.60 | 2.60 |
| 14 | 12 | -5 | 14.30 | 2.30 |
| 13 | -12 | -5 | 0.50 | 2.10 |
| 13 | 12 | -5 | 1.30 | 1.40 |
| 12 | -12 | -5 | 91.89 | 7.30 |
| 12 | 12 | -5 | 85.79 | 6.20 |
| 11 | -12 | -5 | 660.53 | 37.20 |
| 10 | -12 | -5 | 9.20 | 3.90 |
| 10 | 12 | -5 | 2.30 | 2.10 |
| 9 | -12 | -5 | 200.38 | 15.00 |

| | | | | |
|---|---|---|---|---|
| 9 | 12 | -5 | 226.48 | 13.80 |
| -8 | -12 | 5 | 291.07 | 19.10 |
| 8 | -12 | -5 | 274.17 | 19.60 |
| 8 | 12 | -5 | 300.77 | 18.00 |
| 7 | -12 | -5 | 309.37 | 21.70 |
| -7 | -12 | 5 | 322.57 | 20.70 |
| 7 | 12 | -5 | 355.36 | 24.10 |
| -6 | -12 | 5 | 234.68 | 15.80 |
| 6 | -12 | -5 | 214.78 | 17.80 |
| 6 | 12 | -5 | 241.08 | 19.10 |
| 5 | -12 | -5 | 1835.02 | 101.49 |
| -5 | -12 | 5 | 1896.21 | 101.09 |
| 5 | -12 | -5 | 1856.91 | 101.49 |
| -5 | 12 | 5 | 1882.41 | 102.19 |
| 5 | 12 | -5 | 1775.62 | 103.49 |
| -4 | -12 | 5 | 24.00 | 5.40 |
| 4 | -12 | -5 | 56.49 | 11.70 |
| -4 | 12 | 5 | 43.40 | 9.00 |
| 4 | 12 | -5 | 29.00 | 7.90 |
| 3 | -12 | -5 | 17.40 | 5.90 |
| -3 | -12 | 5 | 11.60 | 4.40 |
| -3 | -12 | 5 | 5.60 | 3.20 |
| 3 | 12 | -5 | 1.00 | 5.00 |
| -3 | 12 | 5 | 4.80 | 4.80 |
| -3 | 12 | 5 | 4.60 | 3.90 |
| 2 | -12 | -5 | 191.18 | 14.70 |
| -2 | -12 | 5 | 174.78 | 12.10 |
| -2 | -12 | 5 | 173.88 | 13.00 |
| 2 | 12 | -5 | 153.38 | 15.10 |
| -2 | 12 | 5 | 171.38 | 12.50 |
| -2 | 12 | 5 | 181.98 | 13.90 |
| -1 | -12 | 5 | 2791.62 | 147.39 |
| 1 | -12 | -5 | 2792.12 | 148.29 |
| -1 | -12 | 5 | 2651.23 | 147.79 |
| 1 | 12 | -5 | 2768.42 | 149.79 |
| -1 | 12 | 5 | 2846.92 | 149.09 |
| -1 | 12 | 5 | 2482.75 | 147.59 |
| 0 | -12 | 5 | 0.30 | 4.10 |
| 0 | -12 | -5 | -1.40 | 4.50 |
| 0 | -12 | 5 | 5.70 | 3.00 |
| 0 | 12 | -5 | 0.50 | 4.20 |
| 0 | 12 | 5 | 6.20 | 2.80 |
| 0 | 12 | 5 | 15.70 | 5.50 |
| -1 | -12 | -5 | 360.36 | 21.80 |

| | | | | |
|---|---|---|---|---|
| 1 | -12 | 5 | 362.66 | 22.50 |
| 1 | -12 | 5 | 360.16 | 20.80 |
| -1 | 12 | -5 | 332.27 | 22.40 |
| 1 | 12 | 5 | 325.27 | 20.70 |
| 1 | 12 | 5 | 346.57 | 22.80 |
| 2 | -12 | 5 | 57.29 | 7.90 |
| 2 | -12 | 5 | 71.69 | 11.10 |
| -2 | -12 | -5 | 78.59 | 9.40 |
| 2 | 12 | 5 | 81.79 | 10.20 |
| -2 | 12 | -5 | 76.29 | 9.00 |
| 2 | 12 | 5 | 78.19 | 7.00 |
| 3 | -12 | 5 | 924.11 | 50.39 |
| -3 | -12 | -5 | 904.01 | 50.79 |
| 3 | 12 | 5 | 905.81 | 52.99 |
| 3 | 12 | 5 | 844.82 | 50.29 |
| -3 | 12 | -5 | 959.00 | 51.99 |
| 4 | -12 | 5 | 332.97 | 23.20 |
| 4 | -12 | 5 | 317.57 | 19.40 |
| -4 | -12 | -5 | 329.27 | 19.80 |
| 4 | 12 | 5 | 298.97 | 23.20 |
| -4 | 12 | -5 | 305.57 | 22.90 |
| 4 | 12 | 5 | 330.47 | 19.20 |
| 5 | -12 | 5 | 1921.91 | 103.69 |
| 5 | -12 | 5 | 1974.20 | 105.39 |
| -5 | -12 | -5 | 1933.61 | 103.99 |
| 5 | 12 | 5 | 1799.12 | 107.29 |
| -5 | 12 | -5 | 1980.40 | 105.39 |
| 5 | 12 | 5 | 1834.32 | 103.69 |
| -6 | -12 | -5 | 20.40 | 4.00 |
| 6 | -12 | 5 | 15.50 | 3.50 |
| 6 | 12 | 5 | 23.80 | 4.40 |
| 6 | 12 | 5 | 18.40 | 11.00 |
| -7 | -12 | -5 | 227.78 | 14.90 |
| 7 | -12 | 5 | 211.38 | 14.40 |
| 7 | 12 | 5 | 172.28 | 25.60 |
| 7 | 12 | 5 | 268.97 | 15.20 |
| 8 | -12 | 5 | 50.89 | 6.40 |
| -8 | -12 | -5 | 63.79 | 7.20 |
| 8 | -12 | 5 | 43.40 | 8.20 |
| 8 | 12 | 5 | 57.59 | 6.00 |
| 9 | -12 | 5 | 694.53 | 38.70 |
| 9 | -12 | 5 | 636.34 | 39.40 |
| 9 | 12 | 5 | 745.33 | 38.90 |
| 10 | -12 | 5 | 25.90 | 4.80 |

| | | | | |
|---|---|---|---|---|
| 10 | 12 | 5 | 20.30 | 4.50 |
| 11 | -12 | 5 | 125.79 | 8.50 |
| 11 | 12 | 5 | 117.99 | 8.90 |
| 12 | -12 | 5 | 112.69 | 7.20 |
| 12 | 12 | 5 | 90.29 | 7.50 |
| 13 | -13 | -5 | 63.49 | 5.40 |
| 13 | 13 | -5 | 75.19 | 4.80 |
| 12 | -13 | -5 | 112.89 | 8.10 |
| 12 | 13 | -5 | 121.49 | 7.40 |
| 11 | -13 | -5 | 2.40 | 2.90 |
| 11 | 13 | -5 | 0.50 | 1.70 |
| 10 | -13 | -5 | 353.86 | 21.50 |
| 10 | 13 | -5 | 353.16 | 20.40 |
| 9 | -13 | -5 | 86.39 | 9.40 |
| 9 | 13 | -5 | 77.69 | 6.90 |
| -8 | -13 | 5 | 9.90 | 4.50 |
| 8 | -13 | -5 | 10.10 | 5.00 |
| 8 | 13 | -5 | 7.20 | 2.80 |
| -7 | -13 | 5 | 10.30 | 4.20 |
| 7 | -13 | -5 | 11.10 | 5.80 |
| 7 | 13 | -5 | 9.20 | 8.10 |
| -6 | -13 | 5 | 808.32 | 45.20 |
| 6 | -13 | -5 | 745.73 | 45.90 |
| 6 | 13 | -5 | 853.11 | 48.70 |
| 5 | -13 | -5 | 92.99 | 11.80 |
| 5 | -13 | -5 | 85.99 | 12.80 |
| -5 | -13 | 5 | 67.79 | 8.70 |
| -5 | 13 | 5 | 105.69 | 13.70 |
| 4 | -13 | -5 | 101.99 | 11.70 |
| -4 | -13 | 5 | 104.89 | 9.70 |
| 4 | -13 | -5 | 81.39 | 15.30 |
| 4 | 13 | -5 | 83.29 | 13.90 |
| -4 | 13 | 5 | 122.79 | 13.90 |
| -4 | 13 | 5 | 93.79 | 9.80 |
| -3 | -13 | 5 | 183.38 | 12.90 |
| 3 | -13 | -5 | 168.98 | 14.60 |
| -3 | -13 | 5 | 168.68 | 11.80 |
| 3 | 13 | -5 | 145.89 | 16.60 |
| -3 | 13 | 5 | 186.18 | 16.10 |
| -3 | 13 | 5 | 179.88 | 13.00 |
| 2 | -13 | -5 | 327.57 | 21.80 |
| -2 | -13 | 5 | 325.07 | 20.10 |
| -2 | -13 | 5 | 339.27 | 20.90 |
| -2 | 13 | 5 | 330.67 | 20.80 |

| | | | | |
|---|---|---|---|---|
| -2 | 13 | 5 | 349.27 | 22.60 |
| -1 | -13 | 5 | 197.28 | 14.60 |
| -1 | -13 | 5 | 196.18 | 13.30 |
| 1 | -13 | -5 | 227.88 | 15.50 |
| -1 | 13 | 5 | 199.78 | 16.00 |
| 1 | 13 | -5 | 212.28 | 21.10 |
| -1 | 13 | 5 | 192.58 | 13.60 |
| 0 | -13 | 5 | 1701.43 | 94.09 |
| 0 | -13 | -5 | 1746.73 | 94.19 |
| 0 | -13 | 5 | 1724.93 | 93.39 |
| 0 | 13 | 5 | 1550.74 | 93.59 |
| 0 | 13 | -5 | 1855.51 | 100.39 |
| 1 | -13 | 5 | 511.55 | 30.50 |
| -1 | -13 | -5 | 520.05 | 30.10 |
| 1 | -13 | 5 | 475.45 | 29.10 |
| 1 | 13 | 5 | 505.55 | 29.40 |
| 1 | 13 | 5 | 530.95 | 32.20 |
| 2 | -13 | 5 | 60.69 | 10.20 |
| -2 | -13 | -5 | 47.20 | 7.30 |
| 2 | -13 | 5 | 44.90 | 6.50 |
| 2 | 13 | 5 | 63.79 | 10.50 |
| -2 | 13 | -5 | 45.20 | 8.90 |
| 2 | 13 | 5 | 44.60 | 5.80 |
| 3 | -13 | 5 | 123.59 | 9.70 |
| -3 | -13 | -5 | 135.89 | 10.40 |
| 3 | -13 | 5 | 126.69 | 14.10 |
| -3 | 13 | -5 | 137.69 | 12.70 |
| 3 | 13 | 5 | 124.19 | 9.30 |
| 3 | 13 | 5 | 120.49 | 14.20 |
| 4 | -13 | 5 | 1334.17 | 71.89 |
| -4 | -13 | -5 | 1303.97 | 70.29 |
| 4 | -13 | 5 | 1263.67 | 69.89 |
| 4 | 13 | 5 | 1165.68 | 69.89 |
| -4 | 13 | -5 | 1349.36 | 71.99 |
| 4 | 13 | 5 | 1239.78 | 73.59 |
| 5 | -13 | 5 | 2.40 | 6.50 |
| -5 | -13 | -5 | 0.40 | 3.30 |
| 5 | -13 | 5 | 1.40 | 2.80 |
| 5 | 13 | 5 | -0.60 | 9.30 |
| 5 | 13 | 5 | 2.70 | 2.50 |
| 6 | -13 | 5 | 117.69 | 9.10 |
| -6 | -13 | -5 | 128.39 | 9.60 |
| 6 | 13 | 5 | 118.49 | 8.90 |
| 6 | 13 | 5 | 83.59 | 21.60 |

| | | | | |
|---|---|---|---|---|
| 7 | -13 | 5 | 320.37 | 19.20 |
| -7 | -13 | -5 | 337.67 | 19.40 |
| 7 | 13 | 5 | 312.47 | 19.10 |
| 8 | -13 | 5 | 611.54 | 32.50 |
| -8 | -13 | -5 | 565.24 | 33.40 |
| 8 | 13 | 5 | 549.65 | 32.40 |
| 9 | -13 | 5 | 9.20 | 2.60 |
| 9 | 13 | 5 | 9.20 | 2.50 |
| 10 | -13 | 5 | 350.16 | 20.30 |
| 10 | 13 | 5 | 355.96 | 20.60 |
| 11 | -13 | 5 | 78.69 | 6.10 |
| 11 | 13 | 5 | 78.39 | 6.60 |
| 12 | -14 | -5 | 53.39 | 4.90 |
| 12 | 14 | -5 | 56.79 | 4.10 |
| 11 | -14 | -5 | 188.98 | 12.40 |
| 11 | 14 | -5 | 213.88 | 11.90 |
| 10 | -14 | -5 | 13.90 | 3.50 |
| 10 | 14 | -5 | 7.30 | 1.90 |
| 9 | -14 | -5 | 127.29 | 11.00 |
| 9 | 14 | -5 | 137.89 | 10.00 |
| 8 | -14 | -5 | 27.70 | 5.40 |
| -8 | -14 | 5 | 38.90 | 7.70 |
| 8 | 14 | -5 | 37.10 | 5.20 |
| 7 | -14 | -5 | 30.00 | 6.00 |
| -7 | -14 | 5 | 42.50 | 7.20 |
| -6 | -14 | 5 | -1.00 | 3.40 |
| 6 | 14 | -5 | -12.10 | 8.90 |
| -6 | 14 | 5 | 4.80 | 4.20 |
| 5 | -14 | -5 | 473.65 | 31.30 |
| 5 | -14 | -5 | 552.34 | 30.50 |
| -5 | -14 | 5 | 534.45 | 30.20 |
| 5 | 14 | -5 | 529.25 | 34.80 |
| -5 | 14 | 5 | 498.95 | 31.10 |
| 4 | -14 | -5 | 182.78 | 13.30 |
| -4 | -14 | 5 | 164.18 | 12.00 |
| 4 | 14 | -5 | 137.59 | 17.60 |
| -4 | 14 | 5 | 142.89 | 16.00 |
| -4 | 14 | 5 | 169.48 | 12.80 |
| -3 | -14 | 5 | 60.39 | 6.90 |
| 3 | -14 | -5 | 49.20 | 9.10 |
| -3 | -14 | 5 | 51.19 | 7.70 |
| 3 | 14 | -5 | 54.79 | 13.80 |
| -3 | 14 | 5 | 64.59 | 11.70 |
| -3 | 14 | 5 | 65.99 | 7.80 |

| | | | | |
|---|---|---|---|---|
| -2 | -14 | 5 | 159.18 | 11.90 |
| 2 | -14 | -5 | 145.39 | 12.90 |
| -2 | -14 | 5 | 166.38 | 11.10 |
| 2 | 14 | -5 | 137.09 | 16.20 |
| -2 | 14 | 5 | 157.58 | 11.50 |
| -2 | 14 | 5 | 155.38 | 15.00 |
| -1 | -14 | 5 | 431.36 | 24.30 |
| -1 | -14 | 5 | 388.36 | 24.90 |
| 1 | -14 | -5 | 408.26 | 25.60 |
| -1 | 14 | 5 | 435.56 | 27.20 |
| 1 | 14 | -5 | 408.76 | 28.00 |
| -1 | 14 | 5 | 415.66 | 24.70 |
| 0 | -14 | 5 | 13.80 | 3.30 |
| 0 | -14 | -5 | 19.10 | 4.60 |
| 0 | -14 | 5 | 9.10 | 4.60 |
| 0 | 14 | -5 | 11.00 | 6.70 |
| 0 | 14 | 5 | 21.20 | 6.70 |
| 0 | 14 | 5 | 17.10 | 3.50 |
| 1 | -14 | 5 | 433.56 | 26.80 |
| 1 | -14 | 5 | 445.96 | 25.40 |
| -1 | -14 | -5 | 446.76 | 26.30 |
| 1 | 14 | 5 | 422.26 | 28.30 |
| 1 | 14 | 5 | 436.36 | 25.50 |
| -1 | 14 | -5 | 436.46 | 28.00 |
| 2 | -14 | 5 | 187.28 | 15.50 |
| -2 | -14 | -5 | 198.88 | 13.70 |
| 2 | -14 | 5 | 207.78 | 12.90 |
| 2 | 14 | 5 | 179.68 | 12.70 |
| 2 | 14 | 5 | 194.28 | 17.20 |
| -2 | 14 | -5 | 204.08 | 16.00 |
| -3 | -14 | -5 | 140.59 | 11.00 |
| 3 | -14 | 5 | 143.59 | 10.40 |
| 3 | -14 | 5 | 176.28 | 14.10 |
| 3 | 14 | 5 | 142.39 | 16.40 |
| -3 | 14 | -5 | 160.08 | 14.70 |
| 3 | 14 | 5 | 143.09 | 10.30 |
| 4 | -14 | 5 | 68.59 | 6.40 |
| -4 | -14 | -5 | 46.70 | 6.60 |
| 4 | -14 | 5 | 52.29 | 7.90 |
| 4 | 14 | 5 | 37.30 | 8.40 |
| -4 | 14 | -5 | 55.79 | 13.60 |
| 4 | 14 | 5 | 63.79 | 15.40 |
| 5 | -14 | 5 | 770.82 | 41.40 |
| -5 | -14 | -5 | 788.62 | 41.70 |

| | | | | |
|---|---|---|---|---|
| 5 | -14 | 5 | 772.02 | 43.00 |
| 5 | 14 | 5 | 698.93 | 41.30 |
| 5 | 14 | 5 | 668.23 | 48.00 |
| -6 | -14 | -5 | 290.57 | 17.70 |
| 6 | -14 | 5 | 293.57 | 17.50 |
| 6 | 14 | 5 | 292.47 | 17.40 |
| 7 | -14 | 5 | 8.10 | 2.80 |
| 7 | 14 | 5 | 10.50 | 2.50 |
| 8 | -14 | 5 | 69.59 | 6.20 |
| -8 | -14 | -5 | 56.49 | 6.60 |
| 8 | 14 | 5 | 56.39 | 5.50 |
| 9 | -14 | 5 | 348.77 | 19.90 |
| 9 | 14 | 5 | 343.17 | 20.00 |
| 10 | -14 | 5 | 21.30 | 3.80 |
| 10 | 14 | 5 | 13.20 | 2.90 |
| 11 | -15 | -5 | 0.90 | 2.40 |
| -11 | 15 | 5 | 4.10 | 2.00 |
| 11 | 15 | -5 | 3.30 | 1.40 |
| 10 | -15 | -5 | 193.98 | 11.90 |
| 10 | 15 | -5 | 187.68 | 11.10 |
| -10 | 15 | 5 | 181.68 | 11.70 |
| 9 | -15 | -5 | 10.60 | 3.60 |
| 9 | 15 | -5 | 6.40 | 2.30 |
| -9 | 15 | 5 | 11.50 | 2.80 |
| -8 | -15 | 5 | 6.30 | 3.90 |
| 8 | -15 | -5 | 2.80 | 4.40 |
| -8 | 15 | 5 | 10.90 | 3.30 |
| 8 | 15 | -5 | -1.00 | 2.70 |
| 7 | -15 | -5 | 154.78 | 19.90 |
| -7 | -15 | 5 | 214.08 | 14.10 |
| -7 | 15 | 5 | 226.28 | 14.60 |
| 7 | 15 | -5 | 226.18 | 13.70 |
| 6 | -15 | -5 | 411.96 | 27.30 |
| -6 | -15 | 5 | 476.15 | 26.40 |
| -6 | 15 | 5 | 458.55 | 27.30 |
| 5 | -15 | -5 | -7.20 | 5.70 |
| 5 | -15 | -5 | -2.60 | 4.10 |
| -5 | -15 | 5 | -0.20 | 3.40 |
| -5 | 15 | 5 | 3.70 | 3.60 |
| 5 | 15 | -5 | 22.30 | 12.70 |
| -4 | -15 | 5 | 204.08 | 14.60 |
| 4 | -15 | -5 | 236.68 | 15.50 |
| -4 | 15 | 5 | 233.78 | 15.80 |
| -4 | 15 | 5 | 209.88 | 21.00 |

| | | | | |
|---|---|---|---|---|
| -3 | -15 | 5 | 42.80 | 6.70 |
| 3 | -15 | -5 | 41.60 | 8.00 |
| 3 | 15 | -5 | 19.00 | 9.90 |
| -3 | 15 | 5 | 51.99 | 13.10 |
| -2 | -15 | 5 | 190.98 | 13.90 |
| 2 | -15 | -5 | 210.68 | 14.70 |
| -2 | -15 | 5 | 201.88 | 13.10 |
| -2 | 15 | 5 | 220.38 | 14.20 |
| -2 | 15 | 5 | 202.88 | 17.60 |
| 2 | 15 | -5 | 176.78 | 19.10 |
| 1 | -15 | -5 | 70.69 | 9.20 |
| -1 | -15 | 5 | 78.89 | 8.80 |
| -1 | -15 | 5 | 77.89 | 7.30 |
| -1 | 15 | 5 | 89.19 | 7.90 |
| 1 | 15 | -5 | 85.79 | 14.30 |
| -1 | 15 | 5 | 91.89 | 13.10 |
| 0 | -15 | 5 | 787.12 | 44.00 |
| 0 | -15 | -5 | 791.92 | 44.00 |
| 0 | -15 | 5 | 781.42 | 43.10 |
| 0 | 15 | 5 | 727.43 | 45.90 |
| 0 | 15 | -5 | 762.22 | 46.50 |
| 0 | 15 | 5 | 781.42 | 43.60 |
| 1 | -15 | 5 | 246.48 | 14.50 |
| -1 | -15 | -5 | 228.88 | 15.30 |
| 1 | -15 | 5 | 248.58 | 16.50 |
| 1 | 15 | 5 | 214.48 | 18.70 |
| -1 | 15 | -5 | 233.58 | 18.50 |
| 1 | 15 | 5 | 196.48 | 15.50 |
| 2 | -15 | 5 | 4.10 | 4.90 |
| -2 | -15 | -5 | 2.60 | 2.90 |
| 2 | -15 | 5 | 3.00 | 2.50 |
| 2 | 15 | 5 | 12.20 | 7.80 |
| -2 | 15 | -5 | 8.80 | 6.80 |
| 2 | 15 | 5 | 3.70 | 2.70 |
| 3 | -15 | 5 | 179.28 | 11.40 |
| 3 | -15 | 5 | 196.18 | 15.10 |
| -3 | -15 | -5 | 167.78 | 11.90 |
| 3 | 15 | 5 | 162.28 | 19.00 |
| -3 | 15 | -5 | 175.18 | 17.90 |
| 3 | 15 | 5 | 164.98 | 11.40 |
| -4 | -15 | -5 | 357.96 | 21.80 |
| 4 | -15 | 5 | 374.16 | 21.70 |
| 4 | -15 | 5 | 374.36 | 24.00 |
| 4 | 15 | 5 | 380.76 | 21.70 |

| | | | | |
|---|---|---|---|---|
| 5 | -15 | 5 | 0.90 | 2.50 |
| -5 | -15 | -5 | 0.40 | 2.70 |
| 5 | -15 | 5 | 9.10 | 5.30 |
| 6 | -15 | 5 | 458.15 | 26.70 |
| 6 | 15 | 5 | 464.85 | 26.20 |
| -6 | 15 | -5 | 463.25 | 27.60 |
| -7 | -15 | -5 | 250.37 | 14.80 |
| 7 | -15 | 5 | 247.68 | 14.70 |
| 7 | 15 | 5 | 234.78 | 14.60 |
| -8 | -15 | -5 | 103.99 | 7.70 |
| 8 | -15 | 5 | 98.69 | 7.10 |
| 8 | 15 | 5 | 84.09 | 6.90 |
| 9 | -15 | 5 | 0.30 | 2.00 |
| 9 | 15 | 5 | 2.10 | 2.50 |
| 10 | -15 | 5 | 323.17 | 18.00 |
| 10 | 15 | 5 | 309.07 | 18.20 |
| 10 | -16 | -5 | 5.20 | 3.20 |
| 10 | 16 | -5 | 17.20 | 2.50 |
| -10 | 16 | 5 | 11.80 | 2.40 |
| 9 | -16 | -5 | 116.19 | 8.90 |
| -9 | 16 | 5 | 127.79 | 8.60 |
| 9 | 16 | -5 | 123.99 | 7.80 |
| 8 | -16 | -5 | 62.69 | 7.70 |
| -8 | -16 | 5 | 58.99 | 7.20 |
| -8 | 16 | 5 | 70.29 | 6.40 |
| 8 | 16 | -5 | 66.59 | 5.50 |
| -7 | -16 | 5 | 109.79 | 9.60 |
| 7 | -16 | -5 | 136.39 | 11.20 |
| 7 | 16 | -5 | 131.79 | 8.80 |
| -7 | 16 | 5 | 127.89 | 9.80 |
| -6 | -16 | 5 | 16.70 | 4.40 |
| 6 | -16 | -5 | 21.90 | 5.60 |
| -6 | 16 | 5 | 35.70 | 6.20 |
| 5 | -16 | -5 | 483.45 | 28.10 |
| -5 | -16 | 5 | 438.26 | 25.30 |
| -5 | 16 | 5 | 381.96 | 25.80 |
| -4 | -16 | 5 | 102.19 | 9.10 |
| 4 | -16 | -5 | 108.19 | 9.80 |
| -4 | 16 | 5 | 116.29 | 9.60 |
| 3 | -16 | -5 | 17.40 | 4.10 |
| -3 | -16 | 5 | 33.30 | 6.00 |
| -3 | 16 | 5 | 29.90 | 6.20 |
| 2 | -16 | -5 | 349.67 | 21.80 |
| -2 | -16 | 5 | 348.47 | 21.40 |

| | | | | |
|---|---|---|---|---|
| -2 | -16 | 5 | 346.97 | 20.70 |
| -2 | 16 | 5 | 368.96 | 21.70 |
| -1 | -16 | 5 | 497.55 | 29.00 |
| -1 | -16 | 5 | 516.15 | 28.20 |
| 1 | -16 | -5 | 486.25 | 29.20 |
| -1 | 16 | 5 | 477.35 | 28.70 |
| 0 | -16 | -5 | 39.30 | 7.00 |
| 0 | -16 | 5 | 28.50 | 5.10 |
| 0 | -16 | 5 | 30.60 | 5.00 |
| 0 | 16 | 5 | 39.60 | 5.50 |
| -1 | -16 | -5 | 373.46 | 22.80 |
| 1 | -16 | 5 | 400.46 | 23.80 |
| 1 | -16 | 5 | 360.76 | 22.10 |
| 1 | 16 | 5 | 393.16 | 22.50 |
| 2 | -16 | 5 | 242.38 | 16.50 |
| -2 | -16 | -5 | 229.98 | 14.80 |
| 2 | -16 | 5 | 229.98 | 14.30 |
| 2 | 16 | 5 | 226.58 | 14.30 |
| 3 | -16 | 5 | 98.89 | 7.70 |
| 3 | -16 | 5 | 97.89 | 11.40 |
| -3 | -16 | -5 | 115.89 | 8.30 |
| 3 | 16 | 5 | 99.09 | 7.70 |
| -4 | -16 | -5 | 15.10 | 3.20 |
| 4 | -16 | 5 | 27.30 | 4.50 |
| 4 | -16 | 5 | 17.60 | 5.60 |
| 4 | 16 | 5 | 12.80 | 2.70 |
| 5 | -16 | 5 | 587.14 | 34.90 |
| -5 | -16 | -5 | 624.24 | 34.40 |
| 5 | 16 | 5 | 627.24 | 34.20 |
| 6 | -16 | 5 | 300.27 | 16.80 |
| -6 | -16 | -5 | 284.77 | 16.80 |
| 6 | 16 | 5 | 270.27 | 16.60 |
| 7 | -16 | 5 | 0.20 | 1.80 |
| -7 | -16 | -5 | -0.30 | 2.20 |
| 7 | 16 | 5 | -0.50 | 2.00 |
| -8 | -16 | -5 | 9.70 | 2.80 |
| 8 | -16 | 5 | 12.40 | 2.30 |
| 8 | 16 | 5 | 11.40 | 2.60 |
| 9 | 16 | 5 | 210.08 | 12.60 |
| 9 | 17 | -5 | 34.20 | 3.10 |
| -9 | 17 | 5 | 29.30 | 3.90 |
| -8 | -17 | 5 | 7.40 | 3.00 |
| 8 | 17 | -5 | 11.10 | 2.00 |
| -8 | 17 | 5 | 6.50 | 3.40 |

| | | | | |
|---|---|---|---|---|
| -7 | -17 | 5 | 208.68 | 13.90 |
| -7 | 17 | 5 | 238.78 | 14.30 |
| 7 | 17 | -5 | 229.98 | 13.40 |
| -6 | -17 | 5 | 488.45 | 28.50 |
| 6 | 17 | -5 | 497.65 | 28.20 |
| -6 | 17 | 5 | 516.35 | 29.10 |
| 5 | -17 | -5 | 103.39 | 8.90 |
| -5 | -17 | 5 | 102.09 | 8.30 |
| -5 | 17 | 5 | 97.49 | 8.70 |
| 4 | -17 | -5 | 194.48 | 13.00 |
| -4 | -17 | 5 | 202.98 | 13.10 |
| -4 | 17 | 5 | 185.68 | 13.30 |
| -3 | -17 | 5 | 139.39 | 13.30 |
| 3 | -17 | -5 | 159.08 | 11.60 |
| -3 | 17 | 5 | 166.88 | 11.40 |
| 2 | -17 | -5 | 168.28 | 14.00 |
| -2 | -17 | 5 | 180.18 | 12.50 |
| -2 | 17 | 5 | 189.78 | 12.40 |
| -1 | -17 | 5 | 30.00 | 6.50 |
| 1 | -17 | -5 | 20.70 | 4.00 |
| -1 | -17 | 5 | 25.30 | 4.60 |
| -1 | 17 | 5 | 32.00 | 5.20 |
| 0 | -17 | 5 | 575.64 | 31.70 |
| 0 | -17 | -5 | 573.04 | 32.50 |
| 0 | -17 | 5 | 566.94 | 32.70 |
| 0 | 17 | 5 | 541.55 | 32.00 |
| -1 | -17 | -5 | 257.37 | 16.30 |
| 1 | -17 | 5 | 247.98 | 15.60 |
| 1 | -17 | 5 | 272.57 | 20.10 |
| 1 | 17 | 5 | 245.88 | 15.40 |
| 2 | -17 | 5 | 2.10 | 2.70 |
| 2 | -17 | 5 | 0.40 | 4.20 |
| 2 | 17 | 5 | 2.60 | 2.30 |
| 3 | -17 | 5 | 52.39 | 5.20 |
| -3 | -17 | -5 | 50.49 | 5.80 |
| 3 | -17 | 5 | 47.90 | 8.50 |
| 3 | 17 | 5 | 51.99 | 5.00 |
| 4 | -17 | 5 | 482.35 | 28.10 |
| 4 | -17 | 5 | 473.65 | 27.00 |
| -4 | -17 | -5 | 485.85 | 27.30 |
| 4 | 17 | 5 | 480.95 | 27.00 |
| 5 | -17 | 5 | 22.60 | 3.50 |
| -5 | -17 | -5 | 13.50 | 2.60 |
| 5 | -17 | 5 | 8.10 | 3.90 |

| | | | | |
|---|---|---|---|---|
| 5 | 17 | 5 | 20.20 | 3.50 |
| -6 | -17 | -5 | 148.49 | 9.80 |
| 6 | -17 | 5 | 150.48 | 9.60 |
| 6 | 17 | 5 | 166.28 | 10.20 |
| 7 | -17 | 5 | 184.28 | 11.00 |
| -7 | -17 | -5 | 181.28 | 11.10 |
| 7 | 17 | 5 | 186.08 | 11.20 |
| -7 | -18 | 5 | 106.19 | 8.00 |
| 7 | 18 | -5 | 115.49 | 7.20 |
| -7 | 18 | 5 | 118.89 | 8.20 |
| -6 | -18 | 5 | 8.20 | 2.60 |
| -6 | 18 | 5 | 6.70 | 3.20 |
| 6 | 18 | -5 | 11.40 | 2.20 |
| -5 | -18 | 5 | 394.96 | 22.20 |
| 5 | -18 | -5 | 360.26 | 23.00 |
| -5 | 18 | 5 | 394.66 | 22.70 |
| -4 | -18 | 5 | 91.29 | 7.60 |
| 4 | -18 | -5 | 102.09 | 8.00 |
| -4 | 18 | 5 | 96.39 | 8.10 |
| -3 | -18 | 5 | -1.80 | 2.10 |
| 3 | -18 | -5 | -0.10 | 2.60 |
| -3 | 18 | 5 | 1.70 | 2.80 |
| 2 | -18 | -5 | 181.38 | 12.00 |
| -2 | -18 | 5 | 172.58 | 11.70 |
| -2 | 18 | 5 | 178.08 | 11.80 |
| -1 | -18 | 5 | 547.15 | 30.90 |
| 1 | -18 | -5 | 525.65 | 30.70 |
| -1 | 18 | 5 | 543.95 | 30.70 |
| 0 | -18 | 5 | 94.39 | 8.80 |
| 0 | -18 | 5 | 101.09 | 7.00 |
| 0 | -18 | -5 | 96.69 | 8.20 |
| 0 | 18 | 5 | 93.49 | 7.40 |
| 1 | -18 | 5 | 140.99 | 9.00 |
| 1 | -18 | 5 | 143.09 | 10.70 |
| -1 | -18 | -5 | 129.69 | 9.90 |
| 1 | 18 | 5 | 141.69 | 9.30 |
| 2 | -18 | 5 | 150.18 | 10.80 |
| -2 | -18 | -5 | 134.49 | 9.50 |
| 2 | -18 | 5 | 138.49 | 8.90 |
| 2 | 18 | 5 | 133.99 | 9.10 |
| 3 | -18 | 5 | 187.48 | 11.10 |
| -3 | -18 | -5 | 181.78 | 11.40 |
| 3 | -18 | 5 | 181.48 | 12.50 |
| 3 | 18 | 5 | 181.68 | 11.20 |

| | | | | |
|---|---|---|---|---|
| -4 | -18 | -5 | 56.89 | 5.10 |
| 4 | -18 | 5 | 57.39 | 7.00 |
| 4 | -18 | 5 | 59.59 | 4.80 |
| 4 | 18 | 5 | 62.19 | 5.90 |
| -5 | -18 | -5 | 309.97 | 18.50 |
| 5 | -18 | 5 | 335.87 | 18.40 |
| 5 | 18 | 5 | 329.17 | 18.50 |
| 6 | -18 | 5 | 112.29 | 7.10 |
| -6 | -18 | -5 | 106.79 | 7.10 |
| 6 | 18 | 5 | 109.69 | 7.30 |
| -5 | -19 | 5 | 32.80 | 4.40 |
| -5 | 19 | 5 | 39.50 | 5.20 |
| 5 | 19 | -5 | 38.60 | 3.80 |
| -4 | -19 | 5 | 93.39 | 7.30 |
| 4 | -19 | -5 | 112.99 | 9.60 |
| -4 | 19 | 5 | 103.29 | 8.00 |
| 3 | -19 | -5 | 56.39 | 5.70 |
| -3 | -19 | 5 | 56.79 | 5.50 |
| -3 | 19 | 5 | 62.99 | 7.40 |
| -2 | -19 | 5 | 101.69 | 7.70 |
| 2 | -19 | -5 | 115.49 | 7.80 |
| -2 | 19 | 5 | 92.19 | 8.60 |
| -1 | -19 | 5 | 54.69 | 6.10 |
| 1 | -19 | -5 | 66.39 | 6.10 |
| -1 | 19 | 5 | 66.39 | 7.30 |
| 0 | -19 | 5 | 379.76 | 21.30 |
| 0 | -19 | -5 | 372.06 | 21.00 |
| 0 | 19 | 5 | 332.77 | 21.10 |
| -1 | -19 | -5 | 88.19 | 6.90 |
| 1 | -19 | 5 | 94.49 | 7.70 |
| 1 | 19 | 5 | 84.49 | 6.70 |
| -2 | -19 | -5 | 4.40 | 2.00 |
| 2 | -19 | 5 | 4.30 | 2.90 |
| 2 | -19 | 5 | 7.40 | 1.70 |
| 2 | 19 | 5 | 7.40 | 2.20 |
| 3 | -19 | 5 | 99.69 | 7.40 |
| 3 | -19 | 5 | 88.39 | 6.10 |
| -3 | -19 | -5 | 95.19 | 6.50 |
| 3 | 19 | 5 | 84.39 | 6.40 |
| 4 | -19 | 5 | 232.38 | 13.10 |
| -4 | -19 | -5 | 223.68 | 13.20 |
| 4 | -19 | 5 | 233.08 | 13.70 |
| 4 | 19 | 5 | 218.88 | 13.20 |
| 17 | 0 | -6 | -1.10 | 1.40 |

| | | | | |
|---|---|---|---|---|
| 16 | 0 | -6 | 274.07 | 15.90 |
| 14 | 0 | -6 | 177.58 | 11.60 |
| 13 | 0 | -6 | -3.80 | 2.40 |
| 13 | 0 | -6 | 2.20 | 5.10 |
| 12 | 0 | -6 | 808.22 | 45.50 |
| 12 | 0 | -6 | 826.22 | 46.20 |
| 11 | 0 | -6 | -1.60 | 3.30 |
| 11 | 0 | -6 | -6.30 | 4.90 |
| 11 | 0 | -6 | 4.50 | 5.70 |
| 10 | 0 | -6 | 1235.28 | 71.49 |
| 10 | 0 | -6 | 1378.96 | 72.79 |
| 10 | 0 | -6 | 1278.27 | 70.99 |
| 9 | 0 | -6 | 9.80 | 3.60 |
| 9 | 0 | -6 | 4.40 | 6.70 |
| 9 | 0 | -6 | 1.10 | 5.90 |
| -6 | 0 | 6 | 4372.16 | 235.08 |
| -5 | 0 | 6 | -1.10 | 2.40 |
| -4 | 0 | 6 | 54.19 | 5.60 |
| -3 | 0 | 6 | 3.20 | 2.40 |
| -2 | 0 | 6 | 1104.99 | 61.09 |
| -1 | 0 | 6 | 8.00 | 3.20 |
| 0 | 0 | 6 | 6534.05 | 352.06 |
| 1 | 0 | 6 | -1.10 | 3.10 |
| 2 | 0 | 6 | 1141.59 | 64.09 |
| 3 | 0 | 6 | -3.50 | 4.00 |
| 4 | 0 | 6 | 2169.68 | 119.09 |
| 5 | 0 | 6 | -7.80 | 3.90 |
| 5 | 0 | 6 | -5.00 | 5.00 |
| 6 | 0 | 6 | 61.59 | 8.70 |
| 6 | 0 | 6 | 49.50 | 8.40 |
| 7 | 0 | 6 | 0.00 | 5.20 |
| 7 | 0 | 6 | 4.00 | 5.50 |
| 7 | 0 | 6 | 3.30 | 4.20 |
| 8 | 0 | 6 | 1247.08 | 74.09 |
| 8 | 0 | 6 | 1423.36 | 73.69 |
| 9 | 0 | 6 | 1.30 | 4.20 |
| 10 | 0 | 6 | 1430.06 | 76.49 |
| 10 | 0 | 6 | 1342.97 | 76.89 |
| 11 | 0 | 6 | -3.10 | 3.60 |
| 11 | 0 | 6 | 4.80 | 4.00 |
| 12 | 0 | 6 | 2.50 | 3.60 |
| 12 | 0 | 6 | 10.10 | 3.60 |
| 13 | 0 | 6 | -1.10 | 4.20 |
| 13 | 0 | 6 | 4.20 | 3.30 |

| | | | | |
|---|---|---|---|---|
| 14 | 0 | 6 | 505.05 | 29.50 |
| 14 | 0 | 6 | 524.65 | 29.30 |
| 15 | 0 | 6 | 1.80 | 2.30 |
| 15 | 0 | 6 | 4.80 | 2.40 |
| 17 | -1 | -6 | 115.79 | 7.40 |
| 17 | 1 | -6 | 119.69 | 7.40 |
| 16 | -1 | -6 | 3.50 | 2.00 |
| 16 | 1 | -6 | 3.90 | 1.90 |
| 15 | -1 | -6 | 361.96 | 22.40 |
| 15 | 1 | -6 | 326.57 | 21.80 |
| 14 | -1 | -6 | -0.50 | 2.40 |
| 14 | -1 | -6 | -2.00 | 4.70 |
| 14 | 1 | -6 | 1.60 | 2.40 |
| 13 | -1 | -6 | 51.09 | 8.50 |
| 13 | -1 | -6 | 36.80 | 5.90 |
| 13 | 1 | -6 | 40.40 | 5.60 |
| 12 | -1 | -6 | -1.60 | 3.00 |
| 12 | -1 | -6 | 7.30 | 5.00 |
| 12 | 1 | -6 | -0.60 | 2.60 |
| 11 | -1 | -6 | 1185.38 | 69.79 |
| 11 | -1 | -6 | 1172.88 | 69.69 |
| 11 | 1 | -6 | 1391.26 | 71.19 |
| 11 | 1 | -6 | 1312.87 | 71.19 |
| 10 | -1 | -6 | 43.80 | 7.20 |
| 10 | -1 | -6 | 68.19 | 11.00 |
| 10 | -1 | -6 | 58.99 | 8.80 |
| 10 | 1 | -6 | 54.09 | 7.00 |
| 10 | 1 | -6 | 60.19 | 11.50 |
| 9 | -1 | -6 | 677.63 | 39.10 |
| 9 | -1 | -6 | 658.83 | 37.80 |
| 9 | -1 | -6 | 648.74 | 37.20 |
| 9 | 1 | -6 | 699.03 | 39.30 |
| 9 | 1 | -6 | 607.64 | 36.90 |
| 8 | -1 | -6 | 84.29 | 10.90 |
| -6 | 1 | 6 | 221.58 | 13.60 |
| -5 | -1 | 6 | 4637.34 | 242.58 |
| -5 | 1 | 6 | 4372.06 | 242.38 |
| -4 | -1 | 6 | 16.00 | 3.20 |
| -4 | 1 | 6 | 9.10 | 3.00 |
| -3 | -1 | 6 | 42.90 | 5.20 |
| -3 | 1 | 6 | 45.00 | 5.60 |
| -2 | -1 | 6 | 68.09 | 6.30 |
| -2 | 1 | 6 | 60.79 | 6.40 |
| -1 | -1 | 6 | 4392.36 | 227.68 |

| | | | | |
|---|---|---|---|---|
| -1 | 1 | 6 | 4037.80 | 227.58 |
| 0 | -1 | 6 | 48.30 | 6.10 |
| 0 | 1 | 6 | 43.40 | 6.30 |
| 1 | -1 | 6 | 1084.29 | 62.29 |
| 1 | 1 | 6 | 1146.19 | 62.39 |
| 2 | -1 | 6 | 190.58 | 14.70 |
| 2 | 1 | 6 | 239.08 | 15.30 |
| 3 | -1 | 6 | 1295.37 | 72.29 |
| 3 | 1 | 6 | 1304.87 | 72.89 |
| 4 | -1 | 6 | 301.97 | 18.00 |
| 4 | 1 | 6 | 244.28 | 18.90 |
| 5 | -1 | 6 | 5375.36 | 330.97 |
| 5 | -1 | 6 | 6360.66 | 331.37 |
| 5 | 1 | 6 | 6740.33 | 330.77 |
| 5 | 1 | 6 | 6113.99 | 332.17 |
| 6 | -1 | 6 | 11.40 | 4.40 |
| 6 | -1 | 6 | 3.90 | 5.30 |
| 6 | 1 | 6 | 9.10 | 5.90 |
| 6 | 1 | 6 | 13.70 | 4.70 |
| 7 | -1 | 6 | 43.40 | 9.20 |
| 7 | -1 | 6 | 59.79 | 8.10 |
| 7 | 1 | 6 | 35.30 | 7.80 |
| 7 | 1 | 6 | 58.89 | 9.20 |
| 7 | 1 | 6 | 60.89 | 8.30 |
| 8 | -1 | 6 | 17.70 | 5.20 |
| 8 | -1 | 6 | 12.20 | 4.30 |
| 8 | 1 | 6 | 19.80 | 5.00 |
| 8 | 1 | 6 | 19.90 | 4.90 |
| 9 | -1 | 6 | 1552.34 | 82.19 |
| 9 | 1 | 6 | 1413.96 | 81.99 |
| 10 | -1 | 6 | 1.60 | 4.60 |
| 10 | -1 | 6 | 7.10 | 4.70 |
| 10 | 1 | 6 | -4.60 | 4.20 |
| 10 | 1 | 6 | 2.10 | 4.20 |
| 11 | -1 | 6 | 653.53 | 36.90 |
| 11 | -1 | 6 | 638.84 | 36.70 |
| 11 | 1 | 6 | 610.24 | 36.80 |
| 11 | 1 | 6 | 645.34 | 36.60 |
| 12 | -1 | 6 | 1.20 | 3.50 |
| 12 | -1 | 6 | 2.30 | 3.60 |
| 12 | 1 | 6 | -5.30 | 3.10 |
| 12 | 1 | 6 | 2.60 | 3.70 |
| 13 | -1 | 6 | 411.26 | 27.90 |
| 13 | -1 | 6 | 441.16 | 25.20 |

| | | | | |
|---|---|---|---|---|
| 13 | 1 | 6 | 432.76 | 25.10 |
| 14 | -1 | 6 | 2.30 | 3.10 |
| 14 | -1 | 6 | -1.40 | 2.50 |
| 14 | 1 | 6 | 7.40 | 2.80 |
| 14 | 1 | 6 | 3.90 | 2.90 |
| 15 | -1 | 6 | 258.67 | 15.10 |
| 15 | -1 | 6 | 252.67 | 14.90 |
| 15 | 1 | 6 | 242.98 | 15.00 |
| 15 | 1 | 6 | 255.47 | 14.90 |
| 17 | -2 | -6 | -0.60 | 1.50 |
| 17 | 2 | -6 | -1.70 | 1.30 |
| 16 | -2 | -6 | 405.96 | 22.90 |
| 16 | 2 | -6 | 404.66 | 22.80 |
| 15 | -2 | -6 | 37.60 | 5.30 |
| 15 | -2 | -6 | 25.00 | 5.30 |
| 15 | 2 | -6 | 29.70 | 5.00 |
| 14 | -2 | -6 | 433.86 | 26.20 |
| 14 | -2 | -6 | 452.15 | 25.30 |
| 14 | 2 | -6 | 431.06 | 25.10 |
| 13 | -2 | -6 | 5.90 | 4.60 |
| 13 | -2 | -6 | -3.50 | 2.60 |
| 13 | 2 | -6 | 4.30 | 2.50 |
| 12 | -2 | -6 | 900.51 | 49.60 |
| 12 | -2 | -6 | 878.81 | 49.00 |
| 12 | 2 | -6 | 869.01 | 48.90 |
| 11 | -2 | -6 | -0.70 | 4.80 |
| 11 | 2 | -6 | 8.70 | 4.80 |
| 11 | 2 | -6 | 6.20 | 5.90 |
| 10 | -2 | -6 | 898.51 | 56.29 |
| 10 | -2 | -6 | 1061.29 | 56.09 |
| 10 | 2 | -6 | 1067.29 | 57.79 |
| 10 | 2 | -6 | 1024.30 | 55.79 |
| 9 | -2 | -6 | 11.00 | 4.30 |
| 9 | -2 | -6 | 15.00 | 5.20 |
| 9 | 2 | -6 | 10.30 | 3.70 |
| 9 | 2 | -6 | 4.10 | 7.10 |
| 8 | -2 | -6 | 70.99 | 10.40 |
| 7 | -2 | -6 | 9.10 | 8.20 |
| 7 | 2 | -6 | 2.50 | 3.00 |
| -6 | 2 | 6 | 4864.11 | 261.47 |
| -5 | 2 | 6 | 95.69 | 8.00 |
| -4 | -2 | 6 | 2006.50 | 100.99 |
| -4 | 2 | 6 | 1696.93 | 100.69 |
| -3 | -2 | 6 | 923.51 | 47.70 |

| | | | | |
|---|---|---|---|---|
| -3 | 2 | 6 | 781.52 | 47.40 |
| -2 | -2 | 6 | 1489.45 | 78.49 |
| -2 | 2 | 6 | 1364.96 | 78.19 |
| -1 | -2 | 6 | 272.27 | 17.50 |
| -1 | 2 | 6 | 292.17 | 17.50 |
| 0 | -2 | 6 | 3752.42 | 204.08 |
| 0 | 2 | 6 | 3790.32 | 203.98 |
| 1 | -2 | 6 | 2.40 | 3.50 |
| 1 | 2 | 6 | 6.20 | 3.60 |
| 2 | -2 | 6 | 185.28 | 13.20 |
| 2 | 2 | 6 | 185.88 | 13.40 |
| 3 | -2 | 6 | 447.96 | 25.80 |
| 3 | 2 | 6 | 406.06 | 26.40 |
| 4 | -2 | 6 | 3281.37 | 155.78 |
| 4 | 2 | 6 | 2613.24 | 154.58 |
| 4 | 2 | 6 | 2700.53 | 156.58 |
| 5 | -2 | 6 | 37.20 | 6.70 |
| 5 | -2 | 6 | 50.49 | 10.70 |
| 5 | 2 | 6 | 51.29 | 10.70 |
| 5 | 2 | 6 | 50.99 | 6.60 |
| 6 | -2 | 6 | 2093.09 | 111.39 |
| 6 | -2 | 6 | 2213.18 | 110.79 |
| 6 | 2 | 6 | 2016.90 | 112.09 |
| 6 | 2 | 6 | 1807.02 | 110.29 |
| 6 | 2 | 6 | 2017.40 | 110.39 |
| 7 | -2 | 6 | 34.60 | 6.10 |
| 7 | -2 | 6 | 23.40 | 6.00 |
| 7 | 2 | 6 | 29.90 | 5.10 |
| 7 | 2 | 6 | 36.20 | 7.40 |
| 7 | 2 | 6 | 32.30 | 6.70 |
| 8 | -2 | 6 | 551.34 | 33.20 |
| 8 | -2 | 6 | 549.65 | 33.80 |
| 8 | 2 | 6 | 616.24 | 33.60 |
| 8 | 2 | 6 | 574.04 | 33.10 |
| 9 | -2 | 6 | 71.39 | 9.50 |
| 9 | -2 | 6 | 96.69 | 11.80 |
| 9 | 2 | 6 | 98.19 | 8.90 |
| 9 | 2 | 6 | 82.59 | 9.20 |
| 10 | -2 | 6 | 1538.85 | 85.39 |
| 10 | -2 | 6 | 1530.15 | 84.69 |
| 10 | -2 | 6 | 1595.54 | 85.09 |
| 10 | 2 | 6 | 1588.04 | 84.89 |
| 10 | 2 | 6 | 1473.25 | 85.39 |
| 11 | -2 | 6 | 3.20 | 4.10 |

| | | | | |
|---|---|---|---|---|
| 11 | -2 | 6 | -3.10 | 4.20 |
| 11 | -2 | 6 | 9.40 | 5.10 |
| 11 | 2 | 6 | 2.20 | 4.00 |
| 11 | 2 | 6 | 0.40 | 3.70 |
| 12 | -2 | 6 | 2.60 | 4.00 |
| 12 | -2 | 6 | 5.80 | 3.70 |
| 12 | -2 | 6 | -1.80 | 4.80 |
| 12 | 2 | 6 | 5.10 | 3.80 |
| 12 | 2 | 6 | 1.10 | 3.60 |
| 13 | -2 | 6 | 38.70 | 6.10 |
| 13 | -2 | 6 | 43.60 | 7.30 |
| 13 | 2 | 6 | 37.60 | 5.60 |
| 13 | 2 | 6 | 38.40 | 6.60 |
| 14 | -2 | 6 | 353.66 | 21.00 |
| 14 | -2 | 6 | 374.46 | 20.70 |
| 14 | 2 | 6 | 369.36 | 20.70 |
| 14 | 2 | 6 | 317.37 | 20.80 |
| 15 | -2 | 6 | 0.80 | 2.40 |
| 15 | -2 | 6 | 4.00 | 2.40 |
| 15 | 2 | 6 | 1.20 | 2.10 |
| 15 | 2 | 6 | 5.30 | 2.40 |
| 17 | -3 | -6 | 110.49 | 7.20 |
| 17 | 3 | -6 | 118.39 | 7.10 |
| 16 | -3 | -6 | -1.60 | 1.80 |
| 16 | 3 | -6 | 3.70 | 1.70 |
| 15 | -3 | -6 | 239.48 | 15.50 |
| 15 | -3 | -6 | 226.28 | 14.40 |
| 15 | 3 | -6 | 239.68 | 14.20 |
| 14 | -3 | -6 | -0.30 | 2.60 |
| 14 | -3 | -6 | 7.00 | 4.40 |
| 14 | 3 | -6 | 1.90 | 2.30 |
| 13 | -3 | -6 | 85.99 | 7.70 |
| 13 | 3 | -6 | 80.89 | 7.00 |
| 12 | -3 | -6 | -6.10 | 2.90 |
| 12 | -3 | -6 | -1.50 | 4.20 |
| 12 | 3 | -6 | 3.90 | 2.70 |
| 11 | -3 | -6 | 1762.02 | 98.79 |
| 11 | -3 | -6 | 1880.11 | 99.29 |
| 11 | 3 | -6 | 1801.32 | 98.59 |
| 10 | -3 | -6 | 0.00 | 4.20 |
| 10 | -3 | -6 | -3.70 | 3.80 |
| 10 | 3 | -6 | -0.20 | 6.60 |
| 10 | 3 | -6 | -0.40 | 3.20 |
| 9 | -3 | -6 | 1236.58 | 72.09 |

| | | | | |
|---|---|---|---|---|
| 9 | -3 | -6 | 1350.66 | 72.39 |
| 9 | 3 | -6 | 1340.57 | 73.69 |
| 9 | 3 | -6 | 1322.17 | 71.79 |
| 8 | -3 | -6 | 3.60 | 4.80 |
| 8 | 3 | -6 | 8.40 | 3.90 |
| 7 | -3 | -6 | 531.45 | 32.60 |
| 7 | 3 | -6 | 571.44 | 31.30 |
| -6 | 3 | 6 | 8.90 | 3.50 |
| -5 | 3 | 6 | 3363.86 | 181.78 |
| -4 | -3 | 6 | 128.29 | 9.70 |
| -4 | 3 | 6 | 125.29 | 9.60 |
| -3 | -3 | 6 | 475.35 | 26.30 |
| -3 | 3 | 6 | 420.06 | 25.90 |
| -2 | -3 | 6 | 246.08 | 16.30 |
| -2 | 3 | 6 | 267.07 | 16.00 |
| -1 | -3 | 6 | 3696.73 | 207.98 |
| -1 | 3 | 6 | 3994.10 | 207.78 |
| 0 | -3 | 6 | 688.73 | 44.40 |
| 0 | 3 | 6 | 888.51 | 44.60 |
| 1 | -3 | 6 | 4682.93 | 276.97 |
| 1 | 3 | 6 | 5585.94 | 277.17 |
| 2 | -3 | 6 | 354.76 | 22.10 |
| 2 | 3 | 6 | 373.16 | 22.50 |
| 3 | -3 | 6 | 116.09 | 9.30 |
| 3 | 3 | 6 | 106.39 | 10.20 |
| 4 | -3 | 6 | 45.20 | 6.00 |
| 4 | 3 | 6 | 47.10 | 9.70 |
| 4 | 3 | 6 | 41.40 | 6.30 |
| 5 | -3 | 6 | 5513.35 | 314.07 |
| 5 | -3 | 6 | 6362.46 | 313.97 |
| 5 | 3 | 6 | 5075.09 | 315.17 |
| 5 | 3 | 6 | 6350.46 | 313.37 |
| 6 | -3 | 6 | 6.60 | 5.60 |
| 6 | -3 | 6 | -2.00 | 5.30 |
| 6 | 3 | 6 | -0.90 | 5.70 |
| 6 | 3 | 6 | 7.10 | 4.80 |
| 6 | 3 | 6 | 7.40 | 3.40 |
| 7 | -3 | 6 | 21.40 | 5.70 |
| 7 | -3 | 6 | 17.90 | 5.50 |
| 7 | 3 | 6 | 11.50 | 6.50 |
| 7 | 3 | 6 | 18.00 | 3.90 |
| 7 | 3 | 6 | 21.60 | 5.00 |
| 8 | -3 | 6 | 82.69 | 9.70 |
| 8 | -3 | 6 | 67.89 | 9.30 |

| | | | | |
|---|---|---|---|---|
| 8 | -3 | 6 | 78.89 | 11.50 |
| 8 | 3 | 6 | 72.09 | 8.30 |
| 8 | 3 | 6 | 57.79 | 7.80 |
| 9 | -3 | 6 | 915.31 | 50.29 |
| 9 | -3 | 6 | 905.31 | 50.29 |
| 9 | -3 | 6 | 985.70 | 49.80 |
| 9 | 3 | 6 | 796.62 | 49.30 |
| 9 | 3 | 6 | 820.22 | 49.80 |
| 10 | -3 | 6 | 9.40 | 5.10 |
| 10 | -3 | 6 | 1.70 | 4.60 |
| 10 | -3 | 6 | -7.10 | 4.10 |
| 10 | 3 | 6 | 5.40 | 4.10 |
| 10 | 3 | 6 | -0.80 | 3.80 |
| 11 | -3 | 6 | 822.52 | 46.30 |
| 11 | -3 | 6 | 817.32 | 46.80 |
| 11 | 3 | 6 | 829.92 | 46.40 |
| 12 | -3 | 6 | 96.69 | 8.60 |
| 12 | -3 | 6 | 97.49 | 10.10 |
| 12 | -3 | 6 | 94.29 | 9.70 |
| 12 | 3 | 6 | 85.09 | 8.70 |
| 12 | 3 | 6 | 100.49 | 8.60 |
| 13 | -3 | 6 | 231.28 | 16.00 |
| 13 | -3 | 6 | 272.37 | 16.00 |
| 13 | -3 | 6 | 248.68 | 15.30 |
| 13 | 3 | 6 | 225.18 | 15.10 |
| 13 | 3 | 6 | 233.78 | 15.50 |
| 14 | -3 | 6 | 6.20 | 2.90 |
| 14 | -3 | 6 | 1.40 | 2.60 |
| 14 | 3 | 6 | -0.80 | 2.40 |
| 14 | 3 | 6 | 2.10 | 2.70 |
| 15 | -3 | 6 | 275.47 | 15.70 |
| 15 | -3 | 6 | 270.37 | 15.60 |
| 15 | 3 | 6 | 260.57 | 15.50 |
| 15 | 3 | 6 | 251.77 | 15.60 |
| 16 | -4 | -6 | 245.88 | 14.40 |
| 16 | 4 | -6 | 245.28 | 14.20 |
| 15 | -4 | -6 | 2.70 | 4.00 |
| 15 | -4 | -6 | -1.60 | 2.10 |
| 15 | 4 | -6 | -1.30 | 1.90 |
| 14 | -4 | -6 | 152.78 | 11.20 |
| 14 | -4 | -6 | 130.49 | 9.80 |
| 14 | 4 | -6 | 135.19 | 9.20 |
| 13 | -4 | -6 | 4.20 | 4.60 |
| 13 | -4 | -6 | 3.60 | 3.10 |

| | | | | |
|---|---|---|---|---|
| 13 | 4 | -6 | -2.60 | 2.10 |
| 12 | -4 | -6 | 263.17 | 17.70 |
| 12 | -4 | -6 | 314.57 | 18.50 |
| 12 | 4 | -6 | 280.67 | 17.20 |
| 11 | -4 | -6 | 12.80 | 3.90 |
| 11 | -4 | -6 | 6.70 | 4.20 |
| 11 | 4 | -6 | 15.20 | 3.30 |
| 10 | -4 | -6 | 593.44 | 34.70 |
| 10 | -4 | -6 | 576.34 | 34.70 |
| 10 | 4 | -6 | 641.94 | 34.20 |
| 9 | -4 | -6 | 10.40 | 4.90 |
| 9 | -4 | -6 | 6.00 | 4.60 |
| 9 | 4 | -6 | 15.00 | 3.90 |
| 8 | -4 | -6 | 22.40 | 5.30 |
| 8 | 4 | -6 | 31.40 | 4.80 |
| 7 | -4 | -6 | 106.49 | 11.50 |
| 7 | 4 | -6 | 105.69 | 8.70 |
| 6 | -4 | -6 | 3992.00 | 235.88 |
| -6 | 4 | 6 | 4479.45 | 235.78 |
| 6 | 4 | -6 | 4645.94 | 235.88 |
| -5 | 4 | 6 | 1.30 | 3.30 |
| -4 | 4 | 6 | 50.89 | 6.30 |
| -3 | 4 | 6 | 189.78 | 12.60 |
| -2 | -4 | 6 | 214.78 | 14.90 |
| -2 | 4 | 6 | 229.38 | 14.20 |
| -1 | -4 | 6 | 58.19 | 7.40 |
| -1 | 4 | 6 | 90.09 | 7.10 |
| 0 | -4 | 6 | 3401.06 | 211.48 |
| 0 | 4 | 6 | 4420.36 | 211.58 |
| 1 | -4 | 6 | 344.47 | 22.30 |
| 1 | 4 | 6 | 385.66 | 22.30 |
| 2 | -4 | 6 | 319.77 | 20.30 |
| 2 | 4 | 6 | 341.57 | 20.70 |
| 3 | -4 | 6 | 232.08 | 14.60 |
| 3 | 4 | 6 | 209.58 | 13.90 |
| 3 | 4 | 6 | 224.18 | 16.00 |
| 4 | -4 | 6 | 2032.00 | 103.89 |
| 4 | 4 | 6 | 1775.72 | 104.99 |
| 4 | 4 | 6 | 1887.51 | 102.99 |
| 5 | -4 | 6 | 191.58 | 15.80 |
| 5 | 4 | 6 | 221.08 | 12.80 |
| 5 | 4 | 6 | 160.78 | 15.80 |
| 6 | -4 | 6 | 1081.29 | 62.99 |
| 6 | 4 | 6 | 1149.78 | 62.19 |

| | | | | |
|---|---|---|---|---|
| 6 | 4 | 6 | 1201.98 | 63.99 |
| 6 | 4 | 6 | 1062.09 | 64.29 |
| 7 | -4 | 6 | 61.39 | 11.30 |
| 7 | -4 | 6 | 43.20 | 7.40 |
| 7 | -4 | 6 | 79.79 | 9.60 |
| 7 | 4 | 6 | 60.29 | 6.70 |
| 7 | 4 | 6 | 62.59 | 7.90 |
| 7 | 4 | 6 | 64.09 | 12.30 |
| 8 | -4 | 6 | 690.83 | 40.20 |
| 8 | -4 | 6 | 719.63 | 40.80 |
| 8 | -4 | 6 | 732.83 | 40.70 |
| 8 | 4 | 6 | 713.23 | 39.90 |
| 8 | 4 | 6 | 661.93 | 39.80 |
| 9 | -4 | 6 | 54.59 | 10.00 |
| 9 | -4 | 6 | 38.20 | 8.10 |
| 9 | 4 | 6 | 41.80 | 7.00 |
| 9 | 4 | 6 | 51.79 | 7.20 |
| 10 | -4 | 6 | 1728.83 | 90.79 |
| 10 | -4 | 6 | 1662.63 | 90.79 |
| 10 | -4 | 6 | 1733.13 | 89.99 |
| 10 | 4 | 6 | 1458.15 | 90.39 |
| 10 | 4 | 6 | 1647.54 | 90.09 |
| 11 | -4 | 6 | 0.60 | 4.50 |
| 11 | -4 | 6 | -2.90 | 4.20 |
| 11 | -4 | 6 | 13.00 | 4.50 |
| 11 | 4 | 6 | 5.10 | 3.80 |
| 11 | 4 | 6 | 0.20 | 4.30 |
| 12 | -4 | 6 | 3.30 | 4.70 |
| 12 | -4 | 6 | 0.40 | 4.40 |
| 12 | -4 | 6 | 7.70 | 3.60 |
| 12 | 4 | 6 | 1.30 | 3.20 |
| 12 | 4 | 6 | 6.30 | 3.50 |
| 13 | -4 | 6 | 9.10 | 4.20 |
| 13 | -4 | 6 | 10.50 | 3.40 |
| 13 | -4 | 6 | 5.30 | 3.80 |
| 13 | 4 | 6 | 9.30 | 3.20 |
| 13 | 4 | 6 | 8.00 | 3.10 |
| 14 | -4 | 6 | 312.37 | 17.70 |
| 14 | -4 | 6 | 296.27 | 17.60 |
| 14 | 4 | 6 | 282.37 | 17.20 |
| 14 | 4 | 6 | 276.47 | 17.40 |
| 16 | -5 | -6 | 2.20 | 6.40 |
| 16 | -5 | -6 | -1.90 | 1.60 |
| 16 | 5 | -6 | 0.10 | 1.40 |

| | | | | |
|---|---|---|---|---|
| 15 | -5 | -6 | 333.97 | 19.40 |
| 15 | -5 | -6 | 330.97 | 20.10 |
| 15 | 5 | -6 | 339.47 | 19.10 |
| 14 | -5 | -6 | 39.70 | 6.90 |
| 14 | -5 | -6 | 40.10 | 5.40 |
| 14 | 5 | -6 | 30.20 | 4.30 |
| 13 | -5 | -6 | 4.40 | 3.90 |
| 13 | -5 | -6 | 8.00 | 3.30 |
| 13 | 5 | -6 | 4.60 | 2.30 |
| 12 | -5 | -6 | 37.90 | 7.20 |
| 12 | 5 | -6 | 35.90 | 5.20 |
| 11 | -5 | -6 | 1227.98 | 69.69 |
| 11 | -5 | -6 | 1307.27 | 70.09 |
| 11 | 5 | -6 | 1266.47 | 69.29 |
| 10 | -5 | -6 | 72.69 | 9.50 |
| 10 | -5 | -6 | 87.49 | 8.90 |
| 10 | 5 | -6 | 75.79 | 7.40 |
| 9 | -5 | -6 | 140.29 | 13.20 |
| 9 | -5 | -6 | 159.18 | 11.70 |
| 9 | 5 | -6 | 145.39 | 10.80 |
| 8 | -5 | -6 | 159.68 | 11.80 |
| 8 | -5 | -6 | 125.39 | 14.60 |
| 8 | 5 | -6 | 116.19 | 10.70 |
| 7 | -5 | -6 | 1408.96 | 80.69 |
| 7 | 5 | -6 | 1417.56 | 80.49 |
| 7 | 5 | -6 | 1561.64 | 80.39 |
| 6 | -5 | -6 | 12.10 | 5.50 |
| 6 | 5 | -6 | 10.60 | 3.60 |
| -6 | 5 | 6 | 5.30 | 4.30 |
| 5 | -5 | -6 | 1954.20 | 121.89 |
| -5 | 5 | 6 | 2524.75 | 121.99 |
| -4 | 5 | 6 | 562.74 | 32.20 |
| -3 | 5 | 6 | 0.00 | 2.80 |
| -2 | 5 | 6 | 9.60 | 3.50 |
| -1 | 5 | 6 | 6271.67 | 337.67 |
| 0 | -5 | 6 | 827.42 | 49.00 |
| 0 | 5 | 6 | 902.41 | 48.70 |
| 1 | -5 | 6 | 24.30 | 6.00 |
| 1 | 5 | 6 | 24.40 | 5.90 |
| 2 | -5 | 6 | 286.27 | 16.70 |
| 2 | 5 | 6 | 257.07 | 17.50 |
| 2 | 5 | 6 | 213.38 | 15.30 |
| 3 | -5 | 6 | 2562.44 | 136.29 |
| 3 | 5 | 6 | 2498.75 | 136.89 |

| | | | | |
|---|---|---|---|---|
| 3 | 5 | 6 | 2444.26 | 135.19 |
| 4 | -5 | 6 | 2.60 | 5.50 |
| 4 | 5 | 6 | 7.60 | 4.80 |
| 4 | 5 | 6 | 2.80 | 2.80 |
| 5 | -5 | 6 | 1920.91 | 105.29 |
| 5 | 5 | 6 | 1759.92 | 106.59 |
| 5 | 5 | 6 | 2083.99 | 104.49 |
| 6 | -5 | 6 | 255.77 | 17.50 |
| 6 | -5 | 6 | 260.97 | 17.90 |
| 6 | 5 | 6 | 257.67 | 16.60 |
| 6 | 5 | 6 | 235.18 | 19.60 |
| 6 | 5 | 6 | 252.07 | 15.70 |
| 7 | -5 | 6 | 321.57 | 21.10 |
| 7 | -5 | 6 | 334.47 | 22.50 |
| 7 | -5 | 6 | 325.77 | 21.10 |
| 7 | 5 | 6 | 360.16 | 20.30 |
| 7 | 5 | 6 | 315.47 | 23.80 |
| 7 | 5 | 6 | 301.77 | 19.60 |
| 8 | -5 | 6 | 18.60 | 5.20 |
| 8 | -5 | 6 | 17.10 | 7.10 |
| 8 | -5 | 6 | 7.70 | 4.80 |
| 8 | 5 | 6 | 5.80 | 3.20 |
| 8 | 5 | 6 | 10.00 | 4.00 |
| 9 | -5 | 6 | 828.12 | 48.30 |
| 9 | -5 | 6 | 879.81 | 48.50 |
| 9 | -5 | 6 | 874.61 | 47.50 |
| 9 | 5 | 6 | 815.92 | 47.40 |
| 9 | 5 | 6 | 839.62 | 47.70 |
| 10 | -5 | 6 | 25.10 | 5.40 |
| 10 | -5 | 6 | 23.80 | 5.60 |
| 10 | -5 | 6 | 22.80 | 4.70 |
| 10 | 5 | 6 | 26.30 | 4.50 |
| 10 | 5 | 6 | 15.90 | 4.10 |
| 11 | -5 | 6 | 213.98 | 14.10 |
| 11 | -5 | 6 | 212.78 | 14.50 |
| 11 | -5 | 6 | 185.28 | 15.30 |
| 11 | 5 | 6 | 221.48 | 13.90 |
| 11 | 5 | 6 | 186.58 | 14.10 |
| 12 | -5 | 6 | 35.30 | 6.10 |
| 12 | -5 | 6 | 39.10 | 8.50 |
| 12 | -5 | 6 | 37.40 | 6.40 |
| 12 | 5 | 6 | 23.70 | 4.60 |
| 13 | -5 | 6 | 483.35 | 28.80 |
| 13 | -5 | 6 | 495.35 | 28.70 |

| | | | | |
|---|---|---|---|---|
| 13 | -5 | 6 | 499.75 | 29.10 |
| 13 | 5 | 6 | 522.05 | 28.90 |
| 13 | 5 | 6 | 510.85 | 28.80 |
| 14 | -5 | 6 | 6.40 | 3.10 |
| 14 | -5 | 6 | 2.20 | 2.80 |
| 14 | 5 | 6 | 5.50 | 2.80 |
| 16 | -6 | -6 | 237.78 | 14.30 |
| -16 | 6 | 6 | 254.27 | 14.40 |
| 16 | 6 | -6 | 247.58 | 14.10 |
| 15 | -6 | -6 | 0.60 | 2.10 |
| 15 | -6 | -6 | 3.30 | 3.60 |
| 15 | 6 | -6 | -0.90 | 1.50 |
| 14 | -6 | -6 | 315.37 | 20.00 |
| 14 | 6 | -6 | 341.07 | 19.20 |
| 13 | -6 | -6 | 39.20 | 5.60 |
| 13 | -6 | -6 | 34.80 | 5.70 |
| 13 | 6 | -6 | 31.80 | 4.70 |
| 12 | -6 | -6 | 299.97 | 18.20 |
| 12 | -6 | -6 | 289.67 | 18.50 |
| 12 | 6 | -6 | 292.67 | 17.50 |
| 11 | -6 | -6 | 2.80 | 3.70 |
| 11 | -6 | -6 | 4.90 | 3.50 |
| 11 | 6 | -6 | 1.20 | 2.60 |
| 10 | -6 | -6 | 1074.19 | 61.99 |
| 10 | 6 | -6 | 1158.28 | 61.39 |
| 9 | -6 | -6 | 125.79 | 12.20 |
| 9 | -6 | -6 | 119.49 | 10.30 |
| 9 | 6 | -6 | 114.99 | 9.40 |
| 8 | -6 | -6 | 169.58 | 15.90 |
| 8 | -6 | -6 | 187.08 | 13.60 |
| 8 | 6 | -6 | 175.88 | 12.80 |
| 8 | 6 | -6 | 174.08 | 13.90 |
| 7 | -6 | -6 | 32.80 | 6.00 |
| 7 | 6 | -6 | 25.00 | 4.90 |
| 7 | 6 | -6 | 25.50 | 5.40 |
| 6 | -6 | -6 | 2885.71 | 157.68 |
| 6 | 6 | -6 | 2973.20 | 157.58 |
| -6 | 6 | 6 | 2845.72 | 157.18 |
| 5 | -6 | -6 | 298.77 | 20.60 |
| -5 | 6 | 6 | 356.66 | 19.80 |
| 5 | 6 | -6 | 295.47 | 21.60 |
| -4 | 6 | 6 | 95.39 | 8.30 |
| -3 | 6 | 6 | 477.65 | 27.90 |
| -2 | 6 | 6 | 2752.12 | 149.49 |

| | | | | |
|---|---|---|---|---|
| 0 | 6 | 6 | 1702.03 | 92.79 |
| 1 | 6 | 6 | 926.81 | 53.29 |
| 1 | 6 | 6 | 968.00 | 52.39 |
| 2 | 6 | 6 | 588.44 | 32.20 |
| 2 | 6 | 6 | 550.54 | 33.40 |
| 3 | 6 | 6 | 53.29 | 7.80 |
| 3 | 6 | 6 | 49.80 | 5.90 |
| 4 | -6 | 6 | 2892.51 | 159.88 |
| 4 | 6 | 6 | 2981.10 | 161.78 |
| 4 | 6 | 6 | 2992.30 | 159.58 |
| 5 | -6 | 6 | 186.38 | 14.40 |
| 5 | 6 | 6 | 176.88 | 13.30 |
| 5 | 6 | 6 | 172.88 | 15.60 |
| 5 | 6 | 6 | 190.38 | 13.70 |
| 6 | -6 | 6 | 342.27 | 20.50 |
| 6 | -6 | 6 | 327.67 | 22.00 |
| 6 | 6 | 6 | 278.37 | 19.70 |
| 6 | 6 | 6 | 342.87 | 19.20 |
| 6 | 6 | 6 | 309.87 | 23.20 |
| 7 | -6 | 6 | 131.49 | 16.20 |
| 7 | -6 | 6 | 173.68 | 12.70 |
| 7 | -6 | 6 | 133.79 | 12.70 |
| 7 | 6 | 6 | 149.79 | 11.50 |
| 7 | 6 | 6 | 177.48 | 17.20 |
| 7 | 6 | 6 | 141.69 | 10.50 |
| 8 | -6 | 6 | 618.04 | 38.40 |
| 8 | -6 | 6 | 675.93 | 37.60 |
| 8 | -6 | 6 | 731.73 | 39.40 |
| 8 | 6 | 6 | 655.83 | 37.40 |
| 8 | 6 | 6 | 618.94 | 37.90 |
| 9 | -6 | 6 | 7.30 | 4.10 |
| 9 | -6 | 6 | 4.00 | 4.60 |
| 9 | -6 | 6 | 8.60 | 6.40 |
| 9 | 6 | 6 | 2.90 | 3.30 |
| 9 | 6 | 6 | 3.30 | 3.90 |
| 10 | -6 | 6 | 584.94 | 33.50 |
| 10 | -6 | 6 | 525.65 | 33.70 |
| 10 | -6 | 6 | 579.24 | 32.40 |
| 10 | 6 | 6 | 565.24 | 32.50 |
| 10 | 6 | 6 | 566.44 | 32.80 |
| 11 | -6 | 6 | 7.30 | 4.00 |
| 11 | -6 | 6 | 13.90 | 3.70 |
| 11 | -6 | 6 | 4.70 | 5.10 |
| 11 | 6 | 6 | 8.70 | 3.80 |

| | | | | |
|---|---|---|---|---|
| 11 | 6 | 6 | 6.20 | 3.90 |
| 12 | -6 | 6 | 5.80 | 3.70 |
| 12 | -6 | 6 | -1.90 | 3.20 |
| 12 | -6 | 6 | 1.60 | 4.40 |
| 12 | 6 | 6 | -6.00 | 6.30 |
| 12 | 6 | 6 | 1.40 | 3.00 |
| 13 | -6 | 6 | 12.70 | 4.10 |
| 13 | -6 | 6 | 13.40 | 3.70 |
| 13 | -6 | 6 | 15.50 | 3.70 |
| 13 | 6 | 6 | 10.70 | 3.20 |
| 13 | 6 | 6 | 11.70 | 3.10 |
| 14 | -6 | 6 | 218.88 | 12.90 |
| 14 | -6 | 6 | 181.08 | 15.50 |
| 14 | -6 | 6 | 220.28 | 13.20 |
| 14 | 6 | 6 | 194.78 | 12.50 |
| 14 | 6 | 6 | 202.78 | 12.80 |
| 16 | -7 | -6 | 1.00 | 1.80 |
| -16 | 7 | 6 | 2.20 | 1.80 |
| 16 | 7 | -6 | 4.60 | 1.40 |
| 15 | -7 | -6 | 399.66 | 22.60 |
| -15 | 7 | 6 | 406.46 | 22.80 |
| 15 | 7 | -6 | 393.66 | 22.40 |
| 14 | -7 | -6 | 6.20 | 2.60 |
| 14 | -7 | -6 | 4.30 | 3.50 |
| 14 | 7 | -6 | 6.50 | 2.30 |
| 13 | -7 | -6 | 1.80 | 2.70 |
| 13 | -7 | -6 | 2.20 | 3.50 |
| 13 | 7 | -6 | 2.80 | 2.00 |
| 12 | -7 | -6 | -0.70 | 3.10 |
| 12 | -7 | -6 | -3.30 | 3.70 |
| 12 | 7 | -6 | 1.90 | 2.30 |
| 11 | -7 | -6 | 1561.94 | 86.99 |
| 11 | -7 | -6 | 1594.64 | 87.19 |
| 11 | 7 | -6 | 1609.04 | 86.49 |
| 10 | -7 | -6 | 1.90 | 4.00 |
| 10 | -7 | -6 | 2.80 | 4.90 |
| 10 | 7 | -6 | 2.60 | 2.80 |
| 9 | -7 | -6 | 489.15 | 29.00 |
| 9 | -7 | -6 | 499.85 | 29.80 |
| 9 | 7 | -6 | 479.15 | 28.20 |
| 8 | -7 | -6 | 71.99 | 13.20 |
| 8 | -7 | -6 | 50.09 | 8.00 |
| 8 | 7 | -6 | 48.80 | 8.80 |
| 8 | 7 | -6 | 56.99 | 7.80 |

| | | | | |
|---|---|---|---|---|
| 7 | -7 | -6 | 822.02 | 45.10 |
| 7 | -7 | -6 | 672.73 | 46.30 |
| 7 | 7 | -6 | 854.31 | 45.00 |
| 6 | -7 | -6 | 25.00 | 5.50 |
| 6 | 7 | -6 | 26.80 | 5.20 |
| -6 | 7 | 6 | 33.60 | 6.20 |
| -5 | 7 | 6 | 3811.52 | 205.48 |
| 5 | 7 | -6 | 3788.12 | 206.08 |
| 4 | 7 | -6 | 84.79 | 9.00 |
| -4 | 7 | 6 | 78.69 | 11.90 |
| -3 | 7 | 6 | 21.00 | 4.40 |
| -2 | 7 | 6 | 22.60 | 4.70 |
| -2 | 7 | 6 | 34.60 | 6.00 |
| -1 | 7 | 6 | 4815.82 | 267.77 |
| -1 | 7 | 6 | 5129.79 | 268.67 |
| 0 | 7 | 6 | 155.98 | 11.40 |
| 0 | 7 | 6 | 148.29 | 10.90 |
| 1 | 7 | 6 | 922.61 | 51.49 |
| 1 | 7 | 6 | 938.01 | 52.59 |
| 2 | 7 | 6 | 113.69 | 9.80 |
| 2 | 7 | 6 | 102.29 | 8.40 |
| 3 | -7 | 6 | 1151.68 | 67.09 |
| 3 | 7 | 6 | 1235.78 | 68.59 |
| 3 | 7 | 6 | 1265.07 | 66.79 |
| 4 | -7 | 6 | 12.00 | 5.00 |
| 4 | 7 | 6 | 9.10 | 4.20 |
| 4 | 7 | 6 | 1.80 | 4.90 |
| 5 | -7 | 6 | 1989.40 | 107.09 |
| 5 | -7 | 6 | 2000.80 | 107.39 |
| 5 | 7 | 6 | 2022.30 | 107.19 |
| 5 | 7 | 6 | 1980.30 | 106.89 |
| 5 | 7 | 6 | 1837.82 | 109.39 |
| 6 | -7 | 6 | 162.68 | 12.00 |
| 6 | -7 | 6 | 126.89 | 11.90 |
| 6 | 7 | 6 | 136.39 | 11.10 |
| -6 | 7 | -6 | 126.49 | 11.10 |
| 6 | 7 | 6 | 113.39 | 15.20 |
| 6 | 7 | 6 | 136.89 | 9.60 |
| 7 | -7 | 6 | 126.09 | 11.00 |
| 7 | -7 | 6 | 131.69 | 12.20 |
| 7 | 7 | 6 | 134.29 | 9.70 |
| 7 | 7 | 6 | 166.08 | 17.40 |
| 7 | 7 | 6 | 122.59 | 10.80 |
| 8 | -7 | 6 | 73.79 | 9.20 |

| | | | | |
|---|---|---|---|---|
| 8 | -7 | 6 | 66.39 | 8.30 |
| 8 | 7 | 6 | 61.89 | 7.90 |
| 8 | 7 | 6 | 56.19 | 6.50 |
| 9 | -7 | 6 | 717.83 | 40.30 |
| 9 | -7 | 6 | 672.13 | 41.00 |
| 9 | -7 | 6 | 706.93 | 39.10 |
| 9 | 7 | 6 | 704.53 | 39.30 |
| 9 | 7 | 6 | 664.43 | 39.20 |
| 10 | -7 | 6 | 23.60 | 7.00 |
| 10 | -7 | 6 | 46.30 | 7.10 |
| 10 | -7 | 6 | 31.30 | 6.10 |
| 10 | 7 | 6 | 33.80 | 6.70 |
| 10 | 7 | 6 | 33.80 | 6.50 |
| 11 | -7 | 6 | 249.97 | 15.20 |
| 11 | -7 | 6 | 225.78 | 15.60 |
| 11 | -7 | 6 | 216.58 | 17.00 |
| 11 | 7 | 6 | 214.38 | 15.20 |
| 11 | 7 | 6 | 256.47 | 15.20 |
| 12 | -7 | 6 | 116.19 | 9.10 |
| 12 | -7 | 6 | 98.69 | 10.20 |
| 12 | -7 | 6 | 107.79 | 8.80 |
| 12 | 7 | 6 | 104.29 | 8.60 |
| 12 | 7 | 6 | 94.69 | 8.80 |
| 13 | -7 | 6 | 232.78 | 14.40 |
| 13 | -7 | 6 | 238.28 | 14.70 |
| 13 | -7 | 6 | 239.48 | 15.20 |
| 13 | 7 | 6 | 227.28 | 14.60 |
| 13 | 7 | 6 | 226.38 | 14.40 |
| 14 | -7 | 6 | 6.60 | 3.70 |
| 14 | -7 | 6 | 4.30 | 3.10 |
| 14 | 7 | 6 | 2.30 | 2.40 |
| 14 | 7 | 6 | 3.90 | 2.90 |
| 15 | -8 | -6 | -1.80 | 2.00 |
| -15 | 8 | 6 | 0.30 | 2.10 |
| 15 | 8 | -6 | 0.80 | 1.40 |
| 14 | -8 | -6 | 294.67 | 18.30 |
| 14 | 8 | -6 | 310.97 | 18.00 |
| -14 | 8 | 6 | 338.17 | 18.60 |
| 13 | -8 | -6 | 45.40 | 6.70 |
| 13 | -8 | -6 | 37.10 | 5.80 |
| 13 | 8 | -6 | 40.80 | 4.50 |
| 12 | -8 | -6 | 183.28 | 12.50 |
| 12 | -8 | -6 | 190.48 | 12.90 |
| 12 | 8 | -6 | 181.58 | 11.60 |

| | | | | |
|---|---|---|---|---|
| 11 | -8 | -6 | 2.30 | 3.70 |
| 11 | -8 | -6 | -0.70 | 3.80 |
| 11 | 8 | -6 | -0.40 | 2.20 |
| 10 | -8 | -6 | 1025.80 | 56.19 |
| 10 | -8 | -6 | 921.31 | 55.79 |
| 10 | 8 | -6 | 1062.19 | 55.39 |
| 9 | -8 | -6 | 1.60 | 3.80 |
| 9 | 8 | -6 | 1.70 | 3.00 |
| 8 | -8 | -6 | 107.49 | 14.00 |
| 8 | -8 | -6 | 107.29 | 10.10 |
| 8 | 8 | -6 | 95.09 | 9.40 |
| 8 | 8 | -6 | 120.19 | 11.70 |
| 7 | -8 | -6 | 22.90 | 8.40 |
| 7 | -8 | -6 | 28.60 | 5.60 |
| -7 | 8 | 6 | 28.60 | 7.00 |
| 7 | 8 | -6 | 35.60 | 7.90 |
| -6 | -8 | 6 | 2717.33 | 161.68 |
| -6 | 8 | 6 | 2914.51 | 162.88 |
| -6 | 8 | 6 | 3104.29 | 162.18 |
| 6 | 8 | -6 | 3202.78 | 162.78 |
| -5 | -8 | 6 | -4.30 | 8.40 |
| -5 | 8 | 6 | 5.20 | 3.90 |
| 5 | 8 | -6 | 12.80 | 4.90 |
| 4 | 8 | -6 | 1413.06 | 81.99 |
| -4 | 8 | 6 | 1528.25 | 81.19 |
| -3 | 8 | 6 | 16.10 | 4.30 |
| 3 | 8 | -6 | 2.60 | 4.20 |
| -2 | 8 | 6 | 359.06 | 21.40 |
| 2 | 8 | -6 | 328.97 | 22.40 |
| -2 | 8 | 6 | 356.96 | 21.60 |
| -1 | 8 | 6 | 406.96 | 24.30 |
| -1 | 8 | 6 | 413.16 | 24.90 |
| 0 | 8 | 6 | 3135.69 | 170.28 |
| 0 | 8 | 6 | 3161.88 | 171.28 |
| 1 | 8 | 6 | 245.78 | 15.30 |
| 1 | 8 | 6 | 241.48 | 16.40 |
| 2 | 8 | 6 | 50.79 | 6.00 |
| 2 | 8 | 6 | 53.19 | 7.50 |
| 3 | -8 | 6 | 2.30 | 4.80 |
| 3 | -8 | 6 | 5.20 | 5.70 |
| 3 | -8 | 6 | 30.00 | 9.20 |
| 3 | 8 | 6 | 14.40 | 4.50 |
| 4 | -8 | 6 | 1678.63 | 94.59 |
| 4 | -8 | 6 | 1599.84 | 96.69 |

| | | | | |
|---|---|---|---|---|
| 4 | -8 | 6 | 1718.53 | 94.79 |
| -4 | 8 | -6 | 1945.51 | 95.49 |
| 4 | 8 | 6 | 1774.42 | 94.49 |
| 4 | 8 | 6 | 1761.82 | 94.99 |
| 4 | 8 | 6 | 1658.73 | 96.69 |
| 5 | -8 | 6 | 70.99 | 8.50 |
| 5 | -8 | 6 | 56.09 | 8.80 |
| 5 | -8 | 6 | 66.79 | 10.30 |
| -5 | 8 | -6 | 56.29 | 8.00 |
| 5 | 8 | 6 | 46.80 | 9.10 |
| 5 | 8 | 6 | 61.59 | 6.10 |
| 5 | 8 | 6 | 63.69 | 11.20 |
| 6 | -8 | 6 | 687.03 | 37.30 |
| 6 | -8 | 6 | 619.24 | 37.70 |
| 6 | 8 | 6 | 678.93 | 37.40 |
| 6 | 8 | 6 | 675.73 | 37.00 |
| 6 | 8 | 6 | 586.84 | 40.60 |
| -6 | 8 | -6 | 680.83 | 38.00 |
| 7 | -8 | 6 | 4.90 | 4.50 |
| 7 | -8 | 6 | 7.50 | 4.40 |
| -7 | -8 | -6 | 12.10 | 4.90 |
| 7 | 8 | 6 | 4.30 | 2.80 |
| 7 | 8 | 6 | 5.30 | 9.30 |
| 8 | -8 | 6 | 376.16 | 22.90 |
| 8 | -8 | 6 | 381.56 | 24.00 |
| 8 | 8 | 6 | 388.16 | 22.60 |
| 8 | 8 | 6 | 387.86 | 23.10 |
| 9 | -8 | 6 | 2.50 | 3.30 |
| 9 | -8 | 6 | -10.20 | 4.50 |
| 9 | 8 | 6 | 1.80 | 3.80 |
| 9 | 8 | 6 | 3.50 | 3.20 |
| 10 | -8 | 6 | 825.12 | 44.60 |
| 10 | -8 | 6 | 832.32 | 45.60 |
| 10 | 8 | 6 | 725.83 | 44.80 |
| 10 | 8 | 6 | 812.72 | 44.80 |
| 11 | -8 | 6 | 65.69 | 8.10 |
| 11 | -8 | 6 | 56.09 | 10.70 |
| 11 | 8 | 6 | 55.29 | 10.70 |
| 11 | 8 | 6 | 62.09 | 6.90 |
| 12 | -8 | 6 | 13.10 | 3.20 |
| 12 | -8 | 6 | 13.30 | 3.90 |
| 12 | 8 | 6 | 12.40 | 3.70 |
| 12 | 8 | 6 | 6.30 | 3.20 |
| 13 | -8 | 6 | 16.30 | 3.70 |

| | | | | |
|---|---|---|---|---|
| 13 | -8 | 6 | 11.30 | 3.30 |
| 13 | 8 | 6 | 24.60 | 4.70 |
| 13 | 8 | 6 | 12.00 | 3.70 |
| 15 | -9 | -6 | 273.67 | 15.80 |
| -15 | 9 | 6 | 284.77 | 16.00 |
| 15 | 9 | -6 | 268.87 | 15.50 |
| 14 | -9 | -6 | 2.40 | 2.30 |
| 14 | 9 | -6 | 3.40 | 1.60 |
| -14 | 9 | 6 | 3.70 | 2.30 |
| 13 | -9 | -6 | 22.20 | 3.70 |
| -13 | 9 | 6 | 30.70 | 5.40 |
| 13 | 9 | -6 | 27.60 | 4.40 |
| 12 | -9 | -6 | 131.59 | 10.00 |
| 12 | 9 | -6 | 133.39 | 8.90 |
| -12 | 9 | 6 | 134.89 | 10.00 |
| 11 | -9 | -6 | 659.43 | 38.50 |
| 11 | -9 | -6 | 679.03 | 38.30 |
| 11 | 9 | -6 | 685.53 | 37.60 |
| -11 | 9 | 6 | 671.73 | 38.20 |
| 10 | -9 | -6 | 0.90 | 3.40 |
| 10 | -9 | -6 | 7.40 | 4.60 |
| 10 | 9 | -6 | 3.10 | 2.70 |
| -10 | 9 | 6 | 5.10 | 3.80 |
| 9 | -9 | -6 | 409.56 | 26.20 |
| 9 | -9 | -6 | 408.26 | 25.00 |
| 9 | 9 | -6 | 395.66 | 24.40 |
| -9 | 9 | 6 | 451.65 | 25.70 |
| 8 | -9 | -6 | 12.60 | 6.70 |
| 8 | 9 | -6 | 12.00 | 3.80 |
| -8 | 9 | 6 | 11.90 | 6.10 |
| 8 | 9 | -6 | 23.70 | 6.40 |
| -7 | -9 | 6 | 381.66 | 26.70 |
| 7 | -9 | -6 | 401.16 | 27.90 |
| 7 | 9 | -6 | 464.95 | 26.60 |
| -7 | 9 | 6 | 434.16 | 27.50 |
| 6 | -9 | -6 | 3.70 | 9.20 |
| -6 | -9 | 6 | -0.10 | 5.50 |
| -6 | 9 | 6 | 1.70 | 7.40 |
| 6 | 9 | -6 | 15.50 | 5.50 |
| -6 | 9 | 6 | 13.20 | 5.50 |
| -5 | -9 | 6 | 1041.30 | 62.39 |
| -5 | 9 | 6 | 1083.99 | 64.29 |
| -5 | 9 | 6 | 1210.08 | 62.99 |
| 5 | 9 | -6 | 1157.18 | 63.89 |

| | | | | |
|---|---|---|---|---|
| -4 | -9 | 6 | 19.20 | 5.40 |
| -4 | -9 | 6 | 22.60 | 4.70 |
| 4 | 9 | -6 | 19.00 | 6.00 |
| -4 | 9 | 6 | 18.80 | 4.80 |
| -3 | -9 | 6 | 52.09 | 9.30 |
| -3 | -9 | 6 | 64.29 | 8.90 |
| -3 | 9 | 6 | 65.39 | 7.90 |
| 3 | 9 | -6 | 56.59 | 8.90 |
| -2 | -9 | 6 | 42.10 | 5.80 |
| -2 | -9 | 6 | 31.10 | 5.80 |
| 2 | 9 | -6 | 25.00 | 5.70 |
| -2 | 9 | 6 | 41.10 | 6.70 |
| -2 | 9 | 6 | 37.00 | 6.70 |
| -1 | -9 | 6 | 1910.91 | 108.69 |
| -1 | -9 | 6 | 2003.80 | 108.19 |
| -1 | 9 | 6 | 2074.89 | 109.49 |
| 1 | 9 | -6 | 2012.00 | 110.09 |
| -1 | 9 | 6 | 1951.50 | 108.49 |
| 0 | -9 | 6 | 193.18 | 14.60 |
| 0 | -9 | 6 | 223.38 | 15.60 |
| 0 | 9 | 6 | 214.88 | 15.00 |
| 0 | 9 | -6 | 235.68 | 15.70 |
| 0 | 9 | 6 | 205.48 | 14.00 |
| 1 | -9 | 6 | 1062.29 | 57.89 |
| 1 | -9 | 6 | 965.40 | 56.39 |
| 1 | 9 | 6 | 1029.30 | 57.79 |
| -1 | 9 | -6 | 1029.80 | 57.59 |
| 2 | -9 | 6 | 83.89 | 9.00 |
| 2 | -9 | 6 | 82.09 | 13.70 |
| 2 | 9 | 6 | 71.29 | 8.80 |
| -2 | 9 | -6 | 70.89 | 7.90 |
| 2 | 9 | 6 | 68.79 | 6.90 |
| 3 | -9 | 6 | 560.34 | 31.50 |
| 3 | -9 | 6 | 510.85 | 34.20 |
| 3 | -9 | 6 | 553.64 | 35.30 |
| 3 | 9 | 6 | 552.64 | 31.10 |
| -3 | 9 | -6 | 611.94 | 32.30 |
| 3 | 9 | 6 | 488.35 | 32.50 |
| 3 | 9 | 6 | 545.35 | 33.30 |
| 4 | -9 | 6 | 110.39 | 9.30 |
| 4 | -9 | 6 | 86.89 | 10.40 |
| 4 | -9 | 6 | 94.19 | 17.00 |
| -4 | 9 | -6 | 83.69 | 8.90 |
| 4 | 9 | 6 | 96.59 | 11.60 |

| | | | | |
|---|---|---|---|---|
| 4 | 9 | 6 | 91.89 | 7.60 |
| 4 | 9 | 6 | 78.19 | 11.00 |
| 5 | -9 | 6 | 2105.09 | 119.79 |
| 5 | -9 | 6 | 2331.47 | 118.39 |
| 5 | -9 | 6 | 2111.09 | 118.59 |
| -5 | -9 | -6 | 2258.27 | 118.79 |
| 5 | 9 | 6 | 1983.90 | 121.09 |
| 5 | 9 | 6 | 2206.68 | 118.39 |
| 5 | 9 | 6 | 2190.28 | 118.49 |
| -5 | 9 | -6 | 2249.38 | 119.39 |
| -6 | -9 | -6 | 280.97 | 18.30 |
| 6 | -9 | 6 | 296.47 | 18.40 |
| 6 | 9 | 6 | 236.38 | 22.20 |
| -6 | 9 | -6 | 287.47 | 19.20 |
| 6 | 9 | 6 | 271.87 | 17.00 |
| 6 | 9 | 6 | 296.17 | 18.10 |
| 7 | -9 | 6 | 102.99 | 9.90 |
| -7 | -9 | -6 | 67.09 | 9.30 |
| 7 | -9 | 6 | 89.49 | 8.50 |
| 7 | 9 | 6 | 64.39 | 14.70 |
| 7 | 9 | 6 | 84.49 | 9.00 |
| 7 | 9 | 6 | 93.49 | 7.40 |
| -8 | -9 | -6 | 80.79 | 9.80 |
| 8 | -9 | 6 | 90.29 | 10.10 |
| 8 | -9 | 6 | 82.09 | 8.20 |
| 8 | 9 | 6 | 95.79 | 9.20 |
| 8 | 9 | 6 | 85.49 | 7.50 |
| 9 | -9 | 6 | 869.81 | 49.50 |
| 9 | -9 | 6 | 838.92 | 48.30 |
| 9 | 9 | 6 | 892.01 | 48.60 |
| 9 | 9 | 6 | 876.41 | 48.50 |
| 10 | -9 | 6 | 7.00 | 3.20 |
| 10 | -9 | 6 | -5.80 | 4.20 |
| 10 | 9 | 6 | -0.60 | 2.70 |
| 10 | 9 | 6 | -3.00 | 3.90 |
| 11 | -9 | 6 | 319.97 | 17.50 |
| 11 | -9 | 6 | 278.87 | 18.20 |
| 11 | 9 | 6 | 287.67 | 17.90 |
| 11 | 9 | 6 | 259.07 | 17.50 |
| 12 | -9 | 6 | 107.59 | 7.90 |
| 12 | -9 | 6 | 97.59 | 8.60 |
| 12 | 9 | 6 | 104.29 | 8.90 |
| 12 | 9 | 6 | 84.79 | 7.80 |
| 13 | -9 | 6 | 175.28 | 11.70 |

| | | | | |
|---|---|---|---|---|
| 13 | -9 | 6 | 185.38 | 11.50 |
| 13 | 9 | 6 | 154.58 | 11.80 |
| 13 | 9 | 6 | 177.78 | 11.10 |
| 15 | -10 | -6 | 0.40 | 2.10 |
| -15 | 10 | 6 | 4.20 | 2.30 |
| 15 | 10 | -6 | 4.20 | 1.30 |
| 14 | -10 | -6 | 195.68 | 11.80 |
| 14 | 10 | -6 | 185.88 | 11.30 |
| -14 | 10 | 6 | 200.58 | 12.00 |
| 13 | -10 | -6 | 92.29 | 7.40 |
| 13 | 10 | -6 | 97.09 | 6.50 |
| -13 | 10 | 6 | 90.19 | 7.50 |
| 12 | 10 | -6 | 256.07 | 15.70 |
| -12 | 10 | 6 | 283.87 | 18.20 |
| 11 | -10 | -6 | 44.50 | 7.20 |
| 11 | 10 | -6 | 40.80 | 4.80 |
| -11 | 10 | 6 | 33.60 | 6.50 |
| 10 | -10 | -6 | 795.62 | 45.10 |
| -10 | 10 | 6 | 811.52 | 45.00 |
| 10 | 10 | -6 | 783.02 | 44.20 |
| 9 | -10 | -6 | 49.00 | 7.10 |
| 9 | 10 | -6 | 54.49 | 6.50 |
| -9 | 10 | 6 | 56.49 | 9.30 |
| 8 | -10 | -6 | 112.79 | 13.60 |
| 8 | 10 | -6 | 122.09 | 10.10 |
| 8 | 10 | -6 | 145.39 | 14.60 |
| -8 | 10 | 6 | 113.79 | 13.30 |
| -7 | -10 | 6 | 146.49 | 14.20 |
| 7 | -10 | -6 | 163.28 | 16.60 |
| 7 | 10 | -6 | 194.68 | 14.90 |
| -7 | 10 | 6 | 179.78 | 17.00 |
| -6 | -10 | 6 | 2209.48 | 121.59 |
| 6 | -10 | -6 | 2142.79 | 122.39 |
| 6 | 10 | -6 | 2360.26 | 123.29 |
| -6 | 10 | 6 | 2222.38 | 123.09 |
| -5 | -10 | 6 | 289.17 | 19.70 |
| 5 | -10 | -6 | 295.97 | 21.90 |
| 5 | 10 | -6 | 314.27 | 21.20 |
| -5 | 10 | 6 | 312.57 | 20.20 |
| -5 | 10 | 6 | 298.27 | 23.20 |
| -4 | -10 | 6 | 408.76 | 24.80 |
| 4 | -10 | -6 | 431.36 | 27.20 |
| -4 | 10 | 6 | 409.46 | 27.40 |
| -4 | 10 | 6 | 434.76 | 25.30 |

| | | | | |
|---|---|---|---|---|
| 4 | 10 | -6 | 355.46 | 26.20 |
| 3 | -10 | -6 | 83.09 | 13.60 |
| -3 | -10 | 6 | 93.69 | 9.00 |
| -3 | -10 | 6 | 68.49 | 9.70 |
| -3 | 10 | 6 | 87.29 | 8.80 |
| 3 | 10 | -6 | 64.29 | 10.70 |
| -3 | 10 | 6 | 90.09 | 9.20 |
| -2 | -10 | 6 | 1068.39 | 55.59 |
| -2 | -10 | 6 | 953.80 | 55.79 |
| 2 | -10 | -6 | 926.91 | 56.89 |
| -2 | 10 | 6 | 1089.29 | 56.89 |
| 2 | 10 | -6 | 903.81 | 57.49 |
| -2 | 10 | 6 | 1062.79 | 56.09 |
| 1 | -10 | -6 | 7.60 | 6.10 |
| -1 | -10 | 6 | -0.20 | 4.80 |
| -1 | -10 | 6 | 3.90 | 3.70 |
| 1 | 10 | -6 | 9.30 | 4.90 |
| -1 | 10 | 6 | 6.70 | 3.70 |
| -1 | 10 | 6 | 7.40 | 3.30 |
| 0 | -10 | 6 | 2828.32 | 147.59 |
| 0 | -10 | 6 | 2633.34 | 146.69 |
| 0 | 10 | -6 | 2886.81 | 148.79 |
| 0 | 10 | 6 | 2508.35 | 146.99 |
| 0 | 10 | 6 | 2708.73 | 148.29 |
| 1 | -10 | 6 | 126.99 | 12.80 |
| 1 | -10 | 6 | 102.99 | 10.10 |
| 1 | 10 | 6 | 123.39 | 9.40 |
| 1 | 10 | 6 | 119.79 | 11.00 |
| -1 | 10 | -6 | 142.59 | 11.00 |
| 2 | -10 | 6 | 1.10 | 6.10 |
| 2 | -10 | 6 | 0.30 | 3.90 |
| -2 | 10 | -6 | -0.40 | 3.40 |
| 2 | 10 | 6 | -0.30 | 4.20 |
| 2 | 10 | 6 | 3.80 | 3.00 |
| 3 | -10 | 6 | 110.79 | 9.70 |
| -3 | -10 | -6 | 97.29 | 10.60 |
| 3 | -10 | 6 | 126.79 | 15.90 |
| 3 | 10 | 6 | 113.89 | 8.70 |
| -3 | 10 | -6 | 122.89 | 10.40 |
| 3 | 10 | 6 | 128.59 | 12.30 |
| -4 | -10 | -6 | 1743.23 | 91.39 |
| 4 | -10 | 6 | 1613.84 | 93.09 |
| 4 | -10 | 6 | 1693.23 | 91.19 |
| 4 | -10 | 6 | 1741.03 | 90.99 |

| | | | | |
|---|---|---|---|---|
| -4 | 10 | -6 | 1718.93 | 92.19 |
| 4 | 10 | 6 | 1644.44 | 93.69 |
| 4 | 10 | 6 | 1517.85 | 90.89 |
| 5 | -10 | 6 | 15.70 | 5.80 |
| 5 | -10 | 6 | 20.20 | 8.30 |
| 5 | -10 | 6 | 9.90 | 4.60 |
| 5 | 10 | 6 | 13.40 | 5.50 |
| 5 | 10 | 6 | 9.10 | 2.80 |
| 5 | 10 | 6 | 11.60 | 7.20 |
| -5 | 10 | -6 | 6.60 | 5.00 |
| -6 | -10 | -6 | 857.31 | 46.10 |
| 6 | -10 | 6 | 845.52 | 45.70 |
| 6 | -10 | 6 | 826.32 | 46.30 |
| 6 | 10 | 6 | 743.43 | 50.09 |
| 6 | 10 | 6 | 867.71 | 46.10 |
| 6 | 10 | 6 | 762.62 | 45.50 |
| 7 | -10 | 6 | 252.87 | 15.90 |
| -7 | -10 | -6 | 248.78 | 16.50 |
| 7 | -10 | 6 | 249.18 | 16.90 |
| 7 | 10 | 6 | 246.58 | 15.50 |
| 7 | 10 | 6 | 244.78 | 26.00 |
| 7 | 10 | 6 | 260.47 | 16.70 |
| 8 | -10 | 6 | 607.44 | 39.00 |
| -8 | -10 | -6 | 580.74 | 33.90 |
| 8 | -10 | 6 | 581.64 | 33.70 |
| 8 | 10 | 6 | 588.84 | 34.10 |
| 8 | 10 | 6 | 622.64 | 35.50 |
| 9 | -10 | 6 | 0.30 | 4.50 |
| 9 | -10 | 6 | 5.40 | 3.20 |
| 9 | 10 | 6 | 8.60 | 2.80 |
| 9 | 10 | 6 | 5.90 | 4.70 |
| 10 | -10 | 6 | 926.51 | 47.80 |
| 10 | -10 | 6 | 735.83 | 45.80 |
| 10 | 10 | 6 | 786.42 | 45.40 |
| 10 | 10 | 6 | 816.62 | 45.60 |
| 11 | -10 | 6 | 100.89 | 7.80 |
| 11 | -10 | 6 | 89.49 | 9.40 |
| 11 | 10 | 6 | 104.19 | 8.10 |
| 11 | 10 | 6 | 103.59 | 9.70 |
| 12 | -10 | 6 | 2.40 | 3.90 |
| 12 | -10 | 6 | -3.10 | 2.50 |
| 12 | 10 | 6 | -2.70 | 2.60 |
| 14 | -11 | -6 | 13.10 | 2.40 |
| 14 | 11 | -6 | 12.90 | 2.40 |

| | | | | |
|---|---|---|---|---|
| -14 | 11 | 6 | 9.70 | 3.10 |
| 13 | -11 | -6 | 2.60 | 2.40 |
| -13 | 11 | 6 | 2.70 | 2.80 |
| 13 | 11 | -6 | 1.90 | 1.50 |
| 12 | -11 | -6 | 157.58 | 10.20 |
| -12 | 11 | 6 | 140.89 | 10.50 |
| 12 | 11 | -6 | 143.79 | 9.30 |
| 11 | -11 | -6 | 1098.69 | 60.19 |
| 11 | 11 | -6 | 1115.29 | 59.69 |
| -11 | 11 | 6 | 1064.39 | 61.39 |
| 10 | -11 | -6 | 22.70 | 4.80 |
| 10 | 11 | -6 | 27.40 | 4.50 |
| -10 | 11 | 6 | 26.30 | 5.10 |
| 9 | -11 | -6 | 208.18 | 15.40 |
| 9 | 11 | -6 | 209.18 | 13.60 |
| -9 | 11 | 6 | 210.18 | 16.10 |
| -8 | -11 | 6 | 238.58 | 18.00 |
| 8 | -11 | -6 | 253.67 | 18.80 |
| -8 | 11 | 6 | 276.57 | 20.00 |
| 8 | 11 | -6 | 265.07 | 16.50 |
| -7 | -11 | 6 | 548.15 | 31.60 |
| 7 | -11 | -6 | 518.45 | 32.80 |
| -7 | 11 | 6 | 539.35 | 34.10 |
| 6 | -11 | -6 | 4.70 | 7.60 |
| 6 | 11 | -6 | -1.10 | 5.40 |
| -6 | 11 | 6 | -9.70 | 8.90 |
| 5 | -11 | -6 | 2279.77 | 120.59 |
| -5 | -11 | 6 | 2121.39 | 119.99 |
| 5 | 11 | -6 | 2179.88 | 122.19 |
| -5 | 11 | 6 | 2239.18 | 121.09 |
| -4 | -11 | 6 | 139.59 | 12.00 |
| 4 | -11 | -6 | 159.78 | 15.30 |
| -4 | 11 | 6 | 147.99 | 12.70 |
| 4 | 11 | -6 | 132.59 | 14.20 |
| -3 | -11 | 6 | 15.50 | 4.10 |
| -3 | -11 | 6 | 13.80 | 4.30 |
| 3 | -11 | -6 | 12.80 | 6.70 |
| 3 | 11 | -6 | 12.50 | 5.90 |
| -3 | 11 | 6 | 19.00 | 4.20 |
| -3 | 11 | 6 | 15.40 | 5.10 |
| 2 | -11 | -6 | 137.19 | 13.10 |
| -2 | -11 | 6 | 134.59 | 11.50 |
| -2 | -11 | 6 | 125.49 | 10.10 |
| 2 | 11 | -6 | 105.09 | 12.70 |

| | | | | |
|---|---|---|---|---|
| -2 | 11 | 6 | 128.89 | 11.60 |
| -2 | 11 | 6 | 130.59 | 10.40 |
| -1 | -11 | 6 | 2761.82 | 147.79 |
| -1 | -11 | 6 | 2714.23 | 147.19 |
| 1 | -11 | -6 | 2739.93 | 148.19 |
| -1 | 11 | 6 | 2774.02 | 148.89 |
| 1 | 11 | -6 | 2585.64 | 149.49 |
| -1 | 11 | 6 | 2743.03 | 147.79 |
| 0 | -11 | 6 | 1.50 | 3.30 |
| 0 | -11 | 6 | 2.90 | 4.60 |
| 0 | -11 | -6 | 5.80 | 4.90 |
| 0 | 11 | 6 | 6.00 | 3.30 |
| 0 | 11 | 6 | 1.20 | 4.50 |
| 0 | 11 | -6 | 1.10 | 4.40 |
| 1 | -11 | 6 | 258.47 | 17.60 |
| -1 | -11 | -6 | 241.68 | 16.60 |
| 1 | -11 | 6 | 246.28 | 15.30 |
| 1 | 11 | 6 | 235.38 | 17.20 |
| 1 | 11 | 6 | 212.78 | 15.10 |
| -1 | 11 | -6 | 243.28 | 17.10 |
| -2 | -11 | -6 | 101.69 | 9.70 |
| 2 | -11 | 6 | 108.29 | 12.80 |
| 2 | -11 | 6 | 97.69 | 8.60 |
| 2 | 11 | 6 | 96.09 | 10.90 |
| 2 | 11 | 6 | 82.49 | 7.80 |
| -2 | 11 | -6 | 89.29 | 9.90 |
| 3 | -11 | 6 | 983.70 | 54.99 |
| 3 | -11 | 6 | 949.51 | 52.49 |
| -3 | -11 | -6 | 917.21 | 53.19 |
| -3 | 11 | -6 | 934.91 | 53.89 |
| 3 | 11 | 6 | 952.40 | 55.19 |
| 3 | 11 | 6 | 954.60 | 52.59 |
| -4 | -11 | -6 | 74.99 | 8.10 |
| 4 | -11 | 6 | 68.59 | 7.60 |
| 4 | 11 | 6 | 66.69 | 12.30 |
| -4 | 11 | -6 | 73.49 | 9.50 |
| 4 | 11 | 6 | 57.89 | 6.40 |
| 5 | -11 | 6 | 1871.21 | 103.29 |
| 5 | -11 | 6 | 1949.60 | 104.59 |
| -5 | -11 | -6 | 1989.90 | 103.49 |
| 5 | -11 | 6 | 2011.50 | 103.09 |
| 5 | 11 | 6 | 1721.93 | 106.39 |
| 5 | 11 | 6 | 1816.22 | 103.09 |
| 6 | -11 | 6 | 190.98 | 12.40 |

| | | | | |
|---|---|---|---|---|
| 6 | -11 | 6 | 176.68 | 13.30 |
| -6 | -11 | -6 | 191.68 | 12.90 |
| 6 | 11 | 6 | 173.38 | 11.80 |
| 6 | 11 | 6 | 153.88 | 21.20 |
| 7 | -11 | 6 | 56.09 | 9.00 |
| 7 | -11 | 6 | 61.19 | 6.70 |
| -7 | -11 | -6 | 60.79 | 7.90 |
| 7 | 11 | 6 | 65.49 | 6.40 |
| 7 | 11 | 6 | 54.29 | 15.10 |
| -8 | -11 | -6 | 20.30 | 4.10 |
| 8 | -11 | 6 | 27.80 | 5.50 |
| 8 | -11 | 6 | 22.10 | 5.20 |
| 8 | 11 | 6 | 20.40 | 3.50 |
| 9 | -11 | 6 | 287.07 | 19.30 |
| 9 | -11 | 6 | 308.47 | 18.30 |
| 9 | 11 | 6 | 317.67 | 18.40 |
| 10 | -11 | 6 | 3.60 | 4.20 |
| 10 | -11 | 6 | 9.30 | 2.80 |
| 10 | 11 | 6 | 7.70 | 2.80 |
| 11 | -11 | 6 | 174.58 | 14.00 |
| 11 | -11 | 6 | 176.28 | 10.90 |
| 11 | 11 | 6 | 156.58 | 11.20 |
| 12 | -11 | 6 | 92.09 | 6.60 |
| 12 | 11 | 6 | 79.39 | 6.90 |
| 13 | -12 | -6 | 81.79 | 6.50 |
| 13 | 12 | -6 | 94.79 | 5.90 |
| 12 | -12 | -6 | 214.28 | 13.40 |
| 12 | 12 | -6 | 222.28 | 12.80 |
| 11 | -12 | -6 | -0.40 | 3.00 |
| 11 | 12 | -6 | 3.00 | 2.20 |
| -11 | 12 | 6 | 11.00 | 4.40 |
| 10 | -12 | -6 | 792.42 | 44.30 |
| 10 | 12 | -6 | 752.02 | 43.60 |
| -10 | 12 | 6 | 831.92 | 46.30 |
| 9 | -12 | -6 | 67.39 | 9.40 |
| -9 | 12 | 6 | 66.99 | 11.30 |
| 9 | 12 | -6 | 78.29 | 6.80 |
| 8 | -12 | -6 | -10.80 | 5.20 |
| -8 | -12 | 6 | 5.50 | 4.90 |
| 8 | 12 | -6 | -2.30 | 2.70 |
| -7 | -12 | 6 | 226.18 | 15.50 |
| 7 | -12 | -6 | 178.98 | 17.30 |
| 7 | 12 | -6 | 239.58 | 19.10 |
| -6 | -12 | 6 | 2550.34 | 140.39 |

| | | | | |
|---|---|---|---|---|
| 6 | -12 | -6 | 2447.35 | 141.19 |
| 6 | 12 | -6 | 2759.52 | 142.79 |
| 5 | -12 | -6 | 31.90 | 10.20 |
| -5 | -12 | 6 | 23.60 | 4.90 |
| 5 | 12 | -6 | 54.09 | 12.60 |
| -5 | 12 | 6 | 48.40 | 11.50 |
| 4 | -12 | -6 | 404.26 | 26.80 |
| -4 | -12 | 6 | 401.56 | 23.90 |
| 4 | 12 | -6 | 357.46 | 26.70 |
| -4 | 12 | 6 | 402.56 | 25.10 |
| -4 | 12 | 6 | 376.96 | 24.20 |
| -3 | -12 | 6 | 113.49 | 12.40 |
| 3 | -12 | -6 | 103.19 | 11.90 |
| -3 | -12 | 6 | 102.39 | 9.60 |
| -3 | 12 | 6 | 123.99 | 11.50 |
| -3 | 12 | 6 | 100.99 | 9.90 |
| 3 | 12 | -6 | 95.29 | 13.50 |
| 2 | -12 | -6 | 823.22 | 48.80 |
| -2 | -12 | 6 | 843.92 | 49.20 |
| -2 | -12 | 6 | 890.71 | 48.30 |
| -2 | 12 | 6 | 899.11 | 48.40 |
| 2 | 12 | -6 | 774.12 | 50.39 |
| -2 | 12 | 6 | 879.71 | 49.40 |
| -1 | -12 | 6 | 111.49 | 11.10 |
| -1 | -12 | 6 | 101.19 | 9.30 |
| 1 | -12 | -6 | 124.19 | 12.20 |
| 1 | 12 | -6 | 124.79 | 13.10 |
| -1 | 12 | 6 | 126.99 | 11.90 |
| -1 | 12 | 6 | 115.49 | 9.70 |
| 0 | -12 | 6 | 2160.98 | 116.99 |
| 0 | -12 | 6 | 1953.80 | 116.39 |
| 0 | -12 | -6 | 1966.00 | 117.19 |
| 0 | 12 | -6 | 2238.38 | 117.99 |
| 0 | 12 | 6 | 2144.89 | 115.99 |
| 0 | 12 | 6 | 2302.87 | 117.79 |
| -1 | -12 | -6 | 109.59 | 12.20 |
| 1 | -12 | 6 | 96.19 | 11.40 |
| 1 | 12 | 6 | 107.19 | 11.50 |
| -1 | 12 | -6 | 122.29 | 11.40 |
| 1 | 12 | 6 | 98.99 | 8.70 |
| 2 | -12 | 6 | 357.26 | 22.70 |
| 2 | -12 | 6 | 327.37 | 20.20 |
| -2 | -12 | -6 | 320.07 | 20.90 |
| 2 | 12 | 6 | 324.47 | 23.50 |

| | | | | |
|---|---|---|---|---|
| 2 | 12 | 6 | 352.56 | 20.30 |
| -2 | 12 | -6 | 336.07 | 22.10 |
| -3 | -12 | -6 | 99.99 | 9.50 |
| 3 | -12 | 6 | 102.59 | 8.80 |
| 3 | -12 | 6 | 135.19 | 13.70 |
| 3 | 12 | 6 | 94.19 | 8.10 |
| 3 | 12 | 6 | 97.59 | 13.00 |
| -3 | 12 | -6 | 102.99 | 13.20 |
| 4 | -12 | 6 | 950.10 | 57.49 |
| -4 | -12 | -6 | 1008.50 | 55.89 |
| 4 | -12 | 6 | 1029.60 | 55.49 |
| -4 | 12 | -6 | 1047.90 | 57.39 |
| 4 | 12 | 6 | 1009.00 | 55.59 |
| 4 | 12 | 6 | 974.30 | 59.09 |
| -5 | -12 | -6 | 16.40 | 4.00 |
| 5 | -12 | 6 | 8.50 | 6.80 |
| 5 | -12 | 6 | 16.40 | 3.40 |
| 5 | 12 | 6 | 20.70 | 9.80 |
| -5 | 12 | -6 | 16.50 | 7.20 |
| 5 | 12 | 6 | 14.90 | 3.20 |
| 6 | -12 | 6 | 12.10 | 3.00 |
| -6 | -12 | -6 | 15.40 | 3.60 |
| 6 | 12 | 6 | 12.00 | 2.90 |
| 6 | 12 | 6 | 7.20 | 13.00 |
| 7 | -12 | 6 | 95.99 | 7.90 |
| -7 | -12 | -6 | 101.39 | 8.60 |
| 7 | 12 | 6 | 100.09 | 7.60 |
| 7 | 12 | 6 | 49.40 | 21.10 |
| 8 | -12 | 6 | 298.97 | 18.00 |
| -8 | -12 | -6 | 323.37 | 18.40 |
| 8 | 12 | 6 | 285.37 | 18.00 |
| 9 | -12 | 6 | 27.80 | 5.50 |
| 9 | 12 | 6 | 29.50 | 4.60 |
| 10 | -12 | 6 | 271.87 | 15.60 |
| 10 | 12 | 6 | 251.57 | 15.70 |
| 11 | -12 | 6 | 38.60 | 4.60 |
| 11 | 12 | 6 | 42.10 | 5.60 |
| 13 | -13 | -6 | 2.10 | 2.10 |
| 13 | 13 | -6 | -0.90 | 1.10 |
| 12 | -13 | -6 | 58.89 | 5.60 |
| 12 | 13 | -6 | 61.49 | 4.50 |
| 11 | -13 | -6 | 472.95 | 27.10 |
| 11 | 13 | -6 | 486.15 | 26.70 |
| 10 | -13 | -6 | 0.40 | 3.70 |

| | | | | |
|---|---|---|---|---|
| 10 | 13 | -6 | 3.20 | 3.70 |
| 9 | -13 | -6 | 184.88 | 13.50 |
| 9 | 13 | -6 | 184.78 | 11.90 |
| -8 | -13 | 6 | 126.09 | 10.90 |
| 8 | -13 | -6 | 116.99 | 11.90 |
| 8 | 13 | -6 | 114.69 | 9.00 |
| 7 | -13 | -6 | 322.57 | 23.60 |
| -7 | -13 | 6 | 383.46 | 22.40 |
| 7 | 13 | -6 | 392.76 | 26.60 |
| -6 | -13 | 6 | 87.19 | 9.30 |
| 6 | 13 | -6 | 66.79 | 14.20 |
| 5 | -13 | -6 | 659.43 | 36.40 |
| -5 | -13 | 6 | 608.24 | 35.90 |
| -5 | 13 | 6 | 588.94 | 36.60 |
| 5 | 13 | -6 | 644.94 | 39.50 |
| -4 | -13 | 6 | 194.38 | 14.20 |
| 4 | -13 | -6 | 228.48 | 15.60 |
| -4 | 13 | 6 | 203.08 | 14.70 |
| -4 | 13 | 6 | 211.48 | 17.20 |
| 4 | 13 | -6 | 166.48 | 18.30 |
| -3 | -13 | 6 | 82.99 | 9.20 |
| 3 | -13 | -6 | 85.09 | 10.50 |
| -3 | -13 | 6 | 94.99 | 8.00 |
| -3 | 13 | 6 | 80.59 | 9.00 |
| -3 | 13 | 6 | 83.79 | 11.90 |
| -2 | -13 | 6 | 201.48 | 14.50 |
| 2 | -13 | -6 | 215.28 | 15.70 |
| -2 | -13 | 6 | 210.78 | 13.40 |
| -2 | 13 | 6 | 220.08 | 16.60 |
| 2 | 13 | -6 | 175.68 | 17.80 |
| -2 | 13 | 6 | 205.78 | 14.20 |
| -1 | -13 | 6 | 1674.13 | 94.09 |
| -1 | -13 | 6 | 1659.93 | 93.39 |
| 1 | -13 | -6 | 1800.52 | 94.69 |
| -1 | 13 | 6 | 1761.72 | 94.19 |
| 1 | 13 | -6 | 1698.23 | 96.59 |
| -1 | 13 | 6 | 1702.43 | 95.59 |
| 0 | -13 | 6 | 49.90 | 8.40 |
| 0 | -13 | -6 | 34.10 | 5.70 |
| 0 | -13 | 6 | 41.70 | 6.10 |
| 0 | 13 | 6 | 42.10 | 9.80 |
| 0 | 13 | 6 | 45.40 | 6.00 |
| 0 | 13 | -6 | 30.60 | 6.80 |
| -1 | -13 | -6 | 35.40 | 7.80 |

| | | | | |
|---|---|---|---|---|
| 1 | -13 | 6 | 28.70 | 5.70 |
| 1 | -13 | 6 | 37.10 | 6.10 |
| 1 | 13 | 6 | 48.10 | 5.90 |
| 1 | 13 | 6 | 47.30 | 10.10 |
| -1 | 13 | -6 | 49.10 | 9.70 |
| 2 | -13 | 6 | 312.87 | 21.10 |
| 2 | -13 | 6 | 299.07 | 18.90 |
| -2 | -13 | -6 | 308.97 | 19.70 |
| 2 | 13 | 6 | 328.87 | 19.00 |
| -2 | 13 | -6 | 324.77 | 21.50 |
| 2 | 13 | 6 | 296.27 | 22.10 |
| 3 | -13 | 6 | 919.61 | 50.89 |
| 3 | -13 | 6 | 984.00 | 52.99 |
| -3 | -13 | -6 | 881.71 | 51.29 |
| 3 | 13 | 6 | 888.31 | 54.19 |
| 3 | 13 | 6 | 930.01 | 50.99 |
| -3 | 13 | -6 | 909.01 | 52.89 |
| -4 | -13 | -6 | 30.40 | 5.90 |
| 4 | -13 | 6 | 17.80 | 6.60 |
| 4 | -13 | 6 | 19.50 | 3.50 |
| 4 | 13 | 6 | 17.60 | 8.70 |
| 4 | 13 | 6 | 26.70 | 4.70 |
| -4 | 13 | -6 | 18.10 | 7.20 |
| 5 | -13 | 6 | 460.85 | 28.10 |
| 5 | -13 | 6 | 473.65 | 26.10 |
| -5 | -13 | -6 | 468.35 | 26.40 |
| 5 | 13 | 6 | 402.76 | 27.00 |
| 5 | 13 | 6 | 451.85 | 33.30 |
| -6 | -13 | -6 | 370.56 | 21.50 |
| 6 | -13 | 6 | 367.36 | 21.20 |
| 6 | 13 | 6 | 348.07 | 21.10 |
| 7 | -13 | 6 | 243.18 | 15.20 |
| -7 | -13 | -6 | 250.67 | 15.50 |
| 7 | 13 | 6 | 254.27 | 15.20 |
| -8 | -13 | -6 | 20.60 | 3.70 |
| 8 | -13 | 6 | 31.80 | 5.30 |
| 8 | 13 | 6 | 13.70 | 2.90 |
| 9 | -13 | 6 | 286.17 | 16.60 |
| 9 | 13 | 6 | 278.77 | 16.80 |
| 10 | -13 | 6 | -1.20 | 2.20 |
| 10 | 13 | 6 | 3.60 | 2.50 |
| 11 | -13 | 6 | 57.09 | 5.00 |
| 11 | 13 | 6 | 55.69 | 5.40 |
| 12 | -14 | -6 | 33.90 | 4.40 |

| | | | | |
|---|---|---|---|---|
| -12 | 14 | 6 | 31.10 | 3.80 |
| 12 | 14 | -6 | 29.70 | 3.00 |
| 11 | -14 | -6 | 0.80 | 2.60 |
| -11 | 14 | 6 | 1.20 | 2.00 |
| 11 | 14 | -6 | 1.20 | 1.50 |
| 10 | -14 | -6 | 223.58 | 14.40 |
| 10 | 14 | -6 | 238.68 | 13.60 |
| 9 | -14 | -6 | 44.90 | 7.90 |
| 9 | 14 | -6 | 57.09 | 7.00 |
| 8 | -14 | -6 | 44.70 | 10.50 |
| -8 | -14 | 6 | 56.79 | 11.30 |
| -8 | 14 | 6 | 51.39 | 6.30 |
| 8 | 14 | -6 | 47.90 | 5.60 |
| 7 | -14 | -6 | 118.59 | 12.90 |
| -7 | -14 | 6 | 140.59 | 10.70 |
| -7 | 14 | 6 | 146.99 | 11.00 |
| -6 | -14 | 6 | 830.32 | 47.80 |
| 6 | 14 | -6 | 849.52 | 51.59 |
| -6 | 14 | 6 | 881.21 | 48.70 |
| -5 | -14 | 6 | 99.89 | 9.30 |
| 5 | 14 | -6 | 82.39 | 16.00 |
| -5 | 14 | 6 | 91.99 | 13.20 |
| -4 | -14 | 6 | 387.06 | 23.60 |
| 4 | -14 | -6 | 452.75 | 24.20 |
| -4 | 14 | 6 | 396.16 | 24.30 |
| 4 | 14 | -6 | 315.57 | 27.90 |
| -4 | 14 | 6 | 383.86 | 27.20 |
| -3 | -14 | 6 | 303.97 | 18.10 |
| 3 | -14 | -6 | 303.57 | 18.70 |
| 3 | 14 | -6 | 222.48 | 22.40 |
| -3 | 14 | 6 | 293.17 | 21.40 |
| -3 | 14 | 6 | 258.97 | 18.10 |
| 2 | -14 | -6 | 336.17 | 21.80 |
| -2 | -14 | 6 | 357.56 | 20.40 |
| -2 | -14 | 6 | 350.36 | 21.30 |
| -2 | 14 | 6 | 343.97 | 24.00 |
| -2 | 14 | 6 | 376.06 | 21.50 |
| 2 | 14 | -6 | 277.77 | 25.10 |
| 1 | -14 | -6 | -0.70 | 4.50 |
| -1 | -14 | 6 | 5.80 | 3.10 |
| -1 | -14 | 6 | -5.00 | 3.50 |
| -1 | 14 | 6 | 5.20 | 3.10 |
| -1 | 14 | 6 | 8.00 | 6.60 |
| 1 | 14 | -6 | 1.20 | 7.40 |

| | | | | |
|---|---|---|---|---|
| 0 | -14 | 6 | 1180.98 | 65.99 |
| 0 | -14 | -6 | 1177.88 | 65.89 |
| 0 | -14 | 6 | 1179.98 | 65.09 |
| 0 | 14 | -6 | 1170.58 | 68.09 |
| 0 | 14 | 6 | 1165.98 | 67.59 |
| 0 | 14 | 6 | 1227.88 | 65.69 |
| 1 | -14 | 6 | 712.93 | 39.50 |
| -1 | -14 | -6 | 705.53 | 40.40 |
| 1 | -14 | 6 | 731.93 | 40.90 |
| 1 | 14 | 6 | 688.63 | 42.40 |
| -1 | 14 | -6 | 693.03 | 42.20 |
| 1 | 14 | 6 | 697.23 | 39.80 |
| 2 | -14 | 6 | 57.79 | 9.90 |
| 2 | -14 | 6 | 41.60 | 5.80 |
| -2 | -14 | -6 | 47.00 | 7.00 |
| 2 | 14 | 6 | 34.60 | 7.80 |
| -2 | 14 | -6 | 44.40 | 11.10 |
| 2 | 14 | 6 | 49.60 | 5.80 |
| -3 | -14 | -6 | 40.70 | 6.50 |
| 3 | -14 | 6 | 41.80 | 5.80 |
| 3 | -14 | 6 | 52.89 | 11.00 |
| 3 | 14 | 6 | 39.80 | 7.80 |
| -3 | 14 | -6 | 58.99 | 11.60 |
| 3 | 14 | 6 | 74.89 | 14.10 |
| 4 | -14 | 6 | 706.03 | 40.70 |
| 4 | -14 | 6 | 697.53 | 38.80 |
| -4 | -14 | -6 | 700.53 | 39.10 |
| 4 | 14 | 6 | 638.94 | 44.30 |
| -4 | 14 | -6 | 684.13 | 42.40 |
| 4 | 14 | 6 | 728.33 | 39.00 |
| -5 | -14 | -6 | 113.99 | 9.00 |
| 5 | -14 | 6 | 109.79 | 11.90 |
| 5 | -14 | 6 | 120.19 | 8.70 |
| 5 | 14 | 6 | 122.09 | 8.50 |
| -6 | -14 | -6 | 337.87 | 18.80 |
| 6 | -14 | 6 | 300.97 | 18.50 |
| 6 | 14 | 6 | 304.47 | 18.50 |
| -7 | -14 | -6 | 196.88 | 12.20 |
| 7 | -14 | 6 | 193.48 | 12.10 |
| 7 | 14 | 6 | 173.58 | 11.70 |
| 8 | -14 | 6 | 226.78 | 13.60 |
| -8 | -14 | -6 | 229.28 | 13.70 |
| 8 | 14 | 6 | 212.08 | 13.50 |
| 9 | -14 | 6 | 1.80 | 2.10 |

| | | | | |
|---|---|---|---|---|
| 9 | 14 | 6 | 0.40 | 2.10 |
| 10 | -14 | 6 | 317.27 | 17.50 |
| 10 | 14 | 6 | 292.97 | 17.70 |
| 11 | -15 | -6 | 296.27 | 16.20 |
| 11 | 15 | -6 | 244.28 | 14.40 |
| -11 | 15 | 6 | 236.98 | 14.80 |
| 10 | -15 | -6 | 11.40 | 3.40 |
| 10 | 15 | -6 | 16.00 | 2.80 |
| -10 | 15 | 6 | 22.20 | 3.60 |
| 9 | -15 | -6 | 182.48 | 12.70 |
| -9 | 15 | 6 | 203.48 | 12.10 |
| 9 | 15 | -6 | 182.28 | 11.40 |
| -8 | -15 | 6 | 168.38 | 12.60 |
| -8 | 15 | 6 | 196.48 | 12.20 |
| 8 | 15 | -6 | 175.78 | 11.50 |
| -7 | -15 | 6 | 37.50 | 7.10 |
| 7 | 15 | -6 | 51.49 | 6.00 |
| -7 | 15 | 6 | 47.60 | 6.30 |
| -6 | -15 | 6 | 26.90 | 4.50 |
| -6 | 15 | 6 | 33.90 | 6.60 |
| 5 | -15 | -6 | 563.24 | 31.40 |
| -5 | -15 | 6 | 535.75 | 31.40 |
| -5 | 15 | 6 | 532.35 | 32.20 |
| -4 | -15 | 6 | 79.39 | 8.40 |
| 4 | -15 | -6 | 96.69 | 9.20 |
| 4 | 15 | -6 | 85.49 | 18.00 |
| -4 | 15 | 6 | 69.19 | 8.50 |
| -3 | -15 | 6 | 119.69 | 10.00 |
| 3 | -15 | -6 | 133.89 | 10.80 |
| -3 | 15 | 6 | 117.29 | 16.30 |
| 3 | 15 | -6 | 96.99 | 18.30 |
| -3 | 15 | 6 | 107.29 | 10.40 |
| -2 | -15 | 6 | 213.08 | 14.40 |
| 2 | -15 | -6 | 219.68 | 14.80 |
| -2 | -15 | 6 | 209.98 | 13.30 |
| -2 | 15 | 6 | 195.28 | 18.50 |
| -2 | 15 | 6 | 217.08 | 14.60 |
| 2 | 15 | -6 | 176.38 | 20.40 |
| 1 | -15 | -6 | 738.33 | 41.80 |
| -1 | -15 | 6 | 749.92 | 41.70 |
| -1 | -15 | 6 | 717.03 | 40.80 |
| -1 | 15 | 6 | 739.83 | 44.20 |
| 1 | 15 | -6 | 665.63 | 45.00 |
| -1 | 15 | 6 | 768.62 | 41.70 |

| | | | | |
|---|---|---|---|---|
| 0 | -15 | 6 | 51.69 | 6.40 |
| 0 | -15 | 6 | 61.79 | 8.50 |
| 0 | -15 | -6 | 56.49 | 8.30 |
| 0 | 15 | -6 | 79.69 | 13.30 |
| 0 | 15 | 6 | 60.09 | 7.10 |
| 0 | 15 | 6 | 61.49 | 13.10 |
| 1 | -15 | 6 | 639.04 | 35.80 |
| -1 | -15 | -6 | 667.23 | 36.70 |
| 1 | -15 | 6 | 668.63 | 37.30 |
| -1 | 15 | -6 | 589.34 | 39.10 |
| 1 | 15 | 6 | 674.63 | 36.30 |
| 1 | 15 | 6 | 581.44 | 39.10 |
| -2 | -15 | -6 | 145.09 | 10.70 |
| 2 | -15 | 6 | 154.78 | 12.90 |
| 2 | -15 | 6 | 142.19 | 9.80 |
| 2 | 15 | 6 | 126.29 | 16.50 |
| -2 | 15 | -6 | 132.09 | 17.70 |
| 2 | 15 | 6 | 133.69 | 9.80 |
| 3 | -15 | 6 | 269.77 | 16.30 |
| -3 | -15 | -6 | 270.67 | 16.80 |
| 3 | -15 | 6 | 268.37 | 18.70 |
| 3 | 15 | 6 | 273.67 | 16.50 |
| -4 | -15 | -6 | 0.80 | 2.60 |
| 4 | -15 | 6 | -3.90 | 4.90 |
| 4 | -15 | 6 | -1.90 | 2.20 |
| 4 | 15 | 6 | -1.10 | 2.00 |
| 5 | -15 | 6 | 856.41 | 49.50 |
| 5 | -15 | 6 | 938.11 | 48.80 |
| -5 | -15 | -6 | 893.11 | 49.00 |
| 5 | 15 | 6 | 850.61 | 48.80 |
| 6 | -15 | 6 | 112.09 | 8.30 |
| -6 | -15 | -6 | 111.89 | 8.40 |
| 6 | 15 | 6 | 118.39 | 8.10 |
| -7 | -15 | -6 | 1.50 | 2.30 |
| 7 | -15 | 6 | 1.80 | 2.00 |
| 7 | 15 | 6 | 1.00 | 2.20 |
| -8 | -15 | -6 | 3.40 | 2.60 |
| 8 | -15 | 6 | 6.00 | 2.10 |
| 8 | 15 | 6 | 6.60 | 2.30 |
| 9 | -15 | 6 | 223.08 | 13.00 |
| 9 | 15 | 6 | 218.38 | 13.20 |
| -10 | 16 | 6 | 297.47 | 18.00 |
| 10 | 16 | -6 | 333.27 | 17.70 |
| -9 | 16 | 6 | 35.30 | 4.10 |

| | | | | |
|---|---|---|---|---|
| 9 | 16 | -6 | 29.00 | 3.30 |
| -8 | -16 | 6 | 14.90 | 3.50 |
| -8 | 16 | 6 | 11.40 | 2.80 |
| 8 | 16 | -6 | 10.00 | 2.20 |
| -7 | -16 | 6 | 123.09 | 9.50 |
| -7 | 16 | 6 | 132.39 | 9.70 |
| 7 | 16 | -6 | 129.39 | 8.80 |
| -6 | -16 | 6 | 735.73 | 42.00 |
| -6 | 16 | 6 | 765.72 | 42.60 |
| 5 | -16 | -6 | 163.88 | 11.70 |
| -5 | -16 | 6 | 138.59 | 11.70 |
| -5 | 16 | 6 | 162.38 | 11.60 |
| 4 | -16 | -6 | 290.77 | 17.30 |
| -4 | -16 | 6 | 254.47 | 16.70 |
| -4 | 16 | 6 | 252.97 | 17.20 |
| -3 | -16 | 6 | 88.49 | 8.60 |
| 3 | -16 | -6 | 102.79 | 9.20 |
| -3 | 16 | 6 | 91.09 | 8.60 |
| -2 | -16 | 6 | 282.07 | 18.30 |
| 2 | -16 | -6 | 294.67 | 18.70 |
| -2 | 16 | 6 | 305.27 | 18.50 |
| 1 | -16 | -6 | 12.20 | 4.10 |
| -1 | -16 | 6 | 13.00 | 2.70 |
| -1 | -16 | 6 | 8.30 | 3.50 |
| -1 | 16 | 6 | 13.30 | 3.80 |
| 0 | -16 | 6 | 963.40 | 52.69 |
| 0 | -16 | -6 | 917.71 | 53.39 |
| 0 | -16 | 6 | 968.40 | 53.69 |
| 0 | 16 | 6 | 977.70 | 53.19 |
| -1 | -16 | -6 | 232.98 | 14.80 |
| 1 | -16 | 6 | 212.48 | 15.60 |
| 1 | -16 | 6 | 226.38 | 13.90 |
| 1 | 16 | 6 | 224.58 | 14.10 |
| 2 | -16 | 6 | 26.00 | 4.40 |
| 2 | -16 | 6 | 29.20 | 5.70 |
| -2 | -16 | -6 | 19.90 | 3.80 |
| 2 | 16 | 6 | 28.10 | 4.50 |
| -3 | -16 | -6 | 8.20 | 2.80 |
| 3 | -16 | 6 | 1.60 | 2.30 |
| 3 | -16 | 6 | 1.30 | 6.30 |
| 3 | 16 | 6 | 6.90 | 2.50 |
| 4 | -16 | 6 | 554.74 | 31.30 |
| -4 | -16 | -6 | 569.94 | 31.60 |
| 4 | -16 | 6 | 580.24 | 32.50 |

| | | | | |
|---|---|---|---|---|
| 4 | 16 | 6 | 529.55 | 31.30 |
| 5 | -16 | 6 | 23.20 | 4.40 |
| 5 | -16 | 6 | 29.60 | 4.20 |
| -5 | -16 | -6 | 27.70 | 4.70 |
| 5 | 16 | 6 | 33.20 | 4.20 |
| -6 | -16 | -6 | 315.57 | 18.80 |
| 6 | -16 | 6 | 323.67 | 18.70 |
| 6 | 16 | 6 | 338.77 | 18.80 |
| 7 | -16 | 6 | 112.39 | 7.60 |
| -7 | -16 | -6 | 112.49 | 7.80 |
| 7 | 16 | 6 | 122.09 | 8.40 |
| 8 | -16 | 6 | 123.99 | 7.80 |
| -8 | -16 | -6 | 114.39 | 8.20 |
| 8 | 16 | 6 | 127.09 | 8.10 |
| -9 | 17 | 6 | 124.09 | 7.90 |
| 9 | 17 | -6 | 118.89 | 7.30 |
| -8 | -17 | 6 | 118.49 | 9.30 |
| 8 | 17 | -6 | 123.69 | 7.80 |
| -8 | 17 | 6 | 131.69 | 8.50 |
| -7 | -17 | 6 | 152.68 | 10.90 |
| 7 | 17 | -6 | 179.98 | 10.30 |
| -6 | -17 | 6 | -1.30 | 2.60 |
| -6 | 17 | 6 | 0.50 | 2.60 |
| 6 | 17 | -6 | 4.30 | 2.30 |
| -5 | -17 | 6 | 466.45 | 27.00 |
| 5 | -17 | -6 | 468.25 | 26.80 |
| -5 | 17 | 6 | 473.75 | 27.50 |
| 4 | -17 | -6 | 202.58 | 11.90 |
| -4 | -17 | 6 | 168.98 | 11.90 |
| -4 | 17 | 6 | 154.98 | 12.20 |
| -3 | -17 | 6 | 7.90 | 3.50 |
| 3 | -17 | -6 | 7.00 | 3.10 |
| -3 | 17 | 6 | 13.70 | 3.70 |
| -2 | -17 | 6 | 95.89 | 9.50 |
| 2 | -17 | -6 | 107.19 | 9.30 |
| -2 | 17 | 6 | 101.79 | 8.40 |
| -1 | -17 | 6 | 517.25 | 31.70 |
| 1 | -17 | -6 | 463.05 | 28.40 |
| -1 | 17 | 6 | 477.05 | 28.00 |
| 0 | -17 | 6 | 140.29 | 10.30 |
| 0 | -17 | -6 | 130.19 | 10.60 |
| 0 | -17 | 6 | 126.09 | 11.10 |
| 0 | 17 | 6 | 138.79 | 9.70 |
| 1 | -17 | 6 | 209.48 | 13.20 |

| | | | | |
|---|---|---|---|---|
| -1 | -17 | -6 | 214.18 | 14.20 |
| 1 | -17 | 6 | 211.08 | 14.90 |
| 1 | 17 | 6 | 221.08 | 13.40 |
| 2 | -17 | 6 | 195.08 | 13.40 |
| 2 | -17 | 6 | 172.08 | 11.10 |
| -2 | -17 | -6 | 176.28 | 11.80 |
| 2 | 17 | 6 | 172.08 | 11.30 |
| 3 | -17 | 6 | 115.89 | 9.90 |
| -3 | -17 | -6 | 102.49 | 8.00 |
| 3 | -17 | 6 | 106.19 | 7.40 |
| 3 | 17 | 6 | 99.19 | 7.40 |
| -4 | -17 | -6 | 19.20 | 4.30 |
| 4 | -17 | 6 | 32.60 | 3.70 |
| 4 | -17 | 6 | 20.20 | 4.10 |
| 4 | 17 | 6 | 29.00 | 3.80 |
| -5 | -17 | -6 | 352.46 | 20.30 |
| 5 | -17 | 6 | 326.47 | 20.60 |
| 5 | 17 | 6 | 387.56 | 20.30 |
| 6 | -17 | 6 | 191.08 | 10.60 |
| -6 | -17 | -6 | 180.48 | 10.70 |
| 6 | 17 | 6 | 154.28 | 10.50 |
| -7 | -17 | -6 | 0.70 | 1.90 |
| 7 | -17 | 6 | -1.30 | 1.50 |
| 7 | 17 | 6 | 0.40 | 1.90 |
| -7 | -18 | 6 | 144.19 | 9.60 |
| 7 | 18 | -6 | 139.09 | 8.70 |
| -7 | 18 | 6 | 150.98 | 9.40 |
| -6 | -18 | 6 | 356.56 | 21.30 |
| 6 | 18 | -6 | 387.06 | 21.00 |
| -6 | 18 | 6 | 376.46 | 21.70 |
| -5 | -18 | 6 | 61.89 | 6.10 |
| -5 | 18 | 6 | 57.29 | 6.10 |
| 4 | -18 | -6 | 149.19 | 9.40 |
| -4 | -18 | 6 | 127.49 | 9.20 |
| -4 | 18 | 6 | 124.19 | 9.70 |
| 3 | -18 | -6 | 69.29 | 6.40 |
| -3 | -18 | 6 | 61.79 | 6.10 |
| -3 | 18 | 6 | 59.99 | 6.50 |
| -2 | -18 | 6 | 189.18 | 12.70 |
| 2 | -18 | -6 | 199.98 | 12.80 |
| -2 | 18 | 6 | 209.58 | 13.00 |
| 1 | -18 | -6 | 39.10 | 5.40 |
| -1 | -18 | 6 | 27.30 | 5.40 |
| -1 | 18 | 6 | 27.80 | 5.20 |

| | | | | |
|---|---|---|---|---|
| 0 | -18 | 6 | 507.35 | 28.90 |
| 0 | -18 | -6 | 488.95 | 28.60 |
| 0 | 18 | 6 | 511.15 | 28.60 |
| 1 | -18 | 6 | 226.38 | 14.10 |
| -1 | -18 | -6 | 215.18 | 13.40 |
| 1 | -18 | 6 | 205.08 | 12.60 |
| 1 | 18 | 6 | 206.48 | 13.10 |
| 2 | -18 | 6 | 34.40 | 3.90 |
| 2 | -18 | 6 | 31.90 | 5.90 |
| -2 | -18 | -6 | 31.70 | 4.60 |
| 2 | 18 | 6 | 26.90 | 4.10 |
| 3 | -18 | 6 | 94.69 | 6.50 |
| 3 | -18 | 6 | 94.49 | 7.90 |
| -3 | -18 | -6 | 99.49 | 7.00 |
| 3 | 18 | 6 | 94.69 | 6.70 |
| -4 | -18 | -6 | 329.47 | 18.30 |
| 4 | -18 | 6 | 305.07 | 18.60 |
| 4 | -18 | 6 | 333.57 | 18.10 |
| 4 | 18 | 6 | 309.77 | 18.20 |
| -5 | -18 | -6 | 6.70 | 1.90 |
| 5 | -18 | 6 | 9.70 | 1.80 |
| 5 | 18 | 6 | 8.20 | 2.20 |
| -5 | -19 | 6 | 258.97 | 15.30 |
| 5 | 19 | -6 | 262.67 | 15.00 |
| -5 | 19 | 6 | 268.27 | 15.70 |
| 4 | -19 | -6 | 113.49 | 8.20 |
| -4 | -19 | 6 | 104.79 | 7.60 |
| -4 | 19 | 6 | 117.29 | 8.20 |
| 3 | -19 | -6 | 2.30 | 2.30 |
| -3 | -19 | 6 | 0.50 | 2.00 |
| -3 | 19 | 6 | 2.50 | 2.80 |
| -2 | -19 | 6 | 179.48 | 11.30 |
| 2 | -19 | -6 | 182.28 | 11.20 |
| -2 | 19 | 6 | 178.58 | 11.60 |
| -1 | -19 | 6 | 291.87 | 17.50 |
| 1 | -19 | -6 | 294.67 | 17.40 |
| -1 | 19 | 6 | 306.27 | 17.60 |
| 0 | -19 | 6 | 24.30 | 4.60 |
| 0 | 19 | 6 | 36.90 | 4.50 |
| -1 | -19 | -6 | 87.29 | 6.70 |
| 1 | -19 | 6 | 86.49 | 7.10 |
| 1 | 19 | 6 | 83.39 | 6.40 |
| 2 | -19 | 6 | 121.99 | 8.40 |
| -2 | -19 | -6 | 111.99 | 7.90 |

| | | | | |
|---|---|---|---|---|
| 2 | 19 | 6 | 119.99 | 7.80 |
| -3 | -19 | -6 | 71.19 | 5.30 |
| 3 | -19 | 6 | 65.29 | 4.80 |
| 3 | -19 | 6 | 63.19 | 6.10 |
| 3 | 19 | 6 | 68.29 | 5.30 |
| 17 | 0 | -7 | 276.77 | 16.30 |
| -17 | 0 | 7 | 303.47 | 16.60 |
| 16 | 0 | -7 | 1.50 | 1.90 |
| 15 | 0 | -7 | 505.15 | 28.50 |
| 14 | 0 | -7 | 0.60 | 2.30 |
| 13 | 0 | -7 | 12.80 | 3.20 |
| 12 | 0 | -7 | -2.90 | 2.80 |
| 11 | 0 | -7 | 1905.31 | 103.69 |
| 10 | 0 | -7 | -7.70 | 3.40 |
| 9 | 0 | -7 | 87.49 | 8.90 |
| -6 | 0 | 7 | 5.00 | 3.00 |
| -5 | 0 | 7 | 2112.09 | 114.59 |
| -4 | 0 | 7 | 1.40 | 2.50 |
| -3 | 0 | 7 | 2494.45 | 135.49 |
| -2 | 0 | 7 | 0.80 | 2.80 |
| -1 | 0 | 7 | 8830.42 | 474.95 |
| 0 | 0 | 7 | 4.20 | 3.40 |
| 1 | 0 | 7 | 22.20 | 5.40 |
| 2 | 0 | 7 | -3.60 | 3.80 |
| 3 | 0 | 7 | 972.90 | 55.79 |
| 4 | 0 | 7 | 3.80 | 5.80 |
| 4 | 0 | 7 | -6.30 | 6.50 |
| 5 | 0 | 7 | 2850.61 | 149.39 |
| 5 | 0 | 7 | 2612.34 | 148.39 |
| 6 | 0 | 7 | 2.30 | 4.10 |
| 6 | 0 | 7 | -10.20 | 5.20 |
| 6 | 0 | 7 | -1.90 | 4.50 |
| 7 | 0 | 7 | 78.59 | 10.00 |
| 7 | 0 | 7 | 96.49 | 10.40 |
| 7 | 0 | 7 | 91.69 | 9.10 |
| 8 | 0 | 7 | -1.70 | 3.80 |
| 8 | 0 | 7 | -9.30 | 4.40 |
| 9 | 0 | 7 | 1122.79 | 61.29 |
| 9 | 0 | 7 | 1054.39 | 60.39 |
| 10 | 0 | 7 | -1.40 | 4.10 |
| 10 | 0 | 7 | 0.50 | 3.90 |
| 11 | 0 | 7 | 367.16 | 22.40 |
| 11 | 0 | 7 | 349.87 | 21.80 |
| 12 | 0 | 7 | -7.20 | 3.30 |

| | | | | |
|---|---|---|---|---|
| 12 | 0 | 7 | -4.00 | 3.50 |
| 13 | 0 | 7 | 344.17 | 21.20 |
| 13 | 0 | 7 | 361.86 | 21.00 |
| 14 | 0 | 7 | 0.10 | 2.90 |
| 14 | 0 | 7 | 2.20 | 2.40 |
| 17 | -1 | -7 | 3.30 | 1.50 |
| -17 | -1 | 7 | 3.10 | 1.70 |
| 17 | 1 | -7 | 2.10 | 1.50 |
| -17 | 1 | 7 | 3.60 | 1.80 |
| 16 | -1 | -7 | 516.55 | 28.70 |
| 16 | 1 | -7 | 514.55 | 28.70 |
| 15 | -1 | -7 | 1.60 | 2.50 |
| 15 | 1 | -7 | 1.90 | 2.20 |
| 14 | -1 | -7 | 306.47 | 18.60 |
| 14 | 1 | -7 | 320.37 | 18.50 |
| 13 | -1 | -7 | 1.40 | 2.60 |
| 13 | 1 | -7 | 2.20 | 2.60 |
| 12 | -1 | -7 | 1104.79 | 60.89 |
| 12 | 1 | -7 | 1109.89 | 60.89 |
| 11 | -1 | -7 | 8.50 | 3.60 |
| 11 | 1 | -7 | 10.90 | 3.40 |
| 10 | -1 | -7 | 1319.77 | 69.99 |
| 10 | 1 | -7 | 1231.58 | 69.79 |
| 9 | -1 | -7 | 3.70 | 4.00 |
| 9 | 1 | -7 | 2.10 | 4.00 |
| -6 | -1 | 7 | 6604.14 | 360.96 |
| -6 | 1 | 7 | 6831.32 | 360.86 |
| -5 | -1 | 7 | 259.17 | 16.10 |
| -5 | 1 | 7 | 262.77 | 16.10 |
| -4 | -1 | 7 | 108.39 | 7.80 |
| -4 | 1 | 7 | 87.09 | 8.20 |
| -3 | -1 | 7 | 177.58 | 11.50 |
| -3 | 1 | 7 | 162.38 | 11.80 |
| -2 | -1 | 7 | 3948.41 | 204.48 |
| -2 | 1 | 7 | 3613.44 | 204.48 |
| -1 | -1 | 7 | 546.75 | 31.00 |
| -1 | 1 | 7 | 513.35 | 31.00 |
| 0 | -1 | 7 | 5456.95 | 280.27 |
| 0 | 1 | 7 | 4915.61 | 280.07 |
| 1 | -1 | 7 | 27.00 | 6.80 |
| 1 | 1 | 7 | 30.70 | 7.10 |
| 2 | -1 | 7 | 497.65 | 31.00 |
| 2 | 1 | 7 | 541.15 | 31.60 |
| 3 | -1 | 7 | 94.49 | 10.10 |

| | | | | |
|---|---|---|---|---|
| 3 | 1 | 7 | 101.59 | 11.60 |
| 4 | -1 | 7 | 3552.04 | 195.98 |
| 4 | -1 | 7 | 3684.53 | 198.78 |
| 5 | -1 | 7 | 68.19 | 9.10 |
| 5 | -1 | 7 | 66.09 | 11.40 |
| 5 | 1 | 7 | 68.59 | 10.70 |
| 5 | 1 | 7 | 71.09 | 8.70 |
| 6 | -1 | 7 | 694.43 | 40.00 |
| 6 | -1 | 7 | 762.22 | 39.80 |
| 6 | -1 | 7 | 621.24 | 40.30 |
| 6 | 1 | 7 | 708.83 | 39.60 |
| 6 | 1 | 7 | 685.73 | 39.90 |
| 6 | 1 | 7 | 701.43 | 40.90 |
| 7 | -1 | 7 | -1.50 | 4.80 |
| 7 | -1 | 7 | 2.80 | 4.40 |
| 7 | -1 | 7 | -1.00 | 4.70 |
| 7 | 1 | 7 | 1.50 | 4.20 |
| 7 | 1 | 7 | 8.30 | 5.50 |
| 7 | 1 | 7 | -0.10 | 4.60 |
| 8 | -1 | 7 | 950.00 | 52.09 |
| 8 | -1 | 7 | 971.50 | 52.99 |
| 8 | 1 | 7 | 936.91 | 52.79 |
| 8 | 1 | 7 | 866.11 | 51.79 |
| 9 | -1 | 7 | 4.40 | 4.60 |
| 9 | -1 | 7 | 4.10 | 4.10 |
| 9 | 1 | 7 | 4.40 | 4.40 |
| 9 | 1 | 7 | 8.20 | 4.00 |
| 10 | -1 | 7 | 1250.57 | 68.49 |
| 10 | -1 | 7 | 1233.98 | 67.89 |
| 10 | 1 | 7 | 1191.28 | 68.39 |
| 10 | 1 | 7 | 1232.78 | 67.79 |
| 11 | -1 | 7 | 19.20 | 5.00 |
| 11 | -1 | 7 | 26.20 | 5.00 |
| 11 | 1 | 7 | 14.30 | 4.70 |
| 11 | 1 | 7 | 30.10 | 6.40 |
| 12 | -1 | 7 | 12.70 | 3.70 |
| 12 | -1 | 7 | 12.00 | 4.10 |
| 12 | 1 | 7 | 10.20 | 3.60 |
| 12 | 1 | 7 | 16.50 | 3.90 |
| 13 | -1 | 7 | 1.10 | 2.70 |
| 13 | -1 | 7 | 1.70 | 3.10 |
| 13 | 1 | 7 | -1.00 | 2.80 |
| 13 | 1 | 7 | -1.20 | 2.90 |
| 14 | -1 | 7 | 296.47 | 19.20 |

| | | | | |
|---|---|---|---|---|
| 14 | -1 | 7 | 371.16 | 21.00 |
| 14 | 1 | 7 | 312.67 | 19.20 |
| 14 | 1 | 7 | 334.27 | 20.10 |
| -17 | -2 | 7 | 186.68 | 12.10 |
| 17 | -2 | -7 | 200.18 | 11.70 |
| 17 | 2 | -7 | 194.48 | 11.60 |
| -17 | 2 | 7 | 223.48 | 12.00 |
| 16 | -2 | -7 | 7.80 | 2.10 |
| 16 | 2 | -7 | 8.50 | 2.00 |
| 15 | -2 | -7 | 537.15 | 29.20 |
| 15 | 2 | -7 | 498.95 | 29.00 |
| 14 | -2 | -7 | -1.50 | 2.40 |
| 14 | 2 | -7 | 0.20 | 2.20 |
| 13 | -2 | -7 | 1.20 | 2.90 |
| 13 | 2 | -7 | 4.20 | 2.40 |
| 12 | -2 | -7 | -6.20 | 4.90 |
| 12 | -2 | -7 | 0.20 | 3.00 |
| 12 | 2 | -7 | -1.50 | 2.70 |
| 11 | -2 | -7 | 2051.19 | 108.59 |
| 11 | -2 | -7 | 1829.62 | 108.89 |
| 11 | 2 | -7 | 2113.79 | 108.49 |
| 10 | -2 | -7 | 33.80 | 6.20 |
| 10 | -2 | -7 | 11.70 | 4.30 |
| 10 | 2 | -7 | 23.10 | 4.10 |
| 9 | -2 | -7 | 1216.98 | 65.79 |
| 9 | -2 | -7 | 1142.19 | 65.09 |
| 9 | 2 | -7 | 1190.78 | 64.89 |
| 8 | 2 | -7 | 3.50 | 4.20 |
| -6 | 2 | 7 | 355.16 | 21.10 |
| -5 | -2 | 7 | 7024.70 | 364.46 |
| -5 | 2 | 7 | 6529.75 | 364.46 |
| -4 | -2 | 7 | 24.50 | 4.90 |
| -4 | 2 | 7 | 20.60 | 4.10 |
| -3 | -2 | 7 | 492.55 | 26.90 |
| -3 | 2 | 7 | 427.26 | 27.00 |
| -2 | -2 | 7 | 64.49 | 6.40 |
| -2 | 2 | 7 | 50.59 | 6.80 |
| -1 | -2 | 7 | 5290.27 | 272.37 |
| -1 | 2 | 7 | 4797.62 | 272.17 |
| 0 | -2 | 7 | 49.00 | 7.30 |
| 0 | 2 | 7 | 63.39 | 7.50 |
| 1 | -2 | 7 | 4353.56 | 244.88 |
| 1 | 2 | 7 | 4702.13 | 245.18 |
| 2 | -2 | 7 | 12.00 | 4.30 |

| | | | | |
|---|---|---|---|---|
| 2 | 2 | 7 | 9.10 | 4.80 |
| 3 | -2 | 7 | 1569.44 | 84.89 |
| 3 | 2 | 7 | 1491.55 | 85.69 |
| 4 | -2 | 7 | 254.37 | 18.00 |
| 4 | -2 | 7 | 209.78 | 17.90 |
| 4 | 2 | 7 | 207.98 | 17.30 |
| 5 | -2 | 7 | 5101.79 | 295.77 |
| 5 | -2 | 7 | 5830.12 | 295.97 |
| 5 | 2 | 7 | 5916.61 | 295.17 |
| 5 | 2 | 7 | 5082.89 | 297.17 |
| 6 | -2 | 7 | 124.29 | 10.80 |
| 6 | -2 | 7 | 128.89 | 11.90 |
| 6 | -2 | 7 | 109.99 | 12.60 |
| 6 | 2 | 7 | 141.99 | 10.70 |
| 6 | 2 | 7 | 120.69 | 12.60 |
| 6 | 2 | 7 | 133.99 | 13.40 |
| 7 | -2 | 7 | 89.19 | 10.30 |
| 7 | -2 | 7 | 103.39 | 10.00 |
| 7 | -2 | 7 | 79.09 | 10.90 |
| 7 | 2 | 7 | 92.29 | 12.00 |
| 7 | 2 | 7 | 101.39 | 9.10 |
| 7 | 2 | 7 | 98.29 | 9.40 |
| 8 | -2 | 7 | 76.49 | 9.90 |
| 8 | -2 | 7 | 69.29 | 8.90 |
| 8 | 2 | 7 | 78.79 | 9.00 |
| 8 | 2 | 7 | 72.49 | 8.20 |
| 9 | -2 | 7 | 1232.38 | 62.09 |
| 9 | -2 | 7 | 1142.09 | 61.29 |
| 9 | 2 | 7 | 979.30 | 61.49 |
| 9 | 2 | 7 | 1050.59 | 60.89 |
| 10 | -2 | 7 | 1.80 | 4.20 |
| 10 | -2 | 7 | -1.70 | 4.40 |
| 10 | 2 | 7 | 3.40 | 4.10 |
| 10 | 2 | 7 | 3.10 | 3.70 |
| 11 | -2 | 7 | 816.72 | 43.40 |
| 11 | -2 | 7 | 727.03 | 43.00 |
| 11 | 2 | 7 | 724.93 | 43.10 |
| 11 | 2 | 7 | 764.12 | 42.80 |
| 12 | -2 | 7 | 65.99 | 6.90 |
| 12 | -2 | 7 | 45.00 | 7.10 |
| 12 | 2 | 7 | 43.50 | 6.90 |
| 12 | 2 | 7 | 48.10 | 6.70 |
| 13 | -2 | 7 | 308.97 | 18.80 |
| 13 | -2 | 7 | 317.17 | 18.50 |

| | | | | |
|---|---|---|---|---|
| 13 | 2 | 7 | 301.57 | 18.50 |
| 13 | 2 | 7 | 290.77 | 18.30 |
| 14 | -2 | 7 | -0.30 | 2.50 |
| 14 | -2 | 7 | -1.30 | 2.60 |
| 14 | 2 | 7 | -0.40 | 2.80 |
| 14 | 2 | 7 | 0.50 | 2.40 |
| 16 | -3 | -7 | 426.66 | 24.10 |
| 16 | 3 | -7 | 434.86 | 24.10 |
| 15 | -3 | -7 | 3.50 | 2.50 |
| 15 | 3 | -7 | 5.30 | 2.10 |
| 14 | -3 | -7 | 384.06 | 24.60 |
| 14 | -3 | -7 | 355.16 | 21.70 |
| 14 | 3 | -7 | 383.86 | 21.60 |
| 13 | -3 | -7 | 5.50 | 5.50 |
| 13 | -3 | -7 | 9.40 | 3.20 |
| 13 | 3 | -7 | -1.00 | 2.10 |
| 12 | -3 | -7 | 960.30 | 51.49 |
| 12 | -3 | -7 | 870.21 | 50.69 |
| 12 | 3 | -7 | 918.11 | 50.59 |
| 11 | -3 | -7 | 1.70 | 3.60 |
| 11 | -3 | -7 | 2.50 | 4.80 |
| 11 | 3 | -7 | 4.30 | 3.10 |
| 10 | -3 | -7 | 1153.78 | 63.39 |
| 10 | -3 | -7 | 1065.69 | 63.69 |
| 10 | 3 | -7 | 1228.98 | 63.19 |
| 9 | -3 | -7 | 65.39 | 9.60 |
| 9 | -3 | -7 | 60.49 | 9.10 |
| 9 | 3 | -7 | 56.29 | 7.50 |
| 8 | -3 | -7 | 353.56 | 22.80 |
| 8 | 3 | -7 | 362.16 | 21.60 |
| 7 | -3 | -7 | 93.09 | 12.00 |
| -6 | 3 | 7 | 5040.70 | 271.47 |
| -5 | 3 | 7 | 38.40 | 6.80 |
| -4 | -3 | 7 | 2571.34 | 132.29 |
| -4 | 3 | 7 | 2289.17 | 132.09 |
| -3 | -3 | 7 | 23.90 | 5.40 |
| -3 | 3 | 7 | 23.90 | 5.60 |
| -2 | -3 | 7 | 1825.02 | 96.89 |
| -2 | 3 | 7 | 1704.23 | 96.69 |
| -1 | -3 | 7 | 843.02 | 48.30 |
| -1 | 3 | 7 | 864.11 | 48.20 |
| 0 | -3 | 7 | 9343.87 | 520.15 |
| 0 | 3 | 7 | 9999.00 | 520.15 |
| 1 | -3 | 7 | 231.58 | 16.50 |

| | | | | |
|---|---|---|---|---|
| 1 | 3 | 7 | 263.97 | 16.90 |
| 2 | -3 | 7 | 9.80 | 4.30 |
| 2 | 3 | 7 | 10.30 | 4.30 |
| 3 | -3 | 7 | 243.68 | 20.00 |
| 3 | 3 | 7 | 299.27 | 17.00 |
| 3 | 3 | 7 | 262.37 | 19.00 |
| 4 | -3 | 7 | 2587.24 | 143.89 |
| 4 | -3 | 7 | 2757.42 | 144.09 |
| 4 | 3 | 7 | 2471.25 | 145.29 |
| 4 | 3 | 7 | 2762.52 | 143.09 |
| 5 | -3 | 7 | 5.10 | 6.20 |
| 5 | 3 | 7 | 8.20 | 4.70 |
| 5 | 3 | 7 | 8.80 | 6.70 |
| 5 | 3 | 7 | 8.20 | 3.70 |
| 6 | -3 | 7 | 1550.54 | 82.69 |
| 6 | -3 | 7 | 1456.75 | 83.29 |
| 6 | -3 | 7 | 1569.24 | 82.89 |
| 6 | 3 | 7 | 1542.55 | 82.49 |
| 6 | 3 | 7 | 1521.65 | 82.39 |
| 6 | 3 | 7 | 1374.86 | 84.49 |
| 7 | -3 | 7 | 125.59 | 11.50 |
| 7 | -3 | 7 | 135.09 | 12.20 |
| 7 | 3 | 7 | 116.29 | 10.30 |
| 7 | 3 | 7 | 133.29 | 10.20 |
| 7 | 3 | 7 | 118.09 | 14.30 |
| 8 | -3 | 7 | 706.93 | 38.10 |
| 8 | -3 | 7 | 677.83 | 38.60 |
| 8 | 3 | 7 | 609.54 | 37.90 |
| 8 | 3 | 7 | 641.74 | 39.50 |
| 9 | -3 | 7 | 20.50 | 5.40 |
| 9 | -3 | 7 | 25.00 | 5.50 |
| 9 | 3 | 7 | 18.40 | 4.30 |
| 9 | 3 | 7 | 16.10 | 4.70 |
| 10 | -3 | 7 | 1156.68 | 67.69 |
| 10 | -3 | 7 | 1297.77 | 68.29 |
| 10 | 3 | 7 | 1183.28 | 67.39 |
| 10 | 3 | 7 | 1249.38 | 68.19 |
| 11 | -3 | 7 | -3.30 | 3.60 |
| 11 | -3 | 7 | 5.40 | 4.30 |
| 11 | 3 | 7 | -0.50 | 3.20 |
| 11 | 3 | 7 | 4.30 | 3.50 |
| 12 | -3 | 7 | 3.20 | 4.10 |
| 12 | -3 | 7 | 1.00 | 3.50 |
| 12 | 3 | 7 | 0.50 | 3.40 |

| | | | | |
|---|---|---|---|---|
| 12 | 3 | 7 | 0.10 | 3.10 |
| 13 | -3 | 7 | 1.40 | 3.00 |
| 13 | -3 | 7 | 5.10 | 3.40 |
| 13 | 3 | 7 | 4.40 | 2.80 |
| 13 | 3 | 7 | 1.80 | 2.80 |
| 14 | -3 | 7 | 259.87 | 14.80 |
| 14 | -3 | 7 | 245.48 | 14.80 |
| 14 | 3 | 7 | 227.78 | 14.70 |
| 14 | 3 | 7 | 237.98 | 14.60 |
| -16 | -4 | 7 | 4.10 | 2.40 |
| 16 | -4 | -7 | -2.70 | 1.90 |
| 16 | 4 | -7 | -0.80 | 1.50 |
| 15 | -4 | -7 | 291.27 | 17.70 |
| 15 | 4 | -7 | 312.97 | 17.50 |
| 14 | -4 | -7 | -2.90 | 2.70 |
| 14 | -4 | -7 | -0.20 | 4.60 |
| 14 | 4 | -7 | 3.10 | 2.10 |
| 13 | -4 | -7 | 56.59 | 8.60 |
| 13 | -4 | -7 | 56.19 | 6.30 |
| 13 | 4 | -7 | 43.20 | 5.50 |
| 12 | -4 | -7 | 2.90 | 3.40 |
| 12 | -4 | -7 | 12.30 | 4.60 |
| 12 | 4 | -7 | 0.00 | 2.80 |
| 11 | -4 | -7 | 1765.92 | 97.19 |
| 11 | -4 | -7 | 1801.02 | 96.79 |
| 11 | 4 | -7 | 1760.12 | 96.49 |
| 10 | -4 | -7 | 54.49 | 8.80 |
| 10 | -4 | -7 | 58.49 | 8.70 |
| 10 | 4 | -7 | 61.69 | 6.80 |
| 9 | -4 | -7 | 195.98 | 14.70 |
| 9 | -4 | -7 | 203.08 | 15.20 |
| 9 | 4 | -7 | 215.28 | 13.60 |
| 8 | -4 | -7 | 27.70 | 7.00 |
| 8 | -4 | -7 | 25.50 | 5.70 |
| 8 | 4 | -7 | 14.80 | 4.10 |
| 7 | -4 | -7 | 1136.69 | 64.49 |
| 7 | 4 | -7 | 1179.98 | 63.89 |
| 6 | -4 | -7 | 27.30 | 6.30 |
| -6 | 4 | 7 | 38.00 | 7.40 |
| -5 | 4 | 7 | 2997.60 | 162.58 |
| -4 | 4 | 7 | 116.29 | 10.00 |
| -3 | -4 | 7 | 20.30 | 4.50 |
| -3 | 4 | 7 | 23.00 | 5.80 |
| -2 | -4 | 7 | 12.40 | 4.00 |

| | | | | |
|---|---|---|---|---|
| -2 | 4 | 7 | 18.50 | 4.10 |
| -1 | -4 | 7 | 6745.52 | 378.86 |
| -1 | 4 | 7 | 7315.37 | 378.66 |
| 0 | -4 | 7 | 3.90 | 3.90 |
| 0 | 4 | 7 | 3.50 | 3.80 |
| 1 | -4 | 7 | 494.45 | 31.10 |
| 1 | 4 | 7 | 554.64 | 31.60 |
| 2 | 4 | 7 | 94.09 | 8.60 |
| 3 | -4 | 7 | 616.54 | 35.10 |
| 3 | 4 | 7 | 621.44 | 34.10 |
| 3 | 4 | 7 | 572.64 | 36.00 |
| 4 | -4 | 7 | 168.48 | 14.50 |
| 4 | -4 | 7 | 168.58 | 12.40 |
| 4 | 4 | 7 | 165.38 | 11.40 |
| 4 | 4 | 7 | 171.08 | 14.70 |
| 5 | -4 | 7 | 4333.57 | 226.58 |
| 5 | -4 | 7 | 4327.67 | 226.58 |
| 5 | 4 | 7 | 4012.70 | 225.78 |
| 5 | 4 | 7 | 3599.94 | 227.98 |
| 5 | 4 | 7 | 4665.43 | 225.88 |
| 6 | -4 | 7 | 1.00 | 5.80 |
| 6 | -4 | 7 | -9.10 | 5.20 |
| 6 | 4 | 7 | 0.80 | 5.70 |
| 6 | 4 | 7 | 0.00 | 3.80 |
| 6 | 4 | 7 | 5.90 | 6.90 |
| 6 | 4 | 7 | -2.40 | 3.20 |
| 7 | -4 | 7 | 139.59 | 12.60 |
| 7 | -4 | 7 | 138.69 | 12.60 |
| 7 | 4 | 7 | 138.39 | 10.40 |
| 7 | 4 | 7 | 132.79 | 16.20 |
| 7 | 4 | 7 | 132.69 | 10.70 |
| 8 | -4 | 7 | 17.50 | 5.80 |
| 8 | -4 | 7 | 9.20 | 5.50 |
| 8 | -4 | 7 | 19.10 | 5.80 |
| 8 | 4 | 7 | 10.00 | 3.70 |
| 8 | 4 | 7 | 15.70 | 4.40 |
| 9 | -4 | 7 | 918.41 | 51.39 |
| 9 | -4 | 7 | 931.81 | 50.59 |
| 9 | -4 | 7 | 923.01 | 50.99 |
| 9 | 4 | 7 | 853.51 | 50.29 |
| 9 | 4 | 7 | 886.11 | 50.89 |
| 10 | -4 | 7 | -0.10 | 4.50 |
| 10 | -4 | 7 | -5.10 | 4.60 |
| 10 | -4 | 7 | 3.40 | 4.90 |

| | | | | |
|---|---|---|---|---|
| 10 | 4 | 7 | 0.60 | 3.60 |
| 10 | 4 | 7 | 0.10 | 3.70 |
| 11 | -4 | 7 | 465.25 | 26.50 |
| 11 | -4 | 7 | 430.56 | 25.80 |
| 11 | -4 | 7 | 440.86 | 26.60 |
| 11 | 4 | 7 | 439.26 | 25.80 |
| 11 | 4 | 7 | 418.76 | 26.20 |
| 12 | -4 | 7 | 22.30 | 5.40 |
| 12 | -4 | 7 | 14.40 | 3.80 |
| 12 | 4 | 7 | 27.00 | 5.20 |
| 12 | 4 | 7 | 32.80 | 5.80 |
| 13 | -4 | 7 | 338.37 | 18.80 |
| 13 | -4 | 7 | 302.17 | 18.90 |
| 13 | 4 | 7 | 304.97 | 18.50 |
| 13 | 4 | 7 | 298.37 | 18.60 |
| 14 | -4 | 7 | 1.90 | 2.80 |
| 14 | -4 | 7 | -1.10 | 2.70 |
| 14 | 4 | 7 | 0.70 | 2.30 |
| 14 | 4 | 7 | -0.60 | 2.10 |
| 16 | -5 | -7 | 277.57 | 16.00 |
| 16 | 5 | -7 | 282.17 | 15.80 |
| -16 | 5 | 7 | 275.07 | 16.10 |
| 15 | -5 | -7 | 10.70 | 2.60 |
| 15 | 5 | -7 | 12.00 | 2.20 |
| 14 | -5 | -7 | 303.77 | 20.50 |
| 14 | -5 | -7 | 323.97 | 19.60 |
| 14 | 5 | -7 | 316.07 | 18.30 |
| 13 | -5 | -7 | 55.99 | 8.50 |
| 13 | -5 | -7 | 50.29 | 6.20 |
| 13 | 5 | -7 | 48.40 | 5.30 |
| 12 | -5 | -7 | 356.66 | 21.50 |
| 12 | -5 | -7 | 353.46 | 22.20 |
| 12 | 5 | -7 | 370.16 | 21.00 |
| 11 | -5 | -7 | 2.10 | 3.90 |
| 11 | -5 | -7 | 3.50 | 4.10 |
| 11 | 5 | -7 | 1.30 | 2.90 |
| 10 | -5 | -7 | 651.33 | 38.30 |
| 10 | -5 | -7 | 683.93 | 38.60 |
| 10 | 5 | -7 | 682.53 | 37.90 |
| 9 | -5 | -7 | 84.89 | 10.00 |
| 9 | -5 | -7 | 97.19 | 11.50 |
| 9 | 5 | -7 | 108.89 | 8.60 |
| 8 | -5 | -7 | -0.80 | 6.70 |
| 8 | -5 | -7 | 15.40 | 5.70 |

| | | | | |
|---|---|---|---|---|
| 8 | 5 | -7 | 11.10 | 3.70 |
| 7 | -5 | -7 | 80.49 | 10.50 |
| 7 | 5 | -7 | 88.09 | 8.40 |
| 7 | 5 | -7 | 78.69 | 9.60 |
| 6 | -5 | -7 | 3208.38 | 185.58 |
| -6 | 5 | 7 | 3427.76 | 185.68 |
| 6 | 5 | -7 | 3653.93 | 186.38 |
| -5 | 5 | 7 | 24.00 | 4.70 |
| -4 | 5 | 7 | 82.29 | 8.20 |
| -3 | 5 | 7 | 2.70 | 3.70 |
| -2 | 5 | 7 | 3255.57 | 176.58 |
| -1 | 5 | 7 | 152.98 | 11.70 |
| 0 | -5 | 7 | 2235.88 | 133.99 |
| 0 | 5 | 7 | 2662.73 | 134.09 |
| 1 | -5 | 7 | 4.20 | 3.90 |
| 1 | 5 | 7 | 0.50 | 3.20 |
| 1 | 5 | 7 | 5.80 | 3.70 |
| 2 | -5 | 7 | 1114.09 | 60.29 |
| 2 | 5 | 7 | 1075.19 | 60.49 |
| 2 | 5 | 7 | 1028.10 | 58.89 |
| 3 | -5 | 7 | 349.77 | 20.90 |
| 3 | 5 | 7 | 333.17 | 21.90 |
| 3 | 5 | 7 | 319.07 | 19.80 |
| 4 | -5 | 7 | 2902.91 | 156.48 |
| 4 | 5 | 7 | 2845.22 | 156.08 |
| 4 | 5 | 7 | 2920.91 | 158.68 |
| 5 | -5 | 7 | 59.99 | 10.30 |
| 5 | 5 | 7 | 60.09 | 6.50 |
| 5 | 5 | 7 | 49.70 | 11.60 |
| 5 | 5 | 7 | 57.49 | 8.40 |
| 5 | 5 | 7 | 56.59 | 9.90 |
| 6 | -5 | 7 | 374.46 | 23.30 |
| 6 | -5 | 7 | 343.67 | 22.60 |
| 6 | -5 | 7 | 335.57 | 22.30 |
| 6 | 5 | 7 | 371.06 | 20.90 |
| 6 | 5 | 7 | 360.66 | 21.40 |
| 6 | 5 | 7 | 302.77 | 24.90 |
| 7 | -5 | 7 | 111.89 | 11.40 |
| 7 | -5 | 7 | 94.09 | 12.20 |
| 7 | -5 | 7 | 86.69 | 10.50 |
| 7 | 5 | 7 | 89.79 | 8.60 |
| 7 | 5 | 7 | 75.79 | 7.70 |
| 7 | 5 | 7 | 67.99 | 14.90 |
| 8 | -5 | 7 | 742.53 | 43.80 |

| | | | | |
|---|---|---|---|---|
| 8 | -5 | 7 | 803.12 | 43.30 |
| 8 | 5 | 7 | 739.03 | 42.90 |
| 8 | 5 | 7 | 757.92 | 42.90 |
| 9 | -5 | 7 | 2.50 | 4.60 |
| 9 | -5 | 7 | 6.70 | 4.70 |
| 9 | -5 | 7 | 2.30 | 5.50 |
| 9 | 5 | 7 | 1.20 | 3.70 |
| 9 | 5 | 7 | 3.70 | 3.70 |
| 10 | -5 | 7 | 618.54 | 37.40 |
| 10 | -5 | 7 | 672.83 | 36.70 |
| 10 | 5 | 7 | 610.94 | 39.00 |
| 10 | 5 | 7 | 668.13 | 36.70 |
| 11 | -5 | 7 | 12.20 | 5.00 |
| 11 | -5 | 7 | 19.80 | 4.60 |
| 11 | -5 | 7 | 7.00 | 4.10 |
| 11 | 5 | 7 | 5.70 | 3.50 |
| 11 | 5 | 7 | 15.70 | 3.90 |
| 12 | -5 | 7 | -2.60 | 4.00 |
| 12 | -5 | 7 | -0.30 | 3.50 |
| 12 | -5 | 7 | -7.60 | 4.00 |
| 12 | 5 | 7 | 4.10 | 3.10 |
| 12 | 5 | 7 | -3.80 | 3.10 |
| 13 | -5 | 7 | 14.40 | 3.90 |
| 13 | -5 | 7 | 14.90 | 3.60 |
| 13 | 5 | 7 | 12.60 | 3.00 |
| 13 | 5 | 7 | 15.50 | 3.20 |
| 14 | -5 | 7 | 328.17 | 19.00 |
| 14 | -5 | 7 | 324.17 | 18.60 |
| 14 | 5 | 7 | 313.67 | 18.30 |
| 14 | 5 | 7 | 300.67 | 18.40 |
| 16 | -6 | -7 | 1.00 | 1.90 |
| -16 | 6 | 7 | 4.50 | 1.70 |
| 16 | 6 | -7 | 2.40 | 1.40 |
| 15 | -6 | -7 | 356.16 | 19.90 |
| -15 | 6 | 7 | 345.67 | 20.00 |
| 15 | 6 | -7 | 339.77 | 19.60 |
| 14 | -6 | -7 | 67.39 | 7.00 |
| 14 | -6 | -7 | 74.39 | 8.30 |
| 14 | 6 | -7 | 83.39 | 7.70 |
| 13 | -6 | -7 | 19.00 | 4.50 |
| 13 | -6 | -7 | 26.30 | 3.80 |
| 13 | 6 | -7 | 13.40 | 2.60 |
| 12 | -6 | -7 | 6.70 | 3.50 |
| 12 | 6 | -7 | 1.60 | 2.30 |

| | | | | |
|---|---|---|---|---|
| 11 | -6 | -7 | 1541.55 | 88.39 |
| 11 | -6 | -7 | 1682.23 | 88.19 |
| 11 | 6 | -7 | 1614.74 | 87.79 |
| 10 | -6 | -7 | 13.40 | 4.30 |
| 10 | -6 | -7 | 23.20 | 5.20 |
| 10 | 6 | -7 | 11.20 | 3.00 |
| 9 | -6 | -7 | 562.74 | 32.90 |
| 9 | -6 | -7 | 583.64 | 33.50 |
| 9 | 6 | -7 | 545.35 | 32.00 |
| 8 | -6 | -7 | 30.40 | 5.40 |
| 8 | -6 | -7 | 33.70 | 8.00 |
| 8 | 6 | -7 | 30.40 | 7.40 |
| 8 | 6 | -7 | 28.90 | 4.80 |
| 7 | -6 | -7 | 1334.57 | 71.59 |
| 7 | 6 | -7 | 1292.57 | 71.29 |
| 7 | 6 | -7 | 1237.58 | 70.89 |
| -6 | 6 | 7 | 20.40 | 5.50 |
| -6 | 6 | 7 | 34.00 | 7.30 |
| 6 | 6 | -7 | 21.70 | 4.60 |
| -5 | 6 | 7 | 2695.23 | 146.59 |
| -4 | 6 | 7 | 297.07 | 22.40 |
| -3 | 6 | 7 | 203.88 | 14.20 |
| -2 | 6 | 7 | 98.39 | 9.00 |
| -2 | 6 | 7 | 93.89 | 9.80 |
| -1 | 6 | 7 | 2662.53 | 141.19 |
| -1 | 6 | 7 | 2515.15 | 140.29 |
| 0 | 6 | 7 | 2.10 | 3.60 |
| 0 | 6 | 7 | 4.10 | 3.20 |
| 1 | 6 | 7 | 63.59 | 7.60 |
| 1 | 6 | 7 | 62.29 | 7.10 |
| 2 | 6 | 7 | 3.80 | 3.60 |
| 2 | 6 | 7 | -0.30 | 2.90 |
| 3 | 6 | 7 | 388.26 | 25.60 |
| 3 | 6 | 7 | 422.16 | 23.70 |
| 4 | -6 | 7 | 14.00 | 5.70 |
| 4 | 6 | 7 | 8.00 | 4.40 |
| 4 | 6 | 7 | 7.20 | 3.20 |
| 4 | 6 | 7 | 10.80 | 6.10 |
| 5 | -6 | 7 | 1241.48 | 75.39 |
| 5 | -6 | 7 | 1390.86 | 74.69 |
| 5 | -6 | 7 | 1425.16 | 74.89 |
| 5 | 6 | 7 | 1492.25 | 74.39 |
| 5 | 6 | 7 | 1312.37 | 77.39 |
| 5 | 6 | 7 | 1273.77 | 74.49 |

| | | | | |
|---|---|---|---|---|
| 6 | -6 | 7 | 37.30 | 6.30 |
| 6 | -6 | 7 | 70.29 | 10.40 |
| 6 | 6 | 7 | 32.60 | 8.90 |
| 6 | 6 | 7 | 60.29 | 6.20 |
| 6 | 6 | 7 | 57.99 | 7.60 |
| 7 | -6 | 7 | 160.78 | 12.60 |
| 7 | -6 | 7 | 112.89 | 15.70 |
| 7 | -6 | 7 | 140.69 | 12.80 |
| 7 | 6 | 7 | 168.78 | 10.90 |
| 7 | 6 | 7 | 146.89 | 18.50 |
| 7 | 6 | 7 | 155.28 | 11.40 |
| 8 | -6 | 7 | 119.09 | 14.00 |
| 8 | -6 | 7 | 115.99 | 11.40 |
| 8 | -6 | 7 | 110.29 | 10.70 |
| 8 | 6 | 7 | 104.29 | 8.90 |
| 8 | 6 | 7 | 105.09 | 9.30 |
| 9 | -6 | 7 | 861.61 | 47.60 |
| 9 | -6 | 7 | 847.22 | 46.50 |
| 9 | -6 | 7 | 813.72 | 47.50 |
| 9 | 6 | 7 | 799.42 | 46.40 |
| 9 | 6 | 7 | 834.12 | 46.80 |
| 10 | -6 | 7 | 12.70 | 4.20 |
| 10 | -6 | 7 | 8.20 | 5.70 |
| 10 | -6 | 7 | 4.20 | 4.80 |
| 10 | 6 | 7 | 8.50 | 3.90 |
| 10 | 6 | 7 | 11.40 | 3.50 |
| 11 | -6 | 7 | 225.98 | 17.30 |
| 11 | -6 | 7 | 266.17 | 18.30 |
| 11 | -6 | 7 | 271.67 | 16.60 |
| 11 | 6 | 7 | 264.77 | 15.70 |
| 11 | 6 | 7 | 213.48 | 15.90 |
| 12 | -6 | 7 | 93.89 | 8.30 |
| 12 | -6 | 7 | 75.29 | 8.10 |
| 12 | -6 | 7 | 75.99 | 8.90 |
| 12 | 6 | 7 | 69.19 | 7.30 |
| 12 | 6 | 7 | 89.09 | 7.40 |
| 13 | -6 | 7 | 245.38 | 15.90 |
| 13 | -6 | 7 | 247.58 | 15.30 |
| 13 | 6 | 7 | 223.58 | 14.50 |
| 13 | 6 | 7 | 240.78 | 14.70 |
| 14 | -6 | 7 | -0.80 | 3.60 |
| 14 | 6 | 7 | 2.90 | 2.20 |
| 16 | -7 | -7 | 331.17 | 18.80 |
| -16 | 7 | 7 | 342.67 | 18.90 |

| | | | | |
|---|---|---|---|---|
| 16 | 7 | -7 | 329.77 | 18.60 |
| 15 | -7 | -7 | 3.20 | 2.10 |
| -15 | 7 | 7 | 3.70 | 2.00 |
| 15 | 7 | -7 | 5.40 | 1.60 |
| 14 | -7 | -7 | 286.47 | 17.60 |
| -14 | 7 | 7 | 322.57 | 17.90 |
| 14 | 7 | -7 | 293.87 | 17.30 |
| 13 | -7 | -7 | 95.79 | 8.90 |
| 13 | -7 | -7 | 93.29 | 8.00 |
| 13 | 7 | -7 | 93.19 | 7.00 |
| 12 | -7 | -7 | 535.85 | 31.30 |
| 12 | -7 | -7 | 577.74 | 31.90 |
| 12 | 7 | -7 | 535.85 | 30.80 |
| 11 | -7 | -7 | 20.90 | 4.60 |
| 11 | -7 | -7 | 9.90 | 4.10 |
| 11 | 7 | -7 | 17.40 | 3.20 |
| 10 | -7 | -7 | 1052.19 | 58.29 |
| 10 | -7 | -7 | 1028.80 | 58.19 |
| 10 | 7 | -7 | 1057.49 | 57.59 |
| 9 | -7 | -7 | 251.47 | 17.10 |
| 9 | -7 | -7 | 198.58 | 15.10 |
| 9 | 7 | -7 | 223.08 | 14.30 |
| 8 | -7 | -7 | -2.10 | 7.40 |
| 8 | -7 | -7 | 14.40 | 4.50 |
| 8 | 7 | -7 | 7.80 | 5.00 |
| 8 | 7 | -7 | 4.40 | 3.50 |
| 7 | 7 | -7 | 42.10 | 6.10 |
| -7 | 7 | 7 | 36.70 | 7.20 |
| 7 | 7 | -7 | 40.90 | 7.60 |
| -6 | 7 | 7 | 3761.82 | 196.28 |
| 6 | 7 | -7 | 3813.22 | 196.48 |
| -6 | 7 | 7 | 3286.67 | 196.48 |
| -5 | 7 | 7 | 33.40 | 7.50 |
| -5 | 7 | 7 | 43.70 | 7.90 |
| 5 | 7 | -7 | 39.20 | 7.60 |
| -4 | 7 | 7 | 369.56 | 22.80 |
| -3 | 7 | 7 | 16.00 | 3.90 |
| -2 | 7 | 7 | 1229.78 | 68.09 |
| -2 | 7 | 7 | 1211.18 | 67.49 |
| -1 | 7 | 7 | 31.40 | 6.80 |
| -1 | 7 | 7 | 29.40 | 6.30 |
| 0 | 7 | 7 | 2656.93 | 145.69 |
| 0 | 7 | 7 | 2725.43 | 146.99 |
| 1 | 7 | 7 | 179.48 | 13.20 |

| | | | | |
|---|---|---|---|---|
| 1 | 7 | 7 | 170.68 | 11.90 |
| 2 | 7 | 7 | 74.09 | 8.90 |
| 2 | 7 | 7 | 91.19 | 7.60 |
| 3 | -7 | 7 | 32.10 | 9.70 |
| 3 | 7 | 7 | 52.89 | 8.40 |
| 3 | 7 | 7 | 45.90 | 6.10 |
| 3 | 7 | 7 | 46.70 | 9.20 |
| 4 | -7 | 7 | 1112.19 | 67.39 |
| 4 | -7 | 7 | 1166.58 | 65.49 |
| 4 | -7 | 7 | 1190.78 | 66.19 |
| 4 | 7 | 7 | 1213.68 | 68.09 |
| 4 | 7 | 7 | 1211.38 | 65.29 |
| 4 | 7 | 7 | 1233.28 | 65.79 |
| 5 | -7 | 7 | 2.20 | 7.00 |
| 5 | -7 | 7 | -3.70 | 7.50 |
| 5 | -7 | 7 | -4.70 | 4.70 |
| 5 | 7 | 7 | 1.50 | 6.20 |
| 5 | 7 | 7 | 3.80 | 4.30 |
| 5 | 7 | 7 | 0.10 | 2.70 |
| 6 | -7 | 7 | 589.14 | 34.30 |
| 6 | -7 | 7 | 657.73 | 34.00 |
| 6 | 7 | 7 | 573.44 | 33.70 |
| 6 | 7 | 7 | 526.95 | 33.20 |
| 6 | 7 | 7 | 555.14 | 37.60 |
| -6 | 7 | -7 | 623.24 | 34.50 |
| 7 | -7 | 7 | 69.49 | 9.80 |
| 7 | 7 | 7 | 52.29 | 11.60 |
| 7 | 7 | 7 | 76.69 | 6.90 |
| 7 | 7 | 7 | 69.09 | 7.90 |
| 8 | -7 | 7 | 188.78 | 13.90 |
| 8 | -7 | 7 | 197.28 | 14.90 |
| 8 | 7 | 7 | 219.08 | 13.40 |
| 8 | 7 | 7 | 193.78 | 13.50 |
| 9 | -7 | 7 | 4.50 | 5.30 |
| 9 | -7 | 7 | 13.20 | 7.20 |
| 9 | -7 | 7 | 17.80 | 4.00 |
| 9 | 7 | 7 | 17.00 | 3.90 |
| 9 | 7 | 7 | 16.10 | 3.80 |
| 10 | -7 | 7 | 651.83 | 38.30 |
| 10 | -7 | 7 | 587.04 | 38.70 |
| 10 | -7 | 7 | 705.03 | 37.30 |
| 10 | 7 | 7 | 657.73 | 37.80 |
| 10 | 7 | 7 | 705.63 | 37.50 |
| 11 | -7 | 7 | 51.19 | 8.00 |

| | | | | |
|---|---|---|---|---|
| 11 | -7 | 7 | 65.99 | 7.60 |
| 11 | -7 | 7 | 48.00 | 10.30 |
| 11 | 7 | 7 | 50.09 | 6.70 |
| 12 | -7 | 7 | -5.50 | 4.80 |
| 12 | -7 | 7 | -2.00 | 3.60 |
| 12 | -7 | 7 | 2.30 | 3.70 |
| 12 | 7 | 7 | 2.50 | 2.80 |
| 12 | 7 | 7 | -3.20 | 2.90 |
| 13 | -7 | 7 | 9.80 | 4.00 |
| 13 | 7 | 7 | 13.60 | 3.80 |
| 13 | 7 | 7 | 15.30 | 3.30 |
| 15 | -8 | -7 | 360.26 | 20.80 |
| 15 | 8 | -7 | 364.86 | 20.60 |
| -15 | 8 | 7 | 380.86 | 21.00 |
| 14 | -8 | -7 | 0.30 | 2.40 |
| 14 | 8 | -7 | 2.50 | 1.70 |
| -14 | 8 | 7 | -2.70 | 2.10 |
| 13 | -8 | -7 | 32.10 | 6.60 |
| 13 | 8 | -7 | 35.90 | 4.30 |
| 12 | -8 | -7 | 45.70 | 6.40 |
| 12 | 8 | -7 | 25.90 | 4.50 |
| -12 | 8 | 7 | 42.40 | 5.90 |
| 11 | -8 | -7 | 1068.59 | 62.39 |
| 11 | 8 | -7 | 1168.58 | 62.09 |
| -11 | 8 | 7 | 1159.38 | 62.69 |
| 10 | -8 | -7 | 1.40 | 4.70 |
| -10 | 8 | 7 | 9.10 | 3.90 |
| 10 | 8 | -7 | 2.30 | 2.60 |
| 9 | -8 | -7 | 316.27 | 21.40 |
| 9 | 8 | -7 | 310.47 | 19.20 |
| -9 | 8 | 7 | 324.67 | 20.20 |
| 8 | -8 | -7 | 1.50 | 7.40 |
| -8 | 8 | 7 | 8.80 | 5.20 |
| 8 | 8 | -7 | 1.80 | 4.50 |
| 8 | 8 | -7 | 9.50 | 5.50 |
| -7 | -8 | 7 | 1044.60 | 63.19 |
| -7 | 8 | 7 | 1197.78 | 63.79 |
| 7 | 8 | -7 | 1142.39 | 63.29 |
| -6 | -8 | 7 | 40.20 | 7.20 |
| 6 | 8 | -7 | 37.40 | 7.90 |
| -6 | 8 | 7 | 31.30 | 7.60 |
| -6 | 8 | 7 | 40.30 | 6.60 |
| -5 | -8 | 7 | 1847.62 | 106.89 |
| -5 | 8 | 7 | 1846.92 | 107.89 |

| | | | | |
|---|---|---|---|---|
| -5 | 8 | 7 | 2070.09 | 107.39 |
| 5 | 8 | -7 | 2055.19 | 107.89 |
| -4 | -8 | 7 | 157.58 | 13.90 |
| -4 | 8 | 7 | 169.38 | 12.40 |
| 4 | 8 | -7 | 163.68 | 13.40 |
| -4 | 8 | 7 | 140.59 | 15.80 |
| -3 | -8 | 7 | 7.90 | 4.90 |
| -3 | -8 | 7 | 10.60 | 5.80 |
| -3 | 8 | 7 | 8.40 | 4.00 |
| -3 | 8 | 7 | 7.30 | 4.00 |
| 3 | 8 | -7 | 5.90 | 4.40 |
| -2 | -8 | 7 | 234.18 | 16.90 |
| -2 | 8 | 7 | 252.37 | 16.30 |
| -2 | 8 | 7 | 228.28 | 16.00 |
| -1 | -8 | 7 | 3919.91 | 230.18 |
| -1 | 8 | 7 | 4331.47 | 231.08 |
| -1 | 8 | 7 | 4536.05 | 230.18 |
| 0 | -8 | 7 | 392.66 | 25.30 |
| 0 | 8 | 7 | 388.16 | 23.70 |
| 0 | 8 | 7 | 423.06 | 25.00 |
| 1 | -8 | 7 | 554.44 | 35.40 |
| 1 | 8 | 7 | 629.04 | 33.40 |
| 1 | 8 | 7 | 565.84 | 34.60 |
| 2 | -8 | 7 | 156.78 | 17.00 |
| 2 | -8 | 7 | 135.69 | 12.60 |
| 2 | 8 | 7 | 165.78 | 13.00 |
| 2 | 8 | 7 | 162.48 | 11.50 |
| 2 | 8 | 7 | 155.58 | 13.60 |
| 3 | -8 | 7 | 1034.80 | 57.79 |
| 3 | -8 | 7 | 1016.40 | 55.29 |
| 3 | 8 | 7 | 956.30 | 55.59 |
| 3 | 8 | 7 | 1006.80 | 57.29 |
| 3 | 8 | 7 | 966.80 | 54.99 |
| 4 | -8 | 7 | 52.79 | 9.20 |
| 4 | -8 | 7 | 92.49 | 15.80 |
| 4 | 8 | 7 | 69.79 | 9.60 |
| 4 | 8 | 7 | 79.49 | 11.60 |
| 4 | 8 | 7 | 84.89 | 7.10 |
| 5 | -8 | 7 | 2301.97 | 128.89 |
| 5 | -8 | 7 | 2519.25 | 128.89 |
| 5 | -8 | 7 | 2336.07 | 129.79 |
| 5 | 8 | 7 | 2322.47 | 128.79 |
| -5 | 8 | -7 | 2490.65 | 129.89 |
| 5 | 8 | 7 | 2381.66 | 128.89 |

| | | | | |
|---|---|---|---|---|
| 5 | 8 | 7 | 2272.27 | 131.89 |
| 6 | -8 | 7 | 228.38 | 16.40 |
| 6 | -8 | 7 | 257.07 | 15.90 |
| 6 | 8 | 7 | 221.58 | 15.00 |
| 6 | 8 | 7 | 267.97 | 16.30 |
| -6 | 8 | -7 | 231.58 | 17.20 |
| -7 | -8 | -7 | 70.99 | 9.60 |
| 7 | -8 | 7 | 83.29 | 8.70 |
| 7 | -8 | 7 | 81.69 | 9.80 |
| 7 | 8 | 7 | 73.59 | 8.50 |
| 7 | 8 | 7 | 83.29 | 7.20 |
| 7 | 8 | 7 | 99.29 | 23.20 |
| 8 | -8 | 7 | 85.19 | 9.80 |
| 8 | -8 | 7 | 62.29 | 8.00 |
| -8 | -8 | -7 | 74.49 | 10.00 |
| 8 | 8 | 7 | 65.79 | 6.90 |
| 8 | 8 | 7 | 79.69 | 8.00 |
| 9 | -8 | 7 | 745.63 | 42.80 |
| 9 | -8 | 7 | 755.32 | 41.60 |
| 9 | 8 | 7 | 788.72 | 41.80 |
| 9 | 8 | 7 | 684.33 | 41.90 |
| 10 | -8 | 7 | 4.20 | 4.40 |
| 10 | -8 | 7 | 8.30 | 3.50 |
| 10 | 8 | 7 | 5.90 | 3.30 |
| 10 | 8 | 7 | 3.00 | 3.60 |
| 11 | -8 | 7 | 160.38 | 12.60 |
| 11 | -8 | 7 | 179.18 | 11.60 |
| 11 | 8 | 7 | 155.18 | 11.70 |
| 11 | 8 | 7 | 187.48 | 12.00 |
| 12 | -8 | 7 | 60.09 | 9.20 |
| 12 | -8 | 7 | 65.69 | 6.90 |
| 12 | 8 | 7 | 69.89 | 6.90 |
| 12 | 8 | 7 | 61.99 | 6.40 |
| 13 | -8 | 7 | 169.58 | 12.10 |
| 13 | 8 | 7 | 170.78 | 11.40 |
| 13 | 8 | 7 | 186.48 | 11.20 |
| 15 | -9 | -7 | 7.30 | 2.20 |
| -15 | 9 | 7 | 5.90 | 2.10 |
| 15 | 9 | -7 | 8.40 | 1.50 |
| 14 | -9 | -7 | 150.88 | 9.80 |
| 14 | 9 | -7 | 153.58 | 9.20 |
| -14 | 9 | 7 | 149.29 | 9.80 |
| 13 | -9 | -7 | 110.79 | 8.30 |
| 13 | 9 | -7 | 110.99 | 7.50 |

| | | | | |
|---|---|---|---|---|
| -13 | 9 | 7 | 105.69 | 8.20 |
| 12 | -9 | -7 | 209.88 | 14.00 |
| 12 | 9 | -7 | 216.48 | 13.10 |
| -12 | 9 | 7 | 225.38 | 14.00 |
| 11 | -9 | -7 | -1.80 | 3.70 |
| 11 | 9 | -7 | 2.60 | 2.20 |
| -11 | 9 | 7 | 1.10 | 3.20 |
| 10 | -9 | -7 | 524.45 | 30.40 |
| -10 | 9 | 7 | 478.05 | 30.00 |
| 10 | 9 | -7 | 542.95 | 29.30 |
| 9 | -9 | -7 | 77.99 | 11.50 |
| -9 | 9 | 7 | 68.19 | 9.00 |
| 9 | 9 | -7 | 70.39 | 7.30 |
| 8 | -9 | -7 | 48.70 | 8.70 |
| 8 | 9 | -7 | 23.80 | 7.70 |
| -8 | 9 | 7 | 28.50 | 6.20 |
| 8 | 9 | -7 | 46.80 | 6.80 |
| -7 | -9 | 7 | 438.16 | 28.20 |
| -7 | 9 | 7 | 447.96 | 28.80 |
| 7 | 9 | -7 | 474.75 | 28.20 |
| -6 | -9 | 7 | 1741.43 | 98.69 |
| 6 | 9 | -7 | 1856.61 | 99.79 |
| -6 | 9 | 7 | 1668.73 | 99.79 |
| -6 | 9 | 7 | 1941.11 | 99.79 |
| -5 | -9 | 7 | 125.79 | 13.10 |
| 5 | -9 | -7 | 146.99 | 17.00 |
| -5 | 9 | 7 | 155.78 | 12.80 |
| 5 | 9 | -7 | 165.08 | 13.50 |
| -5 | 9 | 7 | 156.58 | 16.30 |
| -4 | -9 | 7 | 508.55 | 30.80 |
| 4 | 9 | -7 | 555.84 | 32.30 |
| -4 | 9 | 7 | 539.75 | 31.10 |
| -4 | 9 | 7 | 467.95 | 32.10 |
| -3 | -9 | 7 | 167.28 | 12.60 |
| -3 | -9 | 7 | 171.28 | 13.60 |
| -3 | 9 | 7 | 143.99 | 12.40 |
| -3 | 9 | 7 | 178.28 | 13.00 |
| 3 | 9 | -7 | 157.48 | 14.10 |
| -3 | 9 | 7 | 165.48 | 15.60 |
| -2 | -9 | 7 | 1823.22 | 96.99 |
| -2 | -9 | 7 | 1686.03 | 97.39 |
| -2 | 9 | 7 | 1777.72 | 97.39 |
| -2 | 9 | 7 | 1761.52 | 99.09 |
| -2 | 9 | 7 | 1838.32 | 98.29 |

| | | | | |
|---|---|---|---|---|
| -1 | -9 | 7 | 315.17 | 20.20 |
| -1 | -9 | 7 | 294.87 | 21.00 |
| 1 | 9 | -7 | 314.27 | 21.80 |
| -1 | 9 | 7 | 351.46 | 21.30 |
| -1 | 9 | 7 | 346.57 | 20.30 |
| 0 | -9 | 7 | 3534.95 | 198.28 |
| 0 | -9 | 7 | 3556.14 | 197.38 |
| 0 | 9 | 7 | 4059.99 | 199.28 |
| 0 | 9 | 7 | 3525.95 | 197.78 |
| 0 | 9 | -7 | 3705.93 | 199.48 |
| 0 | 9 | 7 | 3584.14 | 198.98 |
| 1 | -9 | 7 | 59.09 | 9.40 |
| 1 | -9 | 7 | 62.39 | 12.00 |
| 1 | 9 | 7 | 88.69 | 11.50 |
| -1 | 9 | -7 | 90.59 | 9.30 |
| 1 | 9 | 7 | 87.79 | 9.40 |
| 1 | 9 | 7 | 95.99 | 7.90 |
| 2 | -9 | 7 | 167.28 | 12.80 |
| 2 | -9 | 7 | 199.08 | 16.30 |
| 2 | 9 | 7 | 181.38 | 14.60 |
| 2 | 9 | 7 | 167.18 | 11.90 |
| -2 | 9 | -7 | 182.58 | 13.30 |
| 2 | 9 | 7 | 166.48 | 14.00 |
| 3 | -9 | 7 | 128.89 | 16.10 |
| 3 | -9 | 7 | 111.89 | 10.00 |
| -3 | 9 | -7 | 100.59 | 10.10 |
| 3 | 9 | 7 | 132.89 | 11.80 |
| 3 | 9 | 7 | 100.89 | 11.90 |
| 3 | 9 | 7 | 112.09 | 8.70 |
| 4 | -9 | 7 | 2308.57 | 122.89 |
| 4 | -9 | 7 | 2292.57 | 124.69 |
| 4 | 9 | 7 | 2261.17 | 125.79 |
| 4 | 9 | 7 | 2267.27 | 123.19 |
| -4 | 9 | -7 | 2324.07 | 124.19 |
| 4 | 9 | 7 | 2146.69 | 122.99 |
| 5 | -9 | 7 | 86.89 | 10.80 |
| 5 | -9 | 7 | 120.99 | 9.80 |
| 5 | -9 | 7 | 84.49 | 13.80 |
| -5 | 9 | -7 | 111.79 | 10.80 |
| 5 | 9 | 7 | 83.69 | 14.60 |
| 5 | 9 | 7 | 116.79 | 8.30 |
| 5 | 9 | 7 | 94.59 | 10.10 |
| -6 | -9 | -7 | 335.47 | 20.30 |
| 6 | -9 | 7 | 327.77 | 19.80 |

| | | | | |
|---|---|---|---|---|
| 6 | -9 | 7 | 314.27 | 20.60 |
| 6 | 9 | 7 | 315.57 | 19.40 |
| 6 | 9 | 7 | 328.07 | 20.20 |
| 7 | -9 | 7 | 373.86 | 22.90 |
| 7 | -9 | 7 | 358.16 | 21.80 |
| -7 | -9 | -7 | 372.86 | 22.30 |
| 7 | 9 | 7 | 376.76 | 21.70 |
| 7 | 9 | 7 | 350.96 | 22.20 |
| 7 | 9 | 7 | 374.86 | 31.00 |
| 8 | -9 | 7 | 664.53 | 37.40 |
| 8 | -9 | 7 | 636.74 | 39.00 |
| -8 | -9 | -7 | 619.24 | 37.60 |
| 8 | 9 | 7 | 685.53 | 38.10 |
| 8 | 9 | 7 | 712.53 | 37.70 |
| 9 | -9 | 7 | 3.70 | 3.20 |
| 9 | -9 | 7 | 4.40 | 4.70 |
| 9 | 9 | 7 | -3.10 | 3.50 |
| 9 | 9 | 7 | 0.60 | 2.60 |
| 10 | -9 | 7 | 703.83 | 43.80 |
| 10 | -9 | 7 | 815.02 | 43.90 |
| 10 | 9 | 7 | 762.12 | 43.40 |
| 10 | 9 | 7 | 817.02 | 44.40 |
| 11 | -9 | 7 | 99.49 | 8.00 |
| 11 | -9 | 7 | 110.29 | 9.80 |
| 11 | 9 | 7 | 87.49 | 8.10 |
| 11 | 9 | 7 | 110.19 | 8.70 |
| 12 | -9 | 7 | 4.80 | 4.90 |
| 12 | -9 | 7 | 6.70 | 3.10 |
| 12 | 9 | 7 | 5.70 | 3.60 |
| 14 | -10 | -7 | 5.60 | 2.10 |
| -14 | 10 | 7 | 7.80 | 2.40 |
| 14 | 10 | -7 | 7.00 | 1.50 |
| 13 | -10 | -7 | 6.30 | 2.70 |
| -13 | 10 | 7 | 8.90 | 2.70 |
| 13 | 10 | -7 | 11.10 | 2.00 |
| 12 | -10 | -7 | 38.50 | 6.30 |
| -12 | 10 | 7 | 38.40 | 6.10 |
| 12 | 10 | -7 | 47.00 | 5.30 |
| 11 | -10 | -7 | 887.71 | 49.90 |
| 11 | 10 | -7 | 931.81 | 49.50 |
| -11 | 10 | 7 | 877.51 | 49.99 |
| 10 | -10 | -7 | 94.59 | 10.40 |
| -10 | 10 | 7 | 103.09 | 9.30 |
| 10 | 10 | -7 | 100.79 | 7.80 |

| | | | | |
|---|---|---|---|---|
| 9 | -10 | -7 | 433.16 | 28.00 |
| 9 | 10 | -7 | 453.35 | 26.70 |
| -9 | 10 | 7 | 497.15 | 28.00 |
| -8 | -10 | 7 | 77.19 | 13.30 |
| 8 | -10 | -7 | 59.69 | 8.50 |
| -8 | 10 | 7 | 66.69 | 10.90 |
| 8 | 10 | -7 | 56.39 | 7.30 |
| 7 | 10 | -7 | 618.64 | 36.20 |
| -7 | 10 | 7 | 578.04 | 36.80 |
| -6 | -10 | 7 | -3.00 | 4.70 |
| 6 | 10 | -7 | 6.80 | 5.20 |
| -6 | 10 | 7 | 8.00 | 7.30 |
| 5 | -10 | -7 | 1865.21 | 98.49 |
| -5 | -10 | 7 | 1742.73 | 97.79 |
| -5 | 10 | 7 | 1814.22 | 98.69 |
| -5 | 10 | 7 | 1673.03 | 98.79 |
| 5 | 10 | -7 | 1830.42 | 99.49 |
| 4 | -10 | -7 | 6.40 | 6.80 |
| -4 | -10 | 7 | 14.70 | 5.00 |
| -4 | 10 | 7 | 16.30 | 4.90 |
| -4 | 10 | 7 | 15.30 | 5.00 |
| 4 | 10 | -7 | 20.40 | 6.60 |
| -4 | 10 | 7 | 19.60 | 7.60 |
| -3 | -10 | 7 | -2.40 | 4.20 |
| -3 | -10 | 7 | -0.40 | 3.90 |
| 3 | -10 | -7 | -1.60 | 6.50 |
| -3 | 10 | 7 | 1.50 | 4.20 |
| -3 | 10 | 7 | 1.00 | 6.90 |
| -3 | 10 | 7 | 5.50 | 4.00 |
| 3 | 10 | -7 | 9.40 | 5.70 |
| -2 | -10 | 7 | 219.68 | 15.40 |
| -2 | -10 | 7 | 216.78 | 14.30 |
| -2 | 10 | 7 | 223.08 | 15.70 |
| -2 | 10 | 7 | 185.68 | 16.60 |
| 2 | 10 | -7 | 181.38 | 16.50 |
| -2 | 10 | 7 | 234.68 | 14.80 |
| -1 | -10 | 7 | 3156.18 | 167.48 |
| -1 | -10 | 7 | 3233.48 | 166.78 |
| -1 | 10 | 7 | 3200.08 | 168.58 |
| -1 | 10 | 7 | 2813.82 | 167.08 |
| 1 | 10 | -7 | 2955.60 | 169.08 |
| -1 | 10 | 7 | 3155.48 | 167.38 |
| 0 | -10 | 7 | 34.50 | 6.70 |
| 0 | -10 | 7 | 15.40 | 5.60 |

| | | | | |
|---|---|---|---|---|
| 0 | 10 | 7 | 21.50 | 6.90 |
| 0 | 10 | 7 | 23.60 | 4.40 |
| 0 | 10 | 7 | 35.30 | 7.60 |
| 0 | 10 | -7 | 27.20 | 6.20 |
| 1 | -10 | 7 | 727.13 | 41.90 |
| 1 | -10 | 7 | 712.13 | 40.30 |
| 1 | 10 | 7 | 731.73 | 42.30 |
| 1 | 10 | 7 | 718.23 | 41.50 |
| 1 | 10 | 7 | 663.83 | 40.40 |
| -1 | 10 | -7 | 757.32 | 42.20 |
| 2 | -10 | 7 | 353.26 | 22.80 |
| 2 | -10 | 7 | 321.37 | 19.70 |
| 2 | 10 | 7 | 305.97 | 19.60 |
| 2 | 10 | 7 | 333.87 | 22.10 |
| -2 | 10 | -7 | 317.77 | 21.00 |
| 2 | 10 | 7 | 323.37 | 21.50 |
| 3 | -10 | 7 | 1072.79 | 59.09 |
| 3 | -10 | 7 | 1127.69 | 61.59 |
| 3 | 10 | 7 | 1104.79 | 59.29 |
| 3 | 10 | 7 | 1039.20 | 61.69 |
| 3 | 10 | 7 | 1009.60 | 59.69 |
| -3 | 10 | -7 | 1079.29 | 60.49 |
| -4 | -10 | -7 | 215.18 | 17.90 |
| 4 | -10 | 7 | 281.97 | 20.50 |
| 4 | 10 | 7 | 237.78 | 16.90 |
| 4 | 10 | 7 | 259.87 | 15.50 |
| -4 | 10 | -7 | 244.08 | 17.40 |
| 4 | 10 | 7 | 251.37 | 19.90 |
| -5 | -10 | -7 | 2214.88 | 117.79 |
| 5 | -10 | 7 | 2390.26 | 117.49 |
| 5 | -10 | 7 | 1826.22 | 118.79 |
| 5 | -10 | 7 | 2130.19 | 118.39 |
| 5 | 10 | 7 | 2215.88 | 117.49 |
| -5 | 10 | -7 | 2386.26 | 119.29 |
| 5 | 10 | 7 | 1958.80 | 120.99 |
| 5 | 10 | 7 | 2166.18 | 117.49 |
| 6 | -10 | 7 | 222.58 | 20.00 |
| 6 | -10 | 7 | 201.08 | 14.90 |
| -6 | -10 | -7 | 204.08 | 14.20 |
| 6 | -10 | 7 | 224.68 | 14.00 |
| 6 | 10 | 7 | 179.08 | 22.70 |
| 6 | 10 | 7 | 207.28 | 13.40 |
| 6 | 10 | 7 | 216.48 | 14.70 |
| 7 | -10 | 7 | 91.89 | 8.40 |

| | | | | |
|---|---|---|---|---|
| 7 | -10 | 7 | 91.09 | 10.40 |
| -7 | -10 | -7 | 112.99 | 9.30 |
| 7 | 10 | 7 | 128.89 | 23.40 |
| 7 | 10 | 7 | 99.99 | 9.70 |
| 7 | 10 | 7 | 85.69 | 7.80 |
| 8 | -10 | 7 | 34.50 | 6.00 |
| -8 | -10 | -7 | 35.10 | 7.40 |
| 8 | -10 | 7 | 46.30 | 6.10 |
| 8 | 10 | 7 | 40.30 | 7.40 |
| 8 | 10 | 7 | 33.20 | 5.30 |
| 9 | -10 | 7 | 538.45 | 32.30 |
| 9 | -10 | 7 | 560.64 | 31.30 |
| 9 | 10 | 7 | 558.74 | 32.00 |
| 9 | 10 | 7 | 559.24 | 31.60 |
| 10 | -10 | 7 | -1.80 | 2.90 |
| 10 | -10 | 7 | -0.30 | 4.60 |
| 10 | 10 | 7 | -1.80 | 2.60 |
| 10 | 10 | 7 | -2.10 | 4.00 |
| 11 | -10 | 7 | 222.98 | 15.50 |
| 11 | -10 | 7 | 245.88 | 13.70 |
| 11 | 10 | 7 | 213.48 | 14.00 |
| 11 | 10 | 7 | 218.08 | 14.60 |
| 12 | -10 | 7 | 111.59 | 9.90 |
| 12 | -10 | 7 | 109.99 | 10.70 |
| 12 | 10 | 7 | 109.59 | 8.00 |
| 14 | -11 | -7 | 144.99 | 10.80 |
| 14 | 11 | -7 | 183.78 | 9.70 |
| -14 | 11 | 7 | 161.08 | 10.30 |
| 13 | -11 | -7 | 146.49 | 9.70 |
| 13 | 11 | -7 | 149.29 | 9.10 |
| -13 | 11 | 7 | 159.28 | 10.10 |
| 12 | -11 | -7 | 276.47 | 16.10 |
| 12 | 11 | -7 | 274.77 | 15.50 |
| -12 | 11 | 7 | 252.37 | 16.30 |
| 11 | -11 | -7 | 7.50 | 4.30 |
| -11 | 11 | 7 | 6.00 | 5.40 |
| 11 | 11 | -7 | 3.50 | 2.00 |
| 10 | -11 | -7 | 777.92 | 45.50 |
| 10 | 11 | -7 | 830.12 | 45.00 |
| -10 | 11 | 7 | 830.62 | 45.80 |
| 9 | -11 | -7 | 17.20 | 5.70 |
| -9 | 11 | 7 | 19.20 | 5.40 |
| 9 | 11 | -7 | 24.90 | 4.00 |
| 8 | -11 | -7 | 42.40 | 7.20 |

| | | | | |
|---|---|---|---|---|
| -8 | -11 | 7 | 30.30 | 6.30 |
| 8 | 11 | -7 | 38.20 | 6.10 |
| -8 | 11 | 7 | 55.49 | 11.40 |
| -7 | -11 | 7 | 128.99 | 12.10 |
| 7 | 11 | -7 | 122.59 | 14.20 |
| -7 | 11 | 7 | 113.69 | 14.80 |
| -6 | -11 | 7 | 2414.26 | 129.49 |
| -6 | 11 | 7 | 2341.07 | 131.09 |
| 5 | -11 | -7 | -2.60 | 6.00 |
| -5 | -11 | 7 | 1.30 | 5.00 |
| -5 | 11 | 7 | 13.80 | 7.60 |
| 5 | 11 | -7 | -4.60 | 5.80 |
| -5 | 11 | 7 | -3.40 | 5.90 |
| -4 | -11 | 7 | 510.55 | 29.90 |
| 4 | -11 | -7 | 561.74 | 31.20 |
| -4 | 11 | 7 | 514.05 | 31.10 |
| 4 | 11 | -7 | 501.95 | 32.30 |
| -4 | 11 | 7 | 431.26 | 31.30 |
| -4 | 11 | 7 | 497.25 | 30.10 |
| -3 | -11 | 7 | 217.48 | 14.50 |
| 3 | -11 | -7 | 222.28 | 16.90 |
| -3 | -11 | 7 | 215.28 | 15.60 |
| -3 | 11 | 7 | 224.08 | 16.60 |
| -3 | 11 | 7 | 227.48 | 15.60 |
| 2 | -11 | -7 | 229.48 | 15.80 |
| -2 | -11 | 7 | 188.28 | 13.00 |
| -2 | -11 | 7 | 171.78 | 14.10 |
| -2 | 11 | 7 | 206.88 | 13.80 |
| 2 | 11 | -7 | 164.28 | 16.50 |
| -2 | 11 | 7 | 193.28 | 15.10 |
| -1 | -11 | 7 | 302.97 | 18.30 |
| 1 | -11 | -7 | 324.27 | 20.60 |
| -1 | -11 | 7 | 281.57 | 19.50 |
| -1 | 11 | 7 | 303.47 | 18.80 |
| -1 | 11 | 7 | 306.37 | 20.40 |
| 1 | 11 | -7 | 264.97 | 21.30 |
| 0 | -11 | 7 | 2167.68 | 115.69 |
| 0 | -11 | 7 | 2147.79 | 114.69 |
| 0 | -11 | -7 | 2057.99 | 115.69 |
| 0 | 11 | 7 | 2044.00 | 115.19 |
| 0 | 11 | -7 | 2108.39 | 117.19 |
| 0 | 11 | 7 | 2153.78 | 116.79 |
| 1 | -11 | 7 | 341.47 | 22.90 |
| 1 | -11 | 7 | 339.27 | 21.10 |

| | | | | |
|---|---|---|---|---|
| -1 | -11 | -7 | 351.46 | 22.30 |
| 1 | 11 | 7 | 373.26 | 21.50 |
| -1 | 11 | -7 | 364.16 | 23.30 |
| 1 | 11 | 7 | 352.26 | 23.40 |
| 2 | -11 | 7 | 27.60 | 6.30 |
| 2 | -11 | 7 | 31.10 | 9.30 |
| 2 | 11 | 7 | 40.00 | 9.50 |
| 2 | 11 | 7 | 36.20 | 5.70 |
| -2 | 11 | -7 | 38.80 | 8.10 |
| 3 | -11 | 7 | 162.58 | 11.60 |
| 3 | -11 | 7 | 168.88 | 16.20 |
| -3 | -11 | -7 | 162.88 | 12.50 |
| 3 | 11 | 7 | 142.19 | 19.30 |
| 3 | 11 | 7 | 179.38 | 11.50 |
| -3 | 11 | -7 | 164.38 | 13.90 |
| 4 | -11 | 7 | 753.92 | 45.40 |
| 4 | -11 | 7 | 791.22 | 43.40 |
| -4 | -11 | -7 | 789.62 | 43.90 |
| 4 | 11 | 7 | 764.12 | 47.10 |
| 4 | 11 | 7 | 735.03 | 43.40 |
| -4 | 11 | -7 | 825.22 | 45.40 |
| -5 | -11 | -7 | 4.10 | 3.40 |
| 5 | -11 | 7 | -0.20 | 6.00 |
| 5 | -11 | 7 | 4.10 | 3.30 |
| -5 | 11 | -7 | 5.00 | 6.30 |
| 5 | 11 | 7 | 2.20 | 9.80 |
| 5 | 11 | 7 | 3.50 | 2.30 |
| 5 | 11 | 7 | 9.70 | 5.20 |
| -6 | -11 | -7 | 283.17 | 17.20 |
| 6 | -11 | 7 | 267.37 | 16.60 |
| 6 | -11 | 7 | 275.17 | 18.80 |
| 6 | 11 | 7 | 295.47 | 27.20 |
| 6 | 11 | 7 | 260.77 | 16.50 |
| 6 | 11 | 7 | 252.97 | 17.60 |
| 7 | -11 | 7 | 185.58 | 12.00 |
| -7 | -11 | -7 | 181.38 | 12.40 |
| 7 | -11 | 7 | 176.98 | 13.80 |
| 7 | 11 | 7 | 177.18 | 13.20 |
| 7 | 11 | 7 | 180.18 | 12.00 |
| 7 | 11 | 7 | 182.98 | 28.80 |
| -8 | -11 | -7 | 108.39 | 9.40 |
| 8 | -11 | 7 | 99.89 | 10.30 |
| 8 | -11 | 7 | 107.29 | 8.30 |
| 8 | 11 | 7 | 101.39 | 8.10 |

| | | | | |
|---|---|---|---|---|
| 8 | 11 | 7 | 114.79 | 10.10 |
| 9 | -11 | 7 | 29.50 | 5.50 |
| 9 | -11 | 7 | 18.50 | 3.50 |
| 9 | 11 | 7 | 20.50 | 4.30 |
| 9 | 11 | 7 | 13.00 | 5.20 |
| 10 | -11 | 7 | 471.45 | 25.10 |
| 10 | -11 | 7 | 392.36 | 26.60 |
| 10 | 11 | 7 | 462.05 | 25.60 |
| 11 | -11 | 7 | 108.39 | 7.60 |
| 11 | 11 | 7 | 102.89 | 8.20 |
| 13 | -12 | -7 | -0.90 | 2.70 |
| 13 | 12 | -7 | 0.20 | 1.20 |
| 12 | -12 | -7 | 77.59 | 7.10 |
| 12 | 12 | -7 | 83.99 | 5.70 |
| -12 | 12 | 7 | 89.19 | 8.00 |
| 11 | -12 | -7 | 617.94 | 35.10 |
| -11 | 12 | 7 | 652.73 | 35.80 |
| 11 | 12 | -7 | 617.04 | 34.80 |
| 10 | -12 | -7 | 5.30 | 5.60 |
| 10 | 12 | -7 | 3.20 | 2.00 |
| -10 | 12 | 7 | -1.40 | 5.40 |
| 9 | -12 | -7 | 41.00 | 6.00 |
| -9 | 12 | 7 | 40.80 | 6.70 |
| 9 | 12 | -7 | 35.60 | 5.30 |
| -8 | -12 | 7 | 49.10 | 9.60 |
| 8 | -12 | -7 | 76.39 | 14.60 |
| 8 | 12 | -7 | 59.79 | 6.60 |
| -8 | 12 | 7 | 68.49 | 12.50 |
| -7 | -12 | 7 | 615.34 | 35.50 |
| -7 | 12 | 7 | 579.54 | 38.20 |
| 7 | 12 | -7 | 650.63 | 38.30 |
| -6 | -12 | 7 | 5.40 | 4.30 |
| 6 | 12 | -7 | 8.60 | 6.70 |
| -5 | -12 | 7 | 690.83 | 39.90 |
| 5 | -12 | -7 | 727.33 | 40.40 |
| 5 | 12 | -7 | 663.93 | 42.30 |
| -5 | 12 | 7 | 720.33 | 42.10 |
| -5 | 12 | 7 | 678.63 | 40.20 |
| 4 | -12 | -7 | 26.50 | 5.90 |
| -4 | -12 | 7 | 13.30 | 4.40 |
| 4 | 12 | -7 | 23.20 | 7.70 |
| -4 | 12 | 7 | 24.70 | 5.40 |
| 3 | -12 | -7 | 405.66 | 26.30 |
| -3 | -12 | 7 | 412.66 | 25.50 |

| | | | | |
|---|---|---|---|---|
| -3 | -12 | 7 | 418.66 | 24.50 |
| -3 | 12 | 7 | 459.05 | 27.20 |
| -3 | 12 | 7 | 399.86 | 25.50 |
| -2 | -12 | 7 | 144.49 | 10.40 |
| -2 | -12 | 7 | 132.69 | 11.70 |
| 2 | -12 | -7 | 134.59 | 13.10 |
| -2 | 12 | 7 | 133.29 | 11.20 |
| 2 | 12 | -7 | 110.09 | 15.90 |
| -2 | 12 | 7 | 167.68 | 13.50 |
| -1 | -12 | 7 | 1690.73 | 91.99 |
| -1 | -12 | 7 | 1666.93 | 91.09 |
| 1 | -12 | -7 | 1726.33 | 92.49 |
| -1 | 12 | 7 | 1737.63 | 91.99 |
| -1 | 12 | 7 | 1728.23 | 93.49 |
| 1 | 12 | -7 | 1476.65 | 95.69 |
| 0 | -12 | 7 | -9.10 | 4.40 |
| 0 | -12 | -7 | 3.00 | 4.50 |
| 0 | -12 | 7 | 0.90 | 3.00 |
| 0 | 12 | 7 | -0.70 | 2.70 |
| 0 | 12 | 7 | 6.60 | 7.80 |
| 0 | 12 | -7 | -3.60 | 7.90 |
| 1 | -12 | 7 | 13.40 | 5.50 |
| 1 | -12 | 7 | 7.90 | 3.00 |
| -1 | -12 | -7 | 23.30 | 4.90 |
| -1 | 12 | -7 | 10.10 | 5.50 |
| 1 | 12 | 7 | 10.10 | 5.40 |
| -2 | -12 | -7 | 112.79 | 10.10 |
| 2 | -12 | 7 | 115.29 | 9.00 |
| 2 | -12 | 7 | 132.99 | 12.60 |
| 2 | 12 | 7 | 111.59 | 8.80 |
| 2 | 12 | 7 | 100.49 | 12.60 |
| -2 | 12 | -7 | 105.49 | 11.70 |
| 3 | -12 | 7 | 287.67 | 17.40 |
| 3 | -12 | 7 | 307.97 | 21.00 |
| -3 | -12 | -7 | 253.27 | 17.90 |
| 3 | 12 | 7 | 262.37 | 21.40 |
| 3 | 12 | 7 | 294.77 | 17.40 |
| -3 | 12 | -7 | 295.47 | 19.90 |
| -4 | -12 | -7 | 162.88 | 12.00 |
| 4 | -12 | 7 | 172.48 | 11.40 |
| 4 | -12 | 7 | 177.78 | 15.50 |
| 4 | 12 | 7 | 167.88 | 11.20 |
| 4 | 12 | 7 | 145.49 | 17.40 |
| -4 | 12 | -7 | 154.58 | 14.70 |

| | | | | |
|---|---|---|---|---|
| 5 | -12 | 7 | 633.64 | 34.60 |
| 5 | -12 | 7 | 623.54 | 36.10 |
| -5 | -12 | -7 | 594.04 | 34.90 |
| 5 | 12 | 7 | 654.63 | 34.70 |
| 5 | 12 | 7 | 540.35 | 40.50 |
| -6 | -12 | -7 | 121.49 | 9.70 |
| 6 | -12 | 7 | 122.89 | 12.10 |
| 6 | -12 | 7 | 128.59 | 9.50 |
| 6 | 12 | 7 | 136.09 | 9.30 |
| 6 | 12 | 7 | 108.89 | 24.70 |
| -7 | -12 | -7 | 55.49 | 6.90 |
| 7 | -12 | 7 | 51.59 | 5.70 |
| 7 | 12 | 7 | 44.50 | 5.50 |
| -8 | -12 | -7 | 9.20 | 3.40 |
| 8 | 12 | 7 | 11.50 | 2.80 |
| 9 | -12 | 7 | 175.08 | 11.20 |
| 9 | 12 | 7 | 177.38 | 11.40 |
| 10 | -12 | 7 | 5.80 | 2.40 |
| 10 | 12 | 7 | 2.20 | 2.40 |
| 11 | -12 | 7 | 67.79 | 5.40 |
| 11 | 12 | 7 | 51.89 | 5.50 |
| 12 | -13 | -7 | 273.27 | 16.60 |
| 12 | 13 | -7 | 273.57 | 15.40 |
| -12 | 13 | 7 | 267.87 | 15.80 |
| 11 | -13 | -7 | 4.90 | 3.20 |
| 11 | 13 | -7 | 1.10 | 1.50 |
| 10 | -13 | -7 | 317.37 | 19.50 |
| 10 | 13 | -7 | 329.57 | 18.70 |
| 9 | -13 | -7 | 101.19 | 11.10 |
| 9 | 13 | -7 | 103.39 | 7.70 |
| -8 | -13 | 7 | 18.10 | 4.60 |
| 8 | 13 | -7 | 16.80 | 3.20 |
| -7 | -13 | 7 | 195.38 | 14.50 |
| -7 | 13 | 7 | 215.48 | 14.40 |
| 7 | 13 | -7 | 221.68 | 20.10 |
| -6 | -13 | 7 | 1192.28 | 68.79 |
| 6 | 13 | -7 | 1278.17 | 71.69 |
| -6 | 13 | 7 | 1269.07 | 69.49 |
| -5 | -13 | 7 | 177.08 | 13.50 |
| 5 | -13 | -7 | 204.68 | 14.30 |
| -5 | 13 | 7 | 186.28 | 13.70 |
| 5 | 13 | -7 | 156.88 | 17.80 |
| -4 | -13 | 7 | 69.29 | 8.70 |
| 4 | -13 | -7 | 68.99 | 9.50 |

| | | | | |
|---|---|---|---|---|
| -4 | 13 | 7 | 54.69 | 12.60 |
| 4 | 13 | -7 | 58.19 | 13.80 |
| -4 | 13 | 7 | 64.29 | 8.30 |
| -3 | -13 | 7 | 195.88 | 12.80 |
| 3 | -13 | -7 | 213.48 | 15.00 |
| -3 | -13 | 7 | 191.68 | 14.20 |
| -3 | 13 | 7 | 206.68 | 16.80 |
| 3 | 13 | -7 | 169.68 | 18.60 |
| -3 | 13 | 7 | 179.98 | 13.90 |
| -2 | -13 | 7 | 1148.19 | 62.79 |
| 2 | -13 | -7 | 1110.09 | 63.89 |
| -2 | -13 | 7 | 1183.28 | 63.69 |
| 2 | 13 | -7 | 1017.10 | 66.39 |
| -2 | 13 | 7 | 1170.38 | 63.89 |
| -2 | 13 | 7 | 1222.28 | 65.59 |
| -1 | -13 | 7 | -4.90 | 2.80 |
| 1 | -13 | -7 | 0.40 | 4.40 |
| -1 | -13 | 7 | -1.50 | 4.20 |
| -1 | 13 | 7 | 2.90 | 5.60 |
| 1 | 13 | -7 | 0.30 | 6.00 |
| -1 | 13 | 7 | 4.40 | 3.60 |
| 0 | -13 | 7 | 795.12 | 45.10 |
| 0 | -13 | -7 | 795.32 | 45.20 |
| 0 | -13 | 7 | 768.12 | 43.90 |
| 0 | 13 | -7 | 800.82 | 47.20 |
| 0 | 13 | 7 | 773.32 | 46.60 |
| 0 | 13 | 7 | 792.32 | 44.50 |
| 1 | -13 | 7 | 462.35 | 28.00 |
| 1 | -13 | 7 | 445.56 | 26.20 |
| -1 | -13 | -7 | 471.15 | 27.40 |
| -1 | 13 | -7 | 467.85 | 29.40 |
| 1 | 13 | 7 | 422.16 | 29.40 |
| 1 | 13 | 7 | 451.75 | 26.60 |
| 2 | -13 | 7 | 310.17 | 17.90 |
| 2 | -13 | 7 | 302.27 | 20.60 |
| -2 | -13 | -7 | 292.07 | 18.80 |
| 2 | 13 | 7 | 288.97 | 18.00 |
| -2 | 13 | -7 | 285.97 | 21.10 |
| 2 | 13 | 7 | 286.07 | 21.80 |
| 3 | -13 | 7 | 50.29 | 10.80 |
| 3 | -13 | 7 | 23.80 | 3.90 |
| -3 | -13 | -7 | 18.80 | 3.90 |
| -3 | 13 | -7 | 27.00 | 6.70 |
| 3 | 13 | 7 | 25.70 | 8.50 |

| | | | | |
|---|---|---|---|---|
| 3 | 13 | 7 | 22.80 | 5.10 |
| 4 | -13 | 7 | 597.04 | 33.00 |
| -4 | -13 | -7 | 581.34 | 33.40 |
| 4 | -13 | 7 | 609.04 | 34.90 |
| 4 | 13 | 7 | 524.45 | 38.10 |
| -4 | 13 | -7 | 604.04 | 36.40 |
| -5 | -13 | -7 | 34.60 | 5.80 |
| 5 | -13 | 7 | 27.70 | 5.80 |
| 5 | -13 | 7 | 46.10 | 5.60 |
| 5 | 13 | 7 | 27.30 | 14.10 |
| 5 | 13 | 7 | 37.70 | 5.00 |
| 6 | -13 | 7 | 130.59 | 9.60 |
| -6 | -13 | -7 | 154.78 | 10.00 |
| 6 | 13 | 7 | 124.09 | 9.40 |
| -7 | -13 | -7 | 120.79 | 9.60 |
| 7 | 13 | 7 | 125.89 | 8.60 |
| 8 | -13 | 7 | 255.87 | 14.90 |
| -8 | -13 | -7 | 253.47 | 15.30 |
| 8 | 13 | 7 | 242.28 | 15.10 |
| 9 | -13 | 7 | 10.50 | 2.40 |
| 9 | 13 | 7 | 9.90 | 2.70 |
| 10 | -13 | 7 | 353.36 | 19.50 |
| 10 | 13 | 7 | 333.67 | 19.80 |
| 12 | 14 | -7 | 80.89 | 5.10 |
| -12 | 14 | 7 | 77.09 | 5.90 |
| -11 | 14 | 7 | 305.37 | 18.00 |
| 11 | 14 | -7 | 314.27 | 17.40 |
| 10 | 14 | -7 | 37.30 | 3.80 |
| -10 | 14 | 7 | 33.30 | 4.50 |
| 9 | 14 | -7 | 203.18 | 12.20 |
| -9 | 14 | 7 | 193.88 | 12.60 |
| -8 | -14 | 7 | 307.57 | 20.60 |
| -8 | 14 | 7 | 358.06 | 20.20 |
| 8 | 14 | -7 | 338.97 | 19.60 |
| -7 | -14 | 7 | 308.07 | 19.40 |
| 7 | 14 | -7 | 300.87 | 18.80 |
| -7 | 14 | 7 | 336.77 | 19.50 |
| -6 | -14 | 7 | -3.70 | 3.50 |
| -6 | 14 | 7 | 12.50 | 3.70 |
| -5 | -14 | 7 | 565.44 | 34.00 |
| 5 | -14 | -7 | 589.94 | 33.90 |
| -5 | 14 | 7 | 622.74 | 34.90 |
| 5 | 14 | -7 | 587.94 | 38.50 |
| 4 | -14 | -7 | 420.26 | 23.60 |

| | | | | |
|---|---|---|---|---|
| -4 | -14 | 7 | 374.36 | 23.40 |
| -4 | 14 | 7 | 371.06 | 27.80 |
| 4 | 14 | -7 | 372.26 | 28.30 |
| -3 | -14 | 7 | 12.40 | 4.20 |
| 3 | -14 | -7 | 11.50 | 4.60 |
| -3 | 14 | 7 | 21.40 | 4.60 |
| 3 | 14 | -7 | 18.30 | 9.30 |
| -3 | 14 | 7 | 18.40 | 8.20 |
| 2 | -14 | -7 | 154.28 | 12.40 |
| -2 | -14 | 7 | 150.38 | 11.80 |
| -2 | -14 | 7 | 149.39 | 10.30 |
| 2 | 14 | -7 | 122.69 | 17.10 |
| -2 | 14 | 7 | 150.48 | 11.50 |
| -2 | 14 | 7 | 130.99 | 15.40 |
| -1 | -14 | 7 | 585.74 | 34.50 |
| -1 | -14 | 7 | 595.54 | 33.50 |
| 1 | -14 | -7 | 615.44 | 35.00 |
| 1 | 14 | -7 | 528.25 | 37.80 |
| -1 | 14 | 7 | 603.24 | 36.90 |
| -1 | 14 | 7 | 630.24 | 34.50 |
| 0 | -14 | 7 | 141.29 | 9.70 |
| 0 | -14 | 7 | 128.99 | 11.60 |
| 0 | -14 | -7 | 134.59 | 11.50 |
| 0 | 14 | 7 | 140.69 | 10.40 |
| 0 | 14 | -7 | 120.09 | 15.40 |
| 0 | 14 | 7 | 131.09 | 15.10 |
| -1 | -14 | -7 | 168.38 | 12.50 |
| 1 | -14 | 7 | 159.38 | 13.50 |
| 1 | -14 | 7 | 164.88 | 11.00 |
| -1 | 14 | -7 | 178.38 | 16.00 |
| 1 | 14 | 7 | 162.38 | 11.80 |
| 1 | 14 | 7 | 145.59 | 16.10 |
| 2 | -14 | 7 | 191.08 | 12.10 |
| 2 | -14 | 7 | 195.58 | 15.10 |
| -2 | -14 | -7 | 179.78 | 13.00 |
| 2 | 14 | 7 | 176.88 | 17.70 |
| -2 | 14 | -7 | 192.18 | 16.70 |
| 2 | 14 | 7 | 170.78 | 12.50 |
| 3 | -14 | 7 | 249.28 | 16.80 |
| 3 | -14 | 7 | 217.98 | 13.80 |
| -3 | -14 | -7 | 220.18 | 14.40 |
| 3 | 14 | 7 | 203.18 | 13.90 |
| -3 | 14 | -7 | 211.58 | 19.30 |
| 4 | -14 | 7 | 15.00 | 3.00 |

| | | | | |
|---|---|---|---|---|
| -4 | -14 | -7 | 12.20 | 3.10 |
| 4 | -14 | 7 | 8.50 | 5.00 |
| 4 | 14 | 7 | 12.70 | 3.00 |
| 5 | -14 | 7 | 718.13 | 41.20 |
| 5 | -14 | 7 | 700.43 | 39.90 |
| -5 | -14 | -7 | 702.73 | 40.20 |
| 5 | 14 | 7 | 753.42 | 40.10 |
| -6 | -14 | -7 | 147.89 | 10.60 |
| 6 | 14 | 7 | 146.29 | 9.70 |
| -7 | -14 | -7 | 11.30 | 2.70 |
| 7 | -14 | 7 | 3.80 | 2.40 |
| 7 | 14 | 7 | 8.30 | 2.30 |
| -8 | -14 | -7 | 14.30 | 3.00 |
| 8 | -14 | 7 | 22.80 | 4.00 |
| 8 | 14 | 7 | 12.80 | 2.70 |
| 9 | -14 | 7 | 181.08 | 10.50 |
| 9 | 14 | 7 | 162.58 | 10.70 |
| -11 | 15 | 7 | 0.30 | 1.90 |
| 11 | 15 | -7 | -1.00 | 1.20 |
| -10 | 15 | 7 | 298.77 | 17.40 |
| 10 | 15 | -7 | 295.87 | 16.80 |
| -9 | 15 | 7 | 25.50 | 4.10 |
| 9 | 15 | -7 | 26.60 | 3.40 |
| -8 | -15 | 7 | 76.99 | 8.00 |
| 8 | 15 | -7 | 88.09 | 6.50 |
| -8 | 15 | 7 | 81.99 | 7.00 |
| -7 | -15 | 7 | 114.89 | 9.90 |
| -7 | 15 | 7 | 123.09 | 9.20 |
| -6 | -15 | 7 | 681.33 | 41.70 |
| -6 | 15 | 7 | 805.52 | 42.30 |
| -5 | -15 | 7 | 124.39 | 10.50 |
| -5 | 15 | 7 | 132.59 | 10.40 |
| -4 | -15 | 7 | 592.44 | 33.20 |
| 4 | -15 | -7 | 599.74 | 33.10 |
| -4 | 15 | 7 | 529.95 | 33.50 |
| 3 | -15 | -7 | 131.29 | 10.30 |
| -3 | -15 | 7 | 121.09 | 10.10 |
| -3 | 15 | 7 | 110.49 | 10.10 |
| -2 | -15 | 7 | 237.68 | 15.50 |
| 2 | -15 | -7 | 226.48 | 15.60 |
| -2 | -15 | 7 | 226.48 | 14.30 |
| -2 | 15 | 7 | 227.38 | 15.40 |
| -2 | 15 | 7 | 238.38 | 24.90 |
| 1 | -15 | -7 | 3.60 | 3.80 |

| | | | | |
|---|---|---|---|---|
| -1 | -15 | 7 | 6.60 | 2.60 |
| -1 | -15 | 7 | 2.80 | 3.90 |
| -1 | 15 | 7 | 4.10 | 9.00 |
| 1 | 15 | -7 | 3.70 | 8.30 |
| -1 | 15 | 7 | 2.90 | 3.00 |
| 0 | -15 | -7 | 980.60 | 57.09 |
| 0 | -15 | 7 | 1012.90 | 56.19 |
| 0 | -15 | 7 | 1087.49 | 57.39 |
| 0 | 15 | -7 | 1025.90 | 63.39 |
| 0 | 15 | 7 | 951.10 | 65.79 |
| 0 | 15 | 7 | 1056.29 | 56.79 |
| 1 | -15 | 7 | 163.38 | 11.30 |
| -1 | -15 | -7 | 189.18 | 12.80 |
| 1 | -15 | 7 | 183.58 | 13.80 |
| 1 | 15 | 7 | 165.18 | 11.70 |
| 2 | -15 | 7 | 3.90 | 4.40 |
| -2 | -15 | -7 | 6.90 | 3.20 |
| 2 | -15 | 7 | -0.70 | 2.30 |
| 2 | 15 | 7 | -2.90 | 2.50 |
| 3 | -15 | 7 | 11.20 | 2.80 |
| -3 | -15 | -7 | 9.50 | 3.00 |
| 3 | -15 | 7 | 16.20 | 5.10 |
| 3 | 15 | 7 | 11.70 | 2.90 |
| -4 | -15 | -7 | 492.35 | 28.40 |
| 4 | -15 | 7 | 532.15 | 28.20 |
| 4 | -15 | 7 | 491.65 | 30.00 |
| 4 | 15 | 7 | 472.25 | 28.20 |
| 5 | -15 | 7 | -1.00 | 3.80 |
| -5 | -15 | -7 | 2.20 | 2.40 |
| 5 | -15 | 7 | 0.20 | 2.20 |
| 5 | 15 | 7 | 1.70 | 2.00 |
| 6 | -15 | 7 | 191.78 | 11.70 |
| -6 | -15 | -7 | 194.48 | 12.00 |
| 6 | 15 | 7 | 188.58 | 11.90 |
| 7 | -15 | 7 | 62.29 | 5.10 |
| -7 | -15 | -7 | 58.09 | 5.60 |
| 7 | 15 | 7 | 59.99 | 5.30 |
| 8 | -15 | 7 | 96.69 | 6.50 |
| 8 | 15 | 7 | 91.89 | 6.80 |
| -10 | 16 | 7 | 53.79 | 4.80 |
| 10 | 16 | -7 | 62.39 | 4.10 |
| -9 | 16 | 7 | 159.08 | 10.50 |
| 9 | 16 | -7 | 180.88 | 10.10 |
| -8 | -16 | 7 | 92.99 | 7.70 |

| | | | | |
|---|---|---|---|---|
| -8 | 16 | 7 | 88.59 | 7.10 |
| 8 | 16 | -7 | 104.59 | 6.70 |
| -7 | -16 | 7 | 128.69 | 9.90 |
| 7 | 16 | -7 | 137.59 | 9.10 |
| -7 | 16 | 7 | 142.99 | 9.70 |
| -6 | -16 | 7 | -2.40 | 3.10 |
| -6 | 16 | 7 | 2.50 | 2.80 |
| -5 | -16 | 7 | 560.84 | 34.30 |
| 5 | -16 | -7 | 617.94 | 34.00 |
| -5 | 16 | 7 | 631.14 | 34.80 |
| -4 | -16 | 7 | 164.48 | 12.70 |
| 4 | -16 | -7 | 182.88 | 12.10 |
| -4 | 16 | 7 | 172.48 | 12.40 |
| -3 | -16 | 7 | 19.00 | 4.00 |
| 3 | -16 | -7 | 18.80 | 4.10 |
| -3 | 16 | 7 | 12.20 | 3.70 |
| 2 | -16 | -7 | 123.99 | 9.80 |
| -2 | -16 | 7 | 108.99 | 9.70 |
| -2 | 16 | 7 | 107.89 | 9.30 |
| 1 | -16 | -7 | 773.72 | 42.50 |
| -1 | -16 | 7 | 739.03 | 41.60 |
| -1 | 16 | 7 | 738.33 | 42.40 |
| 0 | -16 | 7 | 117.59 | 8.20 |
| 0 | -16 | 7 | 105.59 | 10.10 |
| 0 | -16 | -7 | 105.39 | 9.60 |
| 0 | 16 | 7 | 115.19 | 9.00 |
| 1 | -16 | 7 | 334.67 | 21.20 |
| 1 | -16 | 7 | 329.27 | 19.60 |
| -1 | -16 | -7 | 340.67 | 20.50 |
| 1 | 16 | 7 | 342.87 | 20.10 |
| -2 | -16 | -7 | 85.09 | 9.00 |
| 2 | -16 | 7 | 98.09 | 10.60 |
| 2 | -16 | 7 | 117.59 | 13.30 |
| 2 | 16 | 7 | 93.39 | 7.40 |
| -3 | -16 | -7 | 114.79 | 9.10 |
| 3 | -16 | 7 | 125.89 | 8.50 |
| 3 | -16 | 7 | 131.29 | 10.50 |
| 3 | 16 | 7 | 115.79 | 8.40 |
| 4 | -16 | 7 | 5.60 | 2.20 |
| -4 | -16 | -7 | 3.20 | 2.40 |
| 4 | -16 | 7 | 0.80 | 3.50 |
| 4 | 16 | 7 | 4.60 | 2.10 |
| -5 | -16 | -7 | 498.85 | 27.30 |
| 5 | -16 | 7 | 475.15 | 27.60 |

| | | | | |
|---|---|---|---|---|
| 5 | -16 | 7 | 486.55 | 27.10 |
| 5 | 16 | 7 | 480.35 | 27.30 |
| 6 | -16 | 7 | 198.48 | 11.80 |
| -6 | -16 | -7 | 195.38 | 12.00 |
| 6 | 16 | 7 | 198.78 | 12.00 |
| 7 | -16 | 7 | 0.90 | 1.70 |
| -7 | -16 | -7 | 3.60 | 2.10 |
| 7 | 16 | 7 | -1.70 | 1.90 |
| -8 | -17 | 7 | 2.40 | 2.60 |
| 8 | 17 | -7 | 2.50 | 1.40 |
| -8 | 17 | 7 | -0.20 | 2.00 |
| -7 | -17 | 7 | 169.78 | 11.00 |
| 7 | 17 | -7 | 180.38 | 10.60 |
| -7 | 17 | 7 | 170.18 | 11.30 |
| -6 | -17 | 7 | 360.66 | 22.10 |
| -6 | 17 | 7 | 399.96 | 22.40 |
| 6 | 17 | -7 | 388.86 | 21.80 |
| -5 | -17 | 7 | 79.09 | 7.40 |
| 5 | -17 | -7 | 92.79 | 8.10 |
| -5 | 17 | 7 | 97.99 | 7.70 |
| -4 | -17 | 7 | 149.79 | 10.60 |
| 4 | -17 | -7 | 143.09 | 10.20 |
| -4 | 17 | 7 | 156.18 | 10.90 |
| -3 | -17 | 7 | 88.79 | 7.60 |
| 3 | -17 | -7 | 81.59 | 7.30 |
| -3 | 17 | 7 | 72.39 | 7.70 |
| -2 | -17 | 7 | 117.59 | 9.20 |
| 2 | -17 | -7 | 127.39 | 9.40 |
| -2 | 17 | 7 | 110.79 | 9.30 |
| -1 | -17 | 7 | 11.80 | 3.50 |
| 1 | -17 | -7 | 11.60 | 3.30 |
| -1 | 17 | 7 | 12.50 | 3.50 |
| 0 | -17 | -7 | 500.65 | 28.90 |
| 0 | -17 | 7 | 513.65 | 29.20 |
| 0 | -17 | 7 | 498.15 | 28.20 |
| 0 | 17 | 7 | 497.65 | 28.70 |
| 1 | -17 | 7 | 249.08 | 15.50 |
| -1 | -17 | -7 | 218.48 | 14.70 |
| 1 | -17 | 7 | 228.38 | 13.80 |
| 1 | 17 | 7 | 228.58 | 14.20 |
| 2 | -17 | 7 | -7.10 | 3.20 |
| 2 | -17 | 7 | -0.90 | 1.80 |
| -2 | -17 | -7 | 4.30 | 2.60 |
| 2 | 17 | 7 | 1.10 | 2.20 |

| | | | | |
|---|---|---|---|---|
| -3 | -17 | -7 | 72.79 | 6.20 |
| 3 | -17 | 7 | 65.49 | 5.30 |
| 3 | -17 | 7 | 74.19 | 7.70 |
| 3 | 17 | 7 | 68.39 | 5.70 |
| -4 | -17 | -7 | 307.47 | 18.00 |
| 4 | -17 | 7 | 305.17 | 17.70 |
| 4 | -17 | 7 | 307.77 | 18.40 |
| 4 | 17 | 7 | 322.27 | 18.00 |
| -5 | -17 | -7 | 1.40 | 2.00 |
| 5 | -17 | 7 | 3.90 | 1.60 |
| 5 | 17 | 7 | 5.50 | 2.10 |
| -6 | -17 | -7 | 104.99 | 7.10 |
| 6 | -17 | 7 | 111.59 | 6.90 |
| 6 | 17 | 7 | 104.69 | 7.10 |
| -7 | -18 | 7 | 149.99 | 9.80 |
| -7 | 18 | 7 | 173.38 | 10.10 |
| -6 | -18 | 7 | 9.10 | 2.50 |
| 6 | 18 | -7 | 9.90 | 1.90 |
| -5 | -18 | 7 | 244.28 | 15.10 |
| 5 | 18 | -7 | 257.67 | 14.90 |
| -5 | 18 | 7 | 263.77 | 15.60 |
| 4 | -18 | -7 | 160.78 | 10.10 |
| -4 | -18 | 7 | 150.58 | 10.10 |
| -4 | 18 | 7 | 152.38 | 10.60 |
| 3 | -18 | -7 | 3.50 | 2.50 |
| -3 | -18 | 7 | 2.90 | 2.20 |
| -3 | 18 | 7 | 4.40 | 2.90 |
| 2 | -18 | -7 | 224.78 | 12.60 |
| -2 | -18 | 7 | 185.38 | 12.50 |
| -2 | 18 | 7 | 189.98 | 12.90 |
| -1 | -18 | 7 | 332.07 | 19.40 |
| 1 | -18 | -7 | 316.07 | 19.20 |
| -1 | 18 | 7 | 334.07 | 19.40 |
| 0 | -18 | -7 | 77.99 | 6.70 |
| 0 | -18 | 7 | 73.69 | 6.80 |
| 0 | 18 | 7 | 69.69 | 6.40 |
| -1 | -18 | -7 | 64.89 | 6.10 |
| 1 | -18 | 7 | 64.39 | 5.20 |
| 1 | -18 | 7 | 77.99 | 6.80 |
| 1 | 18 | 7 | 67.19 | 5.80 |
| 2 | -18 | 7 | 177.08 | 11.20 |
| 2 | -18 | 7 | 154.18 | 10.10 |
| -2 | -18 | -7 | 168.28 | 10.70 |
| 2 | 18 | 7 | 173.78 | 11.20 |

| | | | | |
|---|---|---|---|---|
| -3 | -18 | -7 | 86.89 | 6.50 |
| 3 | -18 | 7 | 87.69 | 6.00 |
| 3 | -18 | 7 | 90.59 | 7.30 |
| 3 | 18 | 7 | 88.09 | 6.40 |
| 4 | -18 | 7 | 17.90 | 2.80 |
| -4 | -18 | -7 | 16.30 | 3.20 |
| 4 | -18 | 7 | 8.80 | 3.00 |
| 4 | 18 | 7 | 17.80 | 3.30 |
| -4 | -19 | 7 | 49.20 | 4.50 |
| 4 | -19 | -7 | 49.20 | 5.60 |
| 4 | 19 | -7 | 51.99 | 4.30 |
| -4 | 19 | 7 | 49.10 | 5.40 |
| -3 | -19 | 7 | 157.48 | 10.00 |
| -3 | 19 | 7 | 165.78 | 10.60 |
| 2 | -19 | -7 | 96.69 | 6.60 |
| -2 | -19 | 7 | 91.79 | 6.70 |
| -2 | 19 | 7 | 81.39 | 7.10 |
| -1 | -19 | 7 | 49.00 | 4.80 |
| 1 | -19 | -7 | 48.80 | 4.90 |
| -1 | 19 | 7 | 45.30 | 5.30 |
| 0 | -19 | 7 | 231.48 | 13.40 |
| 0 | -19 | -7 | 214.68 | 13.30 |
| 0 | 19 | 7 | 220.28 | 13.50 |
| -1 | -19 | -7 | 146.59 | 9.60 |
| 1 | -19 | 7 | 162.98 | 9.80 |
| 1 | 19 | 7 | 142.59 | 9.60 |
| 16 | 0 | -8 | 438.56 | 24.50 |
| 15 | 0 | -8 | -2.30 | 2.10 |
| 14 | 0 | -8 | 381.76 | 22.10 |
| 12 | 0 | -8 | 1410.46 | 77.09 |
| 11 | 0 | -8 | -4.50 | 3.20 |
| 10 | 0 | -8 | 1300.97 | 71.19 |
| 9 | 0 | -8 | 1.60 | 4.00 |
| 8 | 0 | -8 | 47.50 | 9.20 |
| -6 | 0 | 8 | 6098.99 | 328.07 |
| -5 | 0 | 8 | 3.30 | 2.90 |
| -4 | 0 | 8 | 1988.30 | 108.39 |
| -3 | 0 | 8 | -0.10 | 2.80 |
| -2 | 0 | 8 | 1862.81 | 102.09 |
| -1 | 0 | 8 | 2.50 | 3.80 |
| 0 | 0 | 8 | 8263.27 | 445.16 |
| 1 | 0 | 8 | 1.40 | 4.10 |
| 2 | 0 | 8 | 75.79 | 9.80 |
| 3 | 0 | 8 | -2.10 | 4.70 |

| | | | | |
|---|---|---|---|---|
| 4 | 0 | 8 | 5305.27 | 294.87 |
| 4 | 0 | 8 | 5583.04 | 293.57 |
| 5 | 0 | 8 | -7.80 | 6.20 |
| 5 | 0 | 8 | -8.60 | 4.60 |
| 6 | 0 | 8 | 1038.20 | 63.09 |
| 6 | 0 | 8 | 1017.50 | 62.39 |
| 6 | 0 | 8 | 1245.68 | 62.49 |
| 6 | 0 | 8 | 1197.88 | 63.69 |
| 7 | 0 | 8 | -0.10 | 5.40 |
| 7 | 0 | 8 | -1.80 | 3.90 |
| 7 | 0 | 8 | -1.80 | 4.90 |
| 8 | 0 | 8 | 779.42 | 41.60 |
| 8 | 0 | 8 | 682.13 | 42.40 |
| 9 | 0 | 8 | -0.70 | 3.70 |
| 9 | 0 | 8 | 0.60 | 4.60 |
| 10 | 0 | 8 | 1116.19 | 63.19 |
| 10 | 0 | 8 | 1110.49 | 61.69 |
| 11 | 0 | 8 | -3.30 | 3.20 |
| 11 | 0 | 8 | -1.00 | 3.90 |
| 12 | 0 | 8 | 7.90 | 4.60 |
| 12 | 0 | 8 | 4.90 | 3.60 |
| 13 | 0 | 8 | 1.30 | 2.80 |
| 13 | 0 | 8 | -3.60 | 2.70 |
| 14 | 0 | 8 | 237.68 | 15.20 |
| 14 | 0 | 8 | 274.47 | 15.40 |
| -16 | -1 | 8 | 1.00 | 1.90 |
| 16 | -1 | -8 | 0.10 | 1.80 |
| 16 | 1 | -8 | -2.20 | 1.70 |
| 15 | -1 | -8 | 455.25 | 25.90 |
| 15 | 1 | -8 | 460.05 | 25.80 |
| 14 | -1 | -8 | 26.30 | 5.00 |
| 14 | 1 | -8 | 30.20 | 4.80 |
| 13 | -1 | -8 | 6.20 | 2.70 |
| 13 | 1 | -8 | 4.00 | 2.70 |
| 12 | -1 | -8 | 1.90 | 3.20 |
| 12 | 1 | -8 | 2.90 | 2.90 |
| 11 | -1 | -8 | 1915.31 | 105.79 |
| 11 | 1 | -8 | 1978.60 | 105.79 |
| 10 | -1 | -8 | -2.20 | 3.30 |
| 10 | 1 | -8 | -2.60 | 3.50 |
| 9 | -1 | -8 | 496.35 | 29.30 |
| 9 | 1 | -8 | 518.15 | 29.20 |
| 8 | 1 | -8 | 89.79 | 9.90 |
| -7 | 1 | 8 | 2486.95 | 135.19 |

| | | | | |
|---|---|---|---|---|
| -6 | -1 | 8 | 182.58 | 12.90 |
| -6 | 1 | 8 | 201.18 | 12.80 |
| -5 | -1 | 8 | 4543.85 | 245.48 |
| -5 | 1 | 8 | 4563.54 | 245.58 |
| -4 | -1 | 8 | 95.89 | 7.90 |
| -4 | 1 | 8 | 89.59 | 8.40 |
| -3 | -1 | 8 | 80.19 | 7.40 |
| -3 | 1 | 8 | 79.19 | 8.00 |
| -2 | -1 | 8 | 463.45 | 26.10 |
| -2 | 1 | 8 | 413.96 | 26.20 |
| -1 | -1 | 8 | 8368.16 | 427.26 |
| -1 | 1 | 8 | 7500.45 | 427.16 |
| 0 | -1 | 8 | -4.00 | 3.50 |
| 0 | 1 | 8 | -6.40 | 3.90 |
| 1 | -1 | 8 | 819.42 | 46.40 |
| 1 | 1 | 8 | 749.03 | 49.70 |
| 2 | -1 | 8 | 34.90 | 7.70 |
| 2 | 1 | 8 | 42.80 | 8.50 |
| 3 | -1 | 8 | 878.11 | 50.49 |
| 3 | 1 | 8 | 951.40 | 51.29 |
| 3 | 1 | 8 | 801.22 | 49.10 |
| 4 | -1 | 8 | 12.70 | 7.50 |
| 4 | -1 | 8 | 1.70 | 4.90 |
| 4 | 1 | 8 | 5.40 | 5.60 |
| 4 | 1 | 8 | 17.50 | 5.50 |
| 5 | -1 | 8 | 3537.65 | 205.18 |
| 5 | -1 | 8 | 3866.61 | 206.58 |
| 5 | -1 | 8 | 3774.52 | 206.18 |
| 5 | 1 | 8 | 3780.52 | 207.28 |
| 5 | 1 | 8 | 4054.89 | 205.88 |
| 6 | -1 | 8 | 0.00 | 4.50 |
| 6 | -1 | 8 | 0.50 | 5.20 |
| 6 | -1 | 8 | -0.80 | 4.60 |
| 6 | 1 | 8 | -3.60 | 4.80 |
| 6 | 1 | 8 | 4.70 | 4.10 |
| 6 | 1 | 8 | -1.20 | 5.90 |
| 6 | 1 | 8 | 1.50 | 5.30 |
| 7 | -1 | 8 | 170.98 | 14.10 |
| 7 | -1 | 8 | 167.68 | 12.30 |
| 7 | -1 | 8 | 167.98 | 14.00 |
| 7 | 1 | 8 | 167.48 | 15.00 |
| 7 | 1 | 8 | 144.39 | 13.50 |
| 7 | 1 | 8 | 158.18 | 11.90 |
| 8 | -1 | 8 | 4.10 | 3.90 |

| | | | | |
|---|---|---|---|---|
| 8 | -1 | 8 | -7.70 | 4.50 |
| 8 | 1 | 8 | 7.00 | 5.20 |
| 8 | 1 | 8 | -1.80 | 3.70 |
| 9 | -1 | 8 | 1257.37 | 71.59 |
| 9 | -1 | 8 | 1362.96 | 72.59 |
| 9 | 1 | 8 | 1222.08 | 72.39 |
| 9 | 1 | 8 | 1353.36 | 71.69 |
| 10 | -1 | 8 | -3.10 | 3.70 |
| 10 | -1 | 8 | -0.20 | 5.40 |
| 10 | 1 | 8 | -2.00 | 5.50 |
| 10 | 1 | 8 | -0.40 | 3.40 |
| 11 | -1 | 8 | 483.45 | 29.00 |
| 11 | -1 | 8 | 514.85 | 28.80 |
| 11 | 1 | 8 | 520.15 | 28.60 |
| 11 | 1 | 8 | 444.96 | 28.90 |
| 12 | -1 | 8 | 3.90 | 3.70 |
| 12 | -1 | 8 | 3.80 | 3.50 |
| 12 | 1 | 8 | 4.90 | 3.20 |
| 12 | 1 | 8 | 0.30 | 3.60 |
| 13 | -1 | 8 | 217.28 | 14.50 |
| 13 | -1 | 8 | 241.88 | 14.60 |
| 13 | 1 | 8 | 232.18 | 14.50 |
| 13 | 1 | 8 | 230.08 | 14.30 |
| 14 | -1 | 8 | 5.80 | 2.50 |
| 14 | -1 | 8 | 3.50 | 3.00 |
| 14 | 1 | 8 | 1.50 | 2.40 |
| 14 | 1 | 8 | 0.80 | 2.50 |
| -16 | -2 | 8 | 389.96 | 21.70 |
| 16 | -2 | -8 | 378.16 | 21.60 |
| -16 | 2 | 8 | 389.06 | 21.80 |
| 16 | 2 | -8 | 378.76 | 21.50 |
| 15 | -2 | -8 | 5.00 | 2.50 |
| 15 | 2 | -8 | 6.00 | 2.10 |
| 14 | -2 | -8 | 359.26 | 21.20 |
| 14 | 2 | -8 | 366.86 | 21.00 |
| 13 | -2 | -8 | 15.00 | 3.40 |
| 13 | 2 | -8 | 10.90 | 3.10 |
| 12 | -2 | -8 | 429.86 | 24.90 |
| 12 | 2 | -8 | 423.26 | 24.60 |
| 11 | -2 | -8 | 16.80 | 3.80 |
| 11 | 2 | -8 | 14.90 | 3.40 |
| 10 | -2 | -8 | 1792.12 | 98.99 |
| 10 | 2 | -8 | 1846.12 | 98.89 |
| 9 | -2 | -8 | 76.29 | 9.50 |

| | | | | |
|---|---|---|---|---|
| 9 | 2 | -8 | 80.39 | 8.40 |
| 8 | -2 | -8 | 199.48 | 19.50 |
| 8 | 2 | -8 | 151.18 | 12.40 |
| -6 | 2 | 8 | 5921.81 | 318.67 |
| -5 | -2 | 8 | 157.88 | 10.90 |
| -5 | 2 | 8 | 145.69 | 11.50 |
| -4 | -2 | 8 | 4178.48 | 224.18 |
| -4 | 2 | 8 | 4124.99 | 224.48 |
| -3 | -2 | 8 | 354.96 | 20.80 |
| -3 | 2 | 8 | 327.37 | 21.20 |
| -2 | -2 | 8 | 1671.93 | 88.49 |
| -2 | 2 | 8 | 1541.95 | 88.59 |
| -1 | -2 | 8 | 157.28 | 11.70 |
| -1 | 2 | 8 | 133.99 | 11.80 |
| 0 | -2 | 8 | 9202.08 | 467.75 |
| 0 | 2 | 8 | 8169.88 | 467.65 |
| 1 | -2 | 8 | 387.06 | 25.20 |
| 1 | 2 | 8 | 398.56 | 24.90 |
| 2 | -2 | 8 | 13.70 | 4.50 |
| 2 | 2 | 8 | 33.10 | 7.80 |
| 3 | -2 | 8 | 1.30 | 3.50 |
| 3 | 2 | 8 | -2.20 | 4.80 |
| 3 | 2 | 8 | 15.10 | 5.00 |
| 4 | -2 | 8 | 1580.14 | 89.89 |
| 4 | -2 | 8 | 1644.24 | 90.19 |
| 4 | 2 | 8 | 1703.53 | 90.59 |
| 4 | 2 | 8 | 1417.56 | 91.09 |
| 4 | 2 | 8 | 1789.02 | 89.19 |
| 5 | -2 | 8 | 20.80 | 5.80 |
| 5 | -2 | 8 | 6.20 | 4.40 |
| 5 | -2 | 8 | 6.30 | 9.70 |
| 5 | 2 | 8 | 17.30 | 4.30 |
| 5 | 2 | 8 | 11.90 | 4.50 |
| 5 | 2 | 8 | 3.60 | 6.30 |
| 6 | -2 | 8 | 1071.49 | 64.49 |
| 6 | -2 | 8 | 1146.59 | 64.29 |
| 6 | -2 | 8 | 1319.77 | 63.99 |
| 6 | 2 | 8 | 1060.59 | 63.69 |
| 6 | 2 | 8 | 1141.79 | 63.89 |
| 6 | 2 | 8 | 1092.09 | 65.69 |
| 6 | 2 | 8 | 1205.18 | 63.69 |
| 7 | -2 | 8 | 77.79 | 10.60 |
| 7 | -2 | 8 | 63.99 | 8.80 |
| 7 | -2 | 8 | 75.89 | 10.80 |

| | | | | |
|---|---|---|---|---|
| 7 | 2 | 8 | 78.19 | 12.20 |
| 7 | 2 | 8 | 67.09 | 9.10 |
| 7 | 2 | 8 | 73.59 | 8.10 |
| 8 | -2 | 8 | 649.74 | 35.90 |
| 8 | -2 | 8 | 638.44 | 36.80 |
| 8 | 2 | 8 | 558.14 | 36.30 |
| 8 | 2 | 8 | 646.54 | 35.70 |
| 9 | -2 | 8 | -1.20 | 4.10 |
| 9 | -2 | 8 | -2.70 | 4.30 |
| 9 | 2 | 8 | -3.50 | 3.40 |
| 9 | 2 | 8 | -3.00 | 4.10 |
| 10 | -2 | 8 | 1365.86 | 68.39 |
| 10 | 2 | 8 | 1073.99 | 71.99 |
| 10 | 2 | 8 | 1242.68 | 68.09 |
| 11 | -2 | 8 | 11.90 | 4.10 |
| 11 | -2 | 8 | 16.60 | 4.30 |
| 11 | 2 | 8 | 10.10 | 3.70 |
| 11 | 2 | 8 | 12.20 | 3.80 |
| 12 | -2 | 8 | 12.30 | 3.90 |
| 12 | -2 | 8 | 9.70 | 4.00 |
| 12 | 2 | 8 | 12.00 | 3.50 |
| 13 | -2 | 8 | 1.30 | 2.90 |
| 13 | -2 | 8 | 0.30 | 3.30 |
| 13 | 2 | 8 | 3.40 | 2.60 |
| 13 | 2 | 8 | 1.30 | 2.40 |
| 14 | -2 | 8 | 277.97 | 16.80 |
| 14 | -2 | 8 | 288.57 | 16.90 |
| 14 | 2 | 8 | 303.87 | 16.80 |
| 14 | 2 | 8 | 272.17 | 16.70 |
| 16 | -3 | -8 | -2.70 | 1.90 |
| -16 | -3 | 8 | 2.70 | 2.10 |
| -16 | 3 | 8 | 2.60 | 1.70 |
| 16 | 3 | -8 | -3.00 | 1.60 |
| 15 | -3 | -8 | 557.84 | 31.20 |
| 15 | 3 | -8 | 559.64 | 31.10 |
| 14 | -3 | -8 | 36.00 | 5.30 |
| 14 | 3 | -8 | 32.10 | 4.50 |
| 13 | -3 | -8 | 121.39 | 9.30 |
| 13 | 3 | -8 | 121.49 | 8.70 |
| 12 | -3 | -8 | 21.10 | 3.80 |
| 12 | 3 | -8 | 31.50 | 5.20 |
| 11 | -3 | -8 | 2136.19 | 114.39 |
| 11 | 3 | -8 | 2077.69 | 114.19 |
| 10 | -3 | -8 | 1.10 | 4.00 |

| | | | | |
|---|---|---|---|---|
| 10 | 3 | -8 | -5.20 | 3.10 |
| 9 | -3 | -8 | 1290.17 | 71.89 |
| 9 | 3 | -8 | 1325.47 | 71.59 |
| 8 | -3 | -8 | -4.20 | 6.00 |
| 8 | 3 | -8 | -3.80 | 4.00 |
| -6 | 3 | 8 | 409.36 | 24.70 |
| -5 | 3 | 8 | 4423.96 | 239.08 |
| -4 | -3 | 8 | 637.14 | 34.70 |
| -4 | 3 | 8 | 564.94 | 34.90 |
| -3 | -3 | 8 | 618.04 | 34.10 |
| -3 | 3 | 8 | 556.64 | 34.30 |
| -2 | -3 | 8 | 933.01 | 50.89 |
| -2 | 3 | 8 | 866.91 | 50.99 |
| -1 | -3 | 8 | 4900.21 | 273.47 |
| -1 | 3 | 8 | 5151.98 | 271.87 |
| 0 | -3 | 8 | 421.76 | 26.40 |
| 0 | 3 | 8 | 441.16 | 26.50 |
| 1 | -3 | 8 | 756.22 | 45.50 |
| 1 | 3 | 8 | 831.42 | 46.00 |
| 2 | -3 | 8 | 112.79 | 10.80 |
| 2 | 3 | 8 | 139.29 | 10.90 |
| 2 | 3 | 8 | 126.19 | 11.70 |
| 3 | -3 | 8 | 957.10 | 54.49 |
| 3 | 3 | 8 | 1021.70 | 53.39 |
| 3 | 3 | 8 | 898.61 | 55.39 |
| 4 | -3 | 8 | 62.39 | 11.80 |
| 4 | -3 | 8 | 47.60 | 7.70 |
| 4 | 3 | 8 | 69.59 | 7.70 |
| 4 | 3 | 8 | 51.99 | 8.80 |
| 4 | 3 | 8 | 47.80 | 8.60 |
| 5 | -3 | 8 | 3124.09 | 154.68 |
| 5 | -3 | 8 | 2913.21 | 155.08 |
| 5 | -3 | 8 | 2992.00 | 155.38 |
| 5 | 3 | 8 | 2631.34 | 155.28 |
| 5 | 3 | 8 | 2784.92 | 154.38 |
| 5 | 3 | 8 | 2655.53 | 154.38 |
| 6 | -3 | 8 | 280.07 | 18.10 |
| 6 | -3 | 8 | 269.87 | 18.00 |
| 6 | -3 | 8 | 253.87 | 19.00 |
| 6 | 3 | 8 | 279.77 | 17.20 |
| 6 | 3 | 8 | 297.77 | 17.40 |
| 6 | 3 | 8 | 237.48 | 18.20 |
| 6 | 3 | 8 | 245.48 | 20.80 |
| 7 | -3 | 8 | 61.49 | 8.70 |

| | | | | |
|---|---|---|---|---|
| 7 | -3 | 8 | 50.29 | 7.50 |
| 7 | 3 | 8 | 67.49 | 13.80 |
| 7 | 3 | 8 | 60.79 | 8.20 |
| 7 | 3 | 8 | 69.69 | 7.70 |
| 8 | -3 | 8 | 11.80 | 5.20 |
| 8 | -3 | 8 | 9.90 | 4.70 |
| 8 | 3 | 8 | 9.10 | 3.90 |
| 8 | 3 | 8 | 1.70 | 4.10 |
| 9 | -3 | 8 | 682.53 | 36.60 |
| 9 | -3 | 8 | 660.33 | 37.00 |
| 9 | 3 | 8 | 563.54 | 36.60 |
| 9 | 3 | 8 | 616.94 | 36.00 |
| 10 | -3 | 8 | 19.60 | 5.70 |
| 10 | -3 | 8 | 34.40 | 7.80 |
| 10 | 3 | 8 | 39.00 | 8.30 |
| 10 | 3 | 8 | 36.30 | 6.10 |
| 11 | -3 | 8 | 464.15 | 27.90 |
| 11 | -3 | 8 | 481.35 | 28.10 |
| 11 | 3 | 8 | 469.45 | 27.90 |
| 11 | 3 | 8 | 475.85 | 27.40 |
| 12 | -3 | 8 | 4.70 | 3.60 |
| 12 | -3 | 8 | 0.60 | 3.40 |
| 12 | 3 | 8 | 4.60 | 3.30 |
| 12 | 3 | 8 | 2.80 | 3.30 |
| 13 | -3 | 8 | 242.38 | 16.50 |
| 13 | -3 | 8 | 290.77 | 17.40 |
| 13 | 3 | 8 | 277.47 | 16.30 |
| 13 | 3 | 8 | 270.87 | 16.30 |
| 14 | -3 | 8 | 6.00 | 2.70 |
| 14 | -3 | 8 | 1.70 | 3.10 |
| 14 | 3 | 8 | 7.80 | 2.40 |
| 14 | 3 | 8 | 4.90 | 2.50 |
| 16 | -4 | -8 | 480.55 | 26.90 |
| -16 | 4 | 8 | 495.45 | 27.10 |
| 16 | 4 | -8 | 480.95 | 26.90 |
| -15 | -4 | 8 | 22.70 | 4.60 |
| 15 | -4 | -8 | 11.80 | 2.70 |
| 15 | 4 | -8 | 8.70 | 2.10 |
| 14 | -4 | -8 | 217.98 | 16.50 |
| 14 | 4 | -8 | 255.17 | 14.40 |
| 13 | -4 | -8 | 3.20 | 2.90 |
| 13 | 4 | -8 | 2.40 | 2.40 |
| 12 | -4 | -8 | 765.12 | 43.40 |
| 12 | -4 | -8 | 761.32 | 42.70 |

| | | | | |
|---|---|---|---|---|
| 12 | 4 | -8 | 764.72 | 42.40 |
| 11 | -4 | -8 | 94.19 | 10.00 |
| 11 | -4 | -8 | 52.99 | 8.10 |
| 11 | 4 | -8 | 72.39 | 7.10 |
| 10 | -4 | -8 | 876.01 | 49.30 |
| 10 | -4 | -8 | 871.31 | 49.80 |
| 10 | 4 | -8 | 900.81 | 49.00 |
| 9 | -4 | -8 | 20.80 | 5.80 |
| 9 | -4 | -8 | 19.40 | 5.80 |
| 8 | -4 | -8 | 47.00 | 8.00 |
| 8 | -4 | -8 | 48.30 | 10.10 |
| 8 | 4 | -8 | 48.30 | 7.50 |
| 7 | 4 | -8 | 333.77 | 20.30 |
| 7 | 4 | -8 | 328.37 | 20.90 |
| -6 | 4 | 8 | 5584.24 | 302.97 |
| -6 | 4 | 8 | 5642.04 | 302.67 |
| -5 | 4 | 8 | 10.40 | 4.70 |
| -4 | 4 | 8 | 159.18 | 12.90 |
| -3 | -4 | 8 | 56.59 | 7.20 |
| -3 | 4 | 8 | 49.99 | 7.90 |
| -2 | -4 | 8 | 2084.29 | 109.89 |
| -2 | 4 | 8 | 1915.51 | 111.69 |
| -1 | -4 | 8 | 5.00 | 3.60 |
| -1 | 4 | 8 | 3.30 | 3.70 |
| 0 | -4 | 8 | 2867.61 | 162.68 |
| 0 | 4 | 8 | 3102.99 | 162.78 |
| 1 | -4 | 8 | 148.59 | 12.40 |
| 1 | 4 | 8 | 170.58 | 12.40 |
| 1 | 4 | 8 | 168.48 | 12.80 |
| 2 | -4 | 8 | 234.48 | 16.70 |
| 2 | 4 | 8 | 236.38 | 15.80 |
| 2 | 4 | 8 | 266.07 | 17.60 |
| 3 | -4 | 8 | 159.08 | 12.70 |
| 3 | 4 | 8 | 163.88 | 14.30 |
| 3 | 4 | 8 | 178.38 | 12.00 |
| 4 | -4 | 8 | 2703.23 | 141.79 |
| 4 | 4 | 8 | 2457.05 | 140.89 |
| 4 | 4 | 8 | 2715.63 | 140.59 |
| 4 | 4 | 8 | 2749.02 | 140.89 |
| 4 | 4 | 8 | 2347.97 | 143.09 |
| 5 | -4 | 8 | 3.80 | 6.80 |
| 5 | -4 | 8 | 0.80 | 4.20 |
| 5 | -4 | 8 | 13.90 | 6.70 |
| 5 | -4 | 8 | 6.90 | 6.10 |

| | | | | |
|---|---|---|---|---|
| 5 | 4 | 8 | 4.20 | 4.00 |
| 5 | 4 | 8 | 4.90 | 5.00 |
| 5 | 4 | 8 | 2.50 | 6.70 |
| 5 | 4 | 8 | -3.70 | 3.20 |
| 6 | -4 | 8 | 611.84 | 33.80 |
| 6 | -4 | 8 | 571.44 | 33.40 |
| 6 | 4 | 8 | 560.64 | 32.60 |
| 6 | 4 | 8 | 501.85 | 36.20 |
| 6 | 4 | 8 | 562.24 | 33.20 |
| 6 | 4 | 8 | 584.44 | 32.60 |
| 7 | -4 | 8 | 51.89 | 10.20 |
| 7 | -4 | 8 | 68.89 | 9.60 |
| 7 | 4 | 8 | 66.09 | 11.30 |
| 7 | 4 | 8 | 78.89 | 7.80 |
| 8 | -4 | 8 | 564.74 | 31.80 |
| 8 | -4 | 8 | 510.65 | 32.20 |
| 8 | 4 | 8 | 542.15 | 31.00 |
| 8 | 4 | 8 | 533.15 | 31.60 |
| 9 | -4 | 8 | 4.90 | 4.70 |
| 9 | -4 | 8 | -7.30 | 4.80 |
| 9 | 4 | 8 | -0.40 | 4.20 |
| 9 | 4 | 8 | -3.00 | 3.20 |
| 10 | -4 | 8 | 859.11 | 46.80 |
| 10 | -4 | 8 | 767.72 | 46.20 |
| 10 | 4 | 8 | 911.81 | 45.90 |
| 10 | 4 | 8 | 729.33 | 46.30 |
| 11 | -4 | 8 | 18.10 | 4.60 |
| 11 | -4 | 8 | 17.90 | 4.60 |
| 11 | 4 | 8 | 19.60 | 3.90 |
| 11 | 4 | 8 | 23.20 | 4.50 |
| 12 | -4 | 8 | -3.40 | 3.60 |
| 12 | -4 | 8 | 0.40 | 3.50 |
| 12 | 4 | 8 | -1.40 | 3.00 |
| 12 | 4 | 8 | -2.20 | 2.70 |
| 13 | -4 | 8 | 5.50 | 3.30 |
| 13 | -4 | 8 | 8.50 | 3.60 |
| 13 | 4 | 8 | 2.80 | 2.40 |
| 13 | 4 | 8 | 5.90 | 2.40 |
| 16 | -5 | -8 | 2.10 | 2.00 |
| 16 | 5 | -8 | -0.40 | 1.40 |
| -16 | 5 | 8 | -1.80 | 1.40 |
| 15 | -5 | -8 | 262.67 | 15.20 |
| 15 | 5 | -8 | 256.97 | 14.90 |
| -15 | 5 | 8 | 250.87 | 15.20 |

| | | | | |
|---|---|---|---|---|
| 14 | -5 | -8 | -1.90 | 2.90 |
| 14 | 5 | -8 | 1.40 | 3.70 |
| 13 | -5 | -8 | 28.10 | 5.70 |
| 13 | -5 | -8 | 26.00 | 5.60 |
| 13 | 5 | -8 | 28.90 | 4.40 |
| 12 | -5 | -8 | 4.00 | 3.40 |
| 12 | 5 | -8 | 2.40 | 2.30 |
| 11 | -5 | -8 | 1656.93 | 86.99 |
| 11 | -5 | -8 | 1540.25 | 86.49 |
| 11 | 5 | -8 | 1555.44 | 86.19 |
| 10 | -5 | -8 | 5.00 | 4.70 |
| 10 | -5 | -8 | 11.50 | 5.30 |
| 10 | 5 | -8 | 1.90 | 2.90 |
| 9 | -5 | -8 | 164.98 | 13.20 |
| 9 | -5 | -8 | 137.39 | 13.90 |
| 9 | 5 | -8 | 173.08 | 11.30 |
| 8 | -5 | -8 | -2.20 | 5.40 |
| 8 | -5 | -8 | 3.90 | 11.10 |
| 8 | 5 | -8 | 6.30 | 3.90 |
| 7 | 5 | -8 | 1569.14 | 84.79 |
| -7 | 5 | 8 | 1622.94 | 85.59 |
| 7 | 5 | -8 | 1443.86 | 84.49 |
| -6 | 5 | 8 | 11.00 | 5.70 |
| 6 | 5 | -8 | 7.10 | 7.80 |
| -6 | 5 | 8 | 25.90 | 6.40 |
| -5 | 5 | 8 | 1549.94 | 89.39 |
| -4 | 5 | 8 | 8.10 | 4.20 |
| -3 | 5 | 8 | 406.06 | 22.30 |
| -3 | 5 | 8 | 289.37 | 22.20 |
| -2 | -5 | 8 | 613.74 | 36.90 |
| -2 | 5 | 8 | 652.43 | 36.90 |
| -1 | -5 | 8 | 3397.76 | 191.88 |
| -1 | 5 | 8 | 3346.37 | 190.48 |
| -1 | 5 | 8 | 3844.72 | 191.98 |
| 0 | -5 | 8 | 1.20 | 3.70 |
| 0 | 5 | 8 | 6.50 | 4.10 |
| 0 | 5 | 8 | 0.00 | 3.60 |
| 1 | -5 | 8 | 311.27 | 22.50 |
| 1 | 5 | 8 | 405.96 | 21.80 |
| 1 | 5 | 8 | 364.86 | 22.90 |
| 2 | -5 | 8 | 13.00 | 4.60 |
| 2 | 5 | 8 | 17.70 | 4.10 |
| 2 | 5 | 8 | 9.00 | 4.40 |
| 3 | -5 | 8 | 1077.09 | 59.69 |

| | | | | |
|---|---|---|---|---|
| 3 | 5 | 8 | 1091.49 | 58.39 |
| 3 | 5 | 8 | 1041.30 | 59.09 |
| 3 | 5 | 8 | 977.50 | 58.79 |
| 3 | 5 | 8 | 1105.89 | 60.99 |
| 4 | -5 | 8 | 7.10 | 4.20 |
| 4 | -5 | 8 | -4.70 | 6.90 |
| 4 | 5 | 8 | 6.10 | 4.80 |
| 4 | 5 | 8 | 5.60 | 5.80 |
| 4 | 5 | 8 | 9.50 | 4.10 |
| 5 | -5 | 8 | 2015.60 | 125.79 |
| 5 | -5 | 8 | 2363.76 | 124.59 |
| 5 | -5 | 8 | 2304.97 | 124.79 |
| 5 | 5 | 8 | 2164.28 | 127.49 |
| 5 | 5 | 8 | 2297.37 | 124.49 |
| 5 | 5 | 8 | 2464.55 | 124.49 |
| 5 | 5 | 8 | 2398.86 | 124.29 |
| 6 | -5 | 8 | 3.70 | 5.30 |
| 6 | -5 | 8 | 2.00 | 6.50 |
| 6 | 5 | 8 | 1.20 | 7.90 |
| 6 | 5 | 8 | 5.70 | 3.60 |
| 6 | 5 | 8 | 4.60 | 3.00 |
| 7 | -5 | 8 | 22.10 | 5.90 |
| 7 | -5 | 8 | 27.20 | 6.60 |
| 7 | 5 | 8 | 32.50 | 6.30 |
| 7 | 5 | 8 | 15.90 | 3.70 |
| 7 | 5 | 8 | 13.70 | 9.40 |
| 8 | -5 | 8 | 16.50 | 6.10 |
| 8 | -5 | 8 | 20.80 | 5.80 |
| 8 | 5 | 8 | 28.70 | 4.60 |
| 8 | 5 | 8 | 22.10 | 4.60 |
| 9 | -5 | 8 | 888.61 | 50.19 |
| 9 | -5 | 8 | 869.21 | 49.90 |
| 9 | 5 | 8 | 892.31 | 49.80 |
| 9 | 5 | 8 | 877.11 | 49.20 |
| 10 | -5 | 8 | -4.70 | 4.70 |
| 10 | -5 | 8 | 1.60 | 4.40 |
| 10 | 5 | 8 | -2.20 | 3.10 |
| 10 | 5 | 8 | 0.60 | 3.60 |
| 11 | -5 | 8 | 412.46 | 29.80 |
| 11 | -5 | 8 | 468.05 | 26.90 |
| 11 | 5 | 8 | 429.56 | 26.60 |
| 11 | 5 | 8 | 485.05 | 26.30 |
| 12 | -5 | 8 | -1.80 | 3.80 |
| 12 | -5 | 8 | 7.80 | 3.90 |

| | | | | |
|---|---|---|---|---|
| 12 | 5 | 8 | 5.30 | 2.90 |
| 12 | 5 | 8 | 5.40 | 3.00 |
| 13 | -5 | 8 | 232.48 | 15.10 |
| 13 | -5 | 8 | 248.08 | 15.30 |
| 13 | 5 | 8 | 224.88 | 14.60 |
| 13 | 5 | 8 | 265.67 | 15.30 |
| 16 | -6 | -8 | 258.27 | 15.10 |
| 16 | 6 | -8 | 265.97 | 14.90 |
| -16 | 6 | 8 | 268.57 | 15.20 |
| 15 | -6 | -8 | 1.80 | 2.30 |
| -15 | 6 | 8 | 0.70 | 1.80 |
| 15 | 6 | -8 | -0.30 | 1.50 |
| 14 | -6 | -8 | 197.38 | 12.70 |
| -14 | 6 | 8 | 198.28 | 12.80 |
| 14 | 6 | -8 | 212.78 | 12.30 |
| 13 | -6 | -8 | 98.49 | 8.10 |
| 13 | 6 | -8 | 96.59 | 7.10 |
| 12 | -6 | -8 | 456.15 | 25.70 |
| 12 | 6 | -8 | 430.36 | 25.20 |
| 11 | -6 | -8 | 12.50 | 4.60 |
| 11 | -6 | -8 | 5.10 | 4.10 |
| 11 | 6 | -8 | 10.40 | 3.10 |
| 10 | -6 | -8 | 626.94 | 38.10 |
| 10 | -6 | -8 | 692.13 | 38.10 |
| 10 | 6 | -8 | 676.83 | 37.30 |
| 9 | -6 | -8 | 25.70 | 6.60 |
| 8 | 6 | -8 | 115.99 | 9.90 |
| -8 | 6 | 8 | 108.49 | 10.70 |
| 7 | 6 | -8 | 81.39 | 8.80 |
| -7 | 6 | 8 | 109.29 | 11.10 |
| 7 | 6 | -8 | 81.49 | 9.40 |
| -6 | 6 | 8 | 2240.38 | 126.29 |
| -6 | 6 | 8 | 2306.07 | 126.09 |
| 6 | 6 | -8 | 2369.16 | 125.79 |
| -5 | 6 | 8 | 32.20 | 7.20 |
| -5 | 6 | 8 | 25.20 | 5.60 |
| -4 | 6 | 8 | 109.79 | 10.10 |
| -3 | 6 | 8 | 190.68 | 14.40 |
| -3 | 6 | 8 | 186.18 | 13.80 |
| -2 | 6 | 8 | 173.18 | 13.40 |
| -2 | 6 | 8 | 178.28 | 13.20 |
| -1 | 6 | 8 | 179.48 | 13.10 |
| -1 | 6 | 8 | 172.08 | 13.20 |
| 0 | 6 | 8 | 3255.47 | 181.18 |

| | | | | |
|---|---|---|---|---|
| 0 | 6 | 8 | 3400.26 | 179.88 |
| 1 | 6 | 8 | 34.80 | 6.60 |
| 1 | 6 | 8 | 34.60 | 7.20 |
| 2 | -6 | 8 | 0.40 | 7.00 |
| 2 | 6 | 8 | 9.50 | 5.20 |
| 2 | 6 | 8 | 5.40 | 4.10 |
| 2 | 6 | 8 | 0.20 | 3.10 |
| 2 | 6 | 8 | 1.00 | 4.50 |
| 3 | -6 | 8 | 74.49 | 13.70 |
| 3 | 6 | 8 | 53.09 | 9.00 |
| 3 | 6 | 8 | 51.49 | 9.50 |
| 3 | 6 | 8 | 45.00 | 6.30 |
| 3 | 6 | 8 | 49.99 | 8.40 |
| 4 | -6 | 8 | 1393.96 | 76.99 |
| 4 | -6 | 8 | 1422.06 | 78.19 |
| 4 | 6 | 8 | 1383.06 | 79.79 |
| 4 | 6 | 8 | 1303.67 | 76.89 |
| 4 | 6 | 8 | 1498.05 | 76.79 |
| 5 | -6 | 8 | 19.50 | 7.00 |
| 5 | -6 | 8 | 27.00 | 7.20 |
| 5 | -6 | 8 | 26.10 | 6.60 |
| 5 | 6 | 8 | 35.80 | 5.30 |
| 5 | 6 | 8 | 33.00 | 5.50 |
| 5 | 6 | 8 | 29.30 | 8.50 |
| 6 | -6 | 8 | 362.96 | 22.70 |
| 6 | -6 | 8 | 379.56 | 22.70 |
| 6 | 6 | 8 | 338.97 | 27.20 |
| 6 | 6 | 8 | 379.96 | 21.80 |
| 6 | 6 | 8 | 331.47 | 21.40 |
| 7 | -6 | 8 | 106.89 | 10.90 |
| 7 | -6 | 8 | 95.49 | 13.50 |
| 7 | 6 | 8 | 97.39 | 8.70 |
| 7 | 6 | 8 | 98.39 | 19.10 |
| 7 | 6 | 8 | 87.89 | 8.30 |
| 8 | -6 | 8 | 317.57 | 21.90 |
| 8 | -6 | 8 | 321.07 | 21.00 |
| 8 | -6 | 8 | 320.07 | 20.30 |
| 8 | 6 | 8 | 321.87 | 19.90 |
| 8 | 6 | 8 | 333.67 | 19.80 |
| 9 | -6 | 8 | 7.10 | 6.00 |
| 9 | -6 | 8 | -2.70 | 4.30 |
| 9 | 6 | 8 | -0.50 | 3.00 |
| 9 | 6 | 8 | -1.90 | 7.00 |
| 10 | -6 | 8 | 687.33 | 42.90 |

| | | | | |
|---|---|---|---|---|
| 10 | -6 | 8 | 683.23 | 41.90 |
| 10 | -6 | 8 | 756.22 | 42.80 |
| 10 | 6 | 8 | 815.62 | 42.80 |
| 10 | 6 | 8 | 808.12 | 42.10 |
| 11 | -6 | 8 | 20.10 | 4.80 |
| 11 | -6 | 8 | 22.90 | 5.60 |
| 11 | 6 | 8 | 39.60 | 6.30 |
| 11 | 6 | 8 | 29.00 | 6.10 |
| 12 | -6 | 8 | 6.00 | 4.20 |
| 12 | -6 | 8 | -0.80 | 4.40 |
| 12 | 6 | 8 | 5.00 | 2.90 |
| 12 | 6 | 8 | 4.10 | 2.60 |
| 13 | -6 | 8 | -6.40 | 3.90 |
| 13 | -6 | 8 | 1.10 | 4.30 |
| 13 | 6 | 8 | 0.30 | 2.50 |
| 13 | 6 | 8 | 1.90 | 2.50 |
| 15 | -7 | -8 | 323.57 | 17.80 |
| -15 | 7 | 8 | 291.47 | 17.80 |
| 15 | 7 | -8 | 315.07 | 17.50 |
| 14 | -7 | -8 | 8.20 | 2.70 |
| -14 | 7 | 8 | 8.20 | 2.50 |
| 14 | 7 | -8 | 6.80 | 1.90 |
| 13 | -7 | -8 | 9.20 | 5.40 |
| 13 | 7 | -8 | 4.60 | 2.10 |
| -13 | 7 | 8 | 2.60 | 2.60 |
| 12 | -7 | -8 | 2.20 | 3.50 |
| 12 | 7 | -8 | 2.50 | 2.30 |
| -12 | 7 | 8 | 2.60 | 2.80 |
| 11 | -7 | -8 | 933.31 | 53.59 |
| -11 | 7 | 8 | 989.80 | 53.89 |
| 11 | 7 | -8 | 978.20 | 53.19 |
| 10 | -7 | -8 | 7.00 | 5.10 |
| -10 | 7 | 8 | 26.30 | 4.50 |
| 10 | 7 | -8 | 20.50 | 3.60 |
| 9 | -7 | -8 | 314.87 | 24.80 |
| -9 | 7 | 8 | 352.46 | 21.40 |
| 9 | 7 | -8 | 346.07 | 20.30 |
| -8 | 7 | 8 | 166.98 | 13.10 |
| 8 | 7 | -8 | 149.79 | 11.50 |
| -7 | 7 | 8 | 987.80 | 53.79 |
| 7 | 7 | -8 | 920.11 | 52.59 |
| 7 | 7 | -8 | 922.51 | 53.19 |
| -6 | -7 | 8 | -2.90 | 6.50 |
| -6 | 7 | 8 | 1.70 | 6.00 |

| | | | | |
|---|---|---|---|---|
| -6 | 7 | 8 | 6.20 | 5.40 |
| 6 | 7 | -8 | 2.00 | 3.90 |
| -5 | -7 | 8 | 1193.68 | 74.19 |
| 5 | 7 | -8 | 1481.65 | 74.79 |
| -5 | 7 | 8 | 1438.46 | 74.69 |
| -5 | 7 | 8 | 1237.68 | 74.89 |
| -4 | -7 | 8 | 50.89 | 11.30 |
| -4 | 7 | 8 | 85.09 | 12.30 |
| -4 | 7 | 8 | 72.09 | 8.80 |
| -3 | -7 | 8 | 57.49 | 10.50 |
| -3 | 7 | 8 | 37.70 | 8.80 |
| -3 | 7 | 8 | 35.40 | 7.20 |
| -3 | 7 | 8 | 40.20 | 8.20 |
| -2 | -7 | 8 | 14.00 | 5.60 |
| -2 | 7 | 8 | 10.30 | 4.50 |
| -2 | 7 | 8 | 15.30 | 4.40 |
| -1 | -7 | 8 | 2181.58 | 129.49 |
| -1 | 7 | 8 | 2549.65 | 129.29 |
| -1 | 7 | 8 | 2395.16 | 130.29 |
| 0 | -7 | 8 | 227.68 | 17.60 |
| 0 | 7 | 8 | 216.18 | 15.80 |
| 0 | 7 | 8 | 229.08 | 15.00 |
| 1 | -7 | 8 | 422.46 | 26.80 |
| 1 | 7 | 8 | 449.16 | 24.40 |
| 1 | 7 | 8 | 375.16 | 25.10 |
| 1 | 7 | 8 | 400.46 | 25.80 |
| 2 | -7 | 8 | 197.18 | 16.50 |
| 2 | 7 | 8 | 136.69 | 13.10 |
| 2 | 7 | 8 | 180.88 | 13.90 |
| 3 | -7 | 8 | 330.77 | 23.20 |
| 3 | -7 | 8 | 294.97 | 20.70 |
| 3 | 7 | 8 | 312.77 | 19.40 |
| 3 | 7 | 8 | 349.87 | 20.60 |
| 3 | 7 | 8 | 324.77 | 22.30 |
| 4 | -7 | 8 | 9.00 | 6.90 |
| 4 | -7 | 8 | 12.30 | 5.60 |
| 4 | 7 | 8 | 18.60 | 4.70 |
| 4 | 7 | 8 | 8.60 | 6.00 |
| 4 | 7 | 8 | 18.20 | 3.80 |
| 5 | -7 | 8 | 2273.97 | 126.99 |
| 5 | -7 | 8 | 2434.56 | 126.39 |
| 5 | 7 | 8 | 2154.68 | 129.79 |
| 5 | 7 | 8 | 2488.95 | 126.69 |
| 5 | 7 | 8 | 2286.77 | 126.39 |

| | | | | |
|---|---|---|---|---|
| 6 | -7 | 8 | 120.09 | 16.40 |
| 6 | -7 | 8 | 102.99 | 11.20 |
| 6 | -7 | 8 | 116.09 | 11.10 |
| 6 | 7 | 8 | 113.69 | 8.60 |
| 6 | 7 | 8 | 93.59 | 9.10 |
| 7 | -7 | 8 | 12.30 | 4.70 |
| 7 | 7 | 8 | 11.80 | 11.40 |
| 7 | 7 | 8 | 13.40 | 3.60 |
| 7 | 7 | 8 | 18.20 | 4.00 |
| 8 | -7 | 8 | 2.70 | 4.20 |
| 8 | -7 | 8 | -1.40 | 4.90 |
| 8 | 7 | 8 | 2.60 | 3.30 |
| 8 | 7 | 8 | -1.50 | 3.20 |
| 9 | -7 | 8 | 745.53 | 41.90 |
| 9 | -7 | 8 | 720.53 | 40.90 |
| 9 | 7 | 8 | 694.33 | 41.30 |
| 9 | 7 | 8 | 760.72 | 41.00 |
| 10 | -7 | 8 | -0.50 | 4.50 |
| 10 | 7 | 8 | 4.20 | 3.30 |
| 10 | 7 | 8 | 3.00 | 3.20 |
| 11 | -7 | 8 | 211.28 | 15.80 |
| 11 | -7 | 8 | 239.68 | 18.80 |
| 11 | -7 | 8 | 220.08 | 15.40 |
| 11 | 7 | 8 | 248.28 | 14.40 |
| 11 | 7 | 8 | 216.28 | 14.70 |
| 12 | -7 | 8 | 21.40 | 4.90 |
| 12 | 7 | 8 | 26.70 | 5.00 |
| 12 | 7 | 8 | 26.60 | 5.00 |
| 13 | -7 | 8 | 148.69 | 11.50 |
| 13 | 7 | 8 | 157.38 | 10.20 |
| 13 | 7 | 8 | 159.78 | 10.10 |
| 15 | -8 | -8 | -1.80 | 2.20 |
| 15 | 8 | -8 | 0.60 | 1.30 |
| -15 | 8 | 8 | 2.50 | 1.90 |
| 14 | -8 | -8 | 167.08 | 10.70 |
| 14 | 8 | -8 | 160.78 | 10.10 |
| -14 | 8 | 8 | 176.08 | 10.70 |
| 13 | -8 | -8 | 81.39 | 7.50 |
| -13 | 8 | 8 | 94.09 | 7.70 |
| 13 | 8 | -8 | 90.09 | 6.80 |
| 12 | -8 | -8 | 537.65 | 32.20 |
| 12 | 8 | -8 | 559.74 | 31.80 |
| -12 | 8 | 8 | 611.64 | 32.60 |
| 11 | -8 | -8 | 4.20 | 4.00 |

| | | | | |
|---|---|---|---|---|
| 11 | 8 | -8 | 5.50 | 2.30 |
| -11 | 8 | 8 | 11.10 | 3.40 |
| 10 | -8 | -8 | 578.64 | 32.90 |
| -10 | 8 | 8 | 532.85 | 32.70 |
| 10 | 8 | -8 | 587.64 | 32.00 |
| 9 | -8 | -8 | 56.29 | 7.60 |
| 9 | 8 | -8 | 45.30 | 6.40 |
| -9 | 8 | 8 | 44.60 | 7.80 |
| -8 | 8 | 8 | 12.10 | 4.60 |
| 8 | 8 | -8 | 16.70 | 4.20 |
| -7 | -8 | 8 | 109.79 | 13.90 |
| 7 | 8 | -8 | 122.19 | 11.70 |
| 7 | 8 | -8 | 126.09 | 10.80 |
| -7 | 8 | 8 | 137.49 | 12.90 |
| -6 | -8 | 8 | 2066.99 | 120.59 |
| -6 | 8 | 8 | 2282.17 | 121.49 |
| -6 | 8 | 8 | 2210.88 | 121.59 |
| 6 | 8 | -8 | 2282.47 | 121.29 |
| -5 | -8 | 8 | 279.97 | 19.50 |
| -5 | 8 | 8 | 255.27 | 20.50 |
| 5 | 8 | -8 | 310.97 | 19.10 |
| -5 | 8 | 8 | 267.97 | 19.00 |
| -4 | -8 | 8 | 353.56 | 23.60 |
| 4 | 8 | -8 | 413.06 | 24.10 |
| -4 | 8 | 8 | 335.17 | 23.10 |
| -4 | 8 | 8 | 356.26 | 24.60 |
| -4 | 8 | 8 | 390.56 | 23.60 |
| -3 | -8 | 8 | 76.79 | 10.00 |
| -3 | -8 | 8 | 68.19 | 10.00 |
| -3 | 8 | 8 | 54.09 | 9.00 |
| -3 | 8 | 8 | 72.59 | 8.90 |
| -3 | 8 | 8 | 78.89 | 11.80 |
| -2 | -8 | 8 | 1448.85 | 75.99 |
| -2 | -8 | 8 | 1258.47 | 76.29 |
| -2 | 8 | 8 | 1433.16 | 76.29 |
| -2 | 8 | 8 | 1480.15 | 77.39 |
| -2 | 8 | 8 | 1281.97 | 76.39 |
| -1 | -8 | 8 | 10.10 | 4.90 |
| -1 | -8 | 8 | 16.30 | 5.50 |
| -1 | 8 | 8 | 5.20 | 5.30 |
| -1 | 8 | 8 | 2.90 | 4.10 |
| -1 | 8 | 8 | 12.30 | 4.20 |
| 0 | -8 | 8 | 2668.13 | 137.49 |
| 0 | -8 | 8 | 2524.95 | 138.49 |

| | | | | |
|---|---|---|---|---|
| 0 | 8 | 8 | 2625.44 | 139.29 |
| 0 | 8 | 8 | 2331.27 | 137.79 |
| 1 | -8 | 8 | 429.86 | 26.60 |
| 1 | -8 | 8 | 468.55 | 28.40 |
| 1 | 8 | 8 | 484.35 | 26.30 |
| 1 | 8 | 8 | 394.96 | 26.90 |
| 1 | 8 | 8 | 460.05 | 28.00 |
| 2 | -8 | 8 | 172.08 | 13.30 |
| 2 | -8 | 8 | 168.58 | 16.30 |
| 2 | 8 | 8 | 185.38 | 13.60 |
| 2 | 8 | 8 | 155.08 | 11.70 |
| 2 | 8 | 8 | 182.38 | 14.30 |
| 3 | -8 | 8 | 101.89 | 10.70 |
| 3 | -8 | 8 | 138.59 | 14.80 |
| 3 | 8 | 8 | 128.39 | 11.00 |
| 3 | 8 | 8 | 118.09 | 9.20 |
| 3 | 8 | 8 | 96.89 | 12.40 |
| 4 | -8 | 8 | 2110.19 | 114.39 |
| 4 | -8 | 8 | 2140.89 | 113.09 |
| 4 | 8 | 8 | 2113.39 | 116.39 |
| 4 | 8 | 8 | 2036.70 | 113.29 |
| 4 | 8 | 8 | 2008.70 | 113.09 |
| 5 | -8 | 8 | 251.77 | 17.70 |
| 5 | -8 | 8 | 233.68 | 15.40 |
| 5 | 8 | 8 | 218.58 | 14.00 |
| 5 | 8 | 8 | 199.78 | 14.70 |
| 5 | 8 | 8 | 193.18 | 20.20 |
| -5 | 8 | -8 | 220.08 | 16.00 |
| 6 | -8 | 8 | 261.67 | 17.40 |
| 6 | -8 | 8 | 267.57 | 18.00 |
| 6 | -8 | 8 | 272.17 | 19.20 |
| 6 | 8 | 8 | 281.77 | 16.80 |
| 6 | 8 | 8 | 233.48 | 24.90 |
| -6 | 8 | -8 | 273.27 | 21.50 |
| 6 | 8 | 8 | 281.17 | 17.10 |
| 7 | -8 | 8 | 162.98 | 13.30 |
| 7 | -8 | 8 | 154.68 | 12.20 |
| -7 | -8 | -8 | 164.78 | 12.40 |
| 7 | 8 | 8 | 162.28 | 11.30 |
| 7 | 8 | 8 | 173.08 | 11.80 |
| 7 | 8 | 8 | 185.78 | 24.50 |
| 8 | -8 | 8 | 420.76 | 24.60 |
| -8 | -8 | -8 | 406.66 | 25.00 |
| 8 | 8 | 8 | 432.96 | 24.80 |

| | | | | |
|---|---|---|---|---|
| 9 | -8 | 8 | 6.10 | 3.70 |
| 9 | -8 | 8 | -7.60 | 4.60 |
| 9 | 8 | 8 | 4.70 | 3.30 |
| 9 | 8 | 8 | 4.70 | 3.40 |
| 10 | -8 | 8 | 679.63 | 40.50 |
| 10 | -8 | 8 | 741.03 | 39.60 |
| 10 | 8 | 8 | 712.63 | 40.20 |
| 11 | -8 | 8 | 21.30 | 4.10 |
| 11 | -8 | 8 | 16.20 | 5.00 |
| 11 | 8 | 8 | 12.70 | 3.40 |
| 12 | -8 | 8 | 2.20 | 5.00 |
| 12 | 8 | 8 | -7.30 | 4.00 |
| 12 | 8 | 8 | 1.80 | 2.70 |
| 15 | -9 | -8 | 252.67 | 14.10 |
| 15 | 9 | -8 | 239.28 | 13.80 |
| -15 | 9 | 8 | 241.58 | 14.10 |
| 14 | -9 | -8 | 3.90 | 2.50 |
| 14 | 9 | -8 | -0.20 | 1.30 |
| -14 | 9 | 8 | 0.00 | 2.00 |
| 13 | -9 | -8 | 4.20 | 2.90 |
| 13 | 9 | -8 | 3.50 | 1.80 |
| -13 | 9 | 8 | 1.90 | 2.50 |
| 12 | -9 | -8 | 64.39 | 8.10 |
| -12 | 9 | 8 | 66.59 | 6.90 |
| 12 | 9 | -8 | 64.99 | 5.60 |
| 11 | -9 | -8 | 1112.19 | 63.59 |
| -11 | 9 | 8 | 1205.08 | 63.99 |
| 11 | 9 | -8 | 1157.18 | 63.29 |
| 10 | -9 | -8 | 8.50 | 4.90 |
| -10 | 9 | 8 | 9.30 | 3.80 |
| 10 | 9 | -8 | 9.30 | 2.90 |
| 9 | -9 | -8 | 276.57 | 18.80 |
| 9 | 9 | -8 | 262.17 | 16.30 |
| -9 | 9 | 8 | 249.68 | 17.50 |
| -8 | 9 | 8 | 45.00 | 9.30 |
| 8 | 9 | -8 | 39.10 | 6.70 |
| -7 | -9 | 8 | 1179.88 | 68.79 |
| 7 | 9 | -8 | 1299.27 | 69.69 |
| -6 | -9 | 8 | -2.90 | 5.00 |
| -6 | 9 | 8 | 2.70 | 10.50 |
| 6 | 9 | -8 | 8.70 | 5.10 |
| -6 | 9 | 8 | 1.30 | 6.50 |
| 5 | -9 | -8 | 2024.00 | 113.19 |
| -5 | -9 | 8 | 2002.10 | 112.79 |

| | | | | |
|---|---|---|---|---|
| 5 | 9 | -8 | 2246.98 | 114.19 |
| -5 | 9 | 8 | 1934.31 | 113.59 |
| -5 | 9 | 8 | 2109.69 | 113.69 |
| -4 | -9 | 8 | 169.38 | 13.60 |
| -4 | 9 | 8 | 168.18 | 13.50 |
| -4 | 9 | 8 | 142.49 | 12.50 |
| -4 | 9 | 8 | 150.98 | 15.10 |
| 4 | 9 | -8 | 174.18 | 14.00 |
| -3 | -9 | 8 | 333.37 | 21.60 |
| -3 | -9 | 8 | 393.26 | 21.00 |
| -3 | 9 | 8 | 290.77 | 20.90 |
| -3 | 9 | 8 | 311.37 | 22.30 |
| -3 | 9 | 8 | 349.47 | 22.10 |
| 3 | 9 | -8 | 330.57 | 22.80 |
| -2 | -9 | 8 | 325.07 | 22.30 |
| -2 | -9 | 8 | 384.26 | 21.80 |
| -2 | 9 | 8 | 366.46 | 22.00 |
| 2 | 9 | -8 | 313.47 | 23.60 |
| -2 | 9 | 8 | 376.46 | 23.10 |
| -1 | -9 | 8 | 3747.43 | 188.28 |
| -1 | -9 | 8 | 3509.05 | 188.98 |
| -1 | 9 | 8 | 3612.04 | 190.08 |
| -1 | 9 | 8 | 3329.57 | 188.48 |
| 1 | 9 | -8 | 3116.19 | 190.28 |
| -1 | 9 | 8 | 3595.84 | 188.68 |
| 0 | -9 | 8 | 77.69 | 9.10 |
| 0 | -9 | 8 | 67.49 | 10.80 |
| 0 | 9 | 8 | 91.59 | 9.80 |
| 0 | 9 | 8 | 79.59 | 8.00 |
| 0 | 9 | 8 | 60.69 | 10.40 |
| 1 | -9 | 8 | 315.87 | 19.10 |
| 1 | -9 | 8 | 311.17 | 20.90 |
| 1 | 9 | 8 | 306.17 | 18.90 |
| 1 | 9 | 8 | 296.67 | 19.80 |
| 1 | 9 | 8 | 298.97 | 20.80 |
| 2 | -9 | 8 | 34.20 | 5.20 |
| 2 | -9 | 8 | 19.50 | 6.70 |
| 2 | 9 | 8 | 17.90 | 3.90 |
| 2 | 9 | 8 | 17.00 | 5.50 |
| 2 | 9 | 8 | 24.10 | 5.50 |
| 3 | -9 | 8 | 1088.79 | 59.89 |
| 3 | -9 | 8 | 959.00 | 57.59 |
| -3 | 9 | -8 | 1081.99 | 59.29 |
| 3 | 9 | 8 | 1056.39 | 57.79 |

| | | | | |
|---|---|---|---|---|
| 3 | 9 | 8 | 1093.99 | 58.19 |
| 3 | 9 | 8 | 987.90 | 60.29 |
| 4 | -9 | 8 | -2.50 | 6.30 |
| 4 | -9 | 8 | -4.10 | 3.80 |
| 4 | 9 | 8 | 5.20 | 4.50 |
| 4 | 9 | 8 | -0.70 | 2.60 |
| 4 | 9 | 8 | -4.80 | 6.40 |
| -4 | 9 | -8 | -1.30 | 4.80 |
| 5 | -9 | 8 | 2310.17 | 120.89 |
| 5 | -9 | 8 | 2259.67 | 121.79 |
| 5 | 9 | 8 | 2053.29 | 120.89 |
| -5 | 9 | -8 | 2417.66 | 122.69 |
| 5 | 9 | 8 | 2247.28 | 121.09 |
| 5 | 9 | 8 | 2082.09 | 124.99 |
| 6 | -9 | 8 | 73.19 | 10.90 |
| -6 | -9 | -8 | 69.89 | 8.30 |
| 6 | -9 | 8 | 79.19 | 8.30 |
| 6 | 9 | 8 | 79.29 | 19.30 |
| 6 | 9 | 8 | 73.89 | 6.90 |
| 6 | 9 | 8 | 72.79 | 7.90 |
| 7 | -9 | 8 | 84.89 | 8.50 |
| -7 | -9 | -8 | 103.29 | 9.10 |
| 7 | -9 | 8 | 76.09 | 10.40 |
| 7 | 9 | 8 | 84.59 | 7.70 |
| 7 | 9 | 8 | 80.79 | 8.40 |
| 7 | 9 | 8 | 102.89 | 24.90 |
| 8 | -9 | 8 | 6.20 | 3.30 |
| 8 | -9 | 8 | -7.40 | 5.10 |
| -8 | -9 | -8 | -3.70 | 3.70 |
| 8 | 9 | 8 | -0.20 | 2.60 |
| 8 | 9 | 8 | -0.60 | 3.30 |
| 9 | -9 | 8 | 1021.60 | 53.39 |
| 9 | -9 | 8 | 943.11 | 53.99 |
| 9 | 9 | 8 | 964.90 | 53.39 |
| 9 | 9 | 8 | 931.11 | 53.59 |
| 10 | -9 | 8 | -7.10 | 3.00 |
| 10 | -9 | 8 | 4.80 | 5.10 |
| 10 | 9 | 8 | 2.20 | 3.50 |
| 10 | 9 | 8 | 3.10 | 3.00 |
| 11 | -9 | 8 | 260.77 | 15.50 |
| 11 | -9 | 8 | 254.87 | 17.00 |
| 11 | 9 | 8 | 281.27 | 15.80 |
| 11 | 9 | 8 | 230.28 | 15.70 |
| 12 | 9 | 8 | 56.59 | 5.50 |

| | | | | |
|---|---|---|---|---|
| 12 | 9 | 8 | 46.70 | 6.20 |
| 14 | -10 | -8 | 86.89 | 8.10 |
| 14 | 10 | -8 | 95.89 | 6.20 |
| -14 | 10 | 8 | 105.69 | 7.00 |
| 13 | -10 | -8 | 39.90 | 5.70 |
| 13 | 10 | -8 | 46.30 | 4.00 |
| -13 | 10 | 8 | 52.19 | 5.40 |
| 12 | -10 | -8 | 424.66 | 26.80 |
| 12 | 10 | -8 | 495.75 | 26.50 |
| -12 | 10 | 8 | 494.55 | 27.10 |
| -11 | 10 | 8 | 3.80 | 3.10 |
| 11 | 10 | -8 | 6.40 | 2.10 |
| 10 | -10 | -8 | 640.54 | 37.20 |
| -10 | 10 | 8 | 679.33 | 37.30 |
| 10 | 10 | -8 | 639.24 | 36.50 |
| 9 | 10 | -8 | 19.00 | 3.60 |
| -9 | 10 | 8 | 25.80 | 5.20 |
| -8 | -10 | 8 | 53.29 | 10.80 |
| -8 | 10 | 8 | 63.39 | 10.00 |
| 8 | 10 | -8 | 52.69 | 7.00 |
| -7 | -10 | 8 | 567.54 | 35.30 |
| -7 | 10 | 8 | 680.03 | 37.00 |
| 7 | 10 | -8 | 567.64 | 40.40 |
| -6 | -10 | 8 | 2400.06 | 135.79 |
| 6 | 10 | -8 | 2580.84 | 136.89 |
| -5 | -10 | 8 | 19.60 | 5.60 |
| 5 | -10 | -8 | 30.40 | 6.80 |
| -5 | 10 | 8 | 30.60 | 5.30 |
| -5 | 10 | 8 | 26.30 | 7.40 |
| -5 | 10 | 8 | 28.00 | 7.10 |
| 5 | 10 | -8 | 14.70 | 5.60 |
| 4 | -10 | -8 | 679.03 | 37.00 |
| -4 | -10 | 8 | 581.94 | 36.00 |
| -4 | 10 | 8 | 618.04 | 37.10 |
| 4 | 10 | -8 | 627.34 | 37.80 |
| -4 | 10 | 8 | 633.74 | 37.20 |
| -4 | 10 | 8 | 579.24 | 36.00 |
| -3 | -10 | 8 | 226.18 | 15.70 |
| -3 | -10 | 8 | 242.38 | 15.00 |
| -3 | 10 | 8 | 214.28 | 17.30 |
| 3 | 10 | -8 | 211.58 | 17.60 |
| -3 | 10 | 8 | 218.58 | 16.60 |
| -3 | 10 | 8 | 210.88 | 15.40 |
| -2 | -10 | 8 | 822.72 | 48.70 |

| | | | | |
|---|---|---|---|---|
| -2 | -10 | 8 | 910.31 | 48.10 |
| -2 | 10 | 8 | 825.62 | 48.90 |
| -2 | 10 | 8 | 881.51 | 48.80 |
| -2 | 10 | 8 | 862.01 | 49.90 |
| -1 | -10 | 8 | 70.99 | 8.20 |
| -1 | -10 | 8 | 72.49 | 10.00 |
| -1 | 10 | 8 | 70.09 | 8.20 |
| -1 | 10 | 8 | 70.79 | 10.90 |
| -1 | 10 | 8 | 87.69 | 10.20 |
| 1 | 10 | -8 | 67.99 | 11.00 |
| 0 | -10 | 8 | 2684.93 | 148.99 |
| 0 | -10 | 8 | 2655.93 | 147.89 |
| 0 | 10 | -8 | 2625.14 | 150.38 |
| 0 | 10 | 8 | 2790.22 | 148.59 |
| 0 | 10 | 8 | 2846.12 | 148.49 |
| 0 | 10 | 8 | 2808.82 | 150.28 |
| 1 | -10 | 8 | 707.93 | 41.60 |
| 1 | -10 | 8 | 769.92 | 43.30 |
| -1 | 10 | -8 | 748.33 | 43.90 |
| 1 | 10 | 8 | 749.33 | 42.00 |
| 1 | 10 | 8 | 745.13 | 43.90 |
| 1 | 10 | 8 | 738.03 | 42.40 |
| 2 | -10 | 8 | 210.88 | 15.20 |
| 2 | -10 | 8 | 258.97 | 18.40 |
| -2 | 10 | -8 | 240.88 | 17.30 |
| 2 | 10 | 8 | 236.38 | 18.00 |
| 2 | 10 | 8 | 230.68 | 16.30 |
| 2 | 10 | 8 | 241.58 | 15.20 |
| 3 | -10 | 8 | 110.79 | 15.60 |
| 3 | 10 | 8 | 103.59 | 12.90 |
| 3 | 10 | 8 | 90.99 | 10.20 |
| 3 | 10 | 8 | 96.49 | 8.50 |
| -3 | 10 | -8 | 89.39 | 10.60 |
| 4 | -10 | 8 | 860.31 | 48.50 |
| 4 | -10 | 8 | 871.11 | 50.19 |
| 4 | 10 | 8 | 779.52 | 52.19 |
| -4 | 10 | -8 | 952.80 | 52.19 |
| 4 | 10 | 8 | 928.21 | 49.10 |
| 4 | 10 | 8 | 850.61 | 48.70 |
| 5 | -10 | 8 | 27.90 | 4.40 |
| 5 | -10 | 8 | 32.80 | 6.20 |
| -5 | -10 | -8 | 31.80 | 4.90 |
| 5 | 10 | 8 | 20.90 | 10.30 |
| -5 | 10 | -8 | 26.30 | 7.00 |

| | | | | |
|---|---|---|---|---|
| 5 | 10 | 8 | 28.40 | 4.80 |
| 5 | 10 | 8 | 32.40 | 5.20 |
| -6 | -10 | -8 | 354.46 | 21.10 |
| 6 | -10 | 8 | 358.46 | 20.60 |
| 6 | -10 | 8 | 331.17 | 21.80 |
| 6 | 10 | 8 | 326.77 | 30.00 |
| 6 | 10 | 8 | 321.97 | 20.40 |
| 6 | 10 | 8 | 364.06 | 21.00 |
| -7 | -10 | -8 | 174.38 | 12.50 |
| 7 | -10 | 8 | 187.58 | 12.10 |
| 7 | 10 | 8 | 172.48 | 12.50 |
| 7 | 10 | 8 | 177.08 | 12.10 |
| 8 | -10 | 8 | 108.69 | 8.50 |
| -8 | -10 | -8 | 101.59 | 9.30 |
| 8 | 10 | 8 | 96.79 | 8.10 |
| 8 | 10 | 8 | 114.69 | 9.30 |
| 9 | -10 | 8 | 10.20 | 3.00 |
| 9 | 10 | 8 | 4.10 | 2.80 |
| 9 | 10 | 8 | 5.00 | 3.90 |
| 10 | -10 | 8 | 633.74 | 34.60 |
| 10 | 10 | 8 | 618.14 | 35.00 |
| 10 | 10 | 8 | 619.94 | 35.20 |
| 11 | -10 | 8 | 77.59 | 6.90 |
| 11 | 10 | 8 | 64.99 | 6.50 |
| 11 | 10 | 8 | 70.59 | 7.20 |
| 14 | 11 | -8 | 5.90 | 1.30 |
| -14 | 11 | 8 | 6.40 | 2.50 |
| 13 | -11 | -8 | 1.10 | 2.80 |
| 13 | 11 | -8 | 1.40 | 1.40 |
| -13 | 11 | 8 | 2.90 | 2.60 |
| 12 | -11 | -8 | 52.99 | 6.70 |
| 12 | 11 | -8 | 66.29 | 5.00 |
| -12 | 11 | 8 | 62.89 | 6.30 |
| 11 | -11 | -8 | 647.34 | 38.00 |
| -11 | 11 | 8 | 715.93 | 38.40 |
| 10 | -11 | -8 | 1.30 | 5.50 |
| -10 | 11 | 8 | 4.60 | 3.80 |
| 10 | 11 | -8 | 4.00 | 2.10 |
| 9 | 11 | -8 | 132.49 | 9.70 |
| -9 | 11 | 8 | 143.29 | 11.90 |
| -8 | -11 | 8 | 59.59 | 9.80 |
| -8 | 11 | 8 | 51.39 | 10.30 |
| 8 | 11 | -8 | 58.69 | 6.70 |
| -7 | -11 | 8 | 498.45 | 30.70 |

| | | | | |
|---|---|---|---|---|
| 7 | 11 | -8 | 587.34 | 33.20 |
| -7 | 11 | 8 | 489.95 | 32.40 |
| -6 | -11 | 8 | 96.99 | 10.80 |
| -6 | 11 | 8 | 98.49 | 13.50 |
| 6 | 11 | -8 | 97.09 | 12.90 |
| -6 | 11 | 8 | 110.89 | 9.80 |
| -5 | -11 | 8 | 905.81 | 51.49 |
| 5 | -11 | -8 | 944.11 | 51.79 |
| -5 | 11 | 8 | 920.01 | 51.69 |
| -5 | 11 | 8 | 908.61 | 53.29 |
| -5 | 11 | 8 | 863.61 | 52.59 |
| 5 | 11 | -8 | 941.31 | 53.89 |
| -4 | -11 | 8 | 102.79 | 10.50 |
| -4 | 11 | 8 | 95.89 | 12.70 |
| 4 | 11 | -8 | 113.69 | 13.50 |
| -4 | 11 | 8 | 91.99 | 13.20 |
| -4 | 11 | 8 | 95.09 | 10.00 |
| 3 | -11 | -8 | 22.20 | 8.10 |
| -3 | -11 | 8 | 4.50 | 3.90 |
| -3 | -11 | 8 | -0.10 | 4.10 |
| -3 | 11 | 8 | 0.70 | 3.80 |
| -3 | 11 | 8 | -1.60 | 5.90 |
| 3 | 11 | -8 | -2.20 | 5.80 |
| -3 | 11 | 8 | 5.10 | 5.50 |
| 2 | -11 | -8 | 402.16 | 28.20 |
| -2 | -11 | 8 | 385.26 | 24.50 |
| -2 | 11 | 8 | 400.86 | 23.80 |
| -2 | 11 | 8 | 375.36 | 24.50 |
| -2 | 11 | 8 | 413.06 | 25.50 |
| 2 | 11 | -8 | 337.67 | 26.40 |
| -1 | -11 | 8 | 1015.60 | 57.49 |
| -1 | -11 | 8 | 985.90 | 57.89 |
| -1 | 11 | 8 | 1071.99 | 58.59 |
| -1 | 11 | 8 | 951.60 | 56.69 |
| 1 | 11 | -8 | 960.10 | 59.19 |
| -1 | 11 | 8 | 1099.99 | 56.99 |
| 0 | -11 | 8 | 4.50 | 4.80 |
| 0 | -11 | -8 | 1.90 | 4.90 |
| 0 | 11 | 8 | 8.00 | 5.40 |
| 0 | 11 | -8 | 8.00 | 5.80 |
| 0 | 11 | 8 | 12.80 | 3.70 |
| 0 | 11 | 8 | 12.50 | 5.50 |
| -1 | -11 | -8 | 126.99 | 11.60 |
| 1 | -11 | 8 | 127.69 | 12.80 |

| | | | | |
|---|---|---|---|---|
| 1 | -11 | 8 | 129.09 | 9.90 |
| 1 | 11 | 8 | 142.79 | 10.10 |
| 1 | 11 | 8 | 127.59 | 12.20 |
| -1 | 11 | -8 | 124.79 | 12.90 |
| 1 | 11 | 8 | 88.49 | 16.10 |
| -2 | -11 | -8 | 148.89 | 12.60 |
| 2 | -11 | 8 | 187.58 | 14.90 |
| 2 | -11 | 8 | 167.58 | 11.60 |
| -2 | 11 | -8 | 163.38 | 14.60 |
| 2 | 11 | 8 | 153.18 | 11.30 |
| 2 | 11 | 8 | 155.98 | 15.00 |
| 3 | -11 | 8 | 13.70 | 3.80 |
| 3 | -11 | 8 | 11.50 | 6.20 |
| -3 | -11 | -8 | 12.10 | 4.30 |
| 3 | 11 | 8 | 10.30 | 3.50 |
| 3 | 11 | 8 | 8.00 | 5.10 |
| -3 | 11 | -8 | 7.10 | 5.30 |
| 3 | 11 | 8 | 17.60 | 7.70 |
| 4 | -11 | 8 | 80.89 | 12.10 |
| -4 | -11 | -8 | 87.59 | 9.20 |
| 4 | -11 | 8 | 109.79 | 8.70 |
| -4 | 11 | -8 | 92.69 | 12.60 |
| 4 | 11 | 8 | 86.39 | 15.50 |
| 4 | 11 | 8 | 100.89 | 9.90 |
| 4 | 11 | 8 | 99.29 | 8.00 |
| 5 | -11 | 8 | 866.51 | 48.10 |
| 5 | -11 | 8 | 849.41 | 49.20 |
| -5 | -11 | -8 | 842.82 | 48.50 |
| 5 | 11 | 8 | 774.22 | 53.89 |
| 5 | 11 | 8 | 947.21 | 48.50 |
| -5 | 11 | -8 | 890.71 | 50.99 |
| 5 | 11 | 8 | 897.11 | 48.50 |
| 6 | -11 | 8 | 158.08 | 10.40 |
| -6 | -11 | -8 | 148.89 | 10.70 |
| 6 | -11 | 8 | 139.59 | 12.30 |
| 6 | 11 | 8 | 139.79 | 10.30 |
| 6 | 11 | 8 | 140.59 | 25.50 |
| 6 | 11 | 8 | 128.99 | 11.10 |
| -7 | -11 | -8 | 2.90 | 3.20 |
| 7 | -11 | 8 | 5.30 | 3.00 |
| 7 | 11 | 8 | 5.60 | 2.60 |
| 7 | 11 | 8 | 1.60 | 4.00 |
| 8 | -11 | 8 | 16.40 | 4.60 |
| -8 | -11 | -8 | 12.80 | 3.90 |

| | | | | |
|---|---|---|---|---|
| 8 | 11 | 8 | 8.90 | 2.90 |
| 8 | 11 | 8 | 9.60 | 4.60 |
| 9 | -11 | 8 | 262.27 | 15.10 |
| 9 | 11 | 8 | 266.17 | 16.10 |
| 9 | 11 | 8 | 230.68 | 15.40 |
| 10 | -11 | 8 | 1.60 | 2.60 |
| 10 | 11 | 8 | 6.70 | 2.80 |
| 10 | 11 | 8 | 11.60 | 7.00 |
| 11 | -11 | 8 | 86.49 | 7.10 |
| 11 | 11 | 8 | 80.59 | 6.80 |
| -13 | 12 | 8 | 102.59 | 8.50 |
| 13 | 12 | -8 | 111.59 | 6.50 |
| -13 | 12 | 8 | 97.39 | 7.50 |
| -12 | 12 | 8 | 577.54 | 34.50 |
| 12 | 12 | -8 | 604.04 | 32.90 |
| -12 | 12 | 8 | 617.34 | 33.50 |
| -11 | 12 | 8 | 8.20 | 3.60 |
| 11 | 12 | -8 | 9.30 | 2.00 |
| 10 | 12 | -8 | 303.47 | 17.90 |
| -10 | 12 | 8 | 310.57 | 19.30 |
| 9 | 12 | -8 | 10.10 | 2.50 |
| -9 | 12 | 8 | 10.80 | 5.20 |
| -8 | -12 | 8 | 127.89 | 11.50 |
| 8 | 12 | -8 | 124.29 | 9.80 |
| -8 | 12 | 8 | 158.58 | 13.80 |
| -7 | -12 | 8 | 238.08 | 15.70 |
| 7 | 12 | -8 | 221.58 | 19.40 |
| -7 | 12 | 8 | 208.68 | 18.40 |
| -6 | -12 | 8 | 759.32 | 43.50 |
| 6 | 12 | -8 | 857.01 | 46.20 |
| -6 | 12 | 8 | 652.73 | 44.80 |
| -6 | 12 | 8 | 793.72 | 43.70 |
| 5 | -12 | -8 | -5.90 | 4.90 |
| -5 | -12 | 8 | -0.20 | 4.10 |
| -5 | 12 | 8 | 13.20 | 7.40 |
| 5 | 12 | -8 | 4.30 | 6.60 |
| -5 | 12 | 8 | 5.00 | 9.10 |
| -5 | 12 | 8 | 2.60 | 4.10 |
| 4 | -12 | -8 | 2.70 | 4.90 |
| -4 | -12 | 8 | 13.90 | 4.70 |
| -4 | 12 | 8 | 2.00 | 4.00 |
| -4 | 12 | 8 | 12.00 | 6.90 |
| 4 | 12 | -8 | 8.70 | 6.30 |
| 3 | -12 | -8 | 216.78 | 15.40 |

| | | | | |
|---|---|---|---|---|
| -3 | -12 | 8 | 192.88 | 14.80 |
| -3 | -12 | 8 | 197.38 | 13.30 |
| 3 | 12 | -8 | 189.18 | 18.30 |
| -3 | 12 | 8 | 223.88 | 17.40 |
| -3 | 12 | 8 | 198.88 | 14.40 |
| -2 | -12 | 8 | 353.96 | 22.30 |
| 2 | -12 | -8 | 405.46 | 24.20 |
| -2 | -12 | 8 | 375.96 | 23.60 |
| -2 | 12 | 8 | 393.36 | 25.40 |
| -2 | 12 | 8 | 374.26 | 23.40 |
| -1 | -12 | 8 | 23.70 | 5.00 |
| -1 | -12 | 8 | 36.20 | 6.00 |
| 1 | -12 | -8 | 47.70 | 9.00 |
| -1 | 12 | 8 | 53.39 | 10.30 |
| -1 | 12 | 8 | 41.40 | 6.70 |
| 1 | 12 | -8 | 24.30 | 7.70 |
| 0 | -12 | -8 | 724.93 | 41.70 |
| 0 | -12 | 8 | 698.73 | 40.20 |
| 0 | -12 | 8 | 703.03 | 41.50 |
| 0 | 12 | -8 | 691.73 | 43.70 |
| 0 | 12 | 8 | 738.23 | 43.20 |
| 0 | 12 | 8 | 762.12 | 41.10 |
| 1 | -12 | 8 | 143.59 | 13.10 |
| 1 | -12 | 8 | 128.19 | 10.20 |
| -1 | -12 | -8 | 152.88 | 12.20 |
| 1 | 12 | 8 | 147.39 | 14.40 |
| 1 | 12 | 8 | 145.19 | 10.70 |
| -1 | 12 | -8 | 132.79 | 14.40 |
| 2 | -12 | 8 | 114.79 | 11.90 |
| 2 | -12 | 8 | 92.19 | 8.30 |
| -2 | -12 | -8 | 113.49 | 9.90 |
| -2 | 12 | -8 | 92.89 | 12.60 |
| 2 | 12 | 8 | 96.59 | 8.40 |
| 2 | 12 | 8 | 112.69 | 13.60 |
| 3 | -12 | 8 | 56.29 | 10.60 |
| 3 | -12 | 8 | 51.99 | 6.70 |
| -3 | -12 | -8 | 37.40 | 7.10 |
| 3 | 12 | 8 | 56.99 | 13.00 |
| -3 | 12 | -8 | 56.39 | 11.20 |
| 3 | 12 | 8 | 58.19 | 6.20 |
| 4 | -12 | 8 | 624.74 | 35.30 |
| 4 | -12 | 8 | 656.43 | 36.90 |
| -4 | -12 | -8 | 608.94 | 35.80 |
| 4 | 12 | 8 | 576.74 | 40.50 |

| | | | | |
|---|---|---|---|---|
| -4 | 12 | -8 | 623.44 | 38.40 |
| 4 | 12 | 8 | 665.33 | 35.60 |
| 5 | -12 | 8 | -2.00 | 2.80 |
| 5 | -12 | 8 | -6.70 | 4.40 |
| -5 | -12 | -8 | -0.60 | 2.90 |
| 5 | 12 | 8 | 15.20 | 11.90 |
| 5 | 12 | 8 | 7.00 | 2.80 |
| 6 | -12 | 8 | 115.89 | 8.10 |
| 6 | -12 | 8 | 99.49 | 10.40 |
| -6 | -12 | -8 | 92.19 | 8.30 |
| 6 | 12 | 8 | 102.79 | 8.00 |
| 7 | -12 | 8 | 178.68 | 13.40 |
| 7 | 12 | 8 | 164.78 | 11.20 |
| -8 | -12 | -8 | 232.98 | 15.20 |
| 8 | -12 | 8 | 259.27 | 15.00 |
| 8 | 12 | 8 | 255.97 | 15.30 |
| 9 | -12 | 8 | 37.40 | 4.90 |
| 9 | 12 | 8 | 36.30 | 4.80 |
| 10 | -12 | 8 | 326.07 | 18.00 |
| 10 | 12 | 8 | 299.37 | 18.30 |
| 12 | 13 | -8 | 28.50 | 2.90 |
| -12 | 13 | 8 | 27.10 | 4.20 |
| -11 | 13 | 8 | 489.15 | 27.60 |
| 11 | 13 | -8 | 493.65 | 27.20 |
| 10 | 13 | -8 | 72.69 | 5.60 |
| -10 | 13 | 8 | 75.19 | 6.10 |
| -9 | 13 | 8 | 14.00 | 3.10 |
| 9 | 13 | -8 | 6.10 | 2.50 |
| -8 | -13 | 8 | 180.58 | 13.60 |
| -8 | 13 | 8 | 205.88 | 12.70 |
| 8 | 13 | -8 | 187.68 | 12.30 |
| -7 | -13 | 8 | 801.12 | 47.80 |
| 7 | 13 | -8 | 854.11 | 47.60 |
| -7 | 13 | 8 | 911.21 | 48.10 |
| -6 | 13 | 8 | 8.70 | 3.90 |
| 6 | 13 | -8 | 13.10 | 8.30 |
| 5 | -13 | -8 | 388.36 | 22.10 |
| -5 | -13 | 8 | 352.76 | 21.90 |
| 5 | 13 | -8 | 356.56 | 25.90 |
| -5 | 13 | 8 | 312.07 | 22.40 |
| 4 | -13 | -8 | 122.19 | 11.60 |
| -4 | -13 | 8 | 130.49 | 11.40 |
| -4 | 13 | 8 | 148.99 | 17.10 |
| -4 | 13 | 8 | 141.49 | 11.40 |

| | | | | |
|---|---|---|---|---|
| 4 | 13 | -8 | 143.79 | 16.30 |
| 3 | -13 | -8 | 211.88 | 14.50 |
| -3 | -13 | 8 | 195.28 | 14.10 |
| 3 | 13 | -8 | 175.18 | 18.90 |
| -3 | 13 | 8 | 193.98 | 18.00 |
| -3 | 13 | 8 | 182.78 | 13.80 |
| -2 | -13 | 8 | 179.18 | 12.20 |
| 2 | -13 | -8 | 191.38 | 14.20 |
| -2 | -13 | 8 | 187.18 | 13.70 |
| -2 | 13 | 8 | 184.08 | 16.80 |
| -2 | 13 | 8 | 172.18 | 13.20 |
| -1 | -13 | 8 | 683.73 | 37.10 |
| 1 | -13 | -8 | 660.33 | 38.50 |
| -1 | -13 | 8 | 659.13 | 38.20 |
| -1 | 13 | 8 | 721.53 | 40.60 |
| -1 | 13 | 8 | 660.53 | 38.00 |
| 1 | 13 | -8 | 567.74 | 41.00 |
| 0 | -13 | 8 | 77.49 | 6.80 |
| 0 | -13 | 8 | 53.89 | 9.20 |
| 0 | -13 | -8 | 66.99 | 9.10 |
| 0 | 13 | 8 | 69.79 | 7.50 |
| 0 | 13 | 8 | 52.29 | 12.10 |
| 0 | 13 | -8 | 50.59 | 12.80 |
| 1 | -13 | 8 | 74.39 | 9.70 |
| 1 | -13 | 8 | 71.79 | 6.90 |
| -1 | -13 | -8 | 56.59 | 8.50 |
| -1 | 13 | -8 | 84.39 | 12.50 |
| 1 | 13 | 8 | 62.89 | 12.40 |
| 2 | -13 | 8 | 74.19 | 10.40 |
| -2 | -13 | -8 | 74.49 | 8.00 |
| 2 | -13 | 8 | 59.19 | 6.70 |
| 2 | 13 | 8 | 63.99 | 6.90 |
| -2 | 13 | -8 | 51.79 | 11.80 |
| 2 | 13 | 8 | 75.19 | 13.50 |
| 3 | -13 | 8 | 371.26 | 21.90 |
| -3 | -13 | -8 | 328.37 | 20.30 |
| 3 | -13 | 8 | 348.97 | 19.70 |
| -3 | 13 | -8 | 335.47 | 23.90 |
| 3 | 13 | 8 | 298.77 | 20.00 |
| 3 | 13 | 8 | 302.47 | 25.20 |
| 4 | -13 | 8 | 47.20 | 9.20 |
| 4 | -13 | 8 | 35.90 | 5.80 |
| -4 | -13 | -8 | 53.99 | 6.40 |
| 4 | 13 | 8 | 30.70 | 12.40 |

| | | | | |
|---|---|---|---|---|
| 4 | 13 | 8 | 46.10 | 5.30 |
| 5 | -13 | 8 | 735.93 | 43.20 |
| 5 | -13 | 8 | 780.72 | 42.60 |
| -5 | -13 | -8 | 758.62 | 42.80 |
| 5 | 13 | 8 | 791.22 | 42.80 |
| -6 | -13 | -8 | 60.19 | 7.90 |
| 6 | -13 | 8 | 65.39 | 7.40 |
| 6 | 13 | 8 | 66.89 | 6.00 |
| -7 | -13 | -8 | 10.90 | 2.90 |
| 7 | -13 | 8 | 13.20 | 2.80 |
| -8 | -13 | -8 | 6.70 | 2.90 |
| 8 | -13 | 8 | 10.40 | 2.50 |
| 8 | 13 | 8 | 8.60 | 2.40 |
| 9 | -13 | 8 | 307.07 | 16.50 |
| 9 | 13 | 8 | 259.67 | 17.80 |
| 10 | -13 | 8 | 2.90 | 2.50 |
| 10 | 13 | 8 | -1.40 | 2.40 |
| 11 | 14 | -8 | 12.60 | 2.40 |
| -11 | 14 | 8 | 9.00 | 2.40 |
| -10 | 14 | 8 | 244.18 | 14.00 |
| 10 | 14 | -8 | 222.88 | 13.50 |
| 9 | 14 | -8 | 81.19 | 6.20 |
| -9 | 14 | 8 | 86.09 | 6.70 |
| -8 | -14 | 8 | 2.50 | 3.60 |
| 8 | 14 | -8 | 3.60 | 2.90 |
| -8 | 14 | 8 | 0.00 | 2.70 |
| -7 | -14 | 8 | 20.00 | 4.70 |
| 7 | 14 | -8 | 28.80 | 5.10 |
| -7 | 14 | 8 | 13.20 | 3.30 |
| -6 | -14 | 8 | 707.83 | 42.30 |
| -6 | 14 | 8 | 795.02 | 42.70 |
| 5 | -14 | -8 | 130.79 | 10.20 |
| -5 | -14 | 8 | 98.49 | 9.70 |
| -5 | 14 | 8 | 105.09 | 9.50 |
| -4 | -14 | 8 | 79.09 | 8.90 |
| 4 | -14 | -8 | 98.49 | 9.40 |
| -4 | 14 | 8 | 90.99 | 9.20 |
| 4 | 14 | -8 | 97.49 | 16.10 |
| -4 | 14 | 8 | 109.79 | 20.90 |
| 3 | -14 | -8 | 42.50 | 7.90 |
| -3 | -14 | 8 | 24.60 | 4.50 |
| -3 | 14 | 8 | 20.20 | 4.50 |
| -3 | 14 | 8 | 36.40 | 9.70 |
| 3 | 14 | -8 | 33.60 | 10.50 |

| | | | | |
|---|---|---|---|---|
| -2 | -14 | 8 | 294.17 | 19.40 |
| -2 | -14 | 8 | 298.17 | 18.20 |
| 2 | -14 | -8 | 335.87 | 19.90 |
| -2 | 14 | 8 | 291.17 | 23.40 |
| -2 | 14 | 8 | 305.47 | 19.70 |
| -1 | -14 | 8 | 18.80 | 4.80 |
| -1 | -14 | 8 | 25.90 | 5.00 |
| 1 | -14 | -8 | 12.00 | 4.30 |
| 1 | 14 | -8 | 6.70 | 7.90 |
| -1 | 14 | 8 | 24.40 | 7.70 |
| -1 | 14 | 8 | 13.50 | 3.90 |
| 0 | -14 | 8 | 617.84 | 35.70 |
| 0 | -14 | 8 | 654.73 | 37.10 |
| 0 | -14 | -8 | 628.54 | 36.90 |
| 0 | 14 | 8 | 630.04 | 39.60 |
| 0 | 14 | -8 | 580.64 | 40.10 |
| 0 | 14 | 8 | 705.73 | 36.80 |
| -1 | -14 | -8 | 78.49 | 8.60 |
| 1 | -14 | 8 | 76.19 | 6.90 |
| 1 | -14 | 8 | 79.99 | 9.50 |
| 1 | 14 | 8 | 72.79 | 7.50 |
| 1 | 14 | 8 | 74.39 | 14.30 |
| -1 | 14 | -8 | 83.59 | 14.40 |
| 2 | -14 | 8 | 6.40 | 4.60 |
| -2 | -14 | -8 | 16.20 | 3.70 |
| 2 | -14 | 8 | 25.10 | 5.00 |
| 2 | 14 | 8 | 24.60 | 4.90 |
| 2 | 14 | 8 | 20.60 | 12.80 |
| 3 | -14 | 8 | 45.30 | 5.60 |
| 3 | -14 | 8 | 47.30 | 8.90 |
| -3 | -14 | -8 | 45.70 | 6.40 |
| 3 | 14 | 8 | 50.29 | 5.70 |
| 4 | -14 | 8 | 504.65 | 29.90 |
| 4 | -14 | 8 | 522.55 | 28.70 |
| -4 | -14 | -8 | 478.25 | 29.00 |
| 4 | 14 | 8 | 522.35 | 28.90 |
| 5 | -14 | 8 | -7.90 | 3.70 |
| 5 | -14 | 8 | 2.60 | 4.60 |
| 5 | 14 | 8 | 0.40 | 2.10 |
| -6 | -14 | -8 | 182.28 | 11.20 |
| 6 | -14 | 8 | 166.78 | 10.80 |
| 6 | 14 | 8 | 165.98 | 11.00 |
| 7 | -14 | 8 | 95.89 | 7.00 |
| -7 | -14 | -8 | 105.39 | 7.40 |

| | | | | |
|---|---|---|---|---|
| 7 | 14 | 8 | 96.39 | 7.10 |
| 8 | -14 | 8 | 132.39 | 8.40 |
| -8 | -14 | -8 | 130.59 | 8.90 |
| 8 | 14 | 8 | 123.79 | 9.40 |
| 9 | -14 | 8 | 5.80 | 2.50 |
| 9 | 14 | 8 | 4.10 | 2.50 |
| -10 | 15 | 8 | 61.89 | 5.00 |
| 10 | 15 | -8 | 54.59 | 4.20 |
| -9 | 15 | 8 | 180.18 | 11.00 |
| 9 | 15 | -8 | 174.58 | 10.60 |
| -8 | -15 | 8 | 66.99 | 7.00 |
| 8 | 15 | -8 | 69.09 | 5.80 |
| -8 | 15 | 8 | 72.79 | 6.00 |
| -7 | -15 | 8 | 258.97 | 16.90 |
| -7 | 15 | 8 | 280.27 | 16.80 |
| 7 | 15 | -8 | 282.87 | 16.30 |
| -6 | -15 | 8 | 7.50 | 3.80 |
| -6 | 15 | 8 | 6.00 | 2.90 |
| 5 | -15 | -8 | 543.15 | 31.20 |
| -5 | -15 | 8 | 517.35 | 31.20 |
| -5 | 15 | 8 | 563.84 | 31.50 |
| -4 | -15 | 8 | 35.50 | 6.90 |
| -4 | 15 | 8 | 47.90 | 6.90 |
| -3 | -15 | 8 | 9.90 | 3.40 |
| 3 | -15 | -8 | 18.70 | 3.90 |
| -3 | 15 | 8 | 8.00 | 3.30 |
| 2 | -15 | -8 | 34.20 | 7.00 |
| -2 | -15 | 8 | 39.30 | 6.80 |
| 1 | -15 | -8 | 810.62 | 46.80 |
| -1 | -15 | 8 | 837.22 | 46.90 |
| -1 | -15 | 8 | 824.42 | 45.90 |
| -1 | 15 | 8 | 847.32 | 46.80 |
| 0 | -15 | 8 | 27.90 | 5.00 |
| 0 | -15 | -8 | 21.70 | 4.40 |
| 0 | -15 | 8 | 28.80 | 4.80 |
| 0 | 15 | 8 | 32.70 | 5.90 |
| -1 | -15 | -8 | 319.67 | 20.30 |
| 1 | -15 | 8 | 323.07 | 19.20 |
| 1 | -15 | 8 | 359.46 | 21.00 |
| 1 | 15 | 8 | 316.77 | 19.80 |
| 2 | -15 | 8 | 30.90 | 4.90 |
| -2 | -15 | -8 | 35.10 | 6.10 |
| 2 | -15 | 8 | 18.10 | 4.50 |
| 2 | 15 | 8 | 31.30 | 5.20 |

| | | | | |
|---|---|---|---|---|
| 3 | -15 | 8 | 86.09 | 14.30 |
| -3 | -15 | -8 | 89.99 | 8.10 |
| 3 | -15 | 8 | 111.39 | 10.20 |
| 3 | 15 | 8 | 90.39 | 7.30 |
| -4 | -15 | -8 | 38.80 | 5.90 |
| 4 | -15 | 8 | 39.60 | 4.90 |
| 4 | 15 | 8 | 37.80 | 4.70 |
| -5 | -15 | -8 | 404.86 | 22.90 |
| 5 | -15 | 8 | 383.86 | 22.50 |
| 5 | -15 | 8 | 388.96 | 23.20 |
| 5 | 15 | 8 | 414.96 | 22.80 |
| -6 | -15 | -8 | 120.29 | 8.30 |
| 6 | -15 | 8 | 114.29 | 7.80 |
| 6 | 15 | 8 | 119.69 | 8.00 |
| 7 | -15 | 8 | -1.40 | 1.70 |
| -7 | -15 | -8 | -0.10 | 2.10 |
| 8 | -15 | 8 | 46.60 | 4.20 |
| 8 | 15 | 8 | 39.10 | 4.50 |
| 9 | 16 | -8 | 49.40 | 3.90 |
| -9 | 16 | 8 | 47.10 | 4.50 |
| -8 | -16 | 8 | 29.50 | 4.90 |
| 8 | 16 | -8 | 22.90 | 3.30 |
| -7 | -16 | 8 | 111.19 | 8.40 |
| 7 | 16 | -8 | 103.29 | 7.50 |
| -7 | 16 | 8 | 116.89 | 8.00 |
| -6 | -16 | 8 | 558.14 | 32.80 |
| 6 | 16 | -8 | 592.34 | 32.60 |
| -6 | 16 | 8 | 597.54 | 33.10 |
| -5 | -16 | 8 | 109.09 | 9.20 |
| 5 | -16 | -8 | 130.99 | 9.30 |
| -5 | 16 | 8 | 117.79 | 9.10 |
| -4 | -16 | 8 | 201.38 | 14.00 |
| 4 | -16 | -8 | 216.48 | 13.60 |
| -4 | 16 | 8 | 216.48 | 14.10 |
| -3 | -16 | 8 | 122.59 | 13.10 |
| 3 | -16 | -8 | 113.69 | 9.20 |
| -3 | 16 | 8 | 93.69 | 8.80 |
| 2 | -16 | -8 | 169.38 | 15.00 |
| -2 | -16 | 8 | 181.98 | 14.90 |
| -2 | 16 | 8 | 172.88 | 12.20 |
| -1 | -16 | 8 | 12.70 | 4.60 |
| -1 | 16 | 8 | 26.20 | 5.60 |
| 0 | -16 | 8 | 439.06 | 29.20 |
| 0 | -16 | 8 | 444.56 | 28.00 |

| | | | | |
|---|---|---|---|---|
| 0 | 16 | 8 | 475.25 | 26.50 |
| -1 | -16 | -8 | 136.29 | 11.00 |
| 1 | -16 | 8 | 145.49 | 9.90 |
| 1 | -16 | 8 | 159.28 | 12.00 |
| 1 | 16 | 8 | 156.18 | 10.50 |
| -2 | -16 | -8 | 1.50 | 2.80 |
| 2 | -16 | 8 | 0.30 | 2.00 |
| 2 | -16 | 8 | 0.60 | 3.30 |
| 2 | 16 | 8 | 0.00 | 2.40 |
| 3 | -16 | 8 | 51.49 | 5.00 |
| -3 | -16 | -8 | 53.49 | 5.80 |
| 3 | -16 | 8 | 62.29 | 7.20 |
| 3 | 16 | 8 | 49.90 | 5.20 |
| 4 | -16 | 8 | 447.16 | 25.80 |
| 4 | -16 | 8 | 450.35 | 25.20 |
| -4 | -16 | -8 | 449.26 | 25.50 |
| 4 | 16 | 8 | 452.95 | 25.50 |
| -5 | -16 | -8 | -0.10 | 2.00 |
| 5 | -16 | 8 | 0.80 | 3.30 |
| 5 | -16 | 8 | 5.90 | 1.90 |
| 5 | 16 | 8 | 6.40 | 2.10 |
| -6 | -16 | -8 | 136.89 | 8.90 |
| 6 | -16 | 8 | 136.99 | 8.50 |
| 6 | 16 | 8 | 140.49 | 8.90 |
| -7 | -16 | -8 | 100.59 | 6.80 |
| 7 | 16 | 8 | 101.99 | 7.00 |
| -8 | -17 | 8 | 160.08 | 10.30 |
| 8 | 17 | -8 | 179.28 | 10.00 |
| -8 | 17 | 8 | 172.68 | 10.40 |
| -7 | -17 | 8 | 142.99 | 9.60 |
| -7 | 17 | 8 | 149.59 | 9.70 |
| 7 | 17 | -8 | 165.48 | 9.40 |
| -6 | -17 | 8 | 4.00 | 2.50 |
| 6 | 17 | -8 | 0.80 | 1.80 |
| -6 | 17 | 8 | 3.00 | 2.90 |
| -5 | -17 | 8 | 338.37 | 19.70 |
| 5 | -17 | -8 | 334.27 | 19.60 |
| -5 | 17 | 8 | 339.87 | 19.90 |
| -4 | -17 | 8 | 102.29 | 7.70 |
| 4 | -17 | -8 | 104.39 | 7.70 |
| -4 | 17 | 8 | 85.49 | 7.70 |
| -3 | -17 | 8 | 3.80 | 2.50 |
| 3 | -17 | -8 | 7.30 | 2.50 |
| -3 | 17 | 8 | 6.30 | 3.00 |

| | | | | |
|---|---|---|---|---|
| 2 | -17 | -8 | 144.29 | 9.80 |
| -2 | -17 | 8 | 133.79 | 9.80 |
| -2 | 17 | 8 | 131.09 | 10.00 |
| 1 | -17 | -8 | 430.06 | 24.50 |
| -1 | -17 | 8 | 414.86 | 24.40 |
| -1 | 17 | 8 | 416.46 | 24.50 |
| 0 | -17 | 8 | 60.09 | 6.70 |
| 0 | -17 | -8 | 66.89 | 6.60 |
| 0 | 17 | 8 | 70.29 | 6.40 |
| 1 | -17 | 8 | 47.70 | 6.30 |
| 1 | -17 | 8 | 46.00 | 4.50 |
| -1 | -17 | -8 | 51.39 | 6.00 |
| 1 | 17 | 8 | 51.39 | 5.30 |
| 2 | -17 | 8 | 126.79 | 8.50 |
| 2 | -17 | 8 | 155.88 | 10.00 |
| -2 | -17 | -8 | 130.09 | 9.30 |
| 2 | 17 | 8 | 127.29 | 9.00 |
| 3 | -17 | 8 | 108.59 | 7.40 |
| -3 | -17 | -8 | 111.79 | 8.00 |
| 3 | -17 | 8 | 114.39 | 8.50 |
| 3 | 17 | 8 | 119.59 | 7.90 |
| -4 | -17 | -8 | 30.50 | 4.10 |
| 4 | -17 | 8 | 39.00 | 3.60 |
| 4 | -17 | 8 | 31.50 | 5.50 |
| 4 | 17 | 8 | 32.60 | 4.20 |
| 5 | -17 | 8 | 263.37 | 15.30 |
| -5 | -17 | -8 | 259.17 | 15.50 |
| 5 | 17 | 8 | 286.97 | 15.70 |
| -6 | -18 | 8 | 277.37 | 16.70 |
| 6 | 18 | -8 | 309.57 | 16.70 |
| -6 | 18 | 8 | 296.47 | 17.00 |
| -5 | -18 | 8 | 88.09 | 6.70 |
| -5 | 18 | 8 | 102.89 | 7.20 |
| 5 | 18 | -8 | 94.59 | 6.40 |
| -4 | -18 | 8 | 53.49 | 5.10 |
| 4 | -18 | -8 | 52.59 | 5.80 |
| -4 | 18 | 8 | 67.59 | 5.80 |
| 3 | -18 | -8 | 113.19 | 8.00 |
| -3 | -18 | 8 | 116.89 | 8.00 |
| -3 | 18 | 8 | 122.69 | 9.10 |
| -2 | -18 | 8 | 130.19 | 8.80 |
| 2 | -18 | -8 | 132.39 | 8.60 |
| -2 | 18 | 8 | 119.29 | 10.20 |
| 1 | -18 | -8 | 30.00 | 4.60 |

| | | | | |
|---|---|---|---|---|
| -1 | -18 | 8 | 31.70 | 4.60 |
| -1 | 18 | 8 | 25.80 | 4.40 |
| 0 | -18 | 8 | 417.86 | 21.40 |
| 0 | -18 | -8 | 366.96 | 21.30 |
| 0 | 18 | 8 | 321.07 | 21.80 |
| 1 | -18 | 8 | 202.08 | 11.50 |
| 1 | 18 | 8 | 160.78 | 11.30 |
| -2 | -18 | -8 | 9.10 | 2.30 |
| 2 | -18 | 8 | 6.60 | 2.40 |
| 2 | 18 | 8 | 5.50 | 2.30 |
| 3 | -18 | 8 | 21.10 | 2.80 |
| -3 | -18 | -8 | 20.70 | 3.50 |
| 3 | -18 | 8 | 14.50 | 3.50 |
| 3 | 18 | 8 | 21.60 | 3.70 |
| -16 | 0 | 9 | 2.20 | 1.90 |
| 16 | 0 | -9 | 1.50 | 1.80 |
| 15 | 0 | -9 | 437.96 | 24.50 |
| -15 | 0 | 9 | 429.76 | 24.70 |
| 14 | 0 | -9 | 0.50 | 2.50 |
| 13 | 0 | -9 | 12.70 | 3.10 |
| 11 | 0 | -9 | 1171.48 | 64.29 |
| 10 | 0 | -9 | -1.70 | 3.50 |
| 9 | 0 | -9 | 851.31 | 47.30 |
| 8 | 0 | -9 | -3.30 | 4.90 |
| -7 | 0 | 9 | 3523.45 | 190.38 |
| -6 | 0 | 9 | 3.00 | 3.80 |
| -5 | 0 | 9 | 4086.09 | 220.88 |
| -3 | 0 | 9 | 18.60 | 4.30 |
| -2 | 0 | 9 | -1.30 | 3.00 |
| -1 | 0 | 9 | 3592.64 | 195.08 |
| 0 | 0 | 9 | -2.00 | 3.70 |
| 1 | 0 | 9 | 331.87 | 21.80 |
| 2 | 0 | 9 | 5.80 | 4.50 |
| 3 | 0 | 9 | 1629.04 | 88.19 |
| 3 | 0 | 9 | 1539.25 | 88.69 |
| 4 | 0 | 9 | -0.40 | 7.40 |
| 4 | 0 | 9 | -6.60 | 6.00 |
| 4 | 0 | 9 | 8.20 | 4.70 |
| 4 | 0 | 9 | -2.00 | 4.50 |
| 5 | 0 | 9 | 2289.37 | 130.09 |
| 5 | 0 | 9 | 2270.27 | 130.79 |
| 5 | 0 | 9 | 2293.77 | 131.69 |
| 5 | 0 | 9 | 2715.43 | 130.19 |
| 6 | 0 | 9 | -7.50 | 5.80 |

| | | | | |
|---|---|---|---|---|
| 6 | 0 | 9 | 9.00 | 4.20 |
| 6 | 0 | 9 | 3.80 | 5.90 |
| 6 | 0 | 9 | 4.50 | 5.30 |
| 7 | 0 | 9 | 78.49 | 10.90 |
| 7 | 0 | 9 | 71.89 | 8.70 |
| 7 | 0 | 9 | 80.59 | 12.40 |
| 8 | 0 | 9 | -2.90 | 3.60 |
| 8 | 0 | 9 | 1.00 | 5.00 |
| 9 | 0 | 9 | 634.74 | 40.10 |
| 9 | 0 | 9 | 745.13 | 39.40 |
| 10 | 0 | 9 | -2.80 | 3.30 |
| 10 | 0 | 9 | 0.10 | 4.40 |
| 11 | 0 | 9 | 361.16 | 21.80 |
| 11 | 0 | 9 | 369.16 | 22.40 |
| 12 | 0 | 9 | 3.50 | 3.10 |
| 12 | 0 | 9 | 3.90 | 3.40 |
| 13 | 0 | 9 | 334.27 | 19.80 |
| 13 | 0 | 9 | 339.37 | 19.80 |
| 16 | -1 | -9 | 383.16 | 21.30 |
| -16 | -1 | 9 | 388.66 | 21.50 |
| 16 | 1 | -9 | 386.06 | 21.30 |
| -16 | 1 | 9 | 362.76 | 21.50 |
| -15 | -1 | 9 | -1.60 | 2.10 |
| 15 | -1 | -9 | -2.90 | 2.20 |
| 15 | 1 | -9 | -1.10 | 2.00 |
| -15 | 1 | 9 | -0.60 | 2.10 |
| 14 | -1 | -9 | 227.38 | 14.50 |
| 14 | 1 | -9 | 241.18 | 14.40 |
| 13 | -1 | -9 | -5.60 | 2.50 |
| 13 | 1 | -9 | -0.60 | 2.70 |
| 12 | -1 | -9 | 875.11 | 48.20 |
| 12 | 1 | -9 | 862.51 | 48.10 |
| 11 | -1 | -9 | 2.30 | 3.50 |
| 11 | 1 | -9 | 0.00 | 3.20 |
| 10 | -1 | -9 | 1271.87 | 71.09 |
| 10 | 1 | -9 | 1325.67 | 71.09 |
| 9 | 1 | -9 | 47.90 | 8.00 |
| 8 | -1 | -9 | 54.09 | 6.70 |
| 8 | 1 | -9 | 39.10 | 5.90 |
| -7 | 1 | 9 | 80.89 | 8.70 |
| -6 | 1 | 9 | 4555.34 | 245.68 |
| -5 | -1 | 9 | 98.59 | 11.70 |
| -5 | 1 | 9 | 102.89 | 9.50 |
| -4 | -1 | 9 | 612.94 | 35.30 |

| | | | | |
|---|---|---|---|---|
| -3 | -1 | 9 | 47.00 | 6.50 |
| -3 | 1 | 9 | 48.00 | 7.10 |
| -2 | -1 | 9 | 1663.63 | 90.99 |
| -2 | 1 | 9 | 1643.04 | 91.29 |
| -1 | -1 | 9 | 21.10 | 4.70 |
| -1 | 1 | 9 | 15.50 | 4.50 |
| 0 | -1 | 9 | 5382.96 | 283.17 |
| 0 | 1 | 9 | 5085.09 | 283.17 |
| 1 | -1 | 9 | 6.60 | 4.40 |
| 1 | 1 | 9 | 9.40 | 4.70 |
| 2 | -1 | 9 | 18.50 | 5.30 |
| 2 | 1 | 9 | 27.40 | 6.30 |
| 3 | -1 | 9 | 13.50 | 5.40 |
| 3 | 1 | 9 | 22.20 | 6.30 |
| 3 | 1 | 9 | 19.30 | 5.50 |
| 3 | 1 | 9 | 31.00 | 6.50 |
| 4 | -1 | 9 | 2360.06 | 120.29 |
| 4 | -1 | 9 | 2138.09 | 121.29 |
| 4 | -1 | 9 | 2407.46 | 119.89 |
| 4 | -1 | 9 | 2084.29 | 120.39 |
| 4 | 1 | 9 | 2218.28 | 121.49 |
| 4 | 1 | 9 | 2282.67 | 120.09 |
| 4 | 1 | 9 | 1902.11 | 121.59 |
| 5 | -1 | 9 | 30.40 | 5.60 |
| 5 | -1 | 9 | 25.90 | 6.30 |
| 5 | -1 | 9 | 29.10 | 6.60 |
| 5 | -1 | 9 | 37.80 | 7.60 |
| 5 | 1 | 9 | 30.10 | 6.00 |
| 5 | 1 | 9 | 30.10 | 5.40 |
| 5 | 1 | 9 | 17.90 | 6.80 |
| 6 | -1 | 9 | 684.03 | 42.40 |
| 6 | -1 | 9 | 744.63 | 42.60 |
| 6 | -1 | 9 | 797.92 | 41.40 |
| 6 | -1 | 9 | 715.13 | 41.50 |
| 6 | 1 | 9 | 717.93 | 41.50 |
| 6 | 1 | 9 | 734.63 | 43.30 |
| 6 | 1 | 9 | 710.13 | 42.30 |
| 6 | 1 | 9 | 708.63 | 41.10 |
| 7 | -1 | 9 | -0.20 | 4.20 |
| 7 | -1 | 9 | 6.70 | 5.60 |
| 7 | -1 | 9 | 7.00 | 6.60 |
| 7 | 1 | 9 | -0.90 | 5.30 |
| 7 | 1 | 9 | 7.80 | 7.00 |
| 7 | 1 | 9 | 4.20 | 3.80 |

| | | | | |
|---|---|---|---|---|
| 8 | -1 | 9 | 381.36 | 23.40 |
| 8 | -1 | 9 | 392.66 | 24.90 |
| 8 | 1 | 9 | 408.06 | 25.10 |
| 8 | 1 | 9 | 385.06 | 23.40 |
| 9 | -1 | 9 | 5.70 | 4.00 |
| 9 | -1 | 9 | 7.30 | 5.20 |
| 9 | 1 | 9 | -2.70 | 4.50 |
| 9 | 1 | 9 | 5.60 | 3.30 |
| 10 | -1 | 9 | 1173.48 | 65.29 |
| 10 | -1 | 9 | 1190.88 | 65.89 |
| 10 | 1 | 9 | 1143.39 | 65.09 |
| 10 | 1 | 9 | 1221.08 | 65.99 |
| 11 | -1 | 9 | 0.90 | 3.80 |
| 11 | -1 | 9 | -5.50 | 3.20 |
| 11 | 1 | 9 | 0.60 | 3.20 |
| 11 | 1 | 9 | -2.60 | 3.60 |
| 12 | -1 | 9 | 12.70 | 3.90 |
| 12 | -1 | 9 | 9.30 | 3.30 |
| 12 | 1 | 9 | 12.70 | 3.60 |
| 12 | 1 | 9 | 13.40 | 3.30 |
| 13 | -1 | 9 | -0.50 | 3.40 |
| 13 | -1 | 9 | -2.00 | 2.60 |
| 13 | 1 | 9 | -3.10 | 2.80 |
| 13 | 1 | 9 | 2.40 | 2.30 |
| 16 | -2 | -9 | 2.60 | 2.00 |
| -16 | -2 | 9 | 3.20 | 1.90 |
| 16 | 2 | -9 | -1.60 | 1.60 |
| -16 | 2 | 9 | 2.00 | 1.70 |
| 15 | -2 | -9 | 605.14 | 33.70 |
| -15 | -2 | 9 | 573.94 | 33.70 |
| -15 | 2 | 9 | 642.34 | 34.00 |
| 15 | 2 | -9 | 606.74 | 33.60 |
| 14 | -2 | -9 | 12.20 | 5.30 |
| 14 | 2 | -9 | 7.40 | 2.50 |
| 13 | -2 | -9 | 19.20 | 3.50 |
| 13 | 2 | -9 | 24.10 | 4.80 |
| 12 | -2 | -9 | 11.20 | 3.40 |
| 12 | 2 | -9 | 13.70 | 3.00 |
| 11 | -2 | -9 | 1909.91 | 108.29 |
| 11 | 2 | -9 | 2080.49 | 108.29 |
| 10 | -2 | -9 | 9.70 | 4.00 |
| 9 | -2 | -9 | 681.23 | 38.80 |
| 9 | 2 | -9 | 684.53 | 38.40 |
| 8 | 2 | -9 | 135.39 | 11.20 |

| | | | | |
|---|---|---|---|---|
| -7 | -2 | 9 | 858.41 | 50.59 |
| -7 | 2 | 9 | 918.41 | 50.39 |
| -6 | -2 | 9 | 23.40 | 4.70 |
| -6 | 2 | 9 | 19.40 | 5.10 |
| -6 | 2 | 9 | 20.80 | 5.80 |
| -5 | -2 | 9 | 2342.47 | 123.29 |
| -5 | 2 | 9 | 2181.78 | 126.39 |
| -4 | -2 | 9 | 288.67 | 18.10 |
| -4 | 2 | 9 | 290.57 | 19.00 |
| -3 | -2 | 9 | 33.30 | 6.00 |
| -3 | 2 | 9 | 38.50 | 7.00 |
| -2 | -2 | 9 | 0.00 | 3.20 |
| -2 | 2 | 9 | -2.50 | 3.20 |
| -1 | -2 | 9 | 4534.55 | 233.68 |
| -1 | 2 | 9 | 4092.99 | 233.78 |
| 0 | -2 | 9 | 27.10 | 5.40 |
| 0 | 2 | 9 | 25.30 | 5.60 |
| 1 | -2 | 9 | 878.01 | 49.40 |
| 1 | 2 | 9 | 838.22 | 49.70 |
| 2 | -2 | 9 | 121.49 | 11.80 |
| 2 | 2 | 9 | 136.09 | 12.80 |
| 2 | 2 | 9 | 161.08 | 12.70 |
| 3 | 2 | 9 | 807.42 | 45.40 |
| 3 | 2 | 9 | 741.43 | 47.30 |
| 3 | 2 | 9 | 792.52 | 45.40 |
| 3 | 2 | 9 | 878.11 | 49.60 |
| 4 | -2 | 9 | 157.58 | 13.60 |
| 4 | -2 | 9 | 170.18 | 15.50 |
| 4 | -2 | 9 | 188.08 | 13.70 |
| 4 | 2 | 9 | 169.08 | 13.60 |
| 4 | 2 | 9 | 143.89 | 15.20 |
| 4 | 2 | 9 | 143.09 | 12.90 |
| 5 | -2 | 9 | 2527.65 | 143.19 |
| 5 | -2 | 9 | 2728.03 | 144.49 |
| 5 | -2 | 9 | 2739.43 | 143.19 |
| 5 | -2 | 9 | 2755.92 | 143.89 |
| 5 | 2 | 9 | 2448.46 | 142.99 |
| 5 | 2 | 9 | 2950.60 | 143.69 |
| 5 | 2 | 9 | 2342.07 | 145.39 |
| 5 | 2 | 9 | 2612.14 | 143.19 |
| 6 | -2 | 9 | 25.60 | 6.30 |
| 6 | -2 | 9 | 38.90 | 6.60 |
| 6 | -2 | 9 | 34.80 | 7.40 |
| 6 | -2 | 9 | 45.10 | 8.20 |

| | | | | |
|---|---|---|---|---|
| 6 | 2 | 9 | 33.70 | 6.30 |
| 6 | 2 | 9 | 30.30 | 5.90 |
| 6 | 2 | 9 | 29.90 | 6.70 |
| 6 | 2 | 9 | 22.80 | 7.60 |
| 7 | -2 | 9 | 109.49 | 13.00 |
| 7 | -2 | 9 | 107.79 | 11.90 |
| 7 | -2 | 9 | 85.69 | 9.80 |
| 7 | 2 | 9 | 94.99 | 14.70 |
| 7 | 2 | 9 | 104.39 | 11.00 |
| 7 | 2 | 9 | 103.09 | 9.00 |
| 7 | 2 | 9 | 98.29 | 11.20 |
| 8 | -2 | 9 | 21.80 | 5.90 |
| 8 | -2 | 9 | 30.50 | 5.00 |
| 8 | 2 | 9 | 21.70 | 4.40 |
| 8 | 2 | 9 | 14.10 | 5.40 |
| 9 | -2 | 9 | 629.14 | 37.10 |
| 9 | -2 | 9 | 626.74 | 36.10 |
| 9 | 2 | 9 | 583.04 | 37.00 |
| 9 | 2 | 9 | 687.43 | 36.10 |
| 10 | -2 | 9 | 20.40 | 5.10 |
| 10 | -2 | 9 | 22.10 | 4.70 |
| 10 | 2 | 9 | 21.80 | 5.20 |
| 10 | 2 | 9 | 30.00 | 5.50 |
| 11 | -2 | 9 | 360.66 | 21.50 |
| 11 | -2 | 9 | 305.27 | 20.30 |
| 11 | 2 | 9 | 328.17 | 20.20 |
| 11 | 2 | 9 | 316.87 | 19.70 |
| 12 | -2 | 9 | 38.50 | 6.50 |
| 12 | -2 | 9 | 26.60 | 5.50 |
| 12 | 2 | 9 | 33.20 | 5.20 |
| 12 | 2 | 9 | 30.40 | 5.40 |
| 13 | -2 | 9 | 281.37 | 16.20 |
| 13 | -2 | 9 | 249.88 | 15.90 |
| 13 | 2 | 9 | 272.67 | 15.90 |
| 13 | 2 | 9 | 248.08 | 15.60 |
| -16 | -3 | 9 | 377.86 | 21.40 |
| 16 | -3 | -9 | 388.26 | 21.40 |
| -16 | 3 | 9 | 377.46 | 21.50 |
| 16 | 3 | -9 | 380.16 | 21.30 |
| -15 | -3 | 9 | -0.30 | 2.30 |
| 15 | -3 | -9 | 0.40 | 2.40 |
| -15 | 3 | 9 | -0.50 | 1.80 |
| 15 | 3 | -9 | -2.50 | 1.80 |
| 14 | -3 | -9 | 453.75 | 26.90 |

| | | | | |
|---|---|---|---|---|
| -14 | -3 | 9 | 487.35 | 27.00 |
| 13 | -3 | -9 | 13.60 | 3.20 |
| 13 | 3 | -9 | 9.90 | 2.60 |
| 12 | -3 | -9 | 370.86 | 21.60 |
| 12 | 3 | -9 | 355.66 | 21.20 |
| 11 | -3 | -9 | 115.09 | 10.20 |
| 11 | 3 | -9 | 126.29 | 9.30 |
| 10 | -3 | -9 | 891.01 | 56.19 |
| 10 | 3 | -9 | 1099.69 | 55.09 |
| 9 | -3 | -9 | 170.58 | 13.70 |
| 9 | 3 | -9 | 162.08 | 11.80 |
| 8 | 3 | -9 | 216.28 | 14.70 |
| -7 | 3 | 9 | 448.86 | 27.20 |
| 7 | 3 | -9 | 443.86 | 26.60 |
| -6 | 3 | 9 | 3944.11 | 213.78 |
| -5 | -3 | 9 | 54.29 | 6.90 |
| -5 | 3 | 9 | 46.80 | 8.10 |
| -4 | -3 | 9 | 888.51 | 47.90 |
| -4 | 3 | 9 | 808.82 | 48.30 |
| -3 | -3 | 9 | 72.99 | 7.50 |
| -3 | 3 | 9 | 69.59 | 8.50 |
| -2 | -3 | 9 | 145.29 | 11.10 |
| -2 | 3 | 9 | 142.69 | 11.70 |
| -1 | -3 | 9 | 489.95 | 29.60 |
| -1 | 3 | 9 | 499.55 | 30.00 |
| 0 | -3 | 9 | 1688.53 | 89.09 |
| 0 | 3 | 9 | 1518.65 | 89.09 |
| 1 | -3 | 9 | 74.49 | 8.60 |
| 1 | 3 | 9 | 76.19 | 9.30 |
| 1 | 3 | 9 | 52.99 | 9.90 |
| 2 | -3 | 9 | 9.30 | 4.10 |
| 2 | 3 | 9 | 9.60 | 4.50 |
| 2 | 3 | 9 | 20.60 | 5.50 |
| 3 | -3 | 9 | 10.30 | 4.80 |
| 3 | -3 | 9 | 12.70 | 6.80 |
| 3 | 3 | 9 | 21.90 | 5.30 |
| 3 | 3 | 9 | 2.00 | 5.60 |
| 3 | 3 | 9 | 6.10 | 6.10 |
| 3 | 3 | 9 | 10.20 | 4.60 |
| 4 | -3 | 9 | 1384.86 | 67.49 |
| 4 | -3 | 9 | 1245.58 | 68.19 |
| 4 | -3 | 9 | 1253.57 | 67.79 |
| 4 | 3 | 9 | 1202.48 | 67.19 |
| 4 | 3 | 9 | 1211.78 | 66.89 |

| | | | | |
|---|---|---|---|---|
| 4 | 3 | 9 | 1130.29 | 66.79 |
| 4 | 3 | 9 | 1041.70 | 69.39 |
| 5 | -3 | 9 | 7.90 | 6.40 |
| 5 | -3 | 9 | -0.30 | 5.80 |
| 5 | -3 | 9 | -6.60 | 4.70 |
| 5 | -3 | 9 | -5.00 | 5.00 |
| 5 | 3 | 9 | -1.50 | 7.00 |
| 5 | 3 | 9 | -2.20 | 4.70 |
| 5 | 3 | 9 | 1.80 | 4.10 |
| 5 | 3 | 9 | 6.70 | 3.60 |
| 6 | -3 | 9 | 897.91 | 49.00 |
| 6 | -3 | 9 | 872.91 | 49.80 |
| 6 | -3 | 9 | 879.01 | 49.70 |
| 6 | -3 | 9 | 800.02 | 49.00 |
| 6 | 3 | 9 | 861.51 | 49.20 |
| 6 | 3 | 9 | 858.81 | 52.09 |
| 6 | 3 | 9 | 794.72 | 49.80 |
| 6 | 3 | 9 | 995.60 | 49.00 |
| 7 | -3 | 9 | 9.00 | 5.90 |
| 7 | -3 | 9 | 13.30 | 4.80 |
| 7 | 3 | 9 | 9.10 | 8.50 |
| 7 | 3 | 9 | 12.60 | 3.70 |
| 7 | 3 | 9 | 9.70 | 4.80 |
| 7 | 3 | 9 | 22.60 | 5.40 |
| 8 | -3 | 9 | 324.07 | 20.10 |
| 8 | -3 | 9 | 296.57 | 21.00 |
| 8 | 3 | 9 | 327.07 | 19.70 |
| 8 | 3 | 9 | 338.37 | 21.00 |
| 9 | -3 | 9 | 102.49 | 9.80 |
| 9 | -3 | 9 | 92.29 | 11.40 |
| 9 | 3 | 9 | 95.99 | 8.50 |
| 9 | 3 | 9 | 91.09 | 11.00 |
| 10 | -3 | 9 | 668.73 | 37.30 |
| 10 | -3 | 9 | 617.04 | 36.60 |
| 10 | 3 | 9 | 637.64 | 37.20 |
| 10 | 3 | 9 | 645.04 | 36.30 |
| 11 | -3 | 9 | 55.69 | 7.90 |
| 11 | -3 | 9 | 53.79 | 8.00 |
| 11 | 3 | 9 | 51.49 | 8.40 |
| 11 | 3 | 9 | 57.59 | 7.20 |
| 12 | -3 | 9 | 9.40 | 3.40 |
| 12 | -3 | 9 | 5.30 | 3.90 |
| 12 | 3 | 9 | 4.10 | 2.60 |
| 12 | 3 | 9 | 8.00 | 3.20 |

| | | | | |
|---|---|---|---|---|
| 13 | -3 | 9 | 12.40 | 4.00 |
| 13 | -3 | 9 | 12.40 | 3.20 |
| 13 | 3 | 9 | 14.70 | 3.20 |
| 13 | 3 | 9 | 19.40 | 4.00 |
| 16 | -4 | -9 | 4.00 | 2.10 |
| -16 | 4 | 9 | 6.80 | 1.80 |
| 16 | 4 | -9 | 6.60 | 1.70 |
| 15 | -4 | -9 | 267.97 | 15.50 |
| -15 | -4 | 9 | 251.37 | 15.60 |
| 15 | 4 | -9 | 270.27 | 15.30 |
| -15 | 4 | 9 | 267.57 | 15.60 |
| 14 | -4 | -9 | 10.40 | 3.10 |
| -14 | -4 | 9 | 13.10 | 3.40 |
| -14 | 4 | 9 | 13.10 | 3.90 |
| 14 | 4 | -9 | 10.20 | 2.70 |
| 13 | -4 | -9 | 4.10 | 3.00 |
| 13 | 4 | -9 | -4.10 | 2.00 |
| 12 | -4 | -9 | 5.70 | 3.60 |
| 12 | 4 | -9 | 8.60 | 2.90 |
| 11 | -4 | -9 | 1744.93 | 95.89 |
| 11 | 4 | -9 | 1778.92 | 95.69 |
| 10 | -4 | -9 | 1.60 | 4.60 |
| 10 | 4 | -9 | 1.80 | 3.10 |
| 9 | -4 | -9 | 387.66 | 24.60 |
| 9 | 4 | -9 | 401.26 | 23.20 |
| 8 | 4 | -9 | 120.19 | 9.80 |
| -8 | 4 | 9 | 106.29 | 10.40 |
| -7 | 4 | 9 | 2449.75 | 125.39 |
| 7 | 4 | -9 | 2135.39 | 124.29 |
| -6 | 4 | 9 | 243.48 | 18.00 |
| -6 | 4 | 9 | 258.77 | 17.40 |
| -5 | 4 | 9 | 2192.58 | 119.29 |
| -5 | 4 | 9 | 2159.88 | 119.59 |
| -4 | -4 | 9 | 68.89 | 7.80 |
| -4 | 4 | 9 | 76.99 | 9.10 |
| -3 | -4 | 9 | 91.29 | 8.70 |
| -3 | 4 | 9 | 98.89 | 9.80 |
| -2 | -4 | 9 | 204.28 | 14.20 |
| -2 | 4 | 9 | 198.18 | 14.80 |
| -1 | -4 | 9 | 4743.63 | 250.57 |
| -1 | 4 | 9 | 4504.35 | 250.57 |
| 0 | -4 | 9 | 22.60 | 5.60 |
| 0 | 4 | 9 | 20.70 | 5.30 |
| 0 | 4 | 9 | 21.20 | 5.50 |

| | | | | |
|---|---|---|---|---|
| 1 | -4 | 9 | 532.75 | 33.70 |
| 1 | 4 | 9 | 542.85 | 32.50 |
| 1 | 4 | 9 | 616.84 | 34.40 |
| 2 | -4 | 9 | 158.18 | 15.80 |
| 2 | -4 | 9 | 153.48 | 13.50 |
| 2 | 4 | 9 | 201.18 | 15.20 |
| 2 | 4 | 9 | 178.28 | 14.80 |
| 2 | 4 | 9 | 176.78 | 13.00 |
| 2 | 4 | 9 | 186.88 | 13.50 |
| 3 | -4 | 9 | 630.04 | 37.60 |
| 3 | -4 | 9 | 632.24 | 38.10 |
| 3 | 4 | 9 | 651.03 | 37.10 |
| 3 | 4 | 9 | 595.94 | 36.40 |
| 3 | 4 | 9 | 657.43 | 36.20 |
| 3 | 4 | 9 | 648.14 | 39.10 |
| 4 | -4 | 9 | 113.29 | 11.60 |
| 4 | -4 | 9 | 126.49 | 14.00 |
| 4 | -4 | 9 | 126.29 | 13.90 |
| 4 | 4 | 9 | 107.09 | 14.40 |
| 4 | 4 | 9 | 133.79 | 11.80 |
| 4 | 4 | 9 | 138.99 | 10.40 |
| 4 | 4 | 9 | 135.79 | 10.70 |
| 5 | -4 | 9 | 2531.45 | 140.19 |
| 5 | -4 | 9 | 2525.95 | 139.39 |
| 5 | -4 | 9 | 2545.95 | 139.09 |
| 5 | 4 | 9 | 2455.15 | 139.19 |
| 5 | 4 | 9 | 2657.93 | 139.19 |
| 5 | 4 | 9 | 2663.13 | 139.19 |
| 6 | -4 | 9 | 21.90 | 6.30 |
| 6 | -4 | 9 | 22.30 | 6.20 |
| 6 | -4 | 9 | 15.80 | 5.90 |
| 6 | 4 | 9 | 16.70 | 4.30 |
| 6 | 4 | 9 | 10.80 | 9.00 |
| 6 | 4 | 9 | 14.70 | 5.00 |
| 6 | 4 | 9 | 14.20 | 4.40 |
| 7 | -4 | 9 | 17.80 | 5.50 |
| 7 | -4 | 9 | 35.90 | 6.50 |
| 7 | 4 | 9 | 15.70 | 10.00 |
| 7 | 4 | 9 | 19.70 | 4.90 |
| 7 | 4 | 9 | 32.20 | 6.10 |
| 8 | -4 | 9 | 5.10 | 4.60 |
| 8 | -4 | 9 | 15.50 | 5.70 |
| 8 | 4 | 9 | 10.20 | 4.80 |
| 8 | 4 | 9 | 12.10 | 3.70 |

| | | | | |
|---|---|---|---|---|
| 9 | -4 | 9 | 852.41 | 46.60 |
| 9 | -4 | 9 | 922.31 | 48.10 |
| 9 | 4 | 9 | 792.22 | 46.10 |
| 9 | 4 | 9 | 734.63 | 48.00 |
| 10 | -4 | 9 | 6.10 | 4.40 |
| 10 | -4 | 9 | -0.30 | 4.60 |
| 10 | 4 | 9 | -7.20 | 3.80 |
| 10 | 4 | 9 | 1.10 | 3.00 |
| 11 | -4 | 9 | 323.67 | 20.60 |
| 11 | -4 | 9 | 333.67 | 20.50 |
| 11 | 4 | 9 | 329.37 | 20.40 |
| 11 | 4 | 9 | 339.57 | 20.00 |
| 12 | -4 | 9 | -4.10 | 3.40 |
| 12 | -4 | 9 | 6.10 | 5.40 |
| 12 | 4 | 9 | -3.30 | 2.40 |
| 12 | 4 | 9 | 2.10 | 3.00 |
| 13 | -4 | 9 | 247.38 | 15.50 |
| 13 | -4 | 9 | 253.97 | 16.10 |
| 13 | 4 | 9 | 286.87 | 15.50 |
| 13 | 4 | 9 | 239.98 | 15.20 |
| 16 | -5 | -9 | 263.17 | 16.60 |
| -16 | 5 | 9 | 287.17 | 16.40 |
| 16 | 5 | -9 | 309.07 | 16.20 |
| 15 | -5 | -9 | 0.50 | 2.30 |
| -15 | 5 | 9 | -0.60 | 1.70 |
| 15 | 5 | -9 | -1.50 | 1.50 |
| 14 | -5 | -9 | 54.69 | 5.90 |
| -14 | 5 | 9 | 44.90 | 5.40 |
| 14 | 5 | -9 | 51.79 | 4.90 |
| 13 | -5 | -9 | 42.80 | 6.20 |
| -13 | 5 | 9 | 47.50 | 5.50 |
| 13 | 5 | -9 | 31.20 | 4.80 |
| 12 | -5 | -9 | 838.32 | 46.90 |
| -12 | -5 | 9 | 789.72 | 47.20 |
| 12 | 5 | -9 | 890.71 | 46.70 |
| -12 | 5 | 9 | 864.01 | 47.40 |
| 11 | -5 | -9 | 1.30 | 4.00 |
| -11 | 5 | 9 | 1.80 | 3.30 |
| 11 | 5 | -9 | -0.50 | 2.50 |
| 10 | -5 | -9 | 383.26 | 23.90 |
| -10 | 5 | 9 | 408.06 | 23.70 |
| 10 | 5 | -9 | 386.56 | 22.80 |
| -9 | 5 | 9 | 5.90 | 3.80 |
| 8 | 5 | -9 | 18.10 | 4.10 |

| | | | | |
|---|---|---|---|---|
| -8 | 5 | 9 | 18.80 | 4.60 |
| 7 | 5 | -9 | 58.99 | 7.70 |
| 7 | 5 | -9 | 55.39 | 8.60 |
| -7 | 5 | 9 | 43.80 | 8.90 |
| -6 | -5 | 9 | 2047.80 | 116.19 |
| -6 | 5 | 9 | 2206.38 | 116.39 |
| -6 | 5 | 9 | 2287.17 | 116.69 |
| 6 | 5 | -9 | 1947.31 | 115.39 |
| -5 | 5 | 9 | 58.99 | 11.10 |
| -5 | 5 | 9 | 77.99 | 9.60 |
| -4 | 5 | 9 | 274.97 | 18.70 |
| -4 | 5 | 9 | 236.68 | 19.10 |
| -4 | 5 | 9 | 283.57 | 18.30 |
| -3 | 5 | 9 | 159.08 | 14.00 |
| -3 | 5 | 9 | 172.58 | 13.20 |
| -2 | -5 | 9 | 574.84 | 33.40 |
| -2 | 5 | 9 | 598.64 | 33.70 |
| -2 | 5 | 9 | 510.45 | 32.80 |
| -1 | -5 | 9 | 13.90 | 4.60 |
| -1 | 5 | 9 | 9.50 | 4.10 |
| -1 | 5 | 9 | 12.10 | 5.00 |
| 0 | -5 | 9 | 5002.80 | 245.68 |
| 0 | -5 | 9 | 4090.39 | 244.88 |
| 0 | 5 | 9 | 4614.14 | 243.28 |
| 0 | 5 | 9 | 4628.74 | 245.28 |
| 0 | 5 | 9 | 4252.67 | 244.98 |
| 1 | -5 | 9 | 127.59 | 12.60 |
| 1 | -5 | 9 | 161.38 | 14.80 |
| 1 | 5 | 9 | 175.58 | 13.50 |
| 1 | 5 | 9 | 172.08 | 12.40 |
| 1 | 5 | 9 | 156.78 | 13.90 |
| 1 | 5 | 9 | 165.58 | 13.00 |
| 2 | -5 | 9 | 130.39 | 13.50 |
| 2 | -5 | 9 | 93.49 | 10.40 |
| 2 | 5 | 9 | 94.89 | 11.30 |
| 2 | 5 | 9 | 103.89 | 9.60 |
| 2 | 5 | 9 | 111.59 | 10.40 |
| 2 | 5 | 9 | 106.19 | 11.60 |
| 3 | -5 | 9 | 62.59 | 9.10 |
| 3 | -5 | 9 | 68.19 | 12.50 |
| 3 | 5 | 9 | 82.89 | 8.70 |
| 3 | 5 | 9 | 64.09 | 10.90 |
| 3 | 5 | 9 | 45.00 | 9.50 |
| 3 | 5 | 9 | 57.49 | 10.40 |

| | | | | |
|---|---|---|---|---|
| 4 | -5 | 9 | 2062.99 | 111.49 |
| 4 | -5 | 9 | 2033.60 | 111.89 |
| 4 | 5 | 9 | 2021.90 | 110.89 |
| 4 | 5 | 9 | 1903.51 | 113.69 |
| 4 | 5 | 9 | 2328.57 | 110.79 |
| 4 | 5 | 9 | 1837.32 | 110.59 |
| 5 | -5 | 9 | 27.90 | 7.50 |
| 5 | -5 | 9 | 33.90 | 6.70 |
| 5 | 5 | 9 | 41.10 | 6.70 |
| 5 | 5 | 9 | 20.10 | 4.40 |
| 5 | 5 | 9 | 20.60 | 8.80 |
| 5 | 5 | 9 | 21.60 | 4.70 |
| 6 | -5 | 9 | 384.26 | 23.20 |
| 6 | -5 | 9 | 334.07 | 22.70 |
| 6 | -5 | 9 | 361.86 | 23.00 |
| 6 | 5 | 9 | 360.86 | 21.90 |
| 6 | 5 | 9 | 291.77 | 27.30 |
| 6 | 5 | 9 | 403.96 | 22.00 |
| 7 | -5 | 9 | 23.60 | 6.20 |
| 7 | -5 | 9 | 47.20 | 9.70 |
| 7 | 5 | 9 | 28.00 | 5.10 |
| 7 | 5 | 9 | 36.80 | 6.40 |
| 7 | 5 | 9 | 25.80 | 13.20 |
| 8 | -5 | 9 | 584.54 | 34.00 |
| 8 | -5 | 9 | 568.74 | 33.70 |
| 8 | 5 | 9 | 579.64 | 33.00 |
| 8 | 5 | 9 | 569.74 | 33.60 |
| 9 | -5 | 9 | 6.60 | 5.20 |
| 9 | -5 | 9 | 9.00 | 5.10 |
| 9 | 5 | 9 | 9.80 | 4.80 |
| 9 | 5 | 9 | 1.30 | 3.00 |
| 10 | -5 | 9 | 782.42 | 45.10 |
| 10 | -5 | 9 | 801.72 | 45.20 |
| 10 | 5 | 9 | 773.82 | 44.40 |
| 10 | 5 | 9 | 825.12 | 45.20 |
| 11 | -5 | 9 | -3.40 | 4.50 |
| 11 | -5 | 9 | 0.10 | 4.20 |
| 11 | 5 | 9 | 3.70 | 3.00 |
| 11 | 5 | 9 | 7.90 | 3.60 |
| 12 | -5 | 9 | -3.30 | 3.80 |
| 12 | -5 | 9 | 4.60 | 4.60 |
| 12 | 5 | 9 | -3.70 | 2.30 |
| 12 | 5 | 9 | 1.90 | 2.80 |
| 13 | -5 | 9 | -4.80 | 3.70 |

| | | | | |
|---|---|---|---|---|
| 13 | 5 | 9 | -1.50 | 2.80 |
| 13 | 5 | 9 | 0.10 | 2.10 |
| 15 | -6 | -9 | 207.98 | 12.80 |
| -15 | 6 | 9 | 219.48 | 12.70 |
| 15 | 6 | -9 | 209.88 | 12.40 |
| 14 | -6 | -9 | 4.00 | 2.70 |
| 14 | 6 | -9 | 2.20 | 1.80 |
| -14 | 6 | 9 | 3.50 | 2.30 |
| 13 | -6 | -9 | 3.10 | 6.00 |
| -13 | 6 | 9 | 3.90 | 2.50 |
| 13 | 6 | -9 | 6.10 | 2.20 |
| 12 | -6 | -9 | 35.60 | 6.60 |
| 12 | 6 | -9 | 34.80 | 5.00 |
| -12 | 6 | 9 | 41.50 | 5.80 |
| 11 | -6 | -9 | 604.14 | 36.70 |
| 11 | 6 | -9 | 657.23 | 36.30 |
| -11 | 6 | 9 | 687.43 | 37.10 |
| 10 | -6 | -9 | 5.80 | 5.10 |
| -10 | 6 | 9 | 3.10 | 3.50 |
| 10 | 6 | -9 | 2.70 | 2.90 |
| 9 | 6 | -9 | 39.20 | 6.30 |
| -9 | 6 | 9 | 44.70 | 7.30 |
| 8 | 6 | -9 | 42.30 | 7.00 |
| -8 | 6 | 9 | 46.00 | 8.00 |
| -7 | 6 | 9 | 440.96 | 27.90 |
| 7 | 6 | -9 | 471.45 | 27.00 |
| 7 | 6 | -9 | 456.45 | 27.40 |
| -6 | -6 | 9 | 10.50 | 5.80 |
| -6 | 6 | 9 | -1.20 | 5.40 |
| -6 | 6 | 9 | 2.90 | 5.20 |
| -5 | -6 | 9 | 1113.59 | 63.69 |
| -5 | 6 | 9 | 1185.08 | 64.49 |
| -5 | 6 | 9 | 1127.79 | 64.59 |
| -4 | -6 | 9 | -0.60 | 5.60 |
| -4 | 6 | 9 | -1.50 | 5.90 |
| -4 | 6 | 9 | 6.60 | 4.60 |
| -4 | 6 | 9 | 3.50 | 5.40 |
| -3 | -6 | 9 | 67.69 | 11.10 |
| -3 | 6 | 9 | 85.59 | 10.40 |
| -3 | 6 | 9 | 71.89 | 9.20 |
| -3 | 6 | 9 | 79.79 | 11.60 |
| -3 | 6 | 9 | 123.09 | 18.60 |
| -2 | -6 | 9 | 101.59 | 10.80 |
| -2 | 6 | 9 | 98.69 | 10.90 |

| | | | | |
|---|---|---|---|---|
| -2 | 6 | 9 | 97.19 | 12.30 |
| -2 | 6 | 9 | 99.19 | 11.90 |
| -2 | 6 | 9 | 106.99 | 10.20 |
| -1 | -6 | 9 | 3364.46 | 178.18 |
| -1 | 6 | 9 | 3612.44 | 179.58 |
| -1 | 6 | 9 | 3071.69 | 177.98 |
| -1 | 6 | 9 | 2937.51 | 178.38 |
| -1 | 6 | 9 | 3444.06 | 177.88 |
| 0 | -6 | 9 | 167.48 | 14.80 |
| 0 | 6 | 9 | 206.38 | 13.30 |
| 0 | 6 | 9 | 182.58 | 15.90 |
| 0 | 6 | 9 | 179.38 | 14.30 |
| 0 | 6 | 9 | 175.68 | 13.80 |
| 1 | -6 | 9 | 157.08 | 13.50 |
| 1 | 6 | 9 | 146.59 | 10.90 |
| 1 | 6 | 9 | 130.79 | 11.30 |
| 1 | 6 | 9 | 119.89 | 13.30 |
| 1 | 6 | 9 | 122.59 | 12.20 |
| 2 | -6 | 9 | 17.40 | 6.20 |
| 2 | 6 | 9 | 18.40 | 4.80 |
| 2 | 6 | 9 | 15.10 | 5.60 |
| 2 | 6 | 9 | 17.60 | 6.00 |
| 2 | 6 | 9 | 14.00 | 4.80 |
| 3 | -6 | 9 | 392.46 | 25.60 |
| 3 | 6 | 9 | 372.76 | 26.30 |
| 3 | 6 | 9 | 399.46 | 24.20 |
| 3 | 6 | 9 | 407.46 | 23.80 |
| 3 | 6 | 9 | 378.86 | 23.10 |
| 4 | -6 | 9 | 16.20 | 6.70 |
| 4 | 6 | 9 | 13.30 | 4.20 |
| 4 | 6 | 9 | 9.80 | 3.70 |
| 4 | 6 | 9 | 10.10 | 7.70 |
| 5 | -6 | 9 | 2894.91 | 142.29 |
| 5 | 6 | 9 | 2568.64 | 141.59 |
| 5 | 6 | 9 | 2590.54 | 141.69 |
| 5 | 6 | 9 | 2386.66 | 145.29 |
| 6 | -6 | 9 | 119.39 | 14.60 |
| 6 | 6 | 9 | 119.59 | 9.50 |
| 6 | 6 | 9 | 111.69 | 9.50 |
| 6 | 6 | 9 | 81.49 | 19.00 |
| 7 | -6 | 9 | 2.80 | 6.90 |
| 7 | -6 | 9 | 14.80 | 5.60 |
| 7 | 6 | 9 | 8.80 | 3.90 |
| 7 | 6 | 9 | 6.80 | 3.40 |

| | | | | |
|---|---|---|---|---|
| 7 | 6 | 9 | 5.50 | 11.80 |
| 8 | -6 | 9 | 14.00 | 5.80 |
| 8 | -6 | 9 | 11.30 | 6.50 |
| 8 | 6 | 9 | 7.00 | 3.60 |
| 8 | 6 | 9 | 5.20 | 4.20 |
| 9 | -6 | 9 | 674.93 | 40.30 |
| 9 | -6 | 9 | 695.93 | 40.20 |
| 9 | 6 | 9 | 700.23 | 39.40 |
| 9 | 6 | 9 | 728.23 | 40.20 |
| 10 | -6 | 9 | 14.90 | 6.10 |
| 10 | -6 | 9 | 15.90 | 5.20 |
| 10 | 6 | 9 | 13.10 | 4.00 |
| 10 | 6 | 9 | 24.90 | 5.40 |
| 11 | -6 | 9 | 162.08 | 13.40 |
| 11 | -6 | 9 | 181.28 | 13.40 |
| 11 | 6 | 9 | 184.28 | 11.90 |
| 11 | 6 | 9 | 184.28 | 12.30 |
| 12 | -6 | 9 | -2.60 | 5.20 |
| 12 | -6 | 9 | 9.70 | 5.30 |
| 12 | 6 | 9 | 5.60 | 2.50 |
| 12 | 6 | 9 | 5.20 | 4.00 |
| 15 | -7 | -9 | -0.30 | 2.50 |
| -15 | 7 | 9 | 1.30 | 1.70 |
| 15 | 7 | -9 | 1.20 | 1.40 |
| 14 | -7 | -9 | 184.78 | 12.20 |
| 14 | 7 | -9 | 193.68 | 11.60 |
| -14 | 7 | 9 | 209.98 | 12.30 |
| 13 | -7 | -9 | -4.00 | 3.00 |
| 13 | 7 | -9 | 4.30 | 2.60 |
| -13 | 7 | 9 | -2.60 | 3.40 |
| 12 | -7 | -9 | 533.65 | 33.10 |
| -12 | 7 | 9 | 638.84 | 33.70 |
| 12 | 7 | -9 | 596.04 | 32.90 |
| 11 | -7 | -9 | 47.10 | 7.90 |
| -11 | 7 | 9 | 51.99 | 6.30 |
| 11 | 7 | -9 | 39.10 | 5.40 |
| 10 | -7 | -9 | 572.14 | 35.20 |
| -10 | 7 | 9 | 635.94 | 35.30 |
| 10 | 7 | -9 | 627.34 | 34.40 |
| 9 | 7 | -9 | 10.50 | 3.30 |
| -9 | 7 | 9 | 18.50 | 4.30 |
| -8 | 7 | 9 | -4.10 | 4.00 |
| 8 | 7 | -9 | 6.30 | 3.70 |
| -7 | -7 | 9 | 79.79 | 11.90 |

| | | | | |
|---|---|---|---|---|
| -7 | 7 | 9 | 119.19 | 11.40 |
| 7 | 7 | -9 | 103.19 | 9.60 |
| 7 | 7 | -9 | 97.59 | 10.40 |
| -6 | -7 | 9 | 1990.30 | 118.09 |
| -6 | 7 | 9 | 2112.09 | 119.19 |
| -6 | 7 | 9 | 2374.26 | 119.49 |
| 6 | 7 | -9 | 2198.78 | 118.79 |
| -5 | -7 | 9 | 148.59 | 14.00 |
| -5 | 7 | 9 | 168.98 | 14.00 |
| -5 | 7 | 9 | 160.68 | 15.10 |
| -5 | 7 | 9 | 171.98 | 14.70 |
| -4 | -7 | 9 | 399.66 | 26.40 |
| -4 | 7 | 9 | 438.76 | 26.20 |
| -4 | 7 | 9 | 422.76 | 26.80 |
| -4 | 7 | 9 | 459.65 | 27.60 |
| -3 | -7 | 9 | 67.59 | 10.80 |
| -3 | 7 | 9 | 97.69 | 10.10 |
| -3 | 7 | 9 | 102.89 | 11.60 |
| -3 | 7 | 9 | 80.89 | 9.80 |
| -2 | -7 | 9 | 890.81 | 53.59 |
| -2 | -7 | 9 | 1077.99 | 54.09 |
| -2 | 7 | 9 | 991.20 | 54.19 |
| -2 | 7 | 9 | 823.02 | 53.39 |
| -2 | 7 | 9 | 1006.20 | 54.99 |
| -1 | -7 | 9 | 23.30 | 5.70 |
| -1 | 7 | 9 | 14.10 | 4.90 |
| -1 | 7 | 9 | 24.90 | 4.90 |
| -1 | 7 | 9 | 16.30 | 5.70 |
| -1 | 7 | 9 | 14.50 | 5.20 |
| 0 | -7 | 9 | 1980.60 | 106.19 |
| 0 | 7 | 9 | 1723.83 | 105.49 |
| 0 | 7 | 9 | 1997.60 | 107.29 |
| 0 | 7 | 9 | 2111.19 | 105.59 |
| 0 | 7 | 9 | 1854.71 | 106.19 |
| 1 | -7 | 9 | 278.97 | 19.90 |
| 1 | -7 | 9 | 315.37 | 20.90 |
| 1 | 7 | 9 | 294.77 | 20.90 |
| 1 | 7 | 9 | 319.37 | 20.80 |
| 1 | 7 | 9 | 327.67 | 19.10 |
| 1 | 7 | 9 | 302.27 | 19.70 |
| 2 | -7 | 9 | 110.59 | 12.70 |
| 2 | -7 | 9 | 152.68 | 13.70 |
| 2 | 7 | 9 | 138.59 | 10.30 |
| 2 | 7 | 9 | 123.09 | 12.70 |

| | | | | |
|---|---|---|---|---|
| 2 | 7 | 9 | 121.19 | 11.20 |
| 3 | -7 | 9 | 86.19 | 11.80 |
| 3 | -7 | 9 | 55.69 | 10.50 |
| 3 | 7 | 9 | 83.89 | 8.90 |
| 3 | 7 | 9 | 61.09 | 11.40 |
| 3 | 7 | 9 | 67.09 | 7.40 |
| 4 | -7 | 9 | 1921.21 | 107.49 |
| 4 | -7 | 9 | 1800.62 | 106.39 |
| 4 | 7 | 9 | 1942.41 | 106.59 |
| 4 | 7 | 9 | 1926.91 | 110.09 |
| 4 | 7 | 9 | 2195.08 | 106.99 |
| 5 | -7 | 9 | 68.29 | 10.80 |
| 5 | -7 | 9 | 56.59 | 9.70 |
| 5 | 7 | 9 | 62.09 | 7.60 |
| 5 | 7 | 9 | 63.49 | 6.90 |
| 6 | -7 | 9 | 76.89 | 11.00 |
| 6 | -7 | 9 | 102.09 | 10.30 |
| 6 | 7 | 9 | 63.79 | 7.80 |
| 6 | 7 | 9 | 83.69 | 7.70 |
| 7 | -7 | 9 | 75.79 | 11.60 |
| 7 | 7 | 9 | 143.69 | 23.50 |
| 7 | 7 | 9 | 81.39 | 7.90 |
| 7 | 7 | 9 | 81.49 | 7.80 |
| -8 | -7 | -9 | 339.67 | 21.20 |
| 8 | -7 | 9 | 349.67 | 22.30 |
| 8 | 7 | 9 | 365.16 | 21.40 |
| 8 | 7 | 9 | 329.57 | 20.70 |
| 9 | -7 | 9 | 2.10 | 5.10 |
| 9 | 7 | 9 | -1.70 | 2.90 |
| 9 | 7 | 9 | 1.10 | 3.20 |
| 10 | -7 | 9 | 502.95 | 30.50 |
| 10 | 7 | 9 | 545.35 | 29.80 |
| 10 | 7 | 9 | 512.95 | 30.20 |
| 11 | -7 | 9 | 45.90 | 9.50 |
| 11 | 7 | 9 | 63.99 | 6.70 |
| 11 | 7 | 9 | 64.99 | 6.40 |
| 12 | 7 | 9 | 7.90 | 2.80 |
| 12 | 7 | 9 | 7.40 | 2.90 |
| 15 | -8 | -9 | 270.77 | 16.20 |
| -15 | 8 | 9 | 289.87 | 16.20 |
| 15 | 8 | -9 | 285.87 | 15.90 |
| 14 | -8 | -9 | 11.60 | 3.00 |
| 14 | 8 | -9 | 21.20 | 2.90 |
| -14 | 8 | 9 | 18.80 | 3.60 |

| | | | | |
|---|---|---|---|---|
| 13 | -8 | -9 | 12.10 | 3.40 |
| -13 | 8 | 9 | 10.40 | 2.70 |
| 13 | 8 | -9 | 20.10 | 3.40 |
| 12 | -8 | -9 | 13.30 | 6.30 |
| 12 | 8 | -9 | 5.50 | 2.30 |
| -12 | 8 | 9 | 6.50 | 2.90 |
| 11 | -8 | -9 | 1046.40 | 66.39 |
| 11 | 8 | -9 | 1258.47 | 66.29 |
| -11 | 8 | 9 | 1337.87 | 67.09 |
| -10 | 8 | 9 | 134.79 | 10.40 |
| 10 | 8 | -9 | 126.99 | 9.20 |
| -9 | 8 | 9 | 608.44 | 34.60 |
| 9 | 8 | -9 | 581.94 | 33.60 |
| -8 | 8 | 9 | 14.50 | 4.80 |
| 8 | 8 | -9 | 14.70 | 3.90 |
| -7 | -8 | 9 | 1167.08 | 68.99 |
| 7 | 8 | -9 | 1296.47 | 69.99 |
| 7 | 8 | -9 | 1195.88 | 69.99 |
| -7 | 8 | 9 | 1342.37 | 72.79 |
| -6 | -8 | 9 | -4.50 | 5.20 |
| 6 | 8 | -9 | 5.00 | 4.30 |
| -6 | 8 | 9 | -2.80 | 5.40 |
| -6 | 8 | 9 | 1.50 | 6.60 |
| -5 | -8 | 9 | 1870.71 | 108.59 |
| -5 | 8 | 9 | 2060.99 | 108.69 |
| 5 | 8 | -9 | 2059.29 | 109.59 |
| -5 | 8 | 9 | 1980.40 | 109.79 |
| -5 | 8 | 9 | 1963.70 | 109.69 |
| -4 | -8 | 9 | 62.79 | 9.90 |
| -4 | 8 | 9 | 54.99 | 9.40 |
| 4 | 8 | -9 | 62.19 | 9.10 |
| -4 | 8 | 9 | 60.89 | 11.30 |
| -4 | 8 | 9 | 56.29 | 8.70 |
| -3 | -8 | 9 | 47.90 | 9.60 |
| -3 | 8 | 9 | 57.59 | 8.40 |
| -3 | 8 | 9 | 53.79 | 7.70 |
| -3 | 8 | 9 | 50.79 | 9.20 |
| -2 | -8 | 9 | 363.96 | 21.80 |
| -2 | -8 | 9 | 346.47 | 22.30 |
| -2 | 8 | 9 | 374.36 | 23.20 |
| -2 | 8 | 9 | 298.77 | 21.50 |
| -2 | 8 | 9 | 361.66 | 22.60 |
| -1 | -8 | 9 | 3114.09 | 174.68 |
| -1 | -8 | 9 | 3221.88 | 173.98 |

| | | | | |
|---|---|---|---|---|
| -1 | 8 | 9 | 3313.67 | 174.58 |
| -1 | 8 | 9 | 3290.57 | 176.18 |
| -1 | 8 | 9 | 3160.98 | 174.38 |
| 0 | -8 | 9 | 77.29 | 9.80 |
| 0 | -8 | 9 | 57.49 | 10.60 |
| 0 | 8 | 9 | 61.19 | 9.60 |
| 0 | 8 | 9 | 79.89 | 8.10 |
| 0 | 8 | 9 | 60.89 | 9.30 |
| 1 | -8 | 9 | 351.56 | 23.10 |
| 1 | -8 | 9 | 422.36 | 24.70 |
| 1 | 8 | 9 | 393.96 | 23.40 |
| 1 | 8 | 9 | 378.76 | 22.80 |
| 1 | 8 | 9 | 353.76 | 24.70 |
| 2 | -8 | 9 | 19.20 | 5.40 |
| 2 | -8 | 9 | 41.80 | 6.90 |
| 2 | 8 | 9 | 44.40 | 8.20 |
| 2 | 8 | 9 | 25.00 | 7.10 |
| 2 | 8 | 9 | 24.80 | 4.60 |
| 3 | -8 | 9 | 578.44 | 33.80 |
| 3 | -8 | 9 | 585.84 | 32.40 |
| 3 | 8 | 9 | 529.05 | 35.20 |
| 3 | 8 | 9 | 536.65 | 32.00 |
| 3 | 8 | 9 | 561.14 | 32.40 |
| 4 | -8 | 9 | 0.80 | 6.20 |
| 4 | -8 | 9 | 10.70 | 5.00 |
| 4 | 8 | 9 | 10.50 | 7.70 |
| 4 | 8 | 9 | 5.00 | 3.30 |
| 5 | -8 | 9 | 1591.84 | 87.59 |
| 5 | -8 | 9 | 1605.84 | 88.39 |
| 5 | 8 | 9 | 1669.53 | 87.89 |
| 5 | 8 | 9 | 1675.43 | 87.89 |
| 5 | 8 | 9 | 1474.35 | 91.99 |
| 6 | -8 | 9 | 112.39 | 12.00 |
| 6 | -8 | 9 | 111.09 | 10.70 |
| 6 | 8 | 9 | 132.69 | 9.50 |
| 6 | 8 | 9 | 102.19 | 21.40 |
| 6 | 8 | 9 | 126.59 | 9.70 |
| -7 | -8 | -9 | 23.50 | 4.60 |
| 7 | -8 | 9 | 32.40 | 4.90 |
| 7 | 8 | 9 | 24.60 | 4.60 |
| 7 | 8 | 9 | 8.10 | 14.90 |
| 7 | 8 | 9 | 26.10 | 4.30 |
| -8 | -8 | -9 | 7.40 | 4.10 |
| 8 | -8 | 9 | 6.40 | 4.00 |

| | | | | |
|---|---|---|---|---|
| 8 | 8 | 9 | 14.70 | 3.40 |
| 8 | 8 | 9 | 13.50 | 3.90 |
| 9 | -8 | 9 | 584.84 | 36.80 |
| 9 | 8 | 9 | 685.73 | 37.10 |
| 9 | 8 | 9 | 678.93 | 36.70 |
| 10 | 8 | 9 | 9.80 | 3.50 |
| 10 | 8 | 9 | 10.20 | 3.30 |
| 11 | 8 | 9 | 226.28 | 15.00 |
| 11 | 8 | 9 | 256.07 | 14.80 |
| 12 | 8 | 9 | 71.39 | 6.30 |
| 12 | 8 | 9 | 69.09 | 6.10 |
| 14 | -9 | -9 | 86.59 | 9.50 |
| -14 | 9 | 9 | 97.09 | 6.90 |
| 14 | 9 | -9 | 100.39 | 6.30 |
| 13 | -9 | -9 | 32.70 | 5.70 |
| 13 | 9 | -9 | 22.50 | 3.40 |
| -13 | 9 | 9 | 26.40 | 4.30 |
| 12 | -9 | -9 | 735.73 | 43.40 |
| -12 | 9 | 9 | 842.12 | 43.90 |
| 12 | 9 | -9 | 774.22 | 43.90 |
| 11 | -9 | -9 | -5.30 | 5.50 |
| -11 | 9 | 9 | 4.10 | 3.00 |
| 11 | 9 | -9 | 0.80 | 2.10 |
| -10 | 9 | 9 | 898.21 | 51.29 |
| 10 | 9 | -9 | 939.41 | 50.69 |
| -9 | 9 | 9 | 8.40 | 3.80 |
| 9 | 9 | -9 | -0.10 | 2.60 |
| -8 | 9 | 9 | 189.88 | 14.20 |
| 8 | 9 | -9 | 169.38 | 12.50 |
| -7 | -9 | 9 | 27.70 | 5.60 |
| 7 | 9 | -9 | 18.40 | 4.20 |
| -7 | 9 | 9 | 20.70 | 6.00 |
| 7 | 9 | -9 | 23.30 | 6.60 |
| -6 | -9 | 9 | 3369.86 | 190.18 |
| 6 | 9 | -9 | 3560.34 | 189.28 |
| -6 | 9 | 9 | 3788.22 | 188.38 |
| -6 | 9 | 9 | 3181.48 | 190.88 |
| -5 | -9 | 9 | 113.49 | 12.20 |
| -5 | 9 | 9 | 128.69 | 14.00 |
| -5 | 9 | 9 | 157.08 | 11.50 |
| 5 | 9 | -9 | 141.89 | 12.70 |
| -5 | 9 | 9 | 133.99 | 13.90 |
| -4 | -9 | 9 | 160.98 | 13.50 |
| -4 | 9 | 9 | 156.98 | 14.30 |

| | | | | |
|---|---|---|---|---|
| 4 | 9 | -9 | 162.08 | 14.10 |
| -4 | 9 | 9 | 165.68 | 12.70 |
| -4 | 9 | 9 | 184.38 | 15.20 |
| -3 | -9 | 9 | 33.70 | 5.70 |
| -3 | 9 | 9 | 18.40 | 5.80 |
| -3 | 9 | 9 | 28.70 | 5.30 |
| -3 | 9 | 9 | 36.40 | 7.00 |
| 3 | 9 | -9 | 49.80 | 9.60 |
| -2 | -9 | 9 | 1273.77 | 65.89 |
| -2 | -9 | 9 | 1141.69 | 66.49 |
| 2 | 9 | -9 | 1148.69 | 68.39 |
| -2 | 9 | 9 | 1180.88 | 66.49 |
| -2 | 9 | 9 | 1260.07 | 67.99 |
| -2 | 9 | 9 | 1160.68 | 66.39 |
| -1 | -9 | 9 | -5.90 | 4.10 |
| -1 | -9 | 9 | -7.10 | 4.60 |
| -1 | 9 | 9 | 4.30 | 4.10 |
| -1 | 9 | 9 | 8.60 | 5.00 |
| -1 | 9 | 9 | -5.20 | 4.70 |
| 0 | -9 | 9 | 3345.27 | 178.38 |
| 0 | -9 | 9 | 3277.67 | 177.38 |
| 0 | 9 | 9 | 3478.15 | 177.98 |
| 0 | 9 | 9 | 3049.29 | 177.68 |
| 0 | 9 | 9 | 3274.67 | 179.78 |
| 1 | -9 | 9 | 217.68 | 16.10 |
| 1 | -9 | 9 | 208.98 | 14.50 |
| 1 | 9 | 9 | 208.38 | 16.60 |
| 1 | 9 | 9 | 194.88 | 13.80 |
| 1 | 9 | 9 | 204.78 | 14.90 |
| 2 | -9 | 9 | 107.39 | 11.90 |
| 2 | -9 | 9 | 99.19 | 10.00 |
| 2 | 9 | 9 | 96.39 | 8.60 |
| 2 | 9 | 9 | 86.59 | 9.70 |
| 2 | 9 | 9 | 94.09 | 12.20 |
| 3 | -9 | 9 | 123.79 | 10.70 |
| 3 | -9 | 9 | 133.89 | 13.90 |
| 3 | 9 | 9 | 130.99 | 14.70 |
| -3 | 9 | -9 | 127.99 | 12.40 |
| 3 | 9 | 9 | 138.29 | 10.10 |
| 4 | -9 | 9 | 1923.81 | 106.89 |
| 4 | -9 | 9 | 1972.30 | 105.69 |
| 4 | 9 | 9 | 1799.32 | 105.99 |
| 4 | 9 | 9 | 1859.91 | 109.59 |
| 4 | 9 | 9 | 1980.70 | 106.09 |

| | | | | |
|---|---|---|---|---|
| -4 | 9 | -9 | 2147.39 | 108.39 |
| 5 | -9 | 9 | -0.30 | 5.00 |
| 5 | -9 | 9 | 2.20 | 4.00 |
| 5 | 9 | 9 | -1.90 | 2.80 |
| -5 | 9 | -9 | 1.00 | 6.30 |
| 5 | 9 | 9 | 0.60 | 3.50 |
| 5 | 9 | 9 | 14.60 | 10.30 |
| 6 | -9 | 9 | 392.16 | 23.70 |
| -6 | -9 | -9 | 402.96 | 23.10 |
| 6 | -9 | 9 | 405.76 | 22.90 |
| 6 | 9 | 9 | 327.77 | 32.70 |
| 6 | 9 | 9 | 375.06 | 22.80 |
| 6 | 9 | 9 | 383.46 | 22.80 |
| -7 | -9 | -9 | 137.89 | 10.70 |
| 7 | -9 | 9 | 139.19 | 10.70 |
| 7 | 9 | 9 | 121.99 | 28.00 |
| 7 | 9 | 9 | 149.49 | 10.40 |
| 7 | 9 | 9 | 137.69 | 10.40 |
| 8 | -9 | 9 | 327.37 | 19.80 |
| -8 | -9 | -9 | 331.27 | 20.10 |
| 8 | 9 | 9 | 328.37 | 19.80 |
| 8 | 9 | 9 | 335.47 | 19.90 |
| 9 | -9 | 9 | 11.00 | 3.50 |
| 9 | 9 | 9 | 8.90 | 3.30 |
| 9 | 9 | 9 | 4.10 | 3.50 |
| 10 | 9 | 9 | 723.53 | 39.30 |
| 10 | 9 | 9 | 688.13 | 39.70 |
| 11 | 9 | 9 | 42.00 | 6.00 |
| 11 | 9 | 9 | 41.30 | 5.50 |
| 14 | 10 | -9 | 14.30 | 2.40 |
| -14 | 10 | 9 | 11.50 | 2.40 |
| -13 | 10 | 9 | 10.00 | 2.60 |
| 13 | 10 | -9 | 11.10 | 1.90 |
| 12 | 10 | -9 | 57.69 | 4.70 |
| -12 | 10 | 9 | 47.70 | 5.80 |
| -11 | 10 | 9 | 651.23 | 43.80 |
| 11 | 10 | -9 | 855.51 | 41.90 |
| 10 | 10 | -9 | 79.49 | 6.20 |
| -10 | 10 | 9 | 59.89 | 7.70 |
| 9 | 10 | -9 | 275.67 | 17.20 |
| -9 | 10 | 9 | 296.67 | 18.60 |
| -8 | -10 | 9 | 150.78 | 13.00 |
| 8 | 10 | -9 | 173.78 | 11.50 |
| -8 | 10 | 9 | 153.98 | 13.40 |

| | | | | |
|---|---|---|---|---|
| -7 | -10 | 9 | 703.63 | 42.80 |
| 7 | 10 | -9 | 774.52 | 42.80 |
| -7 | 10 | 9 | 721.43 | 44.20 |
| 7 | 10 | -9 | 775.42 | 44.70 |
| -7 | 10 | 9 | 808.42 | 42.90 |
| -6 | -10 | 9 | -4.80 | 4.30 |
| 6 | 10 | -9 | -5.10 | 5.20 |
| -6 | 10 | 9 | 7.60 | 3.60 |
| -6 | 10 | 9 | -9.60 | 5.80 |
| -5 | -10 | 9 | 1633.84 | 99.99 |
| -5 | 10 | 9 | 1771.02 | 101.69 |
| -5 | 10 | 9 | 1815.32 | 101.19 |
| -5 | 10 | 9 | 1827.62 | 100.29 |
| 5 | 10 | -9 | 2103.99 | 103.49 |
| -4 | -10 | 9 | 204.78 | 15.20 |
| 4 | -10 | -9 | 224.98 | 16.60 |
| -4 | 10 | 9 | 192.98 | 14.80 |
| -4 | 10 | 9 | 208.08 | 17.30 |
| 4 | 10 | -9 | 228.98 | 17.10 |
| -4 | 10 | 9 | 190.68 | 17.20 |
| -3 | 10 | 9 | 24.50 | 6.30 |
| 3 | 10 | -9 | 26.20 | 6.70 |
| -3 | 10 | 9 | 23.40 | 4.80 |
| -3 | 10 | 9 | 39.60 | 6.80 |
| -2 | -10 | 9 | 52.99 | 7.60 |
| -2 | 10 | 9 | 53.69 | 9.70 |
| -2 | 10 | 9 | 36.40 | 7.60 |
| 2 | 10 | -9 | 51.79 | 11.10 |
| -2 | 10 | 9 | 60.69 | 10.50 |
| -1 | -10 | 9 | 1739.93 | 90.89 |
| 1 | 10 | -9 | 1478.35 | 93.69 |
| -1 | 10 | 9 | 1818.12 | 91.79 |
| -1 | 10 | 9 | 1485.05 | 91.29 |
| -1 | 10 | 9 | 1817.62 | 93.69 |
| 0 | -10 | 9 | 218.28 | 13.90 |
| 0 | 10 | -9 | 206.08 | 17.30 |
| 0 | 10 | 9 | 203.28 | 14.80 |
| 0 | 10 | 9 | 202.18 | 14.20 |
| 0 | 10 | 9 | 203.18 | 16.50 |
| 1 | -10 | 9 | 418.86 | 25.00 |
| 1 | 10 | 9 | 346.17 | 23.70 |
| 1 | 10 | 9 | 408.06 | 26.20 |
| -1 | 10 | -9 | 409.86 | 26.10 |
| 1 | 10 | 9 | 377.86 | 23.40 |

| | | | | |
|---|---|---|---|---|
| 2 | -10 | 9 | 38.10 | 6.70 |
| 2 | -10 | 9 | 31.20 | 4.70 |
| -2 | 10 | -9 | 27.70 | 10.10 |
| 2 | 10 | 9 | 40.50 | 5.90 |
| 2 | 10 | 9 | 32.60 | 6.20 |
| 3 | -10 | 9 | 167.88 | 13.20 |
| 3 | -10 | 9 | 209.78 | 15.80 |
| 3 | 10 | 9 | 200.98 | 14.00 |
| 3 | 10 | 9 | 203.28 | 18.20 |
| 3 | 10 | 9 | 211.68 | 13.30 |
| -3 | 10 | -9 | 190.88 | 15.80 |
| 4 | -10 | 9 | 24.20 | 5.40 |
| 4 | -10 | 9 | 29.50 | 4.50 |
| -4 | 10 | -9 | 23.70 | 6.60 |
| 4 | 10 | 9 | 24.70 | 4.80 |
| 4 | 10 | 9 | 34.90 | 5.40 |
| 4 | 10 | 9 | 29.70 | 9.20 |
| 5 | -10 | 9 | 2080.39 | 112.89 |
| -5 | -10 | -9 | 2094.19 | 113.49 |
| 5 | -10 | 9 | 2139.79 | 113.69 |
| 5 | 10 | 9 | 2075.39 | 113.29 |
| 5 | 10 | 9 | 2178.78 | 113.19 |
| 5 | 10 | 9 | 1839.32 | 117.79 |
| -5 | 10 | -9 | 2164.48 | 115.39 |
| 6 | -10 | 9 | 217.98 | 15.20 |
| -6 | -10 | -9 | 210.38 | 14.20 |
| 6 | -10 | 9 | 214.38 | 13.80 |
| 6 | 10 | 9 | 216.28 | 14.20 |
| 6 | 10 | 9 | 208.58 | 14.00 |
| -7 | -10 | -9 | 3.70 | 3.20 |
| 7 | -10 | 9 | 6.50 | 3.30 |
| 7 | 10 | 9 | 2.90 | 2.90 |
| 7 | 10 | 9 | -0.40 | 3.40 |
| 8 | -10 | 9 | 1.00 | 3.30 |
| -8 | -10 | -9 | 2.10 | 3.70 |
| 8 | 10 | 9 | -1.40 | 2.60 |
| 8 | 10 | 9 | -3.00 | 3.80 |
| 9 | -10 | 9 | 440.36 | 25.70 |
| 9 | 10 | 9 | 462.55 | 25.90 |
| 9 | 10 | 9 | 455.65 | 26.20 |
| 10 | 10 | 9 | 2.30 | 3.20 |
| 10 | 10 | 9 | 1.00 | 2.70 |
| 11 | 10 | 9 | 193.88 | 12.40 |
| 11 | 10 | 9 | 199.98 | 12.40 |

| | | | | |
|---|---|---|---|---|
| -13 | 11 | 9 | 100.69 | 7.60 |
| -13 | 11 | 9 | 115.49 | 8.10 |
| 13 | 11 | -9 | 112.89 | 6.80 |
| 12 | 11 | -9 | 371.86 | 20.40 |
| -12 | 11 | 9 | 353.36 | 20.90 |
| -11 | 11 | 9 | 20.60 | 3.70 |
| 11 | 11 | -9 | 29.80 | 3.90 |
| 10 | 11 | -9 | 456.95 | 23.00 |
| -10 | 11 | 9 | 336.87 | 25.30 |
| -9 | 11 | 9 | 44.90 | 5.70 |
| 9 | 11 | -9 | 37.90 | 5.20 |
| -9 | 11 | 9 | 51.49 | 8.20 |
| -8 | -11 | 9 | 9.30 | 4.30 |
| -8 | 11 | 9 | -0.50 | 4.90 |
| 8 | 11 | -9 | 1.70 | 2.90 |
| -8 | 11 | 9 | 5.60 | 3.50 |
| -7 | -11 | 9 | 299.27 | 20.10 |
| 7 | 11 | -9 | 302.87 | 23.20 |
| -7 | 11 | 9 | 308.67 | 22.00 |
| 7 | 11 | -9 | 343.07 | 19.70 |
| -7 | 11 | 9 | 339.97 | 19.90 |
| -6 | -11 | 9 | 623.04 | 38.10 |
| 6 | 11 | -9 | 727.93 | 40.50 |
| -6 | 11 | 9 | 754.72 | 38.50 |
| -6 | 11 | 9 | 558.24 | 39.40 |
| -5 | -11 | 9 | 144.39 | 12.40 |
| 5 | -11 | -9 | 156.88 | 13.40 |
| -5 | 11 | 9 | 159.18 | 15.10 |
| -5 | 11 | 9 | 131.59 | 17.00 |
| -5 | 11 | 9 | 154.08 | 11.90 |
| 5 | 11 | -9 | 156.38 | 14.80 |
| -4 | -11 | 9 | 64.19 | 8.90 |
| 4 | -11 | -9 | 53.69 | 9.70 |
| -4 | 11 | 9 | 72.99 | 11.60 |
| 4 | 11 | -9 | 54.69 | 11.60 |
| -4 | 11 | 9 | 78.19 | 12.40 |
| -4 | 11 | 9 | 66.69 | 8.10 |
| -3 | -11 | 9 | 228.68 | 16.10 |
| 3 | -11 | -9 | 233.98 | 16.60 |
| -3 | 11 | 9 | 227.08 | 15.70 |
| -3 | 11 | 9 | 249.48 | 18.50 |
| -3 | 11 | 9 | 226.08 | 16.90 |
| 3 | 11 | -9 | 190.38 | 18.70 |
| 2 | -11 | -9 | 211.58 | 15.60 |

| | | | | |
|---|---|---|---|---|
| -2 | -11 | 9 | 225.58 | 13.30 |
| -2 | -11 | 9 | 194.08 | 14.60 |
| -2 | 11 | 9 | 179.18 | 14.90 |
| 2 | 11 | -9 | 175.28 | 17.70 |
| -2 | 11 | 9 | 211.48 | 16.90 |
| -2 | 11 | 9 | 185.18 | 14.20 |
| -1 | -11 | 9 | 95.29 | 8.30 |
| -1 | -11 | 9 | 79.59 | 10.00 |
| -1 | 11 | 9 | 99.29 | 12.40 |
| -1 | 11 | 9 | 94.09 | 9.20 |
| 1 | 11 | -9 | 86.09 | 13.30 |
| -1 | 11 | 9 | 95.39 | 10.50 |
| 0 | -11 | 9 | 1401.36 | 73.69 |
| 0 | -11 | 9 | 1335.87 | 72.39 |
| 0 | 11 | -9 | 1312.27 | 75.59 |
| 0 | 11 | 9 | 1347.57 | 75.29 |
| 0 | 11 | 9 | 1339.17 | 73.09 |
| 0 | 11 | 9 | 1197.68 | 72.69 |
| 1 | -11 | 9 | 236.18 | 15.30 |
| 1 | -11 | 9 | 263.07 | 17.50 |
| 1 | 11 | 9 | 232.58 | 16.50 |
| 1 | 11 | 9 | 217.48 | 18.90 |
| 1 | 11 | 9 | 262.37 | 16.00 |
| -1 | 11 | -9 | 249.28 | 19.00 |
| 2 | -11 | 9 | -3.10 | 4.70 |
| 2 | -11 | 9 | 7.60 | 3.30 |
| 2 | 11 | 9 | 11.30 | 7.40 |
| 2 | 11 | 9 | 20.40 | 4.50 |
| 2 | 11 | 9 | 6.50 | 3.20 |
| -2 | 11 | -9 | 7.80 | 5.60 |
| 3 | -11 | 9 | 88.19 | 10.80 |
| 3 | -11 | 9 | 62.19 | 7.20 |
| -3 | -11 | -9 | 62.29 | 8.20 |
| 3 | 11 | 9 | 68.39 | 7.10 |
| 3 | 11 | 9 | 68.89 | 8.80 |
| -3 | 11 | -9 | 56.89 | 11.70 |
| 4 | -11 | 9 | 910.61 | 51.59 |
| 4 | -11 | 9 | 938.71 | 50.39 |
| -4 | -11 | -9 | 885.21 | 50.99 |
| 4 | 11 | 9 | 841.52 | 55.19 |
| -4 | 11 | -9 | 878.01 | 53.19 |
| 4 | 11 | 9 | 913.01 | 50.69 |
| 4 | 11 | 9 | 1009.20 | 50.99 |
| 5 | -11 | 9 | 15.10 | 4.80 |

| | | | | |
|---|---|---|---|---|
| -5 | -11 | -9 | 0.80 | 3.30 |
| 5 | -11 | 9 | 6.60 | 3.40 |
| 5 | 11 | 9 | 5.50 | 3.80 |
| 5 | 11 | 9 | 14.30 | 12.80 |
| 5 | 11 | 9 | 5.30 | 3.30 |
| -6 | -11 | -9 | 165.48 | 11.40 |
| 6 | -11 | 9 | 166.08 | 11.10 |
| 6 | -11 | 9 | 159.68 | 12.70 |
| 6 | 11 | 9 | 155.78 | 11.50 |
| 6 | 11 | 9 | 161.68 | 11.20 |
| -7 | -11 | -9 | 137.89 | 13.30 |
| 7 | 11 | 9 | 137.49 | 9.80 |
| 7 | 11 | 9 | 147.09 | 13.00 |
| -8 | -11 | -9 | 187.48 | 14.00 |
| 8 | -11 | 9 | 148.59 | 10.30 |
| 8 | 11 | 9 | 153.98 | 11.00 |
| 8 | 11 | 9 | 147.99 | 10.60 |
| 9 | -11 | 9 | 64.09 | 6.20 |
| 9 | 11 | 9 | 47.50 | 7.20 |
| 9 | 11 | 9 | 60.79 | 5.90 |
| 10 | 11 | 9 | 262.37 | 15.90 |
| 13 | 12 | -9 | 60.69 | 4.10 |
| -13 | 12 | 9 | 53.79 | 5.80 |
| 12 | 12 | -9 | 57.39 | 4.30 |
| -12 | 12 | 9 | 59.69 | 5.90 |
| -12 | 12 | 9 | 51.29 | 6.00 |
| -11 | 12 | 9 | 416.66 | 24.10 |
| -11 | 12 | 9 | 424.96 | 23.80 |
| 11 | 12 | -9 | 412.86 | 23.40 |
| 10 | 12 | -9 | 7.00 | 2.20 |
| -10 | 12 | 9 | 9.30 | 4.00 |
| -10 | 12 | 9 | 7.30 | 2.60 |
| -9 | 12 | 9 | 74.29 | 14.60 |
| -9 | 12 | 9 | 71.99 | 6.70 |
| 9 | 12 | -9 | 73.19 | 6.20 |
| -8 | -12 | 9 | 249.58 | 16.40 |
| -8 | 12 | 9 | 257.67 | 16.00 |
| 8 | 12 | -9 | 248.08 | 15.60 |
| -8 | 12 | 9 | 262.57 | 18.20 |
| -7 | -12 | 9 | 345.47 | 21.60 |
| -7 | 12 | 9 | 328.37 | 23.50 |
| 7 | 12 | -9 | 337.67 | 21.00 |
| 7 | 12 | -9 | 362.96 | 25.70 |
| -7 | 12 | 9 | 386.16 | 21.50 |

| | | | | |
|---|---|---|---|---|
| -6 | -12 | 9 | 8.60 | 4.10 |
| -6 | 12 | 9 | 2.50 | 6.60 |
| -6 | 12 | 9 | 6.90 | 5.30 |
| 6 | 12 | -9 | 1.60 | 7.00 |
| -5 | -12 | 9 | 606.24 | 36.20 |
| 5 | -12 | -9 | 632.84 | 36.20 |
| 5 | 12 | -9 | 613.74 | 38.90 |
| -5 | 12 | 9 | 603.74 | 37.50 |
| -5 | 12 | 9 | 682.43 | 36.50 |
| -4 | -12 | 9 | 123.59 | 11.00 |
| 4 | -12 | -9 | 123.19 | 11.60 |
| 4 | 12 | -9 | 126.59 | 15.00 |
| -4 | 12 | 9 | 127.69 | 10.80 |
| -4 | 12 | 9 | 113.49 | 13.10 |
| -4 | 12 | 9 | 138.19 | 16.50 |
| 3 | -12 | -9 | 13.00 | 4.60 |
| -3 | -12 | 9 | 11.50 | 4.20 |
| 3 | 12 | -9 | 13.90 | 6.80 |
| -3 | 12 | 9 | 10.50 | 3.80 |
| -3 | 12 | 9 | 13.10 | 7.20 |
| -3 | 12 | 9 | 12.00 | 5.70 |
| 2 | -12 | -9 | 76.29 | 9.60 |
| -2 | -12 | 9 | 65.89 | 8.80 |
| -2 | -12 | 9 | 74.69 | 7.30 |
| -2 | 12 | 9 | 67.39 | 10.50 |
| -2 | 12 | 9 | 76.99 | 12.80 |
| -2 | 12 | 9 | 57.29 | 8.20 |
| 2 | 12 | -9 | 52.29 | 13.50 |
| 1 | -12 | -9 | 739.33 | 42.40 |
| -1 | -12 | 9 | 773.32 | 42.20 |
| -1 | -12 | 9 | 750.32 | 40.90 |
| 1 | 12 | -9 | 622.34 | 44.80 |
| -1 | 12 | 9 | 713.73 | 41.80 |
| -1 | 12 | 9 | 757.12 | 44.40 |
| -1 | 12 | 9 | 761.92 | 41.80 |
| 0 | -12 | 9 | 14.70 | 3.30 |
| 0 | -12 | -9 | 11.00 | 4.70 |
| 0 | -12 | 9 | 13.70 | 4.90 |
| 0 | 12 | -9 | 15.70 | 8.30 |
| 0 | 12 | 9 | 7.90 | 5.10 |
| 0 | 12 | 9 | 20.70 | 7.20 |
| 0 | 12 | 9 | 22.90 | 4.50 |
| 1 | -12 | 9 | 130.29 | 9.50 |
| -1 | -12 | -9 | 114.59 | 11.20 |

| | | | | |
|---|---|---|---|---|
| 1 | -12 | 9 | 134.59 | 11.80 |
| 1 | 12 | 9 | 96.59 | 14.40 |
| 1 | 12 | 9 | 129.79 | 11.30 |
| -1 | 12 | -9 | 112.49 | 14.60 |
| 1 | 12 | 9 | 135.39 | 10.00 |
| 2 | -12 | 9 | 117.19 | 8.90 |
| -2 | -12 | -9 | 108.09 | 10.10 |
| 2 | -12 | 9 | 133.79 | 11.50 |
| 2 | 12 | 9 | 108.69 | 14.70 |
| -2 | 12 | -9 | 87.29 | 13.60 |
| 2 | 12 | 9 | 124.29 | 9.40 |
| 2 | 12 | 9 | 89.79 | 10.40 |
| 3 | -12 | 9 | 274.07 | 16.80 |
| -3 | -12 | -9 | 260.17 | 17.60 |
| 3 | -12 | 9 | 308.37 | 19.10 |
| -3 | 12 | -9 | 289.57 | 21.10 |
| 3 | 12 | 9 | 273.67 | 23.20 |
| 3 | 12 | 9 | 256.07 | 17.40 |
| -4 | -12 | -9 | 10.00 | 3.30 |
| 4 | -12 | 9 | 12.20 | 4.60 |
| 4 | -12 | 9 | 14.40 | 3.20 |
| 4 | 12 | 9 | 12.00 | 3.30 |
| 4 | 12 | 9 | 12.30 | 4.30 |
| 4 | 12 | 9 | -6.40 | 11.30 |
| -4 | 12 | -9 | 5.50 | 8.60 |
| -5 | -12 | -9 | 505.15 | 29.40 |
| 5 | -12 | 9 | 524.55 | 30.00 |
| 5 | -12 | 9 | 528.35 | 29.00 |
| 5 | 12 | 9 | 500.85 | 29.40 |
| 5 | 12 | 9 | 503.75 | 29.40 |
| 6 | -12 | 9 | 115.79 | 9.70 |
| -6 | -12 | -9 | 99.39 | 8.60 |
| 6 | -12 | 9 | 97.89 | 9.80 |
| 6 | 12 | 9 | 104.39 | 9.70 |
| 6 | 12 | 9 | 109.29 | 8.20 |
| -7 | -12 | -9 | 63.69 | 6.50 |
| 7 | -12 | 9 | 54.59 | 6.00 |
| 7 | 12 | 9 | 50.89 | 5.60 |
| -8 | -12 | -9 | -1.40 | 3.00 |
| 8 | -12 | 9 | -1.90 | 2.50 |
| 8 | 12 | 9 | -0.70 | 2.50 |
| 9 | -12 | 9 | 346.67 | 19.00 |
| 9 | 12 | 9 | 318.57 | 19.80 |
| 10 | 12 | 9 | 0.80 | 2.50 |

| | | | | |
|---|---|---|---|---|
| -12 | 13 | 9 | 400.56 | 23.00 |
| 12 | 13 | -9 | 417.06 | 22.50 |
| 11 | 13 | -9 | 6.70 | 1.60 |
| -11 | 13 | 9 | 7.80 | 2.60 |
| -10 | 13 | 9 | 158.68 | 10.30 |
| 10 | 13 | -9 | 156.18 | 9.70 |
| 9 | 13 | -9 | 8.70 | 2.30 |
| -9 | 13 | 9 | 14.50 | 5.70 |
| -9 | 13 | 9 | 6.10 | 2.70 |
| -8 | -13 | 9 | 56.09 | 8.10 |
| -8 | 13 | 9 | 76.89 | 11.80 |
| 8 | 13 | -9 | 44.00 | 5.80 |
| -8 | 13 | 9 | 65.09 | 6.20 |
| -7 | -13 | 9 | 87.69 | 9.10 |
| -7 | 13 | 9 | 98.39 | 8.20 |
| 7 | 13 | -9 | 98.69 | 7.90 |
| -7 | 13 | 9 | 110.19 | 19.00 |
| -6 | -13 | 9 | 946.31 | 53.79 |
| -6 | 13 | 9 | 988.10 | 53.99 |
| -5 | -13 | 9 | 6.10 | 3.90 |
| 5 | -13 | -9 | 9.70 | 4.30 |
| -5 | 13 | 9 | 13.70 | 3.90 |
| 5 | 13 | -9 | 10.40 | 8.00 |
| 4 | -13 | -9 | 151.58 | 12.10 |
| -4 | -13 | 9 | 156.18 | 12.40 |
| -4 | 13 | 9 | 167.88 | 13.20 |
| -4 | 13 | 9 | 164.38 | 19.40 |
| 4 | 13 | -9 | 161.98 | 17.90 |
| 3 | -13 | -9 | 88.09 | 9.60 |
| -3 | -13 | 9 | 89.99 | 9.30 |
| -3 | 13 | 9 | 88.29 | 11.30 |
| 3 | 13 | -9 | 84.79 | 15.40 |
| -3 | 13 | 9 | 104.89 | 15.80 |
| -2 | -13 | 9 | 358.16 | 21.80 |
| 2 | -13 | -9 | 390.76 | 22.30 |
| -2 | -13 | 9 | 350.16 | 20.60 |
| 2 | 13 | -9 | 279.67 | 25.80 |
| -2 | 13 | 9 | 383.36 | 25.90 |
| -2 | 13 | 9 | 333.57 | 21.80 |
| -1 | -13 | 9 | 16.40 | 3.30 |
| 1 | -13 | -9 | 17.70 | 4.60 |
| -1 | -13 | 9 | 20.60 | 4.80 |
| -1 | 13 | 9 | 14.00 | 3.90 |
| -1 | 13 | 9 | 20.80 | 7.50 |

| | | | | |
|---|---|---|---|---|
| 1 | 13 | -9 | 12.10 | 8.80 |
| 0 | -13 | 9 | 1278.47 | 68.49 |
| 0 | -13 | 9 | 1317.77 | 69.79 |
| 0 | -13 | -9 | 1211.28 | 69.79 |
| 0 | 13 | 9 | 1422.36 | 71.29 |
| 0 | 13 | 9 | 1180.78 | 71.79 |
| 0 | 13 | -9 | 1105.59 | 72.09 |
| 1 | -13 | 9 | 122.49 | 11.30 |
| 1 | -13 | 9 | 131.89 | 9.30 |
| -1 | -13 | -9 | 121.09 | 10.80 |
| 1 | 13 | 9 | 108.29 | 15.70 |
| 1 | 13 | 9 | 127.39 | 10.50 |
| -1 | 13 | -9 | 132.29 | 16.30 |
| 2 | -13 | 9 | 95.49 | 8.00 |
| 2 | -13 | 9 | 98.59 | 10.60 |
| -2 | -13 | -9 | 108.19 | 9.70 |
| 2 | 13 | 9 | 114.39 | 8.80 |
| -2 | 13 | -9 | 100.99 | 15.40 |
| 2 | 13 | 9 | 95.49 | 16.00 |
| 3 | -13 | 9 | 134.69 | 11.20 |
| -3 | -13 | -9 | 126.79 | 9.60 |
| 3 | -13 | 9 | 105.99 | 8.50 |
| 3 | 13 | 9 | 104.79 | 9.00 |
| -4 | -13 | -9 | 1037.00 | 59.79 |
| 4 | -13 | 9 | 1072.39 | 60.19 |
| 4 | -13 | 9 | 1144.99 | 59.39 |
| 4 | 13 | 9 | 1076.99 | 59.69 |
| 5 | -13 | 9 | 1.10 | 4.10 |
| -5 | -13 | -9 | 2.10 | 2.70 |
| 5 | 13 | 9 | -0.20 | 2.50 |
| -6 | -13 | -9 | 190.88 | 11.90 |
| 6 | -13 | 9 | 169.38 | 11.30 |
| 6 | 13 | 9 | 182.48 | 11.60 |
| -7 | -13 | -9 | 53.89 | 5.80 |
| 7 | -13 | 9 | 57.49 | 5.40 |
| 7 | 13 | 9 | 55.59 | 5.50 |
| 8 | -13 | 9 | 210.48 | 11.90 |
| -8 | -13 | -9 | 191.48 | 12.30 |
| 8 | 13 | 9 | 191.48 | 12.30 |
| 9 | -13 | 9 | 11.20 | 2.80 |
| 9 | 13 | 9 | 12.30 | 2.80 |
| 11 | 14 | -9 | 351.86 | 19.70 |
| -11 | 14 | 9 | 360.56 | 20.20 |
| 10 | 14 | -9 | 16.70 | 2.60 |

| | | | | |
|---|---|---|---|---|
| -10 | 14 | 9 | 10.00 | 2.50 |
| 9 | 14 | -9 | 61.39 | 4.90 |
| -9 | 14 | 9 | 67.39 | 5.70 |
| -8 | -14 | 9 | 49.60 | 6.50 |
| -8 | 14 | 9 | 55.99 | 5.50 |
| 8 | 14 | -9 | 50.59 | 5.00 |
| -7 | -14 | 9 | 287.87 | 18.20 |
| -7 | 14 | 9 | 309.37 | 18.00 |
| -6 | -14 | 9 | -1.20 | 3.30 |
| -6 | 14 | 9 | 1.00 | 2.80 |
| -5 | -14 | 9 | 350.16 | 22.00 |
| 5 | -14 | -9 | 383.56 | 22.00 |
| -5 | 14 | 9 | 364.76 | 22.10 |
| -4 | -14 | 9 | 36.00 | 6.80 |
| 4 | -14 | -9 | 49.10 | 7.40 |
| -4 | 14 | 9 | 31.40 | 6.70 |
| 3 | -14 | -9 | 2.80 | 3.60 |
| -3 | -14 | 9 | -1.30 | 3.10 |
| -3 | 14 | 9 | 3.30 | 3.50 |
| -2 | -14 | 9 | 100.19 | 9.20 |
| 2 | -14 | -9 | 106.49 | 9.40 |
| 2 | 14 | -9 | 54.09 | 17.20 |
| -2 | 14 | 9 | 87.89 | 8.70 |
| -1 | -14 | 9 | 916.51 | 51.19 |
| 1 | -14 | -9 | 968.00 | 52.39 |
| -1 | -14 | 9 | 932.81 | 52.09 |
| -1 | 14 | 9 | 897.91 | 51.99 |
| 0 | -14 | -9 | 24.00 | 4.90 |
| 0 | -14 | 9 | 34.20 | 7.00 |
| 0 | -14 | 9 | 25.00 | 5.00 |
| 0 | 14 | 9 | 29.60 | 6.10 |
| 1 | -14 | 9 | 184.68 | 13.10 |
| 1 | -14 | 9 | 167.08 | 11.40 |
| -1 | -14 | -9 | 187.08 | 12.90 |
| 1 | 14 | 9 | 163.48 | 12.10 |
| 2 | -14 | 9 | 71.59 | 6.30 |
| 2 | -14 | 9 | 83.59 | 8.70 |
| -2 | -14 | -9 | 61.59 | 7.30 |
| 2 | 14 | 9 | 58.59 | 6.50 |
| -3 | -14 | -9 | 122.19 | 9.30 |
| 3 | -14 | 9 | 139.79 | 10.90 |
| 3 | -14 | 9 | 105.59 | 8.40 |
| 3 | 14 | 9 | 112.89 | 8.80 |
| 4 | -14 | 9 | 53.89 | 7.60 |

| | | | | |
|---|---|---|---|---|
| -4 | -14 | -9 | 36.60 | 7.30 |
| 4 | -14 | 9 | 39.00 | 5.20 |
| 4 | 14 | 9 | 35.60 | 5.00 |
| 5 | -14 | 9 | 571.94 | 30.90 |
| 5 | -14 | 9 | 556.84 | 31.40 |
| -5 | -14 | -9 | 558.84 | 31.20 |
| 5 | 14 | 9 | 527.95 | 31.20 |
| 6 | -14 | 9 | 91.19 | 6.80 |
| -6 | -14 | -9 | 92.59 | 7.20 |
| 6 | 14 | 9 | 100.49 | 7.20 |
| 7 | -14 | 9 | -1.10 | 1.90 |
| -7 | -14 | -9 | 8.40 | 2.40 |
| 7 | 14 | 9 | 1.60 | 2.40 |
| -8 | -14 | -9 | 37.10 | 5.70 |
| 8 | -14 | 9 | 24.30 | 4.80 |
| 8 | 14 | 9 | 24.70 | 4.20 |
| 10 | 15 | -9 | 251.87 | 14.20 |
| -10 | 15 | 9 | 252.97 | 14.70 |
| -9 | 15 | 9 | 18.20 | 3.70 |
| 9 | 15 | -9 | 19.40 | 2.80 |
| -8 | -15 | 9 | 5.80 | 2.70 |
| -8 | 15 | 9 | 4.50 | 2.30 |
| 8 | 15 | -9 | 3.60 | 1.80 |
| -7 | -15 | 9 | 111.69 | 8.60 |
| 7 | 15 | -9 | 116.39 | 7.90 |
| -7 | 15 | 9 | 110.79 | 8.20 |
| -6 | -15 | 9 | 493.25 | 29.30 |
| -6 | 15 | 9 | 536.15 | 29.50 |
| -5 | -15 | 9 | 14.90 | 3.90 |
| -5 | 15 | 9 | 25.20 | 5.30 |
| -4 | -15 | 9 | 124.79 | 10.20 |
| 4 | -15 | -9 | 134.39 | 10.20 |
| -4 | 15 | 9 | 132.19 | 10.10 |
| -3 | -15 | 9 | 16.70 | 3.60 |
| 3 | -15 | -9 | 30.70 | 6.60 |
| -3 | 15 | 9 | 18.90 | 3.80 |
| -2 | -15 | 9 | 292.77 | 17.60 |
| 2 | -15 | -9 | 285.27 | 17.70 |
| -2 | 15 | 9 | 257.87 | 17.60 |
| -1 | -15 | 9 | 56.79 | 7.20 |
| 1 | -15 | -9 | 48.70 | 7.70 |
| -1 | 15 | 9 | 62.39 | 7.20 |
| 0 | -15 | 9 | 627.84 | 34.50 |
| 0 | -15 | 9 | 594.94 | 33.40 |

| | | | | |
|---|---|---|---|---|
| 0 | -15 | -9 | 560.34 | 34.40 |
| 0 | 15 | 9 | 609.14 | 34.20 |
| 1 | -15 | 9 | 105.29 | 8.90 |
| 1 | -15 | 9 | 104.49 | 9.40 |
| -1 | -15 | -9 | 90.69 | 8.70 |
| 1 | 15 | 9 | 93.19 | 8.10 |
| -2 | -15 | -9 | 8.20 | 4.90 |
| 2 | -15 | 9 | -0.90 | 3.90 |
| 2 | -15 | 9 | -1.80 | 2.40 |
| 2 | 15 | 9 | 0.00 | 2.60 |
| 3 | -15 | 9 | 63.99 | 7.50 |
| 3 | -15 | 9 | 61.29 | 5.60 |
| -3 | -15 | -9 | 54.39 | 6.60 |
| 3 | 15 | 9 | 63.59 | 5.80 |
| 4 | -15 | 9 | 561.14 | 32.40 |
| -4 | -15 | -9 | 567.14 | 32.30 |
| 4 | -15 | 9 | 589.34 | 31.80 |
| 4 | 15 | 9 | 575.04 | 32.20 |
| 5 | -15 | 9 | 18.40 | 3.40 |
| 5 | -15 | 9 | 10.40 | 3.50 |
| -5 | -15 | -9 | 11.10 | 2.40 |
| 5 | 15 | 9 | 12.80 | 2.60 |
| 6 | -15 | 9 | 134.49 | 8.90 |
| -6 | -15 | -9 | 142.89 | 9.30 |
| 6 | 15 | 9 | 158.68 | 11.40 |
| -7 | -15 | -9 | 73.19 | 7.60 |
| 7 | -15 | 9 | 71.49 | 5.30 |
| 7 | 15 | 9 | 69.99 | 5.70 |
| -9 | 16 | 9 | 97.19 | 6.90 |
| 9 | 16 | -9 | 108.09 | 6.40 |
| -8 | -16 | 9 | 65.89 | 7.60 |
| 8 | 16 | -9 | 73.89 | 5.10 |
| -8 | 16 | 9 | 75.09 | 5.70 |
| -7 | -16 | 9 | 215.48 | 13.30 |
| -7 | 16 | 9 | 210.18 | 14.20 |
| 7 | 16 | -9 | 239.08 | 13.10 |
| -6 | -16 | 9 | 2.40 | 2.40 |
| 6 | 16 | -9 | 2.30 | 2.00 |
| -6 | 16 | 9 | 3.90 | 2.40 |
| 5 | -16 | -9 | 409.06 | 24.10 |
| -5 | -16 | 9 | 423.96 | 24.20 |
| -5 | 16 | 9 | 429.66 | 24.30 |
| -4 | -16 | 9 | 42.50 | 6.30 |
| 4 | -16 | -9 | 63.19 | 6.30 |

| | | | | |
|---|---|---|---|---|
| -4 | 16 | 9 | 53.69 | 6.00 |
| 3 | -16 | -9 | -2.00 | 2.60 |
| -3 | -16 | 9 | -0.70 | 2.60 |
| -3 | 16 | 9 | -4.50 | 2.80 |
| -2 | -16 | 9 | 184.98 | 12.20 |
| 2 | -16 | -9 | 179.98 | 12.00 |
| -2 | 16 | 9 | 173.08 | 12.30 |
| -1 | -16 | 9 | 425.96 | 25.80 |
| 1 | -16 | -9 | 459.15 | 26.00 |
| -1 | 16 | 9 | 453.55 | 26.00 |
| 0 | -16 | 9 | 63.49 | 6.50 |
| 0 | -16 | -9 | 45.40 | 6.40 |
| 0 | 16 | 9 | 58.59 | 6.30 |
| 1 | -16 | 9 | 82.19 | 7.90 |
| -1 | -16 | -9 | 72.79 | 7.40 |
| 1 | -16 | 9 | 79.59 | 6.30 |
| 1 | 16 | 9 | 78.29 | 6.80 |
| 2 | -16 | 9 | 126.59 | 8.90 |
| -2 | -16 | -9 | 131.39 | 9.80 |
| 2 | -16 | 9 | 156.38 | 10.20 |
| 2 | 16 | 9 | 140.89 | 9.50 |
| 3 | -16 | 9 | 155.98 | 10.70 |
| -3 | -16 | -9 | 145.09 | 10.20 |
| 3 | -16 | 9 | 148.29 | 9.60 |
| 3 | 16 | 9 | 168.78 | 10.20 |
| 4 | -16 | 9 | 29.60 | 3.50 |
| 4 | -16 | 9 | 20.30 | 3.50 |
| -4 | -16 | -9 | 24.80 | 4.00 |
| 4 | 16 | 9 | 29.90 | 4.10 |
| 5 | -16 | 9 | 396.86 | 22.40 |
| -5 | -16 | -9 | 390.76 | 22.70 |
| 5 | 16 | 9 | 420.06 | 22.90 |
| 6 | -16 | 9 | 115.89 | 8.00 |
| -6 | -16 | -9 | 112.09 | 7.60 |
| 6 | 16 | 9 | 120.99 | 7.80 |
| -7 | -17 | 9 | 72.29 | 7.00 |
| 7 | 17 | -9 | 75.39 | 5.20 |
| -7 | 17 | 9 | 73.69 | 5.70 |
| -6 | -17 | 9 | 409.76 | 22.80 |
| -6 | 17 | 9 | 366.06 | 22.80 |
| 6 | 17 | -9 | 440.86 | 22.80 |
| -5 | -17 | 9 | 110.79 | 8.10 |
| -5 | 17 | 9 | 126.39 | 9.10 |
| 5 | 17 | -9 | 117.19 | 7.80 |

| | | | | |
|---|---|---|---|---|
| 4 | -17 | -9 | 101.09 | 7.50 |
| -4 | -17 | 9 | 99.89 | 7.30 |
| -4 | 17 | 9 | 103.69 | 7.70 |
| -3 | -17 | 9 | 51.49 | 5.30 |
| 3 | -17 | -9 | 49.40 | 5.40 |
| -3 | 17 | 9 | 54.99 | 5.80 |
| 2 | -17 | -9 | 216.78 | 12.70 |
| -2 | -17 | 9 | 179.18 | 12.40 |
| -2 | 17 | 9 | 207.38 | 12.90 |
| 1 | -17 | -9 | -0.30 | 2.40 |
| -1 | -17 | 9 | 0.20 | 2.30 |
| -1 | 17 | 9 | -3.30 | 2.70 |
| 0 | -17 | -9 | 491.65 | 28.60 |
| 0 | -17 | 9 | 526.15 | 28.50 |
| 0 | 17 | 9 | 489.75 | 28.60 |
| -1 | -17 | -9 | 101.89 | 7.90 |
| 1 | -17 | 9 | 100.09 | 7.80 |
| 1 | 17 | 9 | 109.59 | 7.80 |
| 2 | -17 | 9 | 8.10 | 1.90 |
| -2 | -17 | -9 | 9.00 | 2.30 |
| 2 | -17 | 9 | 10.10 | 2.50 |
| 2 | 17 | 9 | 6.70 | 3.50 |
| 3 | -17 | 9 | 17.70 | 3.20 |
| -3 | -17 | -9 | 24.20 | 4.00 |
| 3 | -17 | 9 | 25.40 | 4.50 |
| 3 | 17 | 9 | 21.90 | 3.80 |
| -4 | -17 | -9 | 319.97 | 17.90 |
| 4 | -17 | 9 | 294.27 | 18.00 |
| 4 | -17 | 9 | 317.27 | 17.60 |
| 4 | 17 | 9 | 320.17 | 18.10 |
| 4 | -18 | -9 | 96.99 | 7.30 |
| -4 | -18 | 9 | 101.49 | 6.80 |
| -4 | 18 | 9 | 104.29 | 7.30 |
| 4 | 18 | -9 | 104.49 | 6.90 |
| 3 | -18 | -9 | -4.00 | 2.00 |
| -3 | -18 | 9 | -1.00 | 1.60 |
| -3 | 18 | 9 | 0.60 | 2.40 |
| 2 | -18 | -9 | 65.39 | 5.40 |
| -2 | -18 | 9 | 63.49 | 5.10 |
| -2 | 18 | 9 | 61.59 | 5.90 |
| 1 | -18 | -9 | 335.07 | 19.10 |
| -1 | -18 | 9 | 340.07 | 19.10 |
| -1 | 18 | 9 | 324.37 | 19.30 |
| 0 | -18 | -9 | 69.89 | 5.90 |

| | | | | |
|---|---|---|---|---|
| 0 | -18 | 9 | 74.99 | 5.80 |
| 0 | 18 | 9 | 73.49 | 6.30 |
| -1 | -18 | -9 | 97.19 | 7.30 |
| 1 | -18 | 9 | 106.79 | 7.30 |
| 1 | 18 | 9 | 106.99 | 7.50 |
| 16 | 0 | -10 | 322.47 | 17.20 |
| -16 | 0 | 10 | 282.77 | 17.20 |
| -15 | 0 | 10 | 2.80 | 2.00 |
| 15 | 0 | -10 | -1.70 | 2.20 |
| 14 | 0 | -10 | 377.96 | 21.10 |
| -14 | 0 | 10 | 350.26 | 21.50 |
| 13 | 0 | -10 | -0.90 | 2.60 |
| -12 | 0 | 10 | 743.33 | 41.70 |
| 12 | 0 | -10 | 735.23 | 41.20 |
| -10 | 0 | 10 | 530.85 | 30.70 |
| 10 | 0 | -10 | 522.55 | 30.10 |
| -9 | 0 | 10 | -0.10 | 4.10 |
| 9 | 0 | -10 | 0.00 | 4.20 |
| -8 | 0 | 10 | 158.08 | 12.90 |
| -7 | 0 | 10 | 3.70 | 5.10 |
| -7 | 0 | 10 | -3.30 | 4.00 |
| -6 | 0 | 10 | 2810.92 | 142.19 |
| -6 | 0 | 10 | 2413.16 | 142.39 |
| -5 | 0 | 10 | 2.40 | 3.10 |
| -4 | 0 | 10 | 687.53 | 39.40 |
| -3 | 0 | 10 | 1.90 | 3.40 |
| -2 | 0 | 10 | 2003.90 | 109.99 |
| -1 | 0 | 10 | -3.10 | 3.20 |
| 0 | 0 | 10 | 2243.28 | 123.29 |
| 1 | 0 | 10 | -5.20 | 6.20 |
| 1 | 0 | 10 | -1.60 | 4.20 |
| 2 | 0 | 10 | 259.47 | 20.50 |
| 2 | 0 | 10 | 288.27 | 18.80 |
| 2 | 0 | 10 | 286.17 | 19.60 |
| 3 | 0 | 10 | 3.70 | 4.50 |
| 3 | 0 | 10 | 1.60 | 6.20 |
| 3 | 0 | 10 | -0.90 | 5.00 |
| 4 | 0 | 10 | 881.41 | 53.69 |
| 4 | 0 | 10 | 999.10 | 53.39 |
| 5 | 0 | 10 | 6.80 | 4.20 |
| 5 | 0 | 10 | 5.80 | 7.00 |
| 5 | 0 | 10 | -12.00 | 6.20 |
| 5 | 0 | 10 | -6.30 | 4.80 |
| 6 | 0 | 10 | 238.08 | 16.50 |

| | | | | |
|---|---|---|---|---|
| 6 | 0 | 10 | 247.88 | 16.60 |
| 6 | 0 | 10 | 235.48 | 18.10 |
| 6 | 0 | 10 | 228.48 | 18.70 |
| 7 | 0 | 10 | -5.20 | 8.20 |
| 7 | 0 | 10 | -3.90 | 3.40 |
| 7 | 0 | 10 | -1.70 | 5.60 |
| 7 | 0 | 10 | -8.90 | 4.80 |
| 8 | 0 | 10 | 457.25 | 26.70 |
| 8 | 0 | 10 | 452.85 | 28.20 |
| 9 | 0 | 10 | 3.70 | 3.40 |
| 9 | 0 | 10 | -6.60 | 4.80 |
| 10 | 0 | 10 | 392.16 | 24.10 |
| 10 | 0 | 10 | 395.06 | 23.30 |
| 11 | 0 | 10 | -6.80 | 3.90 |
| 11 | 0 | 10 | -0.60 | 2.90 |
| 12 | 0 | 10 | 150.18 | 10.40 |
| 12 | 0 | 10 | 154.18 | 11.60 |
| 13 | 0 | 10 | 6.40 | 3.90 |
| 13 | 0 | 10 | -0.90 | 2.10 |
| 16 | -1 | -10 | 0.10 | 1.80 |
| -16 | -1 | 10 | -0.30 | 1.80 |
| 16 | 1 | -10 | -4.30 | 1.70 |
| -16 | 1 | 10 | 0.90 | 1.70 |
| -15 | -1 | 10 | 433.16 | 25.10 |
| 15 | -1 | -10 | 449.86 | 25.00 |
| 15 | 1 | -10 | 458.15 | 25.00 |
| -15 | 1 | 10 | 439.46 | 25.20 |
| 14 | -1 | -10 | 18.60 | 3.30 |
| -14 | -1 | 10 | 25.60 | 4.90 |
| 14 | 1 | -10 | 16.80 | 3.10 |
| -14 | 1 | 10 | 15.80 | 3.30 |
| 13 | -1 | -10 | 26.50 | 5.30 |
| -13 | -1 | 10 | 28.80 | 5.90 |
| -13 | 1 | 10 | 37.60 | 5.50 |
| 13 | 1 | -10 | 33.70 | 5.10 |
| -12 | -1 | 10 | 31.50 | 6.00 |
| 12 | -1 | -10 | 13.60 | 3.70 |
| -12 | 1 | 10 | 24.90 | 5.60 |
| 12 | 1 | -10 | 14.70 | 3.40 |
| 11 | -1 | -10 | 2068.99 | 113.59 |
| 11 | 1 | -10 | 2119.89 | 113.59 |
| 10 | -1 | -10 | -1.00 | 4.10 |
| -10 | -1 | 10 | 6.80 | 4.00 |
| 10 | 1 | -10 | -0.90 | 3.50 |

| | | | | |
|---|---|---|---|---|
| -10 | 1 | 10 | -0.10 | 3.70 |
| -9 | -1 | 10 | 442.56 | 26.80 |
| 9 | -1 | -10 | 424.16 | 26.50 |
| 9 | 1 | -10 | 434.26 | 26.10 |
| -9 | 1 | 10 | 489.35 | 26.90 |
| -8 | -1 | 10 | -0.40 | 5.00 |
| -8 | 1 | 10 | 3.70 | 4.10 |
| 8 | 1 | -10 | 2.60 | 4.80 |
| -7 | -1 | 10 | 2586.94 | 144.09 |
| -7 | 1 | 10 | 2509.65 | 143.99 |
| -7 | 1 | 10 | 2841.72 | 144.09 |
| -6 | -1 | 10 | 10.20 | 3.80 |
| -6 | -1 | 10 | 14.80 | 6.90 |
| -6 | 1 | 10 | 15.10 | 5.60 |
| -6 | 1 | 10 | 11.00 | 4.20 |
| -5 | -1 | 10 | 2561.04 | 136.19 |
| -5 | 1 | 10 | 2412.26 | 136.79 |
| -5 | 1 | 10 | 2533.25 | 136.39 |
| -4 | -1 | 10 | 3.40 | 3.20 |
| -4 | 1 | 10 | -2.00 | 3.10 |
| -3 | -1 | 10 | 318.67 | 19.50 |
| -3 | 1 | 10 | 299.87 | 19.70 |
| -2 | -1 | 10 | 126.39 | 10.20 |
| -2 | 1 | 10 | 114.29 | 10.40 |
| -1 | -1 | 10 | 4501.85 | 242.58 |
| -1 | 1 | 10 | 4453.35 | 242.78 |
| 0 | -1 | 10 | 72.19 | 8.70 |
| 0 | 1 | 10 | 69.89 | 9.20 |
| 1 | -1 | 10 | 342.37 | 21.30 |
| 1 | -1 | 10 | 303.17 | 21.90 |
| 1 | 1 | 10 | 328.67 | 21.40 |
| 1 | 1 | 10 | 279.97 | 21.30 |
| 2 | -1 | 10 | 1.50 | 5.80 |
| 2 | -1 | 10 | 8.30 | 5.40 |
| 2 | -1 | 10 | 5.70 | 4.70 |
| 2 | 1 | 10 | -3.70 | 4.20 |
| 2 | 1 | 10 | -2.70 | 4.60 |
| 2 | 1 | 10 | 6.10 | 6.10 |
| 3 | -1 | 10 | 690.33 | 41.30 |
| 3 | -1 | 10 | 672.83 | 41.80 |
| 3 | 1 | 10 | 720.53 | 40.10 |
| 3 | 1 | 10 | 707.83 | 42.50 |
| 3 | 1 | 10 | 725.13 | 41.20 |
| 4 | -1 | 10 | 7.80 | 4.80 |

| | | | | |
|---|---|---|---|---|
| 4 | -1 | 10 | -2.00 | 4.90 |
| 4 | -1 | 10 | -5.50 | 5.60 |
| 4 | 1 | 10 | -5.80 | 5.60 |
| 4 | 1 | 10 | -5.20 | 5.10 |
| 4 | 1 | 10 | 5.90 | 4.10 |
| 4 | 1 | 10 | -3.00 | 6.90 |
| 5 | -1 | 10 | 2076.99 | 107.09 |
| 5 | -1 | 10 | 1890.21 | 107.79 |
| 5 | -1 | 10 | 1889.41 | 108.69 |
| 5 | -1 | 10 | 1959.80 | 107.29 |
| 5 | 1 | 10 | 1972.40 | 107.29 |
| 5 | 1 | 10 | 1832.32 | 107.79 |
| 5 | 1 | 10 | 2271.07 | 107.29 |
| 5 | 1 | 10 | 1803.62 | 109.19 |
| 6 | -1 | 10 | 15.00 | 5.10 |
| 6 | -1 | 10 | 13.10 | 6.90 |
| 6 | -1 | 10 | 19.90 | 6.80 |
| 6 | 1 | 10 | 10.00 | 6.10 |
| 6 | 1 | 10 | 14.20 | 4.20 |
| 6 | 1 | 10 | 8.80 | 7.50 |
| 6 | 1 | 10 | 17.00 | 4.80 |
| 7 | -1 | 10 | 384.86 | 25.40 |
| 7 | -1 | 10 | 396.26 | 23.80 |
| 7 | 1 | 10 | 444.26 | 24.00 |
| 7 | 1 | 10 | 378.76 | 24.30 |
| 7 | 1 | 10 | 382.66 | 25.60 |
| 8 | -1 | 10 | 0.90 | 3.30 |
| 8 | -1 | 10 | -2.60 | 5.60 |
| 8 | 1 | 10 | -0.90 | 3.40 |
| 8 | 1 | 10 | 3.90 | 5.30 |
| 9 | -1 | 10 | 860.21 | 43.90 |
| 9 | -1 | 10 | 743.93 | 44.60 |
| 9 | 1 | 10 | 725.33 | 43.60 |
| 9 | 1 | 10 | 782.42 | 44.80 |
| 10 | -1 | 10 | 9.60 | 3.80 |
| 10 | -1 | 10 | 4.00 | 4.60 |
| 10 | 1 | 10 | 7.10 | 4.60 |
| 10 | 1 | 10 | 7.10 | 3.30 |
| 11 | -1 | 10 | 83.49 | 9.10 |
| 11 | -1 | 10 | 94.99 | 8.10 |
| 11 | 1 | 10 | 89.49 | 9.10 |
| 11 | 1 | 10 | 96.09 | 7.90 |
| 12 | -1 | 10 | 0.90 | 2.50 |
| 12 | -1 | 10 | 0.50 | 4.00 |

| | | | | |
|---|---|---|---|---|
| 12 | 1 | 10 | 9.50 | 6.50 |
| 12 | 1 | 10 | -0.10 | 2.30 |
| 13 | -1 | 10 | 323.97 | 19.70 |
| 13 | -1 | 10 | 367.16 | 20.40 |
| 13 | 1 | 10 | 325.47 | 19.60 |
| 13 | 1 | 10 | 353.76 | 20.10 |
| -16 | -2 | 10 | 443.16 | 25.90 |
| 16 | -2 | -10 | 476.65 | 25.80 |
| 16 | 2 | -10 | 480.65 | 25.80 |
| -16 | 2 | 10 | 466.35 | 26.00 |
| -15 | -2 | 10 | 4.10 | 2.20 |
| 15 | -2 | -10 | 6.00 | 2.40 |
| -15 | 2 | 10 | 5.80 | 2.00 |
| 15 | 2 | -10 | 5.10 | 2.10 |
| -14 | -2 | 10 | 275.47 | 17.00 |
| 14 | -2 | -10 | 301.77 | 17.00 |
| 14 | 2 | -10 | 288.27 | 16.80 |
| -14 | 2 | 10 | 273.57 | 17.20 |
| -13 | -2 | 10 | 39.70 | 6.30 |
| 13 | -2 | -10 | 44.40 | 6.30 |
| 13 | 2 | -10 | 49.10 | 5.60 |
| -13 | 2 | 10 | 43.00 | 5.70 |
| -12 | -2 | 10 | 1119.89 | 58.29 |
| 12 | -2 | -10 | 1038.30 | 57.69 |
| 12 | 2 | -10 | 1024.20 | 57.49 |
| -12 | 2 | 10 | 1013.30 | 58.09 |
| 11 | -2 | -10 | 13.60 | 4.10 |
| -11 | -2 | 10 | 13.40 | 4.10 |
| 11 | 2 | -10 | 10.00 | 3.30 |
| -11 | 2 | 10 | 14.60 | 3.80 |
| -10 | -2 | 10 | 829.42 | 43.70 |
| 10 | -2 | -10 | 692.63 | 42.90 |
| 10 | 2 | -10 | 787.72 | 42.80 |
| -10 | 2 | 10 | 763.42 | 43.40 |
| -9 | -2 | 10 | 223.18 | 15.10 |
| 9 | 2 | -10 | 208.68 | 14.20 |
| -9 | 2 | 10 | 191.08 | 17.70 |
| -8 | -2 | 10 | 1.60 | 5.30 |
| -8 | 2 | 10 | 10.70 | 4.40 |
| 8 | 2 | -10 | 0.30 | 4.50 |
| -7 | -2 | 10 | 220.08 | 18.20 |
| -7 | 2 | 10 | 257.57 | 16.70 |
| -6 | -2 | 10 | 3341.17 | 171.58 |
| -6 | -2 | 10 | 2778.22 | 171.78 |

| | | | | |
|---|---|---|---|---|
| -6 | 2 | 10 | 3211.68 | 171.98 |
| -6 | 2 | 10 | 3318.97 | 171.68 |
| -5 | -2 | 10 | -2.60 | 2.90 |
| -5 | 2 | 10 | -5.60 | 6.10 |
| -5 | 2 | 10 | 0.40 | 3.40 |
| -4 | -2 | 10 | 77.09 | 7.50 |
| -4 | 2 | 10 | 61.79 | 8.40 |
| -4 | 2 | 10 | 75.99 | 11.20 |
| -3 | -2 | 10 | 87.89 | 8.40 |
| -3 | 2 | 10 | 96.09 | 9.50 |
| -2 | -2 | 10 | 1053.29 | 59.79 |
| -2 | 2 | 10 | 1079.19 | 60.39 |
| -1 | -2 | 10 | 34.70 | 6.80 |
| -1 | 2 | 10 | 35.30 | 7.50 |
| 0 | -2 | 10 | 2400.56 | 134.99 |
| 0 | 2 | 10 | 2681.83 | 138.69 |
| 0 | 2 | 10 | 2321.37 | 135.19 |
| 1 | -2 | 10 | 97.79 | 9.90 |
| 1 | -2 | 10 | 100.99 | 12.40 |
| 1 | 2 | 10 | 66.69 | 11.90 |
| 1 | 2 | 10 | 83.29 | 10.10 |
| 1 | 2 | 10 | 87.99 | 10.10 |
| 2 | -2 | 10 | 148.69 | 12.80 |
| 2 | -2 | 10 | 158.88 | 14.30 |
| 2 | 2 | 10 | 134.69 | 13.30 |
| 2 | 2 | 10 | 135.29 | 12.00 |
| 2 | 2 | 10 | 157.68 | 14.50 |
| 3 | -2 | 10 | 16.80 | 5.40 |
| 3 | -2 | 10 | 28.90 | 6.20 |
| 3 | -2 | 10 | 32.00 | 7.20 |
| 3 | 2 | 10 | 25.80 | 7.00 |
| 3 | 2 | 10 | 27.40 | 6.40 |
| 3 | 2 | 10 | 38.60 | 7.60 |
| 3 | 2 | 10 | 24.40 | 6.30 |
| 4 | -2 | 10 | 1970.60 | 102.39 |
| 4 | -2 | 10 | 1662.93 | 101.49 |
| 4 | -2 | 10 | 1719.33 | 103.19 |
| 4 | 2 | 10 | 1868.21 | 103.69 |
| 4 | 2 | 10 | 1962.80 | 102.09 |
| 4 | 2 | 10 | 2003.40 | 101.79 |
| 4 | 2 | 10 | 1841.62 | 104.49 |
| 5 | -2 | 10 | 6.40 | 5.70 |
| 5 | -2 | 10 | 19.00 | 4.80 |
| 5 | -2 | 10 | 20.20 | 5.90 |

| | | | | |
|---|---|---|---|---|
| 5 | 2 | 10 | 21.70 | 5.00 |
| 5 | 2 | 10 | 21.10 | 4.10 |
| 5 | 2 | 10 | 20.30 | 6.20 |
| 5 | 2 | 10 | 16.40 | 8.10 |
| 6 | -2 | 10 | 343.07 | 23.10 |
| 6 | -2 | 10 | 348.47 | 22.20 |
| 6 | -2 | 10 | 356.16 | 23.80 |
| 6 | 2 | 10 | 338.17 | 25.20 |
| 6 | 2 | 10 | 312.67 | 22.90 |
| 6 | 2 | 10 | 393.36 | 21.60 |
| 6 | 2 | 10 | 355.06 | 21.80 |
| 7 | -2 | 10 | 100.99 | 12.10 |
| 7 | -2 | 10 | 103.89 | 9.40 |
| 7 | 2 | 10 | 95.29 | 10.20 |
| 7 | 2 | 10 | 112.09 | 9.00 |
| 7 | 2 | 10 | 86.79 | 17.20 |
| 7 | 2 | 10 | 76.19 | 11.10 |
| 8 | -2 | 10 | 534.65 | 32.60 |
| 8 | -2 | 10 | 540.45 | 31.40 |
| 8 | 2 | 10 | 569.44 | 31.40 |
| 8 | 2 | 10 | 526.75 | 32.80 |
| 9 | -2 | 10 | 3.00 | 3.70 |
| 9 | -2 | 10 | -1.30 | 4.90 |
| 9 | 2 | 10 | 2.90 | 4.80 |
| 9 | 2 | 10 | -1.60 | 3.10 |
| 10 | -2 | 10 | 728.33 | 44.30 |
| 10 | -2 | 10 | 798.02 | 44.00 |
| 10 | 2 | 10 | 779.42 | 43.80 |
| 10 | 2 | 10 | 826.02 | 44.60 |
| 11 | -2 | 10 | 44.00 | 7.80 |
| 11 | -2 | 10 | 31.40 | 6.20 |
| 11 | 2 | 10 | 44.80 | 6.90 |
| 11 | 2 | 10 | 34.80 | 5.50 |
| 12 | -2 | 10 | 17.30 | 4.70 |
| 12 | -2 | 10 | 13.30 | 3.20 |
| 12 | 2 | 10 | 26.40 | 5.40 |
| 12 | 2 | 10 | 13.00 | 2.70 |
| 13 | -2 | 10 | 0.40 | 2.70 |
| 13 | 2 | 10 | 3.70 | 2.10 |
| -16 | -3 | 10 | 47.70 | 4.90 |
| 16 | -3 | -10 | 40.20 | 4.90 |
| -16 | 3 | 10 | 38.00 | 3.80 |
| 16 | 3 | -10 | 40.50 | 3.90 |
| 15 | -3 | -10 | 356.96 | 19.30 |

| | | | | |
|---|---|---|---|---|
| -15 | -3 | 10 | 327.97 | 19.30 |
| -15 | 3 | 10 | 303.37 | 19.30 |
| 15 | 3 | -10 | 360.46 | 19.20 |
| 14 | -3 | -10 | 4.50 | 2.90 |
| -14 | -3 | 10 | 6.90 | 2.90 |
| 14 | 3 | -10 | 3.70 | 2.20 |
| -14 | 3 | 10 | 9.80 | 2.70 |
| -13 | -3 | 10 | 10.00 | 3.50 |
| 13 | -3 | -10 | 4.60 | 3.20 |
| 13 | 3 | -10 | 4.00 | 2.40 |
| -13 | 3 | 10 | 7.30 | 2.80 |
| -12 | -3 | 10 | 42.20 | 7.00 |
| 12 | -3 | -10 | 40.10 | 6.80 |
| -12 | 3 | 10 | 47.70 | 6.10 |
| 12 | 3 | -10 | 35.70 | 5.60 |
| 11 | -3 | -10 | 1151.18 | 66.19 |
| -11 | -3 | 10 | 1222.58 | 66.79 |
| 11 | 3 | -10 | 1199.58 | 66.09 |
| -11 | 3 | 10 | 1258.07 | 66.89 |
| 10 | -3 | -10 | 10.50 | 4.70 |
| -10 | -3 | 10 | -6.10 | 4.40 |
| 10 | 3 | -10 | 3.70 | 3.30 |
| -10 | 3 | 10 | 5.80 | 3.60 |
| -9 | -3 | 10 | 153.58 | 13.70 |
| -9 | 3 | 10 | 165.68 | 12.30 |
| 9 | 3 | -10 | 177.38 | 11.90 |
| -8 | -3 | 10 | 15.00 | 6.20 |
| 8 | 3 | -10 | 11.40 | 4.40 |
| -8 | 3 | 10 | 7.20 | 4.00 |
| -7 | -3 | 10 | 1234.08 | 68.29 |
| -7 | 3 | 10 | 1296.77 | 67.89 |
| 7 | 3 | -10 | 1112.39 | 66.69 |
| -6 | 3 | 10 | 80.09 | 10.50 |
| -6 | 3 | 10 | 62.89 | 8.60 |
| -5 | -3 | 10 | 1887.61 | 103.29 |
| -5 | 3 | 10 | 1816.72 | 103.89 |
| -5 | 3 | 10 | 1949.10 | 103.79 |
| -4 | -3 | 10 | 98.09 | 8.70 |
| -4 | 3 | 10 | 94.79 | 10.00 |
| -4 | 3 | 10 | 98.49 | 12.60 |
| -3 | -3 | 10 | 4.90 | 3.50 |
| -3 | 3 | 10 | 3.00 | 5.90 |
| -3 | 3 | 10 | 2.90 | 4.20 |
| -2 | -3 | 10 | 419.96 | 25.40 |

| | | | | |
|---|---|---|---|---|
| -2 | 3 | 10 | 422.46 | 29.50 |
| -2 | 3 | 10 | 410.46 | 26.10 |
| -1 | -3 | 10 | 3645.14 | 198.48 |
| -1 | -3 | 10 | 3559.54 | 197.88 |
| -1 | 3 | 10 | 4091.39 | 198.58 |
| -1 | 3 | 10 | 3316.87 | 198.78 |
| 0 | -3 | 10 | 17.50 | 4.60 |
| 0 | -3 | 10 | 20.30 | 5.80 |
| 0 | 3 | 10 | 16.70 | 5.40 |
| 0 | 3 | 10 | 27.30 | 7.00 |
| 0 | 3 | 10 | 19.60 | 5.50 |
| 1 | -3 | 10 | 253.37 | 17.60 |
| 1 | -3 | 10 | 255.17 | 17.60 |
| 1 | 3 | 10 | 237.48 | 18.00 |
| 1 | 3 | 10 | 232.08 | 18.50 |
| 1 | 3 | 10 | 250.27 | 16.80 |
| 2 | -3 | 10 | 157.48 | 13.90 |
| 2 | -3 | 10 | 166.18 | 15.10 |
| 2 | 3 | 10 | 182.38 | 13.60 |
| 2 | 3 | 10 | 165.48 | 14.80 |
| 2 | 3 | 10 | 175.98 | 14.90 |
| 2 | 3 | 10 | 137.09 | 12.40 |
| 3 | -3 | 10 | 1070.29 | 58.49 |
| 3 | -3 | 10 | 910.51 | 58.59 |
| 3 | -3 | 10 | 979.60 | 57.59 |
| 3 | 3 | 10 | 1071.89 | 57.99 |
| 3 | 3 | 10 | 939.11 | 59.89 |
| 3 | 3 | 10 | 1069.79 | 57.49 |
| 3 | 3 | 10 | 1165.88 | 57.69 |
| 4 | -3 | 10 | 278.37 | 18.40 |
| 4 | -3 | 10 | 248.98 | 18.90 |
| 4 | -3 | 10 | 263.87 | 19.00 |
| 4 | 3 | 10 | 271.97 | 18.00 |
| 4 | 3 | 10 | 280.77 | 18.00 |
| 4 | 3 | 10 | 222.38 | 20.70 |
| 4 | 3 | 10 | 273.07 | 17.10 |
| 5 | -3 | 10 | 1846.92 | 96.99 |
| 5 | -3 | 10 | 1754.92 | 98.09 |
| 5 | -3 | 10 | 1727.53 | 96.49 |
| 5 | 3 | 10 | 1828.72 | 96.79 |
| 5 | 3 | 10 | 1756.72 | 96.59 |
| 5 | 3 | 10 | 1786.42 | 96.99 |
| 5 | 3 | 10 | 1666.13 | 99.79 |
| 6 | -3 | 10 | 20.90 | 7.00 |

| | | | | |
|---|---|---|---|---|
| 6 | -3 | 10 | 9.40 | 5.30 |
| 6 | -3 | 10 | 17.00 | 6.30 |
| 6 | -3 | 10 | 18.90 | 5.10 |
| 6 | 3 | 10 | 22.40 | 5.50 |
| 6 | 3 | 10 | 16.90 | 9.40 |
| 6 | 3 | 10 | 17.90 | 3.90 |
| 7 | -3 | 10 | 173.78 | 12.50 |
| 7 | -3 | 10 | 152.08 | 14.10 |
| 7 | 3 | 10 | 166.98 | 19.40 |
| 7 | 3 | 10 | 184.98 | 14.30 |
| 7 | 3 | 10 | 156.98 | 11.80 |
| 8 | -3 | 10 | 14.20 | 4.10 |
| 8 | -3 | 10 | 7.90 | 5.90 |
| 8 | 3 | 10 | 6.70 | 3.30 |
| 8 | 3 | 10 | 11.70 | 5.80 |
| 9 | -3 | 10 | 680.93 | 41.00 |
| 9 | -3 | 10 | 726.53 | 41.80 |
| 9 | 3 | 10 | 701.73 | 41.80 |
| 9 | 3 | 10 | 792.02 | 40.90 |
| 10 | -3 | 10 | -2.40 | 5.00 |
| 10 | -3 | 10 | 5.00 | 4.10 |
| 10 | 3 | 10 | 7.90 | 4.30 |
| 10 | 3 | 10 | 2.90 | 3.10 |
| 11 | -3 | 10 | 153.18 | 13.00 |
| 11 | -3 | 10 | 170.08 | 11.80 |
| 11 | 3 | 10 | 178.28 | 11.20 |
| 11 | 3 | 10 | 168.38 | 12.00 |
| 12 | -3 | 10 | -5.00 | 2.80 |
| 12 | -3 | 10 | -3.10 | 4.90 |
| 12 | 3 | 10 | 1.10 | 2.40 |
| 12 | 3 | 10 | -2.50 | 3.30 |
| 16 | -4 | -10 | 345.67 | 20.00 |
| 16 | 4 | -10 | 380.36 | 19.90 |
| -16 | 4 | 10 | 347.27 | 20.10 |
| -15 | -4 | 10 | 5.40 | 3.10 |
| 15 | -4 | -10 | 0.20 | 2.50 |
| 15 | 4 | -10 | -0.50 | 1.60 |
| -15 | 4 | 10 | -0.20 | 1.70 |
| 14 | -4 | -10 | 134.89 | 9.60 |
| -14 | -4 | 10 | 124.79 | 9.80 |
| -14 | 4 | 10 | 144.19 | 9.30 |
| 14 | 4 | -10 | 143.59 | 8.90 |
| 13 | -4 | -10 | 184.88 | 14.70 |
| -13 | -4 | 10 | 164.68 | 15.60 |

| | | | | |
|---|---|---|---|---|
| 13 | 4 | -10 | 188.18 | 11.40 |
| -13 | 4 | 10 | 176.48 | 11.90 |
| -12 | -4 | 10 | 652.63 | 37.30 |
| 12 | -4 | -10 | 594.54 | 36.80 |
| -12 | 4 | 10 | 711.73 | 37.40 |
| 12 | 4 | -10 | 669.03 | 36.60 |
| 11 | -4 | -10 | -8.10 | 3.90 |
| -11 | -4 | 10 | -7.50 | 4.30 |
| 11 | 4 | -10 | 5.30 | 2.90 |
| -11 | 4 | 10 | 0.60 | 2.80 |
| -10 | -4 | 10 | 644.44 | 37.50 |
| -10 | 4 | 10 | 666.63 | 37.30 |
| 10 | 4 | -10 | 641.74 | 36.40 |
| -9 | -4 | 10 | 186.88 | 14.80 |
| 9 | 4 | -10 | 161.68 | 13.40 |
| -9 | 4 | 10 | 179.58 | 12.90 |
| -8 | 4 | 10 | 12.10 | 4.10 |
| 8 | 4 | -10 | 14.80 | 4.00 |
| 7 | 4 | -10 | 2.60 | 4.30 |
| -7 | 4 | 10 | -0.80 | 4.00 |
| -6 | -4 | 10 | 2097.79 | 122.79 |
| -6 | 4 | 10 | 2505.55 | 123.89 |
| 6 | 4 | -10 | 2237.88 | 122.79 |
| -6 | 4 | 10 | 2199.08 | 123.79 |
| -5 | -4 | 10 | 83.49 | 10.60 |
| -5 | 4 | 10 | 69.79 | 11.20 |
| -5 | 4 | 10 | 100.69 | 10.30 |
| -4 | -4 | 10 | 976.50 | 49.50 |
| -4 | -4 | 10 | 821.22 | 48.70 |
| -4 | 4 | 10 | 801.22 | 49.80 |
| -4 | 4 | 10 | 871.61 | 49.99 |
| -3 | -4 | 10 | 62.79 | 9.00 |
| -3 | -4 | 10 | 64.39 | 7.60 |
| -3 | 4 | 10 | 51.59 | 10.70 |
| -3 | 4 | 10 | 59.69 | 8.80 |
| -2 | -4 | 10 | 906.41 | 53.89 |
| -2 | -4 | 10 | 1074.99 | 55.19 |
| -2 | 4 | 10 | 955.70 | 54.89 |
| -2 | 4 | 10 | 904.81 | 54.69 |
| -2 | 4 | 10 | 1002.30 | 55.69 |
| -1 | -4 | 10 | 44.60 | 8.40 |
| -1 | -4 | 10 | 35.20 | 7.10 |
| -1 | 4 | 10 | 31.60 | 7.80 |
| -1 | 4 | 10 | 34.80 | 7.40 |

| | | | | |
|---|---|---|---|---|
| -1 | 4 | 10 | 34.40 | 6.40 |
| 0 | -4 | 10 | 4252.27 | 221.38 |
| 0 | -4 | 10 | 4318.27 | 222.78 |
| 0 | 4 | 10 | 3823.42 | 221.78 |
| 0 | 4 | 10 | 4186.28 | 223.08 |
| 0 | 4 | 10 | 3895.11 | 220.88 |
| 1 | -4 | 10 | 59.39 | 8.90 |
| 1 | -4 | 10 | 70.19 | 10.40 |
| 1 | 4 | 10 | 60.29 | 9.60 |
| 1 | 4 | 10 | 67.19 | 9.70 |
| 1 | 4 | 10 | 79.39 | 11.70 |
| 1 | 4 | 10 | 57.49 | 8.70 |
| 2 | -4 | 10 | 19.20 | 6.10 |
| 2 | -4 | 10 | 5.20 | 4.50 |
| 2 | 4 | 10 | 12.40 | 4.20 |
| 2 | 4 | 10 | 8.40 | 5.50 |
| 2 | 4 | 10 | 14.30 | 5.50 |
| 2 | 4 | 10 | 16.90 | 5.80 |
| 3 | -4 | 10 | 62.29 | 11.70 |
| 3 | -4 | 10 | 38.30 | 9.00 |
| 3 | -4 | 10 | 45.60 | 7.50 |
| 3 | 4 | 10 | 39.10 | 8.40 |
| 3 | 4 | 10 | 62.79 | 9.70 |
| 3 | 4 | 10 | 45.60 | 7.70 |
| 3 | 4 | 10 | 24.40 | 7.10 |
| 4 | -4 | 10 | 2325.47 | 123.79 |
| 4 | -4 | 10 | 2209.28 | 124.69 |
| 4 | -4 | 10 | 2352.96 | 123.29 |
| 4 | 4 | 10 | 2372.06 | 123.29 |
| 4 | 4 | 10 | 2340.67 | 123.59 |
| 4 | 4 | 10 | 2171.58 | 123.19 |
| 4 | 4 | 10 | 2077.09 | 126.29 |
| 5 | -4 | 10 | 4.60 | 5.60 |
| 5 | -4 | 10 | 5.80 | 5.70 |
| 5 | -4 | 10 | -7.00 | 5.30 |
| 5 | 4 | 10 | -6.10 | 8.30 |
| 5 | 4 | 10 | 4.60 | 4.40 |
| 5 | 4 | 10 | 5.60 | 4.10 |
| 6 | -4 | 10 | 269.57 | 19.20 |
| 6 | -4 | 10 | 255.47 | 18.20 |
| 6 | 4 | 10 | 276.57 | 17.80 |
| 6 | 4 | 10 | 279.77 | 18.10 |
| 6 | 4 | 10 | 293.07 | 17.40 |
| 6 | 4 | 10 | 255.57 | 26.40 |

| | | | | |
|---|---|---|---|---|
| 7 | -4 | 10 | -4.10 | 5.70 |
| 7 | -4 | 10 | -0.70 | 4.60 |
| 7 | 4 | 10 | 16.30 | 10.50 |
| 7 | 4 | 10 | -3.40 | 4.70 |
| 7 | 4 | 10 | 1.80 | 3.30 |
| 8 | -4 | 10 | 721.03 | 41.90 |
| 8 | 4 | 10 | 752.02 | 44.50 |
| 8 | 4 | 10 | 750.42 | 41.70 |
| 9 | -4 | 10 | -2.30 | 4.20 |
| 9 | -4 | 10 | 6.30 | 5.30 |
| 9 | 4 | 10 | 0.40 | 2.90 |
| 9 | 4 | 10 | -9.00 | 4.40 |
| 10 | -4 | 10 | 589.54 | 34.60 |
| 10 | -4 | 10 | 583.74 | 34.90 |
| 10 | 4 | 10 | 605.74 | 34.80 |
| 10 | 4 | 10 | 628.14 | 34.10 |
| 11 | -4 | 10 | 10.70 | 3.80 |
| 11 | -4 | 10 | 1.20 | 5.30 |
| 11 | 4 | 10 | 6.60 | 2.70 |
| 11 | 4 | 10 | 8.80 | 3.50 |
| 12 | -4 | 10 | 5.10 | 3.50 |
| 12 | -4 | 10 | 4.70 | 5.50 |
| 12 | 4 | 10 | 5.00 | 3.20 |
| 12 | 4 | 10 | 9.20 | 2.60 |
| 15 | -5 | -10 | 144.79 | 9.50 |
| 15 | 5 | -10 | 148.59 | 9.00 |
| -15 | 5 | 10 | 146.09 | 9.30 |
| 14 | -5 | -10 | 24.50 | 3.70 |
| 14 | 5 | -10 | 27.50 | 4.00 |
| -14 | 5 | 10 | 34.00 | 4.40 |
| 13 | -5 | -10 | 4.40 | 3.70 |
| -13 | 5 | 10 | 4.10 | 2.50 |
| 13 | 5 | -10 | 2.40 | 2.20 |
| 12 | -5 | -10 | 50.49 | 7.30 |
| -12 | 5 | 10 | 57.79 | 6.30 |
| 12 | 5 | -10 | 53.89 | 5.50 |
| 11 | -5 | -10 | 919.61 | 55.39 |
| 11 | 5 | -10 | 1038.10 | 55.19 |
| -11 | 5 | 10 | 1056.59 | 55.99 |
| -10 | 5 | 10 | 10.80 | 3.70 |
| 10 | 5 | -10 | 9.30 | 3.20 |
| 9 | 5 | -10 | 175.98 | 12.50 |
| -9 | 5 | 10 | 202.18 | 13.40 |
| 8 | 5 | -10 | 192.88 | 13.00 |

| | | | | |
|---|---|---|---|---|
| 7 | 5 | -10 | 887.31 | 52.39 |
| -7 | 5 | 10 | 1001.00 | 53.59 |
| -6 | -5 | 10 | 31.10 | 5.40 |
| -6 | 5 | 10 | 24.40 | 6.10 |
| 6 | 5 | -10 | 24.00 | 5.70 |
| -6 | 5 | 10 | 17.80 | 5.50 |
| -5 | -5 | 10 | 2202.08 | 120.09 |
| 5 | 5 | -10 | 2074.79 | 120.29 |
| -5 | 5 | 10 | 2414.46 | 121.69 |
| -5 | 5 | 10 | 2212.38 | 120.49 |
| -5 | 5 | 10 | 2138.09 | 121.29 |
| -4 | -5 | 10 | 45.60 | 8.90 |
| -4 | 5 | 10 | 63.49 | 9.20 |
| -4 | 5 | 10 | 45.80 | 7.50 |
| -4 | 5 | 10 | 51.49 | 10.80 |
| -3 | -5 | 10 | 4.40 | 4.30 |
| -3 | 5 | 10 | 6.60 | 5.70 |
| -3 | 5 | 10 | 6.00 | 5.90 |
| -3 | 5 | 10 | 3.90 | 4.50 |
| -2 | -5 | 10 | 79.59 | 9.00 |
| -2 | -5 | 10 | 97.19 | 9.80 |
| -2 | 5 | 10 | 93.29 | 10.80 |
| -2 | 5 | 10 | 74.09 | 9.90 |
| -2 | 5 | 10 | 81.99 | 13.10 |
| -2 | 5 | 10 | 83.59 | 11.60 |
| -1 | -5 | 10 | 4970.70 | 251.37 |
| -1 | -5 | 10 | 4562.84 | 249.68 |
| -1 | 5 | 10 | 4713.63 | 251.57 |
| -1 | 5 | 10 | 4479.45 | 250.27 |
| -1 | 5 | 10 | 4422.56 | 249.28 |
| -1 | 5 | 10 | 4625.04 | 249.88 |
| 0 | -5 | 10 | 47.00 | 8.80 |
| 0 | -5 | 10 | 55.19 | 8.60 |
| 0 | 5 | 10 | 35.90 | 8.30 |
| 0 | 5 | 10 | 47.40 | 9.10 |
| 0 | 5 | 10 | 64.79 | 11.20 |
| 0 | 5 | 10 | 52.79 | 9.50 |
| 1 | -5 | 10 | 512.75 | 29.00 |
| 1 | -5 | 10 | 421.86 | 29.30 |
| 1 | 5 | 10 | 512.95 | 28.40 |
| 1 | 5 | 10 | 492.65 | 28.20 |
| 1 | 5 | 10 | 485.95 | 29.60 |
| 1 | 5 | 10 | 426.16 | 29.80 |
| 2 | -5 | 10 | 15.00 | 5.90 |

| | | | | |
|---|---|---|---|---|
| 2 | -5 | 10 | 9.40 | 5.00 |
| 2 | 5 | 10 | 5.80 | 4.40 |
| 2 | 5 | 10 | 12.40 | 5.60 |
| 2 | 5 | 10 | 5.70 | 5.90 |
| 3 | -5 | 10 | 579.04 | 32.40 |
| 3 | -5 | 10 | 505.25 | 32.40 |
| 3 | 5 | 10 | 483.85 | 33.90 |
| 3 | 5 | 10 | 522.75 | 30.70 |
| 3 | 5 | 10 | 554.14 | 31.60 |
| 4 | -5 | 10 | 12.40 | 6.00 |
| 4 | -5 | 10 | 8.20 | 5.50 |
| 4 | 5 | 10 | 17.60 | 4.50 |
| 4 | 5 | 10 | 6.80 | 7.60 |
| 4 | 5 | 10 | 5.30 | 3.50 |
| 4 | 5 | 10 | 5.40 | 4.20 |
| 5 | -5 | 10 | 2281.97 | 119.99 |
| 5 | -5 | 10 | 2250.77 | 120.19 |
| 5 | 5 | 10 | 2308.27 | 120.09 |
| 5 | 5 | 10 | 2154.88 | 119.79 |
| 5 | 5 | 10 | 2007.20 | 123.59 |
| 6 | -5 | 10 | 20.70 | 5.60 |
| 6 | -5 | 10 | 18.20 | 6.10 |
| 6 | 5 | 10 | 30.90 | 6.10 |
| 6 | 5 | 10 | 31.60 | 5.40 |
| 6 | 5 | 10 | 28.30 | 10.80 |
| 7 | -5 | 10 | 38.10 | 6.40 |
| 7 | 5 | 10 | 34.80 | 6.00 |
| 7 | 5 | 10 | 35.10 | 12.60 |
| 7 | 5 | 10 | 45.70 | 8.30 |
| 8 | -5 | 10 | -4.80 | 5.10 |
| 8 | -5 | 10 | -2.00 | 5.90 |
| 8 | 5 | 10 | -0.50 | 2.90 |
| 8 | 5 | 10 | 0.10 | 4.90 |
| 9 | -5 | 10 | 638.24 | 37.70 |
| 9 | -5 | 10 | 621.84 | 37.30 |
| 9 | 5 | 10 | 641.94 | 37.70 |
| 9 | 5 | 10 | 703.33 | 36.90 |
| 10 | -5 | 10 | 6.50 | 5.50 |
| 10 | -5 | 10 | 1.70 | 4.60 |
| 10 | 5 | 10 | 4.00 | 3.70 |
| 10 | 5 | 10 | 0.00 | 3.30 |
| 11 | -5 | 10 | 173.48 | 12.60 |
| 11 | -5 | 10 | 141.69 | 14.70 |
| 11 | 5 | 10 | 185.38 | 12.20 |

| | | | | |
|---|---|---|---|---|
| 11 | 5 | 10 | 180.68 | 11.30 |
| 12 | -5 | 10 | 5.80 | 9.60 |
| 12 | 5 | 10 | 4.80 | 3.20 |
| 12 | 5 | 10 | 2.60 | 2.20 |
| 15 | 6 | -10 | 23.20 | 3.10 |
| -15 | 6 | 10 | 25.40 | 3.40 |
| 14 | -6 | -10 | 98.29 | 8.10 |
| 14 | 6 | -10 | 103.59 | 6.90 |
| -14 | 6 | 10 | 104.69 | 7.40 |
| 13 | -6 | -10 | 51.59 | 7.00 |
| 13 | 6 | -10 | 42.10 | 4.90 |
| 12 | -6 | -10 | 491.35 | 32.80 |
| -12 | 6 | 10 | 667.93 | 33.40 |
| 12 | 6 | -10 | 590.74 | 32.50 |
| 11 | 6 | -10 | 92.69 | 7.30 |
| -11 | 6 | 10 | 88.19 | 8.10 |
| 10 | 6 | -10 | 800.12 | 46.90 |
| -10 | 6 | 10 | 898.91 | 47.90 |
| 9 | 6 | -10 | 13.30 | 3.40 |
| -9 | 6 | 10 | 13.20 | 4.00 |
| -8 | 6 | 10 | 2.80 | 4.10 |
| 8 | 6 | -10 | 1.60 | 3.10 |
| -7 | -6 | 10 | 21.00 | 5.30 |
| -7 | 6 | 10 | 24.30 | 5.40 |
| 7 | 6 | -10 | 15.40 | 4.50 |
| 7 | 6 | -10 | 20.50 | 5.20 |
| -6 | -6 | 10 | 3183.18 | 180.18 |
| -6 | 6 | 10 | 3530.15 | 181.58 |
| 6 | 6 | -10 | 3326.27 | 180.28 |
| -6 | 6 | 10 | 3556.64 | 181.78 |
| -6 | 6 | 10 | 3074.79 | 180.98 |
| -5 | -6 | 10 | 203.98 | 14.40 |
| -5 | 6 | 10 | 214.78 | 16.30 |
| -5 | 6 | 10 | 163.08 | 14.80 |
| -5 | 6 | 10 | 199.38 | 15.90 |
| -4 | -6 | 10 | 73.29 | 9.70 |
| -4 | 6 | 10 | 88.09 | 10.70 |
| -4 | 6 | 10 | 64.09 | 11.40 |
| -4 | 6 | 10 | 98.39 | 11.60 |
| -4 | 6 | 10 | 75.99 | 10.50 |
| -3 | -6 | 10 | 16.90 | 4.70 |
| -3 | 6 | 10 | 4.40 | 5.30 |
| -3 | 6 | 10 | 8.10 | 5.20 |
| -3 | 6 | 10 | -3.80 | 5.50 |

| | | | | |
|---|---|---|---|---|
| -3 | 6 | 10 | 2.00 | 5.60 |
| -2 | -6 | 10 | 758.82 | 45.60 |
| -2 | 6 | 10 | 780.82 | 46.50 |
| -2 | 6 | 10 | 840.62 | 47.50 |
| -2 | 6 | 10 | 813.72 | 45.90 |
| -2 | 6 | 10 | 851.71 | 46.50 |
| -1 | -6 | 10 | 281.27 | 18.40 |
| -1 | 6 | 10 | 275.27 | 18.90 |
| -1 | 6 | 10 | 278.37 | 20.30 |
| -1 | 6 | 10 | 303.77 | 18.90 |
| -1 | 6 | 10 | 283.27 | 20.00 |
| 0 | -6 | 10 | 1419.56 | 77.89 |
| 0 | 6 | 10 | 1338.37 | 78.59 |
| 0 | 6 | 10 | 1311.67 | 77.39 |
| 0 | 6 | 10 | 1386.06 | 79.49 |
| 0 | 6 | 10 | 1591.74 | 78.09 |
| 1 | -6 | 10 | 34.00 | 5.90 |
| 1 | 6 | 10 | 39.70 | 7.60 |
| 1 | 6 | 10 | 41.60 | 8.00 |
| 1 | 6 | 10 | 46.80 | 9.60 |
| 1 | 6 | 10 | 42.70 | 6.90 |
| 2 | -6 | 10 | 167.58 | 13.70 |
| 2 | 6 | 10 | 151.68 | 13.00 |
| 2 | 6 | 10 | 145.79 | 14.50 |
| 2 | 6 | 10 | 155.48 | 11.90 |
| 2 | 6 | 10 | 131.29 | 11.40 |
| 3 | -6 | 10 | 6.80 | 5.60 |
| 3 | 6 | 10 | 8.60 | 4.40 |
| 3 | 6 | 10 | 7.10 | 3.80 |
| 3 | 6 | 10 | 5.40 | 7.10 |
| 3 | 6 | 10 | 4.40 | 3.80 |
| 4 | -6 | 10 | 2318.17 | 123.09 |
| 4 | 6 | 10 | 2280.57 | 122.49 |
| 4 | 6 | 10 | 2151.08 | 126.09 |
| 5 | -6 | 10 | 83.99 | 11.20 |
| 5 | 6 | 10 | 81.99 | 8.60 |
| 6 | -6 | 10 | 132.29 | 12.80 |
| 6 | 6 | 10 | 153.68 | 10.80 |
| 6 | 6 | 10 | 128.79 | 21.70 |
| 6 | 6 | 10 | 144.59 | 11.30 |
| 7 | 6 | 10 | 8.70 | 4.10 |
| 7 | 6 | 10 | 14.10 | 3.70 |
| 7 | 6 | 10 | 16.00 | 13.90 |
| 8 | -6 | 10 | 533.65 | 33.30 |

| | | | | |
|---|---|---|---|---|
| 8 | 6 | 10 | 552.74 | 32.00 |
| 8 | 6 | 10 | 603.04 | 33.00 |
| 9 | -6 | 10 | 4.40 | 7.50 |
| 9 | 6 | 10 | 6.40 | 4.00 |
| 9 | 6 | 10 | -0.70 | 2.70 |
| 10 | -6 | 10 | 516.45 | 33.50 |
| 10 | 6 | 10 | 579.54 | 33.20 |
| 10 | 6 | 10 | 627.84 | 32.60 |
| 11 | 6 | 10 | 16.90 | 3.80 |
| 12 | 6 | 10 | 16.80 | 3.70 |
| 12 | 6 | 10 | 10.90 | 3.20 |
| 15 | 7 | -10 | 257.67 | 13.90 |
| -15 | 7 | 10 | 231.08 | 14.20 |
| 14 | -7 | -10 | 20.70 | 3.70 |
| -14 | 7 | 10 | 25.00 | 3.80 |
| 14 | 7 | -10 | 28.00 | 3.30 |
| 13 | -7 | -10 | 11.50 | 3.70 |
| 13 | 7 | -10 | 10.60 | 2.20 |
| -13 | 7 | 10 | 10.30 | 2.60 |
| 12 | -7 | -10 | 86.29 | 11.00 |
| 12 | 7 | -10 | 61.29 | 5.70 |
| -12 | 7 | 10 | 60.89 | 6.50 |
| 11 | 7 | -10 | 693.43 | 38.80 |
| -11 | 7 | 10 | 706.13 | 39.60 |
| 10 | 7 | -10 | 129.19 | 8.90 |
| -10 | 7 | 10 | 117.49 | 9.90 |
| -9 | 7 | 10 | 299.97 | 19.50 |
| 9 | 7 | -10 | 313.57 | 18.40 |
| -8 | 7 | 10 | 71.29 | 9.00 |
| 8 | 7 | -10 | 67.99 | 7.60 |
| -7 | -7 | 10 | 708.63 | 44.60 |
| -7 | 7 | 10 | 886.61 | 46.00 |
| 7 | 7 | -10 | 788.52 | 45.50 |
| -6 | -7 | 10 | 24.90 | 5.00 |
| 6 | 7 | -10 | 37.00 | 8.00 |
| -6 | 7 | 10 | 26.50 | 6.10 |
| 6 | 7 | -10 | 28.60 | 5.70 |
| -6 | 7 | 10 | 33.80 | 7.80 |
| -6 | 7 | 10 | 43.80 | 8.20 |
| -5 | -7 | 10 | 1654.63 | 89.79 |
| -5 | 7 | 10 | 1675.03 | 89.89 |
| -5 | 7 | 10 | 1479.85 | 90.79 |
| -5 | 7 | 10 | 1737.73 | 91.49 |
| -4 | -7 | 10 | 34.00 | 5.70 |

| | | | | |
|---|---|---|---|---|
| -4 | 7 | 10 | 55.29 | 9.10 |
| -4 | 7 | 10 | 38.90 | 6.90 |
| -4 | 7 | 10 | 50.79 | 9.80 |
| -3 | -7 | 10 | 13.50 | 4.70 |
| -3 | 7 | 10 | 14.60 | 5.40 |
| -3 | 7 | 10 | 26.00 | 6.20 |
| -3 | 7 | 10 | 18.20 | 5.60 |
| -2 | -7 | 10 | 232.28 | 16.20 |
| -2 | 7 | 10 | 259.57 | 17.10 |
| -2 | 7 | 10 | 221.88 | 16.90 |
| -2 | 7 | 10 | 235.88 | 17.80 |
| -2 | 7 | 10 | 237.88 | 17.40 |
| -1 | -7 | 10 | 3061.69 | 166.88 |
| -1 | -7 | 10 | 3210.98 | 167.58 |
| -1 | 7 | 10 | 3267.97 | 169.58 |
| -1 | 7 | 10 | 3055.89 | 167.58 |
| -1 | 7 | 10 | 2946.01 | 167.18 |
| -1 | 7 | 10 | 2990.80 | 167.98 |
| 0 | -7 | 10 | 117.59 | 11.00 |
| 0 | 7 | 10 | 113.09 | 9.70 |
| 0 | 7 | 10 | 99.19 | 12.00 |
| 0 | 7 | 10 | 98.39 | 10.40 |
| 0 | 7 | 10 | 104.59 | 12.40 |
| 1 | -7 | 10 | 188.98 | 14.60 |
| 1 | 7 | 10 | 176.78 | 15.60 |
| 1 | 7 | 10 | 190.78 | 14.90 |
| 1 | 7 | 10 | 189.48 | 13.60 |
| 1 | 7 | 10 | 173.58 | 13.20 |
| 2 | -7 | 10 | 50.79 | 9.70 |
| 2 | 7 | 10 | 42.50 | 7.30 |
| 2 | 7 | 10 | 46.20 | 7.60 |
| 2 | 7 | 10 | 38.60 | 7.80 |
| 3 | -7 | 10 | 508.75 | 30.10 |
| 3 | 7 | 10 | 465.85 | 32.20 |
| 3 | 7 | 10 | 496.85 | 29.00 |
| 3 | 7 | 10 | 493.85 | 28.70 |
| 4 | -7 | 10 | 5.20 | 5.60 |
| 4 | 7 | 10 | 0.50 | 10.90 |
| 4 | 7 | 10 | 0.00 | 3.70 |
| 4 | 7 | 10 | 2.10 | 3.80 |
| 5 | -7 | 10 | 1395.26 | 77.39 |
| 5 | 7 | 10 | 1539.15 | 77.19 |
| 5 | 7 | 10 | 1432.16 | 77.19 |
| 5 | 7 | 10 | 1226.68 | 81.49 |

| | | | | |
|---|---|---|---|---|
| 6 | -7 | 10 | 205.88 | 14.90 |
| 6 | 7 | 10 | 211.98 | 14.10 |
| 6 | 7 | 10 | 201.68 | 13.50 |
| 6 | 7 | 10 | 183.18 | 25.40 |
| 7 | 7 | 10 | 13.00 | 15.10 |
| 7 | 7 | 10 | 28.00 | 5.80 |
| 7 | 7 | 10 | 22.90 | 5.30 |
| -8 | -7 | -10 | 43.20 | 7.70 |
| 8 | 7 | 10 | 35.20 | 5.60 |
| 8 | 7 | 10 | 36.90 | 6.80 |
| 9 | 7 | 10 | 702.93 | 39.00 |
| 9 | 7 | 10 | 593.94 | 36.80 |
| 10 | 7 | 10 | 43.00 | 5.40 |
| 10 | 7 | 10 | 44.50 | 6.60 |
| 11 | 7 | 10 | 166.38 | 9.90 |
| 11 | 7 | 10 | 130.89 | 10.90 |
| -14 | 8 | 10 | 179.28 | 10.90 |
| 14 | 8 | -10 | 174.78 | 10.40 |
| 13 | 8 | -10 | 41.80 | 4.10 |
| -13 | 8 | 10 | 40.90 | 5.00 |
| 12 | 8 | -10 | 867.51 | 49.20 |
| 11 | 8 | -10 | 27.00 | 4.50 |
| -11 | 8 | 10 | 12.20 | 3.10 |
| -10 | 8 | 10 | 667.43 | 38.20 |
| 10 | 8 | -10 | 676.33 | 37.40 |
| -9 | 8 | 10 | 87.79 | 8.90 |
| 9 | 8 | -10 | 91.89 | 7.70 |
| -8 | 8 | 10 | 113.09 | 10.50 |
| 8 | 8 | -10 | 108.39 | 9.40 |
| -7 | -8 | 10 | 77.09 | 10.20 |
| 7 | 8 | -10 | 107.39 | 9.30 |
| -7 | 8 | 10 | 102.59 | 9.40 |
| -7 | 8 | 10 | 125.09 | 11.40 |
| 7 | 8 | -10 | 90.99 | 11.80 |
| -6 | -8 | 10 | 1702.03 | 101.09 |
| 6 | 8 | -10 | 1904.21 | 100.79 |
| -6 | 8 | 10 | 1731.23 | 99.59 |
| -6 | 8 | 10 | 1958.70 | 101.19 |
| -5 | -8 | 10 | 500.65 | 31.10 |
| -5 | 8 | 10 | 546.65 | 31.20 |
| -5 | 8 | 10 | 506.75 | 32.60 |
| -5 | 8 | 10 | 544.65 | 33.30 |
| 5 | 8 | -10 | 552.74 | 32.20 |
| -4 | -8 | 10 | 289.27 | 19.20 |

| | | | | |
|---|---|---|---|---|
| -4 | 8 | 10 | 310.87 | 19.10 |
| -4 | 8 | 10 | 303.97 | 21.20 |
| -4 | 8 | 10 | 270.27 | 20.30 |
| -3 | -8 | 10 | 195.28 | 14.30 |
| -3 | 8 | 10 | 168.88 | 15.30 |
| -3 | 8 | 10 | 206.08 | 14.50 |
| -3 | 8 | 10 | 212.28 | 16.30 |
| -2 | -8 | 10 | 688.13 | 39.20 |
| -2 | -8 | 10 | 677.23 | 38.70 |
| -2 | 8 | 10 | 660.73 | 39.40 |
| -2 | 8 | 10 | 742.93 | 41.10 |
| -2 | 8 | 10 | 631.04 | 38.80 |
| -1 | -8 | 10 | 10.20 | 4.90 |
| -1 | -8 | 10 | 14.10 | 4.50 |
| -1 | 8 | 10 | 23.80 | 4.80 |
| -1 | 8 | 10 | 25.10 | 6.50 |
| -1 | 8 | 10 | 17.10 | 4.90 |
| 0 | -8 | 10 | 2882.51 | 153.28 |
| 0 | -8 | 10 | 2768.62 | 152.28 |
| 0 | 8 | 10 | 2480.65 | 152.58 |
| 0 | 8 | 10 | 3090.09 | 153.18 |
| 0 | 8 | 10 | 2869.31 | 155.18 |
| 1 | -8 | 10 | 196.48 | 15.00 |
| 1 | -8 | 10 | 196.48 | 14.70 |
| 1 | 8 | 10 | 204.18 | 13.80 |
| 1 | 8 | 10 | 199.88 | 16.60 |
| 1 | 8 | 10 | 188.48 | 14.10 |
| 2 | -8 | 10 | 14.20 | 5.10 |
| 2 | 8 | 10 | 18.60 | 4.50 |
| 2 | 8 | 10 | 16.00 | 6.40 |
| 2 | 8 | 10 | 28.50 | 4.60 |
| 3 | -8 | 10 | 143.09 | 12.60 |
| 3 | -8 | 10 | 130.09 | 11.80 |
| 3 | 8 | 10 | 124.09 | 10.00 |
| 3 | 8 | 10 | 120.19 | 10.70 |
| 4 | -8 | 10 | 1594.14 | 87.79 |
| 4 | 8 | 10 | 1577.44 | 87.09 |
| 4 | 8 | 10 | 1668.73 | 87.19 |
| 4 | 8 | 10 | 1514.85 | 91.09 |
| 5 | -8 | 10 | -3.90 | 4.60 |
| 5 | 8 | 10 | 3.90 | 3.40 |
| 5 | 8 | 10 | 7.70 | 3.60 |
| 5 | 8 | 10 | 2.50 | 10.50 |
| 6 | -8 | 10 | 279.77 | 17.50 |

| | | | | |
|---|---|---|---|---|
| 6 | 8 | 10 | 249.78 | 16.30 |
| 6 | 8 | 10 | 261.97 | 16.90 |
| 6 | 8 | 10 | 247.68 | 29.20 |
| -7 | -8 | -10 | 151.48 | 10.90 |
| 7 | 8 | 10 | 130.19 | 10.10 |
| 7 | 8 | 10 | 135.49 | 10.60 |
| -8 | -8 | -10 | 355.16 | 25.80 |
| 8 | 8 | 10 | 380.16 | 22.00 |
| 8 | 8 | 10 | 377.76 | 22.50 |
| 9 | 8 | 10 | 2.00 | 2.90 |
| 9 | 8 | 10 | 2.10 | 3.50 |
| 10 | 8 | 10 | 453.55 | 27.40 |
| 10 | 8 | 10 | 491.75 | 26.90 |
| 11 | 8 | 10 | 38.00 | 5.00 |
| 11 | 8 | 10 | 47.70 | 5.80 |
| 14 | 9 | -10 | 51.39 | 3.90 |
| -14 | 9 | 10 | 46.70 | 4.50 |
| -13 | 9 | 10 | 18.90 | 3.90 |
| 13 | 9 | -10 | 12.40 | 2.10 |
| 12 | 9 | -10 | 34.00 | 4.20 |
| -12 | 9 | 10 | 35.10 | 5.20 |
| 11 | 9 | -10 | 1259.27 | 67.79 |
| -11 | 9 | 10 | 1230.18 | 68.39 |
| 10 | 9 | -10 | 101.39 | 7.60 |
| -10 | 9 | 10 | 94.39 | 8.60 |
| 9 | 9 | -10 | 344.17 | 20.70 |
| -9 | 9 | 10 | 365.16 | 22.00 |
| -8 | -9 | 10 | 211.68 | 14.90 |
| -8 | 9 | 10 | 231.08 | 14.50 |
| -8 | 9 | 10 | 210.38 | 15.60 |
| 8 | 9 | -10 | 219.08 | 14.30 |
| -7 | -9 | 10 | 999.30 | 57.69 |
| 7 | 9 | -10 | 1080.99 | 59.39 |
| -7 | 9 | 10 | 1017.00 | 58.99 |
| 7 | 9 | -10 | 1054.59 | 57.79 |
| -7 | 9 | 10 | 1055.89 | 57.79 |
| -6 | -9 | 10 | 1.60 | 4.30 |
| -6 | 9 | 10 | -6.40 | 3.80 |
| 6 | 9 | -10 | 6.20 | 5.60 |
| -6 | 9 | 10 | 8.70 | 5.60 |
| -5 | -9 | 10 | 2088.39 | 114.69 |
| -5 | 9 | 10 | 1920.21 | 115.59 |
| -5 | 9 | 10 | 2115.09 | 114.69 |
| -5 | 9 | 10 | 2177.78 | 116.79 |

| | | | | |
|---|---|---|---|---|
| 5 | 9 | -10 | 2210.78 | 116.19 |
| -4 | -9 | 10 | 264.17 | 18.30 |
| -4 | 9 | 10 | 271.97 | 17.90 |
| 4 | 9 | -10 | 281.17 | 19.50 |
| -4 | 9 | 10 | 269.27 | 20.60 |
| -3 | 9 | 10 | 3.90 | 5.40 |
| 3 | 9 | -10 | -0.40 | 5.10 |
| -3 | 9 | 10 | -0.40 | 5.80 |
| -3 | 9 | 10 | -1.30 | 4.20 |
| -2 | -9 | 10 | 6.40 | 6.90 |
| -2 | -9 | 10 | 4.60 | 4.30 |
| -2 | 9 | 10 | -3.20 | 4.60 |
| -2 | 9 | 10 | 2.90 | 4.10 |
| -2 | 9 | 10 | 6.10 | 5.50 |
| -1 | -9 | 10 | 2820.22 | 150.58 |
| -1 | 9 | 10 | 2891.81 | 151.28 |
| -1 | 9 | 10 | 2729.93 | 153.18 |
| -1 | 9 | 10 | 2691.83 | 151.08 |
| 0 | -9 | 10 | 279.27 | 20.30 |
| 0 | -9 | 10 | 298.27 | 19.00 |
| 0 | 9 | 10 | 280.57 | 21.70 |
| 0 | 9 | 10 | 316.27 | 19.60 |
| 0 | 9 | 10 | 338.67 | 19.40 |
| 1 | -9 | 10 | 580.14 | 32.10 |
| 1 | -9 | 10 | 521.45 | 30.90 |
| 1 | 9 | 10 | 537.15 | 35.70 |
| 1 | 9 | 10 | 525.95 | 31.10 |
| 1 | 9 | 10 | 514.55 | 33.40 |
| 2 | -9 | 10 | 139.89 | 12.40 |
| 2 | -9 | 10 | 124.99 | 14.90 |
| 2 | 9 | 10 | 152.28 | 11.40 |
| 2 | 9 | 10 | 134.29 | 10.70 |
| 2 | 9 | 10 | 144.99 | 15.10 |
| 3 | -9 | 10 | 193.18 | 14.50 |
| 3 | -9 | 10 | 154.08 | 12.80 |
| 3 | 9 | 10 | 179.28 | 18.10 |
| 3 | 9 | 10 | 179.28 | 12.80 |
| 3 | 9 | 10 | 181.48 | 12.50 |
| 4 | -9 | 10 | 5.40 | 5.00 |
| 4 | -9 | 10 | 1.90 | 4.40 |
| -4 | 9 | -10 | 0.20 | 6.20 |
| 4 | 9 | 10 | -9.50 | 8.40 |
| 4 | 9 | 10 | 3.10 | 3.50 |
| 4 | 9 | 10 | 3.80 | 3.20 |

| | | | | |
|---|---|---|---|---|
| 5 | -9 | 10 | 1849.71 | 101.49 |
| 5 | -9 | 10 | 1922.31 | 102.29 |
| 5 | 9 | 10 | 1936.11 | 101.89 |
| 5 | 9 | 10 | 1761.62 | 107.09 |
| -5 | 9 | -10 | 1875.81 | 103.99 |
| 5 | 9 | 10 | 1868.41 | 102.09 |
| -6 | -9 | -10 | 391.16 | 22.70 |
| 6 | -9 | 10 | 388.86 | 22.60 |
| 6 | -9 | 10 | 381.76 | 23.20 |
| 6 | 9 | 10 | 297.77 | 34.00 |
| 6 | 9 | 10 | 389.46 | 22.80 |
| 6 | 9 | 10 | 398.06 | 22.60 |
| -7 | -9 | -10 | 6.00 | 3.50 |
| 7 | 9 | 10 | 5.80 | 3.10 |
| 7 | 9 | 10 | 0.90 | 3.20 |
| -8 | -9 | -10 | 18.70 | 4.30 |
| 8 | 9 | 10 | 20.10 | 4.10 |
| 8 | 9 | 10 | 34.90 | 5.80 |
| 9 | 9 | 10 | 547.05 | 30.60 |
| 9 | 9 | 10 | 538.75 | 31.20 |
| 10 | 9 | 10 | 18.10 | 3.80 |
| 10 | 9 | 10 | 22.90 | 4.80 |
| 11 | 9 | 10 | 190.58 | 12.40 |
| 11 | 9 | 10 | 199.08 | 14.40 |
| 13 | 10 | -10 | 158.78 | 9.30 |
| -13 | 10 | 10 | 151.28 | 9.80 |
| -12 | 10 | 10 | 377.66 | 21.40 |
| 12 | 10 | -10 | 363.26 | 20.80 |
| -11 | 10 | 10 | 34.20 | 5.60 |
| 11 | 10 | -10 | 36.60 | 4.60 |
| -11 | 10 | 10 | 34.80 | 6.10 |
| -10 | 10 | 10 | 693.83 | 41.20 |
| -10 | 10 | 10 | 739.73 | 41.80 |
| 10 | 10 | -10 | 791.92 | 41.10 |
| -9 | 10 | 10 | 219.58 | 15.40 |
| -9 | 10 | 10 | 225.78 | 14.60 |
| 9 | 10 | -10 | 237.78 | 14.10 |
| -8 | -10 | 10 | -1.10 | 3.90 |
| 8 | 10 | -10 | 2.60 | 2.80 |
| -8 | 10 | 10 | 6.80 | 4.10 |
| -8 | 10 | 10 | -1.50 | 4.10 |
| -7 | -10 | 10 | 65.19 | 8.70 |
| 7 | 10 | -10 | 69.59 | 7.90 |
| 7 | 10 | -10 | 85.89 | 13.10 |

| | | | | |
|---|---|---|---|---|
| -7 | 10 | 10 | 78.99 | 10.90 |
| -7 | 10 | 10 | 72.99 | 7.90 |
| -6 | -10 | 10 | 1335.17 | 75.09 |
| -6 | 10 | 10 | 1360.36 | 75.19 |
| 6 | 10 | -10 | 1398.56 | 76.79 |
| -6 | 10 | 10 | 1368.86 | 76.49 |
| -5 | -10 | 10 | 271.37 | 18.60 |
| 5 | -10 | -10 | 296.97 | 19.40 |
| 5 | 10 | -10 | 290.77 | 20.50 |
| -5 | 10 | 10 | 295.17 | 22.90 |
| -5 | 10 | 10 | 325.87 | 18.50 |
| -5 | 10 | 10 | 230.28 | 20.20 |
| -4 | -10 | 10 | 771.62 | 46.10 |
| -4 | 10 | 10 | 814.62 | 47.20 |
| -4 | 10 | 10 | 817.62 | 48.60 |
| -4 | 10 | 10 | 824.12 | 46.20 |
| 4 | 10 | -10 | 847.22 | 48.00 |
| -3 | -10 | 10 | 276.97 | 18.10 |
| -3 | 10 | 10 | 252.47 | 17.80 |
| -3 | 10 | 10 | 261.97 | 23.20 |
| 3 | 10 | -10 | 300.47 | 20.70 |
| -3 | 10 | 10 | 277.67 | 19.10 |
| -2 | -10 | 10 | 325.77 | 20.30 |
| -2 | -10 | 10 | 318.87 | 19.30 |
| 2 | 10 | -10 | 322.57 | 23.20 |
| -2 | 10 | 10 | 289.17 | 20.40 |
| -2 | 10 | 10 | 311.47 | 20.00 |
| -2 | 10 | 10 | 333.57 | 27.60 |
| -1 | -10 | 10 | 5.10 | 3.60 |
| -1 | -10 | 10 | 8.90 | 4.40 |
| -1 | 10 | 10 | 8.10 | 5.90 |
| -1 | 10 | 10 | 3.90 | 4.70 |
| -1 | 10 | 10 | 1.60 | 4.10 |
| 1 | 10 | -10 | 15.10 | 7.30 |
| 0 | -10 | 10 | 2645.54 | 142.69 |
| 0 | -10 | 10 | 2783.02 | 143.79 |
| 0 | 10 | 10 | 3010.40 | 143.79 |
| 0 | 10 | 10 | 2560.04 | 143.29 |
| 0 | 10 | -10 | 2324.87 | 145.59 |
| 0 | 10 | 10 | 2512.95 | 145.69 |
| 1 | -10 | 10 | 554.74 | 30.00 |
| 1 | -10 | 10 | 466.55 | 28.50 |
| 1 | 10 | 10 | 476.65 | 32.20 |
| -1 | 10 | -10 | 472.85 | 31.90 |

| | | | | |
|---|---|---|---|---|
| 1 | 10 | 10 | 491.35 | 29.20 |
| 1 | 10 | 10 | 520.85 | 29.20 |
| 2 | -10 | 10 | 10.40 | 4.50 |
| 2 | -10 | 10 | -0.30 | 3.70 |
| 2 | 10 | 10 | 5.50 | 3.40 |
| -2 | 10 | -10 | 10.60 | 6.10 |
| 2 | 10 | 10 | 11.00 | 4.10 |
| 2 | 10 | 10 | 17.30 | 7.70 |
| 3 | -10 | 10 | 69.59 | 9.30 |
| 3 | -10 | 10 | 53.79 | 7.70 |
| 3 | 10 | 10 | 55.09 | 7.40 |
| -3 | 10 | -10 | 51.99 | 12.00 |
| 3 | 10 | 10 | 58.99 | 14.50 |
| 3 | 10 | 10 | 62.99 | 7.00 |
| 4 | -10 | 10 | 1367.66 | 72.89 |
| 4 | -10 | 10 | 1307.27 | 71.79 |
| 4 | 10 | 10 | 1415.46 | 72.49 |
| 4 | 10 | 10 | 1214.98 | 71.99 |
| -4 | 10 | -10 | 1341.67 | 74.59 |
| 4 | 10 | 10 | 1233.98 | 76.79 |
| 5 | -10 | 10 | 62.39 | 8.90 |
| 5 | -10 | 10 | 52.89 | 7.70 |
| -5 | -10 | -10 | 63.49 | 7.30 |
| 5 | 10 | 10 | 65.69 | 7.10 |
| 5 | 10 | 10 | 40.90 | 14.20 |
| 5 | 10 | 10 | 70.69 | 7.30 |
| 6 | -10 | 10 | 263.27 | 16.50 |
| -6 | -10 | -10 | 265.87 | 16.70 |
| 6 | -10 | 10 | 274.47 | 17.30 |
| 6 | 10 | 10 | 266.97 | 16.40 |
| 6 | 10 | 10 | 260.27 | 16.70 |
| 7 | -10 | 10 | 117.79 | 9.30 |
| -7 | -10 | -10 | 102.79 | 10.80 |
| 7 | 10 | 10 | 102.89 | 8.60 |
| -8 | -10 | -10 | 224.08 | 14.10 |
| 8 | 10 | 10 | 199.18 | 13.60 |
| 8 | 10 | 10 | 232.18 | 14.10 |
| 9 | 10 | 10 | 9.30 | 3.20 |
| 9 | 10 | 10 | 3.40 | 3.20 |
| 10 | 10 | 10 | 374.36 | 22.40 |
| 10 | 10 | 10 | 396.46 | 22.00 |
| 13 | 11 | -10 | 2.70 | 1.30 |
| -13 | 11 | 10 | 1.90 | 2.20 |
| 12 | 11 | -10 | 151.98 | 9.10 |

| | | | | |
|---|---|---|---|---|
| -12 | 11 | 10 | 147.79 | 9.80 |
| 11 | 11 | -10 | 260.47 | 14.70 |
| -11 | 11 | 10 | 251.37 | 15.40 |
| -11 | 11 | 10 | 241.68 | 15.30 |
| -10 | 11 | 10 | 62.69 | 6.60 |
| -10 | 11 | 10 | 63.99 | 7.60 |
| 10 | 11 | -10 | 77.89 | 6.90 |
| 9 | 11 | -10 | 387.76 | 22.30 |
| -9 | 11 | 10 | 384.66 | 22.60 |
| -9 | 11 | 10 | 389.46 | 23.50 |
| -8 | -11 | 10 | 135.29 | 10.60 |
| -8 | 11 | 10 | 125.79 | 11.90 |
| 8 | 11 | -10 | 134.09 | 9.80 |
| -8 | 11 | 10 | 146.09 | 10.20 |
| -7 | -11 | 10 | 62.49 | 7.50 |
| 7 | 11 | -10 | 50.09 | 6.80 |
| -7 | 11 | 10 | 56.89 | 9.90 |
| -7 | 11 | 10 | 52.59 | 7.30 |
| -6 | -11 | 10 | -3.90 | 3.60 |
| -6 | 11 | 10 | 1.50 | 5.70 |
| -6 | 11 | 10 | 3.40 | 3.50 |
| 6 | 11 | -10 | 12.50 | 6.90 |
| 5 | -11 | -10 | 849.61 | 47.90 |
| -5 | -11 | 10 | 809.72 | 47.70 |
| -5 | 11 | 10 | 919.81 | 56.29 |
| 5 | 11 | -10 | 874.01 | 49.99 |
| -5 | 11 | 10 | 854.61 | 47.80 |
| -5 | 11 | 10 | 800.92 | 48.90 |
| 4 | -11 | -10 | 101.49 | 10.40 |
| -4 | -11 | 10 | 93.49 | 9.70 |
| -4 | 11 | 10 | 107.59 | 11.60 |
| -4 | 11 | 10 | 83.59 | 9.20 |
| 4 | 11 | -10 | 104.49 | 13.00 |
| -4 | 11 | 10 | 83.79 | 15.20 |
| -3 | -11 | 10 | 19.90 | 4.40 |
| 3 | -11 | -10 | 25.20 | 5.40 |
| -3 | 11 | 10 | 18.20 | 7.40 |
| -3 | 11 | 10 | 26.40 | 5.80 |
| 3 | 11 | -10 | 26.20 | 7.70 |
| -3 | 11 | 10 | 34.80 | 5.40 |
| -2 | -11 | 10 | 241.58 | 15.60 |
| -2 | -11 | 10 | 256.17 | 16.90 |
| -2 | 11 | 10 | 274.57 | 20.30 |
| -2 | 11 | 10 | 242.18 | 16.70 |

| | | | | |
|---|---|---|---|---|
| -2 | 11 | 10 | 253.87 | 17.30 |
| 2 | 11 | -10 | 237.38 | 20.50 |
| -1 | -11 | 10 | 888.81 | 48.70 |
| -1 | -11 | 10 | 843.72 | 47.60 |
| -1 | 11 | 10 | 839.02 | 48.40 |
| -1 | 11 | 10 | 927.81 | 51.39 |
| -1 | 11 | 10 | 920.51 | 48.70 |
| 1 | 11 | -10 | 732.53 | 51.09 |
| 0 | -11 | 10 | 14.50 | 4.10 |
| 0 | -11 | 10 | 16.40 | 3.60 |
| 0 | 11 | -10 | 13.50 | 7.20 |
| 0 | 11 | 10 | 17.50 | 7.80 |
| 0 | 11 | 10 | 21.20 | 5.10 |
| 0 | 11 | 10 | 9.10 | 4.10 |
| 1 | -11 | 10 | 169.38 | 11.80 |
| 1 | -11 | 10 | 190.28 | 13.60 |
| -1 | 11 | -10 | 162.58 | 16.50 |
| 1 | 11 | 10 | 177.78 | 12.60 |
| 1 | 11 | 10 | 168.18 | 12.90 |
| 1 | 11 | 10 | 167.98 | 16.80 |
| 2 | -11 | 10 | 92.99 | 8.80 |
| 2 | -11 | 10 | 111.29 | 10.60 |
| -2 | 11 | -10 | 99.19 | 13.50 |
| 2 | 11 | 10 | 121.79 | 9.40 |
| 2 | 11 | 10 | 106.19 | 15.30 |
| 2 | 11 | 10 | 108.09 | 9.70 |
| 3 | -11 | 10 | 206.68 | 13.80 |
| 3 | -11 | 10 | 241.88 | 15.70 |
| -3 | -11 | -10 | 213.78 | 15.00 |
| 3 | 11 | 10 | 256.37 | 16.00 |
| 3 | 11 | 10 | 208.58 | 20.90 |
| 3 | 11 | 10 | 204.08 | 14.40 |
| -3 | 11 | -10 | 190.08 | 18.30 |
| -4 | -11 | -10 | 24.30 | 4.50 |
| 4 | -11 | 10 | 24.30 | 3.90 |
| 4 | -11 | 10 | 40.60 | 8.40 |
| 4 | 11 | 10 | 41.60 | 12.10 |
| -4 | 11 | -10 | 32.50 | 9.50 |
| 4 | 11 | 10 | 29.10 | 6.20 |
| 4 | 11 | 10 | 35.50 | 5.70 |
| 5 | -11 | 10 | 566.04 | 32.30 |
| 5 | -11 | 10 | 560.74 | 31.50 |
| -5 | -11 | -10 | 542.35 | 31.90 |
| 5 | 11 | 10 | 539.05 | 31.60 |

| | | | | |
|---|---|---|---|---|
| 5 | 11 | 10 | 584.04 | 32.00 |
| -6 | -11 | -10 | 174.28 | 11.40 |
| 6 | -11 | 10 | 158.78 | 12.10 |
| 6 | -11 | 10 | 147.89 | 12.20 |
| 6 | 11 | 10 | 160.48 | 11.10 |
| 6 | 11 | 10 | 155.58 | 14.80 |
| -7 | -11 | -10 | 60.39 | 6.60 |
| 7 | -11 | 10 | 52.59 | 6.30 |
| 7 | 11 | 10 | 59.79 | 6.80 |
| 7 | 11 | 10 | 56.29 | 6.30 |
| 8 | -11 | 10 | 15.60 | 3.40 |
| 8 | 11 | 10 | 16.10 | 3.60 |
| 9 | 11 | 10 | 330.17 | 18.30 |
| 9 | 11 | 10 | 246.48 | 16.70 |
| 10 | 11 | 10 | 5.90 | 3.50 |
| 12 | 12 | -10 | 164.38 | 8.80 |
| -12 | 12 | 10 | 125.99 | 10.20 |
| -11 | 12 | 10 | 0.30 | 2.80 |
| -11 | 12 | 10 | 0.70 | 3.00 |
| 11 | 12 | -10 | -0.30 | 1.40 |
| -10 | 12 | 10 | 230.18 | 14.30 |
| -10 | 12 | 10 | 248.38 | 15.00 |
| 10 | 12 | -10 | 225.78 | 13.80 |
| 9 | 12 | -10 | 37.80 | 6.40 |
| -9 | 12 | 10 | 44.40 | 7.60 |
| -9 | 12 | 10 | 30.70 | 5.20 |
| -8 | -12 | 10 | 10.10 | 5.60 |
| -8 | 12 | 10 | 12.70 | 5.70 |
| 8 | 12 | -10 | 9.20 | 2.80 |
| -8 | 12 | 10 | 6.90 | 3.00 |
| -7 | -12 | 10 | 120.79 | 9.60 |
| -7 | 12 | 10 | 122.39 | 9.20 |
| 7 | 12 | -10 | 122.09 | 9.30 |
| -7 | 12 | 10 | 119.39 | 12.70 |
| -6 | -12 | 10 | 907.71 | 49.50 |
| -6 | 12 | 10 | 941.71 | 49.70 |
| 6 | 12 | -10 | 881.71 | 52.49 |
| -6 | 12 | 10 | 813.32 | 50.69 |
| 5 | -12 | -10 | 22.60 | 4.60 |
| -5 | -12 | 10 | 10.50 | 3.80 |
| -5 | 12 | 10 | 13.30 | 4.30 |
| 5 | 12 | -10 | 15.80 | 7.90 |
| -5 | 12 | 10 | 17.00 | 6.30 |
| -4 | -12 | 10 | 161.38 | 12.10 |

| | | | | |
|---|---|---|---|---|
| 4 | -12 | -10 | 168.18 | 12.10 |
| -4 | 12 | 10 | 167.18 | 19.30 |
| 4 | 12 | -10 | 116.29 | 15.80 |
| -4 | 12 | 10 | 154.18 | 12.60 |
| -4 | 12 | 10 | 161.78 | 14.10 |
| -3 | -12 | 10 | 35.90 | 7.30 |
| 3 | -12 | -10 | 37.30 | 8.10 |
| -3 | 12 | 10 | 48.50 | 7.70 |
| -3 | 12 | 10 | 40.40 | 9.60 |
| 3 | 12 | -10 | 44.00 | 9.40 |
| -3 | 12 | 10 | 50.49 | 9.40 |
| -2 | -12 | 10 | 404.96 | 24.50 |
| 2 | -12 | -10 | 443.56 | 25.00 |
| -2 | 12 | 10 | 363.26 | 24.40 |
| 2 | 12 | -10 | 387.06 | 28.70 |
| -2 | 12 | 10 | 435.26 | 28.30 |
| -2 | 12 | 10 | 389.86 | 24.70 |
| 1 | -12 | -10 | 10.10 | 4.70 |
| -1 | -12 | 10 | 17.20 | 3.30 |
| -1 | -12 | 10 | 12.70 | 4.00 |
| -1 | 12 | 10 | 12.70 | 4.30 |
| -1 | 12 | 10 | 24.50 | 5.20 |
| -1 | 12 | 10 | 14.60 | 7.90 |
| 1 | 12 | -10 | 9.30 | 7.60 |
| 0 | -12 | -10 | 1077.59 | 63.39 |
| 0 | -12 | 10 | 1155.18 | 62.09 |
| 0 | -12 | 10 | 1204.08 | 63.19 |
| 0 | 12 | 10 | 1161.78 | 62.69 |
| 0 | 12 | 10 | 1173.08 | 63.09 |
| 0 | 12 | -10 | 1017.90 | 65.89 |
| 0 | 12 | 10 | 1133.29 | 65.79 |
| 1 | -12 | 10 | 9.00 | 4.20 |
| 1 | -12 | 10 | 10.50 | 3.20 |
| -1 | -12 | -10 | 5.20 | 4.20 |
| -1 | 12 | -10 | 3.80 | 7.50 |
| 1 | 12 | 10 | 17.10 | 6.20 |
| 1 | 12 | 10 | 18.20 | 4.50 |
| 1 | 12 | 10 | 15.60 | 8.40 |
| -2 | -12 | -10 | 34.50 | 7.10 |
| 2 | -12 | 10 | 37.00 | 7.50 |
| 2 | -12 | 10 | 29.30 | 5.50 |
| 2 | 12 | 10 | 32.70 | 7.20 |
| -2 | 12 | -10 | 17.30 | 8.20 |
| 2 | 12 | 10 | 29.80 | 8.90 |

| | | | | |
|---|---|---|---|---|
| 2 | 12 | 10 | 35.80 | 6.80 |
| 3 | -12 | 10 | 34.50 | 7.60 |
| -3 | -12 | -10 | 38.30 | 6.70 |
| 3 | -12 | 10 | 36.20 | 5.80 |
| 3 | 12 | 10 | 31.00 | 5.70 |
| 3 | 12 | 10 | 38.60 | 6.90 |
| 4 | -12 | 10 | 678.13 | 37.50 |
| 4 | -12 | 10 | 639.74 | 36.40 |
| -4 | -12 | -10 | 639.04 | 37.10 |
| 4 | 12 | 10 | 648.24 | 36.70 |
| 4 | 12 | 10 | 663.43 | 37.00 |
| 5 | -12 | 10 | 85.19 | 8.50 |
| 5 | -12 | 10 | 71.79 | 9.70 |
| -5 | -12 | -10 | 76.19 | 7.30 |
| 5 | 12 | 10 | 85.69 | 8.50 |
| 5 | 12 | 10 | 72.79 | 6.90 |
| -6 | -12 | -10 | 108.99 | 8.20 |
| 6 | -12 | 10 | 99.19 | 9.10 |
| 6 | -12 | 10 | 89.49 | 7.60 |
| 6 | 12 | 10 | 99.29 | 7.90 |
| 6 | 12 | 10 | 108.09 | 8.90 |
| 7 | -12 | 10 | 51.79 | 5.80 |
| -7 | -12 | -10 | 49.80 | 5.60 |
| 7 | 12 | 10 | 47.30 | 6.80 |
| 7 | 12 | 10 | 48.20 | 5.60 |
| 8 | -12 | 10 | 260.57 | 16.00 |
| -8 | -12 | -10 | 290.07 | 16.40 |
| 8 | 12 | 10 | 274.57 | 16.60 |
| 9 | 12 | 10 | 7.00 | 3.10 |
| -11 | 13 | 10 | 284.47 | 16.80 |
| 11 | 13 | -10 | 289.77 | 16.10 |
| -10 | 13 | 10 | 3.60 | 2.70 |
| -10 | 13 | 10 | 5.00 | 3.80 |
| 10 | 13 | -10 | 5.90 | 1.70 |
| 9 | 13 | -10 | 85.69 | 6.30 |
| -9 | 13 | 10 | 74.79 | 6.90 |
| -9 | 13 | 10 | 104.49 | 9.30 |
| -8 | -13 | 10 | 66.59 | 7.20 |
| 8 | 13 | -10 | 72.89 | 6.20 |
| -8 | 13 | 10 | 59.29 | 10.30 |
| -8 | 13 | 10 | 69.89 | 6.20 |
| 7 | 13 | -10 | 255.47 | 15.80 |
| -7 | 13 | 10 | 279.47 | 16.00 |
| -7 | 13 | 10 | 235.78 | 19.00 |

| | | | | |
|---|---|---|---|---|
| -6 | -13 | 10 | 3.30 | 3.10 |
| -6 | 13 | 10 | 9.50 | 6.90 |
| -6 | 13 | 10 | 2.40 | 2.90 |
| 5 | -13 | -10 | 783.42 | 43.30 |
| -5 | -13 | 10 | 788.42 | 43.40 |
| -5 | 13 | 10 | 793.72 | 43.50 |
| -5 | 13 | 10 | 717.03 | 44.50 |
| -4 | -13 | 10 | 37.60 | 6.90 |
| 4 | -13 | -10 | 33.90 | 7.10 |
| -4 | 13 | 10 | 36.60 | 6.40 |
| -4 | 13 | 10 | 45.70 | 10.30 |
| -3 | -13 | 10 | -1.40 | 3.20 |
| 3 | -13 | -10 | 3.40 | 3.80 |
| -3 | 13 | 10 | -1.60 | 3.40 |
| 3 | 13 | -10 | 5.40 | 8.20 |
| -3 | 13 | 10 | -6.90 | 11.20 |
| -3 | 13 | 10 | 7.70 | 5.70 |
| -2 | -13 | 10 | 206.58 | 13.90 |
| 2 | -13 | -10 | 211.18 | 14.40 |
| -2 | 13 | 10 | 173.28 | 19.90 |
| -2 | 13 | 10 | 214.18 | 14.20 |
| 2 | 13 | -10 | 170.98 | 20.30 |
| -1 | -13 | 10 | 1083.89 | 59.09 |
| 1 | -13 | -10 | 1044.80 | 59.39 |
| -1 | -13 | 10 | 1044.40 | 58.19 |
| -1 | 13 | 10 | 1103.89 | 59.19 |
| -1 | 13 | 10 | 1026.60 | 62.19 |
| 0 | -13 | -10 | 48.00 | 8.10 |
| 0 | -13 | 10 | 61.19 | 6.40 |
| 0 | -13 | 10 | 65.49 | 7.50 |
| 0 | 13 | 10 | 48.20 | 11.80 |
| 0 | 13 | 10 | 58.59 | 7.30 |
| 1 | -13 | 10 | 88.59 | 8.60 |
| -1 | -13 | -10 | 80.79 | 8.80 |
| 1 | -13 | 10 | 70.39 | 7.00 |
| 1 | 13 | 10 | 79.99 | 7.90 |
| 2 | -13 | 10 | 105.69 | 9.60 |
| 2 | -13 | 10 | 97.99 | 8.10 |
| -2 | -13 | -10 | 109.39 | 9.50 |
| 2 | 13 | 10 | 102.09 | 8.60 |
| 3 | -13 | 10 | 182.38 | 12.20 |
| 3 | -13 | 10 | 207.58 | 13.70 |
| -3 | -13 | -10 | 183.68 | 13.00 |
| 3 | 13 | 10 | 193.88 | 12.70 |

| | | | | |
|---|---|---|---|---|
| -4 | -13 | -10 | 12.30 | 3.60 |
| 4 | -13 | 10 | 17.80 | 3.20 |
| 4 | -13 | 10 | 9.30 | 4.20 |
| 4 | 13 | 10 | 15.10 | 3.30 |
| 5 | -13 | 10 | 549.15 | 31.40 |
| 5 | -13 | 10 | 561.34 | 30.80 |
| -5 | -13 | -10 | 562.44 | 31.30 |
| 5 | 13 | 10 | 537.05 | 31.30 |
| 6 | -13 | 10 | 108.09 | 7.60 |
| -6 | -13 | -10 | 105.69 | 7.90 |
| 6 | 13 | 10 | 107.29 | 8.00 |
| 7 | -13 | 10 | 0.90 | 2.50 |
| -7 | -13 | -10 | 0.30 | 2.30 |
| 7 | 13 | 10 | -1.00 | 2.50 |
| 8 | -13 | 10 | 8.60 | 3.50 |
| 8 | 13 | 10 | 8.70 | 3.10 |
| 10 | 14 | -10 | 243.38 | 13.30 |
| -10 | 14 | 10 | 222.68 | 13.80 |
| 9 | 14 | -10 | 0.00 | 1.50 |
| -9 | 14 | 10 | 6.70 | 2.50 |
| -8 | -14 | 10 | 11.70 | 2.90 |
| 8 | 14 | -10 | 8.70 | 2.10 |
| -7 | -14 | 10 | 106.49 | 8.40 |
| -7 | 14 | 10 | 111.89 | 8.00 |
| 7 | 14 | -10 | 106.19 | 7.80 |
| -6 | -14 | 10 | 708.83 | 39.40 |
| 6 | 14 | -10 | 701.33 | 39.70 |
| -6 | 14 | 10 | 702.53 | 39.40 |
| -5 | -14 | 10 | 16.30 | 3.40 |
| -5 | 14 | 10 | 29.00 | 5.10 |
| -4 | -14 | 10 | 144.69 | 10.80 |
| 4 | -14 | -10 | 151.68 | 11.10 |
| -4 | 14 | 10 | 143.19 | 10.70 |
| -3 | -14 | 10 | 82.89 | 8.10 |
| 3 | -14 | -10 | 93.79 | 8.50 |
| -3 | 14 | 10 | 78.39 | 8.00 |
| -2 | -14 | 10 | 290.27 | 18.70 |
| 2 | -14 | -10 | 314.87 | 19.10 |
| -2 | 14 | 10 | 309.97 | 19.10 |
| 1 | -14 | -10 | 57.69 | 7.60 |
| -1 | -14 | 10 | 49.40 | 6.80 |
| -1 | 14 | 10 | 39.60 | 6.80 |
| 0 | -14 | 10 | 759.52 | 41.60 |
| 0 | -14 | 10 | 757.02 | 40.80 |

| | | | | |
|---|---|---|---|---|
| 0 | -14 | -10 | 699.83 | 41.80 |
| 0 | 14 | 10 | 727.13 | 41.60 |
| -1 | -14 | -10 | 48.30 | 7.20 |
| 1 | -14 | 10 | 48.80 | 7.00 |
| 1 | -14 | 10 | 47.80 | 5.50 |
| 1 | 14 | 10 | 50.49 | 6.40 |
| 2 | -14 | 10 | 13.10 | 3.30 |
| -2 | -14 | -10 | 16.10 | 3.70 |
| 2 | -14 | 10 | 23.90 | 6.40 |
| 2 | 14 | 10 | 11.00 | 3.40 |
| 3 | -14 | 10 | 93.19 | 8.40 |
| 3 | -14 | 10 | 85.39 | 6.80 |
| -3 | -14 | -10 | 85.79 | 8.10 |
| 3 | 14 | 10 | 79.99 | 7.20 |
| -4 | -14 | -10 | 659.53 | 37.50 |
| 4 | -14 | 10 | 692.03 | 37.70 |
| 4 | -14 | 10 | 661.43 | 36.90 |
| 4 | 14 | 10 | 664.63 | 37.50 |
| 5 | -14 | 10 | 34.00 | 4.40 |
| 5 | -14 | 10 | 36.50 | 6.10 |
| -5 | -14 | -10 | 26.00 | 4.90 |
| 5 | 14 | 10 | 26.70 | 4.50 |
| -6 | -14 | -10 | 88.19 | 6.90 |
| 6 | -14 | 10 | 89.69 | 6.50 |
| 6 | 14 | 10 | 92.49 | 7.20 |
| 7 | -14 | 10 | 87.89 | 6.70 |
| -7 | -14 | -10 | 99.09 | 6.90 |
| 7 | 14 | 10 | 88.99 | 7.00 |
| 9 | 15 | -10 | 102.99 | 6.30 |
| -9 | 15 | 10 | 95.49 | 7.00 |
| -8 | -15 | 10 | 56.29 | 5.70 |
| 8 | 15 | -10 | 61.79 | 4.80 |
| -8 | 15 | 10 | 64.29 | 5.40 |
| -7 | -15 | 10 | 199.28 | 12.10 |
| -7 | 15 | 10 | 191.08 | 12.20 |
| 7 | 15 | -10 | 205.18 | 12.00 |
| -6 | -15 | 10 | 8.50 | 2.80 |
| -6 | 15 | 10 | 6.60 | 2.50 |
| 6 | 15 | -10 | 8.90 | 2.30 |
| 5 | -15 | -10 | 447.86 | 25.80 |
| -5 | -15 | 10 | 437.76 | 26.00 |
| -5 | 15 | 10 | 473.05 | 26.00 |
| -4 | -15 | 10 | 88.39 | 8.10 |
| 4 | -15 | -10 | 119.89 | 8.40 |

| | | | | |
|---|---|---|---|---|
| -4 | 15 | 10 | 91.79 | 7.90 |
| 3 | -15 | -10 | 27.20 | 5.70 |
| -3 | -15 | 10 | 9.60 | 3.30 |
| -3 | 15 | 10 | 4.00 | 3.20 |
| 2 | -15 | -10 | 188.88 | 12.30 |
| -2 | -15 | 10 | 146.29 | 12.70 |
| -2 | 15 | 10 | 170.48 | 11.80 |
| 1 | -15 | -10 | 791.12 | 44.50 |
| -1 | 15 | 10 | 788.82 | 44.30 |
| 0 | -15 | -10 | 110.79 | 9.90 |
| 0 | -15 | 10 | 115.49 | 9.70 |
| 0 | 15 | 10 | 126.79 | 9.40 |
| 1 | -15 | 10 | 96.69 | 7.30 |
| -1 | -15 | -10 | 101.29 | 8.80 |
| 1 | -15 | 10 | 107.59 | 8.60 |
| 1 | 15 | 10 | 104.09 | 8.10 |
| 2 | -15 | 10 | 121.79 | 9.00 |
| -2 | -15 | -10 | 107.69 | 8.70 |
| 2 | -15 | 10 | 105.99 | 7.50 |
| 2 | 15 | 10 | 96.49 | 8.20 |
| 3 | -15 | 10 | 288.97 | 16.90 |
| 3 | -15 | 10 | 319.07 | 17.90 |
| -3 | -15 | -10 | 280.57 | 17.70 |
| 3 | 15 | 10 | 289.77 | 17.50 |
| 4 | -15 | 10 | 37.60 | 4.00 |
| 4 | -15 | 10 | 26.80 | 5.50 |
| -4 | -15 | -10 | 27.50 | 4.50 |
| 4 | 15 | 10 | 25.10 | 4.40 |
| 5 | -15 | 10 | 419.66 | 24.40 |
| 5 | -15 | 10 | 456.25 | 26.10 |
| -5 | -15 | -10 | 415.36 | 24.80 |
| 5 | 15 | 10 | 474.85 | 25.40 |
| 6 | -15 | 10 | 127.99 | 9.00 |
| -6 | -15 | -10 | 123.69 | 8.50 |
| 6 | 15 | 10 | 153.08 | 9.10 |
| -8 | -16 | 10 | 2.30 | 2.40 |
| 8 | 16 | -10 | 1.00 | 1.30 |
| -8 | 16 | 10 | 0.20 | 2.20 |
| -7 | -16 | 10 | 110.39 | 7.70 |
| -7 | 16 | 10 | 118.19 | 7.80 |
| 7 | 16 | -10 | 119.79 | 7.40 |
| -6 | -16 | 10 | 499.75 | 26.80 |
| -6 | 16 | 10 | 411.06 | 26.90 |
| 6 | 16 | -10 | 528.55 | 26.80 |

| | | | | |
|---|---|---|---|---|
| 5 | -16 | -10 | 57.99 | 6.40 |
| -5 | -16 | 10 | 43.60 | 5.40 |
| 5 | 16 | -10 | 58.49 | 5.30 |
| -5 | 16 | 10 | 59.69 | 5.60 |
| -4 | -16 | 10 | 121.59 | 9.10 |
| 4 | -16 | -10 | 143.59 | 9.30 |
| -4 | 16 | 10 | 139.09 | 9.50 |
| 3 | -16 | -10 | 99.69 | 7.50 |
| -3 | -16 | 10 | 92.49 | 7.30 |
| -3 | 16 | 10 | 95.19 | 7.70 |
| -2 | -16 | 10 | 141.79 | 9.80 |
| 2 | -16 | -10 | 151.28 | 10.00 |
| -2 | 16 | 10 | 141.69 | 10.10 |
| -1 | -16 | 10 | 22.50 | 4.60 |
| -1 | 16 | 10 | 28.90 | 5.10 |
| 0 | -16 | -10 | 454.15 | 27.40 |
| 0 | -16 | 10 | 515.15 | 27.30 |
| 0 | 16 | 10 | 468.45 | 27.40 |
| 1 | -16 | 10 | 128.09 | 8.70 |
| -1 | -16 | -10 | 105.49 | 8.60 |
| 1 | 16 | 10 | 117.69 | 8.60 |
| 2 | -16 | 10 | 7.60 | 2.60 |
| 2 | -16 | 10 | 9.10 | 2.10 |
| -2 | -16 | -10 | 5.90 | 2.30 |
| 2 | 16 | 10 | 9.00 | 2.70 |
| 3 | -16 | 10 | 35.20 | 5.00 |
| -3 | -16 | -10 | 38.90 | 4.80 |
| 3 | -16 | 10 | 33.30 | 3.90 |
| 3 | 16 | 10 | 32.80 | 5.20 |
| 4 | -16 | 10 | 376.86 | 21.40 |
| 4 | -16 | 10 | 352.66 | 22.50 |
| -4 | -16 | -10 | 394.06 | 21.90 |
| 4 | 16 | 10 | 412.76 | 22.10 |
| -5 | -16 | -10 | 20.50 | 3.50 |
| 5 | 16 | 10 | 21.40 | 4.20 |
| -6 | -17 | 10 | 0.40 | 1.70 |
| -6 | 17 | 10 | 1.40 | 2.20 |
| 6 | 17 | -10 | 1.20 | 1.50 |
| -5 | -17 | 10 | 214.08 | 13.00 |
| -5 | 17 | 10 | 230.38 | 13.30 |
| 5 | 17 | -10 | 223.88 | 13.00 |
| 4 | -17 | -10 | 55.89 | 5.50 |
| -4 | -17 | 10 | 56.59 | 4.90 |
| -4 | 17 | 10 | 62.59 | 5.40 |

| | | | | |
|---|---|---|---|---|
| -3 | -17 | 10 | 19.10 | 3.40 |
| 3 | -17 | -10 | 11.10 | 2.40 |
| -3 | 17 | 10 | 20.60 | 4.20 |
| 2 | -17 | -10 | 68.89 | 5.40 |
| -2 | -17 | 10 | 54.69 | 5.10 |
| -2 | 17 | 10 | 52.19 | 5.90 |
| -1 | -17 | 10 | 494.65 | 28.40 |
| 1 | -17 | -10 | 522.55 | 28.60 |
| -1 | 17 | 10 | 507.65 | 28.80 |
| 0 | -17 | 10 | 51.09 | 5.10 |
| 0 | -17 | -10 | 57.09 | 5.60 |
| 0 | 17 | 10 | 54.99 | 5.60 |
| -1 | -17 | -10 | 46.60 | 5.20 |
| 1 | -17 | 10 | 59.89 | 5.00 |
| 1 | 17 | 10 | 40.70 | 5.10 |
| -2 | -17 | -10 | 35.60 | 4.40 |
| 2 | -17 | 10 | 35.30 | 4.40 |
| 2 | 17 | 10 | 32.10 | 4.60 |
| 3 | -17 | 10 | 107.09 | 8.00 |
| -3 | -17 | -10 | 112.79 | 7.70 |
| 3 | 17 | 10 | 123.39 | 8.00 |
| -16 | 0 | 11 | 1.80 | 1.70 |
| 16 | 0 | -11 | 1.00 | 2.00 |
| -15 | 0 | 11 | 290.47 | 16.90 |
| 15 | 0 | -11 | 289.67 | 16.70 |
| -14 | 0 | 11 | 2.40 | 2.10 |
| 14 | 0 | -11 | -4.20 | 2.40 |
| -13 | 0 | 11 | 91.49 | 7.90 |
| 13 | 0 | -11 | 100.29 | 8.30 |
| -12 | 0 | 11 | 0.00 | 2.80 |
| 12 | 0 | -11 | -1.50 | 3.10 |
| -11 | 0 | 11 | 1231.28 | 66.79 |
| 11 | 0 | -11 | 1183.78 | 66.09 |
| -10 | 0 | 11 | -1.70 | 3.30 |
| 10 | 0 | -11 | 2.80 | 4.10 |
| -9 | 0 | 11 | 247.28 | 16.40 |
| -8 | 0 | 11 | -6.30 | 4.40 |
| -7 | 0 | 11 | 1524.15 | 79.29 |
| -7 | 0 | 11 | 1342.77 | 79.39 |
| -6 | 0 | 11 | 10.20 | 6.30 |
| -6 | 0 | 11 | -1.20 | 4.00 |
| -5 | 0 | 11 | 1244.38 | 72.79 |
| -5 | 0 | 11 | 1260.07 | 73.99 |
| -5 | 0 | 11 | 1456.35 | 73.49 |

| | | | | |
|---|---|---|---|---|
| -4 | 0 | 11 | 1.40 | 4.60 |
| -4 | 0 | 11 | -3.00 | 6.80 |
| -4 | 0 | 11 | 2.30 | 3.40 |
| -3 | 0 | 11 | 217.98 | 17.70 |
| -3 | 0 | 11 | 179.18 | 15.10 |
| -3 | 0 | 11 | 236.68 | 15.10 |
| -2 | 0 | 11 | 6.50 | 6.30 |
| -2 | 0 | 11 | -0.80 | 4.50 |
| -2 | 0 | 11 | 2.20 | 4.00 |
| -1 | 0 | 11 | 2672.93 | 143.69 |
| -1 | 0 | 11 | 2586.24 | 142.59 |
| -1 | 0 | 11 | 2608.34 | 142.79 |
| 0 | 0 | 11 | -4.10 | 5.20 |
| 0 | 0 | 11 | -6.20 | 3.60 |
| 0 | 0 | 11 | -2.00 | 5.20 |
| 1 | 0 | 11 | 71.19 | 9.20 |
| 1 | 0 | 11 | 50.39 | 11.10 |
| 1 | 0 | 11 | 59.79 | 9.50 |
| 2 | 0 | 11 | -1.90 | 5.60 |
| 2 | 0 | 11 | 0.40 | 4.90 |
| 2 | 0 | 11 | -2.60 | 4.40 |
| 3 | 0 | 11 | 1420.56 | 70.69 |
| 3 | 0 | 11 | 1245.28 | 71.39 |
| 3 | 0 | 11 | 1158.78 | 72.49 |
| 4 | 0 | 11 | 0.00 | 5.20 |
| 4 | 0 | 11 | 6.20 | 4.00 |
| 4 | 0 | 11 | -5.40 | 6.40 |
| 5 | 0 | 11 | 1228.58 | 72.69 |
| 5 | 0 | 11 | 1282.97 | 70.29 |
| 5 | 0 | 11 | 1309.27 | 72.09 |
| 6 | 0 | 11 | -6.60 | 3.40 |
| 6 | 0 | 11 | -3.80 | 6.50 |
| 6 | 0 | 11 | -8.60 | 7.80 |
| 6 | 0 | 11 | 2.80 | 4.20 |
| 7 | 0 | 11 | 493.85 | 27.20 |
| 7 | 0 | 11 | 434.96 | 28.80 |
| 7 | 0 | 11 | 459.35 | 27.40 |
| 8 | 0 | 11 | 0.00 | 3.50 |
| 9 | 0 | 11 | 296.97 | 19.90 |
| 9 | 0 | 11 | 304.47 | 18.40 |
| 10 | 0 | 11 | 10.40 | 5.00 |
| 10 | 0 | 11 | 0.80 | 3.40 |
| 11 | 0 | 11 | 78.19 | 12.90 |
| 11 | 0 | 11 | 60.29 | 6.10 |

| | | | | |
|---|---|---|---|---|
| 12 | 0 | 11 | 8.50 | 5.10 |
| 16 | -1 | -11 | 356.66 | 19.60 |
| -16 | -1 | 11 | 364.16 | 20.50 |
| -16 | 1 | 11 | 346.67 | 19.70 |
| 16 | 1 | -11 | 330.97 | 20.00 |
| -15 | -1 | 11 | 1.20 | 2.00 |
| 15 | -1 | -11 | -0.80 | 2.30 |
| -15 | 1 | 11 | 2.10 | 1.90 |
| 15 | 1 | -11 | 0.40 | 2.10 |
| -14 | -1 | 11 | 275.57 | 16.20 |
| 14 | -1 | -11 | 269.37 | 16.00 |
| -14 | 1 | 11 | 263.57 | 16.10 |
| 14 | 1 | -11 | 268.27 | 15.90 |
| -13 | -1 | 11 | 1.40 | 2.80 |
| -13 | 1 | 11 | -1.10 | 2.30 |
| 13 | 1 | -11 | -0.60 | 2.80 |
| 12 | -1 | -11 | 1069.49 | 59.59 |
| -12 | -1 | 11 | 1083.59 | 60.09 |
| -12 | 1 | 11 | 1068.19 | 60.09 |
| 12 | 1 | -11 | 1126.59 | 59.59 |
| 11 | -1 | -11 | 7.00 | 3.80 |
| -11 | -1 | 11 | 7.90 | 3.50 |
| -11 | 1 | 11 | 10.40 | 3.40 |
| 11 | 1 | -11 | 2.10 | 3.40 |
| 10 | -1 | -11 | 772.32 | 44.50 |
| -10 | -1 | 11 | 803.32 | 43.90 |
| -10 | 1 | 11 | 774.32 | 43.80 |
| 10 | 1 | -11 | 750.62 | 43.10 |
| -9 | -1 | 11 | 14.40 | 4.20 |
| -9 | 1 | 11 | 6.50 | 3.50 |
| 9 | 1 | -11 | -1.10 | 4.40 |
| -8 | -1 | 11 | 76.59 | 9.60 |
| -8 | 1 | 11 | 69.39 | 8.80 |
| -7 | -1 | 11 | 3.40 | 5.10 |
| -7 | 1 | 11 | 4.00 | 4.60 |
| -7 | 1 | 11 | 0.20 | 6.10 |
| -6 | -1 | 11 | 2971.30 | 159.88 |
| -6 | -1 | 11 | 2793.22 | 159.78 |
| -6 | -1 | 11 | 2861.11 | 160.18 |
| -6 | 1 | 11 | 3044.70 | 160.28 |
| -6 | 1 | 11 | 3040.60 | 159.98 |
| -5 | -1 | 11 | 2.60 | 7.10 |
| -5 | -1 | 11 | 9.90 | 9.20 |
| -5 | -1 | 11 | -3.90 | 3.50 |

| | | | | |
|---|---|---|---|---|
| -5 | 1 | 11 | -0.40 | 3.80 |
| -5 | 1 | 11 | -5.00 | 5.80 |
| -5 | 1 | 11 | -0.50 | 5.90 |
| -4 | -1 | 11 | 319.47 | 18.90 |
| -4 | -1 | 11 | 281.07 | 22.00 |
| -4 | -1 | 11 | 289.67 | 18.90 |
| -4 | 1 | 11 | 252.57 | 19.30 |
| -4 | 1 | 11 | 337.37 | 19.40 |
| -4 | 1 | 11 | 260.87 | 20.80 |
| -3 | -1 | 11 | 57.79 | 13.20 |
| -3 | -1 | 11 | 67.09 | 9.00 |
| -3 | -1 | 11 | 58.89 | 7.40 |
| -3 | 1 | 11 | 50.99 | 7.70 |
| -3 | 1 | 11 | 64.49 | 8.60 |
| -3 | 1 | 11 | 55.79 | 9.50 |
| -2 | -1 | 11 | 1471.55 | 85.69 |
| -2 | -1 | 11 | 1546.55 | 86.69 |
| -2 | 1 | 11 | 1642.94 | 86.69 |
| -2 | 1 | 11 | 1627.44 | 87.09 |
| -2 | 1 | 11 | 1537.75 | 85.99 |
| -1 | -1 | 11 | 193.98 | 14.30 |
| -1 | -1 | 11 | 188.28 | 14.00 |
| -1 | -1 | 11 | 175.98 | 15.90 |
| -1 | 1 | 11 | 164.98 | 14.70 |
| -1 | 1 | 11 | 195.68 | 14.90 |
| -1 | 1 | 11 | 189.98 | 14.60 |
| 0 | -1 | 11 | 2490.85 | 142.69 |
| 0 | -1 | 11 | 2732.83 | 144.09 |
| 0 | -1 | 11 | 2718.33 | 142.99 |
| 0 | 1 | 11 | 2646.34 | 143.19 |
| 0 | 1 | 11 | 2541.65 | 144.09 |
| 1 | -1 | 11 | 39.50 | 6.50 |
| 1 | -1 | 11 | 42.40 | 8.40 |
| 1 | -1 | 11 | 33.50 | 6.70 |
| 1 | 1 | 11 | 39.30 | 8.50 |
| 1 | 1 | 11 | 60.09 | 10.70 |
| 1 | 1 | 11 | 50.49 | 8.60 |
| 2 | -1 | 11 | 194.78 | 16.00 |
| 2 | -1 | 11 | 233.48 | 17.50 |
| 2 | -1 | 11 | 243.28 | 17.70 |
| 2 | 1 | 11 | 202.68 | 17.50 |
| 2 | 1 | 11 | 254.37 | 15.90 |
| 2 | 1 | 11 | 231.08 | 17.60 |
| 3 | -1 | 11 | 0.90 | 5.50 |

| | | | | |
|---|---|---|---|---|
| 3 | -1 | 11 | 4.90 | 4.40 |
| 3 | -1 | 11 | 2.50 | 5.50 |
| 3 | 1 | 11 | 0.30 | 3.90 |
| 3 | 1 | 11 | 3.50 | 6.20 |
| 3 | 1 | 11 | 5.00 | 5.60 |
| 4 | -1 | 11 | 2592.34 | 129.89 |
| 4 | -1 | 11 | 2024.90 | 131.49 |
| 4 | -1 | 11 | 2441.56 | 130.39 |
| 4 | 1 | 11 | 2580.54 | 129.89 |
| 4 | 1 | 11 | 2213.08 | 132.29 |
| 4 | 1 | 11 | 2469.15 | 130.39 |
| 5 | -1 | 11 | 22.60 | 7.90 |
| 5 | -1 | 11 | 37.10 | 7.30 |
| 5 | -1 | 11 | 33.00 | 8.00 |
| 5 | 1 | 11 | 31.30 | 8.70 |
| 5 | 1 | 11 | 11.40 | 7.40 |
| 5 | 1 | 11 | 21.60 | 4.30 |
| 6 | -1 | 11 | 126.29 | 15.80 |
| 6 | -1 | 11 | 119.99 | 11.80 |
| 6 | -1 | 11 | 141.09 | 15.90 |
| 6 | -1 | 11 | 143.99 | 10.70 |
| 6 | 1 | 11 | 113.29 | 16.30 |
| 6 | 1 | 11 | 139.59 | 11.60 |
| 6 | 1 | 11 | 155.38 | 10.80 |
| 6 | 1 | 11 | 143.39 | 14.70 |
| 7 | -1 | 11 | 1.80 | 4.50 |
| 7 | -1 | 11 | 6.10 | 6.30 |
| 7 | -1 | 11 | 3.60 | 3.50 |
| 7 | 1 | 11 | 1.60 | 4.40 |
| 7 | 1 | 11 | 3.70 | 3.30 |
| 7 | 1 | 11 | 3.20 | 6.10 |
| 8 | -1 | 11 | 732.43 | 41.70 |
| 8 | -1 | 11 | 695.53 | 40.40 |
| 8 | 1 | 11 | 761.22 | 40.50 |
| 8 | 1 | 11 | 680.43 | 41.70 |
| 9 | -1 | 11 | 25.10 | 4.30 |
| 9 | -1 | 11 | 27.90 | 6.00 |
| 9 | 1 | 11 | 30.70 | 5.40 |
| 9 | 1 | 11 | 20.30 | 5.70 |
| 10 | -1 | 11 | 351.16 | 22.80 |
| 10 | -1 | 11 | 385.36 | 22.00 |
| 10 | 1 | 11 | 377.36 | 21.80 |
| 10 | 1 | 11 | 357.86 | 22.80 |
| 11 | -1 | 11 | 6.90 | 5.50 |

| | | | | |
|---|---|---|---|---|
| 11 | -1 | 11 | 0.60 | 2.60 |
| 11 | 1 | 11 | 2.10 | 2.50 |
| 11 | 1 | 11 | -0.70 | 5.40 |
| 12 | -1 | 11 | 68.89 | 6.30 |
| 12 | -1 | 11 | 66.09 | 13.40 |
| 12 | 1 | 11 | 72.89 | 8.40 |
| 12 | 1 | 11 | 76.09 | 6.10 |
| 16 | -2 | -11 | 8.30 | 2.60 |
| -16 | -2 | 11 | 17.30 | 3.70 |
| -16 | 2 | 11 | 15.20 | 2.80 |
| -15 | -2 | 11 | 263.07 | 15.70 |
| 15 | -2 | -11 | 269.67 | 15.70 |
| 15 | 2 | -11 | 281.67 | 15.50 |
| -15 | 2 | 11 | 264.07 | 15.70 |
| -14 | -2 | 11 | -0.80 | 2.40 |
| 14 | -2 | -11 | -2.10 | 2.80 |
| 14 | 2 | -11 | -0.30 | 2.20 |
| -14 | 2 | 11 | -1.00 | 1.90 |
| -13 | -2 | 11 | 22.20 | 4.40 |
| 13 | -2 | -11 | 23.80 | 4.80 |
| 13 | 2 | -11 | 17.30 | 4.40 |
| -13 | 2 | 11 | 20.90 | 4.70 |
| 12 | -2 | -11 | -0.10 | 3.50 |
| -12 | -2 | 11 | -1.20 | 3.10 |
| 12 | 2 | -11 | 1.80 | 2.90 |
| -12 | 2 | 11 | 0.80 | 2.90 |
| -11 | -2 | 11 | 1249.97 | 73.29 |
| 11 | -2 | -11 | 1285.57 | 72.89 |
| -11 | 2 | 11 | 1426.76 | 73.59 |
| 11 | 2 | -11 | 1376.26 | 72.89 |
| -10 | -2 | 11 | 1.80 | 4.10 |
| -10 | 2 | 11 | -0.60 | 2.90 |
| 10 | 2 | -11 | 1.40 | 3.50 |
| -9 | -2 | 11 | 363.06 | 23.20 |
| -9 | 2 | 11 | 384.26 | 22.80 |
| -8 | -2 | 11 | 71.79 | 10.40 |
| -8 | 2 | 11 | 44.60 | 8.60 |
| -7 | -2 | 11 | 1823.02 | 98.69 |
| -7 | 2 | 11 | 1757.32 | 98.49 |
| -6 | -2 | 11 | 140.49 | 13.90 |
| -6 | -2 | 11 | 139.39 | 16.10 |
| -6 | -2 | 11 | 158.58 | 12.50 |
| -6 | 2 | 11 | 172.68 | 12.80 |
| -6 | 2 | 11 | 182.68 | 13.90 |

| | | | | |
|---|---|---|---|---|
| -5 | -2 | 11 | 1979.50 | 105.89 |
| -5 | -2 | 11 | 2090.49 | 105.19 |
| -5 | -2 | 11 | 1709.83 | 104.19 |
| -5 | 2 | 11 | 1809.92 | 105.49 |
| -5 | 2 | 11 | 2161.18 | 105.59 |
| -5 | 2 | 11 | 1743.13 | 104.49 |
| -4 | -2 | 11 | 75.79 | 8.30 |
| -4 | -2 | 11 | 82.09 | 9.50 |
| -4 | -2 | 11 | 97.99 | 14.60 |
| -4 | 2 | 11 | 81.89 | 10.60 |
| -4 | 2 | 11 | 56.69 | 11.60 |
| -4 | 2 | 11 | 87.09 | 9.20 |
| -3 | -2 | 11 | 25.20 | 6.10 |
| -3 | -2 | 11 | 11.60 | 4.50 |
| -3 | 2 | 11 | 14.30 | 5.30 |
| -3 | 2 | 11 | 18.00 | 6.00 |
| -2 | -2 | 11 | 64.49 | 8.10 |
| -2 | -2 | 11 | 66.99 | 8.70 |
| -2 | 2 | 11 | 72.59 | 10.30 |
| -2 | 2 | 11 | 81.99 | 9.30 |
| -2 | 2 | 11 | 71.19 | 10.90 |
| -1 | -2 | 11 | 4318.87 | 240.98 |
| -1 | -2 | 11 | 4388.56 | 239.78 |
| -1 | 2 | 11 | 4514.55 | 240.28 |
| -1 | 2 | 11 | 4560.34 | 241.58 |
| 0 | -2 | 11 | -0.10 | 3.40 |
| 0 | -2 | 11 | -1.70 | 4.70 |
| 0 | -2 | 11 | -2.70 | 6.10 |
| 0 | 2 | 11 | -1.90 | 4.60 |
| 0 | 2 | 11 | -0.30 | 4.70 |
| 0 | 2 | 11 | 1.70 | 5.60 |
| 1 | -2 | 11 | 95.19 | 11.20 |
| 1 | -2 | 11 | 83.09 | 9.90 |
| 1 | -2 | 11 | 100.89 | 11.80 |
| 1 | 2 | 11 | 83.89 | 10.60 |
| 1 | 2 | 11 | 82.09 | 9.40 |
| 1 | 2 | 11 | 52.89 | 11.90 |
| 2 | -2 | 11 | 156.18 | 14.10 |
| 2 | -2 | 11 | 160.08 | 13.70 |
| 2 | -2 | 11 | 178.08 | 14.70 |
| 2 | 2 | 11 | 174.78 | 14.60 |
| 2 | 2 | 11 | 137.89 | 12.40 |
| 2 | 2 | 11 | 164.58 | 14.90 |
| 3 | -2 | 11 | 1637.54 | 90.89 |

| | | | | |
|---|---|---|---|---|
| 3 | -2 | 11 | 1599.94 | 90.19 |
| 3 | 2 | 11 | 1692.33 | 90.99 |
| 3 | 2 | 11 | 1769.62 | 90.29 |
| 3 | 2 | 11 | 1519.85 | 92.69 |
| 4 | -2 | 11 | 18.20 | 5.10 |
| 4 | -2 | 11 | 14.10 | 5.40 |
| 4 | -2 | 11 | 4.90 | 6.10 |
| 4 | 2 | 11 | 9.00 | 7.00 |
| 4 | 2 | 11 | 11.60 | 7.00 |
| 4 | 2 | 11 | 21.40 | 4.60 |
| 4 | 2 | 11 | 12.90 | 4.90 |
| 5 | -2 | 11 | 1568.44 | 92.49 |
| 5 | -2 | 11 | 1752.72 | 94.89 |
| 5 | 2 | 11 | 1812.42 | 93.79 |
| 5 | 2 | 11 | 1612.94 | 95.69 |
| 5 | 2 | 11 | 1726.63 | 93.19 |
| 6 | -2 | 11 | 7.60 | 7.90 |
| 6 | -2 | 11 | 24.70 | 5.10 |
| 6 | -2 | 11 | 20.50 | 4.70 |
| 6 | 2 | 11 | 33.80 | 7.40 |
| 6 | 2 | 11 | 32.30 | 6.10 |
| 6 | 2 | 11 | 28.50 | 4.80 |
| 6 | 2 | 11 | 24.50 | 9.70 |
| 7 | -2 | 11 | 390.16 | 23.80 |
| 7 | -2 | 11 | 389.46 | 24.00 |
| 7 | 2 | 11 | 385.26 | 25.60 |
| 7 | 2 | 11 | 381.36 | 23.80 |
| 7 | 2 | 11 | 440.16 | 23.70 |
| 8 | -2 | 11 | 2.30 | 3.70 |
| 8 | -2 | 11 | 5.40 | 5.90 |
| 8 | 2 | 11 | -5.80 | 5.20 |
| 8 | 2 | 11 | 1.30 | 3.10 |
| 9 | -2 | 11 | 896.81 | 50.49 |
| 9 | -2 | 11 | 902.21 | 49.80 |
| 9 | 2 | 11 | 957.40 | 50.89 |
| 9 | 2 | 11 | 827.82 | 49.60 |
| 10 | -2 | 11 | 2.90 | 3.20 |
| 10 | -2 | 11 | 14.70 | 5.70 |
| 10 | 2 | 11 | 6.00 | 3.20 |
| 10 | 2 | 11 | 7.10 | 4.60 |
| 11 | -2 | 11 | 92.49 | 10.40 |
| 11 | -2 | 11 | 105.29 | 8.00 |
| 11 | 2 | 11 | 84.99 | 12.70 |
| 11 | 2 | 11 | 101.59 | 7.60 |

| | | | | |
|---|---|---|---|---|
| 12 | -2 | 11 | 3.70 | 2.70 |
| 12 | 2 | 11 | 5.80 | 4.30 |
| -15 | -3 | 11 | 0.90 | 2.60 |
| 15 | -3 | -11 | 0.70 | 2.70 |
| 15 | 3 | -11 | 1.40 | 1.90 |
| -15 | 3 | 11 | 1.60 | 1.70 |
| -14 | -3 | 11 | 177.38 | 11.30 |
| 14 | -3 | -11 | 171.88 | 11.30 |
| 14 | 3 | -11 | 184.18 | 10.80 |
| -14 | 3 | 11 | 159.98 | 11.00 |
| 13 | -3 | -11 | 115.39 | 9.40 |
| -13 | -3 | 11 | 103.99 | 8.90 |
| -13 | 3 | 11 | 103.59 | 8.10 |
| 13 | 3 | -11 | 94.49 | 10.60 |
| -12 | -3 | 11 | 493.15 | 27.30 |
| 12 | -3 | -11 | 463.55 | 27.00 |
| 12 | 3 | -11 | 497.15 | 26.70 |
| -12 | 3 | 11 | 423.36 | 27.10 |
| -11 | -3 | 11 | 5.60 | 3.90 |
| -11 | 3 | 11 | 11.30 | 3.50 |
| 11 | 3 | -11 | 17.30 | 3.40 |
| -10 | -3 | 11 | 682.93 | 40.70 |
| -10 | 3 | 11 | 699.23 | 40.70 |
| 10 | 3 | -11 | 766.42 | 40.00 |
| -9 | -3 | 11 | 25.00 | 5.80 |
| 9 | 3 | -11 | 13.40 | 4.20 |
| -9 | 3 | 11 | 25.60 | 4.70 |
| -8 | -3 | 11 | 10.20 | 6.00 |
| 8 | 3 | -11 | -5.90 | 4.30 |
| -8 | 3 | 11 | 2.20 | 4.40 |
| -7 | -3 | 11 | 23.80 | 7.50 |
| -7 | 3 | 11 | 11.70 | 4.60 |
| -6 | -3 | 11 | 2606.64 | 150.78 |
| -6 | -3 | 11 | 2671.13 | 151.98 |
| -6 | 3 | 11 | 2883.01 | 152.08 |
| -6 | 3 | 11 | 2987.30 | 151.98 |
| -5 | -3 | 11 | 60.39 | 7.80 |
| -5 | -3 | 11 | 74.19 | 9.60 |
| -5 | -3 | 11 | 61.19 | 10.10 |
| -5 | 3 | 11 | 62.09 | 7.70 |
| -5 | 3 | 11 | 51.19 | 10.50 |
| -5 | 3 | 11 | 68.59 | 9.00 |
| -4 | -3 | 11 | 322.17 | 20.60 |
| -4 | -3 | 11 | 347.07 | 20.90 |

| | | | | |
|---|---|---|---|---|
| -4 | 3 | 11 | 339.77 | 21.50 |
| -4 | 3 | 11 | 285.47 | 22.10 |
| -4 | 3 | 11 | 357.26 | 21.90 |
| -3 | -3 | 11 | 89.99 | 8.70 |
| -3 | -3 | 11 | 87.29 | 9.20 |
| -3 | 3 | 11 | 96.19 | 10.20 |
| -3 | 3 | 11 | 73.19 | 10.60 |
| -3 | 3 | 11 | 77.89 | 11.40 |
| -2 | -3 | 11 | 1384.16 | 79.39 |
| -2 | -3 | 11 | 1533.45 | 80.59 |
| -2 | 3 | 11 | 1448.66 | 79.99 |
| -2 | 3 | 11 | 1395.76 | 81.09 |
| -2 | 3 | 11 | 1474.95 | 80.29 |
| -1 | -3 | 11 | 3.70 | 4.30 |
| -1 | -3 | 11 | -4.60 | 3.60 |
| -1 | 3 | 11 | 2.20 | 4.70 |
| -1 | 3 | 11 | 2.50 | 5.40 |
| -1 | 3 | 11 | -0.20 | 4.90 |
| 0 | -3 | 11 | 2767.22 | 145.89 |
| 0 | -3 | 11 | 2725.03 | 147.19 |
| 0 | 3 | 11 | 2426.06 | 147.29 |
| 0 | 3 | 11 | 2868.71 | 145.99 |
| 0 | 3 | 11 | 2628.84 | 146.49 |
| 1 | -3 | 11 | 122.49 | 11.80 |
| 1 | -3 | 11 | 104.99 | 13.70 |
| 1 | -3 | 11 | 120.69 | 11.40 |
| 1 | 3 | 11 | 99.99 | 12.00 |
| 1 | 3 | 11 | 108.99 | 12.10 |
| 1 | 3 | 11 | 115.49 | 10.60 |
| 2 | -3 | 11 | 488.95 | 27.80 |
| 2 | -3 | 11 | 391.46 | 28.10 |
| 2 | -3 | 11 | 457.95 | 27.80 |
| 2 | 3 | 11 | 485.35 | 26.90 |
| 2 | 3 | 11 | 407.16 | 27.90 |
| 3 | -3 | 11 | -3.00 | 5.70 |
| 3 | -3 | 11 | -6.60 | 5.20 |
| 3 | -3 | 11 | 0.10 | 5.80 |
| 3 | 3 | 11 | 3.10 | 6.00 |
| 3 | 3 | 11 | 5.20 | 5.00 |
| 3 | 3 | 11 | 2.40 | 6.10 |
| 3 | 3 | 11 | 0.30 | 3.70 |
| 4 | -3 | 11 | 1906.41 | 111.69 |
| 4 | -3 | 11 | 1995.40 | 113.69 |
| 4 | -3 | 11 | 2288.17 | 112.59 |

| | | | | |
|---|---|---|---|---|
| 4 | 3 | 11 | 2111.79 | 115.39 |
| 4 | 3 | 11 | 2184.18 | 112.29 |
| 4 | 3 | 11 | 1752.62 | 112.79 |
| 4 | 3 | 11 | 2122.69 | 112.29 |
| 5 | -3 | 11 | 13.20 | 7.00 |
| 5 | -3 | 11 | 23.10 | 5.10 |
| 5 | -3 | 11 | 16.00 | 5.30 |
| 5 | 3 | 11 | 18.20 | 6.20 |
| 5 | 3 | 11 | 26.80 | 4.90 |
| 5 | 3 | 11 | 28.80 | 6.30 |
| 5 | 3 | 11 | 28.50 | 9.90 |
| 6 | -3 | 11 | 143.49 | 12.90 |
| 6 | -3 | 11 | 159.78 | 12.30 |
| 6 | -3 | 11 | 192.18 | 16.90 |
| 6 | 3 | 11 | 170.78 | 19.00 |
| 6 | 3 | 11 | 153.38 | 14.30 |
| 6 | 3 | 11 | 151.38 | 11.50 |
| 6 | 3 | 11 | 168.88 | 12.20 |
| 7 | -3 | 11 | 12.50 | 4.10 |
| 7 | -3 | 11 | 6.30 | 5.30 |
| 7 | 3 | 11 | 5.50 | 3.10 |
| 7 | 3 | 11 | 11.90 | 6.00 |
| 8 | -3 | 11 | 729.53 | 43.70 |
| 8 | 3 | 11 | 839.72 | 43.90 |
| 8 | 3 | 11 | 774.52 | 45.10 |
| 9 | -3 | 11 | -0.20 | 8.10 |
| 9 | -3 | 11 | 6.50 | 4.00 |
| 9 | 3 | 11 | -5.60 | 4.40 |
| 9 | 3 | 11 | 3.30 | 2.70 |
| 10 | -3 | 11 | 576.14 | 35.40 |
| 10 | 3 | 11 | 647.94 | 35.30 |
| 10 | 3 | 11 | 661.23 | 36.30 |
| 11 | -3 | 11 | 1.80 | 3.20 |
| 11 | 3 | 11 | 4.90 | 6.80 |
| 11 | 3 | 11 | 3.50 | 2.30 |
| 12 | -3 | 11 | 73.39 | 7.10 |
| 12 | 3 | 11 | 86.59 | 8.10 |
| 12 | 3 | 11 | 84.69 | 6.30 |
| 15 | -4 | -11 | 302.87 | 18.70 |
| -15 | 4 | 11 | 305.77 | 17.70 |
| 15 | 4 | -11 | 316.37 | 17.50 |
| -14 | -4 | 11 | 0.00 | 3.70 |
| 14 | -4 | -11 | -3.10 | 3.30 |
| 14 | 4 | -11 | 1.00 | 2.00 |

| | | | | |
|---|---|---|---|---|
| -14 | 4 | 11 | 2.90 | 2.00 |
| -13 | -4 | 11 | 3.10 | 3.60 |
| 13 | -4 | -11 | 7.70 | 3.90 |
| -13 | 4 | 11 | -2.20 | 3.10 |
| 13 | 4 | -11 | 3.60 | 2.50 |
| -12 | -4 | 11 | 36.90 | 7.40 |
| -12 | 4 | 11 | 33.80 | 5.20 |
| 12 | 4 | -11 | 27.30 | 4.70 |
| -11 | -4 | 11 | 1520.95 | 80.99 |
| -11 | 4 | 11 | 1442.86 | 81.09 |
| 11 | 4 | -11 | 1468.45 | 80.29 |
| -10 | -4 | 11 | 87.59 | 10.50 |
| -10 | 4 | 11 | 88.89 | 8.30 |
| 10 | 4 | -11 | 86.79 | 8.00 |
| -9 | -4 | 11 | 504.85 | 28.70 |
| -9 | 4 | 11 | 436.06 | 27.70 |
| 9 | 4 | -11 | 466.85 | 27.00 |
| 8 | 4 | -11 | 40.30 | 5.40 |
| -8 | 4 | 11 | 51.29 | 7.70 |
| -7 | 4 | 11 | 1205.38 | 61.89 |
| 7 | 4 | -11 | 991.70 | 60.39 |
| -6 | -4 | 11 | 3.30 | 4.80 |
| -6 | 4 | 11 | 13.60 | 5.20 |
| -6 | 4 | 11 | 17.50 | 5.50 |
| -5 | -4 | 11 | 1858.01 | 111.99 |
| -5 | 4 | 11 | 1914.61 | 112.39 |
| -5 | 4 | 11 | 2149.19 | 113.69 |
| -5 | 4 | 11 | 2328.77 | 113.79 |
| -4 | -4 | 11 | 20.20 | 4.70 |
| -4 | -4 | 11 | 24.60 | 4.90 |
| -4 | 4 | 11 | 14.50 | 5.10 |
| -4 | 4 | 11 | 16.90 | 5.90 |
| -4 | 4 | 11 | 15.30 | 5.60 |
| -3 | -4 | 11 | 153.48 | 12.70 |
| -3 | -4 | 11 | 187.38 | 13.20 |
| -3 | 4 | 11 | 180.08 | 14.40 |
| -3 | 4 | 11 | 169.48 | 14.80 |
| -3 | 4 | 11 | 185.18 | 14.20 |
| -2 | -4 | 11 | 6.30 | 4.00 |
| -2 | -4 | 11 | -0.60 | 3.70 |
| -2 | 4 | 11 | 1.60 | 4.60 |
| -2 | 4 | 11 | -3.80 | 4.90 |
| -2 | 4 | 11 | 6.80 | 4.70 |
| -1 | -4 | 11 | 3631.84 | 194.58 |

| | | | | |
|---|---|---|---|---|
| -1 | -4 | 11 | 3855.81 | 196.28 |
| -1 | 4 | 11 | 3730.83 | 195.38 |
| -1 | 4 | 11 | 3315.77 | 194.78 |
| -1 | 4 | 11 | 3467.35 | 196.58 |
| 0 | -4 | 11 | 45.50 | 8.30 |
| 0 | -4 | 11 | 37.80 | 8.30 |
| 0 | 4 | 11 | 37.80 | 9.20 |
| 0 | 4 | 11 | 44.30 | 7.10 |
| 0 | 4 | 11 | 51.39 | 8.40 |
| 1 | -4 | 11 | 191.98 | 15.80 |
| 1 | -4 | 11 | 229.58 | 15.50 |
| 1 | 4 | 11 | 171.58 | 16.30 |
| 1 | 4 | 11 | 195.18 | 16.30 |
| 1 | 4 | 11 | 222.68 | 14.70 |
| 2 | -4 | 11 | 28.60 | 6.90 |
| 2 | -4 | 11 | 10.00 | 5.60 |
| 2 | 4 | 11 | 32.10 | 7.20 |
| 2 | 4 | 11 | 33.30 | 7.80 |
| 2 | 4 | 11 | 19.20 | 6.20 |
| 2 | 4 | 11 | 21.60 | 5.70 |
| 3 | -4 | 11 | 632.14 | 42.20 |
| 3 | -4 | 11 | 731.03 | 41.10 |
| 3 | -4 | 11 | 711.93 | 41.40 |
| 3 | 4 | 11 | 669.53 | 43.60 |
| 3 | 4 | 11 | 802.32 | 40.80 |
| 3 | 4 | 11 | 679.33 | 40.70 |
| 3 | 4 | 11 | 747.83 | 41.30 |
| 4 | -4 | 11 | 73.49 | 12.00 |
| 4 | -4 | 11 | 60.99 | 12.30 |
| 4 | -4 | 11 | 81.69 | 10.90 |
| 4 | 4 | 11 | 58.19 | 14.50 |
| 4 | 4 | 11 | 87.79 | 8.40 |
| 4 | 4 | 11 | 96.89 | 10.30 |
| 5 | -4 | 11 | 1629.44 | 88.29 |
| 5 | -4 | 11 | 1531.65 | 87.99 |
| 5 | 4 | 11 | 1635.64 | 88.09 |
| 5 | 4 | 11 | 1633.04 | 88.19 |
| 5 | 4 | 11 | 1739.03 | 88.69 |
| 5 | 4 | 11 | 1466.55 | 91.89 |
| 6 | -4 | 11 | 59.49 | 9.40 |
| 6 | -4 | 11 | 49.80 | 8.70 |
| 6 | 4 | 11 | 66.59 | 9.90 |
| 6 | 4 | 11 | 58.39 | 7.00 |
| 6 | 4 | 11 | 67.89 | 7.70 |

| | | | | |
|---|---|---|---|---|
| 6 | 4 | 11 | 40.70 | 11.70 |
| 7 | -4 | 11 | 71.49 | 9.10 |
| 7 | 4 | 11 | 80.49 | 7.60 |
| 7 | 4 | 11 | 91.49 | 11.70 |
| 8 | -4 | 11 | -0.40 | 4.30 |
| 8 | 4 | 11 | 8.60 | 5.30 |
| 8 | 4 | 11 | -2.00 | 2.70 |
| 9 | -4 | 11 | 838.12 | 49.90 |
| 9 | 4 | 11 | 859.41 | 46.20 |
| 9 | 4 | 11 | 791.42 | 47.20 |
| 10 | -4 | 11 | 5.60 | 4.00 |
| 10 | 4 | 11 | -2.60 | 2.40 |
| 10 | 4 | 11 | -2.50 | 4.10 |
| 11 | -4 | 11 | 102.09 | 9.00 |
| 11 | 4 | 11 | 116.29 | 7.80 |
| 11 | 4 | 11 | 107.99 | 9.20 |
| 12 | -4 | 11 | 0.10 | 3.70 |
| 12 | 4 | 11 | -0.20 | 1.90 |
| 12 | 4 | 11 | -7.20 | 4.20 |
| 15 | 5 | -11 | 1.70 | 1.70 |
| -15 | 5 | 11 | 3.80 | 1.70 |
| -14 | 5 | 11 | 192.88 | 12.10 |
| 14 | 5 | -11 | 198.58 | 11.80 |
| -13 | 5 | 11 | 70.89 | 6.80 |
| 13 | 5 | -11 | 81.19 | 6.20 |
| 12 | 5 | -11 | 765.22 | 43.50 |
| -12 | 5 | 11 | 814.72 | 44.30 |
| 11 | 5 | -11 | 17.40 | 3.10 |
| -11 | 5 | 11 | 31.70 | 5.50 |
| -10 | 5 | 11 | 1115.39 | 60.79 |
| 10 | 5 | -11 | 1072.09 | 59.89 |
| 9 | 5 | -11 | 28.50 | 4.20 |
| -9 | 5 | 11 | 41.60 | 6.90 |
| 8 | 5 | -11 | 12.50 | 3.80 |
| -8 | 5 | 11 | 14.70 | 4.20 |
| -7 | -5 | 11 | 58.29 | 9.70 |
| -7 | 5 | 11 | 71.29 | 9.80 |
| 7 | 5 | -11 | 69.59 | 8.70 |
| -6 | -5 | 11 | 3703.93 | 210.08 |
| -6 | 5 | 11 | 4205.68 | 211.58 |
| -6 | 5 | 11 | 4008.60 | 211.58 |
| 6 | 5 | -11 | 3669.13 | 210.08 |
| -5 | -5 | 11 | -7.70 | 3.80 |
| -5 | 5 | 11 | 6.50 | 5.20 |

| | | | | |
|---|---|---|---|---|
| -5 | 5 | 11 | 5.90 | 5.60 |
| -5 | 5 | 11 | -1.30 | 5.50 |
| -4 | -5 | 11 | 203.18 | 14.80 |
| -4 | 5 | 11 | 226.18 | 16.50 |
| -4 | 5 | 11 | 235.68 | 16.90 |
| -4 | 5 | 11 | 207.88 | 16.80 |
| -3 | -5 | 11 | 36.50 | 5.20 |
| -3 | 5 | 11 | 40.80 | 6.10 |
| -3 | 5 | 11 | 27.10 | 6.10 |
| -3 | 5 | 11 | 33.10 | 6.70 |
| -2 | -5 | 11 | 772.82 | 45.80 |
| -2 | -5 | 11 | 868.61 | 47.30 |
| -2 | 5 | 11 | 845.22 | 46.80 |
| -2 | 5 | 11 | 797.52 | 46.70 |
| -2 | 5 | 11 | 793.72 | 47.90 |
| -1 | -5 | 11 | 14.90 | 5.10 |
| -1 | -5 | 11 | 10.90 | 4.10 |
| -1 | 5 | 11 | 4.70 | 5.30 |
| -1 | 5 | 11 | 4.00 | 4.30 |
| -1 | 5 | 11 | 8.70 | 5.70 |
| 0 | -5 | 11 | 2621.24 | 137.99 |
| 0 | -5 | 11 | 2605.04 | 139.69 |
| 0 | 5 | 11 | 2372.26 | 137.49 |
| 0 | 5 | 11 | 2711.83 | 138.19 |
| 0 | 5 | 11 | 2453.95 | 139.99 |
| 0 | 5 | 11 | 2456.35 | 138.69 |
| 1 | -5 | 11 | 83.69 | 11.10 |
| 1 | -5 | 11 | 108.59 | 10.70 |
| 1 | 5 | 11 | 96.39 | 12.00 |
| 1 | 5 | 11 | 111.89 | 10.80 |
| 1 | 5 | 11 | 96.79 | 9.60 |
| 1 | 5 | 11 | 73.59 | 11.70 |
| 2 | -5 | 11 | 174.28 | 13.60 |
| 2 | -5 | 11 | 135.39 | 13.80 |
| 2 | 5 | 11 | 128.99 | 11.40 |
| 2 | 5 | 11 | 155.48 | 12.50 |
| 2 | 5 | 11 | 145.69 | 14.90 |
| 2 | 5 | 11 | 157.38 | 13.60 |
| 3 | -5 | 11 | 5.00 | 5.80 |
| 3 | -5 | 11 | 20.70 | 5.40 |
| 3 | 5 | 11 | 8.70 | 6.80 |
| 3 | 5 | 11 | 11.60 | 5.60 |
| 3 | 5 | 11 | 11.50 | 5.00 |
| 3 | 5 | 11 | 21.30 | 5.40 |

| | | | | |
|---|---|---|---|---|
| 4 | -5 | 11 | 2014.50 | 103.49 |
| 4 | 5 | 11 | 1612.64 | 107.79 |
| 4 | 5 | 11 | 2018.00 | 103.29 |
| 4 | 5 | 11 | 1768.42 | 102.69 |
| 4 | 5 | 11 | 1984.10 | 103.09 |
| 5 | -5 | 11 | 27.50 | 5.80 |
| 5 | -5 | 11 | 43.10 | 6.70 |
| 5 | 5 | 11 | 35.70 | 7.20 |
| 5 | 5 | 11 | 47.70 | 7.90 |
| 5 | 5 | 11 | 31.20 | 10.30 |
| 5 | 5 | 11 | 37.00 | 6.40 |
| 6 | -5 | 11 | 211.08 | 15.00 |
| 6 | -5 | 11 | 201.18 | 13.90 |
| 6 | 5 | 11 | 155.28 | 23.70 |
| 6 | 5 | 11 | 169.08 | 12.80 |
| 6 | 5 | 11 | 212.08 | 14.70 |
| 7 | -5 | 11 | -9.30 | 5.30 |
| 7 | 5 | 11 | -0.80 | 5.30 |
| 7 | 5 | 11 | 4.20 | 2.90 |
| 8 | -5 | 11 | 279.47 | 18.90 |
| 8 | 5 | 11 | 287.87 | 19.30 |
| 8 | 5 | 11 | 299.47 | 17.60 |
| 9 | -5 | 11 | 0.20 | 4.90 |
| 9 | 5 | 11 | 1.20 | 4.40 |
| 9 | 5 | 11 | -1.20 | 3.50 |
| 10 | -5 | 11 | 313.07 | 21.20 |
| 10 | 5 | 11 | 349.57 | 20.10 |
| 10 | 5 | 11 | 369.86 | 21.30 |
| 11 | -5 | 11 | 23.90 | 5.80 |
| 11 | 5 | 11 | 23.90 | 4.10 |
| 11 | 5 | 11 | 31.30 | 6.30 |
| -15 | 6 | 11 | 201.78 | 12.50 |
| 15 | 6 | -11 | 227.78 | 12.40 |
| -14 | 6 | 11 | -0.20 | 1.70 |
| 14 | 6 | -11 | 1.00 | 1.70 |
| 13 | 6 | -11 | 4.30 | 2.20 |
| -13 | 6 | 11 | 0.50 | 2.00 |
| 12 | 6 | -11 | 29.70 | 4.60 |
| -12 | 6 | 11 | 33.10 | 5.10 |
| -11 | 6 | 11 | 995.30 | 56.39 |
| 11 | 6 | -11 | 1038.10 | 55.69 |
| -10 | 6 | 11 | 9.60 | 3.30 |
| 10 | 6 | -11 | 18.90 | 3.30 |
| -9 | 6 | 11 | 328.87 | 19.90 |

| | | | | |
|---|---|---|---|---|
| 9 | 6 | -11 | 305.07 | 18.70 |
| 8 | 6 | -11 | 63.19 | 6.90 |
| -8 | 6 | 11 | 47.90 | 7.90 |
| -7 | -6 | 11 | 1034.50 | 59.09 |
| 7 | 6 | -11 | 1086.99 | 60.09 |
| -7 | 6 | 11 | 1078.09 | 60.19 |
| -6 | -6 | 11 | 0.30 | 4.10 |
| -6 | 6 | 11 | 3.80 | 6.70 |
| 6 | 6 | -11 | 1.80 | 4.50 |
| -5 | -6 | 11 | 1599.64 | 87.09 |
| 5 | 6 | -11 | 1520.35 | 87.29 |
| -5 | 6 | 11 | 1702.33 | 89.09 |
| -5 | 6 | 11 | 1539.25 | 88.39 |
| -4 | -6 | 11 | 3.80 | 3.90 |
| -4 | 6 | 11 | -3.80 | 5.60 |
| -4 | 6 | 11 | 5.20 | 5.70 |
| -4 | 6 | 11 | -0.70 | 4.90 |
| -3 | -6 | 11 | 12.80 | 3.90 |
| -3 | 6 | 11 | 22.00 | 5.50 |
| -3 | 6 | 11 | 21.20 | 6.40 |
| -3 | 6 | 11 | 21.70 | 6.00 |
| -2 | -6 | 11 | 130.09 | 10.30 |
| -2 | 6 | 11 | 131.49 | 12.90 |
| -2 | 6 | 11 | 102.89 | 11.50 |
| -2 | 6 | 11 | 114.79 | 12.10 |
| -2 | 6 | 11 | 110.19 | 12.10 |
| -1 | -6 | 11 | 3854.61 | 208.08 |
| -1 | 6 | 11 | 3908.41 | 209.38 |
| -1 | 6 | 11 | 3584.34 | 208.28 |
| -1 | 6 | 11 | 3934.91 | 210.58 |
| -1 | 6 | 11 | 3969.60 | 208.88 |
| 0 | -6 | 11 | 33.10 | 5.50 |
| 0 | 6 | 11 | 41.70 | 8.90 |
| 0 | 6 | 11 | 21.70 | 6.20 |
| 0 | 6 | 11 | 53.29 | 10.00 |
| 0 | 6 | 11 | 39.90 | 7.80 |
| 1 | -6 | 11 | 230.48 | 15.00 |
| 1 | 6 | 11 | 162.78 | 15.40 |
| 1 | 6 | 11 | 224.88 | 14.40 |
| 1 | 6 | 11 | 186.58 | 16.80 |
| 1 | 6 | 11 | 179.18 | 14.20 |
| 2 | -6 | 11 | 19.60 | 5.30 |
| 2 | 6 | 11 | 11.60 | 4.80 |
| 2 | 6 | 11 | 21.10 | 5.60 |

| | | | | |
|---|---|---|---|---|
| 2 | 6 | 11 | 21.60 | 6.90 |
| 2 | 6 | 11 | 31.10 | 6.90 |
| 3 | -6 | 11 | 549.35 | 32.30 |
| 3 | 6 | 11 | 495.25 | 35.20 |
| 3 | 6 | 11 | 495.15 | 31.20 |
| 3 | 6 | 11 | 611.64 | 31.60 |
| 3 | 6 | 11 | 532.85 | 32.00 |
| 4 | -6 | 11 | 72.89 | 10.30 |
| 4 | 6 | 11 | 39.20 | 10.00 |
| 4 | 6 | 11 | 71.19 | 8.40 |
| 4 | 6 | 11 | 61.09 | 7.40 |
| 5 | -6 | 11 | 1037.10 | 56.29 |
| 5 | 6 | 11 | 877.31 | 60.99 |
| 5 | 6 | 11 | 1099.39 | 56.09 |
| 5 | 6 | 11 | 998.10 | 56.29 |
| 6 | -6 | 11 | 6.70 | 4.60 |
| 6 | 6 | 11 | 14.00 | 3.30 |
| 6 | 6 | 11 | 24.00 | 13.40 |
| 6 | 6 | 11 | 18.00 | 5.10 |
| 7 | 6 | 11 | 49.50 | 6.20 |
| 7 | 6 | 11 | 55.69 | 8.60 |
| 8 | 6 | 11 | 36.90 | 5.50 |
| 8 | 6 | 11 | 43.20 | 7.80 |
| 9 | 6 | 11 | 494.05 | 27.50 |
| 9 | 6 | 11 | 467.35 | 28.50 |
| 10 | 6 | 11 | 16.40 | 4.30 |
| 10 | 6 | 11 | 14.40 | 3.00 |
| 11 | 6 | 11 | 122.69 | 8.60 |
| 11 | 6 | 11 | 141.49 | 10.00 |
| -14 | 7 | 11 | 157.68 | 10.20 |
| 14 | 7 | -11 | 168.98 | 9.90 |
| -13 | 7 | 11 | 101.79 | 7.50 |
| 13 | 7 | -11 | 99.19 | 6.90 |
| 12 | 7 | -11 | 515.55 | 28.80 |
| -11 | 7 | 11 | 5.90 | 2.90 |
| 11 | 7 | -11 | 5.70 | 2.50 |
| -10 | 7 | 11 | 708.43 | 40.90 |
| 10 | 7 | -11 | 736.83 | 40.10 |
| 9 | 7 | -11 | 45.00 | 6.00 |
| -9 | 7 | 11 | 48.00 | 7.00 |
| 8 | 7 | -11 | 8.10 | 3.40 |
| -8 | 7 | 11 | 29.50 | 5.20 |
| -7 | -7 | 11 | 214.08 | 15.40 |
| -7 | 7 | 11 | 257.37 | 16.90 |

| | | | | |
|---|---|---|---|---|
| 7 | 7 | -11 | 233.88 | 15.30 |
| 7 | 7 | -11 | 221.98 | 21.00 |
| 6 | 7 | -11 | 1718.83 | 95.59 |
| -6 | 7 | 11 | 1630.24 | 95.89 |
| -6 | 7 | 11 | 1854.41 | 98.59 |
| -5 | -7 | 11 | 273.77 | 17.30 |
| -5 | 7 | 11 | 254.57 | 22.90 |
| -5 | 7 | 11 | 259.97 | 20.20 |
| -4 | -7 | 11 | 109.59 | 9.70 |
| -4 | 7 | 11 | 99.09 | 10.70 |
| -4 | 7 | 11 | 103.79 | 12.00 |
| -4 | 7 | 11 | 106.29 | 12.70 |
| -3 | -7 | 11 | 171.28 | 12.50 |
| -3 | 7 | 11 | 148.09 | 14.10 |
| -3 | 7 | 11 | 187.58 | 15.60 |
| -3 | 7 | 11 | 172.08 | 13.50 |
| -2 | -7 | 11 | 512.75 | 31.00 |
| -2 | 7 | 11 | 530.85 | 31.80 |
| -2 | 7 | 11 | 524.75 | 31.70 |
| -2 | 7 | 11 | 566.44 | 33.80 |
| -2 | 7 | 11 | 530.85 | 31.30 |
| -1 | -7 | 11 | 24.40 | 4.90 |
| -1 | 7 | 11 | 21.20 | 5.90 |
| -1 | 7 | 11 | 23.10 | 7.00 |
| -1 | 7 | 11 | 17.20 | 5.00 |
| -1 | 7 | 11 | 20.90 | 5.30 |
| 0 | -7 | 11 | 2173.98 | 113.09 |
| 0 | 7 | 11 | 2162.68 | 112.79 |
| 0 | 7 | 11 | 2051.49 | 115.39 |
| 0 | 7 | 11 | 1935.01 | 112.89 |
| 0 | 7 | 11 | 2021.90 | 113.79 |
| 1 | 7 | 11 | 170.48 | 13.10 |
| 1 | 7 | 11 | 166.78 | 15.40 |
| 1 | 7 | 11 | 194.48 | 16.80 |
| 2 | -7 | 11 | 4.30 | 4.70 |
| 2 | 7 | 11 | 2.50 | 3.70 |
| 2 | 7 | 11 | -1.00 | 4.00 |
| 2 | 7 | 11 | 7.20 | 7.50 |
| 3 | -7 | 11 | 298.97 | 18.40 |
| 3 | 7 | 11 | 259.57 | 17.00 |
| 3 | 7 | 11 | 213.98 | 21.30 |
| 3 | 7 | 11 | 260.77 | 16.90 |
| 4 | -7 | 11 | 1642.84 | 88.49 |
| 4 | 7 | 11 | 1532.45 | 87.99 |

| | | | | |
|---|---|---|---|---|
| 4 | 7 | 11 | 1772.32 | 88.09 |
| 4 | 7 | 11 | 1466.75 | 92.09 |
| 5 | -7 | 11 | 117.79 | 11.40 |
| 5 | 7 | 11 | 104.19 | 21.20 |
| 5 | 7 | 11 | 122.09 | 10.20 |
| 5 | 7 | 11 | 114.89 | 9.50 |
| 6 | -7 | 11 | 205.28 | 14.80 |
| 6 | 7 | 11 | 159.88 | 26.70 |
| 6 | 7 | 11 | 226.78 | 13.80 |
| 6 | 7 | 11 | 210.48 | 15.10 |
| 7 | 7 | 11 | 128.19 | 9.60 |
| 7 | 7 | 11 | 139.39 | 11.10 |
| -8 | -7 | -11 | 372.36 | 21.80 |
| 8 | 7 | 11 | 362.06 | 22.20 |
| 8 | 7 | 11 | 344.77 | 21.10 |
| 9 | 7 | 11 | 10.20 | 4.10 |
| 9 | 7 | 11 | -1.00 | 2.60 |
| 10 | 7 | 11 | 510.25 | 29.50 |
| 10 | 7 | 11 | 473.95 | 27.50 |
| 11 | 7 | 11 | 32.30 | 5.90 |
| 11 | 7 | 11 | 34.20 | 4.40 |
| -14 | 8 | 11 | 27.20 | 3.50 |
| 14 | 8 | -11 | 28.30 | 3.20 |
| 13 | 8 | -11 | 11.70 | 2.10 |
| -13 | 8 | 11 | 17.10 | 3.70 |
| 12 | 8 | -11 | 87.99 | 6.60 |
| -12 | 8 | 11 | 85.89 | 7.20 |
| 11 | 8 | -11 | 553.84 | 30.10 |
| -11 | 8 | 11 | 522.45 | 30.70 |
| 10 | 8 | -11 | 17.10 | 3.10 |
| -10 | 8 | 11 | 11.60 | 3.20 |
| 9 | 8 | -11 | 257.37 | 16.40 |
| -9 | 8 | 11 | 287.37 | 17.60 |
| -8 | 8 | 11 | 154.88 | 12.30 |
| 8 | 8 | -11 | 156.18 | 10.80 |
| -7 | -8 | 11 | 399.36 | 25.40 |
| -7 | 8 | 11 | 454.35 | 25.70 |
| -7 | 8 | 11 | 449.46 | 27.00 |
| 7 | 8 | -11 | 427.16 | 27.60 |
| 7 | 8 | -11 | 436.66 | 25.50 |
| -6 | -8 | 11 | 40.50 | 7.70 |
| 6 | 8 | -11 | 39.10 | 9.20 |
| -6 | 8 | 11 | 35.90 | 6.60 |
| 6 | 8 | -11 | 41.90 | 8.40 |

| | | | | |
|---|---|---|---|---|
| -6 | 8 | 11 | 38.40 | 7.90 |
| -5 | -8 | 11 | 1092.39 | 61.19 |
| -5 | 8 | 11 | 1084.59 | 62.69 |
| 5 | 8 | -11 | 1179.48 | 64.89 |
| -5 | 8 | 11 | 1095.79 | 61.29 |
| -5 | 8 | 11 | 1081.59 | 63.79 |
| -4 | -8 | 11 | 112.99 | 10.10 |
| -4 | 8 | 11 | 129.79 | 10.90 |
| -4 | 8 | 11 | 115.99 | 14.20 |
| -4 | 8 | 11 | 107.99 | 12.40 |
| -3 | -8 | 11 | 2.90 | 4.00 |
| -3 | 8 | 11 | -5.80 | 5.90 |
| -3 | 8 | 11 | 3.30 | 4.50 |
| -3 | 8 | 11 | 7.90 | 8.10 |
| -2 | -8 | 11 | 448.76 | 26.90 |
| -2 | 8 | 11 | 469.45 | 29.90 |
| -2 | 8 | 11 | 445.06 | 27.10 |
| -1 | -8 | 11 | 1543.65 | 87.19 |
| -1 | -8 | 11 | 1663.93 | 87.89 |
| -1 | 8 | 11 | 1713.03 | 90.69 |
| -1 | 8 | 11 | 1481.65 | 87.59 |
| 0 | -8 | 11 | 231.68 | 15.90 |
| 0 | 8 | 11 | 218.08 | 18.80 |
| 0 | 8 | 11 | 247.18 | 16.00 |
| 0 | 8 | 11 | 228.48 | 15.70 |
| 1 | -8 | 11 | 155.18 | 12.00 |
| 1 | 8 | 11 | 149.59 | 11.50 |
| 1 | 8 | 11 | 135.19 | 15.50 |
| 1 | 8 | 11 | 125.89 | 11.10 |
| 2 | -8 | 11 | 207.48 | 15.30 |
| 2 | 8 | 11 | 207.88 | 18.80 |
| 2 | 8 | 11 | 202.98 | 14.10 |
| 2 | 8 | 11 | 205.68 | 14.00 |
| 3 | -8 | 11 | 317.47 | 19.80 |
| 3 | 8 | 11 | 272.77 | 23.70 |
| 3 | 8 | 11 | 274.47 | 18.40 |
| 3 | 8 | 11 | 314.47 | 18.80 |
| 4 | -8 | 11 | 41.90 | 8.80 |
| 4 | 8 | 11 | 12.40 | 9.30 |
| 4 | 8 | 11 | 20.80 | 4.30 |
| 4 | 8 | 11 | 17.20 | 4.30 |
| 5 | -8 | 11 | 1283.87 | 66.39 |
| 5 | 8 | 11 | 1195.68 | 65.89 |
| -5 | 8 | -11 | 1208.88 | 68.39 |

| | | | | |
|---|---|---|---|---|
| 5 | 8 | 11 | 1081.79 | 72.09 |
| 6 | -8 | 11 | 105.59 | 10.40 |
| 6 | 8 | 11 | 105.49 | 9.70 |
| 6 | 8 | 11 | 121.29 | 8.80 |
| -7 | -8 | -11 | 40.80 | 6.80 |
| 7 | 8 | 11 | 35.20 | 5.40 |
| 7 | 8 | 11 | 35.80 | 6.90 |
| -8 | -8 | -11 | 2.60 | 3.60 |
| 8 | 8 | 11 | 3.00 | 3.90 |
| 8 | 8 | 11 | 1.30 | 2.80 |
| 9 | 8 | 11 | 309.37 | 17.90 |
| 9 | 8 | 11 | 298.67 | 19.00 |
| 10 | 8 | 11 | -1.50 | 3.80 |
| 10 | 8 | 11 | -3.10 | 2.30 |
| -14 | 9 | 11 | 196.98 | 11.90 |
| 14 | 9 | -11 | 205.78 | 11.50 |
| 13 | 9 | -11 | 85.49 | 5.80 |
| -13 | 9 | 11 | 83.59 | 6.40 |
| 12 | 9 | -11 | 530.45 | 29.70 |
| -12 | 9 | 11 | 540.75 | 30.20 |
| 11 | 9 | -11 | 6.20 | 2.50 |
| -11 | 9 | 11 | 10.20 | 3.20 |
| 10 | 9 | -11 | 779.02 | 44.00 |
| -10 | 9 | 11 | 817.02 | 44.80 |
| -9 | 9 | 11 | 196.68 | 13.80 |
| 9 | 9 | -11 | 224.28 | 12.90 |
| -9 | 9 | 11 | 182.28 | 14.20 |
| -8 | -9 | 11 | 29.70 | 4.60 |
| -8 | 9 | 11 | 35.70 | 7.40 |
| -8 | 9 | 11 | 22.30 | 6.70 |
| 8 | 9 | -11 | 30.90 | 5.90 |
| -7 | -9 | 11 | 133.39 | 10.50 |
| 7 | 9 | -11 | 140.09 | 10.40 |
| -7 | 9 | 11 | 122.09 | 10.80 |
| 7 | 9 | -11 | 146.09 | 15.00 |
| -7 | 9 | 11 | 134.49 | 12.30 |
| -6 | -9 | 11 | 1740.53 | 102.19 |
| 6 | -9 | -11 | 1896.41 | 103.29 |
| -6 | 9 | 11 | 1814.32 | 102.29 |
| -6 | 9 | 11 | 2053.39 | 103.99 |
| 6 | 9 | -11 | 1875.11 | 103.89 |
| 6 | 9 | -11 | 1894.31 | 102.39 |
| -5 | -9 | 11 | 134.49 | 10.70 |
| -5 | 9 | 11 | 122.09 | 13.40 |

| | | | | |
|---|---|---|---|---|
| -5 | 9 | 11 | 146.39 | 11.00 |
| 5 | 9 | -11 | 127.39 | 13.00 |
| -5 | 9 | 11 | 127.29 | 16.60 |
| -4 | -9 | 11 | 561.74 | 33.10 |
| -4 | 9 | 11 | 572.64 | 33.20 |
| -4 | 9 | 11 | 573.24 | 36.30 |
| -4 | 9 | 11 | 562.84 | 34.60 |
| 4 | 9 | -11 | 603.64 | 35.10 |
| -3 | -9 | 11 | 116.89 | 10.40 |
| -3 | 9 | 11 | 131.39 | 14.70 |
| -3 | 9 | 11 | 115.69 | 12.00 |
| 3 | 9 | -11 | 141.79 | 13.20 |
| -3 | 9 | 11 | 121.49 | 10.90 |
| -2 | -9 | 11 | 650.03 | 37.50 |
| -2 | 9 | 11 | 699.13 | 40.60 |
| -2 | 9 | 11 | 601.44 | 37.40 |
| -2 | 9 | 11 | 672.03 | 38.20 |
| -1 | -9 | 11 | 17.20 | 4.90 |
| -1 | -9 | 11 | 17.70 | 4.30 |
| -1 | 9 | 11 | 37.20 | 8.30 |
| -1 | 9 | 11 | 15.50 | 4.40 |
| -1 | 9 | 11 | 18.10 | 5.00 |
| 0 | -9 | 11 | 2014.50 | 105.29 |
| 0 | -9 | 11 | 1790.52 | 104.29 |
| 0 | 9 | 11 | 1998.20 | 105.19 |
| 0 | 9 | 11 | 1855.41 | 104.89 |
| 0 | 9 | 11 | 1957.30 | 107.99 |
| 1 | -9 | 11 | 355.06 | 22.80 |
| 1 | -9 | 11 | 426.96 | 23.60 |
| 1 | 9 | 11 | 373.46 | 23.10 |
| 1 | 9 | 11 | 372.06 | 23.10 |
| 1 | 9 | 11 | 377.06 | 26.90 |
| 2 | -9 | 11 | 35.10 | 5.60 |
| 2 | -9 | 11 | 11.80 | 4.60 |
| 2 | 9 | 11 | 20.20 | 4.50 |
| 2 | 9 | 11 | 24.40 | 4.50 |
| 2 | 9 | 11 | 14.10 | 7.30 |
| 3 | -9 | 11 | -1.70 | 4.20 |
| -3 | 9 | -11 | -1.90 | 6.90 |
| 3 | 9 | 11 | -4.80 | 3.30 |
| 3 | 9 | 11 | -3.40 | 3.50 |
| 3 | 9 | 11 | 18.10 | 9.00 |
| 4 | -9 | 11 | 1256.47 | 68.59 |
| 4 | 9 | 11 | 1255.27 | 67.99 |

| | | | | |
|---|---|---|---|---|
| 4 | 9 | 11 | 1320.87 | 69.69 |
| 4 | 9 | 11 | 1163.78 | 72.99 |
| -4 | 9 | -11 | 1178.18 | 70.29 |
| 5 | -9 | 11 | 84.59 | 9.30 |
| 5 | 9 | 11 | 93.69 | 8.60 |
| 5 | 9 | 11 | 98.59 | 23.30 |
| 5 | 9 | 11 | 97.29 | 8.20 |
| -6 | -9 | -11 | 202.88 | 13.90 |
| 6 | -9 | 11 | 214.18 | 14.60 |
| 6 | 9 | 11 | 205.98 | 14.10 |
| 6 | 9 | 11 | 219.28 | 13.50 |
| -7 | -9 | -11 | -0.80 | 3.30 |
| 7 | 9 | 11 | 5.80 | 3.50 |
| 7 | 9 | 11 | -6.40 | 5.70 |
| -8 | -9 | -11 | 288.07 | 17.30 |
| 8 | 9 | 11 | 273.67 | 17.70 |
| 8 | 9 | 11 | 286.67 | 16.90 |
| 9 | 9 | 11 | 3.20 | 2.60 |
| 9 | 9 | 11 | 0.00 | 3.60 |
| 10 | 9 | 11 | 374.76 | 20.80 |
| 10 | 9 | 11 | 355.76 | 21.50 |
| 13 | 10 | -11 | 3.20 | 1.50 |
| -13 | 10 | 11 | 5.40 | 2.10 |
| 12 | 10 | -11 | 70.09 | 5.30 |
| -12 | 10 | 11 | 76.29 | 6.10 |
| -11 | 10 | 11 | 453.45 | 27.90 |
| 11 | 10 | -11 | 529.85 | 27.50 |
| -10 | 10 | 11 | 25.10 | 4.70 |
| -9 | 10 | 11 | 391.86 | 22.30 |
| 9 | 10 | -11 | 381.06 | 21.20 |
| -9 | 10 | 11 | 324.77 | 21.40 |
| -8 | -10 | 11 | 241.78 | 16.30 |
| 8 | 10 | -11 | 267.97 | 16.10 |
| -8 | 10 | 11 | 281.77 | 17.70 |
| -8 | 10 | 11 | 259.37 | 16.40 |
| -7 | -10 | 11 | 343.87 | 20.60 |
| -7 | 10 | 11 | 312.37 | 20.90 |
| -7 | 10 | 11 | 363.46 | 22.50 |
| 7 | 10 | -11 | 370.06 | 20.90 |
| 7 | 10 | -11 | 346.07 | 24.50 |
| -6 | -10 | 11 | 0.10 | 3.10 |
| 6 | -10 | -11 | 11.70 | 5.00 |
| 6 | 10 | -11 | 1.30 | 6.80 |
| 6 | 10 | -11 | -3.00 | 3.60 |

| | | | | |
|---|---|---|---|---|
| -6 | 10 | 11 | 2.90 | 3.80 |
| -6 | 10 | 11 | 8.10 | 5.70 |
| -5 | -10 | 11 | 1297.37 | 71.79 |
| 5 | -10 | -11 | 1278.77 | 71.99 |
| -5 | 10 | 11 | 1230.88 | 73.09 |
| -5 | 10 | 11 | 1367.26 | 71.89 |
| -5 | 10 | 11 | 1312.97 | 75.49 |
| 5 | 10 | -11 | 1340.37 | 73.79 |
| -4 | -10 | 11 | 254.17 | 16.40 |
| -4 | 10 | 11 | 254.67 | 18.20 |
| -4 | 10 | 11 | 256.37 | 16.60 |
| 4 | 10 | -11 | 249.68 | 19.00 |
| -4 | 10 | 11 | 244.98 | 21.60 |
| -3 | -10 | 11 | 8.10 | 3.70 |
| -3 | 10 | 11 | 3.10 | 7.20 |
| -3 | 10 | 11 | 1.10 | 4.40 |
| -3 | 10 | 11 | 5.50 | 4.00 |
| 3 | 10 | -11 | 12.70 | 6.60 |
| -2 | -10 | 11 | 186.68 | 13.90 |
| -2 | 10 | 11 | 216.78 | 18.50 |
| -2 | 10 | 11 | 209.98 | 14.10 |
| 2 | 10 | -11 | 200.98 | 17.90 |
| -2 | 10 | 11 | 197.98 | 14.90 |
| -1 | -10 | 11 | 1371.16 | 77.19 |
| -1 | -10 | 11 | 1317.77 | 76.39 |
| -1 | 10 | 11 | 1545.35 | 77.59 |
| 1 | 10 | -11 | 1372.16 | 80.09 |
| -1 | 10 | 11 | 1321.77 | 77.09 |
| -1 | 10 | 11 | 1480.95 | 80.29 |
| 0 | -10 | 11 | 14.00 | 3.90 |
| 0 | -10 | 11 | 9.90 | 3.80 |
| 0 | 10 | 11 | 21.30 | 8.70 |
| 0 | 10 | 11 | 14.90 | 4.30 |
| 0 | 10 | 11 | 16.30 | 4.70 |
| 0 | 10 | -11 | 20.20 | 8.10 |
| 1 | -10 | 11 | 221.48 | 14.60 |
| 1 | -10 | 11 | 206.58 | 13.90 |
| 1 | 10 | 11 | 199.18 | 19.00 |
| 1 | 10 | 11 | 210.28 | 14.20 |
| -1 | 10 | -11 | 192.18 | 18.60 |
| 1 | 10 | 11 | 209.58 | 14.20 |
| 2 | -10 | 11 | 234.08 | 15.00 |
| 2 | -10 | 11 | 252.27 | 16.00 |
| 2 | 10 | 11 | 211.48 | 15.20 |

| | | | | |
|---|---|---|---|---|
| 2 | 10 | 11 | 238.08 | 20.90 |
| -2 | 10 | -11 | 203.38 | 19.20 |
| 2 | 10 | 11 | 233.28 | 15.20 |
| 3 | -10 | 11 | 359.36 | 20.70 |
| 3 | -10 | 11 | 340.87 | 19.40 |
| 3 | 10 | 11 | 260.37 | 22.00 |
| 3 | 10 | 11 | 298.27 | 19.40 |
| -3 | 10 | -11 | 326.37 | 23.90 |
| 3 | 10 | 11 | 323.47 | 26.00 |
| 4 | -10 | 11 | 85.49 | 9.50 |
| 4 | -10 | 11 | 64.79 | 8.20 |
| 4 | 10 | 11 | 98.69 | 19.60 |
| 4 | 10 | 11 | 78.89 | 7.90 |
| 4 | 10 | 11 | 77.19 | 7.40 |
| -5 | -10 | -11 | 521.25 | 30.50 |
| 5 | -10 | 11 | 548.45 | 30.80 |
| 5 | 10 | 11 | 497.35 | 30.00 |
| 5 | 10 | 11 | 549.65 | 30.50 |
| 6 | -10 | 11 | 152.98 | 12.30 |
| -6 | -10 | -11 | 197.78 | 15.20 |
| 6 | 10 | 11 | 163.08 | 11.80 |
| -7 | -10 | -11 | 54.39 | 6.50 |
| 7 | 10 | 11 | 55.29 | 6.30 |
| 7 | 10 | 11 | 56.99 | 6.90 |
| -8 | -10 | -11 | 63.99 | 7.00 |
| 8 | 10 | 11 | 54.99 | 5.90 |
| 8 | 10 | 11 | 69.19 | 7.00 |
| 9 | 10 | 11 | 196.18 | 12.70 |
| 9 | 10 | 11 | 184.48 | 11.70 |
| 12 | 11 | -11 | 137.49 | 8.10 |
| -12 | 11 | 11 | 124.59 | 8.70 |
| 11 | 11 | -11 | 2.50 | 1.80 |
| -11 | 11 | 11 | 2.10 | 2.50 |
| -10 | 11 | 11 | 280.27 | 19.00 |
| 10 | 11 | -11 | 358.76 | 18.40 |
| -10 | 11 | 11 | 318.67 | 19.20 |
| -9 | 11 | 11 | 9.20 | 4.30 |
| -9 | 11 | 11 | 11.70 | 3.50 |
| -8 | -11 | 11 | 22.70 | 4.20 |
| 8 | 11 | -11 | 29.40 | 5.30 |
| -8 | 11 | 11 | 28.50 | 5.40 |
| -8 | 11 | 11 | 30.20 | 4.30 |
| -7 | -11 | 11 | 177.78 | 12.40 |
| 7 | 11 | -11 | 186.38 | 12.20 |

| | | | | |
|---|---|---|---|---|
| -7 | 11 | 11 | 196.38 | 14.60 |
| -7 | 11 | 11 | 171.08 | 12.20 |
| 6 | -11 | -11 | 810.82 | 44.50 |
| -6 | -11 | 11 | 738.63 | 43.80 |
| -6 | 11 | 11 | 839.02 | 44.00 |
| 6 | 11 | -11 | 725.43 | 46.40 |
| 6 | 11 | -11 | 792.72 | 44.00 |
| -6 | 11 | 11 | 798.92 | 45.40 |
| 5 | -11 | -11 | 22.80 | 4.70 |
| -5 | -11 | 11 | 18.30 | 3.80 |
| 5 | 11 | -11 | 7.70 | 7.00 |
| -5 | 11 | 11 | 16.10 | 5.60 |
| 4 | -11 | -11 | 44.80 | 7.90 |
| -4 | -11 | 11 | 35.00 | 6.90 |
| 4 | 11 | -11 | 29.50 | 7.80 |
| -4 | 11 | 11 | 48.60 | 7.40 |
| -4 | 11 | 11 | 68.89 | 16.50 |
| -4 | 11 | 11 | 42.90 | 6.80 |
| -3 | -11 | 11 | 175.38 | 12.50 |
| 3 | -11 | -11 | 187.28 | 13.30 |
| -3 | 11 | 11 | 152.38 | 13.80 |
| 3 | 11 | -11 | 179.58 | 16.90 |
| -3 | 11 | 11 | 161.28 | 18.70 |
| -3 | 11 | 11 | 177.08 | 12.60 |
| -2 | -11 | 11 | 326.07 | 20.10 |
| 2 | 11 | -11 | 321.27 | 24.10 |
| -2 | 11 | 11 | 343.47 | 20.40 |
| -2 | 11 | 11 | 312.97 | 20.80 |
| -2 | 11 | 11 | 324.37 | 25.00 |
| -1 | -11 | 11 | 33.90 | 6.50 |
| 1 | 11 | -11 | 18.80 | 8.30 |
| -1 | 11 | 11 | 36.30 | 7.80 |
| -1 | 11 | 11 | 17.10 | 7.80 |
| -1 | 11 | 11 | 43.10 | 7.20 |
| 0 | -11 | 11 | 380.26 | 22.00 |
| 0 | -11 | 11 | 361.16 | 21.00 |
| 0 | 11 | 11 | 320.87 | 26.00 |
| 0 | 11 | 11 | 364.76 | 21.90 |
| 0 | 11 | 11 | 343.37 | 21.90 |
| 1 | -11 | 11 | 65.99 | 9.00 |
| 1 | -11 | 11 | 60.99 | 7.30 |
| 1 | 11 | 11 | 73.49 | 14.60 |
| 1 | 11 | 11 | 66.49 | 8.20 |
| 2 | -11 | 11 | 104.89 | 9.60 |

| | | | | |
|---|---|---|---|---|
| 2 | -11 | 11 | 79.69 | 8.20 |
| 2 | 11 | 11 | 77.69 | 8.80 |
| 2 | 11 | 11 | 92.69 | 8.70 |
| -2 | 11 | -11 | 99.79 | 14.60 |
| 2 | 11 | 11 | 95.99 | 16.30 |
| 3 | -11 | 11 | 36.50 | 7.60 |
| -3 | -11 | -11 | 17.20 | 4.00 |
| 3 | -11 | 11 | 17.90 | 3.70 |
| 3 | 11 | 11 | 21.10 | 4.10 |
| 3 | 11 | 11 | 25.20 | 4.20 |
| 4 | -11 | 11 | 396.46 | 22.80 |
| -4 | -11 | -11 | 368.66 | 22.50 |
| 4 | -11 | 11 | 368.76 | 21.80 |
| 4 | 11 | 11 | 361.86 | 22.20 |
| 4 | 11 | 11 | 372.86 | 22.00 |
| 5 | -11 | 11 | 7.60 | 3.40 |
| -5 | -11 | -11 | 5.80 | 3.00 |
| 5 | -11 | 11 | 4.20 | 4.00 |
| 5 | 11 | 11 | 9.20 | 4.30 |
| 5 | 11 | 11 | 9.30 | 3.30 |
| -6 | -11 | -11 | 57.89 | 6.40 |
| 6 | -11 | 11 | 50.49 | 7.40 |
| 6 | 11 | 11 | 49.80 | 6.40 |
| 6 | 11 | 11 | 49.50 | 6.10 |
| -7 | -11 | -11 | 63.59 | 6.30 |
| 7 | 11 | 11 | 69.19 | 6.50 |
| 7 | 11 | 11 | 73.19 | 6.90 |
| -8 | -11 | -11 | 289.47 | 17.90 |
| 8 | 11 | 11 | 295.47 | 17.60 |
| 8 | 11 | 11 | 322.07 | 19.30 |
| 9 | 11 | 11 | 14.70 | 3.90 |
| 9 | 11 | 11 | 18.40 | 3.70 |
| -12 | 12 | 11 | 25.60 | 4.20 |
| 12 | 12 | -11 | 24.60 | 2.70 |
| 11 | 12 | -11 | 301.77 | 16.50 |
| -11 | 12 | 11 | 284.77 | 17.10 |
| -10 | 12 | 11 | 1.50 | 3.00 |
| -10 | 12 | 11 | -3.10 | 3.20 |
| 10 | 12 | -11 | -0.50 | 1.60 |
| 9 | 12 | -11 | 148.89 | 9.30 |
| -9 | 12 | 11 | 137.99 | 10.00 |
| -9 | 12 | 11 | 145.99 | 10.80 |
| -8 | -12 | 11 | 71.29 | 7.40 |
| 8 | 12 | -11 | 65.19 | 6.90 |

| | | | | |
|---|---|---|---|---|
| -8 | 12 | 11 | 70.19 | 9.00 |
| -8 | 12 | 11 | 73.29 | 6.80 |
| -7 | -12 | 11 | 213.98 | 14.00 |
| 7 | 12 | -11 | 235.88 | 13.70 |
| -7 | 12 | 11 | 196.28 | 13.60 |
| -7 | 12 | 11 | 220.68 | 16.70 |
| -6 | -12 | 11 | 37.60 | 5.80 |
| 6 | -12 | -11 | 17.40 | 6.20 |
| 6 | 12 | -11 | 28.50 | 4.20 |
| -6 | 12 | 11 | 30.50 | 6.20 |
| -6 | 12 | 11 | 34.10 | 5.80 |
| 5 | -12 | -11 | 509.55 | 28.20 |
| -5 | -12 | 11 | 499.15 | 28.00 |
| -5 | 12 | 11 | 492.15 | 28.10 |
| -5 | 12 | 11 | 431.66 | 29.40 |
| 4 | -12 | -11 | 4.30 | 3.60 |
| -4 | -12 | 11 | 7.50 | 3.30 |
| 4 | 12 | -11 | 8.00 | 7.40 |
| -4 | 12 | 11 | 3.50 | 3.40 |
| -4 | 12 | 11 | 6.70 | 5.40 |
| 3 | -12 | -11 | 50.99 | 7.80 |
| -3 | -12 | 11 | 58.69 | 6.80 |
| -3 | 12 | 11 | 52.79 | 7.20 |
| -3 | 12 | 11 | 36.60 | 6.30 |
| -3 | 12 | 11 | 29.20 | 11.10 |
| 3 | 12 | -11 | 63.69 | 14.30 |
| -2 | -12 | 11 | 133.89 | 9.80 |
| 2 | -12 | -11 | 127.49 | 10.70 |
| -2 | 12 | 11 | 117.29 | 10.90 |
| 2 | 12 | -11 | 114.59 | 16.90 |
| -2 | 12 | 11 | 114.79 | 17.90 |
| -2 | 12 | 11 | 93.19 | 10.10 |
| -1 | -12 | 11 | 991.80 | 51.79 |
| 1 | -12 | -11 | 945.51 | 52.39 |
| 1 | 12 | -11 | 804.72 | 55.29 |
| -1 | 12 | 11 | 904.51 | 51.79 |
| -1 | 12 | 11 | 1008.90 | 51.99 |
| -1 | 12 | 11 | 891.01 | 55.49 |
| 0 | -12 | 11 | 21.00 | 3.60 |
| 0 | -12 | 11 | 32.70 | 6.20 |
| 0 | -12 | -11 | 20.90 | 4.70 |
| 0 | 12 | 11 | 31.40 | 6.50 |
| 0 | 12 | -11 | 6.50 | 9.00 |
| 0 | 12 | 11 | 14.40 | 8.90 |

| | | | | |
|---|---|---|---|---|
| 0 | 12 | 11 | 29.80 | 5.00 |
| 1 | -12 | 11 | 8.80 | 3.70 |
| 1 | -12 | 11 | 6.00 | 3.00 |
| -1 | -12 | -11 | 5.60 | 3.80 |
| 1 | 12 | 11 | 0.50 | 3.80 |
| 1 | 12 | 11 | 0.40 | 3.20 |
| 2 | -12 | 11 | 151.88 | 10.40 |
| 2 | -12 | 11 | 159.88 | 11.40 |
| -2 | -12 | -11 | 142.29 | 11.60 |
| 2 | 12 | 11 | 153.68 | 11.00 |
| 2 | 12 | 11 | 135.89 | 10.80 |
| 3 | -12 | 11 | 337.67 | 19.90 |
| -3 | -12 | -11 | 303.77 | 19.50 |
| 3 | -12 | 11 | 308.97 | 18.80 |
| 3 | 12 | 11 | 313.27 | 19.40 |
| 3 | 12 | 11 | 310.17 | 19.20 |
| 4 | -12 | 11 | 45.10 | 8.70 |
| 4 | -12 | 11 | 55.29 | 6.20 |
| -4 | -12 | -11 | 39.60 | 6.70 |
| 4 | 12 | 11 | 47.50 | 5.90 |
| 4 | 12 | 11 | 37.60 | 6.70 |
| -5 | -12 | -11 | 384.86 | 22.30 |
| 5 | -12 | 11 | 373.46 | 22.30 |
| 5 | -12 | 11 | 371.16 | 21.80 |
| 5 | 12 | 11 | 392.46 | 22.40 |
| 5 | 12 | 11 | 376.06 | 22.00 |
| -6 | -12 | -11 | 136.09 | 9.50 |
| 6 | -12 | 11 | 119.09 | 10.60 |
| 6 | 12 | 11 | 149.19 | 9.90 |
| 6 | 12 | 11 | 141.49 | 9.90 |
| -7 | -12 | -11 | 34.60 | 5.00 |
| 7 | 12 | 11 | 17.70 | 4.60 |
| 7 | 12 | 11 | 24.50 | 4.20 |
| 8 | 12 | 11 | 5.30 | 3.50 |
| 11 | 13 | -11 | 0.30 | 1.30 |
| -10 | 13 | 11 | 200.28 | 16.20 |
| -10 | 13 | 11 | 259.17 | 15.20 |
| 10 | 13 | -11 | 269.37 | 14.10 |
| 9 | 13 | -11 | 14.20 | 2.30 |
| -9 | 13 | 11 | 15.40 | 4.20 |
| -9 | 13 | 11 | 21.10 | 3.40 |
| -8 | -13 | 11 | 3.00 | 2.80 |
| -8 | 13 | 11 | -3.50 | 2.80 |
| 8 | 13 | -11 | -0.90 | 1.90 |

| | | | | |
|---|---|---|---|---|
| -8 | 13 | 11 | 7.70 | 4.30 |
| -7 | -13 | 11 | 128.99 | 9.00 |
| -7 | 13 | 11 | 112.49 | 11.60 |
| -7 | 13 | 11 | 122.49 | 8.90 |
| 7 | 13 | -11 | 126.89 | 8.80 |
| -6 | -13 | 11 | 733.33 | 38.40 |
| -6 | 13 | 11 | 631.64 | 38.60 |
| -6 | 13 | 11 | 626.54 | 37.10 |
| -5 | -13 | 11 | 5.70 | 3.00 |
| -5 | 13 | 11 | 15.60 | 9.50 |
| -5 | 13 | 11 | 14.70 | 3.40 |
| -4 | -13 | 11 | 64.89 | 7.10 |
| 4 | -13 | -11 | 72.19 | 7.50 |
| -4 | 13 | 11 | 57.89 | 11.30 |
| -4 | 13 | 11 | 70.19 | 7.10 |
| -3 | -13 | 11 | 79.59 | 7.70 |
| 3 | -13 | -11 | 89.19 | 8.50 |
| -3 | 13 | 11 | 77.69 | 7.90 |
| -3 | 13 | 11 | 76.09 | 10.40 |
| 2 | -13 | -11 | 161.18 | 12.40 |
| -2 | -13 | 11 | 170.28 | 12.00 |
| -2 | 13 | 11 | 189.88 | 12.20 |
| -2 | 13 | 11 | 168.28 | 13.60 |
| 1 | -13 | -11 | 49.60 | 7.60 |
| -1 | -13 | 11 | 54.29 | 6.60 |
| -1 | 13 | 11 | 64.69 | 8.90 |
| -1 | 13 | 11 | 52.19 | 6.80 |
| 0 | -13 | 11 | 513.25 | 27.70 |
| 0 | -13 | -11 | 466.95 | 28.20 |
| 0 | 13 | 11 | 471.35 | 27.70 |
| 0 | 13 | 11 | 446.46 | 27.90 |
| 1 | -13 | 11 | 47.30 | 6.50 |
| -1 | -13 | -11 | 47.70 | 7.40 |
| 1 | -13 | 11 | 41.90 | 6.00 |
| 1 | 13 | 11 | 51.29 | 11.60 |
| 1 | 13 | 11 | 59.59 | 6.70 |
| 2 | -13 | 11 | 7.10 | 3.30 |
| 2 | -13 | 11 | 5.30 | 3.10 |
| -2 | -13 | -11 | 5.70 | 3.60 |
| 2 | 13 | 11 | -0.80 | 2.90 |
| 2 | 13 | 11 | 10.90 | 4.20 |
| 3 | -13 | 11 | 50.19 | 5.90 |
| -3 | -13 | -11 | 51.79 | 7.80 |
| 3 | -13 | 11 | 64.39 | 7.70 |

| | | | | |
|---|---|---|---|---|
| 3 | 13 | 11 | 54.69 | 6.40 |
| 3 | 13 | 11 | 47.60 | 8.90 |
| 4 | -13 | 11 | 509.05 | 29.50 |
| 4 | -13 | 11 | 491.65 | 28.80 |
| -4 | -13 | -11 | 515.95 | 29.50 |
| 4 | 13 | 11 | 549.35 | 29.50 |
| 5 | -13 | 11 | 37.80 | 6.40 |
| 5 | -13 | 11 | 45.10 | 5.60 |
| -5 | -13 | -11 | 42.10 | 5.50 |
| 5 | 13 | 11 | 52.19 | 5.80 |
| 6 | -13 | 11 | 39.20 | 5.50 |
| -6 | -13 | -11 | 36.50 | 4.90 |
| 6 | 13 | 11 | 38.50 | 6.20 |
| -7 | -13 | -11 | 87.69 | 7.30 |
| 7 | 13 | 11 | 99.09 | 7.60 |
| 10 | 14 | -11 | 18.10 | 2.60 |
| -9 | 14 | 11 | 114.69 | 8.30 |
| 9 | 14 | -11 | 117.19 | 7.30 |
| -8 | -14 | 11 | 85.19 | 7.10 |
| 8 | 14 | -11 | 88.09 | 6.20 |
| -8 | 14 | 11 | 93.89 | 6.90 |
| -7 | -14 | 11 | 241.38 | 14.30 |
| 7 | 14 | -11 | 234.78 | 14.10 |
| -7 | 14 | 11 | 238.88 | 14.30 |
| -6 | -14 | 11 | 7.00 | 2.70 |
| -6 | 14 | 11 | 13.20 | 3.00 |
| 6 | 14 | -11 | 13.20 | 2.70 |
| -5 | -14 | 11 | 434.56 | 24.10 |
| 5 | -14 | -11 | 443.06 | 24.10 |
| -5 | 14 | 11 | 376.46 | 23.90 |
| -4 | -14 | 11 | 63.69 | 7.20 |
| 4 | -14 | -11 | 86.79 | 7.10 |
| -4 | 14 | 11 | 68.49 | 7.10 |
| 3 | -14 | -11 | 9.20 | 3.50 |
| -3 | -14 | 11 | 7.00 | 4.60 |
| -2 | -14 | 11 | 106.69 | 9.00 |
| -2 | 14 | 11 | 111.29 | 8.90 |
| -1 | -14 | 11 | 1038.70 | 54.69 |
| -1 | 14 | 11 | 921.11 | 54.19 |
| 0 | 14 | 11 | 124.49 | 9.70 |
| -1 | -14 | -11 | 89.59 | 9.10 |
| 1 | -14 | 11 | 96.59 | 7.30 |
| 1 | -14 | 11 | 103.39 | 8.30 |
| 1 | 14 | 11 | 91.49 | 8.00 |

| | | | | |
|---|---|---|---|---|
| -2 | -14 | -11 | 50.99 | 6.70 |
| 2 | -14 | 11 | 49.80 | 6.20 |
| 2 | -14 | 11 | 47.50 | 5.20 |
| 2 | 14 | 11 | 45.70 | 6.00 |
| -3 | -14 | -11 | 221.48 | 14.50 |
| 3 | -14 | 11 | 228.78 | 13.50 |
| 3 | -14 | 11 | 234.48 | 14.30 |
| 3 | 14 | 11 | 221.78 | 14.20 |
| 4 | -14 | 11 | -0.60 | 2.80 |
| -4 | -14 | -11 | -1.40 | 2.20 |
| 4 | -14 | 11 | 5.10 | 2.30 |
| 4 | 14 | 11 | -0.10 | 2.70 |
| 5 | -14 | 11 | 412.56 | 23.60 |
| 5 | -14 | 11 | 386.36 | 23.90 |
| -5 | -14 | -11 | 419.26 | 24.00 |
| 5 | 14 | 11 | 477.85 | 25.00 |
| -6 | -14 | -11 | 127.89 | 8.30 |
| 6 | 14 | 11 | 122.59 | 8.80 |
| 9 | 15 | -11 | 24.50 | 2.70 |
| -8 | -15 | 11 | 1.40 | 2.30 |
| -8 | 15 | 11 | 2.30 | 2.60 |
| 8 | 15 | -11 | -0.80 | 1.40 |
| -7 | -15 | 11 | 147.99 | 9.50 |
| -7 | 15 | 11 | 146.19 | 9.60 |
| 7 | 15 | -11 | 159.68 | 9.40 |
| -6 | -15 | 11 | 674.93 | 38.00 |
| 6 | 15 | -11 | 703.13 | 38.00 |
| -5 | -15 | 11 | 71.79 | 6.10 |
| -5 | 15 | 11 | 66.09 | 6.30 |
| 5 | 15 | -11 | 73.79 | 6.10 |
| 4 | -15 | -11 | 189.88 | 11.50 |
| -4 | -15 | 11 | 175.08 | 11.50 |
| -4 | 15 | 11 | 174.38 | 11.60 |
| -3 | -15 | 11 | 65.29 | 6.20 |
| 3 | -15 | -11 | 68.79 | 6.50 |
| -3 | 15 | 11 | 72.19 | 6.50 |
| -2 | -15 | 11 | 186.78 | 12.50 |
| 2 | -15 | -11 | 202.88 | 13.10 |
| -2 | 15 | 11 | 212.18 | 12.90 |
| 1 | -15 | -11 | 0.10 | 2.70 |
| -1 | -15 | 11 | 3.80 | 2.50 |
| -1 | 15 | 11 | 2.10 | 3.00 |
| 0 | -15 | 11 | 598.94 | 32.60 |
| 0 | -15 | -11 | 561.94 | 32.90 |

| | | | | |
|---|---|---|---|---|
| 0 | 15 | 11 | 573.84 | 32.70 |
| 1 | -15 | 11 | 193.88 | 11.70 |
| -1 | -15 | -11 | 176.38 | 12.10 |
| 1 | 15 | 11 | 170.98 | 11.80 |
| -2 | -15 | -11 | 8.90 | 2.90 |
| 2 | -15 | 11 | 10.50 | 2.70 |
| 2 | 15 | 11 | 2.90 | 2.80 |
| 3 | -15 | 11 | 29.70 | 5.20 |
| -3 | -15 | -11 | 29.80 | 4.70 |
| 3 | 15 | 11 | 28.00 | 5.50 |
| -4 | -15 | -11 | 395.96 | 22.10 |
| 4 | -15 | 11 | 393.66 | 21.50 |
| 4 | -15 | 11 | 393.56 | 22.00 |
| 4 | 15 | 11 | 357.06 | 22.00 |
| -5 | -15 | -11 | 26.60 | 3.90 |
| -7 | -16 | 11 | 189.88 | 12.00 |
| 7 | 16 | -11 | 239.68 | 12.00 |
| -7 | 16 | 11 | 192.08 | 12.30 |
| -6 | -16 | 11 | 1.10 | 1.80 |
| 6 | 16 | -11 | 0.80 | 1.70 |
| -6 | 16 | 11 | 0.20 | 2.40 |
| -5 | -16 | 11 | 284.37 | 16.70 |
| -5 | 16 | 11 | 283.87 | 16.90 |
| 5 | 16 | -11 | 306.97 | 16.80 |
| -4 | -16 | 11 | 44.00 | 4.70 |
| -4 | 16 | 11 | 55.99 | 5.10 |
| 3 | -16 | -11 | 26.80 | 4.20 |
| -3 | -16 | 11 | 22.70 | 3.70 |
| -3 | 16 | 11 | 31.90 | 7.00 |
| 2 | -16 | -11 | 100.19 | 7.10 |
| -2 | -16 | 11 | 91.49 | 6.80 |
| -2 | 16 | 11 | 86.19 | 8.30 |
| 1 | -16 | -11 | 725.73 | 40.10 |
| -1 | 16 | 11 | 720.03 | 40.70 |
| 0 | -16 | -11 | 58.59 | 6.10 |
| 0 | -16 | 11 | 74.49 | 5.70 |
| 0 | 16 | 11 | 64.29 | 7.10 |
| 1 | -16 | 11 | 27.50 | 4.20 |
| 1 | 16 | 11 | 27.20 | 4.60 |
| -2 | -16 | -11 | 43.50 | 4.90 |
| 2 | -16 | 11 | 48.20 | 5.00 |
| 2 | 16 | 11 | 39.60 | 5.10 |
| -3 | -16 | -11 | 170.68 | 10.70 |
| 3 | -16 | 11 | 180.08 | 10.70 |

| | | | | |
|---|---|---|---|---|
| 3 | 16 | 11 | 157.58 | 10.80 |
| -4 | -17 | 11 | 28.60 | 3.50 |
| 4 | -17 | -11 | 31.80 | 4.50 |
| -4 | 17 | 11 | 33.60 | 4.20 |
| 4 | 17 | -11 | 36.90 | 3.90 |
| 3 | -17 | -11 | 30.40 | 4.00 |
| -3 | -17 | 11 | 27.50 | 3.30 |
| -3 | 17 | 11 | 26.20 | 4.20 |
| 2 | -17 | -11 | 174.48 | 10.70 |
| -2 | -17 | 11 | 175.68 | 10.60 |
| -2 | 17 | 11 | 179.28 | 11.00 |
| 1 | -17 | -11 | 1.00 | 1.80 |
| -1 | -17 | 11 | 2.00 | 1.60 |
| -1 | 17 | 11 | -0.90 | 2.50 |
| 0 | -17 | 11 | 268.97 | 15.40 |
| 0 | -17 | -11 | 265.27 | 15.60 |
| 0 | 17 | 11 | 265.77 | 15.70 |
| -15 | 0 | 12 | -0.60 | 1.80 |
| 15 | 0 | -12 | -2.00 | 2.70 |
| 14 | 0 | -12 | 59.89 | 6.00 |
| -14 | 0 | 12 | 56.09 | 5.50 |
| 13 | 0 | -12 | -5.40 | 3.30 |
| -13 | 0 | 12 | -0.40 | 2.80 |
| -12 | 0 | 12 | 807.32 | 45.10 |
| 12 | 0 | -12 | 805.52 | 44.70 |
| -11 | 0 | 12 | 0.60 | 3.10 |
| -10 | 0 | 12 | 333.37 | 20.50 |
| -9 | 0 | 12 | -1.00 | 3.60 |
| -8 | 0 | 12 | 41.00 | 8.00 |
| -7 | 0 | 12 | -0.60 | 4.70 |
| -6 | 0 | 12 | 2146.59 | 116.59 |
| -6 | 0 | 12 | 2280.07 | 116.99 |
| -6 | 0 | 12 | 1962.20 | 115.69 |
| -5 | 0 | 12 | -1.70 | 4.00 |
| -5 | 0 | 12 | -0.50 | 5.70 |
| -5 | 0 | 12 | 1.20 | 4.60 |
| -4 | 0 | 12 | 10.80 | 4.60 |
| -4 | 0 | 12 | 15.90 | 6.20 |
| -4 | 0 | 12 | 11.40 | 4.10 |
| -3 | 0 | 12 | -1.10 | 3.80 |
| -3 | 0 | 12 | 1.50 | 4.10 |
| -3 | 0 | 12 | -3.40 | 6.10 |
| -2 | 0 | 12 | 776.92 | 50.59 |
| -2 | 0 | 12 | 927.11 | 49.99 |

| | | | | |
|---|---|---|---|---|
| -2 | 0 | 12 | 961.60 | 51.19 |
| -1 | 0 | 12 | -2.20 | 4.10 |
| -1 | 0 | 12 | 0.00 | 4.40 |
| -1 | 0 | 12 | -6.90 | 5.00 |
| 0 | 0 | 12 | 1380.06 | 77.79 |
| 0 | 0 | 12 | 1386.76 | 76.79 |
| 1 | 0 | 12 | -3.90 | 4.30 |
| 1 | 0 | 12 | 5.60 | 4.60 |
| 1 | 0 | 12 | -2.70 | 5.20 |
| 2 | 0 | 12 | 650.23 | 38.50 |
| 2 | 0 | 12 | 624.74 | 37.20 |
| 2 | 0 | 12 | 672.33 | 39.60 |
| 3 | 0 | 12 | -7.80 | 5.00 |
| 3 | 0 | 12 | -1.50 | 6.00 |
| 3 | 0 | 12 | 2.10 | 4.10 |
| 4 | 0 | 12 | 821.52 | 44.70 |
| 4 | 0 | 12 | 680.13 | 51.29 |
| 4 | 0 | 12 | 856.61 | 45.50 |
| 5 | 0 | 12 | -7.00 | 4.20 |
| 5 | 0 | 12 | 2.50 | 3.50 |
| 5 | 0 | 12 | -2.90 | 7.70 |
| 6 | 0 | 12 | 11.10 | 3.80 |
| 6 | 0 | 12 | -4.80 | 3.70 |
| 6 | 0 | 12 | -3.60 | 9.30 |
| 7 | 0 | 12 | 2.60 | 3.10 |
| 7 | 0 | 12 | -0.50 | 3.90 |
| 8 | 0 | 12 | 210.48 | 12.90 |
| 8 | 0 | 12 | 164.98 | 16.90 |
| 9 | 0 | 12 | -2.90 | 6.00 |
| 9 | 0 | 12 | 1.10 | 3.60 |
| 10 | 0 | 12 | 255.87 | 15.60 |
| 11 | 0 | 12 | -2.60 | 2.20 |
| -15 | -1 | 12 | 253.57 | 14.40 |
| 15 | -1 | -12 | 215.68 | 15.80 |
| -15 | 1 | 12 | 255.07 | 14.40 |
| 15 | 1 | -12 | 247.48 | 14.20 |
| 14 | -1 | -12 | -1.90 | 3.10 |
| -14 | -1 | 12 | 0.50 | 2.20 |
| 14 | 1 | -12 | -1.30 | 2.50 |
| -14 | 1 | 12 | 0.10 | 2.00 |
| 13 | -1 | -12 | 73.49 | 7.70 |
| -13 | -1 | 12 | 71.89 | 7.10 |
| -13 | 1 | 12 | 61.19 | 6.80 |
| 13 | 1 | -12 | 76.59 | 7.00 |

| | | | | |
|---|---|---|---|---|
| 12 | -1 | -12 | 3.60 | 5.20 |
| -12 | -1 | 12 | -0.50 | 2.90 |
| 12 | 1 | -12 | -3.00 | 3.20 |
| -12 | 1 | 12 | 1.80 | 2.80 |
| -11 | -1 | 12 | 882.01 | 51.19 |
| 11 | 1 | -12 | 936.51 | 50.79 |
| -11 | 1 | 12 | 942.21 | 51.39 |
| -10 | -1 | 12 | 15.70 | 4.10 |
| -10 | 1 | 12 | 8.00 | 3.60 |
| -9 | -1 | 12 | 357.06 | 21.40 |
| -9 | 1 | 12 | 328.97 | 21.10 |
| -8 | -1 | 12 | 28.90 | 5.40 |
| -8 | 1 | 12 | 27.90 | 5.20 |
| -7 | -1 | 12 | 1982.20 | 98.09 |
| -7 | 1 | 12 | 1575.04 | 97.69 |
| -6 | -1 | 12 | 3.20 | 5.60 |
| -6 | -1 | 12 | -4.60 | 4.10 |
| -6 | -1 | 12 | -2.40 | 5.10 |
| -6 | 1 | 12 | 10.40 | 4.90 |
| -5 | -1 | 12 | 1068.89 | 61.39 |
| -5 | -1 | 12 | 1225.58 | 62.59 |
| -5 | -1 | 12 | 1012.70 | 62.79 |
| -5 | 1 | 12 | 1220.78 | 62.69 |
| -5 | 1 | 12 | 1082.09 | 61.59 |
| -5 | 1 | 12 | 1052.29 | 62.69 |
| -4 | -1 | 12 | 0.10 | 6.80 |
| -4 | -1 | 12 | 0.60 | 4.10 |
| -4 | -1 | 12 | -6.90 | 3.50 |
| -4 | 1 | 12 | 2.20 | 4.50 |
| -4 | 1 | 12 | 1.70 | 5.50 |
| -4 | 1 | 12 | 2.60 | 4.30 |
| -3 | -1 | 12 | 237.28 | 16.10 |
| -3 | -1 | 12 | 247.78 | 16.80 |
| -3 | -1 | 12 | 246.78 | 19.20 |
| -3 | 1 | 12 | 224.38 | 18.20 |
| -3 | 1 | 12 | 277.57 | 17.40 |
| -3 | 1 | 12 | 235.38 | 16.40 |
| -2 | -1 | 12 | 12.00 | 4.40 |
| -2 | -1 | 12 | 12.40 | 6.80 |
| -2 | -1 | 12 | 4.30 | 3.60 |
| -2 | 1 | 12 | 10.40 | 4.40 |
| -2 | 1 | 12 | 16.70 | 5.70 |
| -2 | 1 | 12 | 2.80 | 4.10 |
| -1 | -1 | 12 | 3716.73 | 202.38 |

| | | | | |
|---|---|---|---|---|
| -1 | -1 | 12 | 3711.53 | 203.68 |
| -1 | -1 | 12 | 3821.32 | 202.68 |
| -1 | 1 | 12 | 3724.93 | 203.88 |
| -1 | 1 | 12 | 3712.73 | 202.58 |
| -1 | 1 | 12 | 3772.22 | 202.68 |
| 0 | -1 | 12 | 33.10 | 7.90 |
| 0 | -1 | 12 | 34.40 | 5.90 |
| 0 | 1 | 12 | 24.90 | 5.20 |
| 0 | 1 | 12 | 26.90 | 5.90 |
| 0 | 1 | 12 | 24.80 | 5.60 |
| 1 | -1 | 12 | 30.30 | 5.90 |
| 1 | -1 | 12 | 38.60 | 6.30 |
| 1 | -1 | 12 | 34.30 | 8.60 |
| 1 | 1 | 12 | 49.20 | 10.10 |
| 1 | 1 | 12 | 20.60 | 4.70 |
| 1 | 1 | 12 | 26.20 | 6.40 |
| 2 | -1 | 12 | 34.70 | 6.20 |
| 2 | -1 | 12 | 30.20 | 5.70 |
| 2 | -1 | 12 | 14.40 | 5.30 |
| 2 | 1 | 12 | 21.10 | 6.00 |
| 2 | 1 | 12 | 27.20 | 6.00 |
| 2 | 1 | 12 | 23.80 | 4.70 |
| 3 | -1 | 12 | 845.02 | 46.20 |
| 3 | -1 | 12 | 869.31 | 44.30 |
| 3 | -1 | 12 | 757.82 | 46.50 |
| 3 | 1 | 12 | 771.62 | 47.00 |
| 3 | 1 | 12 | 680.63 | 45.30 |
| 3 | 1 | 12 | 746.53 | 47.30 |
| 4 | -1 | 12 | 6.60 | 4.20 |
| 4 | -1 | 12 | 6.70 | 4.70 |
| 4 | -1 | 12 | 5.80 | 12.20 |
| 4 | 1 | 12 | 1.30 | 4.50 |
| 4 | 1 | 12 | 10.90 | 4.20 |
| 4 | 1 | 12 | -7.40 | 10.10 |
| 5 | -1 | 12 | 865.01 | 44.50 |
| 5 | -1 | 12 | 722.23 | 47.40 |
| 5 | -1 | 12 | 836.12 | 44.80 |
| 5 | 1 | 12 | 772.12 | 44.30 |
| 5 | 1 | 12 | 710.13 | 47.80 |
| 5 | 1 | 12 | 815.52 | 44.70 |
| 6 | -1 | 12 | 65.09 | 16.20 |
| 6 | -1 | 12 | 38.10 | 6.50 |
| 6 | -1 | 12 | 47.90 | 7.60 |
| 6 | 1 | 12 | 46.70 | 6.40 |

| | | | | |
|---|---|---|---|---|
| 6 | 1 | 12 | 24.00 | 10.40 |
| 6 | 1 | 12 | 38.80 | 7.60 |
| 6 | 1 | 12 | 9.20 | 11.20 |
| 7 | -1 | 12 | 101.19 | 8.40 |
| 7 | -1 | 12 | 87.69 | 9.50 |
| 7 | 1 | 12 | 100.29 | 12.90 |
| 7 | 1 | 12 | 78.59 | 9.10 |
| 7 | 1 | 12 | 100.89 | 8.30 |
| 8 | -1 | 12 | 4.50 | 3.10 |
| 8 | 1 | 12 | 4.80 | 6.00 |
| 8 | 1 | 12 | 7.00 | 3.20 |
| 9 | -1 | 12 | 274.07 | 16.50 |
| 9 | 1 | 12 | 252.77 | 17.10 |
| 9 | 1 | 12 | 271.97 | 18.40 |
| 10 | -1 | 12 | 7.90 | 2.90 |
| 10 | 1 | 12 | 9.50 | 6.00 |
| 10 | 1 | 12 | 8.90 | 2.70 |
| 11 | -1 | 12 | 165.08 | 10.80 |
| 11 | 1 | 12 | 172.38 | 10.70 |
| -15 | -2 | 12 | 0.10 | 2.30 |
| 15 | 2 | -12 | -0.50 | 2.10 |
| -15 | 2 | 12 | 0.90 | 1.70 |
| -14 | -2 | 12 | 376.76 | 21.90 |
| -14 | 2 | 12 | 383.36 | 22.00 |
| 14 | 2 | -12 | 389.06 | 21.70 |
| -13 | -2 | 12 | 1.50 | 2.80 |
| 13 | 2 | -12 | 2.20 | 2.80 |
| -13 | 2 | 12 | 3.00 | 2.50 |
| -12 | -2 | 12 | 626.44 | 35.00 |
| 12 | 2 | -12 | 620.54 | 34.50 |
| -12 | 2 | 12 | 606.24 | 35.00 |
| -11 | -2 | 12 | 14.30 | 3.90 |
| -11 | 2 | 12 | 10.10 | 3.20 |
| 11 | 2 | -12 | 14.10 | 3.60 |
| -10 | -2 | 12 | 813.72 | 43.30 |
| 10 | 2 | -12 | 728.53 | 42.50 |
| -10 | 2 | 12 | 752.22 | 43.20 |
| -9 | -2 | 12 | 13.30 | 4.60 |
| -9 | 2 | 12 | 8.60 | 3.60 |
| -8 | -2 | 12 | 29.70 | 6.10 |
| -8 | 2 | 12 | 32.00 | 7.30 |
| -7 | -2 | 12 | 27.60 | 6.50 |
| -6 | -2 | 12 | 3858.11 | 189.28 |
| -6 | -2 | 12 | 3133.99 | 188.98 |

| | | | | |
|---|---|---|---|---|
| -6 | -2 | 12 | 3364.86 | 188.08 |
| -6 | 2 | 12 | 3740.33 | 189.28 |
| -6 | 2 | 12 | 3463.35 | 189.18 |
| -6 | 2 | 12 | 3335.57 | 188.18 |
| -5 | -2 | 12 | -5.10 | 6.90 |
| -5 | -2 | 12 | -5.50 | 3.80 |
| -5 | -2 | 12 | 3.30 | 4.30 |
| -5 | 2 | 12 | -7.80 | 4.80 |
| -5 | 2 | 12 | 5.30 | 5.40 |
| -5 | 2 | 12 | -2.00 | 4.50 |
| -4 | -2 | 12 | 213.68 | 20.00 |
| -4 | -2 | 12 | 279.67 | 17.20 |
| -4 | -2 | 12 | 259.97 | 16.60 |
| -4 | 2 | 12 | 258.97 | 17.00 |
| -4 | 2 | 12 | 263.37 | 17.70 |
| -4 | 2 | 12 | 222.98 | 18.30 |
| -3 | -2 | 12 | 36.60 | 8.10 |
| -3 | -2 | 12 | 46.00 | 7.60 |
| -3 | -2 | 12 | 55.19 | 7.60 |
| -3 | 2 | 12 | 47.70 | 8.20 |
| -3 | 2 | 12 | 55.69 | 8.30 |
| -2 | -2 | 12 | 1670.83 | 97.89 |
| -2 | -2 | 12 | 1776.02 | 98.29 |
| -2 | -2 | 12 | 1676.33 | 96.99 |
| -2 | 2 | 12 | 1782.92 | 98.79 |
| -2 | 2 | 12 | 1746.73 | 97.29 |
| -2 | 2 | 12 | 2015.90 | 98.29 |
| -1 | -2 | 12 | 5.80 | 4.00 |
| -1 | -2 | 12 | 7.80 | 6.40 |
| -1 | -2 | 12 | -0.10 | 4.60 |
| -1 | 2 | 12 | 5.30 | 4.80 |
| -1 | 2 | 12 | 3.10 | 4.80 |
| -1 | 2 | 12 | 0.90 | 5.00 |
| 0 | -2 | 12 | 2562.34 | 130.59 |
| 0 | -2 | 12 | 2463.55 | 131.99 |
| 0 | 2 | 12 | 2214.48 | 131.99 |
| 0 | 2 | 12 | 2176.68 | 130.29 |
| 0 | 2 | 12 | 2564.14 | 131.19 |
| 1 | -2 | 12 | 85.09 | 11.60 |
| 1 | -2 | 12 | 90.29 | 11.00 |
| 1 | -2 | 12 | 103.89 | 10.50 |
| 1 | 2 | 12 | 96.59 | 11.70 |
| 1 | 2 | 12 | 94.89 | 11.60 |
| 1 | 2 | 12 | 99.09 | 9.80 |

| | | | | |
|---|---|---|---|---|
| 2 | -2 | 12 | 160.18 | 13.40 |
| 2 | -2 | 12 | 132.89 | 13.80 |
| 2 | -2 | 12 | 154.88 | 12.60 |
| 2 | 2 | 12 | 126.19 | 11.40 |
| 2 | 2 | 12 | 158.48 | 14.60 |
| 2 | 2 | 12 | 131.69 | 13.50 |
| 3 | -2 | 12 | -3.90 | 5.50 |
| 3 | -2 | 12 | 1.90 | 4.50 |
| 3 | 2 | 12 | -1.40 | 5.70 |
| 3 | 2 | 12 | -3.30 | 4.10 |
| 4 | -2 | 12 | 2253.27 | 117.59 |
| 4 | -2 | 12 | 2270.87 | 116.99 |
| 4 | 2 | 12 | 1939.51 | 116.79 |
| 4 | 2 | 12 | 2311.37 | 117.59 |
| 4 | 2 | 12 | 1957.40 | 121.39 |
| 5 | -2 | 12 | 8.30 | 4.40 |
| 5 | -2 | 12 | 15.20 | 4.30 |
| 5 | -2 | 12 | 9.30 | 8.00 |
| 5 | 2 | 12 | 19.10 | 8.10 |
| 5 | 2 | 12 | 2.50 | 8.80 |
| 5 | 2 | 12 | 7.50 | 4.30 |
| 5 | 2 | 12 | 19.30 | 4.20 |
| 6 | -2 | 12 | 136.99 | 10.70 |
| 6 | -2 | 12 | 107.19 | 17.90 |
| 6 | -2 | 12 | 140.69 | 11.40 |
| 6 | 2 | 12 | 143.99 | 11.20 |
| 6 | 2 | 12 | 141.59 | 10.30 |
| 6 | 2 | 12 | 147.89 | 19.70 |
| 6 | 2 | 12 | 151.68 | 14.70 |
| 7 | -2 | 12 | 3.00 | 4.10 |
| 7 | -2 | 12 | -2.00 | 3.30 |
| 7 | 2 | 12 | 4.30 | 3.50 |
| 7 | 2 | 12 | 1.40 | 3.00 |
| 7 | 2 | 12 | 3.60 | 6.20 |
| 8 | -2 | 12 | 623.84 | 35.00 |
| 8 | 2 | 12 | 610.44 | 36.30 |
| 8 | 2 | 12 | 617.74 | 34.90 |
| 9 | -2 | 12 | 4.70 | 3.40 |
| 9 | 2 | 12 | 7.60 | 5.50 |
| 9 | 2 | 12 | 3.10 | 3.10 |
| 10 | -2 | 12 | 457.35 | 26.00 |
| 10 | 2 | 12 | 470.15 | 28.50 |
| 10 | 2 | 12 | 436.86 | 25.80 |
| 11 | -2 | 12 | 4.20 | 2.80 |

| | | | | |
|---|---|---|---|---|
| 11 | 2 | 12 | 2.80 | 5.30 |
| 11 | 2 | 12 | 2.80 | 2.20 |
| 15 | 3 | -12 | 237.88 | 13.10 |
| -15 | 3 | 12 | 212.08 | 13.20 |
| -14 | -3 | 12 | 0.30 | 2.90 |
| 14 | 3 | -12 | 4.60 | 2.30 |
| -14 | 3 | 12 | 0.80 | 1.90 |
| -13 | -3 | 12 | 9.40 | 3.30 |
| -13 | 3 | 12 | 6.30 | 2.60 |
| 13 | 3 | -12 | 8.60 | 2.80 |
| -12 | -3 | 12 | 28.80 | 4.80 |
| 12 | 3 | -12 | 37.70 | 5.50 |
| -12 | 3 | 12 | 29.50 | 5.20 |
| -11 | -3 | 12 | 987.30 | 53.19 |
| 11 | 3 | -12 | 985.10 | 52.49 |
| -11 | 3 | 12 | 892.81 | 53.09 |
| -10 | -3 | 12 | 21.80 | 5.00 |
| 10 | 3 | -12 | 28.60 | 4.20 |
| -10 | 3 | 12 | 31.40 | 5.90 |
| -9 | -3 | 12 | 236.48 | 16.40 |
| -9 | 3 | 12 | 233.78 | 15.60 |
| 9 | 3 | -12 | 223.68 | 15.10 |
| -8 | -3 | 12 | 24.30 | 6.20 |
| -8 | 3 | 12 | 14.40 | 4.50 |
| -7 | -3 | 12 | 1074.39 | 60.39 |
| -7 | 3 | 12 | 1062.89 | 60.09 |
| -6 | -3 | 12 | -1.10 | 4.50 |
| -6 | -3 | 12 | 8.60 | 7.40 |
| -6 | 3 | 12 | -1.40 | 7.70 |
| -6 | 3 | 12 | -5.20 | 4.80 |
| -6 | 3 | 12 | -7.20 | 5.10 |
| -5 | -3 | 12 | 1509.95 | 80.59 |
| -5 | -3 | 12 | 1360.46 | 79.49 |
| -5 | 3 | 12 | 1347.47 | 79.59 |
| -5 | 3 | 12 | 1530.35 | 81.09 |
| -5 | 3 | 12 | 1512.05 | 80.99 |
| -4 | -3 | 12 | -7.10 | 8.20 |
| -4 | -3 | 12 | -5.00 | 3.40 |
| -4 | -3 | 12 | -2.60 | 3.60 |
| -4 | 3 | 12 | 2.80 | 4.50 |
| -4 | 3 | 12 | -5.30 | 5.30 |
| -4 | 3 | 12 | 2.60 | 4.20 |
| -3 | -3 | 12 | 257.77 | 21.90 |
| -3 | -3 | 12 | 286.47 | 18.70 |

| | | | | |
|---|---|---|---|---|
| -3 | -3 | 12 | 273.87 | 17.80 |
| -3 | 3 | 12 | 242.48 | 19.40 |
| -3 | 3 | 12 | 291.87 | 18.50 |
| -3 | 3 | 12 | 326.87 | 20.10 |
| -2 | -3 | 12 | 22.90 | 4.50 |
| -2 | -3 | 12 | 33.60 | 6.10 |
| -2 | -3 | 12 | 34.40 | 8.60 |
| -2 | 3 | 12 | 37.00 | 8.90 |
| -2 | 3 | 12 | 48.10 | 9.10 |
| -2 | 3 | 12 | 37.70 | 7.90 |
| -1 | -3 | 12 | 4201.78 | 208.88 |
| -1 | -3 | 12 | 3843.22 | 209.78 |
| -1 | -3 | 12 | 3677.53 | 208.28 |
| -1 | 3 | 12 | 3707.23 | 210.28 |
| -1 | 3 | 12 | 3950.10 | 208.88 |
| -1 | 3 | 12 | 3757.22 | 208.78 |
| 0 | -3 | 12 | 12.10 | 4.60 |
| 0 | -3 | 12 | 24.10 | 7.00 |
| 0 | 3 | 12 | 13.10 | 5.60 |
| 0 | 3 | 12 | 5.00 | 5.10 |
| 0 | 3 | 12 | 10.70 | 4.70 |
| 1 | -3 | 12 | 69.29 | 9.70 |
| 1 | -3 | 12 | 55.29 | 11.80 |
| 1 | -3 | 12 | 69.19 | 10.10 |
| 1 | 3 | 12 | 52.19 | 10.50 |
| 1 | 3 | 12 | 51.19 | 10.60 |
| 1 | 3 | 12 | 65.19 | 8.50 |
| 2 | -3 | 12 | 7.60 | 5.80 |
| 2 | -3 | 12 | 13.30 | 5.30 |
| 2 | -3 | 12 | 13.20 | 6.00 |
| 2 | 3 | 12 | 9.10 | 6.50 |
| 2 | 3 | 12 | 8.90 | 4.00 |
| 2 | 3 | 12 | 5.60 | 5.40 |
| 3 | -3 | 12 | 1211.18 | 67.59 |
| 3 | -3 | 12 | 1196.38 | 68.19 |
| 3 | 3 | 12 | 1225.18 | 70.69 |
| 3 | 3 | 12 | 1219.28 | 67.59 |
| 3 | 3 | 12 | 1248.58 | 68.39 |
| 4 | -3 | 12 | 35.70 | 6.10 |
| 4 | -3 | 12 | 40.80 | 8.50 |
| 4 | 3 | 12 | 52.99 | 11.20 |
| 4 | 3 | 12 | 44.90 | 10.50 |
| 4 | 3 | 12 | 57.39 | 7.20 |
| 4 | 3 | 12 | 60.19 | 9.00 |

| | | | | |
|---|---|---|---|---|
| 5 | -3 | 12 | 1291.37 | 68.79 |
| 5 | -3 | 12 | 1079.09 | 68.09 |
| 5 | -3 | 12 | 1255.77 | 71.19 |
| 5 | 3 | 12 | 1255.77 | 68.69 |
| 5 | 3 | 12 | 1211.78 | 72.29 |
| 5 | 3 | 12 | 1228.28 | 69.39 |
| 5 | 3 | 12 | 1356.96 | 68.59 |
| 6 | -3 | 12 | 0.60 | 4.00 |
| 6 | -3 | 12 | 0.80 | 3.80 |
| 6 | 3 | 12 | 5.10 | 6.70 |
| 6 | 3 | 12 | 0.40 | 3.00 |
| 6 | 3 | 12 | 2.30 | 3.20 |
| 6 | 3 | 12 | 4.50 | 11.40 |
| 7 | -3 | 12 | 345.17 | 20.80 |
| 7 | -3 | 12 | 342.57 | 20.80 |
| 7 | 3 | 12 | 357.06 | 23.00 |
| 7 | 3 | 12 | 326.57 | 20.40 |
| 8 | -3 | 12 | 1.70 | 3.90 |
| 8 | 3 | 12 | 0.10 | 5.60 |
| 8 | 3 | 12 | 2.90 | 2.80 |
| 9 | -3 | 12 | 596.04 | 34.90 |
| 9 | 3 | 12 | 649.93 | 34.80 |
| 9 | 3 | 12 | 605.14 | 35.90 |
| 10 | -3 | 12 | 6.40 | 3.40 |
| 10 | 3 | 12 | 5.20 | 6.10 |
| 11 | -3 | 12 | 29.00 | 5.70 |
| 11 | 3 | 12 | 44.40 | 7.90 |
| 11 | 3 | 12 | 41.70 | 4.50 |
| 15 | 4 | -12 | 4.60 | 2.00 |
| -15 | 4 | 12 | 3.70 | 1.70 |
| -14 | 4 | 12 | 139.59 | 9.30 |
| 14 | 4 | -12 | 147.69 | 9.10 |
| 13 | 4 | -12 | 8.60 | 2.50 |
| -13 | 4 | 12 | 13.50 | 2.80 |
| -12 | -4 | 12 | 585.34 | 34.50 |
| -12 | 4 | 12 | 606.54 | 33.90 |
| -11 | -4 | 12 | 14.40 | 4.60 |
| -11 | 4 | 12 | 15.40 | 3.30 |
| 11 | 4 | -12 | 23.20 | 3.50 |
| -10 | -4 | 12 | 1001.50 | 56.29 |
| 10 | 4 | -12 | 994.00 | 54.79 |
| -10 | 4 | 12 | 997.80 | 55.59 |
| -9 | 4 | 12 | 1.90 | 3.60 |
| 9 | 4 | -12 | -7.60 | 3.40 |

| | | | | |
|---|---|---|---|---|
| -8 | 4 | 12 | 6.30 | 4.10 |
| 8 | 4 | -12 | 0.60 | 4.20 |
| -7 | -4 | 12 | 40.80 | 5.90 |
| -7 | 4 | 12 | 53.79 | 8.40 |
| -6 | -4 | 12 | 2936.41 | 157.68 |
| -6 | 4 | 12 | 3159.38 | 159.18 |
| -6 | 4 | 12 | 2636.84 | 161.48 |
| -5 | -4 | 12 | 9.30 | 3.90 |
| -5 | 4 | 12 | 20.90 | 5.10 |
| -5 | 4 | 12 | 8.80 | 5.50 |
| -5 | 4 | 12 | 13.60 | 5.60 |
| -4 | -4 | 12 | 158.18 | 13.00 |
| -4 | -4 | 12 | 167.28 | 12.40 |
| -4 | 4 | 12 | 181.08 | 14.70 |
| -4 | 4 | 12 | 165.68 | 14.70 |
| -4 | 4 | 12 | 183.68 | 13.40 |
| -3 | -4 | 12 | 34.60 | 7.10 |
| -3 | -4 | 12 | 36.80 | 7.20 |
| -3 | 4 | 12 | 29.30 | 5.20 |
| -3 | 4 | 12 | 40.00 | 6.70 |
| -3 | 4 | 12 | 43.80 | 9.30 |
| -2 | -4 | 12 | 817.22 | 47.00 |
| -2 | -4 | 12 | 789.22 | 45.70 |
| -2 | 4 | 12 | 818.12 | 46.30 |
| -2 | 4 | 12 | 855.91 | 48.10 |
| -2 | 4 | 12 | 795.22 | 46.70 |
| -1 | -4 | 12 | 14.20 | 4.40 |
| -1 | -4 | 12 | 9.80 | 4.60 |
| -1 | 4 | 12 | 12.80 | 5.10 |
| -1 | 4 | 12 | 15.50 | 5.80 |
| -1 | 4 | 12 | 15.30 | 4.90 |
| 0 | -4 | 12 | 2245.88 | 120.79 |
| 0 | -4 | 12 | 2285.17 | 122.49 |
| 0 | 4 | 12 | 2227.98 | 121.49 |
| 0 | 4 | 12 | 2339.07 | 121.09 |
| 0 | 4 | 12 | 1981.20 | 122.69 |
| 1 | -4 | 12 | 10.40 | 5.80 |
| 1 | -4 | 12 | 6.70 | 4.20 |
| 1 | 4 | 12 | 8.70 | 5.50 |
| 1 | 4 | 12 | 12.60 | 6.20 |
| 1 | 4 | 12 | 4.50 | 3.80 |
| 2 | -4 | 12 | 30.40 | 7.40 |
| 2 | -4 | 12 | 28.50 | 7.00 |
| 2 | 4 | 12 | 21.60 | 8.20 |

| | | | | |
|---|---|---|---|---|
| 2 | 4 | 12 | 54.19 | 11.60 |
| 2 | 4 | 12 | 38.60 | 8.10 |
| 3 | -4 | 12 | 4.90 | 4.80 |
| 3 | -4 | 12 | 0.70 | 6.00 |
| 3 | 4 | 12 | -3.20 | 4.70 |
| 3 | 4 | 12 | -3.70 | 3.60 |
| 3 | 4 | 12 | 1.30 | 5.90 |
| 3 | 4 | 12 | 0.00 | 11.00 |
| 4 | -4 | 12 | 1591.74 | 87.79 |
| 4 | -4 | 12 | 1503.65 | 87.19 |
| 4 | -4 | 12 | 1469.45 | 89.29 |
| 4 | 4 | 12 | 1598.94 | 87.69 |
| 4 | 4 | 12 | 1627.64 | 87.29 |
| 4 | 4 | 12 | 1727.13 | 87.89 |
| 4 | 4 | 12 | 1622.84 | 91.19 |
| 5 | -4 | 12 | 9.10 | 4.60 |
| 5 | -4 | 12 | 11.30 | 4.80 |
| 5 | 4 | 12 | -1.10 | 3.70 |
| 5 | 4 | 12 | 8.90 | 6.20 |
| 5 | 4 | 12 | 2.90 | 3.30 |
| 5 | 4 | 12 | -2.50 | 10.60 |
| 6 | -4 | 12 | 75.59 | 9.20 |
| 6 | -4 | 12 | 70.79 | 9.10 |
| 6 | 4 | 12 | 85.59 | 19.60 |
| 6 | 4 | 12 | 78.79 | 7.60 |
| 7 | -4 | 12 | -3.80 | 4.20 |
| 7 | -4 | 12 | -7.10 | 4.00 |
| 7 | 4 | 12 | 0.50 | 6.00 |
| 7 | 4 | 12 | 0.20 | 2.90 |
| 8 | -4 | 12 | 493.55 | 31.40 |
| 8 | 4 | 12 | 515.95 | 28.80 |
| 8 | 4 | 12 | 497.25 | 30.30 |
| 9 | -4 | 12 | 5.20 | 4.00 |
| 9 | 4 | 12 | 7.30 | 2.70 |
| 9 | 4 | 12 | -1.50 | 4.90 |
| 10 | -4 | 12 | 333.47 | 20.10 |
| 10 | 4 | 12 | 341.97 | 20.80 |
| 10 | 4 | 12 | 330.67 | 19.40 |
| 11 | -4 | 12 | 19.50 | 4.40 |
| 11 | 4 | 12 | 8.40 | 4.30 |
| 11 | 4 | 12 | 11.70 | 2.40 |
| 15 | 5 | -12 | 401.06 | 23.90 |
| -15 | 5 | 12 | 448.66 | 23.80 |
| -14 | 5 | 12 | 21.10 | 3.40 |

| | | | | |
|---|---|---|---|---|
| 14 | 5 | -12 | 19.60 | 3.70 |
| -13 | 5 | 12 | 10.50 | 2.60 |
| 13 | 5 | -12 | 4.90 | 2.20 |
| -12 | 5 | 12 | -0.70 | 2.40 |
| 12 | 5 | -12 | 0.30 | 2.70 |
| 11 | 5 | -12 | 1047.90 | 52.69 |
| -11 | 5 | 12 | 871.51 | 53.19 |
| 10 | 5 | -12 | 12.30 | 3.20 |
| -10 | 5 | 12 | 7.80 | 3.30 |
| 9 | 5 | -12 | 127.69 | 9.60 |
| -9 | 5 | 12 | 115.69 | 10.00 |
| -8 | 5 | 12 | 3.40 | 3.90 |
| 8 | 5 | -12 | 5.60 | 3.80 |
| -7 | -5 | 12 | 784.82 | 46.50 |
| -7 | 5 | 12 | 919.71 | 47.90 |
| 7 | 5 | -12 | 795.32 | 46.90 |
| -6 | -5 | 12 | -0.30 | 5.20 |
| -6 | 5 | 12 | 10.70 | 6.10 |
| -6 | 5 | 12 | 7.50 | 4.80 |
| -5 | -5 | 12 | 1215.58 | 69.69 |
| -5 | 5 | 12 | 1371.66 | 71.89 |
| -5 | 5 | 12 | 1219.48 | 69.99 |
| -4 | -5 | 12 | 25.40 | 4.30 |
| -4 | 5 | 12 | 30.70 | 5.70 |
| -4 | 5 | 12 | 27.00 | 6.20 |
| -4 | 5 | 12 | 29.40 | 6.50 |
| -3 | -5 | 12 | 11.20 | 3.80 |
| -3 | 5 | 12 | 19.50 | 4.80 |
| -3 | 5 | 12 | 7.90 | 5.70 |
| -3 | 5 | 12 | 0.10 | 4.90 |
| -2 | -5 | 12 | 0.60 | 3.40 |
| -2 | -5 | 12 | 1.80 | 4.20 |
| -2 | 5 | 12 | 6.70 | 5.70 |
| -2 | 5 | 12 | -0.30 | 4.80 |
| -2 | 5 | 12 | 3.20 | 4.50 |
| -1 | -5 | 12 | 2061.89 | 110.69 |
| -1 | -5 | 12 | 1937.41 | 108.89 |
| -1 | 5 | 12 | 1906.21 | 111.29 |
| -1 | 5 | 12 | 1964.90 | 109.59 |
| -1 | 5 | 12 | 2120.09 | 109.69 |
| 0 | -5 | 12 | 2.00 | 4.00 |
| 0 | -5 | 12 | 4.50 | 5.00 |
| 0 | 5 | 12 | 12.00 | 5.30 |
| 0 | 5 | 12 | -1.10 | 3.90 |

| | | | | |
|---|---|---|---|---|
| 0 | 5 | 12 | -1.80 | 5.70 |
| 1 | -5 | 12 | 6.30 | 5.50 |
| 1 | 5 | 12 | -6.80 | 5.50 |
| 2 | -5 | 12 | 22.70 | 5.00 |
| 2 | -5 | 12 | 10.60 | 6.50 |
| 2 | 5 | 12 | 32.50 | 6.70 |
| 2 | 5 | 12 | 18.10 | 5.40 |
| 2 | 5 | 12 | 20.00 | 5.80 |
| 2 | 5 | 12 | 24.80 | 11.40 |
| 3 | -5 | 12 | 478.85 | 28.40 |
| 3 | 5 | 12 | 479.95 | 28.40 |
| 3 | 5 | 12 | 447.16 | 27.30 |
| 3 | 5 | 12 | 458.45 | 31.80 |
| 3 | 5 | 12 | 453.25 | 27.90 |
| 4 | -5 | 12 | 62.29 | 10.00 |
| 4 | 5 | 12 | 69.79 | 17.00 |
| 4 | 5 | 12 | 89.29 | 8.20 |
| 4 | 5 | 12 | 97.19 | 9.60 |
| 4 | 5 | 12 | 89.69 | 10.50 |
| 5 | -5 | 12 | 957.30 | 51.39 |
| 5 | -5 | 12 | 781.82 | 53.09 |
| 5 | 5 | 12 | 1014.70 | 51.89 |
| 5 | 5 | 12 | 973.40 | 50.99 |
| 5 | 5 | 12 | 812.52 | 56.39 |
| 6 | -5 | 12 | 18.90 | 4.50 |
| 6 | -5 | 12 | 24.90 | 5.80 |
| 6 | 5 | 12 | 15.00 | 3.50 |
| 6 | 5 | 12 | 32.20 | 6.60 |
| 6 | 5 | 12 | 17.20 | 12.60 |
| 7 | -5 | 12 | 98.69 | 11.30 |
| 7 | 5 | 12 | 103.99 | 8.10 |
| 7 | 5 | 12 | 90.99 | 11.20 |
| 8 | -5 | 12 | 12.40 | 5.40 |
| 8 | 5 | 12 | 5.30 | 3.20 |
| 8 | 5 | 12 | 9.30 | 5.40 |
| 9 | -5 | 12 | 344.07 | 21.30 |
| 9 | 5 | 12 | 357.56 | 21.70 |
| 9 | 5 | 12 | 332.37 | 20.00 |
| 10 | -5 | 12 | 15.90 | 8.80 |
| 10 | 5 | 12 | 7.10 | 2.40 |
| 10 | 5 | 12 | 9.40 | 4.90 |
| 11 | 5 | 12 | 79.39 | 8.40 |
| 11 | 5 | 12 | 68.29 | 5.50 |
| 14 | 6 | -12 | 236.08 | 13.60 |

| | | | | |
|---|---|---|---|---|
| -13 | 6 | 12 | 3.20 | 2.00 |
| 13 | 6 | -12 | 2.60 | 2.00 |
| 12 | 6 | -12 | 511.35 | 27.90 |
| -12 | 6 | 12 | 487.05 | 28.70 |
| -11 | 6 | 12 | 6.60 | 3.00 |
| 11 | 6 | -12 | 5.70 | 2.60 |
| 10 | 6 | -12 | 620.84 | 34.30 |
| -10 | 6 | 12 | 608.24 | 35.10 |
| 9 | 6 | -12 | 35.00 | 6.20 |
| -9 | 6 | 12 | 49.80 | 6.80 |
| -8 | 6 | 12 | 8.80 | 3.90 |
| 8 | 6 | -12 | 1.50 | 3.40 |
| -7 | -6 | 12 | 12.80 | 4.10 |
| 7 | 6 | -12 | 9.80 | 6.10 |
| 7 | 6 | -12 | 1.30 | 3.70 |
| -7 | 6 | 12 | 8.00 | 4.70 |
| -6 | -6 | 12 | 1912.01 | 112.69 |
| 6 | 6 | -12 | 2082.99 | 114.79 |
| -6 | 6 | 12 | 1971.60 | 116.99 |
| -6 | 6 | 12 | 2336.17 | 114.49 |
| -5 | -6 | 12 | 18.10 | 4.30 |
| -5 | 6 | 12 | 28.60 | 6.30 |
| -5 | 6 | 12 | 34.10 | 7.50 |
| -4 | 6 | 12 | 104.09 | 10.30 |
| -4 | 6 | 12 | 98.29 | 12.40 |
| -4 | 6 | 12 | 87.09 | 11.20 |
| -3 | -6 | 12 | 4.20 | 3.70 |
| -3 | 6 | 12 | 3.30 | 4.10 |
| -3 | 6 | 12 | -2.50 | 5.80 |
| -2 | -6 | 12 | 886.91 | 53.29 |
| -2 | 6 | 12 | 1010.10 | 55.39 |
| -2 | 6 | 12 | 1000.60 | 53.19 |
| -2 | 6 | 12 | 849.22 | 55.49 |
| -1 | 6 | 12 | 3.90 | 6.20 |
| 0 | -6 | 12 | 1245.58 | 66.99 |
| 0 | 6 | 12 | 1173.08 | 67.09 |
| 0 | 6 | 12 | 1237.48 | 67.89 |
| 0 | 6 | 12 | 1145.19 | 69.49 |
| 0 | 6 | 12 | 1233.98 | 69.49 |
| 1 | -6 | 12 | 113.09 | 10.40 |
| 1 | 6 | 12 | 86.49 | 11.50 |
| 1 | 6 | 12 | 110.89 | 9.50 |
| 1 | 6 | 12 | 79.79 | 10.70 |
| 2 | -6 | 12 | 5.60 | 4.40 |

| | | | | |
|---|---|---|---|---|
| 2 | 6 | 12 | 1.40 | 4.40 |
| 2 | 6 | 12 | 10.30 | 3.70 |
| 2 | 6 | 12 | 4.90 | 7.00 |
| 2 | 6 | 12 | 12.50 | 5.10 |
| 3 | -6 | 12 | 8.80 | 4.80 |
| 3 | 6 | 12 | -3.00 | 4.70 |
| 3 | 6 | 12 | 12.90 | 8.60 |
| 3 | 6 | 12 | 4.80 | 3.60 |
| 3 | 6 | 12 | 6.20 | 4.60 |
| 4 | -6 | 12 | 903.41 | 48.50 |
| 4 | 6 | 12 | 846.42 | 52.99 |
| 4 | 6 | 12 | 789.52 | 47.70 |
| 4 | 6 | 12 | 871.51 | 48.30 |
| 5 | -6 | 12 | 156.18 | 12.60 |
| 5 | 6 | 12 | 153.08 | 17.00 |
| 5 | 6 | 12 | 157.38 | 23.90 |
| 5 | 6 | 12 | 157.98 | 11.10 |
| 6 | -6 | 12 | 102.59 | 10.30 |
| 6 | 6 | 12 | 114.69 | 8.90 |
| 6 | 6 | 12 | 133.29 | 11.50 |
| 7 | 6 | 12 | 3.10 | 2.80 |
| 7 | 6 | 12 | -10.40 | 4.90 |
| -8 | -6 | -12 | 228.78 | 16.60 |
| 8 | 6 | 12 | 216.08 | 15.60 |
| 8 | 6 | 12 | 218.18 | 13.90 |
| 9 | 6 | 12 | 11.10 | 4.60 |
| 9 | 6 | 12 | 12.40 | 2.60 |
| 10 | 6 | 12 | 283.57 | 16.60 |
| 10 | 6 | 12 | 285.47 | 18.00 |
| -14 | 7 | 12 | 8.00 | 1.90 |
| 14 | 7 | -12 | 16.20 | 3.00 |
| 13 | 7 | -12 | 9.90 | 2.10 |
| -13 | 7 | 12 | 5.90 | 2.10 |
| -12 | 7 | 12 | 7.20 | 2.40 |
| 12 | 7 | -12 | 12.10 | 2.40 |
| 11 | 7 | -12 | 707.83 | 44.20 |
| -11 | 7 | 12 | 843.02 | 43.60 |
| -10 | 7 | 12 | 6.50 | 2.80 |
| 10 | 7 | -12 | 4.50 | 2.70 |
| 9 | 7 | -12 | 189.28 | 12.70 |
| -9 | 7 | 12 | 209.68 | 13.80 |
| -8 | 7 | 12 | 24.00 | 4.90 |
| 8 | 7 | -12 | 17.00 | 3.40 |
| -7 | -7 | 12 | 359.36 | 21.80 |

| | | | | |
|---|---|---|---|---|
| 7 | 7 | -12 | 379.46 | 24.50 |
| -7 | 7 | 12 | 350.36 | 23.10 |
| 7 | 7 | -12 | 374.56 | 21.80 |
| -6 | -7 | 12 | 31.90 | 6.90 |
| 6 | 7 | -12 | 15.80 | 6.10 |
| -6 | 7 | 12 | 40.40 | 9.10 |
| 6 | 7 | -12 | 17.70 | 4.70 |
| -5 | -7 | 12 | 1175.18 | 65.09 |
| 5 | 7 | -12 | 1143.59 | 65.19 |
| -5 | 7 | 12 | 1221.98 | 66.79 |
| -4 | -7 | 12 | 20.10 | 4.10 |
| -4 | 7 | 12 | 27.20 | 6.50 |
| -4 | 7 | 12 | 21.00 | 8.10 |
| -3 | -7 | 12 | 10.00 | 3.80 |
| -3 | 7 | 12 | 12.60 | 5.00 |
| -3 | 7 | 12 | 29.50 | 8.00 |
| -2 | -7 | 12 | 129.39 | 10.50 |
| -2 | 7 | 12 | 139.39 | 12.20 |
| -2 | 7 | 12 | 135.09 | 15.30 |
| -1 | -7 | 12 | 2178.08 | 118.89 |
| -1 | 7 | 12 | 2281.97 | 119.69 |
| -1 | 7 | 12 | 2151.48 | 118.69 |
| -1 | 7 | 12 | 2118.49 | 119.29 |
| 0 | -7 | 12 | 77.89 | 8.40 |
| 0 | 7 | 12 | 80.99 | 10.50 |
| 0 | 7 | 12 | 81.89 | 12.70 |
| 0 | 7 | 12 | 73.59 | 9.40 |
| 0 | 7 | 12 | 64.49 | 8.70 |
| 1 | -7 | 12 | 42.00 | 7.90 |
| 1 | 7 | 12 | 53.29 | 12.30 |
| 1 | 7 | 12 | 46.20 | 7.40 |
| 1 | 7 | 12 | 54.59 | 8.60 |
| 2 | -7 | 12 | 4.10 | 4.30 |
| 2 | 7 | 12 | 0.50 | 3.50 |
| 2 | 7 | 12 | 5.80 | 4.50 |
| 2 | 7 | 12 | -0.90 | 7.40 |
| 3 | -7 | 12 | 420.86 | 23.30 |
| 3 | 7 | 12 | 361.06 | 22.60 |
| 3 | 7 | 12 | 346.77 | 22.10 |
| 3 | 7 | 12 | 333.47 | 27.60 |
| 4 | -7 | 12 | 9.80 | 4.60 |
| 4 | 7 | 12 | 8.60 | 4.90 |
| 4 | 7 | 12 | -9.40 | 9.80 |
| 4 | 7 | 12 | 7.20 | 3.60 |

| | | | | |
|---|---|---|---|---|
| 5 | -7 | 12 | 617.24 | 35.30 |
| 5 | 7 | 12 | 589.74 | 34.70 |
| 5 | 7 | 12 | 637.54 | 35.50 |
| 5 | 7 | 12 | 599.14 | 42.60 |
| 6 | -7 | 12 | 25.00 | 4.60 |
| 6 | 7 | 12 | 19.00 | 5.00 |
| 7 | 7 | 12 | 82.89 | 9.90 |
| 7 | 7 | 12 | 101.09 | 7.80 |
| -8 | -7 | -12 | -5.20 | 3.80 |
| 8 | 7 | 12 | 2.20 | 4.50 |
| 9 | 7 | 12 | 223.18 | 12.90 |
| 9 | 7 | 12 | 181.78 | 17.50 |
| 10 | 7 | 12 | 2.30 | 3.90 |
| 10 | 7 | 12 | 3.70 | 2.20 |
| -14 | 8 | 12 | 178.28 | 11.00 |
| 14 | 8 | -12 | 191.38 | 10.90 |
| 13 | 8 | -12 | 73.69 | 5.60 |
| -13 | 8 | 12 | 81.69 | 6.00 |
| 12 | 8 | -12 | 438.86 | 23.20 |
| -12 | 8 | 12 | 389.16 | 23.70 |
| -11 | 8 | 12 | 4.30 | 2.70 |
| 11 | 8 | -12 | -0.80 | 2.10 |
| -10 | 8 | 12 | 507.65 | 30.70 |
| 10 | 8 | -12 | 562.74 | 30.00 |
| -9 | 8 | 12 | 25.50 | 5.80 |
| 9 | 8 | -12 | 30.50 | 5.10 |
| -8 | -8 | 12 | 6.40 | 3.90 |
| -8 | 8 | 12 | 12.60 | 4.00 |
| 8 | 8 | -12 | 13.20 | 3.20 |
| -7 | -8 | 12 | 144.19 | 10.40 |
| -7 | 8 | 12 | 117.29 | 11.50 |
| 7 | 8 | -12 | 126.29 | 14.80 |
| 7 | 8 | -12 | 133.59 | 10.20 |
| -6 | -8 | 12 | 1860.61 | 112.29 |
| 6 | 8 | -12 | 2094.89 | 113.99 |
| -6 | 8 | 12 | 2388.56 | 114.29 |
| 6 | 8 | -12 | 1941.81 | 112.29 |
| -5 | -8 | 12 | 9.30 | 3.40 |
| -5 | 8 | 12 | 5.40 | 8.40 |
| 5 | 8 | -12 | 5.40 | 4.80 |
| -5 | 8 | 12 | 8.60 | 5.60 |
| 5 | 8 | -12 | 16.10 | 6.00 |
| -4 | -8 | 12 | 214.78 | 13.40 |
| -4 | 8 | 12 | 200.28 | 18.50 |

| | | | | |
|---|---|---|---|---|
| -4 | 8 | 12 | 159.98 | 15.10 |
| -3 | -8 | 12 | 10.60 | 3.30 |
| -3 | 8 | 12 | 8.20 | 4.70 |
| -3 | 8 | 12 | -1.40 | 4.70 |
| -3 | 8 | 12 | 9.40 | 6.90 |
| -2 | -8 | 12 | 696.23 | 40.30 |
| -2 | 8 | 12 | 670.83 | 41.10 |
| -2 | 8 | 12 | 740.73 | 43.80 |
| -2 | 8 | 12 | 740.53 | 40.40 |
| -1 | -8 | 12 | 15.30 | 3.80 |
| -1 | 8 | 12 | 7.40 | 4.30 |
| -1 | 8 | 12 | 15.30 | 7.70 |
| -1 | 8 | 12 | 17.70 | 5.00 |
| 0 | -8 | 12 | 1067.09 | 58.29 |
| 0 | 8 | 12 | 1083.09 | 61.69 |
| 0 | 8 | 12 | 1006.70 | 58.39 |
| 0 | 8 | 12 | 1029.20 | 58.09 |
| 1 | -8 | 12 | 32.40 | 5.20 |
| 1 | 8 | 12 | 46.70 | 7.60 |
| 1 | 8 | 12 | 16.80 | 7.50 |
| 1 | 8 | 12 | 33.20 | 7.00 |
| 2 | -8 | 12 | 257.07 | 16.20 |
| 2 | 8 | 12 | 211.08 | 20.50 |
| 2 | 8 | 12 | 227.88 | 15.50 |
| 2 | 8 | 12 | 226.28 | 15.40 |
| 3 | -8 | 12 | 4.90 | 4.20 |
| 3 | 8 | 12 | 4.30 | 3.30 |
| 3 | 8 | 12 | -0.30 | 8.60 |
| 3 | 8 | 12 | 3.00 | 4.20 |
| 4 | -8 | 12 | 1085.79 | 58.29 |
| 4 | 8 | 12 | 1005.50 | 58.09 |
| 4 | 8 | 12 | 1040.40 | 63.59 |
| 4 | 8 | 12 | 1037.60 | 57.69 |
| 5 | -8 | 12 | 61.99 | 8.60 |
| 5 | 8 | 12 | 60.59 | 6.60 |
| 5 | 8 | 12 | 67.39 | 8.30 |
| 6 | 8 | 12 | 47.90 | 7.60 |
| 6 | 8 | 12 | 34.30 | 5.30 |
| -7 | -8 | -12 | 1.50 | 3.20 |
| 7 | 8 | 12 | -1.80 | 2.60 |
| 7 | 8 | 12 | 4.60 | 4.40 |
| -8 | -8 | -12 | 209.18 | 13.60 |
| 8 | 8 | 12 | 196.78 | 12.80 |
| 8 | 8 | 12 | 226.48 | 14.70 |

| | | | | |
|---|---|---|---|---|
| 9 | 8 | 12 | 2.40 | 4.60 |
| 9 | 8 | 12 | 1.70 | 2.20 |
| 10 | 8 | 12 | 222.48 | 13.00 |
| 10 | 8 | 12 | 216.38 | 14.40 |
| 13 | 9 | -12 | 5.40 | 1.80 |
| -13 | 9 | 12 | 2.90 | 1.80 |
| -12 | 9 | 12 | 10.70 | 2.40 |
| 12 | 9 | -12 | 13.50 | 2.20 |
| -11 | 9 | 12 | 684.33 | 38.70 |
| 11 | 9 | -12 | 699.93 | 38.10 |
| 10 | 9 | -12 | 19.10 | 3.10 |
| -9 | 9 | 12 | 488.95 | 27.90 |
| 9 | 9 | -12 | 462.85 | 26.80 |
| -8 | -9 | 12 | 18.30 | 3.90 |
| 8 | 9 | -12 | 28.20 | 5.40 |
| -8 | 9 | 12 | 32.80 | 6.80 |
| -7 | -9 | 12 | 831.02 | 51.69 |
| -7 | 9 | 12 | 938.01 | 53.19 |
| 7 | 9 | -12 | 943.11 | 51.89 |
| 7 | 9 | -12 | 1063.49 | 58.89 |
| -6 | -9 | 12 | 0.30 | 3.10 |
| 6 | -9 | -12 | 3.40 | 5.10 |
| -6 | 9 | 12 | -2.00 | 4.50 |
| 6 | 9 | -12 | -10.30 | 6.40 |
| 6 | 9 | -12 | 3.20 | 3.50 |
| -5 | -9 | 12 | 786.22 | 44.40 |
| -5 | 9 | 12 | 803.52 | 46.20 |
| -5 | 9 | 12 | 785.22 | 44.40 |
| 5 | 9 | -12 | 826.62 | 46.50 |
| -5 | 9 | 12 | 765.12 | 48.90 |
| -4 | -9 | 12 | 92.89 | 8.50 |
| -4 | 9 | 12 | 93.49 | 11.20 |
| -4 | 9 | 12 | 108.59 | 9.60 |
| -4 | 9 | 12 | 88.49 | 15.60 |
| 4 | 9 | -12 | 89.39 | 11.80 |
| -3 | -9 | 12 | 41.00 | 6.50 |
| -3 | 9 | 12 | 53.89 | 13.10 |
| 3 | 9 | -12 | 40.10 | 8.10 |
| -3 | 9 | 12 | 36.50 | 7.70 |
| -2 | -9 | 12 | 16.30 | 3.60 |
| -2 | 9 | 12 | 17.20 | 4.60 |
| -2 | 9 | 12 | 19.50 | 4.60 |
| -2 | 9 | 12 | 28.30 | 8.30 |
| -1 | -9 | 12 | 1654.93 | 89.69 |

| | | | | |
|---|---|---|---|---|
| -1 | 9 | 12 | 1687.33 | 89.59 |
| -1 | 9 | 12 | 1671.73 | 90.09 |
| -1 | 9 | 12 | 1526.75 | 92.69 |
| 0 | -9 | 12 | 7.00 | 3.50 |
| 0 | 9 | 12 | 5.20 | 3.70 |
| 0 | 9 | 12 | -0.50 | 3.90 |
| 0 | 9 | 12 | 15.90 | 7.30 |
| 1 | -9 | 12 | 192.78 | 12.90 |
| 1 | 9 | 12 | 165.68 | 17.90 |
| 1 | 9 | 12 | 176.58 | 12.80 |
| 1 | 9 | 12 | 158.58 | 12.50 |
| 2 | -9 | 12 | 7.10 | 3.90 |
| 2 | 9 | 12 | 6.90 | 8.60 |
| 2 | 9 | 12 | 6.10 | 4.00 |
| 2 | 9 | 12 | 3.90 | 3.40 |
| 3 | -9 | 12 | 530.35 | 27.70 |
| 3 | 9 | 12 | 456.05 | 26.70 |
| 3 | 9 | 12 | 474.75 | 27.20 |
| -3 | 9 | -12 | 416.86 | 30.30 |
| 3 | 9 | 12 | 394.76 | 32.20 |
| 4 | -9 | 12 | 85.39 | 9.00 |
| 4 | 9 | 12 | 57.59 | 6.70 |
| 4 | 9 | 12 | 71.79 | 8.10 |
| 5 | -9 | 12 | 665.93 | 37.30 |
| 5 | 9 | 12 | 655.53 | 37.40 |
| 5 | 9 | 12 | 642.94 | 36.80 |
| 6 | -9 | 12 | -3.00 | 3.70 |
| -6 | -9 | -12 | -0.50 | 3.30 |
| 6 | 9 | 12 | 2.10 | 3.10 |
| 6 | 9 | 12 | -3.10 | 3.60 |
| -7 | -9 | -12 | 165.68 | 11.80 |
| 7 | 9 | 12 | 180.88 | 11.60 |
| 7 | 9 | 12 | 193.38 | 12.90 |
| -8 | -9 | -12 | -2.40 | 3.70 |
| 8 | 9 | 12 | 4.20 | 5.30 |
| 8 | 9 | 12 | 0.70 | 2.30 |
| 9 | 9 | 12 | 242.68 | 14.30 |
| 9 | 9 | 12 | 244.78 | 15.80 |
| -13 | 10 | 12 | 70.09 | 5.60 |
| 13 | 10 | -12 | 83.09 | 5.20 |
| 12 | 10 | -12 | 312.97 | 17.50 |
| -12 | 10 | 12 | 309.77 | 18.00 |
| -11 | 10 | 12 | 0.50 | 2.30 |
| 11 | 10 | -12 | 0.20 | 1.80 |

| | | | | |
|---|---|---|---|---|
| -10 | 10 | 12 | 393.96 | 23.30 |
| 10 | 10 | -12 | 406.86 | 22.60 |
| 9 | 10 | -12 | 10.70 | 3.40 |
| -8 | -10 | 12 | 16.00 | 4.00 |
| 8 | 10 | -12 | 15.00 | 3.20 |
| -8 | 10 | 12 | 20.60 | 4.30 |
| -7 | -10 | 12 | 48.00 | 6.60 |
| -7 | 10 | 12 | 57.39 | 8.70 |
| 7 | 10 | -12 | 64.69 | 6.80 |
| -6 | -10 | 12 | 959.70 | 52.79 |
| 6 | -10 | -12 | 957.00 | 53.29 |
| 6 | 10 | -12 | 960.10 | 55.39 |
| -6 | 10 | 12 | 1010.80 | 54.49 |
| -6 | 10 | 12 | 876.51 | 52.79 |
| 6 | 10 | -12 | 973.40 | 52.99 |
| -5 | -10 | 12 | -0.50 | 2.80 |
| 5 | -10 | -12 | 6.60 | 4.10 |
| -5 | 10 | 12 | 7.10 | 5.90 |
| -5 | 10 | 12 | 7.90 | 5.00 |
| 5 | 10 | -12 | 3.90 | 6.90 |
| -4 | -10 | 12 | 32.90 | 6.20 |
| -4 | 10 | 12 | 18.90 | 5.30 |
| -4 | 10 | 12 | 34.70 | 7.40 |
| 4 | 10 | -12 | 35.30 | 7.90 |
| -4 | 10 | 12 | 28.90 | 10.70 |
| -3 | -10 | 12 | 91.59 | 8.30 |
| 3 | 10 | -12 | 92.29 | 12.90 |
| -3 | 10 | 12 | 96.59 | 10.70 |
| -3 | 10 | 12 | 82.89 | 9.00 |
| -3 | 10 | 12 | 84.39 | 15.90 |
| -2 | -10 | 12 | 743.03 | 43.40 |
| 2 | 10 | -12 | 806.42 | 46.70 |
| -2 | 10 | 12 | 775.42 | 47.60 |
| -2 | 10 | 12 | 792.82 | 43.60 |
| -2 | 10 | 12 | 750.82 | 44.20 |
| -1 | -10 | 12 | 30.30 | 5.90 |
| -1 | 10 | 12 | 32.60 | 5.10 |
| 1 | 10 | -12 | 24.80 | 7.90 |
| -1 | 10 | 12 | 22.40 | 4.50 |
| -1 | 10 | 12 | 9.50 | 7.80 |
| 0 | -10 | 12 | 657.83 | 34.60 |
| 0 | 10 | 12 | 569.84 | 38.50 |
| 0 | 10 | 12 | 548.35 | 34.40 |
| 0 | 10 | -12 | 538.05 | 37.80 |

| | | | | |
|---|---|---|---|---|
| 0 | 10 | 12 | 677.63 | 34.70 |
| 1 | -10 | 12 | 19.70 | 4.00 |
| 1 | 10 | 12 | 24.40 | 5.40 |
| -1 | 10 | -12 | 14.10 | 8.20 |
| 1 | 10 | 12 | 11.10 | 8.20 |
| 1 | 10 | 12 | 15.60 | 4.30 |
| 2 | -10 | 12 | 192.58 | 12.60 |
| 2 | 10 | 12 | 161.68 | 12.00 |
| 2 | 10 | 12 | 146.59 | 19.30 |
| 2 | 10 | 12 | 164.38 | 11.70 |
| -2 | 10 | -12 | 134.59 | 17.40 |
| 3 | -10 | 12 | 8.50 | 4.20 |
| 3 | 10 | 12 | 14.40 | 4.10 |
| 3 | 10 | 12 | 16.40 | 3.60 |
| 4 | -10 | 12 | 582.14 | 31.20 |
| 4 | 10 | 12 | 472.95 | 30.60 |
| 4 | 10 | 12 | 547.25 | 30.40 |
| 5 | -10 | 12 | 4.50 | 3.70 |
| 5 | 10 | 12 | 4.20 | 3.60 |
| 5 | 10 | 12 | 4.40 | 3.60 |
| 6 | -10 | 12 | 66.09 | 7.80 |
| -6 | -10 | -12 | 69.29 | 7.10 |
| 6 | 10 | 12 | 70.39 | 6.60 |
| 6 | 10 | 12 | 76.19 | 8.00 |
| -7 | -10 | -12 | 30.80 | 5.50 |
| 7 | 10 | 12 | 32.70 | 4.90 |
| 7 | 10 | 12 | 40.50 | 6.90 |
| 8 | 10 | 12 | 340.97 | 20.00 |
| 8 | 10 | 12 | 363.16 | 21.20 |
| 12 | 11 | -12 | 44.60 | 3.60 |
| -12 | 11 | 12 | 41.40 | 4.40 |
| 11 | 11 | -12 | 238.98 | 13.40 |
| -11 | 11 | 12 | 227.68 | 14.00 |
| 10 | 11 | -12 | 1.10 | 1.90 |
| -10 | 11 | 12 | 2.60 | 2.60 |
| -9 | 11 | 12 | 15.80 | 3.80 |
| 9 | 11 | -12 | 24.00 | 4.10 |
| -8 | -11 | 12 | 138.19 | 10.30 |
| -8 | 11 | 12 | 132.09 | 11.30 |
| 8 | 11 | -12 | 147.39 | 9.80 |
| -7 | -11 | 12 | 427.86 | 25.40 |
| -7 | 11 | 12 | 427.16 | 25.50 |
| -7 | 11 | 12 | 460.55 | 27.00 |
| 7 | 11 | -12 | 460.55 | 25.50 |

| | | | | |
|---|---|---|---|---|
| 6 | -11 | -12 | 8.30 | 4.30 |
| -6 | -11 | 12 | 1.60 | 2.60 |
| -6 | 11 | 12 | -3.10 | 4.20 |
| -6 | 11 | 12 | 5.20 | 3.60 |
| 6 | 11 | -12 | -0.70 | 2.90 |
| 5 | -11 | -12 | 269.87 | 17.90 |
| -5 | -11 | 12 | 273.87 | 17.20 |
| -5 | 11 | 12 | 305.77 | 17.60 |
| 4 | -11 | -12 | 15.20 | 3.90 |
| -4 | -11 | 12 | 2.30 | 3.20 |
| -4 | 11 | 12 | 1.50 | 4.70 |
| -4 | 11 | 12 | 6.60 | 3.80 |
| 4 | 11 | -12 | 24.60 | 8.00 |
| 3 | -11 | -12 | 139.09 | 11.00 |
| -3 | -11 | 12 | 134.49 | 10.10 |
| 3 | 11 | -12 | 149.79 | 15.90 |
| -3 | 11 | 12 | 127.99 | 19.70 |
| -3 | 11 | 12 | 140.19 | 12.10 |
| -3 | 11 | 12 | 134.49 | 10.70 |
| -2 | -11 | 12 | 133.49 | 9.90 |
| -2 | 11 | 12 | 145.19 | 10.50 |
| 2 | 11 | -12 | 105.99 | 15.50 |
| -2 | 11 | 12 | 118.09 | 18.00 |
| -2 | 11 | 12 | 125.39 | 11.10 |
| -1 | -11 | 12 | 786.82 | 44.80 |
| -1 | 11 | 12 | 857.61 | 45.00 |
| 1 | 11 | -12 | 743.53 | 48.40 |
| -1 | 11 | 12 | 766.12 | 49.10 |
| -1 | 11 | 12 | 831.12 | 45.20 |
| 0 | -11 | 12 | 1.30 | 2.80 |
| 0 | 11 | 12 | 10.00 | 9.30 |
| 0 | 11 | 12 | 4.00 | 3.50 |
| 0 | 11 | 12 | 5.30 | 3.70 |
| 0 | 11 | -12 | 0.60 | 7.80 |
| 1 | -11 | 12 | 17.80 | 3.60 |
| 1 | -11 | 12 | 18.30 | 4.10 |
| 1 | 11 | 12 | 22.40 | 4.30 |
| 1 | 11 | 12 | 26.40 | 4.50 |
| 2 | -11 | 12 | 150.78 | 11.60 |
| 2 | -11 | 12 | 178.38 | 12.00 |
| 2 | 11 | 12 | 139.79 | 11.50 |
| 2 | 11 | 12 | 181.68 | 11.60 |
| -3 | -11 | -12 | 509.15 | 30.60 |
| 3 | -11 | 12 | 570.94 | 30.60 |

| | | | | |
|---|---|---|---|---|
| 3 | 11 | 12 | 501.45 | 29.80 |
| 3 | 11 | 12 | 512.95 | 30.10 |
| 4 | -11 | 12 | 33.50 | 7.30 |
| 4 | 11 | 12 | 33.80 | 6.10 |
| 4 | 11 | 12 | 22.00 | 4.00 |
| 5 | -11 | 12 | 427.76 | 24.90 |
| -5 | -11 | -12 | 463.65 | 25.20 |
| 5 | 11 | 12 | 434.56 | 25.20 |
| 5 | 11 | 12 | 398.76 | 24.50 |
| -6 | -11 | -12 | 84.49 | 7.00 |
| 6 | -11 | 12 | 77.59 | 9.60 |
| 6 | 11 | 12 | 79.89 | 8.00 |
| 6 | 11 | 12 | 86.79 | 7.00 |
| -7 | -11 | -12 | 123.09 | 8.50 |
| 7 | 11 | 12 | 118.19 | 9.50 |
| 7 | 11 | 12 | 116.79 | 8.40 |
| 8 | 11 | 12 | -1.40 | 2.90 |
| 8 | 11 | 12 | 1.10 | 3.80 |
| 11 | 12 | -12 | 3.30 | 1.60 |
| -11 | 12 | 12 | 4.00 | 2.50 |
| 10 | 12 | -12 | 186.98 | 10.40 |
| -10 | 12 | 12 | 161.38 | 11.10 |
| 9 | 12 | -12 | 7.40 | 2.00 |
| -9 | 12 | 12 | 5.70 | 3.10 |
| -8 | -12 | 12 | 26.50 | 5.60 |
| 8 | 12 | -12 | 14.20 | 2.60 |
| -8 | 12 | 12 | 10.40 | 5.10 |
| -8 | 12 | 12 | 17.10 | 4.00 |
| -7 | -12 | 12 | 135.99 | 9.60 |
| -7 | 12 | 12 | 130.89 | 9.80 |
| 7 | 12 | -12 | 136.59 | 9.60 |
| -7 | 12 | 12 | 145.99 | 11.50 |
| -6 | 12 | 12 | 522.15 | 31.30 |
| -6 | 12 | 12 | 596.34 | 35.50 |
| -5 | -12 | 12 | 44.70 | 5.80 |
| 5 | -12 | -12 | 45.90 | 8.60 |
| -5 | 12 | 12 | 42.30 | 6.30 |
| -5 | 12 | 12 | 44.90 | 9.70 |
| 4 | -12 | -12 | 2.20 | 3.30 |
| -4 | -12 | 12 | 2.00 | 3.00 |
| -4 | 12 | 12 | 7.70 | 5.10 |
| -4 | 12 | 12 | 6.20 | 3.30 |
| 3 | -12 | -12 | 59.49 | 7.40 |
| -3 | -12 | 12 | 42.80 | 6.10 |

| | | | | |
|---|---|---|---|---|
| -3 | 12 | 12 | 61.39 | 6.90 |
| -3 | 12 | 12 | 58.89 | 8.90 |
| 2 | -12 | -12 | 317.77 | 19.60 |
| -2 | -12 | 12 | 299.07 | 18.90 |
| -2 | 12 | 12 | 312.97 | 20.10 |
| -2 | 12 | 12 | 326.07 | 19.10 |
| -1 | -12 | 12 | 2.10 | 2.50 |
| 1 | -12 | -12 | -1.10 | 3.90 |
| -1 | 12 | 12 | 14.00 | 4.30 |
| -1 | 12 | 12 | -5.90 | 3.30 |
| 0 | -12 | 12 | 313.47 | 19.40 |
| 0 | -12 | -12 | 302.17 | 20.40 |
| 0 | 12 | 12 | 313.37 | 19.90 |
| 0 | 12 | 12 | 361.66 | 19.80 |
| -1 | -12 | -12 | 79.59 | 9.40 |
| 1 | -12 | 12 | 101.49 | 8.40 |
| 1 | 12 | 12 | 88.39 | 8.90 |
| 1 | 12 | 12 | 108.19 | 8.50 |
| 2 | -12 | 12 | 112.49 | 8.80 |
| -2 | -12 | -12 | 94.69 | 9.20 |
| 2 | -12 | 12 | 85.39 | 8.10 |
| 2 | 12 | 12 | 92.39 | 8.90 |
| 2 | 12 | 12 | 86.29 | 8.30 |
| -3 | -12 | -12 | 34.00 | 7.30 |
| 3 | -12 | 12 | 35.70 | 6.20 |
| 3 | -12 | 12 | 39.20 | 6.70 |
| 3 | 12 | 12 | 37.80 | 6.10 |
| 3 | 12 | 12 | 42.30 | 6.30 |
| 4 | -12 | 12 | 485.95 | 29.70 |
| -4 | -12 | -12 | 514.65 | 29.90 |
| 4 | 12 | 12 | 560.74 | 29.90 |
| 4 | 12 | 12 | 521.25 | 29.40 |
| -5 | -12 | -12 | 53.19 | 6.00 |
| 5 | -12 | 12 | 44.80 | 6.50 |
| 5 | 12 | 12 | 54.39 | 6.10 |
| 5 | 12 | 12 | 54.29 | 6.60 |
| -6 | -12 | -12 | 12.50 | 2.70 |
| 6 | 12 | 12 | 10.70 | 3.40 |
| 6 | 12 | 12 | 13.90 | 3.90 |
| -7 | -12 | -12 | 69.69 | 8.70 |
| 7 | 12 | 12 | 59.89 | 7.20 |
| 7 | 12 | 12 | 65.09 | 7.30 |
| 10 | 13 | -12 | 15.10 | 2.60 |
| -10 | 13 | 12 | 12.20 | 3.20 |

| | | | | |
|---|---|---|---|---|
| -9 | 13 | 12 | 44.60 | 6.20 |
| 9 | 13 | -12 | 59.69 | 4.40 |
| -8 | -13 | 12 | 85.19 | 7.10 |
| -8 | 13 | 12 | 80.59 | 8.10 |
| 8 | 13 | -12 | 85.79 | 6.20 |
| -8 | 13 | 12 | 85.99 | 9.00 |
| -7 | -13 | 12 | 348.37 | 20.20 |
| -7 | 13 | 12 | 361.66 | 21.60 |
| -7 | 13 | 12 | 319.37 | 20.40 |
| 7 | 13 | -12 | 380.86 | 20.30 |
| -6 | -13 | 12 | 2.30 | 2.50 |
| -6 | 13 | 12 | 7.00 | 3.10 |
| -6 | 13 | 12 | 0.30 | 4.60 |
| 6 | 13 | -12 | -1.80 | 2.50 |
| 5 | -13 | -12 | 404.66 | 23.50 |
| -5 | -13 | 12 | 412.56 | 23.40 |
| -5 | 13 | 12 | 384.66 | 23.20 |
| -5 | 13 | 12 | 374.16 | 25.00 |
| 5 | 13 | -12 | 441.46 | 23.50 |
| 4 | -13 | -12 | 113.69 | 8.70 |
| -4 | 13 | 12 | 102.19 | 8.40 |
| -4 | 13 | 12 | 91.29 | 11.40 |
| 3 | -13 | -12 | 7.90 | 5.60 |
| -3 | -13 | 12 | 7.40 | 2.50 |
| -3 | 13 | 12 | 8.50 | 3.30 |
| -3 | 13 | 12 | 7.80 | 5.30 |
| 2 | -13 | -12 | 167.08 | 12.20 |
| -2 | -13 | 12 | 175.48 | 11.00 |
| -2 | 13 | 12 | 146.99 | 10.90 |
| -2 | 13 | 12 | 143.69 | 12.90 |
| -1 | -13 | 12 | 979.90 | 55.79 |
| -1 | 13 | 12 | 1049.09 | 55.99 |
| -1 | 13 | 12 | 1007.10 | 56.29 |
| 0 | -13 | -12 | 145.89 | 15.40 |
| 0 | -13 | 12 | 119.29 | 12.40 |
| 0 | 13 | 12 | 144.39 | 10.10 |
| 0 | 13 | 12 | 124.99 | 11.20 |
| -1 | -13 | -12 | 47.40 | 7.70 |
| 1 | -13 | 12 | 60.89 | 6.80 |
| 1 | 13 | 12 | 50.89 | 8.00 |
| 1 | 13 | 12 | 56.89 | 6.50 |
| -2 | -13 | -12 | 74.49 | 7.60 |
| 2 | -13 | 12 | 78.79 | 7.20 |
| 2 | 13 | 12 | 75.49 | 7.90 |

| | | | | |
|---|---|---|---|---|
| 2 | 13 | 12 | 67.19 | 7.00 |
| 3 | -13 | 12 | 250.37 | 15.70 |
| -3 | -13 | -12 | 237.88 | 15.90 |
| 3 | 13 | 12 | 252.37 | 15.50 |
| 3 | 13 | 12 | 266.97 | 15.80 |
| -4 | -13 | -12 | 3.30 | 2.60 |
| 4 | -13 | 12 | -0.90 | 2.70 |
| 4 | 13 | 12 | -0.40 | 3.00 |
| -5 | -13 | -12 | 325.17 | 20.10 |
| 5 | -13 | 12 | 335.97 | 20.20 |
| 5 | 13 | 12 | 390.26 | 20.70 |
| -6 | -13 | -12 | 101.89 | 7.40 |
| 6 | 13 | 12 | 106.69 | 8.50 |
| 9 | 14 | -12 | 42.90 | 3.60 |
| -8 | -14 | 12 | 8.40 | 4.30 |
| 8 | 14 | -12 | 6.30 | 1.80 |
| -7 | -14 | 12 | 108.99 | 7.40 |
| -7 | 14 | 12 | 84.69 | 7.90 |
| 7 | 14 | -12 | 120.79 | 7.30 |
| -6 | -14 | 12 | 790.72 | 46.20 |
| 6 | 14 | -12 | 817.52 | 46.20 |
| -6 | 14 | 12 | 922.91 | 47.20 |
| 5 | -14 | -12 | 177.18 | 10.90 |
| -5 | -14 | 12 | 157.38 | 10.50 |
| -5 | 14 | 12 | 153.38 | 10.60 |
| 5 | 14 | -12 | 161.58 | 10.60 |
| 4 | -14 | -12 | 17.50 | 3.20 |
| -4 | 14 | 12 | 24.50 | 4.70 |
| -3 | -14 | 12 | 149.59 | 10.00 |
| 3 | -14 | -12 | 163.58 | 10.50 |
| -3 | 14 | 12 | 141.29 | 10.30 |
| -2 | -14 | 12 | 331.77 | 20.10 |
| 2 | -14 | -12 | 334.67 | 20.40 |
| -2 | 14 | 12 | 370.06 | 20.40 |
| -1 | -14 | 12 | 4.10 | 2.20 |
| 1 | -14 | -12 | 4.50 | 2.80 |
| -1 | 14 | 12 | 2.20 | 2.80 |
| 0 | -14 | -12 | 327.27 | 20.90 |
| 0 | -14 | 12 | 345.97 | 20.40 |
| 0 | 14 | 12 | 374.16 | 20.60 |
| 1 | -14 | 12 | 264.97 | 15.90 |
| -1 | -14 | -12 | 237.58 | 16.50 |
| 1 | 14 | 12 | 286.27 | 16.20 |
| -2 | -14 | -12 | 12.80 | 3.10 |

| | | | | |
|---|---|---|---|---|
| 2 | -14 | 12 | 24.00 | 4.60 |
| 2 | 14 | 12 | 18.50 | 3.60 |
| -3 | -14 | -12 | 52.29 | 6.10 |
| 3 | -14 | 12 | 71.89 | 6.30 |
| 3 | 14 | 12 | 57.29 | 9.70 |
| 4 | -14 | 12 | 397.16 | 23.30 |
| -4 | -14 | -12 | 401.96 | 23.40 |
| 4 | 14 | 12 | 429.16 | 23.60 |
| -5 | -14 | -12 | 32.20 | 4.50 |
| 5 | 14 | 12 | 21.70 | 4.00 |
| -7 | -15 | 12 | 195.98 | 11.70 |
| 7 | 15 | -12 | 229.78 | 11.60 |
| -7 | 15 | 12 | 166.58 | 11.90 |
| -6 | -15 | 12 | 3.80 | 1.90 |
| 6 | 15 | -12 | 3.10 | 2.00 |
| -6 | 15 | 12 | -0.80 | 2.80 |
| 5 | -15 | -12 | 434.26 | 25.20 |
| -5 | -15 | 12 | 454.15 | 25.10 |
| -5 | 15 | 12 | 436.66 | 25.20 |
| 5 | 15 | -12 | 469.45 | 25.10 |
| 4 | -15 | -12 | 65.79 | 5.70 |
| -4 | -15 | 12 | 60.99 | 5.30 |
| 4 | 15 | -12 | 65.79 | 5.90 |
| -4 | 15 | 12 | 57.99 | 5.60 |
| -3 | -15 | 12 | 7.40 | 2.10 |
| 3 | -15 | -12 | 9.30 | 2.50 |
| -3 | 15 | 12 | 7.00 | 4.10 |
| -2 | -15 | 12 | 109.39 | 7.60 |
| 2 | -15 | -12 | 110.09 | 7.90 |
| -2 | 15 | 12 | 106.09 | 8.70 |
| 1 | -15 | -12 | 814.02 | 48.40 |
| -1 | 15 | 12 | 944.81 | 49.30 |
| 0 | -15 | 12 | 93.39 | 6.70 |
| 0 | -15 | -12 | 85.89 | 7.30 |
| 0 | 15 | 12 | 83.59 | 9.30 |
| 1 | -15 | 12 | 45.50 | 5.00 |
| -1 | -15 | -12 | 50.89 | 5.70 |
| 1 | 15 | 12 | 53.39 | 5.60 |
| -2 | -15 | -12 | 84.79 | 7.20 |
| 2 | -15 | 12 | 93.89 | 6.90 |
| 2 | 15 | 12 | 83.89 | 7.10 |
| 3 | -15 | 12 | 204.28 | 12.20 |
| -3 | -15 | -12 | 187.78 | 12.30 |
| 3 | 15 | 12 | 207.98 | 12.60 |

| | | | | |
|---|---|---|---|---|
| -5 | -16 | 12 | 123.09 | 7.70 |
| 5 | 16 | -12 | 127.59 | 7.80 |
| -5 | 16 | 12 | 115.79 | 8.20 |
| 4 | -16 | -12 | 9.80 | 3.10 |
| -4 | -16 | 12 | 5.60 | 1.70 |
| 4 | 16 | -12 | 5.30 | 2.10 |
| -4 | 16 | 12 | 0.00 | 2.50 |
| -3 | -16 | 12 | 71.19 | 5.10 |
| 3 | -16 | -12 | 63.29 | 5.30 |
| -3 | 16 | 12 | 57.49 | 5.80 |
| 3 | 16 | -12 | 78.29 | 5.90 |
| -2 | -16 | 12 | 221.68 | 13.50 |
| 2 | -16 | -12 | 227.88 | 13.60 |
| -2 | 16 | 12 | 244.68 | 13.90 |
| -1 | -16 | 12 | -0.30 | 1.60 |
| 1 | -16 | -12 | 3.40 | 2.00 |
| -1 | 16 | 12 | 1.90 | 2.90 |
| 0 | -16 | 12 | 212.58 | 12.20 |
| 0 | -16 | -12 | 205.38 | 12.50 |
| 0 | 16 | 12 | 195.88 | 12.50 |
| -1 | -16 | -12 | 98.99 | 7.70 |
| 1 | -16 | 12 | 110.19 | 7.30 |
| 1 | 16 | 12 | 124.79 | 8.10 |
| -15 | 0 | 13 | 165.98 | 10.00 |
| -14 | 0 | 13 | -1.00 | 2.00 |
| -13 | 0 | 13 | 79.49 | 6.90 |
| -12 | 0 | 13 | 1.60 | 3.20 |
| -11 | 0 | 13 | 1072.59 | 59.39 |
| -10 | 0 | 13 | -0.60 | 3.30 |
| -9 | 0 | 13 | 6.80 | 3.90 |
| -8 | 0 | 13 | 3.30 | 3.70 |
| -7 | 0 | 13 | 1782.42 | 97.99 |
| -6 | 0 | 13 | 0.10 | 5.80 |
| -6 | 0 | 13 | -9.50 | 4.40 |
| -5 | 0 | 13 | 809.72 | 47.30 |
| -5 | 0 | 13 | 877.31 | 48.60 |
| -5 | 0 | 13 | 850.11 | 48.80 |
| -4 | 0 | 13 | 1.90 | 4.40 |
| -4 | 0 | 13 | 7.80 | 6.10 |
| -4 | 0 | 13 | -0.70 | 3.80 |
| -3 | 0 | 13 | 62.99 | 8.60 |
| -3 | 0 | 13 | 48.80 | 7.60 |
| -2 | 0 | 13 | -7.10 | 5.70 |
| -2 | 0 | 13 | -2.80 | 4.40 |

| | | | | |
|---|---|---|---|---|
| -2 | 0 | 13 | 2.80 | 3.80 |
| -1 | 0 | 13 | 2339.67 | 124.49 |
| -1 | 0 | 13 | 2100.79 | 123.19 |
| -1 | 0 | 13 | 2331.67 | 123.09 |
| 0 | 0 | 13 | 0.10 | 4.70 |
| 0 | 0 | 13 | -2.20 | 4.30 |
| 0 | 0 | 13 | -6.50 | 4.30 |
| 1 | 0 | 13 | 6.70 | 4.90 |
| 1 | 0 | 13 | 1.80 | 4.90 |
| 1 | 0 | 13 | 1.70 | 5.40 |
| 2 | 0 | 13 | -3.40 | 4.10 |
| 2 | 0 | 13 | 0.40 | 4.80 |
| 3 | 0 | 13 | 116.19 | 13.70 |
| 3 | 0 | 13 | 97.79 | 9.60 |
| 3 | 0 | 13 | 104.69 | 11.30 |
| 4 | 0 | 13 | -3.10 | 8.20 |
| 4 | 0 | 13 | -3.10 | 3.40 |
| 4 | 0 | 13 | 3.50 | 4.60 |
| 5 | 0 | 13 | 511.35 | 30.30 |
| 5 | 0 | 13 | 535.85 | 29.80 |
| 5 | 0 | 13 | 494.65 | 34.70 |
| 6 | 0 | 13 | -2.10 | 3.10 |
| 6 | 0 | 13 | -5.40 | 3.50 |
| 6 | 0 | 13 | -9.60 | 11.40 |
| 7 | 0 | 13 | 18.10 | 3.60 |
| 7 | 0 | 13 | 11.90 | 3.90 |
| 8 | 0 | 13 | -0.70 | 3.20 |
| 9 | 0 | 13 | 158.58 | 10.60 |
| 10 | 0 | 13 | -1.20 | 2.30 |
| -15 | -1 | 13 | 2.00 | 2.40 |
| -15 | 1 | 13 | 2.70 | 1.70 |
| -14 | -1 | 13 | 190.68 | 11.40 |
| -14 | 1 | 13 | 175.78 | 11.30 |
| -13 | -1 | 13 | 1.50 | 2.50 |
| -13 | 1 | 13 | 4.50 | 2.50 |
| -12 | -1 | 13 | 689.73 | 45.40 |
| -12 | 1 | 13 | 851.31 | 43.50 |
| -11 | -1 | 13 | 3.70 | 3.20 |
| -11 | 1 | 13 | -5.00 | 2.80 |
| -10 | -1 | 13 | 646.84 | 36.20 |
| -10 | 1 | 13 | 619.74 | 36.10 |
| -9 | -1 | 13 | 23.20 | 4.60 |
| -9 | 1 | 13 | 12.80 | 3.90 |
| -8 | -1 | 13 | 36.60 | 5.70 |

| | | | | |
|---|---|---|---|---|
| -8 | 1 | 13 | 48.70 | 7.40 |
| -7 | -1 | 13 | 1.50 | 4.70 |
| -7 | 1 | 13 | 2.60 | 4.40 |
| -6 | -1 | 13 | 2290.17 | 132.79 |
| -6 | -1 | 13 | 2508.55 | 134.19 |
| -6 | 1 | 13 | 2508.05 | 134.29 |
| -6 | 1 | 13 | 2309.17 | 132.99 |
| -6 | 1 | 13 | 2653.53 | 134.29 |
| -5 | -1 | 13 | 38.40 | 8.00 |
| -5 | -1 | 13 | 26.90 | 5.10 |
| -5 | -1 | 13 | 43.10 | 7.90 |
| -5 | 1 | 13 | 41.40 | 6.80 |
| -5 | 1 | 13 | 33.20 | 6.70 |
| -5 | 1 | 13 | 33.20 | 5.20 |
| -4 | -1 | 13 | 37.80 | 7.10 |
| -4 | -1 | 13 | 33.50 | 7.50 |
| -4 | -1 | 13 | 31.60 | 7.60 |
| -4 | 1 | 13 | 27.10 | 6.40 |
| -4 | 1 | 13 | 21.50 | 5.10 |
| -4 | 1 | 13 | 43.80 | 7.50 |
| -3 | -1 | 13 | 21.20 | 4.10 |
| -3 | -1 | 13 | 16.90 | 5.30 |
| -3 | -1 | 13 | 15.60 | 6.30 |
| -3 | 1 | 13 | 15.30 | 5.50 |
| -3 | 1 | 13 | 21.10 | 6.00 |
| -3 | 1 | 13 | 15.50 | 4.10 |
| -2 | -1 | 13 | 774.62 | 43.60 |
| -2 | -1 | 13 | 732.73 | 44.80 |
| -2 | -1 | 13 | 774.72 | 44.90 |
| -2 | 1 | 13 | 753.72 | 44.60 |
| -2 | 1 | 13 | 780.12 | 43.70 |
| -2 | 1 | 13 | 820.32 | 45.30 |
| -1 | -1 | 13 | 141.99 | 12.60 |
| -1 | -1 | 13 | 118.79 | 11.30 |
| -1 | -1 | 13 | 99.79 | 10.20 |
| -1 | 1 | 13 | 91.49 | 11.60 |
| -1 | 1 | 13 | 130.39 | 11.50 |
| -1 | 1 | 13 | 110.69 | 10.70 |
| 0 | -1 | 13 | 426.96 | 27.20 |
| 0 | -1 | 13 | 409.56 | 27.80 |
| 0 | -1 | 13 | 433.36 | 26.80 |
| 0 | 1 | 13 | 517.35 | 27.20 |
| 0 | 1 | 13 | 433.16 | 27.30 |
| 0 | 1 | 13 | 449.36 | 28.40 |

| | | | | |
|---|---|---|---|---|
| 1 | -1 | 13 | 29.00 | 6.60 |
| 1 | -1 | 13 | 16.60 | 5.40 |
| 1 | -1 | 13 | 20.70 | 5.60 |
| 1 | 1 | 13 | 20.70 | 5.60 |
| 1 | 1 | 13 | 5.90 | 5.40 |
| 2 | -1 | 13 | 15.10 | 5.40 |
| 2 | -1 | 13 | 6.90 | 4.20 |
| 2 | 1 | 13 | 15.30 | 9.20 |
| 2 | 1 | 13 | 10.90 | 5.40 |
| 2 | 1 | 13 | 7.00 | 4.20 |
| 3 | -1 | 13 | 5.80 | 4.40 |
| 3 | -1 | 13 | 1.10 | 4.70 |
| 3 | 1 | 13 | -0.70 | 3.70 |
| 3 | 1 | 13 | 4.10 | 4.70 |
| 3 | 1 | 13 | -5.30 | 7.40 |
| 4 | -1 | 13 | 793.82 | 43.00 |
| 4 | -1 | 13 | 678.43 | 45.40 |
| 4 | -1 | 13 | 675.13 | 41.90 |
| 4 | 1 | 13 | 756.62 | 45.90 |
| 4 | 1 | 13 | 778.92 | 42.20 |
| 4 | 1 | 13 | 787.62 | 43.00 |
| 5 | -1 | 13 | 3.10 | 3.50 |
| 5 | -1 | 13 | 7.50 | 4.20 |
| 5 | -1 | 13 | -3.30 | 9.50 |
| 5 | 1 | 13 | -3.10 | 3.80 |
| 5 | 1 | 13 | 12.60 | 10.60 |
| 5 | 1 | 13 | 6.30 | 3.50 |
| 6 | -1 | 13 | 240.58 | 14.50 |
| 6 | -1 | 13 | 190.38 | 25.80 |
| 6 | -1 | 13 | 222.98 | 14.50 |
| 6 | 1 | 13 | 200.88 | 14.10 |
| 6 | 1 | 13 | 221.18 | 14.60 |
| 6 | 1 | 13 | 212.78 | 23.90 |
| 7 | -1 | 13 | 1.10 | 3.10 |
| 7 | -1 | 13 | 5.50 | 3.80 |
| 7 | 1 | 13 | 7.10 | 3.20 |
| 7 | 1 | 13 | 6.50 | 3.60 |
| 8 | -1 | 13 | 305.17 | 18.70 |
| 8 | 1 | 13 | 314.67 | 19.40 |
| 9 | -1 | 13 | 0.10 | 2.70 |
| 9 | 1 | 13 | 2.50 | 2.40 |
| 10 | -1 | 13 | 453.95 | 24.40 |
| 10 | 1 | 13 | 402.16 | 24.20 |
| -15 | -2 | 13 | 267.57 | 15.50 |

| | | | | |
|---|---|---|---|---|
| -15 | 2 | 13 | 266.27 | 15.30 |
| -14 | -2 | 13 | 31.00 | 4.60 |
| -14 | 2 | 13 | 22.40 | 3.70 |
| -13 | -2 | 13 | 72.19 | 6.70 |
| -13 | 2 | 13 | 76.89 | 6.50 |
| 13 | 2 | -13 | 73.19 | 7.10 |
| -12 | -2 | 13 | 6.70 | 3.30 |
| -12 | 2 | 13 | 4.40 | 3.00 |
| 12 | 2 | -13 | 3.50 | 3.70 |
| -11 | -2 | 13 | 820.12 | 48.60 |
| -11 | 2 | 13 | 927.11 | 48.80 |
| -10 | -2 | 13 | 4.20 | 3.60 |
| -10 | 2 | 13 | 1.50 | 3.20 |
| -9 | -2 | 13 | 64.79 | 8.40 |
| -9 | 2 | 13 | 53.29 | 7.40 |
| -8 | -2 | 13 | 68.19 | 9.00 |
| -8 | 2 | 13 | 48.70 | 7.80 |
| -7 | -2 | 13 | 1546.45 | 84.49 |
| -7 | 2 | 13 | 1506.85 | 84.39 |
| -6 | -2 | 13 | -1.40 | 5.10 |
| -6 | -2 | 13 | -2.90 | 6.00 |
| -6 | 2 | 13 | -4.80 | 4.70 |
| -6 | 2 | 13 | -6.70 | 5.20 |
| -6 | 2 | 13 | -1.80 | 4.70 |
| -5 | -2 | 13 | 987.70 | 59.09 |
| -5 | -2 | 13 | 1073.19 | 58.79 |
| -5 | -2 | 13 | 991.40 | 57.49 |
| -5 | 2 | 13 | 1035.50 | 57.59 |
| -5 | 2 | 13 | 1102.79 | 59.09 |
| -4 | -2 | 13 | 110.29 | 9.50 |
| -4 | -2 | 13 | 99.69 | 10.20 |
| -4 | 2 | 13 | 105.19 | 12.00 |
| -4 | 2 | 13 | 99.49 | 11.10 |
| -4 | 2 | 13 | 99.49 | 9.60 |
| -3 | -2 | 13 | 412.96 | 24.90 |
| -3 | -2 | 13 | 347.47 | 28.70 |
| -3 | -2 | 13 | 369.16 | 23.50 |
| -3 | 2 | 13 | 393.46 | 23.80 |
| -3 | 2 | 13 | 418.26 | 25.40 |
| -3 | 2 | 13 | 419.66 | 25.50 |
| -2 | -2 | 13 | 5.20 | 3.50 |
| -2 | -2 | 13 | 1.10 | 4.00 |
| -2 | -2 | 13 | 3.10 | 10.10 |
| -2 | 2 | 13 | 9.00 | 5.30 |

| | | | | |
|---|---|---|---|---|
| -2 | 2 | 13 | -1.00 | 4.70 |
| -2 | 2 | 13 | -0.80 | 3.80 |
| -1 | -2 | 13 | 3198.98 | 168.08 |
| -1 | -2 | 13 | 2906.81 | 166.78 |
| -1 | -2 | 13 | 3047.30 | 166.48 |
| -1 | 2 | 13 | 3127.99 | 166.78 |
| -1 | 2 | 13 | 3086.39 | 168.28 |
| 0 | -2 | 13 | 16.10 | 6.00 |
| 0 | -2 | 13 | 11.90 | 5.10 |
| 0 | 2 | 13 | 5.10 | 4.60 |
| 0 | 2 | 13 | 1.10 | 4.90 |
| 1 | -2 | 13 | 6.00 | 5.10 |
| 1 | -2 | 13 | 6.80 | 5.20 |
| 1 | -2 | 13 | 0.40 | 4.50 |
| 1 | 2 | 13 | 3.00 | 4.40 |
| 1 | 2 | 13 | -2.50 | 4.60 |
| 1 | 2 | 13 | -2.30 | 5.10 |
| 2 | -2 | 13 | 15.70 | 4.90 |
| 2 | -2 | 13 | 6.40 | 5.90 |
| 2 | -2 | 13 | 10.00 | 5.00 |
| 2 | 2 | 13 | 10.40 | 5.10 |
| 2 | 2 | 13 | 14.00 | 3.90 |
| 2 | 2 | 13 | 6.80 | 6.40 |
| 3 | -2 | 13 | 1180.98 | 60.79 |
| 3 | -2 | 13 | 1049.99 | 63.19 |
| 3 | -2 | 13 | 1091.59 | 61.39 |
| 3 | 2 | 13 | 1086.59 | 60.59 |
| 3 | 2 | 13 | 1079.99 | 61.59 |
| 3 | 2 | 13 | 1056.29 | 63.69 |
| 4 | -2 | 13 | -5.00 | 7.90 |
| 4 | -2 | 13 | 4.40 | 4.70 |
| 4 | -2 | 13 | 9.30 | 4.20 |
| 4 | 2 | 13 | 13.80 | 4.80 |
| 4 | 2 | 13 | 8.80 | 3.60 |
| 4 | 2 | 13 | 8.30 | 8.50 |
| 5 | -2 | 13 | 876.91 | 47.50 |
| 5 | -2 | 13 | 816.92 | 50.99 |
| 5 | -2 | 13 | 816.82 | 47.00 |
| 5 | 2 | 13 | 699.13 | 51.39 |
| 5 | 2 | 13 | 908.41 | 47.60 |
| 5 | 2 | 13 | 913.01 | 47.20 |
| 6 | -2 | 13 | 3.10 | 3.50 |
| 6 | -2 | 13 | 7.30 | 3.60 |
| 6 | 2 | 13 | 0.50 | 3.30 |

| | | | | |
|---|---|---|---|---|
| 6 | 2 | 13 | -2.40 | 2.90 |
| 7 | -2 | 13 | 153.78 | 10.60 |
| 7 | -2 | 13 | 145.29 | 13.00 |
| 7 | 2 | 13 | 142.49 | 10.40 |
| 8 | -2 | 13 | 16.40 | 3.60 |
| 8 | 2 | 13 | 22.00 | 4.90 |
| 9 | -2 | 13 | 283.57 | 17.80 |
| 9 | 2 | 13 | 312.67 | 17.60 |
| 10 | -2 | 13 | 12.70 | 3.20 |
| 10 | 2 | 13 | 9.30 | 2.50 |
| -15 | 3 | 13 | 4.30 | 1.60 |
| 14 | 3 | -13 | 83.19 | 6.80 |
| -14 | 3 | 13 | 86.89 | 6.20 |
| -13 | -3 | 13 | 56.59 | 6.40 |
| 13 | 3 | -13 | 43.00 | 5.70 |
| -13 | 3 | 13 | 42.00 | 5.10 |
| -12 | -3 | 13 | 785.52 | 45.40 |
| 12 | 3 | -13 | 850.91 | 45.10 |
| -11 | -3 | 13 | 53.69 | 7.90 |
| 11 | 3 | -13 | 64.19 | 7.20 |
| -11 | 3 | 13 | 67.09 | 6.60 |
| -10 | -3 | 13 | 508.05 | 30.20 |
| -10 | 3 | 13 | 530.15 | 30.20 |
| -9 | -3 | 13 | 121.49 | 11.60 |
| -9 | 3 | 13 | 141.29 | 10.60 |
| -8 | -3 | 13 | 194.38 | 14.30 |
| -8 | 3 | 13 | 156.18 | 12.80 |
| -7 | -3 | 13 | 13.20 | 6.30 |
| -7 | -3 | 13 | 8.20 | 4.50 |
| -7 | 3 | 13 | 9.30 | 4.30 |
| -6 | -3 | 13 | 2285.37 | 132.59 |
| -6 | 3 | 13 | 2443.26 | 133.99 |
| -6 | 3 | 13 | 2616.14 | 133.99 |
| -5 | -3 | 13 | 84.09 | 10.20 |
| -5 | -3 | 13 | 88.89 | 14.40 |
| -5 | 3 | 13 | 80.99 | 10.90 |
| -5 | 3 | 13 | 85.09 | 9.30 |
| -5 | 3 | 13 | 74.69 | 11.00 |
| -4 | -3 | 13 | 14.00 | 3.90 |
| -4 | -3 | 13 | 13.60 | 5.20 |
| -4 | -3 | 13 | 13.00 | 8.10 |
| -4 | 3 | 13 | 9.90 | 4.20 |
| -4 | 3 | 13 | 6.70 | 5.10 |
| -3 | -3 | 13 | 249.08 | 17.60 |

| | | | | |
|---|---|---|---|---|
| -3 | -3 | 13 | 227.68 | 16.20 |
| -3 | -3 | 13 | 278.37 | 20.60 |
| -3 | 3 | 13 | 275.87 | 16.80 |
| -3 | 3 | 13 | 252.57 | 18.40 |
| -3 | 3 | 13 | 252.97 | 18.50 |
| -2 | -3 | 13 | 1717.93 | 92.09 |
| -2 | -3 | 13 | 1479.75 | 91.29 |
| -2 | -3 | 13 | 1686.23 | 90.59 |
| -2 | 3 | 13 | 1696.13 | 90.89 |
| -2 | 3 | 13 | 1696.73 | 92.69 |
| -1 | -3 | 13 | 4.50 | 4.70 |
| -1 | -3 | 13 | 11.90 | 7.00 |
| -1 | 3 | 13 | -7.80 | 5.00 |
| -1 | 3 | 13 | -0.50 | 6.40 |
| -1 | 3 | 13 | 0.40 | 6.50 |
| 0 | -3 | 13 | 1384.26 | 74.39 |
| 0 | -3 | 13 | 1322.77 | 73.89 |
| 0 | -3 | 13 | 1350.46 | 75.49 |
| 0 | 3 | 13 | 1309.07 | 74.49 |
| 0 | 3 | 13 | 1307.67 | 74.39 |
| 0 | 3 | 13 | 1349.16 | 75.99 |
| 1 | -3 | 13 | 21.40 | 6.40 |
| 1 | -3 | 13 | 17.90 | 8.50 |
| 1 | -3 | 13 | 39.10 | 8.30 |
| 1 | 3 | 13 | 31.40 | 7.10 |
| 1 | 3 | 13 | 16.30 | 9.00 |
| 1 | 3 | 13 | 26.60 | 5.60 |
| 2 | -3 | 13 | 768.92 | 43.50 |
| 2 | -3 | 13 | 709.83 | 45.60 |
| 2 | -3 | 13 | 841.72 | 44.20 |
| 2 | 3 | 13 | 761.12 | 43.30 |
| 2 | 3 | 13 | 735.73 | 44.30 |
| 2 | 3 | 13 | 759.52 | 46.10 |
| 3 | -3 | 13 | 5.00 | 6.90 |
| 3 | -3 | 13 | 5.00 | 4.80 |
| 3 | -3 | 13 | 4.50 | 4.70 |
| 3 | 3 | 13 | 11.10 | 5.10 |
| 3 | 3 | 13 | 7.90 | 3.80 |
| 3 | 3 | 13 | 3.70 | 7.50 |
| 4 | -3 | 13 | 1474.75 | 86.49 |
| 4 | -3 | 13 | 1604.04 | 87.29 |
| 4 | 3 | 13 | 1613.34 | 86.69 |
| 4 | 3 | 13 | 1654.13 | 87.39 |
| 4 | 3 | 13 | 1564.74 | 90.29 |

| | | | | |
|---|---|---|---|---|
| 5 | -3 | 13 | 29.80 | 5.20 |
| 5 | -3 | 13 | 44.50 | 7.20 |
| 5 | -3 | 13 | 21.80 | 10.30 |
| 5 | 3 | 13 | 33.00 | 7.10 |
| 5 | 3 | 13 | 30.80 | 5.80 |
| 5 | 3 | 13 | 19.90 | 11.40 |
| 6 | -3 | 13 | 4.70 | 3.70 |
| 6 | -3 | 13 | 3.30 | 3.80 |
| 6 | 3 | 13 | 2.80 | 3.30 |
| 6 | 3 | 13 | 3.30 | 2.90 |
| 7 | -3 | 13 | 51.29 | 10.40 |
| 7 | -3 | 13 | 42.10 | 7.20 |
| 7 | 3 | 13 | 54.99 | 5.80 |
| 8 | -3 | 13 | 569.64 | 32.00 |
| 8 | 3 | 13 | 556.74 | 31.80 |
| 9 | -3 | 13 | 8.70 | 3.40 |
| 9 | 3 | 13 | 5.60 | 2.60 |
| 10 | -3 | 13 | 319.57 | 19.40 |
| 10 | 3 | 13 | 337.87 | 18.90 |
| 14 | 4 | -13 | 2.10 | 2.30 |
| -14 | 4 | 13 | 2.40 | 1.70 |
| 13 | 4 | -13 | 7.10 | 2.60 |
| -13 | 4 | 13 | 9.00 | 2.30 |
| 12 | 4 | -13 | 4.50 | 2.90 |
| -12 | 4 | 13 | 3.40 | 2.80 |
| -11 | 4 | 13 | 1099.59 | 60.79 |
| -10 | 4 | 13 | 3.70 | 2.90 |
| 10 | 4 | -13 | 7.20 | 3.40 |
| -9 | 4 | 13 | 71.89 | 7.70 |
| -8 | 4 | 13 | 46.40 | 7.40 |
| -7 | -4 | 13 | 818.52 | 49.50 |
| -7 | 4 | 13 | 963.70 | 50.79 |
| -6 | -4 | 13 | 48.70 | 7.60 |
| -6 | 4 | 13 | 50.19 | 9.00 |
| -6 | 4 | 13 | 47.60 | 8.30 |
| -6 | 4 | 13 | 48.60 | 9.60 |
| -5 | -4 | 13 | 700.93 | 39.40 |
| -5 | 4 | 13 | 624.84 | 41.00 |
| -5 | 4 | 13 | 732.73 | 41.40 |
| -5 | 4 | 13 | 728.13 | 41.10 |
| -4 | -4 | 13 | 11.20 | 3.80 |
| -4 | 4 | 13 | 16.50 | 6.00 |
| -4 | 4 | 13 | 15.10 | 5.60 |
| -3 | -4 | 13 | 52.29 | 8.10 |

| | | | | |
|---|---|---|---|---|
| -3 | -4 | 13 | 67.19 | 9.20 |
| -3 | 4 | 13 | 51.19 | 9.70 |
| -3 | 4 | 13 | 58.39 | 10.90 |
| -2 | -4 | 13 | 45.60 | 8.30 |
| -2 | -4 | 13 | 34.40 | 6.60 |
| -2 | 4 | 13 | 25.30 | 5.90 |
| -2 | 4 | 13 | 31.80 | 7.00 |
| -1 | -4 | 13 | 2182.38 | 119.69 |
| -1 | -4 | 13 | 2100.49 | 117.89 |
| -1 | 4 | 13 | 2204.58 | 120.49 |
| -1 | 4 | 13 | 2184.38 | 118.49 |
| 0 | -4 | 13 | 71.79 | 12.30 |
| 0 | -4 | 13 | 63.19 | 8.30 |
| 0 | 4 | 13 | 72.29 | 17.00 |
| 0 | 4 | 13 | 73.69 | 8.90 |
| 0 | 4 | 13 | 69.59 | 10.10 |
| 1 | -4 | 13 | 1.90 | 4.30 |
| 1 | -4 | 13 | 7.40 | 5.40 |
| 1 | 4 | 13 | 5.00 | 5.90 |
| 1 | 4 | 13 | -0.60 | 3.80 |
| 1 | 4 | 13 | 1.90 | 4.90 |
| 2 | -4 | 13 | 0.40 | 6.10 |
| 2 | -4 | 13 | 4.00 | 4.20 |
| 2 | 4 | 13 | -0.30 | 3.50 |
| 2 | 4 | 13 | 0.80 | 4.80 |
| 2 | 4 | 13 | -1.00 | 7.50 |
| 3 | -4 | 13 | 556.94 | 40.40 |
| 3 | -4 | 13 | 709.23 | 38.60 |
| 3 | 4 | 13 | 661.23 | 37.70 |
| 3 | 4 | 13 | 680.33 | 38.80 |
| 3 | 4 | 13 | 687.83 | 41.90 |
| 4 | -4 | 13 | 9.10 | 8.70 |
| 4 | -4 | 13 | 10.30 | 4.50 |
| 4 | -4 | 13 | 3.80 | 5.10 |
| 4 | 4 | 13 | 11.70 | 4.10 |
| 4 | 4 | 13 | 8.30 | 3.70 |
| 4 | 4 | 13 | 3.40 | 8.60 |
| 5 | -4 | 13 | 543.15 | 31.00 |
| 5 | -4 | 13 | 556.94 | 31.20 |
| 5 | 4 | 13 | 432.16 | 36.80 |
| 5 | 4 | 13 | 562.64 | 31.10 |
| 5 | 4 | 13 | 542.15 | 30.70 |
| 6 | -4 | 13 | 83.39 | 8.70 |
| 6 | -4 | 13 | 78.59 | 9.20 |

| | | | | |
|---|---|---|---|---|
| 6 | 4 | 13 | 86.79 | 7.30 |
| 6 | 4 | 13 | 60.79 | 11.90 |
| 7 | -4 | 13 | 249.18 | 16.30 |
| 7 | -4 | 13 | 274.47 | 17.10 |
| 7 | 4 | 13 | 261.17 | 15.90 |
| 7 | 4 | 13 | 242.18 | 18.70 |
| 8 | -4 | 13 | -6.30 | 4.10 |
| 8 | 4 | 13 | -2.00 | 2.30 |
| 8 | 4 | 13 | -5.80 | 6.00 |
| 9 | -4 | 13 | 363.16 | 21.30 |
| 9 | 4 | 13 | 303.77 | 22.80 |
| 9 | 4 | 13 | 377.36 | 20.30 |
| 10 | -4 | 13 | -9.30 | 4.70 |
| 10 | 4 | 13 | 4.90 | 2.10 |
| -14 | 5 | 13 | 106.99 | 7.20 |
| 14 | 5 | -13 | 109.09 | 7.90 |
| -13 | 5 | 13 | 2.80 | 2.10 |
| 13 | 5 | -13 | 4.10 | 2.40 |
| -12 | 5 | 13 | 242.58 | 14.90 |
| 12 | 5 | -13 | 242.78 | 14.40 |
| 11 | 5 | -13 | 78.09 | 7.30 |
| -11 | 5 | 13 | 72.39 | 6.80 |
| -10 | 5 | 13 | 464.45 | 27.80 |
| 10 | 5 | -13 | 491.15 | 27.10 |
| -9 | 5 | 13 | 96.79 | 8.40 |
| 9 | 5 | -13 | 80.29 | 8.20 |
| -8 | 5 | 13 | 18.80 | 4.60 |
| -7 | -5 | 13 | 30.10 | 4.80 |
| -7 | 5 | 13 | 36.40 | 7.70 |
| -6 | -5 | 13 | 1527.35 | 84.49 |
| -6 | 5 | 13 | 1568.04 | 85.99 |
| -5 | -5 | 13 | 5.80 | 3.70 |
| -5 | 5 | 13 | 11.80 | 4.30 |
| -5 | 5 | 13 | 9.00 | 5.70 |
| -4 | -5 | 13 | 155.08 | 11.30 |
| -4 | 5 | 13 | 140.79 | 17.60 |
| -4 | 5 | 13 | 156.68 | 12.20 |
| -4 | 5 | 13 | 143.09 | 13.60 |
| -3 | -5 | 13 | 14.80 | 3.70 |
| -3 | 5 | 13 | 6.80 | 4.80 |
| -3 | 5 | 13 | 16.80 | 4.50 |
| -3 | 5 | 13 | 8.30 | 6.80 |
| -2 | -5 | 13 | 418.56 | 24.90 |
| -2 | 5 | 13 | 437.06 | 25.60 |

| | | | | |
|---|---|---|---|---|
| -2 | 5 | 13 | 405.96 | 26.20 |
| -1 | -5 | 13 | 61.09 | 7.70 |
| -1 | -5 | 13 | 81.69 | 11.30 |
| -1 | 5 | 13 | 57.39 | 8.90 |
| -1 | 5 | 13 | 64.79 | 15.10 |
| -1 | 5 | 13 | 56.99 | 8.90 |
| 0 | -5 | 13 | 902.71 | 48.60 |
| 0 | -5 | 13 | 895.01 | 50.69 |
| 0 | 5 | 13 | 776.22 | 50.99 |
| 0 | 5 | 13 | 906.71 | 49.60 |
| 0 | 5 | 13 | 834.52 | 48.90 |
| 1 | -5 | 13 | 16.50 | 4.30 |
| 1 | -5 | 13 | 6.40 | 6.20 |
| 1 | 5 | 13 | 19.30 | 5.60 |
| 1 | 5 | 13 | 19.10 | 7.50 |
| 1 | 5 | 13 | 15.20 | 4.60 |
| 2 | -5 | 13 | 86.39 | 9.70 |
| 2 | 5 | 13 | 66.89 | 8.20 |
| 2 | 5 | 13 | 83.79 | 10.40 |
| 2 | 5 | 13 | 80.09 | 14.30 |
| 3 | -5 | 13 | 20.80 | 5.10 |
| 3 | 5 | 13 | 14.60 | 4.90 |
| 3 | 5 | 13 | 23.30 | 9.90 |
| 3 | 5 | 13 | 23.90 | 4.60 |
| 4 | -5 | 13 | 893.31 | 52.29 |
| 4 | 5 | 13 | 945.31 | 51.79 |
| 4 | 5 | 13 | 902.21 | 56.69 |
| 4 | 5 | 13 | 968.00 | 52.39 |
| 5 | -5 | 13 | 58.19 | 8.10 |
| 5 | 5 | 13 | 37.00 | 5.80 |
| 5 | 5 | 13 | 40.20 | 7.60 |
| 5 | 5 | 13 | 86.69 | 20.10 |
| 6 | -5 | 13 | 19.60 | 4.20 |
| 6 | 5 | 13 | 19.00 | 3.60 |
| 6 | 5 | 13 | 25.30 | 6.70 |
| 7 | 5 | 13 | 15.60 | 6.40 |
| 7 | 5 | 13 | 7.50 | 3.60 |
| 8 | 5 | 13 | 265.77 | 15.80 |
| 8 | 5 | 13 | 257.17 | 18.20 |
| 9 | 5 | 13 | -0.50 | 1.90 |
| 9 | 5 | 13 | 4.20 | 5.90 |
| 10 | 5 | 13 | 308.97 | 17.70 |
| -14 | 6 | 13 | 21.50 | 3.10 |
| 14 | 6 | -13 | 25.30 | 4.00 |

| | | | | |
|---|---|---|---|---|
| -13 | 6 | 13 | 45.00 | 4.50 |
| 13 | 6 | -13 | 40.90 | 4.50 |
| 12 | 6 | -13 | 4.00 | 2.30 |
| -12 | 6 | 13 | 0.60 | 2.10 |
| -11 | 6 | 13 | 688.33 | 37.40 |
| 11 | 6 | -13 | 594.14 | 35.30 |
| -10 | 6 | 13 | 96.09 | 8.50 |
| 10 | 6 | -13 | 118.19 | 8.30 |
| -9 | 6 | 13 | 167.18 | 12.10 |
| 9 | 6 | -13 | 170.18 | 11.30 |
| 8 | 6 | -13 | 4.30 | 3.60 |
| -8 | 6 | 13 | -0.20 | 3.40 |
| -7 | -6 | 13 | 301.77 | 19.00 |
| -7 | 6 | 13 | 321.37 | 20.20 |
| -6 | -6 | 13 | 109.59 | 10.10 |
| -6 | 6 | 13 | 148.29 | 12.10 |
| -5 | -6 | 13 | 471.35 | 28.30 |
| -5 | 6 | 13 | 477.75 | 30.00 |
| -5 | 6 | 13 | 522.85 | 31.70 |
| -4 | -6 | 13 | 63.49 | 7.10 |
| -4 | 6 | 13 | 43.90 | 7.90 |
| -4 | 6 | 13 | 68.09 | 12.60 |
| -4 | 6 | 13 | 42.40 | 9.40 |
| -3 | -6 | 13 | 34.40 | 6.20 |
| -3 | 6 | 13 | 33.00 | 6.30 |
| -3 | 6 | 13 | 24.10 | 4.70 |
| -3 | 6 | 13 | 58.09 | 11.90 |
| -2 | -6 | 13 | 33.80 | 6.30 |
| -2 | 6 | 13 | 19.80 | 6.90 |
| -2 | 6 | 13 | 24.50 | 5.80 |
| -2 | 6 | 13 | 28.10 | 5.10 |
| -1 | -6 | 13 | 1522.55 | 83.59 |
| -1 | 6 | 13 | 1557.74 | 84.29 |
| -1 | 6 | 13 | 1556.84 | 84.29 |
| -1 | 6 | 13 | 1455.25 | 86.39 |
| 0 | -6 | 13 | 333.17 | 20.90 |
| 0 | 6 | 13 | 284.87 | 24.10 |
| 0 | 6 | 13 | 334.47 | 22.10 |
| 0 | 6 | 13 | 392.76 | 21.60 |
| 1 | -6 | 13 | 19.60 | 4.20 |
| 1 | 6 | 13 | 11.50 | 4.20 |
| 1 | 6 | 13 | 13.40 | 7.30 |
| 1 | 6 | 13 | 14.50 | 5.00 |
| 2 | -6 | 13 | 72.39 | 8.70 |

| | | | | |
|---|---|---|---|---|
| 2 | 6 | 13 | 45.50 | 9.70 |
| 2 | 6 | 13 | 56.29 | 9.60 |
| 2 | 6 | 13 | 45.40 | 7.10 |
| 3 | -6 | 13 | 534.35 | 31.40 |
| 3 | 6 | 13 | 553.04 | 31.30 |
| 3 | 6 | 13 | 500.35 | 30.60 |
| 3 | 6 | 13 | 528.35 | 35.90 |
| 4 | -6 | 13 | 112.09 | 10.80 |
| 4 | 6 | 13 | 93.59 | 20.70 |
| 4 | 6 | 13 | 146.09 | 12.80 |
| 4 | 6 | 13 | 112.79 | 9.10 |
| 5 | -6 | 13 | 756.32 | 43.30 |
| 5 | 6 | 13 | 787.52 | 44.10 |
| 5 | 6 | 13 | 759.22 | 43.00 |
| 6 | 6 | 13 | 45.20 | 5.50 |
| 6 | 6 | 13 | 39.90 | 7.20 |
| 7 | 6 | 13 | 131.29 | 9.50 |
| 7 | 6 | 13 | 131.79 | 12.70 |
| 8 | 6 | 13 | 18.20 | 2.90 |
| 9 | 6 | 13 | 338.97 | 19.10 |
| 9 | 6 | 13 | 324.27 | 21.00 |
| -13 | 7 | 13 | 17.70 | 3.40 |
| 13 | 7 | -13 | 20.80 | 3.60 |
| -12 | 7 | 13 | 420.26 | 24.30 |
| 12 | 7 | -13 | 431.56 | 23.80 |
| 11 | 7 | -13 | -2.50 | 2.40 |
| -11 | 7 | 13 | -1.50 | 2.40 |
| -10 | 7 | 13 | 412.16 | 25.10 |
| 10 | 7 | -13 | 449.86 | 25.80 |
| -9 | 7 | 13 | 174.78 | 12.10 |
| 9 | 7 | -13 | 171.08 | 11.30 |
| -8 | 7 | 13 | 34.70 | 6.50 |
| 8 | 7 | -13 | 30.10 | 4.20 |
| -7 | -7 | 13 | 2.30 | 3.40 |
| 7 | 7 | -13 | 4.00 | 3.50 |
| -7 | 7 | 13 | -0.90 | 3.50 |
| -6 | -7 | 13 | 1406.06 | 77.29 |
| 6 | 7 | -13 | 1419.06 | 79.09 |
| -6 | 7 | 13 | 1415.56 | 78.79 |
| -5 | -7 | 13 | 340.77 | 21.70 |
| -5 | 7 | 13 | 363.56 | 26.60 |
| -5 | 7 | 13 | 395.46 | 23.90 |
| -4 | -7 | 13 | 7.70 | 3.20 |
| -4 | 7 | 13 | 1.00 | 4.90 |

| | | | | |
|---|---|---|---|---|
| -4 | 7 | 13 | 3.80 | 8.00 |
| -3 | -7 | 13 | 68.79 | 7.20 |
| -3 | 7 | 13 | 57.99 | 13.10 |
| -3 | 7 | 13 | 45.40 | 9.20 |
| -2 | -7 | 13 | 468.15 | 27.70 |
| -2 | 7 | 13 | 505.95 | 32.00 |
| -2 | 7 | 13 | 462.25 | 29.00 |
| -1 | -7 | 13 | 41.20 | 6.30 |
| -1 | 7 | 13 | 26.00 | 5.30 |
| -1 | 7 | 13 | 19.10 | 8.30 |
| 0 | -7 | 13 | 814.22 | 45.20 |
| 0 | 7 | 13 | 807.62 | 45.60 |
| 0 | 7 | 13 | 781.92 | 48.70 |
| 1 | -7 | 13 | 20.00 | 4.30 |
| 1 | 7 | 13 | 3.70 | 7.30 |
| 1 | 7 | 13 | 11.70 | 3.90 |
| 1 | 7 | 13 | 27.60 | 8.80 |
| 2 | -7 | 13 | 117.49 | 10.10 |
| 2 | 7 | 13 | 105.79 | 9.40 |
| 2 | 7 | 13 | 95.49 | 16.30 |
| 2 | 7 | 13 | 99.49 | 10.70 |
| 3 | -7 | 13 | 3.30 | 4.20 |
| 3 | 7 | 13 | 12.20 | 5.80 |
| 3 | 7 | 13 | 5.20 | 3.10 |
| 3 | 7 | 13 | 4.80 | 9.50 |
| 4 | -7 | 13 | 633.84 | 37.50 |
| 4 | 7 | 13 | 710.13 | 37.80 |
| 4 | 7 | 13 | 649.83 | 36.90 |
| 4 | 7 | 13 | 586.24 | 51.99 |
| 5 | -7 | 13 | 25.70 | 4.60 |
| 5 | 7 | 13 | 32.70 | 5.20 |
| 6 | -7 | 13 | 81.59 | 8.50 |
| 6 | 7 | 13 | 93.89 | 10.70 |
| 6 | 7 | 13 | 86.39 | 8.50 |
| 7 | 7 | 13 | 6.20 | 5.80 |
| 7 | 7 | 13 | -0.40 | 2.40 |
| 8 | 7 | 13 | 152.78 | 12.90 |
| 8 | 7 | 13 | 161.58 | 10.10 |
| 9 | 7 | 13 | -6.00 | 5.50 |
| 9 | 7 | 13 | 0.30 | 1.90 |
| -13 | 8 | 13 | 5.90 | 1.90 |
| 13 | 8 | -13 | 5.90 | 1.80 |
| 12 | 8 | -13 | 4.90 | 2.00 |
| -12 | 8 | 13 | 0.50 | 1.90 |

| | | | | |
|---|---|---|---|---|
| 11 | 8 | -13 | 722.03 | 38.60 |
| -11 | 8 | 13 | 684.03 | 39.20 |
| 10 | 8 | -13 | 26.10 | 4.90 |
| -10 | 8 | 13 | 37.90 | 6.20 |
| 9 | 8 | -13 | 49.70 | 6.30 |
| -9 | 8 | 13 | 61.79 | 6.70 |
| -8 | -8 | 13 | 23.60 | 4.10 |
| -8 | 8 | 13 | 27.70 | 4.70 |
| 8 | 8 | -13 | 33.20 | 5.60 |
| -7 | -8 | 13 | 1226.08 | 72.49 |
| -7 | 8 | 13 | 1517.25 | 74.09 |
| 7 | 8 | -13 | 1247.78 | 72.39 |
| -6 | -8 | 13 | 4.30 | 3.10 |
| 6 | 8 | -13 | 3.80 | 6.80 |
| -6 | 8 | 13 | 6.70 | 4.90 |
| 6 | 8 | -13 | 11.40 | 4.00 |
| -5 | -8 | 13 | 517.45 | 30.00 |
| -5 | 8 | 13 | 521.95 | 31.80 |
| -5 | 8 | 13 | 532.75 | 35.40 |
| 5 | 8 | -13 | 530.25 | 32.60 |
| -4 | -8 | 13 | 59.39 | 6.90 |
| -4 | 8 | 13 | 52.39 | 10.30 |
| -4 | 8 | 13 | 54.79 | 9.40 |
| -3 | -8 | 13 | 120.29 | 9.50 |
| -3 | 8 | 13 | 109.99 | 11.60 |
| -3 | 8 | 13 | 137.09 | 16.00 |
| -2 | -8 | 13 | 4.10 | 3.00 |
| -2 | 8 | 13 | 1.10 | 4.10 |
| -2 | 8 | 13 | 12.40 | 8.50 |
| -1 | -8 | 13 | 1301.57 | 68.89 |
| -1 | 8 | 13 | 1278.47 | 72.69 |
| -1 | 8 | 13 | 1171.08 | 69.39 |
| 0 | -8 | 13 | 171.38 | 11.80 |
| 0 | 8 | 13 | 161.98 | 12.30 |
| 0 | 8 | 13 | 171.58 | 12.60 |
| 0 | 8 | 13 | 130.59 | 17.70 |
| 1 | -8 | 13 | 0.70 | 3.30 |
| 1 | 8 | 13 | 1.10 | 4.80 |
| 1 | 8 | 13 | 3.10 | 3.30 |
| 1 | 8 | 13 | 1.00 | 8.80 |
| 2 | -8 | 13 | 13.00 | 4.40 |
| 2 | 8 | 13 | 7.90 | 8.40 |
| 2 | 8 | 13 | 8.50 | 3.40 |
| 2 | 8 | 13 | 22.20 | 5.60 |

| | | | | |
|---|---|---|---|---|
| 3 | -8 | 13 | 423.06 | 24.30 |
| 3 | 8 | 13 | 394.76 | 24.00 |
| 3 | 8 | 13 | 405.16 | 23.60 |
| 3 | 8 | 13 | 349.67 | 31.50 |
| 4 | -8 | 13 | 6.00 | 4.00 |
| 4 | 8 | 13 | -3.00 | 4.50 |
| 4 | 8 | 13 | 0.10 | 2.60 |
| 5 | -8 | 13 | 480.35 | 34.50 |
| 5 | 8 | 13 | 567.84 | 31.50 |
| 5 | 8 | 13 | 604.14 | 32.50 |
| 6 | -8 | 13 | 9.30 | 3.60 |
| 6 | 8 | 13 | -3.60 | 4.90 |
| 6 | 8 | 13 | 6.10 | 2.60 |
| -7 | -8 | -13 | 172.48 | 11.40 |
| 7 | 8 | 13 | 147.99 | 13.30 |
| 7 | 8 | 13 | 175.78 | 10.80 |
| 8 | 8 | 13 | 12.50 | 5.90 |
| 8 | 8 | 13 | 5.40 | 2.30 |
| 9 | 8 | 13 | 245.78 | 13.60 |
| 9 | 8 | 13 | 207.58 | 17.80 |
| -12 | 9 | 13 | 498.45 | 29.60 |
| 12 | 9 | -13 | 563.34 | 29.20 |
| -11 | 9 | 13 | 3.30 | 2.10 |
| 11 | 9 | -13 | 4.40 | 2.00 |
| 10 | 9 | -13 | 378.96 | 21.40 |
| 9 | 9 | -13 | -0.90 | 2.50 |
| 8 | 9 | -13 | 11.80 | 3.20 |
| -8 | 9 | 13 | 8.90 | 3.50 |
| -7 | -9 | 13 | 3.80 | 2.90 |
| -7 | 9 | 13 | 9.10 | 4.00 |
| 7 | 9 | -13 | 1.40 | 2.90 |
| -6 | -9 | 13 | 1007.40 | 57.09 |
| 6 | -9 | -13 | 1033.20 | 57.49 |
| 6 | 9 | -13 | 1093.09 | 60.99 |
| 6 | 9 | -13 | 1018.10 | 56.99 |
| -5 | -9 | 13 | 63.39 | 6.80 |
| 5 | 9 | -13 | 63.59 | 8.40 |
| -5 | 9 | 13 | 58.69 | 9.60 |
| 5 | 9 | -13 | 85.69 | 12.40 |
| -4 | -9 | 13 | 183.18 | 11.60 |
| 4 | 9 | -13 | 157.18 | 15.50 |
| 4 | 9 | -13 | 145.19 | 14.70 |
| -4 | 9 | 13 | 167.08 | 13.70 |
| -4 | 9 | 13 | 158.68 | 20.10 |

| | | | | |
|---|---|---|---|---|
| -3 | -9 | 13 | 0.90 | 2.70 |
| -3 | 9 | 13 | -4.00 | 8.50 |
| 3 | 9 | -13 | 0.30 | 5.90 |
| -3 | 9 | 13 | 1.20 | 4.50 |
| -2 | -9 | 13 | 1154.48 | 61.19 |
| -2 | 9 | 13 | 1100.89 | 61.99 |
| -2 | 9 | 13 | 1060.49 | 64.99 |
| -1 | -9 | 13 | 20.10 | 3.80 |
| -1 | 9 | 13 | 11.10 | 4.30 |
| -1 | 9 | 13 | 8.60 | 8.50 |
| -1 | 9 | 13 | 27.00 | 6.60 |
| 0 | -9 | 13 | 705.63 | 37.60 |
| 0 | 9 | 13 | 640.44 | 41.80 |
| 0 | 9 | 13 | 692.83 | 38.90 |
| 0 | 9 | 13 | 602.14 | 37.70 |
| 1 | -9 | 13 | 53.39 | 7.10 |
| 1 | 9 | 13 | 52.09 | 7.30 |
| 1 | 9 | 13 | 42.20 | 7.90 |
| 2 | -9 | 13 | 203.18 | 13.70 |
| 2 | 9 | 13 | 199.78 | 13.90 |
| 2 | 9 | 13 | 192.08 | 13.30 |
| 3 | -9 | 13 | 11.30 | 3.80 |
| 3 | 9 | 13 | 4.10 | 3.00 |
| 3 | 9 | 13 | -3.00 | 4.10 |
| 4 | -9 | 13 | 604.04 | 35.20 |
| 4 | 9 | 13 | 587.64 | 34.60 |
| 4 | 9 | 13 | 652.33 | 35.30 |
| 5 | -9 | 13 | 19.70 | 4.10 |
| 5 | 9 | 13 | 36.90 | 7.60 |
| 5 | 9 | 13 | 32.50 | 5.20 |
| 6 | -9 | 13 | 10.60 | 3.70 |
| -6 | -9 | -13 | 17.40 | 3.40 |
| 6 | 9 | 13 | 12.80 | 2.80 |
| 6 | 9 | 13 | 8.00 | 5.00 |
| -7 | -9 | -13 | 7.60 | 3.00 |
| 7 | 9 | 13 | 1.00 | 5.10 |
| 7 | 9 | 13 | 6.10 | 2.40 |
| 8 | 9 | 13 | 334.97 | 18.70 |
| 8 | 9 | 13 | 316.67 | 20.30 |
| -12 | 10 | 13 | 51.29 | 4.80 |
| 12 | 10 | -13 | 58.49 | 4.50 |
| -11 | 10 | 13 | 458.65 | 26.30 |
| 11 | 10 | -13 | 475.65 | 25.80 |
| -10 | 10 | 13 | 5.60 | 2.50 |

| | | | | |
|---|---|---|---|---|
| 10 | 10 | -13 | 6.20 | 2.20 |
| -9 | 10 | 13 | 58.39 | 6.60 |
| 9 | 10 | -13 | 71.49 | 5.80 |
| -8 | -10 | 13 | 139.69 | 10.70 |
| 8 | 10 | -13 | 144.29 | 9.80 |
| -8 | 10 | 13 | 167.88 | 12.70 |
| -7 | -10 | 13 | 572.64 | 34.70 |
| -7 | 10 | 13 | 633.04 | 35.10 |
| -6 | -10 | 13 | 1.30 | 2.60 |
| 6 | -10 | -13 | 8.10 | 4.20 |
| -6 | 10 | 13 | -2.10 | 3.80 |
| 6 | 10 | -13 | 3.80 | 3.20 |
| -5 | -10 | 13 | 506.55 | 28.30 |
| 5 | -10 | -13 | 485.65 | 28.70 |
| -5 | 10 | 13 | 478.05 | 29.90 |
| 5 | 10 | -13 | 508.65 | 28.50 |
| -4 | -10 | 13 | 30.70 | 5.30 |
| 4 | 10 | -13 | 13.60 | 12.60 |
| -4 | 10 | 13 | 15.60 | 5.30 |
| -3 | -10 | 13 | 39.80 | 5.90 |
| -3 | 10 | 13 | 30.70 | 11.20 |
| -3 | 10 | 13 | 33.70 | 7.10 |
| 3 | 10 | -13 | 25.00 | 7.20 |
| -3 | 10 | 13 | 49.20 | 8.20 |
| -2 | -10 | 13 | 25.20 | 5.40 |
| -2 | 10 | 13 | 25.10 | 10.20 |
| 2 | 10 | -13 | 27.60 | 7.90 |
| -1 | -10 | 13 | 1172.78 | 61.49 |
| -1 | 10 | 13 | 1144.49 | 61.99 |
| -1 | 10 | 13 | 1056.99 | 66.09 |
| -1 | 10 | 13 | 1070.19 | 61.19 |
| 0 | -10 | 13 | 4.80 | 2.90 |
| 0 | 10 | 13 | 9.00 | 4.00 |
| 0 | 10 | 13 | 5.80 | 3.50 |
| 1 | -10 | 13 | 74.19 | 7.60 |
| 1 | 10 | 13 | 68.19 | 7.70 |
| 1 | 10 | 13 | 88.59 | 8.20 |
| 2 | -10 | 13 | 23.30 | 4.10 |
| 2 | 10 | 13 | 11.40 | 4.10 |
| 2 | 10 | 13 | 25.50 | 5.80 |
| 3 | -10 | 13 | 789.72 | 44.60 |
| 3 | 10 | 13 | 761.12 | 44.20 |
| 3 | 10 | 13 | 826.22 | 44.50 |
| -4 | -10 | -13 | 7.00 | 3.80 |

| | | | | |
|---|---|---|---|---|
| 4 | -10 | 13 | 0.50 | 3.30 |
| 4 | 10 | 13 | 8.90 | 4.10 |
| 4 | 10 | 13 | 3.40 | 2.90 |
| 5 | -10 | 13 | 528.85 | 30.90 |
| -5 | -10 | -13 | 555.74 | 31.20 |
| 5 | 10 | 13 | 573.94 | 31.50 |
| 5 | 10 | 13 | 528.85 | 30.70 |
| -6 | -10 | -13 | 32.20 | 5.30 |
| 6 | -10 | 13 | 37.40 | 6.50 |
| 6 | 10 | 13 | 35.30 | 7.00 |
| 6 | 10 | 13 | 46.40 | 5.00 |
| -7 | -10 | -13 | 200.08 | 12.80 |
| 7 | 10 | 13 | 216.68 | 12.80 |
| 7 | 10 | 13 | 218.08 | 14.70 |
| -11 | 11 | 13 | -0.60 | 2.00 |
| 11 | 11 | -13 | -0.10 | 1.60 |
| -10 | 11 | 13 | 122.99 | 8.70 |
| 10 | 11 | -13 | 135.29 | 8.10 |
| 9 | 11 | -13 | 22.70 | 3.90 |
| -9 | 11 | 13 | 25.40 | 5.10 |
| -8 | -11 | 13 | 82.89 | 7.40 |
| 8 | 11 | -13 | 77.99 | 6.40 |
| -8 | 11 | 13 | 94.99 | 8.20 |
| -7 | -11 | 13 | 59.69 | 6.50 |
| -7 | 11 | 13 | 68.69 | 8.60 |
| 7 | 11 | -13 | 79.99 | 6.40 |
| 6 | -11 | -13 | 827.02 | 44.40 |
| 6 | 11 | -13 | 762.62 | 44.00 |
| 5 | -11 | -13 | 10.20 | 4.10 |
| -5 | -11 | 13 | 4.00 | 2.60 |
| -5 | 11 | 13 | -1.00 | 4.20 |
| 5 | 11 | -13 | 11.80 | 4.30 |
| -4 | -11 | 13 | 6.60 | 2.60 |
| 4 | -11 | -13 | 4.90 | 3.30 |
| -4 | 11 | 13 | 6.10 | 3.60 |
| -4 | 11 | 13 | 4.60 | 4.40 |
| -3 | -11 | 13 | 106.59 | 8.20 |
| 3 | -11 | -13 | 122.39 | 9.40 |
| -3 | 11 | 13 | 96.19 | 8.90 |
| -3 | 11 | 13 | 93.09 | 10.40 |
| -2 | -11 | 13 | 604.14 | 36.50 |
| -2 | 11 | 13 | 640.84 | 36.60 |
| -2 | 11 | 13 | 709.83 | 37.70 |
| -1 | -11 | 13 | 0.50 | 2.60 |

| | | | | |
|---|---|---|---|---|
| -1 | 11 | 13 | -0.10 | 3.70 |
| -1 | 11 | 13 | 0.10 | 3.60 |
| 0 | -11 | 13 | 353.26 | 20.70 |
| 0 | 11 | 13 | 323.17 | 21.10 |
| 0 | 11 | 13 | 365.16 | 20.90 |
| 1 | -11 | 13 | 77.09 | 7.30 |
| 1 | 11 | 13 | 77.69 | 7.70 |
| 1 | 11 | 13 | 71.19 | 7.80 |
| -2 | -11 | -13 | 212.28 | 15.40 |
| 2 | 11 | 13 | 222.28 | 14.60 |
| 2 | 11 | 13 | 229.98 | 14.50 |
| 3 | -11 | 13 | 10.00 | 3.30 |
| -3 | -11 | -13 | 12.20 | 3.70 |
| 3 | 11 | 13 | 13.90 | 3.90 |
| 3 | 11 | 13 | 17.00 | 3.60 |
| 4 | -11 | 13 | 720.83 | 40.90 |
| -4 | -11 | -13 | 714.33 | 41.20 |
| 4 | 11 | 13 | 774.82 | 41.20 |
| 4 | 11 | 13 | 717.03 | 40.50 |
| 5 | -11 | 13 | 3.50 | 3.00 |
| -5 | -11 | -13 | -0.60 | 2.50 |
| 5 | 11 | 13 | 3.00 | 6.30 |
| 5 | 11 | 13 | 3.20 | 2.50 |
| -6 | -11 | -13 | 29.30 | 4.90 |
| 6 | 11 | 13 | 22.30 | 5.10 |
| 6 | 11 | 13 | 26.70 | 4.50 |
| 7 | 11 | 13 | 10.80 | 4.80 |
| 7 | 11 | 13 | 24.80 | 4.70 |
| -10 | 12 | 13 | 5.80 | 2.40 |
| 10 | 12 | -13 | 6.50 | 1.90 |
| 9 | 12 | -13 | 11.30 | 2.10 |
| -9 | 12 | 13 | 8.80 | 2.80 |
| -8 | -12 | 13 | 90.19 | 7.40 |
| 8 | 12 | -13 | 89.09 | 6.50 |
| -8 | 12 | 13 | 91.39 | 7.80 |
| -7 | -12 | 13 | 380.76 | 22.60 |
| -7 | 12 | 13 | 418.86 | 23.80 |
| 7 | 12 | -13 | 396.66 | 22.50 |
| -6 | -12 | 13 | -0.60 | 2.40 |
| -6 | 12 | 13 | 1.10 | 4.00 |
| 6 | 12 | -13 | 3.40 | 2.60 |
| 5 | -12 | -13 | 328.77 | 20.60 |
| -5 | -12 | 13 | 367.76 | 20.50 |
| -5 | 12 | 13 | 345.17 | 21.50 |

| | | | | |
|---|---|---|---|---|
| 5 | 12 | -13 | 365.46 | 20.60 |
| -5 | 12 | 13 | 341.87 | 21.80 |
| 4 | -12 | -13 | 71.99 | 7.40 |
| -4 | 12 | 13 | 70.99 | 7.30 |
| -4 | 12 | 13 | 50.19 | 9.40 |
| 4 | 12 | -13 | 71.29 | 7.80 |
| -3 | -12 | 13 | 35.50 | 5.30 |
| -3 | 12 | 13 | 47.70 | 8.80 |
| -3 | 12 | 13 | 46.80 | 6.60 |
| 2 | -12 | -13 | 123.19 | 10.40 |
| -2 | -12 | 13 | 129.29 | 8.90 |
| -2 | 12 | 13 | 132.09 | 11.80 |
| -2 | 12 | 13 | 105.69 | 9.40 |
| 1 | -12 | -13 | 962.60 | 56.49 |
| -1 | -12 | 13 | 1022.30 | 55.49 |
| -1 | 12 | 13 | 999.70 | 55.39 |
| -1 | 12 | 13 | 1039.50 | 56.09 |
| 0 | 12 | 13 | 87.29 | 8.00 |
| 0 | 12 | 13 | 97.49 | 8.80 |
| -1 | -12 | -13 | 69.69 | 8.30 |
| 1 | -12 | 13 | 57.09 | 6.50 |
| 1 | 12 | 13 | 54.79 | 7.30 |
| 1 | 12 | 13 | 61.09 | 6.90 |
| -2 | -12 | -13 | 9.40 | 3.10 |
| 2 | -12 | 13 | 9.10 | 3.10 |
| 2 | 12 | 13 | 7.50 | 3.50 |
| 2 | 12 | 13 | 7.10 | 3.30 |
| 3 | -12 | 13 | 316.97 | 18.50 |
| -3 | -12 | -13 | 290.47 | 18.90 |
| 3 | 12 | 13 | 308.77 | 18.30 |
| 3 | 12 | 13 | 323.27 | 18.80 |
| -4 | -12 | -13 | -3.00 | 2.50 |
| 4 | -12 | 13 | 2.50 | 2.70 |
| 4 | 12 | 13 | -7.20 | 4.20 |
| 4 | 12 | 13 | 2.60 | 2.90 |
| -5 | -12 | -13 | 371.16 | 21.70 |
| 5 | -12 | 13 | 361.96 | 21.60 |
| 5 | 12 | 13 | 413.76 | 22.40 |
| 5 | 12 | 13 | 366.86 | 21.50 |
| -6 | -12 | -13 | 87.79 | 6.70 |
| 6 | 12 | 13 | 85.49 | 7.40 |
| 6 | 12 | 13 | 91.89 | 8.90 |
| 9 | 13 | -13 | 32.30 | 3.40 |
| -9 | 13 | 13 | 35.90 | 5.00 |

| | | | | |
|---|---|---|---|---|
| -8 | -13 | 13 | 43.00 | 6.60 |
| 8 | 13 | -13 | 43.30 | 4.00 |
| -8 | 13 | 13 | 32.40 | 5.80 |
| -7 | -13 | 13 | 64.09 | 5.90 |
| -7 | 13 | 13 | 81.29 | 7.60 |
| 7 | 13 | -13 | 75.29 | 5.70 |
| -6 | -13 | 13 | 687.73 | 38.00 |
| -6 | 13 | 13 | 645.54 | 38.80 |
| 6 | 13 | -13 | 728.23 | 37.90 |
| -5 | -13 | 13 | 139.39 | 8.90 |
| 5 | -13 | -13 | 132.69 | 9.50 |
| -5 | 13 | 13 | 115.99 | 11.30 |
| 5 | 13 | -13 | 134.89 | 9.20 |
| 4 | -13 | -13 | 35.80 | 5.20 |
| -4 | -13 | 13 | 37.60 | 4.80 |
| 4 | 13 | -13 | 35.70 | 5.80 |
| -4 | 13 | 13 | 26.40 | 5.50 |
| -4 | 13 | 13 | 20.80 | 3.90 |
| -3 | -13 | 13 | 117.09 | 8.00 |
| 3 | -13 | -13 | 108.99 | 8.40 |
| -3 | 13 | 13 | 107.39 | 8.70 |
| -2 | -13 | 13 | 427.96 | 25.10 |
| 2 | -13 | -13 | 443.76 | 25.50 |
| -2 | 13 | 13 | 453.25 | 25.20 |
| -2 | 13 | 13 | 437.06 | 26.30 |
| -1 | -13 | 13 | 3.20 | 2.40 |
| 1 | -13 | -13 | 4.60 | 2.80 |
| -1 | 13 | 13 | 2.10 | 4.20 |
| -1 | 13 | 13 | -1.30 | 3.20 |
| 0 | -13 | 13 | 394.06 | 22.90 |
| 0 | -13 | -13 | 389.26 | 23.60 |
| 0 | 13 | 13 | 414.46 | 23.10 |
| 0 | 13 | 13 | 393.86 | 23.60 |
| 1 | -13 | 13 | 98.99 | 7.50 |
| -1 | -13 | -13 | 96.39 | 8.60 |
| 1 | 13 | 13 | 103.49 | 8.80 |
| 1 | 13 | 13 | 96.79 | 8.10 |
| -2 | -13 | -13 | 73.99 | 7.50 |
| 2 | -13 | 13 | 82.59 | 7.00 |
| 2 | 13 | 13 | 93.99 | 8.70 |
| 2 | 13 | 13 | 87.19 | 8.00 |
| -3 | -13 | -13 | 34.20 | 5.60 |
| 3 | -13 | 13 | 47.70 | 5.50 |
| 3 | 13 | 13 | 35.70 | 6.10 |

| | | | | |
|---|---|---|---|---|
| 3 | 13 | 13 | 37.30 | 6.40 |
| 4 | -13 | 13 | 516.35 | 27.30 |
| -4 | -13 | -13 | 477.85 | 27.40 |
| 4 | 13 | 13 | 457.45 | 27.50 |
| -5 | -13 | -13 | 24.80 | 4.30 |
| 5 | -13 | 13 | 34.20 | 5.70 |
| 5 | 13 | 13 | 23.00 | 4.80 |
| 8 | 14 | -13 | 143.59 | 9.20 |
| -7 | -14 | 13 | 227.18 | 13.90 |
| 7 | 14 | -13 | 256.47 | 13.90 |
| -6 | -14 | 13 | 5.40 | 2.00 |
| 6 | 14 | -13 | 4.90 | 2.10 |
| -5 | -14 | 13 | 349.96 | 19.50 |
| 5 | -14 | -13 | 357.76 | 19.70 |
| 5 | 14 | -13 | 344.67 | 19.40 |
| -5 | 14 | 13 | 308.07 | 20.50 |
| -4 | -14 | 13 | 67.59 | 5.30 |
| 4 | -14 | -13 | 54.69 | 5.80 |
| -4 | 14 | 13 | 62.69 | 6.40 |
| 4 | 14 | -13 | 62.79 | 6.10 |
| 3 | -14 | -13 | 10.20 | 2.40 |
| -3 | -14 | 13 | 7.10 | 2.00 |
| 3 | 14 | -13 | 15.30 | 3.30 |
| -3 | 14 | 13 | 12.00 | 3.20 |
| 2 | -14 | -13 | 42.30 | 5.00 |
| -2 | -14 | 13 | 40.70 | 4.50 |
| -2 | 14 | 13 | 39.50 | 6.10 |
| -1 | -14 | 13 | 606.44 | 35.00 |
| 1 | -14 | -13 | 636.54 | 35.40 |
| -1 | 14 | 13 | 648.04 | 35.30 |
| 0 | -14 | 13 | 101.39 | 7.60 |
| 0 | -14 | -13 | 122.29 | 8.40 |
| 0 | 14 | 13 | 106.39 | 8.20 |
| -1 | -14 | -13 | 25.00 | 5.00 |
| 1 | -14 | 13 | 33.80 | 4.30 |
| 1 | 14 | 13 | 35.10 | 5.40 |
| 2 | -14 | 13 | 54.09 | 5.20 |
| -2 | -14 | -13 | 51.09 | 5.70 |
| 2 | 14 | 13 | 70.09 | 6.60 |
| -3 | -14 | -13 | 200.78 | 13.80 |
| 3 | -14 | 13 | 228.98 | 13.60 |
| 3 | 14 | 13 | 253.27 | 14.20 |
| -6 | -15 | 13 | 505.25 | 29.00 |
| 6 | 15 | -13 | 552.84 | 29.30 |

| | | | | |
|---|---|---|---|---|
| -5 | -15 | 13 | 62.19 | 5.00 |
| 5 | 15 | -13 | 71.29 | 5.40 |
| -4 | -15 | 13 | 8.80 | 1.90 |
| 4 | -15 | -13 | 12.60 | 2.50 |
| 4 | 15 | -13 | 13.10 | 2.50 |
| -4 | 15 | 13 | 8.30 | 3.00 |
| -3 | -15 | 13 | 149.49 | 8.90 |
| 3 | -15 | -13 | 143.09 | 9.00 |
| 3 | 15 | -13 | 142.29 | 9.10 |
| -3 | 15 | 13 | 130.29 | 9.30 |
| -2 | -15 | 13 | 243.78 | 14.90 |
| 2 | -15 | -13 | 268.57 | 15.10 |
| 2 | 15 | -13 | 263.77 | 15.30 |
| -2 | 15 | 13 | 252.97 | 15.20 |
| -1 | -15 | 13 | 6.30 | 1.70 |
| -1 | 15 | 13 | 9.80 | 3.20 |
| 0 | -15 | 13 | 328.77 | 18.50 |
| 0 | -15 | -13 | 334.47 | 18.90 |
| 0 | 15 | 13 | 313.17 | 18.80 |
| -1 | -15 | -13 | 113.89 | 8.10 |
| 1 | -15 | 13 | 118.89 | 7.70 |
| 1 | 15 | 13 | 113.49 | 8.50 |
| 2 | -15 | 13 | 74.69 | 5.30 |
| -2 | -15 | -13 | 60.49 | 5.60 |
| 2 | 15 | 13 | 60.19 | 6.40 |
| -14 | 0 | 14 | 189.28 | 11.40 |
| -13 | 0 | 14 | -0.60 | 2.00 |
| -12 | 0 | 14 | 509.35 | 28.90 |
| -11 | 0 | 14 | 3.90 | 3.00 |
| -10 | 0 | 14 | 298.77 | 18.40 |
| -9 | 0 | 14 | 1.20 | 3.40 |
| -8 | 0 | 14 | 10.50 | 4.00 |
| -7 | 0 | 14 | -4.50 | 4.20 |
| -6 | 0 | 14 | 1120.89 | 66.69 |
| -6 | 0 | 14 | 1239.58 | 68.39 |
| -6 | 0 | 14 | 1284.57 | 67.99 |
| -5 | 0 | 14 | -0.10 | 5.30 |
| -5 | 0 | 14 | 4.10 | 5.40 |
| -5 | 0 | 14 | 4.30 | 3.70 |
| -4 | 0 | 14 | 77.99 | 14.40 |
| -4 | 0 | 14 | 61.19 | 10.60 |
| -4 | 0 | 14 | 76.69 | 8.10 |
| -3 | 0 | 14 | 2.30 | 3.20 |
| -3 | 0 | 14 | 4.20 | 5.60 |

| | | | | |
|---|---|---|---|---|
| -2 | 0 | 14 | 236.58 | 17.60 |
| -2 | 0 | 14 | 223.68 | 15.80 |
| -2 | 0 | 14 | 275.77 | 20.40 |
| -1 | 0 | 14 | -3.80 | 3.50 |
| -1 | 0 | 14 | -1.40 | 4.80 |
| 0 | 0 | 14 | 1059.69 | 54.69 |
| 0 | 0 | 14 | 948.31 | 54.39 |
| 0 | 0 | 14 | 897.11 | 55.69 |
| 1 | 0 | 14 | 4.20 | 5.50 |
| 1 | 0 | 14 | -3.20 | 4.20 |
| 1 | 0 | 14 | 0.40 | 4.10 |
| 2 | 0 | 14 | 9.70 | 3.80 |
| 2 | 0 | 14 | 10.50 | 4.90 |
| 2 | 0 | 14 | 6.50 | 6.60 |
| 3 | 0 | 14 | -4.00 | 4.10 |
| 3 | 0 | 14 | -0.60 | 3.50 |
| 3 | 0 | 14 | -4.40 | 7.30 |
| 4 | 0 | 14 | 1167.48 | 67.99 |
| 4 | 0 | 14 | 1357.36 | 68.99 |
| 4 | 0 | 14 | 1181.18 | 71.89 |
| 5 | 0 | 14 | -6.20 | 3.40 |
| 5 | 0 | 14 | -4.80 | 2.90 |
| 6 | 0 | 14 | 195.88 | 13.80 |
| 6 | 0 | 14 | 215.08 | 13.50 |
| 7 | 0 | 14 | 3.90 | 3.00 |
| 7 | 0 | 14 | 0.80 | 3.20 |
| 8 | 0 | 14 | 252.17 | 15.20 |
| 9 | 0 | 14 | 0.90 | 2.40 |
| -14 | -1 | 14 | 5.90 | 2.40 |
| -14 | 1 | 14 | 2.90 | 1.90 |
| -13 | -1 | 14 | 49.30 | 5.30 |
| -13 | 1 | 14 | 50.59 | 5.10 |
| -12 | -1 | 14 | 27.60 | 5.20 |
| -12 | 1 | 14 | 16.60 | 3.10 |
| -11 | -1 | 14 | 831.82 | 46.00 |
| -11 | 1 | 14 | 748.93 | 44.00 |
| -10 | -1 | 14 | 13.20 | 3.60 |
| -10 | 1 | 14 | 7.80 | 3.30 |
| -9 | -1 | 14 | 77.69 | 8.50 |
| -9 | 1 | 14 | 81.29 | 8.10 |
| -8 | -1 | 14 | -3.70 | 3.80 |
| -8 | 1 | 14 | -1.10 | 3.60 |
| -7 | -1 | 14 | 803.22 | 46.20 |
| -7 | -1 | 14 | 784.92 | 46.00 |

| | | | | |
|---|---|---|---|---|
| -7 | 1 | 14 | 840.52 | 46.20 |
| -6 | -1 | 14 | -2.00 | 6.00 |
| -6 | -1 | 14 | 5.70 | 4.00 |
| -6 | 1 | 14 | 4.70 | 4.10 |
| -6 | 1 | 14 | 10.80 | 5.00 |
| -6 | 1 | 14 | -7.30 | 6.10 |
| -5 | -1 | 14 | 366.06 | 23.00 |
| -5 | -1 | 14 | 329.87 | 21.00 |
| -5 | -1 | 14 | 355.66 | 22.80 |
| -5 | 1 | 14 | 370.46 | 23.10 |
| -5 | 1 | 14 | 312.57 | 22.50 |
| -5 | 1 | 14 | 344.17 | 21.00 |
| -4 | -1 | 14 | 62.99 | 7.60 |
| -4 | -1 | 14 | 62.89 | 11.50 |
| -4 | -1 | 14 | 57.29 | 9.70 |
| -4 | 1 | 14 | 60.29 | 13.90 |
| -4 | 1 | 14 | 52.99 | 7.60 |
| -3 | -1 | 14 | 14.50 | 6.10 |
| -3 | -1 | 14 | 24.50 | 7.50 |
| -3 | -1 | 14 | 20.80 | 4.00 |
| -3 | 1 | 14 | 13.60 | 5.50 |
| -3 | 1 | 14 | 16.20 | 7.60 |
| -3 | 1 | 14 | 20.40 | 4.10 |
| -2 | -1 | 14 | 12.50 | 5.60 |
| -2 | -1 | 14 | 15.10 | 4.10 |
| -2 | -1 | 14 | 9.40 | 6.00 |
| -2 | 1 | 14 | 20.00 | 5.80 |
| -2 | 1 | 14 | 17.60 | 6.70 |
| -2 | 1 | 14 | 11.10 | 3.70 |
| -1 | -1 | 14 | 1614.94 | 86.49 |
| -1 | -1 | 14 | 1550.64 | 84.79 |
| -1 | -1 | 14 | 1548.15 | 85.29 |
| -1 | 1 | 14 | 1603.04 | 84.99 |
| -1 | 1 | 14 | 1302.67 | 84.99 |
| -1 | 1 | 14 | 1646.34 | 86.69 |
| 0 | -1 | 14 | 30.70 | 5.60 |
| 0 | -1 | 14 | 9.10 | 5.20 |
| 0 | -1 | 14 | 21.70 | 4.90 |
| 0 | 1 | 14 | 12.20 | 5.30 |
| 0 | 1 | 14 | 22.90 | 5.10 |
| 0 | 1 | 14 | 17.10 | 4.70 |
| 1 | -1 | 14 | 241.78 | 16.10 |
| 1 | -1 | 14 | 240.98 | 18.10 |
| 1 | -1 | 14 | 197.98 | 15.50 |

| | | | | |
|---|---|---|---|---|
| 1 | 1 | 14 | 233.78 | 16.40 |
| 1 | 1 | 14 | 236.58 | 18.20 |
| 1 | 1 | 14 | 209.98 | 15.50 |
| 2 | -1 | 14 | 11.10 | 4.40 |
| 2 | -1 | 14 | 12.60 | 7.40 |
| 2 | -1 | 14 | 9.00 | 4.70 |
| 2 | 1 | 14 | -3.30 | 5.90 |
| 2 | 1 | 14 | 5.90 | 3.70 |
| 2 | 1 | 14 | 8.20 | 4.50 |
| 3 | -1 | 14 | 329.37 | 20.50 |
| 3 | -1 | 14 | 309.97 | 23.70 |
| 3 | -1 | 14 | 328.77 | 19.60 |
| 3 | 1 | 14 | 277.97 | 19.20 |
| 3 | 1 | 14 | 287.27 | 23.50 |
| 3 | 1 | 14 | 330.27 | 20.70 |
| 4 | -1 | 14 | -9.40 | 9.30 |
| 4 | -1 | 14 | -1.10 | 3.40 |
| 4 | -1 | 14 | 8.40 | 4.30 |
| 4 | 1 | 14 | 4.80 | 3.40 |
| 4 | 1 | 14 | 10.70 | 9.40 |
| 4 | 1 | 14 | -4.30 | 3.80 |
| 5 | -1 | 14 | 842.92 | 47.10 |
| 5 | -1 | 14 | 817.22 | 47.50 |
| 5 | 1 | 14 | 824.62 | 47.40 |
| 5 | 1 | 14 | 892.31 | 47.20 |
| 6 | -1 | 14 | -3.40 | 3.40 |
| 6 | -1 | 14 | 6.40 | 3.30 |
| 6 | 1 | 14 | 4.60 | 3.40 |
| 6 | 1 | 14 | 3.30 | 2.80 |
| 7 | -1 | 14 | 51.89 | 6.20 |
| 7 | -1 | 14 | 60.09 | 7.10 |
| 7 | 1 | 14 | 63.39 | 6.40 |
| 8 | -1 | 14 | 10.80 | 2.90 |
| 8 | 1 | 14 | 10.40 | 2.70 |
| 9 | -1 | 14 | 294.67 | 17.50 |
| 9 | 1 | 14 | 300.67 | 17.40 |
| -14 | -2 | 14 | 14.70 | 3.20 |
| -13 | -2 | 14 | 20.90 | 3.40 |
| -13 | 2 | 14 | 16.80 | 3.80 |
| -12 | -2 | 14 | 852.11 | 46.10 |
| -12 | 2 | 14 | 814.42 | 46.20 |
| -11 | -2 | 14 | 9.50 | 3.80 |
| -11 | 2 | 14 | 2.60 | 2.90 |
| -10 | -2 | 14 | 609.54 | 33.80 |

| | | | | |
|---|---|---|---|---|
| -10 | 2 | 14 | 574.64 | 33.80 |
| -9 | -2 | 14 | 11.00 | 4.40 |
| -9 | 2 | 14 | 15.50 | 3.70 |
| -8 | -2 | 14 | 263.77 | 17.70 |
| -8 | 2 | 14 | 265.27 | 17.30 |
| -7 | -2 | 14 | 16.00 | 4.40 |
| -7 | -2 | 14 | 11.70 | 5.30 |
| -7 | 2 | 14 | 3.80 | 3.90 |
| -6 | -2 | 14 | 1955.50 | 117.39 |
| -6 | -2 | 14 | 2240.28 | 118.59 |
| -6 | 2 | 14 | 2145.69 | 119.09 |
| -6 | 2 | 14 | 2491.75 | 119.09 |
| -6 | 2 | 14 | 2001.00 | 117.49 |
| -5 | -2 | 14 | 10.00 | 6.30 |
| -5 | -2 | 14 | 10.40 | 5.90 |
| -5 | -2 | 14 | 7.70 | 3.50 |
| -5 | 2 | 14 | 7.00 | 4.00 |
| -5 | 2 | 14 | 11.00 | 5.80 |
| -5 | 2 | 14 | 6.00 | 5.40 |
| -4 | -2 | 14 | 8.60 | 6.40 |
| -4 | -2 | 14 | 8.80 | 3.40 |
| -4 | -2 | 14 | 13.60 | 4.70 |
| -4 | 2 | 14 | 14.60 | 5.40 |
| -4 | 2 | 14 | 9.00 | 3.50 |
| -4 | 2 | 14 | 13.80 | 5.50 |
| -3 | -2 | 14 | 4.30 | 6.60 |
| -3 | -2 | 14 | 1.70 | 3.10 |
| -3 | -2 | 14 | 3.60 | 4.30 |
| -3 | 2 | 14 | 3.90 | 3.40 |
| -3 | 2 | 14 | 2.20 | 5.00 |
| -3 | 2 | 14 | 5.90 | 5.10 |
| -2 | -2 | 14 | 1254.07 | 74.89 |
| -2 | -2 | 14 | 1463.75 | 76.19 |
| -2 | -2 | 14 | 1342.97 | 76.49 |
| -2 | 2 | 14 | 1315.37 | 75.09 |
| -2 | 2 | 14 | 1316.57 | 76.89 |
| -2 | 2 | 14 | 1499.35 | 76.29 |
| -1 | -2 | 14 | 159.58 | 14.00 |
| -1 | -2 | 14 | 142.29 | 11.40 |
| -1 | -2 | 14 | 160.78 | 13.60 |
| -1 | 2 | 14 | 163.78 | 13.00 |
| -1 | 2 | 14 | 141.69 | 14.10 |
| -1 | 2 | 14 | 140.59 | 11.90 |
| 0 | -2 | 14 | 721.13 | 41.40 |

| | | | | |
|---|---|---|---|---|
| 0 | -2 | 14 | 683.03 | 43.00 |
| 0 | -2 | 14 | 788.22 | 42.00 |
| 0 | 2 | 14 | 712.93 | 43.40 |
| 0 | 2 | 14 | 727.23 | 41.80 |
| 1 | -2 | 14 | 24.00 | 5.50 |
| 1 | -2 | 14 | 32.80 | 7.80 |
| 1 | -2 | 14 | 33.20 | 5.60 |
| 1 | 2 | 14 | 26.40 | 7.00 |
| 1 | 2 | 14 | 40.90 | 8.70 |
| 1 | 2 | 14 | 24.90 | 5.00 |
| 2 | -2 | 14 | 245.98 | 17.60 |
| 2 | -2 | 14 | 281.97 | 18.40 |
| 2 | -2 | 14 | 261.37 | 21.00 |
| 2 | 2 | 14 | 286.47 | 18.90 |
| 2 | 2 | 14 | 298.47 | 21.40 |
| 2 | 2 | 14 | 259.87 | 17.50 |
| 3 | -2 | 14 | 106.79 | 10.60 |
| 3 | -2 | 14 | 104.39 | 9.60 |
| 3 | -2 | 14 | 82.39 | 15.10 |
| 3 | 2 | 14 | 94.99 | 15.60 |
| 3 | 2 | 14 | 95.69 | 8.80 |
| 3 | 2 | 14 | 92.89 | 10.70 |
| 4 | -2 | 14 | 1163.48 | 59.29 |
| 4 | -2 | 14 | 929.81 | 62.09 |
| 4 | -2 | 14 | 1024.80 | 58.39 |
| 4 | 2 | 14 | 1000.20 | 58.39 |
| 4 | 2 | 14 | 1203.68 | 59.49 |
| 4 | 2 | 14 | 1003.50 | 62.59 |
| 5 | -2 | 14 | 19.40 | 4.10 |
| 5 | -2 | 14 | 11.50 | 3.80 |
| 5 | 2 | 14 | 13.30 | 3.90 |
| 5 | 2 | 14 | 13.60 | 3.30 |
| 6 | -2 | 14 | 99.29 | 8.90 |
| 6 | -2 | 14 | 104.39 | 11.20 |
| 6 | 2 | 14 | 110.39 | 9.10 |
| 6 | 2 | 14 | 119.39 | 8.60 |
| 7 | -2 | 14 | 10.10 | 3.40 |
| 7 | -2 | 14 | 16.40 | 3.90 |
| 7 | 2 | 14 | 23.40 | 5.10 |
| 8 | -2 | 14 | 365.36 | 20.90 |
| 8 | 2 | 14 | 348.57 | 20.60 |
| 9 | -2 | 14 | 11.50 | 3.20 |
| 9 | 2 | 14 | 4.50 | 2.30 |
| -14 | 3 | 14 | 47.50 | 4.20 |

| | | | | |
|---|---|---|---|---|
| -13 | 3 | 14 | 114.19 | 7.80 |
| -12 | -3 | 14 | 62.09 | 7.10 |
| -12 | 3 | 14 | 60.39 | 5.90 |
| -11 | -3 | 14 | 1004.80 | 55.69 |
| -11 | 3 | 14 | 1017.50 | 56.49 |
| -10 | -3 | 14 | 5.50 | 4.10 |
| -10 | 3 | 14 | 6.00 | 3.00 |
| -9 | -3 | 14 | 8.70 | 4.70 |
| -9 | 3 | 14 | 3.00 | 3.00 |
| -8 | -3 | 14 | 115.39 | 12.00 |
| -8 | 3 | 14 | 136.79 | 11.00 |
| -7 | -3 | 14 | 1691.03 | 90.89 |
| -7 | -3 | 14 | 1539.35 | 89.79 |
| -7 | 3 | 14 | 1721.13 | 90.99 |
| -6 | -3 | 14 | 28.50 | 4.60 |
| -6 | -3 | 14 | 19.80 | 6.90 |
| -6 | 3 | 14 | 41.80 | 8.40 |
| -6 | 3 | 14 | 26.40 | 5.10 |
| -6 | 3 | 14 | 23.50 | 8.50 |
| -5 | -3 | 14 | 513.35 | 31.20 |
| -5 | -3 | 14 | 505.35 | 33.40 |
| -5 | 3 | 14 | 522.15 | 31.50 |
| -5 | 3 | 14 | 609.04 | 33.20 |
| -5 | 3 | 14 | 582.84 | 33.60 |
| -4 | -3 | 14 | 30.90 | 6.40 |
| -4 | -3 | 14 | 30.60 | 6.20 |
| -4 | -3 | 14 | 22.00 | 7.70 |
| -4 | 3 | 14 | 17.70 | 3.80 |
| -4 | 3 | 14 | 20.30 | 6.20 |
| -3 | -3 | 14 | 315.07 | 23.70 |
| -3 | -3 | 14 | 367.46 | 22.80 |
| -3 | -3 | 14 | 342.17 | 20.90 |
| -3 | 3 | 14 | 337.67 | 21.00 |
| -3 | 3 | 14 | 317.27 | 22.50 |
| -3 | 3 | 14 | 391.16 | 23.80 |
| -2 | -3 | 14 | 183.28 | 16.10 |
| -2 | -3 | 14 | 182.28 | 12.40 |
| -2 | -3 | 14 | 185.28 | 14.30 |
| -2 | 3 | 14 | 170.68 | 12.70 |
| -2 | 3 | 14 | 162.38 | 15.00 |
| -2 | 3 | 14 | 154.68 | 14.00 |
| -1 | -3 | 14 | 2261.87 | 129.19 |
| -1 | -3 | 14 | 2342.87 | 127.59 |
| -1 | 3 | 14 | 2378.66 | 129.89 |

| | | | | |
|---|---|---|---|---|
| -1 | 3 | 14 | 2375.96 | 128.29 |
| -1 | 3 | 14 | 2379.16 | 127.99 |
| 0 | -3 | 14 | 3.60 | 5.60 |
| 0 | -3 | 14 | 1.10 | 3.40 |
| 0 | -3 | 14 | 3.50 | 6.10 |
| 0 | 3 | 14 | 2.80 | 4.10 |
| 0 | 3 | 14 | 0.90 | 3.80 |
| 0 | 3 | 14 | -3.00 | 5.70 |
| 1 | -3 | 14 | 26.10 | 4.90 |
| 1 | -3 | 14 | 10.30 | 6.00 |
| 1 | -3 | 14 | 11.30 | 5.80 |
| 1 | 3 | 14 | 9.80 | 4.30 |
| 1 | 3 | 14 | 17.00 | 4.20 |
| 1 | 3 | 14 | 16.00 | 6.30 |
| 2 | -3 | 14 | 297.57 | 22.80 |
| 2 | -3 | 14 | 347.07 | 20.30 |
| 2 | 3 | 14 | 272.77 | 19.30 |
| 2 | 3 | 14 | 322.37 | 20.90 |
| 2 | 3 | 14 | 317.47 | 23.10 |
| 3 | -3 | 14 | 868.71 | 49.50 |
| 3 | -3 | 14 | 958.80 | 50.29 |
| 3 | -3 | 14 | 835.52 | 52.59 |
| 3 | 3 | 14 | 847.62 | 52.99 |
| 3 | 3 | 14 | 969.90 | 50.59 |
| 3 | 3 | 14 | 815.12 | 49.30 |
| 4 | -3 | 14 | 33.90 | 5.50 |
| 4 | -3 | 14 | 26.50 | 11.10 |
| 4 | 3 | 14 | 35.60 | 6.00 |
| 4 | 3 | 14 | 39.80 | 8.00 |
| 4 | 3 | 14 | 36.00 | 11.00 |
| 5 | -3 | 14 | 679.63 | 37.30 |
| 5 | -3 | 14 | 693.93 | 37.10 |
| 5 | 3 | 14 | 659.13 | 37.30 |
| 5 | 3 | 14 | 579.04 | 36.70 |
| 6 | -3 | 14 | 87.89 | 11.50 |
| 6 | -3 | 14 | 57.59 | 11.40 |
| 6 | 3 | 14 | 54.39 | 6.20 |
| 7 | -3 | 14 | 255.97 | 16.30 |
| 7 | -3 | 14 | 253.87 | 16.20 |
| 7 | 3 | 14 | 275.27 | 15.90 |
| 8 | -3 | 14 | 30.90 | 6.30 |
| 8 | 3 | 14 | 29.10 | 4.50 |
| 9 | -3 | 14 | 401.76 | 23.70 |
| 9 | 3 | 14 | 415.36 | 23.10 |

| | | | | |
|---|---|---|---|---|
| -14 | 4 | 14 | 50.39 | 4.20 |
| -13 | 4 | 14 | 5.20 | 2.00 |
| -12 | 4 | 14 | 397.96 | 22.90 |
| -11 | 4 | 14 | 4.00 | 4.70 |
| -10 | 4 | 14 | 364.36 | 21.70 |
| -9 | 4 | 14 | 3.30 | 2.80 |
| -8 | 4 | 14 | 67.39 | 7.70 |
| -7 | -4 | 14 | 62.09 | 7.90 |
| -7 | 4 | 14 | 59.49 | 8.10 |
| -6 | -4 | 14 | 1678.43 | 90.59 |
| -6 | 4 | 14 | 1598.04 | 91.69 |
| -6 | 4 | 14 | 1705.43 | 90.79 |
| -5 | -4 | 14 | 0.90 | 3.10 |
| -5 | 4 | 14 | 9.20 | 6.80 |
| -5 | 4 | 14 | 3.70 | 4.60 |
| -5 | 4 | 14 | 0.10 | 3.90 |
| -4 | -4 | 14 | 29.50 | 6.00 |
| -4 | 4 | 14 | 17.80 | 4.20 |
| -4 | 4 | 14 | 24.40 | 6.40 |
| -4 | 4 | 14 | 21.60 | 5.20 |
| -3 | -4 | 14 | 76.59 | 8.00 |
| -3 | -4 | 14 | 85.69 | 10.50 |
| -3 | 4 | 14 | 86.79 | 10.30 |
| -3 | 4 | 14 | 77.29 | 8.30 |
| -3 | 4 | 14 | 71.89 | 11.60 |
| -2 | -4 | 14 | 1563.14 | 87.29 |
| -2 | -4 | 14 | 1606.24 | 89.09 |
| -2 | 4 | 14 | 1674.83 | 88.59 |
| -2 | 4 | 14 | 1585.04 | 89.69 |
| -2 | 4 | 14 | 1556.04 | 87.59 |
| -1 | -4 | 14 | 17.00 | 3.90 |
| -1 | -4 | 14 | 16.90 | 5.90 |
| -1 | 4 | 14 | 18.10 | 5.20 |
| -1 | 4 | 14 | 9.10 | 5.90 |
| -1 | 4 | 14 | 8.70 | 4.20 |
| 0 | -4 | 14 | 513.15 | 30.90 |
| 0 | -4 | 14 | 497.55 | 28.60 |
| 0 | 4 | 14 | 451.85 | 31.40 |
| 0 | 4 | 14 | 463.15 | 29.00 |
| 0 | 4 | 14 | 501.35 | 29.30 |
| 1 | -4 | 14 | 11.30 | 4.00 |
| 1 | -4 | 14 | 27.50 | 7.50 |
| 1 | 4 | 14 | 23.20 | 4.60 |
| 1 | 4 | 14 | 21.70 | 5.40 |

| | | | | |
|---|---|---|---|---|
| 1 | 4 | 14 | 14.40 | 7.40 |
| 2 | -4 | 14 | 406.96 | 25.50 |
| 2 | -4 | 14 | 449.46 | 24.60 |
| 2 | -4 | 14 | 371.86 | 27.40 |
| 2 | 4 | 14 | 381.16 | 23.80 |
| 2 | 4 | 14 | 392.66 | 27.70 |
| 2 | 4 | 14 | 369.26 | 24.90 |
| 3 | -4 | 14 | 14.70 | 9.00 |
| 3 | -4 | 14 | 24.70 | 5.80 |
| 3 | -4 | 14 | 19.30 | 5.00 |
| 3 | 4 | 14 | 32.50 | 9.30 |
| 3 | 4 | 14 | 22.50 | 4.30 |
| 3 | 4 | 14 | 13.90 | 4.80 |
| 4 | -4 | 14 | 782.52 | 44.40 |
| 4 | -4 | 14 | 743.53 | 44.00 |
| 4 | 4 | 14 | 799.22 | 49.90 |
| 4 | 4 | 14 | 825.42 | 44.60 |
| 4 | 4 | 14 | 759.52 | 43.80 |
| 5 | -4 | 14 | -0.70 | 3.60 |
| 5 | -4 | 14 | -3.50 | 4.60 |
| 5 | 4 | 14 | -2.10 | 2.50 |
| 6 | -4 | 14 | 2.60 | 3.50 |
| 6 | -4 | 14 | 0.80 | 4.50 |
| 6 | 4 | 14 | 3.90 | 2.80 |
| 7 | -4 | 14 | 29.80 | 5.30 |
| 7 | -4 | 14 | 31.00 | 5.40 |
| 8 | -4 | 14 | 345.17 | 20.30 |
| 8 | 4 | 14 | 318.67 | 19.10 |
| 9 | 4 | 14 | 1.70 | 1.90 |
| -14 | 5 | 14 | 3.50 | 1.60 |
| -13 | 5 | 14 | 41.30 | 4.20 |
| -12 | 5 | 14 | 3.60 | 2.00 |
| -11 | 5 | 14 | 574.14 | 32.60 |
| -10 | 5 | 14 | 41.60 | 5.60 |
| -9 | 5 | 14 | 43.70 | 6.00 |
| -8 | 5 | 14 | 58.29 | 7.10 |
| -7 | -5 | 14 | 688.13 | 40.00 |
| -7 | 5 | 14 | 740.93 | 41.10 |
| -6 | -5 | 14 | 63.79 | 7.90 |
| -6 | 5 | 14 | 83.99 | 9.40 |
| -5 | -5 | 14 | 543.15 | 31.10 |
| -5 | 5 | 14 | 540.75 | 34.20 |
| -5 | 5 | 14 | 585.34 | 32.90 |
| -5 | 5 | 14 | 513.35 | 31.40 |

| | | | | |
|---|---|---|---|---|
| -4 | -5 | 14 | 42.20 | 6.50 |
| -4 | 5 | 14 | 68.79 | 11.90 |
| -4 | 5 | 14 | 43.30 | 7.20 |
| -3 | -5 | 14 | 206.08 | 14.20 |
| -3 | 5 | 14 | 202.68 | 16.00 |
| -3 | 5 | 14 | 218.58 | 14.60 |
| -3 | 5 | 14 | 244.28 | 18.80 |
| -2 | -5 | 14 | 2.90 | 3.20 |
| -2 | 5 | 14 | 4.90 | 4.20 |
| -2 | 5 | 14 | 9.20 | 6.70 |
| -2 | 5 | 14 | 4.10 | 3.70 |
| -1 | -5 | 14 | 1817.82 | 102.39 |
| -1 | 5 | 14 | 1921.31 | 102.99 |
| -1 | 5 | 14 | 1929.21 | 105.39 |
| -1 | 5 | 14 | 1842.32 | 103.19 |
| 0 | -5 | 14 | 136.49 | 10.70 |
| 0 | 5 | 14 | 125.89 | 15.50 |
| 0 | 5 | 14 | 124.49 | 11.80 |
| 0 | 5 | 14 | 155.68 | 11.60 |
| 1 | -5 | 14 | -0.60 | 3.50 |
| 1 | 5 | 14 | 7.40 | 7.70 |
| 1 | 5 | 14 | 5.00 | 3.70 |
| 1 | 5 | 14 | -1.30 | 4.00 |
| 2 | -5 | 14 | 51.49 | 7.80 |
| 2 | 5 | 14 | 40.00 | 6.90 |
| 3 | -5 | 14 | 729.93 | 39.20 |
| 3 | 5 | 14 | 662.03 | 42.90 |
| 3 | 5 | 14 | 621.44 | 38.40 |
| 3 | 5 | 14 | 697.63 | 39.50 |
| 4 | -5 | 14 | 5.10 | 4.10 |
| 4 | 5 | 14 | 7.60 | 2.90 |
| 5 | -5 | 14 | 600.64 | 32.10 |
| 5 | 5 | 14 | 506.75 | 31.30 |
| 6 | -5 | 14 | 125.39 | 9.90 |
| 6 | 5 | 14 | 125.29 | 10.50 |
| 7 | 5 | 14 | 259.67 | 15.60 |
| 8 | 5 | 14 | 3.70 | 2.00 |
| 9 | 5 | 14 | 381.36 | 21.60 |
| -13 | 6 | 14 | 7.40 | 2.00 |
| 12 | 6 | -14 | 387.06 | 22.60 |
| -11 | 6 | 14 | 45.60 | 5.10 |
| -10 | 6 | 14 | 279.17 | 19.30 |
| -9 | 6 | 14 | 65.79 | 6.90 |
| -8 | 6 | 14 | 111.19 | 9.30 |

| | | | | |
|---|---|---|---|---|
| -7 | -6 | 14 | 7.50 | 3.20 |
| -7 | 6 | 14 | 2.00 | 3.90 |
| -6 | -6 | 14 | 1051.49 | 54.89 |
| -6 | 6 | 14 | 931.91 | 55.99 |
| -5 | -6 | 14 | 244.08 | 16.00 |
| -5 | 6 | 14 | 269.77 | 18.00 |
| -5 | 6 | 14 | 251.17 | 21.00 |
| -4 | -6 | 14 | 0.50 | 3.00 |
| -4 | 6 | 14 | -1.40 | 4.60 |
| -4 | 6 | 14 | -1.50 | 7.40 |
| -3 | -6 | 14 | 12.10 | 3.10 |
| -3 | 6 | 14 | 11.10 | 3.50 |
| -3 | 6 | 14 | 2.40 | 7.10 |
| -3 | 6 | 14 | 6.80 | 4.60 |
| -2 | -6 | 14 | 541.55 | 31.80 |
| -2 | 6 | 14 | 551.64 | 33.10 |
| -2 | 6 | 14 | 578.84 | 36.00 |
| -2 | 6 | 14 | 555.64 | 32.30 |
| -1 | -6 | 14 | 1.70 | 3.20 |
| -1 | 6 | 14 | 8.20 | 3.90 |
| -1 | 6 | 14 | 2.10 | 4.20 |
| -1 | 6 | 14 | 3.10 | 6.90 |
| 0 | -6 | 14 | 969.60 | 51.89 |
| 0 | 6 | 14 | 887.11 | 52.29 |
| 0 | 6 | 14 | 892.31 | 55.19 |
| 0 | 6 | 14 | 964.90 | 52.69 |
| 1 | -6 | 14 | 197.88 | 13.70 |
| 1 | 6 | 14 | 197.08 | 14.70 |
| 1 | 6 | 14 | 195.28 | 13.70 |
| 1 | 6 | 14 | 189.38 | 18.80 |
| 2 | -6 | 14 | 156.88 | 11.40 |
| 2 | 6 | 14 | 134.09 | 10.70 |
| 2 | 6 | 14 | 128.79 | 12.00 |
| 2 | 6 | 14 | 134.49 | 17.30 |
| 3 | -6 | 14 | 4.60 | 3.80 |
| 3 | 6 | 14 | -1.80 | 12.20 |
| 3 | 6 | 14 | -0.20 | 2.80 |
| 4 | -6 | 14 | 1081.69 | 56.59 |
| 4 | 6 | 14 | 943.81 | 55.89 |
| 5 | -6 | 14 | 47.10 | 10.10 |
| 5 | 6 | 14 | 45.30 | 5.60 |
| 6 | -6 | 14 | 52.99 | 6.80 |
| 6 | 6 | 14 | 43.50 | 5.40 |
| 7 | 6 | 14 | 9.60 | 2.40 |

| | | | | |
|---|---|---|---|---|
| 8 | 6 | 14 | 352.16 | 20.10 |
| -13 | 7 | 14 | 44.80 | 4.00 |
| -12 | 7 | 14 | 3.80 | 1.90 |
| 12 | 7 | -14 | -2.20 | 2.50 |
| -11 | 7 | 14 | 528.15 | 29.80 |
| 11 | 7 | -14 | 525.45 | 29.30 |
| -10 | 7 | 14 | 41.60 | 5.50 |
| 10 | 7 | -14 | 33.40 | 5.50 |
| -9 | 7 | 14 | 42.80 | 5.90 |
| -8 | 7 | 14 | 4.70 | 3.10 |
| -7 | -7 | 14 | 698.23 | 40.90 |
| -7 | 7 | 14 | 773.22 | 42.10 |
| -6 | -7 | 14 | -1.30 | 2.70 |
| 6 | 7 | -14 | 6.20 | 7.50 |
| -6 | 7 | 14 | -1.60 | 3.60 |
| -5 | -7 | 14 | 349.27 | 21.20 |
| -5 | 7 | 14 | 405.96 | 28.60 |
| -5 | 7 | 14 | 336.87 | 22.70 |
| -4 | -7 | 14 | 145.79 | 10.40 |
| -4 | 7 | 14 | 150.58 | 17.70 |
| -4 | 7 | 14 | 132.29 | 12.30 |
| -3 | -7 | 14 | 59.29 | 6.50 |
| -3 | 7 | 14 | 39.50 | 8.40 |
| -2 | -7 | 14 | 13.20 | 3.20 |
| -2 | 7 | 14 | 25.10 | 8.90 |
| -2 | 7 | 14 | 18.20 | 4.80 |
| -1 | -7 | 14 | 1344.17 | 74.79 |
| -1 | 7 | 14 | 1362.56 | 75.59 |
| -1 | 7 | 14 | 1379.56 | 78.39 |
| 0 | -7 | 14 | 241.28 | 15.70 |
| 0 | 7 | 14 | 248.18 | 16.30 |
| 0 | 7 | 14 | 242.88 | 21.00 |
| 1 | -7 | 14 | 12.10 | 3.60 |
| 1 | 7 | 14 | 10.70 | 9.20 |
| 1 | 7 | 14 | 10.70 | 3.70 |
| 2 | -7 | 14 | 23.90 | 4.50 |
| 2 | 7 | 14 | 24.10 | 4.30 |
| 2 | 7 | 14 | 3.70 | 10.10 |
| 3 | -7 | 14 | 446.06 | 24.50 |
| 3 | 7 | 14 | 365.86 | 23.70 |
| 4 | -7 | 14 | 15.60 | 4.20 |
| 4 | 7 | 14 | 24.40 | 4.90 |
| 5 | -7 | 14 | 768.42 | 43.10 |
| 5 | 7 | 14 | 770.32 | 43.00 |

| | | | | |
|---|---|---|---|---|
| 6 | -7 | 14 | 146.19 | 10.70 |
| 6 | 7 | 14 | 154.78 | 10.10 |
| -7 | -7 | -14 | 151.28 | 10.50 |
| 7 | 7 | 14 | 154.98 | 9.80 |
| 8 | 7 | 14 | 4.60 | 2.00 |
| 12 | 8 | -14 | 453.85 | 24.60 |
| -12 | 8 | 14 | 428.36 | 24.80 |
| -11 | 8 | 14 | 31.60 | 4.10 |
| 11 | 8 | -14 | 26.20 | 4.70 |
| -10 | 8 | 14 | 134.49 | 9.40 |
| 10 | 8 | -14 | 132.39 | 9.00 |
| -9 | 8 | 14 | 19.90 | 3.90 |
| 9 | 8 | -14 | 22.40 | 3.60 |
| -8 | -8 | 14 | 37.30 | 7.20 |
| -8 | 8 | 14 | 37.10 | 6.20 |
| -7 | -8 | 14 | 13.20 | 3.20 |
| -7 | 8 | 14 | 10.70 | 3.90 |
| -6 | -8 | 14 | 831.42 | 46.80 |
| -6 | 8 | 14 | 857.91 | 48.10 |
| -5 | -8 | 14 | 42.80 | 6.00 |
| 5 | 8 | -14 | 57.39 | 12.50 |
| -5 | 8 | 14 | 53.19 | 8.60 |
| -4 | -8 | 14 | 18.80 | 3.30 |
| -4 | 8 | 14 | 10.00 | 10.50 |
| -4 | 8 | 14 | 10.40 | 4.80 |
| -3 | -8 | 14 | 1.90 | 2.90 |
| -3 | 8 | 14 | 10.80 | 9.10 |
| -3 | 8 | 14 | -2.70 | 4.20 |
| -2 | -8 | 14 | 650.53 | 36.60 |
| -2 | 8 | 14 | 671.83 | 41.40 |
| -2 | 8 | 14 | 624.94 | 37.70 |
| -1 | -8 | 14 | -2.70 | 2.80 |
| -1 | 8 | 14 | -1.30 | 3.90 |
| -1 | 8 | 14 | -2.00 | 7.90 |
| 0 | -8 | 14 | 562.64 | 30.20 |
| 0 | 8 | 14 | 451.55 | 34.80 |
| 0 | 8 | 14 | 541.45 | 30.70 |
| 1 | -8 | 14 | 40.70 | 6.30 |
| 1 | 8 | 14 | 36.70 | 6.70 |
| 2 | -8 | 14 | 154.48 | 10.80 |
| 2 | 8 | 14 | 126.39 | 10.20 |
| 3 | -8 | 14 | 5.80 | 3.60 |
| 3 | 8 | 14 | 10.00 | 3.20 |
| 4 | -8 | 14 | 617.04 | 33.40 |

| | | | | |
|---|---|---|---|---|
| 4 | 8 | 14 | 536.25 | 34.50 |
| 5 | -8 | 14 | 40.10 | 6.50 |
| 5 | 8 | 14 | 44.70 | 5.10 |
| 5 | 8 | 14 | 35.90 | 9.50 |
| -6 | -8 | -14 | 16.10 | 3.20 |
| 6 | -8 | 14 | 6.00 | 3.30 |
| 6 | 8 | 14 | 10.20 | 2.40 |
| -7 | -8 | -14 | 5.40 | 2.90 |
| 7 | 8 | 14 | 3.10 | 2.10 |
| 8 | 8 | 14 | 188.08 | 11.50 |
| 12 | 9 | -14 | 9.50 | 2.20 |
| -12 | 9 | 14 | 7.10 | 1.90 |
| -11 | 9 | 14 | 466.05 | 28.40 |
| 11 | 9 | -14 | 550.54 | 28.10 |
| 10 | 9 | -14 | 15.50 | 2.80 |
| -10 | 9 | 14 | 10.50 | 2.50 |
| 9 | 9 | -14 | 74.39 | 6.50 |
| -9 | 9 | 14 | 68.79 | 6.80 |
| -8 | -9 | 14 | 32.20 | 6.20 |
| 8 | 9 | -14 | 42.30 | 5.90 |
| -8 | 9 | 14 | 32.90 | 6.10 |
| -7 | -9 | 14 | 816.62 | 46.70 |
| -7 | 9 | 14 | 805.62 | 46.60 |
| 7 | 9 | -14 | 858.71 | 46.40 |
| -6 | -9 | 14 | 31.60 | 5.20 |
| 6 | -9 | -14 | 33.40 | 6.00 |
| -6 | 9 | 14 | 22.60 | 5.10 |
| -5 | -9 | 14 | 198.08 | 13.10 |
| -5 | 9 | 14 | 216.38 | 15.10 |
| -4 | -9 | 14 | 10.00 | 2.90 |
| -4 | 9 | 14 | 0.60 | 4.50 |
| -3 | -9 | 14 | 181.08 | 11.90 |
| -3 | 9 | 14 | 178.38 | 13.70 |
| -2 | -9 | 14 | 14.60 | 3.10 |
| -2 | 9 | 14 | 18.60 | 4.70 |
| -1 | -9 | 14 | 804.92 | 46.10 |
| -1 | 9 | 14 | 851.71 | 47.00 |
| 0 | -9 | 14 | 49.20 | 6.10 |
| 0 | 9 | 14 | 45.40 | 6.90 |
| 1 | -9 | 14 | 63.79 | 6.90 |
| 1 | 9 | 14 | 53.09 | 7.00 |
| 2 | -9 | 14 | 52.79 | 6.60 |
| 2 | 9 | 14 | 33.70 | 5.80 |
| 3 | -9 | 14 | 461.65 | 28.20 |

| | | | | |
|---|---|---|---|---|
| 3 | 9 | 14 | 517.55 | 29.60 |
| 3 | 9 | 14 | 469.35 | 28.60 |
| 4 | -9 | 14 | 0.30 | 3.10 |
| 4 | 9 | 14 | 2.70 | 5.20 |
| 4 | 9 | 14 | 3.80 | 2.50 |
| 5 | -9 | 14 | 478.25 | 27.80 |
| -5 | -9 | -14 | 491.55 | 28.10 |
| 5 | 9 | 14 | 472.25 | 29.00 |
| 5 | 9 | 14 | 513.35 | 27.70 |
| -6 | -9 | -14 | 120.89 | 8.60 |
| 6 | -9 | 14 | 109.79 | 9.00 |
| 6 | 9 | 14 | 93.69 | 16.70 |
| 6 | 9 | 14 | 124.49 | 8.10 |
| -7 | -9 | -14 | 129.89 | 9.00 |
| 7 | 9 | 14 | 130.89 | 8.50 |
| -11 | 10 | 14 | 2.40 | 1.90 |
| 11 | 10 | -14 | 3.20 | 2.10 |
| 10 | 10 | -14 | 227.68 | 13.20 |
| -10 | 10 | 14 | 226.58 | 13.70 |
| -9 | 10 | 14 | 14.00 | 2.90 |
| 9 | 10 | -14 | 13.60 | 2.70 |
| -8 | -10 | 14 | 97.49 | 8.10 |
| 8 | 10 | -14 | 104.69 | 7.70 |
| -8 | 10 | 14 | 99.29 | 8.40 |
| -7 | -10 | 14 | 79.19 | 6.90 |
| 7 | 10 | -14 | 81.99 | 6.90 |
| -7 | 10 | 14 | 70.89 | 8.00 |
| -6 | -10 | 14 | 1051.99 | 64.09 |
| 6 | -10 | -14 | 1222.58 | 64.39 |
| 6 | 10 | -14 | 1244.18 | 64.19 |
| 5 | -10 | -14 | 31.50 | 6.00 |
| -5 | -10 | 14 | 41.60 | 5.20 |
| -5 | 10 | 14 | 26.50 | 5.10 |
| -4 | -10 | 14 | 27.20 | 5.00 |
| -4 | 10 | 14 | 17.20 | 4.90 |
| -3 | -10 | 14 | 67.99 | 6.50 |
| -2 | -10 | 14 | 804.12 | 46.10 |
| -2 | 10 | 14 | 859.11 | 47.20 |
| -1 | -10 | 14 | 1.90 | 2.60 |
| -1 | 10 | 14 | 1.30 | 3.50 |
| 0 | -10 | 14 | 447.66 | 25.70 |
| 0 | 10 | 14 | 440.76 | 26.30 |
| 1 | -10 | 14 | 50.09 | 6.10 |
| 1 | 10 | 14 | 40.00 | 8.20 |

| | | | | |
|---|---|---|---|---|
| 2 | -10 | 14 | 352.66 | 21.20 |
| 2 | 10 | 14 | 355.76 | 21.30 |
| 2 | 10 | 14 | 355.06 | 21.20 |
| 3 | -10 | 14 | 10.40 | 3.10 |
| 3 | 10 | 14 | 12.20 | 3.10 |
| 3 | 10 | 14 | 2.20 | 4.60 |
| 4 | -10 | 14 | 617.04 | 32.80 |
| -4 | -10 | -14 | 591.34 | 33.10 |
| 4 | 10 | 14 | 577.24 | 33.80 |
| 4 | 10 | 14 | 535.45 | 32.40 |
| 5 | -10 | 14 | 6.40 | 3.00 |
| -5 | -10 | -14 | 4.10 | 2.70 |
| 5 | 10 | 14 | 10.60 | 5.70 |
| 5 | 10 | 14 | 9.00 | 2.50 |
| -6 | -10 | -14 | 10.30 | 2.70 |
| 6 | 10 | 14 | 12.30 | 2.60 |
| 10 | 11 | -14 | 5.90 | 2.30 |
| -10 | 11 | 14 | 2.70 | 2.10 |
| 9 | 11 | -14 | 26.10 | 4.00 |
| -9 | 11 | 14 | 29.90 | 4.60 |
| -8 | -11 | 14 | 86.49 | 7.60 |
| 8 | 11 | -14 | 99.69 | 7.00 |
| -8 | 11 | 14 | 96.99 | 7.70 |
| -7 | -11 | 14 | 883.91 | 50.69 |
| 7 | 11 | -14 | 971.10 | 50.59 |
| -7 | 11 | 14 | 926.81 | 51.49 |
| -6 | -11 | 14 | -1.00 | 2.40 |
| 6 | -11 | -14 | -4.00 | 5.60 |
| -6 | 11 | 14 | -1.00 | 3.50 |
| 6 | 11 | -14 | -2.70 | 2.90 |
| 5 | -11 | -14 | 352.96 | 20.80 |
| -5 | -11 | 14 | 342.27 | 20.50 |
| 5 | 11 | -14 | 351.76 | 21.20 |
| -5 | 11 | 14 | 364.96 | 22.00 |
| -4 | -11 | 14 | 39.50 | 5.80 |
| -4 | 11 | 14 | 26.60 | 5.40 |
| -3 | -11 | 14 | 217.78 | 16.70 |
| 3 | -11 | -14 | 256.27 | 15.40 |
| -3 | 11 | 14 | 216.78 | 16.30 |
| -2 | -11 | 14 | 92.59 | 7.80 |
| 2 | -11 | -14 | 76.89 | 9.70 |
| -2 | 11 | 14 | 61.59 | 9.20 |
| -1 | 11 | 14 | 1179.18 | 65.39 |
| 0 | -11 | 14 | 64.89 | 6.70 |

| | | | | |
|---|---|---|---|---|
| 0 | 11 | 14 | 76.59 | 8.00 |
| 1 | -11 | 14 | 1.20 | 2.30 |
| -1 | -11 | -14 | -1.60 | 3.20 |
| 1 | 11 | 14 | -0.40 | 3.40 |
| 1 | 11 | 14 | 1.50 | 4.10 |
| 2 | -11 | 14 | 83.79 | 7.20 |
| -2 | -11 | -14 | 73.59 | 8.10 |
| 2 | 11 | 14 | 73.99 | 7.20 |
| 2 | 11 | 14 | 80.39 | 8.50 |
| 3 | -11 | 14 | 681.23 | 33.50 |
| -3 | -11 | -14 | 520.75 | 33.60 |
| 3 | 11 | 14 | 647.94 | 33.30 |
| 3 | 11 | 14 | 513.65 | 33.50 |
| -4 | -11 | -14 | 3.40 | 2.60 |
| 4 | -11 | 14 | 8.30 | 2.70 |
| 4 | 11 | 14 | 6.30 | 2.70 |
| 4 | 11 | 14 | 1.30 | 4.90 |
| -5 | -11 | -14 | 328.27 | 18.70 |
| 5 | -11 | 14 | 293.27 | 19.10 |
| 5 | 11 | 14 | 332.57 | 19.80 |
| 5 | 11 | 14 | 321.47 | 18.30 |
| -6 | -11 | -14 | 68.39 | 5.90 |
| -9 | 12 | 14 | 31.20 | 4.20 |
| 9 | 12 | -14 | 22.80 | 3.80 |
| 8 | 12 | -14 | 38.90 | 4.30 |
| -8 | 12 | 14 | 38.40 | 5.00 |
| -7 | -12 | 14 | 20.20 | 4.20 |
| -7 | 12 | 14 | 24.00 | 5.10 |
| 7 | 12 | -14 | 16.10 | 2.70 |
| -6 | -12 | 14 | 514.85 | 29.60 |
| 6 | 12 | -14 | 522.75 | 29.50 |
| -6 | 12 | 14 | 559.04 | 30.60 |
| 5 | -12 | -14 | 138.39 | 9.30 |
| -5 | -12 | 14 | 131.79 | 8.70 |
| 5 | 12 | -14 | 125.19 | 9.20 |
| -5 | 12 | 14 | 120.89 | 10.20 |
| -4 | -12 | 14 | 9.50 | 2.40 |
| 4 | -12 | -14 | 7.70 | 2.70 |
| 4 | 12 | -14 | 15.90 | 6.10 |
| -4 | 12 | 14 | 11.00 | 4.30 |
| -3 | -12 | 14 | 49.20 | 5.10 |
| 3 | -12 | -14 | 50.19 | 5.90 |
| -3 | 12 | 14 | 40.00 | 7.80 |
| 2 | -12 | -14 | 565.64 | 31.70 |

| | | | | |
|---|---|---|---|---|
| -2 | 12 | 14 | 553.04 | 32.30 |
| 1 | -12 | -14 | 3.50 | 2.90 |
| -1 | -12 | 14 | 0.90 | 2.20 |
| -1 | 12 | 14 | 3.80 | 3.80 |
| 0 | -12 | 14 | 423.76 | 24.20 |
| 0 | -12 | -14 | 459.85 | 25.10 |
| 0 | 12 | 14 | 422.36 | 24.70 |
| 0 | 12 | 14 | 385.56 | 26.10 |
| 1 | -12 | 14 | 71.39 | 6.10 |
| -1 | -12 | -14 | 80.29 | 7.30 |
| 1 | 12 | 14 | 65.29 | 7.20 |
| 1 | 12 | 14 | 70.69 | 8.10 |
| 2 | -12 | 14 | 83.59 | 6.60 |
| -2 | -12 | -14 | 68.79 | 7.40 |
| 2 | 12 | 14 | 65.99 | 8.10 |
| 2 | 12 | 14 | 88.49 | 7.10 |
| 3 | -12 | 14 | 72.29 | 6.40 |
| -3 | -12 | -14 | 59.19 | 6.50 |
| 3 | 12 | 14 | 71.69 | 6.50 |
| 3 | 12 | 14 | 69.69 | 8.20 |
| 4 | -12 | 14 | 460.45 | 25.50 |
| -4 | -12 | -14 | 426.46 | 25.70 |
| 4 | 12 | 14 | 467.35 | 25.60 |
| 4 | 12 | 14 | 457.55 | 26.20 |
| 5 | -12 | 14 | 50.69 | 6.00 |
| -5 | -12 | -14 | 46.50 | 5.00 |
| 8 | 13 | -14 | 113.79 | 7.10 |
| -8 | 13 | 14 | 107.19 | 7.80 |
| -7 | -13 | 14 | 258.47 | 14.30 |
| -7 | 13 | 14 | 236.08 | 14.90 |
| -6 | -13 | 14 | 3.20 | 2.00 |
| -6 | 13 | 14 | -0.40 | 3.30 |
| 6 | 13 | -14 | 0.80 | 2.30 |
| -5 | -13 | 14 | 276.97 | 16.20 |
| 5 | -13 | -14 | 265.97 | 16.50 |
| 5 | 13 | -14 | 288.37 | 16.20 |
| -5 | 13 | 14 | 286.37 | 17.40 |
| -4 | -13 | 14 | 11.70 | 2.20 |
| 4 | -13 | -14 | 14.10 | 2.60 |
| 4 | 13 | -14 | 15.90 | 3.50 |
| -4 | 13 | 14 | 14.50 | 4.40 |
| 3 | -13 | -14 | 12.60 | 2.50 |
| -3 | -13 | 14 | 18.00 | 3.60 |
| -3 | 13 | 14 | 7.20 | 4.40 |

| | | | | |
|---|---|---|---|---|
| -2 | -13 | 14 | 52.79 | 5.00 |
| 2 | -13 | -14 | 51.59 | 5.50 |
| -2 | 13 | 14 | 49.70 | 7.80 |
| 1 | -13 | -14 | 546.55 | 30.60 |
| -1 | -13 | 14 | 531.85 | 30.20 |
| -1 | 13 | 14 | 543.35 | 31.00 |
| 0 | -13 | 14 | 72.79 | 5.70 |
| 0 | -13 | -14 | 75.19 | 6.50 |
| 0 | 13 | 14 | 60.09 | 7.90 |
| 0 | 13 | 14 | 69.59 | 7.80 |
| -1 | -13 | -14 | 5.90 | 2.60 |
| 1 | -13 | 14 | 9.70 | 2.20 |
| 1 | 13 | 14 | 12.10 | 4.10 |
| 1 | 13 | 14 | 12.30 | 3.80 |
| -2 | -13 | -14 | 32.00 | 4.90 |
| 2 | -13 | 14 | 36.80 | 4.30 |
| 2 | 13 | 14 | 25.50 | 5.60 |
| 2 | 13 | 14 | 18.10 | 4.50 |
| -3 | -13 | -14 | 212.88 | 14.20 |
| 3 | -13 | 14 | 249.38 | 14.10 |
| 3 | 13 | 14 | 229.78 | 15.90 |
| 3 | 13 | 14 | 245.78 | 14.80 |
| -6 | -14 | 14 | 520.85 | 29.70 |
| 6 | 14 | -14 | 562.34 | 29.70 |
| 5 | -14 | -14 | 68.59 | 9.20 |
| -5 | -14 | 14 | 81.59 | 5.70 |
| 5 | 14 | -14 | 83.49 | 6.20 |
| 4 | -14 | -14 | 4.00 | 2.30 |
| -4 | -14 | 14 | 3.80 | 1.80 |
| 4 | 14 | -14 | 5.00 | 3.90 |
| 3 | -14 | -14 | 49.90 | 4.80 |
| -3 | -14 | 14 | 46.70 | 4.30 |
| 2 | -14 | -14 | 324.07 | 18.20 |
| -2 | -14 | 14 | 309.77 | 18.00 |
| -1 | -14 | 14 | 0.60 | 1.50 |
| 1 | -14 | -14 | 4.10 | 2.00 |
| 0 | -14 | 14 | 289.07 | 16.70 |
| 0 | -14 | -14 | 291.47 | 17.00 |
| 1 | -14 | 14 | 133.99 | 8.40 |
| -1 | -14 | -14 | 127.79 | 8.80 |
| -2 | -14 | -14 | 69.99 | 5.90 |
| 2 | -14 | 14 | 75.59 | 5.60 |
| -14 | 0 | 15 | 2.70 | 2.00 |
| -13 | 0 | 15 | 0.90 | 2.10 |

| | | | | |
|---|---|---|---|---|
| -12 | 0 | 15 | -1.20 | 2.20 |
| -11 | 0 | 15 | 243.28 | 15.10 |
| -10 | 0 | 15 | 7.10 | 3.70 |
| -9 | 0 | 15 | 52.29 | 6.90 |
| -8 | 0 | 15 | -5.80 | 3.40 |
| -7 | 0 | 15 | 199.28 | 14.50 |
| -7 | 0 | 15 | 233.48 | 15.10 |
| -6 | 0 | 15 | 4.70 | 4.40 |
| -6 | 0 | 15 | -11.40 | 7.20 |
| -6 | 0 | 15 | -7.40 | 3.50 |
| -5 | 0 | 15 | 168.48 | 14.20 |
| -5 | 0 | 15 | 179.28 | 12.50 |
| -5 | 0 | 15 | 190.58 | 15.60 |
| -4 | 0 | 15 | 4.40 | 5.00 |
| -4 | 0 | 15 | 6.40 | 3.20 |
| -4 | 0 | 15 | 5.10 | 6.10 |
| -3 | 0 | 15 | 190.78 | 15.50 |
| -3 | 0 | 15 | 170.08 | 12.60 |
| -3 | 0 | 15 | 208.38 | 15.10 |
| -2 | 0 | 15 | -3.40 | 4.90 |
| -2 | 0 | 15 | -2.10 | 2.90 |
| -2 | 0 | 15 | 13.20 | 6.30 |
| -1 | 0 | 15 | 1145.59 | 62.79 |
| -1 | 0 | 15 | 1065.39 | 61.99 |
| -1 | 0 | 15 | 1148.39 | 63.99 |
| 0 | 0 | 15 | -5.30 | 5.40 |
| 0 | 0 | 15 | 6.90 | 4.60 |
| 1 | 0 | 15 | 16.30 | 6.70 |
| 1 | 0 | 15 | 14.50 | 4.60 |
| 1 | 0 | 15 | 12.30 | 4.20 |
| 2 | 0 | 15 | 1.60 | 3.70 |
| 2 | 0 | 15 | 3.10 | 4.10 |
| 2 | 0 | 15 | -4.10 | 6.80 |
| 3 | 0 | 15 | 397.06 | 25.40 |
| 3 | 0 | 15 | 466.85 | 26.40 |
| 3 | 0 | 15 | 436.96 | 30.00 |
| 4 | 0 | 15 | -2.50 | 3.50 |
| 4 | 0 | 15 | 3.90 | 3.30 |
| 5 | 0 | 15 | 502.35 | 29.70 |
| 5 | 0 | 15 | 539.25 | 31.20 |
| 6 | 0 | 15 | -2.90 | 2.60 |
| 6 | 0 | 15 | 1.70 | 2.90 |
| 7 | 0 | 15 | 177.68 | 11.40 |
| 8 | 0 | 15 | 0.60 | 2.40 |

| | | | | |
|---|---|---|---|---|
| -14 | -1 | 15 | 29.20 | 4.60 |
| -14 | 1 | 15 | 24.40 | 4.30 |
| -13 | -1 | 15 | 6.70 | 2.60 |
| -13 | 1 | 15 | 3.40 | 2.00 |
| -12 | -1 | 15 | 415.86 | 23.20 |
| -12 | 1 | 15 | 395.56 | 23.20 |
| -11 | -1 | 15 | 2.30 | 2.90 |
| -11 | 1 | 15 | -2.60 | 2.30 |
| -10 | -1 | 15 | 168.78 | 15.10 |
| -10 | 1 | 15 | 214.28 | 13.00 |
| -9 | -1 | 15 | 9.80 | 3.70 |
| -9 | 1 | 15 | 11.80 | 3.30 |
| -8 | -1 | 15 | 9.10 | 4.00 |
| -8 | 1 | 15 | 1.50 | 3.60 |
| -7 | -1 | 15 | 3.60 | 4.30 |
| -7 | -1 | 15 | 0.40 | 3.70 |
| -7 | 1 | 15 | 7.10 | 4.10 |
| -6 | -1 | 15 | 1514.55 | 78.59 |
| -6 | -1 | 15 | 1459.55 | 77.29 |
| -6 | -1 | 15 | 1386.46 | 76.19 |
| -6 | 1 | 15 | 1404.86 | 76.29 |
| -6 | 1 | 15 | 1206.08 | 76.99 |
| -5 | -1 | 15 | 57.19 | 9.90 |
| -5 | -1 | 15 | 55.99 | 11.30 |
| -5 | -1 | 15 | 50.09 | 6.90 |
| -5 | 1 | 15 | 38.80 | 7.80 |
| -5 | 1 | 15 | 50.99 | 7.30 |
| -5 | 1 | 15 | 66.39 | 9.10 |
| -4 | -1 | 15 | 137.69 | 12.10 |
| -4 | -1 | 15 | 107.79 | 9.00 |
| -4 | -1 | 15 | 123.19 | 12.40 |
| -4 | 1 | 15 | 88.69 | 12.50 |
| -4 | 1 | 15 | 108.79 | 9.00 |
| -4 | 1 | 15 | 79.99 | 10.90 |
| -3 | -1 | 15 | 48.80 | 10.00 |
| -3 | -1 | 15 | 41.20 | 6.30 |
| -3 | -1 | 15 | 34.00 | 6.70 |
| -3 | 1 | 15 | 41.00 | 6.40 |
| -3 | 1 | 15 | 29.20 | 6.50 |
| -3 | 1 | 15 | 26.20 | 5.60 |
| -2 | -1 | 15 | 934.91 | 51.09 |
| -2 | -1 | 15 | 874.51 | 49.00 |
| -2 | -1 | 15 | 777.12 | 49.80 |
| -2 | 1 | 15 | 856.41 | 49.00 |

| | | | | |
|---|---|---|---|---|
| -2 | 1 | 15 | 883.91 | 51.09 |
| -2 | 1 | 15 | 944.81 | 50.39 |
| -1 | -1 | 15 | 3.60 | 5.40 |
| -1 | -1 | 15 | 2.10 | 3.30 |
| -1 | -1 | 15 | 2.70 | 5.10 |
| -1 | 1 | 15 | -3.00 | 4.90 |
| -1 | 1 | 15 | 2.90 | 3.50 |
| -1 | 1 | 15 | 6.60 | 4.40 |
| 0 | -1 | 15 | 981.40 | 58.79 |
| 0 | -1 | 15 | 976.30 | 57.19 |
| 0 | -1 | 15 | 1033.50 | 56.99 |
| 0 | 1 | 15 | 1025.90 | 58.99 |
| 0 | 1 | 15 | 1091.39 | 57.29 |
| 1 | -1 | 15 | -0.20 | 6.60 |
| 1 | -1 | 15 | 2.20 | 3.90 |
| 1 | -1 | 15 | 2.60 | 4.00 |
| 1 | 1 | 15 | 2.00 | 4.00 |
| 1 | 1 | 15 | 3.00 | 6.10 |
| 1 | 1 | 15 | 2.60 | 4.10 |
| 2 | -1 | 15 | 81.59 | 14.10 |
| 2 | -1 | 15 | 80.09 | 8.90 |
| 2 | -1 | 15 | 64.69 | 8.30 |
| 2 | 1 | 15 | 73.59 | 9.30 |
| 2 | 1 | 15 | 53.89 | 8.00 |
| 2 | 1 | 15 | 62.39 | 13.20 |
| 3 | -1 | 15 | -2.40 | 8.80 |
| 3 | -1 | 15 | -2.20 | 3.70 |
| 3 | -1 | 15 | -0.70 | 3.50 |
| 3 | 1 | 15 | -2.90 | 3.10 |
| 3 | 1 | 15 | 2.50 | 4.00 |
| 3 | 1 | 15 | 5.30 | 8.70 |
| 4 | -1 | 15 | 820.62 | 46.30 |
| 4 | -1 | 15 | 823.52 | 46.90 |
| 4 | 1 | 15 | 839.32 | 46.90 |
| 4 | 1 | 15 | 829.02 | 46.30 |
| 5 | -1 | 15 | 11.90 | 5.20 |
| 5 | -1 | 15 | -1.90 | 5.60 |
| 5 | 1 | 15 | 8.40 | 4.70 |
| 5 | 1 | 15 | 5.00 | 3.10 |
| 6 | -1 | 15 | 42.00 | 6.20 |
| 6 | -1 | 15 | 26.50 | 5.70 |
| 6 | 1 | 15 | 29.60 | 5.70 |
| 6 | 1 | 15 | 36.50 | 5.20 |
| 7 | -1 | 15 | 4.40 | 2.80 |

| | | | | |
|---|---|---|---|---|
| 7 | 1 | 15 | 2.80 | 2.40 |
| 8 | -1 | 15 | 296.37 | 18.00 |
| 8 | 1 | 15 | 317.57 | 17.90 |
| -13 | -2 | 15 | 112.09 | 9.60 |
| -13 | 2 | 15 | 107.49 | 7.40 |
| -12 | -2 | 15 | 12.80 | 3.20 |
| -12 | 2 | 15 | 4.80 | 2.20 |
| -11 | -2 | 15 | 724.73 | 40.90 |
| -11 | 2 | 15 | 746.43 | 41.00 |
| -10 | -2 | 15 | 66.29 | 7.60 |
| -10 | 2 | 15 | 58.79 | 6.50 |
| -9 | -2 | 15 | 8.70 | 4.00 |
| -9 | 2 | 15 | 11.80 | 3.40 |
| -8 | -2 | 15 | 19.00 | 4.70 |
| -7 | -2 | 15 | 889.71 | 54.19 |
| -7 | -2 | 15 | 1050.99 | 55.29 |
| -7 | 2 | 15 | 1015.60 | 55.29 |
| -6 | -2 | 15 | 43.20 | 7.10 |
| -6 | -2 | 15 | 52.39 | 7.30 |
| -6 | 2 | 15 | 50.89 | 7.90 |
| -6 | 2 | 15 | 73.19 | 8.50 |
| -5 | -2 | 15 | 703.03 | 40.30 |
| -5 | -2 | 15 | 653.53 | 39.30 |
| -5 | -2 | 15 | 638.14 | 37.80 |
| -5 | 2 | 15 | 739.53 | 39.40 |
| -5 | 2 | 15 | 619.24 | 37.90 |
| -5 | 2 | 15 | 685.13 | 40.50 |
| -4 | -2 | 15 | 11.70 | 5.70 |
| -4 | -2 | 15 | 8.90 | 6.10 |
| -4 | -2 | 15 | 8.00 | 3.30 |
| -4 | 2 | 15 | 14.70 | 3.60 |
| -4 | 2 | 15 | 20.90 | 6.40 |
| -4 | 2 | 15 | 14.70 | 5.30 |
| -3 | -2 | 15 | 52.29 | 10.00 |
| -3 | -2 | 15 | 46.10 | 7.60 |
| -3 | -2 | 15 | 55.59 | 6.90 |
| -3 | 2 | 15 | 52.09 | 9.40 |
| -3 | 2 | 15 | 60.69 | 7.00 |
| -3 | 2 | 15 | 69.29 | 10.90 |
| -2 | -2 | 15 | 217.68 | 17.10 |
| -2 | -2 | 15 | 224.88 | 16.90 |
| -2 | -2 | 15 | 205.38 | 14.60 |
| -2 | 2 | 15 | 228.98 | 14.90 |
| -2 | 2 | 15 | 239.88 | 18.00 |

| | | | | |
|---|---|---|---|---|
| -2 | 2 | 15 | 233.58 | 16.40 |
| -1 | -2 | 15 | 1948.71 | 111.89 |
| -1 | -2 | 15 | 2005.40 | 110.19 |
| -1 | -2 | 15 | 1897.21 | 110.49 |
| -1 | 2 | 15 | 2093.79 | 112.39 |
| -1 | 2 | 15 | 2026.80 | 110.39 |
| -1 | 2 | 15 | 2160.18 | 111.09 |
| 0 | -2 | 15 | 59.49 | 10.00 |
| 0 | -2 | 15 | 71.29 | 11.60 |
| 0 | -2 | 15 | 73.09 | 8.00 |
| 0 | 2 | 15 | 65.39 | 12.00 |
| 0 | 2 | 15 | 69.49 | 8.70 |
| 0 | 2 | 15 | 70.29 | 8.60 |
| 1 | -2 | 15 | 127.19 | 10.40 |
| 1 | -2 | 15 | 100.79 | 13.70 |
| 1 | -2 | 15 | 119.59 | 11.10 |
| 1 | 2 | 15 | 121.89 | 10.70 |
| 1 | 2 | 15 | 120.89 | 10.40 |
| 1 | 2 | 15 | 101.59 | 13.60 |
| 2 | -2 | 15 | 49.70 | 8.10 |
| 2 | -2 | 15 | 28.90 | 9.20 |
| 2 | -2 | 15 | 61.79 | 8.00 |
| 2 | 2 | 15 | 42.20 | 8.10 |
| 2 | 2 | 15 | 41.80 | 7.00 |
| 2 | 2 | 15 | 43.40 | 9.10 |
| 3 | -2 | 15 | 508.55 | 30.70 |
| 3 | -2 | 15 | 560.84 | 31.40 |
| 3 | 2 | 15 | 538.55 | 31.60 |
| 3 | 2 | 15 | 444.16 | 42.60 |
| 3 | 2 | 15 | 573.04 | 30.90 |
| 4 | -2 | 15 | 51.39 | 7.40 |
| 4 | -2 | 15 | 59.09 | 8.20 |
| 4 | 2 | 15 | 57.39 | 6.50 |
| 4 | 2 | 15 | 54.19 | 8.00 |
| 5 | -2 | 15 | 898.31 | 49.80 |
| 5 | -2 | 15 | 939.21 | 52.29 |
| 5 | 2 | 15 | 846.72 | 49.50 |
| 6 | -2 | 15 | -4.90 | 2.80 |
| 6 | -2 | 15 | -0.90 | 3.00 |
| 6 | 2 | 15 | 1.40 | 2.30 |
| 7 | -2 | 15 | 166.48 | 11.30 |
| 7 | -2 | 15 | 167.38 | 11.50 |
| 7 | 2 | 15 | 173.28 | 10.90 |
| 8 | -2 | 15 | 7.70 | 3.40 |

| | | | | |
|---|---|---|---|---|
| 8 | 2 | 15 | 6.70 | 2.40 |
| -13 | 3 | 15 | 34.70 | 3.80 |
| -12 | 3 | 15 | 641.54 | 35.70 |
| -11 | 3 | 15 | 4.90 | 2.40 |
| -10 | -3 | 15 | 152.38 | 13.10 |
| -10 | 3 | 15 | 180.38 | 13.50 |
| -9 | -3 | 15 | 5.50 | 7.30 |
| -9 | 3 | 15 | 3.50 | 2.60 |
| -8 | -3 | 15 | 310.27 | 21.80 |
| -8 | 3 | 15 | 401.76 | 21.90 |
| -7 | -3 | 15 | 87.49 | 11.30 |
| -7 | -3 | 15 | 94.79 | 9.00 |
| -7 | 3 | 15 | 115.29 | 9.60 |
| -6 | -3 | 15 | 1742.13 | 101.19 |
| -6 | -3 | 15 | 1913.61 | 102.19 |
| -6 | 3 | 15 | 2070.09 | 102.69 |
| -6 | 3 | 15 | 1733.23 | 101.29 |
| -5 | -3 | 15 | 86.99 | 8.10 |
| -5 | -3 | 15 | 80.79 | 12.50 |
| -5 | 3 | 15 | 79.99 | 13.10 |
| -5 | 3 | 15 | 101.19 | 10.00 |
| -5 | 3 | 15 | 80.99 | 8.60 |
| -4 | -3 | 15 | 1.50 | 6.00 |
| -4 | -3 | 15 | -0.50 | 6.60 |
| -4 | -3 | 15 | -0.30 | 3.00 |
| -4 | 3 | 15 | 2.70 | 3.20 |
| -4 | 3 | 15 | 5.10 | 6.20 |
| -4 | 3 | 15 | 3.60 | 4.90 |
| -3 | -3 | 15 | 27.80 | 5.70 |
| -3 | -3 | 15 | 29.00 | 6.70 |
| -3 | 3 | 15 | 22.20 | 5.50 |
| -3 | 3 | 15 | 23.70 | 4.20 |
| -3 | 3 | 15 | 45.70 | 11.40 |
| -2 | -3 | 15 | 1227.78 | 71.69 |
| -2 | -3 | 15 | 1270.47 | 70.79 |
| -2 | -3 | 15 | 1364.16 | 72.79 |
| -2 | 3 | 15 | 1333.27 | 73.29 |
| -2 | 3 | 15 | 1251.67 | 71.79 |
| -2 | 3 | 15 | 1279.17 | 70.89 |
| -1 | -3 | 15 | 72.09 | 7.50 |
| -1 | -3 | 15 | 79.29 | 11.20 |
| -1 | -3 | 15 | 71.29 | 11.60 |
| -1 | 3 | 15 | 59.89 | 9.00 |
| -1 | 3 | 15 | 72.79 | 12.60 |

| | | | | |
|---|---|---|---|---|
| -1 | 3 | 15 | 69.99 | 8.10 |
| 0 | -3 | 15 | 747.03 | 45.50 |
| 0 | -3 | 15 | 851.81 | 43.50 |
| 0 | -3 | 15 | 683.33 | 43.70 |
| 0 | 3 | 15 | 852.71 | 44.00 |
| 0 | 3 | 15 | 675.93 | 43.60 |
| 0 | 3 | 15 | 796.32 | 46.10 |
| 1 | -3 | 15 | 6.50 | 5.20 |
| 1 | -3 | 15 | -3.40 | 3.50 |
| 1 | -3 | 15 | 8.30 | 7.50 |
| 1 | 3 | 15 | 4.40 | 3.70 |
| 1 | 3 | 15 | -6.10 | 7.10 |
| 1 | 3 | 15 | 2.30 | 4.00 |
| 2 | -3 | 15 | 184.48 | 19.50 |
| 2 | -3 | 15 | 193.48 | 14.80 |
| 2 | -3 | 15 | 228.68 | 14.70 |
| 2 | 3 | 15 | 193.78 | 19.40 |
| 2 | 3 | 15 | 226.78 | 15.10 |
| 2 | 3 | 15 | 197.78 | 14.00 |
| 3 | -3 | 15 | 56.09 | 8.60 |
| 3 | -3 | 15 | 64.99 | 8.50 |
| 3 | 3 | 15 | 61.29 | 8.80 |
| 3 | 3 | 15 | 67.59 | 7.20 |
| 4 | -3 | 15 | 1388.86 | 78.59 |
| 4 | -3 | 15 | 1333.37 | 78.09 |
| 4 | 3 | 15 | 1514.45 | 78.29 |
| 4 | 3 | 15 | 1469.65 | 78.79 |
| 5 | -3 | 15 | 4.70 | 3.80 |
| 5 | -3 | 15 | 12.10 | 4.10 |
| 5 | 3 | 15 | 5.60 | 3.70 |
| 5 | 3 | 15 | 8.40 | 4.30 |
| 6 | -3 | 15 | 16.50 | 4.30 |
| 6 | -3 | 15 | 31.20 | 5.90 |
| 6 | 3 | 15 | 23.30 | 4.40 |
| 7 | -3 | 15 | 19.50 | 4.50 |
| 7 | 3 | 15 | 31.80 | 4.40 |
| 8 | -3 | 15 | 383.56 | 24.30 |
| 8 | 3 | 15 | 411.16 | 22.60 |
| -13 | 4 | 15 | 143.79 | 9.00 |
| -12 | 4 | 15 | 23.50 | 3.90 |
| -11 | 4 | 15 | 522.85 | 29.60 |
| -10 | 4 | 15 | 18.80 | 3.60 |
| -9 | 4 | 15 | 11.30 | 3.20 |
| -8 | 4 | 15 | 53.19 | 6.70 |

| | | | | |
|---|---|---|---|---|
| -7 | -4 | 15 | 796.22 | 45.40 |
| -7 | 4 | 15 | 842.92 | 46.50 |
| -6 | -4 | 15 | 19.80 | 3.80 |
| -6 | 4 | 15 | 15.10 | 4.10 |
| -6 | 4 | 15 | 12.20 | 3.80 |
| -5 | -4 | 15 | 151.58 | 11.10 |
| -5 | 4 | 15 | 171.98 | 16.70 |
| -5 | 4 | 15 | 154.08 | 12.60 |
| -5 | 4 | 15 | 156.68 | 11.60 |
| -4 | -4 | 15 | 13.20 | 3.30 |
| -4 | 4 | 15 | 19.10 | 5.00 |
| -4 | 4 | 15 | 2.10 | 7.00 |
| -4 | 4 | 15 | 16.60 | 3.60 |
| -3 | -4 | 15 | 216.48 | 13.50 |
| -3 | 4 | 15 | 176.18 | 14.90 |
| -3 | 4 | 15 | 190.98 | 13.50 |
| -3 | 4 | 15 | 223.38 | 17.70 |
| -2 | -4 | 15 | 68.29 | 7.10 |
| -2 | 4 | 15 | 58.89 | 8.90 |
| -2 | 4 | 15 | 54.99 | 7.30 |
| -2 | 4 | 15 | 78.79 | 12.40 |
| -1 | -4 | 15 | 880.31 | 49.10 |
| -1 | -4 | 15 | 918.81 | 51.39 |
| -1 | 4 | 15 | 780.12 | 49.70 |
| -1 | 4 | 15 | 909.71 | 51.99 |
| -1 | 4 | 15 | 911.41 | 49.50 |
| 0 | -4 | 15 | -5.90 | 6.40 |
| 0 | -4 | 15 | -1.30 | 3.00 |
| 0 | 4 | 15 | -2.60 | 3.80 |
| 0 | 4 | 15 | -7.20 | 6.70 |
| 0 | 4 | 15 | 1.70 | 3.50 |
| 1 | -4 | 15 | 4.00 | 3.60 |
| 1 | -4 | 15 | -3.80 | 7.10 |
| 1 | 4 | 15 | 1.00 | 4.00 |
| 1 | 4 | 15 | -0.70 | 3.40 |
| 1 | 4 | 15 | 3.40 | 6.80 |
| 2 | -4 | 15 | 88.89 | 8.90 |
| 2 | 4 | 15 | 88.29 | 9.80 |
| 2 | 4 | 15 | 81.99 | 15.10 |
| 2 | 4 | 15 | 84.79 | 8.30 |
| 3 | -4 | 15 | 560.84 | 31.70 |
| 3 | 4 | 15 | 540.55 | 31.20 |
| 3 | 4 | 15 | 524.15 | 32.00 |
| 4 | -4 | 15 | 16.40 | 4.20 |

| | | | | |
|---|---|---|---|---|
| 4 | -4 | 15 | 19.80 | 6.20 |
| 4 | 4 | 15 | 31.80 | 5.20 |
| 4 | 4 | 15 | 17.50 | 4.00 |
| 5 | -4 | 15 | 631.84 | 34.20 |
| 5 | -4 | 15 | 631.14 | 34.50 |
| 5 | 4 | 15 | 534.15 | 33.80 |
| 6 | -4 | 15 | 6.50 | 3.20 |
| 6 | 4 | 15 | 9.30 | 2.60 |
| 7 | 4 | 15 | 297.17 | 17.40 |
| 8 | 4 | 15 | 8.80 | 2.30 |
| -13 | 5 | 15 | 14.80 | 2.90 |
| -12 | 5 | 15 | 376.86 | 21.50 |
| -11 | 5 | 15 | 24.50 | 4.00 |
| -10 | 5 | 15 | 238.18 | 14.80 |
| -9 | 5 | 15 | 10.40 | 3.00 |
| -8 | 5 | 15 | 97.19 | 8.50 |
| -7 | -5 | 15 | 1.30 | 3.20 |
| -7 | 5 | 15 | 6.60 | 3.50 |
| -6 | -5 | 15 | 1156.18 | 66.99 |
| -6 | 5 | 15 | 1295.97 | 68.39 |
| -5 | -5 | 15 | 54.89 | 6.60 |
| -5 | 5 | 15 | 50.89 | 8.30 |
| -5 | 5 | 15 | 56.49 | 7.00 |
| -5 | 5 | 15 | 38.60 | 9.60 |
| -4 | -5 | 15 | 5.30 | 3.00 |
| -4 | 5 | 15 | -4.30 | 4.20 |
| -4 | 5 | 15 | 3.90 | 7.80 |
| -4 | 5 | 15 | 4.40 | 3.10 |
| -3 | -5 | 15 | 15.00 | 3.20 |
| -3 | 5 | 15 | 13.40 | 7.30 |
| -3 | 5 | 15 | 21.40 | 5.30 |
| -2 | -5 | 15 | 943.91 | 54.99 |
| -2 | 5 | 15 | 1053.79 | 58.19 |
| -2 | 5 | 15 | 959.80 | 55.29 |
| -2 | 5 | 15 | 1022.10 | 56.29 |
| -1 | -5 | 15 | -1.80 | 2.70 |
| -1 | 5 | 15 | -8.80 | 7.10 |
| -1 | 5 | 15 | -4.70 | 3.50 |
| -1 | 5 | 15 | 5.30 | 3.30 |
| 0 | -5 | 15 | 560.34 | 34.20 |
| 0 | 5 | 15 | 597.74 | 37.90 |
| 0 | 5 | 15 | 622.74 | 35.00 |
| 0 | 5 | 15 | 622.84 | 35.10 |
| 1 | -5 | 15 | 7.10 | 3.60 |

| | | | | |
|---|---|---|---|---|
| 1 | 5 | 15 | 11.90 | 7.50 |
| 1 | 5 | 15 | 17.30 | 4.30 |
| 1 | 5 | 15 | 4.30 | 3.60 |
| 2 | -5 | 15 | 387.26 | 22.60 |
| 2 | 5 | 15 | 358.96 | 22.30 |
| 2 | 5 | 15 | 362.96 | 23.20 |
| 3 | -5 | 15 | -1.70 | 3.50 |
| 3 | 5 | 15 | 0.60 | 2.90 |
| 4 | 5 | 15 | 1140.69 | 62.89 |
| 5 | -5 | 15 | 7.10 | 3.30 |
| 5 | 5 | 15 | -0.70 | 2.40 |
| 6 | -5 | 15 | 3.20 | 3.00 |
| 6 | 5 | 15 | -0.30 | 2.10 |
| 7 | 5 | 15 | 8.90 | 2.20 |
| 8 | 5 | 15 | 305.77 | 17.50 |
| -12 | 6 | 15 | -1.10 | 1.70 |
| -11 | 6 | 15 | 604.14 | 33.80 |
| -10 | 6 | 15 | 26.80 | 4.50 |
| -9 | 6 | 15 | 11.70 | 3.50 |
| -8 | 6 | 15 | 8.40 | 3.00 |
| -7 | -6 | 15 | 855.91 | 51.69 |
| -7 | 6 | 15 | 1030.00 | 52.99 |
| -6 | -6 | 15 | 3.00 | 2.80 |
| -6 | 6 | 15 | 3.00 | 3.40 |
| -5 | -6 | 15 | 778.52 | 42.40 |
| -5 | 6 | 15 | 737.93 | 43.60 |
| -4 | -6 | 15 | 46.50 | 5.90 |
| -4 | 6 | 15 | 41.90 | 10.30 |
| -4 | 6 | 15 | 39.10 | 8.20 |
| -3 | -6 | 15 | 176.58 | 12.30 |
| -3 | 6 | 15 | 207.98 | 18.40 |
| -2 | -6 | 15 | 41.70 | 6.10 |
| -2 | 6 | 15 | 56.89 | 6.90 |
| -2 | 6 | 15 | 40.10 | 8.10 |
| -2 | 6 | 15 | 70.89 | 14.30 |
| -1 | -6 | 15 | 1824.12 | 97.99 |
| -1 | 6 | 15 | 1705.33 | 101.09 |
| -1 | 6 | 15 | 1854.81 | 98.49 |
| -1 | 6 | 15 | 1805.02 | 98.89 |
| 0 | -6 | 15 | 46.40 | 6.20 |
| 0 | 6 | 15 | 43.20 | 7.40 |
| 0 | 6 | 15 | 29.60 | 8.80 |
| 0 | 6 | 15 | 57.49 | 7.40 |
| 1 | -6 | 15 | 10.70 | 3.30 |

| | | | | |
|---|---|---|---|---|
| 1 | 6 | 15 | 20.60 | 4.20 |
| 2 | -6 | 15 | 92.29 | 8.50 |
| 2 | 6 | 15 | 87.99 | 8.20 |
| 3 | -6 | 15 | 578.54 | 33.10 |
| 3 | 6 | 15 | 565.74 | 32.70 |
| 4 | -6 | 15 | 55.59 | 7.30 |
| 4 | 6 | 15 | 56.79 | 6.90 |
| 5 | -6 | 15 | 756.82 | 43.20 |
| 5 | 6 | 15 | 793.62 | 43.10 |
| 6 | -6 | 15 | 130.69 | 9.90 |
| 6 | 6 | 15 | 146.29 | 9.30 |
| -7 | -6 | -15 | 201.08 | 13.30 |
| 7 | 6 | 15 | 195.38 | 11.90 |
| -12 | 7 | 15 | 401.06 | 22.60 |
| -11 | 7 | 15 | 7.70 | 2.00 |
| -10 | 7 | 15 | 203.58 | 12.70 |
| -9 | 7 | 15 | 13.00 | 3.00 |
| -8 | 7 | 15 | 38.90 | 6.90 |
| -7 | 7 | 15 | 26.40 | 5.70 |
| -6 | -7 | 15 | 1214.48 | 69.49 |
| -6 | 7 | 15 | 1332.97 | 70.79 |
| -5 | -7 | 15 | 18.00 | 3.20 |
| -5 | 7 | 15 | 13.90 | 4.40 |
| -4 | -7 | 15 | 105.09 | 8.00 |
| -4 | 7 | 15 | 89.29 | 10.00 |
| -3 | -7 | 15 | 0.90 | 2.50 |
| -3 | 7 | 15 | -4.50 | 3.60 |
| -2 | -7 | 15 | 747.83 | 41.80 |
| -2 | 7 | 15 | 749.33 | 43.10 |
| -1 | -7 | 15 | 9.10 | 3.00 |
| -1 | 7 | 15 | 3.60 | 3.80 |
| 0 | -7 | 15 | 830.72 | 43.70 |
| 0 | 7 | 15 | 727.03 | 44.10 |
| 1 | -7 | 15 | 43.20 | 6.20 |
| 1 | 7 | 15 | 26.90 | 6.20 |
| 2 | -7 | 15 | 179.58 | 12.40 |
| 2 | 7 | 15 | 173.88 | 12.10 |
| 3 | -7 | 15 | 8.70 | 4.10 |
| 4 | -7 | 15 | 863.11 | 48.70 |
| 4 | 7 | 15 | 885.91 | 48.40 |
| 5 | -7 | 15 | 28.80 | 5.80 |
| 5 | 7 | 15 | 38.60 | 4.60 |
| 6 | -7 | 15 | 14.40 | 3.50 |
| 6 | 7 | 15 | 16.40 | 3.70 |

| | | | | |
|---|---|---|---|---|
| -7 | -7 | -15 | 14.50 | 3.10 |
| -11 | 8 | 15 | 509.85 | 28.50 |
| -10 | 8 | 15 | 3.90 | 2.10 |
| -9 | 8 | 15 | 55.69 | 5.60 |
| -8 | -8 | 15 | 4.70 | 4.00 |
| -8 | 8 | 15 | 3.70 | 2.60 |
| -7 | -8 | 15 | 709.63 | 39.30 |
| -6 | -8 | 15 | -1.10 | 3.20 |
| -6 | 8 | 15 | 1.00 | 3.50 |
| -5 | -8 | 15 | 387.26 | 23.70 |
| -5 | 8 | 15 | 438.36 | 25.20 |
| -4 | -8 | 15 | 17.80 | 3.30 |
| -4 | 8 | 15 | 13.70 | 4.60 |
| -3 | -8 | 15 | 110.39 | 8.00 |
| -3 | 8 | 15 | 91.79 | 9.80 |
| -2 | -8 | 15 | 24.90 | 4.80 |
| -2 | 8 | 15 | 19.00 | 4.70 |
| -1 | -8 | 15 | 1253.67 | 66.59 |
| -1 | 8 | 15 | 1168.18 | 67.29 |
| 0 | -8 | 15 | 12.00 | 3.10 |
| 0 | 8 | 15 | 21.20 | 4.10 |
| 1 | -8 | 15 | 30.90 | 5.50 |
| 1 | 8 | 15 | 28.70 | 6.10 |
| 2 | -8 | 15 | 59.19 | 10.20 |
| 3 | -8 | 15 | 522.75 | 27.80 |
| 3 | 8 | 15 | 434.26 | 27.20 |
| 4 | -8 | 15 | 1.10 | 2.90 |
| 4 | 8 | 15 | 0.40 | 2.10 |
| 5 | -8 | 15 | 513.65 | 30.00 |
| 5 | 8 | 15 | 553.54 | 29.90 |
| -6 | -8 | -15 | 77.79 | 6.80 |
| 6 | 8 | 15 | 77.99 | 5.90 |
| -11 | 9 | 15 | 19.60 | 3.20 |
| -10 | 9 | 15 | 140.59 | 9.10 |
| -9 | 9 | 15 | 12.60 | 2.60 |
| -8 | -9 | 15 | 99.29 | 9.20 |
| -8 | 9 | 15 | 92.99 | 7.60 |
| -7 | -9 | 15 | 36.80 | 5.30 |
| -7 | 9 | 15 | 41.50 | 6.00 |
| -6 | -9 | 15 | 729.33 | 42.20 |
| 6 | -9 | -15 | 760.62 | 42.60 |
| -6 | 9 | 15 | 808.22 | 43.50 |
| -5 | 9 | 15 | 33.30 | 6.50 |
| -4 | -9 | 15 | 2.00 | 2.50 |

| | | | | |
|---|---|---|---|---|
| -4 | 9 | 15 | -3.50 | 3.70 |
| -3 | -9 | 15 | 80.69 | 6.90 |
| -3 | 9 | 15 | 74.29 | 8.80 |
| -2 | -9 | 15 | 608.24 | 31.30 |
| -2 | 9 | 15 | 492.25 | 32.10 |
| -1 | -9 | 15 | -0.80 | 2.50 |
| -1 | 9 | 15 | -1.20 | 3.30 |
| 0 | -9 | 15 | 340.67 | 19.80 |
| 0 | 9 | 15 | 319.67 | 22.50 |
| 1 | -9 | 15 | 57.69 | 6.30 |
| 1 | 9 | 15 | 42.60 | 6.90 |
| 2 | -9 | 15 | 185.48 | 12.00 |
| 2 | 9 | 15 | 176.48 | 12.00 |
| 3 | -9 | 15 | 30.30 | 5.50 |
| 3 | 9 | 15 | 22.10 | 4.80 |
| 4 | -9 | 15 | 452.15 | 26.50 |
| 4 | 9 | 15 | 478.15 | 26.40 |
| -5 | -9 | -15 | 16.70 | 3.20 |
| 5 | -9 | 15 | 12.50 | 3.10 |
| 5 | 9 | 15 | 20.90 | 3.80 |
| -6 | -9 | -15 | -2.40 | 5.20 |
| 6 | 9 | 15 | 1.70 | 2.00 |
| -10 | 10 | 15 | 21.20 | 3.40 |
| -9 | 10 | 15 | 16.90 | 3.70 |
| -8 | 10 | 15 | 38.60 | 5.00 |
| -7 | -10 | 15 | 908.01 | 51.39 |
| -7 | 10 | 15 | 976.90 | 52.19 |
| 6 | -10 | -15 | 10.10 | 4.10 |
| -6 | -10 | 15 | 1.00 | 2.20 |
| -6 | 10 | 15 | 4.90 | 3.20 |
| -5 | -10 | 15 | 342.77 | 20.30 |
| 5 | -10 | -15 | 343.97 | 20.60 |
| -5 | 10 | 15 | 371.66 | 21.60 |
| -4 | -10 | 15 | 12.10 | 2.80 |
| 4 | -10 | -15 | 7.50 | 3.00 |
| -4 | 10 | 15 | 14.70 | 4.40 |
| -3 | -10 | 15 | 139.59 | 9.60 |
| -3 | 10 | 15 | 140.39 | 11.70 |
| -2 | -10 | 15 | 78.69 | 6.70 |
| -2 | 10 | 15 | 69.59 | 8.80 |
| -1 | 10 | 15 | 980.10 | 54.69 |
| 0 | -10 | 15 | 35.60 | 5.00 |
| 0 | 10 | 15 | 38.90 | 6.30 |
| 1 | -10 | 15 | -1.10 | 2.20 |

| | | | | |
|---|---|---|---|---|
| 1 | 10 | 15 | -1.90 | 2.80 |
| 2 | -10 | 15 | 31.20 | 5.00 |
| 2 | 10 | 15 | 25.70 | 5.10 |
| 3 | -10 | 15 | 506.05 | 26.20 |
| -3 | -10 | -15 | 413.76 | 26.40 |
| 3 | 10 | 15 | 449.95 | 25.90 |
| -4 | -10 | -15 | 4.00 | 2.80 |
| 4 | -10 | 15 | 8.60 | 2.70 |
| 4 | 10 | 15 | 8.60 | 2.50 |
| -5 | -10 | -15 | 313.07 | 19.00 |
| 5 | -10 | 15 | 313.27 | 18.80 |
| 5 | 10 | 15 | 354.46 | 18.80 |
| -9 | 11 | 15 | 23.90 | 3.60 |
| -8 | 11 | 15 | 104.79 | 7.50 |
| -7 | -11 | 15 | 43.40 | 4.80 |
| -7 | 11 | 15 | 41.00 | 5.40 |
| -6 | -11 | 15 | 721.83 | 40.30 |
| -6 | 11 | 15 | 745.73 | 41.10 |
| -5 | -11 | 15 | 39.40 | 4.80 |
| -5 | 11 | 15 | 46.80 | 6.70 |
| -4 | -11 | 15 | 5.20 | 2.20 |
| 4 | -11 | -15 | 9.00 | 2.50 |
| -4 | 11 | 15 | 3.70 | 3.50 |
| 3 | -11 | -15 | 65.29 | 6.00 |
| -3 | -11 | 15 | 56.29 | 5.40 |
| -3 | 11 | 15 | 38.20 | 7.00 |
| 2 | -11 | -15 | 690.63 | 37.90 |
| -2 | -11 | 15 | 666.63 | 37.50 |
| -2 | 11 | 15 | 672.13 | 38.40 |
| -1 | -11 | 15 | 1.80 | 1.90 |
| 1 | -11 | -15 | -1.10 | 2.50 |
| -1 | 11 | 15 | 6.30 | 3.60 |
| 0 | -11 | 15 | 311.97 | 19.20 |
| 0 | -11 | -15 | 345.87 | 20.00 |
| 0 | 11 | 15 | 343.67 | 20.00 |
| 1 | -11 | 15 | 88.29 | 6.60 |
| -1 | -11 | -15 | 75.59 | 7.40 |
| 1 | 11 | 15 | 83.99 | 7.40 |
| 2 | -11 | 15 | 61.39 | 5.80 |
| -2 | -11 | -15 | 68.69 | 6.60 |
| 2 | 11 | 15 | 68.99 | 6.30 |
| 3 | -11 | 15 | 71.99 | 6.00 |
| -3 | -11 | -15 | 58.59 | 6.60 |
| 3 | 11 | 15 | 75.59 | 6.20 |

| | | | | |
|---|---|---|---|---|
| -4 | -11 | -15 | 350.86 | 21.90 |
| 4 | -11 | 15 | 384.16 | 21.80 |
| 4 | 11 | 15 | 406.06 | 21.70 |
| -8 | 12 | 15 | 42.30 | 4.60 |
| -7 | -12 | 15 | 393.16 | 23.20 |
| -7 | 12 | 15 | 440.56 | 23.70 |
| -6 | -12 | 15 | 6.20 | 2.00 |
| -6 | 12 | 15 | 6.30 | 2.90 |
| 5 | -12 | -15 | 216.28 | 12.90 |
| -5 | -12 | 15 | 206.78 | 12.50 |
| -5 | 12 | 15 | 206.28 | 13.60 |
| -4 | -12 | 15 | 22.70 | 3.90 |
| 4 | -12 | -15 | 35.20 | 4.50 |
| -4 | 12 | 15 | 33.80 | 6.20 |
| 3 | -12 | -15 | 99.39 | 7.50 |
| -3 | -12 | 15 | 107.49 | 7.10 |
| -3 | 12 | 15 | 97.19 | 9.00 |
| -2 | -12 | 15 | 35.70 | 4.00 |
| 2 | -12 | -15 | 32.30 | 4.80 |
| -2 | 12 | 15 | 43.30 | 6.70 |
| -1 | -12 | 15 | 530.45 | 29.70 |
| 1 | -12 | -15 | 523.55 | 30.10 |
| -1 | 12 | 15 | 546.45 | 30.50 |
| 0 | -12 | 15 | 43.80 | 4.50 |
| 0 | 12 | 15 | 50.79 | 6.40 |
| -1 | -12 | -15 | 3.50 | 2.30 |
| 1 | -12 | 15 | 4.00 | 1.90 |
| 1 | 12 | 15 | 3.50 | 2.90 |
| 2 | -12 | 15 | 29.20 | 4.10 |
| -2 | -12 | -15 | 27.90 | 4.70 |
| 2 | 12 | 15 | 30.70 | 5.10 |
| 3 | -12 | 15 | 241.38 | 13.40 |
| -3 | -12 | -15 | 200.08 | 13.60 |
| 3 | 12 | 15 | 230.18 | 13.70 |
| -6 | -13 | 15 | 484.05 | 27.30 |
| -6 | 13 | 15 | 505.15 | 27.70 |
| -5 | -13 | 15 | 42.60 | 4.10 |
| 5 | -13 | -15 | 42.10 | 5.50 |
| -5 | 13 | 15 | 41.80 | 7.20 |
| 4 | -13 | -15 | 28.10 | 4.20 |
| -4 | -13 | 15 | 25.30 | 3.40 |
| -4 | 13 | 15 | 30.70 | 5.90 |
| 3 | -13 | -15 | 72.09 | 5.50 |
| -3 | -13 | 15 | 71.69 | 5.20 |

| | | | | |
|---|---|---|---|---|
| -3 | 13 | 15 | 56.69 | 7.30 |
| 2 | -13 | -15 | 249.97 | 14.60 |
| -2 | -13 | 15 | 248.08 | 14.40 |
| -2 | 13 | 15 | 246.78 | 15.50 |
| -1 | -13 | 15 | 0.80 | 1.50 |
| 1 | -13 | -15 | -0.10 | 1.90 |
| -1 | 13 | 15 | -5.60 | 3.80 |
| 0 | -13 | 15 | 220.08 | 12.90 |
| 0 | -13 | -15 | 229.58 | 13.30 |
| 0 | 13 | 15 | 206.48 | 14.40 |
| 1 | -13 | 15 | 106.39 | 6.90 |
| -1 | -13 | -15 | 105.19 | 7.40 |
| 1 | 13 | 15 | 97.09 | 8.50 |
| -13 | 0 | 16 | -2.50 | 2.10 |
| -12 | 0 | 16 | 280.17 | 16.30 |
| -11 | 0 | 16 | 1.60 | 2.40 |
| -10 | 0 | 16 | 189.58 | 12.30 |
| -9 | 0 | 16 | 0.00 | 3.20 |
| -8 | 0 | 16 | 12.40 | 3.60 |
| -7 | 0 | 16 | -2.00 | 3.30 |
| -7 | 0 | 16 | -2.00 | 3.60 |
| -6 | 0 | 16 | 1493.45 | 87.49 |
| -6 | 0 | 16 | 1725.43 | 88.49 |
| -5 | 0 | 16 | 2.70 | 4.10 |
| -5 | 0 | 16 | -3.40 | 2.90 |
| -5 | 0 | 16 | -0.60 | 7.80 |
| -4 | 0 | 16 | 91.49 | 9.80 |
| -4 | 0 | 16 | 78.69 | 12.60 |
| -4 | 0 | 16 | 86.79 | 7.90 |
| -3 | 0 | 16 | -2.30 | 6.50 |
| -3 | 0 | 16 | 3.20 | 2.90 |
| -3 | 0 | 16 | 6.40 | 4.50 |
| -2 | 0 | 16 | 888.61 | 48.00 |
| -2 | 0 | 16 | 714.33 | 46.00 |
| -2 | 0 | 16 | 834.12 | 45.40 |
| -1 | 0 | 16 | 6.00 | 4.60 |
| -1 | 0 | 16 | -0.10 | 5.70 |
| 0 | 0 | 16 | 339.67 | 22.70 |
| 0 | 0 | 16 | 359.46 | 25.20 |
| 0 | 0 | 16 | 406.36 | 22.40 |
| 1 | 0 | 16 | 2.60 | 3.80 |
| 1 | 0 | 16 | 1.30 | 3.30 |
| 2 | 0 | 16 | 200.38 | 14.00 |
| 2 | 0 | 16 | 207.08 | 14.30 |

| | | | | |
|---|---|---|---|---|
| 3 | 0 | 16 | -2.20 | 3.20 |
| 3 | 0 | 16 | -2.10 | 3.30 |
| 4 | 0 | 16 | 776.12 | 44.20 |
| 4 | 0 | 16 | 789.72 | 43.70 |
| 5 | 0 | 16 | -3.20 | 2.90 |
| 5 | 0 | 16 | -2.80 | 2.60 |
| 6 | 0 | 16 | 3.30 | 2.70 |
| 6 | 0 | 16 | 5.70 | 2.60 |
| 7 | 0 | 16 | 0.70 | 2.50 |
| -13 | -1 | 16 | 9.60 | 2.80 |
| -13 | 1 | 16 | 8.70 | 2.30 |
| -12 | -1 | 16 | 25.20 | 4.70 |
| -12 | 1 | 16 | 22.20 | 3.90 |
| -11 | -1 | 16 | 531.15 | 28.40 |
| -11 | 1 | 16 | 473.75 | 28.30 |
| -10 | -1 | 16 | 37.70 | 5.40 |
| -10 | 1 | 16 | 33.20 | 5.40 |
| -9 | -1 | 16 | 64.79 | 7.40 |
| -9 | 1 | 16 | 77.79 | 8.00 |
| -8 | -1 | 16 | 57.99 | 7.40 |
| -8 | 1 | 16 | 48.20 | 6.70 |
| -7 | -1 | 16 | 582.64 | 35.00 |
| -7 | -1 | 16 | 678.23 | 35.90 |
| -7 | 1 | 16 | 598.64 | 35.70 |
| -7 | 1 | 16 | 637.34 | 36.60 |
| -6 | -1 | 16 | 31.20 | 5.20 |
| -6 | -1 | 16 | 34.90 | 6.40 |
| -6 | 1 | 16 | 23.30 | 4.20 |
| -6 | 1 | 16 | 43.50 | 7.40 |
| -5 | -1 | 16 | 303.07 | 22.60 |
| -5 | -1 | 16 | 287.47 | 18.20 |
| -5 | -1 | 16 | 351.26 | 20.00 |
| -5 | 1 | 16 | 294.47 | 18.20 |
| -5 | 1 | 16 | 271.17 | 22.60 |
| -5 | 1 | 16 | 305.07 | 19.70 |
| -4 | -1 | 16 | 41.30 | 5.70 |
| -4 | -1 | 16 | 31.60 | 7.10 |
| -4 | -1 | 16 | 18.60 | 5.40 |
| -4 | 1 | 16 | 51.39 | 12.40 |
| -4 | 1 | 16 | 26.00 | 5.30 |
| -3 | -1 | 16 | 141.69 | 14.60 |
| -3 | -1 | 16 | 147.19 | 10.50 |
| -3 | -1 | 16 | 123.69 | 12.40 |
| -3 | 1 | 16 | 157.78 | 12.80 |

| | | | | |
|---|---|---|---|---|
| -3 | 1 | 16 | 150.88 | 14.80 |
| -3 | 1 | 16 | 154.38 | 10.60 |
| -2 | -1 | 16 | 1.80 | 4.90 |
| -2 | -1 | 16 | 10.30 | 6.30 |
| -2 | -1 | 16 | 11.10 | 3.10 |
| -2 | 1 | 16 | 13.30 | 7.40 |
| -2 | 1 | 16 | 23.00 | 5.30 |
| -2 | 1 | 16 | 12.20 | 3.10 |
| -1 | -1 | 16 | 948.91 | 61.59 |
| -1 | -1 | 16 | 1120.39 | 63.39 |
| -1 | -1 | 16 | 1153.18 | 61.29 |
| -1 | 1 | 16 | 1152.58 | 62.09 |
| -1 | 1 | 16 | 1142.09 | 63.59 |
| -1 | 1 | 16 | 1134.19 | 61.39 |
| 0 | -1 | 16 | -1.90 | 4.40 |
| 0 | -1 | 16 | -2.10 | 2.80 |
| 0 | -1 | 16 | -8.60 | 6.40 |
| 0 | 1 | 16 | -1.40 | 3.20 |
| 0 | 1 | 16 | 1.30 | 4.10 |
| 1 | -1 | 16 | 10.30 | 4.20 |
| 1 | -1 | 16 | 10.30 | 3.80 |
| 1 | 1 | 16 | 15.00 | 3.90 |
| 1 | 1 | 16 | 10.00 | 3.80 |
| 2 | -1 | 16 | 21.80 | 4.20 |
| 2 | 1 | 16 | 20.60 | 4.30 |
| 2 | 1 | 16 | 20.00 | 4.50 |
| 3 | -1 | 16 | 536.25 | 31.70 |
| 3 | -1 | 16 | 535.05 | 31.60 |
| 3 | 1 | 16 | 569.34 | 31.20 |
| 3 | 1 | 16 | 522.75 | 31.60 |
| 4 | -1 | 16 | 0.70 | 3.30 |
| 4 | -1 | 16 | -4.90 | 3.40 |
| 4 | 1 | 16 | -3.50 | 3.20 |
| 4 | 1 | 16 | -5.30 | 3.50 |
| 5 | -1 | 16 | 504.65 | 27.80 |
| 5 | -1 | 16 | 442.86 | 27.40 |
| 5 | 1 | 16 | 495.05 | 27.50 |
| 5 | 1 | 16 | 487.25 | 27.70 |
| 6 | -1 | 16 | 3.10 | 2.90 |
| 6 | -1 | 16 | -0.40 | 2.60 |
| 6 | 1 | 16 | 1.40 | 2.40 |
| 7 | -1 | 16 | 197.18 | 12.50 |
| 7 | 1 | 16 | 202.68 | 12.20 |
| -13 | 2 | 16 | 10.30 | 2.10 |

| | | | | |
|---|---|---|---|---|
| -12 | 2 | 16 | 647.44 | 35.80 |
| -11 | -2 | 16 | 65.39 | 6.80 |
| -11 | 2 | 16 | 71.59 | 6.00 |
| -10 | -2 | 16 | 314.87 | 18.80 |
| -10 | 2 | 16 | 318.67 | 18.80 |
| -9 | -2 | 16 | 18.20 | 4.10 |
| -9 | 2 | 16 | 13.10 | 4.00 |
| -8 | 2 | 16 | 53.49 | 6.60 |
| -7 | -2 | 16 | 129.19 | 11.00 |
| -7 | -2 | 16 | 110.19 | 9.70 |
| -7 | 2 | 16 | 148.19 | 10.30 |
| -6 | -2 | 16 | 1774.22 | 93.89 |
| -6 | -2 | 16 | 1828.62 | 94.69 |
| -6 | 2 | 16 | 1538.55 | 94.49 |
| -6 | 2 | 16 | 1762.72 | 93.99 |
| -5 | -2 | 16 | 10.20 | 5.20 |
| -5 | -2 | 16 | 6.60 | 2.90 |
| -5 | 2 | 16 | 10.50 | 4.40 |
| -5 | 2 | 16 | 9.90 | 3.20 |
| -5 | 2 | 16 | 9.50 | 10.10 |
| -4 | -2 | 16 | 97.69 | 8.20 |
| -4 | -2 | 16 | 94.69 | 13.20 |
| -4 | -2 | 16 | 99.59 | 11.00 |
| -4 | 2 | 16 | 100.59 | 14.10 |
| -4 | 2 | 16 | 92.99 | 8.20 |
| -4 | 2 | 16 | 78.39 | 9.90 |
| -3 | -2 | 16 | 52.19 | 9.90 |
| -3 | -2 | 16 | 57.79 | 11.50 |
| -3 | -2 | 16 | 48.70 | 6.40 |
| -3 | 2 | 16 | 50.09 | 11.80 |
| -3 | 2 | 16 | 49.10 | 6.20 |
| -2 | -2 | 16 | 1265.87 | 66.19 |
| -2 | -2 | 16 | 1137.59 | 66.79 |
| -2 | -2 | 16 | 1207.88 | 68.29 |
| -2 | 2 | 16 | 1268.37 | 68.69 |
| -2 | 2 | 16 | 1229.08 | 66.29 |
| -2 | 2 | 16 | 1104.09 | 66.89 |
| -1 | -2 | 16 | 0.90 | 6.00 |
| -1 | -2 | 16 | 5.60 | 5.10 |
| -1 | -2 | 16 | 1.10 | 3.00 |
| -1 | 2 | 16 | 1.90 | 4.10 |
| -1 | 2 | 16 | 3.80 | 3.10 |
| -1 | 2 | 16 | 6.50 | 6.70 |
| 0 | -2 | 16 | 1099.99 | 59.29 |

| | | | | |
|---|---|---|---|---|
| 0 | -2 | 16 | 1092.69 | 58.99 |
| 0 | -2 | 16 | 1041.50 | 61.49 |
| 0 | 2 | 16 | 965.40 | 59.19 |
| 0 | 2 | 16 | 1061.89 | 61.79 |
| 0 | 2 | 16 | 1112.09 | 59.19 |
| 1 | -2 | 16 | -2.90 | 4.30 |
| 1 | -2 | 16 | -3.80 | 3.20 |
| 1 | 2 | 16 | -2.90 | 3.30 |
| 1 | 2 | 16 | 8.90 | 3.80 |
| 2 | -2 | 16 | 300.37 | 18.70 |
| 2 | -2 | 16 | 323.77 | 18.80 |
| 2 | 2 | 16 | 261.67 | 18.10 |
| 2 | 2 | 16 | 292.57 | 18.90 |
| 3 | -2 | 16 | 3.90 | 5.20 |
| 3 | -2 | 16 | 6.20 | 3.60 |
| 3 | 2 | 16 | 5.10 | 3.10 |
| 3 | 2 | 16 | 4.00 | 3.80 |
| 4 | -2 | 16 | 1156.28 | 62.99 |
| 4 | -2 | 16 | 1060.99 | 62.49 |
| 4 | 2 | 16 | 1197.28 | 62.69 |
| 4 | 2 | 16 | 1137.69 | 63.09 |
| 5 | -2 | 16 | 7.20 | 3.50 |
| 5 | -2 | 16 | 1.20 | 2.90 |
| 5 | 2 | 16 | 8.00 | 3.10 |
| 5 | 2 | 16 | 8.10 | 2.80 |
| 6 | -2 | 16 | 0.10 | 2.80 |
| 6 | -2 | 16 | 1.80 | 3.40 |
| 6 | 2 | 16 | 3.50 | 2.40 |
| 7 | -2 | 16 | -8.40 | 3.80 |
| 7 | 2 | 16 | 1.40 | 2.10 |
| -12 | 3 | 16 | 23.30 | 3.50 |
| -11 | 3 | 16 | 638.04 | 35.50 |
| -10 | 3 | 16 | 1.60 | 2.50 |
| -9 | 3 | 16 | 3.30 | 2.80 |
| -8 | 3 | 16 | 98.79 | 8.50 |
| -7 | -3 | 16 | 1131.49 | 67.19 |
| -7 | 3 | 16 | 1335.37 | 68.29 |
| -6 | -3 | 16 | 15.70 | 3.30 |
| -6 | 3 | 16 | 11.20 | 3.80 |
| -6 | 3 | 16 | 16.80 | 3.80 |
| -5 | -3 | 16 | 311.47 | 18.90 |
| -5 | 3 | 16 | 314.07 | 20.10 |
| -5 | 3 | 16 | 315.57 | 18.90 |
| -4 | -3 | 16 | -3.30 | 6.10 |

| | | | | |
|---|---|---|---|---|
| -4 | -3 | 16 | -0.10 | 2.60 |
| -4 | 3 | 16 | 3.50 | 7.60 |
| -4 | 3 | 16 | 0.40 | 2.90 |
| -4 | 3 | 16 | 1.70 | 4.20 |
| -3 | -3 | 16 | 208.48 | 16.10 |
| -3 | -3 | 16 | 209.68 | 13.50 |
| -3 | 3 | 16 | 207.08 | 13.60 |
| -3 | 3 | 16 | 220.38 | 18.50 |
| -3 | 3 | 16 | 205.98 | 15.10 |
| -2 | -3 | 16 | 88.39 | 7.70 |
| -2 | -3 | 16 | 93.39 | 11.80 |
| -2 | -3 | 16 | 106.59 | 13.20 |
| -2 | 3 | 16 | 86.79 | 7.80 |
| -2 | 3 | 16 | 68.99 | 9.20 |
| -2 | 3 | 16 | 76.69 | 13.60 |
| -1 | -3 | 16 | 1466.45 | 88.29 |
| -1 | -3 | 16 | 1590.54 | 86.79 |
| -1 | -3 | 16 | 1614.04 | 86.29 |
| -1 | 3 | 16 | 1566.94 | 88.69 |
| -1 | 3 | 16 | 1653.13 | 86.49 |
| 0 | -3 | 16 | 3.70 | 5.30 |
| 0 | 3 | 16 | 2.50 | 4.00 |
| 0 | 3 | 16 | 2.00 | 3.30 |
| 1 | -3 | 16 | 1.70 | 2.90 |
| 1 | -3 | 16 | 11.10 | 5.40 |
| 1 | 3 | 16 | -4.20 | 3.00 |
| 1 | 3 | 16 | 1.10 | 3.20 |
| 2 | -3 | 16 | 12.20 | 7.50 |
| 2 | -3 | 16 | 9.30 | 3.60 |
| 2 | 3 | 16 | 10.20 | 3.90 |
| 2 | 3 | 16 | 19.10 | 4.10 |
| 3 | -3 | 16 | 888.61 | 45.90 |
| 3 | -3 | 16 | 808.22 | 43.80 |
| 3 | 3 | 16 | 771.42 | 44.50 |
| 3 | 3 | 16 | 662.53 | 43.60 |
| 4 | -3 | 16 | -0.90 | 4.30 |
| 4 | -3 | 16 | -2.70 | 3.10 |
| 4 | 3 | 16 | 5.40 | 2.80 |
| 4 | 3 | 16 | 0.50 | 3.30 |
| 5 | -3 | 16 | 569.44 | 33.60 |
| 5 | -3 | 16 | 613.64 | 33.60 |
| 5 | 3 | 16 | 597.34 | 33.30 |
| 6 | -3 | 16 | 1.60 | 4.50 |
| 6 | -3 | 16 | 1.20 | 2.90 |

| | | | | |
|---|---|---|---|---|
| 6 | 3 | 16 | 5.30 | 2.30 |
| 7 | 3 | 16 | 207.78 | 12.50 |
| -12 | 4 | 16 | 562.54 | 31.20 |
| -11 | 4 | 16 | 3.30 | 1.90 |
| -10 | 4 | 16 | 132.59 | 9.10 |
| -9 | 4 | 16 | 11.10 | 3.10 |
| -8 | 4 | 16 | 276.17 | 17.30 |
| -7 | -4 | 16 | 19.90 | 5.80 |
| -7 | 4 | 16 | 15.20 | 3.60 |
| -6 | -4 | 16 | 714.83 | 37.60 |
| -6 | 4 | 16 | 626.04 | 38.40 |
| -5 | -4 | 16 | -3.10 | 2.60 |
| -5 | 4 | 16 | -3.30 | 3.70 |
| -5 | 4 | 16 | -0.80 | 3.00 |
| -4 | -4 | 16 | -1.40 | 2.70 |
| -4 | 4 | 16 | 1.30 | 2.80 |
| -4 | 4 | 16 | 3.10 | 4.10 |
| -3 | -4 | 16 | 8.40 | 2.90 |
| -3 | 4 | 16 | 16.70 | 11.90 |
| -3 | 4 | 16 | 9.70 | 3.30 |
| -3 | 4 | 16 | 15.60 | 4.70 |
| -2 | -4 | 16 | 1033.10 | 56.49 |
| -2 | 4 | 16 | 1066.59 | 56.69 |
| -2 | 4 | 16 | 967.20 | 57.49 |
| -1 | -4 | 16 | 1.00 | 2.60 |
| -1 | 4 | 16 | -1.70 | 2.80 |
| -1 | 4 | 16 | 8.60 | 4.30 |
| 0 | -4 | 16 | 558.04 | 32.40 |
| 0 | 4 | 16 | 576.24 | 32.80 |
| 0 | 4 | 16 | 566.24 | 33.10 |
| 1 | -4 | 16 | 14.30 | 3.30 |
| 1 | 4 | 16 | 13.90 | 4.10 |
| 1 | 4 | 16 | 12.00 | 3.60 |
| 2 | -4 | 16 | 481.35 | 27.50 |
| 2 | 4 | 16 | 466.85 | 27.40 |
| 2 | 4 | 16 | 455.35 | 27.80 |
| 3 | -4 | 16 | 1.50 | 3.60 |
| 3 | 4 | 16 | -2.10 | 4.50 |
| 3 | 4 | 16 | 4.20 | 4.20 |
| 4 | -4 | 16 | 869.91 | 48.00 |
| 4 | 4 | 16 | 850.21 | 47.70 |
| 5 | -4 | 16 | 3.50 | 2.70 |
| 5 | 4 | 16 | 7.30 | 2.40 |
| 6 | -4 | 16 | -0.60 | 2.90 |

| | | | | |
|---|---|---|---|---|
| 6 | 4 | 16 | 0.30 | 2.10 |
| 7 | 4 | 16 | 7.00 | 2.10 |
| -12 | 5 | 16 | 1.20 | 1.70 |
| -11 | 5 | 16 | 541.15 | 30.20 |
| -10 | 5 | 16 | 20.10 | 3.90 |
| -9 | 5 | 16 | 3.20 | 2.40 |
| -8 | 5 | 16 | 2.80 | 3.40 |
| -7 | -5 | 16 | 849.61 | 47.70 |
| -7 | 5 | 16 | 881.31 | 48.50 |
| -6 | -5 | 16 | 1.50 | 2.80 |
| -6 | 5 | 16 | 4.20 | 3.50 |
| -5 | -5 | 16 | 340.47 | 20.30 |
| -5 | 5 | 16 | 349.77 | 21.60 |
| -4 | -5 | 16 | 12.30 | 3.00 |
| -4 | 5 | 16 | 7.40 | 3.00 |
| -4 | 5 | 16 | 10.30 | 4.20 |
| -3 | -5 | 16 | 120.19 | 9.00 |
| -3 | 5 | 16 | 132.79 | 10.90 |
| -3 | 5 | 16 | 119.19 | 9.20 |
| -2 | -5 | 16 | 4.00 | 2.40 |
| -2 | 5 | 16 | -0.60 | 4.00 |
| -2 | 5 | 16 | -0.10 | 2.60 |
| -1 | -5 | 16 | 1496.75 | 79.59 |
| -1 | 5 | 16 | 1532.45 | 79.89 |
| -1 | 5 | 16 | 1333.47 | 80.19 |
| 0 | -5 | 16 | 1.50 | 2.50 |
| 0 | 5 | 16 | -1.00 | 2.80 |
| 0 | 5 | 16 | -1.80 | 3.20 |
| 1 | -5 | 16 | 3.70 | 3.00 |
| 1 | 5 | 16 | 1.80 | 3.50 |
| 1 | 5 | 16 | 4.70 | 3.10 |
| 2 | -5 | 16 | -0.60 | 3.30 |
| 2 | 5 | 16 | 4.90 | 3.40 |
| 3 | -5 | 16 | 565.14 | 29.50 |
| 3 | 5 | 16 | 448.96 | 28.90 |
| 4 | -5 | 16 | 2.30 | 3.00 |
| 4 | 5 | 16 | 2.80 | 2.50 |
| 5 | -5 | 16 | 580.84 | 32.40 |
| 5 | 5 | 16 | 570.54 | 32.20 |
| 6 | -5 | 16 | 7.60 | 3.80 |
| 6 | 5 | 16 | 9.80 | 2.40 |
| -12 | 6 | 16 | 383.96 | 21.50 |
| -11 | 6 | 16 | 4.60 | 1.80 |
| -10 | 6 | 16 | 382.16 | 21.90 |

| | | | | |
|---|---|---|---|---|
| -9 | 6 | 16 | 11.70 | 2.80 |
| -8 | 6 | 16 | 65.29 | 6.40 |
| -7 | -6 | 16 | 13.00 | 3.20 |
| -6 | -6 | 16 | 1167.78 | 76.69 |
| -6 | 6 | 16 | 1602.64 | 76.79 |
| -5 | -6 | 16 | -0.20 | 2.50 |
| -5 | 6 | 16 | -0.90 | 3.50 |
| -4 | -6 | 16 | 23.90 | 5.00 |
| -4 | 6 | 16 | 14.40 | 4.30 |
| -3 | -6 | 16 | 10.10 | 2.80 |
| -3 | 6 | 16 | 13.10 | 4.50 |
| -2 | -6 | 16 | 870.61 | 45.70 |
| -2 | 6 | 16 | 768.62 | 46.60 |
| -1 | -6 | 16 | 1.00 | 2.50 |
| -1 | 6 | 16 | -2.20 | 3.30 |
| 0 | -6 | 16 | 862.21 | 46.20 |
| 0 | 6 | 16 | 794.92 | 46.80 |
| 1 | -6 | 16 | 76.49 | 7.00 |
| 1 | 6 | 16 | 61.19 | 7.50 |
| 2 | -6 | 16 | 152.68 | 10.50 |
| 2 | 6 | 16 | 125.19 | 10.30 |
| 3 | -6 | 16 | -2.60 | 2.80 |
| 3 | 6 | 16 | 2.00 | 2.60 |
| 4 | -6 | 16 | 942.31 | 52.59 |
| 4 | 6 | 16 | 961.00 | 52.49 |
| 5 | -6 | 16 | 2.50 | 2.80 |
| 5 | 6 | 16 | 0.70 | 2.00 |
| 6 | -6 | 16 | 57.09 | 6.30 |
| 6 | 6 | 16 | 53.99 | 4.90 |
| -11 | 7 | 16 | 501.15 | 27.90 |
| -10 | 7 | 16 | 7.00 | 2.10 |
| -9 | 7 | 16 | 106.99 | 7.70 |
| -8 | 7 | 16 | 76.39 | 6.70 |
| -7 | -7 | 16 | 684.83 | 38.50 |
| -7 | 7 | 16 | 708.53 | 39.40 |
| -6 | -7 | 16 | 4.30 | 2.50 |
| -6 | 7 | 16 | 4.30 | 4.60 |
| -5 | -7 | 16 | 428.36 | 29.40 |
| -5 | 7 | 16 | 548.85 | 29.20 |
| -4 | -7 | 16 | 74.59 | 6.40 |
| -4 | 7 | 16 | 60.39 | 8.10 |
| -3 | -7 | 16 | 68.19 | 6.10 |
| -3 | 7 | 16 | 43.40 | 7.60 |
| -2 | -7 | 16 | 34.00 | 5.00 |

| | | | | |
|---|---|---|---|---|
| -2 | 7 | 16 | 19.60 | 4.70 |
| -1 | -7 | 16 | 1361.26 | 81.39 |
| -1 | 7 | 16 | 1629.24 | 82.49 |
| 0 | -7 | 16 | 50.39 | 6.40 |
| 0 | 7 | 16 | 57.29 | 8.50 |
| 1 | -7 | 16 | 36.40 | 5.80 |
| 1 | 7 | 16 | 34.40 | 6.40 |
| 2 | -7 | 16 | 3.80 | 2.70 |
| 2 | 7 | 16 | 6.20 | 2.90 |
| 3 | -7 | 16 | 371.96 | 22.30 |
| 3 | 7 | 16 | 392.16 | 22.30 |
| 4 | -7 | 16 | 0.90 | 2.50 |
| 4 | 7 | 16 | -1.80 | 2.10 |
| 5 | -7 | 16 | 499.35 | 29.00 |
| 5 | 7 | 16 | 535.15 | 29.00 |
| -6 | -7 | -16 | 36.60 | 5.10 |
| 6 | 7 | 16 | 51.79 | 4.50 |
| -10 | 8 | 16 | 109.39 | 7.40 |
| -9 | 8 | 16 | 10.30 | 2.30 |
| -8 | 8 | 16 | 43.00 | 5.00 |
| -7 | -8 | 16 | 15.30 | 2.90 |
| -7 | 8 | 16 | 22.10 | 4.80 |
| -6 | -8 | 16 | 691.43 | 39.10 |
| -6 | 8 | 16 | 727.83 | 40.10 |
| -5 | -8 | 16 | 26.70 | 4.50 |
| -5 | 8 | 16 | 14.50 | 3.90 |
| -4 | -8 | 16 | 8.10 | 2.80 |
| -4 | 8 | 16 | 11.80 | 4.70 |
| -3 | -8 | 16 | 56.79 | 6.80 |
| -3 | 8 | 16 | 61.09 | 10.60 |
| -2 | -8 | 16 | 686.13 | 40.10 |
| -2 | 8 | 16 | 710.13 | 41.60 |
| -1 | -8 | 16 | -2.10 | 2.50 |
| -1 | 8 | 16 | -3.80 | 3.40 |
| 0 | -8 | 16 | 379.06 | 22.00 |
| 0 | 8 | 16 | 376.66 | 22.70 |
| 1 | -8 | 16 | 92.89 | 7.50 |
| 1 | 8 | 16 | 104.69 | 8.30 |
| 2 | -8 | 16 | 209.08 | 13.30 |
| 2 | 8 | 16 | 214.48 | 13.50 |
| 3 | -8 | 16 | 3.30 | 2.50 |
| 3 | 8 | 16 | -0.20 | 2.30 |
| 4 | -8 | 16 | 479.85 | 27.90 |
| 4 | 8 | 16 | 510.55 | 27.90 |

| | | | | |
|---|---|---|---|---|
| 5 | -8 | 16 | -4.00 | 3.10 |
| -5 | -8 | -16 | 1.70 | 2.60 |
| 5 | 8 | 16 | 3.50 | 2.10 |
| -10 | 9 | 16 | 3.70 | 1.70 |
| -9 | 9 | 16 | 8.00 | 2.10 |
| -8 | 9 | 16 | 42.50 | 4.80 |
| -7 | -9 | 16 | 590.54 | 33.00 |
| -7 | 9 | 16 | 604.64 | 33.60 |
| 6 | -9 | -16 | 9.00 | 3.60 |
| -6 | -9 | 16 | 4.00 | 2.30 |
| -6 | 9 | 16 | 4.60 | 2.50 |
| -5 | -9 | 16 | 232.68 | 14.70 |
| 5 | -9 | -16 | 253.87 | 15.20 |
| -5 | 9 | 16 | 258.27 | 15.90 |
| -4 | -9 | 16 | 11.50 | 2.50 |
| -4 | 9 | 16 | 12.00 | 3.60 |
| -3 | -9 | 16 | 149.88 | 9.40 |
| -3 | 9 | 16 | 131.99 | 10.80 |
| -2 | -9 | 16 | 18.50 | 4.10 |
| -2 | 9 | 16 | 27.00 | 6.10 |
| -1 | -9 | 16 | 869.81 | 48.00 |
| -1 | 9 | 16 | 871.51 | 48.80 |
| 0 | -9 | 16 | 104.09 | 7.70 |
| 0 | 9 | 16 | 103.39 | 8.70 |
| 1 | -9 | 16 | -2.10 | 2.10 |
| 1 | 9 | 16 | -1.20 | 2.70 |
| 2 | -9 | 16 | 33.10 | 4.50 |
| 2 | 9 | 16 | 25.70 | 4.60 |
| 3 | -9 | 16 | 413.06 | 23.60 |
| 3 | 9 | 16 | 415.06 | 23.60 |
| 4 | -9 | 16 | 3.40 | 2.40 |
| -4 | -9 | -16 | -3.10 | 2.40 |
| 4 | 9 | 16 | 0.70 | 2.20 |
| -9 | 10 | 16 | 7.60 | 2.00 |
| -8 | 10 | 16 | 59.39 | 5.10 |
| -7 | -10 | 16 | 65.09 | 5.60 |
| -7 | 10 | 16 | 69.79 | 5.90 |
| -6 | -10 | 16 | 534.75 | 30.40 |
| -6 | 10 | 16 | 563.84 | 31.10 |
| -5 | -10 | 16 | 20.40 | 4.00 |
| 5 | -10 | -16 | 31.60 | 5.20 |
| -5 | 10 | 16 | 27.60 | 5.30 |
| 4 | -10 | -16 | 12.90 | 2.70 |
| -4 | -10 | 16 | 10.50 | 2.20 |

| | | | | |
|---|---|---|---|---|
| -4 | 10 | 16 | 4.10 | 3.10 |
| 3 | -10 | -16 | 96.79 | 7.40 |
| -3 | -10 | 16 | 94.99 | 6.80 |
| -3 | 10 | 16 | 90.69 | 8.30 |
| -2 | -10 | 16 | 461.05 | 25.30 |
| -2 | 10 | 16 | 433.46 | 26.20 |
| -1 | -10 | 16 | -0.40 | 1.70 |
| -1 | 10 | 16 | -1.00 | 3.00 |
| 0 | -10 | 16 | 235.98 | 14.20 |
| 0 | 10 | 16 | 238.18 | 15.00 |
| 1 | -10 | 16 | 67.29 | 5.70 |
| 1 | 10 | 16 | 73.09 | 6.70 |
| -2 | -10 | -16 | 79.29 | 7.00 |
| 2 | -10 | 16 | 83.99 | 6.30 |
| 2 | 10 | 16 | 77.29 | 6.60 |
| -3 | -10 | -16 | 43.60 | 5.50 |
| 3 | -10 | 16 | 47.00 | 5.00 |
| 3 | 10 | 16 | 46.70 | 5.00 |
| -8 | 11 | 16 | 52.49 | 4.60 |
| -7 | -11 | 16 | 426.06 | 24.20 |
| -7 | 11 | 16 | 442.86 | 24.50 |
| -6 | -11 | 16 | 0.20 | 1.90 |
| -6 | 11 | 16 | 1.10 | 2.30 |
| -5 | -11 | 16 | 161.58 | 10.30 |
| 5 | -11 | -16 | 174.98 | 10.90 |
| -5 | 11 | 16 | 173.58 | 11.30 |
| -4 | -11 | 16 | 36.30 | 4.20 |
| 4 | -11 | -16 | 43.30 | 4.90 |
| -4 | 11 | 16 | 39.20 | 5.70 |
| -3 | -11 | 16 | 85.39 | 6.00 |
| 3 | -11 | -16 | 80.59 | 6.40 |
| -3 | 11 | 16 | 79.69 | 7.60 |
| -2 | -11 | 16 | 59.19 | 5.00 |
| 2 | -11 | -16 | 68.19 | 5.70 |
| -2 | 11 | 16 | 63.69 | 6.80 |
| -1 | -11 | 16 | 569.04 | 30.70 |
| 1 | -11 | -16 | 575.54 | 31.00 |
| -1 | 11 | 16 | 512.95 | 31.30 |
| 0 | -11 | -16 | 81.39 | 6.50 |
| 0 | -11 | 16 | 83.39 | 6.00 |
| 0 | 11 | 16 | 77.19 | 7.10 |
| 1 | -11 | 16 | 7.00 | 2.00 |
| -1 | -11 | -16 | 10.30 | 2.60 |
| 1 | 11 | 16 | 12.10 | 3.00 |

| | | | | |
|---|---|---|---|---|
| -2 | -11 | -16 | 12.30 | 2.50 |
| 2 | -11 | 16 | 9.80 | 2.20 |
| 2 | 11 | 16 | 9.60 | 2.80 |
| -6 | -12 | 16 | 439.96 | 24.70 |
| -6 | 12 | 16 | 444.96 | 25.40 |
| -5 | -12 | 16 | 67.89 | 5.00 |
| 5 | -12 | -16 | 62.89 | 6.20 |
| -5 | 12 | 16 | 65.99 | 5.90 |
| -4 | -12 | 16 | 6.30 | 1.70 |
| 4 | -12 | -16 | 6.40 | 2.20 |
| -4 | 12 | 16 | 5.60 | 2.80 |
| 3 | -12 | -16 | 43.40 | 4.50 |
| -3 | -12 | 16 | 54.59 | 4.30 |
| -3 | 12 | 16 | 57.99 | 6.10 |
| 2 | -12 | -16 | 283.17 | 15.60 |
| -2 | -12 | 16 | 265.17 | 15.30 |
| -2 | 12 | 16 | 253.77 | 16.10 |
| 1 | -12 | -16 | 3.80 | 2.00 |
| -1 | -12 | 16 | 3.30 | 1.60 |
| -1 | 12 | 16 | 5.10 | 3.10 |
| 0 | -12 | 16 | 155.38 | 9.10 |
| 0 | -12 | -16 | 150.38 | 9.50 |
| 0 | 12 | 16 | 138.39 | 10.30 |
| -12 | 0 | 17 | 4.20 | 2.40 |
| -11 | 0 | 17 | 496.15 | 27.70 |
| -10 | 0 | 17 | -0.90 | 2.50 |
| -9 | 0 | 17 | 0.20 | 2.70 |
| -8 | 0 | 17 | 1.20 | 2.90 |
| -7 | 0 | 17 | 841.22 | 49.10 |
| -7 | 0 | 17 | 923.61 | 48.70 |
| -6 | 0 | 17 | -1.20 | 3.90 |
| -5 | 0 | 17 | 165.28 | 11.30 |
| -5 | 0 | 17 | 172.08 | 12.50 |
| -4 | 0 | 17 | -0.80 | 3.90 |
| -4 | 0 | 17 | 0.60 | 2.50 |
| -3 | 0 | 17 | 361.36 | 22.00 |
| -3 | 0 | 17 | 393.96 | 23.30 |
| -2 | 0 | 17 | 0.70 | 2.50 |
| -2 | 0 | 17 | 0.30 | 4.10 |
| -1 | 0 | 17 | 1444.66 | 82.39 |
| -1 | 0 | 17 | 1552.54 | 81.99 |
| 0 | 0 | 17 | -1.90 | 2.60 |
| 0 | 0 | 17 | 3.60 | 4.10 |
| 1 | 0 | 17 | 6.00 | 3.00 |

| | | | | |
|---|---|---|---|---|
| 2 | 0 | 17 | 0.70 | 3.10 |
| 2 | 0 | 17 | -1.40 | 3.30 |
| 3 | 0 | 17 | 540.25 | 31.70 |
| 3 | 0 | 17 | 576.04 | 32.10 |
| 4 | 0 | 17 | -3.30 | 2.70 |
| 4 | 0 | 17 | 1.00 | 2.90 |
| 5 | 0 | 17 | 390.96 | 22.40 |
| 5 | 0 | 17 | 387.86 | 22.40 |
| 6 | 0 | 17 | -0.10 | 2.60 |
| -12 | 1 | 17 | 376.86 | 21.70 |
| -11 | -1 | 17 | 4.20 | 2.90 |
| -11 | 1 | 17 | 1.60 | 2.10 |
| -10 | -1 | 17 | 308.47 | 19.00 |
| -10 | 1 | 17 | 346.97 | 19.10 |
| -9 | -1 | 17 | 2.20 | 3.00 |
| -9 | 1 | 17 | -0.90 | 2.40 |
| -8 | -1 | 17 | 93.79 | 8.20 |
| -8 | 1 | 17 | 103.49 | 8.10 |
| -7 | -1 | 17 | -5.20 | 3.30 |
| -7 | -1 | 17 | 3.40 | 3.40 |
| -7 | 1 | 17 | 0.00 | 3.50 |
| -6 | -1 | 17 | 1251.37 | 72.09 |
| -6 | 1 | 17 | 1325.67 | 71.09 |
| -5 | -1 | 17 | -4.00 | 2.60 |
| -5 | -1 | 17 | -3.60 | 4.10 |
| -5 | 1 | 17 | -4.90 | 2.60 |
| -5 | 1 | 17 | -2.30 | 3.40 |
| -4 | -1 | 17 | 8.20 | 2.80 |
| -4 | -1 | 17 | 4.00 | 4.30 |
| -4 | 1 | 17 | 10.60 | 3.00 |
| -4 | 1 | 17 | -0.50 | 3.90 |
| -3 | -1 | 17 | 2.60 | 2.70 |
| -3 | -1 | 17 | -3.80 | 4.20 |
| -3 | 1 | 17 | 0.00 | 3.90 |
| -3 | 1 | 17 | 3.50 | 2.50 |
| -2 | -1 | 17 | 840.92 | 45.40 |
| -2 | -1 | 17 | 684.83 | 45.80 |
| -2 | 1 | 17 | 827.42 | 45.40 |
| -2 | 1 | 17 | 910.01 | 46.50 |
| -1 | -1 | 17 | -0.90 | 4.20 |
| -1 | -1 | 17 | -0.20 | 2.60 |
| -1 | 1 | 17 | 6.70 | 4.00 |
| -1 | 1 | 17 | -2.20 | 2.40 |
| 0 | -1 | 17 | 679.83 | 38.70 |

| | | | | |
|---|---|---|---|---|
| 0 | -1 | 17 | 747.03 | 39.80 |
| 0 | 1 | 17 | 631.94 | 39.10 |
| 0 | 1 | 17 | 691.73 | 38.80 |
| 1 | -1 | 17 | 4.60 | 4.30 |
| 1 | -1 | 17 | 3.70 | 3.10 |
| 1 | 1 | 17 | 4.90 | 3.10 |
| 2 | -1 | 17 | 290.47 | 17.30 |
| 2 | -1 | 17 | 228.98 | 16.80 |
| 2 | 1 | 17 | 286.57 | 17.40 |
| 2 | 1 | 17 | 284.67 | 17.20 |
| 3 | -1 | 17 | 10.10 | 3.50 |
| 3 | -1 | 17 | 9.80 | 3.30 |
| 3 | 1 | 17 | 2.80 | 3.10 |
| 3 | 1 | 17 | 13.00 | 3.30 |
| 4 | -1 | 17 | 827.12 | 47.70 |
| 4 | -1 | 17 | 868.81 | 47.50 |
| 4 | 1 | 17 | 935.01 | 47.60 |
| 4 | 1 | 17 | 806.32 | 47.70 |
| 5 | -1 | 17 | 2.30 | 3.00 |
| 5 | -1 | 17 | 0.80 | 2.50 |
| 5 | 1 | 17 | 1.90 | 2.60 |
| 6 | -1 | 17 | 1.20 | 3.40 |
| 6 | 1 | 17 | 0.60 | 2.30 |
| -12 | 2 | 17 | 43.90 | 4.10 |
| -11 | 2 | 17 | 637.94 | 35.20 |
| -10 | 2 | 17 | 4.20 | 2.10 |
| -9 | -2 | 17 | 152.58 | 11.10 |
| -9 | 2 | 17 | 168.58 | 10.60 |
| -8 | -2 | 17 | 56.29 | 7.40 |
| -8 | 2 | 17 | 65.49 | 6.40 |
| -7 | -2 | 17 | 1186.08 | 61.99 |
| -7 | -2 | 17 | 1065.89 | 62.29 |
| -7 | 2 | 17 | 1149.98 | 62.89 |
| -6 | -2 | 17 | 19.80 | 4.40 |
| -6 | -2 | 17 | 23.60 | 5.00 |
| -6 | 2 | 17 | 22.70 | 4.20 |
| -6 | 2 | 17 | 24.10 | 4.20 |
| -5 | -2 | 17 | 547.45 | 31.20 |
| -5 | -2 | 17 | 498.65 | 34.90 |
| -5 | 2 | 17 | 622.14 | 32.40 |
| -5 | 2 | 17 | 537.65 | 31.30 |
| -4 | -2 | 17 | -2.30 | 5.00 |
| -4 | -2 | 17 | 1.50 | 2.60 |
| -4 | 2 | 17 | -3.50 | 2.50 |

| | | | | |
|---|---|---|---|---|
| -4 | 2 | 17 | -4.80 | 3.50 |
| -3 | -2 | 17 | 208.08 | 15.40 |
| -3 | -2 | 17 | 212.08 | 13.50 |
| -3 | 2 | 17 | 229.98 | 15.10 |
| -3 | 2 | 17 | 208.68 | 13.60 |
| -2 | -2 | 17 | -1.20 | 2.40 |
| -2 | -2 | 17 | -5.00 | 4.80 |
| -2 | 2 | 17 | 1.40 | 3.70 |
| -2 | 2 | 17 | -4.50 | 2.50 |
| -1 | -2 | 17 | 1542.15 | 83.89 |
| -1 | -2 | 17 | 1504.95 | 86.49 |
| -1 | 2 | 17 | 1560.24 | 83.99 |
| 0 | -2 | 17 | 7.20 | 3.00 |
| 0 | -2 | 17 | 11.90 | 5.70 |
| 0 | 2 | 17 | 2.10 | 2.70 |
| 0 | 2 | 17 | 1.50 | 3.60 |
| 1 | -2 | 17 | 2.70 | 3.10 |
| 1 | -2 | 17 | 6.80 | 4.30 |
| 1 | 2 | 17 | 3.30 | 3.20 |
| 2 | -2 | 17 | -2.60 | 2.80 |
| 2 | -2 | 17 | -2.30 | 3.70 |
| 2 | 2 | 17 | 0.20 | 3.40 |
| 2 | 2 | 17 | -2.70 | 3.00 |
| 3 | -2 | 17 | 489.35 | 28.00 |
| 3 | -2 | 17 | 491.85 | 27.90 |
| 3 | 2 | 17 | 473.95 | 27.90 |
| 3 | 2 | 17 | 485.95 | 28.20 |
| 4 | -2 | 17 | 11.70 | 3.90 |
| 4 | -2 | 17 | 11.30 | 3.10 |
| 4 | 2 | 17 | 9.30 | 2.80 |
| 4 | 2 | 17 | 5.30 | 2.80 |
| 5 | -2 | 17 | 378.76 | 22.20 |
| 5 | -2 | 17 | 373.96 | 22.40 |
| 5 | 2 | 17 | 407.26 | 22.20 |
| 6 | -2 | 17 | -1.50 | 4.30 |
| 6 | 2 | 17 | 0.00 | 2.10 |
| -11 | 3 | 17 | -1.30 | 1.70 |
| -10 | 3 | 17 | 239.98 | 14.30 |
| -9 | 3 | 17 | 2.00 | 2.30 |
| -8 | 3 | 17 | 171.98 | 11.50 |
| -7 | -3 | 17 | 1.90 | 3.00 |
| -7 | 3 | 17 | 3.70 | 3.20 |
| -6 | -3 | 17 | 1218.48 | 65.89 |
| -6 | 3 | 17 | 1204.58 | 66.99 |

| | | | | |
|---|---|---|---|---|
| -6 | 3 | 17 | 1194.78 | 65.89 |
| -5 | -3 | 17 | -4.30 | 2.50 |
| -5 | 3 | 17 | -0.60 | 3.60 |
| -5 | 3 | 17 | 3.30 | 4.40 |
| -4 | -3 | 17 | 9.10 | 2.80 |
| -4 | 3 | 17 | 11.10 | 3.10 |
| -4 | 3 | 17 | 17.30 | 4.30 |
| -3 | -3 | 17 | 15.20 | 2.90 |
| -3 | 3 | 17 | 12.20 | 2.90 |
| -3 | 3 | 17 | 19.30 | 4.60 |
| -2 | -3 | 17 | 1098.29 | 65.59 |
| -2 | 3 | 17 | 1130.99 | 65.69 |
| -2 | 3 | 17 | 1372.56 | 66.99 |
| -1 | -3 | 17 | 6.60 | 2.70 |
| -1 | 3 | 17 | 5.80 | 3.70 |
| -1 | 3 | 17 | 12.30 | 3.20 |
| 0 | -3 | 17 | 528.55 | 29.50 |
| 0 | 3 | 17 | 524.85 | 29.70 |
| 0 | 3 | 17 | 491.85 | 30.10 |
| 1 | -3 | 17 | 13.40 | 5.20 |
| 1 | -3 | 17 | 6.10 | 5.50 |
| 1 | 3 | 17 | 9.20 | 4.70 |
| 1 | 3 | 17 | 7.90 | 4.00 |
| 2 | -3 | 17 | 299.37 | 17.50 |
| 2 | -3 | 17 | 258.57 | 18.10 |
| 2 | 3 | 17 | 296.27 | 17.70 |
| 2 | 3 | 17 | 273.87 | 17.30 |
| 3 | -3 | 17 | 5.90 | 6.60 |
| 3 | -3 | 17 | 0.30 | 2.70 |
| 3 | 3 | 17 | 5.20 | 2.70 |
| 3 | 3 | 17 | 4.10 | 3.00 |
| 4 | -3 | 17 | 754.72 | 44.40 |
| 4 | 3 | 17 | 844.22 | 44.40 |
| 5 | -3 | 17 | 2.20 | 2.60 |
| 5 | 3 | 17 | 3.40 | 2.30 |
| 6 | -3 | 17 | 8.80 | 3.10 |
| -11 | 4 | 17 | 385.96 | 21.80 |
| -10 | 4 | 17 | -1.40 | 1.80 |
| -9 | 4 | 17 | 4.80 | 2.30 |
| -8 | 4 | 17 | 32.10 | 4.90 |
| -7 | -4 | 17 | 1157.58 | 66.19 |
| -7 | 4 | 17 | 1275.47 | 66.99 |
| -6 | -4 | 17 | 1.00 | 2.60 |
| -6 | 4 | 17 | -2.60 | 4.20 |

| | | | | |
|---|---|---|---|---|
| -5 | -4 | 17 | 398.86 | 26.10 |
| -5 | 4 | 17 | 445.46 | 25.30 |
| -4 | -4 | 17 | 31.80 | 5.00 |
| -4 | 4 | 17 | 31.60 | 6.70 |
| -3 | -4 | 17 | 521.35 | 28.50 |
| -3 | 4 | 17 | 458.45 | 29.40 |
| -3 | 4 | 17 | 520.35 | 28.50 |
| -2 | -4 | 17 | 15.80 | 3.10 |
| -2 | 4 | 17 | 24.50 | 4.90 |
| -2 | 4 | 17 | 19.90 | 4.80 |
| -1 | -4 | 17 | 1389.36 | 76.29 |
| -1 | 4 | 17 | 1403.66 | 76.49 |
| 0 | -4 | 17 | 17.20 | 4.90 |
| 0 | 4 | 17 | 24.70 | 5.20 |
| 1 | -4 | 17 | 2.40 | 2.70 |
| 1 | 4 | 17 | 4.60 | 3.10 |
| 1 | 4 | 17 | 6.60 | 3.30 |
| 2 | -4 | 17 | 7.80 | 3.00 |
| 2 | 4 | 17 | 1.80 | 2.70 |
| 2 | 4 | 17 | 3.10 | 2.60 |
| 3 | -4 | 17 | 506.25 | 29.50 |
| 3 | 4 | 17 | 527.55 | 29.40 |
| 4 | -4 | 17 | -0.20 | 2.50 |
| 4 | 4 | 17 | 5.50 | 2.50 |
| 5 | -4 | 17 | 369.16 | 21.40 |
| 5 | 4 | 17 | 377.46 | 21.30 |
| -11 | 5 | 17 | -1.70 | 1.50 |
| -10 | 5 | 17 | 125.59 | 8.30 |
| -9 | 5 | 17 | -0.60 | 1.90 |
| -8 | 5 | 17 | 98.89 | 7.50 |
| -7 | -5 | 17 | 0.20 | 2.70 |
| -7 | 5 | 17 | 11.30 | 2.80 |
| -6 | -5 | 17 | 1138.19 | 61.69 |
| -6 | 5 | 17 | 1120.99 | 62.49 |
| -5 | -5 | 17 | -0.30 | 2.60 |
| -5 | 5 | 17 | 4.70 | 4.20 |
| -4 | -5 | 17 | 9.60 | 4.10 |
| -4 | 5 | 17 | 23.50 | 6.80 |
| -3 | -5 | 17 | 4.90 | 4.00 |
| -3 | 5 | 17 | 0.50 | 3.40 |
| -2 | -5 | 17 | 933.01 | 47.90 |
| -2 | 5 | 17 | 760.82 | 48.40 |
| -1 | 5 | 17 | -0.50 | 5.50 |
| 0 | -5 | 17 | 558.14 | 31.60 |

| | | | | |
|---|---|---|---|---|
| 0 | 5 | 17 | 555.04 | 32.30 |
| 1 | -5 | 17 | 50.59 | 5.60 |
| 1 | 5 | 17 | 43.70 | 6.20 |
| 2 | -5 | 17 | 230.18 | 14.50 |
| 2 | 5 | 17 | 238.58 | 14.80 |
| 3 | -5 | 17 | 2.30 | 2.40 |
| 3 | 5 | 17 | 1.50 | 2.30 |
| 4 | -5 | 17 | 833.52 | 47.90 |
| 4 | 5 | 17 | 907.31 | 48.00 |
| 5 | -5 | 17 | 0.80 | 3.20 |
| 5 | 5 | 17 | 5.60 | 2.10 |
| -10 | 6 | 17 | 2.80 | 1.70 |
| -9 | 6 | 17 | 88.99 | 6.50 |
| -8 | 6 | 17 | 47.90 | 5.20 |
| -7 | -6 | 17 | 750.82 | 40.50 |
| -7 | 6 | 17 | 721.23 | 41.10 |
| -6 | -6 | 17 | -0.30 | 2.30 |
| -6 | 6 | 17 | -2.10 | 2.50 |
| -5 | -6 | 17 | 469.45 | 26.70 |
| -5 | 6 | 17 | 473.95 | 27.60 |
| -4 | -6 | 17 | 26.40 | 4.60 |
| -4 | 6 | 17 | 19.70 | 4.30 |
| -3 | -6 | 17 | 65.49 | 6.30 |
| -3 | 6 | 17 | 69.09 | 8.00 |
| -2 | -6 | 17 | -0.10 | 2.20 |
| -2 | 6 | 17 | -6.60 | 3.30 |
| -1 | -6 | 17 | 1069.79 | 54.49 |
| -1 | 6 | 17 | 906.91 | 54.99 |
| 0 | -6 | 17 | 21.40 | 4.60 |
| 0 | 6 | 17 | 31.30 | 5.80 |
| 1 | -6 | 17 | 24.70 | 4.80 |
| 1 | 6 | 17 | 29.10 | 5.70 |
| 2 | -6 | 17 | 10.60 | 2.70 |
| 2 | 6 | 17 | 13.50 | 3.00 |
| 3 | -6 | 17 | 412.46 | 22.90 |
| 3 | 6 | 17 | 379.76 | 22.80 |
| 4 | -6 | 17 | -4.20 | 2.40 |
| 4 | 6 | 17 | 3.00 | 2.10 |
| 5 | -6 | 17 | 356.06 | 21.10 |
| 5 | 6 | 17 | 385.66 | 21.10 |
| -10 | 7 | 17 | 205.58 | 12.10 |
| -9 | 7 | 17 | 3.00 | 1.80 |
| -8 | 7 | 17 | 44.50 | 4.60 |
| -7 | -7 | 17 | 3.30 | 2.40 |

| | | | | |
|---|---|---|---|---|
| -7 | 7 | 17 | 0.70 | 2.20 |
| -6 | -7 | 17 | 729.83 | 40.30 |
| -6 | 7 | 17 | 735.53 | 41.00 |
| -5 | -7 | 17 | 50.79 | 5.30 |
| -5 | 7 | 17 | 54.69 | 6.30 |
| -4 | -7 | 17 | 9.30 | 2.50 |
| -4 | 7 | 17 | 14.20 | 3.50 |
| -3 | -7 | 17 | 51.89 | 5.30 |
| -3 | 7 | 17 | 58.19 | 6.90 |
| -2 | -7 | 17 | 489.65 | 29.60 |
| -2 | 7 | 17 | 570.64 | 30.80 |
| -1 | -7 | 17 | 0.00 | 2.10 |
| -1 | 7 | 17 | -1.30 | 3.00 |
| 0 | -7 | 17 | 324.87 | 19.70 |
| 0 | 7 | 17 | 359.76 | 20.60 |
| 1 | -7 | 17 | 22.80 | 4.00 |
| 1 | 7 | 17 | 16.10 | 3.40 |
| 2 | -7 | 17 | 121.89 | 8.10 |
| 2 | 7 | 17 | 97.69 | 8.00 |
| 3 | -7 | 17 | 3.70 | 2.30 |
| 3 | 7 | 17 | 0.20 | 2.10 |
| 4 | -7 | 17 | 398.36 | 24.20 |
| 4 | 7 | 17 | 463.85 | 24.40 |
| -9 | 8 | 17 | 0.40 | 1.60 |
| -8 | 8 | 17 | 34.90 | 4.00 |
| -7 | -8 | 17 | 611.24 | 33.50 |
| -7 | 8 | 17 | 606.74 | 34.00 |
| 6 | -8 | -17 | 12.40 | 3.70 |
| -6 | -8 | 17 | 12.50 | 2.40 |
| -6 | 8 | 17 | 7.60 | 2.60 |
| -5 | -8 | 17 | 200.38 | 12.00 |
| -5 | 8 | 17 | 193.98 | 12.70 |
| -4 | -8 | 17 | 34.50 | 4.30 |
| -4 | 8 | 17 | 34.00 | 5.60 |
| -3 | -8 | 17 | 121.59 | 7.70 |
| -3 | 8 | 17 | 99.39 | 8.70 |
| -2 | -8 | 17 | -0.20 | 1.70 |
| -2 | 8 | 17 | 1.70 | 2.70 |
| -1 | -8 | 17 | 639.14 | 35.30 |
| -1 | 8 | 17 | 630.84 | 36.00 |
| 0 | -8 | 17 | 82.89 | 6.20 |
| 0 | 8 | 17 | 77.59 | 7.20 |
| 1 | -8 | 17 | 2.80 | 1.90 |
| 1 | 8 | 17 | -1.10 | 2.30 |

| | | | | |
|---|---|---|---|---|
| 2 | -8 | 17 | 2.30 | 2.00 |
| 2 | 8 | 17 | -1.60 | 2.10 |
| 3 | -8 | 17 | 277.17 | 16.00 |
| 3 | 8 | 17 | 273.17 | 16.20 |
| -8 | 9 | 17 | 99.19 | 6.80 |
| -7 | -9 | 17 | 19.40 | 3.90 |
| -7 | 9 | 17 | 20.60 | 3.60 |
| -6 | -9 | 17 | 662.43 | 36.70 |
| -6 | 9 | 17 | 676.23 | 37.20 |
| -5 | -9 | 17 | 30.90 | 4.00 |
| 5 | -9 | -17 | 28.10 | 5.10 |
| -5 | 9 | 17 | 29.50 | 4.60 |
| -4 | -9 | 17 | 3.60 | 1.90 |
| 4 | -9 | -17 | 3.10 | 2.30 |
| -4 | 9 | 17 | 5.40 | 2.60 |
| -3 | -9 | 17 | 5.30 | 1.80 |
| -3 | 9 | 17 | 1.20 | 2.70 |
| -2 | -9 | 17 | 574.54 | 32.10 |
| -2 | 9 | 17 | 582.44 | 32.90 |
| -1 | -9 | 17 | 0.60 | 1.70 |
| -1 | 9 | 17 | -0.90 | 2.60 |
| 0 | -9 | 17 | 233.38 | 13.50 |
| 0 | 9 | 17 | 222.68 | 14.20 |
| 1 | -9 | 17 | 9.60 | 2.10 |
| 1 | 9 | 17 | 7.60 | 2.70 |
| -2 | -9 | -17 | 145.99 | 9.70 |
| 2 | -9 | 17 | 160.28 | 9.30 |
| 2 | 9 | 17 | 129.79 | 9.40 |
| -7 | -10 | 17 | 506.15 | 27.40 |
| -7 | 10 | 17 | 488.25 | 27.60 |
| -6 | -10 | 17 | 9.70 | 2.00 |
| -6 | 10 | 17 | 12.90 | 2.50 |
| 5 | -10 | -17 | 111.79 | 9.40 |
| -5 | -10 | 17 | 87.49 | 6.60 |
| -5 | 10 | 17 | 99.69 | 7.20 |
| -4 | -10 | 17 | 45.70 | 4.10 |
| 4 | -10 | -17 | 48.70 | 4.80 |
| -4 | 10 | 17 | 38.30 | 5.20 |
| 3 | -10 | -17 | 120.99 | 7.80 |
| -3 | -10 | 17 | 116.39 | 7.60 |
| -3 | 10 | 17 | 115.29 | 8.50 |
| 2 | -10 | -17 | 18.90 | 3.70 |
| -2 | -10 | 17 | 17.00 | 3.20 |
| -2 | 10 | 17 | 23.60 | 4.90 |

| | | | | |
|---|---|---|---|---|
| 1 | -10 | -17 | 609.84 | 33.40 |
| -1 | -10 | 17 | 621.14 | 33.20 |
| -1 | 10 | 17 | 574.74 | 33.70 |
| 0 | -10 | -17 | 61.39 | 5.30 |
| 0 | -10 | 17 | 63.29 | 4.80 |
| 0 | 10 | 17 | 52.59 | 5.80 |
| -1 | -10 | -17 | -0.80 | 1.90 |
| 1 | -10 | 17 | -0.60 | 1.60 |
| 1 | 10 | 17 | 1.80 | 2.30 |
| -5 | -11 | 17 | 83.69 | 5.80 |
| 5 | -11 | -17 | 89.79 | 8.80 |
| -5 | 11 | 17 | 88.09 | 6.50 |
| 4 | -11 | -17 | 1.90 | 2.30 |
| -4 | -11 | 17 | 3.00 | 1.50 |
| -4 | 11 | 17 | 2.50 | 2.40 |
| 3 | -11 | -17 | 58.99 | 5.00 |
| -3 | -11 | 17 | 60.09 | 4.50 |
| -3 | 11 | 17 | 57.19 | 5.60 |
| 2 | -11 | -17 | 419.46 | 23.10 |
| -2 | -11 | 17 | 405.76 | 23.00 |
| -2 | 11 | 17 | 414.36 | 23.50 |
| 1 | -11 | -17 | -1.00 | 1.60 |
| -1 | -11 | 17 | 0.10 | 1.30 |
| -1 | 11 | 17 | 0.10 | 2.50 |
| -11 | 0 | 18 | -4.50 | 2.40 |
| -10 | 0 | 18 | 192.58 | 11.90 |
| -9 | 0 | 18 | -2.80 | 2.40 |
| -8 | 0 | 18 | 218.58 | 13.40 |
| -7 | 0 | 18 | 0.30 | 2.70 |
| -6 | 0 | 18 | 939.21 | 51.99 |
| -6 | 0 | 18 | 959.10 | 52.39 |
| -5 | 0 | 18 | -3.50 | 3.10 |
| -5 | 0 | 18 | -1.40 | 2.50 |
| -4 | 0 | 18 | 12.60 | 3.80 |
| -4 | 0 | 18 | 14.70 | 2.80 |
| -3 | 0 | 18 | -1.10 | 2.30 |
| -3 | 0 | 18 | 2.10 | 3.40 |
| -2 | 0 | 18 | 792.62 | 44.40 |
| -2 | 0 | 18 | 790.32 | 43.90 |
| -1 | 0 | 18 | -1.60 | 2.20 |
| -1 | 0 | 18 | -2.30 | 3.50 |
| 0 | 0 | 18 | 297.67 | 19.00 |
| 0 | 0 | 18 | 331.27 | 18.70 |
| 1 | 0 | 18 | -0.10 | 2.20 |

| | | | | |
|---|---|---|---|---|
| 1 | 0 | 18 | -1.20 | 3.10 |
| 2 | 0 | 18 | 181.98 | 12.90 |
| 2 | 0 | 18 | 226.98 | 13.10 |
| 3 | 0 | 18 | -5.20 | 2.70 |
| 3 | 0 | 18 | 2.40 | 2.40 |
| 4 | 0 | 18 | 768.92 | 41.70 |
| 4 | 0 | 18 | 742.93 | 41.80 |
| -10 | -1 | 18 | 11.30 | 3.40 |
| -10 | 1 | 18 | 7.30 | 2.30 |
| -9 | -1 | 18 | 14.40 | 3.30 |
| -9 | 1 | 18 | 17.60 | 3.90 |
| -8 | -1 | 18 | -3.50 | 3.00 |
| -8 | 1 | 18 | 1.80 | 2.30 |
| -7 | -1 | 18 | 833.52 | 45.80 |
| -7 | -1 | 18 | 803.32 | 45.80 |
| -7 | 1 | 18 | 849.02 | 45.90 |
| -6 | -1 | 18 | 2.10 | 2.60 |
| -6 | -1 | 18 | 10.80 | 3.40 |
| -6 | 1 | 18 | 8.50 | 3.00 |
| -6 | 1 | 18 | 3.40 | 2.80 |
| -5 | -1 | 18 | 547.65 | 27.40 |
| -5 | -1 | 18 | 460.75 | 26.70 |
| -5 | 1 | 18 | 456.65 | 26.70 |
| -5 | 1 | 18 | 422.76 | 27.00 |
| -4 | -1 | 18 | 4.10 | 3.70 |
| -4 | -1 | 18 | 3.90 | 2.40 |
| -4 | 1 | 18 | 8.10 | 2.70 |
| -4 | 1 | 18 | 3.00 | 3.10 |
| -3 | -1 | 18 | 162.48 | 10.30 |
| -3 | -1 | 18 | 145.49 | 11.40 |
| -3 | 1 | 18 | 150.48 | 10.20 |
| -3 | 1 | 18 | 138.49 | 11.00 |
| -2 | -1 | 18 | 4.90 | 2.60 |
| -2 | -1 | 18 | -2.90 | 3.70 |
| -2 | 1 | 18 | -1.90 | 3.30 |
| -2 | 1 | 18 | 0.10 | 2.30 |
| -1 | -1 | 18 | 952.20 | 59.29 |
| -1 | -1 | 18 | 1084.29 | 59.19 |
| -1 | 1 | 18 | 1062.59 | 59.19 |
| -1 | 1 | 18 | 1215.28 | 59.89 |
| 0 | -1 | 18 | 4.50 | 2.30 |
| 0 | -1 | 18 | 3.60 | 3.70 |
| 0 | 1 | 18 | 5.50 | 3.40 |
| 0 | 1 | 18 | 9.70 | 2.70 |

| | | | | |
|---|---|---|---|---|
| 1 | -1 | 18 | 0.90 | 2.10 |
| 1 | 1 | 18 | 1.10 | 2.30 |
| 1 | 1 | 18 | 7.30 | 3.10 |
| 2 | -1 | 18 | 3.00 | 3.50 |
| 2 | -1 | 18 | 2.20 | 2.40 |
| 2 | 1 | 18 | -3.50 | 2.20 |
| 2 | 1 | 18 | 0.50 | 2.80 |
| 3 | -1 | 18 | 301.07 | 18.30 |
| 3 | -1 | 18 | 322.37 | 18.30 |
| 3 | 1 | 18 | 306.87 | 18.20 |
| 3 | 1 | 18 | 302.67 | 18.20 |
| 4 | -1 | 18 | 4.40 | 4.10 |
| 4 | -1 | 18 | -2.80 | 2.20 |
| 4 | 1 | 18 | 4.00 | 2.50 |
| -10 | 2 | 18 | 154.98 | 9.60 |
| -9 | 2 | 18 | 0.90 | 2.00 |
| -8 | 2 | 18 | 217.08 | 13.30 |
| -7 | -2 | 18 | -1.30 | 2.80 |
| -7 | 2 | 18 | 4.20 | 2.40 |
| -6 | -2 | 18 | 748.33 | 40.40 |
| -6 | -2 | 18 | 628.54 | 41.00 |
| -6 | 2 | 18 | 809.42 | 41.00 |
| -6 | 2 | 18 | 742.83 | 40.50 |
| -5 | -2 | 18 | 9.10 | 4.10 |
| -5 | -2 | 18 | 10.70 | 2.80 |
| -5 | 2 | 18 | 12.00 | 2.80 |
| -5 | 2 | 18 | 10.80 | 3.10 |
| -4 | -2 | 18 | 3.40 | 4.30 |
| -4 | -2 | 18 | -0.30 | 2.20 |
| -4 | 2 | 18 | 3.40 | 3.10 |
| -4 | 2 | 18 | 3.20 | 2.40 |
| -3 | -2 | 18 | -2.70 | 4.20 |
| -3 | -2 | 18 | 3.30 | 2.30 |
| -3 | 2 | 18 | 4.30 | 2.30 |
| -3 | 2 | 18 | 5.40 | 3.40 |
| -2 | -2 | 18 | 504.95 | 28.40 |
| -2 | -2 | 18 | 562.94 | 29.10 |
| -2 | 2 | 18 | 441.76 | 28.90 |
| -2 | 2 | 18 | 495.95 | 28.40 |
| -1 | -2 | 18 | 7.40 | 4.30 |
| -1 | -2 | 18 | 5.30 | 2.20 |
| -1 | 2 | 18 | 1.30 | 2.30 |
| -1 | 2 | 18 | 3.80 | 3.30 |
| 0 | -2 | 18 | 312.07 | 18.40 |

| | | | | |
|---|---|---|---|---|
| 0 | -2 | 18 | 310.77 | 19.00 |
| 0 | 2 | 18 | 316.77 | 19.00 |
| 0 | 2 | 18 | 306.17 | 18.50 |
| 1 | -2 | 18 | 5.80 | 2.40 |
| 1 | -2 | 18 | 11.50 | 4.20 |
| 1 | 2 | 18 | 7.90 | 2.40 |
| 1 | 2 | 18 | 7.20 | 3.10 |
| 2 | -2 | 18 | 142.29 | 9.40 |
| 2 | -2 | 18 | 136.29 | 10.20 |
| 2 | 2 | 18 | 121.99 | 9.40 |
| 2 | 2 | 18 | 139.79 | 9.50 |
| 3 | -2 | 18 | 8.60 | 4.30 |
| 3 | -2 | 18 | -1.60 | 2.20 |
| 3 | 2 | 18 | 1.80 | 2.30 |
| 3 | 2 | 18 | 0.50 | 2.60 |
| 4 | -2 | 18 | 644.14 | 35.40 |
| 4 | 2 | 18 | 629.64 | 35.30 |
| -10 | 3 | 18 | 6.10 | 1.90 |
| -9 | 3 | 18 | 10.80 | 2.30 |
| -8 | 3 | 18 | 30.20 | 4.20 |
| -7 | -3 | 18 | 1027.20 | 58.09 |
| -7 | 3 | 18 | 1107.89 | 58.59 |
| -6 | -3 | 18 | 4.20 | 2.50 |
| -6 | 3 | 18 | 7.20 | 2.70 |
| -5 | -3 | 18 | 261.87 | 15.40 |
| -5 | 3 | 18 | 258.77 | 15.40 |
| -5 | 3 | 18 | 252.87 | 16.00 |
| -4 | -3 | 18 | 15.80 | 2.80 |
| -4 | 3 | 18 | 20.80 | 4.40 |
| -4 | 3 | 18 | 18.30 | 3.60 |
| -3 | -3 | 18 | 255.67 | 15.20 |
| -3 | 3 | 18 | 249.58 | 16.10 |
| -3 | 3 | 18 | 254.67 | 15.20 |
| -2 | -3 | 18 | -0.60 | 1.90 |
| -2 | 3 | 18 | 3.70 | 3.10 |
| -2 | 3 | 18 | 1.80 | 2.10 |
| -1 | -3 | 18 | 900.11 | 49.10 |
| -1 | 3 | 18 | 873.51 | 49.70 |
| -1 | 3 | 18 | 898.81 | 49.20 |
| 0 | -3 | 18 | 13.00 | 2.90 |
| 0 | 3 | 18 | 11.80 | 3.40 |
| 0 | 3 | 18 | 25.30 | 4.30 |
| 1 | -3 | 18 | 2.70 | 2.10 |
| 1 | 3 | 18 | 1.40 | 2.20 |

| | | | | |
|---|---|---|---|---|
| 1 | 3 | 18 | 0.10 | 2.70 |
| 2 | -3 | 18 | 21.00 | 4.20 |
| 2 | 3 | 18 | 8.30 | 2.70 |
| 3 | -3 | 18 | 353.96 | 20.10 |
| 3 | 3 | 18 | 338.77 | 20.10 |
| 4 | -3 | 18 | -0.80 | 2.20 |
| 4 | 3 | 18 | -1.80 | 2.00 |
| -10 | 4 | 18 | 154.98 | 9.50 |
| -9 | 4 | 18 | 31.10 | 3.70 |
| -8 | 4 | 18 | 189.08 | 11.70 |
| -7 | -4 | 18 | 3.10 | 2.70 |
| -7 | 4 | 18 | 3.30 | 2.20 |
| -6 | -4 | 18 | 722.53 | 41.30 |
| -6 | 4 | 18 | 781.22 | 41.90 |
| -5 | -4 | 18 | 39.80 | 4.90 |
| -5 | 4 | 18 | 34.10 | 5.30 |
| -4 | -4 | 18 | 3.70 | 2.20 |
| -4 | 4 | 18 | 7.20 | 3.20 |
| -3 | -4 | 18 | 11.80 | 2.40 |
| -3 | 4 | 18 | 11.20 | 3.40 |
| -2 | -4 | 18 | 672.23 | 40.10 |
| -2 | 4 | 18 | 813.52 | 41.10 |
| -2 | 4 | 18 | 692.43 | 40.20 |
| -1 | -4 | 18 | 2.10 | 2.00 |
| -1 | 4 | 18 | -3.60 | 3.00 |
| -1 | 4 | 18 | 1.40 | 2.00 |
| 0 | -4 | 18 | 262.97 | 16.20 |
| 0 | 4 | 18 | 287.77 | 17.00 |
| 1 | -4 | 18 | 29.80 | 4.30 |
| 1 | 4 | 18 | 24.50 | 5.00 |
| 2 | -4 | 18 | 212.38 | 12.60 |
| 2 | 4 | 18 | 191.58 | 12.70 |
| 3 | -4 | 18 | 7.00 | 2.30 |
| 3 | 4 | 18 | 11.50 | 2.60 |
| 4 | -4 | 18 | 646.04 | 37.40 |
| 4 | 4 | 18 | 710.13 | 37.50 |
| -9 | 5 | 18 | 23.40 | 3.30 |
| -8 | 5 | 18 | 9.70 | 2.20 |
| -7 | -5 | 18 | 748.83 | 41.30 |
| -7 | 5 | 18 | 759.62 | 41.80 |
| -6 | -5 | 18 | 2.30 | 2.10 |
| -6 | 5 | 18 | 3.70 | 2.40 |
| -5 | -5 | 18 | 259.97 | 15.80 |
| -5 | 5 | 18 | 280.87 | 16.50 |

| | | | | |
|---|---|---|---|---|
| -4 | -5 | 18 | 12.60 | 2.40 |
| -4 | 5 | 18 | 5.50 | 2.70 |
| -3 | -5 | 18 | 85.59 | 6.70 |
| -3 | 5 | 18 | 96.19 | 7.90 |
| -2 | -5 | 18 | 2.60 | 2.00 |
| -2 | 5 | 18 | 0.40 | 2.70 |
| -1 | -5 | 18 | 962.70 | 52.99 |
| -1 | 5 | 18 | 971.70 | 53.69 |
| 0 | -5 | 18 | 24.20 | 3.90 |
| 0 | 5 | 18 | 13.10 | 3.20 |
| 1 | -5 | 18 | 5.00 | 2.10 |
| 1 | 5 | 18 | 3.20 | 2.60 |
| 2 | -5 | 18 | 7.50 | 2.20 |
| 2 | 5 | 18 | 6.30 | 2.40 |
| 3 | -5 | 18 | 361.46 | 20.50 |
| 3 | 5 | 18 | 355.76 | 20.60 |
| -9 | 6 | 18 | 13.10 | 2.80 |
| -8 | 6 | 18 | 74.99 | 5.70 |
| -7 | -6 | 18 | -3.30 | 2.30 |
| -7 | 6 | 18 | -0.50 | 1.90 |
| -6 | -6 | 18 | 782.32 | 41.00 |
| -6 | 6 | 18 | 713.03 | 41.40 |
| -5 | -6 | 18 | 15.20 | 2.50 |
| -5 | 6 | 18 | 21.30 | 4.20 |
| -4 | -6 | 18 | 8.90 | 2.20 |
| -4 | 6 | 18 | 3.30 | 2.50 |
| -3 | -6 | 18 | 5.60 | 2.10 |
| -3 | 6 | 18 | 12.60 | 3.20 |
| -2 | -6 | 18 | 634.64 | 35.50 |
| -2 | 6 | 18 | 649.34 | 36.30 |
| -1 | -6 | 18 | 2.00 | 1.90 |
| -1 | 6 | 18 | 5.50 | 2.80 |
| 0 | -6 | 18 | 251.37 | 15.30 |
| 0 | 6 | 18 | 271.37 | 16.00 |
| 1 | -6 | 18 | 9.30 | 2.10 |
| 1 | 6 | 18 | 11.80 | 2.80 |
| 2 | -6 | 18 | 134.09 | 8.70 |
| 2 | 6 | 18 | 129.99 | 8.90 |
| 3 | -6 | 18 | 2.30 | 2.00 |
| 3 | 6 | 18 | 1.00 | 2.00 |
| -8 | 7 | 18 | 33.80 | 3.60 |
| -7 | -7 | 18 | 451.75 | 26.90 |
| -7 | 7 | 18 | 521.65 | 27.30 |
| -6 | -7 | 18 | 34.50 | 4.20 |

| | | | | |
|---|---|---|---|---|
| -6 | 7 | 18 | 33.10 | 4.20 |
| -5 | -7 | 18 | 248.48 | 15.00 |
| -5 | 7 | 18 | 270.97 | 15.60 |
| -4 | -7 | 18 | 31.60 | 3.80 |
| -4 | 7 | 18 | 27.60 | 4.60 |
| -3 | -7 | 18 | 116.59 | 7.80 |
| -3 | 7 | 18 | 118.59 | 8.60 |
| -2 | -7 | 18 | -2.50 | 1.50 |
| -2 | 7 | 18 | 1.20 | 2.50 |
| -1 | -7 | 18 | 670.43 | 37.00 |
| -1 | 7 | 18 | 672.23 | 37.60 |
| 0 | -7 | 18 | 53.79 | 4.80 |
| 0 | 7 | 18 | 58.99 | 5.80 |
| 1 | -7 | 18 | 2.60 | 1.70 |
| 1 | 7 | 18 | -0.50 | 2.10 |
| 2 | -7 | 18 | 5.60 | 1.80 |
| 2 | 7 | 18 | 6.30 | 2.30 |
| -7 | -8 | 18 | 4.50 | 2.40 |
| -7 | 8 | 18 | 5.80 | 1.80 |
| -6 | -8 | 18 | 575.14 | 32.80 |
| -6 | 8 | 18 | 623.64 | 33.30 |
| -5 | -8 | 18 | 48.10 | 4.50 |
| 5 | -8 | -18 | 56.59 | 5.70 |
| -5 | 8 | 18 | 52.29 | 5.00 |
| -4 | -8 | 18 | 3.20 | 1.70 |
| -4 | 8 | 18 | 3.80 | 2.30 |
| -3 | -8 | 18 | 2.10 | 1.60 |
| -3 | 8 | 18 | 0.60 | 2.30 |
| -2 | -8 | 18 | 528.15 | 30.10 |
| -2 | 8 | 18 | 562.24 | 30.80 |
| -1 | -8 | 18 | 14.50 | 3.00 |
| -1 | 8 | 18 | 13.20 | 2.80 |
| 0 | -8 | 18 | 197.78 | 11.50 |
| 0 | 8 | 18 | 188.88 | 12.10 |
| 1 | -8 | 18 | 14.90 | 2.90 |
| 1 | 8 | 18 | 7.40 | 2.50 |
| 5 | -9 | -18 | 143.39 | 8.90 |
| -5 | -9 | 18 | 126.09 | 8.20 |
| -5 | 9 | 18 | 132.09 | 8.60 |
| -4 | -9 | 18 | 34.10 | 3.50 |
| 4 | -9 | -18 | 26.60 | 4.50 |
| -4 | 9 | 18 | 36.70 | 4.30 |
| -3 | -9 | 18 | 133.19 | 8.00 |
| 3 | -9 | -18 | 128.89 | 8.20 |

| | | | | |
|---|---|---|---|---|
| -3 | 9 | 18 | 122.19 | 8.60 |
| 2 | -9 | -18 | 7.70 | 2.00 |
| -2 | -9 | 18 | 7.50 | 1.70 |
| -2 | 9 | 18 | 9.90 | 2.50 |
| -1 | -9 | 18 | 466.25 | 26.90 |
| 1 | -9 | -18 | 495.85 | 27.10 |
| -1 | 9 | 18 | 499.25 | 27.40 |
| -9 | 0 | 19 | 21.60 | 4.40 |
| -8 | 0 | 19 | 1.60 | 3.10 |
| -7 | 0 | 19 | 592.74 | 32.90 |
| -6 | 0 | 19 | 2.40 | 2.30 |
| -6 | 0 | 19 | 5.70 | 2.70 |
| -5 | 0 | 19 | 347.27 | 20.10 |
| -5 | 0 | 19 | 345.97 | 19.80 |
| -4 | 0 | 19 | 0.30 | 2.00 |
| -4 | 0 | 19 | -2.80 | 2.80 |
| -3 | 0 | 19 | 98.49 | 7.70 |
| -3 | 0 | 19 | 89.59 | 6.80 |
| -2 | 0 | 19 | -1.10 | 1.80 |
| -2 | 0 | 19 | -5.20 | 2.90 |
| -1 | 0 | 19 | 870.81 | 47.30 |
| -1 | 0 | 19 | 843.12 | 47.10 |
| 0 | 0 | 19 | -1.40 | 1.70 |
| 0 | 0 | 19 | -3.20 | 2.80 |
| 1 | 0 | 19 | -0.10 | 1.80 |
| 1 | 0 | 19 | 4.50 | 2.80 |
| 2 | 0 | 19 | -2.40 | 3.10 |
| 2 | 0 | 19 | 1.20 | 1.90 |
| -8 | 1 | 19 | 178.18 | 10.80 |
| -7 | -1 | 19 | 8.50 | 6.90 |
| -7 | 1 | 19 | 4.70 | 2.30 |
| -6 | -1 | 19 | 665.93 | 35.30 |
| -6 | -1 | 19 | 594.94 | 35.00 |
| -6 | 1 | 19 | 649.14 | 35.30 |
| -5 | -1 | 19 | 7.30 | 2.30 |
| -5 | -1 | 19 | 3.50 | 3.30 |
| -5 | 1 | 19 | 5.00 | 2.20 |
| -5 | 1 | 19 | 3.30 | 2.50 |
| -4 | -1 | 19 | 11.50 | 3.60 |
| -4 | -1 | 19 | 11.60 | 2.40 |
| -4 | 1 | 19 | 21.50 | 3.80 |
| -4 | 1 | 19 | 14.30 | 3.00 |
| -3 | -1 | 19 | 0.20 | 3.30 |
| -3 | -1 | 19 | 2.00 | 1.90 |

| | | | | |
|---|---|---|---|---|
| -3 | 1 | 19 | -2.00 | 2.70 |
| -3 | 1 | 19 | -0.90 | 1.80 |
| -2 | -1 | 19 | 524.95 | 29.80 |
| -2 | -1 | 19 | 490.55 | 30.00 |
| -2 | 1 | 19 | 588.84 | 30.30 |
| -2 | 1 | 19 | 534.25 | 29.80 |
| -1 | -1 | 19 | 1.60 | 1.90 |
| -1 | -1 | 19 | 1.30 | 3.40 |
| -1 | 1 | 19 | 0.70 | 1.70 |
| -1 | 1 | 19 | 1.90 | 2.90 |
| 0 | -1 | 19 | 218.28 | 14.00 |
| 0 | -1 | 19 | 246.58 | 14.70 |
| 0 | 1 | 19 | 228.48 | 14.10 |
| 0 | 1 | 19 | 256.37 | 14.50 |
| 1 | -1 | 19 | 0.70 | 3.70 |
| 1 | -1 | 19 | -1.90 | 1.80 |
| 1 | 1 | 19 | 0.60 | 1.80 |
| 1 | 1 | 19 | 1.30 | 2.60 |
| 2 | -1 | 19 | 126.99 | 8.10 |
| 2 | -1 | 19 | 113.69 | 8.90 |
| 2 | 1 | 19 | 130.39 | 8.20 |
| 2 | 1 | 19 | 109.69 | 8.20 |
| -9 | 2 | 19 | 14.10 | 3.20 |
| -8 | 2 | 19 | -1.30 | 1.80 |
| -7 | 2 | 19 | 558.34 | 31.00 |
| -6 | -2 | 19 | -1.00 | 2.10 |
| -6 | 2 | 19 | 2.20 | 2.20 |
| -5 | -2 | 19 | 161.18 | 9.80 |
| -5 | 2 | 19 | 145.69 | 9.90 |
| -4 | -2 | 19 | 1.20 | 1.80 |
| -4 | 2 | 19 | 0.50 | 2.30 |
| -4 | 2 | 19 | 1.30 | 2.00 |
| -3 | -2 | 19 | 143.59 | 9.00 |
| -3 | 2 | 19 | 134.79 | 9.00 |
| -2 | -2 | 19 | -0.20 | 1.80 |
| -2 | 2 | 19 | 1.20 | 2.60 |
| -2 | 2 | 19 | 1.60 | 1.80 |
| -1 | -2 | 19 | 683.43 | 37.50 |
| -1 | 2 | 19 | 685.63 | 37.90 |
| -1 | 2 | 19 | 667.03 | 37.50 |
| 0 | -2 | 19 | 1.30 | 1.70 |
| 0 | 2 | 19 | 0.80 | 1.90 |
| 0 | 2 | 19 | -0.30 | 2.50 |
| 1 | -2 | 19 | 25.30 | 3.80 |

| | | | | |
|---|---|---|---|---|
| 1 | 2 | 19 | 29.40 | 4.50 |
| 1 | 2 | 19 | 26.60 | 3.80 |
| 2 | -2 | 19 | 7.20 | 2.00 |
| 2 | 2 | 19 | 5.60 | 2.40 |
| -8 | 3 | 19 | 212.98 | 12.60 |
| -7 | 3 | 19 | 20.70 | 3.70 |
| -6 | -3 | 19 | 594.94 | 35.80 |
| -6 | 3 | 19 | 711.73 | 36.30 |
| -5 | -3 | 19 | -2.20 | 1.80 |
| -5 | 3 | 19 | 2.20 | 2.20 |
| -4 | -3 | 19 | 7.10 | 2.10 |
| -4 | 3 | 19 | 5.00 | 2.40 |
| -3 | -3 | 19 | 9.70 | 2.10 |
| -3 | 3 | 19 | 8.50 | 2.70 |
| -2 | -3 | 19 | 682.03 | 35.70 |
| -2 | 3 | 19 | 605.94 | 36.00 |
| -2 | 3 | 19 | 648.14 | 35.70 |
| -1 | -3 | 19 | 0.50 | 1.70 |
| -1 | 3 | 19 | -0.60 | 1.60 |
| -1 | 3 | 19 | -0.80 | 2.50 |
| 0 | -3 | 19 | 194.98 | 11.80 |
| 0 | 3 | 19 | 197.98 | 12.30 |
| 1 | -3 | 19 | 17.40 | 3.30 |
| 1 | 3 | 19 | 18.30 | 4.10 |
| 2 | -3 | 19 | 197.48 | 11.50 |
| 2 | 3 | 19 | 184.18 | 11.70 |
| -8 | 4 | 19 | 14.10 | 2.90 |
| -7 | 4 | 19 | 632.14 | 34.90 |
| -6 | -4 | 19 | -1.50 | 2.00 |
| -6 | 4 | 19 | 1.80 | 1.90 |
| -5 | -4 | 19 | 161.48 | 10.60 |
| -5 | 4 | 19 | 186.28 | 11.00 |
| -4 | -4 | 19 | 6.70 | 2.00 |
| -4 | 4 | 19 | 5.80 | 2.40 |
| -3 | -4 | 19 | 124.19 | 8.40 |
| -3 | 4 | 19 | 141.09 | 9.20 |
| -2 | -4 | 19 | 3.20 | 1.60 |
| -2 | 4 | 19 | 8.00 | 2.80 |
| -1 | -4 | 19 | 761.62 | 43.60 |
| -1 | 4 | 19 | 830.02 | 44.10 |
| 0 | -4 | 19 | 35.20 | 3.90 |
| 0 | 4 | 19 | 37.70 | 4.70 |
| 1 | -4 | 19 | 9.30 | 2.00 |
| 1 | 4 | 19 | 8.60 | 2.40 |

| | | | | |
|---|---|---|---|---|
| 2 | -4 | 19 | 15.80 | 3.20 |
| -7 | 5 | 19 | 6.90 | 1.90 |
| -6 | -5 | 19 | 664.23 | 34.10 |
| -6 | 5 | 19 | 572.54 | 34.20 |
| -5 | -5 | 19 | 17.10 | 3.50 |
| -5 | 5 | 19 | 11.30 | 2.40 |
| -4 | -5 | 19 | -2.00 | 1.60 |
| -4 | 5 | 19 | 0.50 | 2.00 |
| -3 | -5 | 19 | 31.90 | 3.70 |
| -3 | 5 | 19 | 28.70 | 4.40 |
| -2 | -5 | 19 | 570.64 | 30.70 |
| -2 | 5 | 19 | 536.05 | 31.10 |
| -1 | -5 | 19 | 4.30 | 1.60 |
| -1 | 5 | 19 | 5.80 | 2.30 |
| 0 | -5 | 19 | 231.18 | 13.60 |
| 0 | 5 | 19 | 236.68 | 14.10 |
| 1 | -5 | 19 | 17.90 | 3.10 |
| 1 | 5 | 19 | 20.80 | 3.60 |
| -6 | -6 | 19 | 1.40 | 1.90 |
| -6 | 6 | 19 | 1.90 | 1.70 |
| -5 | -6 | 19 | 131.29 | 8.60 |
| -5 | 6 | 19 | 148.39 | 9.00 |
| -4 | -6 | 19 | 23.40 | 3.50 |
| -4 | 6 | 19 | 26.50 | 3.90 |
| -3 | -6 | 19 | 118.49 | 7.60 |
| -3 | 6 | 19 | 117.99 | 8.10 |
| -2 | -6 | 19 | 15.00 | 2.90 |
| -2 | 6 | 19 | 9.70 | 2.50 |
| -1 | -6 | 19 | 644.44 | 34.50 |
| -1 | 6 | 19 | 608.74 | 34.80 |
| 0 | -6 | 19 | 17.60 | 2.90 |
| 0 | 6 | 19 | 24.80 | 3.80 |
| 5 | -7 | -19 | 33.70 | 5.00 |
| -5 | -7 | 19 | 36.90 | 5.10 |
| -5 | 7 | 19 | 26.70 | 3.50 |
| -4 | -7 | 19 | 0.70 | 1.60 |
| -4 | 7 | 19 | 2.10 | 1.80 |
| -3 | -7 | 19 | 22.20 | 2.90 |
| -3 | 7 | 19 | 15.90 | 3.40 |
| -2 | -7 | 19 | 360.66 | 19.90 |
| -2 | 7 | 19 | 350.66 | 20.30 |
| -6 | 0 | 20 | 425.56 | 23.80 |
| -5 | 0 | 20 | -1.00 | 2.80 |
| -5 | 0 | 20 | 0.20 | 2.30 |

| | | | | |
|---|---|---|---|---|
| -4 | 0 | 20 | 0.70 | 1.70 |
| -4 | 0 | 20 | -2.30 | 2.70 |
| -3 | 0 | 20 | -0.60 | 3.20 |
| -3 | 0 | 20 | -1.90 | 1.50 |
| -2 | 0 | 20 | 480.35 | 26.40 |
| -2 | 0 | 20 | 464.15 | 26.20 |
| -1 | 0 | 20 | -1.10 | 1.40 |
| -1 | 0 | 20 | 2.50 | 2.80 |
| -6 | 1 | 20 | 8.80 | 2.30 |
| -5 | -1 | 20 | 139.79 | 9.30 |
| -5 | 1 | 20 | 150.18 | 10.10 |
| -4 | -1 | 20 | 2.40 | 1.70 |
| -4 | 1 | 20 | 0.40 | 1.70 |
| -4 | 1 | 20 | 0.60 | 2.40 |
| -3 | -1 | 20 | 98.39 | 6.40 |
| -3 | 1 | 20 | 86.79 | 6.70 |
| -3 | 1 | 20 | 93.59 | 6.30 |
| -2 | -1 | 20 | -1.80 | 1.40 |
| -2 | 1 | 20 | 0.50 | 1.50 |
| -2 | 1 | 20 | 2.90 | 2.60 |
| -1 | -1 | 20 | 578.24 | 32.20 |
| -1 | 1 | 20 | 570.24 | 32.20 |
| -1 | 1 | 20 | 608.84 | 33.10 |
| -6 | -2 | 20 | 374.66 | 20.90 |
| -5 | -2 | 20 | 4.70 | 2.70 |
| -5 | 2 | 20 | -0.30 | 1.90 |
| -4 | -2 | 20 | 1.50 | 1.70 |
| -4 | 2 | 20 | 0.40 | 2.00 |
| -3 | -2 | 20 | 1.20 | 1.60 |
| -3 | 2 | 20 | 0.70 | 2.10 |
| -2 | -2 | 20 | 405.06 | 22.40 |
| -2 | 2 | 20 | 397.66 | 22.60 |
| -1 | -2 | 20 | 4.10 | 1.50 |
| -1 | 2 | 20 | 5.10 | 2.30 |
| -5 | -3 | 20 | 96.09 | 7.40 |
| -5 | 3 | 20 | 95.39 | 6.50 |
| -4 | -3 | 20 | 2.40 | 1.60 |
| -4 | 3 | 20 | -2.30 | 1.80 |
| -3 | -3 | 20 | 115.59 | 7.40 |
| -3 | 3 | 20 | 117.29 | 7.70 |
| -2 | -3 | 20 | 0.30 | 1.40 |
| -2 | 3 | 20 | -2.10 | 2.00 |
| -3 | -4 | 20 | 16.70 | 2.90 |
| 0 | 0 | 0 | 0.00 | 0.00 |

```
_computing_structure_solution     'SHELXT 2014/5 (Sheldrick, 2014)'
;
_shelx_hkl_checksum               55027
_olex2_submission_special_instructions  'No  special  instructions  were
received'
```
