# Supplementary material for: Structure-property relationships of photofunctional diiridium(II) complexes with tetracationic charge and an unsupported Ir–Ir bond
Source: Commun Chem. 2022 Nov 23;5:159. doi: 10.1038/s42004-022-00775-4 (PMC9814866; doi:10.1038/s42004-022-00775-4)
Supplement: Supplementary file 5 — Supplementary Data 2 [file 42004_2022_775_MOESM5_ESM.pdf]

```
data_[Ir(tBu-terpy)(PPh3)(CO)2]2[OTf]4

_audit_creation_method            'SHELXL-2014/7'
_shelx_SHELXL_version_number      '2014/7'
_chemical_name_systematic         ?
_chemical_name_common             ?
_chemical_melting_point           ?
_chemical_formula_moiety          ?
_chemical_formula_sum
 'C50 H53 F6 Ir N4 O7 P S2'
_chemical_formula_weight          1223.25

loop_
 _atom_type_symbol
 _atom_type_description
 _atom_type_scat_dispersion_real
 _atom_type_scat_dispersion_imag
 _atom_type_scat_source
 'C'  'C'   0.0033   0.0016
 'International Tables Vol C Tables 4.2.6.8 and 6.1.1.4'
 'H'  'H'   0.0000   0.0000
 'International Tables Vol C Tables 4.2.6.8 and 6.1.1.4'
 'N'  'N'   0.0061   0.0033
 'International Tables Vol C Tables 4.2.6.8 and 6.1.1.4'
 'O'  'O'   0.0106   0.0060
 'International Tables Vol C Tables 4.2.6.8 and 6.1.1.4'
 'P'  'P'   0.1023   0.0942
 'International Tables Vol C Tables 4.2.6.8 and 6.1.1.4'
 'Ir'  'Ir'  -1.4442   7.9887
 'International Tables Vol C Tables 4.2.6.8 and 6.1.1.4'
 'F'  'F'   0.0171   0.0103
 'International Tables Vol C Tables 4.2.6.8 and 6.1.1.4'
 'S'  'S'   0.1246   0.1234
 'International Tables Vol C Tables 4.2.6.8 and 6.1.1.4'

_space_group_crystal_system       monoclinic
_space_group_IT_number            14
_space_group_name_H-M_alt         'P 21/n'
_space_group_name_Hall            '-P 2yn'

_shelx_space_group_comment
;
The symmetry employed for this shelxl refinement is uniquely defined
```

```
by the following loop, which should always be used as a source of
symmetry information in preference to the above space-group names.
They are only intended as comments.
;

loop_
 _space_group_symop_operation_xyz
 'x, y, z'
 '-x+1/2, y+1/2, -z+1/2'
 '-x, -y, -z'
 'x-1/2, -y-1/2, z-1/2'

_cell_length_a                    16.4301(19)
_cell_length_b                    17.620(2)
_cell_length_c                    17.470(2)
_cell_angle_alpha                 90
_cell_angle_beta                  95.138(4)
_cell_angle_gamma                 90
_cell_volume                      5037.4(10)
_cell_formula_units_Z             4
_cell_measurement_temperature     150(2)
_cell_measurement_reflns_used     9867
_cell_measurement_theta_min       2.31
_cell_measurement_theta_max       27.51

_exptl_crystal_description        orange
_exptl_crystal_colour             block
_exptl_crystal_density_meas       ?
_exptl_crystal_density_method     ?
_exptl_crystal_density_diffrn     1.613
_exptl_crystal_F_000              2460
_exptl_transmission_factor_min    ?
_exptl_transmission_factor_max    ?
_exptl_crystal_size_max           0.210
_exptl_crystal_size_mid           0.190
_exptl_crystal_size_min           0.170
_exptl_absorpt_coefficient_mu     2.842
_shelx_estimated_absorpt_T_min    0.587
_shelx_estimated_absorpt_T_max    0.644
_exptl_absorpt_correction_type    multi-scan
_exptl_absorpt_correction_T_min   0.5830
_exptl_absorpt_correction_T_max   0.7456
_exptl_absorpt_process_details
```

```
;
Bruker APEX2
Empirical absorption correction using spherical harmonics,
 implemented in SCALE3 ABSPACK scaling algorithm.
;

_exptl_absorpt_special_details    ?
_diffrn_ambient_temperature       150(2)
_diffrn_radiation_wavelength      0.710
_diffrn_radiation_type            MoK\a
_diffrn_source                    'Enhance (Mo) X-ray Source'
_diffrn_measurement_device_type   'Bruker APEX-II CCD'
_diffrn_measurement_method        '\f and \w scans'
_diffrn_detector_area_resol_mean  ?
_diffrn_reflns_number             108375
_diffrn_reflns_av_unetI/netI      0.0262
_diffrn_reflns_av_R_equivalents   0.0494
_diffrn_reflns_limit_h_min        -21
_diffrn_reflns_limit_h_max        21
_diffrn_reflns_limit_k_min        -22
_diffrn_reflns_limit_k_max        22
_diffrn_reflns_limit_l_min        -22
_diffrn_reflns_limit_l_max        22
_diffrn_reflns_theta_min          2.123
_diffrn_reflns_theta_max          27.505
_diffrn_reflns_theta_full         25.214
_diffrn_measured_fraction_theta_max   0.998
_diffrn_measured_fraction_theta_full  0.999
_diffrn_reflns_Laue_measured_fraction_max    0.998
_diffrn_reflns_Laue_measured_fraction_full   0.999
_diffrn_reflns_point_group_measured_fraction_max   0.998
_diffrn_reflns_point_group_measured_fraction_full  0.999
_reflns_number_total              11594
_reflns_number_gt                 10061
_reflns_threshold_expression      'I > 2\s(I)'
_reflns_Friedel_coverage          0.000
_reflns_Friedel_fraction_max      .
_reflns_Friedel_fraction_full     .

_reflns_special_details
;
 Reflections were merged by SHELXL according to the crystal
 class for the calculation of statistics and refinement.
```

```
 _reflns_Friedel_fraction is defined as the number of unique
 Friedel pairs measured divided by the number that would be
 possible theoretically, ignoring centric projections and
 systematic absences.
;

_computing_data_collection        'Bruker APEX2'
_computing_cell_refinement        'Bruker SAINT'
_computing_data_reduction         'Bruker SAINT'
_computing_structure_solution     'SHELXS-97 (Sheldrick 2008)'
_computing_structure_refinement 'SHELXL-2014/7 (Sheldrick, 2014)'
_computing_molecular_graphics     'Bruker SHELXTL'
_computing_publication_material   'Bruker SHELXTL'
_refine_special_details           ?
_refine_ls_structure_factor_coef  Fsqd
_refine_ls_matrix_type            full
_refine_ls_weighting_scheme       calc
_refine_ls_weighting_details
'w=1/[\s^2^(Fo^2^)+(0.0251P)^2^+2.9858P] where P=(Fo^2^+2Fc^2^)/3'
_atom_sites_solution_primary      ?
_atom_sites_solution_secondary    ?
_atom_sites_solution_hydrogens    geom
_refine_ls_hydrogen_treatment     constr
_refine_ls_extinction_method      none
_refine_ls_extinction_coef        .
_refine_ls_number_reflns          11594
_refine_ls_number_parameters      641
_refine_ls_number_restraints      0
_refine_ls_R_factor_all           0.0291
_refine_ls_R_factor_gt            0.0206
_refine_ls_wR_factor_ref          0.0513
_refine_ls_wR_factor_gt           0.0485
_refine_ls_goodness_of_fit_ref    1.051
_refine_ls_restrained_S_all       1.051
_refine_ls_shift/su_max           0.008
_refine_ls_shift/su_mean          0.000

loop_
 _atom_site_label
 _atom_site_type_symbol
 _atom_site_fract_x
 _atom_site_fract_y
 _atom_site_fract_z
 _atom_site_U_iso_or_equiv
```

_atom_site_adp_type
_atom_site_occupancy
_atom_site_site_symmetry_order
_atom_site_calc_flag
_atom_site_refinement_flags_posn
_atom_site_refinement_flags_adp
_atom_site_refinement_flags_occupancy
_atom_site_disorder_assembly
_atom_site_disorder_group
Ir1 Ir 1.00447(2) 0.51400(2) 0.58189(2) 0.00961(3) Uani 1 1 d . . . . .
S1 S 0.85794(4) 0.81488(3) 0.51907(4) 0.02143(13) Uani 1 1 d . . . . .
S2 S 1.33454(4) 0.65665(3) 0.56444(3) 0.02084(12) Uani 1 1 d . . . . .
P1 P 1.00639(3) 0.53273(3) 0.72077(3) 0.01287(11) Uani 1 1 d . . . . .
F5 F 1.40059(11) 0.72454(12) 0.68884(10) 0.0567(5) Uani 1 1 d . . . . .
F6 F 1.46533(10) 0.74114(10) 0.58827(10) 0.0465(4) Uani 1 1 d . . . . .
F1 F 0.81809(11) 0.95685(9) 0.54084(11) 0.0479(5) Uani 1 1 d . . . . .
F4 F 1.35267(11) 0.80031(10) 0.59981(12) 0.0566(5) Uani 1 1 d . . . . .
F2 F 0.89412(12) 0.90925(10) 0.63393(9) 0.0497(5) Uani 1 1 d . . . . .
F3 F 0.94563(11) 0.93936(10) 0.52960(11) 0.0524(5) Uani 1 1 d . . . . .
O2 O 0.84802(12) 0.82871(10) 0.43767(10) 0.0312(4) Uani 1 1 d . . . . .
O4 O 0.92936(10) 0.77107(10) 0.54450(10) 0.0282(4) Uani 1 1 d . . . . .
O5 O 1.33792(12) 0.67692(11) 0.48469(10) 0.0344(5) Uani 1 1 d . . . . .
O3 O 0.78507(11) 0.79359(11) 0.55398(12) 0.0362(5) Uani 1 1 d . . . . .
O7 O 1.38089(11) 0.59077(10) 0.58940(12) 0.0375(5) Uani 1 1 d . . . . .
O6 O 1.25453(10) 0.66133(10) 0.59190(11) 0.0319(4) Uani 1 1 d . . . . .
O1 O 1.07365(9) 0.67190(9) 0.56646(9) 0.0181(3) Uani 1 1 d . . . . .
C36 C 0.92921(15) 0.68951(15) 0.86849(14) 0.0261(6) Uani 1 1 d . . . . .
H36A H 0.9232 0.6913 0.9220 0.031 Uiso 1 1 calc R U . . .
N2 N 0.96404(10) 0.40858(10) 0.59333(10) 0.0112(4) Uani 1 1 d . . . . .
C27 C 1.39817(15) 0.37265(16) 0.70367(18) 0.0350(7) Uani 1 1 d . . . . .
H27A H 1.4433 0.3418 0.7273 0.053 Uiso 1 1 calc R U . . .
H27B H 1.4132 0.3935 0.6549 0.053 Uiso 1 1 calc R U . . .
H27C H 1.3868 0.4143 0.7384 0.053 Uiso 1 1 calc R U . . .
C23 C 0.93924(15) 0.12468(13) 0.63155(15) 0.0242(5) Uani 1 1 d . . . . .
H23A H 0.9177 0.0729 0.6264 0.036 Uiso 1 1 calc R U . . .
H23B H 0.9904 0.1280 0.6069 0.036 Uiso 1 1 calc R U . . .
H23C H 0.9496 0.1375 0.6861 0.036 Uiso 1 1 calc R U . . .
N1 N 0.88060(10) 0.53209(10) 0.57261(10) 0.0114(4) Uani 1 1 d . . . . .
N3 N 1.11317(10) 0.45605(10) 0.59780(10) 0.0116(3) Uani 1 1 d . . . . .
C17 C 0.61300(16) 0.61210(17) 0.66036(16) 0.0332(6) Uani 1 1 d . . . . .
H17A H 0.5552 0.6182 0.6686 0.050 Uiso 1 1 calc R U . . .
H17B H 0.6436 0.5965 0.7085 0.050 Uiso 1 1 calc R U . . .
H17C H 0.6347 0.6604 0.6433 0.050 Uiso 1 1 calc R U . . .
C16 C 0.62175(13) 0.55122(13) 0.59867(13) 0.0184(5) Uani 1 1 d . . . . .

C5 C 0.83546(13) 0.46792(12) 0.58282(12) 0.0113(4) Uani 1 1 d . . . . .
C19 C 0.58413(15) 0.47735(15) 0.62551(16) 0.0264(6) Uani 1 1 d . . . . .
H19A H 0.5264 0.4858 0.6328 0.040 Uiso 1 1 calc R U . . .
H19B H 0.5887 0.4378 0.5867 0.040 Uiso 1 1 calc R U . . .
H19C H 0.6132 0.4612 0.6742 0.040 Uiso 1 1 calc R U . . .
C25 C 1.29888(16) 0.29137(16) 0.76599(15) 0.0301(6) Uani 1 1 d . . . . .
H25A H 1.3447 0.2618 0.7903 0.045 Uiso 1 1 calc R U . . .
H25B H 1.2865 0.3334 0.7998 0.045 Uiso 1 1 calc R U . . .
H25C H 1.2508 0.2586 0.7571 0.045 Uiso 1 1 calc R U . . .
C8 C 0.90824(13) 0.26207(12) 0.59947(12) 0.0149(4) Uani 1 1 d . . . . .
C4 C 0.75203(13) 0.47237(12) 0.58944(12) 0.0138(4) Uani 1 1 d . . . . .
H4A H 0.7219 0.4272 0.5963 0.017 Uiso 1 1 calc R U . . .
C10 C 1.01928(13) 0.35201(12) 0.60496(12) 0.0123(4) Uani 1 1 d . . . . .
C50 C 1.04613(12) 0.61326(13) 0.56959(11) 0.0128(4) Uani 1 1 d . . . . .
C31 C 0.82756(17) 0.35302(17) 0.79992(15) 0.0346(7) Uani 1 1 d . . . . .
H31A H 0.7897 0.3160 0.8142 0.041 Uiso 1 1 calc R U . . .
C18 C 0.57641(14) 0.57680(15) 0.52256(14) 0.0249(5) Uani 1 1 d . . . . .
H18A H 0.5182 0.5831 0.5292 0.037 Uiso 1 1 calc R U . . .
H18B H 0.5990 0.6252 0.5068 0.037 Uiso 1 1 calc R U . . .
H18C H 0.5831 0.5384 0.4830 0.037 Uiso 1 1 calc R U . . .
C35 C 0.96005(14) 0.62494(14) 0.83660(13) 0.0213(5) Uani 1 1 d . . . . .
H35A H 0.9757 0.5827 0.8683 0.026 Uiso 1 1 calc R U . . .
C34 C 0.96826(13) 0.62182(13) 0.75738(13) 0.0164(5) Uani 1 1 d . . . . .
C20 C 0.87678(14) 0.18037(13) 0.59275(14) 0.0191(5) Uani 1 1 d . . . . .
C3 C 0.71180(13) 0.54208(13) 0.58619(12) 0.0149(4) Uani 1 1 d . . . . .
C9 C 0.99211(13) 0.27760(12) 0.60756(12) 0.0144(4) Uani 1 1 d . . . . .
H9A H 1.0304 0.2372 0.6148 0.017 Uiso 1 1 calc R U . . .
C32 C 0.80337(16) 0.42767(17) 0.78892(14) 0.0303(6) Uani 1 1 d . . . . .
H32A H 0.7487 0.4419 0.7956 0.036 Uiso 1 1 calc R U . . .
C13 C 1.24956(13) 0.36898(13) 0.65196(12) 0.0160(5) Uani 1 1 d . . . . .
C43 C 1.26778(16) 0.52540(15) 0.83987(16) 0.0310(6) Uani 1 1 d . . . . .
H43A H 1.3222 0.5246 0.8634 0.037 Uiso 1 1 calc R U . . .
C11 C 1.10456(13) 0.37997(12) 0.61354(12) 0.0128(4) Uani 1 1 d . . . . .
C41 C 1.21041(17) 0.47533(15) 0.86352(16) 0.0325(6) Uani 1 1 d . . . . .
H41A H 1.2257 0.4405 0.9038 0.039 Uiso 1 1 calc R U . . .
C12 C 1.17114(13) 0.33692(13) 0.64025(13) 0.0161(5) Uani 1 1 d . . . . .
H12A H 1.1639 0.2846 0.6509 0.019 Uiso 1 1 calc R U . . .
C39 C 0.94701(14) 0.68427(13) 0.71208(13) 0.0189(5) Uani 1 1 d . . . . .
H39A H 0.9537 0.6832 0.6586 0.023 Uiso 1 1 calc R U . . .
C21 C 0.79584(16) 0.17222(14) 0.62958(18) 0.0330(6) Uani 1 1 d . . . . .
H21A H 0.7768 0.1196 0.6247 0.049 Uiso 1 1 calc R U . . .
H21B H 0.8042 0.1859 0.6841 0.049 Uiso 1 1 calc R U . . .
H21C H 0.7549 0.2060 0.6035 0.049 Uiso 1 1 calc R U . . .
C24 C 1.32183(14) 0.32311(13) 0.68887(14) 0.0207(5) Uani 1 1 d . . . . .

C26 C 1.34015(15) 0.25756(15) 0.63551(15) 0.0273(6) Uani 1 1 d . . . . .
H26A H 1.3864 0.2280 0.6590 0.041 Uiso 1 1 calc R U . . .
H26B H 1.2920 0.2247 0.6274 0.041 Uiso 1 1 calc R U . . .
H26C H 1.3538 0.2779 0.5860 0.041 Uiso 1 1 calc R U . . .
C6 C 0.88270(12) 0.39635(12) 0.59004(11) 0.0121(4) Uani 1 1 d . . . . .
C44 C 1.24561(15) 0.57664(15) 0.78179(15) 0.0264(6) Uani 1 1 d . . . . .
H44A H 1.2845 0.6119 0.7659 0.032 Uiso 1 1 calc R U . . .
C45 C 1.16677(14) 0.57655(14) 0.74691(13) 0.0201(5) Uani 1 1 d . . . . .
H45A H 1.1518 0.6117 0.7068 0.024 Uiso 1 1 calc R U . . .
C38 C 0.91579(15) 0.74890(14) 0.74501(15) 0.0259(5) Uani 1 1 d . . . . .
H38A H 0.9004 0.7915 0.7137 0.031 Uiso 1 1 calc R U . . .
C33 C 0.85853(15) 0.48195(15) 0.76823(14) 0.0227(5) Uani 1 1 d . . . . .
H33A H 0.8413 0.5330 0.7605 0.027 Uiso 1 1 calc R U . . .
C40 C 1.10867(13) 0.52535(12) 0.76988(13) 0.0163(5) Uani 1 1 d . . . . .
C37 C 0.90713(15) 0.75123(15) 0.82282(15) 0.0270(6) Uani 1 1 d . . . . .
H37A H 0.8859 0.7955 0.8449 0.032 Uiso 1 1 calc R U . . .
C42 C 1.13098(16) 0.47513(14) 0.82948(14) 0.0249(6) Uani 1 1 d . . . . .
H42A H 1.0919 0.4408 0.8468 0.030 Uiso 1 1 calc R U . . .
C28 C 0.93947(14) 0.46190(13) 0.75869(12) 0.0167(5) Uani 1 1 d . . . . .
C2 C 0.75871(13) 0.60581(13) 0.57126(12) 0.0163(5) Uani 1 1 d . . . . .
H2A H 0.7332 0.6541 0.5654 0.020 Uiso 1 1 calc R U . . .
C47 C 1.39076(16) 0.73464(16) 0.61283(16) 0.0311(6) Uani 1 1 d . . . . .
C46 C 0.88075(17) 0.90960(15) 0.55765(15) 0.0297(6) Uani 1 1 d . . . . .
C7 C 0.85327(13) 0.32314(12) 0.59261(12) 0.0141(4) Uani 1 1 d . . . . .
H7A H 0.7961 0.3141 0.5898 0.017 Uiso 1 1 calc R U . . .
C30 C 0.90728(17) 0.33250(15) 0.79005(14) 0.0291(6) Uani 1 1 d . . . . .
H30A H 0.9239 0.2812 0.7973 0.035 Uiso 1 1 calc R U . . .
C14 C 1.25700(13) 0.44412(13) 0.62955(12) 0.0158(5) Uani 1 1 d . . . . .
H14A H 1.3095 0.4671 0.6320 0.019 Uiso 1 1 calc R U . . .
C1 C 0.84107(13) 0.59930(12) 0.56495(12) 0.0137(4) Uani 1 1 d . . . . .
H1A H 0.8713 0.6436 0.5548 0.016 Uiso 1 1 calc R U . . .
C15 C 1.18863(13) 0.48561(13) 0.60372(12) 0.0140(4) Uani 1 1 d . . . . .
H15A H 1.1954 0.5371 0.5896 0.017 Uiso 1 1 calc R U . . .
C22 C 0.86302(17) 0.16224(15) 0.50644(15) 0.0315(6) Uani 1 1 d . . . . .
H22A H 0.8428 0.1102 0.4996 0.047 Uiso 1 1 calc R U . . .
H22B H 0.8228 0.1976 0.4818 0.047 Uiso 1 1 calc R U . . .
H22C H 0.9147 0.1674 0.4830 0.047 Uiso 1 1 calc R U . . .
C29 C 0.96318(15) 0.38637(14) 0.76957(13) 0.0216(5) Uani 1 1 d . . . . .
H29A H 1.0178 0.3717 0.7630 0.026 Uiso 1 1 calc R U . . .
C49 C 0.8842(2) 0.3922(2) 1.0015(2) 0.0663(11) Uani 1 1 d . . . . .
H49A H 0.8783 0.3844 1.0563 0.099 Uiso 1 1 calc R U . . .
H49B H 0.9324 0.3647 0.9871 0.099 Uiso 1 1 calc R U . . .
H49C H 0.8354 0.3731 0.9712 0.099 Uiso 1 1 calc R U . . .
C48 C 0.8932(4) 0.4695(3) 0.9870(2) 0.0982(19) Uani 1 1 d . . . . .

```
N4 N 0.8975(5) 0.5349(3) 0.9781(3) 0.177(3) Uani 1 1 d . . . . .

loop_
 _atom_site_aniso_label
 _atom_site_aniso_U_11
 _atom_site_aniso_U_22
 _atom_site_aniso_U_33
 _atom_site_aniso_U_23
 _atom_site_aniso_U_13
 _atom_site_aniso_U_12
Ir1 0.00896(4) 0.00856(5) 0.01131(4) 0.00010(3) 0.00087(3) -0.00032(3)
S1 0.0235(3) 0.0140(3) 0.0270(3) 0.0003(2) 0.0036(2) 0.0021(2)
S2 0.0186(3) 0.0181(3) 0.0260(3) -0.0028(2) 0.0034(2) -0.0036(2)
P1 0.0132(3) 0.0139(3) 0.0115(3) 0.0000(2) 0.0008(2) -0.0009(2)
F5 0.0557(12) 0.0816(15) 0.0316(10) -0.0216(10) -0.0019(8) -0.0146(10)
F6 0.0259(9) 0.0532(11) 0.0606(11) -0.0147(9) 0.0056(8) -0.0196(8)
F1 0.0644(12) 0.0196(8) 0.0598(12) -0.0034(8) 0.0063(9) 0.0146(8)
F4 0.0549(12) 0.0213(9) 0.0941(15) -0.0172(9) 0.0088(11) -0.0015(8)
F2 0.0850(14) 0.0303(10) 0.0331(9) -0.0076(8) 0.0002(9) -0.0038(9)
F3 0.0590(12) 0.0322(10) 0.0700(13) -0.0112(9) 0.0272(10) -0.0217(8)
O2 0.0429(11) 0.0236(10) 0.0261(10) 0.0010(8) -0.0021(8) 0.0060(8)
O4 0.0284(10) 0.0230(10) 0.0330(10) 0.0042(8) 0.0013(8) 0.0066(7)
O5 0.0364(11) 0.0429(12) 0.0236(9) -0.0022(8) 0.0011(8) -0.0138(9)
O3 0.0310(10) 0.0256(10) 0.0544(13) 0.0008(9) 0.0168(9) -0.0018(8)
O7 0.0336(11) 0.0227(10) 0.0573(13) 0.0043(9) 0.0111(9) 0.0066(8)
O6 0.0213(9) 0.0303(10) 0.0455(11) 0.0014(9) 0.0106(8) -0.0039(8)
O1 0.0192(8) 0.0127(8) 0.0224(8) -0.0001(7) 0.0026(7) -0.0040(6)
C36 0.0268(13) 0.0321(15) 0.0202(12) -0.0080(11) 0.0069(10) -0.0011(11)
N2 0.0114(9) 0.0103(9) 0.0121(9) -0.0006(7) 0.0020(7) -0.0003(7)
C27 0.0176(13) 0.0307(15) 0.0542(18) 0.0089(14) -0.0110(12) 0.0015(11)
C23 0.0272(13) 0.0115(12) 0.0339(14) 0.0042(10) 0.0028(11) -0.0021(10)
N1 0.0099(8) 0.0123(9) 0.0118(9) -0.0004(7) 0.0000(7) -0.0004(7)
N3 0.0105(8) 0.0113(9) 0.0130(9) -0.0007(7) 0.0006(7) 0.0011(7)
C17 0.0219(13) 0.0423(17) 0.0364(15) -0.0155(13) 0.0089(11) 0.0020(12)
C16 0.0105(10) 0.0228(13) 0.0224(12) -0.0033(10) 0.0041(9) 0.0018(9)
C5 0.0112(10) 0.0122(11) 0.0105(10) -0.0005(8) 0.0014(8) -0.0023(8)
C19 0.0139(12) 0.0325(15) 0.0335(14) 0.0056(11) 0.0067(10) 0.0012(10)
C25 0.0290(14) 0.0333(15) 0.0270(14) 0.0084(12) -0.0025(11) 0.0077(11)
C8 0.0185(11) 0.0137(11) 0.0130(10) 0.0003(9) 0.0034(9) -0.0028(9)
C4 0.0126(10) 0.0156(12) 0.0133(10) -0.0016(8) 0.0010(8) -0.0025(8)
C10 0.0132(10) 0.0117(11) 0.0119(10) 0.0014(8) 0.0011(8) 0.0007(8)
C50 0.0109(10) 0.0189(12) 0.0085(10) -0.0007(8) 0.0015(8) 0.0044(9)
C31 0.0365(16) 0.0436(18) 0.0236(14) 0.0081(12) 0.0030(11) -0.0226(13)
C18 0.0148(11) 0.0292(14) 0.0302(13) 0.0041(11) -0.0007(10) 0.0018(10)
```

C35 0.0227(12) 0.0233(13) 0.0179(12) -0.0013(10) 0.0025(9) -0.0012(10)
C34 0.0140(11) 0.0180(12) 0.0174(11) -0.0040(9) 0.0030(9) -0.0025(9)
C20 0.0182(11) 0.0111(11) 0.0280(13) 0.0011(10) 0.0022(10) -0.0042(9)
C3 0.0114(10) 0.0199(11) 0.0132(10) -0.0024(9) 0.0006(8) 0.0001(9)
C9 0.0154(11) 0.0126(11) 0.0152(11) 0.0011(9) 0.0015(8) 0.0015(8)
C32 0.0211(13) 0.0469(18) 0.0233(13) 0.0019(12) 0.0057(10) -0.0105(12)
C13 0.0147(11) 0.0175(12) 0.0160(11) -0.0009(9) 0.0025(9) 0.0021(9)
C43 0.0205(13) 0.0340(16) 0.0361(15) -0.0109(12) -0.0114(11) 0.0028(11)
C11 0.0140(10) 0.0132(11) 0.0116(10) 0.0009(8) 0.0025(8) -0.0009(8)
C41 0.0337(15) 0.0299(16) 0.0309(15) 0.0045(12) -0.0140(12) 0.0029(11)
C12 0.0160(11) 0.0132(11) 0.0193(11) 0.0023(9) 0.0028(9) 0.0011(8)
C39 0.0200(12) 0.0179(12) 0.0192(12) -0.0016(9) 0.0030(9) 0.0001(9)
C21 0.0250(14) 0.0160(13) 0.0592(19) 0.0065(12) 0.0107(13) -0.0058(10)
C24 0.0137(11) 0.0200(12) 0.0278(13) 0.0038(10) -0.0010(9) 0.0026(9)
C26 0.0224(13) 0.0258(14) 0.0338(14) 0.0055(11) 0.0043(11) 0.0084(10)
C6 0.0118(10) 0.0146(11) 0.0100(10) -0.0004(8) 0.0019(8) 0.0002(8)
C44 0.0200(12) 0.0284(14) 0.0304(14) -0.0078(11) -0.0004(10) -0.0049(10)
C45 0.0203(12) 0.0196(12) 0.0197(12) -0.0027(10) -0.0026(9) -0.0012(9)
C38 0.0284(14) 0.0171(13) 0.0323(14) -0.0009(11) 0.0042(11) 0.0036(10)
C33 0.0222(12) 0.0282(14) 0.0180(12) 0.0004(10) 0.0037(10) -0.0021(10)
C40 0.0155(11) 0.0170(12) 0.0156(11) -0.0048(9) -0.0023(9) 0.0011(8)
C37 0.0277(14) 0.0213(13) 0.0335(14) -0.0107(11) 0.0107(11) 0.0027(10)
C42 0.0257(13) 0.0240(14) 0.0239(13) 0.0032(10) -0.0045(10) -0.0024(10)
C28 0.0200(12) 0.0199(12) 0.0099(10) 0.0006(9) -0.0005(9) -0.0037(9)
C2 0.0158(11) 0.0147(11) 0.0182(11) -0.0020(9) 0.0010(9) 0.0037(9)
C47 0.0267(14) 0.0321(16) 0.0345(15) -0.0097(12) 0.0017(11) -0.0055(11)
C46 0.0419(16) 0.0197(13) 0.0282(14) -0.0011(11) 0.0074(12) -0.0014(11)
C7 0.0129(10) 0.0167(11) 0.0129(10) -0.0006(9) 0.0022(8) -0.0022(8)
C30 0.0408(16) 0.0241(14) 0.0213(13) 0.0076(11) -0.0035(11) -0.0104(11)
C14 0.0114(10) 0.0183(12) 0.0175(11) -0.0009(9) 0.0010(8) -0.0006(8)
C1 0.0162(11) 0.0104(11) 0.0143(10) -0.0009(8) 0.0001(8) -0.0006(8)
C15 0.0140(11) 0.0134(11) 0.0148(10) -0.0005(9) 0.0026(8) -0.0021(8)
C22 0.0436(17) 0.0169(13) 0.0320(15) -0.0046(11) -0.0085(12) -0.0075(11)
C29 0.0263(13) 0.0221(13) 0.0160(11) 0.0022(10) -0.0005(9) -0.0039(10)
C49 0.071(3) 0.074(3) 0.050(2) 0.000(2) -0.0136(19) -0.015(2)
C48 0.156(5) 0.101(4) 0.040(2) 0.007(2) 0.023(3) -0.062(4)
N4 0.336(9) 0.117(4) 0.095(4) 0.007(3) 0.107(5) -0.085(5)

_geom_special_details
;
All esds (except the esd in the dihedral angle between two l.s. planes)
are estimated using the full covariance matrix. The cell esds are taken
into account individually in the estimation of esds in distances,

```
angles
 and torsion angles; correlations between esds in cell parameters are
only
 used when they are defined by crystal symmetry.  An approximate
(isotropic)
 treatment of cell esds is used for estimating esds involving l.s.
planes.
;

loop_
 _geom_bond_atom_site_label_1
 _geom_bond_atom_site_label_2
 _geom_bond_distance
 _geom_bond_site_symmetry_2
 _geom_bond_publ_flag
Ir1 C50 1.897(2) . ?
Ir1 N2 1.9888(17) . ?
Ir1 N1 2.0521(17) . ?
Ir1 N3 2.0544(17) . ?
Ir1 P1 2.4460(6) . ?
Ir1 Ir1 2.8942(4) 3_766 ?
S1 O2 1.4376(18) . ?
S1 O3 1.4412(19) . ?
S1 O4 1.4412(17) . ?
S1 C46 1.826(3) . ?
S2 O7 1.4345(19) . ?
S2 O6 1.4417(18) . ?
S2 O5 1.4442(19) . ?
S2 C47 1.821(3) . ?
P1 C40 1.822(2) . ?
P1 C28 1.826(2) . ?
P1 C34 1.827(2) . ?
F5 C47 1.335(3) . ?
F6 C47 1.339(3) . ?
F1 C46 1.336(3) . ?
F4 C47 1.326(3) . ?
F2 C46 1.331(3) . ?
F3 C46 1.321(3) . ?
O1 C50 1.131(3) . ?
C36 C37 1.378(4) . ?
C36 C35 1.383(3) . ?
C36 H36A 0.9500 . ?
N2 C6 1.350(3) . ?
N2 C10 1.351(3) . ?
```

C27 C24 1.531(3) . ?
C27 H27A 0.9800 . ?
C27 H27B 0.9800 . ?
C27 H27C 0.9800 . ?
C23 C20 1.533(3) . ?
C23 H23A 0.9800 . ?
C23 H23B 0.9800 . ?
C23 H23C 0.9800 . ?
N1 C1 1.352(3) . ?
N1 C5 1.373(3) . ?
N3 C15 1.340(3) . ?
N3 C11 1.378(3) . ?
C17 C16 1.536(3) . ?
C17 H17A 0.9800 . ?
C17 H17B 0.9800 . ?
C17 H17C 0.9800 . ?
C16 C3 1.523(3) . ?
C16 C19 1.532(3) . ?
C16 C18 1.533(3) . ?
C5 C4 1.388(3) . ?
C5 C6 1.480(3) . ?
C19 H19A 0.9800 . ?
C19 H19B 0.9800 . ?
C19 H19C 0.9800 . ?
C25 C24 1.537(3) . ?
C25 H25A 0.9800 . ?
C25 H25B 0.9800 . ?
C25 H25C 0.9800 . ?
C8 C9 1.400(3) . ?
C8 C7 1.403(3) . ?
C8 C20 1.530(3) . ?
C4 C3 1.394(3) . ?
C4 H4A 0.9500 . ?
C10 C9 1.387(3) . ?
C10 C11 1.480(3) . ?
C31 C32 1.382(4) . ?
C31 C30 1.384(4) . ?
C31 H31A 0.9500 . ?
C18 H18A 0.9800 . ?
C18 H18B 0.9800 . ?
C18 H18C 0.9800 . ?
C35 C34 1.404(3) . ?
C35 H35A 0.9500 . ?
C34 C39 1.382(3) . ?

C20 C21 1.535(3) . ?
C20 C22 1.538(3) . ?
C3 C2 1.400(3) . ?
C9 H9A 0.9500 . ?
C32 C33 1.388(3) . ?
C32 H32A 0.9500 . ?
C13 C14 1.389(3) . ?
C13 C12 1.405(3) . ?
C13 C24 1.530(3) . ?
C43 C41 1.381(4) . ?
C43 C44 1.382(4) . ?
C43 H43A 0.9500 . ?
C11 C12 1.378(3) . ?
C41 C42 1.385(4) . ?
C41 H41A 0.9500 . ?
C12 H12A 0.9500 . ?
C39 C38 1.394(3) . ?
C39 H39A 0.9500 . ?
C21 H21A 0.9800 . ?
C21 H21B 0.9800 . ?
C21 H21C 0.9800 . ?
C24 C26 1.531(3) . ?
C26 H26A 0.9800 . ?
C26 H26B 0.9800 . ?
C26 H26C 0.9800 . ?
C6 C7 1.380(3) . ?
C44 C45 1.381(3) . ?
C44 H44A 0.9500 . ?
C45 C40 1.398(3) . ?
C45 H45A 0.9500 . ?
C38 C37 1.380(4) . ?
C38 H38A 0.9500 . ?
C33 C28 1.400(3) . ?
C33 H33A 0.9500 . ?
C40 C42 1.390(3) . ?
C37 H37A 0.9500 . ?
C42 H42A 0.9500 . ?
C28 C29 1.395(3) . ?
C2 C1 1.372(3) . ?
C2 H2A 0.9500 . ?
C7 H7A 0.9500 . ?
C30 C29 1.390(3) . ?
C30 H30A 0.9500 . ?
C14 C15 1.382(3) . ?

```
C14 H14A 0.9500 . ?
C1 H1A 0.9500 . ?
C15 H15A 0.9500 . ?
C22 H22A 0.9800 . ?
C22 H22B 0.9800 . ?
C22 H22C 0.9800 . ?
C29 H29A 0.9500 . ?
C49 C48 1.396(6) . ?
C49 H49A 0.9800 . ?
C49 H49B 0.9800 . ?
C49 H49C 0.9800 . ?
C48 N4 1.166(7) . ?

loop_
 _geom_angle_atom_site_label_1
 _geom_angle_atom_site_label_2
 _geom_angle_atom_site_label_3
 _geom_angle
 _geom_angle_site_symmetry_1
 _geom_angle_site_symmetry_3
 _geom_angle_publ_flag
C50 Ir1 N2 178.12(8) . . ?
C50 Ir1 N1 102.31(8) . . ?
N2 Ir1 N1 79.35(7) . . ?
C50 Ir1 N3 98.95(8) . . ?
N2 Ir1 N3 79.42(7) . . ?
N1 Ir1 N3 158.73(7) . . ?
C50 Ir1 P1 90.87(6) . . ?
N2 Ir1 P1 90.06(5) . . ?
N1 Ir1 P1 88.97(5) . . ?
N3 Ir1 P1 89.95(5) . . ?
C50 Ir1 Ir1 91.84(6) . 3_766 ?
N2 Ir1 Ir1 87.27(5) . 3_766 ?
N1 Ir1 Ir1 89.21(5) . 3_766 ?
N3 Ir1 Ir1 90.89(5) . 3_766 ?
P1 Ir1 Ir1 177.004(14) . 3_766 ?
O2 S1 O3 115.97(12) . . ?
O2 S1 O4 114.37(11) . . ?
O3 S1 O4 114.49(11) . . ?
O2 S1 C46 102.21(11) . . ?
O3 S1 C46 103.45(12) . . ?
O4 S1 C46 103.96(12) . . ?
O7 S2 O6 114.85(11) . . ?
O7 S2 O5 115.35(12) . . ?
```

O6 S2 O5 114.95(12) . . ?
O7 S2 C47 103.66(13) . . ?
O6 S2 C47 104.01(12) . . ?
O5 S2 C47 101.51(12) . . ?
C40 P1 C28 109.89(10) . . ?
C40 P1 C34 103.13(10) . . ?
C28 P1 C34 102.93(10) . . ?
C40 P1 Ir1 112.76(8) . . ?
C28 P1 Ir1 108.23(7) . . ?
C34 P1 Ir1 119.27(7) . . ?
C37 C36 C35 120.2(2) . . ?
C37 C36 H36A 119.9 . . ?
C35 C36 H36A 119.9 . . ?
C6 N2 C10 122.53(18) . . ?
C6 N2 Ir1 118.90(14) . . ?
C10 N2 Ir1 118.57(14) . . ?
C24 C27 H27A 109.5 . . ?
C24 C27 H27B 109.5 . . ?
H27A C27 H27B 109.5 . . ?
C24 C27 H27C 109.5 . . ?
H27A C27 H27C 109.5 . . ?
H27B C27 H27C 109.5 . . ?
C20 C23 H23A 109.5 . . ?
C20 C23 H23B 109.5 . . ?
H23A C23 H23B 109.5 . . ?
C20 C23 H23C 109.5 . . ?
H23A C23 H23C 109.5 . . ?
H23B C23 H23C 109.5 . . ?
C1 N1 C5 118.31(18) . . ?
C1 N1 Ir1 127.47(14) . . ?
C5 N1 Ir1 113.92(14) . . ?
C15 N3 C11 118.26(18) . . ?
C15 N3 Ir1 127.17(15) . . ?
C11 N3 Ir1 114.01(13) . . ?
C16 C17 H17A 109.5 . . ?
C16 C17 H17B 109.5 . . ?
H17A C17 H17B 109.5 . . ?
C16 C17 H17C 109.5 . . ?
H17A C17 H17C 109.5 . . ?
H17B C17 H17C 109.5 . . ?
C3 C16 C19 112.18(19) . . ?
C3 C16 C18 108.04(18) . . ?
C19 C16 C18 109.71(19) . . ?
C3 C16 C17 109.10(19) . . ?

C19 C16 C17 108.2(2) . . ?
C18 C16 C17 109.5(2) . . ?
N1 C5 C4 120.89(19) . . ?
N1 C5 C6 115.31(18) . . ?
C4 C5 C6 123.72(19) . . ?
C16 C19 H19A 109.5 . . ?
C16 C19 H19B 109.5 . . ?
H19A C19 H19B 109.5 . . ?
C16 C19 H19C 109.5 . . ?
H19A C19 H19C 109.5 . . ?
H19B C19 H19C 109.5 . . ?
C24 C25 H25A 109.5 . . ?
C24 C25 H25B 109.5 . . ?
H25A C25 H25B 109.5 . . ?
C24 C25 H25C 109.5 . . ?
H25A C25 H25C 109.5 . . ?
H25B C25 H25C 109.5 . . ?
C9 C8 C7 118.6(2) . . ?
C9 C8 C20 121.0(2) . . ?
C7 C8 C20 120.29(19) . . ?
C5 C4 C3 121.0(2) . . ?
C5 C4 H4A 119.5 . . ?
C3 C4 H4A 119.5 . . ?
N2 C10 C9 119.24(19) . . ?
N2 C10 C11 112.75(18) . . ?
C9 C10 C11 128.01(19) . . ?
O1 C50 Ir1 175.82(19) . . ?
C32 C31 C30 119.6(2) . . ?
C32 C31 H31A 120.2 . . ?
C30 C31 H31A 120.2 . . ?
C16 C18 H18A 109.5 . . ?
C16 C18 H18B 109.5 . . ?
H18A C18 H18B 109.5 . . ?
C16 C18 H18C 109.5 . . ?
H18A C18 H18C 109.5 . . ?
H18B C18 H18C 109.5 . . ?
C36 C35 C34 120.0(2) . . ?
C36 C35 H35A 120.0 . . ?
C34 C35 H35A 120.0 . . ?
C39 C34 C35 119.5(2) . . ?
C39 C34 P1 124.10(17) . . ?
C35 C34 P1 116.43(18) . . ?
C8 C20 C23 111.10(19) . . ?
C8 C20 C21 110.80(19) . . ?

C23 C20 C21 109.0(2) . . ?
C8 C20 C22 106.83(19) . . ?
C23 C20 C22 109.5(2) . . ?
C21 C20 C22 109.5(2) . . ?
C4 C3 C2 116.59(19) . . ?
C4 C3 C16 123.4(2) . . ?
C2 C3 C16 120.0(2) . . ?
C10 C9 C8 119.9(2) . . ?
C10 C9 H9A 120.1 . . ?
C8 C9 H9A 120.1 . . ?
C31 C32 C33 120.4(2) . . ?
C31 C32 H32A 119.8 . . ?
C33 C32 H32A 119.8 . . ?
C14 C13 C12 116.4(2) . . ?
C14 C13 C24 122.5(2) . . ?
C12 C13 C24 121.1(2) . . ?
C41 C43 C44 119.7(2) . . ?
C41 C43 H43A 120.2 . . ?
C44 C43 H43A 120.2 . . ?
C12 C11 N3 120.69(19) . . ?
C12 C11 C10 124.5(2) . . ?
N3 C11 C10 114.63(18) . . ?
C43 C41 C42 121.0(2) . . ?
C43 C41 H41A 119.5 . . ?
C42 C41 H41A 119.5 . . ?
C11 C12 C13 121.2(2) . . ?
C11 C12 H12A 119.4 . . ?
C13 C12 H12A 119.4 . . ?
C34 C39 C38 119.8(2) . . ?
C34 C39 H39A 120.1 . . ?
C38 C39 H39A 120.1 . . ?
C20 C21 H21A 109.5 . . ?
C20 C21 H21B 109.5 . . ?
H21A C21 H21B 109.5 . . ?
C20 C21 H21C 109.5 . . ?
H21A C21 H21C 109.5 . . ?
H21B C21 H21C 109.5 . . ?
C13 C24 C27 111.3(2) . . ?
C13 C24 C26 109.23(19) . . ?
C27 C24 C26 109.3(2) . . ?
C13 C24 C25 108.64(19) . . ?
C27 C24 C25 108.7(2) . . ?
C26 C24 C25 109.6(2) . . ?
C24 C26 H26A 109.5 . . ?

C24 C26 H26B 109.5 . . ?
H26A C26 H26B 109.5 . . ?
C24 C26 H26C 109.5 . . ?
H26A C26 H26C 109.5 . . ?
H26B C26 H26C 109.5 . . ?
N2 C6 C7 119.76(19) . . ?
N2 C6 C5 112.16(18) . . ?
C7 C6 C5 128.08(19) . . ?
C45 C44 C43 119.9(2) . . ?
C45 C44 H44A 120.1 . . ?
C43 C44 H44A 120.1 . . ?
C44 C45 C40 120.8(2) . . ?
C44 C45 H45A 119.6 . . ?
C40 C45 H45A 119.6 . . ?
C37 C38 C39 120.3(2) . . ?
C37 C38 H38A 119.8 . . ?
C39 C38 H38A 119.8 . . ?
C32 C33 C28 120.4(2) . . ?
C32 C33 H33A 119.8 . . ?
C28 C33 H33A 119.8 . . ?
C42 C40 C45 119.0(2) . . ?
C42 C40 P1 124.63(18) . . ?
C45 C40 P1 116.32(17) . . ?
C36 C37 C38 120.1(2) . . ?
C36 C37 H37A 119.9 . . ?
C38 C37 H37A 119.9 . . ?
C41 C42 C40 119.7(2) . . ?
C41 C42 H42A 120.2 . . ?
C40 C42 H42A 120.2 . . ?
C29 C28 C33 118.8(2) . . ?
C29 C28 P1 122.11(18) . . ?
C33 C28 P1 118.67(18) . . ?
C1 C2 C3 120.8(2) . . ?
C1 C2 H2A 119.6 . . ?
C3 C2 H2A 119.6 . . ?
F4 C47 F5 107.5(2) . . ?
F4 C47 F6 107.6(2) . . ?
F5 C47 F6 107.3(2) . . ?
F4 C47 S2 111.53(19) . . ?
F5 C47 S2 111.8(2) . . ?
F6 C47 S2 110.89(18) . . ?
F3 C46 F2 107.9(2) . . ?
F3 C46 F1 107.6(2) . . ?
F2 C46 F1 106.1(2) . . ?

F3 C46 S1 111.82(18) . . ?
F2 C46 S1 112.04(18) . . ?
F1 C46 S1 111.08(19) . . ?
C6 C7 C8 119.7(2) . . ?
C6 C7 H7A 120.2 . . ?
C8 C7 H7A 120.2 . . ?
C31 C30 C29 120.5(3) . . ?
C31 C30 H30A 119.7 . . ?
C29 C30 H30A 119.7 . . ?
C15 C14 C13 120.5(2) . . ?
C15 C14 H14A 119.8 . . ?
C13 C14 H14A 119.8 . . ?
N1 C1 C2 122.3(2) . . ?
N1 C1 H1A 118.9 . . ?
C2 C1 H1A 118.9 . . ?
N3 C15 C14 122.6(2) . . ?
N3 C15 H15A 118.7 . . ?
C14 C15 H15A 118.7 . . ?
C20 C22 H22A 109.5 . . ?
C20 C22 H22B 109.5 . . ?
H22A C22 H22B 109.5 . . ?
C20 C22 H22C 109.5 . . ?
H22A C22 H22C 109.5 . . ?
H22B C22 H22C 109.5 . . ?
C30 C29 C28 120.3(2) . . ?
C30 C29 H29A 119.9 . . ?
C28 C29 H29A 119.9 . . ?
C48 C49 H49A 109.5 . . ?
C48 C49 H49B 109.5 . . ?
H49A C49 H49B 109.5 . . ?
C48 C49 H49C 109.5 . . ?
H49A C49 H49C 109.5 . . ?
H49B C49 H49C 109.5 . . ?
N4 C48 C49 176.0(6) . . ?

loop_
 _geom_torsion_atom_site_label_1
 _geom_torsion_atom_site_label_2
 _geom_torsion_atom_site_label_3
 _geom_torsion_atom_site_label_4
 _geom_torsion
 _geom_torsion_site_symmetry_1
 _geom_torsion_site_symmetry_2
 _geom_torsion_site_symmetry_3

_geom_torsion_site_symmetry_4
_geom_torsion_publ_flag
C1 N1 C5 C4 -3.6(3) . . . . ?
Ir1 N1 C5 C4 170.56(16) . . . . ?
C1 N1 C5 C6 179.44(18) . . . . ?
Ir1 N1 C5 C6 -6.4(2) . . . . ?
N1 C5 C4 C3 -0.2(3) . . . . ?
C6 C5 C4 C3 176.51(19) . . . . ?
C6 N2 C10 C9 5.3(3) . . . . ?
Ir1 N2 C10 C9 -175.43(15) . . . . ?
C6 N2 C10 C11 -174.69(18) . . . . ?
Ir1 N2 C10 C11 4.6(2) . . . . ?
C37 C36 C35 C34 -0.6(4) . . . . ?
C36 C35 C34 C39 1.4(3) . . . . ?
C36 C35 C34 P1 -178.00(18) . . . . ?
C40 P1 C34 C39 117.7(2) . . . . ?
C28 P1 C34 C39 -128.0(2) . . . . ?
Ir1 P1 C34 C39 -8.2(2) . . . . ?
C40 P1 C34 C35 -62.94(19) . . . . ?
C28 P1 C34 C35 51.39(19) . . . . ?
Ir1 P1 C34 C35 171.17(14) . . . . ?
C9 C8 C20 C23 26.1(3) . . . . ?
C7 C8 C20 C23 -158.1(2) . . . . ?
C9 C8 C20 C21 147.4(2) . . . . ?
C7 C8 C20 C21 -36.7(3) . . . . ?
C9 C8 C20 C22 -93.3(2) . . . . ?
C7 C8 C20 C22 82.5(3) . . . . ?
C5 C4 C3 C2 3.8(3) . . . . ?
C5 C4 C3 C16 -175.6(2) . . . . ?
C19 C16 C3 C4 7.6(3) . . . . ?
C18 C16 C3 C4 -113.5(2) . . . . ?
C17 C16 C3 C4 127.5(2) . . . . ?
C19 C16 C3 C2 -171.8(2) . . . . ?
C18 C16 C3 C2 67.2(3) . . . . ?
C17 C16 C3 C2 -51.9(3) . . . . ?
N2 C10 C9 C8 -1.1(3) . . . . ?
C11 C10 C9 C8 178.9(2) . . . . ?
C7 C8 C9 C10 -3.1(3) . . . . ?
C20 C8 C9 C10 172.8(2) . . . . ?
C30 C31 C32 C33 -0.1(4) . . . . ?
C15 N3 C11 C12 4.8(3) . . . . ?
Ir1 N3 C11 C12 -167.20(16) . . . . ?
C15 N3 C11 C10 -179.53(18) . . . . ?
Ir1 N3 C11 C10 8.4(2) . . . . ?

N2 C10 C11 C12 167.0(2) . . . . ?
C9 C10 C11 C12 -13.1(4) . . . . ?
N2 C10 C11 N3 -8.5(3) . . . . ?
C9 C10 C11 N3 171.5(2) . . . . ?
C44 C43 C41 C42 0.6(4) . . . . ?
N3 C11 C12 C13 -0.2(3) . . . . ?
C10 C11 C12 C13 -175.4(2) . . . . ?
C14 C13 C12 C11 -4.9(3) . . . . ?
C24 C13 C12 C11 174.0(2) . . . . ?
C35 C34 C39 C38 -1.6(3) . . . . ?
P1 C34 C39 C38 177.78(18) . . . . ?
C14 C13 C24 C27 5.2(3) . . . . ?
C12 C13 C24 C27 -173.7(2) . . . . ?
C14 C13 C24 C26 -115.5(2) . . . . ?
C12 C13 C24 C26 65.6(3) . . . . ?
C14 C13 C24 C25 124.9(2) . . . . ?
C12 C13 C24 C25 -54.0(3) . . . . ?
C10 N2 C6 C7 -5.1(3) . . . . ?
Ir1 N2 C6 C7 175.61(15) . . . . ?
C10 N2 C6 C5 175.60(18) . . . . ?
Ir1 N2 C6 C5 -3.7(2) . . . . ?
N1 C5 C6 N2 6.6(3) . . . . ?
C4 C5 C6 N2 -170.32(19) . . . . ?
N1 C5 C6 C7 -172.6(2) . . . . ?
C4 C5 C6 C7 10.5(3) . . . . ?
C41 C43 C44 C45 -1.2(4) . . . . ?
C43 C44 C45 C40 0.3(4) . . . . ?
C34 C39 C38 C37 1.0(4) . . . . ?
C31 C32 C33 C28 -0.4(4) . . . . ?
C44 C45 C40 C42 1.1(3) . . . . ?
C44 C45 C40 P1 178.59(19) . . . . ?
C28 P1 C40 C42 -2.4(2) . . . . ?
C34 P1 C40 C42 106.8(2) . . . . ?
Ir1 P1 C40 C42 -123.3(2) . . . . ?
C28 P1 C40 C45 -179.77(17) . . . . ?
C34 P1 C40 C45 -70.58(19) . . . . ?
Ir1 P1 C40 C45 59.39(18) . . . . ?
C35 C36 C37 C38 -0.1(4) . . . . ?
C39 C38 C37 C36 -0.1(4) . . . . ?
C43 C41 C42 C40 0.8(4) . . . . ?
C45 C40 C42 C41 -1.6(4) . . . . ?
P1 C40 C42 C41 -178.9(2) . . . . ?
C32 C33 C28 C29 0.7(3) . . . . ?
C32 C33 C28 P1 173.02(19) . . . . ?

C40 P1 C28 C29 -46.6(2) . . . . ?

C34 P1 C28 C29 -155.87(19) . . . . ?

Ir1 P1 C28 C29 76.99(19) . . . . ?

C40 P1 C28 C33 141.37(18) . . . . ?

C34 P1 C28 C33 32.0(2) . . . . ?

Ir1 P1 C28 C33 -95.10(18) . . . . ?

C4 C3 C2 C1 -3.8(3) . . . . ?

C16 C3 C2 C1 175.6(2) . . . . ?

O7 S2 C47 F4 -175.83(19) . . . . ?

O6 S2 C47 F4 -55.4(2) . . . . ?

O5 S2 C47 F4 64.2(2) . . . . ?

O7 S2 C47 F5 -55.4(2) . . . . ?

O6 S2 C47 F5 65.0(2) . . . . ?

O5 S2 C47 F5 -175.38(19) . . . . ?

O7 S2 C47 F6 64.3(2) . . . . ?

O6 S2 C47 F6 -175.28(19) . . . . ?

O5 S2 C47 F6 -55.6(2) . . . . ?

O2 S1 C46 F3 -57.4(2) . . . . ?

O3 S1 C46 F3 -178.2(2) . . . . ?

O4 S1 C46 F3 61.9(2) . . . . ?

O2 S1 C46 F2 -178.63(19) . . . . ?

O3 S1 C46 F2 60.5(2) . . . . ?

O4 S1 C46 F2 -59.4(2) . . . . ?

O2 S1 C46 F1 62.9(2) . . . . ?

O3 S1 C46 F1 -58.0(2) . . . . ?

O4 S1 C46 F1 -177.87(18) . . . . ?

N2 C6 C7 C8 0.7(3) . . . . ?

C5 C6 C7 C8 179.8(2) . . . . ?

C9 C8 C7 C6 3.3(3) . . . . ?

C20 C8 C7 C6 -172.7(2) . . . . ?

C32 C31 C30 C29 0.3(4) . . . . ?

C12 C13 C14 C15 5.5(3) . . . . ?

C24 C13 C14 C15 -173.4(2) . . . . ?

C5 N1 C1 C2 3.7(3) . . . . ?

Ir1 N1 C1 C2 -169.57(16) . . . . ?

C3 C2 C1 N1 0.1(3) . . . . ?

C11 N3 C15 C14 -4.2(3) . . . . ?

Ir1 N3 C15 C14 166.63(16) . . . . ?

C13 C14 C15 N3 -1.1(3) . . . . ?

C31 C30 C29 C28 0.0(4) . . . . ?

C33 C28 C29 C30 -0.5(3) . . . . ?

P1 C28 C29 C30 -172.54(18) . . . . ?

_refine_diff_density_max   0.405

```
_refine_diff_density_min   -0.700
_refine_diff_density_rms    0.089

_shelx_res_file
;

    z.res created by SHELXL-2014/7


TITL z in P2(1)/n
CELL 0.71 16.4301 17.6202 17.4705 90 95.1376 90
ZERR 4 0.0019 0.0021 0.002 0 0.0037 0
LATT 1
SYMM 0.5-X,0.5+Y,0.5-Z

SFAC C H N O P Ir F S
UNIT 200 212 16 28 4 4 24 8

L.S. 50
PLAN  1
TEMP -123.16
fmap 2
acta
MORE -1
BOND $H
htab
size 0.21 0.19 0.17
CONF
REM
<HKL>C:/Users/Administrator/Desktop/single%20crystal/zfr170216zfr20/wo
rk/z
REM .hkl</HKL>

WGHT    0.025100    2.985800
FVAR       0.05870
IR1    6     1.004475     0.513998     0.581888     11.00000     0.00896
0.00856 =
         0.01131    0.00010    0.00087   -0.00032
S1     8     0.857936     0.814884     0.519074     11.00000     0.02351
0.01402 =
         0.02700    0.00032    0.00363    0.00207
S2     8     1.334544     0.656653     0.564443     11.00000     0.01863
0.01809 =
         0.02604   -0.00280    0.00343   -0.00357
```

P1   5   1.006387   0.532731   0.720767   11.00000   0.01317
0.01387 =
0.01153   0.00002   0.00084   -0.00088
F5   7   1.400589   0.724535   0.688841   11.00000   0.05570
0.08157 =
0.03163   -0.02158   -0.00192   -0.01456
F6   7   1.465332   0.741137   0.588272   11.00000   0.02589
0.05324 =
0.06061   -0.01465   0.00559   -0.01959
F1   7   0.818086   0.956851   0.540843   11.00000   0.06443
0.01960 =
0.05982   -0.00340   0.00631   0.01464
F4   7   1.352667   0.800310   0.599808   11.00000   0.05485
0.02130 =
0.09410   -0.01719   0.00880   -0.00149
F2   7   0.894121   0.909251   0.633930   11.00000   0.08496
0.03027 =
0.03307   -0.00763   0.00016   -0.00384
F3   7   0.945629   0.939365   0.529602   11.00000   0.05900
0.03218 =
0.06997   -0.01120   0.02721   -0.02168
O2   4   0.848019   0.828712   0.437674   11.00000   0.04290
0.02357 =
0.02612   0.00096   -0.00213   0.00597
O4   4   0.929362   0.771068   0.544499   11.00000   0.02838
0.02299 =
0.03299   0.00417   0.00130   0.00665
O5   4   1.337923   0.676923   0.484690   11.00000   0.03637
0.04294 =
0.02356   -0.00220   0.00110   -0.01378
O3   4   0.785067   0.793588   0.553981   11.00000   0.03099
0.02560 =
0.05439   0.00079   0.01679   -0.00183
O7   4   1.380885   0.590774   0.589400   11.00000   0.03365
0.02275 =
0.05728   0.00426   0.01111   0.00662
O6   4   1.254526   0.661335   0.591902   11.00000   0.02126
0.03028 =
0.04550   0.00138   0.01059   -0.00385
O1   4   1.073646   0.671900   0.566456   11.00000   0.01922
0.01269 =
0.02243   -0.00007   0.00263   -0.00397
C36   1   0.929207   0.689511   0.868494   11.00000   0.02681
0.03208 =

```
         0.02023   -0.00796    0.00687   -0.00109
AFIX  43
H36A  2    0.923221    0.691327    0.922047    11.00000   -1.20000
AFIX   0
N2     3     0.964039     0.408581     0.593330     11.00000     0.01144
0.01028 =
         0.01213   -0.00055    0.00202   -0.00031
C27    1     1.398170     0.372650     0.703675     11.00000     0.01755
0.03068 =
         0.05422    0.00895   -0.01096    0.00153
AFIX  33
H27A  2    1.443335    0.341844    0.727266    11.00000   -1.50000
H27B  2    1.413197    0.393514    0.654884    11.00000   -1.50000
H27C  2    1.386844    0.414299    0.738351    11.00000   -1.50000
AFIX   0
C23    1     0.939236     0.124683     0.631549     11.00000     0.02724
0.01152 =
         0.03392    0.00421    0.00283   -0.00205
AFIX  33
H23A  2    0.917663    0.072901    0.626444    11.00000   -1.50000
H23B  2    0.990381    0.128027    0.606869    11.00000   -1.50000
H23C  2    0.949585    0.137524    0.686131    11.00000   -1.50000
AFIX   0
N1     3     0.880602     0.532085     0.572608     11.00000     0.00990
0.01228 =
         0.01179   -0.00041   -0.00003   -0.00039
N3     3     1.113173     0.456046     0.597799     11.00000     0.01051
0.01127 =
         0.01302   -0.00067    0.00055    0.00108
C17    1     0.613000     0.612097     0.660360     11.00000     0.02191
0.04230 =
         0.03643   -0.01551    0.00890    0.00198
AFIX  33
H17A  2    0.555158    0.618226    0.668640    11.00000   -1.50000
H17B  2    0.643565    0.596477    0.708527    11.00000   -1.50000
H17C  2    0.634700    0.660420    0.643260    11.00000   -1.50000
AFIX   0
C16    1     0.621746     0.551220     0.598672     11.00000     0.01046
0.02279 =
         0.02243   -0.00330    0.00410    0.00185
C5     1     0.835459     0.467919     0.582817     11.00000     0.01121
0.01223 =
         0.01046   -0.00047    0.00144   -0.00232
C19    1     0.584130     0.477350     0.625514     11.00000     0.01388
```

```
0.03252 =
         0.03354    0.00562    0.00671    0.00121
AFIX  33
H19A  2    0.526372    0.485750    0.632814    11.00000   -1.50000
H19B  2    0.588747    0.437829    0.586672    11.00000   -1.50000
H19C  2    0.613214    0.461227    0.674224    11.00000   -1.50000
AFIX   0
C25    1     1.298883     0.291365     0.765986     11.00000     0.02898
0.03326 =
         0.02701    0.00836   -0.00254    0.00767
AFIX  33
H25A  2    1.344711    0.261829    0.790255    11.00000   -1.50000
H25B  2    1.286536    0.333420    0.799787    11.00000   -1.50000
H25C  2    1.250767    0.258600    0.757100    11.00000   -1.50000
AFIX   0
C8     1     0.908240     0.262070     0.599473     11.00000     0.01846
0.01374 =
         0.01300    0.00026    0.00342   -0.00284
C4     1     0.752027     0.472370     0.589441     11.00000     0.01261
0.01558 =
         0.01325   -0.00158    0.00096   -0.00254
AFIX  43
H4A   2    0.721909    0.427227    0.596292    11.00000   -1.20000
AFIX   0
C10    1     1.019282     0.352006     0.604964     11.00000     0.01323
0.01170 =
         0.01186    0.00139    0.00107    0.00068
C50    1     1.046128     0.613262     0.569594     11.00000     0.01092
0.01891 =
         0.00854   -0.00066    0.00147    0.00442
C31    1     0.827558     0.353024     0.799917     11.00000     0.03654
0.04356 =
         0.02363    0.00814    0.00302   -0.02255
AFIX  43
H31A  2    0.789673    0.316010    0.814163    11.00000   -1.20000
AFIX   0
C18    1     0.576406     0.576796     0.522556     11.00000     0.01479
0.02923 =
         0.03021    0.00406   -0.00073    0.00175
AFIX  33
H18A  2    0.518200    0.583054    0.529244    11.00000   -1.50000
H18B  2    0.598974    0.625243    0.506785    11.00000   -1.50000
H18C  2    0.583054    0.538446    0.482970    11.00000   -1.50000
AFIX   0
```

```
C35   1    0.960053    0.624941    0.836602    11.00000    0.02266
0.02334 =
         0.01789   -0.00132    0.00253   -0.00122
AFIX  43
H35A  2    0.975697    0.582671    0.868328    11.00000   -1.20000
AFIX   0
C34   1    0.968262    0.621821    0.757379    11.00000    0.01399
0.01800 =
         0.01738   -0.00398    0.00299   -0.00246
C20   1    0.876780    0.180373    0.592751    11.00000    0.01817
0.01115 =
         0.02796    0.00110    0.00220   -0.00424
C3    1    0.711797    0.542082    0.586189    11.00000    0.01144
0.01989 =
         0.01320   -0.00242    0.00056    0.00014
C9    1    0.992109    0.277599    0.607563    11.00000    0.01544
0.01264 =
         0.01517    0.00109    0.00151    0.00147
AFIX  43
H9A   2    1.030364    0.237244    0.614824    11.00000   -1.20000
AFIX   0
C32   1    0.803373    0.427674    0.788925    11.00000    0.02113
0.04695 =
         0.02335    0.00188    0.00569   -0.01047
AFIX  43
H32A  2    0.748679    0.441890    0.795579    11.00000   -1.20000
AFIX   0
C13   1    1.249556    0.368980    0.651965    11.00000    0.01471
0.01749 =
         0.01602   -0.00088    0.00247    0.00209
C43   1    1.267776    0.525401    0.839866    11.00000    0.02048
0.03403 =
         0.03610   -0.01095   -0.01143    0.00283
AFIX  43
H43A  2    1.322190    0.524633    0.863383    11.00000   -1.20000
AFIX   0
C11   1    1.104560    0.379965    0.613536    11.00000    0.01402
0.01316 =
         0.01161    0.00089    0.00249   -0.00093
C41   1    1.210408    0.475330    0.863521    11.00000    0.03371
0.02988 =
         0.03090    0.00452   -0.01404    0.00294
AFIX  43
H41A  2    1.225670    0.440490    0.903765    11.00000   -1.20000
```

```
AFIX   0
C12    1     1.171144     0.336920     0.640245     11.00000     0.01602
0.01322 =
         0.01927    0.00226    0.00283    0.00111
AFIX  43
H12A  2    1.163896    0.284611    0.650929    11.00000   -1.20000
AFIX   0
C39    1     0.947008     0.684265     0.712082     11.00000     0.01996
0.01795 =
         0.01917   -0.00161    0.00302    0.00010
AFIX  43
H39A  2    0.953653    0.683158    0.658633    11.00000   -1.20000
AFIX   0
C21    1     0.795836     0.172224     0.629578     11.00000     0.02499
0.01599 =
         0.05919    0.00651    0.01072   -0.00582
AFIX  33
H21A  2    0.776803    0.119593    0.624652    11.00000   -1.50000
H21B  2    0.804180    0.185873    0.684095    11.00000   -1.50000
H21C  2    0.754851    0.206003    0.603496    11.00000   -1.50000
AFIX   0
C24    1     1.321828     0.323113     0.688869     11.00000     0.01372
0.01998 =
         0.02779    0.00384   -0.00097    0.00265
C26    1     1.340154     0.257558     0.635513     11.00000     0.02242
0.02584 =
         0.03383    0.00550    0.00435    0.00839
AFIX  33
H26A  2    1.386421    0.228043    0.658962    11.00000   -1.50000
H26B  2    1.292004    0.224735    0.627378    11.00000   -1.50000
H26C  2    1.353766    0.277935    0.586047    11.00000   -1.50000
AFIX   0
C6     1     0.882695     0.396350     0.590037     11.00000     0.01181
0.01465 =
         0.01005   -0.00041    0.00195    0.00023
C44    1     1.245613     0.576644     0.781792     11.00000     0.01997
0.02843 =
         0.03036   -0.00776   -0.00045   -0.00493
AFIX  43
H44A  2    1.284527    0.611854    0.765906    11.00000   -1.20000
AFIX   0
C45    1     1.166768     0.576546     0.746907     11.00000     0.02025
0.01956 =
         0.01970   -0.00274   -0.00262   -0.00118
```

```
AFIX  43
H45A  2    1.151850    0.611674    0.706837    11.00000   -1.20000
AFIX   0
C38   1     0.915787     0.748901     0.745007     11.00000     0.02841
0.01714 =
         0.03228   -0.00090    0.00423    0.00357
AFIX  43
H38A  2    0.900402    0.791547    0.713725    11.00000   -1.20000
AFIX   0
C33   1     0.858526     0.481953     0.768229     11.00000     0.02216
0.02823 =
         0.01796    0.00042    0.00373   -0.00208
AFIX  43
H33A  2    0.841262    0.533050    0.760482    11.00000   -1.20000
AFIX   0
C40   1     1.108667     0.525352     0.769884     11.00000     0.01547
0.01704 =
         0.01559   -0.00478   -0.00226    0.00115
C37   1     0.907125     0.751229     0.822825     11.00000     0.02773
0.02132 =
         0.03347   -0.01067    0.01067    0.00267
AFIX  43
H37A  2    0.885901    0.795460    0.844946    11.00000   -1.20000
AFIX   0
C42   1     1.130977     0.475126     0.829481     11.00000     0.02571
0.02399 =
         0.02390    0.00319   -0.00445   -0.00245
AFIX  43
H42A  2    1.091926    0.440839    0.846763    11.00000   -1.20000
AFIX   0
C28   1     0.939468     0.461905     0.758686     11.00000     0.01996
0.01988 =
         0.00985    0.00062   -0.00054   -0.00374
C2    1     0.758709     0.605810     0.571258     11.00000     0.01579
0.01469 =
         0.01823   -0.00197    0.00103    0.00366
AFIX  43
H2A   2    0.733243    0.654123    0.565426    11.00000   -1.20000
AFIX   0
C47   1     1.390764     0.734640     0.612826     11.00000     0.02671
0.03209 =
         0.03447   -0.00975    0.00172   -0.00548
C46   1     0.880747     0.909600     0.557653     11.00000     0.04189
0.01972 =
```

```
        0.02820   -0.00113    0.00741   -0.00138
C7     1     0.853274     0.323135     0.592614     11.00000     0.01287
0.01671 =
        0.01293   -0.00057    0.00218   -0.00220
AFIX  43
H7A   2    0.796076    0.314120    0.589758    11.00000   -1.20000
AFIX   0
C30    1     0.907277     0.332496     0.790055     11.00000     0.04083
0.02407 =
        0.02129    0.00756   -0.00350   -0.01040
AFIX  43
H30A  2    0.923905    0.281170    0.797343    11.00000   -1.20000
AFIX   0
C14    1     1.257000     0.444118     0.629550     11.00000     0.01142
0.01834 =
        0.01755   -0.00088    0.00097   -0.00057
AFIX  43
H14A  2    1.309457    0.467132    0.631998    11.00000   -1.20000
AFIX   0
C1     1     0.841069     0.599301     0.564948     11.00000     0.01619
0.01038 =
        0.01434   -0.00088    0.00015   -0.00059
AFIX  43
H1A   2    0.871342    0.643562    0.554847    11.00000   -1.20000
AFIX   0
C15    1     1.188635     0.485611     0.603719     11.00000     0.01401
0.01338 =
        0.01480   -0.00047    0.00262   -0.00211
AFIX  43
H15A  2    1.195386    0.537141    0.589595    11.00000   -1.20000
AFIX   0
C22    1     0.863018     0.162237     0.506436     11.00000     0.04361
0.01687 =
        0.03200   -0.00460   -0.00853   -0.00752
AFIX  33
H22A  2    0.842753    0.110153    0.499553    11.00000   -1.50000
H22B  2    0.822786    0.197615    0.481755    11.00000   -1.50000
H22C  2    0.914746    0.167390    0.483008    11.00000   -1.50000
AFIX   0
C29    1     0.963183     0.386375     0.769575     11.00000     0.02631
0.02207 =
        0.01600    0.00216   -0.00048   -0.00393
AFIX  43
H29A  2    1.017751    0.371707    0.762967    11.00000   -1.20000
```

```
AFIX   0
C49    1     0.884153     0.392169     1.001472     11.00000     0.07131
0.07427 =
         0.04979    0.00034   -0.01365   -0.01536
AFIX 137
H49A  2    0.878330    0.384446    1.056264    11.00000   -1.50000
H49B  2    0.932392    0.364729    0.987120    11.00000   -1.50000
H49C  2    0.835363    0.373051    0.971165    11.00000   -1.50000
AFIX   0
C48    1     0.893234     0.469510     0.987033     11.00000     0.15600
0.10102 =
         0.04006    0.00690    0.02279   -0.06191
N4     3     0.897482     0.534942     0.978095     11.00000     0.33601
0.11736 =
         0.09460    0.00672    0.10750   -0.08543

HKLF 4

REM  z in P2(1)/n
REM R1 =  0.0206 for   10061 Fo > 4sig(Fo)  and  0.0291 for all   11594
data
REM    641 parameters refined using      0 restraints

END

WGHT      0.0202      2.9958

REM Instructions for potential hydrogen bonds
EQIV $1 -x+2, -y+1, -z+1
HTAB C4 O5_$1
HTAB C35 N4
HTAB C9 O2_$1
HTAB C12 O2_$1
HTAB C39 O4
HTAB C2 O3
HTAB C7 O5_$1
HTAB C14 O7
HTAB C1 O4
HTAB C15 O6
EQIV $2 -x+3/2, y-1/2, -z+3/2
HTAB C49 O3_$2

REM Highest difference peak  0.405,  deepest hole -0.700,  1-sigma
level  0.089
```

```
Q1    1   1.2081  0.3578  0.6606  11.00000  0.05    0.40
;
_shelx_res_checksum   46355

_shelx_hkl_file
;
  -2   0   0  268.35    9.84
  -2   0   0  258.51    9.79
  -2   0   0  265.31    9.82
   2   0   0  264.42    9.83
  -3   0   0    0.05    0.15
  -3   0   0    0.13    0.07
  -3   0   0   -0.05    0.10
  -3   0   0   -0.02    0.11
   3   0   0    0.03    0.15
   3   0   0    0.06    0.10
   3   0   0    0.00    0.10
   3   0   0    0.18    0.07
  -4   0   0   10.77    0.48
  -4   0   0    9.90    0.56
  -4   0   0    9.56    0.60
  -4   0   0    9.71    0.54
   4   0   0   10.65    0.56
   4   0   0   10.79    0.48
   4   0   0   10.16    0.54
   4   0   0   10.49    0.61
  -5   0   0    0.09    0.18
  -5   0   0    0.06    0.24
  -5   0   0    0.20    0.11
  -5   0   0   -0.02    0.17
   5   0   0    0.36    0.25
   5   0   0    0.15    0.16
   5   0   0    0.14    0.16
   5   0   0    0.23    0.11
  -6   0   0  151.63    5.74
  -6   0   0  150.57    5.80
  -6   0   0  149.77    5.85
  -6   0   0  143.02    5.86
   6   0   0  154.88    5.87
   6   0   0  157.52    5.84
   6   0   0  148.51    5.73
   6   0   0  157.68    5.80
  -7   0   0    0.19    0.24
  -7   0   0    0.04    0.14
```

-7	0	0	0.04	0.24
-7	0	0	0.32	0.32
7	0	0	-0.09	0.32
7	0	0	-0.15	0.22
7	0	0	0.00	0.22
7	0	0	0.13	0.14
-8	0	0	356.08	13.04
-8	0	0	366.82	12.96
-8	0	0	336.43	13.09
-8	0	0	331.67	13.08
8	0	0	350.86	13.10
8	0	0	348.66	13.06
8	0	0	322.75	12.94
8	0	0	353.02	13.02
-9	0	0	0.19	0.34
-9	0	0	-0.09	0.42
-9	0	0	0.05	0.34
-9	0	0	-0.08	0.19
9	0	0	0.12	0.19
9	0	0	0.01	0.41
9	0	0	-0.38	0.29
-10	0	0	85.93	3.50
-10	0	0	91.84	3.40
-10	0	0	81.56	3.59
10	0	0	84.47	3.47
10	0	0	84.01	3.58
10	0	0	83.38	3.38
10	0	0	85.22	3.54
-11	0	0	0.01	0.44
-11	0	0	0.03	0.44
-11	0	0	0.33	0.26
-11	0	0	0.34	0.59
11	0	0	0.09	0.25
11	0	0	-0.47	0.37
11	0	0	-0.03	0.37
11	0	0	-0.27	0.52
-12	0	0	167.42	6.98
-12	0	0	175.10	6.96
-12	0	0	181.68	6.70
-12	0	0	166.98	6.86
12	0	0	177.10	6.87
12	0	0	165.26	6.68
12	0	0	181.06	6.77
12	0	0	172.29	6.87

-13	0	0	1.44	0.64
-13	0	0	0.11	0.55
-13	0	0	0.25	0.36
-13	0	0	0.39	0.57
13	0	0	-0.01	0.29
13	0	0	-0.25	0.42
13	0	0	0.03	0.53
13	0	0	-0.37	0.66
-14	0	0	48.47	2.42
-14	0	0	50.83	2.46
-14	0	0	50.61	2.60
14	0	0	53.55	2.33
14	0	0	46.54	2.67
14	0	0	50.00	2.23
-15	0	0	-0.02	0.70
-15	0	0	0.93	0.74
-15	0	0	-1.03	0.77
15	0	0	0.20	0.53
-16	0	0	77.41	3.49
-16	0	0	77.28	3.54
-16	0	0	75.93	3.56
-16	0	0	79.48	3.78
16	0	0	81.57	3.36
16	0	0	78.45	3.39
-17	0	0	0.49	0.88
-17	0	0	-0.25	0.86
17	0	0	0.01	0.52
17	0	0	-0.13	0.66
-18	0	0	67.76	3.65
18	0	0	66.81	3.11
18	0	0	69.82	3.07
-19	0	0	1.43	1.17
19	0	0	1.03	0.62
-20	0	0	59.24	3.66
20	0	0	58.45	2.86
-21	0	0	-1.53	1.43
21	0	0	1.03	0.76
21	0	1	8.22	1.11
-21	0	-1	5.84	1.68
20	0	1	-0.61	0.62
-20	0	-1	-0.81	1.32
19	0	1	4.78	0.81
-19	0	-1	5.20	1.31
18	0	1	-0.21	0.55

18	0	1	-0.82	0.82
-18	0	-1	0.07	1.01
17	0	1	19.89	1.62
17	0	1	24.10	1.46
-17	0	-1	21.00	2.09
16	0	1	-0.19	0.65
16	0	1	-0.49	0.43
-16	0	-1	1.12	0.85
-16	0	-1	0.45	0.59
-16	0	-1	1.54	0.94
15	0	1	84.71	3.51
15	0	1	85.44	3.59
-15	0	-1	84.10	3.87
-15	0	-1	82.76	3.73
-15	0	-1	75.89	3.39
14	0	1	0.11	0.35
14	0	1	-0.65	0.53
-14	0	-1	0.14	0.62
-14	0	-1	0.19	0.72
-14	0	-1	0.27	0.49
13	0	1	7.34	0.70
13	0	1	6.50	0.89
13	0	1	7.94	1.03
-13	0	-1	7.29	0.83
-13	0	-1	7.88	1.11
-13	0	-1	6.79	1.11
12	0	1	-0.19	0.45
12	0	1	-0.10	0.28
12	0	1	0.42	0.44
-12	0	-1	-0.51	0.51
-12	0	-1	-0.73	0.39
-12	0	-1	0.54	0.51
-12	0	-1	-0.72	0.48
-12	0	-1	-0.07	0.30
11	0	1	35.90	1.71
11	0	1	34.65	1.72
11	0	1	35.08	1.67
11	0	1	32.65	1.56
-11	0	-1	33.29	1.76
-11	0	-1	33.31	1.66
-11	0	-1	33.86	1.80
-11	0	-1	33.64	1.55
10	0	1	0.25	0.40
10	0	1	0.29	0.34

10	0	1	-0.09	0.33
10	0	1	-0.24	0.21
-10	0	-1	-0.55	0.42
-10	0	-1	-0.08	0.36
-10	0	-1	0.07	0.53
-10	0	-1	-0.01	0.22
9	0	1	45.82	2.03
9	0	1	52.38	2.18
9	0	1	49.87	2.12
9	0	1	47.40	2.17
-9	0	-1	52.62	2.04
-9	0	-1	48.67	2.21
-9	0	-1	47.18	2.21
-9	0	-1	48.94	2.14
8	0	1	-0.02	0.33
8	0	1	-0.48	0.29
8	0	1	0.05	0.26
8	0	1	0.16	0.18
-8	0	-1	-0.32	0.32
-8	0	-1	0.13	0.17
-8	0	-1	0.00	0.29
-8	0	-1	-0.24	0.32
7	0	1	186.19	6.97
7	0	1	183.50	7.02
7	0	1	177.33	6.92
-7	0	-1	180.78	6.98
-7	0	-1	176.45	7.04
-7	0	-1	186.68	7.06
-7	0	-1	189.82	6.92
6	0	1	-0.43	0.17
6	0	1	-0.04	0.13
6	0	1	0.04	0.22
6	0	1	0.32	0.23
-6	0	-1	-0.17	0.19
-6	0	-1	-0.18	0.23
-6	0	-1	0.21	0.25
5	0	1	380.65	14.74
5	0	1	415.04	14.67
5	0	1	371.00	14.65
5	0	1	401.09	14.73
-5	0	-1	396.17	14.77
-5	0	-1	397.62	14.65
-5	0	-1	402.52	14.69
-5	0	-1	385.61	14.73

4	0	1	-0.10	0.14
4	0	1	0.14	0.15
4	0	1	0.28	0.18
4	0	1	0.12	0.10
-4	0	-1	0.17	0.09
-4	0	-1	0.47	0.17
-4	0	-1	0.45	0.17
-4	0	-1	0.10	0.18
3	0	1	564.37	21.06
3	0	1	563.33	21.10
-3	0	-1	561.22	21.11
-3	0	-1	550.50	21.06
-3	0	-1	598.74	21.02
2	0	1	0.12	0.07
2	0	1	0.00	0.08
2	0	1	0.05	0.04
-2	0	-1	0.11	0.06
-2	0	-1	0.27	0.06
-2	0	-1	0.05	0.10
-2	0	1	0.19	0.13
-2	0	1	-0.02	0.05
-2	0	1	0.16	0.04
2	0	-1	-0.03	0.03
2	0	-1	0.03	0.07
2	0	-1	-0.21	0.12
-3	0	1	10.07	0.46
-3	0	1	11.35	0.56
-3	0	1	12.03	0.66
-3	0	1	10.85	0.48
3	0	-1	11.67	0.55
3	0	-1	11.55	0.66
3	0	-1	10.98	0.48
-4	0	1	-0.11	0.07
-4	0	1	0.16	0.11
-4	0	1	0.06	0.15
-4	0	1	-0.01	0.23
4	0	-1	0.12	0.08
4	0	-1	0.05	0.14
4	0	-1	-0.16	0.24
4	0	-1	0.02	0.08
-5	0	1	143.59	5.18
-5	0	1	138.51	5.18
-5	0	1	138.91	5.29
-5	0	1	126.53	5.35

5	0	-1	133.30	5.18
5	0	-1	136.69	5.17
5	0	-1	137.34	5.27
5	0	-1	144.12	5.39
-6	0	1	-0.31	0.18
-6	0	1	0.03	0.11
-6	0	1	0.23	0.17
-6	0	1	0.20	0.32
6	0	-1	0.05	0.11
6	0	-1	0.04	0.32
6	0	-1	-0.13	0.18
6	0	-1	0.03	0.16
-7	0	1	149.08	5.89
-7	0	1	159.45	5.88
-7	0	1	149.70	6.09
-7	0	1	153.16	6.02
7	0	-1	160.41	6.13
7	0	-1	150.76	5.87
7	0	-1	162.22	5.89
7	0	-1	155.48	5.99
-8	0	1	-0.07	0.28
-8	0	1	-0.09	0.22
-8	0	1	0.19	0.17
8	0	-1	-0.01	0.26
8	0	-1	-0.10	0.23
8	0	-1	0.44	0.43
8	0	-1	-0.04	0.16
-9	0	1	203.53	8.27
-9	0	1	234.73	8.27
-9	0	1	217.11	8.44
-9	0	1	215.47	8.50
9	0	-1	220.94	8.40
9	0	-1	230.93	8.53
9	0	-1	218.60	8.26
9	0	-1	202.99	8.23
-10	0	1	0.33	0.23
-10	0	1	0.05	0.34
-10	0	1	-0.05	0.37
-10	0	1	0.13	0.60
10	0	-1	-0.03	0.57
10	0	-1	-0.17	0.29
10	0	-1	0.17	0.37
10	0	-1	0.25	0.22
-11	0	1	74.81	3.18

-11	0	1	77.19	3.42
-11	0	1	81.79	3.14
-11	0	1	78.35	3.39
11	0	-1	77.87	3.31
11	0	-1	75.70	3.12
11	0	-1	77.58	3.44
-12	0	1	0.43	0.49
-12	0	1	0.29	0.45
-12	0	1	0.20	0.29
-12	0	1	0.91	0.69
12	0	-1	-2.22	1.07
12	0	-1	-0.76	0.39
12	0	-1	-0.10	0.27
12	0	-1	0.39	0.45
-13	0	1	220.41	8.64
-13	0	1	212.79	8.38
-13	0	1	208.50	8.58
-13	0	1	223.70	8.34
13	0	-1	205.35	8.50
13	0	-1	227.64	8.63
13	0	-1	218.01	8.33
13	0	-1	221.80	8.35
-14	0	1	-0.08	0.59
-14	0	1	-0.21	0.57
-14	0	1	1.17	0.80
14	0	-1	-0.59	0.39
14	0	-1	0.09	0.31
14	0	-1	1.59	0.85
-15	0	1	134.76	6.05
-15	0	1	154.90	6.22
15	0	-1	147.65	5.78
15	0	-1	154.22	6.14
15	0	-1	145.17	5.84
-16	0	1	-0.48	0.88
-16	0	1	-0.38	0.74
16	0	-1	-0.84	1.00
16	0	-1	-0.18	0.53
16	0	-1	0.18	0.43
-17	0	1	69.76	3.57
-17	0	1	75.76	3.74
17	0	-1	77.16	3.25
17	0	-1	75.38	3.20
-18	0	1	0.80	1.01
-18	0	1	-0.39	1.05

18	0	-1	-0.29	0.75
18	0	-1	-0.22	0.51
-19	0	1	85.83	4.32
19	0	-1	82.39	3.68
-20	0	1	0.16	1.12
20	0	-1	1.84	0.68
21	0	-1	98.87	4.34
21	0	2	-0.96	1.03
-21	0	-2	0.32	1.50
20	0	2	-0.03	0.72
-20	0	-2	-0.12	1.38
19	0	2	-0.23	0.65
19	0	2	0.60	1.08
-19	0	-2	1.12	1.21
18	0	2	0.26	0.59
18	0	2	0.18	0.88
-18	0	-2	1.22	1.13
17	0	2	-0.70	0.80
17	0	2	0.28	0.53
-17	0	-2	1.68	1.05
16	0	2	2.17	0.58
16	0	2	3.86	0.84
-16	0	-2	2.29	0.91
-16	0	-2	3.10	1.07
15	0	2	-0.32	0.59
15	0	2	0.43	0.42
-15	0	-2	-0.12	0.75
-15	0	-2	-1.17	0.86
14	0	2	8.19	1.09
14	0	2	8.10	0.83
-14	0	-2	10.74	1.32
-14	0	-2	10.50	1.41
13	0	2	-0.26	0.33
13	0	2	0.78	0.56
13	0	2	-0.75	0.49
-13	0	-2	0.12	0.66
-13	0	-2	-0.51	0.54
12	0	2	1.30	0.49
12	0	2	0.28	0.50
12	0	2	-0.12	0.27
-12	0	-2	-0.10	0.50
-12	0	-2	0.60	0.35
-12	0	-2	-0.19	0.29
-12	0	-2	0.47	0.40

-12	0	-2	0.92	0.63
11	0	2	0.28	0.28
11	0	2	-0.18	0.38
11	0	2	0.50	0.45
-11	0	-2	0.54	0.28
-11	0	-2	-0.20	0.27
-11	0	-2	-0.04	0.52
-11	0	-2	-0.53	0.43
10	0	2	-0.39	0.32
10	0	2	0.42	0.25
10	0	2	0.01	0.40
-10	0	-2	0.75	0.47
-10	0	-2	0.05	0.38
-10	0	-2	0.52	0.33
-10	0	-2	0.73	0.26
-10	0	-2	0.33	0.26
9	0	2	-0.09	0.22
9	0	2	0.44	0.37
9	0	2	-0.02	0.30
-9	0	-2	-0.34	0.33
-9	0	-2	-0.19	0.21
-9	0	-2	0.08	0.21
-9	0	-2	0.16	0.42
-9	0	-2	0.05	0.31
8	0	2	44.44	2.00
8	0	2	44.76	1.91
8	0	2	49.30	2.09
-8	0	-2	44.80	1.88
-8	0	-2	48.98	1.90
-8	0	-2	45.11	1.82
-8	0	-2	46.23	2.12
-8	0	-2	43.18	2.03
7	0	2	0.08	0.15
7	0	2	-0.13	0.30
7	0	2	-0.09	0.23
-7	0	-2	-0.04	0.23
-7	0	-2	0.07	0.14
-7	0	-2	0.12	0.26
-7	0	-2	0.10	0.31
6	0	2	15.41	0.75
6	0	2	16.82	0.95
6	0	2	15.81	0.83
-6	0	-2	15.49	0.94
-6	0	-2	16.36	0.74

-6	0	-2	15.57	0.84
5	0	2	0.10	0.16
5	0	2	0.04	0.11
5	0	2	-0.04	0.22
-5	0	-2	0.03	0.23
-5	0	-2	0.01	0.18
-5	0	-2	0.19	0.12
4	0	2	59.68	2.42
4	0	2	54.11	2.32
4	0	2	56.70	2.31
-4	0	-2	64.29	2.45
-4	0	-2	61.26	2.28
-4	0	-2	56.99	2.32
3	0	2	-0.06	0.07
3	0	2	0.04	0.11
3	0	2	0.07	0.17
-3	0	-2	0.07	0.20
-3	0	-2	0.02	0.10
-3	0	-2	0.09	0.08
2	0	2	199.96	7.68
2	0	2	201.39	7.57
2	0	2	203.43	7.57
-2	0	-2	200.62	7.57
-2	0	-2	209.41	7.69
-2	0	-2	207.37	7.57
-2	0	-2	200.71	7.56
1	0	2	0.02	0.07
1	0	2	-0.05	0.08
1	0	2	0.07	0.04
1	0	2	0.05	0.05
-1	0	-2	0.02	0.04
-1	0	-2	-0.02	0.04
-1	0	-2	-0.01	0.10
-1	0	-2	0.04	0.10
0	0	-2	264.61	10.74
0	0	-2	312.33	10.92
0	0	-2	294.44	10.88
-1	0	2	0.02	0.03
-1	0	2	0.03	0.05
-1	0	2	0.06	0.07
-1	0	2	-0.02	0.04
-1	0	2	0.06	0.11
1	0	-2	0.08	0.07
1	0	-2	-0.02	0.03

1	0	-2	0.08	0.14
-2	0	2	50.00	1.90
-2	0	2	46.53	1.85
-2	0	2	47.28	2.03
-2	0	2	48.96	1.84
2	0	-2	48.99	2.04
2	0	-2	52.33	1.90
2	0	-2	46.59	1.84
2	0	-2	49.72	1.85
-3	0	2	0.16	0.07
-3	0	2	-0.08	0.24
-3	0	2	-0.01	0.11
3	0	-2	0.01	0.06
3	0	-2	-0.11	0.25
3	0	-2	-0.01	0.12
-4	0	2	12.61	0.55
-4	0	2	12.50	0.82
-4	0	2	12.15	0.66
4	0	-2	12.91	0.85
4	0	-2	12.11	0.64
4	0	-2	12.29	0.54
-5	0	2	0.10	0.18
-5	0	2	0.07	0.10
-5	0	2	-0.33	0.28
5	0	-2	0.12	0.10
5	0	-2	0.02	0.16
5	0	-2	0.07	0.32
-6	0	2	231.72	8.77
-6	0	2	203.04	9.03
-6	0	2	241.00	8.64
6	0	-2	246.54	8.95
6	0	-2	228.42	8.62
-7	0	2	0.29	0.17
-7	0	2	0.09	0.14
-7	0	2	0.27	0.47
-7	0	2	0.20	0.23
7	0	-2	0.05	0.25
7	0	-2	0.63	0.46
7	0	-2	0.04	0.14
7	0	-2	0.01	0.14
-8	0	2	55.41	2.31
-8	0	2	61.86	2.35
-8	0	2	57.70	2.71
-8	0	2	56.93	2.52

8	0	-2	60.64	2.50
8	0	-2	55.53	2.33
8	0	-2	60.11	2.72
8	0	-2	60.40	2.30
-9	0	2	1.07	0.50
-9	0	2	-0.01	0.25
-9	0	2	0.20	0.18
-9	0	2	0.12	0.29
9	0	-2	0.53	0.33
9	0	-2	-0.18	0.16
9	0	-2	-0.17	0.20
9	0	-2	-0.78	0.55
-10	0	2	2.10	0.35
-10	0	2	2.11	0.32
-10	0	2	1.62	0.48
-10	0	2	1.90	0.67
10	0	-2	1.41	0.26
10	0	-2	1.88	0.32
10	0	-2	1.57	0.41
-11	0	2	0.99	0.70
-11	0	2	-0.04	0.34
-11	0	2	0.34	0.26
-11	0	2	-0.08	0.41
11	0	-2	0.18	0.39
11	0	-2	-0.08	0.69
11	0	-2	-0.06	0.20
11	0	-2	-0.24	0.27
-12	0	2	86.53	3.36
-12	0	2	75.89	3.33
-12	0	2	84.30	3.64
-12	0	2	83.09	3.74
12	0	-2	82.18	3.34
12	0	-2	86.36	3.79
12	0	-2	81.73	3.54
12	0	-2	83.70	3.29
-13	0	2	0.30	0.46
-13	0	2	-0.06	0.52
-13	0	2	0.05	0.75
-13	0	2	-0.05	0.32
13	0	-2	-0.33	0.52
13	0	-2	0.22	0.85
13	0	-2	-0.24	0.35
13	0	-2	0.39	0.31
-14	0	2	94.06	4.04

-14	0	2	87.59	4.07
-14	0	2	83.81	3.65
14	0	-2	91.36	3.67
14	0	-2	92.75	4.19
14	0	-2	91.30	3.64
-15	0	2	-0.43	1.05
-15	0	2	-0.04	0.89
-15	0	2	0.22	0.55
15	0	-2	-1.65	1.00
15	0	-2	0.16	0.35
-16	0	2	69.07	3.53
-16	0	2	72.11	3.51
-16	0	2	64.83	3.02
16	0	-2	73.17	3.62
16	0	-2	73.28	3.04
-17	0	2	1.21	0.89
-17	0	2	-0.14	1.03
17	0	-2	0.54	1.29
17	0	-2	-0.27	0.48
-18	0	2	50.35	3.08
-18	0	2	54.72	3.12
18	0	-2	54.45	2.52
-19	0	2	0.53	1.08
19	0	-2	-0.09	0.58
-20	0	2	21.83	2.41
20	0	-2	18.72	1.49
-21	0	2	0.16	1.27
21	0	-2	0.20	0.75
20	0	3	0.29	0.75
-20	0	-3	0.13	1.45
19	0	3	11.57	1.88
19	0	3	12.36	1.35
-19	0	-3	16.26	2.35
18	0	3	-0.05	0.61
18	0	3	-0.63	0.93
-18	0	-3	2.25	1.22
17	0	3	13.17	1.58
17	0	3	12.70	1.19
-17	0	-3	16.50	2.01
16	0	3	-0.01	0.48
16	0	3	0.84	0.77
-16	0	-3	0.28	0.89
-16	0	-3	0.78	1.10
15	0	3	5.79	0.93

15	0	3	6.16	0.84
-15	0	-3	4.86	1.00
-15	0	-3	5.98	1.18
14	0	3	0.42	0.62
14	0	3	-0.36	0.37
-14	0	-3	-0.44	0.67
-14	0	-3	0.81	0.87
13	0	3	28.86	1.68
13	0	3	28.49	1.72
13	0	3	27.34	1.46
-13	0	-3	27.70	1.80
-13	0	-3	30.88	1.93
12	0	3	-0.35	0.51
12	0	3	0.63	0.50
12	0	3	-0.23	0.29
-12	0	-3	0.38	0.32
-12	0	-3	0.35	0.66
-12	0	-3	-0.05	0.50
11	0	3	12.05	1.11
11	0	3	12.81	1.00
11	0	3	12.78	0.83
-11	0	-3	12.35	1.09
-11	0	-3	14.72	1.29
-11	0	-3	13.90	0.82
10	0	3	-0.20	0.36
10	0	3	0.25	0.24
10	0	3	0.29	0.45
-10	0	-3	0.52	0.39
-10	0	-3	0.06	0.50
-10	0	-3	-0.02	0.24
9	0	3	25.31	1.39
9	0	3	25.10	1.17
9	0	3	23.94	1.27
-9	0	-3	25.04	1.47
-9	0	-3	24.22	1.14
-9	0	-3	21.94	1.30
8	0	3	-0.08	0.38
8	0	3	0.42	0.29
8	0	3	0.20	0.18
-8	0	-3	-0.10	0.18
-8	0	-3	0.13	0.28
-8	0	-3	0.02	0.41
7	0	3	75.27	3.04
7	0	3	75.36	3.23

7	0	3	79.13	3.12
-7	0	-3	68.30	3.38
-7	0	-3	86.46	3.05
-7	0	-3	77.50	3.14
6	0	3	-0.22	0.27
6	0	3	0.02	0.15
6	0	3	0.30	0.21
-6	0	-3	0.25	0.32
-6	0	-3	0.40	0.22
-6	0	-3	-0.03	0.12
5	0	3	49.59	1.97
5	0	3	52.12	2.19
5	0	3	50.25	2.00
-5	0	-3	49.59	2.18
-5	0	-3	48.43	2.01
-5	0	-3	46.20	2.09
4	0	3	-0.08	0.32
4	0	3	0.13	0.15
4	0	3	-0.15	0.11
-4	0	-3	0.02	0.09
-4	0	-3	-0.13	0.25
-4	0	-3	0.13	0.14
3	0	3	79.08	3.28
3	0	3	85.47	3.31
3	0	3	86.94	3.31
3	0	3	87.17	3.48
-3	0	-3	91.66	3.31
-3	0	-3	86.96	3.32
-3	0	-3	92.28	3.51
-3	0	-3	86.80	3.32
2	0	3	-0.17	0.18
2	0	3	-0.02	0.08
2	0	3	0.08	0.07
2	0	3	-0.26	0.12
-2	0	-3	-0.11	0.17
-2	0	-3	0.20	0.09
-2	0	-3	0.05	0.07
-2	0	-3	0.23	0.22
1	0	3	158.12	5.87
1	0	3	156.56	5.77
1	0	3	154.74	5.92
-1	0	-3	152.45	5.78
-1	0	-3	151.71	5.80
-1	0	-3	153.89	5.93

-1	0	-3	152.53	5.89
-1	0	-3	159.59	5.78
0	0	3	-0.03	0.06
0	0	3	0.03	0.06
0	0	3	-0.01	0.12
0	0	3	0.02	0.17
0	0	-3	0.11	0.06
0	0	-3	0.13	0.15
0	0	-3	-0.16	0.18
0	0	-3	-0.03	0.05
-1	0	3	13.76	0.57
-1	0	3	12.88	0.64
-1	0	3	13.24	0.77
-1	0	3	14.28	0.56
1	0	-3	13.08	0.57
1	0	-3	14.32	0.56
1	0	-3	13.86	0.65
1	0	-3	12.73	0.78
-2	0	3	0.17	0.25
-2	0	3	0.08	0.09
-2	0	3	0.10	0.09
-2	0	3	0.09	0.11
-2	0	3	0.10	0.07
2	0	-3	0.01	0.12
2	0	-3	0.32	0.28
2	0	-3	0.10	0.06
-3	0	3	30.86	1.56
-3	0	3	34.10	1.36
-3	0	3	32.09	1.27
-3	0	3	34.41	1.28
3	0	-3	32.59	1.35
3	0	-3	32.97	1.27
3	0	-3	31.07	1.27
3	0	-3	32.79	1.59
-4	0	3	0.14	0.31
-4	0	3	0.17	0.09
4	0	-3	-0.08	0.30
4	0	-3	0.20	0.08
4	0	-3	0.03	0.14
-5	0	3	5.07	0.46
-5	0	3	5.61	0.74
-5	0	3	5.10	0.32
5	0	-3	4.64	0.31
5	0	-3	5.35	0.44

5	0	-3	6.37	0.75
-6	0	3	0.06	0.21
-6	0	3	0.66	0.45
-6	0	3	0.03	0.11
6	0	-3	-0.20	0.20
6	0	-3	0.13	0.11
6	0	-3	-0.12	0.43
-7	0	3	67.30	3.06
-7	0	3	69.91	2.69
-7	0	3	64.43	2.84
7	0	-3	66.95	2.67
7	0	-3	69.59	2.82
7	0	-3	72.25	3.11
-8	0	3	0.23	0.30
-8	0	3	0.17	0.17
-8	0	3	-0.55	0.46
8	0	-3	-0.72	0.99
8	0	-3	0.06	0.27
8	0	-3	-0.29	0.14
-9	0	3	17.92	0.85
-9	0	3	14.58	1.02
-9	0	3	16.56	1.37
9	0	-3	14.40	0.79
9	0	-3	16.24	1.02
9	0	-3	16.97	1.38
-10	0	3	-0.14	0.35
-10	0	3	-0.20	0.22
-10	0	3	1.10	0.68
10	0	-3	-0.17	0.33
10	0	-3	-0.18	0.63
10	0	-3	-0.10	0.19
-11	0	3	3.66	0.60
-11	0	3	5.33	0.54
-11	0	3	4.91	0.92
11	0	-3	4.31	0.59
11	0	-3	4.84	0.50
11	0	-3	6.07	0.99
-12	0	3	0.49	0.29
-12	0	3	-0.09	0.46
-12	0	3	0.10	0.74
12	0	-3	-1.17	0.78
12	0	-3	-0.23	0.24
12	0	-3	0.47	0.46
-13	0	3	21.70	1.51

-13	0	3	18.97	1.79
-13	0	3	20.97	1.16
13	0	-3	22.23	1.93
13	0	-3	20.35	1.11
-14	0	3	0.13	0.83
-14	0	3	-0.40	0.57
14	0	-3	0.83	1.06
14	0	-3	0.15	0.33
-15	0	3	12.08	1.38
-15	0	3	11.77	1.76
15	0	-3	12.10	0.88
15	0	-3	13.01	2.02
-16	0	3	0.08	1.01
-16	0	3	-0.36	0.78
16	0	-3	0.89	0.42
16	0	-3	0.44	1.25
-17	0	3	1.36	1.19
-17	0	3	1.90	0.96
17	0	-3	4.07	1.45
17	0	-3	0.56	0.47
-18	0	3	-0.42	0.94
-18	0	3	-1.35	1.23
18	0	-3	0.26	0.50
-19	0	3	3.46	1.16
-19	0	3	4.11	2.20
19	0	-3	3.51	0.73
-20	0	3	0.56	1.15
20	0	-3	-0.03	0.60
-21	0	3	19.57	2.56
21	0	-3	16.58	1.54
20	0	4	22.15	1.82
-20	0	-4	25.89	2.88
19	0	4	-1.58	1.10
19	0	4	-0.08	0.67
-19	0	-4	0.46	1.34
18	0	4	46.09	2.32
18	0	4	40.94	2.71
-18	0	-4	39.99	3.02
17	0	4	0.34	0.56
17	0	4	0.08	0.91
-17	0	-4	2.20	1.93
-17	0	-4	-0.05	1.03
16	0	4	58.05	2.96
16	0	4	60.51	2.68

-16	0	-4	54.72	3.14
-16	0	-4	55.01	3.33
15	0	4	0.02	0.75
15	0	4	-0.41	0.42
-15	0	-4	-1.19	0.73
-15	0	-4	-2.49	0.99
14	0	4	3.73	0.90
14	0	4	3.95	0.84
-14	0	-4	4.75	1.09
-14	0	-4	5.00	0.96
13	0	4	-1.10	0.52
13	0	4	0.24	0.34
13	0	4	-0.40	0.59
-13	0	-4	-1.21	0.81
-13	0	-4	0.96	0.68
12	0	4	55.19	2.42
12	0	4	51.94	2.67
12	0	4	54.04	2.54
-12	0	-4	55.83	2.70
-12	0	-4	54.52	2.85
-12	0	-4	64.43	2.87
11	0	4	0.03	0.49
11	0	4	-0.05	0.29
11	0	4	0.27	0.41
-11	0	-4	0.10	0.50
-11	0	-4	-0.56	0.26
-11	0	-4	1.22	0.70
10	0	4	57.71	2.50
10	0	4	55.83	2.40
10	0	4	57.77	2.68
-10	0	-4	62.15	2.39
-10	0	-4	56.32	2.76
-10	0	-4	52.21	2.55
9	0	4	0.18	0.31
9	0	4	-0.04	0.42
9	0	4	-0.09	0.21
-9	0	-4	0.31	0.22
-9	0	-4	0.68	0.53
-9	0	-4	0.11	0.38
8	0	4	41.64	1.87
8	0	4	43.76	2.13
8	0	4	44.25	1.96
-8	0	-4	49.64	2.24
-8	0	-4	46.65	1.87

-8	0	-4	42.36	1.99
7	0	4	0.49	0.36
7	0	4	0.17	0.17
7	0	4	0.20	0.24
-7	0	-4	0.45	0.28
-7	0	-4	0.17	0.41
-7	0	-4	-0.19	0.16
6	0	4	7.63	0.51
6	0	4	8.56	0.57
6	0	4	9.20	0.77
-6	0	-4	8.49	0.50
-6	0	-4	8.46	0.83
-6	0	-4	8.03	0.61
5	0	4	0.03	0.30
5	0	4	-0.01	0.14
5	0	4	0.22	0.17
-5	0	-4	0.04	0.13
-5	0	-4	-0.37	0.35
-5	0	-4	0.07	0.25
4	0	4	234.03	9.03
4	0	4	225.39	8.81
4	0	4	234.56	8.81
4	0	4	245.32	8.78
-4	0	-4	246.56	9.07
-4	0	-4	233.34	8.83
-4	0	-4	224.92	8.82
-4	0	-4	237.60	8.81
3	0	4	-0.07	0.11
3	0	4	-0.22	0.27
3	0	4	0.02	0.10
3	0	4	-0.14	0.14
-3	0	-4	0.09	0.10
-3	0	-4	-0.38	0.25
-3	0	-4	0.01	0.12
-3	0	-4	0.22	0.20
2	0	4	51.96	2.07
2	0	4	53.58	2.06
2	0	4	54.81	2.16
-2	0	-4	54.21	2.07
-2	0	-4	53.56	2.07
-2	0	-4	52.54	2.20
-2	0	-4	54.21	2.29
1	0	4	0.17	0.19
1	0	4	0.29	0.20

1	0	4	0.07	0.09
-1	0	-4	-0.08	0.08
-1	0	-4	0.01	0.08
-1	0	-4	-0.01	0.08
-1	0	-4	0.16	0.25
-1	0	-4	-0.13	0.19
0	0	4	216.91	7.63
0	0	4	199.79	7.64
0	0	4	208.20	7.82
0	0	4	199.04	7.78
0	0	-4	206.43	7.62
0	0	-4	200.07	7.78
0	0	-4	196.79	7.63
0	0	-4	210.72	7.86
-1	0	4	0.05	0.07
-1	0	4	-0.08	0.16
-1	0	4	0.09	0.09
-1	0	4	0.22	0.28
1	0	-4	-0.07	0.28
1	0	-4	-0.12	0.06
1	0	-4	0.02	0.08
1	0	-4	-0.07	0.18
-2	0	4	161.32	5.93
-2	0	4	154.45	5.92
-2	0	4	158.04	5.97
-2	0	4	155.63	5.89
2	0	-4	159.91	5.98
2	0	-4	152.36	5.89
2	0	-4	162.34	6.22
-3	0	4	0.45	0.43
-3	0	4	0.06	0.12
-3	0	4	0.08	0.09
-3	0	4	0.14	0.13
-3	0	4	0.17	0.13
3	0	-4	0.07	0.07
3	0	-4	0.13	0.30
3	0	-4	0.12	0.13
3	0	-4	0.01	0.12
-4	0	4	3.48	0.59
-4	0	4	3.76	0.26
-4	0	4	3.88	0.26
-4	0	4	4.05	0.39
4	0	-4	4.05	0.27
4	0	-4	3.93	0.69

4	0	-4	3.64	0.25
4	0	-4	3.90	0.36
-5	0	4	0.11	0.11
-5	0	4	-0.25	0.37
-5	0	4	0.13	0.19
5	0	-4	0.03	0.10
5	0	-4	-0.11	0.17
5	0	-4	0.52	0.41
-6	0	4	0.23	0.14
-6	0	4	0.09	0.43
-6	0	4	0.78	0.25
6	0	-4	0.08	0.11
6	0	-4	0.21	0.22
6	0	-4	-0.14	0.47
-7	0	4	0.11	0.15
-7	0	4	-0.39	0.47
-7	0	4	-0.13	0.25
7	0	-4	-0.39	0.52
7	0	-4	0.03	0.14
7	0	-4	0.20	0.26
-8	0	4	166.10	6.52
-8	0	4	160.14	6.76
-8	0	4	173.97	6.33
8	0	-4	168.36	6.48
8	0	-4	156.12	6.30
8	0	-4	173.91	6.84
-9	0	4	-0.16	0.20
-9	0	4	0.13	0.34
-9	0	4	0.85	0.64
9	0	-4	0.03	0.17
9	0	-4	1.38	0.68
9	0	-4	0.26	0.31
-10	0	4	35.02	2.21
-10	0	4	39.29	1.90
-10	0	4	39.70	1.66
10	0	-4	38.47	2.27
10	0	-4	35.18	1.60
10	0	-4	38.93	1.85
-11	0	4	0.34	0.26
-11	0	4	0.08	0.44
-11	0	4	0.78	0.74
11	0	-4	-1.06	0.36
11	0	-4	0.10	0.23
11	0	-4	-1.04	0.76

-12	0	4	-0.46	0.29
-12	0	4	-0.02	0.44
-12	0	4	0.24	0.83
12	0	-4	0.21	0.26
12	0	-4	0.30	0.90
12	0	-4	-1.12	0.46
-13	0	4	0.29	0.36
-13	0	4	0.47	0.87
-13	0	4	0.01	0.50
13	0	-4	-0.27	0.98
13	0	-4	-0.09	0.29
-14	0	4	2.60	1.07
-14	0	4	1.94	0.71
14	0	-4	1.60	0.39
14	0	-4	3.63	1.17
-15	0	4	0.34	1.03
-15	0	4	0.95	0.71
15	0	-4	-0.92	1.20
15	0	-4	0.42	0.36
-16	0	4	0.66	1.14
-16	0	4	0.09	0.74
16	0	-4	-0.04	0.38
16	0	-4	2.03	1.41
-17	0	4	-0.63	1.15
-17	0	4	-0.53	0.84
17	0	-4	0.44	1.51
17	0	-4	0.16	0.46
-18	0	4	0.68	0.94
-18	0	4	-0.80	1.32
18	0	-4	-0.12	0.51
-19	0	4	-1.78	0.99
-19	0	4	-1.81	1.47
19	0	-4	0.00	0.57
-20	0	4	1.35	1.18
20	0	-4	1.19	0.68
-21	0	4	-0.82	1.31
21	0	-4	0.09	0.70
20	0	5	0.73	0.85
19	0	5	63.57	3.00
-19	0	-5	56.67	3.76
18	0	5	0.25	0.64
18	0	5	0.59	1.13
-18	0	-5	1.40	1.26
17	0	5	54.96	2.66

17	0	5	54.47	3.04
-17	0	-5	53.54	3.21
16	0	5	-1.98	0.88
16	0	5	0.61	0.54
-16	0	-5	-1.60	0.92
15	0	5	37.88	2.38
15	0	5	41.56	2.03
-15	0	-5	40.95	2.81
-15	0	-5	39.04	2.45
14	0	5	0.32	0.76
14	0	5	0.33	0.43
-14	0	-5	0.25	0.93
-14	0	-5	0.69	0.76
13	0	5	63.61	2.69
13	0	5	60.30	2.79
13	0	5	62.14	3.01
-13	0	-5	58.68	2.95
-13	0	-5	57.72	3.17
12	0	5	-0.26	0.49
12	0	5	-0.43	0.35
12	0	5	-0.18	0.61
-12	0	-5	0.93	0.83
-12	0	-5	0.40	0.60
11	0	5	221.39	8.76
11	0	5	219.19	8.46
11	0	5	219.03	8.52
-11	0	-5	213.31	8.61
-11	0	-5	225.85	8.42
-11	0	-5	222.00	8.86
10	0	5	0.30	0.54
10	0	5	1.10	0.42
10	0	5	0.19	0.28
-10	0	-5	0.06	0.42
-10	0	-5	0.27	0.28
-10	0	-5	-1.50	0.61
9	0	5	119.16	4.95
9	0	5	111.33	4.65
9	0	5	121.24	4.73
-9	0	-5	116.40	4.76
-9	0	-5	123.43	5.05
-9	0	-5	121.76	4.64
8	0	5	-0.52	0.44
8	0	5	0.11	0.23
8	0	5	-0.44	0.27

-8	0	-5	-0.60	0.47
-8	0	-5	0.07	0.20
-8	0	-5	0.47	0.36
7	0	5	131.40	5.08
7	0	5	126.56	5.05
7	0	5	124.56	5.31
-7	0	-5	140.51	5.42
-7	0	-5	129.18	5.11
-7	0	-5	133.88	5.03
6	0	5	0.41	0.22
6	0	5	0.02	0.16
6	0	5	0.34	0.38
-6	0	-5	0.40	0.18
-6	0	-5	0.17	0.41
-6	0	-5	0.13	0.25
5	0	5	46.76	1.75
5	0	5	41.56	1.79
5	0	5	44.83	2.08
5	0	5	43.02	1.80
-5	0	-5	42.65	1.85
-5	0	-5	43.28	2.11
-5	0	-5	45.99	1.80
-5	0	-5	43.99	1.84
4	0	5	0.27	0.34
4	0	5	0.05	0.12
4	0	5	-0.11	0.17
4	0	5	0.16	0.16
-4	0	-5	-0.01	0.26
-4	0	-5	0.30	0.37
-4	0	-5	0.05	0.16
3	0	5	118.72	4.61
3	0	5	124.07	4.68
3	0	5	124.40	4.60
3	0	5	115.89	4.84
-3	0	-5	124.50	4.61
-3	0	-5	121.76	4.61
-3	0	-5	116.68	4.74
-3	0	-5	125.34	4.89
2	0	5	0.05	0.11
2	0	5	0.14	0.26
2	0	5	0.07	0.20
-2	0	-5	0.12	0.12
-2	0	-5	-0.03	0.11
-2	0	-5	-0.30	0.27

-2	0	-5	0.12	0.10
-2	0	-5	0.10	0.29
1	0	5	207.43	7.80
1	0	5	211.87	7.61
1	0	5	197.85	7.83
-1	0	-5	200.61	7.62
-1	0	-5	198.94	7.63
-1	0	-5	206.04	7.85
-1	0	-5	205.10	7.60
-1	0	-5	201.80	7.85
0	0	5	0.08	0.23
0	0	5	-0.02	0.28
0	0	5	0.37	0.11
0	0	5	0.49	0.12
0	0	-5	0.47	0.11
0	0	-5	0.28	0.27
0	0	-5	0.31	0.32
0	0	-5	0.48	0.12
-1	0	5	921.81	35.69
1	0	-5	1000.00	35.90
1	0	-5	960.68	35.70
-2	0	5	-0.11	0.11
-2	0	5	0.29	0.19
-2	0	5	0.01	0.09
-2	0	5	0.00	0.11
-2	0	5	-0.16	0.35
2	0	-5	-0.14	0.19
2	0	-5	0.31	0.36
2	0	-5	-0.06	0.09
-3	0	5	36.74	1.54
-3	0	5	35.36	1.85
-3	0	5	37.46	1.53
-3	0	5	37.10	1.48
-3	0	5	34.84	1.55
3	0	-5	37.81	1.47
3	0	-5	39.30	1.93
3	0	-5	40.30	1.58
-4	0	5	-0.27	0.36
-4	0	5	-0.26	0.18
-4	0	5	0.25	0.15
-4	0	5	0.07	0.15
-4	0	5	0.09	0.10
4	0	-5	0.79	0.46
4	0	-5	0.09	0.16

-5	0	5	19.60	1.38
-5	0	5	23.15	1.06
-5	0	5	21.48	0.90
-5	0	5	22.28	0.93
5	0	-5	21.13	0.92
5	0	-5	20.23	1.59
5	0	-5	22.10	0.91
5	0	-5	21.81	1.15
-6	0	5	0.03	0.21
-6	0	5	-0.17	0.51
-6	0	5	0.06	0.14
6	0	-5	-0.01	0.21
6	0	-5	1.11	0.57
6	0	-5	-0.14	0.11
-7	0	5	38.85	1.84
-7	0	5	38.63	2.16
-7	0	5	42.40	1.68
7	0	-5	38.14	1.64
7	0	-5	42.65	2.28
7	0	-5	43.23	1.83
-8	0	5	-0.03	0.61
-8	0	5	-0.26	0.17
-8	0	5	-0.23	0.30
8	0	-5	0.03	0.15
8	0	-5	0.15	0.30
8	0	-5	0.04	0.68
-9	0	5	162.54	6.39
-9	0	5	157.40	6.70
-9	0	5	166.99	6.17
9	0	-5	162.55	6.34
9	0	-5	161.88	6.75
9	0	-5	155.34	6.13
-10	0	5	-0.35	0.38
-10	0	5	0.19	0.77
-10	0	5	-0.09	0.25
10	0	-5	0.35	0.22
10	0	-5	-0.60	0.80
10	0	-5	-0.19	0.34
-11	0	5	115.78	4.20
-11	0	5	103.52	4.43
-11	0	5	103.05	4.76
11	0	-5	105.19	4.38
11	0	-5	102.18	4.13
11	0	-5	104.84	4.84

-12	0	5	0.48	0.33
-12	0	5	1.04	0.90
-12	0	5	0.16	0.44
12	0	-5	-0.09	0.48
12	0	-5	1.02	1.01
12	0	-5	-0.48	0.26
-13	0	5	0.86	0.60
-13	0	5	2.06	0.45
-13	0	5	1.24	0.99
13	0	-5	3.36	1.18
-14	0	5	-0.47	0.58
-14	0	5	-0.17	1.01
14	0	-5	-0.30	0.32
14	0	-5	0.41	1.18
-15	0	5	4.99	1.27
-15	0	5	3.82	0.87
15	0	-5	4.55	0.68
15	0	-5	7.44	1.48
-16	0	5	-0.35	1.17
-16	0	5	0.85	0.87
16	0	-5	0.23	0.40
16	0	-5	0.46	1.47
-17	0	5	29.40	2.22
-17	0	5	26.02	2.64
17	0	-5	28.36	1.57
17	0	-5	30.19	3.07
-18	0	5	-1.56	1.30
-18	0	5	-1.80	0.95
18	0	-5	-0.99	0.49
-19	0	5	4.22	1.63
-19	0	5	4.07	1.19
19	0	-5	3.35	0.69
-20	0	5	0.02	1.21
20	0	-5	-0.44	0.63
-21	0	5	2.67	1.99
21	0	-5	3.96	0.80
-20	0	-6	36.44	3.30
19	0	6	-1.29	1.35
19	0	6	0.67	0.75
-19	0	-6	-0.87	1.40
18	0	6	61.87	2.90
18	0	6	57.59	3.39
-18	0	-6	54.51	3.60
17	0	6	1.04	0.60

17	0	6	-1.28	1.03
-17	0	-6	-0.27	1.18
16	0	6	98.14	4.54
16	0	6	101.62	4.20
-16	0	-6	91.46	4.57
15	0	6	-0.12	0.52
15	0	6	-1.21	0.82
-15	0	-6	-0.86	0.84
14	0	6	58.23	2.55
14	0	6	52.32	2.88
-14	0	-6	52.42	2.87
-14	0	-6	55.33	3.17
13	0	6	-0.39	0.71
13	0	6	0.22	0.49
13	0	6	0.46	0.59
-13	0	-6	-0.32	0.98
-13	0	-6	-0.94	0.65
12	0	6	95.76	3.99
12	0	6	94.96	3.92
12	0	6	93.59	4.26
-12	0	-6	100.90	4.47
-12	0	-6	91.85	4.11
11	0	6	-0.07	0.44
11	0	6	0.42	0.35
11	0	6	-0.39	0.59
-11	0	-6	0.24	0.81
-11	0	-6	-0.16	0.33
-11	0	-6	-0.31	0.49
10	0	6	129.73	5.49
10	0	6	123.43	5.15
10	0	6	134.11	5.22
-10	0	-6	132.33	5.29
-10	0	-6	136.71	5.63
-10	0	-6	130.35	5.12
9	0	6	-0.05	0.50
9	0	6	0.05	0.34
9	0	6	0.19	0.29
-9	0	-6	-0.16	0.41
-9	0	-6	-0.62	0.67
-9	0	-6	0.09	0.25
8	0	6	205.56	7.94
8	0	6	200.62	7.92
8	0	6	204.26	8.24
-8	0	-6	210.69	7.90

-8	0	-6	205.20	7.98
-8	0	-6	220.14	8.36
7	0	6	-0.06	0.43
7	0	6	0.16	0.25
7	0	6	-0.09	0.20
-7	0	-6	-0.09	0.50
-7	0	-6	0.27	0.21
-7	0	-6	0.49	0.32
6	0	6	310.98	11.15
6	0	6	295.58	11.54
6	0	6	298.73	11.22
6	0	6	284.79	11.23
-6	0	-6	302.69	11.26
-6	0	-6	300.12	11.21
-6	0	-6	306.93	11.62
-6	0	-6	289.90	11.24
5	0	6	0.01	0.19
5	0	6	-0.16	0.15
5	0	6	-0.16	0.21
5	0	6	0.07	0.39
-5	0	-6	0.30	0.18
-5	0	-6	0.21	0.42
-5	0	-6	0.17	0.23
-5	0	-6	0.00	0.35
4	0	6	265.72	10.28
4	0	6	282.43	10.32
4	0	6	269.78	10.25
4	0	6	266.09	10.57
-4	0	-6	283.62	10.64
-4	0	-6	266.46	10.42
-4	0	-6	276.02	10.28
-4	0	-6	282.73	10.27
3	0	6	0.04	0.13
3	0	6	-0.03	0.25
3	0	6	0.06	0.14
3	0	6	0.04	0.31
-3	0	-6	-0.03	0.14
-3	0	-6	0.17	0.36
-3	0	-6	-0.57	0.33
-3	0	-6	0.20	0.15
-3	0	-6	-0.04	0.14
2	0	6	195.01	7.65
2	0	6	202.52	7.47
2	0	6	191.71	7.75

-2	0	-6	203.14	7.75
-2	0	-6	203.36	7.52
-2	0	-6	196.18	7.49
-2	0	-6	204.04	7.47
1	0	6	0.14	0.28
1	0	6	-0.17	0.12
1	0	6	0.15	0.12
-1	0	-6	0.07	0.12
-1	0	-6	0.19	0.11
-1	0	-6	0.18	0.14
-1	0	-6	-0.26	0.30
-1	0	-6	-0.03	0.36
0	0	6	927.83	33.58
0	0	6	855.13	33.51
0	0	6	901.35	33.31
0	0	6	923.83	33.30
0	0	-6	946.75	33.55
0	0	-6	927.02	33.66
0	0	-6	893.86	33.28
0	0	-6	818.43	33.35
-1	0	6	0.30	0.34
-1	0	6	0.09	0.37
-1	0	6	0.07	0.13
-1	0	6	0.04	0.12
1	0	-6	0.21	0.12
1	0	-6	-0.07	0.39
1	0	-6	0.12	0.11
1	0	-6	0.39	0.29
-2	0	6	303.26	11.41
-2	0	6	296.96	11.75
-2	0	6	310.86	11.54
-2	0	6	302.60	11.38
-2	0	6	307.26	11.41
2	0	-6	293.95	11.37
2	0	-6	307.60	11.83
2	0	-6	320.91	11.56
-3	0	6	0.00	0.15
-3	0	6	0.19	0.19
-3	0	6	0.12	0.22
-3	0	6	0.14	0.12
-3	0	6	-0.01	0.39
3	0	-6	0.18	0.21
-4	0	6	295.35	11.27
-4	0	6	298.83	11.19

-4	0	6	310.56	11.27
-4	0	6	289.70	11.25
4	0	-6	306.73	11.72
4	0	-6	296.56	11.18
4	0	-6	302.51	11.26
-5	0	6	0.05	0.17
-5	0	6	-0.38	0.51
-5	0	6	-0.09	0.14
-5	0	6	-0.23	0.19
-5	0	6	-0.08	0.21
5	0	-6	-0.56	0.51
5	0	-6	0.19	0.11
5	0	-6	-0.26	0.18
-6	0	6	191.95	7.74
-6	0	6	207.73	8.34
-6	0	6	213.43	7.92
-6	0	6	204.71	7.94
6	0	-6	197.24	7.78
6	0	-6	213.87	8.41
6	0	-6	217.48	7.95
6	0	-6	213.69	7.76
-7	0	6	-0.03	0.28
-7	0	6	0.02	0.17
-7	0	6	0.20	0.60
7	0	-6	-0.11	0.28
7	0	-6	-0.18	0.65
7	0	-6	-0.19	0.13
-8	0	6	229.19	7.88
-8	0	6	204.84	8.06
-8	0	6	205.61	8.45
8	0	-6	207.90	8.04
8	0	-6	194.59	7.83
8	0	-6	204.56	8.51
-9	0	6	0.39	0.73
-9	0	6	0.27	0.22
-9	0	6	-0.21	0.35
9	0	-6	-0.06	0.18
9	0	-6	-0.27	0.34
9	0	-6	-0.48	0.73
-10	0	6	76.40	3.27
-10	0	6	76.30	3.70
-10	0	6	78.36	3.03
10	0	-6	76.45	3.77
10	0	-6	73.70	3.21

10	0	-6	68.78	2.96
-11	0	6	0.18	0.84
-11	0	6	-0.24	0.28
-11	0	6	0.04	0.46
11	0	-6	0.20	0.24
11	0	-6	0.56	0.45
11	0	-6	0.04	0.96
-12	0	6	90.50	3.85
-12	0	6	80.10	4.21
-12	0	6	95.95	3.57
12	0	-6	84.58	3.76
12	0	-6	86.11	3.49
12	0	-6	86.24	4.39
-13	0	6	0.69	0.39
-13	0	6	-0.90	0.53
-13	0	6	-0.53	0.97
13	0	-6	-0.29	0.29
13	0	-6	0.33	1.23
-14	0	6	34.32	2.42
-14	0	6	40.15	2.76
14	0	-6	37.80	1.76
14	0	-6	41.12	3.01
-15	0	6	0.53	1.19
-15	0	6	0.27	0.69
15	0	-6	1.27	1.47
15	0	-6	0.10	0.36
-16	0	6	34.41	2.24
-16	0	6	33.28	2.77
16	0	-6	32.83	1.64
16	0	-6	29.42	3.06
-17	0	6	-1.37	1.40
-17	0	6	-0.07	0.92
17	0	-6	-0.07	0.46
-18	0	6	76.16	3.87
-18	0	6	74.21	4.30
18	0	-6	72.99	3.21
-19	0	6	-0.28	1.09
-19	0	6	-2.04	1.59
19	0	-6	-0.45	0.56
-20	0	6	36.30	2.89
20	0	-6	36.22	2.03
21	0	-6	0.68	0.75
19	0	7	16.93	2.64
19	0	7	16.43	1.62

-19	0	-7	20.26	2.77
18	0	7	0.18	0.68
18	0	7	0.30	1.31
-18	0	-7	0.78	1.34
17	0	7	29.63	1.86
17	0	7	32.60	2.55
-17	0	-7	30.99	2.61
16	0	7	-0.60	0.54
-16	0	-7	0.37	1.03
15	0	7	56.22	2.65
15	0	7	55.93	3.08
-15	0	-7	54.36	3.02
14	0	7	0.02	0.47
14	0	7	-0.15	0.87
-14	0	-7	0.92	0.85
13	0	7	55.16	2.66
13	0	7	55.98	2.56
13	0	7	59.62	3.00
-13	0	-7	56.08	2.85
12	0	7	-0.20	0.73
12	0	7	0.66	0.55
12	0	7	0.06	0.35
-12	0	-7	1.40	1.03
-12	0	-7	-0.43	0.64
11	0	7	55.36	2.40
11	0	7	52.79	2.80
11	0	7	55.23	2.47
-11	0	-7	54.92	2.96
-11	0	-7	51.77	2.58
10	0	7	0.44	0.44
10	0	7	0.12	0.30
10	0	7	0.27	0.66
-10	0	-7	0.33	0.33
-10	0	-7	-0.89	0.78
-10	0	-7	-0.50	0.47
9	0	7	103.42	4.09
9	0	7	100.15	4.10
9	0	7	102.85	4.48
-9	0	-7	106.86	4.61
-9	0	-7	101.99	4.05
-9	0	-7	98.71	4.16
8	0	7	0.01	0.32
8	0	7	0.15	0.53
8	0	7	-0.27	0.23

-8	0	-7	0.30	0.26
-8	0	-7	-0.76	0.62
-8	0	-7	0.09	0.34
7	0	7	66.57	2.78
7	0	7	68.15	3.16
7	0	7	70.69	2.68
7	0	7	67.30	2.79
-7	0	-7	66.29	2.80
-7	0	-7	66.45	2.83
-7	0	-7	68.32	2.76
-7	0	-7	76.69	3.31
6	0	7	0.04	0.42
6	0	7	0.14	0.21
6	0	7	-0.12	0.23
6	0	7	0.08	0.24
-6	0	-7	-0.14	0.26
-6	0	-7	0.14	0.20
-6	0	-7	-0.40	0.39
-6	0	-7	-0.04	0.51
5	0	7	162.16	6.06
5	0	7	156.46	6.00
5	0	7	151.04	6.37
5	0	7	158.04	6.04
-5	0	-7	160.15	6.04
-5	0	-7	164.66	6.47
-5	0	-7	150.44	6.19
-5	0	-7	161.52	6.03
4	0	7	0.28	0.17
4	0	7	0.19	0.19
4	0	7	0.18	0.29
4	0	7	-0.33	0.39
-4	0	-7	-0.07	0.18
-4	0	-7	-0.14	0.15
-4	0	-7	-0.12	0.19
-4	0	-7	0.04	0.42
-4	0	-7	-0.30	0.43
3	0	7	211.10	8.61
3	0	7	219.42	8.27
-3	0	-7	224.87	8.28
-3	0	-7	231.52	8.69
-3	0	-7	226.92	8.30
-3	0	-7	213.81	8.57
-3	0	-7	215.72	8.33
2	0	7	-0.10	0.15

2	0	7	0.35	0.34
2	0	7	0.69	0.40
-2	0	-7	-0.48	0.35
-2	0	-7	-0.43	0.43
-2	0	-7	0.05	0.14
-2	0	-7	0.06	0.15
1	0	7	145.98	5.56
1	0	7	142.86	5.82
1	0	7	150.84	5.52
1	0	7	146.97	5.80
-1	0	-7	144.66	5.85
-1	0	-7	147.04	5.53
-1	0	-7	145.04	5.92
-1	0	-7	145.53	5.51
-1	0	-7	146.35	5.56
0	0	7	0.02	0.31
0	0	7	0.29	0.14
0	0	7	-0.22	0.35
0	0	7	0.39	0.16
0	0	-7	0.30	0.15
0	0	-7	-0.20	0.35
0	0	-7	0.12	0.46
0	0	-7	0.31	0.13
-1	0	7	509.29	18.91
-1	0	7	515.13	19.27
-1	0	7	527.57	18.91
-1	0	7	491.79	19.11
1	0	-7	501.05	18.89
1	0	-7	525.73	19.15
1	0	-7	489.12	18.88
-2	0	7	-0.27	0.14
-2	0	7	0.10	0.13
-2	0	7	-0.19	0.15
-2	0	7	0.10	0.33
-2	0	7	0.97	0.47
2	0	-7	-0.03	0.14
2	0	-7	0.34	0.51
2	0	-7	0.10	0.29
-3	0	7	277.89	10.58
-3	0	7	275.60	10.54
-3	0	7	276.37	10.58
-3	0	7	262.85	10.97
3	0	-7	284.05	10.53
3	0	-7	294.86	11.11

3	0	-7	302.00	10.71
-4	0	7	-0.01	0.19
-4	0	7	0.06	0.19
-4	0	7	-0.05	0.14
-4	0	7	-0.75	0.47
-4	0	7	0.47	0.26
4	0	-7	0.00	0.56
4	0	-7	-0.42	0.24
4	0	-7	0.09	0.13
-5	0	7	116.69	4.54
-5	0	7	117.60	4.52
-5	0	7	112.99	4.47
-5	0	7	113.31	4.98
-5	0	7	120.72	4.43
5	0	-7	113.24	4.48
5	0	-7	114.92	4.40
5	0	-7	116.43	5.06
-6	0	7	0.14	0.20
-6	0	7	0.31	0.26
-6	0	7	0.32	0.17
-6	0	7	0.13	0.63
-6	0	7	0.30	0.26
6	0	-7	-0.05	0.14
6	0	-7	-0.61	0.65
6	0	-7	0.08	0.22
-7	0	7	196.11	7.00
-7	0	7	169.80	6.75
-7	0	7	178.71	7.00
-7	0	7	163.72	7.40
-7	0	7	191.67	6.85
7	0	-7	187.06	7.56
7	0	-7	169.57	6.80
7	0	-7	185.65	6.77
-8	0	7	0.05	0.31
-8	0	7	0.16	0.21
-8	0	7	-0.15	0.67
-8	0	7	-0.24	0.31
8	0	-7	0.49	0.37
8	0	-7	0.51	0.79
8	0	-7	-0.05	0.17
-9	0	7	203.05	7.65
-9	0	7	196.58	8.30
-9	0	7	200.15	7.87
9	0	-7	207.49	7.85

9	0	-7	192.87	7.60
9	0	-7	206.25	8.42
-10	0	7	-0.45	0.83
-10	0	7	0.39	0.27
-10	0	7	-0.32	0.42
10	0	-7	0.10	0.21
10	0	-7	-0.14	0.43
10	0	-7	0.68	0.96
-11	0	7	157.84	5.85
-11	0	7	150.46	6.10
-11	0	7	150.43	6.57
11	0	-7	149.28	6.06
11	0	-7	152.07	6.67
11	0	-7	142.63	5.77
-12	0	7	0.02	0.51
-12	0	7	-0.71	0.32
-12	0	7	-0.01	1.03
12	0	-7	0.07	1.19
12	0	-7	-0.18	0.26
-13	0	7	133.77	6.05
-13	0	7	130.99	5.53
-13	0	7	141.70	5.26
13	0	-7	126.48	5.16
13	0	-7	128.95	6.12
-14	0	7	-0.53	1.20
-14	0	7	0.04	0.70
14	0	-7	0.93	1.58
14	0	-7	0.58	0.35
-15	0	7	106.74	5.27
-15	0	7	113.34	4.83
15	0	-7	108.21	5.47
15	0	-7	105.34	4.33
-16	0	7	1.33	1.44
-16	0	7	0.62	0.83
16	0	-7	0.36	0.43
-17	0	7	92.42	4.95
-17	0	7	96.82	4.53
17	0	-7	95.45	3.93
-18	0	7	1.97	1.61
-18	0	7	-1.01	0.97
18	0	-7	0.09	0.53
-19	0	7	59.57	4.15
-19	0	7	60.39	3.46
19	0	-7	61.01	2.78

-20	0	7	1.11	1.24
-20	0	7	-0.85	1.93
20	0	-7	-0.01	0.63
19	0	8	1.82	1.60
19	0	8	-1.06	0.94
-19	0	-8	-1.72	1.48
18	0	8	3.89	0.89
18	0	8	3.18	1.47
-18	0	-8	0.43	1.46
17	0	8	0.57	0.68
17	0	8	0.14	1.24
-17	0	-8	-1.00	1.23
16	0	8	9.01	1.49
16	0	8	8.66	1.27
-16	0	-8	11.69	1.89
15	0	8	-0.48	0.56
15	0	8	-0.88	0.97
-15	0	-8	1.46	0.93
14	0	8	4.69	1.07
14	0	8	2.17	0.64
-14	0	-8	1.65	0.87
13	0	8	0.55	0.65
13	0	8	-0.55	0.49
13	0	8	0.45	0.84
-13	0	-8	-0.11	0.75
12	0	8	12.20	1.59
12	0	8	12.48	1.15
12	0	8	11.43	1.14
-12	0	-8	11.31	1.30
11	0	8	0.11	0.34
11	0	8	0.27	0.51
11	0	8	-0.07	0.73
-11	0	-8	-0.46	0.53
-11	0	-8	0.47	0.96
10	0	8	6.14	0.99
10	0	8	7.13	0.73
10	0	8	6.26	0.88
-10	0	-8	6.50	0.92
-10	0	-8	8.32	1.27
-10	0	-8	5.12	0.70
9	0	8	-0.58	0.61
9	0	8	0.22	0.31
9	0	8	0.83	0.42
-9	0	-8	0.47	0.44

-9	0	-8	0.05	0.29
-9	0	-8	0.24	0.81
8	0	8	7.42	0.67
8	0	8	8.71	0.73
8	0	8	6.63	1.07
-8	0	-8	8.46	0.77
-8	0	-8	7.51	1.28
-8	0	-8	7.96	0.63
7	0	8	-0.14	0.28
7	0	8	-0.07	0.53
7	0	8	-0.12	0.25
7	0	8	-0.08	0.25
-7	0	-8	0.40	0.46
-7	0	-8	0.05	0.32
-7	0	-8	-0.18	0.22
-7	0	-8	0.63	0.63
6	0	8	9.74	0.65
6	0	8	10.12	0.70
6	0	8	9.51	1.08
6	0	8	9.41	0.65
-6	0	-8	9.16	0.96
-6	0	-8	9.71	1.23
-6	0	-8	9.66	0.69
-6	0	-8	10.78	0.65
5	0	8	-0.43	0.21
5	0	8	0.45	0.45
5	0	8	0.11	0.20
-5	0	-8	-0.27	0.26
-5	0	-8	0.70	0.57
-5	0	-8	0.52	0.49
-5	0	-8	-0.46	0.19
-5	0	-8	-0.06	0.21
4	0	8	67.50	2.70
4	0	8	70.41	2.89
4	0	8	66.94	3.11
-4	0	-8	67.92	2.78
-4	0	-8	72.43	2.74
-4	0	-8	74.50	3.23
-4	0	-8	64.48	3.04
-4	0	-8	65.72	2.72
3	0	8	-0.09	0.19
3	0	8	-0.09	0.36
3	0	8	-0.69	0.40
-3	0	-8	-0.49	0.45

-3	0	-8	-0.06	0.18
-3	0	-8	-0.14	0.50
-3	0	-8	-0.10	0.18
-3	0	-8	0.02	0.17
2	0	8	18.08	1.26
2	0	8	20.43	0.88
2	0	8	17.21	1.14
-2	0	-8	22.46	1.36
-2	0	-8	18.90	0.93
-2	0	-8	19.14	0.88
-2	0	-8	19.72	0.90
-2	0	-8	19.94	1.39
1	0	8	-0.22	0.35
1	0	8	-0.06	0.35
1	0	8	-0.01	0.17
-1	0	-8	-0.08	0.15
-1	0	-8	0.19	0.18
-1	0	-8	0.40	0.42
-1	0	-8	0.02	0.15
0	0	8	32.72	1.36
0	0	8	29.50	1.71
0	0	8	33.00	1.68
0	0	8	32.19	1.38
0	0	-8	31.97	1.90
0	0	-8	33.94	1.70
0	0	-8	32.73	1.33
0	0	-8	33.33	1.37
-1	0	8	-0.55	0.41
-1	0	8	-0.12	0.15
-1	0	8	0.07	0.34
-1	0	8	0.07	0.17
1	0	-8	-0.10	0.51
1	0	-8	0.20	0.36
1	0	-8	-0.07	0.14
1	0	-8	-0.11	0.15
-2	0	8	18.64	0.94
-2	0	8	18.95	1.36
-2	0	8	21.26	0.92
-2	0	8	18.58	0.92
-2	0	8	19.72	1.14
2	0	-8	21.68	0.90
2	0	-8	20.72	1.54
2	0	-8	20.44	1.18
-3	0	8	0.29	0.21

-3	0	8	0.09	0.15
-3	0	8	0.13	0.20
-3	0	8	0.14	0.36
-3	0	8	-0.18	0.46
3	0	-8	0.08	0.14
3	0	-8	-0.28	0.32
3	0	-8	0.05	0.58
-4	0	8	25.61	1.33
-4	0	8	26.75	1.22
-4	0	8	26.30	1.16
-4	0	8	25.54	1.73
4	0	-8	23.70	1.83
4	0	-8	27.71	1.13
4	0	-8	27.02	1.32
-5	0	8	0.12	0.16
-5	0	8	-0.42	0.56
-5	0	8	0.41	0.29
-5	0	8	0.28	0.23
-5	0	8	0.23	0.26
5	0	-8	-0.03	0.29
5	0	-8	0.12	0.15
5	0	-8	-0.47	0.64
-6	0	8	178.32	6.79
-6	0	8	170.45	6.68
-6	0	8	170.04	6.64
-6	0	8	183.62	6.77
-6	0	8	170.94	7.28
6	0	-8	171.44	6.62
6	0	-8	181.43	7.42
6	0	-8	177.06	6.69
-7	0	8	0.50	0.30
-7	0	8	-0.05	0.28
-7	0	8	0.36	0.21
-7	0	8	0.13	0.21
-7	0	8	0.39	0.72
7	0	-8	-1.21	0.77
7	0	-8	0.23	0.26
7	0	-8	-0.06	0.16
-8	0	8	51.94	2.03
-8	0	8	46.71	2.77
-8	0	8	48.83	2.23
-8	0	8	49.87	2.20
8	0	-8	46.34	1.97
8	0	-8	45.57	2.90

-9	0	8	0.69	0.41
-9	0	8	-0.19	0.23
-9	0	8	-0.07	0.40
-9	0	8	-0.66	0.78
9	0	-8	0.12	0.96
9	0	-8	0.19	0.20
-10	0	8	49.62	2.21
-10	0	8	48.67	1.99
-10	0	8	42.60	2.76
-10	0	8	45.74	2.26
10	0	-8	44.90	1.91
10	0	-8	40.05	2.88
-11	0	8	0.07	0.52
-11	0	8	1.11	1.03
-11	0	8	-0.70	0.51
11	0	-8	-0.36	0.54
11	0	-8	-0.02	0.24
11	0	-8	2.02	1.17
-12	0	8	82.69	3.21
-12	0	8	75.17	3.48
-12	0	8	74.61	4.08
12	0	-8	80.38	4.30
12	0	-8	73.81	3.11
-13	0	8	0.87	0.42
-13	0	8	0.12	0.59
-13	0	8	-0.97	1.13
13	0	-8	0.93	1.39
13	0	-8	-0.18	0.30
-14	0	8	19.94	1.60
-14	0	8	20.69	2.47
14	0	-8	21.56	1.17
-15	0	8	0.23	1.28
-15	0	8	0.55	0.73
15	0	-8	0.27	0.37
-16	0	8	109.42	5.45
-16	0	8	112.14	4.90
16	0	-8	103.79	4.36
-17	0	8	0.11	0.92
17	0	-8	0.37	0.49
-18	0	8	85.56	4.29
-18	0	8	93.67	4.97
18	0	-8	82.05	3.65
-19	0	8	0.22	1.10
-19	0	8	-2.06	1.75

19	0	-8	0.16	0.59
-20	0	8	38.66	2.92
-20	0	8	29.45	3.74
20	0	-8	34.59	1.98
18	0	9	-0.16	0.78
18	0	9	0.46	1.45
-18	0	-9	0.70	1.44
17	0	9	17.40	1.56
17	0	9	13.49	2.35
-17	0	-9	18.18	2.41
16	0	9	0.93	0.64
16	0	9	-1.61	1.19
-16	0	-9	1.08	1.10
15	0	9	23.25	2.28
15	0	9	22.54	1.57
-15	0	-9	24.69	2.06
14	0	9	0.44	1.00
14	0	9	0.34	0.55
-14	0	-9	-0.20	0.87
13	0	9	3.15	1.35
13	0	9	4.19	0.69
13	0	9	3.33	0.77
-13	0	-9	3.01	0.85
12	0	9	-0.09	0.82
12	0	9	-0.62	0.38
12	0	9	-0.40	0.60
-12	0	-9	-0.64	0.62
11	0	9	15.97	1.08
11	0	9	13.13	1.17
11	0	9	11.29	1.55
-11	0	-9	14.93	1.26
10	0	9	-0.30	0.34
10	0	9	0.15	0.72
10	0	9	0.19	0.48
-10	0	-9	0.32	0.50
-10	0	-9	-1.15	1.38
9	0	9	7.39	0.75
9	0	9	7.09	0.81
9	0	9	8.76	1.33
-9	0	-9	8.56	0.69
-9	0	-9	9.05	1.58
-9	0	-9	7.90	0.91
8	0	9	0.36	0.30
8	0	9	0.18	0.63

8	0	9	0.24	0.32
8	0	9	-0.38	0.35
-8	0	-9	-0.01	0.28
-8	0	-9	-0.74	0.77
-8	0	-9	-0.30	0.37
-8	0	-9	-0.22	0.49
7	0	9	55.80	2.26
7	0	9	54.79	2.75
7	0	9	51.55	2.24
7	0	9	50.86	2.28
-7	0	-9	56.68	2.90
-7	0	-9	51.36	2.47
-7	0	-9	54.31	2.25
-7	0	-9	54.95	2.31
6	0	9	0.75	0.61
6	0	9	0.26	0.25
6	0	9	0.04	0.37
6	0	9	0.26	0.27
-6	0	-9	-0.32	0.25
-6	0	-9	-0.31	0.58
-6	0	-9	-0.14	0.30
-6	0	-9	0.72	0.68
-6	0	-9	0.29	0.24
5	0	9	19.00	0.92
5	0	9	20.60	1.15
5	0	9	18.23	1.43
-5	0	-9	19.55	0.96
-5	0	-9	16.58	1.41
-5	0	-9	20.42	1.01
-5	0	-9	17.53	1.50
-5	0	-9	17.69	1.02
4	0	9	0.08	0.35
4	0	9	-0.12	0.48
4	0	9	-0.09	0.21
-4	0	-9	0.27	0.20
-4	0	-9	0.46	0.58
-4	0	-9	0.08	0.60
-4	0	-9	0.30	0.22
-4	0	-9	0.07	0.32
3	0	9	144.34	6.31
3	0	9	155.14	5.89
3	0	9	157.39	6.16
-3	0	-9	157.48	5.90
-3	0	-9	154.91	5.97

-3	0	-9	158.91	6.42
-3	0	-9	158.94	5.93
2	0	9	0.05	0.19
2	0	9	0.28	0.46
2	0	9	0.32	0.43
-2	0	-9	0.16	0.19
-2	0	-9	1.12	0.53
-2	0	-9	-0.12	0.17
-2	0	-9	0.24	0.19
-2	0	-9	-0.31	0.57
1	0	9	5.27	0.83
1	0	9	4.39	0.80
1	0	9	4.90	0.43
-1	0	-9	4.08	0.42
-1	0	-9	6.91	0.89
-1	0	-9	5.46	0.42
-1	0	-9	4.86	0.39
-1	0	-9	5.04	0.90
0	0	9	-0.01	0.37
0	0	9	0.29	0.19
0	0	9	0.07	0.41
0	0	9	-0.16	0.17
0	0	-9	0.25	0.17
0	0	-9	0.39	0.60
0	0	-9	0.43	0.45
0	0	-9	0.06	0.17
-1	0	9	6.67	0.97
-1	0	9	5.30	0.76
-1	0	9	6.60	0.47
-1	0	9	6.23	0.48
1	0	-9	9.05	1.16
1	0	-9	6.01	0.43
1	0	-9	5.60	0.81
1	0	-9	5.59	0.44
-2	0	9	0.08	0.38
-2	0	9	0.15	0.22
-2	0	9	-0.28	0.47
-2	0	9	0.14	0.22
-2	0	9	0.12	0.20
2	0	-9	0.18	0.17
2	0	-9	-0.26	0.63
2	0	-9	-0.34	0.39
-3	0	9	77.43	2.94
-3	0	9	73.38	2.95

-3	0	9	65.91	3.42
-3	0	9	72.49	2.95
-3	0	9	72.57	3.14
3	0	-9	74.81	3.65
3	0	-9	74.23	3.31
3	0	-9	80.70	2.91
-4	0	9	-0.03	0.17
-4	0	9	0.09	0.24
-4	0	9	0.78	0.56
-4	0	9	-0.29	0.32
-4	0	9	0.47	0.26
4	0	-9	-0.22	0.36
4	0	-9	-1.00	0.63
4	0	-9	0.12	0.16
-5	0	9	1.69	0.32
-5	0	9	1.51	0.33
-5	0	9	1.11	0.43
-5	0	9	1.59	0.25
-5	0	9	2.33	0.71
5	0	-9	1.39	0.22
5	0	-9	0.71	0.81
5	0	-9	1.63	0.40
-6	0	9	0.15	0.29
-6	0	9	0.18	0.69
-6	0	9	0.20	0.21
-6	0	9	-0.15	0.28
-6	0	9	0.21	0.52
6	0	-9	-0.48	0.31
6	0	-9	0.19	0.17
6	0	-9	0.42	0.79
-7	0	9	0.24	0.30
-7	0	9	0.66	0.36
-7	0	9	0.36	0.24
-7	0	9	0.23	0.71
-7	0	9	0.68	0.36
7	0	-9	0.42	0.94
7	0	-9	0.09	0.18
7	0	-9	0.32	0.32
-8	0	9	0.14	0.25
-8	0	9	1.09	0.81
-8	0	9	-0.11	0.37
-8	0	9	0.43	0.39
-8	0	9	-0.17	0.25
8	0	-9	-0.14	0.29

8	0	-9	0.01	0.19
8	0	-9	-1.82	0.91
-9	0	9	5.12	0.81
-9	0	9	2.69	1.03
-9	0	9	5.87	0.80
-9	0	9	5.39	0.58
9	0	-9	3.79	0.44
9	0	-9	3.92	1.24
-10	0	9	-0.06	0.48
-10	0	9	0.97	0.47
-10	0	9	0.25	0.94
-10	0	9	-0.43	0.30
10	0	-9	-0.22	0.23
10	0	-9	0.55	1.13
-11	0	9	-0.16	0.34
-11	0	9	-1.07	0.94
-11	0	9	0.75	0.52
-11	0	9	1.12	0.56
11	0	-9	2.17	1.33
11	0	-9	0.65	0.27
-12	0	9	-0.25	0.56
-12	0	9	0.20	0.39
-12	0	9	-0.83	1.01
12	0	-9	-0.14	0.29
12	0	-9	-0.40	1.35
-13	0	9	2.93	1.41
-13	0	9	5.20	0.87
-13	0	9	7.09	0.83
13	0	-9	5.11	0.62
-14	0	9	1.84	1.36
-14	0	9	0.21	0.70
14	0	-9	-0.25	0.33
-15	0	9	21.24	1.83
-15	0	9	17.98	2.55
15	0	-9	21.55	1.21
-16	0	9	0.45	1.48
-16	0	9	-0.49	0.82
16	0	-9	-0.16	0.42
-17	0	9	17.49	2.87
-17	0	9	18.69	2.06
17	0	-9	21.03	1.30
-18	0	9	-1.70	1.01
-18	0	9	2.51	1.78
18	0	-9	-1.00	0.48

-19	0	9	3.34	2.02
-19	0	9	4.67	1.38
19	0	-9	8.70	1.14
-20	0	9	-0.28	2.09
-20	0	9	-0.36	1.36
20	0	-9	0.82	0.69
18	0	10	12.40	1.99
18	0	10	6.52	1.19
-18	0	-10	9.54	1.78
17	0	10	-0.92	1.42
17	0	10	-1.04	0.80
-17	0	-10	1.36	1.36
16	0	10	47.40	2.58
16	0	10	48.62	3.29
-16	0	-10	46.49	3.01
15	0	10	0.04	0.65
15	0	10	0.85	1.19
-15	0	-10	0.53	1.01
14	0	10	10.62	1.18
14	0	10	8.56	1.46
-14	0	-10	9.46	1.59
13	0	10	2.05	0.78
13	0	10	0.56	0.54
13	0	10	0.66	0.97
-13	0	-10	-0.77	0.73
12	0	10	8.19	1.13
12	0	10	7.90	1.01
12	0	10	8.60	1.64
-12	0	-10	7.32	1.22
11	0	10	0.52	0.84
11	0	10	0.42	0.56
-11	0	-10	-1.23	0.56
10	0	10	94.25	4.00
10	0	10	98.37	4.03
10	0	10	99.57	4.55
-10	0	-10	98.19	4.07
9	0	10	0.73	0.79
9	0	10	0.44	0.44
9	0	10	0.18	0.36
-9	0	-10	-0.18	0.45
8	0	10	75.72	2.92
8	0	10	72.08	3.01
8	0	10	69.33	2.95
8	0	10	69.50	3.50

-8	0	-10	74.79	2.97
-8	0	-10	70.32	3.07
-8	0	-10	74.03	3.03
-8	0	-10	70.11	3.67
-8	0	-10	68.83	3.12
7	0	10	-1.22	0.59
7	0	10	0.09	0.32
7	0	10	0.19	0.42
7	0	10	0.20	0.31
-7	0	-10	0.69	0.70
-7	0	-10	0.44	0.39
-7	0	-10	0.03	0.35
-7	0	-10	-0.42	0.80
-7	0	-10	-0.08	0.28
6	0	10	47.19	2.64
6	0	10	51.53	2.12
6	0	10	52.65	2.27
-6	0	-10	48.93	2.16
-6	0	-10	51.72	2.25
-6	0	-10	52.21	2.16
-6	0	-10	51.53	2.80
-6	0	-10	51.28	2.58
5	0	10	0.21	0.39
5	0	10	-0.01	0.26
5	0	10	-0.46	0.56
-5	0	-10	0.05	0.69
-5	0	-10	0.15	0.26
-5	0	-10	0.26	0.32
4	0	10	29.07	1.83
4	0	10	30.31	1.30
4	0	10	30.84	1.60
-4	0	-10	29.12	1.33
-4	0	-10	28.62	1.39
-4	0	-10	28.48	1.94
-4	0	-10	29.74	1.95
3	0	10	0.04	0.22
3	0	10	0.36	0.45
3	0	10	0.14	0.53
-3	0	-10	0.16	0.24
-3	0	-10	-0.06	0.23
-3	0	-10	0.30	0.23
-3	0	-10	-1.07	0.57
-3	0	-10	0.09	0.65
2	0	10	22.94	1.04

2	0	10	23.52	1.43
2	0	10	19.51	1.49
-2	0	-10	22.95	1.03
-2	0	-10	25.50	1.63
-2	0	-10	22.66	1.11
-2	0	-10	21.22	1.73
-2	0	-10	23.26	1.06
1	0	10	0.01	0.44
1	0	10	-0.38	0.46
1	0	10	-0.04	0.21
-1	0	-10	-0.60	0.50
-1	0	-10	0.02	0.69
-1	0	-10	-0.07	0.19
-1	0	-10	-0.21	0.19
-1	0	-10	-0.32	0.18
0	0	10	78.40	3.69
0	0	10	76.36	3.61
0	0	10	85.90	3.30
0	0	10	88.17	3.27
0	0	-10	81.93	3.22
0	0	-10	82.74	3.97
0	0	-10	83.19	3.27
0	0	-10	87.35	3.71
-1	0	10	-0.04	0.23
-1	0	10	-0.01	0.20
-1	0	10	-0.04	0.44
-1	0	10	-0.62	0.51
1	0	-10	0.21	0.19
1	0	-10	-0.15	0.47
1	0	-10	-0.84	0.71
1	0	-10	0.40	0.23
-2	0	10	75.90	3.43
-2	0	10	81.98	3.17
-2	0	10	77.93	3.14
-2	0	10	80.68	3.15
-2	0	10	75.80	3.68
2	0	-10	79.25	3.13
2	0	-10	84.73	3.96
2	0	-10	82.64	3.48
-3	0	10	0.63	0.61
-3	0	10	0.15	0.20
-3	0	10	0.23	0.41
-3	0	10	0.28	0.23
3	0	-10	-0.40	0.77

3	0	-10	-0.17	0.43
3	0	-10	0.12	0.19
-4	0	10	0.31	0.21
-4	0	10	0.87	0.28
-4	0	10	0.87	0.67
-4	0	10	0.92	0.31
-4	0	10	0.35	0.44
4	0	-10	1.48	0.48
4	0	-10	1.09	0.85
4	0	-10	0.77	0.21
-5	0	10	-0.17	0.63
-5	0	10	-0.03	0.29
-5	0	10	-0.20	0.27
-5	0	10	0.14	0.37
-5	0	10	-0.37	0.20
5	0	-10	-1.04	0.76
5	0	-10	0.54	0.50
5	0	-10	-0.13	0.16
-6	0	10	6.04	1.07
-6	0	10	6.11	0.74
-6	0	10	6.06	0.52
-6	0	10	5.92	0.67
-6	0	10	5.57	0.65
6	0	-10	6.13	0.73
6	0	-10	5.76	1.18
6	0	-10	4.63	0.44
-7	0	10	-0.51	0.31
-7	0	10	0.39	0.26
-7	0	10	-1.01	0.74
-7	0	10	0.19	0.37
7	0	-10	-0.05	0.20
7	0	-10	0.29	0.37
7	0	-10	-1.40	0.94
-8	0	10	100.68	4.10
-8	0	10	108.05	4.34
-8	0	10	107.18	4.14
-8	0	10	99.74	4.92
-8	0	10	108.59	4.31
8	0	-10	107.80	4.13
8	0	-10	108.23	5.15
8	0	-10	101.50	4.07
-9	0	10	-0.94	0.87
-9	0	10	-0.01	0.45
-9	0	10	0.04	0.44

-9	0	10	0.25	0.31
9	0	-10	-1.20	1.19
9	0	-10	0.32	0.31
9	0	-10	0.04	0.23
-10	0	10	14.93	1.24
-10	0	10	15.88	1.24
-10	0	10	15.47	0.95
-10	0	10	13.75	1.97
10	0	-10	14.37	0.83
10	0	-10	16.53	2.22
-11	0	10	0.74	0.74
-11	0	10	-0.37	1.05
-11	0	10	0.00	0.57
-11	0	10	0.14	0.39
11	0	-10	0.10	0.26
-12	0	10	5.65	0.86
-12	0	10	6.24	0.83
-12	0	10	4.45	1.35
12	0	-10	4.71	0.59
-13	0	10	1.79	1.31
-13	0	10	-0.40	0.62
13	0	-10	-0.14	0.32
-14	0	10	1.44	0.81
14	0	-10	2.13	0.45
-15	0	10	0.35	1.43
-15	0	10	0.37	0.79
-16	0	10	3.60	1.64
-16	0	10	0.66	0.90
16	0	-10	0.15	0.46
-17	0	10	3.77	1.85
-17	0	10	-0.83	0.96
17	0	-10	-1.00	0.48
-18	0	10	-4.16	1.85
-18	0	10	0.61	1.07
18	0	-10	0.53	0.57
-19	0	10	-2.94	1.95
-19	0	10	-1.54	1.16
19	0	-10	0.42	0.63
17	0	11	31.91	3.22
17	0	11	34.76	2.19
-17	0	-11	34.00	2.90
16	0	11	0.27	0.70
16	0	11	-1.30	1.35
-16	0	-11	-1.07	1.17

15	0	11	58.90	2.92
15	0	11	58.10	3.66
-15	0	-11	59.55	3.22
14	0	11	1.03	0.68
14	0	11	-2.90	1.09
-14	0	-11	0.20	0.98
13	0	11	34.27	2.06
13	0	11	39.94	2.03
13	0	11	31.10	2.69
-13	0	-11	35.26	2.19
12	0	11	1.84	0.68
12	0	11	-0.16	0.52
12	0	11	-1.24	0.98
-12	0	-11	-0.59	0.69
11	0	11	6.64	0.82
11	0	11	7.76	1.32
11	0	11	7.77	0.93
-11	0	-11	7.24	1.19
10	0	11	-0.28	0.89
10	0	11	0.39	0.45
10	0	11	0.66	0.56
-10	0	-11	-0.50	0.54
-10	0	-11	0.12	0.47
9	0	11	61.45	2.70
9	0	11	63.06	2.53
9	0	11	61.76	3.27
9	0	11	61.48	2.65
-9	0	-11	58.21	2.71
-9	0	-11	62.40	2.72
-9	0	-11	60.83	2.75
8	0	11	0.08	0.48
8	0	11	0.34	0.42
8	0	11	-0.16	0.77
-8	0	-11	-0.41	0.42
-8	0	-11	1.10	0.72
-8	0	-11	-0.25	0.37
-8	0	-11	0.25	0.42
7	0	11	185.94	6.91
7	0	11	175.31	7.43
7	0	11	177.91	6.81
-7	0	-11	174.59	7.19
-7	0	-11	175.15	6.86
-7	0	-11	172.35	6.96
-7	0	-11	182.75	6.87

6	0	11	0.56	0.44
6	0	11	-0.30	0.36
6	0	11	-0.70	0.64
-6	0	-11	-0.15	0.32
-6	0	-11	0.49	0.35
-6	0	-11	-1.27	0.79
-6	0	-11	0.00	0.29
-6	0	-11	-0.94	0.80
5	0	11	42.34	2.39
5	0	11	42.78	2.06
5	0	11	41.05	1.78
-5	0	-11	46.59	2.57
-5	0	-11	42.06	2.46
-5	0	-11	42.56	1.84
-5	0	-11	40.92	1.90
-5	0	-11	41.72	1.84
4	0	11	-0.25	0.64
4	0	11	0.15	0.25
4	0	11	-0.30	0.47
-4	0	-11	-0.06	0.32
-4	0	-11	0.94	0.83
-4	0	-11	0.33	0.26
-4	0	-11	-1.31	0.70
-4	0	-11	-0.32	0.27
3	0	11	114.68	5.24
3	0	11	125.64	4.73
3	0	11	132.72	5.14
-3	0	-11	122.20	5.42
-3	0	-11	114.82	4.84
-3	0	-11	126.30	4.79
-3	0	-11	125.50	5.37
-3	0	-11	123.55	4.84
2	0	11	-0.07	0.52
2	0	11	-0.28	0.23
2	0	11	0.46	0.57
-2	0	-11	-0.33	0.63
-2	0	-11	-0.04	0.23
-2	0	-11	-0.22	0.23
-2	0	-11	-0.16	0.23
-2	0	-11	0.24	0.86
1	0	11	131.72	4.87
1	0	11	132.85	5.36
1	0	11	124.80	5.34
-1	0	-11	121.46	4.85

-1	0	-11	120.78	5.60
-1	0	-11	125.82	4.92
-1	0	-11	131.25	4.90
-1	0	-11	124.42	5.39
0	0	11	0.34	0.26
0	0	11	0.18	0.58
0	0	11	0.10	0.26
0	0	11	-0.33	0.48
0	0	-11	0.28	0.31
0	0	-11	0.60	0.64
0	0	-11	-0.46	0.83
0	0	-11	0.43	0.24
-1	0	11	16.40	0.86
-1	0	11	14.19	0.82
-1	0	11	14.71	1.40
-1	0	11	13.66	1.23
1	0	-11	15.27	1.30
1	0	-11	15.51	0.80
1	0	-11	16.94	1.81
1	0	-11	14.37	0.80
-2	0	11	0.01	0.23
-2	0	11	-0.06	0.59
-2	0	11	0.47	0.53
-2	0	11	0.05	0.23
-2	0	11	0.16	0.29
2	0	-11	1.03	0.92
2	0	-11	-0.49	0.50
2	0	-11	0.25	0.22
-3	0	11	55.42	2.66
-3	0	11	58.85	2.36
-3	0	11	59.30	2.40
-3	0	11	57.50	2.37
-3	0	11	57.30	3.04
3	0	-11	57.54	2.69
3	0	-11	55.40	2.32
3	0	-11	58.60	3.28
-4	0	11	0.47	0.73
-4	0	11	-0.06	0.45
-4	0	11	-0.10	0.23
-4	0	11	-0.12	0.31
-4	0	11	0.11	0.26
4	0	-11	0.17	0.20
4	0	-11	1.37	0.97
4	0	-11	0.05	0.52

-5	0	11	90.70	3.65
-5	0	11	83.28	3.75
-5	0	11	92.38	4.35
-5	0	11	90.10	3.65
-5	0	11	92.21	3.56
5	0	-11	91.68	3.78
5	0	-11	84.55	4.46
5	0	-11	93.66	3.52
-6	0	11	-0.43	0.30
-6	0	11	0.25	0.26
-6	0	11	0.08	0.38
-6	0	11	0.20	0.41
-6	0	11	0.57	0.78
6	0	-11	0.31	0.50
6	0	-11	-0.03	0.21
6	0	-11	0.46	1.04
-7	0	11	32.25	1.60
-7	0	11	29.86	2.35
-7	0	11	32.60	1.47
-7	0	11	31.99	1.64
-7	0	11	32.48	1.64
7	0	-11	32.72	1.41
7	0	-11	28.70	2.59
7	0	-11	32.78	1.62
-8	0	11	-0.41	0.85
-8	0	11	0.06	0.47
-8	0	11	0.03	0.42
-8	0	11	0.18	0.66
-8	0	11	0.88	0.32
8	0	-11	-0.35	0.44
8	0	-11	-1.51	1.17
8	0	-11	0.51	0.23
-9	0	11	31.98	1.50
-9	0	11	34.28	1.77
-9	0	11	32.01	1.75
-9	0	11	30.04	1.43
-9	0	11	30.26	2.50
9	0	-11	34.13	3.07
9	0	-11	31.72	1.50
9	0	-11	30.18	1.41
-10	0	11	-0.57	0.60
-10	0	11	-0.11	0.50
-10	0	11	1.27	1.08
-10	0	11	-0.31	0.34

10	0	-11	0.05	0.27
-11	0	11	69.11	2.97
-11	0	11	74.12	4.05
-11	0	11	73.12	3.30
11	0	-11	67.68	2.86
-12	0	11	-0.44	1.14
-12	0	11	-0.97	0.49
-12	0	11	-0.67	0.60
12	0	-11	-0.07	0.30
-13	0	11	34.14	3.48
-13	0	11	36.34	2.13
13	0	-11	33.78	1.62
-14	0	11	1.03	0.81
-14	0	11	-0.82	1.30
14	0	-11	-0.34	0.37
-15	0	11	3.22	1.73
-15	0	11	1.54	0.92
15	0	-11	2.50	0.53
-16	0	11	-0.50	1.71
-16	0	11	-0.65	0.90
16	0	-11	-0.54	0.47
-17	0	11	5.61	2.09
-17	0	11	4.59	1.22
17	0	-11	2.60	0.63
-18	0	11	-3.06	1.09
-18	0	11	1.12	2.02
18	0	-11	0.56	0.57
-19	0	11	3.96	2.15
-19	0	11	4.94	1.38
19	0	-11	3.90	0.75
17	0	12	-0.82	1.06
17	0	12	-0.31	1.73
16	0	12	58.84	3.98
16	0	12	62.64	3.14
-16	0	-12	59.48	3.48
-16	0	-12	64.17	3.29
15	0	12	0.57	0.75
15	0	12	1.51	1.36
-15	0	-12	0.37	1.07
-15	0	-12	1.79	0.90
14	0	12	26.35	2.77
14	0	12	31.41	1.94
-14	0	-12	29.40	2.03
-14	0	-12	31.33	2.27

13	0	12	-0.59	1.20
13	0	12	0.20	0.60
-13	0	-12	0.27	0.85
-13	0	-12	0.13	0.70
12	0	12	20.23	2.32
12	0	12	25.24	1.57
12	0	12	22.96	1.60
-12	0	-12	23.42	1.65
-12	0	-12	22.53	1.74
11	0	12	-1.58	0.98
11	0	12	-0.38	0.48
-11	0	-12	0.13	0.58
-11	0	-12	-0.60	0.66
10	0	12	55.30	2.52
10	0	12	53.50	3.24
-10	0	-12	54.61	2.62
-10	0	-12	57.08	2.58
9	0	12	0.53	0.50
9	0	12	-0.47	0.53
9	0	12	-1.93	1.27
-9	0	-12	-1.47	0.74
-9	0	-12	0.17	0.55
-9	0	-12	-0.24	0.44
8	0	12	197.48	8.55
8	0	12	217.16	7.92
8	0	12	207.01	7.86
-8	0	-12	206.23	7.91
-8	0	-12	199.74	8.02
-8	0	-12	201.62	8.18
7	0	12	0.34	0.52
7	0	12	-0.23	0.75
7	0	12	0.16	0.38
-7	0	-12	0.49	0.40
-7	0	-12	0.38	0.88
-7	0	-12	0.15	0.38
-7	0	-12	0.35	0.42
6	0	12	108.55	5.21
6	0	12	121.72	4.80
-6	0	-12	116.87	5.21
-6	0	-12	117.52	4.60
-6	0	-12	120.55	4.65
-6	0	-12	117.83	4.73
5	0	12	-0.39	0.71
5	0	12	0.06	0.29

5	0	12	0.12	0.52
-5	0	-12	0.13	0.34
-5	0	-12	0.05	0.33
-5	0	-12	0.00	0.33
-5	0	-12	-2.05	0.90
-5	0	-12	-0.05	0.95
4	0	12	120.39	4.59
4	0	12	118.22	4.96
4	0	12	112.08	5.20
-4	0	-12	114.10	5.36
-4	0	-12	116.27	4.73
-4	0	-12	124.96	4.68
-4	0	-12	120.66	5.36
-4	0	-12	124.07	4.62
3	0	12	0.24	0.28
3	0	12	-0.28	0.61
3	0	12	0.39	0.61
-3	0	-12	-0.08	0.30
-3	0	-12	-0.72	0.44
-3	0	-12	-0.42	0.76
-3	0	-12	0.24	0.27
-3	0	-12	-0.28	0.92
2	0	12	116.05	5.11
2	0	12	116.35	5.21
2	0	12	118.34	4.65
-2	0	-12	125.94	4.72
-2	0	-12	128.46	5.35
-2	0	-12	118.16	5.51
-2	0	-12	122.34	4.75
1	0	12	0.52	0.29
1	0	12	-0.17	0.58
1	0	12	0.38	0.61
-1	0	-12	-0.14	0.30
-1	0	-12	0.31	0.92
-1	0	-12	-0.72	0.71
-1	0	-12	0.38	0.27
-1	0	-12	0.17	0.27
0	0	12	186.49	7.97
0	0	12	206.41	7.59
0	0	12	198.96	8.08
0	0	12	203.04	7.53
0	0	-12	192.63	7.48
0	0	-12	207.98	7.56
0	0	-12	201.93	8.06

0	0	-12	194.55	7.55
0	0	-12	189.47	8.36
-1	0	12	0.21	0.30
-1	0	12	-0.68	0.64
-1	0	12	0.11	0.29
1	0	-12	0.41	0.97
1	0	-12	0.01	0.27
1	0	-12	-0.72	0.63
1	0	-12	-0.41	0.23
-2	0	12	86.65	3.41
-2	0	12	82.79	4.02
-2	0	12	82.64	3.77
-2	0	12	90.06	3.39
2	0	-12	85.10	3.83
2	0	-12	80.91	4.33
2	0	-12	85.61	3.37
-3	0	12	-0.17	0.30
-3	0	12	-0.40	0.59
-3	0	12	-0.43	0.26
-3	0	12	-0.91	0.67
-3	0	12	-0.28	0.25
3	0	-12	-0.09	0.60
3	0	-12	-0.91	1.00
3	0	-12	0.51	0.24
-4	0	12	37.58	1.65
-4	0	12	37.33	1.69
-4	0	12	36.24	1.99
-4	0	12	36.94	1.73
-4	0	12	37.43	2.45
4	0	-12	38.25	2.75
4	0	-12	36.52	1.60
4	0	-12	37.93	2.04
-5	0	12	0.04	0.27
-5	0	12	-0.11	0.34
-5	0	12	-0.70	0.35
-5	0	12	0.49	0.53
-5	0	12	0.82	0.87
5	0	-12	3.28	1.17
5	0	-12	-0.07	0.22
5	0	-12	0.11	0.57
-6	0	12	98.59	3.81
-6	0	12	92.31	3.66
-6	0	12	90.97	3.78
-6	0	12	87.73	3.85

-6	0	12	88.65	4.50
6	0	-12	93.23	3.88
6	0	-12	90.33	3.60
6	0	-12	94.43	4.83
-7	0	12	0.60	0.43
-7	0	12	-0.48	0.90
-7	0	12	-0.12	0.30
-7	0	12	0.07	0.47
-7	0	12	0.30	0.48
7	0	-12	-1.70	1.18
7	0	-12	0.02	0.54
7	0	-12	-0.19	0.21
-8	0	12	97.34	4.03
-8	0	12	92.61	3.88
-8	0	12	94.93	3.83
-8	0	12	99.39	4.84
-8	0	12	98.50	4.03
8	0	-12	93.44	3.76
8	0	-12	95.39	3.92
-9	0	12	0.23	0.53
-9	0	12	0.69	0.56
-9	0	12	-0.10	0.38
-9	0	12	0.60	1.01
9	0	-12	-0.29	0.50
9	0	-12	0.08	0.26
-10	0	12	15.15	1.08
-10	0	12	14.70	1.38
-10	0	12	14.75	2.19
-10	0	12	17.08	1.38
-10	0	12	16.90	0.91
10	0	-12	16.36	0.93
10	0	-12	18.44	1.02
-11	0	12	-0.14	0.61
-11	0	12	-2.35	1.81
-11	0	12	-0.36	0.45
11	0	-12	0.20	0.31
-12	0	12	56.70	3.02
-12	0	12	53.73	2.44
-12	0	12	52.47	3.59
12	0	-12	51.88	2.28
-13	0	12	-0.28	0.73
-13	0	12	-0.16	1.39
13	0	-12	0.87	0.38
-14	0	12	39.27	3.50

-14	0	12	38.98	2.38
14	0	-12	34.90	1.75
-15	0	12	2.78	1.78
-15	0	12	0.36	0.88
15	0	-12	0.60	0.46
-16	0	12	13.12	1.86
-16	0	12	11.31	2.25
16	0	-12	8.45	1.02
-17	0	12	3.90	2.00
-17	0	12	-0.46	1.02
17	0	-12	0.38	0.56
-18	0	12	31.06	2.64
-18	0	12	32.33	3.92
18	0	-12	27.10	1.69
-19	0	12	1.25	2.17
-19	0	12	-0.49	1.34
19	0	-12	1.04	0.67
16	0	13	-1.36	1.55
16	0	13	0.11	0.84
-16	0	-13	-0.27	1.01
15	0	13	41.82	3.36
15	0	13	41.68	2.42
-15	0	-13	41.37	2.58
-15	0	-13	40.31	2.98
14	0	13	-0.19	1.37
-14	0	-13	0.07	1.00
-14	0	-13	0.27	0.87
13	0	13	57.71	3.69
-13	0	-13	59.31	2.99
-13	0	-13	56.28	2.96
12	0	13	0.56	1.24
-12	0	-13	0.50	0.70
-12	0	-13	1.02	0.82
11	0	13	21.96	2.46
11	0	13	24.45	1.61
-11	0	-13	28.20	1.75
-11	0	-13	27.85	1.76
10	0	13	1.58	1.11
10	0	13	1.32	0.61
-10	0	-13	0.19	0.52
-10	0	-13	-0.52	0.61
9	0	13	111.68	5.25
9	0	13	117.70	4.45
9	0	13	112.32	4.45

-9	0	-13	104.22	4.71
-9	0	-13	107.20	4.49
-9	0	-13	109.32	4.63
8	0	13	-0.07	0.49
8	0	13	0.31	0.91
8	0	13	0.06	0.56
-8	0	-13	0.53	0.43
-8	0	-13	-0.58	0.45
-8	0	-13	-0.24	0.98
7	0	13	86.96	4.84
7	0	13	97.54	3.81
7	0	13	98.41	4.03
-7	0	-13	94.87	4.45
-7	0	-13	98.17	3.86
-7	0	-13	95.96	3.99
6	0	13	1.00	0.85
6	0	13	0.12	0.56
-6	0	-13	-0.09	0.37
-6	0	-13	-0.52	1.08
-6	0	-13	-0.66	0.37
-6	0	-13	0.35	0.39
5	0	13	49.43	2.07
5	0	13	43.02	2.81
5	0	13	49.58	2.44
-5	0	-13	48.94	2.23
-5	0	-13	49.09	3.09
-5	0	-13	52.22	2.18
-5	0	-13	52.02	2.11
4	0	13	0.20	0.64
4	0	13	-1.24	0.72
4	0	13	0.34	0.32
-4	0	-13	-1.10	1.06
-4	0	-13	0.25	0.35
-4	0	-13	-0.31	0.34
-4	0	-13	-0.20	0.35
3	0	13	97.32	3.79
3	0	13	95.79	4.27
3	0	13	93.18	4.48
-3	0	-13	102.12	3.88
-3	0	-13	100.12	3.93
-3	0	-13	93.10	4.57
-3	0	-13	94.08	4.75
-3	0	-13	98.41	3.79
2	0	13	-0.03	0.67

2	0	13	0.09	0.74
2	0	13	-0.28	0.30
-2	0	-13	0.30	0.95
-2	0	-13	0.41	0.30
-2	0	-13	0.16	1.08
-2	0	-13	-0.14	0.29
-2	0	-13	-0.57	0.29
1	0	13	132.74	6.02
1	0	13	137.69	6.02
1	0	13	150.40	5.47
-1	0	-13	147.13	5.51
-1	0	-13	142.21	6.12
-1	0	-13	136.01	6.42
-1	0	-13	143.82	5.42
-1	0	-13	143.54	5.54
0	0	13	-0.23	0.30
0	0	13	-0.64	0.63
0	0	13	0.51	0.35
0	0	13	0.07	0.70
0	0	-13	0.00	0.30
0	0	-13	0.50	0.28
0	0	-13	-0.50	0.79
0	0	-13	-0.09	0.27
0	0	-13	-0.54	1.10
-1	0	13	48.41	2.07
-1	0	13	45.14	2.60
-1	0	13	50.02	2.80
-1	0	13	51.82	2.14
-1	0	13	51.37	2.16
1	0	-13	49.93	3.22
1	0	-13	50.22	2.72
1	0	-13	50.73	2.12
-2	0	13	-0.08	0.64
-2	0	13	0.82	0.37
-2	0	13	-0.26	0.76
-2	0	13	-0.38	0.28
-2	0	13	-0.38	0.29
2	0	-13	0.44	1.17
2	0	-13	0.04	0.75
2	0	-13	-0.06	0.24
-3	0	13	58.25	2.99
-3	0	13	63.49	2.61
-3	0	13	66.60	2.66
-3	0	13	65.17	2.62

-3	0	13	65.56	3.43
3	0	-13	62.81	3.08
3	0	-13	60.71	2.56
3	0	-13	58.00	3.72
-4	0	13	-0.26	0.39
-4	0	13	0.63	0.90
-4	0	13	-0.50	0.59
-4	0	13	-0.15	0.30
-4	0	13	-0.11	0.31
4	0	-13	2.04	1.24
4	0	-13	0.71	0.72
-5	0	13	127.19	5.99
-5	0	13	134.37	5.17
-5	0	13	135.82	5.26
-5	0	13	137.24	5.26
-5	0	13	124.45	5.43
5	0	-13	134.20	5.12
5	0	-13	129.24	6.31
5	0	-13	134.85	5.50
-6	0	13	0.38	0.34
-6	0	13	-0.04	0.94
-6	0	13	0.76	0.47
-6	0	13	-0.27	0.38
-6	0	13	0.09	0.55
6	0	-13	1.13	0.64
6	0	-13	-0.18	0.25
-7	0	13	79.42	4.37
-7	0	13	82.22	3.61
-7	0	13	85.93	3.44
-7	0	13	87.85	3.61
-7	0	13	87.99	3.63
7	0	-13	86.25	3.67
7	0	-13	83.46	3.37
-8	0	13	0.13	0.61
-8	0	13	-0.16	0.96
-8	0	13	0.03	0.52
-8	0	13	0.37	0.37
-8	0	13	-0.54	0.48
8	0	-13	0.32	0.27
8	0	-13	-1.25	0.62
-9	0	13	48.16	2.36
-9	0	13	51.09	3.41
-9	0	13	59.28	2.65
-9	0	13	52.77	2.33

-9	0	13	56.69	2.59
9	0	-13	52.01	2.22
9	0	-13	53.03	2.45
-10	0	13	-0.12	0.44
-10	0	13	-1.37	1.20
-10	0	13	-0.09	0.61
-10	0	13	0.34	0.45
10	0	-13	0.31	0.29
10	0	-13	-0.89	0.55
-11	0	13	152.69	6.28
-11	0	13	167.99	6.61
-11	0	13	167.64	7.49
11	0	-13	154.19	6.17
-12	0	13	-0.18	1.38
-12	0	13	-1.20	0.68
12	0	-13	0.15	0.37
-13	0	13	90.39	3.98
-13	0	13	80.48	4.91
13	0	-13	80.96	3.42
-14	0	13	0.20	0.84
-14	0	13	-1.63	1.60
14	0	-13	0.12	0.54
-15	0	13	45.11	3.85
-15	0	13	45.64	2.72
15	0	-13	42.37	2.05
-16	0	13	0.22	1.86
-16	0	13	-0.31	1.01
16	0	-13	-0.08	0.54
-17	0	13	69.15	5.08
-17	0	13	80.65	3.97
17	0	-13	71.54	3.20
-18	0	13	-3.57	2.10
-18	0	13	-2.51	1.24
18	0	-13	-0.26	0.61
15	0	14	1.03	1.71
-15	0	-14	1.52	1.00
14	0	14	3.21	1.52
-14	0	-14	1.43	0.93
13	0	14	0.10	1.40
-13	0	-14	-0.03	0.93
-13	0	-14	0.57	0.80
12	0	14	9.68	1.71
-12	0	-14	9.68	1.48
-12	0	-14	11.07	1.37

11	0	14	-0.10	1.19
-11	0	-14	-0.83	0.71
-11	0	-14	-0.15	0.66
10	0	14	3.15	1.34
10	0	14	3.84	0.80
10	0	14	4.05	0.72
-10	0	-14	3.34	0.75
-10	0	-14	3.45	0.74
9	0	14	-0.11	0.59
9	0	14	0.10	1.07
9	0	14	-0.64	0.61
-9	0	-14	0.11	0.53
-9	0	-14	-1.21	0.56
-9	0	-14	0.18	1.29
8	0	14	28.22	1.48
8	0	14	27.55	1.66
8	0	14	28.40	2.46
-8	0	-14	26.04	1.78
-8	0	-14	29.94	2.37
-8	0	-14	28.64	1.53
7	0	14	-0.79	0.43
7	0	14	-1.35	0.63
7	0	14	0.58	0.97
-7	0	-14	0.11	0.46
-7	0	-14	-0.07	0.44
-7	0	-14	0.96	1.21
6	0	14	6.88	1.29
6	0	14	5.45	0.86
-6	0	-14	11.74	2.12
-6	0	-14	7.87	0.75
-6	0	-14	6.44	0.86
5	0	14	-0.41	0.64
5	0	14	0.67	0.91
5	0	14	0.14	0.36
-5	0	-14	-0.14	0.38
-5	0	-14	-0.46	0.36
-5	0	-14	-0.03	1.30
-5	0	-14	-0.26	0.40
4	0	14	40.81	1.86
4	0	14	41.45	2.65
4	0	14	44.21	2.41
-4	0	-14	44.48	1.99
-4	0	-14	45.36	1.90
-4	0	-14	45.07	2.05

-4	0	-14	46.00	3.19
3	0	14	-0.75	0.33
3	0	14	-0.22	0.70
3	0	14	0.35	0.82
-3	0	-14	-0.42	0.34
-3	0	-14	1.58	1.30
-3	0	-14	0.13	0.36
2	0	14	10.01	1.50
2	0	14	11.39	1.43
2	0	14	11.18	0.78
-2	0	-14	11.50	0.84
-2	0	-14	10.14	0.83
-2	0	-14	11.87	2.25
-2	0	-14	10.22	0.74
1	0	14	0.39	0.76
1	0	14	0.96	0.79
1	0	14	0.25	0.36
-1	0	-14	-0.39	0.29
-1	0	-14	0.77	0.37
-1	0	-14	0.06	0.91
-1	0	-14	1.14	1.35
-1	0	-14	-0.28	0.30
0	0	14	83.06	3.22
0	0	14	75.21	3.80
0	0	14	77.13	3.89
0	0	-14	79.43	3.23
0	0	-14	78.96	3.14
0	0	-14	78.96	3.93
0	0	-14	79.60	4.40
0	0	-14	81.04	3.23
-1	0	14	-0.12	0.84
-1	0	14	0.21	0.62
-1	0	14	0.07	0.35
-1	0	14	0.00	0.73
-1	0	14	0.29	0.30
1	0	-14	1.00	0.90
1	0	-14	-0.09	1.32
1	0	-14	-0.22	0.29
-2	0	14	29.25	2.01
-2	0	14	29.21	2.27
-2	0	14	28.84	1.39
-2	0	14	30.73	1.47
-2	0	14	29.99	1.48
2	0	-14	28.41	2.13

2	0	-14	27.40	2.86
2	0	-14	31.54	1.41
-3	0	14	-0.29	0.71
-3	0	14	-0.09	0.34
-3	0	14	0.67	0.34
-3	0	14	-0.59	0.41
-3	0	14	0.61	0.90
3	0	-14	0.29	0.29
3	0	-14	-0.25	0.85
3	0	-14	2.04	1.40
-4	0	14	48.58	3.10
-4	0	14	50.04	2.17
-4	0	14	47.26	2.57
-4	0	14	49.64	2.25
-4	0	14	49.76	2.20
4	0	-14	50.45	2.11
4	0	-14	51.53	2.71
-5	0	14	0.25	0.48
-5	0	14	-0.89	0.64
-5	0	14	1.06	0.98
-5	0	14	0.45	0.40
-5	0	14	-0.01	0.36
5	0	-14	0.07	0.27
5	0	-14	-0.51	0.77
-6	0	14	141.74	5.29
-6	0	14	123.08	5.41
-6	0	14	123.90	6.07
-6	0	14	130.60	5.15
-6	0	14	135.10	5.29
6	0	-14	125.41	5.08
6	0	-14	138.23	5.49
-7	0	14	0.79	0.41
-7	0	14	0.10	0.49
-7	0	14	-0.29	1.10
-7	0	14	0.74	0.63
-7	0	14	0.44	0.59
7	0	-14	0.11	0.29
7	0	-14	-0.51	0.76
-8	0	14	36.50	2.01
-8	0	14	28.38	4.40
-8	0	14	38.48	1.83
-8	0	14	40.28	2.08
-8	0	14	40.91	2.04
8	0	-14	40.50	1.72

8	0	-14	38.62	2.13
-9	0	14	1.05	1.25
-9	0	14	0.58	0.57
-9	0	14	0.88	0.63
-9	0	14	-0.11	0.42
-9	0	14	0.26	0.67
9	0	-14	0.33	0.76
9	0	-14	0.07	0.30
-10	0	14	29.23	1.84
-10	0	14	29.43	1.61
-10	0	14	27.79	2.90
-10	0	14	28.44	1.59
10	0	-14	26.45	1.35
10	0	-14	27.41	1.66
-11	0	14	0.12	0.68
-11	0	14	-0.15	0.54
-11	0	14	0.51	1.43
11	0	-14	0.25	0.36
-12	0	14	109.73	4.71
-12	0	14	106.04	5.74
12	0	-14	103.52	4.22
-13	0	14	0.23	1.66
-13	0	14	0.71	0.88
13	0	-14	0.12	0.41
-14	0	14	63.47	3.22
-14	0	14	54.86	4.27
14	0	-14	57.34	2.58
-15	0	14	1.37	1.00
-15	0	14	-3.33	1.78
15	0	-14	0.87	0.52
-16	0	14	33.19	3.96
-16	0	14	40.34	2.72
16	0	-14	33.43	1.83
-17	0	14	-2.52	2.09
-17	0	14	0.07	1.17
17	0	-14	-0.07	0.64
14	0	15	2.72	1.66
-14	0	-15	0.09	0.88
13	0	15	13.95	2.57
-13	0	-15	15.36	1.71
12	0	15	1.41	1.53
-12	0	-15	-0.05	0.77
11	0	15	19.92	2.54
-11	0	-15	21.00	1.68

-11	0	-15	19.67	1.71
10	0	15	-1.07	1.22
-10	0	-15	-1.52	0.61
-10	0	-15	-0.97	0.64
9	0	15	1.04	1.30
9	0	15	0.18	0.70
-9	0	-15	0.90	0.64
-9	0	-15	1.04	0.61
8	0	15	-0.42	0.71
8	0	15	0.90	0.53
8	0	15	-1.44	1.01
-8	0	-15	-0.23	0.50
-8	0	-15	-1.57	1.53
-8	0	-15	0.32	0.53
7	0	15	0.03	0.47
7	0	15	1.32	1.08
7	0	15	0.28	0.75
-7	0	-15	0.19	0.50
-7	0	-15	0.74	0.64
-7	0	-15	1.57	1.43
6	0	15	-0.59	1.01
6	0	15	-0.39	0.71
-6	0	-15	0.71	0.44
-6	0	-15	0.95	0.50
-6	0	-15	0.47	1.47
5	0	15	35.90	1.66
5	0	15	34.85	2.62
5	0	15	36.78	2.19
-5	0	-15	38.16	1.69
-5	0	-15	42.30	3.18
-5	0	-15	37.75	1.87
4	0	15	1.08	0.77
4	0	15	0.70	0.39
-4	0	-15	0.35	1.45
-4	0	-15	0.33	0.41
-4	0	-15	0.22	0.41
3	0	15	8.93	1.33
3	0	15	10.66	1.48
3	0	15	9.99	0.78
-3	0	-15	9.44	0.87
-3	0	-15	9.07	0.76
-3	0	-15	12.30	0.92
-3	0	-15	12.89	2.04
2	0	15	0.13	0.86

2	0	15	0.35	0.82
2	0	15	0.33	0.38
-2	0	-15	-0.58	1.44
-2	0	-15	0.37	0.35
-2	0	-15	-0.12	0.38
-2	0	-15	-0.84	0.36
1	0	15	10.81	1.60
1	0	15	11.92	0.86
1	0	15	12.03	1.68
-1	0	-15	11.63	0.89
-1	0	-15	10.91	0.88
-1	0	-15	12.07	1.96
-1	0	-15	11.57	0.76
0	0	15	1.05	0.87
0	0	15	0.95	0.89
0	0	15	0.55	0.53
0	0	15	0.42	0.33
0	0	-15	1.13	0.36
0	0	-15	-0.04	1.49
0	0	-15	0.19	0.40
-1	0	15	3.50	0.55
-1	0	15	3.84	0.99
-1	0	15	1.60	1.01
-1	0	15	3.22	0.51
-1	0	15	3.54	0.58
1	0	-15	2.31	1.67
1	0	-15	1.60	1.04
1	0	-15	3.57	0.50
1	0	-15	2.85	0.45
-2	0	15	0.85	0.41
-2	0	15	0.64	0.93
-2	0	15	0.65	0.83
-2	0	15	-0.01	0.47
-2	0	15	-0.39	0.34
2	0	-15	-0.99	0.98
2	0	-15	-0.56	0.28
-3	0	15	0.38	0.92
-3	0	15	2.02	0.47
-3	0	15	1.50	0.85
-3	0	15	1.36	0.53
-3	0	15	2.08	0.44
3	0	-15	0.45	0.97
3	0	-15	1.75	0.37
-4	0	15	-0.28	0.75

-4	0	15	0.10	0.41
-4	0	15	-0.35	0.39
-4	0	15	0.62	0.99
-4	0	15	0.14	0.51
4	0	-15	0.16	0.30
4	0	-15	-0.91	0.87
-5	0	15	13.93	1.22
-5	0	15	14.52	2.10
-5	0	15	15.56	1.10
-5	0	15	15.60	1.03
-5	0	15	14.07	1.49
5	0	-15	17.01	1.73
5	0	-15	16.78	0.93
-6	0	15	0.37	0.59
-6	0	15	-0.70	0.47
-6	0	15	-0.20	0.69
-6	0	15	-0.14	0.42
6	0	-15	-0.09	0.87
6	0	-15	-0.23	0.28
-7	0	15	7.79	0.91
-7	0	15	8.09	1.00
-7	0	15	7.63	0.85
-7	0	15	7.99	1.23
-7	0	15	6.54	1.61
7	0	-15	6.94	0.66
7	0	-15	8.23	1.46
-8	0	15	-0.02	1.24
-8	0	15	0.10	0.68
-8	0	15	-0.28	0.52
-8	0	15	0.09	0.47
-8	0	15	0.07	0.71
8	0	-15	-0.05	0.32
8	0	-15	-0.28	0.85
-9	0	15	6.56	0.92
-9	0	15	6.46	1.72
-9	0	15	8.45	1.62
-9	0	15	6.83	1.14
-9	0	15	6.47	0.91
9	0	-15	6.85	1.05
9	0	-15	5.87	0.68
-10	0	15	-0.61	0.52
-10	0	15	0.23	0.72
-10	0	15	-0.27	1.50
-10	0	15	-0.31	0.62

10	0	-15	-0.79	0.93
10	0	-15	0.46	0.36
-11	0	15	1.64	0.92
-11	0	15	3.68	1.70
11	0	-15	1.13	0.41
-12	0	15	0.69	0.82
-12	0	15	-0.70	1.64
12	0	-15	-0.31	0.40
-13	0	15	9.51	2.17
-13	0	15	10.65	1.70
13	0	-15	8.88	1.04
-14	0	15	-0.38	1.86
-14	0	15	-1.03	0.95
14	0	-15	0.18	0.48
-15	0	15	17.80	2.74
-15	0	15	25.82	2.28
15	0	-15	24.03	1.45
-16	0	15	0.50	1.17
-16	0	15	0.41	2.20
16	0	-15	-0.13	0.61
-17	0	15	24.47	2.55
-17	0	15	24.57	4.03
17	0	-15	24.72	1.63
13	0	16	0.25	1.64
-13	0	-16	1.29	0.95
12	0	16	11.88	2.03
-12	0	-16	13.63	1.63
11	0	16	0.11	1.51
-11	0	-16	-1.07	0.72
10	0	16	13.22	2.39
-10	0	-16	14.53	1.48
9	0	16	0.93	0.83
9	0	16	1.16	1.35
-9	0	-16	0.25	0.64
8	0	16	33.61	2.90
8	0	16	42.95	2.27
-8	0	-16	36.10	2.07
-8	0	-16	38.80	1.85
7	0	16	0.21	0.86
7	0	16	-1.95	1.10
-7	0	-16	-0.38	0.53
-7	0	-16	-0.06	0.49
6	0	16	23.47	1.79
6	0	16	17.91	2.37

-6	0	-16	23.35	1.20
-6	0	-16	22.54	1.48
-6	0	-16	17.71	3.00
5	0	16	-0.35	1.05
5	0	16	-0.20	0.85
5	0	16	-0.17	0.42
-5	0	-16	-0.16	0.41
-5	0	-16	0.46	1.74
-5	0	-16	-0.12	0.46
4	0	16	52.46	2.88
4	0	16	50.43	3.24
4	0	16	53.32	2.19
-4	0	-16	52.04	2.19
-4	0	-16	55.54	3.87
-4	0	-16	51.14	2.40
3	0	16	1.05	0.90
3	0	16	0.89	1.00
3	0	16	-0.32	0.40
-3	0	-16	-0.44	0.39
-3	0	-16	0.25	0.44
-3	0	-16	2.09	1.77
2	0	16	9.07	1.66
2	0	16	10.62	0.87
-2	0	-16	11.30	2.09
-2	0	-16	12.24	0.96
-2	0	-16	12.35	0.97
-2	0	-16	12.47	0.82
1	0	16	-0.28	0.91
1	0	16	1.13	1.02
1	0	16	0.43	0.45
-1	0	-16	0.56	2.36
-1	0	-16	-0.04	0.43
-1	0	-16	0.09	0.38
0	0	16	22.99	2.08
0	0	16	26.04	2.19
0	0	16	25.18	1.25
0	0	16	25.24	1.76
0	0	-16	25.00	1.35
0	0	-16	24.62	1.34
-1	0	16	-0.09	0.49
-1	0	16	1.02	0.99
-1	0	16	-0.33	0.92
-1	0	16	-0.42	0.34
1	0	-16	-0.16	0.41

1	0	-16	0.02	0.38
-2	0	16	41.27	2.81
-2	0	16	39.23	1.89
-2	0	16	39.43	1.77
-2	0	16	38.27	2.50
-2	0	16	35.18	1.83
2	0	-16	37.28	1.76
-3	0	16	0.46	0.56
-3	0	16	-0.57	0.44
-3	0	16	0.04	0.42
-3	0	16	-0.39	1.06
-3	0	16	-0.71	0.85
3	0	-16	0.57	0.35
3	0	-16	-0.35	1.04
-4	0	16	21.82	1.91
-4	0	16	25.17	1.37
-4	0	16	24.49	1.34
-4	0	16	23.46	1.46
4	0	-16	21.93	2.11
4	0	-16	22.53	1.22
-5	0	16	-0.24	0.46
-5	0	16	0.97	1.16
-5	0	16	-0.31	0.80
-5	0	16	-1.50	0.60
-5	0	16	-0.67	0.46
5	0	-16	-0.40	0.31
5	0	-16	-0.76	0.98
-6	0	16	0.85	0.57
-6	0	16	1.41	0.70
-6	0	16	2.38	1.32
-6	0	16	1.68	0.84
-6	0	16	1.55	0.51
6	0	-16	1.66	0.38
6	0	-16	2.86	1.07
-7	0	16	-0.26	0.48
-7	0	16	-1.08	1.24
-7	0	16	-0.69	0.81
-7	0	16	0.29	0.71
-7	0	16	-0.47	0.54
7	0	-16	0.69	1.04
7	0	-16	0.00	0.33
-8	0	16	54.93	2.57
-8	0	16	51.63	2.60
-8	0	16	53.05	3.75

-8	0	16	49.94	2.33
-8	0	16	49.38	2.61
8	0	-16	50.93	2.77
8	0	-16	46.12	2.17
-9	0	16	0.29	1.46
-9	0	16	-0.36	0.64
-9	0	16	0.41	0.75
-9	0	16	-0.61	0.54
9	0	-16	0.52	1.03
9	0	-16	0.06	0.36
-10	0	16	17.81	1.62
-10	0	16	19.34	2.90
-10	0	16	15.80	1.40
10	0	-16	15.67	1.04
-11	0	16	-0.26	0.79
-11	0	16	-1.28	1.61
11	0	-16	-1.07	0.38
-12	0	16	3.14	1.00
-12	0	16	2.52	1.89
12	0	-16	2.89	0.53
-13	0	16	-0.96	0.90
-13	0	16	2.87	1.91
13	0	-16	0.06	0.49
-14	0	16	-1.06	2.01
-14	0	16	-0.63	1.02
14	0	-16	0.20	0.54
-15	0	16	-0.60	1.08
-15	0	16	-0.94	2.05
15	0	-16	-0.57	0.59
-16	0	16	2.87	1.29
-16	0	16	0.59	2.34
16	0	-16	0.95	0.67
12	0	17	-0.88	1.64
-12	0	-17	-0.71	0.85
11	0	17	28.07	3.01
-11	0	-17	28.57	2.05
10	0	17	1.40	1.57
-10	0	-17	0.45	0.76
9	0	17	17.72	2.69
-9	0	-17	16.21	1.57
8	0	17	1.09	0.99
8	0	17	1.12	1.40
-8	0	-17	-0.66	0.66
7	0	17	101.17	4.41

7	0	17	88.08	5.00
-7	0	-17	99.11	4.19
6	0	17	-0.14	0.88
6	0	17	-0.33	1.27
-6	0	-17	0.32	0.72
-6	0	-17	0.43	0.57
5	0	17	32.76	2.97
5	0	17	37.52	2.48
-5	0	-17	38.99	1.99
-5	0	-17	33.71	3.88
-5	0	-17	39.75	1.71
4	0	17	-0.24	0.46
4	0	17	1.61	0.99
4	0	17	-0.62	1.18
-4	0	-17	-0.04	0.50
-4	0	-17	0.03	1.91
-4	0	-17	0.61	0.47
3	0	17	34.61	2.76
3	0	17	34.81	2.53
3	0	17	34.55	1.62
-3	0	-17	38.18	1.85
-3	0	-17	35.71	1.61
2	0	17	2.09	1.14
2	0	17	1.38	1.07
-2	0	-17	0.02	0.42
1	0	17	48.70	3.19
1	0	17	57.09	3.25
1	0	17	58.43	2.42
1	0	17	54.03	2.28
-1	0	-17	52.94	2.44
0	0	17	1.17	0.42
0	0	17	-0.38	1.01
0	0	17	-0.11	0.52
0	0	-17	0.72	0.42
0	0	-17	0.62	0.50
-1	0	17	77.30	4.07
-1	0	17	80.43	3.24
-1	0	17	77.80	4.27
-1	0	17	82.83	3.32
1	0	-17	78.09	3.32
-2	0	17	-0.60	0.52
-2	0	17	0.99	0.59
-2	0	17	1.61	1.34
-2	0	17	0.29	0.45

-2	0	17	-0.03	0.93
2	0	-17	-0.96	0.48
2	0	-17	0.07	0.40
-3	0	17	31.52	2.45
-3	0	17	39.54	1.94
-3	0	17	34.92	1.87
-3	0	17	41.43	3.00
-3	0	17	34.95	1.79
3	0	-17	35.13	1.71
-4	0	17	-0.54	0.52
-4	0	17	1.45	0.95
-4	0	17	-0.64	0.46
-4	0	17	-1.14	1.23
-4	0	17	1.15	0.66
4	0	-17	-1.79	1.40
4	0	-17	-0.09	0.37
-5	0	17	19.33	1.35
-5	0	17	18.14	2.56
-5	0	17	20.26	1.93
-5	0	17	21.44	1.39
-5	0	17	23.00	1.56
5	0	-17	20.49	1.16
5	0	-17	20.85	2.24
-6	0	17	1.20	1.44
-6	0	17	-1.50	0.70
-6	0	17	-0.51	0.89
-6	0	17	0.31	0.55
-6	0	17	0.52	0.57
6	0	-17	-0.45	0.36
6	0	-17	1.78	1.12
-7	0	17	25.05	1.99
-7	0	17	26.56	1.69
-7	0	17	21.90	2.95
-7	0	17	25.23	1.83
-7	0	17	25.59	1.56
7	0	-17	26.17	2.26
7	0	-17	26.44	1.34
-8	0	17	-1.96	0.91
-8	0	17	0.42	1.55
-8	0	17	0.87	0.71
-8	0	17	-0.81	0.86
-8	0	17	-0.38	0.63
8	0	-17	-1.38	1.17
8	0	-17	-0.38	0.40

-9	0	17	18.64	1.67
-9	0	17	18.83	2.98
-9	0	17	20.54	1.69
9	0	-17	18.94	1.17
-10	0	17	-0.85	1.72
-10	0	17	0.11	0.98
-10	0	17	-0.90	0.84
10	0	-17	-0.09	0.42
-11	0	17	13.30	2.27
-11	0	17	14.85	1.77
11	0	-17	14.66	1.08
-12	0	17	1.35	1.01
-12	0	17	-0.35	1.83
12	0	-17	0.09	0.57
-13	0	17	13.46	1.95
-13	0	17	9.20	2.28
13	0	-17	12.61	1.12
-14	0	17	-0.66	1.06
-14	0	17	-1.53	2.08
14	0	-17	-1.31	0.54
-15	0	17	7.50	1.51
-15	0	17	5.80	2.36
15	0	-17	8.48	1.18
11	0	18	-0.52	1.83
-11	0	-18	-1.31	0.87
10	0	18	19.94	3.05
-10	0	-18	18.20	1.83
9	0	18	1.53	1.61
-9	0	-18	1.28	0.77
8	0	18	23.16	3.01
-8	0	-18	25.37	1.82
7	0	18	-1.01	1.32
7	0	18	-0.29	1.08
-7	0	-18	-0.64	0.67
6	0	18	66.36	4.14
6	0	18	66.91	3.52
-6	0	-18	63.84	3.06
5	0	18	0.34	1.36
5	0	18	0.71	1.14
-5	0	-18	1.16	0.60
4	0	18	85.42	4.71
4	0	18	90.00	4.36
-4	0	-18	88.26	3.78
3	0	18	0.19	1.05

3	0	18	0.15	1.21
2	0	18	29.00	2.53
2	0	18	29.89	2.69
2	0	18	32.35	1.44
-2	0	-18	29.20	1.67
1	0	18	-0.46	1.13
1	0	18	0.55	0.43
-1	0	-18	-0.47	0.48
0	0	18	64.94	3.84
0	0	18	65.25	3.01
0	0	18	67.21	2.74
0	0	18	61.43	3.71
0	0	-18	68.88	2.88
-1	0	18	-1.43	1.07
-1	0	18	-0.19	0.47
-1	0	18	0.50	0.60
-1	0	18	-0.53	1.20
1	0	-18	-0.26	0.45
-2	0	18	43.84	3.28
-2	0	18	47.56	2.12
-2	0	18	45.74	2.21
2	0	-18	42.48	2.07
-3	0	18	-0.11	1.00
-3	0	18	-2.69	1.25
-3	0	18	0.16	0.52
-3	0	18	-0.27	0.63
-3	0	18	1.33	0.71
3	0	-18	0.51	0.45
-4	0	18	11.52	1.94
-4	0	18	9.72	1.47
-4	0	18	10.90	1.79
-4	0	18	13.21	1.19
-4	0	18	10.71	1.14
4	0	-18	11.32	0.95
-5	0	18	0.61	1.50
-5	0	18	0.63	1.11
-5	0	18	0.05	0.57
-5	0	18	2.03	0.78
-5	0	18	0.25	0.64
5	0	-18	0.28	0.43
-6	0	18	20.50	1.70
-6	0	18	18.17	1.66
-6	0	18	20.70	1.51
-6	0	18	15.43	2.93

-6	0	18	16.32	1.96
6	0	-18	19.02	1.17
6	0	-18	20.36	2.39
-7	0	18	-0.25	0.71
-7	0	18	-2.61	1.50
-7	0	18	-0.80	0.90
-7	0	18	-0.42	0.96
7	0	-18	-2.09	1.39
7	0	-18	-0.10	0.44
-8	0	18	29.56	3.45
-8	0	18	26.58	2.11
-8	0	18	30.93	1.98
8	0	-18	27.60	1.48
-9	0	18	-0.63	0.88
-9	0	18	0.31	0.96
-9	0	18	1.88	1.66
9	0	-18	-0.20	0.43
-10	0	18	24.02	3.41
-10	0	18	25.41	2.05
10	0	-18	24.15	1.39
-11	0	18	1.39	1.02
-11	0	18	-2.00	1.89
11	0	-18	-1.08	0.59
-12	0	18	45.49	4.27
-12	0	18	42.75	2.77
12	0	-18	40.09	2.01
-13	0	18	-0.48	1.15
-13	0	18	1.41	2.17
13	0	-18	-0.31	0.57
-14	0	18	34.32	2.64
-14	0	18	30.40	4.23
14	0	-18	24.94	1.66
-10	0	-19	1.56	1.01
9	0	19	21.78	3.19
-9	0	-19	21.89	1.92
8	0	19	-2.09	1.68
-8	0	-19	-0.25	0.80
7	0	19	45.63	3.13
7	0	19	43.05	3.68
-7	0	-19	49.31	2.56
6	0	19	1.15	1.27
6	0	19	-1.42	1.56
-6	0	-19	-0.27	0.72
5	0	19	40.42	3.40

5	0	19	43.55	2.97
-5	0	-19	39.59	2.24
4	0	19	0.88	1.27
4	0	19	-0.05	1.42
-4	0	-19	-0.68	0.60
3	0	19	34.54	3.27
3	0	19	32.78	3.05
-3	0	-19	31.33	1.86
2	0	19	0.04	1.31
2	0	19	-0.24	1.22
-2	0	-19	0.33	0.56
1	0	19	37.54	2.99
1	0	19	36.10	3.07
0	0	19	2.00	1.34
0	0	19	1.03	1.38
0	0	19	0.71	0.53
0	0	-19	0.60	0.55
-1	0	19	29.65	2.95
-1	0	19	26.71	1.49
-1	0	19	22.64	2.51
1	0	-19	28.36	1.54
-2	0	19	0.24	1.42
-2	0	19	0.17	0.55
-2	0	19	1.30	1.20
2	0	-19	-0.62	0.48
-3	0	19	27.97	1.62
-3	0	19	24.25	2.45
-3	0	19	27.59	3.01
3	0	-19	26.81	1.49
-4	0	19	-0.24	0.62
-4	0	19	1.45	1.58
4	0	-19	-0.74	0.45
-5	0	19	33.62	2.58
-5	0	19	31.91	3.30
-5	0	19	35.40	1.95
5	0	-19	32.96	1.70
-6	0	19	-0.10	1.62
-6	0	19	0.69	0.77
-6	0	19	1.25	1.18
6	0	-19	0.54	0.46
-7	0	19	46.66	2.98
-7	0	19	54.07	2.66
-7	0	19	49.32	4.05
7	0	-19	46.49	2.21

-8	0	19	-0.39	1.78
-8	0	19	0.56	0.90
-8	0	19	-1.04	1.04
8	0	-19	-0.28	0.47
-9	0	19	49.63	4.06
-9	0	19	41.28	2.88
9	0	-19	36.90	2.08
-10	0	19	-0.21	0.99
-10	0	19	-1.27	1.93
10	0	-19	-1.25	0.53
-11	0	19	36.01	4.17
-11	0	19	37.88	2.59
11	0	-19	31.91	1.78
-12	0	19	0.17	1.21
-12	0	19	-2.47	2.05
12	0	-19	-0.14	0.59
-13	0	19	30.74	2.61
-13	0	19	31.44	5.89
13	0	-19	25.73	1.65
8	0	20	7.43	2.50
-8	0	-20	5.00	1.09
7	0	20	1.23	1.75
-7	0	-20	0.57	0.88
6	0	20	18.15	2.51
6	0	20	16.19	2.88
-6	0	-20	19.39	1.73
5	0	20	-0.43	1.55
5	0	20	-0.98	1.44
-5	0	-20	-0.16	0.73
4	0	20	32.55	3.29
4	0	20	29.98	2.90
-4	0	-20	34.04	1.99
3	0	20	0.00	1.46
3	0	20	1.05	1.43
-3	0	-20	-0.04	0.65
2	0	20	31.98	3.18
2	0	20	33.97	3.11
-2	0	-20	34.72	1.96
1	0	20	0.14	1.42
1	0	20	-0.40	1.49
-1	0	-20	-0.72	0.60
0	0	20	34.01	3.14
0	0	20	29.84	3.03
0	0	-20	35.18	1.87

-1	0	20	0.04	1.53
-1	0	20	0.60	1.35
1	0	-20	0.50	0.61
-2	0	20	21.71	2.62
-2	0	20	25.50	3.08
-3	0	20	-2.34	1.27
-3	0	20	1.60	1.64
-3	0	20	1.01	0.73
3	0	-20	0.80	0.64
-4	0	20	19.39	2.41
-4	0	20	15.35	1.49
-4	0	20	18.08	3.15
4	0	-20	19.20	1.32
-5	0	20	-2.74	1.64
-5	0	20	1.82	1.30
-5	0	20	-1.22	0.79
5	0	-20	0.10	0.55
-6	0	20	34.88	2.10
-6	0	20	36.59	3.78
6	0	-20	27.12	1.81
-7	0	20	0.75	1.94
-7	0	20	0.31	0.96
-7	0	20	-1.19	1.21
-8	0	20	55.32	2.92
-8	0	20	51.14	4.35
-8	0	20	54.55	3.47
8	0	-20	50.21	2.43
-9	0	20	-1.95	2.01
-9	0	20	0.58	1.08
9	0	-20	-0.19	0.56
-10	0	20	23.88	2.29
-10	0	20	23.24	3.76
10	0	-20	22.08	1.48
-11	0	20	1.14	1.27
-11	0	20	-4.06	2.26
11	0	-20	0.62	0.61
6	0	21	-2.12	1.90
-6	0	-21	0.74	0.88
5	0	21	-3.18	1.73
5	0	21	0.56	1.61
-5	0	-21	1.70	0.86
4	0	21	-1.56	1.69
4	0	21	-0.33	1.69
-4	0	-21	0.14	0.76

3	0	21	1.69	1.64
3	0	21	3.52	1.80
-3	0	-21	3.92	0.91
2	0	21	0.41	1.68
2	0	21	-0.23	1.59
-2	0	-21	0.06	0.71
1	0	21	5.70	1.83
1	0	21	8.50	1.88
-1	0	-21	5.67	0.90
0	0	21	2.76	1.65
0	0	21	-0.15	1.72
0	0	-21	0.77	0.69
-1	0	21	2.77	1.80
-1	0	21	4.79	1.70
1	0	-21	2.33	0.71
-2	0	21	0.31	1.52
-2	0	21	-1.81	1.77
2	0	-21	-0.24	0.60
-3	0	21	9.16	2.13
-3	0	21	11.43	1.95
3	0	-21	11.30	1.22
-4	0	21	-0.94	1.49
-4	0	21	3.11	1.97
4	0	-21	0.56	0.61
-5	0	21	6.10	1.69
-5	0	21	6.42	2.15
5	0	-21	8.32	1.10
-6	0	21	0.69	1.41
-6	0	21	-2.34	1.84
6	0	-21	0.16	0.59
-7	0	21	8.84	1.57
-7	0	21	4.79	2.21
7	0	-21	9.72	1.15
-8	0	21	-2.37	2.00
8	0	-21	-0.54	0.61
-9	0	21	21.05	3.98
9	0	-21	15.39	1.34
3	0	22	0.17	1.76
3	0	22	0.46	1.89
-3	0	-22	-0.14	0.83
2	0	22	5.46	2.03
2	0	22	6.30	2.00
-2	0	-22	5.73	1.01
1	0	22	-0.58	1.81

1	0	22	0.24	1.73
-1	0	-22	-0.37	0.78
0	0	22	3.04	1.95
0	0	22	9.03	2.11
0	0	-22	3.93	0.88
-1	0	22	-0.06	1.68
-1	0	22	-2.47	1.87
1	0	-22	1.10	0.73
-2	0	22	1.44	1.71
-2	0	22	3.41	2.09
2	0	-22	4.85	0.85
-3	0	22	-1.90	1.60
-3	0	22	-1.23	2.02
3	0	-22	-1.25	0.68
-4	0	22	3.90	2.18
-4	0	22	3.40	1.77
4	0	-22	3.60	0.76
-5	0	22	-0.75	1.57
-5	0	22	1.95	2.24
5	0	-22	0.37	0.67
-6	0	22	-0.50	1.61
-6	0	22	3.59	2.34
6	0	-22	0.33	0.69
-7	0	22	0.04	2.52
7	-1	-22	2.99	0.77
6	-1	-22	1.18	0.71
-6	1	22	0.02	1.58
-6	1	22	2.38	2.27
5	-1	-22	1.25	0.71
-5	1	22	3.04	2.17
-5	1	22	2.44	1.68
4	-1	-22	0.68	0.71
-4	1	22	1.15	1.65
-4	1	22	-1.71	1.99
3	-1	-22	4.01	0.84
-3	1	22	5.69	2.10
-3	1	22	0.16	1.65
2	-1	-22	8.96	1.37
-2	1	22	9.70	2.31
-2	1	22	7.62	1.97
1	-1	-22	14.29	1.50
-1	1	22	12.73	2.39
-1	1	22	16.01	2.97
0	-1	-22	0.87	0.80

0	1	22	1.26	1.82
0	1	22	-0.01	1.76
-1	-1	-22	4.65	0.95
1	1	22	2.71	1.88
1	1	22	1.11	1.77
-2	-1	-22	0.62	0.86
2	1	22	0.63	1.78
2	1	22	1.82	1.97
-3	-1	-22	2.24	0.96
3	1	22	4.54	1.94
3	1	22	2.71	1.96
9	-1	-21	2.90	0.76
-9	1	21	0.80	2.41
8	-1	-21	5.47	0.78
-8	1	21	4.94	2.27
7	-1	-21	-0.15	0.63
-7	1	21	1.14	2.06
-7	1	21	-0.82	1.35
6	-1	-21	11.93	1.23
-6	1	21	10.07	1.74
-6	1	21	12.07	2.40
5	-1	-21	-0.56	0.62
-5	1	21	0.49	1.48
-5	1	21	5.34	2.06
4	-1	-21	7.66	1.15
-4	1	21	7.41	2.05
-4	1	21	3.85	1.66
3	-1	-21	1.10	0.68
-3	1	21	2.44	1.64
-3	1	21	1.43	1.80
2	-1	-21	8.08	1.23
-2	1	21	13.97	2.56
-2	1	21	10.86	2.16
1	-1	-21	1.88	0.73
-1	1	21	3.61	1.81
-1	1	21	2.05	1.56
0	-1	-21	3.33	0.80
0	1	21	6.84	1.79
0	1	21	5.98	1.90
-1	-1	-21	1.41	0.76
1	1	21	-0.02	1.55
1	1	21	2.20	1.69
-2	-1	-21	4.63	0.88
2	1	21	4.79	1.75

2	1	21	5.08	1.81
-3	-1	-21	-0.93	0.77
3	1	21	-2.64	1.50
3	1	21	-2.99	1.57
-4	-1	-21	-0.24	0.80
4	1	21	0.93	1.70
4	1	21	1.99	1.63
-5	-1	-21	1.42	0.85
5	1	21	-0.81	1.58
5	1	21	1.41	1.86
-6	-1	-21	0.42	0.91
6	1	21	0.21	2.06
11	-1	-20	16.69	1.38
-11	1	20	14.20	3.02
-11	1	20	13.69	2.20
10	-1	-20	1.45	0.68
-10	1	20	4.06	1.23
-10	1	20	1.75	2.13
9	-1	-20	53.30	2.40
-9	1	20	46.24	3.23
-9	1	20	48.71	4.50
8	-1	-20	1.16	0.61
-8	1	20	1.22	1.42
-8	1	20	-4.03	1.87
-8	1	20	2.86	1.01
7	-1	-20	43.43	2.13
-7	1	20	47.05	2.52
-7	1	20	48.35	4.04
-7	1	20	39.07	2.92
6	-1	-20	4.13	0.70
-6	1	20	6.33	2.06
-6	1	20	5.24	0.96
-6	1	20	1.12	2.49
5	-1	-20	31.62	1.73
-5	1	20	31.54	3.55
-5	1	20	33.13	1.99
-5	1	20	30.58	2.71
4	-1	-20	1.89	0.60
-4	1	20	1.51	1.77
-4	1	20	3.77	1.39
3	-1	-20	38.68	2.00
-3	1	20	39.62	3.01
-3	1	20	38.87	3.48
2	-1	-20	4.44	0.72

-2	1	20	4.97	1.52
-2	1	20	3.99	1.64
1	-1	-20	24.31	1.53
-1	1	20	18.71	2.62
-1	1	20	23.55	2.97
0	-1	-20	1.55	0.67
0	1	20	0.88	1.53
0	1	20	-0.54	1.45
-1	-1	-20	30.20	1.83
1	1	20	35.74	3.09
1	1	20	27.98	2.98
-2	-1	-20	-0.37	0.63
2	1	20	-1.08	1.42
-3	-1	-20	23.81	1.69
3	1	20	23.46	2.81
3	1	20	25.92	2.76
-4	-1	-20	4.86	0.91
4	1	20	6.63	1.91
4	1	20	4.32	1.55
-5	-1	-20	14.85	1.55
5	1	20	14.30	1.92
5	1	20	13.05	2.13
-6	-1	-20	0.12	0.83
6	1	20	0.78	1.65
-7	-1	-20	20.21	1.83
7	1	20	16.14	3.02
-8	-1	-20	-1.22	0.91
8	1	20	-0.31	2.21
13	-1	-19	-0.12	0.64
-13	1	19	0.04	3.01
-13	1	19	1.43	1.32
12	-1	-19	47.33	2.32
-12	1	19	49.70	4.52
-12	1	19	49.35	3.09
11	-1	-19	-0.74	0.57
-11	1	19	1.54	1.18
-11	1	19	0.75	2.11
10	-1	-19	33.55	1.80
-10	1	19	32.95	3.76
-10	1	19	35.91	2.43
9	-1	-19	-0.42	0.54
-9	1	19	-0.78	0.93
-9	1	19	0.70	1.70
8	-1	-19	45.50	2.22

-8	1	19	45.05	3.97
-8	1	19	46.77	2.85
-8	1	19	50.27	2.63
-7	1	19	-2.54	1.56
-7	1	19	-1.43	1.10
-7	1	19	-1.07	0.82
6	-1	-19	44.04	2.15
-6	1	19	39.73	3.74
-6	1	19	48.54	2.40
-6	1	19	46.39	2.94
5	-1	-19	1.34	0.51
-5	1	19	1.10	0.73
-5	1	19	1.61	1.64
-5	1	19	0.91	1.15
4	-1	-19	35.89	1.84
-4	1	19	38.74	2.00
-4	1	19	35.04	3.33
3	-1	-19	0.80	0.50
-3	1	19	-0.24	1.12
-3	1	19	-1.54	1.45
-3	1	19	1.34	0.65
2	-1	-19	30.24	1.72
-2	1	19	30.21	3.01
-2	1	19	33.54	1.67
-2	1	19	32.76	2.68
-1	1	19	4.28	1.35
-1	1	19	3.57	1.52
0	-1	-19	41.20	2.14
0	1	19	43.73	3.23
0	1	19	43.05	3.17
-1	-1	-19	-0.77	0.55
1	1	19	-1.38	1.22
1	1	19	-0.91	1.33
-2	-1	-19	28.03	1.76
2	1	19	31.87	2.78
2	1	19	29.83	2.71
-3	-1	-19	0.86	0.64
3	1	19	1.23	1.37
3	1	19	-0.30	1.21
-4	-1	-19	72.44	3.19
4	1	19	63.98	4.16
-5	-1	-19	2.95	0.79
5	1	19	2.40	1.50
5	1	19	0.12	1.30

-6	-1	-19	31.90	1.97
6	1	19	30.35	3.04
6	1	19	28.98	2.57
-7	-1	-19	-0.01	0.72
7	1	19	3.82	1.71
7	1	19	0.72	1.33
-8	-1	-19	32.62	2.15
8	1	19	31.30	3.45
-9	-1	-19	0.11	0.89
9	1	19	2.25	1.76
14	-1	-18	0.10	0.65
-14	1	18	1.40	2.21
-14	1	18	1.25	1.30
13	-1	-18	37.55	2.04
-13	1	18	42.85	2.83
-13	1	18	43.92	4.29
12	-1	-18	0.05	0.55
-12	1	18	0.42	1.06
-12	1	18	0.65	2.02
11	-1	-18	39.76	2.01
-11	1	18	49.61	2.71
-11	1	18	32.26	3.92
10	-1	-18	1.32	0.57
-10	1	18	-1.00	1.67
-10	1	18	0.97	0.99
9	-1	-18	12.00	1.28
-9	1	18	18.70	2.63
-9	1	18	15.36	1.79
-9	1	18	18.16	1.67
8	-1	-18	2.69	0.56
-8	1	18	2.85	0.90
-8	1	18	4.99	1.10
-8	1	18	3.38	1.79
7	-1	-18	35.64	2.84
7	-1	-18	37.52	1.79
-7	1	18	35.80	2.13
-7	1	18	35.33	3.42
-7	1	18	38.26	2.39
-7	1	18	36.72	2.45
6	-1	-18	0.41	0.44
6	-1	-18	1.01	1.39
-6	1	18	0.52	1.48
-6	1	18	-0.51	0.68
-6	1	18	2.04	1.06

-6	1	18	1.01	0.81
5	-1	-18	39.44	1.96
-5	1	18	40.89	3.40
-5	1	18	42.11	2.20
-5	1	18	39.97	2.73
-5	1	18	47.93	2.21
4	-1	-18	2.25	0.51
-4	1	18	3.34	1.13
-4	1	18	2.48	1.51
-4	1	18	2.31	0.73
-4	1	18	3.45	0.68
3	-1	-18	30.44	1.56
-3	1	18	29.45	1.61
-3	1	18	30.84	1.70
-3	1	18	26.27	2.33
-3	1	18	30.97	2.94
2	-1	-18	0.13	0.45
-2	1	18	0.20	0.60
-2	1	18	0.42	1.31
-2	1	18	-0.02	0.47
1	-1	-18	35.85	1.82
-1	1	18	37.47	2.75
-1	1	18	39.57	1.73
-1	1	18	32.68	2.88
0	-1	-18	4.07	0.63
0	1	18	5.00	1.39
0	1	18	4.45	0.56
0	1	18	3.03	1.25
-1	-1	-18	44.59	2.12
1	1	18	44.60	3.79
1	1	18	41.48	3.04
2	1	18	-1.26	1.14
2	1	18	-0.41	1.04
-3	-1	-18	48.20	2.28
3	1	18	44.00	3.18
3	1	18	45.21	2.94
-4	-1	-18	0.21	0.58
4	1	18	-1.23	1.32
4	1	18	-1.00	1.08
-5	-1	-18	63.69	2.97
5	1	18	59.15	3.85
5	1	18	65.51	3.52
-6	-1	-18	0.87	0.67
6	1	18	0.53	1.41

6	1	18	-1.11	1.08
-7	-1	-18	55.95	2.81
7	1	18	59.61	3.18
7	1	18	51.73	3.67
-8	-1	-18	0.96	0.76
8	1	18	-1.42	1.43
8	1	18	-0.85	1.77
-9	-1	-18	38.87	2.34
9	1	18	37.99	3.40
-10	-1	-18	-1.54	0.78
10	1	18	4.36	1.71
-11	-1	-18	17.32	1.86
11	1	18	18.10	3.26
15	-1	-17	-0.41	0.63
-15	1	17	1.25	1.30
-15	1	17	1.57	2.18
14	-1	-17	9.99	1.18
-14	1	17	10.70	1.56
-14	1	17	8.14	2.41
13	-1	-17	0.27	0.55
-13	1	17	-0.14	1.07
-13	1	17	-1.84	1.91
12	-1	-17	16.79	1.22
-12	1	17	17.16	1.93
-12	1	17	17.91	2.75
11	-1	-17	0.82	0.51
-11	1	17	-1.05	1.67
-11	1	17	1.14	0.91
10	-1	-17	19.89	1.23
-10	1	17	20.22	3.16
-10	1	17	22.48	1.83
-10	1	17	17.19	1.68
9	-1	-17	6.16	0.81
-9	1	17	5.25	0.95
-9	1	17	5.26	0.98
-9	1	17	7.27	1.75
8	-1	-17	40.99	1.89
8	-1	-17	39.92	2.62
-8	1	17	45.44	2.30
-8	1	17	39.00	2.32
-8	1	17	38.73	2.39
-8	1	17	37.65	3.48
7	-1	-17	1.10	1.13
7	-1	-17	0.95	0.42

-7	1	17	0.87	0.76
-7	1	17	1.06	0.90
-7	1	17	-0.06	1.48
-7	1	17	0.47	0.68
6	-1	-17	15.93	2.03
6	-1	-17	13.98	1.02
-6	1	17	16.86	1.54
-6	1	17	17.31	1.32
-6	1	17	16.45	1.43
-6	1	17	13.63	2.51
-6	1	17	12.82	1.76
5	-1	-17	-0.16	1.16
5	-1	-17	-0.03	0.39
-5	1	17	-0.46	0.93
-5	1	17	-0.02	0.54
-5	1	17	-1.11	1.19
-5	1	17	0.25	0.57
-5	1	17	0.04	0.64
4	-1	-17	28.43	1.45
-4	1	17	25.34	1.57
-4	1	17	31.10	2.77
-4	1	17	29.09	2.18
-4	1	17	28.18	1.55
-4	1	17	26.91	1.63
3	-1	-17	0.31	0.39
-3	1	17	0.76	0.47
-3	1	17	-0.69	0.52
-3	1	17	1.05	0.59
-3	1	17	-0.47	1.02
-3	1	17	-0.38	1.09
2	-1	-17	56.95	2.55
-2	1	17	56.63	3.27
-2	1	17	60.33	3.56
-2	1	17	61.35	2.56
-2	1	17	58.89	2.50
-2	1	17	56.23	2.67
1	-1	-17	1.62	0.48
-1	1	17	2.10	0.46
-1	1	17	1.50	1.76
-1	1	17	0.45	1.01
-1	1	17	1.44	0.57
0	-1	-17	74.60	3.22
0	1	17	75.41	4.03
0	1	17	79.26	3.07

0	1	17	74.79	3.06
-1	-1	-17	7.90	0.93
1	1	17	8.53	1.45
1	1	17	9.95	1.80
-2	-1	-17	37.22	1.87
2	1	17	35.97	2.72
2	1	17	36.24	2.54
-3	-1	-17	0.59	0.49
3	1	17	-0.08	1.11
3	1	17	0.02	0.99
-4	-1	-17	51.95	4.36
-4	-1	-17	45.71	2.50
4	1	17	52.25	3.39
4	1	17	51.92	2.95
-5	-1	-17	0.10	0.55
-5	-1	-17	0.94	2.16
5	1	17	-0.36	1.25
5	1	17	1.10	0.97
-6	-1	-17	48.41	2.45
6	1	17	51.97	2.84
6	1	17	46.17	3.39
-7	-1	-17	3.39	0.76
7	1	17	4.14	1.11
7	1	17	5.05	1.63
-8	-1	-17	63.06	2.97
8	1	17	59.24	3.10
8	1	17	59.91	3.86
-9	-1	-17	-1.12	0.65
9	1	17	-0.79	1.41
-10	-1	-17	27.97	1.98
10	1	17	28.68	2.96
-11	-1	-17	-0.10	0.82
11	1	17	1.05	1.53
-12	-1	-17	14.67	1.77
12	1	17	11.02	2.08
16	-1	-16	-0.40	0.64
-16	1	16	-0.38	1.28
-16	1	16	-3.38	2.18
15	-1	-16	0.04	0.61
-15	1	16	0.03	1.14
-15	1	16	1.51	2.11
14	-1	-16	3.38	0.65
-14	1	16	4.40	2.04
-14	1	16	4.84	1.23

13	-1	-16	0.56	0.50
-13	1	16	0.53	0.95
-13	1	16	3.34	1.85
12	-1	-16	1.20	0.47
-12	1	16	1.57	1.84
-12	1	16	1.88	0.98
11	-1	-16	4.05	0.56
-11	1	16	4.89	1.89
-11	1	16	4.80	0.95
10	-1	-16	0.59	0.40
-10	1	16	0.06	1.49
-10	1	16	0.13	0.75
-10	1	16	0.95	0.73
9	-1	-16	5.02	1.08
9	-1	-16	4.30	0.56
-9	1	16	4.01	0.88
-9	1	16	5.38	1.77
-9	1	16	4.06	0.88
8	-1	-16	1.37	0.94
8	-1	-16	1.48	0.44
-8	1	16	1.02	0.77
-8	1	16	2.64	1.47
-8	1	16	1.34	0.81
-8	1	16	0.65	0.56
-8	1	16	0.09	0.68
7	-1	-16	18.49	1.91
7	-1	-16	22.24	1.18
-7	1	16	23.49	1.53
-7	1	16	20.14	1.35
-7	1	16	19.02	2.58
-7	1	16	20.53	1.54
-7	1	16	21.29	1.75
6	-1	-16	0.78	0.37
6	-1	-16	0.04	1.01
-6	1	16	1.50	1.27
-6	1	16	0.12	0.52
-6	1	16	-0.28	0.78
-6	1	16	0.35	0.52
-6	1	16	0.15	0.63
5	-1	-16	18.23	1.97
5	-1	-16	17.36	1.06
-5	1	16	18.79	1.24
-5	1	16	19.49	1.36
-5	1	16	19.53	1.27

-5	1	16	20.82	2.29
-5	1	16	16.41	1.69
4	-1	-16	0.09	0.37
4	-1	-16	0.44	1.10
-4	1	16	-0.02	0.86
-4	1	16	0.02	1.25
-4	1	16	-0.41	0.53
-4	1	16	-0.37	0.43
-4	1	16	0.75	0.47
3	-1	-16	6.13	1.29
3	-1	-16	7.92	0.76
-3	1	16	7.60	0.95
-3	1	16	9.86	1.85
-3	1	16	8.04	0.92
-3	1	16	6.61	0.80
-3	1	16	8.96	1.55
2	-1	-16	14.66	0.99
-2	1	16	16.79	1.96
-2	1	16	11.66	1.71
-2	1	16	13.88	1.11
-2	1	16	18.04	1.11
-2	1	16	14.19	0.97
1	-1	-16	26.68	1.47
-1	1	16	29.49	2.38
-1	1	16	30.59	1.38
-1	1	16	29.31	1.45
-1	1	16	27.64	1.58
-1	1	16	28.15	2.16
0	-1	-16	3.10	0.53
0	-1	-16	3.01	0.52
0	1	16	3.40	0.51
0	1	16	3.80	1.13
0	1	16	3.70	0.63
0	1	16	3.64	1.06
-1	-1	-16	26.30	1.45
-1	-1	-16	26.32	1.42
1	1	16	27.31	1.24
1	1	16	23.90	2.11
1	1	16	23.36	2.17
-2	-1	-16	3.10	1.76
-2	-1	-16	0.06	0.42
2	1	16	-0.09	0.83
-3	-1	-16	23.49	1.42
-3	-1	-16	25.29	3.31

3	1	16	26.79	2.00
3	1	16	23.16	2.23
-4	-1	-16	0.52	0.47
-4	-1	-16	1.23	1.74
4	1	16	3.12	1.15
4	1	16	-0.07	0.83
-5	-1	-16	25.52	3.24
5	1	16	22.48	1.87
5	1	16	24.33	2.40
-6	-1	-16	0.48	1.78
-6	-1	-16	3.41	0.66
6	1	16	3.27	0.94
6	1	16	4.45	1.41
-7	-1	-16	30.49	4.07
-7	-1	-16	25.16	1.66
7	1	16	22.55	2.53
7	1	16	28.90	1.97
-8	-1	-16	3.37	0.75
8	1	16	4.64	0.95
8	1	16	5.34	1.47
-9	-1	-16	24.39	1.74
9	1	16	26.13	1.80
9	1	16	22.78	2.71
-10	-1	-16	-0.62	0.69
10	1	16	-1.22	1.37
-11	-1	-16	33.73	2.16
11	1	16	33.84	3.12
-12	-1	-16	0.88	0.81
12	1	16	0.03	1.53
-13	-1	-16	24.47	2.07
13	1	16	20.73	3.00
17	-1	-15	-1.38	0.62
-17	1	15	-0.36	1.37
-17	1	15	-0.45	2.23
16	-1	-15	16.24	1.31
-16	1	15	17.55	2.26
-16	1	15	9.53	2.57
15	-1	-15	1.01	0.56
-15	1	15	1.54	1.13
-15	1	15	-0.35	1.93
14	-1	-15	15.83	1.19
-14	1	15	18.23	2.03
-14	1	15	12.48	2.43
13	-1	-15	4.50	0.62

-13	1	15	3.45	1.86
-13	1	15	7.54	1.15
12	-1	-15	11.00	0.91
-12	1	15	10.23	2.05
-12	1	15	11.68	1.59
11	-1	-15	0.95	0.42
-11	1	15	1.61	1.25
-11	1	15	2.09	1.65
10	-1	-15	0.60	0.42
10	-1	-15	0.25	0.83
-10	1	15	0.65	0.67
-10	1	15	1.14	0.66
-10	1	15	-0.01	1.41
9	-1	-15	2.24	0.43
9	-1	-15	3.45	0.88
-9	1	15	2.59	0.76
-9	1	15	4.10	1.50
-9	1	15	2.69	0.56
-9	1	15	2.54	0.71
-9	1	15	4.47	0.81
8	-1	-15	8.24	0.71
8	-1	-15	10.36	1.44
-8	1	15	8.11	1.66
-8	1	15	5.67	0.93
-8	1	15	7.27	1.12
-8	1	15	7.51	1.22
-8	1	15	7.91	1.22
7	-1	-15	3.07	0.46
7	-1	-15	3.09	0.95
-7	1	15	4.75	0.61
-7	1	15	3.75	0.73
-7	1	15	6.27	1.17
-7	1	15	3.72	0.68
-7	1	15	5.15	1.48
6	-1	-15	9.79	1.60
6	-1	-15	12.29	0.83
-6	1	15	14.97	2.79
-6	1	15	14.00	1.10
-6	1	15	10.53	1.35
-6	1	15	12.41	1.00
-6	1	15	12.12	1.16
5	-1	-15	0.58	0.35
5	-1	-15	0.90	0.93
-5	1	15	-0.53	0.43

-5	1	15	0.70	0.73
-5	1	15	-1.19	0.98
-5	1	15	0.75	0.44
-5	1	15	0.27	0.54
4	-1	-15	3.27	0.46
4	-1	-15	4.35	1.07
-4	1	15	3.37	1.18
-4	1	15	4.53	0.59
-4	1	15	5.11	0.98
-4	1	15	3.52	0.56
-4	1	15	2.96	0.60
3	-1	-15	0.14	0.31
3	-1	-15	1.11	0.96
-3	1	15	0.09	0.36
-3	1	15	0.15	0.84
-3	1	15	0.19	0.79
-3	1	15	-0.50	0.38
-3	1	15	1.25	0.47
2	-1	-15	0.27	1.05
2	-1	-15	1.18	0.34
-2	1	15	0.35	0.44
-2	1	15	0.17	0.79
-2	1	15	0.73	0.43
-2	1	15	1.45	0.96
-2	1	15	0.23	0.35
1	-1	-15	4.87	0.66
1	-1	-15	3.92	1.14
1	-1	-15	0.59	1.93
-1	1	15	4.67	0.56
-1	1	15	4.41	1.15
-1	1	15	5.05	0.99
-1	1	15	4.66	0.70
-1	1	15	4.18	0.59
0	-1	-15	2.20	0.44
0	-1	-15	3.17	0.50
0	-1	-15	2.64	1.62
0	1	15	3.85	0.99
0	1	15	3.76	0.62
0	1	15	3.03	0.42
0	1	15	3.44	1.02
-1	-1	-15	-0.35	1.50
-1	-1	-15	-0.20	0.40
-1	-1	-15	-0.51	0.33
1	1	15	0.90	0.33

1	1	15	0.00	0.84
1	1	15	-0.13	0.84
-2	-1	-15	0.15	0.43
-2	-1	-15	0.65	1.45
-2	-1	-15	0.87	0.41
2	1	15	2.78	0.93
2	1	15	1.73	0.90
2	1	15	0.54	0.32
-3	-1	-15	2.45	0.51
-3	-1	-15	1.97	0.54
-3	-1	-15	2.11	1.53
3	1	15	2.41	0.96
3	1	15	3.19	0.91
-4	-1	-15	0.26	0.43
-4	-1	-15	-1.77	1.46
4	1	15	0.88	1.33
4	1	15	-0.77	0.67
4	1	15	-0.24	0.30
-5	-1	-15	2.41	1.58
-5	-1	-15	3.00	0.57
5	1	15	2.13	0.80
5	1	15	3.19	0.54
5	1	15	2.45	1.00
-6	-1	-15	0.55	0.57
-6	-1	-15	3.17	1.62
-6	-1	-15	0.71	0.50
6	1	15	0.90	1.09
6	1	15	0.65	0.80
6	1	15	0.59	0.38
-7	-1	-15	7.73	1.81
-7	-1	-15	11.49	0.90
-7	-1	-15	10.50	1.10
7	1	15	10.95	0.88
7	1	15	10.98	1.36
7	1	15	11.24	1.69
-8	-1	-15	4.87	1.72
-8	-1	-15	6.02	0.79
-8	-1	-15	4.24	0.76
8	1	15	5.12	1.40
8	1	15	6.82	1.21
-9	-1	-15	-0.37	0.54
-9	-1	-15	-0.62	0.58
9	1	15	0.25	0.72
9	1	15	-0.99	1.19

-10	-1	-15	13.32	1.42
-10	-1	-15	12.90	1.24
10	1	15	12.91	2.23
10	1	15	17.24	1.50
-11	-1	-15	0.26	0.70
11	1	15	0.56	1.38
-12	-1	-15	-0.23	0.76
12	1	15	0.18	1.41
-13	-1	-15	1.24	0.88
13	1	15	-0.50	1.47
-14	-1	-15	4.54	1.06
14	1	15	4.80	1.75
17	-1	-14	64.76	3.01
-17	1	14	66.22	3.84
-17	1	14	74.38	5.11
16	-1	-14	-0.75	0.55
-16	1	14	-0.23	1.07
-16	1	14	0.84	1.95
15	-1	-14	74.14	3.34
-15	1	14	80.56	5.07
-15	1	14	84.72	4.00
14	-1	-14	4.37	0.60
-14	1	14	3.52	1.06
-14	1	14	5.02	1.93
-13	1	14	44.07	3.81
-13	1	14	47.38	2.67
12	-1	-14	0.73	0.38
-12	1	14	1.07	0.78
-12	1	14	0.78	1.56
11	-1	-14	60.98	2.55
11	-1	-14	60.12	2.55
11	-1	-14	60.22	2.62
-11	1	14	52.00	3.91
-11	1	14	64.40	2.99
10	-1	-14	0.51	0.69
10	-1	-14	1.15	0.36
-10	1	14	1.55	0.70
-10	1	14	0.31	1.38
-10	1	14	1.28	0.63
-10	1	14	-0.02	0.52
9	-1	-14	85.10	3.49
9	-1	-14	87.06	3.71
-9	1	14	82.29	3.59
-9	1	14	88.66	4.75

-9	1	14	91.18	3.84
-9	1	14	83.15	3.66
-9	1	14	90.17	3.82
8	-1	-14	6.77	1.22
8	-1	-14	6.41	0.65
-8	1	14	5.23	0.80
-8	1	14	4.54	1.64
-8	1	14	5.35	0.82
-8	1	14	6.56	1.04
-8	1	14	7.62	0.88
7	-1	-14	64.35	2.59
7	-1	-14	63.71	2.99
-7	1	14	64.89	2.83
-7	1	14	62.94	2.82
-7	1	14	60.35	2.68
-7	1	14	56.67	2.86
-7	1	14	64.36	3.74
6	-1	-14	0.05	0.82
6	-1	-14	1.02	0.34
-6	1	14	1.72	0.56
-6	1	14	1.28	0.67
-6	1	14	0.73	0.42
-6	1	14	1.19	0.49
-6	1	14	0.25	0.98
5	-1	-14	69.25	3.27
5	-1	-14	65.99	2.75
-5	1	14	65.89	2.82
-5	1	14	63.57	3.13
-5	1	14	65.76	3.70
-5	1	14	68.14	2.88
-5	1	14	70.23	2.88
4	-1	-14	0.75	0.32
4	-1	-14	1.21	0.84
-4	1	14	-0.19	0.95
-4	1	14	0.67	0.38
-4	1	14	1.54	0.49
-4	1	14	1.19	0.75
-4	1	14	1.33	0.40
3	-1	-14	83.05	3.77
3	-1	-14	77.00	3.17
3	-1	-14	77.54	4.50
-3	1	14	81.55	3.22
-3	1	14	70.94	3.59
-3	1	14	75.00	3.98

-3	1	14	78.97	3.23
-3	1	14	81.08	3.20
2	-1	-14	0.25	0.31
2	-1	-14	0.42	1.36
2	-1	-14	1.82	0.92
-2	1	14	0.35	0.73
-2	1	14	0.55	0.36
-2	1	14	0.08	0.32
-2	1	14	-0.83	0.78
1	-1	-14	26.23	2.76
1	-1	-14	29.43	1.37
1	-1	-14	26.12	2.03
-1	1	14	27.72	1.33
-1	1	14	27.31	1.40
-1	1	14	28.81	1.28
-1	1	14	24.80	2.05
-1	1	14	25.20	1.94
0	-1	-14	-0.42	0.93
0	-1	-14	0.37	1.21
0	-1	-14	-0.04	0.34
0	-1	-14	0.10	0.32
0	1	14	1.00	0.40
0	1	14	-0.32	0.28
0	1	14	-0.55	0.72
0	1	14	-0.16	0.32
0	1	14	1.03	0.77
-1	-1	-14	79.59	4.00
-1	-1	-14	83.70	3.35
-1	-1	-14	89.24	3.35
-1	-1	-14	77.11	4.38
1	1	14	84.43	3.19
1	1	14	73.43	3.26
1	1	14	80.69	3.91
1	1	14	80.09	3.87
-2	-1	-14	2.49	0.46
-2	-1	-14	1.91	0.29
-2	-1	-14	2.84	0.49
-2	-1	-14	2.90	1.33
2	1	14	3.03	0.47
2	1	14	1.59	0.77
2	1	14	2.86	0.92
-3	-1	-14	35.46	1.61
-3	-1	-14	33.35	1.64
-3	-1	-14	30.77	2.80

3	1	14	32.63	1.40
3	1	14	32.17	2.25
3	1	14	31.44	2.06
-4	-1	-14	13.83	0.96
-4	-1	-14	13.83	2.39
-4	-1	-14	16.02	1.04
-4	-1	-14	13.72	0.74
4	1	14	13.74	0.77
4	1	14	14.66	1.41
4	1	14	10.70	1.61
-5	-1	-14	27.17	2.69
-5	-1	-14	30.26	1.30
-5	-1	-14	30.08	1.57
5	1	14	29.36	1.29
5	1	14	28.73	1.82
5	1	14	25.29	2.16
-6	-1	-14	1.23	0.46
-6	-1	-14	0.74	1.33
6	1	14	0.88	0.39
6	1	14	1.66	0.70
6	1	14	0.63	0.96
-7	-1	-14	6.26	0.88
-7	-1	-14	9.78	1.63
-7	-1	-14	7.76	0.81
7	1	14	7.73	0.79
7	1	14	7.97	1.40
7	1	14	7.16	1.14
-8	-1	-14	0.87	0.51
-8	-1	-14	1.47	1.32
-8	-1	-14	-0.43	0.47
8	1	14	-0.55	1.02
8	1	14	0.72	0.64
8	1	14	-0.31	0.47
-9	-1	-14	9.55	1.02
-9	-1	-14	9.68	1.13
-9	-1	-14	14.40	2.22
9	1	14	11.38	1.92
9	1	14	11.51	1.18
9	1	14	11.64	1.01
-10	-1	-14	1.64	0.67
-10	-1	-14	1.48	0.69
10	1	14	3.08	1.25
10	1	14	1.85	0.64
-11	-1	-14	7.76	1.23

-11	-1	-14	6.09	0.90
11	1	14	9.54	1.62
-12	-1	-14	1.55	0.75
-12	-1	-14	0.32	0.80
12	1	14	2.08	1.35
-13	-1	-14	11.08	1.55
13	1	14	13.99	2.08
-14	-1	-14	1.57	0.89
14	1	14	2.15	1.47
-15	-1	-14	1.46	0.99
15	1	14	1.05	1.69
18	-1	-13	62.28	2.97
-18	1	13	71.25	3.86
-18	1	13	65.35	5.01
17	-1	-13	0.93	0.64
-17	1	13	-0.36	1.17
-17	1	13	2.06	2.07
16	-1	-13	63.92	2.89
-16	1	13	71.19	4.67
-16	1	13	64.10	3.59
15	-1	-13	-0.20	0.46
-15	1	13	-0.40	0.94
-15	1	13	-0.15	1.79
14	-1	-13	63.87	2.83
-14	1	13	64.42	4.41
-14	1	13	72.25	3.44
13	-1	-13	0.53	0.38
-13	1	13	2.44	1.63
-13	1	13	0.31	0.78
12	-1	-13	84.78	3.48
-12	1	13	86.41	4.86
11	-1	-13	1.80	0.41
-11	1	13	-0.54	1.31
-11	1	13	1.57	0.57
-11	1	13	1.12	0.69
10	-1	-13	56.31	2.56
10	-1	-13	58.71	2.49
-10	1	13	56.71	2.61
-10	1	13	62.85	2.94
-10	1	13	62.00	2.86
-10	1	13	60.98	3.78
9	-1	-13	7.33	0.65
9	-1	-13	5.90	1.00
-9	1	13	6.38	0.85

-9	1	13	6.52	0.88
-9	1	13	6.56	1.10
-9	1	13	6.45	1.45
-9	1	13	5.93	0.91
8	-1	-13	113.49	4.49
8	-1	-13	118.77	4.69
-8	1	13	116.73	5.54
-8	1	13	121.95	4.78
-8	1	13	108.64	4.55
-8	1	13	107.35	4.65
-8	1	13	116.36	4.73
7	-1	-13	1.04	0.68
7	-1	-13	1.48	0.33
-7	1	13	1.41	0.40
-7	1	13	1.84	0.59
-7	1	13	2.08	1.07
-7	1	13	0.71	0.52
-7	1	13	2.32	0.56
6	-1	-13	106.13	4.37
6	-1	-13	99.14	4.02
-6	1	13	104.12	4.19
-6	1	13	103.27	4.98
-6	1	13	97.12	4.29
-6	1	13	99.67	4.08
-6	1	13	107.20	4.19
5	-1	-13	5.60	1.44
5	-1	-13	5.08	0.83
5	-1	-13	3.87	0.53
-5	1	13	4.15	0.69
-5	1	13	4.71	0.59
-5	1	13	3.11	0.72
-5	1	13	3.65	1.07
-5	1	13	3.43	0.47
4	-1	-13	69.13	2.84
4	-1	-13	75.65	3.32
4	-1	-13	74.26	4.10
-4	1	13	71.28	2.91
-4	1	13	66.71	2.88
-4	1	13	66.55	3.22
-4	1	13	66.89	3.64
-4	1	13	71.19	2.92
3	-1	-13	5.85	1.39
3	-1	-13	8.09	1.07
3	-1	-13	6.78	0.62

-3	1	13	6.47	1.10
-3	1	13	5.78	1.16
-3	1	13	8.46	0.70
-3	1	13	7.87	0.71
-3	1	13	8.49	0.74
2	-1	-13	132.96	5.27
2	-1	-13	112.35	5.70
2	-1	-13	118.66	4.71
-2	1	13	113.62	5.12
-2	1	13	122.58	4.70
-2	1	13	122.11	4.75
-2	1	13	116.29	5.37
-2	1	13	123.66	4.68
1	-1	-13	6.97	1.46
1	-1	-13	8.13	1.14
1	-1	-13	7.17	0.66
-1	1	13	8.24	1.36
-1	1	13	8.36	0.61
-1	1	13	7.73	0.65
-1	1	13	6.94	1.21
-1	1	13	6.45	0.70
0	-1	-13	145.32	5.98
0	-1	-13	137.09	5.38
0	-1	-13	131.69	6.28
0	1	13	139.53	5.93
0	1	13	144.78	5.29
0	1	13	139.74	5.42
0	1	13	126.29	5.81
-1	-1	-13	1.07	1.13
-1	-1	-13	-0.19	0.78
-1	-1	-13	0.53	0.34
-1	-1	-13	-0.06	0.29
1	1	13	1.22	0.74
1	1	13	0.70	0.72
1	1	13	0.31	0.28
-2	-1	-13	48.44	3.23
-2	-1	-13	54.49	2.32
-2	-1	-13	56.97	3.04
-2	-1	-13	52.71	2.10
-2	-1	-13	53.99	2.29
2	1	13	51.31	2.12
2	1	13	55.08	2.76
2	1	13	53.07	2.90
-3	-1	-13	3.06	0.41

-3	-1	-13	4.96	1.33
-3	-1	-13	3.33	1.03
-3	-1	-13	3.00	0.47
-3	-1	-13	2.92	0.45
3	1	13	4.54	1.03
3	1	13	3.80	0.76
3	1	13	3.15	0.35
-4	-1	-13	161.81	6.02
-4	-1	-13	171.44	6.21
-4	-1	-13	163.97	6.26
-4	-1	-13	155.36	6.95
4	1	13	145.92	6.74
4	1	13	151.31	6.01
4	1	13	163.75	6.51
-5	-1	-13	2.99	0.52
-5	-1	-13	3.96	0.57
-5	-1	-13	6.05	0.68
-5	-1	-13	3.91	1.26
5	1	13	2.74	0.68
5	1	13	3.40	0.94
5	1	13	3.42	0.50
-6	-1	-13	139.60	5.28
-6	-1	-13	134.85	5.48
-6	-1	-13	135.88	6.08
6	1	13	135.91	5.25
6	1	13	131.62	6.02
6	1	13	142.82	5.61
-7	-1	-13	17.35	1.00
-7	-1	-13	15.14	2.06
-7	-1	-13	17.26	1.16
7	1	13	16.46	1.29
7	1	13	15.74	0.97
7	1	13	10.07	1.94
-8	-1	-13	48.06	2.16
-8	-1	-13	49.12	2.93
-8	-1	-13	45.64	2.30
8	1	13	50.17	2.30
8	1	13	49.98	2.13
8	1	13	46.39	2.95
-9	-1	-13	4.12	0.67
-9	-1	-13	4.50	0.66
-9	-1	-13	4.33	1.80
9	1	13	3.88	0.68
9	1	13	3.61	1.23

9	1	13	5.84	0.86
-10	-1	-13	48.60	2.40
-10	-1	-13	50.75	2.86
-10	-1	-13	52.89	2.57
10	1	13	52.96	2.36
10	1	13	54.18	2.34
10	1	13	50.17	3.19
-11	-1	-13	0.62	0.69
-11	-1	-13	0.54	0.62
11	1	13	-1.98	1.10
11	1	13	1.16	0.63
-12	-1	-13	58.53	2.80
-12	-1	-13	57.39	2.88
12	1	13	56.96	3.58
-13	-1	-13	1.08	0.87
-13	-1	-13	1.73	0.82
13	1	13	3.27	1.42
-14	-1	-13	34.89	2.25
-14	-1	-13	31.46	2.25
14	1	13	30.52	3.02
-15	-1	-13	0.20	0.88
15	1	13	-1.56	1.51
-16	-1	-13	22.58	2.16
16	1	13	27.09	3.12
19	-1	-12	16.54	1.47
-19	1	12	15.32	2.51
18	-1	-12	4.23	0.78
-18	1	12	6.21	1.40
-18	1	12	6.34	2.31
17	-1	-12	28.93	1.72
-17	1	12	32.08	3.68
-17	1	12	34.27	2.62
16	-1	-12	-0.38	0.48
-16	1	12	-1.82	1.69
-16	1	12	0.82	1.05
15	-1	-12	21.59	1.34
-15	1	12	24.15	3.19
-15	1	12	23.68	2.01
-14	1	12	3.72	0.92
-14	1	12	4.35	1.69
13	-1	-12	40.73	1.94
-13	1	12	42.65	2.46
-13	1	12	48.19	3.47
12	-1	-12	-0.19	0.34

-12	1	12	1.12	1.22
-12	1	12	0.34	0.69
11	-1	-12	68.65	2.91
-11	1	12	61.08	4.94
-11	1	12	79.85	3.36
-11	1	12	71.87	3.07
10	-1	-12	6.06	0.74
10	-1	-12	5.41	0.85
10	-1	-12	5.06	0.59
-10	1	12	5.00	0.77
-10	1	12	4.65	0.83
-10	1	12	4.01	0.78
-10	1	12	4.89	1.41
9	-1	-12	38.98	1.78
9	-1	-12	39.92	1.90
-9	1	12	45.49	2.13
-9	1	12	39.31	1.88
-9	1	12	42.78	2.91
-9	1	12	41.25	2.12
8	-1	-12	4.25	0.65
8	-1	-12	3.46	0.50
-8	1	12	4.77	0.70
-8	1	12	4.03	0.65
-8	1	12	3.50	1.22
-8	1	12	4.40	0.79
-8	1	12	4.41	0.65
7	-1	-12	47.76	2.04
7	-1	-12	46.10	3.31
7	-1	-12	50.81	2.30
-7	1	12	47.62	2.10
-7	1	12	48.65	2.24
-7	1	12	52.01	2.28
-7	1	12	44.94	2.96
-7	1	12	47.50	2.25
6	-1	-12	-0.14	0.55
6	-1	-12	0.43	1.19
6	-1	-12	-0.14	0.24
-6	1	12	0.34	0.85
-6	1	12	-0.31	0.35
-6	1	12	-0.16	0.41
-6	1	12	0.27	0.49
-6	1	12	0.41	0.32
5	-1	-12	52.22	2.49
5	-1	-12	51.77	2.14

5	-1	-12	50.17	3.23
-5	1	12	52.21	2.25
-5	1	12	52.58	2.20
-5	1	12	51.84	2.26
-5	1	12	53.55	3.01
-5	1	12	48.00	2.43
4	-1	-12	3.65	0.49
4	-1	-12	5.55	0.86
4	-1	-12	4.49	1.20
-4	1	12	4.11	0.98
-4	1	12	3.81	0.70
-4	1	12	4.53	0.60
-4	1	12	4.53	0.60
-4	1	12	4.20	0.55
3	-1	-12	93.14	4.02
3	-1	-12	86.64	4.58
3	-1	-12	91.63	3.60
-3	1	12	85.88	3.94
-3	1	12	90.83	3.80
-3	1	12	95.06	3.64
-3	1	12	92.95	3.64
-3	1	12	91.63	4.32
2	-1	-12	6.54	0.57
2	-1	-12	7.98	1.18
2	-1	-12	6.22	1.25
-2	1	12	8.14	0.63
-2	1	12	5.63	0.98
-2	1	12	6.59	1.02
-2	1	12	6.07	0.62
-2	1	12	6.61	0.57
1	-1	-12	216.03	8.32
1	-1	-12	200.72	7.82
1	-1	-12	198.74	8.64
-1	1	12	199.44	8.36
-1	1	12	209.08	7.76
-1	1	12	197.47	8.20
-1	1	12	208.85	7.86
-1	1	12	210.86	7.78
0	-1	-12	7.51	0.61
0	-1	-12	11.04	1.72
0	-1	-12	7.59	1.29
0	1	12	6.89	0.66
0	1	12	7.18	0.52
0	1	12	6.85	0.57

0	1	12	7.95	1.22
0	1	12	5.79	1.09
-1	-1	-12	161.79	6.30
-1	-1	-12	159.87	7.21
-1	-1	-12	166.93	6.44
-1	-1	-12	166.05	6.46
-1	-1	-12	173.80	7.01
1	1	12	161.78	6.91
1	1	12	173.51	6.34
1	1	12	175.95	6.92
-2	-1	-12	0.04	0.20
-2	-1	-12	0.22	0.76
-2	-1	-12	-0.12	0.28
-2	-1	-12	1.08	1.02
-2	-1	-12	0.67	0.29
2	1	12	-0.37	0.22
2	1	12	0.52	0.57
2	1	12	-0.61	0.60
-3	-1	-12	117.96	5.17
-3	-1	-12	109.24	5.22
-3	-1	-12	117.81	4.51
-3	-1	-12	115.36	4.35
-3	-1	-12	117.38	4.56
3	1	12	105.62	5.00
3	1	12	118.65	4.86
3	1	12	112.94	4.35
-4	-1	-12	-0.15	0.24
-4	-1	-12	-0.04	0.29
-4	-1	-12	-0.51	0.99
-4	-1	-12	0.75	0.89
-4	-1	-12	0.01	0.30
4	1	12	-0.08	0.24
4	1	12	-0.14	0.52
4	1	12	-0.20	0.68
-5	-1	-12	89.98	3.76
-5	-1	-12	98.36	4.52
-5	-1	-12	94.65	3.70
-5	-1	-12	90.31	3.60
-5	-1	-12	87.17	4.36
5	1	12	92.92	3.91
5	1	12	89.11	4.28
5	1	12	91.07	3.55
-6	-1	-12	2.81	0.46
-6	-1	-12	3.05	1.11

-6	-1	-12	1.17	0.42
-6	-1	-12	2.10	0.42
6	1	12	3.81	0.92
6	1	12	1.99	0.58
6	1	12	3.02	0.40
-7	-1	-12	135.92	5.22
-7	-1	-12	135.18	5.78
-7	-1	-12	129.73	5.37
7	1	12	140.01	5.39
7	1	12	128.18	5.89
7	1	12	134.84	5.19
-8	-1	-12	0.84	0.46
-8	-1	-12	-0.80	1.05
-8	-1	-12	1.06	0.48
8	1	12	0.63	0.51
8	1	12	0.92	0.43
8	1	12	-1.32	0.84
-9	-1	-12	63.29	2.98
-9	-1	-12	66.22	2.85
-9	-1	-12	62.79	3.19
9	1	12	69.39	2.80
9	1	12	65.28	4.48
9	1	12	67.73	2.81
-10	-1	-12	2.68	0.64
-10	-1	-12	1.96	0.63
-10	-1	-12	1.79	0.80
10	1	12	1.92	0.85
10	1	12	2.47	1.04
10	1	12	2.73	0.60
-11	-1	-12	37.73	2.09
-11	-1	-12	41.18	2.16
11	1	12	41.67	1.99
11	1	12	37.31	2.71
-12	-1	-12	0.45	0.68
-12	-1	-12	1.46	0.76
12	1	12	1.85	0.71
12	1	12	-0.69	1.10
-13	-1	-12	45.94	2.48
-13	-1	-12	44.59	2.48
13	1	12	44.05	3.06
13	1	12	45.56	2.36
-14	-1	-12	1.13	0.75
-14	-1	-12	2.19	0.96
14	1	12	-2.12	1.22

14	1	12	-0.63	0.67
-15	-1	-12	44.80	2.67
-15	-1	-12	41.43	2.52
15	1	12	43.21	2.42
15	1	12	39.06	3.24
-16	-1	-12	1.22	1.31
-16	-1	-12	1.16	0.93
16	1	12	-0.02	1.50
16	1	12	1.61	0.89
19	-1	-11	1.22	0.65
-19	1	11	0.57	1.24
-19	1	11	-1.00	2.12
18	-1	-11	6.86	1.13
-18	1	11	13.79	2.09
-18	1	11	10.51	2.29
17	-1	-11	0.68	0.54
-17	1	11	1.11	1.09
-17	1	11	0.90	1.83
16	-1	-11	12.11	1.08
-16	1	11	14.27	1.82
-16	1	11	12.00	2.25
15	-1	-11	0.25	0.44
-15	1	11	-0.38	0.79
-15	1	11	-0.09	1.47
14	-1	-11	12.17	0.92
-14	1	11	10.75	1.91
-14	1	11	13.61	1.53
13	-1	-11	0.05	0.32
-13	1	11	-1.22	1.35
-13	1	11	0.79	0.69
12	-1	-11	2.47	0.41
-12	1	11	2.17	0.75
-12	1	11	1.49	1.24
-12	1	11	1.07	0.54
11	-1	-11	2.44	0.40
-11	1	11	2.51	0.72
-11	1	11	2.24	1.16
-11	1	11	2.11	0.52
10	-1	-11	37.12	1.69
-10	1	11	37.43	2.78
-10	1	11	40.55	2.06
-10	1	11	38.09	2.05
-10	1	11	38.56	1.79
9	-1	-11	27.47	1.37

9	-1	-11	28.02	1.31
9	-1	-11	27.34	3.05
-9	1	11	26.80	1.34
-9	1	11	31.40	1.67
-9	1	11	27.23	1.39
-9	1	11	28.80	1.66
-9	1	11	30.15	2.40
8	-1	-11	71.63	2.98
8	-1	-11	69.22	2.89
8	-1	-11	66.67	4.00
-8	1	11	77.91	3.15
-8	1	11	70.39	3.80
-8	1	11	66.66	3.01
-8	1	11	79.04	3.20
-8	1	11	75.36	2.98
7	-1	-11	16.49	0.88
7	-1	-11	17.74	1.13
7	-1	-11	15.57	2.14
-7	1	11	16.79	1.12
-7	1	11	17.92	1.13
-7	1	11	16.58	1.08
-7	1	11	17.68	1.79
-7	1	11	16.78	0.97
6	-1	-11	33.35	2.60
6	-1	-11	33.42	1.48
6	-1	-11	35.31	1.75
-6	1	11	29.26	1.67
-6	1	11	36.79	1.65
-6	1	11	34.92	1.66
-6	1	11	34.23	2.29
-6	1	11	34.74	1.55
5	-1	-11	0.52	0.95
5	-1	-11	0.19	0.47
5	-1	-11	0.11	0.22
-5	1	11	-0.43	0.33
-5	1	11	-0.03	0.44
-5	1	11	0.93	0.74
-5	1	11	-0.17	0.24
-5	1	11	0.54	0.34
4	-1	-11	34.79	1.84
4	-1	-11	32.36	2.45
4	-1	-11	32.57	1.49
-4	1	11	32.98	1.78
-4	1	11	35.72	1.54

-4	1	11	36.10	1.58
-4	1	11	35.49	1.56
-4	1	11	32.26	2.18
3	-1	-11	50.98	2.40
3	-1	-11	46.37	2.92
3	-1	-11	46.95	2.00
-3	1	11	49.09	2.04
-3	1	11	50.44	2.70
-3	1	11	49.27	2.04
-3	1	11	48.22	2.04
-3	1	11	44.53	2.32
2	-1	-11	106.17	4.48
2	-1	-11	104.06	4.08
2	-1	-11	103.40	4.91
-2	1	11	98.38	4.40
-2	1	11	108.53	4.07
-2	1	11	101.86	4.64
-2	1	11	104.24	4.07
-2	1	11	108.29	4.11
1	-1	-11	57.48	3.18
1	-1	-11	57.15	2.79
1	-1	-11	53.07	2.33
-1	1	11	58.08	2.37
-1	1	11	56.00	2.71
-1	1	11	56.28	2.87
-1	1	11	59.36	2.30
0	-1	-11	283.36	10.75
0	-1	-11	266.17	10.95
0	-1	-11	269.81	10.27
0	1	11	279.92	10.20
0	1	11	272.40	10.72
0	1	11	256.97	10.63
0	1	11	273.98	10.30
-1	-1	-11	4.82	1.08
-1	-1	-11	4.40	0.37
-1	-1	-11	5.21	0.52
-1	-1	-11	5.00	0.90
-1	-1	-11	5.48	0.52
1	1	11	4.65	0.43
1	1	11	4.15	0.75
1	1	11	3.99	0.77
1	1	11	5.24	0.54
-2	-1	-11	25.87	1.78
-2	-1	-11	23.12	1.18

-2	-1	-11	23.29	1.15
-2	-1	-11	26.98	2.02
-2	-1	-11	24.65	1.02
2	1	11	21.12	1.61
2	1	11	22.98	1.03
2	1	11	23.99	1.55
-3	-1	-11	0.37	0.29
-3	-1	-11	0.50	0.20
-3	-1	-11	-0.18	0.87
-3	-1	-11	-0.53	0.72
-3	-1	-11	-0.16	0.24
3	1	11	-0.18	0.47
3	1	11	0.13	0.56
3	1	11	0.28	0.21
-4	-1	-11	70.45	3.56
-4	-1	-11	73.87	2.96
-4	-1	-11	77.25	3.64
-4	-1	-11	74.58	2.85
-4	-1	-11	71.57	3.02
4	1	11	72.01	2.83
4	1	11	73.99	3.21
4	1	11	68.43	3.45
-5	-1	-11	1.28	0.33
-5	-1	-11	1.17	0.39
-5	-1	-11	1.86	0.89
-5	-1	-11	1.86	0.36
-5	-1	-11	3.65	0.95
5	1	11	1.04	0.51
5	1	11	1.85	0.30
5	1	11	2.49	0.77
-6	-1	-11	58.14	2.37
-6	-1	-11	56.34	2.97
-6	-1	-11	55.16	3.14
-6	-1	-11	55.22	2.49
-6	-1	-11	56.03	2.40
6	1	11	58.20	2.32
6	1	11	61.38	2.59
6	1	11	54.91	2.95
-7	-1	-11	0.86	0.37
-7	-1	-11	1.27	0.37
-7	-1	-11	1.15	0.38
7	1	11	0.91	0.37
7	1	11	1.41	0.48
7	1	11	1.23	0.78

-8	-1	-11	40.42	2.00
-8	-1	-11	40.49	2.31
-8	-1	-11	40.80	1.90
8	1	11	39.54	2.52
8	1	11	43.08	1.91
8	1	11	42.20	1.86
-9	-1	-11	2.55	0.54
-9	-1	-11	0.92	0.43
-9	-1	-11	2.22	0.82
9	1	11	1.65	0.82
9	1	11	1.52	0.60
9	1	11	1.78	0.50
-10	-1	-11	63.42	2.84
10	1	11	66.03	2.76
10	1	11	60.87	3.63
-11	-1	-11	3.14	0.68
-11	-1	-11	5.83	0.82
11	1	11	5.78	1.22
11	1	11	5.54	0.76
11	1	11	4.44	0.69
-12	-1	-11	39.77	2.23
-12	-1	-11	41.76	2.23
12	1	11	41.23	2.11
12	1	11	40.11	2.12
12	1	11	44.77	2.85
-13	-1	-11	4.24	0.79
-13	-1	-11	2.00	0.89
13	1	11	3.14	1.26
13	1	11	3.29	0.83
13	1	11	2.40	0.68
-14	-1	-11	24.41	1.95
14	1	11	23.27	1.63
14	1	11	18.09	2.25
-15	-1	-11	0.30	1.07
15	1	11	0.65	1.36
15	1	11	-0.62	0.67
-16	-1	-11	43.50	2.73
16	1	11	41.93	3.19
16	1	11	38.31	2.34
-17	-1	-11	-0.67	1.29
17	1	11	-1.05	1.53
17	1	11	-0.64	0.84
19	-1	-10	0.03	0.60
-19	1	10	-0.89	1.99

-19	1	10	1.53	1.30
18	-1	-10	4.10	0.70
-18	1	10	4.25	1.93
-18	1	10	4.92	1.32
17	-1	-10	2.40	0.60
-17	1	10	2.40	1.08
-17	1	10	4.55	1.80
16	-1	-10	-0.18	0.45
-16	1	10	-1.65	1.52
-16	1	10	-0.94	0.87
15	-1	-10	0.13	0.41
-15	1	10	0.21	1.41
-15	1	10	-0.01	0.75
14	-1	-10	0.11	0.36
-14	1	10	0.70	1.58
-14	1	10	-0.19	0.77
-13	1	10	1.26	1.21
12	-1	-10	6.87	0.67
-12	1	10	6.22	0.87
-12	1	10	8.21	1.14
-12	1	10	10.69	2.01
11	-1	-10	14.10	0.89
-11	1	10	16.40	1.32
-11	1	10	17.05	1.07
-11	1	10	17.93	1.36
-11	1	10	19.28	2.09
10	-1	-10	0.71	0.26
10	-1	-10	-1.35	1.21
-10	1	10	0.42	0.53
-10	1	10	0.65	0.38
-10	1	10	0.32	0.95
-10	1	10	0.41	0.53
9	-1	-10	28.96	1.39
9	-1	-10	31.79	1.37
9	-1	-10	31.43	2.65
9	-1	-10	31.67	1.46
-9	1	10	30.85	1.73
-9	1	10	31.03	1.69
-9	1	10	32.48	1.50
-9	1	10	32.67	2.38
8	-1	-10	0.05	0.22
8	-1	-10	0.75	1.09
8	-1	-10	0.41	0.35
-8	1	10	-0.02	0.42

-8	1	10	0.13	0.43
-8	1	10	-0.31	0.33
-8	1	10	0.19	0.74
-8	1	10	0.34	0.29
7	-1	-10	6.17	0.51
7	-1	-10	8.52	1.45
7	-1	-10	7.21	0.73
-7	1	10	6.86	0.59
-7	1	10	7.19	0.84
-7	1	10	6.02	0.74
-7	1	10	5.50	1.03
-7	1	10	7.13	0.76
6	-1	-10	2.04	0.94
6	-1	-10	0.88	0.24
6	-1	-10	1.80	0.44
-6	1	10	0.93	0.44
-6	1	10	1.08	0.29
-6	1	10	1.29	0.74
-6	1	10	1.39	0.37
-6	1	10	1.04	0.38
5	-1	-10	17.63	1.79
5	-1	-10	14.81	0.77
-5	1	10	14.07	1.44
-5	1	10	15.11	0.91
-5	1	10	13.53	0.97
-5	1	10	15.26	0.82
-5	1	10	14.48	0.87
4	-1	-10	2.26	0.52
4	-1	-10	1.88	0.28
4	-1	-10	2.13	0.87
-4	1	10	1.89	0.50
-4	1	10	2.17	0.31
-4	1	10	2.16	0.71
-4	1	10	2.41	0.36
-4	1	10	2.00	0.36
3	-1	-10	25.20	1.13
3	-1	-10	26.37	1.99
3	-1	-10	25.90	1.48
-3	1	10	24.88	1.17
-3	1	10	23.69	1.40
-3	1	10	25.34	1.16
-3	1	10	23.56	1.14
-3	1	10	23.34	1.75
2	-1	-10	8.92	0.57

2	-1	-10	8.81	0.99
2	-1	-10	10.72	1.48
-2	1	10	7.87	1.09
-2	1	10	9.13	0.59
-2	1	10	8.58	0.92
-2	1	10	8.96	0.60
-2	1	10	8.95	0.60
1	-1	-10	126.33	4.95
1	-1	-10	125.99	5.61
1	-1	-10	130.48	5.33
-1	1	10	132.24	4.98
-1	1	10	131.02	5.43
-1	1	10	120.47	5.25
-1	1	10	131.51	4.91
-1	1	10	129.65	4.90
0	-1	-10	4.41	0.77
0	-1	-10	2.84	0.42
0	-1	-10	4.86	0.95
0	1	10	2.52	0.62
0	1	10	3.78	0.45
0	1	10	3.22	0.68
0	1	10	3.77	0.39
-1	-1	-10	6.33	0.40
-1	-1	-10	5.36	1.00
-1	-1	-10	7.84	1.26
-1	-1	-10	6.67	0.54
-1	-1	-10	7.67	0.54
1	1	10	5.86	0.92
1	1	10	4.51	0.85
1	1	10	6.86	0.45
1	1	10	7.53	0.58
-2	-1	-10	11.60	0.57
-2	-1	-10	12.43	0.70
-2	-1	-10	11.02	0.72
-2	-1	-10	12.11	1.39
-2	-1	-10	12.32	1.24
2	1	10	12.15	1.04
2	1	10	8.71	1.08
2	1	10	11.06	0.58
-3	-1	-10	12.50	1.34
-3	-1	-10	11.06	1.36
-3	-1	-10	11.89	0.62
-3	-1	-10	11.89	0.72
-3	-1	-10	12.30	0.76

3	1	10	10.73	1.20
3	1	10	12.36	1.01
3	1	10	12.91	0.62
-4	-1	-10	12.60	0.69
-4	-1	-10	13.76	0.77
-4	-1	-10	13.89	1.46
-4	-1	-10	13.29	1.40
-4	-1	-10	11.95	0.81
4	1	10	11.36	1.20
4	1	10	13.26	1.01
4	1	10	12.53	0.66
-5	-1	-10	62.43	2.52
-5	-1	-10	63.86	3.15
-5	-1	-10	59.43	2.46
-5	-1	-10	60.04	2.58
-5	-1	-10	60.84	3.00
5	1	10	61.66	2.67
5	1	10	60.20	3.02
5	1	10	60.83	2.43
-6	-1	-10	-0.29	0.33
-6	-1	-10	-0.14	0.25
-6	-1	-10	0.27	0.31
-6	-1	-10	0.61	0.75
-6	-1	-10	0.46	0.72
6	1	10	0.56	0.40
6	1	10	-0.57	0.55
6	1	10	0.05	0.26
-7	-1	-10	43.95	2.11
-7	-1	-10	45.26	2.01
-7	-1	-10	45.10	2.44
-7	-1	-10	52.52	2.81
-7	-1	-10	47.97	2.01
7	1	10	45.88	2.56
7	1	10	43.40	2.00
7	1	10	48.29	2.08
-8	-1	-10	0.79	0.90
-8	-1	-10	0.02	0.70
-8	-1	-10	-0.58	0.39
-8	-1	-10	0.61	0.34
-8	-1	-10	-0.26	0.37
8	1	10	0.06	0.44
8	1	10	0.04	0.71
8	1	10	0.92	0.37
-9	-1	-10	21.35	1.31

-9	-1	-10	21.32	1.48
-9	-1	-10	23.23	1.32
9	1	10	23.02	1.14
9	1	10	22.62	1.25
9	1	10	19.76	1.81
9	1	10	20.20	1.26
-10	-1	-10	0.97	0.51
-10	-1	-10	0.90	0.53
10	1	10	0.90	0.43
10	1	10	1.31	0.79
10	1	10	1.01	0.51
-11	-1	-10	48.89	2.38
11	1	10	51.80	2.31
11	1	10	47.07	2.40
11	1	10	49.46	2.84
-12	-1	-10	4.49	0.80
12	1	10	3.20	0.66
12	1	10	2.47	1.14
12	1	10	2.77	0.68
-13	-1	-10	34.55	2.16
13	1	10	34.50	2.52
13	1	10	36.90	1.97
13	1	10	36.14	1.99
-14	-1	-10	3.27	0.95
14	1	10	2.65	0.68
14	1	10	2.11	1.15
14	1	10	2.89	0.87
-15	-1	-10	25.68	2.15
15	1	10	28.17	1.82
15	1	10	24.11	2.49
-16	-1	-10	0.93	1.10
16	1	10	1.53	0.72
16	1	10	0.68	1.33
-17	-1	-10	28.40	2.67
17	1	10	31.38	2.13
17	1	10	33.34	3.03
-18	-1	-10	0.80	1.47
18	1	10	3.02	1.09
18	1	10	1.37	1.59
20	-1	-9	14.71	1.39
-20	1	9	11.20	2.42
-20	1	9	16.84	2.48
19	-1	-9	0.88	0.59
-19	1	9	0.35	1.11

-19	1	9	0.12	1.94
18	-1	-9	13.87	1.19
-18	1	9	12.57	2.19
-18	1	9	14.12	1.99
17	-1	-9	8.20	0.95
-17	1	9	7.45	1.26
-17	1	9	9.33	1.92
16	-1	-9	14.42	1.11
-16	1	9	19.83	2.96
-16	1	9	17.30	1.87
15	-1	-9	2.13	0.55
-15	1	9	5.65	1.49
-15	1	9	2.74	0.89
14	-1	-9	6.17	0.72
-14	1	9	6.50	0.98
-14	1	9	7.49	1.57
13	-1	-9	0.61	0.33
-13	1	9	2.20	1.22
-13	1	9	0.88	0.60
12	-1	-9	11.12	0.76
12	-1	-9	12.53	2.35
-12	1	9	12.26	0.97
-12	1	9	12.10	1.99
-12	1	9	10.76	1.20
11	-1	-9	8.09	1.05
11	-1	-9	7.18	1.61
11	-1	-9	6.28	0.61
-11	1	9	8.35	1.03
-11	1	9	7.09	0.75
-11	1	9	7.41	1.32
-11	1	9	6.35	1.02
10	-1	-9	6.26	1.37
10	-1	-9	7.24	0.96
10	-1	-9	7.09	0.58
-10	1	9	8.08	1.31
-10	1	9	7.78	0.98
-10	1	9	10.21	0.98
-10	1	9	7.78	0.71
9	-1	-9	4.87	0.49
9	-1	-9	4.82	1.21
-9	1	9	3.91	1.06
-9	1	9	7.71	0.83
-9	1	9	5.30	0.84
-9	1	9	5.07	0.59

8	-1	-9	0.02	0.28
8	-1	-9	1.13	0.96
8	-1	-9	0.49	0.22
-8	1	9	0.51	0.27
-8	1	9	0.57	0.27
-8	1	9	0.48	0.41
-8	1	9	0.59	0.39
-8	1	9	0.74	0.83
7	-1	-9	4.47	1.12
7	-1	-9	7.31	0.51
7	-1	-9	7.50	0.63
-7	1	9	7.30	0.70
-7	1	9	5.38	0.60
-7	1	9	7.50	1.28
-7	1	9	6.13	0.71
-7	1	9	6.62	0.55
6	-1	-9	7.20	0.50
6	-1	-9	6.38	0.68
6	-1	-9	7.73	1.43
-6	1	9	7.11	1.23
-6	1	9	6.30	0.68
-6	1	9	7.99	0.55
-6	1	9	7.22	0.66
5	-1	-9	2.82	0.88
5	-1	-9	3.36	0.63
5	-1	-9	2.66	0.34
-5	1	9	3.11	0.40
-5	1	9	3.06	0.47
-5	1	9	2.97	0.75
-5	1	9	3.14	0.51
-5	1	9	3.09	0.52
4	-1	-9	3.26	0.90
4	-1	-9	1.96	0.27
4	-1	-9	1.99	0.47
-4	1	9	2.48	0.34
-4	1	9	2.37	0.36
-4	1	9	2.75	0.70
-4	1	9	1.89	0.41
3	-1	-9	10.35	1.35
3	-1	-9	8.23	0.55
3	-1	-9	8.66	0.88
-3	1	9	9.54	0.58
-3	1	9	9.55	0.64
-3	1	9	9.35	0.82

-3	1	9	9.59	0.60
-3	1	9	11.33	1.13
2	-1	-9	39.73	1.68
2	-1	-9	39.95	2.33
2	-1	-9	40.71	1.99
-2	1	9	40.75	2.18
-2	1	9	40.39	1.67
-2	1	9	41.59	1.70
-2	1	9	39.32	1.94
1	-1	-9	12.88	0.68
1	-1	-9	11.40	1.02
1	-1	-9	13.13	1.35
-1	1	9	12.33	0.63
-1	1	9	11.89	0.96
-1	1	9	12.94	0.65
-1	1	9	11.95	1.12
-1	1	9	13.14	0.70
0	-1	-9	-0.14	0.16
0	-1	-9	0.13	0.62
0	-1	-9	0.20	0.14
0	-1	-9	0.13	0.47
0	1	9	-0.04	0.41
0	1	9	0.16	0.39
0	1	9	-0.19	0.19
0	1	9	0.22	0.18
-1	-1	-9	64.72	2.63
-1	-1	-9	66.85	3.15
-1	-1	-9	64.61	3.02
-1	-1	-9	65.15	2.51
1	1	9	66.07	2.65
1	1	9	68.16	2.94
1	1	9	59.50	2.94
1	1	9	68.00	2.54
-2	-1	-9	17.69	1.35
-2	-1	-9	16.75	0.73
-2	-1	-9	15.69	0.86
-2	-1	-9	17.85	0.84
-2	-1	-9	16.70	1.39
2	1	9	15.45	0.73
2	1	9	14.66	1.19
2	1	9	15.52	1.13
-3	-1	-9	60.33	2.32
-3	-1	-9	55.39	2.76
-3	-1	-9	54.68	2.35

-3	-1	-9	58.21	2.22
-3	-1	-9	59.57	2.84
3	1	9	54.03	2.20
3	1	9	52.33	2.67
3	1	9	58.28	2.55
-4	-1	-9	24.04	1.21
-4	-1	-9	26.26	1.17
-4	-1	-9	24.49	1.10
-4	-1	-9	26.41	1.72
-4	-1	-9	25.17	1.90
4	1	9	23.99	1.57
4	1	9	24.00	1.07
4	1	9	26.38	1.36
-5	-1	-9	7.28	0.65
-5	-1	-9	8.14	0.59
-5	-1	-9	8.32	1.26
-5	-1	-9	8.17	1.14
-5	-1	-9	8.46	0.59
5	1	9	8.14	0.54
5	1	9	8.22	1.07
5	1	9	9.24	0.78
-6	-1	-9	14.41	0.92
-6	-1	-9	15.01	0.89
-6	-1	-9	14.95	1.49
-6	-1	-9	13.86	0.84
-6	-1	-9	15.42	1.30
6	1	9	15.84	0.81
6	1	9	16.68	1.03
6	1	9	14.61	1.33
-7	-1	-9	0.80	0.73
-7	-1	-9	1.71	0.37
-7	-1	-9	1.45	0.38
-7	-1	-9	1.38	0.66
-7	-1	-9	2.28	0.36
7	1	9	0.90	0.64
7	1	9	1.51	0.35
7	1	9	1.00	0.47
7	1	9	1.14	0.35
-8	-1	-9	8.05	0.67
-8	-1	-9	10.27	1.45
-8	-1	-9	7.61	0.76
-8	-1	-9	7.70	0.76
-8	-1	-9	6.48	1.05
8	1	9	8.22	1.26

8	1	9	7.84	0.68
8	1	9	7.32	0.71
8	1	9	7.21	0.69
-9	-1	-9	-0.09	0.42
-9	-1	-9	0.92	0.91
-9	-1	-9	-0.76	0.29
9	1	9	0.61	0.36
9	1	9	-0.16	0.39
9	1	9	0.04	0.30
9	1	9	-0.23	0.66
-10	-1	-9	36.16	1.90
-10	-1	-9	46.32	4.01
10	1	9	37.35	2.30
10	1	9	39.72	1.84
10	1	9	37.35	1.84
-11	-1	-9	1.08	0.59
11	1	9	1.45	0.46
11	1	9	2.00	0.56
11	1	9	0.74	0.80
-12	-1	-9	5.69	0.85
12	1	9	6.21	1.01
12	1	9	4.83	1.09
12	1	9	4.01	0.64
-13	-1	-9	0.70	0.77
13	1	9	0.10	1.47
13	1	9	0.58	0.66
13	1	9	0.16	0.52
-14	-1	-9	5.85	1.04
14	1	9	6.45	1.05
14	1	9	5.12	1.25
14	1	9	3.90	0.74
-15	-1	-9	0.46	0.98
15	1	9	-0.39	1.07
15	1	9	-0.06	0.60
-16	-1	-9	18.66	2.10
16	1	9	16.57	2.31
16	1	9	18.38	1.60
-17	-1	-9	3.05	1.34
17	1	9	-0.36	0.79
17	1	9	-0.06	1.28
-18	-1	-9	9.94	1.73
18	1	9	12.29	1.67
18	1	9	15.61	2.50
20	-1	-8	2.09	0.75

-20	1	8	0.00	1.31
-20	1	8	2.67	2.08
19	-1	-8	44.17	2.22
-19	1	8	41.84	2.97
-19	1	8	45.88	3.77
18	-1	-8	0.20	0.51
-18	1	8	0.78	1.59
-18	1	8	0.31	0.99
17	-1	-8	54.82	2.55
-17	1	8	61.17	3.21
-17	1	8	52.82	4.38
16	-1	-8	0.05	0.42
-16	1	8	0.87	0.90
-16	1	8	0.46	1.42
-15	1	8	99.98	4.57
-15	1	8	102.63	5.11
14	-1	-8	-0.09	0.33
-14	1	8	2.44	1.29
-14	1	8	-0.96	0.62
13	-1	-8	89.16	3.73
13	-1	-8	86.50	4.84
-13	1	8	95.58	4.14
-13	1	8	107.16	4.08
-13	1	8	85.88	4.64
12	-1	-8	1.10	0.32
12	-1	-8	0.60	1.25
-12	1	8	0.68	0.40
-12	1	8	0.62	1.00
-12	1	8	0.78	0.59
11	-1	-8	82.76	3.40
11	-1	-8	84.94	4.44
11	-1	-8	82.32	3.70
-11	1	8	84.88	4.24
-11	1	8	91.36	3.77
-11	1	8	86.86	3.48
10	-1	-8	0.17	0.23
10	-1	-8	-0.13	0.45
-10	1	8	0.15	0.81
-10	1	8	-0.10	0.29
-10	1	8	0.59	0.47
9	-1	-8	51.91	2.18
9	-1	-8	56.15	2.45
9	-1	-8	49.33	3.08
-9	1	8	49.16	2.91

-9	1	8	57.86	2.26
-9	1	8	54.45	2.47
8	-1	-8	0.76	0.23
8	-1	-8	1.03	0.92
8	-1	-8	1.25	0.43
8	-1	-8	0.75	0.22
-8	1	8	0.52	0.71
-8	1	8	1.09	0.29
-8	1	8	0.60	0.40
7	-1	-8	63.66	3.38
7	-1	-8	64.31	2.58
7	-1	-8	66.46	2.76
7	-1	-8	61.18	2.56
-7	1	8	59.42	3.19
-7	1	8	63.01	2.57
-7	1	8	68.27	2.62
-7	1	8	71.19	2.76
-7	1	8	64.38	2.78
6	-1	-8	3.41	0.34
6	-1	-8	3.86	0.94
6	-1	-8	3.08	0.50
-6	1	8	3.70	0.39
-6	1	8	2.68	0.44
-6	1	8	2.66	0.48
-6	1	8	2.72	0.41
-6	1	8	5.76	1.04
5	-1	-8	35.02	2.14
5	-1	-8	32.89	1.52
5	-1	-8	33.39	1.37
-5	1	8	32.42	1.51
-5	1	8	29.13	1.49
-5	1	8	28.89	1.91
-5	1	8	34.84	1.48
-5	1	8	33.31	1.40
4	-1	-8	1.28	0.22
4	-1	-8	0.81	0.65
4	-1	-8	0.92	0.37
-4	1	8	2.22	0.32
-4	1	8	1.65	0.29
-4	1	8	1.41	0.60
-4	1	8	1.65	0.30
-4	1	8	1.53	0.40
3	-1	-8	101.99	4.00
3	-1	-8	110.08	4.23

3	-1	-8	96.47	4.54
-3	1	8	101.31	4.18
-3	1	8	103.87	4.01
-3	1	8	104.88	4.03
-3	1	8	106.04	4.49
-3	1	8	106.97	4.05
2	-1	-8	0.65	0.19
2	-1	-8	1.06	0.56
2	-1	-8	0.76	0.40
-2	1	8	0.62	0.19
-2	1	8	0.31	0.46
-2	1	8	0.72	0.32
-2	1	8	0.79	0.21
1	-1	-8	26.10	1.64
1	-1	-8	24.76	1.08
1	-1	-8	27.72	1.41
-1	1	8	24.34	1.04
-1	1	8	24.08	1.35
-1	1	8	22.38	1.14
-1	1	8	23.56	1.03
-1	1	8	21.69	1.42
0	-1	-8	5.70	1.01
0	-1	-8	5.73	0.35
0	-1	-8	6.57	0.81
0	-1	-8	6.32	0.46
0	1	8	6.14	0.39
0	1	8	5.70	0.76
0	1	8	5.90	0.45
0	1	8	6.78	0.81
-1	-1	-8	68.27	2.69
-1	-1	-8	74.56	3.28
-1	-1	-8	70.63	2.81
-1	-1	-8	71.62	3.14
1	1	8	72.98	2.82
1	1	8	66.39	3.07
1	1	8	71.21	3.07
1	1	8	70.56	2.71
-2	-1	-8	7.38	0.92
-2	-1	-8	6.06	0.49
-2	-1	-8	6.77	0.47
-2	-1	-8	7.19	0.39
-2	-1	-8	6.38	0.96
2	1	8	6.99	0.39
2	1	8	6.19	0.72

2	1	8	6.94	0.83
-3	-1	-8	43.09	1.68
-3	-1	-8	38.47	1.71
-3	-1	-8	39.52	1.60
-3	-1	-8	43.98	2.15
-3	-1	-8	38.84	2.05
3	1	8	36.92	2.02
3	1	8	38.93	1.58
3	1	8	40.24	1.87
-4	-1	-8	32.78	1.86
-4	-1	-8	32.70	1.38
-4	-1	-8	29.28	1.72
-4	-1	-8	29.74	1.41
-4	-1	-8	31.04	1.31
4	1	8	32.07	1.54
4	1	8	32.43	1.30
4	1	8	29.27	1.72
-5	-1	-8	17.73	1.36
-5	-1	-8	15.17	0.82
-5	-1	-8	16.87	1.22
-5	-1	-8	14.62	0.88
-5	-1	-8	16.70	0.84
5	1	8	16.51	1.24
5	1	8	15.72	0.78
5	1	8	16.68	0.99
-6	-1	-8	1.11	0.30
-6	-1	-8	1.67	0.70
-6	-1	-8	0.75	0.34
-6	-1	-8	1.77	0.54
-6	-1	-8	1.09	0.29
6	1	8	1.20	0.27
6	1	8	1.07	0.47
6	1	8	0.34	0.54
6	1	8	1.00	0.26
-7	-1	-8	13.28	0.78
-7	-1	-8	12.81	0.81
-7	-1	-8	13.56	1.08
-7	-1	-8	14.48	1.40
7	1	8	12.44	0.78
7	1	8	10.68	0.83
7	1	8	12.71	1.21
7	1	8	14.44	0.82
-8	-1	-8	0.87	0.31
-8	-1	-8	0.89	0.75

-8	-1	-8	1.67	0.56
-8	-1	-8	1.32	0.41
8	1	8	1.08	0.30
8	1	8	0.95	0.34
8	1	8	1.30	0.37
8	1	8	0.72	0.64
-9	-1	-8	25.46	1.23
-9	-1	-8	25.76	1.94
-9	-1	-8	22.33	1.33
9	1	8	22.49	1.25
9	1	8	25.20	1.73
9	1	8	23.97	1.28
-10	-1	-8	0.15	0.49
-10	-1	-8	0.86	0.94
10	1	8	0.34	0.35
10	1	8	-0.01	0.43
10	1	8	0.05	0.67
-11	-1	-8	0.87	0.59
-11	-1	-8	0.00	0.96
11	1	8	-0.02	0.50
11	1	8	0.39	0.36
11	1	8	0.60	0.75
-12	-1	-8	4.84	0.84
12	1	8	4.52	1.08
12	1	8	6.18	0.99
-13	-1	-8	3.23	0.86
13	1	8	3.57	0.70
13	1	8	5.31	1.03
13	1	8	3.71	0.61
-14	-1	-8	1.45	0.88
14	1	8	2.65	0.90
14	1	8	2.94	0.97
14	1	8	0.79	0.54
-15	-1	-8	2.65	0.97
15	1	8	1.55	0.63
15	1	8	2.69	1.09
-16	-1	-8	4.72	1.15
16	1	8	-0.10	1.14
16	1	8	1.45	0.67
-17	-1	-8	0.62	1.27
17	1	8	-0.64	1.21
17	1	8	-0.08	0.69
-18	-1	-8	2.86	1.46
18	1	8	2.91	0.86

18	1	8	0.89	1.40
-19	-1	-8	4.45	1.67
19	1	8	2.61	1.24
19	1	8	2.59	1.64
20	-1	-7	67.82	3.15
-20	1	7	70.51	4.54
-20	1	7	69.87	3.89
19	-1	-7	-0.54	0.56
-19	1	7	1.33	1.12
-19	1	7	0.96	2.07
18	-1	-7	105.15	4.32
-18	1	7	96.05	5.30
-18	1	7	111.47	4.96
17	-1	-7	-0.04	0.48
-17	1	7	-1.86	1.31
-17	1	7	0.54	0.92
16	-1	-7	72.94	3.17
-16	1	7	74.87	4.25
-16	1	7	79.74	3.77
15	-1	-7	-2.57	1.53
15	-1	-7	-0.09	0.40
-15	1	7	-0.19	0.72
-15	1	7	0.24	1.21
14	-1	-7	135.09	6.33
14	-1	-7	135.98	5.25
-14	1	7	133.79	6.10
-14	1	7	128.88	6.00
13	-1	-7	1.28	0.35
13	-1	-7	1.04	1.32
-13	1	7	0.59	0.62
-13	1	7	0.26	1.09
-13	1	7	0.75	0.43
12	-1	-7	162.28	6.41
12	-1	-7	163.84	7.31
-12	1	7	167.77	7.17
-12	1	7	168.11	6.48
-12	1	7	170.69	6.76
11	-1	-7	11.34	0.73
11	-1	-7	10.15	1.90
-11	1	7	11.51	0.80
-11	1	7	10.62	1.63
-11	1	7	12.98	1.12
10	-1	-7	120.26	4.82
10	-1	-7	123.04	5.05

10	-1	-7	118.38	5.60
-10	1	7	119.80	5.51
-10	1	7	138.61	4.90
-10	1	7	126.47	5.11
9	-1	-7	11.71	0.67
9	-1	-7	12.05	0.94
9	-1	-7	15.06	1.68
-9	1	7	10.84	0.97
-9	1	7	11.70	0.74
-9	1	7	10.43	1.43
8	-1	-7	134.01	5.24
8	-1	-7	125.67	5.03
8	-1	-7	130.45	5.76
-8	1	7	131.79	5.27
-8	1	7	129.22	5.66
-8	1	7	137.91	5.09
7	-1	-7	2.91	0.83
7	-1	-7	2.06	0.43
7	-1	-7	1.73	0.24
7	-1	-7	1.81	0.34
-7	1	7	1.42	0.41
-7	1	7	2.15	0.30
-7	1	7	2.50	0.70
6	-1	-7	172.29	6.47
6	-1	-7	167.31	6.35
6	-1	-7	170.08	6.98
6	-1	-7	161.42	6.32
-6	1	7	169.34	6.49
-6	1	7	175.58	6.36
-6	1	7	159.60	6.86
-6	1	7	161.67	6.34
5	-1	-7	6.37	0.56
5	-1	-7	6.84	1.09
5	-1	-7	7.32	0.58
5	-1	-7	7.44	0.45
-5	1	7	6.11	0.53
-5	1	7	7.52	1.00
-5	1	7	7.32	0.47
-5	1	7	7.62	0.61
-5	1	7	7.57	0.56
4	-1	-7	96.52	4.35
4	-1	-7	99.03	3.80
4	-1	-7	100.97	3.93
-4	1	7	98.24	3.90

-4	1	7	96.33	3.87
-4	1	7	97.78	4.27
-4	1	7	103.68	3.86
3	-1	-7	-0.31	0.48
3	-1	-7	0.30	0.14
3	-1	-7	-0.11	0.28
-3	1	7	0.19	0.15
-3	1	7	0.25	0.20
-3	1	7	0.30	0.38
-3	1	7	0.08	0.17
2	-1	-7	255.46	9.60
2	-1	-7	236.78	9.13
2	-1	-7	246.49	9.33
-2	1	7	248.30	9.13
-2	1	7	232.84	9.48
-2	1	7	241.71	9.31
-2	1	7	243.07	9.14
-2	1	7	244.26	9.12
1	-1	-7	81.48	3.94
1	-1	-7	92.59	3.65
1	-1	-7	86.89	3.40
-1	1	7	88.24	3.73
-1	1	7	86.45	3.61
-1	1	7	89.30	3.36
-1	1	7	87.69	3.36
-1	1	7	91.18	3.42
0	-1	-7	497.53	18.89
0	-1	-7	506.97	19.25
0	-1	-7	487.15	18.80
0	1	7	528.65	19.17
0	1	7	516.57	18.90
0	1	7	497.45	19.12
0	1	7	519.14	18.83
-1	-1	-7	29.59	1.63
-1	-1	-7	28.97	1.26
-1	-1	-7	30.23	1.17
-1	-1	-7	31.56	1.58
1	1	7	25.62	1.33
1	1	7	28.54	1.53
1	1	7	31.03	1.18
1	1	7	29.78	1.50
-2	-1	-7	217.25	8.07
-2	-1	-7	219.28	8.18
-2	-1	-7	227.26	8.50

-2	-1	-7	226.82	8.15
-2	-1	-7	205.62	8.42
2	1	7	211.67	8.06
2	1	7	217.78	8.34
2	1	7	205.49	8.42
-3	-1	-7	11.86	0.58
-3	-1	-7	12.08	1.05
-3	-1	-7	10.51	0.97
-3	-1	-7	13.44	0.64
-3	-1	-7	12.89	0.68
3	1	7	11.10	0.56
3	1	7	11.72	0.83
3	1	7	10.96	0.95
-4	-1	-7	144.04	5.77
-4	-1	-7	152.96	5.69
-4	-1	-7	147.88	5.94
-4	-1	-7	154.33	5.73
-4	-1	-7	161.32	6.16
4	1	7	148.61	5.66
4	1	7	142.50	6.04
-5	-1	-7	0.65	0.58
-5	-1	-7	0.91	0.26
-5	-1	-7	1.37	0.48
-5	-1	-7	0.60	0.22
-5	-1	-7	0.60	0.23
5	1	7	0.91	0.48
5	1	7	0.85	0.30
5	1	7	0.64	0.23
5	1	7	0.72	0.21
-6	-1	-7	162.68	5.96
-6	-1	-7	145.64	6.07
-6	-1	-7	161.57	5.97
-6	-1	-7	163.55	6.43
6	1	7	147.69	5.97
6	1	7	160.47	5.96
6	1	7	147.55	6.30
6	1	7	155.03	5.93
-7	-1	-7	1.34	0.45
-7	-1	-7	2.36	0.74
-7	-1	-7	1.66	0.35
-7	-1	-7	1.29	0.29
7	1	7	1.39	0.29
7	1	7	0.81	0.57
7	1	7	1.06	0.31

7	1	7	0.75	0.35
-8	-1	-7	191.21	7.49
-8	-1	-7	190.58	7.43
-8	-1	-7	193.25	7.92
-8	-1	-7	195.90	7.43
8	1	7	199.13	7.46
8	1	7	196.20	7.47
8	1	7	199.53	7.34
8	1	7	190.66	7.83
-9	-1	-7	5.97	0.98
-9	-1	-7	5.77	0.79
-9	-1	-7	5.16	0.55
9	1	7	4.87	0.58
9	1	7	3.69	0.81
9	1	7	4.74	0.66
-10	-1	-7	78.90	3.39
-10	-1	-7	86.35	3.84
-10	-1	-7	77.98	3.24
10	1	7	81.15	3.31
10	1	7	77.02	3.28
10	1	7	76.50	3.67
-11	-1	-7	2.90	1.00
-11	-1	-7	3.43	0.71
11	1	7	3.59	0.61
11	1	7	2.11	0.80
11	1	7	2.83	0.49
-12	-1	-7	21.36	1.59
-12	-1	-7	23.74	2.05
12	1	7	20.94	1.74
12	1	7	22.41	1.42
12	1	7	21.94	1.29
-13	-1	-7	5.04	0.94
13	1	7	7.23	1.35
13	1	7	6.10	0.84
13	1	7	4.65	0.73
-14	-1	-7	30.54	2.21
14	1	7	35.27	2.32
14	1	7	36.34	1.85
-15	-1	-7	0.30	0.91
15	1	7	0.31	0.53
15	1	7	1.93	0.98
-16	-1	-7	35.62	2.55
16	1	7	37.74	2.08
-17	-1	-7	1.32	1.21

17	1	7	0.17	0.64
17	1	7	0.43	1.14
-18	-1	-7	33.73	3.00
18	1	7	33.41	2.79
18	1	7	37.57	2.13
-19	-1	-7	-1.04	1.48
19	1	7	-0.59	0.74
19	1	7	-1.02	1.45
21	-1	-6	46.88	2.43
20	-1	-6	-0.66	0.63
-20	1	6	1.05	1.25
19	-1	-6	32.63	1.90
-19	1	6	34.13	3.20
-19	1	6	38.20	2.75
18	-1	-6	0.41	0.53
-18	1	6	-1.26	1.47
-18	1	6	-1.46	0.90
17	-1	-6	59.43	2.78
17	-1	-6	72.39	4.85
-17	1	6	59.87	3.78
-17	1	6	66.82	3.40
16	-1	-6	3.41	0.56
16	-1	-6	5.40	1.66
-16	1	6	3.11	0.98
-16	1	6	5.13	1.40
15	-1	-6	33.08	3.00
15	-1	-6	32.60	1.66
-15	1	6	37.39	2.23
-15	1	6	36.09	2.65
14	-1	-6	3.21	1.39
14	-1	-6	0.57	0.35
-14	1	6	1.60	0.86
-14	1	6	2.35	1.06
13	-1	-6	113.01	5.25
13	-1	-6	106.02	4.31
-13	1	6	105.13	5.05
-13	1	6	108.36	4.38
-13	1	6	113.67	4.71
12	-1	-6	0.40	0.52
12	-1	-6	1.33	0.33
12	-1	-6	2.08	1.11
-12	1	6	1.18	0.93
-12	1	6	0.08	0.52
-12	1	6	0.86	0.35

11	-1	-6	85.57	3.56
11	-1	-6	90.52	4.39
11	-1	-6	89.99	3.81
-11	1	6	95.45	3.64
-11	1	6	92.22	3.90
-11	1	6	87.64	4.22
10	-1	-6	-0.50	0.38
10	-1	-6	-0.01	0.21
10	-1	-6	-0.01	0.90
-10	1	6	0.39	0.26
-10	1	6	0.01	0.70
-10	1	6	-0.35	0.42
9	-1	-6	119.07	4.79
9	-1	-6	116.66	4.58
9	-1	-6	121.31	5.29
-9	1	6	122.28	4.84
-9	1	6	120.20	4.62
-9	1	6	112.74	5.15
8	-1	-6	14.39	0.75
8	-1	-6	14.99	1.51
8	-1	-6	15.53	0.99
-8	1	6	14.51	1.00
-8	1	6	16.18	0.82
-8	1	6	14.94	1.38
7	-1	-6	88.60	3.47
7	-1	-6	87.45	3.63
7	-1	-6	94.92	4.13
-7	1	6	92.47	3.51
-7	1	6	84.21	3.98
-7	1	6	91.53	3.69
6	-1	-6	0.42	0.19
6	-1	-6	1.00	0.61
6	-1	-6	0.13	0.24
6	-1	-6	0.44	0.16
-6	1	6	0.52	0.27
-6	1	6	0.67	0.19
-6	1	6	0.62	0.18
-6	1	6	0.38	0.53
5	-1	-6	220.22	8.41
5	-1	-6	232.86	8.96
5	-1	-6	230.90	8.45
-5	1	6	232.61	8.55
-5	1	6	212.96	8.43
-5	1	6	217.15	8.86

4	-1	-6	5.74	0.90
4	-1	-6	7.11	0.49
4	-1	-6	5.96	0.50
4	-1	-6	6.44	0.39
-4	1	6	6.98	0.52
-4	1	6	6.43	0.85
-4	1	6	5.92	0.40
-4	1	6	5.88	0.48
3	-1	-6	149.46	5.60
3	-1	-6	147.47	5.71
3	-1	-6	155.48	6.29
-3	1	6	153.73	5.62
-3	1	6	151.39	5.66
-3	1	6	143.09	5.70
-3	1	6	149.55	5.60
-3	1	6	136.06	5.94
2	-1	-6	5.08	0.35
2	-1	-6	5.24	0.53
2	-1	-6	6.83	0.82
-2	1	6	5.10	0.51
-2	1	6	5.50	0.67
-2	1	6	5.51	0.37
-2	1	6	5.13	0.35
-2	1	6	4.98	0.34
1	-1	-6	452.62	16.54
1	-1	-6	458.40	16.69
1	-1	-6	421.28	16.34
-1	1	6	432.46	16.62
-1	1	6	437.18	16.54
-1	1	6	437.72	16.31
-1	1	6	442.26	16.31
-1	1	6	434.23	16.35
0	-1	-6	259.15	10.39
0	-1	-6	263.44	10.04
0	-1	-6	277.78	10.34
0	-1	-6	262.28	10.11
0	1	6	277.23	10.36
0	1	6	274.69	10.32
0	1	6	268.72	10.12
0	1	6	280.46	10.06
-1	-1	-6	623.60	23.22
-1	-1	-6	613.72	22.88
-1	-1	-6	652.13	23.22
-1	-1	-6	596.68	22.97

1	1	6	625.01	22.98
1	1	6	593.24	23.17
1	1	6	620.85	23.15
1	1	6	621.89	22.88
-2	-1	-6	3.22	0.31
-2	-1	-6	3.20	0.59
-2	-1	-6	2.70	0.28
-2	-1	-6	3.15	0.23
-2	-1	-6	3.96	0.63
2	1	6	2.96	0.23
2	1	6	3.11	0.55
2	1	6	3.29	0.55
-3	-1	-6	308.01	11.21
-3	-1	-6	283.95	10.91
-3	-1	-6	293.31	10.83
-3	-1	-6	293.67	11.06
-3	-1	-6	286.41	10.87
3	1	6	279.93	10.81
3	1	6	280.96	11.14
3	1	6	298.82	10.98
-4	-1	-6	2.78	0.31
-4	-1	-6	2.79	0.57
-4	-1	-6	2.51	0.35
-4	-1	-6	3.13	0.33
-4	-1	-6	3.24	0.57
4	1	6	3.24	0.43
4	1	6	2.82	0.53
4	1	6	3.32	0.30
4	1	6	2.73	0.31
-5	-1	-6	167.62	6.26
-5	-1	-6	162.19	6.24
-5	-1	-6	176.22	6.66
-5	-1	-6	154.57	6.34
5	1	6	162.46	6.23
5	1	6	157.44	6.55
5	1	6	165.96	6.28
5	1	6	170.90	6.28
-6	-1	-6	0.39	0.32
-6	-1	-6	-0.22	0.49
-6	-1	-6	-0.33	0.28
-6	-1	-6	0.07	0.19
6	1	6	-0.19	0.20
6	1	6	-0.77	0.38
6	1	6	0.28	0.22

6	1	6	0.00	0.22
-7	-1	-6	306.93	11.34
-7	-1	-6	283.99	11.29
-7	-1	-6	306.80	11.30
-7	-1	-6	327.95	11.75
7	1	6	279.65	11.40
7	1	6	286.61	11.31
7	1	6	315.51	11.23
7	1	6	294.93	11.63
-8	-1	-6	4.66	0.47
-8	-1	-6	3.68	0.63
-8	-1	-6	4.73	0.89
8	1	6	3.75	0.50
8	1	6	4.07	0.56
8	1	6	5.56	0.92
-9	-1	-6	102.85	3.95
-9	-1	-6	97.21	4.05
-9	-1	-6	102.78	4.44
9	1	6	95.68	4.30
9	1	6	98.65	3.99
9	1	6	101.74	4.01
-10	-1	-6	0.90	0.49
-10	-1	-6	0.17	0.71
-10	-1	-6	0.55	0.29
10	1	6	0.47	0.41
10	1	6	0.54	0.30
10	1	6	-0.52	0.56
-11	-1	-6	113.56	5.15
-11	-1	-6	121.16	4.73
-11	-1	-6	114.81	4.85
11	1	6	121.34	4.76
11	1	6	121.04	5.07
11	1	6	115.12	4.71
-12	-1	-6	3.01	0.72
-12	-1	-6	3.81	1.03
12	1	6	3.52	0.88
12	1	6	3.84	0.65
12	1	6	3.87	0.56
-13	-1	-6	92.86	4.27
-13	-1	-6	82.22	3.86
13	1	6	84.12	4.07
13	1	6	89.98	4.01
13	1	6	84.84	3.73
-14	-1	-6	5.30	1.28

-14	-1	-6	3.92	0.96
14	1	6	4.78	0.69
14	1	6	4.73	0.98
-15	-1	-6	80.44	3.98
15	1	6	85.49	4.05
15	1	6	87.06	3.67
-16	-1	-6	3.39	1.00
16	1	6	2.98	0.68
16	1	6	2.39	1.07
-17	-1	-6	58.79	3.52
17	1	6	64.13	3.51
17	1	6	65.72	3.01
-18	-1	-6	-1.32	1.30
18	1	6	-0.81	1.21
18	1	6	0.69	0.69
-19	-1	-6	41.03	3.36
19	1	6	34.80	3.00
19	1	6	46.87	2.40
21	-1	-5	-1.00	0.69
-21	1	5	4.21	2.51
20	-1	-5	11.30	1.20
-20	1	5	7.67	1.50
19	-1	-5	0.53	0.59
-19	1	5	1.72	1.19
-19	1	5	-1.50	1.43
18	-1	-5	3.04	0.65
-18	1	5	2.80	1.07
-18	1	5	4.15	1.45
17	-1	-5	4.37	1.69
17	-1	-5	1.84	0.53
-17	1	5	1.41	0.89
-17	1	5	1.18	1.28
16	-1	-5	19.04	1.19
16	-1	-5	22.55	2.68
-16	1	5	20.56	1.82
-16	1	5	19.91	2.23
15	-1	-5	5.39	1.49
15	-1	-5	3.72	0.52
-15	1	5	5.79	1.25
-15	1	5	4.22	0.88
14	-1	-5	5.83	1.38
14	-1	-5	4.37	0.63
-14	1	5	5.84	0.89
-14	1	5	4.85	1.19

13	-1	-5	-0.38	0.28
13	-1	-5	-1.54	1.07
-13	1	5	-0.33	0.36
-13	1	5	-0.88	0.51
-13	1	5	1.67	0.97
12	-1	-5	21.73	1.17
12	-1	-5	28.23	2.17
12	-1	-5	23.21	1.44
-12	1	5	22.53	1.52
-12	1	5	26.08	1.95
-12	1	5	24.32	1.25
11	-1	-5	2.49	0.52
11	-1	-5	3.91	1.04
11	-1	-5	1.61	0.31
-11	1	5	2.35	0.80
-11	1	5	1.93	0.39
-11	1	5	1.59	0.54
10	-1	-5	33.98	1.68
10	-1	-5	32.45	2.21
10	-1	-5	32.56	1.45
-10	1	5	34.83	1.51
-10	1	5	30.45	2.04
-10	1	5	32.88	1.73
9	-1	-5	22.99	1.25
9	-1	-5	20.19	1.00
9	-1	-5	20.58	1.72
-9	1	5	22.81	1.31
-9	1	5	21.44	1.62
-9	1	5	22.22	1.05
8	-1	-5	141.40	5.53
8	-1	-5	148.11	5.71
8	-1	-5	149.36	6.11
-8	1	5	142.32	5.73
-8	1	5	147.25	5.56
-8	1	5	142.68	6.02
7	-1	-5	3.72	0.52
7	-1	-5	3.64	0.77
7	-1	-5	3.17	0.31
-7	1	5	3.08	0.66
-7	1	5	3.76	0.56
-7	1	5	3.01	0.35
6	-1	-5	17.10	0.91
6	-1	-5	15.90	0.75
6	-1	-5	18.66	1.35

-6	1	5	15.84	1.23
-6	1	5	15.96	0.78
-6	1	5	15.81	0.94
5	-1	-5	8.13	0.45
5	-1	-5	8.22	0.46
5	-1	-5	8.65	0.58
5	-1	-5	8.63	0.98
-5	1	5	7.28	0.88
-5	1	5	8.58	0.60
-5	1	5	7.91	0.46
-5	1	5	9.85	0.49
4	-1	-5	49.41	1.97
4	-1	-5	50.32	2.01
4	-1	-5	52.96	2.06
4	-1	-5	50.64	2.39
-4	1	5	49.88	2.07
-4	1	5	51.98	1.98
-4	1	5	48.70	2.32
-4	1	5	47.40	1.99
3	-1	-5	31.16	1.36
3	-1	-5	34.07	1.68
3	-1	-5	32.31	1.32
-3	1	5	30.90	1.33
-3	1	5	30.37	1.34
-3	1	5	31.36	1.28
-3	1	5	29.77	1.59
2	-1	-5	168.01	6.30
2	-1	-5	164.31	6.42
2	-1	-5	169.65	6.62
-2	1	5	163.08	6.40
-2	1	5	165.39	6.31
-2	1	5	178.07	6.61
-2	1	5	164.77	6.30
-2	1	5	170.59	6.32
1	-1	-5	169.47	6.32
1	-1	-5	163.32	6.15
-1	1	5	164.94	6.13
-1	1	5	154.83	6.41
-1	1	5	165.47	6.12
-1	1	5	166.40	6.30
-1	1	5	162.18	6.16
0	-1	-5	906.73	33.80
0	-1	-5	951.52	34.03
0	-1	-5	958.36	33.99

0	1	5	886.67	34.00
0	1	5	859.67	34.07
-1	-1	-5	9.57	0.71
-1	-1	-5	9.53	0.70
-1	-1	-5	8.79	0.40
1	1	5	9.39	0.65
1	1	5	9.29	0.70
1	1	5	9.43	0.41
1	1	5	10.29	0.50
-2	-1	-5	11.62	0.80
-2	-1	-5	10.74	0.48
-2	-1	-5	10.50	0.73
-2	-1	-5	11.14	0.57
-2	-1	-5	10.78	0.53
2	1	5	10.86	0.67
2	1	5	10.94	0.79
2	1	5	10.77	0.48
-3	-1	-5	34.86	1.46
-3	-1	-5	35.04	1.71
-3	-1	-5	36.33	1.43
-3	-1	-5	34.72	1.56
-3	-1	-5	33.89	1.39
3	1	5	35.79	1.50
3	1	5	34.18	1.38
3	1	5	33.84	1.65
-4	-1	-5	90.04	3.55
-4	-1	-5	91.14	3.65
-4	-1	-5	99.54	3.88
-4	-1	-5	92.37	3.57
4	1	5	91.66	3.54
4	1	5	88.21	3.81
4	1	5	91.59	3.58
4	1	5	96.41	3.60
-5	-1	-5	2.34	0.43
-5	-1	-5	2.68	0.54
-5	-1	-5	2.70	0.31
-5	-1	-5	2.38	0.36
5	1	5	2.18	0.32
5	1	5	2.34	0.33
5	1	5	3.20	0.54
5	1	5	2.66	0.30
-6	-1	-5	178.67	6.78
-6	-1	-5	181.96	6.75
-6	-1	-5	175.31	6.75

-6	-1	-5	190.53	7.12
6	1	5	177.34	6.77
6	1	5	167.09	6.75
6	1	5	168.14	7.01
6	1	5	183.77	6.70
-7	-1	-5	12.14	0.66
-7	-1	-5	12.49	0.78
-7	-1	-5	12.73	1.12
-7	-1	-5	11.28	0.67
7	1	5	11.20	0.61
7	1	5	12.07	1.02
7	1	5	10.97	0.67
-8	-1	-5	181.79	7.31
-8	-1	-5	175.52	7.00
-8	-1	-5	181.38	6.92
8	1	5	183.84	6.97
8	1	5	187.14	6.96
8	1	5	178.87	7.22
-9	-1	-5	-0.16	0.22
-9	-1	-5	-0.24	0.37
-9	-1	-5	0.18	0.57
9	1	5	-0.25	0.22
9	1	5	0.32	0.33
9	1	5	-0.67	0.42
-10	-1	-5	67.08	2.75
-10	-1	-5	64.29	2.93
-10	-1	-5	66.98	3.21
10	1	5	69.87	2.86
10	1	5	66.29	3.09
10	1	5	67.88	2.81
-11	-1	-5	0.95	0.55
-11	-1	-5	1.71	0.37
-11	-1	-5	1.45	0.77
11	1	5	0.31	0.45
11	1	5	0.93	0.65
11	1	5	1.83	0.40
-12	-1	-5	64.19	3.17
-12	-1	-5	57.64	2.91
12	1	5	62.36	2.71
12	1	5	63.85	3.02
12	1	5	64.80	2.80
-13	-1	-5	0.57	0.72
-13	-1	-5	0.78	0.97
13	1	5	0.95	0.42

13	1	5	0.70	0.61
13	1	5	0.89	0.72
-14	-1	-5	42.49	2.62
-14	-1	-5	38.03	2.36
14	1	5	42.48	2.02
14	1	5	40.69	2.37
-15	-1	-5	5.72	1.33
-15	-1	-5	3.46	1.00
15	1	5	7.27	1.11
15	1	5	5.96	0.92
-16	-1	-5	78.95	3.90
16	1	5	81.90	3.87
16	1	5	78.70	3.52
-17	-1	-5	1.02	1.13
17	1	5	0.62	0.66
17	1	5	-0.23	0.98
-18	-1	-5	58.97	3.49
18	1	5	56.59	2.77
18	1	5	54.31	3.24
-19	-1	-5	2.63	1.51
19	1	5	0.20	0.75
-20	-1	-5	35.51	3.30
20	1	5	36.30	2.31
21	-1	-4	1.16	0.73
-21	1	4	2.30	1.41
20	-1	-4	-0.01	0.61
-20	1	4	0.03	1.17
19	-1	-4	1.63	0.62
-19	1	4	2.64	1.47
-19	1	4	1.59	1.10
18	-1	-4	-0.69	0.48
-18	1	4	0.68	1.29
-18	1	4	-1.07	0.97
17	-1	-4	1.77	1.53
17	-1	-4	-0.04	0.45
-17	1	4	0.65	1.20
-17	1	4	0.15	0.88
16	-1	-4	3.44	0.53
16	-1	-4	4.45	1.42
-16	1	4	3.09	0.91
-16	1	4	2.46	1.21
15	-1	-4	2.90	0.49
15	-1	-4	4.48	1.42
-15	1	4	3.59	0.84

-15	1	4	1.02	1.07
14	-1	-4	1.99	1.22
14	-1	-4	-0.47	0.29
-14	1	4	0.37	0.58
-14	1	4	0.13	0.91
13	-1	-4	3.80	1.16
-13	1	4	3.29	0.50
-13	1	4	3.27	0.67
-13	1	4	2.83	0.97
12	-1	-4	-0.16	0.49
12	-1	-4	0.24	0.90
12	-1	-4	0.17	0.25
-12	1	4	0.26	0.33
-12	1	4	-0.28	0.74
-12	1	4	0.23	0.48
11	-1	-4	1.85	0.47
11	-1	-4	1.47	0.29
11	-1	-4	1.82	0.86
-11	1	4	2.19	0.37
-11	1	4	0.98	0.71
-11	1	4	0.82	0.47
10	-1	-4	8.96	1.33
10	-1	-4	8.66	0.85
10	-1	-4	9.03	0.62
-10	1	4	11.67	0.68
-10	1	4	8.17	1.20
-10	1	4	8.99	0.90
9	-1	-4	-0.56	0.64
9	-1	-4	0.99	0.37
9	-1	-4	0.38	0.19
-9	1	4	0.00	0.21
-9	1	4	0.04	0.34
-9	1	4	0.38	0.55
8	-1	-4	0.99	0.61
8	-1	-4	-0.08	0.29
8	-1	-4	0.15	0.17
-8	1	4	0.29	0.51
-8	1	4	0.20	0.18
-8	1	4	0.12	0.30
7	-1	-4	23.48	1.56
7	-1	-4	23.37	1.06
7	-1	-4	25.90	1.25
-7	1	4	25.55	1.27
-7	1	4	22.75	1.49

-7	1	4	26.02	1.10
6	-1	-4	0.17	0.23
6	-1	-4	0.05	0.12
6	-1	-4	0.30	0.46
-6	1	4	0.10	0.17
-6	1	4	0.37	0.25
-6	1	4	0.63	0.42
5	-1	-4	46.65	1.79
5	-1	-4	47.20	1.95
5	-1	-4	47.52	2.25
5	-1	-4	45.30	1.83
-5	1	4	46.78	1.85
-5	1	4	47.82	1.98
-5	1	4	44.39	2.17
4	-1	-4	3.08	0.26
4	-1	-4	3.68	0.28
4	-1	-4	3.55	0.64
4	-1	-4	3.55	0.35
-4	1	4	3.88	0.39
-4	1	4	3.17	0.26
-4	1	4	3.18	0.26
-4	1	4	3.46	0.56
3	-1	-4	3.74	0.32
3	-1	-4	3.52	0.26
3	-1	-4	4.57	0.33
3	-1	-4	4.06	0.62
-3	1	4	3.77	0.31
-3	1	4	4.08	0.27
-3	1	4	3.62	0.59
-3	1	4	4.23	0.34
2	-1	-4	9.12	0.46
2	-1	-4	9.79	0.48
2	-1	-4	10.00	0.56
2	-1	-4	9.30	0.74
-2	1	4	10.12	0.55
-2	1	4	9.92	0.47
-2	1	4	10.17	0.73
-2	1	4	10.35	0.50
1	-1	-4	13.06	0.70
1	-1	-4	12.77	0.57
1	-1	-4	13.87	0.83
-1	1	4	12.70	0.78
-1	1	4	12.64	0.55
-1	1	4	13.45	0.56

-1	1	4	12.84	0.58
-1	1	4	12.75	0.69
0	-1	-4	85.72	3.41
0	-1	-4	82.42	3.36
0	-1	-4	82.57	3.23
0	1	4	88.58	3.39
0	1	4	84.46	3.23
0	1	4	83.39	3.36
0	1	4	86.93	3.17
-1	-1	-4	12.74	0.58
-1	-1	-4	13.12	0.76
-1	-1	-4	12.07	0.72
1	1	4	12.53	0.58
1	1	4	11.88	0.75
1	1	4	12.69	0.69
1	1	4	11.94	0.50
-2	-1	-4	1.33	0.17
-2	-1	-4	0.92	0.28
-2	-1	-4	0.86	0.17
-2	-1	-4	1.33	0.15
-2	-1	-4	1.75	0.28
2	1	4	1.34	0.23
2	1	4	1.44	0.15
-3	-1	-4	54.27	2.17
-3	-1	-4	57.21	2.20
-3	-1	-4	60.33	2.44
-3	-1	-4	58.17	2.28
3	1	4	52.46	2.39
3	1	4	55.65	2.20
3	1	4	56.46	2.23
3	1	4	55.74	2.17
-4	-1	-4	18.57	0.85
-4	-1	-4	19.17	1.11
-4	-1	-4	18.84	0.84
-4	-1	-4	20.13	0.91
4	1	4	18.17	0.85
4	1	4	19.16	0.85
4	1	4	19.70	1.08
4	1	4	19.58	0.86
-5	-1	-4	96.69	3.78
-5	-1	-4	102.03	3.76
-5	-1	-4	103.63	3.76
5	1	4	96.19	3.77
5	1	4	99.11	3.72

5	1	4	94.23	3.97
5	1	4	90.37	3.75
-6	-1	-4	0.29	0.19
-6	-1	-4	0.05	0.38
-6	-1	-4	0.12	0.15
-6	-1	-4	0.21	0.25
6	1	4	0.24	0.16
6	1	4	0.23	0.17
6	1	4	0.25	0.21
6	1	4	0.40	0.35
-7	-1	-4	57.63	2.31
-7	-1	-4	58.86	2.28
-7	-1	-4	55.35	2.41
-7	-1	-4	61.34	2.68
-7	-1	-4	62.76	2.35
7	1	4	56.34	2.59
7	1	4	54.52	2.35
-8	-1	-4	6.95	0.65
-8	-1	-4	7.07	0.51
-8	-1	-4	9.13	0.95
8	1	4	6.76	0.53
8	1	4	6.31	0.60
8	1	4	5.69	0.81
-9	-1	-4	18.27	1.32
-9	-1	-4	17.33	0.89
-9	-1	-4	15.07	1.05
9	1	4	15.60	1.20
9	1	4	16.53	0.92
9	1	4	17.87	1.01
-10	-1	-4	4.80	0.80
-10	-1	-4	6.04	0.52
-10	-1	-4	4.78	0.83
10	1	4	4.94	0.57
10	1	4	5.76	0.78
10	1	4	6.14	0.69
-11	-1	-4	50.24	2.46
-11	-1	-4	53.80	2.23
-11	-1	-4	53.37	2.67
11	1	4	52.94	2.28
11	1	4	51.72	2.53
11	1	4	52.42	2.37
-12	-1	-4	20.35	1.45
-12	-1	-4	18.19	1.62
-12	-1	-4	18.72	1.40

12	1	4	18.51	1.39
12	1	4	18.64	1.11
12	1	4	19.72	1.29
-13	-1	-4	30.50	1.88
-13	-1	-4	29.94	2.09
13	1	4	30.52	1.75
13	1	4	31.50	1.89
13	1	4	32.09	1.58
-14	-1	-4	0.44	0.97
-14	-1	-4	0.04	0.72
14	1	4	0.29	0.65
14	1	4	0.82	0.73
-15	-1	-4	34.62	2.23
-15	-1	-4	33.12	2.42
15	1	4	34.00	1.80
15	1	4	34.59	2.13
-16	-1	-4	-1.04	1.17
-16	-1	-4	1.37	0.96
16	1	4	1.03	0.58
16	1	4	2.08	0.86
-17	-1	-4	24.59	2.28
17	1	4	23.76	1.57
17	1	4	26.21	2.04
-18	-1	-4	-1.46	1.13
18	1	4	0.88	0.68
18	1	4	-1.07	1.02
-19	-1	-4	38.53	2.99
19	1	4	35.95	2.13
19	1	4	31.34	2.54
-20	-1	-4	-1.77	1.49
20	1	4	0.50	0.75
21	-1	-3	2.30	0.79
-21	1	3	3.59	1.48
20	-1	-3	4.49	0.81
-20	1	3	4.59	1.28
19	-1	-3	1.97	0.62
-19	1	3	2.64	1.19
18	-1	-3	8.91	1.03
-18	1	3	7.14	1.43
-18	1	3	10.68	1.79
17	-1	-3	2.16	0.55
17	-1	-3	2.93	1.70
-17	1	3	3.06	0.98
-17	1	3	3.27	1.21

16	-1	-3	17.53	1.17
16	-1	-3	20.61	2.29
-16	1	3	20.06	1.79
-16	1	3	18.58	1.99
15	-1	-3	27.44	2.32
15	-1	-3	24.39	1.35
-15	1	3	24.97	2.02
-15	1	3	25.69	1.80
14	-1	-3	1.71	0.38
14	-1	-3	1.47	1.09
-14	1	3	1.18	0.87
-14	1	3	0.77	0.60
13	-1	-3	1.05	0.34
13	-1	-3	1.22	0.96
-13	1	3	1.31	0.36
-13	1	3	0.78	0.81
-13	1	3	0.64	0.58
12	-1	-3	11.99	0.79
12	-1	-3	11.96	1.07
12	-1	-3	12.15	0.67
12	-1	-3	12.82	1.56
-12	1	3	12.17	0.82
-12	1	3	12.90	1.13
-12	1	3	12.78	0.80
-12	1	3	13.40	1.42
11	-1	-3	3.52	0.47
11	-1	-3	3.53	0.38
11	-1	-3	5.14	0.76
11	-1	-3	4.73	0.91
-11	1	3	3.57	0.83
-11	1	3	3.86	0.51
-11	1	3	3.76	0.63
-11	1	3	3.11	0.51
10	-1	-3	4.38	0.36
10	-1	-3	3.64	0.42
10	-1	-3	3.38	0.53
10	-1	-3	3.94	0.78
-10	1	3	3.55	0.55
-10	1	3	4.63	0.48
-10	1	3	4.45	0.77
9	-1	-3	0.25	0.19
9	-1	-3	0.21	0.32
9	-1	-3	-0.09	0.16
9	-1	-3	0.29	0.59

-9	1	3	-0.25	0.30
-9	1	3	-0.51	0.48
-9	1	3	0.35	0.20
8	-1	-3	12.21	1.35
8	-1	-3	11.55	0.63
8	-1	-3	12.28	0.84
-8	1	3	11.97	1.04
-8	1	3	11.68	0.66
-8	1	3	11.57	0.85
7	-1	-3	48.36	2.06
7	-1	-3	47.27	2.31
7	-1	-3	45.77	1.91
-7	1	3	48.75	2.09
-7	1	3	51.46	1.94
-7	1	3	44.13	2.24
6	-1	-3	50.50	2.34
6	-1	-3	48.53	1.96
6	-1	-3	50.85	2.10
-6	1	3	49.15	2.11
-6	1	3	49.52	2.30
5	-1	-3	2.91	0.37
5	-1	-3	3.09	0.51
5	-1	-3	3.18	0.26
-5	1	3	3.00	0.51
-5	1	3	3.83	0.28
-5	1	3	3.32	0.40
4	-1	-3	57.56	2.54
4	-1	-3	68.21	2.60
4	-1	-3	70.09	2.81
4	-1	-3	65.34	2.48
-4	1	3	67.53	2.52
-4	1	3	64.64	2.77
3	-1	-3	93.11	3.39
3	-1	-3	88.86	3.38
3	-1	-3	89.56	3.44
3	-1	-3	84.73	3.60
-3	1	3	89.44	3.45
-3	1	3	87.45	3.38
-3	1	3	90.65	3.39
-3	1	3	90.61	3.61
2	-1	-3	22.50	0.94
2	-1	-3	21.07	1.10
2	-1	-3	21.80	0.92
2	-1	-3	22.96	0.89

-2	1	3	21.32	0.89
-2	1	3	21.93	0.92
-2	1	3	21.46	0.93
-2	1	3	22.45	1.09
1	-1	-3	52.54	2.21
1	-1	-3	52.51	2.05
1	-1	-3	53.63	2.04
1	-1	-3	52.62	2.13
-1	1	3	53.23	2.13
-1	1	3	58.06	2.22
-1	1	3	53.64	2.05
0	-1	-3	16.98	0.83
0	-1	-3	18.14	0.73
0	-1	-3	18.15	0.87
0	1	3	16.29	0.84
0	1	3	17.56	0.68
0	1	3	18.35	0.84
0	1	3	18.07	0.73
-1	-1	-3	66.25	2.59
-1	-1	-3	63.79	2.50
-1	-1	-3	67.61	2.63
1	1	3	62.78	2.61
1	1	3	66.72	2.57
1	1	3	65.37	2.51
-2	-1	-3	73.75	2.97
-2	-1	-3	80.16	3.17
-2	-1	-3	79.92	3.01
-2	-1	-3	82.23	3.08
2	1	3	79.40	3.16
2	1	3	81.49	3.06
2	1	3	79.51	2.98
2	1	3	77.23	3.01
-3	-1	-3	0.07	0.09
-3	-1	-3	0.11	0.11
-3	-1	-3	0.27	0.23
-3	-1	-3	0.04	0.14
3	1	3	0.13	0.11
3	1	3	0.13	0.11
3	1	3	0.02	0.21
3	1	3	0.03	0.12
-4	-1	-3	5.07	0.34
-4	-1	-3	5.23	0.54
-4	-1	-3	5.38	0.33
-4	-1	-3	4.81	0.35

4	1	3	4.48	0.33
4	1	3	4.92	0.32
4	1	3	4.95	0.36
4	1	3	5.15	0.58
-5	-1	-3	11.31	0.67
-5	-1	-3	12.80	0.60
-5	-1	-3	12.82	0.83
5	1	3	12.92	0.86
5	1	3	11.66	0.61
5	1	3	12.39	0.59
5	1	3	12.74	0.65
-6	-1	-3	66.67	2.60
-6	-1	-3	69.36	2.57
-6	-1	-3	63.69	2.60
-6	-1	-3	63.30	2.67
-6	-1	-3	68.22	2.86
6	1	3	68.21	2.67
6	1	3	65.77	2.81
6	1	3	65.20	2.62
-7	-1	-3	5.37	0.49
-7	-1	-3	5.10	0.41
-7	-1	-3	6.05	0.74
-7	-1	-3	5.02	0.54
-7	-1	-3	5.62	0.40
7	1	3	5.44	0.68
7	1	3	5.12	0.43
7	1	3	4.78	0.58
-8	-1	-3	2.47	0.35
-8	-1	-3	2.29	0.36
-8	-1	-3	2.64	0.42
-8	-1	-3	2.46	0.38
-8	-1	-3	2.50	0.53
8	1	3	2.11	0.34
8	1	3	2.02	0.37
8	1	3	2.02	0.50
-9	-1	-3	0.56	0.20
-9	-1	-3	0.32	0.22
-9	-1	-3	-0.21	0.34
-9	-1	-3	0.19	0.34
-9	-1	-3	0.42	0.47
9	1	3	0.03	0.31
9	1	3	0.33	0.41
9	1	3	-0.43	0.18
-10	-1	-3	12.84	0.74

-10	-1	-3	13.35	0.86
-10	-1	-3	13.78	1.15
-10	-1	-3	11.76	0.70
-10	-1	-3	10.38	0.96
10	1	3	11.80	0.76
10	1	3	12.14	1.04
10	1	3	13.11	0.92
-11	-1	-3	0.44	0.28
-11	-1	-3	0.45	0.58
-11	-1	-3	0.70	0.48
11	1	3	0.21	0.49
11	1	3	1.39	0.43
11	1	3	0.79	0.32
-12	-1	-3	20.38	1.09
-12	-1	-3	18.82	1.42
-12	-1	-3	19.14	1.54
12	1	3	20.04	1.31
12	1	3	20.02	1.41
12	1	3	20.53	1.12
-13	-1	-3	23.07	1.64
-13	-1	-3	26.45	1.80
13	1	3	23.19	1.54
13	1	3	22.90	1.50
13	1	3	21.23	1.27
-14	-1	-3	0.89	0.73
-14	-1	-3	1.05	0.87
14	1	3	-0.10	0.62
14	1	3	0.48	0.40
-15	-1	-3	1.07	0.78
-15	-1	-3	1.12	0.97
15	1	3	0.54	0.70
15	1	3	0.53	0.44
-16	-1	-3	32.10	2.50
-16	-1	-3	39.29	2.44
16	1	3	40.42	2.26
16	1	3	39.05	1.97
-17	-1	-3	0.26	1.09
17	1	3	0.36	0.86
17	1	3	-0.51	0.54
-18	-1	-3	7.10	1.38
18	1	3	9.69	1.22
18	1	3	9.73	1.65
-19	-1	-3	0.60	1.28
19	1	3	0.37	1.09

19	1	3	0.94	0.67
-20	-1	-3	11.08	1.82
20	1	3	11.74	1.50
21	-1	-2	43.69	2.36
20	-1	-2	0.61	0.67
-20	1	2	2.23	1.31
19	-1	-2	41.35	2.15
-19	1	2	42.53	2.89
18	-1	-2	0.51	0.51
-18	1	2	1.16	1.13
-18	1	2	0.59	0.98
17	-1	-2	57.65	3.16
17	-1	-2	50.95	2.41
-17	1	2	52.84	3.04
-17	1	2	50.51	2.91
16	-1	-2	0.10	0.41
16	-1	-2	-0.13	1.14
-16	1	2	-1.04	0.95
-16	1	2	-0.01	0.69
-16	1	2	0.60	0.79
15	-1	-2	23.54	2.07
15	-1	-2	20.35	1.33
-15	1	2	21.83	1.41
-15	1	2	21.39	1.84
-15	1	2	21.28	1.80
14	-1	-2	2.83	1.06
-14	1	2	1.56	0.62
-14	1	2	0.69	0.82
-14	1	2	1.19	0.60
13	-1	-2	66.60	2.81
13	-1	-2	65.70	3.29
13	-1	-2	75.92	3.79
13	-1	-2	70.81	2.78
-13	1	2	65.60	3.15
-13	1	2	59.74	2.84
-13	1	2	74.52	2.87
-13	1	2	66.10	3.19
12	-1	-2	4.03	0.58
12	-1	-2	4.73	0.89
12	-1	-2	2.54	0.61
12	-1	-2	3.83	0.46
-12	1	2	4.91	0.83
-12	1	2	3.69	0.69
-12	1	2	4.28	0.60

-12	1	2	4.66	0.58
11	-1	-2	75.23	3.15
11	-1	-2	77.86	3.56
11	-1	-2	82.24	3.13
11	-1	-2	80.72	3.36
-11	1	2	86.71	3.20
-11	1	2	70.62	3.16
-11	1	2	76.32	3.42
-11	1	2	79.04	3.50
10	-1	-2	1.05	0.25
10	-1	-2	1.49	0.42
10	-1	-2	1.06	0.29
-10	1	2	1.14	0.42
-10	1	2	1.60	0.36
-10	1	2	1.09	0.29
-10	1	2	0.72	0.50
9	-1	-2	93.06	3.94
9	-1	-2	93.27	3.53
9	-1	-2	91.00	3.72
9	-1	-2	87.45	3.56
-9	1	2	92.90	3.79
-9	1	2	103.95	3.61
-9	1	2	85.05	3.83
-9	1	2	82.49	3.56
8	-1	-2	27.15	1.15
8	-1	-2	24.49	1.56
8	-1	-2	28.10	1.34
8	-1	-2	26.78	1.17
-8	1	2	28.43	1.19
-8	1	2	25.91	1.36
-8	1	2	28.29	1.55
-8	1	2	24.22	1.18
7	-1	-2	81.34	3.27
7	-1	-2	87.00	3.25
7	-1	-2	88.74	3.61
7	-1	-2	86.48	3.41
-7	1	2	85.61	3.44
-7	1	2	79.99	3.53
-7	1	2	79.30	3.27
-7	1	2	88.70	3.29
6	-1	-2	-0.09	0.23
6	-1	-2	0.07	0.13
6	-1	-2	0.09	0.35
6	-1	-2	0.19	0.13

-6	1	2	-0.11	0.21
-6	1	2	-0.30	0.30
-6	1	2	0.08	0.11
-6	1	2	0.41	0.17
5	-1	-2	34.13	1.36
5	-1	-2	35.31	1.47
5	-1	-2	32.43	1.61
-5	1	2	31.57	1.56
-5	1	2	35.13	1.37
-5	1	2	32.80	1.35
-5	1	2	34.70	1.49
4	-1	-2	115.05	4.64
4	-1	-2	123.45	4.73
4	-1	-2	124.78	4.86
-4	1	2	123.52	4.76
-4	1	2	129.90	4.66
-4	1	2	120.51	4.83
-4	1	2	127.51	4.65
3	-1	-2	209.38	7.83
3	-1	-2	203.06	7.77
3	-1	-2	222.88	7.77
3	-1	-2	217.48	7.95
-3	1	2	198.45	7.77
-3	1	2	201.34	7.92
-3	1	2	207.21	7.83
2	-1	-2	64.59	2.48
2	-1	-2	64.05	2.62
2	-1	-2	64.27	2.52
2	-1	-2	67.83	2.50
-2	1	2	67.73	2.63
-2	1	2	66.64	2.52
-2	1	2	66.45	2.50
-2	1	2	63.19	2.48
1	-1	-2	3.52	0.24
1	-1	-2	3.95	0.31
1	-1	-2	3.68	0.20
-1	1	2	3.59	0.19
-1	1	2	3.32	0.23
-1	1	2	2.77	0.28
0	1	2	2.79	0.23
-1	-1	-2	14.46	0.56
-1	-1	-2	13.80	0.61
-1	-1	-2	15.76	0.71
-1	-1	-2	16.36	0.67

1	1	2	15.61	0.58
1	1	2	11.98	0.67
-2	-1	-2	62.96	2.33
-2	-1	-2	58.71	2.29
-2	-1	-2	58.59	2.29
-2	-1	-2	62.33	2.42
2	1	2	57.37	2.30
2	1	2	60.08	2.41
2	1	2	61.04	2.29
2	1	2	61.55	2.32
-3	-1	-2	18.72	0.83
-3	-1	-2	20.14	0.82
-3	-1	-2	21.64	0.98
-3	-1	-2	19.73	0.83
3	1	2	19.61	0.84
3	1	2	19.40	0.82
3	1	2	18.50	0.82
3	1	2	19.86	0.96
-4	-1	-2	86.99	3.24
-4	-1	-2	82.47	3.05
-4	-1	-2	84.97	3.06
-4	-1	-2	74.98	3.09
4	1	2	79.74	3.05
4	1	2	80.04	3.09
4	1	2	77.55	3.20
4	1	2	73.66	3.07
-5	-1	-2	1.73	0.22
-5	-1	-2	1.59	0.32
-5	-1	-2	1.43	0.24
-5	-1	-2	1.71	0.21
5	1	2	1.31	0.21
5	1	2	1.40	0.22
5	1	2	1.65	0.38
-6	-1	-2	2.38	0.29
-6	-1	-2	2.35	0.32
-6	-1	-2	2.58	0.41
-6	-1	-2	2.18	0.26
-6	-1	-2	1.85	0.32
6	1	2	1.92	0.28
6	1	2	2.00	0.30
6	1	2	2.35	0.41
-7	-1	-2	0.39	0.21
-7	-1	-2	0.44	0.17
-7	-1	-2	1.35	0.32

-7	-1	-2	0.14	0.26
-7	-1	-2	0.72	0.37
7	1	2	0.45	0.19
7	1	2	0.71	0.27
7	1	2	0.75	0.34
-8	-1	-2	11.99	0.64
-8	-1	-2	10.31	0.91
-8	-1	-2	10.64	0.81
-8	-1	-2	10.65	0.79
-8	-1	-2	12.42	0.64
8	1	2	10.62	0.74
8	1	2	10.95	0.84
8	1	2	10.46	0.64
-9	-1	-2	64.15	2.53
-9	-1	-2	61.76	2.64
-9	-1	-2	57.86	2.69
-9	-1	-2	60.35	2.51
-9	-1	-2	62.40	2.77
9	1	2	62.49	2.72
9	1	2	65.09	2.56
9	1	2	65.84	2.66
-10	-1	-2	0.34	0.44
-10	-1	-2	0.29	0.39
-10	-1	-2	0.48	0.24
-10	-1	-2	0.15	0.29
-10	-1	-2	0.30	0.45
10	1	2	0.85	0.38
10	1	2	-0.06	0.21
10	1	2	0.48	0.40
-11	-1	-2	5.44	0.61
-11	-1	-2	5.31	0.55
-11	-1	-2	5.66	0.87
-11	-1	-2	4.92	0.84
-11	-1	-2	6.20	0.97
11	1	2	5.69	0.82
11	1	2	5.06	0.57
11	1	2	5.60	0.79
-12	-1	-2	1.00	0.54
-12	-1	-2	1.50	0.42
-12	-1	-2	2.53	0.44
-12	-1	-2	1.50	0.59
-12	-1	-2	2.28	0.70
12	1	2	1.06	0.53
12	1	2	1.13	0.36

12	1	2	0.59	0.48
-13	-1	-2	3.38	0.64
-13	-1	-2	3.29	0.51
-13	-1	-2	3.56	0.75
-13	-1	-2	5.12	0.86
13	1	2	2.82	0.46
13	1	2	3.48	0.66
13	1	2	4.75	0.72
-14	-1	-2	0.37	0.58
-14	-1	-2	0.60	0.70
-14	-1	-2	-0.23	0.46
-14	-1	-2	-0.70	0.74
14	1	2	0.10	0.59
14	1	2	0.30	0.40
-15	-1	-2	1.82	0.87
-15	-1	-2	0.42	0.76
-15	-1	-2	0.77	0.52
15	1	2	1.44	0.67
15	1	2	-0.31	0.39
-16	-1	-2	1.18	0.87
-16	-1	-2	1.08	1.01
16	1	2	-0.23	0.73
16	1	2	0.24	0.45
-17	-1	-2	0.52	0.97
17	1	2	0.09	0.77
17	1	2	-0.41	0.56
-18	-1	-2	2.73	1.16
18	1	2	2.12	0.67
18	1	2	2.55	0.95
-19	-1	-2	1.90	1.26
19	1	2	0.26	0.64
19	1	2	0.65	1.12
-20	-1	-2	-0.84	1.32
20	1	2	0.64	0.69
-21	-1	-2	-0.19	1.51
21	1	2	0.73	0.94
21	-1	-1	-0.42	0.72
-21	1	1	2.00	1.49
20	-1	-1	72.65	3.27
-20	1	1	68.00	4.02
19	-1	-1	-0.83	0.56
-19	1	1	-0.38	1.10
18	-1	-1	105.16	4.34
18	-1	-1	99.99	4.28

-18	1	1	102.35	4.84
-18	1	1	102.59	4.75
17	-1	-1	1.13	0.52
17	-1	-1	1.43	1.16
17	-1	-1	2.12	0.66
-17	1	1	2.65	1.03
-17	1	1	1.70	0.96
16	-1	-1	107.34	4.41
16	-1	-1	108.32	4.45
16	-1	-1	114.01	4.78
-16	1	1	111.93	4.91
-16	1	1	104.32	4.68
15	-1	-1	2.28	0.53
15	-1	-1	2.11	0.96
-15	1	1	1.72	0.84
14	-1	-1	51.81	2.67
14	-1	-1	50.26	2.21
14	-1	-1	51.23	2.21
-14	1	1	47.60	2.36
-14	1	1	49.66	2.60
-14	1	1	48.67	2.54
13	-1	-1	-0.20	0.30
13	-1	-1	0.13	0.74
13	-1	-1	0.26	0.42
13	-1	-1	0.69	0.58
-13	1	1	-0.09	0.55
-13	1	1	0.04	0.64
-13	1	1	-0.01	0.52
-13	1	1	0.07	0.35
12	-1	-1	244.80	9.15
12	-1	-1	237.07	9.13
12	-1	-1	239.34	9.31
12	-1	-1	228.81	9.61
-12	1	1	250.42	9.43
-12	1	1	249.03	9.15
-12	1	1	235.30	9.34
-12	1	1	232.79	9.20
11	-1	-1	0.74	0.41
11	-1	-1	0.55	0.63
11	-1	-1	0.36	0.59
11	-1	-1	0.66	0.26
-11	1	1	-0.02	0.42
-11	1	1	1.83	0.60
-11	1	1	0.98	0.45

-11	1	1	1.13	0.31
10	-1	-1	161.70	6.27
10	-1	-1	163.56	6.41
10	-1	-1	154.39	6.24
10	-1	-1	173.32	6.55
-10	1	1	160.51	6.46
-10	1	1	175.02	6.27
-10	1	1	154.83	6.45
-10	1	1	161.22	6.31
9	-1	-1	3.00	0.62
9	-1	-1	3.05	0.48
9	-1	-1	2.78	0.36
9	-1	-1	3.65	0.58
-9	1	1	3.11	0.39
-9	1	1	2.84	0.50
-9	1	1	3.07	0.60
-9	1	1	3.61	0.55
8	-1	-1	168.66	6.44
8	-1	-1	160.22	6.19
8	-1	-1	161.20	6.21
8	-1	-1	167.38	6.33
-8	1	1	178.76	6.21
-8	1	1	157.53	6.36
-8	1	1	146.32	6.22
7	-1	-1	26.89	1.30
7	-1	-1	28.12	1.20
7	-1	-1	26.57	1.17
7	-1	-1	27.78	1.45
-7	1	1	26.15	1.36
-7	1	1	25.76	1.22
-7	1	1	26.53	1.32
-7	1	1	29.87	1.19
6	-1	-1	105.81	4.11
6	-1	-1	104.69	3.90
6	-1	-1	104.81	3.92
-6	1	1	95.18	4.04
-6	1	1	99.20	4.02
-6	1	1	101.82	3.94
-6	1	1	102.77	3.97
5	-1	-1	1.32	0.25
5	-1	-1	1.33	0.34
5	-1	-1	1.40	0.24
5	-1	-1	1.05	0.16
-5	1	1	1.25	0.29

-5	1	1	1.15	0.16
-5	1	1	0.95	0.23
-5	1	1	1.29	0.21
4	-1	-1	272.38	9.87
4	-1	-1	271.47	9.94
4	-1	-1	254.04	9.85
4	-1	-1	274.41	10.00
-4	1	1	256.76	9.97
-4	1	1	266.49	9.94
-4	1	1	260.23	9.86
-4	1	1	262.15	9.88
3	-1	-1	0.25	0.19
3	-1	-1	-0.02	0.08
3	-1	-1	0.29	0.08
3	-1	-1	0.28	0.12
-3	1	1	0.27	0.13
-3	1	1	0.26	0.12
-3	1	1	0.20	0.08
-3	1	1	0.23	0.17
2	-1	-1	211.36	7.70
2	-1	-1	214.48	7.75
-2	1	1	204.22	7.66
-2	1	1	176.35	7.69
-2	1	1	216.31	7.75
-2	1	1	214.87	7.70
-1	-1	-1	2.77	0.46
-2	-1	-1	128.15	4.70
-2	-1	-1	125.95	4.69
-2	-1	-1	121.32	4.68
-2	-1	-1	134.93	4.77
2	1	1	112.93	4.68
2	1	1	128.60	4.69
-3	-1	-1	47.96	1.92
-3	-1	-1	48.56	1.92
-3	-1	-1	49.75	1.90
-3	-1	-1	51.69	1.98
-3	-1	-1	46.89	1.87
3	1	1	48.69	1.91
3	1	1	46.15	1.87
3	1	1	50.35	1.97
-4	-1	-1	208.76	7.60
-4	-1	-1	209.81	7.71
-4	-1	-1	210.42	7.62
-4	-1	-1	199.87	7.67

-4	-1	-1	201.63	7.67
4	1	1	187.16	7.60
4	1	1	208.11	7.70
4	1	1	201.57	7.65
-5	-1	-1	5.43	0.32
-5	-1	-1	5.06	0.46
-5	-1	-1	4.32	0.48
-5	-1	-1	4.43	0.37
-5	-1	-1	4.52	0.46
5	1	1	4.60	0.44
5	1	1	4.51	0.39
5	1	1	5.01	0.34
-6	-1	-1	171.85	6.65
-6	-1	-1	169.49	6.57
-6	-1	-1	178.12	6.68
-6	-1	-1	168.87	6.64
6	1	1	174.76	6.63
6	1	1	179.61	6.66
6	1	1	172.07	6.55
-7	-1	-1	0.97	0.26
-7	-1	-1	0.23	0.33
-7	-1	-1	0.66	0.30
-7	-1	-1	0.46	0.33
-7	-1	-1	0.71	0.17
7	1	1	0.62	0.18
7	1	1	0.81	0.27
-8	-1	-1	245.94	9.30
-8	-1	-1	255.49	9.14
-8	-1	-1	234.23	9.17
-8	-1	-1	249.32	9.30
-8	-1	-1	237.53	9.28
8	1	1	232.08	9.14
8	1	1	242.06	9.26
8	1	1	242.88	9.24
-9	-1	-1	-0.02	0.43
-9	-1	-1	0.58	0.39
-9	-1	-1	-0.16	0.19
-9	-1	-1	0.12	0.32
-9	-1	-1	-0.10	0.31
9	1	1	0.24	0.35
9	1	1	0.10	0.20
9	1	1	0.14	0.32
-10	-1	-1	59.71	2.40
-10	-1	-1	57.11	2.62

-10	-1	-1	54.79	2.44
-10	-1	-1	58.53	2.61
10	1	1	60.56	2.54
10	1	1	59.11	2.56
10	1	1	58.68	2.42
-11	-1	-1	0.94	0.27
-11	-1	-1	0.60	0.42
-11	-1	-1	0.70	0.49
-11	-1	-1	-0.05	0.45
11	1	1	0.65	0.28
11	1	1	0.57	0.42
11	1	1	0.87	0.44
-12	-1	-1	71.92	2.79
-12	-1	-1	68.48	3.06
-12	-1	-1	67.35	3.03
-12	-1	-1	59.00	2.81
-12	-1	-1	70.00	3.01
12	1	1	65.65	2.91
12	1	1	67.48	2.81
12	1	1	67.35	2.95
-13	-1	-1	10.02	0.95
-13	-1	-1	12.18	1.21
-13	-1	-1	11.47	1.18
-13	-1	-1	12.90	1.00
-13	-1	-1	10.93	1.35
13	1	1	9.34	1.09
13	1	1	10.90	0.84
13	1	1	13.19	1.05
-14	-1	-1	16.14	1.42
-14	-1	-1	12.75	1.34
-14	-1	-1	13.07	1.09
-14	-1	-1	15.36	1.43
14	1	1	12.12	1.16
14	1	1	12.91	0.93
-15	-1	-1	0.65	0.72
-15	-1	-1	0.26	0.80
-15	-1	-1	-0.41	0.60
-15	-1	-1	1.84	0.78
15	1	1	0.97	0.58
15	1	1	0.59	0.60
-16	-1	-1	15.33	1.31
-16	-1	-1	15.78	1.70
-16	-1	-1	15.71	1.70
-16	-1	-1	16.97	1.53

16	1	1	17.01	1.38
16	1	1	14.43	1.14
-17	-1	-1	1.28	0.93
-17	-1	-1	-0.13	0.87
17	1	1	0.29	0.77
17	1	1	0.62	0.51
-18	-1	-1	15.32	2.03
18	1	1	16.29	1.61
18	1	1	15.27	1.27
-19	-1	-1	0.52	1.12
19	1	1	0.99	0.68
-20	-1	-1	2.23	1.36
20	1	1	1.31	0.69
-21	-1	-1	2.75	1.58
21	1	1	0.26	0.83
21	-1	0	34.52	2.13
20	-1	0	1.33	0.66
-20	1	0	-0.18	1.30
19	-1	0	58.99	2.78
-19	1	0	56.82	3.54
18	-1	0	1.04	0.54
18	-1	0	1.53	0.78
-18	1	0	0.47	1.09
17	-1	0	69.77	3.03
17	-1	0	67.74	3.07
-17	1	0	69.66	3.55
-17	1	0	65.32	3.08
16	-1	0	0.25	0.60
16	-1	0	0.51	0.46
-16	1	0	0.66	0.88
-16	1	0	2.22	0.74
-16	1	0	1.37	0.85
15	-1	0	78.23	3.41
15	-1	0	80.56	3.54
15	-1	0	83.33	3.59
-15	1	0	80.58	3.50
-15	1	0	78.02	3.58
14	-1	0	5.42	0.69
14	-1	0	4.51	0.64
14	-1	0	5.57	0.88
-14	1	0	3.26	0.79
-14	1	0	5.49	0.86
-14	1	0	5.28	0.84
13	-1	0	70.49	3.27

13	-1	0	77.48	3.10
13	-1	0	72.89	3.16
13	-1	0	80.26	3.33
-13	1	0	81.71	3.29
-13	1	0	70.21	3.26
-13	1	0	71.14	3.27
-13	1	0	73.59	3.41
12	-1	0	6.12	1.03
12	-1	0	5.42	0.83
12	-1	0	4.35	0.73
12	-1	0	4.26	0.56
-12	1	0	4.15	0.75
-12	1	0	4.00	0.75
-12	1	0	5.60	0.59
11	-1	0	139.56	5.62
11	-1	0	150.53	5.55
11	-1	0	141.29	5.46
11	-1	0	139.46	5.61
-11	1	0	138.14	5.59
-11	1	0	135.18	5.68
-11	1	0	148.22	5.47
-11	1	0	130.92	6.03
10	-1	0	-0.20	0.35
10	-1	0	-0.01	0.22
10	-1	0	-0.02	0.46
10	-1	0	-0.11	0.37
-10	1	0	-0.37	0.42
-10	1	0	-0.02	0.40
-10	1	0	0.20	0.23
-10	1	0	-0.87	0.34
9	-1	0	169.93	6.71
9	-1	0	173.51	6.62
9	-1	0	178.02	6.79
9	-1	0	176.86	6.76
-9	1	0	168.50	6.79
-9	1	0	188.95	6.64
-9	1	0	160.52	6.70
-9	1	0	172.58	6.73
8	-1	0	12.56	0.84
8	-1	0	14.02	0.93
8	-1	0	12.66	0.80
8	-1	0	13.47	0.69
-8	1	0	13.69	0.70
-8	1	0	12.67	0.82

-8	1	0	11.44	0.82
-8	1	0	11.31	0.84
7	-1	0	350.95	12.82
7	-1	0	360.60	12.84
7	-1	0	356.75	12.77
7	-1	0	325.82	12.69
-7	1	0	322.43	12.76
-7	1	0	329.48	12.82
-7	1	0	354.11	12.70
-7	1	0	316.89	12.77
6	-1	0	4.88	0.57
6	-1	0	4.91	0.34
6	-1	0	4.76	0.50
6	-1	0	5.12	0.45
-6	1	0	5.93	0.48
-6	1	0	4.70	0.47
-6	1	0	5.08	0.48
5	-1	0	373.22	13.26
5	-1	0	335.63	13.15
5	-1	0	371.79	13.22
5	-1	0	364.32	13.24
-5	1	0	331.07	13.20
-5	1	0	350.29	13.25
-5	1	0	356.69	13.16
-5	1	0	344.17	13.22
4	-1	0	2.42	0.30
4	-1	0	3.26	0.24
4	-1	0	3.37	0.31
4	-1	0	3.27	0.36
-4	1	0	3.23	0.33
-4	1	0	2.80	0.33
-4	1	0	3.17	0.23
-4	1	0	3.28	0.31
3	-1	0	496.29	17.73
3	-1	0	492.90	17.72
3	-1	0	468.27	17.73
-3	1	0	468.81	17.73
-3	1	0	472.24	17.73
-3	1	0	477.74	17.71
-3	1	0	464.30	17.68
2	-1	0	63.30	2.41
2	-1	0	63.44	2.41
-2	1	0	64.44	2.38
-2	1	0	63.27	2.40

-2	1	0	61.66	2.43
-2	-1	0	64.54	2.41
-2	-1	0	63.41	2.45
-2	-1	0	62.25	2.37
-2	-1	0	59.50	2.39
2	1	0	63.35	2.40
2	1	0	64.68	2.45
-3	-1	0	476.32	17.76
-3	-1	0	453.02	17.65
-3	-1	0	472.07	17.71
-3	-1	0	485.27	17.71
3	1	0	488.08	17.71
3	1	0	472.09	17.71
3	1	0	490.40	17.76
-4	-1	0	2.58	0.20
-4	-1	0	2.75	0.30
-4	-1	0	2.74	0.41
-4	-1	0	2.83	0.30
4	1	0	3.55	0.40
4	1	0	2.43	0.20
4	1	0	3.20	0.31
4	1	0	2.67	0.30
-5	-1	0	365.29	13.48
-5	-1	0	366.09	13.64
-5	-1	0	363.82	13.58
-5	-1	0	367.45	13.55
5	1	0	340.46	13.47
5	1	0	379.40	13.62
5	1	0	357.87	13.54
5	1	0	357.80	13.56
-6	-1	0	6.05	0.51
-6	-1	0	5.04	0.62
-6	-1	0	6.16	0.37
-6	-1	0	6.26	0.48
6	1	0	5.79	0.58
6	1	0	6.06	0.46
6	1	0	5.82	0.37
6	1	0	5.49	0.48
-7	-1	0	341.58	12.95
-7	-1	0	345.20	13.04
-7	-1	0	348.30	13.17
-7	-1	0	343.68	13.09
7	1	0	350.64	13.03
7	1	0	353.17	13.07

7	1	0	333.80	12.95
7	1	0	358.37	13.12
-8	-1	0	14.22	1.02
-8	-1	0	13.99	0.68
-8	-1	0	12.26	0.85
-8	-1	0	12.59	0.82
8	1	0	11.50	0.79
8	1	0	13.12	0.83
8	1	0	11.93	0.67
8	1	0	13.08	0.94
-9	-1	0	184.46	6.76
-9	-1	0	173.38	6.93
-9	-1	0	176.92	6.86
9	1	0	178.95	6.94
9	1	0	183.37	6.89
9	1	0	168.23	6.75
9	1	0	176.09	6.95
-10	-1	0	-0.28	0.35
-10	-1	0	0.27	0.22
-10	-1	0	0.35	0.40
-10	-1	0	0.96	0.67
10	1	0	-0.05	0.37
10	1	0	-0.07	0.20
10	1	0	0.02	0.33
10	1	0	0.57	0.50
-11	-1	0	148.28	5.57
-11	-1	0	140.34	5.68
-11	-1	0	138.05	5.78
-11	-1	0	141.14	5.84
11	1	0	150.36	5.78
11	1	0	136.31	5.56
11	1	0	140.80	5.72
11	1	0	154.03	5.67
-12	-1	0	4.72	0.79
-12	-1	0	5.34	0.75
-12	-1	0	5.68	0.59
12	1	0	4.85	0.78
12	1	0	4.20	0.56
12	1	0	4.73	0.66
12	1	0	4.96	0.76
-13	-1	0	77.58	3.45
-13	-1	0	77.71	3.27
-13	-1	0	76.39	3.11
-13	-1	0	75.43	3.42

13	1	0	77.80	3.32
13	1	0	72.53	3.12
13	1	0	71.60	3.18
-14	-1	0	4.17	0.78
-14	-1	0	6.25	1.02
-14	-1	0	3.59	0.93
-14	-1	0	4.83	0.88
14	1	0	5.97	0.86
14	1	0	4.82	0.66
-15	-1	0	76.59	3.50
-15	-1	0	78.42	3.63
-15	-1	0	79.97	3.47
-15	-1	0	79.87	3.74
15	1	0	76.25	3.48
15	1	0	83.74	3.42
-16	-1	0	-0.55	0.90
-16	-1	0	0.85	0.75
-16	-1	0	1.59	0.83
16	1	0	1.52	0.48
16	1	0	1.33	0.65
-17	-1	0	67.55	3.28
-17	-1	0	66.75	3.52
17	1	0	70.06	3.02
17	1	0	68.18	3.10
-18	-1	0	1.10	1.08
18	1	0	1.64	0.56
18	1	0	1.24	0.79
-19	-1	0	59.93	3.46
19	1	0	58.47	2.80
-20	-1	0	-0.68	1.22
20	1	0	0.15	0.67
21	1	0	38.86	2.27
21	-1	1	0.01	0.80
-21	1	-1	0.84	1.50
20	-1	1	2.72	0.75
-20	1	-1	2.11	1.39
19	-1	1	0.17	0.62
-19	1	-1	-1.16	1.13
18	-1	1	16.06	1.31
18	-1	1	15.97	1.59
-18	1	-1	15.57	2.15
17	-1	1	0.31	0.54
17	-1	1	0.45	0.78
-17	1	-1	0.86	0.99

16	-1	1	14.27	1.33
16	-1	1	15.69	1.17
-16	1	-1	15.33	1.75
-16	1	-1	15.10	1.76
15	-1	1	0.88	0.62
15	-1	1	1.07	0.43
-15	1	-1	0.29	0.95
-15	1	-1	0.87	0.58
-15	1	-1	1.84	0.87
-15	1	-1	1.57	0.52
14	-1	1	13.53	0.96
14	-1	1	12.94	1.16
-14	1	-1	13.59	0.99
-14	1	-1	12.39	1.04
-14	1	-1	14.95	1.42
-14	1	-1	11.55	1.39
13	-1	1	12.09	1.03
13	-1	1	11.94	1.05
13	-1	1	11.10	0.85
13	-1	1	10.81	1.06
-13	1	-1	11.54	1.25
-13	1	-1	11.76	1.23
-13	1	-1	9.58	0.96
12	-1	1	69.42	2.85
12	-1	1	69.15	2.80
12	-1	1	65.10	2.94
12	-1	1	66.97	2.92
-12	1	-1	66.18	3.01
-12	1	-1	69.55	3.10
-12	1	-1	70.15	2.79
-12	1	-1	60.51	2.79
11	-1	1	0.27	0.26
11	-1	1	0.69	0.40
11	-1	1	0.35	0.44
11	-1	1	0.23	0.40
-11	1	-1	1.37	0.33
-11	1	-1	1.16	0.41
-11	1	-1	0.47	0.51
-11	1	-1	0.45	0.46
10	-1	1	61.02	2.60
10	-1	1	61.19	2.58
10	-1	1	58.87	2.45
10	-1	1	60.91	2.52
-10	1	-1	53.15	2.45

-10	1	-1	63.70	2.46
-10	1	-1	57.52	2.65
9	-1	1	0.02	0.34
9	-1	1	0.22	0.32
9	-1	1	0.40	0.20
9	-1	1	-0.10	0.34
-9	1	-1	0.33	0.22
-9	1	-1	-0.24	0.31
-9	1	-1	0.19	0.39
-9	1	-1	0.28	0.30
8	-1	1	241.48	9.14
8	-1	1	250.39	9.07
8	-1	1	236.66	9.02
8	-1	1	242.55	9.15
-8	1	-1	243.39	9.17
-8	1	-1	215.80	9.00
-8	1	-1	249.88	9.03
-8	1	-1	233.46	9.16
7	-1	1	1.20	0.29
7	-1	1	0.34	0.28
7	-1	1	0.56	0.17
-7	1	-1	0.92	0.19
-7	1	-1	1.07	0.24
-7	1	-1	0.84	0.33
-7	1	-1	1.23	0.32
6	-1	1	173.21	6.59
6	-1	1	180.64	6.53
6	-1	1	179.02	6.62
6	-1	1	164.18	6.50
-6	1	-1	169.82	6.62
-6	1	-1	165.42	6.48
-6	1	-1	166.32	6.59
-6	1	-1	179.01	6.52
5	-1	1	4.09	0.31
5	-1	1	4.75	0.38
5	-1	1	4.99	0.46
5	-1	1	4.49	0.39
-5	1	-1	4.64	0.44
-5	1	-1	4.80	0.32
-5	1	-1	4.23	0.31
-5	1	-1	5.24	0.48
4	-1	1	186.97	7.59
4	-1	1	198.54	7.69
4	-1	1	208.15	7.65

4	-1	1	215.62	7.59
-4	1	-1	207.59	7.66
-4	1	-1	206.97	7.59
-4	1	-1	197.22	7.69
3	-1	1	50.29	1.95
3	-1	1	47.28	1.88
3	-1	1	45.83	1.84
-3	1	-1	49.01	1.85
-3	1	-1	47.18	1.89
-3	1	-1	47.86	1.95
2	-1	1	121.47	4.62
2	-1	1	125.13	4.65
-2	1	-1	122.78	4.62
-2	1	-1	123.12	4.71
-2	1	-1	124.94	4.66
-2	1	-1	125.08	4.60
-1	1	-1	1.54	0.82
-2	-1	1	224.20	8.03
-2	-1	1	214.85	7.91
-2	-1	1	185.82	7.89
-2	-1	1	207.28	7.86
2	1	-1	221.40	8.02
2	1	-1	218.25	7.91
-3	-1	1	0.18	0.07
-3	-1	1	0.32	0.21
-3	-1	1	0.19	0.12
-3	-1	1	0.19	0.10
3	1	-1	-0.02	0.11
3	1	-1	0.36	0.23
3	1	-1	0.22	0.07
-4	-1	1	267.31	10.05
-4	-1	1	272.39	10.26
-4	-1	1	263.97	10.04
-4	-1	1	271.18	10.12
4	1	-1	286.91	10.06
4	1	-1	265.31	10.23
4	1	-1	257.53	10.03
4	1	-1	273.64	10.12
-5	-1	1	0.77	0.35
-5	-1	1	1.00	0.23
-5	-1	1	1.33	0.19
-5	-1	1	1.33	0.25
5	1	-1	1.24	0.19
5	1	-1	1.00	0.33

5	1	-1	1.17	0.21
-6	-1	1	105.21	4.04
-6	-1	1	110.89	4.02
-6	-1	1	103.25	4.14
-6	-1	1	101.15	4.28
6	1	-1	104.46	4.01
6	1	-1	99.03	4.15
6	1	-1	110.17	4.04
6	1	-1	107.62	4.26
-7	-1	1	23.17	1.64
-7	-1	1	25.89	1.34
-7	-1	1	30.58	1.19
-7	-1	1	29.38	1.24
7	1	-1	29.48	1.23
7	1	-1	27.30	1.46
7	1	-1	26.62	1.18
7	1	-1	26.83	1.30
-8	-1	1	159.62	6.38
-8	-1	1	162.90	6.54
-8	-1	1	158.48	6.26
-8	-1	1	175.50	6.23
8	1	-1	165.16	6.25
8	1	-1	165.98	6.49
8	1	-1	167.51	6.36
8	1	-1	154.69	6.21
-9	-1	1	3.30	0.43
-9	-1	1	3.25	0.71
-9	-1	1	3.32	0.40
-9	-1	1	2.72	0.46
9	1	-1	3.27	0.38
9	1	-1	3.03	0.47
9	1	-1	3.19	0.49
9	1	-1	4.07	0.64
-10	-1	1	157.85	6.54
-10	-1	1	160.34	6.38
-10	-1	1	166.43	6.68
-10	-1	1	176.17	6.35
10	1	-1	159.99	6.34
10	1	-1	173.83	6.39
10	1	-1	163.78	6.64
10	1	-1	166.95	6.51
-11	-1	1	0.41	0.42
-11	-1	1	0.60	0.67
-11	-1	1	0.89	0.42

-11	-1	1	0.86	0.28
11	1	-1	0.16	0.39
11	1	-1	1.65	1.22
11	1	-1	1.03	0.27
-12	-1	1	261.05	9.46
-12	-1	1	239.76	9.77
-12	-1	1	244.95	9.71
-12	-1	1	234.33	9.48
12	1	-1	252.58	9.75
12	1	-1	247.70	9.63
12	1	-1	246.69	9.45
12	1	-1	260.28	9.49
-13	-1	1	0.57	0.81
-13	-1	1	0.00	0.33
-13	-1	1	-0.21	0.49
-13	-1	1	-0.22	0.55
13	1	-1	-0.12	0.29
13	1	-1	-0.32	0.45
13	1	-1	-0.56	0.47
13	1	-1	0.42	0.73
-14	-1	1	53.56	2.73
-14	-1	1	50.01	2.38
-14	-1	1	49.56	2.64
14	1	-1	53.24	2.31
14	1	-1	53.09	2.30
14	1	-1	53.14	2.75
-15	-1	1	3.36	1.08
-15	-1	1	2.17	1.05
-15	-1	1	1.99	0.93
15	1	-1	1.33	0.53
15	1	-1	1.93	1.01
15	1	-1	2.47	0.50
-16	-1	1	105.06	4.70
-16	-1	1	110.11	4.85
-16	-1	1	110.46	4.88
16	1	-1	111.45	4.45
16	1	-1	110.24	4.47
-17	-1	1	-0.23	1.05
-17	-1	1	-0.04	0.85
17	1	-1	0.58	0.65
17	1	-1	0.95	0.50
-18	-1	1	94.39	4.51
-18	-1	1	104.71	4.72
18	1	-1	103.75	4.24

18	1	-1	92.55	4.15
-19	-1	1	-0.56	1.05
19	1	-1	0.75	0.64
20	1	-1	74.53	3.40
-21	-1	1	-0.17	1.34
21	1	-1	0.18	0.76
-21	1	-2	-2.11	1.51
20	-1	2	-0.54	0.65
-20	1	-2	-0.44	1.43
19	-1	2	0.33	1.06
19	-1	2	-0.36	0.68
-19	1	-2	1.74	1.30
18	-1	2	1.63	0.67
18	-1	2	1.36	0.89
-18	1	-2	0.65	1.13
17	-1	2	-0.52	0.78
17	-1	2	0.46	0.53
-17	1	-2	-0.80	0.97
16	-1	2	-0.22	0.46
16	-1	2	1.71	0.75
-16	1	-2	1.46	1.09
-16	1	-2	0.65	0.92
15	-1	2	0.87	0.46
15	-1	2	2.33	0.69
-15	1	-2	2.21	0.94
-15	1	-2	0.28	0.86
14	-1	2	-0.65	0.54
14	-1	2	-0.19	0.37
-14	1	-2	-1.00	0.64
-14	1	-2	-0.88	0.75
13	-1	2	2.92	0.65
13	-1	2	2.79	0.59
13	-1	2	2.49	0.47
-13	1	-2	3.52	0.73
-13	1	-2	5.37	0.92
12	-1	2	1.67	0.50
12	-1	2	1.31	0.35
12	-1	2	1.37	0.53
-12	1	-2	1.41	0.55
-12	1	-2	2.53	0.73
-12	1	-2	1.55	0.36
11	-1	2	5.10	0.77
11	-1	2	5.47	0.80
11	-1	2	4.29	0.55

-11	1	-2	5.41	0.95
-11	1	-2	5.27	0.85
-11	1	-2	6.02	0.59
10	-1	2	-0.31	0.35
10	-1	2	0.18	0.24
10	-1	2	0.19	0.40
-10	1	-2	0.67	0.45
-10	1	-2	-0.01	0.24
-10	1	-2	-0.04	0.38
9	-1	2	59.89	2.66
9	-1	2	64.61	2.74
9	-1	2	60.28	2.54
-9	1	-2	64.34	2.77
-9	1	-2	59.89	2.70
-9	1	-2	66.53	2.55
8	-1	2	10.22	0.62
8	-1	2	11.35	0.83
8	-1	2	10.61	0.75
-8	1	-2	9.85	1.02
-8	1	-2	11.75	0.63
-8	1	-2	9.88	0.78
7	-1	2	0.52	0.34
7	-1	2	0.23	0.24
7	-1	2	0.46	0.17
-7	1	-2	0.40	0.17
-7	1	-2	0.43	0.28
-7	1	-2	0.48	0.34
6	-1	2	2.70	0.28
6	-1	2	2.05	0.40
6	-1	2	1.53	0.28
-6	1	-2	1.95	0.30
-6	1	-2	2.43	0.27
-6	1	-2	2.69	0.42
5	-1	2	2.18	0.24
5	-1	2	1.68	0.38
5	-1	2	1.43	0.24
-5	1	-2	2.02	0.23
-5	1	-2	1.59	0.28
-5	1	-2	1.45	0.35
4	-1	2	80.82	3.10
4	-1	2	75.77	3.11
4	-1	2	85.05	3.22
-4	1	-2	78.94	3.11
-4	1	-2	79.21	3.22

-4	1	-2	80.25	3.06
3	-1	2	19.20	0.95
3	-1	2	19.08	0.80
3	-1	2	19.42	0.84
-3	1	-2	19.99	0.97
-3	1	-2	18.71	0.84
-3	1	-2	19.36	0.80
2	-1	2	60.84	2.32
2	-1	2	59.87	2.41
2	-1	2	59.63	2.28
-2	1	-2	59.91	2.32
-2	1	-2	60.81	2.28
-2	1	-2	60.86	2.42
-2	1	-2	59.34	2.30
1	-1	2	12.09	0.67
1	-1	2	15.26	0.62
1	-1	2	14.77	0.58
-1	1	-2	14.93	0.62
-1	1	-2	14.96	0.70
-1	1	-2	14.86	0.68
-1	1	-2	15.10	0.58
0	-1	2	6.10	0.29
0	1	-2	6.10	0.40
0	1	-2	5.19	0.34
0	1	-2	5.64	0.23
0	1	-2	5.26	0.27
-1	-1	2	3.58	0.21
-1	-1	2	3.84	0.16
-1	-1	2	3.94	0.25
-1	-1	2	2.94	0.33
1	1	-2	3.55	0.25
1	1	-2	3.31	0.21
1	1	-2	3.59	0.37
-2	-1	2	67.47	2.43
-2	-1	2	66.18	2.68
-2	-1	2	62.72	2.47
2	1	-2	62.09	2.48
2	1	-2	63.86	2.66
2	1	-2	69.23	2.48
2	1	-2	62.47	2.43
-3	-1	2	202.89	7.98
-3	-1	2	214.53	8.20
-3	-1	2	230.23	7.94
-3	-1	2	202.30	7.91

3	1	-2	215.35	8.00
3	1	-2	219.39	7.94
3	1	-2	216.56	8.18
3	1	-2	203.09	7.91
-4	-1	2	128.38	4.73
-4	-1	2	123.09	5.02
-4	-1	2	123.15	4.70
-4	-1	2	128.36	4.73
4	1	-2	122.47	4.72
4	1	-2	123.12	5.00
4	1	-2	132.60	4.83
-5	-1	2	34.71	1.49
-5	-1	2	34.32	1.40
-5	-1	2	35.27	1.34
-5	-1	2	34.84	1.38
-5	-1	2	33.40	1.70
5	1	-2	31.71	1.41
5	1	-2	35.11	1.70
5	1	-2	35.90	1.49
-6	-1	2	0.24	0.13
-6	-1	2	0.42	0.48
-6	-1	2	0.16	0.21
-6	-1	2	0.22	0.13
6	1	-2	0.38	0.38
6	1	-2	0.07	0.13
6	1	-2	0.09	0.21
6	1	-2	0.09	0.11
-7	-1	2	91.58	3.37
-7	-1	2	84.20	3.51
-7	-1	2	85.24	3.75
-7	-1	2	84.49	3.34
7	1	-2	82.71	3.35
7	1	-2	88.95	3.50
7	1	-2	88.48	3.34
7	1	-2	90.93	3.73
-8	-1	2	28.86	1.20
-8	-1	2	28.38	1.64
-8	-1	2	26.77	1.39
-8	-1	2	25.65	1.17
8	1	-2	24.82	1.17
8	1	-2	28.00	1.18
8	1	-2	27.94	1.35
8	1	-2	28.15	1.60
-9	-1	2	87.27	3.55

-9	-1	2	92.73	3.79
-9	-1	2	88.67	3.98
-9	-1	2	98.02	3.59
9	1	-2	83.95	4.14
9	1	-2	91.36	3.57
9	1	-2	94.66	3.56
9	1	-2	93.86	3.75
-10	-1	2	1.47	0.32
-10	-1	2	1.35	0.71
-10	-1	2	0.66	0.40
-10	-1	2	1.14	0.26
10	1	-2	1.66	0.67
10	1	-2	1.55	0.31
10	1	-2	1.20	0.40
10	1	-2	1.28	0.27
-11	-1	2	79.28	3.66
-11	-1	2	83.95	3.19
-11	-1	2	74.39	3.15
-11	-1	2	76.67	3.42
11	1	-2	82.40	3.16
11	1	-2	76.89	3.36
11	1	-2	77.14	3.17
11	1	-2	83.40	3.62
-12	-1	2	4.11	0.67
-12	-1	2	3.46	0.68
-12	-1	2	3.58	0.94
-12	-1	2	4.76	0.55
12	1	-2	3.65	0.54
12	1	-2	3.64	0.57
12	1	-2	3.80	0.61
12	1	-2	4.09	0.92
-13	-1	2	70.68	3.20
-13	-1	2	64.77	2.85
-13	-1	2	68.83	2.87
-13	-1	2	71.42	3.43
13	1	-2	75.87	3.43
13	1	-2	70.27	2.83
13	1	-2	65.14	2.85
13	1	-2	67.62	3.12
-14	-1	2	2.20	0.70
-14	-1	2	2.16	1.01
-14	-1	2	2.07	0.55
14	1	-2	3.17	1.05
14	1	-2	1.87	0.65

14	1	-2	1.80	0.45
-15	-1	2	19.56	1.31
-15	-1	2	21.55	2.00
-15	-1	2	22.77	2.46
15	1	-2	22.75	1.23
15	1	-2	21.16	2.06
-16	-1	2	-0.60	0.60
-16	-1	2	0.19	0.74
-16	-1	2	-0.40	1.05
16	1	-2	1.09	1.18
16	1	-2	0.39	0.41
-17	-1	2	52.75	3.13
-17	-1	2	56.17	3.04
17	1	-2	53.14	2.49
17	1	-2	56.06	3.25
-18	-1	2	0.43	0.97
-18	-1	2	0.17	1.18
18	1	-2	0.23	0.55
-19	-1	2	38.99	2.75
19	1	-2	40.00	2.13
-20	-1	2	0.07	1.13
20	1	-2	1.11	0.65
-21	-1	2	46.27	3.35
21	1	-2	47.38	2.52
20	-1	3	12.08	1.45
-20	1	-3	8.29	1.75
19	-1	3	-0.28	1.05
19	-1	3	0.89	0.66
-19	1	-3	0.24	1.27
18	-1	3	8.02	1.23
18	-1	3	7.06	0.93
-18	1	-3	8.40	1.46
17	-1	3	0.52	0.56
17	-1	3	-0.03	0.87
-17	1	-3	-0.82	1.02
16	-1	3	36.76	2.14
16	-1	3	36.67	1.91
-16	1	-3	34.00	2.41
-16	1	-3	33.56	2.54
15	-1	3	0.03	0.71
15	-1	3	0.63	0.44
-15	1	-3	0.45	0.85
-15	1	-3	0.73	1.00
14	-1	3	0.55	0.63

14	-1	3	0.65	0.41
-14	1	-3	1.12	0.69
-14	1	-3	1.79	0.88
13	-1	3	21.22	1.43
13	-1	3	22.01	1.51
13	-1	3	22.06	1.25
-13	1	-3	25.47	1.81
-13	1	-3	21.68	1.61
12	-1	3	20.33	1.40
12	-1	3	19.22	1.11
12	-1	3	20.32	1.30
-12	1	-3	18.36	1.44
-12	1	-3	20.85	1.10
-12	1	-3	20.38	1.55
11	-1	3	0.47	0.41
11	-1	3	1.18	0.53
11	-1	3	1.09	0.34
-11	1	-3	1.21	0.61
-11	1	-3	0.79	0.52
-11	1	-3	0.88	0.28
10	-1	3	11.73	0.76
10	-1	3	12.21	0.94
10	-1	3	10.48	1.01
-10	1	-3	13.01	0.75
-10	1	-3	12.90	1.14
-10	1	-3	11.27	0.98
9	-1	3	0.19	0.21
9	-1	3	0.02	0.31
9	-1	3	0.11	0.40
-9	1	-3	0.32	0.48
-9	1	-3	0.45	0.22
-9	1	-3	-0.06	0.35
8	-1	3	2.08	0.37
8	-1	3	2.21	0.35
8	-1	3	2.26	0.52
-8	1	-3	2.58	0.54
-8	1	-3	1.77	0.26
-8	1	-3	2.36	0.43
7	-1	3	4.21	0.50
7	-1	3	4.32	0.41
7	-1	3	5.13	0.65
-7	1	-3	5.36	0.40
-7	1	-3	4.64	0.56
-7	1	-3	5.35	0.72

6	-1	3	65.59	2.64
6	-1	3	67.04	2.69
6	-1	3	71.31	2.86
-6	1	-3	66.89	2.88
-6	1	-3	65.12	2.72
-6	1	-3	66.56	2.63
5	-1	3	11.75	0.61
5	-1	3	12.53	0.86
5	-1	3	12.40	0.66
-5	1	-3	13.11	0.85
-5	1	-3	11.83	0.60
-5	1	-3	12.84	0.71
4	-1	3	4.85	0.58
4	-1	3	4.99	0.36
4	-1	3	4.71	0.33
-4	1	-3	5.07	0.31
-4	1	-3	4.85	0.39
-4	1	-3	5.10	0.54
3	-1	3	0.07	0.13
3	-1	3	0.17	0.09
3	-1	3	0.04	0.11
3	-1	3	0.11	0.22
-3	1	-3	0.15	0.15
-3	1	-3	0.07	0.12
-3	1	-3	0.03	0.23
2	-1	3	76.13	2.97
2	-1	3	79.05	3.05
2	-1	3	81.52	3.00
2	-1	3	78.08	3.14
-2	1	-3	78.13	3.00
-2	1	-3	76.41	2.96
-2	1	-3	80.11	3.06
-2	1	-3	77.89	3.15
1	-1	3	65.51	2.55
1	-1	3	65.95	2.43
1	-1	3	61.82	2.55
1	-1	3	59.38	2.39
-1	1	-3	64.31	2.43
-1	1	-3	65.41	2.56
-1	1	-3	62.63	2.55
-1	1	-3	62.16	2.39
0	-1	3	18.69	0.75
0	-1	3	16.34	0.92
0	-1	3	18.20	0.71

0	-1	3	17.10	0.84
0	1	-3	17.35	0.70
0	1	-3	18.31	0.71
0	1	-3	19.23	0.75
0	1	-3	17.37	0.85
0	1	-3	17.27	0.93
-1	-1	3	52.68	2.14
-1	-1	3	56.24	2.09
-1	-1	3	55.33	2.03
-1	-1	3	55.25	2.31
1	1	-3	53.06	2.14
1	1	-3	54.13	2.08
1	1	-3	51.49	2.30
-2	-1	3	21.69	0.83
-2	-1	3	21.93	1.17
-2	-1	3	21.08	0.90
-2	-1	3	19.89	0.91
2	1	-3	21.92	1.17
2	1	-3	21.53	0.89
2	1	-3	21.18	0.83
2	1	-3	21.65	0.92
-3	-1	3	88.20	3.44
-3	-1	3	86.16	3.69
-3	-1	3	83.74	3.38
-3	-1	3	95.17	3.44
-3	-1	3	89.08	3.36
3	1	-3	85.54	3.35
3	1	-3	91.75	3.70
3	1	-3	95.34	3.40
-4	-1	3	64.97	2.65
-4	-1	3	69.16	2.57
-4	-1	3	66.03	2.55
-4	-1	3	68.07	2.67
4	1	-3	66.51	2.93
4	1	-3	69.06	2.57
4	1	-3	65.57	2.56
4	1	-3	69.06	2.67
-5	-1	3	3.79	0.26
-5	-1	3	2.63	0.54
-5	-1	3	3.99	0.39
5	1	-3	3.38	0.55
5	1	-3	3.59	0.38
5	1	-3	3.26	0.26
-6	-1	3	47.58	2.11

-6	-1	3	50.01	2.43
-6	-1	3	54.09	1.99
6	1	-3	48.45	1.97
6	1	-3	51.06	2.12
6	1	-3	49.63	2.39
-7	-1	3	47.44	2.10
-7	-1	3	48.00	2.39
-7	-1	3	49.30	1.98
7	1	-3	49.15	2.09
7	1	-3	46.36	1.93
7	1	-3	50.96	2.40
-8	-1	3	11.10	0.81
-8	-1	3	13.56	1.23
-8	-1	3	12.04	0.63
8	1	-3	11.22	0.62
8	1	-3	12.29	0.82
8	1	-3	12.92	1.24
-9	-1	3	-0.41	0.28
-9	-1	3	-0.15	0.61
-9	-1	3	-0.17	0.19
9	1	-3	-0.01	0.58
9	1	-3	0.25	0.20
9	1	-3	0.30	0.33
-10	-1	3	4.07	0.87
-10	-1	3	4.56	0.46
-10	-1	3	4.06	0.50
-10	-1	3	4.60	0.73
10	1	-3	4.91	0.86
10	1	-3	3.50	0.54
10	1	-3	3.37	0.41
-11	-1	3	2.69	0.36
-11	-1	3	4.12	0.65
-11	-1	3	4.27	0.90
11	1	-3	4.58	0.94
11	1	-3	2.75	0.50
11	1	-3	3.84	0.46
-12	-1	3	13.19	1.12
-12	-1	3	13.05	0.83
-12	-1	3	12.87	1.60
12	1	-3	14.23	1.61
12	1	-3	12.21	0.78
12	1	-3	11.07	1.06
-13	-1	3	1.25	0.41
-13	-1	3	0.67	0.54

-13	-1	3	1.80	0.98
-13	-1	3	0.52	0.46
13	1	-3	0.96	0.36
13	1	-3	1.22	0.95
-14	-1	3	2.50	1.06
-14	-1	3	2.17	0.67
14	1	-3	1.50	0.38
14	1	-3	2.06	1.15
-15	-1	3	25.95	1.81
-15	-1	3	20.10	2.11
15	1	-3	27.56	2.33
15	1	-3	24.33	1.31
-16	-1	3	15.75	2.14
-16	-1	3	16.46	1.71
16	1	-3	19.64	1.18
16	1	-3	18.44	2.28
-17	-1	3	3.44	1.31
-17	-1	3	5.30	1.06
17	1	-3	3.03	0.60
17	1	-3	0.42	1.41
-18	-1	3	7.09	1.23
-18	-1	3	9.35	1.61
18	1	-3	7.62	1.05
-19	-1	3	2.64	1.09
-19	-1	3	2.37	1.51
19	1	-3	1.55	0.63
-20	-1	3	3.09	1.30
20	1	-3	5.12	0.86
-21	-1	3	1.72	1.38
21	1	-3	1.83	0.79
20	-1	4	0.41	0.77
-20	1	-4	-1.85	1.53
19	-1	4	33.99	2.61
19	-1	4	38.31	2.15
-19	1	-4	37.40	3.07
18	-1	4	0.80	0.58
18	-1	4	0.54	1.08
-18	1	-4	1.78	1.23
17	-1	4	22.91	1.56
17	-1	4	23.13	1.98
-17	1	-4	31.88	4.09
-17	1	-4	19.69	2.27
16	-1	4	0.89	0.53
16	-1	4	0.58	0.82

-16	1	-4	1.45	0.99
-16	1	-4	0.72	1.25
15	-1	4	36.86	1.81
15	-1	4	31.87	2.11
-15	1	-4	36.42	2.51
-15	1	-4	30.72	2.27
14	-1	4	0.26	0.44
14	-1	4	-0.41	0.66
-14	1	-4	-0.21	0.72
-14	1	-4	-2.05	0.93
13	-1	4	29.84	1.53
13	-1	4	30.57	1.72
13	-1	4	29.61	1.86
-13	1	-4	31.25	2.08
-13	1	-4	29.62	1.91
12	-1	4	18.50	1.11
12	-1	4	20.33	1.31
12	-1	4	18.45	1.41
-12	1	-4	18.58	1.43
-12	1	-4	19.41	1.59
11	-1	4	47.57	2.19
11	-1	4	48.47	2.45
11	-1	4	52.64	2.34
-11	1	-4	55.33	2.62
-11	1	-4	49.59	2.43
-11	1	-4	50.30	2.16
10	-1	4	5.15	0.75
10	-1	4	4.11	0.56
10	-1	4	5.31	0.62
-10	1	-4	5.98	0.88
-10	1	-4	4.81	0.66
-10	1	-4	5.78	0.53
9	-1	4	16.18	0.91
9	-1	4	18.19	1.04
9	-1	4	16.87	1.21
-9	1	-4	16.34	1.10
-9	1	-4	18.10	1.35
-9	1	-4	17.31	0.89
8	-1	4	5.90	0.51
8	-1	4	7.05	0.85
8	-1	4	7.07	0.63
-8	1	-4	7.05	0.70
-8	1	-4	6.60	0.50
-8	1	-4	7.82	0.93

7	-1	4	60.28	2.36
7	-1	4	56.43	2.59
7	-1	4	58.63	2.40
-7	1	-4	55.53	2.44
-7	1	-4	57.79	2.65
-7	1	-4	59.01	2.33
6	-1	4	0.33	0.35
6	-1	4	0.12	0.22
6	-1	4	0.22	0.16
-6	1	-4	-0.07	0.15
-6	1	-4	0.14	0.39
-6	1	-4	0.38	0.23
5	-1	4	96.57	4.01
5	-1	4	96.67	3.77
5	-1	4	98.35	3.80
-5	1	-4	100.14	3.76
-5	1	-4	98.23	3.83
4	-1	4	17.82	0.83
4	-1	4	19.36	0.87
4	-1	4	19.15	0.80
4	-1	4	18.89	1.07
-4	1	-4	19.39	1.10
-4	1	-4	18.63	0.89
-4	1	-4	19.39	0.83
-4	1	-4	18.31	0.85
3	-1	4	56.31	2.41
3	-1	4	57.48	2.20
3	-1	4	55.54	2.21
-3	1	-4	54.13	2.39
-3	1	-4	56.31	2.21
-3	1	-4	56.11	2.16
-3	1	-4	54.62	2.26
2	-1	4	1.16	0.16
2	-1	4	1.19	0.27
2	-1	4	1.28	0.18
-2	1	-4	1.30	0.31
-2	1	-4	1.48	0.16
-2	1	-4	1.37	0.18
-2	1	-4	1.36	0.33
1	-1	4	11.73	0.75
1	-1	4	11.14	0.51
1	-1	4	13.29	0.57
1	-1	4	12.02	0.74
-1	1	-4	11.64	0.51

-1	1	-4	11.99	0.56
-1	1	-4	11.89	0.78
-1	1	-4	11.66	0.53
-1	1	-4	11.73	0.72
0	-1	4	85.84	3.19
0	-1	4	87.26	3.23
0	-1	4	82.56	3.45
0	-1	4	79.58	3.35
0	1	-4	86.56	3.23
0	1	-4	85.65	3.19
0	1	-4	81.84	3.46
0	1	-4	87.40	3.37
-1	-1	4	13.83	0.89
-1	-1	4	13.05	0.54
-1	-1	4	13.26	0.60
-1	-1	4	12.15	0.69
1	1	-4	11.13	0.88
1	1	-4	13.18	0.53
1	1	-4	12.06	0.69
1	1	-4	12.66	0.58
-2	-1	4	10.13	0.84
-2	-1	4	10.27	0.57
-2	-1	4	10.08	0.44
-2	-1	4	9.57	0.50
2	1	-4	10.28	0.57
2	1	-4	9.56	0.83
2	1	-4	10.09	0.43
2	1	-4	10.09	0.50
-3	-1	4	4.40	0.24
-3	-1	4	3.80	0.33
-3	-1	4	4.46	0.33
-3	-1	4	3.75	0.32
-3	-1	4	3.70	0.71
3	1	-4	4.10	0.23
3	1	-4	3.96	0.33
3	1	-4	5.33	0.70
-4	-1	4	3.18	0.35
-4	-1	4	3.33	0.33
-4	-1	4	2.79	0.26
-4	-1	4	3.04	0.57
-4	-1	4	3.00	0.22
4	1	-4	3.09	0.21
4	1	-4	2.77	0.53
4	1	-4	3.33	0.29

-5	-1	4	47.63	1.92
-5	-1	4	43.12	1.78
-5	-1	4	43.59	1.74
-5	-1	4	46.80	1.90
-5	-1	4	46.51	2.26
5	1	-4	46.30	2.26
-6	-1	4	0.58	0.48
-6	-1	4	0.16	0.12
-6	-1	4	0.54	0.23
6	1	-4	0.15	0.23
6	1	-4	0.05	0.12
-7	-1	4	26.36	1.25
-7	-1	4	23.54	1.11
-7	-1	4	25.13	1.65
7	1	-4	23.69	1.04
7	1	-4	22.95	1.59
-8	-1	4	0.02	0.54
-8	-1	4	0.13	0.28
-8	-1	4	0.15	0.17
8	1	-4	0.30	0.16
8	1	-4	-0.34	0.58
8	1	-4	-0.05	0.28
-9	-1	4	0.13	0.62
-9	-1	4	0.56	0.34
-9	-1	4	0.00	0.20
9	1	-4	-0.01	0.33
9	1	-4	0.13	0.18
9	1	-4	-0.46	0.65
-10	-1	4	9.46	0.63
-10	-1	4	7.53	0.87
-10	-1	4	7.32	1.36
10	1	-4	10.07	1.38
10	1	-4	9.40	0.59
10	1	-4	9.26	0.84
-11	-1	4	1.73	0.50
-11	-1	4	2.48	0.88
-11	-1	4	1.80	0.32
11	1	-4	1.19	0.28
11	1	-4	1.85	0.83
11	1	-4	0.88	0.46
-12	-1	4	0.10	0.45
-12	-1	4	0.39	0.29
-12	-1	4	1.09	0.88
12	1	-4	0.19	0.26

12	1	-4	1.60	0.93
12	1	-4	-0.58	0.46
-13	-1	4	3.97	0.72
-13	-1	4	3.99	0.51
-13	-1	4	2.56	1.06
13	1	-4	2.49	0.39
13	1	-4	4.13	1.08
-14	-1	4	-0.65	0.53
-14	-1	4	-0.27	1.03
14	1	-4	-0.42	1.10
14	1	-4	0.38	0.33
-15	-1	4	2.78	0.80
-15	-1	4	2.00	1.13
15	1	-4	3.57	1.33
15	1	-4	2.72	0.46
-16	-1	4	4.54	0.95
-16	-1	4	3.08	1.30
16	1	-4	5.11	1.43
16	1	-4	3.73	0.56
-17	-1	4	-0.40	0.90
-17	-1	4	2.38	1.31
17	1	-4	3.43	1.58
-18	-1	4	-0.60	0.93
-18	-1	4	-1.50	1.36
18	1	-4	0.86	0.52
-19	-1	4	1.49	1.11
-19	-1	4	1.69	1.57
19	1	-4	0.43	0.58
-20	-1	4	1.93	1.15
20	1	-4	1.60	0.69
-21	-1	4	1.99	1.39
21	1	-4	-0.35	0.72
20	-1	5	37.28	2.31
-20	1	-5	40.08	3.38
19	-1	5	0.05	0.71
-19	1	-5	2.06	1.44
18	-1	5	55.47	2.77
18	-1	5	60.34	3.28
-18	1	-5	54.24	3.55
17	-1	5	0.35	1.01
17	-1	5	0.54	0.58
-17	1	-5	-0.86	1.12
16	-1	5	86.01	3.93
16	-1	5	81.52	3.53

-16	1	-5	72.83	3.95
15	-1	5	7.15	0.95
15	-1	5	6.96	1.08
-15	1	-5	5.41	1.37
-15	1	-5	4.67	1.10
14	-1	5	40.32	2.37
14	-1	5	40.74	1.99
-14	1	-5	41.71	2.65
-14	1	-5	39.83	2.40
13	-1	5	1.18	0.74
13	-1	5	1.50	0.58
13	-1	5	1.22	0.41
-13	1	-5	0.34	0.68
-13	1	-5	1.30	1.01
12	-1	5	63.97	3.01
12	-1	5	60.51	2.79
12	-1	5	62.89	2.68
-12	1	-5	61.76	2.93
-12	1	-5	60.87	3.14
11	-1	5	1.35	0.37
11	-1	5	1.59	0.52
11	-1	5	1.98	0.69
-11	1	-5	1.50	0.80
-11	1	-5	1.94	0.37
-11	1	-5	1.42	0.59
10	-1	5	65.91	2.82
10	-1	5	68.49	3.14
10	-1	5	67.50	2.91
-10	1	-5	68.46	3.27
-10	1	-5	64.51	2.97
-10	1	-5	73.52	2.81
9	-1	5	0.82	0.49
9	-1	5	-0.23	0.32
9	-1	5	-0.04	0.22
-9	1	-5	0.63	0.59
-9	1	-5	-0.72	0.36
-9	1	-5	0.00	0.24
8	-1	5	181.56	7.33
8	-1	5	180.96	7.07
8	-1	5	176.79	7.02
-8	1	-5	189.99	7.01
-8	1	-5	196.54	7.42
-8	1	-5	177.65	7.11
7	-1	5	11.89	0.68

7	-1	5	11.70	0.99
7	-1	5	10.92	0.72
-7	1	-5	12.15	1.10
-7	1	-5	11.09	0.65
-7	1	-5	12.03	0.81
6	-1	5	169.41	6.58
6	-1	5	155.41	6.69
6	-1	5	171.97	6.87
-6	1	-5	183.46	6.94
-6	1	-5	182.98	6.57
-6	1	-5	176.15	6.65
5	-1	5	2.33	0.48
5	-1	5	2.59	0.31
5	-1	5	2.26	0.36
-5	1	-5	2.28	0.29
-5	1	-5	3.17	0.53
-5	1	-5	2.25	0.34
4	-1	5	93.66	3.54
4	-1	5	94.50	3.55
4	-1	5	91.87	3.80
4	-1	5	85.65	3.52
-4	1	-5	94.20	3.84
-4	1	-5	90.72	3.61
-4	1	-5	91.27	3.53
3	-1	5	34.24	1.48
3	-1	5	33.57	1.65
3	-1	5	33.09	1.39
3	-1	5	35.26	1.42
-3	1	-5	33.29	1.69
-3	1	-5	33.84	1.58
-3	1	-5	34.82	1.38
-3	1	-5	35.07	1.43
2	-1	5	10.99	0.71
2	-1	5	10.90	0.78
2	-1	5	11.23	0.55
2	-1	5	9.76	0.51
-2	1	-5	10.62	0.77
-2	1	-5	10.19	0.50
-2	1	-5	10.85	0.55
-2	1	-5	10.70	0.78
1	-1	5	8.93	0.47
1	-1	5	8.11	0.69
1	-1	5	8.14	0.69
-1	1	-5	9.12	0.77

-1	1	-5	9.00	0.46
-1	1	-5	8.70	0.42
-1	1	-5	8.47	0.43
-1	1	-5	8.23	0.67
0	-1	5	842.82	35.10
0	-1	5	970.83	34.85
0	-1	5	924.87	35.09
0	1	-5	972.33	35.02
0	1	-5	972.48	34.85
0	1	-5	961.99	35.15
-1	-1	5	161.08	6.45
-1	-1	5	170.72	6.33
-1	-1	5	172.25	6.28
1	1	-5	166.68	6.31
1	1	-5	170.14	6.45
1	1	-5	167.75	6.66
1	1	-5	166.29	6.27
-2	-1	5	175.71	6.34
-2	-1	5	164.16	6.27
-2	-1	5	163.46	6.42
2	1	-5	170.14	6.71
2	1	-5	164.29	6.32
2	1	-5	169.97	6.41
2	1	-5	167.81	6.27
-3	-1	5	29.60	1.68
-3	-1	5	31.77	1.23
-3	-1	5	32.28	1.31
-3	-1	5	30.21	1.32
-3	-1	5	30.14	1.34
3	1	-5	28.61	1.70
3	1	-5	33.42	1.34
3	1	-5	31.15	1.22
3	1	-5	29.12	1.36
-4	-1	5	48.98	2.40
-4	-1	5	51.18	2.02
-4	-1	5	49.29	1.92
-4	-1	5	50.58	2.03
-4	-1	5	46.23	1.98
4	1	-5	49.41	2.45
4	1	-5	50.34	1.98
4	1	-5	49.14	1.91
-5	-1	5	8.10	0.45
-5	-1	5	8.53	1.01
-5	-1	5	8.48	0.43

-5	-1	5	7.44	0.61
-5	-1	5	8.41	0.57
5	1	-5	7.60	0.99
5	1	-5	7.70	0.46
5	1	-5	8.27	0.47
-6	-1	5	15.82	0.74
-6	-1	5	16.89	0.91
-6	-1	5	16.12	0.88
-6	-1	5	17.26	1.37
6	1	-5	15.74	1.32
6	1	-5	15.46	0.72
-7	-1	5	3.18	0.51
-7	-1	5	2.88	0.71
-7	-1	5	3.69	0.34
-7	-1	5	3.93	0.53
7	1	-5	3.75	0.80
7	1	-5	3.14	0.31
7	1	-5	4.53	0.56
-8	-1	5	141.46	6.15
-8	-1	5	149.39	5.59
-8	-1	5	144.20	5.77
8	1	-5	152.90	6.21
8	1	-5	150.81	5.78
8	1	-5	140.95	5.56
-9	-1	5	21.04	1.75
-9	-1	5	21.65	1.28
-9	-1	5	23.07	1.06
9	1	-5	21.32	1.02
9	1	-5	22.44	1.81
9	1	-5	23.95	1.29
-10	-1	5	32.69	2.16
-10	-1	5	32.63	1.43
-10	-1	5	30.54	1.67
10	1	-5	31.03	1.65
10	1	-5	31.56	1.38
10	1	-5	31.80	2.17
-11	-1	5	1.86	0.51
-11	-1	5	3.05	0.92
-11	-1	5	2.02	0.35
11	1	-5	1.72	0.50
11	1	-5	1.52	0.31
11	1	-5	2.79	0.98
-12	-1	5	23.51	2.09
-12	-1	5	23.30	1.50

-12	-1	5	24.19	1.21
12	1	-5	24.15	1.46
12	1	-5	21.86	1.14
12	1	-5	23.50	2.09
-13	-1	5	0.30	0.51
-13	-1	5	0.17	0.35
-13	-1	5	-0.64	1.09
13	1	-5	-0.28	0.28
13	1	-5	1.54	1.13
-14	-1	5	4.14	1.25
-14	-1	5	4.65	0.78
14	1	-5	5.07	1.38
14	1	-5	3.93	0.63
-15	-1	5	4.74	0.90
-15	-1	5	2.99	1.24
15	1	-5	4.30	0.69
15	1	-5	5.12	1.41
-16	-1	5	21.37	2.39
-16	-1	5	20.04	1.77
16	1	-5	21.15	2.63
16	1	-5	18.29	1.19
-17	-1	5	0.39	0.89
-17	-1	5	0.35	1.38
17	1	-5	0.61	0.48
17	1	-5	6.66	1.78
-18	-1	5	2.78	1.11
-18	-1	5	2.95	1.58
18	1	-5	2.62	0.61
-19	-1	5	-0.79	1.59
-19	-1	5	-0.98	1.04
19	1	-5	0.24	0.56
-20	-1	5	9.38	1.56
20	1	-5	12.14	1.28
-21	-1	5	0.25	2.35
21	1	-5	0.37	0.76
19	-1	6	41.53	3.10
19	-1	6	40.39	2.39
-19	1	-6	41.76	3.30
18	-1	6	0.16	0.73
18	-1	6	-0.23	1.19
-18	1	-6	-1.11	1.27
17	-1	6	58.01	3.32
17	-1	6	61.62	2.86
-17	1	-6	55.14	3.44

16	-1	6	2.38	0.69
16	-1	6	2.00	1.04
-16	1	-6	1.61	1.06
15	-1	6	80.56	3.81
15	-1	6	81.95	3.45
-15	1	-6	71.66	3.85
14	-1	6	5.32	0.72
14	-1	6	7.45	1.38
-14	1	-6	5.80	1.02
-14	1	-6	6.78	1.29
13	-1	6	84.49	3.64
13	-1	6	71.08	3.75
13	-1	6	90.04	3.94
-13	1	-6	84.81	4.13
-13	1	-6	87.26	3.84
12	-1	6	3.63	0.55
12	-1	6	4.47	0.67
12	-1	6	3.42	0.87
-12	1	-6	4.58	0.81
-12	1	-6	3.82	1.01
11	-1	6	117.92	4.69
11	-1	6	121.92	5.04
11	-1	6	115.08	4.77
-11	1	-6	114.34	4.86
-11	1	-6	121.77	4.65
-11	1	-6	111.32	5.11
10	-1	6	0.55	0.32
10	-1	6	0.87	0.44
10	-1	6	0.18	0.59
-10	1	-6	-0.37	0.73
-10	1	-6	0.91	0.29
-10	1	-6	0.45	0.49
9	-1	6	100.78	4.06
9	-1	6	96.27	4.01
9	-1	6	100.12	4.36
-9	1	-6	106.21	4.50
-9	1	-6	99.42	4.14
-9	1	-6	103.21	3.99
8	-1	6	4.95	0.78
8	-1	6	4.37	0.58
8	-1	6	3.22	0.47
-8	1	-6	2.64	0.35
-8	1	-6	4.00	0.87
-8	1	-6	3.82	0.52

7	-1	6	298.85	11.66
7	-1	6	304.89	11.67
7	-1	6	300.17	11.98
-7	1	-6	311.75	11.73
-7	1	-6	315.54	12.06
-7	1	-6	326.38	11.64
6	-1	6	0.04	0.44
6	-1	6	0.05	0.18
-6	1	-6	0.07	0.17
-6	1	-6	0.25	0.25
-6	1	-6	-0.27	0.46
5	-1	6	167.50	6.25
5	-1	6	157.68	6.27
5	-1	6	166.09	6.29
5	-1	6	165.71	6.60
-5	1	-6	160.85	6.33
-5	1	-6	168.06	6.64
-5	1	-6	163.38	6.32
-5	1	-6	171.51	6.27
4	-1	6	2.72	0.37
4	-1	6	2.72	0.32
4	-1	6	3.20	0.55
4	-1	6	3.08	0.43
-4	1	-6	3.07	0.30
-4	1	-6	3.44	0.56
-4	1	-6	3.09	0.49
-4	1	-6	2.79	0.41
3	-1	6	284.03	11.19
3	-1	6	301.06	10.92
3	-1	6	295.77	11.03
3	-1	6	276.00	10.90
-3	1	-6	294.57	10.93
-3	1	-6	293.36	11.13
-3	1	-6	296.75	11.23
-3	1	-6	292.04	10.89
2	-1	6	3.12	0.33
2	-1	6	3.55	0.48
2	-1	6	3.23	0.58
-2	1	-6	3.84	0.58
-2	1	-6	3.75	0.28
-2	1	-6	3.07	0.33
-2	1	-6	3.24	0.59
-2	1	-6	3.31	0.33
1	-1	6	590.49	22.78

1	-1	6	618.74	22.54
-1	1	-6	579.73	22.53
-1	1	-6	625.19	22.80
-1	1	-6	593.44	22.50
-1	1	-6	621.63	22.54
-1	1	-6	620.46	22.87
0	-1	6	262.96	10.42
0	-1	6	278.30	10.19
0	-1	6	281.76	10.22
0	-1	6	254.48	10.49
0	1	-6	268.02	10.18
0	1	-6	280.49	10.21
0	1	-6	274.38	10.58
0	1	-6	283.02	10.43
-1	-1	6	448.43	16.58
-1	-1	6	427.25	16.76
-1	-1	6	452.83	16.92
-1	-1	6	431.48	16.53
1	1	-6	447.94	16.56
1	1	-6	453.82	16.98
1	1	-6	435.60	16.52
1	1	-6	464.23	16.75
-2	-1	6	5.25	0.40
-2	-1	6	4.78	0.74
-2	-1	6	6.17	0.34
-2	-1	6	5.56	0.56
2	1	-6	5.53	0.32
2	1	-6	5.79	0.54
2	1	-6	5.40	0.39
2	1	-6	5.33	0.86
-3	-1	6	148.80	5.63
-3	-1	6	140.76	5.68
-3	-1	6	139.45	6.01
-3	-1	6	148.03	5.62
-3	-1	6	149.88	5.54
3	1	-6	146.67	6.37
3	1	-6	155.84	5.68
3	1	-6	145.72	5.53
3	1	-6	151.47	5.62
-4	-1	6	6.10	0.47
-4	-1	6	7.04	0.99
-4	-1	6	6.65	0.50
-4	-1	6	7.43	0.38
-4	-1	6	6.64	0.49

4	1	-6	6.26	0.50
4	1	-6	7.32	1.06
4	1	-6	6.01	0.36
-5	-1	6	217.46	9.03
-5	-1	6	234.93	8.49
-5	-1	6	225.55	8.61
-5	-1	6	234.51	8.61
-5	-1	6	212.09	8.52
5	1	-6	234.66	8.53
5	1	-6	217.18	8.47
5	1	-6	237.36	9.12
-6	-1	6	0.18	0.24
-6	-1	6	0.23	0.26
-6	-1	6	0.61	0.17
-6	-1	6	0.40	0.61
-6	-1	6	0.18	0.17
6	1	-6	0.45	0.21
6	1	-6	1.17	0.63
6	1	-6	0.37	0.13
-7	-1	6	85.99	4.08
-7	-1	6	96.14	3.64
-7	-1	6	90.21	3.47
-7	-1	6	86.04	3.63
7	1	-6	86.36	3.44
7	1	-6	93.44	4.17
-8	-1	6	16.77	0.98
-8	-1	6	14.85	0.76
-8	-1	6	14.08	1.48
-8	-1	6	14.13	0.97
8	1	-6	14.24	1.54
8	1	-6	14.45	0.72
-9	-1	6	120.67	4.85
-9	-1	6	128.76	4.64
-9	-1	6	119.09	5.29
-9	-1	6	121.66	4.81
9	1	-6	115.33	5.30
9	1	-6	118.57	4.83
9	1	-6	111.92	4.58
-10	-1	6	-0.69	0.79
-10	-1	6	0.72	0.26
-10	-1	6	0.40	0.40
-10	-1	6	0.03	0.41
10	1	-6	-2.12	0.84
10	1	-6	0.11	0.19

10	1	-6	0.48	0.41
-11	-1	6	96.31	3.75
-11	-1	6	95.86	3.97
-11	-1	6	91.09	4.03
-11	-1	6	99.90	4.56
11	1	-6	93.19	4.54
11	1	-6	89.56	3.69
11	1	-6	93.35	3.98
-12	-1	6	1.30	0.57
-12	-1	6	0.18	1.02
-12	-1	6	0.08	0.55
-12	-1	6	1.22	0.35
12	1	-6	1.95	1.10
12	1	-6	0.67	0.57
12	1	-6	1.08	0.30
-13	-1	6	107.78	4.70
-13	-1	6	121.60	4.45
-13	-1	6	112.92	5.26
13	1	-6	105.99	4.35
13	1	-6	103.72	5.22
-14	-1	6	2.59	1.25
14	1	-6	1.75	1.27
14	1	-6	1.04	0.37
-15	-1	6	33.62	2.82
-15	-1	6	36.25	2.17
15	1	-6	36.88	1.70
15	1	-6	32.68	3.01
-16	-1	6	2.65	0.90
-16	-1	6	4.76	1.49
16	1	-6	3.80	1.60
16	1	-6	3.75	0.55
-17	-1	6	65.21	3.38
-17	-1	6	64.70	3.94
17	1	-6	62.09	2.78
-18	-1	6	0.45	1.58
-18	-1	6	0.02	0.98
18	1	-6	-0.41	0.50
-19	-1	6	36.33	3.39
-19	-1	6	43.61	2.85
19	1	-6	37.35	1.99
-20	-1	6	0.18	1.25
20	1	-6	-0.02	0.68
21	1	-6	47.10	2.46
19	-1	7	0.51	0.79

19	-1	7	-1.01	1.45
-19	1	-7	0.11	1.43
18	-1	7	37.36	2.94
18	-1	7	37.73	2.21
-18	1	-7	37.50	3.00
17	-1	7	-1.17	0.60
17	-1	7	0.29	1.15
-17	1	-7	1.27	1.21
16	-1	7	38.19	2.07
-16	1	-7	34.93	2.58
15	-1	7	-0.36	0.88
15	-1	7	0.17	0.53
-15	1	-7	0.27	1.06
14	-1	7	35.37	2.37
14	-1	7	36.49	1.90
-14	1	-7	36.10	2.26
13	-1	7	5.70	0.87
13	-1	7	5.60	1.07
13	-1	7	6.63	1.11
-13	1	-7	5.72	0.95
12	-1	7	24.47	1.79
12	-1	7	22.02	1.29
12	-1	7	19.46	1.42
-12	1	-7	20.37	2.06
-12	1	-7	21.56	1.60
11	-1	7	3.96	0.58
11	-1	7	2.61	0.79
11	-1	7	1.96	0.58
-11	1	-7	3.11	0.95
-11	1	-7	2.86	0.69
10	-1	7	76.82	3.24
10	-1	7	79.00	3.29
10	-1	7	76.12	3.61
-10	1	-7	81.50	3.20
-10	1	-7	79.72	3.81
-10	1	-7	76.67	3.38
9	-1	7	4.89	0.70
9	-1	7	3.75	0.50
9	-1	7	4.66	0.85
-9	1	-7	4.83	0.91
-9	1	-7	6.26	0.83
-9	1	-7	4.05	0.53
8	-1	7	191.23	7.88
8	-1	7	197.68	7.53

8	-1	7	187.56	7.51
-8	1	-7	200.84	8.00
-8	1	-7	193.83	7.59
-8	1	-7	205.34	7.49
7	-1	7	1.58	0.62
7	-1	7	1.50	0.33
7	-1	7	0.89	0.26
-7	1	-7	1.49	0.28
-7	1	-7	1.02	0.65
-7	1	-7	1.43	0.38
6	-1	7	148.30	6.02
6	-1	7	148.96	5.94
6	-1	7	162.26	5.90
6	-1	7	153.93	6.33
-6	1	-7	149.77	5.98
-6	1	-7	161.76	5.93
-6	1	-7	160.71	6.41
-6	1	-7	155.91	6.00
5	-1	7	0.85	0.26
5	-1	7	0.91	0.22
5	-1	7	0.82	0.30
5	-1	7	0.88	0.47
-5	1	-7	0.49	0.29
-5	1	-7	0.91	0.21
-5	1	-7	0.41	0.41
-5	1	-7	1.04	0.56
4	-1	7	150.99	5.81
4	-1	7	145.90	6.15
4	-1	7	150.87	5.81
4	-1	7	155.27	5.91
-4	1	-7	150.90	5.80
-4	1	-7	154.98	5.85
-4	1	-7	162.55	6.24
-4	1	-7	150.62	6.05
3	-1	7	10.21	0.59
3	-1	7	12.93	0.64
3	-1	7	9.99	0.91
3	-1	7	11.40	0.92
-3	1	-7	11.48	0.58
-3	1	-7	11.88	0.65
-3	1	-7	11.57	0.99
-3	1	-7	10.70	0.63
-3	1	-7	10.43	1.01
2	-1	7	206.46	8.38

2	-1	7	220.07	8.32
2	-1	7	220.35	8.09
-2	1	-7	218.36	8.45
-2	1	-7	212.85	8.10
-2	1	-7	212.62	8.09
-2	1	-7	215.60	8.07
-2	1	-7	213.29	8.41
1	-1	7	28.60	1.55
1	-1	7	27.72	1.60
1	-1	7	32.06	1.29
-1	1	-7	29.97	1.27
-1	1	-7	31.10	1.24
-1	1	-7	30.84	1.27
-1	1	-7	29.40	1.70
-1	1	-7	28.78	1.56
0	-1	7	552.77	19.62
0	-1	7	481.35	19.47
0	-1	7	530.65	19.26
0	-1	7	510.97	19.23
0	1	-7	521.56	19.69
0	1	-7	507.97	19.24
-1	-1	7	83.08	3.61
-1	-1	7	79.07	3.77
-1	-1	7	91.99	3.43
-1	-1	7	91.72	3.38
1	1	-7	91.30	3.64
1	1	-7	87.20	3.41
1	1	-7	91.74	3.38
-2	-1	7	234.95	9.14
-2	-1	7	214.53	9.36
-2	-1	7	253.19	9.00
-2	-1	7	239.10	8.92
2	1	-7	245.17	9.49
2	1	-7	232.51	8.96
2	1	-7	245.15	8.91
2	1	-7	243.28	9.14
-3	-1	7	0.23	0.47
-3	-1	7	0.19	0.12
-3	-1	7	0.58	0.21
3	1	-7	0.09	0.14
3	1	-7	0.65	0.30
3	1	-7	-0.21	0.53
3	1	-7	0.12	0.18
-4	-1	7	97.46	3.81

-4	-1	7	95.84	3.71
-4	-1	7	95.56	3.86
4	1	-7	98.32	3.84
4	1	-7	99.31	3.70
4	1	-7	96.80	4.37
-5	-1	7	7.54	0.43
-5	-1	7	6.42	0.56
-5	-1	7	6.44	1.13
-5	-1	7	6.89	0.56
-5	-1	7	7.44	0.56
5	1	-7	6.81	0.56
5	1	-7	7.90	1.21
5	1	-7	7.28	0.41
-6	-1	7	168.04	6.39
-6	-1	7	157.62	6.25
-6	-1	7	156.86	6.24
-6	-1	7	169.76	6.38
6	1	-7	168.93	6.96
6	1	-7	159.60	6.20
6	1	-7	173.96	6.27
-7	-1	7	2.05	0.33
-7	-1	7	2.17	0.40
-7	-1	7	2.34	0.42
-7	-1	7	2.09	0.31
-7	-1	7	2.10	0.78
7	1	-7	1.98	0.29
7	1	-7	2.32	0.82
7	1	-7	2.47	0.41
-8	-1	7	138.56	5.15
-8	-1	7	128.33	5.82
-8	-1	7	147.07	5.36
-8	-1	7	131.11	5.34
8	1	-7	131.92	5.90
8	1	-7	128.19	5.11
-9	-1	7	12.78	0.99
-9	-1	7	15.00	0.77
-9	-1	7	14.32	0.99
-9	-1	7	10.96	1.56
9	1	-7	12.23	0.69
9	1	-7	11.49	1.67
-10	-1	7	120.07	5.67
-10	-1	7	132.19	5.16
-10	-1	7	133.16	4.95
-10	-1	7	125.92	5.19

10	1	-7	129.67	5.76
10	1	-7	121.27	4.88
-11	-1	7	10.89	1.10
-11	-1	7	12.23	0.81
-11	-1	7	11.47	1.10
-11	-1	7	11.24	1.79
11	1	-7	14.74	1.97
11	1	-7	11.59	0.72
-12	-1	7	159.78	7.20
-12	-1	7	166.42	6.71
-12	-1	7	178.31	6.42
12	1	-7	161.10	7.26
12	1	-7	158.50	6.33
-13	-1	7	2.26	0.44
-13	-1	7	1.34	0.66
-13	-1	7	1.15	1.26
13	1	-7	1.51	1.35
13	1	-7	1.08	0.34
-14	-1	7	134.79	5.77
-14	-1	7	137.77	6.30
14	1	-7	133.42	5.32
14	1	-7	138.28	6.38
-15	-1	7	2.00	1.45
-15	-1	7	-0.21	0.70
15	1	-7	0.24	0.36
-16	-1	7	73.05	3.59
-16	-1	7	67.89	4.16
16	1	-7	74.66	3.09
-17	-1	7	-0.72	0.83
-17	-1	7	1.19	1.45
17	1	-7	0.03	0.45
-18	-1	7	102.78	4.71
-18	-1	7	97.19	5.85
18	1	-7	95.53	4.08
-19	-1	7	0.03	1.08
-19	-1	7	-0.32	1.72
19	1	-7	0.03	0.57
-20	-1	7	69.21	4.62
-20	-1	7	67.38	3.90
20	1	-7	70.95	3.15
19	-1	8	2.09	1.62
19	-1	8	3.56	1.12
-19	1	-8	3.93	1.62
18	-1	8	3.34	1.48

18	-1	8	3.08	0.85
-18	1	-8	2.80	1.48
17	-1	8	-0.45	0.64
17	-1	8	-1.66	1.20
-17	1	-8	-1.87	1.21
16	-1	8	1.17	1.20
16	-1	8	0.54	0.66
-16	1	-8	0.99	1.14
15	-1	8	0.62	0.62
15	-1	8	1.10	1.06
-15	1	-8	0.64	1.52
14	-1	8	0.90	0.57
14	-1	8	1.48	0.91
-14	1	-8	0.92	0.84
13	-1	8	4.74	1.04
13	-1	8	2.77	0.56
13	-1	8	4.18	0.74
-13	1	-8	3.78	0.87
12	-1	8	4.61	0.65
12	-1	8	5.51	1.08
12	-1	8	4.77	0.73
-12	1	-8	4.73	0.86
11	-1	8	0.72	0.54
11	-1	8	0.31	0.75
11	-1	8	-0.50	0.34
-11	1	-8	0.09	0.58
-11	1	-8	0.29	0.98
10	-1	8	0.29	0.69
10	-1	8	0.11	0.34
10	-1	8	-0.61	0.44
-10	1	-8	0.99	0.96
-10	1	-8	0.21	0.32
-10	1	-8	-0.29	0.51
9	-1	8	21.92	1.67
9	-1	8	21.66	1.27
9	-1	8	23.13	1.24
-9	1	-8	25.40	1.93
-9	1	-8	24.15	1.38
-9	1	-8	23.70	1.19
8	-1	8	1.84	0.71
8	-1	8	1.03	0.33
8	-1	8	1.23	0.39
-8	1	-8	1.69	0.44
-8	1	-8	1.23	0.30

-8	1	-8	1.61	0.82
7	-1	8	12.75	0.82
7	-1	8	12.86	0.80
7	-1	8	12.15	1.22
7	-1	8	13.08	0.71
-7	1	-8	11.41	0.85
-7	1	-8	13.47	0.75
-7	1	-8	11.91	0.87
-7	1	-8	12.58	1.33
6	-1	8	1.30	0.33
6	-1	8	1.32	0.30
6	-1	8	1.01	0.26
6	-1	8	0.68	0.55
-6	1	-8	1.04	0.27
-6	1	-8	1.16	0.73
-6	1	-8	1.02	0.35
-6	1	-8	1.41	0.47
5	-1	8	13.76	1.23
5	-1	8	15.89	0.86
5	-1	8	16.61	0.96
5	-1	8	15.89	0.85
-5	1	-8	14.71	1.15
-5	1	-8	16.43	0.91
-5	1	-8	17.18	0.83
-5	1	-8	16.96	1.39
4	-1	8	32.04	1.78
4	-1	8	30.64	1.38
4	-1	8	32.63	1.40
4	-1	8	31.32	1.58
-4	1	-8	32.69	1.77
-4	1	-8	32.33	1.44
-4	1	-8	30.08	1.41
-4	1	-8	32.09	1.84
-4	1	-8	32.28	1.37
3	-1	8	39.73	1.89
3	-1	8	39.38	1.65
3	-1	8	36.27	2.02
-3	1	-8	41.57	1.63
-3	1	-8	39.36	1.67
-3	1	-8	41.37	2.09
-3	1	-8	37.96	2.06
2	-1	8	5.94	0.48
2	-1	8	6.50	0.83
2	-1	8	6.32	0.79

-2	1	-8	7.08	0.45
-2	1	-8	6.44	0.87
-2	1	-8	7.48	0.49
-2	1	-8	6.82	1.01
-2	1	-8	7.19	0.49
1	-1	8	66.81	3.05
1	-1	8	70.35	2.74
1	-1	8	68.14	3.06
-1	1	-8	71.79	2.73
-1	1	-8	72.46	2.71
-1	1	-8	73.59	3.11
-1	1	-8	71.41	2.74
-1	1	-8	54.72	3.86
0	-1	8	6.43	0.77
0	-1	8	5.83	0.42
0	-1	8	6.12	0.46
0	-1	8	5.63	0.94
0	1	-8	5.86	0.42
0	1	-8	5.88	0.44
0	1	-8	7.51	0.78
0	1	-8	7.11	1.06
-1	-1	8	24.08	1.09
-1	-1	8	24.61	1.03
-1	-1	8	22.65	1.52
-1	-1	8	22.69	1.32
1	1	-8	24.48	1.03
1	1	-8	23.86	1.65
1	1	-8	24.33	1.34
1	1	-8	21.73	1.12
-2	-1	8	0.55	0.23
-2	-1	8	1.19	0.56
-2	-1	8	0.28	0.33
-2	-1	8	0.52	0.17
2	1	-8	0.52	0.61
2	1	-8	-0.12	0.36
2	1	-8	0.36	0.16
2	1	-8	0.55	0.23
-3	-1	8	98.74	4.15
-3	-1	8	104.73	4.53
-3	-1	8	102.49	3.93
-3	-1	8	101.66	4.01
3	1	-8	99.10	3.98
3	1	-8	105.66	3.92
3	1	-8	108.34	4.65

3	1	-8	102.56	4.15
-4	-1	8	1.45	0.29
-4	-1	8	1.39	0.39
-4	-1	8	0.65	0.60
-4	-1	8	1.48	0.21
-4	-1	8	1.57	0.29
4	1	-8	1.47	0.20
4	1	-8	1.56	0.38
4	1	-8	0.49	0.71
-5	-1	8	26.67	1.96
-5	-1	8	32.73	1.53
-5	-1	8	35.20	1.52
-5	-1	8	33.56	1.38
-5	-1	8	33.50	1.50
5	1	-8	31.57	2.16
5	1	-8	34.55	1.52
5	1	-8	32.81	1.35
-6	-1	8	3.41	0.55
-6	-1	8	1.71	0.79
-6	-1	8	3.50	0.36
-6	-1	8	2.68	0.43
-6	-1	8	3.34	0.50
6	1	-8	2.91	0.95
6	1	-8	2.63	0.40
6	1	-8	3.04	0.31
-7	-1	8	63.98	2.78
-7	-1	8	63.51	2.75
-7	-1	8	62.56	2.59
-7	-1	8	68.47	2.60
-7	-1	8	66.38	3.39
7	1	-8	68.17	3.45
7	1	-8	63.28	2.55
7	1	-8	66.87	2.62
-8	-1	8	1.48	0.39
-8	-1	8	0.80	0.92
-8	-1	8	1.35	0.27
-8	-1	8	0.42	0.39
8	1	-8	1.10	0.28
8	1	-8	-0.03	0.89
8	1	-8	1.00	0.22
-9	-1	8	53.70	2.48
-9	-1	8	58.03	2.27
-9	-1	8	53.59	3.09
-9	-1	8	60.08	2.50

9	1	-8	52.43	2.20
9	1	-8	47.96	3.12
-10	-1	8	0.97	0.93
-10	-1	8	0.73	0.45
-10	-1	8	0.18	0.29
-10	-1	8	0.28	0.46
10	1	-8	0.98	1.07
10	1	-8	0.12	0.21
-11	-1	8	88.26	4.42
-11	-1	8	85.30	3.72
-11	-1	8	85.98	3.76
-11	-1	8	94.22	3.49
11	1	-8	82.83	3.40
11	1	-8	78.69	4.39
-12	-1	8	1.62	0.38
-12	-1	8	1.55	0.64
-12	-1	8	-0.59	1.11
12	1	-8	1.05	0.31
12	1	-8	0.80	1.32
-13	-1	8	96.28	4.84
-13	-1	8	103.78	3.88
-13	-1	8	94.17	4.17
13	1	-8	90.41	3.75
13	1	-8	85.86	4.89
-14	-1	8	-0.22	0.66
-14	-1	8	-0.04	1.24
14	1	-8	0.03	0.33
-15	-1	8	93.64	4.94
-15	-1	8	99.85	4.38
15	1	-8	94.00	3.87
-16	-1	8	0.64	0.83
-16	-1	8	1.04	1.41
16	1	-8	0.89	0.43
-17	-1	8	57.22	3.16
-17	-1	8	55.24	4.39
17	1	-8	56.88	2.54
-18	-1	8	-0.45	1.69
-18	-1	8	-0.29	0.96
18	1	-8	-0.84	0.49
-19	-1	8	42.80	3.88
-19	-1	8	48.88	3.03
19	1	-8	41.27	2.18
-20	-1	8	2.45	1.32
-20	-1	8	3.32	2.16

20	1	-8	1.89	0.70
18	-1	9	15.49	1.64
18	-1	9	16.42	2.57
-18	1	-9	12.17	1.84
17	-1	9	1.22	1.30
17	-1	9	0.31	0.72
-17	1	-9	-0.01	1.26
16	-1	9	20.62	2.46
16	-1	9	20.75	1.56
-16	1	-9	17.03	2.16
15	-1	9	0.30	0.58
15	-1	9	0.39	1.07
14	-1	9	4.66	0.74
14	-1	9	5.91	1.26
-14	1	-9	3.74	0.98
13	-1	9	0.00	0.47
13	-1	9	1.49	0.68
-13	1	-9	0.99	0.73
12	-1	9	5.95	0.84
12	-1	9	7.76	1.10
12	-1	9	5.35	1.10
-12	1	-9	4.89	0.86
11	-1	9	1.05	0.57
11	-1	9	2.61	0.87
11	-1	9	1.28	0.47
-11	1	-9	1.65	0.62
10	-1	9	35.91	2.27
10	-1	9	39.01	1.86
10	-1	9	36.57	1.81
-10	1	-9	37.63	1.95
9	-1	9	0.12	0.32
9	-1	9	0.24	0.41
9	-1	9	0.36	0.72
-9	1	-9	-0.17	0.31
-9	1	-9	0.33	0.46
-9	1	-9	1.06	0.92
8	-1	9	9.13	0.78
8	-1	9	7.62	0.72
8	-1	9	6.23	1.00
-8	1	-9	9.04	0.85
-8	1	-9	7.55	1.14
-8	1	-9	7.52	0.64
7	-1	9	1.11	0.34
7	-1	9	0.71	0.29

7	-1	9	0.96	0.64
7	-1	9	1.27	0.37
-7	1	-9	1.31	0.31
-7	1	-9	1.52	0.42
-7	1	-9	0.45	0.78
-7	1	-9	1.31	0.54
6	-1	9	15.44	0.86
6	-1	9	15.09	1.33
6	-1	9	14.64	0.86
-6	1	-9	16.40	0.95
-6	1	-9	14.92	0.82
-6	1	-9	12.27	1.16
-6	1	-9	14.65	1.44
5	-1	9	7.86	0.60
5	-1	9	9.29	0.85
5	-1	9	8.53	0.63
5	-1	9	6.62	1.03
-5	1	-9	8.41	0.65
-5	1	-9	7.70	1.17
-5	1	-9	7.94	0.66
-5	1	-9	9.54	0.59
-5	1	-9	8.42	1.05
4	-1	9	26.57	1.40
4	-1	9	25.15	1.65
4	-1	9	25.64	1.20
-4	1	-9	26.51	1.25
-4	1	-9	26.72	1.17
-4	1	-9	24.99	1.23
-4	1	-9	25.67	1.71
-4	1	-9	25.49	1.69
3	-1	9	52.72	2.72
3	-1	9	57.94	2.32
3	-1	9	57.19	2.57
-3	1	-9	56.48	3.04
-3	1	-9	54.81	2.78
-3	1	-9	57.13	2.35
-3	1	-9	61.28	2.31
-3	1	-9	57.95	2.34
2	-1	9	15.65	0.84
2	-1	9	16.66	1.21
2	-1	9	17.46	1.25
-2	1	-9	17.56	1.45
-2	1	-9	15.98	1.28
-2	1	-9	16.72	0.80

-2	1	-9	16.53	0.84
1	-1	9	63.70	2.96
1	-1	9	58.61	2.93
1	-1	9	68.33	2.60
-1	1	-9	67.35	2.56
-1	1	-9	66.85	2.59
-1	1	-9	61.41	3.14
-1	1	-9	65.31	2.99
0	-1	9	-0.10	0.22
0	-1	9	1.12	0.54
0	-1	9	0.10	0.20
0	-1	9	0.24	0.40
0	1	-9	0.34	0.16
0	1	-9	0.09	0.45
0	1	-9	0.72	0.63
0	1	-9	0.01	0.22
-1	-1	9	11.24	0.99
-1	-1	9	13.11	0.66
-1	-1	9	12.16	1.27
-1	-1	9	12.86	0.72
1	1	-9	12.11	0.70
1	1	-9	11.90	0.63
1	1	-9	12.63	1.01
1	1	-9	12.88	1.39
-2	-1	9	40.73	1.67
-2	-1	9	43.65	1.75
-2	-1	9	37.72	1.96
2	1	-9	40.81	1.98
2	1	-9	40.14	1.70
2	1	-9	40.61	1.65
2	1	-9	40.13	2.41
-3	-1	9	9.22	1.26
-3	-1	9	8.65	0.85
-3	-1	9	9.32	0.54
3	1	-9	7.70	1.37
3	1	-9	10.37	0.90
3	1	-9	8.83	0.60
3	1	-9	9.71	0.52
-4	-1	9	3.51	0.61
-4	-1	9	2.15	0.32
-4	-1	9	2.37	0.71
-4	-1	9	2.33	0.37
-4	-1	9	2.24	0.34
4	1	-9	2.16	0.89

4	1	-9	1.82	0.44
4	1	-9	2.43	0.29
-5	-1	9	1.94	0.48
-5	-1	9	2.91	0.39
-5	-1	9	2.49	0.40
-5	-1	9	2.62	0.85
-5	-1	9	2.79	0.43
5	1	-9	2.35	0.95
5	1	-9	2.25	0.45
5	1	-9	2.42	0.29
-6	-1	9	6.97	0.69
-6	-1	9	6.92	1.14
-6	-1	9	6.95	0.49
-6	-1	9	6.71	0.67
6	1	-9	6.01	1.09
6	1	-9	6.83	0.69
6	1	-9	7.33	0.46
-7	-1	9	7.46	0.52
-7	-1	9	5.72	1.10
-7	-1	9	7.63	0.74
-7	-1	9	6.95	0.73
-7	-1	9	6.69	0.60
7	1	-9	6.19	0.66
7	1	-9	6.34	1.18
7	1	-9	6.12	0.45
-8	-1	9	0.40	0.26
-8	-1	9	0.55	0.26
-8	-1	9	0.01	0.35
-8	-1	9	0.73	0.88
-8	-1	9	1.02	0.41
8	1	-9	0.33	0.33
8	1	-9	0.50	0.20
8	1	-9	-0.86	0.92
-9	-1	9	4.37	0.61
-9	-1	9	3.86	0.52
-9	-1	9	5.40	1.23
-9	-1	9	4.85	0.79
9	1	-9	4.59	0.43
9	1	-9	3.60	1.20
-10	-1	9	7.71	0.67
-10	-1	9	6.39	0.96
-10	-1	9	7.99	1.43
-10	-1	9	5.52	0.90
10	1	-9	7.65	1.41

10	1	-9	7.82	0.56
-11	-1	9	4.81	0.75
-11	-1	9	8.34	1.03
-11	-1	9	9.62	1.56
-11	-1	9	7.50	0.70
11	1	-9	6.98	0.58
11	1	-9	4.22	1.47
-12	-1	9	12.09	0.90
-12	-1	9	10.78	1.69
-12	-1	9	11.06	1.19
12	1	-9	10.02	0.73
12	1	-9	16.10	2.93
-13	-1	9	0.71	0.79
-13	-1	9	0.44	0.47
-13	-1	9	-0.76	1.26
13	1	-9	0.58	0.33
-14	-1	9	5.53	0.91
-14	-1	9	7.44	1.63
14	1	-9	6.04	0.70
-15	-1	9	3.10	0.85
-15	-1	9	6.40	1.60
15	1	-9	2.11	0.46
-16	-1	9	15.20	1.76
-16	-1	9	18.26	2.66
16	1	-9	17.05	1.11
-17	-1	9	11.20	1.77
-17	-1	9	8.65	2.02
17	1	-9	7.05	0.93
-18	-1	9	13.59	1.99
-18	-1	9	16.05	2.49
18	1	-9	15.96	1.25
-19	-1	9	-3.30	1.92
-19	-1	9	1.28	1.17
19	1	-9	0.62	0.61
-20	-1	9	16.63	2.48
-20	-1	9	11.90	2.48
20	1	-9	14.52	1.40
18	-1	10	0.24	1.62
18	-1	10	1.10	0.89
-18	1	-10	2.12	1.52
17	-1	10	26.43	2.80
17	-1	10	28.23	1.97
-17	1	-10	25.24	2.65
16	-1	10	-0.31	1.32

16	-1	10	-0.30	0.73
-16	1	-10	-0.61	1.13
15	-1	10	25.39	2.55
15	-1	10	28.09	1.78
14	-1	10	3.42	1.23
14	-1	10	2.49	0.69
-14	1	-10	2.48	0.99
13	-1	10	35.68	1.91
13	-1	10	32.09	1.97
13	-1	10	33.97	2.51
-13	1	-10	36.67	2.18
12	-1	10	4.27	1.27
12	-1	10	2.59	0.59
12	-1	10	3.69	0.73
-12	1	-10	2.15	0.83
11	-1	10	50.19	2.83
11	-1	10	48.52	2.33
11	-1	10	47.30	2.82
-11	1	-10	49.52	2.44
10	-1	10	1.78	0.55
10	-1	10	1.57	0.82
10	-1	10	1.04	0.45
-10	1	-10	0.98	0.60
9	-1	10	22.03	1.25
9	-1	10	22.45	1.81
9	-1	10	21.07	1.26
-9	1	-10	20.69	1.34
8	-1	10	-0.09	0.71
8	-1	10	0.14	0.33
8	-1	10	0.00	0.41
8	-1	10	0.23	0.32
-8	1	-10	-0.36	0.90
-8	1	-10	-0.29	0.50
-8	1	-10	0.16	0.45
-8	1	-10	-0.02	0.30
7	-1	10	47.17	2.06
7	-1	10	45.98	2.08
7	-1	10	46.01	2.61
-7	1	-10	45.67	2.29
-7	1	-10	49.89	2.05
-7	1	-10	48.65	2.14
-7	1	-10	50.61	2.87
6	-1	10	0.55	0.31
6	-1	10	0.37	0.63

6	-1	10	-0.25	0.43
6	-1	10	-0.17	0.25
-6	1	-10	0.12	0.64
-6	1	-10	0.18	0.29
-6	1	-10	0.59	0.37
-6	1	-10	1.14	0.77
-6	1	-10	0.10	0.26
5	-1	10	60.88	2.69
5	-1	10	59.72	3.05
-5	1	-10	62.89	2.98
-5	1	-10	64.42	2.53
-5	1	-10	61.40	2.56
-5	1	-10	59.81	2.59
-5	1	-10	63.72	3.16
4	-1	10	13.25	1.05
4	-1	10	12.33	1.26
4	-1	10	12.79	0.79
-4	1	-10	12.36	1.35
-4	1	-10	14.10	0.81
-4	1	-10	13.59	0.82
-4	1	-10	13.27	0.75
-4	1	-10	13.05	1.39
3	-1	10	11.50	1.06
3	-1	10	12.28	0.73
3	-1	10	10.66	1.19
-3	1	-10	12.32	0.76
-3	1	-10	11.20	1.29
-3	1	-10	11.32	1.41
-3	1	-10	12.12	0.74
-3	1	-10	12.38	0.69
2	-1	10	13.05	0.73
2	-1	10	11.93	1.10
2	-1	10	11.12	1.17
-2	1	-10	11.02	1.40
-2	1	-10	12.12	1.25
-2	1	-10	12.63	0.74
-2	1	-10	12.51	0.68
-2	1	-10	10.53	0.70
1	-1	10	6.56	0.98
1	-1	10	6.80	0.56
1	-1	10	5.92	0.92
-1	1	-10	6.05	0.47
-1	1	-10	6.13	1.00
-1	1	-10	5.88	0.49

-1	1	-10	6.85	0.56
-1	1	-10	8.14	1.11
0	-1	10	4.12	0.42
0	-1	10	3.59	0.49
0	-1	10	2.71	0.63
0	-1	10	4.43	0.80
0	1	-10	4.00	0.97
0	1	-10	3.89	0.41
0	1	-10	3.09	0.71
0	1	-10	4.68	0.50
0	1	-10	4.04	0.39
-1	-1	10	124.69	5.41
-1	-1	10	116.80	5.20
-1	-1	10	129.84	4.87
-1	-1	10	132.60	4.92
1	1	-10	121.99	5.59
1	1	-10	128.97	4.88
1	1	-10	131.44	5.25
1	1	-10	125.69	4.85
-2	-1	10	8.60	0.94
-2	-1	10	6.95	1.23
-2	-1	10	10.00	0.58
-2	-1	10	8.27	0.75
2	1	-10	8.95	0.54
2	1	-10	9.10	0.60
2	1	-10	8.16	0.97
2	1	-10	9.79	1.38
-3	-1	10	23.39	1.10
-3	-1	10	24.06	1.17
-3	-1	10	24.70	1.20
-3	-1	10	21.90	1.81
-3	-1	10	24.63	1.43
3	1	-10	25.32	1.45
3	1	-10	25.20	1.08
3	1	-10	24.58	2.04
-4	-1	10	2.51	0.40
-4	-1	10	2.57	0.54
-4	-1	10	1.86	0.35
-4	-1	10	2.31	0.29
-4	-1	10	2.85	0.93
4	1	-10	1.63	0.25
4	1	-10	2.36	0.96
4	1	-10	1.96	0.48
-5	-1	10	16.57	0.95

-5	-1	10	14.59	1.01
-5	-1	10	11.88	1.58
-5	-1	10	15.52	0.77
-5	-1	10	14.47	0.90
5	1	-10	13.68	0.72
5	1	-10	15.15	1.85
-6	-1	10	0.54	0.89
-6	-1	10	1.39	0.46
-6	-1	10	1.11	0.36
-6	-1	10	1.46	0.27
-6	-1	10	1.98	0.42
6	1	-10	1.26	0.22
6	1	-10	0.78	0.94
6	1	-10	0.92	0.44
-7	-1	10	5.07	0.96
-7	-1	10	7.23	1.24
-7	-1	10	4.47	0.72
-7	-1	10	6.48	0.53
-7	-1	10	6.28	0.76
7	1	-10	6.47	0.46
7	1	-10	6.51	0.75
7	1	-10	5.63	1.23
-8	-1	10	-0.81	0.91
-8	-1	10	-0.05	0.26
-8	-1	10	-0.40	0.35
-8	-1	10	-0.21	0.31
-8	-1	10	-0.14	0.41
8	1	-10	0.94	1.09
8	1	-10	0.06	0.19
8	1	-10	-0.11	0.38
-9	-1	10	34.68	1.75
-9	-1	10	32.98	1.72
-9	-1	10	33.39	2.53
-9	-1	10	29.19	1.34
-9	-1	10	31.31	1.45
9	1	-10	30.06	1.37
9	1	-10	32.28	1.42
9	1	-10	29.35	2.68
-10	-1	10	0.33	0.34
-10	-1	10	1.32	1.20
-10	-1	10	0.37	0.51
-10	-1	10	0.60	0.57
10	1	-10	0.57	1.21
10	1	-10	0.72	0.27

-11	-1	10	15.55	2.22
-11	-1	10	16.23	1.32
-11	-1	10	15.79	0.99
11	1	-10	14.52	0.85
-12	-1	10	6.83	1.10
-12	-1	10	5.62	0.82
-12	-1	10	6.37	1.51
12	1	-10	5.98	0.61
-13	-1	10	-0.79	0.48
-13	-1	10	0.00	0.67
-13	-1	10	-1.79	1.22
13	1	-10	0.17	0.33
-14	-1	10	0.15	0.75
14	1	-10	-0.47	0.35
-15	-1	10	-0.56	0.77
-15	-1	10	-2.01	1.44
15	1	-10	0.12	0.38
-16	-1	10	0.08	1.58
-16	-1	10	-0.38	0.83
16	1	-10	-0.23	0.43
-17	-1	10	3.98	1.12
-17	-1	10	2.71	1.82
17	1	-10	1.50	0.57
-18	-1	10	4.84	1.21
-18	-1	10	7.54	2.20
18	1	-10	5.51	0.75
-19	-1	10	1.19	2.15
-19	-1	10	0.27	1.24
19	1	-10	-0.62	0.65
17	-1	11	0.42	1.56
17	-1	11	-0.85	0.79
-17	1	-11	0.24	1.37
16	-1	11	39.49	3.19
16	-1	11	43.26	2.39
-16	1	-11	40.78	2.93
15	-1	11	-2.25	1.31
15	-1	11	0.11	0.67
14	-1	11	24.71	1.70
14	-1	11	21.01	2.31
-14	1	-11	25.01	2.03
13	-1	11	2.15	0.66
13	-1	11	2.90	0.83
13	-1	11	1.36	1.22
-13	1	-11	2.71	0.89

12	-1	11	41.36	2.17
12	-1	11	40.87	2.11
12	-1	11	40.10	2.81
-12	1	-11	43.00	2.35
11	-1	11	4.40	1.15
11	-1	11	4.32	0.63
11	-1	11	3.62	0.72
-11	1	-11	3.91	0.80
10	-1	11	59.80	3.72
10	-1	11	66.19	2.83
10	-1	11	66.45	2.85
-10	1	-11	62.94	2.91
9	-1	11	0.75	0.44
9	-1	11	2.56	0.55
9	-1	11	1.38	0.82
-9	1	-11	1.21	0.56
8	-1	11	39.99	2.44
8	-1	11	39.70	1.85
8	-1	11	38.64	1.86
-8	1	-11	40.94	1.93
-8	1	-11	37.52	1.91
-8	1	-11	38.17	2.00
-8	1	-11	41.55	1.81
7	-1	11	1.46	0.49
7	-1	11	1.43	0.74
7	-1	11	0.38	0.40
-7	1	-11	1.23	0.46
-7	1	-11	1.07	0.35
-7	1	-11	1.20	0.72
-7	1	-11	0.23	0.36
6	-1	11	58.12	2.51
6	-1	11	56.68	2.36
6	-1	11	54.12	2.96
-6	1	-11	55.71	3.12
-6	1	-11	56.66	2.41
-6	1	-11	54.70	2.45
-6	1	-11	56.89	2.37
5	-1	11	1.41	0.55
5	-1	11	1.24	0.75
5	-1	11	1.60	0.41
-5	1	-11	1.24	0.38
-5	1	-11	2.52	0.87
-5	1	-11	1.37	0.91
-5	1	-11	1.48	0.34

-5	1	-11	1.75	0.40
4	-1	11	75.74	3.26
4	-1	11	72.52	2.97
4	-1	11	70.11	3.50
-4	1	-11	71.05	3.03
-4	1	-11	76.82	3.63
-4	1	-11	74.26	3.60
-4	1	-11	76.45	3.00
-4	1	-11	76.13	2.99
3	-1	11	0.44	0.52
3	-1	11	0.11	0.28
3	-1	11	0.50	0.59
-3	1	-11	-0.21	0.30
-3	1	-11	0.05	0.69
-3	1	-11	-0.95	0.81
-3	1	-11	0.28	0.24
-3	1	-11	0.17	0.24
2	-1	11	24.81	1.16
2	-1	11	23.33	1.58
2	-1	11	20.16	1.61
-2	1	-11	24.38	1.15
-2	1	-11	23.05	1.10
-2	1	-11	23.27	1.98
-2	1	-11	22.51	1.14
-2	1	-11	26.76	1.76
1	-1	11	4.55	0.57
1	-1	11	5.33	0.87
1	-1	11	4.77	0.85
-1	1	-11	5.34	0.50
-1	1	-11	5.23	0.48
-1	1	-11	5.23	0.55
-1	1	-11	5.06	0.87
-1	1	-11	4.02	1.04
0	-1	11	253.71	10.56
0	-1	11	283.52	10.21
0	-1	11	261.00	10.67
0	1	-11	263.06	10.92
0	1	-11	273.06	10.17
0	1	-11	279.10	10.15
0	1	-11	269.38	10.62
0	1	-11	273.39	10.16
-1	-1	11	53.79	2.91
-1	-1	11	58.71	2.40
-1	-1	11	59.57	2.34

-1	-1	11	57.55	2.75
1	1	-11	54.84	2.32
1	1	-11	58.60	2.78
1	1	-11	58.50	2.31
1	1	-11	51.86	3.18
-2	-1	11	99.87	4.39
-2	-1	11	105.03	4.04
-2	-1	11	108.32	4.11
-2	-1	11	102.87	4.69
2	1	-11	106.48	4.42
2	1	-11	100.82	4.01
2	1	-11	100.64	4.05
2	1	-11	105.46	4.96
-3	-1	11	46.61	2.74
-3	-1	11	48.59	2.37
-3	-1	11	47.25	2.06
-3	-1	11	53.25	2.12
-3	-1	11	49.08	2.01
3	1	-11	47.14	1.97
3	1	-11	47.77	2.37
3	1	-11	47.55	2.96
-4	-1	11	35.43	1.61
-4	-1	11	35.62	1.48
-4	-1	11	30.60	1.77
-4	-1	11	34.36	1.57
-4	-1	11	29.23	2.29
4	1	-11	32.24	2.52
4	1	-11	37.18	1.83
4	1	-11	34.17	1.44
-5	-1	11	0.01	0.34
-5	-1	11	-0.19	0.23
-5	-1	11	-0.35	0.29
-5	-1	11	0.05	0.87
-5	-1	11	-0.05	0.45
5	1	-11	0.25	0.99
5	1	-11	0.08	0.19
5	1	-11	0.54	0.47
-6	-1	11	34.11	1.47
-6	-1	11	32.20	1.69
-6	-1	11	33.09	1.61
-6	-1	11	34.40	1.67
-6	-1	11	31.40	2.39
6	1	-11	31.78	2.59
6	1	-11	33.41	1.41

6	1	-11	33.15	1.72
-7	-1	11	18.33	0.94
-7	-1	11	18.78	1.18
-7	-1	11	16.66	1.15
-7	-1	11	14.93	1.95
-7	-1	11	17.50	1.11
7	1	-11	16.33	0.85
7	1	-11	17.74	1.16
7	1	-11	21.43	2.21
-8	-1	11	72.32	3.15
-8	-1	11	66.24	3.06
-8	-1	11	73.58	3.13
-8	-1	11	74.60	3.97
-8	-1	11	72.39	2.92
8	1	-11	71.19	2.86
8	1	-11	78.15	3.04
8	1	-11	67.88	4.05
-9	-1	11	28.98	1.39
-9	-1	11	31.70	1.69
-9	-1	11	29.18	1.65
-9	-1	11	29.06	1.35
-9	-1	11	27.92	2.50
9	1	-11	26.90	3.48
9	1	-11	26.94	1.29
9	1	-11	30.40	1.46
-10	-1	11	36.16	1.67
-10	-1	11	35.13	1.92
-10	-1	11	36.82	1.98
-10	-1	11	36.97	2.78
10	1	-11	33.87	1.54
-11	-1	11	1.81	1.30
-11	-1	11	2.15	0.60
-11	-1	11	2.42	0.47
11	1	-11	2.34	0.36
-12	-1	11	3.43	1.40
-12	-1	11	2.92	0.53
-12	-1	11	2.40	0.73
12	1	-11	2.49	0.41
-13	-1	11	0.75	0.70
-13	-1	11	-0.02	0.53
-13	-1	11	1.63	1.42
13	1	-11	0.22	0.34
-14	-1	11	15.01	2.61
-14	-1	11	11.77	1.58

14	1	-11	11.38	0.88
-15	-1	11	0.53	0.87
-15	-1	11	-0.18	1.57
15	1	-11	0.46	0.43
-16	-1	11	15.76	1.84
-16	-1	11	11.75	2.20
16	1	-11	11.83	1.01
-17	-1	11	0.00	1.94
-17	-1	11	0.75	1.00
17	1	-11	0.57	0.51
-18	-1	11	14.53	2.43
-18	-1	11	8.81	1.48
18	1	-11	10.55	1.15
-19	-1	11	-1.71	2.04
-19	-1	11	-1.86	1.17
19	1	-11	1.47	0.70
17	-1	12	45.43	2.87
-17	1	-12	38.49	3.24
16	-1	12	-0.07	1.48
16	-1	12	0.85	0.81
-16	1	-12	0.46	1.35
15	-1	12	40.52	2.38
15	-1	12	41.93	3.26
-15	1	-12	40.40	3.13
14	-1	12	-0.53	1.29
14	-1	12	-0.32	0.69
-14	1	-12	0.07	0.98
13	-1	12	42.03	2.38
13	-1	12	43.92	2.31
13	-1	12	42.25	3.12
-13	1	-12	47.03	2.57
12	-1	12	0.88	0.60
12	-1	12	2.03	1.19
12	-1	12	1.68	0.75
-12	1	-12	0.44	0.80
11	-1	12	42.48	2.12
11	-1	12	38.42	2.83
11	-1	12	39.81	2.09
-11	1	-12	41.60	2.17
-11	1	-12	42.39	2.22
10	-1	12	4.42	1.19
10	-1	12	2.35	0.62
10	-1	12	2.43	0.59
-10	1	-12	2.09	0.70

-10	1	-12	3.47	0.66
9	-1	12	67.13	2.70
9	-1	12	59.09	3.99
9	-1	12	64.95	2.78
-9	1	-12	64.63	2.88
-9	1	-12	61.74	2.78
-9	1	-12	63.37	2.83
8	-1	12	-0.71	0.84
8	-1	12	0.56	0.51
8	-1	12	0.51	0.49
-8	1	-12	0.24	0.75
-8	1	-12	-0.12	0.48
-8	1	-12	0.89	0.46
7	-1	12	131.09	5.84
7	-1	12	133.55	5.19
7	-1	12	139.67	5.31
-7	1	-12	133.74	5.25
-7	1	-12	116.86	5.98
-7	1	-12	135.14	5.32
-7	1	-12	135.74	5.21
6	-1	12	2.66	0.45
6	-1	12	2.10	0.83
6	-1	12	3.17	0.63
-6	1	-12	2.52	0.42
-6	1	-12	1.98	0.48
-6	1	-12	3.04	0.96
-6	1	-12	2.05	0.45
5	-1	12	87.02	4.27
5	-1	12	97.77	3.96
5	-1	12	88.85	3.65
-5	1	-12	91.95	3.76
-5	1	-12	93.61	3.70
-5	1	-12	87.45	4.48
-5	1	-12	98.30	3.70
-5	1	-12	90.73	4.31
4	-1	12	-0.15	0.54
4	-1	12	-0.23	0.68
-4	1	-12	1.96	0.99
-4	1	-12	0.05	0.32
-4	1	-12	0.32	0.30
-4	1	-12	0.83	0.94
-4	1	-12	-0.44	0.29
3	-1	12	124.23	4.52
3	-1	12	114.05	4.90

3	-1	12	110.94	5.07
-3	1	-12	110.70	5.26
-3	1	-12	115.77	4.56
-3	1	-12	108.89	5.15
-3	1	-12	118.32	4.50
-3	1	-12	118.18	4.51
2	-1	12	-0.11	0.58
2	-1	12	-0.09	0.31
2	-1	12	0.51	0.65
-2	1	-12	0.31	0.77
-2	1	-12	0.30	0.27
-2	1	-12	-0.47	0.29
-2	1	-12	-0.23	0.25
-2	1	-12	-0.35	1.01
1	-1	12	163.10	7.00
1	-1	12	179.02	6.52
1	-1	12	172.00	7.01
-1	1	-12	167.96	7.04
-1	1	-12	167.33	6.51
-1	1	-12	169.97	6.48
-1	1	-12	160.32	7.30
-1	1	-12	175.96	6.49
0	-1	12	6.10	0.97
0	-1	12	8.66	1.31
0	-1	12	7.83	0.71
0	1	-12	7.68	0.60
0	1	-12	6.79	1.26
0	1	-12	8.65	0.61
0	1	-12	6.19	1.32
0	1	-12	7.55	0.59
-1	-1	12	214.88	8.50
-1	-1	12	209.15	7.84
-1	-1	12	209.44	7.90
-1	-1	12	197.06	8.24
1	1	-12	209.39	8.29
1	1	-12	203.84	7.81
1	1	-12	209.70	7.81
1	1	-12	191.81	8.68
-2	-1	12	6.47	1.04
-2	-1	12	6.22	0.69
-2	-1	12	7.10	0.59
-2	-1	12	7.69	1.17
2	1	-12	8.88	1.49
2	1	-12	6.83	0.98

2	1	-12	7.45	0.55
2	1	-12	6.53	0.62
-3	-1	12	92.68	3.55
-3	-1	12	91.98	3.60
-3	-1	12	82.01	4.26
-3	-1	12	90.94	3.63
-3	-1	12	83.55	3.89
3	1	-12	94.43	3.96
3	1	-12	90.72	3.51
3	1	-12	87.87	4.57
-4	-1	12	4.25	0.74
-4	-1	12	5.20	0.71
-4	-1	12	3.86	0.50
-4	-1	12	5.84	1.12
-4	-1	12	4.33	0.59
4	1	-12	4.11	1.24
4	1	-12	3.71	0.43
4	1	-12	4.09	0.76
-5	-1	12	49.81	2.44
-5	-1	12	46.76	3.02
-5	-1	12	51.51	2.24
-5	-1	12	50.23	2.13
-5	-1	12	53.55	2.29
5	1	-12	50.61	3.28
5	1	-12	53.50	2.46
5	1	-12	50.51	2.08
-6	-1	12	-0.11	0.36
-6	-1	12	0.76	0.29
-6	-1	12	-0.24	0.88
-6	-1	12	-0.61	0.43
-6	-1	12	0.93	0.51
6	1	-12	0.89	1.29
6	1	-12	0.20	0.21
6	1	-12	-0.33	0.54
-7	-1	12	49.62	2.30
-7	-1	12	51.71	2.34
-7	-1	12	48.95	2.10
-7	-1	12	48.55	3.10
-7	-1	12	48.22	2.29
7	1	-12	51.26	3.37
7	1	-12	51.25	2.35
7	1	-12	48.02	2.03
-8	-1	12	3.63	0.62
-8	-1	12	4.05	1.23

-8	-1	12	4.26	0.78
-8	-1	12	4.70	0.74
-8	-1	12	3.28	0.47
8	1	-12	3.06	0.63
8	1	-12	4.16	0.47
-9	-1	12	42.55	2.12
-9	-1	12	44.15	3.05
-9	-1	12	40.81	1.86
-9	-1	12	43.21	2.19
9	1	-12	42.12	1.96
9	1	-12	37.85	1.75
-10	-1	12	4.94	0.75
-10	-1	12	5.98	1.19
-10	-1	12	5.71	1.46
-10	-1	12	5.33	0.61
-10	-1	12	5.98	0.83
10	1	-12	5.32	0.80
10	1	-12	6.03	0.56
-11	-1	12	76.82	3.42
-11	-1	12	74.18	3.11
11	1	-12	72.09	2.99
-12	-1	12	0.28	0.50
-12	-1	12	2.26	1.42
12	1	-12	-0.25	0.30
-13	-1	12	44.15	3.54
-13	-1	12	45.16	2.35
-13	-1	12	44.48	2.48
13	1	-12	39.35	1.90
-14	-1	12	2.36	1.70
-14	-1	12	3.20	0.92
14	1	-12	2.42	0.49
-15	-1	12	19.49	3.07
-15	-1	12	19.34	1.89
-16	-1	12	-0.27	1.81
-16	-1	12	-0.13	1.00
16	1	-12	-0.39	0.48
-17	-1	12	34.16	2.53
-17	-1	12	34.90	3.86
17	1	-12	30.96	1.74
-18	-1	12	3.38	2.12
-18	-1	12	4.12	1.29
18	1	-12	8.21	1.09
-19	-1	12	16.58	2.71
19	1	-12	20.63	1.53

16	-1	13	20.49	3.05
16	-1	13	29.24	2.12
-16	1	-13	29.29	2.25
15	-1	13	-2.09	1.50
15	-1	13	-0.55	0.77
-15	1	-13	-0.37	1.17
-15	1	-13	0.13	0.89
14	-1	13	27.72	2.94
14	-1	13	30.13	2.00
-14	1	-13	31.26	2.15
-14	1	-13	32.23	2.31
13	-1	13	1.71	1.38
13	-1	13	-0.18	0.65
-13	1	-13	3.75	1.04
-13	1	-13	0.75	0.73
12	-1	13	56.78	2.83
12	-1	13	57.91	3.59
-12	1	-13	58.77	2.96
-12	1	-13	62.27	2.94
11	-1	13	-0.08	0.72
11	-1	13	-0.07	1.16
-11	1	-13	0.61	0.73
-11	1	-13	0.72	0.61
10	-1	13	55.02	3.22
10	-1	13	51.92	2.39
-10	1	-13	48.92	2.48
-10	1	-13	48.68	2.49
9	-1	13	4.12	0.68
9	-1	13	4.45	1.25
9	-1	13	4.82	0.71
-9	1	-13	4.24	0.73
-9	1	-13	5.45	0.90
8	-1	13	45.40	2.92
8	-1	13	49.86	2.18
8	-1	13	49.45	2.23
-8	1	-13	45.68	2.26
-8	1	-13	45.99	2.24
7	-1	13	13.21	2.29
7	-1	13	16.38	1.07
7	-1	13	16.21	1.22
-7	1	-13	14.77	1.13
-7	1	-13	16.25	1.11
-7	1	-13	13.18	1.74
6	-1	13	140.97	5.64

6	-1	13	134.06	6.08
6	-1	13	134.91	5.38
-6	1	-13	136.93	5.52
-6	1	-13	140.06	6.02
-6	1	-13	144.96	5.45
-6	1	-13	137.09	5.42
5	-1	13	4.15	0.98
5	-1	13	4.10	0.68
5	-1	13	2.86	0.74
-5	1	-13	3.86	0.50
-5	1	-13	0.69	1.09
-5	1	-13	3.12	0.49
-5	1	-13	2.67	0.51
4	-1	13	148.17	6.85
4	-1	13	161.41	6.61
4	-1	13	162.87	6.31
-4	1	-13	169.98	6.27
-4	1	-13	166.20	6.24
-4	1	-13	162.98	6.33
-4	1	-13	160.69	7.02
3	-1	13	3.11	0.82
3	-1	13	3.51	0.96
-3	1	-13	3.96	0.59
-3	1	-13	2.42	1.01
-3	1	-13	0.89	1.16
-3	1	-13	3.97	0.48
-3	1	-13	3.23	0.44
2	-1	13	53.75	2.39
2	-1	13	49.07	2.75
2	-1	13	49.99	2.84
-2	1	-13	54.81	2.24
-2	1	-13	53.95	3.05
-2	1	-13	54.14	2.21
-2	1	-13	47.91	3.24
-2	1	-13	57.17	2.29
1	-1	13	0.98	0.78
1	-1	13	0.79	0.74
-1	1	-13	0.48	1.12
-1	1	-13	0.47	0.31
-1	1	-13	0.82	0.31
-1	1	-13	0.81	0.31
-1	1	-13	0.06	0.83
0	-1	13	132.08	5.84
0	-1	13	134.19	5.93

0	-1	13	146.76	5.40
0	1	-13	142.16	5.94
0	1	-13	133.71	5.32
0	1	-13	124.23	6.28
0	1	-13	144.07	5.32
0	1	-13	139.79	5.31
-1	-1	13	7.77	0.68
-1	-1	13	7.20	0.76
-1	-1	13	5.18	0.96
-1	-1	13	7.95	1.24
1	1	-13	6.76	1.11
1	1	-13	8.13	0.62
1	1	-13	8.13	0.67
1	1	-13	5.49	1.41
-2	-1	13	121.05	4.74
-2	-1	13	116.04	5.41
-2	-1	13	122.74	4.68
-2	-1	13	110.49	5.09
2	1	-13	123.67	4.65
2	1	-13	123.71	5.19
2	1	-13	117.52	5.77
2	1	-13	120.36	4.67
-3	-1	13	8.04	0.71
-3	-1	13	9.98	0.70
-3	-1	13	9.12	0.85
-3	-1	13	8.46	1.22
-3	-1	13	9.42	1.40
3	1	-13	8.25	1.31
3	1	-13	4.68	1.37
3	1	-13	7.98	0.61
-4	-1	13	68.62	2.91
-4	-1	13	69.06	3.74
-4	-1	13	63.96	3.20
-4	-1	13	73.67	2.98
-4	-1	13	70.89	2.84
4	1	-13	68.47	4.13
4	1	-13	71.54	3.25
4	1	-13	70.27	2.79
-5	-1	13	4.19	0.62
-5	-1	13	2.84	0.71
-5	-1	13	3.82	0.56
-5	-1	13	3.96	0.67
-5	-1	13	3.92	1.12
5	1	-13	3.14	0.45

5	1	-13	3.39	0.81
5	1	-13	1.89	1.38
-6	-1	13	97.18	4.34
-6	-1	13	104.36	4.28
-6	-1	13	106.15	5.13
-6	-1	13	107.48	4.26
-6	-1	13	102.89	4.10
6	1	-13	101.96	4.04
6	1	-13	107.55	4.41
-7	-1	13	1.89	1.09
-7	-1	13	1.05	0.38
-7	-1	13	0.84	0.46
-7	-1	13	1.78	0.62
-7	-1	13	0.59	0.55
7	1	-13	1.02	0.70
7	1	-13	1.63	0.30
-8	-1	13	116.93	4.64
-8	-1	13	110.73	5.50
-8	-1	13	111.29	4.42
-8	-1	13	105.19	4.54
-8	-1	13	116.37	4.67
8	1	-13	114.18	4.61
8	1	-13	108.47	4.34
-9	-1	13	8.32	1.57
-9	-1	13	7.40	0.79
-9	-1	13	7.90	0.93
-9	-1	13	8.56	1.18
-9	-1	13	6.89	1.03
9	1	-13	6.34	1.10
9	1	-13	7.20	0.60
-10	-1	13	52.08	3.69
-10	-1	13	64.26	2.83
-10	-1	13	57.98	2.51
-10	-1	13	55.14	2.48
10	1	-13	59.76	2.58
10	1	-13	57.33	2.39
-11	-1	13	1.50	1.36
-11	-1	13	1.24	0.51
-11	-1	13	0.25	0.63
11	1	-13	0.74	0.33
-12	-1	13	91.31	4.03
-12	-1	13	84.54	3.64
-12	-1	13	89.77	5.02
12	1	-13	84.48	3.49

-13	-1	13	0.41	1.58
-13	-1	13	2.11	0.83
13	1	-13	0.37	0.39
-14	-1	13	70.54	3.40
-14	-1	13	60.31	4.44
14	1	-13	63.48	2.75
-15	-1	13	-1.17	1.64
-15	-1	13	0.41	0.92
15	1	-13	-0.13	0.46
-16	-1	13	64.32	4.78
-16	-1	13	65.65	3.54
16	1	-13	65.42	2.85
-17	-1	13	1.18	1.09
-17	-1	13	2.30	2.18
17	1	-13	0.72	0.57
-18	-1	13	66.62	3.73
-18	-1	13	59.09	4.99
18	1	-13	63.69	2.88
15	-1	14	1.52	1.68
-15	1	-14	2.05	1.00
14	-1	14	0.97	1.50
-14	1	-14	0.27	0.94
13	-1	14	12.60	1.94
-13	1	-14	16.32	1.62
-13	1	-14	13.38	1.74
12	-1	14	2.48	1.43
-12	1	-14	-0.61	0.87
-12	1	-14	0.36	0.73
11	-1	14	8.12	1.61
-11	1	-14	8.62	1.31
-11	1	-14	9.20	1.35
10	-1	14	1.94	1.30
10	-1	14	3.76	0.72
-10	1	-14	2.63	0.68
-10	1	-14	1.96	0.73
9	-1	14	12.48	1.08
9	-1	14	9.84	1.14
9	-1	14	11.45	1.67
-9	1	-14	10.30	1.19
-9	1	-14	10.58	1.12
8	-1	14	0.44	1.13
8	-1	14	0.16	0.60
8	-1	14	0.08	0.54
-8	1	-14	3.00	1.06

-8	1	-14	-0.05	0.75
-8	1	-14	0.51	0.58
7	-1	14	7.67	1.11
7	-1	14	6.65	0.90
7	-1	14	7.59	1.34
-7	1	-14	7.73	0.92
-7	1	-14	7.60	1.40
-7	1	-14	6.98	0.92
6	-1	14	2.31	0.48
6	-1	14	0.13	0.62
6	-1	14	0.60	0.95
-6	1	-14	1.04	0.46
-6	1	-14	1.00	1.17
-6	1	-14	0.83	0.45
5	-1	14	27.78	2.24
5	-1	14	29.66	1.47
5	-1	14	26.23	1.83
-5	1	-14	32.07	1.50
-5	1	-14	29.75	2.61
-5	1	-14	32.48	1.58
-5	1	-14	29.65	1.49
4	-1	14	14.52	0.96
4	-1	14	13.20	1.43
4	-1	14	12.57	1.62
-4	1	-14	14.20	1.01
-4	1	-14	15.93	0.98
-4	1	-14	14.77	0.96
-4	1	-14	16.81	2.28
3	-1	14	33.53	2.11
3	-1	14	33.32	2.25
3	-1	14	34.25	1.62
-3	1	-14	32.84	1.58
-3	1	-14	32.39	2.80
-3	1	-14	31.74	1.53
-3	1	-14	35.52	1.63
2	-1	14	2.43	1.01
2	-1	14	2.09	0.90
2	-1	14	2.28	0.52
-2	1	-14	2.16	0.42
-2	1	-14	2.12	0.40
-2	1	-14	3.91	0.61
-2	1	-14	1.00	1.35
1	-1	14	77.26	3.91
1	-1	14	85.99	3.38

1	-1	14	85.72	4.00
-1	1	-14	82.96	4.07
-1	1	-14	85.14	3.34
-1	1	-14	82.80	3.31
-1	1	-14	84.67	3.37
-1	1	-14	75.91	4.46
0	-1	14	-0.90	0.84
0	-1	14	1.20	0.78
0	-1	14	-0.86	0.39
0	1	-14	-1.03	1.28
0	1	-14	-0.01	0.28
0	1	-14	0.23	0.98
0	1	-14	0.57	0.33
0	1	-14	0.15	0.31
-1	-1	14	27.77	1.43
-1	-1	14	25.57	2.19
-1	-1	14	27.46	1.93
1	1	-14	27.11	1.31
1	1	-14	24.06	2.71
1	1	-14	29.55	1.34
1	1	-14	28.05	2.12
1	1	-14	27.02	1.31
-2	-1	14	-0.30	0.72
-2	-1	14	-0.01	0.45
-2	-1	14	0.49	0.34
-2	-1	14	0.30	0.34
-2	-1	14	-0.04	0.85
2	1	-14	0.03	1.37
2	1	-14	0.43	0.83
2	1	-14	-0.06	0.27
-3	-1	14	74.17	4.04
-3	-1	14	73.57	3.61
-3	-1	14	77.82	3.18
-3	-1	14	81.83	3.26
-3	-1	14	77.40	3.15
3	1	-14	77.91	3.68
3	1	-14	78.42	3.10
3	1	-14	74.24	4.47
-4	-1	14	2.17	0.80
-4	-1	14	1.18	0.40
-4	-1	14	0.80	0.39
-4	-1	14	1.59	0.54
-4	-1	14	0.97	1.03
4	1	-14	2.32	0.83

4	1	-14	1.99	0.33
-5	-1	14	68.81	2.80
-5	-1	14	66.33	3.85
-5	-1	14	62.27	3.17
-5	-1	14	68.45	2.97
-5	-1	14	69.93	2.90
5	1	-14	68.66	3.26
5	1	-14	67.73	2.73
-6	-1	14	0.60	0.40
-6	-1	14	1.65	0.61
-6	-1	14	0.73	1.03
-6	-1	14	0.47	0.45
-6	-1	14	0.83	0.67
6	1	-14	1.77	0.82
6	1	-14	0.75	0.29
-7	-1	14	54.70	2.81
-7	-1	14	58.60	3.68
-7	-1	14	65.03	2.78
-7	-1	14	62.47	2.58
-7	-1	14	60.86	2.82
7	1	-14	61.84	2.49
7	1	-14	62.57	2.97
-8	-1	14	7.00	2.11
-8	-1	14	4.94	0.80
-8	-1	14	5.55	0.96
-8	-1	14	7.44	1.19
-8	-1	14	6.11	0.76
8	1	-14	6.81	0.59
8	1	-14	8.22	1.34
-9	-1	14	88.91	3.76
-9	-1	14	80.20	3.59
-9	-1	14	91.00	4.83
-9	-1	14	87.61	4.00
-9	-1	14	82.15	3.49
9	1	-14	86.68	3.71
9	1	-14	83.58	3.39
-10	-1	14	2.99	1.50
-10	-1	14	0.90	0.63
-10	-1	14	1.57	0.50
-10	-1	14	-0.18	0.60
10	1	-14	0.84	0.35
10	1	-14	-0.07	0.72
-11	-1	14	59.54	4.09
-11	-1	14	59.00	2.63

11	1	-14	59.38	2.48
-12	-1	14	1.72	0.79
-12	-1	14	1.43	0.63
-12	-1	14	-0.70	1.45
12	1	-14	1.24	0.40
-13	-1	14	48.25	2.71
-13	-1	14	43.88	3.85
13	1	-14	45.47	2.05
-14	-1	14	2.42	1.04
-14	-1	14	3.80	1.85
14	1	-14	3.44	0.57
-15	-1	14	73.83	5.08
-15	-1	14	80.57	3.89
15	1	-14	74.14	3.21
-16	-1	14	-1.81	1.99
-16	-1	14	-0.35	1.10
16	1	-14	0.04	0.54
-17	-1	14	73.80	3.83
-17	-1	14	68.14	5.12
17	1	-14	66.63	3.05
14	-1	15	5.28	1.85
-14	1	-15	4.76	1.05
-13	1	-15	-0.01	0.81
12	-1	15	-0.02	1.40
-12	1	-15	2.78	0.88
-12	1	-15	-1.10	1.51
11	-1	15	-0.02	1.34
-11	1	-15	0.19	0.72
-11	1	-15	-0.49	0.80
10	-1	15	13.23	2.26
-10	1	-15	14.81	1.38
-10	1	-15	14.07	1.39
9	-1	15	1.42	0.70
9	-1	15	-0.41	1.21
-9	1	-15	-0.17	0.56
-9	1	-15	0.07	0.62
8	-1	15	5.90	0.89
8	-1	15	5.75	1.38
8	-1	15	5.31	0.71
-8	1	-15	3.32	0.69
-8	1	-15	4.76	0.71
7	-1	15	9.44	1.01
7	-1	15	9.54	1.30
7	-1	15	13.39	1.98

-7	1	-15	9.36	1.20
-7	1	-15	10.18	1.00
-7	1	-15	12.55	1.71
6	-1	15	0.71	0.49
6	-1	15	0.86	1.06
6	-1	15	1.10	0.77
-6	1	-15	0.55	0.47
-6	1	-15	1.32	0.50
-6	1	-15	2.15	1.39
5	-1	15	1.27	0.83
5	-1	15	2.40	0.53
5	-1	15	3.04	1.03
-5	1	-15	2.55	0.55
-5	1	-15	2.77	0.53
-5	1	-15	1.72	1.44
4	-1	15	1.07	0.79
4	-1	15	-0.63	1.59
4	-1	15	0.07	0.46
-4	1	-15	-0.21	0.40
-4	1	-15	-0.56	1.37
-4	1	-15	0.76	0.43
-4	1	-15	-0.27	0.38
3	-1	15	2.02	0.49
3	-1	15	1.40	0.90
3	-1	15	0.42	0.90
-3	1	-15	3.60	1.54
-3	1	-15	2.44	0.46
-3	1	-15	2.40	0.44
-3	1	-15	0.71	0.42
2	-1	15	0.62	0.82
2	-1	15	2.20	0.48
2	-1	15	2.02	0.96
-2	1	-15	0.72	0.40
-2	1	-15	-0.64	1.40
-2	1	-15	0.89	0.41
-2	1	-15	1.33	0.39
1	-1	15	0.20	0.45
1	-1	15	0.55	0.87
1	-1	15	-0.18	0.86
-1	1	-15	0.23	0.36
-1	1	-15	0.27	0.33
-1	1	-15	0.01	0.37
-1	1	-15	-1.31	1.43
0	-1	15	2.43	0.97

0	-1	15	3.07	0.58
0	-1	15	4.92	1.13
0	1	-15	2.59	0.44
0	1	-15	3.12	0.48
0	1	-15	2.01	1.58
0	1	-15	3.17	0.45
-1	-1	15	5.34	0.87
-1	-1	15	4.26	1.02
-1	-1	15	3.66	1.17
1	1	-15	4.02	0.62
1	1	-15	4.71	0.61
1	1	-15	5.88	0.70
1	1	-15	9.60	1.61
1	1	-15	3.38	1.68
-2	-1	15	0.72	0.37
-2	-1	15	1.14	0.54
-2	-1	15	1.69	0.87
-2	-1	15	-0.18	0.94
-2	-1	15	0.23	0.39
2	1	-15	0.44	0.32
2	1	-15	-0.72	1.01
-3	-1	15	-0.46	0.36
-3	-1	15	0.01	0.37
-3	-1	15	-0.60	0.94
-3	-1	15	0.11	0.82
-3	-1	15	-0.20	0.50
3	1	-15	1.53	0.93
3	1	-15	0.37	0.30
-4	-1	15	5.01	0.70
-4	-1	15	4.75	0.71
-4	-1	15	4.89	0.72
-4	-1	15	4.28	0.97
-4	-1	15	4.55	1.25
4	1	-15	4.88	1.05
4	1	-15	3.62	0.54
-5	-1	15	-0.91	0.57
-5	-1	15	-0.80	0.69
-5	-1	15	0.58	0.39
-5	-1	15	-0.46	0.41
-5	-1	15	2.94	1.23
5	1	-15	1.16	0.91
5	1	-15	-0.19	0.26
-6	-1	15	12.02	1.08
-6	-1	15	11.10	1.22

-6	-1	15	13.11	1.44
-6	-1	15	11.66	0.92
6	1	-15	13.37	1.65
6	1	-15	12.62	0.79
-7	-1	15	2.50	0.79
-7	-1	15	3.14	0.54
-7	-1	15	3.02	0.64
-7	-1	15	4.09	0.79
-7	-1	15	3.68	1.49
7	1	-15	4.54	1.01
7	1	-15	3.99	0.54
-8	-1	15	8.32	1.21
-8	-1	15	7.76	0.86
-8	-1	15	6.42	1.08
-8	-1	15	7.27	1.75
-8	-1	15	6.34	0.95
8	1	-15	7.94	0.67
8	1	-15	10.64	1.55
-9	-1	15	4.34	1.53
-9	-1	15	2.90	0.56
-9	-1	15	3.39	0.75
-9	-1	15	3.92	0.75
9	1	-15	2.52	0.41
9	1	-15	1.27	0.89
-10	-1	15	0.51	0.69
-10	-1	15	0.41	0.51
-10	-1	15	0.42	1.60
-10	-1	15	0.78	0.63
10	1	-15	0.84	0.36
10	1	-15	0.01	0.89
-11	-1	15	0.74	0.78
-11	-1	15	-1.33	1.50
-11	-1	15	2.04	0.65
11	1	-15	1.70	0.40
-12	-1	15	11.00	1.60
-12	-1	15	15.81	2.99
12	1	-15	12.39	0.94
-13	-1	15	5.14	1.98
-13	-1	15	4.75	1.08
13	1	-15	4.88	0.60
-14	-1	15	18.26	1.96
-14	-1	15	11.76	2.50
-15	-1	15	0.34	1.99
-15	-1	15	0.67	1.07

15	1	-15	0.35	0.54
-16	-1	15	13.64	1.71
-16	-1	15	17.14	2.80
16	1	-15	16.44	1.29
-17	-1	15	2.29	2.30
-17	-1	15	1.83	1.31
17	1	-15	-0.07	0.66
13	-1	16	19.61	3.11
-13	1	-16	22.58	2.00
12	-1	16	1.05	1.55
-12	1	-16	0.79	0.85
11	-1	16	30.58	3.12
-11	1	-16	30.67	2.06
10	-1	16	-0.40	1.35
-10	1	-16	0.78	0.74
9	-1	16	22.53	2.77
9	-1	16	27.64	1.77
-9	1	-16	25.21	1.61
-9	1	-16	24.15	1.72
8	-1	16	3.83	0.92
8	-1	16	5.93	1.54
-8	1	-16	4.22	0.73
-8	1	-16	5.85	0.82
7	-1	16	21.71	2.56
7	-1	16	25.35	1.83
-7	1	-16	20.82	1.50
-7	1	-16	24.10	1.40
6	-1	16	5.40	1.00
6	-1	16	3.24	1.30
6	-1	16	5.29	0.88
-6	1	-16	4.90	0.63
-6	1	-16	3.18	0.72
-6	1	-16	3.03	1.65
5	-1	16	23.93	1.89
5	-1	16	23.28	1.28
5	-1	16	20.36	2.36
-5	1	-16	26.11	3.00
-5	1	-16	22.06	1.39
-5	1	-16	20.59	1.26
4	-1	16	1.03	1.04
4	-1	16	0.03	0.89
4	-1	16	1.35	0.51
-4	1	-16	1.09	0.46
-4	1	-16	1.75	1.65

-4	1	-16	0.66	0.46
3	-1	16	23.23	2.07
3	-1	16	26.90	1.39
3	-1	16	25.48	2.32
-3	1	-16	27.04	1.44
-3	1	-16	26.61	1.33
-3	1	-16	24.70	1.39
-3	1	-16	23.99	3.12
2	-1	16	-0.70	0.89
2	-1	16	0.20	0.49
-2	1	-16	-0.73	0.39
-2	1	-16	-1.62	1.64
-2	1	-16	0.05	0.40
-2	1	-16	0.31	0.45
1	-1	16	26.05	2.23
1	-1	16	26.37	1.44
1	-1	16	27.40	2.24
-1	1	-16	21.63	3.34
-1	1	-16	25.97	1.31
-1	1	-16	26.39	1.37
-1	1	-16	26.60	1.38
0	-1	16	4.21	0.63
0	-1	16	2.72	1.07
0	-1	16	4.18	1.20
0	1	-16	2.37	0.45
0	1	-16	3.55	0.52
0	1	-16	3.43	0.49
-1	-1	16	26.98	2.43
-1	-1	16	32.46	1.64
-1	-1	16	26.96	2.28
-1	-1	16	29.27	1.46
1	1	-16	29.08	1.46
1	1	-16	28.91	1.44
-2	-1	16	16.01	2.07
-2	-1	16	15.91	1.24
-2	-1	16	16.00	1.01
-2	-1	16	13.92	1.04
-2	-1	16	13.83	1.79
2	1	-16	14.63	1.00
2	1	-16	14.10	0.93
-3	-1	16	6.40	0.79
-3	-1	16	7.08	1.07
-3	-1	16	6.80	0.84
-3	-1	16	5.59	1.40

-3	-1	16	7.54	1.22
3	1	-16	7.78	0.69
3	1	-16	10.15	1.82
-4	-1	16	-0.22	0.61
-4	-1	16	-0.09	0.42
-4	-1	16	-0.04	0.43
-4	-1	16	0.78	0.89
4	1	-16	0.28	0.31
4	1	-16	0.89	1.10
-5	-1	16	16.63	1.12
-5	-1	16	18.72	2.39
-5	-1	16	17.00	1.22
-5	-1	16	19.48	1.75
5	1	-16	15.04	1.93
5	1	-16	16.39	0.97
-6	-1	16	-0.25	0.44
-6	-1	16	-0.49	0.52
-6	-1	16	1.74	0.82
-6	-1	16	0.67	0.71
-6	-1	16	1.81	1.43
6	1	-16	0.46	1.02
6	1	-16	0.20	0.32
-7	-1	16	21.20	1.31
-7	-1	16	24.47	1.51
-7	-1	16	20.45	1.82
-7	-1	16	24.37	2.84
-7	-1	16	23.20	1.68
7	1	-16	21.93	1.15
7	1	-16	23.65	2.08
-8	-1	16	2.82	1.53
-8	-1	16	1.28	0.54
-8	-1	16	-0.40	0.90
-8	-1	16	0.36	0.62
-8	-1	16	1.42	0.82
8	1	-16	1.48	0.39
8	1	-16	0.30	1.05
-9	-1	16	4.00	0.87
-9	-1	16	2.74	1.59
-9	-1	16	5.03	0.85
-9	-1	16	3.72	0.65
9	1	-16	3.75	0.49
9	1	-16	4.42	1.16
-10	-1	16	0.37	0.63
-10	-1	16	2.13	1.65

-10	-1	16	-0.44	0.69
10	1	-16	-0.26	0.36
-11	-1	16	7.35	1.88
-11	-1	16	5.53	1.02
11	1	-16	5.59	0.77
-12	-1	16	2.14	0.97
-12	-1	16	1.16	1.85
12	1	-16	1.69	0.46
-13	-1	16	0.89	1.02
-13	-1	16	1.35	1.93
13	1	-16	0.49	0.47
-14	-1	16	8.70	2.38
-14	-1	16	4.05	1.19
14	1	-16	2.94	0.61
-15	-1	16	-1.18	1.16
-15	-1	16	-0.24	2.01
15	1	-16	0.29	0.58
-16	-1	16	-1.68	1.14
-16	-1	16	1.50	2.35
16	1	-16	0.24	0.62
12	-1	17	12.74	2.25
-12	1	-17	11.24	1.68
11	-1	17	-1.44	1.58
-11	1	-17	-0.90	0.83
10	-1	17	26.56	3.03
-10	1	-17	34.05	2.08
9	-1	17	-3.28	1.41
-9	1	-17	-0.67	0.73
8	-1	17	62.04	3.15
8	-1	17	68.92	4.10
-8	1	-17	60.87	3.00
7	-1	17	4.77	1.10
7	-1	17	4.82	1.52
-7	1	-17	4.45	0.79
-7	1	-17	5.09	0.81
6	-1	17	50.00	3.41
6	-1	17	48.84	2.82
-6	1	-17	48.45	2.21
-6	1	-17	52.01	2.44
5	-1	17	1.57	1.00
5	-1	17	2.33	1.34
-5	1	-17	-1.66	1.82
-5	1	-17	1.08	0.49
4	-1	17	50.34	2.21

4	-1	17	50.59	2.95
4	-1	17	48.47	3.35
-4	1	-17	45.52	4.03
-4	1	-17	49.06	2.16
-4	1	-17	51.84	2.35
3	-1	17	-0.05	1.00
3	-1	17	-0.48	0.50
3	-1	17	-0.10	1.18
-3	1	-17	-1.46	2.27
-3	1	-17	-0.24	0.43
-3	1	-17	-0.71	0.44
2	-1	17	40.81	1.88
2	-1	17	41.69	2.75
2	-1	17	33.13	2.79
-2	1	-17	41.06	1.90
-2	1	-17	36.61	1.75
1	-1	17	11.01	1.56
1	-1	17	10.71	1.85
1	-1	17	7.98	1.03
-1	1	-17	9.42	0.89
-1	1	-17	9.64	0.94
-1	1	-17	8.68	0.81
0	-1	17	76.70	3.18
0	-1	17	73.94	3.07
0	-1	17	73.44	4.02
0	1	-17	72.76	3.12
0	1	-17	75.19	3.12
-1	-1	17	2.79	0.67
-1	-1	17	1.91	0.50
-1	-1	17	3.97	1.24
-1	-1	17	0.83	1.11
1	1	-17	2.23	0.50
1	1	-17	2.44	0.49
-2	-1	17	57.49	2.46
-2	-1	17	55.08	3.22
-2	-1	17	48.18	3.92
-2	-1	17	60.97	2.61
2	1	-17	54.75	2.41
2	1	-17	55.31	2.45
-3	-1	17	-0.67	0.48
-3	-1	17	2.57	1.33
-3	-1	17	0.80	0.49
-3	-1	17	1.82	1.06
-3	-1	17	0.67	0.66

3	1	-17	0.22	0.38
-4	-1	17	26.36	1.55
-4	-1	17	26.96	1.50
-4	-1	17	29.03	2.24
-4	-1	17	28.86	1.75
-4	-1	17	23.27	2.80
4	1	-17	27.39	1.36
-5	-1	17	-0.62	1.25
-5	-1	17	0.31	0.58
-5	-1	17	-0.77	0.67
-5	-1	17	0.56	0.95
-5	-1	17	0.26	0.50
5	1	-17	1.10	1.18
5	1	-17	-0.38	0.34
-6	-1	17	13.47	1.47
-6	-1	17	14.49	1.26
-6	-1	17	13.44	1.13
-6	-1	17	14.19	2.16
-6	-1	17	12.65	1.71
6	1	-17	11.87	2.05
6	1	-17	13.62	0.91
-7	-1	17	-1.21	0.81
-7	-1	17	2.16	1.48
-7	-1	17	1.08	0.57
-7	-1	17	0.90	0.66
-7	-1	17	1.11	0.91
7	1	-17	1.36	0.41
7	1	-17	0.75	1.19
-8	-1	17	37.87	2.41
-8	-1	17	43.74	2.28
-8	-1	17	41.25	2.05
-8	-1	17	43.09	3.68
8	1	-17	43.42	2.77
8	1	-17	41.10	1.87
-9	-1	17	5.09	0.92
-9	-1	17	4.56	0.95
-9	-1	17	4.47	0.87
-9	-1	17	4.24	1.68
9	1	-17	4.89	0.73
-10	-1	17	21.26	1.82
-10	-1	17	25.02	3.35
-10	-1	17	20.38	1.68
10	1	-17	19.91	1.19
-11	-1	17	-2.03	1.74

-11	-1	17	0.53	0.93
11	1	-17	1.35	0.46
-12	-1	17	14.01	1.96
-12	-1	17	22.53	3.53
12	1	-17	14.77	1.09
-13	-1	17	0.03	1.07
-13	-1	17	-1.78	1.91
13	1	-17	1.78	0.64
-14	-1	17	10.33	2.36
-14	-1	17	13.00	2.03
14	1	-17	9.95	1.11
-15	-1	17	0.40	1.26
-15	-1	17	0.91	2.28
15	1	-17	-0.53	0.61
11	-1	18	25.96	3.26
-11	1	-18	19.83	1.96
10	-1	18	-1.28	1.62
-10	1	-18	0.64	0.82
9	-1	18	30.86	3.33
-9	1	-18	42.74	2.34
-8	1	-18	-1.12	0.69
7	-1	18	52.22	3.75
7	-1	18	57.92	3.13
-7	1	-18	56.14	2.77
6	-1	18	-0.28	1.13
6	-1	18	0.81	1.52
-6	1	-18	-0.90	0.63
5	-1	18	60.43	3.39
5	-1	18	58.18	3.84
-5	1	-18	63.26	2.85
4	-1	18	2.39	1.20
4	-1	18	1.75	1.40
-4	1	-18	0.53	0.67
-4	1	-18	-0.85	0.51
3	-1	18	47.69	3.41
3	-1	18	49.56	3.08
-3	1	-18	49.35	2.32
-3	1	-18	47.71	2.11
2	-1	18	0.74	1.26
2	-1	18	-0.82	1.19
-2	1	-18	-0.15	0.43
-2	1	-18	-0.13	0.49
1	-1	18	42.02	3.00
1	-1	18	41.09	3.77

-1	1	-18	42.33	2.04
0	-1	18	2.50	1.28
0	-1	18	3.67	1.44
0	-1	18	3.27	0.57
0	-1	18	4.91	0.76
0	1	-18	3.57	0.61
-1	-1	18	36.05	1.75
-1	-1	18	32.12	2.66
-1	-1	18	36.82	1.97
-1	-1	18	33.50	2.92
1	1	-18	36.83	1.79
1	1	-18	36.05	1.74
-2	-1	18	-0.38	1.30
-2	-1	18	0.32	0.50
-2	-1	18	-0.13	0.70
2	1	-18	-0.46	0.53
2	1	-18	-0.02	0.42
-3	-1	18	31.71	1.66
-3	-1	18	28.00	2.40
-3	-1	18	33.06	2.52
-3	-1	18	27.82	2.97
3	1	-18	28.35	1.62
3	1	-18	28.92	1.50
-4	-1	18	2.00	0.63
-4	-1	18	3.77	1.15
-4	-1	18	2.58	0.85
-4	-1	18	2.97	0.68
-4	-1	18	5.88	1.67
4	1	-18	2.28	0.49
-5	-1	18	42.84	2.72
-5	-1	18	38.11	2.02
-5	-1	18	38.74	2.27
-5	-1	18	42.15	3.54
-5	-1	18	40.80	2.12
5	1	-18	40.70	1.86
-6	-1	18	-0.11	0.90
-6	-1	18	1.41	1.05
-6	-1	18	1.79	0.64
-6	-1	18	0.67	0.68
-6	-1	18	0.61	1.54
6	1	-18	2.01	0.44
6	1	-18	-0.81	1.39
-7	-1	18	38.57	2.22
-7	-1	18	40.39	2.53

-7	-1	18	38.29	2.71
-7	-1	18	40.37	3.69
7	1	-18	36.53	2.91
7	1	-18	38.46	1.80
-8	-1	18	3.79	0.89
-8	-1	18	2.41	1.04
-8	-1	18	1.50	1.82
8	1	-18	2.17	0.48
-9	-1	18	13.28	2.31
-9	-1	18	13.15	1.74
9	1	-18	13.90	1.05
-10	-1	18	0.98	0.94
-10	-1	18	2.37	1.80
10	1	-18	1.88	0.49
-11	-1	18	40.85	2.67
-11	-1	18	42.66	4.19
11	1	-18	40.98	1.93
-12	-1	18	0.07	1.97
-12	-1	18	0.88	1.09
-13	-1	18	33.50	4.31
-13	-1	18	42.80	2.84
13	1	-18	37.32	1.92
-14	-1	18	-0.99	1.22
-14	-1	18	0.52	2.23
14	1	-18	1.76	0.64
9	-1	19	1.03	1.76
-9	1	-19	1.42	0.89
8	-1	19	31.97	3.31
-8	1	-19	29.31	2.05
7	-1	19	-0.09	1.62
-7	1	-19	-0.50	0.73
6	-1	19	30.16	3.04
6	-1	19	28.29	2.55
-6	1	-19	28.16	1.86
5	-1	19	6.83	1.47
5	-1	19	-2.10	1.43
-5	1	-19	2.48	0.76
4	-1	19	68.11	4.32
4	-1	19	74.36	3.97
-4	1	-19	71.59	3.23
3	-1	19	2.22	2.59
3	-1	19	0.73	1.43
-3	1	-19	1.26	0.61
2	-1	19	26.43	2.78

-2	1	-19	22.19	1.83
1	-1	19	0.30	0.51
1	-1	19	1.08	1.35
1	-1	19	-0.38	1.38
-1	1	-19	0.61	0.55
0	-1	19	41.47	1.98
0	-1	19	43.52	3.18
0	-1	19	41.51	3.24
0	1	-19	41.66	2.04
-1	-1	19	0.48	1.49
-1	-1	19	2.12	1.33
-1	-1	19	2.18	0.57
1	1	-19	0.75	0.51
-2	-1	19	30.04	2.68
-2	-1	19	32.24	1.72
-2	-1	19	28.74	3.04
2	1	-19	31.21	1.65
-3	-1	19	-1.62	1.52
-3	-1	19	-0.06	1.17
-3	-1	19	-0.21	0.62
3	1	-19	-0.05	0.49
-4	-1	19	41.40	3.55
-4	-1	19	36.70	2.00
4	1	-19	36.08	2.09
4	1	-19	35.20	1.79
-5	-1	19	1.45	1.18
-5	-1	19	1.02	1.57
-5	-1	19	1.47	0.74
5	1	-19	-0.13	0.45
-6	-1	19	50.25	4.00
-6	-1	19	43.74	2.92
-6	-1	19	47.11	2.46
6	1	-19	45.20	2.10
-7	-1	19	-0.47	1.10
-7	-1	19	1.78	1.89
-7	-1	19	1.60	0.79
7	1	-19	0.11	0.47
-8	-1	19	38.53	3.99
-8	-1	19	42.03	2.74
-8	-1	19	49.91	2.57
8	1	-19	43.23	2.04
-9	-1	19	-1.24	0.93
-9	-1	19	2.60	2.01
9	1	-19	0.15	0.50

-10	-1	19	25.45	3.82
-10	-1	19	38.88	2.51
10	1	-19	30.09	1.65
-11	-1	19	-1.68	2.17
-11	-1	19	-2.45	1.11
-12	-1	19	48.48	4.65
-12	-1	19	43.99	3.00
12	1	-19	46.23	2.17
-13	-1	19	0.68	1.32
8	-1	20	2.02	2.33
-8	1	-20	-0.44	0.92
7	-1	20	22.41	3.29
-7	1	-20	23.26	1.87
6	-1	20	1.81	1.69
6	-1	20	0.54	1.43
-6	1	-20	1.75	0.84
5	-1	20	15.27	2.75
-5	1	-20	13.79	1.53
4	-1	20	8.23	1.90
4	-1	20	6.97	1.70
-4	1	-20	4.70	0.87
3	-1	20	28.04	2.86
3	-1	20	19.54	2.91
-3	1	-20	22.60	1.63
2	-1	20	0.78	1.52
2	-1	20	1.09	1.53
-2	1	-20	0.46	0.64
1	-1	20	32.15	3.16
1	-1	20	29.44	3.07
-1	1	-20	31.87	1.81
0	-1	20	-0.35	1.50
0	-1	20	1.86	1.56
-1	-1	20	24.04	3.08
-1	-1	20	22.06	2.73
1	1	-20	24.32	1.49
-2	-1	20	4.46	0.80
-2	-1	20	5.54	1.59
-2	-1	20	5.34	1.81
2	1	-20	4.67	0.70
-3	-1	20	39.12	3.59
-3	-1	20	35.53	2.98
-3	-1	20	35.19	1.98
3	1	-20	35.41	1.83
-4	-1	20	1.74	1.37

-4	-1	20	2.59	1.93
-4	-1	20	0.98	0.75
4	1	-20	1.07	0.57
-5	-1	20	31.92	3.62
-5	-1	20	33.49	2.01
-5	-1	20	29.15	2.72
5	1	-20	30.93	1.65
-6	-1	20	4.18	0.94
-6	-1	20	3.70	1.97
6	1	-20	4.49	0.66
-7	-1	20	37.02	4.15
-7	-1	20	39.46	2.90
-7	-1	20	44.61	2.47
7	1	-20	41.94	1.99
-8	-1	20	1.94	2.10
-8	-1	20	1.61	1.60
-8	-1	20	1.21	1.17
8	1	-20	0.52	0.55
-9	-1	20	59.55	3.14
-9	-1	20	59.75	4.81
9	1	-20	48.60	2.44
-10	-1	20	1.43	1.21
-10	-1	20	-1.50	2.13
10	1	-20	1.04	0.74
-11	-1	20	15.68	3.08
-11	-1	20	18.63	2.34
6	-1	21	-2.54	1.97
-6	1	-21	1.09	0.92
5	-1	21	0.30	1.58
5	-1	21	1.15	1.92
-5	1	-21	2.12	0.87
4	-1	21	4.69	1.83
4	-1	21	0.47	1.62
-4	1	-21	-1.64	0.76
3	-1	21	-3.56	1.61
3	-1	21	0.63	1.66
-3	1	-21	-0.68	0.72
2	-1	21	6.90	1.88
2	-1	21	8.07	1.90
-2	1	-21	3.96	0.88
1	-1	21	-0.18	1.69
1	-1	21	-1.21	1.56
-1	1	-21	0.41	0.66
0	-1	21	5.06	1.92

0	-1	21	3.17	1.66
0	1	-21	2.98	0.76
-1	-1	21	4.70	1.70
-1	-1	21	1.40	1.81
1	1	-21	2.54	0.70
-2	-1	21	8.65	1.81
-2	-1	21	10.43	2.24
2	1	-21	8.23	1.15
-3	-1	21	2.11	1.62
-3	-1	21	0.95	1.80
-4	-1	21	3.43	1.99
-4	-1	21	4.73	1.67
4	1	-21	5.69	0.87
-5	-1	21	-0.34	1.47
-5	-1	21	3.88	2.12
5	1	-21	0.28	0.68
-6	-1	21	13.49	2.53
-6	-1	21	13.13	2.42
6	1	-21	9.87	1.29
-7	-1	21	0.06	1.06
-7	-1	21	-1.25	2.11
-7	-1	21	-0.41	1.35
-8	-1	21	4.49	2.39
-8	-1	21	7.14	1.27
-9	-1	21	2.82	1.30
-9	-1	21	5.48	2.55
3	-1	22	4.54	1.99
3	-1	22	5.67	1.98
-3	1	-22	4.36	0.95
2	-1	22	0.54	1.82
2	-1	22	0.43	1.93
-2	1	-22	0.27	0.81
1	-1	22	0.00	1.75
1	-1	22	1.54	1.94
-1	1	-22	1.49	0.82
0	-1	22	0.16	1.78
0	-1	22	2.04	1.96
0	1	-22	-0.44	0.72
-1	-1	22	10.34	2.05
-1	-1	22	10.76	2.49
1	1	-22	11.49	1.37
-2	-1	22	7.33	2.30
-2	-1	22	6.38	1.89
2	1	-22	8.06	1.28

-3	-1	22	4.79	2.16
-3	-1	22	4.23	1.79
3	1	-22	2.07	0.75
-4	-1	22	1.48	2.18
-4	-1	22	-0.49	1.61
4	1	-22	0.55	0.67
-5	-1	22	-1.10	1.63
-5	-1	22	1.36	2.15
-6	-1	22	1.73	2.24
-6	-1	22	1.79	1.68
6	-2	-22	0.05	0.73
-6	2	22	-2.39	2.03
-6	2	22	1.50	1.67
5	-2	-22	9.17	1.31
-5	2	22	4.29	1.76
-5	2	22	9.36	2.44
4	-2	-22	2.39	0.79
-4	2	22	2.94	1.68
-4	2	22	5.90	2.15
3	-2	-22	3.29	0.84
-3	2	22	-0.06	1.61
-3	2	22	3.31	2.02
2	-2	-22	12.30	1.46
-2	2	22	13.49	2.40
-2	2	22	11.84	2.10
1	-2	-22	2.31	0.83
-1	2	22	3.11	1.95
-1	2	22	2.08	1.72
0	-2	-22	10.54	1.45
0	2	22	9.93	2.21
0	2	22	7.92	2.02
-1	-2	-22	4.76	0.96
1	2	22	4.68	1.99
1	2	22	4.45	1.96
-2	-2	-22	1.05	0.94
2	2	22	0.83	1.92
2	2	22	-0.13	1.78
-3	-2	-22	0.55	0.90
9	-2	-21	10.66	1.31
-9	2	21	8.80	2.92
8	-2	-21	3.02	0.75
-8	2	21	3.79	2.17
7	-2	-21	4.01	0.74
-7	2	21	4.22	1.51

-7	2	21	4.22	2.16
6	-2	-21	-0.67	0.62
-6	2	21	2.98	1.45
-6	2	21	0.65	1.87
5	-2	-21	7.54	0.91
-5	2	21	10.95	2.27
-5	2	21	8.34	1.71
4	-2	-21	1.12	0.70
-4	2	21	0.67	1.48
-4	2	21	1.04	1.84
3	-2	-21	8.61	1.19
-3	2	21	5.51	1.73
-3	2	21	4.50	2.04
2	-2	-21	1.68	0.69
-2	2	21	3.81	1.84
-2	2	21	4.20	1.59
1	-2	-21	2.77	0.75
-1	2	21	2.76	1.61
-1	2	21	1.28	1.60
0	-2	-21	0.70	0.76
0	2	21	2.65	1.65
0	2	21	0.95	1.59
-1	-2	-21	6.40	0.93
1	2	21	3.08	1.72
1	2	21	4.68	1.72
-2	-2	-21	1.71	0.78
2	2	21	4.20	1.71
2	2	21	-0.39	1.57
-3	-2	-21	-0.43	0.76
3	2	21	-1.41	1.68
3	2	21	0.23	1.59
-4	-2	-21	6.04	1.03
4	2	21	4.40	1.88
4	2	21	6.83	1.83
-5	-2	-21	1.32	0.91
5	2	21	-0.05	1.65
5	2	21	-3.91	1.69
-6	-2	-21	0.48	0.89
6	2	21	-2.78	2.46
11	-2	-20	-0.92	0.64
-11	2	20	-2.82	1.23
-11	2	20	1.62	2.63
10	-2	-20	35.58	1.98
-10	2	20	37.35	4.10

-10	2	20	37.36	2.61
9	-2	-20	-0.16	0.62
-9	2	20	0.94	1.02
-9	2	20	1.51	1.98
8	-2	-20	43.13	2.21
-8	2	20	48.75	2.63
-8	2	20	46.61	3.41
-8	2	20	42.66	4.13
7	-2	-20	0.22	0.58
-7	2	20	1.16	0.96
-7	2	20	1.28	1.86
-7	2	20	1.33	1.36
6	-2	-20	26.63	1.54
-6	2	20	22.18	3.35
-6	2	20	19.00	3.24
5	-2	-20	1.03	0.60
-5	2	20	3.52	1.38
-5	2	20	1.80	1.73
4	-2	-20	45.20	2.18
-4	2	20	42.59	3.15
-4	2	20	43.90	3.79
3	-2	-20	-1.09	0.54
-3	2	20	-2.03	1.41
-3	2	20	1.44	1.43
2	-2	-20	35.04	1.91
-2	2	20	36.52	3.31
-2	2	20	33.78	2.96
1	-2	-20	-1.04	0.61
-1	2	20	1.96	1.55
-1	2	20	0.46	1.39
0	-2	-20	39.65	2.03
0	2	20	34.02	3.13
0	2	20	31.85	3.08
-1	-2	-20	0.54	0.69
1	2	20	-0.29	1.41
1	2	20	0.79	1.41
-2	-2	-20	28.15	1.82
2	2	20	25.71	2.83
2	2	20	25.01	2.98
-3	-2	-20	1.85	0.76
3	2	20	0.64	1.40
3	2	20	1.70	1.51
-4	-2	-20	16.07	1.54
4	2	20	16.25	2.91

4	2	20	14.88	2.46
-5	-2	-20	2.13	0.83
5	2	20	-1.58	1.36
5	2	20	1.46	1.58
-6	-2	-20	14.54	1.64
6	2	20	13.22	2.50
6	2	20	14.49	2.24
-7	-2	-20	1.28	0.90
7	2	20	0.65	1.76
-8	-2	-20	18.81	1.88
13	-2	-19	32.37	1.91
-13	2	19	36.35	2.90
12	-2	-19	1.72	0.70
-12	2	19	2.38	2.15
-12	2	19	1.76	1.28
11	-2	-19	42.58	2.19
-11	2	19	40.32	4.34
-11	2	19	50.02	2.86
10	-2	-19	2.42	0.63
-10	2	19	0.57	1.98
-10	2	19	3.05	1.07
9	-2	-19	23.88	1.50
-9	2	19	25.96	2.00
-9	2	19	25.20	3.51
8	-2	-19	-0.05	0.49
-8	2	19	1.55	1.74
-8	2	19	0.01	0.87
-8	2	19	-0.15	1.07
7	-2	-19	45.69	2.14
-7	2	19	42.53	3.82
-7	2	19	42.28	2.88
-7	2	19	46.25	2.40
6	-2	-19	0.13	0.50
-6	2	19	1.52	1.72
-6	2	19	0.78	1.16
-6	2	19	1.27	0.75
5	-2	-19	40.45	1.97
-5	2	19	36.89	2.74
-5	2	19	41.80	2.11
-5	2	19	41.88	3.55
4	-2	-19	-0.02	0.47
-4	2	19	-1.64	1.37
-4	2	19	-1.03	1.39
-4	2	19	1.24	0.65

3	-2	-19	77.17	3.21
-3	2	19	74.46	3.10
-3	2	19	70.83	3.93
-3	2	19	71.59	4.45
2	-2	-19	0.30	0.52
-2	2	19	0.98	0.79
-2	2	19	0.38	1.35
-2	2	19	0.21	1.21
1	-2	-19	44.70	2.22
-1	2	19	41.06	3.11
-1	2	19	52.00	3.45
0	-2	-19	3.76	0.71
0	2	19	4.61	1.41
0	2	19	3.82	1.46
-1	-2	-19	54.42	2.47
1	2	19	46.05	3.46
1	2	19	49.40	3.33
-2	-2	-19	1.60	0.66
2	2	19	3.11	1.42
2	2	19	2.72	1.42
-3	-2	-19	37.22	2.14
3	2	19	42.23	3.27
3	2	19	43.44	2.96
-4	-2	-19	0.62	0.63
4	2	19	0.43	1.42
4	2	19	0.14	1.44
-5	-2	-19	40.58	2.22
5	2	19	38.41	2.90
5	2	19	34.35	3.24
-6	-2	-19	-0.51	0.71
6	2	19	1.30	1.25
6	2	19	0.19	1.52
-7	-2	-19	44.23	2.46
7	2	19	40.46	3.55
7	2	19	45.37	2.98
-8	-2	-19	2.23	0.87
8	2	19	2.49	1.73
-9	-2	-19	38.94	2.34
9	2	19	29.20	3.37
14	-2	-18	15.80	1.45
-14	2	18	20.84	2.53
-14	2	18	31.35	4.14
13	-2	-18	0.37	0.64
-13	2	18	-1.31	1.16

-13	2	18	3.44	2.20
12	-2	-18	35.89	1.90
-12	2	18	38.63	2.61
-12	2	18	34.20	3.97
11	-2	-18	0.56	0.55
-11	2	18	5.34	2.06
-11	2	18	0.99	1.01
10	-2	-18	44.47	2.12
-10	2	18	43.04	3.97
9	-2	-18	1.88	0.55
-9	2	18	2.07	1.06
-9	2	18	3.04	0.90
-9	2	18	0.36	1.71
8	-2	-18	20.38	1.27
-8	2	18	21.32	3.08
-8	2	18	22.13	1.98
-8	2	18	18.11	1.66
7	-2	-18	-1.24	1.39
-7	2	18	0.98	1.03
-7	2	18	-1.36	0.70
-7	2	18	0.32	1.54
6	-2	-18	37.31	1.86
-6	2	18	37.79	3.40
-6	2	18	41.15	2.10
-6	2	18	38.80	2.57
5	-2	-18	-0.06	0.46
-5	2	18	1.33	1.42
-5	2	18	0.36	0.64
-5	2	18	1.26	1.08
4	-2	-18	60.42	2.73
-4	2	18	62.68	4.03
-4	2	18	60.13	3.41
-4	2	18	66.95	2.77
3	-2	-18	5.92	0.84
-3	2	18	4.26	1.21
-3	2	18	5.46	0.87
-3	2	18	6.22	1.56
2	-2	-18	55.56	2.51
-2	2	18	61.44	2.40
-2	2	18	52.27	3.56
-2	2	18	49.81	3.99
1	-2	-18	2.23	0.53
-1	2	18	-0.21	1.15
-1	2	18	1.35	1.17

0	-2	-18	48.60	2.78
0	2	18	49.31	3.19
0	2	18	48.57	3.27
-1	-2	-18	1.42	0.58
1	2	18	1.86	1.13
1	2	18	2.37	1.28
-2	-2	-18	37.36	1.94
2	2	18	35.97	2.94
-3	-2	-18	0.90	0.57
3	2	18	0.66	1.27
3	2	18	1.00	1.09
-4	-2	-18	56.08	2.73
4	2	18	54.60	3.72
4	2	18	60.75	3.32
-5	-2	-18	-0.96	0.63
5	2	18	0.56	1.36
5	2	18	0.94	1.11
-6	-2	-18	37.35	2.12
6	2	18	30.40	3.10
6	2	18	40.42	2.67
-7	-2	-18	-0.84	0.70
7	2	18	-0.50	1.11
7	2	18	1.18	1.43
-8	-2	-18	45.56	2.53
8	2	18	49.30	2.98
8	2	18	45.62	3.54
-9	-2	-18	1.04	0.79
9	2	18	2.37	1.57
-10	-2	-18	30.45	2.15
10	2	18	30.14	3.21
-11	-2	-18	1.96	0.97
11	2	18	-2.01	1.83
15	-2	-17	10.34	1.27
-15	2	17	4.55	2.38
-15	2	17	6.76	1.49
14	-2	-17	1.54	0.63
-14	2	17	3.21	2.30
-14	2	17	0.83	1.23
13	-2	-17	24.16	1.52
-13	2	17	23.88	3.65
-13	2	17	26.79	2.35
12	-2	-17	0.51	0.53
-12	2	17	0.75	1.00
-12	2	17	3.54	1.98

11	-2	-17	18.14	1.23
-11	2	17	19.88	3.11
-11	2	17	16.90	1.77
10	-2	-17	0.76	0.48
-10	2	17	0.38	1.61
-10	2	17	0.71	0.91
-10	2	17	1.11	0.84
9	-2	-17	10.55	1.22
-9	2	17	15.60	1.54
-9	2	17	12.34	1.58
-9	2	17	9.55	1.96
8	-2	-17	3.32	1.15
8	-2	-17	0.12	0.42
-8	2	17	-0.05	0.70
-8	2	17	-0.12	0.90
-8	2	17	-0.78	1.45
-8	2	17	0.56	0.85
7	-2	-17	21.26	1.21
7	-2	-17	22.03	2.15
-7	2	17	15.91	1.56
-7	2	17	20.57	1.86
-7	2	17	21.82	1.53
-7	2	17	14.38	2.67
6	-2	-17	-1.00	1.19
6	-2	-17	-0.37	0.38
-6	2	17	0.91	0.61
-6	2	17	-0.17	1.39
-6	2	17	0.25	0.69
-6	2	17	0.71	0.99
5	-2	-17	43.41	2.85
5	-2	-17	42.42	1.96
-5	2	17	44.41	2.07
-5	2	17	37.77	2.53
-5	2	17	42.24	2.11
-5	2	17	41.66	3.26
4	-2	-17	0.12	0.41
-4	2	17	0.67	0.57
-4	2	17	1.08	1.23
-4	2	17	-0.25	0.50
-4	2	17	1.50	1.03
3	-2	-17	72.43	3.11
-3	2	17	76.15	3.07
-3	2	17	74.23	4.14
-3	2	17	68.47	3.73

-3	2	17	75.95	3.09
2	-2	-17	9.04	0.91
-2	2	17	8.79	0.84
-2	2	17	10.28	1.63
-2	2	17	9.26	1.76
-2	2	17	9.98	0.92
1	-2	-17	68.35	2.96
-1	2	17	71.48	2.84
-1	2	17	68.78	2.78
-1	2	17	65.50	3.66
0	-2	-17	2.68	0.54
0	2	17	4.50	1.72
0	2	17	4.64	1.22
-1	-2	-17	38.53	1.93
1	2	17	35.67	2.74
1	2	17	41.80	2.71
-2	-2	-17	0.41	0.45
2	2	17	-0.96	0.89
-3	-2	-17	33.44	2.05
3	2	17	38.90	2.50
3	2	17	34.07	2.80
-4	-2	-17	0.61	0.55
4	2	17	1.49	1.28
4	2	17	0.65	0.98
-5	-2	-17	34.74	4.01
-5	-2	-17	39.46	2.03
5	2	17	35.14	2.47
5	2	17	37.60	2.95
-6	-2	-17	1.81	3.42
-6	-2	-17	-0.97	0.59
6	2	17	2.70	1.29
6	2	17	1.31	0.96
-7	-2	-17	50.12	2.56
7	2	17	51.53	2.88
7	2	17	50.61	3.54
-8	-2	-17	2.03	0.74
8	2	17	3.43	1.55
8	2	17	1.26	0.98
-9	-2	-17	54.19	2.78
9	2	17	51.91	3.69
-10	-2	-17	-0.30	0.76
10	2	17	1.20	1.51
-11	-2	-17	32.94	2.22
11	2	17	30.66	3.20

-12	-2	-17	1.29	0.92
12	2	17	-0.67	1.62
16	-2	-16	1.19	0.69
-16	2	16	1.82	1.41
-16	2	16	-3.23	2.11
15	-2	-16	-0.18	0.59
-15	2	16	0.27	1.24
-15	2	16	-2.14	2.06
14	-2	-16	0.23	0.56
-14	2	16	1.41	1.98
-14	2	16	1.73	1.14
13	-2	-16	-0.14	0.52
-13	2	16	-0.21	1.70
-13	2	16	-0.01	1.06
12	-2	-16	1.27	0.53
-12	2	16	1.60	0.92
-12	2	16	3.62	1.84
11	-2	-16	3.80	1.38
11	-2	-16	6.14	0.84
-11	2	16	7.47	1.92
-11	2	16	3.73	1.20
10	-2	-16	2.75	0.52
-10	2	16	3.83	0.87
-10	2	16	2.56	0.83
-10	2	16	3.42	1.64
9	-2	-16	9.96	0.85
9	-2	-16	8.48	1.19
-9	2	16	8.16	1.06
-9	2	16	6.31	1.76
-9	2	16	10.02	1.39
-9	2	16	8.74	1.32
8	-2	-16	0.88	0.98
8	-2	-16	2.13	0.44
-8	2	16	1.97	0.70
-8	2	16	2.37	1.47
-8	2	16	0.95	0.74
-8	2	16	0.46	0.82
7	-2	-16	0.06	1.01
7	-2	-16	0.11	0.37
-7	2	16	-0.35	0.57
-7	2	16	1.01	1.23
-7	2	16	0.17	0.82
-7	2	16	0.56	0.69
6	-2	-16	8.19	0.81

6	-2	-16	11.10	1.76
-6	2	16	8.03	1.43
-6	2	16	10.37	1.09
-6	2	16	10.17	1.25
-6	2	16	9.71	1.15
-6	2	16	9.99	1.74
5	-2	-16	7.99	1.32
5	-2	-16	10.73	0.84
-5	2	16	9.91	1.63
-5	2	16	8.74	0.96
-5	2	16	8.12	1.19
-5	2	16	8.09	1.00
-5	2	16	10.89	1.10
4	-2	-16	12.09	0.93
4	-2	-16	14.82	2.03
-4	2	16	13.09	1.04
-4	2	16	16.38	1.13
-4	2	16	15.64	2.05
-4	2	16	12.04	1.58
-4	2	16	11.48	1.08
3	-2	-16	0.90	1.09
3	-2	-16	1.29	0.42
-3	2	16	0.67	1.41
-3	2	16	0.07	0.52
-3	2	16	-0.59	0.84
-3	2	16	1.36	0.49
-3	2	16	1.97	0.52
2	-2	-16	14.32	0.98
-2	2	16	12.14	1.70
-2	2	16	14.91	1.90
-2	2	16	14.46	1.00
-2	2	16	10.88	1.08
-2	2	16	14.41	0.92
1	-2	-16	5.16	0.75
-1	2	16	2.87	0.66
-1	2	16	5.82	1.18
-1	2	16	5.31	1.19
-1	2	16	4.02	0.68
-1	2	16	5.70	0.72
0	-2	-16	40.18	1.90
0	2	16	44.42	2.67
0	2	16	34.75	2.52
-1	-2	-16	6.80	0.83
1	2	16	4.17	1.11

1	2	16	7.07	1.25
-2	-2	-16	10.75	0.95
-2	-2	-16	11.33	2.54
2	2	16	9.48	1.59
2	2	16	8.22	1.57
-3	-2	-16	8.15	1.95
-3	-2	-16	8.65	0.96
3	2	16	10.36	1.80
3	2	16	9.38	1.33
-4	-2	-16	21.94	3.29
-4	-2	-16	18.43	1.27
4	2	16	16.09	2.09
4	2	16	18.83	1.78
-5	-2	-16	0.10	0.50
-5	-2	-16	1.59	1.84
5	2	16	0.86	0.85
5	2	16	0.54	1.07
-6	-2	-16	22.42	3.29
-6	-2	-16	25.15	1.55
6	2	16	22.59	2.42
6	2	16	23.51	1.86
-7	-2	-16	2.07	1.99
-7	-2	-16	0.38	0.56
7	2	16	-0.40	0.79
7	2	16	0.24	1.18
-8	-2	-16	28.17	1.86
8	2	16	32.46	2.74
8	2	16	32.35	2.08
-9	-2	-16	0.32	0.67
9	2	16	-0.02	1.33
9	2	16	0.33	0.88
-10	-2	-16	38.03	2.26
10	2	16	36.21	3.09
-11	-2	-16	1.31	0.85
11	2	16	0.18	1.48
-12	-2	-16	25.59	2.04
12	2	16	28.98	3.06
-13	-2	-16	1.45	0.97
13	2	16	0.94	1.67
17	-2	-15	15.75	1.41
-17	2	15	17.86	2.47
-17	2	15	13.93	2.69
16	-2	-15	1.66	0.67
-16	2	15	5.25	2.37

-16	2	15	1.65	1.27
15	-2	-15	19.67	1.33
-15	2	15	12.35	2.59
-15	2	15	16.84	2.15
14	-2	-15	2.66	0.63
-14	2	15	4.69	1.18
-14	2	15	2.56	1.83
13	-2	-15	13.21	1.08
-13	2	15	16.83	1.83
-13	2	15	13.02	2.27
12	-2	-15	0.03	0.46
-12	2	15	0.81	0.79
-12	2	15	0.27	1.60
11	-2	-15	8.61	1.63
11	-2	-15	10.84	0.89
-11	2	15	8.67	1.90
-11	2	15	13.86	1.43
10	-2	-15	5.88	0.89
10	-2	-15	5.46	0.74
-10	2	15	5.60	1.70
-10	2	15	6.22	1.16
-10	2	15	4.07	0.84
9	-2	-15	17.51	1.04
9	-2	-15	17.13	1.55
-9	2	15	15.10	1.40
-9	2	15	17.83	1.45
-9	2	15	14.65	2.45
-9	2	15	15.69	1.54
8	-2	-15	19.00	1.70
8	-2	-15	19.82	1.11
-8	2	15	18.30	1.50
-8	2	15	18.53	2.41
-8	2	15	18.01	1.53
-8	2	15	20.35	1.42
-8	2	15	17.24	1.46
7	-2	-15	14.57	1.69
7	-2	-15	15.97	1.01
-7	2	15	17.94	2.26
-7	2	15	19.25	1.36
-7	2	15	15.52	1.49
-7	2	15	18.31	1.29
-7	2	15	15.69	1.18
6	-2	-15	-0.09	0.31
6	-2	-15	-0.23	0.92

-6	2	15	0.03	0.77
-6	2	15	0.70	0.55
-6	2	15	0.96	1.24
-6	2	15	-0.32	0.48
-6	2	15	-0.56	0.50
5	-2	-15	1.16	0.37
5	-2	-15	2.49	0.96
-5	2	15	1.30	0.50
-5	2	15	2.07	0.49
-5	2	15	1.48	0.79
-5	2	15	1.93	1.11
-5	2	15	1.04	0.63
4	-2	-15	2.08	0.99
4	-2	-15	1.97	0.42
-4	2	15	1.67	0.50
-4	2	15	3.03	0.88
-4	2	15	2.56	0.52
-4	2	15	0.18	0.96
-4	2	15	2.05	0.50
3	-2	-15	3.10	1.06
3	-2	-15	2.17	0.43
-3	2	15	3.01	0.53
-3	2	15	1.47	0.48
-3	2	15	3.54	0.92
-3	2	15	3.35	0.51
-3	2	15	2.81	1.08
2	-2	-15	21.71	2.09
2	-2	-15	20.38	1.20
-2	2	15	19.51	1.82
-2	2	15	21.95	1.17
-2	2	15	21.83	2.08
-2	2	15	22.50	1.13
-2	2	15	23.91	1.30
1	-2	-15	8.30	1.31
1	-2	-15	6.76	0.77
-1	2	15	7.61	0.87
-1	2	15	10.54	1.65
-1	2	15	8.11	0.72
-1	2	15	7.68	1.17
-1	2	15	8.77	0.70
0	-2	-15	1.42	0.42
0	-2	-15	1.37	0.47
0	-2	-15	1.23	1.50
0	2	15	0.62	0.82

0	2	15	1.51	0.31
0	2	15	0.99	0.48
0	2	15	1.55	0.32
0	2	15	0.68	0.90
-1	-2	-15	7.50	1.70
-1	-2	-15	6.21	0.74
-1	-2	-15	5.41	0.79
1	2	15	5.71	1.10
1	2	15	4.97	1.07
-2	-2	-15	1.98	1.57
-2	-2	-15	0.65	0.41
-2	-2	-15	1.11	0.54
2	2	15	0.28	0.76
2	2	15	-0.44	0.86
-3	-2	-15	3.17	1.61
-3	-2	-15	5.50	0.82
3	2	15	4.60	1.08
3	2	15	3.40	0.91
-4	-2	-15	26.77	1.48
-4	-2	-15	25.57	2.96
4	2	15	26.98	1.89
4	2	15	22.32	2.30
-5	-2	-15	0.54	0.46
-5	-2	-15	1.80	1.52
5	2	15	0.83	0.78
5	2	15	0.98	1.00
-6	-2	-15	0.32	0.56
-6	-2	-15	3.73	1.71
6	2	15	-0.30	1.04
6	2	15	1.52	0.74
-7	-2	-15	0.66	0.55
-7	-2	-15	-0.71	1.62
7	2	15	0.21	0.78
7	2	15	0.11	1.04
-8	-2	-15	-0.70	1.60
-8	-2	-15	0.80	0.55
8	2	15	0.89	1.16
8	2	15	0.06	0.76
-9	-2	-15	4.19	0.78
-9	-2	-15	3.80	0.61
9	2	15	4.59	0.87
9	2	15	4.20	1.53
-10	-2	-15	12.18	1.36
10	2	15	12.40	1.35

10	2	15	12.54	2.33
-11	-2	-15	2.75	0.93
11	2	15	5.52	1.58
-12	-2	-15	1.14	0.85
12	2	15	2.39	1.56
-13	-2	-15	2.97	0.98
13	2	15	2.49	1.65
-14	-2	-15	-0.18	0.88
14	2	15	0.96	1.63
17	-2	-14	2.11	0.71
-17	2	14	5.69	1.41
-17	2	14	2.43	2.19
16	-2	-14	55.79	2.66
-16	2	14	63.04	3.45
-16	2	14	56.04	4.56
15	-2	-14	1.55	0.59
-15	2	14	1.34	1.11
-15	2	14	-0.39	1.78
14	-2	-14	55.54	2.54
-14	2	14	57.67	3.16
-14	2	14	57.70	4.17
13	-2	-14	0.49	0.43
-13	2	14	3.10	1.75
-13	2	14	0.33	0.87
12	-2	-14	40.98	2.16
-12	2	14	49.48	2.56
-12	2	14	46.58	3.60
11	-2	-14	0.63	0.69
11	-2	-14	0.88	0.39
11	-2	-14	0.98	0.61
-11	2	14	-0.40	1.43
-11	2	14	1.00	0.73
10	-2	-14	126.21	5.00
10	-2	-14	126.86	5.14
-10	2	14	133.26	5.33
-10	2	14	119.40	6.16
9	-2	-14	1.14	0.38
9	-2	-14	1.40	0.70
-9	2	14	2.09	0.69
-9	2	14	0.67	0.51
-9	2	14	-0.15	0.60
-9	2	14	2.51	1.32
-9	2	14	1.71	0.71
8	-2	-14	115.97	4.78

8	-2	-14	110.76	4.49
-8	2	14	112.54	4.60
-8	2	14	122.72	4.75
-8	2	14	122.77	4.75
-8	2	14	103.22	5.62
-8	2	14	107.49	4.71
7	-2	-14	0.05	0.78
7	-2	-14	0.47	0.32
-7	2	14	0.70	0.65
-7	2	14	0.60	0.52
-7	2	14	-0.66	0.55
-7	2	14	0.14	0.43
-7	2	14	0.12	1.12
6	-2	-14	35.25	2.08
6	-2	-14	32.06	1.50
-6	2	14	33.20	2.53
-6	2	14	31.99	1.69
-6	2	14	33.98	1.69
-6	2	14	28.51	1.89
-6	2	14	30.73	1.59
5	-2	-14	8.89	1.43
5	-2	-14	7.72	0.68
-5	2	14	7.62	0.87
-5	2	14	7.72	0.85
-5	2	14	6.25	1.31
-5	2	14	7.77	0.80
-5	2	14	7.27	1.22
4	-2	-14	78.54	3.11
4	-2	-14	77.41	3.65
-4	2	14	73.55	3.15
-4	2	14	79.69	3.99
-4	2	14	74.95	3.12
-4	2	14	67.73	3.48
-4	2	14	80.01	3.16
3	-2	-14	1.61	2.00
3	-2	-14	0.05	0.89
3	-2	-14	0.33	0.32
-3	2	14	0.49	0.38
-3	2	14	-0.52	0.58
-3	2	14	0.40	0.38
-3	2	14	0.23	0.87
-3	2	14	0.90	0.73
2	-2	-14	89.71	4.21
2	-2	-14	88.38	4.76

2	-2	-14	87.61	3.59
-2	2	14	86.00	4.07
-2	2	14	82.99	4.27
-2	2	14	94.26	3.54
-2	2	14	92.56	3.54
-2	2	14	88.80	3.65
1	-2	-14	3.02	1.48
1	-2	-14	3.50	1.00
1	-2	-14	1.43	0.39
-1	2	14	0.88	0.84
-1	2	14	0.47	0.40
-1	2	14	1.46	0.80
-1	2	14	1.15	0.33
0	-2	-14	58.58	2.56
0	-2	-14	63.17	3.74
0	-2	-14	60.24	3.33
0	2	14	56.38	3.08
0	2	14	57.57	3.16
0	2	14	60.85	2.62
0	2	14	61.63	2.38
0	2	14	61.28	2.38
-1	-2	-14	0.18	0.36
-1	-2	-14	0.60	0.35
-1	-2	-14	0.87	1.29
-1	-2	-14	-1.03	0.93
1	2	14	-0.66	0.72
1	2	14	-0.59	0.73
-2	-2	-14	28.90	1.47
-2	-2	-14	27.69	1.46
-2	-2	-14	28.58	2.74
2	2	14	28.90	2.06
2	2	14	26.47	1.90
-3	-2	-14	17.52	1.11
-3	-2	-14	12.87	2.33
-3	-2	-14	18.31	1.08
3	2	14	16.88	1.56
3	2	14	15.66	1.67
-4	-2	-14	34.02	2.92
-4	-2	-14	35.70	1.79
4	2	14	31.39	2.31
4	2	14	38.33	2.13
-5	-2	-14	1.37	0.43
-5	-2	-14	0.95	1.39
5	2	14	2.05	0.72

5	2	14	1.73	0.96
-6	-2	-14	17.92	2.46
-6	-2	-14	16.19	1.17
6	2	14	16.74	1.87
6	2	14	16.99	1.43
-7	-2	-14	3.73	1.50
7	2	14	4.58	1.23
7	2	14	5.10	0.62
7	2	14	6.03	1.12
-8	-2	-14	30.69	2.78
-8	-2	-14	30.71	1.75
-8	-2	-14	29.35	1.48
8	2	14	27.87	2.40
8	2	14	31.19	1.44
8	2	14	30.58	1.81
-9	-2	-14	3.05	1.50
-9	-2	-14	1.24	0.62
-9	-2	-14	1.81	0.53
9	2	14	2.09	0.72
9	2	14	3.18	1.15
-10	-2	-14	14.96	1.17
-10	-2	-14	15.29	1.37
10	2	14	14.57	1.35
10	2	14	15.66	2.16
-11	-2	-14	13.01	1.27
-11	-2	-14	11.91	1.33
11	2	14	13.18	2.11
-12	-2	-14	19.46	1.69
12	2	14	18.57	2.56
-13	-2	-14	9.68	1.46
13	2	14	7.74	1.81
-14	-2	-14	6.27	1.09
14	2	14	3.55	1.62
-15	-2	-14	2.06	1.00
15	2	14	2.06	1.72
18	-2	-13	-0.52	0.65
-18	2	13	-4.60	2.09
-18	2	13	-1.54	1.26
17	-2	-13	49.31	2.45
-17	2	13	55.53	3.29
-17	2	13	50.15	4.35
16	-2	-13	-0.23	0.56
-16	2	13	-0.08	1.07
15	-2	-13	67.87	2.96

-15	2	13	65.00	4.57
-15	2	13	70.64	3.62
14	-2	-13	2.32	0.54
-14	2	13	1.05	1.65
-14	2	13	3.30	1.00
13	-2	-13	100.96	4.48
-13	2	13	115.43	4.83
-13	2	13	108.91	5.72
12	-2	-13	3.82	0.54
-12	2	13	5.01	1.59
11	-2	-13	69.17	3.03
-11	2	13	74.11	4.31
-11	2	13	78.55	3.43
10	-2	-13	5.70	0.93
10	-2	-13	5.92	0.68
-10	2	13	6.93	0.92
-10	2	13	6.18	0.92
-10	2	13	4.57	1.39
-10	2	13	5.70	0.87
9	-2	-13	87.83	3.57
-9	2	13	93.17	3.87
-9	2	13	84.49	3.68
-9	2	13	84.07	4.85
-9	2	13	86.55	3.67
-9	2	13	95.56	3.85
8	-2	-13	2.65	0.40
8	-2	-13	1.81	0.68
-8	2	13	2.09	1.14
-8	2	13	2.22	0.48
-8	2	13	1.51	0.57
-8	2	13	1.56	0.63
-8	2	13	2.98	0.63
7	-2	-13	124.80	4.87
7	-2	-13	125.36	5.14
-7	2	13	135.52	5.04
-7	2	13	116.59	4.92
-7	2	13	120.95	5.78
-7	2	13	129.44	5.05
-7	2	13	117.74	5.07
6	-2	-13	9.43	0.71
6	-2	-13	10.18	1.25
-6	2	13	8.83	1.15
-6	2	13	10.29	0.93
-6	2	13	10.65	0.83

-6	2	13	11.90	1.74
-6	2	13	9.87	0.94
5	-2	-13	43.40	2.33
5	-2	-13	44.28	1.88
5	-2	-13	41.26	3.31
-5	2	13	41.09	1.93
-5	2	13	42.42	2.72
-5	2	13	40.71	2.22
-5	2	13	43.73	2.00
-5	2	13	45.60	1.97
4	-2	-13	-0.39	0.72
4	-2	-13	0.58	0.30
4	-2	-13	0.98	1.20
-4	2	13	0.14	0.33
-4	2	13	0.11	0.82
-4	2	13	-0.29	0.59
-4	2	13	-0.37	0.35
-4	2	13	0.42	0.36
3	-2	-13	204.51	8.88
3	-2	-13	209.21	7.85
3	-2	-13	215.20	8.34
-3	2	13	219.42	7.85
-3	2	13	187.01	7.87
-3	2	13	206.21	8.55
-3	2	13	203.43	7.89
-3	2	13	197.26	8.20
2	-2	-13	0.03	1.10
2	-2	-13	-0.13	0.28
2	-2	-13	-0.06	0.83
-2	2	13	-0.40	0.28
-2	2	13	0.21	0.77
-2	2	13	0.93	0.36
-2	2	13	-0.22	0.64
-2	2	13	0.20	0.31
1	-2	-13	128.45	5.26
1	-2	-13	117.07	5.65
1	-2	-13	118.36	4.66
-1	2	13	104.80	4.70
-1	2	13	116.66	5.23
-1	2	13	112.31	5.07
-1	2	13	122.16	4.70
-1	2	13	125.03	4.55
0	-2	-13	5.31	0.62
0	-2	-13	8.18	1.51

0	-2	-13	5.09	1.04
0	2	13	5.79	0.50
0	2	13	6.79	1.25
0	2	13	6.18	0.47
0	2	13	4.14	0.90
0	2	13	6.12	0.68
-1	-2	-13	71.42	3.57
-1	-2	-13	67.84	3.80
-1	-2	-13	71.14	2.88
-1	-2	-13	67.24	2.88
1	2	13	68.88	3.34
1	2	13	65.30	3.38
1	2	13	72.11	2.93
-2	-2	-13	-0.91	1.07
-2	-2	-13	0.41	0.36
-2	-2	-13	-0.69	0.92
-2	-2	-13	1.27	0.38
2	2	13	0.43	0.65
2	2	13	0.23	0.74
-3	-2	-13	105.18	4.05
-3	-2	-13	107.97	4.89
-3	-2	-13	96.39	4.88
-3	-2	-13	100.95	4.09
3	2	13	98.00	4.63
3	2	13	96.08	4.38
-4	-2	-13	12.09	0.88
-4	-2	-13	9.62	1.60
-4	-2	-13	10.96	0.89
4	2	13	10.08	1.20
4	2	13	7.35	1.42
-5	-2	-13	237.31	8.86
-5	-2	-13	227.17	9.54
-5	-2	-13	232.11	8.91
5	2	13	220.18	9.43
5	2	13	232.84	9.08
-6	-2	-13	1.95	1.20
-6	-2	-13	1.53	0.32
-6	-2	-13	0.60	0.47
6	2	13	1.20	0.31
6	2	13	0.17	0.58
6	2	13	1.75	0.91
-7	-2	-13	85.22	4.37
-7	-2	-13	91.39	3.77
7	2	13	90.00	3.82

7	2	13	87.98	3.47
-8	-2	-13	0.01	0.44
-8	-2	-13	-1.65	1.14
8	2	13	-0.45	0.87
8	2	13	1.39	0.61
8	2	13	0.29	0.41
-9	-2	-13	54.01	2.39
-9	-2	-13	54.37	2.59
-9	-2	-13	49.35	3.09
9	2	13	56.24	2.51
9	2	13	50.92	3.18
9	2	13	58.49	2.35
-10	-2	-13	15.45	1.39
-10	-2	-13	15.82	1.27
10	2	13	15.42	2.03
10	2	13	21.68	1.24
10	2	13	18.87	1.25
-11	-2	-13	50.11	2.37
-11	-2	-13	51.03	2.54
11	2	13	45.36	3.22
-12	-2	-13	0.43	0.73
-12	-2	-13	1.21	0.70
12	2	13	-0.99	1.20
-13	-2	-13	44.26	2.52
-13	-2	-13	50.62	2.64
13	2	13	46.76	3.27
-14	-2	-13	1.31	0.82
14	2	13	-1.20	1.34
-15	-2	-13	17.94	1.82
15	2	13	14.38	2.13
-16	-2	-13	2.08	1.14
16	2	13	0.50	1.69
18	-2	-12	51.57	2.53
-18	2	12	57.18	4.45
-18	2	12	50.32	3.35
17	-2	-12	0.94	0.61
-17	2	12	4.65	2.00
-17	2	12	1.93	1.14
16	-2	-12	41.70	2.07
-16	2	12	41.69	3.78
-16	2	12	45.22	2.87
15	-2	-12	3.94	0.64
-15	2	12	4.57	1.14
-15	2	12	3.92	1.82

14	-2	-12	31.16	1.65
-14	2	12	35.51	2.33
-14	2	12	37.13	3.29
13	-2	-12	0.95	0.41
-13	2	12	1.53	0.81
-13	2	12	-0.20	1.37
12	-2	-12	54.66	2.41
-12	2	12	60.47	2.87
-12	2	12	53.89	3.63
11	-2	-12	8.47	0.76
-11	2	12	11.17	1.21
10	-2	-12	76.80	3.14
10	-2	-12	78.47	3.26
10	-2	-12	75.85	3.17
-10	2	12	84.62	3.51
-10	2	12	78.57	4.24
-10	2	12	73.75	3.26
-10	2	12	78.11	3.49
9	-2	-12	4.38	0.58
9	-2	-12	4.45	0.63
-9	2	12	4.73	0.69
-9	2	12	6.94	1.02
-9	2	12	5.48	1.23
-9	2	12	5.03	0.59
-9	2	12	4.98	0.72
8	-2	-12	45.11	2.07
-8	2	12	54.85	2.35
-8	2	12	48.56	2.17
-8	2	12	46.59	3.05
-8	2	12	48.24	2.24
-8	2	12	51.31	2.34
7	-2	-12	1.76	1.47
7	-2	-12	0.78	0.28
7	-2	-12	1.80	0.57
-7	2	12	1.37	0.48
-7	2	12	0.53	0.53
-7	2	12	0.94	0.90
-7	2	12	0.31	0.37
-7	2	12	0.41	0.47
6	-2	-12	33.12	1.52
6	-2	-12	31.92	2.73
6	-2	-12	33.33	1.86
-6	2	12	32.98	1.58
-6	2	12	32.29	2.34

-6	2	12	35.32	1.71
-6	2	12	37.35	1.67
-6	2	12	32.30	1.78
5	-2	-12	-0.14	0.58
5	-2	-12	0.40	1.08
5	-2	-12	0.70	0.30
-5	2	12	0.04	0.53
-5	2	12	0.25	0.38
-5	2	12	0.36	0.37
-5	2	12	1.08	0.35
-5	2	12	0.13	0.76
4	-2	-12	80.31	3.54
4	-2	-12	77.02	3.14
4	-2	-12	76.00	4.12
-4	2	12	74.34	3.43
-4	2	12	81.75	3.19
-4	2	12	75.07	3.84
-4	2	12	77.47	3.15
-4	2	12	81.26	3.20
3	-2	-12	11.38	0.74
3	-2	-12	12.01	1.30
3	-2	-12	13.88	1.90
-3	2	12	10.67	0.75
-3	2	12	11.26	1.47
-3	2	12	11.14	0.80
-3	2	12	12.50	0.77
-3	2	12	11.90	1.18
2	-2	-12	228.79	8.58
2	-2	-12	201.59	8.89
2	-2	-12	212.29	8.10
-2	2	12	201.29	8.43
-2	2	12	208.47	8.67
-2	2	12	212.02	8.03
-2	2	12	213.41	8.13
-2	2	12	220.85	8.05
1	-2	-12	14.11	1.49
1	-2	-12	15.53	0.90
1	-2	-12	14.72	1.90
-1	2	12	16.66	0.81
-1	2	12	15.62	0.94
-1	2	12	13.48	1.29
-1	2	12	16.56	0.81
-1	2	12	14.21	1.45
0	-2	-12	138.71	5.36

0	-2	-12	129.83	6.08
0	-2	-12	136.23	5.87
0	2	12	137.42	5.39
0	2	12	133.05	5.76
0	2	12	137.51	5.84
0	2	12	141.85	5.21
0	2	12	143.33	5.21
-1	-2	-12	12.36	1.51
-1	-2	-12	11.67	0.81
-1	-2	-12	10.17	1.81
1	2	12	11.41	0.81
1	2	12	11.01	1.25
1	2	12	11.06	1.31
-2	-2	-12	32.76	1.57
-2	-2	-12	33.45	2.27
-2	-2	-12	34.37	2.46
-2	-2	-12	34.47	1.56
2	2	12	32.44	1.94
2	2	12	33.57	2.07
-3	-2	-12	4.47	1.19
-3	-2	-12	4.87	1.03
-3	-2	-12	3.82	0.60
-3	-2	-12	5.52	0.61
3	2	12	5.11	0.81
3	2	12	4.92	0.94
-4	-2	-12	74.74	3.95
-4	-2	-12	83.37	3.27
-4	-2	-12	81.81	3.33
-4	-2	-12	83.66	4.01
4	2	12	80.08	3.54
4	2	12	74.82	3.78
-5	-2	-12	1.29	1.03
-5	-2	-12	1.29	0.44
-5	-2	-12	-0.17	1.20
-5	-2	-12	1.23	0.41
5	2	12	0.12	0.79
5	2	12	1.24	0.58
5	2	12	1.72	0.27
-6	-2	-12	139.04	5.25
-6	-2	-12	129.60	5.96
-6	-2	-12	139.02	5.49
-6	-2	-12	137.47	5.40
6	2	12	140.46	5.24
6	2	12	141.11	5.58

6	2	12	134.46	6.01
-7	-2	-12	-0.40	0.39
-7	-2	-12	0.16	0.42
-7	-2	-12	1.46	1.05
7	2	12	0.24	0.75
7	2	12	1.00	0.34
7	2	12	-0.36	0.51
-8	-2	-12	116.69	5.22
-8	-2	-12	116.04	4.87
-8	-2	-12	115.81	4.66
8	2	12	117.69	5.42
8	2	12	122.68	4.66
8	2	12	125.00	4.84
-9	-2	-12	1.66	0.52
9	2	12	1.55	0.56
9	2	12	1.49	0.94
9	2	12	1.79	0.47
-10	-2	-12	46.30	2.18
-10	-2	-12	42.66	2.57
-10	-2	-12	44.62	2.30
10	2	12	46.19	2.86
10	2	12	47.96	2.12
10	2	12	46.97	2.14
-11	-2	-12	0.98	0.65
-11	-2	-12	0.95	0.63
11	2	12	0.02	1.06
11	2	12	0.72	0.58
-12	-2	-12	38.91	2.20
-12	-2	-12	41.96	2.30
12	2	12	39.20	2.84
12	2	12	43.74	2.10
-13	-2	-12	1.28	0.84
-13	-2	-12	-0.69	0.63
13	2	12	-0.73	1.18
-14	-2	-12	61.24	3.02
-14	-2	-12	56.94	3.02
14	2	12	55.61	3.58
-15	-2	-12	1.09	1.13
-15	-2	-12	0.02	0.85
15	2	12	-1.39	1.33
-16	-2	-12	38.73	2.55
16	2	12	37.40	3.30
19	-2	-11	4.04	0.85
-19	2	11	4.03	1.40

-19	2	11	8.08	2.36
18	-2	-11	0.58	0.60
-18	2	11	0.78	1.20
-18	2	11	-2.62	1.85
17	-2	-11	7.49	1.05
-17	2	11	8.67	2.09
-17	2	11	8.71	1.37
16	-2	-11	1.33	0.55
-16	2	11	0.42	0.97
-16	2	11	3.75	1.73
15	-2	-11	6.52	0.85
-15	2	11	5.70	1.11
-15	2	11	2.49	1.62
14	-2	-11	0.50	0.43
-14	2	11	1.20	1.42
-14	2	11	0.33	0.80
13	-2	-11	12.14	0.88
-13	2	11	12.98	2.15
-13	2	11	11.99	1.40
12	-2	-11	2.36	0.44
-12	2	11	2.87	1.29
-12	2	11	3.47	0.77
11	-2	-11	10.68	0.81
-11	2	11	12.84	1.00
-11	2	11	15.18	1.29
-11	2	11	10.27	1.94
10	-2	-11	3.10	0.41
10	-2	-11	2.99	0.40
10	-2	-11	2.73	0.40
-10	2	11	3.56	0.50
-10	2	11	2.27	0.66
-10	2	11	2.10	1.12
-10	2	11	2.84	0.68
9	-2	-11	48.01	2.16
9	-2	-11	49.63	2.32
9	-2	-11	57.46	4.88
9	-2	-11	51.65	2.15
-9	2	11	49.10	2.40
-9	2	11	50.34	3.11
-9	2	11	51.71	2.40
-9	2	11	51.21	2.22
8	-2	-11	6.23	0.55
8	-2	-11	5.89	1.43
8	-2	-11	5.87	0.80

-8	2	11	6.18	0.86
-8	2	11	6.24	0.86
-8	2	11	6.16	0.87
-8	2	11	6.25	0.66
-8	2	11	4.45	1.14
7	-2	-11	27.47	2.53
7	-2	-11	28.00	1.33
7	-2	-11	27.46	1.70
-7	2	11	31.14	1.56
-7	2	11	26.84	1.50
-7	2	11	29.19	1.41
-7	2	11	33.77	1.56
-7	2	11	29.06	2.16
6	-2	-11	34.51	2.58
6	-2	-11	36.42	1.81
6	-2	-11	32.47	1.52
-6	2	11	35.99	1.60
-6	2	11	35.40	1.72
-6	2	11	32.04	2.27
-6	2	11	32.37	1.74
-6	2	11	38.11	1.68
5	-2	-11	29.41	1.65
5	-2	-11	35.62	2.46
5	-2	-11	30.19	1.35
-5	2	11	26.03	1.58
-5	2	11	30.45	2.13
-5	2	11	29.20	1.41
-5	2	11	29.28	1.39
-5	2	11	30.29	1.47
4	-2	-11	8.44	0.60
4	-2	-11	8.00	1.05
4	-2	-11	9.21	1.35
-4	2	11	9.34	0.98
-4	2	11	9.18	0.68
-4	2	11	9.27	0.72
-4	2	11	9.11	0.66
-4	2	11	7.70	1.32
3	-2	-11	50.18	2.91
3	-2	-11	51.30	2.44
3	-2	-11	48.59	2.05
-3	2	11	51.13	2.68
-3	2	11	49.29	2.03
-3	2	11	48.02	2.38
-3	2	11	47.75	2.05

-3	2	11	47.40	2.08
2	-2	-11	0.88	0.87
2	-2	-11	1.34	0.59
2	-2	-11	0.07	0.22
-2	2	11	0.61	0.25
-2	2	11	-0.33	0.47
-2	2	11	0.17	0.61
-2	2	11	0.45	0.28
-2	2	11	-0.29	0.26
1	-2	-11	149.58	6.26
1	-2	-11	135.41	5.50
1	-2	-11	148.86	5.96
-1	2	11	135.61	5.97
-1	2	11	144.17	5.54
-1	2	11	148.54	5.42
-1	2	11	146.09	5.42
-1	2	11	133.25	5.82
0	-2	-11	90.88	4.34
0	-2	-11	94.92	4.13
0	-2	-11	88.95	3.65
0	2	11	86.37	4.01
0	2	11	89.41	4.07
0	2	11	95.39	3.51
0	2	11	94.42	3.68
0	2	11	93.39	3.51
-1	-2	-11	77.89	3.58
-1	-2	-11	74.40	3.05
-1	-2	-11	75.05	3.74
1	2	11	79.06	3.09
1	2	11	71.63	3.45
1	2	11	73.07	3.40
-2	-2	-11	0.31	0.28
-2	-2	-11	1.14	0.86
-2	-2	-11	1.37	0.72
-2	-2	-11	0.63	0.29
2	2	11	1.17	0.56
2	2	11	0.70	0.62
-3	-2	-11	63.47	3.17
-3	-2	-11	63.27	2.53
-3	-2	-11	60.28	3.20
-3	-2	-11	62.31	2.58
3	2	11	58.70	2.81
3	2	11	56.98	2.98
-4	-2	-11	2.55	0.39

-4	-2	-11	2.09	0.94
-4	-2	-11	1.88	0.39
-4	-2	-11	1.78	0.82
4	2	11	0.98	0.51
4	2	11	2.83	0.78
-5	-2	-11	109.74	4.93
-5	-2	-11	114.26	5.09
-5	-2	-11	114.14	4.40
-5	-2	-11	112.21	4.23
-5	-2	-11	110.59	4.46
5	2	11	111.76	4.23
5	2	11	109.96	4.58
5	2	11	104.24	4.88
-6	-2	-11	4.49	0.53
-6	-2	-11	3.25	1.03
-6	-2	-11	4.10	0.64
-6	-2	-11	5.98	1.09
-6	-2	-11	4.27	0.61
6	2	11	4.44	0.66
6	2	11	4.84	0.49
6	2	11	3.38	0.82
-7	-2	-11	20.64	1.16
-7	-2	-11	20.33	1.27
-7	-2	-11	20.86	1.85
-7	-2	-11	22.15	1.18
7	2	11	22.65	1.33
7	2	11	23.07	1.84
7	2	11	23.06	1.09
-8	-2	-11	2.54	0.56
-8	-2	-11	1.99	0.44
8	2	11	0.98	0.50
8	2	11	3.66	0.92
-9	-2	-11	36.82	1.89
-9	-2	-11	37.74	2.24
-9	-2	-11	38.65	1.80
9	2	11	37.47	1.78
9	2	11	34.76	2.36
9	2	11	38.45	1.75
-10	-2	-11	0.36	0.54
-10	-2	-11	-0.63	0.44
-10	-2	-11	-0.32	0.82
10	2	11	-1.14	0.68
10	2	11	0.07	0.48
-11	-2	-11	78.47	3.49

-11	-2	-11	75.53	3.38
11	2	11	77.69	3.95
11	2	11	80.97	3.32
-12	-2	-11	3.35	0.79
-12	-2	-11	5.82	0.91
12	2	11	6.53	1.06
12	2	11	2.77	1.12
-13	-2	-11	40.21	2.29
-13	-2	-11	43.35	2.33
13	2	11	39.83	2.15
13	2	11	42.52	2.20
13	2	11	38.16	2.82
-14	-2	-11	-1.54	0.86
-14	-2	-11	0.06	0.71
14	2	11	-0.46	1.17
14	2	11	1.17	0.69
-15	-2	-11	30.31	2.08
-15	-2	-11	28.88	2.25
15	2	11	27.71	2.77
15	2	11	31.63	1.94
-16	-2	-11	1.14	0.86
-16	-2	-11	4.52	2.20
16	2	11	2.33	0.86
16	2	11	-0.18	1.40
-17	-2	-11	35.44	2.92
-17	-2	-11	32.13	2.45
17	2	11	35.49	2.24
17	2	11	34.23	3.31
19	-2	-10	2.58	0.71
-19	2	10	3.47	2.15
-19	2	10	3.65	1.42
18	-2	-10	0.94	0.62
-18	2	10	-0.71	1.85
-18	2	10	0.72	1.13
17	-2	-10	1.78	0.60
-17	2	10	2.70	1.81
-17	2	10	3.64	1.08
16	-2	-10	9.66	1.00
-16	2	10	9.57	1.34
-16	2	10	11.26	1.94
15	-2	-10	2.54	0.53
-15	2	10	3.00	1.54
-15	2	10	3.88	1.00
-14	2	10	3.50	1.66

-14	2	10	1.96	0.84
13	-2	-10	8.49	0.76
-13	2	10	6.61	1.54
12	-2	-10	0.17	0.34
-12	2	10	0.28	0.56
-12	2	10	0.94	1.15
11	-2	-10	0.01	0.62
11	-2	-10	0.57	0.32
-11	2	10	0.59	0.53
-11	2	10	0.73	1.11
-11	2	10	-0.04	0.42
10	-2	-10	12.71	1.14
10	-2	-10	13.77	2.18
10	-2	-10	11.07	0.78
-10	2	10	13.36	1.18
-10	2	10	12.23	1.83
-10	2	10	11.08	0.91
-10	2	10	15.71	1.19
9	-2	-10	12.30	0.90
9	-2	-10	12.03	1.06
9	-2	-10	11.47	0.74
9	-2	-10	11.99	1.73
9	-2	-10	11.63	0.78
-9	2	10	14.65	1.09
-9	2	10	9.70	1.69
-9	2	10	13.13	0.85
-9	2	10	12.32	1.08
8	-2	-10	6.12	0.54
8	-2	-10	6.93	1.39
8	-2	-10	5.91	0.67
-8	2	10	3.51	0.96
-8	2	10	5.27	0.82
-8	2	10	5.68	0.66
-8	2	10	6.19	0.80
-8	2	10	4.55	0.61
7	-2	-10	0.23	0.23
7	-2	-10	-0.07	0.39
7	-2	-10	-0.04	1.00
-7	2	10	0.37	0.38
-7	2	10	0.58	0.31
-7	2	10	0.41	0.37
-7	2	10	-0.41	0.67
-7	2	10	0.41	0.44
6	-2	-10	0.48	0.45

6	-2	-10	0.97	0.90
6	-2	-10	0.88	0.26
-6	2	10	0.74	0.80
-6	2	10	0.88	0.39
-6	2	10	1.19	0.32
-6	2	10	1.66	0.45
-6	2	10	0.87	0.34
5	-2	-10	1.86	0.30
5	-2	-10	1.62	0.50
5	-2	-10	2.12	0.91
-5	2	10	2.23	0.41
-5	2	10	1.64	0.69
-5	2	10	2.10	0.49
-5	2	10	2.38	0.38
-5	2	10	2.98	0.46
4	-2	-10	41.31	2.06
4	-2	-10	46.61	2.62
4	-2	-10	40.90	1.78
-4	2	10	39.86	2.01
-4	2	10	42.74	2.39
-4	2	10	42.65	1.82
-4	2	10	44.05	1.85
3	-2	-10	0.06	0.20
3	-2	-10	-1.53	0.73
3	-2	-10	0.01	0.46
-3	2	10	0.21	0.41
-3	2	10	0.12	0.25
-3	2	10	-0.34	0.22
-3	2	10	0.28	0.23
-3	2	10	-0.42	0.55
2	-2	-10	35.54	1.87
2	-2	-10	36.12	2.22
2	-2	-10	33.36	1.51
-2	2	10	35.29	1.46
-2	2	10	34.87	1.63
-2	2	10	32.57	1.80
-2	2	10	33.21	2.01
-2	2	10	36.61	1.55
1	-2	-10	8.21	1.35
1	-2	-10	7.86	0.56
1	-2	-10	8.70	1.04
-1	2	10	7.70	0.58
-1	2	10	7.86	0.49
-1	2	10	6.65	0.90

-1	2	10	6.66	1.01
-1	2	10	7.73	0.49
0	-2	-10	14.29	1.50
0	-2	-10	12.55	1.19
0	-2	-10	11.92	0.75
0	2	10	12.38	1.10
0	2	10	14.28	0.80
0	2	10	12.92	0.63
0	2	10	14.19	0.64
0	2	10	13.19	1.18
-1	-2	-10	1.58	0.63
-1	-2	-10	2.21	0.33
-1	-2	-10	2.22	0.82
1	2	10	2.10	0.35
1	2	10	2.30	0.58
1	2	10	2.01	0.58
-2	-2	-10	3.49	0.49
-2	-2	-10	3.92	0.46
-2	-2	-10	3.66	0.86
-2	-2	-10	4.41	0.92
2	2	10	4.80	0.90
2	2	10	4.34	0.82
-3	-2	-10	8.80	1.31
-3	-2	-10	8.74	0.62
-3	-2	-10	7.29	0.63
-3	-2	-10	7.33	1.17
3	2	10	6.92	0.88
3	2	10	6.73	1.01
-4	-2	-10	33.21	1.58
-4	-2	-10	34.86	1.37
-4	-2	-10	37.05	2.14
-4	-2	-10	32.48	1.51
-4	-2	-10	31.38	2.08
4	2	10	32.83	1.98
4	2	10	34.51	1.74
4	2	10	34.36	1.37
-5	-2	-10	9.06	1.24
-5	-2	-10	6.16	0.97
-5	-2	-10	6.60	0.64
-5	-2	-10	6.82	0.49
-5	-2	-10	6.60	0.60
5	2	10	6.83	0.82
5	2	10	6.03	0.97
5	2	10	6.69	0.48

-6	-2	-10	70.89	2.79
-6	-2	-10	65.21	2.84
-6	-2	-10	67.70	3.45
-6	-2	-10	65.97	2.72
-6	-2	-10	62.63	3.18
6	2	10	71.06	2.91
6	2	10	65.24	3.26
6	2	10	68.48	2.67
-7	-2	-10	3.92	0.63
-7	-2	-10	4.00	0.56
-7	-2	-10	3.34	1.03
-7	-2	-10	4.01	0.61
-7	-2	-10	4.10	1.10
7	2	10	3.71	0.55
7	2	10	3.94	0.53
7	2	10	3.51	0.84
-8	-2	-10	12.83	1.43
-8	-2	-10	14.73	0.93
-8	-2	-10	15.84	1.88
-8	-2	-10	12.60	0.99
-8	-2	-10	12.80	0.89
8	2	10	13.05	0.95
8	2	10	13.85	1.48
-9	-2	-10	-0.42	0.41
-9	-2	-10	-0.22	0.74
9	2	10	0.02	0.45
9	2	10	-0.28	0.38
9	2	10	0.25	0.72
-10	-2	-10	43.30	2.22
-10	-2	-10	45.31	2.27
-10	-2	-10	46.37	2.16
10	2	10	42.11	2.63
10	2	10	46.81	2.01
10	2	10	47.68	2.18
10	2	10	47.97	2.14
-11	-2	-10	0.01	0.45
-11	-2	-10	0.41	0.59
11	2	10	0.80	0.47
11	2	10	0.30	0.85
11	2	10	1.52	0.55
-12	-2	-10	52.90	2.63
-12	-2	-10	50.89	2.61
12	2	10	53.69	2.50
12	2	10	57.22	2.55

12	2	10	53.20	3.09
-13	-2	-10	2.02	0.81
13	2	10	0.12	0.67
13	2	10	-0.29	0.56
13	2	10	0.30	0.98
-14	-2	-10	33.25	2.19
14	2	10	31.81	1.94
14	2	10	32.10	1.88
14	2	10	29.08	2.54
-15	-2	-10	3.01	1.09
15	2	10	2.93	1.35
15	2	10	2.85	0.76
16	2	10	20.16	2.50
16	2	10	19.74	1.66
-17	-2	-10	-1.18	1.32
17	2	10	0.57	1.47
17	2	10	0.23	0.82
-18	-2	-10	20.08	2.77
18	2	10	25.34	3.08
20	-2	-9	4.56	0.80
-20	2	9	2.65	2.16
-20	2	9	1.82	1.64
19	-2	-9	15.68	1.31
-19	2	9	10.42	2.20
-19	2	9	11.79	1.69
18	-2	-9	2.60	0.65
-18	2	9	2.93	1.83
-18	2	9	0.70	1.10
17	-2	-9	10.33	1.04
-17	2	9	9.53	1.36
-17	2	9	7.18	1.91
16	-2	-9	5.41	0.83
-16	2	9	5.67	1.89
-16	2	9	5.67	1.12
15	-2	-9	7.48	0.80
-15	2	9	6.62	1.61
-15	2	9	6.42	1.04
14	-2	-9	13.75	0.97
-14	2	9	16.88	1.58
-14	2	9	15.36	2.24
13	-2	-9	10.17	0.77
-13	2	9	11.35	2.13
-13	2	9	8.97	1.28
12	-2	-9	1.50	1.47

12	-2	-9	1.86	0.38
-12	2	9	1.85	0.66
-12	2	9	2.29	1.10
-12	2	9	2.53	0.52
11	-2	-9	12.90	2.34
11	-2	-9	13.98	0.85
11	-2	-9	15.45	1.20
-11	2	9	14.81	1.24
-11	2	9	13.81	1.88
-11	2	9	14.74	1.00
10	-2	-9	4.33	0.53
10	-2	-9	7.16	0.91
10	-2	-9	2.98	1.22
-10	2	9	4.51	0.67
-10	2	9	5.45	0.67
-10	2	9	3.49	1.05
9	-2	-9	0.78	1.12
9	-2	-9	2.22	0.51
9	-2	-9	1.53	0.29
9	-2	-9	1.98	0.30
-9	2	9	1.77	0.39
-9	2	9	2.08	0.52
-9	2	9	1.27	0.86
8	-2	-9	35.27	1.58
8	-2	-9	37.48	1.80
8	-2	-9	37.70	1.72
8	-2	-9	35.26	1.59
8	-2	-9	36.73	2.57
-8	2	9	37.48	1.83
-8	2	9	33.84	2.32
-8	2	9	38.00	1.65
7	-2	-9	7.44	0.76
7	-2	-9	6.38	1.22
7	-2	-9	6.18	0.52
7	-2	-9	5.80	0.65
-7	2	9	8.37	0.62
-7	2	9	8.54	0.68
-7	2	9	7.60	1.29
-7	2	9	6.61	0.76
6	-2	-9	0.34	0.35
6	-2	-9	1.52	0.90
6	-2	-9	0.62	0.22
-6	2	9	0.24	0.32
-6	2	9	0.44	0.38

-6	2	9	1.13	0.70
-6	2	9	1.04	0.27
-6	2	9	0.86	0.33
5	-2	-9	1.65	0.75
5	-2	-9	0.12	0.20
5	-2	-9	0.13	0.37
-5	2	9	0.45	0.24
-5	2	9	0.41	0.31
-5	2	9	0.35	0.27
-5	2	9	0.28	0.37
-5	2	9	1.13	0.61
4	-2	-9	2.42	0.83
4	-2	-9	1.96	0.55
4	-2	-9	1.30	0.24
-4	2	9	1.21	0.31
-4	2	9	2.22	0.31
-4	2	9	1.68	0.64
-4	2	9	0.75	0.39
-4	2	9	1.38	0.26
3	-2	-9	1.32	0.75
3	-2	-9	1.36	0.24
3	-2	-9	1.31	0.45
-3	2	9	1.45	0.58
-3	2	9	1.17	0.31
-3	2	9	0.66	0.42
-3	2	9	1.34	0.25
-3	2	9	0.90	0.23
2	-2	-9	13.23	0.73
2	-2	-9	13.00	1.38
2	-2	-9	14.29	1.06
-2	2	9	13.57	1.18
-2	2	9	14.63	0.72
-2	2	9	13.57	0.68
-2	2	9	13.03	0.99
-2	2	9	13.77	0.75
1	-2	-9	1.24	0.25
1	-2	-9	1.43	0.53
1	-2	-9	1.88	0.72
-1	2	9	0.71	0.52
-1	2	9	1.78	0.23
-1	2	9	1.43	0.26
-1	2	9	1.08	0.45
-1	2	9	2.05	0.29
0	-2	-9	39.02	1.76

0	-2	-9	42.71	2.14
0	-2	-9	44.14	2.31
0	2	9	39.19	2.05
0	2	9	43.33	1.65
0	2	9	41.26	1.64
0	2	9	43.34	2.13
0	2	9	42.24	1.78
-1	-2	-9	27.74	1.74
-1	-2	-9	26.34	1.60
-1	-2	-9	23.81	1.18
1	2	9	25.49	1.48
1	2	9	27.48	1.21
1	2	9	22.64	1.49
-2	-2	-9	11.95	0.68
-2	-2	-9	9.65	0.67
-2	-2	-9	11.13	1.21
-2	-2	-9	12.19	1.17
2	2	9	10.96	0.94
2	2	9	9.72	1.00
2	2	9	11.74	0.72
-3	-2	-9	22.59	1.55
-3	-2	-9	19.60	0.99
-3	-2	-9	19.00	1.02
-3	-2	-9	18.29	1.47
3	2	9	19.54	1.38
3	2	9	20.81	1.24
-4	-2	-9	16.02	0.89
-4	-2	-9	17.33	1.43
-4	-2	-9	14.95	0.71
-4	-2	-9	13.16	1.32
-4	-2	-9	15.23	0.84
4	2	9	15.63	0.70
4	2	9	13.61	1.25
4	2	9	15.45	1.05
-5	-2	-9	5.45	0.46
-5	-2	-9	4.85	0.86
-5	-2	-9	5.06	0.54
-5	-2	-9	5.30	0.56
5	2	9	4.61	0.81
5	2	9	5.84	0.44
5	2	9	6.34	0.70
-6	-2	-9	6.96	0.61
-6	-2	-9	7.98	1.09
-6	-2	-9	7.80	1.13

-6	-2	-9	7.21	0.67
6	2	9	8.11	0.73
6	2	9	7.11	0.54
6	2	9	7.33	1.08
-7	-2	-9	6.54	1.08
-7	-2	-9	5.71	0.68
-7	-2	-9	6.66	0.66
-7	-2	-9	5.85	0.61
-7	-2	-9	6.76	1.11
7	2	9	5.95	0.75
7	2	9	6.05	1.10
7	2	9	7.20	0.58
-8	-2	-9	1.72	0.43
-8	-2	-9	3.84	0.56
-8	-2	-9	3.06	0.95
-8	-2	-9	1.37	0.41
-8	-2	-9	1.87	0.73
8	2	9	1.79	0.38
8	2	9	2.26	0.74
8	2	9	1.70	0.38
8	2	9	2.83	0.49
-9	-2	-9	-0.39	0.34
-9	-2	-9	0.02	0.46
-9	-2	-9	-1.05	0.92
-9	-2	-9	-0.57	0.62
-9	-2	-9	-0.04	0.41
9	2	9	0.14	0.71
9	2	9	0.27	0.34
9	2	9	0.02	0.38
9	2	9	0.45	0.40
-10	-2	-9	0.52	0.61
-10	-2	-9	0.41	0.49
10	2	9	-0.04	0.35
10	2	9	0.88	0.48
10	2	9	0.66	0.39
10	2	9	0.02	0.74
-11	-2	-9	16.77	1.27
11	2	9	14.79	1.61
11	2	9	15.69	1.18
11	2	9	13.54	1.10
-12	-2	-9	7.73	1.21
12	2	9	7.94	1.31
12	2	9	9.37	1.09
12	2	9	8.84	0.95

-13	-2	-9	7.47	1.02
13	2	9	9.22	1.16
13	2	9	7.15	1.00
-14	-2	-9	0.15	0.85
14	2	9	0.76	0.72
14	2	9	1.46	1.08
14	2	9	-0.69	0.57
-15	-2	-9	13.28	1.77
15	2	9	14.30	2.08
15	2	9	12.59	1.36
16	2	9	1.68	0.69
16	2	9	1.96	1.34
-17	-2	-9	14.12	2.23
17	2	9	17.16	2.48
17	2	9	9.17	1.48
-18	-2	-9	2.40	1.46
18	2	9	0.52	0.84
18	2	9	3.92	1.54
20	-2	-8	41.29	2.29
-20	2	8	52.28	3.28
-20	2	8	37.54	3.94
19	-2	-8	0.99	0.62
-19	2	8	1.32	1.76
-19	2	8	0.33	1.12
18	-2	-8	42.75	2.14
-18	2	8	47.10	2.94
-18	2	8	43.60	3.53
17	-2	-8	0.74	0.50
-17	2	8	0.47	1.68
-17	2	8	0.19	0.94
16	-2	-8	63.56	2.74
-16	2	8	64.86	3.31
-16	2	8	58.19	3.80
15	-2	-8	2.57	0.52
-15	2	8	4.64	1.48
-15	2	8	2.30	0.94
14	-2	-8	60.51	2.63
-14	2	8	64.92	3.12
-14	2	8	59.91	3.64
13	-2	-8	1.95	1.48
13	-2	-8	1.03	0.37
-13	2	8	-0.20	1.13
-13	2	8	1.59	0.72
12	-2	-8	71.60	4.15

12	-2	-8	70.55	2.96
-12	2	8	76.76	3.36
-12	2	8	64.32	3.78
-12	2	8	77.45	3.08
11	-2	-8	5.52	0.75
11	-2	-8	5.72	0.57
11	-2	-8	6.46	1.41
-11	2	8	5.99	1.18
-11	2	8	4.82	0.76
-11	2	8	6.24	0.68
10	-2	-8	58.13	2.44
10	-2	-8	60.14	2.70
10	-2	-8	55.41	3.36
-10	2	8	60.80	2.76
-10	2	8	60.56	3.25
-10	2	8	62.03	2.53
9	-2	-8	0.43	0.23
9	-2	-8	1.68	0.94
9	-2	-8	0.47	0.40
-9	2	8	-0.01	0.74
-9	2	8	0.83	0.30
-9	2	8	0.09	0.41
8	-2	-8	141.67	5.66
8	-2	-8	141.79	5.44
8	-2	-8	139.48	6.24
8	-2	-8	137.53	5.48
8	-2	-8	146.88	5.49
-8	2	8	135.61	6.11
-8	2	8	148.32	5.54
-8	2	8	152.18	5.72
7	-2	-8	2.92	0.48
7	-2	-8	1.74	0.28
7	-2	-8	2.01	0.43
7	-2	-8	1.75	0.90
-7	2	8	1.86	0.71
-7	2	8	2.18	0.46
-7	2	8	1.67	0.30
6	-2	-8	190.79	7.12
6	-2	-8	180.41	6.98
6	-2	-8	187.90	7.67
6	-2	-8	189.62	7.06
-6	2	8	179.72	7.04
-6	2	8	184.20	7.01
-6	2	8	171.23	7.51

-6	2	8	189.41	7.13
5	-2	-8	0.31	0.18
5	-2	-8	0.01	0.25
5	-2	-8	0.60	0.66
5	-2	-8	0.46	0.33
-5	2	8	0.58	0.41
-5	2	8	-0.03	0.26
-5	2	8	-0.01	0.19
-5	2	8	-0.13	0.49
4	-2	-8	73.57	2.89
4	-2	-8	77.59	3.52
4	-2	-8	74.69	3.07
-4	2	8	69.30	3.04
-4	2	8	63.49	3.30
-4	2	8	74.76	2.97
-4	2	8	76.23	2.92
-4	2	8	77.22	2.92
3	-2	-8	1.79	0.65
3	-2	-8	2.27	0.46
3	-2	-8	2.15	0.27
-3	2	8	1.77	0.26
-3	2	8	1.58	0.40
-3	2	8	2.53	0.30
-3	2	8	2.01	0.58
-3	2	8	1.16	0.29
2	-2	-8	102.92	3.92
2	-2	-8	100.20	4.39
-2	2	8	96.41	4.11
-2	2	8	107.11	3.90
-2	2	8	103.46	3.93
-2	2	8	98.45	3.86
-2	2	8	99.02	4.30
1	-2	-8	1.17	0.40
1	-2	-8	1.51	0.25
1	-2	-8	1.67	0.65
-1	2	8	2.10	0.32
-1	2	8	1.74	0.27
-1	2	8	1.57	0.51
-1	2	8	1.59	0.24
-1	2	8	0.97	0.42
0	-2	-8	16.59	1.14
0	-2	-8	15.83	0.82
0	-2	-8	16.59	1.31
0	2	8	17.94	0.72

0	2	8	18.29	0.85
0	2	8	13.90	1.09
0	2	8	17.26	0.72
0	2	8	16.17	1.09
-1	-2	-8	77.63	3.38
-1	-2	-8	73.17	3.03
-1	-2	-8	80.27	3.42
1	2	8	73.18	3.27
1	2	8	74.97	3.31
1	2	8	80.56	3.06
-2	-2	-8	5.07	0.75
-2	-2	-8	3.94	0.38
-2	-2	-8	3.29	0.40
-2	-2	-8	3.67	0.67
2	2	8	3.63	0.55
2	2	8	3.65	0.42
2	2	8	3.95	0.65
-3	-2	-8	11.45	1.22
-3	-2	-8	9.79	1.06
-3	-2	-8	11.18	0.64
-3	-2	-8	10.59	0.67
3	2	8	10.33	0.98
3	2	8	11.30	0.83
-4	-2	-8	37.28	1.70
-4	-2	-8	38.31	1.56
-4	-2	-8	38.54	1.97
-4	-2	-8	44.42	1.68
-4	-2	-8	40.54	2.12
4	2	8	38.02	1.80
4	2	8	36.17	2.00
4	2	8	38.12	1.55
-5	-2	-8	7.56	0.51
-5	-2	-8	7.29	0.94
-5	-2	-8	7.95	0.57
-5	-2	-8	7.09	0.59
-5	-2	-8	7.14	1.06
5	2	8	7.51	0.72
5	2	8	7.26	0.49
5	2	8	7.07	0.96
-6	-2	-8	40.60	2.01
-6	-2	-8	39.39	1.85
-6	-2	-8	42.67	1.78
-6	-2	-8	41.65	2.32
6	2	8	42.75	1.87

6	2	8	40.53	2.21
6	2	8	40.99	1.72
-7	-2	-8	11.37	0.73
-7	-2	-8	10.85	1.40
-7	-2	-8	11.09	0.76
-7	-2	-8	11.20	1.04
-7	-2	-8	9.96	0.80
7	2	8	12.39	0.87
7	2	8	12.09	0.72
7	2	8	10.80	1.17
7	2	8	10.73	0.75
-8	-2	-8	24.31	1.85
-8	-2	-8	23.99	1.25
-8	-2	-8	24.47	1.28
-8	-2	-8	20.85	1.38
-8	-2	-8	24.76	1.19
8	2	8	22.81	1.68
8	2	8	23.19	1.21
8	2	8	23.58	1.18
8	2	8	23.23	1.22
-9	-2	-8	0.47	0.34
-9	-2	-8	1.33	0.46
-9	-2	-8	0.35	0.57
-9	-2	-8	1.65	0.83
9	2	8	0.54	0.39
9	2	8	0.99	0.36
9	2	8	1.10	0.67
9	2	8	0.82	0.37
-10	-2	-8	3.67	1.08
-10	-2	-8	3.78	0.65
10	2	8	4.23	0.67
10	2	8	3.64	0.57
10	2	8	3.14	0.84
-11	-2	-8	1.27	0.64
-11	-2	-8	1.20	1.07
11	2	8	1.18	0.53
11	2	8	1.93	0.47
11	2	8	0.05	0.71
-12	-2	-8	0.03	0.64
12	2	8	-0.35	0.80
12	2	8	0.23	0.70
12	2	8	0.69	0.56
-13	-2	-8	6.28	0.98
13	2	8	8.26	1.12

13	2	8	6.96	0.99
13	2	8	10.74	1.52
-14	-2	-8	6.21	1.06
14	2	8	8.27	1.06
14	2	8	6.47	0.85
14	2	8	7.06	1.22
-15	-2	-8	6.25	1.16
15	2	8	5.31	1.22
15	2	8	6.50	0.87
-16	-2	-8	0.68	1.75
16	2	8	0.06	1.15
16	2	8	1.43	0.70
-17	-2	-8	-2.74	1.19
17	2	8	0.78	0.77
17	2	8	4.51	1.40
-18	-2	-8	0.25	1.46
18	2	8	-0.56	1.39
18	2	8	-0.32	0.77
-19	-2	-8	3.38	1.60
19	2	8	1.21	1.62
20	-2	-7	-0.13	0.67
-20	2	7	-1.87	2.48
-20	2	7	1.50	1.31
19	-2	-7	62.71	2.81
-19	2	7	64.35	3.57
-19	2	7	46.94	5.99
18	-2	-7	0.11	0.50
-18	2	7	-0.82	1.08
-18	2	7	-0.81	1.56
17	-2	-7	78.60	3.51
-17	2	7	88.87	4.15
-17	2	7	83.76	4.50
16	-2	-7	0.04	0.43
-16	2	7	-0.37	0.80
-16	2	7	-0.41	1.32
15	-2	-7	52.09	2.38
15	-2	-7	49.36	3.65
-15	2	7	57.27	2.92
-15	2	7	55.14	3.36
14	-2	-7	4.02	1.57
14	-2	-7	2.04	0.43
-14	2	7	1.76	1.17
-14	2	7	2.09	0.83
13	-2	-7	150.90	6.88

13	-2	-7	149.29	5.96
-13	2	7	160.90	6.37
-13	2	7	152.66	6.75
12	-2	-7	1.71	1.31
12	-2	-7	0.14	0.27
-12	2	7	0.34	0.38
-12	2	7	0.81	0.91
-12	2	7	-0.06	0.56
11	-2	-7	176.52	7.68
11	-2	-7	166.77	6.83
11	-2	-7	167.49	7.06
-11	2	7	198.77	6.94
-11	2	7	180.14	7.15
-11	2	7	182.69	7.56
10	-2	-7	-0.77	0.40
10	-2	-7	0.01	0.22
10	-2	-7	0.67	0.92
-10	2	7	0.00	0.28
-10	2	7	0.55	0.79
-10	2	7	0.18	0.44
9	-2	-7	77.41	3.30
9	-2	-7	75.86	3.09
9	-2	-7	81.14	3.87
-9	2	7	79.65	3.15
-9	2	7	71.27	3.68
-9	2	7	81.97	3.36
8	-2	-7	-0.09	0.18
8	-2	-7	0.03	0.78
8	-2	-7	-0.01	0.19
8	-2	-7	-0.49	0.33
-8	2	7	0.31	0.25
-8	2	7	0.77	0.62
-8	2	7	0.32	0.37
7	-2	-7	251.42	9.87
7	-2	-7	269.32	9.86
7	-2	-7	269.52	10.53
7	-2	-7	268.45	9.92
7	-2	-7	260.58	10.02
-7	2	7	270.49	9.92
-7	2	7	247.13	10.39
-7	2	7	264.12	10.05
6	-2	-7	-0.18	0.15
6	-2	-7	0.33	0.64
6	-2	-7	-0.09	0.26

6	-2	-7	0.09	0.27
-6	2	7	0.25	0.20
-6	2	7	0.40	0.25
-6	2	7	-0.50	0.49
-6	2	7	-0.31	0.25
5	-2	-7	172.69	6.69
5	-2	-7	177.86	6.78
5	-2	-7	186.91	7.27
5	-2	-7	177.90	6.80
-5	2	7	166.00	7.15
-5	2	7	178.92	6.81
-5	2	7	175.12	6.77
-5	2	7	181.05	6.72
4	-2	-7	0.98	0.32
4	-2	-7	0.98	0.25
4	-2	-7	1.33	0.62
4	-2	-7	0.87	0.20
-4	2	7	0.61	0.19
-4	2	7	0.98	0.35
-4	2	7	0.78	0.27
-4	2	7	0.74	0.47
3	-2	-7	211.23	8.33
3	-2	-7	232.17	8.51
3	-2	-7	219.65	8.78
-3	2	7	225.31	8.35
-3	2	7	233.45	8.34
-3	2	7	217.24	8.48
-3	2	7	211.55	8.71
2	-2	-7	2.36	0.44
2	-2	-7	2.43	0.30
2	-2	-7	2.58	0.62
-2	2	7	2.29	0.27
-2	2	7	3.13	0.53
-2	2	7	1.96	0.40
-2	2	7	2.08	0.30
1	-2	-7	215.33	8.33
1	-2	-7	225.52	8.57
1	-2	-7	225.36	8.71
-1	2	7	214.50	8.53
-1	2	7	229.26	8.28
-1	2	7	219.03	8.64
-1	2	7	221.49	8.26
0	-2	-7	3.33	0.62
0	-2	-7	2.73	0.33

0	2	7	3.08	0.40
0	2	7	3.57	0.23
0	2	7	3.55	0.23
0	2	7	3.22	0.53
0	2	7	3.53	0.59
-1	-2	-7	295.48	10.86
-1	-2	-7	289.19	10.85
-1	-2	-7	271.16	10.55
1	2	7	280.37	10.76
1	2	7	273.73	10.79
1	2	7	276.18	10.56
-2	-2	-7	14.18	1.01
-2	-2	-7	12.54	0.64
-2	-2	-7	11.59	0.67
-2	-2	-7	12.54	1.01
2	2	7	11.87	0.67
2	2	7	12.56	0.85
2	2	7	11.04	0.93
-3	-2	-7	139.03	5.12
-3	-2	-7	145.16	5.61
-3	-2	-7	135.60	5.46
-3	-2	-7	134.57	5.22
-3	-2	-7	135.30	5.26
3	2	7	134.70	5.11
3	2	7	133.45	5.53
3	2	7	135.16	5.37
-4	-2	-7	4.42	0.41
-4	-2	-7	3.18	0.43
-4	-2	-7	3.74	0.33
-4	-2	-7	4.11	0.67
-4	-2	-7	4.42	0.62
4	2	7	3.09	0.58
4	2	7	3.84	0.32
4	2	7	3.91	0.60
-5	-2	-7	194.37	6.92
-5	-2	-7	177.34	7.07
-5	-2	-7	182.77	6.85
-5	-2	-7	177.75	6.97
-5	-2	-7	194.60	7.35
5	2	7	167.99	7.22
5	2	7	176.53	6.99
5	2	7	181.23	6.85
-6	-2	-7	5.86	0.57
-6	-2	-7	5.61	0.66

-6	-2	-7	7.14	0.54
-6	-2	-7	6.44	0.77
-6	-2	-7	7.34	1.08
6	2	7	7.37	0.52
6	2	7	6.73	0.95
6	2	7	6.18	0.54
6	2	7	8.07	0.80
-7	-2	-7	183.44	7.05
-7	-2	-7	186.73	7.06
-7	-2	-7	189.37	7.55
-7	-2	-7	171.02	7.13
7	2	7	189.61	7.04
7	2	7	195.76	7.11
7	2	7	174.55	7.42
7	2	7	187.09	7.05
-8	-2	-7	1.81	0.50
-8	-2	-7	1.89	0.32
-8	-2	-7	1.98	0.45
-8	-2	-7	1.55	0.76
8	2	7	1.69	0.35
8	2	7	1.70	0.36
8	2	7	1.86	0.66
8	2	7	1.82	0.37
-9	-2	-7	151.44	6.32
-9	-2	-7	169.43	6.34
-9	-2	-7	158.56	6.40
-9	-2	-7	176.00	6.90
9	2	7	161.33	6.26
9	2	7	161.18	6.74
9	2	7	165.46	6.39
9	2	7	170.82	6.37
-10	-2	-7	1.32	0.53
-10	-2	-7	2.15	0.84
-10	-2	-7	1.41	0.34
10	2	7	0.99	0.46
10	2	7	1.44	0.73
10	2	7	1.24	0.39
-11	-2	-7	53.73	2.69
-11	-2	-7	58.84	3.16
11	2	7	63.62	2.64
11	2	7	59.31	2.59
11	2	7	60.27	3.02
-12	-2	-7	3.43	1.06
-12	-2	-7	2.58	0.70

12	2	7	3.47	0.87
12	2	7	1.51	0.47
12	2	7	2.24	0.62
-13	-2	-7	20.28	1.67
13	2	7	21.74	1.82
13	2	7	23.19	1.48
13	2	7	22.14	1.34
-14	-2	-7	1.48	0.85
14	2	7	0.92	0.54
14	2	7	1.80	0.70
14	2	7	0.66	0.89
-15	-2	-7	28.62	2.20
15	2	7	32.47	1.81
15	2	7	32.80	2.33
-16	-2	-7	7.90	1.49
16	2	7	7.35	1.13
16	2	7	6.89	1.51
-17	-2	-7	24.86	2.48
17	2	7	25.62	1.80
17	2	7	30.44	2.49
-18	-2	-7	2.12	1.49
18	2	7	-1.98	1.27
18	2	7	0.60	0.73
-19	-2	-7	6.22	1.73
19	2	7	8.09	1.09
19	2	7	8.02	1.69
20	-2	-6	33.15	1.93
-20	2	6	36.46	2.91
19	-2	-6	2.17	0.63
-19	2	6	-1.14	1.51
-19	2	6	4.16	1.21
18	-2	-6	47.57	2.32
-18	2	6	48.14	2.99
-18	2	6	52.69	3.47
17	-2	-6	0.57	1.87
17	-2	-6	1.10	0.54
-17	2	6	0.47	1.33
-17	2	6	0.03	0.90
16	-2	-6	52.72	3.83
16	-2	-6	56.00	2.40
-16	2	6	52.63	2.93
-16	2	6	49.75	3.30
15	-2	-6	2.52	0.59
15	-2	-6	3.77	1.50

-15	2	6	3.63	1.20
-15	2	6	2.28	0.76
14	-2	-6	52.88	2.36
14	-2	-6	55.45	3.50
-14	2	6	56.92	3.23
13	-2	-6	1.03	0.34
13	-2	-6	1.03	1.21
-13	2	6	2.12	0.67
-13	2	6	1.83	1.02
12	-2	-6	116.37	4.98
12	-2	-6	125.67	5.62
12	-2	-6	115.24	4.73
-12	2	6	125.73	5.11
-12	2	6	122.79	4.82
-12	2	6	121.72	5.41
11	-2	-6	0.75	0.48
11	-2	-6	-0.30	0.24
11	-2	-6	-2.21	0.91
-11	2	6	0.59	0.33
-11	2	6	-0.45	0.71
-11	2	6	0.09	0.48
10	-2	-6	120.23	5.27
10	-2	-6	109.11	4.53
10	-2	-6	118.15	4.76
-10	2	6	129.00	4.62
-10	2	6	116.71	4.81
-10	2	6	108.20	5.09
9	-2	-6	5.33	0.74
9	-2	-6	7.33	1.08
9	-2	-6	5.37	0.49
-9	2	6	5.16	0.93
-9	2	6	5.77	0.76
-9	2	6	6.46	0.55
8	-2	-6	186.45	7.64
8	-2	-6	189.46	7.22
8	-2	-6	181.53	7.03
-8	2	6	197.59	7.09
-8	2	6	186.01	7.25
-8	2	6	172.17	7.52
7	-2	-6	15.96	0.80
7	-2	-6	17.23	1.52
7	-2	-6	16.71	0.99
7	-2	-6	17.26	0.78
7	-2	-6	17.37	0.82

-7	2	6	14.91	1.31
-7	2	6	16.68	0.84
-7	2	6	16.88	1.01
6	-2	-6	195.62	7.20
6	-2	-6	192.50	7.26
6	-2	-6	185.14	7.13
6	-2	-6	187.96	7.63
6	-2	-6	186.04	7.13
-6	2	6	188.41	7.27
-6	2	6	195.30	7.16
-6	2	6	179.42	7.56
5	-2	-6	2.35	0.67
5	-2	-6	2.55	0.41
5	-2	-6	3.33	0.32
-5	2	6	3.09	0.40
-5	2	6	3.94	0.46
-5	2	6	2.35	0.57
-5	2	6	2.88	0.34
4	-2	-6	82.38	3.22
4	-2	-6	86.83	3.57
4	-2	-6	80.96	3.18
4	-2	-6	75.75	3.11
-4	2	6	79.54	3.50
-4	2	6	77.68	3.19
-4	2	6	81.47	3.20
3	-2	-6	10.72	0.72
3	-2	-6	11.16	0.56
3	-2	-6	11.35	0.60
3	-2	-6	11.93	1.03
-3	2	6	12.22	0.58
-3	2	6	10.57	0.61
-3	2	6	10.20	0.70
-3	2	6	9.79	0.89
2	-2	-6	185.26	7.21
2	-2	-6	192.74	7.20
2	-2	-6	196.62	7.38
-2	2	6	194.62	7.21
-2	2	6	195.35	7.23
-2	2	6	185.54	7.51
1	-2	-6	110.86	4.39
1	-2	-6	108.03	4.19
1	-2	-6	112.05	4.51
-1	2	6	109.09	4.14
-1	2	6	110.31	4.46

-1	2	6	114.77	4.21
-1	2	6	104.13	4.36
-1	2	6	108.84	4.12
0	-2	-6	290.24	11.31
0	-2	-6	320.38	11.54
0	-2	-6	304.45	11.58
0	2	6	293.44	11.50
0	2	6	301.18	11.32
0	2	6	305.26	11.53
-1	-2	-6	-0.07	0.12
-1	-2	-6	0.24	0.35
-1	-2	-6	-0.17	0.35
1	2	6	-0.09	0.27
1	2	6	0.11	0.14
1	2	6	0.24	0.30
-2	-2	-6	126.56	4.77
-2	-2	-6	132.10	5.05
-2	-2	-6	124.62	4.96
-2	-2	-6	115.99	4.80
2	2	6	121.99	5.00
2	2	6	123.51	4.85
2	2	6	125.70	4.90
-3	-2	-6	1.12	0.15
-3	-2	-6	0.85	0.37
-3	-2	-6	0.76	0.42
-3	-2	-6	0.82	0.22
-3	-2	-6	0.97	0.19
3	2	6	0.85	0.14
3	2	6	0.67	0.35
-4	-2	-6	152.43	5.81
-4	-2	-6	158.18	6.04
-4	-2	-6	158.62	5.93
-4	-2	-6	161.82	6.24
-4	-2	-6	149.00	5.87
4	2	6	156.41	5.81
4	2	6	147.49	6.16
4	2	6	152.11	5.97
-5	-2	-6	12.80	0.72
-5	-2	-6	14.50	0.81
-5	-2	-6	17.01	0.77
-5	-2	-6	14.80	0.92
-5	-2	-6	17.20	1.19
5	2	6	14.51	0.77
5	2	6	13.07	1.06

5	2	6	13.32	0.71
5	2	6	15.33	0.83
-6	-2	-6	294.37	11.12
-6	-2	-6	290.18	11.02
-6	-2	-6	294.38	11.03
-6	-2	-6	315.12	11.44
6	2	6	282.87	11.34
6	2	6	273.81	11.04
6	2	6	296.27	11.01
6	2	6	299.93	11.04
-7	-2	-6	0.62	0.29
-7	-2	-6	-0.33	0.53
-7	-2	-6	0.95	0.23
-7	-2	-6	0.21	0.36
7	2	6	0.28	0.26
7	2	6	0.49	0.23
7	2	6	-0.01	0.46
7	2	6	0.47	0.28
-8	-2	-6	180.51	7.21
-8	-2	-6	184.52	7.15
-8	-2	-6	198.59	7.63
-8	-2	-6	180.15	7.17
8	2	6	191.17	7.18
8	2	6	192.63	7.11
8	2	6	183.80	7.18
8	2	6	187.89	7.52
-9	-2	-6	0.25	0.35
-9	-2	-6	-0.18	0.37
-9	-2	-6	0.13	0.27
-9	-2	-6	-0.40	0.67
-9	-2	-6	0.41	0.40
9	2	6	-0.28	0.26
9	2	6	0.35	0.54
9	2	6	0.26	0.35
-10	-2	-6	128.37	5.01
-10	-2	-6	134.91	5.53
-10	-2	-6	122.15	5.14
10	2	6	132.22	5.08
10	2	6	126.28	5.05
10	2	6	123.35	5.36
-11	-2	-6	10.76	1.11
-11	-2	-6	10.55	1.54
11	2	6	12.24	0.87
11	2	6	9.27	1.26

11	2	6	11.21	1.00
-12	-2	-6	61.96	3.03
-12	-2	-6	69.38	3.41
12	2	6	64.04	3.20
12	2	6	65.89	2.84
12	2	6	67.12	2.90
-13	-2	-6	6.64	1.01
-13	-2	-6	12.40	1.79
13	2	6	7.90	1.34
13	2	6	9.48	1.10
-14	-2	-6	66.14	3.61
-14	-2	-6	61.16	3.17
14	2	6	63.31	3.00
14	2	6	63.04	2.89
14	2	6	71.37	3.34
-15	-2	-6	-0.82	0.85
15	2	6	0.10	0.52
15	2	6	-0.50	0.91
-16	-2	-6	99.64	4.68
16	2	6	97.44	4.19
16	2	6	96.29	4.56
-17	-2	-6	-0.78	1.12
17	2	6	1.72	1.37
17	2	6	0.66	0.67
-18	-2	-6	67.86	3.90
18	2	6	66.82	3.77
18	2	6	71.44	3.28
-19	-2	-6	-1.53	1.44
19	2	6	-0.89	1.39
19	2	6	0.44	0.83
21	-2	-5	5.19	0.84
20	-2	-5	-0.09	0.64
-20	2	5	-0.98	1.19
19	-2	-5	6.51	1.04
-19	2	5	3.91	1.33
-19	2	5	7.81	1.65
18	-2	-5	0.84	0.55
-18	2	5	2.37	1.14
-18	2	5	1.09	1.38
17	-2	-5	12.84	1.05
17	-2	-5	16.06	2.80
-17	2	5	14.19	1.81
-17	2	5	13.21	2.22
16	-2	-5	0.97	1.54

16	-2	-5	0.22	0.41
-16	2	5	0.14	1.14
-16	2	5	0.76	0.81
15	-2	-5	10.47	1.63
-15	2	5	11.43	1.44
-15	2	5	10.23	1.49
14	-2	-5	1.81	0.40
14	-2	-5	4.91	1.41
-14	2	5	1.87	1.02
-14	2	5	1.98	0.73
13	-2	-5	6.07	0.66
13	-2	-5	7.36	1.43
-13	2	5	7.38	1.11
-13	2	5	8.95	0.96
-13	2	5	7.55	1.21
12	-2	-5	31.71	2.36
12	-2	-5	28.31	1.65
12	-2	-5	28.60	1.39
-12	2	5	27.72	1.74
-12	2	5	32.20	1.48
-12	2	5	30.92	2.12
11	-2	-5	5.85	0.54
11	-2	-5	8.09	1.24
11	-2	-5	5.37	0.83
-11	2	5	6.39	1.01
-11	2	5	5.69	0.91
-11	2	5	5.42	0.60
10	-2	-5	0.31	0.80
10	-2	-5	0.53	0.41
10	-2	-5	0.35	0.23
-10	2	5	0.28	0.25
-10	2	5	-0.32	0.40
-10	2	5	0.85	0.69
9	-2	-5	36.55	1.74
9	-2	-5	32.84	1.49
9	-2	-5	34.54	2.17
-9	2	5	32.59	2.01
-9	2	5	37.24	1.56
-9	2	5	35.34	1.75
8	-2	-5	20.48	1.14
8	-2	-5	19.92	1.55
8	-2	-5	19.64	0.91
-8	2	5	17.67	1.38
-8	2	5	19.89	0.96

-8	2	5	18.25	1.13
7	-2	-5	182.81	6.65
7	-2	-5	164.82	6.47
7	-2	-5	174.49	6.96
7	-2	-5	164.40	6.41
7	-2	-5	174.70	6.41
-7	2	5	167.36	6.89
-7	2	5	171.68	6.65
-7	2	5	168.07	6.50
6	-2	-5	18.81	0.90
6	-2	-5	20.68	1.38
6	-2	-5	20.29	1.02
6	-2	-5	18.61	0.86
6	-2	-5	17.75	0.86
-6	2	5	19.33	1.30
-6	2	5	17.92	1.02
-6	2	5	20.12	0.89
5	-2	-5	85.34	3.31
5	-2	-5	79.87	3.19
5	-2	-5	82.97	3.27
5	-2	-5	86.08	3.61
5	-2	-5	83.11	3.22
-5	2	5	72.47	3.52
-5	2	5	85.79	3.31
-5	2	5	85.54	3.23
4	-2	-5	0.34	0.13
4	-2	-5	0.65	0.22
4	-2	-5	0.83	0.23
4	-2	-5	1.26	0.69
-4	2	5	0.78	0.21
-4	2	5	0.47	0.35
-4	2	5	0.50	0.15
-4	2	5	0.44	0.23
3	-2	-5	20.90	0.91
3	-2	-5	23.08	1.25
3	-2	-5	20.57	0.94
3	-2	-5	20.81	1.01
-3	2	5	21.18	1.21
-3	2	5	20.71	0.95
-3	2	5	19.94	0.99
2	-2	-5	1.05	0.17
2	-2	-5	0.76	0.15
2	-2	-5	0.94	0.35
2	-2	-5	0.46	0.24

-2	2	5	0.76	0.24
-2	2	5	0.69	0.15
-2	2	5	0.81	0.16
-2	2	5	0.68	0.32
1	-2	-5	374.82	13.40
1	-2	-5	344.20	13.18
1	-2	-5	337.96	13.23
1	-2	-5	379.76	13.50
-1	2	5	343.58	13.19
-1	2	5	352.87	13.24
-1	2	5	333.55	13.36
-1	2	5	376.56	13.48
0	-2	-5	34.85	1.61
0	-2	-5	33.65	1.42
0	-2	-5	37.32	1.66
0	2	5	35.83	1.43
0	2	5	33.69	1.64
0	2	5	33.86	1.61
-1	-2	-5	110.06	4.19
-1	-2	-5	117.74	4.40
-1	-2	-5	110.83	4.37
1	2	5	107.86	4.37
1	2	5	110.50	4.19
1	2	5	100.34	4.42
-2	-2	-5	12.24	0.61
-2	-2	-5	15.05	0.90
-2	-2	-5	11.76	0.63
-2	-2	-5	12.84	0.80
2	2	5	13.88	0.65
2	2	5	11.50	0.74
2	2	5	12.32	0.84
-3	-2	-5	117.57	4.30
-3	-2	-5	107.83	4.39
-3	-2	-5	115.79	4.51
3	2	5	117.76	4.46
3	2	5	112.42	4.61
3	2	5	115.18	4.30
-4	-2	-5	0.67	0.37
-4	-2	-5	1.11	0.18
-4	-2	-5	0.97	0.22
-4	-2	-5	1.47	0.25
-4	-2	-5	1.31	0.31
4	2	5	0.89	0.26
4	2	5	0.99	0.37

4	2	5	0.97	0.17
4	2	5	0.55	0.17
-5	-2	-5	83.03	3.28
-5	-2	-5	86.82	3.54
-5	-2	-5	82.04	3.18
-5	-2	-5	80.71	3.19
5	2	5	78.33	3.20
5	2	5	76.57	3.45
5	2	5	83.90	3.22
5	2	5	82.48	3.17
-6	-2	-5	0.70	0.19
-6	-2	-5	0.66	0.29
-6	-2	-5	0.68	0.28
-6	-2	-5	0.56	0.43
6	2	5	0.60	0.23
6	2	5	0.73	0.40
6	2	5	0.73	0.21
6	2	5	0.87	0.25
-7	-2	-5	139.69	5.41
-7	-2	-5	136.69	5.38
-7	-2	-5	150.97	5.77
-7	-2	-5	134.89	5.42
7	2	5	142.93	5.41
7	2	5	142.83	5.36
7	2	5	137.25	5.68
7	2	5	136.39	5.40
-8	-2	-5	6.37	0.64
-8	-2	-5	7.68	1.04
-8	-2	-5	7.07	0.60
-8	-2	-5	7.54	0.54
-8	-2	-5	6.32	0.68
8	2	5	6.99	0.92
8	2	5	6.08	0.54
8	2	5	6.01	0.62
-9	-2	-5	55.34	2.36
-9	-2	-5	52.39	2.49
-9	-2	-5	56.89	2.38
-9	-2	-5	63.01	2.87
-9	-2	-5	56.88	2.41
9	2	5	60.44	2.44
9	2	5	59.82	2.48
9	2	5	58.47	2.73
-10	-2	-5	0.05	0.25
-10	-2	-5	-0.33	0.32

-10	-2	-5	0.20	0.68
-10	-2	-5	-0.19	0.41
10	2	5	-0.07	0.26
10	2	5	0.52	0.55
10	2	5	-0.09	0.36
-11	-2	-5	52.74	2.76
-11	-2	-5	51.53	2.20
-11	-2	-5	47.84	2.41
11	2	5	50.92	2.58
11	2	5	52.91	2.34
11	2	5	51.56	2.25
-12	-2	-5	28.50	1.77
-12	-2	-5	29.85	2.06
12	2	5	31.87	1.67
12	2	5	30.19	1.90
12	2	5	31.20	1.55
-13	-2	-5	66.98	3.15
-13	-2	-5	65.43	3.42
13	2	5	69.01	3.00
13	2	5	65.25	3.23
13	2	5	68.54	2.92
-14	-2	-5	0.42	1.04
-14	-2	-5	0.03	0.76
14	2	5	0.85	0.79
14	2	5	0.11	0.39
14	2	5	0.32	0.77
-15	-2	-5	82.57	3.87
-15	-2	-5	79.41	4.13
15	2	5	83.44	3.55
15	2	5	80.89	3.88
-16	-2	-5	-0.49	0.96
16	2	5	-1.27	0.47
16	2	5	-0.97	0.88
-17	-2	-5	48.31	3.01
17	2	5	55.90	2.60
17	2	5	48.56	2.94
-18	-2	-5	1.17	1.32
18	2	5	2.53	0.76
18	2	5	4.53	1.26
-19	-2	-5	43.28	3.19
19	2	5	42.15	3.41
19	2	5	45.70	2.41
-20	-2	-5	-1.14	1.53
20	2	5	1.39	0.84

21	-2	-4	1.56	0.74
-21	2	4	0.21	2.30
20	-2	-4	0.64	0.62
-20	2	4	-2.45	1.19
19	-2	-4	0.32	0.54
-19	2	4	0.10	1.37
-19	2	4	0.60	1.18
18	-2	-4	0.73	0.51
-18	2	4	-0.45	1.02
-18	2	4	-0.60	1.26
17	-2	-4	0.37	0.48
17	-2	-4	1.69	1.60
-17	2	4	-0.64	1.15
-17	2	4	1.55	0.94
16	-2	-4	0.04	0.39
16	-2	-4	4.95	1.46
-16	2	4	1.71	1.16
-16	2	4	-0.26	0.80
15	-2	-4	6.30	1.51
-15	2	4	4.63	1.11
-15	2	4	4.99	0.92
14	-2	-4	-0.03	1.17
14	-2	-4	0.26	0.32
-14	2	4	0.88	0.89
-14	2	4	0.71	0.64
13	-2	-4	5.71	1.29
13	-2	-4	5.23	0.61
-13	2	4	5.06	0.79
-13	2	4	7.53	1.09
-13	2	4	4.87	1.04
12	-2	-4	0.51	0.51
12	-2	-4	-0.12	0.26
12	-2	-4	0.16	0.90
-12	2	4	1.02	0.53
-12	2	4	0.38	0.73
-12	2	4	0.28	0.34
11	-2	-4	2.90	0.56
11	-2	-4	4.88	1.01
11	-2	-4	3.33	0.48
-11	2	4	2.97	0.82
-11	2	4	3.95	0.55
-11	2	4	2.08	0.56
10	-2	-4	1.48	0.41
10	-2	-4	1.04	0.73

10	-2	-4	1.32	0.27
-10	2	4	0.21	0.61
-10	2	4	0.85	0.29
-10	2	4	1.69	0.50
9	-2	-4	0.74	0.20
9	-2	-4	0.12	0.60
9	-2	-4	0.49	0.33
-9	2	4	0.00	0.21
-9	2	4	0.90	0.55
-9	2	4	0.66	0.37
8	-2	-4	22.46	1.53
8	-2	-4	20.65	1.00
8	-2	-4	22.20	1.18
-8	2	4	20.60	1.21
-8	2	4	23.05	1.04
-8	2	4	21.92	1.43
7	-2	-4	0.15	0.16
7	-2	-4	-0.07	0.14
7	-2	-4	-0.06	0.25
7	-2	-4	0.91	0.52
-7	2	4	0.12	0.45
-7	2	4	0.04	0.27
-7	2	4	0.23	0.19
6	-2	-4	2.88	0.28
6	-2	-4	3.21	0.44
6	-2	-4	2.67	0.32
6	-2	-4	2.99	0.31
-6	2	4	2.48	0.30
-6	2	4	2.38	0.49
-6	2	4	2.66	0.44
5	-2	-4	1.35	0.21
5	-2	-4	1.06	0.25
5	-2	-4	1.18	0.21
5	-2	-4	0.82	0.44
5	-2	-4	1.09	0.23
-5	2	4	0.92	0.17
-5	2	4	0.96	0.23
-5	2	4	1.13	0.37
4	-2	-4	18.42	0.93
4	-2	-4	20.02	0.89
4	-2	-4	20.92	1.19
4	-2	-4	18.74	0.83
4	-2	-4	20.23	0.92
-4	2	4	19.12	0.84

-4	2	4	19.55	1.15
-4	2	4	18.12	0.93
3	-2	-4	37.56	1.73
3	-2	-4	36.01	1.50
3	-2	-4	39.17	1.53
3	-2	-4	35.77	1.46
-3	2	4	35.68	1.51
-3	2	4	35.49	1.50
-3	2	4	34.65	1.71
-3	2	4	36.27	1.46
2	-2	-4	10.34	0.75
2	-2	-4	10.41	0.51
2	-2	-4	11.67	0.64
2	-2	-4	10.05	0.52
-2	2	4	11.05	0.53
-2	2	4	11.42	0.62
-2	2	4	10.91	0.52
1	-2	-4	181.07	6.75
1	-2	-4	173.29	6.54
1	-2	-4	173.27	6.51
1	-2	-4	180.87	6.67
-1	2	4	165.04	6.71
-1	2	4	167.98	6.50
-1	2	4	174.53	6.66
-1	2	4	179.55	6.55
0	-2	-4	21.50	1.06
0	-2	-4	20.36	0.87
0	-2	-4	21.84	1.04
0	2	4	20.86	1.03
0	2	4	19.94	0.87
0	2	4	19.03	1.00
-1	-2	-4	16.95	0.88
-1	-2	-4	16.51	0.90
-1	-2	-4	15.18	0.73
1	2	4	15.82	0.83
1	2	4	16.21	0.99
-2	-2	-4	9.97	0.63
-2	-2	-4	10.09	0.49
-2	-2	-4	9.88	0.70
-2	-2	-4	9.74	0.52
2	2	4	9.17	0.51
2	2	4	10.56	0.73
2	2	4	10.33	0.41
2	2	4	9.47	0.59

-3	-2	-4	46.43	1.91
-3	-2	-4	45.32	1.83
-3	-2	-4	46.99	1.88
-3	-2	-4	48.71	1.98
-3	-2	-4	49.57	2.12
3	2	4	45.73	2.08
3	2	4	48.28	1.84
3	2	4	48.93	1.95
-4	-2	-4	48.76	2.02
-4	-2	-4	52.68	2.11
-4	-2	-4	54.12	2.30
4	2	4	52.93	2.08
4	2	4	49.33	2.25
4	2	4	51.68	2.02
4	2	4	50.17	2.05
-5	-2	-4	2.40	0.38
-5	-2	-4	2.56	0.29
-5	-2	-4	2.59	0.35
-5	-2	-4	2.55	0.47
5	2	4	3.09	0.32
5	2	4	2.70	0.33
5	2	4	2.58	0.35
5	2	4	2.35	0.44
-6	-2	-4	21.72	0.97
-6	-2	-4	20.07	1.03
-6	-2	-4	21.82	1.00
-6	-2	-4	20.77	1.27
6	2	4	20.04	0.97
6	2	4	21.17	0.99
6	2	4	20.20	0.98
6	2	4	21.42	1.23
-7	-2	-4	8.67	0.85
-7	-2	-4	7.37	0.61
-7	-2	-4	8.54	0.52
-7	-2	-4	7.67	0.56
7	2	4	7.20	0.78
7	2	4	7.74	0.67
7	2	4	7.72	0.56
7	2	4	6.80	0.51
-8	-2	-4	35.13	1.73
-8	-2	-4	36.70	1.69
-8	-2	-4	41.36	2.01
-8	-2	-4	37.80	1.63
-8	-2	-4	38.77	1.63

8	2	4	39.37	1.71
8	2	4	36.75	1.91
8	2	4	39.27	1.66
-9	-2	-4	3.74	0.45
-9	-2	-4	3.53	0.64
-9	-2	-4	3.97	0.51
-9	-2	-4	3.74	0.53
-9	-2	-4	3.97	0.74
9	2	4	3.82	0.48
9	2	4	4.48	0.60
9	2	4	3.50	0.63
-10	-2	-4	70.14	3.00
-10	-2	-4	72.23	2.90
-10	-2	-4	75.02	3.35
-10	-2	-4	69.43	3.11
-10	-2	-4	74.71	2.95
10	2	4	72.80	3.25
10	2	4	73.08	2.99
10	2	4	73.55	3.05
-11	-2	-4	1.93	0.36
-11	-2	-4	1.06	0.51
-11	-2	-4	0.75	0.36
-11	-2	-4	0.87	0.51
-11	-2	-4	1.27	0.71
11	2	4	0.87	0.56
11	2	4	1.05	0.36
11	2	4	1.65	0.47
-12	-2	-4	23.65	1.61
-12	-2	-4	26.85	1.84
-12	-2	-4	25.84	1.25
12	2	4	27.02	1.39
12	2	4	26.80	1.67
12	2	4	26.87	1.52
-13	-2	-4	1.24	0.87
-13	-2	-4	1.15	0.65
13	2	4	2.22	0.71
13	2	4	1.65	0.45
13	2	4	0.95	0.56
-14	-2	-4	46.75	2.70
-14	-2	-4	45.93	2.48
14	2	4	46.42	2.44
14	2	4	41.49	2.41
-15	-2	-4	1.56	1.11
-15	-2	-4	0.19	0.78

15	2	4	1.60	0.55
15	2	4	1.05	0.83
-16	-2	-4	50.75	2.84
-16	-2	-4	46.78	3.16
16	2	4	53.48	2.46
16	2	4	50.18	2.76
-17	-2	-4	1.01	1.05
17	2	4	1.60	0.66
17	2	4	0.71	0.94
-18	-2	-4	13.93	2.17
18	2	4	14.53	1.98
18	2	4	14.98	1.36
-19	-2	-4	0.57	1.33
19	2	4	-0.98	1.20
19	2	4	-0.88	0.67
-20	-2	-4	20.68	2.78
20	2	4	22.27	1.80
21	-2	-3	9.51	1.30
-21	2	3	6.20	1.68
20	-2	-3	1.53	0.69
-20	2	3	1.72	1.34
19	-2	-3	9.51	1.12
-19	2	3	6.33	1.32
18	-2	-3	3.57	0.61
-18	2	3	4.37	1.13
-18	2	3	2.50	1.23
17	-2	-3	26.42	1.54
17	-2	-3	29.72	3.06
-17	2	3	28.29	2.29
-17	2	3	26.68	2.21
16	-2	-3	0.68	1.28
16	-2	-3	-0.39	0.40
-16	2	3	-0.25	0.77
-16	2	3	-0.76	0.95
15	-2	-3	16.10	2.06
15	-2	-3	15.66	0.85
-15	2	3	12.59	1.68
-15	2	3	14.65	1.52
14	-2	-3	7.45	0.71
14	-2	-3	6.49	0.62
14	-2	-3	6.27	1.23
-14	2	3	7.24	0.88
-14	2	3	6.42	1.09
-14	2	3	7.08	1.18

13	-2	-3	2.93	0.51
13	-2	-3	2.57	0.42
13	-2	-3	3.32	1.04
-13	2	3	4.42	0.67
-13	2	3	3.30	0.56
-13	2	3	4.01	0.93
-13	2	3	2.79	0.69
12	-2	-3	12.27	1.61
12	-2	-3	13.54	0.84
12	-2	-3	15.20	0.79
12	-2	-3	13.94	1.17
-12	2	3	12.90	0.97
-12	2	3	14.04	1.43
-12	2	3	15.16	1.22
-12	2	3	16.16	0.93
11	-2	-3	18.82	1.19
11	-2	-3	19.10	1.64
11	-2	-3	17.62	0.95
11	-2	-3	18.48	0.89
-11	2	3	17.66	1.44
-11	2	3	16.95	1.26
-11	2	3	16.67	1.02
-11	2	3	19.37	1.02
10	-2	-3	2.77	0.50
10	-2	-3	2.09	0.74
10	-2	-3	2.89	0.41
10	-2	-3	3.40	0.40
-10	2	3	3.23	0.44
-10	2	3	2.27	0.34
-10	2	3	2.87	0.68
-10	2	3	2.40	0.55
9	-2	-3	5.85	0.44
9	-2	-3	5.81	0.66
9	-2	-3	5.42	1.03
9	-2	-3	4.76	0.43
-9	2	3	4.72	0.88
-9	2	3	5.60	0.49
-9	2	3	5.39	0.70
-9	2	3	4.53	0.54
8	-2	-3	0.04	0.28
8	-2	-3	-0.07	0.16
8	-2	-3	0.02	0.18
-8	2	3	0.33	0.32
-8	2	3	0.47	0.43

-8	2	3	0.16	0.27
-8	2	3	0.07	0.18
7	-2	-3	2.36	0.29
7	-2	-3	1.85	0.35
7	-2	-3	1.90	0.28
7	-2	-3	2.18	0.55
-7	2	3	2.03	0.38
-7	2	3	1.84	0.46
-7	2	3	2.37	0.42
-7	2	3	2.63	0.33
6	-2	-3	12.01	0.72
6	-2	-3	11.69	0.94
6	-2	-3	10.86	0.57
6	-2	-3	11.44	0.57
-6	2	3	11.71	0.66
-6	2	3	12.23	0.60
-6	2	3	10.71	0.73
-6	2	3	10.54	0.90
5	-2	-3	48.96	1.99
5	-2	-3	52.72	2.26
5	-2	-3	52.71	2.09
5	-2	-3	49.26	1.97
-5	2	3	48.92	2.24
-5	2	3	48.77	2.09
-5	2	3	50.24	1.99
4	-2	-3	0.37	0.12
4	-2	-3	0.17	0.27
4	-2	-3	0.30	0.15
4	-2	-3	0.35	0.19
4	-2	-3	0.48	0.18
-4	2	3	0.46	0.12
-4	2	3	0.19	0.23
3	-2	-3	6.96	0.59
3	-2	-3	6.51	0.49
3	-2	-3	6.77	0.43
3	-2	-3	6.84	0.43
3	-2	-3	6.39	0.35
-3	2	3	6.49	0.57
-3	2	3	7.42	0.43
2	-2	-3	1.75	0.22
2	-2	-3	1.61	0.30
2	-2	-3	1.61	0.17
2	-2	-3	1.35	0.23
-2	2	3	2.11	0.22

-2	2	3	1.42	0.18
-2	2	3	1.53	0.23
-2	2	3	1.45	0.29
1	-2	-3	79.54	3.18
1	-2	-3	82.12	3.13
1	-2	-3	76.88	3.00
-1	2	3	75.33	3.16
-1	2	3	80.74	3.00
-1	2	3	82.76	3.03
-1	2	3	79.74	3.12
0	-2	-3	31.72	1.39
0	-2	-3	32.11	1.20
0	-2	-3	32.60	1.39
0	-2	-3	31.40	1.26
0	2	3	31.33	1.37
0	2	3	30.62	1.37
-1	-2	-3	0.43	0.22
-1	-2	-3	0.22	0.09
-1	-2	-3	0.21	0.18
1	2	3	0.46	0.18
1	2	3	0.40	0.21
1	2	3	0.27	0.10
-2	-2	-3	0.23	0.11
-2	-2	-3	0.14	0.17
-2	-2	-3	0.13	0.08
-2	-2	-3	0.05	0.11
-2	-2	-3	0.48	0.23
-2	-2	-3	0.13	0.07
2	2	3	0.46	0.23
2	2	3	0.22	0.16
-3	-2	-3	18.66	0.86
-3	-2	-3	21.03	0.91
-3	-2	-3	20.44	0.94
-3	-2	-3	21.78	1.08
-3	-2	-3	20.69	0.87
3	2	3	19.58	0.88
3	2	3	21.05	0.93
3	2	3	21.44	0.85
-4	-2	-3	3.91	0.30
-4	-2	-3	4.63	0.38
-4	-2	-3	4.91	0.54
-4	-2	-3	3.77	0.34
4	2	3	4.31	0.32
4	2	3	4.81	0.33

4	2	3	4.34	0.36
4	2	3	3.99	0.55
-5	-2	-3	7.62	0.43
-5	-2	-3	6.02	0.47
-5	-2	-3	6.85	0.65
-5	-2	-3	7.04	0.47
5	2	3	6.36	0.46
5	2	3	7.01	0.43
5	2	3	6.62	0.45
5	2	3	7.74	0.65
-6	-2	-3	14.90	1.14
-6	-2	-3	15.76	0.86
-6	-2	-3	17.45	0.77
-6	-2	-3	17.07	0.81
6	2	3	16.87	0.83
6	2	3	15.20	0.97
6	2	3	15.06	0.80
6	2	3	15.74	0.78
-7	-2	-3	4.13	0.53
-7	-2	-3	4.38	0.38
-7	-2	-3	3.75	0.43
-7	-2	-3	3.43	0.49
-7	-2	-3	4.23	0.60
7	2	3	3.72	0.65
7	2	3	4.16	0.39
-8	-2	-3	3.28	0.37
-8	-2	-3	2.77	0.44
-8	-2	-3	3.29	0.57
-8	-2	-3	3.53	0.56
-8	-2	-3	2.83	0.45
8	2	3	3.21	0.54
8	2	3	2.88	0.41
8	2	3	2.46	0.39
-9	-2	-3	2.77	0.38
-9	-2	-3	2.09	0.45
-9	-2	-3	1.82	0.58
-9	-2	-3	1.70	0.28
-9	-2	-3	1.72	0.47
9	2	3	2.57	0.54
9	2	3	1.83	0.38
9	2	3	2.78	0.43
-10	-2	-3	0.44	0.42
-10	-2	-3	0.65	0.34
-10	-2	-3	0.53	0.22

-10	-2	-3	0.62	0.49
-10	-2	-3	0.40	0.55
10	2	3	0.42	0.27
10	2	3	0.58	0.49
10	2	3	0.51	0.36
-11	-2	-3	28.72	1.31
-11	-2	-3	25.82	1.32
-11	-2	-3	26.09	1.56
-11	-2	-3	26.84	1.52
-11	-2	-3	27.22	1.73
11	2	3	26.84	1.62
11	2	3	27.76	1.36
11	2	3	28.11	1.48
-12	-2	-3	1.51	0.65
-12	-2	-3	2.20	0.38
-12	-2	-3	1.66	0.44
-12	-2	-3	1.61	0.60
-12	-2	-3	2.72	0.76
12	2	3	2.20	0.43
12	2	3	1.93	0.64
12	2	3	1.76	0.53
-13	-2	-3	3.36	0.75
-13	-2	-3	4.60	0.73
-13	-2	-3	3.45	0.53
-13	-2	-3	3.83	0.91
13	2	3	3.50	0.52
13	2	3	2.12	0.61
13	2	3	3.40	0.74
-14	-2	-3	0.51	0.84
-14	-2	-3	0.79	0.71
-14	-2	-3	0.63	0.46
14	2	3	0.33	0.41
14	2	3	-0.22	0.62
-15	-2	-3	16.30	1.80
-15	-2	-3	15.64	1.57
15	2	3	15.04	1.11
15	2	3	13.50	1.43
-16	-2	-3	13.43	1.66
-16	-2	-3	12.13	1.90
16	2	3	10.20	1.08
16	2	3	11.50	1.41
-17	-2	-3	18.72	1.93
17	2	3	15.64	1.71
17	2	3	15.55	1.34

-18	-2	-3	3.13	1.16
18	2	3	2.08	0.76
18	2	3	2.47	1.06
-19	-2	-3	4.10	1.37
19	2	3	4.92	1.29
19	2	3	6.38	0.92
-20	-2	-3	-0.05	1.46
20	2	3	0.53	0.76
21	-2	-2	4.53	0.86
-21	2	2	4.04	1.55
20	-2	-2	30.24	1.87
-20	2	2	37.16	2.84
19	-2	-2	0.40	0.58
-19	2	2	-0.14	1.17
18	-2	-2	35.02	1.86
-18	2	2	36.51	2.60
-18	2	2	33.39	2.37
17	-2	-2	2.50	0.56
17	-2	-2	3.89	1.36
17	-2	-2	2.55	0.68
-17	2	2	2.46	1.07
-17	2	2	3.67	1.09
16	-2	-2	58.31	2.62
16	-2	-2	61.11	2.58
16	-2	-2	62.24	3.26
-16	2	2	61.23	3.16
-16	2	2	56.98	3.03
-16	2	2	55.65	2.77
15	-2	-2	11.25	0.99
15	-2	-2	9.95	1.82
-15	2	2	9.60	1.20
-15	2	2	12.02	1.39
-15	2	2	10.49	1.51
14	-2	-2	28.92	2.12
14	-2	-2	29.44	1.40
14	-2	-2	27.70	1.45
-14	2	2	29.14	1.84
-14	2	2	25.61	1.59
-14	2	2	24.61	1.82
13	-2	-2	19.86	1.72
13	-2	-2	17.62	1.03
-13	2	2	16.50	1.23
-13	2	2	18.18	1.44
-13	2	2	19.57	1.25

-13	2	2	16.70	1.50
12	-2	-2	62.32	2.60
12	-2	-2	62.43	3.05
12	-2	-2	63.67	2.60
12	-2	-2	63.32	2.81
-12	2	2	61.53	2.90
-12	2	2	65.49	2.64
-12	2	2	59.13	2.92
-12	2	2	60.81	2.70
11	-2	-2	6.12	0.62
11	-2	-2	6.85	0.81
11	-2	-2	5.56	0.91
11	-2	-2	5.46	0.54
-11	2	2	7.19	0.59
-11	2	2	5.90	0.76
-11	2	2	5.05	0.79
-11	2	2	4.89	0.85
10	-2	-2	169.17	6.53
10	-2	-2	169.29	6.71
10	-2	-2	172.68	6.55
-10	2	2	176.72	6.80
-10	2	2	161.78	6.60
-10	2	2	162.08	6.79
-10	2	2	186.53	6.59
9	-2	-2	8.21	0.58
9	-2	-2	8.25	0.75
9	-2	-2	7.66	0.53
9	-2	-2	7.43	1.00
-9	2	2	7.98	0.77
-9	2	2	7.75	0.69
-9	2	2	8.81	0.58
-9	2	2	6.99	0.88
8	-2	-2	24.18	1.46
8	-2	-2	26.01	1.13
8	-2	-2	27.17	1.30
8	-2	-2	24.54	1.11
-8	2	2	23.04	1.40
-8	2	2	24.04	1.30
-8	2	2	25.53	1.13
-8	2	2	23.74	1.19
7	-2	-2	22.04	1.12
7	-2	-2	21.89	0.98
7	-2	-2	21.40	1.29
7	-2	-2	20.06	0.95

-7	2	2	24.31	0.99
-7	2	2	20.52	1.04
-7	2	2	20.05	1.23
-7	2	2	21.32	1.12
6	-2	-2	23.35	1.11
6	-2	-2	21.45	1.23
6	-2	-2	22.24	0.98
6	-2	-2	22.35	0.97
-6	2	2	21.73	1.04
-6	2	2	21.84	1.11
-6	2	2	23.12	0.99
5	-2	-2	43.75	1.85
5	-2	-2	42.83	1.78
5	-2	-2	46.97	1.99
5	-2	-2	43.95	1.76
-5	2	2	44.60	1.83
-5	2	2	42.98	1.95
-5	2	2	44.70	1.87
-5	2	2	46.02	1.77
4	-2	-2	46.17	1.78
4	-2	-2	44.98	1.81
4	-2	-2	39.53	1.76
4	-2	-2	45.71	1.92
-4	2	2	46.21	1.82
-4	2	2	43.70	1.73
-4	2	2	42.54	1.90
3	-2	-2	0.60	0.24
3	-2	-2	0.94	0.21
3	-2	-2	0.64	0.16
3	-2	-2	0.55	0.10
-3	2	2	0.48	0.11
-3	2	2	0.56	0.15
-3	2	2	0.75	0.23
-3	2	2	0.62	0.23
2	-2	-2	243.58	9.11
2	-2	-2	232.54	9.05
2	-2	-2	252.60	9.17
2	-2	-2	249.64	9.19
2	-2	-2	237.21	9.07
-2	2	2	248.93	9.19
-2	2	2	238.91	9.08
1	-2	-2	82.74	3.25
1	-2	-2	86.98	3.25
1	-2	-2	92.77	3.38

1	-2	-2	87.51	3.33
-1	2	2	84.56	3.26
-1	2	2	86.28	3.25
-1	2	2	83.67	3.36
-1	2	2	86.31	3.33
0	-2	-2	28.37	1.23
0	-2	-2	28.75	1.14
0	-2	-2	30.21	1.10
0	-2	-2	28.98	1.23
0	2	2	29.31	1.22
0	2	2	28.12	1.23
0	2	2	27.71	1.13
-1	-2	-2	5.72	0.37
-1	-2	-2	6.23	0.42
-1	-2	-2	5.35	0.25
-1	-2	-2	5.61	0.29
1	2	2	4.97	0.39
1	2	2	5.64	0.30
1	2	2	5.16	0.35
-2	-2	-2	1.73	0.15
-2	-2	-2	1.76	0.17
-2	-2	-2	1.89	0.27
-2	-2	-2	2.08	0.16
-2	-2	-2	2.28	0.35
-2	-2	-2	2.05	0.19
2	2	2	2.08	0.33
2	2	2	2.10	0.26
-3	-2	-2	74.32	2.85
-3	-2	-2	65.76	2.79
-3	-2	-2	77.37	2.95
-3	-2	-2	72.65	2.80
3	2	2	74.76	2.80
3	2	2	72.08	2.84
3	2	2	75.39	2.80
3	2	2	73.00	2.94
-4	-2	-2	21.46	0.92
-4	-2	-2	22.27	0.98
-4	-2	-2	20.68	0.96
-4	-2	-2	23.80	1.11
4	2	2	22.18	0.95
4	2	2	22.34	0.97
4	2	2	21.44	1.09
4	2	2	21.48	0.93
-5	-2	-2	13.25	0.74

-5	-2	-2	13.32	0.69
-5	-2	-2	14.62	0.84
-5	-2	-2	13.37	0.64
5	2	2	13.59	0.69
5	2	2	13.21	0.84
5	2	2	14.32	0.66
5	2	2	13.33	0.70
-6	-2	-2	2.32	0.44
-6	-2	-2	2.07	0.32
-6	-2	-2	2.55	0.27
-6	-2	-2	2.89	0.44
-6	-2	-2	2.39	0.36
6	2	2	2.44	0.43
6	2	2	2.10	0.29
6	2	2	2.60	0.38
-7	-2	-2	8.28	0.50
-7	-2	-2	8.28	0.56
-7	-2	-2	7.20	0.63
-7	-2	-2	7.78	0.75
-7	-2	-2	8.06	0.68
7	2	2	8.30	0.60
7	2	2	7.26	0.50
7	2	2	8.62	0.70
-8	-2	-2	0.58	0.28
-8	-2	-2	0.37	0.39
-8	-2	-2	0.32	0.19
-8	-2	-2	0.59	0.29
8	2	2	0.44	0.27
8	2	2	0.34	0.31
8	2	2	0.70	0.22
-9	-2	-2	3.51	0.58
-9	-2	-2	2.43	0.54
-9	-2	-2	2.52	0.46
-9	-2	-2	2.67	0.38
-9	-2	-2	2.77	0.51
9	2	2	1.98	0.42
9	2	2	2.53	0.40
9	2	2	2.26	0.50
-10	-2	-2	18.03	1.05
-10	-2	-2	20.50	0.99
-10	-2	-2	18.78	1.30
-10	-2	-2	19.07	1.30
-10	-2	-2	18.74	1.20
10	2	2	19.86	1.02

10	2	2	20.21	1.23
10	2	2	20.37	1.16
-11	-2	-2	-0.13	0.26
-11	-2	-2	-0.08	0.46
-11	-2	-2	1.42	0.59
-11	-2	-2	0.43	0.37
11	2	2	0.10	0.40
11	2	2	0.02	0.26
11	2	2	0.05	0.44
-12	-2	-2	0.06	0.42
-12	-2	-2	0.11	0.61
-12	-2	-2	-0.40	0.78
-12	-2	-2	-0.21	0.51
-12	-2	-2	0.30	0.31
12	2	2	-0.46	0.27
12	2	2	0.61	0.52
12	2	2	-0.03	0.46
-13	-2	-2	9.11	1.34
-13	-2	-2	8.29	1.22
-13	-2	-2	6.86	0.87
-13	-2	-2	6.77	1.14
13	2	2	6.69	0.76
13	2	2	8.15	1.01
13	2	2	7.06	1.03
-14	-2	-2	4.35	0.87
-14	-2	-2	5.06	1.00
-14	-2	-2	6.10	0.89
-14	-2	-2	4.90	1.00
14	2	2	4.75	0.72
14	2	2	5.84	0.85
-15	-2	-2	10.54	1.50
-15	-2	-2	10.24	1.40
-15	-2	-2	6.62	1.02
-15	-2	-2	12.81	1.56
15	2	2	8.28	0.90
15	2	2	10.09	1.21
-16	-2	-2	-0.73	0.94
-16	-2	-2	0.59	0.90
-16	-2	-2	0.83	0.85
-16	-2	-2	0.31	0.63
16	2	2	-0.16	0.46
16	2	2	0.15	0.75
-17	-2	-2	-0.49	0.93
17	2	2	0.58	0.83

17	2	2	0.05	0.56
-18	-2	-2	0.45	1.07
18	2	2	0.85	0.94
18	2	2	-0.20	0.59
-19	-2	-2	0.00	1.17
19	2	2	-1.99	1.32
19	2	2	0.60	0.67
-20	-2	-2	2.88	1.39
20	2	2	-0.62	0.72
21	-2	-1	46.11	2.39
-21	2	1	37.85	3.39
20	-2	-1	1.05	0.69
-20	2	1	1.22	1.25
19	-2	-1	78.20	3.46
-19	2	1	79.04	4.19
18	-2	-1	4.20	0.68
18	-2	-1	3.43	0.86
-18	2	1	2.78	1.15
17	-2	-1	129.15	5.29
17	-2	-1	148.94	6.61
17	-2	-1	128.84	5.25
-17	2	1	123.51	5.41
-17	2	1	129.35	5.82
16	-2	-1	2.67	0.54
16	-2	-1	3.02	0.64
16	-2	-1	2.75	1.05
-16	2	1	1.66	0.88
-16	2	1	2.08	0.89
15	-2	-1	45.52	2.11
15	-2	-1	45.44	2.55
15	-2	-1	46.28	2.15
-15	2	1	42.35	2.51
-15	2	1	48.12	2.61
-15	2	1	44.80	2.38
14	-2	-1	9.71	1.46
14	-2	-1	7.51	0.76
14	-2	-1	7.75	0.85
-14	2	1	7.17	1.23
-14	2	1	9.28	1.23
13	-2	-1	72.78	2.94
13	-2	-1	70.91	2.91
13	-2	-1	64.65	3.88
-13	2	1	67.00	3.05
-13	2	1	70.10	3.14

-13	2	1	71.29	3.27
12	-2	-1	8.18	1.26
12	-2	-1	7.03	0.92
12	-2	-1	8.58	0.82
12	-2	-1	7.98	0.66
-12	2	1	5.68	1.00
-12	2	1	8.05	1.02
-12	2	1	8.06	0.98
-12	2	1	9.20	0.72
11	-2	-1	194.49	7.31
11	-2	-1	186.90	7.34
11	-2	-1	190.51	7.48
11	-2	-1	198.66	7.60
-11	2	1	204.33	7.34
-11	2	1	189.37	7.56
-11	2	1	179.44	7.47
-11	2	1	184.10	7.41
10	-2	-1	6.98	0.78
10	-2	-1	8.17	1.05
10	-2	-1	7.90	0.58
-10	2	1	8.04	0.88
-10	2	1	8.04	0.99
-10	2	1	8.80	0.80
-10	2	1	8.72	0.61
9	-2	-1	56.77	2.36
9	-2	-1	58.97	2.42
9	-2	-1	59.59	2.53
9	-2	-1	64.29	2.69
-9	2	1	55.21	2.47
-9	2	1	54.64	2.57
-9	2	1	61.06	2.38
-9	2	1	56.05	2.56
8	-2	-1	3.06	0.52
8	-2	-1	1.61	0.24
8	-2	-1	1.74	0.37
8	-2	-1	2.38	0.35
-8	2	1	2.47	0.40
-8	2	1	2.68	0.35
-8	2	1	2.47	0.58
-8	2	1	1.59	0.38
7	-2	-1	165.94	6.31
7	-2	-1	165.93	6.23
7	-2	-1	160.69	6.10
7	-2	-1	160.78	6.14

-7	2	1	167.18	6.12
-7	2	1	152.47	6.18
-7	2	1	161.07	6.25
-7	2	1	153.96	6.24
6	-2	-1	2.91	0.36
6	-2	-1	2.53	0.27
6	-2	-1	2.64	0.41
6	-2	-1	2.27	0.42
-6	2	1	2.53	0.47
-6	2	1	2.74	0.41
-6	2	1	2.93	0.42
-6	2	1	3.29	0.30
5	-2	-1	360.78	14.57
5	-2	-1	394.99	14.63
5	-2	-1	406.32	14.72
5	-2	-1	400.71	14.67
-5	2	1	392.57	14.59
-5	2	1	391.50	14.70
-5	2	1	388.93	14.66
-5	2	1	400.26	14.69
4	-2	-1	1.51	0.26
4	-2	-1	1.32	0.21
4	-2	-1	1.13	0.22
-4	2	1	1.24	0.18
-4	2	1	1.04	0.24
-4	2	1	1.38	0.26
3	-2	-1	182.07	6.69
3	-2	-1	168.88	6.62
3	-2	-1	180.58	6.72
3	-2	-1	178.66	6.67
-3	2	1	179.63	6.72
-3	2	1	182.57	6.68
-3	2	1	168.16	6.62
-3	2	1	178.18	6.72
2	-2	-1	3.39	0.20
2	-2	-1	3.28	0.24
2	-2	-1	3.18	0.32
2	-2	-1	3.49	0.30
-2	2	1	3.40	0.25
-2	2	1	3.93	0.21
-2	2	1	3.17	0.29
-2	2	1	3.55	0.33
1	-2	-1	149.43	5.53
1	-2	-1	146.08	5.59

-1	2	1	139.03	5.51
-1	2	1	150.34	5.64
-1	2	1	155.00	5.59
0	-2	-1	0.11	0.04
0	-2	-1	0.40	0.12
0	-2	-1	0.19	0.06
0	-2	-1	0.20	0.10
0	-2	-1	-0.07	0.11
0	2	1	0.03	0.07
0	2	1	0.28	0.06
-1	-2	-1	205.29	7.99
-1	-2	-1	215.70	8.05
-1	-2	-1	221.71	7.99
1	2	1	217.27	8.00
-2	-2	-1	42.68	1.70
-2	-2	-1	44.58	1.75
-2	-2	-1	43.19	1.64
-2	-2	-1	40.87	1.64
-2	-2	-1	43.14	1.65
2	2	1	42.51	1.64
2	2	1	43.82	1.75
2	2	1	41.82	1.70
2	2	1	40.83	1.64
-3	-2	-1	186.08	6.87
-3	-2	-1	185.06	6.92
-3	-2	-1	179.86	6.83
-3	-2	-1	176.39	6.80
-3	-2	-1	173.90	6.82
3	2	1	177.10	6.86
3	2	1	186.18	6.92
3	2	1	187.92	6.83
3	2	1	187.63	6.81
-4	-2	-1	102.80	3.88
-4	-2	-1	100.47	3.99
-4	-2	-1	103.69	3.95
-4	-2	-1	107.22	4.01
-4	-2	-1	102.53	3.93
4	2	1	104.96	4.01
4	2	1	106.50	3.93
4	2	1	91.86	3.88
-5	-2	-1	128.79	5.04
-5	-2	-1	136.40	5.05
-5	-2	-1	134.70	4.91
-5	-2	-1	125.60	5.00

-5	-2	-1	125.64	4.98
5	2	1	134.12	4.98
5	2	1	130.02	5.05
5	2	1	124.26	4.92
-6	-2	-1	11.99	0.76
-6	-2	-1	11.85	0.79
-6	-2	-1	11.73	0.71
-6	-2	-1	11.43	0.68
6	2	1	12.49	0.69
6	2	1	11.05	0.84
6	2	1	12.87	0.61
-7	-2	-1	256.32	9.50
-7	-2	-1	252.64	9.42
-7	-2	-1	247.08	9.52
-7	-2	-1	251.86	9.34
-7	-2	-1	239.13	9.46
7	2	1	254.49	9.44
7	2	1	227.73	9.34
7	2	1	260.49	9.50
-8	-2	-1	4.73	0.64
-8	-2	-1	4.58	0.39
-8	-2	-1	3.43	0.53
-8	-2	-1	3.93	0.74
-8	-2	-1	4.40	0.59
8	2	1	4.55	0.55
8	2	1	4.00	0.64
8	2	1	4.15	0.40
-9	-2	-1	49.08	2.24
-9	-2	-1	52.52	2.27
-9	-2	-1	50.98	2.06
-9	-2	-1	47.19	2.14
9	2	1	49.92	2.19
9	2	1	51.31	2.23
9	2	1	48.39	2.07
-10	-2	-1	0.11	0.50
-10	-2	-1	0.07	0.36
-10	-2	-1	0.34	0.38
-10	-2	-1	0.72	0.26
-10	-2	-1	-0.25	0.63
10	2	1	0.38	0.23
10	2	1	0.56	0.37
10	2	1	0.93	0.37
-11	-2	-1	71.96	3.23
-11	-2	-1	73.11	3.09

-11	-2	-1	70.50	2.85
-11	-2	-1	70.42	3.10
-11	-2	-1	64.93	2.94
11	2	1	68.78	2.88
11	2	1	72.42	3.04
11	2	1	69.92	3.02
-12	-2	-1	-0.07	0.51
-12	-2	-1	0.03	0.48
-12	-2	-1	0.37	0.30
-12	-2	-1	0.30	0.70
-12	-2	-1	0.48	0.52
12	2	1	0.06	0.31
12	2	1	0.55	0.49
12	2	1	0.36	0.46
-13	-2	-1	16.20	1.35
-13	-2	-1	16.40	1.34
-13	-2	-1	15.55	1.45
-13	-2	-1	15.31	1.05
-13	-2	-1	14.60	1.15
13	2	1	16.07	1.00
13	2	1	15.75	1.24
13	2	1	14.94	1.17
-14	-2	-1	0.46	0.79
-14	-2	-1	0.31	0.66
-14	-2	-1	0.41	0.75
14	2	1	0.71	0.39
14	2	1	0.13	0.56
-15	-2	-1	1.46	0.77
-15	-2	-1	2.79	0.77
-15	-2	-1	3.16	1.00
-15	-2	-1	1.93	0.84
15	2	1	2.02	0.68
-16	-2	-1	19.85	1.76
-16	-2	-1	15.87	1.71
-16	-2	-1	14.77	1.65
-16	-2	-1	18.91	1.45
16	2	1	16.67	1.23
16	2	1	18.44	1.47
-17	-2	-1	32.61	2.12
-17	-2	-1	31.83	2.31
17	2	1	30.93	2.00
17	2	1	30.33	1.75
-18	-2	-1	1.37	1.01
18	2	1	2.67	0.94

18	2	1	2.85	0.66
-19	-2	-1	18.96	2.22
19	2	1	15.76	1.44
-20	-2	-1	-1.77	1.23
20	2	1	0.26	0.72
-21	-2	-1	3.46	1.51
21	2	1	2.35	0.84
21	-2	0	-1.29	0.76
-21	2	0	-1.71	1.44
20	-2	0	34.11	2.04
-20	2	0	37.17	3.05
19	-2	0	-0.01	0.58
-19	2	0	-1.82	1.20
18	-2	0	65.84	3.17
18	-2	0	68.82	3.10
-18	2	0	75.22	3.81
17	-2	0	0.41	0.53
17	-2	0	-0.19	0.70
-17	2	0	-0.34	0.96
-17	2	0	0.03	0.77
16	-2	0	142.48	5.47
16	-2	0	139.07	5.50
16	-2	0	139.97	5.57
-16	2	0	130.39	5.49
-16	2	0	128.68	5.69
-16	2	0	137.08	5.96
15	-2	0	2.81	0.62
15	-2	0	3.39	0.87
15	-2	0	2.88	0.48
-15	2	0	3.30	0.88
-15	2	0	3.15	0.78
-15	2	0	2.66	1.00
14	-2	0	56.39	2.66
14	-2	0	55.89	2.40
14	-2	0	55.38	2.49
-14	2	0	52.68	2.56
-14	2	0	53.63	2.67
-14	2	0	53.12	2.76
13	-2	0	1.27	0.69
13	-2	0	1.79	0.39
13	-2	0	1.01	0.70
13	-2	0	1.67	0.52
-13	2	0	2.08	0.69
-13	2	0	1.82	0.70

-13	2	0	1.53	0.67
12	-2	0	75.50	3.27
12	-2	0	77.50	3.28
12	-2	0	77.80	3.18
12	-2	0	76.84	3.08
-12	2	0	75.93	3.39
-12	2	0	69.91	3.19
-12	2	0	69.36	3.29
-12	2	0	78.11	3.09
11	-2	0	5.45	0.71
11	-2	0	5.84	0.97
11	-2	0	5.07	0.53
11	-2	0	4.80	0.80
-11	2	0	5.24	0.75
-11	2	0	6.31	0.56
-11	2	0	4.22	0.71
10	-2	0	212.86	7.98
10	-2	0	206.56	8.13
10	-2	0	218.67	8.08
10	-2	0	216.10	8.15
-10	2	0	201.14	8.11
-10	2	0	199.34	8.18
-10	2	0	236.18	8.01
-10	2	0	186.74	8.03
9	-2	0	4.20	0.66
9	-2	0	4.46	0.42
9	-2	0	3.96	0.53
9	-2	0	4.22	0.76
-9	2	0	3.82	0.60
-9	2	0	4.32	0.68
-9	2	0	4.59	0.43
-9	2	0	4.60	0.65
8	-2	0	324.70	11.73
8	-2	0	325.18	11.75
8	-2	0	288.51	11.59
-8	2	0	300.68	11.72
-8	2	0	325.67	11.61
-8	2	0	294.44	11.65
-8	2	0	304.83	11.76
7	-2	0	7.32	0.59
7	-2	0	7.85	0.70
7	-2	0	7.50	0.60
7	-2	0	7.12	0.46
-7	2	0	7.33	0.58

-7	2	0	8.81	0.48
-7	2	0	6.38	0.59
-7	2	0	6.76	0.65
6	-2	0	160.02	6.04
6	-2	0	156.38	6.21
6	-2	0	168.33	6.14
6	-2	0	161.28	6.13
-6	2	0	151.12	6.15
-6	2	0	167.45	6.06
-6	2	0	157.99	6.16
-6	2	0	156.28	6.10
5	-2	0	20.37	0.97
5	-2	0	19.88	0.88
5	-2	0	21.16	0.97
5	-2	0	20.81	0.97
-5	2	0	17.78	0.99
-5	2	0	22.88	0.90
-5	2	0	20.28	0.94
-5	2	0	21.37	1.00
4	-2	0	406.58	15.00
4	-2	0	406.04	15.01
4	-2	0	403.31	15.03
-4	2	0	395.85	15.05
-4	2	0	403.07	14.99
-4	2	0	399.65	14.95
3	-2	0	9.67	0.50
3	-2	0	10.27	0.55
3	-2	0	9.43	0.50
3	-2	0	9.77	0.44
-3	2	0	9.26	0.51
-3	2	0	9.49	0.57
-3	2	0	9.73	0.50
-3	2	0	9.25	0.44
2	-2	0	182.84	7.24
2	-2	0	192.74	7.29
2	-2	0	206.09	7.29
2	-2	0	201.43	7.34
-2	2	0	191.71	7.29
-2	2	0	187.04	7.24
-2	2	0	206.06	7.36
-2	2	0	190.27	7.29
1	-2	0	44.14	1.67
1	-2	0	43.57	1.69
-1	2	0	41.28	1.68

-1	2	0	42.45	1.63
-1	2	0	44.11	1.76
0	-2	0	90.41	3.37
-1	-2	0	44.29	1.73
-1	-2	0	44.63	1.78
-1	-2	0	44.23	1.70
1	2	0	45.81	1.71
1	2	0	46.50	1.85
-2	-2	0	205.28	7.45
-2	-2	0	206.87	7.49
-2	-2	0	177.18	7.31
-2	-2	0	202.19	7.36
-2	-2	0	203.14	7.41
-2	-2	0	197.31	7.41
2	2	0	203.33	7.42
2	2	0	200.94	7.42
2	2	0	181.27	7.34
2	2	0	193.18	7.32
-3	-2	0	9.73	0.44
-3	-2	0	10.71	0.57
-3	-2	0	10.09	0.54
-3	-2	0	9.38	0.60
-3	-2	0	9.46	0.48
-3	-2	0	9.76	0.49
3	2	0	9.65	0.44
3	2	0	10.19	0.55
-4	-2	0	404.14	15.34
-4	-2	0	406.37	15.41
-4	-2	0	424.43	15.43
-4	-2	0	408.31	15.51
4	2	0	416.87	15.41
4	2	0	394.56	15.34
4	2	0	427.48	15.50
4	2	0	424.74	15.44
-5	-2	0	20.51	0.88
-5	-2	0	20.19	0.98
-5	-2	0	20.38	0.99
-5	-2	0	22.08	1.12
5	2	0	20.10	0.89
5	2	0	21.96	0.98
5	2	0	22.32	1.10
5	2	0	20.42	0.99
-6	-2	0	169.98	6.35
-6	-2	0	160.43	6.35

-6	-2	0	169.07	6.25
-6	-2	0	161.12	6.47
6	2	0	166.84	6.26
6	2	0	167.18	6.44
6	2	0	166.57	6.34
6	2	0	164.89	6.35
-7	-2	0	6.98	0.81
-7	-2	0	8.68	0.49
-7	-2	0	7.50	0.63
-7	-2	0	8.53	0.66
7	2	0	8.11	0.74
7	2	0	9.12	0.50
7	2	0	7.36	0.60
7	2	0	7.90	0.62
-8	-2	0	300.67	11.96
-8	-2	0	320.42	11.80
-8	-2	0	325.85	11.93
-8	-2	0	307.13	12.13
8	2	0	304.10	11.81
8	2	0	323.54	11.93
8	2	0	323.19	12.02
-9	-2	0	5.26	0.44
-9	-2	0	3.31	0.61
-9	-2	0	4.25	0.81
-9	-2	0	4.54	0.64
9	2	0	4.45	0.43
9	2	0	4.51	0.80
9	2	0	5.02	0.66
9	2	0	4.50	0.60
-10	-2	0	214.51	8.34
-10	-2	0	210.34	8.41
-10	-2	0	219.00	8.21
-10	-2	0	212.65	8.55
10	2	0	214.93	8.23
10	2	0	220.74	8.45
10	2	0	215.21	8.36
10	2	0	223.12	8.32
-11	-2	0	4.14	0.63
-11	-2	0	5.08	0.84
-11	-2	0	4.87	0.79
-11	-2	0	5.18	0.54
-11	-2	0	5.48	0.79
11	2	0	5.65	0.76
11	2	0	4.30	0.76

11	2	0	4.36	0.53
11	2	0	6.51	0.72
-12	-2	0	77.57	3.54
-12	-2	0	71.46	3.44
-12	-2	0	72.87	3.29
-12	-2	0	78.36	3.11
-12	-2	0	79.05	3.40
12	2	0	74.91	3.13
12	2	0	76.91	3.30
12	2	0	80.51	3.25
-13	-2	0	1.60	0.86
-13	-2	0	1.59	0.63
-13	-2	0	2.10	0.63
-13	-2	0	2.28	0.64
-13	-2	0	2.49	0.43
13	2	0	1.85	0.57
13	2	0	1.93	0.59
13	2	0	1.09	0.38
-14	-2	0	56.41	2.82
-14	-2	0	56.07	2.64
-14	-2	0	52.72	2.63
-14	-2	0	60.24	2.94
14	2	0	55.46	2.55
14	2	0	56.80	2.46
-15	-2	0	3.93	1.08
-15	-2	0	2.36	0.76
-15	-2	0	2.85	0.77
-15	-2	0	2.58	0.83
15	2	0	1.59	0.59
15	2	0	3.26	0.56
-16	-2	0	143.96	5.74
-16	-2	0	136.65	5.90
-16	-2	0	141.27	5.97
-16	-2	0	130.39	5.70
16	2	0	146.94	5.61
16	2	0	138.57	5.64
-17	-2	0	0.23	0.92
-17	-2	0	0.35	1.02
17	2	0	0.89	0.53
17	2	0	0.35	0.73
-18	-2	0	73.51	4.26
-18	-2	0	69.55	3.70
18	2	0	72.25	3.20
18	2	0	70.60	3.30

-19	-2	0	0.52	1.06
19	2	0	-0.55	0.60
-20	-2	0	36.56	2.86
20	2	0	35.76	2.11
-21	-2	0	-0.87	1.39
21	2	0	-0.61	0.77
-21	2	-1	6.89	1.72
20	-2	1	0.53	0.66
-20	2	-1	-0.52	1.45
19	-2	1	14.41	1.33
-19	2	-1	15.79	2.37
18	-2	1	2.70	0.70
18	-2	1	2.35	0.90
-18	2	-1	2.97	1.21
17	-2	1	32.47	1.96
17	-2	1	30.18	1.71
-17	2	-1	28.66	2.38
16	-2	1	15.86	1.39
16	-2	1	15.56	1.17
-16	2	-1	18.62	1.83
-16	2	-1	13.94	1.82
15	-2	1	2.02	0.50
15	-2	1	1.81	0.66
-15	2	-1	3.19	1.40
-15	2	-1	2.75	0.95
14	-2	1	0.24	0.38
14	-2	1	-0.34	0.59
14	-2	1	0.94	0.58
-14	2	-1	-0.82	0.78
-14	2	-1	0.12	0.66
-14	2	-1	0.86	0.49
13	-2	1	14.53	0.96
13	-2	1	16.22	1.15
13	-2	1	15.90	1.26
13	-2	1	15.91	1.18
-13	2	-1	13.45	0.98
-13	2	-1	13.76	1.38
-13	2	-1	17.46	1.43
12	-2	1	0.63	0.32
12	-2	1	0.77	0.47
12	-2	1	-0.13	0.45
12	-2	1	1.12	0.52
-12	2	-1	-0.04	0.50
-12	2	-1	0.55	0.59

-12	2	-1	1.27	0.35
-12	2	-1	0.73	0.40
11	-2	1	69.49	2.97
11	-2	1	69.58	2.98
11	-2	1	70.57	2.87
11	-2	1	66.70	2.81
-11	2	-1	62.68	2.77
-11	2	-1	72.92	2.83
-11	2	-1	70.51	3.06
-11	2	-1	65.17	3.05
10	-2	1	1.16	0.42
10	-2	1	0.27	0.23
10	-2	1	0.37	0.39
10	-2	1	0.84	0.41
-10	2	-1	-0.48	0.37
-10	2	-1	0.29	0.47
-10	2	-1	-0.08	0.23
-10	2	-1	0.49	0.31
9	-2	1	50.76	2.22
9	-2	1	51.11	2.23
9	-2	1	48.58	2.08
9	-2	1	51.78	2.17
-9	2	-1	54.22	2.09
-9	2	-1	47.68	2.06
-9	2	-1	48.56	2.29
-9	2	-1	47.49	2.25
8	-2	1	3.35	0.36
8	-2	1	3.99	0.64
8	-2	1	3.72	0.55
8	-2	1	4.84	0.58
-8	2	-1	3.21	0.41
-8	2	-1	4.81	0.69
-8	2	-1	3.54	0.59
-8	2	-1	4.26	0.39
7	-2	1	262.57	9.56
7	-2	1	254.27	9.50
7	-2	1	254.92	9.44
7	-2	1	249.47	9.41
-7	2	-1	241.20	9.37
-7	2	-1	258.54	9.41
-7	2	-1	241.44	9.53
-7	2	-1	238.98	9.56
6	-2	1	11.28	0.78
6	-2	1	10.94	0.84

6	-2	1	12.44	0.67
6	-2	1	12.18	0.61
-6	2	-1	11.68	0.60
-6	2	-1	13.05	0.62
-6	2	-1	12.80	0.82
-6	2	-1	12.73	0.75
5	-2	1	127.27	4.81
5	-2	1	129.19	4.94
5	-2	1	123.42	4.81
5	-2	1	128.56	4.89
-5	2	-1	124.65	4.96
-5	2	-1	125.95	4.79
-5	2	-1	122.81	4.90
-5	2	-1	134.96	4.82
4	-2	1	108.14	4.04
4	-2	1	103.07	3.91
4	-2	1	101.18	3.97
-4	2	-1	102.77	4.05
-4	2	-1	101.32	3.90
-4	2	-1	103.29	3.91
3	-2	1	180.12	6.74
3	-2	1	184.16	6.86
3	-2	1	183.93	6.80
3	-2	1	171.91	6.74
-3	2	-1	177.64	6.80
-3	2	-1	184.27	6.74
-3	2	-1	185.04	6.88
-3	2	-1	176.56	6.74
2	-2	1	42.10	1.65
2	-2	1	41.65	1.63
2	-2	1	45.43	1.76
2	-2	1	42.98	1.68
-2	2	-1	42.64	1.76
-2	2	-1	41.15	1.69
-2	2	-1	42.75	1.63
-2	2	-1	41.81	1.66
1	-2	1	219.31	7.98
1	-2	1	202.88	7.92
1	-2	1	217.49	7.98
-1	2	-1	204.11	7.92
-1	2	-1	206.68	7.98
-1	2	-1	218.22	7.99
-1	2	-1	227.62	8.06
0	-2	1	0.30	0.11

0	-2	1	0.20	0.08
0	-2	1	0.18	0.10
0	-2	1	0.19	0.03
0	-2	1	-0.04	0.10
0	2	-1	0.20	0.03
0	2	-1	0.00	0.05
0	2	-1	0.10	0.12
0	2	-1	0.12	0.08
-1	-2	1	136.01	5.35
-1	-2	1	153.44	5.50
-1	-2	1	138.26	5.36
-1	-2	1	142.69	5.42
1	2	-1	147.70	5.52
1	2	-1	150.01	5.50
-2	-2	1	2.67	0.15
-2	-2	1	3.57	0.33
-2	-2	1	3.57	0.39
2	2	-1	2.80	0.31
2	2	-1	2.72	0.15
2	2	-1	2.92	0.22
2	2	-1	3.04	0.37
-3	-2	1	176.79	6.57
-3	-2	1	182.78	6.65
-3	-2	1	173.77	6.57
-3	-2	1	170.39	6.56
-3	-2	1	179.27	6.73
-3	-2	1	165.62	6.50
3	2	-1	183.67	6.72
3	2	-1	171.30	6.56
3	2	-1	168.76	6.50
-4	-2	1	1.37	0.29
-4	-2	1	1.26	0.23
-4	-2	1	1.48	0.16
-4	-2	1	1.59	0.24
-4	-2	1	1.22	0.20
-4	-2	1	1.11	0.31
4	2	-1	1.46	0.34
4	2	-1	1.49	0.16
-5	-2	1	383.78	15.07
-5	-2	1	412.09	15.26
-5	-2	1	389.80	14.97
-5	-2	1	397.55	15.03
5	2	-1	413.25	15.08
5	2	-1	418.69	15.23

5	2	-1	407.37	15.05
-6	-2	1	2.71	0.36
-6	-2	1	2.64	0.39
-6	-2	1	3.53	0.28
6	2	-1	3.23	0.60
6	2	-1	3.24	0.28
6	2	-1	3.00	0.37
-7	-2	1	161.81	6.37
-7	-2	1	164.22	6.58
-7	-2	1	162.77	6.28
-7	-2	1	168.38	6.23
7	2	-1	166.41	6.51
7	2	-1	168.76	6.36
7	2	-1	156.36	6.23
7	2	-1	169.67	6.30
-8	-2	1	1.67	0.63
-8	-2	1	1.41	0.36
-8	-2	1	2.35	0.35
-8	-2	1	2.45	0.33
8	2	-1	2.15	0.36
8	2	-1	2.70	0.55
8	2	-1	1.41	0.23
8	2	-1	1.88	0.35
-9	-2	1	57.24	2.81
-9	-2	1	63.97	2.42
-9	-2	1	58.77	2.48
-9	-2	1	58.06	2.61
9	2	-1	57.48	2.72
9	2	-1	62.13	2.58
9	2	-1	57.67	2.41
9	2	-1	62.06	2.50
-10	-2	1	8.66	1.16
-10	-2	1	9.37	0.75
-10	-2	1	9.28	0.87
-10	-2	1	9.26	0.63
10	2	-1	8.64	0.62
10	2	-1	9.50	0.83
-11	-2	1	197.00	7.90
-11	-2	1	193.06	7.53
-11	-2	1	201.75	7.47
-11	-2	1	191.35	7.69
11	2	-1	193.04	7.63
11	2	-1	188.41	7.46
11	2	-1	201.74	7.79

11	2	-1	196.97	7.53
-12	-2	1	7.88	0.97
-12	-2	1	6.79	1.07
-12	-2	1	9.06	0.67
-12	-2	1	6.60	0.86
12	2	-1	7.17	1.22
12	2	-1	7.30	0.66
12	2	-1	7.14	0.84
12	2	-1	6.47	0.89
-13	-2	1	72.87	2.98
-13	-2	1	70.10	3.49
-13	-2	1	72.08	3.29
-13	-2	1	71.51	3.06
13	2	-1	72.19	2.99
13	2	-1	70.11	3.18
13	2	-1	75.27	3.41
13	2	-1	71.31	3.03
-14	-2	1	9.93	1.60
-14	-2	1	8.75	1.03
-14	-2	1	8.04	1.18
14	2	-1	7.38	0.88
14	2	-1	8.44	0.79
14	2	-1	10.97	1.56
-15	-2	1	41.78	2.44
-15	-2	1	39.83	2.93
-15	-2	1	43.05	2.78
15	2	-1	47.97	2.15
15	2	-1	50.47	2.12
-16	-2	1	3.33	0.90
-16	-2	1	2.62	0.92
-16	-2	1	3.92	0.86
-16	-2	1	1.40	1.16
16	2	-1	0.97	0.64
16	2	-1	1.84	0.51
-17	-2	1	132.68	5.74
-17	-2	1	134.84	5.73
17	2	-1	128.45	5.28
17	2	-1	127.74	5.27
-18	-2	1	4.03	1.30
-18	-2	1	2.60	1.10
18	2	-1	2.79	0.66
-19	-2	1	81.13	4.13
19	2	-1	78.00	3.51
-20	-2	1	2.26	1.25

20	2	-1	2.52	0.74
21	2	-1	45.22	2.46
20	-2	2	0.45	0.77
-20	2	-2	-0.62	1.42
19	-2	2	-0.42	0.62
19	-2	2	-0.11	1.16
-19	2	-2	-1.17	1.18
18	-2	2	-0.72	0.55
18	-2	2	0.14	0.90
-18	2	-2	0.09	1.13
17	-2	2	0.18	0.53
17	-2	2	0.86	0.84
-17	2	-2	-0.25	1.03
16	-2	2	0.17	0.52
16	-2	2	0.60	0.74
-16	2	-2	0.61	0.89
-16	2	-2	1.34	1.18
15	-2	2	8.55	1.22
15	-2	2	8.58	0.91
-15	2	-2	9.15	1.66
14	-2	2	5.95	0.74
14	-2	2	5.45	0.83
-14	2	-2	6.53	1.08
-14	2	-2	5.52	0.93
13	-2	2	7.81	0.78
13	-2	2	7.50	1.04
13	-2	2	8.24	1.01
-13	2	-2	7.68	1.06
-13	2	-2	9.56	1.20
12	-2	2	0.15	0.32
12	-2	2	-0.27	0.50
12	-2	2	1.04	0.52
-12	2	-2	0.48	0.31
-12	2	-2	-0.57	0.50
-12	2	-2	1.04	0.68
11	-2	2	-0.23	0.40
11	-2	2	0.08	0.25
11	-2	2	-0.12	0.46
-11	2	-2	1.36	0.51
-11	2	-2	0.72	0.58
-11	2	-2	-0.26	0.27
10	-2	2	20.09	1.18
10	-2	2	18.15	1.00
10	-2	2	19.62	1.24

-10	2	-2	19.37	1.33
-10	2	-2	18.69	1.24
-10	2	-2	20.75	1.02
9	-2	2	2.66	0.43
9	-2	2	3.07	0.54
9	-2	2	2.19	0.32
-9	2	-2	2.11	0.44
-9	2	-2	2.47	0.40
-9	2	-2	4.06	0.64
8	-2	2	0.59	0.30
8	-2	2	0.62	0.35
8	-2	2	0.42	0.20
-8	2	-2	0.14	0.29
-8	2	-2	0.88	0.21
7	-2	2	6.83	0.49
7	-2	2	6.85	0.68
7	-2	2	8.33	0.61
-7	2	-2	8.77	0.78
-7	2	-2	7.77	0.65
-7	2	-2	8.19	0.50
6	-2	2	1.81	0.42
6	-2	2	2.83	0.39
6	-2	2	2.02	0.28
-6	2	-2	2.58	0.28
-6	2	-2	2.70	0.44
-6	2	-2	2.38	0.45
5	-2	2	12.82	0.84
5	-2	2	12.52	0.70
5	-2	2	12.91	0.63
-5	2	-2	14.29	0.64
-5	2	-2	12.81	0.85
-5	2	-2	14.01	0.75
4	-2	2	19.92	0.92
4	-2	2	23.27	1.10
4	-2	2	22.41	0.98
-4	2	-2	21.44	1.09
-4	2	-2	22.03	0.91
-4	2	-2	20.65	1.08
3	-2	2	71.01	2.77
3	-2	2	71.68	2.75
3	-2	2	75.47	2.94
3	-2	2	73.52	2.83
-3	2	-2	72.07	2.84
-3	2	-2	74.42	2.77

-3	2	-2	70.83	2.93
2	-2	2	2.44	0.35
2	-2	2	1.88	0.15
2	-2	2	2.18	0.28
2	-2	2	1.68	0.24
2	-2	2	1.78	0.21
-2	2	-2	1.93	0.15
-2	2	-2	2.12	0.33
-2	2	-2	2.04	0.24
1	-2	2	5.11	0.32
1	-2	2	5.50	0.25
1	-2	2	5.69	0.42
1	-2	2	5.67	0.41
-1	2	-2	5.67	0.38
-1	2	-2	5.52	0.25
-1	2	-2	4.84	0.32
-1	2	-2	5.48	0.44
0	-2	2	27.45	1.23
0	-2	2	27.41	1.12
0	-2	2	27.05	1.03
0	-2	2	27.70	1.19
0	2	-2	26.64	1.21
0	2	-2	28.30	1.20
0	2	-2	28.03	1.03
0	2	-2	26.33	1.11
-1	-2	2	89.59	3.33
-1	-2	2	90.75	3.39
-1	-2	2	85.51	3.47
1	2	-2	83.54	3.46
1	2	-2	86.05	3.33
1	2	-2	88.63	3.39
-2	-2	2	240.38	9.00
-2	-2	2	226.59	8.72
-2	-2	2	230.78	8.80
-2	-2	2	247.61	8.85
2	2	-2	246.89	8.86
2	2	-2	240.76	8.98
2	2	-2	215.86	8.79
-3	-2	2	0.66	0.10
-3	-2	2	0.59	0.31
-3	-2	2	0.69	0.20
-3	-2	2	0.46	0.14
3	2	-2	0.51	0.30
3	2	-2	0.51	0.08

3	2	-2	0.36	0.12
3	2	-2	0.63	0.21
-4	-2	2	43.24	1.83
-4	-2	2	43.47	1.72
-4	-2	2	44.58	1.77
-4	-2	2	43.88	2.05
-4	-2	2	45.12	1.81
-4	-2	2	45.44	1.83
4	2	-2	44.00	1.72
4	2	-2	47.67	2.05
-5	-2	2	42.96	1.82
-5	-2	2	44.15	1.84
-5	-2	2	43.94	1.75
-5	-2	2	43.36	1.83
-5	-2	2	44.36	1.73
5	2	-2	45.00	2.07
5	2	-2	44.66	1.73
-6	-2	2	21.09	1.39
-6	-2	2	22.47	1.10
-6	-2	2	22.63	0.97
-6	-2	2	20.46	1.08
-6	-2	2	24.29	0.96
-6	-2	2	23.40	1.08
6	2	-2	21.30	0.95
6	2	-2	22.51	1.08
6	2	-2	22.31	1.32
-7	-2	2	21.51	0.98
-7	-2	2	21.54	1.11
-7	-2	2	21.48	1.45
-7	-2	2	21.84	1.11
7	2	-2	21.74	0.96
7	2	-2	22.89	1.38
-8	-2	2	26.40	1.34
-8	-2	2	23.04	1.31
-8	-2	2	26.81	1.64
-8	-2	2	27.10	1.18
-8	-2	2	27.13	1.15
8	2	-2	28.47	1.34
8	2	-2	24.73	1.59
8	2	-2	26.30	1.15
-9	-2	2	7.11	0.57
-9	-2	2	8.23	0.52
-9	-2	2	7.40	0.72
-9	-2	2	9.23	1.15

9	2	-2	7.51	0.53
9	2	-2	8.06	0.74
9	2	-2	6.50	1.04
9	2	-2	7.29	0.59
-10	-2	2	167.35	6.95
-10	-2	2	173.22	6.73
-10	-2	2	172.65	7.21
-10	-2	2	183.14	6.73
10	2	-2	174.39	6.73
10	2	-2	179.61	6.92
10	2	-2	183.72	6.75
10	2	-2	176.12	7.14
-11	-2	2	5.76	0.63
-11	-2	2	5.41	1.02
-11	-2	2	5.17	0.82
-11	-2	2	5.62	0.55
11	2	-2	6.43	0.82
11	2	-2	4.87	0.63
11	2	-2	5.41	0.53
11	2	-2	7.96	1.24
-12	-2	2	65.19	2.96
-12	-2	2	63.01	2.69
-12	-2	2	66.88	2.68
-12	-2	2	63.48	3.27
12	2	-2	62.94	2.88
12	2	-2	65.61	2.69
12	2	-2	67.72	3.21
12	2	-2	62.91	2.66
-13	-2	2	19.86	1.06
-13	-2	2	16.45	1.85
-13	-2	2	17.01	1.36
-13	-2	2	18.16	1.08
13	2	-2	16.56	1.01
13	2	-2	19.77	1.51
13	2	-2	19.90	1.77
-14	-2	2	27.37	1.48
-14	-2	2	30.07	2.24
-14	-2	2	28.00	2.76
-14	-2	2	28.60	1.80
14	2	-2	28.93	1.45
14	2	-2	29.40	2.19
-15	-2	2	10.41	1.87
-15	-2	2	10.19	1.67
-15	-2	2	9.35	1.02

15	2	-2	11.23	0.88
15	2	-2	14.53	1.89
-16	-2	2	58.67	3.11
-16	-2	2	56.45	3.33
-16	-2	2	56.34	2.62
-16	-2	2	61.02	3.11
16	2	-2	61.84	2.65
16	2	-2	63.94	3.35
-17	-2	2	3.59	1.00
-17	-2	2	3.10	1.23
-17	-2	2	2.08	1.27
17	2	-2	2.53	0.57
-18	-2	2	35.89	2.77
-18	-2	2	36.11	2.52
18	2	-2	37.00	1.96
-19	-2	2	0.53	1.05
19	2	-2	-0.47	0.58
-20	-2	2	28.83	2.59
20	2	-2	29.23	1.82
-21	-2	2	3.12	1.38
21	2	-2	6.66	0.99
20	-2	3	0.49	0.77
-20	2	-3	-2.05	1.45
19	-2	3	3.85	0.74
19	-2	3	4.54	1.19
-19	2	-3	3.42	1.46
18	-2	3	3.43	0.73
18	-2	3	3.28	1.08
-18	2	-3	0.85	1.17
17	-2	3	19.85	1.79
17	-2	3	17.42	1.35
-17	2	-3	18.09	2.11
16	-2	3	8.85	1.01
16	-2	3	10.52	1.39
-16	2	-3	12.08	1.79
-16	2	-3	12.21	2.01
15	-2	3	16.63	1.48
15	-2	3	12.35	1.07
-15	2	-3	15.64	1.90
14	-2	3	0.89	0.66
-14	2	-3	1.12	0.77
-14	2	-3	-0.16	0.89
13	-2	3	3.77	0.76
13	-2	3	2.77	0.47

13	-2	3	3.66	0.68
-13	2	-3	2.93	0.76
-13	2	-3	4.36	0.97
12	-2	3	2.03	0.41
12	-2	3	2.00	0.55
12	-2	3	1.58	0.61
-12	2	-3	2.80	0.66
-12	2	-3	2.91	0.80
-12	2	-3	1.74	0.38
11	-2	3	26.91	1.50
11	-2	3	27.19	1.34
11	-2	3	27.12	1.62
-11	2	-3	27.15	1.31
-11	2	-3	25.08	1.57
-11	2	-3	28.43	1.76
10	-2	3	0.26	0.46
10	-2	3	0.58	0.39
10	-2	3	0.15	0.24
-10	2	-3	0.66	0.27
-10	2	-3	0.91	0.58
-10	2	-3	0.08	0.41
9	-2	3	2.09	0.40
9	-2	3	1.73	0.49
9	-2	3	2.23	0.33
-9	2	-3	2.22	0.45
-9	2	-3	1.71	0.59
-9	2	-3	2.58	0.34
8	-2	3	3.67	0.60
8	-2	3	2.36	0.40
8	-2	3	2.68	0.37
-8	2	-3	3.45	0.59
-8	2	-3	3.36	0.58
-8	2	-3	2.91	0.39
7	-2	3	4.07	0.51
7	-2	3	3.62	0.68
7	-2	3	4.53	0.40
-7	2	-3	4.66	0.53
-7	2	-3	4.85	0.73
-7	2	-3	4.28	0.39
6	-2	3	16.41	0.77
6	-2	3	14.71	0.99
6	-2	3	15.69	0.84
-6	2	-3	14.25	1.16
-6	2	-3	16.27	0.89

-6	2	-3	17.25	0.77
5	-2	3	7.13	0.42
5	-2	3	7.14	0.50
5	-2	3	6.60	0.66
-5	2	-3	7.50	0.47
-5	2	-3	7.42	0.54
-5	2	-3	5.65	0.65
4	-2	3	3.87	0.53
4	-2	3	4.38	0.31
4	-2	3	4.18	0.37
-4	2	-3	4.00	0.41
-4	2	-3	4.15	0.55
-4	2	-3	4.60	0.30
3	-2	3	19.44	0.90
3	-2	3	19.35	0.83
3	-2	3	19.58	0.89
-3	2	-3	19.45	1.04
-3	2	-3	19.62	0.90
-3	2	-3	20.63	0.83
2	-2	3	0.13	0.22
2	-2	3	0.25	0.14
2	-2	3	0.08	0.21
2	-2	3	0.07	0.07
-2	2	-3	0.26	0.14
-2	2	-3	-0.03	0.17
-2	2	-3	0.22	0.08
-2	2	-3	0.26	0.23
1	-2	3	0.46	0.23
1	-2	3	0.45	0.20
1	-2	3	0.21	0.06
1	-2	3	0.24	0.14
-1	2	-3	0.21	0.21
-1	2	-3	0.31	0.07
-1	2	-3	0.42	0.22
-1	2	-3	0.32	0.13
0	-2	3	31.28	1.24
0	-2	3	31.52	1.42
0	-2	3	29.08	1.32
0	-2	3	27.91	1.14
0	2	-3	29.48	1.14
0	2	-3	30.55	1.23
0	2	-3	30.54	1.33
0	2	-3	29.98	1.39
-1	-2	3	79.72	3.25

-1	-2	3	80.08	3.04
-1	-2	3	76.84	3.09
-1	-2	3	76.05	2.93
1	2	-3	78.27	3.03
1	2	-3	79.41	3.23
1	2	-3	81.74	3.11
-2	-2	3	1.94	0.31
-2	-2	3	1.62	0.10
-2	-2	3	1.81	0.24
-2	-2	3	1.27	0.36
2	2	-3	1.42	0.22
2	2	-3	1.51	0.38
2	2	-3	1.69	0.25
-3	-2	3	7.19	0.74
-3	-2	3	6.05	0.43
-3	-2	3	6.26	0.30
-3	-2	3	6.34	0.41
3	2	-3	6.52	0.30
3	2	-3	7.02	0.46
3	2	-3	6.79	0.41
3	2	-3	7.33	0.72
-4	-2	3	0.52	0.17
-4	-2	3	0.14	0.40
-4	-2	3	0.44	0.09
-4	-2	3	0.36	0.17
-4	-2	3	0.31	0.15
4	2	-3	0.19	0.18
4	2	-3	0.34	0.09
4	2	-3	0.54	0.37
-5	-2	3	52.39	1.96
-5	-2	3	50.90	2.45
-5	-2	3	50.67	2.02
-5	-2	3	49.08	2.08
-5	-2	3	49.90	1.98
5	2	-3	50.19	1.96
5	2	-3	49.56	2.04
5	2	-3	52.35	2.38
-6	-2	3	11.61	0.70
-6	-2	3	11.01	0.68
-6	-2	3	11.79	0.56
-6	-2	3	11.16	0.62
-6	-2	3	11.46	1.05
-6	-2	3	11.81	0.55
6	2	-3	10.80	0.55

6	2	-3	10.91	1.03
-7	-2	3	1.64	0.32
-7	-2	3	2.56	0.42
-7	-2	3	2.25	0.63
-7	-2	3	2.44	0.33
-7	-2	3	2.08	0.39
-7	-2	3	2.56	0.28
7	2	-3	1.92	0.36
7	2	-3	2.18	0.26
7	2	-3	1.92	1.06
-8	-2	3	0.37	0.16
-8	-2	3	0.25	0.18
-8	-2	3	0.84	0.27
-8	-2	3	-0.06	0.27
-8	-2	3	1.38	0.64
8	2	-3	0.11	0.27
8	2	-3	-0.16	0.14
8	2	-3	0.03	0.58
-9	-2	3	4.89	0.66
-9	-2	3	5.92	0.45
-9	-2	3	5.15	0.58
-9	-2	3	5.06	0.86
-9	-2	3	5.55	0.43
9	2	-3	4.67	0.43
9	2	-3	6.39	0.70
9	2	-3	5.62	1.06
-10	-2	3	3.05	0.40
-10	-2	3	3.51	0.43
-10	-2	3	1.78	0.81
-10	-2	3	2.94	0.44
-10	-2	3	2.87	0.49
10	2	-3	2.44	0.40
10	2	-3	2.96	0.51
10	2	-3	2.82	0.80
-11	-2	3	17.98	0.95
-11	-2	3	17.54	0.90
-11	-2	3	17.67	1.06
-11	-2	3	17.90	1.25
-11	-2	3	17.71	1.69
11	2	-3	20.18	1.69
11	2	-3	19.66	1.23
11	2	-3	17.40	0.94
-12	-2	3	14.66	1.18
-12	-2	3	15.72	0.86

-12	-2	3	13.53	0.79
-12	-2	3	12.74	0.97
-12	-2	3	14.83	1.77
12	2	-3	13.71	1.16
12	2	-3	13.60	0.82
12	2	-3	10.79	1.62
-13	-2	3	3.11	0.65
-13	-2	3	3.82	0.58
-13	-2	3	2.83	1.06
-13	-2	3	3.44	0.59
-13	-2	3	3.09	0.41
13	2	-3	4.43	1.12
13	2	-3	3.28	0.45
-14	-2	3	7.81	1.32
-14	-2	3	6.96	0.91
-14	-2	3	6.13	0.90
14	2	-3	6.22	0.70
14	2	-3	8.21	1.37
-15	-2	3	15.76	1.46
-15	-2	3	11.23	2.07
15	2	-3	15.90	1.02
15	2	-3	14.06	2.08
-16	-2	3	-0.64	1.24
-16	-2	3	0.58	0.77
16	2	-3	0.14	0.41
16	2	-3	-1.87	1.22
-17	-2	3	24.57	2.56
-17	-2	3	26.20	2.10
17	2	-3	26.58	2.67
17	2	-3	27.65	1.54
-18	-2	3	4.93	1.17
-18	-2	3	4.41	1.43
18	2	-3	4.25	0.69
-19	-2	3	7.89	1.32
-19	-2	3	6.36	1.63
19	2	-3	8.31	1.15
-20	-2	3	1.11	1.09
20	2	-3	1.80	0.70
-21	-2	3	7.07	1.57
21	2	-3	5.59	0.92
20	-2	4	24.12	1.77
-20	2	-4	17.84	2.85
19	-2	4	-0.90	1.14
19	-2	4	-1.15	0.65

-19	2	-4	0.06	1.37
18	-2	4	12.54	1.29
18	-2	4	15.18	1.97
-18	2	-4	16.60	2.37
17	-2	4	0.93	0.57
17	-2	4	-0.29	0.93
-17	2	-4	-2.03	1.07
16	-2	4	50.59	2.74
16	-2	4	49.30	2.40
-16	2	-4	49.56	3.24
-16	2	-4	49.42	2.95
15	-2	4	0.50	0.48
15	-2	4	1.38	0.80
-15	2	-4	2.29	1.12
14	-2	4	41.18	2.06
14	-2	4	45.60	2.39
-14	2	-4	44.35	2.70
-14	2	-4	42.00	2.45
13	-2	4	1.42	0.43
13	-2	4	2.07	0.62
13	-2	4	2.56	0.69
-13	2	-4	1.42	0.71
-13	2	-4	2.36	0.96
12	-2	4	25.13	1.66
12	-2	4	24.16	1.32
12	-2	4	24.97	1.53
-12	2	-4	27.37	1.71
-12	2	-4	24.98	1.88
11	-2	4	1.33	0.48
11	-2	4	0.71	0.54
11	-2	4	1.14	0.33
-11	2	-4	0.25	0.50
-11	2	-4	1.39	0.34
-11	2	-4	1.19	0.75
10	-2	4	72.40	3.29
10	-2	4	73.77	3.11
10	-2	4	72.52	3.01
-10	2	-4	74.71	3.37
-10	2	-4	75.15	2.99
-10	2	-4	72.09	3.19
9	-2	4	5.14	0.64
9	-2	4	4.83	0.83
9	-2	4	4.52	0.51
-9	2	-4	4.66	0.81

-9	2	-4	4.33	0.59
-9	2	-4	4.31	0.47
8	-2	4	36.57	1.92
8	-2	4	38.48	1.74
8	-2	4	39.53	1.66
-8	2	-4	37.89	1.99
-8	2	-4	36.52	1.79
-8	2	-4	39.47	1.64
7	-2	4	7.65	0.82
7	-2	4	8.48	0.55
7	-2	4	7.66	0.61
-7	2	-4	9.19	0.54
-7	2	-4	7.49	0.88
-7	2	-4	7.38	0.68
6	-2	4	21.06	1.02
6	-2	4	19.86	0.96
6	-2	4	21.65	1.26
-6	2	-4	20.32	1.07
-6	2	-4	21.45	0.95
-6	2	-4	20.06	1.26
5	-2	4	2.52	0.39
5	-2	4	2.36	0.48
5	-2	4	2.29	0.30
-5	2	-4	2.87	0.40
-5	2	-4	3.09	0.29
-5	2	-4	3.86	0.60
4	-2	4	50.05	2.28
4	-2	4	51.98	2.04
4	-2	4	51.25	2.07
4	-2	4	51.05	2.09
-4	2	-4	53.90	2.04
-4	2	-4	49.93	2.03
-4	2	-4	52.65	2.30
-4	2	-4	52.84	2.13
3	-2	4	47.80	1.93
3	-2	4	47.94	1.99
3	-2	4	47.59	2.11
-3	2	-4	46.70	1.95
-3	2	-4	47.62	2.12
-3	2	-4	49.40	1.86
-3	2	-4	46.75	1.94
2	-2	4	9.32	0.69
2	-2	4	10.05	0.73
2	-2	4	10.32	0.55

2	-2	4	10.05	0.46
-2	2	-4	9.94	0.54
-2	2	-4	9.58	0.64
-2	2	-4	10.31	0.46
-2	2	-4	9.55	0.70
1	-2	4	15.84	0.95
1	-2	4	15.68	0.66
1	-2	4	15.87	1.00
1	-2	4	17.07	0.75
-1	2	-4	16.63	0.66
-1	2	-4	15.04	0.73
-1	2	-4	16.77	0.93
-1	2	-4	15.09	0.88
0	-2	4	20.94	0.90
0	-2	4	20.35	1.03
0	-2	4	20.72	1.15
0	2	-4	19.58	1.12
0	2	-4	20.04	0.81
0	2	-4	20.35	0.89
0	2	-4	21.09	1.02
0	2	-4	19.28	0.79
-1	-2	4	176.94	6.75
-1	-2	4	177.21	6.46
-1	-2	4	166.63	6.35
-1	-2	4	167.80	6.54
1	2	-4	172.83	6.71
1	2	-4	168.48	6.45
1	2	-4	166.76	6.35
1	2	-4	172.55	6.54
-2	-2	4	10.89	0.44
-2	-2	4	9.96	0.61
-2	-2	4	10.91	0.56
2	2	-4	10.09	0.84
2	2	-4	10.19	0.55
2	2	-4	11.02	0.63
2	2	-4	11.04	0.44
-3	-2	4	36.05	1.88
-3	-2	4	36.08	1.37
-3	-2	4	36.86	1.49
-3	-2	4	33.77	1.49
3	2	-4	37.40	1.51
3	2	-4	34.99	1.36
3	2	-4	34.27	1.48
3	2	-4	34.94	1.80

-4	-2	4	19.83	0.92
-4	-2	4	20.12	1.32
-4	-2	4	18.74	0.78
-4	-2	4	17.74	0.90
-4	-2	4	19.05	0.88
4	2	-4	19.41	1.29
4	2	-4	20.81	0.91
4	2	-4	18.73	0.78
-5	-2	4	1.52	0.51
-5	-2	4	0.87	0.20
-5	-2	4	1.16	0.18
-5	-2	4	1.31	0.34
-5	-2	4	1.03	0.22
5	2	-4	0.76	0.12
5	2	-4	0.63	0.49
5	2	-4	1.10	0.23
-6	-2	4	1.49	0.61
-6	-2	4	3.08	0.41
-6	-2	4	2.57	0.26
-6	-2	4	2.83	0.40
-6	-2	4	2.81	0.27
-6	-2	4	2.14	0.29
6	2	-4	2.48	0.26
6	2	-4	2.09	0.60
-7	-2	4	0.08	0.17
-7	-2	4	-0.10	0.23
-7	-2	4	0.26	0.13
-7	-2	4	0.01	0.14
-7	-2	4	0.21	0.52
-7	-2	4	0.27	0.23
7	2	-4	0.03	0.13
7	2	-4	0.86	0.60
-8	-2	4	24.11	1.22
-8	-2	4	24.04	1.04
-8	-2	4	22.26	1.21
-8	-2	4	22.94	1.69
8	2	-4	22.73	1.62
8	2	-4	22.39	1.02
-9	-2	4	0.83	0.33
-9	-2	4	0.96	0.22
-9	-2	4	-0.16	0.64
-9	-2	4	0.53	0.34
9	2	-4	0.70	0.66
9	2	-4	0.91	0.36

9	2	-4	0.68	0.19
-10	-2	4	0.51	0.40
-10	-2	4	2.21	0.46
-10	-2	4	1.17	0.81
-10	-2	4	1.25	0.28
10	2	-4	1.72	0.47
10	2	-4	0.89	0.24
10	2	-4	2.04	0.83
-11	-2	4	2.87	1.04
-11	-2	4	2.87	0.58
-11	-2	4	5.13	0.78
-11	-2	4	3.86	0.49
11	2	-4	3.76	0.97
11	2	-4	2.60	0.53
11	2	-4	3.31	0.43
-12	-2	4	0.69	0.49
-12	-2	4	-0.96	0.91
-12	-2	4	0.31	0.48
-12	-2	4	0.98	0.32
12	2	-4	-0.08	0.48
12	2	-4	-0.14	0.92
12	2	-4	0.54	0.28
-13	-2	4	6.06	0.68
-13	-2	4	6.66	1.29
-13	-2	4	7.22	1.07
13	2	-4	6.42	0.64
13	2	-4	5.18	1.25
-14	-2	4	0.06	0.57
-14	-2	4	0.57	0.41
-14	-2	4	-0.58	1.08
14	2	-4	0.25	0.33
14	2	-4	2.13	1.22
-15	-2	4	4.84	0.93
-15	-2	4	7.36	1.48
15	2	-4	5.32	0.71
15	2	-4	7.04	1.41
-16	-2	4	0.77	1.29
-16	-2	4	1.62	0.84
16	2	-4	0.50	0.54
16	2	-4	-1.25	1.32
-17	-2	4	0.18	0.86
-17	-2	4	0.33	1.34
17	2	-4	0.82	0.48
17	2	-4	0.88	1.46

-18	-2	4	0.72	1.02
-18	-2	4	0.58	1.48
18	2	-4	0.10	0.52
-19	-2	4	-0.30	1.01
-19	-2	4	1.21	1.58
19	2	-4	-0.42	0.56
-20	-2	4	-0.65	1.11
20	2	-4	0.50	0.67
-21	-2	4	-0.63	1.41
21	2	-4	0.76	0.75
20	-2	5	-0.92	0.91
-20	2	-5	1.26	1.59
19	-2	5	44.29	2.41
19	-2	5	40.90	3.40
-19	2	-5	45.52	3.35
18	-2	5	5.50	1.27
18	-2	5	3.01	0.74
-18	2	-5	3.62	1.39
17	-2	5	50.50	2.96
17	-2	5	51.86	2.52
-17	2	-5	51.19	3.19
16	-2	5	-1.68	0.87
16	-2	5	-0.69	0.54
-16	2	-5	0.63	1.04
15	-2	5	84.84	3.52
15	-2	5	82.18	3.89
-15	2	-5	76.41	4.17
14	-2	5	0.05	0.74
14	-2	5	0.10	0.41
-14	2	-5	-0.29	0.77
-14	2	-5	1.18	1.08
13	-2	5	64.55	3.07
13	-2	5	65.87	2.89
13	-2	5	67.92	3.22
-13	2	-5	64.75	3.22
-13	2	-5	70.80	3.48
12	-2	5	31.47	1.73
12	-2	5	29.71	1.90
12	-2	5	29.67	1.55
-12	2	-5	31.01	2.16
-12	2	-5	31.24	1.88
11	-2	5	50.78	2.62
11	-2	5	52.13	2.29
11	-2	5	53.46	2.41

-11	2	-5	50.83	2.51
-11	2	-5	53.93	2.25
-11	2	-5	52.75	2.78
10	-2	5	0.35	0.52
10	-2	5	0.19	0.40
10	-2	5	0.21	0.28
-10	2	-5	-0.27	0.45
-10	2	-5	0.22	0.69
-10	2	-5	0.10	0.27
9	-2	5	55.86	2.77
9	-2	5	58.10	2.46
9	-2	5	56.85	2.53
-9	2	-5	61.58	2.89
-9	2	-5	62.10	2.44
-9	2	-5	59.98	2.62
8	-2	5	7.79	0.68
8	-2	5	5.92	0.87
8	-2	5	6.95	0.57
-8	2	-5	6.94	0.75
-8	2	-5	5.85	0.98
-8	2	-5	7.72	0.54
7	-2	5	134.47	5.35
7	-2	5	138.36	5.40
7	-2	5	136.64	5.66
-7	2	-5	139.34	5.70
-7	2	-5	139.09	5.45
-7	2	-5	146.30	5.34
6	-2	5	0.66	0.43
6	-2	5	1.12	0.24
6	-2	5	0.47	0.25
-6	2	-5	0.64	0.30
-6	2	-5	0.93	0.48
-6	2	-5	1.10	0.22
5	-2	5	81.97	3.15
5	-2	5	80.27	3.49
5	-2	5	79.95	3.19
5	-2	5	83.22	3.25
-5	2	-5	85.03	3.19
-5	2	-5	82.84	3.28
-5	2	-5	81.28	3.52
-5	2	-5	81.56	3.16
4	-2	5	1.24	0.29
4	-2	5	0.72	0.38
4	-2	5	0.92	0.16

4	-2	5	0.96	0.24
-4	2	-5	1.21	0.28
-4	2	-5	0.66	0.37
-4	2	-5	1.07	0.28
-4	2	-5	1.02	0.16
3	-2	5	113.71	4.53
3	-2	5	110.32	4.24
3	-2	5	114.98	4.30
3	-2	5	109.22	4.39
-3	2	-5	109.59	4.38
-3	2	-5	111.66	4.23
2	-2	5	11.24	0.82
2	-2	5	12.43	0.84
2	-2	5	12.75	0.64
2	-2	5	11.26	0.55
-2	2	-5	11.68	0.54
-2	2	-5	12.38	0.64
-2	2	-5	11.51	0.82
-2	2	-5	12.43	0.84
1	-2	5	111.13	4.32
1	-2	5	101.73	4.00
1	-2	5	103.01	4.25
1	-2	5	110.46	4.08
-1	2	-5	106.95	4.25
-1	2	-5	108.97	4.31
-1	2	-5	103.18	4.01
-1	2	-5	105.04	3.99
-1	2	-5	105.42	4.07
0	-2	5	36.58	1.44
0	-2	5	30.25	1.59
0	-2	5	32.23	1.75
0	2	-5	34.57	1.34
0	2	-5	34.16	1.71
0	2	-5	34.70	1.43
0	2	-5	36.11	1.35
0	2	-5	36.39	1.61
-1	-2	5	357.94	13.46
-1	-2	5	342.12	13.23
-1	-2	5	345.49	13.01
-1	-2	5	366.61	13.12
1	2	-5	355.43	13.43
1	2	-5	349.64	13.10
1	2	-5	352.60	13.22
1	2	-5	338.26	13.01

-2	-2	5	0.94	0.26
-2	-2	5	1.06	0.47
-2	-2	5	0.81	0.13
-2	-2	5	1.26	0.21
2	2	-5	1.06	0.27
2	2	-5	0.53	0.41
2	2	-5	0.73	0.12
-3	-2	5	19.61	0.94
-3	-2	5	19.56	0.98
-3	-2	5	19.32	1.33
-3	-2	5	20.22	0.82
3	2	-5	20.28	0.94
3	2	-5	20.41	1.34
3	2	-5	22.20	1.00
3	2	-5	19.97	0.80
-4	-2	5	0.59	0.20
-4	-2	5	0.02	0.43
-4	-2	5	0.52	0.21
-4	-2	5	0.45	0.18
-4	-2	5	0.71	0.12
4	2	-5	0.60	0.23
4	2	-5	0.45	0.18
4	2	-5	0.43	0.09
-5	-2	5	84.46	3.29
-5	-2	5	81.09	3.13
-5	-2	5	84.49	3.75
-5	-2	5	77.49	3.19
-5	-2	5	82.60	3.28
5	2	-5	79.17	3.12
5	2	-5	86.81	3.23
5	2	-5	83.11	3.71
-6	-2	5	18.17	0.85
-6	-2	5	18.32	1.45
-6	-2	5	19.62	0.83
-6	-2	5	19.22	1.00
-6	-2	5	18.57	1.00
6	2	-5	18.65	0.82
6	2	-5	20.50	1.45
6	2	-5	19.49	0.91
-7	-2	5	182.57	6.73
-7	-2	5	170.16	6.73
-7	-2	5	168.08	7.17
-7	-2	5	170.38	6.57
7	2	-5	185.59	7.20

7	2	-5	166.57	6.53
-8	-2	5	20.02	1.68
-8	-2	5	19.12	1.10
-8	-2	5	19.34	1.10
-8	-2	5	19.33	0.90
8	2	-5	19.36	0.89
8	2	-5	19.21	1.63
-9	-2	5	37.38	1.53
-9	-2	5	33.57	2.22
-9	-2	5	33.60	1.72
-9	-2	5	35.56	1.72
9	2	-5	34.66	1.49
9	2	-5	38.07	2.29
-10	-2	5	0.94	0.84
-10	-2	5	0.99	0.26
-10	-2	5	0.39	0.44
-10	-2	5	0.85	0.40
10	2	-5	0.37	0.21
10	2	-5	1.33	0.84
-11	-2	5	7.56	0.90
-11	-2	5	6.87	0.60
-11	-2	5	8.12	0.88
-11	-2	5	6.01	1.19
11	2	-5	5.14	0.53
11	2	-5	9.79	1.56
-12	-2	5	31.12	1.44
-12	-2	5	30.52	1.69
-12	-2	5	31.35	2.39
-12	-2	5	27.50	1.73
12	2	-5	28.84	2.32
12	2	-5	30.47	1.39
-13	-2	5	6.20	0.72
-13	-2	5	8.23	1.08
-13	-2	5	9.02	1.45
13	2	-5	7.66	1.37
13	2	-5	6.56	0.65
-14	-2	5	1.61	1.20
-14	-2	5	1.01	0.67
-14	-2	5	1.91	0.47
14	2	-5	1.45	0.38
14	2	-5	1.44	1.25
-15	-2	5	10.42	1.40
-15	-2	5	9.73	1.56
15	2	-5	9.78	0.83

15	2	-5	10.77	1.67
-16	-2	5	0.31	0.80
-16	-2	5	0.57	1.32
16	2	-5	-3.42	1.50
-17	-2	5	17.34	1.76
-17	-2	5	14.45	2.53
17	2	-5	13.09	1.92
17	2	-5	13.77	1.11
-18	-2	5	1.09	0.96
-18	-2	5	1.63	1.59
18	2	-5	0.89	0.56
-19	-2	5	6.67	1.26
-19	-2	5	6.75	1.79
19	2	-5	7.04	1.09
-20	-2	5	-2.05	1.13
20	2	-5	0.55	0.64
21	2	-5	5.15	0.88
19	-2	6	0.06	1.39
19	-2	6	0.10	0.80
-19	2	-6	0.70	1.45
18	-2	6	66.46	3.16
18	-2	6	67.01	3.66
-18	2	-6	62.52	3.83
17	-2	6	0.90	0.64
17	-2	6	0.07	1.48
-17	2	-6	1.81	1.22
16	-2	6	96.17	4.47
16	-2	6	94.62	4.06
-16	2	-6	92.64	4.52
15	-2	6	-0.52	0.54
15	-2	6	0.73	0.93
-15	2	-6	0.07	1.02
14	-2	6	66.17	2.96
14	-2	6	67.58	3.35
-14	2	-6	68.21	3.89
-14	2	-6	67.09	3.34
13	-2	6	6.45	0.99
13	-2	6	7.29	1.33
-13	2	-6	7.21	1.04
-13	2	-6	8.03	1.41
12	-2	6	68.11	3.30
12	-2	6	68.29	3.04
12	-2	6	69.22	2.91
-12	2	-6	64.72	3.16

-12	2	-6	69.22	3.47
11	-2	6	13.05	0.90
11	-2	6	11.07	1.30
11	-2	6	12.70	1.08
-11	2	-6	10.99	1.18
-11	2	-6	10.23	1.54
-11	2	-6	12.27	0.85
10	-2	6	124.49	5.44
10	-2	6	128.41	5.15
10	-2	6	127.59	5.09
-10	2	-6	134.65	5.05
-10	2	-6	130.80	5.26
-10	2	-6	128.02	5.54
9	-2	6	0.01	0.27
9	-2	6	-0.23	0.54
9	-2	6	0.24	0.37
-9	2	-6	0.41	0.40
-9	2	-6	0.54	0.26
-9	2	-6	-0.52	0.64
8	-2	6	184.48	7.48
8	-2	6	184.60	7.14
8	-2	6	188.70	7.18
-8	2	-6	192.28	7.11
-8	2	-6	182.65	7.24
-8	2	-6	184.93	7.55
7	-2	6	0.31	0.28
7	-2	6	0.71	0.48
7	-2	6	0.23	0.20
-7	2	-6	0.36	0.20
-7	2	-6	0.65	0.32
-7	2	-6	1.12	0.60
6	-2	6	290.87	11.02
6	-2	6	293.76	11.34
6	-2	6	278.24	10.98
-6	2	-6	297.70	11.38
-6	2	-6	287.04	11.07
-6	2	-6	305.33	10.97
5	-2	6	13.48	0.71
5	-2	6	14.08	0.81
5	-2	6	14.34	1.10
5	-2	6	14.46	0.76
-5	2	-6	13.45	0.69
-5	2	-6	14.01	0.80
-5	2	-6	14.23	0.82

-5	2	-6	14.53	1.17
4	-2	6	145.31	5.77
4	-2	6	157.33	5.83
4	-2	6	152.80	6.12
4	-2	6	152.10	5.87
-4	2	-6	154.09	5.85
-4	2	-6	152.66	6.13
-4	2	-6	147.47	5.89
-4	2	-6	155.68	5.76
3	-2	6	0.61	0.16
3	-2	6	0.74	0.22
3	-2	6	0.64	0.38
3	-2	6	0.90	0.37
-3	2	-6	0.98	0.39
-3	2	-6	0.36	0.35
-3	2	-6	1.12	0.17
-3	2	-6	0.89	0.26
2	-2	6	130.93	5.04
2	-2	6	120.24	5.05
2	-2	6	130.91	4.81
2	-2	6	119.43	4.75
-2	2	-6	119.76	4.88
-2	2	-6	127.15	5.06
-2	2	-6	126.50	5.04
-2	2	-6	127.50	4.73
1	-2	6	0.26	0.30
1	-2	6	0.39	0.48
1	-2	6	0.35	0.18
-1	2	-6	0.47	0.13
-1	2	-6	0.07	0.16
-1	2	-6	0.21	0.38
-1	2	-6	0.55	0.12
-1	2	-6	0.66	0.38
0	-2	6	301.23	11.03
0	-2	6	282.56	11.34
0	-2	6	284.95	11.21
0	2	-6	306.24	11.38
0	2	-6	295.58	11.02
0	2	-6	286.68	10.93
0	2	-6	305.29	11.22
0	2	-6	290.14	10.94
-1	-2	6	111.78	4.26
-1	-2	6	104.65	4.40
-1	-2	6	113.48	4.16

-1	-2	6	108.89	4.63
1	2	-6	111.52	4.40
1	2	-6	110.46	4.64
1	2	-6	111.66	4.15
-2	-2	6	189.64	7.17
-2	-2	6	206.59	7.30
-2	-2	6	183.18	7.68
2	2	-6	203.04	7.40
2	2	-6	186.35	7.79
2	2	-6	186.57	7.17
2	2	-6	190.13	7.27
-3	-2	6	11.60	0.65
-3	-2	6	10.60	0.73
-3	-2	6	11.75	0.52
-3	-2	6	9.87	1.09
3	2	-6	11.55	0.73
3	2	-6	11.76	0.50
3	2	-6	10.88	1.13
3	2	-6	11.30	0.63
-4	-2	6	77.23	3.14
-4	-2	6	79.03	3.62
-4	-2	6	79.24	2.99
4	2	-6	80.34	3.15
4	2	-6	79.05	3.11
4	2	-6	74.93	2.98
4	2	-6	81.59	3.61
-5	-2	6	2.21	0.72
-5	-2	6	3.00	0.26
-5	-2	6	3.03	0.41
-5	-2	6	2.50	0.42
-5	-2	6	2.47	0.41
5	2	-6	3.04	0.44
5	2	-6	2.59	0.26
5	2	-6	3.36	0.78
-6	-2	6	185.42	7.22
-6	-2	6	185.40	7.06
-6	-2	6	177.90	7.09
-6	-2	6	186.74	7.22
-6	-2	6	192.90	7.76
6	2	-6	180.06	7.04
6	2	-6	193.88	7.74
6	2	-6	202.71	7.15
-7	-2	6	16.27	0.98
-7	-2	6	17.10	1.56

-7	-2	6	16.11	0.86
-7	-2	6	18.12	1.01
-7	-2	6	17.12	0.78
7	2	-6	18.58	0.86
7	2	-6	17.71	1.55
7	2	-6	16.82	0.78
-8	-2	6	176.47	7.60
-8	-2	6	183.66	7.09
-8	-2	6	190.42	6.90
-8	-2	6	179.73	7.10
8	2	-6	176.13	6.87
8	2	-6	186.64	7.61
-9	-2	6	5.34	0.73
-9	-2	6	6.77	0.50
-9	-2	6	5.61	1.07
-9	-2	6	4.67	0.69
9	2	-6	5.65	0.44
9	2	-6	4.67	1.04
-10	-2	6	117.17	4.83
-10	-2	6	115.44	4.81
-10	-2	6	126.26	4.60
-10	-2	6	119.62	5.39
10	2	-6	111.00	4.54
10	2	-6	119.50	5.36
-11	-2	6	-0.75	0.41
-11	-2	6	0.90	0.94
-11	-2	6	-0.06	0.46
-11	-2	6	-0.13	0.28
11	2	-6	0.10	0.23
11	2	-6	1.00	0.96
-12	-2	6	135.71	5.67
-12	-2	6	115.47	5.71
-12	-2	6	133.68	4.95
-12	-2	6	122.04	5.24
12	2	-6	127.88	5.77
12	2	-6	120.09	4.88
-13	-2	6	1.28	1.15
-13	-2	6	0.90	0.38
-13	-2	6	2.25	0.62
13	2	-6	1.23	0.35
13	2	-6	1.85	1.26
-14	-2	6	57.53	2.46
-14	-2	6	54.07	3.49
14	2	-6	58.61	3.49

14	2	-6	53.31	2.37
-15	-2	6	2.10	1.43
-15	-2	6	2.39	0.78
15	2	-6	2.50	1.47
15	2	-6	3.45	0.50
-16	-2	6	60.37	3.75
-16	-2	6	53.82	2.98
-17	-2	6	1.60	1.54
-17	-2	6	0.59	0.86
17	2	-6	-0.67	0.42
-18	-2	6	51.02	2.94
-18	-2	6	43.56	3.57
18	2	-6	48.27	2.29
-19	-2	6	3.85	2.06
-19	-2	6	0.94	1.11
19	2	-6	3.16	0.67
-20	-2	6	34.58	2.79
-20	-2	6	38.00	3.56
20	2	-6	34.05	1.98
19	-2	7	10.50	1.46
19	-2	7	8.04	1.76
-19	2	-7	8.88	1.81
18	-2	7	1.93	1.37
18	-2	7	-0.27	0.70
-18	2	-7	-0.21	1.28
17	-2	7	25.73	1.75
17	-2	7	25.87	2.44
-17	2	-7	24.66	2.49
16	-2	7	9.57	1.66
16	-2	7	8.10	1.17
-16	2	-7	8.37	1.39
15	-2	7	37.06	2.41
15	-2	7	32.11	1.89
-15	2	-7	31.90	2.45
14	-2	7	0.28	0.89
14	-2	7	0.89	0.50
-14	2	-7	1.44	0.87
13	-2	7	18.76	1.78
13	-2	7	24.47	2.31
13	-2	7	23.23	1.35
-13	2	-7	21.00	1.75
12	-2	7	2.60	0.65
12	-2	7	2.43	0.87
12	-2	7	2.51	0.51

-12	2	-7	3.34	0.78
-12	2	-7	1.26	1.00
11	-2	7	58.27	3.03
11	-2	7	62.53	2.73
11	-2	7	57.28	2.61
-11	2	-7	59.66	2.84
-11	2	-7	64.49	3.26
10	-2	7	0.54	0.68
10	-2	7	0.93	0.35
10	-2	7	1.05	0.46
-10	2	-7	0.32	0.82
-10	2	-7	-0.04	0.51
-10	2	-7	0.99	0.36
9	-2	7	163.62	6.34
9	-2	7	156.69	6.28
9	-2	7	156.44	6.69
-9	2	-7	170.91	6.27
-9	2	-7	163.98	6.42
-9	2	-7	160.59	6.76
8	-2	7	1.46	0.34
8	-2	7	1.40	0.64
8	-2	7	1.33	0.40
-8	2	-7	1.32	0.29
-8	2	-7	1.45	0.73
-8	2	-7	2.13	0.48
7	-2	7	177.61	6.97
7	-2	7	179.71	7.00
7	-2	7	182.90	7.36
-7	2	-7	179.47	7.06
-7	2	-7	188.10	6.95
-7	2	-7	185.53	7.45
6	-2	7	6.68	0.94
6	-2	7	6.92	0.53
6	-2	7	6.93	0.59
6	-2	7	6.08	0.52
-6	2	-7	5.89	0.61
-6	2	-7	7.55	1.05
-6	2	-7	7.18	0.51
-6	2	-7	6.37	0.67
5	-2	7	176.76	7.13
5	-2	7	172.98	6.74
5	-2	7	178.05	6.78
-5	2	-7	177.39	6.81
-5	2	-7	181.37	6.72

-5	2	-7	181.57	7.19
-5	2	-7	173.60	6.84
4	-2	7	3.88	0.51
4	-2	7	3.25	0.37
4	-2	7	4.24	0.47
4	-2	7	4.24	0.68
-4	2	-7	4.37	0.50
-4	2	-7	5.84	0.86
-4	2	-7	3.73	0.34
-4	2	-7	4.68	0.73
3	-2	7	141.94	5.20
3	-2	7	130.20	5.49
3	-2	7	130.15	5.13
-3	2	-7	136.67	5.43
-3	2	-7	132.26	5.51
-3	2	-7	138.15	5.21
2	-2	7	12.82	0.71
2	-2	7	11.44	1.05
2	-2	7	11.45	0.96
-2	2	-7	12.62	1.01
-2	2	-7	12.07	1.06
-2	2	-7	12.96	0.65
-2	2	-7	12.25	0.71
-2	2	-7	13.75	0.61
1	-2	7	256.22	10.45
1	-2	7	285.07	10.54
1	-2	7	284.35	10.21
-1	2	-7	265.78	10.21
-1	2	-7	254.94	10.28
-1	2	-7	283.54	10.48
-1	2	-7	275.63	10.57
-1	2	-7	265.40	10.12
0	-2	7	2.73	0.50
0	-2	7	3.79	0.40
0	-2	7	3.79	0.63
0	2	-7	2.70	0.27
0	2	-7	3.59	0.66
0	2	-7	3.03	0.51
0	2	-7	3.05	0.39
0	2	-7	2.71	0.25
-1	-2	7	222.68	8.37
-1	-2	7	208.95	8.86
-1	-2	7	234.70	8.48
-1	-2	7	220.21	8.66

1	2	-7	219.14	8.96
1	2	-7	230.21	8.65
1	2	-7	234.64	8.46
1	2	-7	220.39	8.36
-2	-2	7	2.35	0.39
-2	-2	7	2.69	0.24
-2	-2	7	2.59	0.47
-2	-2	7	2.35	0.62
2	2	-7	2.61	0.37
2	2	-7	2.71	0.23
2	2	-7	3.35	0.56
2	2	-7	3.97	0.76
-3	-2	7	215.93	8.81
-3	-2	7	225.75	8.35
-3	-2	7	219.67	8.19
-3	-2	7	206.53	8.41
3	2	-7	216.17	8.18
3	2	-7	214.95	8.29
3	2	-7	227.32	8.85
3	2	-7	229.10	8.41
-4	-2	7	0.77	0.15
-4	-2	7	0.82	0.32
-4	-2	7	1.82	0.66
-4	-2	7	0.62	0.24
4	2	-7	0.95	0.63
4	2	-7	0.43	0.30
4	2	-7	0.52	0.13
4	2	-7	0.84	0.24
-5	-2	7	174.24	6.68
-5	-2	7	175.78	7.39
-5	-2	7	184.91	6.85
-5	-2	7	172.69	6.82
-5	-2	7	177.12	6.83
5	2	-7	186.78	6.82
5	2	-7	184.11	7.41
5	2	-7	168.13	6.67
-6	-2	7	-0.13	0.23
-6	-2	7	-0.09	0.15
-6	-2	7	-0.27	0.24
-6	-2	7	-0.14	0.26
-6	-2	7	-0.17	0.57
6	2	-7	-0.09	0.30
6	2	-7	0.74	0.71
6	2	-7	0.16	0.13

-7	-2	7	258.83	9.93
-7	-2	7	267.03	9.94
-7	-2	7	250.76	10.48
-7	-2	7	242.28	9.73
7	2	-7	267.39	10.51
7	2	-7	279.37	9.80
7	2	-7	253.10	9.71
-8	-2	7	0.42	0.76
-8	-2	7	0.24	0.19
-8	-2	7	0.35	0.36
-8	-2	7	0.21	0.32
8	2	-7	0.04	0.15
8	2	-7	0.03	0.80
-9	-2	7	79.48	3.37
-9	-2	7	81.91	3.15
-9	-2	7	74.51	3.94
-9	-2	7	83.29	3.38
9	2	-7	81.18	3.99
9	2	-7	75.71	3.10
-10	-2	7	-0.14	0.24
-10	-2	7	-0.49	0.41
-10	-2	7	-0.11	0.91
-10	-2	7	0.41	0.42
10	2	-7	-0.15	0.20
10	2	-7	0.47	0.95
-11	-2	7	177.06	7.13
-11	-2	7	190.22	6.87
-11	-2	7	182.52	7.12
-11	-2	7	176.94	7.72
11	2	-7	173.83	6.80
11	2	-7	170.53	7.67
-12	-2	7	-0.36	0.48
-12	-2	7	1.02	1.14
-12	-2	7	0.21	0.33
12	2	-7	0.71	1.16
12	2	-7	-0.09	0.25
-13	-2	7	153.92	7.01
-13	-2	7	162.36	6.10
-13	-2	7	159.13	6.46
13	2	-7	154.97	6.03
13	2	-7	149.57	6.99
-14	-2	7	2.67	0.50
-14	-2	7	1.49	1.36
-14	-2	7	2.67	0.80

14	2	-7	3.31	1.40
14	2	-7	1.94	0.40
-15	-2	7	55.82	2.88
-15	-2	7	57.01	3.74
15	2	-7	53.08	2.37
-16	-2	7	0.37	1.41
-16	-2	7	0.34	0.80
-17	-2	7	85.67	4.70
-17	-2	7	81.55	4.01
17	2	-7	80.09	3.45
-18	-2	7	0.32	0.98
-18	-2	7	-0.76	1.86
18	2	-7	-0.39	0.47
-19	-2	7	67.23	4.35
-19	-2	7	56.99	3.52
19	2	-7	62.39	2.84
-20	-2	7	-1.40	1.20
-20	-2	7	-0.97	1.96
20	2	-7	0.16	0.63
19	-2	8	0.91	1.08
19	-2	8	2.37	1.66
-19	2	-8	4.27	1.61
18	-2	8	-1.36	0.73
18	-2	8	0.83	1.42
-18	2	-8	-0.36	1.42
17	-2	8	0.40	1.30
17	-2	8	1.34	0.80
-17	2	-8	0.98	1.30
16	-2	8	-0.34	1.16
16	-2	8	-1.06	0.66
-16	2	-8	1.93	1.13
15	-2	8	7.15	0.93
15	-2	8	7.58	1.37
-15	2	-8	7.13	1.18
14	-2	8	7.56	1.26
14	-2	8	5.68	0.77
-14	2	-8	5.08	1.07
13	-2	8	7.62	0.91
13	-2	8	7.94	1.51
-13	2	-8	6.74	1.03
12	-2	8	0.54	0.87
12	-2	8	0.69	0.45
12	-2	8	-0.60	0.61
-12	2	-8	0.32	0.67

11	-2	8	0.97	0.42
11	-2	8	0.97	0.56
11	-2	8	0.32	0.74
-11	2	-8	1.52	1.10
-11	2	-8	1.49	0.64
10	-2	8	3.53	0.53
10	-2	8	4.24	0.60
10	-2	8	3.46	0.89
-10	2	-8	3.92	1.08
-10	2	-8	3.24	0.64
-10	2	-8	3.39	0.47
9	-2	8	1.08	0.46
9	-2	8	0.81	0.35
9	-2	8	0.67	0.68
-9	2	-8	1.44	0.49
-9	2	-8	0.87	0.32
-9	2	-8	2.18	0.87
8	-2	8	26.34	1.76
8	-2	8	22.59	1.27
8	-2	8	24.13	1.23
-8	2	-8	23.19	1.82
-8	2	-8	25.58	1.20
-8	2	-8	23.18	1.37
7	-2	8	9.93	0.73
7	-2	8	10.45	0.78
7	-2	8	11.27	0.65
7	-2	8	12.61	1.26
-7	2	-8	9.95	0.71
-7	2	-8	11.04	0.70
-7	2	-8	13.52	1.34
-7	2	-8	11.07	0.88
6	-2	8	41.44	1.82
6	-2	8	40.40	1.78
6	-2	8	39.83	2.21
6	-2	8	42.56	1.79
-6	2	-8	45.07	1.77
-6	2	-8	44.49	2.34
-6	2	-8	39.58	1.87
-6	2	-8	38.57	1.85
5	-2	8	6.54	0.92
5	-2	8	5.98	0.59
5	-2	8	7.13	0.70
5	-2	8	6.80	0.54
-5	2	-8	7.80	1.09

-5	2	-8	7.86	0.67
-5	2	-8	7.65	0.50
-5	2	-8	6.75	0.85
4	-2	8	35.86	1.56
4	-2	8	37.80	1.61
4	-2	8	36.42	1.96
4	-2	8	37.26	1.80
-4	2	-8	35.90	2.05
-4	2	-8	36.44	1.63
-4	2	-8	38.44	1.55
-4	2	-8	35.40	1.88
3	-2	8	10.03	1.01
3	-2	8	11.17	0.68
3	-2	8	8.53	0.98
-3	2	-8	11.00	1.09
-3	2	-8	10.69	0.64
-3	2	-8	10.82	1.12
-3	2	-8	11.33	0.59
-3	2	-8	11.85	0.72
2	-2	8	4.00	0.47
2	-2	8	5.03	0.81
2	-2	8	4.81	0.78
-2	2	-8	4.11	0.35
-2	2	-8	4.18	0.70
-2	2	-8	5.10	0.94
-2	2	-8	4.50	0.38
-2	2	-8	3.84	0.49
1	-2	8	71.46	3.31
1	-2	8	73.34	3.37
1	-2	8	78.32	3.03
-1	2	-8	79.08	3.34
-1	2	-8	77.28	3.02
-1	2	-8	76.11	2.93
-1	2	-8	75.91	2.96
0	-2	8	14.54	1.30
0	-2	8	14.18	1.11
0	-2	8	17.03	0.87
0	2	-8	17.18	0.75
0	2	-8	17.22	1.15
0	2	-8	17.66	0.88
0	2	-8	16.89	1.40
0	2	-8	17.29	0.78
-1	-2	8	1.79	0.25
-1	-2	8	1.44	0.48

-1	-2	8	1.56	0.29
-1	-2	8	1.83	0.60
1	2	-8	1.15	0.26
1	2	-8	1.62	0.44
1	2	-8	2.34	0.72
1	2	-8	1.24	0.21
-2	-2	8	101.10	3.84
-2	-2	8	94.00	4.12
-2	-2	8	101.35	4.46
-2	-2	8	103.46	3.96
2	2	-8	102.49	4.52
2	2	-8	100.20	3.83
2	2	-8	102.17	3.92
-3	-2	8	1.74	0.44
-3	-2	8	1.59	0.20
-3	-2	8	1.30	0.30
-3	-2	8	1.84	0.69
3	2	-8	1.78	0.41
3	2	-8	1.47	0.19
3	2	-8	2.32	0.74
3	2	-8	1.88	0.29
-4	-2	8	66.22	3.44
-4	-2	8	68.40	2.99
-4	-2	8	71.69	2.75
-4	-2	8	71.55	2.89
4	2	-8	78.18	2.99
4	2	-8	68.97	2.77
4	2	-8	69.25	3.51
4	2	-8	72.63	2.87
-5	-2	8	-0.23	0.23
-5	-2	8	-0.18	0.26
-5	-2	8	0.47	0.65
-5	-2	8	0.34	0.16
5	2	-8	-0.45	0.66
5	2	-8	0.08	0.34
5	2	-8	0.22	0.14
-6	-2	8	183.26	7.70
-6	-2	8	192.89	7.09
-6	-2	8	182.88	6.89
-6	-2	8	177.05	7.00
-6	-2	8	178.27	7.06
6	2	-8	177.98	6.87
6	2	-8	185.36	7.72
6	2	-8	186.04	7.02

-7	-2	8	2.29	0.29
-7	-2	8	2.03	0.34
-7	-2	8	0.67	0.78
-7	-2	8	2.07	0.40
-7	-2	8	2.73	0.45
7	2	-8	2.13	0.29
7	2	-8	1.54	0.40
7	2	-8	2.54	1.00
-8	-2	8	141.68	5.68
-8	-2	8	136.37	5.41
-8	-2	8	142.03	6.32
-8	-2	8	149.66	5.47
-8	-2	8	141.28	5.69
8	2	-8	137.71	5.42
8	2	-8	150.63	5.49
8	2	-8	144.43	6.33
-9	-2	8	0.18	0.37
-9	-2	8	0.95	0.45
-9	-2	8	0.45	0.26
-9	-2	8	0.89	0.93
9	2	-8	2.66	0.99
9	2	-8	0.41	0.19
-10	-2	8	65.76	2.54
-10	-2	8	61.08	2.79
-10	-2	8	62.94	2.80
-10	-2	8	58.94	3.48
10	2	-8	58.97	2.47
10	2	-8	61.83	3.56
-11	-2	8	5.70	0.78
-11	-2	8	5.09	0.74
-11	-2	8	5.80	1.37
-11	-2	8	7.42	0.64
11	2	-8	6.21	0.54
11	2	-8	6.26	1.35
-12	-2	8	73.77	3.32
-12	-2	8	68.16	4.03
-12	-2	8	77.28	3.01
12	2	-8	70.17	4.13
12	2	-8	71.24	2.92
-13	-2	8	1.67	0.65
-13	-2	8	0.53	1.26
-13	-2	8	1.15	0.42
13	2	-8	-0.34	1.47
13	2	-8	1.18	0.34

-14	-2	8	62.90	3.95
-14	-2	8	62.99	2.72
-14	-2	8	62.12	3.09
14	2	-8	61.77	2.61
-15	-2	8	3.81	0.94
-15	-2	8	5.70	1.60
15	2	-8	4.50	0.67
-16	-2	8	64.55	3.30
-16	-2	8	59.85	4.07
-17	-2	8	-0.44	1.60
-17	-2	8	0.59	0.91
17	2	-8	0.12	0.47
-18	-2	8	38.84	3.74
-18	-2	8	48.46	2.88
18	2	-8	44.11	2.15
-19	-2	8	-1.60	1.04
-19	-2	8	-1.20	1.97
19	2	-8	-0.69	0.55
-20	-2	8	43.09	3.13
-20	-2	8	44.57	4.17
20	2	-8	48.29	2.36
18	-2	9	4.93	1.59
18	-2	9	1.35	0.84
-18	2	-9	-0.87	1.42
17	-2	9	12.05	1.77
17	-2	9	13.29	1.45
-17	2	-9	11.50	1.72
16	-2	9	2.08	0.74
16	-2	9	3.62	1.40
-16	2	-9	0.89	1.17
15	-2	9	11.43	1.24
15	-2	9	11.03	1.63
-15	2	-9	13.17	1.79
14	-2	9	0.28	1.05
14	-2	9	-0.87	0.53
-14	2	-9	-0.64	0.86
13	-2	9	6.69	0.98
-13	2	-9	6.44	1.01
12	-2	9	6.74	0.91
12	-2	9	9.67	0.92
12	-2	9	8.54	1.53
-12	2	-9	8.98	1.29
11	-2	9	12.81	1.59
11	-2	9	15.01	1.08

11	-2	9	14.05	1.23
-11	2	-9	15.87	1.38
10	-2	9	0.50	0.80
10	-2	9	0.97	0.42
10	-2	9	1.44	0.55
-10	2	-9	0.33	0.57
9	-2	9	0.09	0.71
9	-2	9	0.13	0.45
9	-2	9	0.22	0.31
-9	2	-9	0.77	0.50
-9	2	-9	0.05	0.32
-9	2	-9	-0.39	0.87
8	-2	9	1.90	0.45
8	-2	9	1.92	0.39
8	-2	9	2.24	0.73
-8	2	-9	3.44	0.98
-8	2	-9	2.23	0.38
-8	2	-9	2.74	0.55
7	-2	9	4.25	0.82
7	-2	9	6.38	0.60
7	-2	9	7.00	0.69
7	-2	9	5.31	0.58
-7	2	-9	8.49	1.34
-7	2	-9	4.80	0.76
-7	2	-9	4.54	0.74
-7	2	-9	6.46	0.56
6	-2	9	9.23	0.72
6	-2	9	7.36	0.62
6	-2	9	8.49	1.12
6	-2	9	6.71	0.78
-6	2	-9	7.83	0.58
-6	2	-9	6.50	1.22
-6	2	-9	6.89	0.90
-6	2	-9	7.68	0.75
5	-2	9	6.44	0.81
5	-2	9	6.09	0.62
5	-2	9	5.46	0.53
5	-2	9	5.40	0.88
-5	2	-9	5.93	0.50
-5	2	-9	6.48	0.97
-5	2	-9	6.24	0.66
-5	2	-9	6.45	1.18
4	-2	9	15.40	0.82
4	-2	9	15.55	1.09

4	-2	9	12.81	1.24
-4	2	-9	15.61	0.91
-4	2	-9	15.23	0.86
-4	2	-9	14.85	1.29
-4	2	-9	17.43	0.82
-4	2	-9	18.22	1.47
3	-2	9	20.89	1.43
3	-2	9	20.88	1.05
3	-2	9	19.93	1.31
-3	2	-9	19.88	1.54
-3	2	-9	20.30	1.00
-3	2	-9	21.10	1.05
-3	2	-9	21.71	0.96
2	-2	9	9.80	1.10
2	-2	9	12.47	0.73
2	-2	9	10.09	1.06
-2	2	-9	11.11	0.59
-2	2	-9	10.04	0.63
-2	2	-9	10.97	0.71
-2	2	-9	11.18	1.14
-2	2	-9	11.73	1.26
1	-2	9	25.34	1.62
1	-2	9	24.07	1.15
1	-2	9	20.20	1.43
-1	2	-9	25.69	1.16
-1	2	-9	25.22	1.55
-1	2	-9	22.76	1.10
-1	2	-9	24.56	1.71
-1	2	-9	24.12	1.07
0	-2	9	42.76	1.79
0	-2	9	40.28	2.22
0	-2	9	38.74	2.08
0	2	-9	42.35	2.11
0	2	-9	42.27	1.69
0	2	-9	40.41	1.75
0	2	-9	40.34	2.35
0	2	-9	42.20	1.67
-1	-2	9	1.56	0.49
-1	-2	9	1.33	0.21
-1	-2	9	2.20	0.34
-1	-2	9	1.38	0.70
1	2	-9	3.18	0.83
1	2	-9	0.96	0.21
1	2	-9	1.83	0.30

1	2	-9	1.09	0.47
-2	-2	9	12.31	1.02
-2	-2	9	14.19	0.67
-2	-2	9	12.90	1.39
-2	-2	9	13.46	0.91
2	2	-9	13.11	1.50
2	2	-9	13.73	1.04
2	2	-9	13.97	0.77
2	2	-9	13.87	0.66
-3	-2	9	1.69	0.34
-3	-2	9	1.04	0.20
-3	-2	9	1.31	0.47
-3	-2	9	0.96	0.69
3	2	-9	1.00	0.75
3	2	-9	0.83	0.28
3	2	-9	0.25	0.40
3	2	-9	1.46	0.21
-4	-2	9	2.44	0.29
-4	-2	9	2.07	0.46
-4	-2	9	1.38	0.70
-4	-2	9	2.31	0.37
4	2	-9	1.67	0.57
4	2	-9	1.33	0.31
4	2	-9	3.66	0.92
4	2	-9	1.91	0.28
-5	-2	9	0.53	0.18
-5	-2	9	0.48	0.32
-5	-2	9	-0.17	0.67
-5	-2	9	0.36	0.40
-5	-2	9	-0.02	0.26
5	2	-9	0.73	0.79
5	2	-9	0.29	0.15
5	2	-9	0.67	0.42
-6	-2	9	0.20	0.33
-6	-2	9	0.91	0.43
-6	-2	9	0.39	0.27
-6	-2	9	0.71	0.85
6	2	-9	0.20	0.39
6	2	-9	0.58	0.16
6	2	-9	0.54	0.85
-7	-2	9	9.13	1.50
-7	-2	9	5.87	0.70
-7	-2	9	8.26	0.53
-7	-2	9	7.93	0.68

-7	-2	9	7.89	0.79
7	2	-9	7.51	1.23
7	2	-9	8.23	0.74
7	2	-9	7.61	0.48
-8	-2	9	34.60	1.74
-8	-2	9	35.20	2.50
-8	-2	9	35.74	1.51
-8	-2	9	31.58	1.50
-8	-2	9	34.94	1.74
8	2	-9	38.23	1.61
8	2	-9	33.61	1.44
8	2	-9	33.75	2.55
-9	-2	9	1.96	0.50
-9	-2	9	0.35	0.95
-9	-2	9	2.45	0.53
-9	-2	9	2.70	0.36
9	2	-9	1.79	1.15
9	2	-9	2.26	0.36
-10	-2	9	4.69	0.73
-10	-2	9	4.20	1.24
-10	-2	9	4.67	0.57
-10	-2	9	4.96	0.84
10	2	-9	6.52	1.37
10	2	-9	4.22	0.46
-11	-2	9	14.58	0.89
-11	-2	9	11.77	1.18
-11	-2	9	10.86	2.01
11	2	-9	16.64	2.28
11	2	-9	13.16	0.77
-12	-2	9	1.61	1.35
-12	-2	9	2.03	0.44
-12	-2	9	3.12	0.64
12	2	-9	1.44	0.34
-13	-2	9	9.87	0.91
-13	-2	9	10.29	1.78
13	2	-9	9.02	0.72
-14	-2	9	14.87	1.52
-14	-2	9	14.39	1.13
-14	-2	9	16.79	2.63
14	2	-9	13.73	0.92
-15	-2	9	5.44	1.08
15	2	-9	8.39	0.75
-16	-2	9	5.16	1.78
-16	-2	9	6.47	1.14

-17	-2	9	9.08	2.10
-17	-2	9	10.15	1.73
17	2	-9	10.46	1.01
-18	-2	9	2.12	1.09
-18	-2	9	1.25	1.96
18	2	-9	0.50	0.55
-19	-2	9	13.83	2.09
-19	-2	9	14.42	2.58
19	2	-9	13.24	1.25
-20	-2	9	3.63	2.34
-20	-2	9	1.70	1.91
20	2	-9	2.91	0.76
18	-2	10	19.42	2.95
18	-2	10	22.64	1.87
-18	2	-10	19.18	2.81
17	-2	10	-0.44	0.74
17	-2	10	-0.20	1.52
-17	2	-10	-1.76	1.36
16	-2	10	21.13	2.50
16	-2	10	18.13	1.63
-16	2	-10	19.58	2.35
15	-2	10	4.18	1.35
15	-2	10	2.86	0.75
-15	2	-10	3.77	1.15
14	-2	10	36.70	2.72
14	-2	10	33.29	1.93
-14	2	-10	33.64	2.31
13	-2	10	1.25	1.01
13	-2	10	-0.06	0.52
-13	2	-10	1.57	0.86
12	-2	10	51.92	3.02
12	-2	10	53.99	2.46
12	-2	10	49.43	2.55
-12	2	-10	53.63	2.69
11	-2	10	0.40	0.59
11	-2	10	2.08	0.89
11	-2	10	0.50	0.50
-11	2	-10	0.91	0.66
10	-2	10	45.71	2.12
10	-2	10	45.28	2.70
10	-2	10	45.02	2.18
-10	2	-10	47.22	2.30
9	-2	10	-0.62	0.47
9	-2	10	-0.22	0.74

9	-2	10	0.26	0.38
-9	2	-10	0.74	0.53
-9	2	-10	-0.59	0.34
8	-2	10	12.14	0.76
8	-2	10	12.21	0.95
8	-2	10	12.27	0.89
8	-2	10	10.78	1.47
-8	2	-10	12.72	1.04
-8	2	-10	12.36	0.85
-8	2	-10	10.31	1.75
7	-2	10	3.75	0.48
7	-2	10	4.12	0.69
7	-2	10	3.78	0.85
7	-2	10	4.13	0.69
-7	2	-10	3.62	0.58
-7	2	-10	3.63	0.66
-7	2	-10	4.83	1.04
-7	2	-10	4.83	0.57
6	-2	10	67.63	3.34
6	-2	10	67.61	2.81
6	-2	10	65.63	2.79
6	-2	10	68.63	2.90
-6	2	-10	66.91	2.86
-6	2	-10	69.90	3.43
-6	2	-10	69.85	2.76
-6	2	-10	66.88	3.03
5	-2	10	6.54	0.66
5	-2	10	6.56	1.13
5	-2	10	6.11	0.81
-5	2	-10	7.90	0.71
-5	2	-10	6.10	1.00
-5	2	-10	6.94	0.55
-5	2	-10	6.89	0.96
-5	2	-10	6.98	0.63
4	-2	10	34.70	1.58
4	-2	10	33.25	2.06
4	-2	10	35.63	1.82
-4	2	-10	32.40	2.11
-4	2	-10	37.89	2.09
-4	2	-10	34.31	1.57
-4	2	-10	36.70	1.52
-4	2	-10	34.80	1.61
3	-2	10	7.24	1.02
3	-2	10	7.21	1.03

-3	2	-10	8.00	1.11
-3	2	-10	8.40	0.54
-3	2	-10	7.67	0.59
-3	2	-10	8.23	0.64
-3	2	-10	7.39	1.24
2	-2	10	5.06	0.83
2	-2	10	4.39	0.55
2	-2	10	4.10	0.78
-2	2	-10	4.13	0.90
-2	2	-10	4.43	0.51
-2	2	-10	3.51	0.43
-2	2	-10	5.87	1.10
-2	2	-10	3.64	0.40
1	-2	10	1.87	0.36
1	-2	10	2.15	0.61
1	-2	10	1.70	0.66
-1	2	-10	2.11	0.33
-1	2	-10	2.35	0.78
-1	2	-10	2.12	0.30
-1	2	-10	2.80	0.35
-1	2	-10	3.55	0.71
0	-2	10	12.46	0.92
0	-2	10	11.79	1.14
0	-2	10	13.26	1.34
0	2	-10	13.93	0.77
0	2	-10	12.59	1.57
0	2	-10	12.79	0.73
0	2	-10	15.04	1.24
0	2	-10	12.70	0.68
-1	-2	10	8.63	0.54
-1	-2	10	8.69	0.68
-1	-2	10	8.04	0.98
-1	-2	10	8.89	1.27
1	2	-10	8.33	0.99
1	2	-10	9.07	1.44
1	2	-10	7.87	0.61
1	2	-10	9.18	0.52
-2	-2	10	36.47	1.61
-2	-2	10	32.75	1.84
-2	-2	10	35.99	1.47
-2	-2	10	33.96	2.20
2	2	-10	36.40	1.57
2	2	-10	34.25	2.37
2	2	-10	36.25	1.86

2	2	-10	33.58	1.44
-3	-2	10	-0.21	0.67
-3	-2	10	0.46	0.19
-3	-2	10	0.20	0.44
-3	-2	10	0.43	0.29
3	2	-10	0.02	0.15
3	2	-10	1.47	0.95
3	2	-10	0.55	0.26
3	2	-10	0.60	0.44
-4	-2	10	41.56	1.86
-4	-2	10	42.20	2.07
-4	-2	10	40.00	2.56
-4	-2	10	42.81	1.73
-4	-2	10	45.21	1.92
4	2	-10	39.82	2.66
4	2	-10	40.67	1.70
4	2	-10	42.26	1.85
4	2	-10	44.27	2.05
-5	-2	10	2.45	0.41
-5	-2	10	0.73	0.87
-5	-2	10	2.52	0.54
-5	-2	10	2.25	0.34
-5	-2	10	2.15	0.43
5	2	-10	2.12	0.29
5	2	-10	3.40	1.06
5	2	-10	2.44	0.63
-6	-2	10	0.89	0.38
-6	-2	10	0.91	0.44
-6	-2	10	1.09	0.34
-6	-2	10	0.83	0.24
-6	-2	10	0.17	0.87
6	2	-10	-0.52	0.93
6	2	-10	1.09	0.21
6	2	-10	1.37	0.50
-7	-2	10	-0.67	0.86
-7	-2	10	0.47	0.23
-7	-2	10	-0.08	0.35
-7	-2	10	0.32	0.39
-7	-2	10	-0.04	0.43
7	2	-10	1.61	1.01
7	2	-10	0.14	0.16
7	2	-10	0.38	0.45
-8	-2	10	6.41	0.84
-8	-2	10	5.54	0.54

-8	-2	10	5.36	0.78
-8	-2	10	5.05	0.65
-8	-2	10	4.75	1.26
8	2	-10	4.97	0.42
8	2	-10	5.46	1.31
8	2	-10	6.04	0.77
-9	-2	10	13.67	1.93
-9	-2	10	14.72	1.09
-9	-2	10	12.92	0.80
-9	-2	10	12.60	1.12
-9	-2	10	12.32	0.74
9	2	-10	12.41	2.18
9	2	-10	12.66	0.90
9	2	-10	12.13	0.70
-10	-2	10	11.87	1.09
-10	-2	10	12.59	0.82
-10	-2	10	11.52	1.17
-10	-2	10	15.98	2.13
10	2	-10	10.78	0.68
10	2	-10	14.34	2.25
-11	-2	10	-0.13	0.38
-11	-2	10	3.17	1.39
-11	-2	10	1.07	0.58
-12	-2	10	0.31	0.41
-12	-2	10	0.60	0.57
-12	-2	10	0.90	1.31
12	2	-10	0.24	0.27
-13	-2	10	8.73	1.80
-13	-2	10	8.60	1.23
-13	-2	10	9.25	0.93
13	2	-10	8.34	0.71
-14	-2	10	1.46	1.72
-14	-2	10	0.74	0.74
-14	-2	10	1.33	0.81
14	2	-10	1.12	0.38
-15	-2	10	2.08	0.87
-15	-2	10	3.99	1.75
15	2	-10	2.79	0.49
-16	-2	10	9.76	1.61
-16	-2	10	10.34	2.08
16	2	-10	8.44	1.01
-17	-2	10	2.50	1.88
-17	-2	10	3.31	1.08
17	2	-10	4.18	0.65

-18	-2	10	-1.66	1.95
-18	-2	10	-0.52	1.02
18	2	-10	-0.12	0.55
-19	-2	10	-0.02	1.17
-19	-2	10	3.77	2.28
19	2	-10	3.68	0.75
17	-2	11	33.66	3.22
17	-2	11	33.94	2.24
-17	2	-11	32.94	2.98
16	-2	11	1.13	1.43
16	-2	11	1.43	0.85
-16	2	-11	4.26	1.33
15	-2	11	28.51	2.81
15	-2	11	29.08	1.90
-15	2	-11	29.46	2.39
14	-2	11	-0.74	1.16
14	-2	11	0.27	0.71
-14	2	-11	1.41	1.02
13	-2	11	40.50	2.23
13	-2	11	42.80	3.01
-13	2	-11	45.50	2.56
12	-2	11	4.94	0.73
12	-2	11	3.97	1.20
12	-2	11	8.24	1.27
-12	2	-11	3.21	0.87
11	-2	11	76.07	3.26
11	-2	11	74.74	3.88
11	-2	11	72.05	3.25
-11	2	-11	74.17	3.38
10	-2	11	-0.04	0.56
10	-2	11	0.46	0.43
10	-2	11	0.21	1.33
-10	2	-11	0.42	0.60
9	-2	11	36.95	1.78
9	-2	11	36.47	1.82
9	-2	11	35.40	2.35
-9	2	-11	33.52	1.88
8	-2	11	2.10	0.46
8	-2	11	1.61	0.50
8	-2	11	1.72	0.86
8	-2	11	2.47	0.52
-8	2	-11	1.37	0.60
-8	2	-11	1.69	0.40
-8	2	-11	1.85	0.55

7	-2	11	21.01	1.18
7	-2	11	23.24	1.30
7	-2	11	22.61	1.82
7	-2	11	22.06	1.24
-7	2	-11	22.62	1.16
-7	2	-11	21.89	1.31
-7	2	-11	18.71	1.21
-7	2	-11	21.57	1.50
6	-2	11	3.09	0.84
6	-2	11	5.22	0.80
6	-2	11	5.46	0.72
-6	2	-11	4.28	0.60
-6	2	-11	3.91	1.07
-6	2	-11	5.02	0.65
-6	2	-11	4.05	0.57
-6	2	-11	6.26	1.13
5	-2	11	105.53	4.89
5	-2	11	106.99	4.52
5	-2	11	113.97	4.34
-5	2	-11	110.75	4.37
-5	2	-11	114.06	4.30
-5	2	-11	109.43	4.37
-5	2	-11	107.53	4.98
-5	2	-11	108.05	4.75
4	-2	11	2.77	0.74
4	-2	11	2.21	0.41
4	-2	11	2.58	0.62
-4	2	-11	1.58	0.82
-4	2	-11	1.86	0.42
-4	2	-11	2.78	0.82
-4	2	-11	1.67	0.34
-4	2	-11	1.59	0.31
3	-2	11	61.26	2.55
3	-2	11	63.60	2.91
3	-2	11	58.68	3.05
-3	2	-11	62.21	2.55
-3	2	-11	59.94	3.17
-3	2	-11	61.14	3.12
-3	2	-11	59.92	2.53
-3	2	-11	63.11	2.49
2	-2	11	0.92	0.41
2	-2	11	0.81	0.65
2	-2	11	0.71	0.61
-2	2	-11	0.95	0.34

-2	2	-11	0.47	0.25
-2	2	-11	1.77	0.88
-2	2	-11	1.11	0.25
-2	2	-11	1.03	0.71
1	-2	11	70.55	3.46
1	-2	11	73.51	3.16
1	-2	11	72.90	3.50
-1	2	-11	73.58	2.94
-1	2	-11	76.65	3.76
-1	2	-11	76.22	3.02
-1	2	-11	77.31	3.52
-1	2	-11	76.70	2.98
0	-2	11	97.35	3.77
0	-2	11	95.50	4.32
0	-2	11	91.30	4.14
0	2	-11	97.40	3.66
0	2	-11	95.67	4.16
0	2	-11	89.96	3.71
0	2	-11	93.61	3.67
0	2	-11	90.71	4.47
-1	-2	11	136.41	5.91
-1	-2	11	148.89	5.61
-1	-2	11	129.12	6.12
-1	-2	11	144.13	5.50
1	2	-11	144.12	5.56
1	2	-11	151.41	5.95
1	2	-11	148.75	5.48
1	2	-11	147.45	5.49
1	2	-11	141.82	6.35
-2	-2	11	0.31	0.33
-2	-2	11	0.31	0.22
-2	-2	11	0.70	0.58
-2	-2	11	0.44	0.75
2	2	-11	1.38	1.01
2	2	-11	0.41	0.20
2	2	-11	0.60	0.30
2	2	-11	1.15	0.58
-3	-2	11	47.72	2.07
-3	-2	11	49.06	2.15
-3	-2	11	50.09	2.85
-3	-2	11	47.19	2.42
3	2	-11	47.05	2.09
3	2	-11	49.38	1.97
3	2	-11	51.00	3.04

3	2	-11	50.69	2.39
-4	-2	11	8.67	0.71
-4	-2	11	8.07	1.00
-4	-2	11	8.90	1.56
-4	-2	11	8.43	0.57
-4	-2	11	8.80	0.79
4	2	-11	8.41	0.52
4	2	-11	12.26	1.82
4	2	-11	10.36	1.04
-5	-2	11	28.92	1.65
-5	-2	11	28.49	1.44
-5	-2	11	31.10	1.51
-5	-2	11	28.76	2.30
-5	-2	11	28.80	1.29
5	2	-11	28.11	2.41
5	2	-11	30.05	1.63
5	2	-11	29.06	1.25
-6	-2	11	37.22	1.73
-6	-2	11	35.12	1.49
-6	-2	11	30.39	2.48
-6	-2	11	33.88	1.77
-6	-2	11	34.63	1.67
6	2	-11	32.64	1.44
6	2	-11	35.94	2.69
6	2	-11	35.74	1.81
-7	-2	11	30.07	1.62
-7	-2	11	29.12	1.35
-7	-2	11	30.41	2.41
-7	-2	11	28.83	1.53
-7	-2	11	30.96	1.57
7	2	-11	29.40	1.29
7	2	-11	30.87	2.62
7	2	-11	31.22	1.64
-8	-2	11	5.18	0.82
-8	-2	11	3.16	1.20
-8	-2	11	6.93	0.96
-8	-2	11	6.15	0.58
-8	-2	11	5.91	0.84
8	2	-11	6.53	0.48
8	2	-11	5.22	1.35
8	2	-11	6.19	0.87
-9	-2	11	51.07	2.38
-9	-2	11	51.91	3.34
-9	-2	11	51.40	2.44

-9	-2	11	50.88	2.11
-9	-2	11	44.93	2.09
9	2	-11	46.49	2.02
9	2	-11	52.55	2.22
-10	-2	11	3.69	0.48
-10	-2	11	2.87	0.63
-10	-2	11	3.64	0.77
-10	-2	11	1.95	1.24
10	2	-11	2.30	0.33
-11	-2	11	12.27	0.93
-11	-2	11	16.08	2.37
-11	-2	11	13.21	1.21
11	2	-11	12.22	0.77
-12	-2	11	2.94	0.55
-12	-2	11	3.56	1.69
-12	-2	11	2.36	0.81
12	2	-11	2.78	0.39
-13	-2	11	13.89	1.12
-13	-2	11	12.05	1.43
-13	-2	11	14.46	2.14
13	2	-11	11.29	0.84
-14	-2	11	-0.50	1.48
-14	-2	11	1.46	0.84
14	2	-11	-0.22	0.37
-15	-2	11	6.53	1.98
-15	-2	11	5.08	1.07
15	2	-11	7.12	0.78
-16	-2	11	2.53	1.79
-16	-2	11	0.94	0.94
16	2	-11	1.81	0.51
-17	-2	11	8.34	1.33
-17	-2	11	14.25	2.46
17	2	-11	8.10	1.01
-18	-2	11	-2.16	0.99
-18	-2	11	-0.34	2.04
18	2	-11	1.64	0.61
-19	-2	11	4.40	2.36
-19	-2	11	4.21	1.36
19	2	-11	3.75	0.79
16	-2	12	41.30	3.47
16	-2	12	41.03	2.46
-16	2	-12	46.50	3.10
15	-2	12	1.55	1.50
15	-2	12	0.79	0.78

-15	2	-12	1.29	1.18
14	-2	12	63.69	3.02
14	-2	12	56.13	3.74
-14	2	-12	62.78	3.28
13	-2	12	0.03	1.28
13	-2	12	0.45	0.66
-13	2	-12	1.10	0.91
12	-2	12	37.11	2.79
12	-2	12	40.72	2.18
12	-2	12	38.20	2.10
-12	2	-12	40.00	2.32
11	-2	12	0.30	1.05
11	-2	12	0.77	0.70
11	-2	12	1.06	0.55
-11	2	-12	-0.14	0.73
10	-2	12	46.07	2.89
10	-2	12	44.70	2.22
-10	2	-12	46.20	2.33
9	-2	12	-0.01	0.87
9	-2	12	1.14	0.58
9	-2	12	1.09	0.45
-9	2	-12	0.10	0.45
-9	2	-12	1.30	0.60
8	-2	12	120.96	4.78
8	-2	12	125.27	4.74
8	-2	12	119.41	4.74
8	-2	12	117.90	5.41
-8	2	-12	118.02	4.81
-8	2	-12	110.06	4.83
-8	2	-12	117.32	4.82
7	-2	12	0.18	0.46
7	-2	12	0.16	0.78
7	-2	12	0.21	0.50
-7	2	-12	0.18	0.37
-7	2	-12	0.47	0.44
-7	2	-12	0.13	0.45
6	-2	12	141.27	5.64
6	-2	12	140.57	5.48
6	-2	12	139.42	6.13
-6	2	-12	146.52	5.46
-6	2	-12	134.51	5.86
-6	2	-12	138.57	5.54
-6	2	-12	138.54	5.52
5	-2	12	0.70	0.42

5	-2	12	-0.20	0.80
5	-2	12	1.56	0.64
-5	2	-12	2.36	1.30
-5	2	-12	1.14	0.36
-5	2	-12	1.47	0.43
-5	2	-12	1.49	0.38
-5	2	-12	2.08	0.95
4	-2	12	73.97	3.84
4	-2	12	82.21	3.27
4	-2	12	79.77	3.57
-4	2	-12	78.36	3.92
-4	2	-12	81.94	3.22
-4	2	-12	83.08	3.93
-4	2	-12	80.24	3.29
-4	2	-12	81.53	3.28
3	-2	12	4.42	0.66
3	-2	12	4.53	0.98
3	-2	12	5.07	0.85
-3	2	-12	5.73	1.16
-3	2	-12	4.35	1.00
-3	2	-12	5.69	0.56
-3	2	-12	4.93	0.65
-3	2	-12	6.17	0.57
2	-2	12	29.64	2.08
2	-2	12	34.10	2.04
2	-2	12	33.55	1.57
-2	2	-12	32.55	2.14
-2	2	-12	33.74	1.51
-2	2	-12	33.63	2.46
-2	2	-12	34.08	1.55
-2	2	-12	33.51	1.48
1	-2	12	9.71	1.35
1	-2	12	13.93	0.90
1	-2	12	9.72	1.32
-1	2	-12	10.52	1.80
-1	2	-12	12.16	1.40
-1	2	-12	14.09	0.74
-1	2	-12	12.80	0.82
-1	2	-12	12.34	0.75
0	-2	12	133.67	5.93
0	-2	12	131.53	5.80
0	-2	12	142.75	5.39
0	2	-12	136.31	5.29
0	2	-12	134.87	5.81

0	2	-12	142.77	5.29
0	2	-12	141.34	6.23
0	2	-12	137.47	5.34
-1	-2	12	15.22	1.69
-1	-2	12	15.41	1.45
-1	-2	12	16.67	1.02
1	2	-12	16.38	0.95
1	2	-12	15.49	1.39
1	2	-12	15.95	2.01
1	2	-12	17.18	0.85
1	2	-12	17.19	0.85
-2	-2	12	219.14	7.92
-2	-2	12	202.41	8.35
-2	-2	12	196.90	8.66
-2	-2	12	222.02	8.05
2	2	-12	206.88	7.97
2	2	-12	195.46	8.83
2	2	-12	211.09	8.35
2	2	-12	212.67	7.89
-3	-2	12	12.47	0.93
-3	-2	12	12.09	0.72
-3	-2	12	13.78	1.78
-3	-2	12	11.42	1.23
3	2	-12	10.68	0.80
3	2	-12	13.09	1.96
3	2	-12	11.51	0.67
3	2	-12	12.70	1.24
-4	-2	12	82.06	3.37
-4	-2	12	82.08	3.20
-4	-2	12	80.86	3.32
-4	-2	12	74.33	3.55
4	2	-12	81.62	3.59
4	2	-12	80.63	3.16
4	2	-12	84.85	4.34
-5	-2	12	0.71	0.36
-5	-2	12	0.69	0.45
-5	-2	12	0.62	0.28
-5	-2	12	-0.14	0.54
-5	-2	12	0.26	1.03
5	2	-12	1.55	1.14
5	2	-12	0.28	0.21
5	2	-12	-0.66	0.60
-6	-2	12	33.80	1.66
-6	-2	12	29.72	1.77

-6	-2	12	33.57	2.59
-6	-2	12	33.13	1.47
-6	-2	12	35.12	1.74
6	2	-12	33.42	1.84
6	2	-12	32.53	1.40
6	2	-12	34.32	2.83
-7	-2	12	0.75	1.12
-7	-2	12	1.18	0.30
-7	-2	12	1.03	0.57
-7	-2	12	1.07	0.45
-7	-2	12	0.86	0.55
7	2	-12	1.76	0.62
7	2	-12	0.58	0.23
7	2	-12	2.67	1.59
-8	-2	12	49.19	2.33
-8	-2	12	47.50	2.19
-8	-2	12	48.93	2.06
-8	-2	12	46.94	3.27
-8	-2	12	53.28	2.40
8	2	-12	44.53	1.97
-9	-2	12	4.65	0.51
-9	-2	12	4.90	1.43
-9	-2	12	5.26	0.82
-9	-2	12	4.60	0.86
-9	-2	12	3.94	0.70
9	2	-12	4.48	0.48
9	2	-12	3.97	0.68
-10	-2	12	71.16	3.04
-10	-2	12	79.00	3.44
-10	-2	12	76.01	4.44
-10	-2	12	77.76	3.14
10	2	-12	80.79	3.19
10	2	-12	74.84	3.04
-11	-2	12	12.26	2.56
-11	-2	12	8.44	1.15
-11	-2	12	8.09	0.86
11	2	-12	8.63	0.66
-12	-2	12	58.26	2.79
-12	-2	12	54.79	2.42
-12	-2	12	54.58	3.80
12	2	-12	51.16	2.28
-13	-2	12	0.84	0.78
-13	-2	12	0.14	0.60
-13	-2	12	-1.11	1.61

13	2	-12	0.12	0.36
-14	-2	12	36.78	3.47
-14	-2	12	37.24	2.32
14	2	-12	30.86	1.61
-15	-2	12	4.34	1.06
-15	-2	12	7.15	2.03
15	2	-12	3.31	0.55
-16	-2	12	41.11	2.71
-16	-2	12	44.68	4.07
16	2	-12	40.02	1.96
-17	-2	12	-1.36	1.93
-17	-2	12	0.70	1.07
17	2	-12	1.04	0.56
-18	-2	12	56.15	4.60
-18	-2	12	57.98	3.38
18	2	-12	50.89	2.52
16	-2	13	0.41	0.88
16	-2	13	0.41	1.76
-16	2	-13	0.58	1.50
15	-2	13	16.46	2.77
15	-2	13	15.99	1.68
-15	2	-13	16.32	2.22
14	-2	13	-0.63	0.70
14	-2	13	-0.26	1.52
-14	2	-13	2.72	1.11
13	-2	13	47.27	2.45
13	-2	13	44.91	3.28
-13	2	-13	49.85	2.60
-13	2	-13	46.57	2.72
12	-2	13	1.25	0.89
12	-2	13	0.22	1.22
12	-2	13	0.06	0.61
-12	2	-13	-0.20	0.67
-12	2	-13	1.26	0.87
11	-2	13	49.13	3.24
11	-2	13	54.11	2.49
11	-2	13	50.09	2.49
-11	2	-13	50.11	2.54
-11	2	-13	48.03	2.59
10	-2	13	18.34	1.39
10	-2	13	20.27	2.21
-10	2	-13	16.29	1.37
-10	2	-13	17.62	1.48
9	-2	13	52.78	2.45

9	-2	13	56.37	3.23
9	-2	13	55.58	2.80
-9	2	-13	49.56	2.44
-9	2	-13	48.77	2.66
-9	2	-13	54.15	2.54
8	-2	13	0.71	0.55
8	-2	13	0.74	0.96
8	-2	13	0.15	0.52
-8	2	-13	-0.18	0.54
-8	2	-13	0.43	0.46
-8	2	-13	1.21	1.07
7	-2	13	87.19	3.64
7	-2	13	86.07	3.52
-7	2	-13	83.20	3.89
-7	2	-13	83.10	3.56
-7	2	-13	86.43	3.60
6	-2	13	0.32	0.62
6	-2	13	0.85	0.83
6	-2	13	0.78	0.52
-6	2	-13	1.43	0.46
-6	2	-13	2.48	0.47
-6	2	-13	1.90	0.51
-6	2	-13	1.70	1.05
5	-2	13	236.24	9.20
5	-2	13	223.82	9.55
5	-2	13	237.35	8.91
-5	2	-13	228.05	8.93
-5	2	-13	230.92	8.97
-5	2	-13	248.15	8.91
-5	2	-13	227.48	9.48
4	-2	13	10.73	0.89
4	-2	13	8.80	1.25
4	-2	13	9.64	1.54
-4	2	-13	11.08	1.83
-4	2	-13	8.91	0.74
-4	2	-13	10.82	0.85
-4	2	-13	8.52	0.79
3	-2	13	102.89	4.06
3	-2	13	102.51	4.50
3	-2	13	91.72	4.62
-3	2	-13	100.85	4.05
-3	2	-13	104.50	4.89
-3	2	-13	106.14	4.02
-3	2	-13	100.71	4.78

-3	2	-13	102.20	4.06
2	-2	13	-0.11	0.39
2	-2	13	-0.67	0.76
2	-2	13	0.31	0.74
-2	2	-13	-0.34	0.91
-2	2	-13	0.51	0.36
-2	2	-13	0.76	0.32
-2	2	-13	1.45	0.33
-2	2	-13	0.01	1.13
1	-2	13	61.24	3.38
1	-2	13	71.98	3.53
1	-2	13	73.24	2.94
-1	2	-13	72.02	3.89
-1	2	-13	70.70	2.85
-1	2	-13	72.15	2.83
-1	2	-13	69.74	2.87
-1	2	-13	67.66	3.51
0	-2	13	6.19	1.02
0	-2	13	5.39	0.76
0	-2	13	5.91	1.15
0	2	-13	4.01	1.29
0	2	-13	5.26	0.55
0	2	-13	7.49	1.33
0	2	-13	6.68	0.57
0	2	-13	6.26	0.65
-1	-2	13	117.63	5.47
-1	-2	13	129.83	4.84
-1	-2	13	111.53	5.20
1	2	-13	120.64	5.25
1	2	-13	120.12	5.77
1	2	-13	120.54	4.75
1	2	-13	127.22	4.70
1	2	-13	120.14	4.69
-2	-2	13	-0.07	0.28
-2	-2	13	-0.58	0.41
-2	-2	13	-0.48	0.66
-2	-2	13	0.37	0.90
2	2	-13	-1.46	1.14
2	2	-13	0.09	0.33
2	2	-13	-0.17	0.23
2	2	-13	0.87	0.80
-3	-2	13	209.53	7.81
-3	-2	13	192.08	8.22
-3	-2	13	196.20	8.67

-3	-2	13	215.37	7.96
3	2	-13	202.11	7.87
3	2	-13	200.30	8.91
3	2	-13	215.42	8.28
3	2	-13	210.11	7.78
-4	-2	13	0.47	0.65
-4	-2	13	-0.25	0.28
-4	-2	13	-0.31	0.47
-4	-2	13	0.52	0.99
-4	-2	13	0.38	0.34
4	2	-13	2.19	1.36
4	2	-13	-0.63	0.72
4	2	-13	0.14	0.22
-5	-2	13	42.84	1.82
-5	-2	13	38.97	2.89
-5	-2	13	40.22	1.96
-5	-2	13	41.13	2.22
-5	-2	13	43.91	2.07
5	2	-13	41.81	1.76
5	2	-13	45.09	2.30
5	2	-13	43.34	3.26
-6	-2	13	13.47	2.09
-6	-2	13	10.09	1.08
-6	-2	13	11.19	0.73
-6	-2	13	10.25	0.91
-6	-2	13	9.18	1.16
6	2	-13	9.89	1.29
6	2	-13	9.28	0.62
-7	-2	13	114.31	4.99
-7	-2	13	122.51	4.77
-7	-2	13	124.43	5.95
-7	-2	13	122.82	5.03
-7	-2	13	124.40	4.98
7	2	-13	131.88	5.10
7	2	-13	117.65	4.70
-8	-2	13	2.56	0.63
-8	-2	13	1.86	0.62
-8	-2	13	1.73	0.39
-8	-2	13	2.20	0.70
-8	-2	13	3.50	1.41
8	2	-13	2.04	0.74
8	2	-13	2.77	0.44
-9	-2	13	90.15	3.76
-9	-2	13	83.98	3.55

-9	-2	13	89.07	3.48
-9	-2	13	76.45	5.26
-9	-2	13	89.03	3.86
9	2	-13	86.83	3.38
9	2	-13	81.22	3.87
-10	-2	13	7.76	1.10
-10	-2	13	5.50	0.77
-10	-2	13	3.14	1.51
10	2	-13	5.87	1.10
10	2	-13	5.36	0.56
-11	-2	13	75.96	4.38
-11	-2	13	67.99	3.37
-11	-2	13	68.76	2.93
11	2	-13	66.74	2.80
-12	-2	13	5.01	1.76
-12	-2	13	4.54	0.64
-12	-2	13	3.59	0.87
12	2	-13	4.27	0.61
-13	-2	13	104.34	4.39
-13	-2	13	115.35	5.89
-13	-2	13	107.61	4.78
13	2	-13	104.54	4.24
-14	-2	13	0.80	0.92
-14	-2	13	-0.19	1.81
-15	-2	13	68.66	3.57
-15	-2	13	72.96	4.75
15	2	-13	66.16	2.90
-16	-2	13	1.21	1.10
-16	-2	13	-2.93	1.87
16	2	-13	-0.25	0.50
-17	-2	13	51.03	3.21
-17	-2	13	46.99	4.55
17	2	-13	53.88	2.43
-18	-2	13	2.71	1.30
-18	-2	13	1.61	2.24
18	2	-13	1.57	0.70
15	-2	14	2.19	0.97
15	-2	14	2.24	1.74
-15	2	-14	2.44	1.03
14	-2	14	2.31	1.69
14	-2	14	6.34	1.01
-14	2	-14	4.43	1.39
-14	2	-14	7.59	1.16
13	-2	14	7.40	1.79

-13	2	-14	7.59	1.12
-13	2	-14	8.40	1.19
12	-2	14	17.58	2.53
-12	2	-14	16.77	1.78
-12	2	-14	19.53	1.64
11	-2	14	12.63	2.29
-11	2	-14	12.56	1.52
-11	2	-14	13.42	1.34
10	-2	14	14.55	1.36
10	-2	14	13.96	2.14
-10	2	-14	15.35	1.44
-10	2	-14	14.48	1.35
9	-2	14	0.66	0.59
9	-2	14	2.87	0.68
9	-2	14	0.01	1.13
-9	2	-14	1.04	0.70
-9	2	-14	0.51	0.55
8	-2	14	29.30	1.75
8	-2	14	30.25	1.69
8	-2	14	29.75	2.51
-8	2	-14	32.54	1.72
-8	2	-14	32.98	2.12
-8	2	-14	30.63	1.73
7	-2	14	5.54	0.83
7	-2	14	3.55	1.18
7	-2	14	4.85	0.66
-7	2	-14	3.93	0.61
-7	2	-14	6.13	1.18
-7	2	-14	4.17	0.69
6	-2	14	14.75	1.84
6	-2	14	15.31	1.42
6	-2	14	18.79	1.19
-6	2	-14	12.53	2.03
-6	2	-14	16.36	1.18
-6	2	-14	19.66	1.27
-6	2	-14	16.53	1.14
5	-2	14	1.86	0.76
5	-2	14	1.91	0.54
5	-2	14	-0.29	0.89
-5	2	-14	2.19	1.29
-5	2	-14	0.90	0.42
-5	2	-14	1.19	0.43
-5	2	-14	1.36	0.52
4	-2	14	38.78	2.53

4	-2	14	38.07	2.20
4	-2	14	38.33	1.79
-4	2	-14	36.15	1.75
-4	2	-14	35.48	1.70
-4	2	-14	36.85	2.77
-4	2	-14	36.44	1.76
3	-2	14	16.74	1.77
3	-2	14	17.16	1.10
3	-2	14	16.35	1.63
-3	2	-14	15.60	1.00
-3	2	-14	17.33	2.32
-3	2	-14	14.79	1.01
-3	2	-14	15.45	0.96
2	-2	14	30.10	1.52
2	-2	14	26.24	2.06
2	-2	14	30.18	2.16
-2	2	-14	26.85	2.61
-2	2	-14	30.86	1.40
-2	2	-14	28.33	1.42
-2	2	-14	29.73	1.43
1	-2	14	0.69	0.81
1	-2	14	0.65	0.47
1	-2	14	0.95	0.83
-1	2	-14	-0.21	0.31
-1	2	-14	-0.90	1.24
-1	2	-14	0.31	0.31
-1	2	-14	0.64	0.37
-1	2	-14	0.96	0.94
0	-2	14	61.11	2.57
0	-2	14	54.91	3.07
0	-2	14	60.72	3.28
0	2	-14	57.55	2.47
0	2	-14	61.91	2.43
0	2	-14	58.52	2.43
0	2	-14	61.29	3.20
0	2	-14	48.35	3.63
-1	-2	14	0.34	0.96
-1	-2	14	2.04	0.52
-1	-2	14	2.61	0.91
1	2	-14	1.38	0.40
1	2	-14	1.17	0.32
1	2	-14	2.31	0.92
1	2	-14	1.36	0.35
1	2	-14	1.54	1.33

-2	-2	14	88.00	4.44
-2	-2	14	87.01	3.55
-2	-2	14	85.59	4.10
-2	-2	14	92.85	3.70
2	2	-14	89.21	4.15
2	2	-14	88.72	3.51
2	2	-14	85.47	3.58
2	2	-14	93.42	3.53
2	2	-14	87.20	4.82
-3	-2	14	0.26	0.36
-3	-2	14	-0.95	1.00
-3	-2	14	-0.22	0.31
-3	-2	14	-0.45	0.71
-3	-2	14	0.19	0.49
3	2	-14	1.00	0.88
3	2	-14	0.23	0.25
3	2	-14	1.55	1.39
-4	-2	14	70.75	3.14
-4	-2	14	69.12	3.51
-4	-2	14	83.01	3.27
-4	-2	14	70.32	4.06
-4	-2	14	77.79	3.05
4	2	-14	74.83	2.99
4	2	-14	78.62	3.60
-5	-2	14	5.81	0.79
-5	-2	14	7.66	0.68
-5	-2	14	7.72	1.27
-5	-2	14	6.04	1.03
-5	-2	14	8.39	1.58
5	2	-14	7.82	1.41
5	2	-14	7.04	0.55
-6	-2	14	30.30	1.90
-6	-2	14	30.95	1.78
-6	-2	14	32.01	2.73
-6	-2	14	31.48	1.47
-6	-2	14	31.22	1.64
6	2	-14	31.02	1.38
6	2	-14	31.33	2.06
-7	-2	14	1.21	1.27
-7	-2	14	1.41	0.69
-7	-2	14	0.08	0.48
-7	-2	14	1.03	0.62
-7	-2	14	0.20	0.35
7	2	-14	-0.57	0.84

7	2	-14	0.37	0.25
-8	-2	14	124.91	6.00
-8	-2	14	108.05	4.76
-8	-2	14	116.17	4.87
-8	-2	14	112.64	4.55
-8	-2	14	117.82	4.82
8	2	-14	121.19	4.89
8	2	-14	111.83	4.47
-9	-2	14	1.61	0.44
-9	-2	14	0.83	0.60
-9	-2	14	2.77	1.45
-9	-2	14	0.17	0.63
9	2	-14	0.49	0.80
9	2	-14	1.10	0.30
-10	-2	14	119.38	4.98
-10	-2	14	129.75	5.26
-10	-2	14	125.16	4.96
-10	-2	14	127.42	6.35
10	2	-14	119.00	4.84
10	2	-14	131.84	5.13
-11	-2	14	1.65	0.56
-11	-2	14	0.42	0.46
-11	-2	14	1.23	1.62
-11	-2	14	1.46	0.72
11	2	-14	2.28	0.71
11	2	-14	1.61	0.38
-12	-2	14	46.16	2.56
-12	-2	14	48.25	3.86
-12	-2	14	44.17	2.20
12	2	-14	45.14	1.98
-13	-2	14	0.49	1.75
-13	-2	14	1.61	0.83
-13	-2	14	2.32	0.89
13	2	-14	0.27	0.42
-14	-2	14	51.76	2.98
-14	-2	14	51.66	4.20
14	2	-14	49.72	2.24
-15	-2	14	-0.31	1.00
-15	-2	14	-1.28	1.92
-16	-2	14	64.57	4.88
-16	-2	14	60.18	3.42
16	2	-14	59.84	2.68
-17	-2	14	2.33	1.28
-17	-2	14	0.31	2.20

17	2	-14	2.17	0.66
14	-2	15	2.06	1.71
-14	2	-15	1.03	0.92
13	-2	15	2.47	1.69
-13	2	-15	2.42	0.94
12	-2	15	2.07	1.53
-12	2	-15	2.99	0.90
-12	2	-15	2.22	0.98
11	-2	15	0.82	1.42
-11	2	-15	2.89	0.95
-11	2	-15	4.25	0.91
10	-2	15	9.54	1.74
-10	2	-15	11.78	1.37
-10	2	-15	10.60	1.32
9	-2	15	4.22	1.44
9	-2	15	6.64	1.19
9	-2	15	4.39	1.23
-9	2	-15	4.49	0.78
-9	2	-15	3.74	0.79
8	-2	15	-0.16	0.71
8	-2	15	-0.77	0.72
8	-2	15	0.32	1.13
-8	2	-15	-0.17	0.53
-8	2	-15	-0.45	0.60
7	-2	15	0.88	1.09
7	-2	15	0.61	0.57
7	-2	15	1.71	0.78
-7	2	-15	1.15	1.25
-7	2	-15	0.06	0.46
-7	2	-15	0.40	0.56
6	-2	15	1.25	1.12
6	-2	15	1.16	0.78
6	-2	15	-0.72	0.52
-6	2	-15	-0.40	0.51
-6	2	-15	-0.36	0.44
-6	2	-15	0.85	1.36
5	-2	15	0.75	0.55
5	-2	15	2.69	1.14
5	-2	15	2.00	0.89
-5	2	-15	1.47	0.53
-5	2	-15	-0.96	1.35
-5	2	-15	0.74	0.48
-5	2	-15	0.69	0.44
4	-2	15	24.84	2.36

4	-2	15	26.60	1.96
4	-2	15	27.31	1.48
-4	2	-15	26.91	1.43
-4	2	-15	27.32	1.45
-4	2	-15	23.99	2.68
-4	2	-15	26.59	1.39
3	-2	15	4.84	1.18
3	-2	15	5.70	1.09
3	-2	15	5.80	0.89
-3	2	-15	3.58	0.50
-3	2	-15	4.29	0.56
-3	2	-15	3.86	0.57
-3	2	-15	4.77	1.54
2	-2	15	0.50	0.92
2	-2	15	1.45	0.90
2	-2	15	0.30	0.51
-2	2	-15	0.47	0.37
-2	2	-15	0.84	0.38
-2	2	-15	0.47	0.41
-2	2	-15	-0.77	1.45
1	-2	15	5.16	1.19
1	-2	15	5.77	0.70
1	-2	15	5.10	1.12
-1	2	-15	5.39	0.67
-1	2	-15	4.79	0.68
-1	2	-15	6.23	1.61
-1	2	-15	6.18	0.72
0	-2	15	1.84	0.98
0	-2	15	1.78	0.56
0	-2	15	1.48	0.99
0	2	-15	1.07	0.38
0	2	-15	1.73	0.43
0	2	-15	-1.08	1.56
0	2	-15	0.69	0.32
-1	-2	15	7.72	0.99
-1	-2	15	8.90	1.40
-1	-2	15	8.12	1.47
1	2	-15	7.11	1.20
1	2	-15	7.61	0.78
1	2	-15	4.58	1.79
1	2	-15	8.65	0.74
1	2	-15	7.56	0.66
-2	-2	15	23.37	1.41
-2	-2	15	18.94	1.88

-2	-2	15	22.56	2.26
2	2	-15	19.64	1.20
2	2	-15	23.73	1.16
2	2	-15	21.26	2.10
2	2	-15	21.21	1.09
-3	-2	15	2.67	0.47
-3	-2	15	2.47	0.92
-3	-2	15	3.79	1.28
-3	-2	15	3.50	0.68
-3	-2	15	3.47	0.53
3	2	-15	4.20	1.03
3	2	-15	3.66	0.53
-4	-2	15	1.44	0.63
-4	-2	15	1.70	0.41
-4	-2	15	1.65	0.84
-4	-2	15	1.73	0.47
-4	-2	15	0.88	1.10
4	2	-15	2.93	1.03
4	2	-15	1.50	0.33
-5	-2	15	1.96	0.52
-5	-2	15	1.71	0.81
-5	-2	15	2.13	0.43
-5	-2	15	2.34	0.69
-5	-2	15	1.70	1.16
5	2	-15	1.17	0.31
5	2	-15	1.31	0.94
-6	-2	15	0.22	0.38
-6	-2	15	0.90	0.48
-6	-2	15	0.60	0.66
-6	-2	15	-0.72	0.75
-6	-2	15	0.95	1.57
6	2	-15	0.22	0.26
6	2	-15	0.20	0.95
-7	-2	15	19.84	2.54
-7	-2	15	14.75	1.02
-7	-2	15	16.27	1.49
-7	-2	15	13.81	1.24
-7	-2	15	16.51	1.48
7	2	-15	13.87	1.75
7	2	-15	17.16	0.89
-8	-2	15	17.86	1.14
-8	-2	15	18.46	2.64
-8	-2	15	16.49	1.49
-8	-2	15	17.88	1.86

-8	-2	15	19.50	1.42
8	2	-15	22.15	1.85
8	2	-15	18.10	0.98
-9	-2	15	16.86	1.40
-9	-2	15	15.42	2.31
-9	-2	15	17.42	1.40
-9	-2	15	16.99	1.14
9	2	-15	17.51	1.69
9	2	-15	16.49	0.93
-10	-2	15	7.39	1.94
-10	-2	15	6.64	1.11
-10	-2	15	6.42	1.21
-10	-2	15	4.99	0.65
10	2	-15	3.52	0.98
10	2	-15	5.99	0.62
-11	-2	15	12.21	1.42
-11	-2	15	9.54	1.08
-11	-2	15	6.42	2.00
11	2	-15	11.02	0.81
-12	-2	15	-0.10	0.86
-12	-2	15	2.24	1.81
12	2	-15	-0.52	0.37
-13	-2	15	16.75	1.81
-13	-2	15	10.70	2.30
13	2	-15	13.57	1.01
-14	-2	15	3.44	1.11
-14	-2	15	4.28	2.03
14	2	-15	3.56	0.60
-15	-2	15	15.50	2.69
-15	-2	15	16.33	2.07
15	2	-15	16.64	1.19
-16	-2	15	4.48	1.27
-16	-2	15	1.84	2.28
16	2	-15	2.57	0.67
-17	-2	15	10.99	1.67
-17	-2	15	23.21	4.21
17	2	-15	16.19	1.34
13	-2	16	0.62	1.72
-13	2	-16	-1.12	0.93
12	-2	16	26.42	3.14
-12	2	-16	28.61	2.07
11	-2	16	1.03	1.49
-11	2	-16	1.43	0.80
10	-2	16	34.72	3.17

-10	2	-16	36.40	2.16
-10	2	-16	40.60	2.24
9	-2	16	0.17	1.40
-9	2	-16	0.39	0.74
-9	2	-16	0.83	0.66
8	-2	16	25.09	2.66
8	-2	16	29.91	1.96
-8	2	-16	33.27	1.83
-8	2	-16	30.56	1.73
7	-2	16	0.07	0.87
7	-2	16	-0.06	1.16
7	-2	16	-0.49	0.73
-7	2	-16	0.63	0.61
-7	2	-16	0.38	0.53
6	-2	16	23.37	1.46
6	-2	16	25.93	1.92
6	-2	16	21.18	2.38
-6	2	-16	22.69	1.42
-6	2	-16	24.93	1.48
-6	2	-16	24.74	2.75
5	-2	16	1.42	0.60
5	-2	16	0.01	0.85
5	-2	16	0.89	1.17
-5	2	-16	-3.14	1.51
-5	2	-16	0.37	0.45
-5	2	-16	0.53	0.54
4	-2	16	21.14	1.92
4	-2	16	20.05	2.10
4	-2	16	17.44	1.26
-4	2	-16	16.62	2.87
-4	2	-16	17.67	1.19
-4	2	-16	16.19	1.17
3	-2	16	7.80	1.37
3	-2	16	9.07	1.35
3	-2	16	8.32	1.03
-3	2	-16	7.98	0.90
-3	2	-16	7.25	1.86
-3	2	-16	12.29	0.91
-3	2	-16	10.94	0.91
2	-2	16	6.45	1.64
2	-2	16	8.90	1.05
2	-2	16	10.17	1.80
-2	2	-16	9.25	0.84
-2	2	-16	9.03	0.84

-2	2	-16	10.04	0.88
-2	2	-16	10.73	1.94
1	-2	16	5.60	0.74
1	-2	16	5.66	1.28
1	-2	16	2.45	1.08
-1	2	-16	4.53	0.76
-1	2	-16	6.67	0.77
-1	2	-16	3.40	1.96
-1	2	-16	5.86	0.71
0	-2	16	42.98	2.04
0	-2	16	45.54	2.89
0	-2	16	37.84	2.59
0	2	-16	37.81	1.86
0	2	-16	39.46	1.83
0	2	-16	40.40	1.83
-1	-2	16	5.97	1.20
-1	-2	16	6.23	1.30
-1	-2	16	6.21	0.79
1	2	-16	4.47	0.59
1	2	-16	5.05	0.73
1	2	-16	5.17	0.65
-2	-2	16	14.17	0.99
-2	-2	16	17.30	2.14
-2	-2	16	13.22	1.76
-2	-2	16	14.70	1.28
2	2	-16	10.89	0.84
2	2	-16	12.66	0.92
-3	-2	16	1.14	0.47
-3	-2	16	-0.05	0.42
-3	-2	16	1.92	0.67
-3	-2	16	1.08	0.92
-3	-2	16	2.41	1.20
3	2	-16	0.78	0.34
3	2	-16	-0.38	1.09
-4	-2	16	14.01	1.07
-4	-2	16	13.77	1.72
-4	-2	16	13.90	2.44
-4	-2	16	14.14	0.99
-4	-2	16	14.97	1.33
4	2	-16	13.26	0.85
4	2	-16	13.23	2.01
-5	-2	16	12.23	1.90
-5	-2	16	9.31	0.87
-5	-2	16	9.73	1.26

-5	-2	16	9.08	1.25
-5	-2	16	9.17	1.00
5	2	-16	9.04	1.37
5	2	-16	9.93	0.71
-6	-2	16	9.02	0.92
-6	-2	16	10.03	1.07
-6	-2	16	12.41	2.03
-6	-2	16	11.90	1.11
-6	-2	16	9.41	1.49
6	2	-16	9.37	1.36
6	2	-16	11.76	0.76
-7	-2	16	-0.76	0.81
-7	-2	16	0.01	0.45
-7	-2	16	-0.15	0.57
-7	-2	16	-1.08	1.47
-7	-2	16	-0.09	0.75
7	2	-16	0.32	0.30
7	2	-16	-0.36	1.10
-8	-2	16	2.15	0.54
-8	-2	16	1.77	0.65
-8	-2	16	2.84	1.63
-8	-2	16	0.88	0.84
8	2	-16	2.11	1.09
8	2	-16	1.71	0.36
-9	-2	16	8.33	1.91
-9	-2	16	10.87	1.01
-9	-2	16	9.74	1.28
-9	-2	16	9.24	1.34
9	2	-16	11.34	1.77
9	2	-16	9.45	0.75
-10	-2	16	4.51	0.71
-10	-2	16	4.15	0.87
-10	-2	16	3.64	0.82
-10	-2	16	6.04	1.89
10	2	-16	3.15	0.46
-11	-2	16	6.85	2.06
-11	-2	16	5.78	1.01
11	2	-16	5.63	0.71
-12	-2	16	2.64	0.94
-12	-2	16	3.46	1.82
12	2	-16	1.38	0.48
-13	-2	16	-1.19	0.99
-13	-2	16	-0.56	1.81
13	2	-16	0.39	0.48

-14	-2	16	-1.07	2.08
-14	-2	16	1.51	1.07
14	2	-16	-0.37	0.57
-15	-2	16	1.26	2.24
-15	-2	16	1.72	1.20
15	2	-16	0.87	0.59
-16	-2	16	0.85	2.34
-16	-2	16	-0.76	1.24
16	2	-16	-0.33	0.63
12	-2	17	-1.79	1.75
-12	2	-17	-1.41	0.86
11	-2	17	25.75	3.26
-11	2	-17	33.11	2.13
10	-2	17	1.87	1.58
-10	2	-17	0.49	0.77
9	-2	17	44.21	3.61
-9	2	-17	50.74	2.55
8	-2	17	0.88	0.96
8	-2	17	2.66	1.52
-8	2	-17	1.04	0.65
-8	2	-17	-0.18	0.85
7	-2	17	51.57	3.59
7	-2	17	49.48	2.77
-7	2	-17	51.32	2.48
-7	2	-17	46.39	2.34
6	-2	17	1.66	1.03
6	-2	17	1.39	1.23
-6	2	-17	0.63	0.57
-6	2	-17	0.11	0.59
5	-2	17	38.29	2.50
5	-2	17	35.29	2.91
-5	2	-17	34.22	1.79
-5	2	-17	34.61	1.87
-5	2	-17	36.23	3.50
4	-2	17	1.65	1.10
4	-2	17	2.31	0.64
4	-2	17	1.33	1.25
-4	2	-17	0.48	1.88
-4	2	-17	0.84	0.51
-4	2	-17	1.29	0.54
3	-2	17	32.03	2.69
3	-2	17	32.57	2.54
3	-2	17	34.15	1.78
-3	2	-17	32.10	1.65

-3	2	-17	36.52	4.25
-3	2	-17	34.07	1.74
2	-2	17	-0.03	1.07
2	-2	17	0.36	0.62
2	-2	17	-1.06	1.00
-2	2	-17	-0.29	0.46
-2	2	-17	0.30	0.45
-2	2	-17	0.82	0.47
1	-2	17	38.45	2.82
1	-2	17	43.30	2.03
1	-2	17	35.35	2.71
-1	2	-17	37.45	1.84
-1	2	-17	40.67	1.87
-1	2	-17	40.45	1.88
0	-2	17	8.07	1.11
0	-2	17	4.83	1.33
0	-2	17	1.53	1.87
0	2	-17	4.91	0.74
0	2	-17	3.96	0.56
0	2	-17	3.32	0.59
-1	-2	17	65.73	3.73
-1	-2	17	76.18	3.10
-1	-2	17	70.95	4.06
1	2	-17	66.21	2.87
1	2	-17	66.04	2.90
1	2	-17	68.26	2.87
-2	-2	17	11.04	1.85
-2	-2	17	10.68	1.90
-2	-2	17	8.57	0.95
-2	-2	17	9.90	1.24
2	2	-17	9.26	0.89
2	2	-17	9.55	0.79
-3	-2	17	75.49	3.14
-3	-2	17	76.94	3.29
-3	-2	17	68.49	3.75
-3	-2	17	76.35	4.29
3	2	-17	68.38	2.98
3	2	-17	73.04	3.04
-4	-2	17	0.35	0.75
-4	-2	17	-0.89	1.32
-4	-2	17	1.37	0.50
-4	-2	17	0.45	1.00
-4	-2	17	0.18	0.51
4	2	-17	1.05	0.38

-5	-2	17	38.28	1.92
-5	-2	17	38.90	2.57
-5	-2	17	41.86	3.37
-5	-2	17	43.24	2.07
-5	-2	17	42.11	2.26
5	2	-17	41.97	2.82
5	2	-17	39.29	1.79
-6	-2	17	0.89	0.85
-6	-2	17	-0.40	1.54
-6	-2	17	-0.43	0.60
-6	-2	17	0.10	0.51
-6	-2	17	0.52	1.00
6	2	-17	2.06	1.32
6	2	-17	-0.21	0.33
-7	-2	17	19.14	1.53
-7	-2	17	19.33	1.27
-7	-2	17	17.96	1.80
-7	-2	17	19.40	2.92
7	2	-17	17.78	1.03
7	2	-17	17.25	2.22
-8	-2	17	0.10	0.66
-8	-2	17	-0.56	0.89
-8	-2	17	0.62	1.60
-8	-2	17	0.15	0.64
8	2	-17	0.85	0.35
8	2	-17	-1.12	1.24
-9	-2	17	13.93	2.37
-9	-2	17	11.98	1.54
9	2	-17	11.48	0.86
-10	-2	17	1.63	0.93
-10	-2	17	1.94	0.87
-10	-2	17	1.08	1.77
10	2	-17	0.78	0.42
-11	-2	17	17.48	3.31
-11	-2	17	19.78	1.86
11	2	-17	16.80	1.10
-12	-2	17	-0.18	0.99
-12	-2	17	0.33	2.03
12	2	-17	0.31	0.47
-13	-2	17	25.63	3.77
-13	-2	17	26.36	2.31
13	2	-17	19.73	1.32
-14	-2	17	-0.02	1.15
-14	-2	17	2.40	2.09

14	2	-17	2.54	0.62
-15	-2	17	7.52	1.45
-15	-2	17	4.29	2.47
11	-2	18	-1.52	2.08
-11	2	-18	1.93	1.05
10	-2	18	27.40	3.30
-10	2	-18	25.79	2.03
9	-2	18	0.55	1.57
-9	2	-18	1.29	0.87
8	-2	18	49.73	3.70
-8	2	-18	51.65	2.66
7	-2	18	0.50	1.45
7	-2	18	-0.73	1.08
-7	2	-18	-0.09	0.70
6	-2	18	37.89	2.65
6	-2	18	34.70	3.25
-6	2	-18	40.35	2.11
5	-2	18	1.47	1.40
5	-2	18	2.73	1.21
-5	2	-18	0.34	0.62
-5	2	-18	0.33	0.58
4	-2	18	64.32	3.48
4	-2	18	53.49	3.65
-4	2	-18	56.79	2.51
-4	2	-18	55.53	2.65
3	-2	18	-0.50	1.16
3	-2	18	0.47	1.38
-3	2	-18	0.49	0.54
-3	2	-18	0.40	0.51
2	-2	18	38.30	2.85
2	-2	18	37.37	3.04
-2	2	-18	38.31	1.89
-2	2	-18	35.99	1.80
1	-2	18	2.46	1.79
1	-2	18	2.04	0.79
1	-2	18	1.50	1.26
-1	2	-18	1.66	0.57
-1	2	-18	1.23	0.53
-1	2	-18	2.54	0.56
0	-2	18	51.53	3.49
0	-2	18	52.17	2.53
0	-2	18	53.17	3.38
0	2	-18	50.65	2.33
0	2	-18	54.02	2.38

0	2	-18	54.93	2.36
-1	-2	18	2.01	0.58
-1	-2	18	1.94	0.79
-1	-2	18	2.64	1.22
-1	-2	18	2.83	1.36
1	2	-18	1.64	0.47
1	2	-18	2.21	0.55
-2	-2	18	55.75	2.73
-2	-2	18	49.48	4.41
-2	-2	18	56.33	2.54
-2	-2	18	59.04	3.80
2	2	-18	55.19	2.43
2	2	-18	58.61	2.48
-3	-2	18	4.15	0.68
-3	-2	18	3.90	1.66
-3	-2	18	4.64	1.27
-3	-2	18	5.43	0.94
3	2	-18	3.97	0.55
3	2	-18	2.20	0.58
-4	-2	18	60.87	2.67
-4	-2	18	59.11	3.35
-4	-2	18	61.88	2.77
-4	-2	18	59.62	2.92
-4	-2	18	59.06	4.09
4	2	-18	58.66	2.61
4	2	-18	60.01	2.54
-5	-2	18	1.21	1.52
-5	-2	18	-1.08	0.58
-5	-2	18	-0.95	1.05
-5	-2	18	-0.68	0.85
-5	-2	18	-0.91	0.60
5	2	-18	0.17	0.37
-6	-2	18	33.53	2.54
-6	-2	18	39.50	2.16
-6	-2	18	34.98	3.52
-6	-2	18	38.31	2.28
6	2	-18	38.13	1.74
6	2	-18	46.96	3.64
-7	-2	18	0.04	0.69
-7	-2	18	-0.33	1.62
-7	-2	18	-0.55	0.98
7	2	-18	-1.07	1.54
7	2	-18	0.48	0.40
-8	-2	18	22.00	1.66

-8	-2	18	26.55	3.31
-8	-2	18	14.75	1.89
8	2	-18	16.89	1.09
-9	-2	18	1.13	1.06
-9	-2	18	2.33	0.91
-9	-2	18	1.78	1.85
9	2	-18	1.85	0.49
-10	-2	18	44.17	2.60
-10	-2	18	41.81	4.12
10	2	-18	42.58	1.95
-11	-2	18	-3.33	1.92
-11	-2	18	0.56	0.99
11	2	-18	0.28	0.48
-12	-2	18	34.93	4.20
-12	-2	18	41.99	2.65
12	2	-18	32.11	1.73
-13	-2	18	2.76	2.24
-13	-2	18	-0.96	1.17
-14	-2	18	19.16	2.42
-14	-2	18	14.40	2.92
9	-2	19	35.33	3.68
-9	2	-19	38.32	2.33
8	-2	19	5.51	1.89
-8	2	-19	2.85	0.89
7	-2	19	42.35	3.70
-7	2	-19	42.12	2.38
6	-2	19	-2.91	1.45
6	-2	19	-0.17	1.28
-6	2	-19	-0.14	0.71
5	-2	19	32.77	3.31
5	-2	19	38.42	2.87
-5	2	-19	38.19	2.08
4	-2	19	-0.15	1.53
4	-2	19	-2.34	1.25
-4	2	-19	-0.89	0.59
3	-2	19	40.15	3.32
2	-2	19	3.58	1.40
2	-2	19	1.71	1.43
-2	2	-19	2.39	0.73
-2	2	-19	2.63	0.62
1	-2	19	51.23	3.50
1	-2	19	52.04	3.58
-1	2	-19	50.08	2.31
-1	2	-19	54.69	2.42

0	-2	19	4.93	1.52
0	-2	19	6.13	1.65
0	-2	19	5.55	0.76
0	2	-19	5.39	0.73
-1	-2	19	47.15	2.23
-1	-2	19	45.09	3.27
-1	-2	19	46.95	3.66
1	2	-19	46.92	2.16
1	2	-19	46.03	2.20
-2	-2	19	1.41	1.34
-2	-2	19	-0.29	0.57
-2	-2	19	0.01	1.50
2	2	-19	-0.84	0.53
2	2	-19	0.04	0.48
-3	-2	19	74.24	3.24
-3	-2	19	68.68	3.96
-3	-2	19	73.59	4.62
3	2	-19	75.19	3.08
-4	-2	19	1.56	0.68
-4	-2	19	-0.59	1.64
4	2	-19	-0.26	0.44
-5	-2	19	38.79	3.67
-5	-2	19	35.33	2.12
-5	-2	19	37.32	2.73
5	2	-19	39.08	1.79
-6	-2	19	1.01	1.77
-6	-2	19	1.92	1.21
-6	-2	19	0.02	0.77
6	2	-19	-0.46	0.42
-7	-2	19	40.37	4.01
-7	-2	19	37.90	2.85
-7	-2	19	48.24	2.44
7	2	-19	43.12	1.96
-8	-2	19	1.37	1.15
-8	-2	19	-2.45	1.90
-8	-2	19	2.11	0.90
8	2	-19	-0.39	0.45
-9	-2	19	24.61	2.01
-9	-2	19	19.77	3.75
9	2	-19	20.71	1.26
-10	-2	19	2.68	2.10
-10	-2	19	2.85	1.05
10	2	-19	1.28	0.54
-11	-2	19	47.62	2.92

-11	-2	19	42.04	4.58
11	2	-19	46.01	2.56
-12	-2	19	1.41	1.31
-12	-2	19	-0.03	2.34
-13	-2	19	34.60	2.86
-8	2	-20	19.24	1.86
7	-2	20	2.00	1.82
-7	2	-20	1.57	0.88
6	-2	20	14.39	2.20
-6	2	-20	13.63	1.57
5	-2	20	-0.33	1.63
-5	2	-20	-0.98	0.72
4	-2	20	12.63	1.95
4	-2	20	16.03	2.99
-4	2	-20	15.97	1.46
3	-2	20	-0.27	1.54
3	-2	20	0.23	1.60
-3	2	-20	2.30	0.72
2	-2	20	29.09	3.17
2	-2	20	30.31	3.12
-2	2	-20	31.59	1.83
1	-2	20	0.80	1.52
1	-2	20	2.23	1.62
-1	2	-20	-0.73	0.58
0	-2	20	43.60	2.35
0	-2	20	37.80	3.48
0	-2	20	31.74	3.25
0	2	-20	40.78	2.01
-1	-2	20	-0.79	0.62
-1	-2	20	0.87	1.68
-1	-2	20	-0.56	1.43
1	2	-20	-0.69	0.53
-2	-2	20	37.65	1.98
-2	-2	20	33.26	3.08
-2	-2	20	33.33	3.56
2	2	-20	36.80	1.84
-3	-2	20	0.51	0.72
-3	-2	20	0.01	1.73
-3	-2	20	-0.95	1.36
3	2	-20	0.47	0.52
-4	-2	20	53.30	4.20
-4	-2	20	42.69	3.21
-4	-2	20	43.64	2.40
4	2	-20	45.60	2.11

-5	-2	20	3.16	1.37
-5	-2	20	1.92	0.85
-5	-2	20	0.26	1.89
5	2	-20	1.26	0.53
-6	-2	20	31.06	3.75
-6	-2	20	26.08	1.90
6	2	-20	24.22	1.41
-7	-2	20	-0.61	1.98
-7	-2	20	3.33	1.35
7	2	-20	0.58	0.51
-8	-2	20	43.69	2.63
-8	-2	20	45.41	3.85
-8	-2	20	44.39	4.38
8	2	-20	42.55	2.02
-9	-2	20	0.26	1.06
-9	-2	20	-0.62	2.17
-10	-2	20	36.33	2.67
-10	-2	20	34.47	4.35
-11	-2	20	-0.22	2.48
-11	-2	20	3.34	1.31
6	-2	21	5.22	2.51
-6	2	-21	1.52	0.91
5	-2	21	1.17	1.84
5	-2	21	-2.49	1.79
-5	2	-21	-0.31	0.83
4	-2	21	2.92	1.87
4	-2	21	8.39	1.92
-4	2	-21	6.96	1.00
3	-2	21	0.60	1.74
3	-2	21	-0.70	1.69
-3	2	-21	-0.46	0.74
2	-2	21	2.56	1.71
2	-2	21	1.95	1.79
-2	2	-21	2.62	0.83
1	-2	21	6.77	1.96
1	-2	21	7.12	2.03
-1	2	-21	10.03	1.27
0	-2	21	0.55	1.81
0	-2	21	2.73	1.63
0	2	-21	1.50	0.77
-1	-2	21	4.54	1.91
-1	-2	21	4.56	1.70
-2	-2	21	0.40	1.92
-2	-2	21	1.95	1.62

2	2	-21	2.77	0.67
-3	-2	21	6.28	2.20
-3	-2	21	3.94	1.68
3	2	-21	6.45	0.76
-4	-2	21	1.98	2.12
-4	-2	21	0.05	1.55
4	2	-21	-0.36	0.56
-5	-2	21	8.76	1.82
-5	-2	21	9.57	1.60
-5	-2	21	5.81	2.39
5	2	-21	9.95	1.02
-6	-2	21	1.23	1.46
-6	-2	21	0.83	2.08
-6	-2	21	1.81	1.00
-7	-2	21	6.11	2.36
-7	-2	21	5.95	1.16
-7	-2	21	3.64	1.51
-8	-2	21	3.61	1.17
-8	-2	21	1.14	2.28
-9	-2	21	14.95	3.10
-9	-2	21	12.62	1.60
-3	2	-22	-0.32	0.81
2	-2	22	2.50	2.00
2	-2	22	1.33	1.96
-2	2	-22	0.05	0.80
1	-2	22	6.39	2.23
1	-2	22	2.48	1.90
-1	2	-22	5.38	0.93
0	-2	22	7.40	2.06
0	-2	22	10.05	2.29
0	2	-22	9.51	1.32
-1	-2	22	5.68	1.92
-1	-2	22	2.37	2.20
1	2	-22	2.86	0.77
-2	-2	22	11.35	2.07
-2	-2	22	11.99	2.46
2	2	-22	11.92	1.84
-3	-2	22	4.08	2.22
-3	-2	22	1.20	1.69
-4	-2	22	0.80	2.33
-4	-2	22	2.36	1.73
-5	-2	22	9.59	2.54
-5	-2	22	7.12	1.88
-6	-2	22	-1.18	2.27

-6	-2	22	0.08	1.66
6	-3	-22	3.96	0.86
-6	3	22	6.06	1.86
-6	3	22	5.65	2.44
5	-3	-22	1.54	0.80
-5	3	22	0.83	1.70
-5	3	22	4.07	2.05
4	-3	-22	11.03	1.44
-4	3	22	5.48	1.79
-4	3	22	10.61	2.29
3	-3	-22	15.07	1.53
-3	3	22	14.85	2.49
-3	3	22	15.71	2.87
2	-3	-22	5.12	0.95
-2	3	22	4.74	1.81
-2	3	22	4.22	2.09
1	-3	-22	5.28	0.94
-1	3	22	4.67	1.88
-1	3	22	2.41	1.88
0	-3	-22	4.18	0.88
0	3	22	6.84	1.98
0	3	22	3.08	1.84
-1	-3	-22	6.00	1.00
1	3	22	5.57	2.10
1	3	22	9.64	2.12
-2	-3	-22	1.40	0.92
2	3	22	2.39	1.95
2	3	22	1.28	1.88
9	-3	-21	2.54	0.79
8	-3	-21	6.63	0.90
-8	3	21	7.52	2.23
7	-3	-21	1.36	0.71
-7	3	21	2.98	2.07
-7	3	21	-0.68	1.48
6	-3	-21	9.57	1.23
-6	3	21	9.92	2.21
-6	3	21	6.83	1.66
5	-3	-21	3.91	0.80
-5	3	21	4.48	1.57
-5	3	21	4.64	2.00
4	-3	-21	6.64	0.90
-4	3	21	8.65	2.06
-4	3	21	9.22	1.80
3	-3	-21	1.20	0.72

-3	3	21	4.97	1.84
-3	3	21	0.64	1.56
2	-3	-21	4.56	0.81
-2	3	21	4.16	1.65
-2	3	21	9.19	1.98
1	-3	-21	1.76	0.75
-1	3	21	5.98	1.77
-1	3	21	1.81	1.69
0	-3	-21	3.69	0.80
0	3	21	2.64	1.66
0	3	21	2.78	1.76
-1	-3	-21	3.48	0.86
1	3	21	1.54	1.68
1	3	21	0.80	1.72
-2	-3	-21	0.36	0.78
2	3	21	4.54	1.77
2	3	21	1.86	1.57
-3	-3	-21	9.33	1.13
3	3	21	8.70	1.94
3	3	21	7.12	2.01
-4	-3	-21	-0.27	0.84
4	3	21	1.87	1.78
4	3	21	1.74	1.67
-5	-3	-21	-0.08	0.86
5	3	21	1.70	1.84
5	3	21	5.08	1.74
11	-3	-20	23.18	1.66
-11	3	20	22.61	3.45
-11	3	20	36.44	6.44
10	-3	-20	-0.70	0.64
-10	3	20	-1.09	1.97
9	-3	-20	43.84	2.25
-9	3	20	46.59	4.19
8	-3	-20	1.10	0.67
-8	3	20	0.91	1.77
-8	3	20	0.72	1.92
7	-3	-20	60.75	2.73
-7	3	20	58.27	4.48
-7	3	20	57.26	3.49
6	-3	-20	0.07	0.58
-6	3	20	-1.04	1.34
-6	3	20	2.27	1.82
5	-3	-20	25.17	1.59
-5	3	20	22.43	3.22

-5	3	20	27.84	2.63
4	-3	-20	-0.46	0.57
-4	3	20	1.69	1.70
-4	3	20	0.79	1.27
3	-3	-20	27.82	1.73
-3	3	20	26.08	3.17
-3	3	20	31.10	2.77
2	-3	-20	0.11	0.60
-2	3	20	-0.02	1.44
-2	3	20	1.01	1.38
1	-3	-20	35.13	2.00
-1	3	20	36.71	3.28
-1	3	20	37.83	3.15
0	-3	-20	-0.54	0.63
0	3	20	-0.58	1.47
0	3	20	-0.78	1.44
-1	-3	-20	30.51	1.88
1	3	20	28.22	2.96
1	3	20	29.69	2.89
-2	-3	-20	1.23	0.73
2	3	20	0.76	1.40
2	3	20	0.45	1.53
-3	-3	-20	6.77	0.98
3	3	20	10.09	1.74
3	3	20	6.68	1.81
-4	-3	-20	1.52	0.77
4	3	20	2.35	1.52
4	3	20	0.92	1.63
-5	-3	-20	26.00	1.89
5	3	20	22.72	2.99
5	3	20	29.52	2.85
-6	-3	-20	2.13	0.93
6	3	20	4.06	1.53
6	3	20	2.58	1.84
-7	-3	-20	29.86	2.18
7	3	20	29.75	3.55
-8	-3	-20	3.17	1.01
12	-3	-19	34.25	1.91
-12	3	19	35.52	2.68
-12	3	19	28.27	4.05
11	-3	-19	0.51	0.62
-11	3	19	-4.38	1.80
-11	3	19	-0.49	1.13
10	-3	-19	42.42	2.14

-10	3	19	41.33	4.03
9	-3	-19	1.56	0.61
-9	3	19	2.08	1.83
-9	3	19	0.83	0.96
8	-3	-19	37.38	1.95
-8	3	19	42.69	2.35
-8	3	19	32.72	2.59
-8	3	19	39.03	3.75
7	-3	-19	-0.34	0.53
-7	3	19	0.58	1.18
-7	3	19	0.27	0.82
-7	3	19	2.72	1.73
6	-3	-19	32.10	1.84
-6	3	19	39.62	3.52
-6	3	19	37.66	2.05
-6	3	19	36.80	2.70
5	-3	-19	2.59	0.63
-5	3	19	2.42	1.29
-5	3	19	0.93	0.69
-5	3	19	2.56	1.57
4	-3	-19	60.77	2.73
-4	3	19	63.68	2.75
-4	3	19	59.82	4.02
-4	3	19	54.89	3.45
3	-3	-19	-0.29	0.52
-3	3	19	-0.29	1.21
-3	3	19	-2.23	1.31
2	-3	-19	61.68	2.79
-2	3	19	57.81	3.63
-2	3	19	65.00	3.98
1	-3	-19	0.91	0.58
-1	3	19	0.42	1.29
-1	3	19	2.38	1.38
0	-3	-19	54.85	2.59
0	3	19	54.94	3.58
0	3	19	53.47	3.56
-1	-3	-19	0.23	0.62
1	3	19	0.30	1.39
1	3	19	0.54	1.20
-2	-3	-19	34.11	1.96
2	3	19	35.66	3.00
2	3	19	32.65	2.81
-3	-3	-19	-0.59	0.61
3	3	19	-0.41	1.40

3	3	19	-2.70	1.15
-4	-3	-19	46.36	2.40
4	3	19	46.05	3.49
4	3	19	41.25	3.09
-5	-3	-19	0.74	0.71
5	3	19	0.50	1.51
5	3	19	0.25	1.46
-6	-3	-19	36.49	2.22
6	3	19	36.23	2.84
6	3	19	36.64	3.36
-7	-3	-19	1.39	0.82
7	3	19	-1.15	1.64
7	3	19	1.39	1.28
-8	-3	-19	42.28	2.46
8	3	19	40.33	3.75
-9	-3	-19	-1.53	0.85
9	3	19	-0.97	1.79
14	-3	-18	1.77	0.72
-14	3	18	-3.98	2.24
-14	3	18	1.67	1.33
13	-3	-18	17.93	1.45
-13	3	18	23.49	2.36
-13	3	18	16.75	2.79
12	-3	-18	-0.04	0.60
-12	3	18	-2.26	1.85
-12	3	18	-0.96	1.11
11	-3	-18	27.13	1.63
-11	3	18	23.19	3.50
-11	3	18	31.15	2.27
10	-3	-18	3.16	0.64
-10	3	18	1.79	1.01
-10	3	18	4.33	2.01
9	-3	-18	34.02	1.78
-9	3	18	38.60	2.28
-9	3	18	35.57	3.51
-9	3	18	30.41	2.34
8	-3	-18	0.02	0.52
-8	3	18	-0.05	0.86
-8	3	18	0.79	1.65
-8	3	18	0.94	1.04
7	-3	-18	62.03	3.75
7	-3	-18	57.52	2.52
-7	3	18	52.18	3.09
-7	3	18	50.19	3.87

-7	3	18	55.75	2.72
6	-3	-18	-0.37	0.44
-6	3	18	0.84	1.10
-6	3	18	0.10	0.68
-6	3	18	1.61	1.42
5	-3	-18	21.42	1.46
-5	3	18	22.23	2.11
-5	3	18	19.30	1.38
-5	3	18	22.37	2.74
4	-3	-18	0.04	0.80
-4	3	18	1.27	1.06
-4	3	18	0.69	0.57
-4	3	18	1.94	1.47
-3	3	18	107.39	3.96
-3	3	18	88.41	5.08
-3	3	18	98.63	5.19
2	-3	-18	2.74	0.67
-2	3	18	2.88	1.21
-2	3	18	3.85	1.38
1	-3	-18	63.52	2.92
-1	3	18	70.77	3.90
-1	3	18	63.91	3.65
0	3	18	3.69	1.33
0	3	18	3.91	1.30
-1	-3	-18	41.92	2.09
1	3	18	39.99	2.88
1	3	18	40.25	3.01
-2	-3	-18	-0.68	0.53
2	3	18	-0.59	1.21
2	3	18	-0.93	0.99
-3	-3	-18	35.22	1.87
3	3	18	28.84	3.06
3	3	18	28.81	2.76
-4	-3	-18	0.42	0.61
4	3	18	-0.65	1.28
4	3	18	-1.06	1.08
-5	-3	-18	54.97	2.66
5	3	18	56.93	3.18
5	3	18	46.53	3.60
-6	-3	-18	1.22	0.69
6	3	18	-0.65	1.35
6	3	18	-0.99	1.07
-7	-3	-18	23.36	1.75
7	3	18	26.50	2.30

7	3	18	20.01	2.75
-8	-3	-18	-0.40	0.76
8	3	18	0.39	1.43
8	3	18	-0.54	1.16
-9	-3	-18	45.87	2.52
9	3	18	41.35	3.58
-10	-3	-18	1.19	0.93
10	3	18	0.50	1.62
-11	-3	-18	27.13	2.17
11	3	18	28.22	4.59
15	-3	-17	0.88	0.70
-15	3	17	-0.93	1.30
-15	3	17	-1.46	2.06
14	-3	-17	2.84	0.69
-14	3	17	5.32	1.33
-14	3	17	-1.32	2.09
13	-3	-17	0.46	0.58
-13	3	17	0.66	1.18
-13	3	17	1.31	1.94
12	-3	-17	20.61	1.40
-12	3	17	20.12	3.40
-12	3	17	24.11	2.16
11	-3	-17	0.08	0.53
-11	3	17	0.61	0.99
-11	3	17	-0.78	1.68
10	-3	-17	25.84	1.51
-10	3	17	28.90	2.03
-10	3	17	28.36	3.28
-10	3	17	24.86	1.99
9	-3	-17	9.10	0.97
-9	3	17	7.90	1.11
-9	3	17	13.05	1.62
-9	3	17	9.90	1.98
-9	3	17	7.88	1.19
8	-3	-17	28.28	2.40
8	-3	-17	31.08	1.77
-8	3	17	28.62	3.08
-8	3	17	33.58	1.93
-8	3	17	31.05	1.98
-8	3	17	29.59	2.16
7	-3	-17	0.14	0.49
7	-3	-17	-0.03	1.17
-7	3	17	-0.28	0.90
-7	3	17	2.15	0.76

-7	3	17	1.03	1.43
-7	3	17	0.48	0.65
6	-3	-17	28.55	1.51
6	-3	-17	29.78	2.53
-6	3	17	28.46	1.70
-6	3	17	31.49	1.65
-6	3	17	25.63	2.15
-6	3	17	22.29	2.72
5	-3	-17	-0.21	0.44
5	-3	-17	-0.71	1.22
-5	3	17	0.06	0.61
-5	3	17	0.41	0.92
-5	3	17	0.17	0.54
-5	3	17	-0.59	1.23
4	-3	-17	19.67	1.27
-4	3	17	21.52	2.43
-4	3	17	20.98	1.26
-4	3	17	22.83	2.06
-4	3	17	21.84	1.30
3	-3	-17	2.31	0.55
-3	3	17	4.50	0.74
-3	3	17	3.07	0.59
-3	3	17	3.13	1.05
-3	3	17	3.27	1.20
2	-3	-17	61.00	2.60
-2	3	17	60.72	3.56
-2	3	17	48.83	2.51
-2	3	17	57.35	3.29
-2	3	17	63.90	2.42
1	-3	-17	3.30	0.57
-1	3	17	1.21	1.22
-1	3	17	5.70	1.34
0	-3	-17	57.50	2.52
0	3	17	52.62	3.30
-1	-3	-17	-0.34	0.72
1	3	17	0.85	1.09
1	3	17	0.87	1.00
-2	-3	-17	23.33	1.42
2	3	17	23.72	2.15
2	3	17	18.70	2.25
-3	-3	-17	0.96	0.53
3	3	17	-0.93	1.13
3	3	17	0.90	0.94
-4	-3	-17	19.00	1.36

4	3	17	15.75	2.25
4	3	17	19.47	1.96
-5	-3	-17	-0.74	0.56
5	3	17	0.33	1.01
5	3	17	-1.57	1.21
-6	-3	-17	48.95	6.50
-6	-3	-17	57.46	2.74
6	3	17	60.02	3.14
6	3	17	53.85	3.57
-7	-3	-17	1.65	0.73
7	3	17	0.67	1.39
7	3	17	1.06	1.01
-8	-3	-17	30.94	1.95
8	3	17	32.29	2.24
8	3	17	24.76	2.77
-9	-3	-17	1.49	0.76
9	3	17	-0.42	1.48
-10	-3	-17	25.76	1.99
10	3	17	27.68	2.97
-11	-3	-17	0.90	0.85
11	3	17	0.33	1.63
-12	-3	-17	40.49	2.54
12	3	17	33.91	3.56
16	-3	-16	1.79	0.71
-16	3	16	2.37	2.24
-16	3	16	2.04	1.42
15	-3	-16	2.34	0.72
-15	3	16	2.09	1.28
-15	3	16	3.28	2.14
14	-3	-16	2.77	0.67
-14	3	16	5.76	1.25
-14	3	16	5.04	2.21
13	-3	-16	-0.60	0.53
-13	3	16	1.53	1.81
-13	3	16	-1.76	0.95
12	-3	-16	2.28	0.60
-12	3	16	5.72	1.91
-12	3	16	3.72	1.07
11	-3	-16	-0.29	0.46
-11	3	16	-0.71	1.51
10	-3	-16	16.36	1.11
-10	3	16	14.24	2.73
-10	3	16	16.97	1.59
9	-3	-16	8.25	0.99

9	-3	-16	11.40	1.66
-9	3	16	7.70	1.40
-9	3	16	9.01	1.31
-9	3	16	10.70	2.05
-9	3	16	9.31	1.47
8	-3	-16	5.07	0.76
8	-3	-16	6.18	1.16
-8	3	16	5.03	0.98
-8	3	16	7.01	0.95
-8	3	16	3.57	1.49
-8	3	16	5.73	0.86
7	-3	-16	5.83	0.76
7	-3	-16	7.37	1.25
-7	3	16	6.29	0.84
-7	3	16	7.36	1.17
-7	3	16	8.65	1.73
-7	3	16	6.13	1.06
6	-3	-16	1.11	1.06
6	-3	-16	1.24	0.44
-6	3	16	1.92	0.59
-6	3	16	1.21	1.37
-6	3	16	2.06	0.88
-6	3	16	2.45	0.68
5	-3	-16	-0.48	0.39
5	-3	-16	1.51	1.11
-5	3	16	-0.29	0.48
-5	3	16	-0.02	0.79
-5	3	16	-1.17	1.07
-5	3	16	0.72	0.56
4	-3	-16	1.27	0.46
4	-3	-16	-0.69	1.12
-4	3	16	2.10	0.56
-4	3	16	2.23	0.92
-4	3	16	2.71	1.16
-4	3	16	1.91	0.48
3	-3	-16	24.15	2.38
3	-3	-16	26.61	1.40
-3	3	16	28.36	1.38
-3	3	16	27.14	1.32
-3	3	16	20.60	2.01
-3	3	16	28.00	3.60
2	-3	-16	3.06	0.54
-2	3	16	3.85	0.66
-2	3	16	2.98	1.14

-2	3	16	2.83	1.03
-2	3	16	4.03	0.50
1	-3	-16	29.81	1.56
-1	3	16	28.07	2.25
-1	3	16	32.00	2.39
0	-3	-16	10.54	0.94
0	3	16	12.94	1.73
0	3	16	11.88	1.68
-1	-3	-16	1.79	0.52
1	3	16	0.48	1.04
1	3	16	1.04	0.91
-2	-3	-16	0.47	0.43
2	3	16	-0.77	0.94
2	3	16	1.62	0.91
-3	-3	-16	30.83	4.12
-3	-3	-16	37.41	1.90
3	3	16	32.85	2.55
3	3	16	37.17	2.39
-4	-3	-16	5.89	0.93
-4	-3	-16	7.30	1.92
4	3	16	5.65	1.09
4	3	16	4.53	1.24
-5	-3	-16	26.21	3.52
-5	-3	-16	25.57	1.57
5	3	16	23.99	2.38
5	3	16	26.86	2.01
-6	-3	-16	-0.69	0.55
-6	-3	-16	2.59	1.93
6	3	16	2.08	0.92
6	3	16	-0.83	1.18
-7	-3	-16	16.62	1.35
-7	-3	-16	13.31	2.62
7	3	16	14.96	2.22
7	3	16	15.15	1.64
-8	-3	-16	-0.16	0.63
8	3	16	-0.15	1.21
8	3	16	-0.78	0.89
-9	-3	-16	42.17	2.35
9	3	16	41.90	3.27
9	3	16	44.84	2.51
-10	-3	-16	3.00	0.84
10	3	16	0.92	1.39
-11	-3	-16	38.02	2.33
11	3	16	40.65	3.26

-12	-3	-16	-1.72	0.82
12	3	16	1.83	1.61
-13	-3	-16	22.14	2.02
13	3	16	23.96	3.16
16	-3	-15	16.50	1.40
-16	3	15	16.86	2.74
-16	3	15	16.90	2.36
15	-3	-15	2.79	0.65
-15	3	15	1.96	2.10
-15	3	15	3.02	1.21
14	-3	-15	16.80	1.29
-14	3	15	21.57	2.16
-14	3	15	19.50	3.24
13	-3	-15	9.63	1.02
-13	3	15	11.83	2.22
-13	3	15	9.05	1.35
12	-3	-15	11.38	0.99
-12	3	15	16.54	2.89
-12	3	15	10.89	1.68
11	-3	-15	5.22	1.03
11	-3	-15	5.42	0.80
-11	3	15	7.67	1.07
-11	3	15	7.97	1.90
10	-3	-15	19.01	1.16
10	-3	-15	17.66	1.83
10	-3	-15	19.79	1.54
-10	3	15	14.17	2.62
-10	3	15	20.18	1.63
9	-3	-15	14.01	1.50
9	-3	-15	9.78	0.88
-9	3	15	13.05	1.30
-9	3	15	9.57	1.35
-9	3	15	9.13	1.40
-9	3	15	10.46	1.77
8	-3	-15	6.20	0.73
8	-3	-15	6.46	1.09
-8	3	15	7.18	1.19
-8	3	15	6.69	1.08
-8	3	15	6.37	0.97
-8	3	15	4.84	1.47
7	-3	-15	-0.84	0.88
7	-3	-15	-0.05	0.35
-7	3	15	-0.29	0.57
-7	3	15	0.06	0.75

-7	3	15	-0.40	0.50
-7	3	15	-0.58	1.10
6	-3	-15	10.30	0.82
6	-3	-15	8.95	1.65
-6	3	15	10.00	1.05
-6	3	15	9.48	1.39
-6	3	15	9.10	1.56
-6	3	15	8.26	1.03
-6	3	15	9.22	0.96
5	-3	-15	7.35	1.20
5	-3	-15	6.80	0.73
-5	3	15	6.36	1.03
-5	3	15	6.31	0.87
-5	3	15	6.32	0.84
-5	3	15	6.17	0.89
4	-3	-15	16.25	1.86
4	-3	-15	15.27	1.01
-4	3	15	14.89	1.93
-4	3	15	16.79	1.02
-4	3	15	16.04	1.05
-4	3	15	14.97	1.12
-4	3	15	16.43	1.61
3	-3	-15	18.90	1.10
3	-3	-15	18.80	2.03
-3	3	15	19.48	1.04
-3	3	15	20.71	1.09
-3	3	15	18.31	1.78
-3	3	15	15.64	1.19
-3	3	15	20.16	1.96
2	-3	-15	1.29	1.11
2	-3	-15	0.21	0.34
-2	3	15	0.08	0.89
-2	3	15	0.91	0.82
-2	3	15	0.72	0.37
-2	3	15	0.10	0.35
-2	3	15	-0.16	0.44
1	-3	-15	3.53	1.34
1	-3	-15	3.80	0.53
-1	3	15	3.97	1.07
-1	3	15	2.97	1.00
-1	3	15	2.58	0.54
0	-3	-15	0.92	0.43
0	3	15	0.38	0.80
0	3	15	1.00	0.90

0	3	15	0.47	0.51
-1	-3	-15	4.52	0.60
-1	-3	-15	4.97	1.61
1	3	15	4.08	0.96
1	3	15	5.81	1.12
-2	-3	-15	11.12	0.95
-2	-3	-15	9.88	1.89
2	3	15	11.19	1.66
2	3	15	9.00	1.47
-3	-3	-15	25.80	1.43
-3	-3	-15	26.82	3.00
3	3	15	24.76	1.89
3	3	15	21.38	2.45
-4	-3	-15	9.93	1.97
-4	-3	-15	7.47	0.88
4	3	15	7.50	1.09
4	3	15	7.96	1.37
-5	-3	-15	9.19	1.89
5	3	15	6.98	1.32
5	3	15	7.75	1.35
-6	-3	-15	16.20	1.20
-6	-3	-15	12.86	2.14
6	3	15	17.07	2.03
6	3	15	13.94	1.52
-7	-3	-15	5.08	0.76
-7	-3	-15	8.40	1.94
7	3	15	7.14	1.30
7	3	15	6.10	1.32
-8	-3	-15	5.91	0.84
-8	-3	-15	7.02	1.96
8	3	15	5.56	0.98
8	3	15	5.47	1.42
-9	-3	-15	1.90	0.69
9	3	15	3.25	1.43
9	3	15	3.42	0.89
-10	-3	-15	2.53	0.78
10	3	15	2.83	1.37
10	3	15	4.12	0.93
-11	-3	-15	5.42	0.94
11	3	15	8.50	1.73
-12	-3	-15	5.68	1.03
12	3	15	6.47	1.71
-13	-3	-15	1.70	0.93
13	3	15	2.65	1.62

-14	-3	-15	1.08	1.01
14	3	15	-0.43	1.67
17	-3	-14	24.67	1.70
-17	3	14	30.18	2.69
-17	3	14	25.86	3.81
16	-3	-14	0.79	0.63
-16	3	14	1.79	1.17
-16	3	14	-0.92	1.91
15	-3	-14	42.71	2.20
-15	3	14	49.28	2.92
-15	3	14	46.90	4.03
14	-3	-14	9.95	1.02
-14	3	14	10.20	2.10
-14	3	14	7.95	1.31
13	-3	-14	64.48	2.81
-13	3	14	61.02	4.38
-13	3	14	68.10	3.37
12	-3	-14	0.62	0.44
-12	3	14	0.17	1.46
-12	3	14	1.51	0.86
11	-3	-14	57.02	2.52
11	-3	-14	55.52	2.50
-11	3	14	53.94	3.86
-11	3	14	61.19	2.92
10	-3	-14	4.52	0.70
10	-3	-14	3.59	0.91
10	-3	-14	4.64	0.92
-10	3	14	2.87	1.52
-10	3	14	2.95	1.04
-10	3	14	3.08	0.85
9	-3	-14	22.44	1.61
9	-3	-14	21.02	1.20
-9	3	14	23.97	1.53
-9	3	14	20.86	1.56
-9	3	14	19.34	2.45
-9	3	14	20.79	1.51
8	-3	-14	0.92	0.77
8	-3	-14	0.86	0.38
-8	3	14	1.22	0.70
-8	3	14	-0.30	1.08
-8	3	14	1.09	0.64
-8	3	14	1.08	0.58
-8	3	14	0.47	0.61
7	-3	-14	144.41	5.74

7	-3	-14	140.47	5.40
-7	3	14	141.32	5.54
-7	3	14	133.51	5.47
-7	3	14	132.91	5.66
-7	3	14	151.67	5.55
-7	3	14	111.23	7.35
6	-3	-14	1.96	0.93
6	-3	-14	3.30	0.47
-6	3	14	3.64	0.61
-6	3	14	2.23	0.79
-6	3	14	4.57	0.64
-6	3	14	5.50	0.80
-6	3	14	2.58	1.07
5	-3	-14	66.20	2.76
5	-3	-14	69.36	3.33
-5	3	14	61.46	3.12
-5	3	14	67.43	2.79
-5	3	14	69.05	2.83
-5	3	14	66.67	2.83
-5	3	14	60.59	3.61
4	-3	-14	4.29	1.04
4	-3	-14	2.74	0.43
-4	3	14	2.33	0.48
-4	3	14	3.85	0.50
-4	3	14	2.99	0.80
-4	3	14	2.86	1.02
-4	3	14	2.53	0.48
3	-3	-14	72.24	3.02
3	-3	-14	74.59	3.66
-3	3	14	73.68	2.95
-3	3	14	76.43	2.96
-3	3	14	67.79	3.44
-3	3	14	72.69	3.08
-3	3	14	71.76	3.79
2	-3	-14	14.78	0.98
2	-3	-14	12.05	1.80
2	-3	-14	15.65	2.43
-2	3	14	16.34	1.62
-2	3	14	16.61	1.08
-2	3	14	16.37	0.92
-2	3	14	15.78	0.88
-2	3	14	17.84	1.76
1	-3	-14	65.70	3.91
1	-3	-14	66.53	3.51

1	-3	-14	65.45	2.78
-1	3	14	63.74	3.38
-1	3	14	64.77	2.82
-1	3	14	70.01	2.59
-1	3	14	65.49	2.58
-1	3	14	63.19	3.29
0	-3	-14	3.04	0.49
0	-3	-14	2.67	1.35
0	-3	-14	2.51	1.10
0	3	14	2.34	0.87
0	3	14	2.41	0.49
0	3	14	3.52	0.91
-1	-3	-14	41.16	1.95
-1	-3	-14	43.20	1.96
-1	-3	-14	46.10	3.33
-1	-3	-14	43.01	3.15
1	3	14	42.34	2.01
1	3	14	42.30	2.45
1	3	14	37.99	2.48
-2	-3	-14	6.55	0.74
-2	-3	-14	4.55	0.73
-2	-3	-14	7.92	1.58
2	3	14	5.42	1.02
2	3	14	7.57	1.40
-3	-3	-14	14.18	2.35
-3	-3	-14	10.26	0.90
3	3	14	8.58	1.26
3	3	14	9.60	1.47
-4	-3	-14	6.31	0.79
-4	-3	-14	6.58	1.55
4	3	14	5.11	0.93
-5	-3	-14	48.35	2.25
-5	-3	-14	46.53	3.29
5	3	14	47.37	2.49
-6	-3	-14	4.01	1.57
-6	-3	-14	5.47	0.84
6	3	14	5.82	0.95
6	3	14	4.80	1.14
-7	-3	-14	26.65	2.84
-7	-3	-14	27.62	1.64
7	3	14	31.11	1.85
7	3	14	27.84	2.34
-8	-3	-14	6.39	1.67
-8	-3	-14	3.90	0.68

8	3	14	6.22	1.17
8	3	14	8.69	1.49
-9	-3	-14	65.60	4.14
-9	-3	-14	56.58	2.74
-9	-3	-14	53.47	2.56
9	3	14	58.32	2.72
9	3	14	53.92	3.39
-10	-3	-14	16.27	1.14
-10	-3	-14	14.96	1.45
10	3	14	15.20	2.24
10	3	14	19.13	1.46
-11	-3	-14	12.14	1.33
11	3	14	10.86	1.75
-12	-3	-14	12.25	1.50
12	3	14	11.32	1.89
-13	-3	-14	2.30	0.92
13	3	14	5.21	1.64
-14	-3	-14	3.21	1.02
14	3	14	4.65	1.72
-15	-3	-14	-1.19	0.97
15	3	14	-1.05	1.57
18	-3	-13	45.18	2.41
-18	3	13	49.84	3.31
-18	3	13	49.82	4.42
17	-3	-13	-1.38	0.55
-17	3	13	0.84	1.21
-17	3	13	-0.37	1.96
16	-3	-13	45.86	2.23
-16	3	13	46.80	2.99
-16	3	13	42.57	3.97
15	-3	-13	-0.50	0.49
-15	3	13	0.48	1.68
-15	3	13	0.82	0.97
14	-3	-13	29.94	1.62
-14	3	13	32.54	2.29
-14	3	13	26.81	3.26
13	-3	-13	0.35	0.42
-13	3	13	-0.02	0.87
-13	3	13	2.10	1.59
-12	3	13	69.17	4.38
-12	3	13	77.53	3.99
11	-3	-13	-0.36	0.58
11	-3	-13	-0.38	0.50
11	-3	-13	-0.49	0.34

-11	3	13	-0.57	1.19
-11	3	13	1.42	0.70
10	-3	-13	140.43	5.67
10	-3	-13	144.16	5.64
10	-3	-13	145.90	5.85
-10	3	13	143.88	5.88
-10	3	13	153.41	5.94
-10	3	13	135.90	6.64
9	-3	-13	5.64	1.04
9	-3	-13	5.27	0.67
-9	3	13	3.87	0.64
-9	3	13	6.60	0.85
-9	3	13	4.51	0.75
8	-3	-13	64.85	2.85
8	-3	-13	57.79	2.56
-8	3	13	59.56	2.67
-8	3	13	58.03	3.54
-8	3	13	66.02	2.81
-8	3	13	65.31	2.80
-8	3	13	56.54	2.77
7	-3	-13	1.91	0.39
7	-3	-13	2.70	0.79
-7	3	13	2.72	0.58
-7	3	13	2.98	1.14
-7	3	13	2.60	0.58
-7	3	13	1.78	0.48
-7	3	13	2.08	0.70
6	-3	-13	174.87	6.77
6	-3	-13	190.19	7.13
-6	3	13	171.08	6.82
-6	3	13	162.86	7.00
-6	3	13	161.98	7.56
-6	3	13	183.08	6.87
-6	3	13	183.68	6.84
5	-3	-13	14.58	0.91
5	-3	-13	18.13	1.56
-5	3	13	13.35	0.98
-5	3	13	14.03	1.80
-5	3	13	14.72	0.99
-5	3	13	12.94	1.29
-5	3	13	14.42	0.97
4	-3	-13	147.86	5.88
4	-3	-13	127.89	6.37
4	-3	-13	138.61	5.42

-4	3	13	131.73	5.73
-4	3	13	140.52	5.38
-4	3	13	133.22	6.12
-4	3	13	135.62	5.46
-4	3	13	152.64	5.43
3	-3	-13	-0.30	0.28
3	-3	-13	1.83	1.22
3	-3	-13	0.51	0.78
-3	3	13	-0.58	0.31
-3	3	13	-0.49	0.71
-3	3	13	-0.17	0.34
-3	3	13	-0.55	0.62
-3	3	13	0.05	0.31
2	-3	-13	80.92	4.36
2	-3	-13	86.72	3.92
2	-3	-13	79.66	3.34
-2	3	13	80.69	3.39
-2	3	13	86.18	3.24
-2	3	13	83.38	3.98
-2	3	13	78.30	3.75
1	-3	-13	2.48	0.89
1	-3	-13	1.82	0.41
1	-3	-13	2.61	1.25
-1	3	13	2.41	0.48
-1	3	13	0.76	0.70
-1	3	13	1.83	0.82
-1	3	13	2.20	0.29
-1	3	13	2.69	0.32
0	-3	-13	83.71	3.94
0	-3	-13	78.34	4.21
0	-3	-13	85.42	3.32
0	3	13	82.22	3.34
0	3	13	78.39	3.76
0	3	13	73.57	3.77
-1	-3	-13	0.23	1.11
-1	-3	-13	0.41	0.35
-1	-3	-13	0.42	0.90
1	3	13	0.80	0.72
1	3	13	1.08	0.41
1	3	13	1.66	0.72
-2	-3	-13	183.12	7.72
-2	-3	-13	190.69	7.08
-2	-3	-13	176.21	7.84
-2	-3	-13	183.12	7.10

2	3	13	180.34	7.59
2	3	13	181.06	7.45
-3	-3	-13	12.46	1.69
-3	-3	-13	13.43	0.96
-3	-3	-13	13.12	0.95
3	3	13	14.96	1.36
3	3	13	12.27	1.53
-4	-3	-13	71.32	3.00
-4	-3	-13	65.63	3.77
-4	-3	-13	71.45	2.93
4	3	13	63.99	3.52
4	3	13	72.67	3.27
-5	-3	-13	6.56	1.47
-5	-3	-13	4.97	0.77
5	3	13	4.52	1.03
5	3	13	5.58	0.85
-6	-3	-13	61.17	2.76
-6	-3	-13	66.32	3.62
6	3	13	58.81	3.30
6	3	13	63.58	2.94
-7	-3	-13	0.68	0.45
-7	-3	-13	-0.46	1.24
7	3	13	0.21	0.59
7	3	13	-0.99	1.08
-8	-3	-13	104.40	4.62
-8	-3	-13	109.10	5.23
-8	-3	-13	114.10	4.68
8	3	13	115.34	4.68
8	3	13	121.63	4.34
8	3	13	106.44	5.22
-9	-3	-13	2.07	0.50
-9	-3	-13	2.10	0.66
-9	-3	-13	3.71	1.31
9	3	13	1.79	0.64
9	3	13	2.16	0.45
9	3	13	1.79	1.11
-10	-3	-13	60.04	2.58
-10	-3	-13	57.45	2.82
10	3	13	53.45	3.48
10	3	13	59.73	2.70
10	3	13	63.35	2.54
-11	-3	-13	5.46	0.78
-11	-3	-13	6.08	0.93
11	3	13	9.44	1.55

11	3	13	6.69	1.06
-12	-3	-13	13.30	1.36
-12	-3	-13	13.35	1.42
12	3	13	13.13	2.21
-13	-3	-13	8.85	1.36
13	3	13	7.62	1.69
-14	-3	-13	46.21	2.66
14	3	13	48.05	3.50
-15	-3	-13	1.41	0.98
15	3	13	0.22	1.51
-16	-3	-13	24.50	3.33
16	3	13	23.69	3.15
18	-3	-12	1.40	0.67
-18	3	12	-1.92	1.15
-18	3	12	0.21	2.00
17	-3	-12	23.28	1.53
-17	3	12	23.08	2.37
-17	3	12	19.35	2.67
16	-3	-12	-0.23	0.53
-16	3	12	-3.21	1.62
-16	3	12	-0.70	0.96
15	-3	-12	22.12	1.40
-15	3	12	22.96	3.14
-15	3	12	27.08	2.19
14	-3	-12	0.49	0.46
-14	3	12	1.33	1.59
-14	3	12	-0.55	0.87
13	-3	-12	62.17	2.92
-13	3	12	75.96	4.31
-13	3	12	75.95	3.47
12	-3	-12	0.90	0.38
-12	3	12	-0.88	1.23
-12	3	12	-1.35	0.68
11	-3	-12	53.17	2.38
-11	3	12	55.31	4.83
-11	3	12	58.71	2.81
10	-3	-12	0.09	0.48
10	-3	-12	-0.31	0.77
10	-3	-12	-0.01	0.30
-10	3	12	0.01	0.55
-10	3	12	-0.08	1.00
-10	3	12	-0.25	0.58
-10	3	12	-0.22	0.61
9	-3	-12	19.69	1.29

9	-3	-12	19.10	1.50
9	-3	-12	20.46	1.12
-9	3	12	21.59	1.28
-9	3	12	22.58	1.43
-9	3	12	20.01	1.43
-9	3	12	22.84	2.19
8	-3	-12	11.79	1.14
8	-3	-12	11.89	0.81
-8	3	12	12.33	1.80
-8	3	12	11.17	1.07
-8	3	12	11.79	0.91
-8	3	12	12.55	1.11
-8	3	12	13.09	1.12
7	-3	-12	137.24	5.64
7	-3	-12	136.20	5.43
-7	3	12	146.92	5.60
-7	3	12	150.12	5.57
-7	3	12	136.64	5.49
-7	3	12	137.92	6.27
-7	3	12	134.18	5.61
6	-3	-12	5.35	0.85
6	-3	-12	7.28	1.50
6	-3	-12	5.69	0.59
-6	3	12	5.61	0.97
-6	3	12	6.99	0.78
-6	3	12	6.44	0.78
-6	3	12	6.02	1.16
-6	3	12	4.86	0.68
5	-3	-12	74.68	3.07
5	-3	-12	81.98	3.46
5	-3	-12	76.89	4.09
-5	3	12	78.78	3.15
-5	3	12	78.19	3.10
-5	3	12	72.74	3.34
-5	3	12	69.24	3.76
-5	3	12	73.08	3.11
4	-3	-12	1.05	1.07
4	-3	-12	2.21	0.73
4	-3	-12	1.99	0.35
-4	3	12	1.83	0.39
-4	3	12	1.67	0.65
-4	3	12	1.88	0.40
-4	3	12	0.92	0.82
-4	3	12	1.49	0.40

3	-3	-12	75.97	3.89
3	-3	-12	70.86	3.34
3	-3	-12	70.04	2.91
-3	3	12	69.58	3.53
-3	3	12	69.58	2.94
-3	3	12	67.06	3.24
-3	3	12	74.32	2.88
-3	3	12	72.04	2.84
2	-3	-12	10.14	0.73
2	-3	-12	9.76	1.32
2	-3	-12	10.66	1.78
-2	3	12	10.19	1.19
-2	3	12	10.99	0.65
-2	3	12	8.52	1.32
-2	3	12	10.32	0.77
-2	3	12	10.65	0.95
1	-3	-12	168.80	6.81
1	-3	-12	157.55	6.32
1	-3	-12	173.54	7.16
-1	3	12	165.96	6.17
-1	3	12	162.42	6.83
-1	3	12	161.69	6.36
-1	3	12	167.79	6.18
-1	3	12	153.29	6.69
0	-3	-12	10.95	1.44
0	-3	-12	13.71	1.82
0	-3	-12	11.59	0.80
0	3	12	12.12	1.32
0	3	12	11.99	0.85
0	3	12	12.36	1.28
-1	-3	-12	107.22	5.13
-1	-3	-12	112.12	4.97
-1	-3	-12	108.80	4.41
1	3	12	113.60	4.44
1	3	12	116.26	4.83
1	3	12	106.50	4.82
-2	-3	-12	0.14	0.32
-2	-3	-12	0.22	0.96
-2	-3	-12	-0.71	0.76
-2	-3	-12	0.41	0.33
2	3	12	-0.85	0.52
2	3	12	-0.23	0.58
2	3	12	0.11	0.35
-3	-3	-12	55.58	3.15

-3	-3	-12	56.05	2.43
-3	-3	-12	56.65	3.09
-3	-3	-12	57.70	2.40
3	3	12	53.22	2.91
3	3	12	55.75	2.71
-4	-3	-12	4.29	0.66
-4	-3	-12	5.59	1.15
-4	-3	-12	3.27	1.11
-4	-3	-12	3.19	0.45
4	3	12	4.19	0.72
4	3	12	2.56	0.86
-5	-3	-12	48.68	2.98
-5	-3	-12	51.20	2.26
-5	-3	-12	51.41	2.34
5	3	12	57.89	2.56
5	3	12	49.28	2.82
-6	-3	-12	7.38	1.31
6	3	12	6.36	1.13
6	3	12	5.67	0.80
-7	-3	-12	78.40	3.41
-7	-3	-12	79.52	3.13
-7	-3	-12	74.50	3.93
7	3	12	83.54	3.49
7	3	12	76.75	3.94
7	3	12	84.22	3.11
-8	-3	-12	0.99	0.45
-8	-3	-12	-0.25	0.45
-8	-3	-12	-1.26	1.03
8	3	12	0.81	0.35
8	3	12	-0.53	0.84
8	3	12	0.21	0.56
-9	-3	-12	91.15	3.59
-9	-3	-12	86.52	3.80
-9	-3	-12	87.44	4.20
9	3	12	91.78	3.73
9	3	12	86.09	4.34
-10	-3	-12	0.93	1.22
-10	-3	-12	0.01	0.54
-10	-3	-12	0.22	0.53
10	3	12	1.51	0.58
10	3	12	-0.37	1.00
10	3	12	-0.19	0.46
-11	-3	-12	78.60	3.59
-11	-3	-12	85.60	3.80

11	3	12	86.29	4.39
11	3	12	87.37	3.56
11	3	12	89.82	3.56
-12	-3	-12	3.11	0.76
-12	-3	-12	3.96	0.79
12	3	12	3.28	1.29
-13	-3	-12	37.80	2.24
-13	-3	-12	41.61	2.32
13	3	12	38.81	2.92
-14	-3	-12	1.77	0.97
-14	-3	-12	3.65	0.86
14	3	12	-0.29	1.34
-15	-3	-12	52.08	2.86
15	3	12	47.08	3.42
-16	-3	-12	2.31	1.03
16	3	12	3.46	1.62
19	-3	-11	0.06	0.67
-19	3	11	1.33	1.35
-19	3	11	0.52	2.18
18	-3	-11	26.38	1.68
-18	3	11	21.67	3.48
-18	3	11	30.25	2.59
17	-3	-11	3.50	0.68
-17	3	11	-0.03	1.86
-17	3	11	3.35	1.19
16	-3	-11	8.57	1.01
-16	3	11	5.30	1.81
-16	3	11	8.86	1.34
15	-3	-11	0.77	0.49
-15	3	11	2.36	1.55
-15	3	11	-0.81	0.89
14	-3	-11	3.50	0.54
-14	3	11	4.19	1.61
-14	3	11	4.48	0.99
13	-3	-11	0.20	0.51
-13	3	11	0.28	1.26
-13	3	11	0.19	0.73
12	-3	-11	23.56	1.27
-12	3	11	22.22	2.45
-12	3	11	26.06	1.76
11	-3	-11	0.50	0.34
-11	3	11	-0.82	0.99
-11	3	11	0.31	0.64
10	-3	-11	9.82	1.20

10	-3	-11	10.29	0.95
10	-3	-11	10.87	0.79
10	-3	-11	12.05	0.84
-10	3	11	11.16	1.84
-10	3	11	11.88	1.18
-10	3	11	10.38	0.97
-10	3	11	11.06	1.15
9	-3	-11	3.26	0.64
9	-3	-11	2.78	0.41
9	-3	-11	3.19	1.11
9	-3	-11	2.82	0.55
-9	3	11	2.53	0.61
-9	3	11	4.36	0.70
-9	3	11	4.53	1.13
-9	3	11	3.14	0.48
8	-3	-11	5.83	0.86
8	-3	-11	4.99	0.57
8	-3	-11	5.77	1.34
-8	3	11	6.60	0.88
-8	3	11	5.57	0.84
-8	3	11	4.05	0.65
-8	3	11	5.45	0.86
-8	3	11	6.15	1.16
7	-3	-11	9.41	1.62
7	-3	-11	7.46	0.62
7	-3	-11	7.81	0.95
-7	3	11	9.20	0.85
-7	3	11	8.00	0.89
-7	3	11	8.09	0.74
-7	3	11	8.25	1.46
-7	3	11	8.59	0.84
6	-3	-11	106.16	5.08
6	-3	-11	106.15	4.17
6	-3	-11	108.06	4.40
-6	3	11	99.85	4.35
-6	3	11	105.27	4.22
-6	3	11	102.95	4.86
-6	3	11	108.45	4.28
-6	3	11	111.95	4.26
5	-3	-11	5.70	0.57
5	-3	-11	6.93	1.23
5	-3	-11	6.46	0.98
-5	3	11	6.60	0.91
-5	3	11	6.80	1.29

-5	3	11	6.74	0.63
-5	3	11	6.88	0.69
-5	3	11	7.75	0.65
4	-3	-11	68.40	3.64
4	-3	-11	69.03	2.81
4	-3	-11	72.56	3.16
-4	3	11	63.32	3.06
-4	3	11	74.21	2.85
-4	3	11	66.43	2.84
-4	3	11	71.02	3.46
3	-3	-11	1.56	0.62
3	-3	-11	0.75	0.29
3	-3	-11	0.11	0.88
-3	3	11	0.74	0.30
-3	3	11	0.84	0.56
-3	3	11	0.56	0.64
-3	3	11	0.77	0.34
-3	3	11	0.84	0.26
2	-3	-11	18.91	1.49
2	-3	-11	19.53	1.07
2	-3	-11	21.24	1.96
-2	3	11	22.34	1.46
-2	3	11	21.61	0.98
-2	3	11	23.73	1.03
-2	3	11	22.00	1.63
-2	3	11	22.26	1.13
1	-3	-11	8.60	1.27
1	-3	-11	5.30	0.57
1	-3	-11	6.08	1.06
-1	3	11	5.96	0.43
-1	3	11	5.62	0.59
-1	3	11	4.74	0.89
-1	3	11	5.86	0.43
-1	3	11	5.88	0.92
0	-3	-11	77.12	3.62
0	-3	-11	83.41	3.88
0	-3	-11	76.30	3.15
0	3	11	74.66	3.52
0	3	11	74.08	3.55
0	3	11	81.18	3.18
-1	-3	-11	0.80	0.68
-1	-3	-11	0.73	0.31
-1	-3	-11	1.87	0.89
1	3	11	1.78	0.67

1	3	11	1.93	0.61
1	3	11	1.16	0.35
-2	-3	-11	55.32	2.30
-2	-3	-11	59.45	2.89
-2	-3	-11	52.03	2.33
-2	-3	-11	52.46	2.95
2	3	11	57.68	2.37
2	3	11	51.12	2.72
2	3	11	53.71	2.62
-3	-3	-11	4.46	0.59
-3	-3	-11	6.54	0.60
-3	-3	-11	5.05	1.09
-3	-3	-11	7.29	1.33
3	3	11	5.86	0.98
3	3	11	6.00	0.92
-4	-3	-11	9.85	1.30
-4	-3	-11	8.65	0.69
-4	-3	-11	7.79	1.38
-4	-3	-11	7.69	0.72
4	3	11	6.49	1.02
4	3	11	7.31	0.99
-5	-3	-11	4.59	0.59
-5	-3	-11	3.64	1.04
-5	-3	-11	3.63	0.54
-5	-3	-11	2.60	1.02
5	3	11	3.65	0.89
5	3	11	3.77	0.65
-6	-3	-11	57.93	3.26
-6	-3	-11	53.85	3.09
-6	-3	-11	59.81	2.53
-6	-3	-11	59.22	2.61
6	3	11	63.82	2.34
6	3	11	57.39	3.06
6	3	11	62.14	2.72
-7	-3	-11	3.74	0.60
-7	-3	-11	3.47	1.09
-7	-3	-11	5.45	0.74
7	3	11	5.08	0.51
7	3	11	6.25	1.05
7	3	11	4.54	0.84
-8	-3	-11	149.20	5.90
-8	-3	-11	142.58	5.68
-8	-3	-11	140.43	6.15
8	3	11	152.05	5.85

8	3	11	154.37	5.66
8	3	11	144.48	6.36
-9	-3	-11	5.34	0.85
-9	-3	-11	6.20	0.82
-9	-3	-11	7.49	1.13
9	3	11	6.93	0.74
9	3	11	5.90	1.19
9	3	11	5.57	0.90
-10	-3	-11	57.99	2.80
-10	-3	-11	59.27	2.66
-10	-3	-11	59.09	3.05
10	3	11	64.00	2.61
10	3	11	63.01	2.65
-11	-3	-11	0.26	0.51
-11	-3	-11	-1.31	0.92
-11	-3	-11	-0.14	0.58
11	3	11	0.63	0.94
11	3	11	-0.01	0.52
11	3	11	-0.54	0.61
-12	-3	-11	29.96	1.86
-12	-3	-11	27.44	1.78
12	3	11	28.63	2.42
12	3	11	32.28	1.69
-13	-3	-11	-0.27	0.75
-13	-3	-11	0.20	0.71
13	3	11	-0.15	1.15
13	3	11	0.38	0.80
-14	-3	-11	53.18	2.75
-14	-3	-11	48.77	2.72
14	3	11	50.16	3.33
-15	-3	-11	4.50	1.14
-15	-3	-11	4.18	1.00
15	3	11	2.52	1.49
-16	-3	-11	52.50	2.98
-16	-3	-11	52.23	3.10
16	3	11	56.64	3.71
-17	-3	-11	1.87	0.99
17	3	11	-0.19	1.56
19	-3	-10	-0.58	0.63
-19	3	10	1.30	2.02
-19	3	10	-1.27	1.26
18	-3	-10	0.23	0.58
-18	3	10	-0.09	1.82
-18	3	10	0.43	1.12

17	-3	-10	0.14	0.53
-17	3	10	-3.11	1.58
-17	3	10	-1.30	1.03
16	-3	-10	2.27	0.56
-16	3	10	1.05	0.94
-16	3	10	0.78	1.57
15	-3	-10	1.58	0.48
-15	3	10	2.28	0.89
-15	3	10	0.58	1.36
14	-3	-10	0.85	0.43
-14	3	10	1.60	0.81
13	-3	-10	1.16	0.41
-13	3	10	1.97	1.33
12	-3	-10	7.63	0.74
-12	3	10	10.36	1.96
-12	3	10	8.97	1.25
11	-3	-10	0.10	0.60
11	-3	-10	0.44	0.32
-11	3	10	1.37	0.66
-11	3	10	1.30	1.05
-11	3	10	1.46	0.48
10	-3	-10	11.36	0.80
10	-3	-10	12.91	0.82
10	-3	-10	12.32	2.21
10	-3	-10	12.41	0.75
10	-3	-10	11.32	1.12
-10	3	10	14.47	1.21
-10	3	10	11.91	0.95
-10	3	10	10.57	1.77
9	-3	-10	5.53	0.85
9	-3	-10	5.75	0.70
9	-3	-10	5.36	0.58
9	-3	-10	6.52	1.47
9	-3	-10	5.51	0.88
-9	3	10	7.27	1.30
-9	3	10	6.86	0.68
-9	3	10	5.34	0.85
8	-3	-10	45.40	1.99
8	-3	-10	46.49	2.95
8	-3	-10	42.45	2.09
8	-3	-10	46.15	1.90
8	-3	-10	44.19	2.20
-8	3	10	38.33	2.64
-8	3	10	44.86	2.12

-8	3	10	42.76	1.95
7	-3	-10	18.78	1.16
7	-3	-10	18.05	0.97
7	-3	-10	18.96	1.16
7	-3	-10	21.30	2.08
-7	3	10	19.99	1.15
-7	3	10	17.30	1.16
-7	3	10	16.95	1.69
-7	3	10	18.03	1.01
6	-3	-10	1.44	0.95
6	-3	-10	0.18	0.24
-6	3	10	0.81	0.36
-6	3	10	0.73	0.29
-6	3	10	0.15	0.42
-6	3	10	0.19	0.67
-6	3	10	0.78	0.37
5	-3	-10	18.84	1.85
5	-3	-10	18.66	1.23
5	-3	-10	16.23	0.92
-5	3	10	19.80	1.61
-5	3	10	19.35	1.04
-5	3	10	17.60	0.98
-5	3	10	19.82	1.00
-5	3	10	17.39	1.19
4	-3	-10	2.14	0.81
4	-3	-10	2.17	0.33
4	-3	-10	2.14	0.58
-4	3	10	1.67	0.31
-4	3	10	1.36	0.49
-4	3	10	1.58	0.68
-4	3	10	1.89	0.33
-4	3	10	1.47	0.32
3	-3	-10	13.55	1.53
3	-3	-10	11.30	0.70
3	-3	-10	11.76	1.11
-3	3	10	11.59	0.68
-3	3	10	12.61	1.04
-3	3	10	12.71	0.76
-3	3	10	12.73	1.29
-3	3	10	13.57	0.77
2	-3	-10	5.56	0.99
2	-3	-10	3.20	0.68
2	-3	-10	4.09	0.46
-2	3	10	3.90	0.49

-2	3	10	3.59	0.77
-2	3	10	3.58	0.38
-2	3	10	4.18	0.43
-2	3	10	2.81	0.60
1	-3	-10	46.05	2.65
1	-3	-10	48.46	2.42
1	-3	-10	43.68	2.00
-1	3	10	50.30	2.05
-1	3	10	47.37	2.34
-1	3	10	49.57	1.88
-1	3	10	47.11	2.42
-1	3	10	47.92	1.88
0	-3	-10	9.14	0.65
0	-3	-10	12.53	1.16
0	-3	-10	11.32	1.42
0	3	10	9.11	1.04
0	3	10	8.39	0.98
0	3	10	10.73	0.70
-1	-3	-10	7.70	1.28
-1	-3	-10	8.47	1.12
-1	-3	-10	7.43	0.61
1	3	10	7.98	0.96
1	3	10	7.32	0.62
1	3	10	8.12	0.97
-2	-3	-10	8.90	0.67
-2	-3	-10	10.03	0.67
-2	-3	-10	8.38	1.32
-2	-3	-10	8.98	1.22
2	3	10	9.64	0.99
2	3	10	9.41	0.72
2	3	10	8.74	1.04
-3	-3	-10	55.17	2.74
-3	-3	-10	52.38	2.21
-3	-3	-10	53.27	2.25
-3	-3	-10	53.91	2.73
3	3	10	50.52	2.45
3	3	10	49.38	2.61
-4	-3	-10	2.09	0.79
-4	-3	-10	2.42	0.37
-4	-3	-10	3.26	0.51
-4	-3	-10	2.82	0.78
4	3	10	3.06	0.58
4	3	10	2.62	0.72
-5	-3	-10	28.02	1.32

-5	-3	-10	29.36	1.97
-5	-3	-10	26.17	1.38
-5	-3	-10	28.79	1.86
5	3	10	26.40	1.52
5	3	10	25.16	1.80
-6	-3	-10	0.15	0.31
-6	-3	-10	0.15	0.82
-6	-3	-10	0.36	0.32
-6	-3	-10	1.36	0.76
-6	-3	-10	0.50	0.25
6	3	10	0.35	0.68
6	3	10	-0.43	0.41
6	3	10	0.21	0.22
-7	-3	-10	17.20	1.32
-7	-3	-10	20.12	1.07
-7	-3	-10	16.82	1.09
-7	-3	-10	14.83	1.76
-7	-3	-10	16.58	1.57
7	3	10	20.18	0.93
7	3	10	18.41	1.58
7	3	10	18.74	1.18
-8	-3	-10	3.71	0.55
-8	-3	-10	2.00	0.99
-8	-3	-10	4.35	2.06
-8	-3	-10	4.08	0.49
8	3	10	4.08	0.58
8	3	10	3.53	0.92
8	3	10	4.19	0.76
-9	-3	-10	37.36	2.28
-9	-3	-10	39.11	1.97
-9	-3	-10	41.15	1.86
9	3	10	43.59	1.83
9	3	10	39.77	1.87
9	3	10	38.49	2.44
-10	-3	-10	0.61	0.52
-10	-3	-10	1.65	0.56
-10	-3	-10	1.54	0.85
10	3	10	1.24	0.54
10	3	10	0.82	0.78
10	3	10	1.39	0.48
-11	-3	-10	48.81	2.47
-11	-3	-10	50.10	2.39
-11	-3	-10	50.29	2.45
11	3	10	48.61	3.05

11	3	10	51.80	2.28
-12	-3	-10	-0.14	0.67
-12	-3	-10	-0.71	0.48
12	3	10	-0.53	0.90
12	3	10	0.34	0.52
12	3	10	1.17	0.62
-13	-3	-10	17.88	1.59
-13	-3	-10	14.41	1.43
13	3	10	17.44	1.35
13	3	10	18.23	1.41
13	3	10	17.89	1.95
-14	-3	-10	1.78	0.90
-14	-3	-10	1.10	0.80
14	3	10	1.19	1.15
14	3	10	0.41	0.80
14	3	10	1.29	0.64
-15	-3	-10	39.79	2.34
-15	-3	-10	38.94	2.53
15	3	10	42.63	2.28
15	3	10	37.30	2.95
-16	-3	-10	4.14	0.99
-16	-3	-10	4.07	1.19
16	3	10	2.03	0.79
16	3	10	2.98	1.44
-17	-3	-10	32.43	2.30
-17	-3	-10	32.49	2.63
17	3	10	26.77	2.94
17	3	10	28.20	2.05
-18	-3	-10	-1.86	1.82
18	3	10	-0.51	1.63
20	-3	-9	14.48	1.45
-20	3	9	15.25	2.55
-20	3	9	16.90	2.52
19	-3	-9	1.99	0.66
-19	3	9	1.07	1.83
-19	3	9	3.46	1.31
18	-3	-9	12.34	1.17
-18	3	9	13.96	2.17
-18	3	9	11.69	1.61
17	-3	-9	1.70	0.58
-17	3	9	3.07	1.16
-17	3	9	0.07	1.52
16	-3	-9	5.89	0.85
-16	3	9	4.02	1.71

-16	3	9	3.98	1.09
15	-3	-9	10.09	0.92
-15	3	9	10.92	1.77
-15	3	9	11.42	1.60
14	-3	-9	16.00	1.33
-14	3	9	16.92	2.37
-14	3	9	19.26	1.65
13	-3	-9	23.60	1.27
-13	3	9	22.22	2.42
-13	3	9	25.90	1.77
12	-3	-9	0.37	0.33
12	-3	-9	1.94	1.74
-12	3	9	0.64	0.62
-12	3	9	0.63	1.08
11	-3	-9	2.92	0.62
11	-3	-9	2.57	1.37
11	-3	-9	1.76	0.35
-11	3	9	2.49	0.63
-11	3	9	2.08	0.49
-11	3	9	3.64	1.12
10	-3	-9	14.58	1.10
10	-3	-9	12.01	0.78
10	-3	-9	17.12	2.13
-10	3	9	11.25	1.14
-10	3	9	9.08	1.65
-10	3	9	11.81	0.88
9	-3	-9	6.61	0.63
9	-3	-9	6.30	0.57
9	-3	-9	6.94	0.73
9	-3	-9	7.31	0.86
9	-3	-9	7.29	1.34
-9	3	9	6.14	1.12
-9	3	9	7.12	0.89
-9	3	9	7.42	0.67
8	-3	-9	21.56	2.09
8	-3	-9	20.42	1.11
8	-3	-9	21.50	1.05
8	-3	-9	20.50	1.25
8	-3	-9	22.23	1.29
-8	3	9	21.99	1.29
-8	3	9	20.34	1.12
-8	3	9	20.69	1.78
7	-3	-9	4.47	1.02
7	-3	-9	4.66	0.66

7	-3	-9	3.38	0.44
7	-3	-9	4.84	0.66
-7	3	9	3.79	0.71
-7	3	9	4.06	0.52
-7	3	9	3.79	0.89
-7	3	9	3.92	0.66
6	-3	-9	3.61	1.00
6	-3	-9	2.46	0.42
6	-3	-9	2.72	0.41
6	-3	-9	2.46	0.51
-6	3	9	2.64	0.50
-6	3	9	2.12	0.73
-6	3	9	3.04	0.45
-6	3	9	3.43	0.48
5	-3	-9	1.63	0.28
5	-3	-9	1.77	0.48
5	-3	-9	2.18	0.38
5	-3	-9	1.94	0.77
-5	3	9	1.89	0.46
-5	3	9	1.66	0.65
-5	3	9	2.11	0.32
-5	3	9	2.64	0.41
4	-3	-9	2.30	0.50
4	-3	-9	3.87	0.84
4	-3	-9	1.18	0.25
-4	3	9	1.42	0.28
-4	3	9	1.96	0.30
-4	3	9	1.61	0.60
-4	3	9	2.39	0.36
-4	3	9	1.45	0.43
3	-3	-9	12.69	1.39
3	-3	-9	11.58	0.69
3	-3	-9	12.79	1.01
-3	3	9	12.39	0.97
-3	3	9	12.11	0.72
-3	3	9	11.45	0.71
-3	3	9	11.71	0.65
-3	3	9	11.32	1.13
2	-3	-9	76.01	3.63
2	-3	-9	80.48	3.10
2	-3	-9	81.31	3.40
-2	3	9	80.12	3.03
-2	3	9	78.83	3.11
-2	3	9	77.10	3.51

-2	3	9	73.29	3.33
-2	3	9	75.41	3.00
1	-3	-9	6.24	0.93
1	-3	-9	6.23	0.85
1	-3	-9	5.29	0.47
-1	3	9	5.45	0.85
-1	3	9	4.43	0.34
-1	3	9	4.58	0.77
-1	3	9	5.48	0.49
-1	3	9	5.07	0.36
0	-3	-9	18.84	1.39
0	-3	-9	20.80	1.52
0	-3	-9	20.15	0.97
0	3	9	19.04	1.32
0	3	9	20.18	0.99
0	3	9	17.59	1.26
-1	-3	-9	40.32	1.78
-1	-3	-9	45.31	2.24
-1	-3	-9	42.40	2.23
1	3	9	38.63	2.04
1	3	9	38.80	2.09
1	3	9	43.49	1.80
-2	-3	-9	20.07	1.42
-2	-3	-9	19.68	1.41
-2	-3	-9	16.87	0.94
2	3	9	17.63	0.96
2	3	9	17.43	1.27
2	3	9	16.86	1.18
-3	-3	-9	37.17	2.07
-3	-3	-9	36.85	1.57
-3	-3	-9	36.03	1.98
-3	-3	-9	33.73	1.59
3	3	9	32.87	1.92
3	3	9	35.85	1.79
-4	-3	-9	9.47	1.11
-4	-3	-9	9.74	0.70
-4	-3	-9	9.56	1.23
-4	-3	-9	9.87	0.67
4	3	9	9.13	1.10
4	3	9	9.75	0.87
-5	-3	-9	6.64	0.45
-5	-3	-9	6.27	0.61
-5	-3	-9	6.49	0.58
-5	-3	-9	7.22	1.06

-5	-3	-9	7.55	1.19
5	3	9	6.56	0.80
5	3	9	6.77	0.42
5	3	9	5.87	1.00
-6	-3	-9	0.20	0.30
-6	-3	-9	1.53	0.74
-6	-3	-9	1.17	0.63
-6	-3	-9	0.99	0.31
-6	-3	-9	1.14	0.27
6	3	9	0.36	0.40
6	3	9	1.43	0.67
6	3	9	1.04	0.24
-7	-3	-9	1.24	1.07
-7	-3	-9	4.17	0.96
-7	-3	-9	2.20	0.41
-7	-3	-9	2.42	0.47
-7	-3	-9	2.36	0.38
7	3	9	3.08	0.79
7	3	9	3.04	0.47
7	3	9	2.49	0.47
-8	-3	-9	10.20	0.86
-8	-3	-9	7.32	1.19
-8	-3	-9	10.30	0.79
-8	-3	-9	10.64	0.76
-8	-3	-9	8.94	1.55
8	3	9	11.04	0.75
8	3	9	10.62	0.85
8	3	9	9.69	1.26
-9	-3	-9	1.39	0.47
-9	-3	-9	2.46	0.48
-9	-3	-9	1.08	1.02
-9	-3	-9	1.45	0.70
9	3	9	2.02	0.80
9	3	9	1.44	0.41
9	3	9	1.24	0.48
-10	-3	-9	2.41	0.55
-10	-3	-9	5.37	0.89
-10	-3	-9	3.71	0.76
10	3	9	3.49	0.54
10	3	9	3.68	0.59
10	3	9	3.21	0.52
10	3	9	4.23	1.01
-11	-3	-9	9.38	1.00
-11	-3	-9	9.21	1.22

-11	-3	-9	9.86	1.10
11	3	9	11.68	1.01
11	3	9	10.99	1.49
-12	-3	-9	9.34	1.11
-12	-3	-9	9.84	1.29
12	3	9	9.73	1.38
12	3	9	11.03	1.10
12	3	9	10.06	1.02
-13	-3	-9	11.45	1.51
13	3	9	16.08	1.32
13	3	9	15.30	2.21
13	3	9	15.08	1.28
-14	-3	-9	12.61	1.68
14	3	9	16.43	1.37
14	3	9	15.54	1.48
14	3	9	14.91	1.97
-15	-3	-9	2.71	1.08
15	3	9	5.87	1.36
15	3	9	4.99	0.87
-16	-3	-9	8.46	1.58
16	3	9	12.87	1.44
16	3	9	10.72	1.74
-17	-3	-9	1.04	1.30
17	3	9	-2.62	1.30
17	3	9	-0.82	0.69
-18	-3	-9	5.99	1.55
18	3	9	3.64	1.63
18	3	9	5.43	1.05
20	-3	-8	1.49	0.69
-20	3	8	1.39	1.27
-20	3	8	0.89	2.13
19	-3	-8	30.56	1.76
-19	3	8	33.83	3.28
-19	3	8	23.09	2.49
18	-3	-8	6.66	0.98
-18	3	8	7.07	1.35
-18	3	8	5.03	1.79
17	-3	-8	80.17	3.42
-17	3	8	80.34	4.05
16	-3	-8	2.82	0.56
-16	3	8	3.71	1.06
-16	3	8	0.15	1.39
15	-3	-8	41.80	2.00
-15	3	8	46.01	2.57

-15	3	8	39.73	3.08
14	-3	-8	13.56	1.10
-14	3	8	16.09	1.54
-14	3	8	14.32	2.19
13	-3	-8	27.76	1.39
13	-3	-8	28.50	2.82
-13	3	8	28.46	1.84
-13	3	8	26.04	2.43
12	-3	-8	0.34	0.30
12	-3	-8	-0.77	1.37
-12	3	8	-0.49	0.98
-12	3	8	0.66	0.65
-12	3	8	0.24	0.60
11	-3	-8	86.91	4.44
11	-3	-8	83.93	3.67
11	-3	-8	82.45	3.41
-11	3	8	83.27	3.49
-11	3	8	87.01	4.21
-11	3	8	88.03	3.76
10	-3	-8	4.42	0.51
10	-3	-8	6.88	1.38
10	-3	-8	5.12	0.68
-10	3	8	5.27	0.63
-10	3	8	2.58	0.98
-10	3	8	3.56	0.66
9	-3	-8	84.33	3.29
9	-3	-8	84.65	4.18
9	-3	-8	84.61	3.33
9	-3	-8	81.60	3.25
9	-3	-8	86.55	3.54
-9	3	8	77.83	3.96
-9	3	8	84.06	3.39
-9	3	8	85.29	3.58
8	-3	-8	33.73	1.47
8	-3	-8	32.56	1.63
8	-3	-8	31.81	1.54
8	-3	-8	32.35	2.26
8	-3	-8	31.26	1.43
-8	3	8	33.25	1.51
-8	3	8	31.05	2.09
-8	3	8	33.58	1.67
7	-3	-8	152.66	5.90
7	-3	-8	148.41	5.87
7	-3	-8	142.38	6.38

7	-3	-8	154.03	5.80
7	-3	-8	153.88	5.75
-7	3	8	135.00	6.27
-7	3	8	158.89	5.79
-7	3	8	151.12	5.90
6	-3	-8	3.59	0.60
6	-3	-8	3.56	0.45
6	-3	-8	3.39	0.38
6	-3	-8	4.24	0.94
-6	3	8	4.02	0.47
-6	3	8	3.20	0.46
-6	3	8	2.78	0.72
5	-3	-8	49.94	2.73
5	-3	-8	53.15	2.15
5	-3	-8	53.44	2.30
5	-3	-8	53.45	2.22
-5	3	8	52.12	2.31
-5	3	8	50.58	2.63
-5	3	8	53.37	2.18
-5	3	8	55.26	2.25
4	-3	-8	13.91	0.75
4	-3	-8	13.97	0.79
4	-3	-8	14.82	0.98
4	-3	-8	13.68	1.38
-4	3	8	15.09	1.23
-4	3	8	14.78	0.78
-4	3	8	14.27	0.80
-4	3	8	14.43	0.95
3	-3	-8	47.24	1.97
3	-3	-8	47.98	1.96
3	-3	-8	48.18	2.51
-3	3	8	49.85	2.00
-3	3	8	49.71	2.42
-3	3	8	46.37	2.15
2	-3	-8	6.43	0.77
2	-3	-8	6.95	1.03
2	-3	-8	6.42	0.48
-2	3	8	6.80	0.52
-2	3	8	6.47	0.86
-2	3	8	7.31	0.45
-2	3	8	5.85	0.73
-2	3	8	6.55	0.41
1	-3	-8	73.13	2.86
1	-3	-8	71.95	3.14

1	-3	-8	75.08	3.30
-1	3	8	68.99	3.18
-1	3	8	73.80	2.76
-1	3	8	70.04	3.09
-1	3	8	67.84	2.74
-1	3	8	74.93	2.88
0	-3	-8	0.90	0.23
0	-3	-8	0.61	0.43
0	-3	-8	1.78	0.56
0	3	8	0.93	0.39
0	3	8	1.13	0.25
0	3	8	0.60	0.39
-1	-3	-8	48.73	2.29
-1	-3	-8	48.41	2.31
-1	-3	-8	45.47	1.94
1	3	8	43.02	2.15
1	3	8	46.71	1.95
1	3	8	46.30	2.24
-2	-3	-8	1.95	0.32
-2	-3	-8	2.37	0.61
-2	-3	-8	2.54	0.62
2	3	8	2.23	0.33
2	3	8	2.25	0.49
2	3	8	2.99	0.61
-3	-3	-8	42.14	2.15
-3	-3	-8	39.81	1.70
-3	-3	-8	38.94	1.74
-3	-3	-8	40.38	2.04
3	3	8	41.39	1.92
3	3	8	37.08	2.02
-4	-3	-8	40.06	2.33
-4	-3	-8	46.57	1.89
-4	-3	-8	44.94	2.35
-4	-3	-8	44.05	1.93
4	3	8	44.49	2.27
4	3	8	46.96	2.06
-5	-3	-8	47.26	1.98
-5	-3	-8	43.49	2.45
-5	-3	-8	46.72	2.04
-5	-3	-8	49.32	2.48
-5	-3	-8	44.95	1.86
5	3	8	47.83	1.86
5	3	8	46.26	2.37
5	3	8	48.99	2.11

-6	-3	-8	38.99	1.63
-6	-3	-8	39.28	1.71
-6	-3	-8	40.43	2.22
-6	-3	-8	38.05	1.96
-6	-3	-8	36.85	1.76
6	3	8	38.95	1.61
6	3	8	40.56	1.80
6	3	8	37.74	2.12
-7	-3	-8	15.37	0.94
-7	-3	-8	16.50	0.99
-7	-3	-8	16.64	0.91
-7	-3	-8	18.93	1.53
-7	-3	-8	13.99	1.20
7	3	8	17.13	0.86
7	3	8	16.48	1.02
7	3	8	15.32	1.37
-8	-3	-8	4.18	0.70
-8	-3	-8	3.38	0.54
-8	-3	-8	3.98	0.63
-8	-3	-8	4.06	0.65
-8	-3	-8	6.30	1.10
8	3	8	2.59	0.42
8	3	8	4.78	0.86
8	3	8	4.07	0.72
-9	-3	-8	3.02	0.53
-9	-3	-8	3.25	0.67
-9	-3	-8	2.14	0.95
-9	-3	-8	2.85	0.47
-9	-3	-8	3.92	0.57
9	3	8	2.08	0.77
9	3	8	3.13	0.47
9	3	8	3.01	0.53
9	3	8	4.12	0.63
-10	-3	-8	10.03	1.06
-10	-3	-8	10.58	1.74
-10	-3	-8	10.05	0.99
10	3	8	10.73	0.89
10	3	8	9.95	1.36
10	3	8	8.71	0.83
-11	-3	-8	24.81	1.56
-11	-3	-8	25.83	1.42
-11	-3	-8	24.70	1.47
-11	-3	-8	32.35	3.63
11	3	8	24.38	1.42

11	3	8	27.84	1.47
11	3	8	25.28	1.93
-12	-3	-8	21.86	1.61
12	3	8	24.27	1.94
12	3	8	26.09	1.49
12	3	8	24.58	1.43
-13	-3	-8	20.00	1.65
13	3	8	20.70	1.90
13	3	8	21.30	1.42
13	3	8	23.04	1.49
-14	-3	-8	21.48	1.82
14	3	8	23.06	1.62
14	3	8	21.85	1.48
-15	-3	-8	4.88	1.08
15	3	8	10.73	1.81
15	3	8	7.19	1.09
-16	-3	-8	7.20	1.92
16	3	8	14.18	1.35
16	3	8	12.84	2.09
-17	-3	-8	0.54	1.31
17	3	8	1.47	0.74
17	3	8	0.75	1.30
-18	-3	-8	-0.31	1.38
18	3	8	0.87	1.43
18	3	8	1.73	0.85
-19	-3	-8	0.83	1.60
20	-3	-7	33.39	1.94
-20	3	7	35.78	2.90
19	-3	-7	1.63	0.62
-19	3	7	4.68	3.19
-19	3	7	2.42	1.25
18	-3	-7	71.62	3.23
-18	3	7	69.67	4.27
-18	3	7	79.54	3.94
17	-3	-7	0.64	0.50
-17	3	7	0.57	1.35
-17	3	7	0.25	0.96
16	-3	-7	98.71	4.17
-16	3	7	100.64	5.09
-16	3	7	108.33	4.76
15	-3	-7	-1.34	1.52
15	-3	-7	0.17	0.40
-15	3	7	-1.76	1.11
-15	3	7	0.77	0.79

14	-3	-7	73.55	3.11
14	-3	-7	71.26	4.34
-14	3	7	75.98	3.97
-14	3	7	78.27	3.60
13	-3	-7	3.03	1.42
13	-3	-7	5.62	0.63
-13	3	7	4.04	0.79
-13	3	7	3.93	1.22
12	-3	-7	83.37	3.49
12	-3	-7	86.05	4.49
-12	3	7	91.84	3.89
-12	3	7	90.14	3.60
-12	3	7	82.51	4.23
11	-3	-7	5.24	1.26
11	-3	-7	2.62	0.59
11	-3	-7	3.89	0.52
-11	3	7	2.28	0.63
-11	3	7	3.08	0.48
-11	3	7	3.36	1.03
10	-3	-7	259.76	11.02
10	-3	-7	267.45	10.33
10	-3	-7	282.94	10.56
-10	3	7	281.86	10.62
-10	3	7	287.05	10.41
-10	3	7	261.16	10.93
9	-3	-7	1.75	0.28
9	-3	-7	1.41	0.45
9	-3	-7	1.99	0.32
9	-3	-7	1.81	0.90
-9	3	7	1.83	0.37
-9	3	7	0.51	0.72
-9	3	7	1.57	0.47
8	-3	-7	148.23	5.58
8	-3	-7	149.41	5.62
8	-3	-7	152.10	5.79
8	-3	-7	151.86	6.27
8	-3	-7	142.23	5.60
-8	3	7	134.85	6.12
-8	3	7	148.32	5.82
-8	3	7	142.42	5.65
7	-3	-7	10.22	0.80
7	-3	-7	10.24	0.68
7	-3	-7	9.81	1.35
7	-3	-7	10.65	0.79

7	-3	-7	9.28	0.60
-7	3	7	9.93	0.64
-7	3	7	8.00	0.77
-7	3	7	8.61	1.10
6	-3	-7	194.54	7.42
6	-3	-7	201.82	7.83
6	-3	-7	190.46	7.25
6	-3	-7	191.80	7.32
6	-3	-7	184.27	7.35
-6	3	7	196.20	7.38
-6	3	7	188.74	7.74
-6	3	7	184.77	7.28
5	-3	-7	-0.69	0.57
5	-3	-7	0.25	0.31
5	-3	-7	0.17	0.17
5	-3	-7	-0.07	0.23
-5	3	7	0.52	0.38
-5	3	7	-0.15	0.24
-5	3	7	0.29	0.47
-5	3	7	0.16	0.19
4	-3	-7	243.38	9.31
4	-3	-7	254.48	9.64
4	-3	-7	240.99	9.20
4	-3	-7	244.96	9.17
-4	3	7	243.10	9.18
-4	3	7	237.28	9.29
-4	3	7	238.34	9.57
-4	3	7	246.42	9.21
3	-3	-7	0.66	0.20
3	-3	-7	0.43	0.35
3	-3	-7	0.59	0.19
3	-3	-7	0.81	0.50
-3	3	7	0.61	0.35
-3	3	7	0.78	0.26
-3	3	7	0.86	0.21
-3	3	7	-0.31	0.40
2	-3	-7	148.90	5.93
2	-3	-7	136.92	5.53
2	-3	-7	146.45	5.49
2	-3	-7	152.52	5.77
-2	3	7	147.67	5.55
-2	3	7	133.52	5.83
-2	3	7	149.13	5.50
-2	3	7	146.42	5.73

1	-3	-7	8.22	1.20
1	-3	-7	9.04	0.54
1	-3	-7	9.34	0.93
-1	3	7	7.62	0.82
-1	3	7	9.22	0.77
-1	3	7	9.07	0.55
-1	3	7	9.31	0.45
-1	3	7	9.11	0.43
0	-3	-7	149.27	5.90
0	-3	-7	146.63	5.84
0	-3	-7	149.01	5.58
0	3	7	140.40	5.81
0	3	7	144.06	5.81
-1	-3	-7	0.23	0.39
-1	-3	-7	0.29	0.39
-1	-3	-7	0.01	0.15
1	3	7	-0.15	0.30
1	3	7	0.53	0.36
1	3	7	0.23	0.17
-2	-3	-7	96.96	3.89
-2	-3	-7	85.38	3.55
2	3	7	87.94	3.71
2	3	7	87.69	3.82
2	3	7	94.94	3.57
-3	-3	-7	15.41	0.78
-3	-3	-7	13.96	0.80
-3	-3	-7	16.71	1.09
-3	-3	-7	15.05	1.17
3	3	7	15.45	0.95
3	3	7	13.82	1.08
-4	-3	-7	159.00	6.34
-4	-3	-7	164.14	6.30
-4	-3	-7	174.52	6.72
-4	-3	-7	158.59	6.18
-4	-3	-7	170.95	6.54
4	3	7	167.05	6.43
4	3	7	169.17	6.18
4	3	7	158.26	6.62
-5	-3	-7	18.02	0.96
-5	-3	-7	19.02	0.92
-5	-3	-7	20.75	1.43
-5	-3	-7	19.47	1.19
-5	-3	-7	16.14	0.83
5	3	7	16.28	1.25

5	3	7	18.37	1.05
5	3	7	18.93	0.83
-6	-3	-7	137.35	5.53
-6	-3	-7	142.50	5.64
-6	-3	-7	122.32	5.40
-6	-3	-7	155.05	5.96
-6	-3	-7	147.56	5.47
6	3	7	138.40	5.83
6	3	7	146.00	5.40
6	3	7	145.24	5.54
-7	-3	-7	5.00	0.62
-7	-3	-7	5.17	0.78
-7	-3	-7	4.85	0.51
-7	-3	-7	4.36	0.57
-7	-3	-7	6.64	1.14
7	3	7	4.36	0.51
7	3	7	3.57	0.73
7	3	7	5.78	0.68
7	3	7	4.29	0.51
-8	-3	-7	170.78	6.86
-8	-3	-7	170.45	6.37
-8	-3	-7	157.69	6.36
-8	-3	-7	159.89	6.45
-8	-3	-7	160.33	6.46
8	3	7	160.18	6.76
8	3	7	160.55	6.39
8	3	7	173.55	6.37
8	3	7	171.54	6.38
-9	-3	-7	3.30	0.45
-9	-3	-7	2.98	0.59
-9	-3	-7	3.27	0.55
-9	-3	-7	2.09	0.89
9	3	7	3.28	0.46
9	3	7	2.90	0.46
9	3	7	3.44	0.80
-10	-3	-7	79.59	3.24
-10	-3	-7	76.79	3.29
-10	-3	-7	84.53	3.88
-10	-3	-7	78.65	3.29
-10	-3	-7	72.23	3.32
10	3	7	77.57	3.68
10	3	7	84.95	3.29
10	3	7	77.95	3.28
-11	-3	-7	5.87	0.79

-11	-3	-7	5.63	1.15
-11	-3	-7	5.42	0.86
-11	-3	-7	6.02	0.91
11	3	7	5.49	0.99
11	3	7	7.68	0.90
11	3	7	6.96	0.77
-12	-3	-7	46.38	2.97
-12	-3	-7	45.51	2.43
12	3	7	49.93	2.73
12	3	7	52.19	2.33
12	3	7	53.08	2.33
-13	-3	-7	21.21	1.68
13	3	7	22.85	1.47
13	3	7	28.28	1.59
13	3	7	26.48	1.98
-14	-3	-7	27.43	2.07
14	3	7	34.44	1.89
14	3	7	32.01	2.26
14	3	7	33.14	1.80
-15	-3	-7	2.39	0.95
15	3	7	1.84	0.97
15	3	7	1.04	0.61
16	3	7	17.54	1.49
16	3	7	15.79	2.00
-17	-3	-7	3.14	1.27
17	3	7	1.82	0.80
17	3	7	4.00	1.33
-18	-3	-7	22.59	2.66
18	3	7	25.42	2.70
18	3	7	27.39	1.92
-19	-3	-7	3.64	1.53
19	3	7	2.55	0.90
19	3	7	4.49	1.62
20	-3	-6	1.74	0.68
-20	3	6	-1.87	1.28
19	-3	-6	41.21	2.14
-19	3	6	43.62	2.90
-19	3	6	37.09	3.17
18	-3	-6	-0.95	0.50
-18	3	6	0.37	1.37
-18	3	6	0.36	1.01
17	-3	-6	68.40	3.05
17	-3	-6	73.18	4.62
-17	3	6	72.67	3.69

-17	3	6	68.17	3.99
16	-3	-6	1.70	0.50
16	-3	-6	3.47	1.78
-16	3	6	2.69	1.28
-16	3	6	2.62	0.94
15	-3	-6	41.30	3.16
15	-3	-6	37.80	1.82
-15	3	6	35.52	2.70
-15	3	6	37.31	2.32
14	-3	-6	1.17	1.40
14	-3	-6	0.81	0.38
-14	3	6	-0.19	1.02
13	-3	-6	79.44	4.21
13	-3	-6	77.99	3.19
-13	3	6	79.05	3.58
-13	3	6	74.02	3.89
12	-3	-6	1.06	1.16
12	-3	-6	0.78	0.64
12	-3	-6	0.17	0.28
-12	3	6	1.01	0.39
-12	3	6	1.07	0.59
-12	3	6	0.01	0.88
11	-3	-6	119.86	4.97
11	-3	-6	131.29	5.76
11	-3	-6	128.99	5.23
-11	3	6	134.67	5.06
-11	3	6	121.98	5.57
-11	3	6	132.03	5.30
10	-3	-6	-0.29	0.21
10	-3	-6	0.13	0.85
10	-3	-6	0.06	0.42
-10	3	6	1.03	0.73
-10	3	6	-0.64	0.38
-10	3	6	-0.24	0.27
9	-3	-6	116.29	4.47
9	-3	-6	111.59	4.56
9	-3	-6	119.11	5.21
9	-3	-6	122.42	4.78
-9	3	6	112.83	5.08
-9	3	6	121.50	4.83
-9	3	6	122.21	4.63
8	-3	-6	15.02	0.96
8	-3	-6	14.07	1.45
8	-3	-6	14.18	0.78

8	-3	-6	13.43	0.75
8	-3	-6	15.30	0.75
-8	3	6	13.65	0.80
-8	3	6	12.92	0.98
-8	3	6	12.83	1.28
7	-3	-6	96.96	4.39
7	-3	-6	102.67	3.89
7	-3	-6	95.81	3.87
7	-3	-6	101.05	4.02
7	-3	-6	99.87	3.93
-7	3	6	100.11	4.05
-7	3	6	95.40	4.32
-7	3	6	106.27	3.92
6	-3	-6	90.56	4.00
6	-3	-6	90.70	3.64
6	-3	-6	90.76	3.52
6	-3	-6	92.86	3.65
6	-3	-6	93.77	3.58
-6	3	6	88.48	3.95
-6	3	6	89.23	3.65
-6	3	6	88.28	3.54
5	-3	-6	144.06	5.56
5	-3	-6	147.01	5.74
5	-3	-6	145.94	5.64
5	-3	-6	151.74	6.02
-5	3	6	152.84	5.59
-5	3	6	136.66	5.94
-5	3	6	144.91	5.65
4	-3	-6	16.87	1.24
4	-3	-6	17.39	0.89
4	-3	-6	16.85	0.81
4	-3	-6	18.38	0.96
-4	3	6	18.62	0.91
-4	3	6	16.90	0.83
-4	3	6	16.24	0.94
-4	3	6	17.70	1.19
3	-3	-6	208.73	8.20
3	-3	-6	237.92	8.36
3	-3	-6	220.00	8.19
3	-3	-6	230.09	8.57
-3	3	6	218.79	8.20
-3	3	6	222.63	8.20
-3	3	6	217.65	8.52
-3	3	6	185.69	8.40

2	-3	-6	0.01	0.29
2	-3	-6	-0.05	0.15
2	-3	-6	0.01	0.36
2	-3	-6	0.04	0.13
-2	3	6	-0.14	0.32
-2	3	6	-0.11	0.25
-2	3	6	0.20	0.27
-2	3	6	0.20	0.15
1	-3	-6	142.51	5.60
1	-3	-6	145.80	5.52
1	-3	-6	152.77	5.81
-1	3	6	154.42	5.52
-1	3	6	144.18	5.78
-1	3	6	144.36	5.85
0	-3	-6	14.51	0.73
0	-3	-6	14.92	1.02
0	-3	-6	15.89	0.99
0	3	6	16.31	0.96
0	3	6	13.88	0.92
-1	-3	-6	173.95	6.71
-1	-3	-6	177.96	6.66
-1	-3	-6	161.46	6.41
1	3	6	165.88	6.58
1	3	6	166.70	6.64
-2	-3	-6	7.03	0.74
-2	-3	-6	5.89	0.70
-2	-3	-6	5.12	0.42
2	3	6	5.51	0.68
2	3	6	5.32	0.61
2	3	6	5.26	0.43
-3	-3	-6	143.25	5.50
-3	-3	-6	135.14	5.17
-3	-3	-6	133.12	5.20
-3	-3	-6	138.12	5.38
3	3	6	132.96	5.30
3	3	6	129.33	5.44
-4	-3	-6	3.21	0.49
-4	-3	-6	3.21	0.60
-4	-3	-6	2.40	0.33
-4	-3	-6	2.41	0.27
-4	-3	-6	2.55	0.33
4	3	6	2.47	0.44
4	3	6	3.15	0.27
4	3	6	2.84	0.55

-5	-3	-6	378.45	13.46
-5	-3	-6	381.78	13.84
-5	-3	-6	343.26	13.50
-5	-3	-6	361.46	13.60
-5	-3	-6	332.78	13.38
5	3	6	372.74	13.53
5	3	6	365.21	13.38
5	3	6	337.43	13.75
-6	-3	-6	7.15	1.03
-6	-3	-6	8.20	0.75
-6	-3	-6	7.19	0.59
-6	-3	-6	8.14	0.55
6	3	6	7.63	0.70
6	3	6	7.54	0.56
6	3	6	8.12	0.90
6	3	6	8.16	0.53
-7	-3	-6	140.66	5.79
-7	-3	-6	159.21	5.81
-7	-3	-6	152.79	5.90
-7	-3	-6	157.03	6.24
7	3	6	146.34	6.14
7	3	6	155.31	5.78
7	3	6	144.14	5.82
-8	-3	-6	18.68	1.48
-8	-3	-6	16.56	1.02
-8	-3	-6	17.31	0.93
-8	-3	-6	16.15	1.05
8	3	6	18.42	0.97
8	3	6	18.10	0.97
8	3	6	17.51	1.37
-9	-3	-6	118.46	4.54
-9	-3	-6	126.72	5.07
-9	-3	-6	113.91	4.58
-9	-3	-6	109.62	4.60
9	3	6	110.65	4.90
9	3	6	107.44	4.58
9	3	6	123.69	4.58
9	3	6	114.34	4.58
-10	-3	-6	4.84	0.96
-10	-3	-6	4.49	0.67
-10	-3	-6	4.77	0.81
-10	-3	-6	4.89	0.60
-10	-3	-6	3.94	0.57
10	3	6	5.09	0.63

10	3	6	4.42	0.84
10	3	6	4.43	0.71
-11	-3	-6	134.38	5.52
-11	-3	-6	134.95	5.69
-11	-3	-6	139.28	5.59
-11	-3	-6	148.29	6.13
11	3	6	143.54	5.62
11	3	6	149.54	5.64
11	3	6	144.31	5.97
-12	-3	-6	0.74	0.93
-12	-3	-6	0.34	0.58
12	3	6	0.94	0.74
12	3	6	0.20	0.51
12	3	6	0.80	0.40
-13	-3	-6	67.08	3.55
-13	-3	-6	62.16	3.11
13	3	6	65.07	3.11
13	3	6	71.04	3.01
13	3	6	69.37	3.34
-14	-3	-6	0.26	0.82
14	3	6	1.02	0.88
14	3	6	1.97	0.57
14	3	6	1.26	0.65
-15	-3	-6	41.23	2.56
15	3	6	45.24	2.23
15	3	6	44.82	2.68
16	3	6	0.52	0.61
16	3	6	-1.54	0.98
-17	-3	-6	105.76	4.79
17	3	6	97.37	4.37
-18	-3	-6	-0.53	1.31
18	3	6	-2.06	1.20
18	3	6	0.97	0.74
-19	-3	-6	47.62	3.39
19	3	6	48.63	2.67
19	3	6	50.71	3.42
20	-3	-5	0.00	0.61
-20	3	5	1.76	1.32
19	-3	-5	3.35	0.74
-19	3	5	3.18	1.28
-19	3	5	3.25	1.53
18	-3	-5	16.15	1.29
-18	3	5	19.24	2.38
-18	3	5	23.42	2.14

17	-3	-5	5.25	1.87
17	-3	-5	6.02	0.86
-17	3	5	6.49	1.43
-17	3	5	4.59	1.16
16	-3	-5	60.34	3.82
16	-3	-5	54.73	2.56
-16	3	5	58.16	3.15
-16	3	5	61.73	3.43
15	-3	-5	0.41	0.39
15	-3	-5	-1.67	1.38
-15	3	5	1.14	0.75
-15	3	5	0.32	1.02
14	-3	-5	2.81	0.44
14	-3	-5	1.82	1.36
-14	3	5	3.37	1.11
-14	3	5	3.66	0.99
13	-3	-5	0.89	1.27
13	-3	-5	2.53	0.42
-13	3	5	2.72	1.00
-13	3	5	2.77	0.75
12	-3	-5	4.55	1.19
12	-3	-5	6.05	0.93
12	-3	-5	4.14	0.57
-12	3	5	4.47	0.57
-12	3	5	3.54	0.93
-12	3	5	5.14	0.79
11	-3	-5	1.80	0.97
11	-3	-5	0.57	0.44
11	-3	-5	0.90	0.29
-11	3	5	1.91	0.79
-11	3	5	1.12	0.35
-11	3	5	0.38	0.51
10	-3	-5	87.62	3.87
10	-3	-5	75.74	3.17
10	-3	-5	78.79	3.38
-10	3	5	80.72	3.46
-10	3	5	75.74	3.69
-10	3	5	80.97	3.23
9	-3	-5	0.60	0.24
9	-3	-5	0.31	0.22
9	-3	-5	-0.93	0.71
9	-3	-5	0.45	0.20
9	-3	-5	0.38	0.37
-9	3	5	0.37	0.39

-9	3	5	0.10	0.25
-9	3	5	0.84	0.62
8	-3	-5	16.56	0.83
8	-3	-5	15.26	1.41
8	-3	-5	18.38	1.05
8	-3	-5	15.21	0.81
8	-3	-5	16.59	0.82
-8	3	5	15.53	1.05
-8	3	5	19.09	0.89
-8	3	5	14.79	1.28
7	-3	-5	38.56	2.03
7	-3	-5	37.34	1.52
7	-3	-5	37.12	1.66
7	-3	-5	34.37	1.53
7	-3	-5	31.93	1.49
-7	3	5	32.84	1.66
-7	3	5	34.16	1.91
-7	3	5	39.41	1.55
6	-3	-5	18.69	0.91
6	-3	-5	20.10	0.97
6	-3	-5	21.12	1.06
6	-3	-5	19.66	1.03
6	-3	-5	20.46	1.39
-6	3	5	18.17	1.29
-6	3	5	20.41	1.07
-6	3	5	21.81	0.96
5	-3	-5	35.70	1.57
5	-3	-5	32.66	1.41
5	-3	-5	35.56	1.50
5	-3	-5	32.25	1.60
5	-3	-5	34.95	1.81
-5	3	5	33.69	1.78
-5	3	5	34.09	1.52
4	-3	-5	128.56	5.08
4	-3	-5	125.38	4.92
4	-3	-5	123.11	4.79
4	-3	-5	127.58	4.74
4	-3	-5	123.25	4.83
-4	3	5	125.85	4.80
-4	3	5	119.86	4.76
-4	3	5	121.00	5.04
3	-3	-5	1.31	0.21
3	-3	-5	1.25	0.19
3	-3	-5	1.38	0.30

3	-3	-5	1.11	0.46
-3	3	5	1.42	0.23
-3	3	5	1.18	0.29
-3	3	5	0.99	0.35
-3	3	5	1.46	0.21
2	-3	-5	79.88	3.16
2	-3	-5	78.40	3.02
2	-3	-5	68.89	2.84
-2	3	5	69.56	3.09
-2	3	5	73.40	2.84
-2	3	5	72.39	3.02
1	-3	-5	24.57	1.12
1	-3	-5	29.01	1.33
1	-3	-5	28.19	1.40
1	-3	-5	24.94	1.07
-1	3	5	26.95	1.36
-1	3	5	25.20	1.32
-1	3	5	28.28	1.07
-1	3	5	27.16	1.14
0	-3	-5	195.83	7.30
0	-3	-5	196.18	7.52
0	-3	-5	197.96	7.50
0	3	5	189.35	7.48
0	3	5	191.35	7.48
0	3	5	192.38	7.30
-1	-3	-5	0.82	0.17
-1	-3	-5	0.89	0.32
-1	-3	-5	0.77	0.35
1	3	5	0.58	0.29
1	3	5	0.79	0.30
1	3	5	0.91	0.19
-2	-3	-5	90.27	3.50
-2	-3	-5	99.70	3.68
-2	-3	-5	84.23	3.86
2	3	5	90.54	3.62
2	3	5	85.34	3.69
-3	-3	-5	1.59	0.19
-3	-3	-5	1.59	0.17
-3	-3	-5	1.75	0.26
-3	-3	-5	1.43	0.22
-3	-3	-5	1.93	0.37
-3	-3	-5	1.62	0.44
3	3	5	1.65	0.38
3	3	5	1.70	0.40

-4	-3	-5	15.20	0.80
-4	-3	-5	17.29	0.93
-4	-3	-5	14.81	0.76
-4	-3	-5	15.90	1.06
-4	-3	-5	15.28	0.72
4	3	5	16.15	0.71
4	3	5	15.61	1.05
-5	-3	-5	1.39	0.23
-5	-3	-5	2.30	0.33
-5	-3	-5	2.08	0.55
-5	-3	-5	1.41	0.24
-5	-3	-5	2.03	0.38
5	3	5	1.49	0.44
5	3	5	1.46	0.24
5	3	5	1.53	0.23
5	3	5	1.40	0.36
-6	-3	-5	111.22	4.36
-6	-3	-5	105.86	4.37
-6	-3	-5	123.29	4.75
-6	-3	-5	118.40	4.47
6	3	5	115.51	4.35
6	3	5	108.06	4.65
6	3	5	114.73	4.39
6	3	5	106.50	4.42
-7	-3	-5	14.33	1.23
-7	-3	-5	14.05	0.89
-7	-3	-5	13.18	0.76
-7	-3	-5	13.76	0.83
7	3	5	12.78	1.08
7	3	5	14.27	0.79
7	3	5	14.45	0.86
7	3	5	15.02	0.80
-8	-3	-5	105.41	4.58
-8	-3	-5	123.15	4.54
-8	-3	-5	120.76	4.60
-8	-3	-5	121.38	4.95
8	3	5	120.15	4.56
8	3	5	113.50	4.56
8	3	5	112.84	4.84
8	3	5	113.61	4.56
-9	-3	-5	7.09	0.57
-9	-3	-5	7.09	1.09
-9	-3	-5	5.74	0.76
-9	-3	-5	6.13	0.79

-9	-3	-5	7.34	0.71
9	3	5	6.55	0.98
9	3	5	7.36	0.69
9	3	5	5.83	0.59
-10	-3	-5	101.35	4.23
-10	-3	-5	107.57	4.60
-10	-3	-5	106.79	4.13
-10	-3	-5	100.40	4.26
-10	-3	-5	103.28	4.15
10	3	5	101.44	4.47
10	3	5	111.83	4.22
10	3	5	102.04	4.17
-11	-3	-5	2.04	0.38
-11	-3	-5	2.73	0.89
-11	-3	-5	2.78	0.65
-11	-3	-5	3.69	0.54
-11	-3	-5	2.48	0.59
11	3	5	3.91	0.56
11	3	5	2.75	0.74
11	3	5	3.09	0.47
-12	-3	-5	103.11	4.56
-12	-3	-5	97.33	4.10
-12	-3	-5	94.80	3.98
-12	-3	-5	94.34	4.21
12	3	5	100.22	4.08
12	3	5	105.06	4.14
12	3	5	103.33	4.41
-13	-3	-5	-0.62	0.91
-13	-3	-5	-1.09	0.63
-13	-3	-5	0.43	0.63
13	3	5	0.26	0.69
13	3	5	-0.06	0.41
13	3	5	0.22	0.55
-14	-3	-5	75.77	3.97
-14	-3	-5	79.96	3.70
14	3	5	82.78	3.81
14	3	5	81.36	3.56
14	3	5	82.04	3.46
-15	-3	-5	-0.61	1.16
-15	-3	-5	-1.67	0.74
15	3	5	0.10	0.52
15	3	5	-0.11	0.85
-16	-3	-5	47.10	2.85
16	3	5	42.73	2.64

16	3	5	44.18	2.29
-17	-3	-5	5.43	1.28
17	3	5	3.34	1.19
17	3	5	3.49	0.79
-18	-3	-5	57.16	3.53
18	3	5	62.90	3.47
18	3	5	61.30	2.95
-19	-3	-5	3.01	1.42
19	3	5	3.21	1.46
19	3	5	1.03	0.77
20	3	5	49.58	2.80
21	-3	-4	0.31	0.74
20	-3	-4	1.80	0.71
-20	3	4	2.45	1.42
19	-3	-4	0.17	0.58
-19	3	4	1.49	1.24
18	-3	-4	3.40	0.65
-18	3	4	4.75	1.35
-18	3	4	4.92	1.23
17	-3	-4	4.72	1.57
17	-3	-4	1.58	0.54
-17	3	4	0.01	0.88
-17	3	4	0.19	1.14
16	-3	-4	2.82	0.52
16	-3	-4	2.84	1.50
-16	3	4	2.41	1.15
-16	3	4	2.60	0.98
15	-3	-4	1.39	1.32
15	-3	-4	0.15	0.35
-15	3	4	-0.65	0.65
-15	3	4	-0.22	0.95
14	-3	-4	0.59	0.35
14	-3	-4	0.90	1.24
-14	3	4	0.69	0.63
-14	3	4	0.87	0.92
13	-3	-4	13.82	1.96
13	-3	-4	11.69	0.83
-13	3	4	13.22	1.28
-13	3	4	12.93	1.55
12	-3	-4	6.97	0.91
12	-3	-4	6.03	0.51
12	-3	-4	6.44	0.62
12	-3	-4	9.16	1.65
-12	3	4	6.88	1.02

-12	3	4	6.15	0.73
-12	3	4	5.46	0.98
11	-3	-4	2.27	0.51
11	-3	-4	1.52	0.28
11	-3	-4	2.38	0.93
11	-3	-4	1.16	0.31
-11	3	4	1.52	0.45
-11	3	4	0.52	0.49
-11	3	4	0.90	0.72
-11	3	4	1.37	0.33
10	-3	-4	5.77	0.51
10	-3	-4	7.45	0.78
10	-3	-4	6.32	0.50
10	-3	-4	6.70	1.02
-10	3	4	6.67	0.70
-10	3	4	6.27	1.09
-10	3	4	6.56	0.58
-10	3	4	6.13	0.83
9	-3	-4	14.17	0.79
9	-3	-4	15.50	1.01
9	-3	-4	14.61	0.77
9	-3	-4	17.82	1.44
-9	3	4	15.03	0.93
-9	3	4	14.95	1.06
-9	3	4	16.45	0.86
-9	3	4	16.18	1.29
8	-3	-4	40.75	1.66
8	-3	-4	39.81	2.13
8	-3	-4	39.43	1.67
8	-3	-4	40.58	1.85
-8	3	4	38.86	1.87
-8	3	4	40.35	1.71
-8	3	4	40.70	2.09
7	-3	-4	1.88	0.31
7	-3	-4	2.34	0.39
7	-3	-4	2.12	0.32
7	-3	-4	2.93	0.62
7	-3	-4	1.96	0.37
-7	3	4	2.17	0.57
-7	3	4	2.18	0.39
-7	3	4	2.13	0.34
6	-3	-4	17.42	0.94
6	-3	-4	19.14	0.93
6	-3	-4	19.03	1.28

6	-3	-4	17.62	0.86
6	-3	-4	18.95	1.00
-6	3	4	19.02	1.02
-6	3	4	19.87	1.23
-6	3	4	20.97	0.90
5	-3	-4	70.03	2.86
5	-3	-4	73.95	2.88
5	-3	-4	66.65	2.76
5	-3	-4	73.90	2.83
5	-3	-4	66.93	3.09
-5	3	4	76.17	2.88
-5	3	4	73.86	2.79
-5	3	4	66.58	3.04
4	-3	-4	5.45	0.53
4	-3	-4	5.46	0.66
4	-3	-4	4.90	0.34
4	-3	-4	5.66	0.45
4	-3	-4	5.57	0.47
-4	3	4	5.39	0.63
-4	3	4	5.94	0.47
-4	3	4	4.85	0.36
3	-3	-4	70.41	2.72
3	-3	-4	74.32	2.96
3	-3	-4	68.60	2.88
3	-3	-4	68.35	2.79
3	-3	-4	65.16	2.68
-3	3	4	69.08	2.94
-3	3	4	70.02	2.72
2	-3	-4	38.70	1.61
2	-3	-4	42.83	1.75
2	-3	-4	42.38	1.84
2	-3	-4	40.92	1.61
-2	3	4	38.01	1.60
-2	3	4	39.83	1.82
-2	3	4	38.85	1.72
1	-3	-4	4.20	0.30
1	-3	-4	3.92	0.26
1	-3	-4	3.97	0.51
1	-3	-4	4.07	0.45
-1	3	4	4.10	0.29
-1	3	4	3.84	0.50
-1	3	4	3.77	0.26
-1	3	4	3.44	0.44
0	-3	-4	39.26	1.45

0	-3	-4	37.58	1.70
0	-3	-4	36.22	1.54
0	-3	-4	38.75	1.72
0	3	4	38.71	1.54
-1	-3	-4	1.84	0.36
-1	-3	-4	2.59	0.40
-1	-3	-4	1.66	0.24
1	3	4	1.45	0.29
1	3	4	1.74	0.34
1	3	4	1.04	0.17
-2	-3	-4	71.25	2.92
-2	-3	-4	69.09	2.72
-2	-3	-4	69.46	2.83
-2	-3	-4	69.38	2.61
-2	-3	-4	67.76	2.61
2	3	4	72.57	2.73
2	3	4	68.09	2.90
2	3	4	68.24	2.80
-3	-3	-4	138.51	5.25
-3	-3	-4	137.17	5.13
-3	-3	-4	133.88	5.16
-3	-3	-4	129.02	5.08
-3	-3	-4	140.08	5.37
-3	-3	-4	133.66	5.06
3	3	4	133.80	5.35
3	3	4	135.13	5.22
-4	-3	-4	7.69	0.50
-4	-3	-4	7.71	0.42
-4	-3	-4	7.40	0.46
-4	-3	-4	8.47	0.60
-4	-3	-4	7.58	0.45
4	3	4	8.13	0.73
4	3	4	8.44	0.44
4	3	4	7.89	0.56
-5	-3	-4	32.81	1.50
-5	-3	-4	35.54	1.50
-5	-3	-4	37.51	1.80
-5	-3	-4	37.75	1.60
5	3	4	38.49	1.50
5	3	4	36.02	1.77
5	3	4	37.20	1.58
5	3	4	32.76	1.50
-6	-3	-4	12.03	0.75
-6	-3	-4	12.49	0.65

-6	-3	-4	11.27	0.73
-6	-3	-4	13.07	0.97
6	3	4	11.93	0.67
6	3	4	12.53	0.74
6	3	4	11.26	0.89
6	3	4	11.85	0.66
-7	-3	-4	11.18	0.75
-7	-3	-4	10.81	0.74
-7	-3	-4	11.65	0.66
-7	-3	-4	13.34	1.06
7	3	4	11.95	0.67
7	3	4	10.85	0.94
7	3	4	12.00	0.72
7	3	4	11.70	0.75
-8	-3	-4	1.39	0.36
-8	-3	-4	1.06	0.26
-8	-3	-4	1.20	0.54
-8	-3	-4	1.65	0.37
-8	-3	-4	1.22	0.42
8	3	4	1.48	0.32
8	3	4	0.73	0.26
8	3	4	1.01	0.48
-9	-3	-4	35.48	1.81
-9	-3	-4	38.98	1.71
-9	-3	-4	39.63	2.04
-9	-3	-4	37.68	1.63
-9	-3	-4	35.38	1.78
9	3	4	36.71	1.97
9	3	4	36.18	1.66
9	3	4	39.61	1.73
-10	-3	-4	0.45	0.61
-10	-3	-4	-0.03	0.36
-10	-3	-4	0.52	0.26
-10	-3	-4	0.00	0.41
-10	-3	-4	0.38	0.57
10	3	4	0.36	0.37
10	3	4	-0.32	0.46
10	3	4	0.17	0.26
-11	-3	-4	70.25	2.97
-11	-3	-4	69.17	3.67
-11	-3	-4	70.87	3.17
-11	-3	-4	72.48	3.40
-11	-3	-4	71.52	2.99
11	3	4	77.49	3.11

11	3	4	71.14	3.29
11	3	4	75.70	3.03
-12	-3	-4	7.15	1.07
-12	-3	-4	7.08	0.81
-12	-3	-4	8.90	1.41
12	3	4	7.82	0.94
12	3	4	6.17	0.71
12	3	4	9.21	1.18
-13	-3	-4	18.43	2.24
-13	-3	-4	17.73	1.82
-13	-3	-4	18.02	1.18
-13	-3	-4	22.96	1.59
13	3	4	21.16	1.40
13	3	4	20.35	1.20
13	3	4	18.73	1.55
-14	-3	-4	5.66	1.12
-14	-3	-4	4.30	0.90
-14	-3	-4	4.21	0.88
-14	-3	-4	4.18	0.62
14	3	4	5.53	0.95
14	3	4	4.79	0.71
14	3	4	5.49	0.96
-15	-3	-4	25.88	2.18
-15	-3	-4	22.16	1.85
15	3	4	24.75	1.46
15	3	4	23.07	1.80
-16	-3	-4	4.34	1.05
-16	-3	-4	7.13	1.47
16	3	4	4.33	1.07
16	3	4	5.62	0.82
-17	-3	-4	37.45	2.63
17	3	4	39.80	2.61
17	3	4	42.69	2.15
-18	-3	-4	3.55	1.25
18	3	4	2.61	0.77
18	3	4	0.86	1.16
-19	-3	-4	17.49	2.55
19	3	4	18.34	1.63
19	3	4	20.58	2.33
-20	-3	-4	3.73	1.55
20	3	4	3.01	0.88
21	-3	-3	6.35	0.91
-21	3	3	6.47	2.82
20	-3	-3	15.35	1.37

-20	3	3	14.62	2.45
19	-3	-3	2.50	0.65
-19	3	3	4.35	1.32
18	-3	-3	4.85	0.69
-18	3	3	5.25	1.31
-18	3	3	4.40	1.30
17	-3	-3	-1.73	1.39
17	-3	-3	1.25	0.50
-17	3	3	0.26	1.06
-17	3	3	-0.50	0.95
16	-3	-3	9.75	0.92
16	-3	-3	9.51	1.56
16	-3	-3	11.96	0.83
-16	3	3	9.16	1.30
-16	3	3	9.89	1.54
-16	3	3	9.11	1.15
15	-3	-3	6.33	0.76
15	-3	-3	7.10	0.74
15	-3	-3	7.46	1.37
-15	3	3	7.53	1.12
-15	3	3	6.63	1.12
-15	3	3	7.00	1.09
14	-3	-3	23.36	1.20
14	-3	-3	23.48	2.22
14	-3	-3	21.86	1.22
-14	3	3	24.22	1.69
-14	3	3	23.50	1.43
-14	3	3	21.64	1.83
13	-3	-3	0.24	0.37
13	-3	-3	0.78	0.96
13	-3	-3	0.48	0.33
-13	3	3	0.79	0.56
-13	3	3	0.66	0.60
-13	3	3	0.44	0.76
12	-3	-3	-0.48	0.84
12	-3	-3	1.09	0.30
12	-3	-3	1.13	0.36
12	-3	-3	0.62	0.50
-12	3	3	1.25	0.35
-12	3	3	0.99	0.52
-12	3	3	1.53	0.72
-12	3	3	1.45	0.50
11	-3	-3	0.89	0.28
11	-3	-3	1.13	0.49

11	-3	-3	2.71	0.83
11	-3	-3	1.47	0.36
-11	3	3	1.35	0.35
-11	3	3	1.28	0.51
-11	3	3	1.22	0.63
-11	3	3	1.04	0.48
10	-3	-3	0.22	0.21
10	-3	-3	1.16	0.68
10	-3	-3	1.17	0.43
10	-3	-3	0.80	0.29
-10	3	3	0.82	0.46
-10	3	3	0.04	0.56
-10	3	3	0.58	0.26
-10	3	3	0.91	0.43
9	-3	-3	0.97	0.28
9	-3	-3	0.35	0.36
9	-3	-3	1.80	0.64
9	-3	-3	0.57	0.21
-9	3	3	0.94	0.26
-9	3	3	1.33	0.55
-9	3	3	0.68	0.39
-9	3	3	1.51	0.42
8	-3	-3	81.02	3.31
8	-3	-3	79.80	3.15
8	-3	-3	83.60	3.55
8	-3	-3	78.65	3.14
-8	3	3	81.94	3.35
-8	3	3	85.57	3.19
-8	3	3	76.24	3.25
-8	3	3	73.79	3.43
7	-3	-3	59.54	2.47
7	-3	-3	59.44	2.44
7	-3	-3	63.76	2.79
7	-3	-3	64.02	2.60
-7	3	3	65.44	2.48
-7	3	3	61.44	2.61
-7	3	3	60.80	2.73
-7	3	3	59.33	2.56
6	-3	-3	2.42	0.27
6	-3	-3	1.95	0.45
6	-3	-3	1.70	0.31
6	-3	-3	2.42	0.39
-6	3	3	1.50	0.36
-6	3	3	2.65	0.29

-6	3	3	1.87	0.44
-6	3	3	1.44	0.33
5	-3	-3	17.61	0.90
5	-3	-3	17.16	0.79
5	-3	-3	17.93	1.06
5	-3	-3	17.68	0.89
-5	3	3	16.88	0.89
-5	3	3	17.22	0.97
-5	3	3	18.61	1.07
4	-3	-3	41.26	1.79
4	-3	-3	41.99	1.74
4	-3	-3	41.33	1.90
4	-3	-3	43.04	1.68
-4	3	3	43.61	1.87
-4	3	3	39.23	1.89
-4	3	3	42.43	1.68
-4	3	3	43.13	1.76
3	-3	-3	31.10	1.35
3	-3	-3	29.69	1.40
3	-3	-3	31.42	1.29
3	-3	-3	34.98	1.48
3	-3	-3	26.84	1.24
-3	3	3	30.69	1.29
2	-3	-3	24.87	1.19
2	-3	-3	25.67	1.13
2	-3	-3	23.96	1.01
2	-3	-3	23.97	1.20
2	-3	-3	21.43	1.03
-2	3	3	24.60	1.02
-2	3	3	24.75	1.20
1	-3	-3	0.24	0.11
1	-3	-3	0.32	0.21
1	-3	-3	0.40	0.12
1	-3	-3	0.33	0.22
-1	3	3	0.24	0.11
-1	3	3	0.38	0.21
-1	3	3	0.34	0.11
-1	3	3	0.15	0.20
0	-3	-3	7.81	0.56
0	-3	-3	7.91	0.56
0	-3	-3	8.13	0.36
0	-3	-3	7.41	0.40
0	3	3	6.85	0.55
0	3	3	7.93	0.55

0	3	3	7.92	0.36
-1	-3	-3	82.84	3.08
-1	-3	-3	81.99	3.20
-1	-3	-3	80.53	3.23
-1	-3	-3	77.75	3.01
1	3	3	79.32	3.08
1	3	3	77.92	3.17
1	3	3	81.00	3.24
-2	-3	-3	3.40	0.21
-2	-3	-3	2.69	0.38
-2	-3	-3	4.13	0.47
-2	-3	-3	3.41	0.22
2	3	3	3.76	0.38
2	3	3	3.59	0.28
-3	-3	-3	26.44	1.10
-3	-3	-3	27.08	1.32
-3	-3	-3	29.59	1.24
-3	-3	-3	27.05	1.16
-3	-3	-3	26.30	1.09
-3	-3	-3	26.70	1.12
3	3	3	26.49	1.33
3	3	3	26.29	1.21
-4	-3	-3	0.11	0.14
-4	-3	-3	0.20	0.27
-4	-3	-3	0.14	0.12
-4	-3	-3	0.34	0.13
-4	-3	-3	0.20	0.19
-4	-3	-3	0.01	0.13
4	3	3	0.02	0.11
4	3	3	0.07	0.25
4	3	3	0.10	0.19
-5	-3	-3	16.89	0.78
-5	-3	-3	16.70	0.83
-5	-3	-3	17.88	1.04
-5	-3	-3	16.08	0.87
5	3	3	16.98	1.02
5	3	3	15.95	0.86
5	3	3	16.46	0.79
5	3	3	18.03	0.81
-6	-3	-3	4.15	0.62
-6	-3	-3	4.04	0.47
-6	-3	-3	3.85	0.35
-6	-3	-3	3.68	0.49
6	3	3	3.65	0.35

6	3	3	4.25	0.48
6	3	3	4.39	0.42
6	3	3	3.36	0.59
-7	-3	-3	13.21	0.70
-7	-3	-3	12.67	0.89
-7	-3	-3	13.82	0.80
-7	-3	-3	12.25	0.80
-7	-3	-3	16.19	1.04
7	3	3	12.53	0.72
7	3	3	12.53	0.97
-8	-3	-3	0.47	0.44
-8	-3	-3	0.77	0.21
-8	-3	-3	0.99	0.30
-8	-3	-3	0.04	0.40
-8	-3	-3	0.72	0.32
8	3	3	0.45	0.22
8	3	3	0.25	0.25
8	3	3	0.43	0.43
-9	-3	-3	0.41	0.49
-9	-3	-3	0.61	0.24
-9	-3	-3	0.57	0.32
-9	-3	-3	0.70	0.39
9	3	3	0.23	0.33
9	3	3	1.32	0.47
9	3	3	0.60	0.28
-10	-3	-3	6.19	0.56
-10	-3	-3	6.43	0.94
-10	-3	-3	6.35	0.80
-10	-3	-3	6.94	1.00
-10	-3	-3	6.29	0.70
10	3	3	5.03	0.87
10	3	3	7.03	0.73
10	3	3	6.86	0.60
-11	-3	-3	7.39	0.79
-11	-3	-3	8.13	1.15
-11	-3	-3	7.36	1.19
-11	-3	-3	6.86	0.94
-11	-3	-3	8.20	0.64
11	3	3	6.71	0.84
11	3	3	6.65	0.97
11	3	3	7.11	0.67
-12	-3	-3	13.68	0.86
-12	-3	-3	12.80	1.20
-12	-3	-3	15.00	1.48

-12	-3	-3	15.70	1.43
-12	-3	-3	10.70	0.98
12	3	3	12.34	1.21
12	3	3	12.92	1.09
12	3	3	13.43	0.92
-13	-3	-3	9.14	1.47
-13	-3	-3	6.01	0.91
-13	-3	-3	8.56	1.14
-13	-3	-3	6.92	1.16
13	3	3	7.99	0.80
13	3	3	8.31	1.13
13	3	3	7.82	1.04
-14	-3	-3	11.11	1.58
-14	-3	-3	11.07	1.06
-14	-3	-3	11.64	1.60
-14	-3	-3	9.66	1.32
14	3	3	11.94	0.96
14	3	3	10.11	1.26
-15	-3	-3	2.20	0.66
-15	-3	-3	3.10	1.06
-15	-3	-3	3.31	1.05
-15	-3	-3	3.74	0.90
15	3	3	3.23	0.85
15	3	3	2.89	0.57
-16	-3	-3	8.66	1.22
-16	-3	-3	10.16	1.45
-16	-3	-3	10.42	1.43
16	3	3	9.50	1.10
16	3	3	8.67	1.46
-17	-3	-3	-0.09	0.96
17	3	3	1.45	0.96
17	3	3	0.50	0.57
-18	-3	-3	10.85	1.51
18	3	3	12.99	1.90
18	3	3	13.39	1.33
-19	-3	-3	-0.22	1.22
19	3	3	-0.83	1.15
19	3	3	0.31	0.69
-20	-3	-3	4.78	1.55
20	3	3	8.78	1.13
21	-3	-2	29.47	1.92
-21	3	2	31.03	3.43
20	-3	-2	1.94	0.69
-20	3	2	3.36	1.34

19	-3	-2	21.70	1.50
-19	3	2	21.71	2.41
18	-3	-2	8.38	1.04
-18	3	2	4.89	1.18
-18	3	2	5.23	1.29
17	-3	-2	40.36	1.93
17	-3	-2	41.61	3.59
17	-3	-2	40.54	1.99
-17	3	2	42.60	2.74
-17	3	2	36.35	2.41
16	-3	-2	0.35	0.51
16	-3	-2	0.62	0.45
16	-3	-2	0.91	1.17
-16	3	2	0.37	0.89
-16	3	2	0.34	0.86
-16	3	2	0.57	0.91
15	-3	-2	29.94	1.55
15	-3	-2	30.57	1.57
15	-3	-2	32.38	2.40
-15	3	2	30.70	2.02
-15	3	2	29.88	2.04
-15	3	2	30.69	1.88
14	-3	-2	18.91	1.89
14	-3	-2	16.26	1.10
14	-3	-2	14.97	1.00
-14	3	2	18.44	1.50
-14	3	2	16.65	1.48
-14	3	2	15.44	1.40
13	-3	-2	49.30	2.13
13	-3	-2	47.51	2.65
13	-3	-2	48.18	2.10
-13	3	2	46.88	2.43
-13	3	2	48.30	2.49
-13	3	2	45.43	2.30
12	-3	-2	15.24	0.92
12	-3	-2	16.91	1.03
12	-3	-2	14.07	1.17
12	-3	-2	16.36	1.59
-12	3	2	16.05	1.36
-12	3	2	14.70	1.25
-12	3	2	19.65	1.04
-12	3	2	14.86	1.21
11	-3	-2	37.93	1.78
11	-3	-2	44.09	2.30

11	-3	-2	41.70	2.01
-11	3	2	41.08	2.09
-11	3	2	39.99	1.97
-11	3	2	38.43	2.11
-11	3	2	46.19	1.86
10	-3	-2	11.69	0.88
10	-3	-2	9.06	0.62
10	-3	-2	9.94	0.74
-10	3	2	8.11	0.86
-10	3	2	9.80	0.92
-10	3	2	10.19	0.68
-10	3	2	6.88	0.98
9	-3	-2	39.40	2.02
9	-3	-2	39.30	1.71
9	-3	-2	39.44	1.84
9	-3	-2	37.48	1.65
-9	3	2	35.98	1.90
-9	3	2	38.51	1.83
-9	3	2	38.88	1.88
-9	3	2	42.25	1.70
8	-3	-2	5.45	0.62
8	-3	-2	3.19	0.47
8	-3	-2	4.33	0.80
8	-3	-2	3.29	0.35
-8	3	2	3.12	0.60
-8	3	2	4.23	0.60
-8	3	2	4.92	0.74
-8	3	2	4.10	0.41
7	-3	-2	186.71	7.35
7	-3	-2	198.47	7.48
7	-3	-2	195.74	7.40
7	-3	-2	197.09	7.59
-7	3	2	184.22	7.47
-7	3	2	192.31	7.57
-7	3	2	204.85	7.38
-7	3	2	198.61	7.52
6	-3	-2	18.27	0.95
6	-3	-2	18.31	0.83
6	-3	-2	18.66	0.91
6	-3	-2	19.79	1.09
-6	3	2	16.18	0.99
-6	3	2	17.82	0.96
-6	3	2	17.46	1.07
-6	3	2	18.82	0.85

5	-3	-2	14.25	0.76
5	-3	-2	15.06	0.78
5	-3	-2	12.88	0.86
5	-3	-2	14.87	0.67
-5	3	2	13.42	0.76
-5	3	2	14.85	0.84
-5	3	2	14.88	0.69
4	-3	-2	1.29	0.21
4	-3	-2	1.36	0.30
4	-3	-2	2.08	0.30
4	-3	-2	1.80	0.29
-4	3	2	2.08	0.36
-4	3	2	2.15	0.40
-4	3	2	1.45	0.25
-4	3	2	1.78	0.21
3	-3	-2	1.95	0.26
3	-3	-2	2.33	0.40
3	-3	-2	1.45	0.30
-3	3	2	1.50	0.33
-3	3	2	1.01	0.20
-3	3	2	1.26	0.27
-3	3	2	1.22	0.18
2	-3	-2	43.67	1.82
2	-3	-2	40.50	1.69
2	-3	-2	43.49	1.67
2	-3	-2	43.31	1.84
-2	3	2	43.14	1.87
-2	3	2	39.56	1.69
-2	3	2	45.03	1.82
1	-3	-2	7.30	0.38
1	-3	-2	7.46	0.51
1	-3	-2	7.78	0.52
1	-3	-2	7.39	0.60
-1	3	2	8.08	0.52
-1	3	2	7.03	0.38
-1	3	2	7.29	0.37
0	-3	-2	33.84	1.40
0	-3	-2	31.16	1.24
0	-3	-2	32.33	1.38
0	-3	-2	31.31	1.26
0	3	2	31.02	1.37
0	3	2	31.26	1.24
0	3	2	32.12	1.40
0	3	2	30.59	1.26

-1	-3	-2	422.27	15.67
-1	-3	-2	433.40	15.79
-1	-3	-2	412.17	15.69
1	3	2	422.61	15.78
1	3	2	431.63	15.84
1	3	2	416.09	15.66
1	3	2	420.48	15.69
-2	-3	-2	5.64	0.44
-2	-3	-2	5.48	0.33
-2	-3	-2	6.29	0.51
-2	-3	-2	5.64	0.31
2	3	2	5.63	0.30
2	3	2	5.81	0.43
2	3	2	6.36	0.53
2	3	2	5.59	0.34
-3	-3	-2	5.32	0.32
-3	-3	-2	5.66	0.51
-3	-3	-2	5.21	0.43
-3	-3	-2	5.74	0.32
-3	-3	-2	5.48	0.33
3	3	2	5.58	0.42
3	3	2	5.29	0.54
-4	-3	-2	2.45	0.36
-4	-3	-2	2.63	0.29
-4	-3	-2	2.58	0.27
-4	-3	-2	2.48	0.45
-4	-3	-2	3.18	0.25
4	3	2	2.68	0.46
4	3	2	3.10	0.27
4	3	2	3.19	0.38
-5	-3	-2	103.80	4.06
-5	-3	-2	97.34	3.88
-5	-3	-2	95.32	3.86
-5	-3	-2	100.83	3.95
5	3	2	97.70	3.87
5	3	2	104.37	3.91
5	3	2	102.79	3.96
5	3	2	106.32	4.08
-6	-3	-2	80.86	3.35
-6	-3	-2	77.56	3.21
-6	-3	-2	78.77	3.29
-6	-3	-2	81.66	3.22
-6	-3	-2	80.53	3.13
6	3	2	82.89	3.20

6	3	2	82.29	3.35
6	3	2	80.75	3.15
-7	-3	-2	33.84	1.56
-7	-3	-2	34.12	1.53
-7	-3	-2	33.99	1.43
-7	-3	-2	34.28	1.65
-7	-3	-2	29.52	2.12
7	3	2	35.60	1.52
7	3	2	33.53	1.45
7	3	2	34.70	1.66
-8	-3	-2	21.60	1.18
-8	-3	-2	21.92	1.15
-8	-3	-2	22.19	1.31
-8	-3	-2	22.91	1.04
8	3	2	22.38	1.06
8	3	2	22.01	1.29
8	3	2	24.43	1.16
-9	-3	-2	1.18	0.26
-9	-3	-2	1.12	0.44
-9	-3	-2	0.64	0.38
-9	-3	-2	0.45	0.56
-9	-3	-2	1.15	0.38
9	3	2	1.18	0.36
9	3	2	1.00	0.43
9	3	2	1.03	0.28
-10	-3	-2	39.95	1.74
-10	-3	-2	40.27	2.12
-10	-3	-2	39.14	1.84
-10	-3	-2	37.16	1.92
-10	-3	-2	40.64	2.04
10	3	2	41.38	1.78
10	3	2	43.25	1.90
10	3	2	40.58	2.01
-11	-3	-2	2.38	0.38
-11	-3	-2	3.20	0.54
-11	-3	-2	2.88	0.78
-11	-3	-2	3.38	0.68
-11	-3	-2	2.17	0.56
11	3	2	2.41	0.41
11	3	2	2.79	0.59
11	3	2	2.57	0.51
-12	-3	-2	3.17	0.81
-12	-3	-2	3.58	0.69
-12	-3	-2	2.66	0.56

-12	-3	-2	3.78	0.76
-12	-3	-2	1.82	0.38
12	3	2	2.74	0.64
12	3	2	2.81	0.46
12	3	2	1.26	0.52
-13	-3	-2	14.12	1.48
-13	-3	-2	12.09	1.40
-13	-3	-2	12.94	1.16
-13	-3	-2	14.35	1.35
13	3	2	16.22	1.00
13	3	2	14.75	1.24
13	3	2	15.73	1.26
-14	-3	-2	13.88	1.52
-14	-3	-2	13.39	1.43
-14	-3	-2	12.69	1.55
14	3	2	12.82	0.97
14	3	2	12.51	1.27
-15	-3	-2	2.87	0.86
-15	-3	-2	1.46	0.97
-15	-3	-2	2.42	0.93
15	3	2	2.41	0.55
15	3	2	3.04	0.78
-16	-3	-2	16.23	1.72
-16	-3	-2	16.76	1.79
-16	-3	-2	16.39	1.89
16	3	2	17.02	1.22
16	3	2	12.82	1.51
-17	-3	-2	2.12	1.03
-17	-3	-2	2.29	0.98
17	3	2	2.54	0.94
17	3	2	0.84	0.61
-18	-3	-2	3.48	1.12
18	3	2	7.33	1.17
18	3	2	6.89	0.89
-19	-3	-2	-1.56	1.12
19	3	2	0.41	1.38
19	3	2	-0.20	0.66
-20	-3	-2	-0.29	1.32
20	3	2	0.35	0.76
21	-3	-1	3.41	0.88
-21	3	1	-0.24	1.61
20	-3	-1	52.62	2.62
-20	3	1	53.16	3.51
19	-3	-1	0.06	0.56

-19	3	1	0.77	1.22
18	-3	-1	32.21	1.85
18	-3	-1	35.61	2.05
-18	3	1	36.06	2.68
17	-3	-1	0.98	0.66
17	-3	-1	0.35	0.50
-17	3	1	0.51	0.85
-17	3	1	0.95	1.03
16	-3	-1	81.21	3.43
16	-3	-1	84.12	3.79
16	-3	-1	80.79	3.41
-16	3	1	76.67	3.71
-16	3	1	74.95	3.59
-16	3	1	86.29	4.00
15	-3	-1	3.69	0.54
15	-3	-1	4.68	1.09
15	-3	-1	4.00	0.68
-15	3	1	4.43	0.91
-15	3	1	4.60	0.96
-15	3	1	4.49	0.90
14	-3	-1	188.79	7.31
14	-3	-1	194.24	7.35
14	-3	-1	198.95	7.64
-14	3	1	185.36	7.69
-14	3	1	180.93	7.50
-14	3	1	182.95	7.47
13	-3	-1	16.04	2.01
13	-3	-1	12.31	0.98
13	-3	-1	10.29	0.83
-13	3	1	13.05	1.20
-13	3	1	12.16	1.29
12	-3	-1	87.60	3.55
12	-3	-1	85.71	3.75
12	-3	-1	92.77	3.63
12	-3	-1	88.29	4.06
-12	3	1	84.10	3.75
-12	3	1	97.39	3.60
-12	3	1	80.20	3.71
-12	3	1	88.71	3.86
11	-3	-1	13.78	1.05
11	-3	-1	12.49	1.33
11	-3	-1	12.80	0.81
11	-3	-1	12.47	0.96
-11	3	1	13.21	1.13

-11	3	1	13.72	1.14
-11	3	1	13.47	1.10
-11	3	1	15.49	0.86
10	-3	-1	142.16	5.43
10	-3	-1	148.38	5.64
10	-3	-1	131.05	5.50
10	-3	-1	134.57	5.33
-10	3	1	136.18	5.51
-10	3	1	136.10	5.57
-10	3	1	147.47	5.37
-10	3	1	129.06	5.49
9	-3	-1	3.93	0.63
9	-3	-1	4.97	0.85
9	-3	-1	3.32	0.40
9	-3	-1	3.61	0.62
-9	3	1	3.96	0.44
-9	3	1	4.13	0.75
-9	3	1	3.10	0.54
-9	3	1	2.96	0.51
8	-3	-1	210.68	8.22
8	-3	-1	213.24	8.13
8	-3	-1	223.14	8.33
8	-3	-1	219.91	8.27
-8	3	1	238.83	8.17
-8	3	1	210.03	8.30
-8	3	1	210.00	8.28
-8	3	1	194.91	8.40
7	-3	-1	0.69	0.18
7	-3	-1	0.28	0.25
7	-3	-1	0.65	0.29
7	-3	-1	0.65	0.35
-7	3	1	0.80	0.32
-7	3	1	0.41	0.18
-7	3	1	0.61	0.35
-7	3	1	0.83	0.28
6	-3	-1	212.55	8.06
6	-3	-1	202.96	7.97
6	-3	-1	219.74	8.13
6	-3	-1	214.32	8.08
-6	3	1	208.96	8.11
-6	3	1	213.15	8.11
-6	3	1	220.50	8.00
-6	3	1	206.12	8.12
5	-3	-1	3.98	0.47

5	-3	-1	3.57	0.28
5	-3	-1	3.89	0.40
5	-3	-1	3.78	0.43
-5	3	1	4.14	0.30
-5	3	1	4.11	0.50
-5	3	1	3.86	0.44
-5	3	1	3.10	0.39
4	-3	-1	141.43	5.44
4	-3	-1	140.98	5.45
4	-3	-1	141.98	5.40
4	-3	-1	133.70	5.33
-4	3	1	138.34	5.42
-4	3	1	147.39	5.46
-4	3	1	143.49	5.49
-4	3	1	145.80	5.34
3	-3	-1	111.36	4.38
3	-3	-1	115.47	4.44
3	-3	-1	114.12	4.51
3	-3	-1	116.27	4.50
-3	3	1	117.41	4.39
-3	3	1	123.66	4.51
-3	3	1	118.64	4.46
-3	3	1	114.77	4.55
2	-3	-1	381.23	14.30
2	-3	-1	342.02	14.25
2	-3	-1	403.28	14.37
2	-3	-1	397.44	14.42
-2	3	1	378.31	14.31
-2	3	1	371.69	14.26
-2	3	1	399.18	14.45
-2	3	1	401.75	14.37
1	-3	-1	89.76	3.46
1	-3	-1	91.04	3.64
1	-3	-1	96.10	3.57
1	-3	-1	97.61	3.50
-1	3	1	89.58	3.50
-1	3	1	86.30	3.56
-1	3	1	93.63	3.67
0	-3	-1	193.60	7.50
0	-3	-1	203.34	7.52
0	-3	-1	206.90	7.60
0	-3	-1	210.38	7.72
0	3	1	194.82	7.50
0	3	1	197.13	7.60

0	3	1	194.08	7.52
0	3	1	211.90	7.72
-1	-3	-1	23.51	0.94
-1	-3	-1	22.56	0.92
-1	-3	-1	23.16	1.06
-1	-3	-1	23.58	1.02
1	3	1	21.56	0.92
1	3	1	22.30	1.02
1	3	1	23.45	0.93
-2	-3	-1	47.10	1.78
-2	-3	-1	44.99	1.86
-2	-3	-1	46.06	1.78
-2	-3	-1	44.09	1.76
2	3	1	43.13	1.86
2	3	1	43.60	1.77
2	3	1	49.40	1.78
2	3	1	45.97	1.92
-3	-3	-1	5.63	0.46
-3	-3	-1	5.58	0.43
-3	-3	-1	5.68	0.34
-3	-3	-1	5.46	0.30
3	3	1	5.02	0.31
3	3	1	5.63	0.42
3	3	1	4.85	0.46
3	3	1	4.95	0.30
-4	-3	-1	53.32	2.12
-4	-3	-1	52.99	2.17
-4	-3	-1	54.10	2.24
-4	-3	-1	55.82	2.28
-4	-3	-1	54.25	2.16
4	3	1	55.98	2.30
4	3	1	56.91	2.25
-5	-3	-1	6.21	0.43
-5	-3	-1	5.89	0.53
-5	-3	-1	6.53	0.51
-5	-3	-1	6.38	0.47
-5	-3	-1	6.11	0.37
-5	-3	-1	5.84	0.59
5	3	1	5.96	0.38
-6	-3	-1	73.70	3.01
-6	-3	-1	73.66	3.13
-6	-3	-1	80.74	3.10
-6	-3	-1	76.08	3.05
-6	-3	-1	71.05	2.95

-6	-3	-1	75.23	2.99
6	3	1	69.94	2.92
6	3	1	77.89	3.12
6	3	1	79.75	3.01
-7	-3	-1	108.02	4.09
-7	-3	-1	106.39	4.26
-7	-3	-1	101.28	4.20
-7	-3	-1	106.25	4.22
7	3	1	108.88	4.19
7	3	1	104.05	4.10
7	3	1	109.81	4.29
-8	-3	-1	138.77	5.72
-8	-3	-1	146.34	5.63
-8	-3	-1	141.44	5.57
-8	-3	-1	139.53	5.59
-8	-3	-1	145.30	5.45
8	3	1	144.00	5.56
8	3	1	140.93	5.46
8	3	1	143.60	5.63
-9	-3	-1	19.87	1.17
-9	-3	-1	18.46	1.40
-9	-3	-1	21.42	1.15
-9	-3	-1	13.48	1.67
-9	-3	-1	20.46	0.95
9	3	1	19.55	1.16
9	3	1	19.55	1.10
9	3	1	19.14	0.96
-10	-3	-1	85.32	3.78
-10	-3	-1	83.25	3.57
-10	-3	-1	85.52	3.64
-10	-3	-1	86.71	3.64
-10	-3	-1	88.21	3.44
10	3	1	88.57	3.59
10	3	1	88.09	3.64
10	3	1	87.60	3.47
-11	-3	-1	3.27	0.74
-11	-3	-1	2.53	0.54
-11	-3	-1	2.67	0.36
-11	-3	-1	3.06	0.79
-11	-3	-1	2.58	0.63
11	3	1	3.15	0.51
11	3	1	3.75	0.60
11	3	1	2.77	0.53
-12	-3	-1	2.68	0.66

-12	-3	-1	1.83	0.83
-12	-3	-1	0.88	0.52
-12	-3	-1	1.18	0.57
-12	-3	-1	1.86	0.35
12	3	1	1.06	0.36
12	3	1	1.35	0.53
12	3	1	1.06	0.48
-13	-3	-1	0.60	0.39
-13	-3	-1	0.88	0.59
-13	-3	-1	2.75	0.89
-13	-3	-1	1.49	0.69
-13	-3	-1	1.12	0.60
13	3	1	2.01	0.57
13	3	1	0.67	0.38
13	3	1	1.34	0.57
-14	-3	-1	61.74	3.14
-14	-3	-1	65.70	2.93
-14	-3	-1	60.61	2.97
-14	-3	-1	56.73	2.76
14	3	1	64.46	2.86
14	3	1	55.97	2.61
-15	-3	-1	3.26	0.80
-15	-3	-1	1.92	0.85
-15	-3	-1	2.16	1.07
-15	-3	-1	3.91	0.91
15	3	1	4.39	0.79
-16	-3	-1	40.37	2.41
-16	-3	-1	39.50	2.48
-16	-3	-1	35.86	2.36
-16	-3	-1	37.26	2.16
16	3	1	40.01	2.16
16	3	1	38.98	1.97
-17	-3	-1	2.95	1.06
-17	-3	-1	5.32	1.22
17	3	1	4.35	0.72
17	3	1	4.77	0.95
-18	-3	-1	7.26	1.32
18	3	1	6.65	1.10
18	3	1	8.17	1.14
-19	-3	-1	1.74	1.17
19	3	1	3.56	0.79
-20	-3	-1	8.08	1.52
20	3	1	9.68	1.43
21	3	1	3.22	0.87

21	-3	0	34.41	2.91
-21	3	0	26.97	3.20
20	-3	0	1.16	0.71
-20	3	0	2.41	1.38
19	-3	0	69.05	3.13
-19	3	0	67.92	3.90
18	-3	0	-0.70	0.78
18	-3	0	1.33	0.58
-18	3	0	-0.26	1.13
17	-3	0	69.60	3.18
17	-3	0	71.31	3.09
-17	3	0	68.56	3.70
16	-3	0	1.66	0.50
16	-3	0	1.49	0.68
16	-3	0	3.43	0.92
-16	3	0	1.82	0.95
-16	3	0	3.18	1.09
-16	3	0	1.71	0.79
15	-3	0	97.52	4.03
15	-3	0	97.51	4.13
15	-3	0	97.73	3.94
-15	3	0	100.99	4.41
-15	3	0	90.18	4.00
-15	3	0	90.33	4.26
14	-3	0	0.96	0.51
14	-3	0	1.34	0.38
14	-3	0	2.52	0.81
-14	3	0	0.98	0.72
-14	3	0	1.63	0.69
-14	3	0	2.24	0.80
13	-3	0	103.83	4.27
13	-3	0	101.27	4.07
13	-3	0	105.54	4.19
-13	3	0	95.14	4.15
-13	3	0	103.27	4.45
-13	3	0	97.89	4.32
12	-3	0	-0.74	0.45
12	-3	0	-0.40	0.26
12	-3	0	-0.10	0.61
12	-3	0	-0.85	0.48
-12	3	0	0.47	0.54
-12	3	0	1.07	0.64
-12	3	0	0.19	0.54
-12	3	0	0.57	0.33

11	-3	0	110.95	4.45
11	-3	0	115.38	4.54
11	-3	0	110.22	4.33
11	-3	0	107.50	4.52
-11	3	0	102.42	4.64
-11	3	0	95.52	4.77
-11	3	0	114.05	4.63
-11	3	0	121.24	4.37
10	-3	0	0.71	0.40
10	-3	0	0.10	0.36
10	-3	0	-0.12	0.51
10	-3	0	0.20	0.22
-10	3	0	0.18	0.43
-10	3	0	0.15	0.39
-10	3	0	-0.07	0.22
-10	3	0	-0.74	0.46
9	-3	0	213.21	8.29
9	-3	0	228.91	8.43
9	-3	0	224.21	8.45
9	-3	0	221.81	8.43
-9	3	0	217.08	8.50
-9	3	0	201.94	8.46
-9	3	0	234.81	8.32
-9	3	0	208.47	8.35
8	-3	0	1.10	0.40
8	-3	0	0.61	0.32
8	-3	0	0.83	0.22
8	-3	0	0.85	0.39
-8	3	0	0.74	0.22
-8	3	0	1.03	0.33
-8	3	0	0.27	0.32
-8	3	0	1.12	0.42
7	-3	0	414.27	15.22
7	-3	0	391.08	15.11
7	-3	0	393.60	15.36
7	-3	0	418.54	15.21
-7	3	0	400.29	15.26
-7	3	0	382.43	15.27
-7	3	0	423.51	15.13
-7	3	0	415.08	15.18
6	-3	0	13.19	0.64
6	-3	0	13.70	0.79
6	-3	0	12.81	0.75
6	-3	0	13.56	0.75

-6	3	0	10.80	0.75
-6	3	0	12.02	0.84
-6	3	0	12.52	0.72
-6	3	0	13.09	0.64
5	-3	0	221.18	8.68
5	-3	0	240.06	8.76
5	-3	0	229.52	8.80
5	-3	0	241.16	8.77
-5	3	0	235.75	8.69
-5	3	0	227.92	8.85
-5	3	0	231.47	8.75
-5	3	0	228.22	8.79
4	-3	0	54.93	2.19
4	-3	0	53.15	2.12
4	-3	0	51.24	2.12
4	-3	0	53.06	2.05
-4	3	0	51.46	2.05
-4	3	0	53.70	2.12
-4	3	0	53.52	2.23
-4	3	0	50.17	2.14
3	-3	0	4.63	0.28
3	-3	0	5.60	0.39
3	-3	0	5.33	0.40
3	-3	0	5.19	0.46
-3	3	0	5.97	0.40
-3	3	0	4.60	0.50
-3	3	0	5.57	0.39
-3	3	0	4.73	0.29
2	-3	0	44.41	1.74
2	-3	0	45.11	1.81
2	-3	0	46.28	1.91
2	-3	0	45.74	1.83
-2	3	0	47.30	1.94
-2	3	0	44.70	1.83
-2	3	0	43.79	1.81
-2	3	0	45.47	1.74
1	-3	0	481.23	18.09
1	-3	0	487.79	18.21
1	-3	0	466.09	18.12
-1	3	0	497.92	18.23
-1	3	0	494.07	18.13
-1	3	0	495.86	18.10
0	-3	0	-0.14	0.08
0	-3	0	-0.10	0.16

0	-3	0	0.07	0.05
0	-3	0	0.01	0.12
0	3	0	-0.03	0.11
0	3	0	0.03	0.05
0	3	0	0.21	0.19
0	3	0	0.12	0.08
-1	-3	0	498.72	18.15
-1	-3	0	479.38	17.98
-1	-3	0	479.69	18.04
1	3	0	465.17	17.97
1	3	0	488.91	18.05
1	3	0	495.08	18.14
-2	-3	0	46.12	1.88
-2	-3	0	47.23	1.99
-2	-3	0	45.51	1.88
-2	-3	0	45.11	1.79
-2	-3	0	45.72	1.75
-2	-3	0	47.09	1.74
2	3	0	43.11	1.78
2	3	0	44.60	1.87
-3	-3	0	4.95	0.27
-3	-3	0	5.45	0.35
-3	-3	0	4.85	0.43
-3	-3	0	5.27	0.50
-3	-3	0	4.73	0.42
-3	-3	0	5.20	0.57
3	3	0	5.65	0.34
3	3	0	5.50	0.28
-4	-3	0	52.68	2.29
-4	-3	0	56.75	2.08
-4	-3	0	53.51	2.14
-4	-3	0	57.30	2.24
-4	-3	0	47.32	2.17
-4	-3	0	54.71	2.20
4	3	0	51.75	2.08
4	3	0	55.32	2.23
-5	-3	0	256.64	9.02
-5	-3	0	234.32	8.84
-5	-3	0	227.82	8.92
-5	-3	0	241.33	8.97
-5	-3	0	233.02	9.08
-5	-3	0	231.72	8.91
5	3	0	243.14	8.99
5	3	0	225.09	8.84

-6	-3	0	14.49	0.65
-6	-3	0	14.51	0.80
-6	-3	0	11.94	0.75
-6	-3	0	13.73	0.85
6	3	0	13.92	0.83
6	3	0	13.90	0.66
-7	-3	0	400.49	15.58
-7	-3	0	411.38	15.39
-7	-3	0	398.00	15.41
-7	-3	0	421.32	15.47
-7	-3	0	433.65	15.44
-7	-3	0	406.10	15.29
7	3	0	426.67	15.42
7	3	0	384.88	15.30
-8	-3	0	1.27	0.21
-8	-3	0	0.18	0.32
-8	-3	0	1.09	0.31
-8	-3	0	1.11	0.56
-8	-3	0	1.23	0.40
8	3	0	1.42	0.35
8	3	0	1.06	0.23
8	3	0	1.19	0.42
-9	-3	0	229.23	8.66
-9	-3	0	219.87	8.69
-9	-3	0	239.52	8.52
-9	-3	0	220.01	8.87
-9	-3	0	224.31	8.70
9	3	0	225.95	8.67
9	3	0	232.81	8.68
9	3	0	210.96	8.52
-10	-3	0	-0.01	0.40
-10	-3	0	0.53	0.65
-10	-3	0	0.40	0.37
-10	-3	0	0.37	0.24
-10	-3	0	0.79	0.70
10	3	0	0.59	0.27
10	3	0	0.43	0.37
10	3	0	0.11	0.36
-11	-3	0	113.47	4.67
-11	-3	0	117.12	4.44
-11	-3	0	110.43	4.89
-11	-3	0	110.85	4.63
11	3	0	108.36	4.45
11	3	0	117.18	4.59

11	3	0	114.53	4.62
-12	-3	0	1.03	0.82
-12	-3	0	-0.53	0.28
-12	-3	0	0.70	0.56
-12	-3	0	0.10	0.53
-12	-3	0	-0.30	0.46
12	3	0	0.04	0.30
12	3	0	0.35	0.50
12	3	0	0.03	0.48
-13	-3	0	97.57	4.32
-13	-3	0	107.07	4.12
-13	-3	0	102.25	4.60
-13	-3	0	99.67	4.39
-13	-3	0	106.63	4.31
13	3	0	105.07	4.26
13	3	0	104.31	4.14
13	3	0	99.14	4.31
-14	-3	0	1.22	0.73
-14	-3	0	1.69	0.69
-14	-3	0	1.79	1.00
-14	-3	0	1.70	0.68
14	3	0	1.48	0.41
14	3	0	1.72	0.60
-15	-3	0	93.28	4.12
-15	-3	0	89.37	4.13
-15	-3	0	92.98	4.44
-15	-3	0	94.83	4.29
15	3	0	103.64	3.96
15	3	0	98.72	4.06
-16	-3	0	2.57	1.17
-16	-3	0	3.15	0.91
-16	-3	0	3.06	0.96
-16	-3	0	2.87	0.95
16	3	0	2.50	0.74
16	3	0	3.33	0.60
-17	-3	0	63.84	3.55
-17	-3	0	70.34	3.47
17	3	0	63.76	2.95
17	3	0	66.21	3.09
-18	-3	0	0.32	1.26
-18	-3	0	-1.04	0.92
18	3	0	-0.02	0.85
18	3	0	0.86	0.58
-19	-3	0	66.24	3.68

19	3	0	68.54	3.13
-20	-3	0	-0.64	1.23
20	3	0	-0.41	0.72
-21	-3	0	27.75	2.77
21	3	0	25.11	1.92
20	-3	1	10.95	1.33
-20	3	-1	9.91	1.77
19	-3	1	3.54	0.80
-19	3	-1	3.24	1.39
18	-3	1	8.89	1.48
18	-3	1	6.85	0.86
-18	3	-1	6.81	1.37
17	-3	1	3.82	0.68
17	-3	1	2.75	0.88
-17	3	-1	4.19	1.16
16	-3	1	40.39	1.95
16	-3	1	41.16	2.14
-16	3	-1	34.04	2.54
-16	3	-1	37.94	2.54
15	-3	1	2.61	0.51
15	-3	1	4.43	0.75
-15	3	-1	2.57	0.91
-15	3	-1	3.66	1.01
14	-3	1	61.98	2.64
14	-3	1	59.68	2.60
14	-3	1	58.47	2.76
-14	3	-1	61.27	3.05
-14	3	-1	58.42	3.01
13	-3	1	0.05	0.56
13	-3	1	1.86	0.60
13	-3	1	1.60	0.41
-13	3	-1	1.78	0.67
-13	3	-1	2.31	0.79
12	-3	1	1.74	0.55
12	-3	1	0.80	0.34
12	-3	1	1.10	0.53
12	-3	1	1.41	0.55
-12	3	-1	0.65	0.55
-12	3	-1	1.00	0.42
-12	3	-1	1.47	0.68
-12	3	-1	1.17	0.35
11	-3	1	2.76	0.56
11	-3	1	1.93	0.35
11	-3	1	2.94	0.53

11	-3	1	4.33	0.79
-11	3	-1	3.38	0.50
-11	3	-1	3.71	0.58
-11	3	-1	2.97	0.71
-11	3	-1	3.03	0.61
10	-3	1	88.98	3.46
10	-3	1	88.47	3.56
10	-3	1	80.66	3.35
10	-3	1	82.68	3.50
-10	3	-1	82.46	3.33
-10	3	-1	81.61	3.61
-10	3	-1	82.50	3.35
-10	3	-1	84.19	3.59
9	-3	1	18.46	0.95
9	-3	1	19.43	1.11
9	-3	1	19.53	1.15
9	-3	1	19.78	1.10
-9	3	-1	20.88	0.96
-9	3	-1	17.02	1.15
-9	3	-1	18.76	0.96
-9	3	-1	16.84	1.36
8	-3	1	141.30	5.54
8	-3	1	137.04	5.48
8	-3	1	138.10	5.43
8	-3	1	139.29	5.58
-8	3	-1	142.14	5.42
-8	3	-1	155.83	5.46
-8	3	-1	140.75	5.65
-8	3	-1	139.13	5.60
7	-3	1	101.82	3.99
7	-3	1	101.87	4.09
7	-3	1	107.02	4.16
7	-3	1	103.65	4.02
-7	3	-1	100.91	3.97
-7	3	-1	109.44	4.00
-7	3	-1	99.50	4.19
-7	3	-1	101.13	4.13
6	-3	1	73.16	2.91
6	-3	1	75.07	2.94
6	-3	1	76.39	3.01
6	-3	1	75.35	3.08
-6	3	-1	80.49	2.93
-6	3	-1	68.88	3.10
-6	3	-1	73.64	2.92

-6	3	-1	75.27	3.04
5	-3	1	6.00	0.47
5	-3	1	5.89	0.37
5	-3	1	5.78	0.42
-5	3	-1	6.14	0.49
-5	3	-1	6.20	0.60
-5	3	-1	5.29	0.36
-5	3	-1	5.88	0.40
4	-3	1	57.37	2.31
4	-3	1	55.85	2.22
4	-3	1	55.89	2.17
4	-3	1	52.55	2.14
-4	3	-1	55.31	2.14
-4	3	-1	53.84	2.33
-4	3	-1	55.56	2.24
-4	3	-1	53.52	2.16
3	-3	1	5.96	0.41
3	-3	1	5.76	0.31
3	-3	1	5.56	0.51
3	-3	1	5.17	0.37
-3	3	-1	4.79	0.40
-3	3	-1	6.13	0.31
-3	3	-1	5.76	0.38
-3	3	-1	5.31	0.51
2	-3	1	45.84	1.79
2	-3	1	46.91	1.87
2	-3	1	48.14	1.86
2	-3	1	46.41	1.96
-2	3	-1	45.84	1.87
-2	3	-1	47.06	1.79
-2	3	-1	47.40	1.99
-2	3	-1	45.87	1.86
1	-3	1	23.96	1.11
1	-3	1	22.66	1.00
1	-3	1	22.64	0.91
1	-3	1	24.29	1.02
-1	3	-1	24.23	1.13
-1	3	-1	23.94	0.92
-1	3	-1	22.32	1.00
-1	3	-1	24.31	1.03
0	-3	1	188.58	7.43
0	-3	1	206.22	7.63
0	-3	1	202.12	7.52
0	3	-1	202.40	7.57

0	3	-1	213.26	7.65
0	3	-1	189.45	7.43
0	3	-1	202.24	7.52
-1	-3	1	89.78	3.53
-1	-3	1	85.82	3.33
-1	-3	1	93.21	3.49
-1	-3	1	92.14	3.54
1	3	-1	90.69	3.41
1	3	-1	83.33	3.33
1	3	-1	90.10	3.54
-2	-3	1	365.83	14.04
-2	-3	1	359.72	13.95
-2	-3	1	400.52	14.18
-2	-3	1	368.59	14.18
-2	-3	1	389.26	14.13
-2	-3	1	368.91	13.96
2	3	-1	371.14	14.03
2	3	-1	366.51	13.95
2	3	-1	402.76	14.17
-3	-3	1	111.05	4.32
-3	-3	1	116.37	4.47
-3	-3	1	116.02	4.24
-3	-3	1	113.76	4.50
3	3	-1	108.68	4.31
3	3	-1	118.69	4.46
3	3	-1	115.39	4.49
3	3	-1	106.49	4.24
-4	-3	1	144.16	5.28
-4	-3	1	140.17	5.39
-4	-3	1	140.57	5.37
-4	-3	1	141.06	5.57
-4	-3	1	136.75	5.50
4	3	-1	146.38	5.55
4	3	-1	139.60	5.36
4	3	-1	138.99	5.28
-5	-3	1	3.39	0.36
-5	-3	1	4.03	0.41
-5	-3	1	3.38	0.25
-5	-3	1	3.28	0.62
-5	-3	1	4.27	0.56
-5	-3	1	4.02	0.38
5	3	-1	4.02	0.61
5	3	-1	3.56	0.39
-6	-3	1	205.09	8.37

-6	-3	1	204.95	8.22
-6	-3	1	215.85	8.47
-6	-3	1	221.32	8.13
-6	-3	1	208.24	8.24
-6	-3	1	220.58	8.22
6	3	-1	222.93	8.42
6	3	-1	226.37	8.26
6	3	-1	225.96	8.14
-7	-3	1	0.26	0.24
-7	-3	1	0.53	0.50
-7	-3	1	0.29	0.23
-7	-3	1	0.22	0.25
-7	-3	1	0.42	0.16
7	3	-1	0.45	0.28
7	3	-1	0.88	0.46
7	3	-1	0.35	0.16
-8	-3	1	217.84	8.75
-8	-3	1	225.30	8.45
-8	-3	1	227.06	8.36
-8	-3	1	216.31	8.51
8	3	-1	228.53	8.51
8	3	-1	212.98	8.36
8	3	-1	223.58	8.48
8	3	-1	222.68	8.68
-9	-3	1	3.08	0.47
-9	-3	1	3.64	0.40
-9	-3	1	3.26	0.73
-9	-3	1	3.58	0.56
-9	-3	1	3.20	0.45
9	3	-1	2.24	0.31
9	3	-1	3.44	0.49
9	3	-1	3.18	0.65
-10	-3	1	135.06	5.96
-10	-3	1	141.42	5.58
-10	-3	1	148.10	5.49
-10	-3	1	139.55	5.71
10	3	-1	141.36	5.50
10	3	-1	143.97	5.85
10	3	-1	146.57	5.62
10	3	-1	144.61	5.68
-11	-3	1	14.95	1.53
-11	-3	1	13.28	0.99
-11	-3	1	12.65	1.10
-11	-3	1	14.45	0.82

11	3	-1	14.45	1.34
11	3	-1	13.39	1.06
11	3	-1	13.61	1.01
11	3	-1	14.10	0.82
-12	-3	1	89.40	3.77
-12	-3	1	89.72	3.93
-12	-3	1	91.51	3.65
-12	-3	1	89.53	4.23
12	3	-1	90.48	3.86
12	3	-1	93.38	3.77
12	3	-1	93.76	3.68
12	3	-1	92.02	4.07
-13	-3	1	13.49	0.88
-13	-3	1	11.17	1.33
-13	-3	1	11.80	1.35
-13	-3	1	13.13	1.22
-13	-3	1	14.00	1.74
13	3	-1	13.28	1.04
13	3	-1	12.09	0.85
13	3	-1	12.48	1.16
-14	-3	1	192.34	7.53
-14	-3	1	184.98	7.80
-14	-3	1	189.71	7.56
-14	-3	1	165.63	7.52
14	3	-1	192.55	7.27
14	3	-1	196.35	7.33
-15	-3	1	6.99	1.12
-15	-3	1	3.71	0.95
-15	-3	1	4.13	1.24
15	3	-1	4.26	0.73
15	3	-1	5.49	0.77
-16	-3	1	84.64	3.98
-16	-3	1	81.68	3.58
-16	-3	1	80.24	4.13
-16	-3	1	77.67	3.82
-16	-3	1	85.71	3.92
16	3	-1	85.62	3.51
-17	-3	1	0.68	1.11
-17	-3	1	-1.71	1.32
-17	-3	1	-0.41	0.84
17	3	-1	0.70	0.49
-18	-3	1	30.57	2.74
-18	-3	1	34.36	2.51
18	3	-1	37.14	1.92

-19	-3	1	1.47	1.12
19	3	-1	-1.49	0.59
-20	-3	1	59.82	3.46
20	3	-1	52.90	2.75
-21	-3	1	-1.30	1.35
21	3	-1	2.46	0.88
20	-3	2	-0.17	0.67
-20	3	-2	-0.37	1.47
19	-3	2	0.22	1.28
19	-3	2	-0.71	0.59
-19	3	-2	-1.38	1.31
18	-3	2	5.36	1.11
18	-3	2	6.04	0.80
-18	3	-2	6.53	1.42
17	-3	2	1.77	0.60
17	-3	2	1.87	0.90
-17	3	-2	0.24	1.05
16	-3	2	18.09	1.57
16	-3	2	17.06	1.20
-16	3	-2	14.96	1.86
-16	3	-2	13.24	2.06
15	-3	2	2.75	0.77
15	-3	2	1.20	0.50
-15	3	-2	1.46	0.92
-15	3	-2	3.19	1.11
14	-3	2	11.19	1.23
14	-3	2	13.40	1.04
-14	3	-2	12.02	1.68
-14	3	-2	14.36	1.51
13	-3	2	14.29	1.24
13	-3	2	14.97	0.95
-13	3	-2	14.60	1.42
-13	3	-2	9.63	1.52
12	-3	2	2.22	0.62
12	-3	2	2.24	0.41
12	-3	2	1.70	0.56
-12	3	-2	2.20	0.67
-12	3	-2	1.77	0.78
-12	3	-2	1.94	0.42
11	-3	2	2.59	0.60
11	-3	2	2.53	0.41
11	-3	2	2.26	0.51
-11	3	-2	3.12	0.43
-11	3	-2	2.98	0.75

-11	3	-2	2.49	0.60
10	-3	2	41.76	1.94
10	-3	2	40.21	2.02
10	-3	2	40.56	1.79
-10	3	-2	38.99	2.02
-10	3	-2	39.71	2.12
-10	3	-2	43.61	1.80
9	-3	2	0.40	0.40
9	-3	2	0.82	0.37
9	-3	2	0.50	0.23
-9	3	-2	0.88	0.48
-9	3	-2	1.34	0.29
-9	3	-2	0.61	0.39
8	-3	2	21.05	1.03
8	-3	2	22.18	1.17
8	-3	2	21.47	1.27
-8	3	-2	20.88	1.21
-8	3	-2	23.90	1.05
-8	3	-2	21.19	1.32
7	-3	2	34.88	1.60
7	-3	2	36.89	1.72
7	-3	2	32.93	1.48
7	-3	2	35.21	1.46
-7	3	-2	33.98	1.64
-7	3	-2	37.48	1.50
6	-3	2	80.40	3.31
6	-3	2	77.48	3.06
6	-3	2	78.60	3.19
6	-3	2	79.86	3.11
-6	3	-2	79.51	3.05
-6	3	-2	78.65	3.23
-6	3	-2	77.12	3.33
-6	3	-2	85.92	3.11
5	-3	2	98.48	3.99
5	-3	2	98.26	3.80
5	-3	2	103.29	3.78
5	-3	2	96.74	3.90
-5	3	-2	105.06	3.81
-5	3	-2	94.43	4.01
-5	3	-2	100.58	3.91
-5	3	-2	96.76	3.77
4	-3	2	4.13	0.51
4	-3	2	3.20	0.37
4	-3	2	2.74	0.25

4	-3	2	2.77	0.26
-4	3	-2	2.91	0.24
-4	3	-2	2.59	0.41
-4	3	-2	3.14	0.28
-4	3	-2	2.77	0.39
3	-3	2	5.06	0.52
3	-3	2	5.58	0.41
3	-3	2	5.19	0.30
3	-3	2	5.18	0.35
-3	3	-2	5.51	0.30
-3	3	-2	5.65	0.37
-3	3	-2	4.93	0.54
2	-3	2	5.66	0.39
2	-3	2	5.13	0.50
2	-3	2	5.74	0.40
2	-3	2	5.90	0.29
-2	3	-2	5.73	0.40
-2	3	-2	5.85	0.29
-2	3	-2	6.11	0.55
-2	3	-2	5.63	0.40
1	-3	2	417.16	15.70
1	-3	2	391.07	15.60
1	-3	2	433.71	15.81
1	-3	2	432.86	15.78
1	-3	2	433.42	15.72
-1	3	-2	433.19	15.82
-1	3	-2	409.35	15.69
0	-3	2	30.00	1.40
0	-3	2	31.11	1.28
0	-3	2	32.48	1.40
0	-3	2	29.77	1.34
0	-3	2	30.36	1.16
0	3	-2	30.67	1.38
0	3	-2	32.51	1.41
0	3	-2	31.43	1.28
0	3	-2	30.13	1.16
-1	-3	2	7.66	0.54
-1	-3	2	7.23	0.60
-1	-3	2	6.99	0.43
1	3	-2	7.26	0.59
1	3	-2	7.21	0.55
1	3	-2	7.27	0.42
1	3	-2	7.01	0.29
-2	-3	2	42.43	1.92

-2	-3	2	42.12	1.82
-2	-3	2	44.70	1.73
-2	-3	2	41.01	1.60
2	3	-2	43.91	1.83
2	3	-2	43.47	1.90
2	3	-2	41.67	1.72
-3	-3	2	1.62	0.38
-3	-3	2	0.66	0.16
-3	-3	2	1.24	0.12
-3	-3	2	1.52	0.28
3	3	-2	1.12	0.12
3	3	-2	1.04	0.28
3	3	-2	1.52	0.36
-4	-3	2	1.45	0.22
-4	-3	2	1.65	0.48
-4	-3	2	2.00	0.33
-4	-3	2	1.57	0.16
4	3	-2	1.74	0.32
4	3	-2	1.18	0.39
4	3	-2	1.64	0.28
4	3	-2	1.49	0.16
-5	-3	2	14.65	0.85
-5	-3	2	13.39	1.08
-5	-3	2	14.26	0.75
-5	-3	2	14.14	0.75
-5	-3	2	13.85	0.62
-5	-3	2	14.14	0.73
5	3	-2	14.14	0.63
5	3	-2	15.78	1.04
-6	-3	2	18.11	0.87
-6	-3	2	18.12	1.02
-6	-3	2	16.23	0.91
-6	-3	2	18.31	1.25
-6	-3	2	17.76	0.92
-6	-3	2	19.06	0.79
6	3	-2	18.42	1.22
6	3	-2	17.41	0.79
-7	-3	2	195.04	7.65
-7	-3	2	194.70	7.24
-7	-3	2	191.45	7.34
-7	-3	2	195.26	7.33
-7	-3	2	181.26	7.37
7	3	-2	183.90	7.78
7	3	-2	193.96	7.19

-8	-3	2	3.96	0.46
-8	-3	2	3.66	0.54
-8	-3	2	4.45	0.70
-8	-3	2	3.23	0.54
-8	-3	2	3.11	0.74
-8	-3	2	3.67	0.36
8	3	-2	3.82	0.36
8	3	-2	5.12	0.91
-9	-3	2	35.77	1.91
-9	-3	2	39.46	1.87
-9	-3	2	43.38	1.68
-9	-3	2	38.65	1.72
-9	-3	2	38.77	2.26
-9	-3	2	40.39	1.84
9	3	-2	40.00	1.68
9	3	-2	40.87	2.14
-10	-3	2	9.64	0.61
-10	-3	2	6.73	1.30
-10	-3	2	8.26	0.99
-10	-3	2	8.66	0.80
-10	-3	2	8.70	0.69
-10	-3	2	7.96	0.81
10	3	-2	9.14	1.19
10	3	-2	8.47	0.85
10	3	-2	8.71	0.59
-11	-3	2	40.64	2.48
-11	-3	2	43.15	2.03
-11	-3	2	39.84	1.86
-11	-3	2	42.88	1.81
-11	-3	2	40.02	2.07
11	3	-2	42.38	1.82
11	3	-2	43.04	2.05
11	3	-2	43.86	2.38
-12	-3	2	17.61	1.51
-12	-3	2	16.55	1.28
-12	-3	2	16.09	1.05
-12	-3	2	15.98	1.19
-12	-3	2	18.25	0.96
-12	-3	2	16.54	1.78
12	3	-2	14.71	0.93
12	3	-2	13.62	1.63
12	3	-2	18.79	1.27
-13	-3	2	46.30	2.88
-13	-3	2	48.23	2.46

-13	-3	2	45.23	2.16
-13	-3	2	52.45	2.15
-13	-3	2	47.82	2.54
13	3	-2	51.62	2.78
13	3	-2	50.12	2.15
-14	-3	2	16.11	1.13
-14	-3	2	15.62	1.64
-14	-3	2	17.46	2.03
-14	-3	2	16.47	1.41
14	3	-2	15.69	1.04
14	3	-2	16.62	1.92
-15	-3	2	30.27	2.13
-15	-3	2	29.47	1.61
-15	-3	2	30.47	2.51
-15	-3	2	31.01	2.12
15	3	-2	31.35	1.58
15	3	-2	31.46	2.42
-16	-3	2	0.89	1.35
-16	-3	2	0.09	0.70
-16	-3	2	0.90	0.96
-16	-3	2	1.10	0.66
-16	-3	2	0.90	0.99
16	3	-2	1.32	0.48
-17	-3	2	38.66	2.46
-17	-3	2	39.80	2.87
-17	-3	2	43.40	3.04
-17	-3	2	37.13	2.39
17	3	-2	37.15	1.96
-18	-3	2	7.05	1.27
-18	-3	2	7.11	1.66
18	3	-2	7.10	1.01
-19	-3	2	23.42	2.31
19	3	-2	24.75	1.62
-20	-3	2	1.26	1.18
20	3	-2	2.72	0.75
-21	-3	2	31.24	2.83
21	3	-2	29.36	1.98
20	-3	3	11.82	1.40
-20	3	-3	9.53	1.87
19	-3	3	0.66	0.69
19	-3	3	1.62	1.16
-19	3	-3	-1.31	1.32
18	-3	3	13.62	1.88
18	-3	3	14.24	1.31

-18	3	-3	13.71	2.22
17	-3	3	0.18	0.56
17	-3	3	-0.27	0.90
-17	3	-3	0.43	1.16
16	-3	3	8.86	0.99
16	-3	3	10.20	1.41
-16	3	-3	5.86	1.26
-16	3	-3	8.16	1.47
15	-3	3	3.66	0.59
15	-3	3	3.04	0.86
-15	3	-3	0.92	0.89
-15	3	-3	1.62	1.12
14	-3	3	9.29	1.25
14	-3	3	12.07	1.30
-14	3	-3	10.29	1.44
-14	3	-3	11.24	1.39
13	-3	3	9.15	1.15
13	-3	3	7.46	0.77
-13	3	-3	8.54	1.26
-13	3	-3	9.17	1.53
12	-3	3	14.36	0.95
12	-3	3	15.91	1.22
12	-3	3	14.91	1.28
-12	3	-3	14.94	1.51
-12	3	-3	15.42	1.35
-12	3	-3	15.73	1.06
11	-3	3	7.03	0.91
11	-3	3	7.76	1.00
11	-3	3	7.87	0.68
-11	3	-3	7.32	1.22
-11	3	-3	9.07	0.69
-11	3	-3	8.63	1.03
10	-3	3	6.42	0.77
10	-3	3	4.99	0.74
10	-3	3	5.13	0.55
-10	3	-3	7.07	0.94
-10	3	-3	6.67	0.88
-10	3	-3	7.03	0.57
9	-3	3	0.39	0.24
9	-3	3	0.30	0.33
9	-3	3	1.00	0.44
-9	3	-3	0.78	0.42
-9	3	-3	0.70	0.28
-9	3	-3	0.87	0.55

8	-3	3	0.58	0.34
8	-3	3	0.29	0.41
8	-3	3	0.27	0.19
-8	3	-3	0.81	0.22
-8	3	-3	1.48	0.51
-8	3	-3	0.45	0.33
7	-3	3	13.82	0.98
7	-3	3	12.34	0.81
7	-3	3	12.36	0.70
-7	3	-3	11.97	0.85
-7	3	-3	13.21	1.05
-7	3	-3	14.65	0.71
6	-3	3	4.16	0.65
6	-3	3	3.51	0.35
6	-3	3	4.10	0.47
-6	3	-3	4.38	0.71
-6	3	-3	4.48	0.36
-6	3	-3	4.07	0.52
5	-3	3	16.66	0.72
5	-3	3	16.48	0.77
5	-3	3	17.40	1.02
5	-3	3	17.16	0.79
-5	3	-3	16.55	0.89
-5	3	-3	15.66	1.08
4	-3	3	0.05	0.13
4	-3	3	-0.02	0.18
4	-3	3	0.00	0.12
4	-3	3	0.18	0.20
4	-3	3	0.09	0.27
-4	3	-3	0.17	0.11
-4	3	-3	-0.09	0.29
-4	3	-3	0.05	0.18
3	-3	3	27.11	1.10
3	-3	3	27.40	1.23
3	-3	3	26.95	1.34
3	-3	3	26.53	1.14
3	-3	3	27.05	1.20
-3	3	-3	26.81	1.21
-3	3	-3	27.04	1.35
-3	3	-3	27.01	1.09
2	-3	3	3.42	0.34
2	-3	3	3.34	0.34
2	-3	3	3.36	0.45
2	-3	3	3.29	0.21

-2	3	-3	4.10	0.50
-2	3	-3	2.93	0.34
-2	3	-3	3.23	0.21
1	-3	3	80.28	3.14
1	-3	3	72.75	2.90
1	-3	3	77.35	3.02
1	-3	3	78.04	3.14
-1	3	-3	76.90	3.10
-1	3	-3	76.75	3.02
-1	3	-3	81.61	3.16
-1	3	-3	75.98	2.90
0	-3	3	7.08	0.57
0	-3	3	7.21	0.64
0	-3	3	8.65	0.46
0	-3	3	6.59	0.29
0	3	-3	6.26	0.29
0	3	-3	6.80	0.59
0	3	-3	7.23	0.58
0	3	-3	7.63	0.44
-1	-3	3	0.11	0.22
-1	-3	3	0.30	0.30
-1	-3	3	0.26	0.14
1	3	-3	0.42	0.14
1	3	-3	0.69	0.24
1	3	-3	0.52	0.29
-2	-3	3	23.59	1.04
-2	-3	3	23.31	0.88
-2	-3	3	20.34	1.40
-2	-3	3	24.05	1.12
2	3	-3	25.11	1.14
2	3	-3	23.64	1.04
2	3	-3	23.18	1.26
-3	-3	3	29.29	1.32
-3	-3	3	27.03	1.25
-3	-3	3	29.61	1.59
-3	-3	3	29.26	1.12
3	3	-3	30.07	1.52
3	3	-3	28.63	1.12
3	3	-3	28.24	1.25
3	3	-3	31.30	1.35
-4	-3	3	40.99	1.78
-4	-3	3	40.64	1.60
-4	-3	3	43.22	2.09
-4	-3	3	41.35	1.74

4	3	-3	41.37	1.79
4	3	-3	43.43	1.73
4	3	-3	40.24	1.60
4	3	-3	43.11	2.03
-5	-3	3	19.63	0.97
-5	-3	3	17.26	0.91
-5	-3	3	18.83	0.77
-5	-3	3	17.62	1.24
-5	-3	3	17.72	1.00
5	3	-3	17.75	1.22
5	3	-3	18.53	0.77
5	3	-3	18.54	0.96
-6	-3	3	1.74	0.23
-6	-3	3	2.06	0.32
-6	-3	3	2.31	0.34
-6	-3	3	2.58	0.38
-6	-3	3	2.24	0.37
-6	-3	3	1.63	0.55
6	3	-3	1.71	0.24
-7	-3	3	63.07	3.02
-7	-3	3	62.29	2.48
-7	-3	3	62.78	2.62
-7	-3	3	58.03	2.58
-7	-3	3	65.19	2.45
-7	-3	3	61.45	2.60
7	3	-3	63.99	2.94
7	3	-3	63.58	2.45
-8	-3	3	77.56	3.37
-8	-3	3	78.22	3.32
-8	-3	3	88.36	3.39
-8	-3	3	80.49	3.77
-8	-3	3	86.39	3.20
-8	-3	3	82.32	3.20
8	3	-3	77.88	3.19
8	3	-3	85.91	3.72
-9	-3	3	1.43	0.28
-9	-3	3	1.25	0.24
-9	-3	3	0.49	0.40
-9	-3	3	1.23	0.36
-9	-3	3	0.75	0.34
-9	-3	3	1.41	0.69
9	3	-3	0.58	0.63
9	3	-3	1.12	0.23
-10	-3	3	0.51	0.35

-10	-3	3	0.46	0.45
-10	-3	3	0.66	0.37
-10	-3	3	1.77	0.82
-10	-3	3	0.48	0.28
-10	-3	3	0.50	0.24
10	3	-3	0.49	0.22
10	3	-3	0.18	0.67
-11	-3	3	0.57	0.88
-11	-3	3	0.95	0.47
-11	-3	3	0.53	0.48
-11	-3	3	1.20	0.29
-11	-3	3	0.94	0.35
-11	-3	3	0.72	0.44
11	3	-3	0.97	0.28
11	3	-3	0.73	0.83
-12	-3	3	1.01	0.57
-12	-3	3	-0.89	0.87
-12	-3	3	1.02	0.38
-12	-3	3	0.98	0.30
-12	-3	3	0.56	0.52
-12	-3	3	0.73	0.49
12	3	-3	0.59	0.87
12	3	-3	0.68	0.28
-13	-3	3	1.48	0.35
-13	-3	3	-1.10	0.96
-13	-3	3	0.90	0.61
-13	-3	3	0.47	0.39
-13	-3	3	1.58	0.59
13	3	-3	1.46	1.02
13	3	-3	0.71	0.33
-14	-3	3	23.97	1.67
-14	-3	3	25.97	1.33
-14	-3	3	27.42	2.38
-14	-3	3	22.92	1.23
-14	-3	3	23.29	1.50
14	3	-3	25.87	2.26
14	3	-3	24.04	1.30
-15	-3	3	6.36	0.80
-15	-3	3	7.27	1.08
-15	-3	3	7.25	1.47
-15	-3	3	6.23	0.97
15	3	-3	11.43	2.00
15	3	-3	7.37	0.76
-16	-3	3	11.06	1.46

-16	-3	3	9.63	1.69
16	3	-3	8.78	1.35
16	3	-3	12.82	2.21
-17	-3	3	-0.30	1.47
-17	-3	3	1.04	0.89
17	3	-3	2.27	1.46
17	3	-3	0.71	0.49
-18	-3	3	6.10	1.22
-18	-3	3	5.99	1.70
18	3	-3	5.05	0.74
-19	-3	3	1.61	1.66
-19	-3	3	4.24	1.16
19	3	-3	3.56	0.75
-20	-3	3	10.34	1.58
20	3	-3	13.88	1.35
-21	-3	3	5.75	1.52
21	3	-3	5.94	0.93
20	-3	4	2.13	0.86
-20	3	-4	1.58	1.65
19	-3	4	16.61	1.50
19	-3	4	20.60	2.25
-19	3	-4	18.32	2.61
18	-3	4	2.11	0.71
18	-3	4	2.30	1.15
-18	3	-4	1.86	1.29
17	-3	4	42.83	2.12
17	-3	4	36.43	2.55
-17	3	-4	38.95	2.81
16	-3	4	3.88	1.05
16	-3	4	4.16	0.70
-16	3	-4	4.61	1.45
-16	3	-4	4.45	1.12
15	-3	4	24.24	1.47
15	-3	4	22.71	1.81
-15	3	-4	24.83	2.05
-15	3	-4	25.90	2.33
14	-3	4	5.74	0.95
14	-3	4	3.95	0.57
-14	3	-4	4.60	0.95
-14	3	-4	5.71	1.28
13	-3	4	19.98	1.22
13	-3	4	19.79	1.57
-13	3	-4	20.31	1.88
-13	3	-4	22.20	1.69

12	-3	4	6.28	0.72
12	-3	4	8.57	1.15
12	-3	4	10.19	1.05
-12	3	-4	8.03	1.43
-12	3	-4	6.78	1.16
11	-3	4	73.92	3.00
11	-3	4	71.38	3.28
11	-3	4	70.83	3.12
-11	3	-4	70.63	3.44
-11	3	-4	75.73	2.99
-11	3	-4	69.87	3.24
10	-3	4	0.60	0.42
10	-3	4	0.18	0.25
10	-3	4	-0.26	0.50
-10	3	-4	0.20	0.63
-10	3	-4	0.06	0.45
-10	3	-4	0.30	0.26
9	-3	4	37.89	2.04
9	-3	4	40.71	1.85
9	-3	4	38.36	1.72
-9	3	-4	40.16	1.71
-9	3	-4	39.02	2.13
-9	3	-4	38.74	1.91
8	-3	4	1.85	0.37
8	-3	4	1.14	0.26
8	-3	4	0.65	0.47
-8	3	-4	1.14	0.38
-8	3	-4	1.65	0.61
-8	3	-4	1.12	0.25
7	-3	4	10.55	0.64
7	-3	4	12.25	0.77
7	-3	4	11.11	0.97
-7	3	-4	12.04	0.64
-7	3	-4	12.19	1.09
-7	3	-4	10.31	0.81
6	-3	4	12.40	0.74
6	-3	4	11.16	0.91
6	-3	4	10.58	0.63
-6	3	-4	11.75	0.78
-6	3	-4	12.59	1.04
-6	3	-4	12.82	0.64
5	-3	4	33.96	1.47
5	-3	4	35.74	1.42
5	-3	4	33.64	1.63

5	-3	4	36.14	1.52
5	-3	4	36.81	1.77
-5	3	-4	35.09	1.59
-5	3	-4	37.48	1.47
-5	3	-4	35.37	1.80
4	-3	4	8.84	0.57
4	-3	4	7.99	0.61
4	-3	4	8.29	0.73
4	-3	4	8.45	0.48
4	-3	4	7.88	0.45
-4	3	-4	7.74	0.57
-4	3	-4	8.54	0.45
3	-3	4	134.86	5.32
3	-3	4	138.71	5.42
3	-3	4	138.53	5.24
-3	3	-4	136.73	5.43
-3	3	-4	140.27	5.15
-3	3	-4	130.79	5.24
-3	3	-4	136.81	5.26
2	-3	4	69.08	2.70
2	-3	4	67.45	2.84
2	-3	4	66.17	2.59
2	-3	4	69.67	2.87
-2	3	-4	68.26	2.88
-2	3	-4	69.01	2.59
-2	3	-4	67.23	2.77
1	-3	4	1.75	0.36
1	-3	4	1.97	0.30
1	-3	4	1.76	0.15
1	-3	4	1.79	0.41
-1	3	-4	1.49	0.34
-1	3	-4	2.16	0.29
-1	3	-4	1.83	0.37
-1	3	-4	1.80	0.15
0	-3	4	37.70	1.55
0	-3	4	37.43	1.80
0	-3	4	35.56	1.41
0	3	-4	36.70	1.41
0	3	-4	35.95	1.54
0	3	-4	39.30	1.69
0	3	-4	38.15	1.75
-1	-3	4	3.74	0.47
-1	-3	4	4.21	0.36
1	3	-4	3.80	0.47

1	3	-4	3.37	0.38
1	3	-4	3.82	0.17
1	3	-4	4.31	0.61
-2	-3	4	40.81	1.66
-2	-3	4	38.68	1.72
-2	-3	4	39.29	1.49
-2	-3	4	40.16	1.99
2	3	-4	41.17	1.75
2	3	-4	39.43	1.65
2	3	-4	38.88	1.92
-3	-3	4	70.43	2.69
-3	-3	4	65.04	2.72
-3	-3	4	67.85	3.04
-3	-3	4	66.03	2.52
3	3	-4	66.70	2.99
3	3	-4	68.11	2.67
3	3	-4	65.32	2.52
3	3	-4	69.82	2.75
-4	-3	4	4.64	0.80
-4	-3	4	5.60	0.50
-4	-3	4	5.53	0.28
4	3	-4	5.62	0.43
4	3	-4	7.39	0.85
4	3	-4	5.21	0.27
4	3	-4	4.69	0.51
-5	-3	4	67.83	2.81
-5	-3	4	73.51	2.83
-5	-3	4	67.98	2.79
-5	-3	4	66.78	3.22
-5	-3	4	71.13	2.66
5	3	-4	71.01	3.23
5	3	-4	69.28	2.66
5	3	-4	71.38	2.83
5	3	-4	69.19	2.80
-6	-3	4	19.70	1.00
-6	-3	4	18.46	1.47
-6	-3	4	18.73	0.94
-6	-3	4	18.64	0.97
-6	-3	4	18.73	0.81
6	3	-4	17.24	0.85
6	3	-4	19.82	1.39
6	3	-4	19.66	0.98
-7	-3	4	2.60	0.34
-7	-3	4	1.21	0.64

-7	-3	4	2.27	0.36
-7	-3	4	2.27	0.28
-7	-3	4	2.45	0.43
-7	-3	4	1.81	0.35
7	3	-4	1.75	0.25
7	3	-4	1.70	0.64
-8	-3	4	39.48	1.62
-8	-3	4	37.47	1.69
-8	-3	4	42.02	1.64
-8	-3	4	40.79	1.84
-8	-3	4	36.97	2.30
-8	-3	4	39.81	1.84
8	3	-4	41.76	2.27
8	3	-4	40.21	1.63
-9	-3	4	15.39	0.78
-9	-3	4	15.05	1.01
-9	-3	4	15.59	0.75
-9	-3	4	15.37	0.85
-9	-3	4	16.73	1.63
-9	-3	4	14.96	0.99
9	3	-4	15.09	0.76
9	3	-4	13.68	1.46
-10	-3	4	7.28	0.53
-10	-3	4	6.42	0.77
-10	-3	4	7.21	0.78
-10	-3	4	5.71	1.10
-10	-3	4	7.21	0.61
-10	-3	4	7.38	0.50
10	3	-4	5.63	0.50
10	3	-4	7.36	1.09
-11	-3	4	1.75	0.50
-11	-3	4	1.98	0.34
-11	-3	4	2.28	0.53
-11	-3	4	1.38	0.94
-11	-3	4	1.87	0.29
-11	-3	4	1.76	0.39
11	3	-4	1.03	0.28
11	3	-4	3.66	1.02
-12	-3	4	7.99	0.98
-12	-3	4	6.57	0.62
-12	-3	4	5.77	1.22
-12	-3	4	8.84	0.96
12	3	-4	7.40	1.22
12	3	-4	6.01	0.59

-13	-3	4	13.32	0.88
-13	-3	4	11.67	1.23
-13	-3	4	12.42	2.04
13	3	-4	13.24	0.86
13	3	-4	13.64	1.94
-14	-3	4	1.43	0.64
-14	-3	4	0.45	1.12
-14	-3	4	1.31	0.42
14	3	-4	0.41	0.34
14	3	-4	-1.63	1.14
-15	-3	4	0.36	0.69
-15	-3	4	1.09	1.30
15	3	-4	-0.64	1.25
15	3	-4	-0.19	0.35
-16	-3	4	4.74	1.51
-16	-3	4	2.02	0.83
16	3	-4	3.91	1.42
-17	-3	4	0.92	1.57
-17	-3	4	0.89	0.91
17	3	-4	1.36	0.54
17	3	-4	2.54	1.68
-18	-3	4	3.92	1.05
-18	-3	4	6.87	1.71
18	3	-4	4.17	0.72
-19	-3	4	0.88	1.67
-19	-3	4	0.69	1.03
19	3	-4	0.34	0.60
-20	-3	4	4.40	1.21
20	3	-4	2.20	0.74
-21	-3	4	0.17	1.59
21	3	-4	-0.78	0.71
-20	3	-5	49.71	3.71
19	-3	5	1.94	1.34
19	-3	5	1.55	0.74
-19	3	-5	2.16	1.44
18	-3	5	59.33	3.40
18	-3	5	60.37	2.87
-18	3	-5	56.98	3.60
17	-3	5	3.69	1.16
17	-3	5	4.67	0.81
-17	3	-5	6.51	1.36
16	-3	5	44.33	2.72
16	-3	5	46.93	2.27
-16	3	-5	42.59	2.86

15	-3	5	-0.36	0.47
15	-3	5	0.36	0.88
-15	3	-5	0.06	0.88
-15	3	-5	-0.76	1.29
14	-3	5	80.51	3.82
14	-3	5	82.70	3.47
-14	3	-5	78.28	4.11
-14	3	-5	83.33	3.85
13	-3	5	-0.14	0.39
13	-3	5	0.22	0.72
-13	3	-5	0.32	0.98
-13	3	-5	0.93	0.74
12	-3	5	97.69	4.01
12	-3	5	95.89	4.15
12	-3	5	102.84	4.40
-12	3	-5	99.84	4.30
-12	3	-5	96.23	4.52
11	-3	5	2.29	0.71
11	-3	5	2.84	0.58
11	-3	5	2.09	0.42
-11	3	-5	3.51	0.93
-11	3	-5	3.21	0.68
-11	3	-5	2.93	0.42
10	-3	5	105.81	4.30
10	-3	5	103.27	4.52
10	-3	5	101.47	4.20
-10	3	-5	104.61	4.65
-10	3	-5	111.32	4.18
-10	3	-5	105.86	4.40
9	-3	5	6.72	0.96
9	-3	5	7.45	0.76
9	-3	5	6.20	0.59
-9	3	-5	7.09	1.15
-9	3	-5	6.09	0.79
-9	3	-5	6.98	0.56
8	-3	5	117.67	4.65
8	-3	5	113.80	4.90
8	-3	5	113.43	4.57
-8	3	-5	122.59	5.00
-8	3	-5	121.92	4.56
-8	3	-5	113.89	4.71
7	-3	5	13.96	1.16
7	-3	5	14.75	0.78
7	-3	5	13.97	0.87

-7	3	-5	13.92	0.93
-7	3	-5	13.37	1.25
-7	3	-5	15.76	0.77
6	-3	5	106.89	4.29
6	-3	5	115.02	4.39
6	-3	5	112.42	4.63
-6	3	-5	112.90	4.67
-6	3	-5	107.40	4.41
-6	3	-5	112.74	4.28
5	-3	5	2.17	0.34
5	-3	5	1.65	0.31
5	-3	5	1.95	0.50
5	-3	5	2.11	0.30
5	-3	5	1.73	0.33
-5	3	-5	1.60	0.22
-5	3	-5	1.69	0.32
-5	3	-5	1.73	0.51
4	-3	5	15.88	1.06
4	-3	5	16.85	0.85
4	-3	5	15.83	0.93
4	-3	5	14.76	0.72
-4	3	-5	15.66	1.09
-4	3	-5	15.33	0.84
-4	3	-5	15.08	0.72
-4	3	-5	15.66	0.84
3	-3	5	1.55	0.41
3	-3	5	1.50	0.41
3	-3	5	1.79	0.28
3	-3	5	1.96	0.24
-3	3	-5	1.50	0.44
-3	3	-5	1.82	0.34
-3	3	-5	1.57	0.30
-3	3	-5	1.94	0.22
2	-3	5	85.43	3.35
2	-3	5	91.88	3.47
2	-3	5	90.94	3.67
2	-3	5	91.06	3.67
-2	3	-5	89.77	3.47
-2	3	-5	90.16	3.35
-2	3	-5	87.78	3.59
-2	3	-5	78.99	3.79
1	-3	5	0.63	0.10
1	-3	5	0.50	0.20
1	-3	5	0.56	0.32

1	-3	5	0.91	0.39
-1	3	-5	0.70	0.35
-1	3	-5	0.99	0.36
-1	3	-5	0.51	0.21
-1	3	-5	0.65	0.10
0	-3	5	194.11	7.06
0	-3	5	191.16	7.37
0	-3	5	184.24	7.20
0	3	-5	171.25	7.11
0	3	-5	190.67	7.30
0	3	-5	184.26	6.92
0	3	-5	189.73	7.20
0	3	-5	181.45	6.91
-1	-3	5	27.72	1.19
-1	-3	5	24.37	1.33
-1	-3	5	25.51	1.55
1	3	-5	27.07	1.31
1	3	-5	26.38	1.48
1	3	-5	26.65	1.02
1	3	-5	26.95	1.02
1	3	-5	26.67	1.18
-2	-3	5	71.53	3.25
-2	-3	5	71.74	2.72
-2	-3	5	74.24	2.89
-2	-3	5	70.93	2.99
2	3	-5	70.46	2.71
2	3	-5	71.44	2.87
2	3	-5	74.88	3.22
2	3	-5	75.71	2.98
-3	-3	5	1.50	0.52
-3	-3	5	1.51	0.13
-3	-3	5	1.27	0.31
-3	-3	5	0.92	0.23
3	3	-5	1.35	0.12
3	3	-5	1.37	0.32
3	3	-5	1.16	0.23
-4	-3	5	122.14	4.47
-4	-3	5	114.62	4.64
-4	-3	5	116.93	4.65
-4	-3	5	127.51	5.13
4	3	-5	122.93	5.04
4	3	-5	113.35	4.46
4	3	-5	124.78	4.68
4	3	-5	113.90	4.61

-5	-3	5	33.95	1.53
-5	-3	5	36.13	1.36
-5	-3	5	32.03	2.01
-5	-3	5	35.22	1.51
5	3	-5	33.29	1.50
5	3	-5	34.86	1.96
5	3	-5	35.33	1.56
5	3	-5	33.10	1.35
-6	-3	5	19.07	0.84
-6	-3	5	21.45	1.05
-6	-3	5	18.29	1.01
-6	-3	5	19.22	0.96
-6	-3	5	19.00	1.53
6	3	-5	21.23	1.03
6	3	-5	18.42	1.50
-7	-3	5	37.40	1.69
-7	-3	5	35.84	1.53
-7	-3	5	34.46	1.65
-7	-3	5	37.70	2.22
-7	-3	5	38.23	1.48
7	3	-5	34.25	1.46
7	3	-5	37.13	2.15
7	3	-5	35.63	1.60
-8	-3	5	17.92	0.82
-8	-3	5	17.70	1.04
-8	-3	5	16.74	0.79
-8	-3	5	15.51	1.59
-8	-3	5	16.72	1.03
-8	-3	5	17.99	0.85
8	3	-5	16.35	0.80
8	3	-5	19.45	1.60
-9	-3	5	0.38	0.34
-9	-3	5	0.48	0.36
-9	-3	5	0.96	0.23
-9	-3	5	0.70	0.78
-9	-3	5	0.78	0.22
9	3	-5	1.40	0.77
9	3	-5	0.22	0.18
-10	-3	5	87.36	3.25
-10	-3	5	82.81	3.47
-10	-3	5	76.30	3.98
-10	-3	5	79.61	3.47
10	3	-5	79.87	3.93
10	3	-5	82.20	3.22

-11	-3	5	0.95	0.30
-11	-3	5	0.83	0.46
-11	-3	5	1.05	1.01
-11	-3	5	0.17	0.46
11	3	-5	1.43	0.93
11	3	-5	0.96	0.26
-12	-3	5	4.08	0.69
-12	-3	5	3.63	1.22
-12	-3	5	4.21	0.72
-12	-3	5	5.31	0.60
12	3	-5	6.02	1.32
12	3	-5	5.07	0.53
-13	-3	5	3.27	1.30
-13	-3	5	2.90	0.70
-13	-3	5	2.63	0.45
13	3	-5	2.28	0.40
13	3	-5	1.51	1.26
-14	-3	5	2.16	1.31
-14	-3	5	2.98	0.50
-14	-3	5	3.31	1.04
14	3	-5	4.05	0.61
14	3	-5	3.05	1.29
-15	-3	5	0.29	1.31
-15	-3	5	-1.39	0.60
15	3	-5	-0.54	0.34
15	3	-5	0.86	1.34
-16	-3	5	57.66	3.06
-16	-3	5	60.91	3.77
16	3	-5	59.36	3.68
16	3	-5	54.77	2.58
-17	-3	5	2.91	1.01
-17	-3	5	6.06	1.87
17	3	-5	6.89	0.90
-18	-3	5	17.24	2.90
-18	-3	5	20.86	2.00
18	3	-5	17.03	1.28
-19	-3	5	2.98	1.80
-19	-3	5	1.84	1.14
19	3	-5	3.45	0.69
-20	-3	5	3.49	1.34
20	3	-5	-0.68	0.64
19	-3	6	50.97	2.65
19	-3	6	51.07	3.43
-19	3	-6	45.24	3.57

18	-3	6	-1.38	1.22
18	-3	6	-0.68	0.70
-18	3	-6	-0.06	1.28
17	-3	6	110.64	4.52
-17	3	-6	99.87	5.06
16	-3	6	-1.12	0.99
16	-3	6	0.49	0.54
-16	3	-6	0.24	1.04
15	-3	6	45.50	2.69
15	-3	6	46.39	2.24
-15	3	-6	44.07	2.74
14	-3	6	1.55	0.93
14	-3	6	1.32	0.51
-14	3	-6	1.95	0.88
13	-3	6	65.50	3.30
-13	3	-6	62.89	3.58
-13	3	-6	69.89	3.27
12	-3	6	0.22	0.34
12	-3	6	0.84	0.65
12	-3	6	-0.10	0.71
-12	3	-6	0.23	0.65
-12	3	-6	0.61	0.94
11	-3	6	143.87	5.71
11	-3	6	145.06	6.09
11	-3	6	140.75	5.79
-11	3	-6	151.68	5.70
-11	3	-6	144.12	5.93
-11	3	-6	144.20	6.23
10	-3	6	2.83	0.56
10	-3	6	4.89	0.87
10	-3	6	2.67	0.42
-10	3	-6	5.17	0.94
-10	3	-6	4.61	0.70
-10	3	-6	4.31	0.57
9	-3	6	116.92	4.63
9	-3	6	117.57	4.70
9	-3	6	111.94	4.98
-9	3	-6	123.13	4.61
-9	3	-6	115.08	5.10
-9	3	-6	119.38	4.80
8	-3	6	17.06	0.91
8	-3	6	15.75	1.00
8	-3	6	14.84	1.33
-8	3	-6	18.29	1.48

-8	3	-6	15.31	1.07
-8	3	-6	18.35	0.90
7	-3	6	156.19	5.84
7	-3	6	142.57	5.75
7	-3	6	145.16	6.12
-7	3	-6	151.43	6.20
-7	3	-6	145.38	5.88
-7	3	-6	157.17	5.74
6	-3	6	7.11	0.89
6	-3	6	7.77	0.64
6	-3	6	6.66	0.50
6	-3	6	7.51	0.55
-6	3	-6	7.12	1.00
-6	3	-6	6.09	0.65
-6	3	-6	7.75	0.50
5	-3	6	368.76	13.25
5	-3	6	353.38	13.54
5	-3	6	356.49	13.24
5	-3	6	341.45	13.16
-5	3	-6	349.84	13.15
-5	3	-6	347.43	13.55
-5	3	-6	353.40	13.27
-5	3	-6	344.61	13.19
4	-3	6	2.36	0.48
4	-3	6	2.82	0.31
4	-3	6	1.81	0.47
-4	3	-6	2.97	0.43
-4	3	-6	1.85	0.50
-4	3	-6	2.63	0.33
-4	3	-6	3.37	0.47
3	-3	6	137.87	5.37
3	-3	6	139.30	5.19
3	-3	6	126.91	5.07
3	-3	6	130.86	5.42
-3	3	-6	132.05	5.19
-3	3	-6	131.61	5.29
-3	3	-6	135.19	5.45
-3	3	-6	138.99	5.07
2	-3	6	5.50	0.49
2	-3	6	4.97	0.38
2	-3	6	5.58	0.71
-2	3	-6	5.59	0.70
-2	3	-6	5.44	0.49
-2	3	-6	6.07	0.34

-2	3	-6	5.98	0.74
1	-3	6	164.38	6.62
1	-3	6	161.78	6.51
1	-3	6	168.12	6.31
-1	3	-6	164.54	6.55
-1	3	-6	170.90	6.31
-1	3	-6	160.58	6.19
-1	3	-6	165.68	6.17
-1	3	-6	164.44	6.54
0	-3	6	14.50	0.96
0	-3	6	14.15	1.18
0	-3	6	15.31	0.79
0	3	-6	14.95	0.62
0	3	-6	14.73	1.11
0	3	-6	14.71	0.78
0	3	-6	15.72	0.65
0	3	-6	14.51	0.96
-1	-3	6	139.31	5.72
-1	-3	6	153.13	5.59
-1	-3	6	148.10	5.98
1	3	-6	142.61	5.56
1	3	-6	152.75	5.73
1	3	-6	146.16	5.94
1	3	-6	143.26	5.42
1	3	-6	137.92	5.42
-2	-3	6	0.30	0.31
-2	-3	6	0.14	0.08
-2	-3	6	0.26	0.21
-2	-3	6	0.36	0.51
2	3	-6	0.42	0.32
2	3	-6	0.23	0.07
2	3	-6	0.16	0.49
2	3	-6	0.10	0.17
-3	-3	6	210.64	8.60
-3	-3	6	214.36	7.96
3	3	-6	213.51	8.11
3	3	-6	207.61	7.95
3	3	-6	214.78	8.57
3	3	-6	223.74	8.22
-4	-3	6	19.14	0.93
-4	-3	6	17.08	0.72
-4	-3	6	16.58	1.40
-4	-3	6	16.51	0.96
4	3	-6	17.91	0.90

4	3	-6	18.76	0.99
4	3	-6	16.78	0.71
4	3	-6	16.27	1.37
-5	-3	6	149.87	5.62
-5	-3	6	139.46	5.58
-5	-3	6	142.24	5.42
-5	-3	6	144.91	6.15
-5	-3	6	142.21	5.60
5	3	-6	139.27	5.57
5	3	-6	158.31	5.64
5	3	-6	140.62	5.41
5	3	-6	143.95	6.08
-6	-3	6	86.85	3.58
-6	-3	6	87.20	4.13
-6	-3	6	91.41	3.39
-6	-3	6	86.96	3.57
-6	-3	6	87.11	3.49
6	3	-6	86.28	3.41
6	3	-6	93.83	3.56
6	3	-6	88.69	4.11
-7	-3	6	92.81	3.88
-7	-3	6	98.77	4.61
-7	-3	6	105.20	3.84
-7	-3	6	99.14	4.03
-7	-3	6	102.22	4.05
7	3	-6	101.62	3.96
7	3	-6	104.66	4.58
7	3	-6	98.28	3.82
-8	-3	6	14.90	0.72
-8	-3	6	14.31	0.71
-8	-3	6	12.84	1.52
-8	-3	6	13.48	0.95
-8	-3	6	14.37	0.70
-8	-3	6	11.84	0.91
8	3	-6	13.97	0.69
8	3	-6	14.84	1.58
-9	-3	6	115.99	5.33
-9	-3	6	116.42	4.75
-9	-3	6	116.40	4.73
-9	-3	6	123.99	4.51
9	3	-6	116.87	5.26
9	3	-6	111.00	4.47
-10	-3	6	-0.12	0.40
-10	-3	6	0.64	0.91

-10	-3	6	0.21	0.24
-10	-3	6	-0.27	0.38
10	3	-6	0.08	0.20
10	3	-6	1.52	0.97
-11	-3	6	126.72	5.26
-11	-3	6	137.42	5.02
-11	-3	6	127.43	5.91
-11	-3	6	124.72	5.26
11	3	-6	125.27	4.97
11	3	-6	131.14	5.81
-12	-3	6	-0.06	0.48
-12	-3	6	-0.41	1.06
-12	-3	6	1.09	0.34
12	3	-6	0.03	1.11
12	3	-6	0.51	0.27
-13	-3	6	77.75	3.61
-13	-3	6	84.70	3.30
-13	-3	6	79.83	4.31
13	3	-6	77.07	3.23
13	3	-6	81.44	4.25
-14	-3	6	-0.25	0.60
-14	-3	6	0.17	0.41
-14	-3	6	-0.24	1.29
14	3	-6	-0.45	0.32
14	3	-6	1.64	1.46
-15	-3	6	35.14	3.11
-15	-3	6	38.56	2.30
15	3	-6	38.80	1.79
15	3	-6	40.56	3.14
-16	-3	6	4.76	1.70
-16	-3	6	2.24	0.90
16	3	-6	2.12	0.50
-17	-3	6	72.05	3.63
-17	-3	6	71.07	4.41
17	3	-6	71.09	3.07
-18	-3	6	-1.28	1.71
-18	-3	6	-0.35	0.96
18	3	-6	-0.24	0.51
-19	-3	6	43.94	2.93
-19	-3	6	50.06	3.89
19	3	-6	44.11	2.27
-20	-3	6	3.34	2.09
-20	-3	6	2.54	1.31
20	3	-6	2.21	0.74

19	-3	7	3.28	0.93
19	-3	7	4.51	1.66
-19	3	-7	3.08	1.65
18	-3	7	29.85	1.91
18	-3	7	26.59	2.69
-18	3	-7	28.14	2.87
17	-3	7	1.60	1.25
17	-3	7	1.50	0.69
-17	3	-7	1.99	1.26
16	-3	7	17.01	1.39
16	-3	7	14.30	2.04
-16	3	-7	18.67	2.14
15	-3	7	1.68	0.62
15	-3	7	2.30	1.01
-15	3	-7	2.22	0.98
14	-3	7	35.62	2.31
14	-3	7	31.87	1.77
-14	3	-7	31.54	2.20
13	-3	7	26.93	1.50
13	-3	7	25.13	2.00
-13	3	-7	26.98	1.91
12	-3	7	50.55	2.27
12	-3	7	51.73	2.77
12	-3	7	46.68	2.42
-12	3	-7	49.45	2.99
-12	3	-7	49.86	2.59
11	-3	7	5.39	0.95
11	-3	7	6.45	0.98
11	-3	7	5.76	0.73
-11	3	-7	5.68	1.17
-11	3	-7	6.51	1.09
10	-3	7	77.19	3.76
10	-3	7	80.25	3.34
10	-3	7	82.86	3.44
-10	3	-7	82.07	3.30
-10	3	-7	82.96	3.95
-10	3	-7	80.50	3.53
9	-3	7	3.03	0.44
9	-3	7	1.69	0.70
9	-3	7	1.61	0.48
-9	3	-7	2.94	0.41
-9	3	-7	2.94	0.58
-9	3	-7	2.18	0.84
8	-3	7	167.10	6.84

8	-3	7	166.31	6.47
8	-3	7	165.00	6.41
-8	3	-7	165.38	6.55
-8	3	-7	173.50	6.39
-8	3	-7	160.18	6.88
7	-3	7	5.02	0.85
7	-3	7	3.82	0.60
7	-3	7	4.46	0.50
-7	3	-7	4.03	0.84
-7	3	-7	4.89	0.47
-7	3	-7	3.83	0.55
6	-3	7	138.75	5.44
6	-3	7	140.21	5.87
6	-3	7	141.32	5.45
6	-3	7	144.29	5.52
-6	3	-7	149.63	5.44
-6	3	-7	141.72	5.92
-6	3	-7	135.93	5.55
5	-3	7	16.59	0.84
5	-3	7	18.04	1.04
5	-3	7	17.08	1.27
5	-3	7	17.47	0.96
-5	3	-7	17.23	1.01
-5	3	-7	17.66	1.01
-5	3	-7	16.39	1.36
-5	3	-7	17.15	0.83
4	-3	7	166.72	6.22
4	-3	7	156.90	6.53
4	-3	7	165.38	6.35
4	-3	7	156.96	6.12
-4	3	-7	161.47	6.56
-4	3	-7	157.84	6.31
-4	3	-7	166.14	6.12
-4	3	-7	160.35	6.23
3	-3	7	14.34	0.70
3	-3	7	14.74	1.16
3	-3	7	14.15	1.12
3	-3	7	14.79	0.85
-3	3	-7	14.87	0.83
-3	3	-7	14.40	1.06
-3	3	-7	14.59	0.69
-3	3	-7	16.16	1.16
2	-3	7	91.85	3.48
2	-3	7	88.00	3.85

2	-3	7	92.18	3.92
2	-3	7	94.27	3.60
-2	3	-7	89.40	3.85
-2	3	-7	92.55	3.47
-2	3	-7	87.55	3.86
-2	3	-7	90.49	3.59
1	-3	7	0.23	0.23
1	-3	7	-0.39	0.43
1	-3	7	0.13	0.34
-1	3	-7	-0.10	0.51
-1	3	-7	0.00	0.13
-1	3	-7	-0.13	0.21
-1	3	-7	0.31	0.11
-1	3	-7	0.06	0.40
0	-3	7	139.91	5.89
0	-3	7	147.21	5.50
0	-3	7	137.62	5.70
0	3	-7	140.94	5.36
0	3	-7	143.35	5.35
0	3	-7	143.20	5.70
0	3	-7	145.68	5.49
-1	-3	7	10.08	0.64
-1	-3	7	8.28	1.10
-1	-3	7	9.33	0.81
1	3	-7	8.92	0.43
1	3	-7	8.84	0.44
1	3	-7	10.55	1.12
1	3	-7	9.30	0.60
-2	-3	7	146.18	5.44
-2	-3	7	138.60	6.08
-2	-3	7	140.86	5.75
2	3	-7	148.97	5.59
2	3	-7	147.55	6.07
2	3	-7	149.64	5.74
2	3	-7	145.03	5.43
-3	-3	7	0.97	0.58
-3	-3	7	0.38	0.37
-3	-3	7	0.22	0.11
-3	-3	7	0.64	0.25
3	3	-7	0.12	0.33
3	3	-7	0.61	0.23
3	3	-7	0.83	0.61
-4	-3	7	252.73	9.12
-4	-3	7	235.95	8.92

-4	-3	7	235.06	9.17
-4	-3	7	237.64	9.66
4	3	-7	245.66	9.16
4	3	-7	235.83	9.07
4	3	-7	227.80	8.90
4	3	-7	245.03	9.64
-5	-3	7	0.26	0.12
-5	-3	7	0.20	0.64
-5	-3	7	-0.47	0.23
-5	-3	7	0.41	0.33
5	3	-7	-0.13	0.21
5	3	-7	0.08	0.10
5	3	-7	-0.65	0.60
5	3	-7	0.36	0.35
-6	-3	7	186.32	7.24
-6	-3	7	186.15	7.85
-6	-3	7	185.03	7.04
-6	-3	7	199.60	7.27
-6	-3	7	180.19	7.17
6	3	-7	196.39	7.22
6	3	-7	182.60	7.02
6	3	-7	184.81	7.81
-7	-3	7	9.12	0.65
-7	-3	7	9.75	0.78
-7	-3	7	9.17	0.52
-7	-3	7	10.16	1.45
-7	-3	7	9.03	0.73
7	3	-7	8.82	0.75
7	3	-7	11.12	1.48
7	3	-7	8.65	0.49
-8	-3	7	150.92	5.53
-8	-3	7	141.32	6.38
-8	-3	7	145.62	5.75
-8	-3	7	145.43	5.76
-8	-3	7	136.54	5.52
8	3	-7	149.12	6.36
8	3	-7	149.59	5.60
8	3	-7	142.59	5.50
-9	-3	7	0.11	0.84
-9	-3	7	1.23	0.44
-9	-3	7	1.56	0.28
-9	-3	7	1.85	0.50
9	3	-7	1.57	0.24
9	3	-7	2.44	0.93

-10	-3	7	284.45	11.64
-10	-3	7	307.02	11.01
-10	-3	7	296.63	10.73
-10	-3	7	270.11	10.96
10	3	-7	273.80	10.68
10	3	-7	275.88	11.52
-11	-3	7	3.23	0.51
-11	-3	7	3.88	1.29
-11	-3	7	3.42	0.68
-11	-3	7	3.53	0.63
11	3	-7	2.95	1.18
11	3	-7	2.28	0.34
-12	-3	7	94.59	3.56
-12	-3	7	87.77	3.86
-12	-3	7	90.56	4.64
12	3	-7	88.67	3.50
12	3	-7	76.53	4.44
-13	-3	7	4.80	1.47
-13	-3	7	4.55	0.70
-13	-3	7	3.28	0.70
13	3	-7	3.29	0.43
13	3	-7	4.28	1.49
-14	-3	7	75.37	3.61
-14	-3	7	78.12	4.42
-14	-3	7	79.21	3.24
14	3	-7	72.78	3.14
14	3	-7	82.57	4.95
-15	-3	7	0.36	1.48
-15	-3	7	0.58	0.72
15	3	-7	0.04	0.37
-16	-3	7	106.32	4.83
-16	-3	7	110.70	5.61
16	3	-7	104.91	4.30
-17	-3	7	-0.64	0.86
-17	-3	7	1.77	2.09
17	3	-7	0.61	0.48
-18	-3	7	69.73	4.59
-18	-3	7	69.38	3.70
18	3	-7	72.93	3.11
-19	-3	7	2.78	1.22
-19	-3	7	4.07	2.04
19	3	-7	3.97	0.72
-20	-3	7	35.32	3.94
-20	-3	7	39.21	2.91

20	3	-7	34.42	2.02
-19	3	-8	0.45	1.60
18	-3	8	-0.84	1.44
18	-3	8	0.57	0.81
-18	3	-8	-1.50	1.38
17	-3	8	2.26	1.39
17	-3	8	0.10	0.73
-17	3	-8	0.80	1.26
16	-3	8	13.93	1.43
16	-3	8	12.45	2.20
-16	3	-8	16.77	2.13
15	-3	8	8.99	1.19
15	-3	8	9.54	1.43
-15	3	-8	7.11	1.21
14	-3	8	24.34	1.53
-14	3	-8	24.55	1.97
13	-3	8	21.31	1.34
13	-3	8	19.06	1.88
-13	3	-8	21.56	1.74
12	-3	8	25.71	1.44
12	-3	8	20.83	1.55
12	-3	8	25.02	1.98
-12	3	-8	25.74	1.75
11	-3	8	28.05	1.41
11	-3	8	23.17	1.52
11	-3	8	23.13	1.94
-11	3	-8	25.65	1.68
10	-3	8	8.40	1.36
10	-3	8	11.07	0.85
10	-3	8	9.83	1.01
-10	3	-8	10.18	0.79
-10	3	-8	8.99	1.65
-10	3	-8	9.07	1.11
9	-3	8	3.07	0.56
9	-3	8	3.61	0.50
9	-3	8	2.10	0.76
-9	3	-8	3.77	0.65
-9	3	-8	3.11	0.97
-9	3	-8	3.42	0.51
8	-3	8	4.28	0.54
8	-3	8	3.24	0.84
8	-3	8	3.49	0.53
-8	3	-8	3.33	0.61
-8	3	-8	4.77	0.93

-8	3	-8	3.55	0.52
7	-3	8	15.29	0.83
7	-3	8	15.30	0.88
7	-3	8	15.92	0.99
7	-3	8	15.91	1.38
-7	3	-8	12.00	1.44
-7	3	-8	15.92	0.84
-7	3	-8	15.14	1.06
6	-3	8	40.37	1.75
6	-3	8	37.06	1.65
6	-3	8	37.65	1.70
6	-3	8	37.02	2.15
-6	3	-8	37.24	1.73
-6	3	-8	37.48	1.79
-6	3	-8	40.06	1.63
-6	3	-8	36.58	2.20
5	-3	8	47.70	2.44
5	-3	8	44.10	2.21
5	-3	8	47.43	2.04
5	-3	8	47.15	1.96
-5	3	-8	46.36	2.07
-5	3	-8	48.02	2.47
-5	3	-8	50.06	1.94
-5	3	-8	46.04	2.15
4	-3	8	45.77	2.14
4	-3	8	42.71	1.81
4	-3	8	42.22	2.26
4	-3	8	46.06	1.92
-4	3	-8	41.29	2.26
-4	3	-8	43.18	1.93
-4	3	-8	45.20	1.79
-4	3	-8	43.40	2.10
3	-3	8	37.36	2.01
3	-3	8	37.91	1.59
3	-3	8	38.69	1.98
3	-3	8	36.43	1.79
-3	3	-8	38.25	2.06
-3	3	-8	38.96	1.70
-3	3	-8	40.13	1.57
-3	3	-8	36.93	1.97
2	-3	8	1.73	0.41
2	-3	8	2.87	0.65
2	-3	8	1.08	0.48
-2	3	-8	2.20	0.34

-2	3	-8	1.77	0.25
-2	3	-8	1.72	0.27
-2	3	-8	1.89	0.65
-2	3	-8	2.48	0.56
1	-3	8	46.85	1.97
1	-3	8	45.25	2.37
1	-3	8	44.20	2.24
-1	3	-8	47.26	1.85
-1	3	-8	44.81	2.23
-1	3	-8	45.28	1.81
-1	3	-8	46.24	2.39
-1	3	-8	46.52	1.95
0	-3	8	1.12	0.64
0	-3	8	1.01	0.29
0	-3	8	0.84	0.45
0	3	-8	0.42	0.45
0	3	-8	1.27	0.63
0	3	-8	0.88	0.17
0	3	-8	1.02	0.15
0	3	-8	0.83	0.26
-1	-3	8	64.31	3.02
-1	-3	8	69.58	3.31
1	3	-8	69.19	2.79
1	3	-8	71.75	2.65
1	3	-8	69.79	2.65
1	3	-8	70.50	3.03
1	3	-8	67.45	3.29
-2	-3	8	6.48	0.57
-2	-3	8	6.05	0.37
-2	-3	8	5.45	0.92
-2	-3	8	6.73	0.79
2	3	-8	6.83	0.55
2	3	-8	6.43	1.14
2	3	-8	6.24	0.78
2	3	-8	6.80	0.36
-3	-3	8	43.66	2.59
-3	-3	8	45.11	2.16
-3	-3	8	49.45	2.05
-3	-3	8	47.19	1.84
3	3	-8	48.38	1.83
3	3	-8	48.88	2.64
3	3	-8	46.87	2.00
-4	-3	8	13.33	0.62
-4	-3	8	13.15	1.47

-4	-3	8	14.16	0.86
-4	-3	8	12.31	0.95
4	3	-8	14.04	1.50
4	3	-8	13.00	0.60
4	3	-8	14.18	0.81
4	3	-8	14.66	0.99
-5	-3	8	52.33	2.05
-5	-3	8	52.39	2.33
-5	-3	8	52.34	2.26
-5	-3	8	50.01	2.89
5	3	-8	55.08	2.32
5	3	-8	53.79	2.90
5	3	-8	50.41	2.22
-6	-3	8	4.59	0.98
-6	-3	8	3.45	0.49
-6	-3	8	2.79	0.43
-6	-3	8	3.70	0.33
6	3	-8	3.56	0.31
6	3	-8	3.65	0.98
6	3	-8	3.08	0.52
-7	-3	8	146.22	6.53
-7	-3	8	149.20	5.86
-7	-3	8	145.46	5.83
-7	-3	8	149.21	5.62
-7	-3	8	143.67	5.73
7	3	-8	159.33	5.81
7	3	-8	150.02	6.52
7	3	-8	143.42	5.59
-8	-3	8	31.58	1.34
-8	-3	8	30.88	1.58
-8	-3	8	31.71	1.62
-8	-3	8	30.62	1.39
-8	-3	8	31.49	2.34
8	3	-8	29.58	1.30
8	3	-8	27.39	2.30
8	3	-8	34.92	1.52
-9	-3	8	84.02	4.37
-9	-3	8	87.13	3.64
-9	-3	8	82.94	3.62
-9	-3	8	90.28	3.38
9	3	-8	83.15	3.33
9	3	-8	88.36	4.36
9	3	-8	84.87	3.40
-10	-3	8	4.18	0.45

-10	-3	8	5.36	0.83
-10	-3	8	4.84	0.69
-10	-3	8	4.70	1.30
10	3	-8	5.39	1.13
10	3	-8	3.95	0.44
-11	-3	8	92.30	3.44
-11	-3	8	83.15	3.71
-11	-3	8	83.36	4.51
-11	-3	8	91.75	4.02
11	3	-8	79.27	4.42
11	3	-8	82.56	3.37
-12	-3	8	-0.20	0.47
-12	-3	8	-0.06	0.35
-12	-3	8	-0.71	1.14
12	3	-8	0.61	1.23
12	3	-8	0.27	0.26
-13	-3	8	29.60	1.45
-13	-3	8	24.26	2.49
-13	-3	8	27.87	2.81
13	3	-8	28.38	1.34
-14	-3	8	15.93	2.51
-14	-3	8	16.42	1.56
-14	-3	8	16.55	1.14
14	3	-8	16.02	0.97
-15	-3	8	41.97	3.42
-15	-3	8	43.27	2.56
15	3	-8	45.28	1.99
-16	-3	8	1.20	0.90
-17	-3	8	81.90	4.79
-17	-3	8	74.44	3.90
17	3	-8	80.02	3.33
-18	-3	8	7.14	1.30
-18	-3	8	4.34	2.00
18	3	-8	6.36	0.99
-19	-3	8	30.96	2.50
-19	-3	8	26.86	3.80
19	3	-8	29.41	1.72
-20	-3	8	0.17	1.34
-20	-3	8	0.71	2.24
20	3	-8	0.52	0.71
18	-3	9	5.21	1.67
18	-3	9	6.34	1.04
-18	3	-9	4.57	1.53
17	-3	9	-0.66	0.72

17	-3	9	0.64	1.48
-17	3	-9	-0.14	1.34
16	-3	9	10.39	1.28
16	-3	9	10.52	1.73
-16	3	-9	14.09	2.11
15	-3	9	3.90	0.76
15	-3	9	6.16	1.38
-15	3	-9	3.50	1.15
14	-3	9	15.73	1.27
14	-3	9	15.25	1.99
-14	3	-9	14.34	1.84
13	-3	9	15.71	1.90
13	-3	9	12.34	1.18
-13	3	-9	14.03	1.60
12	-3	9	9.73	1.00
12	-3	9	12.81	1.61
12	-3	9	9.11	1.21
-12	3	-9	9.21	1.39
11	-3	9	11.76	1.49
11	-3	9	9.68	0.88
11	-3	9	8.42	1.10
-11	3	-9	8.08	1.25
10	-3	9	3.67	0.64
10	-3	9	4.55	1.03
10	-3	9	2.74	0.49
-10	3	-9	4.89	0.75
9	-3	9	1.25	0.51
9	-3	9	1.25	0.76
9	-3	9	1.65	0.42
-9	3	-9	2.29	0.42
-9	3	-9	-0.23	0.88
-9	3	-9	1.29	0.58
8	-3	9	10.01	0.90
8	-3	9	8.58	1.26
8	-3	9	10.07	0.77
-8	3	-9	10.52	1.46
-8	3	-9	11.04	0.75
-8	3	-9	10.86	1.00
7	-3	9	3.44	0.58
7	-3	9	2.53	0.50
7	-3	9	2.00	0.76
7	-3	9	2.37	0.40
-7	3	-9	2.03	0.88
-7	3	-9	2.61	0.52

-7	3	-9	2.73	0.48
-7	3	-9	2.90	0.40
6	-3	9	0.83	0.28
6	-3	9	0.10	0.57
6	-3	9	0.79	0.39
-6	3	-9	0.97	0.42
-6	3	-9	-0.15	0.68
-6	3	-9	1.24	0.48
-6	3	-9	0.81	0.27
5	-3	9	5.99	0.90
5	-3	9	6.18	0.55
5	-3	9	6.36	1.06
5	-3	9	6.62	0.68
-5	3	-9	7.31	1.17
-5	3	-9	6.52	0.92
-5	3	-9	7.23	0.52
-5	3	-9	6.40	0.70
4	-3	9	9.97	1.00
4	-3	9	8.33	1.10
4	-3	9	8.80	0.59
4	-3	9	9.44	0.74
-4	3	-9	10.32	0.58
-4	3	-9	10.34	1.22
-4	3	-9	8.06	1.08
-4	3	-9	8.67	0.74
3	-3	9	33.15	1.96
3	-3	9	32.13	1.86
3	-3	9	35.69	1.58
-3	3	-9	34.75	1.49
-3	3	-9	36.41	2.01
-3	3	-9	34.83	1.59
-3	3	-9	35.81	1.45
-3	3	-9	28.33	2.46
2	-3	9	19.28	0.98
2	-3	9	17.98	1.39
2	-3	9	15.54	1.32
-2	3	-9	18.18	1.37
-2	3	-9	14.06	1.43
-2	3	-9	16.87	0.94
-2	3	-9	17.34	0.81
-2	3	-9	17.14	0.84
1	-3	9	38.49	2.15
1	-3	9	41.02	1.83
1	-3	9	40.55	2.27

-1	3	-9	42.35	2.15
-1	3	-9	42.80	1.81
-1	3	-9	39.12	2.29
-1	3	-9	43.16	1.67
-1	3	-9	42.05	1.70
0	-3	9	18.63	1.33
0	-3	9	21.92	1.09
0	-3	9	19.35	1.59
0	3	-9	17.84	1.58
0	3	-9	20.40	0.88
0	3	-9	20.11	1.38
0	3	-9	21.32	1.05
0	3	-9	20.97	0.92
-1	-3	9	5.48	0.85
-1	-3	9	3.98	0.55
-1	-3	9	5.16	0.94
1	3	-9	4.91	0.53
1	3	-9	4.51	0.67
1	3	-9	5.61	0.96
1	3	-9	4.57	0.33
-2	-3	9	78.27	2.99
-2	-3	9	79.37	3.16
-2	-3	9	73.05	3.36
2	3	-9	76.13	2.97
2	3	-9	80.76	3.80
2	3	-9	78.10	3.35
2	3	-9	77.36	3.12
-3	-3	9	12.63	0.61
-3	-3	9	11.08	1.41
-3	-3	9	12.24	1.06
-3	-3	9	13.32	0.83
3	3	-9	12.24	0.78
3	3	-9	13.68	1.54
3	3	-9	12.52	0.59
3	3	-9	12.83	0.99
-4	-3	9	1.61	0.48
-4	-3	9	1.71	0.22
-4	-3	9	1.58	0.39
-4	-3	9	1.97	0.79
4	3	-9	1.34	0.49
4	3	-9	2.12	0.36
4	3	-9	2.18	0.83
-5	-3	9	1.91	0.23
-5	-3	9	1.66	0.46

-5	-3	9	2.28	0.84
-5	-3	9	2.38	0.41
5	3	-9	2.05	0.26
5	3	-9	2.11	0.90
5	3	-9	1.48	0.33
5	3	-9	1.38	0.50
-6	-3	9	2.58	0.33
-6	-3	9	3.29	0.51
-6	-3	9	2.18	0.42
-6	-3	9	2.14	0.88
-6	-3	9	2.19	0.54
6	3	-9	2.18	0.28
6	3	-9	2.54	0.95
6	3	-9	2.84	0.51
-7	-3	9	3.41	0.53
-7	-3	9	2.44	1.04
-7	-3	9	4.11	0.62
7	3	-9	3.82	0.32
7	3	-9	3.33	1.04
7	3	-9	3.63	0.57
-8	-3	9	19.09	2.12
-8	-3	9	21.83	1.00
-8	-3	9	19.55	1.27
-8	-3	9	21.31	1.11
-8	-3	9	21.18	1.24
8	3	-9	22.96	1.23
8	3	-9	23.27	2.18
8	3	-9	19.52	0.94
-9	-3	9	5.97	0.83
-9	-3	9	7.07	0.91
-9	-3	9	7.19	0.58
-9	-3	9	7.44	0.57
-9	-3	9	6.75	1.38
9	3	-9	6.43	0.47
9	3	-9	5.29	0.74
9	3	-9	11.38	1.90
-10	-3	9	13.29	1.19
-10	-3	9	13.92	0.79
-10	-3	9	13.81	2.04
-10	-3	9	12.38	1.08
10	3	-9	11.67	0.70
10	3	-9	13.05	2.24
-11	-3	9	1.13	1.20
-11	-3	9	2.59	0.61

-11	-3	9	2.80	0.42
11	3	-9	2.48	0.35
11	3	-9	3.50	1.33
-12	-3	9	0.43	1.31
-12	-3	9	1.12	0.40
-12	-3	9	0.47	0.53
12	3	-9	0.63	0.28
-13	-3	9	27.18	1.77
-13	-3	9	27.22	1.40
-13	-3	9	25.30	2.79
13	3	-9	23.99	1.25
-14	-3	9	15.75	1.58
-14	-3	9	18.85	1.20
-14	-3	9	13.60	2.14
14	3	-9	16.17	0.98
-15	-3	9	11.14	1.53
-15	-3	9	13.69	2.28
15	3	-9	9.90	0.87
-16	-3	9	5.27	1.08
-16	-3	9	5.87	1.99
16	3	-9	6.73	0.80
-17	-3	9	1.33	0.98
-17	-3	9	3.05	1.88
17	3	-9	0.87	0.49
-18	-3	9	12.32	2.54
-18	-3	9	12.19	1.89
18	3	-9	12.68	1.15
-19	-3	9	4.27	1.26
-19	-3	9	1.42	2.28
19	3	-9	0.96	0.66
-20	-3	9	22.10	3.91
20	3	-9	19.69	1.56
18	-3	10	1.99	1.00
18	-3	10	3.38	1.73
-18	3	-10	3.70	1.59
17	-3	10	30.82	2.03
17	-3	10	32.51	3.06
-17	3	-10	31.18	2.92
16	-3	10	3.03	0.80
16	-3	10	2.21	1.40
-16	3	-10	1.83	1.25
15	-3	10	41.22	3.03
15	-3	10	42.23	2.26
-15	3	-10	39.92	2.73

14	-3	10	0.26	1.15
14	-3	10	0.60	0.63
13	-3	10	15.70	1.30
13	-3	10	15.97	1.94
-13	3	-10	18.67	1.70
12	-3	10	0.34	0.96
12	-3	10	0.22	0.71
12	-3	10	1.04	0.52
-12	3	-10	0.34	0.77
11	-3	10	45.23	3.07
11	-3	10	50.50	2.42
11	-3	10	49.76	2.34
-11	3	-10	52.13	2.57
10	-3	10	1.28	0.45
10	-3	10	1.61	0.61
10	-3	10	1.97	0.93
-10	3	-10	0.84	0.62
9	-3	10	37.53	1.83
9	-3	10	37.39	1.91
9	-3	10	39.65	2.48
-9	3	-10	39.13	1.76
-9	3	-10	39.23	2.03
8	-3	10	4.42	0.60
8	-3	10	3.60	0.57
8	-3	10	4.00	0.52
8	-3	10	2.92	0.89
-8	3	-10	3.88	0.67
-8	3	-10	4.58	0.60
7	-3	10	18.29	1.16
7	-3	10	18.59	1.65
7	-3	10	18.57	1.06
-7	3	-10	18.79	1.20
-7	3	-10	19.95	1.01
-7	3	-10	17.05	1.21
-7	3	-10	20.12	1.83
6	-3	10	0.61	0.41
6	-3	10	1.06	0.68
6	-3	10	0.49	0.52
6	-3	10	-0.35	0.27
-6	3	-10	0.06	0.26
-6	3	-10	-1.10	0.72
-6	3	-10	0.27	0.58
-6	3	-10	-0.11	0.38
5	-3	10	27.76	1.40

5	-3	10	26.58	1.29
5	-3	10	27.74	1.86
5	-3	10	28.83	1.55
-5	3	-10	29.09	1.28
-5	3	-10	26.65	1.43
-5	3	-10	27.15	1.94
-5	3	-10	28.05	1.72
4	-3	10	3.03	0.46
4	-3	10	2.31	0.72
4	-3	10	2.27	0.68
-4	3	-10	2.92	0.46
-4	3	-10	3.50	0.41
-4	3	-10	2.53	0.83
-4	3	-10	2.56	0.38
-4	3	-10	2.92	0.76
3	-3	10	51.85	2.62
3	-3	10	51.86	2.70
3	-3	10	54.95	2.29
-3	3	-10	53.11	2.21
-3	3	-10	54.65	2.16
-3	3	-10	55.12	2.29
-3	3	-10	50.95	2.73
-3	3	-10	53.15	2.74
2	-3	10	8.45	1.20
2	-3	10	8.67	0.73
2	-3	10	8.85	1.11
-2	3	-10	10.52	0.56
-2	3	-10	8.84	1.35
-2	3	-10	9.96	1.23
-2	3	-10	8.26	0.58
-2	3	-10	8.76	0.71
1	-3	10	7.88	1.08
1	-3	10	7.32	1.24
1	-3	10	8.07	0.71
-1	3	-10	9.23	0.70
-1	3	-10	9.60	1.15
-1	3	-10	8.15	0.51
-1	3	-10	7.39	1.31
-1	3	-10	7.85	0.53
0	-3	10	7.83	1.05
0	-3	10	10.71	0.77
0	-3	10	9.65	1.31
0	3	-10	9.71	0.54
0	3	-10	9.70	0.56

0	3	-10	10.80	0.74
0	3	-10	9.83	1.44
0	3	-10	10.24	1.12
-1	-3	10	49.42	2.10
-1	-3	10	44.50	2.66
-1	-3	10	44.03	2.37
1	3	-10	48.15	1.92
1	3	-10	53.04	2.82
1	3	-10	47.04	2.05
1	3	-10	47.38	1.91
1	3	-10	51.11	2.41
-2	-3	10	3.61	0.94
-2	-3	10	3.55	0.59
-2	-3	10	3.76	0.35
-2	-3	10	3.25	0.67
2	3	-10	4.13	0.97
2	3	-10	4.11	0.70
2	3	-10	3.28	0.43
2	3	-10	3.58	0.35
-3	-3	10	11.94	1.11
-3	-3	10	11.89	0.85
-3	-3	10	12.07	0.62
-3	-3	10	13.35	1.60
3	3	-10	12.89	0.61
3	3	-10	11.19	1.64
3	3	-10	11.45	0.78
3	3	-10	10.31	1.06
-4	-3	10	1.98	0.39
-4	-3	10	1.54	0.23
-4	-3	10	3.72	0.99
-4	-3	10	2.09	0.55
4	3	-10	1.80	0.37
4	3	-10	1.25	0.21
4	3	-10	2.63	0.96
4	3	-10	1.48	0.54
-5	-3	10	16.23	1.07
-5	-3	10	17.12	0.82
-5	-3	10	17.85	1.87
-5	-3	10	17.92	1.04
-5	-3	10	17.23	1.22
5	3	-10	17.03	1.91
5	3	-10	17.40	0.79
5	3	-10	20.62	1.26
5	3	-10	17.20	1.01

-6	-3	10	1.02	0.44
-6	-3	10	1.32	0.43
-6	-3	10	0.84	0.37
-6	-3	10	1.22	0.87
-6	-3	10	0.75	0.22
6	3	-10	0.82	1.01
6	3	-10	0.68	0.17
-7	-3	10	19.29	1.13
-7	-3	10	20.20	1.21
-7	-3	10	17.11	0.87
-7	-3	10	17.20	1.18
-7	-3	10	18.08	2.09
7	3	-10	16.71	0.82
7	3	-10	19.03	1.23
7	3	-10	20.99	2.09
-8	-3	10	45.13	1.84
-8	-3	10	44.56	2.14
-8	-3	10	43.61	2.97
-8	-3	10	43.96	2.09
8	3	-10	46.36	2.07
8	3	-10	40.83	3.02
8	3	-10	42.40	1.78
-9	-3	10	7.34	0.96
-9	-3	10	5.82	0.85
-9	-3	10	5.29	0.56
-9	-3	10	5.32	0.63
-9	-3	10	6.53	1.37
9	3	-10	2.25	1.32
9	3	-10	4.73	0.44
9	3	-10	6.19	0.85
-10	-3	10	11.68	1.11
-10	-3	10	11.86	1.23
-10	-3	10	13.75	2.11
-10	-3	10	12.90	0.82
10	3	-10	13.15	0.88
10	3	-10	11.83	0.70
-11	-3	10	-1.63	1.18
-11	-3	10	0.44	0.53
-11	-3	10	1.30	0.40
11	3	-10	0.58	0.26
-12	-3	10	8.09	0.80
-12	-3	10	7.30	1.66
-12	-3	10	8.02	1.10
12	3	-10	7.14	0.62

-13	-3	10	1.97	0.51
-13	-3	10	1.36	0.66
-13	-3	10	1.55	1.68
13	3	-10	1.09	0.34
-14	-3	10	-0.43	0.51
-14	-3	10	2.27	0.75
-14	-3	10	0.03	1.54
14	3	-10	0.66	0.38
-15	-3	10	2.14	1.80
-15	-3	10	1.74	0.89
15	3	-10	1.24	0.43
-16	-3	10	6.43	2.01
-16	-3	10	2.31	1.00
16	3	-10	2.25	0.54
-17	-3	10	-2.68	1.89
-17	-3	10	0.46	1.00
17	3	-10	-0.17	0.51
-18	-3	10	0.43	1.04
-18	-3	10	2.08	2.15
18	3	-10	0.64	0.57
-19	-3	10	0.77	2.17
-19	-3	10	1.46	1.18
19	3	-10	-0.17	0.64
17	-3	11	0.77	1.58
17	-3	11	0.01	0.83
-17	3	-11	0.65	1.50
16	-3	11	55.49	3.68
16	-3	11	54.16	2.80
-16	3	-11	52.50	3.43
15	-3	11	2.18	0.77
15	-3	11	4.29	1.48
-15	3	-11	4.81	1.23
14	-3	11	44.88	3.26
14	-3	11	51.61	2.55
-14	3	-11	54.82	4.06
13	-3	11	0.93	1.15
13	-3	11	-0.14	0.61
-13	3	-11	-0.97	0.84
12	-3	11	26.10	2.37
12	-3	11	27.94	1.64
-12	3	-11	25.59	1.94
11	-3	11	-0.02	1.01
11	-3	11	0.82	0.68
11	-3	11	-0.45	0.48

-11	3	-11	0.79	0.71
10	-3	11	61.00	2.73
10	-3	11	57.96	2.77
10	-3	11	67.92	4.65
-10	3	-11	61.95	2.89
9	-3	11	6.78	0.84
9	-3	11	4.57	1.07
9	-3	11	7.78	0.99
-9	3	-11	6.61	1.06
8	-3	11	143.78	6.33
8	-3	11	147.03	5.76
8	-3	11	150.93	5.67
8	-3	11	142.47	5.72
-8	3	-11	141.05	5.69
-8	3	-11	146.05	5.84
-8	3	-11	147.05	5.68
7	-3	11	5.50	0.86
7	-3	11	5.39	1.06
7	-3	11	5.86	0.85
7	-3	11	4.63	0.67
-7	3	-11	4.24	0.78
-7	3	-11	4.48	0.63
-7	3	-11	5.05	0.86
6	-3	11	60.25	2.60
6	-3	11	62.05	2.69
6	-3	11	61.27	3.18
6	-3	11	58.58	2.54
-6	3	-11	62.59	2.51
-6	3	-11	59.73	2.65
-6	3	-11	58.14	3.27
-6	3	-11	59.21	2.58
5	-3	11	3.62	0.53
5	-3	11	4.40	0.89
5	-3	11	4.11	0.74
-5	3	-11	2.75	1.04
-5	3	-11	4.19	0.59
-5	3	-11	4.17	0.89
-5	3	-11	3.44	0.54
-5	3	-11	4.72	0.53
4	-3	11	8.68	1.24
4	-3	11	6.99	1.09
4	-3	11	7.19	0.77
-4	3	-11	7.18	0.62
-4	3	-11	9.73	1.37

-4	3	-11	8.10	1.33
-4	3	-11	7.72	0.59
-4	3	-11	8.19	0.78
3	-3	11	7.07	1.15
3	-3	11	4.10	0.83
3	-3	11	4.72	0.69
-3	3	-11	5.25	0.54
-3	3	-11	5.51	0.65
-3	3	-11	5.85	1.05
-3	3	-11	5.94	0.50
-3	3	-11	6.06	0.99
2	-3	11	56.48	2.33
2	-3	11	51.36	2.75
2	-3	11	49.74	2.76
-2	3	-11	55.92	2.22
-2	3	-11	52.67	2.96
-2	3	-11	54.17	2.30
-2	3	-11	53.72	2.17
-2	3	-11	52.91	2.80
1	-3	11	1.48	0.77
1	-3	11	1.19	0.40
1	-3	11	1.77	0.67
-1	3	-11	2.09	0.29
-1	3	-11	2.37	0.89
-1	3	-11	1.07	0.73
-1	3	-11	1.23	0.25
-1	3	-11	1.47	0.36
0	-3	11	78.76	3.78
0	-3	11	74.71	3.57
0	-3	11	82.02	3.22
0	3	-11	76.27	3.03
0	3	-11	82.04	3.17
0	3	-11	74.94	3.87
0	3	-11	75.63	3.05
0	3	-11	77.03	3.57
-1	-3	11	4.97	0.94
-1	-3	11	5.80	0.70
-1	-3	11	5.58	1.10
1	3	-11	6.37	0.64
1	3	-11	5.56	1.15
1	3	-11	5.84	0.43
1	3	-11	5.97	0.51
1	3	-11	5.27	0.87
-2	-3	11	21.05	1.52

-2	-3	11	22.33	1.01
-2	-3	11	22.73	1.22
-2	-3	11	22.42	1.94
2	3	-11	21.94	0.97
2	3	-11	21.81	2.03
2	3	-11	21.80	1.14
2	3	-11	21.31	1.49
-3	-3	11	0.68	0.59
-3	-3	11	0.91	0.39
-3	-3	11	0.93	0.23
-3	-3	11	1.08	0.84
3	3	-11	0.99	0.21
3	3	-11	0.35	0.32
3	3	-11	1.46	0.95
3	3	-11	0.97	0.60
-4	-3	11	66.48	3.68
-4	-3	11	66.65	3.11
-4	-3	11	71.06	2.72
-4	-3	11	73.83	2.96
4	3	-11	66.64	3.74
4	3	-11	70.16	3.12
4	3	-11	65.69	2.87
4	3	-11	70.27	2.68
-5	-3	11	7.60	0.96
-5	-3	11	6.90	1.34
-5	-3	11	6.61	0.48
-5	-3	11	5.40	0.77
-5	-3	11	5.97	0.68
5	3	-11	5.24	1.26
5	3	-11	6.37	0.42
5	3	-11	6.62	0.69
5	3	-11	5.93	1.01
-6	-3	11	106.26	4.28
-6	-3	11	99.46	4.32
-6	-3	11	109.79	5.13
-6	-3	11	101.84	4.23
6	3	-11	108.06	4.34
6	3	-11	107.79	5.14
6	3	-11	99.20	3.98
-7	-3	11	5.90	0.91
-7	-3	11	7.93	0.57
-7	-3	11	7.56	0.89
-7	-3	11	8.25	0.81
-7	-3	11	7.62	1.34

7	3	-11	7.49	1.44
7	3	-11	7.61	0.49
7	3	-11	8.20	1.15
-8	-3	11	4.66	1.28
-8	-3	11	5.32	0.53
-8	-3	11	7.88	0.97
-8	-3	11	4.60	0.65
-8	-3	11	4.57	0.78
8	3	-11	2.67	1.36
8	3	-11	4.99	0.43
8	3	-11	6.01	0.97
-9	-3	11	2.93	1.26
-9	-3	11	3.88	0.72
-9	-3	11	3.91	0.65
-9	-3	11	3.40	0.42
9	3	-11	3.43	0.65
9	3	-11	3.15	0.40
-10	-3	11	10.72	0.73
-10	-3	11	11.23	0.79
-10	-3	11	12.19	2.16
-10	-3	11	12.06	1.16
10	3	-11	10.61	0.66
10	3	-11	11.73	1.00
-11	-3	11	-1.09	0.48
-11	-3	11	0.33	0.40
-11	-3	11	-1.40	1.22
11	3	-11	-0.07	0.25
-12	-3	11	24.79	1.82
-12	-3	11	24.16	1.32
12	3	-11	22.13	1.15
-13	-3	11	-0.18	0.68
-13	-3	11	2.08	1.56
-13	-3	11	0.37	0.50
13	3	-11	0.34	0.32
-14	-3	11	1.62	0.62
-14	-3	11	4.79	0.95
-14	-3	11	3.90	1.83
14	3	-11	2.47	0.45
-15	-3	11	1.30	1.92
-15	-3	11	0.66	0.92
15	3	-11	0.55	0.42
-16	-3	11	7.52	1.31
-16	-3	11	10.93	2.29
16	3	-11	8.23	1.06

-17	-3	11	5.03	2.14
-17	-3	11	3.46	1.15
17	3	-11	3.14	0.62
-18	-3	11	23.30	3.87
-18	-3	11	22.28	2.37
18	3	-11	23.46	1.51
-19	-3	11	1.65	1.28
-19	-3	11	0.30	2.27
19	3	-11	0.81	0.65
16	-3	12	0.50	0.85
16	-3	12	2.71	1.63
-16	3	-12	-0.26	1.35
15	-3	12	53.84	3.70
15	-3	12	50.72	2.69
-15	3	-12	52.88	3.24
14	-3	12	1.43	1.43
14	-3	12	1.61	0.72
-14	3	-12	1.98	1.10
13	-3	12	40.80	3.00
13	-3	12	42.76	2.24
-13	3	-12	40.50	2.50
12	-3	12	4.17	1.31
12	-3	12	3.51	0.75
-12	3	-12	3.23	0.94
11	-3	12	87.15	3.66
11	-3	12	80.84	4.36
11	-3	12	81.79	3.69
-11	3	-12	88.30	3.83
10	-3	12	0.21	0.98
10	-3	12	0.43	0.66
-10	3	-12	-0.10	0.66
9	-3	12	90.19	3.68
9	-3	12	86.15	4.36
9	-3	12	86.76	3.68
-9	3	-12	86.69	3.78
8	-3	12	-1.56	0.89
8	-3	12	0.46	0.55
8	-3	12	-0.17	0.55
8	-3	12	-0.05	0.42
-8	3	-12	0.75	0.57
-8	3	-12	0.87	0.62
-8	3	-12	0.37	0.46
7	-3	12	81.53	3.37
7	-3	12	76.94	4.00

7	-3	12	83.83	3.41
-7	3	-12	77.54	3.41
-7	3	-12	78.09	3.36
-7	3	-12	80.62	3.27
6	-3	12	5.83	1.09
6	-3	12	4.88	0.84
6	-3	12	4.76	0.77
-6	3	-12	5.09	0.70
-6	3	-12	4.89	0.63
-6	3	-12	4.78	0.97
-6	3	-12	4.95	0.67
5	-3	12	52.96	2.98
5	-3	12	51.58	2.61
5	-3	12	56.73	2.41
-5	3	-12	53.50	2.41
-5	3	-12	53.07	2.34
-5	3	-12	55.84	2.29
-5	3	-12	55.69	2.88
4	-3	12	3.72	0.83
4	-3	12	2.41	0.85
4	-3	12	3.90	0.57
-4	3	-12	2.78	0.41
-4	3	-12	4.42	0.73
-4	3	-12	3.25	0.51
-4	3	-12	1.64	0.87
-4	3	-12	3.92	1.02
3	-3	12	57.10	2.42
3	-3	12	54.63	2.80
3	-3	12	51.79	2.90
-3	3	-12	58.67	2.28
-3	3	-12	53.18	2.99
-3	3	-12	54.26	3.09
-3	3	-12	54.75	2.32
-3	3	-12	55.60	2.40
2	-3	12	-0.69	0.60
2	-3	12	-0.36	0.40
2	-3	12	-1.17	0.64
-2	3	-12	0.86	0.81
-2	3	-12	-0.48	0.21
-2	3	-12	-1.06	0.96
-2	3	-12	-0.03	0.37
-2	3	-12	0.02	0.24
1	-3	12	118.34	4.57
1	-3	12	119.92	5.15

1	-3	12	108.36	4.98
-1	3	-12	110.49	5.24
-1	3	-12	116.33	4.53
-1	3	-12	111.82	5.01
-1	3	-12	114.27	4.40
-1	3	-12	113.30	4.43
0	-3	12	11.90	1.65
0	-3	12	11.82	0.94
0	-3	12	10.57	1.32
0	3	-12	12.83	0.72
0	3	-12	10.05	1.51
0	3	-12	11.90	1.41
0	3	-12	13.14	0.87
0	3	-12	14.16	0.71
-1	-3	12	156.50	6.98
-1	-3	12	151.17	6.71
-1	-3	12	174.34	6.40
1	3	-12	172.32	6.20
1	3	-12	164.58	6.20
1	3	-12	165.33	6.73
1	3	-12	156.05	7.12
1	3	-12	162.90	6.32
-2	-3	12	11.17	1.71
-2	-3	12	10.50	1.27
-2	-3	12	11.71	0.94
2	3	-12	11.48	0.85
2	3	-12	9.60	1.30
2	3	-12	11.06	0.64
2	3	-12	12.46	0.65
2	3	-12	11.54	1.93
-3	-3	12	76.17	3.04
-3	-3	12	67.89	3.77
-3	-3	12	69.69	3.29
3	3	-12	71.11	3.29
3	3	-12	70.08	2.77
3	3	-12	69.37	3.89
3	3	-12	69.92	2.94
-4	-3	12	0.10	0.93
-4	-3	12	0.79	0.49
-4	-3	12	0.88	0.68
4	3	-12	0.92	0.23
4	3	-12	2.16	1.13
4	3	-12	1.97	0.71
4	3	-12	1.32	0.39

-5	-3	12	79.76	3.22
-5	-3	12	82.86	4.14
-5	-3	12	65.99	3.30
-5	-3	12	72.69	2.94
-5	-3	12	72.44	3.13
5	3	-12	75.03	4.16
5	3	-12	71.47	2.88
5	3	-12	77.25	3.39
-6	-3	12	5.72	0.76
-6	-3	12	10.76	1.85
-6	-3	12	5.66	0.74
-6	-3	12	5.99	0.53
-6	-3	12	5.75	1.05
6	3	-12	5.95	0.88
6	3	-12	5.10	0.44
6	3	-12	6.59	1.46
-7	-3	12	137.14	5.40
-7	-3	12	138.74	5.47
-7	-3	12	127.38	5.41
-7	-3	12	135.76	6.34
-7	-3	12	135.00	5.17
7	3	-12	136.96	5.47
7	3	-12	130.30	5.11
-8	-3	12	11.78	1.05
-8	-3	12	9.66	1.67
-8	-3	12	12.74	1.07
-8	-3	12	12.02	1.21
-8	-3	12	12.97	0.77
8	3	-12	11.38	0.66
8	3	-12	12.88	1.27
-9	-3	12	21.07	1.53
-9	-3	12	21.32	1.40
-9	-3	12	17.68	2.41
-9	-3	12	20.97	1.08
-9	-3	12	20.44	1.30
9	3	-12	19.00	0.97
9	3	-12	22.36	1.47
-10	-3	12	-0.56	0.50
-10	-3	12	0.49	0.42
-10	-3	12	-0.53	0.35
-10	-3	12	-0.12	1.31
10	3	-12	0.07	0.24
10	3	-12	-0.36	0.61
-11	-3	12	57.87	2.39

-11	-3	12	57.64	2.73
-11	-3	12	53.72	3.80
11	3	-12	51.22	2.26
-12	-3	12	0.17	0.47
-12	-3	12	2.34	1.69
-12	-3	12	0.41	0.63
12	3	-12	1.12	0.34
-13	-3	12	68.32	4.51
-13	-3	12	68.27	2.89
-13	-3	12	66.68	3.27
13	3	-12	63.96	2.73
-14	-3	12	0.89	0.84
-14	-3	12	2.21	1.88
-14	-3	12	0.42	0.65
14	3	-12	-0.12	0.39
-15	-3	12	18.71	3.49
-15	-3	12	29.25	2.17
15	3	-12	23.76	1.33
-16	-3	12	0.01	1.01
-16	-3	12	2.12	2.01
-17	-3	12	24.49	3.85
-17	-3	12	20.71	2.25
17	3	-12	24.45	1.46
-18	-3	12	1.61	1.25
-18	-3	12	1.67	2.23
18	3	-12	-0.27	0.61
16	-3	13	17.13	3.09
16	-3	13	20.44	1.89
-16	3	-13	18.12	2.65
15	-3	13	-0.27	1.51
15	-3	13	0.13	0.84
-15	3	-13	2.89	1.29
14	-3	13	50.89	2.60
14	-3	13	42.44	3.51
-14	3	-13	48.89	2.97
13	-3	13	7.23	1.63
13	-3	13	6.22	0.93
-13	3	-13	6.62	1.18
12	-3	13	15.45	1.38
12	-3	13	16.65	2.22
-12	3	-13	11.92	1.71
11	-3	13	4.38	1.45
11	-3	13	4.93	0.78
11	-3	13	4.57	0.89

-11	3	-13	5.67	0.97
10	-3	13	52.70	3.46
10	-3	13	59.17	2.67
10	-3	13	55.80	2.71
-10	3	-13	60.22	2.76
-10	3	-13	61.44	2.86
9	-3	13	1.54	0.64
9	-3	13	2.52	1.13
9	-3	13	1.69	0.56
-9	3	-13	1.34	0.68
-9	3	-13	2.41	0.58
8	-3	13	117.86	4.60
8	-3	13	112.47	5.31
8	-3	13	119.05	4.57
-8	3	-13	105.48	4.75
-8	3	-13	109.22	4.66
-8	3	-13	110.90	4.63
7	-3	13	1.51	1.25
7	-3	13	0.64	0.61
7	-3	13	-0.76	0.53
-7	3	-13	0.10	0.41
-7	3	-13	0.36	0.84
-7	3	-13	0.27	0.51
-7	3	-13	1.07	0.44
6	-3	13	61.65	2.92
6	-3	13	62.39	2.75
6	-3	13	59.23	3.39
-6	3	-13	64.05	2.78
-6	3	-13	61.43	2.73
-6	3	-13	69.56	2.66
-6	3	-13	60.91	3.17
5	-3	13	5.99	0.93
5	-3	13	5.82	0.71
5	-3	13	7.77	1.47
-5	3	-13	6.95	0.70
-5	3	-13	7.91	0.86
-5	3	-13	5.55	1.21
4	-3	13	71.11	3.00
4	-3	13	69.02	3.65
4	-3	13	70.45	3.35
-4	3	-13	72.90	2.89
-4	3	-13	70.30	2.99
-4	3	-13	67.83	3.65
-4	3	-13	71.41	2.95

3	-3	13	15.08	1.06
3	-3	13	13.89	1.64
3	-3	13	14.71	1.51
-3	3	-13	15.10	0.85
-3	3	-13	12.27	0.86
-3	3	-13	10.45	1.96
-3	3	-13	18.62	3.08
-3	3	-13	12.10	0.97
2	-3	13	196.78	7.18
2	-3	13	166.59	7.64
2	-3	13	180.74	7.63
-2	3	-13	188.62	7.13
-2	3	-13	187.02	7.07
-2	3	-13	181.07	7.89
-2	3	-13	175.68	7.71
-2	3	-13	194.73	7.03
1	-3	13	1.57	0.52
1	-3	13	1.16	0.94
1	-3	13	0.79	0.76
-1	3	-13	1.22	0.32
-1	3	-13	0.97	1.14
-1	3	-13	1.74	0.92
-1	3	-13	1.33	0.45
-1	3	-13	1.25	0.31
0	-3	13	74.09	3.82
0	-3	13	86.66	3.40
0	-3	13	82.81	4.07
0	3	-13	78.42	3.30
0	3	-13	82.07	3.20
0	3	-13	81.20	3.18
0	3	-13	79.55	3.84
0	3	-13	78.64	4.23
-1	-3	13	1.69	1.06
-1	-3	13	1.74	0.51
-1	-3	13	0.65	0.75
1	3	-13	1.70	0.30
1	3	-13	2.03	0.92
1	3	-13	1.61	0.43
1	3	-13	1.37	1.18
1	3	-13	1.71	0.31
-2	-3	13	89.78	3.55
-2	-3	13	76.95	3.87
-2	-3	13	82.59	4.33
2	3	-13	84.94	3.31

2	3	-13	83.59	3.30
2	3	-13	82.89	4.48
2	3	-13	89.38	3.95
2	3	-13	83.83	3.45
-3	-3	13	0.75	0.99
-3	-3	13	-0.48	0.66
-3	-3	13	0.06	0.46
-3	-3	13	-0.40	0.26
3	3	-13	0.83	1.21
3	3	-13	-0.07	0.22
3	3	-13	0.53	0.79
3	3	-13	0.26	0.38
-4	-3	13	144.98	5.62
-4	-3	13	132.83	5.84
-4	-3	13	140.06	5.38
-4	-3	13	149.83	6.49
4	3	-13	133.81	6.55
4	3	-13	144.37	5.34
4	3	-13	140.26	5.87
4	3	-13	134.87	5.51
-5	-3	13	11.75	1.33
-5	-3	13	12.37	0.77
-5	-3	13	15.62	2.12
-5	-3	13	14.00	1.01
-5	-3	13	12.64	1.15
5	3	-13	17.48	3.20
5	3	-13	12.78	0.69
5	3	-13	15.41	1.50
-6	-3	13	170.89	6.82
-6	-3	13	175.85	7.80
-6	-3	13	169.63	6.97
-6	-3	13	176.77	6.62
-6	-3	13	179.73	6.91
6	3	-13	161.35	6.56
6	3	-13	182.47	7.02
-7	-3	13	2.17	0.64
-7	-3	13	1.75	1.23
-7	-3	13	2.29	0.71
-7	-3	13	3.89	0.53
-7	-3	13	2.91	0.57
7	3	-13	1.64	0.85
7	3	-13	2.39	0.31
-8	-3	13	60.86	2.76
-8	-3	13	62.43	3.86

-8	-3	13	55.93	2.72
-8	-3	13	58.86	2.85
-8	-3	13	59.73	2.47
8	3	-13	57.83	2.39
8	3	-13	65.57	2.88
-9	-3	13	4.53	0.77
-9	-3	13	4.51	0.70
-9	-3	13	5.10	0.67
9	3	-13	5.39	0.49
9	3	-13	4.25	0.95
-10	-3	13	141.40	5.73
-10	-3	13	133.00	5.44
-10	-3	13	138.82	5.40
-10	-3	13	140.68	6.80
10	3	-13	147.71	5.60
10	3	-13	130.45	5.30
-11	-3	13	-0.47	0.66
-11	-3	13	-2.27	1.42
-11	-3	13	0.57	0.39
-11	-3	13	-0.25	0.44
11	3	-13	-0.60	0.62
11	3	-13	0.62	0.30
-12	-3	13	80.82	4.83
-12	-3	13	71.67	3.10
-12	-3	13	73.57	3.49
12	3	-13	72.14	2.97
-13	-3	13	1.14	0.61
-13	-3	13	0.56	1.83
-13	-3	13	-0.71	0.73
13	3	-13	0.13	0.37
-14	-3	13	33.54	2.28
-14	-3	13	33.37	3.75
14	3	-13	30.00	1.54
-15	-3	13	0.33	1.90
-15	-3	13	0.03	0.94
15	3	-13	-0.06	0.43
-16	-3	13	50.05	4.37
-16	-3	13	47.64	2.92
16	3	-13	40.39	2.07
-17	-3	13	2.67	2.34
-17	-3	13	0.95	1.10
17	3	-13	0.37	0.57
-18	-3	13	42.21	4.54
-18	-3	13	48.11	3.15

18	3	-13	43.23	2.22
15	-3	14	0.96	0.88
15	-3	14	1.27	1.76
-15	3	-14	2.38	1.81
-15	3	-14	2.68	1.00
14	-3	14	6.81	1.08
14	-3	14	4.74	1.75
-14	3	-14	5.09	1.30
-14	3	-14	11.49	1.57
13	-3	14	3.54	1.59
13	-3	14	6.33	0.99
-13	3	-14	1.70	1.09
-13	3	-14	5.73	1.01
12	-3	14	10.79	1.86
12	-3	14	12.71	1.40
-12	3	-14	13.26	1.73
-12	3	-14	14.43	1.49
11	-3	14	13.35	2.23
-11	3	-14	8.28	1.25
-11	3	-14	8.66	1.47
10	-3	14	19.04	2.30
10	-3	14	16.82	1.52
-10	3	-14	19.17	1.46
-10	3	-14	16.03	1.56
9	-3	14	54.06	3.49
9	-3	14	59.21	2.55
9	-3	14	54.91	2.64
-9	3	-14	54.17	2.71
-9	3	-14	59.78	2.69
8	-3	14	5.96	0.85
8	-3	14	6.55	0.82
8	-3	14	5.64	1.36
-8	3	-14	6.05	0.92
-8	3	-14	5.12	0.84
-8	3	-14	7.52	1.11
7	-3	14	26.81	1.72
7	-3	14	27.28	2.34
7	-3	14	23.99	1.52
-7	3	-14	23.91	1.57
-7	3	-14	27.55	1.51
-7	3	-14	28.51	2.10
6	-3	14	5.43	0.74
6	-3	14	5.56	0.93
6	-3	14	4.45	1.19

-6	3	-14	6.23	0.93
-6	3	-14	5.98	0.69
-6	3	-14	5.74	0.76
-6	3	-14	4.45	1.19
5	-3	14	47.21	2.20
5	-3	14	46.64	2.52
-5	3	-14	44.04	2.94
-5	3	-14	47.80	2.15
-5	3	-14	44.13	2.16
-5	3	-14	50.07	2.09
4	-3	14	5.05	0.69
4	-3	14	5.14	1.13
4	-3	14	6.10	1.03
-4	3	-14	6.77	1.39
-4	3	-14	4.16	0.67
-4	3	-14	4.85	0.84
-4	3	-14	4.71	0.69
3	-3	14	6.75	0.97
3	-3	14	10.28	1.60
3	-3	14	9.67	1.50
-3	3	-14	9.71	0.77
-3	3	-14	11.03	1.70
-3	3	-14	8.47	0.89
-3	3	-14	8.48	0.74
2	-3	14	6.03	1.14
2	-3	14	5.32	0.91
2	-3	14	4.62	1.05
-2	3	-14	4.75	0.77
-2	3	-14	5.32	0.62
-2	3	-14	6.20	1.48
-2	3	-14	5.22	0.62
1	-3	14	42.45	1.99
1	-3	14	38.67	2.60
1	-3	14	41.21	2.53
-1	3	-14	40.35	1.79
-1	3	-14	45.38	2.99
-1	3	-14	40.66	1.77
-1	3	-14	38.09	1.87
-1	3	-14	38.40	3.00
0	-3	14	4.14	1.14
0	-3	14	3.53	1.03
0	-3	14	3.76	0.62
0	3	-14	3.29	1.41
0	3	-14	2.98	0.40

0	3	-14	3.18	0.49
0	3	-14	3.67	1.05
0	3	-14	3.19	0.53
-1	-3	14	59.30	3.29
-1	-3	14	67.35	2.83
-1	-3	14	61.21	3.60
1	3	-14	63.25	2.71
1	3	-14	56.88	3.83
1	3	-14	63.87	2.58
1	3	-14	69.46	2.60
1	3	-14	64.05	3.36
-2	-3	14	15.19	1.20
-2	-3	14	13.80	2.10
-2	-3	14	11.10	1.61
2	3	-14	15.61	0.85
2	3	-14	18.25	2.49
2	3	-14	12.19	1.73
2	3	-14	16.01	1.03
2	3	-14	14.71	0.81
-3	-3	14	73.05	2.92
-3	-3	14	70.77	3.54
-3	-3	14	74.92	3.17
3	3	-14	72.19	2.87
3	3	-14	66.33	3.03
3	3	-14	73.84	4.90
3	3	-14	77.13	3.58
-4	-3	14	1.21	0.86
-4	-3	14	4.69	1.46
-4	-3	14	2.92	0.41
-4	-3	14	3.52	0.54
-4	-3	14	3.13	0.66
4	3	-14	3.12	1.02
4	3	-14	2.46	0.52
4	3	-14	3.19	0.43
-5	-3	14	65.29	2.64
-5	-3	14	64.25	2.94
-5	-3	14	61.37	3.14
-5	-3	14	59.34	3.85
-5	-3	14	63.44	2.83
5	3	-14	71.86	3.28
5	3	-14	65.78	2.58
-6	-3	14	4.11	0.58
-6	-3	14	4.26	0.81
-6	-3	14	3.37	0.59

-6	-3	14	3.64	0.74
-6	-3	14	5.21	1.51
6	3	-14	4.16	0.45
6	3	-14	2.52	0.98
-7	-3	14	138.81	5.63
-7	-3	14	139.02	5.30
-7	-3	14	139.78	5.54
-7	-3	14	125.51	5.60
-7	-3	14	129.92	7.25
7	3	-14	135.05	5.22
7	3	-14	147.56	5.76
-8	-3	14	2.12	1.43
-8	-3	14	-0.20	0.83
-8	-3	14	-0.14	0.68
-8	-3	14	0.44	0.37
-8	-3	14	0.85	0.55
8	3	-14	1.31	0.85
8	3	-14	0.29	0.25
-9	-3	14	21.68	1.49
-9	-3	14	20.29	1.14
-9	-3	14	19.44	1.43
-9	-3	14	21.04	2.92
9	3	-14	18.12	1.71
9	3	-14	20.57	0.99
-10	-3	14	1.89	1.67
-10	-3	14	3.52	0.53
-10	-3	14	3.94	0.66
-10	-3	14	4.82	0.85
10	3	-14	3.08	0.40
-11	-3	14	59.72	2.87
-11	-3	14	55.34	2.48
-11	-3	14	56.14	4.21
11	3	-14	58.36	2.65
11	3	-14	53.54	2.32
-12	-3	14	0.94	0.59
-12	-3	14	0.43	1.81
-12	-3	14	0.29	0.73
12	3	-14	0.43	0.37
-13	-3	14	65.67	4.53
-13	-3	14	61.92	3.20
13	3	-14	58.21	2.55
-14	-3	14	10.69	1.70
-14	-3	14	7.44	2.23
14	3	-14	9.11	0.90

-15	-3	14	46.54	4.35
-15	-3	14	49.63	3.00
15	3	-14	44.51	2.10
-16	-3	14	0.59	1.11
-16	-3	14	1.48	2.26
16	3	-14	2.23	0.58
-17	-3	14	29.03	2.57
-17	-3	14	21.67	4.04
17	3	-14	25.57	1.58
14	-3	15	0.73	1.77
-14	3	-15	-0.38	0.95
13	-3	15	3.67	1.71
-13	3	-15	1.75	1.14
-13	3	-15	0.64	0.95
12	-3	15	8.59	1.85
-12	3	-15	7.94	1.15
-12	3	-15	7.80	1.18
11	-3	15	6.84	1.61
-11	3	-15	6.14	1.06
-11	3	-15	7.26	1.01
10	-3	15	4.23	1.52
-10	3	-15	2.81	0.76
-10	3	-15	3.10	0.87
9	-3	15	3.88	1.38
9	-3	15	4.22	0.89
9	-3	15	2.91	0.77
-9	3	-15	3.70	0.83
-9	3	-15	1.16	0.66
8	-3	15	6.16	0.92
8	-3	15	6.80	1.43
8	-3	15	7.11	1.15
-8	3	-15	5.24	0.84
-8	3	-15	5.24	0.89
7	-3	15	4.45	1.32
7	-3	15	8.36	1.11
7	-3	15	7.14	1.04
-7	3	-15	8.21	1.14
-7	3	-15	7.14	0.98
-7	3	-15	9.62	1.44
6	-3	15	11.79	1.99
6	-3	15	18.46	1.57
6	-3	15	14.39	1.25
-6	3	-15	13.92	1.09
-6	3	-15	12.90	1.18

-6	3	-15	14.70	2.18
5	-3	15	7.80	1.09
5	-3	15	7.59	1.14
5	-3	15	7.06	1.43
-5	3	-15	8.47	1.58
-5	3	-15	9.35	0.84
-5	3	-15	7.50	0.98
-5	3	-15	6.96	0.85
4	-3	15	7.63	1.02
4	-3	15	6.51	1.14
4	-3	15	8.63	1.43
-4	3	-15	7.44	0.80
-4	3	-15	12.74	2.21
-4	3	-15	6.73	0.93
-4	3	-15	5.33	0.76
3	-3	15	22.04	2.43
3	-3	15	24.04	2.06
3	-3	15	29.26	1.55
-3	3	-15	27.08	1.38
-3	3	-15	25.42	1.45
-3	3	-15	20.53	2.72
-3	3	-15	27.81	1.35
2	-3	15	9.94	1.74
2	-3	15	10.01	1.43
2	-3	15	10.36	1.09
-2	3	-15	10.54	0.81
-2	3	-15	10.47	0.79
-2	3	-15	11.99	1.89
-2	3	-15	9.11	0.91
1	-3	15	5.80	1.28
1	-3	15	4.56	1.09
1	-3	15	5.94	0.77
-1	3	-15	4.66	0.77
-1	3	-15	6.24	0.67
-1	3	-15	4.18	0.60
-1	3	-15	5.65	1.61
0	-3	15	2.50	1.08
0	-3	15	1.27	0.61
0	-3	15	0.49	0.96
0	3	-15	1.34	0.34
0	3	-15	-0.85	1.45
0	3	-15	1.45	0.45
0	3	-15	0.89	0.35
-1	-3	15	1.41	1.15

-1	-3	15	3.43	0.68
-1	-3	15	3.14	1.04
1	3	-15	4.40	1.22
1	3	-15	3.79	0.56
1	3	-15	2.96	0.44
1	3	-15	3.32	0.43
1	3	-15	5.01	2.10
-2	-3	15	0.57	1.13
-2	-3	15	-0.03	0.58
-2	-3	15	0.83	0.96
2	3	-15	-1.28	1.06
2	3	-15	1.02	0.35
2	3	-15	-0.08	0.42
2	3	-15	-0.50	0.25
-3	-3	15	18.08	1.41
-3	-3	15	19.55	1.84
-3	-3	15	18.34	2.32
3	3	-15	17.01	1.17
3	3	-15	17.82	0.96
3	3	-15	21.00	1.04
3	3	-15	19.62	1.95
-4	-3	15	15.71	1.71
-4	-3	15	17.26	1.34
-4	-3	15	14.86	1.12
-4	-3	15	14.91	0.95
-4	-3	15	18.79	2.36
4	3	-15	15.96	0.85
4	3	-15	16.74	1.88
-5	-3	15	5.41	0.84
-5	-3	15	5.74	0.86
-5	-3	15	5.89	0.68
-5	-3	15	6.84	1.68
-5	-3	15	5.73	1.02
5	3	-15	6.61	1.16
5	3	-15	5.43	0.53
-6	-3	15	7.73	1.12
-6	-3	15	9.76	1.01
-6	-3	15	10.18	0.79
-6	-3	15	8.54	1.24
-6	-3	15	11.52	1.96
6	3	-15	9.38	1.34
6	3	-15	9.25	0.63
-7	-3	15	0.56	0.40
-7	-3	15	0.04	0.50

-7	-3	15	0.96	0.70
-7	-3	15	-0.34	1.46
-7	-3	15	0.48	0.79
7	3	-15	-0.20	1.02
7	3	-15	0.46	0.24
-8	-3	15	5.98	0.97
-8	-3	15	7.96	1.07
-8	-3	15	7.28	0.79
-8	-3	15	6.61	1.79
8	3	-15	7.27	1.18
8	3	-15	5.94	0.56
-9	-3	15	8.99	0.89
-9	-3	15	12.06	2.24
-9	-3	15	8.88	1.31
-9	-3	15	10.44	1.26
9	3	-15	11.12	1.68
9	3	-15	9.60	0.69
-10	-3	15	18.08	1.16
-10	-3	15	16.69	1.36
-10	-3	15	17.34	1.53
-10	-3	15	15.92	2.99
10	3	-15	19.94	1.79
10	3	-15	16.32	0.94
-11	-3	15	6.86	1.96
-11	-3	15	6.17	1.06
-11	-3	15	5.90	0.97
11	3	-15	5.48	0.64
-12	-3	15	10.20	1.56
-12	-3	15	13.12	2.30
12	3	-15	11.61	0.87
-13	-3	15	12.32	2.35
-13	-3	15	10.50	1.69
13	3	-15	9.81	0.89
-14	-3	15	19.43	2.06
-14	-3	15	19.22	3.58
14	3	-15	18.72	1.18
-15	-3	15	0.88	2.11
-15	-3	15	4.24	1.28
15	3	-15	3.91	0.61
-16	-3	15	16.66	2.27
-16	-3	15	11.92	2.73
16	3	-15	18.63	1.28
13	-3	16	21.09	3.19
-13	3	-16	21.19	2.01

12	-3	16	0.68	1.61
-12	3	-16	-0.40	0.84
11	-3	16	42.38	3.37
-11	3	-16	33.53	2.52
-11	3	-16	38.17	2.28
10	-3	16	-1.21	1.37
-10	3	-16	0.62	0.87
-10	3	-16	2.61	0.79
9	-3	16	37.50	3.20
-9	3	-16	42.19	2.19
-9	3	-16	38.39	2.20
8	-3	16	0.54	1.22
8	-3	16	-0.22	0.84
-8	3	-16	0.24	0.61
-8	3	-16	0.24	0.67
7	-3	16	19.75	1.76
7	-3	16	15.84	1.46
7	-3	16	18.78	2.37
-7	3	-16	17.60	1.40
6	-3	16	1.14	0.92
6	-3	16	1.12	1.21
6	-3	16	-0.23	0.79
-6	3	-16	0.39	0.48
-6	3	-16	1.98	1.49
-6	3	-16	0.11	0.58
5	-3	16	22.65	2.44
5	-3	16	26.02	2.09
5	-3	16	27.99	1.62
-5	3	-16	27.55	2.87
-5	3	-16	27.78	1.52
-5	3	-16	25.77	1.56
4	-3	16	5.17	1.17
4	-3	16	4.65	0.75
4	-3	16	5.47	1.36
-4	3	-16	10.78	1.73
-4	3	-16	6.50	0.95
-4	3	-16	5.72	0.83
-4	3	-16	5.33	0.61
3	-3	16	32.73	2.62
3	-3	16	39.17	1.92
3	-3	16	35.47	2.52
-3	3	-16	29.16	3.46
-3	3	-16	37.08	1.75
-3	3	-16	36.92	1.71

-3	3	-16	34.32	1.78
2	-3	16	-0.29	0.99
2	-3	16	-1.23	0.96
2	-3	16	2.51	0.67
-2	3	-16	0.86	0.42
-2	3	-16	-1.46	1.67
-2	3	-16	0.65	0.51
-2	3	-16	1.70	0.41
1	-3	16	1.99	0.68
1	-3	16	2.58	1.20
1	-3	16	0.13	1.05
-1	3	-16	1.38	0.40
-1	3	-16	3.38	2.51
-1	3	-16	2.39	0.55
-1	3	-16	2.03	0.45
0	-3	16	9.44	1.22
0	-3	16	13.48	1.88
0	-3	16	11.64	1.70
0	3	-16	11.23	0.85
0	3	-16	8.49	0.93
0	3	-16	11.10	0.80
-1	-3	16	30.67	2.39
-1	-3	16	34.86	1.87
-1	-3	16	29.33	2.65
1	3	-16	32.05	1.50
1	3	-16	30.40	1.48
1	3	-16	32.07	1.63
-2	-3	16	3.20	0.79
-2	-3	16	3.93	1.13
-2	-3	16	3.69	1.34
2	3	-16	3.64	0.60
2	3	-16	3.88	0.56
2	3	-16	4.21	0.51
-3	-3	16	22.89	2.60
-3	-3	16	23.43	2.11
-3	-3	16	24.58	1.69
3	3	-16	23.80	2.36
3	3	-16	28.30	1.30
3	3	-16	25.35	1.44
3	3	-16	25.64	1.22
-4	-3	16	2.03	0.73
-4	-3	16	2.03	0.56
-4	-3	16	3.73	1.50
-4	-3	16	1.56	0.44

-4	-3	16	2.24	1.00
4	3	-16	1.66	0.34
4	3	-16	-1.22	1.20
-5	-3	16	1.98	0.70
-5	-3	16	-0.30	0.88
-5	-3	16	0.40	0.40
-5	-3	16	0.09	0.49
-5	-3	16	1.34	1.39
5	3	-16	0.17	0.28
5	3	-16	1.57	1.16
-6	-3	16	2.32	0.59
-6	-3	16	1.16	1.42
-6	-3	16	1.13	0.89
-6	-3	16	2.04	0.46
-6	-3	16	1.57	0.78
6	3	-16	1.98	0.34
6	3	-16	0.77	1.13
-7	-3	16	9.35	1.88
-7	-3	16	6.95	0.83
-7	-3	16	6.96	1.09
-7	-3	16	5.75	1.07
7	3	-16	9.03	1.36
7	3	-16	6.02	0.60
-8	-3	16	5.71	0.65
-8	-3	16	6.69	1.84
-8	-3	16	6.61	1.03
-8	-3	16	6.41	0.90
8	3	-16	4.88	0.59
8	3	-16	3.82	1.22
-9	-3	16	8.39	1.34
-9	-3	16	7.48	0.96
-9	-3	16	6.95	2.01
9	3	-16	7.55	0.67
9	3	-16	7.85	1.29
-10	-3	16	17.13	1.56
-10	-3	16	18.79	3.21
-10	-3	16	14.64	1.45
10	3	-16	14.66	0.92
-11	-3	16	-1.06	1.73
-11	-3	16	-0.54	0.82
11	3	-16	-0.40	0.38
-12	-3	16	4.74	2.02
-12	-3	16	3.85	1.06
12	3	-16	5.08	0.74

-13	-3	16	-1.64	1.85
-13	-3	16	-0.54	0.96
13	3	-16	0.27	0.45
-14	-3	16	3.02	1.17
-14	-3	16	1.84	2.09
14	3	-16	3.01	0.57
-15	-3	16	0.52	1.21
-15	-3	16	0.17	2.17
-16	-3	16	4.11	2.44
-16	-3	16	5.15	1.38
12	-3	17	38.26	3.70
-12	3	-17	48.37	2.71
11	-3	17	-0.89	1.70
-11	3	-17	0.43	0.86
10	-3	17	26.47	3.18
-10	3	-17	32.03	2.07
9	-3	17	-0.43	1.48
-9	3	-17	-0.10	0.73
8	-3	17	34.54	2.27
8	-3	17	30.38	3.01
-8	3	-17	30.86	1.94
-8	3	-17	33.08	1.97
7	-3	17	0.43	1.00
7	-3	17	1.14	1.39
-7	3	-17	0.67	0.72
-7	3	-17	0.96	0.65
6	-3	17	62.39	3.27
6	-3	17	56.04	3.76
-6	3	-17	60.40	2.75
5	-3	17	1.72	1.11
5	-3	17	-0.21	1.28
-5	3	-17	0.70	0.52
-5	3	-17	-0.03	0.59
-5	3	-17	-2.10	1.68
4	-3	17	16.46	2.07
4	-3	17	19.23	1.45
4	-3	17	16.59	2.44
-4	3	-17	19.24	1.30
-4	3	-17	18.35	3.10
-4	3	-17	19.65	1.29
3	-3	17	-1.00	0.68
3	-3	17	0.20	1.18
3	-3	17	1.67	1.21
-3	3	-17	0.88	0.47

-3	3	-17	-0.45	0.52
-3	3	-17	-0.67	2.55
-3	3	-17	0.19	0.46
2	-3	17	23.80	2.44
2	-3	17	23.67	2.34
2	-3	17	23.25	1.58
-2	3	-17	22.16	1.31
-2	3	-17	24.42	1.32
-2	3	-17	21.55	1.38
1	-3	17	0.17	1.13
1	-3	17	0.91	0.71
1	-3	17	-0.65	1.15
-1	3	-17	1.08	0.48
-1	3	-17	0.34	0.42
-1	3	-17	0.81	0.52
0	-3	17	54.24	3.52
0	-3	17	54.96	3.47
0	-3	17	65.33	2.78
0	3	-17	56.44	2.56
0	3	-17	57.23	2.49
0	3	-17	59.44	2.49
-1	-3	17	3.51	1.30
-1	-3	17	5.41	0.85
-1	-3	17	1.33	1.55
1	3	-17	3.44	0.63
1	3	-17	3.05	0.52
1	3	-17	3.46	0.50
-2	-3	17	53.82	3.41
-2	-3	17	65.30	3.98
-2	-3	17	63.27	2.93
2	3	-17	61.88	2.73
2	3	-17	65.73	2.62
2	3	-17	61.24	2.58
-3	-3	17	4.15	0.66
-3	-3	17	2.80	1.47
-3	-3	17	4.02	1.18
3	3	-17	3.00	0.45
3	3	-17	3.41	0.52
-4	-3	17	18.60	2.05
-4	-3	17	20.97	1.64
-4	-3	17	20.98	1.37
-4	-3	17	24.51	2.84
4	3	-17	20.10	1.16
4	3	-17	18.73	1.06

-5	-3	17	-0.09	1.44
-5	-3	17	-1.55	0.78
-5	-3	17	0.08	0.46
-5	-3	17	-0.11	1.00
5	3	-17	0.32	0.33
5	3	-17	2.78	1.34
-6	-3	17	29.26	2.29
-6	-3	17	25.98	3.14
-6	-3	17	27.74	1.73
-6	-3	17	27.32	2.22
-6	-3	17	27.47	1.49
6	3	-17	30.09	2.62
6	3	-17	29.30	1.34
-7	-3	17	0.67	0.96
-7	-3	17	0.95	0.81
-7	-3	17	1.97	1.76
-7	-3	17	0.75	0.65
7	3	-17	-0.92	1.27
7	3	-17	0.56	0.34
-8	-3	17	31.81	2.18
-8	-3	17	30.54	3.52
-8	-3	17	32.14	1.97
8	3	-17	31.53	2.62
8	3	-17	30.64	1.45
-9	-3	17	9.10	2.12
-9	-3	17	7.63	1.12
-9	-3	17	10.80	1.42
9	3	-17	7.63	0.74
-10	-3	17	30.55	2.03
-10	-3	17	25.77	2.00
-10	-3	17	30.23	3.59
10	3	-17	25.79	1.33
-11	-3	17	0.63	1.89
-11	-3	17	-1.18	0.85
11	3	-17	0.76	0.39
-12	-3	17	23.49	2.04
-12	-3	17	26.77	3.77
12	3	-17	17.50	1.16
-13	-3	17	2.40	1.10
-13	-3	17	-0.94	2.02
13	3	-17	0.28	0.50
-14	-3	17	4.66	1.32
-14	-3	17	0.96	2.33
-15	-3	17	0.85	1.28

-15	-3	17	5.55	2.54
11	-3	18	24.47	4.21
-11	3	-18	28.92	2.16
10	-3	18	1.04	1.71
-10	3	-18	1.36	0.89
9	-3	18	42.91	3.65
-9	3	-18	41.35	2.41
8	-3	18	1.99	1.59
-8	3	-18	1.17	0.79
7	-3	18	24.05	2.23
7	-3	18	20.73	2.78
-7	3	-18	25.08	1.69
6	-3	18	-1.39	1.46
6	-3	18	1.17	1.20
-6	3	-18	-0.20	0.61
-6	3	-18	0.33	0.72
5	-3	18	59.56	3.29
5	-3	18	50.23	3.61
-5	3	-18	51.97	2.46
-5	3	-18	47.97	3.29
4	-3	18	2.52	1.47
4	-3	18	-0.91	1.22
-4	3	-18	1.16	0.58
-4	3	-18	0.33	0.61
3	-3	18	33.55	2.71
3	-3	18	32.14	2.97
-3	3	-18	31.30	1.72
-3	3	-18	32.45	1.75
2	-3	18	1.86	1.34
2	-3	18	-1.04	1.24
-2	3	-18	-1.35	0.52
-2	3	-18	-0.18	0.54
-2	3	-18	-0.87	0.47
1	-3	18	41.73	2.17
1	-3	18	36.76	3.00
-1	3	-18	37.74	1.95
-1	3	-18	40.54	1.92
-1	3	-18	40.16	1.91
0	-3	18	3.63	1.46
0	-3	18	3.67	1.36
0	-3	18	1.55	0.81
0	3	-18	2.18	0.64
0	3	-18	2.33	0.53
0	3	-18	2.71	0.54

-1	-3	18	65.44	4.11
-1	-3	18	65.30	3.76
-1	-3	18	67.20	3.11
1	3	-18	63.03	2.86
1	3	-18	66.46	2.78
1	3	-18	67.73	2.81
-2	-3	18	3.22	0.67
-2	-3	18	2.74	0.88
-2	-3	18	4.21	1.59
-2	-3	18	1.72	1.20
2	3	-18	3.69	0.56
2	3	-18	2.34	0.47
-3	-3	18	92.39	4.75
-3	-3	18	96.01	4.07
-3	-3	18	94.41	4.23
-3	-3	18	99.21	5.31
3	3	-18	98.41	3.89
3	3	-18	99.21	3.87
-4	-3	18	0.22	0.92
-4	-3	18	0.92	1.13
-4	-3	18	0.15	0.60
-4	-3	18	2.04	1.59
4	3	-18	0.35	0.40
4	3	-18	0.49	0.50
-5	-3	18	18.33	3.13
-5	-3	18	19.26	1.49
-5	-3	18	22.08	1.81
-5	-3	18	20.06	2.17
5	3	-18	20.21	1.37
5	3	-18	18.52	1.09
-6	-3	18	1.22	0.67
-6	-3	18	-1.48	1.66
-6	-3	18	1.15	1.11
6	3	-18	0.15	0.37
-7	-3	18	51.89	4.18
-7	-3	18	56.62	2.75
-7	-3	18	52.94	3.09
7	3	-18	58.04	4.17
7	3	-18	52.65	2.28
-8	-3	18	1.63	0.85
-8	-3	18	-1.80	1.78
-8	-3	18	-0.97	0.99
8	3	-18	0.48	0.37
-9	-3	18	36.89	2.19

-9	-3	18	26.93	2.25
-9	-3	18	36.66	3.80
9	3	-18	32.06	1.53
-10	-3	18	2.86	2.16
-10	-3	18	0.31	0.99
10	3	-18	2.87	0.49
-11	-3	18	31.62	2.28
-11	-3	18	30.35	4.07
11	3	-18	24.80	1.41
-12	-3	18	0.31	2.14
-12	-3	18	0.34	1.04
-13	-3	18	22.33	3.97
-13	-3	18	16.85	2.25
-14	-3	18	-0.83	3.42
-14	-3	18	-1.05	1.29
-9	3	-19	-0.33	0.85
8	-3	19	37.31	3.76
-8	3	-19	44.09	2.42
7	-3	19	-0.69	1.67
-7	3	-19	-0.36	0.75
6	-3	19	36.97	2.84
6	-3	19	35.94	3.40
-6	3	-19	34.39	2.08
5	-3	19	-0.87	1.36
5	-3	19	1.93	1.58
-5	3	-19	1.18	0.70
4	-3	19	43.24	3.52
4	-3	19	47.98	3.24
-4	3	-19	44.26	2.50
3	-3	19	1.17	1.50
-3	3	-19	-0.89	0.63
-3	3	-19	0.99	0.59
2	-3	19	36.10	3.01
2	-3	19	26.83	2.86
-2	3	-19	32.04	1.76
-2	3	-19	35.50	1.78
1	-3	19	2.36	1.53
1	-3	19	0.28	1.41
-1	3	-19	-0.97	0.57
-1	3	-19	-1.33	0.51
0	-3	19	55.01	3.76
0	-3	19	51.68	3.60
0	3	-19	50.11	2.40
0	3	-19	54.64	2.40

-1	-3	19	0.42	1.42
-1	-3	19	1.39	1.63
1	3	-19	1.34	0.52
-2	-3	19	61.27	3.72
-2	-3	19	58.85	4.16
-2	-3	19	59.71	2.83
2	3	-19	65.14	2.66
-3	-3	19	1.58	1.29
-3	-3	19	-0.10	0.63
-3	-3	19	-0.27	1.66
3	3	-19	0.54	0.46
-4	-3	19	63.04	2.86
-4	-3	19	61.26	3.63
-4	-3	19	60.26	4.35
4	3	-19	56.89	2.54
-5	-3	19	4.15	1.30
-5	-3	19	1.35	0.78
-5	-3	19	3.37	1.81
5	3	-19	2.26	0.46
-6	-3	19	37.40	3.85
-6	-3	19	35.21	2.13
-6	-3	19	33.35	2.67
6	3	-19	35.67	1.64
-7	-3	19	-1.04	1.20
-7	-3	19	-0.96	1.83
7	3	-19	0.53	0.44
-8	-3	19	36.20	2.61
-8	-3	19	38.18	2.38
-8	-3	19	43.89	4.09
8	3	-19	37.12	1.75
-9	-3	19	3.27	1.06
-9	-3	19	3.77	2.19
-10	-3	19	42.77	4.40
-10	-3	19	49.30	2.86
-11	-3	19	-1.93	2.17
-11	-3	19	0.00	1.07
-12	-3	19	37.71	2.71
-12	-3	19	27.20	4.49
7	-3	20	34.23	3.64
-7	3	-20	30.18	2.14
6	-3	20	0.97	1.78
-6	3	-20	1.50	0.84
5	-3	20	28.16	3.30
-5	3	-20	27.80	1.89

4	-3	20	3.28	1.81
4	-3	20	2.62	1.63
-4	3	-20	1.36	0.74
3	-3	20	5.19	1.76
3	-3	20	5.90	1.78
-3	3	-20	8.82	1.20
2	-3	20	2.16	1.62
2	-3	20	1.43	1.54
-2	3	-20	2.33	0.68
1	-3	20	27.16	3.22
1	-3	20	30.49	3.11
-1	3	-20	32.52	1.75
0	-3	20	0.43	1.60
0	-3	20	1.87	1.72
0	3	-20	0.04	0.58
-1	-3	20	41.50	2.11
-1	-3	20	36.99	3.69
-1	-3	20	37.65	3.31
1	3	-20	37.86	1.92
-2	-3	20	0.01	1.44
-2	-3	20	2.44	1.80
-2	-3	20	-0.34	0.68
2	3	-20	1.94	0.54
-3	-3	20	30.41	3.49
-3	-3	20	26.69	1.77
-3	-3	20	26.11	2.82
3	3	-20	26.18	1.46
-4	-3	20	2.44	1.42
-4	-3	20	-0.38	0.76
-4	-3	20	0.19	1.94
4	3	-20	-0.58	0.44
-5	-3	20	27.88	1.88
-5	-3	20	25.68	2.70
-5	-3	20	28.00	3.86
5	3	-20	24.89	1.40
-6	-3	20	-0.70	1.97
-6	-3	20	0.84	1.54
-6	-3	20	0.43	1.03
6	3	-20	0.41	0.46
-7	-3	20	55.24	3.46
-7	-3	20	60.29	4.80
-7	-3	20	61.51	3.09
-8	-3	20	-2.44	2.01
-8	-3	20	1.74	1.03

-9	-3	20	33.60	4.49
-9	-3	20	43.67	2.70
-10	-3	20	-0.50	2.33
-10	-3	20	1.02	1.16
-11	-3	20	20.67	2.40
5	-3	21	3.87	1.96
-5	3	-21	-0.46	0.85
4	-3	21	-0.64	1.72
4	-3	21	-0.05	1.84
-4	3	-21	0.08	0.76
3	-3	21	6.39	1.93
3	-3	21	11.77	2.19
-3	3	-21	7.22	0.97
2	-3	21	-1.34	1.71
2	-3	21	-0.01	1.83
-2	3	-21	2.26	0.77
1	-3	21	1.08	1.79
1	-3	21	1.06	1.77
0	-3	21	1.77	1.89
0	-3	21	2.30	1.72
0	3	-21	3.30	0.71
-1	-3	21	-1.28	1.65
-1	-3	21	4.57	2.02
1	3	-21	3.43	0.68
-2	-3	21	6.95	2.14
-2	-3	21	5.92	1.78
2	3	-21	4.88	0.73
-3	-3	21	1.09	1.62
-3	-3	21	1.23	2.02
-3	-3	21	0.85	1.00
3	3	-21	1.49	0.70
-4	-3	21	7.40	1.06
-4	-3	21	6.12	2.29
-4	-3	21	4.47	1.69
-5	-3	21	3.59	2.21
-5	-3	21	5.07	1.06
-5	-3	21	5.06	1.65
-6	-3	21	7.84	1.21
-6	-3	21	9.61	1.73
-6	-3	21	10.44	2.52
-7	-3	21	2.06	1.09
-7	-3	21	2.06	1.55
-7	-3	21	0.07	2.34
-8	-3	21	2.86	2.38

-8	-3	21	5.91	1.30
-9	-3	21	1.02	3.44
-9	-3	21	4.37	1.28
2	-3	22	5.92	2.18
2	-3	22	1.00	1.96
1	-3	22	8.11	2.11
1	-3	22	8.93	2.28
0	-3	22	3.57	1.95
0	-3	22	3.63	2.15
-1	-3	22	7.07	2.44
-1	-3	22	5.61	2.00
-2	-3	22	4.54	1.87
-2	-3	22	3.66	2.23
-3	-3	22	10.83	2.68
-3	-3	22	15.70	2.95
-4	-3	22	7.03	2.49
-4	-3	22	5.51	1.82
-5	-3	22	-0.49	1.62
-5	-3	22	3.13	2.45
-6	-3	22	3.41	1.81
-6	-3	22	9.55	2.74
5	-4	-22	-0.67	0.78
-5	4	22	-1.34	1.92
-5	4	22	0.21	1.62
4	-4	-22	0.53	0.80
-4	4	22	1.63	1.98
-4	4	22	-1.04	1.63
3	-4	-22	15.81	1.54
-3	4	22	13.73	2.13
-3	4	22	10.18	2.29
2	-4	-22	2.85	0.87
-2	4	22	2.30	1.74
-2	4	22	4.33	2.04
1	-4	-22	15.11	1.57
-1	4	22	20.96	3.27
-1	4	22	11.27	2.14
0	-4	-22	14.51	1.63
0	4	22	12.31	2.19
0	4	22	17.94	3.19
-1	-4	-22	0.42	0.85
1	4	22	3.02	1.91
1	4	22	1.09	1.84
8	-4	-21	1.78	0.78
-8	4	21	3.52	2.17

7	-4	-21	12.14	1.36
-7	4	21	13.06	2.58
-7	4	21	13.88	2.62
6	-4	-21	3.30	0.76
-6	4	21	2.30	2.01
-6	4	21	3.62	1.62
5	-4	-21	12.88	1.33
-5	4	21	11.32	1.88
-5	4	21	10.67	2.19
4	-4	-21	2.07	0.75
-4	4	21	3.41	1.62
-4	4	21	1.68	1.81
3	-4	-21	4.78	0.85
-3	4	21	6.63	1.86
-3	4	21	5.81	1.72
2	-4	-21	9.35	1.32
-2	4	21	8.28	1.86
-2	4	21	7.93	1.95
1	-4	-21	4.09	0.84
-1	4	21	6.77	1.89
-1	4	21	2.55	1.67
0	-4	-21	3.58	0.89
0	4	21	5.72	1.74
0	4	21	5.44	1.75
-1	-4	-21	2.20	0.82
1	4	21	6.13	1.85
1	4	21	1.61	1.60
-2	-4	-21	5.33	0.96
2	4	21	6.01	1.83
2	4	21	3.62	1.78
-3	-4	-21	-0.34	0.82
3	4	21	-0.27	1.64
3	4	21	1.09	1.59
-4	-4	-21	9.48	1.20
4	4	21	15.51	2.88
4	4	21	8.78	2.18
-5	-4	-21	-0.91	0.86
5	4	21	-1.67	1.64
5	4	21	2.02	1.96
11	-4	-20	-1.08	0.69
10	-4	-20	46.07	2.39
-10	4	20	49.08	4.50
9	-4	-20	1.05	0.65
-9	4	20	-1.65	1.89

8	-4	-20	32.14	1.82
-8	4	20	25.85	3.73
7	-4	-20	0.19	0.62
-7	4	20	-1.13	2.41
-7	4	20	-3.92	1.68
6	-4	-20	30.81	1.81
-6	4	20	31.09	2.74
-6	4	20	31.52	3.49
5	-4	-20	1.81	0.65
-5	4	20	-1.38	1.56
-5	4	20	1.70	1.43
4	-4	-20	34.83	1.95
-4	4	20	37.08	3.48
-4	4	20	32.72	2.99
3	-4	-20	4.61	0.82
-3	4	20	5.19	1.59
-3	4	20	4.85	1.71
2	-4	-20	29.46	1.77
-2	4	20	25.40	2.81
-2	4	20	29.34	3.11
1	-4	-20	3.88	0.79
-1	4	20	1.55	1.51
-1	4	20	4.62	1.62
0	-4	-20	27.80	1.81
0	4	20	27.30	2.96
0	4	20	33.60	3.00
-1	-4	-20	1.65	0.74
1	4	20	-0.45	1.51
1	4	20	0.54	1.45
-2	-4	-20	21.98	1.61
2	4	20	15.65	2.61
2	4	20	15.86	2.27
-3	-4	-20	6.74	1.00
3	4	20	3.95	1.80
3	4	20	5.82	1.64
-4	-4	-20	2.97	0.82
4	4	20	5.40	1.78
4	4	20	7.89	1.72
-5	-4	-20	1.87	0.84
5	4	20	1.53	1.69
5	4	20	1.20	1.55
-6	-4	-20	21.79	1.80
6	4	20	15.46	2.44
6	4	20	21.28	2.73

-7	-4	-20	-0.17	0.86
7	4	20	1.58	1.96
12	-4	-19	0.01	0.69
-12	4	19	2.68	2.29
-12	4	19	-1.09	1.26
11	-4	-19	40.04	2.10
-11	4	19	39.32	4.12
-11	4	19	38.77	2.72
10	-4	-19	0.87	0.64
-10	4	19	-1.23	1.05
-10	4	19	2.77	1.91
9	-4	-19	35.32	1.89
-9	4	19	38.00	2.35
-9	4	19	27.97	3.61
8	-4	-19	-0.12	0.58
-8	4	19	0.03	0.87
-8	4	19	1.61	1.19
-8	4	19	-1.84	1.71
7	-4	-19	62.94	2.90
-7	4	19	71.03	3.05
-7	4	19	58.42	3.55
-7	4	19	66.67	4.47
6	-4	-19	-0.06	0.54
-6	4	19	-0.83	1.18
-6	4	19	0.69	0.75
-6	4	19	-0.54	1.60
5	-4	-19	38.98	1.99
-5	4	19	36.72	3.43
4	-4	-19	-0.95	0.56
-4	4	19	0.90	1.22
-4	4	19	-2.95	1.34
3	-4	-19	68.64	2.97
-3	4	19	60.44	3.69
-3	4	19	66.88	4.14
2	-4	-19	0.38	0.60
-2	4	19	0.10	1.35
-2	4	19	0.33	1.28
1	-4	-19	28.71	1.72
-1	4	19	26.89	2.77
-1	4	19	29.05	2.72
0	-4	-19	-0.62	0.59
0	4	19	-0.68	1.31
0	4	19	1.38	1.35
-1	-4	-19	28.72	1.73

1	4	19	28.28	2.73
1	4	19	25.72	2.85
-2	-4	-19	0.02	0.63
2	4	19	-1.61	1.29
2	4	19	0.73	1.29
-3	-4	-19	36.99	2.05
3	4	19	32.99	3.05
3	4	19	30.08	2.82
-4	-4	-19	1.13	0.74
4	4	19	-0.06	1.20
4	4	19	1.79	1.43
-5	-4	-19	46.19	2.46
5	4	19	44.42	3.46
5	4	19	43.66	3.12
-6	-4	-19	7.81	1.06
6	4	19	6.41	1.93
6	4	19	7.80	1.65
-7	-4	-19	34.05	2.17
7	4	19	27.64	3.34
7	4	19	31.26	2.82
-8	-4	-19	2.78	0.90
8	4	19	0.89	1.79
-9	-4	-19	24.49	2.05
9	4	19	26.34	3.90
13	-4	-18	1.13	0.64
-13	4	18	0.25	1.37
-13	4	18	-2.18	1.96
12	-4	-18	33.85	1.94
-12	4	18	33.81	2.66
-12	4	18	44.97	4.05
11	-4	-18	0.93	0.64
-11	4	18	2.41	1.98
-11	4	18	0.91	1.07
10	-4	-18	39.57	2.09
-10	4	18	42.39	3.91
-10	4	18	43.39	2.52
9	-4	-18	-0.37	0.49
-9	4	18	0.07	1.08
-9	4	18	0.89	0.91
-9	4	18	-1.18	1.57
8	-4	-18	29.41	1.67
-8	4	18	29.86	3.22
-8	4	18	30.07	1.95
-8	4	18	32.60	2.36

7	-4	-18	5.97	0.94
-7	4	18	6.87	0.91
-7	4	18	8.57	1.92
-7	4	18	5.90	1.29
6	-4	-18	37.59	1.97
-6	4	18	37.08	2.68
-6	4	18	40.42	3.41
-6	4	18	44.06	2.08
5	-4	-18	0.52	0.53
-5	4	18	-0.78	1.37
-5	4	18	0.79	0.59
-5	4	18	-0.05	1.12
4	-4	-18	50.18	2.46
-4	4	18	56.28	2.33
-4	4	18	56.97	3.74
-4	4	18	50.64	3.16
3	-4	-18	0.35	0.51
-3	4	18	-0.14	1.20
2	-4	-18	38.74	1.86
-2	4	18	28.91	2.57
-2	4	18	36.86	2.95
1	-4	-18	4.47	0.71
-1	4	18	3.40	1.28
-1	4	18	7.27	1.38
0	-4	-18	51.22	2.45
0	4	18	49.26	3.27
0	4	18	53.81	3.34
-1	-4	-18	2.16	0.68
1	4	18	2.58	1.32
1	4	18	0.42	1.14
-2	-4	-18	48.46	2.41
2	4	18	50.40	3.36
2	4	18	46.70	3.08
-3	-4	-18	2.84	0.72
3	4	18	1.00	1.31
3	4	18	3.69	1.27
-4	-4	-18	36.73	2.02
4	4	18	33.25	2.91
-5	-4	-18	-0.24	0.66
5	4	18	1.06	1.18
5	4	18	0.07	1.36
-6	-4	-18	41.30	2.25
6	4	18	42.34	2.80
6	4	18	33.72	3.23

-7	-4	-18	1.03	0.75
7	4	18	1.11	1.20
7	4	18	-0.45	1.40
-8	-4	-18	31.51	2.13
8	4	18	33.28	2.64
8	4	18	37.35	3.28
-9	-4	-18	-1.26	0.81
9	4	18	2.04	1.70
-10	-4	-18	14.74	1.74
10	4	18	19.08	3.00
15	-4	-17	4.09	0.83
-15	4	17	5.61	1.46
14	-4	-17	2.26	0.70
-14	4	17	-1.57	1.96
-14	4	17	0.50	1.29
13	-4	-17	10.74	1.23
-13	4	17	11.66	2.31
-13	4	17	12.35	2.09
12	-4	-17	0.41	0.56
-12	4	17	1.65	1.12
-12	4	17	-0.25	1.74
11	-4	-17	27.58	1.62
-11	4	17	25.91	2.45
-11	4	17	29.32	3.37
10	-4	-17	1.72	1.32
10	-4	-17	0.16	0.53
-10	4	17	0.10	0.82
-10	4	17	1.71	1.68
9	-4	-17	27.20	1.47
-9	4	17	22.67	2.02
-9	4	17	21.78	2.98
-9	4	17	26.07	1.84
8	-4	-17	-0.07	0.47
8	-4	-17	-1.74	1.39
-8	4	17	1.41	1.37
-8	4	17	0.54	0.71
-8	4	17	1.17	0.83
-8	4	17	0.64	0.95
7	-4	-17	28.69	2.47
7	-4	-17	27.11	1.57
-7	4	17	31.32	3.01
-7	4	17	29.11	2.20
-7	4	17	31.75	1.79
-7	4	17	30.74	1.81

6	-4	-17	18.98	2.28
6	-4	-17	18.57	1.25
-6	4	17	20.29	2.58
-6	4	17	15.08	1.90
-6	4	17	17.28	1.38
-6	4	17	20.11	1.34
-5	4	17	6.16	1.03
-5	4	17	5.39	1.23
-5	4	17	5.71	0.93
-5	4	17	9.37	1.77
-4	4	17	3.12	1.09
-4	4	17	2.99	0.57
-4	4	17	2.44	0.64
-4	4	17	2.07	1.28
3	-4	-17	25.06	1.87
-3	4	17	34.92	1.48
-3	4	17	33.05	1.50
-3	4	17	26.28	2.28
-3	4	17	33.36	2.73
2	-4	-17	2.12	0.88
-2	4	17	2.18	1.07
-2	4	17	0.98	1.16
-1	4	17	42.45	2.91
0	-4	-17	0.69	0.53
0	4	17	0.46	1.05
0	4	17	1.60	1.05
-1	-4	-17	46.99	2.20
1	4	17	40.35	2.95
-2	-4	-17	0.27	0.49
2	4	17	-0.05	0.92
2	4	17	0.14	1.09
-3	-4	-17	18.27	1.40
3	4	17	23.69	2.04
3	4	17	19.04	2.27
-4	-4	-17	2.72	0.66
4	4	17	3.79	1.15
4	4	17	3.90	1.39
-5	-4	-17	24.78	1.65
5	4	17	22.77	2.61
5	4	17	28.69	2.23
-6	-4	-17	1.54	0.66
6	4	17	1.32	1.45
6	4	17	1.16	1.12
-7	-4	-17	26.69	1.75

7	4	17	19.55	2.77
7	4	17	26.16	2.22
-8	-4	-17	-0.05	0.70
8	4	17	1.39	1.13
8	4	17	-0.81	1.37
-9	-4	-17	46.12	2.53
9	4	17	41.32	3.47
9	4	17	45.51	2.90
-10	-4	-17	0.60	0.79
10	4	17	0.51	1.50
-11	-4	-17	36.80	2.28
11	4	17	27.44	3.26
-12	-4	-17	1.04	0.93
12	4	17	1.42	1.97
15	-4	-16	4.60	0.80
-15	4	16	4.70	2.15
-15	4	16	5.87	1.36
14	-4	-16	0.35	0.61
-14	4	16	1.42	1.92
-14	4	16	0.27	1.14
13	-4	-16	10.31	1.15
-13	4	16	8.81	2.24
-13	4	16	8.95	1.40
12	-4	-16	0.66	0.51
-12	4	16	-0.87	1.65
-12	4	16	1.17	0.98
11	-4	-16	2.12	0.57
-11	4	16	2.42	1.74
-11	4	16	3.84	1.13
10	-4	-16	-0.96	1.14
10	-4	-16	0.25	0.50
-10	4	16	-0.77	1.44
-10	4	16	0.45	0.73
9	-4	-16	16.37	1.12
9	-4	-16	15.20	1.87
-9	4	16	14.48	1.66
-9	4	16	13.35	1.62
-9	4	16	18.12	2.67
-9	4	16	16.69	1.51
8	-4	-16	4.03	0.75
8	-4	-16	3.82	1.09
-8	4	16	3.90	0.82
-8	4	16	2.11	1.42
-8	4	16	4.10	0.77

-8	4	16	2.70	0.97
7	-4	-16	27.54	2.24
7	-4	-16	23.80	1.44
-7	4	16	27.93	2.72
-7	4	16	27.99	1.67
-7	4	16	30.63	1.66
-7	4	16	26.08	1.98
6	-4	-16	22.10	1.26
6	-4	-16	22.83	2.12
-6	4	16	18.66	1.38
-6	4	16	24.62	2.56
-6	4	16	17.53	1.84
-6	4	16	23.72	1.37
5	-4	-16	9.52	0.89
5	-4	-16	13.45	2.08
-5	4	16	9.30	1.00
-5	4	16	8.20	1.54
-5	4	16	7.97	1.53
-5	4	16	10.75	0.95
4	-4	-16	0.75	0.40
4	-4	-16	-1.76	1.08
-4	4	16	1.58	0.50
-4	4	16	1.78	0.92
-4	4	16	0.12	0.44
-4	4	16	1.99	1.10
3	-4	-16	0.32	0.42
-3	4	16	0.22	0.40
-3	4	16	0.65	0.40
-3	4	16	1.43	0.94
-3	4	16	0.04	0.99
2	-4	-16	2.43	0.51
-2	4	16	3.85	1.10
1	-4	-16	10.26	0.94
-1	4	16	11.58	1.73
-1	4	16	10.14	1.73
0	-4	-16	27.10	1.51
0	4	16	31.62	2.26
0	4	16	25.19	2.25
-1	-4	-16	2.65	0.54
1	4	16	1.48	0.91
2	4	16	19.20	1.86
2	4	16	18.58	2.00
-3	-4	-16	4.90	0.72
3	4	16	4.89	1.09

3	4	16	5.26	1.32
-4	-4	-16	31.01	1.73
4	4	16	29.44	2.51
4	4	16	32.44	2.25
-5	-4	-16	3.78	1.97
-5	-4	-16	0.88	0.56
5	4	16	2.26	0.99
5	4	16	-0.22	1.12
-6	-4	-16	28.58	3.90
-6	-4	-16	32.95	1.83
6	4	16	31.12	2.22
6	4	16	29.03	2.68
-7	-4	-16	5.49	0.82
-7	-4	-16	3.78	2.72
7	4	16	3.87	1.47
7	4	16	6.69	1.19
-8	-4	-16	12.21	1.33
8	4	16	10.54	1.78
8	4	16	13.18	1.73
-9	-4	-16	-0.03	0.70
9	4	16	1.08	1.36
9	4	16	1.95	1.03
-10	-4	-16	26.65	1.90
10	4	16	24.81	2.89
-11	-4	-16	0.64	0.82
11	4	16	5.52	1.84
-12	-4	-16	35.57	2.32
12	4	16	35.37	3.46
-13	-4	-16	0.23	0.98
13	4	16	1.26	1.77
16	-4	-15	8.82	1.27
-16	4	15	9.80	2.38
-16	4	15	11.43	1.69
15	-4	-15	21.36	1.48
-15	4	15	22.81	2.41
-15	4	15	15.08	2.56
14	-4	-15	9.99	1.11
-14	4	15	9.74	2.27
-14	4	15	11.98	2.01
13	-4	-15	10.33	1.09
-13	4	15	15.61	2.34
-13	4	15	12.87	1.89
12	-4	-15	10.11	1.04
-12	4	15	15.40	1.71

-12	4	15	14.18	2.19
11	-4	-15	17.10	1.70
11	-4	-15	13.88	1.10
-11	4	15	12.52	2.08
-11	4	15	17.88	1.64
10	-4	-15	12.17	0.96
10	-4	-15	11.06	1.50
10	-4	-15	9.44	1.77
-10	4	15	12.34	2.05
-10	4	15	14.79	1.51
9	-4	-15	5.38	1.03
9	-4	-15	5.06	0.78
-9	4	15	7.57	1.21
-9	4	15	6.64	0.94
-9	4	15	4.56	1.57
-9	4	15	8.28	1.37
8	-4	-15	17.77	1.77
8	-4	-15	15.62	1.04
-8	4	15	13.12	1.54
-8	4	15	17.07	1.32
-8	4	15	14.79	2.29
-8	4	15	14.42	1.36
7	-4	-15	32.91	2.22
7	-4	-15	28.70	1.54
-7	4	15	30.75	2.05
-7	4	15	30.61	1.73
-7	4	15	33.09	1.73
-7	4	15	32.02	2.71
6	-4	-15	27.06	2.16
6	-4	-15	26.39	1.43
-6	4	15	31.19	1.57
-6	4	15	28.66	1.52
-6	4	15	26.89	2.50
-6	4	15	25.67	1.90
5	-4	-15	4.46	1.22
5	-4	-15	3.70	0.55
-5	4	15	4.52	0.61
-5	4	15	5.33	0.77
-5	4	15	3.47	0.96
-5	4	15	5.79	1.37
4	-4	-15	9.45	0.82
4	-4	-15	6.08	1.27
-4	4	15	10.25	1.64
-4	4	15	7.99	1.45

-4	4	15	7.82	0.80
-4	4	15	7.47	0.80
3	-4	-15	5.89	0.74
3	-4	-15	4.69	1.30
-3	4	15	3.85	1.11
-3	4	15	4.49	0.65
-3	4	15	5.32	1.09
-3	4	15	4.68	0.67
2	-4	-15	8.31	1.36
2	-4	-15	8.48	0.83
-2	4	15	7.39	1.21
-2	4	15	6.75	0.57
-2	4	15	10.40	1.51
1	-4	-15	0.94	0.45
-1	4	15	-0.69	0.71
-1	4	15	-0.56	0.82
0	-4	-15	38.05	1.83
0	4	15	33.99	2.42
0	4	15	37.50	2.43
-1	-4	-15	0.61	0.41
1	4	15	0.42	0.81
1	4	15	0.51	0.89
-2	-4	-15	12.00	2.02
-2	-4	-15	15.17	1.08
2	4	15	14.17	1.62
2	4	15	17.40	1.82
-3	-4	-15	5.71	0.84
-3	-4	-15	5.37	1.84
3	4	15	7.25	1.10
-4	-4	-15	1.04	1.62
4	4	15	1.64	1.05
4	4	15	2.05	0.87
-5	-4	-15	1.00	0.51
-5	-4	-15	0.43	1.71
5	4	15	1.18	0.84
5	4	15	2.29	1.04
-6	-4	-15	7.81	1.96
-6	-4	-15	5.68	0.78
6	4	15	7.63	1.10
6	4	15	7.88	1.44
-7	-4	-15	-1.37	0.53
-7	-4	-15	-1.70	1.71
7	4	15	-0.14	1.16
7	4	15	0.11	0.79

-8	-4	-15	23.85	1.63
-8	-4	-15	23.92	3.19
8	4	15	24.27	2.50
8	4	15	23.77	1.83
-9	-4	-15	0.04	0.63
9	4	15	-1.80	1.18
9	4	15	2.23	0.93
-10	-4	-15	3.72	0.86
10	4	15	4.19	0.97
10	4	15	3.19	1.41
-11	-4	-15	2.02	0.85
11	4	15	2.70	1.48
-12	-4	-15	10.07	1.49
12	4	15	7.34	1.78
-13	-4	-15	5.50	1.09
13	4	15	3.20	1.66
-14	-4	-15	15.24	1.98
14	4	15	12.36	2.14
17	-4	-14	0.27	0.71
-17	4	14	2.22	2.15
-17	4	14	0.24	1.33
16	-4	-14	36.43	2.00
-16	4	14	40.05	2.81
-16	4	14	40.36	3.97
15	-4	-14	10.43	1.10
-15	4	14	5.67	2.10
-15	4	14	12.29	1.97
14	-4	-14	36.36	1.95
-14	4	14	38.06	3.61
-14	4	14	43.78	2.63
13	-4	-14	7.99	0.95
-13	4	14	7.78	1.18
-13	4	14	5.70	1.90
12	-4	-14	23.64	1.38
-12	4	14	23.44	1.90
-12	4	14	25.24	2.98
11	-4	-14	-0.31	0.90
11	-4	-14	0.17	0.69
11	-4	-14	0.43	0.42
-11	4	14	0.33	1.39
-11	4	14	0.85	0.71
10	-4	-14	64.31	2.97
10	-4	-14	71.71	3.38
10	-4	-14	68.07	3.13

-10	4	14	78.19	3.31
-10	4	14	74.46	3.31
-10	4	14	63.14	4.20
9	-4	-14	2.04	0.46
9	-4	-14	1.03	0.99
9	-4	-14	1.58	0.83
-9	4	14	2.54	0.73
-9	4	14	-0.53	1.25
-9	4	14	2.85	0.73
8	-4	-14	47.60	2.05
8	-4	-14	48.71	2.49
-8	4	14	46.52	2.26
-8	4	14	45.98	2.27
-8	4	14	43.95	3.19
-8	4	14	38.76	2.35
7	-4	-14	31.45	1.54
7	-4	-14	32.67	2.12
-7	4	14	27.70	1.89
-7	4	14	30.16	2.97
-7	4	14	31.52	1.70
-7	4	14	31.96	1.71
6	-4	-14	64.35	2.69
6	-4	-14	62.88	3.21
-6	4	14	62.12	2.80
-6	4	14	68.58	2.77
-6	4	14	67.43	2.77
-6	4	14	56.57	3.51
-6	4	14	59.68	3.04
5	-4	-14	7.25	0.75
5	-4	-14	6.49	1.12
-5	4	14	9.29	1.63
-5	4	14	7.69	0.83
-5	4	14	6.86	1.28
-5	4	14	7.12	0.85
-5	4	14	8.64	0.83
4	-4	-14	65.67	3.22
4	-4	-14	59.67	2.57
-4	4	14	59.16	2.56
-4	4	14	57.85	3.33
-4	4	14	55.24	2.62
-4	4	14	64.75	2.53
-4	4	14	57.10	2.98
3	-4	-14	5.32	0.69
3	-4	-14	5.36	1.17

-3	4	14	5.26	0.61
-3	4	14	4.22	1.08
-3	4	14	5.46	0.62
-3	4	14	5.54	0.79
-3	4	14	5.84	1.00
2	-4	-14	59.71	3.15
2	-4	-14	54.55	2.43
-2	4	14	51.74	2.49
-2	4	14	57.17	2.25
-2	4	14	61.38	2.25
-2	4	14	54.72	3.12
-2	4	14	52.61	2.92
1	-4	-14	10.62	0.85
1	-4	-14	11.74	1.85
1	-4	-14	9.85	1.74
-1	4	14	8.41	1.45
-1	4	14	9.48	1.47
-1	4	14	9.58	0.93
0	-4	-14	5.16	0.72
0	-4	-14	3.85	1.15
0	-4	-14	5.42	1.55
0	4	14	5.35	1.04
0	4	14	5.45	0.78
0	4	14	6.76	1.12
-1	-4	-14	7.30	1.64
-1	-4	-14	4.36	0.68
1	4	14	5.30	1.04
1	4	14	4.36	0.63
1	4	14	5.97	1.02
-2	-4	-14	39.45	1.89
-2	-4	-14	39.47	3.03
2	4	14	38.59	2.51
2	4	14	37.69	2.30
-3	-4	-14	15.33	1.08
-3	-4	-14	14.33	2.45
3	4	14	13.93	1.48
3	4	14	15.25	1.70
-4	-4	-14	63.34	3.98
-4	-4	-14	68.38	2.94
4	4	14	66.22	3.24
4	4	14	68.45	3.59
-5	-4	-14	18.81	1.26
-5	-4	-14	16.48	2.61
5	4	14	19.83	1.60

5	4	14	17.82	1.99
-6	-4	-14	32.49	2.95
-6	-4	-14	34.56	1.97
6	4	14	33.58	2.04
6	4	14	33.53	2.48
-7	-4	-14	9.99	1.05
-7	-4	-14	10.14	1.84
7	4	14	10.30	1.33
7	4	14	8.04	1.44
-8	-4	-14	25.31	2.98
-8	-4	-14	28.58	1.71
8	4	14	28.02	1.85
8	4	14	28.08	2.45
-9	-4	-14	8.43	2.70
-9	-4	-14	9.57	1.18
9	4	14	9.98	1.38
9	4	14	9.01	1.56
-10	-4	-14	8.96	1.23
10	4	14	6.03	0.98
10	4	14	8.85	1.64
-11	-4	-14	12.72	1.38
11	4	14	11.06	1.55
11	4	14	9.98	1.70
-12	-4	-14	1.45	0.77
12	4	14	1.29	1.45
-13	-4	-14	5.59	1.00
13	4	14	5.88	1.75
-14	-4	-14	9.16	1.32
14	4	14	10.66	2.07
-15	-4	-14	7.53	1.60
15	4	14	10.96	2.07
18	-4	-13	1.01	0.73
-18	4	13	1.02	2.13
-18	4	13	2.08	1.45
17	-4	-13	31.08	1.85
-17	4	13	29.87	3.81
-17	4	13	35.55	2.79
16	-4	-13	2.56	0.64
-16	4	13	0.63	1.86
-16	4	13	1.66	1.16
15	-4	-13	30.27	1.67
-15	4	13	26.21	3.27
-15	4	13	29.29	2.41
14	-4	-13	1.52	0.51

-14	4	13	0.55	0.97
-14	4	13	0.52	1.61
13	-4	-13	44.82	2.10
-13	4	13	46.57	2.63
-13	4	13	42.42	3.58
12	-4	-13	0.60	0.41
-12	4	13	0.67	0.88
-12	4	13	0.55	1.46
11	-4	-13	73.28	3.57
11	-4	-13	85.51	3.46
-11	4	13	93.83	3.86
-11	4	13	84.50	4.71
10	-4	-13	1.04	0.74
10	-4	-13	0.83	0.39
-10	4	13	0.18	0.69
-10	4	13	-0.50	1.17
-10	4	13	-0.56	0.60
9	-4	-13	125.87	5.09
9	-4	-13	127.86	5.44
9	-4	-13	131.44	5.27
-9	4	13	117.82	6.52
-9	4	13	135.55	5.31
-9	4	13	133.46	5.32
8	-4	-13	1.54	0.40
8	-4	-13	2.43	0.80
-8	4	13	1.43	0.66
-8	4	13	1.46	1.08
-8	4	13	2.26	0.64
-8	4	13	1.74	0.61
7	-4	-13	145.19	5.81
7	-4	-13	137.97	5.50
-7	4	13	147.68	5.64
-7	4	13	133.43	6.35
-7	4	13	134.21	5.56
-7	4	13	128.00	5.71
-7	4	13	157.70	5.63
6	-4	-13	3.58	0.89
6	-4	-13	2.07	0.42
-6	4	13	2.97	1.04
-6	4	13	1.66	0.68
-6	4	13	3.10	0.55
-6	4	13	2.41	0.53
-6	4	13	1.30	0.47
5	-4	-13	68.79	3.28

5	-4	-13	65.23	2.82
-5	4	13	71.05	3.64
-5	4	13	64.17	3.15
-5	4	13	70.57	2.82
-5	4	13	70.33	2.85
-5	4	13	64.64	2.87
4	-4	-13	2.54	1.98
4	-4	-13	0.64	0.33
4	-4	-13	1.64	0.83
-4	4	13	1.63	0.69
-4	4	13	0.82	0.40
-4	4	13	0.71	0.37
-4	4	13	0.58	0.90
3	-4	-13	102.11	5.40
3	-4	-13	118.77	5.05
3	-4	-13	114.55	4.54
-3	4	13	114.66	4.58
-3	4	13	113.76	4.42
-3	4	13	123.16	4.45
-3	4	13	114.22	5.20
-3	4	13	110.77	4.91
2	-4	-13	-0.05	0.32
2	-4	-13	-0.08	0.83
2	-4	-13	1.49	1.22
-2	4	13	0.23	0.67
-2	4	13	-0.01	0.24
-2	4	13	-0.84	0.68
-2	4	13	-0.24	0.26
-2	4	13	-0.09	0.36
1	-4	-13	105.60	4.31
1	-4	-13	115.53	4.92
1	-4	-13	104.59	5.20
-1	4	13	111.87	4.88
-1	4	13	100.27	4.74
-1	4	13	109.16	4.36
0	-4	-13	19.91	2.27
0	-4	-13	20.09	1.16
0	-4	-13	20.70	1.89
0	4	13	19.89	1.18
0	4	13	20.09	1.71
0	4	13	20.45	1.67
-1	-4	-13	100.39	4.15
-1	-4	-13	104.99	4.82
-1	-4	-13	106.56	5.01

1	4	13	95.75	4.60
1	4	13	109.52	4.62
1	4	13	100.77	4.19
-2	-4	-13	0.07	0.36
-2	-4	-13	-0.05	1.15
-2	-4	-13	-0.07	0.34
-2	-4	-13	-1.09	0.92
2	4	13	0.33	0.61
2	4	13	0.83	0.81
2	4	13	0.22	0.41
-3	-4	-13	72.88	3.00
-3	-4	-13	71.30	3.87
-3	-4	-13	68.86	2.94
3	4	13	68.55	3.31
3	4	13	67.36	3.55
-4	-4	-13	11.47	1.67
-4	-4	-13	9.52	0.91
4	4	13	10.65	1.26
4	4	13	8.27	1.43
-5	-4	-13	69.85	3.00
-5	-4	-13	63.78	3.84
5	4	13	69.19	3.57
5	4	13	70.39	3.24
-6	-4	-13	15.62	1.91
-6	-4	-13	19.56	1.25
6	4	13	17.05	2.04
6	4	13	17.70	1.44
-7	-4	-13	39.42	2.98
7	4	13	38.44	2.61
7	4	13	45.78	2.25
-8	-4	-13	3.72	1.39
-8	-4	-13	1.88	0.60
8	4	13	1.69	0.71
8	4	13	1.41	1.01
-9	-4	-13	44.68	3.23
-9	-4	-13	47.32	1.98
-9	-4	-13	48.79	2.40
9	4	13	50.98	2.43
9	4	13	47.95	3.05
-10	-4	-13	0.56	0.49
-10	-4	-13	-0.42	0.60
10	4	13	0.14	0.69
10	4	13	0.37	1.10
-11	-4	-13	36.18	1.89

-11	-4	-13	39.64	2.17
11	4	13	38.34	2.05
11	4	13	40.60	2.92
-12	-4	-13	6.43	1.17
-12	-4	-13	3.87	0.88
12	4	13	2.35	1.33
-13	-4	-13	29.23	1.97
13	4	13	24.06	2.82
-14	-4	-13	17.98	1.76
14	4	13	15.92	2.70
-15	-4	-13	20.64	1.98
15	4	13	17.94	2.84
18	-4	-12	19.46	1.52
-18	4	12	18.17	2.76
-18	4	12	19.91	2.46
17	-4	-12	8.68	1.10
-17	4	12	7.67	1.49
-17	4	12	7.87	2.18
16	-4	-12	24.46	1.53
-16	4	12	25.98	2.29
-16	4	12	23.71	3.29
15	-4	-12	0.55	0.50
-15	4	12	-0.10	1.01
-15	4	12	-0.50	1.56
14	-4	-12	19.96	1.28
-14	4	12	18.66	1.88
-14	4	12	21.13	2.82
13	-4	-12	1.83	0.47
-13	4	12	1.89	0.85
-13	4	12	2.33	1.49
-12	4	12	67.81	3.16
-12	4	12	60.52	3.94
11	-4	-12	6.08	0.93
11	-4	-12	4.84	0.67
11	-4	-12	4.39	0.59
-11	4	12	5.94	0.92
-11	4	12	5.75	1.48
10	-4	-12	97.09	3.97
10	-4	-12	94.67	3.92
10	-4	-12	89.94	4.15
10	-4	-12	97.97	4.13
-10	4	12	93.64	4.96
-10	4	12	103.34	4.20
-10	4	12	103.62	4.23

9	-4	-12	1.66	0.73
9	-4	-12	1.19	0.71
9	-4	-12	0.72	0.34
-9	4	12	0.00	0.58
-9	4	12	1.11	0.61
-9	4	12	-0.89	0.52
-9	4	12	1.00	1.02
8	-4	-12	56.70	2.81
8	-4	-12	54.55	2.37
8	-4	-12	56.47	2.62
-8	4	12	52.27	3.23
-8	4	12	50.28	2.44
-8	4	12	60.30	2.57
-8	4	12	57.48	2.58
7	-4	-12	0.61	0.63
7	-4	-12	0.41	0.31
-7	4	12	0.19	0.45
-7	4	12	0.65	0.59
-7	4	12	0.19	0.88
-7	4	12	-0.10	0.38
-7	4	12	-0.58	0.42
6	-4	-12	84.06	3.53
6	-4	-12	74.61	4.38
6	-4	-12	74.85	3.17
-6	4	12	76.42	3.95
-6	4	12	72.28	3.43
-6	4	12	80.03	3.27
-6	4	12	83.01	3.24
-6	4	12	78.01	3.24
5	-4	-12	13.80	2.03
5	-4	-12	16.17	1.42
5	-4	-12	13.98	0.86
-5	4	12	12.09	1.25
-5	4	12	12.71	0.93
-5	4	12	13.40	0.94
-5	4	12	14.17	0.90
-5	4	12	12.45	1.54
4	-4	-12	83.41	4.24
4	-4	-12	80.52	3.30
4	-4	-12	87.56	3.74
-4	4	12	77.37	3.33
-4	4	12	78.95	3.94
-4	4	12	76.88	3.60
-4	4	12	85.37	3.28

3	-4	-12	-0.53	0.68
3	-4	-12	0.52	0.31
3	-4	-12	-0.50	0.97
-3	4	12	0.33	0.72
-3	4	12	0.61	0.33
-3	4	12	1.28	0.37
-3	4	12	0.07	0.27
-3	4	12	0.22	0.57
2	-4	-12	86.41	3.81
2	-4	-12	76.94	3.30
2	-4	-12	79.58	4.14
-2	4	12	81.51	3.15
-2	4	12	85.88	3.16
-2	4	12	79.92	3.86
-2	4	12	81.07	3.35
-2	4	12	78.20	3.67
1	-4	-12	3.65	0.90
1	-4	-12	3.48	1.19
1	-4	-12	2.40	0.40
-1	4	12	3.32	0.83
-1	4	12	3.15	0.78
-1	4	12	1.96	0.42
0	-4	-12	95.16	4.25
0	-4	-12	87.10	3.69
0	-4	-12	86.00	4.40
0	4	12	96.95	4.18
0	4	12	92.15	4.13
0	4	12	91.07	3.73
-1	-4	-12	7.78	1.36
-1	-4	-12	8.51	1.41
-1	-4	-12	9.29	0.75
1	4	12	8.78	1.19
1	4	12	9.81	1.23
1	4	12	7.35	0.75
-2	-4	-12	92.00	3.77
-2	-4	-12	93.22	3.75
-2	-4	-12	88.52	4.43
-2	-4	-12	93.42	4.37
2	4	12	89.12	4.22
2	4	12	96.26	3.80
2	4	12	94.10	4.12
-3	-4	-12	1.40	0.39
-3	-4	-12	1.39	0.90
-3	-4	-12	1.97	1.06

-3	-4	-12	0.50	0.35
3	4	12	1.23	0.61
3	4	12	0.75	0.69
-4	-4	-12	62.63	2.60
-4	-4	-12	59.43	2.65
-4	-4	-12	64.05	3.43
-4	-4	-12	63.04	3.35
4	4	12	59.73	2.88
4	4	12	56.93	3.14
-5	-4	-12	10.16	1.38
-5	-4	-12	6.74	0.90
-5	-4	-12	6.68	0.78
5	4	12	5.55	0.84
5	4	12	7.51	1.35
-6	-4	-12	56.93	2.48
-6	-4	-12	54.06	3.12
6	4	12	54.87	2.62
6	4	12	49.85	2.94
-7	-4	-12	0.29	0.44
-7	-4	-12	0.99	1.12
7	4	12	-0.19	0.57
7	4	12	-0.15	0.80
-8	-4	-12	66.73	3.72
-8	-4	-12	69.81	2.80
-8	-4	-12	73.59	2.85
8	4	12	75.25	3.21
-9	-4	-12	2.91	1.25
-9	-4	-12	1.47	0.55
-9	-4	-12	2.44	0.52
-9	-4	-12	1.93	0.41
9	4	12	1.67	1.02
9	4	12	0.55	0.61
-10	-4	-12	62.97	2.61
-10	-4	-12	58.92	3.41
-10	-4	-12	61.75	2.69
-10	-4	-12	64.80	2.95
10	4	12	60.21	3.51
10	4	12	65.89	2.84
-11	-4	-12	2.68	0.64
-11	-4	-12	0.54	0.60
11	4	12	2.22	0.72
11	4	12	1.13	1.13
-12	-4	-12	50.77	2.49
-12	-4	-12	49.12	2.65

12	4	12	52.25	3.34
12	4	12	54.88	2.41
-13	-4	-12	0.16	0.74
-13	-4	-12	1.10	0.85
13	4	12	2.73	1.32
-14	-4	-12	71.77	3.79
-14	-4	-12	73.30	3.52
14	4	12	72.67	4.24
-15	-4	-12	0.19	0.84
15	4	12	-0.34	1.44
-16	-4	-12	40.79	2.63
16	4	12	42.39	3.45
19	-4	-11	9.54	1.32
-19	4	11	10.14	1.64
-19	4	11	8.14	2.27
18	-4	-11	3.87	0.74
-18	4	11	2.99	1.38
-18	4	11	5.33	1.97
17	-4	-11	13.04	1.26
-17	4	11	14.21	2.09
-17	4	11	11.60	2.28
16	-4	-11	7.19	0.99
-16	4	11	6.60	1.90
-16	4	11	6.12	1.27
15	-4	-11	9.89	0.97
-15	4	11	19.11	2.70
-15	4	11	10.64	1.80
14	-4	-11	0.53	0.45
-14	4	11	-1.04	1.32
-14	4	11	-1.95	0.80
13	-4	-11	22.78	1.29
-13	4	11	22.97	1.81
-13	4	11	24.19	2.64
12	-4	-11	2.80	0.47
-12	4	11	0.40	1.85
-12	4	11	2.44	0.76
11	-4	-11	8.59	0.77
11	-4	-11	10.09	0.79
-11	4	11	12.79	1.26
-11	4	11	15.86	2.04
10	-4	-11	18.46	1.68
10	-4	-11	23.74	1.27
10	-4	-11	21.25	1.50
10	-4	-11	22.80	1.21

-10	4	11	21.70	2.23
-10	4	11	23.22	1.54
9	-4	-11	19.41	1.40
9	-4	-11	17.80	1.01
9	-4	-11	17.10	1.28
9	-4	-11	19.79	1.22
-9	4	11	17.56	1.30
-9	4	11	16.40	1.97
-9	4	11	14.72	1.11
8	-4	-11	0.90	0.47
8	-4	-11	0.10	0.58
8	-4	-11	0.74	0.64
8	-4	-11	0.35	1.26
8	-4	-11	0.22	0.29
-8	4	11	0.47	0.49
-8	4	11	-0.05	0.88
-8	4	11	-0.48	0.38
7	-4	-11	63.26	3.74
7	-4	-11	66.92	2.74
7	-4	-11	68.82	2.96
7	-4	-11	67.26	2.87
-7	4	11	69.82	2.89
-7	4	11	66.70	2.80
-7	4	11	62.78	2.91
-7	4	11	63.93	3.49
6	-4	-11	0.33	0.59
6	-4	-11	0.02	1.04
6	-4	-11	0.25	0.26
-6	4	11	0.38	0.37
-6	4	11	-0.31	0.50
-6	4	11	0.05	0.39
-6	4	11	0.10	0.32
-6	4	11	-0.57	0.71
5	-4	-11	14.82	1.87
5	-4	-11	19.50	1.37
5	-4	-11	16.03	0.95
-5	4	11	16.20	1.27
-5	4	11	17.68	1.03
-5	4	11	19.87	0.99
-5	4	11	19.29	1.66
-5	4	11	15.11	0.99
4	-4	-11	56.43	3.09
4	-4	-11	49.56	2.22
4	-4	-11	56.73	2.63

-4	4	11	53.50	2.22
-4	4	11	53.42	2.85
-4	4	11	53.73	2.21
-4	4	11	52.22	2.27
-4	4	11	48.60	2.52
3	-4	-11	21.28	1.99
3	-4	-11	24.04	1.62
3	-4	-11	19.95	1.12
-3	4	11	22.39	1.48
-3	4	11	23.08	1.04
-3	4	11	21.95	1.73
-3	4	11	21.85	1.15
-3	4	11	23.69	1.10
2	-4	-11	0.23	0.63
2	-4	-11	0.67	0.31
2	-4	-11	-1.21	0.83
-2	4	11	0.89	0.23
-2	4	11	-0.29	0.28
-2	4	11	1.28	0.61
-2	4	11	0.26	0.23
-2	4	11	0.26	0.57
1	-4	-11	81.95	3.61
1	-4	-11	79.10	3.83
1	-4	-11	73.88	3.14
-1	4	11	79.21	3.18
-1	4	11	73.31	3.48
-1	4	11	77.63	3.60
0	-4	-11	-0.86	0.75
0	-4	-11	-0.38	0.68
0	-4	-11	0.50	0.28
0	4	11	0.29	0.59
0	4	11	0.43	0.62
0	4	11	-0.13	0.30
-1	-4	-11	29.37	1.41
-1	-4	-11	30.14	2.11
-1	-4	-11	29.69	2.00
1	4	11	30.22	1.45
1	4	11	29.47	1.79
1	4	11	27.38	1.82
-2	-4	-11	4.17	0.94
-2	-4	-11	2.77	0.83
-2	-4	-11	2.14	0.40
2	4	11	3.67	0.57
2	4	11	2.93	0.67

2	4	11	2.61	0.75
-3	-4	-11	29.37	1.47
-3	-4	-11	29.61	2.13
-3	-4	-11	30.89	1.45
-3	-4	-11	30.93	2.14
3	4	11	28.72	1.93
3	4	11	30.42	1.76
-4	-4	-11	6.35	0.69
-4	-4	-11	8.10	1.21
-4	-4	-11	8.83	1.28
-4	-4	-11	5.50	0.65
4	4	11	5.01	0.91
4	4	11	7.47	0.99
-5	-4	-11	80.84	3.41
-5	-4	-11	84.82	4.06
-5	-4	-11	81.59	3.88
-5	-4	-11	81.88	3.35
5	4	11	77.26	3.84
5	4	11	84.02	3.58
-6	-4	-11	4.93	0.69
-6	-4	-11	2.65	0.44
-6	-4	-11	4.15	1.02
6	4	11	4.39	0.99
6	4	11	3.99	0.68
-7	-4	-11	41.34	1.66
-7	-4	-11	41.15	1.97
-7	-4	-11	34.72	2.40
7	4	11	40.63	2.02
7	4	11	39.74	2.44
-8	-4	-11	1.13	0.47
-8	-4	-11	0.90	0.30
-8	-4	-11	-0.30	0.96
-8	-4	-11	0.84	0.53
8	4	11	-0.27	0.55
8	4	11	0.37	0.84
-9	-4	-11	70.67	3.52
-9	-4	-11	73.04	2.97
9	4	11	68.59	3.66
9	4	11	75.22	3.15
-10	-4	-11	3.95	1.16
-10	-4	-11	4.50	0.69
-10	-4	-11	4.09	0.62
-10	-4	-11	4.63	0.74
10	4	11	4.67	1.25

10	4	11	4.96	0.74
-11	-4	-11	97.83	4.26
-11	-4	-11	98.12	4.01
-11	-4	-11	97.54	4.09
11	4	11	98.44	4.79
11	4	11	103.37	4.09
-12	-4	-11	1.00	0.66
-12	-4	-11	0.41	0.61
-12	-4	-11	0.50	0.84
-12	-4	-11	0.55	0.62
12	4	11	-0.50	1.03
-13	-4	-11	56.12	2.80
-13	-4	-11	54.31	2.74
13	4	11	55.57	3.44
-14	-4	-11	-0.29	0.89
-14	-4	-11	-0.13	0.74
14	4	11	2.16	1.38
-15	-4	-11	42.34	2.51
-15	-4	-11	40.75	2.56
15	4	11	39.99	3.18
-16	-4	-11	0.09	0.90
16	4	11	-0.51	1.42
-17	-4	-11	44.19	2.92
17	4	11	39.19	3.43
19	-4	-10	10.88	1.27
-19	4	10	4.16	2.06
-19	4	10	16.07	2.39
18	-4	-10	-0.02	0.59
-18	4	10	-0.61	1.18
-18	4	10	-2.27	1.69
17	-4	-10	7.95	1.03
-17	4	10	4.83	1.88
-17	4	10	9.79	1.45
16	-4	-10	1.10	0.57
-16	4	10	2.25	1.07
-16	4	10	3.09	1.67
15	-4	-10	0.99	0.50
-15	4	10	0.78	0.88
-15	4	10	-0.06	1.39
14	-4	-10	6.97	0.85
-14	4	10	7.61	1.93
-14	4	10	9.78	1.50
-13	4	10	4.41	1.38
12	-4	-10	1.00	0.37

-12	4	10	2.84	1.18
-12	4	10	0.77	0.69
11	-4	-10	8.11	1.18
11	-4	-10	8.99	0.73
-11	4	10	9.83	1.20
-11	4	10	7.91	1.41
10	-4	-10	3.45	0.54
10	-4	-10	3.76	0.65
10	-4	-10	3.86	0.68
10	-4	-10	3.36	1.75
10	-4	-10	3.03	0.60
-10	4	10	3.90	0.54
-10	4	10	5.95	0.98
-10	4	10	5.33	1.18
9	-4	-10	6.94	0.65
9	-4	-10	9.38	1.47
9	-4	-10	8.58	0.85
9	-4	-10	11.64	2.07
9	-4	-10	8.74	0.95
-9	4	10	8.73	0.79
-9	4	10	8.80	0.99
-9	4	10	8.33	1.57
8	-4	-10	8.11	1.38
8	-4	-10	6.17	0.87
8	-4	-10	7.99	0.88
8	-4	-10	7.26	0.62
8	-4	-10	6.96	1.06
-8	4	10	6.52	1.17
-8	4	10	7.89	0.72
-8	4	10	9.39	0.94
7	-4	-10	8.19	0.64
7	-4	-10	9.45	1.10
7	-4	-10	8.19	0.83
7	-4	-10	10.31	1.71
-7	4	10	8.13	1.40
-7	4	10	9.62	0.84
-7	4	10	7.54	0.71
6	-4	-10	17.67	1.76
6	-4	-10	17.56	0.92
6	-4	-10	16.58	1.20
6	-4	-10	16.30	1.03
-6	4	10	16.90	1.62
-6	4	10	16.42	1.04
-6	4	10	16.69	1.17

-6	4	10	16.38	0.96
5	-4	-10	10.72	0.68
5	-4	-10	10.45	0.73
5	-4	-10	8.01	1.01
5	-4	-10	8.94	1.54
-5	4	10	8.59	1.26
-5	4	10	8.17	0.92
-5	4	10	8.62	0.69
-5	4	10	9.98	0.75
4	-4	-10	50.62	2.35
4	-4	-10	54.04	2.84
4	-4	-10	46.21	2.02
-4	4	10	48.38	2.01
-4	4	10	43.34	2.26
-4	4	10	48.73	2.00
-4	4	10	46.84	2.05
-4	4	10	44.91	2.53
3	-4	-10	5.62	0.54
3	-4	-10	6.03	0.94
3	-4	-10	5.68	1.02
-3	4	10	5.92	0.60
-3	4	10	6.44	1.04
-3	4	10	5.98	0.48
-3	4	10	5.55	0.54
-3	4	10	6.25	0.87
2	-4	-10	24.20	1.89
2	-4	-10	23.61	1.21
2	-4	-10	27.49	1.64
-2	4	10	25.74	1.11
-2	4	10	25.88	1.09
-2	4	10	25.05	1.53
-2	4	10	24.53	1.68
-2	4	10	26.49	1.26
1	-4	-10	2.88	0.39
1	-4	-10	3.39	0.72
1	-4	-10	3.20	0.85
-1	4	10	2.15	0.58
-1	4	10	2.96	0.70
-1	4	10	2.86	0.28
-1	4	10	4.02	0.51
0	-4	-10	36.21	1.65
0	-4	-10	38.28	2.13
0	-4	-10	36.68	2.23
0	4	10	38.73	1.69

0	4	10	36.14	2.02
0	4	10	35.73	2.01
-1	-4	-10	6.87	0.60
-1	-4	-10	8.73	1.22
-1	-4	-10	8.65	1.11
1	4	10	5.04	0.84
1	4	10	5.98	0.90
1	4	10	7.45	0.64
-2	-4	-10	42.09	2.27
-2	-4	-10	40.60	2.28
-2	-4	-10	38.38	1.75
2	4	10	35.81	2.00
2	4	10	39.43	1.77
2	4	10	37.69	2.14
-3	-4	-10	14.00	0.83
-3	-4	-10	16.08	1.51
-3	-4	-10	12.93	0.86
-3	-4	-10	14.42	1.43
3	4	10	10.84	1.22
3	4	10	13.71	1.11
-4	-4	-10	16.81	1.03
-4	-4	-10	18.32	1.57
-4	-4	-10	20.44	1.64
-4	-4	-10	18.13	1.00
4	4	10	16.29	1.44
4	4	10	17.08	1.24
-5	-4	-10	1.08	0.36
-5	-4	-10	2.39	0.84
-5	-4	-10	1.23	0.31
-5	-4	-10	2.05	0.78
5	4	10	0.37	0.62
5	4	10	1.89	0.53
-6	-4	-10	44.29	2.47
-6	-4	-10	45.95	1.81
-6	-4	-10	42.84	2.07
-6	-4	-10	45.67	2.01
-6	-4	-10	49.92	2.71
6	4	10	46.46	2.19
6	4	10	43.97	2.50
-7	-4	-10	1.18	0.40
-7	-4	-10	0.20	0.25
-7	-4	-10	0.42	0.30
-7	-4	-10	0.40	0.33
-7	-4	-10	1.29	0.78

-7	-4	-10	0.67	0.90
7	4	10	0.67	0.71
7	4	10	0.37	0.48
-8	-4	-10	32.46	1.43
-8	-4	-10	30.83	2.02
-8	-4	-10	32.95	1.52
-8	-4	-10	33.46	1.69
8	4	10	32.35	1.70
8	4	10	32.89	2.18
-9	-4	-10	1.43	0.86
-9	-4	-10	0.61	0.36
-9	-4	-10	-0.07	0.42
-9	-4	-10	0.25	0.42
9	4	10	0.70	0.50
9	4	10	0.07	0.51
9	4	10	1.36	0.82
-10	-4	-10	21.35	1.29
-10	-4	-10	14.68	2.04
10	4	10	22.48	1.33
10	4	10	21.26	1.23
10	4	10	20.44	1.91
-11	-4	-10	6.93	1.06
-11	-4	-10	4.83	0.74
-11	-4	-10	5.98	1.07
11	4	10	6.69	0.95
11	4	10	8.85	0.97
-12	-4	-10	46.09	2.47
-12	-4	-10	50.37	2.43
-12	-4	-10	48.18	2.48
-12	-4	-10	47.05	2.58
12	4	10	51.83	2.33
12	4	10	48.87	2.94
-13	-4	-10	1.05	0.70
-13	-4	-10	-0.02	0.63
-13	-4	-10	1.33	0.83
13	4	10	2.28	1.14
13	4	10	1.48	0.70
-14	-4	-10	31.15	2.10
-14	-4	-10	36.15	2.22
14	4	10	35.81	2.76
-15	-4	-10	1.95	1.06
-15	-4	-10	-0.21	0.70
15	4	10	-1.20	1.22
-16	-4	-10	16.14	1.73

-16	-4	-10	15.83	2.11
16	4	10	15.52	1.53
16	4	10	13.21	2.52
-17	-4	-10	2.23	1.08
-17	-4	-10	2.28	1.33
17	4	10	2.85	0.91
17	4	10	2.19	1.56
19	-4	-9	15.31	1.32
-19	4	9	11.92	2.26
-19	4	9	17.71	2.43
18	-4	-9	11.00	1.17
-18	4	9	8.42	1.49
-18	4	9	7.41	1.94
17	-4	-9	20.59	1.38
-17	4	9	22.54	2.25
-17	4	9	21.81	2.88
16	-4	-9	15.36	1.18
-16	4	9	16.44	1.89
-16	4	9	13.65	2.50
15	-4	-9	7.16	0.85
-15	4	9	7.11	1.59
-15	4	9	6.78	1.10
14	-4	-9	12.00	0.94
-14	4	9	13.76	1.52
-14	4	9	11.28	1.74
13	-4	-9	9.96	0.82
-13	4	9	10.77	1.41
-13	4	9	9.76	1.55
12	-4	-9	5.00	0.62
-12	4	9	3.27	1.16
-12	4	9	2.95	0.73
11	-4	-9	2.87	0.44
11	-4	-9	0.52	1.25
11	-4	-9	2.17	0.60
-11	4	9	2.44	0.91
-11	4	9	4.18	0.71
-11	4	9	3.03	1.05
10	-4	-9	34.67	1.55
10	-4	-9	34.70	1.59
10	-4	-9	34.00	2.76
10	-4	-9	32.35	1.58
10	-4	-9	35.38	1.84
-10	4	9	35.41	1.71
-10	4	9	35.35	1.91

-10	4	9	36.50	2.46
9	-4	-9	2.25	0.55
9	-4	-9	2.24	0.44
9	-4	-9	2.54	1.10
9	-4	-9	1.71	0.34
9	-4	-9	2.54	0.55
-9	4	9	2.64	0.92
-9	4	9	2.75	0.45
-9	4	9	2.69	0.54
8	-4	-9	3.41	0.48
8	-4	-9	3.60	0.68
8	-4	-9	3.49	0.57
8	-4	-9	4.88	0.73
8	-4	-9	4.54	1.14
-8	4	9	3.53	0.94
-8	4	9	4.22	0.56
-8	4	9	4.73	0.77
7	-4	-9	6.22	0.72
7	-4	-9	6.50	1.17
7	-4	-9	6.69	1.01
7	-4	-9	6.47	0.55
7	-4	-9	5.72	0.76
-7	4	9	6.67	0.77
-7	4	9	6.96	0.63
-7	4	9	6.08	1.04
6	-4	-9	21.90	1.84
6	-4	-9	21.44	1.40
6	-4	-9	19.27	1.01
6	-4	-9	19.84	1.12
-6	4	9	19.67	1.61
-6	4	9	20.19	1.20
-6	4	9	22.23	1.16
-6	4	9	20.04	1.07
5	-4	-9	2.51	0.39
5	-4	-9	1.55	0.51
5	-4	-9	1.91	0.81
5	-4	-9	1.40	0.29
-5	4	9	2.14	0.35
-5	4	9	2.05	0.39
-5	4	9	1.67	0.47
-5	4	9	2.14	0.70
4	-4	-9	48.48	2.07
4	-4	-9	49.15	2.08
4	-4	-9	49.41	2.70

4	-4	-9	52.86	2.36
-4	4	9	49.67	2.11
-4	4	9	49.19	2.58
-4	4	9	51.31	2.09
-4	4	9	48.87	2.31
3	-4	-9	0.25	0.23
3	-4	-9	0.21	0.47
3	-4	-9	0.08	0.22
3	-4	-9	0.23	0.64
-3	4	9	0.10	0.26
-3	4	9	0.14	0.47
-3	4	9	-0.02	0.39
-3	4	9	0.37	0.25
2	-4	-9	18.91	0.97
2	-4	-9	20.52	1.58
2	-4	-9	19.82	1.33
-2	4	9	19.49	1.01
-2	4	9	18.68	0.86
-2	4	9	19.91	1.27
-2	4	9	19.22	1.39
-2	4	9	20.96	0.90
1	-4	-9	-0.09	0.19
1	-4	-9	0.28	0.59
1	-4	-9	0.43	0.49
-1	4	9	-0.23	0.40
-1	4	9	0.21	0.45
-1	4	9	0.28	0.25
-1	4	9	-0.02	0.13
-1	4	9	-0.03	0.14
0	-4	-9	9.44	0.63
0	-4	-9	8.85	1.17
0	-4	-9	9.78	1.08
0	4	9	9.64	0.66
0	4	9	8.89	0.95
0	4	9	8.88	0.97
-1	-4	-9	3.80	0.47
-1	-4	-9	4.94	0.82
-1	-4	-9	3.60	0.74
1	4	9	4.24	0.72
1	4	9	4.51	0.78
1	4	9	3.63	0.48
-2	-4	-9	31.15	1.48
-2	-4	-9	33.17	1.92
-2	-4	-9	33.38	1.93

2	4	9	32.01	1.73
2	4	9	36.87	1.53
2	4	9	27.74	1.77
-3	-4	-9	9.66	0.69
-3	-4	-9	9.28	1.16
-3	-4	-9	10.54	0.69
-3	-4	-9	9.85	1.15
3	4	9	9.53	0.91
3	4	9	11.44	0.74
3	4	9	6.56	1.02
-4	-4	-9	16.60	1.40
-4	-4	-9	18.92	1.54
-4	-4	-9	18.03	1.01
-4	-4	-9	17.07	0.95
4	4	9	19.49	1.21
4	4	9	16.91	1.38
-5	-4	-9	5.86	0.88
-5	-4	-9	4.07	0.85
-5	-4	-9	3.40	0.49
-5	-4	-9	3.36	0.54
5	4	9	3.88	0.62
5	4	9	4.30	0.78
-6	-4	-9	0.84	0.21
-6	-4	-9	0.36	0.23
-6	-4	-9	0.54	0.75
-6	-4	-9	0.01	0.64
-6	-4	-9	0.41	0.32
-6	-4	-9	0.07	0.31
6	4	9	-0.12	0.38
6	4	9	0.11	0.59
-7	-4	-9	4.50	0.46
-7	-4	-9	3.70	0.47
-7	-4	-9	5.32	0.86
-7	-4	-9	5.04	0.98
-7	-4	-9	4.09	0.55
7	4	9	4.76	0.92
7	4	9	4.78	0.77
-8	-4	-9	13.19	1.38
-8	-4	-9	16.21	0.93
-8	-4	-9	12.50	1.74
-8	-4	-9	13.74	0.91
-8	-4	-9	13.26	0.97
8	4	9	11.44	1.38
8	4	9	14.72	0.85

8	4	9	16.69	1.07
-9	-4	-9	6.59	0.81
-9	-4	-9	6.01	0.80
-9	-4	-9	8.83	1.54
-9	-4	-9	5.88	1.16
9	4	9	5.95	0.84
9	4	9	5.99	1.09
9	4	9	7.07	0.73
-10	-4	-9	22.84	1.36
-10	-4	-9	22.29	1.39
-10	-4	-9	22.55	1.66
10	4	9	25.50	1.30
10	4	9	22.97	1.32
10	4	9	18.38	1.80
-11	-4	-9	9.33	1.10
-11	-4	-9	9.96	1.03
-11	-4	-9	8.26	1.31
11	4	9	9.45	1.01
11	4	9	9.48	1.00
11	4	9	8.99	1.45
-12	-4	-9	2.51	0.80
-12	-4	-9	4.41	1.06
-12	-4	-9	1.33	0.74
-12	-4	-9	2.46	0.61
12	4	9	3.09	0.62
12	4	9	3.45	1.07
12	4	9	2.35	0.64
-13	-4	-9	9.79	1.18
-13	-4	-9	8.03	1.38
13	4	9	8.24	1.19
13	4	9	10.23	1.48
13	4	9	8.80	1.06
-14	-4	-9	8.15	1.13
-14	-4	-9	6.50	1.04
-14	-4	-9	5.50	0.97
14	4	9	6.72	0.94
14	4	9	7.92	1.10
14	4	9	6.96	1.36
-15	-4	-9	13.64	1.48
-15	-4	-9	11.23	1.86
15	4	9	11.55	1.34
15	4	9	11.37	2.11
-16	-4	-9	2.76	0.94
-16	-4	-9	3.52	1.27

16	4	9	3.85	0.90
16	4	9	5.25	1.46
-17	-4	-9	10.92	1.66
-17	-4	-9	10.98	1.58
17	4	9	11.82	1.87
17	4	9	12.18	1.45
-18	-4	-9	2.80	1.17
-18	-4	-9	2.84	1.50
18	4	9	2.15	0.96
18	4	9	0.72	1.59
20	-4	-8	32.46	1.98
-20	4	8	35.77	3.21
19	-4	-8	9.20	1.18
-19	4	8	8.54	2.00
-19	4	8	9.10	1.58
18	-4	-8	42.99	2.21
-18	4	8	39.98	3.50
-18	4	8	48.26	2.98
17	-4	-8	0.26	0.51
-17	4	8	-1.47	1.00
16	-4	-8	50.14	2.33
-16	4	8	55.12	2.99
-16	4	8	44.87	3.38
15	-4	-8	12.96	1.04
-15	4	8	14.72	1.68
-15	4	8	13.20	2.29
14	-4	-8	35.36	1.76
-14	4	8	38.41	2.83
-14	4	8	37.57	2.26
13	-4	-8	14.95	2.50
13	-4	-8	10.60	0.82
-13	4	8	10.60	1.33
-13	4	8	10.46	1.91
12	-4	-8	26.32	2.69
12	-4	-8	21.32	1.19
-12	4	8	21.07	2.14
-12	4	8	25.05	1.67
11	-4	-8	16.57	0.95
11	-4	-8	12.54	2.20
11	-4	-8	18.17	1.27
-11	4	8	18.55	1.11
-11	4	8	14.71	1.87
-11	4	8	16.69	1.34
10	-4	-8	79.28	4.30

10	-4	-8	82.46	3.35
10	-4	-8	85.03	3.33
10	-4	-8	85.73	3.41
10	-4	-8	82.05	3.61
-10	4	8	93.09	3.72
-10	4	8	80.34	4.10
-10	4	8	90.07	3.50
9	-4	-8	0.71	0.36
9	-4	-8	0.65	0.27
9	-4	-8	0.63	0.41
9	-4	-8	1.31	0.94
9	-4	-8	0.78	0.46
-9	4	8	0.16	0.31
-9	4	8	-0.06	0.42
-9	4	8	0.25	0.69
8	-4	-8	81.70	4.06
8	-4	-8	88.58	3.53
8	-4	-8	84.66	3.50
8	-4	-8	83.60	3.39
8	-4	-8	85.10	3.36
-8	4	8	83.52	3.40
-8	4	8	82.23	3.97
-8	4	8	83.45	3.53
7	-4	-8	8.91	0.79
7	-4	-8	8.57	0.60
7	-4	-8	8.19	0.99
7	-4	-8	9.09	0.78
7	-4	-8	9.91	1.47
-7	4	8	9.07	0.67
-7	4	8	6.22	1.20
-7	4	8	9.47	0.81
6	-4	-8	39.80	1.81
6	-4	-8	40.34	2.06
6	-4	-8	38.46	2.37
6	-4	-8	38.70	1.70
6	-4	-8	43.34	1.88
-6	4	8	38.11	2.22
-6	4	8	39.87	1.82
-6	4	8	40.33	1.75
5	-4	-8	49.73	2.14
5	-4	-8	50.30	2.19
5	-4	-8	54.19	2.33
5	-4	-8	54.46	2.75
-5	4	8	51.20	2.63

-5	4	8	51.87	2.18
-5	4	8	52.39	2.21
-5	4	8	53.27	2.33
4	-4	-8	41.70	2.24
4	-4	-8	40.82	1.71
4	-4	-8	37.99	1.67
-4	4	8	40.22	1.71
-4	4	8	37.23	1.86
-4	4	8	39.55	1.71
-4	4	8	39.78	2.13
3	-4	-8	127.51	5.08
3	-4	-8	126.27	4.81
3	-4	-8	126.59	4.85
3	-4	-8	131.83	5.34
-3	4	8	116.48	5.04
-3	4	8	128.26	4.87
-3	4	8	121.48	5.24
2	-4	-8	120.04	4.80
2	-4	-8	111.38	4.44
2	-4	-8	113.59	4.52
2	-4	-8	121.39	4.98
-2	4	8	113.70	4.76
-2	4	8	114.66	4.88
-2	4	8	122.02	4.45
-2	4	8	118.76	4.55
1	-4	-8	55.76	2.52
1	-4	-8	56.11	2.66
1	-4	-8	54.96	2.21
-1	4	8	52.83	2.07
-1	4	8	50.69	2.07
-1	4	8	54.91	2.23
-1	4	8	52.52	2.45
-1	4	8	54.33	2.55
0	-4	-8	23.41	1.43
0	-4	-8	21.05	1.04
0	-4	-8	21.28	1.44
0	4	8	22.07	1.06
0	4	8	21.44	1.33
0	4	8	20.14	1.31
-1	-4	-8	0.54	0.50
-1	-4	-8	0.14	0.22
-1	-4	-8	0.17	0.48
1	4	8	0.82	0.40
1	4	8	0.78	0.27

1	4	8	0.48	0.42
-2	-4	-8	51.91	2.49
-2	-4	-8	50.45	2.11
-2	-4	-8	48.70	2.42
2	4	8	51.65	2.12
2	4	8	47.97	2.41
2	4	8	49.67	2.31
-3	-4	-8	22.99	1.56
-3	-4	-8	22.96	1.14
-3	-4	-8	25.86	1.14
-3	-4	-8	21.90	1.45
3	4	8	24.09	1.17
3	4	8	23.31	1.33
3	4	8	21.47	1.45
-4	-4	-8	68.68	3.16
-4	-4	-8	65.64	2.69
-4	-4	-8	66.62	2.75
-4	-4	-8	68.95	3.02
4	4	8	65.92	2.86
4	4	8	61.97	3.07
-5	-4	-8	73.26	3.30
-5	-4	-8	69.58	3.08
-5	-4	-8	66.27	2.83
-5	-4	-8	68.81	2.78
-5	-4	-8	68.29	2.63
-5	-4	-8	65.72	2.61
5	4	8	64.07	3.16
5	4	8	70.32	2.94
-6	-4	-8	8.61	0.70
-6	-4	-8	9.68	1.27
-6	-4	-8	8.79	0.64
-6	-4	-8	8.46	0.51
-6	-4	-8	7.93	0.57
-6	-4	-8	7.29	1.01
6	4	8	8.96	0.82
6	4	8	8.31	1.07
-7	-4	-8	10.17	1.09
-7	-4	-8	11.81	1.39
-7	-4	-8	9.55	0.75
-7	-4	-8	9.76	0.80
-7	-4	-8	9.07	0.62
-7	-4	-8	11.01	0.73
7	4	8	10.67	0.87
7	4	8	8.51	1.22

-8	-4	-8	21.15	1.23
-8	-4	-8	23.45	1.82
-8	-4	-8	22.35	1.16
-8	-4	-8	21.66	1.15
8	4	8	21.23	1.24
8	4	8	19.64	1.61
8	4	8	22.97	1.11
-9	-4	-8	21.90	1.23
-9	-4	-8	21.16	1.28
-9	-4	-8	22.60	1.19
-9	-4	-8	21.39	1.85
-9	-4	-8	18.70	1.45
9	4	8	19.73	1.65
9	4	8	21.84	1.15
9	4	8	21.70	1.23
-10	-4	-8	12.49	1.25
-10	-4	-8	9.50	1.76
-10	-4	-8	11.84	1.07
-10	-4	-8	13.26	1.02
10	4	8	13.21	1.47
10	4	8	14.35	0.98
10	4	8	13.31	0.99
10	4	8	13.27	0.97
-11	-4	-8	50.28	2.51
-11	-4	-8	51.35	2.52
-11	-4	-8	51.48	2.47
-11	-4	-8	54.30	2.51
11	4	8	57.55	2.42
11	4	8	52.08	2.89
11	4	8	54.48	2.43
-12	-4	-8	-0.22	1.01
-12	-4	-8	0.39	0.70
-12	-4	-8	1.63	0.72
-12	-4	-8	1.22	0.55
12	4	8	1.06	0.57
12	4	8	-0.04	0.81
12	4	8	1.72	0.53
-13	-4	-8	11.45	1.44
13	4	8	12.71	1.74
13	4	8	15.89	1.32
13	4	8	16.47	1.22
-14	-4	-8	4.35	1.01
14	4	8	6.35	1.49
14	4	8	6.12	0.92

14	4	8	6.83	1.06
-15	-4	-8	5.69	1.10
15	4	8	5.20	0.88
15	4	8	7.49	1.37
-16	-4	-8	0.65	1.13
16	4	8	0.67	0.67
16	4	8	0.11	1.18
-17	-4	-8	9.93	1.55
17	4	8	8.79	1.41
17	4	8	9.04	1.68
-18	-4	-8	-1.39	1.34
18	4	8	3.33	1.65
18	4	8	-1.08	0.83
20	-4	-7	-0.39	0.65
-20	4	7	1.88	1.47
19	-4	-7	54.24	2.59
-19	4	7	53.16	4.32
-19	4	7	55.29	3.49
18	-4	-7	1.90	0.62
-18	4	7	1.01	1.09
-18	4	7	1.78	1.54
17	-4	-7	60.73	2.76
-17	4	7	63.55	3.44
-17	4	7	60.56	3.72
16	-4	-7	0.58	0.47
-16	4	7	-0.88	0.86
-16	4	7	-0.86	1.24
15	-4	-7	61.24	4.10
15	-4	-7	58.37	2.53
-15	4	7	50.66	3.40
-15	4	7	57.47	3.04
14	-4	-7	2.69	1.64
14	-4	-7	-0.94	0.56
-14	4	7	-0.34	0.69
-14	4	7	0.91	1.11
13	-4	-7	106.88	4.18
13	-4	-7	96.50	5.14
-13	4	7	109.12	4.59
-13	4	7	103.33	4.95
12	-4	-7	-0.11	0.30
12	-4	-7	0.02	1.19
-12	4	7	0.61	0.95
-12	4	7	0.56	0.56
11	-4	-7	207.93	8.80

11	-4	-7	209.02	8.25
11	-4	-7	206.34	8.00
-11	4	7	196.45	8.61
-11	4	7	218.69	8.10
-11	4	7	217.92	8.32
10	-4	-7	5.60	0.86
10	-4	-7	3.58	0.49
10	-4	-7	2.35	1.07
10	-4	-7	4.50	0.52
-10	4	7	3.97	0.96
-10	4	7	4.29	0.70
-10	4	7	4.77	0.60
9	-4	-7	110.79	4.35
9	-4	-7	106.77	5.00
9	-4	-7	109.29	4.31
9	-4	-7	110.86	4.34
9	-4	-7	114.94	4.54
-9	4	7	118.77	4.42
-9	4	7	110.57	4.57
-9	4	7	105.72	4.92
8	-4	-7	5.70	0.73
8	-4	-7	4.76	0.59
8	-4	-7	4.80	0.96
8	-4	-7	4.34	0.70
8	-4	-7	4.04	0.47
-8	4	7	4.90	0.56
-8	4	7	4.70	0.62
-8	4	7	4.88	0.86
7	-4	-7	212.81	8.40
7	-4	-7	219.17	8.56
7	-4	-7	234.38	8.49
7	-4	-7	228.58	8.55
7	-4	-7	232.30	9.00
-7	4	7	229.59	8.57
-7	4	7	208.21	8.88
-7	4	7	212.30	8.44
6	-4	-7	24.49	1.23
6	-4	-7	26.34	1.27
6	-4	-7	21.31	1.57
6	-4	-7	22.94	1.70
6	-4	-7	24.78	1.12
-6	4	7	23.50	1.24
-6	4	7	24.10	1.60
5	-4	-7	194.43	7.55

5	-4	-7	194.90	7.33
5	-4	-7	178.52	7.69
5	-4	-7	178.91	7.25
5	-4	-7	205.97	7.42
-5	4	7	185.30	7.68
-5	4	7	196.10	7.29
-5	4	7	196.64	7.33
4	-4	-7	-0.07	0.49
4	-4	-7	0.01	0.35
4	-4	-7	0.01	0.18
4	-4	-7	0.29	0.24
-4	4	7	0.16	0.20
-4	4	7	0.20	0.24
-4	4	7	-0.06	0.33
-4	4	7	0.14	0.44
3	-4	-7	77.34	3.24
3	-4	-7	75.86	3.00
3	-4	-7	76.55	3.01
3	-4	-7	76.60	3.42
-3	4	7	76.47	3.19
-3	4	7	74.54	3.36
2	-4	-7	35.08	1.47
2	-4	-7	36.90	1.91
2	-4	-7	35.77	1.52
2	-4	-7	36.23	1.79
-2	4	7	35.83	1.74
-2	4	7	35.15	1.53
-2	4	7	32.89	1.81
-2	4	7	37.14	1.46
1	-4	-7	225.47	8.71
1	-4	-7	243.40	8.99
1	-4	-7	245.36	9.09
1	-4	-7	225.85	8.61
-1	4	7	225.32	8.61
-1	4	7	236.77	8.74
-1	4	7	216.99	8.98
-1	4	7	229.44	8.95
0	-4	-7	0.07	0.43
0	-4	-7	0.40	0.18
0	-4	-7	-0.24	0.37
0	4	7	1.00	0.25
0	4	7	0.52	0.38
0	4	7	0.07	0.31
-1	-4	-7	215.51	8.15

-1	-4	-7	204.62	7.83
-1	-4	-7	218.23	8.15
1	4	7	196.84	8.03
1	4	7	207.81	7.85
1	4	7	196.15	8.08
-2	-4	-7	4.05	0.43
-2	-4	-7	4.77	0.67
-2	-4	-7	3.38	0.64
2	4	7	2.74	0.54
2	4	7	4.95	0.45
2	4	7	3.35	0.62
-3	-4	-7	71.71	3.08
-3	-4	-7	72.02	2.81
-3	-4	-7	65.83	2.83
-3	-4	-7	74.29	3.20
3	4	7	70.01	2.84
3	4	7	66.60	3.11
3	4	7	70.71	2.99
-4	-4	-7	10.00	0.95
-4	-4	-7	9.37	0.61
-4	-4	-7	11.01	1.08
-4	-4	-7	8.96	0.64
4	4	7	9.77	0.96
4	4	7	10.51	0.82
-5	-4	-7	120.26	4.95
-5	-4	-7	112.47	4.41
-5	-4	-7	124.86	4.53
-5	-4	-7	112.08	4.39
-5	-4	-7	113.91	4.57
5	4	7	109.05	4.86
5	4	7	119.81	4.65
-6	-4	-7	37.22	1.66
-6	-4	-7	32.91	1.99
-6	-4	-7	34.65	1.48
-6	-4	-7	37.20	1.59
-6	-4	-7	33.56	1.51
-6	-4	-7	38.71	2.10
6	4	7	35.29	1.99
6	4	7	37.46	1.70
-7	-4	-7	47.76	2.50
-7	-4	-7	47.53	1.97
-7	-4	-7	47.72	2.18
-7	-4	-7	42.99	2.03
-7	-4	-7	42.57	1.94

-7	-4	-7	44.75	1.89
7	4	7	44.34	2.38
7	4	7	46.16	2.04
7	4	7	47.66	1.91
-8	-4	-7	24.99	1.11
-8	-4	-7	20.67	1.32
-8	-4	-7	18.71	1.62
-8	-4	-7	19.94	1.11
-8	-4	-7	18.83	1.16
8	4	7	19.67	1.50
8	4	7	20.16	1.16
-9	-4	-7	78.11	3.37
-9	-4	-7	73.47	3.24
-9	-4	-7	83.42	3.80
-9	-4	-7	79.54	3.32
-9	-4	-7	80.43	3.22
9	4	7	81.29	3.22
9	4	7	78.69	3.26
9	4	7	79.49	3.67
9	4	7	74.95	3.24
-10	-4	-7	18.91	1.77
-10	-4	-7	12.61	1.13
-10	-4	-7	14.43	1.19
-10	-4	-7	16.11	1.10
10	4	7	17.16	1.53
10	4	7	17.46	1.08
10	4	7	17.58	1.13
10	4	7	16.24	1.03
-11	-4	-7	77.69	3.45
-11	-4	-7	83.27	4.03
-11	-4	-7	79.40	3.51
-11	-4	-7	73.39	3.44
11	4	7	85.78	3.42
11	4	7	85.24	3.42
11	4	7	82.86	3.86
-12	-4	-7	9.48	1.16
-12	-4	-7	12.30	2.64
-12	-4	-7	7.79	1.08
-12	-4	-7	8.84	1.43
12	4	7	10.56	1.09
12	4	7	10.52	1.46
12	4	7	8.96	1.17
-13	-4	-7	48.31	2.67
-13	-4	-7	50.92	2.81

-13	-4	-7	50.47	2.44
13	4	7	56.95	2.99
13	4	7	59.79	2.54
13	4	7	56.46	2.57
-14	-4	-7	10.34	1.53
14	4	7	10.56	1.64
14	4	7	11.82	1.34
14	4	7	10.65	1.11
-15	-4	-7	30.88	2.26
15	4	7	36.93	2.00
-16	-4	-7	4.89	1.38
16	4	7	5.57	0.85
16	4	7	4.56	1.30
-17	-4	-7	30.68	2.46
17	4	7	26.48	1.92
17	4	7	29.30	2.63
-18	-4	-7	2.97	1.43
18	4	7	9.11	1.72
18	4	7	5.99	0.97
-19	-4	-7	13.36	1.97
19	4	7	20.98	2.84
19	4	7	22.03	1.88
20	-4	-6	20.90	1.60
-20	4	6	23.07	2.69
19	-4	-6	2.62	0.69
-19	4	6	1.47	1.49
-19	4	6	3.01	1.28
18	-4	-6	38.19	1.96
-18	4	6	34.53	2.75
-18	4	6	35.14	3.00
17	-4	-6	-0.32	2.33
17	-4	-6	2.25	0.54
-17	4	6	0.16	1.31
-17	4	6	0.69	0.95
16	-4	-6	72.53	3.15
16	-4	-6	77.66	4.54
-16	4	6	72.57	3.70
-16	4	6	73.92	3.96
15	-4	-6	9.54	1.80
15	-4	-6	8.55	0.87
-15	4	6	7.47	1.42
-15	4	6	8.81	1.48
14	-4	-6	46.71	3.35
-14	4	6	48.18	2.60

-14	4	6	42.38	2.84
13	-4	-6	-0.07	0.30
13	-4	-6	-1.80	1.24
-13	4	6	-0.73	0.87
-13	4	6	-0.66	0.59
12	-4	-6	66.43	3.65
12	-4	-6	63.55	2.67
-12	4	6	65.96	3.05
-12	4	6	60.53	3.34
11	-4	-6	1.45	1.05
11	-4	-6	1.03	0.30
11	-4	-6	0.54	0.52
-11	4	6	1.14	0.85
-11	4	6	1.44	0.37
-11	4	6	0.70	0.56
10	-4	-6	101.59	4.19
10	-4	-6	108.43	4.40
10	-4	-6	108.68	4.14
10	-4	-6	105.18	4.13
10	-4	-6	108.01	4.88
-10	4	6	112.77	4.28
-10	4	6	103.95	4.46
-10	4	6	108.14	4.77
9	-4	-6	45.86	2.60
9	-4	-6	44.13	2.08
9	-4	-6	45.66	1.89
9	-4	-6	44.05	1.90
9	-4	-6	44.49	1.89
-9	4	6	36.89	2.37
-9	4	6	47.85	1.96
-9	4	6	47.15	2.16
8	-4	-6	73.68	3.08
8	-4	-6	70.04	2.97
8	-4	-6	74.97	3.50
8	-4	-6	71.34	2.91
8	-4	-6	75.26	2.95
-8	4	6	73.27	2.96
-8	4	6	74.69	3.13
-8	4	6	71.91	3.40
7	-4	-6	23.48	1.16
7	-4	-6	25.85	1.33
7	-4	-6	25.23	1.26
7	-4	-6	26.17	1.35
7	-4	-6	29.60	1.78

-7	4	6	25.55	1.36
-7	4	6	26.47	1.67
-7	4	6	27.25	1.22
6	-4	-6	203.67	7.80
6	-4	-6	208.33	7.71
6	-4	-6	215.11	8.18
6	-4	-6	205.12	7.82
6	-4	-6	206.13	7.88
-6	4	6	205.07	7.74
-6	4	6	196.75	7.81
-6	4	6	189.91	8.10
5	-4	-6	0.06	0.47
5	-4	-6	0.09	0.30
5	-4	-6	0.17	0.26
5	-4	-6	0.25	0.18
-5	4	6	0.11	0.18
-5	4	6	0.05	0.23
-5	4	6	0.30	0.44
4	-4	-6	242.02	9.08
4	-4	-6	236.91	8.97
4	-4	-6	237.12	9.30
4	-4	-6	233.77	8.93
4	-4	-6	240.78	9.21
-4	4	6	242.61	8.96
-4	4	6	229.24	9.07
-4	4	6	239.86	8.94
-4	4	6	231.16	9.28
3	-4	-6	1.83	0.30
3	-4	-6	3.02	0.36
3	-4	-6	2.35	0.51
3	-4	-6	2.78	0.46
-3	4	6	2.26	0.44
-3	4	6	2.30	0.33
-3	4	6	3.21	0.42
-3	4	6	2.39	0.45
2	-4	-6	218.31	8.45
2	-4	-6	234.77	8.83
2	-4	-6	222.67	8.49
2	-4	-6	241.99	8.71
-2	4	6	200.54	8.73
-2	4	6	241.33	8.45
-2	4	6	227.18	8.50
-2	4	6	215.73	8.66
1	-4	-6	0.13	0.13

1	-4	-6	0.45	0.38
1	-4	-6	0.37	0.34
1	-4	-6	0.08	0.16
-1	4	6	0.06	0.12
-1	4	6	0.13	0.30
-1	4	6	0.33	0.34
-1	4	6	-0.10	0.15
0	-4	-6	229.72	8.63
0	-4	-6	214.64	8.38
0	-4	-6	246.44	8.69
0	4	6	223.16	8.61
0	4	6	209.77	8.38
0	4	6	210.50	8.59
-1	-4	-6	0.06	0.39
-1	-4	-6	0.32	0.38
-1	-4	-6	0.21	0.17
1	4	6	-0.42	0.29
1	4	6	0.02	0.29
1	4	6	-0.02	0.16
-2	-4	-6	174.79	6.49
-2	-4	-6	159.79	6.19
-2	-4	-6	151.33	6.56
2	4	6	160.88	6.44
2	4	6	168.11	6.20
2	4	6	157.14	6.33
-3	-4	-6	22.41	1.32
-3	-4	-6	21.38	1.21
-3	-4	-6	18.76	0.98
-3	-4	-6	19.81	0.96
3	4	6	19.58	1.27
3	4	6	20.02	1.12
3	4	6	21.52	1.01
-4	-4	-6	97.95	3.73
-4	-4	-6	108.70	4.08
-4	-4	-6	94.50	3.84
-4	-4	-6	96.48	3.89
-4	-4	-6	91.22	3.73
-4	-4	-6	104.20	4.23
4	4	6	96.91	4.17
4	4	6	100.73	3.98
-5	-4	-6	2.11	0.34
-5	-4	-6	2.63	0.62
-5	-4	-6	1.87	0.28
-5	-4	-6	2.01	0.29

-5	-4	-6	2.07	0.33
-5	-4	-6	2.98	0.51
5	4	6	2.24	0.57
5	4	6	2.49	0.41
-6	-4	-6	105.55	4.24
-6	-4	-6	101.31	3.82
-6	-4	-6	92.81	3.87
-6	-4	-6	94.84	3.74
-6	-4	-6	98.43	3.98
6	4	6	98.48	3.91
6	4	6	88.37	4.13
-7	-4	-6	5.80	0.75
-7	-4	-6	7.74	1.11
-7	-4	-6	5.10	0.50
-7	-4	-6	4.98	0.50
-7	-4	-6	5.74	0.58
-7	-4	-6	4.70	0.60
7	4	6	5.53	0.67
7	4	6	5.27	0.90
7	4	6	4.74	0.52
-8	-4	-6	164.24	6.31
-8	-4	-6	148.48	6.22
-8	-4	-6	158.44	6.65
-8	-4	-6	164.37	6.34
-8	-4	-6	167.39	6.21
8	4	6	149.46	6.56
8	4	6	159.12	6.24
8	4	6	169.06	6.21
8	4	6	168.28	6.28
-9	-4	-6	0.83	0.30
-9	-4	-6	0.10	0.46
-9	-4	-6	-0.20	0.68
-9	-4	-6	0.14	0.39
9	4	6	0.48	0.59
9	4	6	-0.14	0.28
9	4	6	0.58	0.44
9	4	6	0.14	0.34
-10	-4	-6	154.61	6.53
-10	-4	-6	175.16	6.97
-10	-4	-6	166.46	6.59
-10	-4	-6	160.12	6.44
-10	-4	-6	167.28	6.55
10	4	6	175.72	6.51
10	4	6	164.64	6.88

10	4	6	170.57	6.51
-11	-4	-6	5.61	0.88
-11	-4	-6	4.35	1.04
-11	-4	-6	4.54	0.70
-11	-4	-6	4.69	0.84
11	4	6	5.00	0.69
11	4	6	3.83	0.63
11	4	6	4.54	0.90
-12	-4	-6	82.37	3.63
-12	-4	-6	87.65	3.83
-12	-4	-6	83.45	3.78
-12	-4	-6	96.18	4.29
12	4	6	87.11	4.06
12	4	6	91.79	3.73
12	4	6	87.53	3.67
-13	-4	-6	0.80	1.07
-13	-4	-6	1.06	0.59
-13	-4	-6	0.72	0.67
13	4	6	-0.46	0.78
13	4	6	0.93	0.49
13	4	6	-0.64	0.60
-14	-4	-6	107.00	4.72
-14	-4	-6	107.55	4.61
14	4	6	113.28	4.59
14	4	6	109.94	4.92
14	4	6	107.16	4.53
-15	-4	-6	0.30	0.85
15	4	6	0.12	0.94
15	4	6	-0.31	0.57
16	4	6	71.35	3.66
16	4	6	69.80	3.24
-17	-4	-6	3.45	1.26
17	4	6	3.58	0.79
17	4	6	6.67	1.44
-18	-4	-6	28.18	2.75
18	4	6	35.62	2.81
18	4	6	34.87	2.09
-19	-4	-6	1.71	1.40
19	4	6	0.35	1.51
19	4	6	0.85	0.84
20	-4	-5	5.45	0.87
-20	4	5	5.25	1.49
19	-4	-5	14.82	1.30
-19	4	5	13.42	2.52

-19	4	5	14.18	2.25
18	-4	-5	-0.22	0.56
-18	4	5	0.04	1.30
-18	4	5	1.23	1.09
17	-4	-5	8.84	1.99
17	-4	-5	7.25	0.92
-17	4	5	7.05	1.33
-17	4	5	8.59	1.48
16	-4	-5	2.00	1.70
16	-4	-5	2.94	0.59
-16	4	5	3.08	0.95
-16	4	5	4.05	1.22
15	-4	-5	23.75	2.75
15	-4	-5	25.36	1.40
-15	4	5	28.77	2.20
-15	4	5	26.32	1.97
14	-4	-5	12.63	0.97
14	-4	-5	21.36	2.41
-14	4	5	13.69	1.74
13	-4	-5	19.58	1.09
13	-4	-5	20.95	2.27
-13	4	5	20.35	1.51
-13	4	5	16.41	1.81
12	-4	-5	4.95	0.55
12	-4	-5	5.22	0.58
12	-4	-5	5.98	1.24
12	-4	-5	4.10	0.72
12	-4	-5	5.14	0.53
-12	4	5	4.43	1.05
-12	4	5	4.94	0.72
-12	4	5	6.53	1.04
11	-4	-5	27.78	1.25
11	-4	-5	28.41	1.27
11	-4	-5	27.05	1.31
11	-4	-5	30.68	2.23
11	-4	-5	26.13	1.55
-11	4	5	27.67	1.66
-11	4	5	27.61	1.39
-11	4	5	24.87	1.89
10	-4	-5	67.74	3.31
10	-4	-5	68.09	2.65
10	-4	-5	64.93	2.88
10	-4	-5	64.07	2.63
10	-4	-5	64.77	2.67

-10	4	5	63.66	2.94
-10	4	5	60.18	3.14
-10	4	5	70.09	2.74
9	-4	-5	36.99	1.67
9	-4	-5	36.67	1.65
9	-4	-5	43.46	2.28
9	-4	-5	39.68	1.69
9	-4	-5	39.28	1.87
-9	4	5	38.13	2.16
-9	4	5	38.84	1.91
-9	4	5	38.03	1.71
8	-4	-5	50.91	2.21
8	-4	-5	53.72	2.20
8	-4	-5	53.64	2.33
8	-4	-5	55.10	2.67
8	-4	-5	49.41	2.14
-8	4	5	54.12	2.20
-8	4	5	50.27	2.58
-8	4	5	52.86	2.35
7	-4	-5	94.97	3.76
7	-4	-5	95.65	4.18
7	-4	-5	98.69	3.87
7	-4	-5	100.20	3.83
7	-4	-5	97.37	3.91
-7	4	5	101.24	3.80
-7	4	5	93.06	3.91
-7	4	5	91.85	4.14
6	-4	-5	3.54	0.52
6	-4	-5	3.36	0.59
6	-4	-5	3.47	0.66
6	-4	-5	2.98	0.51
6	-4	-5	3.39	0.35
-6	4	5	2.29	0.38
-6	4	5	2.87	0.49
-6	4	5	3.32	0.61
5	-4	-5	29.47	1.59
5	-4	-5	28.51	1.44
5	-4	-5	29.87	1.36
5	-4	-5	29.15	1.22
5	-4	-5	24.60	1.38
-5	4	5	27.62	1.55
-5	4	5	27.43	1.24
-5	4	5	28.62	1.32
4	-4	-5	0.54	0.23

4	-4	-5	0.38	0.14
4	-4	-5	0.00	0.38
4	-4	-5	0.58	0.28
4	-4	-5	-0.25	0.39
-4	4	5	0.34	0.16
-4	4	5	0.72	0.36
-4	4	5	0.16	0.20
3	-4	-5	117.77	4.98
3	-4	-5	120.79	4.70
3	-4	-5	124.37	4.71
3	-4	-5	128.52	4.86
-3	4	5	124.44	4.71
-3	4	5	118.19	4.96
-3	4	5	117.37	4.87
-3	4	5	132.15	4.74
2	-4	-5	4.31	0.75
2	-4	-5	4.67	0.34
2	-4	-5	3.69	0.54
2	-4	-5	4.11	0.34
-2	4	5	3.66	0.33
-2	4	5	4.54	0.57
-2	4	5	4.42	0.57
1	-4	-5	155.04	5.69
1	-4	-5	148.26	5.41
1	-4	-5	154.62	5.87
1	-4	-5	139.29	5.46
-1	4	5	129.94	5.53
-1	4	5	145.49	5.69
-1	4	5	142.72	5.66
-1	4	5	136.47	5.40
0	-4	-5	39.24	1.46
0	-4	-5	39.90	1.81
0	-4	-5	39.22	1.79
0	4	5	38.62	1.76
0	4	5	37.02	1.76
0	4	5	33.81	1.62
0	4	5	37.03	1.46
-1	-4	-5	2.65	0.31
-1	-4	-5	2.20	0.41
1	4	5	3.75	0.50
1	4	5	3.08	0.53
-2	-4	-5	28.12	1.37
-2	-4	-5	26.07	1.17
-2	-4	-5	26.32	1.42

2	4	5	27.53	1.18
2	4	5	25.67	1.31
2	4	5	25.77	1.39
-3	-4	-5	33.10	1.35
-3	-4	-5	34.85	1.45
-3	-4	-5	36.31	1.76
-3	-4	-5	34.70	1.63
-3	-4	-5	34.53	1.48
-3	-4	-5	34.15	1.35
3	4	5	35.35	1.48
3	4	5	34.04	1.60
3	4	5	32.27	1.71
-4	-4	-5	32.41	1.40
-4	-4	-5	36.84	1.65
-4	-4	-5	37.41	1.81
-4	-4	-5	32.19	1.49
-4	-4	-5	33.97	1.38
-4	-4	-5	36.63	1.47
4	4	5	31.57	1.73
4	4	5	34.85	1.60
-5	-4	-5	109.69	4.46
-5	-4	-5	115.49	4.54
-5	-4	-5	117.48	4.49
-5	-4	-5	121.34	4.66
-5	-4	-5	112.02	4.43
-5	-4	-5	126.15	4.85
5	4	5	116.18	4.60
5	4	5	114.06	4.80
-6	-4	-5	2.22	0.60
-6	-4	-5	1.96	0.32
-6	-4	-5	1.50	0.26
-6	-4	-5	2.29	0.31
-6	-4	-5	2.16	0.35
-6	-4	-5	1.84	0.44
6	4	5	2.22	0.58
6	4	5	1.92	0.41
-7	-4	-5	120.73	4.69
-7	-4	-5	115.26	4.77
-7	-4	-5	124.75	4.71
-7	-4	-5	115.94	4.73
-7	-4	-5	122.55	4.84
-7	-4	-5	124.27	5.09
7	4	5	123.46	4.80
7	4	5	128.39	4.72

7	4	5	117.66	5.05
7	4	5	121.44	4.74
-8	-4	-5	14.03	1.29
-8	-4	-5	13.80	0.95
-8	-4	-5	13.54	0.89
-8	-4	-5	13.77	0.78
8	4	5	13.04	0.93
8	4	5	13.67	1.18
8	4	5	11.77	0.79
8	4	5	13.98	0.84
-9	-4	-5	150.31	5.64
-9	-4	-5	142.37	5.73
-9	-4	-5	152.51	6.09
-9	-4	-5	142.25	5.79
-9	-4	-5	136.13	5.72
9	4	5	145.74	5.68
9	4	5	145.66	6.01
9	4	5	149.84	5.69
-10	-4	-5	1.72	0.36
-10	-4	-5	1.45	0.55
-10	-4	-5	1.93	0.54
-10	-4	-5	2.82	0.75
-10	-4	-5	1.92	0.67
10	4	5	1.89	0.45
10	4	5	1.04	0.61
10	4	5	1.18	0.36
-11	-4	-5	144.46	5.76
-11	-4	-5	141.71	5.68
-11	-4	-5	145.15	6.15
-11	-4	-5	141.02	5.84
-11	-4	-5	147.74	5.89
11	4	5	143.66	6.07
11	4	5	154.26	5.80
11	4	5	148.95	5.75
-12	-4	-5	-1.42	0.53
-12	-4	-5	-0.22	0.53
-12	-4	-5	-0.99	0.83
-12	-4	-5	-0.67	0.76
12	4	5	0.46	0.38
12	4	5	-0.37	0.54
12	4	5	-0.03	0.67
-13	-4	-5	128.30	5.50
-13	-4	-5	111.64	4.91
-13	-4	-5	121.16	5.15

-13	-4	-5	126.54	5.17
13	4	5	128.12	5.06
13	4	5	123.41	5.31
13	4	5	122.69	4.99
-14	-4	-5	11.37	1.14
-14	-4	-5	11.69	1.70
-14	-4	-5	13.61	2.04
-14	-4	-5	13.62	1.49
14	4	5	10.82	1.49
14	4	5	13.20	1.08
14	4	5	10.84	1.30
-15	-4	-5	71.86	3.60
-15	-4	-5	73.43	3.76
15	4	5	81.11	3.40
15	4	5	79.04	3.76
-16	-4	-5	1.86	1.10
16	4	5	1.38	1.00
16	4	5	0.81	0.63
-17	-4	-5	72.48	3.73
17	4	5	70.10	3.31
17	4	5	73.99	3.79
-18	-4	-5	-0.53	1.21
18	4	5	-0.72	0.66
-19	-4	-5	24.75	2.77
19	4	5	26.51	1.86
19	4	5	26.76	2.70
20	4	5	2.40	1.10
20	-4	-4	1.21	0.66
-20	4	4	3.01	1.51
-19	4	4	1.20	1.23
18	-4	-4	0.86	0.56
-18	4	4	-0.51	1.09
-18	4	4	0.55	1.20
17	-4	-4	5.66	1.76
17	-4	-4	3.85	0.65
-17	4	4	2.83	1.23
-17	4	4	5.88	1.24
16	-4	-4	0.21	0.42
16	-4	-4	2.20	1.52
-16	4	4	0.05	1.04
-16	4	4	-0.69	0.85
15	-4	-4	10.90	0.92
15	-4	-4	11.66	1.69
-15	4	4	10.07	1.73

-15	4	4	13.97	1.56
14	-4	-4	1.84	1.29
14	-4	-4	0.20	0.36
14	-4	-4	0.50	0.35
-14	4	4	-0.13	0.80
-14	4	4	0.24	0.59
-14	4	4	0.54	1.16
13	-4	-4	15.15	0.93
13	-4	-4	13.99	0.89
13	-4	-4	17.05	2.05
-13	4	4	14.29	1.54
-13	4	4	16.88	1.39
-13	4	4	13.91	1.15
12	-4	-4	-0.59	0.47
12	-4	-4	0.08	0.96
12	-4	-4	0.70	0.35
12	-4	-4	-0.07	0.28
-12	4	4	0.52	0.37
-12	4	4	0.64	0.71
-12	4	4	-0.35	0.55
-12	4	4	-0.19	0.51
11	-4	-4	1.95	0.90
11	-4	-4	2.75	0.56
11	-4	-4	1.41	0.31
11	-4	-4	2.51	0.42
-11	4	4	1.89	0.36
-11	4	4	2.44	0.78
-11	4	4	2.44	0.61
-11	4	4	3.44	0.60
10	-4	-4	0.25	0.39
10	-4	-4	0.57	0.25
10	-4	-4	0.82	0.32
10	-4	-4	0.86	0.73
-10	4	4	0.71	0.49
-10	4	4	1.31	0.49
-10	4	4	0.65	0.30
-10	4	4	-0.54	0.55
9	-4	-4	7.97	1.17
9	-4	-4	6.75	0.63
9	-4	-4	5.93	0.50
9	-4	-4	7.12	0.77
-9	4	4	5.66	0.76
-9	4	4	6.80	1.00
-9	4	4	5.38	0.81

-9	4	4	6.57	0.57
8	-4	-4	0.27	0.58
8	-4	-4	1.42	0.39
8	-4	-4	0.94	0.34
8	-4	-4	1.15	0.25
-8	4	4	0.69	0.45
-8	4	4	0.51	0.50
-8	4	4	1.25	0.27
-8	4	4	0.66	0.35
7	-4	-4	13.68	0.74
7	-4	-4	17.13	0.95
7	-4	-4	15.98	1.20
7	-4	-4	15.74	0.90
-7	4	4	13.42	1.11
-7	4	4	14.47	1.03
-7	4	4	15.18	0.79
-7	4	4	15.14	0.95
6	-4	-4	104.88	4.00
6	-4	-4	104.72	4.23
6	-4	-4	98.53	4.01
6	-4	-4	100.61	4.00
6	-4	-4	97.41	3.89
-6	4	4	99.12	4.02
-6	4	4	105.11	3.92
-6	4	4	97.12	4.20
5	-4	-4	3.59	0.68
5	-4	-4	4.38	0.51
5	-4	-4	3.74	0.57
5	-4	-4	3.92	0.33
-5	4	4	3.65	0.35
-5	4	4	4.35	0.46
-5	4	4	3.47	0.65
4	-4	-4	80.43	3.30
4	-4	-4	76.72	3.09
4	-4	-4	81.30	3.15
4	-4	-4	82.51	3.23
-4	4	4	76.01	3.34
-4	4	4	81.42	3.16
3	-4	-4	78.27	3.04
3	-4	-4	80.07	3.16
3	-4	-4	81.21	3.02
3	-4	-4	77.37	3.27
3	-4	-4	78.65	3.27
-3	4	4	74.63	3.05

-3	4	4	74.56	3.24
2	-4	-4	3.04	0.28
2	-4	-4	2.82	0.52
2	-4	-4	2.56	0.27
2	-4	-4	2.74	0.40
-2	4	4	3.75	0.51
-2	4	4	2.56	0.27
-2	4	4	2.96	0.29
1	-4	-4	0.26	0.13
1	-4	-4	0.42	0.28
1	-4	-4	0.32	0.12
1	-4	-4	0.71	0.30
-1	4	4	0.47	0.13
-1	4	4	0.61	0.16
-1	4	4	0.72	0.29
-1	4	4	0.35	0.26
0	-4	-4	47.26	1.86
0	-4	-4	49.32	2.12
0	-4	-4	43.99	2.08
0	4	4	53.97	2.13
0	4	4	46.75	1.86
0	4	4	47.31	2.12
0	4	4	49.49	1.93
-1	-4	-4	2.07	0.19
-1	-4	-4	1.90	0.38
-1	-4	-4	2.20	0.36
-1	-4	-4	1.61	0.25
1	4	4	1.67	0.34
1	4	4	2.08	0.26
1	4	4	2.37	0.43
1	4	4	2.35	0.18
-2	-4	-4	48.20	1.86
-2	-4	-4	48.29	1.95
-2	-4	-4	49.15	2.17
-2	-4	-4	48.43	2.10
2	4	4	48.36	2.17
2	4	4	47.10	1.85
2	4	4	49.50	2.09
-3	-4	-4	3.13	0.33
-3	-4	-4	3.43	0.26
-3	-4	-4	4.09	0.64
-3	-4	-4	3.91	0.49
-3	-4	-4	2.95	0.36
-3	-4	-4	3.18	0.26

3	4	4	3.55	0.56
3	4	4	3.34	0.39
3	4	4	3.17	0.43
-4	-4	-4	9.52	0.68
-4	-4	-4	7.88	0.53
-4	-4	-4	9.04	0.50
-4	-4	-4	9.13	0.48
-4	-4	-4	7.94	0.79
-4	-4	-4	8.80	0.51
4	4	4	9.58	0.68
4	4	4	9.63	0.81
-5	-4	-4	86.23	3.36
-5	-4	-4	87.01	3.40
-5	-4	-4	85.03	3.39
-5	-4	-4	95.11	3.55
-5	-4	-4	89.50	3.70
-5	-4	-4	83.67	3.44
5	4	4	84.99	3.67
5	4	4	85.91	3.51
-6	-4	-4	49.97	2.08
-6	-4	-4	46.35	2.00
-6	-4	-4	49.89	2.28
-6	-4	-4	45.34	1.99
-6	-4	-4	44.18	1.92
-6	-4	-4	47.55	1.93
6	4	4	46.03	1.95
6	4	4	48.45	2.06
6	4	4	46.37	2.22
-7	-4	-4	6.42	0.60
-7	-4	-4	6.78	0.49
-7	-4	-4	7.78	0.69
-7	-4	-4	7.54	0.92
7	4	4	6.38	0.52
7	4	4	6.65	0.71
7	4	4	6.69	0.85
-8	-4	-4	60.30	2.70
-8	-4	-4	65.93	2.74
-8	-4	-4	61.85	2.61
-8	-4	-4	69.49	3.02
-8	-4	-4	62.85	2.65
-8	-4	-4	63.84	2.83
8	4	4	64.01	2.65
8	4	4	61.78	2.93
8	4	4	67.52	2.76

8	4	4	68.72	2.68
-9	-4	-4	0.06	0.57
-9	-4	-4	1.03	0.43
-9	-4	-4	1.05	0.26
-9	-4	-4	1.08	0.56
-9	-4	-4	1.09	0.39
9	4	4	0.67	0.27
9	4	4	0.72	0.52
9	4	4	1.08	0.36
-10	-4	-4	56.47	2.71
-10	-4	-4	58.78	2.53
-10	-4	-4	55.77	2.57
-10	-4	-4	60.07	2.86
-10	-4	-4	56.56	2.40
10	4	4	63.06	2.54
10	4	4	55.32	2.75
10	4	4	55.43	2.44
-11	-4	-4	15.30	1.16
-11	-4	-4	18.08	1.02
-11	-4	-4	18.61	1.54
-11	-4	-4	16.49	1.40
-11	-4	-4	16.43	1.24
11	4	4	18.71	1.19
11	4	4	17.81	1.40
11	4	4	17.41	1.04
-12	-4	-4	50.77	2.71
-12	-4	-4	45.13	2.40
-12	-4	-4	50.68	2.50
-12	-4	-4	48.05	2.27
12	4	4	50.92	2.55
12	4	4	49.95	2.23
12	4	4	50.27	2.34
-13	-4	-4	13.87	1.71
-13	-4	-4	12.27	1.48
-13	-4	-4	13.71	1.11
-13	-4	-4	13.75	1.36
13	4	4	13.72	1.42
13	4	4	12.49	0.96
13	4	4	12.79	1.21
-14	-4	-4	31.75	2.10
-14	-4	-4	28.36	1.96
-14	-4	-4	30.16	1.72
-14	-4	-4	30.79	2.32
14	4	4	32.32	2.15

14	4	4	30.89	2.01
14	4	4	33.09	1.68
-15	-4	-4	2.86	0.75
-15	-4	-4	5.61	1.24
-15	-4	-4	3.98	0.92
-15	-4	-4	2.58	1.09
15	4	4	4.48	1.00
15	4	4	3.38	0.67
-16	-4	-4	11.16	1.63
-16	-4	-4	11.70	1.61
-16	-4	-4	11.38	1.78
16	4	4	13.42	1.72
16	4	4	10.05	1.12
-17	-4	-4	1.08	1.00
17	4	4	2.77	0.74
17	4	4	1.69	1.15
-18	-4	-4	26.60	2.44
18	4	4	25.91	2.32
18	4	4	25.10	1.73
-19	-4	-4	5.28	1.36
19	4	4	4.18	1.41
19	4	4	3.80	0.88
-20	-4	-4	23.45	2.81
20	4	4	22.53	1.88
21	-4	-3	3.15	1.33
20	-4	-3	4.01	0.78
-20	4	3	4.89	1.49
19	-4	-3	7.97	1.11
-19	4	3	5.87	1.49
18	-4	-3	3.09	0.65
-18	4	3	2.53	1.23
-18	4	3	2.22	1.17
17	-4	-3	9.35	1.00
17	-4	-3	12.33	2.62
-17	4	3	9.59	1.40
-17	4	3	11.03	1.84
16	-4	-3	9.03	1.45
16	-4	-3	15.14	2.28
16	-4	-3	7.08	0.87
-16	4	3	7.56	1.23
-16	4	3	8.82	1.11
-16	4	3	7.60	1.24
15	-4	-3	8.02	0.82
15	-4	-3	8.20	0.96

15	-4	-3	11.31	2.11
-15	4	3	10.61	1.49
-15	4	3	8.54	1.52
-15	4	3	9.36	1.28
14	-4	-3	38.63	1.78
14	-4	-3	39.87	2.68
14	-4	-3	37.30	1.77
-14	4	3	36.74	2.02
-14	4	3	34.96	2.32
-14	4	3	35.89	2.24
13	-4	-3	5.71	1.20
13	-4	-3	3.44	0.56
13	-4	-3	2.78	0.44
-13	4	3	3.69	0.87
-13	4	3	4.68	0.79
-13	4	3	3.31	0.75
12	-4	-3	1.71	0.36
12	-4	-3	2.82	0.95
12	-4	-3	1.61	0.59
12	-4	-3	2.23	0.54
-12	4	3	1.62	0.74
-12	4	3	1.87	0.39
-12	4	3	1.46	0.64
-12	4	3	1.65	0.56
11	-4	-3	20.21	1.06
11	-4	-3	19.38	1.29
11	-4	-3	23.56	1.76
-11	4	3	19.10	1.35
-11	4	3	23.26	1.13
-11	4	3	19.79	1.48
-11	4	3	20.38	1.37
10	-4	-3	48.35	2.23
10	-4	-3	46.74	2.01
10	-4	-3	51.66	2.52
10	-4	-3	42.90	2.09
-10	4	3	48.88	2.30
-10	4	3	46.85	2.37
-10	4	3	52.39	2.08
-10	4	3	46.15	2.23
9	-4	-3	8.84	0.71
9	-4	-3	7.79	0.57
9	-4	-3	11.31	1.13
9	-4	-3	8.48	0.81
-9	4	3	9.32	0.97

-9	4	3	7.87	0.82
-9	4	3	9.42	0.64
-9	4	3	8.59	0.89
8	-4	-3	8.08	0.54
8	-4	-3	8.87	0.99
8	-4	-3	8.72	0.75
8	-4	-3	8.92	0.70
-8	4	3	8.90	0.76
-8	4	3	8.74	0.59
-8	4	3	9.23	0.87
-8	4	3	9.94	0.94
7	-4	-3	0.16	0.28
7	-4	-3	0.15	0.17
7	-4	-3	0.82	0.31
7	-4	-3	0.64	0.46
-7	4	3	0.22	0.37
-7	4	3	0.56	0.41
-7	4	3	0.47	0.28
-7	4	3	0.54	0.20
6	-4	-3	16.69	0.80
6	-4	-3	17.84	0.97
6	-4	-3	18.27	0.94
6	-4	-3	17.11	1.15
-6	4	3	17.57	0.84
-6	4	3	16.44	0.95
-6	4	3	17.40	1.08
-6	4	3	16.92	1.08
5	-4	-3	5.95	0.41
5	-4	-3	6.33	0.62
5	-4	-3	7.58	0.74
-5	4	3	6.96	0.55
-5	4	3	6.17	0.43
-5	4	3	7.45	0.72
-5	4	3	8.34	0.71
4	-4	-3	5.88	0.46
4	-4	-3	6.12	0.38
4	-4	-3	7.03	0.67
4	-4	-3	6.27	0.61
-4	4	3	5.63	0.54
-4	4	3	6.28	0.63
-4	4	3	6.65	0.51
-4	4	3	6.02	0.68
3	-4	-3	5.18	0.35
3	-4	-3	5.65	0.63

3	-4	-3	5.83	0.41
3	-4	-3	6.15	0.54
3	-4	-3	5.86	0.58
-3	4	3	6.54	0.62
-3	4	3	5.67	0.48
2	-4	-3	90.09	3.51
2	-4	-3	91.45	3.52
2	-4	-3	91.48	3.70
2	-4	-3	94.46	3.67
2	-4	-3	87.26	3.75
-2	4	3	92.89	3.53
-2	4	3	95.05	3.71
1	-4	-3	3.16	0.58
1	-4	-3	3.00	0.42
1	-4	-3	2.97	0.26
1	-4	-3	2.29	0.26
1	-4	-3	3.22	0.43
-1	4	3	2.98	0.26
-1	4	3	2.55	0.25
-1	4	3	3.38	0.46
0	-4	-3	9.35	0.62
0	-4	-3	7.85	0.63
0	-4	-3	7.19	0.46
0	-4	-3	8.28	0.41
0	4	3	8.12	0.41
0	4	3	7.74	0.61
-1	-4	-3	19.11	1.02
-1	-4	-3	20.27	1.07
-1	-4	-3	19.43	0.89
-1	-4	-3	20.65	0.84
1	4	3	20.14	0.84
1	4	3	21.17	1.10
1	4	3	21.12	0.89
-2	-4	-3	20.22	0.90
-2	-4	-3	20.21	1.03
-2	-4	-3	20.41	0.85
-2	-4	-3	20.36	1.09
2	4	3	21.20	1.12
2	4	3	19.47	0.83
-3	-4	-3	19.86	1.10
-3	-4	-3	19.49	0.84
-3	-4	-3	21.67	0.87
-3	-4	-3	19.03	0.83
-3	-4	-3	18.98	0.89

-3	-4	-3	20.09	1.01
3	4	3	19.22	1.09
3	4	3	18.19	0.82
-4	-4	-3	12.33	0.73
-4	-4	-3	11.85	0.61
-4	-4	-3	10.91	0.84
-4	-4	-3	10.41	0.56
-4	-4	-3	10.75	0.57
4	4	3	11.56	0.83
4	4	3	9.73	0.80
-5	-4	-3	9.66	0.70
-5	-4	-3	9.74	0.54
-5	-4	-3	10.12	0.56
-5	-4	-3	10.25	0.83
-5	-4	-3	9.53	0.58
5	4	3	9.79	0.70
5	4	3	9.36	0.55
5	4	3	10.01	0.83
-6	-4	-3	50.48	2.17
-6	-4	-3	50.97	2.32
-6	-4	-3	48.95	2.06
-6	-4	-3	49.05	2.10
-6	-4	-3	53.97	2.05
6	4	3	49.62	2.05
6	4	3	50.98	2.32
6	4	3	49.40	2.17
-7	-4	-3	12.65	0.82
-7	-4	-3	11.45	0.70
-7	-4	-3	11.20	0.65
-7	-4	-3	10.95	0.75
-7	-4	-3	12.49	1.00
7	4	3	12.85	0.88
7	4	3	10.19	0.67
7	4	3	12.23	0.96
-8	-4	-3	39.28	1.91
-8	-4	-3	37.21	1.86
-8	-4	-3	35.06	1.70
-8	-4	-3	36.24	1.57
-8	-4	-3	37.01	1.64
-8	-4	-3	35.45	1.70
8	4	3	36.76	1.87
8	4	3	37.45	1.65
8	4	3	35.07	1.59
-9	-4	-3	12.53	0.73

-9	-4	-3	12.03	0.91
-9	-4	-3	10.72	0.88
-9	-4	-3	14.49	1.17
-9	-4	-3	10.85	0.81
-9	-4	-3	12.28	1.11
9	4	3	11.65	0.75
9	4	3	11.34	1.05
9	4	3	13.42	0.86
-10	-4	-3	1.04	0.46
-10	-4	-3	-0.31	0.58
-10	-4	-3	0.90	0.44
-10	-4	-3	1.23	0.30
-10	-4	-3	1.43	0.63
10	4	3	0.83	0.31
10	4	3	1.34	0.57
10	4	3	0.88	0.40
-11	-4	-3	8.57	1.15
-11	-4	-3	6.81	0.88
-11	-4	-3	6.66	0.91
-11	-4	-3	6.93	0.90
-11	-4	-3	7.19	0.61
11	4	3	7.01	1.00
11	4	3	6.43	0.85
11	4	3	5.25	0.65
-12	-4	-3	0.47	0.54
-12	-4	-3	0.98	0.34
-12	-4	-3	-1.19	0.68
-12	-4	-3	-0.88	0.68
12	4	3	0.14	0.32
12	4	3	-0.47	0.56
12	4	3	0.86	0.53
-13	-4	-3	-0.87	0.83
-13	-4	-3	0.01	0.60
-13	-4	-3	0.33	0.88
-13	-4	-3	0.27	0.81
-13	-4	-3	0.89	0.63
13	4	3	0.73	0.40
13	4	3	0.39	0.58
13	4	3	0.62	0.69
-14	-4	-3	28.75	1.88
-14	-4	-3	31.70	2.21
-14	-4	-3	31.73	2.00
-14	-4	-3	30.56	2.17
14	4	3	29.75	1.96

14	4	3	33.56	1.73
-15	-4	-3	5.22	0.97
-15	-4	-3	9.14	1.34
-15	-4	-3	7.07	1.27
-15	-4	-3	7.09	1.21
15	4	3	7.71	1.34
15	4	3	5.58	0.91
-16	-4	-3	-0.67	0.82
-16	-4	-3	1.00	1.09
-16	-4	-3	3.86	1.20
16	4	3	1.90	0.60
16	4	3	-0.11	0.87
-17	-4	-3	16.18	1.81
17	4	3	13.34	1.79
17	4	3	11.05	1.20
-18	-4	-3	3.66	1.17
18	4	3	3.05	0.81
18	4	3	3.17	1.17
-19	-4	-3	5.38	1.39
19	4	3	3.83	0.82
19	4	3	4.38	1.37
-20	-4	-3	4.56	1.47
20	4	3	5.26	0.96
20	-4	-2	22.39	1.61
-20	4	2	23.54	2.76
19	-4	-2	2.09	0.68
-19	4	2	2.38	1.25
18	-4	-2	40.93	2.08
-18	4	2	39.24	2.87
17	-4	-2	3.17	0.72
17	-4	-2	3.25	0.66
-17	4	2	2.69	1.04
-17	4	2	2.30	1.06
16	-4	-2	41.26	2.00
16	-4	-2	41.55	2.85
16	-4	-2	40.20	1.97
-16	4	2	43.31	2.64
-16	4	2	38.88	2.40
15	-4	-2	11.37	1.97
15	-4	-2	11.96	0.92
15	-4	-2	12.64	1.04
-15	4	2	9.54	1.46
-15	4	2	12.88	1.60
14	-4	-2	26.88	1.43

14	-4	-2	29.58	1.50
14	-4	-2	30.74	2.25
-14	4	2	28.31	1.85
-14	4	2	24.35	1.88
-14	4	2	30.05	1.96
13	-4	-2	29.21	2.16
13	-4	-2	29.35	1.47
13	-4	-2	27.73	1.40
-13	4	2	25.24	1.75
-13	4	2	29.19	1.85
-13	4	2	30.28	1.80
12	-4	-2	26.42	1.30
12	-4	-2	26.87	1.40
12	-4	-2	25.58	1.90
12	-4	-2	27.47	1.58
-12	4	2	27.42	1.66
-12	4	2	25.79	1.66
-12	4	2	24.06	1.63
-12	4	2	27.01	1.36
11	-4	-2	0.61	0.72
11	-4	-2	1.14	0.45
11	-4	-2	0.68	0.26
11	-4	-2	0.30	0.46
-11	4	2	1.08	0.57
-11	4	2	0.73	0.57
-11	4	2	0.75	0.48
-11	4	2	1.23	0.34
10	-4	-2	58.06	2.45
10	-4	-2	58.94	2.55
10	-4	-2	54.74	2.34
10	-4	-2	59.03	2.73
-10	4	2	62.98	2.40
-10	4	2	52.62	2.60
-10	4	2	50.96	2.58
-10	4	2	55.83	2.58
9	-4	-2	20.08	0.96
9	-4	-2	18.58	1.10
9	-4	-2	20.75	1.18
-9	4	2	17.56	1.18
-9	4	2	18.96	1.26
-9	4	2	18.45	1.24
-9	4	2	22.60	1.03
8	-4	-2	9.96	0.59
8	-4	-2	10.41	0.96

8	-4	-2	10.35	0.78
-8	4	2	10.29	0.82
-8	4	2	10.41	0.87
-8	4	2	9.21	0.91
-8	4	2	11.73	0.66
7	-4	-2	27.55	1.31
7	-4	-2	27.09	1.17
7	-4	-2	25.26	1.29
7	-4	-2	25.54	1.42
-7	4	2	25.34	1.40
-7	4	2	26.06	1.35
-7	4	2	29.09	1.20
-7	4	2	26.40	1.41
6	-4	-2	44.00	1.98
6	-4	-2	41.62	1.73
6	-4	-2	44.17	1.88
6	-4	-2	43.12	1.85
-6	4	2	44.38	1.76
-6	4	2	41.56	1.95
-6	4	2	42.90	1.97
-6	4	2	40.54	1.86
5	-4	-2	51.77	2.21
5	-4	-2	53.76	2.27
5	-4	-2	46.93	2.04
-5	4	2	52.25	2.30
-5	4	2	49.98	2.16
-5	4	2	52.55	2.26
-5	4	2	55.85	2.08
4	-4	-2	115.06	4.49
4	-4	-2	115.87	4.38
4	-4	-2	118.56	4.51
4	-4	-2	107.61	4.31
-4	4	2	115.72	4.56
-4	4	2	115.81	4.32
-4	4	2	109.19	4.49
-4	4	2	110.07	4.39
3	-4	-2	45.84	1.94
3	-4	-2	42.57	1.76
3	-4	-2	45.11	1.82
3	-4	-2	44.15	1.96
-3	4	2	46.01	2.03
-3	4	2	44.14	1.77
2	-4	-2	45.81	1.84
2	-4	-2	47.58	2.06

2	-4	-2	46.58	1.97
-2	4	2	43.22	2.06
-2	4	2	49.66	2.10
-2	4	2	43.80	1.85
1	-4	-2	162.01	6.24
1	-4	-2	168.45	6.37
1	-4	-2	166.26	6.49
1	-4	-2	171.23	6.40
1	-4	-2	163.68	6.22
-1	4	2	168.63	6.25
-1	4	2	159.54	6.22
-1	4	2	171.07	6.52
-1	4	2	162.33	6.37
0	-4	-2	15.45	0.72
0	-4	-2	15.92	0.86
0	-4	-2	17.39	1.04
0	-4	-2	16.44	0.72
0	4	2	17.06	0.87
0	4	2	16.02	0.72
0	4	2	17.36	0.73
-1	-4	-2	55.75	2.30
-1	-4	-2	57.33	2.35
-1	-4	-2	56.54	2.17
-1	-4	-2	55.75	2.16
1	4	2	51.97	2.47
1	4	2	56.99	2.16
1	4	2	57.60	2.18
1	4	2	53.86	2.29
-2	-4	-2	74.98	3.04
-2	-4	-2	76.64	2.91
-2	-4	-2	76.37	2.95
-2	-4	-2	78.84	3.11
2	4	2	77.03	2.93
2	4	2	74.97	2.90
2	4	2	76.55	3.05
2	4	2	71.63	3.24
-3	-4	-2	93.03	3.54
-3	-4	-2	89.78	3.43
-3	-4	-2	89.22	3.40
-3	-4	-2	86.58	3.40
-3	-4	-2	91.67	3.60
3	4	2	84.36	3.39
3	4	2	84.42	3.63
3	4	2	92.82	3.64

-4	-4	-2	136.15	5.21
-4	-4	-2	135.37	5.23
-4	-4	-2	133.62	5.35
-4	-4	-2	140.66	5.26
-4	-4	-2	132.84	5.23
-4	-4	-2	146.52	5.44
4	4	2	140.74	5.45
-5	-4	-2	114.65	4.37
-5	-4	-2	109.87	4.17
-5	-4	-2	105.84	4.27
-5	-4	-2	108.73	4.18
5	4	2	105.92	4.14
5	4	2	101.49	4.29
5	4	2	110.77	4.38
-6	-4	-2	28.92	1.16
-6	-4	-2	25.17	1.28
-6	-4	-2	24.16	1.18
-6	-4	-2	26.05	1.22
-6	-4	-2	25.67	1.41
6	4	2	26.45	1.40
6	4	2	27.61	1.17
6	4	2	26.78	1.33
-7	-4	-2	0.74	0.47
-7	-4	-2	1.21	0.42
-7	-4	-2	1.11	0.36
-7	-4	-2	0.79	0.28
-7	-4	-2	0.87	0.21
-7	-4	-2	0.31	0.24
7	4	2	0.86	0.39
7	4	2	0.83	0.22
-8	-4	-2	4.76	0.86
-8	-4	-2	4.84	0.60
-8	-4	-2	4.95	0.54
-8	-4	-2	5.76	0.44
-8	-4	-2	4.86	0.77
-8	-4	-2	4.48	0.65
8	4	2	4.03	0.43
8	4	2	4.42	0.73
-9	-4	-2	10.00	0.93
-9	-4	-2	7.61	0.70
-9	-4	-2	7.51	0.80
-9	-4	-2	8.15	0.58
-9	-4	-2	8.25	0.97
-9	-4	-2	6.96	0.80

9	4	2	8.17	0.74
9	4	2	8.06	0.89
9	4	2	7.37	0.58
-10	-4	-2	9.05	1.14
-10	-4	-2	10.08	0.91
-10	-4	-2	8.43	1.06
-10	-4	-2	8.52	0.82
-10	-4	-2	11.59	0.70
10	4	2	11.89	1.04
10	4	2	10.54	0.72
10	4	2	11.57	0.88
-11	-4	-2	57.27	2.69
-11	-4	-2	54.42	2.77
-11	-4	-2	54.47	2.48
-11	-4	-2	56.67	2.58
-11	-4	-2	53.67	2.57
-11	-4	-2	57.62	2.34
11	4	2	56.02	2.63
11	4	2	57.52	2.40
11	4	2	54.39	2.50
-12	-4	-2	1.25	0.56
-12	-4	-2	-0.07	0.64
-12	-4	-2	2.75	0.86
-12	-4	-2	0.67	0.48
-12	-4	-2	1.16	0.59
-12	-4	-2	1.48	0.34
12	4	2	1.41	0.53
12	4	2	1.86	0.64
12	4	2	1.28	0.39
-13	-4	-2	46.25	2.41
-13	-4	-2	53.24	2.72
-13	-4	-2	52.68	2.49
-13	-4	-2	53.07	2.58
-13	-4	-2	52.66	2.83
13	4	2	54.32	2.34
13	4	2	51.42	2.51
13	4	2	53.24	2.60
-14	-4	-2	0.09	0.67
-14	-4	-2	0.61	0.67
-14	-4	-2	1.54	0.90
-14	-4	-2	1.36	0.98
14	4	2	0.26	0.64
14	4	2	0.77	0.40
-15	-4	-2	-0.03	0.94

-15	-4	-2	0.82	0.75
-15	-4	-2	1.26	0.77
-15	-4	-2	2.20	1.08
15	4	2	1.24	0.51
15	4	2	0.49	0.70
-16	-4	-2	3.10	1.09
-16	-4	-2	1.17	1.23
-16	-4	-2	-0.52	1.12
-16	-4	-2	0.65	0.84
16	4	2	-0.53	0.77
16	4	2	0.14	0.48
-17	-4	-2	2.88	1.03
-17	-4	-2	3.70	1.25
17	4	2	3.35	0.74
17	4	2	5.04	1.04
-18	-4	-2	1.88	1.16
18	4	2	3.62	0.79
18	4	2	2.07	1.01
-19	-4	-2	3.18	1.26
19	4	2	2.94	0.78
-20	-4	-2	0.07	1.28
20	4	2	-1.00	0.75
-21	4	1	40.75	5.27
20	-4	-1	0.74	0.69
-20	4	1	0.07	1.39
19	-4	-1	54.61	2.61
-19	4	1	53.15	3.47
18	-4	-1	1.11	0.57
18	-4	-1	-0.76	0.78
-18	4	1	-0.25	1.11
17	-4	-1	79.53	3.39
17	-4	-1	78.63	3.42
-17	4	1	69.84	3.50
-17	4	1	86.48	4.09
16	-4	-1	8.33	1.30
16	-4	-1	9.34	0.96
16	-4	-1	8.19	1.13
-16	4	1	11.12	1.75
-16	4	1	7.31	1.37
-16	4	1	10.83	1.77
15	-4	-1	61.80	2.72
15	-4	-1	60.68	2.64
15	-4	-1	59.87	3.15
-15	4	1	60.97	3.16

-15	4	1	58.80	3.02
-15	4	1	58.25	2.86
14	-4	-1	1.01	0.55
14	-4	-1	2.66	0.99
14	-4	-1	1.54	0.39
-14	4	1	1.23	0.80
-14	4	1	1.16	0.73
-14	4	1	0.24	0.69
13	-4	-1	95.22	3.81
13	-4	-1	97.48	3.90
13	-4	-1	96.03	4.20
-13	4	1	86.66	3.97
-13	4	1	92.18	4.10
-13	4	1	96.83	4.18
12	-4	-1	0.31	0.46
12	-4	-1	-0.01	0.25
12	-4	-1	-1.04	0.47
-12	4	1	0.43	0.66
-12	4	1	0.92	0.56
-12	4	1	0.26	0.32
-12	4	1	0.07	0.54
11	-4	-1	296.03	11.19
11	-4	-1	295.12	11.31
11	-4	-1	292.00	11.09
11	-4	-1	287.15	10.98
-11	4	1	256.13	11.44
-11	4	1	277.76	11.14
-11	4	1	298.43	11.28
-11	4	1	320.34	11.04
10	-4	-1	11.33	0.94
10	-4	-1	10.76	0.85
10	-4	-1	11.07	0.71
10	-4	-1	11.31	1.15
-10	4	1	10.63	1.00
-10	4	1	11.49	0.99
-10	4	1	12.50	0.75
-10	4	1	10.21	1.08
9	-4	-1	167.65	6.38
9	-4	-1	155.82	6.15
9	-4	-1	163.38	6.28
9	-4	-1	157.21	6.30
-9	4	1	150.62	6.37
-9	4	1	161.62	6.37
-9	4	1	158.29	6.32

-9	4	1	172.00	6.20
8	-4	-1	11.03	0.79
8	-4	-1	10.43	0.61
8	-4	-1	10.66	0.89
8	-4	-1	9.33	0.82
-8	4	1	9.15	0.91
-8	4	1	11.49	0.65
-8	4	1	11.34	0.90
-8	4	1	10.10	0.82
7	-4	-1	219.77	8.49
7	-4	-1	223.64	8.46
7	-4	-1	222.75	8.49
7	-4	-1	212.66	8.33
-7	4	1	225.71	8.50
-7	4	1	203.36	8.53
-7	4	1	240.07	8.37
-7	4	1	220.95	8.50
6	-4	-1	7.29	0.63
6	-4	-1	4.41	0.81
6	-4	-1	6.31	0.53
6	-4	-1	6.33	0.41
-6	4	1	5.43	0.41
-6	4	1	6.14	0.70
-6	4	1	6.08	0.57
5	-4	-1	256.78	9.41
5	-4	-1	236.29	9.25
5	-4	-1	263.85	9.43
-5	4	1	226.00	9.32
-5	4	1	245.56	9.37
-5	4	1	250.65	9.43
-5	4	1	252.06	9.49
4	-4	-1	18.46	0.86
4	-4	-1	17.05	0.96
4	-4	-1	17.03	0.76
4	-4	-1	18.57	0.92
-4	4	1	16.68	0.77
-4	4	1	18.10	0.92
-4	4	1	15.13	0.86
-4	4	1	17.59	1.03
3	-4	-1	185.16	7.11
3	-4	-1	191.01	7.06
3	-4	-1	171.89	6.92
3	-4	-1	197.04	6.99
-3	4	1	161.09	7.06

-3	4	1	191.51	7.06
-3	4	1	189.66	6.92
-3	4	1	189.06	7.16
2	-4	-1	0.73	0.29
2	-4	-1	0.28	0.22
2	-4	-1	0.08	0.08
2	-4	-1	0.02	0.14
-2	4	1	0.08	0.15
-2	4	1	0.10	0.09
-2	4	1	-0.01	0.18
-2	4	1	0.47	0.29
1	-4	-1	131.28	5.14
1	-4	-1	130.83	5.03
1	-4	-1	124.62	4.93
-1	4	1	125.79	5.02
-1	4	1	133.91	4.95
-1	4	1	135.00	5.17
0	-4	-1	8.77	0.54
0	-4	-1	8.13	0.38
0	-4	-1	7.03	0.42
0	-4	-1	7.81	0.69
0	4	1	7.00	0.42
0	4	1	9.06	0.71
0	4	1	6.31	0.50
0	4	1	7.60	0.38
-1	-4	-1	55.81	2.34
-1	-4	-1	56.38	2.17
-1	-4	-1	54.90	2.20
-1	-4	-1	56.63	2.31
1	4	1	57.00	2.31
1	4	1	56.61	2.20
1	4	1	56.98	2.17
-2	-4	-1	6.60	0.53
-2	-4	-1	8.02	0.44
-2	-4	-1	8.54	0.75
-2	-4	-1	8.80	0.60
-2	-4	-1	8.43	0.42
2	4	1	8.57	0.42
2	4	1	8.05	0.43
2	4	1	7.50	0.55
-3	-4	-1	88.05	3.42
-3	-4	-1	88.87	3.60
-3	-4	-1	92.99	3.47
-3	-4	-1	91.29	3.44

-3	-4	-1	96.23	3.59
3	4	1	82.20	3.44
3	4	1	90.64	3.59
-4	-4	-1	11.92	0.53
-4	-4	-1	11.76	0.70
-4	-4	-1	10.92	0.56
-4	-4	-1	11.47	0.72
-4	-4	-1	9.64	0.57
-4	-4	-1	9.63	0.62
4	4	1	10.06	0.53
4	4	1	11.38	0.78
-5	-4	-1	171.35	6.48
-5	-4	-1	177.42	6.68
-5	-4	-1	174.11	6.64
-5	-4	-1	170.96	6.72
-5	-4	-1	171.16	6.54
-5	-4	-1	174.53	6.54
5	4	1	178.09	6.71
5	4	1	159.64	6.48
-6	-4	-1	189.72	7.38
-6	-4	-1	195.94	7.30
-6	-4	-1	190.38	7.45
-6	-4	-1	195.43	7.56
-6	-4	-1	205.17	7.51
6	4	1	183.10	7.30
6	4	1	196.88	7.52
-7	-4	-1	33.52	1.57
-7	-4	-1	35.99	1.62
-7	-4	-1	35.63	1.68
-7	-4	-1	37.40	1.49
-7	-4	-1	34.86	1.83
-7	-4	-1	35.53	1.71
7	4	1	38.20	1.75
7	4	1	34.58	1.50
-8	-4	-1	42.74	1.97
-8	-4	-1	40.92	1.91
-8	-4	-1	44.40	1.89
-8	-4	-1	44.48	1.78
-8	-4	-1	42.48	2.15
-8	-4	-1	41.19	2.10
8	4	1	44.54	2.03
8	4	1	41.79	1.80
-9	-4	-1	28.92	1.73
-9	-4	-1	29.18	1.56

-9	-4	-1	29.48	1.45
-9	-4	-1	28.12	1.49
-9	-4	-1	30.73	1.32
9	4	1	30.60	1.60
9	4	1	28.98	1.34
-10	-4	-1	12.50	1.03
-10	-4	-1	14.12	1.37
-10	-4	-1	15.05	0.79
-10	-4	-1	13.52	1.04
-10	-4	-1	14.18	0.97
-10	-4	-1	13.41	1.10
10	4	1	13.87	1.07
10	4	1	14.37	0.83
-11	-4	-1	36.58	2.18
-11	-4	-1	35.71	1.87
-11	-4	-1	36.64	1.63
-11	-4	-1	37.49	1.94
-11	-4	-1	35.44	1.89
-11	-4	-1	37.02	1.82
11	4	1	38.26	1.86
11	4	1	36.75	1.68
11	4	1	39.00	1.93
-12	-4	-1	55.18	2.64
-12	-4	-1	59.15	2.61
-12	-4	-1	54.47	2.67
-12	-4	-1	55.88	2.68
-12	-4	-1	55.78	2.93
-12	-4	-1	58.73	2.42
12	4	1	55.24	2.44
12	4	1	58.98	2.64
12	4	1	61.07	2.70
-13	-4	-1	61.60	3.03
-13	-4	-1	67.44	3.14
-13	-4	-1	65.81	3.41
-13	-4	-1	72.08	3.18
-13	-4	-1	70.11	3.05
-13	-4	-1	70.48	2.94
13	4	1	64.95	2.86
13	4	1	70.94	3.09
13	4	1	66.50	3.06
-14	-4	-1	18.72	1.60
-14	-4	-1	19.25	1.53
-14	-4	-1	18.94	1.95
-14	-4	-1	15.41	1.45

14	4	1	19.06	1.15
14	4	1	17.84	1.40
-15	-4	-1	35.22	2.12
-15	-4	-1	31.89	2.46
-15	-4	-1	33.91	2.03
-15	-4	-1	34.73	2.16
15	4	1	33.20	1.98
-16	-4	-1	0.30	0.81
-16	-4	-1	0.85	1.25
-16	-4	-1	-0.27	0.93
-16	-4	-1	0.50	0.87
16	4	1	0.49	0.49
16	4	1	-0.15	0.77
-17	-4	-1	35.63	2.45
-17	-4	-1	35.44	2.67
17	4	1	38.70	1.98
17	4	1	32.84	2.21
-18	-4	-1	13.13	1.98
18	4	1	15.83	1.32
18	4	1	12.71	1.70
-19	-4	-1	9.55	1.48
19	4	1	10.20	1.31
-20	-4	-1	8.35	1.59
20	4	1	10.05	1.44
20	-4	0	34.95	2.03
-20	4	0	33.36	3.10
19	-4	0	1.98	0.68
-19	4	0	1.93	1.29
18	-4	0	38.31	2.23
18	-4	0	39.35	2.01
-18	4	0	36.52	2.90
17	-4	0	3.30	0.60
17	-4	0	3.11	0.84
-17	4	0	4.31	1.03
-17	4	0	1.56	1.11
16	-4	0	66.69	2.90
16	-4	0	65.56	3.03
16	-4	0	69.02	3.15
-16	4	0	67.06	3.53
-16	4	0	68.22	3.47
-16	4	0	60.73	2.97
15	-4	0	3.45	0.75
15	-4	0	4.65	0.63
15	-4	0	3.10	0.93

-15	4	0	3.04	0.77
-15	4	0	4.95	1.09
-15	4	0	3.56	0.94
14	-4	0	150.29	5.94
14	-4	0	156.32	6.14
14	-4	0	151.24	6.05
-14	4	0	147.88	6.26
-14	4	0	141.54	5.96
-14	4	0	156.70	6.35
13	-4	0	2.04	0.56
13	-4	0	1.25	0.81
13	-4	0	1.26	0.37
-13	4	0	3.39	0.86
-13	4	0	1.85	0.73
-13	4	0	1.89	0.63
12	-4	0	105.93	4.59
12	-4	0	112.70	4.55
12	-4	0	117.62	4.65
12	-4	0	109.81	4.39
-12	4	0	114.36	4.42
-12	4	0	110.61	4.71
-12	4	0	107.26	4.70
-12	4	0	106.03	4.48
11	-4	0	12.95	1.05
11	-4	0	14.17	1.07
11	-4	0	11.47	0.77
11	-4	0	13.31	1.20
-11	4	0	14.29	0.83
-11	4	0	11.62	1.11
-11	4	0	9.77	1.01
-11	4	0	13.17	1.26
10	-4	0	266.37	10.09
10	-4	0	269.33	10.25
10	-4	0	271.51	10.25
10	-4	0	279.68	10.24
-10	4	0	240.92	10.18
-10	4	0	261.51	10.32
-10	4	0	263.18	10.35
-10	4	0	279.69	10.11
9	-4	0	0.58	0.45
9	-4	0	0.35	0.37
9	-4	0	0.67	0.35
9	-4	0	0.14	0.21
-9	4	0	0.45	0.62

-9	4	0	0.64	0.24
-9	4	0	0.20	0.37
8	-4	0	128.93	5.19
8	-4	0	141.58	5.36
8	-4	0	134.89	5.31
8	-4	0	139.48	5.34
-8	4	0	144.34	5.22
-8	4	0	132.41	5.28
-8	4	0	127.46	5.43
-8	4	0	133.91	5.38
7	-4	0	33.29	1.52
7	-4	0	34.76	1.59
7	-4	0	31.45	1.39
7	-4	0	33.97	1.53
-7	4	0	30.17	1.64
-7	4	0	33.90	1.41
-7	4	0	30.26	1.54
-7	4	0	32.13	1.49
6	-4	0	142.64	5.49
6	-4	0	140.93	5.49
6	-4	0	136.11	5.38
6	-4	0	147.20	5.57
-6	4	0	145.21	5.40
-6	4	0	144.04	5.48
-6	4	0	140.76	5.63
-6	4	0	137.47	5.52
5	-4	0	22.46	1.15
5	-4	0	23.78	1.12
5	-4	0	23.97	1.02
5	-4	0	24.42	1.36
-5	4	0	23.94	1.12
-5	4	0	24.36	1.15
-5	4	0	22.87	1.27
4	-4	0	200.94	8.04
4	-4	0	194.29	7.83
4	-4	0	212.77	7.93
4	-4	0	218.35	7.95
-4	4	0	208.95	7.84
-4	4	0	212.45	7.94
-4	4	0	213.88	7.95
-4	4	0	212.80	8.08
3	-4	0	0.44	0.13
3	-4	0	0.64	0.20
3	-4	0	0.50	0.29

3	-4	0	0.71	0.21
-3	4	0	1.02	0.22
-3	4	0	0.61	0.12
-3	4	0	0.54	0.20
-3	4	0	0.60	0.29
2	-4	0	239.93	8.70
2	-4	0	231.38	8.55
2	-4	0	218.08	8.46
2	-4	0	224.65	8.58
-2	4	0	239.18	8.73
-2	4	0	207.41	8.46
-2	4	0	223.44	8.58
-2	4	0	234.57	8.56
1	-4	0	0.80	0.13
1	-4	0	0.57	0.30
1	-4	0	0.81	0.23
1	-4	0	0.52	0.16
-1	4	0	0.96	0.14
-1	4	0	0.83	0.30
-1	4	0	0.80	0.21
-1	4	0	0.65	0.17
0	-4	0	196.98	7.41
0	-4	0	192.47	7.34
0	-4	0	196.73	7.48
0	-4	0	203.29	7.60
0	4	0	195.79	7.34
0	4	0	192.20	7.48
0	4	0	205.83	7.62
0	4	0	193.92	7.41
-1	-4	0	0.76	0.16
-1	-4	0	0.95	0.34
-1	-4	0	0.73	0.10
-1	-4	0	0.97	0.24
1	4	0	0.47	0.09
1	4	0	0.84	0.23
1	4	0	0.94	0.33
1	4	0	0.64	0.15
-2	-4	0	236.12	8.90
-2	-4	0	232.06	8.66
-2	-4	0	230.20	8.75
-2	-4	0	217.02	8.60
2	4	0	237.90	8.66
2	4	0	236.81	8.88
2	4	0	226.47	8.60

2	4	0	230.11	8.75
-3	-4	0	0.43	0.23
-3	-4	0	0.93	0.23
-3	-4	0	0.61	0.11
-3	-4	0	1.01	0.36
-3	-4	0	0.52	0.16
-3	-4	0	0.67	0.12
3	4	0	0.37	0.15
3	4	0	1.02	0.31
-4	-4	0	206.43	8.17
-4	-4	0	216.06	8.06
-4	-4	0	215.91	7.87
-4	-4	0	211.31	8.03
-4	-4	0	209.09	7.94
-4	-4	0	198.27	8.22
4	4	0	213.38	7.87
4	4	0	213.23	7.93
-5	-4	0	23.20	1.09
-5	-4	0	24.72	1.19
-5	-4	0	22.59	1.20
-5	-4	0	23.89	1.34
-5	-4	0	23.57	1.09
-5	-4	0	24.80	1.01
5	4	0	24.60	1.02
-6	-4	0	138.48	5.66
-6	-4	0	136.91	5.43
-6	-4	0	146.83	5.55
-6	-4	0	137.74	5.43
-6	-4	0	143.86	5.33
-6	-4	0	142.40	5.50
6	4	0	149.54	5.56
6	4	0	131.50	5.34
-7	-4	0	35.39	1.65
-7	-4	0	32.18	1.52
-7	-4	0	36.06	1.60
-7	-4	0	32.17	1.75
-7	-4	0	33.44	1.52
-7	-4	0	33.00	1.39
7	4	0	32.34	1.41
7	4	0	33.25	1.64
-8	-4	0	141.36	5.23
-8	-4	0	141.65	5.40
-8	-4	0	135.13	5.35
-8	-4	0	132.49	5.59

-8	-4	0	130.84	5.36
8	4	0	133.76	5.24
8	4	0	141.78	5.45
-9	-4	0	0.36	0.30
-9	-4	0	0.03	0.29
-9	-4	0	0.67	0.40
-9	-4	0	0.55	0.43
-9	-4	0	-0.55	0.56
-9	-4	0	0.32	0.20
9	4	0	-0.19	0.37
9	4	0	0.55	0.23
-10	-4	0	264.80	10.18
-10	-4	0	268.29	10.41
-10	-4	0	263.99	10.62
-10	-4	0	263.80	10.33
-10	-4	0	279.21	10.40
-10	-4	0	263.45	10.39
10	4	0	275.16	10.22
10	4	0	280.87	10.40
-11	-4	0	13.26	1.14
-11	-4	0	13.15	1.09
-11	-4	0	11.73	1.49
-11	-4	0	13.73	1.11
-11	-4	0	14.42	0.80
-11	-4	0	11.10	0.99
11	4	0	13.75	0.82
11	4	0	13.80	1.10
-12	-4	0	109.96	4.67
-12	-4	0	119.92	4.63
-12	-4	0	105.69	4.65
-12	-4	0	114.67	5.01
-12	-4	0	112.52	4.40
-12	-4	0	104.13	4.58
12	4	0	115.67	4.63
12	4	0	111.98	4.44
-13	-4	0	2.35	0.62
-13	-4	0	2.55	0.41
-13	-4	0	2.27	1.07
-13	-4	0	2.05	0.69
-13	-4	0	2.13	0.71
-13	-4	0	1.43	0.66
13	4	0	3.59	0.66
13	4	0	2.69	0.44
-14	-4	0	146.32	6.24

-14	-4	0	156.59	6.56
-14	-4	0	148.05	6.15
-14	-4	0	144.23	6.18
14	4	0	158.04	6.15
14	4	0	157.20	5.99
-15	-4	0	3.22	1.24
-15	-4	0	3.51	0.89
-15	-4	0	2.73	0.94
-15	-4	0	4.02	1.02
15	4	0	3.27	0.55
15	4	0	2.02	0.75
-16	-4	0	68.13	3.35
-16	-4	0	71.23	3.28
-16	-4	0	63.73	3.30
-16	-4	0	67.27	3.71
16	4	0	67.79	2.96
16	4	0	67.05	3.15
-17	-4	0	5.11	1.44
-17	-4	0	4.08	1.40
-17	-4	0	3.89	1.08
17	4	0	3.81	0.67
-18	-4	0	35.15	3.00
-18	-4	0	38.96	3.21
-18	-4	0	36.11	2.58
18	4	0	39.38	2.05
-19	-4	0	2.93	1.21
19	4	0	4.66	0.85
-20	-4	0	32.28	2.84
20	4	0	37.95	2.13
20	-4	1	12.00	1.34
-20	4	-1	8.20	1.76
19	-4	1	9.72	1.19
-19	4	-1	10.29	1.72
18	-4	1	13.88	1.25
18	-4	1	13.62	1.65
-18	4	-1	11.41	1.65
17	-4	1	38.41	2.28
17	-4	1	38.64	1.98
-17	4	-1	40.08	2.77
16	-4	1	0.67	0.73
16	-4	1	0.17	0.46
-16	4	-1	0.24	0.88
-16	4	-1	1.32	1.18
15	-4	1	33.19	1.96

15	-4	1	34.55	1.75
-15	4	-1	36.11	2.30
-15	4	-1	33.44	2.41
14	-4	1	16.76	1.35
14	-4	1	17.98	1.30
14	-4	1	17.45	1.22
-14	4	-1	16.41	1.62
-14	4	-1	15.88	1.75
13	-4	1	68.87	2.84
13	-4	1	69.01	2.93
13	-4	1	69.17	3.03
-13	4	-1	66.87	3.22
-13	4	-1	60.18	2.75
-13	4	-1	67.53	3.26
12	-4	1	52.85	2.59
12	-4	1	57.36	2.39
12	-4	1	56.81	2.61
12	-4	1	56.75	2.53
-12	4	-1	54.41	2.73
-12	4	-1	53.25	2.35
-12	4	-1	57.54	2.40
-12	4	-1	55.46	2.79
11	-4	1	36.83	1.84
11	-4	1	35.88	1.87
11	-4	1	37.26	1.82
11	-4	1	36.81	1.64
-11	4	-1	34.02	1.63
-11	4	-1	34.47	2.02
-11	4	-1	36.58	1.95
-11	4	-1	36.35	1.64
10	-4	1	15.40	1.03
10	-4	1	11.43	0.98
10	-4	1	15.02	1.04
10	-4	1	13.25	0.79
-10	4	-1	12.50	0.79
-10	4	-1	13.76	0.84
-10	4	-1	12.18	1.21
-10	4	-1	12.21	1.06
9	-4	1	29.97	1.54
9	-4	1	29.35	1.47
9	-4	1	29.80	1.44
9	-4	1	28.75	1.32
-9	4	-1	28.51	1.55
-9	4	-1	26.92	1.34

-9	4	-1	26.46	1.63
-9	4	-1	31.94	1.35
8	-4	1	40.22	1.76
8	-4	1	43.32	1.91
8	-4	1	43.63	1.98
8	-4	1	41.78	1.83
-8	4	-1	39.52	1.94
-8	4	-1	44.47	1.78
-8	4	-1	40.30	2.07
-8	4	-1	42.19	1.79
7	-4	1	37.01	1.72
7	-4	1	34.56	1.52
7	-4	1	37.77	1.66
7	-4	1	34.60	1.59
-7	4	-1	36.79	1.57
-7	4	-1	35.13	1.83
-7	4	-1	35.31	1.70
-7	4	-1	39.51	1.55
6	-4	1	192.96	7.26
6	-4	1	192.87	7.31
6	-4	1	186.40	7.21
6	-4	1	195.79	7.41
-6	4	-1	195.01	7.22
-6	4	-1	190.35	7.35
-6	4	-1	189.63	7.25
-6	4	-1	185.92	7.46
5	-4	1	167.21	6.44
5	-4	1	173.73	6.64
5	-4	1	174.49	6.50
5	-4	1	176.27	6.55
-5	4	-1	164.26	6.68
-5	4	-1	171.91	6.49
-5	4	-1	167.38	6.56
4	-4	1	10.33	0.51
4	-4	1	11.10	0.78
4	-4	1	11.06	0.63
4	-4	1	10.69	0.60
-4	4	-1	9.90	0.79
-4	4	-1	10.91	0.61
-4	4	-1	10.04	0.51
-4	4	-1	9.77	0.63
3	-4	1	84.27	3.30
3	-4	1	87.52	3.38
3	-4	1	87.59	3.40

-3	4	-1	87.73	3.30
-3	4	-1	86.46	3.41
-3	4	-1	84.30	3.39
-3	4	-1	89.88	3.57
2	-4	1	7.64	0.37
2	-4	1	7.66	0.65
2	-4	1	8.07	0.51
2	-4	1	8.34	0.49
-2	4	-1	7.19	0.56
-2	4	-1	7.74	0.37
-2	4	-1	8.73	0.67
-2	4	-1	8.06	0.51
1	-4	1	54.49	2.27
1	-4	1	58.89	2.40
1	-4	1	54.07	2.13
1	-4	1	58.15	2.25
-1	4	-1	58.48	2.42
-1	4	-1	53.74	2.28
-1	4	-1	57.84	2.25
-1	4	-1	54.96	2.14
0	-4	1	7.56	0.34
0	-4	1	7.59	0.52
0	-4	1	7.94	0.65
0	4	-1	7.86	0.34
0	4	-1	7.47	0.53
0	4	-1	7.56	0.45
0	4	-1	7.38	0.66
-1	-4	1	125.94	4.86
-1	-4	1	130.12	4.97
-1	-4	1	135.29	5.15
1	4	-1	135.54	5.16
1	4	-1	124.04	4.87
1	4	-1	126.37	4.96
1	4	-1	137.08	5.07
-2	-4	1	0.25	0.07
-2	-4	1	0.22	0.08
-2	-4	1	-0.07	0.18
-2	-4	1	0.24	0.14
-2	-4	1	0.49	0.30
-2	-4	1	0.60	0.26
2	4	-1	0.30	0.14
2	4	-1	0.32	0.29
-3	-4	1	176.40	6.70
-3	-4	1	186.64	6.91

-3	-4	1	191.47	7.00
-3	-4	1	185.17	6.80
-3	-4	1	175.76	7.15
-3	-4	1	176.53	6.68
3	4	-1	155.16	6.83
3	4	-1	175.98	6.68
3	4	-1	191.16	6.98
-4	-4	1	17.37	0.90
-4	-4	1	17.30	0.83
-4	-4	1	16.58	0.95
-4	-4	1	16.35	0.71
-4	-4	1	17.62	1.05
-4	-4	1	17.54	1.12
4	4	-1	16.77	0.72
4	4	-1	16.86	0.82
-5	-4	1	245.51	9.43
-5	-4	1	244.33	9.44
-5	-4	1	244.76	9.31
-5	-4	1	250.26	9.69
-5	-4	1	248.56	9.56
-5	-4	1	258.15	9.47
-5	-4	1	258.52	9.65
5	4	-1	248.11	9.41
-6	-4	1	5.95	0.94
-6	-4	1	5.72	0.55
-6	-4	1	6.22	0.52
-6	-4	1	6.77	0.53
-6	-4	1	6.71	0.70
-6	-4	1	6.44	0.38
-6	-4	1	5.88	0.80
6	4	-1	7.41	0.82
6	4	-1	5.61	0.39
-7	-4	1	229.50	8.43
-7	-4	1	219.99	8.42
-7	-4	1	224.80	8.44
-7	-4	1	225.38	8.29
-7	-4	1	204.08	8.80
-7	-4	1	221.69	8.71
7	4	-1	219.13	8.30
-8	-4	1	11.37	0.57
-8	-4	1	9.20	1.11
-8	-4	1	8.83	0.75
-8	-4	1	10.17	0.75
-8	-4	1	9.02	1.13

-8	-4	1	9.82	0.74
-8	-4	1	10.97	0.93
8	4	-1	9.57	0.57
8	4	-1	10.56	1.05
-9	-4	1	153.97	6.62
-9	-4	1	166.38	6.41
-9	-4	1	169.78	6.24
-9	-4	1	163.18	6.73
-9	-4	1	169.66	6.38
-9	-4	1	158.26	6.51
-9	-4	1	161.38	6.42
9	4	-1	161.13	6.25
9	4	-1	170.94	6.63
-10	-4	1	12.11	0.70
-10	-4	1	11.50	0.94
-10	-4	1	13.63	0.92
-10	-4	1	10.47	1.28
-10	-4	1	11.68	1.12
-10	-4	1	11.34	1.40
-10	-4	1	9.82	0.87
10	4	-1	9.94	0.68
10	4	-1	11.77	0.93
-11	-4	1	272.98	11.48
-11	-4	1	299.05	11.79
-11	-4	1	298.90	11.47
-11	-4	1	303.39	11.38
-11	-4	1	308.79	11.43
-11	-4	1	311.71	11.23
-11	-4	1	278.64	11.60
11	4	-1	312.06	11.40
11	4	-1	292.60	11.24
-12	-4	1	0.11	0.48
-12	-4	1	-0.12	0.64
-12	-4	1	-0.35	0.78
-12	-4	1	0.26	0.92
-12	-4	1	0.92	0.51
-12	-4	1	-0.04	0.30
12	4	-1	-0.18	0.27
12	4	-1	-0.01	0.50
-13	-4	1	100.53	3.91
-13	-4	1	92.85	4.36
-13	-4	1	98.14	4.21
-13	-4	1	99.29	4.26
-13	-4	1	99.44	4.06

-13	-4	1	99.31	4.60
13	4	-1	90.13	3.90
-14	-4	1	1.50	1.12
-14	-4	1	0.22	0.60
-14	-4	1	2.13	0.99
-14	-4	1	0.66	0.63
-14	-4	1	0.90	0.83
14	4	-1	1.46	0.43
-15	-4	1	61.16	3.51
-15	-4	1	60.55	3.08
-15	-4	1	63.08	2.85
-15	-4	1	60.47	3.25
15	4	-1	62.11	2.69
-16	-4	1	12.30	2.24
-16	-4	1	8.90	1.46
-16	-4	1	7.71	1.35
-16	-4	1	8.28	1.35
-16	-4	1	10.60	1.69
16	4	-1	8.73	0.94
-17	-4	1	74.03	3.89
-17	-4	1	78.10	3.83
-17	-4	1	78.91	4.22
17	4	-1	80.45	3.40
-18	-4	1	-1.76	0.95
-18	-4	1	-1.17	1.49
18	4	-1	-0.17	0.58
-19	-4	1	57.78	3.28
19	4	-1	53.75	2.71
-20	-4	1	0.05	1.26
20	4	-1	1.51	0.75
-21	-4	1	33.26	2.90
21	4	-1	31.62	2.07
20	-4	2	1.48	0.70
-20	4	-2	-1.12	1.48
19	-4	2	2.97	1.57
19	-4	2	2.27	0.74
-19	4	-2	1.42	1.42
18	-4	2	2.10	0.71
18	-4	2	3.13	1.01
-18	4	-2	1.76	1.25
17	-4	2	2.20	0.62
17	-4	2	3.24	1.01
-17	4	-2	3.16	1.26
16	-4	2	-0.87	0.44

16	-4	2	-0.08	0.74
-16	4	-2	-1.11	0.94
-16	4	-2	-2.72	1.14
15	-4	2	0.74	0.71
15	-4	2	1.25	0.48
-15	4	-2	0.66	0.86
-15	4	-2	0.58	1.06
14	-4	2	-0.71	0.61
-14	4	-2	0.96	0.75
-14	4	-2	2.04	1.00
13	-4	2	48.07	2.40
13	-4	2	46.79	2.13
-13	4	-2	46.46	2.65
-13	4	-2	49.24	2.56
12	-4	2	1.33	0.54
12	-4	2	1.53	0.39
12	-4	2	1.45	0.60
-12	4	-2	1.36	0.41
-12	4	-2	0.08	0.53
-12	4	-2	-0.07	0.70
11	-4	2	55.42	2.30
11	-4	2	51.87	2.54
11	-4	2	53.81	2.47
-11	4	-2	52.41	2.71
-11	4	-2	51.60	2.58
-11	4	-2	56.82	2.31
10	-4	2	9.98	1.02
10	-4	2	9.75	0.69
10	-4	2	11.65	0.82
10	-4	2	10.09	0.92
-10	4	-2	10.17	1.21
-10	4	-2	10.58	1.02
-10	4	-2	11.48	0.72
9	-4	2	8.14	0.78
9	-4	2	7.66	0.68
9	-4	2	7.48	0.57
9	-4	2	7.20	0.86
-9	4	-2	7.61	0.84
-9	4	-2	8.99	1.08
-9	4	-2	8.30	0.59
-9	4	-2	8.07	0.55
8	-4	2	3.96	0.42
8	-4	2	4.85	0.52
8	-4	2	4.13	0.61

8	-4	2	5.94	0.63
-8	4	-2	4.63	0.44
-8	4	-2	4.91	0.45
-8	4	-2	6.13	0.87
-8	4	-2	4.26	0.66
7	-4	2	0.50	0.24
7	-4	2	0.92	0.31
7	-4	2	1.30	0.41
7	-4	2	0.86	0.21
-7	4	-2	0.99	0.34
-7	4	-2	1.17	0.25
-7	4	-2	0.68	0.50
-7	4	-2	0.28	0.18
6	-4	2	27.04	1.36
6	-4	2	25.84	1.13
6	-4	2	25.76	1.23
6	-4	2	24.18	1.11
-6	4	-2	24.30	1.25
-6	4	-2	26.45	1.12
-6	4	-2	24.89	1.12
5	-4	2	106.13	4.08
5	-4	2	106.58	4.06
5	-4	2	107.40	4.17
5	-4	2	110.64	4.30
-5	4	-2	102.48	4.33
-5	4	-2	104.34	4.08
-5	4	-2	105.42	4.19
4	-4	2	130.57	5.14
4	-4	2	135.22	5.21
4	-4	2	133.02	5.10
4	-4	2	140.85	5.35
-4	4	-2	133.78	5.16
-4	4	-2	137.98	5.11
-4	4	-2	133.22	5.23
-4	4	-2	136.53	5.38
3	-4	2	89.81	3.48
3	-4	2	91.44	3.61
3	-4	2	86.66	3.36
3	-4	2	89.76	3.44
-3	4	-2	87.42	3.36
-3	4	-2	86.37	3.48
-3	4	-2	88.72	3.64
-3	4	-2	88.16	3.46
2	-4	2	73.69	2.85

2	-4	2	76.28	2.97
2	-4	2	72.47	3.24
2	-4	2	74.72	2.96
-2	4	-2	74.31	2.98
-2	4	-2	74.18	3.00
-2	4	-2	76.67	2.85
-2	4	-2	79.26	3.15
1	-4	2	52.34	2.45
1	-4	2	55.19	2.08
1	-4	2	51.15	2.23
1	-4	2	56.17	2.21
-1	4	-2	58.65	2.39
-1	4	-2	56.53	2.08
-1	4	-2	58.26	2.27
-1	4	-2	50.28	2.27
0	-4	2	16.27	0.85
0	-4	2	15.70	0.78
0	-4	2	15.60	0.94
0	-4	2	15.96	0.93
0	-4	2	16.02	0.63
0	4	-2	16.04	0.63
0	4	-2	17.34	0.95
-1	-4	2	163.59	6.22
-1	-4	2	152.81	5.98
-1	-4	2	161.76	6.30
-1	-4	2	170.95	6.14
-1	-4	2	167.73	6.28
1	4	-2	154.25	6.12
1	4	-2	167.90	6.29
1	4	-2	153.22	5.99
-2	-4	2	43.75	2.24
-2	-4	2	44.82	1.75
-2	-4	2	47.19	2.04
-2	-4	2	44.26	1.74
-2	-4	2	46.20	1.89
-2	-4	2	47.87	2.01
2	4	-2	49.76	2.06
2	4	-2	47.12	2.07
-3	-4	2	41.88	2.05
-3	-4	2	45.62	1.80
-3	-4	2	43.00	1.65
-3	-4	2	44.06	1.95
3	4	-2	42.70	1.65
3	4	-2	41.96	1.99

3	4	-2	42.74	1.78
3	4	-2	43.88	1.94
-4	-4	2	107.99	4.23
-4	-4	2	111.90	4.37
-4	-4	2	116.77	4.52
-4	-4	2	114.52	4.65
4	4	-2	117.11	4.51
4	4	-2	111.38	4.35
4	4	-2	118.49	4.62
4	4	-2	105.39	4.23
-5	-4	2	51.40	2.44
-5	-4	2	52.96	2.31
-5	-4	2	51.64	2.01
-5	-4	2	51.78	2.16
5	4	-2	54.09	2.41
5	4	-2	50.24	2.12
5	4	-2	51.56	2.01
-6	-4	2	44.55	1.85
-6	-4	2	42.88	2.02
-6	-4	2	43.03	1.71
-6	-4	2	43.00	2.18
-6	-4	2	41.43	1.85
-6	-4	2	43.26	1.86
6	4	-2	43.28	1.72
-7	-4	2	26.15	1.17
-7	-4	2	24.54	1.44
-7	-4	2	24.99	1.69
-7	-4	2	27.40	1.29
-7	-4	2	25.25	1.27
-7	-4	2	26.47	1.24
7	4	-2	26.59	1.12
7	4	-2	28.23	1.59
-8	-4	2	11.77	0.74
-8	-4	2	8.90	1.22
-8	-4	2	10.64	0.79
-8	-4	2	10.89	1.21
-8	-4	2	10.63	0.58
-8	-4	2	10.94	0.78
8	4	-2	10.64	1.12
8	4	-2	10.67	0.61
-9	-4	2	19.38	1.05
-9	-4	2	20.01	1.14
-9	-4	2	18.83	1.58
-9	-4	2	20.23	0.92

-9	-4	2	20.72	1.13
-9	-4	2	15.22	1.54
9	4	-2	18.31	0.92
9	4	-2	18.27	1.46
-10	-4	2	56.11	2.74
-10	-4	2	57.53	2.43
-10	-4	2	56.20	3.00
-10	-4	2	55.78	2.56
-10	-4	2	63.58	2.35
-10	-4	2	56.18	2.53
10	4	-2	57.28	2.85
10	4	-2	54.28	2.33
-11	-4	2	1.75	0.45
-11	-4	2	0.24	0.87
-11	-4	2	-0.16	0.43
-11	-4	2	1.11	0.78
-11	-4	2	0.76	0.29
-11	-4	2	0.49	0.39
11	4	-2	1.16	0.31
11	4	-2	0.98	0.76
-12	-4	2	25.72	1.60
-12	-4	2	26.79	1.63
-12	-4	2	24.84	2.19
-12	-4	2	29.17	1.32
-12	-4	2	27.30	1.45
-12	-4	2	25.82	1.80
12	4	-2	28.67	2.04
12	4	-2	27.29	1.31
-13	-4	2	30.77	1.45
-13	-4	2	27.76	1.92
-13	-4	2	30.56	1.60
-13	-4	2	30.77	2.43
-13	-4	2	27.90	1.82
13	4	-2	33.83	2.27
13	4	-2	32.04	1.50
-14	-4	2	34.10	1.97
-14	-4	2	26.88	2.44
-14	-4	2	26.93	1.54
-14	-4	2	27.44	2.03
-14	-4	2	27.17	1.79
-14	-4	2	27.38	1.93
14	4	-2	27.47	1.43
-15	-4	2	11.42	1.18
-15	-4	2	8.89	1.29

-15	-4	2	9.38	1.64
-15	-4	2	11.01	1.33
-15	-4	2	10.55	1.56
15	4	-2	11.51	0.92
-16	-4	2	41.29	2.49
-16	-4	2	44.53	2.51
-16	-4	2	44.05	2.69
-16	-4	2	42.94	2.37
-16	-4	2	43.26	3.14
16	4	-2	40.81	2.04
-17	-4	2	3.75	1.61
-17	-4	2	5.24	1.61
-17	-4	2	4.75	1.13
-17	-4	2	3.56	1.00
17	4	-2	3.14	0.64
-18	-4	2	36.67	3.30
-18	-4	2	40.94	2.67
18	4	-2	41.94	2.10
-19	-4	2	4.74	2.29
-19	-4	2	0.81	1.07
19	4	-2	1.39	0.68
-20	-4	2	24.71	2.50
20	4	-2	26.53	1.79
-21	-4	2	8.46	1.62
21	4	-2	10.08	1.45
20	-4	3	4.74	0.91
-20	4	-3	7.93	1.74
19	-4	3	5.42	0.84
19	-4	3	3.81	1.35
-19	4	-3	4.05	1.46
18	-4	3	2.30	1.16
18	-4	3	2.94	0.73
-18	4	-3	3.49	1.36
17	-4	3	13.53	1.75
17	-4	3	13.92	1.23
-17	4	-3	17.34	2.15
16	-4	3	0.98	0.52
16	-4	3	0.69	0.85
-16	4	-3	-1.08	0.99
-16	4	-3	3.46	1.30
15	-4	3	5.83	1.00
15	-4	3	6.25	0.87
-15	4	-3	6.16	1.10
-15	4	-3	7.85	1.39

14	-4	3	31.03	1.62
14	-4	3	33.40	2.00
-14	4	-3	32.16	2.11
-14	4	-3	29.85	2.37
13	-4	3	1.02	0.64
13	-4	3	0.23	0.35
-13	4	-3	1.02	0.72
-13	4	-3	-0.09	0.93
12	-4	3	0.27	0.53
12	-4	3	0.56	0.37
12	-4	3	0.63	0.61
-12	4	-3	1.14	0.83
-12	4	-3	1.38	0.64
11	-4	3	6.62	0.64
11	-4	3	8.40	1.06
11	-4	3	6.19	0.87
-11	4	-3	7.29	1.03
-11	4	-3	7.32	1.04
-11	4	-3	7.68	0.68
10	-4	3	0.59	0.28
10	-4	3	0.93	0.56
10	-4	3	0.32	0.41
-10	4	-3	0.11	0.68
-10	4	-3	1.53	0.33
-10	4	-3	1.26	0.53
9	-4	3	11.36	0.91
9	-4	3	11.81	1.07
9	-4	3	11.57	0.73
-9	4	-3	10.50	0.96
-9	4	-3	13.18	0.74
-9	4	-3	11.94	1.21
8	-4	3	34.93	1.55
8	-4	3	34.43	1.83
8	-4	3	36.38	1.68
-8	4	-3	38.49	1.56
-8	4	-3	36.25	1.74
-8	4	-3	33.09	1.92
7	-4	3	12.29	0.97
7	-4	3	12.13	0.63
7	-4	3	12.11	0.81
7	-4	3	12.82	0.68
-7	4	-3	11.20	1.07
-7	4	-3	12.78	0.68
-7	4	-3	11.70	0.86

6	-4	3	49.14	2.04
6	-4	3	49.96	2.01
6	-4	3	50.35	2.33
6	-4	3	51.79	2.16
-6	4	-3	46.09	2.35
-6	4	-3	56.12	2.05
-6	4	-3	49.43	2.19
5	-4	3	10.49	0.67
5	-4	3	10.56	0.56
5	-4	3	11.20	0.85
5	-4	3	10.56	0.69
5	-4	3	10.35	0.58
-5	4	-3	11.69	0.93
-5	4	-3	10.69	0.57
-5	4	-3	10.49	0.72
4	-4	3	10.76	0.70
4	-4	3	11.34	0.59
4	-4	3	8.25	0.89
4	-4	3	10.55	0.54
4	-4	3	10.82	0.80
-4	4	-3	11.87	0.54
-4	4	-3	10.42	0.67
-4	4	-3	9.00	1.00
3	-4	3	18.99	0.82
3	-4	3	19.29	0.95
3	-4	3	19.68	0.91
3	-4	3	19.25	1.11
3	-4	3	20.18	1.02
-3	4	-3	18.92	0.96
-3	4	-3	19.63	1.14
-3	4	-3	19.50	0.82
2	-4	3	20.57	0.98
2	-4	3	20.02	1.07
2	-4	3	20.68	0.97
2	-4	3	20.99	1.13
2	-4	3	20.05	0.84
-2	4	-3	21.22	0.84
-2	4	-3	20.54	1.16
-2	4	-3	20.37	0.98
1	-4	3	21.46	1.01
1	-4	3	18.62	1.09
1	-4	3	20.44	0.95
1	-4	3	19.66	0.81
1	-4	3	21.77	1.13

-1	4	-3	20.85	0.97
-1	4	-3	20.31	0.80
-1	4	-3	19.84	1.11
0	-4	3	7.91	0.33
0	-4	3	7.59	0.70
0	-4	3	8.50	0.51
0	4	-3	7.63	0.64
0	4	-3	7.75	0.58
0	4	-3	8.04	0.33
0	4	-3	8.34	0.68
-1	-4	3	3.03	0.48
-1	-4	3	2.35	0.53
-1	-4	3	2.70	0.33
1	4	-3	3.35	0.53
1	4	-3	2.75	0.43
1	4	-3	2.76	0.33
-2	-4	3	88.88	3.77
-2	-4	3	87.89	3.62
-2	-4	3	89.36	3.51
2	4	-3	92.53	3.64
2	4	-3	88.78	3.71
2	4	-3	91.02	3.51
-3	-4	3	6.05	0.27
-3	-4	3	5.60	0.46
-3	-4	3	5.96	0.58
-3	-4	3	5.97	0.76
3	4	-3	5.88	0.71
3	4	-3	6.09	0.27
3	4	-3	5.58	0.59
3	4	-3	5.69	0.44
-4	-4	3	6.47	0.62
-4	-4	3	4.77	0.53
-4	-4	3	6.45	0.30
-4	-4	3	6.78	0.82
4	4	-3	6.00	0.44
4	4	-3	5.47	0.29
4	4	-3	5.45	0.61
4	4	-3	5.58	0.76
-5	-4	3	6.46	0.34
-5	-4	3	6.30	0.66
-5	-4	3	5.91	0.92
5	4	-3	7.26	0.67
5	4	-3	6.38	0.49
5	4	-3	6.60	0.95

5	4	-3	6.37	0.35
-6	-4	3	18.03	0.95
-6	-4	3	16.10	1.36
-6	-4	3	17.45	1.07
-6	-4	3	17.86	0.92
-6	-4	3	18.31	0.78
-6	-4	3	18.28	0.94
6	4	-3	17.65	1.30
6	4	-3	17.19	0.83
-7	-4	3	0.62	0.28
-7	-4	3	0.42	0.27
-7	-4	3	0.33	0.36
-7	-4	3	0.06	0.23
-7	-4	3	0.12	0.53
-7	-4	3	0.25	0.15
7	4	-3	-0.11	0.52
7	4	-3	0.32	0.15
-8	-4	3	7.84	1.19
-8	-4	3	9.36	0.85
-8	-4	3	8.70	0.73
-8	-4	3	9.59	0.53
-8	-4	3	8.94	0.64
-8	-4	3	8.28	0.70
8	4	-3	9.48	0.54
8	4	-3	8.17	1.13
-9	-4	3	10.08	0.68
-9	-4	3	8.07	0.92
-9	-4	3	8.51	0.79
-9	-4	3	10.04	0.57
-9	-4	3	8.39	0.79
-9	-4	3	9.15	1.35
9	4	-3	8.73	0.56
9	4	-3	7.33	1.23
-10	-4	3	43.03	2.20
-10	-4	3	41.14	2.69
-10	-4	3	49.29	1.95
-10	-4	3	46.93	2.18
-10	-4	3	47.87	2.01
-10	-4	3	48.21	2.17
10	4	-3	48.25	2.57
10	4	-3	47.13	1.95
-11	-4	3	19.70	1.10
-11	-4	3	18.49	1.91
-11	-4	3	22.67	1.04

-11	-4	3	19.48	1.30
-11	-4	3	18.97	1.51
-11	-4	3	21.17	1.31
11	4	-3	21.22	1.78
11	4	-3	19.93	1.03
-12	-4	3	1.35	0.50
-12	-4	3	0.90	0.52
-12	-4	3	2.16	0.44
-12	-4	3	0.44	0.96
-12	-4	3	1.86	0.37
12	4	-3	1.51	0.34
12	4	-3	0.26	0.90
-13	-4	3	3.12	0.73
-13	-4	3	4.18	0.60
-13	-4	3	3.31	0.53
-13	-4	3	2.91	1.23
13	4	-3	3.84	1.15
13	4	-3	3.25	0.46
-14	-4	3	37.51	2.17
-14	-4	3	35.01	2.83
-14	-4	3	39.54	1.79
-14	-4	3	36.19	1.76
14	4	-3	37.63	1.77
14	4	-3	42.30	2.72
-15	-4	3	10.18	1.35
-15	-4	3	6.77	1.57
-15	-4	3	10.39	0.94
15	4	-3	9.83	0.86
15	4	-3	8.65	1.45
-16	-4	3	8.54	1.44
-16	-4	3	8.39	1.43
-16	-4	3	8.62	0.98
-16	-4	3	8.01	1.72
-17	-4	3	11.76	1.93
-17	-4	3	18.54	2.22
-17	-4	3	11.28	1.63
17	4	-3	10.58	1.08
-18	-4	3	-0.37	1.72
-18	-4	3	1.67	1.01
18	4	-3	1.81	0.61
-19	-4	3	11.45	2.02
-19	-4	3	6.74	1.35
19	4	-3	10.87	1.22
-20	-4	3	3.89	1.33

20	4	-3	3.28	0.75
-21	-4	3	3.70	1.54
21	4	-3	3.79	0.89
20	-4	4	26.86	2.00
-20	4	-4	28.15	3.10
19	-4	4	4.90	1.38
19	-4	4	2.87	0.76
-19	4	-4	4.95	1.54
18	-4	4	20.90	1.58
18	-4	4	25.24	2.25
-18	4	-4	24.51	2.58
17	-4	4	3.15	1.16
17	-4	4	1.75	0.65
-17	4	-4	3.49	1.21
16	-4	4	10.97	1.66
16	-4	4	10.45	1.10
-16	4	-4	7.83	1.69
-16	4	-4	11.37	1.93
15	-4	4	2.70	0.58
15	-4	4	3.26	0.92
-15	4	-4	5.27	1.44
-15	4	-4	2.80	1.00
14	-4	4	29.09	2.00
14	-4	4	30.00	1.60
-14	4	-4	29.73	2.06
-14	4	-4	33.21	2.48
13	-4	4	11.33	0.91
13	-4	4	12.59	1.40
-13	4	-4	14.13	1.47
-13	4	-4	11.58	1.82
12	-4	4	46.29	2.29
12	-4	4	48.49	2.13
12	-4	4	44.68	2.44
-12	4	-4	47.10	2.69
-12	4	-4	48.73	2.46
11	-4	4	18.67	1.23
11	-4	4	16.96	1.42
11	-4	4	17.07	1.03
-11	4	-4	17.42	1.35
-11	4	-4	19.45	1.04
-11	4	-4	15.57	1.63
10	-4	4	56.13	2.77
10	-4	4	56.77	2.43
10	-4	4	58.57	2.58

-10	4	-4	56.42	2.92
-10	4	-4	58.02	2.68
-10	4	-4	60.28	2.43
9	-4	4	0.78	0.28
9	-4	4	0.39	0.50
9	-4	4	1.25	0.43
-9	4	-4	1.48	0.46
-9	4	-4	1.37	0.69
-9	4	-4	1.07	0.29
8	-4	4	65.94	2.75
8	-4	4	63.59	2.95
8	-4	4	63.19	2.62
-8	4	-4	68.04	2.63
-8	4	-4	64.67	3.02
-8	4	-4	62.18	2.80
7	-4	4	6.37	0.65
7	-4	4	6.69	0.86
7	-4	4	6.30	0.50
-7	4	-4	5.96	0.68
-7	4	-4	7.64	0.52
-7	4	-4	5.97	0.93
6	-4	4	49.98	2.07
6	-4	4	47.95	2.25
6	-4	4	46.16	1.94
6	-4	4	46.25	1.98
6	-4	4	50.20	1.89
-6	4	-4	50.19	1.95
-6	4	-4	44.40	2.08
-6	4	-4	44.89	2.31
5	-4	4	86.73	3.50
5	-4	4	87.14	3.71
5	-4	4	88.11	3.40
5	-4	4	87.92	3.40
5	-4	4	91.17	3.52
-5	4	-4	81.97	3.73
-5	4	-4	94.65	3.41
-5	4	-4	85.14	3.54
4	-4	4	9.46	0.56
4	-4	4	8.83	0.63
4	-4	4	8.38	0.70
4	-4	4	9.64	0.84
4	-4	4	9.05	0.49
-4	4	-4	9.05	0.49
-4	4	-4	10.78	0.89

-4	4	-4	8.62	0.64
3	-4	4	3.88	0.43
3	-4	4	3.16	0.41
3	-4	4	3.55	0.58
3	-4	4	3.65	0.57
-3	4	-4	3.60	0.65
-3	4	-4	3.46	0.42
-3	4	-4	3.92	0.27
2	-4	4	48.72	2.03
2	-4	4	48.83	2.18
2	-4	4	48.42	2.19
2	-4	4	51.48	2.06
2	-4	4	48.01	1.89
-2	4	-4	46.39	2.08
-2	4	-4	50.33	2.23
-2	4	-4	47.19	2.03
-2	4	-4	50.14	1.89
1	-4	4	1.80	0.16
1	-4	4	1.76	0.38
1	-4	4	1.93	0.43
1	-4	4	1.78	0.28
-1	4	-4	1.74	0.28
-1	4	-4	2.21	0.39
-1	4	-4	1.88	0.43
-1	4	-4	1.99	0.16
0	-4	4	44.21	1.76
0	-4	4	47.49	1.94
0	-4	4	47.45	2.09
0	-4	4	47.59	2.23
0	4	-4	46.91	1.94
0	4	-4	46.49	1.76
0	4	-4	46.45	2.11
0	4	-4	48.51	2.10
-1	-4	4	0.55	0.20
-1	-4	4	0.90	0.35
-1	-4	4	0.32	0.41
1	4	-4	0.64	0.22
1	4	-4	0.79	0.30
1	4	-4	0.60	0.33
-2	-4	4	2.36	0.35
-2	-4	4	2.37	0.50
2	4	-4	3.11	0.35
2	4	-4	2.16	0.47
2	4	-4	2.99	0.52

-3	-4	4	75.29	2.82
-3	-4	4	78.65	3.03
-3	-4	4	75.64	3.37
-3	-4	4	78.16	3.12
3	4	-4	77.70	3.38
3	4	-4	77.78	3.13
3	4	-4	76.20	3.00
3	4	-4	66.71	2.82
-4	-4	4	76.87	3.52
-4	-4	4	78.59	3.13
-4	-4	4	79.35	3.21
-4	-4	4	77.89	2.94
4	4	-4	81.12	2.95
4	4	-4	78.05	3.48
4	4	-4	75.60	3.11
4	4	-4	78.95	3.23
-5	-4	4	6.42	0.95
-5	-4	4	4.14	0.25
-5	-4	4	4.09	0.47
-5	-4	4	3.05	0.55
5	4	-4	3.63	0.70
5	4	-4	3.52	0.42
5	4	-4	3.92	0.25
5	4	-4	4.02	0.59
-6	-4	4	104.48	3.89
-6	-4	4	96.21	3.86
-6	-4	4	93.20	3.88
-6	-4	4	98.85	4.33
-6	-4	4	99.42	3.68
6	4	-4	86.70	3.71
6	4	-4	97.33	4.26
6	4	-4	99.86	3.92
-7	-4	4	14.13	0.95
-7	-4	4	16.37	0.71
-7	-4	4	14.09	0.89
-7	-4	4	15.84	0.93
-7	-4	4	13.42	1.38
-7	-4	4	14.77	0.82
7	4	-4	15.26	0.71
7	4	-4	16.39	1.40
-8	-4	4	0.82	0.73
-8	-4	4	1.28	0.21
-8	-4	4	0.85	0.31
-8	-4	4	1.39	0.31

-8	-4	4	1.16	0.38
-8	-4	4	0.90	0.40
8	4	-4	1.12	0.20
8	4	-4	1.43	0.68
-9	-4	4	5.42	1.08
-9	-4	4	7.46	0.57
-9	-4	4	7.45	0.49
-9	-4	4	6.94	0.74
-9	-4	4	6.96	0.77
-9	-4	4	6.84	0.73
9	4	-4	5.28	0.96
9	4	-4	5.68	0.46
-10	-4	4	-0.51	0.81
-10	-4	4	0.79	0.45
-10	-4	4	0.93	0.30
-10	-4	4	1.09	0.41
-10	-4	4	1.09	0.25
-10	-4	4	0.69	0.41
10	4	-4	0.49	0.23
10	4	-4	1.87	0.82
-11	-4	4	2.71	0.55
-11	-4	4	2.01	0.52
-11	-4	4	2.29	0.36
-11	-4	4	2.44	1.01
-11	-4	4	3.01	0.54
-11	-4	4	2.66	0.40
11	4	-4	3.19	1.01
11	4	-4	2.64	0.35
-12	-4	4	0.90	0.31
-12	-4	4	-0.53	0.45
-12	-4	4	0.77	0.48
-12	-4	4	0.19	0.51
-12	-4	4	1.16	0.36
-12	-4	4	-1.03	0.95
12	4	-4	1.08	1.01
12	4	-4	-0.07	0.25
-13	-4	4	15.08	1.29
-13	-4	4	14.34	0.87
-13	-4	4	14.59	2.20
-13	-4	4	15.91	0.95
-13	-4	4	16.08	1.09
13	4	-4	14.04	2.00
13	4	-4	15.06	0.92
-14	-4	4	-0.06	0.38

-14	-4	4	0.02	0.59
-14	-4	4	0.39	1.24
-14	-4	4	1.04	0.53
14	4	-4	0.27	1.19
14	4	-4	1.00	0.38
-15	-4	4	13.15	2.37
-15	-4	4	12.08	1.42
15	4	-4	13.16	1.72
15	4	-4	12.49	0.95
-16	-4	4	0.68	1.46
-16	-4	4	0.41	0.81
16	4	-4	3.63	1.51
16	4	-4	-0.38	0.44
-17	-4	4	5.07	1.05
-17	-4	4	3.21	1.71
17	4	-4	5.01	0.69
17	4	-4	4.88	1.65
-18	-4	4	2.75	1.85
-18	-4	4	1.25	1.03
18	4	-4	1.84	0.59
-19	-4	4	1.78	1.08
-19	-4	4	9.45	2.04
19	4	-4	4.51	0.74
-20	-4	4	0.20	1.15
20	4	-4	1.96	0.75
19	-4	5	25.56	1.85
19	-4	5	30.01	2.66
-19	4	-5	28.44	2.91
18	-4	5	-0.33	0.61
-18	4	-5	0.33	1.21
17	-4	5	69.88	3.23
17	-4	5	71.41	3.68
-17	4	-5	69.34	3.86
16	-4	5	0.64	0.54
16	-4	5	1.05	0.99
-16	4	-5	1.83	1.13
15	-4	5	84.49	3.54
15	-4	5	79.52	3.90
-15	4	-5	80.80	4.00
14	-4	5	10.87	1.51
14	-4	5	12.23	1.06
-14	4	-5	11.01	1.57
-14	4	-5	12.33	1.67
13	-4	5	117.45	4.97

13	-4	5	126.78	5.36
-13	4	-5	122.32	5.30
-13	4	-5	128.36	5.61
12	-4	5	-0.25	0.37
12	-4	5	0.92	0.72
12	-4	5	0.06	0.55
-12	4	-5	0.05	0.59
-12	4	-5	-0.78	0.93
11	-4	5	144.81	5.72
11	-4	5	141.69	6.08
11	-4	5	142.54	5.83
-11	4	-5	145.98	5.99
-11	4	-5	144.28	6.21
-11	4	-5	155.31	5.71
10	-4	5	1.73	0.38
10	-4	5	1.58	0.52
10	-4	5	2.18	0.68
-10	4	-5	2.18	0.40
-10	4	-5	2.06	0.55
-10	4	-5	1.87	0.79
9	-4	5	133.88	5.78
9	-4	5	139.86	5.45
9	-4	5	141.89	5.55
-9	4	-5	137.01	5.88
-9	4	-5	146.42	5.44
-9	4	-5	140.31	5.64
8	-4	5	14.28	0.82
8	-4	5	14.25	0.96
8	-4	5	13.54	1.22
-8	4	-5	13.52	1.01
-8	4	-5	15.19	1.38
-8	4	-5	15.24	0.81
7	-4	5	117.42	4.70
7	-4	5	120.11	5.06
7	-4	5	122.50	4.82
-7	4	-5	118.86	5.12
-7	4	-5	120.86	4.87
-7	4	-5	129.34	4.71
6	-4	5	1.43	0.24
6	-4	5	1.91	0.52
6	-4	5	1.98	0.26
6	-4	5	1.96	0.36
6	-4	5	1.92	0.41
-6	4	-5	2.18	0.67

-6	4	-5	1.38	0.36
-6	4	-5	1.74	0.25
5	-4	5	117.72	4.45
5	-4	5	112.86	4.45
5	-4	5	116.08	4.59
5	-4	5	114.14	4.79
5	-4	5	116.31	4.56
-5	4	-5	116.99	4.59
-5	4	-5	111.58	4.81
-5	4	-5	121.81	4.44
4	-4	5	36.00	1.47
4	-4	5	35.94	1.71
4	-4	5	33.51	1.80
4	-4	5	36.25	1.54
4	-4	5	37.45	1.61
-4	4	-5	34.59	1.61
-4	4	-5	36.46	1.46
-4	4	-5	34.16	1.84
3	-4	5	34.44	1.41
3	-4	5	33.66	1.75
3	-4	5	34.89	1.72
-3	4	-5	33.98	1.59
-3	4	-5	34.89	1.55
-3	4	-5	36.17	1.40
-3	4	-5	34.95	1.78
2	-4	5	26.89	1.43
2	-4	5	25.62	1.66
2	-4	5	26.89	1.26
2	-4	5	26.32	1.10
-2	4	-5	28.50	1.10
-2	4	-5	29.09	1.49
-2	4	-5	27.18	1.37
-2	4	-5	26.59	1.25
1	-4	5	3.03	0.48
1	-4	5	2.51	0.39
1	-4	5	2.88	0.21
1	-4	5	3.06	0.60
-1	4	-5	3.17	0.40
-1	4	-5	2.97	0.20
-1	4	-5	2.81	0.48
0	-4	5	38.60	1.97
0	-4	5	38.89	1.79
0	-4	5	40.74	1.64
0	-4	5	36.58	1.44

0	4	-5	36.18	1.43
0	4	-5	35.49	1.61
0	4	-5	39.10	1.81
0	4	-5	36.79	1.85
-1	-4	5	143.46	5.67
-1	-4	5	140.52	5.86
-1	-4	5	148.25	5.55
1	4	-5	141.02	5.52
1	4	-5	145.47	5.82
1	4	-5	138.65	5.33
1	4	-5	149.96	5.69
-2	-4	5	4.31	0.79
-2	-4	5	3.40	0.61
-2	-4	5	3.96	0.44
2	4	-5	3.92	0.40
2	4	-5	5.34	0.61
-3	-4	5	118.91	4.29
-3	-4	5	101.12	4.71
-3	-4	5	118.00	4.94
-3	-4	5	124.34	4.55
3	4	-5	103.82	4.29
3	4	-5	122.51	4.63
3	4	-5	119.83	4.87
3	4	-5	116.59	4.49
-4	-4	5	0.70	0.24
-4	-4	5	0.34	0.33
-4	-4	5	0.31	0.08
-4	-4	5	-0.19	0.50
4	4	-5	0.43	0.32
4	4	-5	0.31	0.08
4	4	-5	0.51	0.22
4	4	-5	-0.08	0.46
-5	-4	5	27.60	1.80
-5	-4	5	27.17	1.30
-5	-4	5	27.10	1.35
-5	-4	5	28.16	1.09
5	4	-5	26.84	1.27
5	4	-5	26.59	1.08
5	4	-5	27.06	1.38
5	4	-5	28.52	1.73
-6	-4	5	3.51	0.28
-6	-4	5	3.24	0.79
-6	-4	5	5.05	0.56
-6	-4	5	3.02	0.55

-6	-4	5	3.60	0.50
6	4	-5	3.97	0.77
6	4	-5	3.71	0.63
6	4	-5	4.21	0.29
6	4	-5	4.47	0.49
-7	-4	5	96.22	3.91
-7	-4	5	99.26	4.48
-7	-4	5	96.64	3.89
-7	-4	5	91.92	3.83
-7	-4	5	98.10	3.69
7	4	-5	96.18	3.89
7	4	-5	94.79	3.68
7	4	-5	99.76	4.37
-8	-4	5	51.38	2.09
-8	-4	5	51.73	2.26
-8	-4	5	49.30	2.08
-8	-4	5	48.36	2.82
-8	-4	5	49.10	2.24
-8	-4	5	49.96	2.15
8	4	-5	51.97	2.75
8	4	-5	51.92	2.02
-9	-4	5	36.18	1.85
-9	-4	5	40.05	1.60
-9	-4	5	36.62	1.68
-9	-4	5	37.53	1.83
-9	-4	5	38.69	1.61
-9	-4	5	38.41	2.48
9	4	-5	38.18	1.59
9	4	-5	38.79	2.40
-10	-4	5	69.42	2.98
-10	-4	5	65.62	2.74
-10	-4	5	66.98	2.67
-10	-4	5	66.75	2.98
-10	-4	5	65.62	3.59
-10	-4	5	72.58	2.72
10	4	-5	67.12	2.70
10	4	-5	65.96	3.47
-11	-4	5	30.43	1.33
-11	-4	5	27.17	2.35
-11	-4	5	27.25	1.23
-11	-4	5	27.28	1.62
-11	-4	5	29.83	1.32
-11	-4	5	26.88	1.57
11	4	-5	27.40	1.29

11	4	-5	32.13	2.29
-12	-4	5	5.75	1.39
-12	-4	5	6.72	0.62
-12	-4	5	5.44	0.80
12	4	-5	7.41	1.33
12	4	-5	5.69	0.58
-13	-4	5	17.89	2.34
-13	-4	5	20.15	1.45
-13	-4	5	21.00	1.12
13	4	-5	18.67	1.07
13	4	-5	20.21	2.27
-14	-4	5	15.71	1.03
-14	-4	5	14.30	1.82
-14	-4	5	12.53	2.37
14	4	-5	16.18	2.29
14	4	-5	15.99	0.97
-15	-4	5	28.22	1.92
-15	-4	5	25.25	2.81
15	4	-5	27.51	1.43
15	4	-5	26.05	2.72
-16	-4	5	4.50	1.00
-16	-4	5	2.84	1.59
16	4	-5	3.38	0.56
-17	-4	5	6.53	1.16
-17	-4	5	6.11	1.74
17	4	-5	8.54	0.96
-18	-4	5	-0.89	0.89
-18	-4	5	-1.25	1.82
18	4	-5	1.26	0.56
-19	-4	5	14.03	2.64
-19	-4	5	12.93	1.95
19	4	-5	14.11	1.32
-20	-4	5	5.87	2.89
-20	-4	5	2.66	1.31
20	4	-5	4.06	0.77
19	-4	6	1.31	1.52
19	-4	6	-0.40	0.79
-19	4	-6	-1.56	1.45
18	-4	6	30.00	2.67
18	-4	6	29.15	1.89
-18	4	-6	33.65	2.87
17	-4	6	4.71	1.32
17	-4	6	3.15	0.79
-17	4	-6	2.81	1.29

16	-4	6	72.96	3.18
16	-4	6	71.56	3.66
-16	4	-6	64.87	3.71
15	-4	6	-0.09	0.95
15	-4	6	0.12	0.52
-15	4	-6	-0.07	0.88
14	-4	6	109.12	4.62
14	-4	6	116.45	5.09
13	-4	6	-0.11	0.77
-13	4	-6	1.21	0.76
-13	4	-6	-2.51	1.11
12	-4	6	84.25	3.68
12	-4	6	83.35	3.96
12	-4	6	86.09	3.55
-12	4	-6	80.66	4.17
-12	4	-6	90.16	3.87
11	-4	6	3.94	0.88
11	-4	6	3.37	0.64
11	-4	6	4.29	0.65
-11	4	-6	4.20	0.65
-11	4	-6	3.02	0.74
-11	4	-6	5.96	1.13
10	-4	6	167.18	6.49
10	-4	6	168.16	6.89
10	-4	6	164.48	6.58
-10	4	-6	163.69	6.69
-10	4	-6	175.18	6.47
-10	4	-6	162.00	6.99
9	-4	6	-0.02	0.40
9	-4	6	0.40	0.30
9	-4	6	-0.02	0.59
-9	4	-6	0.48	0.27
-9	4	-6	2.34	0.80
-9	4	-6	-0.29	0.44
8	-4	6	162.88	6.31
8	-4	6	163.10	6.63
8	-4	6	158.90	6.21
-8	4	-6	160.51	6.39
-8	4	-6	159.10	6.69
-8	4	-6	163.46	6.19
7	-4	6	5.95	0.94
7	-4	6	6.14	0.71
7	-4	6	5.91	0.51
-7	4	-6	4.57	0.70

-7	4	-6	6.59	1.13
-7	4	-6	7.07	0.51
6	-4	6	95.04	4.13
6	-4	6	98.12	3.86
6	-4	6	95.90	3.70
6	-4	6	92.03	3.75
6	-4	6	95.38	3.83
-6	4	-6	102.59	3.74
-6	4	-6	93.95	3.90
-6	4	-6	94.58	4.20
5	-4	6	2.70	0.33
5	-4	6	2.54	0.49
5	-4	6	2.11	0.61
5	-4	6	2.52	0.44
5	-4	6	2.07	0.38
-5	4	-6	2.64	0.33
-5	4	-6	2.38	0.42
-5	4	-6	2.58	0.61
4	-4	6	96.35	3.98
4	-4	6	94.50	3.70
4	-4	6	101.76	3.84
4	-4	6	95.54	4.09
-4	4	-6	95.68	3.85
-4	4	-6	94.57	4.12
-4	4	-6	94.55	3.85
3	-4	6	18.29	0.85
3	-4	6	19.49	1.01
3	-4	6	19.60	1.25
-3	4	-6	18.03	1.26
-3	4	-6	18.35	1.10
-3	4	-6	19.58	0.83
-3	4	-6	19.65	1.01
2	-4	6	167.05	6.57
2	-4	6	150.42	6.13
2	-4	6	163.35	6.52
-2	4	-6	166.20	6.29
-2	4	-6	170.67	6.13
-2	4	-6	164.86	6.52
-2	4	-6	161.54	6.44
1	-4	6	0.36	0.36
1	-4	6	0.52	0.48
1	-4	6	0.02	0.09
1	-4	6	0.25	0.23
-1	4	-6	0.03	0.09

-1	4	-6	0.39	0.40
-1	4	-6	-0.04	0.19
-1	4	-6	-0.23	0.37
0	-4	6	226.96	8.36
0	-4	6	216.70	8.53
0	-4	6	224.36	8.72
0	4	-6	221.01	8.63
0	4	-6	218.67	8.34
0	4	-6	213.32	8.16
0	4	-6	212.04	8.18
0	4	-6	225.17	8.55
-1	-4	6	-0.80	0.41
-1	-4	6	0.04	0.30
1	4	-6	0.38	0.39
1	4	-6	0.11	0.19
1	4	-6	0.02	0.06
1	4	-6	0.03	0.06
1	4	-6	0.04	0.46
-2	-4	6	225.07	8.94
-2	-4	6	208.41	8.59
-2	-4	6	235.80	8.51
2	4	-6	229.55	8.87
2	4	-6	228.95	8.48
2	4	-6	210.93	8.26
2	4	-6	239.11	8.65
2	4	-6	209.45	8.27
-3	-4	6	3.06	0.45
-3	-4	6	3.00	0.75
-3	-4	6	2.94	0.17
-3	-4	6	2.49	0.48
3	4	-6	2.86	0.48
3	4	-6	2.41	0.15
3	4	-6	2.51	0.61
3	4	-6	2.63	0.42
-4	-4	6	230.96	8.80
-4	-4	6	224.42	8.51
-4	-4	6	221.97	8.73
-4	-4	6	225.53	9.25
4	4	-6	243.00	8.83
4	4	-6	219.34	8.51
4	4	-6	230.15	8.72
4	4	-6	239.31	9.21
-5	-4	6	0.39	0.12
-5	-4	6	0.58	0.72

-5	-4	6	0.12	0.27
5	4	-6	0.10	0.61
5	4	-6	0.47	0.11
5	4	-6	0.11	0.36
5	4	-6	0.37	0.25
-6	-4	6	200.53	8.38
-6	-4	6	207.30	7.80
-6	-4	6	194.64	7.74
-6	-4	6	203.89	7.56
-6	-4	6	198.76	7.77
6	4	-6	213.49	7.82
6	4	-6	206.58	8.29
6	4	-6	191.09	7.55
6	4	-6	204.03	7.76
-7	-4	6	24.24	1.27
-7	-4	6	24.95	1.32
-7	-4	6	25.53	1.36
-7	-4	6	24.28	1.93
-7	-4	6	28.16	1.10
7	4	-6	25.63	1.88
7	4	-6	25.56	1.09
7	4	-6	28.01	1.37
-8	-4	6	75.51	3.14
-8	-4	6	72.91	3.11
-8	-4	6	72.32	3.74
-8	-4	6	72.97	2.97
8	4	-6	75.28	3.67
8	4	-6	73.10	3.05
8	4	-6	74.05	2.86
-9	-4	6	44.18	2.14
-9	-4	6	43.98	2.14
-9	-4	6	46.18	1.90
-9	-4	6	45.09	1.93
-9	-4	6	49.04	1.90
-9	-4	6	49.22	2.90
9	4	-6	45.72	1.88
9	4	-6	47.12	2.75
-10	-4	6	104.53	4.13
-10	-4	6	103.39	4.45
-10	-4	6	116.48	4.20
-10	-4	6	105.28	4.11
-10	-4	6	106.45	5.11
-10	-4	6	105.86	4.45
10	4	-6	109.60	5.00

10	4	-6	108.72	4.16
-11	-4	6	1.66	1.09
-11	-4	6	1.88	0.58
-11	-4	6	1.74	0.33
-11	-4	6	1.31	0.50
11	4	-6	1.50	1.08
11	4	-6	1.06	0.28
-12	-4	6	64.55	3.04
-12	-4	6	70.05	2.75
-12	-4	6	66.93	3.88
12	4	-6	65.09	2.70
12	4	-6	65.51	3.77
-13	-4	6	0.49	1.28
-13	-4	6	0.20	0.50
-13	-4	6	0.85	0.38
13	4	-6	-0.84	1.27
13	4	-6	-0.05	0.28
-14	-4	6	46.09	2.50
-14	-4	6	46.45	3.44
-14	-4	6	50.25	2.15
14	4	-6	41.78	3.20
14	4	-6	47.56	2.07
-15	-4	6	5.75	1.69
-15	-4	6	7.71	1.11
15	4	-6	7.85	0.79
-16	-4	6	80.74	3.75
-16	-4	6	71.42	4.43
16	4	-6	73.06	3.16
-17	-4	6	2.47	0.91
-17	-4	6	5.08	1.80
17	4	-6	2.69	0.59
-18	-4	6	27.86	5.01
-18	-4	6	40.96	2.63
18	4	-6	40.85	2.03
-19	-4	6	3.09	1.16
-19	-4	6	1.99	2.02
19	4	-6	2.61	0.69
-20	-4	6	23.70	2.46
-20	-4	6	27.12	3.73
20	4	-6	25.66	1.74
19	-4	7	22.09	2.80
19	-4	7	15.59	1.63
-19	4	-7	17.12	2.81
18	-4	7	8.59	1.08

18	-4	7	5.35	1.58
-18	4	-7	4.74	1.53
17	-4	7	26.84	1.78
17	-4	7	35.06	2.63
-17	4	-7	20.21	2.57
16	-4	7	4.92	1.22
16	-4	7	3.81	0.76
-16	4	-7	4.32	1.27
15	-4	7	30.95	1.76
-15	4	-7	28.77	2.28
14	-4	7	9.76	1.02
14	-4	7	9.02	1.66
13	-4	7	55.36	2.53
13	-4	7	54.60	3.06
-13	4	-7	57.57	2.94
12	-4	7	9.81	1.48
12	-4	7	9.48	0.89
12	-4	7	10.21	1.47
-12	4	-7	11.43	1.36
11	-4	7	81.69	3.36
11	-4	7	81.54	3.83
11	-4	7	80.41	3.50
-11	4	-7	83.40	3.63
-11	4	-7	73.58	4.02
10	-4	7	17.36	1.20
10	-4	7	15.15	0.98
10	-4	7	14.40	1.51
-10	4	-7	14.56	1.26
-10	4	-7	13.27	1.77
-10	4	-7	15.20	0.96
9	-4	7	79.63	3.73
9	-4	7	80.24	3.25
9	-4	7	77.48	3.34
-9	4	-7	82.89	3.22
-9	4	-7	81.61	3.47
-9	4	-7	74.05	3.79
8	-4	7	19.70	1.06
8	-4	7	18.86	1.53
8	-4	7	21.83	1.22
-8	4	-7	18.78	1.25
-8	4	-7	20.77	1.03
-8	4	-7	18.86	1.66
7	-4	7	44.73	2.00
7	-4	7	44.56	2.35

7	-4	7	42.75	1.87
7	-4	7	41.94	1.85
7	-4	7	43.26	1.78
-7	4	-7	45.90	1.87
-7	4	-7	46.52	2.47
-7	4	-7	43.77	2.06
6	-4	7	35.84	2.03
6	-4	7	34.32	1.54
6	-4	7	36.20	1.67
6	-4	7	35.34	1.67
6	-4	7	36.03	1.54
-6	4	-7	34.53	1.71
-6	4	-7	36.08	1.52
-6	4	-7	35.94	2.11
5	-4	7	121.22	4.76
5	-4	7	113.32	4.52
5	-4	7	117.90	4.64
5	-4	7	115.50	4.96
-5	4	-7	112.37	4.63
-5	4	-7	118.56	4.68
-5	4	-7	115.33	5.00
-5	4	-7	125.55	4.52
4	-4	7	8.78	0.71
4	-4	7	8.73	0.55
4	-4	7	9.14	0.97
4	-4	7	9.87	1.24
-4	4	-7	10.18	1.10
-4	4	-7	8.53	0.82
-4	4	-7	9.94	0.73
-4	4	-7	10.05	0.54
3	-4	7	62.91	2.60
3	-4	7	69.38	2.77
3	-4	7	65.54	3.03
3	-4	7	66.90	3.06
-3	4	-7	67.32	2.77
-3	4	-7	69.44	2.60
-3	4	-7	65.60	3.06
-3	4	-7	67.06	2.92
2	-4	7	4.15	0.52
2	-4	7	3.42	0.33
2	-4	7	3.42	0.73
2	-4	7	3.96	0.68
-2	4	-7	4.92	0.80
-2	4	-7	4.30	0.30

-2	4	-7	3.34	0.50
-2	4	-7	3.19	0.65
1	-4	7	204.54	7.72
1	-4	7	196.03	8.06
1	-4	7	193.93	7.92
-1	4	-7	201.27	7.54
-1	4	-7	208.55	7.94
-1	4	-7	201.68	7.68
-1	4	-7	201.18	7.51
0	-4	7	0.27	0.39
0	-4	7	-0.13	0.55
0	-4	7	0.88	0.29
0	4	-7	0.27	0.24
0	4	-7	0.84	0.50
0	4	-7	0.18	0.11
0	4	-7	0.33	0.10
0	4	-7	0.08	0.40
-1	-4	7	216.97	9.08
-1	-4	7	239.87	8.65
-1	-4	7	224.21	8.83
1	4	-7	219.53	8.42
1	4	-7	236.95	8.63
1	4	-7	227.63	9.02
1	4	-7	214.92	8.42
1	4	-7	233.22	8.82
-2	-4	7	32.13	1.70
-2	-4	7	36.34	1.59
-2	-4	7	33.84	2.09
2	4	-7	34.71	1.55
2	4	-7	35.54	1.32
2	4	-7	34.00	1.88
2	4	-7	33.15	1.32
2	4	-7	35.91	2.04
-3	-4	7	77.98	2.92
-3	-4	7	77.11	3.72
-3	-4	7	73.80	3.25
-3	-4	7	81.36	3.16
3	4	-7	81.39	3.29
3	4	-7	78.96	3.13
3	4	-7	77.02	3.62
3	4	-7	72.65	2.90
-4	-4	7	-0.16	0.27
-4	-4	7	0.57	0.37
-4	-4	7	0.11	0.10

-4	-4	7	0.29	0.63
4	4	-7	0.09	0.08
4	4	-7	0.08	0.37
4	4	-7	0.37	0.25
4	4	-7	0.45	0.63
-5	-4	7	185.65	7.03
-5	-4	7	190.75	7.90
-5	-4	7	180.57	7.29
-5	-4	7	194.59	7.29
5	4	-7	181.69	7.02
5	4	-7	201.28	7.35
5	4	-7	188.28	7.80
5	4	-7	185.13	7.23
-6	-4	7	23.93	1.26
-6	-4	7	23.96	1.23
-6	-4	7	23.33	0.98
-6	-4	7	25.12	1.94
6	4	-7	22.67	0.97
6	4	-7	22.34	1.19
6	4	-7	23.99	1.31
6	4	-7	24.36	1.85
-7	-4	7	213.60	8.39
-7	-4	7	216.66	9.15
-7	-4	7	215.05	8.24
-7	-4	7	216.94	8.48
-7	-4	7	230.54	8.52
7	4	-7	222.13	9.07
7	4	-7	230.19	8.48
7	4	-7	215.76	8.23
-8	-4	7	5.64	1.21
-8	-4	7	3.86	0.63
-8	-4	7	4.83	0.73
-8	-4	7	4.26	0.60
8	4	-7	5.67	0.73
8	4	-7	4.61	0.36
8	4	-7	3.47	1.03
-9	-4	7	107.58	4.55
-9	-4	7	113.38	5.31
-9	-4	7	119.04	4.31
-9	-4	7	109.18	4.31
-9	-4	7	107.57	4.33
9	4	-7	114.86	5.18
9	4	-7	110.89	4.28
-10	-4	7	2.72	1.07

-10	-4	7	4.08	0.48
-10	-4	7	2.98	0.60
-10	-4	7	3.20	0.62
10	4	-7	3.92	1.16
10	4	-7	4.01	0.41
-11	-4	7	215.32	9.11
-11	-4	7	210.07	8.36
-11	-4	7	226.16	8.07
-11	-4	7	216.30	8.43
11	4	-7	201.21	8.02
11	4	-7	202.27	8.90
-12	-4	7	0.67	0.33
-12	-4	7	-0.23	0.48
-12	-4	7	0.48	1.24
12	4	-7	0.73	1.27
12	4	-7	-0.18	0.26
-13	-4	7	104.93	5.45
-13	-4	7	103.31	4.65
-13	-4	7	113.19	4.25
13	4	-7	105.21	5.28
13	4	-7	103.87	4.19
-14	-4	7	-0.16	0.43
-14	-4	7	-0.83	1.43
-14	-4	7	0.74	0.64
14	4	-7	-0.55	0.31
-15	-4	7	57.82	3.03
-15	-4	7	55.53	3.83
15	4	-7	58.12	2.47
-16	-4	7	-0.47	0.78
-16	-4	7	-1.02	1.60
16	4	-7	0.53	0.43
-17	-4	7	62.95	4.36
-17	-4	7	63.03	3.36
17	4	-7	62.59	2.76
-18	-4	7	1.61	2.02
-18	-4	7	0.97	1.03
18	4	-7	1.64	0.62
-19	-4	7	49.38	3.30
-19	-4	7	60.09	4.55
19	4	-7	60.90	2.70
-20	-4	7	-1.48	2.18
-20	-4	7	0.82	1.21
20	4	-7	-1.00	0.68
18	-4	8	0.57	0.83

18	-4	8	0.78	1.55
-18	4	-8	1.25	1.43
17	-4	8	12.47	1.40
17	-4	8	8.50	1.70
-17	4	-8	8.23	1.55
16	-4	8	-0.58	1.23
16	-4	8	1.11	0.67
-16	4	-8	0.77	1.14
15	-4	8	5.35	1.36
15	-4	8	6.65	0.90
-15	4	-8	4.01	1.16
14	-4	8	7.47	0.86
14	-4	8	3.90	1.30
-14	4	-8	4.00	1.19
13	-4	8	13.20	1.75
13	-4	8	13.31	1.15
-13	4	-8	15.75	1.62
12	-4	8	2.06	0.93
12	-4	8	0.99	0.51
-12	4	-8	0.73	0.74
11	-4	8	60.09	3.02
11	-4	8	54.39	2.43
11	-4	8	50.85	2.58
-11	4	-8	53.29	2.71
10	-4	8	12.03	0.92
10	-4	8	13.22	1.15
10	-4	8	11.51	1.47
-10	4	-8	13.51	1.87
-10	4	-8	12.96	0.87
-10	4	-8	13.37	1.25
9	-4	8	22.36	1.75
9	-4	8	20.62	1.33
9	-4	8	19.56	1.16
-9	4	-8	23.01	1.13
-9	4	-8	22.75	1.95
-9	4	-8	20.74	1.44
8	-4	8	22.18	1.27
8	-4	8	21.37	1.14
8	-4	8	19.48	1.70
-8	4	-8	23.22	1.10
-8	4	-8	20.64	1.34
-8	4	-8	21.96	1.84
7	-4	8	10.09	1.30
7	-4	8	9.36	0.69

7	-4	8	8.20	0.65
7	-4	8	10.12	0.74
7	-4	8	9.11	0.85
-7	4	-8	10.23	1.35
-7	4	-8	9.92	0.91
-7	4	-8	10.59	0.65
6	-4	8	7.98	1.17
6	-4	8	8.24	0.78
6	-4	8	7.39	0.59
6	-4	8	8.43	0.87
-6	4	-8	8.62	0.84
-6	4	-8	8.39	0.57
-6	4	-8	7.56	1.25
5	-4	8	68.32	3.00
5	-4	8	64.32	2.68
5	-4	8	69.62	2.82
5	-4	8	63.48	3.16
-5	4	-8	67.33	2.85
-5	4	-8	66.62	2.86
-5	4	-8	71.46	2.68
-5	4	-8	65.37	3.22
4	-4	8	64.68	3.09
4	-4	8	62.90	2.60
4	-4	8	67.81	2.75
4	-4	8	66.74	3.00
-4	4	-8	64.18	2.76
-4	4	-8	63.25	2.88
-4	4	-8	69.14	2.60
-4	4	-8	66.97	3.14
3	-4	8	21.94	1.47
3	-4	8	22.43	1.48
3	-4	8	22.00	0.99
3	-4	8	23.19	1.17
-3	4	-8	24.41	1.45
-3	4	-8	22.91	1.54
-3	4	-8	20.47	1.02
-3	4	-8	21.15	1.15
2	-4	8	46.53	1.91
2	-4	8	47.98	2.48
2	-4	8	46.39	2.37
2	-4	8	49.24	2.08
-2	4	-8	44.32	2.06
-2	4	-8	47.28	2.40
-2	4	-8	49.26	2.43

-2	4	-8	52.28	1.91
-2	4	-8	47.96	1.94
1	-4	8	0.05	0.28
1	-4	8	0.54	0.61
1	-4	8	0.11	0.42
-1	4	-8	0.04	0.54
-1	4	-8	0.47	0.28
-1	4	-8	0.51	0.14
-1	4	-8	0.25	0.50
0	-4	8	22.45	1.12
0	-4	8	20.22	1.33
0	-4	8	18.05	1.56
0	4	-8	21.34	1.09
0	4	-8	21.28	0.87
0	4	-8	19.08	1.52
0	4	-8	22.83	1.40
0	4	-8	21.10	0.89
-1	-4	8	51.27	2.78
-1	-4	8	48.70	2.45
-1	-4	8	55.50	2.28
1	4	-8	55.98	2.25
1	4	-8	53.02	2.04
1	4	-8	51.49	2.72
1	4	-8	54.43	2.48
1	4	-8	52.98	2.04
-2	-4	8	107.96	4.64
-2	-4	8	121.04	4.49
-2	-4	8	111.49	5.04
2	4	-8	109.47	4.43
2	4	-8	117.06	4.65
2	4	-8	108.75	4.23
2	4	-8	114.42	4.97
2	4	-8	113.29	4.24
-3	-4	8	122.03	5.03
-3	-4	8	125.21	4.65
-3	-4	8	116.17	5.46
-3	-4	8	130.30	4.90
3	4	-8	126.43	5.03
3	4	-8	121.83	4.63
3	4	-8	124.23	4.86
3	4	-8	124.44	5.42
-4	-4	8	37.92	1.91
-4	-4	8	38.64	2.42
-4	-4	8	40.49	1.55

-4	-4	8	41.71	1.83
4	4	-8	41.69	1.78
4	4	-8	37.08	1.53
4	4	-8	41.01	2.39
-5	-4	8	51.62	2.32
-5	-4	8	47.00	2.88
-5	-4	8	50.87	1.99
-5	-4	8	55.23	2.27
5	4	-8	51.65	2.87
5	4	-8	48.31	2.19
5	4	-8	56.01	2.36
5	4	-8	48.77	1.97
-6	-4	8	39.11	1.84
-6	-4	8	39.25	1.91
-6	-4	8	39.77	1.87
-6	-4	8	40.29	1.59
-6	-4	8	38.46	2.61
6	4	-8	42.77	1.94
6	4	-8	39.04	2.51
6	4	-8	40.00	1.82
-7	-4	8	7.86	0.49
-7	-4	8	8.55	0.76
-7	-4	8	8.19	1.55
-7	-4	8	8.66	0.83
-7	-4	8	7.50	1.00
7	4	-8	8.81	1.61
7	4	-8	8.82	0.89
7	4	-8	8.38	0.47
-8	-4	8	80.67	4.20
-8	-4	8	80.17	3.41
-8	-4	8	80.56	3.44
-8	-4	8	80.63	3.28
8	4	-8	83.53	3.13
8	4	-8	84.56	3.39
8	4	-8	75.44	4.06
-9	-4	8	-0.53	0.99
-9	-4	8	0.03	0.22
-9	-4	8	0.17	0.43
-9	-4	8	-0.05	0.36
-9	-4	8	0.40	0.32
9	4	-8	-0.01	1.03
9	4	-8	0.16	0.45
9	4	-8	-0.04	0.18
-10	-4	8	77.29	3.17

-10	-4	8	81.70	3.58
-10	-4	8	81.11	3.54
-10	-4	8	81.73	3.17
-10	-4	8	80.66	4.38
-10	-4	8	88.84	3.25
10	4	-8	82.09	4.26
10	4	-8	81.07	3.20
-11	-4	8	16.24	2.28
-11	-4	8	16.75	0.91
-11	-4	8	16.71	1.28
11	4	-8	13.69	2.26
11	4	-8	15.61	0.84
-12	-4	8	25.04	2.66
-12	-4	8	22.65	1.55
-12	-4	8	24.72	1.23
12	4	-8	23.49	2.91
12	4	-8	22.99	1.15
-13	-4	8	11.62	0.87
-13	-4	8	8.41	1.23
-13	-4	8	12.39	1.93
13	4	-8	9.80	0.72
-14	-4	8	33.47	2.15
-14	-4	8	36.13	1.75
-14	-4	8	37.36	3.32
14	4	-8	36.13	1.65
-15	-4	8	12.18	2.09
-15	-4	8	14.81	1.60
15	4	-8	11.83	0.92
-16	-4	8	50.36	3.83
-16	-4	8	53.77	2.94
16	4	-8	50.43	2.28
-17	-4	8	0.37	0.89
-17	-4	8	-0.57	1.97
17	4	-8	-0.30	0.48
-18	-4	8	44.65	2.88
-18	-4	8	44.38	4.34
18	4	-8	43.56	2.17
-19	-4	8	6.07	1.30
-19	-4	8	7.26	2.41
19	4	-8	7.31	1.17
-20	-4	8	32.44	3.75
-20	-4	8	47.44	4.40
20	4	-8	36.63	2.12
18	-4	9	-0.07	1.66

18	-4	9	0.78	0.83
-18	4	-9	1.21	1.49
17	-4	9	11.10	1.40
17	-4	9	8.19	1.75
-17	4	-9	9.94	1.63
16	-4	9	4.78	0.88
16	-4	9	4.74	1.50
-16	4	-9	2.35	1.25
15	-4	9	10.60	1.23
15	-4	9	13.09	2.13
-15	4	-9	9.16	1.42
14	-4	9	7.01	1.39
14	-4	9	8.59	1.09
-14	4	-9	11.50	1.62
13	-4	9	8.74	1.42
13	-4	9	8.09	1.01
-13	4	-9	7.27	1.12
12	-4	9	2.50	0.60
12	-4	9	3.22	1.05
-12	4	-9	2.98	0.83
11	-4	9	11.88	0.98
11	-4	9	12.34	1.27
11	-4	9	10.92	1.56
-11	4	-9	12.59	1.39
10	-4	9	23.48	1.33
10	-4	9	24.12	1.91
10	-4	9	22.50	1.46
-10	4	-9	23.00	1.60
9	-4	9	5.58	0.73
9	-4	9	6.25	1.08
9	-4	9	4.98	0.71
-9	4	-9	6.46	0.69
-9	4	-9	8.16	1.93
-9	4	-9	5.92	1.00
8	-4	9	12.80	0.90
8	-4	9	13.58	1.05
8	-4	9	14.36	1.50
-8	4	-9	15.61	0.89
-8	4	-9	13.72	1.16
-8	4	-9	13.39	1.72
7	-4	9	4.36	0.63
7	-4	9	4.91	0.71
7	-4	9	4.89	0.64
7	-4	9	3.42	0.85

7	-4	9	3.97	0.54
-7	4	-9	3.77	0.50
-7	4	-9	4.77	1.08
-7	4	-9	6.52	0.86
6	-4	9	0.72	0.62
6	-4	9	1.04	0.29
6	-4	9	1.47	0.44
6	-4	9	1.45	0.57
-6	4	-9	0.63	0.43
-6	4	-9	2.06	0.50
-6	4	-9	-0.30	0.73
-6	4	-9	0.78	0.26
5	-4	9	4.42	0.67
5	-4	9	3.53	0.86
5	-4	9	3.86	0.74
5	-4	9	3.34	0.47
-5	4	-9	3.99	0.67
-5	4	-9	3.11	0.79
-5	4	-9	4.58	0.69
-5	4	-9	5.10	0.45
4	-4	9	20.79	1.38
4	-4	9	16.93	1.43
4	-4	9	19.62	1.07
4	-4	9	15.99	0.88
-4	4	-9	18.41	0.87
-4	4	-9	15.34	1.51
-4	4	-9	14.98	1.49
-4	4	-9	20.43	1.10
3	-4	9	8.39	1.08
3	-4	9	9.53	0.76
3	-4	9	7.92	0.55
3	-4	9	10.21	1.24
-3	4	-9	8.57	1.09
-3	4	-9	9.25	0.76
-3	4	-9	9.50	0.54
-3	4	-9	9.46	1.18
-3	4	-9	9.38	0.59
2	-4	9	33.58	1.54
2	-4	9	33.54	2.00
2	-4	9	30.49	1.89
-2	4	-9	30.74	1.52
-2	4	-9	33.79	1.94
-2	4	-9	32.40	1.38
-2	4	-9	32.25	1.35

-2	4	-9	32.21	1.90
1	-4	9	2.94	0.66
1	-4	9	4.49	0.91
1	-4	9	4.23	0.58
-1	4	-9	3.73	0.32
-1	4	-9	3.52	0.71
-1	4	-9	4.66	0.85
-1	4	-9	3.50	0.54
-1	4	-9	3.58	0.38
0	-4	9	10.91	0.77
0	-4	9	9.64	1.02
0	-4	9	7.58	1.26
0	4	-9	7.51	1.25
0	4	-9	9.79	1.11
0	4	-9	10.15	0.49
0	4	-9	8.64	0.69
-1	-4	9	0.34	0.31
-1	-4	9	0.95	0.48
-1	-4	9	1.08	0.76
1	4	-9	0.03	0.12
1	4	-9	0.25	0.51
1	4	-9	0.43	0.28
1	4	-9	0.57	0.67
-2	-4	9	19.69	1.11
-2	-4	9	17.57	1.29
-2	-4	9	20.38	1.78
2	4	-9	20.57	0.84
2	4	-9	21.27	1.39
2	4	-9	20.29	1.07
2	4	-9	19.22	1.74
2	4	-9	20.55	0.84
-3	-4	9	0.54	0.45
-3	-4	9	0.27	0.13
-3	-4	9	-0.11	0.32
-3	-4	9	0.18	0.74
3	4	-9	1.22	0.79
3	4	-9	0.40	0.47
3	4	-9	0.30	0.12
3	4	-9	0.15	0.26
-4	-4	9	49.63	2.89
-4	-4	9	45.16	2.29
-4	-4	9	52.28	2.19
-4	-4	9	48.39	1.90
4	4	-9	48.65	2.12

4	4	-9	46.33	1.87
4	4	-9	47.67	2.83
4	4	-9	51.47	2.31
-5	-4	9	1.25	0.42
-5	-4	9	1.74	0.21
-5	-4	9	1.02	0.47
-5	-4	9	1.54	0.85
5	4	-9	1.84	0.22
5	4	-9	1.73	0.38
5	4	-9	1.43	0.54
5	4	-9	2.71	0.90
-6	-4	9	20.63	0.90
-6	-4	9	19.69	1.99
-6	-4	9	20.76	1.18
-6	-4	9	21.44	1.25
-6	-4	9	20.22	1.22
6	4	-9	19.57	0.88
6	4	-9	21.02	1.33
6	4	-9	20.73	1.94
6	4	-9	21.48	1.16
-7	-4	9	6.49	0.86
-7	-4	9	5.47	0.79
-7	-4	9	6.38	0.72
-7	-4	9	6.85	0.45
-7	-4	9	5.42	1.16
7	4	-9	5.89	0.89
7	4	-9	5.83	0.40
7	4	-9	4.94	1.10
-8	-4	9	4.34	1.13
-8	-4	9	4.36	0.61
-8	-4	9	4.33	0.63
-8	-4	9	4.34	0.54
8	4	-9	4.68	0.65
8	4	-9	3.95	0.37
8	4	-9	3.99	1.22
-9	-4	9	2.15	0.61
-9	-4	9	2.41	0.33
-9	-4	9	2.02	0.49
-9	-4	9	2.12	0.42
-9	-4	9	0.98	1.13
9	4	-9	0.72	1.11
9	4	-9	2.23	0.35
9	4	-9	2.37	0.58
-10	-4	9	32.01	1.49

-10	-4	9	33.28	2.81
-10	-4	9	36.02	1.85
-10	-4	9	36.13	1.53
-10	-4	9	38.52	1.95
10	4	-9	31.91	2.73
10	4	-9	35.97	1.66
10	4	-9	32.26	1.46
-11	-4	9	1.85	0.57
-11	-4	9	2.92	0.41
-11	-4	9	3.23	1.40
11	4	-9	2.84	0.43
-12	-4	9	3.60	1.51
-12	-4	9	3.44	0.48
-12	-4	9	4.13	0.75
12	4	-9	3.20	0.48
-13	-4	9	12.04	0.92
-13	-4	9	11.48	1.96
-13	-4	9	10.97	1.26
13	4	-9	9.58	0.75
-14	-4	9	13.73	1.04
-14	-4	9	11.68	1.43
14	4	-9	11.37	0.86
-15	-4	9	5.86	1.10
-15	-4	9	7.53	1.93
15	4	-9	6.49	0.77
-16	-4	9	21.11	3.19
-16	-4	9	14.20	1.81
16	4	-9	14.10	1.04
-17	-4	9	24.40	3.70
-17	-4	9	25.45	2.15
17	4	-9	23.11	1.40
-18	-4	9	7.88	1.36
-18	-4	9	11.88	2.58
18	4	-9	10.70	1.13
-19	-4	9	10.91	2.62
-19	-4	9	14.75	2.12
19	4	-9	13.18	1.36
17	-4	10	1.79	1.59
17	-4	10	-0.02	0.81
-17	4	-10	3.61	1.45
16	-4	10	13.12	1.53
16	-4	10	15.47	2.51
-16	4	-10	15.25	2.35
15	-4	10	1.52	1.33

15	-4	10	-0.44	0.64
-15	4	-10	-0.43	1.07
14	-4	10	34.70	2.75
14	-4	10	32.88	1.98
-14	4	-10	37.89	2.44
13	-4	10	1.44	1.12
13	-4	10	0.49	0.58
-13	4	-10	0.74	0.82
12	-4	10	47.04	2.31
12	-4	10	49.37	3.02
-12	4	-10	48.18	2.64
11	-4	10	5.60	0.89
11	-4	10	6.05	0.84
-11	4	-10	7.61	1.00
10	-4	10	20.76	1.30
10	-4	10	22.52	1.45
10	-4	10	24.16	1.96
-10	4	-10	21.29	1.60
9	-4	10	0.60	0.39
9	-4	10	0.69	0.53
9	-4	10	1.18	0.80
-9	4	-10	0.17	0.57
-9	4	-10	0.37	0.37
8	-4	10	32.38	1.75
8	-4	10	35.31	1.59
8	-4	10	36.81	1.56
8	-4	10	33.39	2.25
8	-4	10	32.83	1.65
-8	4	-10	35.29	1.60
-8	4	-10	34.09	1.87
7	-4	10	0.50	0.58
7	-4	10	0.27	0.33
7	-4	10	0.64	0.50
7	-4	10	0.53	0.78
-7	4	-10	0.32	0.31
-7	4	-10	0.68	0.95
-7	4	-10	0.68	0.45
6	-4	10	49.32	2.15
6	-4	10	45.67	2.59
6	-4	10	43.25	1.99
6	-4	10	45.44	2.20
-6	4	-10	47.99	2.16
-6	4	-10	40.54	2.70
-6	4	-10	49.41	1.97

-6	4	-10	45.97	2.22
5	-4	10	1.70	0.34
5	-4	10	2.31	0.48
5	-4	10	1.94	0.80
5	-4	10	1.20	0.65
-5	4	-10	1.65	0.49
-5	4	-10	-0.02	0.75
-5	4	-10	1.31	0.30
4	-4	10	18.34	1.44
4	-4	10	17.80	1.09
4	-4	10	17.27	1.52
4	-4	10	15.97	0.90
-4	4	-10	16.88	1.40
-4	4	-10	17.64	1.09
-4	4	-10	16.98	0.93
-4	4	-10	20.04	0.90
-4	4	-10	16.00	1.56
3	-4	10	13.24	0.95
3	-4	10	13.64	1.34
3	-4	10	15.55	1.36
-3	4	-10	13.78	0.93
-3	4	-10	13.09	1.39
-3	4	-10	14.35	0.73
-3	4	-10	14.96	0.79
-3	4	-10	11.38	1.37
2	-4	10	41.83	1.87
2	-4	10	38.42	2.26
2	-4	10	38.24	2.34
-2	4	-10	42.26	2.33
-2	4	-10	38.79	1.83
-2	4	-10	39.59	1.69
-2	4	-10	41.35	1.66
-2	4	-10	42.86	2.32
1	-4	10	7.43	1.33
1	-4	10	5.65	1.03
1	-4	10	7.74	0.72
-1	4	-10	6.55	0.45
-1	4	-10	8.63	1.19
-1	4	-10	6.29	0.67
-1	4	-10	7.56	0.48
-1	4	-10	8.52	1.19
0	-4	10	38.29	1.77
0	-4	10	36.72	2.37
0	-4	10	35.02	2.05

0	4	-10	38.99	1.74
0	4	-10	37.03	2.34
0	4	-10	37.78	1.56
0	4	-10	38.93	2.13
0	4	-10	38.72	1.53
-1	-4	10	3.54	0.70
-1	-4	10	3.25	0.94
-1	-4	10	3.01	0.49
1	4	-10	3.11	0.69
1	4	-10	2.55	0.29
1	4	-10	2.59	0.92
1	4	-10	3.00	0.43
-2	-4	10	23.90	1.60
-2	-4	10	23.58	2.03
-2	-4	10	26.12	1.35
2	4	-10	25.16	1.06
2	4	-10	24.55	2.05
2	4	-10	26.37	1.30
2	4	-10	26.99	1.62
2	4	-10	25.90	1.06
-3	-4	10	5.60	0.95
-3	-4	10	5.26	1.10
-3	-4	10	5.62	0.39
-3	-4	10	6.62	0.72
3	4	-10	5.40	0.63
3	4	-10	6.08	0.99
3	4	-10	7.89	1.22
3	4	-10	5.73	0.36
-4	-4	10	44.59	1.77
-4	-4	10	44.29	2.24
-4	-4	10	46.07	2.06
-4	-4	10	45.16	2.85
4	4	-10	48.28	2.26
4	4	-10	42.01	1.74
4	4	-10	42.81	2.76
4	4	-10	43.84	1.98
-5	-4	10	10.53	1.04
-5	-4	10	7.14	1.31
-5	-4	10	9.02	0.86
-5	-4	10	9.37	0.51
5	4	-10	9.52	1.05
5	4	-10	8.48	1.29
5	4	-10	9.36	0.76
-6	-4	10	18.70	1.99

-6	-4	10	15.73	0.76
-6	-4	10	14.96	1.02
-6	-4	10	16.50	1.11
-6	-4	10	15.87	1.19
6	4	-10	15.55	0.73
6	4	-10	17.89	1.26
6	4	-10	14.04	1.97
6	4	-10	16.56	1.03
-7	-4	10	8.95	1.52
-7	-4	10	9.23	0.93
-7	-4	10	8.73	0.95
-7	-4	10	9.57	0.55
-7	-4	10	8.73	0.84
7	4	-10	12.67	1.88
7	4	-10	7.93	0.48
-8	-4	10	8.31	0.55
-8	-4	10	5.97	0.93
-8	-4	10	7.52	0.91
-8	-4	10	8.88	0.84
-8	-4	10	5.30	1.35
8	4	-10	6.32	1.34
8	4	-10	8.28	1.03
8	4	-10	6.92	0.47
-9	-4	10	6.67	1.05
-9	-4	10	7.64	0.91
-9	-4	10	6.80	1.46
-9	-4	10	8.67	0.61
9	4	-10	7.91	0.51
9	4	-10	8.06	1.59
9	4	-10	8.16	1.02
-10	-4	10	3.52	0.59
-10	-4	10	3.72	0.55
-10	-4	10	5.57	1.49
-10	-4	10	4.39	0.69
10	4	-10	2.69	0.42
10	4	-10	5.56	0.88
-11	-4	10	7.57	0.73
-11	-4	10	6.49	1.01
-11	-4	10	7.62	1.70
11	4	-10	8.42	0.59
-12	-4	10	0.50	1.39
-12	-4	10	0.80	0.42
12	4	-10	1.20	0.33
-13	-4	10	4.50	0.84

-13	-4	10	6.47	2.25
-13	-4	10	3.53	0.56
13	4	-10	2.95	0.42
-14	-4	10	8.12	0.98
-14	-4	10	7.26	1.05
-14	-4	10	10.12	1.98
14	4	-10	7.77	0.74
-15	-4	10	1.32	0.82
-15	-4	10	0.68	1.68
15	4	-10	1.22	0.44
-16	-4	10	0.42	2.02
-16	-4	10	1.42	1.03
16	4	-10	1.64	0.49
-17	-4	10	10.76	2.48
-17	-4	10	11.17	1.84
17	4	-10	8.70	1.00
-18	-4	10	0.44	1.06
-18	-4	10	-3.22	2.12
18	4	-10	-0.04	0.59
-19	-4	10	10.44	2.50
-19	-4	10	9.40	1.53
19	4	-10	7.75	1.24
17	-4	11	42.99	2.58
17	-4	11	49.70	3.66
-17	4	-11	41.93	3.38
16	-4	11	3.09	1.53
16	-4	11	2.19	0.85
-16	4	-11	-1.38	1.37
15	-4	11	44.26	3.29
15	-4	11	46.19	2.46
-15	4	-11	45.49	3.06
14	-4	11	0.61	0.63
14	-4	11	1.44	1.27
-14	4	-11	1.17	1.00
13	-4	11	55.01	3.43
13	-4	11	58.00	2.73
-13	4	-11	56.96	3.07
12	-4	11	0.18	0.53
12	-4	11	-1.68	1.06
-12	4	-11	-0.01	0.79
11	-4	11	97.28	4.76
11	-4	11	100.01	4.06
11	-4	11	88.16	4.11
-11	4	-11	100.83	4.28

10	-4	11	3.96	0.63
10	-4	11	4.12	1.18
10	-4	11	5.68	0.81
-10	4	-11	8.62	1.23
9	-4	11	76.24	3.13
9	-4	11	73.39	3.78
9	-4	11	69.28	3.17
-9	4	-11	73.03	3.29
8	-4	11	0.18	0.55
8	-4	11	1.02	0.86
8	-4	11	0.40	0.47
8	-4	11	0.68	0.41
-8	4	-11	1.10	0.41
-8	4	-11	0.92	0.61
7	-4	11	39.74	1.90
7	-4	11	41.61	2.00
7	-4	11	42.95	2.05
7	-4	11	39.89	2.53
-7	4	-11	42.97	2.08
-7	4	-11	44.63	1.86
-7	4	-11	41.27	2.07
6	-4	11	3.59	0.48
6	-4	11	5.18	0.93
6	-4	11	3.06	0.96
6	-4	11	4.42	0.64
-6	4	-11	4.22	0.64
-6	4	-11	5.05	1.08
-6	4	-11	3.95	0.47
5	-4	11	84.45	3.45
5	-4	11	81.18	3.97
5	-4	11	82.46	3.33
5	-4	11	85.27	3.67
-5	4	-11	83.54	3.37
-5	4	-11	87.79	3.30
-5	4	-11	81.60	3.47
-5	4	-11	78.13	4.02
-5	4	-11	79.47	3.72
4	-4	11	6.49	0.99
4	-4	11	6.35	0.81
4	-4	11	5.97	1.03
-4	4	-11	6.69	0.61
-4	4	-11	8.27	0.82
-4	4	-11	8.59	1.33
-4	4	-11	4.95	1.04

-4	4	-11	6.23	0.55
3	-4	11	31.85	2.04
3	-4	11	29.80	2.00
3	-4	11	31.03	1.57
-3	4	-11	32.12	1.36
-3	4	-11	30.20	1.40
-3	4	-11	32.26	2.09
-3	4	-11	29.10	2.05
-3	4	-11	30.00	1.53
2	-4	11	2.10	0.48
2	-4	11	3.62	0.82
2	-4	11	3.51	0.92
-2	4	-11	3.44	0.42
-2	4	-11	3.17	0.39
-2	4	-11	2.96	0.47
-2	4	-11	2.66	0.79
-2	4	-11	2.28	0.91
1	-4	11	30.09	1.53
1	-4	11	32.20	2.10
1	-4	11	30.09	1.92
-1	4	-11	30.35	1.31
-1	4	-11	27.25	1.97
-1	4	-11	26.72	1.46
-1	4	-11	31.42	1.28
-1	4	-11	32.14	2.16
0	-4	11	0.60	0.85
0	-4	11	-0.18	0.63
0	-4	11	0.61	0.39
0	4	-11	0.55	0.84
0	4	-11	0.28	0.34
0	4	-11	0.58	0.71
0	4	-11	0.61	0.20
0	4	-11	0.46	0.20
-1	-4	11	65.93	3.37
-1	-4	11	80.38	3.12
-1	-4	11	71.40	3.80
1	4	-11	73.64	2.84
1	4	-11	72.14	3.79
1	4	-11	75.59	3.44
1	4	-11	74.25	2.86
1	4	-11	72.98	3.04
-2	-4	11	-0.80	0.59
-2	-4	11	-0.17	0.88
-2	-4	11	0.25	0.39

2	4	-11	-0.97	0.94
2	4	-11	0.13	0.33
2	4	-11	0.72	0.18
2	4	-11	1.05	0.67
2	4	-11	0.29	0.19
-3	-4	11	24.20	1.30
-3	-4	11	22.46	1.00
-3	-4	11	21.28	1.57
-3	-4	11	21.79	2.08
3	4	-11	21.54	2.17
3	4	-11	22.55	1.23
3	4	-11	22.96	1.62
3	4	-11	22.94	0.98
-4	-4	11	49.76	2.58
-4	-4	11	52.46	2.08
-4	-4	11	54.57	2.39
-4	-4	11	52.54	3.22
4	4	-11	51.71	2.30
4	4	-11	57.74	2.61
4	4	-11	45.98	3.14
4	4	-11	52.48	2.05
-5	-4	11	17.96	1.18
-5	-4	11	16.86	2.10
-5	-4	11	17.22	0.82
-5	-4	11	15.88	1.34
5	4	-11	14.53	2.02
5	4	-11	20.15	1.40
5	4	-11	15.70	0.77
5	4	-11	19.64	1.10
-6	-4	11	0.97	0.41
-6	-4	11	-1.50	0.93
-6	-4	11	0.03	0.21
-6	-4	11	-0.80	0.43
-6	-4	11	1.05	0.56
6	4	-11	0.09	0.16
6	4	-11	-0.55	0.59
6	4	-11	1.37	1.18
-7	-4	11	61.23	2.57
-7	-4	11	68.97	2.93
-7	-4	11	63.43	2.88
-7	-4	11	66.12	2.85
-7	-4	11	67.68	3.86
7	4	-11	62.69	3.79
7	4	-11	61.80	2.51

7	4	-11	67.27	2.97
-8	-4	11	0.06	0.49
-8	-4	11	0.59	0.27
-8	-4	11	0.98	0.43
-8	-4	11	0.30	0.56
-8	-4	11	0.50	1.12
8	4	-11	0.75	0.20
-9	-4	11	16.30	0.88
-9	-4	11	16.72	1.22
-9	-4	11	15.56	1.18
-9	-4	11	13.36	2.31
-9	-4	11	15.85	1.35
9	4	-11	15.41	0.79
9	4	-11	17.11	1.35
-10	-4	11	20.87	2.55
-10	-4	11	22.47	1.13
-10	-4	11	22.61	1.47
-10	-4	11	21.11	1.19
10	4	-11	21.58	1.03
10	4	-11	24.16	1.45
-11	-4	11	10.10	0.82
-11	-4	11	9.33	1.13
-11	-4	11	14.51	2.41
11	4	-11	9.09	0.65
-12	-4	11	4.24	0.78
-12	-4	11	1.67	0.48
-12	-4	11	1.35	1.48
12	4	-11	1.53	0.34
-13	-4	11	21.66	1.66
-13	-4	11	22.37	1.29
-13	-4	11	19.70	3.03
13	4	-11	18.81	1.08
-14	-4	11	0.93	0.60
-14	-4	11	1.71	1.74
-14	-4	11	-0.29	0.75
14	4	-11	0.01	0.35
-15	-4	11	13.53	1.69
-15	-4	11	12.49	2.37
15	4	-11	11.56	0.97
-16	-4	11	7.73	1.28
-16	-4	11	8.54	2.36
16	4	-11	7.68	0.90
-17	-4	11	17.96	2.76
-17	-4	11	14.96	1.98

17	4	-11	14.03	1.17
-18	-4	11	6.96	2.48
-18	-4	11	4.51	1.31
18	4	-11	5.73	0.78
-19	-4	11	12.83	2.38
-19	-4	11	10.27	2.58
19	4	-11	10.67	1.30
16	-4	12	41.17	2.46
16	-4	12	38.68	3.50
-16	4	-12	38.83	3.10
15	-4	12	1.66	0.84
15	-4	12	0.97	1.48
-15	4	-12	2.22	1.37
14	-4	12	71.72	4.18
14	-4	12	78.51	3.50
-14	4	-12	72.52	3.79
13	-4	12	2.69	1.36
13	-4	12	2.32	0.78
-13	4	-12	1.45	1.81
12	-4	12	53.07	2.55
12	-4	12	48.50	3.27
-12	4	-12	52.53	2.84
11	-4	12	1.10	0.59
11	-4	12	-1.54	1.10
-11	4	-12	0.38	0.79
10	-4	12	63.50	2.95
10	-4	12	65.39	3.68
10	-4	12	62.42	3.04
-10	4	-12	64.72	3.06
9	-4	12	0.11	0.94
9	-4	12	2.15	0.54
9	-4	12	0.30	0.60
-9	4	-12	1.51	0.70
8	-4	12	70.19	3.19
8	-4	12	74.71	3.14
8	-4	12	76.72	3.15
-8	4	-12	74.63	3.14
-8	4	-12	72.68	3.28
7	-4	12	-0.77	0.77
7	-4	12	-0.56	0.58
7	-4	12	-0.37	0.54
7	-4	12	-0.09	0.38
-7	4	-12	0.70	0.37
-7	4	-12	0.14	0.38

-7	4	-12	-0.45	0.55
6	-4	12	52.34	2.59
6	-4	12	53.36	3.02
6	-4	12	57.59	2.50
-6	4	-12	53.07	2.50
-6	4	-12	53.49	2.39
-6	4	-12	57.64	2.32
-6	4	-12	53.44	2.72
5	-4	12	6.45	1.16
5	-4	12	8.64	0.95
5	-4	12	6.62	1.16
-5	4	-12	7.84	1.12
-5	4	-12	5.16	0.88
-5	4	-12	7.05	0.71
-5	4	-12	6.65	0.65
4	-4	12	67.58	2.79
4	-4	12	59.12	3.27
4	-4	12	64.52	3.11
-4	4	-12	62.07	3.24
-4	4	-12	67.70	2.60
-4	4	-12	61.63	2.64
-4	4	-12	63.05	3.47
-4	4	-12	61.68	2.75
3	-4	12	1.68	0.85
3	-4	12	1.37	0.87
3	-4	12	1.71	0.51
-3	4	-12	0.86	0.90
-3	4	-12	0.26	0.30
-3	4	-12	1.27	0.29
-3	4	-12	1.46	0.47
-3	4	-12	1.84	0.96
2	-4	12	91.47	4.35
2	-4	12	88.71	4.34
2	-4	12	98.71	3.88
-2	4	-12	90.20	4.46
-2	4	-12	97.88	3.67
-2	4	-12	93.27	4.36
-2	4	-12	95.16	3.84
-2	4	-12	94.73	3.71
1	-4	12	8.23	0.90
1	-4	12	7.78	1.35
1	-4	12	8.65	1.59
-1	4	-12	9.00	1.39
-1	4	-12	8.51	1.42

-1	4	-12	10.27	0.62
-1	4	-12	8.94	0.84
-1	4	-12	9.17	0.58
0	-4	12	87.11	4.23
0	-4	12	90.50	4.49
0	-4	12	96.05	3.85
0	4	-12	97.75	4.28
0	4	-12	94.29	3.60
0	4	-12	93.58	3.62
0	4	-12	94.63	3.79
0	4	-12	89.11	4.56
-1	-4	12	1.85	0.79
-1	-4	12	3.05	0.55
-1	-4	12	2.77	1.03
1	4	-12	3.08	0.37
1	4	-12	2.67	0.48
1	4	-12	2.37	1.10
1	4	-12	1.07	0.80
-2	-4	12	82.45	3.42
-2	-4	12	75.50	3.72
-2	-4	12	79.41	4.17
2	4	-12	82.05	3.14
2	4	-12	79.32	4.25
2	4	-12	85.36	3.14
2	4	-12	79.25	3.34
2	4	-12	82.08	3.73
-3	-4	12	0.35	0.45
-3	-4	12	1.18	0.68
-3	-4	12	1.09	1.03
3	4	-12	0.09	0.19
3	4	-12	0.31	0.75
3	4	-12	-0.90	1.03
3	4	-12	0.78	0.39
-4	-4	12	79.58	4.25
-4	-4	12	82.10	3.39
-4	-4	12	79.99	3.09
-4	-4	12	76.17	3.60
4	4	-12	74.20	4.29
4	4	-12	82.90	3.64
4	4	-12	79.92	3.30
4	4	-12	78.22	3.05
-5	-4	12	12.07	1.32
-5	-4	12	15.29	1.16
-5	-4	12	10.41	1.96

-5	-4	12	14.11	0.73
5	4	-12	14.41	0.66
5	4	-12	10.28	1.68
5	4	-12	13.13	0.98
5	4	-12	13.51	1.42
-6	-4	12	76.32	2.97
-6	-4	12	75.96	3.24
-6	-4	12	73.73	3.39
-6	-4	12	75.58	3.32
6	4	-12	82.34	3.48
6	4	-12	73.78	2.92
6	4	-12	71.04	4.30
-7	-4	12	0.44	0.45
-7	-4	12	-1.05	0.53
-7	-4	12	-0.07	0.28
-7	-4	12	-0.53	0.56
-7	-4	12	-0.43	1.07
7	4	-12	0.50	0.19
7	4	-12	0.28	0.70
-8	-4	12	53.08	2.51
-8	-4	12	55.12	2.65
-8	-4	12	53.61	2.21
-8	-4	12	52.35	2.53
8	4	-12	51.88	2.15
8	4	-12	60.73	2.70
-9	-4	12	0.95	0.51
-9	-4	12	0.51	0.32
-9	-4	12	0.32	0.55
-9	-4	12	1.67	1.44
9	4	-12	0.24	0.23
-10	-4	12	92.88	3.89
-10	-4	12	96.09	4.10
-10	-4	12	96.68	3.75
10	4	-12	98.01	4.01
10	4	-12	89.49	3.67
-11	-4	12	4.82	0.55
-11	-4	12	3.20	1.63
11	4	-12	4.40	0.75
11	4	-12	4.07	0.53
-12	-4	12	60.30	4.16
-12	-4	12	56.36	2.53
-12	-4	12	62.12	2.95
12	4	-12	58.76	2.43
-13	-4	12	0.89	0.56

-13	-4	12	0.71	0.76
-13	-4	12	0.44	1.78
13	4	-12	1.67	0.39
-14	-4	12	28.45	3.58
-14	-4	12	18.90	1.84
-14	-4	12	20.10	1.50
14	4	-12	19.20	1.12
-15	-4	12	2.45	2.18
-15	-4	12	1.93	0.93
15	4	-12	0.50	0.46
-16	-4	12	26.35	3.89
-16	-4	12	23.93	2.22
16	4	-12	24.80	1.42
-17	-4	12	7.82	1.42
-17	-4	12	5.94	2.41
17	4	-12	6.30	1.01
-18	-4	12	26.01	2.41
-18	-4	12	14.75	2.82
18	4	-12	17.37	1.40
15	-4	13	20.40	2.86
15	-4	13	20.53	1.79
-15	4	-13	21.12	2.53
14	-4	13	15.40	2.65
14	-4	13	14.93	1.59
-14	4	-13	17.24	2.19
13	-4	13	29.46	2.83
13	-4	13	32.81	1.89
-13	4	-13	24.21	2.59
12	-4	13	4.59	1.46
12	-4	13	5.19	0.86
-12	4	-13	5.01	1.04
11	-4	13	36.31	2.01
11	-4	13	41.90	3.04
-11	4	-13	35.68	2.29
10	-4	13	-1.04	1.10
10	-4	13	0.58	0.49
10	-4	13	0.55	0.75
-10	4	-13	0.18	0.75
9	-4	13	52.59	3.28
9	-4	13	51.47	2.25
9	-4	13	50.54	2.35
9	-4	13	47.52	2.42
-9	4	-13	49.89	2.39
-9	4	-13	50.15	2.53

8	-4	13	1.10	0.62
8	-4	13	1.85	0.62
8	-4	13	1.76	0.53
8	-4	13	1.19	1.15
-8	4	-13	2.48	0.55
-8	4	-13	1.33	0.67
-8	4	-13	1.20	1.00
7	-4	13	42.40	2.77
7	-4	13	40.13	2.13
7	-4	13	44.29	2.10
-7	4	-13	40.75	2.00
-7	4	-13	40.90	1.89
-7	4	-13	41.02	2.11
-7	4	-13	40.54	2.29
6	-4	13	19.37	1.56
6	-4	13	20.76	1.39
6	-4	13	16.64	2.08
-6	4	-13	20.38	1.83
-6	4	-13	20.12	1.20
-6	4	-13	20.32	1.12
-6	4	-13	20.94	1.37
5	-4	13	74.75	3.09
5	-4	13	69.74	3.32
5	-4	13	67.64	3.62
-5	4	-13	69.46	2.96
-5	4	-13	67.78	3.50
-5	4	-13	68.61	3.06
-5	4	-13	74.78	2.90
4	-4	13	9.99	1.43
4	-4	13	8.52	1.58
4	-4	13	10.60	1.05
-4	4	-13	11.68	0.76
-4	4	-13	8.29	0.93
-4	4	-13	10.85	0.80
-4	4	-13	9.15	1.73
3	-4	13	69.20	3.65
3	-4	13	77.96	3.13
3	-4	13	69.80	3.55
-3	4	-13	68.42	2.93
-3	4	-13	70.17	3.06
-3	4	-13	75.84	2.90
-3	4	-13	70.51	3.74
2	-4	13	0.32	0.92
2	-4	13	0.03	0.50

2	-4	13	0.12	0.74
-2	4	-13	-0.95	0.94
-2	4	-13	0.41	0.45
-2	4	-13	0.47	1.08
-2	4	-13	0.12	0.26
-2	4	-13	1.08	0.31
1	-4	13	95.77	4.63
1	-4	13	105.97	4.18
1	-4	13	99.31	4.75
-1	4	-13	103.08	4.71
-1	4	-13	99.37	4.91
-1	4	-13	105.15	3.94
-1	4	-13	100.29	4.10
-1	4	-13	99.26	3.96
0	-4	13	15.38	1.97
0	-4	13	18.67	1.71
0	-4	13	20.97	1.33
0	4	-13	23.11	1.81
0	4	-13	19.95	1.19
0	4	-13	21.27	0.97
0	4	-13	18.73	0.96
0	4	-13	13.91	2.17
-1	-4	13	114.24	4.46
-1	-4	13	98.88	4.78
-1	-4	13	110.94	5.19
1	4	-13	99.29	5.25
1	4	-13	109.17	4.17
1	4	-13	111.24	4.84
1	4	-13	108.65	4.16
1	4	-13	109.62	4.36
-2	-4	13	-0.88	0.49
-2	-4	13	-0.41	1.04
-2	-4	13	0.06	0.72
2	4	-13	-0.59	0.85
2	4	-13	0.44	1.18
2	4	-13	0.22	0.38
2	4	-13	0.08	0.21
2	4	-13	0.02	0.23
-3	-4	13	115.79	5.54
-3	-4	13	108.79	4.95
-3	-4	13	118.82	4.68
3	4	-13	102.92	5.61
3	4	-13	114.01	4.58
3	4	-13	117.73	4.99

3	4	-13	116.74	4.36
-4	-4	13	0.66	0.30
-4	-4	13	2.14	1.16
-4	-4	13	1.21	0.78
-4	-4	13	0.09	0.53
4	4	-13	1.02	0.22
4	4	-13	0.67	0.44
4	4	-13	-1.34	1.31
4	4	-13	1.09	0.86
-5	-4	13	55.76	2.81
-5	-4	13	68.57	3.95
-5	-4	13	62.63	3.14
-5	-4	13	69.29	2.99
5	4	-13	67.59	2.86
5	4	-13	71.80	3.27
5	4	-13	65.82	2.57
-6	-4	13	1.96	0.54
-6	-4	13	2.59	0.77
-6	-4	13	3.03	0.69
-6	-4	13	0.61	1.25
-6	-4	13	2.36	0.37
6	4	-13	1.76	0.83
6	4	-13	2.42	0.36
-7	-4	13	136.69	5.53
-7	-4	13	138.89	5.63
-7	-4	13	135.44	6.53
-7	-4	13	138.08	5.24
-7	-4	13	129.64	5.61
7	4	-13	133.36	5.18
7	4	-13	145.09	5.72
-8	-4	13	2.36	0.36
-8	-4	13	1.83	0.56
-8	-4	13	1.59	0.86
-8	-4	13	1.78	1.42
-8	-4	13	2.34	0.72
8	4	-13	1.98	0.28
8	4	-13	0.77	0.86
-9	-4	13	116.46	5.09
-9	-4	13	127.07	5.23
-9	-4	13	123.39	4.88
-9	-4	13	141.30	6.46
9	4	-13	117.84	4.81
9	4	-13	136.09	5.28
-10	-4	13	0.68	0.57

-10	-4	13	0.73	0.58
-10	-4	13	0.08	0.38
-10	-4	13	0.60	1.51
10	4	-13	0.13	0.95
10	4	-13	0.12	0.26
-11	-4	13	83.70	3.33
-11	-4	13	80.98	4.96
-11	-4	13	88.27	3.75
-11	-4	13	70.80	3.29
11	4	-13	85.27	3.52
11	4	-13	81.21	3.22
-12	-4	13	0.19	0.69
-12	-4	13	-0.97	1.68
-12	-4	13	1.30	0.52
12	4	-13	-0.04	0.31
-13	-4	13	45.35	2.57
-13	-4	13	44.69	4.05
13	4	-13	44.40	1.95
-14	-4	13	0.41	0.84
-14	-4	13	3.80	2.16
14	4	-13	0.88	0.41
-15	-4	13	30.48	4.05
-15	-4	13	30.76	2.34
15	4	-13	29.77	1.54
-16	-4	13	2.50	1.17
-16	-4	13	-2.56	2.06
16	4	-13	1.91	0.56
-17	-4	13	30.18	4.40
-17	-4	13	35.44	2.71
17	4	-13	33.92	1.80
-18	-4	13	-1.18	1.31
-18	-4	13	-2.65	2.38
18	4	-13	0.54	0.69
15	-4	14	8.30	1.16
15	-4	14	12.16	2.16
-15	4	-14	6.60	1.55
14	-4	14	6.81	1.05
14	-4	14	8.01	1.85
-14	4	-14	7.78	1.43
13	-4	14	7.90	1.03
13	-4	14	5.95	1.68
-13	4	-14	9.46	1.37
-13	4	-14	8.15	1.16
12	-4	14	1.91	1.48

12	-4	14	2.29	0.73
-12	4	-14	2.97	0.86
-12	4	-14	4.09	1.09
11	-4	14	10.46	1.27
11	-4	14	10.37	1.77
-11	4	-14	11.90	1.61
-11	4	-14	11.12	1.32
10	-4	14	6.57	0.95
10	-4	14	7.15	1.51
-10	4	-14	8.84	1.15
-10	4	-14	5.30	0.98
9	-4	14	10.15	1.66
9	-4	14	8.62	1.32
9	-4	14	10.36	1.12
-9	4	-14	6.88	0.90
-9	4	-14	8.46	1.33
8	-4	14	31.81	1.78
8	-4	14	25.72	1.74
8	-4	14	28.64	2.63
-8	4	-14	31.25	1.67
-8	4	-14	27.67	1.80
-8	4	-14	27.63	1.92
7	-4	14	8.82	1.07
7	-4	14	9.40	1.17
7	-4	14	8.78	1.52
-7	4	-14	9.15	1.64
-7	4	-14	8.64	0.95
-7	4	-14	8.71	1.15
6	-4	14	35.69	2.12
6	-4	14	33.83	1.88
6	-4	14	33.65	2.59
-6	4	-14	35.10	1.89
-6	4	-14	34.86	1.75
-6	4	-14	34.93	2.47
-6	4	-14	37.52	1.70
5	-4	14	21.57	1.38
5	-4	14	18.11	2.20
5	-4	14	16.75	1.63
-5	4	-14	16.79	1.28
-5	4	-14	16.91	1.12
-5	4	-14	19.27	1.08
-5	4	-14	18.42	2.07
4	-4	14	69.46	2.99
4	-4	14	65.28	3.61

4	-4	14	65.99	3.37
-4	4	-14	65.83	2.85
-4	4	-14	66.73	2.95
-4	4	-14	66.15	3.66
-4	4	-14	72.77	2.80
3	-4	14	15.11	1.23
3	-4	14	12.45	1.74
3	-4	14	14.39	1.72
-3	4	-14	13.47	0.90
-3	4	-14	14.76	2.16
-3	4	-14	12.30	1.08
-3	4	-14	14.33	0.89
2	-4	14	38.13	2.58
2	-4	14	36.05	2.60
2	-4	14	39.95	2.04
-2	4	-14	39.90	1.95
-2	4	-14	42.11	1.80
-2	4	-14	42.82	2.97
-2	4	-14	42.00	1.77
1	-4	14	6.55	1.28
1	-4	14	4.23	1.07
1	-4	14	6.06	0.77
-1	4	-14	5.09	0.57
-1	4	-14	3.86	0.55
-1	4	-14	4.95	0.79
-1	4	-14	5.15	1.43
0	-4	14	2.91	1.22
0	-4	14	6.19	0.99
0	-4	14	2.50	0.98
0	4	-14	2.61	1.04
0	4	-14	4.01	0.51
0	4	-14	2.17	1.37
0	4	-14	4.84	0.62
0	4	-14	4.47	0.54
-1	-4	14	12.81	2.04
-1	-4	14	10.71	1.11
-1	-4	14	8.98	1.55
1	4	-14	8.92	0.91
1	4	-14	8.00	1.76
1	4	-14	9.83	0.64
1	4	-14	9.26	1.41
1	4	-14	9.97	0.67
-2	-4	14	57.18	2.58
-2	-4	14	51.41	2.95

-2	-4	14	52.83	3.41
2	4	-14	55.09	3.05
2	4	-14	56.48	2.21
2	4	-14	57.50	2.23
2	4	-14	46.35	3.61
2	4	-14	54.39	2.44
-3	-4	14	4.47	1.00
-3	-4	14	6.35	1.02
-3	-4	14	5.07	1.46
3	4	-14	3.76	0.51
3	4	-14	5.15	0.68
3	4	-14	4.33	0.46
3	4	-14	3.08	1.04
-4	-4	14	58.94	2.72
-4	-4	14	64.12	3.77
-4	-4	14	59.24	2.36
-4	-4	14	53.66	3.00
4	4	-14	59.46	3.11
4	4	-14	53.52	2.56
4	4	-14	58.28	2.30
-5	-4	14	6.79	0.87
-5	-4	14	5.75	0.59
-5	-4	14	7.11	1.62
-5	-4	14	4.58	0.96
-5	-4	14	10.25	1.16
5	4	-14	9.02	1.63
5	4	-14	6.82	0.89
5	4	-14	6.10	0.48
-6	-4	14	56.91	2.83
-6	-4	14	61.57	2.45
-6	-4	14	56.43	2.98
-6	-4	14	60.73	2.75
-6	-4	14	63.47	3.93
6	4	-14	60.01	2.38
6	4	-14	63.94	3.13
-7	-4	14	29.83	1.68
-7	-4	14	31.83	4.79
-7	-4	14	29.88	1.91
-7	-4	14	29.34	1.36
-7	-4	14	28.55	1.86
7	4	-14	28.25	1.27
7	4	-14	32.74	2.16
-8	-4	14	44.93	1.86
-8	-4	14	36.69	3.33

-8	-4	14	41.83	2.30
-8	-4	14	45.25	2.20
8	4	-14	42.07	1.76
8	4	-14	43.30	2.52
-9	-4	14	1.28	0.73
-9	-4	14	2.15	1.69
-9	-4	14	2.71	0.44
-9	-4	14	2.34	0.67
9	4	-14	1.49	0.30
9	4	-14	2.49	1.01
-10	-4	14	68.46	3.28
-10	-4	14	62.50	4.45
-10	-4	14	68.15	2.76
-10	-4	14	61.38	2.93
10	4	-14	71.95	3.23
10	4	-14	64.85	2.65
-11	-4	14	0.52	0.71
-11	-4	14	-2.54	1.66
-11	-4	14	0.48	0.66
-11	-4	14	0.64	0.48
11	4	-14	0.69	0.93
11	4	-14	0.52	0.32
-12	-4	14	25.77	3.55
-12	-4	14	24.08	1.84
12	4	-14	22.67	1.18
-13	-4	14	6.71	1.13
-13	-4	14	6.20	2.15
13	4	-14	8.15	0.77
-14	-4	14	37.74	4.26
-14	-4	14	39.02	2.52
14	4	-14	37.80	1.77
-15	-4	14	9.14	1.41
-15	-4	14	11.35	2.40
15	4	-14	8.16	0.90
-16	-4	14	37.81	4.37
-16	-4	14	40.67	2.81
16	4	-14	35.11	2.00
-17	-4	14	-0.28	2.43
-17	-4	14	0.60	1.23
14	-4	15	16.25	2.99
-14	4	-15	17.01	1.96
13	-4	15	4.85	1.73
-13	4	-15	5.18	1.11
-13	4	-15	3.78	1.23

12	-4	15	13.58	2.54
-12	4	-15	8.36	1.16
-12	4	-15	8.72	1.28
11	-4	15	0.94	1.45
-11	4	-15	2.15	0.84
-11	4	-15	2.99	0.98
10	-4	15	5.31	1.54
-10	4	-15	5.29	0.89
-10	4	-15	5.23	0.96
9	-4	15	0.14	0.72
9	-4	15	-0.95	1.30
9	-4	15	0.83	0.85
-9	4	-15	1.65	0.76
-9	4	-15	0.10	0.60
8	-4	15	28.12	1.76
8	-4	15	19.74	1.66
8	-4	15	26.81	2.55
-8	4	-15	24.31	1.68
7	-4	15	-0.98	1.18
7	-4	15	0.25	0.70
7	-4	15	0.66	0.85
-7	4	-15	0.02	0.47
-7	4	-15	0.10	1.12
-7	4	-15	-0.30	0.63
6	-4	15	7.13	1.21
6	-4	15	7.61	1.58
6	-4	15	7.94	1.19
-6	4	-15	7.79	0.91
-6	4	-15	8.55	1.13
-6	4	-15	9.52	1.56
5	-4	15	1.60	0.93
5	-4	15	2.55	0.70
5	-4	15	2.15	1.12
-5	4	-15	1.79	1.34
-5	4	-15	2.28	0.49
-5	4	-15	0.99	0.48
-5	4	-15	1.50	0.63
4	-4	15	0.66	0.94
4	-4	15	2.71	0.69
4	-4	15	-0.11	1.06
-4	4	-15	3.10	1.32
-4	4	-15	1.09	0.57
-4	4	-15	0.99	0.42
-4	4	-15	1.08	0.43

3	-4	15	6.05	1.26
3	-4	15	7.99	1.12
-3	4	-15	6.37	1.59
-3	4	-15	7.22	0.95
-3	4	-15	6.17	0.72
-3	4	-15	6.06	0.73
2	-4	15	11.35	1.88
2	-4	15	16.27	1.32
2	-4	15	14.27	1.83
-2	4	-15	15.74	0.96
-2	4	-15	16.06	1.15
-2	4	-15	15.73	0.95
-2	4	-15	13.48	1.97
1	-4	15	1.02	0.64
1	-4	15	1.53	0.98
1	-4	15	1.08	1.07
-1	4	-15	0.42	0.31
-1	4	-15	0.51	0.47
-1	4	-15	0.56	0.35
-1	4	-15	1.00	1.45
0	-4	15	34.09	2.48
0	-4	15	35.13	2.77
0	-4	15	40.01	2.00
0	4	-15	35.28	1.62
0	4	-15	40.04	1.64
0	4	-15	30.07	3.15
0	4	-15	37.65	1.83
-1	-4	15	0.77	1.19
-1	-4	15	0.08	0.92
-1	-4	15	0.97	0.63
1	4	-15	-0.01	0.45
1	4	-15	-0.21	0.27
1	4	-15	0.34	0.45
-2	-4	15	6.53	1.51
-2	-4	15	7.74	1.30
-2	-4	15	8.13	1.18
2	4	-15	8.67	1.35
2	4	-15	7.38	0.89
2	4	-15	7.28	0.60
2	4	-15	8.33	0.66
-3	-4	15	6.54	1.11
-3	-4	15	2.84	1.37
-3	-4	15	5.95	1.17
3	4	-15	4.07	1.28

3	4	-15	4.15	0.65
3	4	-15	5.13	0.50
3	4	-15	4.76	0.58
-4	-4	15	7.03	0.90
-4	-4	15	8.19	1.65
-4	-4	15	5.72	1.10
4	4	-15	8.21	0.66
4	4	-15	7.14	0.54
4	4	-15	12.14	1.75
4	4	-15	6.69	0.91
-5	-4	15	4.27	0.61
-5	-4	15	4.08	1.00
-5	-4	15	2.94	0.74
-5	-4	15	4.52	0.70
5	4	-15	3.38	0.42
5	4	-15	3.61	1.17
-6	-4	15	25.26	1.54
-6	-4	15	27.52	1.26
-6	-4	15	26.16	2.93
-6	-4	15	23.97	1.91
-6	-4	15	25.40	1.75
6	4	-15	24.94	1.15
6	4	-15	27.65	2.19
-7	-4	15	30.99	1.77
-7	-4	15	32.23	1.46
-7	-4	15	32.21	3.18
-7	-4	15	28.96	2.05
7	4	-15	30.76	1.34
7	4	-15	31.99	2.28
-8	-4	15	16.80	1.33
-8	-4	15	16.82	2.87
-8	-4	15	17.84	0.99
-8	-4	15	11.86	1.50
8	4	-15	13.73	0.80
8	4	-15	15.79	1.94
-9	-4	15	4.69	0.92
-9	-4	15	6.39	0.77
-9	-4	15	5.41	1.93
9	4	-15	6.06	0.56
9	4	-15	9.59	1.76
-10	-4	15	11.43	1.16
-10	-4	15	7.19	2.17
-10	-4	15	12.02	1.29
-10	-4	15	13.12	1.43

10	4	-15	10.71	0.73
10	4	-15	9.52	1.82
-11	-4	15	9.30	2.35
-11	-4	15	11.80	1.50
-11	-4	15	13.24	1.19
11	4	-15	14.29	0.86
-12	-4	15	11.63	1.53
-12	-4	15	15.01	2.63
12	4	-15	10.51	0.82
-13	-4	15	13.79	1.70
-13	-4	15	7.49	2.43
13	4	-15	9.47	0.84
-14	-4	15	11.51	1.88
-14	-4	15	13.06	2.48
14	4	-15	10.47	0.94
-15	-4	15	20.01	2.20
-15	-4	15	22.03	4.00
15	4	-15	21.15	1.27
-16	-4	15	6.83	1.45
-16	-4	15	2.68	2.42
13	-4	16	-1.01	1.79
-13	4	-16	0.82	1.00
12	-4	16	38.05	3.54
-12	4	-16	39.48	2.43
11	-4	16	2.89	1.64
-11	4	-16	2.80	1.03
-11	4	-16	1.68	0.83
10	-4	16	27.27	2.96
-10	4	-16	25.10	1.98
-10	4	-16	25.33	1.84
9	-4	16	-0.57	1.36
-9	4	-16	0.99	0.69
-9	4	-16	2.42	0.85
8	-4	16	13.25	1.59
8	-4	16	11.44	1.91
-8	4	-16	14.06	1.32
-8	4	-16	13.95	1.46
7	-4	16	6.24	1.14
7	-4	16	7.13	0.93
7	-4	16	5.96	1.55
-7	4	-16	4.73	0.78
-7	4	-16	5.44	0.90
6	-4	16	32.47	2.89
6	-4	16	29.93	1.87

6	-4	16	31.83	2.26
-6	4	-16	29.11	1.68
-6	4	-16	35.43	2.92
-6	4	-16	29.99	1.81
5	-4	16	-1.81	1.15
5	-4	16	1.60	1.06
5	-4	16	-1.15	0.70
-5	4	-16	1.83	0.52
-5	4	-16	1.00	0.65
-5	4	-16	0.39	1.45
4	-4	16	31.98	1.85
4	-4	16	28.71	2.58
4	-4	16	33.14	2.37
-4	4	-16	31.40	2.94
-4	4	-16	32.64	1.60
-4	4	-16	31.07	1.76
-4	4	-16	30.98	1.62
3	-4	16	5.90	0.84
3	-4	16	3.33	1.17
3	-4	16	3.81	1.34
-3	4	-16	4.21	0.58
-3	4	-16	4.80	0.75
-3	4	-16	5.19	1.83
-3	4	-16	5.39	0.75
2	-4	16	16.99	2.02
2	-4	16	15.88	1.41
2	-4	16	15.77	2.07
-2	4	-16	18.39	1.05
-2	4	-16	16.95	1.04
-2	4	-16	14.61	1.22
-2	4	-16	15.83	2.82
1	-4	16	1.84	1.22
1	-4	16	3.73	0.74
-1	4	-16	0.84	0.56
-1	4	-16	0.97	0.37
-1	4	-16	0.93	0.40
0	-4	16	28.04	2.42
0	-4	16	28.73	1.84
0	-4	16	28.28	2.62
0	4	-16	29.50	1.41
0	4	-16	29.90	1.61
0	4	-16	30.28	1.41
-1	-4	16	11.50	1.86
-1	-4	16	8.46	1.66

-1	-4	16	11.90	1.32
1	4	-16	12.62	0.82
1	4	-16	9.68	1.02
1	4	-16	10.69	0.76
-2	-4	16	3.03	1.18
-2	-4	16	4.46	1.49
-2	-4	16	4.00	0.78
2	4	-16	3.94	0.54
2	4	-16	3.27	0.45
2	4	-16	3.95	0.65
-3	-4	16	0.98	0.73
-3	-4	16	1.14	0.99
3	4	-16	1.46	0.38
3	4	-16	0.37	0.53
3	4	-16	0.61	0.31
-4	-4	16	1.29	0.73
-4	-4	16	-0.14	1.48
-4	-4	16	1.21	0.96
4	4	-16	0.49	0.29
4	4	-16	0.57	1.21
4	4	-16	1.32	0.38
4	4	-16	0.51	0.52
-5	-4	16	9.21	1.05
-5	-4	16	9.07	1.33
-5	-4	16	9.35	1.39
-5	-4	16	9.13	1.98
-5	-4	16	10.19	0.80
5	4	-16	9.85	1.49
5	4	-16	9.20	0.64
-6	-4	16	20.29	1.15
-6	-4	16	19.62	2.99
-6	-4	16	22.46	1.49
-6	-4	16	23.77	1.95
6	4	-16	22.26	2.24
6	4	-16	20.11	1.01
-7	-4	16	25.44	1.30
-7	-4	16	23.95	1.60
-7	-4	16	32.75	3.32
-7	-4	16	21.93	1.95
7	4	-16	24.95	1.16
7	4	-16	25.60	2.25
-8	-4	16	3.56	1.00
-8	-4	16	3.83	0.61
-8	-4	16	3.94	1.86

-8	-4	16	5.23	0.80
8	4	-16	3.86	1.23
8	4	-16	2.40	0.37
-9	-4	16	13.19	1.44
-9	-4	16	19.16	2.78
-9	-4	16	16.23	1.61
9	4	-16	14.10	0.85
9	4	-16	15.06	2.25
-10	-4	16	0.75	0.81
-10	-4	16	1.59	0.82
-10	-4	16	-0.44	1.84
10	4	-16	1.19	0.35
-11	-4	16	2.47	2.08
-11	-4	16	0.72	0.85
11	4	-16	1.82	0.41
-12	-4	16	0.68	0.95
-12	-4	16	0.92	2.07
12	4	-16	0.52	0.41
-13	-4	16	10.54	1.72
-13	-4	16	4.78	2.36
13	4	-16	7.45	0.84
-14	-4	16	-1.54	2.06
-14	-4	16	0.07	1.07
-15	-4	16	5.65	2.55
-15	-4	16	3.88	1.30
12	-4	17	-0.39	1.95
-12	4	-17	0.52	0.97
11	-4	17	33.03	3.39
-11	4	-17	32.97	2.22
10	-4	17	-0.46	1.57
-10	4	-17	-0.37	0.76
9	-4	17	41.82	3.51
-9	4	-17	40.72	2.38
-9	4	-17	46.58	2.40
8	-4	17	-0.59	1.34
-8	4	-17	0.63	0.81
-8	4	-17	2.11	0.73
7	-4	17	26.06	2.19
7	-4	17	28.07	2.81
-7	4	-17	27.07	1.69
-7	4	-17	27.08	1.80
6	-4	17	0.07	1.13
6	-4	17	1.75	1.42
-6	4	-17	0.16	0.57

-6	4	-17	1.51	0.71
5	-4	17	23.82	2.08
5	-4	17	23.50	2.63
5	-4	17	25.63	2.29
-5	4	-17	22.47	3.38
-5	4	-17	26.19	1.49
-5	4	-17	22.73	1.59
4	-4	17	5.17	0.86
4	-4	17	5.30	1.30
4	-4	17	4.12	1.43
-4	4	-17	1.95	1.74
-4	4	-17	2.65	0.72
-4	4	-17	3.63	0.73
-4	4	-17	4.42	0.66
3	-4	17	20.81	2.39
3	-4	17	18.63	2.31
3	-4	17	21.93	1.60
-3	4	-17	18.65	1.22
-3	4	-17	19.89	1.40
-3	4	-17	20.02	1.24
2	-4	17	-0.22	1.16
2	-4	17	-0.03	0.76
2	-4	17	0.38	1.23
-2	4	-17	-1.09	0.54
-2	4	-17	0.55	0.44
-2	4	-17	0.39	0.45
1	-4	17	46.54	2.36
1	-4	17	41.29	3.02
1	-4	17	48.03	3.22
-1	4	-17	41.90	2.16
-1	4	-17	46.84	2.00
-1	4	-17	43.90	2.01
0	-4	17	0.03	1.21
0	-4	17	0.74	1.13
0	-4	17	0.11	0.75
0	4	-17	1.02	0.40
0	4	-17	0.87	0.57
0	4	-17	0.73	0.44
-1	-4	17	45.82	2.41
1	4	-17	44.82	1.97
1	4	-17	47.58	2.00
1	4	-17	42.67	2.17
-2	-4	17	2.49	1.42
-2	-4	17	0.77	0.82

-2	-4	17	3.83	1.26
2	4	-17	1.09	0.35
2	4	-17	1.62	0.44
2	4	-17	2.20	0.62
-3	-4	17	35.19	2.08
-3	-4	17	32.20	2.58
-3	-4	17	36.28	3.26
3	4	-17	34.74	1.58
3	4	-17	32.47	1.82
3	4	-17	34.24	1.53
-4	-4	17	3.22	1.16
-4	-4	17	4.33	0.72
-4	-4	17	3.17	0.90
-4	-4	17	1.80	1.59
4	4	-17	3.52	0.52
4	4	-17	2.82	0.39
-5	-4	17	5.41	1.86
-5	-4	17	7.68	1.30
-5	-4	17	7.44	1.31
-5	-4	17	7.03	1.12
5	4	-17	7.03	0.62
5	4	-17	8.20	0.80
-6	-4	17	19.37	1.46
-6	-4	17	21.34	3.15
-6	-4	17	17.85	1.99
6	4	-17	19.45	0.99
6	4	-17	20.80	2.46
-7	-4	17	28.05	3.51
-7	-4	17	31.84	1.86
-7	-4	17	29.98	2.29
7	4	-17	29.31	2.65
7	4	-17	29.04	1.35
-8	-4	17	1.21	0.98
-8	-4	17	0.54	1.92
-8	-4	17	0.66	0.72
8	4	-17	-0.02	0.31
8	4	-17	2.94	1.89
-9	-4	17	27.69	3.53
-9	-4	17	27.00	1.88
-9	-4	17	22.49	1.98
9	4	-17	23.89	1.20
-10	-4	17	-0.27	0.83
-10	-4	17	1.02	1.33
-10	-4	17	1.43	1.94

10	4	-17	0.01	0.36
-11	-4	17	30.37	2.13
-11	-4	17	34.72	3.96
11	4	-17	27.04	1.39
-12	-4	17	-1.10	2.03
-12	-4	17	0.48	0.95
-13	-4	17	14.83	2.74
-13	-4	17	10.53	1.46
-14	-4	17	0.81	1.19
-14	-4	17	-1.97	2.29
-15	-4	17	5.13	1.51
10	-4	18	12.11	2.15
-10	4	-18	14.54	1.70
9	-4	18	-1.27	1.56
-9	4	-18	0.35	0.80
8	-4	18	31.32	3.15
-8	4	-18	33.36	2.04
7	-4	18	-0.27	1.54
7	-4	18	-0.12	1.24
-7	4	-18	-0.71	0.96
-7	4	-18	0.83	0.72
6	-4	18	38.94	2.82
6	-4	18	40.42	3.31
-6	4	-18	40.22	2.11
-6	4	-18	38.77	2.17
5	-4	18	-0.06	1.28
5	-4	18	-0.48	1.41
-5	4	-18	-0.58	0.56
-5	4	-18	1.10	0.70
4	-4	18	39.82	2.91
4	-4	18	32.73	3.07
-4	4	-18	36.22	1.87
-4	4	-18	33.26	1.92
3	-4	18	2.96	1.50
3	-4	18	5.81	1.45
-3	4	-18	4.00	0.65
-3	4	-18	1.75	0.69
2	-4	18	43.21	3.38
2	-4	18	46.58	3.31
-2	4	-18	45.94	2.26
-2	4	-18	48.10	2.17
-2	4	-18	47.25	2.15
1	-4	18	1.52	0.90
1	-4	18	1.89	2.22

1	-4	18	2.28	1.38
-1	4	-18	2.16	0.67
-1	4	-18	2.66	0.55
-1	4	-18	2.43	0.53
0	-4	18	52.02	2.72
0	-4	18	52.49	3.63
0	-4	18	54.96	3.52
0	4	-18	48.09	2.45
0	4	-18	53.05	2.31
0	4	-18	56.19	2.32
-1	-4	18	8.88	1.61
-1	-4	18	5.43	1.03
-1	-4	18	5.97	1.76
1	4	-18	4.97	0.60
1	4	-18	5.70	0.79
1	4	-18	7.45	0.85
-2	-4	18	39.77	3.48
-2	-4	18	41.50	2.37
-2	-4	18	38.34	2.85
2	4	-18	43.26	1.86
2	4	-18	36.94	2.03
2	4	-18	40.72	1.82
-3	-4	18	-1.01	0.91
-3	-4	18	-0.97	1.59
-3	-4	18	-0.62	0.60
3	4	-18	-0.28	0.50
3	4	-18	0.03	0.37
-4	-4	18	50.58	2.49
-4	-4	18	48.85	3.10
-4	-4	18	51.38	3.97
-4	-4	18	51.26	2.91
4	4	-18	48.78	2.11
-5	-4	18	0.64	0.63
-5	-4	18	0.86	1.14
-5	-4	18	-1.80	1.63
5	4	-18	0.24	0.36
-6	-4	18	36.52	2.05
-6	-4	18	38.11	3.71
-6	-4	18	35.12	2.63
6	4	-18	34.36	1.59
-7	-4	18	4.34	1.26
-7	-4	18	6.34	1.08
-7	-4	18	8.30	2.16
7	4	-18	6.18	0.67

-8	-4	18	33.57	2.06
-8	-4	18	35.73	3.84
-8	-4	18	29.12	2.36
8	4	-18	28.23	1.40
-9	-4	18	-0.66	0.88
-9	-4	18	0.01	1.03
-9	-4	18	0.51	1.89
9	4	-18	0.11	0.36
-10	-4	18	42.22	4.35
-10	-4	18	40.83	2.59
-11	-4	18	0.58	0.99
-11	-4	18	0.15	2.11
-12	-4	18	35.48	2.55
-12	-4	18	29.10	4.35
-13	-4	18	2.44	2.37
-13	-4	18	1.00	1.18
9	-4	19	23.62	3.60
-9	4	-19	27.27	2.07
8	-4	19	-0.42	1.86
-8	4	-19	2.33	0.91
7	-4	19	29.52	3.38
-7	4	-19	26.29	1.88
6	-4	19	4.12	1.71
6	-4	19	4.41	1.56
-6	4	-19	4.11	0.83
5	-4	19	48.19	3.23
5	-4	19	40.74	3.45
-5	4	-19	45.65	2.31
4	-4	19	0.83	1.51
4	-4	19	1.29	1.40
-4	4	-19	0.62	0.84
-4	4	-19	1.77	0.65
3	-4	19	31.48	3.19
3	-4	19	29.19	3.58
-3	4	-19	33.72	1.89
-3	4	-19	34.52	1.80
2	-4	19	-1.42	1.38
2	-4	19	-2.54	1.44
-2	4	-19	0.61	0.56
-2	4	-19	-0.76	0.63
1	-4	19	28.50	3.10
1	-4	19	22.02	2.88
-1	4	-19	26.32	1.53
-1	4	-19	28.66	1.66

0	-4	19	0.70	1.42
0	-4	19	-0.11	1.54
0	4	-19	-0.76	0.48
0	4	-19	0.87	0.64
-1	-4	19	22.53	2.69
-1	-4	19	24.58	3.20
1	4	-19	24.26	1.58
1	4	-19	25.67	1.37
-2	-4	19	-1.66	1.29
-2	-4	19	1.16	1.67
2	4	-19	0.60	0.64
2	4	-19	0.03	0.41
-3	-4	19	68.13	3.15
-3	-4	19	62.68	3.89
-3	-4	19	73.94	4.79
3	4	-19	69.48	2.83
-4	-4	19	1.83	1.81
-4	-4	19	0.84	0.71
-4	-4	19	-0.56	1.20
4	4	-19	0.34	0.40
-5	-4	19	42.35	3.84
-5	-4	19	37.08	2.16
5	4	-19	36.76	1.70
-6	-4	19	1.52	1.93
-6	-4	19	1.13	1.30
-7	-4	19	72.22	4.92
-7	-4	19	68.11	3.23
-7	-4	19	60.12	3.60
7	4	-19	63.34	2.75
-8	-4	19	1.86	0.95
-8	-4	19	1.05	1.23
-8	-4	19	1.26	2.06
-9	-4	19	26.07	4.11
-9	-4	19	33.94	2.31
-10	-4	19	-0.12	0.99
-10	-4	19	-0.96	2.14
-11	-4	19	34.15	4.50
-11	-4	19	40.15	2.72
-12	-4	19	0.71	1.15
-12	-4	19	-1.53	2.55
7	-4	20	-3.43	1.87
-7	4	-20	1.02	0.90
6	-4	20	21.22	3.11
-6	4	-20	18.86	1.72

5	-4	20	-2.64	3.07
5	-4	20	3.71	1.75
-5	4	-20	2.33	0.83
4	-4	20	3.25	1.84
4	-4	20	4.80	1.75
-4	4	-20	6.38	0.89
3	-4	20	5.84	1.86
3	-4	20	7.99	1.87
-3	4	-20	6.52	0.89
2	-4	20	18.21	2.99
2	-4	20	17.37	2.83
-2	4	-20	15.50	1.32
1	-4	20	-0.69	1.52
1	-4	20	3.64	1.75
-1	4	-20	0.32	0.58
0	-4	20	25.24	3.03
0	-4	20	26.85	3.44
0	4	-20	27.42	1.53
0	4	-20	24.88	1.66
-1	-4	20	0.72	1.61
-1	-4	20	2.33	1.88
1	4	-20	3.30	0.77
1	4	-20	3.38	0.60
-2	-4	20	25.52	1.81
-2	-4	20	24.72	2.93
-2	-4	20	30.96	3.63
2	4	-20	27.23	1.46
-3	-4	20	5.58	1.71
-3	-4	20	2.18	1.95
-3	-4	20	4.68	0.90
3	4	-20	4.97	0.60
-4	-4	20	34.16	3.10
-4	-4	20	40.73	4.06
-4	-4	20	37.42	2.18
4	4	-20	38.65	1.92
-5	-4	20	0.94	1.53
-5	-4	20	0.22	1.45
-5	-4	20	1.19	2.01
-6	-4	20	33.83	2.19
-6	-4	20	32.65	2.84
-6	-4	20	33.71	4.12
-7	-4	20	-1.73	2.07
-7	-4	20	0.45	1.56
-7	-4	20	-0.34	0.92

-8	-4	20	32.17	4.27
-8	-4	20	35.89	2.48
-9	-4	20	1.71	1.11
-9	-4	20	4.82	2.42
-10	-4	20	45.41	2.91
-10	-4	20	40.85	4.78
-11	-4	20	2.84	1.29
5	-4	21	-3.67	1.85
-5	4	-21	-1.35	0.81
4	-4	21	10.76	2.22
4	-4	21	10.67	2.20
-4	4	-21	10.07	1.45
3	-4	21	2.76	1.87
3	-4	21	0.01	1.82
-3	4	-21	-0.85	0.71
2	-4	21	1.20	1.90
2	-4	21	7.34	2.17
1	-4	21	3.83	2.02
1	-4	21	3.45	1.91
-1	4	-21	2.04	0.70
0	-4	21	3.27	1.97
0	-4	21	1.68	1.79
0	4	-21	2.85	1.14
-1	-4	21	3.93	1.82
-1	-4	21	2.23	2.10
-2	-4	21	5.92	1.91
-2	-4	21	10.39	1.57
-2	-4	21	8.61	2.20
-3	-4	21	2.13	2.22
-3	-4	21	7.11	1.01
-3	-4	21	3.92	1.76
-4	-4	21	6.71	2.35
-4	-4	21	4.49	1.72
-4	-4	21	2.40	1.17
-5	-4	21	7.99	1.80
-5	-4	21	7.53	2.48
-5	-4	21	10.95	1.66
-6	-4	21	5.05	2.43
-6	-4	21	3.30	1.13
-6	-4	21	2.56	1.58
-7	-4	21	10.66	2.16
-7	-4	21	12.01	2.68
-7	-4	21	14.61	2.00
-8	-4	21	3.09	1.19

-8	-4	21	2.17	2.40
1	-4	22	4.32	2.16
1	-4	22	-2.58	1.85
0	-4	22	9.69	2.17
0	-4	22	18.98	2.80
-1	-4	22	11.89	2.26
-1	-4	22	16.37	2.91
-2	-4	22	8.42	2.40
-2	-4	22	5.43	1.95
-3	-4	22	12.07	2.11
-3	-4	22	13.10	2.72
-4	-4	22	-1.88	2.24
-4	-4	22	1.85	1.74
-5	-4	22	-0.56	2.42
-5	-4	22	1.23	1.76
4	-5	-22	10.35	1.50
3	-5	-22	0.29	0.84
-3	5	22	1.05	1.81
-3	5	22	4.76	2.05
2	-5	-22	5.89	0.98
-2	5	22	5.59	1.89
-2	5	22	4.00	2.02
1	-5	-22	6.57	1.07
-1	5	22	3.33	2.06
-1	5	22	10.42	2.03
0	-5	-22	6.26	1.07
0	5	22	7.64	2.02
0	5	22	6.51	2.08
8	-5	-21	1.25	0.76
-8	5	21	4.05	3.05
7	-5	-21	11.00	1.33
-7	5	21	9.98	2.39
-7	5	21	11.89	2.45
6	-5	-21	3.63	0.81
-6	5	21	6.04	1.72
-6	5	21	3.55	2.06
5	-5	-21	9.43	1.32
-5	5	21	10.24	2.35
-5	5	21	8.55	1.84
4	-5	-21	9.57	1.33
-4	5	21	11.23	1.97
-4	5	21	8.64	2.10
3	-5	-21	9.05	1.34
-3	5	21	9.13	2.08

-3	5	21	10.03	1.89
2	-5	-21	4.84	0.86
-2	5	21	5.78	1.78
-2	5	21	2.12	1.65
1	-5	-21	14.98	1.51
-1	5	21	15.42	2.30
-1	5	21	17.03	2.77
0	-5	-21	1.20	0.77
0	5	21	0.57	1.71
0	5	21	1.58	1.64
-1	-5	-21	11.82	1.55
1	5	21	14.81	2.74
1	5	21	15.85	2.91
-2	-5	-21	2.98	0.86
2	5	21	2.62	1.74
2	5	21	1.49	1.75
-3	-5	-21	28.37	2.08
3	5	21	27.79	3.46
3	5	21	30.63	3.27
-4	-5	-21	-0.76	0.82
4	5	21	0.13	1.69
4	5	21	2.49	1.88
10	-5	-20	0.30	0.70
-10	5	20	2.41	2.50
9	-5	-20	25.29	1.70
-9	5	20	25.42	3.60
8	-5	-20	0.05	0.66
-8	5	20	-1.19	1.90
7	-5	-20	24.05	1.62
-7	5	20	22.83	3.29
-7	5	20	23.81	2.58
6	-5	-20	5.64	0.82
-6	5	20	5.81	1.52
-6	5	20	8.12	1.99
5	-5	-20	26.82	1.68
-5	5	20	22.33	2.73
-5	5	20	24.23	3.28
4	-5	-20	4.38	0.82
-4	5	20	8.42	2.06
-4	5	20	4.91	1.58
3	-5	-20	37.74	2.00
-3	5	20	29.90	3.02
-3	5	20	33.28	3.34
2	-5	-20	1.59	0.71

-2	5	20	2.64	1.61
-2	5	20	1.13	1.53
1	-5	-20	18.40	1.53
-1	5	20	18.61	2.78
-1	5	20	18.82	2.70
0	-5	-20	11.77	1.35
0	5	20	9.04	1.82
0	5	20	10.58	1.86
-1	-5	-20	25.46	1.83
1	5	20	27.22	2.98
1	5	20	25.67	2.85
-2	-5	-20	4.69	0.90
2	5	20	4.92	1.84
2	5	20	5.37	1.68
-3	-5	-20	15.15	1.53
3	5	20	17.28	2.88
3	5	20	16.50	2.63
-4	-5	-20	0.92	0.80
4	5	20	-1.35	1.65
4	5	20	-0.24	1.42
-5	-5	-20	6.36	1.01
5	5	20	3.04	1.76
5	5	20	5.02	1.69
-6	-5	-20	3.05	0.97
6	5	20	4.10	1.67
6	5	20	5.58	1.91
-7	-5	-20	7.19	1.15
7	5	20	5.20	3.30
12	-5	-19	49.83	2.60
-12	5	19	55.53	6.09
-12	5	19	57.27	3.29
11	-5	-19	0.83	0.70
-11	5	19	0.14	1.16
-11	5	19	5.81	2.15
10	-5	-19	47.18	2.39
-10	5	19	48.97	4.33
-10	5	19	50.40	2.87
9	-5	-19	1.07	0.64
-9	5	19	1.55	1.07
-9	5	19	-1.39	1.66
8	-5	-19	31.01	1.79
-8	5	19	30.42	2.58
-8	5	19	30.55	3.41
7	-5	-19	-0.08	0.59

-7	5	19	-2.32	1.50
-7	5	19	1.78	1.26
6	-5	-19	36.41	1.93
-6	5	19	29.94	3.34
-6	5	19	41.20	3.28
5	-5	-19	0.59	0.59
-5	5	19	3.37	1.31
-5	5	19	1.22	1.52
4	-5	-19	49.13	2.35
-4	5	19	47.07	3.20
-4	5	19	44.39	3.63
3	-5	-19	1.55	0.65
-3	5	19	0.34	1.39
-3	5	19	2.78	1.38
2	-5	-19	41.47	2.15
-2	5	19	40.61	3.08
-2	5	19	42.00	3.30
1	-5	-19	-0.17	0.60
-1	5	19	-0.21	1.31
-1	5	19	1.01	1.39
0	-5	-19	31.68	1.82
0	5	19	27.24	2.79
0	5	19	27.38	2.70
-1	-5	-19	-0.99	0.63
1	5	19	-1.08	1.22
1	5	19	0.20	1.43
-2	-5	-19	41.67	2.24
2	5	19	42.01	3.25
2	5	19	37.60	3.09
3	5	19	2.82	1.52
3	5	19	2.27	1.27
-4	-5	-19	24.79	1.82
4	5	19	26.10	2.63
4	5	19	29.32	3.01
-5	-5	-19	1.37	0.79
5	5	19	-0.02	1.30
5	5	19	0.94	1.55
-6	-5	-19	20.01	1.77
6	5	19	22.42	2.51
6	5	19	18.38	2.79
-7	-5	-19	0.09	0.82
7	5	19	-0.13	1.72
7	5	19	1.09	1.40
-8	-5	-19	33.99	2.25

8	5	19	32.11	3.54
-9	-5	-19	1.40	0.94
13	-5	-18	31.73	1.90
-13	5	18	29.43	3.95
-13	5	18	33.70	2.77
12	-5	-18	0.40	0.64
-12	5	18	0.69	1.21
-12	5	18	-3.10	1.86
11	-5	-18	52.91	2.64
-11	5	18	55.98	4.41
-11	5	18	60.45	3.16
10	-5	-18	1.45	0.61
-10	5	18	2.13	1.03
-10	5	18	1.11	1.81
9	-5	-18	20.95	1.45
-9	5	18	22.60	2.25
-9	5	18	23.50	1.90
-9	5	18	26.15	3.24
8	-5	-18	0.02	0.54
-8	5	18	-0.66	1.46
-8	5	18	-0.05	1.10
-8	5	18	1.92	0.80
7	-5	-18	23.88	1.55
-7	5	18	30.67	1.73
-7	5	18	23.95	3.06
-7	5	18	24.22	2.35
6	-5	-18	-0.72	0.53
-6	5	18	0.98	1.11
-6	5	18	-1.32	1.37
-6	5	18	0.81	0.63
5	-5	-18	46.06	2.27
-5	5	18	47.62	2.15
-5	5	18	47.01	3.47
-5	5	18	45.82	2.96
4	-5	-18	-0.55	0.50
-4	5	18	0.25	0.48
-4	5	18	0.46	1.30
3	-5	-18	52.54	2.47
-3	5	18	53.29	3.61
-3	5	18	50.73	3.22
2	-5	-18	0.95	0.57
-2	5	18	1.31	1.28
-2	5	18	2.21	1.25
1	-5	-18	34.38	1.83

-1	5	18	30.12	2.64
-1	5	18	31.46	2.79
0	-5	-18	0.70	0.58
0	5	18	-3.01	1.20
0	5	18	-1.11	1.16
-1	-5	-18	44.80	2.28
1	5	18	44.48	3.08
1	5	18	47.39	3.16
-2	-5	-18	0.77	0.63
2	5	18	0.25	1.27
2	5	18	0.95	1.14
-3	-5	-18	36.39	2.07
3	5	18	38.74	2.79
3	5	18	37.77	3.02
-4	-5	-18	0.21	0.66
4	5	18	1.39	1.19
4	5	18	0.82	1.36
-5	-5	-18	31.33	1.98
5	5	18	28.86	3.36
5	5	18	33.38	2.98
-6	-5	-18	0.04	0.77
6	5	18	0.05	1.38
6	5	18	-0.03	1.22
7	5	18	16.54	2.22
7	5	18	16.58	2.78
-8	-5	-18	-0.06	0.77
8	5	18	0.44	1.52
8	5	18	0.97	1.25
-9	-5	-18	30.11	2.12
9	5	18	32.68	3.31
-10	-5	-18	-0.92	0.94
10	5	18	0.34	1.69
14	-5	-17	3.78	0.80
-14	5	17	6.34	1.48
-14	5	17	6.13	2.24
13	-5	-17	4.83	0.79
-13	5	17	5.14	1.32
-13	5	17	0.29	1.96
12	-5	-17	25.83	1.62
-12	5	17	28.52	2.29
-12	5	17	24.75	3.51
11	-5	-17	1.04	0.59
-11	5	17	0.95	1.75
10	-5	-17	7.13	1.58

10	-5	-17	7.58	1.03
-10	5	17	9.84	1.56
-10	5	17	9.83	2.11
9	-5	-17	4.81	0.70
9	-5	-17	4.55	1.59
-9	5	17	6.95	1.01
-9	5	17	4.02	1.77
8	-5	-17	13.29	1.12
-8	5	17	14.90	1.44
-8	5	17	10.92	2.11
-8	5	17	12.00	1.83
7	-5	-17	10.62	1.03
7	-5	-17	9.79	1.60
-7	5	17	11.59	1.90
-7	5	17	13.16	1.80
-7	5	17	10.93	1.26
6	-5	-17	38.30	3.38
6	-5	-17	35.57	1.86
-6	5	17	35.50	2.48
-6	5	17	39.04	1.88
-6	5	17	32.88	3.03
5	-5	-17	0.60	0.49
-5	5	17	1.75	0.67
-5	5	17	1.01	1.21
-5	5	17	1.96	0.56
-5	5	17	1.77	1.10
4	-5	-17	48.83	2.25
-4	5	17	47.26	2.10
-4	5	17	48.05	2.06
-4	5	17	48.83	3.34
-4	5	17	45.34	2.89
3	-5	-17	0.93	0.50
-3	5	17	-0.75	1.02
-3	5	17	0.57	1.16
2	-5	-17	20.08	1.35
-2	5	17	22.05	2.87
-2	5	17	22.62	2.33
1	-5	-17	3.77	0.66
-1	5	17	4.85	1.24
-1	5	17	6.82	1.36
0	-5	-17	33.61	1.78
0	5	17	33.84	2.57
0	5	17	32.42	2.55
-1	-5	-17	0.43	0.56

1	5	17	1.78	1.14
1	5	17	1.49	1.17
-2	-5	-17	16.33	1.30
2	5	17	13.47	2.17
-3	-5	-17	1.30	0.58
3	5	17	-0.55	1.25
3	5	17	0.39	0.99
-4	-5	-17	24.61	1.66
4	5	17	25.49	2.61
4	5	17	27.26	2.26
-5	-5	-17	1.86	0.69
5	5	17	-0.02	1.27
5	5	17	0.02	1.02
-6	-5	-17	35.55	2.00
6	5	17	31.84	2.44
6	5	17	31.60	3.02
-7	-5	-17	-0.40	0.66
7	5	17	-0.02	1.15
7	5	17	1.58	1.40
-8	-5	-17	27.06	1.94
8	5	17	25.67	2.95
8	5	17	29.05	2.35
-9	-5	-17	0.20	0.81
9	5	17	-1.51	1.49
9	5	17	0.33	1.19
-10	-5	-17	33.31	2.17
10	5	17	35.31	3.39
-11	-5	-17	3.54	1.02
11	5	17	2.71	1.71
15	-5	-16	2.01	0.73
-15	5	16	4.25	2.15
-15	5	16	2.73	1.38
14	-5	-16	5.18	0.77
-14	5	16	3.48	1.31
-14	5	16	6.94	2.21
13	-5	-16	0.56	0.57
-13	5	16	-2.32	1.78
-13	5	16	-0.45	1.11
12	-5	-16	11.09	1.13
-12	5	16	7.52	2.00
-12	5	16	12.11	1.79
11	-5	-16	1.50	0.57
-11	5	16	2.77	0.99
-11	5	16	2.64	1.81

10	-5	-16	32.19	2.54
10	-5	-16	30.25	1.69
-10	5	16	31.29	3.20
-10	5	16	34.22	2.12
9	-5	-16	-0.30	0.47
9	-5	-16	-0.43	1.22
-9	5	16	-0.72	0.83
-9	5	16	-0.45	0.72
-9	5	16	-1.22	1.31
8	-5	-16	0.85	1.15
8	-5	-16	2.63	0.53
-8	5	16	2.95	0.81
-8	5	16	2.16	0.73
-8	5	16	1.91	0.99
-8	5	16	3.62	1.45
7	-5	-16	7.36	1.38
7	-5	-16	7.49	0.87
-7	5	16	11.16	1.13
-7	5	16	7.09	1.19
-7	5	16	5.80	1.61
-7	5	16	8.12	1.17
6	-5	-16	9.58	1.45
6	-5	-16	9.60	1.12
-6	5	16	13.09	1.69
-6	5	16	9.44	1.69
-6	5	16	11.76	1.12
-6	5	16	10.33	1.03
5	-5	-16	9.21	1.35
5	-5	-16	11.43	2.12
-5	5	16	10.69	1.00
-5	5	16	10.82	2.05
-5	5	16	11.89	0.95
-5	5	16	10.06	1.69
4	-5	-16	26.95	1.50
-4	5	16	25.21	2.13
-4	5	16	29.85	1.36
-4	5	16	25.04	1.40
-4	5	16	26.81	2.48
3	-5	-16	0.55	0.45
-3	5	16	-0.08	0.99
-3	5	16	0.55	0.37
-3	5	16	-0.25	0.96
2	-5	-16	10.02	0.95
-2	5	16	11.80	1.86

-2	5	16	10.73	1.72
1	-5	-16	13.41	1.07
-1	5	16	10.69	1.73
0	5	16	4.04	1.41
0	5	16	4.35	1.14
-1	-5	-16	24.99	1.58
1	5	16	21.50	2.07
1	5	16	22.26	2.13
-2	-5	-16	9.28	0.98
2	5	16	9.76	1.62
2	5	16	6.22	1.35
-3	-5	-16	12.59	1.13
3	5	16	12.34	1.93
3	5	16	9.62	1.63
-4	-5	-16	3.12	0.68
4	5	16	4.02	1.09
4	5	16	2.59	1.23
-5	-5	-16	15.36	1.30
5	5	16	17.92	1.86
5	5	16	14.04	2.26
-6	-5	-16	2.61	2.28
-6	-5	-16	3.39	0.76
6	5	16	5.23	1.12
6	5	16	0.74	1.22
-7	-5	-16	22.01	1.61
7	5	16	20.46	1.98
7	5	16	23.49	2.62
-8	-5	-16	14.79	1.46
8	5	16	13.81	2.41
8	5	16	14.87	1.85
-9	-5	-16	20.93	1.68
9	5	16	17.23	2.62
9	5	16	22.01	2.02
-10	-5	-16	7.21	1.06
10	5	16	7.16	1.74
-11	-5	-16	32.17	2.15
11	5	16	29.76	3.06
-12	-5	-16	-0.16	0.86
12	5	16	-0.33	1.69
16	-5	-15	16.73	1.45
-16	5	15	19.58	2.48
-16	5	15	13.62	2.63
15	-5	-15	19.47	1.45
-15	5	15	19.77	2.37

-15	5	15	23.90	3.55
14	-5	-15	12.03	1.15
-14	5	15	7.61	1.40
-14	5	15	8.41	2.12
13	-5	-15	13.63	1.22
-13	5	15	18.55	1.96
-13	5	15	12.48	2.27
12	-5	-15	9.56	1.04
-12	5	15	8.40	2.00
-12	5	15	10.81	1.62
11	-5	-15	9.98	1.79
11	-5	-15	12.10	1.08
-11	5	15	13.48	1.55
-11	5	15	13.02	2.16
10	-5	-15	22.33	2.13
10	-5	-15	23.58	1.35
-10	5	15	23.02	1.75
-10	5	15	21.78	2.84
9	-5	-15	30.78	2.40
9	-5	-15	28.01	2.16
9	-5	-15	31.18	1.61
-9	5	15	32.24	1.95
-9	5	15	28.60	2.93
-9	5	15	34.65	1.91
8	-5	-15	25.77	2.00
-8	5	15	21.31	1.49
-8	5	15	21.21	1.55
-8	5	15	21.20	1.80
-8	5	15	16.06	2.47
7	-5	-15	32.11	2.36
7	-5	-15	33.36	1.65
-7	5	15	31.51	2.81
-7	5	15	32.92	1.78
-7	5	15	36.17	1.82
-7	5	15	29.76	2.14
6	-5	-15	-0.08	0.38
6	-5	-15	0.96	1.00
-6	5	15	-0.97	0.99
-6	5	15	1.60	0.85
-6	5	15	-1.00	0.46
-6	5	15	0.57	0.55
5	-5	-15	15.43	1.02
5	-5	-15	14.80	1.93
-5	5	15	13.42	1.63

-5	5	15	12.94	1.93
-5	5	15	15.58	1.07
-5	5	15	14.85	1.01
4	-5	-15	5.78	1.25
4	-5	-15	5.17	0.73
-4	5	15	6.12	1.32
-4	5	15	5.00	0.99
-4	5	15	5.69	0.70
-4	5	15	4.86	0.74
3	-5	-15	38.80	1.79
3	-5	-15	39.04	2.63
-3	5	15	36.46	1.60
-3	5	15	35.01	2.68
-3	5	15	34.52	2.37
2	-5	-15	12.98	0.97
-2	5	15	11.87	1.66
-2	5	15	11.77	1.68
1	-5	-15	18.67	1.14
-1	5	15	17.03	1.80
-1	5	15	14.93	1.71
0	-5	-15	4.01	0.58
0	5	15	5.65	1.18
0	5	15	5.68	1.03
-1	-5	-15	12.06	0.99
1	5	15	12.23	1.64
1	5	15	13.44	1.74
-2	-5	-15	1.27	0.45
2	5	15	2.24	0.89
2	5	15	2.33	1.28
-3	-5	-15	42.24	3.78
3	5	15	30.56	2.21
3	5	15	34.54	2.54
-4	-5	-15	5.81	0.93
-4	-5	-15	3.82	1.76
4	5	15	6.18	1.06
4	5	15	3.73	1.21
-5	-5	-15	15.61	2.36
-5	-5	-15	15.89	1.26
5	5	15	14.22	2.00
5	5	15	15.87	1.67
-6	-5	-15	-0.99	0.52
-6	-5	-15	1.09	1.79
6	5	15	0.92	0.82
6	5	15	0.19	1.16

-7	-5	-15	21.26	3.17
-7	-5	-15	17.13	1.42
7	5	15	20.84	1.78
7	5	15	15.18	2.18
8	5	15	3.89	0.94
8	5	15	3.34	1.32
-9	-5	-15	14.03	1.37
9	5	15	15.51	1.71
9	5	15	14.62	2.36
-10	-5	-15	1.43	0.74
10	5	15	2.92	1.47
10	5	15	1.99	1.01
-11	-5	-15	19.61	1.67
11	5	15	13.98	2.47
-12	-5	-15	5.83	1.01
12	5	15	5.66	1.68
-13	-5	-15	4.05	0.97
13	5	15	4.20	1.69
17	-5	-14	48.94	2.53
-17	5	14	53.65	4.51
-17	5	14	56.21	3.44
16	-5	-14	3.49	0.76
-16	5	14	1.64	2.02
-16	5	14	4.53	1.34
15	-5	-14	31.50	1.77
-15	5	14	26.60	2.49
-15	5	14	31.88	3.59
14	-5	-14	1.49	0.57
-14	5	14	1.21	1.06
-14	5	14	1.30	1.79
13	-5	-14	26.22	1.50
-13	5	14	25.67	3.14
-13	5	14	22.33	2.05
12	-5	-14	5.47	0.85
-12	5	14	7.12	1.13
-12	5	14	6.16	1.82
11	-5	-14	61.69	2.83
11	-5	-14	68.65	3.14
-11	5	14	68.40	3.19
-11	5	14	61.29	4.11
10	-5	-14	11.91	1.67
10	-5	-14	7.76	1.42
10	-5	-14	10.59	0.95
-10	5	14	9.94	1.83

-10	5	14	12.76	1.38
-10	5	14	9.03	1.39
9	-5	-14	62.88	3.23
9	-5	-14	73.38	3.38
9	-5	-14	75.21	3.60
-9	5	14	75.25	3.28
-9	5	14	77.72	3.27
-9	5	14	65.30	4.13
8	-5	-14	0.83	0.40
-8	5	14	2.10	1.23
-8	5	14	1.30	0.80
-8	5	14	2.24	0.66
-8	5	14	0.55	0.60
7	-5	-14	19.47	1.86
7	-5	-14	18.44	1.17
-7	5	14	22.51	1.39
-7	5	14	22.48	1.36
-7	5	14	19.50	1.62
-7	5	14	22.75	2.26
6	-5	-14	20.32	1.85
6	-5	-14	22.11	1.23
-6	5	14	21.77	1.47
-6	5	14	21.47	1.27
-6	5	14	20.65	1.69
5	-5	-14	15.34	1.03
5	-5	-14	17.28	1.80
-5	5	14	15.67	1.02
-5	5	14	16.87	1.05
-5	5	14	17.15	1.96
-5	5	14	13.74	1.55
4	-5	-14	43.58	2.59
4	-5	-14	40.86	1.92
-4	5	14	35.60	1.96
-4	5	14	41.01	2.69
-4	5	14	45.51	1.84
-4	5	14	41.93	2.44
3	-5	-14	80.64	3.81
3	-5	-14	78.53	3.17
-3	5	14	77.45	2.99
-3	5	14	71.73	3.80
-3	5	14	68.03	3.01
-3	5	14	78.06	3.68
2	-5	-14	8.65	1.35
2	-5	-14	7.80	0.79

-2	5	14	8.10	1.18
-2	5	14	10.74	1.58
1	-5	-14	49.21	2.16
1	-5	-14	46.80	2.89
-1	5	14	46.75	2.64
-1	5	14	43.89	2.74
0	-5	-14	20.32	1.22
0	-5	-14	18.72	2.71
0	5	14	20.38	1.79
0	5	14	20.99	1.84
-1	-5	-14	43.57	3.26
-1	-5	-14	44.00	2.13
1	5	14	47.03	2.65
1	5	14	48.82	2.77
-2	-5	-14	40.48	3.10
-2	-5	-14	34.69	1.77
2	5	14	34.14	2.41
2	5	14	35.78	2.24
-3	-5	-14	13.74	2.50
-3	-5	-14	14.19	1.08
3	5	14	13.64	1.52
3	5	14	14.00	1.74
-4	-5	-14	2.62	1.52
-4	-5	-14	5.15	0.69
4	5	14	5.22	0.96
-5	-5	-14	13.96	2.01
5	5	14	13.22	1.84
5	5	14	15.34	1.50
-6	-5	-14	21.23	2.92
-6	-5	-14	25.89	1.59
6	5	14	27.30	2.28
6	5	14	29.26	1.92
-7	-5	-14	16.23	1.33
-7	-5	-14	17.82	2.19
7	5	14	17.86	1.60
7	5	14	16.46	2.08
-8	-5	-14	14.59	2.10
-8	-5	-14	15.06	1.34
8	5	14	14.59	2.08
8	5	14	15.49	1.55
-9	-5	-14	4.95	0.79
9	5	14	5.77	1.37
9	5	14	6.97	1.04
-10	-5	-14	2.59	0.70

10	5	14	2.04	0.90
10	5	14	1.68	1.23
-11	-5	-14	5.42	0.87
11	5	14	6.62	1.00
11	5	14	4.35	1.52
-12	-5	-14	9.43	1.44
12	5	14	14.24	2.49
-13	-5	-14	4.58	1.02
13	5	14	5.97	1.78
-14	-5	-14	19.54	1.90
14	5	14	19.52	2.83
17	-5	-13	4.49	0.83
-17	5	13	8.47	1.44
-17	5	13	4.99	2.17
16	-5	-13	28.34	1.74
-16	5	13	33.74	2.60
-16	5	13	26.88	3.53
15	-5	-13	1.97	0.60
-15	5	13	3.87	1.80
-15	5	13	1.79	1.17
14	-5	-13	33.09	1.78
-14	5	13	33.29	2.41
-14	5	13	34.97	3.38
13	-5	-13	-0.83	0.45
-13	5	13	1.44	1.55
-13	5	13	-1.42	0.85
12	-5	-13	52.27	2.37
-12	5	13	53.49	3.74
11	-5	-13	0.31	0.72
11	-5	-13	0.56	0.40
11	-5	-13	-0.26	0.79
-11	5	13	0.45	0.69
-11	5	13	-0.51	1.32
10	-5	-13	90.96	3.84
10	-5	-13	81.91	3.90
10	-5	-13	92.41	4.02
10	-5	-13	78.55	3.77
-10	5	13	90.20	4.78
-10	5	13	100.33	3.97
-10	5	13	94.09	3.96
9	-5	-13	0.36	0.38
9	-5	-13	0.88	0.77
9	-5	-13	1.40	0.86
-9	5	13	1.52	1.10

-9	5	13	1.22	0.64
-9	5	13	0.51	0.57
8	-5	-13	103.95	4.86
8	-5	-13	109.32	4.41
8	-5	-13	104.83	4.88
-8	5	13	119.22	4.57
-8	5	13	113.29	4.56
7	-5	-13	8.33	0.77
7	-5	-13	7.45	1.05
-7	5	13	8.55	1.24
-7	5	13	6.86	1.35
-7	5	13	5.95	0.97
-7	5	13	7.49	0.92
6	-5	-13	49.39	2.24
6	-5	-13	52.36	2.73
-6	5	13	51.71	3.08
-6	5	13	56.06	2.31
-6	5	13	52.83	2.30
-6	5	13	48.37	2.34
-6	5	13	48.87	2.59
5	-5	-13	4.09	1.00
5	-5	-13	1.61	0.39
-5	5	13	1.59	0.96
-5	5	13	1.24	0.46
-5	5	13	2.66	0.48
-5	5	13	3.57	0.82
-5	5	13	1.69	0.45
4	-5	-13	114.24	4.49
4	-5	-13	116.77	4.98
-4	5	13	110.72	4.87
-4	5	13	112.45	5.20
-4	5	13	108.35	4.53
-4	5	13	103.69	4.45
-4	5	13	120.44	4.41
3	-5	-13	42.18	3.79
3	-5	-13	37.29	2.32
3	-5	-13	33.85	1.63
-3	5	13	33.40	2.13
-3	5	13	31.70	2.29
-3	5	13	34.27	1.48
-3	5	13	32.88	1.69
-3	5	13	33.31	1.50
2	-5	-13	135.50	6.94
2	-5	-13	149.78	6.16

2	-5	-13	169.14	6.77
-2	5	13	161.94	6.24
-2	5	13	166.26	6.80
-2	5	13	157.25	6.60
-2	5	13	163.45	5.97
-2	5	13	160.24	5.96
1	-5	-13	2.54	0.99
1	-5	-13	1.28	0.41
1	-5	-13	1.55	1.27
-1	5	13	1.37	0.78
-1	5	13	1.15	0.73
-1	5	13	0.85	0.45
0	-5	-13	63.69	3.33
0	-5	-13	58.26	3.57
0	-5	-13	61.76	2.59
0	5	13	58.92	3.07
0	5	13	54.57	3.05
0	5	13	59.87	2.63
-1	-5	-13	12.49	1.69
-1	-5	-13	11.53	1.73
-1	-5	-13	13.37	0.92
1	5	13	12.87	1.55
1	5	13	10.85	1.41
1	5	13	12.07	0.96
-2	-5	-13	45.07	2.20
-2	-5	-13	47.42	2.12
-2	-5	-13	44.93	3.02
2	5	13	45.59	2.54
2	5	13	47.18	2.25
2	5	13	41.90	2.65
-3	-5	-13	5.57	0.78
-3	-5	-13	5.29	1.41
3	5	13	3.66	1.08
3	5	13	4.85	0.88
-4	-5	-13	62.88	2.78
-4	-5	-13	66.17	3.60
4	5	13	61.12	3.32
4	5	13	61.78	3.06
-5	-5	-13	5.56	1.42
-5	-5	-13	6.01	0.82
5	5	13	5.90	1.18
5	5	13	6.71	0.96
-6	-5	-13	85.31	3.96
-6	-5	-13	89.39	4.54

6	5	13	96.92	4.06
6	5	13	91.40	5.18
-7	-5	-13	0.06	0.44
-7	-5	-13	2.22	1.37
7	5	13	0.72	1.00
7	5	13	1.04	0.67
-8	-5	-13	20.20	1.38
-8	-5	-13	15.76	2.56
8	5	13	20.33	1.56
8	5	13	18.44	2.15
-9	-5	-13	5.14	0.79
-9	-5	-13	7.36	1.68
9	5	13	7.08	1.42
9	5	13	9.08	1.28
-10	-5	-13	19.63	1.52
-10	-5	-13	21.83	1.30
10	5	13	18.51	1.60
10	5	13	20.81	2.31
-11	-5	-13	1.44	1.00
-11	-5	-13	1.68	0.70
11	5	13	2.73	0.86
11	5	13	0.58	1.25
-12	-5	-13	40.73	2.26
12	5	13	32.59	2.88
-13	-5	-13	1.64	0.85
13	5	13	2.01	1.49
-14	-5	-13	39.08	2.47
14	5	13	39.80	3.34
-15	-5	-13	10.70	1.71
15	5	13	6.98	1.81
18	-5	-12	-0.26	0.69
-18	5	12	-1.61	1.29
-18	5	12	2.53	2.06
17	-5	-12	24.14	1.61
-17	5	12	29.93	3.57
-17	5	12	27.09	2.51
16	-5	-12	7.56	1.08
-16	5	12	5.75	1.33
-16	5	12	7.16	1.99
15	-5	-12	24.29	1.54
-15	5	12	26.84	3.16
-15	5	12	27.55	2.25
14	-5	-12	8.33	0.96
-14	5	12	9.52	1.34

-14	5	12	9.95	1.93
13	-5	-12	32.02	1.71
-13	5	12	36.30	2.28
-13	5	12	35.45	3.11
12	-5	-12	1.73	0.49
12	-5	-12	1.82	0.47
-12	5	12	0.76	1.31
-12	5	12	2.79	0.81
11	-5	-12	109.70	4.79
11	-5	-12	108.06	4.38
-11	5	12	103.15	5.36
-11	5	12	114.69	4.73
10	-5	-12	0.95	0.39
10	-5	-12	1.87	0.74
10	-5	-12	0.39	0.70
-10	5	12	0.99	0.64
9	-5	-12	90.58	3.91
9	-5	-12	90.39	3.72
9	-5	-12	85.13	3.53
9	-5	-12	78.50	3.71
-9	5	12	90.12	3.77
-9	5	12	83.96	4.47
8	-5	-12	8.85	1.09
8	-5	-12	10.54	1.27
8	-5	-12	10.31	0.81
8	-5	-12	10.77	1.43
-8	5	12	11.00	1.73
-8	5	12	9.93	1.05
7	-5	-12	71.00	3.12
7	-5	-12	67.05	2.76
7	-5	-12	65.23	2.87
-7	5	12	59.81	3.01
-7	5	12	62.41	3.53
-7	5	12	70.11	2.88
-7	5	12	62.86	2.81
6	-5	-12	10.06	1.31
6	-5	-12	8.45	0.73
-6	5	12	9.42	0.84
-6	5	12	7.23	1.15
-6	5	12	7.31	0.84
-6	5	12	9.19	0.85
-6	5	12	11.00	1.59
5	-5	-12	64.70	2.84
5	-5	-12	66.27	3.86

5	-5	-12	73.45	3.30
-5	5	12	72.26	2.85
-5	5	12	72.86	2.83
-5	5	12	63.08	2.87
-5	5	12	66.58	3.54
-5	5	12	66.74	3.17
4	-5	-12	4.39	0.95
4	-5	-12	3.10	0.47
4	-5	-12	4.61	1.30
-4	5	12	5.11	0.56
-4	5	12	3.23	0.50
-4	5	12	2.89	0.74
-4	5	12	4.26	0.95
-4	5	12	3.94	0.56
3	-5	-12	92.77	4.12
3	-5	-12	89.54	3.65
3	-5	-12	87.85	4.51
-3	5	12	85.52	4.01
-3	5	12	95.57	3.54
-3	5	12	90.83	3.70
-3	5	12	88.32	4.22
-3	5	12	90.83	3.51
2	-5	-12	4.38	1.16
2	-5	-12	3.52	0.48
2	-5	-12	4.81	0.98
-2	5	12	3.65	0.51
-2	5	12	3.52	0.82
-2	5	12	3.44	0.40
-2	5	12	3.94	0.90
-2	5	12	3.89	0.43
1	-5	-12	85.92	4.22
1	-5	-12	84.58	3.47
1	-5	-12	92.49	4.05
-1	5	12	79.58	3.92
-1	5	12	79.28	3.85
-1	5	12	88.28	3.52
0	-5	-12	0.82	0.81
0	-5	-12	-0.76	0.95
0	-5	-12	0.44	0.34
0	5	12	0.43	0.35
0	5	12	-0.31	0.59
0	5	12	-0.68	0.63
-1	-5	-12	44.98	2.79
-1	-5	-12	49.40	2.71

-1	-5	-12	42.88	2.01
1	5	12	39.79	2.44
1	5	12	44.62	2.04
1	5	12	45.65	2.44
-2	-5	-12	1.42	1.00
-2	-5	-12	-0.12	0.30
-2	-5	-12	0.80	0.96
2	5	12	0.55	0.63
2	5	12	-0.04	0.37
2	5	12	0.67	0.75
-3	-5	-12	36.56	2.45
-3	-5	-12	34.79	1.70
-3	-5	-12	37.19	2.47
-3	-5	-12	34.60	1.67
3	5	12	33.74	2.00
3	5	12	31.65	2.18
-4	-5	-12	8.04	0.78
-4	-5	-12	7.02	1.24
-4	-5	-12	7.08	0.87
4	5	12	6.34	1.10
4	5	12	6.35	1.14
-5	-5	-12	62.23	2.80
-5	-5	-12	62.54	3.44
5	5	12	69.08	3.04
5	5	12	61.01	3.30
-6	-5	-12	2.15	0.48
-6	-5	-12	0.25	1.06
6	5	12	3.87	1.02
6	5	12	2.04	0.69
-7	-5	-12	61.87	3.62
-7	-5	-12	69.02	3.07
7	5	12	75.84	3.22
7	5	12	69.68	3.69
-8	-5	-12	1.20	1.18
-8	-5	-12	0.90	0.52
8	5	12	0.70	0.88
8	5	12	2.59	0.67
-9	-5	-12	54.85	2.65
-9	-5	-12	54.60	2.26
-9	-5	-12	54.83	3.26
9	5	12	57.28	3.27
9	5	12	57.70	2.68
-10	-5	-12	5.17	0.82
-10	-5	-12	3.49	0.78

-10	-5	-12	3.82	1.42
10	5	12	3.91	1.25
10	5	12	4.57	0.83
-11	-5	-12	39.27	1.95
-11	-5	-12	40.25	2.21
11	5	12	40.90	2.17
11	5	12	39.94	2.91
-12	-5	-12	-0.44	0.67
-12	-5	-12	0.85	0.69
12	5	12	-0.05	0.77
12	5	12	-0.10	1.17
-13	-5	-12	63.15	3.07
-13	-5	-12	59.28	2.95
13	5	12	59.86	3.70
-14	-5	-12	-0.05	0.74
14	5	12	0.28	1.43
-15	-5	-12	58.13	3.20
15	5	12	65.29	4.00
-16	-5	-12	-0.37	0.90
16	5	12	-1.16	1.63
18	-5	-11	3.17	0.74
-18	5	11	2.77	1.33
-18	5	11	6.76	2.10
17	-5	-11	4.26	0.75
-17	5	11	5.91	1.31
-17	5	11	6.13	1.96
16	-5	-11	13.31	1.13
-16	5	11	9.75	1.99
-16	5	11	8.29	1.39
15	-5	-11	4.68	0.68
-15	5	11	4.30	1.61
-15	5	11	2.16	1.10
14	-5	-11	14.59	1.15
-14	5	11	16.51	2.61
-14	5	11	19.86	1.84
13	-5	-11	16.29	1.11
-13	5	11	14.65	1.63
-13	5	11	16.61	2.40
12	-5	-11	30.60	1.58
-12	5	11	31.03	3.06
-12	5	11	33.07	2.01
11	-5	-11	6.02	0.87
11	-5	-11	6.00	0.88
11	-5	-11	6.27	0.70

-11	5	11	10.43	1.92
-11	5	11	5.82	0.90
10	-5	-11	36.19	1.96
10	-5	-11	36.66	1.97
10	-5	-11	35.74	1.68
10	-5	-11	35.15	1.81
-10	5	11	37.12	2.00
-10	5	11	33.17	2.63
9	-5	-11	2.70	0.68
9	-5	-11	3.15	0.73
9	-5	-11	1.90	0.59
9	-5	-11	2.29	0.41
-9	5	11	2.92	0.64
-9	5	11	1.58	0.95
8	-5	-11	35.08	1.63
8	-5	-11	34.56	2.07
8	-5	-11	36.19	1.89
8	-5	-11	33.23	1.79
-8	5	11	32.06	1.70
-8	5	11	37.71	1.86
-8	5	11	38.42	2.55
7	-5	-11	39.88	2.92
7	-5	-11	39.35	1.85
7	-5	-11	40.61	2.35
7	-5	-11	42.20	1.99
7	-5	-11	43.59	2.15
-7	5	11	43.77	2.00
-7	5	11	41.64	2.64
-7	5	11	42.02	1.94
6	-5	-11	19.88	1.10
6	-5	-11	23.28	2.20
6	-5	-11	23.15	1.22
6	-5	-11	21.44	1.50
-6	5	11	18.09	1.43
-6	5	11	17.75	1.78
-6	5	11	22.53	1.21
5	-5	-11	9.60	1.22
5	-5	-11	11.98	0.78
5	-5	-11	10.52	1.54
5	-5	-11	12.07	0.83
-5	5	11	11.03	0.81
-5	5	11	13.14	1.46
-5	5	11	9.93	1.13
-5	5	11	10.72	0.84

4	-5	-11	19.63	1.08
4	-5	-11	17.41	1.93
4	-5	-11	21.12	1.54
-4	5	11	22.00	1.05
-4	5	11	18.89	1.45
-4	5	11	22.54	1.05
-4	5	11	20.12	1.69
-4	5	11	19.96	1.14
3	-5	-11	0.51	0.66
3	-5	-11	-0.01	0.25
3	-5	-11	0.14	0.86
-3	5	11	0.14	0.52
-3	5	11	0.13	0.28
-3	5	11	-1.01	0.58
-3	5	11	-0.15	0.30
-3	5	11	0.01	0.23
2	-5	-11	38.87	2.39
2	-5	-11	38.10	2.11
2	-5	-11	34.28	1.60
-2	5	11	34.93	1.64
-2	5	11	33.01	1.98
-2	5	11	34.57	1.43
-2	5	11	33.86	1.44
-2	5	11	33.20	2.10
1	-5	-11	22.23	1.69
1	-5	-11	22.47	1.16
1	-5	-11	22.52	1.93
-1	5	11	22.63	1.20
-1	5	11	21.06	1.58
-1	5	11	22.76	1.63
0	-5	-11	65.75	3.20
0	-5	-11	62.71	2.68
0	-5	-11	66.16	3.35
0	5	11	60.68	3.07
0	5	11	67.23	2.73
0	5	11	62.64	3.07
-1	-5	-11	4.19	0.94
-1	-5	-11	1.95	0.85
-1	-5	-11	2.38	0.42
1	5	11	2.52	0.75
1	5	11	3.10	0.70
1	5	11	2.70	0.46
-2	-5	-11	16.81	1.65
-2	-5	-11	13.72	0.93

-2	-5	-11	13.72	1.61
2	5	11	14.88	1.30
2	5	11	13.36	1.37
2	5	11	16.04	1.00
-3	-5	-11	14.29	1.64
-3	-5	-11	15.22	0.94
-3	-5	-11	15.21	0.98
-3	-5	-11	14.31	1.63
3	5	11	13.17	1.43
3	5	11	15.60	0.99
3	5	11	16.91	1.34
-4	-5	-11	149.12	6.38
-4	-5	-11	146.74	5.70
-4	-5	-11	143.62	5.75
-4	-5	-11	148.55	6.24
4	5	11	136.83	6.19
4	5	11	147.84	5.97
-5	-5	-11	0.51	0.35
-5	-5	-11	0.50	0.92
-5	-5	-11	0.65	0.36
-5	-5	-11	1.16	0.95
5	5	11	0.09	0.53
5	5	11	1.18	0.73
-6	-5	-11	83.27	3.51
-6	-5	-11	83.56	3.99
6	5	11	85.57	3.65
6	5	11	79.96	4.00
-7	-5	-11	9.45	0.91
-7	-5	-11	9.40	1.64
7	5	11	9.87	1.08
7	5	11	8.34	1.48
-8	-5	-11	20.19	1.30
-8	-5	-11	19.02	0.95
-8	-5	-11	20.69	1.92
-8	-5	-11	19.45	1.01
8	5	11	18.92	1.86
8	5	11	20.56	1.39
-9	-5	-11	3.22	0.66
-9	-5	-11	3.53	0.46
-9	-5	-11	4.21	1.16
-9	-5	-11	2.94	0.53
9	5	11	4.02	0.73
-10	-5	-11	60.08	2.63
-10	-5	-11	56.27	3.21

-10	-5	-11	62.03	2.56
-10	-5	-11	61.67	2.86
10	5	11	63.24	2.82
10	5	11	63.90	3.48
-11	-5	-11	11.74	1.46
-11	-5	-11	11.27	1.20
-11	-5	-11	11.24	1.12
-11	-5	-11	10.30	1.01
11	5	11	11.66	1.25
11	5	11	13.53	1.89
-12	-5	-11	69.26	3.23
-12	-5	-11	73.85	3.42
12	5	11	74.08	4.03
12	5	11	77.89	3.30
-13	-5	-11	-1.35	0.73
-13	-5	-11	1.23	0.71
13	5	11	-0.39	1.20
-14	-5	-11	53.46	2.85
-14	-5	-11	57.09	2.88
14	5	11	55.28	3.56
-15	-5	-11	-1.32	0.80
-15	-5	-11	1.44	1.20
15	5	11	0.80	1.37
-16	-5	-11	28.74	2.25
16	5	11	29.47	3.02
17	5	11	-0.78	1.68
19	-5	-10	0.12	0.70
-19	5	10	1.70	1.45
-19	5	10	-2.84	1.89
18	-5	-10	3.20	0.71
-18	5	10	5.01	1.95
-18	5	10	1.93	1.34
17	-5	-10	-0.11	0.55
-17	5	10	1.51	1.15
-17	5	10	-0.83	1.66
16	-5	-10	24.59	1.53
-16	5	10	27.08	2.98
-16	5	10	30.42	2.29
15	-5	-10	0.96	0.50
-15	5	10	-0.36	1.36
-15	5	10	0.23	0.93
14	-5	-10	5.88	0.81
-14	5	10	6.09	1.12
-14	5	10	8.46	1.61

13	-5	-10	0.26	0.38
-13	5	10	0.02	0.83
-13	5	10	0.64	1.25
12	-5	-10	0.75	0.38
-12	5	10	0.48	1.16
-12	5	10	0.69	0.68
11	-5	-10	0.18	0.33
11	-5	-10	0.18	0.51
11	-5	-10	0.70	0.45
-11	5	10	-0.59	0.59
-11	5	10	0.08	0.98
10	-5	-10	17.81	1.14
10	-5	-10	16.37	1.03
10	-5	-10	18.13	1.42
10	-5	-10	19.43	1.34
-10	5	10	17.10	1.97
-10	5	10	18.09	1.35
9	-5	-10	9.00	0.96
9	-5	-10	6.12	0.90
9	-5	-10	8.39	1.07
9	-5	-10	6.15	0.67
9	-5	-10	8.22	1.53
-9	5	10	7.25	0.95
-9	5	10	8.48	1.38
-9	5	10	7.70	0.79
8	-5	-10	22.03	1.24
8	-5	-10	17.72	2.21
8	-5	-10	19.48	1.41
8	-5	-10	17.01	1.00
8	-5	-10	19.26	1.27
-8	5	10	17.54	1.79
-8	5	10	18.60	1.24
-8	5	10	17.39	1.10
7	-5	-10	3.67	0.51
7	-5	-10	4.78	0.89
7	-5	-10	4.78	0.72
7	-5	-10	3.33	0.76
7	-5	-10	2.70	1.02
-7	5	10	2.87	0.48
-7	5	10	2.51	0.49
-7	5	10	2.93	0.86
6	-5	-10	28.04	2.21
6	-5	-10	28.12	1.42
6	-5	-10	27.88	1.34

6	-5	-10	30.88	1.66
-6	5	10	28.27	1.62
-6	5	10	28.41	1.40
-6	5	10	25.71	1.95
-6	5	10	28.06	1.43
5	-5	-10	17.14	1.28
5	-5	-10	15.24	0.88
5	-5	-10	15.47	1.76
5	-5	-10	14.09	0.91
-5	5	10	15.73	0.95
-5	5	10	15.84	1.52
-5	5	10	14.93	1.20
-5	5	10	16.77	0.94
4	-5	-10	22.09	1.10
4	-5	-10	21.42	1.82
4	-5	-10	24.60	1.54
4	-5	-10	22.13	1.11
-4	5	10	20.16	1.64
-4	5	10	24.09	1.10
-4	5	10	20.62	1.14
-4	5	10	20.74	1.42
3	-5	-10	15.75	0.89
3	-5	-10	16.51	0.83
3	-5	-10	15.99	1.34
3	-5	-10	16.43	1.62
-3	5	10	16.01	0.85
-3	5	10	15.87	1.38
-3	5	10	14.34	1.23
-3	5	10	15.33	0.92
2	-5	-10	3.85	0.49
2	-5	-10	4.02	0.77
2	-5	-10	2.94	0.82
-2	5	10	4.13	0.40
-2	5	10	4.17	0.78
-2	5	10	3.82	0.55
-2	5	10	3.28	0.35
-2	5	10	4.05	0.73
1	-5	-10	17.11	1.43
1	-5	-10	18.85	1.59
1	-5	-10	15.95	0.92
-1	5	10	14.96	1.31
-1	5	10	16.93	0.96
-1	5	10	14.91	1.26
0	-5	-10	0.48	0.72

0	-5	-10	-0.13	0.61
0	-5	-10	0.96	0.29
0	5	10	1.08	0.51
0	5	10	0.86	0.32
0	5	10	0.99	0.55
-1	-5	-10	22.09	1.11
-1	-5	-10	20.42	1.70
-1	-5	-10	20.42	1.56
1	5	10	19.79	1.42
1	5	10	19.29	1.50
1	5	10	21.30	1.12
-2	-5	-10	30.25	1.89
-2	-5	-10	27.54	1.87
-2	-5	-10	25.55	1.30
2	5	10	23.61	1.56
2	5	10	26.18	1.31
2	5	10	23.42	1.66
-3	-5	-10	31.66	2.01
-3	-5	-10	29.55	1.48
-3	-5	-10	33.53	1.47
-3	-5	-10	29.52	2.02
3	5	10	29.69	1.49
3	5	10	32.30	1.75
3	5	10	26.31	1.85
-4	-5	-10	2.72	0.81
-4	-5	-10	1.07	0.38
-4	-5	-10	1.80	0.41
-4	-5	-10	2.04	0.83
4	5	10	1.97	0.68
4	5	10	1.81	0.55
-5	-5	-10	110.21	4.72
-5	-5	-10	110.20	4.89
-5	-5	-10	102.54	4.22
-5	-5	-10	106.57	4.31
5	5	10	102.07	4.72
5	5	10	106.81	4.46
-6	-5	-10	0.44	0.91
-6	-5	-10	0.67	0.35
-6	-5	-10	1.95	0.84
-6	-5	-10	0.41	0.33
6	5	10	1.56	0.77
6	5	10	0.65	0.51
-7	-5	-10	32.27	1.62
-7	-5	-10	29.80	2.09

-7	-5	-10	32.70	1.32
-7	-5	-10	31.18	1.34
7	5	10	30.03	1.71
7	5	10	28.83	2.09
-8	-5	-10	52.50	2.15
-8	-5	-10	50.43	2.41
-8	-5	-10	50.72	2.80
-8	-5	-10	56.69	2.25
8	5	10	50.86	2.93
8	5	10	54.47	2.49
-9	-5	-10	6.58	0.77
-9	-5	-10	6.54	0.85
-9	-5	-10	6.66	1.10
9	5	10	5.00	0.96
9	5	10	6.91	1.13
-10	-5	-10	1.62	0.94
-10	-5	-10	1.70	0.47
-10	-5	-10	1.54	0.55
-10	-5	-10	3.31	0.57
10	5	10	2.47	0.63
10	5	10	0.89	0.90
-11	-5	-10	45.54	2.28
-11	-5	-10	48.43	2.42
-11	-5	-10	49.28	2.18
-11	-5	-10	48.68	2.67
11	5	10	47.84	2.92
11	5	10	48.31	2.32
-12	-5	-10	1.30	0.87
-12	-5	-10	1.29	0.72
-12	-5	-10	0.97	0.61
-12	-5	-10	0.39	0.60
-12	-5	-10	-0.09	1.30
12	5	10	0.38	0.96
-13	-5	-10	68.67	3.21
-13	-5	-10	67.75	3.13
-13	-5	-10	63.47	3.13
13	5	10	66.79	3.75
-14	-5	-10	1.75	0.96
-14	-5	-10	2.85	0.84
14	5	10	3.50	1.36
-15	-5	-10	14.23	1.90
-15	-5	-10	14.67	1.67
15	5	10	18.66	2.42
-16	-5	-10	1.18	1.16

-16	-5	-10	1.81	0.88
16	5	10	1.80	1.45
-17	-5	-10	7.41	1.30
17	5	10	9.17	1.95
19	-5	-9	11.95	1.28
-19	5	9	8.92	2.19
-19	5	9	11.83	1.78
18	-5	-9	21.00	1.51
-18	5	9	21.45	2.44
-18	5	9	21.01	3.15
17	-5	-9	2.36	0.65
-17	5	9	4.06	1.22
-17	5	9	3.17	1.68
16	-5	-9	4.70	0.72
-16	5	9	4.32	1.53
15	-5	-9	29.26	1.59
-15	5	9	31.64	2.22
14	-5	-9	6.42	0.83
-14	5	9	6.28	1.08
-14	5	9	6.60	1.49
13	-5	-9	45.21	2.12
-13	5	9	47.91	3.16
12	-5	-9	7.30	0.74
-12	5	9	7.37	1.49
-12	5	9	8.48	1.21
11	-5	-9	9.25	0.75
11	-5	-9	8.46	0.78
11	-5	-9	7.77	1.10
11	-5	-9	8.72	1.74
11	-5	-9	9.86	0.83
-11	5	9	9.79	1.17
-11	5	9	11.49	1.78
10	-5	-9	11.58	2.05
10	-5	-9	12.36	1.09
10	-5	-9	11.08	0.79
10	-5	-9	10.96	0.89
10	-5	-9	9.13	1.26
-10	5	9	10.41	1.12
-10	5	9	9.73	0.92
-10	5	9	11.42	1.66
9	-5	-9	12.83	0.84
9	-5	-9	17.21	2.03
9	-5	-9	13.69	1.02
9	-5	-9	14.55	1.11

9	-5	-9	13.85	1.22
-9	5	9	11.98	1.64
-9	5	9	13.42	1.11
-9	5	9	14.29	0.98
8	-5	-9	-0.15	0.58
8	-5	-9	2.37	1.06
8	-5	-9	0.63	0.28
8	-5	-9	0.45	0.48
8	-5	-9	1.42	0.49
-8	5	9	0.31	0.33
-8	5	9	0.69	0.78
-8	5	9	0.99	0.48
7	-5	-9	117.43	5.54
7	-5	-9	130.33	4.99
7	-5	-9	130.78	5.18
7	-5	-9	117.48	5.16
7	-5	-9	120.49	4.86
-7	5	9	129.85	4.92
-7	5	9	129.46	4.99
-7	5	9	119.78	5.46
6	-5	-9	2.02	0.35
6	-5	-9	2.41	0.88
6	-5	-9	3.32	0.74
6	-5	-9	1.98	0.55
6	-5	-9	3.73	0.62
-6	5	9	3.77	0.50
-6	5	9	1.30	0.71
-6	5	9	3.71	0.53
5	-5	-9	43.87	1.89
5	-5	-9	44.99	1.94
5	-5	-9	45.38	2.15
5	-5	-9	43.54	2.53
-5	5	9	41.08	2.11
-5	5	9	42.10	2.40
-5	5	9	45.44	1.93
-5	5	9	45.33	1.94
4	-5	-9	1.31	0.53
4	-5	-9	1.29	0.29
4	-5	-9	1.41	0.72
4	-5	-9	1.07	0.31
-4	5	9	1.75	0.64
-4	5	9	0.87	0.31
-4	5	9	0.88	0.46
-4	5	9	1.31	0.30

3	-5	-9	50.63	2.25
3	-5	-9	54.21	2.21
3	-5	-9	54.67	2.83
3	-5	-9	60.07	2.62
-3	5	9	55.49	2.72
-3	5	9	52.39	2.21
-3	5	9	55.79	2.30
-3	5	9	54.13	2.54
2	-5	-9	4.78	0.41
2	-5	-9	6.61	1.15
2	-5	-9	5.37	0.51
2	-5	-9	6.83	0.94
-2	5	9	4.48	0.83
-2	5	9	5.58	0.55
-2	5	9	4.75	0.42
-2	5	9	5.34	0.94
1	-5	-9	2.95	0.67
1	-5	-9	2.86	0.43
1	-5	-9	3.18	0.74
-1	5	9	1.91	0.58
-1	5	9	3.04	0.67
-1	5	9	3.45	0.44
-1	5	9	2.81	0.29
0	-5	-9	2.51	0.70
0	-5	-9	1.71	0.31
0	-5	-9	2.41	0.69
0	5	9	1.82	0.36
0	5	9	3.14	0.59
0	5	9	2.78	0.62
-1	-5	-9	8.32	1.17
-1	-5	-9	7.07	0.60
-1	-5	-9	7.49	1.16
1	5	9	8.09	0.93
1	5	9	7.03	0.93
1	5	9	7.64	0.64
-2	-5	-9	0.30	0.27
-2	-5	-9	-0.45	0.57
-2	-5	-9	0.00	0.61
2	5	9	-0.35	0.48
2	5	9	0.44	0.29
2	5	9	-0.34	0.42
-3	-5	-9	14.26	0.85
-3	-5	-9	12.34	1.30
-3	-5	-9	13.60	0.86

-3	-5	-9	18.25	1.44
3	5	9	12.90	1.10
3	5	9	14.41	0.89
3	5	9	13.81	1.26
-4	-5	-9	42.51	1.94
-4	-5	-9	47.12	2.48
-4	-5	-9	43.22	1.90
-4	-5	-9	44.97	2.35
4	5	9	43.25	2.13
4	5	9	41.12	2.32
-5	-5	-9	14.06	0.85
-5	-5	-9	13.31	0.91
-5	-5	-9	13.99	1.39
-5	-5	-9	17.13	1.50
5	5	9	13.68	1.34
5	5	9	12.78	1.06
-6	-5	-9	1.53	0.25
-6	-5	-9	0.74	0.22
-6	-5	-9	0.90	0.30
-6	-5	-9	1.17	0.80
-6	-5	-9	0.64	0.69
-6	-5	-9	0.66	0.31
6	5	9	0.33	0.62
6	5	9	1.12	0.47
-7	-5	-9	16.15	1.05
-7	-5	-9	16.14	1.48
-7	-5	-9	16.84	0.88
-7	-5	-9	15.78	0.98
-7	-5	-9	16.18	0.82
-7	-5	-9	18.95	1.80
7	5	9	16.67	1.54
7	5	9	15.95	1.18
-8	-5	-9	-0.19	0.34
-8	-5	-9	0.22	0.29
-8	-5	-9	0.33	0.39
-8	-5	-9	-0.18	0.94
-8	-5	-9	0.12	0.75
8	5	9	1.05	0.71
8	5	9	-0.03	0.48
-9	-5	-9	20.39	1.17
-9	-5	-9	18.79	1.24
-9	-5	-9	21.03	1.08
-9	-5	-9	18.84	1.63
9	5	9	19.60	1.30

9	5	9	19.99	1.80
-10	-5	-9	3.19	0.89
-10	-5	-9	1.91	0.55
-10	-5	-9	4.21	0.70
-10	-5	-9	3.07	0.61
10	5	9	3.72	0.95
10	5	9	2.07	0.60
-11	-5	-9	8.01	1.10
-11	-5	-9	6.27	0.88
-11	-5	-9	8.85	0.99
-11	-5	-9	8.22	1.43
11	5	9	6.39	1.07
11	5	9	7.52	1.21
-12	-5	-9	18.08	1.28
-12	-5	-9	17.12	1.36
-12	-5	-9	15.83	1.42
-12	-5	-9	16.68	1.56
-13	-5	-9	12.42	1.23
-13	-5	-9	7.28	1.04
-13	-5	-9	8.54	1.22
-13	-5	-9	10.23	1.51
13	5	9	8.35	1.40
-14	-5	-9	0.01	0.93
-14	-5	-9	2.77	1.08
-14	-5	-9	2.54	0.77
14	5	9	2.35	1.17
-15	-5	-9	6.30	1.16
-15	-5	-9	8.97	1.39
15	5	9	5.76	1.47
-16	-5	-9	6.42	1.26
-16	-5	-9	9.71	1.55
16	5	9	8.37	1.67
-17	-5	-9	7.84	1.25
-17	-5	-9	9.27	1.51
17	5	9	9.31	1.83
17	5	9	9.01	1.46
-18	-5	-9	21.42	2.31
18	5	9	13.42	1.99
18	5	9	20.04	3.00
20	-5	-8	0.62	0.69
19	-5	-8	33.62	1.92
-19	5	8	30.61	3.35
18	-5	-8	9.36	1.17
-18	5	8	7.31	1.51

-18	5	8	7.54	1.93
17	-5	-8	35.56	1.86
-17	5	8	35.63	2.69
-17	5	8	34.56	3.43
16	-5	-8	2.82	0.60
-16	5	8	1.44	1.02
-16	5	8	3.98	1.50
15	-5	-8	33.01	1.71
-15	5	8	34.22	2.75
-15	5	8	33.30	2.27
14	-5	-8	20.18	1.22
-14	5	8	23.29	1.79
-14	5	8	20.34	2.29
13	-5	-8	50.98	2.76
-13	5	8	57.93	2.89
-13	5	8	57.61	3.37
12	-5	-8	2.45	1.42
12	-5	-8	1.51	0.37
-12	5	8	1.90	0.72
-12	5	8	1.69	1.03
11	-5	-8	46.69	3.21
11	-5	-8	48.12	2.38
11	-5	-8	50.63	2.12
11	-5	-8	44.62	2.06
11	-5	-8	51.02	2.06
-11	5	8	46.86	2.91
-11	5	8	51.75	2.47
10	-5	-8	38.58	1.71
10	-5	-8	37.33	2.73
10	-5	-8	38.83	1.95
10	-5	-8	36.78	1.79
10	-5	-8	38.88	1.73
-10	5	8	36.65	1.80
-10	5	8	36.27	2.47
-10	5	8	39.06	2.01
9	-5	-8	85.18	4.14
9	-5	-8	81.43	3.36
9	-5	-8	86.29	3.78
9	-5	-8	81.14	3.50
9	-5	-8	81.93	3.33
-9	5	8	85.92	3.57
-9	5	8	76.33	3.95
-9	5	8	83.80	3.40
8	-5	-8	42.53	1.85

8	-5	-8	38.66	1.88
8	-5	-8	39.05	1.72
8	-5	-8	39.25	2.49
8	-5	-8	39.04	2.00
-8	5	8	39.23	1.90
-8	5	8	38.33	1.76
-8	5	8	33.50	2.30
7	-5	-8	87.57	3.65
7	-5	-8	82.76	4.01
7	-5	-8	87.71	3.52
7	-5	-8	79.04	3.35
7	-5	-8	87.85	3.50
-7	5	8	84.11	3.41
-7	5	8	88.50	3.51
-7	5	8	77.08	3.90
6	-5	-8	47.14	2.08
6	-5	-8	49.48	2.41
6	-5	-8	50.26	2.71
6	-5	-8	53.03	2.19
-6	5	8	50.85	2.19
-6	5	8	47.61	2.12
-6	5	8	51.72	2.62
5	-5	-8	82.06	3.60
5	-5	-8	85.42	3.43
5	-5	-8	77.68	3.73
5	-5	-8	79.69	3.24
5	-5	-8	80.07	3.20
-5	5	8	77.39	3.22
-5	5	8	82.12	3.25
-5	5	8	77.28	3.65
4	-5	-8	36.81	1.67
4	-5	-8	41.43	1.94
4	-5	-8	39.45	1.70
4	-5	-8	35.55	2.16
-4	5	8	38.65	2.11
-4	5	8	40.56	1.72
-4	5	8	40.78	1.70
-4	5	8	38.79	1.93
3	-5	-8	31.07	1.41
3	-5	-8	32.76	1.70
3	-5	-8	31.61	1.38
3	-5	-8	32.45	1.93
-3	5	8	31.94	1.66
-3	5	8	29.21	1.39

-3	5	8	32.06	1.44
-3	5	8	32.29	1.83
2	-5	-8	102.37	4.31
2	-5	-8	105.34	4.17
2	-5	-8	95.66	3.76
2	-5	-8	95.56	3.84
-2	5	8	95.42	3.75
-2	5	8	95.79	4.10
-2	5	8	99.84	3.87
-2	5	8	93.54	4.18
1	-5	-8	21.69	0.93
1	-5	-8	22.31	1.07
1	-5	-8	21.11	1.52
1	-5	-8	23.29	1.42
-1	5	8	23.04	1.41
-1	5	8	22.62	0.94
-1	5	8	22.71	1.10
-1	5	8	20.56	1.37
0	-5	-8	10.43	1.06
0	-5	-8	9.48	0.64
0	-5	-8	10.53	1.13
0	5	8	10.20	0.67
0	5	8	9.81	0.94
0	5	8	10.17	0.97
-1	-5	-8	43.78	1.92
-1	-5	-8	47.14	2.31
-1	-5	-8	46.43	2.32
1	5	8	43.51	2.24
1	5	8	47.05	1.95
1	5	8	42.40	2.16
-2	-5	-8	18.74	1.41
-2	-5	-8	16.34	0.92
-2	-5	-8	17.88	1.34
2	5	8	17.06	1.17
2	5	8	17.17	1.26
2	5	8	18.96	0.97
-3	-5	-8	61.40	2.60
-3	-5	-8	63.46	2.92
-3	-5	-8	64.09	2.57
-3	-5	-8	66.03	3.03
3	5	8	65.88	2.62
3	5	8	60.62	2.77
3	5	8	56.06	2.92
-4	-5	-8	4.56	0.53

-4	-5	-8	5.66	0.53
-4	-5	-8	5.52	0.83
-4	-5	-8	6.61	0.97
4	5	8	5.24	0.75
4	5	8	3.26	0.72
-5	-5	-8	1.08	0.30
-5	-5	-8	1.46	0.58
-5	-5	-8	3.43	0.77
-5	-5	-8	0.86	0.20
-5	-5	-8	1.26	0.32
5	5	8	0.51	0.43
5	5	8	0.83	0.56
-6	-5	-8	60.42	2.66
-6	-5	-8	67.67	2.62
-6	-5	-8	66.73	3.16
-6	-5	-8	60.50	2.45
-6	-5	-8	57.25	2.46
-6	-5	-8	66.56	2.93
6	5	8	62.54	2.75
6	5	8	61.23	3.08
-7	-5	-8	38.44	1.78
-7	-5	-8	45.96	1.87
-7	-5	-8	40.27	2.14
-7	-5	-8	39.46	1.67
-7	-5	-8	37.45	1.85
-7	-5	-8	40.94	2.42
7	5	8	40.81	1.95
7	5	8	36.09	2.24
-8	-5	-8	63.06	2.59
-8	-5	-8	68.41	3.33
-8	-5	-8	60.33	2.61
-8	-5	-8	63.01	2.96
-8	-5	-8	62.86	2.66
-8	-5	-8	59.79	2.74
8	5	8	67.60	2.79
8	5	8	63.26	3.19
-9	-5	-8	10.20	1.64
-9	-5	-8	8.01	1.21
-9	-5	-8	9.57	0.88
-9	-5	-8	9.38	0.75
-9	-5	-8	9.39	0.86
9	5	8	8.32	1.28
9	5	8	10.43	0.95
-10	-5	-8	31.48	1.67

-10	-5	-8	31.62	1.52
-10	-5	-8	27.72	1.59
-10	-5	-8	30.35	1.86
10	5	8	30.68	2.06
10	5	8	31.80	1.67
-11	-5	-8	1.90	0.59
-11	-5	-8	0.86	0.77
-11	-5	-8	1.44	0.76
-11	-5	-8	2.14	0.54
-11	-5	-8	1.17	0.55
11	5	8	2.28	0.91
-12	-5	-8	1.29	0.69
-12	-5	-8	3.32	0.86
-12	-5	-8	2.27	0.57
-12	-5	-8	3.42	0.61
12	5	8	1.68	0.92
-13	-5	-8	1.71	0.84
-13	-5	-8	0.52	0.59
-13	-5	-8	1.01	0.65
-13	-5	-8	0.17	0.77
-13	-5	-8	0.03	0.87
13	5	8	0.03	0.48
13	5	8	0.21	0.96
-14	-5	-8	29.51	2.11
-14	-5	-8	34.92	2.03
-14	-5	-8	35.02	2.25
-14	-5	-8	32.81	2.04
14	5	8	34.86	2.51
14	5	8	33.34	1.93
-15	-5	-8	7.16	1.20
-15	-5	-8	6.60	1.00
15	5	8	6.73	1.41
15	5	8	6.99	0.88
-16	-5	-8	8.44	1.36
-16	-5	-8	9.70	1.48
16	5	8	10.59	1.27
16	5	8	10.86	1.72
-17	-5	-8	2.08	1.27
-17	-5	-8	0.75	0.87
17	5	8	-0.09	0.75
17	5	8	3.52	1.46
-18	-5	-8	2.49	1.48
-18	-5	-8	3.93	1.02
18	5	8	5.56	0.96

18	5	8	9.04	1.79
20	-5	-7	33.65	2.00
19	-5	-7	0.35	0.62
-19	5	7	0.04	1.67
-19	5	7	-1.98	1.14
18	-5	-7	39.89	2.11
-18	5	7	40.06	3.30
-18	5	7	42.69	2.99
17	-5	-7	1.05	0.53
-17	5	7	-0.17	1.09
-17	5	7	1.05	1.40
16	-5	-7	57.88	2.60
-16	5	7	60.24	3.29
-16	5	7	54.82	3.54
15	-5	-7	0.00	0.45
-15	5	7	0.68	1.24
-15	5	7	0.31	0.84
14	-5	-7	73.87	3.02
14	-5	-7	64.37	4.21
-14	5	7	75.02	3.49
-14	5	7	68.20	3.81
13	-5	-7	1.28	0.37
13	-5	-7	2.67	1.52
-13	5	7	1.09	1.04
-13	5	7	1.75	0.71
12	-5	-7	149.87	5.81
12	-5	-7	151.57	5.95
12	-5	-7	155.73	5.81
12	-5	-7	150.88	6.83
-12	5	7	160.31	6.32
-12	5	7	147.80	6.60
11	-5	-7	-0.38	0.50
11	-5	-7	0.48	0.39
11	-5	-7	0.34	0.29
11	-5	-7	-0.18	0.36
-11	5	7	-0.23	0.38
-11	5	7	-0.91	0.52
-11	5	7	-1.32	0.77
10	-5	-7	117.17	4.66
10	-5	-7	118.88	4.83
10	-5	-7	110.99	4.63
10	-5	-7	120.37	5.39
10	-5	-7	121.91	4.64
-10	5	7	123.96	4.93

-10	5	7	124.44	4.73
-10	5	7	107.90	5.21
9	-5	-7	3.26	0.47
9	-5	-7	2.58	0.48
9	-5	-7	2.91	0.53
9	-5	-7	2.61	0.95
-9	5	7	3.16	0.45
-9	5	7	2.23	0.78
-9	5	7	2.83	0.56
8	-5	-7	112.62	4.61
8	-5	-7	109.36	4.43
8	-5	-7	116.08	4.52
8	-5	-7	112.36	4.58
8	-5	-7	121.58	5.10
-8	5	7	111.03	4.97
-8	5	7	115.03	4.62
-8	5	7	110.90	4.47
7	-5	-7	0.83	0.38
7	-5	-7	1.56	0.45
7	-5	-7	1.45	0.56
7	-5	-7	0.72	0.26
7	-5	-7	1.21	0.68
-7	5	7	1.13	0.60
-7	5	7	0.88	0.39
-7	5	7	1.16	0.31
6	-5	-7	128.86	5.26
6	-5	-7	136.24	5.54
6	-5	-7	129.83	5.09
6	-5	-7	124.63	4.99
6	-5	-7	135.38	5.16
-6	5	7	128.07	5.02
-6	5	7	128.29	5.09
-6	5	7	123.43	5.43
5	-5	-7	0.07	0.19
5	-5	-7	0.69	0.57
5	-5	-7	-0.10	0.44
5	-5	-7	0.47	0.28
5	-5	-7	0.71	0.42
-5	5	7	0.21	0.45
-5	5	7	-0.19	0.20
-5	5	7	-0.08	0.28
4	-5	-7	243.24	9.93
4	-5	-7	270.73	10.04
4	-5	-7	259.10	9.57

4	-5	-7	256.38	9.59
4	-5	-7	254.98	9.93
-4	5	7	234.22	9.92
-4	5	7	253.49	9.77
-4	5	7	237.99	9.56
-4	5	7	270.90	9.60
3	-5	-7	2.76	0.62
3	-5	-7	4.43	0.72
3	-5	-7	3.16	0.39
3	-5	-7	3.03	0.41
-3	5	7	3.29	0.42
-3	5	7	2.93	0.57
-3	5	7	2.63	0.35
-3	5	7	2.76	0.53
2	-5	-7	127.50	5.05
2	-5	-7	135.93	5.21
2	-5	-7	119.95	4.78
2	-5	-7	116.36	4.72
-2	5	7	125.40	4.80
-2	5	7	125.43	4.72
-2	5	7	122.05	5.02
-2	5	7	120.04	5.10
1	-5	-7	8.26	0.84
1	-5	-7	7.43	0.53
1	-5	-7	7.91	0.94
1	-5	-7	7.47	0.44
-1	5	7	7.54	0.82
-1	5	7	6.59	0.78
-1	5	7	7.98	0.56
-1	5	7	7.60	0.43
0	-5	-7	129.99	5.10
0	-5	-7	136.88	5.44
0	-5	-7	135.40	5.40
0	5	7	132.04	5.11
0	5	7	126.26	5.36
0	5	7	132.78	5.37
-1	-5	-7	1.16	0.50
-1	-5	-7	0.33	0.22
-1	-5	-7	1.51	0.53
1	5	7	1.22	0.28
1	5	7	0.79	0.47
1	5	7	1.33	0.43
-2	-5	-7	115.19	4.54
-2	-5	-7	102.28	4.15

-2	-5	-7	108.28	4.45
2	5	7	100.95	4.34
2	5	7	102.96	4.46
2	5	7	107.23	4.17
-3	-5	-7	13.78	0.78
-3	-5	-7	15.28	1.21
-3	-5	-7	13.28	1.08
3	5	7	12.16	0.95
3	5	7	13.70	0.79
3	5	7	13.03	1.08
-4	-5	-7	130.52	5.12
-4	-5	-7	140.05	5.41
-4	-5	-7	142.63	5.57
-4	-5	-7	122.89	5.15
4	5	7	132.32	5.30
4	5	7	126.86	5.48
-5	-5	-7	28.94	1.56
-5	-5	-7	27.25	1.24
-5	-5	-7	25.14	1.12
-5	-5	-7	26.01	1.70
-5	-5	-7	24.75	1.10
-5	-5	-7	27.09	1.30
5	5	7	27.39	1.43
5	5	7	26.59	1.66
-6	-5	-7	76.98	3.50
-6	-5	-7	79.55	3.29
-6	-5	-7	70.27	2.93
-6	-5	-7	71.05	2.90
-6	-5	-7	76.34	3.09
-6	-5	-7	75.75	3.02
6	5	7	75.87	3.17
6	5	7	71.10	3.41
-7	-5	-7	35.20	2.13
-7	-5	-7	31.51	1.54
-7	-5	-7	35.30	1.56
-7	-5	-7	33.37	1.64
-7	-5	-7	33.21	1.48
-7	-5	-7	37.72	1.85
7	5	7	35.64	2.03
7	5	7	34.36	1.72
-8	-5	-7	86.01	3.47
-8	-5	-7	80.11	3.47
-8	-5	-7	91.90	3.73
-8	-5	-7	79.23	3.46

-8	-5	-7	87.08	3.59
-8	-5	-7	90.07	4.05
8	5	7	84.00	3.92
8	5	7	93.14	3.62
-9	-5	-7	25.24	1.57
-9	-5	-7	25.91	1.28
-9	-5	-7	23.75	1.27
-9	-5	-7	24.84	1.40
-9	-5	-7	29.41	2.03
-9	-5	-7	23.90	1.35
9	5	7	27.11	1.44
9	5	7	26.85	1.85
-10	-5	-7	38.26	1.75
-10	-5	-7	34.38	1.82
-10	-5	-7	39.95	2.50
-10	-5	-7	34.31	1.95
-10	-5	-7	35.74	1.85
10	5	7	40.06	1.87
10	5	7	35.88	2.25
-11	-5	-7	10.80	1.25
-11	-5	-7	7.62	1.04
-11	-5	-7	9.26	1.25
-11	-5	-7	10.04	1.40
-11	-5	-7	9.55	0.94
-11	-5	-7	10.89	1.00
11	5	7	10.33	1.46
11	5	7	11.72	0.91
-12	-5	-7	21.81	1.53
-12	-5	-7	22.05	1.67
-12	-5	-7	20.87	1.62
-12	-5	-7	23.08	1.41
12	5	7	24.12	1.57
12	5	7	23.01	1.86
-13	-5	-7	27.07	1.62
-13	-5	-7	27.42	1.65
-13	-5	-7	22.35	1.74
-13	-5	-7	25.16	1.83
-13	-5	-7	27.03	1.72
13	5	7	24.37	1.50
13	5	7	27.02	2.07
-14	-5	-7	42.07	2.41
-14	-5	-7	49.03	2.57
-14	-5	-7	48.88	2.63
14	5	7	50.00	2.37

14	5	7	48.95	2.86
-15	-5	-7	16.77	1.77
15	5	7	10.34	1.92
15	5	7	16.22	1.40
16	5	7	49.62	2.55
16	5	7	48.95	3.15
-17	-5	-7	2.35	1.31
17	5	7	5.76	0.94
17	5	7	7.11	1.50
-18	-5	-7	3.45	1.41
18	5	7	4.63	0.93
18	5	7	4.18	1.58
-19	-5	-7	2.55	1.56
19	5	7	2.29	1.77
19	5	7	0.72	1.03
20	-5	-6	13.62	1.37
-20	5	6	12.91	1.93
19	-5	-6	27.33	1.66
-19	5	6	28.08	2.87
-19	5	6	24.67	2.64
18	-5	-6	1.24	0.62
-18	5	6	3.81	1.51
-18	5	6	3.48	1.21
17	-5	-6	26.60	1.52
-17	5	6	24.79	2.61
-17	5	6	25.09	2.31
16	-5	-6	5.21	1.90
16	-5	-6	4.86	0.67
-16	5	6	7.19	1.25
-16	5	6	6.39	1.44
15	-5	-6	40.00	1.93
15	-5	-6	41.90	3.48
-15	5	6	40.20	2.52
-15	5	6	39.52	2.78
14	-5	-6	24.21	1.32
14	-5	-6	23.27	2.73
-14	5	6	23.84	2.18
-14	5	6	24.86	1.86
13	-5	-6	92.42	4.56
13	-5	-6	86.52	3.36
13	-5	-6	86.38	3.37
13	-5	-6	83.81	3.51
-13	5	6	79.90	4.16
-13	5	6	88.98	3.92

12	-5	-6	9.91	0.78
12	-5	-6	12.66	2.10
12	-5	-6	11.15	0.79
12	-5	-6	10.44	0.78
-12	5	6	12.09	1.24
-12	5	6	11.31	1.58
11	-5	-6	124.17	5.33
11	-5	-6	136.49	5.10
11	-5	-6	126.99	5.11
11	-5	-6	135.01	5.87
11	-5	-6	128.21	5.08
-11	5	6	131.51	5.43
-11	5	6	140.01	5.21
-11	5	6	125.79	5.67
10	-5	-6	6.20	0.87
10	-5	-6	6.72	0.71
10	-5	-6	5.86	0.58
10	-5	-6	7.12	1.15
10	-5	-6	5.67	0.74
-10	5	6	5.73	0.80
-10	5	6	7.42	1.28
-10	5	6	6.47	0.66
9	-5	-6	103.76	3.97
9	-5	-6	97.81	4.08
9	-5	-6	93.90	3.89
9	-5	-6	100.34	4.48
-9	5	6	110.31	3.98
-9	5	6	95.34	4.12
-9	5	6	90.48	4.37
8	-5	-6	3.24	0.52
8	-5	-6	3.71	0.64
8	-5	-6	2.53	0.35
8	-5	-6	3.39	0.87
-8	5	6	3.58	0.52
-8	5	6	4.02	0.69
-8	5	6	3.57	0.72
7	-5	-6	297.55	11.11
7	-5	-6	278.88	10.99
7	-5	-6	294.04	11.18
7	-5	-6	293.93	11.13
7	-5	-6	302.00	11.48
-7	5	6	304.19	11.05
-7	5	6	277.06	11.41
-7	5	6	289.67	11.14

6	-5	-6	6.77	1.04
6	-5	-6	7.58	0.93
6	-5	-6	6.99	0.74
6	-5	-6	7.00	0.66
6	-5	-6	6.37	0.50
-6	5	6	7.46	0.68
-6	5	6	5.89	0.92
-6	5	6	6.21	0.53
5	-5	-6	103.92	4.39
5	-5	-6	104.12	4.10
5	-5	-6	112.81	4.29
5	-5	-6	110.26	4.54
5	-5	-6	105.23	4.17
-5	5	6	99.09	4.46
-5	5	6	104.30	4.17
-5	5	6	105.47	4.12
4	-5	-6	-0.42	0.43
4	-5	-6	0.70	0.50
4	-5	-6	-0.19	0.31
4	-5	-6	0.08	0.18
4	-5	-6	0.27	0.24
-4	5	6	0.14	0.23
-4	5	6	0.54	0.42
-4	5	6	-0.29	0.17
3	-5	-6	56.55	2.32
3	-5	-6	59.60	2.70
3	-5	-6	59.23	2.32
3	-5	-6	55.26	2.68
-3	5	6	58.82	2.33
-3	5	6	54.43	2.62
-3	5	6	58.61	2.54
2	-5	-6	30.33	1.27
2	-5	-6	30.85	1.55
2	-5	-6	26.90	1.22
2	-5	-6	29.22	1.63
-2	5	6	27.83	1.49
-2	5	6	28.30	1.22
-2	5	6	27.56	1.55
-2	5	6	29.91	1.29
1	-5	-6	81.06	3.29
1	-5	-6	85.90	3.21
1	-5	-6	85.43	3.55
1	-5	-6	85.93	3.61
-1	5	6	84.14	3.54

-1	5	6	84.32	3.56
-1	5	6	81.19	3.21
0	-5	-6	10.45	0.88
0	-5	-6	10.74	0.90
0	-5	-6	10.24	0.49
0	5	6	10.47	0.63
0	5	6	11.47	0.86
0	5	6	10.18	0.88
0	5	6	10.27	0.49
-1	-5	-6	299.34	11.59
-1	-5	-6	311.19	11.85
-1	-5	-6	324.98	11.90
1	5	6	302.27	11.86
1	5	6	297.51	11.78
1	5	6	316.11	11.61
-2	-5	-6	2.43	0.38
-2	-5	-6	3.55	0.60
-2	-5	-6	2.63	0.54
2	5	6	2.63	0.39
2	5	6	3.05	0.60
2	5	6	2.29	0.46
-3	-5	-6	116.55	4.72
-3	-5	-6	118.98	4.61
-3	-5	-6	105.46	4.22
-3	-5	-6	104.80	4.37
3	5	6	116.68	4.40
3	5	6	111.03	4.68
3	5	6	114.37	4.55
-4	-5	-6	21.03	0.91
-4	-5	-6	19.68	0.92
-4	-5	-6	20.59	1.05
-4	-5	-6	21.65	1.43
-4	-5	-6	22.57	1.27
-4	-5	-6	21.62	1.02
4	5	6	20.71	1.37
4	5	6	22.61	1.23
-5	-5	-6	103.64	4.03
-5	-5	-6	96.63	4.04
-5	-5	-6	107.61	4.18
-5	-5	-6	113.14	4.54
-5	-5	-6	107.41	4.12
-5	-5	-6	112.06	4.37
5	5	6	102.91	4.27
5	5	6	102.36	4.46

-6	-5	-6	1.22	0.45
-6	-5	-6	1.02	0.27
-6	-5	-6	0.35	0.57
-6	-5	-6	1.00	0.26
-6	-5	-6	0.74	0.24
6	5	6	0.77	0.41
6	5	6	1.40	0.52
-7	-5	-6	113.41	4.56
-7	-5	-6	119.69	4.52
-7	-5	-6	125.00	5.03
-7	-5	-6	107.75	4.55
-7	-5	-6	121.31	4.78
-7	-5	-6	114.95	4.64
7	5	6	118.88	4.69
7	5	6	113.97	4.95
-8	-5	-6	0.25	0.29
-8	-5	-6	0.14	0.47
-8	-5	-6	0.71	0.35
-8	-5	-6	-0.16	0.30
-8	-5	-6	0.54	0.29
-8	-5	-6	0.48	0.66
8	5	6	0.17	0.58
8	5	6	0.32	0.40
-9	-5	-6	120.12	5.03
-9	-5	-6	112.79	4.50
-9	-5	-6	113.89	4.62
-9	-5	-6	117.93	4.69
-9	-5	-6	106.83	4.55
-9	-5	-6	115.63	4.50
9	5	6	114.06	4.94
9	5	6	116.00	4.62
9	5	6	109.71	4.54
-10	-5	-6	0.58	0.40
-10	-5	-6	0.07	0.49
-10	-5	-6	0.56	0.41
-10	-5	-6	0.62	0.33
-10	-5	-6	-0.10	0.57
-10	-5	-6	1.26	0.65
-10	-5	-6	0.85	0.84
10	5	6	0.90	0.68
10	5	6	0.76	0.35
-11	-5	-6	59.49	2.60
-11	-5	-6	56.92	2.80
-11	-5	-6	57.46	2.70

-11	-5	-6	58.77	2.78
-11	-5	-6	60.32	3.16
11	5	6	59.41	3.02
11	5	6	61.72	2.61
-12	-5	-6	1.02	0.78
-12	-5	-6	1.61	0.99
-12	-5	-6	-0.28	0.98
-12	-5	-6	-0.98	0.57
-12	-5	-6	0.32	0.55
12	5	6	0.01	0.42
12	5	6	0.38	0.73
-13	-5	-6	104.37	4.53
-13	-5	-6	99.17	4.39
-13	-5	-6	105.62	4.58
13	5	6	113.39	4.42
13	5	6	108.54	4.82
-14	-5	-6	4.82	1.05
-14	-5	-6	3.34	0.90
-14	-5	-6	3.60	0.83
-14	-5	-6	4.95	0.94
14	5	6	3.45	1.05
14	5	6	3.42	0.66
-15	-5	-6	115.66	5.26
-15	-5	-6	124.81	5.25
15	5	6	124.52	5.54
15	5	6	126.57	5.10
16	5	6	0.89	0.64
-17	-5	-6	55.48	3.17
17	5	6	54.40	3.41
17	5	6	57.74	2.79
-18	-5	-6	3.22	1.35
18	5	6	1.41	0.79
18	5	6	1.78	1.43
-19	-5	-6	25.80	2.80
19	5	6	31.41	2.06
19	5	6	27.29	2.96
20	-5	-5	4.26	0.86
-20	5	5	1.49	1.39
19	-5	-5	3.94	0.72
-19	5	5	4.56	1.39
18	-5	-5	1.76	0.62
-18	5	5	1.76	1.34
-18	5	5	3.16	1.20
17	-5	-5	22.80	3.44

17	-5	-5	18.07	1.23
-17	5	5	16.27	2.20
-17	5	5	14.82	2.01
16	-5	-5	11.71	1.94
16	-5	-5	11.81	1.00
-16	5	5	13.74	1.84
-16	5	5	10.11	1.53
15	-5	-5	9.43	1.84
15	-5	-5	9.82	0.92
-15	5	5	12.30	1.61
-15	5	5	9.74	1.79
14	-5	-5	36.71	1.60
14	-5	-5	35.01	2.87
14	-5	-5	32.31	1.68
14	-5	-5	33.73	1.55
-14	5	5	33.49	2.39
-14	5	5	38.08	2.26
13	-5	-5	18.82	1.10
13	-5	-5	19.54	1.10
13	-5	-5	24.99	2.41
13	-5	-5	20.18	1.14
-13	5	5	18.48	1.60
-13	5	5	19.58	1.85
12	-5	-5	49.76	3.06
12	-5	-5	53.22	2.17
12	-5	-5	49.40	2.14
12	-5	-5	48.28	2.16
-12	5	5	50.89	2.57
-12	5	5	48.13	2.77
11	-5	-5	24.74	2.02
11	-5	-5	24.80	1.48
11	-5	-5	21.37	1.13
11	-5	-5	19.43	1.23
11	-5	-5	23.79	1.21
-11	5	5	23.25	1.49
-11	5	5	20.59	1.72
-11	5	5	21.26	1.20
10	-5	-5	35.61	1.63
10	-5	-5	37.74	2.33
10	-5	-5	37.76	1.87
10	-5	-5	36.72	1.71
-10	5	5	36.52	1.93
-10	5	5	34.89	2.12
-10	5	5	39.76	1.72

9	-5	-5	15.57	1.08
9	-5	-5	15.27	0.84
9	-5	-5	16.20	0.99
9	-5	-5	14.77	1.51
-9	5	5	15.16	1.35
-9	5	5	15.05	0.89
-9	5	5	14.51	1.12
8	-5	-5	104.41	4.35
8	-5	-5	93.32	3.86
8	-5	-5	101.40	4.05
8	-5	-5	102.67	3.98
-8	5	5	96.41	3.91
-8	5	5	96.64	4.07
-8	5	5	97.17	4.29
7	-5	-5	64.82	2.96
7	-5	-5	70.38	3.09
7	-5	-5	64.08	2.61
7	-5	-5	64.26	2.74
7	-5	-5	67.38	2.76
-7	5	5	66.53	2.65
-7	5	5	58.26	2.97
-7	5	5	65.20	2.78
6	-5	-5	67.42	2.65
6	-5	-5	69.00	2.89
6	-5	-5	67.27	2.75
6	-5	-5	64.57	3.03
6	-5	-5	68.16	2.79
-6	5	5	61.80	2.98
-6	5	5	64.67	2.75
5	-5	-5	80.72	3.66
5	-5	-5	82.77	3.31
5	-5	-5	85.20	3.59
5	-5	-5	85.50	3.39
5	-5	-5	91.39	3.50
-5	5	5	82.97	3.40
-5	5	5	84.32	3.66
4	-5	-5	27.35	1.23
4	-5	-5	28.43	1.44
4	-5	-5	27.71	1.57
4	-5	-5	29.58	1.29
4	-5	-5	28.80	1.59
-4	5	5	29.08	1.55
-4	5	5	27.69	1.28
-4	5	5	28.05	1.25

3	-5	-5	0.69	0.21
3	-5	-5	0.51	0.39
3	-5	-5	1.18	0.47
3	-5	-5	0.71	0.36
3	-5	-5	1.09	0.21
-3	5	5	0.64	0.24
-3	5	5	0.99	0.22
-3	5	5	0.78	0.38
2	-5	-5	54.34	2.06
2	-5	-5	53.24	2.31
2	-5	-5	54.49	2.39
2	-5	-5	52.91	2.47
2	-5	-5	50.85	2.09
-2	5	5	52.88	2.07
-2	5	5	50.04	2.30
-2	5	5	49.31	2.34
-2	5	5	47.87	2.09
1	-5	-5	7.73	0.74
1	-5	-5	7.00	0.41
1	-5	-5	6.26	0.45
-1	5	5	6.77	0.46
-1	5	5	6.49	0.69
-1	5	5	7.41	0.69
-1	5	5	6.87	0.42
0	-5	-5	106.38	4.22
0	-5	-5	108.85	4.13
0	-5	-5	110.97	4.47
0	-5	-5	106.02	4.45
0	5	5	114.16	4.24
0	5	5	108.74	4.14
0	5	5	106.85	4.46
0	5	5	114.71	4.45
-1	-5	-5	82.21	3.25
-1	-5	-5	90.98	3.63
-1	-5	-5	81.14	3.35
-1	-5	-5	91.33	3.64
1	5	5	85.56	3.25
1	5	5	83.59	3.54
1	5	5	87.15	3.62
1	5	5	86.86	3.37
-2	-5	-5	143.47	5.49
-2	-5	-5	135.37	5.21
-2	-5	-5	134.10	5.56
-2	-5	-5	137.34	5.09

2	5	5	130.27	5.08
2	5	5	134.58	5.48
2	5	5	138.55	5.22
2	5	5	132.40	5.36
-3	-5	-5	1.79	0.51
-3	-5	-5	0.97	0.22
-3	-5	-5	1.30	0.17
-3	-5	-5	1.36	0.19
3	5	5	1.45	0.25
3	5	5	1.24	0.43
3	5	5	1.34	0.34
-4	-5	-5	112.74	4.45
-4	-5	-5	112.33	4.49
-4	-5	-5	120.95	4.80
-4	-5	-5	113.82	4.39
-4	-5	-5	116.39	4.39
4	5	5	118.14	4.62
4	5	5	114.18	4.79
-5	-5	-5	0.37	0.22
-5	-5	-5	0.37	0.21
-5	-5	-5	-0.08	0.47
-5	-5	-5	0.35	0.22
-5	-5	-5	0.08	0.19
5	5	5	0.23	0.32
5	5	5	0.03	0.42
-6	-5	-5	91.81	3.58
-6	-5	-5	87.80	3.59
-6	-5	-5	89.27	3.64
-6	-5	-5	92.29	3.54
-6	-5	-5	96.51	3.98
6	5	5	91.38	3.94
6	5	5	88.58	3.71
-7	-5	-5	1.78	0.29
-7	-5	-5	1.47	0.33
-7	-5	-5	1.99	0.37
-7	-5	-5	2.22	0.63
-7	-5	-5	2.35	0.38
7	5	5	1.42	0.57
7	5	5	2.08	0.45
-8	-5	-5	80.77	3.23
-8	-5	-5	82.91	3.66
-8	-5	-5	78.34	3.31
-8	-5	-5	79.20	3.56
-8	-5	-5	77.59	3.29

-8	-5	-5	79.45	3.23
8	5	5	80.75	3.26
8	5	5	85.59	3.38
8	5	5	79.69	3.61
-9	-5	-5	3.42	0.50
-9	-5	-5	2.77	0.50
-9	-5	-5	1.98	0.57
-9	-5	-5	4.79	0.85
-9	-5	-5	2.66	0.47
-9	-5	-5	2.59	0.44
9	5	5	2.88	0.54
9	5	5	3.68	0.78
9	5	5	2.85	0.43
-10	-5	-5	109.09	4.66
-10	-5	-5	112.83	4.78
-10	-5	-5	117.82	5.04
-10	-5	-5	117.27	4.56
-10	-5	-5	117.69	4.73
-10	-5	-5	118.82	4.61
10	5	5	115.33	4.95
10	5	5	114.01	4.60
-11	-5	-5	8.79	1.21
-11	-5	-5	9.66	1.05
-11	-5	-5	8.37	1.01
-11	-5	-5	10.17	1.28
-11	-5	-5	9.79	0.91
11	5	5	7.91	0.80
11	5	5	10.77	1.26
-12	-5	-5	95.50	4.16
-12	-5	-5	98.34	4.13
-12	-5	-5	104.94	4.60
-12	-5	-5	92.70	4.21
-12	-5	-5	102.46	4.35
12	5	5	101.69	4.48
12	5	5	103.21	4.11
-13	-5	-5	1.61	0.62
-13	-5	-5	2.13	1.16
-13	-5	-5	1.88	0.95
-13	-5	-5	0.66	0.68
-13	-5	-5	0.70	0.69
13	5	5	1.57	0.81
-14	-5	-5	112.68	5.14
-14	-5	-5	104.22	4.80
-14	-5	-5	113.82	5.23

-14	-5	-5	106.88	4.80
-14	-5	-5	115.44	4.91
14	5	5	118.68	4.66
14	5	5	111.41	4.99
-15	-5	-5	-0.51	0.78
-15	-5	-5	0.80	1.12
15	5	5	1.45	0.61
15	5	5	-0.05	0.94
-16	-5	-5	52.56	3.21
16	5	5	50.98	2.54
16	5	5	50.26	3.01
-17	-5	-5	0.71	1.03
17	5	5	2.37	1.24
17	5	5	2.38	0.73
-18	-5	-5	34.45	2.76
18	5	5	37.08	2.82
18	5	5	34.30	2.13
-19	-5	-5	3.80	1.34
19	5	5	2.31	1.52
19	5	5	2.74	0.86
20	-5	-4	2.54	0.72
-20	5	4	2.11	1.45
19	-5	-4	0.40	0.65
-19	5	4	1.81	1.27
18	-5	-4	8.48	1.07
-18	5	4	7.24	1.49
-18	5	4	8.98	1.43
17	-5	-4	-0.29	0.47
-17	5	4	1.38	1.17
-17	5	4	-0.74	1.00
16	-5	-4	4.95	1.65
16	-5	-4	6.52	1.03
16	-5	-4	5.14	0.83
-16	5	4	5.26	1.26
-16	5	4	4.61	1.09
15	-5	-4	1.24	1.51
15	-5	-4	1.19	0.48
-15	5	4	1.86	0.80
-15	5	4	1.87	0.85
-15	5	4	1.61	1.02
14	-5	-4	34.20	2.71
14	-5	-4	28.02	1.76
14	-5	-4	31.31	1.98
-14	5	4	29.35	2.16

-14	5	4	34.37	2.23
-14	5	4	31.96	1.85
13	-5	-4	6.83	0.68
13	-5	-4	10.57	1.93
13	-5	-4	5.89	0.83
-13	5	4	6.35	1.01
-13	5	4	6.08	0.91
-13	5	4	7.76	1.15
12	-5	-4	2.00	1.07
12	-5	-4	0.67	0.48
12	-5	-4	1.17	0.35
-12	5	4	0.75	0.61
-12	5	4	0.71	0.69
-12	5	4	1.09	0.74
11	-5	-4	1.10	0.30
11	-5	-4	1.42	0.54
11	-5	-4	1.22	0.90
11	-5	-4	1.43	0.45
-11	5	4	1.34	0.71
-11	5	4	0.46	0.51
-11	5	4	2.01	0.70
-11	5	4	1.17	0.36
10	-5	-4	9.07	0.64
10	-5	-4	8.55	1.36
10	-5	-4	9.30	0.85
10	-5	-4	9.63	0.93
-10	5	4	7.59	1.07
-10	5	4	8.81	1.01
-10	5	4	7.54	1.13
-10	5	4	10.45	0.73
9	-5	-4	0.69	0.22
9	-5	-4	0.58	0.37
9	-5	-4	0.54	0.69
9	-5	-4	0.49	0.46
-9	5	4	1.56	0.47
-9	5	4	0.43	0.56
-9	5	4	0.53	0.56
-9	5	4	0.88	0.27
8	-5	-4	18.32	1.26
8	-5	-4	20.70	1.42
8	-5	-4	17.40	0.90
8	-5	-4	18.45	1.11
-8	5	4	17.90	1.13
-8	5	4	17.80	1.31

-8	5	4	20.57	0.98
-8	5	4	15.96	1.27
7	-5	-4	18.91	1.09
7	-5	-4	20.98	1.37
7	-5	-4	19.29	0.92
7	-5	-4	18.02	1.30
-7	5	4	19.35	1.29
-7	5	4	17.68	1.28
-7	5	4	19.53	0.96
-7	5	4	19.49	1.11
6	-5	-4	23.61	1.18
6	-5	-4	24.17	1.36
6	-5	-4	22.94	1.06
6	-5	-4	24.25	1.43
-6	5	4	22.91	1.20
-6	5	4	25.02	1.10
-6	5	4	21.69	1.42
-6	5	4	22.69	1.37
5	-5	-4	3.10	0.54
5	-5	-4	3.50	0.56
5	-5	-4	3.55	0.46
5	-5	-4	3.10	0.33
5	-5	-4	3.79	0.57
-5	5	4	2.21	0.34
-5	5	4	3.52	0.49
-5	5	4	3.60	0.65
4	-5	-4	13.89	0.88
4	-5	-4	14.03	1.03
4	-5	-4	14.56	1.01
4	-5	-4	13.27	0.74
4	-5	-4	12.48	0.66
-4	5	4	12.60	0.75
-4	5	4	14.47	0.98
3	-5	-4	1.88	0.42
3	-5	-4	1.27	0.20
3	-5	-4	1.14	0.46
3	-5	-4	1.05	0.34
3	-5	-4	1.28	0.24
-3	5	4	1.83	0.28
-3	5	4	1.25	0.37
-3	5	4	1.63	0.28
2	-5	-4	24.25	1.39
2	-5	-4	24.04	1.01
2	-5	-4	21.52	1.00

2	-5	-4	23.91	1.22
2	-5	-4	21.99	1.62
-2	5	4	22.25	1.23
-2	5	4	22.27	1.07
-2	5	4	21.59	1.01
1	-5	-4	39.80	1.55
1	-5	-4	35.65	1.56
1	-5	-4	42.01	1.82
1	-5	-4	40.85	1.98
1	-5	-4	34.88	1.92
-1	5	4	39.05	1.78
-1	5	4	33.56	1.63
-1	5	4	39.17	1.56
-1	5	4	40.03	1.81
0	-5	-4	22.39	1.24
0	-5	-4	23.30	1.03
0	-5	-4	23.41	0.98
0	5	4	23.75	1.05
0	5	4	23.34	1.21
0	5	4	23.95	1.27
0	5	4	23.53	0.99
-1	-5	-4	156.46	6.03
-1	-5	-4	159.61	5.96
-1	-5	-4	170.44	6.24
1	5	4	156.41	6.26
1	5	4	153.76	6.19
1	5	4	156.18	5.96
-2	-5	-4	72.49	2.85
-2	-5	-4	77.32	3.11
-2	-5	-4	77.88	3.18
-2	-5	-4	73.15	2.92
2	5	4	71.67	2.83
2	5	4	75.18	3.07
2	5	4	73.18	2.94
2	5	4	74.13	3.17
-3	-5	-4	1.30	0.23
-3	-5	-4	1.12	0.34
-3	-5	-4	1.03	0.18
-3	-5	-4	1.09	0.41
3	5	4	0.87	0.30
3	5	4	1.88	0.45
3	5	4	1.18	0.22
3	5	4	1.16	0.17
-4	-5	-4	11.94	0.83

-4	-5	-4	12.64	0.94
-4	-5	-4	10.50	0.57
-4	-5	-4	10.58	0.64
-4	-5	-4	10.80	0.60
-4	-5	-4	10.97	0.58
4	5	4	10.97	0.78
4	5	4	12.23	0.94
-5	-5	-4	122.65	5.56
-5	-5	-4	141.15	5.44
-5	-5	-4	150.54	5.63
-5	-5	-4	139.21	5.49
-5	-5	-4	143.63	5.42
-5	-5	-4	156.63	5.78
5	5	4	146.26	5.77
5	5	4	139.49	5.58
-6	-5	-4	4.42	0.65
-6	-5	-4	4.25	0.44
-6	-5	-4	4.84	0.44
-6	-5	-4	5.32	0.81
-6	-5	-4	4.60	0.49
-6	-5	-4	4.01	0.52
6	5	4	4.11	0.70
6	5	4	4.91	0.61
-7	-5	-4	107.09	4.22
-7	-5	-4	94.55	3.82
-7	-5	-4	99.40	3.86
-7	-5	-4	91.15	3.88
-7	-5	-4	97.62	3.89
7	5	4	95.44	4.18
7	5	4	101.95	3.98
7	5	4	95.48	3.85
-8	-5	-4	10.25	0.72
-8	-5	-4	11.47	1.15
-8	-5	-4	10.43	0.82
-8	-5	-4	10.42	0.67
8	5	4	10.36	0.86
8	5	4	11.14	1.09
8	5	4	10.12	0.70
-9	-5	-4	28.11	1.31
-9	-5	-4	27.36	1.63
-9	-5	-4	28.07	1.39
-9	-5	-4	27.98	1.46
-9	-5	-4	27.04	1.53
-9	-5	-4	29.43	1.78

9	5	4	28.08	1.35
9	5	4	27.70	1.71
-10	-5	-4	36.02	1.76
-10	-5	-4	32.58	1.91
-10	-5	-4	31.67	1.70
-10	-5	-4	36.39	2.04
-10	-5	-4	33.81	1.64
-10	-5	-4	32.26	1.52
10	5	4	32.68	1.95
10	5	4	31.76	1.57
-11	-5	-4	35.09	2.09
-11	-5	-4	31.64	1.74
-11	-5	-4	32.82	1.99
-11	-5	-4	32.91	1.76
-11	-5	-4	31.80	1.65
-11	-5	-4	32.14	1.52
11	5	4	33.25	2.00
11	5	4	30.11	1.56
-12	-5	-4	1.53	0.62
-12	-5	-4	0.46	0.56
-12	-5	-4	1.75	0.87
-12	-5	-4	0.44	0.78
-12	-5	-4	0.75	0.52
12	5	4	-1.19	0.62
12	5	4	1.15	0.42
-13	-5	-4	41.60	2.22
-13	-5	-4	43.74	2.53
-13	-5	-4	38.43	2.06
-13	-5	-4	36.41	2.06
-13	-5	-4	40.60	2.43
13	5	4	41.23	2.35
13	5	4	40.12	1.95
-14	-5	-4	1.84	0.72
-14	-5	-4	1.47	0.79
-14	-5	-4	2.35	1.05
-14	-5	-4	2.12	1.09
14	5	4	3.23	0.59
14	5	4	0.31	0.82
-15	-5	-4	44.81	2.45
-15	-5	-4	46.58	2.65
-15	-5	-4	51.50	3.14
-15	-5	-4	52.24	2.92
15	5	4	49.28	2.78
15	5	4	51.47	2.39

-16	-5	-4	6.31	1.07
-16	-5	-4	8.88	1.46
16	5	4	7.33	1.26
16	5	4	6.12	0.83
-17	-5	-4	28.92	2.32
17	5	4	30.63	2.40
17	5	4	29.04	1.77
-18	-5	-4	0.95	1.16
18	5	4	0.68	1.20
18	5	4	0.72	0.70
-19	-5	-4	21.09	2.47
19	5	4	20.61	1.71
19	5	4	21.24	3.21
-20	-5	-4	0.61	1.41
20	5	4	1.41	0.93
20	-5	-3	4.99	0.88
-20	5	3	4.03	1.61
19	-5	-3	13.32	1.24
-19	5	3	11.52	1.78
18	-5	-3	21.80	1.46
-18	5	3	26.55	2.40
-18	5	3	19.00	2.18
17	-5	-3	9.40	1.01
17	-5	-3	10.25	1.07
-17	5	3	11.12	1.83
-17	5	3	8.54	1.38
16	-5	-3	18.73	1.24
16	-5	-3	18.95	1.25
16	-5	-3	22.53	2.55
-16	5	3	19.78	1.98
-16	5	3	19.32	1.93
15	-5	-3	11.68	0.99
15	-5	-3	9.68	0.87
15	-5	-3	9.81	1.61
-15	5	3	8.95	1.57
-15	5	3	9.90	1.53
-15	5	3	9.44	1.26
14	-5	-3	2.00	0.53
14	-5	-3	0.02	1.17
-14	5	3	2.48	0.85
-14	5	3	2.65	0.90
13	-5	-3	47.57	2.80
13	-5	-3	46.47	2.06
13	-5	-3	43.53	2.00

-13	5	3	43.69	2.38
-13	5	3	45.82	2.42
-13	5	3	42.94	2.45
12	-5	-3	-0.14	0.62
12	-5	-3	-0.01	0.92
12	-5	-3	-0.07	0.40
12	-5	-3	0.29	0.28
-12	5	3	0.56	0.70
-12	5	3	0.13	0.57
-12	5	3	0.08	0.67
-12	5	3	-0.23	0.69
11	-5	-3	7.68	0.64
11	-5	-3	6.93	1.08
11	-5	-3	6.85	0.92
11	-5	-3	8.90	0.85
-11	5	3	7.19	1.13
-11	5	3	8.46	0.70
-11	5	3	9.22	1.19
-11	5	3	8.81	1.03
10	-5	-3	10.96	0.70
10	-5	-3	12.59	1.36
10	-5	-3	11.32	0.97
10	-5	-3	11.86	0.93
-10	5	3	11.44	0.76
-10	5	3	10.53	1.15
-10	5	3	9.81	1.12
-10	5	3	10.73	1.03
9	-5	-3	4.66	0.47
9	-5	-3	4.40	0.71
9	-5	-3	4.59	0.74
9	-5	-3	4.45	0.77
-9	5	3	5.28	0.91
-9	5	3	3.32	0.58
-9	5	3	5.25	0.52
-9	5	3	4.71	0.72
8	-5	-3	9.48	0.79
8	-5	-3	9.86	0.60
8	-5	-3	9.45	1.01
8	-5	-3	9.70	0.87
-8	5	3	10.17	0.65
-8	5	3	8.96	0.83
-8	5	3	9.72	1.01
-8	5	3	10.23	0.97
7	-5	-3	46.06	1.93

7	-5	-3	51.75	2.29
7	-5	-3	44.63	2.12
7	-5	-3	49.68	2.09
-7	5	3	50.31	1.98
-7	5	3	44.92	2.10
-7	5	3	44.81	2.23
-7	5	3	46.24	2.25
6	-5	-3	1.04	0.46
6	-5	-3	1.93	0.49
6	-5	-3	1.31	0.31
6	-5	-3	1.57	0.24
-6	5	3	1.89	0.51
-6	5	3	1.01	0.42
-6	5	3	1.37	0.34
-6	5	3	1.44	0.25
5	-5	-3	43.13	1.81
5	-5	-3	40.96	2.13
5	-5	-3	41.66	1.99
5	-5	-3	39.65	1.71
-5	5	3	42.72	1.84
-5	5	3	43.46	2.00
-5	5	3	42.57	2.06
-5	5	3	42.76	1.74
4	-5	-3	14.16	0.68
4	-5	-3	14.44	0.76
4	-5	-3	12.60	1.06
4	-5	-3	14.44	0.98
-4	5	3	13.86	0.92
-4	5	3	13.41	1.04
-4	5	3	14.93	0.70
-4	5	3	13.97	0.77
3	-5	-3	58.56	2.29
3	-5	-3	57.37	2.47
3	-5	-3	57.29	2.24
3	-5	-3	57.46	2.54
-3	5	3	53.15	2.44
-3	5	3	59.27	2.61
-3	5	3	54.38	2.30
2	-5	-3	2.70	0.44
2	-5	-3	2.44	0.50
2	-5	-3	2.99	0.49
2	-5	-3	3.05	0.33
2	-5	-3	2.84	0.27
-2	5	3	2.46	0.33

-2	5	3	2.81	0.43
-2	5	3	3.06	0.28
1	-5	-3	94.08	3.78
1	-5	-3	94.65	3.94
1	-5	-3	94.19	3.58
1	-5	-3	94.35	3.78
-1	5	3	92.78	3.58
-1	5	3	91.65	3.76
-1	5	3	90.72	3.58
0	-5	-3	18.70	0.86
0	-5	-3	19.63	0.85
0	-5	-3	18.86	1.26
0	-5	-3	20.09	1.08
0	-5	-3	19.64	1.05
0	5	3	19.09	0.86
0	5	3	18.52	1.02
0	5	3	18.82	0.85
-1	-5	-3	0.81	0.17
-1	-5	-3	1.00	0.18
-1	-5	-3	0.74	0.26
-1	-5	-3	1.08	0.32
1	5	3	0.85	0.16
1	5	3	1.06	0.34
1	5	3	1.33	0.22
1	5	3	0.58	0.25
-2	-5	-3	49.84	2.21
-2	-5	-3	48.73	2.13
-2	-5	-3	47.56	1.97
-2	-5	-3	48.20	1.94
2	5	3	49.41	1.92
2	5	3	49.11	1.97
2	5	3	48.87	2.21
2	5	3	50.38	2.13
-3	-5	-3	29.88	1.34
-3	-5	-3	26.99	1.16
-3	-5	-3	27.09	1.13
-3	-5	-3	27.22	1.54
-3	-5	-3	22.92	1.17
3	5	3	27.97	1.31
3	5	3	22.73	1.21
3	5	3	26.50	1.11
3	5	3	28.42	1.43
-4	-5	-3	10.88	0.59
-4	-5	-3	11.94	0.80

-4	-5	-3	11.08	0.59
-4	-5	-3	10.58	0.86
-4	-5	-3	11.66	0.59
-4	-5	-3	10.53	0.62
4	5	3	10.67	0.88
4	5	3	11.69	0.77
-5	-5	-3	50.82	2.14
-5	-5	-3	47.04	1.97
-5	-5	-3	46.32	1.95
-5	-5	-3	47.82	1.95
-5	-5	-3	46.44	1.99
-5	-5	-3	48.37	2.24
5	5	3	49.15	2.11
5	5	3	48.66	2.25
-6	-5	-3	14.44	0.71
-6	-5	-3	12.76	1.01
-6	-5	-3	13.65	0.93
-6	-5	-3	13.17	0.76
-6	-5	-3	13.31	0.78
-6	-5	-3	13.99	0.75
6	5	3	13.04	0.72
6	5	3	14.55	1.06
6	5	3	13.82	0.90
-7	-5	-3	22.79	1.09
-7	-5	-3	21.94	1.12
-7	-5	-3	23.80	1.04
-7	-5	-3	19.29	1.35
-7	-5	-3	24.84	1.40
7	5	3	19.69	1.35
7	5	3	21.50	1.05
7	5	3	21.06	1.20
-8	-5	-3	0.89	0.31
-8	-5	-3	-0.22	0.49
-8	-5	-3	0.60	0.50
-8	-5	-3	0.39	0.43
-8	-5	-3	0.37	0.21
-8	-5	-3	0.47	0.33
8	5	3	-0.44	0.43
8	5	3	0.05	0.22
-9	-5	-3	10.15	1.13
-9	-5	-3	10.71	0.69
-9	-5	-3	10.47	0.94
-9	-5	-3	10.37	0.91
-9	-5	-3	12.43	1.14

-9	-5	-3	11.26	0.82
9	5	3	10.83	0.73
9	5	3	11.58	1.09
-10	-5	-3	1.80	0.72
-10	-5	-3	1.74	0.69
-10	-5	-3	0.98	0.29
-10	-5	-3	1.50	0.46
-10	-5	-3	1.73	0.43
-10	-5	-3	1.23	0.52
10	5	3	1.34	0.33
10	5	3	1.67	0.62
-11	-5	-3	11.75	1.31
-11	-5	-3	9.63	1.06
-11	-5	-3	10.15	0.77
-11	-5	-3	10.19	0.94
-11	-5	-3	10.53	1.06
-11	-5	-3	10.84	1.33
11	5	3	11.06	0.80
11	5	3	10.63	1.20
-12	-5	-3	2.06	0.58
-12	-5	-3	3.48	0.88
-12	-5	-3	1.92	0.84
-12	-5	-3	2.46	0.66
-12	-5	-3	2.09	0.44
-12	-5	-3	3.25	0.68
12	5	3	3.57	0.80
12	5	3	2.77	0.48
-13	-5	-3	4.22	0.70
-13	-5	-3	2.81	0.72
-13	-5	-3	2.89	1.00
-13	-5	-3	4.66	1.00
-13	-5	-3	1.80	0.72
13	5	3	3.64	0.85
13	5	3	3.89	0.56
-14	-5	-3	12.76	1.70
-14	-5	-3	8.71	1.29
-14	-5	-3	11.93	1.79
-14	-5	-3	8.57	1.31
14	5	3	12.70	1.43
-15	-5	-3	5.11	0.99
-15	-5	-3	5.07	0.98
-15	-5	-3	7.34	1.33
-15	-5	-3	7.58	1.37
15	5	3	6.04	1.05

15	5	3	8.17	0.97
-16	-5	-3	13.64	2.10
-16	-5	-3	14.48	2.20
-16	-5	-3	13.60	1.66
16	5	3	16.00	1.28
16	5	3	15.39	1.73
-17	-5	-3	10.23	1.78
17	5	3	13.50	1.85
17	5	3	12.35	1.21
-18	-5	-3	3.62	1.24
18	5	3	7.35	0.97
18	5	3	7.83	1.41
-19	-5	-3	2.41	1.34
19	5	3	1.19	1.33
19	5	3	1.47	0.79
-20	-5	-3	8.64	1.61
20	5	3	8.39	1.10
20	-5	-2	2.65	0.76
-20	5	2	3.10	1.50
19	-5	-2	29.54	1.75
-19	5	2	28.73	2.72
18	-5	-2	10.62	1.10
-18	5	2	12.71	2.15
17	-5	-2	46.94	2.33
17	-5	-2	49.51	2.33
-17	5	2	51.63	3.07
-17	5	2	46.93	2.72
16	-5	-2	10.01	1.60
16	-5	-2	11.76	1.16
16	-5	-2	11.49	0.98
-16	5	2	8.64	1.57
-16	5	2	10.82	1.76
-16	5	2	11.54	2.18
15	-5	-2	38.25	1.93
15	-5	-2	37.06	2.67
15	-5	-2	36.29	1.81
-15	5	2	37.53	2.36
-15	5	2	34.69	2.23
-15	5	2	41.90	2.43
14	-5	-2	0.72	1.11
14	-5	-2	1.12	0.61
-14	5	2	1.11	0.82
-14	5	2	0.57	0.86
13	-5	-2	32.95	1.71

13	-5	-2	33.90	1.57
13	-5	-2	35.12	2.32
-13	5	2	35.43	2.08
-13	5	2	27.75	1.92
-13	5	2	31.91	1.99
12	-5	-2	3.33	0.95
12	-5	-2	2.14	0.39
12	-5	-2	2.36	0.69
12	-5	-2	4.24	0.74
-12	5	2	3.13	0.67
-12	5	2	3.81	0.63
-12	5	2	2.40	0.73
11	-5	-2	83.34	3.46
11	-5	-2	82.73	3.64
11	-5	-2	80.30	3.23
11	-5	-2	84.52	3.35
-11	5	2	65.85	3.69
-11	5	2	80.47	3.55
-11	5	2	84.15	3.30
-11	5	2	76.43	3.51
10	-5	-2	63.71	2.85
10	-5	-2	60.01	2.59
10	-5	-2	60.42	2.48
10	-5	-2	58.80	2.66
-10	5	2	68.08	2.55
-10	5	2	58.73	2.81
-10	5	2	57.16	2.75
-10	5	2	53.93	2.74
9	-5	-2	34.47	1.66
9	-5	-2	33.50	1.82
9	-5	-2	32.29	1.48
9	-5	-2	33.22	1.66
-9	5	2	37.42	1.54
-9	5	2	33.11	1.78
-9	5	2	32.47	1.84
-9	5	2	33.30	1.73
8	-5	-2	19.01	1.12
8	-5	-2	17.66	1.04
8	-5	-2	16.72	0.86
8	-5	-2	18.20	1.21
-8	5	2	15.38	1.23
-8	5	2	16.24	1.15
-8	5	2	19.28	0.92
-8	5	2	17.70	1.10

7	-5	-2	39.72	1.69
7	-5	-2	40.75	1.84
7	-5	-2	40.90	1.90
-7	5	2	39.88	1.87
-7	5	2	44.10	1.73
-7	5	2	39.29	2.01
-7	5	2	40.46	1.93
6	-5	-2	118.99	4.58
6	-5	-2	111.23	4.47
6	-5	-2	104.94	4.36
6	-5	-2	112.99	4.60
-6	5	2	112.65	4.51
-6	5	2	120.30	4.40
-6	5	2	115.08	4.68
-6	5	2	114.78	4.59
5	-5	-2	96.78	3.89
5	-5	-2	101.07	3.90
5	-5	-2	91.92	3.66
5	-5	-2	92.08	3.75
-5	5	2	89.87	3.85
-5	5	2	92.25	3.96
-5	5	2	94.65	3.78
-5	5	2	102.05	3.69
4	-5	-2	61.53	2.52
4	-5	-2	61.16	2.44
4	-5	-2	64.30	2.65
4	-5	-2	66.11	2.72
-4	5	2	65.07	2.78
-4	5	2	60.33	2.53
-4	5	2	61.74	2.64
-4	5	2	59.37	2.51
3	-5	-2	73.14	2.92
3	-5	-2	75.41	3.23
3	-5	-2	76.47	2.99
3	-5	-2	80.97	3.13
-3	5	2	74.21	3.00
-3	5	2	77.14	3.27
-3	5	2	73.33	3.11
2	-5	-2	85.03	3.54
2	-5	-2	89.04	3.29
2	-5	-2	87.56	3.42
2	-5	-2	83.69	3.23
-2	5	2	81.77	3.41
-2	5	2	84.28	3.57

-2	5	2	78.55	3.35
1	-5	-2	12.16	0.74
1	-5	-2	11.46	0.93
1	-5	-2	11.43	0.56
1	-5	-2	11.13	0.60
-1	5	2	11.98	0.77
-1	5	2	11.48	0.61
-1	5	2	12.74	0.94
-1	5	2	11.79	0.57
0	-5	-2	95.10	3.87
0	-5	-2	95.60	3.67
0	-5	-2	91.11	3.51
0	-5	-2	95.22	3.70
0	-5	-2	84.02	3.54
0	5	2	88.14	3.65
0	5	2	90.34	3.51
-1	-5	-2	16.73	0.80
-1	-5	-2	19.45	0.98
-1	-5	-2	17.27	0.81
-1	-5	-2	18.94	1.22
1	5	2	17.49	0.79
1	5	2	17.35	0.96
1	5	2	18.04	0.80
-2	-5	-2	101.95	3.99
-2	-5	-2	105.95	4.40
-2	-5	-2	104.83	3.99
-2	-5	-2	102.30	4.13
2	5	2	101.96	3.98
2	5	2	109.00	4.16
2	5	2	104.58	4.23
2	5	2	103.39	3.98
-3	-5	-2	133.81	4.97
-3	-5	-2	136.17	5.22
-3	-5	-2	133.03	5.00
-3	-5	-2	129.43	5.13
-3	-5	-2	122.31	4.98
3	5	2	132.61	5.23
3	5	2	130.54	5.14
3	5	2	136.98	5.00
3	5	2	125.31	4.97
-4	-5	-2	0.67	0.18
-4	-5	-2	0.98	0.21
-4	-5	-2	0.70	0.31
-4	-5	-2	0.45	0.15

-4	-5	-2	0.47	0.39
4	5	2	1.10	0.35
4	5	2	0.92	0.31
4	5	2	0.86	0.22
-5	-5	-2	31.27	1.51
-5	-5	-2	28.48	1.28
-5	-5	-2	29.09	1.28
-5	-5	-2	28.37	1.23
-5	-5	-2	28.79	1.43
-5	-5	-2	27.59	1.28
5	5	2	31.12	1.54
5	5	2	28.09	1.41
-6	-5	-2	13.10	0.97
-6	-5	-2	12.91	0.73
-6	-5	-2	13.98	0.76
-6	-5	-2	13.36	0.68
-6	-5	-2	14.00	0.91
6	5	2	13.23	0.70
6	5	2	14.28	0.91
6	5	2	13.80	1.03
-7	-5	-2	1.03	0.44
-7	-5	-2	1.79	0.51
-7	-5	-2	1.64	0.31
-7	-5	-2	1.42	0.33
-7	-5	-2	1.64	0.26
7	5	2	1.42	0.45
7	5	2	1.59	0.26
-8	-5	-2	25.41	1.39
-8	-5	-2	20.67	1.41
-8	-5	-2	22.99	1.05
-8	-5	-2	23.39	1.28
-8	-5	-2	20.34	1.12
-8	-5	-2	21.39	1.17
8	5	2	21.39	1.06
8	5	2	20.62	1.36
-9	-5	-2	24.00	1.43
-9	-5	-2	20.49	1.51
-9	-5	-2	21.19	1.05
-9	-5	-2	20.49	1.28
-9	-5	-2	21.24	1.17
-9	-5	-2	21.68	1.23
9	5	2	23.10	1.41
9	5	2	20.11	1.07
-10	-5	-2	29.06	1.59

-10	-5	-2	31.25	1.54
-10	-5	-2	29.79	1.38
-10	-5	-2	33.62	1.78
-10	-5	-2	29.29	1.62
-10	-5	-2	29.03	1.86
10	5	2	28.96	1.72
10	5	2	30.12	1.42
-11	-5	-2	22.03	1.52
-11	-5	-2	18.91	1.24
-11	-5	-2	19.89	1.06
-11	-5	-2	21.64	1.36
-11	-5	-2	22.04	1.67
-11	-5	-2	20.27	1.36
11	5	2	19.35	1.42
11	5	2	19.58	1.10
-12	-5	-2	0.56	0.54
-12	-5	-2	1.48	0.69
-12	-5	-2	1.66	0.90
-12	-5	-2	0.85	0.35
-12	-5	-2	0.62	0.50
-12	-5	-2	2.25	0.66
12	5	2	0.53	0.61
12	5	2	1.61	0.41
-13	-5	-2	1.81	0.71
-13	-5	-2	1.33	0.83
-13	-5	-2	0.04	0.57
-13	-5	-2	0.77	0.65
-13	-5	-2	1.72	0.94
13	5	2	1.07	0.43
13	5	2	1.38	0.66
-14	-5	-2	65.66	3.42
-14	-5	-2	60.41	2.97
-14	-5	-2	65.10	3.20
-14	-5	-2	64.56	3.06
14	5	2	67.38	3.10
14	5	2	58.16	2.74
-15	-5	-2	0.87	1.01
-15	-5	-2	0.47	0.87
-15	-5	-2	-0.11	1.20
-15	-5	-2	-1.08	0.71
15	5	2	-0.81	0.72
15	5	2	-0.19	0.43
-16	-5	-2	10.84	1.96
-16	-5	-2	19.31	1.73

-16	-5	-2	13.92	1.76
16	5	2	16.66	1.68
16	5	2	19.18	1.31
-17	-5	-2	0.73	0.98
-17	-5	-2	-0.18	1.42
17	5	2	-0.85	0.92
17	5	2	1.19	0.62
-18	-5	-2	3.59	1.21
18	5	2	4.25	1.14
18	5	2	4.48	0.80
-19	-5	-2	3.40	1.28
19	5	2	2.64	0.76
-20	-5	-2	3.41	1.42
20	5	2	3.99	0.94
20	-5	-1	31.95	1.91
-20	5	1	29.56	3.03
19	-5	-1	5.67	0.86
-19	5	1	4.29	1.46
18	-5	-1	65.67	3.01
18	-5	-1	69.95	3.53
-18	5	1	65.66	3.80
17	-5	-1	1.09	0.54
17	-5	-1	1.75	0.76
-17	5	1	0.87	1.01
-17	5	1	0.87	0.88
16	-5	-1	86.95	3.62
16	-5	-1	85.86	4.11
16	-5	-1	84.92	3.71
-16	5	1	91.61	4.25
-16	5	1	79.93	3.77
-16	5	1	86.92	4.13
15	-5	-1	0.10	1.10
15	-5	-1	1.77	0.67
15	-5	-1	1.67	0.47
-15	5	1	0.97	0.78
-15	5	1	2.45	1.04
-15	5	1	2.67	0.90
14	-5	-1	42.10	2.49
14	-5	-1	40.77	2.04
-14	5	1	38.83	2.41
-14	5	1	38.01	2.13
13	-5	-1	5.04	1.04
13	-5	-1	4.11	0.67
13	-5	-1	4.27	0.54

-13	5	1	4.53	0.81
-13	5	1	5.31	0.86
-13	5	1	5.57	0.99
12	-5	-1	164.28	6.49
12	-5	-1	164.94	6.74
12	-5	-1	171.32	6.82
12	-5	-1	169.00	6.63
-12	5	1	183.57	6.70
-12	5	1	161.94	6.67
-12	5	1	156.85	6.81
-12	5	1	171.59	6.85
11	-5	-1	2.58	0.75
11	-5	-1	3.50	0.59
11	-5	-1	3.02	0.58
11	-5	-1	3.77	0.51
-11	5	1	3.96	0.50
-11	5	1	3.13	0.65
-11	5	1	4.55	0.83
-11	5	1	3.22	0.64
10	-5	-1	224.71	8.55
10	-5	-1	211.78	8.36
10	-5	-1	228.27	8.52
-10	5	1	209.62	8.53
-10	5	1	216.48	8.62
-10	5	1	214.15	8.71
-10	5	1	234.77	8.42
9	-5	-1	0.06	0.20
9	-5	-1	-0.44	0.34
9	-5	-1	0.01	0.34
9	-5	-1	-0.09	0.47
-9	5	1	0.00	0.38
-9	5	1	0.33	0.22
-9	5	1	-0.43	0.37
8	-5	-1	97.83	4.06
8	-5	-1	99.16	4.09
8	-5	-1	94.91	3.89
8	-5	-1	100.88	4.05
-8	5	1	101.09	4.10
-8	5	1	102.34	4.09
-8	5	1	107.75	3.94
-8	5	1	96.04	4.20
7	-5	-1	38.73	1.79
7	-5	-1	38.27	1.72
7	-5	-1	36.65	1.78

7	-5	-1	36.35	1.57
-7	5	1	34.16	1.89
-7	5	1	40.62	1.62
-7	5	1	35.20	1.75
-7	5	1	39.93	1.80
6	-5	-1	194.10	7.43
6	-5	-1	179.94	7.24
6	-5	-1	190.46	7.36
6	-5	-1	195.57	7.45
-6	5	1	190.14	7.55
-6	5	1	195.72	7.43
-6	5	1	196.43	7.27
-6	5	1	194.74	7.41
5	-5	-1	16.83	0.81
5	-5	-1	19.29	1.02
5	-5	-1	15.97	0.91
5	-5	-1	19.01	1.11
-5	5	1	18.11	0.83
-5	5	1	16.86	0.96
-5	5	1	18.33	1.17
-5	5	1	20.23	1.06
4	-5	-1	186.78	7.46
4	-5	-1	187.04	7.35
4	-5	-1	203.05	7.53
4	-5	-1	203.86	7.61
-4	5	1	198.76	7.67
-4	5	1	197.33	7.53
-4	5	1	194.05	7.38
-4	5	1	196.73	7.47
3	-5	-1	1.19	0.17
3	-5	-1	0.99	0.27
3	-5	-1	0.80	0.38
3	-5	-1	0.91	0.22
-3	5	1	1.07	0.38
-3	5	1	1.07	0.17
-3	5	1	1.24	0.25
-3	5	1	0.67	0.30
2	-5	-1	190.18	6.89
2	-5	-1	187.35	6.98
2	-5	-1	159.76	6.84
2	-5	-1	186.46	7.14
-2	5	1	172.45	6.95
-2	5	1	191.21	7.15
-2	5	1	183.32	6.81

-2	5	1	182.34	6.98
1	-5	-1	153.68	5.87
1	-5	-1	154.00	6.16
1	-5	-1	163.03	5.98
-1	5	1	157.12	5.82
-1	5	1	146.60	5.87
-1	5	1	158.39	6.15
-1	5	1	149.98	5.98
0	-5	-1	48.89	1.91
0	-5	-1	48.49	2.08
0	-5	-1	50.57	1.97
0	-5	-1	49.17	2.27
0	5	1	47.96	2.09
0	5	1	50.74	2.27
0	5	1	49.55	1.92
0	5	1	47.10	1.96
-1	-5	-1	0.76	0.24
-1	-5	-1	0.75	0.40
-1	-5	-1	0.62	0.12
-1	-5	-1	0.95	0.30
-1	-5	-1	0.46	0.17
1	5	1	0.74	0.16
1	5	1	0.56	0.12
1	5	1	0.76	0.39
1	5	1	1.38	0.32
-2	-5	-1	270.48	10.22
-2	-5	-1	255.73	10.02
-2	-5	-1	261.26	9.99
-2	-5	-1	258.91	9.82
-2	-5	-1	268.80	9.87
2	5	1	265.92	9.83
2	5	1	260.19	9.86
2	5	1	263.20	9.99
-3	-5	-1	2.79	0.47
-3	-5	-1	2.49	0.57
-3	-5	-1	2.45	0.31
-3	-5	-1	2.54	0.24
-3	-5	-1	2.77	0.33
-3	-5	-1	3.80	0.46
3	5	1	2.60	0.40
-4	-5	-1	227.10	8.27
-4	-5	-1	216.56	8.64
-4	-5	-1	239.88	8.42
-4	-5	-1	197.67	8.18

-4	-5	-1	225.55	8.43
-4	-5	-1	212.80	8.23
4	5	1	213.26	8.24
4	5	1	210.80	8.38
4	5	1	226.60	8.48
-5	-5	-1	34.81	1.48
-5	-5	-1	32.27	1.46
-5	-5	-1	34.35	1.61
-5	-5	-1	33.82	1.44
-5	-5	-1	34.74	1.40
-5	-5	-1	33.62	1.62
5	5	1	34.94	1.68
5	5	1	35.04	1.41
5	5	1	32.83	1.44
5	5	1	33.70	1.62
-6	-5	-1	86.00	3.65
-6	-5	-1	89.85	3.34
-6	-5	-1	83.46	3.42
-6	-5	-1	88.59	3.43
-6	-5	-1	84.02	3.58
6	5	1	89.48	3.63
6	5	1	82.44	3.35
-7	-5	-1	28.31	1.17
-7	-5	-1	26.77	1.54
-7	-5	-1	24.98	1.42
-7	-5	-1	26.37	1.28
-7	-5	-1	25.77	1.54
-7	-5	-1	25.97	1.28
7	5	1	28.01	1.49
7	5	1	26.48	1.18
-8	-5	-1	28.91	1.43
-8	-5	-1	27.81	1.72
-8	-5	-1	30.99	1.30
-8	-5	-1	29.86	1.43
-8	-5	-1	29.84	1.55
-8	-5	-1	29.78	1.55
8	5	1	29.41	1.32
8	5	1	28.08	1.59
-9	-5	-1	31.12	1.62
-9	-5	-1	33.12	1.94
-9	-5	-1	34.19	1.71
-9	-5	-1	31.94	1.59
-9	-5	-1	33.02	1.74
-9	-5	-1	33.69	1.46

9	5	1	33.91	1.76
9	5	1	34.83	1.49
-10	-5	-1	59.18	2.61
-10	-5	-1	55.62	2.31
-10	-5	-1	57.02	2.57
-10	-5	-1	52.43	2.50
-10	-5	-1	54.68	2.46
-10	-5	-1	55.59	2.81
10	5	1	56.61	2.35
10	5	1	56.41	2.62
-11	-5	-1	86.69	3.64
-11	-5	-1	92.33	3.79
-11	-5	-1	79.92	3.70
-11	-5	-1	87.04	3.47
-11	-5	-1	85.25	3.70
-11	-5	-1	88.70	4.03
11	5	1	85.94	3.51
11	5	1	88.46	3.77
-12	-5	-1	56.98	2.34
-12	-5	-1	54.27	2.96
-12	-5	-1	56.91	2.69
-12	-5	-1	52.41	2.52
-12	-5	-1	55.47	2.63
-12	-5	-1	49.40	2.60
12	5	1	54.93	2.64
12	5	1	58.75	2.40
-13	-5	-1	57.34	2.78
-13	-5	-1	52.62	2.65
-13	-5	-1	53.11	2.74
-13	-5	-1	58.74	2.77
-13	-5	-1	59.73	3.16
13	5	1	58.66	2.76
13	5	1	59.60	2.51
-14	-5	-1	15.95	1.50
-14	-5	-1	15.34	1.41
-14	-5	-1	14.31	1.53
-14	-5	-1	18.34	2.10
14	5	1	11.39	1.35
14	5	1	13.83	1.05
-15	-5	-1	16.98	1.76
-15	-5	-1	14.52	1.53
-15	-5	-1	13.16	2.14
-15	-5	-1	17.36	1.62
15	5	1	16.40	1.35

15	5	1	15.75	1.54
-16	-5	-1	12.97	1.62
-16	-5	-1	12.76	1.83
-16	-5	-1	13.90	2.29
-16	-5	-1	15.55	1.75
16	5	1	16.20	1.25
16	5	1	16.06	1.61
-17	-5	-1	18.64	1.94
-17	-5	-1	15.40	2.44
-17	-5	-1	15.58	2.06
17	5	1	17.14	1.82
17	5	1	18.34	1.36
-18	-5	-1	21.25	2.98
-18	-5	-1	22.51	2.11
18	5	1	18.96	1.50
-19	-5	-1	3.37	1.25
19	5	1	3.17	0.81
-20	-5	-1	6.98	1.52
20	5	1	9.89	1.44
20	-5	0	12.53	1.34
-20	5	0	8.65	1.76
19	-5	0	42.24	2.23
-19	5	0	44.72	3.20
18	-5	0	0.69	0.56
18	-5	0	2.53	0.95
-18	5	0	4.28	1.31
17	-5	0	78.51	3.45
17	-5	0	81.53	3.64
-17	5	0	80.80	4.18
16	-5	0	2.30	1.03
16	-5	0	3.66	0.61
16	-5	0	4.06	0.82
-16	5	0	0.68	0.93
-16	5	0	3.47	0.80
-16	5	0	2.55	1.30
15	-5	0	111.64	4.43
15	-5	0	113.57	4.59
15	-5	0	110.32	4.69
-15	5	0	115.57	4.96
-15	5	0	101.39	4.89
-15	5	0	99.22	4.44
14	-5	0	27.76	1.94
14	-5	0	30.08	1.71
-14	5	0	26.35	1.61

-14	5	0	27.37	2.14
13	-5	0	86.51	3.79
13	-5	0	101.09	3.96
13	-5	0	87.54	3.60
-13	5	0	87.37	4.05
-13	5	0	81.86	3.69
-13	5	0	85.84	3.98
12	-5	0	0.37	0.53
12	-5	0	0.14	0.30
12	-5	0	0.46	0.69
12	-5	0	0.43	0.58
-12	5	0	-0.24	0.50
-12	5	0	0.31	0.43
-12	5	0	1.07	0.77
-12	5	0	0.27	0.58
11	-5	0	154.24	6.25
11	-5	0	158.85	6.27
11	-5	0	154.84	6.25
11	-5	0	156.68	6.08
-11	5	0	164.98	6.11
-11	5	0	161.61	6.38
-11	5	0	152.29	6.43
-11	5	0	151.95	6.17
10	-5	0	1.05	0.56
10	-5	0	0.97	0.48
10	-5	0	0.61	0.41
10	-5	0	1.23	0.32
-10	5	0	0.91	0.27
-10	5	0	1.46	0.51
-10	5	0	1.07	0.50
-10	5	0	1.36	0.63
9	-5	0	180.23	7.14
9	-5	0	184.74	7.17
9	-5	0	183.95	7.13
9	-5	0	178.46	6.98
-9	5	0	186.58	7.23
-9	5	0	176.99	7.30
-9	5	0	179.08	7.10
-9	5	0	194.43	7.02
8	-5	0	73.50	3.16
8	-5	0	72.14	3.02
8	-5	0	75.91	3.23
8	-5	0	78.63	3.18
-8	5	0	72.67	3.38

-8	5	0	76.17	3.24
-8	5	0	84.83	3.07
-8	5	0	76.83	3.17
7	-5	0	148.95	5.73
7	-5	0	148.67	5.93
7	-5	0	151.96	5.86
7	-5	0	152.44	5.87
-7	5	0	138.95	5.88
-7	5	0	162.15	5.76
-7	5	0	148.24	5.88
6	-5	0	3.57	0.33
6	-5	0	3.84	0.51
6	-5	0	4.42	0.63
6	-5	0	3.45	0.52
-6	5	0	4.03	0.37
-6	5	0	3.72	0.53
-6	5	0	4.62	0.67
5	-5	0	37.33	1.59
5	-5	0	35.71	1.71
5	-5	0	35.53	1.60
5	-5	0	34.08	1.45
-5	5	0	37.26	1.47
-5	5	0	34.18	1.60
-5	5	0	34.34	1.79
4	-5	0	55.72	2.44
4	-5	0	58.35	2.32
4	-5	0	52.79	2.17
-4	5	0	57.55	2.19
-4	5	0	53.73	2.30
-4	5	0	54.88	2.50
3	-5	0	320.32	11.68
3	-5	0	309.38	11.71
3	-5	0	315.72	11.84
3	-5	0	291.24	11.56
-3	5	0	322.41	11.90
-3	5	0	326.85	11.70
-3	5	0	295.88	11.57
-3	5	0	304.02	11.82
2	-5	0	27.07	1.40
2	-5	0	25.97	1.09
2	-5	0	26.00	1.19
2	-5	0	28.35	1.26
-2	5	0	25.39	1.21
-2	5	0	27.93	1.10

-2	5	0	28.12	1.43
1	-5	0	227.43	9.01
1	-5	0	244.98	9.36
1	-5	0	250.29	9.18
1	-5	0	237.88	9.10
-1	5	0	257.74	9.37
-1	5	0	233.98	9.01
0	-5	0	0.29	0.36
0	-5	0	0.41	0.21
0	-5	0	0.20	0.15
0	-5	0	0.21	0.09
0	5	0	-0.03	0.25
0	5	0	0.48	0.33
0	5	0	0.08	0.16
0	5	0	0.24	0.09
-1	-5	0	248.85	9.47
-1	-5	0	242.80	9.26
-1	-5	0	234.78	9.07
-1	-5	0	253.01	9.17
1	5	0	244.69	9.27
1	5	0	235.67	9.15
1	5	0	258.87	9.44
1	5	0	230.68	9.07
-2	-5	0	27.82	1.15
-2	-5	0	24.31	1.23
-2	-5	0	25.49	1.05
-2	-5	0	26.85	1.46
2	5	0	25.03	1.05
2	5	0	25.14	1.40
-3	-5	0	322.74	11.88
-3	-5	0	320.75	12.07
-3	-5	0	294.31	11.67
-3	-5	0	305.97	11.87
-3	-5	0	314.57	11.76
-3	-5	0	311.65	11.66
3	5	0	312.26	11.74
3	5	0	325.69	11.90
-4	-5	0	53.97	2.42
-4	-5	0	52.21	2.31
-4	-5	0	55.01	2.54
-4	-5	0	55.13	2.09
-4	-5	0	51.90	2.18
-4	-5	0	53.79	2.31
4	5	0	57.53	2.18

4	5	0	50.24	2.31
-5	-5	0	35.44	1.89
-5	-5	0	37.99	1.71
-5	-5	0	35.45	1.42
-5	-5	0	35.16	1.65
-5	-5	0	34.14	1.76
5	5	0	35.06	1.50
5	5	0	33.27	1.43
-6	-5	0	4.22	0.60
-6	-5	0	4.69	0.75
-6	-5	0	4.01	0.47
-6	-5	0	4.56	0.78
-6	-5	0	4.79	0.48
6	5	0	4.31	0.35
-7	-5	0	154.84	5.75
-7	-5	0	142.53	5.73
-7	-5	0	147.53	6.03
-7	-5	0	148.26	5.87
-7	-5	0	142.87	6.08
-7	-5	0	150.44	5.61
-7	-5	0	145.23	5.84
7	5	0	152.08	5.89
7	5	0	145.30	5.62
-8	-5	0	72.83	3.34
-8	-5	0	74.88	3.26
-8	-5	0	79.46	2.99
-8	-5	0	77.05	3.13
-8	-5	0	74.00	3.44
-8	-5	0	71.16	3.48
-8	-5	0	75.91	3.14
8	5	0	77.22	3.01
8	5	0	80.37	3.29
-9	-5	0	176.34	7.15
-9	-5	0	190.04	6.99
-9	-5	0	174.64	7.24
-9	-5	0	194.79	7.21
-9	-5	0	184.74	7.46
-9	-5	0	186.64	7.14
9	5	0	187.64	7.26
9	5	0	175.66	7.00
-10	-5	0	1.61	0.29
-10	-5	0	0.35	0.78
-10	-5	0	0.54	0.39
-10	-5	0	1.17	0.43

-10	-5	0	0.96	0.53
-10	-5	0	1.36	0.51
10	5	0	0.61	0.28
10	5	0	1.47	0.53
-11	-5	0	146.53	6.46
-11	-5	0	160.54	6.16
-11	-5	0	149.76	5.91
-11	-5	0	149.97	6.15
-11	-5	0	153.55	6.21
-11	-5	0	154.39	6.11
11	5	0	158.97	5.96
11	5	0	154.14	6.18
-12	-5	0	-0.40	0.65
-12	-5	0	0.00	0.29
-12	-5	0	0.89	0.51
-12	-5	0	-1.50	0.89
-12	-5	0	-1.05	0.47
-12	-5	0	0.56	0.59
12	5	0	0.27	0.58
12	5	0	-0.06	0.30
-13	-5	0	96.78	3.96
-13	-5	0	87.14	4.34
-13	-5	0	88.49	3.67
-13	-5	0	91.15	4.40
-13	-5	0	94.23	4.02
-13	-5	0	82.71	4.00
-13	-5	0	92.21	4.22
13	5	0	93.50	3.74
13	5	0	95.20	3.98
-14	-5	0	27.50	1.81
-14	-5	0	27.34	1.82
-14	-5	0	28.71	2.39
-14	-5	0	24.25	1.90
-14	-5	0	26.32	2.39
14	5	0	28.78	1.47
14	5	0	29.34	1.77
-15	-5	0	119.99	4.93
-15	-5	0	114.74	4.98
-15	-5	0	112.44	5.39
-15	-5	0	110.23	5.32
15	5	0	115.97	4.70
-16	-5	0	6.29	1.50
-16	-5	0	0.95	1.28
-16	-5	0	5.09	1.14

-16	-5	0	2.93	0.90
-16	-5	0	2.46	0.97
16	5	0	2.63	0.59
-17	-5	0	74.33	4.08
-17	-5	0	73.22	3.71
-17	-5	0	69.14	4.20
17	5	0	80.08	3.34
-18	-5	0	2.05	1.98
-18	-5	0	1.91	1.57
-18	-5	0	4.50	1.09
18	5	0	1.72	0.68
-19	-5	0	43.89	2.92
19	5	0	42.31	2.31
-20	-5	0	6.05	1.53
20	5	0	11.55	1.44
20	-5	1	8.82	1.26
-20	5	-1	7.68	1.76
19	-5	1	2.64	0.72
-19	5	-1	3.08	1.42
18	-5	1	19.79	1.90
18	-5	1	21.16	1.45
-18	5	-1	18.92	2.43
17	-5	1	14.53	1.19
17	-5	1	15.90	1.65
-17	5	-1	16.07	2.16
16	-5	1	16.32	1.18
16	-5	1	14.02	1.56
-16	5	-1	19.22	1.98
-16	5	-1	18.57	2.29
15	-5	1	16.12	1.49
15	-5	1	16.24	1.12
-15	5	-1	15.82	1.80
-15	5	-1	14.90	2.04
14	-5	1	12.30	1.07
14	-5	1	13.04	1.34
14	-5	1	14.46	1.35
-14	5	-1	14.07	1.82
-14	5	-1	11.58	1.02
13	-5	1	59.30	2.72
13	-5	1	60.04	2.81
13	-5	1	59.83	2.54
-13	5	-1	57.21	3.08
-13	5	-1	58.01	2.95
12	-5	1	50.26	2.21

12	-5	1	52.19	2.51
12	-5	1	54.11	2.42
12	-5	1	50.21	2.45
-12	5	-1	52.76	2.72
-12	5	-1	41.08	2.42
-12	5	-1	54.06	2.61
11	-5	1	85.19	3.62
11	-5	1	80.15	3.38
11	-5	1	86.47	3.54
11	-5	1	84.08	3.58
-11	5	-1	86.09	3.71
-11	5	-1	72.86	3.48
-11	5	-1	91.87	3.43
-11	5	-1	85.40	3.83
10	-5	1	53.58	2.36
10	-5	1	54.02	2.41
10	-5	1	53.91	2.23
10	-5	1	53.42	2.48
-10	5	-1	52.05	2.65
-10	5	-1	49.54	2.29
-10	5	-1	54.79	2.25
-10	5	-1	51.74	2.50
9	-5	1	30.27	1.66
9	-5	1	32.59	1.53
9	-5	1	30.09	1.58
9	-5	1	31.15	1.41
-9	5	-1	30.43	1.66
-9	5	-1	33.11	1.43
-9	5	-1	31.34	1.83
-9	5	-1	30.65	1.48
8	-5	1	29.29	1.55
8	-5	1	29.22	1.45
8	-5	1	29.01	1.30
8	-5	1	29.52	1.41
-8	5	-1	26.66	1.67
-8	5	-1	30.91	1.33
-8	5	-1	28.92	1.38
-8	5	-1	28.79	1.52
7	-5	1	25.43	1.41
7	-5	1	26.32	1.30
7	-5	1	25.02	1.15
7	-5	1	26.42	1.25
-7	5	-1	27.57	1.17
-7	5	-1	23.32	1.32

-7	5	-1	24.43	1.53
-7	5	-1	25.64	1.24
6	-5	1	87.03	3.41
6	-5	1	80.56	3.31
6	-5	1	84.49	3.56
6	-5	1	86.24	3.45
-6	5	-1	83.73	3.49
-6	5	-1	91.53	3.34
-6	5	-1	83.91	3.40
5	-5	1	34.38	1.53
5	-5	1	34.83	1.66
5	-5	1	32.31	1.39
5	-5	1	35.36	1.51
-5	5	-1	32.94	1.73
-5	5	-1	33.27	1.55
-5	5	-1	34.08	1.52
-5	5	-1	33.96	1.41
4	-5	1	224.26	8.48
4	-5	1	210.06	8.22
4	-5	1	222.85	8.36
4	-5	1	225.16	8.33
-4	5	-1	217.75	8.36
-4	5	-1	220.66	8.55
-4	5	-1	219.95	8.23
-4	5	-1	214.97	8.34
3	-5	1	2.23	0.32
3	-5	1	3.11	0.49
3	-5	1	2.81	0.38
3	-5	1	2.28	0.23
-3	5	-1	2.04	0.31
-3	5	-1	3.39	0.61
-3	5	-1	2.58	0.23
-3	5	-1	2.11	0.33
2	-5	1	276.66	9.89
2	-5	1	268.90	9.91
2	-5	1	274.11	10.07
2	-5	1	243.65	9.75
-2	5	-1	275.71	10.11
-2	5	-1	240.69	9.75
-2	5	-1	262.57	9.89
-2	5	-1	255.43	9.92
1	-5	1	0.39	0.32
1	-5	1	0.88	0.25
1	-5	1	1.01	0.12

1	-5	1	0.72	0.20
-1	5	-1	0.86	0.12
-1	5	-1	0.87	0.29
-1	5	-1	0.68	0.20
-1	5	-1	0.95	0.38
0	-5	1	50.12	1.97
0	-5	1	50.75	2.20
0	-5	1	46.59	1.83
0	5	-1	46.95	1.98
0	5	-1	42.59	2.14
0	5	-1	49.39	1.84
0	5	-1	49.07	2.19
-1	-5	1	147.75	5.55
-1	-5	1	150.45	5.94
-1	-5	1	151.42	5.77
-1	-5	1	152.14	5.68
1	5	-1	145.94	5.78
1	5	-1	148.28	5.56
1	5	-1	145.31	5.69
-2	-5	1	185.16	7.13
-2	-5	1	190.60	7.03
-2	-5	1	176.85	6.90
-2	-5	1	188.28	7.29
2	5	-1	182.05	7.01
2	5	-1	187.62	7.24
2	5	-1	184.17	6.90
-3	-5	1	0.88	0.21
-3	-5	1	0.90	0.38
-3	-5	1	0.91	0.12
-3	-5	1	1.10	0.30
-3	-5	1	0.68	0.43
-3	-5	1	0.88	0.13
3	5	-1	0.95	0.23
3	5	-1	0.43	0.34
-4	-5	1	198.38	7.39
-4	-5	1	201.43	7.66
-4	-5	1	197.68	7.53
-4	-5	1	184.92	7.25
-4	-5	1	192.59	7.47
-4	-5	1	199.90	7.64
-4	-5	1	189.62	7.28
4	5	-1	180.40	7.26
4	5	-1	193.29	7.37
4	5	-1	205.23	7.63

-5	-5	1	18.15	0.99
-5	-5	1	18.62	0.92
-5	-5	1	18.77	0.77
-5	-5	1	18.71	1.22
-5	-5	1	18.19	1.28
5	5	-1	16.89	0.88
5	5	-1	15.80	0.78
-6	-5	1	190.09	7.41
-6	-5	1	195.35	7.32
-6	-5	1	194.82	7.12
-6	-5	1	186.36	7.25
-6	-5	1	190.00	7.26
-6	-5	1	188.58	7.54
-6	-5	1	183.50	7.55
6	5	-1	186.22	7.12
-7	-5	1	37.37	1.68
-7	-5	1	35.77	1.63
-7	-5	1	37.41	1.82
-7	-5	1	33.79	1.96
-7	-5	1	36.59	2.00
-7	-5	1	35.55	1.48
-7	-5	1	34.68	1.63
7	5	-1	36.48	1.50
-8	-5	1	100.38	4.44
-8	-5	1	99.79	4.10
-8	-5	1	92.07	4.35
-8	-5	1	106.07	4.29
-8	-5	1	105.77	4.13
-8	-5	1	101.82	4.13
-8	-5	1	105.75	3.95
8	5	-1	105.37	3.97
-9	-5	1	0.00	0.20
-9	-5	1	0.99	0.60
-9	-5	1	0.30	0.49
-9	-5	1	0.44	0.43
-9	-5	1	0.38	0.34
-9	-5	1	-0.34	0.66
-9	-5	1	0.21	0.32
9	5	-1	0.35	0.21
-10	-5	1	234.16	8.32
-10	-5	1	208.03	8.76
-10	-5	1	206.64	8.61
-10	-5	1	216.66	8.50
-10	-5	1	218.31	8.65

-10	-5	1	217.07	8.52
-10	-5	1	220.38	8.91
10	5	-1	232.66	8.35
-11	-5	1	2.99	0.59
-11	-5	1	3.59	1.01
-11	-5	1	3.44	0.61
-11	-5	1	5.22	0.81
-11	-5	1	3.99	0.92
-11	-5	1	2.79	0.58
-11	-5	1	4.24	0.51
11	5	-1	3.61	0.52
-12	-5	1	170.84	6.47
-12	-5	1	169.11	7.16
-12	-5	1	168.50	6.75
-12	-5	1	161.21	6.96
-12	-5	1	170.21	6.83
-12	-5	1	161.47	6.62
-12	-5	1	165.30	6.70
12	5	-1	177.16	6.52
-13	-5	1	7.06	1.01
-13	-5	1	6.41	1.12
-13	-5	1	5.12	0.99
-13	-5	1	4.09	0.66
-13	-5	1	4.99	0.79
-13	-5	1	3.67	1.23
13	5	-1	4.77	0.66
-14	-5	1	39.85	2.42
-14	-5	1	41.17	2.16
-14	-5	1	42.43	2.28
-14	-5	1	40.33	2.88
-14	-5	1	42.39	2.62
14	5	-1	42.50	1.96
-15	-5	1	2.41	1.32
-15	-5	1	2.68	0.97
-15	-5	1	1.16	0.80
-15	-5	1	1.62	0.79
-15	-5	1	1.42	1.09
15	5	-1	2.20	0.49
-16	-5	1	89.89	4.71
-16	-5	1	89.11	4.23
-16	-5	1	90.53	3.94
-16	-5	1	88.24	4.36
-16	-5	1	82.98	4.11
16	5	-1	95.84	3.79

-17	-5	1	0.96	1.55
-17	-5	1	1.18	0.90
-17	-5	1	1.55	1.32
17	5	-1	1.39	0.57
-18	-5	1	61.69	3.37
-18	-5	1	62.34	3.98
18	5	-1	64.19	2.94
-19	-5	1	6.60	1.34
19	5	-1	4.40	0.85
-20	-5	1	31.45	2.72
20	5	-1	30.42	1.98
20	-5	2	2.59	0.81
-20	5	-2	3.54	1.59
19	-5	2	4.52	0.79
-19	5	-2	6.49	1.59
18	-5	2	4.79	0.80
18	-5	2	9.74	1.74
-18	5	-2	5.34	1.38
17	-5	2	-0.53	0.50
17	-5	2	0.53	0.91
-17	5	-2	0.27	1.09
16	-5	2	14.36	1.62
16	-5	2	14.75	1.15
-16	5	-2	11.94	1.89
-16	5	-2	17.55	2.37
15	-5	2	0.18	0.73
15	-5	2	-0.31	0.40
-15	5	-2	-0.41	0.85
-15	5	-2	0.23	1.11
14	-5	2	60.81	2.98
14	-5	2	62.15	2.70
-14	5	-2	63.24	3.36
13	-5	2	1.37	0.41
13	-5	2	0.68	0.66
-13	5	-2	1.06	0.90
-13	5	-2	-0.54	0.62
12	-5	2	1.09	0.35
12	-5	2	0.04	0.66
12	-5	2	0.48	0.60
-12	5	-2	0.88	0.63
-12	5	-2	0.46	0.75
11	-5	2	20.37	1.08
11	-5	2	19.58	1.40
11	-5	2	20.89	1.21

11	-5	2	19.35	1.31
-11	5	-2	19.98	1.65
-11	5	-2	18.98	1.02
-11	5	-2	20.75	1.11
-11	5	-2	19.48	1.43
10	-5	2	28.85	1.43
10	-5	2	28.41	1.55
10	-5	2	28.52	1.36
10	-5	2	29.75	1.69
-10	5	-2	30.60	1.38
-10	5	-2	27.86	1.33
-10	5	-2	28.32	1.88
-10	5	-2	28.91	1.66
9	-5	2	20.68	1.22
9	-5	2	20.90	1.05
9	-5	2	22.95	1.39
9	-5	2	20.38	1.10
-9	5	-2	21.81	1.06
-9	5	-2	20.83	1.54
-9	5	-2	21.30	1.31
-9	5	-2	19.62	1.05
8	-5	2	22.04	1.08
8	-5	2	20.13	1.02
8	-5	2	21.14	1.20
8	-5	2	21.94	1.34
-8	5	-2	22.91	1.04
-8	5	-2	19.52	1.23
-8	5	-2	20.63	1.06
-8	5	-2	22.86	1.48
7	-5	2	1.80	0.37
7	-5	2	1.44	0.32
7	-5	2	1.50	0.27
7	-5	2	1.77	0.46
-7	5	-2	1.96	0.32
-7	5	-2	0.93	0.21
-7	5	-2	1.42	0.37
-7	5	-2	1.47	0.53
6	-5	2	12.39	0.66
6	-5	2	13.36	0.98
6	-5	2	14.08	0.83
6	-5	2	12.87	0.75
-6	5	-2	12.27	0.85
-6	5	-2	11.88	1.03
-6	5	-2	13.29	0.76

-6	5	-2	13.46	0.68
5	-5	2	29.12	1.53
5	-5	2	30.17	1.31
5	-5	2	27.79	1.23
5	-5	2	30.11	1.37
-5	5	-2	27.71	1.58
-5	5	-2	28.18	1.32
-5	5	-2	30.23	1.23
-5	5	-2	29.38	1.40
4	-5	2	0.59	0.23
4	-5	2	0.87	0.17
4	-5	2	0.87	0.25
4	-5	2	0.61	0.36
-4	5	-2	0.49	0.24
-4	5	-2	0.67	0.25
-4	5	-2	0.75	0.16
3	-5	2	132.60	5.04
3	-5	2	133.40	5.22
3	-5	2	123.12	5.07
-3	5	-2	129.74	5.07
-3	5	-2	135.95	4.93
-3	5	-2	126.99	5.06
-3	5	-2	132.87	5.27
2	-5	2	96.80	3.83
2	-5	2	101.83	4.14
2	-5	2	104.57	3.99
2	-5	2	100.21	3.97
-2	5	-2	101.05	3.83
-2	5	-2	100.86	3.98
-2	5	-2	104.83	4.19
-2	5	-2	103.19	4.01
1	-5	2	17.08	0.73
1	-5	2	17.63	1.09
1	-5	2	18.90	0.90
1	-5	2	20.16	0.95
-1	5	-2	17.38	0.73
-1	5	-2	17.33	0.90
-1	5	-2	18.82	1.11
0	-5	2	92.25	3.67
0	-5	2	92.42	3.78
0	-5	2	88.02	3.44
0	-5	2	93.28	3.79
0	-5	2	92.46	3.60
0	5	-2	90.82	3.70

0	5	-2	89.51	3.60
0	5	-2	93.09	3.45
0	5	-2	96.39	3.82
-1	-5	2	10.47	0.71
-1	-5	2	10.31	0.45
-1	-5	2	11.21	0.88
-1	-5	2	11.59	0.64
-1	-5	2	10.75	0.85
1	5	-2	10.53	0.46
1	5	-2	11.69	0.64
1	5	-2	11.08	0.84
-2	-5	2	82.99	3.51
-2	-5	2	78.75	3.11
-2	-5	2	90.88	3.30
-2	-5	2	81.48	3.12
-2	-5	2	84.79	3.50
2	5	-2	83.96	3.48
2	5	-2	79.69	3.27
-3	-5	2	73.77	2.86
-3	-5	2	71.76	2.88
-3	-5	2	76.38	3.29
-3	-5	2	73.49	3.16
-3	-5	2	80.43	3.05
-3	-5	2	75.04	3.28
3	5	-2	78.79	3.03
3	5	-2	80.31	3.24
-4	-5	2	61.72	2.34
-4	-5	2	62.86	2.77
-4	-5	2	55.13	2.58
-4	-5	2	64.33	2.82
4	5	-2	65.85	2.72
4	5	-2	63.03	2.35
4	5	-2	58.10	2.48
-5	-5	2	97.88	3.76
-5	-5	2	93.72	3.59
-5	-5	2	100.79	3.96
-5	-5	2	91.18	3.80
-5	-5	2	94.10	4.08
-5	-5	2	94.29	3.99
5	5	-2	95.30	3.94
5	5	-2	96.82	3.60
5	5	-2	88.56	3.72
5	5	-2	96.22	4.02
-6	-5	2	114.30	4.49

-6	-5	2	116.65	4.67
-6	-5	2	112.92	4.81
-6	-5	2	111.12	4.47
-6	-5	2	115.44	4.30
-6	-5	2	110.01	4.69
-6	-5	2	110.68	4.46
6	5	-2	118.44	4.77
6	5	-2	112.16	4.31
-7	-5	2	38.76	1.76
-7	-5	2	39.69	1.75
-7	-5	2	37.78	2.04
-7	-5	2	37.17	1.63
-7	-5	2	40.34	2.17
-7	-5	2	38.89	1.76
7	5	-2	39.73	2.07
7	5	-2	38.55	1.58
-8	-5	2	19.28	0.86
-8	-5	2	19.23	1.06
-8	-5	2	17.98	1.53
-8	-5	2	16.20	1.40
-8	-5	2	20.13	1.09
-8	-5	2	17.11	1.02
8	5	-2	17.95	0.87
8	5	-2	17.54	1.41
-9	-5	2	33.30	1.67
-9	-5	2	33.56	1.43
-9	-5	2	35.13	1.89
-9	-5	2	32.55	2.14
-9	-5	2	36.68	1.62
-9	-5	2	32.08	1.93
-9	-5	2	33.03	1.65
9	5	-2	34.30	2.01
9	5	-2	31.40	1.44
-10	-5	2	56.61	3.12
-10	-5	2	56.86	2.82
-10	-5	2	62.33	2.42
-10	-5	2	59.09	2.63
-10	-5	2	59.09	2.57
-10	-5	2	58.16	2.64
-10	-5	2	58.68	2.86
10	5	-2	61.76	3.00
10	5	-2	60.52	2.43
-11	-5	2	85.73	3.26
-11	-5	2	79.44	3.50

-11	-5	2	82.88	3.50
-11	-5	2	82.66	3.71
-11	-5	2	77.99	3.68
-11	-5	2	80.78	3.39
-11	-5	2	78.83	4.01
11	5	-2	82.49	3.27
-12	-5	2	3.34	1.14
-12	-5	2	3.04	0.80
-12	-5	2	2.35	0.58
-12	-5	2	3.51	0.42
-12	-5	2	2.56	0.63
-12	-5	2	2.63	0.84
12	5	-2	2.98	0.42
-13	-5	2	34.83	1.94
-13	-5	2	29.79	2.11
-13	-5	2	33.12	2.15
-13	-5	2	33.97	1.57
-13	-5	2	32.71	2.23
-13	-5	2	36.39	2.67
13	5	-2	32.93	1.58
-14	-5	2	1.54	0.68
-14	-5	2	1.22	0.61
-14	-5	2	3.35	0.98
-14	-5	2	1.50	1.21
-14	-5	2	0.82	0.50
-14	-5	2	1.79	1.01
14	5	-2	0.12	0.36
-15	-5	2	38.11	2.06
-15	-5	2	37.82	2.28
-15	-5	2	38.63	2.50
-15	-5	2	40.86	3.01
-15	-5	2	38.98	2.52
15	5	-2	42.85	1.90
-16	-5	2	10.43	1.94
-16	-5	2	13.14	1.84
-16	-5	2	13.02	1.76
-16	-5	2	18.36	2.59
-16	-5	2	13.51	1.57
16	5	-2	10.72	1.09
-17	-5	2	47.14	3.58
-17	-5	2	47.59	2.90
-17	-5	2	49.91	2.85
17	5	-2	50.89	2.35
-18	-5	2	7.84	1.27

-18	-5	2	10.13	2.03
18	5	-2	8.76	1.13
-19	-5	2	30.60	2.46
-19	-5	2	26.72	3.71
19	5	-2	31.46	1.86
-20	-5	2	3.04	1.27
20	5	-2	2.40	0.82
20	-5	3	5.81	1.00
-20	5	-3	10.75	1.93
19	-5	3	3.19	1.27
19	-5	3	1.26	0.71
-19	5	-3	1.43	1.37
18	-5	3	6.33	1.28
18	-5	3	6.97	1.11
-18	5	-3	8.06	1.55
17	-5	3	14.68	1.23
17	-5	3	9.25	1.75
-17	5	-3	14.32	2.10
16	-5	3	13.74	1.70
16	-5	3	15.49	1.22
-16	5	-3	17.93	2.03
-16	5	-3	13.95	1.95
15	-5	3	7.41	1.38
15	-5	3	7.44	0.89
-15	5	-3	6.78	1.40
-15	5	-3	6.17	1.13
14	-5	3	12.28	1.39
14	-5	3	10.33	0.91
-14	5	-3	13.68	1.96
13	-5	3	2.65	0.46
13	-5	3	1.99	0.77
-13	5	-3	3.81	0.79
-13	5	-3	4.35	1.14
12	-5	3	1.88	0.39
12	-5	3	1.59	0.75
-12	5	-3	2.71	0.73
-12	5	-3	2.77	0.91
11	-5	3	9.15	1.04
11	-5	3	11.69	1.22
11	-5	3	10.03	0.79
-11	5	-3	11.78	1.18
-11	5	-3	9.25	1.42
-11	5	-3	12.19	0.81
10	-5	3	1.23	0.48

10	-5	3	1.13	0.34
10	-5	3	1.56	0.61
-10	5	-3	1.60	0.55
-10	5	-3	1.33	0.77
-10	5	-3	1.64	0.33
9	-5	3	10.26	0.72
9	-5	3	11.37	0.91
9	-5	3	11.80	0.72
9	-5	3	10.72	1.08
-9	5	-3	11.70	1.31
-9	5	-3	12.01	0.73
-9	5	-3	11.53	1.01
8	-5	3	0.24	0.20
8	-5	3	0.71	0.29
8	-5	3	-0.03	0.46
8	-5	3	0.39	0.36
-8	5	-3	1.65	0.64
-8	5	-3	0.65	0.28
-8	5	-3	0.02	0.36
-8	5	-3	0.66	0.25
7	-5	3	22.14	1.36
7	-5	3	21.42	1.03
7	-5	3	21.95	1.18
7	-5	3	20.85	1.02
-7	5	-3	20.79	1.05
-7	5	-3	22.34	1.47
-7	5	-3	21.91	1.22
-7	5	-3	23.44	1.04
6	-5	3	13.54	0.71
6	-5	3	14.24	1.04
6	-5	3	13.74	0.88
6	-5	3	13.40	0.75
-6	5	-3	14.17	1.16
-6	5	-3	12.86	0.89
-6	5	-3	15.05	0.72
-6	5	-3	13.45	0.78
5	-5	3	49.54	2.11
5	-5	3	49.49	2.02
5	-5	3	47.34	1.97
5	-5	3	50.84	2.29
-5	5	-3	50.15	1.97
-5	5	-3	50.51	2.14
-5	5	-3	46.02	2.32
-5	5	-3	49.00	2.06

4	-5	3	11.32	0.57
4	-5	3	11.61	0.68
4	-5	3	11.54	0.72
4	-5	3	10.62	0.88
-4	5	-3	10.97	0.94
-4	5	-3	11.47	0.72
-4	5	-3	10.45	0.73
-4	5	-3	12.00	0.56
3	-5	3	27.30	1.43
3	-5	3	26.37	1.36
3	-5	3	27.81	1.25
3	-5	3	27.83	1.37
-3	5	-3	26.78	1.63
-3	5	-3	27.14	1.29
-3	5	-3	26.80	1.11
2	-5	3	41.37	2.23
2	-5	3	48.17	2.17
2	-5	3	48.90	2.00
2	-5	3	49.88	2.02
2	-5	3	46.22	1.84
-2	5	-3	47.43	2.24
-2	5	-3	50.37	2.02
-2	5	-3	47.50	1.84
1	-5	3	1.00	0.40
1	-5	3	0.44	0.32
1	-5	3	1.41	0.30
1	-5	3	1.03	0.13
1	-5	3	0.90	0.23
-1	5	-3	0.57	0.23
-1	5	-3	0.89	0.10
-1	5	-3	0.69	0.36
0	-5	3	18.77	0.75
0	-5	3	18.74	1.18
0	-5	3	20.27	1.14
0	-5	3	19.36	0.95
0	-5	3	18.78	1.03
0	5	-3	19.51	0.95
0	5	-3	19.74	1.15
0	5	-3	19.24	0.76
-1	-5	3	93.16	3.75
-1	-5	3	94.84	3.82
-1	-5	3	91.22	3.42
-1	-5	3	91.66	3.63
-1	-5	3	90.80	3.86

1	5	-3	89.38	3.43
1	5	-3	89.79	3.61
1	5	-3	96.54	3.83
-2	-5	3	2.94	0.52
-2	-5	3	2.48	0.13
-2	-5	3	2.29	0.37
-2	-5	3	2.48	0.14
-2	-5	3	2.62	0.48
2	5	-3	2.81	0.36
2	5	-3	2.92	0.49
2	5	-3	2.28	0.44
-3	-5	3	53.76	2.05
-3	-5	3	53.10	2.06
-3	-5	3	57.99	2.46
-3	-5	3	54.61	2.56
3	5	-3	51.39	2.23
3	5	-3	57.04	2.44
-4	-5	3	13.94	0.58
-4	-5	3	12.47	0.99
-4	-5	3	14.61	1.16
-4	-5	3	13.60	0.79
4	5	-3	14.85	0.99
4	5	-3	13.41	0.58
4	5	-3	15.08	1.09
4	5	-3	14.61	0.78
-5	-5	3	40.90	2.05
-5	-5	3	44.03	1.85
-5	-5	3	42.91	1.65
-5	-5	3	41.04	2.20
5	5	-3	40.20	1.80
5	5	-3	44.66	2.02
5	5	-3	41.75	2.15
5	5	-3	42.49	1.65
-6	-5	3	0.95	0.31
-6	-5	3	1.14	0.17
-6	-5	3	1.43	0.53
-6	-5	3	1.18	0.57
-6	-5	3	1.01	0.34
6	5	-3	1.14	0.26
6	5	-3	1.28	0.58
6	5	-3	1.38	0.46
-7	-5	3	45.24	2.27
-7	-5	3	48.09	1.88
-7	-5	3	44.30	2.06

-7	-5	3	48.64	2.10
-7	-5	3	45.91	2.05
-7	-5	3	47.53	2.56
7	5	-3	51.58	2.48
7	5	-3	48.32	1.89
-8	-5	3	8.63	0.73
-8	-5	3	10.38	0.79
-8	-5	3	9.35	0.53
-8	-5	3	9.71	0.75
-8	-5	3	9.08	1.09
-8	-5	3	8.11	1.26
8	5	-3	8.69	0.54
8	5	-3	10.94	1.23
-9	-5	3	4.65	0.99
-9	-5	3	4.96	0.44
-9	-5	3	5.05	0.66
-9	-5	3	5.94	0.65
-9	-5	3	4.54	0.81
-9	-5	3	6.58	0.72
9	5	-3	3.75	0.83
9	5	-3	4.87	0.44
-10	-5	3	9.69	0.92
-10	-5	3	10.44	1.25
-10	-5	3	10.33	0.95
-10	-5	3	10.80	1.59
-10	-5	3	11.04	0.84
-10	-5	3	11.93	0.68
10	5	-3	9.96	1.40
10	5	-3	10.34	0.67
-11	-5	3	8.97	0.94
-11	-5	3	7.36	1.03
-11	-5	3	6.86	0.87
-11	-5	3	8.23	0.82
-11	-5	3	7.65	1.31
-11	-5	3	9.02	0.63
11	5	-3	7.75	0.61
11	5	-3	11.66	1.56
-12	-5	3	0.81	0.55
-12	-5	3	0.69	0.45
-12	-5	3	0.24	0.28
-12	-5	3	0.09	0.71
-12	-5	3	0.45	0.48
-12	-5	3	0.61	1.11
12	5	-3	0.27	0.29

12	5	-3	1.26	0.96
-13	-5	3	47.66	3.17
-13	-5	3	46.65	2.49
-13	-5	3	49.08	2.17
-13	-5	3	48.04	2.07
-13	-5	3	46.26	2.40
13	5	-3	46.15	2.92
13	5	-3	46.53	2.07
-14	-5	3	3.59	0.64
-14	-5	3	2.00	0.46
-14	-5	3	2.11	0.72
-14	-5	3	3.49	1.04
-14	-5	3	2.14	0.88
-14	-5	3	1.66	1.33
14	5	-3	3.06	1.25
14	5	-3	1.80	0.42
-15	-5	3	8.62	1.73
-15	-5	3	8.52	1.50
-15	-5	3	8.59	1.04
-15	-5	3	10.72	1.39
-15	-5	3	10.45	1.43
15	5	-3	9.49	0.86
-16	-5	3	26.13	2.35
-16	-5	3	18.94	1.81
-16	-5	3	15.69	2.73
-16	-5	3	19.91	1.80
-17	-5	3	7.39	1.46
-17	-5	3	9.11	1.29
-17	-5	3	10.32	1.91
17	5	-3	8.20	1.01
-18	-5	3	20.83	2.04
-18	-5	3	20.34	3.16
18	5	-3	21.05	1.45
-19	-5	3	14.52	2.06
-19	-5	3	10.33	2.21
19	5	-3	14.05	1.34
-20	-5	3	4.58	1.33
20	5	-3	4.57	0.87
-20	5	-4	0.73	1.63
19	-5	4	18.54	1.63
-19	5	-4	21.16	2.69
18	-5	4	1.57	0.67
18	-5	4	0.80	1.17
-18	5	-4	-0.44	1.20

17	-5	4	28.45	1.73
17	-5	4	29.15	2.29
-17	5	-4	26.75	2.49
16	-5	4	5.48	1.21
16	-5	4	7.05	1.04
-16	5	-4	6.82	1.30
15	-5	4	45.99	2.71
15	-5	4	50.68	2.29
-15	5	-4	49.61	2.84
-15	5	-4	40.93	3.16
14	-5	4	1.57	0.47
14	-5	4	1.47	0.86
-14	5	-4	1.89	1.02
-14	5	-4	1.40	1.19
13	-5	4	38.59	1.94
13	-5	4	43.80	2.43
-13	5	-4	41.09	2.34
-13	5	-4	43.33	2.76
12	-5	4	0.67	0.68
12	-5	4	0.79	0.37
-12	5	-4	0.78	0.64
-12	5	-4	1.05	0.95
11	-5	4	32.86	1.80
11	-5	4	33.90	2.01
11	-5	4	32.75	1.57
-11	5	-4	33.51	1.58
-11	5	-4	31.86	1.91
-11	5	-4	33.85	2.24
10	-5	4	35.12	2.00
10	-5	4	33.45	1.59
10	-5	4	34.40	1.77
-10	5	-4	36.73	1.60
-10	5	-4	33.43	2.18
-10	5	-4	33.20	1.86
9	-5	4	27.13	1.30
9	-5	4	27.84	1.47
9	-5	4	26.74	1.68
-9	5	-4	27.70	1.89
-9	5	-4	26.55	1.54
-9	5	-4	29.40	1.31
8	-5	4	10.64	0.86
8	-5	4	9.84	0.67
8	-5	4	11.24	1.08
-8	5	-4	11.59	0.68

-8	5	-4	9.52	1.26
-8	5	-4	10.31	0.89
7	-5	4	98.24	3.95
7	-5	4	96.83	3.86
7	-5	4	96.47	3.81
7	-5	4	99.39	3.77
7	-5	4	96.62	4.16
-7	5	-4	90.06	4.21
-7	5	-4	107.18	3.82
-7	5	-4	92.97	3.98
6	-5	4	4.86	0.61
6	-5	4	4.74	0.46
6	-5	4	4.66	0.81
6	-5	4	5.26	0.64
6	-5	4	4.24	0.40
-6	5	-4	4.55	0.41
-6	5	-4	5.33	0.81
-6	5	-4	5.60	0.65
5	-5	4	148.06	5.74
5	-5	4	150.09	5.94
5	-5	4	144.31	5.59
5	-5	4	149.37	5.76
5	-5	4	148.96	5.63
-5	5	-4	153.86	5.60
-5	5	-4	141.46	5.75
-5	5	-4	141.33	5.98
4	-5	4	12.01	0.75
4	-5	4	11.58	0.58
4	-5	4	11.49	0.69
4	-5	4	12.90	0.89
4	-5	4	10.92	0.94
-4	5	-4	11.73	0.77
-4	5	-4	10.74	1.01
-4	5	-4	11.35	0.58
3	-5	4	0.96	0.28
3	-5	4	1.44	0.31
3	-5	4	0.80	0.41
3	-5	4	1.51	0.44
3	-5	4	0.99	0.17
-3	5	-4	2.15	0.50
-3	5	-4	1.68	0.21
-3	5	-4	1.26	0.31
2	-5	4	72.36	3.10
2	-5	4	72.71	2.94

2	-5	4	69.48	2.74
2	-5	4	72.74	3.14
-2	5	-4	70.48	2.92
-2	5	-4	72.64	3.13
-2	5	-4	73.80	2.74
1	-5	4	147.35	5.73
1	-5	4	156.82	5.99
1	-5	4	160.16	6.11
1	-5	4	154.92	6.14
1	-5	4	149.65	5.94
-1	5	-4	152.91	5.92
-1	5	-4	154.45	5.73
0	-5	4	25.45	1.16
0	-5	4	23.98	1.30
0	-5	4	22.80	0.92
0	-5	4	23.17	1.40
0	5	-4	23.72	1.33
0	5	-4	24.44	1.31
0	5	-4	23.30	0.92
0	5	-4	24.01	1.12
-1	-5	4	38.71	1.72
-1	-5	4	38.32	1.86
-1	-5	4	39.49	1.98
1	5	-4	38.17	1.68
1	5	-4	41.15	1.90
1	5	-4	40.67	1.93
-2	-5	4	22.24	1.27
-2	-5	4	22.74	1.42
2	5	-4	21.25	1.08
2	5	-4	24.99	1.32
-3	-5	4	1.63	0.28
-3	-5	4	0.98	0.52
-3	-5	4	1.88	0.45
3	5	-4	1.22	0.43
3	5	-4	1.67	0.51
3	5	-4	1.42	0.28
-4	-5	4	13.02	0.54
-4	-5	4	12.86	0.78
-4	-5	4	12.67	0.99
-4	-5	4	13.21	1.16
4	5	-4	13.25	0.75
4	5	-4	13.89	0.96
4	5	-4	12.65	0.54
4	5	-4	13.02	1.10

-5	-5	4	3.06	0.22
-5	-5	4	3.44	0.48
-5	-5	4	3.60	0.60
-5	-5	4	3.10	0.71
5	5	-4	2.90	0.22
5	5	-4	3.81	0.72
5	5	-4	3.25	0.43
5	5	-4	4.53	0.69
-6	-5	4	24.34	0.99
-6	-5	4	23.55	1.22
-6	-5	4	20.40	1.45
-6	-5	4	24.14	1.71
6	5	-4	23.89	1.63
6	5	-4	27.20	1.39
6	5	-4	23.80	1.00
-7	-5	4	19.24	1.61
-7	-5	4	18.88	1.05
-7	-5	4	19.55	1.06
-7	-5	4	15.01	1.38
-7	-5	4	19.14	0.85
7	5	-4	20.02	1.55
7	5	-4	20.42	0.87
7	5	-4	19.72	1.22
-8	-5	4	16.39	0.87
-8	-5	4	17.51	1.62
-8	-5	4	16.71	1.03
-8	-5	4	17.12	1.07
-8	-5	4	17.80	0.99
8	5	-4	17.98	1.54
8	5	-4	18.79	0.83
-9	-5	4	0.77	0.41
-9	-5	4	1.15	0.23
-9	-5	4	0.38	0.76
-9	-5	4	0.45	0.34
-9	-5	4	0.61	0.36
9	5	-4	2.89	0.77
9	5	-4	0.66	0.21
-10	-5	4	9.23	0.74
-10	-5	4	9.63	0.88
-10	-5	4	10.73	0.95
-10	-5	4	8.98	1.63
-10	-5	4	9.72	0.63
10	5	-4	9.73	0.62
10	5	-4	11.13	1.50

-11	-5	4	1.55	0.42
-11	-5	4	0.66	0.52
-11	-5	4	1.02	0.45
-11	-5	4	0.60	1.01
-11	-5	4	1.57	0.32
11	5	-4	1.20	0.30
11	5	-4	1.40	0.91
-12	-5	4	1.07	0.32
-12	-5	4	0.40	0.52
-12	-5	4	0.86	0.41
-12	-5	4	-0.13	1.10
12	5	-4	1.22	1.05
12	5	-4	1.10	0.33
-13	-5	4	5.79	1.43
-13	-5	4	5.87	0.70
-13	-5	4	7.18	1.05
-13	-5	4	7.99	0.79
13	5	-4	9.89	1.46
13	5	-4	7.53	0.68
-14	-5	4	31.73	1.61
-14	-5	4	34.97	1.63
-14	-5	4	32.48	2.91
14	5	-4	35.68	1.63
14	5	-4	33.93	2.65
-15	-5	4	2.54	0.83
-15	-5	4	1.08	1.42
-15	-5	4	2.05	0.72
-15	-5	4	3.08	0.53
15	5	-4	1.59	0.46
15	5	-4	1.89	1.42
-16	-5	4	3.84	1.44
-16	-5	4	4.85	0.99
-16	-5	4	4.82	1.73
16	5	-4	4.65	1.59
16	5	-4	5.10	0.78
-17	-5	4	0.57	1.71
-17	-5	4	0.29	0.92
17	5	-4	-0.08	0.49
-18	-5	4	8.53	2.08
-18	-5	4	7.88	1.36
18	5	-4	6.60	1.04
-19	-5	4	0.47	1.99
-19	-5	4	0.50	1.02
19	5	-4	0.20	0.64

-20	-5	4	2.97	1.27
20	5	-4	3.34	0.84
19	-5	5	4.06	0.84
19	-5	5	6.30	1.61
-19	5	-5	5.18	1.71
18	-5	5	36.32	2.05
18	-5	5	34.20	2.74
-18	5	-5	34.20	2.95
17	-5	5	3.68	1.21
17	-5	5	1.58	0.65
-17	5	-5	2.06	1.24
16	-5	5	48.89	2.35
16	-5	5	46.07	2.85
-16	5	-5	46.68	2.97
15	-5	5	-0.92	0.97
15	-5	5	-0.79	0.52
-15	5	-5	0.18	0.92
14	-5	5	115.46	4.57
14	-5	5	107.81	4.95
-14	5	-5	115.91	5.04
-14	5	-5	104.14	5.35
13	-5	5	0.90	0.82
-13	5	-5	1.78	0.80
-13	5	-5	-0.13	1.26
12	-5	5	100.40	4.04
12	-5	5	99.13	4.46
-12	5	-5	101.32	4.46
-12	5	-5	94.69	4.63
11	-5	5	10.57	1.10
11	-5	5	8.90	1.29
11	-5	5	8.36	0.78
-11	5	-5	9.88	0.80
-11	5	-5	8.93	1.14
-11	5	-5	9.65	1.33
10	-5	5	115.25	4.60
10	-5	5	115.36	5.01
10	-5	5	119.62	4.74
-10	5	-5	106.01	5.08
-10	5	-5	125.64	4.60
-10	5	-5	113.68	4.84
9	-5	5	3.06	0.54
9	-5	5	3.13	0.49
9	-5	5	3.82	0.79
-9	5	-5	1.87	0.85

-9	5	-5	2.10	0.52
-9	5	-5	4.31	0.52
8	-5	5	78.66	3.61
8	-5	5	79.29	3.22
8	-5	5	80.74	3.36
-8	5	-5	86.09	3.23
-8	5	-5	80.10	3.44
-8	5	-5	77.87	3.73
7	-5	5	2.69	0.47
7	-5	5	2.21	0.43
7	-5	5	2.08	0.32
7	-5	5	2.09	0.39
7	-5	5	2.20	0.61
-7	5	-5	2.29	0.74
-7	5	-5	2.51	0.37
-7	5	-5	1.51	0.42
6	-5	5	92.48	3.61
6	-5	5	94.46	3.75
6	-5	5	90.73	3.60
6	-5	5	89.11	3.74
6	-5	5	88.02	3.97
-6	5	-5	93.42	3.80
-6	5	-5	91.52	4.06
-6	5	-5	99.30	3.61
5	-5	5	0.45	0.19
5	-5	5	0.26	0.42
5	-5	5	0.55	0.26
5	-5	5	1.11	0.32
5	-5	5	0.73	0.48
-5	5	-5	1.35	0.55
-5	5	-5	0.70	0.19
-5	5	-5	0.47	0.29
4	-5	5	114.40	4.76
4	-5	5	113.97	4.67
4	-5	5	117.28	4.55
4	-5	5	116.72	4.50
4	-5	5	110.88	4.39
-4	5	-5	124.37	4.40
-4	5	-5	110.42	4.56
-4	5	-5	112.77	4.82
3	-5	5	0.95	0.31
3	-5	5	0.98	0.18
3	-5	5	1.41	0.34
3	-5	5	1.09	0.52

3	-5	5	1.29	0.45
-3	5	-5	1.45	0.53
-3	5	-5	1.39	0.33
-3	5	-5	1.49	0.23
2	-5	5	135.95	5.49
2	-5	5	133.41	5.11
2	-5	5	141.62	5.34
2	-5	5	137.26	5.52
2	-5	5	137.99	5.29
-2	5	-5	137.67	5.11
-2	5	-5	137.22	5.53
-2	5	-5	131.49	5.29
-2	5	-5	130.92	5.38
1	-5	5	84.77	3.68
1	-5	5	86.26	3.42
1	-5	5	86.16	3.62
1	-5	5	83.27	3.22
-1	5	-5	84.54	3.41
-1	5	-5	86.80	3.64
-1	5	-5	85.79	3.59
-1	5	-5	85.31	3.21
0	-5	5	108.54	4.48
0	-5	5	107.32	4.36
0	-5	5	108.48	4.21
0	-5	5	96.26	3.94
0	5	-5	105.60	4.17
0	5	-5	109.79	4.39
0	5	-5	105.77	4.38
0	5	-5	106.41	3.95
-1	-5	5	6.58	0.73
-1	-5	5	5.43	0.87
-1	-5	5	8.10	0.58
1	5	-5	6.62	0.54
1	5	-5	6.44	0.87
1	5	-5	6.56	0.95
-2	-5	5	53.06	2.33
-2	-5	5	49.44	2.56
-2	-5	5	53.29	2.19
2	5	-5	50.51	2.34
2	5	-5	51.22	2.47
2	5	-5	50.78	2.16
-3	-5	5	0.82	0.27
-3	-5	5	0.33	0.46
-3	-5	5	0.82	0.43

3	5	-5	1.12	0.42
3	5	-5	0.64	0.23
3	5	-5	0.68	0.52
-4	-5	5	28.40	1.50
-4	-5	5	27.66	1.83
-4	-5	5	28.03	1.35
-4	-5	5	26.98	1.08
4	5	-5	28.39	1.08
4	5	-5	29.18	1.33
4	5	-5	28.69	1.51
4	5	-5	28.54	1.74
-5	-5	5	85.40	3.25
-5	-5	5	88.65	4.02
-5	-5	5	84.45	3.60
5	5	-5	86.76	3.48
5	5	-5	88.71	3.62
5	5	-5	85.64	3.91
5	5	-5	85.85	3.26
-6	-5	5	64.82	3.35
-6	-5	5	66.38	2.90
-6	-5	5	66.15	2.58
-6	-5	5	65.65	2.82
6	5	-5	69.16	3.30
6	5	-5	69.32	2.93
6	5	-5	70.92	2.80
6	5	-5	66.61	2.58
-7	-5	5	67.79	3.40
-7	-5	5	63.72	2.81
-7	-5	5	66.80	2.83
-7	-5	5	67.57	2.58
7	5	-5	67.54	3.30
7	5	-5	66.03	2.89
7	5	-5	67.21	2.58
-8	-5	5	97.88	3.89
-8	-5	5	96.68	4.06
-8	-5	5	98.43	4.67
-8	-5	5	97.57	3.96
-8	-5	5	102.53	4.11
8	5	-5	101.86	3.84
8	5	-5	105.70	4.59
-9	-5	5	15.12	1.10
-9	-5	5	17.73	0.82
-9	-5	5	15.62	1.15
-9	-5	5	15.83	1.06

-9	-5	5	15.19	1.80
-9	-5	5	16.70	0.96
9	5	-5	17.83	1.73
9	5	-5	17.60	0.82
-10	-5	5	38.70	2.70
-10	-5	5	38.67	1.60
-10	-5	5	36.25	1.66
-10	-5	5	37.73	1.82
-10	-5	5	35.26	1.86
-10	-5	5	35.35	1.86
10	5	-5	42.65	2.51
10	5	-5	36.70	1.59
-11	-5	5	21.84	1.35
-11	-5	5	25.05	1.14
-11	-5	5	22.05	1.47
-11	-5	5	24.53	1.46
-11	-5	5	21.25	2.27
-11	-5	5	24.57	1.18
11	5	-5	23.05	1.12
11	5	-5	24.52	2.15
-12	-5	5	50.61	2.28
-12	-5	5	50.25	2.14
-12	-5	5	53.84	2.19
-12	-5	5	52.45	2.50
-12	-5	5	48.29	3.33
12	5	-5	51.93	3.13
12	5	-5	51.72	2.17
-13	-5	5	19.66	1.05
-13	-5	5	20.39	1.49
-13	-5	5	18.99	1.11
-13	-5	5	19.73	2.55
-13	-5	5	20.57	1.20
13	5	-5	24.14	2.30
13	5	-5	20.86	1.10
-14	-5	5	37.79	2.12
-14	-5	5	34.02	3.08
-14	-5	5	35.76	1.74
14	5	-5	38.02	1.72
14	5	-5	35.63	2.91
-15	-5	5	11.40	1.46
-15	-5	5	8.28	1.75
15	5	-5	10.37	2.09
15	5	-5	11.78	0.91
-16	-5	5	12.19	2.12

-16	-5	5	14.18	1.66
16	5	-5	11.94	1.02
-17	-5	5	12.93	2.24
-17	-5	5	17.88	1.85
17	5	-5	17.96	1.24
-18	-5	5	1.99	1.93
-18	-5	5	1.10	0.98
18	5	-5	2.58	0.67
-19	-5	5	4.98	2.14
-19	-5	5	4.05	1.25
19	5	-5	5.53	0.83
-20	-5	5	8.41	2.83
-20	-5	5	4.43	1.32
20	5	-5	6.18	0.90
19	-5	6	27.06	2.92
19	-5	6	28.44	1.88
-19	5	-6	21.76	2.90
18	-5	6	2.78	1.39
18	-5	6	2.31	0.78
-18	5	-6	0.21	1.35
17	-5	6	55.58	3.35
17	-5	6	58.60	2.73
-17	5	-6	51.46	3.36
16	-5	6	1.47	0.64
-16	5	-6	-1.34	1.04
15	-5	6	123.90	5.59
15	-5	6	129.91	5.15
-15	5	-6	120.89	5.58
14	-5	6	2.68	0.61
14	-5	6	3.81	1.05
-14	5	-6	4.38	1.01
13	-5	6	103.18	4.74
13	-5	6	100.29	4.30
-13	5	-6	110.03	4.68
12	-5	6	0.63	0.80
12	-5	6	-0.47	0.39
-12	5	-6	-1.13	1.06
-12	5	-6	0.29	0.70
11	-5	6	61.78	3.08
11	-5	6	59.61	2.58
11	-5	6	59.74	2.74
-11	5	-6	56.21	3.25
-11	5	-6	58.93	2.86
10	-5	6	0.15	0.49

10	-5	6	0.99	0.74
10	-5	6	0.19	0.34
-10	5	-6	1.36	0.37
-10	5	-6	0.42	0.51
-10	5	-6	0.49	0.91
9	-5	6	110.70	4.47
9	-5	6	111.14	4.92
9	-5	6	115.57	4.60
-9	5	-6	111.70	4.70
-9	5	-6	108.30	5.02
-9	5	-6	120.41	4.47
8	-5	6	0.01	0.27
8	-5	6	1.47	0.62
8	-5	6	0.65	0.41
-8	5	-6	-0.46	0.41
-8	5	-6	-0.62	0.75
-8	5	-6	0.02	0.25
7	-5	6	119.11	4.72
7	-5	6	114.80	4.58
7	-5	6	121.19	5.02
7	-5	6	116.79	4.66
7	-5	6	115.96	4.51
-7	5	-6	113.02	5.08
-7	5	-6	126.41	4.58
-7	5	-6	114.85	4.77
6	-5	6	0.54	0.50
6	-5	6	0.74	0.50
6	-5	6	1.20	0.38
6	-5	6	0.64	0.24
6	-5	6	0.81	0.30
-6	5	-6	0.41	0.21
-6	5	-6	0.71	0.35
-6	5	-6	1.85	0.70
5	-5	6	109.30	4.20
5	-5	6	107.14	4.27
5	-5	6	107.93	4.40
5	-5	6	105.87	4.54
5	-5	6	104.71	4.12
-5	5	-6	101.86	4.58
-5	5	-6	105.08	4.31
-5	5	-6	112.07	4.12
4	-5	6	18.84	1.41
4	-5	6	20.46	0.95
4	-5	6	21.73	1.14

4	-5	6	22.66	1.13
4	-5	6	20.62	1.38
-4	5	-6	21.60	1.01
-4	5	-6	21.00	1.15
-4	5	-6	22.85	1.49
3	-5	6	103.87	4.17
3	-5	6	114.59	4.36
3	-5	6	112.05	4.61
3	-5	6	108.57	4.59
-3	5	-6	113.97	4.17
-3	5	-6	108.65	4.36
-3	5	-6	107.68	4.63
-3	5	-6	107.60	4.43
2	-5	6	2.22	0.37
2	-5	6	2.41	0.25
2	-5	6	2.57	0.59
2	-5	6	2.59	0.64
-2	5	-6	2.87	0.39
-2	5	-6	2.21	0.23
-2	5	-6	3.28	0.54
-2	5	-6	3.39	0.64
1	-5	6	301.19	11.57
1	-5	6	304.49	11.37
1	-5	6	305.18	11.69
1	-5	6	285.79	11.15
-1	5	-6	294.76	11.15
-1	5	-6	302.89	11.36
-1	5	-6	293.68	11.59
-1	5	-6	314.26	11.61
0	-5	6	10.39	1.12
0	-5	6	11.53	0.74
0	-5	6	10.14	0.90
0	5	-6	10.46	1.02
0	5	-6	9.13	0.69
0	5	-6	10.56	0.45
0	5	-6	11.60	0.94
-1	-5	6	80.89	3.49
-1	-5	6	83.03	3.73
-1	-5	6	81.45	3.31
1	5	-6	80.60	3.04
1	5	-6	81.71	3.30
1	5	-6	82.39	3.62
1	5	-6	83.08	3.49
-2	-5	6	29.32	1.34

-2	-5	6	27.85	1.50
-2	-5	6	26.25	1.77
2	5	-6	27.62	1.72
2	5	-6	26.93	1.31
2	5	-6	28.02	1.68
-3	-5	6	57.47	2.57
-3	-5	6	57.97	2.94
-3	-5	6	60.07	2.46
3	5	-6	57.71	2.87
3	5	-6	57.76	2.42
3	5	-6	56.10	2.62
-4	-5	6	0.15	0.07
-4	-5	6	0.28	0.38
-4	-5	6	0.51	0.62
-4	-5	6	0.69	0.32
4	5	-6	0.32	0.58
4	5	-6	0.30	0.08
4	5	-6	0.18	0.25
4	5	-6	0.58	0.40
-5	-5	6	100.99	4.28
-5	-5	6	104.30	3.93
-5	-5	6	110.40	4.23
-5	-5	6	102.93	4.76
5	5	-6	102.79	4.17
5	5	-6	108.08	4.70
5	5	-6	106.45	4.32
5	5	-6	103.72	3.93
-6	-5	6	7.12	0.71
-6	-5	6	6.92	0.88
-6	-5	6	7.88	0.39
-6	-5	6	9.47	1.33
6	5	-6	7.28	0.39
6	5	-6	7.15	1.31
6	5	-6	7.96	0.86
6	5	-6	5.76	0.61
-7	-5	6	288.04	10.64
-7	-5	6	271.59	10.89
-7	-5	6	284.48	10.89
-7	-5	6	286.32	11.53
-7	-5	6	280.80	10.91
7	5	-6	283.77	10.64
7	5	-6	288.97	11.43
7	5	-6	298.81	10.96
-8	-5	6	3.26	0.35

-8	-5	6	3.20	0.51
-8	-5	6	3.58	0.56
-8	-5	6	2.44	0.94
8	5	-6	2.87	0.99
8	5	-6	4.74	0.79
8	5	-6	3.69	0.33
-9	-5	6	100.10	4.78
-9	-5	6	96.41	4.03
-9	-5	6	102.26	3.75
-9	-5	6	97.21	3.90
-9	-5	6	93.72	4.00
-9	-5	6	93.75	3.83
9	5	-6	96.29	4.59
9	5	-6	93.63	3.72
-10	-5	6	6.17	1.31
-10	-5	6	6.07	0.82
-10	-5	6	7.61	0.77
-10	-5	6	7.18	0.67
-10	-5	6	7.91	0.56
-10	-5	6	7.22	0.91
10	5	-6	6.33	0.51
10	5	-6	5.07	1.15
-11	-5	6	126.51	5.03
-11	-5	6	134.37	6.15
-11	-5	6	128.48	5.37
-11	-5	6	127.20	5.09
-11	-5	6	127.10	5.41
-11	-5	6	142.24	5.08
11	5	-6	133.96	5.96
11	5	-6	128.46	5.05
-12	-5	6	10.65	1.74
-12	-5	6	9.79	1.08
-12	-5	6	10.72	0.78
-12	-5	6	10.64	0.68
-12	-5	6	11.80	0.79
12	5	-6	10.38	1.60
12	5	-6	10.63	0.73
-13	-5	6	88.36	4.87
-13	-5	6	92.38	4.04
-13	-5	6	94.48	3.66
13	5	-6	90.39	4.67
13	5	-6	86.71	3.61
-14	-5	6	23.09	1.73
-14	-5	6	23.41	2.88

-14	-5	6	25.07	1.34
14	5	-6	24.20	1.28
-15	-5	6	41.88	3.52
-15	-5	6	45.51	2.55
15	5	-6	44.36	2.02
-16	-5	6	5.78	1.12
-16	-5	6	6.41	1.80
16	5	-6	7.92	0.86
-17	-5	6	25.68	2.12
17	5	-6	25.41	1.50
-18	-5	6	2.46	2.01
-18	-5	6	0.77	1.01
18	5	-6	2.10	0.66
-19	-5	6	20.31	3.78
-19	-5	6	23.97	2.31
19	5	-6	27.90	1.65
-20	-5	6	12.57	2.74
-20	-5	6	14.98	2.25
20	5	-6	15.54	1.48
19	-5	7	3.76	1.74
19	-5	7	2.25	1.00
-19	5	-7	-2.43	1.48
18	-5	7	3.99	0.89
18	-5	7	4.11	1.56
-18	5	-7	4.48	1.48
17	-5	7	5.46	1.50
17	-5	7	4.49	0.84
-17	5	-7	3.38	1.37
16	-5	7	43.40	2.94
16	-5	7	45.10	2.32
-16	5	-7	43.00	2.96
15	-5	7	15.22	1.96
15	-5	7	13.21	1.23
-15	5	-7	11.07	1.85
14	-5	7	50.82	2.34
14	-5	7	43.82	2.83
-14	5	-7	48.27	2.80
13	-5	7	24.88	2.08
13	-5	7	26.13	1.47
-13	5	-7	26.34	1.92
12	-5	7	25.92	1.41
12	-5	7	21.70	1.94
-12	5	-7	26.35	1.84
11	-5	7	11.35	1.15

11	-5	7	9.61	1.48
11	-5	7	9.64	0.89
-11	5	-7	10.52	1.29
-11	5	-7	9.27	1.49
10	-5	7	37.26	2.33
10	-5	7	39.02	1.95
10	-5	7	37.97	1.78
-10	5	-7	33.49	2.56
-10	5	-7	39.00	1.75
-10	5	-7	39.04	2.09
9	-5	7	25.20	1.29
9	-5	7	25.53	1.47
9	-5	7	22.81	1.83
-9	5	-7	25.47	1.57
-9	5	-7	27.02	1.28
-9	5	-7	25.03	2.03
8	-5	7	88.64	3.70
8	-5	7	88.88	3.57
8	-5	7	89.44	4.07
-8	5	-7	84.67	4.12
-8	5	-7	93.47	3.55
-8	5	-7	87.73	3.78
7	-5	7	35.70	1.69
7	-5	7	34.05	1.70
7	-5	7	35.02	2.04
7	-5	7	32.88	1.55
7	-5	7	34.33	1.50
-7	5	-7	35.27	1.78
-7	5	-7	32.39	2.16
-7	5	-7	38.59	1.55
6	-5	7	72.87	3.45
6	-5	7	73.06	2.98
6	-5	7	75.51	3.13
6	-5	7	75.93	3.01
6	-5	7	77.04	3.23
-6	5	-7	79.56	2.98
-6	5	-7	72.10	3.50
-6	5	-7	71.51	3.17
5	-5	7	26.79	1.31
5	-5	7	26.62	1.36
5	-5	7	27.80	1.69
5	-5	7	27.16	1.69
5	-5	7	25.64	1.17
-5	5	-7	27.79	1.17

-5	5	-7	24.26	1.37
-5	5	-7	22.90	1.72
4	-5	7	126.57	4.95
4	-5	7	125.89	5.41
4	-5	7	133.28	5.37
4	-5	7	132.04	5.12
-4	5	-7	129.64	5.45
-4	5	-7	125.75	5.16
-4	5	-7	137.54	4.94
-4	5	-7	125.63	5.12
3	-5	7	12.84	1.19
3	-5	7	12.47	0.66
3	-5	7	12.97	1.13
3	-5	7	13.27	0.87
-3	5	-7	13.50	1.23
-3	5	-7	13.96	0.65
-3	5	-7	12.97	0.87
-3	5	-7	14.75	1.05
2	-5	7	95.97	4.01
2	-5	7	101.50	4.47
2	-5	7	108.86	4.15
2	-5	7	100.72	4.40
-2	5	-7	108.36	3.94
-2	5	-7	105.79	4.44
-2	5	-7	104.13	4.14
1	-5	7	0.51	0.60
1	-5	7	1.05	0.51
1	-5	7	1.37	0.33
1	-5	7	0.93	0.15
-1	5	-7	1.17	0.31
-1	5	-7	1.27	0.53
-1	5	-7	2.13	0.59
-1	5	-7	1.48	0.20
0	-5	7	120.58	5.16
0	-5	7	133.83	4.99
0	-5	7	127.84	5.41
0	5	-7	126.57	4.74
0	5	-7	122.84	4.94
0	5	-7	128.11	5.20
0	5	-7	126.13	4.72
0	5	-7	124.27	5.28
-1	-5	7	7.14	0.83
-1	-5	7	7.05	0.65
-1	-5	7	6.13	1.10

1	5	-7	7.61	0.33
1	5	-7	8.35	0.92
1	5	-7	7.69	0.61
1	5	-7	7.55	0.34
1	5	-7	8.00	1.10
-2	-5	7	126.25	4.82
-2	-5	7	121.10	5.32
-2	-5	7	116.25	4.96
2	5	-7	121.21	4.78
2	5	-7	124.30	4.98
2	5	-7	120.89	5.21
-3	-5	7	2.36	0.39
-3	-5	7	2.58	0.78
-3	-5	7	2.29	0.52
3	5	-7	2.15	0.35
3	5	-7	4.23	0.86
3	5	-7	2.29	0.55
-4	-5	7	258.05	10.18
-4	-5	7	264.57	9.59
-4	-5	7	249.03	9.27
-4	-5	7	235.44	9.64
4	5	-7	243.63	9.27
4	5	-7	254.43	9.54
4	5	-7	246.74	10.03
-5	-5	7	0.18	0.11
-5	-5	7	-0.21	0.68
-5	-5	7	-0.12	0.37
-5	-5	7	0.17	0.34
5	5	-7	-0.46	0.64
5	5	-7	0.53	0.46
5	5	-7	0.16	0.26
5	5	-7	0.28	0.10
-6	-5	7	125.92	4.75
-6	-5	7	129.42	5.06
-6	-5	7	123.41	5.69
-6	-5	7	122.09	5.06
6	5	-7	129.76	5.60
6	5	-7	133.78	5.14
6	5	-7	125.85	4.74
6	5	-7	120.63	4.99
-7	-5	7	0.66	0.17
-7	-5	7	-0.27	0.85
-7	-5	7	0.83	0.51
-7	-5	7	1.00	0.36

-7	-5	7	1.71	0.41
7	5	-7	1.14	0.87
7	5	-7	0.88	0.51
7	5	-7	0.41	0.33
7	5	-7	0.66	0.19
-8	-5	7	108.05	4.40
-8	-5	7	111.19	4.12
-8	-5	7	91.21	4.49
-8	-5	7	108.64	4.43
-8	-5	7	111.46	5.19
8	5	-7	110.57	4.11
8	5	-7	106.84	5.00
8	5	-7	114.32	4.43
-9	-5	7	3.28	0.40
-9	-5	7	2.69	1.12
-9	-5	7	3.00	0.53
-9	-5	7	2.57	0.56
-9	-5	7	3.42	0.53
9	5	-7	3.53	1.10
9	5	-7	2.99	0.60
9	5	-7	3.31	0.37
-10	-5	7	119.55	4.86
-10	-5	7	125.99	4.57
-10	-5	7	118.94	4.90
-10	-5	7	116.38	4.58
-10	-5	7	114.26	5.64
-10	-5	7	113.49	4.61
10	5	-7	119.07	5.53
10	5	-7	115.52	4.53
-11	-5	7	-0.39	0.31
-11	-5	7	0.55	0.47
-11	-5	7	0.37	0.34
-11	-5	7	0.32	0.29
-11	-5	7	-0.01	1.13
11	5	-7	-1.19	1.19
11	5	-7	0.17	0.24
-12	-5	7	157.80	7.26
-12	-5	7	150.01	6.40
-12	-5	7	166.31	6.10
12	5	-7	156.34	7.04
12	5	-7	158.38	6.06
-13	-5	7	-0.74	1.38
-13	-5	7	0.61	0.98
-13	-5	7	1.86	0.43

13	5	-7	0.97	0.32
-14	-5	7	73.20	4.57
-14	-5	7	81.99	3.26
-14	-5	7	77.08	3.64
14	5	-7	78.42	3.20
-15	-5	7	0.19	1.57
-15	-5	7	-0.91	0.59
-15	-5	7	-0.23	0.71
15	5	-7	-0.31	0.37
-16	-5	7	58.46	3.20
16	5	-7	60.15	2.61
-17	-5	7	-0.25	0.91
-17	-5	7	-0.44	1.80
17	5	-7	1.87	0.53
-18	-5	7	44.35	4.17
-18	-5	7	46.24	2.88
18	5	-7	44.28	2.21
-19	-5	7	-0.39	2.35
-19	-5	7	1.62	1.16
19	5	-7	0.26	0.70
-20	-5	7	39.04	4.44
-20	-5	7	33.62	2.98
20	5	-7	41.01	2.17
18	-5	8	4.42	0.92
18	-5	8	4.10	1.72
-18	5	-8	4.25	1.62
17	-5	8	1.65	1.43
17	-5	8	-1.46	0.66
-17	5	-8	0.92	1.37
16	-5	8	10.36	1.28
16	-5	8	12.62	2.28
-16	5	-8	15.84	2.21
15	-5	8	4.58	1.36
15	-5	8	5.41	0.84
-15	5	-8	6.08	1.26
14	-5	8	33.79	1.87
14	-5	8	30.51	2.49
-14	5	-8	36.60	2.41
13	-5	8	-0.23	0.46
13	-5	8	0.40	1.13
-13	5	-8	0.68	0.86
12	-5	8	2.21	0.89
12	-5	8	4.16	1.02
-12	5	-8	3.06	0.85

11	-5	8	2.53	0.71
11	-5	8	0.63	0.81
11	-5	8	1.72	0.47
-11	5	-8	1.97	0.73
10	-5	8	31.43	1.55
10	-5	8	31.86	2.18
10	-5	8	30.70	1.76
-10	5	-8	30.53	1.88
-10	5	-8	31.09	1.53
9	-5	8	10.55	1.41
9	-5	8	8.52	1.00
9	-5	8	9.47	0.81
-9	5	-8	9.20	1.63
-9	5	-8	9.08	1.09
-9	5	-8	10.69	0.78
8	-5	8	62.86	2.69
8	-5	8	62.33	2.84
8	-5	8	62.85	3.25
-8	5	-8	66.21	3.39
-8	5	-8	64.08	2.91
-8	5	-8	68.79	2.67
7	-5	8	40.13	1.93
7	-5	8	38.83	1.76
7	-5	8	38.98	2.34
7	-5	8	39.55	1.90
7	-5	8	39.94	1.73
-7	5	-8	43.29	1.75
-7	5	-8	38.57	1.97
-7	5	-8	40.04	2.42
6	-5	8	61.54	3.10
6	-5	8	64.31	2.62
6	-5	8	58.44	3.17
6	-5	8	64.37	2.72
6	-5	8	60.53	2.55
-6	5	-8	64.06	2.76
-6	5	-8	67.77	2.55
-6	5	-8	58.28	3.12
5	-5	8	0.70	0.59
5	-5	8	0.60	0.63
5	-5	8	0.95	0.42
5	-5	8	0.60	0.24
5	-5	8	0.83	0.36
-5	5	-8	0.65	0.23
-5	5	-8	0.93	0.66

-5	5	-8	0.39	0.38
4	-5	8	5.00	0.43
4	-5	8	5.30	0.68
4	-5	8	4.00	0.76
4	-5	8	5.38	0.82
-4	5	-8	5.00	0.68
-4	5	-8	5.11	0.82
-4	5	-8	4.71	0.41
-4	5	-8	7.30	1.16
3	-5	8	61.42	2.95
3	-5	8	61.47	2.91
3	-5	8	61.72	2.59
3	-5	8	60.47	2.39
-3	5	-8	57.45	2.95
-3	5	-8	60.54	2.59
-3	5	-8	59.95	2.93
2	-5	8	18.53	1.05
2	-5	8	17.53	0.82
2	-5	8	17.63	1.31
2	-5	8	17.34	1.51
-2	5	-8	18.33	1.34
-2	5	-8	17.40	1.02
-2	5	-8	16.34	1.41
-2	5	-8	18.25	0.79
1	-5	8	45.66	2.01
1	-5	8	41.83	2.47
1	-5	8	47.24	2.30
-1	5	-8	42.99	2.33
-1	5	-8	44.27	2.30
-1	5	-8	45.95	2.00
-1	5	-8	45.09	1.76
-1	5	-8	45.67	1.79
0	-5	8	9.74	0.76
0	-5	8	8.56	0.99
0	-5	8	9.58	1.33
0	5	-8	9.41	0.47
0	5	-8	10.20	0.73
0	5	-8	8.76	1.07
0	5	-8	9.88	1.19
0	5	-8	9.95	0.45
-1	-5	8	23.16	1.20
-1	-5	8	22.52	1.81
-1	-5	8	22.12	1.41
1	5	-8	22.80	1.16

1	5	-8	21.72	0.88
1	5	-8	20.75	1.63
1	5	-8	21.98	0.89
1	5	-8	22.00	1.45
-2	-5	8	98.72	4.56
-2	-5	8	97.69	3.95
-2	-5	8	96.01	4.14
2	5	-8	95.80	3.65
2	5	-8	97.32	3.65
2	5	-8	103.09	4.17
2	5	-8	99.57	4.44
2	5	-8	95.45	3.91
-3	-5	8	30.03	2.17
-3	-5	8	29.81	1.66
-3	-5	8	32.18	1.52
3	5	-8	34.33	1.72
3	5	-8	29.10	1.17
3	5	-8	30.04	1.18
3	5	-8	30.15	1.47
3	5	-8	32.66	2.08
-4	-5	8	35.79	1.87
-4	-5	8	36.58	2.42
-4	-5	8	37.63	1.43
-4	-5	8	38.13	1.76
4	5	-8	37.83	1.71
4	5	-8	35.59	1.42
4	5	-8	39.71	1.94
4	5	-8	35.66	2.33
-5	-5	8	77.28	3.99
-5	-5	8	79.31	3.30
-5	-5	8	77.02	2.97
-5	-5	8	75.31	3.35
5	5	-8	75.90	2.96
5	5	-8	82.61	3.43
5	5	-8	78.21	3.24
5	5	-8	85.55	3.95
-6	-5	8	47.92	2.89
-6	-5	8	50.28	2.18
-6	-5	8	43.22	2.15
-6	-5	8	48.35	1.82
6	5	-8	46.87	2.48
6	5	-8	47.76	1.81
6	5	-8	45.25	2.78
6	5	-8	43.81	2.08

-7	-5	8	82.39	3.45
-7	-5	8	82.52	3.16
-7	-5	8	84.26	3.46
-7	-5	8	82.53	4.27
-7	-5	8	83.66	3.49
7	5	-8	80.88	3.42
7	5	-8	79.50	4.08
7	5	-8	77.32	3.19
7	5	-8	89.08	3.57
-8	-5	8	37.43	1.87
-8	-5	8	37.70	1.56
-8	-5	8	37.59	1.86
-8	-5	8	38.06	2.75
-8	-5	8	42.19	1.95
8	5	-8	39.08	1.93
8	5	-8	36.01	1.54
8	5	-8	37.89	2.62
-9	-5	8	86.26	3.20
-9	-5	8	81.70	4.37
-9	-5	8	82.34	3.56
-9	-5	8	78.67	3.48
9	5	-8	83.65	3.48
9	5	-8	81.02	3.18
9	5	-8	78.15	4.18
-10	-5	8	38.29	1.59
-10	-5	8	36.74	1.69
-10	-5	8	36.22	1.91
-10	-5	8	39.07	2.94
-10	-5	8	35.84	1.65
-10	-5	8	33.21	1.96
10	5	-8	38.05	1.56
10	5	-8	38.66	2.84
-11	-5	8	49.45	2.07
-11	-5	8	53.56	3.45
-11	-5	8	53.57	2.13
-11	-5	8	47.04	2.07
-11	-5	8	53.21	2.49
11	5	-8	50.61	2.08
11	5	-8	48.26	3.31
-12	-5	8	1.21	0.55
-12	-5	8	1.23	1.43
-12	-5	8	1.17	0.37
12	5	-8	1.44	0.31
-13	-5	8	54.25	2.77

-13	-5	8	59.09	2.41
-13	-5	8	55.82	3.87
13	5	-8	54.41	2.34
-14	-5	8	26.14	3.13
-14	-5	8	25.51	1.73
-14	-5	8	21.16	1.26
14	5	-8	20.41	1.17
-15	-5	8	34.01	2.25
-15	-5	8	31.21	3.71
-15	-5	8	35.19	1.78
15	5	-8	34.41	1.65
-16	-5	8	2.14	0.92
-16	-5	8	4.33	1.88
16	5	-8	2.26	0.51
-17	-5	8	35.75	2.47
-17	-5	8	35.64	3.86
17	5	-8	33.54	1.81
-18	-5	8	9.98	2.49
-18	-5	8	8.48	1.35
18	5	-8	8.80	1.13
-19	-5	8	31.14	2.71
-19	-5	8	35.60	4.48
19	5	-8	35.06	1.93
20	5	-8	0.73	0.77
18	-5	9	16.08	2.93
18	-5	9	14.53	1.71
-18	5	-9	13.30	1.99
17	-5	9	5.93	1.67
17	-5	9	8.23	1.13
-17	5	-9	8.46	1.65
16	-5	9	8.20	1.67
16	-5	9	10.59	1.33
-16	5	-9	8.35	1.58
15	-5	9	7.27	1.14
15	-5	9	5.53	1.49
-15	5	-9	4.61	1.25
14	-5	9	1.34	1.20
14	-5	9	2.43	0.69
-14	5	-9	3.62	1.06
13	-5	9	10.21	1.77
13	-5	9	10.36	1.13
12	-5	9	16.34	2.67
12	-5	9	17.43	1.23
-12	5	-9	20.65	1.70

11	-5	9	8.67	0.92
11	-5	9	7.88	1.26
-11	5	-9	6.63	1.02
10	-5	9	3.91	0.74
10	-5	9	3.32	0.55
10	-5	9	3.60	0.98
-10	5	-9	3.39	0.77
9	-5	9	19.80	1.18
9	-5	9	18.33	1.78
9	-5	9	22.78	1.41
-9	5	-9	21.87	1.18
-9	5	-9	21.28	1.49
8	-5	9	0.15	0.53
8	-5	9	0.09	0.39
8	-5	9	0.27	0.33
8	-5	9	0.01	0.74
8	-5	9	0.05	0.34
-8	5	-9	0.06	0.32
-8	5	-9	-0.46	0.95
-8	5	-9	0.58	0.52
7	-5	9	17.11	1.19
7	-5	9	17.73	1.01
7	-5	9	16.88	1.55
7	-5	9	16.54	0.97
-7	5	-9	17.69	0.95
-7	5	-9	16.10	1.24
-7	5	-9	18.08	1.86
6	-5	9	1.38	0.46
6	-5	9	0.77	0.28
6	-5	9	0.28	0.67
6	-5	9	1.65	0.71
6	-5	9	1.51	0.49
-6	5	-9	1.06	0.48
-6	5	-9	1.12	0.28
-6	5	-9	0.89	0.84
5	-5	9	14.07	1.41
5	-5	9	13.44	0.78
5	-5	9	15.09	1.27
5	-5	9	12.93	0.99
-5	5	-9	15.33	0.77
-5	5	-9	13.46	1.01
-5	5	-9	13.46	1.49
-5	5	-9	12.67	1.12
4	-5	9	44.99	2.02

4	-5	9	43.06	2.34
4	-5	9	40.32	2.37
4	-5	9	41.94	1.81
-4	5	-9	45.35	2.03
-4	5	-9	45.69	1.80
-4	5	-9	40.47	2.42
-4	5	-9	44.82	2.19
3	-5	9	12.92	0.95
3	-5	9	13.24	0.72
3	-5	9	14.41	1.43
3	-5	9	13.71	1.32
-3	5	-9	14.26	1.25
-3	5	-9	13.12	0.95
-3	5	-9	14.16	1.37
-3	5	-9	15.27	0.71
2	-5	9	0.07	0.54
2	-5	9	0.13	0.68
2	-5	9	0.15	0.35
-2	5	-9	-0.01	0.31
-2	5	-9	0.21	0.66
-2	5	-9	-0.29	0.60
-2	5	-9	0.02	0.17
-2	5	-9	0.14	0.15
1	-5	9	7.09	1.33
1	-5	9	7.43	1.01
1	-5	9	7.86	0.76
-1	5	-9	7.30	0.70
-1	5	-9	8.37	0.44
-1	5	-9	8.44	1.19
-1	5	-9	8.07	1.14
-1	5	-9	7.38	0.48
0	-5	9	1.28	0.81
0	-5	9	1.67	0.58
0	-5	9	2.99	0.45
0	5	-9	2.16	0.27
0	5	-9	2.46	0.25
0	5	-9	2.73	0.80
0	5	-9	2.45	0.43
0	5	-9	2.31	0.67
-1	-5	9	3.13	0.49
-1	-5	9	2.63	0.92
-1	-5	9	2.54	0.65
1	5	-9	3.43	0.57
1	5	-9	3.17	0.24

1	5	-9	2.94	0.65
1	5	-9	3.41	0.93
1	5	-9	3.07	0.26
-2	-5	9	4.99	0.94
-2	-5	9	6.79	0.71
-2	-5	9	5.98	1.15
2	5	-9	6.49	0.86
2	5	-9	5.24	0.30
2	5	-9	6.14	0.63
2	5	-9	5.59	0.31
2	5	-9	6.12	1.00
-3	-5	9	54.18	2.59
-3	-5	9	55.78	2.40
-3	-5	9	55.15	3.10
3	5	-9	54.63	2.58
3	5	-9	55.08	2.34
3	5	-9	52.61	2.05
3	5	-9	54.81	3.00
3	5	-9	51.36	2.04
-4	-5	9	2.06	0.57
-4	-5	9	1.18	0.40
-4	-5	9	1.31	0.19
-4	-5	9	0.13	0.82
4	5	-9	1.24	0.16
4	5	-9	1.52	0.36
4	5	-9	1.13	0.82
4	5	-9	-0.05	0.56
-5	-5	9	42.64	1.68
-5	-5	9	41.60	2.78
-5	-5	9	43.51	2.16
-5	-5	9	45.16	2.05
5	5	-9	41.63	1.66
5	5	-9	42.12	1.96
5	5	-9	48.98	2.81
5	5	-9	42.72	2.17
-6	-5	9	3.50	0.62
-6	-5	9	2.35	0.54
-6	-5	9	2.49	1.01
-6	-5	9	2.53	0.29
6	5	-9	3.78	1.04
6	5	-9	3.07	0.65
6	5	-9	2.83	0.26
6	5	-9	2.85	0.47
-7	-5	9	134.79	6.00

-7	-5	9	127.55	5.10
-7	-5	9	128.12	5.06
-7	-5	9	122.71	5.06
-7	-5	9	122.10	4.75
7	5	-9	119.84	5.72
7	5	-9	125.59	5.03
7	5	-9	124.91	4.74
-8	-5	9	0.60	0.38
-8	-5	9	-0.19	1.08
-8	-5	9	0.42	0.21
-8	-5	9	0.74	0.48
-8	-5	9	-0.28	0.49
8	5	-9	1.51	0.60
8	5	-9	2.91	1.07
8	5	-9	0.96	0.19
-9	-5	9	13.94	0.74
-9	-5	9	13.67	1.03
-9	-5	9	12.68	1.20
-9	-5	9	12.87	1.05
-9	-5	9	13.64	2.11
9	5	-9	13.14	1.18
9	5	-9	14.01	2.14
9	5	-9	12.97	0.68
-10	-5	9	9.78	1.71
-10	-5	9	9.41	1.02
-10	-5	9	9.91	0.86
-10	-5	9	11.24	0.70
10	5	-9	11.69	2.04
10	5	-9	10.78	1.08
10	5	-9	10.15	0.61
-11	-5	9	7.93	0.69
-11	-5	9	10.46	0.71
-11	-5	9	9.59	1.80
-11	-5	9	8.02	0.68
-11	-5	9	10.24	1.08
11	5	-9	10.10	0.63
-12	-5	9	9.15	1.75
-12	-5	9	8.33	0.76
12	5	-9	7.46	0.64
-13	-5	9	45.59	2.44
-13	-5	9	46.48	2.04
-13	-5	9	48.12	3.68
13	5	-9	43.12	1.94
-14	-5	9	4.91	0.97

-14	-5	9	7.03	0.88
-14	-5	9	5.03	1.91
14	5	-9	5.61	0.67
-15	-5	9	29.93	1.60
-15	-5	9	31.82	3.42
-15	-5	9	24.04	2.02
15	5	-9	27.51	1.44
-16	-5	9	6.68	2.16
-16	-5	9	5.01	1.10
16	5	-9	5.75	0.81
-17	-5	9	3.13	2.18
-17	-5	9	1.67	1.03
17	5	-9	1.52	0.68
-18	-5	9	15.11	2.75
-18	-5	9	13.49	2.05
18	5	-9	18.23	1.32
-19	-5	9	8.73	1.47
-19	-5	9	10.35	2.81
19	5	-9	12.71	1.35
17	-5	10	11.31	1.57
17	-5	10	12.84	2.01
-17	5	-10	9.47	1.70
16	-5	10	0.45	0.73
16	-5	10	1.25	1.43
-16	5	-10	0.67	1.31
15	-5	10	13.52	2.42
15	-5	10	15.03	1.44
-15	5	-10	17.74	2.21
14	-5	10	3.15	0.76
14	-5	10	3.17	1.33
-14	5	-10	1.07	1.05
13	-5	10	71.00	3.11
13	-5	10	68.81	3.87
-13	5	-10	63.77	3.77
12	-5	10	0.90	1.07
12	-5	10	-0.49	0.48
-12	5	-10	1.38	0.86
11	-5	10	45.83	2.27
11	-5	10	50.00	2.96
-11	5	-10	48.19	2.59
10	-5	10	2.49	0.69
10	-5	10	1.81	0.96
10	-5	10	1.55	0.48
-10	5	-10	2.51	0.78

9	-5	10	7.28	1.19
9	-5	10	4.88	0.72
9	-5	10	5.14	0.72
-9	5	-10	6.58	0.87
-9	5	-10	4.42	0.69
8	-5	10	54.57	2.54
8	-5	10	53.84	3.02
8	-5	10	55.77	2.30
8	-5	10	54.57	2.38
8	-5	10	54.08	2.37
-8	5	-10	57.75	2.34
-8	5	-10	52.28	2.59
7	-5	10	34.75	1.63
7	-5	10	33.43	2.25
7	-5	10	32.01	1.74
7	-5	10	31.65	1.78
7	-5	10	30.54	1.54
-7	5	-10	35.14	1.53
-7	5	-10	32.45	1.79
6	-5	10	0.58	0.32
6	-5	10	1.48	0.80
6	-5	10	0.37	0.50
6	-5	10	0.66	0.64
-6	5	-10	0.61	0.56
-6	5	-10	1.35	0.32
-6	5	-10	1.03	0.89
-6	5	-10	0.63	0.49
5	-5	10	105.64	4.53
5	-5	10	104.60	4.76
5	-5	10	109.27	4.31
5	-5	10	102.42	4.13
-5	5	-10	100.97	4.80
-5	5	-10	107.28	4.33
-5	5	-10	112.84	4.12
-5	5	-10	100.22	4.41
4	-5	10	0.61	0.69
4	-5	10	1.52	0.29
4	-5	10	1.64	0.80
4	-5	10	1.02	0.46
-4	5	-10	1.50	0.82
-4	5	-10	1.26	0.44
-4	5	-10	1.50	0.70
-4	5	-10	1.76	0.28
3	-5	10	29.51	1.96

3	-5	10	28.61	1.52
3	-5	10	32.07	2.03
-3	5	-10	30.40	1.27
-3	5	-10	27.56	1.89
-3	5	-10	27.67	1.49
-3	5	-10	28.53	2.00
-3	5	-10	29.77	1.32
2	-5	10	21.99	1.89
2	-5	10	25.56	1.38
2	-5	10	26.58	1.77
-2	5	-10	26.94	1.81
-2	5	-10	22.95	1.80
-2	5	-10	24.51	1.35
-2	5	-10	25.42	1.15
-2	5	-10	26.10	1.14
1	-5	10	20.55	1.23
1	-5	10	19.34	1.83
1	-5	10	20.62	1.56
-1	5	-10	22.33	0.93
-1	5	-10	20.88	0.96
-1	5	-10	21.61	1.19
-1	5	-10	20.94	1.72
-1	5	-10	20.42	1.60
0	-5	10	1.84	0.65
0	-5	10	0.97	0.84
0	-5	10	1.20	0.42
0	5	-10	0.77	0.31
0	5	-10	0.32	0.76
0	5	-10	1.61	0.41
0	5	-10	1.10	0.19
0	5	-10	0.74	0.72
-1	-5	10	16.70	1.82
-1	-5	10	14.60	1.32
-1	-5	10	17.43	1.07
1	5	-10	17.08	1.41
1	5	-10	16.37	0.73
1	5	-10	16.53	1.72
1	5	-10	15.78	0.71
1	5	-10	15.61	1.00
-2	-5	10	4.65	0.70
-2	-5	10	4.42	1.08
-2	-5	10	3.73	0.72
2	5	-10	3.60	0.28
2	5	-10	4.82	1.12

2	5	-10	3.99	0.29
2	5	-10	4.28	0.82
2	5	-10	4.01	0.60
-3	-5	10	15.66	1.07
-3	-5	10	14.13	1.82
-3	-5	10	17.06	1.33
3	5	-10	14.95	0.68
3	5	-10	17.87	1.02
3	5	-10	15.45	0.67
3	5	-10	17.13	1.35
3	5	-10	16.40	1.82
-4	-5	10	22.72	1.52
-4	-5	10	20.18	2.11
-4	-5	10	20.97	1.27
-4	-5	10	22.73	0.91
4	5	-10	22.70	1.22
4	5	-10	22.15	1.50
4	5	-10	22.47	2.05
4	5	-10	20.27	0.88
-5	-5	10	16.92	2.01
-5	-5	10	15.83	0.71
-5	-5	10	17.09	1.30
-5	-5	10	17.49	1.13
5	5	-10	14.82	1.90
5	5	-10	14.90	1.01
5	5	-10	14.75	0.67
5	5	-10	17.16	1.35
-6	-5	10	27.62	1.55
-6	-5	10	26.59	2.42
-6	-5	10	27.92	1.16
-6	-5	10	28.99	1.65
6	5	-10	26.30	1.13
6	5	-10	31.53	1.74
6	5	-10	29.99	1.46
6	5	-10	23.58	2.29
-7	-5	10	2.28	0.63
-7	-5	10	2.89	0.59
-7	-5	10	3.93	1.24
-7	-5	10	4.68	0.72
-7	-5	10	2.95	0.37
7	5	-10	3.43	0.71
7	5	-10	3.62	0.34
7	5	-10	4.43	1.27
-8	-5	10	18.22	1.20

-8	-5	10	18.75	0.87
-8	-5	10	16.52	1.23
-8	-5	10	17.50	2.20
-8	-5	10	19.39	1.36
8	5	-10	15.84	2.20
8	5	-10	18.23	0.82
-9	-5	10	8.45	0.58
-9	-5	10	6.64	0.96
-9	-5	10	7.13	1.07
-9	-5	10	7.22	0.89
-9	-5	10	7.23	1.59
9	5	-10	7.35	0.50
9	5	-10	7.31	1.15
-10	-5	10	15.69	1.26
-10	-5	10	16.37	2.37
-10	-5	10	18.43	0.92
10	5	-10	16.23	1.34
10	5	-10	17.09	0.84
-11	-5	10	0.55	0.56
-11	-5	10	-0.43	1.25
-11	-5	10	0.57	0.39
-11	-5	10	0.34	0.37
-11	-5	10	0.00	0.33
11	5	-10	0.30	0.24
-12	-5	10	0.88	0.38
-12	-5	10	-0.06	0.54
-12	-5	10	2.61	2.27
12	5	-10	0.50	0.30
-13	-5	10	0.84	0.47
-13	-5	10	0.93	1.60
-13	-5	10	0.04	0.62
13	5	-10	-0.06	0.32
-14	-5	10	6.20	1.87
-14	-5	10	8.78	1.36
-14	-5	10	6.03	0.92
14	5	-10	7.23	0.72
-15	-5	10	1.44	0.60
-15	-5	10	2.78	2.01
-15	-5	10	-0.21	0.80
15	5	-10	0.63	0.41
-16	-5	10	25.73	2.10
-16	-5	10	20.65	3.60
16	5	-10	21.43	1.30
-17	-5	10	0.99	2.25

-17	-5	10	-1.41	0.97
17	5	-10	0.49	0.67
-18	-5	10	3.75	2.56
-18	-5	10	4.44	1.26
18	5	-10	3.15	0.72
-19	-5	10	-0.20	2.42
-19	-5	10	-1.12	1.25
19	5	-10	1.29	0.70
17	-5	11	3.71	1.83
17	-5	11	1.90	0.91
-17	5	-11	1.18	1.50
16	-5	11	33.66	2.11
16	-5	11	29.68	3.04
-16	5	-11	24.27	2.81
15	-5	11	0.96	1.47
15	-5	11	1.04	0.74
-15	5	-11	-0.96	1.25
14	-5	11	59.44	2.78
14	-5	11	52.88	3.55
-14	5	-11	53.28	3.20
13	-5	11	0.40	1.23
13	-5	11	1.28	0.64
-13	5	-11	0.78	0.92
12	-5	11	75.62	4.22
12	-5	11	78.16	3.43
-12	5	-11	80.65	3.73
11	-5	11	10.48	1.99
11	-5	11	11.28	1.08
-11	5	-11	12.45	1.55
10	-5	11	59.03	2.83
10	-5	11	62.36	3.42
10	-5	11	64.16	2.71
-10	5	-11	57.72	2.95
9	-5	11	3.22	0.57
9	-5	11	2.20	0.67
-9	5	-11	2.89	0.75
8	-5	11	20.88	1.97
8	-5	11	19.04	1.42
8	-5	11	20.14	1.18
8	-5	11	20.90	1.36
8	-5	11	19.95	1.20
-8	5	-11	19.97	1.14
-8	5	-11	17.25	1.43
7	-5	11	9.11	0.80

7	-5	11	8.64	1.09
7	-5	11	7.94	1.28
7	-5	11	11.43	1.03
7	-5	11	8.09	1.02
-7	5	-11	7.78	1.08
-7	5	-11	10.69	0.78
6	-5	11	85.38	3.76
6	-5	11	87.06	3.61
6	-5	11	82.75	3.43
6	-5	11	86.56	4.15
-6	5	-11	90.48	3.42
-6	5	-11	82.17	3.63
-6	5	-11	81.28	3.69
5	-5	11	1.04	0.74
5	-5	11	1.69	0.78
5	-5	11	0.24	0.30
5	-5	11	0.25	0.47
-5	5	-11	0.44	0.49
-5	5	-11	-1.03	0.98
-5	5	-11	0.76	0.29
-5	5	-11	0.34	0.70
4	-5	11	152.80	5.84
4	-5	11	141.99	6.28
4	-5	11	148.64	6.16
-4	5	-11	133.03	6.31
-4	5	-11	145.13	5.82
-4	5	-11	158.56	5.63
-4	5	-11	145.99	5.68
-4	5	-11	144.78	6.10
3	-5	11	13.40	1.55
3	-5	11	16.61	1.14
3	-5	11	15.90	1.60
-3	5	-11	15.79	1.71
-3	5	-11	13.26	1.04
-3	5	-11	14.22	1.57
-3	5	-11	14.42	0.83
-3	5	-11	15.23	0.78
2	-5	11	14.82	1.09
2	-5	11	13.37	1.49
2	-5	11	14.24	1.67
-2	5	-11	14.63	1.03
-2	5	-11	14.88	0.80
-2	5	-11	16.30	1.64
-2	5	-11	15.63	1.58

-2	5	-11	15.78	0.76
1	-5	11	2.95	0.54
1	-5	11	3.10	0.94
1	-5	11	1.68	0.75
-1	5	-11	1.64	0.95
-1	5	-11	2.73	0.80
-1	5	-11	2.27	0.49
-1	5	-11	2.35	0.37
0	-5	11	65.95	2.78
0	-5	11	55.76	3.07
0	-5	11	63.48	3.46
0	5	-11	64.69	2.47
0	5	-11	62.85	2.72
0	5	-11	61.17	3.36
0	5	-11	67.18	3.17
0	5	-11	64.09	2.49
-1	-5	11	20.56	1.67
-1	-5	11	24.74	2.12
-1	-5	11	22.65	1.34
1	5	-11	24.26	1.11
1	5	-11	22.42	1.26
1	5	-11	23.17	1.73
1	5	-11	22.65	2.09
1	5	-11	23.64	0.98
-2	-5	11	34.42	2.07
-2	-5	11	33.52	2.54
-2	-5	11	38.06	1.79
2	5	-11	34.22	1.69
2	5	-11	36.16	1.42
2	5	-11	35.07	2.09
2	5	-11	33.42	2.53
2	5	-11	35.37	1.42
-3	-5	11	-0.12	0.42
-3	-5	11	-0.26	0.96
-3	-5	11	0.08	0.58
3	5	-11	0.31	0.35
3	5	-11	0.73	0.98
3	5	-11	0.06	0.15
3	5	-11	-0.03	0.71
3	5	-11	0.25	0.15
-4	-5	11	20.58	0.89
-4	-5	11	22.40	1.59
-4	-5	11	22.26	1.30
-4	-5	11	18.87	2.19

4	5	-11	19.87	1.20
4	5	-11	21.29	1.60
4	5	-11	19.68	0.85
4	5	-11	18.75	2.13
-5	-5	11	11.00	1.99
-5	-5	11	11.70	0.57
-5	-5	11	10.19	1.00
-5	-5	11	10.77	1.25
5	5	-11	12.17	1.29
5	5	-11	9.44	1.44
5	5	-11	10.56	0.52
5	5	-11	10.48	0.89
-6	-5	11	23.72	2.32
-6	-5	11	20.58	1.50
-6	-5	11	21.17	0.93
-6	-5	11	21.47	1.39
6	5	-11	22.46	2.35
6	5	-11	18.82	1.58
6	5	-11	21.65	1.26
-7	-5	11	41.56	2.05
-7	-5	11	41.02	2.15
-7	-5	11	40.30	3.08
-7	-5	11	39.98	2.12
-7	-5	11	41.68	1.70
7	5	-11	42.21	1.67
7	5	-11	43.18	3.15
7	5	-11	45.70	2.30
-8	-5	11	35.23	2.94
-8	-5	11	34.99	1.84
-8	-5	11	35.38	1.90
-8	-5	11	36.22	1.95
-8	-5	11	33.59	1.48
8	5	-11	33.44	1.44
8	5	-11	40.75	2.09
-9	-5	11	2.02	1.35
-9	-5	11	2.50	0.60
-9	-5	11	1.94	0.54
-9	-5	11	2.31	0.35
9	5	-11	2.93	0.39
-10	-5	11	34.06	1.81
-10	-5	11	37.68	1.98
-10	-5	11	37.35	1.58
-10	-5	11	34.44	3.24
10	5	-11	36.85	2.03

10	5	-11	34.76	1.51
-11	-5	11	6.62	0.69
-11	-5	11	5.99	0.75
-11	-5	11	8.39	2.76
11	5	-11	5.67	0.54
11	5	-11	6.01	0.86
-12	-5	11	28.63	1.89
-12	-5	11	29.22	1.47
-12	-5	11	30.34	3.23
12	5	-11	29.51	1.35
-13	-5	11	16.81	1.15
-13	-5	11	17.13	3.14
-13	-5	11	20.12	1.60
13	5	-11	15.40	0.95
-14	-5	11	15.41	1.20
-14	-5	11	17.70	3.33
-14	-5	11	16.47	1.66
14	5	-11	13.08	0.94
-15	-5	11	4.58	1.00
-15	-5	11	5.89	2.19
15	5	-11	3.62	0.54
-16	-5	11	6.33	2.33
-16	-5	11	11.85	1.80
16	5	-11	9.42	0.95
-17	-5	11	8.03	2.55
-17	-5	11	5.81	1.31
17	5	-11	4.75	0.82
-18	-5	11	6.28	1.34
-18	-5	11	4.71	2.63
18	5	-11	3.30	0.73
16	-5	12	0.69	1.65
16	-5	12	-0.82	0.84
-16	5	-12	1.90	1.39
15	-5	12	60.37	3.08
15	-5	12	61.83	4.04
-15	5	-12	63.46	3.66
14	-5	12	0.36	1.40
14	-5	12	0.49	0.71
-14	5	-12	0.50	1.13
13	-5	12	62.65	2.98
13	-5	12	61.30	3.79
-13	5	-12	66.77	3.41
12	-5	12	0.96	0.64
12	-5	12	-0.13	1.25

-12	5	-12	-0.23	0.88
11	-5	12	38.23	2.02
11	-5	12	39.33	2.85
-11	5	-12	38.81	2.33
10	-5	12	6.89	1.08
10	-5	12	4.46	1.20
-10	5	-12	5.50	0.99
9	-5	12	54.51	2.69
9	-5	12	57.58	2.54
9	-5	12	57.67	3.33
-9	5	-12	54.97	2.76
8	-5	12	1.36	0.47
8	-5	12	1.00	0.61
8	-5	12	1.54	0.64
8	-5	12	0.86	0.97
8	-5	12	1.13	0.68
-8	5	-12	1.09	0.62
-8	5	-12	1.89	0.45
7	-5	12	75.37	3.13
7	-5	12	75.92	3.31
7	-5	12	70.88	3.90
7	-5	12	75.23	3.28
-7	5	-12	72.48	3.32
-7	5	-12	73.24	3.33
-7	5	-12	79.54	3.09
6	-5	12	1.10	0.74
6	-5	12	2.54	0.46
6	-5	12	2.22	0.65
6	-5	12	1.75	0.93
-6	5	-12	2.56	0.48
-6	5	-12	4.35	0.88
-6	5	-12	2.86	0.44
-6	5	-12	2.15	0.64
5	-5	12	67.93	2.94
5	-5	12	65.73	3.22
5	-5	12	66.01	3.49
-5	5	-12	63.33	2.78
-5	5	-12	64.85	3.23
-5	5	-12	71.20	2.73
-5	5	-12	65.86	2.95
4	-5	12	6.66	1.18
4	-5	12	6.70	0.98
4	-5	12	7.77	1.14
-4	5	-12	6.06	0.89

-4	5	-12	6.70	1.18
-4	5	-12	6.54	0.61
-4	5	-12	7.48	0.66
3	-5	12	36.94	1.86
3	-5	12	37.18	2.37
3	-5	12	33.85	2.33
-3	5	-12	38.11	1.57
-3	5	-12	35.62	2.37
-3	5	-12	34.97	1.80
-3	5	-12	35.32	2.44
-3	5	-12	36.01	1.61
2	-5	12	-0.17	0.78
2	-5	12	0.19	0.84
2	-5	12	0.60	0.50
-2	5	-12	-0.03	0.92
-2	5	-12	0.36	0.89
-2	5	-12	0.31	0.24
-2	5	-12	0.32	0.43
-2	5	-12	-0.18	0.24
1	-5	12	45.18	2.11
1	-5	12	38.92	2.52
1	-5	12	44.03	2.80
-1	5	-12	41.61	2.02
-1	5	-12	43.28	1.81
-1	5	-12	46.86	2.60
-1	5	-12	45.46	1.78
-1	5	-12	39.76	2.72
0	-5	12	0.79	0.74
0	-5	12	0.79	1.01
0	-5	12	-0.16	0.47
0	5	-12	0.09	0.40
0	5	-12	0.20	0.25
0	5	-12	-0.73	1.00
0	5	-12	-1.38	0.77
0	5	-12	-0.13	0.20
-1	-5	12	83.04	4.38
-1	-5	12	79.12	3.94
-1	-5	12	91.36	3.64
1	5	-12	77.45	4.24
1	5	-12	89.28	4.02
1	5	-12	87.73	3.54
1	5	-12	84.51	3.29
1	5	-12	86.04	3.27
-2	-5	12	2.61	1.20

-2	-5	12	5.03	0.96
-2	-5	12	3.95	0.66
2	5	-12	4.83	0.38
2	5	-12	4.75	0.39
2	5	-12	2.70	0.52
2	5	-12	5.67	1.00
2	5	-12	2.33	1.18
-3	-5	12	88.67	4.68
-3	-5	12	92.90	3.82
-3	-5	12	89.25	4.11
3	5	-12	83.38	4.57
3	5	-12	92.10	3.73
3	5	-12	91.75	3.45
3	5	-12	91.44	4.10
-4	-5	12	2.84	1.12
-4	-5	12	4.25	0.85
-4	-5	12	4.57	0.70
4	5	-12	4.34	0.91
4	5	-12	2.22	1.37
4	5	-12	3.82	0.57
-5	-5	12	62.56	3.87
-5	-5	12	67.86	2.58
-5	-5	12	62.61	3.15
-5	-5	12	70.19	3.01
5	5	-12	69.03	2.90
5	5	-12	64.60	2.62
5	5	-12	63.80	3.88
5	5	-12	67.11	3.21
-6	-5	12	9.82	0.55
-6	-5	12	8.89	1.27
-6	-5	12	5.64	1.49
-6	-5	12	9.68	1.09
6	5	-12	11.95	1.45
6	5	-12	8.26	0.91
6	5	-12	8.22	0.49
-7	-5	12	61.59	3.96
-7	-5	12	64.93	2.86
-7	-5	12	59.92	2.98
-7	-5	12	64.50	2.51
-7	-5	12	66.12	2.99
7	5	-12	65.97	3.12
7	5	-12	62.28	2.47
-8	-5	12	7.99	0.76
-8	-5	12	10.77	1.16

-8	-5	12	9.50	1.78
-8	-5	12	11.10	1.03
8	5	-12	10.57	1.43
8	5	-12	9.59	0.56
-9	-5	12	86.51	3.34
-9	-5	12	85.28	3.70
-9	-5	12	78.67	3.62
-9	-5	12	91.55	5.01
9	5	-12	82.78	3.29
9	5	-12	88.45	3.83
-10	-5	12	1.07	0.37
-10	-5	12	1.79	0.61
-10	-5	12	0.95	0.57
-10	-5	12	0.09	1.43
10	5	-12	1.41	1.44
10	5	-12	1.66	0.28
-11	-5	12	110.81	4.58
-11	-5	12	103.85	4.14
-11	-5	12	98.90	5.65
11	5	-12	104.29	4.07
11	5	-12	106.12	4.39
-12	-5	12	4.31	1.88
-12	-5	12	2.89	0.51
-12	-5	12	2.81	0.78
12	5	-12	2.58	0.39
-13	-5	12	35.06	1.73
-13	-5	12	34.02	3.61
-13	-5	12	32.94	2.14
13	5	-12	33.27	1.55
-14	-5	12	8.35	2.31
-14	-5	12	9.10	1.25
14	5	-12	11.28	0.85
-15	-5	12	22.45	3.62
-15	-5	12	22.97	2.06
15	5	-12	22.56	1.28
-16	-5	12	5.85	1.25
-16	-5	12	7.65	2.40
16	5	-12	6.08	0.68
-17	-5	12	26.79	2.41
-17	-5	12	27.07	4.41
17	5	-12	19.61	1.58
-18	-5	12	1.36	1.31
-18	-5	12	4.76	2.53
18	5	-12	-0.38	0.62

15	-5	13	7.46	1.08
15	-5	13	8.10	1.93
-15	5	-13	7.18	1.55
14	-5	13	35.93	3.37
14	-5	13	43.91	2.37
-14	5	-13	40.87	2.90
13	-5	13	0.62	1.48
13	-5	13	0.71	0.72
-13	5	-13	2.04	1.09
12	-5	13	35.11	3.06
12	-5	13	37.04	2.07
11	-5	13	1.12	1.40
11	-5	13	1.95	0.65
-11	5	-13	2.44	0.91
10	-5	13	21.77	1.42
10	-5	13	19.81	2.34
-10	5	-13	22.08	1.78
9	-5	13	7.28	1.01
9	-5	13	5.94	0.90
9	-5	13	7.13	0.94
9	-5	13	10.70	1.96
9	-5	13	10.16	1.22
-9	5	-13	8.22	1.29
8	-5	13	17.84	1.50
8	-5	13	20.57	1.42
8	-5	13	17.51	2.10
8	-5	13	18.14	1.24
-8	5	-13	19.94	1.29
-8	5	-13	20.19	1.53
7	-5	13	1.26	0.73
7	-5	13	0.70	0.65
7	-5	13	0.98	1.00
-7	5	-13	1.71	0.86
-7	5	-13	1.58	0.47
-7	5	-13	0.92	0.41
-7	5	-13	1.50	0.65
6	-5	13	99.20	5.48
6	-5	13	94.68	4.03
6	-5	13	96.51	4.22
-6	5	-13	93.03	3.91
-6	5	-13	98.91	3.83
-6	5	-13	91.23	4.27
-6	5	-13	96.42	4.06
5	-5	13	3.62	1.10

5	-5	13	6.12	1.06
5	-5	13	6.72	1.05
-5	5	-13	7.77	1.21
-5	5	-13	5.46	0.71
-5	5	-13	4.90	0.65
-5	5	-13	6.01	0.79
4	-5	13	63.53	3.49
4	-5	13	64.01	3.36
4	-5	13	69.03	2.97
-4	5	-13	65.93	2.75
-4	5	-13	64.17	3.48
-4	5	-13	62.35	2.91
-4	5	-13	70.92	2.70
3	-5	13	4.24	0.71
3	-5	13	5.88	1.20
3	-5	13	4.75	1.18
-3	5	-13	3.69	0.54
-3	5	-13	5.26	0.59
-3	5	-13	5.24	1.21
-3	5	-13	4.80	0.66
2	-5	13	48.55	2.91
2	-5	13	44.00	2.76
2	-5	13	47.63	2.24
-2	5	-13	45.71	1.89
-2	5	-13	44.64	1.93
-2	5	-13	44.96	2.15
-2	5	-13	41.20	2.89
1	-5	13	14.13	1.94
1	-5	13	11.41	1.59
1	-5	13	12.36	1.19
-1	5	-13	13.41	0.73
-1	5	-13	12.65	1.04
-1	5	-13	12.09	1.99
-1	5	-13	10.67	1.68
-1	5	-13	12.84	0.74
0	-5	13	56.85	3.25
0	-5	13	61.91	3.55
0	-5	13	66.76	2.84
0	5	-13	61.17	3.62
0	5	-13	61.33	2.48
0	5	-13	62.92	2.47
0	5	-13	61.46	2.72
0	5	-13	63.97	3.33
-1	-5	13	1.59	0.59

-1	-5	13	1.12	0.85
-1	-5	13	2.53	1.19
1	5	-13	2.09	0.28
1	5	-13	2.10	1.20
1	5	-13	1.97	0.51
1	5	-13	2.42	1.00
-2	-5	13	151.22	6.79
-2	-5	13	168.52	6.47
-2	-5	13	158.06	7.28
2	5	-13	168.02	6.39
2	5	-13	168.18	6.11
2	5	-13	161.32	6.81
2	5	-13	162.18	6.11
2	5	-13	153.38	7.24
-3	-5	13	35.88	2.85
-3	-5	13	34.43	1.88
-3	-5	13	32.88	2.26
3	5	-13	38.85	2.33
3	5	-13	35.04	1.43
3	5	-13	34.68	1.76
3	5	-13	29.41	2.86
3	5	-13	33.96	1.42
-4	-5	13	110.31	4.63
-4	-5	13	105.86	4.91
-4	-5	13	115.84	5.64
4	5	-13	109.38	4.22
4	5	-13	111.50	4.24
4	5	-13	120.16	4.96
4	5	-13	106.72	4.52
-5	-5	13	3.28	1.41
-5	-5	13	2.69	0.31
-5	-5	13	0.92	0.78
-5	-5	13	1.56	0.67
5	5	-13	2.42	0.34
5	5	-13	2.35	0.54
5	5	-13	2.52	0.96
-6	-5	13	49.10	3.50
-6	-5	13	48.87	1.94
-6	-5	13	45.40	2.41
-6	-5	13	47.27	2.56
6	5	-13	47.12	1.88
6	5	-13	51.27	2.68
6	5	-13	45.70	2.26
-7	-5	13	5.85	0.95

-7	-5	13	7.97	1.27
-7	-5	13	5.38	1.61
-7	-5	13	7.79	0.56
-7	-5	13	7.28	1.24
7	5	-13	7.51	0.46
7	5	-13	8.98	1.56
-8	-5	13	111.00	4.34
-8	-5	13	106.09	4.46
-8	-5	13	100.34	4.49
8	5	-13	114.14	4.76
8	5	-13	100.55	4.05
-9	-5	13	0.46	0.69
-9	-5	13	1.62	1.62
-9	-5	13	0.75	0.32
-9	-5	13	-0.07	0.53
9	5	-13	0.00	0.22
9	5	-13	-0.02	0.94
-10	-5	13	90.44	3.47
-10	-5	13	83.80	3.69
-10	-5	13	84.53	4.07
10	5	-13	93.00	3.95
10	5	-13	84.38	3.39
-11	-5	13	0.22	0.42
-11	-5	13	-0.20	0.64
-11	-5	13	-1.27	1.73
-11	-5	13	-0.31	0.64
11	5	-13	2.30	0.92
11	5	-13	0.09	0.27
-12	-5	13	54.80	4.31
-12	-5	13	55.34	2.85
-12	-5	13	55.87	2.47
12	5	-13	52.66	2.25
-13	-5	13	0.81	1.97
-13	-5	13	0.80	0.83
13	5	-13	0.31	0.35
-14	-5	13	34.34	2.33
-14	-5	13	34.84	3.95
14	5	-13	33.69	1.61
-15	-5	13	-0.82	2.15
-15	-5	13	2.65	1.05
15	5	-13	1.43	0.50
-16	-5	13	26.92	4.25
-16	-5	13	31.20	2.49
16	5	-13	27.45	1.52

-17	-5	13	6.26	1.46
-17	-5	13	10.74	2.71
17	5	-13	5.48	0.94
14	-5	14	24.38	2.99
14	-5	14	22.56	1.84
-14	5	-14	15.04	2.37
13	-5	14	6.03	1.73
13	-5	14	5.43	0.93
-13	5	-14	7.36	1.31
12	-5	14	14.23	1.42
12	-5	14	9.98	1.91
-12	5	-14	11.83	1.82
11	-5	14	6.61	1.71
11	-5	14	7.88	1.19
-11	5	-14	3.82	1.00
-11	5	-14	4.31	0.90
10	-5	14	1.43	1.25
10	-5	14	1.22	0.61
-10	5	-14	0.24	0.87
-10	5	-14	0.61	0.63
9	-5	14	6.36	0.82
9	-5	14	6.02	1.48
9	-5	14	4.15	0.95
-9	5	-14	7.58	1.02
-9	5	-14	6.54	0.99
8	-5	14	17.10	2.21
8	-5	14	15.78	1.51
8	-5	14	16.05	1.53
8	-5	14	14.69	1.42
-8	5	-14	15.73	1.45
-8	5	-14	15.26	1.21
7	-5	14	14.84	1.53
7	-5	14	17.41	2.21
7	-5	14	16.50	1.43
-7	5	-14	15.72	1.41
-7	5	-14	19.43	1.79
-7	5	-14	16.89	1.19
6	-5	14	25.73	1.89
6	-5	14	25.43	1.69
6	-5	14	23.85	2.32
-6	5	-14	26.09	2.17
-6	5	-14	28.29	1.40
-6	5	-14	28.26	1.46
-6	5	-14	25.55	1.65

5	-5	14	17.44	1.69
5	-5	14	14.01	1.87
5	-5	14	12.20	1.31
-5	5	-14	15.38	1.02
-5	5	-14	12.80	1.95
-5	5	-14	13.34	1.24
-5	5	-14	17.44	0.99
4	-5	14	5.43	1.15
4	-5	14	4.35	0.76
-4	5	-14	5.40	0.76
-4	5	-14	4.34	0.64
-4	5	-14	3.57	1.29
-4	5	-14	6.09	0.68
3	-5	14	14.86	1.94
3	-5	14	14.28	1.80
3	-5	14	15.71	1.33
-3	5	-14	14.06	1.20
-3	5	-14	17.46	0.93
-3	5	-14	14.41	0.94
-3	5	-14	16.51	2.11
2	-5	14	31.01	2.41
2	-5	14	33.86	2.60
2	-5	14	37.55	1.93
-2	5	-14	28.11	2.69
-2	5	-14	37.68	1.55
-2	5	-14	33.86	1.79
-2	5	-14	33.93	1.56
1	-5	14	48.76	2.96
1	-5	14	50.15	2.40
1	-5	14	47.24	3.10
-1	5	-14	47.66	2.04
-1	5	-14	44.52	2.26
-1	5	-14	51.32	2.03
-1	5	-14	51.17	3.29
0	-5	14	22.63	2.34
0	-5	14	21.44	1.46
0	-5	14	17.34	1.88
0	5	-14	14.44	2.44
0	5	-14	20.30	1.28
0	5	-14	20.88	0.98
0	5	-14	17.80	0.96
-1	-5	14	44.68	2.81
-1	-5	14	48.15	3.25
-1	-5	14	48.13	2.36

1	5	-14	47.18	1.94
1	5	-14	39.57	3.23
1	5	-14	48.87	2.88
1	5	-14	47.39	2.22
1	5	-14	49.56	1.94
-2	-5	14	6.39	1.17
-2	-5	14	10.09	1.16
-2	-5	14	6.39	1.55
2	5	-14	6.18	1.32
2	5	-14	7.26	0.58
2	5	-14	7.76	0.54
2	5	-14	8.22	0.94
2	5	-14	6.81	2.14
-3	-5	14	79.01	3.47
-3	-5	14	70.88	3.80
-3	-5	14	81.93	4.47
3	5	-14	81.56	3.86
3	5	-14	79.67	3.12
3	5	-14	75.99	3.33
3	5	-14	79.41	3.04
-4	-5	14	43.42	2.18
-4	-5	14	40.92	3.21
-4	-5	14	38.53	2.47
4	5	-14	41.14	2.02
4	5	-14	42.97	1.70
4	5	-14	40.29	1.65
4	5	-14	38.45	2.58
-5	-5	14	12.26	1.54
-5	-5	14	15.02	0.79
-5	-5	14	15.52	2.46
-5	-5	14	15.24	1.39
5	5	-14	15.00	1.16
5	5	-14	11.79	1.81
5	5	-14	14.96	0.70
-6	-5	14	19.13	0.96
-6	-5	14	21.30	1.36
-6	-5	14	20.50	3.26
-6	-5	14	20.32	1.74
-6	-5	14	18.99	1.55
6	5	-14	17.91	1.94
6	5	-14	19.59	0.88
6	5	-14	19.88	1.36
-7	-5	14	19.65	1.37
-7	-5	14	18.37	1.66

-7	-5	14	26.15	2.91
7	5	-14	19.39	0.91
7	5	-14	23.31	2.02
-8	-5	14	1.55	0.58
-8	-5	14	2.07	1.53
-8	-5	14	0.89	0.33
-8	-5	14	1.68	0.83
8	5	-14	1.40	0.26
8	5	-14	1.07	1.01
-9	-5	14	65.47	3.16
-9	-5	14	64.03	3.79
-9	-5	14	65.99	4.48
-9	-5	14	70.17	2.76
9	5	-14	71.74	3.46
9	5	-14	68.82	2.68
-10	-5	14	8.67	0.79
-10	-5	14	10.45	2.19
-10	-5	14	10.26	1.27
-10	-5	14	8.04	1.19
10	5	-14	10.71	1.79
10	5	-14	9.41	0.64
-11	-5	14	61.76	3.01
-11	-5	14	54.79	2.62
-11	-5	14	61.23	4.52
11	5	-14	59.87	2.39
-12	-5	14	4.94	2.13
-12	-5	14	6.24	1.07
12	5	-14	6.38	0.66
-13	-5	14	23.71	1.95
-13	-5	14	27.57	3.77
13	5	-14	24.33	1.24
-14	-5	14	-1.00	2.09
-14	-5	14	1.05	0.95
14	5	-14	1.87	0.46
-15	-5	14	30.42	2.44
-15	-5	14	34.57	4.28
15	5	-14	31.99	1.60
-16	-5	14	3.67	1.20
-16	-5	14	5.25	2.72
-17	-5	14	48.58	5.19
-17	-5	14	53.24	3.41
14	-5	15	1.45	0.96
-14	5	-15	2.33	1.53
13	-5	15	3.73	0.96

13	-5	15	3.41	1.71
-13	5	-15	3.78	1.29
-13	5	-15	4.74	1.07
12	-5	15	2.53	1.63
-12	5	-15	5.07	1.17
-12	5	-15	8.03	1.13
11	-5	15	21.06	2.69
-11	5	-15	19.12	1.70
-11	5	-15	19.17	1.93
10	-5	15	1.48	1.40
-10	5	-15	1.07	0.76
-10	5	-15	0.36	0.87
9	-5	15	14.89	2.43
9	-5	15	18.71	1.90
-9	5	-15	14.18	1.63
-9	5	-15	17.56	1.42
8	-5	15	2.25	0.90
8	-5	15	2.24	0.86
8	-5	15	-0.18	1.27
-8	5	-15	2.83	0.82
-8	5	-15	1.68	0.69
7	-5	15	17.14	1.70
7	-5	15	17.98	1.58
7	-5	15	16.71	2.25
-7	5	-15	18.84	1.27
-7	5	-15	16.52	1.50
6	-5	15	-0.34	0.75
6	-5	15	-1.07	0.91
6	-5	15	0.71	1.17
-6	5	-15	0.16	1.18
-6	5	-15	0.41	0.48
-6	5	-15	0.35	0.65
-6	5	-15	-0.26	0.44
5	-5	15	15.28	1.40
5	-5	15	15.11	2.20
5	-5	15	14.02	1.78
-5	5	-15	12.78	1.28
-5	5	-15	11.63	2.21
-5	5	-15	16.06	1.07
-5	5	-15	15.96	1.02
4	-5	15	5.84	0.87
4	-5	15	7.28	1.27
4	-5	15	4.62	1.31
-4	5	-15	5.01	0.72

-4	5	-15	5.51	0.77
-4	5	-15	6.98	1.54
-4	5	-15	5.15	0.75
3	-5	15	35.73	2.66
3	-5	15	35.97	2.00
3	-5	15	35.43	2.57
-3	5	-15	35.30	1.66
-3	5	-15	28.11	2.81
-3	5	-15	37.98	1.63
-3	5	-15	35.93	1.89
2	-5	15	1.19	1.20
2	-5	15	2.79	1.29
2	-5	15	1.37	0.73
-2	5	-15	0.58	0.36
-2	5	-15	1.11	1.44
-2	5	-15	1.49	0.56
-2	5	-15	1.11	0.35
1	-5	15	13.13	2.20
1	-5	15	13.51	1.34
1	-5	15	12.00	1.84
-1	5	-15	13.17	0.82
-1	5	-15	12.05	1.11
-1	5	-15	12.78	0.83
-1	5	-15	17.15	2.56
0	-5	15	5.96	1.27
0	-5	15	9.38	1.20
0	-5	15	6.52	1.53
0	5	-15	6.17	0.61
0	5	-15	5.03	0.59
0	5	-15	4.95	0.70
0	5	-15	4.61	1.83
-1	-5	15	17.50	2.50
-1	-5	15	13.91	1.44
-1	-5	15	14.30	1.85
1	5	-15	14.67	1.21
1	5	-15	17.68	0.88
1	5	-15	17.09	0.89
-2	-5	15	11.09	1.77
-2	-5	15	11.25	1.85
-2	-5	15	10.55	1.34
2	5	-15	13.34	2.59
2	5	-15	12.63	0.75
2	5	-15	12.56	0.71
2	5	-15	12.92	1.11

-3	-5	15	35.41	2.45
-3	-5	15	33.70	2.01
-3	-5	15	34.72	3.05
3	5	-15	36.17	1.52
3	5	-15	37.46	2.60
3	5	-15	36.04	1.51
3	5	-15	34.77	1.85
-4	-5	15	4.30	1.10
-4	-5	15	4.58	1.67
-4	-5	15	5.30	0.85
4	5	-15	5.64	0.90
4	5	-15	5.49	1.31
4	5	-15	5.75	0.56
4	5	-15	5.00	0.47
-5	-5	15	16.87	2.66
-5	-5	15	16.58	1.46
-5	-5	15	13.30	1.72
5	5	-15	13.09	0.72
5	5	-15	15.61	1.21
5	5	-15	14.18	1.92
5	5	-15	13.98	0.85
-6	-5	15	1.98	1.52
-6	-5	15	-0.47	0.52
-6	-5	15	0.95	0.91
6	5	-15	0.14	0.23
6	5	-15	-0.13	1.10
-7	-5	15	30.24	2.17
-7	-5	15	35.12	3.42
-7	-5	15	31.63	1.41
-7	-5	15	32.46	1.82
7	5	-15	32.89	2.41
7	5	-15	28.96	1.31
-8	-5	15	17.34	3.01
-8	-5	15	19.66	1.05
-8	-5	15	19.96	1.47
-8	-5	15	19.56	1.78
8	5	-15	18.04	2.13
8	5	-15	20.40	0.94
-9	-5	15	33.78	1.61
-9	-5	15	29.90	1.87
-9	-5	15	27.88	3.41
-9	-5	15	25.76	1.93
9	5	-15	29.59	1.31
9	5	-15	30.54	2.38

-10	-5	15	16.41	2.71
-10	-5	15	18.82	1.59
-10	-5	15	23.18	1.70
10	5	-15	19.86	1.00
-11	-5	15	12.45	1.55
-11	-5	15	6.71	2.16
-11	-5	15	10.33	1.30
11	5	-15	11.95	0.77
-12	-5	15	9.94	2.50
-12	-5	15	8.58	1.21
12	5	-15	8.85	0.75
-13	-5	15	15.47	1.78
-13	-5	15	15.27	2.78
13	5	-15	12.77	0.92
-14	-5	15	4.44	2.52
-14	-5	15	9.19	1.31
14	5	-15	9.76	1.19
-15	-5	15	19.78	3.14
-15	-5	15	18.82	2.22
-16	-5	15	18.92	2.39
12	-5	16	1.23	1.81
-12	5	-16	0.92	0.88
-12	5	-16	-1.34	1.13
11	-5	16	26.05	3.17
-11	5	-16	32.25	2.07
10	-5	16	7.72	1.83
-10	5	-16	8.49	1.06
-10	5	-16	10.27	1.66
9	-5	16	22.64	2.79
-9	5	-16	22.40	1.62
-9	5	-16	21.37	1.87
8	-5	16	15.27	1.71
8	-5	16	16.23	2.56
-8	5	-16	14.35	1.33
-8	5	-16	19.08	1.63
7	-5	16	18.38	1.92
7	-5	16	22.06	2.59
7	-5	16	21.76	1.77
-7	5	-16	22.86	1.45
-7	5	-16	18.32	1.64
6	-5	16	2.75	1.12
6	-5	16	3.77	1.41
6	-5	16	3.66	0.88
-6	5	-16	2.41	0.61

-6	5	-16	1.98	0.80
5	-5	16	14.29	1.47
5	-5	16	16.68	1.98
5	-5	16	16.23	2.40
-5	5	-16	13.68	2.53
-5	5	-16	15.04	1.35
-5	5	-16	16.33	1.08
-5	5	-16	13.78	1.13
4	-5	16	2.64	1.16
4	-5	16	5.71	0.92
4	-5	16	2.52	1.34
-4	5	-16	2.67	0.52
-4	5	-16	3.11	0.75
-4	5	-16	4.66	1.60
-4	5	-16	3.55	0.51
3	-5	16	12.38	1.43
3	-5	16	12.09	2.06
3	-5	16	18.73	2.19
-3	5	-16	11.70	0.95
-3	5	-16	13.03	1.98
-3	5	-16	10.49	1.22
-3	5	-16	14.11	0.94
2	-5	16	9.62	1.60
2	-5	16	6.89	1.48
2	-5	16	7.70	1.30
-2	5	-16	9.26	2.01
-2	5	-16	8.87	1.08
-2	5	-16	9.50	0.83
-2	5	-16	9.12	0.80
1	-5	16	26.15	1.72
1	-5	16	21.39	2.57
1	-5	16	21.39	2.33
-1	5	-16	25.26	1.23
-1	5	-16	22.60	1.46
-1	5	-16	22.44	1.20
0	-5	16	4.77	1.65
0	-5	16	5.81	0.95
0	-5	16	4.55	1.50
0	5	-16	6.34	0.68
0	5	-16	5.68	0.97
0	5	-16	4.77	0.62
-1	-5	16	14.85	1.47
-1	-5	16	12.99	1.98
-1	-5	16	10.50	1.92

1	5	-16	14.07	1.17
1	5	-16	13.01	0.81
1	5	-16	13.31	0.84
-2	-5	16	13.01	1.39
-2	-5	16	8.22	1.43
-2	-5	16	11.98	1.98
2	5	-16	9.30	0.73
2	5	-16	10.01	0.70
2	5	-16	11.83	1.10
-3	-5	16	0.77	1.52
-3	-5	16	2.27	0.85
-3	-5	16	-0.63	0.98
3	5	-16	0.44	0.28
3	5	-16	0.07	0.32
3	5	-16	-0.12	0.51
-4	-5	16	24.37	2.22
-4	-5	16	26.60	1.88
-4	-5	16	27.03	3.09
4	5	-16	29.92	1.29
4	5	-16	28.44	1.63
4	5	-16	29.52	3.13
4	5	-16	27.33	1.23
-5	-5	16	8.29	1.34
-5	-5	16	9.22	1.13
-5	-5	16	15.94	2.85
5	5	-16	10.01	0.61
5	5	-16	11.54	2.09
5	5	-16	9.05	0.72
5	5	-16	9.64	1.13
-6	-5	16	12.01	2.15
-6	-5	16	9.66	1.40
-6	-5	16	12.24	1.20
6	5	-16	10.56	0.66
6	5	-16	17.74	2.12
-7	-5	16	9.45	1.98
-7	-5	16	8.63	1.62
-7	-5	16	8.36	1.16
7	5	-16	7.71	1.46
7	5	-16	7.19	0.56
-8	-5	16	2.17	0.73
-8	-5	16	2.95	1.93
-8	-5	16	2.12	1.00
8	5	-16	2.49	0.33
8	5	-16	0.98	1.26

-9	-5	16	-1.24	1.76
-9	-5	16	0.57	0.72
-9	-5	16	0.35	0.90
9	5	-16	-0.20	0.28
-10	-5	16	37.56	3.91
-10	-5	16	26.75	2.03
-10	-5	16	36.51	2.16
10	5	-16	32.33	1.43
-11	-5	16	3.23	0.91
-11	-5	16	6.97	2.36
11	5	-16	2.33	0.41
-12	-5	16	7.08	2.40
-12	-5	16	10.54	1.66
12	5	-16	10.05	0.94
-13	-5	16	2.67	2.30
-13	-5	16	1.12	1.00
-14	-5	16	4.71	2.39
-14	-5	16	6.22	1.25
-15	-5	16	-2.03	2.50
-15	-5	16	2.55	1.24
11	-5	17	4.46	1.84
-11	5	-17	3.15	0.98
10	-5	17	35.62	3.38
-10	5	-17	32.67	2.58
-10	5	-17	36.00	2.20
9	-5	17	-1.10	1.45
-9	5	-17	-0.73	0.90
-9	5	-17	-0.40	0.75
8	-5	17	27.79	3.17
-8	5	-17	29.30	2.02
-8	5	-17	30.18	1.85
7	-5	17	3.64	1.56
7	-5	17	-0.69	1.08
-7	5	-17	-0.78	0.75
-7	5	-17	-0.01	0.63
6	-5	17	29.97	2.49
6	-5	17	30.30	2.97
-6	5	-17	31.35	1.93
-6	5	-17	31.99	1.76
5	-5	17	-0.21	0.97
5	-5	17	1.68	1.31
5	-5	17	0.44	1.21
-5	5	-17	0.16	0.55
-5	5	-17	0.77	0.77

4	-5	17	29.87	2.62
4	-5	17	29.10	1.92
4	-5	17	26.07	2.84
-4	5	-17	28.45	1.77
-4	5	-17	29.21	3.30
-4	5	-17	28.29	1.50
-4	5	-17	28.83	1.58
3	-5	17	1.85	1.31
3	-5	17	1.53	1.36
3	-5	17	1.74	0.83
-3	5	-17	-0.05	0.45
-3	5	-17	0.77	0.65
-3	5	-17	0.24	0.46
2	-5	17	16.10	1.60
2	-5	17	19.06	2.38
2	-5	17	14.82	2.39
-2	5	-17	15.66	1.06
-2	5	-17	14.71	1.30
-2	5	-17	17.78	1.08
1	-5	17	0.71	0.83
1	-5	17	-1.65	1.22
1	-5	17	0.62	1.33
-1	5	-17	0.55	0.39
-1	5	-17	0.10	0.60
-1	5	-17	0.73	0.42
0	-5	17	32.81	2.05
0	-5	17	29.85	2.62
0	-5	17	29.14	3.05
0	5	-17	35.29	1.56
0	5	-17	33.31	1.55
0	5	-17	33.82	1.84
-1	-5	17	5.46	0.99
-1	-5	17	4.90	1.42
-1	-5	17	4.10	1.76
1	5	-17	4.87	0.63
1	5	-17	4.22	0.77
1	5	-17	5.77	0.70
-2	-5	17	25.04	3.05
-2	-5	17	24.65	1.92
-2	-5	17	20.62	2.67
2	5	-17	24.74	1.55
2	5	-17	24.60	1.23
2	5	-17	24.67	1.19
-3	-5	17	-0.13	1.13

-3	-5	17	-2.02	0.79
-3	-5	17	2.43	1.76
3	5	-17	0.75	0.38
3	5	-17	0.92	0.31
3	5	-17	-0.40	0.55
-4	-5	17	49.45	3.96
-4	-5	17	44.17	2.92
4	5	-17	48.29	1.99
4	5	-17	45.93	2.32
4	5	-17	46.44	1.93
-5	-5	17	1.77	1.20
-5	-5	17	3.03	0.71
-5	-5	17	-0.58	1.75
5	5	-17	1.81	0.43
5	5	-17	1.87	0.32
-6	-5	17	34.49	2.01
-6	-5	17	42.81	3.90
-6	-5	17	33.61	2.52
6	5	-17	35.91	2.93
6	5	-17	34.52	1.50
-7	-5	17	8.07	1.14
-7	-5	17	9.14	1.40
-7	-5	17	10.85	2.34
7	5	-17	10.84	0.67
7	5	-17	10.54	1.71
-8	-5	17	14.30	1.47
-8	-5	17	16.22	1.86
-8	-5	17	18.12	3.43
8	5	-17	13.79	0.81
-9	-5	17	6.15	1.17
-9	-5	17	6.69	2.30
-9	-5	17	6.99	1.03
9	5	-17	5.83	0.62
-10	-5	17	5.97	2.33
-10	-5	17	9.04	1.20
10	5	-17	9.94	0.91
-11	-5	17	-2.62	2.21
-11	-5	17	0.88	0.95
-12	-5	17	23.00	4.17
-12	-5	17	25.86	2.15
-13	-5	17	5.36	1.26
-13	-5	17	3.51	2.48
-14	-5	17	6.36	1.40
-14	-5	17	5.81	2.67

10	-5	18	-2.66	1.72
-10	5	-18	0.94	0.95
9	-5	18	31.16	3.36
-9	5	-18	33.48	2.16
8	-5	18	-0.99	1.53
-8	5	-18	-0.11	0.71
7	-5	18	17.69	2.98
-7	5	-18	21.22	1.60
-7	5	-18	22.03	1.79
6	-5	18	1.99	1.39
6	-5	18	-1.59	1.43
-6	5	-18	1.50	0.83
-6	5	-18	-1.20	0.73
5	-5	18	34.82	3.24
5	-5	18	28.53	2.77
-5	5	-18	32.11	1.79
-5	5	-18	32.09	1.96
4	-5	18	-0.40	1.43
4	-5	18	1.38	1.38
-4	5	-18	-0.65	0.55
-4	5	-18	-0.24	0.71
3	-5	18	30.16	3.13
3	-5	18	35.67	2.98
-3	5	-18	33.87	1.77
-3	5	-18	35.25	1.96
2	-5	18	-0.19	1.59
2	-5	18	-0.21	1.43
2	-5	18	1.08	1.24
-2	5	-18	1.43	0.73
-2	5	-18	0.61	0.49
1	-5	18	48.40	2.66
1	-5	18	44.47	3.38
1	-5	18	49.91	3.54
-1	5	-18	48.10	2.13
-1	5	-18	45.44	2.35
0	-5	18	-3.50	1.32
0	-5	18	0.86	1.53
0	-5	18	2.27	0.98
0	5	-18	0.34	0.43
0	5	-18	-0.40	0.63
-1	-5	18	36.52	2.23
-1	-5	18	29.91	3.19
-1	-5	18	31.39	2.80
1	5	-18	35.26	1.59

1	5	-18	32.11	1.88
-2	-5	18	1.25	1.72
-2	-5	18	2.44	1.03
-2	-5	18	1.93	1.33
2	5	-18	1.33	0.65
-3	-5	18	50.87	4.10
-3	-5	18	47.14	3.20
3	5	-18	47.39	2.40
3	5	-18	49.40	2.05
-4	-5	18	0.28	0.67
-4	-5	18	-2.00	1.78
-4	-5	18	0.72	1.43
4	5	-18	0.35	0.32
4	5	-18	0.18	0.65
-5	-5	18	47.81	2.41
-5	-5	18	46.48	3.04
-5	-5	18	46.24	4.16
5	5	-18	43.63	1.91
-6	-5	18	0.69	1.17
-6	-5	18	0.84	0.80
-6	-5	18	-1.39	1.91
6	5	-18	0.12	0.33
-7	-5	18	28.40	2.38
-7	-5	18	25.15	1.82
-7	-5	18	26.54	3.81
7	5	-18	25.13	1.19
-8	-5	18	0.34	1.15
-8	-5	18	-0.03	2.03
-8	-5	18	2.12	0.86
-9	-5	18	23.75	1.91
-9	-5	18	17.04	2.11
-9	-5	18	20.58	3.81
-10	-5	18	3.59	1.11
-10	-5	18	2.36	2.15
-11	-5	18	51.52	3.06
-11	-5	18	58.98	4.90
-12	-5	18	1.24	2.50
-12	-5	18	-0.44	1.02
-13	-5	18	32.77	4.62
-13	-5	18	30.00	2.55
-9	5	-19	1.38	0.96
8	-5	19	36.96	3.71
-8	5	-19	34.73	2.23
7	-5	19	1.92	1.82

-7	5	-19	1.51	0.83
6	-5	19	21.13	2.70
6	-5	19	18.49	3.03
-6	5	-19	25.42	1.70
5	-5	19	-1.03	1.55
5	-5	19	4.25	1.55
-5	5	-19	1.29	1.06
4	-5	19	23.36	3.07
4	-5	19	24.91	2.93
-4	5	-19	24.40	1.74
-4	5	-19	26.79	1.63
3	-5	19	-0.37	1.55
3	-5	19	-1.62	1.59
-3	5	-19	-0.21	0.56
-3	5	-19	0.30	0.74
2	-5	19	46.33	3.49
2	-5	19	38.77	3.24
-2	5	-19	37.00	2.11
-2	5	-19	38.45	1.93
1	-5	19	2.19	1.51
1	-5	19	-1.54	1.54
-1	5	-19	-1.49	0.65
-1	5	-19	-1.00	0.47
0	-5	19	30.32	2.95
0	-5	19	33.54	3.40
0	5	-19	30.73	1.56
0	5	-19	29.47	1.85
-1	-5	19	-0.15	1.74
-1	-5	19	-2.57	1.37
1	5	-19	-0.52	0.65
1	5	-19	-0.24	0.43
-2	-5	19	37.98	3.20
-2	-5	19	44.32	3.90
2	5	-19	39.91	2.17
2	5	-19	40.97	1.83
-3	-5	19	0.14	1.93
-3	-5	19	-0.19	1.40
3	5	-19	1.70	0.72
3	5	-19	1.32	0.42
-4	-5	19	50.40	3.34
-4	-5	19	54.16	4.31
-4	-5	19	48.53	2.59
4	5	-19	49.49	2.10
-5	-5	19	1.14	1.29

-5	-5	19	2.08	2.05
-5	-5	19	1.53	1.00
-6	-5	19	34.13	2.17
-6	-5	19	33.47	2.79
-6	-5	19	40.92	4.23
-7	-5	19	0.58	2.06
-7	-5	19	0.12	1.28
-7	-5	19	1.05	0.87
-8	-5	19	32.77	2.20
-8	-5	19	37.40	4.31
-8	-5	19	25.01	2.57
-9	-5	19	1.95	1.07
-9	-5	19	0.87	2.30
-10	-5	19	47.84	2.93
-10	-5	19	49.51	4.84
-11	-5	19	2.79	1.14
-11	-5	19	1.69	2.40
-12	-5	19	46.30	3.11
7	-5	20	6.17	3.32
-7	5	-20	7.40	1.09
6	-5	20	-1.91	1.72
-6	5	-20	3.42	0.90
5	-5	20	4.70	1.95
5	-5	20	4.00	2.08
-5	5	-20	6.64	0.95
4	-5	20	3.67	1.78
4	-5	20	3.31	1.83
3	-5	20	14.11	2.28
3	-5	20	10.68	2.07
-3	5	-20	12.07	1.26
2	-5	20	2.59	1.86
2	-5	20	3.82	1.81
-2	5	-20	5.58	0.78
1	-5	20	25.16	3.08
1	-5	20	27.72	3.44
-1	5	-20	26.70	1.52
-1	5	-20	23.27	1.73
0	-5	20	10.79	2.01
0	-5	20	13.03	2.33
0	5	-20	8.41	1.33
0	5	-20	10.68	1.04
-1	-5	20	20.48	3.39
-1	-5	20	17.29	2.90
1	5	-20	20.22	1.46

1	5	-20	17.51	1.55
-2	-5	20	4.14	2.05
-2	-5	20	2.35	1.61
2	5	-20	2.49	0.78
-3	-5	20	36.68	4.08
-3	-5	20	38.68	2.19
-3	-5	20	29.34	3.08
-4	-5	20	3.92	1.13
-4	-5	20	8.80	2.36
-4	-5	20	5.39	1.71
-5	-5	20	25.74	2.85
-5	-5	20	24.81	1.93
-5	-5	20	28.30	4.03
-6	-5	20	6.07	2.43
-6	-5	20	6.30	1.63
-6	-5	20	6.66	1.09
-7	-5	20	20.08	3.05
-7	-5	20	26.54	2.12
-7	-5	20	30.06	4.22
-8	-5	20	0.48	1.09
-8	-5	20	2.54	2.48
-9	-5	20	24.27	4.32
-9	-5	20	28.46	2.40
-10	-5	20	-0.92	1.10
-10	-5	20	3.33	2.88
4	-5	21	0.70	1.89
4	-5	21	-0.83	1.99
3	-5	21	25.49	3.49
3	-5	21	26.24	3.59
2	-5	21	-0.14	2.09
2	-5	21	2.77	1.86
1	-5	21	13.16	2.51
1	-5	21	18.20	3.15
0	-5	21	0.59	2.08
0	-5	21	2.00	1.83
-1	-5	21	12.16	2.59
-1	-5	21	15.37	2.90
-2	-5	21	3.11	1.87
-2	-5	21	3.24	2.17
-2	-5	21	2.91	1.15
-3	-5	21	9.69	2.56
-3	-5	21	9.64	1.62
-3	-5	21	6.84	1.85
-4	-5	21	17.43	2.93

-4	-5	21	12.59	1.68
-4	-5	21	9.96	2.66
-5	-5	21	7.41	1.86
-5	-5	21	6.61	2.52
-5	-5	21	7.75	1.17
-6	-5	21	4.90	1.12
-6	-5	21	4.01	1.77
-6	-5	21	5.49	2.61
-7	-5	21	14.81	2.87
-7	-5	21	12.12	1.50
-8	-5	21	0.43	1.15
-8	-5	21	1.21	2.79
0	-5	22	6.59	2.14
0	-5	22	7.69	2.55
-1	-5	22	9.94	2.25
-1	-5	22	8.53	2.63
-2	-5	22	5.40	1.97
-2	-5	22	4.43	2.60
-3	-5	22	-3.15	1.78
-3	-5	22	-0.87	2.36
-4	-5	22	11.19	2.87
7	-6	-21	2.46	0.81
-7	6	21	3.94	2.34
6	-6	-21	21.56	1.66
-6	6	21	17.32	2.83
-6	6	21	25.07	3.60
5	-6	-21	1.80	0.79
-5	6	21	5.00	2.05
-5	6	21	1.69	1.65
4	-6	-21	11.87	1.45
-4	6	21	13.02	2.09
-4	6	21	10.06	2.21
3	-6	-21	4.35	0.88
-3	6	21	9.20	1.98
-3	6	21	7.82	1.90
2	-6	-21	18.88	1.60
-2	6	21	18.30	3.11
-2	6	21	13.90	2.24
1	-6	-21	2.36	0.83
-1	6	21	-0.20	1.64
-1	6	21	2.32	1.70
0	-6	-21	16.80	1.66
0	6	21	15.16	2.31
0	6	21	19.63	3.11

-1	-6	-21	5.88	0.97
1	6	21	4.92	1.85
1	6	21	0.39	1.84
-2	-6	-21	28.38	2.08
2	6	21	32.22	3.31
2	6	21	28.02	3.46
-3	-6	-21	1.00	0.89
3	6	21	-1.40	1.82
3	6	21	-0.99	1.70
-4	-6	-21	12.93	1.71
4	6	21	9.71	2.24
10	-6	-20	25.74	1.80
9	-6	-20	2.15	0.76
-9	6	20	0.86	2.04
8	-6	-20	29.74	1.84
-8	6	20	27.07	3.72
7	-6	-20	5.02	0.85
-7	6	20	5.31	1.57
-7	6	20	3.63	1.93
6	-6	-20	25.29	1.67
-6	6	20	19.44	3.27
-6	6	20	28.75	2.76
5	-6	-20	11.93	1.32
-5	6	20	10.18	1.88
-5	6	20	11.73	2.21
4	-6	-20	11.21	1.32
-4	6	20	13.92	2.27
-4	6	20	9.88	1.90
3	-6	-20	3.00	0.78
-3	6	20	5.48	1.73
-3	6	20	4.05	1.65
2	-6	-20	33.71	1.96
-2	6	20	33.84	3.30
-2	6	20	30.62	3.10
1	-6	-20	2.31	0.80
-1	6	20	1.67	1.58
-1	6	20	2.75	1.62
0	-6	-20	19.21	1.62
0	6	20	28.52	2.90
0	6	20	16.64	2.84
-1	-6	-20	3.47	0.87
1	6	20	3.03	1.52
1	6	20	2.66	1.59
-2	-6	-20	13.56	1.51

2	6	20	13.47	2.00
2	6	20	10.86	2.09
-3	-6	-20	12.56	1.52
3	6	20	13.94	2.64
3	6	20	12.71	2.15
-4	-6	-20	3.83	0.96
4	6	20	7.71	1.96
4	6	20	3.26	1.68
-5	-6	-20	2.74	0.86
5	6	20	1.78	1.70
5	6	20	0.25	1.57
-6	-6	-20	11.53	1.62
6	6	20	11.78	2.30
6	6	20	9.35	1.85
11	-6	-19	44.87	2.31
-11	6	19	37.76	4.24
10	-6	-19	-0.89	0.63
-10	6	19	1.17	1.94
9	-6	-19	35.28	1.97
-9	6	19	36.38	3.76
8	-6	-19	0.96	0.61
-8	6	19	2.00	1.31
-8	6	19	1.48	1.72
7	-6	-19	30.90	1.79
-7	6	19	30.23	2.68
-7	6	19	29.08	3.41
6	-6	-19	-1.13	0.59
-6	6	19	1.58	1.39
-6	6	19	-1.27	1.52
5	-6	-19	47.61	2.38
-5	6	19	54.25	3.83
-5	6	19	42.06	3.14
4	-6	-19	-0.20	0.63
-4	6	19	1.91	1.51
-4	6	19	-1.07	1.30
3	-6	-19	37.86	1.97
-3	6	19	32.46	3.12
-3	6	19	30.94	2.85
2	-6	-19	-0.16	0.61
-2	6	19	-1.51	1.30
-2	6	19	0.05	1.38
1	-6	-19	30.37	1.82
-1	6	19	32.31	2.93
-1	6	19	26.73	2.85

0	-6	-19	1.95	0.66
0	6	19	3.13	1.46
0	6	19	1.81	1.38
-1	-6	-19	37.43	2.15
1	6	19	35.62	3.06
1	6	19	41.80	3.19
-2	-6	-19	-0.22	0.67
2	6	19	0.37	1.42
2	6	19	-1.09	1.25
-3	-6	-19	31.66	1.93
3	6	19	27.33	2.79
3	6	19	28.45	3.07
-4	-6	-19	1.64	0.76
4	6	19	0.91	1.47
4	6	19	0.54	1.35
-5	-6	-19	15.84	1.60
5	6	19	13.04	1.87
5	6	19	14.27	2.21
-6	-6	-19	3.16	0.88
6	6	19	3.31	1.67
6	6	19	4.76	1.54
-7	-6	-19	34.73	2.22
7	6	19	33.01	3.48
7	6	19	31.69	3.01
-8	-6	-19	1.96	0.95
8	6	19	0.36	1.89
-13	6	18	3.66	2.32
-13	6	18	3.15	1.52
12	-6	-18	35.28	2.07
-12	6	18	35.65	3.98
-12	6	18	42.01	2.92
11	-6	-18	-0.11	0.60
-11	6	18	-2.67	1.80
-11	6	18	1.76	1.22
10	-6	-18	32.56	1.85
-10	6	18	34.75	2.36
-10	6	18	28.40	3.55
9	-6	-18	3.51	0.72
9	-6	-18	4.40	1.80
-9	6	18	4.34	1.87
-9	6	18	3.54	1.04
8	-6	-18	45.97	2.32
-8	6	18	47.68	3.05
-8	6	18	50.32	2.52

-8	6	18	44.48	3.74
7	-6	-18	2.50	0.67
-7	6	18	4.99	1.27
-7	6	18	5.19	1.68
-7	6	18	4.66	0.83
6	-6	-18	35.96	1.93
-6	6	18	36.11	2.66
-6	6	18	37.88	1.91
-6	6	18	34.44	3.33
5	-6	-18	-0.52	0.56
-5	6	18	0.67	1.33
-5	6	18	-2.22	1.27
4	-6	-18	39.44	1.90
-4	6	18	31.06	3.01
-4	6	18	29.95	2.60
3	-6	-18	-0.24	0.54
-3	6	18	-1.22	1.13
-3	6	18	-0.36	1.25
2	-6	-18	41.37	2.06
-2	6	18	37.08	2.90
-2	6	18	35.88	3.05
1	-6	-18	0.64	0.59
-1	6	18	-2.17	1.16
-1	6	18	0.28	1.28
0	-6	-18	35.39	1.91
0	6	18	28.89	2.70
0	6	18	32.64	2.82
-1	-6	-18	-0.59	0.59
1	6	18	-0.37	1.26
1	6	18	1.36	1.26
-2	-6	-18	19.45	1.57
2	6	18	20.98	2.51
2	6	18	21.68	2.34
-3	-6	-18	0.37	0.65
3	6	18	0.52	1.30
3	6	18	-1.83	1.09
-4	-6	-18	40.44	2.22
4	6	18	41.62	2.92
4	6	18	35.37	3.22
-5	-6	-18	0.48	0.71
5	6	18	0.38	1.17
5	6	18	-1.82	1.40
-6	-6	-18	26.74	1.96
6	6	18	29.55	3.18

6	6	18	30.84	2.57
-7	-6	-18	0.43	0.75
7	6	18	0.54	1.58
7	6	18	-0.40	2.51
-8	-6	-18	31.22	2.15
8	6	18	29.74	3.18
8	6	18	33.69	2.76
-9	-6	-18	-0.87	0.85
9	6	18	-3.56	1.47
-10	-6	-18	26.31	2.13
10	6	18	25.86	4.50
14	-6	-17	4.65	0.83
-14	6	17	6.48	2.30
-14	6	17	8.36	1.62
13	-6	-17	9.39	1.25
-13	6	17	9.05	2.38
-13	6	17	14.77	2.35
12	-6	-17	0.59	0.60
-12	6	17	2.64	1.24
-12	6	17	0.71	1.89
11	-6	-17	15.77	1.28
-11	6	17	13.77	2.29
10	-6	-17	13.71	2.53
10	-6	-17	15.09	1.27
-10	6	17	17.26	1.85
-10	6	17	10.34	2.07
9	-6	-17	12.02	1.15
9	-6	-17	11.93	1.84
-9	6	17	15.19	1.67
-9	6	17	9.94	1.97
8	-6	-17	3.07	0.62
-8	6	17	2.09	1.54
-8	6	17	3.78	1.21
-8	6	17	3.30	0.82
7	-6	-17	42.02	3.15
7	-6	-17	32.92	1.82
-7	6	17	36.86	1.96
-7	6	17	33.17	3.12
-7	6	17	32.19	2.52
6	-6	-17	4.07	0.65
-6	6	17	4.76	1.25
-6	6	17	5.62	1.55
-6	6	17	5.38	0.75
5	-6	-17	26.70	1.56

-5	6	17	25.32	1.46
-5	6	17	29.51	2.31
-5	6	17	25.10	2.73
4	-6	-17	0.53	0.50
-4	6	17	0.96	0.49
-4	6	17	0.77	1.05
-4	6	17	1.51	1.29
3	-6	-17	12.49	1.11
-3	6	17	14.72	2.28
-3	6	17	17.17	2.25
2	-6	-17	4.35	0.67
-2	6	17	5.17	1.31
-2	6	17	6.38	1.33
1	-6	-17	47.59	2.32
-1	6	17	52.34	3.21
-1	6	17	45.62	3.10
0	-6	-17	1.10	0.61
0	6	17	-0.19	1.05
0	6	17	-1.36	1.09
-1	-6	-17	27.89	1.73
1	6	17	31.33	2.62
1	6	17	31.92	2.52
-2	-6	-17	1.68	0.62
2	6	17	1.98	1.22
2	6	17	1.97	1.10
-3	-6	-17	9.83	1.18
3	6	17	11.40	1.76
3	6	17	14.67	1.91
-4	-6	-17	0.39	0.65
4	6	17	-0.86	1.32
-5	-6	-17	44.40	2.29
5	6	17	36.67	3.17
5	6	17	45.92	2.88
-6	-6	-17	-1.01	0.66
6	6	17	0.28	1.40
-7	-6	-17	37.34	2.30
7	6	17	42.21	2.79
7	6	17	41.70	3.26
-8	-6	-17	-0.02	0.71
8	6	17	0.70	1.17
8	6	17	-1.72	1.57
-9	-6	-17	9.83	1.54
9	6	17	12.28	2.14
9	6	17	8.61	1.81

-10	-6	-17	5.34	1.09
10	6	17	4.97	1.86
-11	-6	-17	18.93	1.89
11	6	17	20.12	3.09
15	-6	-16	13.00	1.36
-15	6	16	17.44	2.63
-15	6	16	12.65	2.58
14	-6	-16	-0.84	0.61
-14	6	16	0.67	1.91
-14	6	16	0.62	1.31
13	-6	-16	15.15	1.30
-13	6	16	10.22	2.23
-13	6	16	18.01	2.19
12	-6	-16	0.05	0.57
-12	6	16	-0.02	1.07
-12	6	16	-1.34	1.50
11	-6	-16	15.28	1.22
-11	6	16	12.12	2.16
-11	6	16	20.81	1.86
10	-6	-16	1.19	1.35
10	-6	-16	0.12	0.51
-10	6	16	1.17	0.92
-10	6	16	0.52	1.51
9	-6	-16	38.50	1.98
9	-6	-16	45.66	3.07
-9	6	16	38.28	3.28
-9	6	16	39.19	2.31
-9	6	16	40.02	2.27
8	-6	-16	15.06	1.14
8	-6	-16	17.61	2.19
8	-6	-16	13.02	2.34
-8	6	16	16.04	2.50
-8	6	16	14.85	1.41
-8	6	16	13.12	1.44
7	-6	-16	23.31	2.25
7	-6	-16	14.84	1.16
-7	6	16	16.19	2.45
-7	6	16	16.81	1.33
-7	6	16	14.96	1.88
-7	6	16	15.98	1.77
6	-6	-16	1.81	1.24
6	-6	-16	1.85	0.51
-6	6	16	3.05	0.65
-6	6	16	1.70	1.22

-6	6	16	1.30	1.02
-6	6	16	2.47	0.60
5	-6	-16	24.11	1.39
-5	6	16	21.41	1.29
-5	6	16	22.30	1.30
-5	6	16	18.87	2.39
-5	6	16	23.99	2.09
4	-6	-16	5.47	0.80
-4	6	16	4.43	0.55
-4	6	16	4.65	1.28
-4	6	16	5.49	1.24
-4	6	16	5.05	0.56
3	-6	-16	9.76	0.92
-3	6	16	8.69	1.34
-3	6	16	6.57	1.41
2	-6	-16	6.17	0.82
-2	6	16	4.12	1.15
-2	6	16	4.50	1.22
1	-6	-16	14.31	1.10
-1	6	16	14.75	2.04
-1	6	16	10.93	1.84
0	-6	-16	7.07	0.91
0	6	16	6.86	1.38
0	6	16	7.40	1.47
-1	-6	-16	7.46	0.94
1	6	16	10.03	1.99
1	6	16	5.32	1.31
-2	-6	-16	8.17	1.00
2	6	16	6.98	1.37
2	6	16	6.79	1.19
-3	-6	-16	23.27	1.46
3	6	16	22.27	2.07
3	6	16	18.38	2.20
-4	-6	-16	13.27	1.23
4	6	16	14.42	1.78
4	6	16	15.55	2.17
-5	-6	-16	11.08	1.24
5	6	16	12.83	1.72
5	6	16	8.74	1.64
-6	-6	-16	8.30	1.24
6	6	16	11.01	1.84
6	6	16	11.39	1.75
-7	-6	-16	3.06	0.80
7	6	16	3.40	1.42

7	6	16	2.89	1.17
-8	-6	-16	27.08	1.85
8	6	16	23.04	2.75
8	6	16	28.02	2.27
-9	-6	-16	6.56	0.97
9	6	16	7.03	1.75
9	6	16	7.26	1.28
-10	-6	-16	9.97	1.48
10	6	16	11.32	1.98
-11	-6	-16	17.99	1.80
11	6	16	19.35	2.88
-12	-6	-16	21.58	1.93
12	6	16	16.86	2.93
16	-6	-15	22.33	1.68
-16	6	15	25.55	2.87
-16	6	15	26.54	3.81
15	-6	-15	13.10	1.29
-15	6	15	11.61	1.71
-15	6	15	10.27	2.44
14	-6	-15	18.25	1.40
-14	6	15	23.55	3.28
-14	6	15	16.44	2.27
13	-6	-15	12.45	1.17
-13	6	15	12.57	2.17
-13	6	15	11.69	1.95
12	-6	-15	17.98	1.32
-12	6	15	22.34	1.93
-12	6	15	15.39	2.87
11	-6	-15	10.41	1.85
11	-6	-15	11.60	1.05
-11	6	15	13.98	1.57
-11	6	15	9.62	1.94
10	-6	-15	36.90	2.61
10	-6	-15	38.14	1.91
-10	6	15	34.99	3.24
-10	6	15	40.63	2.27
9	-6	-15	4.93	1.20
9	-6	-15	3.64	1.24
9	-6	-15	2.48	0.54
-9	6	15	2.59	1.40
-9	6	15	3.16	0.87
-9	6	15	2.40	0.79
8	-6	-15	15.52	1.94
8	-6	-15	16.11	1.13

8	-6	-15	15.53	2.18
-8	6	15	19.74	1.43
-8	6	15	12.96	1.89
-8	6	15	16.88	1.38
7	-6	-15	-0.84	1.04
7	-6	-15	1.29	0.48
-7	6	15	2.33	0.65
-7	6	15	1.59	1.20
-7	6	15	2.00	0.94
-7	6	15	2.16	0.64
6	-6	-15	15.48	1.96
-6	6	15	12.69	1.14
-6	6	15	18.08	1.18
-6	6	15	15.74	1.72
-6	6	15	14.62	2.10
5	-6	-15	11.66	0.96
-5	6	15	11.68	1.63
-5	6	15	13.52	1.99
-5	6	15	11.16	0.94
-5	6	15	10.57	0.93
4	-6	-15	67.43	3.64
4	-6	-15	70.11	2.98
-4	6	15	65.06	3.76
-4	6	15	69.36	2.81
-4	6	15	73.78	2.82
-4	6	15	66.54	3.50
3	-6	-15	2.60	0.51
-3	6	15	2.81	0.37
-3	6	15	1.56	1.01
-3	6	15	1.24	1.06
2	-6	-15	32.95	1.65
-2	6	15	29.24	2.91
-2	6	15	31.54	2.31
1	-6	-15	0.29	0.41
-1	6	15	0.50	0.97
-1	6	15	1.41	0.89
0	-6	-15	36.31	1.83
0	6	15	39.12	2.47
0	6	15	33.42	2.44
-1	-6	-15	2.62	0.83
1	6	15	2.41	1.04
1	6	15	2.49	0.94
-2	-6	-15	37.93	2.04
2	6	15	36.65	2.67

2	6	15	41.06	2.47
-3	-6	-15	8.52	0.96
3	6	15	6.94	1.17
3	6	15	5.65	1.36
-4	-6	-15	44.20	2.16
4	6	15	39.22	2.87
4	6	15	43.94	2.61
-5	-6	-15	5.54	0.81
-5	-6	-15	4.09	2.33
5	6	15	6.24	1.48
5	6	15	3.96	1.06
-6	-6	-15	24.22	1.60
-6	-6	-15	20.30	3.40
6	6	15	24.09	2.49
6	6	15	25.34	1.98
-7	-6	-15	-0.70	0.58
-7	-6	-15	-1.48	1.98
7	6	15	-0.63	1.10
7	6	15	-0.65	0.91
-8	-6	-15	16.84	1.48
8	6	15	18.37	1.86
8	6	15	23.08	2.43
-9	-6	-15	0.04	0.62
9	6	15	1.68	1.03
9	6	15	0.88	1.35
-10	-6	-15	15.96	1.59
10	6	15	18.53	2.52
10	6	15	15.72	1.85
-11	-6	-15	5.82	0.97
11	6	15	8.55	1.81
-12	-6	-15	13.73	1.62
12	6	15	15.03	2.71
-13	-6	-15	3.04	1.03
13	6	15	4.27	1.79
16	-6	-14	10.65	1.31
-16	6	14	16.87	2.53
-16	6	14	9.85	2.35
15	-6	-14	4.84	0.74
-15	6	14	6.22	1.98
-15	6	14	3.26	1.36
14	-6	-14	34.68	1.87
-14	6	14	34.72	2.63
-14	6	14	39.20	3.62
13	-6	-14	2.28	0.59

-13	6	14	2.79	1.68
-13	6	14	2.53	1.07
12	-6	-14	79.27	3.51
-12	6	14	79.57	4.82
-12	6	14	91.00	3.97
11	-6	-14	10.12	1.00
11	-6	-14	6.95	1.16
-11	6	14	8.45	1.72
-11	6	14	8.66	1.39
10	-6	-14	41.26	2.41
10	-6	-14	42.10	2.61
10	-6	-14	41.10	2.02
-10	6	14	35.85	3.16
-10	6	14	44.71	2.35
-10	6	14	47.00	2.42
9	-6	-14	5.14	1.05
9	-6	-14	5.23	1.16
9	-6	-14	6.51	0.82
-9	6	14	5.40	1.41
-9	6	14	7.04	1.21
-9	6	14	8.12	1.16
8	-6	-14	24.84	2.01
8	-6	-14	28.20	2.29
-8	6	14	23.27	2.50
-8	6	14	28.96	1.67
-8	6	14	28.53	1.64
7	-6	-14	20.17	1.86
7	-6	-14	16.66	1.09
-7	6	14	15.30	1.66
-7	6	14	15.99	1.26
-7	6	14	19.00	1.30
-7	6	14	17.76	2.14
6	-6	-14	60.96	2.80
6	-6	-14	68.61	3.39
-6	6	14	70.87	2.84
-6	6	14	63.19	3.67
-6	6	14	68.48	2.83
-6	6	14	61.83	3.22
5	-6	-14	22.91	1.31
5	-6	-14	24.33	2.10
-5	6	14	23.85	1.89
-5	6	14	24.85	1.29
-5	6	14	24.37	1.30
4	-6	-14	14.02	1.85

4	-6	-14	13.79	0.97
-4	6	14	15.16	1.79
-4	6	14	12.18	0.84
-4	6	14	12.68	0.85
-4	6	14	11.25	1.57
3	-6	-14	55.23	3.17
3	-6	-14	52.02	2.39
-3	6	14	51.09	2.29
-3	6	14	56.18	3.12
-3	6	14	55.77	2.19
-3	6	14	54.29	2.94
2	-6	-14	25.22	2.25
2	-6	-14	25.63	1.38
-2	6	14	25.10	2.04
-2	6	14	24.69	2.00
1	-6	-14	26.58	1.43
-1	6	14	25.54	2.05
-1	6	14	25.71	2.05
0	-6	-14	68.42	2.97
0	6	14	70.17	3.59
0	6	14	69.14	3.53
-1	-6	-14	21.46	1.28
1	6	14	19.32	1.90
1	6	14	20.95	1.83
-2	-6	-14	63.68	2.74
-2	-6	-14	56.39	3.79
2	6	14	62.98	3.17
2	6	14	59.93	3.31
-3	-6	-14	19.61	1.26
-3	-6	-14	20.17	2.45
3	6	14	20.00	1.72
3	6	14	17.12	2.16
-4	-6	-14	7.36	1.25
-4	-6	-14	11.93	1.95
4	6	14	10.08	1.45
4	6	14	10.64	1.73
-5	-6	-14	10.90	1.83
-5	-6	-14	10.08	1.01
5	6	14	9.13	1.69
5	6	14	7.87	1.38
-6	-6	-14	4.12	1.53
-6	-6	-14	2.80	0.61
6	6	14	1.44	0.84
6	6	14	1.21	1.03

-7	-6	-14	8.64	1.79
-7	-6	-14	8.79	1.11
7	6	14	7.39	1.45
7	6	14	7.90	1.37
-8	-6	-14	3.67	1.78
-8	-6	-14	4.08	0.71
8	6	14	1.63	1.20
8	6	14	3.53	0.97
-9	-6	-14	18.79	1.52
9	6	14	18.17	1.77
9	6	14	17.52	2.28
-10	-6	-14	12.18	1.38
10	6	14	8.76	1.20
10	6	14	12.85	2.23
-11	-6	-14	12.50	1.49
11	6	14	14.12	1.73
11	6	14	13.47	2.04
-12	-6	-14	3.36	0.87
12	6	14	2.36	1.46
-13	-6	-14	15.39	1.68
13	6	14	14.14	2.66
-14	-6	-14	2.09	0.97
14	6	14	4.99	1.74
17	-6	-13	45.29	2.38
-17	6	13	51.97	3.45
-17	6	13	44.97	4.23
16	-6	-13	2.32	0.72
-16	6	13	2.51	1.88
-16	6	13	1.04	1.29
15	-6	-13	41.43	2.16
-15	6	13	46.00	2.97
-15	6	13	40.27	3.77
14	-6	-13	5.10	0.72
-14	6	13	7.43	1.27
-14	6	13	5.03	1.73
13	-6	-13	60.95	2.75
-13	6	13	60.52	4.12
-13	6	13	64.32	3.25
12	-6	-13	-1.25	0.82
12	-6	-13	-0.68	0.43
-12	6	13	-1.04	1.37
11	-6	-13	73.65	3.25
11	-6	-13	70.93	3.11
11	-6	-13	74.59	3.41

-11	6	13	68.75	4.23
-11	6	13	77.04	3.43
10	-6	-13	2.51	0.79
10	-6	-13	1.94	0.45
10	-6	-13	0.18	0.92
10	-6	-13	0.96	0.80
-10	6	13	2.14	1.29
-10	6	13	1.70	0.66
9	-6	-13	38.36	2.40
9	-6	-13	33.33	2.02
9	-6	-13	38.04	2.21
-9	6	13	37.74	2.87
-9	6	13	41.70	2.06
8	-6	-13	0.62	0.64
8	-6	-13	0.36	0.37
8	-6	-13	0.02	0.91
8	-6	-13	-0.09	0.86
-8	6	13	0.93	0.58
-8	6	13	0.44	1.03
7	-6	-13	42.52	2.68
7	-6	-13	43.82	2.45
7	-6	-13	38.55	1.91
-7	6	13	41.83	2.05
-7	6	13	44.21	2.06
6	-6	-13	0.31	0.37
6	-6	-13	0.04	0.87
-6	6	13	0.09	0.90
-6	6	13	-0.09	0.48
-6	6	13	-0.35	0.72
-6	6	13	0.13	0.46
5	-6	-13	163.66	6.86
5	-6	-13	187.08	7.34
-5	6	13	181.54	7.62
-5	6	13	168.78	6.81
-5	6	13	172.74	7.23
-5	6	13	188.44	6.82
4	-6	-13	12.97	1.65
4	-6	-13	11.70	0.93
-4	6	13	11.18	1.64
-4	6	13	12.62	1.45
-4	6	13	13.39	0.84
-4	6	13	13.50	0.84
3	-6	-13	80.61	3.38
3	-6	-13	83.81	3.97

-3	6	13	85.10	3.64
-3	6	13	83.88	4.05
-3	6	13	80.33	3.20
-3	6	13	78.22	3.82
2	-6	-13	1.30	0.96
2	-6	-13	0.94	0.43
-2	6	13	1.42	0.81
-2	6	13	1.29	0.50
-2	6	13	0.04	0.78
1	-6	-13	48.16	3.09
1	-6	-13	45.48	2.86
1	-6	-13	49.24	2.13
-1	6	13	46.18	2.32
-1	6	13	43.03	2.62
-1	6	13	44.66	2.64
0	-6	-13	0.07	0.35
0	-6	-13	1.03	1.11
0	-6	-13	0.13	0.96
0	6	13	0.49	0.69
0	6	13	-0.13	0.74
-1	-6	-13	128.74	5.90
-1	-6	-13	128.74	5.11
1	6	13	126.78	5.52
1	6	13	124.77	5.60
-2	-6	-13	1.61	0.44
-2	-6	-13	2.35	1.22
2	6	13	0.26	0.80
2	6	13	1.60	0.76
-3	-6	-13	76.02	3.24
-3	-6	-13	77.03	4.03
3	6	13	74.38	3.59
3	6	13	76.34	3.82
-4	-6	-13	5.50	0.82
-4	-6	-13	6.31	1.38
4	6	13	4.61	1.12
4	6	13	5.51	0.93
-5	-6	-13	7.39	1.53
-5	-6	-13	5.05	0.69
5	6	13	5.11	0.93
-6	-6	-13	1.47	0.52
-6	-6	-13	0.34	1.26
6	6	13	1.83	0.74
6	6	13	1.23	0.89
-7	-6	-13	57.07	3.49

-7	-6	-13	61.52	2.72
7	6	13	55.95	2.87
7	6	13	54.52	3.34
-8	-6	-13	-0.31	0.50
-8	-6	-13	2.44	1.46
8	6	13	-0.01	1.05
8	6	13	0.09	0.76
-9	-6	-13	54.81	2.65
-9	-6	-13	50.58	3.54
9	6	13	52.88	3.35
9	6	13	55.53	2.76
-10	-6	-13	4.81	0.83
10	6	13	7.52	1.08
10	6	13	5.60	1.52
-11	-6	-13	17.92	1.58
11	6	13	17.01	2.33
11	6	13	17.93	1.70
-12	-6	-13	2.19	0.82
12	6	13	0.88	1.31
-13	-6	-13	15.18	1.64
13	6	13	19.53	2.58
-14	-6	-13	14.81	1.73
14	6	13	11.75	2.05
-15	-6	-13	25.50	2.19
15	6	13	23.22	2.90
18	-6	-12	9.91	1.38
-18	6	12	10.11	2.32
-18	6	12	15.82	2.63
17	-6	-12	5.10	0.81
-17	6	12	6.21	1.55
-17	6	12	9.15	2.22
16	-6	-12	18.52	1.39
-16	6	12	20.96	3.25
-16	6	12	22.22	2.46
15	-6	-12	7.31	1.03
-15	6	12	6.92	1.36
-15	6	12	7.94	1.88
14	-6	-12	40.09	2.05
-14	6	12	39.92	3.49
-14	6	12	46.69	2.74
13	-6	-12	13.37	1.10
-13	6	12	15.35	1.69
-13	6	12	13.31	2.53
12	-6	-12	90.69	3.97

12	-6	-12	95.21	3.95
-12	6	12	95.37	5.12
-12	6	12	109.34	4.45
11	-6	-12	-0.11	0.76
11	-6	-12	0.16	0.40
-11	6	12	-0.42	1.13
-11	6	12	0.70	0.66
10	-6	-12	30.65	1.83
10	-6	-12	25.24	1.86
10	-6	-12	31.69	1.82
10	-6	-12	32.04	1.99
-10	6	12	31.01	1.84
-10	6	12	27.38	2.53
9	-6	-12	18.71	1.37
9	-6	-12	17.54	1.53
9	-6	-12	18.01	1.65
9	-6	-12	15.84	1.05
-9	6	12	14.42	2.56
-9	6	12	19.41	1.35
8	-6	-12	93.41	3.94
8	-6	-12	100.19	4.13
8	-6	-12	90.57	3.80
8	-6	-12	88.75	4.27
-8	6	12	87.40	4.60
-8	6	12	101.58	3.98
7	-6	-12	23.89	1.87
7	-6	-12	18.31	1.15
7	-6	-12	21.24	1.33
7	-6	-12	22.90	1.67
-7	6	12	20.50	1.31
-7	6	12	16.59	1.92
6	-6	-12	123.37	4.77
6	-6	-12	116.59	4.79
6	-6	-12	123.72	5.14
-6	6	12	116.99	5.47
-6	6	12	115.07	5.06
-6	6	12	119.01	4.86
-6	6	12	124.96	4.80
5	-6	-12	4.82	0.92
5	-6	-12	4.42	0.69
5	-6	-12	2.54	0.43
-5	6	12	5.94	1.12
-5	6	12	3.05	0.52
-5	6	12	3.42	0.80

-5	6	12	3.93	0.66
4	-6	-12	80.02	4.27
4	-6	-12	73.81	3.17
4	-6	-12	80.72	3.66
-4	6	12	80.15	3.10
-4	6	12	74.15	3.23
-4	6	12	75.39	3.83
-4	6	12	71.28	3.53
-4	6	12	80.79	3.10
3	-6	-12	1.74	1.06
3	-6	-12	2.39	0.87
3	-6	-12	1.85	0.40
-3	6	12	1.37	0.42
-3	6	12	1.75	0.76
-3	6	12	1.53	0.46
-3	6	12	1.65	0.32
-3	6	12	1.30	0.74
2	-6	-12	31.54	2.09
2	-6	-12	27.04	1.42
2	-6	-12	30.76	2.42
-2	6	12	29.28	1.20
-2	6	12	27.47	1.48
-2	6	12	25.14	1.86
-2	6	12	26.99	1.93
-2	6	12	28.69	1.19
1	-6	-12	9.83	0.80
1	-6	-12	12.94	1.83
1	-6	-12	11.02	1.57
-1	6	12	8.47	1.24
-1	6	12	7.93	0.84
-1	6	12	11.45	1.33
0	-6	-12	84.96	3.40
0	-6	-12	82.18	4.13
0	-6	-12	83.83	3.98
0	6	12	80.60	3.43
0	6	12	81.26	3.84
0	6	12	78.77	3.85
-1	-6	-12	13.91	0.96
-1	-6	-12	16.23	1.85
-1	-6	-12	15.37	1.79
1	6	12	15.75	1.43
1	6	12	13.08	1.03
1	6	12	15.45	1.52
-2	-6	-12	68.64	3.51

-2	-6	-12	67.71	3.58
-2	-6	-12	68.66	2.88
2	6	12	66.52	3.35
2	6	12	63.62	2.89
2	6	12	67.25	3.24
-3	-6	-12	1.47	1.07
-3	-6	-12	1.52	0.44
3	6	12	1.37	0.80
3	6	12	2.22	0.67
-4	-6	-12	63.09	3.55
-4	-6	-12	68.23	2.92
4	6	12	66.87	3.47
4	6	12	68.65	3.19
-5	-6	-12	1.21	0.46
-5	-6	-12	1.47	1.11
5	6	12	-0.18	0.61
5	6	12	0.78	0.81
-6	-6	-12	39.75	1.91
-6	-6	-12	35.60	2.64
6	6	12	38.87	2.15
6	6	12	34.50	2.46
-7	-6	-12	2.29	1.21
7	6	12	2.16	1.49
7	6	12	1.74	0.71
-8	-6	-12	79.38	3.52
-8	-6	-12	76.62	4.09
8	6	12	85.34	3.65
8	6	12	80.27	4.13
-9	-6	-12	-0.31	0.50
-9	-6	-12	0.15	1.25
9	6	12	0.41	0.99
9	6	12	1.05	0.75
-10	-6	-12	33.48	1.64
-10	-6	-12	33.59	1.95
-10	-6	-12	31.13	2.77
10	6	12	33.73	2.70
10	6	12	34.45	2.00
-11	-6	-12	5.28	0.73
-11	-6	-12	4.49	0.81
11	6	12	6.33	1.00
11	6	12	6.59	1.41
-12	-6	-12	20.28	1.63
-12	-6	-12	20.34	1.51
12	6	12	18.46	2.29

12	6	12	20.12	1.74
-13	-6	-12	0.33	0.70
13	6	12	-1.27	1.31
-14	-6	-12	44.53	2.60
14	6	12	43.59	3.39
-15	-6	-12	-1.58	0.84
15	6	12	-0.49	1.53
16	6	12	61.55	4.21
18	-6	-11	0.67	0.66
-18	6	11	3.48	1.94
-18	6	11	-0.85	1.35
17	-6	-11	8.02	1.15
-17	6	11	9.79	2.16
-17	6	11	12.26	1.74
16	-6	-11	20.45	1.43
-16	6	11	16.32	3.07
-16	6	11	19.12	2.28
15	-6	-11	11.15	1.09
-15	6	11	8.16	1.98
-15	6	11	9.89	1.44
14	-6	-11	20.94	1.35
-14	6	11	24.85	2.07
-14	6	11	20.29	2.68
13	-6	-11	9.56	0.93
-13	6	11	13.33	2.30
-13	6	11	11.19	1.55
12	-6	-11	8.47	0.96
12	-6	-11	8.11	0.85
-12	6	11	8.64	1.56
-12	6	11	10.91	1.36
11	-6	-11	50.65	2.35
11	-6	-11	48.30	2.46
-11	6	11	53.37	2.60
10	-6	-11	10.56	1.17
10	-6	-11	9.62	1.23
10	-6	-11	11.58	1.32
10	-6	-11	11.82	0.88
-10	6	11	10.62	1.86
-10	6	11	13.56	1.25
9	-6	-11	25.44	1.78
9	-6	-11	25.13	1.33
9	-6	-11	26.41	1.57
9	-6	-11	24.28	1.60
-9	6	11	28.64	1.61

-9	6	11	24.43	2.22
8	-6	-11	21.10	1.52
8	-6	-11	19.62	1.64
8	-6	-11	19.15	1.12
8	-6	-11	18.83	1.29
-8	6	11	20.71	1.33
-8	6	11	20.61	2.00
7	-6	-11	55.80	2.55
7	-6	-11	48.76	2.68
7	-6	-11	47.51	2.17
7	-6	-11	49.51	2.27
-7	6	11	46.19	2.90
-7	6	11	53.76	2.32
-7	6	11	47.10	2.24
6	-6	-11	3.86	0.85
6	-6	-11	3.67	0.54
6	-6	-11	1.13	1.16
6	-6	-11	3.58	0.96
6	-6	-11	3.97	0.55
-6	6	11	3.84	0.98
-6	6	11	4.70	0.80
-6	6	11	3.44	0.55
-6	6	11	3.54	0.51
5	-6	-11	31.86	2.51
5	-6	-11	38.22	2.10
5	-6	-11	34.37	1.62
5	-6	-11	35.93	1.62
-5	6	11	37.08	1.69
-5	6	11	35.82	1.63
-5	6	11	33.00	1.98
-5	6	11	28.65	2.15
4	-6	-11	1.97	0.80
4	-6	-11	1.82	0.39
4	-6	-11	3.22	1.03
-4	6	11	1.18	0.61
-4	6	11	1.85	0.38
-4	6	11	2.44	0.41
-4	6	11	2.78	0.83
3	-6	-11	14.84	1.78
3	-6	-11	16.46	0.97
3	-6	-11	19.76	1.61
-3	6	11	17.12	1.55
-3	6	11	15.66	1.38
-3	6	11	17.49	1.03

-3	6	11	16.76	0.90
2	-6	-11	1.12	0.88
2	-6	-11	1.96	0.75
2	-6	-11	1.16	0.32
-2	6	11	1.56	0.25
-2	6	11	1.60	0.27
-2	6	11	1.85	0.39
-2	6	11	2.33	0.72
-2	6	11	1.33	0.67
1	-6	-11	36.00	2.31
1	-6	-11	33.02	1.61
1	-6	-11	34.10	2.15
-1	6	11	34.39	2.08
-1	6	11	35.60	1.67
-1	6	11	32.76	2.03
0	-6	-11	3.78	0.91
0	-6	-11	4.93	1.07
0	-6	-11	3.90	0.60
0	6	11	4.15	0.85
0	6	11	2.63	0.79
0	6	11	4.41	0.66
-1	-6	-11	212.03	8.30
-1	-6	-11	197.38	8.31
-1	-6	-11	199.33	7.78
1	6	11	191.23	8.09
1	6	11	204.03	8.22
1	6	11	205.20	7.81
-2	-6	-11	1.43	0.88
-2	-6	-11	2.41	0.88
-2	-6	-11	0.78	0.36
2	6	11	1.76	0.79
2	6	11	0.79	0.60
2	6	11	1.80	0.42
-3	-6	-11	60.11	2.64
-3	-6	-11	61.20	3.24
-3	-6	-11	64.55	3.30
-3	-6	-11	62.03	2.62
3	6	11	59.57	3.09
3	6	11	60.44	2.93
3	6	11	59.31	2.66
-4	-6	-11	8.84	1.57
-4	-6	-11	8.74	1.56
-4	-6	-11	9.37	0.83
-4	-6	-11	10.32	0.81

4	6	11	9.87	1.41
4	6	11	9.87	1.17
-5	-6	-11	4.64	0.70
-5	-6	-11	6.31	1.16
5	6	11	5.40	1.03
5	6	11	5.62	1.05
-6	-6	-11	11.59	0.94
-6	-6	-11	12.24	1.74
6	6	11	9.85	1.46
6	6	11	11.53	1.20
-7	-6	-11	43.93	2.13
-7	-6	-11	44.34	2.74
7	6	11	47.55	2.31
7	6	11	40.52	2.67
-8	-6	-11	7.37	1.31
8	6	11	4.22	0.81
8	6	11	3.85	1.14
-9	-6	-11	85.22	4.24
-9	-6	-11	90.12	3.54
-9	-6	-11	90.71	3.83
-9	-6	-11	84.47	3.47
9	6	11	91.78	3.87
9	6	11	88.73	4.39
-10	-6	-11	1.16	0.49
-10	-6	-11	0.75	0.55
-10	-6	-11	0.10	1.21
-10	-6	-11	0.52	0.51
10	6	11	0.16	1.05
10	6	11	1.39	0.70
-11	-6	-11	16.24	1.39
-11	-6	-11	12.85	1.28
-11	-6	-11	17.69	1.25
-11	-6	-11	14.59	2.14
11	6	11	16.01	1.45
11	6	11	16.84	1.97
-12	-6	-11	3.59	0.76
-12	-6	-11	3.69	0.77
12	6	11	4.19	0.91
12	6	11	1.75	1.18
-13	-6	-11	41.75	2.34
-13	-6	-11	46.07	2.47
13	6	11	45.24	3.18
-14	-6	-11	4.37	1.06
-14	-6	-11	5.69	0.99

14	6	11	7.00	1.58
-15	-6	-11	54.33	2.94
15	6	11	51.72	3.65
-16	-6	-11	1.85	0.95
16	6	11	1.64	1.66
19	-6	-10	2.87	0.75
-19	6	10	1.14	2.01
-19	6	10	1.37	1.50
18	-6	-10	0.47	0.67
-18	6	10	1.43	1.93
17	-6	-10	13.41	1.26
-17	6	10	13.84	2.24
-17	6	10	17.37	3.05
16	-6	-10	0.71	0.55
-16	6	10	2.09	1.22
-16	6	10	2.70	1.60
15	-6	-10	40.46	1.99
-15	6	10	42.87	2.72
-15	6	10	36.79	3.22
14	-6	-10	6.61	0.89
-14	6	10	5.38	1.13
-14	6	10	6.63	1.57
13	-6	-10	18.21	1.18
-13	6	10	16.43	2.63
-13	6	10	19.93	1.71
12	-6	-10	7.73	0.85
12	-6	-10	5.04	0.95
12	-6	-10	6.55	0.78
-12	6	10	8.84	1.30
-12	6	10	7.43	1.39
11	-6	-10	7.40	1.19
11	-6	-10	8.80	0.98
11	-6	-10	9.02	0.79
-11	6	10	10.24	1.82
-11	6	10	12.31	1.25
10	-6	-10	3.37	0.71
10	-6	-10	3.00	0.47
10	-6	-10	3.03	0.69
-10	6	10	1.86	1.05
-10	6	10	3.08	0.69
9	-6	-10	10.59	0.84
9	-6	-10	13.28	1.17
9	-6	-10	12.32	1.32
9	-6	-10	11.13	1.10

-9	6	10	9.66	1.69
-9	6	10	13.62	1.15
8	-6	-10	6.23	0.64
8	-6	-10	5.51	0.86
8	-6	-10	7.05	1.37
8	-6	-10	6.11	0.78
8	-6	-10	7.32	0.89
-8	6	10	5.65	1.09
-8	6	10	6.02	0.91
-8	6	10	5.76	0.75
7	-6	-10	21.03	2.20
7	-6	-10	23.93	1.18
7	-6	-10	23.08	1.49
7	-6	-10	21.19	1.67
7	-6	-10	23.76	1.32
-7	6	10	19.74	1.83
-7	6	10	20.91	1.24
-7	6	10	22.81	1.32
6	-6	-10	6.55	1.22
6	-6	-10	4.94	1.09
6	-6	-10	7.01	0.76
6	-6	-10	5.51	0.59
6	-6	-10	5.77	1.03
-6	6	10	5.61	0.71
-6	6	10	7.14	1.27
-6	6	10	5.12	0.69
5	-6	-10	5.32	0.65
5	-6	-10	6.16	1.12
5	-6	-10	4.32	0.54
5	-6	-10	5.66	1.05
-5	6	10	4.72	0.76
-5	6	10	4.57	0.64
-5	6	10	4.42	0.63
-5	6	10	5.30	0.97
4	-6	-10	4.41	1.04
4	-6	-10	2.88	0.41
4	-6	-10	3.37	0.74
4	-6	-10	3.50	0.65
-4	6	10	3.32	0.70
-4	6	10	3.23	0.46
-4	6	10	3.04	0.76
-4	6	10	3.87	0.43
3	-6	-10	9.72	1.18
3	-6	-10	8.49	0.60

3	-6	-10	9.29	1.39
3	-6	-10	8.13	0.67
-3	6	10	6.79	1.01
-3	6	10	8.50	0.72
-3	6	10	7.88	1.16
-3	6	10	8.24	0.64
2	-6	-10	16.44	0.96
2	-6	-10	18.50	0.84
2	-6	-10	18.77	1.46
2	-6	-10	15.05	1.61
-2	6	10	17.61	0.84
-2	6	10	18.05	1.00
-2	6	10	17.33	1.44
-2	6	10	17.68	1.38
1	-6	-10	14.65	0.89
1	-6	-10	14.96	1.40
1	-6	-10	14.18	1.54
-1	6	10	14.50	1.27
-1	6	10	15.37	1.31
-1	6	10	15.37	0.93
0	-6	-10	31.10	2.00
0	-6	-10	29.41	1.91
0	-6	-10	26.22	1.36
0	6	10	29.34	1.41
0	6	10	26.14	1.73
0	6	10	27.15	1.79
-1	-6	-10	57.89	2.54
-1	-6	-10	64.09	3.05
-1	-6	-10	59.93	3.07
1	6	10	58.71	2.87
1	6	10	61.35	2.57
1	6	10	58.19	2.96
-2	-6	-10	7.54	1.27
-2	-6	-10	8.65	1.32
-2	-6	-10	6.75	0.67
2	6	10	8.36	0.72
2	6	10	7.20	1.13
2	6	10	8.28	1.04
-3	-6	-10	7.01	0.67
-3	-6	-10	7.35	1.34
-3	-6	-10	8.29	1.36
-3	-6	-10	6.85	0.69
3	6	10	5.48	0.97
3	6	10	5.60	0.91

3	6	10	7.21	0.71
-4	-6	-10	27.88	2.00
-4	-6	-10	25.45	1.34
-4	-6	-10	26.62	1.39
-4	-6	-10	27.18	1.93
4	6	10	27.81	1.66
4	6	10	24.48	1.78
-5	-6	-10	7.52	1.36
-5	-6	-10	7.30	0.74
-5	-6	-10	6.92	1.15
-5	-6	-10	6.36	0.67
5	6	10	5.69	0.96
5	6	10	6.36	0.98
-6	-6	-10	37.93	2.38
-6	-6	-10	38.62	2.68
-6	-6	-10	38.96	1.87
-6	-6	-10	39.11	1.90
6	6	10	37.16	2.02
6	6	10	37.97	2.34
-7	-6	-10	19.19	1.78
-7	-6	-10	17.65	1.18
7	6	10	18.02	1.77
7	6	10	19.07	1.38
-8	-6	-10	25.37	2.03
-8	-6	-10	27.74	1.49
-8	-6	-10	24.30	1.18
8	6	10	26.56	1.97
8	6	10	26.61	1.60
-9	-6	-10	8.65	1.61
-9	-6	-10	6.76	0.73
-9	-6	-10	7.88	0.81
9	6	10	8.16	1.47
9	6	10	6.74	1.04
-10	-6	-10	33.08	1.65
-10	-6	-10	35.13	1.90
-10	-6	-10	37.87	1.78
-10	-6	-10	33.93	2.38
10	6	10	36.91	1.97
-11	-6	-10	2.42	0.59
-11	-6	-10	3.28	0.70
-11	-6	-10	2.90	1.15
-11	-6	-10	3.98	0.61
11	6	10	1.59	0.73
11	6	10	2.57	1.05

-12	-6	-10	24.28	1.51
-12	-6	-10	23.77	1.68
-12	-6	-10	23.75	1.63
12	6	10	28.75	2.41
-13	-6	-10	0.02	0.65
-13	-6	-10	-0.11	0.95
-13	-6	-10	0.45	0.82
13	6	10	2.22	1.21
-14	-6	-10	16.14	1.75
-14	-6	-10	16.81	1.54
14	6	10	11.77	2.24
-15	-6	-10	0.01	1.06
-15	-6	-10	0.04	0.79
15	6	10	1.99	1.49
-16	-6	-10	40.40	2.55
16	6	10	41.64	3.35
-17	-6	-10	4.89	1.06
17	6	10	5.39	1.72
19	-6	-9	7.56	0.99
-19	6	9	4.47	1.58
-19	6	9	6.91	2.05
18	-6	-9	11.41	1.28
-18	6	9	10.62	2.13
-18	6	9	8.45	1.60
17	-6	-9	9.61	1.10
-17	6	9	8.41	1.46
-17	6	9	6.20	1.84
16	-6	-9	20.89	1.39
-16	6	9	24.26	2.74
-16	6	9	22.86	2.27
15	-6	-9	14.81	1.14
-15	6	9	18.24	2.41
-15	6	9	14.86	1.91
14	-6	-9	28.31	1.56
-14	6	9	31.12	2.18
-14	6	9	29.11	2.68
13	-6	-9	19.37	1.16
-13	6	9	18.43	2.27
-13	6	9	18.02	1.86
12	-6	-9	7.87	0.89
12	-6	-9	8.02	0.87
-12	6	9	9.30	1.31
-12	6	9	11.25	1.88
11	-6	-9	5.85	1.44

11	-6	-9	5.91	0.87
11	-6	-9	5.66	0.65
11	-6	-9	7.43	1.04
-11	6	9	5.94	1.25
-11	6	9	7.97	1.16
10	-6	-9	57.65	2.42
10	-6	-9	53.68	2.64
10	-6	-9	60.22	2.52
10	-6	-9	56.50	3.48
10	-6	-9	55.75	2.59
-10	6	9	53.59	3.16
-10	6	9	56.74	2.70
9	-6	-9	29.18	2.45
9	-6	-9	28.64	1.65
9	-6	-9	26.43	1.55
9	-6	-9	28.63	1.34
-9	6	9	22.86	2.03
-9	6	9	26.44	1.42
-9	6	9	26.49	1.59
8	-6	-9	40.81	1.94
8	-6	-9	35.93	1.70
8	-6	-9	40.09	2.08
8	-6	-9	37.77	2.62
8	-6	-9	38.29	1.89
-8	6	9	36.53	2.42
-8	6	9	36.21	1.92
-8	6	9	39.58	1.80
7	-6	-9	2.25	0.99
7	-6	-9	3.42	0.50
7	-6	-9	3.81	0.66
7	-6	-9	3.06	0.54
7	-6	-9	2.76	0.74
-7	6	9	3.13	0.53
-7	6	9	2.74	0.86
-7	6	9	2.81	0.44
6	-6	-9	19.67	1.54
6	-6	-9	18.93	1.03
6	-6	-9	22.94	1.39
6	-6	-9	19.36	1.15
6	-6	-9	21.22	1.86
-6	6	9	18.52	1.59
-6	6	9	20.00	1.09
-6	6	9	17.40	1.12
5	-6	-9	17.25	1.26

5	-6	-9	14.57	0.88
5	-6	-9	16.68	1.61
5	-6	-9	15.97	0.95
5	-6	-9	14.73	1.43
-5	6	9	15.34	0.93
-5	6	9	17.45	0.96
-5	6	9	15.19	1.43
4	-6	-9	51.18	2.45
4	-6	-9	54.80	2.90
4	-6	-9	57.97	2.65
4	-6	-9	58.28	2.32
-4	6	9	52.89	2.77
-4	6	9	51.77	2.27
-4	6	9	59.35	2.36
-4	6	9	54.00	2.60
3	-6	-9	2.80	0.81
3	-6	-9	1.50	0.31
3	-6	-9	1.10	0.55
3	-6	-9	1.63	0.34
-3	6	9	1.99	0.62
-3	6	9	1.19	0.56
-3	6	9	1.27	0.32
-3	6	9	1.60	0.31
2	-6	-9	47.74	1.94
2	-6	-9	46.40	2.04
2	-6	-9	53.20	2.64
2	-6	-9	52.78	2.46
-2	6	9	45.19	2.35
-2	6	9	44.87	1.92
-2	6	9	49.09	2.08
-2	6	9	45.22	2.44
1	-6	-9	7.76	1.26
1	-6	-9	6.85	0.43
1	-6	-9	8.82	1.13
1	-6	-9	8.37	0.64
-1	6	9	7.74	0.45
-1	6	9	7.61	0.99
-1	6	9	7.46	0.96
-1	6	9	8.49	0.66
0	-6	-9	101.33	4.39
0	-6	-9	101.77	4.32
0	-6	-9	94.91	3.90
0	6	9	96.55	4.22
0	6	9	96.99	3.92

0	6	9	96.91	4.26
-1	-6	-9	6.59	0.60
-1	-6	-9	7.83	1.20
-1	-6	-9	7.45	1.18
1	6	9	7.13	0.65
1	6	9	7.34	0.96
1	6	9	7.69	1.02
-2	-6	-9	73.62	3.04
-2	-6	-9	78.12	3.51
-2	-6	-9	74.54	3.47
2	6	9	77.23	3.07
2	6	9	69.33	3.41
2	6	9	70.19	3.29
-3	-6	-9	10.37	0.78
-3	-6	-9	9.54	1.35
-3	-6	-9	9.87	1.27
3	6	9	11.68	0.81
3	6	9	12.18	1.21
3	6	9	11.45	1.04
-4	-6	-9	5.77	0.92
-4	-6	-9	6.73	1.24
-4	-6	-9	4.76	0.62
-4	-6	-9	4.64	0.59
4	6	9	6.17	0.96
4	6	9	5.59	0.87
-5	-6	-9	0.93	0.79
-5	-6	-9	1.08	0.37
-5	-6	-9	1.27	0.69
-5	-6	-9	0.47	0.30
5	6	9	0.70	0.52
5	6	9	0.56	0.62
-6	-6	-9	36.10	1.77
-6	-6	-9	36.69	2.18
-6	-6	-9	36.40	1.72
-6	-6	-9	40.28	2.40
6	6	9	34.79	2.18
6	6	9	39.01	1.96
-7	-6	-9	21.04	1.00
-7	-6	-9	19.70	1.19
-7	-6	-9	18.80	1.84
-7	-6	-9	19.49	0.95
-7	-6	-9	22.14	1.68
-7	-6	-9	17.13	1.10
7	6	9	18.92	1.63

7	6	9	21.93	1.38
-8	-6	-9	8.19	0.87
-8	-6	-9	8.29	0.66
-8	-6	-9	8.75	0.79
-8	-6	-9	8.12	1.39
8	6	9	9.17	1.03
8	6	9	9.78	1.41
-9	-6	-9	2.91	0.45
-9	-6	-9	3.00	0.54
-9	-6	-9	2.86	0.93
-9	-6	-9	3.33	0.53
9	6	9	3.04	0.66
9	6	9	2.73	0.90
-10	-6	-9	12.99	1.08
-10	-6	-9	15.51	0.99
-10	-6	-9	12.07	1.61
10	6	9	14.15	1.26
10	6	9	14.12	1.68
-11	-6	-9	0.45	0.53
-11	-6	-9	-0.06	0.92
-11	-6	-9	0.39	0.50
-11	-6	-9	0.38	0.60
11	6	9	1.40	1.05
11	6	9	0.75	0.65
-12	-6	-9	1.84	1.05
-12	-6	-9	2.29	0.69
-12	-6	-9	1.67	0.93
-12	-6	-9	2.17	0.65
-12	-6	-9	2.41	0.73
12	6	9	3.35	1.12
-13	-6	-9	4.64	1.07
-13	-6	-9	2.36	0.77
-13	-6	-9	3.71	0.81
-13	-6	-9	5.11	0.93
13	6	9	5.11	1.26
-14	-6	-9	18.37	1.80
-14	-6	-9	22.90	1.67
14	6	9	21.21	2.35
-15	-6	-9	11.83	1.87
-15	-6	-9	14.22	1.53
15	6	9	12.27	2.22
-16	-6	-9	5.89	1.16
-16	-6	-9	5.38	1.29
16	6	9	8.12	1.72

-17	-6	-9	10.57	1.53
-17	-6	-9	6.58	1.22
17	6	9	14.62	2.71
19	-6	-8	3.00	0.80
-19	6	8	6.18	1.63
-19	6	8	0.79	1.79
18	-6	-8	54.14	2.59
-18	6	8	49.69	3.77
-18	6	8	57.14	3.50
17	-6	-8	3.71	0.67
-17	6	8	5.23	1.73
-17	6	8	3.88	1.25
16	-6	-8	55.26	2.58
-16	6	8	57.39	3.22
15	-6	-8	20.81	1.35
-15	6	8	24.85	2.47
-15	6	8	22.65	2.07
14	-6	-8	35.11	1.73
-14	6	8	33.56	2.73
-14	6	8	35.97	2.32
13	-6	-8	22.06	1.14
13	-6	-8	21.65	1.27
13	-6	-8	24.11	1.16
-13	6	8	20.51	2.29
12	-6	-8	113.56	4.51
12	-6	-8	115.92	4.45
12	-6	-8	110.03	5.60
-12	6	8	101.72	5.21
-12	6	8	121.18	4.91
11	-6	-8	12.52	2.21
11	-6	-8	10.09	1.20
11	-6	-8	13.10	1.13
11	-6	-8	12.59	0.87
11	-6	-8	15.27	1.01
-11	6	8	16.04	1.82
-11	6	8	12.95	1.31
10	-6	-8	64.64	2.95
10	-6	-8	70.18	2.85
10	-6	-8	67.52	3.66
10	-6	-8	64.64	2.75
10	-6	-8	62.43	2.91
-10	6	8	61.16	3.45
-10	6	8	70.01	3.09
-10	6	8	69.74	3.22

9	-6	-8	16.40	1.17
9	-6	-8	16.75	0.96
9	-6	-8	15.60	1.89
9	-6	-8	17.59	1.19
9	-6	-8	17.26	1.25
-9	6	8	17.67	1.08
-9	6	8	14.73	1.22
-9	6	8	17.75	1.71
8	-6	-8	34.30	2.37
8	-6	-8	28.18	2.16
8	-6	-8	34.36	1.85
8	-6	-8	36.01	1.72
8	-6	-8	35.10	1.56
-8	6	8	32.31	2.17
-8	6	8	35.74	1.78
-8	6	8	33.21	1.60
7	-6	-8	57.20	2.45
7	-6	-8	52.66	2.22
7	-6	-8	52.24	2.89
7	-6	-8	51.38	2.56
7	-6	-8	53.36	2.34
-7	6	8	46.51	2.74
-7	6	8	51.19	2.26
-7	6	8	55.71	2.37
6	-6	-8	52.39	2.61
6	-6	-8	50.09	2.82
6	-6	-8	52.52	2.30
6	-6	-8	57.21	2.48
6	-6	-8	52.63	2.21
-6	6	8	49.93	2.29
-6	6	8	52.75	2.74
-6	6	8	54.33	2.26
5	-6	-8	15.06	0.91
5	-6	-8	14.73	1.16
5	-6	-8	16.96	1.49
5	-6	-8	15.28	0.85
5	-6	-8	15.09	1.33
-5	6	8	15.58	1.35
-5	6	8	16.23	0.93
-5	6	8	14.32	0.87
4	-6	-8	60.89	2.57
4	-6	-8	63.98	3.10
4	-6	-8	65.53	3.08
4	-6	-8	60.16	2.67

4	-6	-8	66.05	2.87
-4	6	8	60.38	2.84
-4	6	8	59.86	2.99
-4	6	8	65.08	2.61
-4	6	8	62.96	2.56
3	-6	-8	103.39	4.06
3	-6	-8	102.08	4.02
3	-6	-8	114.74	4.40
3	-6	-8	114.46	4.60
-3	6	8	101.22	4.33
-3	6	8	96.67	4.00
-3	6	8	95.60	4.42
-3	6	8	99.56	4.08
2	-6	-8	49.11	2.05
2	-6	-8	52.77	2.41
2	-6	-8	45.48	1.96
2	-6	-8	48.98	2.52
-2	6	8	49.63	1.96
-2	6	8	47.84	2.06
-2	6	8	47.04	2.41
-2	6	8	47.76	2.36
1	-6	-8	37.19	2.07
1	-6	-8	38.67	1.98
1	-6	-8	37.98	1.62
1	-6	-8	33.60	1.48
-1	6	8	34.74	1.48
-1	6	8	35.39	1.94
-1	6	8	37.47	1.97
-1	6	8	38.56	1.64
0	-6	-8	58.00	2.77
0	-6	-8	57.21	2.40
0	-6	-8	64.33	2.86
0	6	8	59.61	2.43
0	6	8	51.95	2.66
0	6	8	55.81	2.71
-1	-6	-8	74.20	3.19
-1	-6	-8	64.64	2.73
-1	-6	-8	68.17	3.13
1	6	8	68.78	2.76
1	6	8	61.81	2.98
1	6	8	63.45	3.08
-2	-6	-8	18.99	1.41
-2	-6	-8	18.76	1.44
-2	-6	-8	17.91	0.99

2	6	8	20.46	1.28
2	6	8	17.39	1.35
2	6	8	18.90	1.02
-3	-6	-8	19.08	1.43
-3	-6	-8	17.79	1.01
-3	-6	-8	20.36	1.51
3	6	8	19.50	1.05
3	6	8	17.40	1.24
3	6	8	17.67	1.41
-4	-6	-8	5.47	0.55
-4	-6	-8	5.72	0.84
-4	-6	-8	5.30	0.91
-4	-6	-8	4.98	0.57
4	6	8	4.33	0.76
4	6	8	4.35	0.81
-5	-6	-8	60.83	3.00
-5	-6	-8	64.55	2.86
-5	-6	-8	53.67	2.50
-5	-6	-8	58.34	2.46
5	6	8	60.86	2.70
5	6	8	56.05	2.90
-6	-6	-8	27.83	1.70
-6	-6	-8	25.27	1.89
-6	-6	-8	28.83	1.29
-6	-6	-8	22.57	1.12
-6	-6	-8	25.09	1.32
-6	-6	-8	25.10	1.12
6	6	8	26.00	1.50
6	6	8	24.12	1.74
-7	-6	-8	31.45	1.40
-7	-6	-8	30.92	1.60
-7	-6	-8	37.16	1.68
-7	-6	-8	32.76	1.94
-7	-6	-8	37.40	2.19
-7	-6	-8	29.53	1.53
7	6	8	30.87	1.71
7	6	8	29.36	2.02
-8	-6	-8	5.33	0.91
-8	-6	-8	4.92	0.72
-8	-6	-8	5.28	0.60
-8	-6	-8	4.51	0.65
-8	-6	-8	5.77	0.66
-8	-6	-8	6.51	1.14
8	6	8	4.52	0.93

8	6	8	4.73	0.88
-9	-6	-8	5.42	1.19
-9	-6	-8	5.22	0.66
-9	-6	-8	7.14	0.80
-9	-6	-8	4.80	0.75
-9	-6	-8	4.97	0.91
9	6	8	4.03	0.95
9	6	8	6.66	0.94
-10	-6	-8	0.29	0.49
-10	-6	-8	-0.05	0.78
-10	-6	-8	1.63	0.51
-10	-6	-8	0.68	0.44
10	6	8	-0.10	0.73
10	6	8	0.17	0.60
-11	-6	-8	22.60	1.77
-11	-6	-8	23.90	1.53
-11	-6	-8	21.85	1.44
-11	-6	-8	24.22	1.38
11	6	8	23.21	1.99
11	6	8	23.75	1.54
-12	-6	-8	8.77	1.11
-12	-6	-8	9.91	1.49
-12	-6	-8	10.49	1.26
-12	-6	-8	8.84	1.51
-12	-6	-8	12.62	1.18
12	6	8	10.38	1.58
-13	-6	-8	39.13	2.24
-13	-6	-8	41.36	2.25
-13	-6	-8	42.33	2.15
-13	-6	-8	42.92	2.46
-13	-6	-8	40.88	2.44
13	6	8	39.84	2.93
-14	-6	-8	-0.03	0.69
-14	-6	-8	0.97	0.85
-14	-6	-8	-0.24	1.07
-14	-6	-8	-1.62	0.78
14	6	8	1.14	1.12
-15	-6	-8	41.24	2.64
-15	-6	-8	43.74	2.50
15	6	8	46.82	3.00
-16	-6	-8	0.17	0.79
-16	-6	-8	-0.47	1.09
16	6	8	1.58	1.36
-17	-6	-8	14.72	1.72

-17	-6	-8	14.65	2.19
17	6	8	15.55	2.61
-18	-6	-8	2.69	1.06
-18	-6	-8	2.92	1.46
18	6	8	3.55	1.69
20	-6	-7	4.94	0.90
19	-6	-7	25.59	1.67
-19	6	7	28.02	2.85
-19	6	7	30.33	3.14
18	-6	-7	0.19	0.57
-18	6	7	1.72	1.28
17	-6	-7	50.39	2.50
-17	6	7	50.21	3.50
-17	6	7	61.63	3.39
16	-6	-7	0.63	0.53
-16	6	7	0.22	1.28
-16	6	7	-0.81	0.98
15	-6	-7	97.05	4.02
-15	6	7	97.74	4.88
14	-6	-7	5.82	2.17
14	-6	-7	4.36	0.62
-14	6	7	4.91	1.00
-14	6	7	3.74	1.33
13	-6	-7	53.41	2.53
13	-6	-7	54.82	3.69
13	-6	-7	55.52	2.34
13	-6	-7	56.96	2.35
-13	6	7	56.03	2.95
-13	6	7	55.68	3.26
12	-6	-7	4.88	0.77
12	-6	-7	3.91	0.60
12	-6	-7	4.21	1.39
-12	6	7	4.86	0.89
-12	6	7	3.31	1.10
11	-6	-7	101.33	4.28
11	-6	-7	107.81	4.09
11	-6	-7	98.83	4.05
11	-6	-7	102.20	4.12
11	-6	-7	101.22	4.89
-11	6	7	105.31	4.40
-11	6	7	92.92	4.68
10	-6	-7	6.74	1.32
10	-6	-7	6.89	0.64
10	-6	-7	7.89	0.95

10	-6	-7	7.90	0.87
10	-6	-7	5.53	0.96
-10	6	7	8.13	0.77
-10	6	7	8.13	1.05
-10	6	7	6.57	1.14
9	-6	-7	145.54	5.91
9	-6	-7	155.12	6.45
9	-6	-7	149.30	5.94
9	-6	-7	144.91	5.76
9	-6	-7	154.17	5.87
-9	6	7	136.05	6.28
-9	6	7	154.75	5.84
-9	6	7	150.78	5.99
8	-6	-7	0.35	0.27
8	-6	-7	0.43	0.48
8	-6	-7	0.39	0.77
8	-6	-7	0.24	0.50
8	-6	-7	1.18	0.45
-8	6	7	0.41	0.63
-8	6	7	0.76	0.33
-8	6	7	0.97	0.44
7	-6	-7	61.67	2.64
7	-6	-7	59.25	3.08
7	-6	-7	62.93	2.80
7	-6	-7	60.81	2.50
-7	6	7	58.78	3.00
-7	6	7	58.02	2.63
-7	6	7	64.30	2.56
6	-6	-7	3.62	0.65
6	-6	-7	3.73	0.45
6	-6	-7	4.53	0.76
6	-6	-7	3.61	0.50
6	-6	-7	3.61	0.83
-6	6	7	4.25	0.52
-6	6	7	4.52	0.63
-6	6	7	3.46	0.74
5	-6	-7	94.51	3.84
5	-6	-7	98.26	4.29
5	-6	-7	98.31	4.02
5	-6	-7	97.55	3.80
5	-6	-7	97.49	4.19
-5	6	7	97.64	3.85
-5	6	7	95.93	3.82
-5	6	7	91.50	4.20

4	-6	-7	16.37	1.01
4	-6	-7	18.75	1.19
4	-6	-7	18.00	1.38
4	-6	-7	16.50	1.40
4	-6	-7	17.46	0.88
-4	6	7	17.35	0.91
-4	6	7	16.34	0.91
-4	6	7	16.14	1.27
3	-6	-7	196.08	7.90
3	-6	-7	194.28	7.42
3	-6	-7	207.34	7.74
3	-6	-7	197.07	7.86
3	-6	-7	187.40	7.44
-3	6	7	204.23	7.43
-3	6	7	190.15	7.80
-3	6	7	188.03	7.69
2	-6	-7	35.01	1.52
2	-6	-7	36.19	1.98
2	-6	-7	37.20	1.86
2	-6	-7	36.28	1.47
-2	6	7	33.34	1.47
-2	6	7	31.74	1.83
-2	6	7	33.34	1.81
-2	6	7	35.86	1.55
1	-6	-7	83.57	3.33
1	-6	-7	84.12	3.71
1	-6	-7	91.07	3.67
1	-6	-7	81.86	3.22
-1	6	7	82.26	3.22
-1	6	7	80.97	3.60
-1	6	7	80.12	3.61
-1	6	7	86.43	3.35
0	-6	-7	33.39	1.81
0	-6	-7	30.47	1.42
0	-6	-7	33.84	1.78
0	-6	-7	32.64	1.28
0	6	7	33.39	1.45
0	6	7	29.36	1.68
0	6	7	30.74	1.28
0	6	7	30.97	1.75
-1	-6	-7	160.48	6.10
-1	-6	-7	143.29	5.71
-1	-6	-7	148.04	6.03
1	6	7	142.30	5.94

1	6	7	146.43	6.03
-2	-6	-7	9.81	1.09
-2	-6	-7	10.82	1.04
-2	-6	-7	9.68	0.67
2	6	7	11.43	1.03
2	6	7	10.22	0.92
2	6	7	11.60	0.71
-3	-6	-7	204.89	8.02
-3	-6	-7	216.12	8.41
-3	-6	-7	220.46	8.32
3	6	7	199.65	8.35
3	6	7	200.07	8.20
3	6	7	219.17	8.05
-4	-6	-7	1.06	0.19
-4	-6	-7	0.44	0.25
-4	-6	-7	0.33	0.25
-4	-6	-7	2.02	0.65
-4	-6	-7	0.60	0.19
-4	-6	-7	0.48	0.49
4	6	7	0.89	0.43
4	6	7	1.33	0.51
-5	-6	-7	61.81	2.55
-5	-6	-7	65.74	2.87
-5	-6	-7	57.65	2.43
-5	-6	-7	62.96	3.04
-5	-6	-7	62.28	2.61
-5	-6	-7	61.50	2.42
5	6	7	62.35	2.76
5	6	7	61.38	3.00
-6	-6	-7	43.74	1.86
-6	-6	-7	49.14	2.30
-6	-6	-7	43.19	1.95
-6	-6	-7	42.79	1.88
-6	-6	-7	46.34	2.04
-6	-6	-7	50.12	2.53
6	6	7	47.76	2.18
6	6	7	43.13	2.39
-7	-6	-7	38.12	1.68
-7	-6	-7	40.30	1.85
-7	-6	-7	43.38	2.33
-7	-6	-7	36.28	1.74
-7	-6	-7	43.71	2.10
-7	-6	-7	39.17	1.76
7	6	7	39.84	1.96

7	6	7	37.32	2.25
-8	-6	-7	24.54	1.33
-8	-6	-7	25.10	1.21
-8	-6	-7	22.34	1.25
-8	-6	-7	22.94	1.22
-8	-6	-7	23.92	1.82
-8	-6	-7	26.68	1.61
8	6	7	26.17	1.46
8	6	7	23.21	1.72
-9	-6	-7	67.92	3.23
-9	-6	-7	54.83	2.58
-9	-6	-7	59.50	2.67
-9	-6	-7	62.51	2.88
-9	-6	-7	61.22	2.54
-9	-6	-7	58.72	2.57
9	6	7	61.49	2.74
9	6	7	59.02	3.10
-10	-6	-7	4.67	0.79
-10	-6	-7	6.42	0.81
-10	-6	-7	6.62	0.92
-10	-6	-7	4.80	1.11
-10	-6	-7	5.65	0.72
10	6	7	5.27	0.95
10	6	7	6.59	1.07
-11	-6	-7	20.09	1.40
-11	-6	-7	21.94	1.40
-11	-6	-7	20.75	1.66
-11	-6	-7	20.84	1.61
-11	-6	-7	21.80	1.31
11	6	7	20.06	1.83
-12	-6	-7	8.19	0.96
-12	-6	-7	5.94	1.03
-12	-6	-7	6.15	0.76
-12	-6	-7	5.08	0.85
-12	-6	-7	5.97	0.96
12	6	7	4.97	1.02
-13	-6	-7	17.17	1.39
-13	-6	-7	14.25	1.48
-13	-6	-7	17.02	1.79
-13	-6	-7	14.88	1.34
-13	-6	-7	14.20	1.59
13	6	7	18.36	1.85
13	6	7	16.36	1.23
-14	-6	-7	32.22	2.31

-14	-6	-7	35.91	2.04
-14	-6	-7	33.35	2.13
-14	-6	-7	33.51	2.03
14	6	7	36.12	1.94
-15	-6	-7	36.64	2.33
-15	-6	-7	38.28	3.14
-15	-6	-7	33.81	2.16
15	6	7	36.73	2.64
15	6	7	38.45	2.09
-16	-6	-7	8.95	1.55
-16	-6	-7	13.10	1.54
16	6	7	12.16	2.13
16	6	7	9.51	1.28
-17	-6	-7	7.52	1.38
-17	-6	-7	6.84	1.13
17	6	7	8.26	1.69
17	6	7	11.06	1.37
-18	-6	-7	15.85	1.86
-18	-6	-7	10.17	1.70
18	6	7	10.52	1.95
18	6	7	15.44	1.61
20	-6	-6	21.39	1.62
-20	6	6	22.73	3.25
19	-6	-6	6.66	0.85
-19	6	6	5.67	2.46
-19	6	6	6.10	1.60
18	-6	-6	35.11	1.94
-18	6	6	41.78	2.93
-18	6	6	34.69	3.07
17	-6	-6	20.89	1.40
-17	6	6	23.05	2.36
-17	6	6	19.34	2.52
16	-6	-6	37.81	4.34
16	-6	-6	32.77	1.84
-16	6	6	39.05	2.59
-16	6	6	37.38	2.84
15	-6	-6	30.58	1.56
15	-6	-6	28.95	3.19
-15	6	6	25.15	2.38
-15	6	6	27.95	2.20
14	-6	-6	68.48	4.13
14	-6	-6	69.51	2.83
14	-6	-6	67.51	2.84
14	-6	-6	67.48	2.93

-14	6	6	69.36	3.68
-14	6	6	71.45	3.45
13	-6	-6	19.72	2.52
13	-6	-6	23.23	1.26
-13	6	6	24.34	1.75
-13	6	6	17.47	1.99
12	-6	-6	133.81	5.39
12	-6	-6	135.27	5.40
12	-6	-6	141.56	6.22
12	-6	-6	137.41	5.40
-12	6	6	126.21	5.95
-12	6	6	152.23	5.82
11	-6	-6	4.23	0.58
11	-6	-6	3.34	0.64
11	-6	-6	4.33	0.80
11	-6	-6	5.79	0.95
11	-6	-6	5.07	1.19
-11	6	6	4.37	1.05
-11	6	6	4.08	0.77
10	-6	-6	76.12	3.29
10	-6	-6	82.64	3.28
10	-6	-6	74.89	3.37
10	-6	-6	72.06	3.15
10	-6	-6	83.81	3.91
-10	6	6	82.83	3.27
-10	6	6	74.12	3.70
-10	6	6	80.02	3.46
9	-6	-6	3.93	0.89
9	-6	-6	1.43	0.56
9	-6	-6	1.41	0.46
9	-6	-6	1.29	0.31
9	-6	-6	0.96	0.48
-9	6	6	2.23	0.75
-9	6	6	1.35	0.51
-9	6	6	2.05	0.40
8	-6	-6	119.33	4.66
8	-6	-6	112.44	5.02
8	-6	-6	111.20	4.65
8	-6	-6	122.35	4.73
8	-6	-6	115.16	4.51
-8	6	6	117.33	4.55
-8	6	6	115.07	4.70
-8	6	6	108.84	4.95
7	-6	-6	36.66	1.74

7	-6	-6	37.59	1.89
7	-6	-6	37.53	1.60
7	-6	-6	37.32	1.81
7	-6	-6	38.50	2.15
-7	6	6	35.84	1.76
-7	6	6	33.02	2.02
-7	6	6	36.07	1.64
6	-6	-6	83.89	4.25
6	-6	-6	92.96	3.61
6	-6	-6	87.97	3.51
6	-6	-6	89.67	3.82
6	-6	-6	91.31	3.99
-6	6	6	90.40	3.94
-6	6	6	86.55	3.61
5	-6	-6	21.60	1.07
5	-6	-6	21.79	1.38
5	-6	-6	21.36	1.27
5	-6	-6	20.34	1.45
5	-6	-6	19.36	0.97
-5	6	6	17.48	1.33
-5	6	6	20.05	1.01
-5	6	6	18.97	1.06
4	-6	-6	113.82	4.94
4	-6	-6	114.28	4.57
4	-6	-6	125.16	4.84
4	-6	-6	125.93	5.00
-4	6	6	114.77	4.60
-4	6	6	115.60	4.59
-4	6	6	118.76	4.94
3	-6	-6	15.53	1.20
3	-6	-6	13.71	0.78
3	-6	-6	15.42	0.79
3	-6	-6	16.34	1.09
3	-6	-6	16.00	1.29
-3	6	6	14.45	0.81
-3	6	6	14.51	0.80
-3	6	6	14.20	1.12
2	-6	-6	180.94	7.48
2	-6	-6	189.55	7.29
2	-6	-6	189.64	6.98
2	-6	-6	179.12	7.03
2	-6	-6	196.13	7.40
-2	6	6	179.87	7.32
-2	6	6	192.44	7.06

-2	6	6	181.10	7.29
-2	6	6	174.77	7.00
1	-6	-6	17.27	0.88
1	-6	-6	19.11	1.25
1	-6	-6	19.32	1.20
1	-6	-6	16.73	0.80
-1	6	6	16.86	0.82
-1	6	6	17.37	1.16
-1	6	6	18.02	1.18
-1	6	6	17.99	0.90
0	-6	-6	97.99	3.87
0	-6	-6	103.63	4.19
0	-6	-6	90.92	3.75
0	-6	-6	98.74	4.16
0	6	6	103.69	4.15
0	6	6	99.11	4.17
0	6	6	98.74	3.77
0	6	6	100.10	3.89
-1	-6	-6	3.06	0.38
-1	-6	-6	2.11	0.50
-1	-6	-6	2.00	0.25
-1	-6	-6	2.92	0.57
1	6	6	2.79	0.26
1	6	6	2.88	0.40
1	6	6	1.95	0.46
1	6	6	2.65	0.55
-2	-6	-6	70.25	2.87
-2	-6	-6	71.01	2.71
-2	-6	-6	78.25	3.23
-2	-6	-6	71.78	3.13
2	6	6	68.76	3.17
2	6	6	73.02	2.88
2	6	6	68.48	2.70
2	6	6	67.60	3.05
-3	-6	-6	41.01	1.66
-3	-6	-6	41.46	1.67
-3	-6	-6	43.83	2.20
-3	-6	-6	44.44	2.09
-3	-6	-6	39.85	1.81
3	6	6	42.79	2.16
3	6	6	43.59	1.84
3	6	6	42.08	2.00
-4	-6	-6	136.89	5.41
-4	-6	-6	120.60	5.03

-4	-6	-6	140.22	5.02
-4	-6	-6	122.56	4.89
-4	-6	-6	126.32	4.89
-4	-6	-6	137.60	5.28
4	6	6	124.71	5.18
4	6	6	125.34	5.36
-5	-6	-6	0.20	0.22
-5	-6	-6	0.98	0.48
-5	-6	-6	0.92	0.28
-5	-6	-6	0.34	0.22
-5	-6	-6	0.51	0.21
-5	-6	-6	1.66	0.56
5	6	6	1.13	0.40
5	6	6	0.40	0.53
-6	-6	-6	99.53	3.75
-6	-6	-6	99.48	4.21
-6	-6	-6	100.21	4.01
-6	-6	-6	93.79	3.81
-6	-6	-6	93.04	3.67
-6	-6	-6	90.85	3.73
6	6	6	90.02	3.91
6	6	6	90.17	4.15
-7	-6	-6	-0.14	0.26
-7	-6	-6	0.94	0.68
-7	-6	-6	0.22	0.32
-7	-6	-6	0.02	0.29
-7	-6	-6	0.08	0.26
-7	-6	-6	0.08	0.50
7	6	6	0.74	0.59
7	6	6	0.14	0.42
-8	-6	-6	60.50	2.76
-8	-6	-6	56.47	2.52
-8	-6	-6	60.02	2.48
-8	-6	-6	54.41	2.56
-8	-6	-6	58.17	2.48
-8	-6	-6	64.02	3.01
8	6	6	65.07	2.71
8	6	6	59.40	2.96
-9	-6	-6	0.27	0.32
-9	-6	-6	-0.14	0.40
-9	-6	-6	0.16	0.34
-9	-6	-6	0.54	0.38
-9	-6	-6	-0.75	0.69
-9	-6	-6	0.41	0.58

9	6	6	0.72	0.69
9	6	6	0.14	0.49
-10	-6	-6	95.75	4.13
-10	-6	-6	97.93	4.18
-10	-6	-6	101.72	4.30
-10	-6	-6	105.38	4.63
-10	-6	-6	98.13	4.07
10	6	6	105.51	4.59
10	6	6	103.31	4.23
-11	-6	-6	3.87	0.80
-11	-6	-6	2.48	0.53
-11	-6	-6	2.88	0.78
-11	-6	-6	2.50	0.56
-11	-6	-6	2.14	1.01
-11	-6	-6	1.90	0.64
11	6	6	1.13	0.39
11	6	6	1.63	0.86
-12	-6	-6	83.14	3.56
-12	-6	-6	80.25	3.71
-12	-6	-6	80.73	3.64
-12	-6	-6	85.62	3.85
-12	-6	-6	81.07	3.69
-12	-6	-6	90.61	4.28
12	6	6	86.56	4.03
12	6	6	82.84	3.56
-13	-6	-6	7.67	1.32
-13	-6	-6	6.11	0.83
-13	-6	-6	6.44	0.94
-13	-6	-6	6.57	0.87
-13	-6	-6	4.78	1.10
13	6	6	6.30	1.18
13	6	6	4.79	0.68
-14	-6	-6	50.38	2.72
-14	-6	-6	58.45	2.98
-14	-6	-6	51.13	2.72
-14	-6	-6	52.94	2.66
-14	-6	-6	51.15	2.68
14	6	6	54.12	2.56
14	6	6	55.95	3.08
-15	-6	-6	1.81	0.75
-15	-6	-6	1.48	1.14
-15	-6	-6	2.08	0.90
15	6	6	1.33	0.63
15	6	6	2.86	1.13

16	6	6	66.32	3.53
16	6	6	58.90	2.95
-17	-6	-6	1.05	1.08
17	6	6	1.41	0.76
17	6	6	0.82	1.36
-18	-6	-6	38.03	2.85
18	6	6	35.92	2.25
18	6	6	36.61	3.11
-19	-6	-6	2.39	1.45
-19	-6	-6	2.10	1.04
19	6	6	0.87	1.62
19	6	6	1.26	0.94
20	-6	-5	11.20	1.29
-20	6	5	17.04	2.76
19	-6	-5	1.91	0.64
-19	6	5	1.52	1.36
18	-6	-5	7.07	1.01
-18	6	5	4.56	1.43
-18	6	5	5.58	1.41
17	-6	-5	16.57	1.27
-17	6	5	19.54	2.19
-17	6	5	18.10	2.27
16	-6	-5	13.07	1.11
16	-6	-5	16.81	3.05
16	-6	-5	13.21	0.99
-16	6	5	13.12	2.11
-16	6	5	13.30	1.88
15	-6	-5	9.72	1.78
15	-6	-5	9.80	0.90
15	-6	-5	10.92	1.00
-15	6	5	7.19	1.18
-15	6	5	9.52	1.46
14	-6	-5	26.06	1.52
14	-6	-5	28.56	1.52
14	-6	-5	27.78	1.47
14	-6	-5	27.64	2.80
-14	6	5	28.42	2.23
-14	6	5	30.33	2.02
13	-6	-5	8.24	0.97
13	-6	-5	6.90	0.91
13	-6	-5	6.47	0.74
13	-6	-5	8.19	1.47
-13	6	5	5.73	1.15
-13	6	5	8.07	1.26

12	-6	-5	8.18	0.96
12	-6	-5	6.15	1.35
12	-6	-5	7.92	0.87
12	-6	-5	7.41	0.69
-12	6	5	6.04	1.13
-12	6	5	6.58	0.95
11	-6	-5	67.02	3.07
11	-6	-5	67.79	2.83
11	-6	-5	71.21	2.95
11	-6	-5	73.38	3.55
11	-6	-5	67.57	2.91
-11	6	5	70.33	2.94
-11	6	5	63.37	3.33
-11	6	5	66.49	3.15
10	-6	-5	34.00	1.79
10	-6	-5	34.86	1.73
10	-6	-5	33.30	2.25
10	-6	-5	32.53	1.53
10	-6	-5	35.03	1.72
-10	6	5	34.64	1.88
-10	6	5	30.97	2.04
-10	6	5	34.31	1.61
9	-6	-5	61.73	3.06
9	-6	-5	62.88	2.72
9	-6	-5	62.11	2.70
9	-6	-5	59.27	2.52
9	-6	-5	64.55	2.69
-9	6	5	59.82	2.57
-9	6	5	55.31	2.94
-9	6	5	63.62	2.78
8	-6	-5	35.11	1.55
8	-6	-5	35.13	2.07
8	-6	-5	37.19	1.76
8	-6	-5	36.13	1.73
8	-6	-5	35.17	1.78
-8	6	5	33.88	1.59
-8	6	5	32.89	1.97
-8	6	5	35.03	1.77
7	-6	-5	45.49	2.10
7	-6	-5	47.67	2.38
7	-6	-5	45.83	2.04
7	-6	-5	42.91	1.89
7	-6	-5	45.36	2.15
-7	6	5	43.18	2.29

-7	6	5	44.84	2.07
-7	6	5	46.67	1.94
6	-6	-5	37.26	1.78
6	-6	-5	34.48	1.64
6	-6	-5	33.05	1.52
6	-6	-5	38.27	2.01
6	-6	-5	37.33	1.85
-6	6	5	35.25	1.67
-6	6	5	36.48	1.58
-6	6	5	34.58	1.92
5	-6	-5	55.83	2.65
5	-6	-5	64.26	2.77
5	-6	-5	56.57	2.34
5	-6	-5	56.81	2.70
5	-6	-5	58.29	2.42
-5	6	5	56.83	2.69
-5	6	5	54.38	2.42
-5	6	5	61.17	2.38
4	-6	-5	7.56	0.92
4	-6	-5	7.13	0.49
4	-6	-5	6.96	0.89
-4	6	5	7.79	0.54
-4	6	5	6.98	0.84
-4	6	5	6.96	0.57
3	-6	-5	21.46	1.06
3	-6	-5	22.71	1.47
3	-6	-5	24.19	1.09
3	-6	-5	24.84	1.37
3	-6	-5	25.19	1.43
-3	6	5	23.35	1.11
-3	6	5	23.24	1.08
-3	6	5	23.01	1.37
2	-6	-5	115.10	4.48
2	-6	-5	120.21	4.79
2	-6	-5	121.09	4.75
2	-6	-5	114.66	4.89
2	-6	-5	110.51	4.45
-2	6	5	112.19	4.74
-2	6	5	120.27	4.50
1	-6	-5	69.21	3.24
1	-6	-5	67.16	2.75
1	-6	-5	74.35	3.07
1	-6	-5	66.62	2.70
1	-6	-5	72.04	3.04

-1	6	5	65.97	2.72
-1	6	5	71.67	3.02
-1	6	5	69.43	3.05
0	-6	-5	0.37	0.17
0	-6	-5	0.57	0.17
0	-6	-5	0.48	0.41
0	-6	-5	0.14	0.37
0	6	5	0.55	0.35
0	6	5	0.06	0.30
0	6	5	0.24	0.19
0	6	5	0.46	0.17
-1	-6	-5	105.85	4.42
-1	-6	-5	107.70	4.09
-1	-6	-5	107.82	4.19
-1	-6	-5	115.08	4.49
1	6	5	108.59	4.19
1	6	5	107.86	4.09
1	6	5	108.50	4.49
1	6	5	100.85	4.37
-2	-6	-5	31.57	1.67
-2	-6	-5	33.66	1.44
-2	-6	-5	35.53	1.76
-2	-6	-5	32.61	1.34
2	6	5	33.76	1.45
2	6	5	32.79	1.63
2	6	5	30.12	1.72
2	6	5	31.87	1.32
-3	-6	-5	107.96	4.29
-3	-6	-5	112.53	4.60
-3	-6	-5	112.79	4.50
-3	-6	-5	111.04	4.19
3	6	5	106.92	4.45
3	6	5	106.41	4.16
3	6	5	107.12	4.59
3	6	5	114.52	4.30
-4	-6	-5	16.73	1.11
-4	-6	-5	15.68	0.78
-4	-6	-5	17.83	0.81
-4	-6	-5	17.56	0.86
-4	-6	-5	18.74	1.27
-4	-6	-5	16.12	0.89
4	6	5	17.60	1.08
4	6	5	15.37	1.22
-5	-6	-5	176.98	6.70

-5	-6	-5	189.09	7.00
-5	-6	-5	176.62	6.80
-5	-6	-5	151.05	6.75
-5	-6	-5	191.14	7.14
-5	-6	-5	174.11	6.74
5	6	5	181.46	7.15
5	6	5	176.26	6.93
-6	-6	-5	4.93	0.48
-6	-6	-5	5.45	0.78
-6	-6	-5	5.14	0.80
-6	-6	-5	4.97	0.56
-6	-6	-5	4.62	0.52
-6	-6	-5	4.90	0.49
6	6	5	3.97	0.69
6	6	5	5.24	0.80
-7	-6	-5	115.10	5.31
-7	-6	-5	138.19	5.43
-7	-6	-5	135.34	5.20
-7	-6	-5	145.23	5.65
-7	-6	-5	134.90	5.22
-7	-6	-5	129.70	5.27
7	6	5	137.26	5.63
7	6	5	138.19	5.39
-8	-6	-5	37.35	1.66
-8	-6	-5	36.83	1.64
-8	-6	-5	37.93	1.90
-8	-6	-5	36.66	2.13
-8	-6	-5	38.16	1.75
-8	-6	-5	35.52	1.74
8	6	5	35.56	2.11
8	6	5	38.34	1.86
-9	-6	-5	33.90	2.15
-9	-6	-5	37.08	1.64
-9	-6	-5	37.15	1.69
-9	-6	-5	34.43	1.75
-9	-6	-5	35.24	1.74
-9	-6	-5	36.47	1.91
9	6	5	34.40	2.09
9	6	5	39.38	1.88
-10	-6	-5	25.02	1.39
-10	-6	-5	24.89	1.27
-10	-6	-5	23.96	1.43
-10	-6	-5	22.24	1.83
-10	-6	-5	25.58	1.58

-10	-6	-5	26.59	1.73
-10	-6	-5	24.29	1.35
10	6	5	26.41	1.81
10	6	5	24.76	1.30
-11	-6	-5	49.16	2.35
-11	-6	-5	49.85	2.30
-11	-6	-5	54.77	2.84
-11	-6	-5	47.28	2.37
-11	-6	-5	47.69	2.45
-11	-6	-5	50.92	2.62
11	6	5	51.17	2.72
11	6	5	52.52	2.28
-12	-6	-5	0.82	0.62
-12	-6	-5	1.91	0.87
-12	-6	-5	0.94	0.99
-12	-6	-5	1.01	0.69
-12	-6	-5	1.80	0.58
12	6	5	1.40	0.44
12	6	5	2.02	0.87
-13	-6	-5	57.21	3.05
-13	-6	-5	54.36	2.76
-13	-6	-5	58.60	2.80
-13	-6	-5	58.21	3.21
-13	-6	-5	59.99	2.70
13	6	5	52.53	2.70
13	6	5	58.22	3.05
-14	-6	-5	1.34	1.12
-14	-6	-5	0.23	0.76
-14	-6	-5	0.07	0.84
14	6	5	0.39	0.49
14	6	5	-0.44	0.90
-15	-6	-5	64.59	3.43
-15	-6	-5	60.37	3.14
15	6	5	63.91	3.41
15	6	5	65.72	2.95
-16	-6	-5	5.06	1.67
-16	-6	-5	3.28	1.04
16	6	5	2.69	1.20
16	6	5	2.76	0.70
-17	-6	-5	49.55	2.96
17	6	5	43.89	4.65
17	6	5	50.59	2.58
-18	-6	-5	4.01	1.36
18	6	5	8.72	1.60

18	6	5	3.69	0.91
-19	-6	-5	34.01	2.94
19	6	5	35.50	2.29
19	6	5	34.25	3.16
20	-6	-4	0.46	0.67
-20	6	4	0.16	1.44
19	-6	-4	5.64	0.82
-19	6	4	6.00	1.47
18	-6	-4	0.26	0.51
-18	6	4	1.71	1.31
-18	6	4	0.70	1.22
17	-6	-4	8.26	0.97
-17	6	4	9.44	1.49
-17	6	4	8.36	1.50
16	-6	-4	0.58	1.63
16	-6	-4	1.07	0.62
16	-6	-4	-0.37	0.45
-16	6	4	2.57	1.15
-16	6	4	0.59	0.97
15	-6	-4	7.04	0.95
15	-6	-4	10.20	1.70
15	-6	-4	5.58	0.81
-15	6	4	6.41	1.23
-15	6	4	6.49	1.19
-15	6	4	7.11	1.15
14	-6	-4	8.30	1.54
14	-6	-4	9.18	0.93
14	-6	-4	6.85	0.76
-14	6	4	7.06	1.23
-14	6	4	9.10	1.42
-14	6	4	6.25	1.10
13	-6	-4	32.21	2.58
13	-6	-4	31.08	1.54
13	-6	-4	32.50	1.61
-13	6	4	30.81	2.01
-13	6	4	31.74	2.01
-13	6	4	27.98	2.12
12	-6	-4	1.59	0.50
12	-6	-4	3.49	1.13
12	-6	-4	2.10	0.37
-12	6	4	2.78	0.74
-12	6	4	2.03	0.85
-12	6	4	1.85	0.85
11	-6	-4	41.28	1.88

11	-6	-4	41.78	2.46
11	-6	-4	37.41	1.75
11	-6	-4	38.93	2.04
-11	6	4	42.22	1.85
-11	6	4	39.25	2.12
-11	6	4	37.62	2.21
-11	6	4	36.56	2.24
10	-6	-4	1.87	0.33
10	-6	-4	3.61	0.89
10	-6	-4	1.50	0.52
10	-6	-4	1.98	0.51
-10	6	4	1.96	0.55
-10	6	4	1.55	0.70
-10	6	4	1.61	0.76
-10	6	4	1.45	0.35
9	-6	-4	16.76	1.13
9	-6	-4	18.51	1.16
9	-6	-4	17.37	0.92
9	-6	-4	19.85	1.53
-9	6	4	18.01	1.20
-9	6	4	17.11	1.40
-9	6	4	15.08	1.40
-9	6	4	16.80	0.96
8	-6	-4	0.62	0.37
8	-6	-4	0.68	0.22
8	-6	-4	0.57	0.43
8	-6	-4	0.45	0.58
-8	6	4	0.90	0.28
-8	6	4	0.69	0.52
-8	6	4	0.30	0.38
-8	6	4	0.87	0.64
7	-6	-4	53.32	2.22
7	-6	-4	58.55	2.65
7	-6	-4	54.04	2.46
7	-6	-4	52.84	2.35
-7	6	4	51.20	2.63
-7	6	4	53.38	2.59
-7	6	4	55.06	2.27
-7	6	4	57.35	2.42
6	-6	-4	2.22	0.57
6	-6	-4	1.33	0.33
6	-6	-4	1.48	0.48
6	-6	-4	0.82	0.21
-6	6	4	1.49	0.59

-6	6	4	1.03	0.49
-6	6	4	0.53	0.33
-6	6	4	1.18	0.25
5	-6	-4	23.60	1.35
5	-6	-4	21.96	1.26
5	-6	-4	22.02	1.10
5	-6	-4	20.44	1.35
5	-6	-4	21.95	1.01
-5	6	4	24.01	1.05
-5	6	4	20.80	1.49
-5	6	4	22.31	1.34
-5	6	4	21.18	1.12
4	-6	-4	25.95	1.38
4	-6	-4	23.88	1.13
4	-6	-4	24.96	1.48
4	-6	-4	25.98	1.46
-4	6	4	26.65	1.23
-4	6	4	26.50	1.16
-4	6	4	26.87	1.45
3	-6	-4	12.85	0.68
3	-6	-4	13.25	0.74
3	-6	-4	13.92	1.00
3	-6	-4	15.56	1.12
3	-6	-4	13.75	1.06
-3	6	4	13.82	0.71
-3	6	4	13.20	0.96
-3	6	4	13.12	0.75
2	-6	-4	2.58	0.31
2	-6	-4	3.49	0.59
2	-6	-4	3.06	0.59
2	-6	-4	2.82	0.37
-2	6	4	2.99	0.33
-2	6	4	2.55	0.39
-2	6	4	2.76	0.48
1	-6	-4	6.37	0.46
1	-6	-4	8.00	0.75
1	-6	-4	7.72	0.47
1	-6	-4	8.06	0.93
1	-6	-4	7.61	0.72
-1	6	4	6.89	0.47
-1	6	4	6.84	0.48
-1	6	4	6.80	0.69
0	-6	-4	18.74	1.13
0	-6	-4	17.26	0.85

0	-6	-4	16.85	1.09
0	-6	-4	19.12	1.36
0	-6	-4	17.67	0.82
0	6	4	18.01	0.86
0	6	4	19.01	1.16
0	6	4	17.38	0.83
0	6	4	18.38	1.08
-1	-6	-4	45.81	2.08
-1	-6	-4	42.28	1.78
-1	-6	-4	45.14	2.01
-1	-6	-4	43.12	1.73
1	6	4	40.62	1.97
1	6	4	42.05	2.06
1	6	4	43.82	1.79
1	6	4	42.02	1.73
-2	-6	-4	22.34	1.01
-2	-6	-4	22.75	1.28
-2	-6	-4	24.45	1.35
-2	-6	-4	22.21	1.06
2	6	4	24.32	1.28
2	6	4	23.66	1.00
2	6	4	23.16	1.35
2	6	4	23.43	1.07
-3	-6	-4	59.23	2.31
-3	-6	-4	56.22	2.35
-3	-6	-4	59.56	2.57
-3	-6	-4	61.01	2.66
3	6	4	56.17	2.28
3	6	4	61.88	2.38
3	6	4	57.22	2.65
3	6	4	54.10	2.51
-4	-6	-4	262.01	9.72
-4	-6	-4	259.50	9.82
-4	-6	-4	247.92	9.49
-4	-6	-4	243.97	9.52
-4	-6	-4	256.87	9.47
-4	-6	-4	249.02	9.45
4	6	4	244.23	9.45
4	6	4	249.16	9.68
4	6	4	255.61	9.84
-5	-6	-4	9.44	0.61
-5	-6	-4	9.83	0.64
-5	-6	-4	9.66	0.61
-5	-6	-4	9.44	0.86

-5	-6	-4	11.33	0.62
-5	-6	-4	10.23	0.97
5	6	4	10.28	0.84
5	6	4	9.69	0.98
-6	-6	-4	64.27	2.55
-6	-6	-4	63.60	2.90
-6	-6	-4	61.31	2.55
-6	-6	-4	59.43	2.59
-6	-6	-4	66.92	2.79
-6	-6	-4	62.74	2.57
6	6	4	62.01	2.92
6	6	4	64.36	2.75
-7	-6	-4	38.31	2.04
-7	-6	-4	40.08	1.90
-7	-6	-4	41.33	1.66
-7	-6	-4	36.50	1.69
-7	-6	-4	35.07	1.69
-7	-6	-4	37.99	1.67
7	6	4	37.32	1.85
7	6	4	36.57	2.04
-8	-6	-4	6.26	0.55
-8	-6	-4	5.65	0.65
-8	-6	-4	5.90	0.63
-8	-6	-4	5.11	0.61
-8	-6	-4	6.15	1.07
-8	-6	-4	5.03	0.86
8	6	4	5.33	0.84
8	6	4	5.15	0.77
-9	-6	-4	30.96	1.55
-9	-6	-4	33.54	1.74
-9	-6	-4	33.32	1.96
-9	-6	-4	30.60	1.54
-9	-6	-4	32.23	1.46
-9	-6	-4	30.09	1.59
-9	-6	-4	31.23	1.87
9	6	4	31.72	1.49
9	6	4	31.31	1.91
-10	-6	-4	49.57	2.19
-10	-6	-4	51.13	2.61
-10	-6	-4	51.38	2.46
-10	-6	-4	49.61	2.37
-10	-6	-4	51.60	2.33
-10	-6	-4	53.33	2.30
-10	-6	-4	53.96	2.70

10	6	4	51.64	2.26
10	6	4	52.93	2.68
-11	-6	-4	27.74	1.92
-11	-6	-4	28.14	1.62
-11	-6	-4	28.85	1.38
-11	-6	-4	26.86	1.51
-11	-6	-4	25.07	1.93
-11	-6	-4	26.90	1.68
11	6	4	27.45	1.43
11	6	4	28.77	1.87
-12	-6	-4	77.02	3.34
-12	-6	-4	74.32	3.41
-12	-6	-4	81.86	3.78
-12	-6	-4	77.07	3.45
-12	-6	-4	74.11	3.65
12	6	4	77.68	3.26
12	6	4	78.29	3.65
-13	-6	-4	0.50	1.00
-13	-6	-4	-0.35	0.97
-13	-6	-4	0.23	0.64
-13	-6	-4	1.45	0.74
-13	-6	-4	-0.19	0.60
13	6	4	0.32	0.76
13	6	4	0.46	0.45
-14	-6	-4	19.21	1.65
-14	-6	-4	18.86	2.08
-14	-6	-4	19.37	2.09
-14	-6	-4	15.76	1.61
14	6	4	20.63	1.81
14	6	4	22.71	1.34
-15	-6	-4	1.91	1.28
-15	-6	-4	1.58	0.84
-15	-6	-4	1.78	1.05
-15	-6	-4	4.34	1.48
15	6	4	2.79	1.06
15	6	4	2.68	0.65
-16	-6	-4	47.88	2.76
-16	-6	-4	47.89	3.16
16	6	4	51.35	2.98
16	6	4	50.26	2.49
-17	-6	-4	6.38	1.20
17	6	4	7.89	1.24
17	6	4	6.90	1.35
-18	-6	-4	21.25	2.32

18	6	4	25.49	1.77
18	6	4	25.37	2.45
-19	-6	-4	4.44	1.40
19	6	4	2.80	1.51
19	6	4	4.91	0.98
20	-6	-3	1.00	0.67
-20	6	3	2.15	1.55
19	-6	-3	6.70	0.91
-19	6	3	8.39	1.58
18	-6	-3	10.43	1.11
-18	6	3	10.01	1.57
17	-6	-3	1.77	0.75
17	-6	-3	3.80	0.69
-17	6	3	0.46	1.13
-17	6	3	2.43	1.11
16	-6	-3	12.38	1.02
16	-6	-3	15.19	2.55
16	-6	-3	11.82	1.21
-16	6	3	10.64	1.43
-16	6	3	13.14	1.81
15	-6	-3	26.19	1.47
15	-6	-3	28.38	1.62
15	-6	-3	28.15	2.67
-15	6	3	28.03	2.13
-15	6	3	24.15	2.07
-15	6	3	27.54	2.10
14	-6	-3	23.75	2.40
14	-6	-3	25.17	1.47
14	-6	-3	23.01	1.28
-14	6	3	20.90	1.80
-14	6	3	22.94	1.86
-14	6	3	22.28	1.92
13	-6	-3	40.01	1.94
13	-6	-3	42.85	2.69
13	-6	-3	38.42	1.79
-13	6	3	38.50	2.33
-13	6	3	35.26	2.24
-13	6	3	38.51	2.26
12	-6	-3	1.16	0.38
12	-6	-3	2.69	1.01
12	-6	-3	1.33	0.51
-12	6	3	1.60	0.76
-12	6	3	0.83	0.86
-12	6	3	0.92	0.61

11	-6	-3	3.24	0.66
11	-6	-3	2.45	0.91
11	-6	-3	3.35	0.59
11	-6	-3	3.09	0.41
-11	6	3	3.12	0.76
-11	6	3	3.62	0.70
-11	6	3	3.63	0.50
10	-6	-3	51.46	2.39
10	-6	-3	49.75	2.18
10	-6	-3	53.48	2.36
10	-6	-3	52.06	2.66
-10	6	3	46.92	2.52
-10	6	3	53.62	2.49
-10	6	3	54.76	2.25
9	-6	-3	10.56	0.69
9	-6	-3	11.59	0.93
9	-6	-3	12.61	1.24
9	-6	-3	11.11	0.95
-9	6	3	11.06	0.99
-9	6	3	11.63	1.10
-9	6	3	11.00	1.25
-9	6	3	13.12	0.78
8	-6	-3	44.65	2.02
8	-6	-3	42.81	2.18
8	-6	-3	42.56	1.96
8	-6	-3	40.62	1.79
-8	6	3	43.53	1.84
-8	6	3	41.83	2.23
-8	6	3	41.19	2.02
-8	6	3	43.07	2.14
7	-6	-3	32.70	1.73
7	-6	-3	32.45	1.53
7	-6	-3	29.54	1.36
7	-6	-3	33.40	1.64
-7	6	3	31.45	1.67
-7	6	3	30.78	1.56
-7	6	3	30.66	1.40
-7	6	3	29.31	1.80
6	-6	-3	27.67	1.29
6	-6	-3	25.07	1.42
6	-6	-3	25.15	1.15
6	-6	-3	28.84	1.53
-6	6	3	26.00	1.32
-6	6	3	25.79	1.61

-6	6	3	25.36	1.18
-6	6	3	24.29	1.45
5	-6	-3	1.69	0.32
5	-6	-3	1.33	0.44
5	-6	-3	1.91	0.47
5	-6	-3	1.08	0.23
-5	6	3	2.01	0.46
-5	6	3	1.64	0.30
-5	6	3	2.04	0.57
-5	6	3	1.26	0.23
4	-6	-3	12.03	0.63
4	-6	-3	12.90	0.93
4	-6	-3	12.77	0.75
4	-6	-3	11.72	0.98
-4	6	3	11.94	1.11
-4	6	3	11.65	0.74
-4	6	3	13.23	0.67
-4	6	3	12.75	0.93
3	-6	-3	0.02	0.36
3	-6	-3	0.50	0.17
3	-6	-3	0.73	0.23
3	-6	-3	0.63	0.31
-3	6	3	0.78	0.36
-3	6	3	0.25	0.44
-3	6	3	0.39	0.16
-3	6	3	0.41	0.22
2	-6	-3	7.81	0.51
2	-6	-3	6.69	0.44
2	-6	-3	8.60	0.72
2	-6	-3	7.05	0.72
2	-6	-3	6.47	0.79
-2	6	3	7.51	0.55
-2	6	3	6.75	0.45
-2	6	3	7.92	0.70
-2	6	3	7.39	0.92
1	-6	-3	31.49	1.53
1	-6	-3	28.59	1.66
1	-6	-3	31.31	1.69
1	-6	-3	31.42	1.30
-1	6	3	31.03	1.50
-1	6	3	27.14	1.36
0	-6	-3	5.27	0.60
0	-6	-3	5.10	0.40
0	-6	-3	4.48	0.75

0	-6	-3	5.18	0.37
0	6	3	4.56	0.37
0	6	3	4.50	0.57
0	6	3	4.47	0.38
-1	-6	-3	28.24	1.70
-1	-6	-3	29.25	1.47
-1	-6	-3	28.39	1.21
-1	-6	-3	28.44	1.54
-1	-6	-3	29.12	1.21
1	6	3	28.85	1.42
1	6	3	27.67	1.20
1	6	3	25.75	1.28
-2	-6	-3	198.74	7.78
-2	-6	-3	206.33	8.28
-2	-6	-3	209.40	8.04
-2	-6	-3	199.66	7.76
-2	-6	-3	213.31	8.03
2	6	3	207.04	8.08
2	6	3	207.11	7.97
2	6	3	208.13	7.76
-3	-6	-3	2.26	0.41
-3	-6	-3	2.30	0.33
-3	-6	-3	2.02	0.32
-3	-6	-3	1.69	0.39
3	6	3	2.24	0.33
3	6	3	2.46	0.43
3	6	3	2.17	0.30
3	6	3	1.82	0.46
-4	-6	-3	54.50	2.18
-4	-6	-3	51.59	2.15
-4	-6	-3	51.03	2.19
-4	-6	-3	56.31	2.47
-4	-6	-3	54.71	2.39
4	6	3	54.91	2.51
4	6	3	53.42	2.15
4	6	3	54.39	2.37
-5	-6	-3	25.32	1.20
-5	-6	-3	24.73	1.19
-5	-6	-3	27.76	1.20
-5	-6	-3	27.60	1.41
-5	-6	-3	26.78	1.50
-5	-6	-3	25.72	1.16
5	6	3	26.60	1.40
5	6	3	25.45	1.50

-6	-6	-3	8.34	0.55
-6	-6	-3	8.56	0.60
-6	-6	-3	9.51	0.84
-6	-6	-3	8.76	0.60
-6	-6	-3	9.78	0.94
6	6	3	8.28	0.81
6	6	3	8.40	0.91
-7	-6	-3	3.44	0.54
-7	-6	-3	3.47	0.47
-7	-6	-3	2.90	0.40
-7	-6	-3	3.89	0.69
-7	-6	-3	4.51	0.71
-7	-6	-3	4.14	0.50
7	6	3	4.08	0.69
7	6	3	3.72	0.71
-8	-6	-3	21.25	1.45
-8	-6	-3	19.31	1.14
-8	-6	-3	20.39	1.43
-8	-6	-3	22.62	1.05
-8	-6	-3	21.83	1.33
-8	-6	-3	20.51	1.12
-8	-6	-3	22.24	1.13
8	6	3	19.51	1.42
8	6	3	22.37	1.08
-9	-6	-3	0.91	0.43
-9	-6	-3	1.93	0.69
-9	-6	-3	2.04	0.33
-9	-6	-3	1.71	0.55
-9	-6	-3	0.66	0.64
-9	-6	-3	1.08	0.39
9	6	3	1.49	0.57
9	6	3	1.41	0.34
-10	-6	-3	37.13	1.68
-10	-6	-3	43.43	2.19
-10	-6	-3	38.78	2.21
-10	-6	-3	37.30	1.89
-10	-6	-3	38.25	1.98
-10	-6	-3	36.88	1.83
10	6	3	36.62	2.11
10	6	3	38.08	1.75
-11	-6	-3	1.44	0.71
-11	-6	-3	0.05	0.34
-11	-6	-3	0.36	0.76
-11	-6	-3	0.56	0.45

-11	-6	-3	-1.34	0.55
-11	-6	-3	0.03	0.51
11	6	3	0.51	0.33
11	6	3	-0.13	0.62
-12	-6	-3	0.82	0.88
-12	-6	-3	0.27	0.55
-12	-6	-3	1.42	0.68
-12	-6	-3	1.13	0.55
-12	-6	-3	1.92	0.85
12	6	3	0.89	0.41
12	6	3	1.35	0.73
-13	-6	-3	16.04	1.51
-13	-6	-3	17.71	1.47
-13	-6	-3	20.50	1.99
-13	-6	-3	18.56	1.81
-13	-6	-3	18.88	1.42
13	6	3	18.51	1.63
13	6	3	18.77	1.17
-14	-6	-3	12.34	1.92
-14	-6	-3	12.96	1.75
-14	-6	-3	11.45	1.39
-14	-6	-3	9.26	1.46
14	6	3	12.69	1.30
14	6	3	9.39	1.44
-15	-6	-3	3.87	1.23
-15	-6	-3	2.22	0.81
-15	-6	-3	1.24	0.96
-15	-6	-3	3.57	1.34
15	6	3	1.42	0.92
15	6	3	3.21	0.59
-16	-6	-3	17.83	2.34
-16	-6	-3	16.82	1.75
-16	-6	-3	14.87	2.32
16	6	3	14.20	1.32
16	6	3	16.55	1.86
-17	-6	-3	15.93	1.95
-17	-6	-3	13.28	2.32
17	6	3	18.29	1.40
17	6	3	15.26	2.06
-18	-6	-3	0.78	1.18
18	6	3	2.62	0.79
18	6	3	3.37	1.28
-19	-6	-3	18.44	2.43
19	6	3	28.15	1.95

19	6	3	33.71	3.40
-20	-6	-3	0.20	1.32
20	6	3	-0.57	0.83
20	-6	-2	28.37	1.83
-20	6	2	33.91	3.08
19	-6	-2	5.33	0.85
-19	6	2	5.51	1.64
18	-6	-2	29.97	1.76
-18	6	2	34.89	2.75
17	-6	-2	3.51	0.83
17	-6	-2	6.55	0.92
-17	6	2	3.69	1.13
-17	6	2	4.35	1.23
16	-6	-2	22.27	2.56
16	-6	-2	26.14	1.44
16	-6	-2	25.39	1.64
-16	6	2	24.26	2.17
-16	6	2	21.01	1.89
15	-6	-2	2.49	0.70
15	-6	-2	5.18	1.41
15	-6	-2	1.71	0.45
-15	6	2	3.39	1.16
-15	6	2	3.85	0.99
-15	6	2	0.89	0.91
14	-6	-2	71.08	3.64
14	-6	-2	69.41	3.08
14	-6	-2	70.82	2.96
-14	6	2	73.66	3.45
-14	6	2	64.54	3.30
-14	6	2	68.92	3.46
13	-6	-2	21.68	2.03
13	-6	-2	23.08	1.38
13	-6	-2	21.20	1.17
-13	6	2	22.56	1.65
-13	6	2	21.04	1.84
-13	6	2	17.24	1.59
12	-6	-2	105.52	4.37
12	-6	-2	116.06	4.85
12	-6	-2	107.86	4.51
-12	6	2	117.49	4.76
-12	6	2	111.81	4.80
-12	6	2	102.78	4.64
11	-6	-2	31.86	1.77
11	-6	-2	32.38	1.52

11	-6	-2	32.74	1.71
11	-6	-2	35.31	2.05
-11	6	2	29.53	1.86
-11	6	2	33.43	1.89
-11	6	2	34.46	2.06
-11	6	2	33.97	1.59
10	-6	-2	27.54	1.50
10	-6	-2	26.36	1.70
10	-6	-2	25.17	1.26
10	-6	-2	26.80	1.50
-10	6	2	29.28	1.34
-10	6	2	25.29	1.58
-10	6	2	26.97	1.62
-10	6	2	26.89	1.78
9	-6	-2	43.67	1.87
9	-6	-2	44.00	2.20
9	-6	-2	41.15	2.03
9	-6	-2	44.23	2.08
-9	6	2	44.42	2.33
-9	6	2	42.48	2.13
-9	6	2	44.74	2.18
-9	6	2	47.46	1.93
8	-6	-2	59.98	2.65
8	-6	-2	55.90	2.42
8	-6	-2	57.65	2.71
8	-6	-2	62.96	2.61
-8	6	2	68.69	2.50
-8	6	2	56.97	2.64
-8	6	2	56.86	2.69
7	-6	-2	15.36	0.81
7	-6	-2	16.75	0.99
7	-6	-2	17.75	1.15
7	-6	-2	17.80	1.10
-7	6	2	14.40	1.44
-7	6	2	15.37	1.12
-7	6	2	15.97	1.02
-7	6	2	17.01	0.85
6	-6	-2	111.76	4.62
6	-6	-2	127.11	4.90
6	-6	-2	122.66	4.89
6	-6	-2	124.30	4.75
-6	6	2	118.77	5.03
-6	6	2	122.99	4.88
-6	6	2	117.23	4.78

-6	6	2	120.11	4.65
5	-6	-2	34.80	1.74
5	-6	-2	35.78	1.58
5	-6	-2	37.52	1.77
5	-6	-2	33.04	1.46
-5	6	2	35.32	1.72
-5	6	2	35.36	1.89
-5	6	2	32.90	1.60
-5	6	2	36.95	1.49
4	-6	-2	91.70	3.84
4	-6	-2	90.83	3.63
4	-6	-2	86.90	3.53
-4	6	2	92.59	3.95
-4	6	2	92.43	3.78
-4	6	2	91.35	3.66
-4	6	2	97.45	3.57
3	-6	-2	103.50	3.90
3	-6	-2	87.88	4.15
3	-6	-2	102.25	3.82
3	-6	-2	101.83	4.16
-3	6	2	91.86	3.89
-3	6	2	101.04	4.04
-3	6	2	108.08	4.26
2	-6	-2	38.92	1.95
2	-6	-2	38.78	1.85
2	-6	-2	38.21	1.67
2	-6	-2	36.55	1.59
-2	6	2	42.97	1.62
-2	6	2	37.99	1.68
-2	6	2	40.61	1.82
-2	6	2	43.67	2.05
1	-6	-2	25.42	1.17
1	-6	-2	27.30	1.13
1	-6	-2	26.47	1.35
1	-6	-2	27.46	1.51
-1	6	2	27.33	1.56
-1	6	2	26.09	1.32
-1	6	2	24.54	1.20
-1	6	2	28.17	1.14
0	-6	-2	98.98	3.82
0	-6	-2	97.63	4.21
0	-6	-2	103.03	3.86
0	-6	-2	98.92	4.05
0	-6	-2	103.01	4.08

0	6	2	103.51	3.83
0	6	2	99.65	4.02
0	6	2	89.51	3.91
0	6	2	102.91	4.27
-1	-6	-2	63.62	2.47
-1	-6	-2	63.65	2.44
-1	-6	-2	62.98	2.87
-1	-6	-2	58.14	2.65
1	6	2	64.54	2.44
1	6	2	60.27	2.62
1	6	2	59.84	2.46
-2	-6	-2	31.64	1.32
-2	-6	-2	32.15	1.30
-2	-6	-2	29.10	1.75
-2	-6	-2	30.06	1.69
2	6	2	31.33	1.30
2	6	2	32.34	1.50
2	6	2	27.30	1.36
-3	-6	-2	3.19	0.69
-3	-6	-2	3.19	0.32
-3	-6	-2	3.57	0.59
-3	-6	-2	3.45	0.38
3	6	2	3.35	0.53
3	6	2	3.23	0.36
3	6	2	3.03	0.33
-4	-6	-2	20.82	1.16
-4	-6	-2	21.94	1.24
-4	-6	-2	18.62	0.94
-4	-6	-2	20.27	0.92
-4	-6	-2	20.21	1.51
-4	-6	-2	20.36	0.98
4	6	2	20.85	1.15
4	6	2	19.43	0.95
4	6	2	20.73	1.28
-5	-6	-2	22.20	1.03
-5	-6	-2	21.77	1.23
-5	-6	-2	22.11	0.97
-5	-6	-2	21.49	1.05
-5	-6	-2	22.94	1.29
5	6	2	21.26	1.30
5	6	2	21.49	1.23
5	6	2	21.13	1.01
-6	-6	-2	41.05	1.73
-6	-6	-2	42.56	1.68

-6	-6	-2	41.69	1.93
-6	-6	-2	39.81	1.74
-6	-6	-2	39.42	1.73
-6	-6	-2	39.45	1.97
6	6	2	40.08	2.02
6	6	2	37.23	1.90
-7	-6	-2	156.06	6.37
-7	-6	-2	157.57	6.08
-7	-6	-2	156.32	6.10
-7	-6	-2	157.81	6.31
-7	-6	-2	168.32	6.28
-7	-6	-2	151.81	6.01
-7	-6	-2	152.95	6.09
7	6	2	167.08	6.40
7	6	2	152.57	6.03
-8	-6	-2	25.41	1.55
-8	-6	-2	24.98	1.32
-8	-6	-2	26.36	1.31
-8	-6	-2	24.87	1.60
-8	-6	-2	27.24	1.19
-8	-6	-2	25.86	1.47
8	6	2	25.65	1.57
8	6	2	24.92	1.21
-9	-6	-2	36.89	1.62
-9	-6	-2	37.82	2.12
-9	-6	-2	40.82	2.02
-9	-6	-2	37.88	1.93
-9	-6	-2	35.60	1.77
-9	-6	-2	37.79	1.76
9	6	2	36.51	2.02
9	6	2	35.80	1.65
-10	-6	-2	0.21	0.51
-10	-6	-2	-0.22	0.41
-10	-6	-2	0.04	0.40
-10	-6	-2	0.12	0.26
-10	-6	-2	0.38	0.72
-10	-6	-2	0.34	0.60
10	6	2	0.16	0.54
10	6	2	0.07	0.28
-11	-6	-2	7.42	1.14
-11	-6	-2	4.03	0.69
-11	-6	-2	6.39	0.60
-11	-6	-2	6.64	0.85
-11	-6	-2	7.50	1.42

-11	-6	-2	5.34	0.83
11	6	2	5.09	0.86
11	6	2	5.22	0.62
-12	-6	-2	6.96	0.95
-12	-6	-2	5.96	0.98
-12	-6	-2	5.97	0.66
-12	-6	-2	7.31	1.15
-12	-6	-2	6.27	1.07
-12	-6	-2	6.75	0.97
12	6	2	9.13	1.21
12	6	2	5.92	0.72
-13	-6	-2	11.88	1.27
-13	-6	-2	10.29	1.21
-13	-6	-2	11.64	1.38
-13	-6	-2	14.14	1.59
-13	-6	-2	13.64	1.85
13	6	2	12.31	0.95
13	6	2	12.53	1.39
-14	-6	-2	0.81	0.69
-14	-6	-2	1.12	0.85
-14	-6	-2	-1.65	1.04
-14	-6	-2	1.90	0.93
14	6	2	0.07	0.72
14	6	2	1.26	0.45
-15	-6	-2	21.87	1.79
-15	-6	-2	20.47	1.90
-15	-6	-2	25.99	2.16
-15	-6	-2	20.78	2.38
15	6	2	23.69	1.42
15	6	2	23.36	1.88
-16	-6	-2	2.13	1.20
-16	-6	-2	-0.01	1.36
-16	-6	-2	0.34	0.83
16	6	2	-0.70	0.50
16	6	2	-1.29	0.88
-17	-6	-2	22.32	2.08
-17	-6	-2	18.92	2.74
-17	-6	-2	23.21	3.07
17	6	2	22.25	2.13
17	6	2	21.91	1.52
-18	-6	-2	2.33	1.19
-18	-6	-2	3.67	1.90
18	6	2	1.83	0.74
-19	-6	-2	1.12	1.24

19	6	2	1.99	0.81
-20	-6	-2	1.42	1.34
20	6	2	1.51	0.84
20	-6	-1	0.33	0.71
-20	6	1	-0.77	1.43
19	-6	-1	30.96	1.83
-19	6	1	32.20	2.91
18	-6	-1	0.77	0.58
-18	6	1	0.11	1.19
17	-6	-1	39.73	2.22
17	-6	-1	36.73	1.96
-17	6	1	43.67	2.82
-17	6	1	36.09	2.27
16	-6	-1	0.50	0.47
16	-6	-1	0.25	0.71
16	-6	-1	0.18	1.24
-16	6	1	0.67	0.86
-16	6	1	2.30	2.02
-16	6	1	2.29	1.07
15	-6	-1	110.83	4.44
15	-6	-1	116.57	4.97
15	-6	-1	108.53	4.59
-15	6	1	101.59	4.60
-15	6	1	105.67	4.98
-15	6	1	109.99	4.98
14	-6	-1	2.33	0.71
14	-6	-1	4.97	1.13
14	-6	-1	3.65	0.55
-14	6	1	4.20	1.15
-14	6	1	3.04	0.90
-14	6	1	3.17	0.83
13	-6	-1	89.02	3.73
13	-6	-1	102.76	4.23
13	-6	-1	89.51	3.91
-13	6	1	91.51	4.24
-13	6	1	87.64	3.94
-13	6	1	91.94	4.16
12	-6	-1	37.28	2.10
12	-6	-1	33.73	1.59
12	-6	-1	32.91	1.82
-12	6	1	30.47	1.84
-12	6	1	32.53	2.17
-12	6	1	35.53	2.02
11	-6	-1	68.61	3.01

11	-6	-1	65.60	3.01
11	-6	-1	67.80	3.14
11	-6	-1	68.93	2.81
-11	6	1	75.34	2.88
-11	6	1	63.21	3.02
-11	6	1	67.52	3.27
-11	6	1	65.89	3.12
10	-6	-1	40.44	1.75
10	-6	-1	38.53	1.95
10	-6	-1	40.28	2.05
10	-6	-1	37.82	1.93
-10	6	1	43.53	1.81
-10	6	1	37.43	1.98
-10	6	1	38.50	2.21
-10	6	1	40.07	2.05
9	-6	-1	111.44	4.61
9	-6	-1	110.57	4.58
9	-6	-1	112.78	4.55
9	-6	-1	108.96	4.37
-9	6	1	109.82	4.61
-9	6	1	115.21	4.80
-9	6	1	112.25	4.59
-9	6	1	115.84	4.42
8	-6	-1	13.57	1.06
8	-6	-1	13.52	0.94
8	-6	-1	12.37	1.18
8	-6	-1	12.75	0.74
-8	6	1	11.11	1.20
-8	6	1	14.68	1.02
-8	6	1	16.54	0.81
-8	6	1	12.25	0.97
7	-6	-1	190.84	7.29
7	-6	-1	178.64	7.04
7	-6	-1	183.56	7.18
-7	6	1	186.36	7.24
-7	6	1	183.00	7.48
-7	6	1	190.13	7.08
-7	6	1	188.16	7.26
6	-6	-1	1.66	0.42
6	-6	-1	1.41	0.23
6	-6	-1	1.36	0.43
6	-6	-1	1.14	0.32
-6	6	1	1.12	0.38
-6	6	1	1.39	0.34

-6	6	1	1.78	0.28
5	-6	-1	129.08	5.03
5	-6	-1	121.30	4.82
5	-6	-1	132.25	4.96
5	-6	-1	130.88	5.12
-5	6	1	130.22	5.04
-5	6	1	122.48	4.84
-5	6	1	124.14	4.98
-5	6	1	122.88	5.25
4	-6	-1	76.96	3.13
4	-6	-1	80.93	3.24
4	-6	-1	83.13	3.34
4	-6	-1	81.59	3.44
-4	6	1	82.75	3.34
-4	6	1	81.85	3.53
-4	6	1	77.97	3.26
-4	6	1	83.33	3.15
3	-6	-1	99.58	3.88
3	-6	-1	91.00	3.74
3	-6	-1	101.48	3.96
3	-6	-1	97.75	4.08
-3	6	1	96.90	3.76
-3	6	1	102.33	4.17
-3	6	1	101.26	3.97
-3	6	1	95.96	3.87
2	-6	-1	0.24	0.13
2	-6	-1	0.03	0.18
2	-6	-1	0.29	0.25
2	-6	-1	0.17	0.34
-2	6	1	0.41	0.40
-2	6	1	0.18	0.12
-2	6	1	0.19	0.20
-2	6	1	0.01	0.24
1	-6	-1	214.12	8.36
1	-6	-1	220.62	8.20
1	-6	-1	202.42	7.98
1	-6	-1	208.72	8.07
-1	6	1	221.92	8.42
-1	6	1	219.00	8.20
-1	6	1	210.27	8.00
-1	6	1	210.51	8.08
0	-6	-1	2.37	0.29
0	-6	-1	1.54	0.40
0	-6	-1	2.32	0.39

0	-6	-1	1.87	0.26
0	6	1	2.21	0.35
0	6	1	2.29	0.25
0	6	1	2.08	0.37
0	6	1	2.94	0.56
-1	-6	-1	127.31	5.10
-1	-6	-1	129.60	5.32
-1	-6	-1	133.23	4.98
1	6	1	130.68	4.91
1	6	1	134.50	5.34
1	6	1	127.63	5.10
1	6	1	124.59	4.98
-2	-6	-1	226.90	8.81
-2	-6	-1	244.24	9.00
-2	-6	-1	236.82	8.75
-2	-6	-1	244.03	9.22
2	6	1	234.66	8.80
2	6	1	233.50	8.75
2	6	1	214.81	8.93
-3	-6	-1	65.19	2.50
-3	-6	-1	59.21	2.40
-3	-6	-1	61.32	2.89
-3	-6	-1	61.69	2.43
3	6	1	60.10	2.47
3	6	1	64.00	2.43
3	6	1	60.07	2.63
-4	-6	-1	0.57	0.35
-4	-6	-1	0.68	0.16
-4	-6	-1	0.61	0.18
-4	-6	-1	0.52	0.22
-4	-6	-1	0.16	0.51
-4	-6	-1	0.71	0.33
4	6	1	0.85	0.34
4	6	1	0.29	0.19
-5	-6	-1	58.05	2.55
-5	-6	-1	63.95	2.66
-5	-6	-1	59.43	2.40
-5	-6	-1	56.79	2.42
-5	-6	-1	58.96	2.92
-5	-6	-1	64.89	2.65
5	6	1	59.30	2.43
5	6	1	59.03	2.62
-6	-6	-1	36.63	1.47
-6	-6	-1	34.65	1.52

-6	-6	-1	35.05	1.88
-6	-6	-1	34.74	1.74
-6	-6	-1	36.55	1.75
-6	-6	-1	35.37	1.57
-6	-6	-1	33.23	1.56
-6	-6	-1	34.35	2.08
6	6	1	35.80	1.83
-7	-6	-1	95.55	3.69
-7	-6	-1	97.35	3.97
-7	-6	-1	92.52	3.79
-7	-6	-1	98.15	3.81
-7	-6	-1	89.87	4.07
-7	-6	-1	98.55	3.97
-7	-6	-1	87.34	4.26
7	6	1	99.68	3.72
7	6	1	95.97	4.05
-8	-6	-1	94.79	3.85
-8	-6	-1	93.18	3.85
-8	-6	-1	89.09	3.55
-8	-6	-1	90.50	3.69
-8	-6	-1	92.27	3.69
-8	-6	-1	84.30	3.97
8	6	1	89.51	3.89
8	6	1	92.50	3.58
-9	-6	-1	117.04	4.83
-9	-6	-1	115.73	4.81
-9	-6	-1	120.17	4.67
-9	-6	-1	123.88	4.97
-9	-6	-1	112.55	5.14
-9	-6	-1	125.23	4.98
9	6	1	124.90	5.04
9	6	1	125.94	4.71
-10	-6	-1	39.96	2.10
-10	-6	-1	40.52	2.36
-10	-6	-1	40.30	1.98
-10	-6	-1	42.12	1.96
-10	-6	-1	42.49	1.78
-10	-6	-1	42.80	2.12
10	6	1	38.35	1.79
10	6	1	40.00	2.13
-11	-6	-1	14.49	1.62
-11	-6	-1	13.43	1.29
-11	-6	-1	13.84	1.27
-11	-6	-1	14.44	0.82

-11	-6	-1	13.69	1.08
-11	-6	-1	12.60	1.10
11	6	1	12.74	0.87
11	6	1	13.36	1.26
-12	-6	-1	1.54	1.03
-12	-6	-1	2.48	0.60
-12	-6	-1	3.76	0.71
-12	-6	-1	3.53	0.78
-12	-6	-1	4.17	0.61
-12	-6	-1	4.32	0.82
12	6	1	3.30	0.49
12	6	1	3.88	0.78
-13	-6	-1	13.60	1.52
-13	-6	-1	14.24	1.49
-13	-6	-1	10.66	1.83
-13	-6	-1	11.55	1.41
-13	-6	-1	11.79	1.31
13	6	1	12.22	0.94
13	6	1	13.61	1.34
-14	-6	-1	31.75	1.98
-14	-6	-1	26.93	1.82
-14	-6	-1	27.29	2.53
-14	-6	-1	20.07	1.87
-14	-6	-1	25.87	2.40
14	6	1	26.98	1.86
14	6	1	26.44	1.46
-15	-6	-1	46.65	2.68
-15	-6	-1	46.36	3.20
-15	-6	-1	48.38	2.72
-15	-6	-1	46.22	3.33
15	6	1	47.88	2.28
15	6	1	48.04	2.62
-16	-6	-1	32.55	2.96
-16	-6	-1	30.81	2.15
-16	-6	-1	30.67	2.32
-16	-6	-1	26.77	2.84
-16	-6	-1	31.04	2.35
16	6	1	30.20	1.69
-17	-6	-1	4.61	1.13
-17	-6	-1	3.65	1.64
-17	-6	-1	3.21	1.68
17	6	1	3.79	0.70
-18	-6	-1	26.09	2.30
-18	-6	-1	23.07	3.18

18	6	1	24.11	1.66
-19	-6	-1	0.86	1.20
19	6	1	1.14	0.75
-20	-6	-1	14.10	2.31
20	6	1	10.74	1.47
20	-6	0	25.80	1.74
-20	6	0	24.45	2.92
19	-6	0	0.18	0.61
-19	6	0	1.72	1.35
18	-6	0	31.48	2.20
18	-6	0	34.85	1.88
-18	6	0	32.27	2.77
17	-6	0	6.25	0.96
17	-6	0	6.55	1.05
-17	6	0	3.49	1.22
16	-6	0	52.31	2.41
16	-6	0	51.40	2.65
-16	6	0	53.09	3.32
-16	6	0	55.61	3.14
-16	6	0	47.49	2.47
15	-6	0	15.95	1.12
15	-6	0	16.58	1.48
-15	6	0	16.84	1.41
-15	6	0	19.15	1.89
-15	6	0	14.99	2.16
14	-6	0	110.54	4.53
14	-6	0	119.54	4.77
-14	6	0	109.41	5.07
-14	6	0	106.26	4.62
-14	6	0	117.00	5.02
13	-6	0	19.88	1.49
13	-6	0	22.29	1.17
-13	6	0	19.38	1.39
-13	6	0	19.58	1.87
-13	6	0	20.72	1.64
12	-6	0	130.97	5.26
12	-6	0	124.82	5.02
-12	6	0	129.14	5.50
-12	6	0	121.23	5.13
-12	6	0	131.81	5.41
11	-6	0	0.14	0.52
11	-6	0	-0.26	0.52
11	-6	0	-0.34	0.27
11	-6	0	0.75	0.76

-11	6	0	0.36	0.48
-11	6	0	-0.16	0.49
-11	6	0	0.60	0.34
-11	6	0	-0.94	0.72
10	-6	0	36.59	1.85
10	-6	0	37.03	1.88
10	-6	0	34.41	1.61
10	-6	0	37.90	1.89
-10	6	0	35.12	2.12
-10	6	0	37.64	1.67
-10	6	0	35.69	1.79
-10	6	0	34.94	1.92
9	-6	0	3.63	0.54
9	-6	0	4.77	0.48
9	-6	0	5.42	0.83
9	-6	0	4.41	0.65
-9	6	0	4.39	0.83
-9	6	0	4.17	0.74
-9	6	0	5.08	0.53
-9	6	0	4.24	0.67
8	-6	0	97.09	3.99
8	-6	0	99.91	4.07
8	-6	0	101.08	3.99
8	-6	0	95.13	3.82
-8	6	0	100.51	3.85
-8	6	0	100.07	3.99
-8	6	0	91.58	4.20
-8	6	0	95.12	4.03
7	-6	0	3.62	0.64
7	-6	0	3.97	0.74
7	-6	0	3.49	0.37
7	-6	0	3.66	0.59
-7	6	0	3.63	0.41
-7	6	0	3.68	0.70
-7	6	0	3.69	0.65
-7	6	0	2.77	0.44
6	-6	0	440.82	15.52
6	-6	0	425.98	15.61
6	-6	0	418.81	15.48
6	-6	0	384.14	15.33
-6	6	0	416.99	15.36
-6	6	0	400.71	15.49
-6	6	0	405.80	15.51
-6	6	0	394.89	15.71

5	-6	0	52.75	2.17
5	-6	0	51.08	2.28
5	-6	0	47.74	1.97
5	-6	0	49.22	2.11
-5	6	0	46.71	2.54
-5	6	0	51.01	1.99
-5	6	0	43.68	2.11
-5	6	0	51.37	2.18
4	-6	0	102.88	3.87
4	-6	0	103.20	4.05
4	-6	0	106.67	4.01
4	-6	0	109.00	4.21
-4	6	0	87.36	3.92
-4	6	0	107.92	4.08
-4	6	0	92.48	4.45
-4	6	0	100.03	4.02
3	-6	0	12.00	0.58
3	-6	0	12.84	0.79
3	-6	0	12.65	0.93
-3	6	0	10.98	0.80
-3	6	0	12.18	0.74
-3	6	0	12.03	0.59
-3	6	0	14.26	1.05
2	-6	0	20.32	0.99
2	-6	0	20.62	1.06
2	-6	0	21.03	1.21
2	-6	0	19.63	0.85
-2	6	0	19.30	0.86
-2	6	0	18.96	0.98
-2	6	0	19.79	1.08
-2	6	0	20.26	1.27
1	-6	0	20.55	0.93
1	-6	0	19.06	1.18
1	-6	0	19.80	1.01
1	-6	0	16.61	0.78
-1	6	0	17.54	0.91
-1	6	0	18.70	1.21
-1	6	0	17.29	0.99
-1	6	0	17.41	0.79
0	-6	0	307.69	11.34
0	-6	0	327.78	11.46
0	-6	0	281.08	11.22
0	-6	0	301.25	11.61
0	6	0	292.56	11.23

0	6	0	304.41	11.63
0	6	0	295.87	11.33
-1	-6	0	20.12	0.82
-1	-6	0	18.50	1.04
-1	-6	0	19.10	0.93
-1	-6	0	19.18	1.24
1	6	0	22.26	1.28
1	6	0	19.61	0.82
1	6	0	18.73	1.05
1	6	0	17.10	0.92
-2	-6	0	20.47	0.99
-2	-6	0	21.71	1.33
-2	-6	0	20.57	0.88
-2	-6	0	20.64	1.12
2	6	0	19.09	0.97
2	6	0	19.94	1.31
2	6	0	21.34	0.88
2	6	0	20.83	1.12
-3	-6	0	11.69	0.65
-3	-6	0	13.34	1.07
-3	-6	0	13.08	0.59
-3	-6	0	12.02	0.72
-3	-6	0	12.69	0.88
3	6	0	14.05	1.08
3	6	0	12.37	0.86
3	6	0	12.74	0.70
3	6	0	13.72	0.62
-4	-6	0	117.28	3.95
-4	-6	0	89.25	4.30
-4	-6	0	103.38	3.97
-4	-6	0	107.18	4.44
-4	-6	0	94.78	4.14
-4	-6	0	103.73	4.21
4	6	0	106.69	4.04
4	6	0	103.95	4.22
-5	-6	0	48.67	2.38
-5	-6	0	48.95	2.49
-5	-6	0	49.92	2.25
-5	-6	0	49.86	2.09
-5	-6	0	51.53	1.96
-5	-6	0	49.75	2.00
5	6	0	47.17	2.05
-6	-6	0	424.62	15.11
-6	-6	0	399.36	14.91

-6	-6	0	379.14	14.78
-6	-6	0	402.40	15.21
-6	-6	0	389.85	15.31
-6	-6	0	396.84	14.84
-6	-6	0	415.74	15.06
-6	-6	0	389.83	14.92
6	6	0	377.14	14.88
6	6	0	394.83	14.81
-7	-6	0	4.58	0.87
-7	-6	0	3.27	0.67
-7	-6	0	3.28	0.49
-7	-6	0	3.78	0.70
-7	-6	0	4.05	0.56
-7	-6	0	3.63	0.35
-7	-6	0	4.54	0.64
7	6	0	3.84	0.39
-8	-6	0	98.43	3.96
-8	-6	0	92.45	4.29
-8	-6	0	89.91	4.33
-8	-6	0	102.77	3.80
-8	-6	0	100.30	4.05
-8	-6	0	95.15	3.93
-8	-6	0	96.30	4.11
8	6	0	104.03	3.82
-9	-6	0	5.04	0.70
-9	-6	0	4.99	0.48
-9	-6	0	5.05	0.93
-9	-6	0	4.24	0.89
-9	-6	0	5.02	0.66
-9	-6	0	4.21	0.95
-9	-6	0	5.49	0.84
9	6	0	5.61	0.51
-10	-6	0	36.27	1.58
-10	-6	0	35.76	2.28
-10	-6	0	36.24	1.80
-10	-6	0	37.38	1.81
-10	-6	0	35.44	1.99
-10	-6	0	37.30	1.89
-10	-6	0	34.16	2.29
10	6	0	36.12	1.62
-11	-6	0	0.50	0.45
-11	-6	0	0.09	0.59
-11	-6	0	-0.34	0.85
-11	-6	0	-0.33	0.57

-11	-6	0	1.18	0.50
-11	-6	0	0.91	0.89
-11	-6	0	0.36	0.27
11	6	0	-0.13	0.28
-12	-6	0	125.96	5.39
-12	-6	0	130.96	5.44
-12	-6	0	123.02	5.83
-12	-6	0	129.85	5.53
-12	-6	0	130.86	5.87
-12	-6	0	141.42	5.19
-12	-6	0	142.51	5.48
12	6	0	134.54	5.22
-13	-6	0	22.22	1.73
-13	-6	0	21.35	1.58
-13	-6	0	19.18	2.19
-13	-6	0	22.28	1.18
-13	-6	0	23.05	1.62
-13	-6	0	17.69	2.11
13	6	0	24.96	1.27
-14	-6	0	113.93	5.53
-14	-6	0	123.36	5.15
-14	-6	0	126.81	5.21
-14	-6	0	120.52	5.66
-14	-6	0	114.15	5.20
14	6	0	129.64	4.90
-15	-6	0	15.47	1.71
-15	-6	0	13.09	1.80
-15	-6	0	18.13	2.28
-15	-6	0	16.99	1.59
-15	-6	0	13.84	2.46
15	6	0	16.40	1.20
-16	-6	0	55.93	2.96
-16	-6	0	53.88	3.60
-16	-6	0	49.80	3.41
-16	-6	0	54.59	3.03
-16	-6	0	49.65	2.84
16	6	0	54.12	2.47
-17	-6	0	6.30	1.15
-17	-6	0	6.42	1.68
-17	-6	0	6.39	1.84
17	6	0	7.92	1.01
-18	-6	0	29.79	3.37
-18	-6	0	35.66	2.53
18	6	0	36.20	1.95

-19	-6	0	1.52	1.14
19	6	0	1.43	0.71
-20	-6	0	19.80	2.49
20	6	0	24.21	1.83
20	-6	1	13.99	1.53
-20	6	-1	18.55	2.79
19	-6	1	0.80	0.65
-19	6	-1	3.05	1.45
18	-6	1	25.22	1.57
18	-6	1	22.29	2.07
-18	6	-1	24.79	2.56
17	-6	1	4.36	0.73
17	-6	1	5.65	1.10
-17	6	-1	5.45	1.29
16	-6	1	30.71	2.02
16	-6	1	30.79	1.67
-16	6	-1	31.83	2.44
-16	6	-1	24.55	2.71
15	-6	1	47.90	2.38
15	-6	1	45.65	2.16
15	-6	1	46.67	2.48
-15	6	-1	45.55	2.77
-15	6	-1	40.03	2.99
14	-6	1	24.73	1.76
14	-6	1	24.58	1.38
14	-6	1	25.43	1.77
-14	6	-1	22.56	2.25
-14	6	-1	24.43	1.80
-14	6	-1	27.17	1.94
13	-6	1	12.96	0.88
13	-6	1	10.47	1.26
13	-6	1	11.95	1.37
-13	6	-1	10.85	1.14
-13	6	-1	11.56	1.41
-13	6	-1	12.66	1.78
12	-6	1	2.16	0.40
12	-6	1	3.38	0.75
12	-6	1	3.74	0.73
-12	6	-1	2.56	0.65
-12	6	-1	2.70	1.00
-12	6	-1	2.53	0.73
11	-6	1	11.57	0.82
11	-6	1	11.38	1.10
11	-6	1	13.09	1.14

11	-6	1	12.51	1.20
-11	6	-1	13.98	1.54
-11	6	-1	13.39	1.08
-11	6	-1	13.53	1.24
-11	6	-1	14.25	0.89
10	-6	1	39.14	1.78
10	-6	1	40.99	1.95
10	-6	1	38.63	2.07
10	-6	1	40.43	2.00
-10	6	-1	39.72	2.08
-10	6	-1	40.73	1.93
-10	6	-1	38.81	2.30
-10	6	-1	43.52	1.81
9	-6	1	122.42	4.95
9	-6	1	125.25	4.80
9	-6	1	116.13	4.82
9	-6	1	117.09	4.67
-9	6	-1	121.25	4.78
-9	6	-1	111.99	5.09
-9	6	-1	126.39	4.71
-9	6	-1	118.91	4.92
8	-6	1	93.01	3.66
8	-6	1	87.02	3.53
8	-6	1	89.65	3.81
8	-6	1	88.44	3.68
-8	6	-1	88.36	3.76
-8	6	-1	96.82	3.57
-8	6	-1	86.50	3.65
-8	6	-1	86.81	3.96
7	-6	1	94.54	3.83
7	-6	1	94.97	3.98
7	-6	1	91.49	3.68
7	-6	1	97.47	3.81
-7	6	-1	94.04	3.82
-7	6	-1	89.43	4.08
-7	6	-1	100.47	3.72
-7	6	-1	93.34	3.89
6	-6	1	34.68	1.60
6	-6	1	32.25	1.44
6	-6	1	34.49	1.61
6	-6	1	35.26	1.78
-6	6	-1	34.62	1.63
-6	6	-1	33.11	1.87
-6	6	-1	36.66	1.47

-6	6	-1	33.42	1.64
5	-6	1	62.86	2.60
5	-6	1	62.56	2.58
5	-6	1	62.82	2.76
5	-6	1	58.05	2.43
-5	6	-1	65.43	2.88
-5	6	-1	64.42	2.46
-5	6	-1	59.87	2.60
-5	6	-1	60.33	2.62
4	-6	1	0.37	0.28
4	-6	1	0.31	0.13
4	-6	1	0.47	0.36
4	-6	1	0.40	0.26
-4	6	-1	0.44	0.47
-4	6	-1	0.12	0.16
-4	6	-1	0.46	0.33
-4	6	-1	0.39	0.25
3	-6	1	58.92	2.41
3	-6	1	65.62	2.77
3	-6	1	62.79	2.57
3	-6	1	62.00	2.59
-3	6	-1	63.25	2.42
-3	6	-1	62.94	2.62
-3	6	-1	61.57	2.58
2	-6	1	195.54	8.74
2	-6	1	240.87	8.89
2	-6	1	241.77	8.73
-2	6	-1	222.62	8.67
-2	6	-1	231.22	8.73
-2	6	-1	232.36	8.53
-2	6	-1	233.54	8.98
1	-6	1	131.90	4.92
1	-6	1	127.87	4.98
1	-6	1	126.43	4.76
-1	6	-1	128.18	4.78
-1	6	-1	121.13	4.99
-1	6	-1	122.40	4.91
-1	6	-1	129.92	5.19
0	-6	1	1.71	0.28
0	-6	1	1.83	0.18
0	-6	1	2.47	0.40
0	-6	1	1.99	0.47
0	6	-1	1.82	0.19
0	6	-1	2.25	0.38

0	6	-1	2.75	0.53
0	6	-1	1.87	0.29
-1	-6	1	218.48	8.08
-1	-6	1	216.45	8.34
-1	-6	1	211.32	8.17
-1	-6	1	204.01	7.92
1	6	-1	204.31	8.05
1	6	-1	205.56	7.93
1	6	-1	219.89	8.35
1	6	-1	221.11	8.20
-2	-6	1	0.23	0.21
-2	-6	1	0.78	0.35
-2	-6	1	0.17	0.37
-2	-6	1	0.19	0.09
2	6	-1	0.21	0.18
2	6	-1	0.27	0.26
2	6	-1	0.57	0.40
2	6	-1	0.16	0.10
-3	-6	1	95.13	4.05
-3	-6	1	99.89	3.99
-3	-6	1	93.84	3.65
-3	-6	1	91.17	3.90
-3	-6	1	94.37	3.66
-3	-6	1	104.34	4.13
3	6	-1	95.90	3.78
3	6	-1	98.41	4.09
-4	-6	1	76.53	3.04
-4	-6	1	78.56	3.48
-4	-6	1	82.80	3.37
-4	-6	1	82.79	3.21
-4	-6	1	78.02	3.32
-4	-6	1	80.36	3.07
-4	-6	1	79.75	3.51
4	6	-1	77.20	3.18
4	6	-1	83.21	3.50
-5	-6	1	124.89	5.23
-5	-6	1	122.66	5.00
-5	-6	1	126.55	4.79
-5	-6	1	126.21	4.91
-5	-6	1	121.74	5.19
-5	-6	1	122.27	4.74
-5	-6	1	130.79	5.08
5	6	-1	129.25	4.88
-6	-6	1	1.75	0.50

-6	-6	1	2.22	0.27
-6	-6	1	1.58	0.34
-6	-6	1	1.76	0.58
-6	-6	1	1.71	0.55
6	6	-1	1.78	0.27
6	6	-1	1.25	0.27
-7	-6	1	176.96	7.47
-7	-6	1	185.97	7.32
-7	-6	1	190.82	6.98
-7	-6	1	179.22	7.50
-7	-6	1	186.55	7.40
-7	-6	1	178.61	7.13
-7	-6	1	192.29	7.16
7	6	-1	189.62	7.00
-8	-6	1	13.77	0.98
-8	-6	1	12.49	1.34
-8	-6	1	13.82	1.12
-8	-6	1	13.14	0.91
-8	-6	1	13.52	0.87
-8	-6	1	12.64	1.34
-8	-6	1	12.82	0.68
8	6	-1	12.74	0.70
-9	-6	1	108.95	4.93
-9	-6	1	117.61	4.72
-9	-6	1	101.46	4.83
-9	-6	1	117.08	4.56
-9	-6	1	118.47	4.33
-9	-6	1	113.35	4.52
-9	-6	1	110.67	4.51
9	6	-1	104.76	4.33
-10	-6	1	38.01	2.42
-10	-6	1	37.98	2.10
-10	-6	1	38.45	1.91
-10	-6	1	42.36	1.68
-10	-6	1	41.25	1.93
-10	-6	1	36.79	1.90
-10	-6	1	37.29	2.32
10	6	-1	37.73	1.69
-11	-6	1	67.62	3.19
-11	-6	1	67.05	3.00
-11	-6	1	66.68	3.02
-11	-6	1	69.95	2.77
-11	-6	1	65.18	3.39
-11	-6	1	70.29	3.56

-11	-6	1	67.63	3.02
11	6	-1	70.71	2.82
-12	-6	1	30.81	2.02
-12	-6	1	30.99	1.83
-12	-6	1	33.35	1.83
-12	-6	1	33.40	1.54
-12	-6	1	34.34	2.49
-12	-6	1	37.06	1.87
-12	-6	1	32.65	2.30
12	6	-1	34.17	1.59
-13	-6	1	96.26	4.71
-13	-6	1	97.64	3.84
-13	-6	1	94.63	4.16
-13	-6	1	86.02	4.44
-13	-6	1	101.44	4.09
-13	-6	1	89.29	4.27
13	6	-1	100.12	3.88
-14	-6	1	1.77	1.16
-14	-6	1	3.73	0.83
-14	-6	1	3.42	0.79
-14	-6	1	1.80	1.29
-14	-6	1	2.84	1.04
14	6	-1	3.48	0.54
-15	-6	1	107.37	5.02
-15	-6	1	112.28	4.73
-15	-6	1	106.56	5.30
-15	-6	1	112.26	4.60
-15	-6	1	95.86	4.77
15	6	-1	104.79	4.35
-16	-6	1	1.17	1.22
-16	-6	1	1.32	1.43
-16	-6	1	-0.69	0.79
-16	-6	1	0.64	1.15
16	6	-1	0.66	0.49
-17	-6	1	39.25	2.97
-17	-6	1	39.83	2.59
-17	-6	1	39.57	3.46
17	6	-1	44.53	2.14
-18	-6	1	2.13	1.82
-18	-6	1	2.92	1.08
18	6	-1	0.10	0.63
-19	-6	1	34.60	2.68
19	6	-1	37.09	2.07
-20	-6	1	-0.56	1.22

20	6	-1	-0.07	0.76
20	-6	2	0.30	0.80
-20	6	-2	-0.40	1.55
19	-6	2	2.28	0.73
-19	6	-2	3.17	1.36
18	-6	2	1.00	0.66
18	-6	2	2.60	1.19
-18	6	-2	1.84	1.20
17	-6	2	21.37	2.05
17	-6	2	22.91	1.49
-17	6	-2	25.04	2.36
16	-6	2	0.04	0.90
16	-6	2	0.39	0.48
-16	6	-2	3.19	1.48
-16	6	-2	1.71	1.03
15	-6	2	23.21	1.83
15	-6	2	22.55	1.40
-15	6	-2	25.32	2.48
-15	6	-2	24.64	2.05
14	-6	2	-0.88	0.71
14	-6	2	0.34	0.42
-14	6	-2	-0.92	0.68
-14	6	-2	1.39	1.11
13	-6	2	12.64	1.37
13	-6	2	13.14	0.95
13	-6	2	12.02	1.18
-13	6	-2	16.34	1.49
-13	6	-2	12.62	1.86
12	-6	2	6.64	1.10
12	-6	2	6.07	1.00
12	-6	2	5.65	0.69
-12	6	-2	5.34	0.73
-12	6	-2	6.04	0.91
-12	6	-2	6.50	1.17
11	-6	2	4.57	0.65
11	-6	2	5.04	0.58
11	-6	2	4.84	0.80
11	-6	2	3.38	0.64
-11	6	-2	3.88	0.71
-11	6	-2	4.17	0.72
-11	6	-2	3.17	0.46
-11	6	-2	4.21	1.03
10	-6	2	-0.23	0.43
10	-6	2	-0.03	0.27

10	-6	2	0.03	0.45
10	-6	2	-0.40	0.50
-10	6	-2	0.38	0.47
-10	6	-2	-0.26	0.39
-10	6	-2	-0.02	0.29
-10	6	-2	0.76	0.77
9	-6	2	34.49	1.56
9	-6	2	36.90	1.76
9	-6	2	33.45	1.89
9	-6	2	35.79	1.64
-9	6	-2	33.98	1.64
-9	6	-2	34.31	2.09
-9	6	-2	34.82	1.84
-9	6	-2	35.97	1.58
8	-6	2	26.93	1.31
8	-6	2	25.57	1.38
8	-6	2	26.97	1.56
8	-6	2	24.69	1.20
-8	6	-2	26.50	1.75
-8	6	-2	26.12	1.43
-8	6	-2	26.47	1.23
-8	6	-2	25.67	1.45
7	-6	2	150.84	6.30
7	-6	2	154.16	6.01
7	-6	2	161.00	6.18
7	-6	2	160.27	6.09
-7	6	-2	167.74	6.03
-7	6	-2	152.33	6.21
-7	6	-2	155.36	6.45
6	-6	2	40.36	2.00
6	-6	2	39.01	1.69
6	-6	2	41.49	1.86
6	-6	2	40.93	1.80
-6	6	-2	44.48	1.71
-6	6	-2	39.49	1.89
-6	6	-2	39.20	2.12
5	-6	2	23.38	1.30
5	-6	2	20.63	0.94
5	-6	2	19.97	1.10
5	-6	2	21.13	1.08
-5	6	-2	18.53	1.11
-5	6	-2	22.67	0.96
-5	6	-2	19.61	1.24
-5	6	-2	21.38	1.42

4	-6	2	21.92	1.07
4	-6	2	19.79	0.88
4	-6	2	20.15	1.25
4	-6	2	19.58	1.03
-4	6	-2	21.00	1.08
-4	6	-2	20.17	0.89
-4	6	-2	19.59	1.09
-4	6	-2	19.68	1.32
3	-6	2	3.17	0.45
3	-6	2	3.22	0.29
3	-6	2	3.73	0.67
3	-6	2	4.90	0.50
-3	6	-2	3.32	0.55
-3	6	-2	3.06	0.26
-3	6	-2	3.18	0.56
-3	6	-2	3.66	0.49
2	-6	2	31.57	1.62
2	-6	2	30.95	1.23
2	-6	2	29.62	1.42
2	-6	2	31.44	1.45
-2	6	-2	30.26	1.23
-2	6	-2	29.77	1.40
-2	6	-2	31.24	1.85
-2	6	-2	31.23	1.48
1	-6	2	64.67	2.72
1	-6	2	59.45	2.54
1	-6	2	60.38	2.31
1	-6	2	56.62	2.58
-1	6	-2	60.90	2.49
-1	6	-2	59.22	2.57
-1	6	-2	61.95	2.32
0	-6	2	101.06	4.13
0	-6	2	96.38	3.72
0	-6	2	99.74	3.91
0	6	-2	103.24	3.73
0	6	-2	95.64	4.00
0	6	-2	97.43	3.90
0	6	-2	100.58	4.16
-1	-6	2	26.67	1.45
-1	-6	2	27.54	1.46
-1	-6	2	25.80	1.23
-1	-6	2	25.42	1.03
1	6	-2	27.55	1.49
1	6	-2	26.28	1.21

1	6	-2	26.03	1.04
-2	-6	2	40.23	2.01
-2	-6	2	42.35	2.00
-2	-6	2	40.53	1.55
-2	-6	2	39.63	1.99
2	6	-2	39.97	1.99
2	6	-2	39.14	1.72
2	6	-2	39.95	1.56
-3	-6	2	94.98	3.85
-3	-6	2	102.99	4.29
-3	-6	2	100.35	3.84
-3	-6	2	106.55	4.05
-3	-6	2	98.01	4.30
3	6	-2	105.64	4.28
3	6	-2	108.69	4.03
-4	-6	2	85.94	3.34
-4	-6	2	90.64	3.79
-4	-6	2	85.05	3.32
-4	-6	2	86.16	3.82
-4	-6	2	91.41	3.53
-4	-6	2	87.50	3.68
4	6	-2	91.35	3.77
4	6	-2	85.28	3.48
-5	-6	2	35.03	1.87
-5	-6	2	37.08	1.81
-5	-6	2	34.75	1.39
-5	-6	2	34.90	1.59
-5	-6	2	33.55	1.42
-5	-6	2	34.69	1.93
5	6	-2	35.32	1.85
5	6	-2	35.34	1.56
5	6	-2	34.94	1.41
-6	-6	2	116.08	4.98
-6	-6	2	114.47	4.86
-6	-6	2	111.14	4.78
-6	-6	2	119.24	4.41
-6	-6	2	118.77	4.66
-6	-6	2	118.87	4.60
6	6	-2	113.81	4.56
6	6	-2	116.53	4.42
-7	-6	2	16.20	0.96
-7	-6	2	15.13	1.28
-7	-6	2	15.88	1.03
-7	-6	2	16.70	1.00

-7	-6	2	18.31	1.26
-7	-6	2	17.33	1.45
7	6	-2	16.17	0.77
-8	-6	2	61.93	2.86
-8	-6	2	63.32	2.41
-8	-6	2	58.47	2.59
-8	-6	2	60.59	2.63
-8	-6	2	60.84	3.07
-8	-6	2	55.07	2.88
8	6	-2	64.13	2.43
-9	-6	2	41.99	2.15
-9	-6	2	41.30	1.99
-9	-6	2	45.72	1.78
-9	-6	2	46.63	2.29
-9	-6	2	41.63	2.51
-9	-6	2	41.34	2.01
-9	-6	2	40.49	2.33
9	6	-2	42.68	1.80
-10	-6	2	25.68	1.44
-10	-6	2	25.09	1.44
-10	-6	2	25.37	2.04
-10	-6	2	25.38	1.72
-10	-6	2	25.67	1.43
-10	-6	2	27.67	1.19
-10	-6	2	22.71	1.81
10	6	-2	25.01	1.20
-11	-6	2	35.23	1.50
-11	-6	2	33.40	2.43
-11	-6	2	31.47	1.76
-11	-6	2	33.83	1.73
-11	-6	2	32.73	2.04
-11	-6	2	30.28	2.14
-11	-6	2	32.42	1.77
11	6	-2	34.04	1.52
-12	-6	2	111.88	4.59
-12	-6	2	111.43	5.20
-12	-6	2	102.37	4.56
-12	-6	2	110.85	4.47
-12	-6	2	102.39	4.79
-12	-6	2	106.40	4.77
-12	-6	2	112.34	4.29
12	6	-2	112.33	4.32
-13	-6	2	22.01	1.92
-13	-6	2	22.09	1.43

-13	-6	2	20.78	2.34
-13	-6	2	22.45	1.17
-13	-6	2	22.00	1.53
-13	-6	2	17.57	1.83
13	6	-2	21.43	1.19
-14	-6	2	81.20	3.19
-14	-6	2	70.27	4.07
-14	-6	2	70.19	3.36
-14	-6	2	70.85	3.19
-14	-6	2	66.11	3.54
-14	-6	2	66.42	3.58
14	6	-2	74.98	3.03
-15	-6	2	4.53	1.16
-15	-6	2	3.27	0.84
-15	-6	2	3.27	1.12
-15	-6	2	2.87	0.79
-15	-6	2	1.88	1.41
15	6	-2	2.56	0.55
-16	-6	2	24.68	2.40
-16	-6	2	24.13	2.27
-16	-6	2	25.48	3.05
-16	-6	2	27.93	2.02
16	6	-2	23.84	1.54
-17	-6	2	5.24	1.34
-17	-6	2	5.15	1.11
-17	-6	2	6.42	1.79
17	6	-2	5.14	0.78
-18	-6	2	32.88	2.38
-18	-6	2	25.44	3.50
18	6	-2	31.32	1.82
-19	-6	2	6.11	1.30
-19	-6	2	4.15	2.27
19	6	-2	6.49	0.93
-20	-6	2	25.76	2.53
20	6	-2	27.69	1.89
-20	6	-3	0.33	1.64
19	-6	3	25.05	2.47
19	-6	3	24.90	1.74
-19	6	-3	24.24	2.86
18	-6	3	1.21	1.15
18	-6	3	0.75	0.65
-18	6	-3	0.95	1.24
17	-6	3	14.90	1.30
17	-6	3	14.91	1.98

-17	6	-3	14.96	2.17
16	-6	3	13.19	1.17
16	-6	3	15.11	1.77
-16	6	-3	11.76	1.91
-16	6	-3	17.25	2.69
15	-6	3	1.62	0.55
15	-6	3	3.04	0.98
-15	6	-3	0.95	1.42
-15	6	-3	1.44	0.96
14	-6	3	11.60	0.96
14	-6	3	9.23	1.45
-14	6	-3	10.00	1.47
-14	6	-3	12.01	1.61
13	-6	3	18.15	1.12
13	-6	3	19.72	1.63
-13	6	-3	17.73	1.61
-13	6	-3	16.74	2.05
12	-6	3	0.83	0.34
12	-6	3	0.98	0.73
-12	6	-3	0.81	0.66
-12	6	-3	0.14	0.88
11	-6	3	0.99	0.65
11	-6	3	-0.74	0.50
11	-6	3	-0.02	0.28
-11	6	-3	2.93	0.93
-11	6	-3	-0.59	0.30
-11	6	-3	0.28	0.55
10	-6	3	40.02	1.92
10	-6	3	36.44	1.71
10	-6	3	37.37	1.71
10	-6	3	38.41	2.11
-10	6	-3	40.92	1.73
-10	6	-3	39.38	2.04
-10	6	-3	37.19	2.31
-10	6	-3	35.53	1.66
9	-6	3	2.36	0.42
9	-6	3	1.46	0.45
9	-6	3	1.11	0.29
9	-6	3	1.62	0.60
-9	6	-3	1.78	0.35
-9	6	-3	1.84	0.51
-9	6	-3	3.07	0.79
-9	6	-3	1.60	0.39
8	-6	3	20.86	1.43

8	-6	3	20.53	1.23
8	-6	3	20.89	1.05
8	-6	3	22.34	1.11
-8	6	-3	23.29	1.07
-8	6	-3	22.07	1.31
-8	6	-3	23.37	1.64
-8	6	-3	20.85	1.12
7	-6	3	2.84	0.40
7	-6	3	3.86	0.63
7	-6	3	3.31	0.52
7	-6	3	4.11	0.69
-7	6	-3	3.27	0.46
-7	6	-3	4.11	0.42
-7	6	-3	2.52	0.47
-7	6	-3	5.42	0.99
6	-6	3	8.85	0.94
6	-6	3	9.66	0.66
6	-6	3	8.52	0.55
6	-6	3	8.44	0.75
-6	6	-3	9.43	1.06
-6	6	-3	8.44	0.71
-6	6	-3	9.24	0.55
-6	6	-3	9.41	0.76
5	-6	3	27.13	1.24
5	-6	3	26.06	1.32
5	-6	3	25.19	1.13
5	-6	3	25.46	1.48
-5	6	-3	23.30	1.57
-5	6	-3	28.00	1.13
-5	6	-3	24.97	1.33
4	-6	3	54.77	2.28
4	-6	3	56.78	2.52
4	-6	3	54.17	2.32
4	-6	3	54.75	2.15
-4	6	-3	55.16	2.61
-4	6	-3	54.91	2.35
-4	6	-3	54.42	2.15
-4	6	-3	51.50	2.33
3	-6	3	1.99	0.36
3	-6	3	2.34	0.27
3	-6	3	2.28	0.50
3	-6	3	1.78	0.32
-3	6	-3	3.21	0.60
-3	6	-3	2.00	0.35

-3	6	-3	2.03	0.23
-3	6	-3	2.07	0.42
2	-6	3	202.90	7.64
2	-6	3	196.39	7.66
2	-6	3	193.33	7.45
2	-6	3	204.60	7.84
2	-6	3	204.32	7.80
-2	6	-3	204.58	7.89
-2	6	-3	197.82	7.64
-2	6	-3	192.35	7.45
1	-6	3	27.43	1.12
1	-6	3	29.34	1.54
1	-6	3	28.68	1.40
1	-6	3	28.87	1.33
1	-6	3	28.35	1.52
-1	6	-3	27.85	1.12
-1	6	-3	28.64	1.33
-1	6	-3	29.17	1.58
0	-6	3	4.31	0.23
0	-6	3	4.90	0.59
0	-6	3	5.27	0.69
0	-6	3	4.88	0.49
0	-6	3	4.48	0.67
0	6	-3	4.65	0.45
0	6	-3	4.49	0.25
-1	-6	3	33.05	1.44
-1	-6	3	29.40	1.96
-1	-6	3	31.90	1.64
-1	-6	3	31.33	1.68
-1	-6	3	30.63	1.19
1	6	-3	30.14	1.85
1	6	-3	31.35	1.41
1	6	-3	31.44	1.20
-2	-6	3	6.96	0.88
-2	-6	3	7.13	0.31
-2	-6	3	7.06	0.78
-2	-6	3	7.03	0.57
-2	-6	3	6.76	0.30
-2	-6	3	6.35	0.70
2	6	-3	6.97	0.54
2	6	-3	7.51	0.81
-3	-6	3	0.30	0.34
-3	-6	3	0.39	0.08
-3	-6	3	0.39	0.09

-3	-6	3	0.13	0.41
-3	-6	3	0.37	0.38
-3	-6	3	0.48	0.24
3	6	-3	0.15	0.39
3	6	-3	0.35	0.22
-4	-6	3	11.71	0.54
-4	-6	3	12.93	1.04
-4	-6	3	13.58	1.13
-4	-6	3	12.82	0.99
-4	-6	3	11.83	0.52
-4	-6	3	11.84	0.77
4	6	-3	13.18	1.04
4	6	-3	11.94	0.73
4	6	-3	12.18	1.06
-5	-6	3	1.30	0.50
-5	-6	3	1.09	0.32
-5	-6	3	1.99	0.50
-5	-6	3	1.50	0.19
-5	-6	3	1.18	0.58
5	6	-3	1.33	0.21
5	6	-3	1.42	0.29
5	6	-3	2.05	0.58
5	6	-3	2.33	0.57
-6	-6	3	26.14	1.05
-6	-6	3	25.96	1.73
-6	-6	3	24.14	1.28
-6	-6	3	25.01	1.55
6	6	-3	25.93	1.65
6	6	-3	24.06	1.23
6	6	-3	25.92	1.12
6	6	-3	25.93	1.52
-7	-6	3	29.96	1.47
-7	-6	3	30.73	2.00
-7	-6	3	30.13	1.52
-7	-6	3	28.35	1.73
-7	-6	3	31.60	1.27
-7	-6	3	30.14	1.51
7	6	-3	33.49	1.93
7	6	-3	29.77	1.27
7	6	-3	30.54	1.45
-8	-6	3	41.84	1.97
-8	-6	3	38.56	2.43
-8	-6	3	42.34	1.94
-8	-6	3	39.08	2.16

-8	-6	3	44.47	1.72
-8	-6	3	42.81	1.94
8	6	-3	43.54	2.35
8	6	-3	42.36	1.73
-9	-6	3	12.09	0.95
-9	-6	3	10.65	0.88
-9	-6	3	11.68	1.55
-9	-6	3	10.71	1.20
-9	-6	3	13.06	0.68
-9	-6	3	12.06	0.92
9	6	-3	12.68	0.69
9	6	-3	12.21	1.44
-10	-6	3	51.00	2.37
-10	-6	3	47.46	2.41
-10	-6	3	49.93	2.65
-10	-6	3	51.11	2.40
-10	-6	3	49.40	2.96
-10	-6	3	50.87	2.59
-10	-6	3	56.30	2.13
10	6	-3	55.13	2.86
10	6	-3	51.25	2.13
-11	-6	3	4.61	0.71
-11	-6	3	4.84	0.52
-11	-6	3	4.55	1.22
-11	-6	3	2.98	0.58
-11	-6	3	4.59	0.90
-11	-6	3	4.76	0.89
11	6	-3	4.61	0.52
11	6	-3	6.21	1.10
-12	-6	3	2.09	0.83
-12	-6	3	1.14	1.09
-12	-6	3	1.46	0.52
-12	-6	3	1.47	0.35
-12	-6	3	1.45	0.89
12	6	-3	1.15	0.33
-13	-6	3	37.92	2.19
-13	-6	3	38.61	3.00
-13	-6	3	36.59	2.07
-13	-6	3	37.97	2.44
-13	-6	3	35.86	2.29
-13	-6	3	42.03	1.76
13	6	-3	37.98	1.76
-14	-6	3	24.68	2.63
-14	-6	3	23.87	1.29

-14	-6	3	20.46	1.72
-14	-6	3	25.29	2.10
-14	-6	3	23.06	1.94
14	6	-3	24.41	1.29
-15	-6	3	25.11	1.93
-15	-6	3	26.03	2.08
-15	-6	3	25.15	2.15
-15	-6	3	29.13	2.93
-15	-6	3	27.54	2.43
15	6	-3	28.10	1.46
-16	-6	3	17.89	2.22
-16	-6	3	12.89	2.15
-16	-6	3	12.81	1.80
-16	-6	3	17.40	1.72
16	6	-3	11.85	1.35
-17	-6	3	4.02	1.85
-17	-6	3	2.37	0.98
17	6	-3	2.27	0.61
-18	-6	3	12.31	2.18
-18	-6	3	15.11	1.92
18	6	-3	10.24	1.21
-19	-6	3	6.88	2.20
-19	-6	3	7.16	1.36
19	6	-3	8.21	1.31
-20	-6	3	1.25	1.24
20	6	-3	1.84	0.81
19	-6	4	5.84	1.52
19	-6	4	3.58	0.85
-19	6	-4	4.33	1.59
18	-6	4	20.90	1.58
18	-6	4	21.21	2.70
-18	6	-4	21.95	2.53
17	-6	4	6.49	1.32
17	-6	4	6.02	0.85
-17	6	-4	6.87	1.37
16	-6	4	45.81	2.79
16	-6	4	46.34	2.27
-16	6	-4	43.86	2.97
15	-6	4	3.44	1.05
15	-6	4	3.44	0.63
-15	6	-4	3.43	1.07
14	-6	4	20.04	1.80
14	-6	4	17.61	1.21
-14	6	-4	19.01	2.43

-14	6	-4	20.45	1.80
13	-6	4	2.17	0.81
13	-6	4	-0.15	0.40
-13	6	-4	1.03	0.75
-13	6	-4	-0.82	1.11
12	-6	4	75.36	3.62
12	-6	4	77.46	3.21
-12	6	-4	74.85	3.89
-12	6	-4	79.78	3.60
11	-6	4	28.11	1.39
11	-6	4	25.96	1.80
11	-6	4	26.44	1.61
-11	6	-4	25.50	2.07
-11	6	-4	28.98	1.39
-11	6	-4	27.74	1.74
10	-6	4	48.15	2.56
10	-6	4	47.98	2.34
10	-6	4	48.80	2.18
-10	6	-4	47.61	2.78
-10	6	-4	51.29	2.49
-10	6	-4	57.77	2.21
9	-6	4	32.89	1.92
9	-6	4	32.02	1.42
9	-6	4	29.41	1.45
9	-6	4	31.91	1.65
9	-6	4	31.76	1.52
-9	6	-4	31.29	1.73
-9	6	-4	30.50	2.08
-9	6	-4	32.64	1.47
8	-6	4	5.61	0.73
8	-6	4	5.78	0.58
8	-6	4	5.71	0.75
8	-6	4	6.09	0.99
8	-6	4	5.35	0.51
-8	6	-4	5.37	0.82
-8	6	-4	7.28	1.08
-8	6	-4	5.52	0.54
7	-6	4	37.80	1.78
7	-6	4	35.65	2.00
7	-6	4	37.02	1.79
7	-6	4	37.15	1.63
7	-6	4	36.14	1.60
-7	6	-4	41.92	1.62
-7	6	-4	36.16	1.82

-7	6	-4	35.37	2.13
6	-6	4	61.76	2.51
6	-6	4	65.56	2.58
6	-6	4	59.33	2.88
6	-6	4	64.16	2.73
6	-6	4	64.25	2.70
-6	6	-4	66.12	2.52
-6	6	-4	60.11	2.98
-6	6	-4	61.06	2.72
5	-6	4	9.64	0.67
5	-6	4	9.47	0.75
5	-6	4	7.66	0.52
5	-6	4	8.25	0.95
-5	6	-4	8.27	1.06
-5	6	-4	9.72	0.53
-5	6	-4	9.65	0.78
4	-6	4	259.99	9.76
4	-6	4	261.01	9.87
4	-6	4	265.93	9.72
4	-6	4	256.59	9.97
4	-6	4	246.65	9.58
-4	6	-4	250.30	9.58
-4	6	-4	247.90	10.01
-4	6	-4	257.74	9.78
3	-6	4	59.46	2.65
3	-6	4	57.83	2.28
3	-6	4	55.44	2.65
3	-6	4	59.64	2.46
3	-6	4	58.88	2.47
-3	6	-4	60.02	2.27
-3	6	-4	56.71	2.73
-3	6	-4	58.38	2.48
2	-6	4	22.07	0.95
2	-6	4	23.49	1.21
2	-6	4	23.25	1.17
2	-6	4	22.35	1.36
2	-6	4	23.11	1.37
-2	6	-4	24.18	0.95
-2	6	-4	23.29	1.17
-2	6	-4	22.59	1.43
1	-6	4	43.29	2.11
1	-6	4	43.05	2.08
1	-6	4	43.49	1.95
1	-6	4	43.37	1.87

1	-6	4	41.47	1.65
-1	6	-4	40.94	1.86
-1	6	-4	45.41	2.14
-1	6	-4	42.28	1.65
0	-6	4	16.92	1.15
0	-6	4	18.79	0.96
0	-6	4	17.19	1.24
0	-6	4	16.90	0.69
0	6	-4	17.66	0.94
0	6	-4	17.35	0.70
0	6	-4	18.07	1.21
-1	-6	4	7.14	0.59
-1	-6	4	6.58	0.95
-1	-6	4	7.31	0.80
-1	-6	4	7.30	0.74
1	6	-4	7.45	0.85
1	6	-4	6.42	0.29
1	6	-4	7.79	0.58
-2	-6	4	2.03	0.61
-2	-6	4	4.22	0.48
-2	-6	4	3.58	0.58
2	6	-4	2.49	0.42
2	6	-4	4.48	0.74
-3	-6	4	12.67	1.01
-3	-6	4	13.86	0.83
-3	-6	4	14.08	1.20
3	6	-4	14.53	1.16
3	6	-4	13.39	0.79
3	6	-4	12.84	1.17
-4	-6	4	28.14	1.34
-4	-6	4	27.52	1.52
-4	-6	4	25.53	1.05
-4	-6	4	26.30	1.04
-4	-6	4	24.94	1.71
4	6	-4	28.09	1.66
4	6	-4	28.47	1.55
-5	-6	4	20.38	1.60
-5	-6	4	22.07	0.90
-5	-6	4	21.40	1.17
-5	-6	4	24.91	1.41
5	6	-4	22.52	0.91
5	6	-4	22.12	1.37
5	6	-4	23.14	1.57
-6	-6	4	1.21	0.48

-6	-6	4	0.68	0.34
-6	-6	4	0.97	0.16
-6	-6	4	0.94	0.62
6	6	-4	1.12	0.31
6	6	-4	1.52	0.22
6	6	-4	0.75	0.51
6	6	-4	1.49	0.63
-7	-6	4	57.80	2.43
-7	-6	4	51.43	2.89
-7	-6	4	53.38	2.38
-7	-6	4	55.79	2.15
-7	-6	4	50.70	2.54
7	6	-4	56.39	2.84
7	6	-4	55.01	2.16
7	6	-4	53.82	2.35
7	6	-4	58.74	2.59
-8	-6	4	0.51	0.32
-8	-6	4	0.78	0.57
-8	-6	4	0.28	0.40
-8	-6	4	1.21	0.42
-8	-6	4	0.70	0.70
8	6	-4	0.41	0.68
8	6	-4	0.88	0.19
-9	-6	4	18.17	0.83
-9	-6	4	16.19	1.33
-9	-6	4	16.26	1.10
-9	-6	4	16.59	1.05
-9	-6	4	15.46	1.07
-9	-6	4	15.53	1.76
9	6	-4	16.68	0.82
9	6	-4	16.65	1.64
-10	-6	4	2.73	0.52
-10	-6	4	3.09	0.49
-10	-6	4	1.62	0.30
-10	-6	4	2.62	1.02
-10	-6	4	2.08	0.69
-10	-6	4	1.72	0.46
10	6	-4	2.02	0.92
10	6	-4	2.48	0.33
-11	-6	4	38.00	2.13
-11	-6	4	41.70	1.74
-11	-6	4	44.48	2.86
-11	-6	4	38.91	1.87
-11	-6	4	37.44	2.03

11	6	-4	39.74	2.64
11	6	-4	40.93	1.74
-12	-6	4	2.50	0.40
-12	-6	4	2.75	0.59
-12	-6	4	2.62	0.53
-12	-6	4	1.23	1.26
-12	-6	4	3.40	0.75
12	6	-4	4.89	1.21
12	6	-4	2.03	0.38
-13	-6	4	31.64	1.98
-13	-6	4	32.75	1.66
-13	-6	4	31.23	2.87
-13	-6	4	33.38	1.56
-13	-6	4	35.23	1.94
13	6	-4	32.78	2.58
13	6	-4	32.62	1.55
-14	-6	4	7.65	1.07
-14	-6	4	6.90	1.16
-14	-6	4	7.69	0.81
-14	-6	4	5.45	1.30
-14	-6	4	7.01	1.64
-14	-6	4	7.03	0.97
14	6	-4	6.18	0.74
14	6	-4	7.50	1.53
-15	-6	4	6.60	1.60
-15	-6	4	7.64	1.04
-15	-6	4	8.32	1.34
-15	-6	4	8.90	1.49
-15	-6	4	7.04	1.73
15	6	-4	8.58	0.86
-16	-6	4	-0.31	1.65
-16	-6	4	0.47	0.95
-16	-6	4	1.69	0.92
16	6	-4	0.07	0.46
-17	-6	4	12.87	1.71
-17	-6	4	8.65	1.97
17	6	-4	8.33	1.03
-18	-6	4	0.68	0.99
-18	-6	4	2.18	1.98
18	6	-4	2.20	0.65
-19	-6	4	5.13	2.58
-19	-6	4	4.76	1.28
19	6	-4	5.74	0.86
-20	-6	4	1.88	1.25

20	6	-4	0.77	0.76
19	-6	5	39.29	3.16
19	-6	5	39.45	2.26
-19	6	-5	36.28	3.23
18	-6	5	5.30	1.51
18	-6	5	5.31	0.89
-18	6	-5	2.56	1.48
17	-6	5	51.09	2.48
17	-6	5	39.43	3.47
-17	6	-5	52.74	3.26
16	-6	5	4.06	1.22
16	-6	5	2.74	0.69
-16	6	-5	1.58	1.15
15	-6	5	61.79	2.78
15	-6	5	59.67	3.25
-15	6	-5	59.90	3.34
14	-6	5	-0.09	0.87
14	-6	5	0.43	0.45
-14	6	-5	1.07	0.88
13	-6	5	57.13	2.99
13	-6	5	51.24	2.72
-13	6	-5	52.79	3.37
-13	6	-5	55.21	2.93
12	-6	5	0.69	0.41
12	-6	5	-0.12	0.74
-12	6	-5	-0.72	1.06
-12	6	-5	1.87	0.69
11	-6	5	50.99	2.48
11	-6	5	52.36	2.74
11	-6	5	49.92	2.23
-11	6	-5	48.95	2.54
-11	6	-5	51.29	2.50
-11	6	-5	48.66	2.94
10	-6	5	25.05	1.54
10	-6	5	26.24	1.31
10	-6	5	26.07	1.80
-10	6	-5	28.82	1.32
-10	6	-5	21.75	2.02
-10	6	-5	24.95	1.63
9	-6	5	36.47	1.65
9	-6	5	35.47	2.11
9	-6	5	36.50	1.85
-9	6	-5	32.68	2.31
-9	6	-5	36.65	1.95

-9	6	-5	40.69	1.67
8	-6	5	34.71	2.11
8	-6	5	36.94	1.84
8	-6	5	38.39	1.61
8	-6	5	36.55	1.65
8	-6	5	36.97	1.76
-8	6	-5	36.72	1.89
-8	6	-5	42.25	1.67
-8	6	-5	35.29	2.26
7	-6	5	141.82	5.34
7	-6	5	134.82	5.34
7	-6	5	137.92	5.77
7	-6	5	141.10	5.51
7	-6	5	136.72	5.48
-7	6	-5	136.42	5.56
-7	6	-5	131.76	5.84
-7	6	-5	147.99	5.35
6	-6	5	5.93	0.94
6	-6	5	4.98	0.70
6	-6	5	5.44	0.46
6	-6	5	5.38	0.86
6	-6	5	5.58	0.57
-6	6	-5	5.85	0.95
-6	6	-5	5.84	0.46
-6	6	-5	4.54	0.67
5	-6	5	179.81	6.82
5	-6	5	174.55	6.72
5	-6	5	173.70	7.04
5	-6	5	182.36	6.90
5	-6	5	180.55	7.14
-5	6	-5	185.61	6.72
-5	6	-5	172.51	7.21
-5	6	-5	170.08	6.92
4	-6	5	17.36	0.80
4	-6	5	16.77	1.01
4	-6	5	16.80	1.23
4	-6	5	17.65	0.99
4	-6	5	17.07	1.29
-4	6	-5	16.40	1.35
-4	6	-5	16.94	1.03
3	-6	5	106.99	4.57
3	-6	5	110.66	4.57
3	-6	5	111.26	4.37
3	-6	5	106.25	4.16

3	-6	5	111.40	4.36
-3	6	-5	111.69	4.15
-3	6	-5	108.33	4.63
-3	6	-5	108.30	4.37
2	-6	5	30.34	1.72
2	-6	5	32.27	1.57
2	-6	5	32.53	1.29
2	-6	5	32.77	1.52
2	-6	5	32.78	1.77
-2	6	-5	29.74	1.50
-2	6	-5	33.66	1.29
-2	6	-5	33.02	1.79
1	-6	5	103.94	4.46
1	-6	5	103.73	4.38
1	-6	5	100.73	3.95
1	-6	5	107.31	4.19
-1	6	-5	101.16	4.17
-1	6	-5	113.69	4.48
-1	6	-5	106.36	3.95
0	-6	5	1.08	0.43
0	-6	5	0.40	0.08
0	-6	5	0.60	0.29
0	-6	5	0.34	0.51
0	6	-5	0.56	0.23
0	6	-5	0.51	0.08
0	6	-5	0.81	0.46
0	6	-5	0.05	0.41
-1	-6	5	65.32	3.17
-1	-6	5	68.61	3.03
-1	-6	5	72.83	2.86
1	6	-5	69.60	3.08
1	6	-5	68.84	3.10
1	6	-5	67.03	2.82
-2	-6	5	108.14	4.82
-2	-6	5	114.16	4.63
-2	-6	5	114.74	4.46
2	6	-5	108.65	4.42
2	6	-5	115.59	4.66
2	6	-5	111.38	4.73
-3	-6	5	22.82	1.63
-3	-6	5	24.22	1.42
-3	-6	5	25.59	1.24
3	6	-5	24.43	1.47
3	6	-5	21.88	1.18

3	6	-5	25.08	1.59
-4	-6	5	7.91	0.87
-4	-6	5	7.72	0.66
-4	-6	5	6.78	1.06
4	6	-5	8.64	1.10
4	6	-5	6.72	0.61
-5	-6	5	61.06	2.51
-5	-6	5	54.68	2.97
-5	-6	5	57.57	2.63
-5	-6	5	56.89	2.20
5	6	-5	56.17	2.89
5	6	-5	59.25	2.71
5	6	-5	57.76	2.46
5	6	-5	59.76	2.21
-6	-6	5	34.23	1.82
-6	-6	5	34.39	1.69
-6	-6	5	35.11	1.39
-6	-6	5	33.60	2.22
6	6	-5	34.22	1.63
6	6	-5	38.14	1.88
6	6	-5	35.68	2.15
6	6	-5	36.11	1.40
-7	-6	5	46.52	2.11
-7	-6	5	46.31	1.82
-7	-6	5	45.42	2.69
-7	-6	5	44.36	2.22
7	6	-5	47.55	2.27
7	6	-5	44.62	1.83
7	6	-5	48.05	2.60
-8	-6	5	35.25	2.43
-8	-6	5	34.49	1.72
-8	-6	5	37.36	1.47
-8	-6	5	35.87	1.90
-8	-6	5	33.28	1.76
-8	-6	5	36.59	1.72
8	6	-5	35.82	1.93
8	6	-5	35.15	1.46
8	6	-5	36.70	2.27
-9	-6	5	66.62	2.50
-9	-6	5	64.98	3.49
-9	-6	5	62.17	2.80
-9	-6	5	62.13	2.87
-9	-6	5	62.38	2.77
-9	-6	5	62.79	2.69

9	6	-5	59.92	3.27
9	6	-5	62.84	2.50
-10	-6	5	35.97	1.69
-10	-6	5	34.98	1.93
-10	-6	5	30.37	2.55
-10	-6	5	34.37	1.80
-10	-6	5	32.54	1.82
-10	-6	5	36.72	1.51
10	6	-5	35.24	2.40
10	6	-5	36.76	1.52
-11	-6	5	75.17	2.83
-11	-6	5	66.59	3.21
-11	-6	5	68.59	3.13
-11	-6	5	70.01	2.92
-11	-6	5	71.91	3.16
-11	-6	5	66.44	3.96
11	6	-5	71.64	3.72
11	6	-5	69.74	2.83
-12	-6	5	8.29	0.82
-12	-6	5	7.85	0.65
-12	-6	5	4.14	1.47
-12	-6	5	8.12	1.01
-12	-6	5	7.28	0.97
12	6	-5	8.17	1.42
12	6	-5	6.84	0.63
-13	-6	5	7.82	0.72
-13	-6	5	7.18	0.81
-13	-6	5	7.36	1.06
-13	-6	5	7.29	1.34
-13	-6	5	7.37	1.74
13	6	-5	6.26	1.51
13	6	-5	6.67	0.67
-14	-6	5	26.58	1.42
-14	-6	5	25.97	2.97
-14	-6	5	28.48	1.83
-14	-6	5	29.47	1.45
14	6	-5	23.43	3.09
14	6	-5	28.68	1.40
-15	-6	5	9.98	1.45
-15	-6	5	11.20	2.12
-15	-6	5	9.50	1.07
-15	-6	5	11.04	0.90
15	6	-5	9.42	0.86
-16	-6	5	22.52	3.02

-16	-6	5	15.05	1.79
16	6	-5	17.60	1.18
-17	-6	5	16.43	1.86
-17	-6	5	18.81	3.28
17	6	-5	16.10	1.23
-18	-6	5	7.26	1.23
-18	-6	5	6.45	2.17
18	6	-5	6.26	0.84
-19	-6	5	6.81	2.29
-19	-6	5	2.87	1.19
19	6	-5	2.43	0.74
-20	-6	5	10.09	2.80
-20	-6	5	19.47	2.33
20	6	-5	12.82	1.48
19	-6	6	2.81	1.60
19	-6	6	0.71	0.78
-19	6	-6	-0.02	1.55
18	-6	6	31.82	2.93
18	-6	6	31.37	1.94
-18	6	-6	29.76	2.92
17	-6	6	1.24	0.73
17	-6	6	-0.38	1.25
-17	6	-6	1.99	1.34
16	-6	6	60.42	2.80
16	-6	6	58.19	3.40
-16	6	-6	58.59	3.47
15	-6	6	0.52	0.59
15	-6	6	-1.44	1.06
-15	6	-6	2.51	1.08
14	-6	6	53.64	3.07
14	-6	6	55.51	2.56
-14	6	-6	56.40	3.05
13	-6	6	5.44	0.82
13	-6	6	4.69	1.11
12	-6	6	85.13	3.71
12	-6	6	89.62	4.25
-12	6	-6	89.80	4.08
-12	6	-6	97.52	5.11
11	-6	6	1.25	0.87
11	-6	6	1.40	0.42
-11	6	-6	2.65	1.17
-11	6	-6	2.00	0.74
10	-6	6	100.86	4.29
10	-6	6	103.35	4.15

10	-6	6	102.85	4.64
-10	6	-6	102.49	4.42
-10	6	-6	107.15	4.13
-10	6	-6	100.85	4.81
9	-6	6	-0.30	0.47
9	-6	6	0.06	0.65
9	-6	6	0.04	0.29
-9	6	-6	0.46	0.31
-9	6	-6	1.87	0.96
-9	6	-6	0.13	0.50
8	-6	6	60.06	2.62
8	-6	6	55.89	2.44
8	-6	6	56.40	2.89
8	-6	6	60.94	2.54
8	-6	6	59.37	2.36
-8	6	-6	52.90	3.05
-8	6	-6	62.01	2.43
-8	6	-6	58.86	2.68
7	-6	6	-0.75	0.39
7	-6	6	0.10	0.30
7	-6	6	0.18	0.24
7	-6	6	0.15	0.52
7	-6	6	-0.05	0.55
-7	6	-6	0.01	0.79
-7	6	-6	0.13	0.23
-7	6	-6	0.27	0.44
6	-6	6	93.26	3.72
6	-6	6	95.02	3.77
6	-6	6	95.96	4.20
6	-6	6	95.98	3.90
-6	6	-6	100.06	3.71
-6	6	-6	88.30	4.25
-6	6	-6	95.39	3.94
5	-6	6	0.20	0.33
5	-6	6	-0.34	0.57
5	-6	6	0.53	0.55
5	-6	6	0.19	0.32
5	-6	6	0.49	0.20
-5	6	-6	0.46	0.34
-5	6	-6	0.34	0.19
-5	6	-6	0.89	0.64
4	-6	6	133.12	5.07
4	-6	6	129.21	5.03
4	-6	6	126.26	5.29

4	-6	6	125.91	4.86
4	-6	6	125.34	5.25
-4	6	-6	129.71	4.86
-4	6	-6	121.73	5.06
-4	6	-6	128.12	5.39
3	-6	6	42.68	2.19
3	-6	6	43.75	1.99
3	-6	6	42.71	1.74
3	-6	6	43.24	2.22
3	-6	6	45.75	1.97
-3	6	-6	44.63	1.73
-3	6	-6	43.81	2.29
-3	6	-6	40.97	1.95
2	-6	6	71.32	3.24
2	-6	6	70.14	3.17
2	-6	6	68.45	2.69
2	-6	6	66.61	3.13
2	-6	6	72.80	2.93
-2	6	-6	73.56	2.69
-2	6	-6	67.63	2.92
-2	6	-6	69.24	3.21
1	-6	6	2.84	0.63
1	-6	6	2.61	0.41
1	-6	6	2.90	0.69
1	-6	6	3.00	0.22
-1	6	-6	2.76	0.22
-1	6	-6	2.16	0.38
-1	6	-6	3.43	0.66
-1	6	-6	2.74	0.60
0	-6	6	89.56	3.56
0	-6	6	94.54	4.21
0	-6	6	95.28	4.05
0	-6	6	96.74	3.85
0	6	-6	94.57	3.56
0	6	-6	98.44	3.84
0	6	-6	93.48	4.08
0	6	-6	101.21	4.12
-1	-6	6	18.29	1.01
-1	-6	6	16.22	1.16
-1	-6	6	17.48	1.44
1	6	-6	16.39	1.23
1	6	-6	16.10	0.96
1	6	-6	17.76	1.31
-2	-6	6	181.84	7.30

-2	-6	6	196.86	7.16
-2	-6	6	183.63	7.61
2	6	-6	170.84	7.09
2	6	-6	182.98	7.46
2	6	-6	190.79	7.35
-3	-6	6	14.25	1.45
-3	-6	6	13.77	1.12
-3	-6	6	15.64	0.95
3	6	-6	14.42	1.35
3	6	-6	15.74	1.15
3	6	-6	14.93	0.89
-4	-6	6	113.29	5.14
-4	-6	6	110.16	4.75
-4	-6	6	118.64	4.64
4	6	-6	121.60	4.82
4	6	-6	116.66	5.05
4	6	-6	115.15	4.60
-5	-6	6	21.17	1.72
-5	-6	6	19.80	1.32
-5	-6	6	21.01	0.82
-5	-6	6	21.95	1.19
5	6	-6	20.46	0.82
5	6	-6	21.04	1.12
5	6	-6	18.26	1.49
5	6	-6	21.62	1.67
-6	-6	6	83.36	3.68
-6	-6	6	89.10	3.29
-6	-6	6	86.97	3.62
-6	-6	6	86.24	4.23
6	6	-6	91.39	3.30
6	6	-6	87.29	3.56
6	6	-6	80.19	3.93
6	6	-6	91.05	4.14
-7	-6	6	36.58	1.80
-7	-6	6	35.92	1.86
-7	-6	6	37.35	2.50
-7	-6	6	37.59	1.47
7	6	-6	34.91	1.54
7	6	-6	40.48	2.40
7	6	-6	37.58	1.95
7	6	-6	34.21	1.71
-8	-6	6	108.15	4.65
-8	-6	6	117.96	4.39
-8	-6	6	110.53	4.70

-8	-6	6	111.91	4.69
-8	-6	6	119.77	5.42
8	6	-6	121.83	4.79
8	6	-6	119.13	4.39
8	6	-6	111.64	5.20
-9	-6	6	1.91	0.26
-9	-6	6	1.53	0.44
-9	-6	6	1.54	0.52
-9	-6	6	1.36	0.46
-9	-6	6	1.84	0.64
-9	-6	6	0.76	0.99
9	6	-6	1.86	0.26
9	6	-6	-0.36	0.89
-10	-6	6	76.63	3.41
-10	-6	6	82.87	3.06
-10	-6	6	75.53	3.32
-10	-6	6	76.06	3.34
-10	-6	6	72.60	4.16
-10	-6	6	75.88	3.18
10	6	-6	79.02	4.00
10	6	-6	78.06	3.05
-11	-6	6	4.75	0.80
-11	-6	6	5.21	0.85
-11	-6	6	5.37	0.72
-11	-6	6	4.31	0.53
-11	-6	6	2.23	1.38
11	6	-6	4.29	0.51
11	6	-6	5.53	1.26
-12	-6	6	138.21	5.76
-12	-6	6	145.76	5.42
-12	-6	6	137.05	5.42
-12	-6	6	137.60	6.61
12	6	-6	142.95	5.41
12	6	-6	135.57	6.35
-13	-6	6	23.18	1.22
-13	-6	6	27.53	2.82
-13	-6	6	23.12	1.19
-13	-6	6	21.62	1.31
13	6	-6	21.63	1.16
-14	-6	6	69.91	2.87
-14	-6	6	69.90	3.36
-14	-6	6	70.08	2.99
-14	-6	6	74.34	4.45
14	6	-6	72.66	2.96

-15	-6	6	34.00	2.18
-15	-6	6	36.52	1.88
-15	-6	6	24.16	3.18
15	6	-6	30.40	1.59
-16	-6	6	33.05	4.03
-16	-6	6	33.59	2.34
16	6	-6	36.01	1.73
-17	-6	6	28.56	3.48
-17	-6	6	22.70	2.15
17	6	-6	23.57	1.44
-18	-6	6	38.28	2.71
-18	-6	6	34.54	4.03
18	6	-6	40.41	2.03
-19	-6	6	11.16	2.60
-19	-6	6	7.57	1.32
19	6	-6	10.42	1.28
-20	-6	6	23.39	4.28
-20	-6	6	26.12	2.55
20	6	-6	21.48	1.73
18	-6	7	11.30	1.94
18	-6	7	13.08	1.50
-18	6	-7	17.16	2.70
17	-6	7	9.68	1.32
17	-6	7	6.37	1.60
-17	6	-7	12.61	1.74
16	-6	7	13.26	1.30
16	-6	7	9.49	1.61
-16	6	-7	12.50	2.11
15	-6	7	34.00	1.95
15	-6	7	36.17	2.59
-15	6	-7	37.29	2.66
14	-6	7	35.43	1.87
-14	6	-7	32.46	2.41
13	-6	7	17.15	1.84
13	-6	7	18.08	1.25
-13	6	-7	18.28	2.10
12	-6	7	6.26	0.84
12	-6	7	6.88	1.16
-12	6	-7	6.44	1.04
11	-6	7	19.79	1.90
11	-6	7	21.77	1.26
-11	6	-7	20.47	1.63
10	-6	7	3.72	0.70
10	-6	7	4.42	0.95

10	-6	7	4.34	0.56
-10	6	-7	5.99	1.26
-10	6	-7	4.76	0.82
9	-6	7	61.19	2.77
9	-6	7	65.82	3.23
9	-6	7	58.45	2.61
-9	6	-7	61.40	2.89
-9	6	-7	55.33	3.33
-9	6	-7	68.16	2.61
8	-6	7	23.68	1.23
8	-6	7	24.85	1.16
8	-6	7	24.51	1.80
8	-6	7	26.04	1.44
8	-6	7	23.33	1.38
-8	6	-7	23.98	1.52
-8	6	-7	21.98	1.92
-8	6	-7	26.16	1.21
7	-6	7	40.50	1.94
7	-6	7	41.13	2.32
7	-6	7	42.99	2.07
7	-6	7	38.94	1.75
7	-6	7	39.73	1.76
-7	6	-7	41.28	2.02
-7	6	-7	41.28	1.74
-7	6	-7	35.75	2.37
6	-6	7	46.78	2.00
6	-6	7	46.42	2.12
6	-6	7	45.81	2.33
6	-6	7	45.18	2.43
6	-6	7	44.55	1.92
-6	6	-7	47.62	1.91
-6	6	-7	43.23	2.51
-6	6	-7	45.06	2.15
5	-6	7	63.22	2.72
5	-6	7	62.54	2.95
5	-6	7	60.66	3.01
5	-6	7	62.57	2.51
5	-6	7	65.13	2.66
-5	6	-7	54.75	3.04
-5	6	-7	66.64	2.50
-5	6	-7	62.00	2.73
4	-6	7	1.72	0.44
4	-6	7	1.17	0.22
4	-6	7	1.67	0.42

4	-6	7	1.03	0.59
4	-6	7	0.59	0.50
-4	6	-7	0.50	0.34
-4	6	-7	1.09	0.22
-4	6	-7	0.21	0.62
3	-6	7	212.15	7.75
3	-6	7	197.50	8.02
3	-6	7	195.13	7.81
3	-6	7	187.88	7.51
3	-6	7	205.32	8.05
-3	6	-7	194.79	7.74
-3	6	-7	196.41	8.06
-3	6	-7	211.22	7.52
2	-6	7	10.00	1.02
2	-6	7	10.65	1.26
2	-6	7	10.32	0.82
-2	6	-7	10.05	0.50
-2	6	-7	9.96	1.15
-2	6	-7	9.21	0.79
-2	6	-7	9.30	0.99
1	-6	7	135.05	5.39
1	-6	7	146.28	6.07
1	-6	7	143.69	5.91
1	-6	7	154.14	5.68
-1	6	-7	140.75	5.88
-1	6	-7	138.70	5.64
-1	6	-7	148.81	5.96
-1	6	-7	145.47	5.39
0	-6	7	32.26	1.52
0	-6	7	28.71	1.94
0	-6	7	28.10	1.70
0	6	-7	30.04	1.18
0	6	-7	30.35	1.47
0	6	-7	30.43	1.80
0	6	-7	32.92	1.79
0	6	-7	30.86	1.20
-1	-6	7	82.54	3.85
-1	-6	7	86.67	3.36
-1	-6	7	76.73	3.51
1	6	-7	80.50	3.68
1	6	-7	76.87	3.30
1	6	-7	81.25	3.58
-2	-6	7	32.52	1.85
-2	-6	7	36.68	1.69

-2	-6	7	33.61	2.19
2	6	-7	35.41	2.09
2	6	-7	35.38	1.63
2	6	-7	35.37	1.91
-3	-6	7	178.35	7.53
-3	-6	7	183.79	7.94
-3	-6	7	199.16	7.43
3	6	-7	197.52	7.39
3	6	-7	196.58	7.60
3	6	-7	189.94	7.84
-4	-6	7	18.08	1.69
-4	-6	7	17.35	1.07
-4	-6	7	18.43	1.29
4	6	-7	17.80	1.59
4	6	-7	18.30	1.28
4	6	-7	16.00	1.00
-5	-6	7	92.82	3.83
-5	-6	7	92.32	3.47
-5	-6	7	86.28	3.89
-5	-6	7	90.99	4.45
5	6	-7	88.89	3.77
5	6	-7	96.28	3.47
5	6	-7	99.31	4.00
5	6	-7	95.38	4.36
-6	-6	7	3.55	0.64
-6	-6	7	3.79	1.06
-6	-6	7	3.75	0.27
-6	-6	7	4.86	0.62
6	6	-7	2.44	0.89
6	6	-7	3.93	0.27
6	6	-7	3.47	0.59
6	6	-7	4.27	0.73
-7	-6	7	56.98	3.32
-7	-6	7	58.42	2.63
-7	-6	7	57.41	2.64
-7	-6	7	60.14	2.28
7	6	-7	56.17	3.22
7	6	-7	59.16	2.56
7	6	-7	61.44	2.28
-8	-6	7	0.42	0.93
-8	-6	7	1.03	0.49
-8	-6	7	0.91	0.39
-8	-6	7	0.67	0.19
-8	-6	7	0.85	0.53

8	6	-7	-0.90	0.85
8	6	-7	-0.18	0.58
8	6	-7	0.27	0.20
-9	-6	7	141.73	5.87
-9	-6	7	141.26	5.58
-9	-6	7	145.09	5.78
-9	-6	7	142.71	6.68
-9	-6	7	146.08	5.83
9	6	-7	146.98	5.52
9	6	-7	146.78	6.47
9	6	-7	152.06	5.90
-10	-6	7	6.36	1.01
-10	-6	7	7.90	0.56
-10	-6	7	7.47	1.50
-10	-6	7	5.47	0.86
-10	-6	7	6.42	0.78
-10	-6	7	7.04	1.00
10	6	-7	6.62	0.51
10	6	-7	8.94	1.51
-11	-6	7	101.25	4.15
-11	-6	7	98.91	4.40
-11	-6	7	108.29	4.09
-11	-6	7	110.54	5.38
11	6	-7	108.55	4.08
11	6	-7	100.52	5.08
-12	-6	7	4.46	0.68
-12	-6	7	4.89	0.82
-12	-6	7	5.70	0.62
-12	-6	7	5.40	1.51
-12	-6	7	5.31	0.78
12	6	-7	5.79	0.57
-13	-6	7	51.89	2.30
-13	-6	7	59.98	2.43
-13	-6	7	58.93	3.92
-13	-6	7	55.65	2.79
13	6	-7	58.31	2.38
-14	-6	7	5.07	0.78
-14	-6	7	5.66	0.92
-14	-6	7	6.11	1.83
14	6	-7	5.10	0.70
-15	-6	7	109.77	4.36
-15	-6	7	107.61	4.82
15	6	-7	106.23	4.32
-16	-6	7	1.14	1.87

-16	-6	7	-0.12	0.82
16	6	-7	0.61	0.48
-17	-6	7	47.29	4.21
-17	-6	7	49.61	2.96
17	6	-7	46.92	2.44
-18	-6	7	-2.65	2.20
-18	-6	7	-0.35	0.98
18	6	-7	-0.02	0.62
-19	-6	7	22.64	2.41
-19	-6	7	24.66	4.16
19	6	-7	23.94	1.66
-20	-6	7	3.47	2.69
20	6	-7	4.12	0.91
18	-6	8	1.53	0.85
18	-6	8	1.57	1.63
-18	6	-8	4.24	1.62
17	-6	8	15.04	2.57
17	-6	8	15.03	1.55
-17	6	-8	19.23	2.54
16	-6	8	-0.14	1.36
16	-6	8	-0.29	0.68
-16	6	-8	-2.12	1.20
15	-6	8	43.45	2.31
15	-6	8	51.48	3.20
-15	6	-8	43.54	2.92
14	-6	8	-0.42	1.10
14	-6	8	-0.95	0.52
-14	6	-8	0.61	0.93
13	-6	8	42.52	2.14
13	-6	8	41.85	2.75
-13	6	-8	44.01	2.59
12	-6	8	9.12	1.42
-12	6	-8	10.71	1.48
11	-6	8	24.82	2.04
11	-6	8	21.60	1.32
-11	6	-8	24.84	1.79
10	-6	8	0.02	0.58
10	-6	8	0.69	0.87
10	-6	8	0.29	0.40
-10	6	-8	-0.02	0.61
-10	6	-8	0.60	0.39
9	-6	8	6.09	1.12
9	-6	8	5.56	0.76
9	-6	8	6.33	0.69

-9	6	-8	4.57	1.30
-9	6	-8	5.55	0.69
-9	6	-8	5.44	0.81
8	-6	8	5.36	0.98
8	-6	8	5.38	0.75
8	-6	8	4.67	0.61
8	-6	8	5.17	0.92
8	-6	8	5.01	0.61
-8	6	-8	5.06	0.61
-8	6	-8	6.36	1.28
-8	6	-8	5.71	0.80
7	-6	8	31.62	1.50
7	-6	8	33.58	1.52
7	-6	8	30.65	2.40
7	-6	8	33.78	2.11
7	-6	8	33.47	1.73
-7	6	-8	36.01	1.50
-7	6	-8	29.37	2.23
-7	6	-8	31.22	1.77
6	-6	8	24.86	1.76
6	-6	8	26.33	1.34
6	-6	8	23.26	1.39
6	-6	8	26.98	1.72
6	-6	8	24.41	1.19
-6	6	-8	26.33	1.17
-6	6	-8	23.87	1.45
-6	6	-8	22.02	1.94
5	-6	8	58.94	2.53
5	-6	8	58.10	2.81
5	-6	8	55.76	2.54
5	-6	8	56.15	2.34
5	-6	8	57.19	2.92
-5	6	-8	60.15	2.33
-5	6	-8	55.49	2.98
-5	6	-8	57.93	2.58
4	-6	8	5.52	0.80
4	-6	8	5.07	0.45
4	-6	8	3.43	0.75
4	-6	8	6.10	1.14
4	-6	8	5.24	0.73
-4	6	-8	4.75	0.73
-4	6	-8	5.32	0.42
-4	6	-8	7.45	1.21
3	-6	8	16.45	0.80

3	-6	8	17.60	1.11
3	-6	8	15.90	1.41
3	-6	8	18.76	1.56
-3	6	-8	15.90	1.46
-3	6	-8	16.38	1.06
-3	6	-8	18.37	0.83
-3	6	-8	18.47	1.30
2	-6	8	19.25	1.57
2	-6	8	17.17	0.82
2	-6	8	21.41	1.15
2	-6	8	16.70	1.39
-2	6	-8	18.76	0.81
-2	6	-8	16.71	1.47
-2	6	-8	17.99	1.09
-2	6	-8	18.09	1.36
1	-6	8	66.75	2.80
1	-6	8	64.00	3.26
1	-6	8	67.32	3.08
-1	6	-8	66.99	2.51
-1	6	-8	62.41	2.77
-1	6	-8	64.36	3.10
-1	6	-8	65.12	3.08
0	-6	8	51.36	2.94
0	-6	8	52.19	2.63
0	-6	8	55.43	2.43
0	6	-8	55.20	2.13
0	6	-8	57.31	2.81
0	6	-8	58.16	2.73
0	6	-8	55.82	2.11
0	6	-8	57.23	2.41
-1	-6	8	35.12	2.28
-1	-6	8	34.67	1.94
-1	-6	8	37.59	1.72
1	6	-8	32.88	2.12
1	6	-8	35.10	1.34
1	6	-8	34.24	1.66
1	6	-8	35.48	1.35
1	6	-8	36.13	1.98
-2	-6	8	44.57	2.73
-2	-6	8	47.86	2.39
-2	-6	8	51.18	2.19
2	6	-8	47.23	2.67
2	6	-8	49.51	2.43
2	6	-8	47.57	2.13

-3	-6	8	107.75	4.16
-3	-6	8	94.88	4.29
-3	-6	8	99.50	4.77
3	6	-8	100.95	4.10
3	6	-8	104.59	4.36
3	6	-8	102.70	4.67
-4	-6	8	62.35	2.68
-4	-6	8	60.57	3.35
-4	-6	8	57.26	2.79
4	6	-8	64.69	2.87
4	6	-8	59.21	2.61
4	6	-8	61.91	3.25
-5	-6	8	15.63	1.06
-5	-6	8	14.16	1.71
-5	-6	8	14.98	1.17
5	6	-8	15.20	1.70
5	6	-8	14.43	1.26
5	6	-8	14.36	0.97
-6	-6	8	53.34	3.18
-6	-6	8	52.52	1.99
-6	-6	8	49.65	2.44
-6	-6	8	50.80	2.37
6	6	-8	51.23	1.98
6	6	-8	56.26	2.56
6	6	-8	50.34	2.97
6	6	-8	50.95	2.30
-7	-6	8	53.42	2.39
-7	-6	8	48.73	3.15
-7	-6	8	51.27	1.98
-7	-6	8	49.69	2.38
7	6	-8	49.75	1.97
7	6	-8	49.79	2.28
7	6	-8	53.50	2.50
7	6	-8	48.93	3.07
-8	-6	8	36.31	1.83
-8	-6	8	36.12	2.68
-8	-6	8	35.48	1.79
-8	-6	8	34.23	1.43
-8	-6	8	32.15	1.81
8	6	-8	35.21	1.43
8	6	-8	35.26	2.59
-9	-6	8	15.43	1.16
-9	-6	8	17.09	1.20
-9	-6	8	15.72	2.20

-9	-6	8	15.79	1.27
9	6	-8	17.88	1.38
9	6	-8	19.73	2.27
9	6	-8	16.37	0.79
-10	-6	8	68.88	2.61
-10	-6	8	67.28	3.96
-10	-6	8	63.03	2.94
-10	-6	8	64.35	2.85
-10	-6	8	65.08	2.77
10	6	-8	65.30	2.99
10	6	-8	64.00	2.59
10	6	-8	64.18	3.74
-11	-6	8	14.17	1.41
-11	-6	8	11.86	1.13
-11	-6	8	11.83	0.93
-11	-6	8	15.44	2.44
-11	-6	8	13.94	0.80
11	6	-8	12.88	0.75
-12	-6	8	116.20	4.41
-12	-6	8	112.02	5.79
-12	-6	8	105.61	4.38
-12	-6	8	110.93	4.36
12	6	-8	116.51	4.38
-13	-6	8	24.51	1.27
-13	-6	8	26.71	3.04
-13	-6	8	22.24	1.69
13	6	-8	24.05	1.20
-14	-6	8	35.69	1.71
-14	-6	8	35.56	2.17
14	6	-8	34.10	1.63
-15	-6	8	19.93	1.80
-15	-6	8	20.75	1.31
-15	-6	8	20.76	3.25
15	6	-8	20.01	1.19
-16	-6	8	55.66	3.08
-16	-6	8	59.93	4.41
16	6	-8	52.77	2.42
-17	-6	8	4.74	2.26
-17	-6	8	5.35	1.18
-18	-6	8	52.60	3.26
18	6	-8	54.54	2.52
-19	-6	8	0.86	2.46
-19	-6	8	3.57	1.30
19	6	-8	4.19	0.81

17	-6	9	11.59	1.52
17	-6	9	10.36	1.97
-17	6	-9	11.26	1.83
16	-6	9	6.14	1.63
16	-6	9	9.00	1.26
-16	6	-9	4.91	1.47
15	-6	9	14.68	2.34
15	-6	9	14.56	1.33
-15	6	-9	14.55	2.16
14	-6	9	21.14	2.40
14	-6	9	21.36	1.51
-14	6	-9	19.94	2.11
13	-6	9	2.67	1.21
13	-6	9	3.78	0.68
-13	6	-9	5.14	1.08
12	-6	9	2.51	1.06
12	-6	9	1.80	0.56
-12	6	-9	2.04	0.85
11	-6	9	0.54	0.47
11	-6	9	0.46	1.09
-11	6	-9	0.43	0.71
10	-6	9	14.72	1.05
10	-6	9	14.46	1.79
10	-6	9	15.07	1.43
-10	6	-9	13.93	1.44
9	-6	9	1.87	0.93
9	-6	9	3.13	0.49
9	-6	9	2.28	0.70
-9	6	-9	4.32	0.67
-9	6	-9	3.02	0.71
8	-6	9	9.62	1.54
8	-6	9	10.08	1.10
8	-6	9	8.56	1.03
8	-6	9	9.54	0.77
8	-6	9	8.15	0.77
-8	6	-9	10.88	0.78
-8	6	-9	10.34	1.16
7	-6	9	21.93	1.20
7	-6	9	19.91	1.10
7	-6	9	19.50	1.72
7	-6	9	20.98	1.57
7	-6	9	21.50	1.38
-7	6	-9	19.60	1.39
-7	6	-9	17.70	1.97

-7	6	-9	21.72	1.09
6	-6	9	38.80	2.13
6	-6	9	39.85	1.86
6	-6	9	37.75	1.92
6	-6	9	40.99	2.38
6	-6	9	37.99	1.71
-6	6	-9	33.11	2.45
-6	6	-9	37.99	1.97
-6	6	-9	42.89	1.70
5	-6	9	0.24	0.47
5	-6	9	0.33	0.72
5	-6	9	0.58	0.25
5	-6	9	0.97	0.65
5	-6	9	0.48	0.45
-5	6	-9	0.22	0.83
-5	6	-9	1.13	0.44
-5	6	-9	0.58	0.26
4	-6	9	5.76	0.98
4	-6	9	6.77	1.06
4	-6	9	5.78	0.81
4	-6	9	5.17	0.49
-4	6	-9	6.66	0.97
-4	6	-9	6.29	1.11
-4	6	-9	6.25	0.48
-4	6	-9	5.00	0.77
3	-6	9	12.28	0.95
3	-6	9	10.86	1.25
3	-6	9	10.74	1.44
3	-6	9	11.32	0.64
-3	6	-9	10.35	1.21
-3	6	-9	12.18	0.62
-3	6	-9	12.45	0.92
-3	6	-9	11.04	1.37
2	-6	9	67.44	2.83
2	-6	9	70.32	3.61
2	-6	9	70.67	3.44
2	-6	9	76.79	3.12
-2	6	-9	74.38	3.48
-2	6	-9	73.50	3.39
-2	6	-9	71.28	3.08
-2	6	-9	78.24	2.83
1	-6	9	8.11	0.82
1	-6	9	7.81	1.26
1	-6	9	7.62	1.13

-1	6	-9	6.82	0.74
-1	6	-9	5.63	0.94
-1	6	-9	8.04	0.42
-1	6	-9	6.97	1.21
0	-6	9	101.69	4.01
0	-6	9	93.75	4.59
0	-6	9	92.66	4.27
0	6	-9	95.11	3.96
0	6	-9	101.88	3.68
0	6	-9	95.58	4.40
0	6	-9	97.03	4.31
0	6	-9	99.90	3.70
-1	-6	9	7.33	1.02
-1	-6	9	8.18	1.50
-1	-6	9	6.04	0.76
1	6	-9	7.40	0.36
1	6	-9	10.60	1.43
1	6	-9	7.39	0.36
1	6	-9	6.93	0.69
1	6	-9	8.37	1.17
-2	-6	9	48.88	2.20
-2	-6	9	44.42	2.38
-2	-6	9	47.18	2.88
2	6	-9	48.60	2.76
2	6	-9	47.28	2.13
2	6	-9	49.51	2.46
-3	-6	9	1.67	0.45
-3	-6	9	1.37	0.60
-3	-6	9	0.41	0.92
3	6	-9	1.08	0.63
3	6	-9	1.62	0.41
3	6	-9	2.19	0.91
-4	-6	9	58.36	3.36
-4	-6	9	60.39	2.58
-4	-6	9	51.45	2.69
4	6	-9	60.40	2.79
4	6	-9	57.18	2.50
4	6	-9	56.66	3.16
-5	-6	9	14.74	1.91
-5	-6	9	15.66	1.10
-5	-6	9	14.50	1.23
5	6	-9	15.97	1.02
5	6	-9	15.58	0.63
5	6	-9	15.26	1.81

5	6	-9	16.25	1.38
-6	-6	9	18.44	0.79
-6	-6	9	18.55	1.33
-6	-6	9	18.66	1.25
-6	-6	9	18.25	2.06
6	6	-9	19.73	0.78
6	6	-9	18.96	1.49
6	6	-9	20.67	2.05
6	6	-9	18.98	1.16
-7	-6	9	1.60	1.12
-7	-6	9	2.84	0.32
-7	-6	9	2.68	0.65
-7	-6	9	3.54	0.62
7	6	-9	3.75	1.10
7	6	-9	2.27	0.47
7	6	-9	4.59	0.80
7	6	-9	2.91	0.29
-8	-6	9	37.09	1.51
-8	-6	9	36.38	2.85
-8	-6	9	36.85	1.98
-8	-6	9	34.61	1.90
-8	-6	9	38.04	1.89
8	6	-9	37.06	2.79
8	6	-9	38.37	2.07
8	6	-9	37.63	1.50
-9	-6	9	25.16	1.15
-9	-6	9	25.96	1.69
-9	-6	9	22.69	2.57
-9	-6	9	26.73	1.49
-9	-6	9	24.99	1.50
9	6	-9	26.18	1.12
9	6	-9	28.72	2.62
-10	-6	9	53.30	3.67
-10	-6	9	55.90	2.53
-10	-6	9	57.08	2.63
-10	-6	9	55.40	2.25
10	6	-9	54.09	2.67
10	6	-9	56.00	2.22
-11	-6	9	5.67	0.83
-11	-6	9	4.95	1.53
-11	-6	9	6.47	0.62
-11	-6	9	6.72	0.80
11	6	-9	5.44	0.52
-12	-6	9	3.47	1.57

-12	-6	9	7.00	0.93
-12	-6	9	6.67	0.74
-12	-6	9	9.32	0.74
-12	-6	9	8.28	0.71
12	6	-9	7.11	0.60
-13	-6	9	17.50	1.56
-13	-6	9	18.94	1.13
-13	-6	9	18.92	3.37
13	6	-9	19.75	1.04
-14	-6	9	30.08	2.03
-14	-6	9	32.21	3.40
-14	-6	9	31.39	1.56
14	6	-9	27.77	1.44
-15	-6	9	16.96	1.23
-15	-6	9	16.04	1.68
-15	-6	9	17.70	3.28
15	6	-9	14.32	1.05
-16	-6	9	20.93	2.00
-16	-6	9	16.50	2.71
16	6	-9	21.95	1.30
-17	-6	9	12.37	1.89
-17	-6	9	5.50	2.44
17	6	-9	7.66	0.92
-18	-6	9	14.57	2.84
-18	-6	9	15.50	2.07
18	6	-9	10.70	1.22
-19	-6	9	1.96	2.62
-19	-6	9	6.95	1.47
19	6	-9	5.55	0.83
17	-6	10	3.12	1.01
17	-6	10	4.96	1.74
-17	6	-10	5.42	1.63
16	-6	10	37.07	3.29
16	-6	10	38.77	2.29
-16	6	-10	46.26	3.20
15	-6	10	0.93	0.73
15	-6	10	3.18	1.47
-15	6	-10	0.01	1.22
14	-6	10	17.39	1.49
14	-6	10	14.80	2.37
-14	6	-10	16.62	2.10
13	-6	10	-1.35	1.14
13	-6	10	0.01	0.60
-13	6	-10	0.06	0.95

12	-6	10	26.82	1.55
12	-6	10	26.46	2.43
-12	6	-10	20.12	2.72
11	-6	10	3.00	0.68
11	-6	10	1.92	1.08
-11	6	-10	3.20	0.86
10	-6	10	42.24	3.85
10	-6	10	34.87	1.78
-10	6	-10	34.15	2.18
9	-6	10	6.72	0.81
9	-6	10	5.62	1.21
9	-6	10	7.57	1.18
-9	6	-10	6.52	0.91
8	-6	10	26.46	1.61
8	-6	10	27.44	2.08
8	-6	10	25.46	1.35
8	-6	10	23.94	1.71
8	-6	10	25.09	1.35
-8	6	-10	28.09	1.33
-8	6	-10	25.42	1.68
7	-6	10	21.15	1.12
7	-6	10	19.43	1.45
7	-6	10	17.30	1.83
7	-6	10	20.97	1.41
7	-6	10	21.10	1.26
-7	6	-10	19.80	1.42
-7	6	-10	19.87	1.08
6	-6	10	37.68	1.97
6	-6	10	40.24	1.94
6	-6	10	37.18	1.71
6	-6	10	39.85	2.20
6	-6	10	37.94	2.39
-6	6	-10	40.46	1.70
-6	6	-10	37.38	2.91
-6	6	-10	36.89	1.97
5	-6	10	7.52	1.32
5	-6	10	6.30	1.04
5	-6	10	6.56	0.61
5	-6	10	10.05	0.96
-5	6	-10	6.19	0.91
-5	6	-10	7.70	0.59
-5	6	-10	7.86	1.10
-5	6	-10	6.45	1.30
4	-6	10	26.77	1.26

4	-6	10	28.05	1.99
4	-6	10	27.32	1.93
4	-6	10	29.55	1.56
-4	6	-10	30.38	1.25
-4	6	-10	26.07	1.51
-4	6	-10	27.17	1.75
-4	6	-10	24.27	2.05
3	-6	10	6.60	1.14
3	-6	10	6.46	0.86
3	-6	10	6.63	0.52
3	-6	10	8.10	1.20
-3	6	-10	7.04	1.21
-3	6	-10	6.72	0.80
-3	6	-10	6.48	1.09
-3	6	-10	7.23	0.49
2	-6	10	6.44	1.20
2	-6	10	6.55	0.84
2	-6	10	7.11	1.19
-2	6	-10	7.79	1.31
-2	6	-10	6.64	0.49
-2	6	-10	7.47	0.47
-2	6	-10	7.20	1.08
-2	6	-10	6.28	0.78
1	-6	10	58.82	3.05
1	-6	10	59.51	3.29
1	-6	10	64.77	2.72
-1	6	-10	64.15	2.43
-1	6	-10	62.43	2.69
-1	6	-10	59.08	3.10
-1	6	-10	56.91	3.10
-1	6	-10	63.70	2.39
0	-6	10	26.09	2.19
0	-6	10	28.87	1.52
0	-6	10	27.21	1.86
0	6	-10	28.47	1.14
0	6	-10	28.42	1.47
0	6	-10	29.64	1.90
0	6	-10	28.04	1.16
0	6	-10	25.20	2.06
-1	-6	10	15.56	1.09
-1	-6	10	14.09	1.38
-1	-6	10	15.03	1.85
1	6	-10	16.95	1.03
1	6	-10	14.96	0.66

1	6	-10	14.44	1.44
1	6	-10	14.45	0.65
1	6	-10	16.39	1.74
-2	-6	10	14.87	1.39
-2	-6	10	18.63	1.18
-2	-6	10	20.55	2.01
2	6	-10	17.44	0.72
2	6	-10	17.94	1.82
2	6	-10	18.26	1.52
2	6	-10	17.61	1.09
2	6	-10	17.29	0.72
-3	-6	10	7.11	1.12
-3	-6	10	9.23	0.89
-3	-6	10	8.95	1.42
3	6	-10	8.40	0.81
3	6	-10	8.80	1.20
3	6	-10	8.05	0.36
3	6	-10	10.07	1.66
-4	-6	10	3.73	0.77
-4	-6	10	4.16	0.61
-4	-6	10	5.07	1.22
4	6	-10	4.01	0.67
4	6	-10	5.38	0.88
4	6	-10	5.27	1.17
4	6	-10	4.04	0.23
-5	-6	10	4.32	0.63
-5	-6	10	4.47	0.80
-5	-6	10	3.62	1.22
5	6	-10	5.34	0.27
5	6	-10	4.66	0.85
5	6	-10	4.48	1.12
5	6	-10	4.38	0.67
-6	-6	10	5.77	0.73
-6	-6	10	5.24	1.29
-6	-6	10	5.43	0.83
-6	-6	10	5.28	0.34
6	6	-10	6.17	0.91
6	6	-10	5.44	1.21
6	6	-10	5.45	0.32
6	6	-10	6.69	0.77
-7	-6	10	19.09	2.31
-7	-6	10	22.25	0.95
-7	-6	10	22.03	1.51
-7	-6	10	21.02	1.45

7	6	-10	21.94	2.28
7	6	-10	25.82	1.70
7	6	-10	23.22	1.34
7	6	-10	22.74	0.94
-8	-6	10	5.93	0.45
-8	-6	10	5.48	1.02
-8	-6	10	6.28	1.02
-8	-6	10	5.36	1.40
-8	-6	10	6.92	0.88
8	6	-10	5.67	0.42
8	6	-10	7.60	1.23
-9	-6	10	10.15	1.77
-9	-6	10	11.72	1.07
-9	-6	10	11.11	0.66
-9	-6	10	11.71	1.13
9	6	-10	11.73	0.61
9	6	-10	12.39	1.40
-10	-6	10	2.28	0.62
-10	-6	10	3.06	0.37
-10	-6	10	3.08	0.60
-10	-6	10	1.86	1.37
10	6	-10	2.77	0.95
10	6	-10	2.73	0.32
-11	-6	10	7.31	1.71
-11	-6	10	7.17	0.68
11	6	-10	8.37	0.59
11	6	-10	9.05	1.36
-12	-6	10	5.82	0.71
-12	-6	10	5.48	0.70
-12	-6	10	6.54	1.75
-12	-6	10	5.85	0.91
-12	-6	10	6.93	0.72
12	6	-10	5.51	0.56
-13	-6	10	16.33	1.12
-13	-6	10	21.08	1.63
-13	-6	10	18.36	3.07
13	6	-10	17.49	1.00
-14	-6	10	6.06	0.99
-14	-6	10	6.84	2.09
-14	-6	10	6.73	0.94
14	6	-10	7.62	0.74
-15	-6	10	42.75	2.55
15	6	-10	39.64	1.88
-16	-6	10	-2.24	2.03

-16	-6	10	0.57	0.87
16	6	-10	0.93	0.52
-17	-6	10	15.92	2.01
-17	-6	10	16.95	2.80
17	6	-10	14.80	1.20
-18	-6	10	-1.07	2.39
-18	-6	10	1.35	1.18
18	6	-10	-0.12	0.62
-19	-6	10	0.72	2.59
19	6	-10	1.74	0.75
16	-6	11	0.37	1.62
16	-6	11	0.83	0.85
-16	6	-11	3.29	1.48
15	-6	11	50.33	2.66
15	-6	11	51.67	3.66
-15	6	-11	56.20	3.38
14	-6	11	3.88	1.51
14	-6	11	4.76	0.84
-14	6	-11	7.16	1.34
13	-6	11	44.79	3.17
13	-6	11	43.18	2.29
-13	6	-11	43.48	2.74
12	-6	11	3.96	0.71
12	-6	11	4.02	1.41
-12	6	-11	3.82	1.18
11	-6	11	19.98	2.19
11	-6	11	18.51	1.30
-11	6	-11	15.05	1.69
10	-6	11	3.10	1.15
10	-6	11	0.12	0.46
-10	6	-11	1.28	0.81
9	-6	11	91.63	4.52
9	-6	11	89.22	3.92
9	-6	11	92.57	3.66
9	-6	11	88.08	3.75
-9	6	-11	91.81	4.02
8	-6	11	9.12	1.19
8	-6	11	7.37	1.36
8	-6	11	6.85	0.93
8	-6	11	6.08	0.85
8	-6	11	6.67	0.78
-8	6	-11	6.27	0.90
-8	6	-11	6.24	0.77
7	-6	11	45.01	2.33

7	-6	11	44.57	2.01
7	-6	11	44.59	2.24
7	-6	11	47.06	2.78
7	-6	11	39.14	2.31
-7	6	-11	44.52	2.27
-7	6	-11	47.48	1.98
6	-6	11	13.43	1.21
6	-6	11	12.66	1.44
6	-6	11	11.81	0.86
6	-6	11	12.53	1.23
6	-6	11	9.95	1.55
-6	6	-11	14.00	0.84
-6	6	-11	13.48	1.18
5	-6	11	5.86	0.96
5	-6	11	3.55	1.00
5	-6	11	4.97	1.03
5	-6	11	3.88	0.57
-5	6	-11	7.40	1.21
-5	6	-11	5.05	0.55
-5	6	-11	4.15	0.68
4	-6	11	10.21	1.54
4	-6	11	12.12	1.52
4	-6	11	9.72	1.05
4	-6	11	10.90	0.72
-4	6	-11	11.86	1.03
-4	6	-11	11.65	0.69
-4	6	-11	9.72	0.71
-4	6	-11	7.75	1.29
-4	6	-11	10.43	1.40
3	-6	11	59.72	3.20
3	-6	11	66.03	2.79
3	-6	11	63.82	3.31
-3	6	-11	64.34	2.47
-3	6	-11	60.83	2.74
-3	6	-11	57.28	3.30
-3	6	-11	60.26	2.51
-3	6	-11	59.97	3.11
2	-6	11	-0.49	0.89
2	-6	11	1.12	0.53
2	-6	11	2.25	0.87
-2	6	-11	2.83	0.91
-2	6	-11	1.87	0.26
-2	6	-11	1.22	0.27
-2	6	-11	1.30	0.47

-2	6	-11	3.14	0.95
1	-6	11	201.70	8.53
1	-6	11	190.57	8.25
1	-6	11	205.75	7.91
-1	6	-11	212.49	8.34
-1	6	-11	212.85	7.60
-1	6	-11	205.27	7.63
-1	6	-11	189.81	8.34
-1	6	-11	198.10	7.86
0	-6	11	3.62	0.90
0	-6	11	4.17	0.63
0	-6	11	4.13	1.11
0	6	-11	3.99	0.60
0	6	-11	4.36	1.09
0	6	-11	3.96	0.34
0	6	-11	4.04	0.42
0	6	-11	4.27	0.94
-1	-6	11	35.73	2.60
-1	-6	11	36.05	1.80
-1	-6	11	33.22	2.10
1	6	-11	35.22	1.71
1	6	-11	32.59	2.39
1	6	-11	33.31	2.15
1	6	-11	34.55	1.36
1	6	-11	32.51	1.37
-2	-6	11	1.55	0.51
-2	-6	11	1.99	0.76
-2	-6	11	1.48	1.09
2	6	-11	2.49	1.02
2	6	-11	3.00	0.81
2	6	-11	1.05	0.45
2	6	-11	2.08	0.24
2	6	-11	1.58	0.23
-3	-6	11	17.34	1.22
-3	-6	11	15.95	1.48
-3	-6	11	16.73	2.09
3	6	-11	15.60	0.67
3	6	-11	15.80	1.54
3	6	-11	16.47	1.98
3	6	-11	17.25	1.12
3	6	-11	16.75	0.69
-4	-6	11	2.10	0.79
-4	-6	11	2.09	1.17
-4	-6	11	2.09	0.54

4	6	-11	1.91	0.19
4	6	-11	2.33	0.22
4	6	-11	1.70	0.46
4	6	-11	1.15	1.05
4	6	-11	2.78	0.86
-5	-6	11	35.96	2.78
-5	-6	11	35.05	2.04
-5	-6	11	34.86	1.84
5	6	-11	34.17	2.75
5	6	-11	35.10	2.09
5	6	-11	32.73	1.71
5	6	-11	32.28	1.31
-6	-6	11	3.90	0.80
-6	-6	11	3.74	0.31
-6	-6	11	2.40	0.63
-6	-6	11	3.82	1.30
6	6	-11	4.15	0.94
6	6	-11	2.71	0.54
6	6	-11	3.38	0.27
6	6	-11	1.34	1.13
-7	-6	11	47.37	2.48
-7	-6	11	48.42	3.43
-7	-6	11	49.64	1.96
-7	-6	11	51.63	2.49
7	6	-11	51.71	2.33
7	6	-11	48.75	1.94
7	6	-11	52.78	2.67
-8	-6	11	19.86	2.56
-8	-6	11	18.11	1.30
-8	-6	11	18.02	1.42
-8	-6	11	19.05	0.88
8	6	-11	18.63	0.86
8	6	-11	22.06	1.74
-9	-6	11	22.98	1.59
-9	-6	11	24.77	1.52
-9	-6	11	26.33	2.79
-9	-6	11	25.00	1.11
9	6	-11	24.64	1.06
9	6	-11	23.93	1.83
-10	-6	11	10.29	1.13
-10	-6	11	10.96	1.13
-10	-6	11	10.62	0.71
10	6	-11	11.01	0.64
10	6	-11	12.37	1.58

-11	-6	11	47.57	3.80
-11	-6	11	48.15	2.33
-11	-6	11	51.94	2.55
-11	-6	11	49.21	2.10
11	6	-11	51.79	2.55
11	6	-11	49.61	2.04
-12	-6	11	8.29	0.81
-12	-6	11	7.47	1.94
-12	-6	11	8.75	0.84
-12	-6	11	9.09	0.83
-12	-6	11	7.98	1.30
12	6	-11	9.34	0.67
-13	-6	11	10.06	1.40
-13	-6	11	12.17	0.99
-13	-6	11	9.15	2.22
13	6	-11	10.19	0.77
-14	-6	11	25.63	3.52
-14	-6	11	23.03	1.85
14	6	-11	20.29	1.17
-15	-6	11	9.71	2.34
-15	-6	11	10.88	1.66
15	6	-11	9.92	0.91
-16	-6	11	21.55	2.03
-16	-6	11	19.17	2.95
16	6	-11	19.20	1.28
-17	-6	11	9.02	2.69
-17	-6	11	7.08	1.33
17	6	-11	12.02	1.09
-18	-6	11	0.06	1.19
-18	-6	11	-3.84	2.57
18	6	-11	1.81	0.69
16	-6	12	58.35	4.19
16	-6	12	63.49	3.19
-16	6	-12	62.96	3.87
15	-6	12	0.68	1.59
15	-6	12	0.64	0.83
-15	6	-12	1.94	1.42
14	-6	12	45.21	2.45
14	-6	12	46.13	3.57
-14	6	-12	49.02	3.13
13	-6	12	0.02	0.66
13	-6	12	0.07	1.48
-13	6	-12	0.25	1.07
12	-6	12	17.93	2.38

12	-6	12	18.18	1.45
-12	6	-12	19.95	1.90
11	-6	12	6.73	1.01
11	-6	12	6.70	1.48
-11	6	-12	6.21	1.09
10	-6	12	31.48	2.63
10	-6	12	32.75	1.81
-10	6	-12	31.98	2.08
9	-6	12	0.21	0.47
9	-6	12	0.60	0.53
9	-6	12	0.14	1.02
-9	6	-12	-0.01	0.72
8	-6	12	77.35	3.59
8	-6	12	92.40	3.82
8	-6	12	82.29	3.42
8	-6	12	81.19	3.51
8	-6	12	78.88	4.24
-8	6	-12	80.27	3.35
-8	6	-12	80.10	3.66
7	-6	12	1.49	0.75
7	-6	12	1.44	0.66
7	-6	12	0.59	0.41
7	-6	12	1.27	1.25
7	-6	12	0.62	0.69
-7	6	-12	0.68	0.65
-7	6	-12	0.87	0.39
6	-6	12	37.91	2.03
6	-6	12	40.17	2.28
6	-6	12	37.87	2.62
6	-6	12	37.88	1.75
-6	6	-12	34.74	2.17
-6	6	-12	37.57	2.04
-6	6	-12	38.84	1.73
5	-6	12	0.49	0.85
5	-6	12	1.24	0.64
5	-6	12	0.47	0.88
-5	6	-12	1.56	0.36
-5	6	-12	2.23	0.88
-5	6	-12	0.50	0.55
-5	6	-12	1.40	0.39
4	-6	12	69.49	3.52
4	-6	12	66.86	3.60
4	-6	12	69.84	3.08
-4	6	-12	68.93	3.05

-4	6	-12	66.42	2.82
-4	6	-12	64.88	3.38
-4	6	-12	74.55	2.79
3	-6	12	2.35	0.64
3	-6	12	2.44	1.00
3	-6	12	1.60	0.91
-3	6	-12	1.77	0.56
-3	6	-12	2.52	0.96
-3	6	-12	2.16	0.37
-3	6	-12	2.38	0.33
2	-6	12	66.99	3.62
2	-6	12	59.71	3.36
2	-6	12	70.01	2.99
-2	6	-12	61.97	3.49
-2	6	-12	66.50	2.92
-2	6	-12	70.94	2.63
-2	6	-12	65.76	2.66
-2	6	-12	65.41	3.49
1	-6	12	12.66	1.55
1	-6	12	15.49	1.23
1	-6	12	14.75	1.94
-1	6	-12	15.01	0.75
-1	6	-12	12.10	1.85
-1	6	-12	13.08	0.90
-1	6	-12	14.78	1.92
-1	6	-12	13.83	1.10
0	-6	12	81.65	4.31
0	-6	12	76.25	3.95
0	-6	12	81.31	3.57
0	6	-12	83.73	3.22
0	6	-12	84.34	4.18
0	6	-12	84.06	3.52
0	6	-12	87.98	3.20
0	6	-12	82.81	3.98
-1	-6	12	8.92	1.46
-1	-6	12	9.46	1.06
-1	-6	12	12.06	2.03
1	6	-12	10.90	0.54
1	6	-12	9.47	0.94
1	6	-12	9.79	1.50
1	6	-12	9.20	1.63
-2	-6	12	29.39	1.68
-2	-6	12	25.89	1.98
-2	-6	12	30.11	2.62

2	6	-12	29.59	1.18
2	6	-12	28.82	1.18
2	6	-12	29.26	2.12
2	6	-12	28.27	2.46
2	6	-12	30.10	1.57
-3	-6	12	0.82	0.82
-3	-6	12	1.31	0.56
-3	-6	12	0.15	1.05
3	6	-12	1.64	0.23
3	6	-12	1.56	0.19
3	6	-12	1.72	0.50
3	6	-12	2.49	0.92
3	6	-12	1.67	1.22
-4	-6	12	78.03	3.33
-4	-6	12	73.98	3.57
-4	-6	12	81.29	4.30
4	6	-12	76.66	2.84
4	6	-12	71.25	2.83
4	6	-12	75.79	3.59
4	6	-12	65.22	4.08
4	6	-12	76.57	3.24
-5	-6	12	4.48	0.73
-5	-6	12	2.32	1.35
-5	-6	12	4.09	0.91
5	6	-12	4.75	1.01
5	6	-12	4.20	0.33
5	6	-12	3.89	0.28
5	6	-12	4.08	0.61
-6	-6	12	120.58	4.48
-6	-6	12	118.47	5.94
-6	-6	12	120.21	5.09
-6	-6	12	118.96	4.95
6	6	-12	116.41	4.84
6	6	-12	114.06	4.45
6	6	-12	122.16	5.17
-7	-6	12	20.02	1.37
-7	-6	12	20.77	1.66
-7	-6	12	21.95	1.66
-7	-6	12	21.76	0.95
-7	-6	12	22.43	2.62
7	6	-12	19.84	1.37
7	6	-12	24.68	1.90
-8	-6	12	90.50	3.50
-8	-6	12	94.72	5.15

-8	-6	12	86.82	3.90
-8	-6	12	86.63	3.98
8	6	-12	98.58	4.20
8	6	-12	90.61	3.48
-9	-6	12	17.99	1.35
-9	-6	12	17.22	0.90
-9	-6	12	17.84	1.47
-9	-6	12	19.19	3.25
9	6	-12	21.84	1.86
9	6	-12	18.30	0.84
-10	-6	12	26.76	1.90
-10	-6	12	28.07	1.30
-10	-6	12	30.13	3.18
-10	-6	12	28.39	1.74
10	6	-12	28.50	1.24
10	6	-12	30.05	2.05
-11	-6	12	-0.35	0.63
-11	-6	12	0.59	0.62
-11	-6	12	2.42	1.68
-11	-6	12	0.23	0.36
11	6	-12	0.24	0.27
-12	-6	12	102.32	5.69
-12	-6	12	93.58	3.77
-12	-6	12	87.07	3.75
-12	-6	12	98.76	4.29
12	6	-12	95.19	3.70
-13	-6	12	9.77	2.33
-13	-6	12	11.86	1.56
13	6	-12	13.21	0.85
-14	-6	12	40.32	2.49
-14	-6	12	44.43	4.26
14	6	-12	40.97	1.84
-15	-6	12	7.63	2.40
-15	-6	12	7.34	1.18
15	6	-12	8.97	0.88
-16	-6	12	24.22	4.00
-16	-6	12	22.70	2.12
16	6	-12	18.36	1.28
-17	-6	12	6.23	2.74
-17	-6	12	7.22	1.37
17	6	-12	9.30	1.14
-18	-6	12	8.80	1.76
-18	-6	12	9.43	2.92
15	-6	13	29.31	2.09

15	-6	13	31.91	3.25
-15	6	-13	21.24	2.64
14	-6	13	18.09	1.65
14	-6	13	12.78	2.06
-14	6	-13	10.36	1.65
13	-6	13	20.71	1.64
13	-6	13	15.34	2.75
-13	6	-13	16.69	2.18
12	-6	13	1.72	0.70
12	-6	13	2.85	1.52
-12	6	-13	2.49	1.04
11	-6	13	15.85	2.36
11	-6	13	19.05	1.41
10	-6	13	3.98	0.68
10	-6	13	4.56	1.44
-10	6	-13	4.83	0.95
9	-6	13	53.23	3.44
9	-6	13	55.32	2.50
9	-6	13	53.62	2.53
9	-6	13	55.39	2.47
-9	6	-13	53.60	2.81
8	-6	13	0.20	0.72
8	-6	13	1.20	1.03
8	-6	13	0.29	0.74
8	-6	13	1.18	0.78
8	-6	13	0.90	0.49
-8	6	-13	0.65	0.74
7	-6	13	55.83	2.86
7	-6	13	58.24	2.54
7	-6	13	55.95	3.40
7	-6	13	61.93	2.84
7	-6	13	61.24	2.81
-7	6	-13	56.81	2.57
-7	6	-13	58.56	2.50
-7	6	-13	54.87	2.78
6	-6	13	-0.22	0.97
6	-6	13	0.80	0.87
6	-6	13	1.80	0.69
-6	6	-13	1.43	0.41
-6	6	-13	0.51	0.42
-6	6	-13	1.81	0.66
-6	6	-13	0.53	0.91
5	-6	13	6.80	0.86
5	-6	13	8.38	1.55

-5	6	-13	6.04	0.70
-5	6	-13	6.62	1.06
-5	6	-13	5.71	1.14
-5	6	-13	7.40	0.71
4	-6	13	4.53	1.12
4	-6	13	3.70	1.08
4	-6	13	5.18	0.80
-4	6	-13	4.62	0.75
-4	6	-13	4.58	0.60
-4	6	-13	4.82	1.17
-4	6	-13	3.81	0.61
3	-6	13	75.85	3.99
3	-6	13	80.93	4.02
3	-6	13	79.74	3.43
-3	6	-13	81.03	3.10
-3	6	-13	77.42	3.14
-3	6	-13	74.81	3.36
-3	6	-13	73.55	3.91
2	-6	13	1.79	0.65
2	-6	13	1.62	0.95
2	-6	13	2.47	1.17
-2	6	-13	1.09	0.30
-2	6	-13	1.45	0.55
-2	6	-13	1.74	0.33
-2	6	-13	2.37	1.19
1	-6	13	121.97	5.69
1	-6	13	129.30	5.24
1	-6	13	124.53	5.94
-1	6	-13	127.29	5.16
-1	6	-13	136.26	4.88
-1	6	-13	126.61	4.90
-1	6	-13	124.34	5.84
0	-6	13	1.09	1.14
0	-6	13	0.27	0.60
0	-6	13	0.59	0.87
0	6	-13	0.70	0.23
0	6	-13	0.22	0.48
0	6	-13	1.33	1.16
0	6	-13	0.54	1.02
-1	-6	13	48.64	2.33
-1	-6	13	42.88	3.21
-1	-6	13	43.04	2.75
1	6	-13	46.87	1.84
1	6	-13	47.44	2.86

1	6	-13	47.21	1.86
1	6	-13	47.14	2.20
1	6	-13	41.28	3.09
-2	-6	13	1.75	0.67
-2	-6	13	3.19	1.30
-2	-6	13	1.35	0.90
2	6	-13	1.18	0.51
2	6	-13	-0.33	1.19
2	6	-13	-0.52	0.96
2	6	-13	1.15	0.22
2	6	-13	1.52	0.34
-3	-6	13	81.39	3.53
-3	-6	13	79.70	4.51
-3	-6	13	76.71	3.86
3	6	-13	79.57	3.41
3	6	-13	74.81	3.03
3	6	-13	79.70	3.04
3	6	-13	84.66	4.92
3	6	-13	82.94	3.89
-4	-6	13	14.35	1.28
-4	-6	13	10.58	1.80
-4	-6	13	12.98	1.60
4	6	-13	11.49	0.58
4	6	-13	12.39	1.08
4	6	-13	11.18	0.55
4	6	-13	12.72	1.70
-5	-6	13	176.46	8.01
-5	-6	13	173.99	6.99
-5	-6	13	169.83	7.19
5	6	-13	181.69	7.25
5	6	-13	163.83	6.48
5	6	-13	178.90	6.51
5	6	-13	172.31	6.87
-6	-6	13	-0.37	0.66
-6	-6	13	0.68	0.84
-6	-6	13	0.17	0.21
-6	-6	13	-0.33	1.26
6	6	-13	-0.05	0.17
6	6	-13	0.52	0.49
6	6	-13	0.00	1.00
-7	-6	13	39.57	2.34
-7	-6	13	41.77	2.11
-7	-6	13	34.66	4.22
-7	-6	13	41.00	1.64

7	6	-13	40.96	2.09
7	6	-13	43.56	2.59
7	6	-13	38.61	1.69
-8	-6	13	-0.06	0.52
-8	-6	13	0.53	0.26
-8	-6	13	0.55	0.78
-8	-6	13	0.81	1.50
8	6	-13	0.68	0.22
8	6	-13	0.13	1.02
-9	-6	13	33.50	1.51
-9	-6	13	29.03	3.48
-9	-6	13	35.06	2.05
9	6	-13	35.98	1.45
9	6	-13	39.27	2.43
-10	-6	13	2.62	0.75
-10	-6	13	1.78	0.70
-10	-6	13	0.29	1.62
-10	-6	13	1.08	0.35
10	6	-13	1.55	0.28
10	6	-13	3.14	1.09
-11	-6	13	71.74	4.86
-11	-6	13	72.13	2.89
-11	-6	13	70.33	3.35
-11	-6	13	69.74	3.09
11	6	-13	70.15	2.79
11	6	-13	69.15	3.40
-12	-6	13	1.10	1.95
-12	-6	13	1.11	0.81
12	6	-13	0.06	0.31
-13	-6	13	52.21	4.55
-13	-6	13	66.25	3.18
13	6	-13	59.50	2.49
-14	-6	13	5.89	2.30
-14	-6	13	3.87	1.02
14	6	-13	3.99	0.52
-15	-6	13	46.81	4.50
-15	-6	13	42.32	2.76
15	6	-13	42.81	1.97
-16	-6	13	2.90	2.61
-16	-6	13	4.29	1.13
-17	-6	13	51.84	3.27
14	-6	14	3.57	0.99
14	-6	14	3.72	1.77
-14	6	-14	4.43	1.43

13	-6	14	17.61	2.88
13	-6	14	15.66	1.61
-13	6	-14	13.97	2.19
12	-6	14	3.77	0.83
12	-6	14	3.62	1.59
-12	6	-14	5.23	1.19
11	-6	14	13.08	1.34
11	-6	14	16.35	2.52
-11	6	-14	12.27	1.43
10	-6	14	9.70	1.17
10	-6	14	8.36	1.70
-10	6	-14	9.35	1.56
9	-6	14	17.20	2.38
9	-6	14	20.58	1.49
-9	6	-14	22.05	1.73
8	-6	14	3.63	1.27
8	-6	14	4.08	1.00
8	-6	14	3.34	0.89
-8	6	-14	3.85	0.65
-8	6	-14	2.34	0.81
7	-6	14	7.82	1.48
7	-6	14	10.74	1.37
7	-6	14	8.80	1.63
-7	6	-14	9.59	0.98
-7	6	-14	8.41	1.06
-7	6	-14	8.61	1.28
6	-6	14	2.42	0.79
6	-6	14	1.90	1.12
6	-6	14	2.04	0.95
-6	6	-14	2.58	0.49
-6	6	-14	0.48	1.09
-6	6	-14	1.81	0.69
-6	6	-14	1.52	0.49
5	-6	14	9.75	1.42
5	-6	14	8.17	1.46
5	-6	14	9.35	1.25
-5	6	-14	10.81	1.85
-5	6	-14	8.75	1.12
-5	6	-14	9.46	0.85
-5	6	-14	9.56	0.80
4	-6	14	8.26	1.25
4	-6	14	11.20	1.84
4	-6	14	10.08	1.45
-4	6	-14	8.46	1.12

-4	6	-14	7.78	0.78
-4	6	-14	11.83	1.98
-4	6	-14	9.76	0.75
3	-6	14	15.99	2.19
3	-6	14	17.28	1.48
3	-6	14	19.67	2.14
-3	6	-14	20.89	1.02
-3	6	-14	13.79	2.23
-3	6	-14	18.28	1.35
-3	6	-14	16.93	1.01
2	-6	14	57.36	3.41
2	-6	14	61.86	2.91
2	-6	14	61.59	3.62
-2	6	-14	62.19	2.55
-2	6	-14	62.37	2.83
-2	6	-14	67.57	2.90
-2	6	-14	59.43	3.69
1	-6	14	22.76	2.42
1	-6	14	19.17	1.54
1	-6	14	18.55	1.98
-1	6	-14	21.27	1.05
-1	6	-14	21.76	1.40
-1	6	-14	22.26	1.03
-1	6	-14	18.76	2.37
0	-6	14	65.99	3.63
0	-6	14	69.72	3.14
0	-6	14	68.18	3.97
0	6	-14	67.32	2.70
0	6	-14	72.40	2.69
0	6	-14	62.98	3.94
0	6	-14	66.21	3.00
-1	-6	14	24.11	2.20
-1	-6	14	27.81	1.74
-1	-6	14	31.00	2.78
1	6	-14	26.87	2.82
1	6	-14	23.55	1.34
1	6	-14	31.35	2.88
1	6	-14	26.24	1.16
1	6	-14	26.38	1.56
-2	-6	14	24.84	2.18
-2	-6	14	32.21	2.91
-2	-6	14	27.33	1.76
2	6	-14	26.56	1.16
2	6	-14	25.59	1.55

2	6	-14	28.20	1.17
2	6	-14	27.14	2.37
-3	-6	14	49.49	3.08
-3	-6	14	54.00	3.74
-3	-6	14	56.90	2.71
3	6	-14	56.58	2.57
3	6	-14	55.52	2.17
3	6	-14	57.94	3.19
3	6	-14	54.58	2.16
-4	-6	14	11.46	1.39
-4	-6	14	11.65	1.73
-4	-6	14	13.13	2.49
4	6	-14	11.06	1.14
4	6	-14	13.60	0.67
4	6	-14	16.83	1.89
-5	-6	14	20.38	1.62
-5	-6	14	22.31	2.80
-5	-6	14	22.43	1.98
5	6	-14	17.73	2.08
5	6	-14	21.42	0.95
5	6	-14	24.25	1.45
-6	-6	14	57.94	3.17
-6	-6	14	61.22	4.37
6	6	-14	65.81	2.89
6	6	-14	64.89	3.38
-7	-6	14	15.67	1.31
-7	-6	14	16.09	1.71
-7	-6	14	21.62	2.90
-7	-6	14	16.30	0.81
7	6	-14	15.67	0.76
7	6	-14	15.18	2.03
-8	-6	14	24.63	3.21
-8	-6	14	29.28	1.95
-8	-6	14	25.65	1.20
-8	-6	14	27.80	1.75
8	6	-14	26.46	1.14
8	6	-14	28.93	2.29
-9	-6	14	4.71	0.73
-9	-6	14	5.15	0.99
-9	-6	14	6.18	2.07
-9	-6	14	6.03	0.87
9	6	-14	5.09	1.24
9	6	-14	6.10	0.49
-10	-6	14	43.97	2.32

-10	-6	14	42.02	3.93
-10	-6	14	36.51	2.25
10	6	-14	45.81	2.72
10	6	-14	39.43	1.69
-11	-6	14	9.96	2.22
-11	-6	14	10.07	1.27
-11	-6	14	6.74	1.05
11	6	-14	7.31	0.62
-12	-6	14	80.91	3.91
12	6	-14	83.81	3.27
-13	-6	14	2.99	0.97
-13	-6	14	5.57	2.31
13	6	-14	2.23	0.45
-14	-6	14	33.76	4.31
-14	-6	14	34.22	2.43
14	6	-14	35.77	1.71
-15	-6	14	7.87	2.62
-16	-6	14	11.30	1.62
-16	-6	14	11.64	3.00
13	-6	15	5.70	1.86
13	-6	15	5.17	0.97
-13	6	-15	4.90	1.09
-13	6	-15	4.42	1.34
12	-6	15	11.35	2.03
12	-6	15	12.71	1.56
-12	6	-15	10.54	1.50
-12	6	-15	10.44	1.63
11	-6	15	8.52	1.33
11	-6	15	5.10	1.67
-11	6	-15	10.83	1.45
-11	6	-15	5.77	1.25
10	-6	15	18.08	2.73
-10	6	-15	16.10	2.10
-10	6	-15	16.12	1.55
9	-6	15	-1.42	1.41
-9	6	-15	-0.46	0.82
-9	6	-15	0.80	0.63
8	-6	15	20.60	2.58
8	-6	15	19.69	1.76
-8	6	-15	18.53	1.65
-8	6	-15	18.65	1.38
7	-6	15	1.22	1.12
7	-6	15	1.21	1.04
7	-6	15	1.47	1.33

-7	6	-15	0.03	0.71
-7	6	-15	0.69	0.55
6	-6	15	25.22	2.53
6	-6	15	23.10	1.80
6	-6	15	25.34	2.11
-6	6	-15	26.48	1.72
-6	6	-15	23.82	1.44
-6	6	-15	27.58	1.39
-6	6	-15	24.97	2.34
5	-6	15	6.59	1.29
5	-6	15	4.71	1.51
5	-6	15	4.86	0.93
-5	6	-15	6.00	0.89
-5	6	-15	5.94	0.79
-5	6	-15	4.70	0.76
-5	6	-15	8.43	1.48
4	-6	15	42.33	2.23
4	-6	15	39.96	2.77
4	-6	15	38.94	2.92
-4	6	-15	37.66	2.11
-4	6	-15	38.90	1.84
-4	6	-15	37.69	2.96
-4	6	-15	42.19	1.80
3	-6	15	7.82	1.00
3	-6	15	7.32	1.57
3	-6	15	7.36	1.39
-3	6	-15	6.25	0.72
-3	6	-15	9.11	1.84
-3	6	-15	6.62	0.72
-3	6	-15	6.37	0.86
2	-6	15	42.71	2.99
2	-6	15	41.02	3.03
2	-6	15	41.17	2.24
-2	6	-15	45.23	1.82
-2	6	-15	42.14	3.29
-2	6	-15	40.10	2.11
-2	6	-15	39.60	1.82
1	-6	15	2.52	0.77
1	-6	15	0.32	1.05
1	-6	15	3.63	1.37
-1	6	-15	3.53	1.53
-1	6	-15	3.14	0.41
-1	6	-15	1.74	0.64
-1	6	-15	2.67	0.39

0	-6	15	37.77	2.07
0	-6	15	32.73	3.05
0	-6	15	32.99	2.63
0	6	-15	36.74	1.54
0	6	-15	35.64	1.54
0	6	-15	33.96	1.88
-1	-6	15	1.05	1.03
-1	-6	15	3.00	1.42
-1	-6	15	1.49	0.73
1	6	-15	0.46	0.29
1	6	-15	1.06	0.29
1	6	-15	1.13	0.56
-2	-6	15	32.45	3.03
-2	-6	15	30.79	2.46
-2	-6	15	29.66	1.96
2	6	-15	30.54	1.74
2	6	-15	32.85	1.35
-3	-6	15	1.09	1.05
-3	-6	15	2.05	1.51
-3	-6	15	2.49	0.78
3	6	-15	1.74	0.26
3	6	-15	2.14	0.31
3	6	-15	1.68	1.59
3	6	-15	1.00	0.58
-4	-6	15	59.31	4.93
-4	-6	15	68.71	3.61
-4	-6	15	63.87	3.25
4	6	-15	67.57	3.05
4	6	-15	67.43	2.62
4	6	-15	65.95	3.66
4	6	-15	74.15	2.65
-5	-6	15	11.56	1.67
-5	-6	15	10.53	1.97
5	6	-15	9.07	1.11
5	6	-15	8.02	1.45
5	6	-15	10.29	0.58
5	6	-15	11.61	0.63
-6	-6	15	16.49	2.89
-6	-6	15	14.33	1.79
6	6	-15	17.01	1.31
6	6	-15	14.81	0.74
6	6	-15	21.29	2.17
6	6	-15	15.46	0.80
-7	-6	15	1.23	0.31

-7	-6	15	2.13	0.66
-7	-6	15	-0.01	0.93
-7	-6	15	4.51	1.91
7	6	-15	0.18	1.26
7	6	-15	1.93	0.26
-8	-6	15	18.78	1.13
-8	-6	15	14.25	1.81
-8	-6	15	16.92	1.42
-8	-6	15	20.68	3.23
8	6	-15	18.83	2.25
8	6	-15	15.93	0.80
-9	-6	15	3.69	0.98
-9	-6	15	2.10	1.90
-9	-6	15	2.88	0.77
9	6	-15	3.10	0.46
9	6	-15	0.54	2.28
-10	-6	15	37.36	3.97
-10	-6	15	36.12	2.16
-10	-6	15	31.98	2.11
10	6	-15	33.80	1.45
-11	-6	15	7.36	2.26
-11	-6	15	11.23	1.49
-11	-6	15	9.08	1.54
11	6	-15	10.56	0.72
-12	-6	15	20.28	3.72
-12	-6	15	17.40	1.84
12	6	-15	15.91	0.94
-13	-6	15	6.54	2.38
-13	-6	15	13.05	1.80
-14	-6	15	20.52	2.09
-14	-6	15	11.52	2.79
-15	-6	15	11.61	2.84
-15	-6	15	11.50	1.58
-16	-6	15	21.26	3.47
-16	-6	15	25.06	2.49
12	-6	16	22.10	3.19
-12	6	-16	17.46	2.25
-12	6	-16	16.42	1.88
11	-6	16	19.45	2.97
-11	6	-16	18.95	2.01
-11	6	-16	15.04	1.77
10	-6	16	11.42	2.07
-10	6	-16	12.37	1.47
-10	6	-16	8.94	1.49

9	-6	16	5.41	1.72
-9	6	-16	7.92	1.17
-9	6	-16	8.07	1.00
8	-6	16	25.63	2.05
8	-6	16	26.87	2.85
-8	6	-16	25.54	1.70
-8	6	-16	30.40	2.01
7	-6	16	3.67	1.22
7	-6	16	3.74	1.62
-7	6	-16	3.82	0.95
-7	6	-16	4.77	0.73
6	-6	16	9.55	1.46
6	-6	16	8.50	1.76
6	-6	16	10.75	1.46
-6	6	-16	7.86	1.05
-6	6	-16	9.29	1.06
5	-6	16	10.76	1.85
5	-6	16	11.78	1.63
5	-6	16	10.38	1.51
-5	6	-16	14.84	1.94
-5	6	-16	11.33	1.03
-5	6	-16	12.07	1.04
-5	6	-16	12.42	1.40
4	-6	16	14.29	2.15
4	-6	16	16.74	1.67
4	-6	16	16.43	2.33
-4	6	-16	14.35	1.44
-4	6	-16	18.33	1.08
-4	6	-16	13.94	2.64
-4	6	-16	15.75	1.09
3	-6	16	18.58	2.42
3	-6	16	22.67	2.53
3	-6	16	21.83	1.77
-3	6	-16	23.92	1.20
-3	6	-16	20.35	1.55
-3	6	-16	21.06	1.22
-3	6	-16	18.38	2.88
2	-6	16	5.33	1.45
2	-6	16	6.81	1.03
2	-6	16	8.45	1.66
-2	6	-16	5.74	0.83
-2	6	-16	6.31	0.75
-2	6	-16	7.22	0.73
1	-6	16	8.39	1.76

1	-6	16	9.62	1.44
1	-6	16	7.38	1.43
-1	6	-16	7.03	1.12
-1	6	-16	6.58	0.69
-1	6	-16	7.53	0.71
0	-6	16	6.12	1.47
0	-6	16	7.77	1.69
0	-6	16	6.31	1.05
0	6	-16	7.05	0.65
0	6	-16	5.77	0.81
0	6	-16	5.60	0.62
-1	-6	16	15.93	2.24
-1	-6	16	16.05	2.06
-1	-6	16	17.04	1.63
1	6	-16	14.25	1.29
1	6	-16	14.31	0.84
1	6	-16	16.37	0.88
-2	-6	16	7.75	2.82
-2	-6	16	5.57	0.96
-2	-6	16	10.03	1.80
2	6	-16	7.34	0.59
2	6	-16	5.12	0.76
2	6	-16	6.12	0.56
-3	-6	16	11.04	1.76
-3	-6	16	7.87	1.34
-3	-6	16	3.90	1.72
3	6	-16	7.56	0.54
3	6	-16	6.34	1.08
-4	-6	16	1.79	1.12
-4	-6	16	4.01	1.74
4	6	-16	4.83	0.77
4	6	-16	4.00	0.41
4	6	-16	4.85	0.55
-5	-6	16	23.10	3.17
-5	-6	16	18.31	2.09
5	6	-16	19.17	2.51
5	6	-16	19.72	1.46
5	6	-16	23.43	1.02
5	6	-16	21.38	0.95
-6	-6	16	1.04	1.07
-6	-6	16	2.93	1.85
-6	-6	16	2.68	0.69
6	6	-16	1.45	0.67
6	6	-16	4.26	1.45

6	6	-16	1.53	0.25
6	6	-16	2.07	0.35
-7	-6	16	17.30	2.57
-7	-6	16	15.26	1.90
-7	-6	16	14.11	1.40
7	6	-16	15.16	0.74
7	6	-16	13.32	2.34
-8	-6	16	14.82	1.42
-8	-6	16	14.18	1.85
-8	-6	16	14.22	2.49
8	6	-16	13.39	2.41
8	6	-16	13.76	0.74
-9	-6	16	36.83	3.93
-9	-6	16	35.10	2.36
-9	-6	16	36.95	2.18
9	6	-16	35.22	1.51
-10	-6	16	0.96	0.84
-10	-6	16	0.48	0.95
-10	-6	16	1.95	2.08
10	6	-16	0.74	0.37
-11	-6	16	8.76	2.55
-11	-6	16	14.42	1.70
-12	-6	16	-0.76	0.93
-12	-6	16	1.96	2.35
-13	-6	16	22.70	4.11
-13	-6	16	15.88	1.96
-14	-6	16	1.43	1.15
-14	-6	16	4.32	2.62
-15	-6	16	10.29	3.13
-15	-6	16	16.63	2.24
11	-6	17	13.81	2.40
-11	6	-17	15.03	1.82
10	-6	17	5.64	1.88
-10	6	-17	4.99	1.17
-10	6	-17	7.69	1.09
9	-6	17	18.37	2.83
-9	6	-17	15.98	1.53
-9	6	-17	12.37	1.60
8	-6	17	-1.90	1.55
-8	6	-17	-0.27	0.89
-8	6	-17	1.33	0.74
7	-6	17	39.91	3.37
7	-6	17	37.56	2.67
-7	6	-17	39.85	2.32

6	-6	17	-0.46	1.21
6	-6	17	0.59	1.51
-6	6	-17	0.45	0.57
-6	6	-17	-1.85	0.77
5	-6	17	38.44	3.37
5	-6	17	41.18	3.02
-5	6	-17	46.56	2.41
-5	6	-17	44.75	2.14
4	-6	17	1.20	1.41
4	-6	17	0.70	1.45
4	-6	17	-0.06	0.93
-4	6	-17	0.78	0.52
-4	6	-17	0.66	0.74
-4	6	-17	0.57	0.52
3	-6	17	13.30	1.92
3	-6	17	14.40	1.63
3	-6	17	13.22	2.35
-3	6	-17	14.77	1.02
-3	6	-17	12.31	1.02
-3	6	-17	12.53	1.37
2	-6	17	-1.04	1.40
2	-6	17	2.18	0.91
2	-6	17	1.86	1.49
-2	6	-17	1.91	0.47
-2	6	-17	2.35	0.74
-2	6	-17	2.08	0.48
1	-6	17	32.65	3.07
1	-6	17	29.27	2.79
1	-6	17	30.75	2.08
-1	6	-17	29.10	1.81
-1	6	-17	32.24	1.48
-1	6	-17	29.03	1.47
0	-6	17	-0.23	2.52
0	-6	17	0.29	1.23
0	-6	17	0.42	0.87
0	6	-17	0.30	0.63
0	6	-17	0.59	0.39
0	6	-17	0.64	0.38
-1	-6	17	47.51	3.77
-1	-6	17	46.61	2.86
-1	-6	17	43.80	3.16
1	6	-17	47.83	2.38
1	6	-17	49.50	2.02
1	6	-17	48.54	2.02

-2	-6	17	6.77	1.95
-2	-6	17	4.09	1.46
2	6	-17	3.88	0.56
2	6	-17	4.22	0.80
-3	-6	17	13.71	2.12
-3	-6	17	20.89	3.16
3	6	-17	14.82	1.36
3	6	-17	14.62	0.81
3	6	-17	15.83	0.96
-4	-6	17	-0.07	1.14
-4	-6	17	1.84	1.87
4	6	-17	0.02	0.60
4	6	-17	0.33	0.27
-5	-6	17	32.34	3.70
-5	-6	17	25.69	2.34
5	6	-17	25.46	1.16
5	6	-17	27.54	1.71
-6	-6	17	4.12	1.34
-6	-6	17	3.52	1.97
-6	-6	17	4.57	0.85
6	6	-17	5.01	0.47
-7	-6	17	40.02	4.00
-7	-6	17	38.75	2.17
-7	-6	17	34.89	2.60
7	6	-17	34.27	1.51
7	6	-17	40.42	3.07
-8	-6	17	4.50	0.90
-8	-6	17	5.02	2.15
-8	-6	17	1.80	1.16
-9	-6	17	9.04	1.35
-9	-6	17	14.39	1.59
-9	-6	17	13.35	2.65
-10	-6	17	13.71	2.83
-10	-6	17	12.24	1.67
-11	-6	17	16.35	1.88
-11	-6	17	17.66	3.03
-12	-6	17	0.21	0.98
-12	-6	17	0.32	2.42
-13	-6	17	13.89	1.98
-13	-6	17	10.75	2.93
-14	-6	17	3.14	2.79
-14	-6	17	4.41	1.26
-10	6	-18	28.13	2.15
9	-6	18	0.57	1.73

-9	6	-18	0.81	0.90
8	-6	18	29.89	3.45
-8	6	-18	30.34	2.16
-8	6	-18	29.59	1.99
7	-6	18	0.59	1.68
-7	6	-18	0.12	0.88
-7	6	-18	0.27	0.74
6	-6	18	24.67	3.07
6	-6	18	27.92	2.68
-6	6	-18	28.42	1.97
-6	6	-18	25.50	1.90
5	-6	18	0.53	1.54
5	-6	18	-0.64	1.43
-5	6	-18	0.66	0.62
-5	6	-18	-0.71	0.82
4	-6	18	45.20	3.24
4	-6	18	37.02	3.45
-4	6	-18	39.10	2.31
-4	6	-18	42.94	2.06
3	-6	18	2.96	1.59
3	-6	18	0.06	1.49
-3	6	-18	1.19	0.78
-3	6	-18	0.38	0.52
2	-6	18	19.43	2.79
-2	6	-18	19.48	1.61
-2	6	-18	22.78	1.29
1	-6	18	1.67	1.47
1	-6	18	-0.81	1.60
-1	6	-18	0.68	0.44
-1	6	-18	1.11	0.72
0	-6	18	36.60	3.02
0	-6	18	34.16	3.44
0	6	-18	35.77	1.64
0	6	-18	32.46	2.00
-1	-6	18	0.12	1.76
-1	-6	18	-0.38	1.38
1	6	-18	0.03	0.36
1	6	-18	-0.81	0.66
-2	-6	18	39.13	3.87
-2	-6	18	39.32	3.06
2	6	-18	37.42	2.08
2	6	-18	38.24	1.68
-3	-6	18	-1.26	1.29
-3	-6	18	-0.41	1.92

3	6	-18	0.08	0.65
3	6	-18	0.31	0.32
-4	-6	18	35.79	4.05
-4	-6	18	32.20	2.88
4	6	-18	34.62	1.97
4	6	-18	33.34	1.45
-5	-6	18	-1.92	1.18
-5	-6	18	0.68	0.71
-5	-6	18	-0.78	1.91
5	6	-18	-0.03	0.38
-6	-6	18	31.45	4.13
-6	-6	18	40.09	2.25
-6	-6	18	37.00	2.79
-7	-6	18	2.37	1.29
-7	-6	18	4.44	0.91
-7	-6	18	3.54	2.21
-8	-6	18	40.60	2.89
-8	-6	18	45.01	2.56
-8	-6	18	56.51	4.78
-9	-6	18	7.55	2.63
-9	-6	18	2.63	0.96
-9	-6	18	2.73	1.25
-10	-6	18	29.39	2.21
-10	-6	18	22.79	3.34
-11	-6	18	-2.42	2.46
-11	-6	18	1.35	1.10
-12	-6	18	35.88	2.65
-12	-6	18	43.07	4.99
-13	-6	18	1.40	1.25
8	-6	19	1.81	2.02
-8	6	-19	0.84	0.87
7	-6	19	29.75	3.65
-7	6	-19	38.47	2.17
6	-6	19	3.90	1.83
-6	6	-19	3.72	0.81
5	-6	19	20.46	3.12
5	-6	19	15.06	2.13
-5	6	-19	14.94	1.37
-5	6	-19	13.65	1.62
4	-6	19	1.39	3.22
4	-6	19	1.14	1.65
-4	6	-19	-1.78	0.78
-4	6	-19	0.43	0.61
3	-6	19	33.74	3.27

3	-6	19	25.27	3.22
-3	6	-19	28.14	1.93
-3	6	-19	28.12	1.62
2	-6	19	3.57	1.78
2	-6	19	0.84	1.56
-2	6	-19	0.60	0.77
-2	6	-19	-0.05	0.51
1	-6	19	48.77	3.74
1	-6	19	36.19	3.30
-1	6	-19	38.38	1.82
-1	6	-19	34.15	2.13
0	-6	19	2.29	2.00
0	-6	19	-1.09	1.48
0	6	-19	1.42	0.76
0	6	-19	1.19	0.50
-1	-6	19	28.09	3.59
-1	-6	19	33.45	3.07
1	6	-19	31.73	1.53
1	6	-19	32.32	1.94
-2	-6	19	-1.45	1.42
-2	-6	19	-0.23	1.97
2	6	-19	0.86	0.75
-3	-6	19	34.42	3.86
-3	-6	19	37.88	3.13
3	6	-19	35.77	2.09
-4	-6	19	-1.99	1.38
-4	-6	19	-1.53	2.06
4	6	-19	0.43	0.87
-5	-6	19	47.25	3.27
-5	-6	19	35.33	4.39
-5	-6	19	44.49	2.49
-6	-6	19	0.19	0.82
-6	-6	19	0.27	2.15
-6	-6	19	-0.55	1.51
-7	-6	19	33.26	2.17
-7	-6	19	28.41	4.27
-7	-6	19	27.08	2.66
-8	-6	19	-0.74	1.30
-8	-6	19	-1.70	2.45
-8	-6	19	-0.59	0.87
-9	-6	19	37.51	2.48
-9	-6	19	32.31	4.71
-10	-6	19	-0.06	1.06
-10	-6	19	-0.34	2.45

-11	-6	19	39.23	2.84
-11	-6	19	47.37	4.94
6	-6	20	8.05	2.19
-6	6	-20	14.84	1.56
5	-6	20	7.08	2.42
5	-6	20	-1.40	1.80
-5	6	-20	0.64	0.79
4	-6	20	5.76	1.98
4	-6	20	2.35	1.94
-4	6	-20	2.72	1.05
3	-6	20	12.94	2.30
3	-6	20	12.18	2.16
2	-6	20	12.20	2.34
2	-6	20	18.59	3.13
-2	6	-20	13.51	1.50
1	-6	20	5.18	1.81
1	-6	20	3.81	2.02
-1	6	-20	2.04	0.82
0	-6	20	27.01	3.63
0	-6	20	18.47	3.02
0	6	-20	22.96	1.73
-1	-6	20	4.29	2.16
-1	-6	20	1.51	1.68
1	6	-20	2.08	0.81
-2	-6	20	32.84	3.31
-2	-6	20	35.52	4.07
-3	-6	20	3.77	1.78
-3	-6	20	2.87	2.25
3	6	-20	3.97	0.90
-4	-6	20	15.52	1.69
-4	-6	20	10.65	1.95
-4	-6	20	12.42	2.70
-5	-6	20	10.48	2.82
-5	-6	20	8.71	1.89
-5	-6	20	10.69	1.30
-6	-6	20	28.32	2.10
-6	-6	20	23.88	4.17
-6	-6	20	23.72	2.84
-7	-6	20	6.55	1.19
-7	-6	20	5.40	1.63
-7	-6	20	7.43	2.64
-8	-6	20	28.90	2.34
-8	-6	20	37.67	4.71
-9	-6	20	5.15	2.65

-9	-6	20	1.93	1.20
-10	-6	20	24.37	2.47
4	-6	21	6.78	2.24
3	-6	21	-1.77	2.07
3	-6	21	1.42	1.94
2	-6	21	27.56	3.92
2	-6	21	26.98	3.58
1	-6	21	5.97	2.00
1	-6	21	7.11	2.35
0	-6	21	19.07	3.25
0	-6	21	21.49	3.91
-1	-6	21	1.95	2.24
-1	-6	21	3.38	1.81
-2	-6	21	13.61	2.26
-2	-6	21	15.01	2.83
2	6	-21	16.02	1.61
-3	-6	21	2.22	1.82
-3	-6	21	6.57	1.15
-3	-6	21	4.63	2.47
-4	-6	21	16.50	2.95
-4	-6	21	10.29	1.37
-4	-6	21	11.59	2.05
-5	-6	21	-0.80	1.73
-5	-6	21	1.31	1.02
-5	-6	21	1.55	2.50
-6	-6	21	24.99	3.50
-6	-6	21	25.20	2.16
-6	-6	21	21.56	3.06
-7	-6	21	-0.65	2.58
-7	-6	21	-0.04	1.07
6	-7	-21	4.07	0.90
-6	7	21	1.27	1.78
-6	7	21	7.83	2.33
5	-7	-21	15.71	1.54
-5	7	21	12.87	2.07
-5	7	21	16.36	2.55
4	-7	-21	0.71	0.79
-4	7	21	-1.79	1.71
-4	7	21	-2.51	1.70
3	-7	-21	20.88	1.75
-3	7	21	22.10	3.01
-3	7	21	17.89	3.11
2	-7	-21	5.49	1.01
-2	7	21	3.46	1.80

-2	7	21	9.59	2.09
1	-7	-21	21.52	1.74
-1	7	21	20.02	3.19
-1	7	21	20.71	3.13
0	-7	-21	2.20	0.93
0	7	21	3.62	1.71
0	7	21	4.66	1.96
-1	-7	-21	23.45	1.92
1	7	21	22.94	3.34
1	7	21	31.18	3.39
-2	-7	-21	0.61	0.86
2	7	21	1.74	1.85
2	7	21	0.38	1.69
-3	-7	-21	21.35	1.90
9	-7	-20	20.74	1.65
8	-7	-20	8.14	1.30
-8	7	20	8.07	2.11
7	-7	-20	16.92	1.53
-7	7	20	21.14	3.35
-7	7	20	21.52	2.75
6	-7	-20	1.68	0.75
-6	7	20	-1.17	1.53
-6	7	20	4.60	1.95
5	-7	-20	15.49	1.39
-5	7	20	10.13	1.90
-5	7	20	13.08	2.27
4	-7	-20	10.46	1.34
-4	7	20	14.31	2.10
-4	7	20	11.51	2.15
3	-7	-20	11.52	1.42
-3	7	20	16.34	2.75
-3	7	20	14.46	2.22
2	-7	-20	7.84	0.99
-2	7	20	4.86	1.76
-2	7	20	8.78	1.95
1	-7	-20	10.29	1.36
-1	7	20	9.88	1.97
-1	7	20	14.86	2.67
0	-7	-20	1.38	0.74
0	7	20	-1.72	1.46
0	7	20	0.89	1.56
-1	-7	-20	22.16	1.71
1	7	20	23.28	2.89
1	7	20	17.82	2.98

-2	-7	-20	3.84	0.91
2	7	20	4.53	1.86
2	7	20	2.91	1.62
-3	-7	-20	14.66	1.57
3	7	20	8.00	2.01
3	7	20	14.49	2.71
-4	-7	-20	12.56	1.61
4	7	20	17.11	3.15
4	7	20	12.44	1.97
-5	-7	-20	5.32	1.02
5	7	20	7.91	1.86
5	7	20	7.17	2.14
-6	-7	-20	13.53	1.73
11	-7	-19	1.53	0.73
10	-7	-19	22.02	1.68
-10	7	19	34.58	3.82
9	-7	-19	-0.01	0.66
-9	7	19	0.02	1.81
8	-7	-19	49.02	4.13
8	-7	-19	36.75	2.10
-8	7	19	38.30	3.75
7	-7	-19	0.34	0.66
-7	7	19	0.84	1.38
-7	7	19	-1.30	1.64
6	-7	-19	30.45	1.79
-6	7	19	30.10	2.84
-6	7	19	22.84	3.16
5	-7	-19	-0.77	0.61
-5	7	19	0.95	1.37
-5	7	19	-0.61	1.51
4	-7	-19	23.92	1.61
-4	7	19	21.41	3.01
-4	7	19	23.90	2.73
3	-7	-19	1.19	0.66
-3	7	19	2.08	1.52
-3	7	19	0.64	1.45
2	-7	-19	44.87	2.22
-2	7	19	36.58	3.19
-2	7	19	37.58	3.33
1	-7	-19	0.42	0.64
-1	7	19	0.09	1.40
-1	7	19	-0.34	1.39
0	-7	-19	22.22	1.62
0	7	19	22.69	2.68

0	7	19	16.51	2.57
-1	-7	-19	0.30	0.71
1	7	19	0.54	1.53
1	7	19	1.66	1.48
-2	-7	-19	33.20	2.00
2	7	19	26.76	2.88
2	7	19	27.58	2.99
-3	-7	-19	0.76	0.76
3	7	19	2.14	1.42
3	7	19	2.71	1.66
-4	-7	-19	24.72	1.83
4	7	19	20.17	2.64
4	7	19	27.63	3.07
-5	-7	-19	5.95	1.01
5	7	19	7.00	1.66
5	7	19	7.65	1.91
-6	-7	-19	25.13	1.91
6	7	19	21.41	3.19
6	7	19	23.57	2.82
-7	-7	-19	4.19	1.06
7	7	19	0.06	1.57
7	7	19	3.26	1.90
-8	-7	-19	13.66	1.81
12	-7	-18	2.52	0.78
-12	7	18	2.02	2.04
-12	7	18	0.44	1.35
11	-7	-18	26.49	1.76
-11	7	18	25.95	3.73
-11	7	18	28.76	2.47
10	-7	-18	0.60	0.63
-10	7	18	2.04	1.88
-10	7	18	0.95	1.09
9	-7	-18	25.46	3.39
9	-7	-18	28.30	1.73
-9	7	18	31.39	3.43
-9	7	18	28.81	2.09
8	-7	-18	4.07	0.71
8	-7	-18	-0.81	1.87
-8	7	18	2.60	1.04
-8	7	18	1.61	1.66
7	-7	-18	22.63	1.51
-7	7	18	19.50	2.39
-7	7	18	25.23	3.02
6	-7	-18	9.09	1.10

-6	7	18	10.34	1.86
-6	7	18	5.74	1.67
5	-7	-18	46.32	2.23
-5	7	18	38.67	3.01
-5	7	18	45.11	3.50
4	-7	-18	1.20	0.62
-4	7	18	0.23	1.29
-4	7	18	3.44	1.27
3	-7	-18	29.70	1.75
-3	7	18	28.01	2.83
-3	7	18	27.05	2.59
2	-7	-18	0.17	0.59
-2	7	18	0.12	1.27
-2	7	18	-2.40	1.21
1	-7	-18	37.08	2.08
-1	7	18	36.52	3.01
-1	7	18	41.90	3.15
0	-7	-18	-0.69	0.60
0	7	18	0.03	1.21
0	7	18	-1.14	1.32
-1	-7	-18	38.07	2.05
1	7	18	31.11	3.01
1	7	18	40.08	2.94
-2	-7	-18	0.72	0.71
2	7	18	-0.68	1.19
2	7	18	0.68	1.40
-3	-7	-18	26.32	1.82
3	7	18	29.51	2.66
3	7	18	27.62	2.86
-4	-7	-18	-0.51	0.71
4	7	18	1.44	1.51
4	7	18	0.38	1.31
-5	-7	-18	31.55	2.04
5	7	18	31.80	2.80
5	7	18	35.30	3.14
-6	-7	-18	-0.40	0.69
6	7	18	-2.52	1.23
6	7	18	-0.28	1.48
-7	-7	-18	48.20	2.66
7	7	18	49.72	3.26
7	7	18	45.56	3.79
-8	-7	-18	0.35	0.82
8	7	18	-0.89	1.65
8	7	18	-2.35	1.37

-9	-7	-18	24.09	2.00
9	7	18	16.54	2.57
13	-7	-17	12.19	1.35
-13	7	17	10.47	1.64
-13	7	17	9.59	2.41
12	-7	-17	20.96	1.50
-12	7	17	16.87	2.31
-12	7	17	21.04	3.39
11	-7	-17	1.05	0.64
-11	7	17	0.73	1.65
10	-7	-17	7.38	1.10
10	-7	-17	6.61	1.85
-10	7	17	10.17	1.72
-10	7	17	8.32	1.99
9	-7	-17	9.07	1.09
9	-7	-17	6.60	1.74
-9	7	17	10.12	1.20
-9	7	17	10.85	2.03
8	-7	-17	28.19	3.25
8	-7	-17	32.12	1.81
-8	7	17	40.74	3.38
-8	7	17	32.13	2.00
7	-7	-17	4.86	0.74
-7	7	17	5.87	1.75
-7	7	17	4.91	1.37
-7	7	17	8.21	1.21
6	-7	-17	13.27	1.19
-6	7	17	17.22	2.54
-6	7	17	11.33	1.95
-6	7	17	14.62	1.21
5	-7	-17	2.72	0.65
-5	7	17	2.72	0.58
-5	7	17	2.28	1.16
-5	7	17	3.03	1.36
4	-7	-17	17.28	1.31
-4	7	17	18.17	2.49
-4	7	17	15.77	2.39
3	-7	-17	0.31	0.55
-3	7	17	0.14	1.09
-3	7	17	-0.46	1.14
2	-7	-17	23.39	1.53
-2	7	17	27.51	2.45
-2	7	17	26.83	2.55
1	-7	-17	2.49	0.63

-1	7	17	2.56	1.32
-1	7	17	1.85	1.24
0	-7	-17	26.07	1.57
0	7	17	19.93	2.43
0	7	17	24.84	2.40
-1	-7	-17	4.64	0.73
1	7	17	6.39	1.36
1	7	17	3.45	1.47
-2	-7	-17	22.35	1.58
2	7	17	20.17	2.26
2	7	17	22.84	2.51
-3	-7	-17	3.58	0.72
3	7	17	2.36	1.31
3	7	17	3.22	1.23
-4	-7	-17	44.85	2.27
4	7	17	39.37	3.24
4	7	17	41.31	2.86
-5	-7	-17	0.48	0.68
5	7	17	2.77	1.44
5	7	17	0.24	1.11
-6	-7	-17	27.85	1.85
6	7	17	25.18	2.77
6	7	17	25.17	2.89
-7	-7	-17	4.06	0.90
7	7	17	2.56	1.51
7	7	17	5.95	1.75
-8	-7	-17	28.43	1.98
8	7	17	25.45	2.99
8	7	17	30.51	2.57
-9	-7	-17	0.78	0.81
9	7	17	2.77	1.67
9	7	17	1.29	1.34
-10	-7	-17	17.83	1.86
10	7	17	16.18	2.92
-11	-7	-17	-0.61	1.25
14	-7	-16	26.77	1.77
-14	7	16	23.93	3.73
-14	7	16	31.11	2.78
13	-7	-16	0.34	0.65
-13	7	16	4.37	1.35
-13	7	16	-1.29	1.83
12	-7	-16	6.08	0.81
-12	7	16	6.55	1.33
-12	7	16	7.65	2.06

11	-7	-16	3.22	0.65
-11	7	16	3.31	1.67
-11	7	16	3.95	1.14
10	-7	-16	21.71	1.44
10	-7	-16	24.80	2.63
-10	7	16	15.42	2.85
-10	7	16	22.08	1.90
9	-7	-16	0.27	0.53
9	-7	-16	-3.28	1.34
-9	7	16	0.00	0.80
-9	7	16	1.08	1.45
8	-7	-16	40.74	2.91
8	-7	-16	39.18	2.01
8	-7	-16	40.44	3.21
-8	7	16	45.18	2.24
-8	7	16	36.25	3.26
7	-7	-16	6.55	1.39
7	-7	-16	4.63	0.71
7	-7	-16	5.76	1.69
-7	7	16	7.48	1.29
-7	7	16	7.42	1.71
-7	7	16	6.94	0.88
-7	7	16	8.76	1.01
6	-7	-16	16.51	1.24
-6	7	16	19.20	1.28
-6	7	16	15.84	1.25
-6	7	16	16.58	2.46
-6	7	16	17.30	2.02
5	-7	-16	6.81	0.95
-5	7	16	7.56	0.87
-5	7	16	6.45	0.87
-5	7	16	4.07	1.23
-5	7	16	4.99	1.42
4	-7	-16	6.16	0.89
-4	7	16	5.18	0.73
-4	7	16	7.06	0.80
-4	7	16	6.45	1.29
-4	7	16	6.01	1.42
3	-7	-16	1.67	0.53
-3	7	16	1.50	1.11
-3	7	16	1.03	0.99
2	-7	-16	16.30	1.19
-2	7	16	14.93	2.09
-2	7	16	17.17	2.11

1	-7	-16	8.94	0.98
-1	7	16	10.75	1.87
-1	7	16	7.63	1.46
0	-7	-16	20.67	1.39
0	7	16	21.26	2.14
0	7	16	20.82	2.21
-1	-7	-16	10.33	1.12
1	7	16	9.74	1.61
1	7	16	13.08	1.85
-2	-7	-16	22.93	1.51
2	7	16	22.88	2.31
2	7	16	25.11	2.44
-3	-7	-16	2.80	0.66
3	7	16	6.35	1.42
-4	-7	-16	16.07	1.35
4	7	16	17.08	1.90
4	7	16	13.39	2.18
-5	-7	-16	13.59	1.30
5	7	16	13.76	1.85
5	7	16	13.35	2.26
-6	-7	-16	9.78	1.24
6	7	16	9.49	1.61
6	7	16	11.83	1.85
-7	-7	-16	14.89	1.50
7	7	16	14.93	1.95
7	7	16	17.99	2.58
-8	-7	-16	5.55	0.88
8	7	16	4.26	1.46
8	7	16	8.06	1.42
-9	-7	-16	-0.68	0.76
9	7	16	1.26	1.49
9	7	16	0.27	1.21
-10	-7	-16	13.59	1.58
10	7	16	12.70	2.03
-11	-7	-16	12.05	1.61
11	7	16	7.09	1.90
-12	-7	-16	2.97	1.07
12	7	16	0.97	1.76
15	-7	-15	20.31	1.51
-15	7	15	19.13	2.60
-15	7	15	15.11	2.63
14	-7	-15	3.96	0.76
-14	7	15	7.22	2.10
-14	7	15	4.09	1.37

13	-7	-15	15.47	1.31
-13	7	15	13.67	2.10
-13	7	15	17.84	3.12
12	-7	-15	9.30	1.08
-12	7	15	9.70	2.01
-12	7	15	5.63	1.21
11	-7	-15	12.10	1.15
11	-7	-15	15.60	2.08
-11	7	15	13.06	1.75
-11	7	15	9.90	1.99
10	-7	-15	8.93	1.47
10	-7	-15	8.33	0.95
-10	7	15	6.38	1.11
-10	7	15	4.50	1.59
9	-7	-15	32.69	2.64
9	-7	-15	22.43	2.25
9	-7	-15	27.17	1.56
-9	7	15	26.45	2.91
-9	7	15	28.19	1.86
-9	7	15	30.33	1.91
8	-7	-15	8.84	1.37
8	-7	-15	9.72	1.01
8	-7	-15	13.18	2.15
-8	7	15	8.10	1.62
-8	7	15	9.54	1.29
-8	7	15	11.57	1.25
7	-7	-15	51.55	2.97
7	-7	-15	44.64	2.24
7	-7	-15	52.83	3.31
-7	7	15	51.84	2.34
-7	7	15	48.79	3.37
-7	7	15	45.07	2.31
6	-7	-15	11.45	2.09
6	-7	-15	8.67	0.96
-6	7	15	11.45	2.06
-6	7	15	10.00	1.04
-6	7	15	9.82	1.67
-6	7	15	12.19	1.05
5	-7	-15	31.64	1.69
5	-7	-15	35.35	2.57
-5	7	15	32.95	1.59
-5	7	15	31.45	2.29
-5	7	15	33.28	2.70
-5	7	15	31.76	1.59

4	-7	-15	-0.49	0.40
-4	7	15	0.71	0.42
-4	7	15	0.89	0.93
-4	7	15	1.10	0.40
-4	7	15	1.49	1.05
3	-7	-15	24.47	1.50
-3	7	15	24.22	2.16
-3	7	15	25.82	2.25
2	-7	-15	4.75	0.71
-2	7	15	5.30	1.17
-2	7	15	5.80	1.23
1	-7	-15	31.48	1.67
-1	7	15	28.39	2.32
0	-7	-15	6.00	0.86
0	7	15	5.35	1.19
0	7	15	2.91	1.16
-1	-7	-15	94.66	3.83
1	7	15	91.33	4.34
1	7	15	82.50	4.47
-2	-7	-15	3.14	0.62
2	7	15	1.85	1.02
2	7	15	1.32	0.92
-3	-7	-15	60.92	2.87
3	7	15	63.80	3.35
3	7	15	64.83	3.61
-4	-7	-15	0.64	0.56
4	7	15	0.36	0.82
4	7	15	1.49	1.14
-5	-7	-15	23.23	1.54
5	7	15	20.22	1.90
5	7	15	21.97	2.35
-6	-7	-15	-0.46	0.54
6	7	15	-1.02	0.95
6	7	15	-0.75	1.16
-7	-7	-15	41.82	2.25
7	7	15	41.98	3.06
7	7	15	40.03	2.64
-8	-7	-15	0.34	0.70
8	7	15	2.60	1.07
8	7	15	1.68	1.35
-9	-7	-15	10.50	1.37
9	7	15	9.09	1.39
9	7	15	13.06	2.46
-10	-7	-15	1.57	0.78

10	7	15	0.84	1.11
10	7	15	-2.07	1.34
-11	-7	-15	16.83	1.72
11	7	15	18.99	2.83
-12	-7	-15	-1.25	0.92
12	7	15	0.23	1.58
-13	-7	-15	18.69	2.12
13	7	15	26.51	3.17
16	-7	-14	2.01	0.74
-16	7	14	2.58	1.48
-16	7	14	-1.84	1.95
15	-7	-14	24.55	1.69
-15	7	14	32.48	2.72
-15	7	14	28.51	3.71
14	-7	-14	8.59	1.10
-14	7	14	9.44	2.12
-14	7	14	9.76	1.49
13	-7	-14	30.84	1.70
-13	7	14	28.30	2.35
-13	7	14	28.24	3.28
12	-7	-14	3.28	0.61
-12	7	14	2.43	0.96
-12	7	14	-1.17	1.45
11	-7	-14	39.21	2.55
11	-7	-14	40.35	2.02
-11	7	14	39.37	3.41
-11	7	14	44.37	2.45
10	-7	-14	2.42	1.05
10	-7	-14	3.06	1.20
10	-7	-14	3.27	0.56
-10	7	14	5.99	1.67
-10	7	14	3.40	0.88
9	-7	-14	42.23	2.74
9	-7	-14	41.47	1.98
9	-7	-14	42.69	2.53
9	-7	-14	38.43	2.22
-9	7	14	34.87	3.07
-9	7	14	43.38	2.24
8	-7	-14	21.37	2.29
8	-7	-14	22.30	1.36
8	-7	-14	27.55	2.15
-8	7	14	24.65	1.60
-8	7	14	22.56	2.49
-8	7	14	24.74	1.57

7	-7	-14	26.32	1.44
7	-7	-14	25.37	2.38
7	-7	-14	23.96	2.15
-7	7	14	25.98	1.55
-7	7	14	28.97	1.59
-7	7	14	24.17	2.47
6	-7	-14	14.49	1.87
-6	7	14	13.09	1.09
-6	7	14	14.10	1.66
-6	7	14	15.88	2.02
5	-7	-14	48.80	2.41
5	-7	-14	58.42	3.09
-5	7	14	54.89	3.27
-5	7	14	52.68	2.33
-5	7	14	50.47	2.92
-5	7	14	58.89	2.34
4	-7	-14	23.42	1.34
4	-7	-14	27.68	2.34
-4	7	14	21.32	2.73
-4	7	14	24.84	1.21
-4	7	14	22.09	1.20
-4	7	14	26.22	2.02
3	-7	-14	32.79	1.64
3	-7	-14	31.22	2.43
-3	7	14	32.94	2.27
-3	7	14	33.39	1.40
-3	7	14	28.27	2.34
2	-7	-14	38.22	1.86
-2	7	14	40.08	2.56
-2	7	14	35.70	2.44
1	-7	-14	21.78	1.30
-1	7	14	20.33	2.00
-1	7	14	21.72	1.93
0	-7	-14	39.96	1.95
0	7	14	38.00	2.57
0	7	14	40.92	2.49
-1	-7	-14	6.90	0.87
1	7	14	6.77	1.13
1	7	14	6.37	1.20
2	7	14	19.13	2.07
2	7	14	18.40	1.77
-3	-7	-14	25.17	1.48
3	7	14	22.70	1.91
3	7	14	24.24	2.20

-4	-7	-14	18.08	1.25
4	7	14	17.99	2.01
4	7	14	18.64	1.74
-5	-7	-14	0.42	1.51
-5	-7	-14	1.78	0.55
5	7	14	1.99	0.85
5	7	14	2.55	1.16
-6	-7	-14	24.57	1.53
-6	-7	-14	22.21	2.82
6	7	14	21.15	2.32
6	7	14	21.90	1.89
-7	-7	-14	15.64	1.34
-7	-7	-14	15.95	2.90
7	7	14	15.51	1.72
7	7	14	15.08	2.10
-8	-7	-14	20.65	3.14
-8	-7	-14	26.97	1.72
8	7	14	22.02	2.55
8	7	14	27.75	2.05
-9	-7	-14	12.58	1.30
9	7	14	9.76	1.66
9	7	14	14.85	1.75
-10	-7	-14	21.05	1.71
10	7	14	22.61	1.96
10	7	14	21.35	2.69
-11	-7	-14	1.20	0.72
11	7	14	4.83	1.51
11	7	14	1.04	1.07
-12	-7	-14	13.63	1.59
12	7	14	12.13	1.99
-13	-7	-14	5.24	1.09
13	7	14	5.16	1.83
-14	-7	-14	42.23	3.59
14	7	14	31.00	3.57
17	-7	-13	5.96	0.92
-17	7	13	6.72	2.21
-17	7	13	8.84	1.70
16	-7	-13	37.04	2.05
-16	7	13	33.09	3.85
-16	7	13	42.47	3.03
15	-7	-13	3.60	0.68
-15	7	13	4.86	1.34
-15	7	13	5.11	1.95
14	-7	-13	37.60	2.01

-14	7	13	41.54	2.75
-14	7	13	39.47	3.60
13	-7	-13	4.41	0.68
-13	7	13	1.00	1.49
-13	7	13	5.54	1.19
12	-7	-13	91.37	3.79
12	-7	-13	88.16	3.97
-12	7	13	88.49	4.99
-12	7	13	92.59	4.33
11	-7	-13	2.82	1.02
11	-7	-13	2.21	0.95
11	-7	-13	1.46	0.53
-11	7	13	2.51	1.37
-11	7	13	1.94	0.85
10	-7	-13	56.51	2.85
10	-7	-13	57.67	3.06
10	-7	-13	55.63	2.52
10	-7	-13	55.72	3.03
-10	7	13	59.93	2.84
-10	7	13	51.67	3.57
9	-7	-13	1.59	0.47
9	-7	-13	1.50	0.68
9	-7	-13	1.61	0.94
9	-7	-13	1.06	0.98
-9	7	13	1.85	1.23
-9	7	13	1.33	0.72
8	-7	-13	60.64	3.10
8	-7	-13	56.04	2.78
8	-7	-13	64.81	3.38
-8	7	13	65.81	2.84
-8	7	13	60.25	3.64
7	-7	-13	3.90	1.04
7	-7	-13	3.06	0.52
7	-7	-13	4.12	0.69
7	-7	-13	4.09	1.17
-7	7	13	5.81	1.24
-7	7	13	5.03	0.72
6	-7	-13	45.12	2.91
6	-7	-13	46.76	2.67
6	-7	-13	45.80	2.12
6	-7	-13	46.19	2.09
-6	7	13	44.66	2.56
-6	7	13	45.06	2.10
5	-7	-13	2.25	0.49

5	-7	-13	1.12	0.97
-5	7	13	3.40	1.03
-5	7	13	2.78	0.50
-5	7	13	3.56	0.52
-5	7	13	2.58	0.90
4	-7	-13	59.56	2.62
4	-7	-13	59.03	3.20
-4	7	13	64.60	3.34
-4	7	13	55.90	3.07
-4	7	13	60.87	2.49
-4	7	13	61.80	2.49
3	-7	-13	0.68	0.94
3	-7	-13	0.36	0.35
-3	7	13	0.56	0.81
-3	7	13	-0.04	0.28
-3	7	13	-0.18	0.72
2	-7	-13	56.96	3.05
2	-7	-13	47.67	2.23
-2	7	13	47.60	2.79
-2	7	13	46.89	2.75
1	-7	-13	2.83	1.04
1	-7	-13	-0.17	0.38
-1	7	13	1.12	0.78
-1	7	13	0.65	0.80
0	-7	-13	59.91	2.51
0	7	13	51.12	3.07
0	7	13	55.62	3.00
-1	-7	-13	14.99	1.05
-1	-7	-13	15.96	2.26
1	7	13	16.11	1.61
1	7	13	14.16	1.75
-2	-7	-13	26.81	2.53
-2	-7	-13	32.90	1.65
2	7	13	29.48	2.24
2	7	13	34.94	2.15
-3	-7	-13	4.95	0.80
-3	-7	-13	3.46	1.35
3	7	13	3.84	0.88
3	7	13	5.21	1.18
-4	-7	-13	58.59	3.47
-4	-7	-13	54.72	2.64
4	7	13	53.40	2.84
4	7	13	48.71	3.77
-5	-7	-13	2.36	0.55

-5	-7	-13	3.89	1.43
5	7	13	1.96	0.95
5	7	13	3.84	0.91
-6	-7	-13	47.35	2.27
-6	-7	-13	47.24	3.15
6	7	13	43.46	2.87
6	7	13	45.66	2.55
-7	-7	-13	19.81	1.42
-7	-7	-13	24.04	2.57
7	7	13	18.80	2.13
7	7	13	19.60	1.71
-8	-7	-13	33.81	1.90
-8	-7	-13	27.06	2.90
8	7	13	32.41	2.70
8	7	13	35.67	2.15
-9	-7	-13	4.45	1.69
-9	-7	-13	3.55	0.82
9	7	13	2.69	0.95
9	7	13	2.53	1.34
-10	-7	-13	24.38	1.70
10	7	13	22.65	2.57
10	7	13	26.44	1.98
-11	-7	-13	2.63	0.83
11	7	13	3.02	1.03
11	7	13	3.02	1.42
-12	-7	-13	20.06	1.75
12	7	13	20.42	2.61
-13	-7	-13	23.00	1.87
13	7	13	16.05	2.79
-14	-7	-13	10.63	1.65
14	7	13	12.61	2.23
15	7	13	1.04	1.82
17	-7	-12	19.67	1.50
-17	7	12	25.15	3.47
-17	7	12	25.31	2.75
16	-7	-12	16.43	1.34
-16	7	12	16.31	2.44
-16	7	12	13.87	2.28
15	-7	-12	43.92	2.18
-15	7	12	39.20	3.70
-15	7	12	43.22	2.90
14	-7	-12	7.45	0.96
-14	7	12	6.50	1.88
-14	7	12	6.83	1.28

13	-7	-12	25.41	1.49
-13	7	12	28.85	2.15
-13	7	12	22.99	2.92
12	-7	-12	7.52	1.30
12	-7	-12	3.95	0.64
-12	7	12	3.23	1.51
11	-7	-12	62.98	2.95
11	-7	-12	60.48	3.10
11	-7	-12	64.77	2.79
-11	7	12	70.90	3.16
-11	7	12	58.71	3.81
10	-7	-12	1.87	0.72
10	-7	-12	1.74	0.94
10	-7	-12	2.50	0.82
10	-7	-12	2.87	0.53
-10	7	12	1.57	0.71
-10	7	12	3.50	1.23
9	-7	-12	38.40	2.15
9	-7	-12	41.27	2.54
9	-7	-12	44.12	2.35
-9	7	12	48.16	2.27
-9	7	12	43.63	2.97
8	-7	-12	3.46	0.96
8	-7	-12	3.57	0.67
8	-7	-12	2.46	0.46
8	-7	-12	3.69	0.93
-8	7	12	3.12	0.64
-8	7	12	2.34	1.30
7	-7	-12	49.77	2.54
7	-7	-12	42.16	2.69
7	-7	-12	40.90	2.09
7	-7	-12	43.92	2.02
-7	7	12	44.42	2.86
-7	7	12	46.38	2.14
6	-7	-12	29.59	1.53
6	-7	-12	33.30	2.11
6	-7	-12	25.74	2.23
6	-7	-12	29.35	1.47
-6	7	12	29.83	1.53
-6	7	12	26.23	2.17
5	-7	-12	82.10	3.33
5	-7	-12	87.71	3.87
5	-7	-12	80.58	3.37
-5	7	12	76.84	4.01

-5	7	12	84.90	3.33
-5	7	12	75.94	3.74
4	-7	-12	9.00	0.82
4	-7	-12	9.18	1.52
-4	7	12	10.85	0.75
-4	7	12	11.11	1.39
-4	7	12	10.77	1.51
3	-7	-12	88.58	3.76
3	-7	-12	95.30	4.33
-3	7	12	96.34	3.64
-3	7	12	86.92	4.34
-3	7	12	97.00	3.60
-3	7	12	91.59	3.85
-3	7	12	89.16	4.19
2	-7	-12	5.58	1.12
2	-7	-12	5.05	1.28
2	-7	-12	5.09	0.67
-2	7	12	5.00	1.01
-2	7	12	4.87	0.45
-2	7	12	3.11	0.53
-2	7	12	4.92	0.41
-2	7	12	2.85	0.88
1	-7	-12	56.25	2.43
1	-7	-12	59.29	3.06
1	-7	-12	57.55	3.22
-1	7	12	52.75	2.90
-1	7	12	55.11	2.91
-1	7	12	51.34	2.46
0	-7	-12	0.23	0.39
0	-7	-12	0.27	0.93
0	-7	-12	-0.30	1.05
0	7	12	0.60	0.76
0	7	12	1.13	0.47
0	7	12	0.24	0.65
-1	-7	-12	67.36	3.62
-1	-7	-12	67.92	3.50
-1	-7	-12	66.52	2.84
1	7	12	63.89	3.34
1	7	12	63.39	3.24
-2	-7	-12	0.28	0.36
-2	-7	-12	-0.22	0.97
2	7	12	0.55	0.69
2	7	12	0.17	0.77
-3	-7	-12	77.71	4.05

-3	-7	-12	75.27	3.36
3	7	12	79.19	3.95
3	7	12	86.44	3.75
-4	-7	-12	3.09	0.53
-4	-7	-12	3.16	1.21
4	7	12	2.52	0.89
4	7	12	3.93	0.83
-5	-7	-12	56.57	2.56
-5	-7	-12	54.06	3.28
5	7	12	54.80	3.13
5	7	12	57.18	2.84
-6	-7	-12	2.01	1.20
6	7	12	2.00	0.79
-7	-7	-12	65.66	2.87
-7	-7	-12	57.29	3.54
7	7	12	59.04	3.43
7	7	12	67.18	3.10
-8	-7	-12	5.45	1.41
-8	-7	-12	2.95	0.66
8	7	12	3.94	1.15
8	7	12	2.66	0.84
-9	-7	-12	59.03	2.77
-9	-7	-12	52.52	3.48
9	7	12	60.16	2.91
9	7	12	55.35	3.44
-10	-7	-12	6.04	0.91
-10	-7	-12	5.16	1.63
10	7	12	3.30	0.96
10	7	12	4.66	1.31
-11	-7	-12	18.69	1.55
-11	-7	-12	19.12	1.49
11	7	12	21.80	1.81
11	7	12	17.35	2.28
-12	-7	-12	2.47	0.78
12	7	12	1.55	1.02
12	7	12	5.38	1.49
-13	-7	-12	27.74	2.00
13	7	12	25.25	2.81
-14	-7	-12	-0.13	0.88
14	7	12	0.32	1.51
-15	-7	-12	33.07	2.38
15	7	12	29.82	3.29
18	-7	-11	1.51	0.80
-18	7	11	2.49	2.04

-18	7	11	0.54	1.45
17	-7	-11	15.44	1.41
-17	7	11	15.22	2.46
-17	7	11	17.05	2.46
16	-7	-11	4.73	0.75
-16	7	11	4.17	1.36
-16	7	11	6.60	1.93
15	-7	-11	13.31	1.16
-15	7	11	11.95	2.09
-15	7	11	12.80	2.08
14	-7	-11	22.02	1.39
-14	7	11	21.43	2.14
-14	7	11	19.44	2.83
13	-7	-11	1.21	0.49
-13	7	11	0.44	1.33
-13	7	11	1.09	0.93
12	-7	-11	33.54	1.89
12	-7	-11	31.95	1.69
-12	7	11	32.81	2.89
-12	7	11	35.30	2.15
11	-7	-11	2.21	0.74
11	-7	-11	2.92	0.53
11	-7	-11	2.27	0.88
-11	7	11	2.96	0.82
-11	7	11	3.68	1.32
10	-7	-11	10.16	1.40
10	-7	-11	7.97	1.23
10	-7	-11	12.44	1.34
-10	7	11	11.33	1.28
-10	7	11	9.90	2.30
9	-7	-11	62.29	3.16
9	-7	-11	60.40	2.89
9	-7	-11	67.42	3.02
9	-7	-11	63.23	2.71
-9	7	11	64.34	2.95
-9	7	11	62.03	3.58
8	-7	-11	46.46	2.05
8	-7	-11	39.95	2.54
8	-7	-11	49.92	2.46
8	-7	-11	42.87	2.20
-8	7	11	46.17	2.23
-8	7	11	48.65	2.92
7	-7	-11	6.68	0.90
7	-7	-11	6.46	0.69

7	-7	-11	3.52	1.00
7	-7	-11	6.37	0.93
-7	7	11	6.53	1.15
-7	7	11	6.69	0.89
6	-7	-11	58.64	2.58
6	-7	-11	59.86	3.20
6	-7	-11	65.32	3.03
6	-7	-11	57.24	2.62
-6	7	11	60.06	2.62
-6	7	11	63.84	3.31
5	-7	-11	1.53	0.38
5	-7	-11	1.83	0.86
5	-7	-11	0.55	1.09
5	-7	-11	2.82	0.84
5	-7	-11	1.49	0.44
-5	7	11	2.40	0.83
-5	7	11	1.61	0.72
-5	7	11	0.55	0.41
-5	7	11	1.32	0.40
4	-7	-11	43.86	2.29
4	-7	-11	32.81	1.95
4	-7	-11	36.06	1.71
4	-7	-11	31.88	2.48
-4	7	11	37.51	1.65
-4	7	11	40.08	2.34
-4	7	11	37.07	1.79
-4	7	11	37.15	2.17
3	-7	-11	6.68	1.07
3	-7	-11	7.70	0.60
3	-7	-11	8.61	1.32
3	-7	-11	6.83	0.68
-3	7	11	6.80	0.63
-3	7	11	5.80	0.76
-3	7	11	6.00	0.97
-3	7	11	6.02	0.96
2	-7	-11	43.95	2.82
2	-7	-11	50.20	2.67
2	-7	-11	49.60	2.11
-2	7	11	44.46	2.53
-2	7	11	47.50	2.14
-2	7	11	47.14	2.61
-2	7	11	47.26	1.92
1	-7	-11	1.39	0.91
1	-7	-11	2.08	0.43

1	-7	-11	3.21	0.88
-1	7	11	1.43	0.69
-1	7	11	1.57	0.69
-1	7	11	1.84	0.45
0	-7	-11	17.54	1.79
0	-7	-11	14.88	1.68
0	-7	-11	13.64	0.95
0	7	11	11.89	1.40
0	7	11	14.09	1.38
0	7	11	16.55	1.04
-1	-7	-11	0.84	0.39
-1	-7	-11	0.97	0.90
-1	-7	-11	0.90	0.88
1	7	11	1.01	0.65
1	7	11	-0.05	0.67
1	7	11	-0.16	0.39
-2	-7	-11	29.41	2.20
-2	-7	-11	27.08	1.43
-2	-7	-11	26.68	2.14
2	7	11	27.36	1.82
2	7	11	27.56	1.48
2	7	11	29.36	1.98
-3	-7	-11	59.89	3.16
-3	-7	-11	56.26	2.50
-3	-7	-11	56.01	3.17
3	7	11	55.62	2.52
3	7	11	57.43	2.82
3	7	11	53.95	3.02
-4	-7	-11	90.48	4.27
-4	-7	-11	87.94	3.70
4	7	11	91.08	3.97
4	7	11	85.34	4.23
-5	-7	-11	7.60	1.26
-5	-7	-11	5.13	0.74
5	7	11	5.59	1.03
5	7	11	6.98	1.11
-6	-7	-11	48.44	2.29
-6	-7	-11	50.98	2.95
6	7	11	49.11	2.53
6	7	11	48.43	2.82
-7	-7	-11	0.20	1.04
-7	-7	-11	1.00	0.46
7	7	11	-0.01	0.66
7	7	11	-0.25	0.86

-8	-7	-11	38.02	2.63
-8	-7	-11	36.08	1.92
8	7	11	32.53	2.60
8	7	11	38.76	2.12
-9	-7	-11	7.22	0.70
-9	-7	-11	10.33	1.57
-9	-7	-11	9.04	1.03
9	7	11	7.70	1.25
9	7	11	7.05	1.29
-10	-7	-11	28.35	1.74
-10	-7	-11	28.51	1.46
-10	-7	-11	26.25	2.52
10	7	11	29.20	1.90
10	7	11	28.51	2.37
-11	-7	-11	26.49	1.54
-11	-7	-11	29.22	3.38
-11	-7	-11	28.02	1.80
11	7	11	24.82	2.40
11	7	11	26.79	1.87
-12	-7	-11	32.23	2.00
-12	-7	-11	34.09	1.86
12	7	11	30.07	2.10
12	7	11	30.19	2.79
-13	-7	-11	-0.38	0.68
-13	-7	-11	1.08	0.85
13	7	11	2.38	1.37
-14	-7	-11	24.93	1.92
14	7	11	26.37	2.84
-15	-7	-11	8.65	1.24
15	7	11	7.19	1.74
-16	-7	-11	31.39	2.36
16	7	11	32.66	3.35
18	-7	-10	14.57	1.44
-18	7	10	15.87	2.53
-18	7	10	11.96	2.41
17	-7	-10	1.42	0.65
-17	7	10	0.70	1.24
-17	7	10	1.75	1.85
16	-7	-10	5.70	0.80
-16	7	10	8.32	1.44
-16	7	10	4.81	1.73
15	-7	-10	0.59	0.50
-15	7	10	-0.65	1.02
-15	7	10	0.56	1.43

14	-7	-10	13.40	1.10
-14	7	10	17.05	2.47
-14	7	10	16.68	1.92
13	-7	-10	0.19	0.43
13	-7	-10	0.69	0.56
-13	7	10	1.90	1.27
-13	7	10	0.05	0.83
12	-7	-10	26.48	1.59
12	-7	-10	25.11	1.63
12	-7	-10	26.44	1.45
-12	7	10	28.21	1.93
11	-7	-10	14.68	1.36
11	-7	-10	16.25	1.26
11	-7	-10	11.99	0.96
-11	7	10	12.88	2.05
-11	7	10	12.82	1.44
10	-7	-10	47.90	2.53
10	-7	-10	43.93	2.42
10	-7	-10	50.21	2.19
10	-7	-10	54.48	2.43
-10	7	10	51.17	2.51
-10	7	10	45.46	3.01
9	-7	-10	0.29	0.56
9	-7	-10	-0.18	0.69
9	-7	-10	1.09	0.69
9	-7	-10	0.11	0.33
-9	7	10	0.94	0.56
-9	7	10	0.07	0.93
8	-7	-10	16.39	1.58
8	-7	-10	16.26	1.22
8	-7	-10	17.10	1.03
8	-7	-10	19.81	1.45
-8	7	10	17.87	1.27
-8	7	10	18.57	1.86
7	-7	-10	6.29	1.17
7	-7	-10	5.69	0.97
7	-7	-10	4.97	0.60
7	-7	-10	7.56	1.40
7	-7	-10	6.06	0.85
-7	7	10	5.97	0.84
-7	7	10	5.68	0.74
-7	7	10	6.75	1.16
6	-7	-10	6.38	1.17
6	-7	-10	7.30	1.36

6	-7	-10	7.75	0.67
6	-7	-10	7.04	1.28
6	-7	-10	8.91	0.84
-6	7	10	6.26	0.76
-6	7	10	5.69	1.02
-6	7	10	6.34	0.78
5	-7	-10	6.46	0.71
5	-7	-10	4.09	1.06
5	-7	-10	4.38	0.58
5	-7	-10	7.75	1.19
5	-7	-10	6.04	1.02
-5	7	10	6.04	0.67
-5	7	10	5.25	0.95
-5	7	10	5.88	0.68
4	-7	-10	0.95	0.70
4	-7	-10	1.84	0.82
4	-7	-10	2.13	0.39
4	-7	-10	1.94	0.42
-4	7	10	2.12	0.70
-4	7	10	1.76	0.43
-4	7	10	2.24	0.39
-4	7	10	1.33	0.68
3	-7	-10	2.64	0.38
3	-7	-10	3.12	0.81
3	-7	-10	2.43	0.42
3	-7	-10	3.71	0.93
-3	7	10	1.92	0.41
-3	7	10	2.22	0.68
-3	7	10	2.26	0.69
-3	7	10	3.47	0.54
2	-7	-10	33.86	2.03
2	-7	-10	31.27	1.38
2	-7	-10	31.75	1.53
2	-7	-10	38.72	2.29
-2	7	10	32.55	1.57
-2	7	10	31.35	1.39
-2	7	10	30.77	1.99
-2	7	10	31.85	1.95
1	-7	-10	5.34	0.92
1	-7	-10	5.30	0.61
1	-7	-10	7.14	1.12
-1	7	10	4.83	0.64
-1	7	10	6.08	1.09
-1	7	10	4.72	0.85

0	-7	-10	10.59	1.42
0	-7	-10	9.14	1.30
0	-7	-10	10.07	0.75
0	7	10	8.58	1.16
0	7	10	10.46	1.23
0	7	10	9.84	0.79
-1	-7	-10	19.07	1.09
-1	-7	-10	21.15	1.73
-1	-7	-10	20.91	1.71
1	7	10	19.15	1.46
1	7	10	19.86	1.13
1	7	10	16.55	1.52
-2	-7	-10	25.55	1.81
-2	-7	-10	20.01	1.15
-2	-7	-10	18.15	1.74
2	7	10	21.83	1.50
2	7	10	19.27	1.17
2	7	10	21.41	1.64
-3	-7	-10	13.69	1.56
-3	-7	-10	14.46	1.56
-3	-7	-10	11.77	0.89
3	7	10	10.50	1.20
3	7	10	11.98	0.92
3	7	10	12.29	1.39
-4	-7	-10	50.40	2.28
-4	-7	-10	49.97	2.24
-4	-7	-10	54.19	2.84
-4	-7	-10	53.79	2.91
4	7	10	46.43	2.71
4	7	10	51.79	2.56
-5	-7	-10	19.85	1.79
-5	-7	-10	19.20	1.17
-5	-7	-10	17.06	1.11
-5	-7	-10	17.96	1.87
5	7	10	19.70	1.44
5	7	10	17.22	1.62
-6	-7	-10	4.53	1.08
-6	-7	-10	5.03	0.72
6	7	10	3.47	0.75
6	7	10	2.91	0.89
-7	-7	-10	44.98	2.72
-7	-7	-10	46.34	2.18
7	7	10	47.97	2.38
7	7	10	43.87	2.68

-8	-7	-10	4.40	1.10
-8	-7	-10	1.82	0.48
-8	-7	-10	2.63	0.39
-8	-7	-10	2.67	0.56
8	7	10	2.89	0.73
8	7	10	3.34	1.00
-9	-7	-10	13.59	0.90
-9	-7	-10	15.33	1.15
-9	-7	-10	12.80	1.86
-9	-7	-10	12.26	0.99
9	7	10	15.03	1.37
-10	-7	-10	24.25	2.23
-10	-7	-10	26.74	1.34
-10	-7	-10	23.66	1.58
-10	-7	-10	25.20	1.44
10	7	10	26.13	1.74
10	7	10	24.46	2.21
-11	-7	-10	19.39	1.54
-11	-7	-10	20.35	1.45
-11	-7	-10	24.26	1.37
-11	-7	-10	22.57	2.28
11	7	10	25.16	2.27
11	7	10	22.30	1.69
-12	-7	-10	6.20	0.89
-12	-7	-10	6.68	0.98
12	7	10	8.57	1.45
12	7	10	8.97	1.51
-13	-7	-10	29.46	2.30
-13	-7	-10	31.66	1.92
-13	-7	-10	26.02	1.91
13	7	10	29.19	2.62
-14	-7	-10	1.74	0.77
-14	-7	-10	-0.28	0.95
14	7	10	0.95	1.37
-15	-7	-10	13.30	1.58
-15	-7	-10	15.62	2.10
15	7	10	15.10	2.56
-16	-7	-10	11.15	1.73
16	7	10	7.21	1.85
17	7	10	15.01	2.29
19	-7	-9	5.01	0.82
-19	7	9	6.58	2.06
-19	7	9	4.44	1.63
18	-7	-9	22.27	1.48

-18	7	9	21.52	2.64
-18	7	9	14.72	2.34
17	-7	-9	17.75	1.40
-17	7	9	15.76	2.93
-17	7	9	17.22	2.38
16	-7	-9	17.07	1.28
-16	7	9	16.68	2.11
-16	7	9	14.24	2.64
15	-7	-9	26.60	1.52
-15	7	9	20.70	2.66
-15	7	9	26.30	2.25
14	-7	-9	5.43	0.82
-14	7	9	3.43	1.85
-14	7	9	4.98	1.12
13	-7	-9	10.74	0.96
13	-7	-9	10.75	1.44
13	-7	-9	11.00	1.04
-13	7	9	13.09	2.14
-13	7	9	12.98	1.57
12	-7	-9	14.66	1.23
12	-7	-9	14.98	1.01
12	-7	-9	13.35	1.14
-12	7	9	11.39	1.69
-12	7	9	14.49	1.52
11	-7	-9	40.14	2.47
11	-7	-9	51.88	2.38
11	-7	-9	50.03	2.19
-11	7	9	52.42	2.57
-11	7	9	49.32	3.05
10	-7	-9	11.59	0.83
10	-7	-9	13.83	1.18
10	-7	-9	12.43	1.32
10	-7	-9	11.67	1.21
-10	7	9	11.62	1.80
-10	7	9	11.26	1.24
9	-7	-9	45.06	2.16
9	-7	-9	42.73	2.09
9	-7	-9	41.67	2.26
9	-7	-9	41.33	2.93
9	-7	-9	40.74	1.89
-9	7	9	37.14	2.56
-9	7	9	43.48	2.13
8	-7	-9	6.97	1.21
8	-7	-9	7.85	1.10

8	-7	-9	9.91	0.96
8	-7	-9	8.00	1.34
8	-7	-9	7.40	0.66
-8	7	9	9.02	0.98
-8	7	9	7.59	0.80
-8	7	9	5.66	1.06
7	-7	-9	30.64	1.78
7	-7	-9	27.06	1.89
7	-7	-9	33.39	1.57
7	-7	-9	28.75	1.40
7	-7	-9	30.08	2.31
-7	7	9	29.00	1.48
-7	7	9	28.96	1.56
-7	7	9	26.26	2.02
6	-7	-9	8.78	1.28
6	-7	-9	8.82	1.13
6	-7	-9	11.03	1.55
6	-7	-9	10.28	0.84
6	-7	-9	9.00	0.70
-6	7	9	8.29	1.31
-6	7	9	9.43	0.76
-6	7	9	7.23	0.80
5	-7	-9	54.46	2.54
5	-7	-9	49.11	2.15
5	-7	-9	50.63	2.82
5	-7	-9	48.98	2.71
5	-7	-9	51.80	2.20
-5	7	9	49.60	2.69
-5	7	9	49.22	2.17
-5	7	9	48.27	2.19
4	-7	-9	16.10	1.58
4	-7	-9	12.68	0.85
4	-7	-9	12.99	0.82
4	-7	-9	16.46	1.32
4	-7	-9	13.36	1.53
-4	7	9	12.86	1.34
-4	7	9	12.02	0.86
-4	7	9	13.54	0.82
3	-7	-9	69.09	3.36
3	-7	-9	67.76	2.73
3	-7	-9	63.36	2.78
3	-7	-9	74.56	3.21
-3	7	9	68.77	2.82
-3	7	9	66.80	3.25

-3	7	9	64.71	2.72
-3	7	9	66.32	3.13
2	-7	-9	8.21	0.58
2	-7	-9	7.97	1.26
2	-7	-9	10.70	1.20
2	-7	-9	8.48	0.65
-2	7	9	7.87	1.06
-2	7	9	8.58	0.69
-2	7	9	7.23	1.04
-2	7	9	8.74	0.59
1	-7	-9	32.28	1.41
1	-7	-9	33.62	1.57
1	-7	-9	39.08	2.05
1	-7	-9	34.55	2.12
-1	7	9	32.16	1.95
-1	7	9	34.47	1.42
-1	7	9	36.46	1.62
-1	7	9	32.81	1.94
0	-7	-9	8.19	0.67
0	-7	-9	10.51	1.21
0	-7	-9	7.24	1.27
0	7	9	8.23	0.71
0	7	9	9.04	1.05
0	7	9	8.74	1.12
-1	-7	-9	53.36	2.74
-1	-7	-9	53.69	2.74
-1	-7	-9	52.63	2.25
1	7	9	54.08	2.28
1	7	9	48.87	2.65
1	7	9	49.39	2.55
-2	-7	-9	1.35	0.72
-2	-7	-9	0.61	0.68
-2	-7	-9	-0.22	0.26
2	7	9	0.14	0.57
2	7	9	-0.09	0.28
2	7	9	-0.06	0.48
-3	-7	-9	162.20	6.65
-3	-7	-9	161.10	6.72
-3	-7	-9	158.32	6.24
3	7	9	155.07	6.48
3	7	9	158.11	6.68
3	7	9	164.71	6.27
-4	-7	-9	16.36	0.99
-4	-7	-9	18.78	1.57

-4	-7	-9	20.85	1.72
-4	-7	-9	16.43	1.02
4	7	9	15.32	1.28
4	7	9	15.22	1.47
4	7	9	18.93	1.08
-5	-7	-9	26.63	1.37
-5	-7	-9	29.30	2.12
-5	-7	-9	27.77	1.43
-5	-7	-9	27.25	1.92
5	7	9	29.26	1.67
5	7	9	24.76	1.90
-6	-7	-9	2.23	0.85
-6	-7	-9	2.55	0.83
-6	-7	-9	1.46	0.44
6	7	9	2.72	0.69
6	7	9	2.39	0.77
-7	-7	-9	8.94	0.87
-7	-7	-9	9.34	0.68
-7	-7	-9	10.28	1.39
-7	-7	-9	9.55	0.64
7	7	9	8.95	1.09
7	7	9	9.13	1.38
-8	-7	-9	5.36	0.79
-8	-7	-9	4.39	0.73
-8	-7	-9	5.25	0.61
-8	-7	-9	7.49	1.39
8	7	9	3.81	0.98
8	7	9	4.51	0.78
-9	-7	-9	41.88	2.42
-9	-7	-9	38.69	1.96
-9	-7	-9	40.75	1.75
-9	-7	-9	36.02	1.79
9	7	9	38.50	2.49
9	7	9	38.49	2.10
-10	-7	-9	8.15	1.22
-10	-7	-9	5.02	0.62
-10	-7	-9	6.96	0.91
-10	-7	-9	5.28	0.73
10	7	9	5.01	0.85
10	7	9	6.56	1.23
-11	-7	-9	24.49	2.16
-11	-7	-9	28.37	1.66
-11	-7	-9	23.92	1.58
-11	-7	-9	26.69	1.52

11	7	9	28.72	1.80
11	7	9	32.15	2.34
-12	-7	-9	3.47	1.09
-12	-7	-9	2.98	1.16
-12	-7	-9	1.12	0.74
-12	-7	-9	3.13	0.74
-12	-7	-9	2.84	0.71
12	7	9	3.20	1.15
-13	-7	-9	4.59	0.95
-13	-7	-9	4.27	0.83
-13	-7	-9	7.33	1.25
13	7	9	5.75	1.41
-14	-7	-9	3.24	1.02
-14	-7	-9	3.29	0.84
-14	-7	-9	3.06	1.71
14	7	9	5.43	1.41
-15	-7	-9	11.36	1.54
15	7	9	13.66	2.40
-16	-7	-9	10.72	1.56
-16	-7	-9	9.44	1.43
16	7	9	12.31	1.96
-17	-7	-9	15.69	1.80
17	7	9	17.72	2.84
19	-7	-8	19.30	1.58
-19	7	8	19.54	3.27
-19	7	8	18.23	2.77
18	-7	-8	12.96	1.22
-18	7	8	9.65	2.00
-18	7	8	7.96	1.60
17	-7	-8	35.36	1.93
-17	7	8	35.61	2.82
-17	7	8	35.96	3.25
16	-7	-8	3.33	0.65
-16	7	8	4.57	1.58
-16	7	8	3.60	1.22
15	-7	-8	63.69	2.78
-15	7	8	64.50	3.40
-15	7	8	58.10	3.78
14	-7	-8	7.52	0.87
14	-7	-8	6.98	0.88
14	-7	-8	6.48	0.98
-14	7	8	8.11	1.48
-14	7	8	6.43	1.11
13	-7	-8	36.29	1.78

13	-7	-8	38.14	1.88
13	-7	-8	39.69	1.81
-13	7	8	28.73	2.64
-13	7	8	38.72	2.32
12	-7	-8	36.24	1.92
12	-7	-8	39.33	1.92
12	-7	-8	36.56	1.94
-12	7	8	39.64	2.73
-12	7	8	41.45	2.26
11	-7	-8	34.01	1.89
11	-7	-8	38.67	2.90
11	-7	-8	35.84	1.69
-11	7	8	37.37	2.11
-11	7	8	34.93	2.54
10	-7	-8	75.80	3.45
10	-7	-8	80.19	3.49
10	-7	-8	76.20	4.14
10	-7	-8	79.61	3.26
10	-7	-8	86.70	3.44
-10	7	8	72.19	3.91
-10	7	8	83.87	3.55
9	-7	-8	65.01	3.61
9	-7	-8	67.48	3.00
9	-7	-8	69.47	2.99
9	-7	-8	67.19	3.08
9	-7	-8	69.19	2.81
-9	7	8	70.87	3.06
-9	7	8	67.11	2.92
-9	7	8	62.67	3.43
8	-7	-8	23.61	2.04
8	-7	-8	25.70	1.63
8	-7	-8	21.86	1.19
8	-7	-8	26.70	1.51
8	-7	-8	23.32	1.40
-8	7	8	22.98	1.28
-8	7	8	24.05	1.44
-8	7	8	21.51	1.84
7	-7	-8	34.76	2.28
7	-7	-8	34.36	1.56
7	-7	-8	36.81	1.73
7	-7	-8	35.27	1.85
7	-7	-8	35.77	2.01
-7	7	8	30.72	1.69
-7	7	8	32.94	1.60

-7	7	8	32.73	2.14
6	-7	-8	9.08	1.45
6	-7	-8	10.94	1.26
6	-7	-8	9.09	1.07
6	-7	-8	9.15	0.68
6	-7	-8	11.30	0.87
-6	7	8	9.60	1.26
-6	7	8	10.08	0.83
-6	7	8	8.89	0.75
5	-7	-8	36.82	1.60
5	-7	-8	34.68	2.18
5	-7	-8	36.39	1.67
5	-7	-8	34.44	2.09
5	-7	-8	39.23	1.97
-5	7	8	33.86	2.08
-5	7	8	34.52	1.62
-5	7	8	33.73	1.63
4	-7	-8	58.14	3.04
4	-7	-8	61.81	2.86
4	-7	-8	59.41	2.51
4	-7	-8	61.58	3.07
4	-7	-8	62.05	2.53
-4	7	8	60.90	2.98
-4	7	8	61.31	2.52
3	-7	-8	76.53	3.29
3	-7	-8	72.93	2.89
3	-7	-8	74.68	3.53
3	-7	-8	70.15	3.41
3	-7	-8	66.29	2.91
-3	7	8	70.56	3.26
-3	7	8	72.87	2.89
-3	7	8	68.78	3.32
-3	7	8	74.72	2.96
2	-7	-8	54.42	2.28
2	-7	-8	54.39	2.20
2	-7	-8	55.76	2.67
2	-7	-8	56.95	2.80
-2	7	8	53.43	2.62
-2	7	8	52.52	2.65
1	-7	-8	95.00	4.23
1	-7	-8	94.83	3.79
1	-7	-8	95.20	3.66
1	-7	-8	102.20	4.20
-1	7	8	95.83	4.15

-1	7	8	92.42	4.13
-1	7	8	93.47	3.66
-1	7	8	93.09	3.80
0	-7	-8	49.52	2.53
0	-7	-8	47.09	1.91
0	-7	-8	51.90	2.54
0	-7	-8	48.53	2.09
0	7	8	49.39	2.12
0	7	8	46.72	2.44
0	7	8	50.36	2.44
0	7	8	47.80	1.91
-1	-7	-8	6.69	1.06
-1	-7	-8	6.42	0.57
-1	-7	-8	5.98	1.06
1	7	8	5.23	0.98
1	7	8	4.68	0.87
1	7	8	4.87	0.58
-2	-7	-8	83.56	3.76
-2	-7	-8	88.72	3.82
-2	-7	-8	81.62	3.37
2	7	8	83.88	3.39
2	7	8	79.62	3.75
2	7	8	81.91	3.64
-3	-7	-8	29.79	1.48
-3	-7	-8	32.63	1.89
-3	-7	-8	34.23	2.01
3	7	8	28.61	1.87
3	7	8	32.57	1.52
3	7	8	29.20	1.73
-4	-7	-8	69.52	3.23
-4	-7	-8	65.06	3.09
-4	-7	-8	64.34	2.75
-4	-7	-8	67.48	2.71
4	7	8	62.27	3.17
4	7	8	63.33	2.95
4	7	8	64.47	2.75
-5	-7	-8	4.81	0.63
-5	-7	-8	4.53	0.42
-5	-7	-8	5.18	0.42
-5	-7	-8	5.41	0.96
-5	-7	-8	5.83	1.11
-5	-7	-8	5.73	0.59
5	7	8	4.86	0.87
5	7	8	4.62	0.85

-6	-7	-8	35.50	1.69
-6	-7	-8	31.99	1.47
-6	-7	-8	33.76	1.47
-6	-7	-8	36.07	2.23
-6	-7	-8	36.63	2.08
-6	-7	-8	34.95	1.62
6	7	8	32.70	2.13
6	7	8	36.68	1.88
-7	-7	-8	1.05	0.90
-7	-7	-8	1.44	0.44
-7	-7	-8	1.63	0.34
-7	-7	-8	2.10	0.44
-7	-7	-8	1.81	0.73
-7	-7	-8	1.61	0.37
7	7	8	2.30	0.63
7	7	8	1.98	0.77
-8	-7	-8	36.85	1.75
-8	-7	-8	40.02	2.26
-8	-7	-8	35.06	1.98
-8	-7	-8	43.01	2.58
-8	-7	-8	39.51	1.92
8	7	8	40.64	2.41
8	7	8	40.38	2.06
-9	-7	-8	2.42	0.94
-9	-7	-8	4.73	0.68
-9	-7	-8	4.88	0.79
-9	-7	-8	6.08	0.76
9	7	8	6.62	1.13
9	7	8	5.25	0.78
-10	-7	-8	41.13	2.44
-10	-7	-8	43.92	2.13
-10	-7	-8	44.60	1.99
-10	-7	-8	37.51	2.01
10	7	8	44.58	2.65
10	7	8	45.97	2.24
-11	-7	-8	2.81	0.68
-11	-7	-8	3.03	0.63
-11	-7	-8	4.02	0.71
-11	-7	-8	4.49	1.03
11	7	8	4.71	1.11
11	7	8	3.67	0.79
-12	-7	-8	34.52	1.88
-12	-7	-8	28.48	1.73
-12	-7	-8	33.23	2.22

-12	-7	-8	28.94	1.82
-12	-7	-8	29.83	2.20
-13	-7	-8	2.67	1.12
-13	-7	-8	3.73	0.80
-13	-7	-8	1.29	0.80
-13	-7	-8	1.89	0.67
13	7	8	5.34	1.24
-14	-7	-8	43.69	2.87
-14	-7	-8	51.48	2.55
-14	-7	-8	44.40	2.56
14	7	8	47.75	3.15
-15	-7	-8	3.78	1.01
-15	-7	-8	2.21	0.87
15	7	8	3.14	1.33
-16	-7	-8	35.07	2.26
16	7	8	34.92	3.03
-17	-7	-8	2.70	1.28
-17	-7	-8	0.41	0.88
17	7	8	1.56	1.52
-18	-7	-8	28.67	3.20
-18	-7	-8	32.35	2.47
18	7	8	33.97	3.39
19	-7	-7	1.25	0.71
-19	7	7	-0.46	1.82
-19	7	7	-0.57	1.36
18	-7	-7	41.91	2.22
-18	7	7	45.58	3.20
-18	7	7	46.68	3.41
17	-7	-7	1.78	0.61
-17	7	7	1.87	1.15
-17	7	7	3.75	1.85
16	-7	-7	54.50	2.54
-16	7	7	61.43	3.33
-16	7	7	51.25	3.51
15	-7	-7	5.55	0.90
15	-7	-7	6.20	0.85
-15	7	7	5.65	1.14
-15	7	7	7.26	1.43
14	-7	-7	65.33	2.87
14	-7	-7	59.91	2.62
14	-7	-7	60.58	2.69
-14	7	7	57.36	3.52
-14	7	7	63.37	3.25
13	-7	-7	3.61	0.55

13	-7	-7	6.15	1.69
13	-7	-7	3.13	0.63
13	-7	-7	2.66	0.63
-13	7	7	2.47	1.19
-13	7	7	2.59	1.16
12	-7	-7	95.98	3.87
12	-7	-7	89.04	3.89
12	-7	-7	97.87	3.92
12	-7	-7	88.57	4.78
-12	7	7	102.86	4.23
-12	7	7	87.04	4.49
11	-7	-7	31.64	2.70
11	-7	-7	34.89	1.59
11	-7	-7	34.76	1.77
-11	7	7	29.51	2.30
-11	7	7	34.24	1.97
10	-7	-7	59.47	2.68
10	-7	-7	56.38	3.25
10	-7	-7	55.84	2.43
10	-7	-7	60.54	2.62
10	-7	-7	55.49	2.65
-10	7	7	56.58	2.73
-10	7	7	56.05	3.09
9	-7	-7	-0.28	0.93
9	-7	-7	1.32	0.62
9	-7	-7	1.72	0.37
9	-7	-7	1.12	0.51
9	-7	-7	2.46	0.60
-9	7	7	1.98	0.81
-9	7	7	1.51	0.59
-9	7	7	1.84	0.45
8	-7	-7	56.10	2.47
8	-7	-7	57.82	2.64
8	-7	-7	60.08	2.69
8	-7	-7	62.69	2.82
8	-7	-7	61.83	3.16
-8	7	7	57.81	2.52
-8	7	7	60.94	2.68
-8	7	7	57.93	3.02
7	-7	-7	0.14	0.39
7	-7	-7	0.00	0.57
7	-7	-7	0.39	0.26
7	-7	-7	0.56	0.72
7	-7	-7	0.35	0.54

-7	7	7	-0.25	0.58
-7	7	7	0.43	0.38
-7	7	7	0.53	0.30
6	-7	-7	174.58	6.63
6	-7	-7	170.99	6.92
6	-7	-7	168.37	7.06
6	-7	-7	182.04	6.82
6	-7	-7	172.04	6.56
-6	7	7	167.40	7.02
-6	7	7	168.17	6.58
-6	7	7	160.92	6.63
5	-7	-7	0.87	0.36
5	-7	-7	0.74	0.58
5	-7	-7	1.17	0.54
5	-7	-7	1.38	0.68
5	-7	-7	0.48	0.26
-5	7	7	0.02	0.28
-5	7	7	0.60	0.56
-5	7	7	0.81	0.34
4	-7	-7	190.81	7.27
4	-7	-7	199.44	7.56
4	-7	-7	193.06	7.28
4	-7	-7	194.69	7.73
4	-7	-7	185.11	7.72
-4	7	7	191.60	7.31
-4	7	7	180.88	7.28
-4	7	7	187.83	7.67
3	-7	-7	159.94	5.94
3	-7	-7	152.80	5.96
3	-7	-7	149.61	6.46
3	-7	-7	168.55	6.31
3	-7	-7	160.57	6.41
-3	7	7	152.62	5.99
-3	7	7	146.07	5.93
-3	7	7	150.11	6.32
2	-7	-7	96.00	3.70
2	-7	-7	95.41	4.27
2	-7	-7	92.54	3.62
2	-7	-7	88.44	4.07
2	-7	-7	97.26	4.04
-2	7	7	87.76	4.02
-2	7	7	96.79	3.72
-2	7	7	91.66	4.02
-2	7	7	92.87	3.66

1	-7	-7	15.56	1.24
1	-7	-7	14.01	1.24
1	-7	-7	14.89	0.72
1	-7	-7	14.58	0.82
-1	7	7	13.29	1.14
-1	7	7	14.53	0.75
-1	7	7	14.00	1.14
0	-7	-7	127.89	5.02
0	-7	-7	137.55	5.53
0	-7	-7	134.16	5.53
0	-7	-7	131.79	5.17
0	7	7	128.72	5.51
0	7	7	131.55	5.04
0	7	7	139.62	5.49
0	7	7	134.37	5.19
-1	-7	-7	7.06	0.42
-1	-7	-7	7.69	0.99
-1	-7	-7	7.07	0.58
-1	-7	-7	7.18	1.01
1	7	7	6.96	0.42
1	7	7	6.29	0.90
1	7	7	7.51	0.60
1	7	7	7.33	0.87
-2	-7	-7	11.83	0.56
-2	-7	-7	13.99	1.23
-2	-7	-7	13.07	0.77
-2	-7	-7	13.06	1.14
2	7	7	11.23	0.77
2	7	7	10.80	0.54
2	7	7	10.78	1.14
2	7	7	10.61	1.00
-3	-7	-7	25.69	1.08
-3	-7	-7	26.31	1.28
-3	-7	-7	24.56	1.67
-3	-7	-7	25.14	1.06
-3	-7	-7	27.14	1.62
3	7	7	25.62	1.50
3	7	7	24.30	1.65
3	7	7	28.07	1.30
-4	-7	-7	81.02	3.37
-4	-7	-7	79.34	3.17
-4	-7	-7	84.72	3.80
-4	-7	-7	87.65	3.69
-4	-7	-7	81.24	3.18

-4	-7	-7	82.01	3.32
4	7	7	85.29	3.38
4	7	7	83.59	3.78
4	7	7	80.89	3.56
-5	-7	-7	8.48	1.06
-5	-7	-7	7.78	0.69
-5	-7	-7	7.03	0.62
-5	-7	-7	9.50	1.24
-5	-7	-7	8.34	0.56
-5	-7	-7	7.45	0.53
5	7	7	8.20	1.12
5	7	7	7.13	0.90
-6	-7	-7	24.76	1.76
-6	-7	-7	22.27	1.23
-6	-7	-7	24.02	1.10
-6	-7	-7	24.87	1.19
-6	-7	-7	22.73	1.59
6	7	7	21.76	1.70
6	7	7	22.38	1.44
-7	-7	-7	17.54	0.95
-7	-7	-7	19.81	1.03
-7	-7	-7	17.18	1.09
-7	-7	-7	17.68	1.44
-7	-7	-7	17.14	1.01
-7	-7	-7	19.48	1.65
7	7	7	15.57	1.24
7	7	7	17.20	1.56
-8	-7	-7	37.80	1.68
-8	-7	-7	33.96	1.71
-8	-7	-7	38.09	1.73
-8	-7	-7	37.70	2.13
-8	-7	-7	36.18	2.32
-8	-7	-7	35.74	1.80
8	7	7	36.39	2.25
8	7	7	41.73	2.00
-9	-7	-7	48.30	2.29
-9	-7	-7	51.34	2.25
-9	-7	-7	55.56	2.69
-9	-7	-7	49.77	2.91
-9	-7	-7	51.84	2.39
9	7	7	54.72	2.86
9	7	7	53.22	2.51
-10	-7	-7	34.93	1.75
-10	-7	-7	31.95	2.04

-10	-7	-7	32.44	1.63
-10	-7	-7	29.52	1.69
10	7	7	34.30	2.26
10	7	7	33.18	1.87
-11	-7	-7	3.16	0.94
-11	-7	-7	2.43	0.61
-11	-7	-7	2.25	0.67
-11	-7	-7	1.40	0.62
-11	-7	-7	3.37	0.95
11	7	7	2.76	0.99
-12	-7	-7	23.39	1.68
-12	-7	-7	28.21	1.63
-12	-7	-7	23.60	1.95
-12	-7	-7	26.41	2.05
-12	-7	-7	29.35	1.61
12	7	7	25.62	2.16
-13	-7	-7	23.62	2.13
-13	-7	-7	23.08	2.03
-13	-7	-7	24.53	1.65
-13	-7	-7	22.60	1.62
-13	-7	-7	22.33	1.72
13	7	7	29.73	2.31
-14	-7	-7	18.91	1.71
-14	-7	-7	18.09	1.66
-14	-7	-7	21.74	1.62
-14	-7	-7	18.83	2.14
14	7	7	20.75	2.13
-15	-7	-7	12.86	2.31
-15	-7	-7	9.76	1.25
-15	-7	-7	13.97	1.45
15	7	7	11.81	1.66
-16	-7	-7	10.14	1.65
-16	-7	-7	13.93	1.58
16	7	7	11.96	2.25
-17	-7	-7	25.33	2.02
-17	-7	-7	25.55	2.37
17	7	7	25.83	2.86
-18	-7	-7	6.07	1.46
-18	-7	-7	8.43	1.30
18	7	7	10.64	1.91
19	-7	-6	16.29	1.45
-19	7	6	15.73	2.56
18	-7	-6	8.70	1.15
-18	7	6	6.42	1.44

-18	7	6	7.18	1.70
17	-7	-6	31.61	1.83
-17	7	6	34.38	2.74
-17	7	6	35.39	2.89
16	-7	-6	27.95	1.64
-16	7	6	30.56	2.45
-16	7	6	32.35	2.69
15	-7	-6	58.21	2.63
15	-7	-6	56.77	4.12
15	-7	-6	60.89	2.79
15	-7	-6	59.04	2.63
-15	7	6	56.03	3.47
-15	7	6	63.04	3.30
14	-7	-6	14.69	1.08
14	-7	-6	16.28	1.19
14	-7	-6	14.52	2.05
14	-7	-6	16.75	1.23
-14	7	6	11.28	1.99
-14	7	6	17.80	1.78
13	-7	-6	12.51	1.20
13	-7	-6	13.77	2.39
13	-7	-6	12.78	1.08
13	-7	-6	14.51	0.97
-13	7	6	12.52	1.82
12	-7	-6	0.27	0.32
12	-7	-6	3.14	1.32
12	-7	-6	-0.10	0.56
-12	7	6	0.51	0.90
-12	7	6	0.30	0.63
11	-7	-6	74.03	3.16
11	-7	-6	79.20	3.32
11	-7	-6	81.22	4.02
11	-7	-6	76.99	3.52
-11	7	6	72.22	3.77
-11	7	6	80.31	3.53
10	-7	-6	22.75	1.20
10	-7	-6	22.58	2.07
10	-7	-6	25.99	1.47
10	-7	-6	22.77	1.45
10	-7	-6	22.54	1.47
-10	7	6	22.99	1.84
-10	7	6	23.41	1.54
-10	7	6	23.47	1.31
9	-7	-6	128.12	5.01

9	-7	-6	121.21	4.99
9	-7	-6	116.51	4.81
9	-7	-6	126.89	5.44
9	-7	-6	128.96	5.07
-9	7	6	121.41	4.88
-9	7	6	121.09	5.05
-9	7	6	121.59	5.35
8	-7	-6	9.02	0.86
8	-7	-6	8.76	0.96
8	-7	-6	8.05	0.63
8	-7	-6	8.67	1.08
8	-7	-6	7.67	1.30
-8	7	6	7.32	0.69
-8	7	6	8.23	0.91
-8	7	6	7.99	1.21
7	-7	-6	91.75	3.80
7	-7	-6	91.51	3.91
7	-7	-6	83.14	3.55
7	-7	-6	88.92	4.07
7	-7	-6	91.37	3.69
-7	7	6	91.61	3.61
-7	7	6	87.84	4.03
-7	7	6	91.69	3.73
6	-7	-6	1.68	0.61
6	-7	-6	1.98	0.69
6	-7	-6	1.11	0.26
6	-7	-6	1.73	0.40
6	-7	-6	1.93	0.53
-6	7	6	1.06	0.39
-6	7	6	1.82	0.32
-6	7	6	1.30	0.58
5	-7	-6	126.54	5.13
5	-7	-6	123.22	4.71
5	-7	-6	107.07	4.64
5	-7	-6	126.03	4.93
5	-7	-6	120.45	5.06
-5	7	6	122.10	5.07
-5	7	6	114.73	4.72
4	-7	-6	0.86	0.49
4	-7	-6	1.16	0.59
4	-7	-6	1.29	0.55
4	-7	-6	1.67	0.32
4	-7	-6	1.38	0.29
-4	7	6	0.91	0.48

-4	7	6	1.02	0.30
-4	7	6	0.63	0.31
3	-7	-6	154.69	6.02
3	-7	-6	151.97	5.71
3	-7	-6	145.54	5.72
3	-7	-6	150.75	6.11
3	-7	-6	149.39	6.20
-3	7	6	151.26	5.73
-3	7	6	137.96	6.03
2	-7	-6	39.17	2.22
2	-7	-6	37.18	1.66
2	-7	-6	40.57	2.02
2	-7	-6	38.49	2.05
2	-7	-6	39.32	1.63
-2	7	6	37.08	1.98
-2	7	6	39.89	1.70
-2	7	6	38.78	1.67
1	-7	-6	111.14	4.43
1	-7	-6	110.79	4.98
1	-7	-6	115.68	4.36
1	-7	-6	122.22	4.77
1	-7	-6	117.62	4.79
-1	7	6	112.09	4.38
-1	7	6	109.67	4.71
-1	7	6	110.76	4.76
0	-7	-6	32.00	1.50
0	-7	-6	35.37	1.87
0	-7	-6	38.18	1.89
0	-7	-6	35.26	1.42
0	7	6	31.89	1.84
0	7	6	34.15	1.43
0	7	6	32.82	1.79
-1	-7	-6	96.49	3.85
-1	-7	-6	99.46	4.21
-1	-7	-6	96.13	3.73
-1	-7	-6	102.97	4.18
1	7	6	96.95	4.11
1	7	6	98.20	3.87
1	7	6	95.58	3.73
1	7	6	96.72	4.20
-2	-7	-6	10.48	1.07
-2	-7	-6	9.84	0.57
-2	-7	-6	10.78	0.68
-2	-7	-6	10.91	1.03

2	7	6	10.38	0.54
2	7	6	11.54	0.71
2	7	6	10.26	1.02
2	7	6	10.97	0.94
-3	-7	-6	140.64	5.64
-3	-7	-6	145.87	5.73
-3	-7	-6	135.13	5.35
-3	-7	-6	136.54	5.22
3	7	6	139.83	5.74
3	7	6	126.62	5.53
3	7	6	133.60	5.19
3	7	6	144.33	5.37
-4	-7	-6	0.35	0.49
-4	-7	-6	0.18	0.22
-4	-7	-6	0.28	0.25
-4	-7	-6	0.01	0.21
-4	-7	-6	0.47	0.57
-4	-7	-6	-0.09	0.19
4	7	6	0.05	0.51
4	7	6	0.16	0.27
4	7	6	0.11	0.40
-5	-7	-6	79.39	3.28
-5	-7	-6	80.59	3.15
-5	-7	-6	82.78	3.23
-5	-7	-6	75.59	3.15
-5	-7	-6	86.00	3.55
-5	-7	-6	80.19	3.65
5	7	6	81.60	3.46
5	7	6	75.86	3.64
-6	-7	-6	-0.46	0.58
-6	-7	-6	0.58	0.28
-6	-7	-6	0.19	0.31
-6	-7	-6	-0.07	0.26
-6	-7	-6	0.10	0.25
-6	-7	-6	-0.05	0.52
6	7	6	-0.54	0.42
6	7	6	0.80	0.63
-7	-7	-6	53.44	2.31
-7	-7	-6	58.61	2.85
-7	-7	-6	58.54	2.68
-7	-7	-6	55.22	2.40
-7	-7	-6	51.10	2.30
7	7	6	54.29	2.80
7	7	6	54.42	2.54

-8	-7	-6	0.29	0.29
-8	-7	-6	0.01	0.32
-8	-7	-6	1.24	0.66
-8	-7	-6	0.65	0.41
-8	-7	-6	-0.40	0.31
-8	-7	-6	0.65	0.73
8	7	6	-0.01	0.47
8	7	6	0.24	0.69
-9	-7	-6	120.83	5.07
-9	-7	-6	135.27	5.59
-9	-7	-6	127.50	5.13
-9	-7	-6	131.40	5.03
-9	-7	-6	127.27	5.03
-9	-7	-6	129.10	5.33
9	7	6	124.85	5.24
9	7	6	125.95	5.53
-10	-7	-6	0.64	0.45
-10	-7	-6	1.49	0.74
-10	-7	-6	0.70	0.41
-10	-7	-6	0.20	0.92
-10	-7	-6	0.76	0.52
10	7	6	0.37	0.78
10	7	6	0.86	0.59
-11	-7	-6	33.05	2.11
-11	-7	-6	32.47	1.70
-11	-7	-6	30.88	2.37
-11	-7	-6	30.47	1.71
-11	-7	-6	32.17	1.76
-11	-7	-6	29.49	1.98
11	7	6	33.95	2.22
-12	-7	-6	2.06	0.69
-12	-7	-6	4.91	0.73
-12	-7	-6	4.41	1.11
-12	-7	-6	3.42	1.07
-12	-7	-6	4.54	0.98
12	7	6	3.89	1.08
-13	-7	-6	44.78	2.77
-13	-7	-6	44.17	2.34
-13	-7	-6	44.13	2.25
-13	-7	-6	38.72	2.49
-13	-7	-6	47.38	2.31
13	7	6	43.31	2.72
-14	-7	-6	-0.33	0.59
-14	-7	-6	1.01	1.46

-14	-7	-6	0.28	1.17
-14	-7	-6	-0.17	0.78
-14	-7	-6	2.32	1.04
14	7	6	1.19	0.97
14	7	6	0.15	0.54
-15	-7	-6	57.92	3.37
-15	-7	-6	57.05	2.89
-15	-7	-6	52.45	2.92
15	7	6	57.40	3.68
15	7	6	58.44	2.74
-16	-7	-6	4.70	1.35
-16	-7	-6	3.20	0.95
16	7	6	4.62	1.33
16	7	6	3.63	0.82
-17	-7	-6	23.67	2.25
-17	-7	-6	21.76	1.93
17	7	6	22.21	2.54
17	7	6	21.12	1.75
-18	-7	-6	3.19	1.40
-18	-7	-6	4.52	1.08
18	7	6	6.73	0.99
18	7	6	5.48	1.72
20	-7	-5	3.39	0.99
19	-7	-5	2.95	0.76
-19	7	5	4.80	1.60
18	-7	-5	9.51	1.13
-18	7	5	9.98	1.65
-18	7	5	9.23	1.63
17	-7	-5	14.08	1.22
-17	7	5	15.12	2.33
-17	7	5	18.26	2.25
16	-7	-5	6.37	0.91
16	-7	-5	17.11	3.11
16	-7	-5	8.47	1.06
-16	7	5	5.44	1.26
-16	7	5	6.70	1.45
15	-7	-5	46.17	2.18
15	-7	-5	48.24	2.21
15	-7	-5	51.09	2.52
15	-7	-5	46.95	3.61
-15	7	5	43.50	2.96
-15	7	5	41.81	2.76
14	-7	-5	24.88	1.38
14	-7	-5	27.56	1.49

14	-7	-5	19.33	2.71
-14	7	5	25.34	2.21
-14	7	5	24.32	1.94
13	-7	-5	10.43	1.71
13	-7	-5	9.93	0.92
13	-7	-5	10.43	1.04
-13	7	5	10.92	1.73
-13	7	5	9.17	1.34
12	-7	-5	32.04	2.50
12	-7	-5	30.46	1.46
12	-7	-5	29.74	1.64
-12	7	5	24.62	2.09
-12	7	5	29.97	1.89
11	-7	-5	0.78	1.05
11	-7	-5	1.89	0.61
11	-7	-5	1.72	0.61
11	-7	-5	0.56	0.30
-11	7	5	2.31	0.94
-11	7	5	0.45	0.58
-11	7	5	1.30	0.82
10	-7	-5	10.49	1.64
10	-7	-5	13.13	1.11
10	-7	-5	12.55	1.12
10	-7	-5	11.90	0.79
-10	7	5	12.58	1.49
-10	7	5	13.23	0.91
-10	7	5	13.73	1.54
-10	7	5	13.86	1.20
9	-7	-5	43.71	2.15
9	-7	-5	45.39	2.19
9	-7	-5	44.67	2.50
9	-7	-5	43.75	2.10
9	-7	-5	44.64	1.91
-9	7	5	41.08	2.41
-9	7	5	45.71	1.97
-9	7	5	42.28	2.17
8	-7	-5	51.89	2.50
8	-7	-5	53.71	2.46
8	-7	-5	49.72	2.20
8	-7	-5	53.27	2.37
8	-7	-5	57.75	2.78
-8	7	5	52.08	2.26
-8	7	5	52.36	2.67
-8	7	5	52.60	2.44

7	-7	-5	27.83	1.81
7	-7	-5	25.49	1.24
7	-7	-5	28.61	1.42
7	-7	-5	28.10	1.54
7	-7	-5	26.58	1.60
-7	7	5	27.83	1.31
-7	7	5	26.78	1.44
-7	7	5	25.42	1.67
6	-7	-5	79.88	3.64
6	-7	-5	86.55	3.31
6	-7	-5	88.21	3.75
6	-7	-5	86.24	3.57
6	-7	-5	83.37	3.40
-6	7	5	80.22	3.33
-6	7	5	83.35	3.69
-6	7	5	78.42	3.42
5	-7	-5	35.57	1.58
5	-7	-5	35.41	1.98
5	-7	-5	37.86	2.02
5	-7	-5	39.27	1.89
5	-7	-5	36.24	1.66
-5	7	5	35.10	1.95
-5	7	5	37.18	1.70
-5	7	5	38.28	1.62
4	-7	-5	29.16	1.73
4	-7	-5	28.49	1.55
4	-7	-5	27.54	1.64
4	-7	-5	26.75	1.24
4	-7	-5	27.24	1.30
-4	7	5	27.44	1.28
-4	7	5	29.17	1.34
-4	7	5	27.86	1.61
3	-7	-5	8.03	0.54
3	-7	-5	9.75	0.94
3	-7	-5	8.25	1.02
3	-7	-5	6.91	0.56
3	-7	-5	7.94	0.84
-3	7	5	7.28	0.87
-3	7	5	8.17	0.60
-3	7	5	8.37	0.57
2	-7	-5	198.28	7.80
2	-7	-5	190.43	7.93
2	-7	-5	199.54	7.46
2	-7	-5	201.82	7.77

2	-7	-5	193.69	7.47
-2	7	5	195.63	7.76
-2	7	5	192.46	7.48
-2	7	5	202.75	7.50
1	-7	-5	85.18	3.26
1	-7	-5	88.62	3.58
1	-7	-5	78.46	3.53
1	-7	-5	82.84	3.78
1	-7	-5	80.40	3.21
-1	7	5	81.89	3.26
-1	7	5	78.70	3.52
0	-7	-5	121.71	4.86
0	-7	-5	126.31	4.85
0	-7	-5	114.57	4.52
0	-7	-5	113.68	5.09
0	-7	-5	114.23	4.47
0	7	5	116.47	4.79
0	7	5	114.97	4.87
0	7	5	118.05	4.54
0	7	5	112.35	4.48
-1	-7	-5	6.72	0.82
-1	-7	-5	6.90	0.50
-1	-7	-5	6.96	0.47
-1	-7	-5	6.75	0.85
1	7	5	6.25	0.75
1	7	5	6.44	0.45
1	7	5	6.56	0.52
1	7	5	6.27	0.84
-2	-7	-5	12.64	0.75
-2	-7	-5	12.76	0.68
-2	-7	-5	15.34	1.06
-2	-7	-5	14.19	1.11
2	7	5	13.24	0.66
2	7	5	12.32	0.97
2	7	5	13.57	0.77
2	7	5	12.37	1.09
-3	-7	-5	29.70	1.37
-3	-7	-5	29.78	1.73
-3	-7	-5	29.02	1.28
-3	-7	-5	32.32	1.65
3	7	5	29.71	1.59
3	7	5	28.81	1.72
3	7	5	29.75	1.26
3	7	5	30.70	1.38

-4	-7	-5	75.92	3.30
-4	-7	-5	67.51	2.92
-4	-7	-5	71.58	2.84
-4	-7	-5	72.78	2.89
-4	-7	-5	73.42	3.17
4	7	5	69.80	3.12
4	7	5	68.44	3.27
4	7	5	76.99	2.95
-5	-7	-5	43.87	1.79
-5	-7	-5	45.88	2.26
-5	-7	-5	41.05	1.77
-5	-7	-5	41.35	1.81
-5	-7	-5	43.16	2.10
-5	-7	-5	40.27	1.85
5	7	5	41.26	2.23
5	7	5	43.23	2.06
-6	-7	-5	82.34	3.01
-6	-7	-5	73.53	2.97
-6	-7	-5	72.37	2.97
-6	-7	-5	73.22	3.26
-6	-7	-5	69.82	3.03
-6	-7	-5	78.13	3.43
6	7	5	73.02	3.42
6	7	5	70.78	3.21
-7	-7	-5	12.05	0.83
-7	-7	-5	12.84	0.80
-7	-7	-5	12.24	0.85
-7	-7	-5	12.15	1.14
-7	-7	-5	11.99	0.78
-7	-7	-5	14.09	1.34
7	7	5	12.94	1.30
7	7	5	12.89	1.07
-8	-7	-5	42.66	2.15
-8	-7	-5	42.01	1.92
-8	-7	-5	43.49	1.83
-8	-7	-5	41.12	2.31
-8	-7	-5	40.60	1.89
-8	-7	-5	40.63	1.85
8	7	5	41.21	2.30
8	7	5	39.77	2.06
-9	-7	-5	23.69	1.55
-9	-7	-5	22.77	1.25
-9	-7	-5	21.20	1.77
-9	-7	-5	20.76	1.30

-9	-7	-5	22.73	1.17
-9	-7	-5	21.33	1.26
9	7	5	24.40	1.48
9	7	5	22.48	1.72
-10	-7	-5	78.27	3.65
-10	-7	-5	77.40	3.51
-10	-7	-5	78.91	3.73
-10	-7	-5	75.37	3.32
-10	-7	-5	80.37	3.21
-10	-7	-5	75.23	3.32
-10	-7	-5	79.61	3.27
10	7	5	77.72	3.71
-11	-7	-5	44.62	2.15
-11	-7	-5	45.03	2.14
-11	-7	-5	44.48	2.65
-11	-7	-5	43.15	2.49
-11	-7	-5	41.60	2.32
-11	-7	-5	44.89	2.20
11	7	5	45.52	2.56
-12	-7	-5	101.93	4.87
-12	-7	-5	99.25	4.57
-12	-7	-5	102.07	4.46
-12	-7	-5	106.88	4.46
-12	-7	-5	111.61	4.43
-12	-7	-5	109.84	4.78
12	7	5	110.09	4.83
12	7	5	109.34	4.38
-13	-7	-5	0.44	0.69
-13	-7	-5	-0.14	0.85
-13	-7	-5	0.76	0.68
-13	-7	-5	-0.67	0.99
-13	-7	-5	0.03	0.54
13	7	5	1.02	0.47
13	7	5	0.12	0.86
-14	-7	-5	28.72	1.77
-14	-7	-5	29.22	2.41
-14	-7	-5	22.94	2.00
-14	-7	-5	27.30	1.93
14	7	5	30.52	2.23
14	7	5	29.78	1.68
-15	-7	-5	1.03	0.87
-15	-7	-5	2.54	1.31
-15	-7	-5	0.62	0.67
15	7	5	1.00	0.58

15	7	5	2.99	1.10
-16	-7	-5	39.07	2.34
-16	-7	-5	39.61	3.03
-16	-7	-5	39.49	2.51
16	7	5	41.74	2.18
-17	-7	-5	-0.27	1.08
-17	-7	-5	0.10	0.77
17	7	5	0.14	0.73
17	7	5	-1.67	1.27
-18	-7	-5	28.67	2.55
-18	-7	-5	26.46	2.13
18	7	5	27.25	2.83
18	7	5	28.86	1.95
-19	-7	-5	4.31	1.47
-19	-7	-5	0.75	1.04
19	7	5	2.92	1.74
19	7	5	2.49	0.93
20	-7	-4	1.57	0.75
-20	7	4	2.18	2.06
19	-7	-4	-0.22	0.61
-19	7	4	1.00	1.36
18	-7	-4	9.58	1.11
-18	7	4	8.67	1.55
-18	7	4	6.60	1.53
17	-7	-4	2.32	0.62
-17	7	4	1.61	1.23
-17	7	4	0.40	1.22
16	-7	-4	15.90	1.19
16	-7	-4	21.23	3.02
16	-7	-4	16.42	1.41
-16	7	4	16.48	2.08
-16	7	4	15.37	2.08
15	-7	-4	3.56	1.63
15	-7	-4	1.93	0.56
15	-7	-4	2.97	0.71
-15	7	4	1.84	1.07
-15	7	4	1.66	0.95
-15	7	4	2.12	1.15
14	-7	-4	13.22	2.38
14	-7	-4	14.35	1.19
14	-7	-4	14.35	0.98
-14	7	4	13.81	1.65
-14	7	4	11.77	1.79
-14	7	4	12.81	1.74

13	-7	-4	2.96	1.30
13	-7	-4	3.98	0.70
-13	7	4	4.27	0.93
-13	7	4	3.66	1.02
-13	7	4	1.11	1.03
12	-7	-4	17.31	1.23
12	-7	-4	15.83	0.96
12	-7	-4	18.77	2.05
-12	7	4	17.66	1.45
-12	7	4	13.28	1.60
-12	7	4	15.47	1.73
11	-7	-4	8.02	1.06
11	-7	-4	9.04	1.64
11	-7	-4	7.84	0.67
11	-7	-4	8.15	0.96
-11	7	4	6.83	1.08
-11	7	4	6.19	1.10
-11	7	4	6.94	1.02
10	-7	-4	69.89	2.90
10	-7	-4	75.74	3.15
10	-7	-4	75.34	3.49
10	-7	-4	68.88	3.11
-10	7	4	68.66	3.35
-10	7	4	72.73	3.44
-10	7	4	72.76	2.99
-10	7	4	65.17	3.19
9	-7	-4	13.43	1.16
9	-7	-4	13.63	0.82
9	-7	-4	14.18	1.06
9	-7	-4	13.64	1.44
-9	7	4	12.78	1.46
-9	7	4	14.66	1.32
-9	7	4	16.40	0.92
-9	7	4	13.88	1.14
8	-7	-4	38.47	1.95
8	-7	-4	35.99	1.63
8	-7	-4	36.94	1.80
8	-7	-4	41.74	2.16
-8	7	4	38.82	1.69
-8	7	4	37.07	1.87
-8	7	4	34.76	2.17
-8	7	4	35.14	2.05
7	-7	-4	11.38	1.18
7	-7	-4	12.47	0.87

7	-7	-4	10.05	0.66
7	-7	-4	11.22	1.07
-7	7	4	10.76	0.89
-7	7	4	10.93	1.32
-7	7	4	11.21	0.71
-7	7	4	10.16	1.08
6	-7	-4	22.90	1.21
6	-7	-4	23.04	1.48
6	-7	-4	22.83	1.08
6	-7	-4	25.01	1.51
-6	7	4	22.15	1.45
-6	7	4	24.23	1.66
-6	7	4	22.78	1.12
-6	7	4	22.72	1.24
5	-7	-4	30.47	1.70
5	-7	-4	29.62	1.40
5	-7	-4	29.97	1.72
5	-7	-4	29.44	1.30
-5	7	4	28.99	1.33
-5	7	4	29.26	1.87
-5	7	4	28.37	1.42
-5	7	4	29.85	1.65
4	-7	-4	5.03	0.43
4	-7	-4	5.25	0.78
4	-7	-4	4.53	0.50
4	-7	-4	5.40	0.89
4	-7	-4	5.47	0.79
-4	7	4	4.32	0.53
-4	7	4	4.87	0.76
-4	7	4	3.98	0.43
3	-7	-4	7.58	0.79
3	-7	-4	6.24	0.47
3	-7	-4	7.07	0.82
3	-7	-4	7.85	0.98
-3	7	4	5.72	0.76
-3	7	4	5.88	0.54
-3	7	4	6.08	0.49
2	-7	-4	21.94	1.27
2	-7	-4	19.99	1.39
2	-7	-4	19.76	1.24
2	-7	-4	19.27	0.98
2	-7	-4	19.95	0.94
-2	7	4	20.14	1.01
-2	7	4	20.34	1.23

-2	7	4	19.86	0.97
1	-7	-4	6.65	0.96
1	-7	-4	5.80	0.76
1	-7	-4	5.61	0.77
1	-7	-4	5.50	0.43
1	-7	-4	5.66	0.47
-1	7	4	5.54	0.69
-1	7	4	5.57	0.51
0	-7	-4	26.13	1.67
0	-7	-4	24.74	1.14
0	-7	-4	24.86	1.41
0	-7	-4	25.27	1.14
0	-7	-4	27.95	1.49
0	7	4	25.71	1.16
0	7	4	24.77	1.39
-1	-7	-4	9.17	1.12
-1	-7	-4	9.21	0.89
-1	-7	-4	7.93	0.53
-1	-7	-4	8.12	0.80
-1	-7	-4	8.34	0.53
1	7	4	8.86	0.56
1	7	4	8.86	0.53
1	7	4	7.71	0.77
-2	-7	-4	8.44	0.90
-2	-7	-4	8.05	0.82
-2	-7	-4	8.11	0.54
-2	-7	-4	8.63	0.56
-2	-7	-4	7.55	1.15
2	7	4	9.45	0.54
2	7	4	8.63	0.57
2	7	4	8.88	0.91
2	7	4	8.48	0.79
-3	-7	-4	16.14	0.81
-3	-7	-4	17.97	1.12
-3	-7	-4	15.03	0.83
-3	-7	-4	16.13	1.16
3	7	4	15.85	1.05
3	7	4	15.19	1.18
3	7	4	14.94	0.83
-4	-7	-4	63.25	2.75
-4	-7	-4	62.64	2.83
-4	-7	-4	61.05	2.47
-4	-7	-4	59.57	2.50
-4	-7	-4	64.36	2.48

4	7	4	60.81	2.71
4	7	4	60.97	2.51
4	7	4	58.52	2.43
4	7	4	59.50	2.86
-5	-7	-4	19.51	0.97
-5	-7	-4	18.99	0.94
-5	-7	-4	18.56	0.94
-5	-7	-4	17.07	0.97
-5	-7	-4	19.38	1.21
-5	-7	-4	21.53	1.39
5	7	4	18.63	1.34
5	7	4	18.56	1.20
-6	-7	-4	55.91	2.30
-6	-7	-4	50.79	2.39
-6	-7	-4	58.67	2.59
-6	-7	-4	55.91	2.70
-6	-7	-4	58.19	2.29
-6	-7	-4	54.77	2.34
6	7	4	56.81	2.73
6	7	4	54.08	2.53
-7	-7	-4	63.21	2.63
-7	-7	-4	63.32	2.67
-7	-7	-4	68.36	3.05
-7	-7	-4	64.21	2.89
-7	-7	-4	62.74	2.60
-7	-7	-4	63.92	2.65
7	7	4	63.62	2.86
7	7	4	61.92	3.04
-8	-7	-4	3.03	0.45
-8	-7	-4	3.23	0.78
-8	-7	-4	4.06	0.62
-8	-7	-4	3.02	0.39
-8	-7	-4	3.45	0.71
-8	-7	-4	3.01	0.46
8	7	4	2.69	0.69
8	7	4	3.14	0.62
-9	-7	-4	41.79	1.80
-9	-7	-4	40.85	2.28
-9	-7	-4	41.97	2.12
-9	-7	-4	40.45	1.88
-9	-7	-4	39.24	1.91
-9	-7	-4	39.09	1.89
-9	-7	-4	37.14	2.25
9	7	4	42.92	2.28

-10	-7	-4	20.96	1.69
-10	-7	-4	19.42	1.23
-10	-7	-4	18.84	1.28
-10	-7	-4	20.72	1.25
-10	-7	-4	19.62	1.46
-10	-7	-4	21.55	1.69
-10	-7	-4	21.14	1.09
10	7	4	20.00	1.60
-11	-7	-4	32.24	2.15
-11	-7	-4	31.32	1.82
-11	-7	-4	34.72	2.18
-11	-7	-4	30.99	1.69
-11	-7	-4	29.47	1.77
-11	-7	-4	30.64	1.67
-11	-7	-4	32.52	1.94
11	7	4	32.53	2.08
11	7	4	33.19	1.60
-12	-7	-4	19.69	1.85
-12	-7	-4	15.51	1.82
-12	-7	-4	17.95	1.33
-12	-7	-4	14.53	1.35
-12	-7	-4	18.51	1.22
-12	-7	-4	17.95	1.55
12	7	4	17.09	1.12
12	7	4	14.50	1.60
-13	-7	-4	28.20	2.30
-13	-7	-4	26.69	1.87
-13	-7	-4	29.31	1.99
-13	-7	-4	30.57	2.46
-13	-7	-4	33.03	1.86
-13	-7	-4	32.27	1.89
13	7	4	31.19	1.80
13	7	4	31.54	2.16
-14	-7	-4	12.72	2.12
-14	-7	-4	13.95	1.49
-14	-7	-4	15.67	2.08
-14	-7	-4	14.00	1.69
14	7	4	14.29	1.15
14	7	4	11.41	1.66
-15	-7	-4	20.88	1.80
-15	-7	-4	24.83	2.47
15	7	4	24.26	2.18
15	7	4	26.69	1.61
-16	-7	-4	9.01	1.74

-16	-7	-4	9.35	1.59
16	7	4	8.65	1.22
16	7	4	12.88	1.93
-17	-7	-4	29.40	2.36
17	7	4	32.28	2.57
17	7	4	29.13	1.93
-18	-7	-4	13.04	1.65
18	7	4	15.59	1.56
18	7	4	18.93	2.44
-19	-7	-4	21.96	2.54
19	7	4	29.99	1.98
19	7	4	27.74	2.97
20	-7	-3	1.96	0.83
-20	7	3	3.98	1.82
19	-7	-3	0.69	0.59
-19	7	3	3.09	1.42
18	-7	-3	17.74	1.37
-18	7	3	24.91	2.54
17	-7	-3	16.03	1.20
17	-7	-3	15.32	1.44
-17	7	3	11.24	1.99
-17	7	3	14.25	2.12
16	-7	-3	8.44	1.85
16	-7	-3	7.18	0.94
16	-7	-3	5.80	0.93
-16	7	3	4.78	1.18
-16	7	3	8.69	1.36
15	-7	-3	37.62	2.96
15	-7	-3	35.46	1.95
15	-7	-3	35.04	1.78
-15	7	3	29.48	2.36
-15	7	3	36.35	2.60
-15	7	3	39.16	2.51
14	-7	-3	19.46	1.37
14	-7	-3	14.13	2.32
14	-7	-3	18.26	1.12
-14	7	3	15.57	1.78
-14	7	3	17.14	1.72
-14	7	3	20.12	2.04
13	-7	-3	4.37	1.25
13	-7	-3	3.01	0.58
13	-7	-3	3.01	0.69
-13	7	3	3.80	1.09
-13	7	3	4.93	0.99

-13	7	3	4.63	0.94
12	-7	-3	12.14	1.14
12	-7	-3	16.18	1.83
12	-7	-3	14.43	0.90
-12	7	3	11.38	1.41
-12	7	3	13.08	1.36
-12	7	3	17.76	1.72
11	-7	-3	45.35	2.03
11	-7	-3	41.87	2.26
11	-7	-3	47.74	2.25
11	-7	-3	50.25	2.65
-11	7	3	47.44	2.41
-11	7	3	46.33	2.63
-11	7	3	40.40	2.41
-11	7	3	54.65	2.33
10	-7	-3	5.33	0.92
10	-7	-3	5.28	0.91
10	-7	-3	6.64	0.89
10	-7	-3	5.42	0.56
-10	7	3	5.95	0.66
-10	7	3	8.41	1.34
-10	7	3	5.66	0.90
-10	7	3	7.02	0.98
9	-7	-3	59.13	2.86
9	-7	-3	59.64	2.69
9	-7	-3	56.57	2.43
9	-7	-3	60.54	2.62
-9	7	3	59.56	2.82
-9	7	3	58.71	2.49
-9	7	3	60.26	2.72
-9	7	3	57.13	2.93
8	-7	-3	8.89	0.94
8	-7	-3	7.23	0.55
8	-7	-3	7.69	0.80
8	-7	-3	8.00	1.06
-8	7	3	7.84	0.61
-8	7	3	5.88	0.96
-8	7	3	5.94	0.81
-8	7	3	7.49	1.04
7	-7	-3	4.89	0.93
7	-7	-3	5.39	0.45
7	-7	-3	3.81	0.67
7	-7	-3	4.11	0.65
-7	7	3	4.65	0.84

-7	7	3	4.50	0.83
-7	7	3	5.82	0.52
-7	7	3	4.10	0.68
6	-7	-3	12.56	0.71
6	-7	-3	11.96	1.08
6	-7	-3	14.66	0.89
6	-7	-3	14.25	1.13
-6	7	3	14.91	1.33
-6	7	3	11.28	1.02
-6	7	3	13.08	0.90
-6	7	3	13.87	0.76
5	-7	-3	1.33	0.45
5	-7	-3	1.57	0.33
5	-7	-3	1.12	0.23
5	-7	-3	1.63	0.53
-5	7	3	1.37	0.25
-5	7	3	2.24	0.49
-5	7	3	1.47	0.65
-5	7	3	1.35	0.33
4	-7	-3	19.99	1.35
4	-7	-3	18.44	0.90
4	-7	-3	18.16	1.00
4	-7	-3	20.09	1.22
-4	7	3	19.44	1.21
-4	7	3	19.51	0.93
-4	7	3	19.00	1.04
-4	7	3	19.51	1.46
3	-7	-3	120.37	5.35
3	-7	-3	136.54	5.62
3	-7	-3	146.35	5.52
3	-7	-3	134.56	5.21
-3	7	3	136.81	5.49
-3	7	3	138.47	5.23
-3	7	3	144.19	5.75
-3	7	3	136.51	5.30
2	-7	-3	0.53	0.17
2	-7	-3	0.25	0.33
2	-7	-3	-0.09	0.40
2	-7	-3	-0.20	0.21
2	-7	-3	0.27	0.47
-2	7	3	0.75	0.54
-2	7	3	0.08	0.17
-2	7	3	0.04	0.21
-2	7	3	0.10	0.32

1	-7	-3	83.41	3.85
1	-7	-3	85.44	3.65
1	-7	-3	95.51	3.46
1	-7	-3	89.93	3.70
-1	7	3	85.70	3.45
-1	7	3	84.13	3.64
-1	7	3	88.51	3.42
0	-7	-3	11.85	1.10
0	-7	-3	12.70	0.89
0	-7	-3	10.61	0.65
0	-7	-3	10.89	0.61
0	-7	-3	11.66	0.93
0	7	3	11.49	0.85
0	7	3	10.37	0.66
0	7	3	11.26	0.62
-1	-7	-3	73.69	3.45
-1	-7	-3	71.85	2.92
-1	-7	-3	77.75	3.18
-1	-7	-3	76.50	3.23
-1	-7	-3	73.09	2.94
1	7	3	69.55	2.94
1	7	3	75.16	3.16
1	7	3	77.48	2.94
-2	-7	-3	6.82	0.48
-2	-7	-3	6.02	0.80
-2	-7	-3	6.87	1.02
-2	-7	-3	7.72	0.74
-2	-7	-3	6.69	0.51
2	7	3	6.55	0.70
2	7	3	6.64	0.50
2	7	3	6.98	0.48
-3	-7	-3	175.65	7.33
-3	-7	-3	187.19	6.99
-3	-7	-3	169.02	6.73
-3	-7	-3	175.56	6.74
-3	-7	-3	180.94	7.04
3	7	3	172.15	6.95
3	7	3	177.40	6.72
3	7	3	179.65	6.75
-4	-7	-3	20.32	0.99
-4	-7	-3	20.24	1.62
-4	-7	-3	22.79	1.26
-4	-7	-3	19.96	1.29
-4	-7	-3	20.89	0.95

-4	-7	-3	18.55	0.97
4	7	3	19.68	1.20
4	7	3	18.98	0.95
4	7	3	19.33	1.34
-5	-7	-3	9.52	0.62
-5	-7	-3	7.86	0.71
-5	-7	-3	9.15	0.56
-5	-7	-3	9.31	0.87
-5	-7	-3	8.92	0.95
5	7	3	11.20	1.01
5	7	3	9.62	0.85
5	7	3	9.22	0.57
-6	-7	-3	2.56	0.38
-6	-7	-3	3.14	0.45
-6	-7	-3	3.35	0.60
-6	-7	-3	3.25	0.64
-6	-7	-3	2.83	0.39
-6	-7	-3	2.83	0.46
6	7	3	3.32	0.68
6	7	3	3.25	0.56
-7	-7	-3	15.38	1.12
-7	-7	-3	12.44	0.81
-7	-7	-3	12.70	0.74
-7	-7	-3	15.01	1.22
-7	-7	-3	12.14	0.81
-7	-7	-3	14.37	0.85
7	7	3	13.28	1.17
7	7	3	12.37	1.06
-8	-7	-3	16.01	1.38
-8	-7	-3	16.18	1.32
-8	-7	-3	16.82	1.26
-8	-7	-3	16.83	1.02
-8	-7	-3	15.97	0.96
-8	-7	-3	17.64	0.89
-8	-7	-3	15.79	1.00
8	7	3	16.66	1.23
8	7	3	14.99	1.34
-9	-7	-3	25.72	1.23
-9	-7	-3	23.24	1.35
-9	-7	-3	26.86	1.71
-9	-7	-3	26.64	1.37
-9	-7	-3	25.44	1.32
-9	-7	-3	23.32	1.72
-9	-7	-3	25.03	1.58

9	7	3	24.36	1.70
-10	-7	-3	20.56	1.59
-10	-7	-3	18.67	1.01
-10	-7	-3	17.75	1.12
-10	-7	-3	18.19	1.18
-10	-7	-3	17.25	1.65
-10	-7	-3	16.37	1.19
-10	-7	-3	16.21	1.39
10	7	3	18.17	1.53
10	7	3	19.09	1.06
-11	-7	-3	17.54	1.34
-11	-7	-3	19.35	1.23
-11	-7	-3	21.08	1.87
-11	-7	-3	18.72	1.29
-11	-7	-3	19.75	1.09
-11	-7	-3	17.70	1.50
-11	-7	-3	22.79	1.72
11	7	3	21.30	1.65
11	7	3	20.98	1.16
-12	-7	-3	26.23	1.64
-12	-7	-3	23.26	2.03
-12	-7	-3	26.79	1.96
-12	-7	-3	22.90	1.74
-12	-7	-3	23.68	1.52
12	7	3	24.17	1.83
12	7	3	25.52	1.38
-13	-7	-3	28.17	1.74
-13	-7	-3	29.87	1.82
-13	-7	-3	29.11	2.30
-13	-7	-3	26.12	1.94
-13	-7	-3	25.63	2.15
13	7	3	29.11	1.54
13	7	3	28.05	2.00
-14	-7	-3	9.82	1.58
-14	-7	-3	12.02	2.06
-14	-7	-3	8.47	1.35
-14	-7	-3	9.26	1.22
14	7	3	11.65	1.02
14	7	3	9.92	1.57
-15	-7	-3	12.21	1.48
-15	-7	-3	11.46	1.72
-15	-7	-3	10.26	1.53
-15	-7	-3	10.09	1.76
15	7	3	11.29	1.12

15	7	3	12.51	1.74
-16	-7	-3	12.90	1.65
-16	-7	-3	12.49	1.89
16	7	3	11.74	1.54
16	7	3	12.67	1.28
-17	-7	-3	2.54	1.80
-17	-7	-3	2.17	1.07
-17	-7	-3	3.51	1.91
17	7	3	1.99	1.25
17	7	3	1.81	0.74
-18	-7	-3	5.50	1.26
-18	-7	-3	5.80	2.22
18	7	3	3.26	1.44
18	7	3	2.71	0.84
-19	-7	-3	5.75	1.41
19	7	3	7.48	1.06
20	-7	-2	3.68	0.84
-20	7	2	5.39	1.75
19	-7	-2	21.11	1.53
-19	7	2	17.17	2.61
18	-7	-2	8.38	1.06
-18	7	2	5.74	1.49
17	-7	-2	35.45	2.08
17	-7	-2	33.77	1.85
-17	7	2	33.68	2.40
-17	7	2	36.96	2.77
16	-7	-2	10.55	1.85
16	-7	-2	11.74	1.05
16	-7	-2	12.25	1.40
-16	7	2	11.58	1.90
-16	7	2	11.20	1.64
15	-7	-2	57.52	3.34
15	-7	-2	51.65	2.65
15	-7	-2	52.74	2.45
-15	7	2	48.68	2.85
-15	7	2	59.43	3.28
-15	7	2	58.61	3.11
14	-7	-2	37.62	1.79
14	-7	-2	39.09	2.70
14	-7	-2	36.93	2.01
-14	7	2	38.15	2.57
-14	7	2	33.34	2.22
-14	7	2	35.12	2.32
13	-7	-2	62.60	2.66

13	-7	-2	64.59	3.29
13	-7	-2	57.42	2.81
-13	7	2	60.58	3.11
-13	7	2	64.20	3.30
-13	7	2	58.92	2.98
12	-7	-2	22.55	1.90
12	-7	-2	21.84	1.43
12	-7	-2	20.94	1.14
-12	7	2	20.87	1.60
-12	7	2	18.53	1.86
-12	7	2	19.55	1.56
11	-7	-2	21.86	1.36
11	-7	-2	16.72	1.02
11	-7	-2	18.40	1.63
11	-7	-2	18.08	1.33
-11	7	2	16.25	1.71
-11	7	2	18.25	1.10
-11	7	2	21.75	1.46
-11	7	2	19.22	1.49
10	-7	-2	24.45	1.63
10	-7	-2	21.84	1.34
10	-7	-2	20.05	1.10
10	-7	-2	21.70	1.43
-10	7	2	20.13	1.76
-10	7	2	22.35	1.51
-10	7	2	25.29	1.21
-10	7	2	21.66	1.46
9	-7	-2	61.45	2.62
9	-7	-2	64.42	2.88
9	-7	-2	63.94	2.81
9	-7	-2	67.90	3.01
-9	7	2	61.86	2.93
-9	7	2	61.68	3.12
-9	7	2	62.48	2.90
-9	7	2	69.07	2.68
8	-7	-2	47.94	2.14
8	-7	-2	54.53	2.50
8	-7	-2	54.20	2.33
8	-7	-2	54.53	2.46
-8	7	2	52.63	2.47
-8	7	2	51.39	2.39
-8	7	2	50.85	2.66
-8	7	2	50.89	2.19
7	-7	-2	47.30	1.99

7	-7	-2	46.57	2.30
7	-7	-2	47.82	2.15
7	-7	-2	49.83	2.31
-7	7	2	46.09	2.19
-7	7	2	47.14	2.29
-7	7	2	48.79	2.51
-7	7	2	51.25	2.04
6	-7	-2	28.36	1.43
6	-7	-2	29.85	1.64
6	-7	-2	29.07	1.29
6	-7	-2	28.90	1.58
-6	7	2	27.46	1.46
-6	7	2	31.16	1.85
-6	7	2	31.37	1.33
-6	7	2	29.04	1.57
5	-7	-2	43.58	1.97
5	-7	-2	38.90	1.78
5	-7	-2	37.24	1.66
5	-7	-2	41.51	2.04
-5	7	2	39.20	1.69
-5	7	2	39.96	1.94
-5	7	2	41.77	2.19
-5	7	2	40.89	1.83
4	-7	-2	41.57	2.09
4	-7	-2	42.02	1.98
4	-7	-2	41.13	1.71
4	-7	-2	44.94	1.84
-4	7	2	37.63	1.82
-4	7	2	43.59	2.23
-4	7	2	40.33	1.97
3	-7	-2	44.76	2.04
3	-7	-2	41.18	1.78
3	-7	-2	46.29	1.90
3	-7	-2	43.38	2.21
-3	7	2	41.20	1.89
-3	7	2	46.50	2.32
-3	7	2	44.90	2.05
2	-7	-2	250.07	9.35
2	-7	-2	251.50	9.51
2	-7	-2	250.23	9.26
2	-7	-2	241.37	9.66
-2	7	2	253.92	9.76
-2	7	2	231.39	9.48
-2	7	2	245.15	9.35

-2	7	2	250.22	9.27
1	-7	-2	72.85	2.93
1	-7	-2	76.58	3.11
1	-7	-2	69.98	2.85
-1	7	2	77.17	3.38
-1	7	2	70.91	2.93
-1	7	2	73.37	3.11
0	-7	-2	43.25	2.01
0	-7	-2	42.34	2.23
0	-7	-2	42.80	2.07
0	-7	-2	43.56	1.77
0	-7	-2	41.68	1.83
0	7	2	42.21	1.83
0	7	2	47.07	2.04
0	7	2	47.05	2.32
0	7	2	43.60	1.79
-1	-7	-2	31.89	1.83
-1	-7	-2	32.63	1.39
-1	-7	-2	32.17	1.33
-1	-7	-2	33.02	1.64
-1	-7	-2	32.00	1.58
1	7	2	31.05	1.34
1	7	2	28.61	1.54
1	7	2	30.43	1.38
-2	-7	-2	6.44	0.42
-2	-7	-2	6.93	0.74
-2	-7	-2	7.17	0.69
-2	-7	-2	6.49	0.97
-2	-7	-2	5.90	0.51
2	7	2	6.06	0.50
2	7	2	5.99	0.65
-3	-7	-2	56.03	2.25
-3	-7	-2	52.52	2.20
-3	-7	-2	54.63	2.49
-3	-7	-2	51.47	2.42
-3	-7	-2	56.90	2.78
3	7	2	53.85	2.42
3	7	2	55.03	2.23
3	7	2	56.67	2.21
-4	-7	-2	109.15	4.25
-4	-7	-2	111.59	4.50
-4	-7	-2	105.88	4.21
-4	-7	-2	110.80	4.81
-4	-7	-2	109.31	4.18

-4	-7	-2	110.52	4.45
4	7	2	105.83	4.21
4	7	2	107.02	4.44
-5	-7	-2	16.10	0.85
-5	-7	-2	12.22	1.34
-5	-7	-2	15.40	1.09
-5	-7	-2	14.47	1.04
-5	-7	-2	15.19	0.74
-5	-7	-2	14.29	0.78
5	7	2	14.37	1.03
5	7	2	14.31	0.79
-6	-7	-2	71.75	3.58
-6	-7	-2	74.16	3.16
-6	-7	-2	72.43	2.96
-6	-7	-2	71.28	2.94
-6	-7	-2	69.48	2.91
-6	-7	-2	74.49	3.21
-6	-7	-2	73.85	2.87
6	7	2	73.78	3.31
6	7	2	73.43	3.16
6	7	2	70.98	2.91
-7	-7	-2	16.00	1.32
-7	-7	-2	15.51	0.79
-7	-7	-2	15.97	0.93
-7	-7	-2	16.17	1.19
-7	-7	-2	14.69	1.55
-7	-7	-2	15.50	0.87
-7	-7	-2	14.28	1.13
-7	-7	-2	13.90	0.88
7	7	2	15.26	1.26
7	7	2	14.76	1.13
-8	-7	-2	16.47	1.02
-8	-7	-2	15.73	1.03
-8	-7	-2	18.37	1.32
-8	-7	-2	18.64	0.91
-8	-7	-2	17.65	1.47
-8	-7	-2	16.75	1.26
-8	-7	-2	16.22	0.96
8	7	2	17.79	1.35
-9	-7	-2	2.80	0.67
-9	-7	-2	1.18	0.70
-9	-7	-2	2.23	0.42
-9	-7	-2	1.91	0.65
-9	-7	-2	1.42	0.42

-9	-7	-2	2.20	0.46
-9	-7	-2	2.25	0.32
9	7	2	1.60	0.34
9	7	2	2.29	0.64
-10	-7	-2	52.56	2.38
-10	-7	-2	50.01	2.37
-10	-7	-2	49.12	2.78
-10	-7	-2	52.78	2.56
-10	-7	-2	51.49	2.19
-10	-7	-2	54.31	2.62
10	7	2	53.46	2.25
10	7	2	51.98	2.64
-11	-7	-2	7.06	0.97
-11	-7	-2	6.97	1.03
-11	-7	-2	5.84	1.13
-11	-7	-2	6.85	1.17
-11	-7	-2	7.80	0.95
-11	-7	-2	6.23	0.65
11	7	2	8.12	0.74
11	7	2	8.06	1.21
-12	-7	-2	25.87	2.14
-12	-7	-2	23.38	1.58
-12	-7	-2	24.04	1.55
-12	-7	-2	25.39	1.29
-12	-7	-2	23.81	1.85
-12	-7	-2	24.92	1.78
12	7	2	24.67	1.34
12	7	2	24.29	1.77
-13	-7	-2	1.97	0.93
-13	-7	-2	-1.04	1.03
-13	-7	-2	0.77	0.87
-13	-7	-2	0.36	0.66
-13	-7	-2	0.78	0.80
13	7	2	0.08	0.39
13	7	2	-0.05	0.75
-14	-7	-2	32.55	2.26
-14	-7	-2	35.80	2.11
-14	-7	-2	35.34	2.41
-14	-7	-2	38.00	3.08
-14	-7	-2	32.89	2.70
14	7	2	35.16	2.29
14	7	2	37.56	1.85
-15	-7	-2	4.97	1.19
-15	-7	-2	4.99	1.55

-15	-7	-2	2.99	1.66
-15	-7	-2	6.46	1.19
15	7	2	3.69	1.04
15	7	2	4.61	0.68
-16	-7	-2	16.88	2.80
-16	-7	-2	10.39	1.62
-16	-7	-2	15.82	2.22
-16	-7	-2	13.77	2.57
16	7	2	10.03	1.17
-17	-7	-2	-0.46	1.65
-17	-7	-2	1.62	1.00
-17	-7	-2	0.14	1.78
17	7	2	0.14	0.61
-18	-7	-2	24.62	2.27
-18	-7	-2	26.27	3.43
18	7	2	20.97	1.71
-19	-7	-2	-0.86	1.23
19	7	2	2.22	0.81
20	7	2	13.66	1.73
20	-7	-1	34.63	2.22
-20	7	1	42.00	3.51
19	-7	-1	-0.05	0.59
-19	7	1	-0.61	1.35
18	-7	-1	45.41	2.34
-18	7	1	48.23	3.25
17	-7	-1	3.24	0.97
17	-7	-1	0.38	0.53
-17	7	1	1.79	1.12
16	-7	-1	68.20	3.13
16	-7	-1	67.57	2.88
-16	7	1	56.17	3.09
-16	7	1	64.55	3.56
15	-7	-1	1.03	0.77
15	-7	-1	0.35	1.24
15	-7	-1	0.47	0.43
-15	7	1	1.65	0.89
-15	7	1	0.81	0.93
-15	7	1	0.40	1.28
14	-7	-1	42.27	1.95
14	-7	-1	41.20	2.69
14	-7	-1	40.10	2.18
-14	7	1	37.98	2.27
-14	7	1	45.85	2.53
-14	7	1	37.24	2.72

13	-7	-1	5.33	0.68
13	-7	-1	4.61	0.82
13	-7	-1	4.07	1.06
-13	7	1	4.40	0.88
-13	7	1	4.20	0.82
-13	7	1	5.22	1.20
12	-7	-1	134.48	5.43
12	-7	-1	123.72	5.04
12	-7	-1	133.49	5.30
-12	7	1	128.62	5.62
-12	7	1	128.37	5.44
-12	7	1	120.75	5.28
11	-7	-1	58.08	2.62
11	-7	-1	53.32	2.57
11	-7	-1	56.75	2.71
11	-7	-1	53.30	2.33
-11	7	1	52.81	2.95
-11	7	1	53.19	2.61
-11	7	1	57.10	2.40
-11	7	1	54.61	2.70
10	-7	-1	64.83	2.82
10	-7	-1	57.94	2.68
10	-7	-1	58.03	2.73
10	-7	-1	54.89	2.47
-10	7	1	61.85	3.07
-10	7	1	59.85	2.80
-10	7	1	57.42	2.74
-10	7	1	62.65	2.53
9	-7	-1	24.58	1.50
9	-7	-1	27.00	1.55
9	-7	-1	24.30	1.18
9	-7	-1	23.62	1.38
-9	7	1	23.69	1.48
-9	7	1	24.25	1.46
-9	7	1	23.84	1.79
-9	7	1	24.83	1.23
8	-7	-1	36.62	1.92
8	-7	-1	38.90	1.81
8	-7	-1	40.65	1.93
8	-7	-1	36.34	1.61
-8	7	1	35.62	2.14
-8	7	1	36.30	1.64
-8	7	1	37.05	1.86
-8	7	1	36.67	1.88

7	-7	-1	19.70	0.96
7	-7	-1	20.33	1.32
7	-7	-1	20.27	1.15
7	-7	-1	20.72	1.27
-7	7	1	19.39	1.53
-7	7	1	18.52	1.18
-7	7	1	20.26	1.27
-7	7	1	21.60	1.01
6	-7	-1	145.44	5.87
6	-7	-1	155.77	6.20
6	-7	-1	154.04	6.03
6	-7	-1	160.08	6.13
-6	7	1	156.26	6.13
-6	7	1	147.18	6.05
-6	7	1	153.41	6.34
-6	7	1	162.18	5.91
5	-7	-1	16.46	1.06
5	-7	-1	17.51	1.18
5	-7	-1	18.02	0.99
5	-7	-1	16.50	0.80
-5	7	1	17.59	0.83
-5	7	1	15.57	1.30
-5	7	1	15.96	1.07
-5	7	1	15.53	0.98
4	-7	-1	158.29	6.20
4	-7	-1	167.62	6.33
4	-7	-1	169.22	6.44
4	-7	-1	159.04	6.55
-4	7	1	155.88	6.34
-4	7	1	164.51	6.22
-4	7	1	169.34	6.70
-4	7	1	167.25	6.45
3	-7	-1	177.76	6.69
3	-7	-1	174.90	6.80
3	-7	-1	167.22	6.56
3	-7	-1	175.79	6.97
-3	7	1	175.73	6.58
-3	7	1	175.00	6.81
-3	7	1	179.64	7.06
-3	7	1	167.04	6.70
2	-7	-1	42.39	2.15
2	-7	-1	40.87	1.71
2	-7	-1	42.44	1.95
-2	7	1	44.65	1.75

-2	7	1	42.74	1.85
-2	7	1	41.43	1.96
-2	7	1	45.45	2.23
1	-7	-1	14.67	0.85
1	-7	-1	15.83	1.19
1	-7	-1	16.15	0.99
1	-7	-1	17.00	0.74
-1	7	1	16.23	1.24
-1	7	1	15.34	0.84
-1	7	1	16.82	0.76
0	-7	-1	246.41	9.23
0	-7	-1	244.83	9.47
0	-7	-1	241.60	9.08
0	-7	-1	220.97	8.98
0	7	1	243.63	9.48
0	7	1	241.29	9.00
0	7	1	233.02	9.07
0	7	1	248.45	9.25
-1	-7	-1	57.55	2.19
-1	-7	-1	53.20	2.66
-1	-7	-1	54.28	2.28
-1	-7	-1	54.32	2.42
1	7	1	57.93	2.70
1	7	1	58.27	2.20
1	7	1	51.97	2.26
1	7	1	55.17	2.43
-2	-7	-1	57.32	2.57
-2	-7	-1	57.04	2.78
-2	-7	-1	57.46	2.52
-2	-7	-1	55.61	2.36
-2	-7	-1	60.25	2.28
2	7	1	56.13	2.35
2	7	1	59.90	2.80
2	7	1	57.34	2.28
2	7	1	57.00	2.52
-3	-7	-1	97.03	4.36
-3	-7	-1	98.50	4.11
-3	-7	-1	104.68	4.10
-3	-7	-1	98.86	3.83
-3	-7	-1	98.11	3.91
3	7	1	104.06	3.84
3	7	1	96.18	3.88
3	7	1	98.09	4.08
-4	-7	-1	55.13	2.32

-4	-7	-1	53.13	2.22
-4	-7	-1	54.67	2.48
-4	-7	-1	54.41	2.77
-4	-7	-1	57.83	2.52
4	7	1	56.90	2.28
4	7	1	55.59	2.48
-5	-7	-1	21.91	1.06
-5	-7	-1	23.96	1.02
-5	-7	-1	23.75	1.37
-5	-7	-1	22.99	1.62
-5	-7	-1	23.06	1.14
-5	-7	-1	24.98	1.37
5	7	1	22.22	1.10
5	7	1	20.08	1.30
-6	-7	-1	50.44	2.36
-6	-7	-1	51.01	2.04
-6	-7	-1	50.38	2.49
-6	-7	-1	50.65	2.09
-6	-7	-1	50.43	2.17
-6	-7	-1	47.94	2.68
-6	-7	-1	50.19	2.36
6	7	1	48.97	2.11
6	7	1	54.49	2.40
-7	-7	-1	12.61	1.08
-7	-7	-1	12.38	1.24
-7	-7	-1	14.72	0.72
-7	-7	-1	12.34	0.82
-7	-7	-1	12.74	1.06
-7	-7	-1	13.02	1.46
-7	-7	-1	13.28	0.87
-7	-7	-1	12.19	0.76
-8	-7	-1	47.04	2.36
-8	-7	-1	50.91	2.19
-8	-7	-1	42.31	2.73
-8	-7	-1	49.64	2.57
-8	-7	-1	50.70	2.18
-8	-7	-1	49.74	2.03
-8	-7	-1	52.18	2.40
-8	-7	-1	49.58	2.11
8	7	1	47.99	2.05
-9	-7	-1	27.55	1.63
-9	-7	-1	26.55	1.63
-9	-7	-1	26.84	1.22
-9	-7	-1	22.89	2.03

-9	-7	-1	25.25	1.41
-9	-7	-1	25.01	1.38
-9	-7	-1	26.14	1.88
9	7	1	27.86	1.26
-10	-7	-1	19.86	1.47
-10	-7	-1	18.81	1.25
-10	-7	-1	19.52	1.26
-10	-7	-1	17.33	1.94
-10	-7	-1	21.33	1.04
-10	-7	-1	20.62	1.47
-10	-7	-1	21.39	1.79
10	7	1	20.60	1.08
-11	-7	-1	5.69	0.90
-11	-7	-1	5.47	1.10
-11	-7	-1	5.67	0.92
-11	-7	-1	6.82	0.62
-11	-7	-1	4.54	0.70
-11	-7	-1	2.69	1.16
-11	-7	-1	9.01	1.19
11	7	1	5.57	0.66
-12	-7	-1	28.48	1.96
-12	-7	-1	30.96	2.36
-12	-7	-1	26.29	2.48
-12	-7	-1	32.57	1.80
-12	-7	-1	28.55	1.75
-12	-7	-1	32.19	1.50
-12	-7	-1	34.34	1.99
12	7	1	30.16	1.53
-13	-7	-1	29.08	1.99
-13	-7	-1	30.52	2.05
-13	-7	-1	25.10	2.43
-13	-7	-1	27.51	2.53
-13	-7	-1	28.51	1.81
13	7	1	32.43	1.56
-14	-7	-1	31.24	2.18
-14	-7	-1	25.02	2.11
-14	-7	-1	30.47	1.95
-14	-7	-1	28.42	2.63
-14	-7	-1	28.41	2.71
14	7	1	34.68	1.61
-15	-7	-1	43.32	3.31
-15	-7	-1	47.20	2.71
-15	-7	-1	43.46	2.70
-15	-7	-1	44.67	2.66

-15	-7	-1	44.12	3.28
15	7	1	46.77	2.19
-16	-7	-1	6.78	1.78
-16	-7	-1	3.42	1.67
-16	-7	-1	9.43	1.53
-16	-7	-1	10.57	1.94
16	7	1	6.04	0.80
-17	-7	-1	35.83	2.44
-17	-7	-1	31.77	3.35
-17	-7	-1	28.52	3.26
17	7	1	40.48	2.01
-18	-7	-1	8.97	2.06
-18	-7	-1	7.82	1.37
18	7	1	8.49	1.27
-19	-7	-1	23.77	2.37
19	7	1	20.97	1.75
-20	-7	-1	6.66	1.50
20	7	1	6.14	1.04
20	-7	0	4.12	0.95
-20	7	0	5.60	1.64
19	-7	0	14.38	1.35
-19	7	0	15.77	2.51
18	-7	0	5.78	0.87
18	-7	0	7.55	1.22
-18	7	0	7.92	1.56
17	-7	0	29.77	1.72
17	-7	0	27.76	2.10
-17	7	0	32.20	2.63
16	-7	0	11.01	1.56
16	-7	0	10.01	1.03
-16	7	0	10.57	1.42
-16	7	0	12.57	1.93
15	-7	0	64.24	2.96
15	-7	0	55.32	2.61
15	-7	0	62.04	3.19
-15	7	0	54.66	2.81
-15	7	0	58.75	3.24
-15	7	0	58.47	3.51
14	-7	0	7.74	1.24
14	-7	0	5.79	0.95
14	-7	0	6.13	0.78
-14	7	0	5.31	1.08
-14	7	0	6.85	1.24
-14	7	0	7.46	1.42

13	-7	0	27.41	1.75
13	-7	0	26.67	1.39
13	-7	0	29.71	1.95
-13	7	0	27.15	2.33
-13	7	0	25.70	1.98
-13	7	0	23.85	1.68
12	-7	0	19.27	1.48
12	-7	0	21.19	1.64
12	-7	0	19.01	1.11
-12	7	0	19.56	1.43
-12	7	0	23.36	1.98
-12	7	0	17.76	1.52
11	-7	0	57.09	2.73
11	-7	0	63.33	2.80
11	-7	0	57.06	2.47
11	-7	0	57.94	2.75
-11	7	0	57.18	2.81
-11	7	0	60.44	2.53
-11	7	0	58.31	2.70
-11	7	0	55.87	3.08
10	-7	0	9.13	1.07
10	-7	0	9.37	0.94
10	-7	0	8.62	0.67
10	-7	0	8.43	1.06
-10	7	0	7.53	1.00
-10	7	0	12.10	1.49
-10	7	0	9.16	0.73
-10	7	0	8.11	1.01
9	-7	0	143.47	5.92
9	-7	0	151.74	5.85
9	-7	0	146.40	5.81
9	-7	0	139.36	5.63
-9	7	0	135.55	6.11
-9	7	0	152.69	5.68
-9	7	0	147.21	5.92
-9	7	0	151.29	5.84
8	-7	0	11.51	0.96
8	-7	0	9.49	0.63
8	-7	0	9.86	1.05
8	-7	0	9.10	0.85
-8	7	0	10.29	1.28
-8	7	0	9.59	0.90
-8	7	0	10.48	0.94
-8	7	0	10.78	0.69

7	-7	0	66.18	2.78
7	-7	0	60.82	2.68
7	-7	0	65.42	2.86
7	-7	0	59.66	2.51
-7	7	0	59.45	2.73
-7	7	0	63.24	3.04
-7	7	0	64.62	2.55
-7	7	0	62.50	2.73
6	-7	0	4.61	0.41
6	-7	0	4.95	0.79
6	-7	0	4.12	0.78
6	-7	0	4.35	0.62
-6	7	0	5.33	0.44
-6	7	0	4.96	0.69
-6	7	0	4.95	0.61
-6	7	0	6.63	1.04
5	-7	0	79.30	3.34
5	-7	0	72.59	2.97
5	-7	0	78.49	3.14
-5	7	0	75.88	3.19
-5	7	0	77.01	3.48
-5	7	0	79.42	3.00
-5	7	0	70.74	3.15
4	-7	0	27.07	1.35
4	-7	0	30.41	1.61
4	-7	0	24.72	1.80
4	-7	0	28.26	1.20
-4	7	0	27.88	1.44
-4	7	0	29.29	1.22
-4	7	0	27.37	1.38
-4	7	0	30.77	1.73
3	-7	0	125.14	5.01
3	-7	0	136.99	5.43
3	-7	0	135.07	5.17
-3	7	0	134.49	5.50
-3	7	0	135.32	5.26
-3	7	0	128.36	5.18
2	-7	0	10.14	0.51
2	-7	0	9.41	0.67
2	-7	0	8.84	0.84
2	-7	0	10.26	0.97
-2	7	0	10.94	1.04
-2	7	0	8.90	0.66
-2	7	0	10.21	0.52

-2	7	0	9.78	0.77
1	-7	0	338.24	12.64
1	-7	0	325.97	12.43
1	-7	0	312.90	12.18
-1	7	0	333.52	12.20
-1	7	0	325.36	12.33
-1	7	0	338.97	12.69
-1	7	0	316.04	12.43
0	-7	0	0.07	0.22
0	-7	0	0.13	0.44
0	-7	0	0.43	0.30
0	-7	0	0.24	0.16
0	7	0	0.15	0.12
0	7	0	0.61	0.45
0	7	0	0.22	0.20
0	7	0	0.34	0.29
-1	-7	0	331.70	12.57
-1	-7	0	317.11	12.24
-1	-7	0	333.44	12.36
1	7	0	346.07	12.61
1	7	0	311.86	12.35
1	7	0	324.89	12.09
1	7	0	306.66	12.20
-2	-7	0	7.99	0.55
-2	-7	0	7.57	0.86
-2	-7	0	9.69	0.79
-2	-7	0	9.84	1.04
2	7	0	9.86	0.50
2	7	0	10.73	1.04
2	7	0	9.67	0.62
2	7	0	10.48	0.81
-3	-7	0	133.35	5.40
-3	-7	0	133.00	5.42
-3	-7	0	138.04	5.27
-3	-7	0	131.31	5.12
-3	-7	0	134.55	5.65
3	7	0	141.12	5.43
3	7	0	135.73	5.22
3	7	0	136.27	5.63
3	7	0	133.29	5.13
-4	-7	0	30.16	1.57
-4	-7	0	30.35	1.23
-4	-7	0	30.84	1.57
-4	-7	0	30.38	1.27

-4	-7	0	30.62	1.83
-4	-7	0	29.44	1.39
4	7	0	30.38	1.57
4	7	0	26.78	1.34
-5	-7	0	75.33	3.43
-5	-7	0	75.32	3.54
-5	-7	0	80.56	3.33
-5	-7	0	78.16	3.02
-5	-7	0	77.93	3.29
-5	-7	0	74.85	2.98
-5	-7	0	79.06	3.13
5	7	0	73.02	3.08
-6	-7	0	4.28	0.84
-6	-7	0	3.92	0.75
-6	-7	0	5.70	0.40
-6	-7	0	5.66	0.61
-6	-7	0	4.40	0.75
-6	-7	0	6.24	0.47
-6	-7	0	5.48	0.81
6	7	0	5.11	0.52
-7	-7	0	63.25	2.59
-7	-7	0	60.90	3.05
-7	-7	0	65.97	2.90
-7	-7	0	65.03	2.84
-7	-7	0	67.19	2.71
-7	-7	0	65.32	2.53
-7	-7	0	61.24	2.67
-7	-7	0	59.92	3.15
7	7	0	63.40	2.55
-8	-7	0	9.89	0.59
-8	-7	0	9.54	0.75
-8	-7	0	9.38	1.27
-8	-7	0	9.02	0.81
-8	-7	0	11.57	1.43
-8	-7	0	8.69	0.94
-8	-7	0	10.54	1.06
-8	-7	0	8.56	0.65
8	7	0	8.90	0.61
-9	-7	0	146.33	6.19
-9	-7	0	132.35	6.20
-9	-7	0	144.74	5.75
-9	-7	0	143.67	5.94
-9	-7	0	149.93	5.57
-9	-7	0	158.24	5.91

-9	-7	0	142.25	5.76
9	7	0	143.06	5.60
-10	-7	0	9.27	1.61
-10	-7	0	7.89	0.91
-10	-7	0	9.76	0.93
-10	-7	0	9.67	1.53
-10	-7	0	10.91	1.24
-10	-7	0	10.96	1.11
-10	-7	0	10.22	0.68
10	7	0	10.63	0.73
-11	-7	0	57.54	2.71
-11	-7	0	56.83	3.22
-11	-7	0	61.05	2.73
-11	-7	0	60.71	2.82
-11	-7	0	60.36	2.47
-11	-7	0	55.93	3.26
-11	-7	0	58.40	2.91
11	7	0	63.72	2.52
-12	-7	0	20.81	1.10
-12	-7	0	18.19	1.42
-12	-7	0	20.44	1.42
-12	-7	0	19.04	1.57
-12	-7	0	22.01	2.15
-12	-7	0	20.82	1.70
-12	-7	0	19.82	2.08
12	7	0	21.33	1.14
-13	-7	0	28.74	1.39
-13	-7	0	28.63	1.87
-13	-7	0	21.46	1.94
-13	-7	0	27.94	1.77
-13	-7	0	25.75	2.38
-13	-7	0	25.19	2.43
13	7	0	28.09	1.43
-14	-7	0	6.95	1.15
-14	-7	0	5.83	1.44
-14	-7	0	5.79	1.51
-14	-7	0	5.39	0.94
-14	-7	0	7.08	1.09
14	7	0	7.20	0.79
-15	-7	0	62.58	3.13
-15	-7	0	57.75	3.70
-15	-7	0	61.69	3.05
-15	-7	0	61.71	3.80
-15	-7	0	54.54	3.19

-16	-7	0	15.80	2.00
-16	-7	0	11.60	1.65
-16	-7	0	11.76	1.97
-16	-7	0	10.02	1.81
16	7	0	11.14	1.09
-17	-7	0	28.51	3.28
-17	-7	0	28.47	2.29
-17	-7	0	32.79	3.02
17	7	0	29.04	1.76
-18	-7	0	6.71	1.27
-18	-7	0	3.40	2.04
18	7	0	5.73	0.85
-19	-7	0	16.33	2.18
19	7	0	19.03	1.56
-20	-7	0	6.91	1.48
20	7	0	7.34	1.04
-20	7	-1	4.94	1.77
19	-7	1	17.85	1.45
-19	7	-1	18.58	2.55
18	-7	1	7.90	1.17
18	-7	1	10.09	1.41
-18	7	-1	10.70	1.64
17	-7	1	34.53	1.94
17	-7	1	39.26	2.47
-17	7	-1	37.15	2.82
16	-7	1	6.93	1.14
16	-7	1	6.39	0.94
-16	7	-1	8.32	1.89
-16	7	-1	7.37	1.28
15	-7	1	43.26	2.43
15	-7	1	40.80	2.03
15	-7	1	42.46	2.40
-15	7	-1	38.14	2.12
-15	7	-1	40.81	3.00
-15	7	-1	42.38	2.68
14	-7	1	31.99	2.00
14	-7	1	31.74	2.04
14	-7	1	29.86	1.55
-14	7	-1	27.54	2.49
-14	7	-1	26.20	1.73
-14	7	-1	32.52	2.19
13	-7	1	27.24	1.89
13	-7	1	30.01	1.47
13	-7	1	28.79	1.81

-13	7	-1	28.91	2.36
-13	7	-1	21.39	2.10
-13	7	-1	27.34	1.60
12	-7	1	32.02	1.92
12	-7	1	32.53	1.53
12	-7	1	30.37	1.85
-12	7	-1	29.45	1.68
-12	7	-1	30.55	2.31
-12	7	-1	31.63	1.95
11	-7	1	4.69	0.62
11	-7	1	5.23	0.79
11	-7	1	6.06	0.86
11	-7	1	4.05	0.77
-11	7	-1	5.02	0.88
-11	7	-1	7.24	1.18
-11	7	-1	5.74	0.69
-11	7	-1	7.14	1.06
10	-7	1	19.16	1.39
10	-7	1	19.50	1.31
10	-7	1	19.72	1.28
10	-7	1	17.87	1.02
-10	7	-1	18.19	1.70
-10	7	-1	20.40	1.09
-10	7	-1	20.92	1.40
-10	7	-1	18.80	1.22
9	-7	1	25.05	1.25
9	-7	1	26.04	1.47
9	-7	1	26.73	1.47
9	-7	1	24.21	1.62
-9	7	-1	26.20	1.55
-9	7	-1	26.52	1.44
-9	7	-1	28.74	1.29
-9	7	-1	25.84	1.88
8	-7	1	48.37	2.04
8	-7	1	50.15	2.22
8	-7	1	48.70	2.39
8	-7	1	47.63	2.23
-8	7	-1	47.41	2.28
-8	7	-1	46.37	2.57
-8	7	-1	50.10	2.22
-8	7	-1	51.13	2.08
7	-7	1	13.64	1.12
7	-7	1	12.56	0.92
7	-7	1	11.75	0.69

7	-7	1	12.74	0.93
-7	7	-1	12.70	0.72
-7	7	-1	11.95	0.92
-7	7	-1	12.35	0.94
-7	7	-1	11.81	1.26
6	-7	1	54.52	2.26
6	-7	1	53.24	2.26
6	-7	1	52.18	2.44
6	-7	1	48.00	2.06
-6	7	-1	51.81	2.27
-6	7	-1	50.72	2.09
-6	7	-1	48.16	2.27
-6	7	-1	49.47	2.57
5	-7	1	21.13	1.18
5	-7	1	23.26	1.21
5	-7	1	21.10	0.98
5	-7	1	22.90	1.39
-5	7	-1	21.74	1.21
-5	7	-1	22.09	0.99
-5	7	-1	20.69	1.19
-5	7	-1	22.68	1.55
4	-7	1	53.12	2.35
4	-7	1	52.85	2.15
4	-7	1	54.27	2.53
4	-7	1	57.30	2.33
-4	7	-1	54.30	2.16
-4	7	-1	53.76	2.64
-4	7	-1	54.69	2.37
-4	7	-1	54.15	2.35
3	-7	1	86.19	3.98
3	-7	1	92.31	3.65
3	-7	1	99.69	3.83
3	-7	1	96.88	4.06
-3	7	-1	97.44	3.90
-3	7	-1	95.64	3.66
-3	7	-1	98.27	3.85
-3	7	-1	98.10	4.15
2	-7	1	59.19	2.37
2	-7	1	55.38	2.19
2	-7	1	57.02	2.61
-2	7	-1	58.09	2.20
-2	7	-1	53.04	2.36
-2	7	-1	53.35	2.43
-2	7	-1	58.16	2.70

1	-7	1	54.68	2.56
1	-7	1	54.04	2.52
1	-7	1	56.31	2.30
-1	7	-1	53.36	2.60
-1	7	-1	56.64	2.42
-1	7	-1	53.74	2.29
-1	7	-1	53.57	2.13
0	-7	1	225.56	8.82
0	-7	1	235.81	9.29
0	-7	1	234.73	9.00
0	-7	1	242.80	9.11
0	7	-1	230.61	9.11
0	7	-1	232.62	8.99
0	7	-1	242.48	8.84
0	7	-1	249.71	9.35
-1	-7	1	15.53	0.87
-1	-7	1	18.18	1.26
-1	-7	1	14.27	0.66
1	7	-1	14.07	0.98
1	7	-1	15.16	0.68
1	7	-1	17.11	1.21
1	7	-1	14.03	0.82
-2	-7	1	42.83	2.20
-2	-7	1	43.84	1.98
-2	-7	1	42.90	1.84
-2	-7	1	41.84	1.66
2	7	-1	40.40	1.66
2	7	-1	42.12	1.82
2	7	-1	39.58	1.97
2	7	-1	44.58	2.18
-3	-7	1	170.10	6.38
-3	-7	1	175.76	6.55
-3	-7	1	168.10	6.88
-3	-7	1	168.72	6.68
-3	-7	1	159.79	6.36
-3	-7	1	173.50	6.82
3	7	-1	165.59	6.70
3	7	-1	180.02	6.89
3	7	-1	174.83	6.38
3	7	-1	161.22	6.51
-4	-7	1	138.96	5.94
-4	-7	1	158.43	6.43
-4	-7	1	171.43	6.25
-4	-7	1	165.38	6.06

-4	-7	1	157.05	6.36
-4	-7	1	142.05	5.86
4	7	-1	154.75	6.02
4	7	-1	162.44	6.40
-5	-7	1	17.67	1.42
-5	-7	1	16.92	1.32
-5	-7	1	16.80	0.76
-5	-7	1	17.70	0.82
-5	-7	1	16.57	1.10
-5	-7	1	16.11	1.15
-5	-7	1	16.88	0.97
5	7	-1	16.61	0.92
-6	-7	1	140.87	5.73
-6	-7	1	149.88	6.23
-6	-7	1	153.95	5.88
-6	-7	1	149.20	5.72
-6	-7	1	148.96	6.26
-6	-7	1	154.28	5.99
-6	-7	1	161.41	6.10
6	7	-1	144.01	5.81
-7	-7	1	22.01	1.40
-7	-7	1	20.71	1.27
-7	-7	1	19.70	0.99
-7	-7	1	19.43	1.56
-7	-7	1	19.21	1.59
-7	-7	1	20.92	1.12
-7	-7	1	21.56	1.16
-7	-7	1	20.86	0.92
7	7	-1	20.39	0.95
7	7	-1	18.40	1.07
-8	-7	1	36.46	1.82
-8	-7	1	36.94	1.50
-8	-7	1	34.08	2.19
-8	-7	1	34.54	1.72
-8	-7	1	37.88	1.98
-8	-7	1	33.71	1.68
-8	-7	1	34.66	2.18
8	7	-1	35.73	1.53
-9	-7	1	23.86	1.86
-9	-7	1	24.45	1.47
-9	-7	1	25.75	1.12
-9	-7	1	23.10	1.32
-9	-7	1	25.72	1.63
-9	-7	1	24.27	1.90

-9	-7	1	23.99	1.38
9	7	-1	22.91	1.15
-10	-7	1	60.04	2.89
-10	-7	1	57.46	2.65
-10	-7	1	55.04	3.11
-10	-7	1	57.98	3.18
-10	-7	1	58.14	2.63
-10	-7	1	60.03	2.71
-10	-7	1	61.83	2.41
10	7	-1	58.88	2.43
-11	-7	1	52.10	2.76
-11	-7	1	56.48	2.56
-11	-7	1	54.55	2.26
-11	-7	1	49.57	3.10
-11	-7	1	56.39	2.59
-11	-7	1	54.35	2.55
-11	-7	1	52.10	3.03
11	7	-1	56.39	2.31
-12	-7	1	133.42	5.09
-12	-7	1	129.70	5.37
-12	-7	1	129.41	5.96
-12	-7	1	135.41	5.39
-12	-7	1	126.06	5.40
-12	-7	1	124.11	5.55
-12	-7	1	124.98	5.78
12	7	-1	139.69	5.15
-13	-7	1	5.80	1.30
-13	-7	1	4.54	1.05
-13	-7	1	6.18	0.90
-13	-7	1	4.28	0.76
-13	-7	1	4.77	0.70
-13	-7	1	3.36	1.39
13	7	-1	4.74	0.69
-14	-7	1	46.92	2.38
-14	-7	1	40.45	2.86
-14	-7	1	44.66	2.39
-14	-7	1	42.15	3.15
-14	-7	1	38.48	2.60
14	7	-1	43.18	2.01
-15	-7	1	0.13	0.75
-15	-7	1	2.76	1.51
-15	-7	1	0.36	0.88
-15	-7	1	0.33	1.07
-15	-7	1	1.70	1.26

15	7	-1	0.26	0.43
-16	-7	1	68.44	3.36
-16	-7	1	62.40	3.64
-16	-7	1	64.47	3.75
-16	-7	1	64.32	4.19
16	7	-1	67.94	2.94
-17	-7	1	3.87	1.54
-17	-7	1	1.44	1.72
-17	-7	1	1.42	0.97
17	7	-1	1.88	0.65
-18	-7	1	47.85	2.93
-18	-7	1	50.02	4.01
18	7	-1	48.05	2.43
-19	-7	1	-0.39	1.13
19	7	-1	0.71	0.71
-20	-7	1	32.94	2.89
20	7	-1	36.67	2.22
19	-7	2	1.18	0.74
-19	7	-2	1.07	1.42
18	-7	2	22.16	1.54
18	-7	2	20.18	2.24
-18	7	-2	18.67	2.54
17	-7	2	-0.57	1.07
17	-7	2	0.36	0.60
-17	7	-2	1.18	1.24
16	-7	2	11.16	1.73
16	-7	2	12.63	1.14
-16	7	-2	9.76	1.51
-16	7	-2	13.66	2.03
15	-7	2	4.85	0.69
15	-7	2	4.38	1.05
-15	7	-2	2.43	1.49
-15	7	-2	5.08	1.15
14	-7	2	32.35	2.21
14	-7	2	35.23	1.79
-14	7	-2	36.03	2.79
-14	7	-2	38.62	2.40
13	-7	2	0.30	0.73
13	-7	2	0.74	0.75
13	-7	2	0.12	0.43
-13	7	-2	1.45	1.14
12	-7	2	23.25	1.69
12	-7	2	25.80	1.52
12	-7	2	23.32	1.27

-12	7	-2	21.98	1.64
-12	7	-2	24.91	1.73
-12	7	-2	24.84	2.21
11	-7	2	7.42	0.97
11	-7	2	7.07	0.68
11	-7	2	6.20	1.14
-11	7	-2	7.14	1.20
-11	7	-2	8.65	1.13
-11	7	-2	7.73	0.98
-11	7	-2	8.14	0.73
10	-7	2	49.99	2.17
10	-7	2	53.99	2.58
10	-7	2	53.10	2.31
10	-7	2	49.81	2.40
-10	7	-2	48.36	2.30
-10	7	-2	55.48	2.22
-10	7	-2	46.27	2.78
-10	7	-2	49.04	2.48
9	-7	2	1.55	0.33
9	-7	2	2.02	0.53
9	-7	2	2.34	0.53
9	-7	2	2.06	0.62
-9	7	-2	1.84	0.84
-9	7	-2	1.93	0.34
-9	7	-2	1.87	0.57
-9	7	-2	2.52	0.57
8	-7	2	16.08	1.29
8	-7	2	18.34	1.08
8	-7	2	15.76	0.89
8	-7	2	15.15	1.09
-8	7	-2	16.77	1.11
-8	7	-2	18.40	0.92
-8	7	-2	17.11	1.55
-8	7	-2	16.77	1.16
7	-7	2	16.51	1.05
7	-7	2	14.93	0.80
7	-7	2	15.96	1.23
7	-7	2	15.69	1.00
-7	7	-2	16.87	0.84
-7	7	-2	14.65	1.04
-7	7	-2	14.99	1.08
-7	7	-2	15.31	1.44
6	-7	2	70.93	3.21
6	-7	2	75.62	2.99

6	-7	2	71.31	2.84
6	-7	2	69.79	3.01
-6	7	-2	74.66	2.85
-6	7	-2	71.54	3.02
-6	7	-2	70.17	3.05
-6	7	-2	68.94	3.35
5	-7	2	14.14	0.72
5	-7	2	13.94	0.91
5	-7	2	14.95	1.13
5	-7	2	15.18	0.94
-5	7	-2	15.50	1.31
-5	7	-2	14.41	0.96
-5	7	-2	15.61	0.98
-5	7	-2	14.83	0.74
4	-7	2	111.47	4.31
4	-7	2	117.10	4.33
4	-7	2	109.66	4.53
4	-7	2	104.31	4.12
-4	7	-2	109.03	4.13
-4	7	-2	101.23	4.33
-4	7	-2	105.87	4.61
-4	7	-2	104.47	4.32
3	-7	2	53.82	2.27
3	-7	2	50.57	2.06
3	-7	2	54.51	2.48
3	-7	2	53.01	2.29
-3	7	-2	51.15	2.30
-3	7	-2	53.61	2.28
-3	7	-2	52.48	2.58
-3	7	-2	51.71	2.07
2	-7	2	6.21	0.80
2	-7	2	5.58	0.57
2	-7	2	5.59	0.35
-2	7	-2	5.50	0.35
-2	7	-2	7.46	0.90
-2	7	-2	6.34	0.56
-2	7	-2	4.59	0.61
1	-7	2	31.45	1.70
1	-7	2	31.07	1.55
1	-7	2	28.74	1.43
1	-7	2	30.29	1.24
-1	7	-2	32.66	1.25
-1	7	-2	33.04	1.78
-1	7	-2	30.52	1.55

-1	7	-2	30.53	1.44
0	-7	2	44.04	1.90
0	-7	2	43.34	2.17
0	-7	2	42.99	1.98
0	-7	2	41.41	1.68
0	7	-2	46.47	2.22
0	7	-2	43.99	2.03
0	7	-2	40.97	1.87
0	7	-2	44.51	1.69
-1	-7	2	73.65	2.79
-1	-7	2	73.65	3.27
-1	-7	2	70.22	3.10
-1	-7	2	74.13	3.00
-1	-7	2	74.13	3.25
1	7	-2	70.24	3.13
1	7	-2	72.62	2.98
1	7	-2	80.01	3.34
1	7	-2	73.77	2.80
-2	-7	2	244.07	9.62
-2	-7	2	235.21	9.13
-2	-7	2	255.79	9.35
-2	-7	2	249.47	9.64
2	7	-2	257.30	9.65
2	7	-2	238.25	9.14
2	7	-2	238.86	9.32
-3	-7	2	45.57	2.08
-3	-7	2	43.03	1.68
-3	-7	2	43.62	1.90
-3	-7	2	42.22	2.19
-3	-7	2	38.21	1.66
-3	-7	2	44.44	2.24
3	7	-2	46.82	2.22
3	7	-2	41.89	1.86
-4	-7	2	41.81	1.65
-4	-7	2	42.48	2.04
-4	-7	2	41.34	2.19
-4	-7	2	42.76	1.87
-4	-7	2	40.22	1.63
-4	-7	2	43.07	2.21
4	7	-2	42.46	2.17
4	7	-2	40.13	1.82
-5	-7	2	40.82	2.21
-5	-7	2	39.69	1.58
-5	-7	2	41.47	2.02

-5	-7	2	38.53	2.15
-5	-7	2	41.59	1.82
5	7	-2	40.29	2.11
5	7	-2	37.22	1.75
-6	-7	2	29.15	1.44
-6	-7	2	28.09	1.18
-6	-7	2	28.90	1.86
-6	-7	2	30.54	1.69
-6	-7	2	27.64	1.24
-6	-7	2	26.98	1.79
-6	-7	2	28.66	1.52
6	7	-2	29.02	1.38
-7	-7	2	47.68	2.36
-7	-7	2	46.44	2.50
-7	-7	2	50.51	2.14
-7	-7	2	49.52	2.20
-7	-7	2	47.77	2.58
-7	-7	2	43.11	1.93
7	7	-2	47.08	1.90
7	7	-2	45.53	2.06
-8	-7	2	49.69	2.51
-8	-7	2	53.75	2.04
-8	-7	2	49.15	2.31
-8	-7	2	49.05	2.28
-8	-7	2	49.81	2.66
-8	-7	2	53.05	2.79
-8	-7	2	50.60	2.26
8	7	-2	48.64	2.05
-9	-7	2	59.09	2.98
-9	-7	2	63.04	2.77
-9	-7	2	61.10	2.72
-9	-7	2	65.45	2.51
-9	-7	2	62.49	2.76
-9	-7	2	64.20	3.29
-9	-7	2	59.97	3.11
9	7	-2	64.18	2.53
-10	-7	2	20.89	1.32
-10	-7	2	21.60	1.86
-10	-7	2	22.83	1.38
-10	-7	2	23.02	1.06
-10	-7	2	21.03	1.38
-10	-7	2	21.94	1.66
-10	-7	2	19.99	2.01
10	7	-2	22.54	1.08

-11	-7	2	20.39	1.36
-11	-7	2	20.06	1.40
-11	-7	2	17.03	1.66
-11	-7	2	18.94	1.82
-11	-7	2	18.51	1.29
-11	-7	2	20.09	1.01
-11	-7	2	18.83	2.12
11	7	-2	18.84	1.02
-12	-7	2	20.60	2.26
-12	-7	2	19.51	1.79
-12	-7	2	23.11	1.49
-12	-7	2	18.90	1.94
-12	-7	2	23.08	1.13
-12	-7	2	21.85	1.47
12	7	-2	20.50	1.12
-13	-7	2	67.30	2.68
-13	-7	2	63.41	2.93
-13	-7	2	58.14	3.28
-13	-7	2	61.99	2.99
-13	-7	2	68.71	3.84
-13	-7	2	57.72	3.34
13	7	-2	66.90	2.71
-14	-7	2	38.92	2.51
-14	-7	2	34.72	2.64
-14	-7	2	39.50	2.01
-14	-7	2	37.88	2.13
-14	-7	2	41.19	3.12
14	7	-2	37.11	1.81
-15	-7	2	52.91	3.23
-15	-7	2	55.31	2.81
-15	-7	2	56.33	3.83
-15	-7	2	56.48	3.28
-15	-7	2	55.79	2.91
15	7	-2	58.47	2.51
-16	-7	2	11.01	1.70
-16	-7	2	12.50	2.14
-16	-7	2	13.21	1.71
-16	-7	2	13.08	2.09
16	7	-2	13.26	1.09
-17	-7	2	36.04	2.42
-17	-7	2	31.81	3.65
-17	-7	2	38.12	2.80
17	7	-2	33.34	1.85
-18	-7	2	17.42	3.22

-18	-7	2	6.57	1.27
18	7	-2	8.20	1.20
-19	-7	2	21.58	2.31
-19	-7	2	28.89	3.82
19	7	-2	22.22	1.70
-20	-7	2	3.55	1.33
20	7	-2	9.58	1.44
19	-7	3	4.30	0.84
-19	7	-3	10.47	1.84
18	-7	3	6.39	1.39
18	-7	3	4.16	0.80
-18	7	-3	3.05	1.46
17	-7	3	0.90	0.59
17	-7	3	1.61	1.16
-17	7	-3	1.54	1.25
16	-7	3	10.60	1.06
16	-7	3	10.10	1.86
-16	7	-3	10.62	1.55
15	-7	3	10.39	1.03
15	-7	3	11.49	1.69
-15	7	-3	15.51	2.55
-15	7	-3	10.97	1.76
14	-7	3	8.62	0.93
14	-7	3	9.34	1.47
-14	7	-3	12.03	1.80
-14	7	-3	9.30	1.27
13	-7	3	28.30	2.00
-13	7	-3	27.29	2.46
12	-7	3	23.09	1.77
12	-7	3	24.61	1.31
12	-7	3	24.64	1.42
-12	7	-3	24.12	1.74
-12	7	-3	24.78	2.22
11	-7	3	19.31	1.58
11	-7	3	20.96	1.22
11	-7	3	19.27	1.11
-11	7	-3	20.84	1.55
-11	7	-3	18.08	1.97
-11	7	-3	21.10	1.17
-11	7	-3	18.44	1.16
10	-7	3	17.02	0.98
10	-7	3	18.44	1.48
10	-7	3	17.77	1.10
10	-7	3	16.79	1.28

-10	7	-3	15.69	1.18
-10	7	-3	19.12	1.02
-10	7	-3	16.58	1.80
-10	7	-3	14.64	1.31
9	-7	3	23.32	1.62
9	-7	3	24.56	1.27
9	-7	3	22.27	1.39
9	-7	3	22.68	1.17
-9	7	-3	21.57	1.87
-9	7	-3	23.54	1.49
-9	7	-3	22.60	1.70
-9	7	-3	26.98	1.21
8	-7	3	17.19	1.31
8	-7	3	17.28	1.01
8	-7	3	16.21	0.89
8	-7	3	15.13	1.09
-8	7	-3	15.41	1.57
-8	7	-3	17.34	0.91
-8	7	-3	16.68	1.10
-8	7	-3	16.34	1.17
7	-7	3	12.54	1.17
7	-7	3	11.41	0.70
7	-7	3	13.56	0.98
7	-7	3	13.92	0.89
-7	7	-3	13.04	0.99
-7	7	-3	12.62	0.99
-7	7	-3	14.20	0.74
-7	7	-3	11.37	1.32
6	-7	3	2.87	0.64
6	-7	3	2.93	0.38
6	-7	3	3.06	0.46
6	-7	3	2.64	0.47
-6	7	-3	3.77	0.39
-6	7	-3	2.89	0.51
-6	7	-3	2.89	0.56
-6	7	-3	5.64	1.04
5	-7	3	9.20	0.55
5	-7	3	9.62	0.79
5	-7	3	8.87	0.97
5	-7	3	10.40	0.75
-5	7	-3	9.55	0.84
-5	7	-3	9.40	0.79
-5	7	-3	11.09	1.18
-5	7	-3	9.93	0.56

4	-7	3	18.57	0.89
4	-7	3	21.15	1.37
4	-7	3	20.94	1.11
4	-7	3	19.96	1.11
-4	7	-3	18.86	0.94
-4	7	-3	20.92	1.17
-4	7	-3	19.70	1.47
-4	7	-3	19.70	1.13
3	-7	3	180.40	6.84
3	-7	3	171.48	6.81
3	-7	3	170.31	6.61
3	-7	3	175.55	7.04
-3	7	-3	178.62	6.88
-3	7	-3	180.92	7.14
-3	7	-3	175.15	6.84
-3	7	-3	175.91	6.61
2	-7	3	6.57	0.63
2	-7	3	7.55	0.85
-2	7	-3	6.41	0.60
-2	7	-3	7.35	0.98
-2	7	-3	6.50	0.37
-2	7	-3	7.34	0.73
1	-7	3	74.16	3.06
1	-7	3	75.25	3.28
1	-7	3	77.17	3.30
1	-7	3	79.07	3.14
1	-7	3	71.30	2.82
-1	7	-3	74.33	3.35
-1	7	-3	70.65	3.04
-1	7	-3	73.92	2.83
0	-7	3	10.31	0.84
0	-7	3	10.42	0.47
0	-7	3	11.66	1.01
0	-7	3	10.36	0.99
0	-7	3	10.89	0.74
0	7	-3	11.44	1.08
0	7	-3	9.96	0.70
0	7	-3	11.24	0.49
-1	-7	3	81.24	3.54
-1	-7	3	81.82	3.18
-1	-7	3	87.90	3.73
-1	-7	3	86.81	3.44
-1	-7	3	89.03	3.70
1	7	-3	83.13	3.19

1	7	-3	88.90	3.74
1	7	-3	80.73	3.40
-2	-7	3	0.05	0.37
-2	-7	3	0.15	0.08
-2	-7	3	0.17	0.46
-2	-7	3	0.26	0.43
-2	-7	3	-0.07	0.25
2	7	-3	0.09	0.24
2	7	-3	0.10	0.48
2	7	-3	0.03	0.08
-3	-7	3	140.16	5.83
-3	-7	3	137.83	5.73
-3	-7	3	128.09	5.22
-3	-7	3	138.14	5.23
-3	-7	3	154.79	5.70
-3	-7	3	145.39	5.50
3	7	-3	142.66	5.76
3	7	-3	134.87	5.45
-4	-7	3	20.32	1.42
-4	-7	3	18.10	1.46
-4	-7	3	19.30	0.81
-4	-7	3	20.45	1.07
-4	-7	3	19.45	1.30
-4	-7	3	19.17	0.79
4	7	-3	21.08	1.38
4	7	-3	18.07	1.02
-5	-7	3	1.56	0.20
-5	-7	3	1.23	0.49
-5	-7	3	1.55	0.38
-5	-7	3	1.56	0.24
-5	-7	3	0.89	0.63
-5	-7	3	1.15	0.58
5	7	-3	0.98	0.57
5	7	-3	1.29	0.30
-6	-7	3	13.35	0.61
-6	-7	3	13.78	1.28
-6	-7	3	11.42	1.32
-6	-7	3	11.99	0.88
-6	-7	3	13.45	1.16
-6	-7	3	13.23	0.68
6	7	-3	13.26	0.64
6	7	-3	12.79	0.83
6	7	-3	13.13	1.19
-7	-7	3	5.45	0.88

-7	-7	3	2.91	0.79
-7	-7	3	5.34	0.83
-7	-7	3	4.52	0.35
-7	-7	3	4.80	0.63
-7	-7	3	4.00	0.67
7	7	-3	3.77	0.35
7	7	-3	4.50	0.57
-8	-7	3	9.39	1.14
-8	-7	3	7.39	0.74
-8	-7	3	8.40	0.50
-8	-7	3	6.33	0.78
-8	-7	3	8.09	1.22
-8	-7	3	7.12	0.84
-8	-7	3	8.15	1.35
8	7	-3	7.92	0.51
-9	-7	3	58.18	2.63
-9	-7	3	55.72	2.96
-9	-7	3	59.34	2.96
-9	-7	3	63.43	2.71
-9	-7	3	60.23	3.31
-9	-7	3	59.91	2.39
-9	-7	3	60.40	2.65
9	7	-3	61.69	2.42
-10	-7	3	5.04	1.23
-10	-7	3	5.93	0.51
-10	-7	3	7.10	1.31
-10	-7	3	5.39	0.88
-10	-7	3	5.69	0.80
-10	-7	3	7.14	0.88
-10	-7	3	6.33	0.97
10	7	-3	5.94	0.53
-11	-7	3	49.59	1.99
-11	-7	3	44.03	2.25
-11	-7	3	44.84	2.57
-11	-7	3	48.85	2.24
-11	-7	3	46.70	2.64
-11	-7	3	43.29	3.00
-11	-7	3	47.29	2.32
11	7	-3	48.10	2.00
-12	-7	3	14.35	0.89
-12	-7	3	14.38	1.23
-12	-7	3	14.67	1.66
-12	-7	3	14.94	1.17
-12	-7	3	12.26	1.59

-12	-7	3	13.36	2.18
12	7	-3	14.59	0.89
-13	-7	3	4.55	1.10
-13	-7	3	5.38	0.66
-13	-7	3	3.67	0.70
-13	-7	3	3.68	1.04
-13	-7	3	3.26	1.45
-13	-7	3	3.52	0.72
13	7	-3	3.48	0.51
-14	-7	3	18.20	1.93
-14	-7	3	20.33	1.54
-14	-7	3	17.10	1.41
-14	-7	3	18.41	2.06
-14	-7	3	16.77	1.09
-14	-7	3	17.33	2.63
14	7	-3	15.85	1.08
-15	-7	3	35.16	2.04
-15	-7	3	35.40	2.49
-15	-7	3	34.87	3.32
-15	-7	3	36.45	2.26
-15	-7	3	42.28	3.36
15	7	-3	37.71	1.82
-16	-7	3	5.79	1.11
-16	-7	3	8.05	1.97
-16	-7	3	8.13	1.36
16	7	-3	6.30	0.97
-17	-7	3	13.84	2.34
-17	-7	3	17.32	1.85
17	7	-3	13.68	1.22
-18	-7	3	19.85	2.13
-18	-7	3	23.76	3.53
18	7	-3	22.04	1.54
-19	-7	3	1.26	1.13
19	7	-3	0.47	0.72
-20	-7	3	4.09	1.33
20	7	-3	2.35	0.84
19	-7	4	29.75	1.93
19	-7	4	23.08	2.79
-19	7	-4	35.18	3.18
18	-7	4	13.20	2.30
18	-7	4	12.56	1.37
-18	7	-4	11.71	1.77
17	-7	4	30.77	1.86
17	-7	4	32.18	2.58

-17	7	-4	34.25	2.80
16	-7	4	11.27	1.18
16	-7	4	11.20	1.90
-16	7	-4	9.82	1.47
15	-7	4	24.93	1.54
15	-7	4	29.70	2.26
-15	7	-4	27.57	2.26
14	-7	4	14.01	1.06
14	-7	4	12.12	1.68
-14	7	-4	12.93	1.69
-14	7	-4	11.90	2.61
13	-7	4	30.81	2.15
13	-7	4	27.88	1.70
-13	7	-4	32.55	2.29
-13	7	-4	30.76	2.72
12	-7	4	16.75	1.06
12	-7	4	16.39	1.65
-12	7	-4	13.21	2.05
-12	7	-4	19.03	1.55
11	-7	4	32.15	2.01
11	-7	4	30.82	1.47
11	-7	4	31.11	1.52
-11	7	-4	30.16	1.91
-11	7	-4	30.53	2.37
10	-7	4	20.28	1.15
10	-7	4	20.08	1.61
10	-7	4	20.68	1.26
10	-7	4	19.83	1.12
10	-7	4	18.30	1.34
-10	7	-4	19.55	1.88
-10	7	-4	24.13	1.16
-10	7	-4	20.26	1.45
9	-7	4	42.89	1.83
9	-7	4	39.42	1.99
9	-7	4	40.06	2.26
9	-7	4	39.30	1.79
9	-7	4	39.03	1.97
-9	7	-4	45.47	1.82
-9	7	-4	37.63	2.08
-9	7	-4	40.22	2.43
8	-7	4	2.12	0.52
8	-7	4	2.97	0.46
8	-7	4	3.31	0.79
8	-7	4	3.40	0.47

-8	7	-4	2.52	0.54
-8	7	-4	2.63	0.36
-8	7	-4	2.55	0.85
7	-7	4	63.09	3.01
7	-7	4	63.89	2.79
7	-7	4	66.20	2.88
7	-7	4	62.02	2.59
7	-7	4	66.37	2.67
-7	7	-4	68.43	2.60
-7	7	-4	61.28	2.83
-7	7	-4	59.35	3.17
6	-7	4	56.35	2.74
6	-7	4	58.88	2.51
6	-7	4	57.28	2.41
6	-7	4	54.91	2.30
6	-7	4	57.68	2.63
-6	7	-4	54.82	2.86
-6	7	-4	54.65	2.54
-6	7	-4	58.77	2.30
5	-7	4	18.65	0.90
5	-7	4	21.04	1.15
5	-7	4	18.45	1.30
5	-7	4	17.84	1.35
5	-7	4	20.68	1.08
-5	7	-4	17.96	1.52
-5	7	-4	18.28	1.14
-5	7	-4	20.23	0.91
4	-7	4	61.41	2.75
4	-7	4	60.22	2.56
4	-7	4	58.27	2.82
4	-7	4	64.63	2.61
4	-7	4	58.94	2.37
-4	7	-4	59.06	2.60
-4	7	-4	57.08	2.42
-4	7	-4	63.18	2.95
3	-7	4	14.60	1.17
3	-7	4	15.22	0.71
3	-7	4	15.83	1.18
3	-7	4	13.13	1.06
3	-7	4	16.55	0.97
-3	7	-4	15.71	1.28
-3	7	-4	15.28	0.70
-3	7	-4	14.98	0.96
2	-7	4	8.67	0.79

2	-7	4	8.60	0.96
2	-7	4	8.44	0.44
2	-7	4	8.53	0.91
2	-7	4	9.07	0.72
-2	7	-4	8.65	0.43
-2	7	-4	7.67	0.69
-2	7	-4	9.47	1.07
1	-7	4	8.19	0.69
1	-7	4	7.71	0.40
1	-7	4	9.45	1.00
1	-7	4	8.78	0.78
1	-7	4	7.77	0.93
-1	7	-4	9.40	0.70
-1	7	-4	8.40	0.41
-1	7	-4	9.91	1.05
0	-7	4	26.03	1.40
0	-7	4	25.58	1.29
0	-7	4	26.29	1.52
0	-7	4	24.92	1.01
0	-7	4	26.01	1.60
0	7	-4	26.12	1.61
0	7	-4	24.37	1.26
0	7	-4	25.99	1.01
-1	-7	4	5.16	0.87
-1	-7	4	5.47	0.81
-1	-7	4	6.17	0.28
-1	-7	4	6.89	0.62
-1	-7	4	7.07	0.79
1	7	-4	6.06	0.29
1	7	-4	5.95	0.56
1	7	-4	5.80	0.92
-2	-7	4	18.99	0.76
-2	-7	4	20.50	1.35
-2	-7	4	20.06	1.26
-2	-7	4	20.67	1.10
-2	-7	4	19.89	1.47
2	7	-4	21.74	1.41
2	7	-4	18.25	1.03
-3	-7	4	7.02	0.94
-3	-7	4	6.53	0.28
-3	-7	4	6.00	0.28
-3	-7	4	6.15	0.62
-3	-7	4	6.60	0.84
-3	-7	4	6.60	1.03

3	7	-4	5.82	0.57
3	7	-4	7.50	0.93
-4	-7	4	5.14	0.26
-4	-7	4	5.09	0.92
-4	-7	4	4.30	0.82
-4	-7	4	3.56	0.58
-4	-7	4	4.60	0.86
-4	-7	4	4.81	0.27
4	7	-4	7.21	0.93
4	7	-4	4.99	0.94
4	7	-4	5.06	0.54
-5	-7	4	28.68	1.91
-5	-7	4	29.92	1.73
-5	-7	4	28.58	1.72
-5	-7	4	30.01	1.48
-5	-7	4	29.31	1.15
-5	-7	4	26.91	1.17
5	7	-4	32.79	1.88
5	7	-4	30.91	1.73
5	7	-4	27.68	1.39
-6	-7	4	23.02	0.99
-6	-7	4	23.07	0.96
-6	-7	4	22.74	1.55
-6	-7	4	23.03	1.27
-6	-7	4	24.22	1.78
-6	-7	4	23.10	1.56
6	7	-4	23.20	1.21
6	7	-4	22.90	0.97
6	7	-4	21.19	1.66
6	7	-4	24.80	1.55
-7	-7	4	9.46	1.39
-7	-7	4	10.57	1.20
-7	-7	4	10.96	0.88
-7	-7	4	10.80	0.54
7	7	-4	9.68	1.15
7	7	-4	9.62	1.36
7	7	-4	11.07	0.57
7	7	-4	10.41	0.79
-8	-7	4	37.86	2.44
-8	-7	4	37.25	1.79
-8	-7	4	37.82	1.82
-8	-7	4	35.91	1.82
-8	-7	4	34.10	2.07
8	7	-4	38.67	1.52

8	7	-4	34.94	2.27
8	7	-4	35.16	1.73
-9	-7	4	14.81	1.08
-9	-7	4	13.39	1.39
-9	-7	4	15.23	1.11
-9	-7	4	13.42	1.03
-9	-7	4	15.30	1.85
-9	-7	4	15.75	0.76
9	7	-4	14.75	1.61
9	7	-4	14.68	0.76
-10	-7	4	78.01	2.90
-10	-7	4	72.22	3.89
-10	-7	4	70.71	3.22
-10	-7	4	66.70	3.37
-10	-7	4	72.80	3.53
-10	-7	4	74.52	3.13
-10	-7	4	70.03	3.16
10	7	-4	74.33	2.91
10	7	-4	73.67	3.69
-11	-7	4	7.13	1.06
-11	-7	4	8.16	0.61
-11	-7	4	5.82	0.97
-11	-7	4	7.69	1.13
-11	-7	4	7.08	1.45
-11	-7	4	6.45	0.92
-11	-7	4	7.47	0.95
11	7	-4	7.25	0.62
11	7	-4	11.77	1.76
-12	-7	4	17.45	1.35
-12	-7	4	15.70	1.62
-12	-7	4	17.61	0.96
-12	-7	4	14.95	1.84
-12	-7	4	13.21	2.27
-12	-7	4	17.28	1.23
12	7	-4	17.57	0.98
-13	-7	4	2.28	1.48
-13	-7	4	2.57	0.99
-13	-7	4	3.83	0.75
-13	-7	4	4.34	0.67
-13	-7	4	3.77	1.13
-13	-7	4	3.72	0.48
13	7	-4	3.17	0.48
-14	-7	4	13.31	2.07
-14	-7	4	11.72	1.21

-14	-7	4	14.18	1.40
-14	-7	4	13.71	0.98
-14	-7	4	11.79	1.67
14	7	-4	14.07	0.97
-15	-7	4	4.59	1.73
-15	-7	4	1.07	0.88
-15	-7	4	1.89	0.80
-15	-7	4	1.21	1.02
15	7	-4	1.96	0.50
-16	-7	4	15.98	2.98
-16	-7	4	16.31	1.77
-16	-7	4	16.65	1.77
16	7	-4	16.97	1.20
-17	-7	4	1.53	1.90
-17	-7	4	0.64	0.96
17	7	-4	1.71	0.57
-18	-7	4	7.26	2.35
-18	-7	4	11.43	1.89
18	7	-4	8.21	1.16
-19	-7	4	-0.45	2.24
-19	-7	4	0.19	1.12
19	7	-4	1.40	0.72
-20	-7	4	-1.05	1.18
20	7	-4	2.17	0.83
19	-7	5	1.67	0.81
19	-7	5	-0.27	1.53
-19	7	-5	4.24	1.62
18	-7	5	22.44	2.81
18	-7	5	31.68	1.91
-18	7	-5	30.15	2.82
17	-7	5	0.14	0.63
17	-7	5	0.62	1.32
-17	7	-5	0.27	1.21
16	-7	5	38.54	2.02
-16	7	-5	35.48	2.73
15	-7	5	1.08	1.06
15	-7	5	1.08	0.56
-15	7	-5	0.26	0.99
14	-7	5	29.23	2.28
14	-7	5	31.83	1.69
-14	7	-5	31.58	2.27
13	-7	5	-0.12	0.41
13	-7	5	-0.21	0.83
-13	7	-5	0.41	0.73

12	-7	5	108.92	4.89
12	-7	5	109.57	4.40
-12	7	-5	106.56	4.77
-12	7	-5	108.46	5.18
11	-7	5	40.32	2.49
11	-7	5	45.35	2.00
-11	7	-5	40.73	2.87
-11	7	-5	47.35	2.43
10	-7	5	76.25	3.70
10	-7	5	78.08	3.21
10	-7	5	73.76	3.39
10	-7	5	81.43	3.12
-10	7	-5	73.34	3.89
-10	7	-5	84.01	3.23
-10	7	-5	78.41	3.54
9	-7	5	24.33	1.19
9	-7	5	22.84	1.19
9	-7	5	22.53	1.38
9	-7	5	20.51	1.39
9	-7	5	22.68	1.72
-9	7	-5	25.62	1.21
-9	7	-5	21.68	1.97
-9	7	-5	23.77	1.54
8	-7	5	42.63	1.84
8	-7	5	42.30	2.35
8	-7	5	36.39	2.55
8	-7	5	44.28	1.86
8	-7	5	42.71	2.06
-8	7	-5	40.23	2.10
-8	7	-5	45.13	1.84
-8	7	-5	40.11	2.51
7	-7	5	13.23	0.86
7	-7	5	13.96	1.02
7	-7	5	12.81	1.21
7	-7	5	13.43	1.28
7	-7	5	12.40	0.77
-7	7	-5	14.84	0.78
-7	7	-5	11.98	1.06
-7	7	-5	12.44	1.50
6	-7	5	69.39	2.91
6	-7	5	74.30	3.27
6	-7	5	70.47	3.36
6	-7	5	73.68	3.02
6	-7	5	75.95	3.14

-6	7	-5	73.14	3.17
-6	7	-5	70.67	3.50
-6	7	-5	79.00	2.92
5	-7	5	43.73	2.18
5	-7	5	43.46	1.79
5	-7	5	44.66	1.95
5	-7	5	42.50	2.26
5	-7	5	44.29	2.02
-5	7	-5	45.63	1.79
-5	7	-5	40.44	2.02
-5	7	-5	41.80	2.37
4	-7	5	69.24	2.75
4	-7	5	71.37	3.16
4	-7	5	67.82	3.20
4	-7	5	73.06	2.98
4	-7	5	67.67	2.97
-4	7	-5	71.28	3.33
-4	7	-5	71.98	2.75
-4	7	-5	69.10	2.99
3	-7	5	29.39	1.55
3	-7	5	30.22	1.26
3	-7	5	31.00	1.51
3	-7	5	29.21	1.73
3	-7	5	31.08	1.77
-3	7	-5	30.06	1.85
-3	7	-5	32.01	1.25
-3	7	-5	28.92	1.50
2	-7	5	14.11	1.23
2	-7	5	12.58	0.60
2	-7	5	13.51	0.95
2	-7	5	13.48	1.13
2	-7	5	13.63	0.91
-2	7	-5	11.77	1.20
-2	7	-5	12.13	0.85
-2	7	-5	13.19	0.59
1	-7	5	5.70	0.74
1	-7	5	7.93	0.69
1	-7	5	5.95	0.90
1	-7	5	6.32	1.03
1	-7	5	6.89	0.34
-1	7	-5	5.80	0.61
-1	7	-5	6.32	0.33
-1	7	-5	6.31	1.01
0	-7	5	115.72	4.79

0	-7	5	113.37	4.89
0	-7	5	112.64	4.67
0	-7	5	107.92	4.26
0	-7	5	119.78	4.57
0	7	-5	118.64	4.85
0	7	-5	112.04	4.53
0	7	-5	113.27	4.26
-1	-7	5	79.77	3.74
-1	-7	5	82.50	3.61
-1	-7	5	83.34	3.38
-1	-7	5	80.05	3.53
1	7	-5	84.28	3.64
1	7	-5	81.79	3.35
-2	-7	5	201.65	7.52
-2	-7	5	189.88	7.70
-2	-7	5	198.35	7.70
-2	-7	5	184.31	7.88
2	7	-5	185.86	7.47
2	7	-5	195.03	7.79
2	7	-5	202.30	7.77
-3	-7	5	10.41	1.01
-3	-7	5	8.43	1.17
-3	-7	5	9.06	0.74
3	7	-5	8.45	1.11
3	7	-5	8.56	0.68
3	7	-5	7.90	1.03
-4	-7	5	29.83	1.48
-4	-7	5	28.36	1.67
-4	-7	5	28.76	1.94
4	7	-5	29.16	1.42
4	7	-5	29.80	1.88
4	7	-5	29.95	1.72
-5	-7	5	36.89	2.26
-5	-7	5	35.36	1.40
-5	-7	5	36.35	1.39
-5	-7	5	37.97	1.76
-5	-7	5	35.06	1.94
5	7	-5	36.44	1.98
5	7	-5	34.25	2.15
-6	-7	5	83.69	4.03
-6	-7	5	82.03	3.44
-6	-7	5	84.10	3.11
-6	-7	5	80.45	3.63
6	7	-5	79.38	3.37

6	7	-5	81.37	3.12
6	7	-5	84.05	3.65
6	7	-5	81.33	3.87
-7	-7	5	23.85	2.07
-7	-7	5	27.29	1.46
-7	-7	5	29.31	1.13
-7	-7	5	26.29	1.66
7	7	-5	25.13	1.95
7	7	-5	28.30	1.72
7	7	-5	26.69	1.39
-8	-7	5	50.38	3.04
-8	-7	5	49.90	2.39
-8	-7	5	51.33	2.61
-8	-7	5	52.98	2.43
-8	-7	5	55.37	2.12
-8	-7	5	56.45	2.47
8	7	-5	53.36	2.38
8	7	-5	53.55	2.65
8	7	-5	54.83	2.12
8	7	-5	53.91	2.95
-9	-7	5	41.68	2.30
-9	-7	5	47.43	1.81
-9	-7	5	42.11	2.13
-9	-7	5	45.44	2.09
-9	-7	5	41.52	2.82
-9	-7	5	43.59	2.09
9	7	-5	42.94	1.80
9	7	-5	45.27	2.70
-10	-7	5	14.02	1.07
-10	-7	5	14.22	0.76
-10	-7	5	14.33	2.03
-10	-7	5	14.53	1.39
-10	-7	5	14.12	1.10
-10	-7	5	14.08	1.19
10	7	-5	10.80	1.83
10	7	-5	14.21	0.77
-11	-7	5	2.02	1.23
-11	-7	5	0.38	0.72
-11	-7	5	1.15	0.49
-11	-7	5	1.98	0.85
-11	-7	5	0.95	0.30
11	7	-5	2.04	1.13
11	7	-5	1.27	0.30
-12	-7	5	28.74	2.81

-12	-7	5	32.64	1.91
-12	-7	5	29.97	1.41
-12	-7	5	29.86	1.79
12	7	-5	31.01	1.42
12	7	-5	26.17	2.62
-13	-7	5	12.77	0.85
-13	-7	5	10.78	1.21
-13	-7	5	11.68	1.41
-13	-7	5	14.83	2.55
13	7	-5	10.53	0.81
13	7	-5	10.44	2.33
-14	-7	5	26.78	3.02
-14	-7	5	26.53	1.83
-14	-7	5	23.91	1.67
-14	-7	5	25.23	1.49
-14	-7	5	24.70	1.78
14	7	-5	26.85	1.35
-15	-7	5	45.28	3.79
-15	-7	5	48.78	2.72
-15	-7	5	49.31	2.56
15	7	-5	52.92	2.26
-16	-7	5	5.60	1.07
-16	-7	5	6.14	2.07
16	7	-5	6.09	0.87
-17	-7	5	12.55	1.87
-17	-7	5	16.70	2.58
17	7	-5	13.89	1.19
-18	-7	5	11.78	1.91
-18	-7	5	11.07	2.58
18	7	-5	10.10	1.22
-19	-7	5	1.82	1.23
-19	-7	5	1.53	2.33
19	7	-5	2.83	0.77
-20	-7	5	6.11	2.99
-20	-7	5	1.78	1.37
20	7	-5	5.86	0.94
18	-7	6	3.20	0.85
18	-7	6	4.85	1.66
-18	7	-6	6.39	1.67
17	-7	6	20.78	2.57
17	-7	6	18.03	1.54
-17	7	-6	17.56	2.51
16	-7	6	3.40	1.30
16	-7	6	3.75	0.78

-16	7	-6	1.78	1.18
15	-7	6	57.25	2.70
15	-7	6	58.77	3.35
-15	7	-6	55.87	3.32
14	-7	6	1.52	1.06
14	-7	6	1.05	0.57
-14	7	-6	1.15	0.97
13	-7	6	45.16	2.87
13	-7	6	49.13	2.28
-13	7	-6	49.80	2.76
12	-7	6	2.73	0.51
12	-7	6	3.21	1.01
-12	7	-6	4.31	0.87
11	-7	6	31.41	1.56
11	-7	6	30.81	2.17
-11	7	-6	30.54	1.95
-11	7	-6	26.44	2.67
10	-7	6	-0.51	0.72
10	-7	6	-0.19	0.58
10	-7	6	0.94	0.37
-10	7	-6	0.58	0.60
-10	7	-6	0.35	0.35
-10	7	-6	1.95	1.07
9	-7	6	131.83	5.60
9	-7	6	128.68	5.00
9	-7	6	126.12	5.07
9	-7	6	131.88	5.16
9	-7	6	127.82	5.28
-9	7	-6	138.41	5.08
-9	7	-6	129.17	5.37
-9	7	-6	120.09	5.69
8	-7	6	-0.18	0.34
8	-7	6	-0.82	0.68
8	-7	6	-0.31	0.66
8	-7	6	0.42	0.30
8	-7	6	0.73	0.50
-8	7	-6	0.78	0.84
-8	7	-6	0.21	0.27
-8	7	-6	0.33	0.52
7	-7	6	52.74	2.79
7	-7	6	56.06	2.35
7	-7	6	56.15	2.52
7	-7	6	53.67	2.29
7	-7	6	57.05	2.67

-7	7	-6	60.23	2.30
-7	7	-6	51.72	2.54
-7	7	-6	54.13	2.98
6	-7	6	0.70	0.25
6	-7	6	-0.12	0.61
6	-7	6	0.78	0.44
6	-7	6	0.64	0.59
6	-7	6	0.54	0.38
-6	7	-6	0.76	0.24
-6	7	-6	0.24	0.44
-6	7	-6	2.02	0.72
5	-7	6	81.79	3.34
5	-7	6	83.17	3.41
5	-7	6	80.88	3.57
5	-7	6	81.37	3.68
5	-7	6	78.52	3.17
-5	7	-6	86.34	3.18
-5	7	-6	79.93	3.43
-5	7	-6	75.09	3.74
4	-7	6	0.33	0.37
4	-7	6	0.14	0.18
4	-7	6	-0.17	0.47
4	-7	6	0.29	0.56
4	-7	6	0.37	0.45
-4	7	-6	-0.50	0.58
-4	7	-6	-0.03	0.15
-4	7	-6	-0.35	0.29
3	-7	6	135.87	5.24
3	-7	6	137.94	5.75
3	-7	6	138.68	5.77
3	-7	6	142.87	5.50
3	-7	6	140.13	5.51
-3	7	-6	137.31	5.82
-3	7	-6	133.37	5.49
-3	7	-6	141.94	5.24
2	-7	6	9.64	0.52
2	-7	6	10.96	1.07
2	-7	6	11.47	0.86
2	-7	6	10.15	1.20
2	-7	6	10.29	0.90
-2	7	-6	11.29	1.20
-2	7	-6	10.19	0.81
-2	7	-6	10.73	0.51
1	-7	6	91.11	3.60

1	-7	6	99.27	4.02
1	-7	6	95.59	4.15
1	-7	6	100.85	3.92
1	-7	6	93.70	4.24
-1	7	-6	91.96	3.88
-1	7	-6	94.65	3.60
-1	7	-6	99.21	4.23
0	-7	6	33.81	1.62
0	-7	6	32.61	1.83
0	-7	6	31.57	1.26
0	-7	6	32.80	2.03
0	-7	6	35.25	1.78
0	7	-6	31.46	1.91
0	7	-6	33.46	1.26
0	7	-6	32.99	1.85
0	7	-6	32.62	1.58
-1	-7	6	111.33	4.80
-1	-7	6	118.34	4.61
-1	-7	6	115.79	5.04
1	7	-6	120.06	4.88
1	7	-6	113.60	4.89
1	7	-6	108.70	4.55
-2	-7	6	39.28	2.08
-2	-7	6	39.57	2.36
-2	-7	6	40.66	1.88
2	7	-6	39.94	1.84
2	7	-6	41.04	2.23
2	7	-6	41.35	2.12
-3	-7	6	141.12	6.34
-3	-7	6	155.46	5.89
-3	-7	6	145.77	6.05
3	7	-6	144.55	5.83
3	7	-6	154.27	6.12
3	7	-6	152.78	6.26
-4	-7	6	1.21	0.74
-4	-7	6	1.03	0.40
-4	-7	6	0.53	0.51
4	7	-6	1.49	0.62
4	7	-6	1.77	0.67
4	7	-6	0.94	0.31
-5	-7	6	108.69	4.82
-5	-7	6	113.67	5.28
-5	-7	6	119.68	4.71
5	7	-6	117.19	5.14

5	7	-6	115.99	4.65
5	7	-6	122.40	4.92
-6	-7	6	2.11	0.82
-6	-7	6	2.07	0.64
-6	-7	6	1.84	0.21
-6	-7	6	2.30	0.46
6	7	-6	2.01	0.40
6	7	-6	2.16	0.22
6	7	-6	1.52	0.61
6	7	-6	2.28	0.90
-7	-7	6	86.57	3.70
-7	-7	6	89.30	4.39
-7	-7	6	84.16	3.81
-7	-7	6	88.82	3.34
7	7	-6	87.86	4.19
7	7	-6	86.75	3.34
7	7	-6	86.31	3.63
7	7	-6	93.58	3.90
-8	-7	6	8.68	0.49
-8	-7	6	7.86	0.81
-8	-7	6	8.07	0.91
-8	-7	6	7.80	1.11
-8	-7	6	7.83	1.32
8	7	-6	8.90	1.18
8	7	-6	10.19	1.58
8	7	-6	7.63	0.78
8	7	-6	8.04	0.51
-9	-7	6	118.76	4.94
-9	-7	6	120.26	5.70
-9	-7	6	115.00	4.96
-9	-7	6	117.99	4.89
-9	-7	6	118.81	4.64
-9	-7	6	124.28	4.86
9	7	-6	125.72	4.59
9	7	-6	116.27	5.46
-10	-7	6	22.87	1.57
-10	-7	6	23.62	1.45
-10	-7	6	24.93	1.59
-10	-7	6	22.05	2.35
-10	-7	6	24.91	1.11
10	7	-6	24.48	1.10
10	7	-6	23.09	2.21
-11	-7	6	77.36	3.39
-11	-7	6	81.51	3.07

-11	-7	6	80.84	4.42
-11	-7	6	77.10	3.41
-11	-7	6	64.72	3.44
11	7	-6	74.80	4.09
11	7	-6	81.32	3.07
-12	-7	6	0.59	1.35
-12	-7	6	1.19	0.34
-12	-7	6	1.41	0.67
-12	-7	6	0.89	0.51
-12	-7	6	1.13	0.54
12	7	-6	0.79	0.31
-13	-7	6	14.41	2.10
-13	-7	6	12.98	1.30
-13	-7	6	13.53	1.40
-13	-7	6	12.92	1.07
-13	-7	6	15.40	0.94
13	7	-6	14.26	0.89
-14	-7	6	16.96	1.06
-14	-7	6	14.48	1.59
-14	-7	6	15.09	2.25
-14	-7	6	17.61	1.53
-14	-7	6	16.50	1.15
14	7	-6	16.00	1.03
-15	-7	6	53.32	4.56
-15	-7	6	61.44	2.65
-15	-7	6	65.83	2.74
-15	-7	6	58.67	3.11
15	7	-6	60.10	2.63
-16	-7	6	26.98	2.09
-16	-7	6	22.52	3.39
16	7	-6	27.74	1.48
-17	-7	6	33.59	2.42
-17	-7	6	33.09	3.85
17	7	-6	31.30	1.93
-18	-7	6	7.63	1.37
-18	-7	6	7.22	2.51
18	7	-6	7.37	1.16
-19	-7	6	16.38	2.24
-19	-7	6	10.76	2.72
19	7	-6	16.14	1.50
18	-7	7	10.92	1.44
18	-7	7	11.43	1.93
-18	7	-7	6.92	1.75
17	-7	7	24.50	1.72

17	-7	7	22.84	2.82
-17	7	-7	26.33	2.75
16	-7	7	12.42	2.30
16	-7	7	14.88	1.38
-16	7	-7	13.28	2.21
15	-7	7	10.93	1.22
15	-7	7	16.07	2.11
-15	7	-7	12.07	2.04
14	-7	7	21.40	2.17
14	-7	7	20.32	1.41
-14	7	-7	22.71	2.13
13	-7	7	26.37	2.53
13	-7	7	25.96	1.53
-13	7	-7	24.22	2.03
12	-7	7	24.97	1.60
12	-7	7	27.81	2.17
-12	7	-7	23.09	2.03
11	-7	7	1.87	0.91
11	-7	7	1.70	0.48
-11	7	-7	1.76	0.73
10	-7	7	33.17	2.23
10	-7	7	32.34	1.66
-10	7	-7	36.34	1.65
-10	7	-7	34.87	2.07
9	-7	7	52.85	2.57
9	-7	7	54.22	2.24
9	-7	7	53.42	2.34
9	-7	7	53.67	2.90
-9	7	-7	51.46	3.12
-9	7	-7	58.08	2.34
-9	7	-7	54.20	2.66
8	-7	7	35.63	1.73
8	-7	7	37.44	1.96
8	-7	7	39.01	2.31
8	-7	7	38.83	1.72
-8	7	-7	37.07	2.57
-8	7	-7	37.78	2.05
-8	7	-7	42.39	1.74
7	-7	7	17.94	1.06
7	-7	7	17.15	1.24
7	-7	7	18.18	0.98
7	-7	7	17.83	1.55
7	-7	7	17.73	1.53
-7	7	-7	16.37	1.27

-7	7	-7	20.67	0.98
-7	7	-7	17.41	1.84
6	-7	7	23.63	1.40
6	-7	7	23.70	1.72
6	-7	7	22.54	1.15
6	-7	7	24.29	1.31
6	-7	7	24.37	1.65
-6	7	-7	22.32	1.42
-6	7	-7	26.02	1.92
-6	7	-7	26.13	1.15
5	-7	7	6.70	1.12
5	-7	7	7.47	0.56
5	-7	7	6.22	1.17
5	-7	7	8.60	1.16
5	-7	7	7.96	0.85
-5	7	-7	9.46	0.56
-5	7	-7	7.18	1.34
-5	7	-7	7.88	0.86
4	-7	7	80.49	3.76
4	-7	7	85.97	3.48
4	-7	7	80.83	3.75
4	-7	7	82.41	3.23
4	-7	7	82.79	3.48
-4	7	-7	80.35	3.87
-4	7	-7	86.54	3.22
-4	7	-7	81.82	3.49
3	-7	7	27.20	1.43
3	-7	7	27.97	1.48
3	-7	7	27.70	1.81
3	-7	7	25.65	1.66
3	-7	7	24.43	1.12
-3	7	-7	26.98	1.12
-3	7	-7	24.53	1.38
-3	7	-7	26.13	1.80
2	-7	7	10.82	1.00
2	-7	7	12.61	0.93
2	-7	7	11.66	1.35
2	-7	7	11.64	1.15
-2	7	-7	11.50	0.55
-2	7	-7	11.74	0.87
-2	7	-7	11.05	1.32
1	-7	7	7.27	1.09
1	-7	7	6.78	0.75
1	-7	7	7.64	0.98

1	-7	7	6.02	0.35
-1	7	-7	7.17	1.01
-1	7	-7	5.54	0.65
-1	7	-7	6.36	0.34
-1	7	-7	6.89	1.13
0	-7	7	129.16	5.42
0	-7	7	138.09	5.19
0	-7	7	131.26	5.66
0	7	-7	127.56	5.45
0	7	-7	125.65	4.83
0	7	-7	131.24	5.48
0	7	-7	124.94	5.14
-1	-7	7	13.71	1.52
-1	-7	7	14.79	1.00
-1	-7	7	15.69	1.26
1	7	-7	14.90	1.33
1	7	-7	14.38	1.37
1	7	-7	14.09	0.94
-2	-7	7	95.53	3.83
-2	-7	7	90.19	4.35
-2	-7	7	91.08	4.04
2	7	-7	93.46	3.79
2	7	-7	94.32	4.09
2	7	-7	91.34	4.18
-3	-7	7	152.51	5.89
-3	-7	7	143.52	6.05
-3	-7	7	146.56	6.46
3	7	-7	155.81	6.12
3	7	-7	145.46	5.83
3	7	-7	147.11	6.28
-4	-7	7	184.23	7.55
-4	-7	7	191.84	7.40
-4	-7	7	192.05	8.02
4	7	-7	184.87	7.82
4	7	-7	187.73	7.34
4	7	-7	194.14	7.61
-5	-7	7	0.39	0.56
-5	-7	7	0.73	0.39
-5	-7	7	0.22	0.75
5	7	-7	1.16	0.75
5	7	-7	-0.03	0.62
5	7	-7	0.59	0.32
-6	-7	7	172.53	7.42
-6	-7	7	163.33	6.31

-6	-7	7	166.99	6.70
-6	-7	7	168.00	6.80
6	7	-7	165.64	6.63
6	7	-7	172.92	6.88
6	7	-7	174.31	7.23
6	7	-7	173.31	6.31
-7	-7	7	0.10	0.57
-7	-7	7	0.05	0.42
-7	-7	7	0.66	0.15
-7	-7	7	0.00	0.89
7	7	-7	0.35	0.15
7	7	-7	0.12	0.83
7	7	-7	0.48	0.64
7	7	-7	0.56	0.38
-8	-7	7	60.01	2.31
-8	-7	7	59.47	2.71
-8	-7	7	56.67	2.77
-8	-7	7	58.32	3.50
-8	-7	7	60.27	2.67
8	7	-7	61.84	2.32
8	7	-7	58.63	2.86
8	7	-7	56.57	3.29
-9	-7	7	2.43	1.18
-9	-7	7	2.26	0.70
-9	-7	7	2.39	0.36
-9	-7	7	2.30	0.59
-9	-7	7	1.14	0.46
9	7	-7	1.70	0.25
9	7	-7	1.88	0.78
9	7	-7	2.70	1.17
-10	-7	7	58.45	3.69
-10	-7	7	58.79	2.32
-10	-7	7	54.65	2.66
-10	-7	7	57.74	2.68
10	7	-7	57.54	2.31
10	7	-7	59.69	3.41
-11	-7	7	32.87	3.88
-11	-7	7	34.00	1.73
-11	-7	7	34.29	1.51
-11	-7	7	33.65	1.87
-11	-7	7	33.70	1.85
11	7	-7	35.50	1.50
-12	-7	7	91.62	5.19
-12	-7	7	95.38	4.03

-12	-7	7	99.87	3.80
-12	-7	7	92.35	3.88
12	7	-7	98.79	3.78
-13	-7	7	2.58	1.06
-13	-7	7	4.83	0.67
-13	-7	7	4.39	0.79
-13	-7	7	2.77	0.73
-13	-7	7	2.36	1.56
13	7	-7	3.20	0.45
-14	-7	7	60.64	3.04
-14	-7	7	62.59	2.51
-14	-7	7	59.71	2.55
-14	-7	7	60.50	2.58
-14	-7	7	44.46	4.81
14	7	-7	63.92	2.57
-15	-7	7	7.37	0.97
-15	-7	7	7.29	2.01
-15	-7	7	5.51	1.02
15	7	-7	6.47	0.81
-16	-7	7	56.22	3.09
-16	-7	7	55.53	4.44
16	7	-7	57.19	2.51
-17	-7	7	-0.14	2.20
-17	-7	7	1.89	0.98
17	7	-7	1.49	1.14
-18	-7	7	43.45	2.93
-18	-7	7	45.23	4.64
18	7	-7	43.02	2.17
-19	-7	7	-0.48	2.48
-19	-7	7	1.12	1.19
19	7	-7	-0.03	0.71
18	-7	8	39.09	3.32
18	-7	8	29.89	2.15
-18	7	-8	32.92	3.17
17	-7	8	0.74	1.52
17	-7	8	0.41	0.72
-17	7	-8	1.87	1.46
16	-7	8	28.84	2.93
16	-7	8	34.00	2.01
-16	7	-8	38.21	2.88
15	-7	8	2.60	0.71
15	-7	8	5.07	1.47
-15	7	-8	3.22	1.28
14	-7	8	44.72	2.31

14	-7	8	47.39	3.08
-14	7	-8	48.39	2.96
13	-7	8	2.04	1.11
13	-7	8	2.57	0.60
-13	7	-8	4.32	1.05
12	-7	8	28.84	1.69
11	-7	8	4.25	1.05
11	-7	8	3.29	0.57
-11	7	-8	3.16	0.85
10	-7	8	40.54	2.63
10	-7	8	44.01	2.01
-10	7	-8	44.21	2.40
9	-7	8	5.53	0.96
9	-7	8	5.32	0.65
9	-7	8	5.65	0.80
9	-7	8	4.94	0.70
9	-7	8	6.66	1.14
-9	7	-8	7.63	0.73
-9	7	-8	7.58	1.11
8	-7	8	40.78	2.54
8	-7	8	40.64	1.83
8	-7	8	41.43	2.09
8	-7	8	40.68	1.85
8	-7	8	40.67	2.46
-8	7	-8	44.14	1.83
-8	7	-8	39.71	2.17
-8	7	-8	35.95	2.71
7	-7	8	1.51	0.77
7	-7	8	1.57	0.82
7	-7	8	1.87	0.48
7	-7	8	1.29	0.35
7	-7	8	1.08	0.56
-7	7	-8	2.06	0.36
-7	7	-8	0.88	0.52
-7	7	-8	0.17	1.02
6	-7	8	35.71	1.57
6	-7	8	35.87	2.22
6	-7	8	33.96	1.81
6	-7	8	36.47	2.08
-6	7	-8	34.75	1.86
-6	7	-8	32.68	2.36
-6	7	-8	38.41	1.57
5	-7	8	4.28	0.93
5	-7	8	5.54	0.99

5	-7	8	5.71	0.83
5	-7	8	5.09	0.49
5	-7	8	4.27	0.62
-5	7	-8	5.36	0.48
-5	7	-8	6.29	1.07
-5	7	-8	5.16	0.83
4	-7	8	64.55	2.60
4	-7	8	65.46	2.88
4	-7	8	63.86	2.86
4	-7	8	62.90	3.20
4	-7	8	65.16	3.21
-4	7	-8	65.73	2.88
-4	7	-8	64.69	3.31
-4	7	-8	70.13	2.60
3	-7	8	29.85	1.30
3	-7	8	29.41	2.05
3	-7	8	31.69	1.70
3	-7	8	29.59	1.61
3	-7	8	30.61	1.93
-3	7	-8	31.74	1.60
-3	7	-8	32.45	2.01
-3	7	-8	30.90	1.30
2	-7	8	83.00	3.42
2	-7	8	77.62	3.70
2	-7	8	79.27	3.11
2	-7	8	82.16	3.89
-2	7	-8	78.80	3.78
-2	7	-8	84.47	3.11
-2	7	-8	80.74	3.40
-2	7	-8	81.60	3.64
1	-7	8	5.37	0.35
1	-7	8	5.38	0.99
1	-7	8	6.73	0.78
1	-7	8	5.16	1.03
-1	7	-8	5.34	0.67
-1	7	-8	6.08	0.90
-1	7	-8	5.84	0.34
-1	7	-8	6.08	0.93
0	-7	8	50.41	2.20
0	-7	8	49.47	2.77
0	-7	8	46.71	2.44
0	7	-8	45.28	2.50
0	7	-8	47.25	1.83
0	7	-8	45.98	1.80

0	7	-8	45.27	2.13
0	7	-8	50.15	2.53
-1	-7	8	86.49	4.04
-1	-7	8	95.26	3.84
-1	-7	8	91.16	4.40
1	7	-8	93.11	4.21
1	7	-8	91.72	3.80
1	7	-8	97.44	4.16
-2	-7	8	51.05	2.58
-2	-7	8	56.60	2.41
-2	-7	8	50.96	3.00
2	7	-8	50.83	2.32
2	7	-8	54.22	2.68
2	7	-8	52.20	2.84
-3	-7	8	69.17	3.05
-3	-7	8	71.39	3.74
-3	-7	8	69.75	3.27
3	7	-8	75.02	3.35
3	7	-8	68.27	3.51
3	7	-8	72.11	3.01
-4	-7	8	60.51	2.66
-4	-7	8	54.59	2.80
-4	-7	8	60.21	3.35
4	7	-8	62.34	2.89
4	7	-8	56.46	3.15
4	7	-8	61.42	2.60
-5	-7	8	33.13	1.78
-5	-7	8	35.63	2.56
-5	-7	8	34.42	1.97
5	7	-8	37.19	2.06
5	7	-8	35.75	1.73
5	7	-8	36.76	2.40
-6	-7	8	9.45	1.16
-6	-7	8	11.42	1.80
-6	-7	8	11.76	1.01
6	7	-8	9.46	0.85
6	7	-8	9.71	1.64
6	7	-8	9.05	1.27
-7	-7	8	30.40	1.76
-7	-7	8	32.77	1.32
-7	-7	8	35.40	2.61
-7	-7	8	31.78	1.89
7	7	-8	37.69	2.04
7	7	-8	34.61	1.32

7	7	-8	32.21	1.68
7	7	-8	33.59	2.45
-8	-7	8	24.15	1.39
-8	-7	8	22.78	2.40
-8	-7	8	22.36	1.55
-8	-7	8	21.54	1.50
-8	-7	8	23.94	0.99
8	7	-8	21.78	2.27
8	7	-8	24.29	1.71
8	7	-8	23.58	1.37
-9	-7	8	70.89	3.61
-9	-7	8	67.13	3.05
-9	-7	8	59.25	3.94
-9	-7	8	69.44	3.08
-9	-7	8	69.60	2.67
9	7	-8	65.66	3.79
9	7	-8	68.51	3.22
9	7	-8	71.16	2.66
-10	-7	8	77.97	3.48
-10	-7	8	81.45	3.17
-10	-7	8	78.27	3.52
-10	-7	8	82.14	4.62
10	7	-8	83.20	3.17
10	7	-8	83.18	3.71
-11	-7	8	34.49	1.82
-11	-7	8	41.35	3.25
-11	-7	8	37.31	1.92
-11	-7	8	37.09	2.00
-11	-7	8	36.82	1.61
11	7	-8	38.10	1.60
-12	-7	8	39.42	1.89
-12	-7	8	39.29	2.05
-12	-7	8	41.85	1.77
-12	-7	8	40.10	2.19
-12	-7	8	38.09	3.43
12	7	-8	40.92	1.74
-13	-7	8	37.15	1.70
-13	-7	8	36.61	1.71
-13	-7	8	34.35	2.17
-13	-7	8	35.43	1.81
13	7	-8	38.98	1.67
-14	-7	8	5.37	0.83
-14	-7	8	5.76	0.94
-14	-7	8	6.85	1.96

14	7	-8	6.51	0.71
-15	-7	8	60.93	2.78
-15	-7	8	68.44	4.65
-15	-7	8	63.97	3.30
15	7	-8	64.72	2.72
-16	-7	8	2.44	2.11
-16	-7	8	3.06	1.04
16	7	-8	5.70	0.90
-17	-7	8	38.11	2.60
-17	-7	8	27.56	4.16
17	7	-8	35.23	1.83
-18	-7	8	11.41	2.81
-18	-7	8	8.73	1.50
18	7	-8	8.81	1.20
-19	-7	8	25.80	2.51
-19	-7	8	18.83	3.26
19	7	-8	20.85	1.59
17	-7	9	14.90	1.57
17	-7	9	15.96	2.22
-17	7	-9	12.59	1.99
16	-7	9	11.35	1.45
16	-7	9	9.28	1.91
-16	7	-9	7.88	1.58
15	-7	9	12.07	1.89
15	-7	9	9.45	1.33
-15	7	-9	13.05	2.20
14	-7	9	3.52	0.77
14	-7	9	2.06	1.31
-14	7	-9	4.50	1.19
13	-7	9	5.28	0.77
13	-7	9	4.49	1.32
-13	7	-9	5.63	1.14
12	-7	9	1.61	1.18
12	-7	9	3.07	0.61
-12	7	-9	3.33	0.98
11	-7	9	31.51	2.39
11	-7	9	28.44	1.59
-11	7	-9	29.88	2.05
10	-7	9	6.22	1.20
10	-7	9	6.61	0.82
-10	7	-9	5.11	0.88
9	-7	9	39.35	2.57
9	-7	9	37.19	1.95
9	-7	9	37.75	2.19

9	-7	9	40.69	1.77
9	-7	9	39.64	1.87
-9	7	-9	41.23	2.24
-9	7	-9	43.79	1.87
8	-7	9	4.14	0.57
8	-7	9	4.45	0.77
8	-7	9	4.94	0.93
8	-7	9	5.60	1.09
8	-7	9	3.83	0.53
-8	7	-9	3.96	0.50
-8	7	-9	3.03	0.72
7	-7	9	10.11	1.11
7	-7	9	12.45	1.49
7	-7	9	10.19	1.31
7	-7	9	11.58	0.96
7	-7	9	8.12	0.74
-7	7	-9	10.21	0.75
-7	7	-9	7.77	1.10
6	-7	9	2.04	0.37
6	-7	9	2.58	0.85
6	-7	9	1.82	0.58
6	-7	9	3.00	0.85
6	-7	9	2.45	0.58
-6	7	-9	2.39	0.37
-6	7	-9	1.89	0.59
-6	7	-9	2.19	1.05
5	-7	9	27.11	1.94
5	-7	9	30.64	2.04
5	-7	9	27.07	1.31
5	-7	9	27.79	1.60
5	-7	9	30.42	1.62
-5	7	-9	30.35	1.30
-5	7	-9	28.65	1.62
-5	7	-9	26.61	2.20
4	-7	9	16.55	0.86
4	-7	9	14.90	1.53
4	-7	9	18.31	1.29
4	-7	9	19.16	1.22
4	-7	9	18.35	1.68
-4	7	-9	13.16	1.63
-4	7	-9	17.43	0.85
-4	7	-9	17.00	1.21
3	-7	9	157.83	6.15
3	-7	9	152.92	6.51

3	-7	9	148.98	5.85
3	-7	9	156.32	6.59
-3	7	-9	153.10	6.14
-3	7	-9	148.39	6.54
-3	7	-9	148.34	6.32
-3	7	-9	168.57	5.88
2	-7	9	0.58	0.64
2	-7	9	-0.22	0.40
2	-7	9	0.12	0.20
2	-7	9	0.49	0.84
-2	7	-9	0.04	0.16
-2	7	-9	-0.03	0.38
-2	7	-9	0.28	0.65
-2	7	-9	0.77	0.81
1	-7	9	54.14	2.39
1	-7	9	51.42	2.97
1	-7	9	49.10	2.68
-1	7	-9	51.56	2.76
-1	7	-9	54.45	2.74
-1	7	-9	51.78	2.05
-1	7	-9	53.60	2.02
-1	7	-9	49.73	2.34
0	-7	9	8.90	0.89
0	-7	9	8.79	1.16
0	-7	9	9.26	1.40
0	7	-9	8.38	0.79
0	7	-9	8.47	1.30
0	7	-9	8.31	1.14
0	7	-9	7.95	0.43
0	7	-9	8.52	0.40
-1	-7	9	34.91	1.73
-1	-7	9	34.12	2.46
-1	-7	9	32.07	1.96
1	7	-9	34.15	2.07
1	7	-9	32.16	1.65
1	7	-9	33.42	2.20
-2	-7	9	8.13	0.88
-2	-7	9	9.59	1.10
-2	-7	9	9.56	1.45
2	7	-9	7.87	1.24
2	7	-9	7.95	0.79
2	7	-9	8.13	1.46
-3	-7	9	66.04	3.65
-3	-7	9	65.52	2.91

-3	-7	9	63.91	3.12
3	7	-9	66.23	2.85
3	7	-9	65.13	3.45
3	7	-9	69.38	3.19
-4	-7	9	9.70	1.85
-4	-7	9	12.83	1.25
-4	-7	9	12.18	1.02
4	7	-9	12.06	1.37
4	7	-9	13.83	0.96
4	7	-9	13.37	1.73
-5	-7	9	49.15	2.34
-5	-7	9	50.21	3.20
-5	-7	9	47.57	2.50
5	7	-9	53.72	2.63
5	7	-9	47.88	2.98
5	7	-9	48.06	2.25
-6	-7	9	8.09	1.15
-6	-7	9	5.79	1.35
-6	-7	9	10.39	1.01
6	7	-9	8.08	0.82
6	7	-9	10.30	1.34
6	7	-9	10.47	1.76
-7	-7	9	28.17	1.67
-7	-7	9	27.19	2.57
-7	-7	9	27.87	1.13
-7	-7	9	27.04	1.74
7	7	-9	27.55	1.91
7	7	-9	28.66	2.47
7	7	-9	29.40	1.13
7	7	-9	29.21	1.55
-8	-7	9	6.49	1.55
-8	-7	9	7.96	0.45
-8	-7	9	8.79	1.10
-8	-7	9	6.66	1.06
-8	-7	9	7.54	0.90
8	7	-9	6.86	0.85
8	7	-9	7.80	0.45
8	7	-9	8.81	1.35
8	7	-9	5.61	1.37
-9	-7	9	43.97	2.20
-9	-7	9	39.91	2.15
-9	-7	9	43.43	1.76
-9	-7	9	44.83	3.37
9	7	-9	43.15	1.75

9	7	-9	46.65	2.43
-10	-7	9	11.13	1.17
-10	-7	9	12.38	1.98
-10	-7	9	11.09	1.10
-10	-7	9	10.49	0.68
10	7	-9	11.19	0.65
10	7	-9	13.06	1.50
-11	-7	9	49.31	2.32
-11	-7	9	43.35	2.35
-11	-7	9	50.78	2.05
-11	-7	9	52.47	3.73
11	7	-9	51.06	2.02
-12	-7	9	12.29	1.30
-12	-7	9	11.99	1.23
-12	-7	9	13.77	0.87
-12	-7	9	13.71	1.06
12	7	-9	13.75	0.81
-13	-7	9	10.34	0.87
-13	-7	9	11.91	0.89
-13	-7	9	10.79	0.90
-13	-7	9	10.08	1.37
-13	-7	9	8.32	2.08
13	7	-9	10.08	0.78
-14	-7	9	5.38	2.04
-14	-7	9	4.44	0.97
-14	-7	9	5.60	0.86
14	7	-9	6.59	0.72
-15	-7	9	26.01	3.75
-15	-7	9	25.36	2.01
15	7	-9	24.78	1.36
-16	-7	9	12.28	1.79
-16	-7	9	12.07	2.57
16	7	-9	16.35	1.14
-17	-7	9	19.44	2.95
-17	-7	9	17.18	2.11
17	7	-9	17.86	1.29
-18	-7	9	20.06	2.37
18	7	-9	20.95	1.46
-19	-7	9	4.91	2.64
19	7	-9	4.98	0.87
17	-7	10	16.60	1.71
17	-7	10	10.21	2.09
-17	7	-10	12.23	1.95
16	-7	10	5.08	1.75

16	-7	10	5.05	0.95
-16	7	-10	5.58	1.56
15	-7	10	12.47	1.43
15	-7	10	8.73	1.78
-15	7	-10	14.48	2.27
14	-7	10	2.93	0.72
14	-7	10	1.36	1.36
-14	7	-10	0.60	1.07
13	-7	10	28.57	2.72
13	-7	10	31.72	1.84
-13	7	-10	32.63	2.41
12	-7	10	7.82	1.00
12	-7	10	6.84	1.47
-12	7	-10	7.28	1.15
11	-7	10	24.61	2.28
-11	7	-10	22.01	1.94
10	-7	10	28.14	2.30
10	-7	10	27.56	1.49
-10	7	-10	22.22	1.81
9	-7	10	13.64	1.53
9	-7	10	16.85	1.03
9	-7	10	15.89	1.10
-9	7	-10	16.36	1.49
-9	7	-10	15.08	1.03
8	-7	10	2.80	0.61
8	-7	10	3.21	0.82
8	-7	10	2.90	1.08
8	-7	10	3.07	0.73
8	-7	10	3.49	0.52
-8	7	-10	3.10	0.50
-8	7	-10	2.31	0.73
7	-7	10	46.03	2.48
7	-7	10	45.81	2.04
7	-7	10	42.63	2.24
7	-7	10	47.30	2.80
7	-7	10	45.54	2.32
-7	7	-10	46.84	2.36
-7	7	-10	49.83	2.03
6	-7	10	2.74	0.93
6	-7	10	5.78	0.98
6	-7	10	4.79	0.75
6	-7	10	3.85	0.56
6	-7	10	3.50	0.94
-6	7	-10	4.71	0.59

-6	7	-10	4.35	0.74
5	-7	10	18.24	0.98
5	-7	10	20.38	1.83
5	-7	10	19.58	1.73
5	-7	10	16.91	1.33
5	-7	10	17.86	1.38
-5	7	-10	18.13	1.32
-5	7	-10	16.32	2.03
-5	7	-10	21.43	0.99
4	-7	10	50.29	2.83
4	-7	10	51.09	2.14
4	-7	10	52.84	2.94
4	-7	10	50.16	2.45
4	-7	10	55.40	2.57
-4	7	-10	51.37	2.63
-4	7	-10	51.15	2.45
-4	7	-10	54.67	2.14
-4	7	-10	52.52	3.01
3	-7	10	11.06	1.39
3	-7	10	13.48	1.13
3	-7	10	13.11	1.75
3	-7	10	12.39	0.70
-3	7	-10	12.30	1.05
-3	7	-10	11.90	1.41
-3	7	-10	12.84	0.68
-3	7	-10	14.76	1.77
2	-7	10	21.23	1.33
2	-7	10	19.74	1.66
2	-7	10	19.75	1.96
-2	7	-10	22.39	0.93
-2	7	-10	20.58	0.97
-2	7	-10	21.08	1.84
-2	7	-10	19.29	1.28
-2	7	-10	19.12	1.70
1	-7	10	18.94	1.24
1	-7	10	18.97	1.62
1	-7	10	17.45	1.97
-1	7	-10	19.80	1.21
-1	7	-10	19.56	0.81
-1	7	-10	17.18	1.65
-1	7	-10	14.34	1.63
-1	7	-10	17.42	0.96
0	-7	10	9.14	1.33
0	-7	10	9.43	1.79

0	-7	10	9.76	0.98
0	7	-10	13.14	1.50
0	7	-10	10.20	0.49
0	7	-10	11.17	0.52
0	7	-10	9.95	0.93
0	7	-10	7.74	1.49
-1	-7	10	7.34	1.21
-1	-7	10	7.01	0.88
-1	-7	10	5.51	1.27
1	7	-10	4.40	1.04
1	7	-10	5.90	0.98
1	7	-10	5.33	0.30
1	7	-10	5.64	0.30
1	7	-10	5.95	0.78
-2	-7	10	34.12	1.74
-2	-7	10	36.52	2.62
-2	-7	10	28.99	2.00
2	7	-10	31.79	2.06
2	7	-10	31.98	1.65
2	7	-10	30.57	2.32
-3	-7	10	1.75	1.13
-3	-7	10	2.21	0.78
-3	-7	10	2.80	0.56
3	7	-10	2.56	0.82
3	7	-10	2.72	0.48
3	7	-10	2.71	1.01
-4	-7	10	2.77	0.59
-4	-7	10	1.30	1.03
-4	-7	10	1.95	0.74
4	7	-10	2.21	0.84
4	7	-10	2.75	1.04
4	7	-10	2.07	0.48
-5	-7	10	4.91	0.90
-5	-7	10	4.61	0.71
-5	-7	10	4.51	1.28
5	7	-10	6.44	1.30
5	7	-10	6.57	1.00
5	7	-10	5.13	0.77
-6	-7	10	5.85	0.98
-6	-7	10	7.17	1.20
-6	-7	10	6.35	1.45
6	7	-10	5.88	1.33
6	7	-10	6.80	0.83
6	7	-10	5.90	1.02

-7	-7	10	5.60	1.53
-7	-7	10	5.63	0.93
-7	-7	10	5.57	0.79
7	7	-10	7.96	0.88
7	7	-10	6.48	0.35
7	7	-10	8.13	1.44
-8	-7	10	16.84	1.40
-8	-7	10	16.04	0.74
-8	-7	10	17.25	2.39
-8	-7	10	15.47	1.21
8	7	-10	17.29	1.68
8	7	-10	16.78	0.74
8	7	-10	16.60	1.21
-9	-7	10	0.35	0.23
-9	-7	10	-0.93	1.26
-9	-7	10	0.05	0.64
-9	-7	10	0.72	0.44
9	7	-10	0.51	0.23
9	7	-10	1.19	0.94
-10	-7	10	48.52	2.44
-10	-7	10	54.94	3.78
-10	-7	10	49.43	2.46
10	7	-10	46.42	2.67
10	7	-10	51.54	2.01
-11	-7	10	12.57	1.26
-11	-7	10	12.98	1.22
-11	-7	10	13.46	0.82
-11	-7	10	17.26	2.99
11	7	-10	12.87	0.75
11	7	-10	14.09	1.81
-12	-7	10	28.49	1.30
-12	-7	10	25.58	1.46
-12	-7	10	24.56	3.20
-12	-7	10	24.00	1.57
-12	-7	10	25.44	1.75
12	7	-10	26.37	1.24
-13	-7	10	-0.16	0.45
-13	-7	10	1.45	0.72
-13	-7	10	-2.86	1.71
13	7	-10	-0.07	0.34
-14	-7	10	13.31	2.45
-14	-7	10	15.14	1.66
14	7	-10	14.16	0.96
-15	-7	10	0.35	0.86

-15	-7	10	2.66	2.30
15	7	-10	0.82	0.46
-16	-7	10	7.21	2.37
-16	-7	10	6.80	1.23
16	7	-10	7.97	0.95
-17	-7	10	0.58	0.98
-17	-7	10	0.41	2.45
17	7	-10	1.89	0.61
-18	-7	10	12.61	2.15
-18	-7	10	13.57	2.95
18	7	-10	12.48	1.27
16	-7	11	31.85	2.13
16	-7	11	28.20	3.27
-16	7	-11	33.35	2.97
15	-7	11	5.23	1.71
15	-7	11	6.09	0.97
-15	7	-11	8.46	1.56
14	-7	11	26.80	2.82
14	-7	11	26.24	1.79
-14	7	-11	26.86	2.49
13	-7	11	0.85	0.67
13	-7	11	0.44	1.30
-13	7	-11	0.54	1.07
12	-7	11	33.33	1.84
12	-7	11	30.70	2.80
-12	7	-11	32.40	2.33
11	-7	11	28.01	1.63
11	-7	11	29.61	2.62
10	-7	11	29.24	1.58
10	-7	11	27.22	2.43
-10	7	-11	28.42	1.99
9	-7	11	8.86	1.49
9	-7	11	8.77	1.43
9	-7	11	8.86	0.90
9	-7	11	7.46	0.92
-9	7	-11	8.08	1.35
8	-7	11	36.62	2.71
8	-7	11	37.30	1.81
8	-7	11	35.32	2.17
8	-7	11	39.41	2.09
8	-7	11	39.11	2.24
-8	7	-11	39.95	1.80
-8	7	-11	38.79	2.19
7	-7	11	1.57	1.01

7	-7	11	0.77	0.38
7	-7	11	1.83	0.90
7	-7	11	-0.56	0.60
7	-7	11	1.36	0.72
-7	7	-11	0.70	0.67
-7	7	-11	1.09	0.39
6	-7	11	46.98	2.13
6	-7	11	49.25	2.44
6	-7	11	49.74	2.92
6	-7	11	47.11	2.74
6	-7	11	51.61	2.46
-6	7	-11	54.29	2.15
-6	7	-11	49.79	2.47
5	-7	11	8.31	1.24
5	-7	11	7.46	1.19
5	-7	11	6.49	0.63
5	-7	11	7.09	1.08
5	-7	11	7.09	1.19
-5	7	-11	6.68	0.63
-5	7	-11	7.16	1.03
4	-7	11	85.03	3.52
4	-7	11	90.92	3.84
4	-7	11	93.92	4.33
4	-7	11	88.88	4.29
-4	7	-11	85.14	4.04
-4	7	-11	85.11	3.80
-4	7	-11	96.68	3.52
3	-7	11	57.25	2.65
3	-7	11	57.04	3.22
3	-7	11	55.67	3.07
-3	7	-11	55.03	3.22
-3	7	-11	53.41	2.59
-3	7	-11	60.31	2.28
-3	7	-11	57.00	2.96
-3	7	-11	55.37	2.31
2	-7	11	26.63	2.32
2	-7	11	28.49	1.65
2	-7	11	24.18	2.00
-2	7	-11	30.18	2.07
-2	7	-11	29.82	2.30
-2	7	-11	27.17	1.55
-2	7	-11	27.26	1.22
1	-7	11	0.03	0.99
1	-7	11	0.91	0.81

1	-7	11	0.48	0.54
-1	7	-11	1.45	0.89
-1	7	-11	0.75	0.20
-1	7	-11	0.40	0.47
-1	7	-11	0.48	0.23
-1	7	-11	1.59	0.93
0	-7	11	15.58	1.21
0	-7	11	15.44	2.03
0	-7	11	13.45	1.52
0	7	-11	14.91	0.70
0	7	-11	17.28	1.73
0	7	-11	14.83	1.11
0	7	-11	14.77	0.67
0	7	-11	14.40	1.82
-1	-7	11	1.28	0.54
-1	-7	11	1.69	1.14
-1	-7	11	1.37	0.78
1	7	-11	1.56	0.48
1	7	-11	2.03	0.24
1	7	-11	1.76	0.23
1	7	-11	2.01	1.00
1	7	-11	2.24	0.90
-2	-7	11	52.34	3.26
-2	-7	11	51.60	2.35
-2	-7	11	46.78	2.65
2	7	-11	45.10	2.97
2	7	-11	48.21	2.69
2	7	-11	45.30	2.22
-3	-7	11	7.48	1.33
-3	-7	11	6.16	1.00
-3	-7	11	8.27	1.57
3	7	-11	7.22	1.31
3	7	-11	7.09	0.85
3	7	-11	7.73	1.13
-4	-7	11	39.09	2.98
-4	-7	11	36.85	2.31
-4	-7	11	40.24	2.04
4	7	-11	39.20	1.93
4	7	-11	38.04	2.38
4	7	-11	40.83	2.96
-5	-7	11	1.40	1.25
-5	-7	11	2.61	0.80
-5	-7	11	2.09	0.62
5	7	-11	1.71	0.50

5	7	-11	1.18	0.90
5	7	-11	1.98	1.25
-6	-7	11	64.61	3.93
-6	-7	11	58.92	2.85
-6	-7	11	56.30	2.98
6	7	-11	63.75	3.14
6	7	-11	61.48	2.74
-7	-7	11	6.97	1.63
-7	-7	11	5.57	0.95
7	7	-11	8.55	0.93
7	7	-11	7.30	0.37
7	7	-11	8.39	1.19
-8	-7	11	45.78	2.25
-8	-7	11	50.96	3.52
-8	-7	11	42.35	1.77
-8	-7	11	43.89	2.39
8	7	-11	45.79	2.23
8	7	-11	48.46	2.66
8	7	-11	44.11	1.77
-9	-7	11	54.86	4.29
-9	-7	11	62.85	2.92
-9	-7	11	61.43	2.45
-9	-7	11	59.20	2.94
9	7	-11	66.79	2.45
9	7	-11	66.12	3.22
-10	-7	11	10.15	1.24
-10	-7	11	12.38	1.29
-10	-7	11	11.84	1.94
-10	-7	11	10.38	0.70
10	7	-11	12.50	1.71
10	7	-11	12.18	0.64
-11	-7	11	4.03	1.77
-11	-7	11	2.93	0.69
-11	-7	11	2.05	0.74
-11	-7	11	3.17	0.41
11	7	-11	3.00	1.05
11	7	-11	2.16	0.33
-12	-7	11	32.06	1.56
-12	-7	11	30.88	1.82
-12	-7	11	33.25	2.05
-12	-7	11	33.83	1.82
-12	-7	11	38.48	3.72
12	7	-11	35.09	1.49
-13	-7	11	2.92	1.93

-13	-7	11	1.90	0.84
13	7	-11	2.07	0.41
-14	-7	11	23.61	1.92
-14	-7	11	23.64	3.84
14	7	-11	24.62	1.28
-15	-7	11	13.11	1.71
-15	-7	11	7.86	2.29
15	7	-11	14.28	0.99
-16	-7	11	4.03	1.11
-16	-7	11	5.10	2.47
16	7	-11	5.14	0.69
-17	-7	11	12.37	2.87
-17	-7	11	15.12	2.14
17	7	-11	17.40	1.32
-18	-7	11	3.55	2.71
-18	-7	11	1.35	1.28
15	-7	12	35.28	2.20
15	-7	12	34.26	3.36
-15	7	-12	32.70	2.95
14	-7	12	1.05	0.74
14	-7	12	0.91	1.64
-14	7	-12	-0.10	1.23
13	-7	12	30.09	1.88
13	-7	12	30.10	2.98
-13	7	-12	28.32	2.41
12	-7	12	3.10	0.74
12	-7	12	2.60	1.36
-12	7	-12	2.78	1.07
11	-7	12	18.51	2.44
11	-7	12	19.38	1.37
-11	7	-12	17.29	1.83
10	-7	12	4.01	1.32
10	-7	12	5.25	0.77
-10	7	-12	4.31	0.97
9	-7	12	58.20	3.59
9	-7	12	59.12	2.73
9	-7	12	59.98	2.60
9	-7	12	59.58	2.64
-9	7	-12	59.47	2.97
8	-7	12	3.73	0.57
8	-7	12	5.07	1.33
8	-7	12	4.09	0.76
8	-7	12	4.06	0.94
-8	7	-12	3.72	0.86

-8	7	-12	4.84	0.75
7	-7	12	71.15	3.03
7	-7	12	64.37	2.76
7	-7	12	64.37	3.17
7	-7	12	62.62	3.03
7	-7	12	61.78	3.60
-7	7	-12	66.29	2.73
-7	7	-12	64.94	3.08
6	-7	12	0.47	0.77
6	-7	12	-0.11	0.92
6	-7	12	0.40	0.37
6	-7	12	2.16	0.76
-6	7	-12	0.90	0.61
-6	7	-12	1.58	0.40
5	-7	12	58.18	3.20
5	-7	12	57.26	2.74
5	-7	12	54.69	2.42
5	-7	12	59.72	3.33
-5	7	-12	62.26	2.42
-5	7	-12	57.93	2.97
-5	7	-12	56.88	2.75
4	-7	12	3.15	1.11
4	-7	12	3.64	1.00
4	-7	12	2.96	0.74
-4	7	-12	3.28	1.02
-4	7	-12	2.43	0.67
-4	7	-12	5.36	0.56
-4	7	-12	3.51	0.44
3	-7	12	81.47	4.07
3	-7	12	84.62	3.59
3	-7	12	82.55	4.18
-3	7	-12	81.29	3.25
-3	7	-12	77.22	3.53
-3	7	-12	77.15	3.91
-3	7	-12	86.50	3.22
2	-7	12	-0.10	0.57
2	-7	12	-0.21	0.79
2	-7	12	1.01	1.01
-2	7	-12	-0.11	0.24
-2	7	-12	0.37	0.48
-2	7	-12	0.01	0.23
-2	7	-12	1.35	0.99
1	-7	12	63.21	3.73
1	-7	12	68.79	3.04

1	-7	12	66.79	3.46
-1	7	-12	65.12	3.53
-1	7	-12	62.78	3.53
-1	7	-12	63.33	2.93
-1	7	-12	69.32	2.58
-1	7	-12	66.04	2.60
0	-7	12	0.52	0.59
0	-7	12	1.06	1.14
0	-7	12	0.07	0.77
0	7	-12	-1.06	0.95
0	7	-12	0.53	0.28
0	7	-12	-0.87	1.04
0	7	-12	0.71	0.20
0	7	-12	0.97	0.51
-1	-7	12	53.80	2.65
-1	-7	12	52.91	3.52
-1	-7	12	56.65	3.06
1	7	-12	56.40	3.11
1	7	-12	56.01	3.37
1	7	-12	58.21	2.16
1	7	-12	56.82	2.16
1	7	-12	53.98	2.55
-2	-7	12	5.96	0.82
-2	-7	12	4.20	1.00
-2	-7	12	5.44	1.41
2	7	-12	4.11	1.10
2	7	-12	5.07	0.69
2	7	-12	4.81	1.37
2	7	-12	4.90	0.30
2	7	-12	5.34	0.31
-3	-7	12	92.34	4.30
-3	-7	12	92.63	3.97
-3	-7	12	92.15	4.93
3	7	-12	93.22	4.33
3	7	-12	92.19	3.88
3	7	-12	90.64	4.76
-4	-7	12	8.42	1.73
-4	-7	12	8.93	1.18
-4	-7	12	11.43	1.50
4	7	-12	10.93	1.67
4	7	-12	9.88	1.00
-5	-7	12	84.69	4.70
-5	-7	12	76.67	3.84
-5	-7	12	79.61	3.62

5	7	-12	85.85	3.98
5	7	-12	84.06	3.53
-6	-7	12	31.66	2.30
-6	-7	12	31.14	2.93
-6	-7	12	28.38	1.99
6	7	-12	26.94	2.17
6	7	-12	28.30	1.65
-7	-7	12	41.26	2.40
-7	-7	12	43.92	3.43
7	7	-12	44.51	2.72
7	7	-12	43.95	2.18
7	7	-12	44.19	1.68
-8	-7	12	3.19	0.87
-8	-7	12	3.32	0.35
-8	-7	12	3.68	0.70
8	7	-12	3.36	0.65
8	7	-12	3.89	1.15
8	7	-12	3.39	0.32
-9	-7	12	41.15	1.69
-9	-7	12	39.01	2.31
-9	-7	12	44.78	3.68
9	7	-12	43.15	1.69
9	7	-12	42.57	2.63
-10	-7	12	3.49	0.86
-10	-7	12	1.53	1.73
-10	-7	12	2.62	0.35
-10	-7	12	2.85	0.67
10	7	-12	3.42	1.12
10	7	-12	2.72	0.40
-11	-7	12	62.50	3.05
-11	-7	12	61.99	4.59
-11	-7	12	61.60	2.92
-11	-7	12	62.85	2.55
11	7	-12	63.83	3.24
11	7	-12	64.15	2.51
-12	-7	12	3.73	0.87
-12	-7	12	3.82	1.93
-12	-7	12	4.72	0.76
12	7	-12	4.33	0.58
-13	-7	12	25.20	1.95
-13	-7	12	24.37	3.92
13	7	-12	24.25	1.23
-14	-7	12	5.74	1.10
-14	-7	12	11.17	2.33

14	7	-12	7.37	0.79
-15	-7	12	39.89	4.53
-15	-7	12	43.74	2.72
15	7	-12	43.49	1.97
-16	-7	12	9.67	2.83
-16	-7	12	15.93	1.93
-17	-7	12	22.73	2.30
-17	-7	12	14.18	3.10
15	-7	13	-0.07	1.77
15	-7	13	1.33	0.88
-15	7	-13	-0.73	1.37
14	-7	13	10.25	2.06
14	-7	13	12.05	1.52
-14	7	-13	8.17	1.58
13	-7	13	18.60	2.75
13	-7	13	20.03	1.65
-13	7	-13	18.37	2.31
12	-7	13	20.38	1.60
12	-7	13	17.44	2.76
-12	7	-13	20.31	2.15
11	-7	13	1.28	1.44
11	-7	13	3.33	0.74
-11	7	-13	4.44	1.08
10	-7	13	25.20	2.67
10	-7	13	24.51	1.54
9	-7	13	3.01	0.81
9	-7	13	2.24	0.58
9	-7	13	1.24	1.22
-9	7	-13	3.67	0.91
8	-7	13	32.32	1.70
8	-7	13	34.25	2.80
8	-7	13	34.79	1.96
8	-7	13	31.96	2.03
-8	7	-13	31.32	2.06
7	-7	13	19.94	1.21
7	-7	13	20.72	2.32
7	-7	13	20.69	1.68
7	-7	13	18.01	1.84
-7	7	-13	20.55	1.63
-7	7	-13	20.66	1.20
6	-7	13	49.92	3.07
6	-7	13	43.59	2.69
6	-7	13	47.63	2.41
6	-7	13	48.00	2.54

-6	7	-13	44.87	2.11
-6	7	-13	47.61	2.04
-6	7	-13	43.61	2.39
5	-7	13	2.56	1.08
5	-7	13	3.64	0.80
5	-7	13	3.73	1.16
-5	7	-13	1.66	0.69
-5	7	-13	3.35	1.08
-5	7	-13	2.77	0.44
-5	7	-13	2.22	0.45
4	-7	13	57.32	3.25
4	-7	13	46.57	3.47
4	-7	13	53.48	2.65
-4	7	-13	53.81	2.63
-4	7	-13	53.80	2.31
-4	7	-13	47.95	2.99
-4	7	-13	61.89	2.28
3	-7	13	6.22	1.41
3	-7	13	3.62	0.76
3	-7	13	3.42	1.14
-3	7	-13	6.52	1.29
-3	7	-13	3.77	0.72
-3	7	-13	4.44	0.56
-3	7	-13	5.02	0.58
2	-7	13	35.18	1.93
2	-7	13	34.22	2.70
2	-7	13	32.15	2.43
-2	7	-13	35.75	1.44
-2	7	-13	32.48	1.46
-2	7	-13	27.92	2.49
-2	7	-13	31.83	1.82
1	-7	13	16.49	1.39
1	-7	13	16.86	2.25
1	-7	13	14.44	1.83
-1	7	-13	13.31	2.11
-1	7	-13	14.26	1.24
-1	7	-13	15.68	0.81
-1	7	-13	16.48	0.85
0	-7	13	60.29	3.53
0	-7	13	59.42	2.78
0	-7	13	59.94	3.68
0	7	-13	60.03	2.28
0	7	-13	57.28	2.30
0	7	-13	57.48	2.67

0	7	-13	50.76	3.45
-1	-7	13	0.16	0.92
-1	-7	13	1.04	1.20
-1	-7	13	1.57	0.66
1	7	-13	0.34	0.19
1	7	-13	0.91	0.53
1	7	-13	-1.06	1.24
1	7	-13	0.78	1.04
1	7	-13	0.35	0.25
-2	-7	13	48.51	2.42
-2	-7	13	44.83	3.37
-2	-7	13	46.07	2.81
2	7	-13	47.61	2.29
2	7	-13	46.74	2.94
2	7	-13	50.30	1.84
2	7	-13	45.89	1.84
2	7	-13	47.73	3.47
-3	-7	13	2.46	1.41
-3	-7	13	0.17	0.90
-3	-7	13	-1.03	0.66
3	7	-13	1.00	1.01
3	7	-13	0.88	0.52
-4	-7	13	58.69	3.87
-4	-7	13	56.80	2.78
-4	-7	13	54.78	3.08
4	7	-13	60.85	2.68
4	7	-13	55.46	3.19
-5	-7	13	2.01	0.96
-5	-7	13	3.06	1.07
-5	-7	13	5.73	1.66
5	7	-13	2.60	0.63
5	7	-13	2.08	1.10
-6	-7	13	44.22	3.75
-6	-7	13	43.53	2.61
6	7	-13	48.77	2.88
6	7	-13	47.35	2.30
-7	-7	13	6.54	1.83
-7	-7	13	4.85	1.04
7	7	-13	2.50	1.13
7	7	-13	4.48	0.30
7	7	-13	4.50	0.73
-8	-7	13	66.26	4.33
-8	-7	13	57.52	3.01
-8	-7	13	56.42	2.95

-8	-7	13	56.45	2.27
8	7	-13	60.46	2.27
8	7	-13	60.44	3.29
-9	-7	13	1.25	0.60
-9	-7	13	0.24	0.82
-9	-7	13	-0.03	1.72
-9	-7	13	1.01	0.27
9	7	-13	3.10	1.25
9	7	-13	1.47	0.34
-10	-7	13	52.11	2.77
-10	-7	13	55.00	4.38
-10	-7	13	57.97	2.80
-10	-7	13	54.75	2.24
10	7	-13	57.31	2.21
10	7	-13	57.34	3.17
-11	-7	13	2.11	0.76
-11	-7	13	1.35	0.72
-11	-7	13	-0.69	1.88
11	7	-13	3.35	1.32
11	7	-13	1.96	0.33
-12	-7	13	91.42	4.07
-12	-7	13	95.06	5.91
-12	-7	13	96.10	4.32
12	7	-13	95.25	3.68
-13	-7	13	5.87	1.08
-13	-7	13	3.48	2.20
13	7	-13	4.71	0.66
-14	-7	13	35.12	4.46
-14	-7	13	40.97	2.61
14	7	-13	43.96	1.93
-15	-7	13	6.59	1.23
-15	-7	13	3.99	2.45
-16	-7	13	34.81	2.64
-16	-7	13	36.65	4.77
-17	-7	13	2.90	1.32
-17	-7	13	4.28	2.76
14	-7	14	33.63	3.59
14	-7	14	34.99	2.29
-14	7	-14	37.23	2.94
13	-7	14	4.68	0.97
13	-7	14	5.86	1.81
-13	7	-14	6.83	1.51
12	-7	14	13.19	1.46
12	-7	14	13.69	2.18

-12	7	-14	13.67	2.11
11	-7	14	2.89	0.76
11	-7	14	3.82	1.49
-11	7	-14	2.60	1.05
10	-7	14	22.96	2.79
10	-7	14	25.09	1.57
-10	7	-14	20.91	1.96
9	-7	14	11.76	1.46
9	-7	14	14.16	1.48
9	-7	14	8.40	1.64
-9	7	-14	9.31	1.51
8	-7	14	23.74	2.68
8	-7	14	25.40	1.48
8	-7	14	24.42	1.92
-8	7	-14	27.30	1.55
-8	7	-14	25.57	1.88
7	-7	14	17.62	1.84
7	-7	14	13.51	2.25
7	-7	14	20.05	1.78
-7	7	-14	18.64	1.14
-7	7	-14	14.56	1.21
-7	7	-14	18.21	1.60
6	-7	14	25.65	2.23
6	-7	14	21.32	2.50
6	-7	14	24.96	2.01
-6	7	-14	26.69	1.76
-6	7	-14	25.59	1.41
-6	7	-14	24.11	1.32
5	-7	14	3.78	1.33
5	-7	14	2.97	1.18
5	-7	14	0.98	0.76
-5	7	-14	2.87	0.47
-5	7	-14	2.28	0.45
-5	7	-14	4.39	1.27
-5	7	-14	2.33	0.71
4	-7	14	16.58	1.59
4	-7	14	18.02	2.16
4	-7	14	14.93	2.19
-4	7	-14	21.26	1.06
-4	7	-14	18.14	2.09
-4	7	-14	17.77	1.08
-4	7	-14	18.86	1.47
3	-7	14	21.97	2.22
3	-7	14	22.06	2.38

3	-7	14	20.87	1.69
-3	7	-14	25.64	1.14
-3	7	-14	21.10	1.14
-3	7	-14	20.59	1.51
-3	7	-14	21.60	2.32
2	-7	14	18.59	2.41
2	-7	14	15.47	1.56
2	-7	14	16.30	2.05
-2	7	-14	18.10	2.32
-2	7	-14	17.93	0.95
-2	7	-14	16.37	1.36
-2	7	-14	18.39	0.93
1	-7	14	5.19	1.25
1	-7	14	5.22	1.52
1	-7	14	7.20	0.95
-1	7	-14	5.80	0.56
-1	7	-14	5.89	0.53
-1	7	-14	5.59	0.80
-1	7	-14	6.21	1.54
0	-7	14	39.78	2.69
0	-7	14	42.71	3.21
0	-7	14	43.35	2.20
0	7	-14	34.02	1.64
0	7	-14	36.79	1.56
0	7	-14	32.05	2.99
0	7	-14	38.03	2.00
-1	-7	14	23.22	2.78
-1	-7	14	17.55	1.64
-1	-7	14	21.86	2.13
1	7	-14	22.11	0.99
1	7	-14	22.21	1.46
1	7	-14	22.79	0.97
-2	-7	14	36.69	2.15
-2	-7	14	36.30	2.61
-2	-7	14	37.16	3.12
2	7	-14	39.06	1.48
2	7	-14	36.06	1.95
2	7	-14	35.05	1.48
-3	-7	14	33.96	2.47
-3	-7	14	32.10	3.12
-3	-7	14	30.98	1.98
3	7	-14	33.50	2.56
3	7	-14	32.90	1.82
3	7	-14	31.14	1.29

3	7	-14	35.14	1.31
-4	-7	14	24.83	2.99
-4	-7	14	25.26	2.19
4	7	-14	28.30	2.42
4	7	-14	23.72	1.58
-5	-7	14	50.62	3.04
-5	-7	14	51.44	4.62
5	7	-14	55.54	2.60
5	7	-14	56.57	3.20
-6	-7	14	21.79	2.96
-6	-7	14	13.69	1.79
6	7	-14	12.56	2.09
6	7	-14	14.02	1.26
-7	-7	14	24.51	2.06
-7	-7	14	28.50	3.31
7	7	-14	28.64	2.38
7	7	-14	26.68	1.68
-8	-7	14	17.45	3.13
-8	-7	14	20.01	1.53
-8	-7	14	19.30	1.93
8	7	-14	20.89	0.88
8	7	-14	22.28	2.34
-9	-7	14	39.73	2.22
-9	-7	14	38.87	2.38
-9	-7	14	40.32	3.97
9	7	-14	37.23	1.73
9	7	-14	43.28	2.79
-10	-7	14	4.15	0.83
-10	-7	14	3.58	2.05
-10	-7	14	4.01	0.94
10	7	-14	3.82	1.35
10	7	-14	3.82	0.45
-11	-7	14	39.25	2.25
-11	-7	14	37.95	4.13
-11	-7	14	41.74	2.38
11	7	-14	40.49	1.68
-12	-7	14	2.73	0.94
-12	-7	14	1.63	2.03
12	7	-14	2.48	0.39
-13	-7	14	31.20	4.14
-13	-7	14	25.64	2.22
-14	-7	14	12.35	1.85
-14	-7	14	12.05	2.76
-15	-7	14	27.81	2.31

-15	-7	14	28.39	4.56
-16	-7	14	-3.39	2.53
-16	-7	14	1.51	1.20
13	-7	15	18.38	1.72
13	-7	15	14.08	2.33
-13	7	-15	14.89	2.41
12	-7	15	1.80	0.84
12	-7	15	0.05	1.67
-12	7	-15	2.01	1.23
-12	7	-15	0.46	0.93
11	-7	15	20.61	1.61
11	-7	15	15.10	2.76
-11	7	-15	15.34	1.68
-11	7	-15	15.62	2.08
10	-7	15	0.65	1.57
-10	7	-15	0.33	0.71
-10	7	-15	0.52	1.00
9	-7	15	15.35	2.61
-9	7	-15	11.57	1.32
8	-7	15	2.07	1.07
8	-7	15	2.83	1.41
-8	7	-15	1.23	0.86
-8	7	-15	2.33	0.68
7	-7	15	44.58	3.20
7	-7	15	39.99	2.68
-7	7	-15	42.13	2.41
-7	7	-15	46.81	2.17
6	-7	15	1.22	1.13
6	-7	15	-1.13	1.29
-6	7	-15	1.17	1.29
-6	7	-15	0.30	0.74
-6	7	-15	0.42	0.49
-6	7	-15	-0.02	0.48
5	-7	15	20.21	2.48
5	-7	15	23.11	2.41
-5	7	-15	22.59	1.70
-5	7	-15	24.69	1.34
-5	7	-15	24.55	1.28
-5	7	-15	18.74	2.35
4	-7	15	-0.36	1.20
4	-7	15	-0.53	1.25
4	-7	15	0.60	0.83
-4	7	-15	-0.28	0.38
-4	7	-15	0.05	1.31

-4	7	-15	-0.53	0.36
-4	7	-15	0.61	0.69
3	-7	15	57.05	3.65
3	-7	15	62.25	3.03
3	-7	15	62.83	3.78
-3	7	-15	62.66	2.62
-3	7	-15	61.53	3.80
-3	7	-15	62.31	2.94
-3	7	-15	66.49	2.59
2	-7	15	1.72	0.80
2	-7	15	2.07	1.43
2	-7	15	0.10	1.12
-2	7	-15	1.50	1.47
-2	7	-15	2.22	0.41
-2	7	-15	2.11	0.70
-2	7	-15	2.53	0.39
1	-7	15	95.12	5.06
1	-7	15	94.32	4.14
1	-7	15	86.65	5.05
-1	7	-15	102.54	3.68
-1	7	-15	89.61	4.02
-1	7	-15	93.61	3.69
0	-7	15	4.57	0.87
0	-7	15	4.25	1.34
0	-7	15	5.69	1.60
0	7	-15	5.22	0.53
0	7	-15	5.11	0.80
0	7	-15	4.32	0.53
-1	-7	15	31.54	2.08
-1	-7	15	35.42	3.25
-1	-7	15	34.76	2.62
1	7	-15	37.65	1.45
1	7	-15	33.19	1.91
1	7	-15	33.23	1.44
-2	-7	15	6.81	1.21
-2	-7	15	6.66	1.87
-2	-7	15	5.88	1.33
2	7	-15	6.09	0.84
2	7	-15	6.24	0.51
2	7	-15	5.88	0.48
-3	-7	15	25.59	2.39
3	7	-15	22.88	1.04
3	7	-15	25.97	1.63
3	7	-15	26.91	1.06

-4	-7	15	-0.14	1.62
-4	-7	15	-1.52	1.05
4	7	-15	-0.04	0.15
4	7	-15	0.67	0.61
4	7	-15	0.32	0.19
-5	-7	15	26.34	2.38
-5	-7	15	26.74	3.42
5	7	-15	28.99	1.79
5	7	-15	35.81	2.67
5	7	-15	32.18	1.23
-6	-7	15	9.91	1.77
-6	-7	15	10.52	2.17
6	7	-15	9.35	1.14
6	7	-15	5.44	1.53
-7	-7	15	47.34	2.43
-7	-7	15	45.35	4.04
-7	-7	15	42.93	2.81
7	7	-15	46.75	2.44
7	7	-15	49.24	3.17
-8	-7	15	13.60	1.36
-8	-7	15	6.26	1.99
-8	-7	15	9.85	1.75
8	7	-15	11.50	0.58
8	7	-15	12.39	1.72
-9	-7	15	30.72	1.92
-9	-7	15	27.02	2.12
-9	-7	15	23.07	3.69
9	7	-15	26.91	2.67
-10	-7	15	8.69	1.58
-10	-7	15	3.65	2.11
-10	-7	15	6.90	1.03
10	7	-15	7.96	0.62
-11	-7	15	11.67	1.59
-11	-7	15	10.80	2.50
-11	-7	15	11.61	1.91
-12	-7	15	9.72	2.48
-12	-7	15	6.66	1.18
-13	-7	15	10.73	1.80
-13	-7	15	16.94	2.93
-14	-7	15	1.83	2.39
-14	-7	15	4.60	1.17
-15	-7	15	15.92	3.12
-15	-7	15	16.75	2.24
12	-7	16	2.09	1.88

-12	7	-16	3.36	1.06
-12	7	-16	1.55	1.38
11	-7	16	7.59	2.03
-11	7	-16	8.07	1.39
-11	7	-16	9.00	1.20
10	-7	16	15.53	2.94
-10	7	-16	13.03	1.56
-10	7	-16	11.08	1.91
9	-7	16	2.90	1.67
-9	7	-16	2.21	0.81
-9	7	-16	0.48	0.97
8	-7	16	6.42	1.72
-8	7	-16	6.05	1.27
-8	7	-16	7.13	0.93
7	-7	16	15.58	2.09
7	-7	16	13.83	2.64
-7	7	-16	20.92	1.71
-7	7	-16	16.28	1.33
6	-7	16	10.51	1.62
6	-7	16	5.48	1.61
-6	7	-16	8.91	1.40
-6	7	-16	10.91	1.10
5	-7	16	16.44	2.33
5	-7	16	12.86	1.99
-5	7	-16	15.84	2.08
-5	7	-16	16.85	1.56
-5	7	-16	16.76	1.13
-5	7	-16	14.52	1.15
4	-7	16	13.82	2.49
4	-7	16	14.76	2.45
-4	7	-16	14.88	2.04
-4	7	-16	15.99	1.09
-4	7	-16	16.98	1.07
-4	7	-16	15.20	1.54
3	-7	16	3.76	1.37
3	-7	16	6.59	1.70
-3	7	-16	4.98	0.75
-3	7	-16	4.49	0.84
-3	7	-16	4.78	0.56
2	-7	16	26.17	2.55
2	-7	16	23.85	2.80
-2	7	-16	22.66	1.23
-2	7	-16	23.06	1.65
-2	7	-16	24.65	1.22

1	-7	16	8.15	1.79
1	-7	16	9.88	1.70
-1	7	-16	12.80	0.78
-1	7	-16	9.91	0.76
-1	7	-16	9.66	1.25
0	-7	16	19.47	2.82
0	-7	16	20.24	2.37
0	7	-16	20.26	1.05
0	7	-16	18.56	1.51
0	7	-16	23.89	1.07
-1	-7	16	7.50	1.61
1	7	-16	11.57	0.68
1	7	-16	10.57	0.75
1	7	-16	9.91	1.22
-2	-7	16	16.96	2.19
-2	-7	16	16.46	2.86
2	7	-16	15.03	0.79
2	7	-16	18.27	1.39
2	7	-16	15.85	0.81
-3	-7	16	0.75	1.12
-3	-7	16	2.13	1.72
3	7	-16	2.21	0.68
3	7	-16	0.36	0.38
3	7	-16	1.15	0.26
-4	-7	16	6.61	1.98
-4	-7	16	6.46	1.34
4	7	-16	4.76	0.85
4	7	-16	4.44	0.40
4	7	-16	6.82	0.46
-5	-7	16	8.26	2.16
-5	-7	16	5.35	1.36
5	7	-16	5.91	0.43
5	7	-16	4.84	0.36
5	7	-16	7.08	1.11
-6	-7	16	18.13	3.31
-6	-7	16	19.77	2.17
6	7	-16	19.80	3.09
6	7	-16	19.08	1.51
-7	-7	16	6.91	2.29
-7	-7	16	5.94	1.27
-7	-7	16	6.70	0.97
7	7	-16	5.79	1.63
-8	-7	16	39.22	2.66
-8	-7	16	43.85	2.37

-8	-7	16	46.27	4.32
8	7	-16	36.63	3.05
-9	-7	16	1.43	1.99
-9	-7	16	-0.61	1.01
-9	-7	16	-0.60	0.73
-10	-7	16	18.71	2.91
-10	-7	16	14.91	1.92
-10	-7	16	21.33	1.80
-11	-7	16	3.48	0.99
-11	-7	16	-0.42	2.21
-12	-7	16	5.92	1.19
-12	-7	16	5.19	2.51
-13	-7	16	-4.18	2.35
-13	-7	16	0.17	0.99
-14	-7	16	20.56	3.46
-14	-7	16	27.28	2.34
-11	7	-17	1.94	0.98
-11	7	-17	-4.31	1.60
10	-7	17	25.94	3.36
-10	7	-17	20.10	1.86
-10	7	-17	20.04	2.19
9	-7	17	1.00	1.67
-9	7	-17	1.97	0.83
-9	7	-17	2.35	1.04
8	-7	17	30.91	3.31
-8	7	-17	25.68	2.13
-8	7	-17	28.15	1.83
7	-7	17	3.38	1.57
7	-7	17	3.30	1.36
-7	7	-17	3.89	0.89
-7	7	-17	5.49	1.13
6	-7	17	26.41	2.61
6	-7	17	27.54	3.06
-6	7	-17	27.26	1.64
-6	7	-17	27.17	1.97
5	-7	17	1.23	1.39
5	-7	17	-1.05	1.45
-5	7	-17	-0.14	0.54
-5	7	-17	0.41	0.82
4	-7	17	44.02	3.35
4	-7	17	41.04	3.28
-4	7	-17	42.54	2.02
-4	7	-17	42.11	2.35
3	-7	17	6.03	1.69

3	-7	17	0.82	1.46
-3	7	-17	3.37	0.82
-3	7	-17	5.19	0.82
2	-7	17	16.01	2.45
2	-7	17	19.48	2.93
-2	7	-17	19.86	1.15
-2	7	-17	20.39	1.59
1	-7	17	7.53	1.63
-1	7	-17	4.97	0.54
-1	7	-17	5.64	0.90
0	-7	17	25.43	2.65
0	-7	17	27.76	3.16
0	7	-17	21.39	1.64
0	7	-17	23.29	1.18
-1	-7	17	2.47	1.86
-1	-7	17	2.82	1.34
1	7	-17	2.84	0.74
1	7	-17	1.61	0.36
-2	-7	17	23.45	3.44
-2	-7	17	28.46	2.61
2	7	-17	27.35	1.22
2	7	-17	28.72	1.77
-3	-7	17	-0.38	1.81
-3	-7	17	-0.24	1.19
3	7	-17	0.19	0.26
3	7	-17	1.19	0.69
-4	-7	17	16.26	2.58
-4	-7	17	16.69	2.27
4	7	-17	17.20	1.68
-5	-7	17	4.54	1.31
-5	-7	17	-0.92	1.96
5	7	-17	2.65	0.74
-6	-7	17	13.19	2.09
-6	-7	17	14.18	2.63
-6	-7	17	14.48	1.51
6	7	-17	16.20	1.47
-7	-7	17	5.12	0.97
-7	-7	17	2.99	1.34
-7	-7	17	5.15	2.25
-8	-7	17	35.93	4.30
-8	-7	17	34.50	2.16
-8	-7	17	28.70	2.58
-9	-7	17	9.42	1.50
-9	-7	17	8.58	1.42

-9	-7	17	6.27	2.32
-10	-7	17	3.99	2.52
-10	-7	17	6.32	1.15
-11	-7	17	2.17	1.01
-11	-7	17	1.21	2.38
-12	-7	17	18.21	2.08
-12	-7	17	17.86	3.17
-13	-7	17	13.15	1.99
-13	-7	17	12.50	3.00
9	-7	18	24.38	3.41
-9	7	-18	19.37	1.85
8	-7	18	-0.35	1.84
-8	7	-18	-0.16	0.78
-8	7	-18	-1.06	1.00
7	-7	18	45.68	3.90
-7	7	-18	42.63	2.66
-7	7	-18	47.96	2.39
6	-7	18	1.41	1.70
6	-7	18	-0.02	1.48
-6	7	-18	0.68	1.08
-6	7	-18	1.18	0.65
5	-7	18	27.88	2.97
5	-7	18	32.54	3.21
-5	7	-18	31.66	1.76
-5	7	-18	30.07	2.09
4	-7	18	0.75	1.51
4	-7	18	3.20	1.65
-4	7	-18	0.31	0.56
-4	7	-18	0.86	0.84
3	-7	18	22.38	3.07
3	-7	18	30.24	5.34
-3	7	-18	26.74	1.48
-3	7	-18	26.74	1.86
2	-7	18	0.09	1.69
2	-7	18	-0.35	1.44
-2	7	-18	-0.37	0.76
-2	7	-18	-0.21	0.44
1	-7	18	30.67	3.01
1	-7	18	39.36	3.78
-1	7	-18	36.70	2.12
-1	7	-18	36.98	1.70
0	-7	18	-1.04	1.78
0	-7	18	-1.07	1.39
0	7	-18	-0.75	0.38

0	7	-18	0.83	0.73
-1	-7	18	42.93	4.14
-1	-7	18	40.05	3.23
1	7	-18	39.46	1.73
1	7	-18	40.66	2.22
-2	-7	18	2.45	2.01
-2	-7	18	0.03	1.40
2	7	-18	0.37	0.43
2	7	-18	0.16	0.71
-3	-7	18	23.40	2.69
-3	-7	18	30.08	3.88
-4	-7	18	4.01	2.15
-4	-7	18	2.36	1.33
4	7	-18	0.69	0.73
-5	-7	18	43.07	3.41
-5	-7	18	48.68	4.45
5	7	-18	45.26	2.43
-6	-7	18	8.61	1.66
-6	-7	18	9.17	2.67
-6	-7	18	7.73	1.10
-7	-7	18	24.53	1.96
-7	-7	18	23.71	2.49
-7	-7	18	26.59	4.06
-8	-7	18	2.81	1.40
-8	-7	18	4.82	1.02
-8	-7	18	10.07	2.54
-9	-7	18	26.77	4.34
-9	-7	18	35.01	3.15
-9	-7	18	27.51	2.20
-10	-7	18	1.31	0.96
-10	-7	18	-1.70	2.41
-11	-7	18	32.14	4.76
-11	-7	18	30.80	2.43
-12	-7	18	-0.86	2.86
-12	-7	18	0.56	1.15
7	-7	19	4.32	1.99
-7	7	-19	3.05	0.90
6	-7	19	26.83	3.42
-6	7	-19	21.34	2.06
-6	7	-19	23.57	1.70
5	-7	19	6.19	2.04
5	-7	19	6.97	1.98
-5	7	-19	6.80	0.83
4	-7	19	27.12	3.17

4	-7	19	23.63	3.14
-4	7	-19	23.54	1.54
-4	7	-19	20.50	1.89
3	-7	19	2.10	1.81
3	-7	19	0.61	1.72
-3	7	-19	-0.17	0.86
-3	7	-19	0.71	0.61
2	-7	19	34.97	3.60
2	-7	19	27.71	3.23
-2	7	-19	31.32	2.07
-2	7	-19	35.51	2.20
1	-7	19	1.01	1.60
1	-7	19	-0.69	1.89
-1	7	-19	0.61	0.83
0	-7	19	25.14	3.04
0	-7	19	20.46	2.93
0	7	-19	21.70	1.74
-1	-7	19	0.00	1.52
-1	-7	19	1.18	2.05
-2	-7	19	46.05	4.48
-2	-7	19	39.51	3.43
2	7	-19	40.63	2.35
-3	-7	19	-0.48	1.56
-3	-7	19	2.87	2.19
3	7	-19	0.56	0.82
-4	-7	19	22.38	2.88
-4	-7	19	22.00	4.07
4	7	-19	25.51	1.81
-5	-7	19	-0.99	0.83
-5	-7	19	0.21	1.44
-5	-7	19	-3.56	2.38
5	7	-19	0.30	0.79
-6	-7	19	26.97	4.45
-6	-7	19	32.35	3.02
-6	-7	19	33.31	2.24
-7	-7	19	0.69	2.50
-7	-7	19	1.04	0.92
-8	-7	19	41.68	3.05
-8	-7	19	41.74	4.95
-8	-7	19	41.14	2.55
-9	-7	19	-2.29	2.69
-9	-7	19	0.19	0.98
-10	-7	19	24.85	2.23
-10	-7	19	20.20	3.42

-11	-7	19	-2.07	1.11
5	-7	20	3.57	2.02
4	-7	20	8.33	2.20
4	-7	20	10.08	2.29
3	-7	20	12.32	2.23
3	-7	20	11.93	2.39
-3	7	-20	9.39	1.47
2	-7	20	2.14	1.86
2	-7	20	2.30	2.11
-2	7	-20	3.50	1.09
1	-7	20	22.99	3.01
1	-7	20	19.46	3.28
-1	7	-20	19.99	1.77
0	-7	20	0.71	2.07
0	-7	20	3.13	1.81
0	7	-20	0.77	0.89
-1	-7	20	14.59	2.66
-1	-7	20	11.66	2.16
1	7	-20	9.30	1.53
-2	-7	20	7.80	1.90
-2	-7	20	6.79	2.48
2	7	-20	6.01	1.02
-3	-7	20	7.81	2.56
-3	-7	20	9.42	2.01
3	7	-20	11.01	1.52
-4	-7	20	12.03	2.84
-4	-7	20	13.27	2.15
4	7	-20	13.38	1.64
-5	-7	20	10.94	1.75
-5	-7	20	9.25	2.01
-5	-7	20	11.59	2.94
-6	-7	20	0.85	1.58
-6	-7	20	2.16	1.04
-6	-7	20	-2.74	2.44
-7	-7	20	18.57	2.01
-7	-7	20	19.94	2.82
-7	-7	20	16.35	3.14
-8	-7	20	7.26	1.30
-8	-7	20	2.81	2.77
-9	-7	20	23.69	2.30
-9	-7	20	24.67	4.98
2	-7	21	-1.03	2.23
2	-7	21	4.35	2.15
1	-7	21	29.13	4.16

1	-7	21	24.59	3.39
0	-7	21	4.40	2.02
0	-7	21	6.16	2.46
-1	-7	21	31.84	4.25
-1	-7	21	18.18	3.33
1	7	-21	21.63	1.90
-2	-7	21	2.98	1.93
-2	-7	21	5.35	2.56
2	7	-21	4.42	1.03
-3	-7	21	22.04	3.29
-3	-7	21	19.61	3.21
3	7	-21	20.05	1.88
-4	-7	21	-3.45	2.29
-4	-7	21	-0.53	1.74
-4	-7	21	-0.09	1.05
-5	-7	21	19.03	2.03
-5	-7	21	14.20	2.20
-5	-7	21	11.93	2.97
-6	-7	21	5.61	2.84
-6	-7	21	-0.72	1.83
-6	-7	21	2.78	1.19
5	-8	-21	4.11	0.94
4	-8	-21	19.00	1.72
-4	8	21	12.79	2.17
-4	8	21	14.77	2.43
3	-8	-21	1.96	0.89
-3	8	21	1.27	1.65
-3	8	21	3.49	1.91
2	-8	-21	21.65	1.81
-2	8	21	25.76	3.25
-2	8	21	20.74	3.24
1	-8	-21	-0.14	0.78
-1	8	21	-1.52	1.77
-1	8	21	-0.61	1.71
0	-8	-21	19.87	1.83
0	8	21	19.24	3.22
0	8	21	17.41	3.19
-1	-8	-21	2.40	0.92
1	8	21	2.16	1.86
1	8	21	2.11	1.76
8	-8	-20	13.27	1.48
7	-8	-20	1.86	2.59
7	-8	-20	1.91	0.78
-7	8	20	1.32	1.94

6	-8	-20	13.32	1.41
-6	8	20	15.70	2.75
-6	8	20	13.49	2.30
5	-8	-20	9.46	1.38
-5	8	20	11.29	2.13
-5	8	20	10.23	2.01
4	-8	-20	5.48	0.97
-4	8	20	10.16	2.04
-4	8	20	9.19	1.93
3	-8	-20	3.18	0.82
-3	8	20	1.10	1.70
-3	8	20	3.47	1.68
2	-8	-20	9.81	1.39
-2	8	20	15.11	2.82
-2	8	20	7.58	1.88
1	-8	-20	4.64	0.89
-1	8	20	5.12	1.76
-1	8	20	5.05	1.74
0	-8	-20	4.50	0.93
0	8	20	8.57	1.92
0	8	20	7.04	1.98
-1	-8	-20	16.94	1.69
1	8	20	18.93	3.09
1	8	20	20.13	2.89
-2	-8	-20	10.88	1.60
2	8	20	18.15	2.82
2	8	20	15.53	2.39
-3	-8	-20	13.20	1.67
3	8	20	16.72	3.16
3	8	20	16.61	2.84
-4	-8	-20	7.18	1.10
4	8	20	3.41	1.92
4	8	20	6.39	1.81
-5	-8	-20	13.91	1.68
5	8	20	8.43	2.40
10	-8	-19	1.27	0.73
-10	8	19	-0.81	2.64
9	-8	-19	22.84	1.66
-9	8	19	21.58	3.56
8	-8	-19	0.62	0.68
8	-8	-19	2.99	2.34
-8	8	19	1.71	1.86
7	-8	-19	16.78	1.43
7	-8	-19	23.46	3.88

-7	8	19	16.37	2.46
6	-8	-19	4.06	0.78
-6	8	19	1.40	1.55
-6	8	19	0.80	1.57
5	-8	-19	30.20	1.85
-5	8	19	28.35	3.24
-5	8	19	30.29	3.01
4	-8	-19	1.57	0.70
-4	8	19	4.55	1.68
-4	8	19	-0.99	1.51
3	-8	-19	29.35	1.80
-3	8	19	24.12	2.89
-3	8	19	28.31	3.08
2	-8	-19	0.02	0.67
-2	8	19	-0.23	1.48
-2	8	19	1.11	1.50
1	-8	-19	21.72	1.61
-1	8	19	18.07	2.70
-1	8	19	20.83	2.74
0	-8	-19	-1.06	0.69
0	8	19	-0.97	1.41
0	8	19	0.50	1.50
-1	-8	-19	24.45	1.83
1	8	19	30.03	2.92
1	8	19	26.38	2.97
-2	-8	-19	2.95	0.85
2	8	19	3.99	1.67
2	8	19	4.58	1.52
-3	-8	-19	39.21	2.31
3	8	19	38.17	3.09
3	8	19	34.56	3.46
-4	-8	-19	2.64	0.85
4	8	19	3.40	1.59
4	8	19	0.52	1.53
-5	-8	-19	12.09	1.52
5	8	19	12.20	1.99
5	8	19	10.78	2.18
-6	-8	-19	1.78	0.88
6	8	19	2.71	1.82
6	8	19	1.07	1.60
-7	-8	-19	8.55	1.24
7	8	19	9.78	3.08
7	8	19	14.53	2.03
12	-8	-18	17.21	1.56

-12	8	18	16.22	3.68
11	-8	-18	0.70	0.72
-11	8	18	0.88	1.88
10	-8	-18	13.39	1.34
-10	8	18	12.68	2.50
9	-8	-18	7.56	1.18
9	-8	-18	10.04	2.18
-9	8	18	8.48	2.06
8	-8	-18	28.48	3.56
8	-8	-18	21.88	1.58
-8	8	18	23.91	3.19
7	-8	-18	3.96	2.26
7	-8	-18	0.95	0.63
-7	8	18	1.41	1.54
6	-8	-18	35.81	1.98
-6	8	18	39.13	3.37
-6	8	18	32.31	2.84
5	-8	-18	1.62	0.64
-5	8	18	1.41	1.33
-5	8	18	3.57	1.55
4	-8	-18	43.06	2.17
-4	8	18	39.49	3.03
-4	8	18	34.80	3.24
3	-8	-18	-0.38	0.57
-3	8	18	0.28	1.32
-3	8	18	0.94	1.30
2	-8	-18	28.55	1.72
-2	8	18	25.11	2.73
-2	8	18	29.32	2.83
1	-8	-18	1.36	0.68
-1	8	18	-1.04	1.33
-1	8	18	-1.43	1.15
0	-8	-18	16.75	1.42
0	8	18	16.89	2.49
0	8	18	16.07	2.43
-1	-8	-18	1.62	0.75
1	8	18	0.73	1.30
1	8	18	2.19	1.42
-2	-8	-18	35.71	2.08
2	8	18	30.27	3.07
2	8	18	34.84	2.91
-3	-8	-18	0.55	0.74
3	8	18	1.06	1.28
3	8	18	-3.44	1.38

-4	-8	-18	45.02	2.40
4	8	18	43.75	3.17
4	8	18	38.05	3.37
-5	-8	-18	1.76	0.83
5	8	18	3.07	1.43
5	8	18	2.24	1.63
-6	-8	-18	16.38	1.67
6	8	18	16.18	2.51
6	8	18	14.57	2.26
-7	-8	-18	-0.43	0.76
7	8	18	1.49	1.46
7	8	18	-0.27	1.70
-8	-8	-18	23.50	1.91
8	8	18	15.64	2.70
8	8	18	20.32	3.27
-9	-8	-18	0.16	1.15
13	-8	-17	9.28	1.33
-13	8	17	8.54	2.44
-13	8	17	10.00	1.72
12	-8	-17	3.04	0.76
-12	8	17	2.07	1.41
-12	8	17	6.41	2.12
11	-8	-17	11.86	1.29
-11	8	17	11.83	2.20
10	-8	-17	9.47	1.17
-10	8	17	9.10	2.08
-10	8	17	15.38	1.84
9	-8	-17	15.89	2.95
9	-8	-17	11.93	1.24
-9	8	17	15.30	1.69
-9	8	17	14.53	2.34
8	-8	-17	15.27	1.29
8	-8	-17	18.62	3.11
-8	8	17	12.92	1.52
-8	8	17	18.92	2.78
7	-8	-17	7.24	1.11
7	-8	-17	8.95	1.97
-7	8	17	10.15	1.22
-7	8	17	8.53	1.80
6	-8	-17	10.97	1.16
-6	8	17	9.97	1.10
-6	8	17	11.40	2.08
-6	8	17	9.50	1.89
5	-8	-17	22.34	1.48

-5	8	17	22.31	2.66
4	-8	-17	1.10	0.60
-4	8	17	-1.98	1.18
-4	8	17	1.60	1.12
3	-8	-17	8.75	1.12
-3	8	17	8.94	1.53
-3	8	17	10.18	1.72
2	-8	-17	6.73	1.06
-2	8	17	5.18	1.47
-2	8	17	4.68	1.40
1	-8	-17	21.26	1.49
-1	8	17	17.32	2.29
-1	8	17	21.50	2.37
0	-8	-17	0.48	0.62
0	8	17	1.54	1.25
0	8	17	2.74	1.38
-1	-8	-17	20.82	1.48
1	8	17	16.14	2.25
1	8	17	20.59	2.60
-2	-8	-17	2.23	0.73
2	8	17	1.77	1.21
2	8	17	0.73	1.34
-3	-8	-17	32.43	1.91
3	8	17	30.93	2.53
3	8	17	29.61	2.84
-4	-8	-17	2.94	0.77
4	8	17	3.42	1.34
4	8	17	2.14	1.52
-5	-8	-17	20.95	1.61
5	8	17	18.11	2.56
5	8	17	18.28	2.28
-6	-8	-17	1.20	0.69
6	8	17	2.85	1.60
6	8	17	1.65	1.29
-7	-8	-17	15.95	1.59
7	8	17	16.18	2.47
-8	-8	-17	2.02	0.84
8	8	17	1.93	1.43
8	8	17	-0.98	1.65
-9	-8	-17	38.60	2.32
9	8	17	29.78	3.37
9	8	17	31.43	3.48
-10	-8	-17	1.35	0.97
10	8	17	1.67	1.83

14	-8	-16	0.00	0.70
-14	8	16	1.33	2.08
-14	8	16	-2.55	1.41
13	-8	-16	8.89	1.23
-13	8	16	9.59	1.59
-13	8	16	4.01	2.02
12	-8	-16	2.22	0.71
-12	8	16	2.23	1.23
-12	8	16	3.19	1.77
11	-8	-16	32.57	1.89
-11	8	16	44.27	3.90
-11	8	16	30.35	3.42
10	-8	-16	-1.23	1.50
10	-8	-16	0.11	0.54
-10	8	16	0.66	0.98
-10	8	16	-0.08	1.55
9	-8	-16	37.04	3.11
9	-8	-16	33.95	1.83
-9	8	16	30.29	3.25
-9	8	16	34.83	2.18
8	-8	-16	-0.50	0.52
8	-8	-16	-2.05	1.47
8	-8	-16	1.53	1.80
-8	8	16	1.98	1.46
-8	8	16	-1.13	0.70
7	-8	-16	12.87	1.20
7	-8	-16	14.48	2.18
-7	8	16	14.12	1.29
-7	8	16	17.24	2.47
6	-8	-16	4.72	0.73
6	-8	-16	8.57	1.76
-6	8	16	8.64	1.61
-6	8	16	6.85	1.02
-6	8	16	8.14	1.44
5	-8	-16	20.22	1.38
-5	8	16	22.34	1.26
-5	8	16	18.66	2.14
-5	8	16	16.78	2.39
4	-8	-16	7.07	0.97
-4	8	16	6.74	1.45
-4	8	16	6.67	1.35
3	-8	-16	17.12	1.29
-3	8	16	19.45	2.27
2	-8	-16	1.61	0.60

-2	8	16	0.83	1.08
-2	8	16	5.39	1.30
1	-8	-16	21.07	1.39
-1	8	16	18.43	2.19
-1	8	16	18.30	2.13
0	-8	-16	2.44	0.68
0	8	16	1.76	1.20
0	8	16	2.48	1.15
-1	-8	-16	26.20	1.59
1	8	16	22.91	2.51
1	8	16	25.35	2.28
-2	-8	-16	2.66	0.67
2	8	16	3.29	1.15
2	8	16	4.38	1.32
-3	-8	-16	28.66	1.72
3	8	16	24.92	2.58
3	8	16	27.73	2.32
-4	-8	-16	11.17	1.25
4	8	16	15.41	1.88
4	8	16	10.71	1.75
-5	-8	-16	7.45	1.16
5	8	16	5.39	1.56
5	8	16	9.42	1.52
-6	-8	-16	3.62	0.79
6	8	16	3.67	1.67
-7	-8	-16	26.56	1.82
7	8	16	25.62	2.90
7	8	16	24.10	2.40
-8	-8	-16	8.95	1.37
8	8	16	7.33	1.83
8	8	16	8.86	1.52
-9	-8	-16	15.64	1.69
9	8	16	14.94	2.75
9	8	16	21.13	2.34
-10	-8	-16	8.08	1.14
10	8	16	10.26	2.04
-11	-8	-16	6.43	1.10
11	8	16	8.67	2.06
15	-8	-15	13.44	1.37
-15	8	15	10.58	1.75
-15	8	15	7.05	2.46
14	-8	-15	18.65	1.49
-14	8	15	21.47	2.47
-14	8	15	17.30	2.74

13	-8	-15	2.78	0.66
-13	8	15	-0.55	1.74
-13	8	15	1.62	1.21
12	-8	-15	14.44	1.30
-12	8	15	14.44	2.30
-12	8	15	14.77	2.04
11	-8	-15	0.19	1.26
11	-8	-15	-0.27	0.51
-11	8	15	1.06	1.56
-11	8	15	0.99	0.97
10	-8	-15	33.41	2.82
10	-8	-15	33.70	1.84
-10	8	15	33.26	3.24
-10	8	15	36.57	2.30
9	-8	-15	5.72	1.51
9	-8	-15	6.53	1.86
9	-8	-15	7.42	0.95
-9	8	15	6.76	1.62
-9	8	15	5.33	1.00
-9	8	15	8.32	1.39
8	-8	-15	37.90	3.13
8	-8	-15	44.11	2.90
8	-8	-15	42.34	2.08
-8	8	15	39.71	3.24
-8	8	15	45.31	2.25
-8	8	15	42.05	2.22
7	-8	-15	10.25	1.04
7	-8	-15	11.03	1.61
7	-8	-15	9.58	1.70
-7	8	15	10.51	1.17
-7	8	15	11.59	2.20
-7	8	15	15.66	1.38
6	-8	-15	33.32	3.04
6	-8	-15	30.41	1.73
6	-8	-15	32.07	2.72
-6	8	15	33.80	2.81
-6	8	15	33.32	1.71
-6	8	15	38.21	1.78
-6	8	15	31.72	2.40
5	-8	-15	2.23	0.59
-5	8	15	1.70	1.03
-5	8	15	1.47	0.53
-5	8	15	1.01	1.17
-5	8	15	2.45	0.54

4	-8	-15	25.74	1.49
-4	8	15	24.17	1.29
-4	8	15	24.60	2.36
-4	8	15	26.16	1.25
-4	8	15	24.17	2.19
3	-8	-15	11.57	1.02
-3	8	15	10.40	1.81
-3	8	15	10.28	1.58
2	-8	-15	64.76	2.85
-2	8	15	64.61	3.57
-2	8	15	62.08	3.47
1	-8	-15	14.67	1.15
-1	8	15	14.93	2.26
-1	8	15	14.22	1.81
0	-8	-15	39.42	2.03
0	8	15	41.51	2.67
0	8	15	38.29	2.73
-1	-8	-15	0.60	0.54
1	8	15	-0.74	1.00
1	8	15	0.56	1.13
-2	-8	-15	43.69	2.17
2	8	15	40.23	2.88
-3	-8	-15	-0.05	0.51
3	8	15	-0.64	1.11
3	8	15	-0.96	0.83
-4	-8	-15	65.82	3.02
4	8	15	63.62	3.84
4	8	15	66.27	3.46
-5	-8	-15	0.35	0.57
5	8	15	0.75	1.19
5	8	15	3.35	1.06
-6	-8	-15	37.90	2.15
6	8	15	40.52	2.57
6	8	15	38.70	3.09
-7	-8	-15	1.23	0.64
7	8	15	-0.19	1.33
7	8	15	1.96	1.09
-8	-8	-15	22.34	1.69
8	8	15	24.01	2.18
8	8	15	21.10	2.75
-9	-8	-15	3.45	0.85
9	8	15	2.80	1.24
9	8	15	2.49	1.62
-10	-8	-15	39.98	2.43

10	8	15	45.48	2.88
10	8	15	41.35	3.55
-11	-8	-15	0.25	0.81
11	8	15	0.34	1.55
-12	-8	-15	13.69	1.71
12	8	15	13.80	2.32
16	-8	-14	35.93	2.12
-16	8	14	32.67	3.94
-16	8	14	41.77	3.23
15	-8	-14	0.01	0.66
-15	8	14	-1.58	1.86
-15	8	14	-1.06	1.35
14	-8	-14	16.95	1.41
-14	8	14	21.92	2.36
-14	8	14	15.36	2.50
13	-8	-14	-0.63	0.57
-13	8	14	0.12	1.58
-13	8	14	-1.76	1.05
12	-8	-14	6.15	0.80
-12	8	14	6.79	1.82
-12	8	14	6.51	1.22
11	-8	-14	10.26	1.08
11	-8	-14	14.75	2.03
-11	8	14	13.67	2.56
-11	8	14	15.96	1.69
10	-8	-14	13.56	1.14
10	-8	-14	15.56	2.20
-10	8	14	12.95	2.03
-10	8	14	13.87	1.64
9	-8	-14	7.65	1.01
9	-8	-14	6.66	1.42
9	-8	-14	5.67	0.87
9	-8	-14	4.30	1.17
-9	8	14	8.15	1.01
-9	8	14	3.76	1.39
8	-8	-14	61.02	2.71
8	-8	-14	54.59	2.82
8	-8	-14	64.40	3.32
8	-8	-14	61.13	3.56
-8	8	14	63.14	2.84
-8	8	14	62.14	3.80
7	-8	-14	19.41	1.38
7	-8	-14	18.14	2.05
7	-8	-14	19.38	1.24

7	-8	-14	16.96	2.29
-7	8	14	17.69	1.32
-7	8	14	16.15	2.24
6	-8	-14	27.57	2.66
6	-8	-14	28.19	2.38
6	-8	-14	28.52	1.56
-6	8	14	27.80	2.50
-6	8	14	25.84	1.53
-6	8	14	32.94	1.60
5	-8	-14	38.69	1.90
5	-8	-14	40.87	2.69
-5	8	14	34.32	2.47
-5	8	14	41.64	1.81
-5	8	14	37.02	1.78
-5	8	14	36.50	2.74
4	-8	-14	28.57	1.56
4	-8	-14	28.11	2.42
-4	8	14	26.36	1.33
-4	8	14	24.25	2.25
-4	8	14	28.15	1.33
-4	8	14	27.16	2.16
-3	8	14	18.85	1.97
-3	8	14	19.23	0.95
-3	8	14	19.06	1.92
-2	8	14	23.69	2.09
-1	8	14	17.60	1.82
-1	8	14	15.21	1.84
0	-8	-14	10.59	1.14
0	8	14	11.19	1.62
0	8	14	10.10	1.65
-1	-8	-14	41.85	2.03
1	8	14	35.80	2.96
1	8	14	44.70	2.61
-2	-8	-14	4.05	0.62
2	8	14	2.00	0.99
2	8	14	2.02	0.90
-3	-8	-14	48.34	2.35
3	8	14	47.81	3.07
3	8	14	49.61	2.81
-4	-8	-14	11.37	1.08
4	8	14	7.98	1.51
4	8	14	10.00	1.90
-5	-8	-14	27.21	1.66
5	8	14	28.43	2.13

5	8	14	27.03	2.45
-6	-8	-14	22.87	1.54
6	8	14	22.88	1.99
6	8	14	20.33	2.39
-7	-8	-14	55.13	2.76
7	8	14	54.58	3.57
7	8	14	59.49	3.12
-8	-8	-14	-2.36	2.29
-8	-8	-14	1.00	0.63
8	8	14	2.05	1.41
8	8	14	1.19	1.06
-9	-8	-14	20.43	1.65
9	8	14	21.18	2.58
9	8	14	21.02	2.07
-10	-8	-14	0.11	0.64
10	8	14	2.05	1.15
10	8	14	-1.17	1.33
-11	-8	-14	9.30	1.42
11	8	14	8.86	1.40
11	8	14	10.75	1.83
-12	-8	-14	6.15	1.07
12	8	14	10.05	1.86
-13	-8	-14	47.34	2.79
13	8	14	49.66	3.82
16	-8	-13	7.67	1.19
-16	8	13	7.85	1.66
-16	8	13	8.55	2.31
15	-8	-13	34.98	2.00
-15	8	13	32.45	3.65
-15	8	13	44.34	2.99
14	-8	-13	2.49	0.66
-14	8	13	3.97	1.84
-14	8	13	6.34	1.37
13	-8	-13	25.00	1.58
-13	8	13	21.59	3.01
-13	8	13	33.72	2.39
12	-8	-13	-0.67	1.03
12	-8	-13	-0.37	0.48
-12	8	13	-2.42	1.40
-12	8	13	1.70	0.96
11	-8	-13	24.48	2.14
11	-8	-13	23.00	1.45
-11	8	13	18.73	2.64
-11	8	13	25.69	1.89

10	-8	-13	-0.97	0.91
10	-8	-13	-1.03	1.06
10	-8	-13	0.53	0.51
-10	8	13	-1.18	1.15
-10	8	13	-0.25	0.73
9	-8	-13	85.77	3.81
9	-8	-13	82.89	4.06
9	-8	-13	74.49	3.53
9	-8	-13	79.28	3.39
-9	8	13	79.53	4.40
-9	8	13	79.44	3.58
8	-8	-13	-0.82	0.60
8	-8	-13	0.17	0.96
8	-8	-13	0.89	0.42
8	-8	-13	1.62	1.07
-8	8	13	0.43	0.61
-8	8	13	1.14	1.12
7	-8	-13	36.76	2.39
7	-8	-13	34.44	1.75
7	-8	-13	32.23	2.59
7	-8	-13	35.65	1.85
-7	8	13	33.17	2.64
-7	8	13	36.69	1.86
6	-8	-13	8.44	1.47
6	-8	-13	9.14	0.98
6	-8	-13	8.13	1.32
-6	8	13	10.01	0.94
-6	8	13	11.62	1.80
5	-8	-13	27.90	1.49
5	-8	-13	32.25	2.57
5	-8	-13	32.79	2.31
5	-8	-13	26.36	1.45
-5	8	13	26.46	2.88
-5	8	13	27.91	1.42
-5	8	13	26.25	2.09
4	-8	-13	11.37	0.95
4	-8	-13	12.88	1.96
-4	8	13	11.34	1.61
-4	8	13	11.35	1.69
-4	8	13	11.23	0.81
3	-8	-13	48.25	2.86
3	-8	-13	43.42	2.04
-3	8	13	44.03	1.82
-3	8	13	41.95	2.71

-3	8	13	41.89	2.63
2	-8	-13	4.60	0.71
2	-8	-13	2.66	1.20
-2	8	13	4.34	0.96
-2	8	13	3.56	0.98
1	-8	-13	38.96	1.91
-1	8	13	42.05	2.56
-1	8	13	38.20	2.46
0	-8	-13	0.59	0.45
0	8	13	0.83	0.79
0	8	13	2.59	0.97
-1	-8	-13	15.44	1.14
1	8	13	17.90	1.69
1	8	13	14.42	1.74
-2	-8	-13	0.79	0.44
2	8	13	-0.33	0.79
2	8	13	0.98	0.89
3	8	13	36.39	2.29
3	8	13	36.31	3.60
-4	-8	-13	19.75	1.29
-4	-8	-13	19.58	2.93
4	8	13	16.69	1.70
4	8	13	17.89	1.94
-5	-8	-13	55.38	2.55
-5	-8	-13	48.73	3.47
5	8	13	52.59	2.86
5	8	13	57.15	3.26
-6	-8	-13	14.52	2.40
-6	-8	-13	9.23	1.08
6	8	13	10.93	1.69
6	8	13	10.35	1.49
-7	-8	-13	16.38	2.67
-7	-8	-13	19.52	1.44
7	8	13	20.70	1.83
7	8	13	18.61	2.25
-8	-8	-13	6.57	1.79
-8	-8	-13	5.69	0.85
8	8	13	8.79	1.57
8	8	13	4.18	1.36
-9	-8	-13	12.33	2.08
-9	-8	-13	10.70	1.28
9	8	13	10.78	1.67
9	8	13	10.23	1.69
-10	-8	-13	12.24	1.41

10	8	13	13.35	1.81
10	8	13	13.09	2.25
-11	-8	-13	32.93	2.06
11	8	13	33.53	2.37
11	8	13	33.61	3.03
-12	-8	-13	1.77	0.86
12	8	13	3.78	1.66
-13	-8	-13	20.26	1.90
13	8	13	23.89	3.02
-14	-8	-13	13.18	1.78
14	8	13	17.36	2.87
17	-8	-12	4.38	0.88
-17	8	12	4.18	1.59
-17	8	12	1.33	2.03
16	-8	-12	36.43	2.02
-16	8	12	37.81	2.94
-16	8	12	35.30	3.68
15	-8	-12	29.96	1.74
-15	8	12	31.09	2.62
-15	8	12	30.74	3.43
14	-8	-12	16.76	1.31
-14	8	12	19.26	2.12
-14	8	12	14.33	2.22
13	-8	-12	8.53	1.01
-13	8	12	9.20	1.89
-13	8	12	6.85	1.19
12	-8	-12	16.14	1.20
12	-8	-12	14.33	1.67
-12	8	12	17.14	2.56
-12	8	12	13.67	1.72
11	-8	-12	1.16	0.90
11	-8	-12	0.18	0.46
11	-8	-12	0.39	0.97
-11	8	12	0.57	0.77
-11	8	12	-0.44	1.28
10	-8	-12	24.54	2.18
10	-8	-12	22.36	1.82
10	-8	-12	21.72	1.31
10	-8	-12	20.00	1.96
-10	8	12	20.30	2.39
-10	8	12	23.32	1.69
9	-8	-12	21.65	1.55
9	-8	-12	22.91	1.29
9	-8	-12	24.39	1.89

9	-8	-12	21.59	1.98
-9	8	12	19.98	1.57
-9	8	12	22.80	2.35
8	-8	-12	58.53	3.02
8	-8	-12	56.42	2.82
8	-8	-12	57.33	3.20
8	-8	-12	57.30	2.68
-8	8	12	58.45	3.42
-8	8	12	57.87	2.68
7	-8	-12	0.57	0.40
7	-8	-12	0.44	0.89
7	-8	-12	1.40	0.58
7	-8	-12	0.69	1.01
-7	8	12	0.72	0.53
6	-8	-12	26.51	1.98
6	-8	-12	23.30	1.39
6	-8	-12	24.95	2.21
6	-8	-12	23.22	1.30
-6	8	12	27.34	2.20
-6	8	12	23.56	1.36
5	-8	-12	14.15	1.68
5	-8	-12	11.20	0.87
5	-8	-12	14.93	1.88
5	-8	-12	11.95	0.94
-5	8	12	11.36	1.63
-5	8	12	11.64	0.90
4	-8	-12	66.03	2.86
4	-8	-12	64.35	2.77
4	-8	-12	70.35	3.49
-4	8	12	63.98	3.34
-4	8	12	70.97	3.55
-4	8	12	67.78	2.76
3	-8	-12	0.59	0.33
3	-8	-12	0.35	0.37
3	-8	-12	0.12	0.95
-3	8	12	1.44	0.80
-3	8	12	0.26	0.37
-3	8	12	1.35	0.80
2	-8	-12	103.59	4.24
2	-8	-12	104.02	4.85
-2	8	12	102.75	4.78
-2	8	12	100.77	4.73
-2	8	12	109.67	3.99
1	-8	-12	6.24	1.16

1	-8	-12	4.66	0.61
-1	8	12	6.33	1.11
-1	8	12	3.48	0.94
0	-8	-12	88.29	3.68
0	-8	-12	87.61	4.89
0	-8	-12	92.67	4.44
0	8	12	86.53	4.20
0	8	12	88.68	4.16
-1	-8	-12	0.23	1.05
-1	-8	-12	-0.09	0.38
1	8	12	0.05	0.82
1	8	12	0.02	0.67
-2	-8	-12	38.96	1.92
-2	-8	-12	40.80	2.78
2	8	12	40.20	2.34
2	8	12	38.73	2.49
-3	-8	-12	15.29	1.09
-3	-8	-12	14.07	2.11
3	8	12	14.88	1.70
3	8	12	13.09	1.49
-4	-8	-12	122.98	5.88
-4	-8	-12	134.40	5.24
4	8	12	127.02	5.79
4	8	12	130.29	5.55
-5	-8	-12	1.60	1.24
5	8	12	2.08	0.79
-6	-8	-12	40.61	2.95
-6	-8	-12	41.40	2.08
6	8	12	41.84	2.43
6	8	12	42.51	2.73
-7	-8	-12	-0.58	0.45
-7	-8	-12	3.04	1.41
7	8	12	0.71	0.98
7	8	12	0.62	0.78
-8	-8	-12	30.68	1.75
-8	-8	-12	24.57	2.66
8	8	12	28.26	2.47
8	8	12	29.53	2.01
-9	-8	-12	6.67	1.62
-9	-8	-12	6.54	0.89
9	8	12	4.09	1.33
9	8	12	6.17	1.08
-10	-8	-12	46.88	2.42
-10	-8	-12	44.89	3.92

10	8	12	48.05	2.62
10	8	12	41.50	3.13
-11	-8	-12	1.01	0.70
11	8	12	-0.25	1.22
11	8	12	-0.94	0.94
-12	-8	-12	40.02	2.42
12	8	12	48.35	3.32
12	8	12	44.48	2.66
-13	-8	-12	4.26	0.97
13	8	12	2.70	1.55
-14	-8	-12	15.18	1.77
14	8	12	16.66	2.86
-15	-8	-12	4.60	1.15
15	8	12	2.53	1.71
17	-8	-11	4.01	0.75
-17	8	11	6.13	2.11
-17	8	11	2.20	1.46
16	-8	-11	5.77	0.83
-16	8	11	6.60	1.55
-16	8	11	5.18	1.92
15	-8	-11	11.72	1.15
-15	8	11	10.74	2.06
-15	8	11	10.98	1.58
14	-8	-11	14.10	1.19
-14	8	11	13.16	1.96
-14	8	11	14.82	2.64
13	-8	-11	5.58	0.74
13	-8	-11	5.13	0.88
-13	8	11	5.79	1.13
-13	8	11	8.17	1.85
12	-8	-11	16.67	1.44
12	-8	-11	14.32	1.07
12	-8	-11	11.28	1.51
-12	8	11	12.50	2.30
11	-8	-11	20.00	1.65
11	-8	-11	20.34	1.78
11	-8	-11	19.96	1.25
-11	8	11	18.87	2.38
-11	8	11	23.23	1.73
10	-8	-11	10.86	0.92
10	-8	-11	9.95	1.61
10	-8	-11	12.60	1.50
10	-8	-11	12.96	1.36
-10	8	11	11.57	1.92

-10	8	11	9.92	1.33
9	-8	-11	22.43	1.86
9	-8	-11	19.63	1.49
9	-8	-11	24.10	1.77
-9	8	11	20.51	1.52
8	-8	-11	4.27	1.06
8	-8	-11	5.37	0.77
8	-8	-11	4.25	0.69
8	-8	-11	9.70	1.40
-8	8	11	5.53	1.18
-8	8	11	5.80	0.80
7	-8	-11	36.22	1.73
7	-8	-11	40.54	2.27
7	-8	-11	35.97	2.42
7	-8	-11	35.47	1.87
-7	8	11	35.47	1.84
-7	8	11	35.42	2.48
6	-8	-11	8.20	0.76
6	-8	-11	8.75	1.50
6	-8	-11	8.63	0.93
6	-8	-11	6.77	1.20
-6	8	11	9.09	0.85
-6	8	11	10.96	1.52
5	-8	-11	39.59	1.88
5	-8	-11	40.58	2.40
5	-8	-11	40.12	1.86
5	-8	-11	42.39	2.68
-5	8	11	41.29	2.55
-5	8	11	37.87	1.83
4	-8	-11	3.84	0.54
4	-8	-11	6.28	1.08
4	-8	-11	5.16	1.15
4	-8	-11	5.02	0.68
-4	8	11	5.82	0.64
-4	8	11	5.91	1.03
-4	8	11	4.27	0.99
-4	8	11	3.98	0.97
3	-8	-11	80.22	3.69
3	-8	-11	74.58	3.12
3	-8	-11	74.11	3.98
3	-8	-11	72.81	2.98
-3	8	11	71.94	3.16
-3	8	11	77.28	3.69
-3	8	11	72.32	3.55

-3	8	11	74.66	3.00
2	-8	-11	26.93	1.96
2	-8	-11	22.09	2.01
2	-8	-11	20.44	1.16
2	-8	-11	20.66	0.98
-2	8	11	19.15	1.23
-2	8	11	21.04	1.70
-2	8	11	21.13	1.73
-2	8	11	20.02	1.00
1	-8	-11	93.80	4.41
1	-8	-11	102.59	4.62
1	-8	-11	94.19	3.87
-1	8	11	94.90	4.35
-1	8	11	95.15	3.91
-1	8	11	90.04	4.31
0	-8	-11	38.90	2.64
0	-8	-11	44.72	2.63
0	-8	-11	39.17	1.89
0	8	11	42.14	1.95
0	8	11	38.75	2.31
0	8	11	39.96	2.43
-1	-8	-11	14.59	1.74
-1	-8	-11	13.11	1.78
-1	-8	-11	13.45	0.95
1	8	11	14.05	1.05
1	8	11	11.88	1.47
1	8	11	13.30	1.42
-2	-8	-11	0.45	0.91
-2	-8	-11	0.18	0.98
-2	-8	-11	0.23	0.36
2	8	11	0.90	0.71
2	8	11	1.31	0.80
-3	-8	-11	4.47	0.71
-3	-8	-11	5.50	1.23
3	8	11	6.47	1.11
3	8	11	5.33	0.88
-4	-8	-11	-0.72	1.00
-4	-8	-11	0.03	0.36
4	8	11	0.48	0.63
4	8	11	0.93	0.87
-5	-8	-11	109.16	4.48
-5	-8	-11	109.56	5.07
5	8	11	111.85	4.77
5	8	11	104.94	5.03

-6	-8	-11	14.80	1.09
-6	-8	-11	13.60	1.67
6	8	11	14.03	1.45
6	8	11	11.93	1.70
-7	-8	-11	38.67	2.70
-7	-8	-11	35.59	1.91
7	8	11	36.51	2.71
7	8	11	37.96	2.19
-8	-8	-11	5.12	1.39
-8	-8	-11	3.12	0.63
8	8	11	3.83	0.90
8	8	11	4.98	1.18
-9	-8	-11	36.70	1.93
-9	-8	-11	28.39	2.70
9	8	11	32.94	2.11
9	8	11	36.64	2.66
-10	-8	-11	11.87	1.02
-10	-8	-11	10.91	1.23
-10	-8	-11	14.87	1.82
10	8	11	10.83	1.51
10	8	11	11.69	1.69
-11	-8	-11	40.02	2.06
-11	-8	-11	43.87	2.32
11	8	11	45.23	2.53
11	8	11	42.33	3.06
-12	-8	-11	7.22	1.00
-12	-8	-11	5.91	0.93
12	8	11	6.66	1.18
12	8	11	11.48	2.16
-13	-8	-11	30.67	2.01
13	8	11	28.07	2.87
-14	-8	-11	-0.54	0.82
14	8	11	2.03	1.51
-15	-8	-11	14.55	1.82
15	8	11	14.07	2.21
16	8	11	-1.36	1.71
18	-8	-10	-0.92	0.64
-18	8	10	-0.43	1.42
-18	8	10	3.83	1.97
17	-8	-10	23.14	1.60
-17	8	10	23.61	2.65
-17	8	10	23.83	3.39
16	-8	-10	-1.09	0.55
-16	8	10	0.76	1.74

-16	8	10	-1.44	1.17
15	-8	-10	25.51	1.58
-15	8	10	27.49	2.37
-15	8	10	23.88	2.99
14	-8	-10	-0.20	0.48
-14	8	10	-1.29	0.91
-14	8	10	1.16	1.43
13	-8	-10	3.90	0.83
13	-8	-10	3.29	0.59
-13	8	10	5.73	1.06
-13	8	10	4.62	1.48
12	-8	-10	-0.04	0.42
12	-8	-10	-0.20	0.72
12	-8	-10	-1.82	0.78
-12	8	10	-0.38	1.13
-12	8	10	-1.75	0.79
11	-8	-10	5.35	0.76
11	-8	-10	5.33	0.93
11	-8	-10	5.66	0.89
-11	8	10	6.76	0.97
10	-8	-10	29.68	1.78
10	-8	-10	26.91	1.63
10	-8	-10	32.20	1.90
10	-8	-10	28.68	2.00
-10	8	10	27.77	2.37
-10	8	10	32.82	1.89
9	-8	-10	46.91	2.36
9	-8	-10	42.65	2.14
9	-8	-10	40.39	2.45
9	-8	-10	42.95	1.97
-9	8	10	42.01	2.22
-9	8	10	42.96	2.79
8	-8	-10	1.41	0.55
8	-8	-10	1.33	0.36
8	-8	-10	0.02	0.79
8	-8	-10	2.43	0.80
-8	8	10	2.64	1.01
-8	8	10	2.21	0.59
7	-8	-10	24.01	1.44
7	-8	-10	24.85	1.30
7	-8	-10	25.69	1.94
7	-8	-10	26.27	1.83
-7	8	10	24.25	1.44
-7	8	10	22.49	2.03

6	-8	-10	1.84	0.82
6	-8	-10	2.47	0.42
6	-8	-10	2.12	0.50
6	-8	-10	1.32	0.87
-6	8	10	2.16	0.63
-6	8	10	2.82	0.52
-6	8	10	2.37	0.88
5	-8	-10	17.77	1.60
5	-8	-10	18.67	1.01
5	-8	-10	15.98	1.78
5	-8	-10	17.05	1.06
5	-8	-10	15.79	1.82
-5	8	10	15.81	1.02
-5	8	10	15.33	1.61
-5	8	10	16.50	1.07
4	-8	-10	9.75	1.63
4	-8	-10	10.93	1.38
4	-8	-10	8.21	0.76
4	-8	-10	9.54	1.36
4	-8	-10	8.51	0.73
-4	8	10	9.87	0.75
-4	8	10	9.20	0.83
-4	8	10	10.59	1.31
3	-8	-10	11.82	0.84
3	-8	-10	13.14	0.82
3	-8	-10	12.84	1.45
3	-8	-10	10.19	1.61
-3	8	10	13.51	0.94
-3	8	10	12.82	1.39
-3	8	10	12.88	1.37
-3	8	10	12.58	0.80
2	-8	-10	21.65	1.63
2	-8	-10	17.28	1.04
2	-8	-10	15.87	1.74
2	-8	-10	18.34	0.93
-2	8	10	18.37	1.10
-2	8	10	18.78	1.61
-2	8	10	18.90	1.56
1	-8	-10	15.23	1.54
1	-8	-10	10.36	0.80
1	-8	-10	11.47	0.64
1	-8	-10	10.74	1.51
-1	8	10	11.74	1.33
-1	8	10	11.68	0.64

-1	8	10	11.02	0.88
-1	8	10	12.38	1.32
0	-8	-10	1.39	0.37
0	-8	-10	2.14	0.87
0	-8	-10	1.77	0.87
0	8	10	1.42	0.62
0	8	10	1.40	0.70
0	8	10	0.95	0.37
-1	-8	-10	35.12	1.69
-1	-8	-10	34.24	2.33
-1	-8	-10	36.95	2.29
1	8	10	36.25	1.75
1	8	10	34.45	2.19
1	8	10	35.44	2.09
-2	-8	-10	11.72	0.86
-2	-8	-10	14.12	1.63
-2	-8	-10	12.22	1.58
2	8	10	10.94	1.37
2	8	10	11.33	1.28
2	8	10	11.11	0.92
-3	-8	-10	62.76	3.31
-3	-8	-10	63.42	3.24
-3	-8	-10	59.02	2.66
3	8	10	62.36	3.20
3	8	10	61.46	3.00
-4	-8	-10	22.40	1.93
-4	-8	-10	21.24	1.44
-4	-8	-10	22.42	1.30
-4	-8	-10	26.66	2.75
4	8	10	22.03	1.85
4	8	10	23.50	1.63
-5	-8	-10	2.39	0.48
-5	-8	-10	2.33	1.00
5	8	10	3.66	0.95
5	8	10	4.10	0.80
-6	-8	-10	25.27	1.45
-6	-8	-10	25.70	2.09
6	8	10	27.77	2.03
6	8	10	25.83	1.75
-7	-8	-10	28.01	2.24
-7	-8	-10	28.07	1.57
7	8	10	31.08	1.88
7	8	10	26.50	2.19
-8	-8	-10	4.38	0.52

-8	-8	-10	4.11	1.20
-8	-8	-10	3.16	0.65
-8	-8	-10	6.27	0.62
8	8	10	5.20	1.88
8	8	10	4.86	0.86
-9	-8	-10	21.90	2.23
-9	-8	-10	23.53	1.22
-9	-8	-10	26.60	1.51
-9	-8	-10	20.65	1.27
9	8	10	25.54	2.20
9	8	10	21.93	1.71
-10	-8	-10	24.38	1.32
-10	-8	-10	24.83	1.56
-10	-8	-10	18.84	2.28
-10	-8	-10	21.22	1.36
10	8	10	23.79	1.79
10	8	10	24.94	2.24
-11	-8	-10	12.92	1.26
-11	-8	-10	14.41	1.82
-11	-8	-10	17.63	1.39
11	8	10	18.35	1.66
11	8	10	16.34	2.18
-12	-8	-10	38.85	2.19
12	8	10	35.47	2.93
12	8	10	39.49	2.39
-13	-8	-10	1.24	0.83
-13	-8	-10	0.27	0.85
-13	-8	-10	-0.62	1.23
13	8	10	2.02	1.41
-14	-8	-10	12.89	1.56
-14	-8	-10	9.21	1.30
14	8	10	11.69	1.92
-15	-8	-10	7.59	1.19
15	8	10	6.40	1.70
-16	-8	-10	13.99	1.76
16	8	10	9.00	2.04
18	-8	-9	18.86	1.50
-18	8	9	17.90	3.19
-18	8	9	13.82	1.90
17	-8	-9	2.87	0.70
-17	8	9	4.04	1.46
-17	8	9	6.74	1.92
16	-8	-9	15.05	1.25
-16	8	9	13.06	2.16

-16	8	9	12.93	1.70
15	-8	-9	8.28	1.03
-15	8	9	8.67	1.39
-15	8	9	3.44	1.60
14	-8	-9	28.89	1.60
14	-8	-9	30.19	1.69
-14	8	9	27.38	2.71
-14	8	9	29.41	2.30
13	-8	-9	7.17	1.18
13	-8	-9	9.64	0.89
13	-8	-9	6.29	1.09
-13	8	9	9.47	1.57
12	-8	-9	36.37	2.10
12	-8	-9	39.15	2.02
12	-8	-9	37.66	1.83
-12	8	9	39.95	2.28
-12	8	9	36.02	2.78
11	-8	-9	10.80	1.29
11	-8	-9	11.48	1.35
11	-8	-9	10.35	0.90
-11	8	9	10.75	1.86
-11	8	9	12.09	1.33
10	-8	-9	31.01	1.97
10	-8	-9	30.11	1.53
10	-8	-9	31.13	1.86
10	-8	-9	30.83	1.79
-10	8	9	31.16	1.89
-10	8	9	30.73	2.39
9	-8	-9	4.03	0.60
9	-8	-9	2.45	0.62
9	-8	-9	6.48	1.19
9	-8	-9	2.89	0.88
-9	8	9	3.14	0.67
-9	8	9	2.85	0.95
8	-8	-9	30.30	2.60
8	-8	-9	31.11	1.70
8	-8	-9	30.17	1.53
8	-8	-9	29.95	2.03
8	-8	-9	35.18	1.97
-8	8	9	31.37	1.73
-8	8	9	33.82	2.28
7	-8	-9	24.78	1.66
7	-8	-9	21.83	1.16
7	-8	-9	20.66	1.75

7	-8	-9	19.99	2.02
7	-8	-9	23.26	1.34
-7	8	9	22.96	1.32
-7	8	9	22.44	1.85
-7	8	9	19.68	1.25
6	-8	-9	76.80	3.11
6	-8	-9	77.91	3.18
6	-8	-9	78.06	3.86
6	-8	-9	75.17	3.46
6	-8	-9	75.02	3.63
-6	8	9	71.86	3.16
-6	8	9	74.33	3.16
-6	8	9	74.58	3.70
5	-8	-9	16.27	1.04
5	-8	-9	18.84	1.51
5	-8	-9	17.55	0.97
5	-8	-9	14.70	1.59
5	-8	-9	16.62	1.76
-5	8	9	15.06	1.51
-5	8	9	16.53	1.03
-5	8	9	14.70	0.99
4	-8	-9	54.75	2.68
4	-8	-9	51.01	2.23
4	-8	-9	49.02	2.82
4	-8	-9	52.82	2.89
4	-8	-9	50.70	2.23
-4	8	9	51.73	2.27
-4	8	9	54.66	2.22
-4	8	9	49.49	2.70
3	-8	-9	7.72	1.23
3	-8	-9	8.08	1.13
3	-8	-9	7.44	0.67
3	-8	-9	6.83	1.19
3	-8	-9	7.15	0.64
-3	8	9	6.92	0.70
-3	8	9	6.63	1.14
-3	8	9	6.79	0.61
2	-8	-9	36.28	2.23
2	-8	-9	35.22	2.06
2	-8	-9	33.90	1.51
2	-8	-9	33.49	1.60
-2	8	9	32.67	2.04
-2	8	9	37.67	2.10
-2	8	9	34.74	1.53

-2	8	9	34.22	1.64
1	-8	-9	2.60	0.33
1	-8	-9	2.78	0.43
1	-8	-9	2.83	0.83
1	-8	-9	3.47	0.86
-1	8	9	3.32	0.44
-1	8	9	3.01	0.73
-1	8	9	2.23	0.69
-1	8	9	2.97	0.45
0	-8	-9	69.64	2.99
0	-8	-9	72.24	3.51
0	-8	-9	77.74	3.51
0	8	9	72.15	3.46
0	8	9	65.06	3.32
0	8	9	75.02	3.04
0	8	9	74.81	2.79
-1	-8	-9	2.10	0.80
-1	-8	-9	2.72	0.44
-1	-8	-9	1.43	0.76
1	8	9	1.80	0.60
1	8	9	1.91	0.44
1	8	9	1.88	0.64
-2	-8	-9	75.55	3.18
-2	-8	-9	79.44	3.71
-2	-8	-9	78.18	3.67
2	8	9	75.86	3.50
2	8	9	76.95	3.66
2	8	9	74.86	3.20
-3	-8	-9	0.34	0.73
-3	-8	-9	0.28	0.74
-3	-8	-9	0.26	0.32
3	8	9	0.26	0.34
3	8	9	0.43	0.53
3	8	9	-0.11	0.67
-4	-8	-9	62.01	3.35
-4	-8	-9	65.56	2.78
-4	-8	-9	67.68	3.22
-4	-8	-9	69.24	2.75
4	8	9	60.65	3.23
4	8	9	62.88	2.78
4	8	9	63.13	3.04
-5	-8	-9	4.17	0.66
-5	-8	-9	5.90	1.07
-5	-8	-9	4.70	0.71

-5	-8	-9	6.68	1.03
5	8	9	7.84	1.25
5	8	9	5.54	1.03
-6	-8	-9	41.09	2.42
-6	-8	-9	40.58	1.69
-6	-8	-9	40.68	1.97
6	8	9	41.32	2.51
6	8	9	42.78	2.25
-7	-8	-9	1.23	0.87
-7	-8	-9	-0.16	0.40
-7	-8	-9	-0.56	0.29
-7	-8	-9	0.30	0.33
7	8	9	0.00	0.59
7	8	9	0.31	0.83
-8	-8	-9	24.70	1.22
-8	-8	-9	23.03	2.00
-8	-8	-9	23.20	1.28
-8	-8	-9	25.24	1.45
8	8	9	25.70	1.69
8	8	9	24.46	2.01
-9	-8	-9	0.31	0.48
-9	-8	-9	0.15	0.96
-9	-8	-9	1.62	0.46
9	8	9	3.03	0.99
9	8	9	0.55	0.72
-10	-8	-9	28.73	1.64
-10	-8	-9	27.34	2.19
-10	-8	-9	23.73	1.49
-10	-8	-9	27.44	1.45
10	8	9	26.92	2.57
10	8	9	28.11	1.85
-11	-8	-9	18.12	1.41
-11	-8	-9	21.25	1.37
-11	-8	-9	21.43	2.15
-11	-8	-9	22.63	1.52
11	8	9	21.80	2.22
11	8	9	24.35	1.78
-12	-8	-9	16.18	1.48
-12	-8	-9	21.20	1.58
-12	-8	-9	24.58	1.54
-12	-8	-9	18.33	2.20
-12	-8	-9	15.37	2.06
12	8	9	22.29	2.30
-13	-8	-9	4.27	0.90

-13	-8	-9	5.48	1.34
-13	-8	-9	4.36	0.79
13	8	9	4.17	1.41
-14	-8	-9	29.22	2.18
-14	-8	-9	37.11	2.91
-14	-8	-9	38.46	2.20
14	8	9	36.41	2.99
-15	-8	-9	-0.12	0.78
-15	-8	-9	-0.56	1.04
15	8	9	2.14	1.43
-16	-8	-9	17.62	1.88
16	8	9	22.94	2.89
-17	-8	-9	1.55	1.06
17	8	9	4.45	1.83
19	-8	-8	1.45	0.77
-19	8	8	1.84	1.65
-19	8	8	3.96	2.19
18	-8	-8	23.29	1.63
-18	8	8	29.93	2.87
-18	8	8	23.31	3.24
17	-8	-8	14.82	1.33
-17	8	8	15.73	2.41
-17	8	8	17.12	2.87
16	-8	-8	30.88	1.79
-16	8	8	37.32	2.69
-16	8	8	32.63	3.03
15	-8	-8	12.54	1.10
-15	8	8	14.86	1.97
-15	8	8	13.09	2.26
14	-8	-8	37.24	1.90
14	-8	-8	38.67	1.93
-14	8	8	38.21	2.92
-14	8	8	37.42	2.49
13	-8	-8	-0.08	0.68
13	-8	-8	1.80	0.48
13	-8	-8	2.12	0.73
-13	8	8	-1.05	1.11
-13	8	8	-0.46	0.76
12	-8	-8	26.28	1.39
12	-8	-8	26.46	1.69
12	-8	-8	27.31	1.66
-12	8	8	25.91	1.87
-12	8	8	23.88	2.30
11	-8	-8	70.17	3.06

11	-8	-8	66.29	3.10
-11	8	8	72.68	3.26
-11	8	8	60.36	3.60
10	-8	-8	11.52	1.22
10	-8	-8	8.84	1.24
10	-8	-8	6.49	0.74
10	-8	-8	7.87	1.12
10	-8	-8	8.20	1.58
-10	8	8	8.06	1.21
-10	8	8	6.04	1.15
9	-8	-8	23.42	2.21
9	-8	-8	23.27	1.67
9	-8	-8	24.94	1.47
9	-8	-8	22.54	1.58
9	-8	-8	22.75	1.21
-9	8	8	20.77	1.92
-9	8	8	22.46	1.52
8	-8	-8	73.74	3.84
8	-8	-8	76.74	3.10
8	-8	-8	74.68	3.25
8	-8	-8	74.51	3.37
8	-8	-8	79.43	3.53
-8	8	8	73.74	3.18
-8	8	8	75.87	3.27
-8	8	8	71.37	3.71
7	-8	-8	19.14	1.13
7	-8	-8	22.77	1.71
7	-8	-8	24.88	1.58
7	-8	-8	23.00	1.32
7	-8	-8	20.71	1.91
-7	8	8	22.04	1.22
-7	8	8	21.85	1.79
-7	8	8	20.71	1.30
6	-8	-8	37.69	2.28
6	-8	-8	37.50	1.79
6	-8	-8	36.07	1.69
6	-8	-8	39.39	2.09
6	-8	-8	39.21	2.38
-6	8	8	39.18	2.28
-6	8	8	37.09	1.74
-6	8	8	34.43	1.77
5	-8	-8	77.08	3.06
5	-8	-8	75.58	3.42
5	-8	-8	75.25	3.09

5	-8	-8	71.87	3.58
5	-8	-8	78.13	3.67
-5	8	8	74.54	3.56
-5	8	8	70.58	3.09
-5	8	8	76.36	3.11
4	-8	-8	14.97	1.57
4	-8	-8	20.68	1.45
4	-8	-8	19.59	1.00
4	-8	-8	17.26	1.57
4	-8	-8	16.39	0.96
-4	8	8	15.74	1.45
-4	8	8	17.49	1.00
-4	8	8	16.88	0.99
3	-8	-8	127.38	4.99
3	-8	-8	126.79	5.49
3	-8	-8	133.51	5.38
3	-8	-8	128.22	5.60
3	-8	-8	120.88	5.02
-3	8	8	130.11	4.95
-3	8	8	124.51	5.40
2	-8	-8	10.83	0.68
2	-8	-8	11.01	1.50
2	-8	-8	11.56	1.26
2	-8	-8	12.01	0.75
2	-8	-8	9.92	1.28
-2	8	8	10.46	0.77
-2	8	8	10.30	0.74
-2	8	8	12.19	1.16
-2	8	8	10.62	1.14
1	-8	-8	31.44	1.37
1	-8	-8	32.83	1.98
1	-8	-8	32.90	1.50
1	-8	-8	36.42	2.00
-1	8	8	33.12	1.52
-1	8	8	29.28	1.39
-1	8	8	32.16	1.88
-1	8	8	30.33	1.90
0	-8	-8	24.81	1.06
0	-8	-8	24.03	1.68
0	-8	-8	23.57	1.21
0	-8	-8	25.67	1.71
0	8	8	23.74	1.58
0	8	8	24.39	1.26
0	8	8	25.07	1.67

0	8	8	23.61	1.07
-1	-8	-8	123.24	5.09
-1	-8	-8	111.97	4.62
-1	-8	-8	117.55	5.04
-1	-8	-8	122.19	4.43
1	8	8	114.41	5.05
1	8	8	120.82	4.66
1	8	8	117.54	4.43
1	8	8	108.98	4.92
-2	-8	-8	31.25	1.48
-2	-8	-8	33.34	1.98
-2	-8	-8	29.44	1.89
2	8	8	28.89	1.21
2	8	8	29.23	1.89
2	8	8	30.41	1.49
2	8	8	31.30	1.80
-3	-8	-8	60.61	2.57
-3	-8	-8	60.64	3.04
-3	-8	-8	59.28	2.95
3	8	8	59.48	3.01
3	8	8	61.03	2.57
3	8	8	58.02	2.83
-4	-8	-8	15.28	0.74
-4	-8	-8	14.71	0.93
-4	-8	-8	16.29	0.76
-4	-8	-8	17.50	1.56
-4	-8	-8	16.45	1.44
-4	-8	-8	15.73	0.99
4	8	8	17.30	1.03
4	8	8	17.29	1.50
4	8	8	15.00	1.27
-5	-8	-8	27.33	1.85
-5	-8	-8	26.51	1.20
-5	-8	-8	26.30	1.20
-5	-8	-8	27.21	1.43
-5	-8	-8	31.94	2.01
-5	-8	-8	29.17	1.37
5	8	8	26.85	1.90
5	8	8	24.89	1.66
-6	-8	-8	11.94	1.37
-6	-8	-8	9.99	0.68
-6	-8	-8	10.39	0.71
-6	-8	-8	10.15	1.54
-6	-8	-8	9.81	0.85

-6	-8	-8	10.99	0.80
6	8	8	11.54	1.39
6	8	8	8.98	1.13
-7	-8	-8	104.99	4.49
-7	-8	-8	127.05	5.09
-7	-8	-8	113.17	4.47
-7	-8	-8	120.23	5.03
-7	-8	-8	118.54	5.29
-7	-8	-8	116.64	4.69
7	8	8	113.09	4.86
7	8	8	111.33	5.15
-8	-8	-8	6.46	1.02
-8	-8	-8	3.66	0.64
-8	-8	-8	4.79	0.74
-8	-8	-8	4.45	0.55
8	8	8	4.83	1.06
8	8	8	3.19	0.74
-9	-8	-8	56.62	2.54
-9	-8	-8	54.01	2.35
-9	-8	-8	55.67	2.92
-9	-8	-8	50.99	2.39
9	8	8	53.53	3.02
9	8	8	57.70	2.73
-10	-8	-8	3.49	0.65
-10	-8	-8	5.33	1.01
-10	-8	-8	4.94	1.08
-10	-8	-8	4.44	0.62
10	8	8	2.92	0.81
10	8	8	3.65	1.05
-11	-8	-8	30.31	2.24
-11	-8	-8	28.91	1.72
-11	-8	-8	29.81	1.63
-11	-8	-8	27.35	1.68
11	8	8	31.87	1.97
-12	-8	-8	5.56	0.90
-12	-8	-8	5.84	0.85
-12	-8	-8	4.77	1.23
-12	-8	-8	8.47	1.10
-12	-8	-8	7.21	1.31
12	8	8	7.82	1.42
-13	-8	-8	60.34	2.85
-13	-8	-8	54.98	3.23
-13	-8	-8	55.98	2.74
-13	-8	-8	51.15	2.75

13	8	8	60.92	3.46
-14	-8	-8	1.65	1.33
-14	-8	-8	1.83	0.86
-14	-8	-8	1.17	0.73
14	8	8	-1.03	1.26
-15	-8	-8	43.11	2.38
-15	-8	-8	35.88	2.47
15	8	8	39.25	3.00
-16	-8	-8	1.02	0.89
-16	-8	-8	-0.07	1.08
16	8	8	-0.47	1.48
-17	-8	-8	17.69	1.95
-17	-8	-8	17.44	2.25
17	8	8	20.08	2.87
19	-8	-7	38.64	2.20
-19	8	7	45.95	3.39
18	-8	-7	0.11	0.67
-18	8	7	-1.53	1.54
-18	8	7	0.56	1.42
17	-8	-7	20.62	1.48
-17	8	7	22.06	2.53
-17	8	7	20.22	2.82
16	-8	-7	2.38	0.65
-16	8	7	2.94	1.49
-16	8	7	4.33	1.25
15	-8	-7	32.28	1.71
15	-8	-7	30.55	1.68
-15	8	7	31.34	2.46
-15	8	7	31.45	2.73
14	-8	-7	0.03	0.64
14	-8	-7	0.70	1.00
14	-8	-7	0.97	0.47
-14	8	7	1.98	1.16
-14	8	7	-0.07	0.90
13	-8	-7	50.09	2.29
13	-8	-7	50.34	2.44
13	-8	-7	51.54	2.43
-13	8	7	50.75	2.78
-13	8	7	47.66	3.15
12	-8	-7	0.06	0.68
12	-8	-7	3.44	1.68
12	-8	-7	-1.44	0.65
12	-8	-7	-0.36	0.36
-12	8	7	-1.91	0.93

-12	8	7	0.36	0.70
11	-8	-7	75.74	3.37
11	-8	-7	83.96	3.40
11	-8	-7	79.92	3.17
11	-8	-7	77.00	4.09
-11	8	7	71.62	3.49
-11	8	7	64.77	3.79
10	-8	-7	11.19	0.80
10	-8	-7	12.29	1.27
10	-8	-7	10.78	1.53
10	-8	-7	11.60	1.25
10	-8	-7	8.04	1.16
-10	8	7	10.81	1.21
-10	8	7	9.44	1.60
9	-8	-7	52.16	2.47
9	-8	-7	51.00	2.98
9	-8	-7	51.02	2.36
9	-8	-7	50.06	2.52
9	-8	-7	44.74	2.13
-9	8	7	49.70	2.42
-9	8	7	48.05	2.80
8	-8	-7	7.07	0.63
8	-8	-7	6.82	1.19
8	-8	-7	9.18	1.14
8	-8	-7	6.99	1.15
8	-8	-7	7.68	0.89
-8	8	7	6.27	0.90
-8	8	7	7.57	1.10
-8	8	7	5.00	0.71
7	-8	-7	89.93	3.68
7	-8	-7	92.42	3.89
7	-8	-7	84.85	3.95
7	-8	-7	92.08	4.19
7	-8	-7	86.41	3.57
-7	8	7	88.70	3.72
-7	8	7	88.92	4.10
-7	8	7	88.95	3.62
6	-8	-7	3.75	0.74
6	-8	-7	3.43	0.51
6	-8	-7	6.12	1.13
6	-8	-7	5.04	0.94
6	-8	-7	4.51	0.69
-6	8	7	3.82	0.78
-6	8	7	4.07	0.60

-6	8	7	4.89	0.70
5	-8	-7	75.23	3.13
5	-8	-7	79.99	3.68
5	-8	-7	81.99	3.50
5	-8	-7	76.66	3.65
5	-8	-7	80.54	3.19
-5	8	7	73.68	3.16
-5	8	7	76.92	3.61
-5	8	7	76.69	3.20
4	-8	-7	6.04	1.12
4	-8	-7	6.09	1.00
4	-8	-7	4.70	0.51
4	-8	-7	4.30	0.92
4	-8	-7	4.66	0.59
-4	8	7	5.31	0.58
-4	8	7	5.04	0.60
-4	8	7	5.16	0.97
3	-8	-7	44.55	2.32
3	-8	-7	44.24	2.22
3	-8	-7	41.75	1.84
3	-8	-7	41.79	2.45
-3	8	7	42.26	1.87
-3	8	7	42.71	2.24
-3	8	7	40.10	1.82
2	-8	-7	15.88	1.28
2	-8	-7	12.90	0.81
2	-8	-7	15.16	1.33
2	-8	-7	14.86	0.79
2	-8	-7	14.50	1.51
-2	8	7	13.06	0.83
-2	8	7	14.85	0.87
-2	8	7	13.83	1.22
1	-8	-7	35.62	1.63
1	-8	-7	36.32	1.53
1	-8	-7	35.88	2.03
1	-8	-7	36.95	2.32
1	-8	-7	40.28	2.08
-1	8	7	33.79	1.95
-1	8	7	36.79	2.03
-1	8	7	36.54	1.65
-1	8	7	35.29	1.64
0	-8	-7	24.07	1.62
0	-8	-7	24.24	1.04
0	-8	-7	22.38	1.57

0	-8	-7	22.62	1.15
0	8	7	24.96	1.52
0	8	7	21.84	1.56
0	8	7	22.93	1.06
0	8	7	22.00	1.17
-1	-8	-7	90.29	3.73
-1	-8	-7	101.59	4.16
-1	-8	-7	88.78	3.59
-1	-8	-7	96.03	4.16
1	8	7	100.69	3.78
1	8	7	95.03	4.17
1	8	7	85.42	4.01
1	8	7	92.28	3.60
-2	-8	-7	16.74	0.81
-2	-8	-7	17.38	0.98
-2	-8	-7	19.49	1.47
-2	-8	-7	18.95	1.39
2	8	7	17.28	0.99
2	8	7	20.52	1.34
2	8	7	14.17	1.35
2	8	7	17.10	0.80
-3	-8	-7	38.16	2.06
-3	-8	-7	32.27	1.38
-3	-8	-7	33.05	1.92
-3	-8	-7	33.35	1.56
3	8	7	31.18	1.96
3	8	7	33.55	1.57
3	8	7	33.52	1.84
3	8	7	31.83	1.35
-4	-8	-7	12.68	0.67
-4	-8	-7	13.97	0.72
-4	-8	-7	12.55	0.86
-4	-8	-7	14.51	1.30
-4	-8	-7	13.98	1.39
-4	-8	-7	12.28	0.82
4	8	7	12.36	1.13
4	8	7	11.88	1.28
4	8	7	13.16	0.88
-5	-8	-7	50.20	2.09
-5	-8	-7	46.27	1.97
-5	-8	-7	47.25	1.97
-5	-8	-7	48.31	2.14
-5	-8	-7	46.87	2.60
-5	-8	-7	50.20	2.50

5	8	7	49.46	2.62
5	8	7	47.22	2.38
-6	-8	-7	31.50	1.58
-6	-8	-7	32.64	1.45
-6	-8	-7	35.38	2.12
-6	-8	-7	31.71	1.51
-6	-8	-7	31.43	1.47
-6	-8	-7	34.16	2.01
6	8	7	30.53	2.08
6	8	7	31.86	1.83
-7	-8	-7	51.81	2.64
-7	-8	-7	48.57	2.28
-7	-8	-7	51.06	2.21
-7	-8	-7	50.34	2.17
-7	-8	-7	50.86	2.84
-7	-8	-7	51.84	2.28
7	8	7	49.51	2.76
7	8	7	51.94	2.50
-8	-8	-7	33.42	1.67
-8	-8	-7	37.75	1.79
-8	-8	-7	35.97	2.12
-8	-8	-7	34.64	2.31
-8	-8	-7	36.20	1.64
-8	-8	-7	32.33	1.74
8	8	7	35.64	1.96
8	8	7	36.05	2.29
-9	-8	-7	64.41	2.66
-9	-8	-7	61.66	2.78
-9	-8	-7	63.53	3.12
-9	-8	-7	59.51	2.68
9	8	7	64.55	2.96
9	8	7	60.84	3.29
-10	-8	-7	31.67	2.10
-10	-8	-7	29.46	1.60
-10	-8	-7	29.95	1.65
-10	-8	-7	30.68	1.69
10	8	7	31.90	2.24
10	8	7	31.83	1.91
-11	-8	-7	12.99	1.13
-11	-8	-7	14.36	1.69
-11	-8	-7	10.26	1.67
-11	-8	-7	12.05	1.12
-11	-8	-7	9.70	1.14
11	8	7	11.65	1.39

11	8	7	14.25	1.72
-12	-8	-7	16.71	1.86
-12	-8	-7	16.57	1.87
-12	-8	-7	13.14	1.32
-12	-8	-7	14.64	1.41
-12	-8	-7	15.96	1.31
12	8	7	15.44	1.87
-13	-8	-7	15.51	2.06
-13	-8	-7	19.48	1.51
-13	-8	-7	14.81	1.40
-13	-8	-7	14.89	1.99
-13	-8	-7	14.87	1.51
13	8	7	17.21	2.04
-14	-8	-7	25.36	2.49
-14	-8	-7	22.34	1.86
-14	-8	-7	24.73	1.90
-14	-8	-7	26.12	1.78
14	8	7	25.79	2.47
-15	-8	-7	25.48	1.86
-15	-8	-7	21.10	2.60
-15	-8	-7	26.32	2.02
15	8	7	20.69	2.53
-16	-8	-7	33.85	2.23
16	8	7	35.65	3.00
-17	-8	-7	6.79	1.37
-17	-8	-7	6.19	1.13
17	8	7	9.01	1.87
-18	-8	-7	15.17	1.89
-18	-8	-7	15.58	2.42
18	8	7	14.99	2.25
19	-8	-6	1.95	0.69
-19	8	6	2.52	1.49
18	-8	-6	11.02	1.23
-18	8	6	11.50	1.91
-18	8	6	8.77	1.63
17	-8	-6	11.81	1.15
-17	8	6	10.85	1.64
-17	8	6	11.26	1.80
16	-8	-6	22.72	1.45
-16	8	6	25.76	2.59
-16	8	6	24.02	2.31
15	-8	-6	30.73	1.62
15	-8	-6	28.52	1.73
-15	8	6	28.66	2.38

-15	8	6	23.44	2.61
14	-8	-6	51.04	4.12
14	-8	-6	54.10	2.50
14	-8	-6	48.41	2.32
14	-8	-6	50.65	2.44
-14	8	6	48.45	3.15
-14	8	6	54.83	2.96
13	-8	-6	24.25	2.78
13	-8	-6	29.98	1.71
13	-8	-6	29.80	1.52
13	-8	-6	31.73	1.79
-13	8	6	27.34	2.04
-13	8	6	25.98	2.30
12	-8	-6	59.15	3.20
12	-8	-6	67.94	3.90
12	-8	-6	71.80	3.08
12	-8	-6	71.18	3.13
-12	8	6	64.82	3.57
-12	8	6	73.68	3.35
11	-8	-6	6.79	1.18
11	-8	-6	6.39	1.08
11	-8	-6	6.67	1.33
11	-8	-6	6.77	0.66
-11	8	6	7.60	1.21
-11	8	6	4.65	1.12
10	-8	-6	39.90	1.83
10	-8	-6	43.24	2.72
10	-8	-6	41.73	2.15
10	-8	-6	38.62	2.08
10	-8	-6	42.23	2.15
-10	8	6	38.58	2.14
-10	8	6	38.56	2.47
9	-8	-6	13.29	1.27
9	-8	-6	13.87	1.08
9	-8	-6	11.35	0.79
9	-8	-6	10.39	1.20
9	-8	-6	14.04	1.73
-9	8	6	10.59	1.43
-9	8	6	11.97	1.15
-9	8	6	13.44	0.91
8	-8	-6	18.93	1.25
8	-8	-6	21.40	1.53
8	-8	-6	21.89	1.46
8	-8	-6	21.97	1.78

8	-8	-6	19.40	1.07
-8	8	6	20.37	1.31
-8	8	6	20.69	1.64
-8	8	6	19.93	1.13
7	-8	-6	14.74	1.37
7	-8	-6	14.05	0.86
7	-8	-6	16.34	1.54
7	-8	-6	16.16	1.04
7	-8	-6	14.11	1.24
-7	8	6	15.21	1.42
-7	8	6	14.76	1.06
-7	8	6	15.26	0.92
6	-8	-6	80.90	3.29
6	-8	-6	78.83	3.61
6	-8	-6	82.60	3.51
6	-8	-6	82.23	3.71
6	-8	-6	80.46	3.20
-6	8	6	76.46	3.63
-6	8	6	80.81	3.24
-6	8	6	72.65	3.29
5	-8	-6	-0.15	0.31
5	-8	-6	0.50	0.63
5	-8	-6	0.32	0.54
5	-8	-6	-0.05	0.60
5	-8	-6	0.12	0.22
-5	8	6	-0.15	0.50
-5	8	6	0.11	0.27
-5	8	6	-0.08	0.32
4	-8	-6	104.17	4.32
4	-8	-6	101.45	4.01
4	-8	-6	97.47	4.48
4	-8	-6	100.99	4.42
4	-8	-6	97.91	3.99
-4	8	6	97.64	4.37
-4	8	6	100.38	4.04
-4	8	6	110.47	4.04
3	-8	-6	-0.33	0.53
3	-8	-6	0.10	0.21
3	-8	-6	0.02	0.54
3	-8	-6	0.40	0.28
3	-8	-6	-0.29	0.54
-3	8	6	0.25	0.29
-3	8	6	-0.24	0.44
-3	8	6	-0.07	0.24

2	-8	-6	116.46	5.18
2	-8	-6	129.28	4.99
2	-8	-6	115.33	4.98
2	-8	-6	115.83	4.57
-2	8	6	119.37	4.96
-2	8	6	113.53	4.60
1	-8	-6	29.24	1.28
1	-8	-6	30.50	1.73
1	-8	-6	28.43	1.95
1	-8	-6	27.17	1.32
1	-8	-6	29.89	1.72
-1	8	6	28.37	1.65
-1	8	6	29.03	1.36
-1	8	6	29.20	1.32
0	-8	-6	95.75	4.39
0	-8	-6	95.05	4.07
0	-8	-6	93.88	4.06
0	-8	-6	90.08	3.70
0	-8	-6	100.05	3.64
0	8	6	87.34	3.99
0	8	6	93.20	3.73
-1	-8	-6	0.10	0.23
-1	-8	-6	0.23	0.22
-1	-8	-6	0.45	0.55
-1	-8	-6	-0.20	0.50
1	8	6	-0.10	0.24
1	8	6	-0.89	0.47
1	8	6	-0.41	0.39
1	8	6	-0.32	0.20
-2	-8	-6	164.33	6.41
-2	-8	-6	167.55	6.52
-2	-8	-6	174.33	6.92
-2	-8	-6	167.91	6.84
2	8	6	169.92	6.95
2	8	6	168.57	6.54
2	8	6	166.38	6.40
2	8	6	177.22	6.82
-3	-8	-6	8.02	0.67
-3	-8	-6	9.56	1.04
-3	-8	-6	9.48	1.11
-3	-8	-6	10.25	0.60
3	8	6	9.60	0.69
3	8	6	9.84	0.97
3	8	6	9.22	1.13

3	8	6	9.06	0.56
-4	-8	-6	118.20	4.54
-4	-8	-6	113.80	4.64
-4	-8	-6	120.42	4.95
-4	-8	-6	117.93	4.61
-4	-8	-6	122.83	5.07
4	8	6	120.43	4.67
4	8	6	115.20	4.88
4	8	6	113.41	5.07
-5	-8	-6	1.24	0.37
-5	-8	-6	-0.07	0.62
-5	-8	-6	0.73	0.28
-5	-8	-6	1.35	0.31
-5	-8	-6	1.86	0.32
-5	-8	-6	0.85	0.61
5	8	6	1.61	0.69
5	8	6	1.16	0.51
-6	-8	-6	115.73	4.91
-6	-8	-6	131.49	5.36
-6	-8	-6	123.06	4.82
-6	-8	-6	120.13	4.81
-6	-8	-6	125.53	5.21
-6	-8	-6	127.74	4.86
6	8	6	127.91	5.14
6	8	6	121.85	5.37
-7	-8	-6	0.25	0.70
-7	-8	-6	0.77	0.33
-7	-8	-6	0.23	0.61
-7	-8	-6	0.38	0.33
-7	-8	-6	0.31	0.34
-7	-8	-6	0.23	0.31
7	8	6	0.34	0.64
7	8	6	0.68	0.51
-8	-8	-6	88.43	3.53
-8	-8	-6	83.54	3.61
-8	-8	-6	84.54	3.53
-8	-8	-6	86.49	3.89
-8	-8	-6	89.13	3.53
-8	-8	-6	85.74	4.07
8	8	6	88.96	3.81
8	8	6	88.15	4.10
-9	-8	-6	-0.14	0.46
-9	-8	-6	1.40	0.91
-9	-8	-6	0.41	0.42

-9	-8	-6	-0.51	0.42
-9	-8	-6	1.09	0.45
-9	-8	-6	0.56	0.73
9	8	6	0.23	0.72
9	8	6	-0.25	0.60
-10	-8	-6	47.74	2.29
-10	-8	-6	48.96	2.60
-10	-8	-6	48.42	2.23
-10	-8	-6	49.80	2.95
-10	-8	-6	45.52	2.26
10	8	6	47.75	2.48
10	8	6	53.50	2.85
-11	-8	-6	11.43	1.61
-11	-8	-6	7.77	1.08
-11	-8	-6	9.60	0.99
-11	-8	-6	8.30	1.50
-11	-8	-6	10.20	1.08
11	8	6	10.31	1.60
-12	-8	-6	32.83	1.79
-12	-8	-6	30.21	2.31
-12	-8	-6	30.24	1.74
-12	-8	-6	29.07	1.76
-12	-8	-6	24.86	2.09
12	8	6	33.59	2.33
-13	-8	-6	1.45	1.18
-13	-8	-6	1.90	0.69
-13	-8	-6	1.12	0.75
-13	-8	-6	2.73	1.08
-13	-8	-6	3.55	0.83
13	8	6	1.99	1.01
-14	-8	-6	23.48	1.69
-14	-8	-6	23.76	1.84
-14	-8	-6	19.38	2.38
14	8	6	27.76	3.41
-15	-8	-6	-0.44	0.86
-15	-8	-6	0.13	1.43
-15	-8	-6	0.63	0.74
15	8	6	1.39	1.24
-16	-8	-6	15.85	1.72
-16	-8	-6	16.99	1.95
16	8	6	18.46	2.42
-17	-8	-6	1.49	1.14
-17	-8	-6	2.99	0.98
17	8	6	0.40	1.44

-18	-8	-6	14.51	1.87
-18	-8	-6	13.77	2.27
18	8	6	12.40	2.04
19	-8	-5	6.37	0.91
-19	8	5	7.89	1.71
18	-8	-5	16.77	1.37
-18	8	5	19.65	2.56
-18	8	5	19.14	2.63
17	-8	-5	10.96	1.12
-17	8	5	14.72	2.43
-17	8	5	11.42	1.72
16	-8	-5	16.77	3.19
16	-8	-5	19.70	1.34
16	-8	-5	16.89	1.49
-16	8	5	17.39	2.31
-16	8	5	21.09	2.16
15	-8	-5	19.97	1.31
15	-8	-5	20.02	3.12
15	-8	-5	19.75	1.49
-15	8	5	20.69	2.05
-15	8	5	23.97	2.30
14	-8	-5	26.39	2.80
14	-8	-5	22.00	1.53
14	-8	-5	21.48	1.29
-14	8	5	20.69	2.16
-14	8	5	23.91	1.99
13	-8	-5	32.32	1.82
13	-8	-5	32.84	1.60
13	-8	-5	29.30	2.71
-13	8	5	32.09	2.13
-13	8	5	33.16	2.41
-13	8	5	29.93	2.33
12	-8	-5	6.67	1.09
12	-8	-5	9.57	0.82
12	-8	-5	10.55	1.57
-12	8	5	6.07	1.19
-12	8	5	9.09	1.31
-12	8	5	9.02	1.36
11	-8	-5	7.81	1.39
11	-8	-5	8.90	0.74
11	-8	-5	9.64	1.14
-11	8	5	8.69	1.48
-11	8	5	9.56	1.71
-11	8	5	9.74	1.24

10	-8	-5	36.25	1.96
10	-8	-5	33.37	1.88
10	-8	-5	38.84	2.42
10	-8	-5	35.35	1.63
-10	8	5	35.94	1.97
-10	8	5	33.71	2.24
-10	8	5	33.72	2.38
9	-8	-5	35.44	1.63
9	-8	-5	38.45	2.00
9	-8	-5	36.82	2.00
9	-8	-5	36.30	1.85
9	-8	-5	36.59	2.31
-9	8	5	36.14	2.19
-9	8	5	31.59	2.31
-9	8	5	33.95	1.71
-9	8	5	38.62	1.96
8	-8	-5	25.98	1.84
8	-8	-5	28.01	1.63
8	-8	-5	23.77	1.64
8	-8	-5	25.50	1.43
8	-8	-5	26.08	1.25
-8	8	5	27.31	1.31
-8	8	5	25.62	1.77
-8	8	5	23.96	1.48
7	-8	-5	32.36	1.88
7	-8	-5	32.43	1.63
7	-8	-5	32.61	2.00
7	-8	-5	31.57	1.47
7	-8	-5	32.88	1.82
-7	8	5	32.15	1.93
-7	8	5	31.09	1.51
-7	8	5	34.56	1.67
6	-8	-5	25.24	1.53
6	-8	-5	23.05	1.15
6	-8	-5	23.75	1.66
6	-8	-5	26.16	1.69
6	-8	-5	23.72	1.28
-6	8	5	24.63	1.60
-6	8	5	24.84	1.21
-6	8	5	23.56	1.32
5	-8	-5	28.36	1.36
5	-8	-5	29.14	1.63
5	-8	-5	26.68	1.73
5	-8	-5	26.38	1.70

5	-8	-5	25.66	1.25
-5	8	5	28.59	1.31
-5	8	5	27.11	1.38
-5	8	5	26.19	1.65
4	-8	-5	11.12	1.09
4	-8	-5	11.54	1.23
4	-8	-5	13.24	1.15
4	-8	-5	13.08	0.79
4	-8	-5	11.22	0.69
-4	8	5	13.29	1.11
-4	8	5	11.00	0.74
-4	8	5	11.69	0.80
3	-8	-5	43.38	2.17
3	-8	-5	43.96	1.85
3	-8	-5	40.09	1.81
3	-8	-5	43.20	2.35
3	-8	-5	44.94	2.23
-3	8	5	41.17	2.15
-3	8	5	43.74	1.88
2	-8	-5	14.82	1.15
2	-8	-5	14.28	0.77
2	-8	-5	13.54	1.38
2	-8	-5	14.17	0.79
2	-8	-5	14.16	1.15
-2	8	5	13.27	0.79
-2	8	5	13.20	1.07
-2	8	5	14.20	0.82
1	-8	-5	158.36	6.36
1	-8	-5	157.64	6.61
1	-8	-5	150.46	6.02
1	-8	-5	166.63	6.39
1	-8	-5	158.37	6.00
-1	8	5	150.49	6.31
-1	8	5	155.54	6.02
-1	8	5	159.78	6.05
0	-8	-5	86.85	3.54
0	-8	-5	91.19	4.20
0	-8	-5	97.98	3.94
0	-8	-5	95.29	3.96
0	-8	-5	88.32	3.58
0	8	5	92.26	3.60
0	8	5	84.48	3.84
0	8	5	89.77	3.56
-1	-8	-5	159.90	6.31

-1	-8	-5	151.89	5.94
-1	-8	-5	153.40	6.64
-1	-8	-5	157.00	6.36
-1	-8	-5	153.92	6.00
1	8	5	156.72	6.28
1	8	5	161.38	6.02
1	8	5	154.31	5.97
-2	-8	-5	42.08	1.84
-2	-8	-5	47.29	2.24
-2	-8	-5	41.12	2.51
-2	-8	-5	43.92	2.18
-2	-8	-5	42.97	1.79
2	8	5	42.36	1.86
2	8	5	41.90	2.25
2	8	5	41.73	1.85
2	8	5	42.50	2.10
-3	-8	-5	89.50	3.84
-3	-8	-5	85.25	3.46
-3	-8	-5	87.85	3.76
-3	-8	-5	86.03	3.40
3	8	5	84.02	3.86
3	8	5	84.78	3.38
3	8	5	88.38	3.48
3	8	5	87.61	3.72
-4	-8	-5	14.74	1.20
-4	-8	-5	15.17	0.84
-4	-8	-5	15.88	0.87
-4	-8	-5	16.46	1.32
-4	-8	-5	15.53	0.83
4	8	5	14.46	0.78
4	8	5	14.69	0.87
4	8	5	12.01	1.08
4	8	5	14.92	1.30
-5	-8	-5	48.34	2.05
-5	-8	-5	50.08	2.09
-5	-8	-5	48.67	2.14
-5	-8	-5	50.32	2.55
-5	-8	-5	47.97	2.08
-5	-8	-5	52.69	2.45
5	8	5	50.64	2.38
5	8	5	48.52	2.58
-6	-8	-5	22.39	1.19
-6	-8	-5	21.96	1.16
-6	-8	-5	22.20	1.12

-6	-8	-5	22.78	1.50
-6	-8	-5	22.08	1.11
-6	-8	-5	22.79	1.64
6	8	5	24.38	1.65
6	8	5	22.99	1.43
-7	-8	-5	96.97	3.95
-7	-8	-5	105.75	4.44
-7	-8	-5	101.93	3.95
-7	-8	-5	96.47	4.01
-7	-8	-5	104.78	3.97
-7	-8	-5	105.04	4.31
7	8	5	99.43	4.47
7	8	5	84.80	4.16
-8	-8	-5	12.99	0.82
-8	-8	-5	12.78	0.92
-8	-8	-5	11.47	0.91
-8	-8	-5	14.54	0.93
-8	-8	-5	15.31	1.31
-8	-8	-5	12.20	1.48
8	8	5	11.49	1.16
8	8	5	12.98	1.44
-9	-8	-5	73.36	3.21
-9	-8	-5	75.85	3.16
-9	-8	-5	76.12	3.49
-9	-8	-5	76.75	3.12
-9	-8	-5	79.47	3.65
-9	-8	-5	75.45	3.20
9	8	5	71.01	3.39
9	8	5	78.85	3.68
-10	-8	-5	25.00	2.05
-10	-8	-5	24.02	1.78
-10	-8	-5	23.29	1.92
-10	-8	-5	22.33	1.45
-10	-8	-5	25.15	1.43
-10	-8	-5	26.93	1.45
10	8	5	25.82	1.89
-11	-8	-5	40.54	2.55
-11	-8	-5	46.17	2.68
-11	-8	-5	40.49	2.41
-11	-8	-5	41.95	2.09
-11	-8	-5	43.67	2.17
-11	-8	-5	43.06	2.11
11	8	5	43.42	2.60
-12	-8	-5	11.62	1.25

-12	-8	-5	10.00	1.78
-12	-8	-5	11.69	1.59
-12	-8	-5	11.41	1.23
-12	-8	-5	13.49	1.21
12	8	5	14.85	1.68
-13	-8	-5	25.96	1.65
-13	-8	-5	25.28	1.72
-13	-8	-5	23.12	1.74
-13	-8	-5	28.58	2.41
-13	-8	-5	21.68	2.00
13	8	5	28.18	2.18
-14	-8	-5	2.38	0.86
-14	-8	-5	1.76	1.32
-14	-8	-5	1.58	1.10
-14	-8	-5	2.77	0.82
14	8	5	2.16	1.07
-15	-8	-5	36.25	2.13
-15	-8	-5	35.34	2.92
-15	-8	-5	33.29	2.23
15	8	5	35.14	3.34
-16	-8	-5	1.52	1.01
-16	-8	-5	2.11	0.82
-16	-8	-5	0.79	1.62
16	8	5	2.08	1.25
-17	-8	-5	29.48	2.07
-17	-8	-5	24.43	2.35
17	8	5	25.68	2.67
-18	-8	-5	7.33	1.42
-18	-8	-5	5.85	1.16
18	8	5	5.93	1.02
18	8	5	7.00	1.78
19	-8	-4	4.06	0.77
-19	8	4	4.69	1.57
18	-8	-4	-0.42	0.56
-18	8	4	0.86	1.36
17	-8	-4	11.20	1.09
-17	8	4	10.94	1.64
-17	8	4	15.27	2.18
16	-8	-4	-0.19	0.78
16	-8	-4	0.06	0.48
16	-8	-4	0.94	1.87
-16	8	4	-1.03	1.01
-16	8	4	2.03	1.23
15	-8	-4	52.79	3.64

15	-8	-4	50.64	2.34
15	-8	-4	53.00	2.51
-15	8	4	52.44	3.03
-15	8	4	41.91	2.92
14	-8	-4	3.11	0.56
14	-8	-4	1.66	1.47
14	-8	-4	1.71	0.71
-14	8	4	2.16	1.05
-14	8	4	2.68	0.93
-14	8	4	3.03	1.23
13	-8	-4	10.36	1.59
13	-8	-4	11.95	0.88
13	-8	-4	8.93	1.18
-13	8	4	11.26	1.90
-13	8	4	11.97	1.45
-13	8	4	8.40	1.59
12	-8	-4	3.85	0.75
12	-8	-4	5.25	1.32
12	-8	-4	4.32	0.61
-12	8	4	3.45	0.77
-12	8	4	5.19	1.21
-12	8	4	2.22	0.89
11	-8	-4	0.89	1.01
11	-8	-4	1.31	0.65
11	-8	-4	0.50	0.32
-11	8	4	1.67	0.66
-11	8	4	0.78	1.02
-11	8	4	1.03	0.77
10	-8	-4	0.31	0.27
10	-8	-4	0.34	0.60
10	-8	-4	0.22	0.82
10	-8	-4	-0.23	0.54
-10	8	4	-0.50	0.50
-10	8	4	0.13	0.37
-10	8	4	1.54	0.94
-10	8	4	-0.03	0.66
9	-8	-4	79.23	3.42
9	-8	-4	81.18	3.77
9	-8	-4	81.82	3.55
9	-8	-4	81.42	3.25
-9	8	4	83.08	3.53
-9	8	4	80.62	3.87
-9	8	4	81.03	3.31
-9	8	4	74.76	3.66

8	-8	-4	-0.13	0.67
8	-8	-4	1.35	0.46
8	-8	-4	0.33	0.24
8	-8	-4	1.07	0.61
-8	8	4	0.87	0.60
-8	8	4	0.74	0.43
-8	8	4	1.18	0.86
-8	8	4	0.65	0.30
7	-8	-4	21.12	1.51
7	-8	-4	18.03	0.95
7	-8	-4	19.27	1.40
7	-8	-4	18.26	1.14
-7	8	4	20.15	1.72
-7	8	4	17.80	1.38
-7	8	4	18.86	1.01
-7	8	4	17.04	1.18
6	-8	-4	24.95	1.63
6	-8	-4	25.85	1.32
6	-8	-4	25.00	1.18
6	-8	-4	26.10	1.62
-6	8	4	24.02	1.34
-6	8	4	23.70	1.86
-6	8	4	26.03	1.23
-6	8	4	25.60	1.58
5	-8	-4	15.66	1.23
5	-8	-4	15.51	0.84
5	-8	-4	17.13	0.97
5	-8	-4	15.55	1.31
-5	8	4	14.83	1.18
-5	8	4	16.05	1.52
-5	8	4	16.79	0.88
-5	8	4	14.61	0.97
4	-8	-4	34.04	1.51
4	-8	-4	35.36	1.89
4	-8	-4	34.19	1.86
4	-8	-4	34.15	1.98
4	-8	-4	37.50	1.61
-4	8	4	34.38	1.62
-4	8	4	36.22	1.55
-4	8	4	33.76	2.15
-4	8	4	33.89	1.85
3	-8	-4	63.89	2.76
3	-8	-4	56.87	2.45
3	-8	-4	61.05	2.76

3	-8	-4	59.96	2.40
3	-8	-4	56.87	2.88
-3	8	4	56.28	2.71
-3	8	4	59.95	2.43
-3	8	4	59.38	2.49
2	-8	-4	19.92	1.01
2	-8	-4	25.61	1.42
2	-8	-4	20.32	1.55
2	-8	-4	23.84	1.39
-2	8	4	22.10	1.05
-2	8	4	21.55	1.09
-2	8	4	19.44	1.32
1	-8	-4	8.95	1.14
1	-8	-4	8.54	0.62
1	-8	-4	8.46	0.89
1	-8	-4	9.67	0.94
1	-8	-4	8.88	0.58
-1	8	4	9.17	0.62
-1	8	4	7.90	0.84
-1	8	4	9.24	0.59
0	-8	-4	87.80	3.52
0	-8	-4	89.54	3.53
0	-8	-4	91.07	4.13
0	-8	-4	91.23	3.87
0	-8	-4	94.07	3.85
0	8	4	83.87	3.79
0	8	4	91.30	3.55
0	8	4	89.99	3.54
-1	-8	-4	79.62	3.21
-1	-8	-4	79.77	3.84
-1	-8	-4	83.15	3.58
-1	-8	-4	81.33	3.22
-1	-8	-4	86.16	3.53
1	8	4	78.76	3.21
1	8	4	77.07	3.47
1	8	4	84.20	3.24
-2	-8	-4	24.74	1.17
-2	-8	-4	24.68	1.82
-2	-8	-4	25.32	1.54
-2	-8	-4	25.04	1.47
-2	-8	-4	24.47	1.16
2	8	4	25.11	1.44
2	8	4	24.61	1.18
2	8	4	25.89	1.17

-3	-8	-4	215.64	8.26
-3	-8	-4	221.64	8.56
-3	-8	-4	218.22	8.97
-3	-8	-4	213.26	8.28
-3	-8	-4	222.57	8.63
3	8	4	228.26	8.30
3	8	4	210.10	8.52
3	8	4	214.19	8.23
-4	-8	-4	7.39	0.59
-4	-8	-4	6.72	1.00
-4	-8	-4	7.30	0.54
-4	-8	-4	8.07	0.91
-4	-8	-4	6.46	1.11
-4	-8	-4	6.46	0.55
4	8	4	6.86	1.03
4	8	4	6.16	0.82
4	8	4	7.01	0.53
4	8	4	7.25	0.59
-5	-8	-4	2.91	0.40
-5	-8	-4	2.52	0.41
-5	-8	-4	2.79	0.67
-5	-8	-4	2.71	0.60
5	8	4	3.52	0.41
5	8	4	3.37	0.59
5	8	4	3.42	0.74
-6	-8	-4	30.78	1.43
-6	-8	-4	31.33	1.46
-6	-8	-4	29.89	1.41
-6	-8	-4	30.24	1.47
-6	-8	-4	35.01	1.81
-6	-8	-4	32.08	1.90
6	8	4	29.76	1.71
6	8	4	31.36	1.93
-7	-8	-4	19.25	1.10
-7	-8	-4	20.64	1.09
-7	-8	-4	19.59	1.03
-7	-8	-4	19.01	1.40
-7	-8	-4	22.16	1.57
-7	-8	-4	21.21	1.12
7	8	4	19.16	1.35
7	8	4	18.52	1.54
-8	-8	-4	19.24	1.56
-8	-8	-4	20.23	1.50
-8	-8	-4	18.94	1.15

-8	-8	-4	19.96	1.16
-8	-8	-4	20.29	1.04
-8	-8	-4	20.91	1.14
8	8	4	22.38	1.42
8	8	4	17.44	1.58
-9	-8	-4	33.81	2.02
-9	-8	-4	37.10	1.73
-9	-8	-4	34.08	1.70
-9	-8	-4	36.02	2.15
-9	-8	-4	34.54	2.18
-9	-8	-4	38.75	1.64
-9	-8	-4	34.46	1.73
9	8	4	34.60	2.18
9	8	4	32.54	1.95
-10	-8	-4	27.05	1.33
-10	-8	-4	25.04	1.42
-10	-8	-4	26.65	1.49
-10	-8	-4	24.76	1.48
-10	-8	-4	24.18	1.92
-10	-8	-4	26.37	1.78
-10	-8	-4	28.62	1.98
10	8	4	26.23	1.94
-11	-8	-4	52.07	2.43
-11	-8	-4	49.02	2.46
-11	-8	-4	50.96	2.88
-11	-8	-4	53.54	2.45
-11	-8	-4	55.03	2.94
-11	-8	-4	48.36	2.69
11	8	4	53.41	2.86
-12	-8	-4	32.88	1.81
-12	-8	-4	34.89	2.46
-12	-8	-4	34.69	1.96
-12	-8	-4	32.60	2.38
-12	-8	-4	35.10	2.19
-12	-8	-4	34.05	1.88
12	8	4	33.22	2.28
-13	-8	-4	3.94	1.07
-13	-8	-4	3.42	0.95
-13	-8	-4	1.51	0.74
-13	-8	-4	2.95	0.81
-13	-8	-4	3.23	1.23
-13	-8	-4	3.41	1.42
13	8	4	3.26	1.03
-14	-8	-4	19.16	1.65

-14	-8	-4	20.70	1.51
-14	-8	-4	20.02	2.43
-14	-8	-4	20.58	1.99
14	8	4	19.36	2.02
14	8	4	19.01	1.35
-15	-8	-4	22.59	1.71
-15	-8	-4	20.95	2.66
-15	-8	-4	23.75	1.87
15	8	4	22.71	1.54
15	8	4	24.96	2.18
-16	-8	-4	31.74	3.09
-16	-8	-4	30.94	2.28
-16	-8	-4	36.81	2.15
16	8	4	34.30	2.00
16	8	4	36.92	2.71
-17	-8	-4	3.07	0.93
-17	-8	-4	10.03	2.41
-17	-8	-4	4.79	1.20
17	8	4	5.21	0.92
17	8	4	5.63	1.55
-18	-8	-4	19.15	2.28
-18	-8	-4	20.87	3.89
-18	-8	-4	17.25	1.90
18	8	4	15.92	1.60
-19	-8	-4	4.86	1.46
-19	-8	-4	7.36	1.30
19	8	4	7.23	1.06
19	-8	-3	9.75	1.19
-19	8	3	7.31	1.67
18	-8	-3	27.57	1.68
-18	8	3	25.29	2.67
17	-8	-3	6.51	1.10
17	-8	-3	7.30	0.99
-17	8	3	7.39	1.48
-17	8	3	7.52	1.46
16	-8	-3	16.30	1.56
16	-8	-3	17.22	1.21
-16	8	3	17.33	2.07
-16	8	3	14.86	1.98
15	-8	-3	0.06	1.53
15	-8	-3	1.21	0.77
15	-8	-3	2.15	0.56
-15	8	3	0.77	0.97
-15	8	3	1.80	1.42

-15	8	3	1.03	1.08
14	-8	-3	34.41	1.98
14	-8	-3	32.69	1.67
14	-8	-3	37.48	2.84
-14	8	3	32.70	2.63
-14	8	3	33.67	2.29
-14	8	3	29.99	2.30
13	-8	-3	2.38	0.69
13	-8	-3	1.07	0.40
13	-8	-3	2.29	1.26
-13	8	3	1.91	1.16
-13	8	3	0.27	0.91
-13	8	3	1.06	0.78
12	-8	-3	23.03	1.24
12	-8	-3	24.37	2.13
12	-8	-3	23.60	1.57
-12	8	3	22.38	1.81
-12	8	3	25.63	1.75
-12	8	3	22.27	2.11
11	-8	-3	39.64	2.41
11	-8	-3	37.00	1.75
11	-8	-3	39.29	2.06
-11	8	3	38.33	2.51
-11	8	3	37.76	2.24
-11	8	3	37.93	2.15
10	-8	-3	32.47	1.84
10	-8	-3	29.59	1.75
10	-8	-3	31.67	1.49
10	-8	-3	35.69	2.12
-10	8	3	31.61	1.56
-10	8	3	31.04	2.23
-10	8	3	32.25	1.83
-10	8	3	32.22	1.98
9	-8	-3	17.58	0.96
9	-8	-3	18.92	1.24
9	-8	-3	17.30	1.34
9	-8	-3	20.03	1.59
-9	8	3	19.02	1.74
-9	8	3	19.26	1.05
-9	8	3	16.54	1.25
-9	8	3	17.00	1.43
8	-8	-3	32.75	1.86
8	-8	-3	36.11	1.98
8	-8	-3	33.08	1.49

8	-8	-3	33.51	1.68
-8	8	3	33.71	1.91
-8	8	3	33.69	1.75
-8	8	3	30.50	1.52
-8	8	3	32.67	2.16
7	-8	-3	21.97	1.28
7	-8	-3	24.96	1.56
7	-8	-3	23.14	1.54
7	-8	-3	22.85	1.09
-7	8	3	22.28	1.50
-7	8	3	22.09	1.32
-7	8	3	22.98	1.14
-7	8	3	22.81	1.77
6	-8	-3	44.77	1.91
6	-8	-3	48.08	2.30
6	-8	-3	45.22	2.30
6	-8	-3	48.02	2.07
-6	8	3	45.17	2.54
-6	8	3	43.92	2.24
-6	8	3	41.46	2.06
-6	8	3	48.96	1.96
5	-8	-3	0.61	0.48
5	-8	-3	0.73	0.54
5	-8	-3	0.68	0.35
5	-8	-3	0.88	0.21
-5	8	3	0.90	0.26
-5	8	3	0.46	0.41
-5	8	3	0.52	0.68
-5	8	3	0.56	0.34
4	-8	-3	12.67	1.21
4	-8	-3	14.63	1.10
4	-8	-3	14.24	0.88
4	-8	-3	13.48	0.73
-4	8	3	14.75	1.41
-4	8	3	14.07	0.78
-4	8	3	13.60	1.06
-4	8	3	14.52	0.90
3	-8	-3	13.56	1.24
3	-8	-3	12.94	0.71
3	-8	-3	14.63	1.07
3	-8	-3	13.82	0.84
-3	8	3	14.85	0.76
-3	8	3	12.92	1.04
-3	8	3	13.11	0.84

-3	8	3	15.09	1.41
2	-8	-3	45.68	1.95
2	-8	-3	52.50	2.28
2	-8	-3	48.06	2.30
2	-8	-3	45.82	2.02
2	-8	-3	46.43	2.44
-2	8	3	51.08	2.60
-2	8	3	47.00	2.04
-2	8	3	45.36	2.24
1	-8	-3	11.51	0.84
1	-8	-3	11.60	0.97
1	-8	-3	11.65	1.26
1	-8	-3	12.05	0.67
1	-8	-3	12.90	1.06
-1	8	3	13.94	0.71
-1	8	3	11.96	0.96
-1	8	3	13.39	0.75
0	-8	-3	151.90	6.03
0	-8	-3	161.77	6.61
0	-8	-3	161.42	6.32
0	-8	-3	160.75	6.08
0	-8	-3	159.33	6.37
0	8	3	151.07	6.30
0	8	3	160.68	6.08
0	8	3	160.32	6.05
-1	-8	-3	10.57	0.91
-1	-8	-3	9.12	0.57
-1	-8	-3	9.92	0.65
-1	-8	-3	8.84	1.18
-1	-8	-3	9.06	0.93
1	8	3	9.12	0.58
1	8	3	9.57	0.63
1	8	3	8.85	0.85
-2	-8	-3	60.11	2.55
-2	-8	-3	54.62	2.27
-2	-8	-3	53.60	2.24
-2	-8	-3	56.20	2.59
-2	-8	-3	52.41	2.85
2	8	3	54.92	2.25
2	8	3	55.83	2.25
2	8	3	51.92	2.50
-3	-8	-3	0.13	0.24
-3	-8	-3	0.27	0.46
-3	-8	-3	-0.21	0.38

-3	-8	-3	-0.07	0.20
-3	-8	-3	0.26	0.66
3	8	3	0.11	0.37
3	8	3	-0.27	0.20
3	8	3	0.29	0.21
-4	-8	-3	21.83	1.43
-4	-8	-3	22.45	1.02
-4	-8	-3	23.52	1.37
-4	-8	-3	21.70	1.05
-4	-8	-3	19.80	1.73
4	8	3	20.43	1.02
4	8	3	19.55	1.29
4	8	3	21.35	1.05
-5	-8	-3	3.03	0.50
-5	-8	-3	3.63	0.41
-5	-8	-3	3.27	0.65
-5	-8	-3	2.75	0.62
-5	-8	-3	2.60	0.36
-5	-8	-3	3.11	0.92
5	8	3	1.90	0.53
5	8	3	2.90	0.44
-6	-8	-3	26.15	2.04
-6	-8	-3	27.91	1.25
-6	-8	-3	27.31	1.31
-6	-8	-3	29.62	1.66
-6	-8	-3	28.79	1.69
-6	-8	-3	28.40	1.35
-6	-8	-3	27.44	1.30
6	8	3	24.54	1.55
6	8	3	25.17	1.26
6	8	3	29.19	1.78
-7	-8	-3	29.26	1.40
-7	-8	-3	27.34	1.27
-7	-8	-3	27.18	1.35
-7	-8	-3	28.90	1.66
-7	-8	-3	25.98	1.33
-7	-8	-3	28.48	1.75
-7	-8	-3	25.61	2.12
7	8	3	27.12	1.62
7	8	3	28.40	1.81
-8	-8	-3	44.97	2.04
-8	-8	-3	46.95	2.09
-8	-8	-3	44.78	2.06
-8	-8	-3	45.20	1.95

-8	-8	-3	46.63	2.35
-8	-8	-3	47.73	2.44
8	8	3	45.73	2.33
8	8	3	46.96	2.51
-9	-8	-3	7.70	1.32
-9	-8	-3	7.17	0.65
-9	-8	-3	8.06	1.20
-9	-8	-3	10.22	1.19
-9	-8	-3	8.65	0.83
-9	-8	-3	7.11	0.72
-9	-8	-3	6.98	0.82
9	8	3	7.18	1.21
-10	-8	-3	11.11	0.94
-10	-8	-3	10.71	1.32
-10	-8	-3	13.84	1.58
-10	-8	-3	11.92	1.02
-10	-8	-3	10.72	1.00
-10	-8	-3	11.11	1.43
-10	-8	-3	12.42	0.81
10	8	3	12.10	1.38
-11	-8	-3	6.21	0.70
-11	-8	-3	7.84	1.01
-11	-8	-3	6.58	0.85
-11	-8	-3	6.65	1.04
-11	-8	-3	7.74	1.01
-11	-8	-3	8.41	1.15
-11	-8	-3	6.99	1.24
11	8	3	7.38	1.34
-12	-8	-3	38.31	2.44
-12	-8	-3	39.58	1.97
-12	-8	-3	33.85	2.25
-12	-8	-3	40.96	2.05
-12	-8	-3	35.87	2.59
-12	-8	-3	37.99	2.08
12	8	3	37.00	1.83
12	8	3	39.41	2.38
-13	-8	-3	9.51	1.06
-13	-8	-3	8.59	1.55
-13	-8	-3	7.68	1.27
-13	-8	-3	7.18	1.31
-13	-8	-3	8.47	1.60
-13	-8	-3	7.80	1.42
13	8	3	9.86	1.57
13	8	3	9.41	0.88

-14	-8	-3	29.98	2.63
-14	-8	-3	26.95	1.74
-14	-8	-3	30.18	2.41
-14	-8	-3	28.75	2.23
-14	-8	-3	31.31	1.96
-14	-8	-3	30.33	3.08
14	8	3	29.06	2.30
14	8	3	31.54	1.68
-15	-8	-3	19.54	2.62
-15	-8	-3	17.03	2.10
-15	-8	-3	13.69	1.44
-15	-8	-3	16.92	2.43
15	8	3	17.38	2.05
15	8	3	16.30	1.32
-16	-8	-3	13.68	2.04
-16	-8	-3	12.74	1.69
-16	-8	-3	14.54	2.28
-16	-8	-3	10.63	1.46
16	8	3	14.95	1.30
-17	-8	-3	5.24	1.20
-17	-8	-3	4.76	0.95
-17	-8	-3	6.75	2.10
-17	-8	-3	3.98	2.01
17	8	3	5.74	0.89
-18	-8	-3	22.15	2.30
-18	-8	-3	18.16	4.75
-18	-8	-3	22.62	1.94
18	8	3	17.67	1.66
-19	-8	-3	1.51	1.27
-19	-8	-3	0.55	1.01
19	8	3	1.14	0.83
19	-8	-2	6.70	0.92
-19	8	2	5.56	1.63
18	-8	-2	31.75	1.80
-18	8	2	28.25	2.81
17	-8	-2	14.62	1.18
17	-8	-2	11.97	1.66
-17	8	2	11.57	1.92
-17	8	2	14.22	2.14
16	-8	-2	39.61	2.02
16	-8	-2	41.93	2.37
-16	8	2	40.71	2.77
-16	8	2	40.60	2.58
15	-8	-2	25.84	2.65

15	-8	-2	23.57	1.41
15	-8	-2	25.13	1.77
-15	8	2	24.88	2.70
-15	8	2	23.35	2.13
-15	8	2	24.32	2.05
14	-8	-2	13.85	2.14
14	-8	-2	12.41	1.37
14	-8	-2	13.38	1.00
-14	8	2	13.77	1.67
-14	8	2	12.93	2.20
-14	8	2	12.54	1.62
13	-8	-2	0.40	0.63
13	-8	-2	1.27	1.14
13	-8	-2	1.51	0.40
-13	8	2	1.87	1.17
-13	8	2	1.03	0.81
-13	8	2	-0.33	0.76
12	-8	-2	41.34	1.93
12	-8	-2	45.77	2.60
12	-8	-2	39.85	2.20
-12	8	2	43.29	2.73
-12	8	2	42.36	2.35
-12	8	2	44.46	2.38
11	-8	-2	101.99	4.24
11	-8	-2	94.65	3.95
11	-8	-2	100.74	4.41
-11	8	2	98.11	4.32
-11	8	2	97.52	4.60
-11	8	2	99.95	4.34
10	-8	-2	91.24	3.86
10	-8	-2	82.67	3.70
10	-8	-2	81.45	3.40
10	-8	-2	82.34	3.62
-10	8	2	82.28	4.04
-10	8	2	83.84	3.76
-10	8	2	90.66	3.48
-10	8	2	80.82	3.70
9	-8	-2	123.70	4.80
9	-8	-2	121.31	5.10
9	-8	-2	122.68	5.16
9	-8	-2	125.01	4.99
-9	8	2	122.82	5.40
-9	8	2	118.85	5.12
-9	8	2	126.57	4.85

-9	8	2	121.14	5.07
8	-8	-2	39.02	2.12
8	-8	-2	39.92	2.10
8	-8	-2	39.40	1.73
8	-8	-2	41.07	1.93
-8	8	2	43.19	1.80
-8	8	2	39.01	1.99
-8	8	2	37.19	2.37
-8	8	2	41.52	2.11
7	-8	-2	1.91	0.61
7	-8	-2	2.77	0.40
7	-8	-2	2.13	0.45
7	-8	-2	2.27	0.60
-7	8	2	3.16	0.46
-7	8	2	2.37	0.80
-7	8	2	2.33	0.47
-7	8	2	2.11	0.55
6	-8	-2	31.98	1.60
6	-8	-2	32.76	1.75
6	-8	-2	33.35	1.85
6	-8	-2	32.39	1.42
-6	8	2	32.40	1.63
-6	8	2	33.81	1.47
-6	8	2	32.41	1.77
-6	8	2	32.12	2.06
5	-8	-2	19.70	1.11
5	-8	-2	19.36	1.26
5	-8	-2	19.35	0.94
5	-8	-2	19.64	1.38
-5	8	2	20.57	0.97
-5	8	2	20.11	1.26
-5	8	2	18.81	1.55
-5	8	2	18.96	1.12
4	-8	-2	71.63	2.90
4	-8	-2	70.26	2.77
4	-8	-2	71.53	3.08
4	-8	-2	68.07	3.19
-4	8	2	72.92	3.35
-4	8	2	72.85	2.80
-4	8	2	64.85	2.90
-4	8	2	67.32	3.06
3	-8	-2	192.24	7.29
3	-8	-2	185.14	7.10
3	-8	-2	179.56	7.43

3	-8	-2	177.54	6.98
-3	8	2	179.23	7.27
-3	8	2	180.23	7.12
-3	8	2	194.18	7.02
-3	8	2	189.67	7.58
2	-8	-2	29.05	1.39
2	-8	-2	30.76	1.59
2	-8	-2	30.67	1.78
2	-8	-2	29.67	1.28
-2	8	2	28.64	1.41
-2	8	2	29.98	1.58
-2	8	2	30.48	1.31
-2	8	2	31.15	1.91
1	-8	-2	161.04	6.24
1	-8	-2	156.99	6.47
1	-8	-2	157.07	6.06
1	-8	-2	156.31	5.95
-1	8	2	142.66	6.20
-1	8	2	158.57	6.06
-1	8	2	159.62	6.53
-1	8	2	161.70	5.98
0	-8	-2	5.58	0.79
0	-8	-2	5.06	0.96
0	-8	-2	5.26	0.70
0	-8	-2	4.78	0.41
0	8	2	3.94	0.76
0	8	2	5.37	0.71
0	8	2	5.49	0.42
0	8	2	5.47	0.50
-1	-8	-2	243.76	9.57
-1	-8	-2	246.41	9.35
-1	-8	-2	246.18	9.82
-1	-8	-2	246.56	9.53
-1	-8	-2	241.95	9.24
1	8	2	252.48	9.26
1	8	2	248.53	9.54
1	8	2	241.36	9.31
-2	-8	-2	13.78	0.95
-2	-8	-2	16.44	1.08
-2	-8	-2	16.75	1.15
-2	-8	-2	16.08	0.78
-2	-8	-2	14.55	1.39
2	8	2	14.52	0.83
2	8	2	15.67	0.78

2	8	2	16.09	1.07
-3	-8	-2	45.02	1.83
-3	-8	-2	43.37	2.17
-3	-8	-2	44.64	2.47
-3	-8	-2	41.20	1.98
-3	-8	-2	45.63	2.13
3	8	2	44.29	1.87
3	8	2	45.59	1.84
3	8	2	41.98	2.09
-4	-8	-2	109.65	4.38
-4	-8	-2	115.02	5.09
-4	-8	-2	118.24	4.49
-4	-8	-2	117.67	4.72
-4	-8	-2	120.45	4.78
-4	-8	-2	110.95	4.42
4	8	2	96.02	4.64
4	8	2	123.75	4.44
4	8	2	115.66	4.45
-5	-8	-2	1.83	0.77
-5	-8	-2	1.72	0.30
-5	-8	-2	1.53	0.56
-5	-8	-2	1.81	0.26
-5	-8	-2	1.39	0.35
-5	-8	-2	1.50	0.47
5	8	2	1.36	0.49
5	8	2	1.45	0.29
-6	-8	-2	5.41	0.90
-6	-8	-2	3.15	0.94
-6	-8	-2	4.44	0.60
-6	-8	-2	3.61	0.64
-6	-8	-2	4.18	0.44
-6	-8	-2	4.34	0.48
6	8	2	3.67	0.63
6	8	2	3.90	0.48
-7	-8	-2	62.47	2.70
-7	-8	-2	66.51	2.99
-7	-8	-2	61.17	2.67
-7	-8	-2	67.48	2.58
-7	-8	-2	62.75	3.39
-7	-8	-2	65.62	2.95
-7	-8	-2	63.50	2.64
7	8	2	60.33	2.91
-8	-8	-2	0.44	0.35
-8	-8	-2	0.28	0.24

-8	-8	-2	0.15	0.58
-8	-8	-2	-0.07	0.28
-8	-8	-2	-0.73	0.79
-8	-8	-2	0.61	0.56
-8	-8	-2	1.03	0.70
-8	-8	-2	0.33	0.37
-9	-8	-2	132.88	6.33
-9	-8	-2	145.23	5.65
-9	-8	-2	144.94	5.50
-9	-8	-2	139.48	5.63
-9	-8	-2	139.97	5.59
-9	-8	-2	144.01	5.87
-9	-8	-2	145.93	5.91
-9	-8	-2	143.66	6.05
-10	-8	-2	7.29	1.17
-10	-8	-2	7.78	1.03
-10	-8	-2	9.21	0.68
-10	-8	-2	8.70	0.81
-10	-8	-2	6.55	0.93
-10	-8	-2	6.09	1.38
-10	-8	-2	5.20	0.73
-10	-8	-2	8.33	1.22
-11	-8	-2	3.91	0.67
-11	-8	-2	4.00	0.88
-11	-8	-2	4.37	1.11
-11	-8	-2	3.84	0.90
-11	-8	-2	3.53	0.66
-11	-8	-2	2.30	0.50
-11	-8	-2	2.23	1.23
-11	-8	-2	4.04	0.58
11	8	2	3.17	0.49
-12	-8	-2	3.56	0.95
-12	-8	-2	5.69	1.48
-12	-8	-2	3.49	0.74
-12	-8	-2	3.99	0.74
-12	-8	-2	3.56	0.99
-12	-8	-2	4.56	1.23
-12	-8	-2	4.08	0.66
12	8	2	3.24	0.53
-13	-8	-2	42.44	3.22
-13	-8	-2	42.65	2.56
-13	-8	-2	42.37	2.88
-13	-8	-2	44.42	2.14
-13	-8	-2	40.77	2.28

-13	-8	-2	37.49	2.47
13	8	2	42.59	2.02
-14	-8	-2	2.99	1.39
-14	-8	-2	-0.38	1.01
-14	-8	-2	0.16	0.76
-14	-8	-2	-0.71	0.98
-14	-8	-2	-2.26	1.40
-15	-8	-2	50.40	3.14
-15	-8	-2	47.35	2.98
-15	-8	-2	52.35	3.50
-15	-8	-2	46.19	2.73
-15	-8	-2	51.66	3.78
15	8	2	52.58	2.41
-16	-8	-2	3.76	1.67
-16	-8	-2	4.27	1.02
-16	-8	-2	5.50	1.88
16	8	2	3.17	0.68
-17	-8	-2	16.57	3.14
-17	-8	-2	18.60	1.98
-17	-8	-2	18.36	2.59
17	8	2	19.16	1.53
-18	-8	-2	5.11	2.01
-18	-8	-2	3.60	1.20
18	8	2	3.19	0.84
-19	-8	-2	9.17	1.50
19	8	2	7.68	1.10
19	-8	-1	20.70	1.61
-19	8	1	28.86	2.89
18	-8	-1	1.64	0.68
-18	8	1	0.40	1.26
17	-8	-1	49.82	2.35
17	-8	-1	47.44	2.73
-17	8	1	47.11	3.15
16	-8	-1	1.08	0.93
16	-8	-1	0.97	0.49
-16	8	1	0.97	0.98
-16	8	1	1.10	1.02
15	-8	-1	35.36	2.11
15	-8	-1	31.03	1.69
15	-8	-1	39.05	2.75
-15	8	1	30.55	2.85
-15	8	1	30.70	2.35
-15	8	1	31.04	2.15
14	-8	-1	8.47	1.36

14	-8	-1	7.11	0.82
14	-8	-1	9.71	1.37
-14	8	1	6.90	1.12
-14	8	1	8.37	1.19
-14	8	1	10.89	1.65
13	-8	-1	36.33	1.93
13	-8	-1	41.08	2.50
13	-8	-1	37.01	2.17
-13	8	1	39.37	2.33
-13	8	1	36.04	2.21
-13	8	1	39.92	2.76
12	-8	-1	4.44	0.57
12	-8	-1	4.79	0.80
12	-8	-1	4.97	1.02
-12	8	1	5.28	0.85
-12	8	1	3.17	0.82
-12	8	1	4.34	1.20
11	-8	-1	131.96	5.25
11	-8	-1	143.71	5.61
11	-8	-1	132.46	5.54
-11	8	1	132.99	5.88
-11	8	1	135.47	5.61
-11	8	1	127.17	5.51
10	-8	-1	13.06	1.28
10	-8	-1	13.26	0.86
10	-8	-1	15.92	1.40
10	-8	-1	14.81	1.17
-10	8	1	14.73	1.30
-10	8	1	13.20	1.24
-10	8	1	14.46	0.94
-10	8	1	16.70	1.73
9	-8	-1	62.63	2.89
9	-8	-1	64.95	2.71
9	-8	-1	70.25	3.07
9	-8	-1	65.98	3.07
-9	8	1	64.01	3.00
-9	8	1	61.41	3.32
-9	8	1	66.98	3.02
-9	8	1	72.85	2.78
8	-8	-1	24.07	1.57
8	-8	-1	23.03	1.17
8	-8	-1	25.77	1.54
8	-8	-1	22.75	1.36
-8	8	1	25.87	1.22

-8	8	1	24.86	1.47
-8	8	1	27.26	1.89
-8	8	1	26.06	1.54
7	-8	-1	40.43	1.91
7	-8	-1	37.70	1.98
7	-8	-1	36.93	1.76
7	-8	-1	35.98	1.57
-7	8	1	34.62	1.80
-7	8	1	35.35	1.60
-7	8	1	35.60	1.89
-7	8	1	34.92	2.20
6	-8	-1	14.50	0.78
6	-8	-1	18.12	1.16
6	-8	-1	15.13	1.25
6	-8	-1	15.66	1.00
-6	8	1	16.45	0.84
-6	8	1	15.43	1.03
-6	8	1	15.82	1.12
-6	8	1	14.07	1.43
5	-8	-1	90.22	3.93
5	-8	-1	87.37	3.51
5	-8	-1	92.66	3.80
5	-8	-1	98.34	3.69
-5	8	1	85.93	3.69
-5	8	1	89.34	4.08
-5	8	1	86.77	3.53
-5	8	1	90.89	3.80
4	-8	-1	72.25	2.91
4	-8	-1	66.82	2.74
4	-8	-1	71.42	3.02
4	-8	-1	67.86	3.17
-4	8	1	70.33	3.32
-4	8	1	69.85	2.93
-4	8	1	68.93	3.03
3	-8	-1	138.32	5.44
3	-8	-1	142.92	5.32
3	-8	-1	127.53	5.16
3	-8	-1	134.01	5.62
-3	8	1	140.39	5.75
-3	8	1	141.31	5.19
-3	8	1	132.01	5.32
-3	8	1	129.47	5.43
2	-8	-1	100.29	4.40
2	-8	-1	101.96	4.05

2	-8	-1	98.29	3.91
2	-8	-1	103.43	4.20
-2	8	1	101.47	4.20
-2	8	1	100.65	4.06
-2	8	1	104.80	3.93
-2	8	1	104.07	4.49
1	-8	-1	44.05	2.33
1	-8	-1	46.77	1.96
1	-8	-1	45.79	1.82
1	-8	-1	47.07	2.12
-1	8	1	43.23	1.95
-1	8	1	46.96	2.13
-1	8	1	45.96	2.40
-1	8	1	41.01	1.97
0	-8	-1	23.72	1.59
0	-8	-1	24.53	1.36
0	-8	-1	24.65	1.20
0	-8	-1	24.39	1.07
0	8	1	24.30	1.09
0	8	1	25.56	1.36
0	8	1	26.01	1.69
0	8	1	23.83	1.19
-1	-8	-1	28.80	1.54
-1	-8	-1	28.32	1.82
-1	-8	-1	30.75	1.39
-1	-8	-1	29.82	1.25
1	8	1	30.53	1.27
1	8	1	28.57	1.36
1	8	1	26.88	1.53
1	8	1	32.66	1.89
-2	-8	-1	13.18	0.80
-2	-8	-1	13.11	0.96
-2	-8	-1	14.54	1.06
-2	-8	-1	12.92	0.66
-2	-8	-1	11.50	1.26
2	8	1	14.78	1.30
2	8	1	12.72	0.78
2	8	1	12.26	0.96
2	8	1	12.32	0.68
-3	-8	-1	127.31	5.07
-3	-8	-1	122.58	5.33
-3	-8	-1	122.95	4.72
-3	-8	-1	122.73	4.83
-3	-8	-1	126.31	5.02

3	8	1	116.68	5.00
3	8	1	124.44	4.81
-4	-8	-1	32.63	1.74
-4	-8	-1	32.26	1.71
-4	-8	-1	31.54	2.01
-4	-8	-1	31.30	1.38
-4	-8	-1	32.60	1.50
-4	-8	-1	31.17	1.34
4	8	1	31.41	1.45
4	8	1	32.89	1.71
-5	-8	-1	78.43	3.40
-5	-8	-1	77.69	3.08
-5	-8	-1	73.70	3.37
-5	-8	-1	79.92	3.18
-5	-8	-1	75.04	3.72
-5	-8	-1	80.36	3.04
5	8	1	80.17	3.40
5	8	1	74.54	3.12
-6	-8	-1	52.30	2.92
-6	-8	-1	57.30	2.20
-6	-8	-1	56.63	2.35
-6	-8	-1	53.76	2.24
-6	-8	-1	54.63	2.56
-6	-8	-1	48.64	2.53
6	8	1	54.64	2.29
6	8	1	54.67	2.57
-7	-8	-1	32.97	1.56
-7	-8	-1	35.23	1.83
-7	-8	-1	31.08	1.59
-7	-8	-1	32.91	1.49
-7	-8	-1	30.78	2.21
-7	-8	-1	32.91	2.02
-7	-8	-1	32.45	1.41
-7	-8	-1	34.73	1.85
7	8	1	31.21	1.52
-8	-8	-1	35.53	1.72
-8	-8	-1	35.30	1.64
-8	-8	-1	38.36	1.58
-8	-8	-1	35.34	2.43
-8	-8	-1	35.83	2.00
-8	-8	-1	37.42	1.77
-8	-8	-1	34.69	2.18
-8	-8	-1	35.85	1.97
-9	-8	-1	53.66	2.56

-9	-8	-1	48.77	2.20
-9	-8	-1	48.53	2.77
-9	-8	-1	51.62	2.31
-9	-8	-1	49.78	2.28
-9	-8	-1	51.09	2.55
-9	-8	-1	48.86	2.10
-9	-8	-1	50.40	3.00
-10	-8	-1	58.79	2.72
-10	-8	-1	49.33	3.15
-10	-8	-1	51.33	2.70
-10	-8	-1	52.53	3.01
-10	-8	-1	54.00	2.25
-10	-8	-1	51.63	2.44
-10	-8	-1	54.92	2.37
-10	-8	-1	53.43	2.47
10	8	1	55.11	2.30
-11	-8	-1	35.53	1.89
-11	-8	-1	37.46	1.93
-11	-8	-1	36.06	2.54
-11	-8	-1	34.99	2.16
-11	-8	-1	33.94	2.69
-11	-8	-1	39.02	2.16
-11	-8	-1	35.58	1.78
-11	-8	-1	35.27	1.64
11	8	1	36.63	1.71
-12	-8	-1	12.67	1.62
-12	-8	-1	14.02	2.14
-12	-8	-1	14.35	0.94
-12	-8	-1	15.10	1.60
-12	-8	-1	13.95	1.26
-12	-8	-1	10.87	1.97
-12	-8	-1	13.52	1.28
12	8	1	15.45	1.00
-13	-8	-1	46.19	2.62
-13	-8	-1	43.73	3.00
-13	-8	-1	47.52	3.22
-13	-8	-1	46.00	2.69
-13	-8	-1	46.32	2.43
13	8	1	48.28	2.16
-14	-8	-1	36.48	2.36
-14	-8	-1	34.78	3.01
-14	-8	-1	33.80	2.11
-14	-8	-1	35.79	2.94
-14	-8	-1	31.79	2.37

14	8	1	36.97	1.82
-15	-8	-1	12.46	1.90
-15	-8	-1	13.26	1.55
-15	-8	-1	9.87	1.85
-15	-8	-1	12.03	1.52
-15	-8	-1	12.54	2.02
15	8	1	13.33	1.15
-16	-8	-1	30.94	2.23
-16	-8	-1	30.42	3.23
-16	-8	-1	31.12	3.22
16	8	1	33.08	1.85
-17	-8	-1	25.43	2.18
-17	-8	-1	23.96	3.30
-17	-8	-1	25.27	3.18
17	8	1	24.64	1.70
-18	-8	-1	31.63	4.04
-18	-8	-1	26.45	2.36
-18	-8	-1	22.94	4.06
18	8	1	26.16	1.82
-19	-8	-1	1.11	1.26
19	-8	0	3.46	0.77
-19	8	0	3.49	1.55
18	-8	0	27.32	2.32
18	-8	0	24.74	1.69
-18	8	0	25.66	2.67
17	-8	0	3.87	0.68
17	-8	0	5.94	1.15
-17	8	0	4.70	1.29
16	-8	0	15.03	1.75
16	-8	0	14.03	1.14
-16	8	0	12.99	1.55
-16	8	0	12.16	1.92
15	-8	0	23.37	1.86
15	-8	0	23.53	2.25
15	-8	0	22.36	1.36
-15	8	0	19.83	1.71
-15	8	0	24.53	2.09
-15	8	0	21.04	2.72
14	-8	0	6.46	0.81
14	-8	0	6.31	1.25
14	-8	0	6.84	1.29
-14	8	0	4.51	1.47
-14	8	0	7.17	1.14
-14	8	0	6.94	1.00

13	-8	0	28.59	2.02
13	-8	0	22.50	1.56
13	-8	0	22.98	1.73
-13	8	0	23.50	1.69
-13	8	0	25.68	1.86
-13	8	0	24.28	2.45
12	-8	0	51.65	2.60
12	-8	0	48.25	2.18
12	-8	0	52.80	2.58
-12	8	0	46.83	3.02
-12	8	0	47.66	2.45
-12	8	0	49.34	2.62
11	-8	0	38.16	1.79
11	-8	0	41.53	2.18
11	-8	0	37.27	2.16
-11	8	0	41.28	2.20
-11	8	0	41.63	2.61
-11	8	0	38.67	2.08
10	-8	0	113.12	4.78
10	-8	0	116.63	4.72
10	-8	0	111.18	4.44
10	-8	0	108.46	4.64
-10	8	0	111.58	4.68
-10	8	0	118.68	4.50
-10	8	0	112.22	4.76
-10	8	0	107.82	5.05
9	-8	0	7.41	1.03
9	-8	0	6.08	0.85
9	-8	0	7.63	1.00
9	-8	0	5.21	0.55
-9	8	0	6.38	0.95
-9	8	0	5.51	0.63
-9	8	0	4.77	0.71
-9	8	0	6.61	1.11
8	-8	0	93.12	3.81
8	-8	0	94.73	4.00
8	-8	0	92.91	3.87
8	-8	0	88.38	3.61
-8	8	0	90.77	3.88
-8	8	0	89.03	3.86
-8	8	0	95.24	3.66
-8	8	0	87.15	4.20
7	-8	0	23.78	1.60
7	-8	0	26.24	1.46

7	-8	0	24.63	1.18
7	-8	0	24.07	1.39
-7	8	0	24.78	1.44
-7	8	0	28.02	1.22
-7	8	0	27.12	1.88
-7	8	0	26.83	1.48
6	-8	0	35.55	1.68
6	-8	0	32.05	1.45
6	-8	0	36.06	1.91
6	-8	0	35.69	1.75
-6	8	0	33.76	2.11
-6	8	0	29.92	1.67
-6	8	0	34.97	1.49
-6	8	0	35.72	1.76
5	-8	0	1.87	0.39
5	-8	0	1.37	0.58
5	-8	0	2.96	0.52
5	-8	0	2.04	0.27
-5	8	0	1.76	0.42
-5	8	0	2.03	0.51
-5	8	0	2.80	0.77
-5	8	0	1.92	0.27
4	-8	0	166.62	6.83
4	-8	0	172.77	6.61
4	-8	0	171.63	7.03
4	-8	0	176.83	6.78
-4	8	0	171.89	6.87
-4	8	0	177.99	6.63
-4	8	0	180.86	7.18
-4	8	0	179.18	6.82
3	-8	0	17.40	1.10
3	-8	0	17.09	0.82
3	-8	0	17.28	1.30
3	-8	0	18.17	1.01
-3	8	0	18.23	0.84
-3	8	0	18.22	1.03
-3	8	0	16.66	1.10
-3	8	0	19.12	1.44
2	-8	0	307.83	11.92
2	-8	0	329.44	11.84
2	-8	0	325.93	12.17
2	-8	0	291.89	11.65
-2	8	0	317.16	11.95
-2	8	0	302.49	11.83

1	-8	0	0.36	0.27
1	-8	0	0.50	0.37
1	-8	0	0.22	0.44
1	-8	0	0.23	0.14
-1	8	0	0.08	0.25
-1	8	0	-0.02	0.33
-1	8	0	0.15	0.14
-1	8	0	0.54	0.52
0	-8	0	43.12	1.86
0	-8	0	42.14	1.69
0	-8	0	41.06	2.21
0	-8	0	41.83	1.99
0	8	0	40.72	1.84
0	8	0	42.20	2.27
0	8	0	43.10	1.71
0	8	0	42.32	2.01
-1	-8	0	0.29	0.49
-1	-8	0	0.42	0.17
-1	-8	0	-0.03	0.25
-1	-8	0	0.20	0.35
1	8	0	0.67	0.17
1	8	0	1.25	0.61
1	8	0	0.19	0.36
1	8	0	0.11	0.23
-2	-8	0	314.54	11.79
-2	-8	0	323.18	11.95
-2	-8	0	316.60	12.11
-2	-8	0	316.61	12.36
2	8	0	310.57	12.11
2	8	0	307.53	11.80
2	8	0	329.23	12.38
2	8	0	308.98	11.92
-3	-8	0	17.12	1.45
-3	-8	0	16.19	0.81
-3	-8	0	18.09	0.98
-3	-8	0	17.44	1.15
3	8	0	15.63	0.80
3	8	0	18.04	1.43
3	8	0	19.54	1.19
3	8	0	17.55	0.94
-4	-8	0	174.40	6.78
-4	-8	0	181.01	6.76
-4	-8	0	189.55	6.94
-4	-8	0	181.28	7.40

-4	-8	0	181.73	7.12
-4	-8	0	176.34	7.10
4	8	0	174.27	7.11
4	8	0	176.45	6.89
-5	-8	0	1.76	0.63
-5	-8	0	2.36	0.55
-5	-8	0	2.36	0.35
-5	-8	0	2.11	0.55
-5	-8	0	0.65	0.61
-5	-8	0	1.86	0.39
-5	-8	0	1.60	0.23
5	8	0	1.02	0.50
5	8	0	1.66	0.35
-6	-8	0	32.02	1.46
-6	-8	0	32.18	2.15
-6	-8	0	33.35	1.99
-6	-8	0	34.62	1.41
-6	-8	0	35.80	1.63
-6	-8	0	31.44	1.80
-6	-8	0	34.18	1.80
6	8	0	32.89	1.54
-7	-8	0	26.06	1.13
-7	-8	0	24.61	1.57
-7	-8	0	25.43	1.20
-7	-8	0	24.39	1.73
-7	-8	0	24.85	1.34
-7	-8	0	26.03	1.95
-7	-8	0	25.68	1.54
7	8	0	24.17	1.26
-8	-8	0	96.43	4.06
-8	-8	0	95.72	3.97
-8	-8	0	92.44	3.79
-8	-8	0	91.17	3.69
-8	-8	0	92.49	3.82
-8	-8	0	85.16	4.35
-8	-8	0	94.89	3.62
-8	-8	0	91.31	4.25
-9	-8	0	4.14	0.72
-9	-8	0	5.86	1.10
-9	-8	0	6.82	1.07
-9	-8	0	5.60	0.81
-9	-8	0	5.98	0.90
-9	-8	0	6.78	0.64
-9	-8	0	6.69	1.21

-9	-8	0	6.22	0.54
9	8	0	6.16	0.57
-10	-8	0	116.05	4.75
-10	-8	0	108.82	4.58
-10	-8	0	104.06	5.16
-10	-8	0	120.95	5.19
-10	-8	0	116.77	4.38
-10	-8	0	115.30	4.49
-10	-8	0	102.27	4.79
-10	-8	0	108.90	4.60
10	8	0	113.83	4.42
-11	-8	0	37.58	2.65
-11	-8	0	37.07	2.03
-11	-8	0	40.45	2.16
-11	-8	0	41.42	2.32
-11	-8	0	36.71	2.68
-11	-8	0	38.76	2.03
-11	-8	0	40.64	1.74
11	8	0	38.83	1.78
-12	-8	0	51.18	3.20
-12	-8	0	53.13	2.80
-12	-8	0	49.93	3.19
-12	-8	0	53.50	2.67
-12	-8	0	49.54	2.49
-12	-8	0	47.78	2.78
-12	-8	0	53.87	2.22
12	8	0	51.84	2.27
-13	-8	0	28.87	1.94
-13	-8	0	27.74	1.53
-13	-8	0	22.20	2.57
-13	-8	0	21.49	2.02
-13	-8	0	28.15	2.51
-13	-8	0	25.64	1.70
13	8	0	28.12	1.43
-14	-8	0	5.49	1.11
-14	-8	0	8.29	1.66
-14	-8	0	8.75	1.28
-14	-8	0	5.69	1.52
-14	-8	0	6.64	0.98
14	8	0	7.35	0.82
-15	-8	0	25.57	2.10
-15	-8	0	23.38	1.87
-15	-8	0	22.12	2.26
-15	-8	0	23.49	2.88

-15	-8	0	19.10	2.66
-16	-8	0	14.79	1.81
-16	-8	0	15.60	2.25
-16	-8	0	16.46	2.37
-16	-8	0	20.44	2.69
16	8	0	16.75	1.31
-17	-8	0	4.56	1.17
-17	-8	0	4.26	1.77
-17	-8	0	4.25	2.00
17	8	0	4.30	0.75
-18	-8	0	24.30	2.31
-18	-8	0	27.01	3.61
18	8	0	29.34	1.83
-19	-8	0	3.51	1.33
19	8	0	3.63	0.88
19	-8	1	3.94	0.84
-19	8	-1	5.36	1.64
18	-8	1	25.07	1.63
18	-8	1	24.33	2.36
-18	8	-1	24.37	2.65
17	-8	1	25.95	2.27
17	-8	1	22.86	1.57
-17	8	-1	23.70	2.47
16	-8	1	30.29	2.27
16	-8	1	30.51	1.75
-16	8	-1	34.66	2.57
15	-8	1	13.24	1.69
15	-8	1	12.28	1.04
-15	8	-1	9.45	1.28
-15	8	-1	13.98	1.95
-15	8	-1	13.84	1.83
14	-8	1	39.29	2.28
14	-8	1	36.08	2.39
14	-8	1	34.22	1.80
-14	8	-1	42.78	2.99
-14	8	-1	30.67	1.96
-14	8	-1	38.10	2.41
13	-8	1	41.41	2.11
13	-8	1	46.15	2.46
13	-8	1	43.09	2.44
-13	8	-1	50.74	2.59
-13	8	-1	42.83	3.03
-13	8	-1	40.59	2.23
12	-8	1	13.75	0.92

12	-8	1	12.35	1.38
12	-8	1	13.16	1.40
-12	8	-1	14.35	1.39
-12	8	-1	11.96	1.25
-12	8	-1	14.01	2.05
11	-8	1	36.74	1.70
11	-8	1	38.34	2.14
11	-8	1	38.05	2.02
-11	8	-1	34.89	2.52
-11	8	-1	36.02	2.09
-11	8	-1	35.90	1.94
10	-8	1	58.04	2.51
10	-8	1	54.25	2.67
10	-8	1	50.93	2.46
10	-8	1	52.19	2.24
-10	8	-1	49.76	2.45
-10	8	-1	48.34	2.92
-10	8	-1	50.22	2.56
-10	8	-1	55.47	2.30
9	-8	1	48.77	2.14
9	-8	1	52.34	2.57
9	-8	1	50.55	2.37
9	-8	1	50.43	2.35
-9	8	-1	51.77	2.40
-9	8	-1	51.51	2.18
-9	8	-1	50.74	2.45
-9	8	-1	49.35	2.85
8	-8	1	33.34	1.97
8	-8	1	35.91	1.80
8	-8	1	38.56	1.84
8	-8	1	36.97	1.59
-8	8	-1	35.97	1.85
-8	8	-1	37.97	2.30
-8	8	-1	37.95	1.62
-8	8	-1	31.30	1.83
7	-8	1	31.15	1.65
7	-8	1	35.55	1.71
7	-8	1	31.25	1.42
7	-8	1	32.40	1.88
-7	8	-1	31.75	1.71
-7	8	-1	33.62	1.46
-7	8	-1	32.89	2.09
-7	8	-1	33.56	1.71
6	-8	1	50.99	2.19

6	-8	1	55.51	2.40
6	-8	1	56.18	2.43
6	-8	1	53.68	2.62
-6	8	-1	52.45	2.80
-6	8	-1	52.34	2.43
-6	8	-1	54.99	2.47
-6	8	-1	55.71	2.22
5	-8	1	75.04	3.39
5	-8	1	71.76	2.96
5	-8	1	77.19	3.20
5	-8	1	75.43	3.16
-5	8	-1	74.35	3.19
-5	8	-1	74.34	3.22
-5	8	-1	78.36	2.99
-5	8	-1	78.99	3.59
4	-8	1	30.54	1.32
4	-8	1	29.23	1.52
4	-8	1	33.26	1.59
4	-8	1	30.80	1.79
-4	8	-1	31.32	1.97
-4	8	-1	32.73	1.63
-4	8	-1	31.17	1.55
-4	8	-1	32.31	1.34
3	-8	1	117.07	4.57
3	-8	1	124.22	4.78
3	-8	1	123.18	4.84
3	-8	1	122.69	5.05
-3	8	-1	119.99	4.86
-3	8	-1	123.86	5.16
-3	8	-1	116.83	4.78
-3	8	-1	114.82	4.60
2	-8	1	12.12	1.10
2	-8	1	11.64	0.60
2	-8	1	13.33	0.92
2	-8	1	13.15	0.84
-2	8	-1	13.08	0.63
-2	8	-1	13.22	0.84
-2	8	-1	12.60	0.93
-2	8	-1	12.05	1.21
1	-8	1	28.82	1.22
1	-8	1	31.21	1.53
1	-8	1	30.63	1.73
1	-8	1	28.02	1.41
-1	8	-1	29.48	1.41

-1	8	-1	29.16	1.23
-1	8	-1	31.32	1.86
0	-8	1	23.80	1.03
0	-8	1	24.25	1.35
0	-8	1	24.98	1.59
0	-8	1	23.45	1.23
0	8	-1	23.77	1.21
0	8	-1	25.05	1.64
0	8	-1	26.01	1.38
0	8	-1	25.17	1.04
-1	-8	1	51.56	2.06
-1	-8	1	46.27	2.16
-1	-8	1	44.98	2.38
1	8	-1	46.19	2.40
1	8	-1	44.56	1.83
1	8	-1	45.12	2.18
1	8	-1	45.93	2.00
-2	-8	1	105.56	4.20
-2	-8	1	100.29	4.42
-2	-8	1	101.25	4.04
2	8	-1	100.60	4.22
2	8	-1	100.21	4.02
2	8	-1	102.51	4.43
2	8	-1	97.10	3.85
-3	-8	1	128.11	5.07
-3	-8	1	136.86	5.25
-3	-8	1	139.30	5.43
-3	-8	1	136.22	5.65
3	8	-1	132.72	5.06
3	8	-1	126.24	5.42
3	8	-1	133.97	5.22
3	8	-1	135.61	5.64
-4	-8	1	71.93	3.08
-4	-8	1	66.57	2.65
-4	-8	1	68.70	2.69
-4	-8	1	70.32	3.30
-4	-8	1	72.01	2.88
-4	-8	1	64.75	3.19
4	8	-1	65.29	2.82
4	8	-1	70.32	3.28
-5	-8	1	94.27	3.89
-5	-8	1	88.94	4.04
-5	-8	1	89.95	3.82
-5	-8	1	88.13	3.49

-5	-8	1	92.01	3.46
-5	-8	1	89.28	4.12
-5	-8	1	90.89	3.67
5	8	-1	87.15	3.62
-6	-8	1	15.70	1.16
-6	-8	1	15.61	0.76
-6	-8	1	13.95	1.21
-6	-8	1	15.56	1.47
-6	-8	1	16.26	0.81
-6	-8	1	16.30	1.01
-6	-8	1	17.22	1.40
6	8	-1	16.29	0.95
-7	-8	1	35.93	1.51
-7	-8	1	32.15	2.21
-7	-8	1	36.92	1.90
-7	-8	1	37.89	2.03
-7	-8	1	38.61	1.59
-7	-8	1	36.50	1.77
-7	-8	1	37.18	2.20
7	8	-1	35.50	1.69
-8	-8	1	21.83	1.93
-8	-8	1	23.05	1.63
-8	-8	1	23.91	1.31
-8	-8	1	23.74	1.88
-8	-8	1	23.02	1.35
-8	-8	1	24.83	1.09
-8	-8	1	26.30	1.53
-8	-8	1	22.90	1.16
-9	-8	1	70.22	3.48
-9	-8	1	67.74	3.17
-9	-8	1	58.52	3.39
-9	-8	1	64.01	2.89
-9	-8	1	67.85	2.65
-9	-8	1	64.87	3.00
-9	-8	1	65.34	2.86
-9	-8	1	66.24	2.74
9	8	-1	68.39	2.68
-10	-8	1	12.15	1.75
-10	-8	1	14.81	1.29
-10	-8	1	15.42	0.82
-10	-8	1	13.16	1.47
-10	-8	1	17.61	1.21
-10	-8	1	13.75	1.08
-10	-8	1	15.01	1.81

10	8	-1	13.68	0.85
-11	-8	1	128.99	5.65
-11	-8	1	128.67	5.43
-11	-8	1	137.76	5.50
-11	-8	1	123.47	5.89
-11	-8	1	138.51	5.43
-11	-8	1	130.49	6.02
-11	-8	1	135.86	5.15
11	8	-1	135.73	5.19
-12	-8	1	3.51	0.47
-12	-8	1	4.43	0.74
-12	-8	1	3.29	1.30
-12	-8	1	3.97	1.00
-12	-8	1	2.74	1.19
-12	-8	1	5.16	0.87
12	8	-1	5.01	0.66
-13	-8	1	40.66	2.14
-13	-8	1	34.29	2.41
-13	-8	1	36.27	2.69
-13	-8	1	39.49	2.22
-13	-8	1	38.81	2.94
-13	-8	1	36.85	1.75
13	8	-1	38.15	1.81
-14	-8	1	9.04	1.50
-14	-8	1	7.73	1.59
-14	-8	1	8.72	1.72
-14	-8	1	12.33	1.90
14	8	-1	7.96	0.90
-15	-8	1	32.59	3.12
-15	-8	1	30.38	2.27
-15	-8	1	29.03	2.85
-15	-8	1	34.63	2.18
-15	-8	1	29.89	2.53
15	8	-1	37.66	1.76
-16	-8	1	-0.52	1.44
-16	-8	1	-0.34	0.86
-16	-8	1	1.66	1.79
16	8	-1	0.10	0.51
-17	-8	1	49.68	2.94
-17	-8	1	49.86	3.57
-17	-8	1	50.51	4.09
17	8	-1	52.42	2.48
-18	-8	1	3.03	2.05
-18	-8	1	1.69	1.04

18	8	-1	2.62	0.79
-19	-8	1	29.14	2.51
19	8	-1	27.07	1.90
20	8	-1	2.20	0.92
19	-8	2	5.91	0.86
-19	8	-2	7.45	1.65
18	-8	2	2.59	1.27
18	-8	2	3.11	0.79
-18	8	-2	3.54	1.42
17	-8	2	19.32	2.17
17	-8	2	17.78	1.40
-17	8	-2	17.39	2.32
16	-8	2	4.96	1.19
16	-8	2	4.25	0.69
-16	8	-2	3.14	1.12
15	-8	2	47.64	2.88
15	-8	2	53.74	2.38
-15	8	-2	48.76	3.57
-15	8	-2	50.11	3.04
14	-8	2	0.29	0.81
14	-8	2	2.65	0.92
14	-8	2	0.37	0.43
-14	8	-2	1.33	0.86
-14	8	-2	2.57	1.43
-14	8	-2	0.34	0.73
13	-8	2	42.14	1.98
13	-8	2	41.05	2.42
13	-8	2	47.57	2.29
-13	8	-2	43.88	2.50
-13	8	-2	36.92	2.91
-13	8	-2	37.89	2.12
12	-8	2	4.39	0.79
12	-8	2	2.99	0.48
12	-8	2	3.00	0.82
-12	8	-2	4.93	1.31
-12	8	-2	3.51	0.69
-12	8	-2	4.64	0.89
11	-8	2	2.86	0.95
11	-8	2	3.73	0.85
11	-8	2	2.46	0.43
-11	8	-2	3.96	0.68
-11	8	-2	3.57	0.74
-11	8	-2	2.54	1.12
10	-8	2	6.63	1.15

10	-8	2	7.88	0.66
10	-8	2	4.98	0.74
-10	8	-2	8.17	0.74
-10	8	-2	8.22	1.28
-10	8	-2	7.37	1.03
-10	8	-2	7.20	0.92
9	-8	2	148.13	5.93
9	-8	2	138.20	5.50
9	-8	2	144.15	5.71
-9	8	-2	137.12	5.64
-9	8	-2	152.14	5.54
-9	8	-2	138.00	5.80
-9	8	-2	134.47	6.10
8	-8	2	-1.13	0.40
8	-8	2	-0.07	0.22
8	-8	2	0.59	0.62
8	-8	2	0.00	0.45
-8	8	-2	0.78	0.29
-8	8	-2	0.10	0.75
-8	8	-2	0.34	0.47
-8	8	-2	0.31	0.42
7	-8	2	63.80	2.60
7	-8	2	65.92	2.78
7	-8	2	64.11	2.81
7	-8	2	63.48	3.01
-7	8	-2	70.35	2.64
-7	8	-2	59.70	2.84
-7	8	-2	61.49	3.21
-7	8	-2	64.62	2.83
6	-8	2	3.91	0.56
6	-8	2	4.60	0.79
6	-8	2	3.05	0.51
6	-8	2	3.54	0.39
-6	8	-2	3.67	0.41
-6	8	-2	4.58	0.75
-6	8	-2	3.54	0.56
-6	8	-2	3.53	0.82
5	-8	2	1.00	0.58
5	-8	2	1.14	0.38
5	-8	2	1.86	0.25
5	-8	2	1.56	0.42
-5	8	-2	1.56	0.24
-5	8	-2	1.97	0.75
-5	8	-2	1.81	0.43

-5	8	-2	1.80	0.48
4	-8	2	111.15	4.40
4	-8	2	117.08	4.87
4	-8	2	115.89	4.62
4	-8	2	113.98	4.63
-4	8	-2	115.40	4.68
-4	8	-2	119.26	4.42
-4	8	-2	110.85	4.63
-4	8	-2	118.80	5.01
3	-8	2	46.02	2.01
3	-8	2	41.59	1.71
3	-8	2	44.60	1.97
3	-8	2	42.61	2.20
-3	8	-2	42.22	1.96
-3	8	-2	39.22	1.77
-3	8	-2	45.97	2.38
-3	8	-2	42.19	2.03
2	-8	2	14.94	1.22
2	-8	2	15.39	0.94
2	-8	2	15.24	1.01
2	-8	2	15.26	0.70
-2	8	-2	17.09	1.37
-2	8	-2	16.14	0.71
-2	8	-2	13.69	0.92
-2	8	-2	14.93	1.04
1	-8	2	261.30	9.73
1	-8	2	260.21	9.64
1	-8	2	245.44	9.90
-1	8	-2	256.09	10.00
-1	8	-2	243.32	9.62
-1	8	-2	247.48	9.75
-1	8	-2	251.82	9.42
0	-8	2	6.04	0.90
0	-8	2	6.02	0.72
0	-8	2	5.57	0.33
0	-8	2	5.08	0.59
0	8	-2	5.61	0.56
0	8	-2	7.12	0.99
0	8	-2	4.22	0.69
0	8	-2	5.80	0.35
-1	-8	2	160.25	6.14
-1	-8	2	153.95	6.31
-1	-8	2	150.53	5.76
-1	-8	2	158.81	6.01

1	8	-2	151.32	5.78
1	8	-2	155.97	6.36
1	8	-2	151.03	5.97
1	8	-2	148.42	6.15
-2	-8	2	30.53	1.76
-2	-8	2	28.84	1.40
-2	-8	2	28.51	1.15
-2	-8	2	27.17	1.69
2	8	-2	28.39	1.77
2	8	-2	27.39	1.37
2	8	-2	28.13	1.16
-3	-8	2	178.87	7.48
-3	-8	2	185.92	6.91
-3	-8	2	188.05	7.33
-3	-8	2	180.53	6.92
-3	-8	2	188.29	7.16
-3	-8	2	190.43	7.52
3	8	-2	189.09	7.51
3	8	-2	178.13	7.12
-4	-8	2	67.67	3.06
-4	-8	2	68.87	2.61
-4	-8	2	67.38	3.23
-4	-8	2	70.27	2.87
-4	-8	2	65.89	2.64
-4	-8	2	67.57	3.22
4	8	-2	71.39	3.24
4	8	-2	64.47	2.81
-5	-8	2	19.49	0.89
-5	-8	2	22.17	1.38
-5	-8	2	19.17	1.12
-5	-8	2	19.02	1.51
-5	-8	2	18.86	0.83
-5	-8	2	19.41	1.52
5	8	-2	19.07	1.49
5	8	-2	17.92	1.04
-6	-8	2	33.23	1.39
-6	-8	2	32.70	1.73
-6	-8	2	32.86	1.35
-6	-8	2	30.47	2.02
-6	-8	2	32.88	1.87
-6	-8	2	33.17	2.02
-6	-8	2	32.28	1.63
6	8	-2	33.94	1.56
-7	-8	2	2.74	0.66

-7	-8	2	2.09	0.80
-7	-8	2	2.23	0.30
-7	-8	2	2.52	0.32
-7	-8	2	3.01	0.49
-7	-8	2	2.58	0.58
-7	-8	2	1.17	0.80
7	8	-2	2.40	0.42
-8	-8	2	39.48	1.72
-8	-8	2	41.83	1.66
-8	-8	2	39.97	1.95
-8	-8	2	36.17	2.38
-8	-8	2	41.45	2.04
-8	-8	2	39.37	1.89
-8	-8	2	40.45	2.49
-8	-8	2	41.14	2.21
8	8	-2	40.73	1.68
8	8	-2	38.12	1.85
-9	-8	2	121.57	4.92
-9	-8	2	128.66	5.03
-9	-8	2	121.60	5.51
-9	-8	2	131.57	5.25
-9	-8	2	116.44	4.94
-9	-8	2	106.47	5.30
-9	-8	2	123.17	4.68
9	8	-2	122.39	4.71
-10	-8	2	81.81	3.56
-10	-8	2	86.60	3.31
-10	-8	2	82.75	4.21
-10	-8	2	81.77	3.60
-10	-8	2	85.82	3.64
-10	-8	2	88.30	3.89
-10	-8	2	78.33	4.03
10	8	-2	82.38	3.33
-11	-8	2	89.27	4.53
-11	-8	2	86.80	4.33
-11	-8	2	93.76	4.10
-11	-8	2	97.84	4.82
-11	-8	2	100.86	3.80
-11	-8	2	102.01	4.14
-11	-8	2	100.00	4.09
11	8	-2	97.09	3.84
-12	-8	2	45.11	1.91
-12	-8	2	39.06	2.55
-12	-8	2	43.94	2.27

-12	-8	2	42.55	2.25
-12	-8	2	40.94	2.75
-12	-8	2	46.63	3.15
12	8	-2	45.80	1.95
-13	-8	2	1.67	1.03
-13	-8	2	1.60	0.75
-13	-8	2	-0.25	0.37
-13	-8	2	0.21	1.05
-13	-8	2	1.15	0.59
-13	-8	2	-1.07	1.20
13	8	-2	0.53	0.37
-14	-8	2	14.54	1.52
-14	-8	2	9.96	1.44
-14	-8	2	12.49	1.98
-14	-8	2	14.34	2.10
-14	-8	2	12.84	1.41
14	8	-2	12.25	0.97
-15	-8	2	27.77	2.00
-15	-8	2	24.64	3.15
-15	-8	2	27.70	2.54
-15	-8	2	28.24	2.00
15	8	-2	28.06	1.52
-16	-8	2	38.27	3.63
-16	-8	2	41.76	3.01
-16	-8	2	40.61	2.59
16	8	-2	43.32	2.06
-17	-8	2	13.34	1.98
-17	-8	2	13.73	1.84
-17	-8	2	17.33	2.51
17	8	-2	13.64	1.27
-18	-8	2	33.73	3.90
-18	-8	2	35.98	2.61
18	8	-2	37.36	2.07
-19	-8	2	6.29	1.41
-19	-8	2	8.51	2.86
19	8	-2	9.76	1.41
-20	-8	2	14.84	2.37
20	8	-2	18.51	1.70
19	-8	3	2.18	0.75
-19	8	-3	1.18	1.65
18	-8	3	22.03	1.56
-18	8	-3	19.24	2.52
17	-8	3	10.96	2.01
17	-8	3	3.81	0.73

-17	8	-3	4.62	1.33
16	-8	3	13.92	1.25
16	-8	3	15.92	1.99
-16	8	-3	14.49	2.06
15	-8	3	17.76	1.27
15	-8	3	16.51	1.98
-15	8	-3	19.35	2.02
14	-8	3	28.86	1.57
14	-8	3	28.65	2.17
-14	8	-3	27.86	2.16
-14	8	-3	29.66	2.92
13	-8	3	8.62	1.19
13	-8	3	8.48	1.49
13	-8	3	8.22	0.85
-13	8	-3	9.53	1.70
-13	8	-3	9.06	1.41
12	-8	3	39.60	1.85
12	-8	3	39.63	2.40
12	-8	3	39.30	1.98
-12	8	-3	38.96	2.81
-12	8	-3	40.26	2.31
11	-8	3	7.43	0.71
11	-8	3	6.46	1.03
11	-8	3	6.82	0.99
-11	8	-3	6.10	0.82
-11	8	-3	8.01	1.39
-11	8	-3	7.63	1.14
10	-8	3	11.88	1.07
10	-8	3	11.29	0.82
10	-8	3	11.02	1.36
10	-8	3	11.94	1.20
-10	8	-3	13.64	1.28
-10	8	-3	12.00	1.09
-10	8	-3	13.32	0.90
-10	8	-3	12.23	1.80
9	-8	3	7.06	0.95
9	-8	3	9.48	1.15
9	-8	3	7.78	0.66
9	-8	3	9.12	0.91
-9	8	-3	8.48	0.70
-9	8	-3	7.57	1.21
-9	8	-3	7.88	1.00
-9	8	-3	8.45	0.90
8	-8	3	45.37	1.99

8	-8	3	44.16	2.45
8	-8	3	45.94	2.22
8	-8	3	47.79	2.18
-8	8	-3	52.02	2.03
-8	8	-3	46.79	2.18
-8	8	-3	44.94	2.69
-8	8	-3	48.12	2.30
7	-8	3	27.26	1.26
7	-8	3	28.51	1.51
7	-8	3	27.67	1.76
-7	8	-3	23.69	1.96
-7	8	-3	27.97	1.50
-7	8	-3	27.34	1.55
-7	8	-3	29.08	1.29
6	-8	3	27.13	1.24
6	-8	3	28.84	1.73
6	-8	3	26.66	1.57
6	-8	3	27.03	1.47
-6	8	-3	28.33	1.52
-6	8	-3	30.69	1.27
-6	8	-3	26.70	1.92
-6	8	-3	26.41	1.52
5	-8	3	2.80	0.63
5	-8	3	3.77	0.63
5	-8	3	2.35	0.47
5	-8	3	2.24	0.34
-5	8	-3	2.62	0.50
-5	8	-3	2.98	0.53
-5	8	-3	2.81	0.36
-5	8	-3	3.11	0.83
4	-8	3	19.91	1.42
4	-8	3	22.00	1.20
4	-8	3	20.74	0.95
4	-8	3	21.09	1.19
-4	8	-3	22.22	1.61
-4	8	-3	20.76	0.94
-4	8	-3	19.36	1.17
-4	8	-3	21.82	1.30
3	-8	3	0.11	0.50
3	-8	3	0.29	0.32
3	-8	3	0.45	0.37
3	-8	3	-0.19	0.13
-3	8	-3	0.16	0.36
-3	8	-3	0.14	0.14

-3	8	-3	-0.58	0.26
-3	8	-3	0.22	0.54
2	-8	3	54.36	2.44
2	-8	3	52.89	2.13
2	-8	3	55.83	2.39
2	-8	3	56.75	2.66
-2	8	-3	51.28	2.37
-2	8	-3	57.76	2.78
-2	8	-3	55.50	2.50
-2	8	-3	54.85	2.14
1	-8	3	9.97	0.78
1	-8	3	10.54	1.02
1	-8	3	8.46	0.99
1	-8	3	8.93	0.84
1	-8	3	9.07	0.46
-1	8	-3	9.31	0.86
-1	8	-3	10.24	1.13
-1	8	-3	10.36	0.49
-1	8	-3	8.88	0.72
0	-8	3	158.46	6.59
0	-8	3	176.21	6.35
0	-8	3	159.27	6.60
0	-8	3	159.90	6.42
0	-8	3	153.29	6.05
0	8	-3	168.31	6.69
0	8	-3	159.61	6.07
0	8	-3	161.97	6.31
-1	-8	3	13.62	1.02
-1	-8	3	12.43	1.17
-1	-8	3	12.76	0.55
-1	-8	3	12.27	0.87
-1	-8	3	13.70	1.18
1	8	-3	12.83	0.57
1	8	-3	14.63	1.25
1	8	-3	11.53	0.81
-2	-8	3	50.60	2.15
-2	-8	3	52.30	2.33
-2	-8	3	47.63	2.44
-2	-8	3	46.69	1.85
-2	-8	3	46.52	2.47
2	8	-3	46.66	1.86
2	8	-3	47.54	2.47
2	8	-3	48.28	2.11
-3	-8	3	12.58	0.91

-3	-8	3	14.49	1.23
-3	-8	3	12.92	1.25
-3	-8	3	13.93	0.60
-3	-8	3	14.23	1.10
-3	-8	3	13.23	0.59
3	8	-3	14.75	1.30
3	8	-3	14.26	0.87
-4	-8	3	13.35	1.12
-4	-8	3	13.55	0.58
-4	-8	3	14.55	0.92
-4	-8	3	11.76	1.26
-4	-8	3	12.84	0.60
-4	-8	3	11.32	1.25
4	8	-3	12.62	0.84
4	8	-3	14.74	1.29
-5	-8	3	0.67	0.35
-5	-8	3	1.32	0.62
-5	-8	3	0.67	0.15
-5	-8	3	0.91	0.59
-5	-8	3	0.76	0.17
-5	-8	3	0.50	0.51
5	8	-3	0.68	0.59
5	8	-3	0.39	0.30
-6	-8	3	44.08	2.44
-6	-8	3	47.42	2.35
-6	-8	3	41.33	2.53
-6	-8	3	45.86	1.77
-6	-8	3	44.49	2.07
-6	-8	3	45.35	1.81
6	8	-3	45.50	2.41
6	8	-3	43.06	2.00
-7	-8	3	23.63	1.87
-7	-8	3	23.82	1.34
-7	-8	3	20.94	1.72
-7	-8	3	23.56	1.41
-7	-8	3	23.50	1.01
-7	-8	3	23.05	1.06
-7	-8	3	22.72	1.62
7	8	-3	24.51	1.27
-8	-8	3	34.09	1.68
-8	-8	3	33.43	1.46
-8	-8	3	33.34	1.78
-8	-8	3	31.74	1.71
-8	-8	3	33.31	2.33

-8	-8	3	31.27	2.00
-8	-8	3	34.37	1.39
-8	-8	3	30.12	2.10
8	8	-3	33.74	1.42
8	8	-3	34.69	1.64
-9	-8	3	20.43	2.00
-9	-8	3	18.66	1.19
-9	-8	3	18.48	0.89
-9	-8	3	17.56	1.73
-9	-8	3	18.39	1.26
-9	-8	3	17.42	1.55
-9	-8	3	17.43	1.31
9	8	-3	18.45	0.91
-10	-8	3	28.64	1.77
-10	-8	3	35.82	1.42
-10	-8	3	30.12	2.53
-10	-8	3	33.70	2.12
-10	-8	3	31.08	1.77
-10	-8	3	29.27	2.19
-10	-8	3	31.52	1.70
10	8	-3	31.66	1.43
-11	-8	3	38.50	1.68
-11	-8	3	38.37	2.90
-11	-8	3	38.42	2.00
-11	-8	3	33.13	2.44
-11	-8	3	40.07	2.41
-11	-8	3	37.94	2.05
11	8	-3	42.01	1.73
-12	-8	3	24.09	1.60
-12	-8	3	24.32	2.11
-12	-8	3	25.15	2.12
-12	-8	3	25.04	1.26
-12	-8	3	25.51	1.64
-12	-8	3	25.35	2.63
12	8	-3	26.84	1.29
-13	-8	3	1.10	0.63
-13	-8	3	2.67	1.47
-13	-8	3	1.77	1.09
-13	-8	3	1.54	0.73
-13	-8	3	2.68	0.45
-13	-8	3	1.81	1.01
13	8	-3	1.13	0.39
-14	-8	3	32.19	2.55
-14	-8	3	34.85	1.68

-14	-8	3	32.86	2.04
-14	-8	3	35.17	3.26
-14	-8	3	35.71	2.12
14	8	-3	37.21	1.72
-15	-8	3	1.68	1.65
-15	-8	3	1.83	1.25
-15	-8	3	2.56	0.94
-15	-8	3	1.87	0.87
15	8	-3	3.43	0.57
-16	-8	3	15.95	2.23
-16	-8	3	14.69	1.77
-16	-8	3	16.12	2.47
-17	-8	3	6.96	2.20
-17	-8	3	5.69	1.20
17	8	-3	6.69	1.07
-18	-8	3	24.68	3.82
-18	-8	3	26.66	2.31
18	8	-3	30.19	1.77
-19	-8	3	7.75	1.49
-19	-8	3	14.82	2.73
19	8	-3	11.57	1.39
19	-8	4	4.06	1.74
19	-8	4	2.54	0.87
-19	8	-4	2.43	1.64
18	-8	4	15.51	1.39
18	-8	4	13.18	2.46
-18	8	-4	13.63	1.87
17	-8	4	4.13	1.39
17	-8	4	5.39	0.86
-17	8	-4	4.33	1.33
16	-8	4	33.84	2.67
16	-8	4	34.75	1.90
-16	8	-4	35.25	2.68
15	-8	4	25.87	2.25
15	-8	4	23.61	1.49
-15	8	-4	24.01	2.25
14	-8	4	21.85	2.07
14	-8	4	21.52	1.35
-14	8	-4	21.44	1.97
13	-8	4	2.97	0.55
13	-8	4	3.18	0.97
-13	8	-4	2.66	0.90
12	-8	4	33.21	1.66
12	-8	4	35.51	1.71

12	-8	4	36.45	2.24
-12	8	-4	30.00	2.35
-12	8	-4	30.65	2.71
11	-8	4	50.13	3.06
11	-8	4	53.73	2.34
11	-8	4	51.11	2.82
11	-8	4	55.56	2.33
-11	8	-4	48.52	3.20
-11	8	-4	56.03	2.74
10	-8	4	26.13	1.36
10	-8	4	27.37	1.68
10	-8	4	26.92	1.46
10	-8	4	25.90	1.88
-10	8	-4	25.93	2.30
-10	8	-4	27.12	1.76
-10	8	-4	30.36	1.41
9	-8	4	35.67	1.93
9	-8	4	35.31	2.16
9	-8	4	37.44	1.73
9	-8	4	33.32	1.61
9	-8	4	34.68	1.89
-9	8	-4	37.63	1.65
-9	8	-4	31.28	2.39
-9	8	-4	35.27	1.96
8	-8	4	20.71	1.32
8	-8	4	21.48	1.22
8	-8	4	20.14	1.58
8	-8	4	19.50	1.05
8	-8	4	21.57	1.44
-8	8	-4	22.03	1.08
-8	8	-4	19.77	1.37
-8	8	-4	18.32	1.83
7	-8	4	20.32	1.30
7	-8	4	19.88	1.53
7	-8	4	20.21	1.21
7	-8	4	20.96	1.40
7	-8	4	20.14	1.03
-7	8	-4	22.53	1.05
-7	8	-4	20.20	1.79
-7	8	-4	21.10	1.35
6	-8	4	34.19	1.79
6	-8	4	32.29	1.65
6	-8	4	31.25	1.88
6	-8	4	29.78	1.38

-6	8	-4	29.74	2.09
-6	8	-4	29.62	1.65
-6	8	-4	33.74	1.40
5	-8	4	3.81	0.39
5	-8	4	2.56	0.67
5	-8	4	3.86	0.66
5	-8	4	3.00	0.52
5	-8	4	3.80	0.71
-5	8	-4	2.67	0.36
-5	8	-4	2.33	0.50
-5	8	-4	3.51	0.88
4	-8	4	7.20	1.00
4	-8	4	6.55	0.47
4	-8	4	7.51	0.77
4	-8	4	7.22	0.78
4	-8	4	7.35	1.02
-4	8	-4	7.47	0.47
-4	8	-4	7.48	1.07
-4	8	-4	7.29	0.75
3	-8	4	206.86	8.07
3	-8	4	216.72	8.31
3	-8	4	213.79	8.56
3	-8	4	221.80	8.55
3	-8	4	221.08	8.35
-3	8	-4	218.92	8.69
-3	8	-4	206.16	8.32
-3	8	-4	216.42	8.07
2	-8	4	25.05	1.36
2	-8	4	24.20	1.55
2	-8	4	24.26	1.30
2	-8	4	22.55	1.52
2	-8	4	22.37	1.01
-2	8	-4	24.18	1.00
-2	8	-4	24.11	1.28
-2	8	-4	25.06	1.69
1	-8	4	83.94	3.30
1	-8	4	79.07	3.56
1	-8	4	80.29	3.38
1	-8	4	76.44	3.56
1	-8	4	76.80	3.00
-1	8	-4	79.84	3.65
-1	8	-4	79.73	3.01
-1	8	-4	76.27	3.27
0	-8	4	88.68	3.44

0	-8	4	93.55	4.01
0	-8	4	86.60	4.02
0	-8	4	95.65	3.87
0	-8	4	93.84	3.75
0	8	-4	87.29	3.71
0	8	-4	93.40	4.09
0	8	-4	91.84	3.45
-1	-8	4	8.66	0.89
-1	-8	4	9.31	0.76
-1	-8	4	8.61	1.02
-1	-8	4	8.46	0.38
-1	-8	4	8.16	1.12
1	8	-4	8.76	0.71
1	8	-4	7.48	1.05
1	8	-4	8.74	0.40
-2	-8	4	20.84	0.83
-2	-8	4	22.07	1.60
-2	-8	4	22.45	1.39
-2	-8	4	22.04	1.19
-2	-8	4	20.71	1.48
2	8	-4	20.82	1.51
2	8	-4	21.22	1.15
2	8	-4	20.99	0.85
-3	-8	4	58.64	2.53
-3	-8	4	57.70	2.95
-3	-8	4	56.88	2.19
-3	-8	4	59.68	2.82
-3	-8	4	56.84	2.72
-3	-8	4	57.97	2.19
3	8	-4	57.75	2.49
3	8	-4	59.58	2.84
-4	-8	4	37.21	1.96
-4	-8	4	37.12	1.72
-4	-8	4	33.37	1.35
-4	-8	4	35.21	1.36
-4	-8	4	31.55	2.11
-4	-8	4	35.80	2.00
4	8	-4	37.72	2.04
4	8	-4	32.86	1.63
-5	-8	4	15.65	0.70
-5	-8	4	16.31	1.37
-5	-8	4	18.01	1.32
-5	-8	4	15.52	1.03
-5	-8	4	14.64	1.51

-5	-8	4	16.51	0.68
5	8	-4	17.09	1.40
5	8	-4	16.00	0.98
-6	-8	4	25.26	1.41
-6	-8	4	26.12	1.10
-6	-8	4	25.78	1.06
-6	-8	4	25.44	1.72
-6	-8	4	25.51	1.95
-6	-8	4	26.83	1.75
6	8	-4	25.86	1.36
6	8	-4	28.11	1.78
-7	-8	4	19.49	1.21
-7	-8	4	18.82	0.91
-7	-8	4	15.90	1.77
-7	-8	4	18.14	1.57
-7	-8	4	20.15	1.54
-7	-8	4	19.59	0.85
[truncated: 328,627 more chars]
