# Supplementary material for: Structure-property relationships of photofunctional diiridium(II) complexes with tetracationic charge and an unsupported Ir–Ir bond
Source: Commun Chem. 2022 Nov 23;5:159. doi: 10.1038/s42004-022-00775-4 (PMC9814866; doi:10.1038/s42004-022-00775-4)
Supplement: Supplementary file 7 — Supplementary Data 4 [file 42004_2022_775_MOESM7_ESM.pdf]

```
data_[Ir(II)(terpyr)(PPh3)(CO)]2[OTf]4

_audit_creation_method            'SHELXL-2014/7'
_shelx_SHELXL_version_number      '2014/7'
_chemical_name_systematic         ?
_chemical_name_common             ?
_chemical_melting_point           ?
_chemical_formula_moiety            'C34 H16 Ir N3 O P, C F3 O3 S, C
F1.59 O3 S, 0.5(C2 N), 0.5(C2 N)'
_chemical_formula_sum
 'C38 H16 F6 Ir N4 O7 P S2'
_chemical_formula_weight          1041.84

loop_
 _atom_type_symbol
 _atom_type_description
 _atom_type_scat_dispersion_real
 _atom_type_scat_dispersion_imag
 _atom_type_scat_source
 'C'  'C'   0.0033   0.0016
 'International Tables Vol C Tables 4.2.6.8 and 6.1.1.4'
 'H'  'H'   0.0000   0.0000
 'International Tables Vol C Tables 4.2.6.8 and 6.1.1.4'
 'N'  'N'   0.0061   0.0033
 'International Tables Vol C Tables 4.2.6.8 and 6.1.1.4'
 'O'  'O'   0.0106   0.0060
 'International Tables Vol C Tables 4.2.6.8 and 6.1.1.4'
 'F'  'F'   0.0171   0.0103
 'International Tables Vol C Tables 4.2.6.8 and 6.1.1.4'
 'P'  'P'   0.1023   0.0942
 'International Tables Vol C Tables 4.2.6.8 and 6.1.1.4'
 'S'  'S'   0.1246   0.1234
 'International Tables Vol C Tables 4.2.6.8 and 6.1.1.4'
 'Ir'  'Ir'  -1.4442   7.9887
 'International Tables Vol C Tables 4.2.6.8 and 6.1.1.4'

_space_group_crystal_system       triclinic
_space_group_IT_number            2
_space_group_name_H-M_alt         'P -1'
_space_group_name_Hall            '-P 1'

_shelx_space_group_comment
;
```

```
The symmetry employed for this shelxl refinement is uniquely defined
by the following loop, which should always be used as a source of
symmetry information in preference to the above space-group names.
They are only intended as comments.
;

loop_
 _space_group_symop_operation_xyz
 'x, y, z'
 '-x, -y, -z'

_cell_length_a                    12.732(3)
_cell_length_b                    13.152(3)
_cell_length_c                    14.553(4)
_cell_angle_alpha                 72.951(7)
_cell_angle_beta                  81.397(8)
_cell_angle_gamma                 66.186(8)
_cell_volume                      2130.1(9)
_cell_formula_units_Z             2
_cell_measurement_temperature     141(2)
_cell_measurement_reflns_used     9951
_cell_measurement_theta_min       2.321
_cell_measurement_theta_max       27.11

_exptl_crystal_description        slice
_exptl_crystal_colour             orange
_exptl_crystal_density_meas       ?
_exptl_crystal_density_method     ?
_exptl_crystal_density_diffrn     1.624
_exptl_crystal_F_000              1012
_exptl_transmission_factor_min    ?
_exptl_transmission_factor_max    ?
_exptl_crystal_size_max           0.210
_exptl_crystal_size_mid           0.190
_exptl_crystal_size_min           0.170
_exptl_absorpt_coefficient_mu     3.346
_shelx_estimated_absorpt_T_min    0.540
_shelx_estimated_absorpt_T_max    0.600
_exptl_absorpt_correction_type    multi-scan
_exptl_absorpt_correction_T_min   0.4376
_exptl_absorpt_correction_T_max   0.7455
_exptl_absorpt_process_details
'SADABS-2014/5 (Bruker,2014/5) was used for absorption correction.
wR2(int) was 0.1271 before and 0.0718 after correction. The Ratio of
```

```
minimum to maximum transmission is 0.5870. The \l/2 correction factor
is Not present.'
_exptl_absorpt_special_details    ?
_diffrn_ambient_temperature       141(2)
_diffrn_radiation_wavelength      0.710
_diffrn_radiation_type            MoK\a
_diffrn_source                    ?
_diffrn_measurement_device_type   'Bruker APEX-II CCD'
_diffrn_measurement_method        '\f and \w scans'
_diffrn_detector_area_resol_mean  ?
_diffrn_reflns_number             41064
_diffrn_reflns_av_unetI/netI      0.0567
_diffrn_reflns_av_R_equivalents   0.0689
_diffrn_reflns_limit_h_min        -16
_diffrn_reflns_limit_h_max        15
_diffrn_reflns_limit_k_min        -16
_diffrn_reflns_limit_k_max        16
_diffrn_reflns_limit_l_min        -18
_diffrn_reflns_limit_l_max        18
_diffrn_reflns_theta_min          2.239
_diffrn_reflns_theta_max          26.702
_diffrn_reflns_theta_full         25.214
_diffrn_measured_fraction_theta_max   0.986
_diffrn_measured_fraction_theta_full  0.989
_diffrn_reflns_Laue_measured_fraction_max    0.986
_diffrn_reflns_Laue_measured_fraction_full   0.989
_diffrn_reflns_point_group_measured_fraction_max   0.986
_diffrn_reflns_point_group_measured_fraction_full  0.989
_reflns_number_total              8923
_reflns_number_gt                 7442
_reflns_threshold_expression      'I > 2\s(I)'
_reflns_Friedel_coverage          0.000
_reflns_Friedel_fraction_max      .
_reflns_Friedel_fraction_full     .

_reflns_special_details
;
 Reflections were merged by SHELXL according to the crystal
 class for the calculation of statistics and refinement.

 _reflns_Friedel_fraction is defined as the number of unique
 Friedel pairs measured divided by the number that would be
 possible theoretically, ignoring centric projections and
 systematic absences.
```

```
;

_computing_data_collection        'Bruker APEX2'
_computing_cell_refinement        'Bruker SAINT'
_computing_data_reduction         'Bruker SAINT'
_computing_structure_solution     'SHELXS-97 (Sheldrick 2008)'
_computing_structure_refinement 'SHELXL-2014/7 (Sheldrick, 2014)'
_computing_molecular_graphics     'Bruker SHELXTL'
_computing_publication_material   'Bruker SHELXTL'
_refine_special_details           ?
_refine_ls_structure_factor_coef  Fsqd
_refine_ls_matrix_type            full
_refine_ls_weighting_scheme       calc
_refine_ls_weighting_details
'w=1/[\s^2^(Fo^2^)+(0.0562P)^2^+23.9954P] where P=(Fo^2^+2Fc^2^)/3'
_atom_sites_solution_primary      ?
_atom_sites_solution_secondary    ?
_atom_sites_solution_hydrogens    geom
_refine_ls_hydrogen_treatment     constr
_refine_ls_extinction_method      none
_refine_ls_extinction_coef        .
_refine_ls_number_reflns          8923
_refine_ls_number_parameters      767
_refine_ls_number_restraints      258
_refine_ls_R_factor_all           0.0680
_refine_ls_R_factor_gt            0.0496
_refine_ls_wR_factor_ref          0.1354
_refine_ls_wR_factor_gt           0.1197
_refine_ls_goodness_of_fit_ref    1.042
_refine_ls_restrained_S_all       1.047
_refine_ls_shift/su_max           0.002
_refine_ls_shift/su_mean          0.000

loop_
 _atom_site_label
 _atom_site_type_symbol
 _atom_site_fract_x
 _atom_site_fract_y
 _atom_site_fract_z
 _atom_site_U_iso_or_equiv
 _atom_site_adp_type
 _atom_site_occupancy
 _atom_site_site_symmetry_order
 _atom_site_calc_flag
```

```
 _atom_site_refinement_flags_posn
 _atom_site_refinement_flags_adp
 _atom_site_refinement_flags_occupancy
 _atom_site_disorder_assembly
 _atom_site_disorder_group
Ir1 Ir 0.50186(2) 1.00522(2) 0.39743(2) 0.01804(10) Uani 1 1 d . . . . .
N2 N 0.6090(5) 0.8408(5) 0.4181(4) 0.0161(11) Uani 1 1 d . . . . .
N1 N 0.3859(5) 0.9264(5) 0.4269(4) 0.0179(11) Uani 1 1 d . . . . .
C1 C 0.3987(6) 1.1608(6) 0.3832(4) 0.0175(13) Uani 1 1 d . . . . .
O1 O 0.3383(5) 1.2529(4) 0.3722(4) 0.0250(11) Uani 1 1 d . . . . .
N3 N 0.6600(6) 1.0201(5) 0.3737(4) 0.0248(13) Uani 1 1 d . . . . .
P1 P 0.49378(17) 1.02302(14) 0.22571(14) 0.0224(4) Uani 1 1 d . . . . .
C7 C 0.5633(6) 0.7593(6) 0.4412(5) 0.0212(14) Uani 1 1 d . . . . .
C3 C 0.2032(7) 0.9138(7) 0.4396(5) 0.0307(17) Uani 1 1 d . . . . .
H3 H 0.1220 0.9517 0.4380 0.037 Uiso 1 1 calc R U . . .
C6 C 0.4374(6) 0.8086(6) 0.4439(5) 0.0216(14) Uani 1 1 d . . . . .
C8 C 0.6355(7) 0.6442(6) 0.4570(5) 0.0258(16) Uani 1 1 d . . . . .
H8 H 0.6049 0.5859 0.4730 0.031 Uiso 1 1 calc R U . . .
C11 C 0.7226(7) 0.8143(6) 0.4076(5) 0.0244(15) Uani 1 1 d . . . . .
C2 C 0.2708(6) 0.9775(6) 0.4258(5) 0.0249(15) Uani 1 1 d . . . . .
H2 H 0.2352 1.0587 0.4153 0.030 Uiso 1 1 calc R U . . .
C17 C 0.4767(8) 1.1604(6) 0.1429(5) 0.0316(18) Uani 1 1 d . . . . .
C18 C 0.4887(8) 1.2479(7) 0.1684(6) 0.0361(18) Uani 1 1 d . U . . .
H18 H 0.5035 1.2386 0.2330 0.043 Uiso 1 1 calc R U . . .
C10 C 0.7977(7) 0.7007(7) 0.4232(6) 0.0304(17) Uani 1 1 d . . . . .
H10 H 0.8784 0.6812 0.4164 0.037 Uiso 1 1 calc R U . . .
C5 C 0.3705(7) 0.7438(6) 0.4586(5) 0.0285(16) Uani 1 1 d . . . . .
H5 H 0.4062 0.6625 0.4709 0.034 Uiso 1 1 calc R U . . .
C16 C 0.6812(7) 1.1178(7) 0.3505(5) 0.0291(17) Uani 1 1 d . . . . .
H16 H 0.6191 1.1893 0.3492 0.035 Uiso 1 1 calc R U . . .
C4 C 0.2524(7) 0.7967(7) 0.4556(6) 0.0323(18) Uani 1 1 d . . . . .
H4 H 0.2063 0.7528 0.4645 0.039 Uiso 1 1 calc R U . . .
C12 C 0.7507(7) 0.9173(7) 0.3793(5) 0.0273(16) Uani 1 1 d . . . . .
C13 C 0.8633(7) 0.9123(8) 0.3587(6) 0.0363(19) Uani 1 1 d . . . . .
H13 H 0.9255 0.8404 0.3631 0.044 Uiso 1 1 calc R U . . .
C9 C 0.7529(8) 0.6161(7) 0.4490(6) 0.0340(18) Uani 1 1 d . . . . .
H9 H 0.8034 0.5375 0.4613 0.041 Uiso 1 1 calc R U . . .
C23 C 0.3695(9) 1.0009(9) 0.2030(5) 0.041(2) Uani 1 1 d . . . . .
C15 C 0.7914(8) 1.1153(8) 0.3290(6) 0.040(2) Uani 1 1 d . . . . .
H15 H 0.8042 1.1850 0.3118 0.048 Uiso 1 1 calc R U . . .
C14 C 0.8829(8) 1.0121(9) 0.3322(7) 0.044(2) Uani 1 1 d . . . . .
H14 H 0.9587 1.0104 0.3161 0.053 Uiso 1 1 calc R U . . .
C22 C 0.4533(10) 1.1747(8) 0.0482(7) 0.048(2) Uani 1 1 d . U . . .
H22 H 0.4423 1.1155 0.0310 0.057 Uiso 1 1 calc R U . . .
```

C19 C 0.4791(11) 1.3499(9) 0.0996(7) 0.057(3) Uani 1 1 d . U . . .
H19 H 0.4868 1.4106 0.1172 0.068 Uiso 1 1 calc R U . . .
C24 C 0.2635(11) 1.0921(11) 0.2048(9) 0.064(3) Uani 1 1 d . U . . .
C20 C 0.4584(12) 1.3624(10) 0.0058(8) 0.065(3) Uani 1 1 d . U . . .
H20 H 0.4524 1.4319 -0.0412 0.077 Uiso 1 1 calc R U . . .
C21 C 0.4462(11) 1.2755(9) -0.0207(8) 0.060(3) Uani 1 1 d . U . . .
H21 H 0.4330 1.2846 -0.0858 0.072 Uiso 1 1 calc R U . . .
S2 S 1.00509(18) 0.27580(19) 0.40675(18) 0.0388(5) Uani 1 1 d . . . . .
S1 S 0.5052(2) 0.4897(2) 0.29996(15) 0.0457(6) Uani 1 1 d . . . . .
O5 O 0.9437(7) 0.3314(6) 0.4808(6) 0.063(2) Uani 1 1 d . . . . .
O4 O 0.4394(14) 0.5631(11) 0.2145(11) 0.060(4) Uani 0.531(6) 1 d . . P
A 1
C35 C 0.6441(18) 0.4712(17) 0.2780(14) 0.051(5) Uani 0.531(6) 1 d . U P
A 1
O3 O 0.4681(15) 0.5342(14) 0.3836(11) 0.054(4) Uani 0.531(6) 1 d . U P
A 1
O3' O 0.533(2) 0.505(2) 0.3836(16) 0.082(7) Uani 0.469(6) 1 d . U P A 2
C35' C 0.3632(19) 0.5682(17) 0.2711(14) 0.041(4) Uani 0.469(6) 1 d . U
P A 2
O4' O 0.575(2) 0.506(2) 0.2134(18) 0.112(8) Uani 0.469(6) 1 d . U P A 2
F1 F 0.6533(13) 0.5732(11) 0.2476(9) 0.072(4) Uani 0.531(6) 1 d . U P A
1
F3 F 0.7068(12) 0.4089(12) 0.3530(9) 0.071(4) Uani 0.531(6) 1 d . U P A
1
F2 F 0.6889(13) 0.4168(12) 0.2048(10) 0.081(4) Uani 0.531(6) 1 d . . P
A 1
F3* F 0.355(2) 0.597(2) 0.1734(18) 0.049(6) Uani 0.235(3) 1 d . U P A 2
F3' F 0.300(2) 0.565(2) 0.3484(16) 0.048(6) Uani 0.235(3) 1 d . U P A 2
F2' F 0.353(2) 0.683(2) 0.2437(18) 0.051(6) Uani 0.235(3) 1 d . U P A 2
F1* F 0.300(3) 0.650(3) 0.301(2) 0.075(8) Uani 0.235(3) 1 d . U P A 2
F2* F 0.325(3) 0.479(3) 0.295(3) 0.100(11) Uani 0.235(3) 1 d . U P A 2
F1' F 0.334(2) 0.544(2) 0.2043(18) 0.048(6) Uani 0.235(3) 1 d . U P A 2
F6 F 0.9249(8) 0.4692(7) 0.2787(6) 0.093(2) Uani 1 1 d . U . . .
F4 F 0.9401(15) 0.3190(13) 0.2374(10) 0.090(5) Uani 0.531(6) 1 d . U P
A 1
C36 C 0.916(2) 0.3644(15) 0.3115(15) 0.054(5) Uani 0.531(6) 1 d . . P A
1
F5 F 0.8068(10) 0.3893(11) 0.3355(11) 0.077(4) Uani 0.531(6) 1 d . . P
A 1
O7 O 0.9835(10) 0.1646(10) 0.4261(11) 0.044(3) Uani 0.531(6) 1 d . . P
A 1
O6 O 1.1193(15) 0.2601(13) 0.3791(16) 0.059(5) Uani 0.531(6) 1 d . . P
A 1
O7' O 0.9634(15) 0.2262(18) 0.3578(16) 0.070(5) Uani 0.469(6) 1 d . . P

A 2
C36' C 1.018(2) 0.406(2) 0.313(2) 0.066(7) Uani 0.469(6) 1 d . U P A 2
O6' O 1.1259(16) 0.2194(15) 0.4254(13) 0.043(4) Uani 0.469(6) 1 d . . P
A 2
F4' F 1.0915(17) 0.3711(16) 0.2391(13) 0.099(6) Uani 0.469(6) 1 d . U P
A 2
F5' F 1.050(2) 0.471(2) 0.3480(17) 0.123(7) Uani 0.469(6) 1 d . U P A 2
C29 C 0.6257(9) 0.9252(9) 0.1842(6) 0.046(2) Uani 1 1 d . U . . .
C30 C 0.6680(14) 0.8133(12) 0.2039(10) 0.023(3) Uani 0.531(6) 1 d . U P
A 1
C31 C 0.771(3) 0.744(2) 0.1711(17) 0.046(7) Uani 0.531(6) 1 d . . P A 1
C34 C 0.7020(18) 0.9901(19) 0.1238(16) 0.043(5) Uani 0.531(6) 1 d . . P
A 1
C32 C 0.849(2) 0.795(3) 0.1162(19) 0.060(7) Uani 0.531(6) 1 d . . P A 1
C33 C 0.8120(19) 0.920(2) 0.0918(15) 0.056(5) Uani 0.531(6) 1 d . U P A
1
C31' C 0.717(3) 0.706(3) 0.194(2) 0.066(7) Uani 0.469(6) 1 d . U P A 2
C30' C 0.6165(19) 0.7953(17) 0.2208(14) 0.035(4) Uani 0.469(6) 1 d . U
P A 2
C34' C 0.707(3) 0.942(3) 0.143(2) 0.057(8) Uani 0.469(6) 1 d . U P A 2
C32' C 0.809(4) 0.739(5) 0.151(3) 0.077(15) Uani 0.469(6) 1 d . U P A 2
C33' C 0.814(3) 0.843(3) 0.121(2) 0.067(9) Uani 0.469(6) 1 d . U P A 2
O2 O 0.5064(8) 0.3713(6) 0.3241(6) 0.065(2) Uani 1 1 d . . . . .
C37 C 0.120(3) 0.626(3) 0.277(2) 0.083(8) Uani 0.5 1 d . U P . .
C39 C 0.839(3) 0.297(3) 0.032(2) 0.097(10) Uani 0.5 1 d . U P . .
N5 N 0.851(3) 0.200(3) 0.035(2) 0.120(10) Uani 0.5 1 d . U P . .
C38 C 0.1505(18) 0.4599(18) 0.2981(13) 0.046(4) Uani 0.5 1 d . U P . .
C40 C 0.858(4) 0.389(4) -0.014(3) 0.119(12) Uani 0.5 1 d . U P . .
N4 N 0.074(3) 0.723(4) 0.261(3) 0.145(13) Uani 0.5 1 d . U P . .
C25 C 0.1723(18) 1.059(2) 0.1838(15) 0.046(5) Uani 0.531(6) 1 d . U P A
1
C26 C 0.187(2) 0.947(2) 0.1801(17) 0.050(5) Uani 0.531(6) 1 d . . P A 1
C27 C 0.294(2) 0.859(2) 0.1907(19) 0.057(5) Uani 0.531(6) 1 d . . P A 1
C28 C 0.3910(17) 0.8776(17) 0.2013(14) 0.041(4) Uani 0.531(6) 1 d . U P
A 1
C28' C 0.3591(19) 0.930(2) 0.1643(15) 0.041(5) Uani 0.469(6) 1 d . U P
A 2
C27' C 0.254(3) 0.933(3) 0.140(2) 0.058(6) Uani 0.469(6) 1 d . . P A 2
C26' C 0.152(3) 1.023(3) 0.154(2) 0.062(7) Uani 0.469(6) 1 d . U P A 2
C25' C 0.151(2) 1.117(3) 0.1857(18) 0.057(6) Uani 0.469(6) 1 d . U P A
2

loop_
 _atom_site_aniso_label

_atom_site_aniso_U_11
_atom_site_aniso_U_22
_atom_site_aniso_U_33
_atom_site_aniso_U_23
_atom_site_aniso_U_13
_atom_site_aniso_U_12
Ir1 0.02346(16) 0.01130(13) 0.02085(14) -0.00603(9) -0.00043(10) -
0.00691(10)
N2 0.020(3) 0.015(3) 0.014(2) -0.007(2) -0.002(2) -0.006(2)
N1 0.017(3) 0.020(3) 0.017(3) -0.007(2) 0.000(2) -0.006(2)
C1 0.025(4) 0.019(3) 0.014(3) -0.007(2) 0.001(3) -0.013(3)
O1 0.029(3) 0.014(2) 0.030(3) -0.008(2) -0.004(2) -0.004(2)
N3 0.033(4) 0.031(3) 0.018(3) -0.009(2) -0.001(2) -0.017(3)
P1 0.0256(10) 0.0082(8) 0.0354(10) -0.0046(7) -0.0061(8) -0.0075(7)
C7 0.031(4) 0.015(3) 0.022(3) -0.007(3) -0.009(3) -0.008(3)
C3 0.025(4) 0.045(5) 0.030(4) -0.016(3) 0.002(3) -0.018(4)
C6 0.032(4) 0.018(3) 0.018(3) -0.005(3) -0.004(3) -0.010(3)
C8 0.035(4) 0.017(3) 0.028(4) -0.011(3) -0.004(3) -0.008(3)
C11 0.027(4) 0.026(4) 0.024(3) -0.011(3) -0.002(3) -0.011(3)
C2 0.024(4) 0.023(4) 0.029(4) -0.013(3) -0.001(3) -0.005(3)
C17 0.058(6) 0.021(4) 0.017(3) -0.007(3) 0.006(3) -0.018(4)
C18 0.053(4) 0.035(3) 0.028(3) -0.010(3) -0.002(3) -0.022(3)
C10 0.021(4) 0.035(4) 0.034(4) -0.019(3) 0.000(3) -0.003(3)
C5 0.037(5) 0.022(4) 0.032(4) -0.005(3) -0.004(3) -0.017(3)
C16 0.040(5) 0.031(4) 0.026(4) -0.001(3) -0.005(3) -0.026(4)
C4 0.036(5) 0.039(5) 0.037(4) -0.013(3) -0.001(4) -0.027(4)
C12 0.035(4) 0.029(4) 0.023(3) -0.010(3) 0.000(3) -0.015(3)
C13 0.028(4) 0.055(6) 0.032(4) -0.015(4) 0.003(3) -0.021(4)
C9 0.040(5) 0.021(4) 0.034(4) -0.013(3) -0.005(4) 0.001(3)
C23 0.054(6) 0.065(6) 0.020(4) -0.018(4) 0.007(4) -0.037(5)
C15 0.048(6) 0.053(6) 0.034(4) -0.008(4) 0.001(4) -0.040(5)
C14 0.037(5) 0.065(6) 0.042(5) -0.013(4) 0.001(4) -0.033(5)
C22 0.069(5) 0.039(4) 0.039(4) -0.015(3) -0.005(3) -0.020(3)
C19 0.084(5) 0.045(4) 0.048(4) -0.017(3) 0.001(4) -0.030(4)
C24 0.056(5) 0.070(5) 0.061(5) -0.006(4) -0.012(4) -0.024(4)
C20 0.090(5) 0.053(4) 0.051(4) -0.013(4) 0.002(4) -0.030(4)
C21 0.085(5) 0.049(4) 0.047(4) -0.014(3) -0.003(4) -0.026(4)
S2 0.0248(11) 0.0306(11) 0.0621(14) -0.0204(10) -0.0061(10) -0.0041(8)
S1 0.0727(18) 0.0329(11) 0.0280(10) -0.0123(8) -0.0101(11) -0.0105(11)
O5 0.063(5) 0.043(4) 0.084(6) -0.034(4) 0.018(4) -0.016(4)
O4 0.066(10) 0.037(7) 0.075(10) -0.006(7) -0.021(8) -0.019(7)
C35 0.052(6) 0.049(6) 0.048(6) -0.009(4) -0.002(4) -0.019(4)
O3 0.068(8) 0.053(7) 0.055(7) -0.035(6) 0.007(6) -0.026(6)
O3' 0.086(10) 0.093(10) 0.063(9) -0.044(7) -0.016(8) -0.011(8)

C35' 0.042(6) 0.040(6) 0.038(6) -0.007(4) 0.000(4) -0.014(4)
O4' 0.102(11) 0.130(12) 0.100(11) -0.025(8) -0.008(8) -0.043(9)
F1 0.099(8) 0.052(6) 0.068(6) 0.000(5) -0.009(6) -0.040(6)
F3 0.067(5) 0.072(5) 0.070(5) -0.004(4) -0.010(4) -0.029(4)
F2 0.085(10) 0.071(9) 0.076(9) -0.029(7) 0.016(8) -0.017(8)
F3* 0.049(7) 0.050(7) 0.048(7) -0.014(5) -0.005(5) -0.018(5)
F3' 0.047(7) 0.047(7) 0.043(7) -0.011(5) 0.000(5) -0.012(5)
F2' 0.053(7) 0.046(7) 0.050(7) -0.009(5) -0.002(5) -0.017(5)
F1* 0.075(10) 0.077(9) 0.074(9) -0.024(5) -0.004(5) -0.026(6)
F2* 0.101(12) 0.099(12) 0.103(12) -0.023(6) -0.004(5) -0.042(6)
F1' 0.049(7) 0.050(7) 0.047(7) -0.014(5) -0.009(5) -0.019(5)
F6 0.089(4) 0.072(4) 0.092(4) -0.002(3) 0.001(3) -0.020(3)
F4 0.109(9) 0.084(8) 0.071(7) -0.028(6) -0.019(6) -0.020(6)
C36 0.071(15) 0.023(8) 0.062(12) -0.016(8) -0.015(10) -0.006(9)
F5 0.032(7) 0.068(9) 0.117(12) -0.018(8) -0.029(7) -0.001(6)
O7 0.030(7) 0.021(6) 0.078(10) -0.005(6) 0.002(6) -0.012(5)
O6 0.029(8) 0.020(8) 0.122(18) -0.007(8) -0.014(10) -0.008(7)
O7' 0.051(11) 0.074(13) 0.110(16) -0.049(12) 0.003(11) -0.035(10)
C36' 0.065(8) 0.066(8) 0.066(8) -0.016(5) -0.001(5) -0.023(5)
O6' 0.028(8) 0.032(10) 0.059(11) -0.011(7) -0.015(8) 0.001(8)
F4' 0.099(9) 0.089(9) 0.089(9) -0.014(7) 0.017(7) -0.029(7)
F5' 0.126(9) 0.117(8) 0.128(9) -0.031(5) 0.002(5) -0.051(5)
C29 0.049(4) 0.048(4) 0.034(3) -0.021(3) -0.006(3) -0.004(3)
C30 0.027(5) 0.020(5) 0.019(4) -0.008(4) 0.002(4) -0.005(4)
C31 0.050(16) 0.046(12) 0.032(10) -0.023(9) -0.003(11) 0.001(12)
C34 0.041(11) 0.037(11) 0.054(12) -0.015(10) 0.015(9) -0.022(10)
C32 0.044(13) 0.061(16) 0.070(15) -0.049(14) 0.000(11) 0.008(12)
C33 0.057(7) 0.054(7) 0.055(7) -0.017(5) 0.004(5) -0.020(5)
C31' 0.068(9) 0.065(8) 0.064(9) -0.019(5) -0.004(5) -0.022(5)
C30' 0.036(6) 0.034(6) 0.034(6) -0.014(4) -0.005(4) -0.008(4)
C34' 0.057(9) 0.057(9) 0.055(9) -0.017(5) -0.001(5) -0.019(5)
C32' 0.077(16) 0.077(16) 0.075(16) -0.020(6) -0.002(5) -0.028(8)
C33' 0.067(10) 0.067(10) 0.064(10) -0.017(5) -0.002(5) -0.023(6)
O2 0.095(6) 0.036(4) 0.067(5) -0.015(3) -0.016(4) -0.023(4)
C37 0.083(9) 0.083(9) 0.084(9) -0.021(5) -0.002(5) -0.032(6)
C39 0.098(11) 0.098(11) 0.096(11) -0.024(5) -0.006(5) -0.038(6)
N5 0.116(13) 0.131(13) 0.136(14) -0.031(9) 0.005(9) -0.076(10)
C38 0.048(6) 0.052(6) 0.037(6) -0.017(4) -0.004(4) -0.013(4)
C40 0.125(15) 0.122(15) 0.112(15) -0.042(10) -0.007(9) -0.041(10)
N4 0.135(16) 0.144(15) 0.152(16) -0.043(10) 0.002(9) -0.050(10)
C25 0.047(6) 0.046(6) 0.046(6) -0.012(5) -0.004(4) -0.018(5)
C26 0.059(14) 0.049(13) 0.059(13) -0.037(11) -0.012(12) -0.017(11)
C27 0.058(14) 0.052(13) 0.082(16) -0.034(12) 0.004(12) -0.033(11)
C28 0.042(6) 0.040(6) 0.046(6) -0.012(4) -0.004(4) -0.019(4)

```
C28' 0.044(6) 0.044(6) 0.042(6) -0.016(4) -0.006(4) -0.018(5)
C27' 0.060(18) 0.063(18) 0.062(17) -0.018(14) -0.007(14) -0.030(15)
C26' 0.060(8) 0.064(8) 0.061(8) -0.016(5) -0.004(5) -0.024(5)
C25' 0.059(8) 0.057(8) 0.054(7) -0.018(5) -0.006(5) -0.019(5)

_geom_special_details
;
 All esds (except the esd in the dihedral angle between two l.s. planes)
 are estimated using the full covariance matrix.  The cell esds are taken
 into account individually in the estimation of esds in distances, angles
 and torsion angles; correlations between esds in cell parameters are only
 used when they are defined by crystal symmetry.  An approximate (isotropic)
 treatment of cell esds is used for estimating esds involving l.s. planes.
;

loop_
 _geom_bond_atom_site_label_1
 _geom_bond_atom_site_label_2
 _geom_bond_distance
 _geom_bond_site_symmetry_2
 _geom_bond_publ_flag
Ir1 C1 1.902(7) . ?
Ir1 N2 1.997(5) . ?
Ir1 N1 2.055(6) . ?
Ir1 N3 2.069(6) . ?
Ir1 P1 2.455(2) . ?
Ir1 Ir1 2.9421(9) 2_676 ?
N2 C11 1.339(10) . ?
N2 C7 1.354(9) . ?
N1 C2 1.344(9) . ?
N1 C6 1.376(9) . ?
C1 O1 1.121(8) . ?
N3 C16 1.355(9) . ?
N3 C12 1.368(10) . ?
P1 C29 1.802(10) . ?
P1 C17 1.802(7) . ?
P1 C23 1.812(9) . ?
C7 C8 1.388(10) . ?
C7 C6 1.465(11) . ?
```

C3 C4 1.369(12) . ?
C3 C2 1.383(11) . ?
C3 H3 0.9500 . ?
C6 C5 1.386(10) . ?
C8 C9 1.382(12) . ?
C8 H8 0.9500 . ?
C11 C10 1.383(11) . ?
C11 C12 1.467(10) . ?
C2 H2 0.9500 . ?
C17 C18 1.375(11) . ?
C17 C22 1.397(11) . ?
C18 C19 1.390(13) . ?
C18 H18 0.9500 . ?
C10 C9 1.382(12) . ?
C10 H10 0.9500 . ?
C5 C4 1.380(12) . ?
C5 H5 0.9500 . ?
C16 C15 1.380(12) . ?
C16 H16 0.9500 . ?
C4 H4 0.9500 . ?
C12 C13 1.398(11) . ?
C13 C14 1.369(13) . ?
C13 H13 0.9500 . ?
C9 H9 0.9500 . ?
C23 C28' 1.27(2) . ?
C23 C24 1.400(16) . ?
C23 C28 1.54(2) . ?
C15 C14 1.380(14) . ?
C15 H15 0.9500 . ?
C14 H14 0.9500 . ?
C22 C21 1.386(14) . ?
C22 H22 0.9500 . ?
C19 C20 1.379(15) . ?
C19 H19 0.9500 . ?
C24 C25' 1.38(3) . ?
C24 C25 1.49(2) . ?
C20 C21 1.378(15) . ?
C20 H20 0.9500 . ?
C21 H21 0.9500 . ?
S2 O7' 1.372(16) . ?
S2 O6 1.396(18) . ?
S2 O5 1.434(8) . ?
S2 O6' 1.441(18) . ?
S2 O7 1.534(11) . ?

S2 C36 1.74(2) . ?
S2 C36' 1.90(3) . ?
S1 O3' 1.41(2) . ?
S1 O3 1.439(15) . ?
S1 O4' 1.44(3) . ?
S1 O4 1.461(14) . ?
S1 O2 1.486(7) . ?
S1 C35 1.68(2) . ?
S1 C35' 1.73(2) . ?
C35 F3 1.31(2) . ?
C35 F1 1.33(2) . ?
C35 F2 1.38(2) . ?
C35' F1* 1.21(4) . ?
C35' F1' 1.25(3) . ?
C35' F3' 1.28(3) . ?
C35' F3* 1.37(3) . ?
C35' F2* 1.37(4) . ?
C35' F2' 1.40(3) . ?
F3 F5 1.195(17) . ?
F3* F1' 0.83(3) . ?
F3* F2' 1.73(3) . ?
F3' F1* 1.14(4) . ?
F3' F2* 1.46(4) . ?
F2' F1* 1.09(4) . ?
F2* F1' 1.36(4) . ?
F6 C36' 1.22(3) . ?
F6 C36 1.37(2) . ?
F4 C36 1.32(2) . ?
C36 F5 1.31(3) . ?
C36' F5' 1.32(3) . ?
C36' F4' 1.38(3) . ?
C36' C38 2.04(4) 1_655 ?
F5' C38 1.35(3) 1_655 ?
C29 C34' 1.18(3) . ?
C29 C30 1.302(17) . ?
C29 C34 1.55(2) . ?
C29 C30' 1.68(2) . ?
C30 C31 1.37(3) . ?
C31 C32 1.44(5) . ?
C34 C33 1.43(3) . ?
C32 C33 1.45(4) . ?
C31' C32' 1.40(6) . ?
C31' C30' 1.44(4) . ?
C34' C33' 1.53(5) . ?

```
C32' C33' 1.33(6) . ?
C37 N4 1.14(4) . ?
C37 C38 1.99(4) . ?
C39 N5 1.21(4) . ?
C39 C40 1.30(5) . ?
C38 F5' 1.35(3) 1_455 ?
C38 C36' 2.04(4) 1_455 ?
C25 C26 1.42(3) . ?
C26 C27 1.39(3) . ?
C27 C28 1.39(3) . ?
C28' C27' 1.42(3) . ?
C27' C26' 1.39(4) . ?
C26' C25' 1.45(4) . ?

loop_
 _geom_angle_atom_site_label_1
 _geom_angle_atom_site_label_2
 _geom_angle_atom_site_label_3
 _geom_angle
 _geom_angle_site_symmetry_1
 _geom_angle_site_symmetry_3
 _geom_angle_publ_flag
C1 Ir1 N2 177.7(2) . . ?
C1 Ir1 N1 99.7(3) . . ?
N2 Ir1 N1 79.7(2) . . ?
C1 Ir1 N3 102.1(3) . . ?
N2 Ir1 N3 78.5(2) . . ?
N1 Ir1 N3 158.2(2) . . ?
C1 Ir1 P1 91.21(19) . . ?
N2 Ir1 P1 91.04(15) . . ?
N1 Ir1 P1 89.14(15) . . ?
N3 Ir1 P1 90.34(16) . . ?
C1 Ir1 Ir1 85.77(18) . 2_676 ?
N2 Ir1 Ir1 91.97(14) . 2_676 ?
N1 Ir1 Ir1 89.94(15) . 2_676 ?
N3 Ir1 Ir1 91.72(15) . 2_676 ?
P1 Ir1 Ir1 176.64(5) . 2_676 ?
C11 N2 C7 122.1(6) . . ?
C11 N2 Ir1 119.7(5) . . ?
C7 N2 Ir1 118.2(5) . . ?
C2 N1 C6 119.6(6) . . ?
C2 N1 Ir1 127.2(5) . . ?
C6 N1 Ir1 113.1(5) . . ?
O1 C1 Ir1 178.1(6) . . ?
```

C16 N3 C12 118.8(7) . . ?
C16 N3 Ir1 127.6(6) . . ?
C12 N3 Ir1 113.5(5) . . ?
C29 P1 C17 103.8(5) . . ?
C29 P1 C23 112.0(5) . . ?
C17 P1 C23 102.2(4) . . ?
C29 P1 Ir1 110.2(3) . . ?
C17 P1 Ir1 117.7(2) . . ?
C23 P1 Ir1 110.7(3) . . ?
N2 C7 C8 119.6(7) . . ?
N2 C7 C6 112.5(6) . . ?
C8 C7 C6 127.9(7) . . ?
C4 C3 C2 120.6(8) . . ?
C4 C3 H3 119.7 . . ?
C2 C3 H3 119.7 . . ?
N1 C6 C5 119.9(7) . . ?
N1 C6 C7 116.4(6) . . ?
C5 C6 C7 123.6(7) . . ?
C9 C8 C7 118.6(7) . . ?
C9 C8 H8 120.7 . . ?
C7 C8 H8 120.7 . . ?
N2 C11 C10 120.2(7) . . ?
N2 C11 C12 111.9(7) . . ?
C10 C11 C12 127.8(7) . . ?
N1 C2 C3 121.0(7) . . ?
N1 C2 H2 119.5 . . ?
C3 C2 H2 119.5 . . ?
C18 C17 C22 119.9(7) . . ?
C18 C17 P1 123.3(6) . . ?
C22 C17 P1 116.8(6) . . ?
C17 C18 C19 120.1(8) . . ?
C17 C18 H18 120.0 . . ?
C19 C18 H18 120.0 . . ?
C11 C10 C9 118.5(8) . . ?
C11 C10 H10 120.7 . . ?
C9 C10 H10 120.7 . . ?
C4 C5 C6 120.4(7) . . ?
C4 C5 H5 119.8 . . ?
C6 C5 H5 119.8 . . ?
N3 C16 C15 121.2(8) . . ?
N3 C16 H16 119.4 . . ?
C15 C16 H16 119.4 . . ?
C3 C4 C5 118.5(7) . . ?
C3 C4 H4 120.8 . . ?

C5 C4 H4 120.8 . . ?
N3 C12 C13 121.1(7) . . ?
N3 C12 C11 116.2(7) . . ?
C13 C12 C11 122.7(8) . . ?
C14 C13 C12 119.4(9) . . ?
C14 C13 H13 120.3 . . ?
C12 C13 H13 120.3 . . ?
C8 C9 C10 120.9(7) . . ?
C8 C9 H9 119.5 . . ?
C10 C9 H9 119.5 . . ?
C28' C23 C24 109.7(13) . . ?
C24 C23 C28 127.6(11) . . ?
C28' C23 P1 132.2(13) . . ?
C24 C23 P1 116.0(8) . . ?
C28 C23 P1 115.5(10) . . ?
C14 C15 C16 120.3(8) . . ?
C14 C15 H15 119.9 . . ?
C16 C15 H15 119.9 . . ?
C13 C14 C15 119.2(8) . . ?
C13 C14 H14 120.4 . . ?
C15 C14 H14 120.4 . . ?
C21 C22 C17 119.9(9) . . ?
C21 C22 H22 120.0 . . ?
C17 C22 H22 120.0 . . ?
C20 C19 C18 119.6(10) . . ?
C20 C19 H19 120.2 . . ?
C18 C19 H19 120.2 . . ?
C25' C24 C23 136.3(16) . . ?
C23 C24 C25 108.3(13) . . ?
C21 C20 C19 121.0(11) . . ?
C21 C20 H20 119.5 . . ?
C19 C20 H20 119.5 . . ?
C20 C21 C22 119.5(10) . . ?
C20 C21 H21 120.3 . . ?
C22 C21 H21 120.3 . . ?
O7' S2 O5 125.5(9) . . ?
O6 S2 O5 123.2(10) . . ?
O7' S2 O6' 115.6(11) . . ?
O5 S2 O6' 110.5(9) . . ?
O6 S2 O7 112.3(8) . . ?
O5 S2 O7 107.2(6) . . ?
O6 S2 C36 110.4(12) . . ?
O5 S2 C36 99.3(8) . . ?
O7 S2 C36 101.6(8) . . ?

O7' S2 C36' 104.7(13) . . ?
O5 S2 C36' 98.3(9) . . ?
O6' S2 C36' 95.1(11) . . ?
O3' S1 O4' 118.5(16) . . ?
O3 S1 O4 114.4(10) . . ?
O3' S1 O2 107.3(11) . . ?
O3 S1 O2 109.3(7) . . ?
O4' S1 O2 108.7(12) . . ?
O4 S1 O2 111.6(6) . . ?
O3 S1 C35 106.9(10) . . ?
O4 S1 C35 110.0(9) . . ?
O2 S1 C35 104.1(8) . . ?
O3' S1 C35' 113.0(12) . . ?
O4' S1 C35' 107.9(13) . . ?
O2 S1 C35' 99.8(8) . . ?
F3 C35 F1 109.9(17) . . ?
F3 C35 F2 107.7(17) . . ?
F1 C35 F2 106.7(16) . . ?
F3 C35 S1 112.7(14) . . ?
F1 C35 S1 109.6(15) . . ?
F2 C35 S1 109.9(14) . . ?
F1* C35' F1' 121(3) . . ?
F1* C35' F3' 54(2) . . ?
F1' C35' F3' 116(2) . . ?
F1* C35' F3* 109(2) . . ?
F1' C35' F3* 36.5(14) . . ?
F3' C35' F3* 141(2) . . ?
F1* C35' F2* 114(3) . . ?
F1' C35' F2* 62(2) . . ?
F3' C35' F2* 66(2) . . ?
F3* C35' F2* 98(2) . . ?
F1* C35' F2' 48.6(19) . . ?
F1' C35' F2' 110(2) . . ?
F3' C35' F2' 102(2) . . ?
F3* C35' F2' 77.3(18) . . ?
F2* C35' F2' 157(3) . . ?
F1* C35' S1 125(2) . . ?
F1' C35' S1 113.7(19) . . ?
F3' C35' S1 109.2(16) . . ?
F3* C35' S1 109.1(18) . . ?
F2* C35' S1 99(2) . . ?
F2' C35' S1 104.5(16) . . ?
F5 F3 C35 112.7(16) . . ?
F1' F3* C35' 64(3) . . ?

F1' F3* F2' 112(3) . . ?
C35' F3* F2' 52.2(14) . . ?
F1* F3' C35' 60(2) . . ?
F1* F3' F2* 113(3) . . ?
C35' F3' F2* 60(2) . . ?
F1* F2' C35' 57(2) . . ?
F1* F2' F3* 93(3) . . ?
C35' F2' F3* 50.6(14) . . ?
F2' F1* F3' 139(4) . . ?
F2' F1* C35' 75(3) . . ?
F3' F1* C35' 66(2) . . ?
F1' F2* C35' 55(2) . . ?
F1' F2* F3' 100(3) . . ?
C35' F2* F3' 54(2) . . ?
F3* F1' C35' 79(3) . . ?
F3* F1' F2* 143(4) . . ?
C35' F1' F2* 63(2) . . ?
F5 C36 F4 108.8(18) . . ?
F5 C36 F6 104.1(15) . . ?
F4 C36 F6 106.8(17) . . ?
F5 C36 S2 113.0(16) . . ?
F4 C36 S2 111.9(14) . . ?
F6 C36 S2 111.8(13) . . ?
F3 F5 C36 175.6(18) . . ?
F6 C36' F5' 105(2) . . ?
F6 C36' F4' 107(2) . . ?
F5' C36' F4' 111(2) . . ?
F6 C36' S2 109.9(19) . . ?
F5' C36' S2 114(2) . . ?
F4' C36' S2 109.5(19) . . ?
F6 C36' C38 122(2) . 1_655 ?
F5' C36' C38 40.7(14) . 1_655 ?
F4' C36' C38 70.3(16) . 1_655 ?
S2 C36' C38 125.9(16) . 1_655 ?
C36' F5' C38 100(2) . 1_655 ?
C30 C29 C34 118.1(14) . . ?
C34' C29 C30' 124.1(18) . . ?
C34' C29 P1 130.0(17) . . ?
C30 C29 P1 130.4(11) . . ?
C34 C29 P1 111.1(10) . . ?
C30' C29 P1 105.9(10) . . ?
C29 C30 C31 127.1(19) . . ?
C30 C31 C32 119(2) . . ?
C33 C34 C29 115.8(18) . . ?

```
C31 C32 C33 118(2) . . ?
C34 C33 C32 121(2) . . ?
C32' C31' C30' 116(3) . . ?
C31' C30' C29 113(2) . . ?
C29 C34' C33' 121(3) . . ?
C33' C32' C31' 130(4) . . ?
C32' C33' C34' 116(3) . . ?
N4 C37 C38 162(3) . . ?
N5 C39 C40 148(4) . . ?
F5' C38 C37 91.8(17) 1_455 . ?
F5' C38 C36' 39.8(14) 1_455 1_455 ?
C37 C38 C36' 120.5(16) . 1_455 ?
C26 C25 C24 125.6(19) . . ?
C27 C26 C25 121(2) . . ?
C28 C27 C26 121.1(19) . . ?
C27 C28 C23 115.5(17) . . ?
C23 C28' C27' 126(2) . . ?
C26' C27' C28' 119(2) . . ?
C27' C26' C25' 122(3) . . ?
C24 C25' C26' 107(2) . . ?

_refine_diff_density_max    2.151
_refine_diff_density_min   -2.974
_refine_diff_density_rms    0.201

_shelx_res_file
;

    z.res created by SHELXL-2014/7


TITL z in P-1 #2
REM reset to P-1 #2
CELL 0.71 12.7319 13.1519 14.5528 72.9512 81.3972 66.1863
ZERR 2 0.0031 0.0032 0.0036 0.0073 0.0083 0.0075
LATT 1
SFAC C H N O F P S Ir
UNIT 76 32 8 14 12 2 4 2
ISOR 0.005 0.01 C18 C19 C20 C21 C22 C24 C25 C25' C26' C28 C28'
ISOR 0.005 0.01 C29 C30 C30' C31' C32' C33' C33 C34'
ISOR 0.005 0.01 C35 C35' C36' F1 F3 F4 F6 F4' F5'
ISOR 0.005 0.01 O4' O3 O3'
ISOR 0.005 0.01 F1' F2' F3' F1* F2* F3*
ISOR 0.005 0.01 C37 C38 N4 C39 C40 N5
```

```
L.S. 20
PLAN  1
TEMP -132.33
BOND $h
htab
size 0.21 0.19 0.17
fmap 2
acta
REM <olex2.extras>
REM <HklSrc "%.\\z.hkl">
REM </olex2.extras>

WGHT    0.056200   23.995399
FVAR       0.09492   0.53054
IR1   8    0.501859    1.005217    0.397430    11.00000    0.02346
0.01130 =
         0.02085   -0.00603   -0.00043   -0.00691
N2    3    0.609011    0.840799    0.418100    11.00000    0.02043
0.01512 =
         0.01418   -0.00666   -0.00195   -0.00566
N1    3    0.385932    0.926367    0.426872    11.00000    0.01724
0.02026 =
         0.01650   -0.00652   -0.00033   -0.00623
C1    1    0.398700    1.160779    0.383249    11.00000    0.02491
0.01856 =
         0.01441   -0.00657    0.00055   -0.01255
O1    4    0.338257    1.252949    0.372247    11.00000    0.02878
0.01441 =
         0.02985   -0.00773   -0.00393   -0.00396
N3    3    0.660007    1.020105    0.373680    11.00000    0.03286
0.03073 =
         0.01764   -0.00945   -0.00140   -0.01657
P1    6    0.493781    1.023023    0.225710    11.00000    0.02560
0.00816 =
         0.03545   -0.00461   -0.00606   -0.00755
C7    1    0.563305    0.759298    0.441170    11.00000    0.03073
0.01489 =
         0.02225   -0.00748   -0.00884   -0.00828
C3    1    0.203225    0.913805    0.439647    11.00000    0.02537
0.04501 =
         0.02976   -0.01609    0.00214   -0.01807
AFIX  43
H3    2   0.122000   0.951659   0.438048   11.00000  -1.20000
```

```
AFIX   0
C6    1    0.437437    0.808609    0.443856    11.00000    0.03169
0.01788 =
         0.01752   -0.00506   -0.00403   -0.01026
C8    1    0.635484    0.644167    0.456995    11.00000    0.03527
0.01665 =
         0.02752   -0.01068   -0.00358   -0.00764
AFIX  43
H8    2    0.604890    0.585898    0.473013    11.00000   -1.20000
AFIX   0
C11   1    0.722585    0.814344    0.407621    11.00000    0.02706
0.02639 =
         0.02396   -0.01062   -0.00222   -0.01099
C2    1    0.270757    0.977537    0.425789    11.00000    0.02397
0.02256 =
         0.02902   -0.01276   -0.00133   -0.00534
AFIX  43
H2    2    0.235209    1.058691    0.415349    11.00000   -1.20000
AFIX   0
C17   1    0.476749    1.160401    0.142936    11.00000    0.05806
0.02099 =
         0.01729   -0.00665    0.00552   -0.01770
C18   1    0.488674    1.247885    0.168372    11.00000    0.05277
0.03459 =
         0.02826   -0.01039   -0.00221   -0.02222
AFIX  43
H18   2    0.503480    1.238603    0.233013    11.00000   -1.20000
AFIX   0
C10   1    0.797746    0.700701    0.423204    11.00000    0.02128
0.03454 =
         0.03429   -0.01858    0.00044   -0.00315
AFIX  43
H10   2    0.878423    0.681225    0.416366    11.00000   -1.20000
AFIX   0
C5    1    0.370539    0.743821    0.458640    11.00000    0.03678
0.02199 =
         0.03247   -0.00533   -0.00389   -0.01727
AFIX  43
H5    2    0.406247    0.662482    0.470917    11.00000   -1.20000
AFIX   0
C16   1    0.681212    1.117801    0.350548    11.00000    0.03983
0.03086 =
         0.02596   -0.00094   -0.00511   -0.02594
AFIX  43
```

```
H16   2    0.619124    1.189270    0.349175    11.00000   -1.20000
AFIX   0
C4     1     0.252396     0.796692     0.455643     11.00000     0.03591
0.03903 =
         0.03725   -0.01314   -0.00128   -0.02738
AFIX  43
H4    2    0.206267    0.752793    0.464481    11.00000   -1.20000
AFIX   0
C12    1     0.750722     0.917282     0.379304     11.00000     0.03493
0.02919 =
         0.02257   -0.00990    0.00027   -0.01513
C13    1     0.863271     0.912347     0.358711     11.00000     0.02797
0.05525 =
         0.03207   -0.01530    0.00260   -0.02076
AFIX  43
H13   2    0.925547    0.840364    0.363116    11.00000   -1.20000
AFIX   0
C9     1     0.752865     0.616088     0.448951     11.00000     0.03969
0.02133 =
         0.03423   -0.01347   -0.00464    0.00059
AFIX  43
H9    2    0.803391    0.537535    0.461291    11.00000   -1.20000
AFIX   0
C23    1     0.369466     1.000922     0.203031     11.00000     0.05433
0.06470 =
         0.01962   -0.01759    0.00651   -0.03704
C15    1     0.791354     1.115340     0.328956     11.00000     0.04824
0.05349 =
         0.03446   -0.00755    0.00066   -0.03998
AFIX  43
H15   2    0.804215    1.184966    0.311818    11.00000   -1.20000
AFIX   0
C14    1     0.882925     1.012135     0.332152     11.00000     0.03651
0.06514 =
         0.04198   -0.01318    0.00076   -0.03257
AFIX  43
H14   2    0.958652    1.010387    0.316117    11.00000   -1.20000
AFIX   0
C22    1     0.453329     1.174732     0.048192     11.00000     0.06942
0.03853 =
         0.03925   -0.01531   -0.00459   -0.02015
AFIX  43
H22   2    0.442305    1.115481    0.031012    11.00000   -1.20000
AFIX   0
```

```
C19   1    0.479093    1.349885    0.099619    11.00000    0.08385
0.04512 =
         0.04824   -0.01733    0.00108   -0.02957
AFIX  43
H19   2    0.486769    1.410608    0.117156    11.00000   -1.20000
AFIX   0
C24   1    0.263544    1.092129    0.204823    11.00000    0.05649
0.06992 =
         0.06065   -0.00625   -0.01203   -0.02374
C20   1    0.458373    1.362368    0.005790    11.00000    0.09018
0.05302 =
         0.05128   -0.01285    0.00242   -0.03032
AFIX  43
H20   2    0.452416    1.431882   -0.041228    11.00000   -1.20000
AFIX   0
C21   1    0.446189    1.275496   -0.020745    11.00000    0.08494
0.04917 =
         0.04727   -0.01427   -0.00262   -0.02553
AFIX  43
H21   2    0.433002    1.284582   -0.085753    11.00000   -1.20000
AFIX   0

S2    7    1.005093    0.275803    0.406746    11.00000    0.02479
0.03061 =
         0.06211   -0.02044   -0.00607   -0.00405
S1    7    0.505222    0.489708    0.299961    11.00000    0.07267
0.03286 =
         0.02797   -0.01233   -0.01007   -0.01048
O5    4    0.943729    0.331441    0.480767    11.00000    0.06266
0.04335 =
         0.08360   -0.03381    0.01754   -0.01631
PART 1
O4    4    0.439430    0.563052    0.214501    21.00000    0.06590
0.03685 =
         0.07547   -0.00629   -0.02111   -0.01852
C35   1    0.644082    0.471201    0.277968    21.00000    0.05247
0.04854 =
         0.04812   -0.00925   -0.00213   -0.01889
O3    4    0.468059    0.534226    0.383627    21.00000    0.06803
0.05253 =
         0.05457   -0.03543    0.00743   -0.02583
PART 0
PART 2
O3'   4    0.532937    0.505365    0.383600   -21.00000    0.08647
```

```
0.09268 =
         0.06322   -0.04357   -0.01603   -0.01063
C35'  1     0.363175     0.568216     0.271057    -21.00000     0.04199
0.03987 =
         0.03773   -0.00654   -0.00013   -0.01389
O4'   4     0.575060     0.506383     0.213388    -21.00000     0.10198
0.13047 =
         0.10031   -0.02466   -0.00787   -0.04303
PART 0
PART 1
F1    5     0.653262     0.573163     0.247600     21.00000     0.09874
0.05226 =
         0.06769   -0.00003   -0.00869   -0.04047
F3    5     0.706788     0.408904     0.352961     21.00000     0.06739
0.07211 =
         0.07027   -0.00411   -0.00997   -0.02940
F2    5     0.688872     0.416792     0.204782     21.00000     0.08462
0.07075 =
         0.07624   -0.02912    0.01591   -0.01712
PART 0
PART 2
F3*   5     0.354935     0.596682     0.173387    -20.50000     0.04926
0.04971 =
         0.04784   -0.01399   -0.00467   -0.01766
F3'   5     0.299866     0.564683     0.348371    -20.50000     0.04654
0.04654 =
         0.04271   -0.01089   -0.00012   -0.01181
F2'   5     0.353339     0.682890     0.243676    -20.50000     0.05289
0.04637 =
         0.04987   -0.00923   -0.00226   -0.01697
F1*   5     0.300229     0.650386     0.300668    -20.50000     0.07530
0.07705 =
         0.07428   -0.02427   -0.00393   -0.02581
F2*   5     0.325439     0.479268     0.294607    -20.50000     0.10072
0.09860 =
         0.10267   -0.02319   -0.00418   -0.04160
F1'   5     0.333579     0.544108     0.204290    -20.50000     0.04927
0.05039 =
         0.04699   -0.01403   -0.00861   -0.01904
PART 0
F6    5     0.924924     0.469205     0.278717     11.00000     0.08949
0.07164 =
         0.09238   -0.00216    0.00061   -0.02000
PART 1
```

```
F4     5     0.940094     0.318953     0.237390     21.00000     0.10862
0.08443 =
         0.07056   -0.02766   -0.01851   -0.01972
C36    1     0.915505     0.364441     0.311457     21.00000     0.07143
0.02332 =
         0.06177   -0.01577   -0.01488   -0.00575
F5     5     0.806756     0.389338     0.335531     21.00000     0.03212
0.06823 =
         0.11679   -0.01758   -0.02940   -0.00090
O7     4     0.983533     0.164634     0.426098     21.00000     0.03006
0.02143 =
         0.07763   -0.00484    0.00189   -0.01232
O6     4     1.119254     0.260133     0.379094     21.00000     0.02891
0.01952 =
         0.12223   -0.00696   -0.01355   -0.00796
PART 0
PART 2
O7'    4     0.963374     0.226172     0.357796    -21.00000     0.05105
0.07395 =
         0.11046   -0.04873    0.00257   -0.03495
C36'   1     1.018181     0.406091     0.313460    -21.00000     0.06495
0.06563 =
         0.06558   -0.01641   -0.00087   -0.02342
O6'    4     1.125923     0.219383     0.425442    -21.00000     0.02813
0.03215 =
         0.05947   -0.01082   -0.01526    0.00135
F4'    5     1.091544     0.371129     0.239144    -21.00000     0.09904
0.08928 =
         0.08898   -0.01426    0.01720   -0.02918
F5'    5     1.050256     0.471136     0.348000    -21.00000     0.12611
0.11687 =
         0.12845   -0.03066    0.00187   -0.05101

PART 0
C29    1     0.625714     0.925192     0.184221     11.00000     0.04921
0.04755 =
         0.03408   -0.02087   -0.00570   -0.00354
PART 1
C30    1     0.667954     0.813307     0.203880     21.00000     0.02675
0.01998 =
         0.01945   -0.00771    0.00189   -0.00529
C31    1     0.771118     0.744195     0.171138     21.00000     0.04992
0.04587 =
         0.03225   -0.02284   -0.00272    0.00133
```

```
C34   1    0.701979    0.990061    0.123834    21.00000    0.04139
0.03709 =
         0.05379   -0.01498    0.01513   -0.02247
C32   1    0.848957    0.795356    0.116230    21.00000    0.04363
0.06149 =
         0.06955   -0.04851    0.00000    0.00774
C33   1    0.811996    0.919559    0.091796    21.00000    0.05718
0.05403 =
         0.05520   -0.01676    0.00407   -0.01982
PART 0
PART 2
C31'  1    0.717439    0.706493    0.194309   -21.00000    0.06776
0.06480 =
         0.06391   -0.01895   -0.00390   -0.02217
C30'  1    0.616495    0.795299    0.220774   -21.00000    0.03618
0.03411 =
         0.03421   -0.01375   -0.00471   -0.00760
C34'  1    0.706794    0.941954    0.143096   -21.00000    0.05706
0.05668 =
         0.05465   -0.01671   -0.00051   -0.01874
C32'  1    0.808963    0.738856    0.150707   -21.00000    0.07655
0.07688 =
         0.07537   -0.01976   -0.00225   -0.02768
C33'  1    0.813754    0.842707    0.120567   -21.00000    0.06692
0.06720 =
         0.06435   -0.01748   -0.00203   -0.02303

PART 0
O2    4    0.506392    0.371347    0.324115    11.00000    0.09468
0.03589 =
         0.06685   -0.01463   -0.01554   -0.02270

C37   1    0.119917    0.625868    0.276737    10.50000    0.08297
0.08321 =
         0.08446   -0.02111   -0.00247   -0.03244
C39   1    0.839481    0.297002    0.032232    10.50000    0.09837
0.09757 =
         0.09584   -0.02429   -0.00566   -0.03753
N5    3    0.850930    0.200345    0.035165    10.50000    0.11562
0.13133 =
         0.13588   -0.03086    0.00462   -0.07647
C38   1    0.150524    0.459878    0.298121    10.50000    0.04786
0.05225 =
         0.03671   -0.01686   -0.00362   -0.01343
```

```
C40   1     0.858196    0.389450   -0.014295    10.50000    0.12524
0.12195 =
         0.11212   -0.04214   -0.00676   -0.04126
N4    3     0.074397    0.723084    0.260806    10.50000    0.13545
0.14401 =
         0.15168   -0.04324    0.00229   -0.04950
PART 1
C25   1     0.172256    1.058920    0.183846    21.00000    0.04678
0.04606 =
         0.04637   -0.01230   -0.00448   -0.01815
C26   1     0.186965    0.947342    0.180117    21.00000    0.05899
0.04880 =
         0.05910   -0.03663   -0.01217   -0.01699
C27   1     0.294388    0.858763    0.190724    21.00000    0.05767
0.05225 =
         0.08158   -0.03394    0.00354   -0.03271
C28   1     0.390991    0.877567    0.201332    21.00000    0.04197
0.03950 =
         0.04624   -0.01174   -0.00415   -0.01909
PART 0
PART 2
C28'  1     0.359125    0.930420    0.164326   -21.00000    0.04446
0.04403 =
         0.04219   -0.01600   -0.00648   -0.01815
C27'  1     0.253726    0.933129    0.139693   -21.00000    0.05963
0.06272 =
         0.06164   -0.01812   -0.00727   -0.03012
C26'  1     0.152019    1.022777    0.153873   -21.00000    0.06022
0.06396 =
         0.06140   -0.01640   -0.00394   -0.02364
C25'  1     0.151207    1.117400    0.185655   -21.00000    0.05871
0.05746 =
         0.05366   -0.01764   -0.00598   -0.01869
HKLF 4

REM  z in P-1 #2
REM R1 =  0.0496 for    7442 Fo > 4sig(Fo)  and  0.0680 for all    8923
data
REM    767 parameters refined using    258 restraints

END

WGHT      0.0531     24.3798
```

```
REM Instructions for potential hydrogen bonds
EQIV $1 x-1, y+1, z
HTAB C3 O7_$1
EQIV $2 -x+1, -y+1, -z+1
HTAB C8 O3_$2
HTAB C8 O3'
HTAB C2 O6_$1
HTAB C2 O6'_$1
EQIV $3 x, y+1, z
HTAB C18 O2_$3
HTAB C5 O3
HTAB C5 O3'
HTAB C5 O3'_$2
HTAB C16 O2_$3
EQIV $4 x+1, y, z
HTAB C13 N4_$4
HTAB C15 O7'_$3

REM  Highest  difference  peak   2.151,   deepest  hole  -2.974,   1-sigma
level  0.201
Q1    1   0.5153  0.9990  0.1823  11.00000  0.05    2.15
;
_shelx_res_checksum   55337

_shelx_hkl_file
;
  -2   0   0  283.26   13.83
  -2   0   0  291.43   13.86
   2   0   0  277.39   13.78
   2   0   0  308.13   13.86
   2   0   0  273.29   13.83
  -3   0   0  259.57   12.92
  -3   0   0  269.07   12.79
  -3   0   0  259.32   12.87
   3   0   0  275.04   12.92
   3   0   0  265.64   12.80
  -4   0   0  208.45    9.72
  -4   0   0  184.48    9.81
  -4   0   0  204.58    9.90
   4   0   0  219.28    9.91
   4   0   0  189.57    9.72
  -5   0   0  199.43    9.09
  -5   0   0  178.60    9.22
  -5   0   0  177.68    9.32
```

5	0	0	189.03	9.10
5	0	0	193.45	9.33
-6	0	0	217.54	11.50
-6	0	0	241.63	11.20
-6	0	0	223.73	11.36
6	0	0	242.59	11.47
6	0	0	230.12	11.21
-7	0	0	140.40	7.16
-7	0	0	136.99	7.38
-7	0	0	147.43	6.95
7	0	0	146.73	7.31
7	0	0	136.93	6.95
-8	0	0	152.72	8.23
-8	0	0	160.84	7.68
-8	0	0	153.15	7.91
8	0	0	158.15	7.69
-9	0	0	73.99	3.73
-9	0	0	72.88	4.08
-9	0	0	63.94	4.68
9	0	0	75.12	3.75
-10	0	0	76.99	5.51
10	0	0	87.89	4.36
-11	0	0	21.44	1.84
-11	0	0	28.46	3.96
-12	0	0	24.90	2.07
-13	0	0	21.31	2.09
-14	0	0	33.37	6.24
-14	1	0	6.91	4.82
-14	1	0	3.34	1.35
-13	1	0	8.91	1.29
-13	1	0	18.97	4.26
-12	1	0	19.30	2.11
-12	1	0	26.53	2.88
-12	1	0	28.35	4.98
-11	1	0	32.30	2.26
-11	1	0	37.55	4.50
10	-1	0	61.00	3.57
-10	1	0	55.27	4.95
-10	1	0	56.00	3.16
9	-1	0	52.73	2.94
-9	1	0	54.96	2.86
-9	1	0	51.73	3.29
-9	1	0	49.94	4.12
8	-1	0	104.82	5.30

-8	1	0	106.37	5.53
-8	1	0	103.48	5.95
-8	1	0	104.73	5.23
7	-1	0	79.79	4.17
-7	1	0	82.41	4.12
-7	1	0	86.49	4.71
-7	1	0	80.48	4.37
6	-1	0	53.98	2.90
-6	1	0	55.28	3.39
-6	1	0	58.45	3.11
-6	1	0	57.16	2.85
5	-1	0	81.21	4.18
-5	1	0	82.43	4.33
-5	1	0	84.23	4.15
-5	1	0	89.22	4.50
4	-1	0	35.97	2.05
4	-1	0	35.88	1.90
-4	1	0	37.71	2.20
-4	1	0	36.41	1.87
-4	1	0	37.77	2.05
3	-1	0	112.72	5.78
3	-1	0	110.49	5.70
-3	1	0	112.47	5.77
-3	1	0	133.31	5.85
-3	1	0	117.07	5.70
2	-1	0	2.90	0.32
2	-1	0	2.49	0.50
-2	1	0	3.04	0.30
-2	1	0	2.42	0.49
1	-1	0	176.00	8.39
-1	1	0	171.76	8.38
-2	-1	0	74.42	3.79
-2	-1	0	73.30	3.76
2	1	0	81.16	3.77
2	1	0	77.23	3.80
-3	-1	0	141.77	7.31
-3	-1	0	141.31	7.30
-3	-1	0	156.53	7.37
3	1	0	149.51	7.31
3	1	0	159.89	7.38
-4	-1	0	222.60	10.50
-4	-1	0	217.30	10.60
-4	-1	0	207.41	10.51
-4	-1	0	214.04	10.52

-4	-1	0	207.58	10.42
4	1	0	221.01	10.51
4	1	0	220.24	10.41
4	1	0	217.82	10.59
-5	-1	0	69.19	3.76
-5	-1	0	61.96	3.73
-5	-1	0	79.35	3.95
5	1	0	81.58	3.94
-6	-1	0	143.15	7.60
-6	-1	0	157.81	7.61
-6	-1	0	162.21	7.80
-6	-1	0	138.44	7.54
6	1	0	163.25	7.77
-7	-1	0	56.61	3.24
-7	-1	0	58.81	3.60
7	1	0	61.49	3.59
7	1	0	58.51	2.96
-8	-1	0	130.15	6.90
-8	-1	0	118.32	7.11
8	1	0	136.96	7.25
8	1	0	128.66	6.32
-9	-1	0	110.81	5.89
-9	-1	0	106.43	6.33
9	1	0	115.42	5.59
9	1	0	115.45	6.26
-10	-1	0	45.27	3.03
-10	-1	0	42.20	3.92
10	1	0	46.48	2.52
10	1	0	44.90	3.63
-11	-1	0	47.80	4.50
11	1	0	51.55	2.86
-12	-1	0	30.59	4.59
12	1	0	25.06	2.05
-13	-1	0	28.63	3.66
-14	-1	0	23.02	4.87
-15	-1	0	14.46	5.13
-13	2	0	25.74	2.53
-12	2	0	16.33	2.04
-12	2	0	19.35	4.16
-11	2	0	47.23	5.43
-11	2	0	50.69	3.56
-11	2	0	51.26	2.97
-10	2	0	25.84	4.11
-10	2	0	30.06	1.95

-9	2	0	105.91	5.27
-9	2	0	100.79	6.30
-9	2	0	105.38	5.65
8	-2	0	91.73	4.68
-8	2	0	88.87	5.42
-8	2	0	89.13	4.88
-8	2	0	91.79	4.58
7	-2	0	139.71	6.78
-7	2	0	133.50	6.97
-7	2	0	137.32	7.24
-7	2	0	133.52	6.70
6	-2	0	92.16	4.58
-6	2	0	90.18	4.72
-6	2	0	91.14	4.99
-6	2	0	89.41	4.51
5	-2	0	235.77	11.49
5	-2	0	227.11	11.58
-5	2	0	231.12	11.56
-5	2	0	235.27	11.45
-5	2	0	250.71	11.72
4	-2	0	143.95	7.34
-4	2	0	161.09	7.52
-4	2	0	154.78	7.32
-4	2	0	140.16	7.39
3	-2	0	180.40	8.56
3	-2	0	161.61	8.59
-3	2	0	185.60	8.54
2	-2	0	130.90	6.73
-2	2	0	150.66	6.84
-2	2	0	133.58	6.72
1	-2	0	229.04	11.28
-1	2	0	231.79	11.23
-1	2	0	236.89	11.30
0	-2	0	247.89	12.02
0	2	0	247.92	12.02
-1	-2	0	501.25	21.42
-1	-2	0	399.65	21.35
1	2	0	505.27	21.42
1	2	0	390.09	21.35
1	2	0	389.66	21.33
1	2	0	478.44	21.38
-2	-2	0	90.41	4.71
-2	-2	0	99.37	4.75
-2	-2	0	96.37	4.74

2	2	0	99.41	4.76
2	2	0	95.73	4.64
2	2	0	88.99	4.74
2	2	0	101.80	4.74
-3	-2	0	419.66	19.00
-3	-2	0	354.89	18.96
-3	-2	0	362.09	19.00
3	2	0	378.53	19.02
3	2	0	450.62	19.01
-4	-2	0	106.42	5.23
-4	-2	0	97.71	5.13
-4	-2	0	100.81	5.19
4	2	0	107.13	5.24
4	2	0	105.06	5.19
-5	-2	0	286.85	14.26
-5	-2	0	293.65	14.21
-5	-2	0	288.51	14.16
-5	-2	0	295.87	14.18
5	2	0	292.99	14.20
5	2	0	292.01	14.27
-6	-2	0	228.03	10.99
-6	-2	0	224.87	10.88
-6	-2	0	214.91	10.82
6	2	0	230.43	11.00
6	2	0	233.68	10.87
6	2	0	192.70	10.99
-7	-2	0	167.59	8.62
-7	-2	0	163.01	8.52
-7	-2	0	171.60	8.55
-7	-2	0	177.69	8.78
7	2	0	180.00	8.76
-8	-2	0	61.74	3.48
-8	-2	0	65.71	3.64
-8	-2	0	61.21	3.44
-8	-2	0	61.25	3.95
8	2	0	61.34	3.93
-9	-2	0	125.16	6.33
-9	-2	0	114.89	6.15
-9	-2	0	115.23	6.60
9	2	0	117.57	6.53
-10	-2	0	79.33	4.79
-10	-2	0	79.51	5.21
-10	-2	0	80.03	4.47
10	2	0	77.19	5.04

-11	-2	0	53.47	3.52
-11	-2	0	53.83	4.94
11	2	0	55.15	5.54
-12	-2	0	47.13	4.99
-13	-2	0	36.44	3.41
-13	-2	0	43.57	5.30
-14	-2	0	25.01	4.47
-15	-2	0	20.70	5.05
-15	-2	0	23.78	5.75
-13	3	0	11.73	1.62
-13	3	0	18.29	5.09
-12	3	0	17.33	4.64
-12	3	0	17.86	2.02
-11	3	0	31.16	3.10
-11	3	0	33.86	5.46
-10	3	0	29.59	2.72
-10	3	0	30.98	4.58
-10	3	0	30.43	2.02
-9	3	0	22.30	2.30
-9	3	0	23.56	1.67
-9	3	0	24.04	3.54
-8	3	0	94.38	5.21
-8	3	0	101.85	5.82
-8	3	0	94.10	4.89
-7	3	0	119.44	6.23
-7	3	0	122.79	6.65
-7	3	0	119.55	5.99
6	-3	0	114.23	6.04
-6	3	0	115.97	5.99
-6	3	0	125.63	6.35
-6	3	0	113.54	5.81
5	-3	0	90.87	4.72
-5	3	0	91.92	4.81
-5	3	0	96.58	5.08
-5	3	0	95.66	4.68
4	-3	0	103.11	5.36
4	-3	0	103.67	5.45
-4	3	0	119.45	5.64
-4	3	0	110.72	5.34
-4	3	0	102.86	5.41
3	-3	0	111.83	5.83
-3	3	0	112.48	5.81
-3	3	0	119.24	5.77
-3	3	0	125.83	5.97

-2	3	0	192.72	9.13
-2	3	0	183.01	9.25
1	-3	0	166.06	8.16
-1	3	0	164.49	8.07
-1	3	0	167.16	8.18
0	-3	0	151.88	8.01
0	-3	0	183.38	7.89
0	3	0	152.87	8.02
-1	-3	0	21.08	1.19
-1	-3	0	21.51	1.40
1	3	0	20.71	1.17
1	3	0	20.68	1.39
1	3	0	23.72	1.24
1	3	0	19.82	1.10
-2	-3	0	60.89	3.30
-2	-3	0	61.24	3.38
-2	-3	0	76.32	3.49
2	3	0	64.30	3.30
2	3	0	71.58	3.43
2	3	0	63.40	3.38
2	3	0	76.66	3.49
-3	-3	0	91.85	4.72
-3	-3	0	101.27	4.88
-3	-3	0	92.05	4.83
-3	-3	0	96.70	4.88
3	3	0	98.55	4.84
3	3	0	103.09	4.89
3	3	0	97.01	4.90
3	3	0	98.40	4.72
-4	-3	0	167.87	8.63
-4	-3	0	160.44	8.68
-4	-3	0	190.44	8.68
4	3	0	178.75	8.75
4	3	0	194.95	8.69
4	3	0	165.78	8.71
-5	-3	0	188.64	9.43
-5	-3	0	170.96	9.33
-5	-3	0	191.06	9.39
5	3	0	187.34	9.34
5	3	0	210.23	9.42
5	3	0	194.14	9.47
-6	-3	0	128.60	6.39
-6	-3	0	125.98	6.31
-6	-3	0	121.18	6.45

6	3	0	122.74	6.49
6	3	0	129.52	6.39
-7	-3	0	95.69	5.05
-7	-3	0	94.68	4.92
-7	-3	0	94.69	5.21
7	3	0	96.07	5.24
7	3	0	95.93	5.02
-8	-3	0	59.47	3.55
-8	-3	0	69.23	4.09
-8	-3	0	66.78	3.57
-8	-3	0	65.85	3.82
8	3	0	56.56	3.88
8	3	0	67.39	4.08
8	3	0	66.66	3.75
-9	-3	0	97.64	5.69
-9	-3	0	98.11	5.16
-9	-3	0	96.78	5.17
-9	-3	0	100.02	5.41
9	3	0	98.14	5.11
9	3	0	93.91	5.62
-10	-3	0	76.58	5.10
-10	-3	0	81.17	4.68
-10	-3	0	75.20	4.26
-10	-3	0	75.07	4.31
10	3	0	72.21	4.95
-11	-3	0	17.25	2.17
-11	-3	0	15.17	2.15
11	3	0	10.78	3.67
-12	-3	0	42.88	4.87
-13	-3	0	31.99	2.99
-13	-3	0	35.72	5.24
13	3	0	21.41	3.73
-14	-3	0	29.15	5.72
-14	-3	0	28.17	4.83
-15	-3	0	15.40	4.67
-15	-3	0	19.73	4.88
-12	4	0	46.88	6.68
-12	4	0	36.73	2.81
-11	4	0	38.60	5.91
-11	4	0	24.83	2.10
-10	4	0	42.76	4.17
-10	4	0	45.35	3.38
-10	4	0	37.39	2.73
-10	4	0	54.08	5.31

-9	4	0	30.13	4.16
-9	4	0	29.47	1.98
-9	4	0	29.55	2.65
-9	4	0	31.44	3.44
-8	4	0	81.03	4.85
-8	4	0	72.00	3.89
-8	4	0	71.43	4.27
-7	4	0	56.49	3.39
-7	4	0	64.24	3.98
-7	4	0	51.88	4.13
-7	4	0	56.40	3.06
6	-4	0	115.00	6.03
-6	4	0	118.85	6.40
-6	4	0	107.49	6.00
-6	4	0	126.35	6.32
-6	4	0	113.44	5.80
-5	4	0	106.12	5.47
-5	4	0	113.26	5.88
-4	4	0	123.26	6.37
-4	4	0	121.80	6.07
-3	4	0	190.66	9.25
-3	4	0	187.80	9.46
2	-4	0	284.86	13.99
-2	4	0	281.74	13.86
-2	4	0	291.15	14.03
1	-4	0	148.73	7.58
-1	4	0	157.78	7.43
-1	4	0	149.04	7.59
0	-4	0	258.54	13.02
0	-4	0	266.73	12.86
0	4	0	275.93	12.86
0	4	0	262.25	13.03
-1	-4	0	174.09	8.83
-1	-4	0	183.99	9.00
1	4	0	184.99	8.99
1	4	0	193.05	8.86
1	4	0	171.56	8.82
-2	-4	0	314.27	15.08
-2	-4	0	297.18	15.07
-2	-4	0	314.19	15.17
2	4	0	294.32	15.00
2	4	0	300.60	15.07
2	4	0	329.48	15.19
2	4	0	329.53	15.10

-3	-4	0	95.12	5.04
-3	-4	0	103.87	5.14
-3	-4	0	87.49	4.93
-3	-4	0	113.92	5.11
3	4	0	100.40	5.04
3	4	0	105.28	5.16
3	4	0	96.01	4.93
3	4	0	109.79	5.10
-4	-4	0	212.14	10.70
-4	-4	0	229.65	10.70
-4	-4	0	204.26	10.52
-4	-4	0	207.33	10.65
4	4	0	234.53	10.72
4	4	0	220.03	10.52
4	4	0	220.78	10.66
4	4	0	215.74	10.74
-5	-4	0	227.57	11.46
-5	-4	0	214.46	11.51
-5	-4	0	248.02	11.52
5	4	0	218.33	11.57
5	4	0	254.88	11.54
5	4	0	242.74	11.48
-6	-4	0	290.08	14.82
-6	-4	0	285.97	14.73
-6	-4	0	312.97	14.81
6	4	0	309.44	14.76
6	4	0	295.40	14.88
6	4	0	318.43	14.82
-7	-4	0	129.28	6.81
-7	-4	0	129.22	6.63
-7	-4	0	131.24	6.75
7	4	0	131.46	6.88
7	4	0	128.85	6.76
7	4	0	135.86	6.77
-8	-4	0	125.40	6.51
-8	-4	0	123.56	6.60
-8	-4	0	121.59	6.35
8	4	0	120.67	6.32
8	4	0	127.95	6.49
8	4	0	124.11	6.68
-9	-4	0	55.22	3.38
-9	-4	0	59.27	3.90
-9	-4	0	59.04	3.73
9	4	0	62.37	3.69

9	4	0	54.04	4.04
-10	-4	0	45.89	2.99
-10	-4	0	50.49	3.51
-10	-4	0	45.83	2.99
-10	-4	0	44.17	3.87
10	4	0	48.98	3.01
10	4	0	42.21	3.34
-11	-4	0	40.26	3.53
-11	-4	0	36.54	2.81
-11	-4	0	37.35	2.81
-11	-4	0	44.92	4.24
11	4	0	26.46	4.86
-12	-4	0	46.30	4.15
-12	-4	0	44.44	4.67
-12	-4	0	37.95	3.23
-12	-4	0	51.81	5.10
-13	-4	0	45.80	5.23
-13	-4	0	48.51	5.73
-13	-4	0	38.24	3.46
-13	-4	0	42.12	3.50
-13	-4	0	46.28	4.70
13	4	0	27.96	5.70
-14	-4	0	32.67	5.61
-14	-4	0	39.05	6.68
-14	-4	0	31.25	5.24
-15	-4	0	22.43	4.86
-15	-4	0	23.30	4.52
-12	5	0	18.12	2.34
-12	5	0	27.76	5.55
-11	5	0	24.93	2.22
-11	5	0	32.21	6.06
-10	5	0	30.61	3.10
-10	5	0	30.28	2.25
-10	5	0	30.57	5.28
-9	5	0	35.39	3.88
-9	5	0	38.68	4.69
-9	5	0	39.98	3.03
-9	5	0	36.73	2.38
-8	5	0	39.72	3.67
-8	5	0	36.80	4.08
-8	5	0	37.45	2.31
-7	5	0	30.41	3.11
-7	5	0	28.45	1.84
-7	5	0	22.69	3.46

-7	5	0	27.79	2.29
-6	5	0	45.41	2.62
-6	5	0	49.77	3.56
-5	5	0	122.15	6.48
-5	5	0	116.94	5.99
-4	5	0	84.09	4.33
-4	5	0	86.26	4.79
-3	5	0	113.90	5.71
-3	5	0	115.28	6.04
2	-5	0	146.36	7.80
-2	5	0	165.06	7.63
-2	5	0	153.12	7.88
1	-5	0	212.75	10.65
-1	5	0	217.56	10.46
-1	5	0	213.16	10.68
0	-5	0	117.30	6.40
0	5	0	129.18	6.18
0	5	0	125.78	6.43
0	5	0	129.44	6.18
-1	-5	0	222.42	10.83
-1	-5	0	219.02	11.04
1	5	0	218.19	11.02
1	5	0	231.50	10.85
1	5	0	221.31	10.82
-2	-5	0	41.62	2.35
-2	-5	0	44.04	2.62
2	5	0	42.81	2.23
2	5	0	46.06	2.42
2	5	0	42.95	2.33
2	5	0	46.46	2.63
-3	-5	0	98.33	4.71
-3	-5	0	86.66	4.64
-3	-5	0	92.61	4.79
3	5	0	87.33	4.51
3	5	0	93.22	4.70
3	5	0	95.68	4.82
3	5	0	90.64	4.64
-4	-5	0	72.37	3.83
-4	-5	0	73.06	3.95
-4	-5	0	79.37	3.91
-4	-5	0	68.22	3.65
4	5	0	77.12	3.84
4	5	0	71.66	4.01
4	5	0	78.17	3.91

4	5	0	71.62	3.64
-5	-5	0	178.51	9.32
-5	-5	0	196.48	9.39
-5	-5	0	172.50	9.14
-5	-5	0	183.12	9.40
5	5	0	203.55	9.43
5	5	0	191.35	9.14
5	5	0	190.84	9.48
5	5	0	195.20	9.34
-6	-5	0	103.56	5.84
-6	-5	0	110.68	5.73
-6	-5	0	117.78	5.83
6	5	0	117.70	5.75
6	5	0	118.12	5.86
6	5	0	106.66	5.96
-7	-5	0	95.54	5.14
-7	-5	0	95.14	5.20
-7	-5	0	91.94	5.01
7	5	0	101.90	5.05
7	5	0	92.60	5.31
7	5	0	100.03	5.18
-8	-5	0	74.62	4.31
-8	-5	0	69.96	4.01
-8	-5	0	74.11	4.23
8	5	0	75.67	4.07
8	5	0	75.42	4.24
8	5	0	59.49	5.60
-9	-5	0	69.09	4.28
-9	-5	0	70.23	3.87
-9	-5	0	68.33	4.19
9	5	0	66.34	4.46
9	5	0	60.16	5.00
-10	-5	0	15.27	2.86
-10	-5	0	13.78	2.53
-10	-5	0	14.62	1.90
10	5	0	13.63	1.83
10	5	0	10.29	2.44
10	5	0	16.06	2.49
-11	-5	0	35.87	2.73
-11	-5	0	34.51	2.84
-11	-5	0	37.53	3.47
11	5	0	35.74	3.38
-12	-5	0	28.80	2.78
-12	-5	0	28.50	4.30

-12	-5	0	30.36	3.69
-12	-5	0	27.87	4.24
12	5	0	33.08	2.75
-13	-5	0	37.04	3.33
-13	-5	0	40.52	5.33
-13	-5	0	42.12	4.63
-13	-5	0	40.25	5.30
-14	-5	0	14.67	4.03
-14	-5	0	19.15	3.75
-14	-5	0	16.53	3.11
-14	-5	0	16.75	3.11
-14	-5	0	18.12	3.13
-15	-5	0	46.65	7.39
-15	-5	0	33.62	6.09
-15	-5	0	37.48	5.81
-11	6	0	36.97	6.32
-11	6	0	24.61	2.42
-10	6	0	25.63	2.20
-10	6	0	24.52	4.29
-9	6	0	45.83	4.88
-9	6	0	49.64	2.95
-9	6	0	49.48	5.29
-8	6	0	31.05	2.14
-8	6	0	29.83	3.92
-8	6	0	30.91	2.65
-8	6	0	32.77	4.37
-7	6	0	72.24	4.89
-7	6	0	59.32	4.27
-7	6	0	71.78	5.00
-6	6	0	53.75	4.00
-6	6	0	49.10	2.86
-5	6	0	122.14	6.26
-5	6	0	126.55	6.85
-4	6	0	124.29	6.29
-4	6	0	126.59	6.78
-3	6	0	103.49	6.04
-3	6	0	109.35	5.38
2	-6	0	169.33	9.04
-2	6	0	182.70	8.82
-2	6	0	185.57	9.15
1	-6	0	150.58	7.84
-1	6	0	152.91	7.87
-1	6	0	155.43	7.55
0	-6	0	118.59	6.36

0	6	0	120.37	6.38
0	6	0	127.51	6.07
-1	-6	0	189.88	9.37
-1	-6	0	193.36	9.65
1	6	0	185.29	9.62
1	6	0	196.06	9.40
1	6	0	191.30	9.36
-2	-6	0	331.19	16.20
-2	-6	0	327.65	15.99
2	6	0	323.49	15.97
2	6	0	339.61	16.02
2	6	0	320.20	15.91
2	6	0	332.45	16.18
-3	-6	0	202.53	10.19
-3	-6	0	214.28	10.39
-3	-6	0	198.85	10.24
3	6	0	218.62	10.40
3	6	0	211.93	10.24
3	6	0	200.99	10.08
3	6	0	206.91	10.18
-4	-6	0	133.87	6.91
-4	-6	0	134.90	7.05
-4	-6	0	146.62	6.97
4	6	0	143.20	7.13
4	6	0	136.16	6.90
4	6	0	133.79	6.74
4	6	0	138.86	6.98
-5	-6	0	202.95	10.55
-5	-6	0	222.82	10.51
-5	-6	0	204.47	10.45
5	6	0	216.49	10.46
5	6	0	208.52	10.63
5	6	0	207.24	10.26
5	6	0	220.61	10.53
-6	-6	0	108.66	5.73
-6	-6	0	99.40	5.46
-6	-6	0	107.99	5.85
-6	-6	0	117.32	5.83
6	6	0	116.60	5.75
6	6	0	115.74	5.86
6	6	0	113.77	6.01
6	6	0	113.72	5.46
-7	-6	0	119.06	6.71
-7	-6	0	124.51	6.58

-7	-6	0	135.22	6.69
7	6	0	134.85	6.62
7	6	0	133.28	6.74
7	6	0	125.19	6.91
-8	-6	0	103.27	5.64
-8	-6	0	102.24	5.50
-8	-6	0	99.23	5.67
8	6	0	110.18	5.70
8	6	0	98.51	5.86
8	6	0	108.95	5.50
-9	-6	0	101.83	5.72
-9	-6	0	106.54	6.02
-9	-6	0	107.75	5.94
9	6	0	106.52	6.18
9	6	0	113.03	5.74
-10	-6	0	80.79	5.04
-10	-6	0	76.45	4.55
-10	-6	0	77.51	4.85
10	6	0	79.21	4.88
10	6	0	78.65	5.26
10	6	0	83.63	4.65
-11	-6	0	54.37	3.63
-11	-6	0	55.31	4.73
-11	-6	0	55.28	4.16
11	6	0	52.76	4.20
-12	-6	0	46.95	4.81
-12	-6	0	48.23	3.60
-12	-6	0	56.69	4.80
12	6	0	43.58	4.24
-13	-6	0	28.90	3.14
-13	-6	0	33.95	4.32
-13	-6	0	27.99	4.84
-13	-6	0	31.85	4.91
13	6	0	25.00	3.82
-14	-6	0	28.38	5.39
-14	-6	0	18.79	3.20
-14	-6	0	18.57	3.25
-15	-6	0	27.49	4.66
-15	-6	0	18.35	4.27
-15	-6	0	19.53	3.76
-10	7	0	20.68	2.20
-10	7	0	20.89	4.20
-9	7	0	18.77	3.81
-9	7	0	22.77	2.79

-9	7	0	23.38	2.06
-8	7	0	36.11	2.41
-8	7	0	31.74	4.76
-8	7	0	33.19	4.54
-7	7	0	20.32	1.83
-7	7	0	25.53	3.79
-6	7	0	84.24	5.39
-6	7	0	72.30	4.14
-5	7	0	61.30	4.43
-5	7	0	58.10	3.29
-4	7	0	55.20	3.00
-4	7	0	52.77	3.97
-3	7	0	44.93	2.56
-3	7	0	46.32	3.38
2	-7	0	66.70	4.19
-2	7	0	75.92	4.50
-2	7	0	74.59	3.77
1	-7	0	77.67	4.55
-1	7	0	81.35	4.07
-1	7	0	77.97	4.63
0	-7	0	90.87	4.24
0	-7	0	73.83	4.62
0	7	0	83.77	4.20
0	7	0	79.49	4.64
-1	-7	0	65.32	3.29
-1	-7	0	59.16	3.72
1	7	0	58.54	3.70
1	7	0	63.22	3.17
1	7	0	61.89	3.33
1	7	0	61.01	3.20
1	7	0	61.37	3.24
-2	-7	0	130.03	6.54
-2	-7	0	133.56	6.89
2	7	0	131.36	6.58
2	7	0	134.58	6.83
2	7	0	126.99	6.42
2	7	0	126.95	6.51
-3	-7	0	12.82	1.89
-3	-7	0	16.45	1.38
-3	-7	0	14.43	2.39
3	7	0	16.02	1.49
3	7	0	14.96	1.02
3	7	0	13.49	1.91
3	7	0	17.27	1.31

-4	-7	0	108.61	5.61
-4	-7	0	110.45	5.84
-4	-7	0	111.65	5.68
4	7	0	110.41	5.59
4	7	0	114.37	5.88
4	7	0	106.19	5.68
4	7	0	107.06	5.41
-5	-7	0	40.94	2.53
-5	-7	0	42.21	2.67
-5	-7	0	40.99	2.81
5	7	0	43.26	2.53
5	7	0	41.51	2.18
5	7	0	43.77	2.98
5	7	0	40.81	2.66
-6	-7	0	96.81	5.09
-6	-7	0	92.08	5.27
-6	-7	0	98.07	5.16
6	7	0	92.76	5.40
6	7	0	100.54	5.09
6	7	0	99.04	5.22
6	7	0	96.40	4.77
-7	-7	0	45.10	3.28
-7	-7	0	51.79	3.20
-7	-7	0	42.45	2.55
-7	-7	0	50.39	3.03
7	7	0	52.45	3.24
7	7	0	55.80	3.05
7	7	0	50.22	2.54
-8	-7	0	49.48	3.03
-8	-7	0	41.16	3.29
-8	-7	0	46.15	3.25
8	7	0	41.74	3.71
8	7	0	50.32	3.01
8	7	0	49.84	3.28
-9	-7	0	11.79	2.48
-9	-7	0	10.78	1.63
-9	-7	0	16.01	2.01
9	7	0	17.84	3.00
9	7	0	14.80	2.30
9	7	0	16.70	1.99
-10	-7	0	95.54	5.88
-10	-7	0	97.42	5.99
-10	-7	0	95.69	5.62
10	7	0	104.02	5.98

10	7	0	108.43	5.62
10	7	0	108.57	6.54
-11	-7	0	69.74	4.86
-11	-7	0	63.51	4.61
-11	-7	0	60.26	4.15
11	7	0	69.77	4.09
11	7	0	61.18	4.72
-12	-7	0	49.79	3.75
-12	-7	0	52.20	4.84
12	7	0	41.49	4.43
12	7	0	54.08	3.83
-13	-7	0	13.60	3.48
-13	-7	0	14.31	2.63
-13	-7	0	11.18	1.94
13	7	0	9.80	2.47
13	7	0	15.08	2.87
-14	-7	0	23.27	4.14
-14	-7	0	27.29	4.82
-14	-7	0	22.89	3.38
-15	-7	0	12.18	3.64
-15	-7	0	12.21	4.43
-9	8	0	19.83	2.18
-9	8	0	20.64	3.99
-8	8	0	37.12	2.61
-8	8	0	34.92	5.06
-7	8	0	48.14	5.09
-7	8	0	39.01	2.73
-6	8	0	73.52	4.18
-6	8	0	80.12	5.70
-5	8	0	57.76	4.57
-5	8	0	54.10	3.19
-4	8	0	70.23	4.87
-3	8	0	66.47	3.58
-3	8	0	66.34	4.56
2	-8	0	102.71	5.98
-2	8	0	113.41	6.18
-2	8	0	106.57	5.48
1	-8	0	91.22	5.53
-1	8	0	101.97	5.62
-1	8	0	100.57	5.01
0	-8	0	122.81	6.86
0	8	0	128.54	6.39
0	8	0	130.81	6.88
-1	-8	0	108.93	6.65

-1	-8	0	124.09	6.79
1	8	0	134.07	6.27
1	8	0	128.81	6.33
1	8	0	130.70	6.30
1	8	0	126.26	6.40
-2	-8	0	160.50	7.75
-2	-8	0	153.43	8.15
2	8	0	155.94	7.63
2	8	0	153.62	8.09
2	8	0	150.68	7.79
2	8	0	154.58	7.71
-3	-8	0	180.26	8.95
-3	-8	0	183.70	9.30
-3	-8	0	184.72	8.95
3	8	0	176.29	8.79
3	8	0	172.30	8.98
3	8	0	182.37	9.27
3	8	0	177.37	8.91
-4	-8	0	148.99	7.59
-4	-8	0	152.54	7.88
-4	-8	0	156.27	7.63
4	8	0	143.29	7.38
4	8	0	145.30	7.64
4	8	0	151.77	7.57
4	8	0	155.48	7.90
-5	-8	0	199.00	10.03
-5	-8	0	202.78	9.85
-5	-8	0	196.06	9.81
5	8	0	186.87	9.57
5	8	0	193.35	9.87
5	8	0	200.68	9.79
-6	-8	0	100.69	5.60
-6	-8	0	103.16	5.40
-6	-8	0	102.88	5.45
6	8	0	97.22	5.48
6	8	0	98.09	5.05
6	8	0	109.16	5.38
6	8	0	101.64	5.81
-7	-8	0	132.55	6.84
-7	-8	0	124.47	6.74
-7	-8	0	125.42	6.92
7	8	0	128.08	6.38
7	8	0	130.56	7.14
7	8	0	128.18	6.87

7	8	0	138.81	6.75
-8	-8	0	101.68	5.83
-8	-8	0	102.95	5.70
-8	-8	0	102.73	5.96
8	8	0	113.83	5.71
8	8	0	105.77	5.89
8	8	0	107.86	6.23
-9	-8	0	36.18	3.11
-9	-8	0	36.39	2.84
-9	-8	0	35.62	3.27
9	8	0	41.15	3.34
9	8	0	38.71	2.81
9	8	0	32.31	3.86
-10	-8	0	80.26	4.78
-10	-8	0	75.20	5.08
10	8	0	85.02	4.74
10	8	0	84.01	5.21
-11	-8	0	64.51	4.36
-11	-8	0	66.01	5.01
-11	-8	0	63.97	4.71
11	8	0	71.02	4.25
11	8	0	68.81	4.92
-12	-8	0	48.28	4.81
-12	-8	0	46.52	3.76
-12	-8	0	46.84	5.24
12	8	0	45.21	4.57
12	8	0	49.50	3.58
-13	-8	0	39.76	3.83
-13	-8	0	38.18	4.64
-13	-8	0	46.64	5.23
13	8	0	35.73	4.70
13	8	0	38.90	5.93
13	8	0	46.40	3.70
-14	-8	0	20.17	3.56
-14	-8	0	24.21	4.08
-14	-8	0	16.90	3.36
14	8	0	21.04	4.02
14	8	0	13.71	3.94
-15	-8	0	16.90	4.42
-15	-8	0	16.17	3.87
-8	9	0	7.82	1.23
-8	9	0	3.49	3.42
-7	9	0	26.97	2.34
-6	9	0	24.47	2.18

-6	9	0	32.43	4.77
-5	9	0	62.96	5.10
-5	9	0	53.03	3.34
-4	9	0	15.71	1.65
-4	9	0	16.27	2.66
-3	9	0	37.84	2.39
-3	9	0	38.36	3.89
2	-9	0	23.69	2.90
-2	9	0	23.18	1.71
-2	9	0	20.99	3.17
1	-9	0	53.11	3.89
-1	9	0	55.87	4.09
-1	9	0	58.34	3.10
0	-9	0	31.05	3.13
0	9	0	34.41	2.05
0	9	0	34.22	2.22
0	9	0	34.96	3.16
0	9	0	33.88	2.07
-1	-9	0	56.90	4.08
1	9	0	57.75	3.99
1	9	0	58.43	3.47
1	9	0	62.47	3.18
1	9	0	60.66	3.31
1	9	0	60.73	3.22
-2	-9	0	65.19	3.49
-2	-9	0	59.05	4.16
2	9	0	64.18	3.32
2	9	0	59.92	4.06
2	9	0	60.65	3.57
2	9	0	63.23	3.27
2	9	0	62.56	3.42
-3	-9	0	86.66	5.42
-3	-9	0	96.19	4.92
3	9	0	91.00	4.88
3	9	0	96.57	5.40
3	9	0	88.67	4.95
3	9	0	92.59	4.82
-4	-9	0	56.18	3.41
-4	-9	0	50.75	3.77
-4	-9	0	57.59	3.62
4	9	0	59.21	3.20
4	9	0	53.40	3.34
4	9	0	53.83	3.81
4	9	0	53.99	2.90

-5	-9	0	122.24	6.33
-5	-9	0	123.31	6.35
-5	-9	0	125.26	6.66
5	9	0	114.93	6.40
5	9	0	114.51	6.02
5	9	0	125.87	6.29
5	9	0	124.74	6.75
-6	-9	0	53.78	3.18
-6	-9	0	51.94	3.30
-6	-9	0	46.80	3.59
6	9	0	45.86	2.64
6	9	0	46.78	3.87
6	9	0	51.63	3.33
6	9	0	53.22	3.10
-7	-9	0	91.74	5.40
-7	-9	0	88.70	5.15
-7	-9	0	91.81	5.09
7	9	0	92.21	5.68
7	9	0	91.74	5.26
7	9	0	88.09	4.62
7	9	0	100.67	5.09
-8	-9	0	35.61	2.73
-8	-9	0	33.61	3.14
-8	-9	0	29.68	2.91
8	9	0	32.57	3.02
8	9	0	31.08	1.89
8	9	0	35.23	3.77
8	9	0	35.86	2.65
-9	-9	0	42.85	3.52
-9	-9	0	42.54	5.31
-9	-9	0	45.77	3.26
9	9	0	45.71	3.65
9	9	0	47.65	3.21
-10	-9	0	15.31	2.60
-10	-9	0	20.86	2.54
-10	-9	0	18.32	3.01
10	9	0	20.78	3.20
10	9	0	20.72	2.40
-11	-9	0	48.81	4.23
-11	-9	0	47.19	3.77
-11	-9	0	39.09	4.41
11	9	0	53.56	3.53
11	9	0	47.06	4.46
-12	-9	0	21.20	4.22

-12	-9	0	22.42	3.21
-12	-9	0	22.41	3.78
12	9	0	28.28	4.32
12	9	0	24.00	2.82
-13	-9	0	30.32	4.97
-13	-9	0	33.36	3.74
-13	-9	0	28.54	4.41
13	9	0	30.24	3.38
-14	-9	0	11.70	2.39
-14	-9	0	12.30	3.16
-14	-9	0	11.41	3.96
-15	-9	0	19.67	3.96
-15	-9	0	17.70	4.53
-7	10	0	13.52	2.10
-6	10	0	28.60	2.38
-5	10	0	41.65	2.85
-4	10	0	53.26	4.97
-4	10	0	45.64	3.03
-3	10	0	46.67	2.94
-3	10	0	49.74	4.57
2	-10	0	45.68	4.04
-2	10	0	48.72	2.89
-2	10	0	52.41	4.31
1	-10	0	70.28	4.94
-1	10	0	79.47	4.10
-1	10	0	78.09	5.17
0	-10	0	51.58	4.50
0	10	0	53.86	3.14
0	10	0	55.30	3.11
0	10	0	60.11	3.34
0	10	0	55.33	4.20
-1	-10	0	22.87	3.02
-1	-10	0	25.07	2.09
1	10	0	25.05	1.69
1	10	0	24.66	1.94
1	10	0	21.02	2.23
1	10	0	23.85	3.10
1	10	0	24.11	1.71
-2	-10	0	25.42	3.23
-2	-10	0	30.01	2.20
2	10	0	27.27	2.28
2	10	0	27.62	1.79
2	10	0	29.13	1.87
2	10	0	25.70	3.14

2	10	0	28.12	2.08
-3	-10	0	82.63	5.25
-3	-10	0	87.37	4.59
3	10	0	81.38	4.66
3	10	0	83.46	5.24
3	10	0	81.53	4.29
3	10	0	83.93	4.49
3	10	0	83.82	4.36
-4	-10	0	110.75	6.41
-4	-10	0	114.48	5.88
4	10	0	106.72	5.93
4	10	0	110.04	6.38
4	10	0	113.09	5.79
4	10	0	108.29	5.55
-5	-10	0	50.25	3.08
-5	-10	0	46.57	3.13
-5	-10	0	46.79	3.66
5	10	0	43.63	2.53
5	10	0	50.70	2.99
5	10	0	42.89	3.80
5	10	0	43.88	3.19
-6	-10	0	46.54	3.29
-6	-10	0	49.99	3.76
-6	-10	0	49.44	3.23
6	10	0	46.90	3.35
6	10	0	48.35	4.02
6	10	0	45.34	2.73
6	10	0	51.66	3.13
-7	-10	0	36.39	3.43
-7	-10	0	37.72	3.02
-7	-10	0	38.24	3.34
7	10	0	35.46	2.13
7	10	0	36.68	3.95
7	10	0	38.43	2.77
7	10	0	39.31	3.15
-8	-10	0	56.09	4.04
8	10	0	61.82	3.24
8	10	0	66.87	3.87
8	10	0	60.49	4.18
-9	-10	0	36.95	3.82
-9	-10	0	39.18	3.27
-9	-10	0	41.63	3.44
9	10	0	46.36	3.17
9	10	0	39.28	3.61

9	10	0	41.05	2.31
-10	-10	0	50.55	3.78
-10	-10	0	46.31	3.96
-10	-10	0	46.53	4.37
10	10	0	53.99	3.61
10	10	0	49.19	4.16
-11	-10	0	36.05	4.36
-11	-10	0	35.39	3.92
-11	-10	0	39.57	3.60
11	10	0	41.68	3.32
11	10	0	42.28	4.43
-12	-10	0	25.55	3.68
-12	-10	0	24.39	3.49
-12	-10	0	26.21	3.85
12	10	0	29.51	4.60
12	10	0	19.29	5.25
12	10	0	31.44	3.13
-13	-10	0	20.75	3.70
-13	-10	0	24.55	3.73
-13	-10	0	21.60	4.20
13	10	0	17.36	3.34
13	10	0	23.80	3.32
-14	-10	0	7.86	3.28
-14	-10	0	17.98	4.10
-14	-10	0	11.79	2.61
-15	-10	0	20.76	4.65
-15	-10	0	10.74	2.62
-15	-10	0	13.94	3.71
-6	11	0	22.62	2.34
-5	11	0	17.15	2.12
-4	11	0	35.04	2.60
-3	11	0	31.50	4.58
-3	11	0	27.53	2.21
-3	11	0	22.13	2.14
2	-11	0	55.54	4.75
-2	11	0	57.00	4.92
-2	11	0	57.07	3.35
1	-11	0	46.38	4.32
-1	11	0	52.64	4.59
-1	11	0	52.24	3.04
0	-11	0	80.87	5.44
0	11	0	82.24	4.21
0	11	0	68.48	5.22
-1	-11	0	29.61	3.57

1	11	0	27.41	1.97
1	11	0	31.24	2.00
1	11	0	27.91	2.58
-2	-11	0	45.20	3.99
2	11	0	44.21	2.53
2	11	0	43.39	3.03
2	11	0	40.25	2.90
-3	-11	0	34.65	2.59
-3	-11	0	35.96	4.86
3	11	0	36.16	2.18
3	11	0	31.26	2.66
3	11	0	32.65	2.09
3	11	0	33.05	2.42
-4	-11	0	42.79	3.07
-4	-11	0	48.51	3.11
4	11	0	44.86	2.90
4	11	0	44.59	2.56
4	11	0	47.57	2.68
4	11	0	43.73	3.19
-5	-11	0	41.51	3.07
-5	-11	0	44.36	3.10
5	11	0	44.33	2.94
5	11	0	42.15	2.43
5	11	0	41.33	3.18
-6	-11	0	34.98	3.73
-6	-11	0	35.72	2.98
-6	-11	0	43.49	3.01
6	11	0	40.20	2.83
6	11	0	37.24	2.24
6	11	0	37.74	3.13
-7	-11	0	36.59	3.72
-7	-11	0	33.07	3.04
-7	-11	0	36.49	2.95
7	11	0	34.04	3.21
7	11	0	37.40	2.79
7	11	0	31.80	2.10
-8	-11	0	46.46	3.45
-8	-11	0	47.44	4.11
-8	-11	0	43.19	3.48
8	11	0	47.90	3.29
8	11	0	41.17	2.52
8	11	0	40.04	3.70
-9	-11	0	18.11	3.03
-9	-11	0	21.43	2.83

-9	-11	0	17.97	3.70
9	11	0	23.92	2.60
9	11	0	17.13	3.20
-10	-11	0	29.53	3.52
-10	-11	0	29.39	3.33
-10	-11	0	29.80	4.12
10	11	0	32.28	3.84
10	11	0	33.05	2.07
10	11	0	36.07	3.15
-11	-11	0	21.42	3.20
-11	-11	0	15.06	2.44
-11	-11	0	17.00	3.23
11	11	0	15.85	2.74
11	11	0	19.33	2.84
-12	-11	0	25.19	4.82
-12	-11	0	25.57	3.89
-12	-11	0	23.02	3.57
12	11	0	17.54	3.22
12	11	0	26.32	3.32
-13	-11	0	12.91	3.71
-13	-11	0	8.43	2.43
-13	-11	0	9.97	2.72
13	11	0	6.08	2.10
13	11	0	10.19	3.40
-14	-11	0	15.35	4.11
-14	-11	0	9.44	3.21
-5	12	0	21.30	2.37
-4	12	0	24.73	2.39
-3	12	0	24.89	2.35
-2	12	0	38.56	2.74
1	-12	0	47.34	4.71
-1	12	0	48.72	3.14
-1	12	0	54.18	5.09
-1	12	0	53.93	3.17
0	-12	0	54.27	4.99
0	12	0	61.25	3.43
0	12	0	58.02	5.01
0	12	0	56.44	3.37
0	12	0	60.78	3.96
-1	-12	0	61.07	5.10
1	12	0	58.25	4.81
1	12	0	60.86	3.69
1	12	0	60.39	3.53
1	12	0	62.77	3.51

-2	-12	0	52.25	4.55
2	12	0	44.43	3.38
2	12	0	48.83	3.21
2	12	0	50.24	2.94
-3	-12	0	43.38	3.08
-3	-12	0	40.33	4.38
3	12	0	42.86	2.95
3	12	0	41.97	3.14
3	12	0	43.89	2.66
-4	-12	0	30.56	2.65
-4	-12	0	23.72	3.89
4	12	0	29.51	2.53
4	12	0	30.41	2.24
4	12	0	28.15	1.92
4	12	0	29.97	2.74
-5	-12	0	36.79	4.19
-5	-12	0	32.02	3.18
-5	-12	0	37.06	2.99
5	12	0	35.45	3.08
5	12	0	34.00	2.23
5	12	0	39.81	2.43
5	12	0	41.01	2.95
-6	-12	0	48.04	4.48
-6	-12	0	51.90	3.52
-6	-12	0	43.47	3.36
6	12	0	46.88	2.73
6	12	0	45.78	3.59
6	12	0	50.67	3.28
-7	-12	0	62.95	4.13
-7	-12	0	60.59	4.95
-7	-12	0	59.55	4.03
7	12	0	62.61	3.91
7	12	0	58.76	3.32
7	12	0	60.60	4.27
-8	-12	0	48.23	3.70
-8	-12	0	43.85	3.65
-8	-12	0	43.87	4.57
8	12	0	52.60	3.53
8	12	0	44.68	3.94
8	12	0	44.71	2.71
-9	-12	0	50.21	4.89
-9	-12	0	51.19	4.13
-9	-12	0	53.74	4.08
9	12	0	55.97	3.82

9	12	0	45.56	4.62
9	12	0	51.32	2.99
-10	-12	0	36.92	4.62
-10	-12	0	39.04	3.77
-10	-12	0	32.80	3.87
10	12	0	36.18	2.36
10	12	0	30.08	4.92
10	12	0	38.27	4.11
10	12	0	36.74	3.44
-11	-12	0	26.45	4.08
-11	-12	0	29.82	3.85
-11	-12	0	25.53	3.72
11	12	0	35.05	3.45
11	12	0	32.86	4.55
-12	-12	0	14.40	2.77
-12	-12	0	13.83	2.57
-12	-12	0	14.45	3.59
12	12	0	15.27	2.32
12	12	0	18.12	3.42
-13	-12	0	19.51	3.88
-13	-12	0	22.27	4.37
-13	-12	0	26.01	4.90
13	12	0	22.67	3.77
13	12	0	16.37	3.71
-14	-12	0	16.54	3.45
-14	-12	0	8.23	4.38
-3	13	0	23.44	2.45
-2	13	0	14.29	2.08
1	-13	0	22.21	3.46
-1	13	0	25.13	2.30
0	-13	0	21.70	4.37
0	13	0	26.87	2.27
-1	-13	0	33.29	4.62
1	13	0	34.71	5.61
1	13	0	26.77	4.97
1	13	0	30.16	2.74
1	13	0	31.15	2.31
1	13	0	26.03	2.20
-2	-13	0	27.14	4.34
2	13	0	25.18	2.83
2	13	0	29.39	2.23
2	13	0	26.98	2.11
2	13	0	28.22	4.35
2	13	0	27.55	2.65

-3	-13	0	40.52	4.71
-3	-13	0	40.51	3.14
-3	-13	0	37.60	3.16
3	13	0	42.79	4.72
3	13	0	39.51	3.06
3	13	0	42.81	2.66
3	13	0	36.84	3.21
3	13	0	37.20	2.53
-4	-13	0	22.67	2.70
-4	-13	0	26.23	4.35
4	13	0	26.10	2.84
4	13	0	19.26	3.93
4	13	0	30.50	2.18
4	13	0	28.10	2.61
-5	-13	0	27.40	4.49
-5	-13	0	36.70	4.21
-5	-13	0	30.46	3.06
5	13	0	32.07	3.12
5	13	0	37.23	2.42
5	13	0	23.35	4.05
5	13	0	34.57	2.83
-6	-13	0	24.54	4.39
-6	-13	0	19.78	2.78
-6	-13	0	23.92	2.91
6	13	0	20.26	4.20
6	13	0	27.64	3.08
6	13	0	26.96	2.64
6	13	0	29.98	2.18
-7	-13	0	31.77	3.08
-7	-13	0	34.93	3.33
-7	-13	0	31.39	4.58
7	13	0	35.84	4.85
7	13	0	35.74	2.98
7	13	0	36.20	2.57
7	13	0	32.61	2.26
7	13	0	34.34	3.55
-8	-13	0	30.03	3.31
-8	-13	0	26.16	3.36
-8	-13	0	26.73	4.50
8	13	0	28.25	3.55
8	13	0	23.33	3.91
8	13	0	27.16	2.09
-9	-13	0	35.92	3.82
-9	-13	0	38.42	4.93

-9	-13	0	34.82	3.78
9	13	0	32.92	6.11
9	13	0	42.17	3.48
9	13	0	37.95	2.51
-10	-13	0	13.68	2.40
-10	-13	0	19.37	3.49
-10	-13	0	14.98	2.40
10	13	0	19.07	3.07
10	13	0	10.82	1.85
10	13	0	13.63	2.61
-11	-13	0	20.72	4.04
-11	-13	0	24.51	4.96
-11	-13	0	17.38	2.81
11	13	0	24.65	4.58
11	13	0	20.27	3.50
-12	-13	0	19.42	3.12
-12	-13	0	12.53	2.87
-12	-13	0	16.76	3.79
12	13	0	15.86	3.67
12	13	0	19.34	3.76
-13	-13	0	12.16	3.98
-13	-13	0	15.05	3.58
-13	-13	0	14.29	3.23
13	13	0	5.37	3.92
13	13	0	14.91	2.64
1	-14	0	12.12	3.55
-1	14	0	14.49	2.22
0	-14	0	19.45	3.54
0	14	0	23.21	2.32
-1	-14	0	29.55	5.17
1	14	0	32.47	2.62
-2	-14	0	32.75	5.07
2	14	0	31.51	3.37
2	14	0	39.32	2.71
-3	-14	0	30.30	4.72
3	14	0	30.82	2.98
3	14	0	23.68	3.04
3	14	0	31.36	2.74
3	14	0	30.52	2.45
-4	-14	0	35.55	5.00
4	14	0	34.56	2.57
4	14	0	37.55	3.40
4	14	0	37.89	3.23
4	14	0	43.15	2.79

-5	-14	0	26.92	3.44
-5	-14	0	24.02	4.74
5	14	0	29.29	2.98
5	14	0	24.15	3.13
5	14	0	26.78	2.12
5	14	0	27.50	2.38
-6	-14	0	25.13	3.34
-6	-14	0	26.87	4.92
-6	-14	0	26.08	3.34
6	14	0	29.96	3.47
6	14	0	25.15	2.15
6	14	0	29.83	3.02
-7	-14	0	15.03	2.99
-7	-14	0	19.71	3.45
-7	-14	0	19.44	3.52
7	14	0	17.67	3.44
7	14	0	20.64	2.24
7	14	0	17.89	2.94
-8	-14	0	37.26	5.13
-8	-14	0	31.07	3.88
-8	-14	0	33.14	3.54
8	14	0	32.84	4.00
8	14	0	38.93	2.78
8	14	0	36.64	3.42
-9	-14	0	19.50	3.57
-9	-14	0	20.16	3.90
-9	-14	0	25.72	5.02
9	14	0	21.56	3.32
9	14	0	25.32	2.38
-10	-14	0	30.02	5.19
-10	-14	0	32.53	4.30
-10	-14	0	29.00	4.19
10	14	0	25.93	2.45
10	14	0	29.34	4.56
10	14	0	29.89	3.68
10	14	0	32.26	2.62
-11	-14	0	10.36	2.73
-11	-14	0	9.18	2.70
-11	-14	0	8.72	3.63
11	14	0	10.35	2.35
11	14	0	9.83	3.15
11	14	0	13.25	2.07
-12	-14	0	17.92	3.34
-12	-14	0	28.85	4.82

-12	-14	0	29.72	5.65
12	14	0	14.65	4.01
12	14	0	23.35	2.90
-1	-15	0	10.49	3.54
1	15	0	5.97	1.33
-2	-15	0	15.39	3.85
2	15	0	19.33	2.29
-3	-15	0	16.59	3.54
3	15	0	15.64	2.08
3	15	0	16.88	2.28
-4	-15	0	23.18	3.76
4	15	0	22.24	3.16
4	15	0	24.79	2.48
-5	-15	0	16.29	3.66
5	15	0	18.33	2.37
5	15	0	18.24	3.11
-6	-15	0	14.78	3.46
6	15	0	9.59	1.57
6	15	0	15.91	2.28
6	15	0	12.19	2.05
-7	-15	0	18.94	3.72
7	15	0	17.21	2.07
7	15	0	18.08	3.32
7	15	0	18.45	2.47
-8	-15	0	20.33	3.86
8	15	0	19.79	2.25
8	15	0	18.61	2.58
8	15	0	22.61	3.62
-9	-15	0	15.22	3.84
9	15	0	13.89	2.42
9	15	0	13.62	2.21
9	15	0	19.92	2.49
-10	-15	0	13.22	3.76
10	15	0	9.03	1.50
10	15	0	9.42	2.35
10	15	0	10.28	1.43
9	15	-1	-0.49	2.31
9	15	-1	1.41	1.16
9	15	-1	0.01	1.21
-9	-15	1	-3.73	3.60
8	15	-1	-0.54	1.15
8	15	-1	-2.24	2.08
8	15	-1	1.04	1.10
-8	-15	1	-2.24	3.43

7	15	-1	-0.60	1.99
7	15	-1	1.02	1.18
7	15	-1	1.02	1.02
-7	-15	1	3.75	3.20
6	15	-1	-0.78	1.07
6	15	-1	0.06	1.91
-6	-15	1	0.69	3.03
5	15	-1	1.03	1.16
5	15	-1	0.02	1.81
-5	-15	1	0.58	3.08
4	15	-1	0.76	1.81
4	15	-1	0.72	1.20
-4	-15	1	1.76	3.09
3	15	-1	3.64	1.24
-3	-15	1	3.23	3.36
2	15	-1	0.75	1.83
2	15	-1	0.04	1.17
-2	-15	1	1.59	3.45
11	14	-1	-0.76	1.09
11	14	-1	-0.61	2.32
11	14	-1	3.59	3.29
-11	-14	1	-1.69	2.57
-11	-14	1	0.55	3.83
-11	-14	1	2.46	2.67
10	14	-1	6.19	2.18
10	14	-1	3.91	1.32
10	14	-1	1.96	1.13
10	14	-1	2.44	2.86
-10	-14	1	1.19	2.15
-10	-14	1	3.89	3.39
-10	-14	1	2.37	2.40
9	14	-1	0.73	1.02
9	14	-1	0.59	2.00
9	14	-1	-0.03	2.41
9	14	-1	-0.26	0.88
-9	-14	1	0.36	2.27
-9	-14	1	1.55	2.01
-9	-14	1	2.43	3.19
8	14	-1	3.10	2.22
8	14	-1	2.33	1.02
8	14	-1	3.14	1.89
-8	-14	1	-0.19	3.25
-8	-14	1	0.47	1.96
-8	-14	1	0.81	2.11

7	14	-1	-0.32	1.67
7	14	-1	0.73	0.98
7	14	-1	0.91	1.94
-7	-14	1	-0.99	1.87
-7	-14	1	-0.94	3.00
-7	-14	1	-1.23	1.74
6	14	-1	1.72	1.03
6	14	-1	2.79	1.77
6	14	-1	2.62	0.92
6	14	-1	2.51	1.65
-6	-14	1	3.27	1.75
-6	-14	1	3.60	1.84
-6	-14	1	6.49	2.94
5	14	-1	3.47	1.71
5	14	-1	3.00	0.94
5	14	-1	1.98	1.62
5	14	-1	1.37	0.96
-5	-14	1	1.68	2.47
-5	-14	1	1.52	2.85
4	14	-1	8.05	1.81
4	14	-1	6.65	1.05
4	14	-1	8.98	1.21
4	14	-1	8.83	1.72
-4	-14	1	10.12	3.00
3	14	-1	2.88	1.07
3	14	-1	1.70	1.64
3	14	-1	3.64	1.52
-3	-14	1	0.77	2.84
2	14	-1	5.36	1.15
-2	-14	1	6.13	3.08
1	14	-1	1.05	1.08
-1	-14	1	2.31	3.05
0	14	-1	4.47	1.15
-1	14	-1	-0.38	1.09
1	-14	1	0.19	3.35
13	13	-1	2.97	4.05
13	13	-1	-2.12	2.37
-13	-13	1	-1.92	3.88
-13	-13	1	2.85	3.30
12	13	-1	1.77	2.24
12	13	-1	0.70	3.29
-12	-13	1	-1.59	3.39
-12	-13	1	3.52	3.62
-12	-13	1	0.16	2.63

11	13	-1	1.09	2.74
11	13	-1	-0.93	1.97
-11	-13	1	4.86	3.39
-11	-13	1	2.94	2.29
-11	-13	1	-1.42	2.43
10	13	-1	1.19	2.37
10	13	-1	0.36	0.98
10	13	-1	-0.24	1.76
10	13	-1	1.57	0.91
-10	-13	1	-0.26	3.08
-10	-13	1	1.08	2.09
-10	-13	1	-1.40	2.03
9	13	-1	-1.61	2.07
9	13	-1	1.80	0.90
9	13	-1	0.38	0.90
9	13	-1	0.28	1.60
-9	-13	1	1.90	1.94
-9	-13	1	3.40	3.02
-9	-13	1	-1.18	1.83
8	13	-1	1.00	0.89
8	13	-1	0.79	0.81
8	13	-1	2.90	1.97
-8	-13	1	1.11	1.61
-8	-13	1	-0.66	2.80
-8	-13	1	2.65	1.77
7	13	-1	1.20	0.96
7	13	-1	1.71	3.07
7	13	-1	2.10	1.72
7	13	-1	-0.21	1.40
7	13	-1	0.42	0.78
-7	-13	1	-1.45	1.55
-7	-13	1	-1.47	1.49
-7	-13	1	-0.02	2.72
6	13	-1	-0.74	0.77
6	13	-1	0.94	3.43
6	13	-1	0.73	1.56
6	13	-1	0.31	0.67
6	13	-1	0.78	1.36
-6	-13	1	0.73	2.62
-6	-13	1	-0.75	1.49
-6	-13	1	0.30	1.51
5	13	-1	1.64	1.51
5	13	-1	-1.45	1.33
5	13	-1	-0.74	0.78

5	13	-1	-0.84	3.30
-5	-13	1	0.72	1.43
-5	-13	1	-2.63	2.52
-5	-13	1	-0.93	1.81
4	13	-1	2.39	1.38
4	13	-1	2.03	0.86
4	13	-1	-0.95	2.66
4	13	-1	2.95	1.48
-4	-13	1	2.72	1.36
-4	-13	1	-0.18	2.60
3	13	-1	6.24	0.98
3	13	-1	7.38	1.58
3	13	-1	10.78	1.61
3	13	-1	7.95	2.83
3	13	-1	7.70	1.47
-3	-13	1	7.37	1.65
-3	-13	1	7.50	2.87
2	13	-1	5.99	1.02
2	13	-1	5.98	3.93
2	13	-1	5.17	1.48
2	13	-1	4.31	0.91
2	13	-1	4.39	1.46
-2	-13	1	6.07	2.64
1	13	-1	2.51	1.47
1	13	-1	1.59	0.94
1	13	-1	1.33	0.90
1	13	-1	2.22	1.45
-1	-13	1	-0.23	2.81
0	13	-1	2.67	1.83
0	13	-1	0.77	0.92
0	-13	1	1.02	2.65
-1	13	-1	3.31	1.06
1	-13	1	1.10	2.84
-2	13	-1	0.50	1.02
-3	13	-1	5.29	1.24
13	12	-1	-1.51	2.22
13	12	-1	2.28	3.84
-13	-12	1	0.17	2.96
-13	-12	1	-2.68	2.61
-13	-12	1	4.44	3.69
12	12	-1	-0.17	2.03
12	12	-1	5.06	3.05
-12	-12	1	0.60	3.26
-12	-12	1	1.52	3.53

-12	-12	1	1.26	2.33
11	12	-1	5.55	1.90
11	12	-1	6.53	2.72
-11	-12	1	6.10	2.12
-11	-12	1	3.81	2.42
-11	-12	1	8.13	3.19
10	12	-1	4.20	1.67
10	12	-1	7.39	4.69
10	12	-1	0.45	2.18
-10	-12	1	2.41	2.05
-10	-12	1	1.97	1.88
-10	-12	1	3.56	2.96
9	12	-1	9.53	2.16
9	12	-1	12.23	1.61
9	12	-1	7.53	0.99
9	12	-1	9.75	1.75
-9	-12	1	11.82	3.00
-9	-12	1	8.20	1.90
-9	-12	1	8.08	1.89
8	12	-1	2.96	0.78
8	12	-1	3.44	1.44
8	12	-1	5.27	0.85
8	12	-1	4.52	1.83
-8	-12	1	2.67	1.64
-8	-12	1	2.43	2.56
-8	-12	1	3.14	1.59
7	12	-1	5.28	1.62
7	12	-1	5.25	1.31
7	12	-1	5.47	0.88
7	12	-1	4.80	0.79
-7	-12	1	3.95	1.48
-7	-12	1	5.87	2.48
-7	-12	1	4.46	1.54
6	12	-1	0.21	0.68
6	12	-1	0.06	0.60
6	12	-1	0.46	1.15
6	12	-1	0.03	1.42
-6	-12	1	-1.66	2.32
-6	-12	1	2.02	1.36
-6	-12	1	-1.19	1.33
5	12	-1	1.37	1.30
5	12	-1	1.86	0.63
5	12	-1	1.13	2.29
5	12	-1	1.33	0.71

-5	-12	1	1.24	1.32
-5	-12	1	0.45	2.36
-5	-12	1	1.49	1.27
4	12	-1	-0.24	0.64
4	12	-1	-0.86	1.11
4	12	-1	0.36	0.61
4	12	-1	1.73	1.28
-4	-12	1	-0.06	1.16
-4	-12	1	-1.42	2.30
-4	-12	1	-0.39	1.55
3	12	-1	2.65	0.76
3	12	-1	1.01	0.63
3	12	-1	2.70	1.25
3	12	-1	2.62	1.17
-3	-12	1	1.23	2.33
-3	-12	1	3.45	1.19
2	12	-1	8.47	1.45
2	12	-1	7.16	1.40
2	12	-1	9.08	1.37
-2	-12	1	8.90	2.50
1	12	-1	6.45	0.93
1	12	-1	7.49	1.36
1	12	-1	6.92	1.49
1	12	-1	7.16	1.00
-1	-12	1	9.30	2.60
0	12	-1	6.53	1.02
0	12	-1	8.19	1.06
0	12	-1	9.43	2.78
0	-12	1	10.01	2.68
-1	12	-1	19.36	4.27
-1	12	-1	13.32	1.81
-1	12	-1	10.49	1.76
1	-12	1	10.95	2.80
-2	12	-1	6.13	1.10
-3	12	-1	5.91	1.15
-4	12	-1	3.63	1.12
-14	-11	1	1.77	3.96
-14	-11	1	1.31	3.25
13	11	-1	-1.08	2.04
13	11	-1	0.51	3.43
-13	-11	1	-0.57	3.56
-13	-11	1	2.47	2.74
-13	-11	1	-1.98	2.27
12	11	-1	5.05	2.94

12	11	-1	2.91	1.87
-12	-11	1	-0.33	3.13
-12	-11	1	2.24	3.91
-12	-11	1	1.60	2.01
11	11	-1	1.73	1.61
11	11	-1	0.95	3.71
11	11	-1	1.18	2.38
-11	-11	1	2.86	1.81
-11	-11	1	1.63	2.30
-11	-11	1	3.13	2.82
10	11	-1	5.19	2.11
10	11	-1	4.98	1.55
-10	-11	1	4.03	1.73
-10	-11	1	5.88	2.68
-10	-11	1	5.60	1.98
9	11	-1	5.12	1.43
9	11	-1	3.95	1.95
9	11	-1	3.02	0.75
-9	-11	1	3.26	1.54
-9	-11	1	6.36	2.54
-9	-11	1	3.70	1.75
8	11	-1	12.13	2.29
8	11	-1	8.35	1.25
8	11	-1	9.28	1.90
-8	-11	1	10.17	1.79
-8	-11	1	11.63	1.71
-8	-11	1	11.02	2.51
7	11	-1	2.00	1.15
7	11	-1	1.02	1.49
7	11	-1	2.14	0.69
7	11	-1	0.64	0.58
-7	-11	1	3.26	1.35
-7	-11	1	-0.88	1.36
-7	-11	1	1.44	2.11
6	11	-1	3.69	1.11
6	11	-1	3.61	1.43
6	11	-1	3.48	0.73
6	11	-1	2.88	0.60
-6	-11	1	1.78	1.22
-6	-11	1	2.44	1.27
-6	-11	1	3.08	2.07
5	11	-1	6.06	1.18
5	11	-1	5.30	0.79
5	11	-1	4.81	0.66

5	11	-1	5.10	1.35
-5	-11	1	5.08	1.27
-5	-11	1	3.68	1.29
-5	-11	1	5.19	2.12
4	11	-1	11.99	1.84
4	11	-1	11.90	1.33
4	11	-1	11.58	1.22
4	11	-1	10.26	1.51
-4	-11	1	9.00	1.73
-4	-11	1	11.71	2.05
-4	-11	1	14.03	2.41
3	11	-1	5.16	0.74
3	11	-1	4.88	0.72
3	11	-1	5.39	1.24
3	11	-1	5.04	1.08
-3	-11	1	5.78	2.12
-3	-11	1	5.63	1.18
2	11	-1	4.19	0.70
2	11	-1	3.40	1.73
2	11	-1	2.72	1.17
-2	-11	1	2.40	1.12
-2	-11	1	2.74	2.05
-2	-11	1	2.37	1.18
1	11	-1	-0.14	1.04
1	11	-1	1.05	0.62
1	11	-1	-0.38	1.13
1	11	-1	1.02	0.61
-1	-11	1	-1.34	1.33
-1	-11	1	0.97	2.02
0	11	-1	6.87	1.72
0	11	-1	8.50	1.26
0	11	-1	6.38	0.92
0	-11	1	5.81	2.35
-1	11	-1	5.71	0.92
1	-11	1	4.47	2.27
-2	11	-1	8.84	2.61
-2	11	-1	7.55	1.06
2	-11	1	6.91	2.51
-3	11	-1	3.25	0.94
-4	11	-1	5.20	1.09
-5	11	-1	0.63	0.99
-6	11	-1	4.16	1.19
-15	-10	1	1.45	4.24
-15	-10	1	5.27	3.46

14	10	-1	0.49	1.70
-14	-10	1	1.35	3.62
-14	-10	1	1.82	2.99
13	10	-1	2.09	1.83
13	10	-1	0.77	2.87
-13	-10	1	5.72	3.42
-13	-10	1	0.51	2.08
-13	-10	1	0.95	2.56
12	10	-1	4.04	1.70
12	10	-1	3.06	2.65
12	10	-1	6.88	4.19
-12	-10	1	1.19	2.96
-12	-10	1	3.16	1.97
11	10	-1	2.44	2.16
11	10	-1	-1.11	1.45
-11	-10	1	-2.18	1.63
-11	-10	1	-1.16	1.94
-11	-10	1	-0.14	2.74
10	10	-1	7.97	2.14
10	10	-1	13.06	1.74
-10	-10	1	12.79	2.64
-10	-10	1	10.72	1.85
-10	-10	1	9.58	1.99
9	10	-1	3.71	1.27
9	10	-1	4.12	1.91
-9	-10	1	2.55	1.62
-9	-10	1	1.66	2.16
-9	-10	1	2.96	1.46
8	10	-1	11.46	2.09
8	10	-1	8.73	1.71
8	10	-1	8.78	1.09
-8	-10	1	13.67	2.23
-8	-10	1	6.63	1.63
7	10	-1	7.73	1.23
7	10	-1	6.10	1.46
7	10	-1	4.49	0.67
-7	-10	1	5.68	1.40
-7	-10	1	8.13	1.41
6	10	-1	6.30	0.96
6	10	-1	7.21	1.14
6	10	-1	6.40	1.41
6	10	-1	6.89	2.29
-6	-10	1	7.27	1.26
-6	-10	1	6.89	1.94

-6	-10	1	6.37	1.34
5	10	-1	22.40	1.54
5	10	-1	19.73	2.32
5	10	-1	19.17	1.38
5	10	-1	19.82	3.16
5	10	-1	21.16	2.00
-5	-10	1	21.13	2.18
-5	-10	1	15.97	2.14
-5	-10	1	19.03	2.87
4	10	-1	79.50	4.08
4	10	-1	72.92	4.48
4	10	-1	78.77	4.29
4	10	-1	77.56	5.04
4	10	-1	74.38	4.00
-4	-10	1	80.32	4.43
-4	-10	1	77.86	4.45
-4	-10	1	80.48	5.04
3	10	-1	35.78	2.73
3	10	-1	39.23	2.56
3	10	-1	39.53	2.27
3	10	-1	38.70	3.54
3	10	-1	37.45	2.18
-3	-10	1	31.36	2.59
-3	-10	1	39.01	3.51
-3	-10	1	39.67	2.67
2	10	-1	27.51	2.10
2	10	-1	25.20	1.70
2	10	-1	21.35	2.29
2	10	-1	23.41	3.10
2	10	-1	26.16	1.74
-2	-10	1	25.62	3.18
-2	-10	1	26.04	2.19
-2	-10	1	25.98	3.21
1	10	-1	2.79	0.64
1	10	-1	2.64	0.60
1	10	-1	3.28	0.94
1	10	-1	2.07	1.80
1	10	-1	2.22	1.06
-1	-10	1	2.48	0.89
-1	-10	1	3.67	1.87
0	10	-1	10.88	2.13
0	10	-1	17.81	2.18
0	-10	1	17.27	3.02
-1	10	-1	25.71	2.49

-1	10	-1	24.00	1.83
1	-10	1	21.13	3.19
-2	10	-1	19.88	1.73
2	-10	1	17.63	3.32
-3	10	-1	1.37	2.05
-4	10	-1	22.97	4.26
-4	10	-1	16.69	1.88
-5	10	-1	9.25	1.23
-6	10	-1	8.93	1.24
-7	10	-1	4.17	1.09
-15	-9	1	6.87	3.55
-15	-9	1	1.18	4.23
14	9	-1	1.23	1.58
-14	-9	1	-0.38	2.25
-14	-9	1	1.12	2.92
-14	-9	1	-2.45	1.94
-14	-9	1	-0.87	3.69
13	9	-1	1.88	2.72
13	9	-1	1.75	1.72
-13	-9	1	3.78	3.33
-13	-9	1	1.44	2.50
-13	-9	1	1.55	1.99
12	9	-1	-1.82	2.48
12	9	-1	-1.63	1.40
12	9	-1	-3.70	3.61
-12	-9	1	-3.77	2.83
-12	-9	1	-0.74	1.72
11	9	-1	0.68	2.17
11	9	-1	1.42	1.35
-11	-9	1	-0.05	1.86
-11	-9	1	2.57	2.58
-11	-9	1	1.01	1.54
10	9	-1	1.78	1.21
10	9	-1	1.02	1.78
-10	-9	1	0.06	1.58
-10	-9	1	2.33	1.41
-10	-9	1	-0.02	2.17
9	9	-1	4.59	2.04
9	9	-1	3.90	1.19
-9	-9	1	2.16	2.49
-9	-9	1	4.57	1.32
-9	-9	1	1.99	1.49
8	9	-1	0.34	1.38
8	9	-1	-0.01	0.98

8	9	-1	0.54	2.23
-8	-9	1	0.99	1.13
-8	-9	1	-2.09	1.36
-8	-9	1	-1.45	1.71
7	9	-1	0.58	0.94
7	9	-1	-0.39	1.99
7	9	-1	0.48	1.22
-7	-9	1	0.49	1.00
-7	-9	1	-0.42	1.57
-7	-9	1	-1.33	1.21
6	9	-1	-0.49	1.07
6	9	-1	-0.35	0.84
6	9	-1	1.22	1.77
-6	-9	1	-0.20	0.95
-6	-9	1	-0.94	1.50
-6	-9	1	-1.49	1.08
5	9	-1	11.53	1.02
5	9	-1	14.00	1.66
5	9	-1	15.86	2.65
5	9	-1	11.44	2.00
-5	-9	1	11.73	1.41
-5	-9	1	11.55	1.84
-5	-9	1	12.72	2.42
4	9	-1	16.70	1.68
4	9	-1	13.77	2.61
4	9	-1	16.72	1.19
4	9	-1	14.52	1.97
4	9	-1	17.94	1.28
-4	-9	1	13.44	1.88
-4	-9	1	15.66	2.58
3	9	-1	25.91	2.88
3	9	-1	28.06	1.68
3	9	-1	22.94	2.17
3	9	-1	26.29	1.99
-3	-9	1	24.28	2.89
-3	-9	1	25.84	2.95
-3	-9	1	25.89	2.07
2	9	-1	10.66	1.27
2	9	-1	10.58	1.06
2	9	-1	11.20	1.11
2	9	-1	11.62	1.85
2	9	-1	10.98	1.46
-2	-9	1	13.64	1.97
-2	-9	1	10.41	1.56

1	9	-1	10.84	1.30
1	9	-1	11.16	1.11
1	9	-1	10.06	1.47
1	9	-1	13.63	1.91
-1	-9	1	10.82	1.91
-1	-9	1	9.97	1.51
-1	-9	1	8.50	1.61
0	9	-1	0.42	0.47
0	9	-1	0.20	1.61
0	9	-1	1.12	0.83
0	-9	1	-1.14	1.52
-1	9	-1	9.46	1.18
-1	9	-1	10.20	1.93
-1	9	-1	8.69	1.30
1	-9	1	7.74	1.80
-2	9	-1	-0.26	1.02
-2	9	-1	0.89	1.62
-3	9	-1	13.46	1.50
-4	9	-1	2.38	2.74
-4	9	-1	3.96	0.85
-5	9	-1	13.11	1.69
-5	9	-1	11.79	2.71
-6	9	-1	2.40	0.94
-7	9	-1	6.00	1.10
-8	9	-1	1.18	1.06
-8	9	-1	-0.53	3.37
-15	-8	1	8.15	3.52
-15	-8	1	3.35	4.14
-14	-8	1	10.08	3.64
-14	-8	1	2.04	2.10
-14	-8	1	3.95	3.08
13	8	-1	3.22	2.56
13	8	-1	9.10	1.91
13	8	-1	17.97	4.02
-13	-8	1	8.88	2.71
-13	-8	1	6.95	1.97
-13	-8	1	13.88	3.36
12	8	-1	9.35	1.54
12	8	-1	7.37	5.39
12	8	-1	5.73	2.32
-12	-8	1	6.88	1.80
-12	-8	1	6.72	2.68
-12	-8	1	8.05	2.91
11	8	-1	10.76	1.60

11	8	-1	9.52	2.20
-11	-8	1	13.68	2.64
-11	-8	1	10.80	1.73
-11	-8	1	9.89	2.05
10	8	-1	7.00	1.33
10	8	-1	8.27	1.84
-10	-8	1	7.21	1.41
-10	-8	1	5.24	2.23
-10	-8	1	6.50	1.72
9	8	-1	9.91	1.90
9	8	-1	7.87	2.51
9	8	-1	7.33	1.87
-9	-8	1	5.51	1.56
-9	-8	1	8.35	2.02
-9	-8	1	7.90	1.34
8	8	-1	2.02	1.33
8	8	-1	4.42	2.03
8	8	-1	3.05	1.00
-8	-8	1	5.39	1.69
-8	-8	1	1.44	1.32
-8	-8	1	4.53	1.13
7	8	-1	26.70	2.53
7	8	-1	26.97	2.19
7	8	-1	27.93	3.10
-7	-8	1	26.87	2.66
-7	-8	1	27.24	2.29
-7	-8	1	25.16	2.43
6	8	-1	3.91	1.72
6	8	-1	5.68	0.92
6	8	-1	7.18	1.17
6	8	-1	4.43	0.74
-6	-8	1	6.41	1.05
-6	-8	1	6.04	1.45
-6	-8	1	5.20	1.16
5	8	-1	33.89	2.18
5	8	-1	34.68	2.90
5	8	-1	32.23	2.40
5	8	-1	27.44	1.74
-5	-8	1	28.41	2.33
-5	-8	1	32.09	2.26
-5	-8	1	31.82	2.63
4	8	-1	6.43	1.01
4	8	-1	7.01	1.27
4	8	-1	4.97	0.56

-4	-8	1	7.00	0.96
-4	-8	1	5.66	1.41
-4	-8	1	6.41	1.02
3	8	-1	23.93	1.45
3	8	-1	26.12	1.78
3	8	-1	24.30	1.51
3	8	-1	22.64	1.96
-3	-8	1	25.48	1.91
-3	-8	1	24.05	2.52
-3	-8	1	19.51	2.09
2	8	-1	22.12	1.39
2	8	-1	20.53	1.34
2	8	-1	18.35	1.80
-2	-8	1	20.31	1.69
-2	-8	1	20.40	2.43
1	8	-1	26.11	1.77
1	8	-1	24.14	1.53
1	8	-1	26.95	2.52
1	8	-1	21.01	1.88
-1	-8	1	23.84	2.55
-1	-8	1	17.76	2.34
-1	-8	1	25.49	1.95
0	8	-1	26.70	2.60
0	8	-1	24.39	1.55
0	8	-1	20.32	1.88
0	8	-1	24.98	1.76
0	-8	1	20.33	2.51
-1	8	-1	21.73	2.64
-1	8	-1	21.16	1.46
-1	8	-1	17.72	2.64
1	-8	1	16.59	2.51
-2	8	-1	16.80	1.40
-2	8	-1	19.78	2.74
2	-8	1	15.62	2.42
-3	8	-1	15.87	1.46
-3	8	-1	18.04	2.82
-6	8	-1	19.66	3.65
-6	8	-1	21.47	1.89
-7	8	-1	29.66	5.90
-7	8	-1	20.63	1.99
-8	8	-1	11.83	1.82
-8	8	-1	11.75	3.37
-9	8	-1	7.96	1.25
-9	8	-1	8.76	3.74

-15	-7	1	3.42	3.37
-15	-7	1	1.93	3.98
-14	-7	1	-0.09	1.91
-14	-7	1	1.44	3.61
-14	-7	1	3.37	2.97
-14	-7	1	-0.54	1.85
13	7	-1	1.31	1.42
13	7	-1	0.86	2.33
13	7	-1	4.77	3.75
-13	-7	1	4.02	3.24
-13	-7	1	-0.98	2.42
-13	-7	1	2.25	1.74
12	7	-1	10.16	2.29
12	7	-1	14.40	2.20
-12	-7	1	9.71	2.31
-12	-7	1	10.81	1.81
-12	-7	1	12.09	3.00
11	7	-1	7.47	1.33
11	7	-1	7.44	2.00
-11	-7	1	6.13	1.93
-11	-7	1	9.09	2.49
-11	-7	1	6.86	1.51
10	7	-1	49.76	3.89
10	7	-1	47.90	4.42
10	7	-1	51.07	3.24
-10	-7	1	50.93	4.07
-10	-7	1	46.15	3.37
-10	-7	1	46.50	3.73
9	7	-1	2.36	0.99
9	7	-1	0.16	2.07
9	7	-1	2.55	1.45
-9	-7	1	1.75	1.37
-9	-7	1	3.31	1.08
-9	-7	1	1.85	1.67
8	7	-1	4.14	1.94
8	7	-1	5.05	1.32
8	7	-1	5.52	0.99
-8	-7	1	5.94	1.29
-8	-7	1	6.64	1.12
-8	-7	1	6.57	1.63
7	7	-1	-0.40	1.57
7	7	-1	1.63	1.04
7	7	-1	0.16	0.73
-7	-7	1	0.38	1.01

-7	-7	1	1.36	0.83
-7	-7	1	0.14	1.25
6	7	-1	25.93	2.23
6	7	-1	25.69	2.73
6	7	-1	28.54	1.98
-6	-7	1	24.47	2.34
-6	-7	1	26.76	2.17
-6	-7	1	28.05	2.07
5	7	-1	9.91	1.31
5	7	-1	7.32	0.75
5	7	-1	10.16	2.03
5	7	-1	9.22	1.56
-5	-7	1	8.45	1.12
-5	-7	1	9.55	1.39
-5	-7	1	10.05	1.36
4	7	-1	53.22	3.19
4	7	-1	58.87	3.50
4	7	-1	50.81	2.80
4	7	-1	54.20	3.04
-4	-7	1	54.48	3.14
-4	-7	1	57.22	3.43
-4	-7	1	57.03	3.14
3	7	-1	7.75	0.98
3	7	-1	8.87	1.43
3	7	-1	7.52	1.10
3	7	-1	8.29	0.74
-3	-7	1	8.46	1.24
-3	-7	1	5.56	1.00
2	7	-1	13.08	1.49
2	7	-1	16.43	1.14
2	7	-1	17.91	1.39
2	7	-1	17.18	2.00
2	7	-1	16.20	1.08
-2	-7	1	16.89	1.43
-2	-7	1	15.68	2.09
1	7	-1	11.26	1.22
1	7	-1	10.03	1.28
1	7	-1	10.84	0.93
1	7	-1	11.78	1.91
-1	-7	1	9.16	1.87
-1	-7	1	11.00	1.20
0	7	-1	27.62	1.66
0	7	-1	26.32	1.86
0	7	-1	30.01	2.44

0	7	-1	27.50	1.82
0	-7	1	30.17	1.95
0	-7	1	24.80	2.43
-1	7	-1	23.10	1.61
-1	7	-1	21.45	1.42
1	-7	1	19.45	2.31
-2	7	-1	9.18	1.00
-2	7	-1	10.63	2.11
2	-7	1	9.55	1.84
-3	7	-1	2.86	0.60
-3	7	-1	5.93	1.52
-4	7	-1	22.20	2.80
-4	7	-1	16.71	1.45
-5	7	-1	9.95	1.64
-7	7	-1	3.21	0.83
-7	7	-1	5.47	2.22
-8	7	-1	10.78	3.03
-8	7	-1	8.63	3.18
-8	7	-1	8.86	1.41
-8	7	-1	10.85	1.61
-9	7	-1	2.32	0.98
-9	7	-1	5.29	3.57
-10	7	-1	7.11	4.05
-10	7	-1	7.02	1.27
-15	-6	1	-0.50	3.74
-15	-6	1	4.62	4.31
-15	-6	1	2.40	3.45
-14	-6	1	1.90	3.84
-14	-6	1	0.43	3.05
-14	-6	1	1.71	1.72
-14	-6	1	2.20	2.92
-14	-6	1	0.31	1.76
13	6	-1	2.23	1.39
13	6	-1	-4.88	3.38
-13	-6	1	-1.61	2.35
-13	-6	1	-0.75	3.32
-13	-6	1	2.74	1.61
-13	-6	1	2.10	2.61
12	6	-1	11.17	2.27
-12	-6	1	19.98	3.16
-12	-6	1	14.81	2.59
-12	-6	1	14.10	2.35
11	6	-1	7.06	1.88
11	6	-1	10.15	2.99

11	6	-1	6.97	1.37
-11	-6	1	5.03	1.82
-11	-6	1	11.78	2.47
-11	-6	1	7.13	1.47
10	6	-1	12.68	1.85
10	6	-1	13.08	2.09
10	6	-1	10.61	2.47
-10	-6	1	15.01	2.85
-10	-6	1	12.99	2.05
-10	-6	1	9.94	1.73
9	6	-1	22.13	2.58
9	6	-1	20.22	3.12
9	6	-1	23.61	2.12
-9	-6	1	18.00	2.50
-9	-6	1	19.57	2.70
-9	-6	1	20.57	2.12
8	6	-1	19.32	1.78
8	6	-1	18.76	2.30
8	6	-1	16.55	2.17
-8	-6	1	15.61	2.26
-8	-6	1	17.47	2.23
-8	-6	1	20.31	1.94
7	6	-1	18.49	2.53
7	6	-1	24.39	1.83
7	6	-1	23.43	2.16
-7	-6	1	23.90	1.91
-7	-6	1	17.86	2.19
-7	-6	1	23.40	2.11
6	6	-1	16.71	2.21
6	6	-1	16.75	1.52
6	6	-1	18.94	1.86
-6	-6	1	16.83	1.60
-6	-6	1	15.06	1.88
-6	-6	1	15.95	1.76
5	6	-1	31.52	1.89
5	6	-1	28.03	2.42
5	6	-1	30.00	2.12
5	6	-1	27.11	1.56
-5	-6	1	27.27	2.21
-5	-6	1	30.78	2.04
-5	-6	1	30.97	1.96
4	6	-1	47.89	2.65
4	6	-1	52.38	2.88
4	6	-1	52.79	3.01

4	6	-1	54.35	3.25
-4	-6	1	53.15	2.93
-4	-6	1	54.00	2.98
-4	-6	1	53.09	3.16
3	6	-1	2.33	0.54
3	6	-1	1.70	0.62
3	6	-1	1.66	0.31
3	6	-1	1.79	1.03
-3	-6	1	0.91	0.69
-3	-6	1	2.75	0.60
-3	-6	1	1.99	0.99
2	6	-1	49.50	2.66
2	6	-1	47.57	2.73
2	6	-1	46.76	3.06
2	6	-1	48.92	2.55
-2	-6	1	49.92	2.70
-2	-6	1	48.23	3.08
-2	-6	1	52.17	2.76
1	6	-1	9.16	1.11
1	6	-1	10.40	1.05
1	6	-1	10.43	1.65
1	6	-1	8.23	0.80
-1	-6	1	9.26	1.66
-1	-6	1	9.57	1.03
0	6	-1	43.53	2.81
0	6	-1	41.73	2.37
0	6	-1	43.01	2.28
0	6	-1	44.18	2.37
0	-6	1	39.81	2.80
-1	6	-1	34.72	1.87
-1	6	-1	34.52	2.52
1	-6	1	29.38	2.50
-2	6	-1	30.24	2.32
-2	6	-1	25.99	1.63
2	-6	1	26.10	2.20
-3	6	-1	38.27	2.96
-3	6	-1	35.07	2.09
-4	6	-1	25.58	2.56
-4	6	-1	24.13	1.64
-5	6	-1	28.06	2.84
-5	6	-1	25.66	1.77
-6	6	-1	4.09	1.63
-7	6	-1	19.67	1.67
-7	6	-1	26.04	3.60

-8	6	-1	16.73	3.15
-8	6	-1	16.26	1.58
-8	6	-1	11.31	2.37
-9	6	-1	21.96	1.95
-9	6	-1	25.42	4.71
-10	6	-1	6.19	1.11
-10	6	-1	9.11	4.00
-11	6	-1	22.86	4.90
-15	-5	1	12.32	3.37
-15	-5	1	10.39	4.30
-15	-5	1	6.75	4.06
-14	-5	1	2.32	1.82
-14	-5	1	0.37	2.78
-14	-5	1	-0.97	3.64
-14	-5	1	4.96	3.28
-14	-5	1	-1.08	2.14
13	5	-1	3.99	3.29
-13	-5	1	6.71	1.61
-13	-5	1	7.85	2.47
-13	-5	1	7.81	3.35
-13	-5	1	7.99	2.86
-13	-5	1	7.24	1.68
12	5	-1	3.62	1.86
-12	-5	1	6.02	2.78
-12	-5	1	4.31	2.30
-12	-5	1	2.53	2.00
-12	-5	1	5.28	1.45
11	5	-1	-1.93	2.49
11	5	-1	1.45	1.65
-11	-5	1	1.23	3.08
-11	-5	1	1.23	1.18
-11	-5	1	-0.38	1.68
10	5	-1	0.71	1.41
10	5	-1	-2.80	2.21
10	5	-1	-0.31	0.75
-10	-5	1	0.16	1.83
-10	-5	1	-0.31	1.03
-10	-5	1	-0.75	1.42
9	5	-1	18.69	3.07
9	5	-1	25.36	2.56
9	5	-1	27.28	2.16
-9	-5	1	25.42	2.16
-9	-5	1	24.23	2.79
-9	-5	1	24.13	2.52

8	5	-1	31.92	2.27
8	5	-1	33.06	2.55
-8	-5	1	28.24	2.62
-8	-5	1	29.92	2.48
-8	-5	1	29.01	2.23
7	5	-1	45.29	2.58
7	5	-1	42.25	2.80
7	5	-1	36.41	3.04
-7	-5	1	39.59	2.82
-7	-5	1	42.64	2.75
-7	-5	1	41.95	2.56
6	5	-1	9.12	1.54
6	5	-1	8.18	1.15
6	5	-1	7.77	1.40
-6	-5	1	6.70	1.20
-6	-5	1	8.22	1.23
-6	-5	1	6.75	0.99
5	5	-1	71.02	4.00
5	5	-1	71.56	3.71
5	5	-1	72.83	3.84
-5	-5	1	71.26	3.81
-5	-5	1	68.75	3.86
-5	-5	1	67.68	3.73
4	5	-1	69.66	3.64
4	5	-1	67.25	3.53
4	5	-1	68.89	3.81
4	5	-1	62.25	3.35
-4	-5	1	69.52	3.58
-4	-5	1	66.20	3.71
-4	-5	1	72.01	3.62
3	5	-1	87.50	4.60
3	5	-1	84.27	4.38
3	5	-1	80.04	4.24
3	5	-1	89.49	4.46
-3	-5	1	88.20	4.56
-3	-5	1	86.45	4.41
-3	-5	1	89.66	4.44
2	5	-1	0.87	0.48
2	5	-1	-0.28	0.75
2	5	-1	1.40	0.42
-2	-5	1	1.36	0.79
-2	-5	1	1.17	0.45
1	5	-1	64.64	3.22
1	5	-1	60.05	3.22

1	5	-1	61.65	3.50
1	5	-1	62.92	3.14
-1	-5	1	61.74	3.21
-1	-5	1	61.17	3.51
0	5	-1	42.96	2.24
0	5	-1	39.79	2.24
0	5	-1	41.37	2.17
0	5	-1	38.21	2.59
0	-5	1	37.19	2.55
0	-5	1	47.49	2.33
0	-5	1	40.15	2.22
-1	5	-1	137.80	6.64
-1	5	-1	137.51	6.63
-1	5	-1	135.53	6.91
1	-5	1	126.83	6.88
-2	5	-1	48.73	2.49
-2	5	-1	48.97	2.93
2	-5	1	42.57	2.82
-3	5	-1	29.31	2.23
-3	5	-1	25.74	1.62
-4	5	-1	24.97	1.56
-5	5	-1	15.74	2.15
-5	5	-1	13.02	1.17
-6	5	-1	1.76	1.55
-6	5	-1	0.93	0.54
-7	5	-1	6.20	1.05
-7	5	-1	9.05	1.12
7	-5	1	7.26	1.21
-8	5	-1	2.82	1.20
-8	5	-1	7.16	2.53
-9	5	-1	15.99	2.56
-9	5	-1	20.44	1.69
-9	5	-1	23.14	4.40
-9	5	-1	17.17	2.32
-10	5	-1	12.37	2.48
-10	5	-1	12.67	1.70
-10	5	-1	20.99	3.99
-11	5	-1	7.52	1.18
-12	5	-1	13.91	5.19
-15	-4	1	6.22	3.24
-15	-4	1	6.72	4.20
-15	-4	1	5.71	4.25
-14	-4	1	4.00	3.67
-14	-4	1	2.29	3.77

13	4	-1	12.03	3.38
-13	-4	1	15.46	2.57
-13	-4	1	9.92	1.83
-13	-4	1	9.61	1.68
-13	-4	1	13.36	3.21
-13	-4	1	12.75	3.53
-12	-4	1	4.10	1.42
-12	-4	1	2.68	2.53
-12	-4	1	4.77	2.82
-12	-4	1	7.55	2.09
11	4	-1	0.75	2.46
11	4	-1	6.20	1.14
-11	-4	1	6.78	2.21
-11	-4	1	7.20	1.27
-11	-4	1	6.84	1.90
-11	-4	1	5.32	1.24
10	4	-1	4.39	2.35
-10	-4	1	9.95	1.61
-10	-4	1	8.36	1.81
-10	-4	1	9.36	1.76
-10	-4	1	10.13	2.19
9	4	-1	27.29	2.40
-9	-4	1	22.38	2.77
-9	-4	1	24.38	2.04
-9	-4	1	19.84	2.82
-9	-4	1	24.70	2.43
8	4	-1	51.78	3.86
8	4	-1	59.67	3.46
-8	-4	1	55.86	3.20
-8	-4	1	58.05	3.42
-8	-4	1	54.33	3.58
-8	-4	1	52.05	3.61
7	4	-1	50.97	3.38
7	4	-1	52.10	2.96
7	4	-1	53.97	3.18
-7	-4	1	53.32	3.16
-7	-4	1	54.56	3.24
-7	-4	1	49.56	2.97
6	4	-1	46.69	2.82
6	4	-1	48.35	2.88
6	4	-1	47.36	3.03
-6	-4	1	45.76	2.69
-6	-4	1	49.24	2.82
-6	-4	1	47.94	2.88

5	4	-1	49.63	3.15
5	4	-1	57.39	3.05
5	4	-1	54.64	2.91
-5	-4	1	52.34	2.91
-5	-4	1	51.74	3.03
-5	-4	1	56.93	3.01
4	4	-1	60.37	3.35
4	4	-1	62.15	3.23
4	4	-1	58.64	3.12
-4	-4	1	58.03	3.15
-4	-4	1	62.36	3.22
-4	-4	1	59.31	3.26
3	4	-1	18.99	1.51
3	4	-1	20.80	1.33
3	4	-1	18.56	1.20
3	4	-1	16.36	1.00
-3	-4	1	18.13	1.43
-3	-4	1	19.41	1.32
-3	-4	1	19.59	1.25
2	4	-1	80.08	3.98
2	4	-1	74.61	3.93
2	4	-1	83.02	4.13
-2	-4	1	77.12	4.09
-2	-4	1	79.53	3.98
-2	-4	1	75.94	3.95
1	4	-1	52.24	2.81
1	4	-1	57.39	2.87
1	4	-1	56.96	3.09
1	4	-1	55.32	2.88
-1	-4	1	58.50	3.11
-1	-4	1	55.94	2.87
0	4	-1	136.71	6.55
0	4	-1	140.84	6.57
0	4	-1	129.54	6.74
0	4	-1	136.65	6.56
0	-4	1	122.63	6.73
0	-4	1	136.57	6.57
-1	4	-1	45.88	2.58
-1	4	-1	44.63	2.27
1	-4	1	41.32	2.56
-2	4	-1	105.00	5.34
-2	4	-1	103.30	5.34
-2	4	-1	106.06	5.10
2	-4	1	97.10	5.29

-3	4	-1	91.48	4.90
-3	4	-1	96.67	4.64
3	-4	1	89.58	4.66
-4	4	-1	39.44	2.13
-4	4	-1	44.36	2.64
4	-4	1	36.83	2.32
4	-4	1	36.54	2.18
-5	4	-1	44.05	3.96
-5	4	-1	52.39	2.70
-5	4	-1	50.66	2.86
5	-4	1	52.78	2.78
-6	4	-1	40.54	2.27
-6	4	-1	41.17	3.00
6	-4	1	39.27	2.39
-7	4	-1	15.35	2.32
-7	4	-1	14.12	1.72
-7	4	-1	14.01	1.22
-7	4	-1	17.86	2.46
7	-4	1	14.30	1.94
-8	4	-1	26.06	3.74
-8	4	-1	22.72	1.64
-8	4	-1	22.00	2.19
8	-4	1	24.83	2.60
-9	4	-1	11.88	2.83
-9	4	-1	9.98	1.49
-9	4	-1	7.47	1.27
-10	4	-1	23.75	1.95
-10	4	-1	25.23	2.72
-10	4	-1	28.89	3.60
-10	4	-1	35.30	4.99
-11	4	-1	11.10	1.71
-11	4	-1	15.33	4.16
-12	4	-1	13.89	2.06
-12	4	-1	21.55	4.96
-15	-3	1	3.76	4.40
-15	-3	1	-0.68	4.17
-14	-3	1	7.39	3.95
-14	-3	1	5.11	3.93
13	3	-1	1.35	3.20
-13	-3	1	10.81	2.46
-13	-3	1	8.73	2.07
-13	-3	1	5.84	3.57
-13	-3	1	5.44	1.60
-13	-3	1	11.79	3.32

-12	-3	1	15.03	2.94
-12	-3	1	15.51	3.32
-12	-3	1	13.89	2.30
11	3	-1	-2.38	2.35
-11	-3	1	1.94	1.23
-11	-3	1	-2.44	3.48
-11	-3	1	0.44	1.08
10	3	-1	14.11	2.43
-10	-3	1	20.33	2.02
-10	-3	1	24.87	3.29
-10	-3	1	17.20	3.22
9	3	-1	19.11	1.73
9	3	-1	15.32	2.26
-9	-3	1	18.81	2.84
-9	-3	1	19.30	1.83
-9	-3	1	17.07	1.83
-9	-3	1	22.96	2.77
-9	-3	1	19.14	2.26
8	3	-1	60.63	3.46
8	3	-1	55.37	3.84
-8	-3	1	57.19	3.77
-8	-3	1	54.98	3.27
-8	-3	1	61.52	3.82
-8	-3	1	59.19	3.50
7	3	-1	9.79	2.00
7	3	-1	13.07	1.65
-7	-3	1	14.06	1.67
-7	-3	1	15.07	1.97
-7	-3	1	12.02	1.85
-7	-3	1	14.99	1.36
6	3	-1	43.00	2.89
6	3	-1	45.38	2.67
-6	-3	1	45.48	2.52
-6	-3	1	43.47	2.77
-6	-3	1	46.74	2.66
5	3	-1	34.63	2.34
5	3	-1	38.40	2.19
-5	-3	1	34.03	2.04
-5	-3	1	35.70	2.24
-5	-3	1	36.99	2.18
4	3	-1	14.15	1.17
4	3	-1	11.61	1.32
-4	-3	1	14.13	1.01
-4	-3	1	13.66	1.12

-4	-3	1	11.44	1.19
3	3	-1	14.96	1.28
3	3	-1	16.05	1.06
3	3	-1	16.57	1.16
-3	-3	1	16.55	1.13
-3	-3	1	16.46	1.20
-3	-3	1	16.15	1.07
2	3	-1	101.62	4.80
2	3	-1	95.82	4.87
2	3	-1	92.54	4.76
-2	-3	1	93.17	4.77
-2	-3	1	99.98	4.79
-2	-3	1	95.31	4.84
1	3	-1	13.61	0.77
1	3	-1	14.08	0.88
1	3	-1	12.93	0.91
1	3	-1	11.74	1.14
-1	-3	1	12.84	1.16
-1	-3	1	13.86	0.88
0	3	-1	59.91	2.97
0	3	-1	57.03	3.13
0	3	-1	58.56	2.94
0	3	-1	63.97	2.97
0	-3	1	55.74	3.13
0	-3	1	59.19	2.97
-1	3	-1	63.02	3.06
-1	3	-1	63.20	3.08
-1	3	-1	60.03	3.24
1	-3	1	58.08	3.22
1	-3	1	61.92	3.08
-2	3	-1	52.13	2.57
-2	3	-1	49.53	2.78
2	-3	1	49.81	2.59
-3	3	-1	12.98	0.89
-3	3	-1	14.94	1.35
3	-3	1	12.23	0.92
-4	3	-1	38.87	2.00
-4	3	-1	39.37	2.41
4	-3	1	33.27	2.18
4	-3	1	37.76	2.04
-5	3	-1	22.99	1.93
-5	3	-1	18.64	1.22
5	-3	1	18.59	1.57
5	-3	1	18.86	1.30

-6	3	-1	75.42	3.97
-6	3	-1	81.96	4.65
6	-3	1	79.05	4.26
6	-3	1	77.57	4.06
-7	3	-1	50.65	2.95
-7	3	-1	47.81	2.59
-7	3	-1	49.45	3.67
7	-3	1	41.76	3.31
7	-3	1	49.70	3.04
-8	3	-1	45.75	2.64
-8	3	-1	48.34	3.11
-8	3	-1	54.26	4.03
8	-3	1	46.27	2.81
-9	3	-1	2.10	1.05
-9	3	-1	6.26	2.51
-9	3	-1	3.43	0.73
9	-3	1	4.70	0.84
-10	3	-1	10.51	2.19
-10	3	-1	9.80	1.36
-10	3	-1	13.35	3.17
-11	3	-1	12.91	1.65
-11	3	-1	15.99	2.64
-11	3	-1	18.12	3.89
-12	3	-1	12.64	4.56
-12	3	-1	6.26	1.19
-13	3	-1	15.50	5.22
-15	-2	1	7.43	4.67
-15	-2	1	4.34	4.91
-14	-2	1	8.26	4.13
-14	-2	1	7.19	4.26
13	2	-1	8.59	3.19
-13	-2	1	12.55	3.74
-13	-2	1	10.86	3.58
-12	-2	1	16.38	3.23
11	2	-1	15.32	2.72
-11	-2	1	20.45	2.37
-11	-2	1	17.78	2.28
-11	-2	1	21.48	3.11
10	2	-1	37.54	3.56
-10	-2	1	38.28	2.80
-10	-2	1	38.90	2.82
-10	-2	1	39.65	3.78
9	2	-1	47.10	3.63
-9	-2	1	47.07	3.70

-9	-2	1	46.25	2.95
8	2	-1	5.98	1.64
-8	-2	1	8.95	1.34
-8	-2	1	6.96	1.61
-8	-2	1	6.24	1.12
-8	-2	1	6.13	0.90
7	2	-1	55.00	3.39
-7	-2	1	50.75	2.94
-7	-2	1	56.65	3.36
-7	-2	1	50.24	2.97
-7	-2	1	54.54	3.11
6	2	-1	33.21	2.44
6	2	-1	33.60	2.11
-6	-2	1	33.08	2.35
-6	-2	1	31.65	1.95
-6	-2	1	32.66	2.13
5	2	-1	158.79	7.90
5	2	-1	157.62	7.81
-5	-2	1	157.23	7.89
-5	-2	1	160.03	7.81
-5	-2	1	154.21	7.75
4	2	-1	110.85	5.50
4	2	-1	116.39	5.43
-4	-2	1	104.30	5.45
-4	-2	1	99.47	5.36
-4	-2	1	109.78	5.41
-4	-2	1	110.49	5.50
3	2	-1	233.33	10.46
3	2	-1	203.44	10.48
-3	-2	1	201.06	10.46
-3	-2	1	207.88	10.42
-3	-2	1	227.87	10.45
2	2	-1	57.06	3.24
2	2	-1	68.73	3.25
2	2	-1	67.40	3.29
-2	-2	1	63.87	3.26
-2	-2	1	62.88	3.21
-2	-2	1	66.23	3.24
1	2	-1	75.90	4.38
1	2	-1	87.30	4.42
1	2	-1	104.61	4.52
1	2	-1	95.17	4.43
-1	-2	1	83.54	4.42
-1	-2	1	85.22	4.42

-1	-2	1	101.21	4.51
0	2	-1	107.53	5.64
0	2	-1	127.23	5.56
0	2	-1	117.38	5.54
0	-2	1	102.92	5.64
0	-2	1	115.12	5.54
-1	2	-1	158.56	7.53
-1	2	-1	159.23	7.50
-1	2	-1	153.86	7.58
1	-2	1	143.83	7.57
1	-2	1	157.33	7.50
-2	2	-1	119.25	5.96
-2	2	-1	120.96	5.86
2	-2	1	120.47	5.87
-3	2	-1	196.08	9.15
3	-2	1	191.04	9.17
3	-2	1	178.53	9.21
-4	2	-1	57.64	3.06
-4	2	-1	61.52	2.95
4	-2	1	56.57	2.97
4	-2	1	57.49	3.09
-5	2	-1	84.67	4.48
-5	2	-1	88.46	4.34
-5	2	-1	93.26	4.75
5	-2	1	83.14	4.52
5	-2	1	87.55	4.39
-6	2	-1	44.04	2.97
-6	2	-1	44.97	2.27
-6	2	-1	41.92	2.53
6	-2	1	42.39	2.33
6	-2	1	41.24	2.60
-7	2	-1	77.62	4.59
-7	2	-1	75.34	4.11
-7	2	-1	75.20	3.85
7	-2	1	76.19	3.94
7	-2	1	75.13	4.19
-8	2	-1	66.07	4.21
-8	2	-1	60.12	3.14
-8	2	-1	56.49	3.48
8	-2	1	56.50	3.26
-9	2	-1	71.54	5.06
-9	2	-1	66.43	3.53
-9	2	-1	64.73	3.95
9	-2	1	66.34	3.68

-10	2	-1	14.81	1.50
-10	2	-1	17.49	3.01
10	-2	1	18.54	1.80
-11	2	-1	25.26	2.64
-11	2	-1	24.23	1.97
-11	2	-1	26.22	4.95
-12	2	-1	15.65	4.05
-12	2	-1	7.32	1.17
-13	2	-1	10.23	1.51
-13	2	-1	20.68	4.81
-15	-1	1	3.56	4.81
-15	-1	1	0.62	5.20
-14	-1	1	0.53	4.63
-14	-1	1	7.47	4.20
-13	-1	1	15.97	3.90
-12	-1	1	7.67	3.21
11	1	-1	21.47	3.27
-11	-1	1	23.54	3.81
10	1	-1	23.04	3.03
-10	-1	1	19.63	3.29
-10	-1	1	19.57	2.18
9	1	-1	32.52	3.06
-9	-1	1	29.97	2.35
-9	-1	1	32.11	3.18
8	1	-1	35.31	3.20
-8	-1	1	33.69	2.90
7	1	-1	12.49	1.95
-7	-1	1	9.28	1.31
-7	-1	1	10.73	1.33
-7	-1	1	10.17	1.90
6	1	-1	10.02	1.67
-6	-1	1	9.74	1.23
-6	-1	1	6.78	1.12
-6	-1	1	9.37	1.64
5	1	-1	15.70	1.56
-5	-1	1	13.97	1.51
4	1	-1	13.88	1.28
-4	-1	1	11.93	1.02
-4	-1	1	11.91	1.22
3	1	-1	39.17	1.97
3	1	-1	32.78	1.87
-3	-1	1	36.14	1.94
-3	-1	1	31.87	1.80
-3	-1	1	32.34	1.86

2	1	-1	1.58	0.41
2	1	-1	1.35	0.38
2	1	-1	1.12	0.28
-2	-1	1	1.03	0.21
-2	-1	1	1.29	0.34
-2	-1	1	1.29	0.39
-2	-1	1	0.68	0.26
1	1	-1	200.00	9.69
1	1	-1	213.41	9.67
-1	-1	1	187.54	9.66
0	1	-1	62.76	3.00
0	1	-1	61.46	3.07
0	-1	1	61.90	3.00
0	-1	1	59.17	2.98
-1	1	-1	147.51	7.22
-1	1	-1	139.87	7.24
-1	1	-1	167.09	7.22
-1	1	-1	150.89	7.20
1	-1	1	140.56	7.22
1	-1	1	149.11	7.21
-2	1	-1	4.83	0.38
-2	1	-1	5.32	0.47
-2	1	-1	4.03	0.52
2	-1	1	4.44	0.38
2	-1	1	4.12	0.55
-3	1	-1	65.54	3.05
-3	1	-1	58.28	3.11
3	-1	1	63.75	3.06
3	-1	1	58.66	3.13
-4	1	-1	9.50	0.92
-4	1	-1	11.31	0.69
-4	1	-1	10.12	1.25
4	-1	1	9.76	0.95
4	-1	1	11.09	0.72
-5	1	-1	29.01	1.89
-5	1	-1	32.81	1.66
-5	1	-1	32.56	2.20
5	-1	1	30.46	1.93
5	-1	1	31.30	1.69
-6	1	-1	14.02	1.34
-6	1	-1	14.91	1.86
-6	1	-1	15.16	0.96
6	-1	1	14.19	1.00
6	-1	1	13.63	1.38

-7	1	-1	61.61	3.43
-7	1	-1	63.36	3.81
-7	1	-1	62.62	3.14
7	-1	1	59.17	3.18
7	-1	1	60.41	3.46
-8	1	-1	40.92	2.66
-8	1	-1	43.12	2.28
-8	1	-1	41.96	3.32
8	-1	1	38.99	2.33
-9	1	-1	32.75	3.50
-9	1	-1	34.98	2.05
-9	1	-1	32.48	2.54
9	-1	1	34.79	2.12
-10	1	-1	14.94	2.77
-10	1	-1	11.10	1.41
10	-1	1	12.57	1.52
-11	1	-1	20.45	1.90
-11	1	-1	22.17	4.40
11	-1	1	27.50	2.10
-12	1	-1	13.10	2.64
-12	1	-1	13.58	3.59
12	-1	1	15.41	2.09
-13	1	-1	16.71	4.36
-13	1	-1	7.81	1.47
-14	1	-1	14.52	5.19
-14	0	1	10.49	4.68
-13	0	1	18.67	4.27
-12	0	1	15.55	3.43
-11	0	1	17.05	3.05
-10	0	1	48.74	4.19
-9	0	1	34.96	2.59
-9	0	1	37.93	3.43
8	0	-1	77.15	4.77
8	0	-1	64.91	3.56
-8	0	1	74.09	4.37
-8	0	1	69.05	3.54
-8	0	1	66.06	3.89
7	0	-1	35.08	1.98
-7	0	1	43.39	2.83
-7	0	1	34.13	2.34
-6	0	1	69.60	3.63
-6	0	1	77.53	4.14
5	0	-1	17.43	0.99
-5	0	1	17.11	1.33

4	0	-1	160.97	7.73
-4	0	1	150.09	7.72
3	0	-1	11.75	1.07
-3	0	1	10.20	1.06
2	0	-1	151.14	7.10
-2	0	1	139.73	7.10
-1	0	-1	112.31	5.52
1	0	1	115.71	5.58
-2	0	-1	82.01	4.22
-2	0	-1	89.40	4.23
-2	0	-1	96.24	4.29
-2	0	-1	88.22	4.19
-2	0	-1	76.68	4.22
2	0	1	90.18	4.23
2	0	1	76.02	4.22
2	0	1	94.41	4.27
2	0	1	84.23	4.19
-3	0	-1	2.65	0.30
-3	0	-1	1.93	0.36
-3	0	-1	2.00	0.60
3	0	1	1.91	0.35
3	0	1	2.04	0.29
3	0	1	1.16	0.32
-4	0	-1	98.33	4.92
-4	0	-1	103.64	5.06
-4	0	-1	96.78	4.93
-4	0	-1	92.35	4.90
-4	0	-1	105.80	4.84
4	0	1	97.05	4.84
4	0	1	104.46	5.03
4	0	1	94.14	4.94
-5	0	-1	45.74	2.52
-5	0	-1	44.78	2.57
-5	0	-1	47.21	2.57
-5	0	-1	45.87	2.78
-5	0	-1	52.13	2.40
5	0	1	48.99	2.73
5	0	1	45.05	2.58
5	0	1	47.87	2.40
-6	0	-1	39.26	1.97
-6	0	-1	36.08	2.22
-6	0	-1	38.29	2.57
6	0	1	36.33	2.24
6	0	1	36.71	1.96

-7	0	-1	74.83	3.62
-7	0	-1	70.43	3.85
-7	0	-1	69.91	4.20
7	0	1	70.28	3.61
-8	0	-1	105.24	5.86
-8	0	-1	102.77	5.45
-8	0	-1	105.39	5.22
8	0	1	103.83	5.22
-9	0	-1	12.80	1.76
-9	0	-1	18.56	2.92
-9	0	-1	15.09	1.31
9	0	1	15.48	1.25
-10	0	-1	32.88	4.44
-10	0	-1	36.18	2.35
10	0	1	42.75	2.36
-11	0	-1	17.80	1.75
-11	0	-1	22.63	3.87
11	0	1	22.34	1.80
-12	0	-1	32.76	4.81
12	0	1	27.96	2.33
-13	0	-1	18.16	4.10
13	0	1	18.45	2.46
-14	0	-1	10.75	2.18
-14	1	1	7.31	4.79
-13	1	1	11.73	4.17
-12	1	1	16.54	1.96
-12	1	1	24.17	2.78
-12	1	1	27.80	4.95
-11	1	1	24.71	4.24
-11	1	1	17.11	1.67
-10	1	1	62.72	6.30
-10	1	1	62.22	3.36
-9	1	1	20.14	3.08
-9	1	1	12.22	1.95
-9	1	1	18.00	1.31
-8	1	1	64.27	3.19
-8	1	1	61.79	4.14
-8	1	1	59.00	3.57
7	-1	-1	55.80	3.02
-7	1	1	60.99	3.68
-7	1	1	58.28	3.31
-7	1	1	57.87	2.96
6	-1	-1	57.16	3.04
-6	1	1	59.15	3.27

-6	1	1	60.10	3.00
-6	1	1	62.24	3.52
5	-1	-1	189.34	9.35
-5	1	1	194.66	9.48
-5	1	1	195.35	9.32
-5	1	1	190.01	9.57
4	-1	-1	165.47	8.36
-4	1	1	169.78	8.53
-4	1	1	173.72	8.35
-4	1	1	180.35	8.48
-3	1	1	119.71	6.16
-3	1	1	139.47	6.31
-3	1	1	122.43	6.26
-2	1	1	314.19	15.02
-2	1	1	307.34	14.96
-2	-1	-1	152.09	7.43
-2	-1	-1	156.32	7.41
-2	-1	-1	135.98	7.39
-2	-1	-1	161.40	7.36
2	1	1	160.47	7.36
2	1	1	144.80	7.42
2	1	1	157.19	7.41
-3	-1	-1	104.91	4.79
-3	-1	-1	85.03	4.68
-3	-1	-1	85.15	4.71
-3	-1	-1	93.78	4.62
3	1	1	106.82	4.62
3	1	1	100.75	4.77
3	1	1	89.61	4.70
-4	-1	-1	84.36	4.16
-4	-1	-1	85.40	4.28
-4	-1	-1	95.68	4.41
-4	-1	-1	76.07	4.24
-4	-1	-1	79.52	4.24
4	1	1	91.27	4.27
4	1	1	94.58	4.38
4	1	1	72.16	4.23
4	1	1	86.79	4.15
-5	-1	-1	10.92	1.25
-5	-1	-1	16.32	1.53
-5	-1	-1	15.18	1.13
-5	-1	-1	10.94	1.12
5	1	1	11.37	1.10
5	1	1	14.44	1.20

5	1	1	17.92	1.51
5	1	1	15.40	0.82
5	1	1	14.43	1.12
-6	-1	-1	121.73	6.38
-6	-1	-1	125.61	6.41
-6	-1	-1	134.79	6.60
6	1	1	122.54	6.21
6	1	1	122.30	6.33
6	1	1	130.38	6.37
6	1	1	133.82	6.56
-7	-1	-1	26.63	2.26
-7	-1	-1	22.96	1.74
-7	-1	-1	22.69	1.92
7	1	1	23.07	1.36
7	1	1	26.38	2.23
-8	-1	-1	66.87	4.03
-8	-1	-1	62.39	3.94
8	1	1	64.34	5.18
8	1	1	63.71	3.28
-9	-1	-1	33.59	3.21
-9	-1	-1	32.38	2.42
9	1	1	35.06	1.98
9	1	1	37.47	2.97
-10	-1	-1	17.22	3.08
-10	-1	-1	13.83	1.90
-10	-1	-1	17.09	1.99
10	1	1	18.00	1.37
-11	-1	-1	37.35	4.11
-11	-1	-1	33.92	2.95
11	1	1	37.25	2.27
-12	-1	-1	30.94	4.48
-12	-1	-1	20.84	1.96
12	1	1	26.46	2.03
-13	-1	-1	19.57	3.54
-13	-1	-1	30.36	5.17
-13	-1	-1	17.51	2.01
13	1	1	25.05	2.21
-14	-1	-1	19.04	4.60
14	1	1	16.52	2.42
-15	-1	-1	11.25	5.20
-13	2	1	22.24	4.75
-12	2	1	6.93	1.14
-12	2	1	11.58	3.98
-11	2	1	33.62	4.97

-11	2	1	28.29	2.84
-11	2	1	33.37	2.18
-10	2	1	19.74	1.56
-10	2	1	19.68	3.91
-9	2	1	52.17	4.07
-9	2	1	44.08	2.48
-9	2	1	38.65	2.99
-8	2	1	23.80	2.16
-8	2	1	28.91	1.64
-7	2	1	84.83	4.30
-7	2	1	93.46	4.96
-7	2	1	79.74	4.67
-6	2	1	30.90	1.67
-6	2	1	29.78	2.05
-5	2	1	122.48	6.24
-5	2	1	139.96	6.55
-5	2	1	120.27	6.40
-4	2	1	185.82	9.46
-4	2	1	190.76	9.36
-4	2	1	201.75	9.55
-3	2	1	118.68	5.80
-2	2	1	59.16	2.94
1	-2	-1	460.25	21.67
-1	2	1	439.11	21.69
0	-2	-1	24.30	1.24
0	-2	-1	25.76	1.36
0	2	1	24.20	1.24
0	2	1	23.84	1.36
-1	-2	-1	166.09	8.14
-1	-2	-1	166.75	8.20
-1	-2	-1	168.26	8.17
1	2	1	171.92	8.18
1	2	1	155.54	8.17
1	2	1	164.03	8.14
1	2	1	189.48	8.20
-2	-2	-1	103.01	4.94
-2	-2	-1	96.04	4.86
-2	-2	-1	96.64	4.95
-2	-2	-1	113.31	5.03
2	2	1	110.78	4.96
2	2	1	96.47	4.86
2	2	1	96.47	4.95
2	2	1	92.05	4.98
-3	-2	-1	574.28	27.64

-3	-2	-1	610.56	27.63
-3	-2	-1	538.44	27.61
3	2	1	563.07	27.54
3	2	1	578.05	27.65
3	2	1	615.15	27.63
3	2	1	533.16	27.61
-4	-2	-1	35.54	2.28
-4	-2	-1	38.83	2.22
-4	-2	-1	43.28	2.32
4	2	1	37.86	2.26
4	2	1	43.17	2.34
4	2	1	47.08	2.06
-5	-2	-1	205.01	10.48
-5	-2	-1	209.14	10.60
-5	-2	-1	212.90	10.47
-5	-2	-1	218.45	10.37
-5	-2	-1	207.24	10.53
5	2	1	215.57	10.59
5	2	1	214.75	10.52
5	2	1	233.29	10.37
-6	-2	-1	184.69	9.25
-6	-2	-1	199.36	9.57
-6	-2	-1	178.36	9.38
-6	-2	-1	185.20	9.45
6	2	1	196.13	9.43
6	2	1	195.79	9.24
6	2	1	195.85	9.54
-7	-2	-1	85.17	4.38
-7	-2	-1	76.97	4.36
-7	-2	-1	77.27	4.50
-7	-2	-1	88.99	4.68
7	2	1	88.57	4.62
7	2	1	83.03	4.12
7	2	1	86.92	4.44
7	2	1	81.84	4.36
-8	-2	-1	51.92	3.22
-8	-2	-1	50.47	3.02
-8	-2	-1	51.64	3.49
-8	-2	-1	51.88	3.03
8	2	1	53.36	3.11
8	2	1	51.69	2.68
8	2	1	54.57	3.47
8	2	1	54.08	3.01
-9	-2	-1	39.08	3.14

-9	-2	-1	48.32	3.48
-9	-2	-1	44.78	2.79
9	2	1	50.08	3.36
9	2	1	39.66	2.87
9	2	1	44.32	2.75
9	2	1	44.71	2.36
-10	-2	-1	13.45	1.95
-10	-2	-1	17.39	1.95
-10	-2	-1	19.40	3.04
10	2	1	21.00	1.34
10	2	1	19.54	2.92
-11	-2	-1	46.43	2.63
-11	-2	-1	48.44	4.45
-11	-2	-1	45.77	3.20
-11	-2	-1	40.44	5.57
11	2	1	47.65	2.61
-12	-2	-1	37.59	4.56
-12	-2	-1	29.59	2.06
-13	-2	-1	27.91	3.12
-13	-2	-1	42.14	5.28
13	2	1	31.93	2.38
-14	-2	-1	19.83	4.36
-15	-2	-1	14.87	4.79
-13	3	1	8.32	1.45
-13	3	1	18.48	4.69
-12	3	1	17.08	4.24
-12	3	1	11.04	1.87
-11	3	1	27.77	2.08
-11	3	1	26.38	3.09
-11	3	1	37.34	5.34
-10	3	1	24.06	2.64
-10	3	1	26.42	1.88
-10	3	1	27.69	4.43
-9	3	1	25.29	2.43
-9	3	1	28.49	1.80
-9	3	1	25.40	4.35
-8	3	1	23.32	2.16
-8	3	1	24.83	1.59
-7	3	1	91.60	4.75
-7	3	1	93.82	5.02
-7	3	1	98.47	5.48
-6	3	1	72.95	3.87
-6	3	1	57.21	3.21
-6	3	1	59.36	3.46

-5	3	1	66.70	3.54
-5	3	1	74.00	3.98
-4	3	1	97.07	4.81
-3	3	1	159.95	8.27
-3	3	1	179.60	8.46
-2	3	1	84.98	4.35
-2	3	1	85.09	4.21
1	-3	-1	68.83	3.50
-1	3	1	68.44	3.54
0	-3	-1	103.39	4.93
0	3	1	95.33	4.93
0	3	1	96.34	4.79
-1	-3	-1	8.66	0.66
-1	-3	-1	7.23	0.87
-1	-3	-1	7.78	0.51
1	3	1	8.57	0.64
1	3	1	7.39	0.50
1	3	1	7.43	0.71
1	3	1	7.53	0.84
-2	-3	-1	94.78	4.61
-2	-3	-1	89.11	4.69
-2	-3	-1	100.44	4.75
2	3	1	92.83	4.72
2	3	1	94.78	4.61
2	3	1	100.18	4.74
2	3	1	91.97	4.70
-3	-3	-1	131.01	6.50
-3	-3	-1	127.73	6.39
-3	-3	-1	123.51	6.51
-3	-3	-1	140.71	6.54
3	3	1	142.53	6.54
3	3	1	128.12	6.38
3	3	1	127.70	6.53
3	3	1	134.99	6.51
-4	-3	-1	135.38	6.96
-4	-3	-1	151.44	7.01
-4	-3	-1	137.96	6.99
-4	-3	-1	129.82	6.81
4	3	1	140.05	7.02
4	3	1	134.42	6.96
4	3	1	139.75	6.82
4	3	1	159.64	7.01
-5	-3	-1	216.74	10.47
-5	-3	-1	206.27	10.63

-5	-3	-1	216.62	10.67
-5	-3	-1	214.02	10.70
5	3	1	222.98	10.68
5	3	1	215.95	10.71
5	3	1	227.05	10.49
-6	-3	-1	103.37	5.39
-6	-3	-1	101.70	5.48
-6	-3	-1	110.52	5.58
6	3	1	112.08	5.19
6	3	1	101.74	5.46
6	3	1	106.99	5.56
-7	-3	-1	89.45	4.60
-7	-3	-1	75.22	4.29
-7	-3	-1	75.84	4.44
7	3	1	79.68	4.41
7	3	1	89.07	4.60
-8	-3	-1	33.90	2.70
-8	-3	-1	23.87	2.39
-8	-3	-1	27.65	2.10
8	3	1	34.20	2.66
8	3	1	29.27	2.30
8	3	1	31.62	1.62
-9	-3	-1	64.88	4.03
-9	-3	-1	75.11	4.47
-9	-3	-1	67.72	4.22
-9	-3	-1	68.51	3.94
9	3	1	68.84	4.10
9	3	1	75.32	3.95
9	3	1	74.87	4.39
-10	-3	-1	43.04	2.90
-10	-3	-1	48.02	3.76
-10	-3	-1	43.04	2.92
-10	-3	-1	39.76	3.42
10	3	1	48.35	3.66
10	3	1	41.79	3.13
-11	-3	-1	18.92	2.14
-11	-3	-1	19.45	3.01
11	3	1	19.75	2.10
-12	-3	-1	45.45	4.57
-12	-3	-1	37.60	4.41
-13	-3	-1	20.22	3.30
-13	-3	-1	25.03	4.90
-13	-3	-1	24.01	2.76
-14	-3	-1	22.74	4.32

-15	-3	-1	12.24	4.64
-12	4	1	26.24	2.45
-12	4	1	36.32	6.43
-11	4	1	16.47	4.17
-11	4	1	14.71	1.73
-10	4	1	28.22	2.85
-10	4	1	35.71	4.81
-10	4	1	24.26	3.70
-10	4	1	29.58	2.10
-9	4	1	9.48	2.20
-9	4	1	10.46	1.28
-9	4	1	9.41	2.05
-9	4	1	11.23	2.71
-8	4	1	38.00	2.33
-8	4	1	42.31	3.99
-8	4	1	38.66	2.85
-8	4	1	44.54	3.58
-7	4	1	25.50	2.15
-7	4	1	29.10	3.01
-7	4	1	26.92	2.86
-7	4	1	26.35	1.70
-6	4	1	80.71	4.70
-6	4	1	84.10	4.69
-6	4	1	73.00	4.03
-5	4	1	70.77	3.96
-5	4	1	62.81	3.42
-4	4	1	88.04	4.69
-4	4	1	83.81	4.31
-3	4	1	79.06	4.03
-3	4	1	81.39	4.32
-2	4	1	235.64	11.55
-2	4	1	233.30	11.40
1	-4	-1	214.37	10.38
-1	4	1	208.26	10.42
0	-4	-1	163.35	7.91
0	4	1	152.11	7.91
0	4	1	163.05	7.75
-1	-4	-1	100.22	5.21
-1	-4	-1	104.44	5.36
1	4	1	121.30	5.25
1	4	1	98.82	5.16
1	4	1	109.56	5.35
1	4	1	100.92	5.20
-2	-4	-1	232.06	11.52

-2	-4	-1	235.29	11.44
-2	-4	-1	225.22	11.38
2	4	1	228.77	11.38
2	4	1	241.41	11.52
2	4	1	239.41	11.45
2	4	1	247.34	11.47
-3	-4	-1	88.46	4.77
-3	-4	-1	93.70	4.66
-3	-4	-1	94.53	4.83
-3	-4	-1	95.94	4.83
3	4	1	94.81	4.78
3	4	1	98.56	4.65
3	4	1	100.64	4.85
3	4	1	96.19	4.83
-4	-4	-1	181.38	9.28
-4	-4	-1	181.20	9.15
-4	-4	-1	182.93	9.32
-4	-4	-1	194.64	9.33
4	4	1	200.87	9.34
4	4	1	188.65	9.14
4	4	1	193.84	9.30
4	4	1	190.03	9.35
-5	-4	-1	256.81	13.28
-5	-4	-1	257.53	13.07
-5	-4	-1	287.30	13.28
-5	-4	-1	268.77	13.23
5	4	1	277.51	13.25
5	4	1	255.54	13.09
5	4	1	267.74	13.32
5	4	1	297.28	13.30
-6	-4	-1	208.00	10.43
-6	-4	-1	200.79	10.36
-6	-4	-1	206.01	10.43
-6	-4	-1	203.93	10.15
6	4	1	212.93	10.48
6	4	1	215.39	10.43
6	4	1	216.27	10.16
6	4	1	216.44	10.38
-7	-4	-1	43.69	3.16
-7	-4	-1	52.22	2.95
-7	-4	-1	54.61	3.18
7	4	1	56.20	3.26
7	4	1	49.48	3.22
7	4	1	48.12	3.13

-8	-4	-1	84.37	4.62
-8	-4	-1	80.02	4.85
-8	-4	-1	89.72	4.92
8	4	1	88.59	4.64
8	4	1	85.74	4.80
8	4	1	90.60	4.97
-9	-4	-1	28.91	2.25
-9	-4	-1	35.42	2.93
-9	-4	-1	25.58	2.72
9	4	1	24.92	3.65
9	4	1	32.02	3.01
-10	-4	-1	20.89	2.80
-10	-4	-1	25.83	3.15
-10	-4	-1	25.00	2.17
-10	-4	-1	24.63	2.10
10	4	1	24.77	3.14
10	4	1	23.89	2.57
-11	-4	-1	11.83	2.03
-11	-4	-1	13.84	1.96
-11	-4	-1	14.64	2.06
11	4	1	11.25	1.75
11	4	1	14.99	2.02
-12	-4	-1	43.41	4.58
-12	-4	-1	31.28	2.92
-12	-4	-1	41.90	3.11
-12	-4	-1	39.61	4.07
12	4	1	41.73	3.07
-13	-4	-1	21.20	3.26
-13	-4	-1	37.59	5.00
-13	-4	-1	30.59	2.99
-14	-4	-1	18.45	3.09
-14	-4	-1	24.62	4.24
-15	-4	-1	14.35	4.98
-15	-4	-1	20.26	4.69
-12	5	1	20.33	2.34
-12	5	1	21.37	5.15
-11	5	1	27.96	5.77
-11	5	1	19.89	2.11
-10	5	1	29.13	2.11
-10	5	1	28.28	5.18
-10	5	1	27.57	3.05
-10	5	1	24.92	4.20
-9	5	1	40.49	4.53
-9	5	1	30.03	2.58

-9	5	1	31.67	2.89
-9	5	1	36.12	3.87
-8	5	1	42.42	4.11
-8	5	1	41.13	2.51
-8	5	1	46.15	3.95
-8	5	1	41.23	2.98
-7	5	1	16.91	1.46
-7	5	1	17.81	1.96
-7	5	1	18.94	3.09
-7	5	1	19.25	2.91
-6	5	1	39.47	3.20
-6	5	1	36.57	2.17
-6	5	1	39.66	3.35
-6	5	1	35.85	2.46
-5	5	1	101.88	5.40
-5	5	1	113.19	5.95
-4	5	1	83.05	4.32
-4	5	1	87.48	4.78
-3	5	1	63.66	3.71
-3	5	1	64.74	3.32
2	-5	-1	110.29	5.59
-2	5	1	111.43	5.40
-2	5	1	103.12	5.66
1	-5	-1	111.92	5.48
-1	5	1	100.38	5.51
0	-5	-1	91.57	4.71
0	5	1	92.96	4.45
0	5	1	85.10	4.71
-1	-5	-1	96.51	4.90
-1	-5	-1	101.46	4.91
-1	-5	-1	97.59	5.14
1	5	1	104.58	4.94
1	5	1	98.23	4.86
1	5	1	96.83	4.90
1	5	1	96.11	5.10
-2	-5	-1	55.38	3.01
-2	-5	-1	59.03	3.22
2	5	1	61.44	3.20
2	5	1	56.60	3.01
2	5	1	58.10	2.91
2	5	1	58.74	3.07
-3	-5	-1	55.25	2.83
-3	-5	-1	55.90	2.97
-3	-5	-1	55.30	3.11

3	5	1	57.69	3.04
3	5	1	57.56	2.98
3	5	1	58.16	3.12
3	5	1	56.01	2.82
-4	-5	-1	69.26	3.82
-4	-5	-1	68.51	3.71
-4	-5	-1	75.12	3.80
-4	-5	-1	66.81	3.53
4	5	1	73.85	3.87
4	5	1	73.84	3.81
4	5	1	72.74	3.53
4	5	1	71.79	3.73
-5	-5	-1	183.08	9.38
-5	-5	-1	184.18	9.15
-5	-5	-1	179.94	9.33
-5	-5	-1	189.74	9.40
5	5	1	192.48	9.14
5	5	1	190.08	9.44
5	5	1	198.29	9.42
5	5	1	192.57	9.35
-6	-5	-1	121.95	6.19
-6	-5	-1	115.39	6.17
-6	-5	-1	112.54	5.84
-6	-5	-1	116.47	6.09
6	5	1	124.93	6.22
6	5	1	121.20	5.84
6	5	1	123.37	6.11
6	5	1	123.08	6.29
-7	-5	-1	103.46	5.68
-7	-5	-1	108.11	5.70
-7	-5	-1	101.91	5.55
7	5	1	109.05	5.71
7	5	1	110.30	5.79
7	5	1	110.73	5.57
-8	-5	-1	82.34	4.66
-8	-5	-1	76.83	4.86
-8	-5	-1	90.58	4.89
8	5	1	93.76	5.08
8	5	1	82.66	4.84
8	5	1	93.36	4.73
-9	-5	-1	56.95	4.08
-9	-5	-1	64.78	3.76
-9	-5	-1	70.69	4.15
9	5	1	67.60	3.93

9	5	1	68.30	4.33
9	5	1	63.11	4.45
-10	-5	-1	16.15	1.98
-10	-5	-1	13.72	2.56
-10	-5	-1	17.98	2.82
10	5	1	15.94	2.51
10	5	1	15.96	2.06
10	5	1	16.53	3.01
-11	-5	-1	26.78	3.26
-11	-5	-1	30.60	2.55
11	5	1	26.25	3.10
-12	-5	-1	24.65	2.63
-12	-5	-1	24.34	4.01
-12	-5	-1	31.18	4.06
12	5	1	22.67	3.31
-13	-5	-1	45.65	4.87
-13	-5	-1	49.69	3.78
-13	-5	-1	54.10	5.30
-14	-5	-1	15.78	3.32
-14	-5	-1	17.40	3.92
-14	-5	-1	17.07	3.02
-14	-5	-1	17.53	4.09
-15	-5	-1	29.01	4.83
-15	-5	-1	22.11	4.20
-15	-5	-1	22.42	4.87
-11	6	1	21.88	4.60
-11	6	1	18.38	2.23
-10	6	1	22.56	4.07
-10	6	1	19.59	1.99
-9	6	1	34.16	4.98
-9	6	1	37.63	2.48
-9	6	1	38.67	4.73
-9	6	1	38.39	3.15
-8	6	1	20.87	3.06
-8	6	1	17.75	1.66
-8	6	1	16.83	2.76
-7	6	1	70.94	4.79
-7	6	1	62.09	3.50
-7	6	1	60.19	4.78
-6	6	1	46.67	2.72
-6	6	1	49.15	3.92
-5	6	1	84.51	4.49
-5	6	1	89.98	5.22
-4	6	1	91.94	5.17

-4	6	1	87.62	4.58
-3	6	1	104.76	5.71
-3	6	1	103.17	5.24
2	-6	-1	111.38	5.71
-2	6	1	108.25	5.47
-2	6	1	107.21	5.82
1	-6	-1	151.22	7.64
-1	6	1	153.51	7.40
-1	6	1	144.39	7.66
0	-6	-1	85.26	4.64
0	6	1	90.89	4.30
0	6	1	80.81	4.63
1	6	1	145.02	6.85
1	6	1	131.31	6.78
1	6	1	136.75	7.05
1	6	1	137.81	6.75
-2	-6	-1	139.41	7.14
-2	-6	-1	138.27	6.91
2	6	1	138.70	7.10
2	6	1	143.56	6.96
2	6	1	136.77	6.90
2	6	1	140.50	6.83
-3	-6	-1	187.23	9.34
-3	-6	-1	199.79	9.26
-3	-6	-1	173.93	9.17
3	6	1	183.23	9.07
3	6	1	193.17	9.34
3	6	1	187.04	9.23
3	6	1	181.46	9.18
-4	-6	-1	67.55	3.76
-4	-6	-1	72.18	3.94
-4	-6	-1	74.04	3.88
-4	-6	-1	69.40	3.57
4	6	1	71.89	3.78
4	6	1	71.40	3.56
4	6	1	73.49	3.87
4	6	1	72.76	3.96
-5	-6	-1	109.23	5.75
-5	-6	-1	110.55	5.54
-5	-6	-1	108.71	5.86
-5	-6	-1	113.76	5.83
5	6	1	112.92	5.94
5	6	1	113.71	5.85
5	6	1	118.57	5.78

5	6	1	115.81	5.53
-6	-6	-1	71.49	3.61
-6	-6	-1	74.51	4.07
-6	-6	-1	69.51	3.93
-6	-6	-1	68.17	4.04
6	6	1	72.47	4.15
6	6	1	69.52	4.07
6	6	1	77.89	3.60
6	6	1	75.90	3.94
-7	-6	-1	104.64	5.91
-7	-6	-1	109.56	5.79
-7	-6	-1	108.96	5.91
7	6	1	115.01	5.82
7	6	1	113.99	5.47
7	6	1	114.72	6.10
7	6	1	113.73	5.96
-8	-6	-1	71.41	3.73
-8	-6	-1	76.93	4.37
-8	-6	-1	72.84	4.17
-8	-6	-1	71.84	4.39
8	6	1	76.35	4.41
8	6	1	79.82	4.20
-9	-6	-1	105.51	5.91
-9	-6	-1	104.28	6.17
-9	-6	-1	115.87	6.20
9	6	1	117.47	5.94
9	6	1	109.35	6.17
9	6	1	116.74	6.40
-10	-6	-1	54.12	3.50
-10	-6	-1	58.64	4.01
-10	-6	-1	49.19	3.92
10	6	1	57.85	4.26
10	6	1	49.73	3.88
10	6	1	60.09	3.60
-11	-6	-1	47.28	3.39
-11	-6	-1	46.94	3.99
-11	-6	-1	51.84	5.93
11	6	1	43.54	3.94
11	6	1	53.81	3.72
-12	-6	-1	26.43	2.78
12	6	1	31.80	2.86
12	6	1	26.41	3.52
-13	-6	-1	36.88	3.40
-13	-6	-1	40.23	4.69

-13	-6	-1	48.32	4.98
13	6	1	35.05	4.16
-14	-6	-1	16.03	3.34
-14	-6	-1	29.19	5.26
-14	-6	-1	18.71	3.79
-14	-6	-1	17.72	3.07
-14	-6	-1	21.49	3.18
14	6	1	19.82	3.42
-15	-6	-1	32.76	6.01
-15	-6	-1	21.07	3.92
-15	-6	-1	20.00	4.45
-16	-6	-1	9.05	4.30
-16	-6	-1	13.75	4.84
-16	-6	-1	11.29	5.00
-10	7	1	19.43	2.19
-10	7	1	23.03	4.24
-9	7	1	38.98	2.61
-9	7	1	32.31	5.19
-8	7	1	39.84	2.55
-8	7	1	35.02	4.77
-8	7	1	36.16	4.78
-7	7	1	44.28	2.72
-7	7	1	46.70	4.39
-7	7	1	41.67	4.39
-6	7	1	70.19	5.11
-5	7	1	60.27	4.43
-5	7	1	56.61	3.21
-4	7	1	61.25	4.23
-4	7	1	61.44	3.32
-3	7	1	103.72	5.31
-3	7	1	105.46	5.90
2	-7	-1	140.24	7.38
-2	7	1	144.66	7.11
1	-7	-1	60.44	3.68
-1	7	1	61.09	3.21
-1	7	1	60.93	3.77
0	-7	-1	68.36	3.95
0	7	1	71.92	3.48
0	7	1	62.56	3.93
-1	-7	-1	52.89	3.46
1	7	1	60.52	2.95
1	7	1	54.36	3.43
1	7	1	58.18	3.00
1	7	1	57.71	3.11

-2	-7	-1	143.16	7.33
-2	-7	-1	137.70	6.98
2	7	1	144.64	7.25
2	7	1	139.11	6.89
2	7	1	135.50	6.96
2	7	1	142.53	7.04
-3	-7	-1	37.16	2.19
-3	-7	-1	36.51	2.60
3	7	1	35.35	1.96
3	7	1	36.99	2.17
3	7	1	36.02	2.28
3	7	1	38.95	2.55
-4	-7	-1	90.70	4.84
-4	-7	-1	92.39	4.94
-4	-7	-1	97.98	5.08
4	7	1	90.11	4.92
4	7	1	97.65	5.08
4	7	1	93.52	4.64
4	7	1	93.50	4.84
-5	-7	-1	35.59	2.28
-5	-7	-1	33.89	2.53
-5	-7	-1	35.20	2.40
5	7	1	35.07	1.90
5	7	1	36.98	2.29
5	7	1	36.29	2.62
5	7	1	37.22	2.47
-6	-7	-1	78.41	4.22
-6	-7	-1	73.64	4.11
-6	-7	-1	74.19	3.81
-6	-7	-1	72.31	4.30
6	7	1	81.73	4.14
6	7	1	76.32	4.26
6	7	1	74.26	4.41
6	7	1	76.21	3.79
-7	-7	-1	72.94	4.27
-7	-7	-1	71.79	4.12
-7	-7	-1	71.66	3.74
-7	-7	-1	74.20	4.30
7	7	1	78.36	4.15
7	7	1	73.66	4.29
7	7	1	73.12	4.85
7	7	1	78.98	3.73
-8	-7	-1	95.10	5.21
-8	-7	-1	87.59	4.79

-8	-7	-1	92.77	5.38
-8	-7	-1	96.82	5.37
8	7	1	100.02	5.22
8	7	1	101.90	5.42
8	7	1	101.52	4.79
8	7	1	97.82	5.67
-9	-7	-1	24.99	2.80
-9	-7	-1	23.41	2.68
-9	-7	-1	26.62	2.34
9	7	1	24.62	3.16
9	7	1	29.50	2.36
9	7	1	28.75	2.78
-10	-7	-1	58.40	4.41
-10	-7	-1	73.48	4.41
-10	-7	-1	60.71	3.99
10	7	1	69.57	4.81
10	7	1	68.67	4.02
10	7	1	60.46	4.46
-11	-7	-1	61.46	3.98
-11	-7	-1	55.87	4.48
-11	-7	-1	66.61	4.56
11	7	1	62.62	3.91
11	7	1	54.30	4.52
-12	-7	-1	45.37	4.45
-12	-7	-1	52.54	4.73
-12	-7	-1	48.51	3.68
12	7	1	54.14	3.73
12	7	1	41.02	4.37
-13	-7	-1	15.35	2.83
-13	-7	-1	19.84	3.57
-13	-7	-1	20.25	2.88
13	7	1	13.87	2.45
-14	-7	-1	31.41	3.51
-14	-7	-1	38.22	5.49
-14	-7	-1	31.29	5.14
-14	-7	-1	31.22	3.51
14	7	1	23.29	4.47
-15	-7	-1	15.12	4.40
-15	-7	-1	10.77	3.71
-16	-7	-1	10.86	4.14
-16	-7	-1	19.40	5.07
-9	8	1	15.30	3.69
-9	8	1	15.24	2.04
-8	8	1	41.06	2.76

-8	8	1	38.66	5.27
-7	8	1	28.99	4.82
-7	8	1	28.61	2.21
-6	8	1	88.72	4.83
-6	8	1	92.27	6.24
-5	8	1	26.62	3.83
-5	8	1	28.96	2.03
-4	8	1	39.67	3.79
-4	8	1	39.60	2.41
-3	8	1	48.96	3.80
-3	8	1	46.89	2.70
2	-8	-1	84.12	4.85
-2	8	1	83.57	5.08
-2	8	1	83.38	4.36
1	-8	-1	89.67	5.31
-1	8	1	103.57	4.87
-1	8	1	91.51	5.45
-1	8	1	95.44	4.87
0	-8	-1	130.16	6.82
0	8	1	130.19	6.38
0	8	1	120.85	6.39
-1	-8	-1	104.88	5.88
1	8	1	112.62	5.36
1	8	1	102.53	5.85
-2	-8	-1	103.54	5.85
-2	-8	-1	108.47	5.41
-2	-8	-1	107.16	5.41
2	8	1	104.19	5.37
2	8	1	103.66	5.77
2	8	1	105.74	5.47
2	8	1	107.83	5.29
-3	-8	-1	84.98	4.55
-3	-8	-1	87.49	4.98
3	8	1	90.13	4.92
3	8	1	88.56	4.64
3	8	1	85.72	4.52
3	8	1	85.52	4.37
-4	-8	-1	141.16	7.15
-4	-8	-1	132.04	7.28
-4	-8	-1	142.20	7.43
4	8	1	149.97	7.43
4	8	1	144.81	7.15
4	8	1	139.50	7.22
4	8	1	141.11	6.96

-5	-8	-1	136.00	6.88
-5	-8	-1	134.08	7.04
-5	-8	-1	126.43	6.79
5	8	1	141.99	7.10
5	8	1	137.62	6.82
5	8	1	131.92	6.56
5	8	1	126.36	6.89
-6	-8	-1	119.27	6.35
-6	-8	-1	115.46	6.26
-6	-8	-1	121.54	6.47
6	8	1	124.67	5.96
6	8	1	117.68	6.39
6	8	1	125.70	6.29
-7	-8	-1	65.56	4.14
-7	-8	-1	64.84	3.47
-7	-8	-1	67.18	4.08
-7	-8	-1	66.50	3.89
7	8	1	68.87	4.09
7	8	1	73.73	3.91
7	8	1	69.08	4.41
7	8	1	69.20	3.46
-8	-8	-1	95.13	5.53
-8	-8	-1	96.02	5.37
-8	-8	-1	95.83	5.58
-8	-8	-1	90.78	4.92
8	8	1	99.53	5.58
8	8	1	106.27	5.90
8	8	1	109.40	5.42
-9	-8	-1	19.03	2.68
-9	-8	-1	16.54	2.17
9	8	1	20.20	3.15
9	8	1	17.91	2.13
9	8	1	18.60	2.71
-10	-8	-1	51.15	4.38
-10	-8	-1	47.51	3.90
-10	-8	-1	49.57	3.54
10	8	1	54.47	4.03
10	8	1	54.24	3.50
-11	-8	-1	49.86	3.63
-11	-8	-1	50.42	4.22
-11	-8	-1	43.54	4.06
11	8	1	50.72	4.31
11	8	1	49.94	3.49
-12	-8	-1	48.46	3.79

-12	-8	-1	59.32	4.74
-12	-8	-1	45.71	4.37
12	8	1	46.54	4.65
12	8	1	51.35	3.85
-13	-8	-1	40.26	4.96
-13	-8	-1	30.53	4.42
-13	-8	-1	35.48	3.59
13	8	1	39.15	3.50
-14	-8	-1	25.46	3.48
-14	-8	-1	28.68	4.82
-14	-8	-1	31.81	5.39
-14	-8	-1	22.15	3.35
14	8	1	26.54	3.89
-15	-8	-1	8.27	3.58
-15	-8	-1	17.31	4.34
-15	-8	-1	6.39	2.43
-8	9	1	10.05	3.59
-8	9	1	17.64	2.12
-7	9	1	45.93	3.03
-6	9	1	56.36	5.64
-6	9	1	49.92	3.21
-5	9	1	90.05	6.20
-5	9	1	86.83	4.76
-4	9	1	35.94	4.11
-4	9	1	38.16	2.43
-3	9	1	61.09	3.35
-3	9	1	57.66	3.33
-3	9	1	61.05	4.61
2	-9	-1	65.10	4.37
-2	9	1	70.63	3.69
-2	9	1	69.46	3.69
-2	9	1	67.01	4.75
1	-9	-1	93.85	5.58
-1	9	1	93.54	5.72
-1	9	1	99.12	5.03
-1	9	1	101.97	5.02
0	-9	-1	133.24	7.40
0	9	1	127.98	7.40
0	9	1	144.39	6.86
0	9	1	141.54	6.90
-1	-9	-1	114.58	6.49
1	9	1	114.06	6.43
1	9	1	122.87	5.88
1	9	1	115.94	5.92

-2	-9	-1	98.88	4.99
-2	-9	-1	90.50	5.53
2	9	1	98.03	4.84
2	9	1	97.59	5.08
2	9	1	94.60	4.94
2	9	1	93.86	5.46
-3	-9	-1	160.05	8.22
-3	-9	-1	157.69	8.52
3	9	1	168.74	8.47
3	9	1	161.16	8.06
3	9	1	162.35	8.05
3	9	1	155.58	8.14
-4	-9	-1	62.19	4.14
-4	-9	-1	59.13	3.46
-4	-9	-1	59.64	3.90
4	9	1	58.28	3.06
4	9	1	57.98	3.31
4	9	1	60.10	3.81
4	9	1	54.09	3.47
-5	-9	-1	151.16	7.63
-5	-9	-1	150.46	7.86
-5	-9	-1	143.93	7.56
5	9	1	141.55	7.65
5	9	1	157.20	7.90
5	9	1	145.14	7.30
5	9	1	148.02	7.53
-6	-9	-1	70.89	4.36
-6	-9	-1	74.31	4.62
-6	-9	-1	77.86	4.28
6	9	1	75.35	3.87
6	9	1	75.72	4.44
6	9	1	81.62	4.28
6	9	1	77.32	4.75
-7	-9	-1	137.04	7.50
-7	-9	-1	146.65	7.76
-7	-9	-1	137.75	7.59
7	9	1	153.69	7.93
7	9	1	154.62	7.55
7	9	1	144.25	7.14
7	9	1	141.37	7.67
-8	-9	-1	62.75	3.84
-8	-9	-1	59.29	4.15
-8	-9	-1	58.10	4.01
8	9	1	60.23	4.11

8	9	1	67.73	3.86
8	9	1	63.03	3.24
8	9	1	68.15	4.63
-9	-9	-1	29.01	3.31
-9	-9	-1	35.79	2.17
-9	-9	-1	39.40	2.96
-9	-9	-1	40.21	3.45
9	9	1	40.09	3.42
9	9	1	43.47	2.99
9	9	1	37.93	2.11
9	9	1	37.55	3.90
-10	-9	-1	14.01	2.43
-10	-9	-1	14.33	2.80
-10	-9	-1	12.77	2.25
10	9	1	17.61	2.34
10	9	1	15.94	3.05
-11	-9	-1	63.00	4.34
-11	-9	-1	68.17	4.95
-11	-9	-1	58.72	4.71
11	9	1	70.98	4.27
11	9	1	63.92	5.04
-12	-9	-1	33.22	4.36
-12	-9	-1	35.95	3.46
-12	-9	-1	29.75	4.15
12	9	1	37.56	3.25
12	9	1	30.79	4.31
-13	-9	-1	63.88	5.48
-13	-9	-1	53.42	4.56
-13	-9	-1	57.52	5.34
13	9	1	60.82	6.10
13	9	1	64.80	4.32
13	9	1	46.34	5.40
-14	-9	-1	21.61	3.63
-14	-9	-1	23.15	4.05
-14	-9	-1	16.43	3.40
14	9	1	25.40	3.59
-15	-9	-1	16.99	4.30
-15	-9	-1	14.40	3.82
-15	-9	-1	10.15	2.08
-7	10	1	18.27	2.22
-6	10	1	24.24	2.28
-5	10	1	42.54	2.98
-5	10	1	47.67	2.99
-4	10	1	41.52	2.73

-4	10	1	38.62	2.69
-4	10	1	45.04	4.70
-3	10	1	63.01	3.60
-3	10	1	64.11	5.14
2	-10	-1	25.53	3.19
-2	10	1	26.81	1.94
-2	10	1	26.40	3.64
-1	10	1	55.63	4.31
-1	10	1	57.79	3.26
0	-10	-1	67.97	4.54
0	10	1	61.80	4.49
0	10	1	64.98	3.53
0	10	1	66.78	3.57
-1	-10	-1	90.70	5.47
1	10	1	85.45	5.45
1	10	1	94.81	4.68
1	10	1	90.48	4.72
-2	-10	-1	52.44	4.05
2	10	1	54.99	3.24
2	10	1	54.67	2.87
2	10	1	47.93	3.85
2	10	1	52.20	3.05
-3	-10	-1	160.04	8.78
-3	-10	-1	163.38	8.22
3	10	1	160.83	8.17
3	10	1	164.27	8.05
3	10	1	168.73	8.70
3	10	1	157.70	8.29
-4	-10	-1	187.47	9.01
-4	-10	-1	154.44	8.96
-4	-10	-1	181.83	9.50
4	10	1	175.12	8.77
4	10	1	190.01	9.41
4	10	1	186.43	8.96
4	10	1	172.42	9.06
-5	-10	-1	98.80	5.23
-5	-10	-1	99.30	5.74
-5	-10	-1	93.81	5.35
5	10	1	94.06	4.88
5	10	1	100.32	5.19
5	10	1	92.59	5.34
5	10	1	99.09	5.72
-6	-10	-1	107.06	5.79
-6	-10	-1	102.01	5.67

-6	-10	-1	106.71	6.13
6	10	1	101.06	5.82
6	10	1	105.33	5.64
6	10	1	111.82	6.25
-7	-10	-1	99.84	6.26
-7	-10	-1	101.22	5.85
-7	-10	-1	106.26	6.15
7	10	1	102.82	5.93
7	10	1	111.28	6.48
7	10	1	115.99	5.78
7	10	1	106.18	5.33
-8	-10	-1	73.02	4.79
-8	-10	-1	66.91	4.46
8	10	1	75.00	4.26
8	10	1	71.24	3.67
8	10	1	70.53	5.07
8	10	1	67.57	4.50
-9	-10	-1	36.36	3.49
-9	-10	-1	43.43	3.71
-9	-10	-1	44.31	3.26
9	10	1	42.08	3.19
9	10	1	40.93	3.58
9	10	1	42.05	2.35
-10	-10	-1	46.86	4.04
-10	-10	-1	52.82	4.38
-10	-10	-1	48.09	3.73
10	10	1	53.02	3.66
10	10	1	52.54	2.81
10	10	1	52.60	4.22
-11	-10	-1	65.83	4.93
-11	-10	-1	62.63	4.62
-11	-10	-1	65.32	5.20
11	10	1	75.89	4.55
11	10	1	71.73	5.33
-12	-10	-1	32.15	4.04
-12	-10	-1	26.64	4.42
-12	-10	-1	38.64	3.64
12	10	1	34.39	3.34
12	10	1	36.02	4.64
-13	-10	-1	38.91	5.01
-13	-10	-1	36.14	4.66
-13	-10	-1	28.31	3.86
13	10	1	29.03	5.08
13	10	1	39.36	3.67

-14	-10	-1	11.21	2.54
-14	-10	-1	17.48	3.38
14	10	1	10.84	3.36
-15	-10	-1	9.37	4.35
-15	-10	-1	19.92	4.15
-6	11	1	43.46	3.09
-5	11	1	30.78	2.53
-4	11	1	45.27	3.02
-3	11	1	41.83	4.97
-3	11	1	37.47	2.64
-3	11	1	39.15	2.65
-2	11	1	72.69	4.05
-2	11	1	72.46	5.43
1	-11	-1	48.51	4.15
-1	11	1	48.60	4.43
-1	11	1	51.78	2.99
0	-11	-1	107.26	6.43
0	11	1	105.39	5.46
0	11	1	100.89	6.40
-1	-11	-1	47.40	4.00
1	11	1	44.83	2.69
1	11	1	41.82	3.89
1	11	1	45.36	2.63
2	11	1	45.64	2.74
2	11	1	45.35	3.92
2	11	1	44.79	3.10
2	11	1	48.05	2.65
-3	-11	-1	33.87	3.81
-3	-11	-1	42.97	2.74
-3	-11	-1	40.76	2.73
3	11	1	39.98	2.60
3	11	1	40.57	2.31
3	11	1	36.86	3.68
3	11	1	37.87	2.84
-4	-11	-1	61.61	4.64
-4	-11	-1	65.79	3.75
4	11	1	61.59	4.44
4	11	1	60.39	3.85
4	11	1	63.38	3.64
4	11	1	60.78	3.33
-5	-11	-1	50.68	4.27
-5	-11	-1	52.77	3.34
-5	-11	-1	47.05	3.59
5	11	1	52.23	2.79

5	11	1	54.09	3.21
5	11	1	49.96	4.07
5	11	1	46.98	3.42
-6	-11	-1	76.50	4.64
-6	-11	-1	82.50	4.61
6	11	1	78.85	5.30
6	11	1	76.40	4.76
6	11	1	78.61	4.10
6	11	1	83.03	4.53
-7	-11	-1	85.76	5.78
-7	-11	-1	83.93	4.84
-7	-11	-1	82.32	4.92
7	11	1	78.07	4.24
7	11	1	84.35	4.75
7	11	1	80.85	4.99
-8	-11	-1	82.60	5.36
-8	-11	-1	93.81	5.33
-8	-11	-1	94.47	5.83
8	11	1	96.56	5.23
8	11	1	92.08	4.68
8	11	1	87.82	5.50
-9	-11	-1	47.36	4.32
-9	-11	-1	44.91	3.81
-9	-11	-1	49.66	3.66
9	11	1	51.08	3.55
9	11	1	49.51	2.72
9	11	1	45.16	4.00
-10	-11	-1	71.71	4.80
-10	-11	-1	76.19	5.41
-10	-11	-1	69.25	4.95
10	11	1	75.09	4.66
10	11	1	74.13	5.23
10	11	1	74.11	3.92
-11	-11	-1	39.53	4.25
-11	-11	-1	45.44	3.94
-11	-11	-1	51.70	4.74
11	11	1	49.50	4.75
11	11	1	50.02	3.78
-12	-11	-1	51.59	5.14
-12	-11	-1	44.35	4.60
-12	-11	-1	47.61	4.30
12	11	1	47.60	3.97
12	11	1	47.47	5.25
12	11	1	43.15	8.09

-13	-11	-1	25.26	4.98
-13	-11	-1	32.84	4.94
-13	-11	-1	33.88	4.11
13	11	1	32.42	3.82
13	11	1	31.99	5.51
-14	-11	-1	32.33	5.42
-14	-11	-1	38.75	5.42
-14	-11	-1	26.84	4.50
14	11	1	34.06	4.31
-15	-11	-1	21.61	3.97
-15	-11	-1	16.13	4.37
-15	-11	-1	11.34	2.46
-5	12	1	22.68	2.44
-4	12	1	24.52	2.36
-3	12	1	29.34	2.43
-2	12	1	62.80	5.76
-2	12	1	60.62	3.64
1	-12	-1	56.17	4.81
-1	12	1	59.13	3.46
-1	12	1	59.08	5.23
0	-12	-1	58.78	4.79
0	12	1	53.07	4.91
0	12	1	59.51	3.40
-1	-12	-1	40.94	4.22
1	12	1	41.81	2.59
1	12	1	35.13	4.00
-2	-12	-1	49.70	4.41
2	12	1	44.94	4.22
2	12	1	48.07	2.89
2	12	1	47.54	3.32
-3	-12	-1	41.97	4.33
3	12	1	44.43	2.59
3	12	1	37.88	2.40
3	12	1	40.25	2.78
3	12	1	35.44	3.96
3	12	1	42.28	3.04
-4	-12	-1	30.49	2.54
-4	-12	-1	25.48	3.80
4	12	1	28.86	2.59
4	12	1	25.35	2.82
4	12	1	25.90	1.84
4	12	1	27.19	3.54
-5	-12	-1	36.60	2.90
-5	-12	-1	42.23	3.42

-5	-12	-1	30.51	4.01
5	12	1	34.62	2.19
5	12	1	34.06	3.76
5	12	1	37.63	2.80
5	12	1	36.94	3.02
-6	-12	-1	55.31	4.64
-6	-12	-1	54.82	3.62
-6	-12	-1	53.64	3.60
6	12	1	52.68	3.43
6	12	1	41.06	4.28
6	12	1	51.16	2.92
6	12	1	55.98	3.79
-7	-12	-1	43.15	3.43
-7	-12	-1	50.68	4.43
-7	-12	-1	44.92	3.50
7	12	1	44.45	3.64
7	12	1	46.72	3.29
7	12	1	46.17	2.64
-8	-12	-1	50.59	4.51
-8	-12	-1	39.67	3.55
-8	-12	-1	39.66	3.50
8	12	1	41.79	3.76
8	12	1	42.43	2.53
8	12	1	43.55	3.31
-9	-12	-1	45.32	4.58
-9	-12	-1	47.55	3.81
-9	-12	-1	45.46	3.88
9	12	1	43.52	4.05
9	12	1	49.07	3.59
9	12	1	46.44	2.74
-10	-12	-1	44.75	4.13
-10	-12	-1	42.29	4.06
-10	-12	-1	51.35	4.95
10	12	1	47.52	2.83
10	12	1	51.08	4.45
10	12	1	53.01	5.56
10	12	1	49.25	3.80
-11	-12	-1	41.96	4.26
-11	-12	-1	41.36	4.09
-11	-12	-1	44.15	4.84
11	12	1	42.37	2.71
11	12	1	44.12	3.77
11	12	1	43.96	4.80
11	12	1	52.89	7.75

-12	-12	-1	34.93	4.13
-12	-12	-1	29.85	4.31
-12	-12	-1	33.50	4.74
12	12	1	31.36	3.81
12	12	1	29.49	5.17
-13	-12	-1	28.11	5.16
-13	-12	-1	17.53	3.88
13	12	1	23.92	3.87
13	12	1	18.83	3.85
-14	-12	-1	5.76	3.78
-14	-12	-1	21.25	4.30
-14	-12	-1	14.95	3.47
14	12	1	13.22	2.57
14	12	1	12.28	4.66
-3	13	1	37.58	2.90
-2	13	1	40.73	2.94
1	-13	-1	60.87	5.46
-1	13	1	62.75	3.73
0	-13	-1	58.93	5.25
0	13	1	62.91	3.67
-1	-13	-1	69.90	5.57
1	13	1	72.74	3.81
1	13	1	54.71	5.41
1	13	1	59.97	3.67
-2	-13	-1	49.70	4.92
2	13	1	55.06	3.24
2	13	1	53.17	3.74
2	13	1	50.02	4.85
2	13	1	50.77	3.09
2	13	1	54.44	3.48
-3	-13	-1	75.68	5.75
3	13	1	76.29	4.41
3	13	1	70.59	4.58
3	13	1	80.23	4.22
3	13	1	74.88	5.60
3	13	1	73.11	4.06
-4	-13	-1	57.15	3.85
-4	-13	-1	58.09	5.08
-4	-13	-1	65.25	3.83
4	13	1	52.05	4.79
4	13	1	61.77	3.48
4	13	1	57.29	3.91
4	13	1	56.89	3.67
-5	-13	-1	78.99	5.77

-5	-13	-1	76.69	4.60
5	13	1	67.61	5.46
5	13	1	75.58	4.45
5	13	1	71.51	4.70
5	13	1	78.64	4.21
-6	-13	-1	58.89	4.08
-6	-13	-1	63.02	5.32
-6	-13	-1	60.79	4.06
6	13	1	62.13	3.90
6	13	1	57.54	4.30
6	13	1	65.60	5.12
-7	-13	-1	61.97	4.27
-7	-13	-1	62.14	4.32
-7	-13	-1	63.74	5.49
7	13	1	69.62	5.36
7	13	1	62.59	3.50
7	13	1	59.62	4.51
7	13	1	64.49	4.19
-8	-13	-1	43.85	3.78
-8	-13	-1	47.23	5.00
-8	-13	-1	46.34	3.81
8	13	1	47.35	4.07
8	13	1	42.19	5.37
8	13	1	47.73	2.81
-9	-13	-1	63.97	5.45
-9	-13	-1	57.39	4.43
-9	-13	-1	52.38	4.37
9	13	1	64.00	4.18
9	13	1	52.54	4.65
9	13	1	55.98	5.76
9	13	1	56.73	3.34
-10	-13	-1	38.08	4.10
-10	-13	-1	33.64	3.97
-10	-13	-1	36.97	5.00
10	13	1	35.67	2.48
10	13	1	35.01	3.55
10	13	1	40.53	4.32
-11	-13	-1	44.67	4.52
-11	-13	-1	43.83	4.61
-11	-13	-1	44.55	5.31
11	13	1	43.15	4.05
11	13	1	45.15	2.94
11	13	1	46.45	5.35
-12	-13	-1	31.76	4.58

-12	-13	-1	26.70	5.09
-12	-13	-1	34.46	4.53
12	13	1	28.46	2.36
12	13	1	19.25	3.74
12	13	1	27.12	3.92
-13	-13	-1	48.51	7.42
-13	-13	-1	45.62	5.71
-13	-13	-1	43.13	5.67
13	13	1	36.13	4.53
13	13	1	37.41	6.60
-2	14	1	28.83	2.67
1	-14	-1	28.01	5.03
-1	14	1	29.48	2.57
0	-14	-1	27.13	4.83
0	14	1	32.45	2.59
-1	-14	-1	43.61	5.24
1	14	1	44.57	3.03
-2	-14	-1	58.29	5.67
2	14	1	58.00	3.60
-3	-14	-1	66.98	5.62
3	14	1	57.97	4.11
3	14	1	62.24	3.62
3	14	1	52.96	3.42
3	14	1	59.30	3.93
-4	-14	-1	53.18	5.35
4	14	1	47.37	3.05
4	14	1	47.27	3.75
4	14	1	54.11	3.28
-5	-14	-1	39.87	3.51
-5	-14	-1	43.15	5.07
5	14	1	38.10	3.58
5	14	1	45.16	2.93
5	14	1	39.65	2.64
5	14	1	41.82	3.31
-6	-14	-1	47.26	3.88
-6	-14	-1	47.12	5.73
-6	-14	-1	52.74	5.42
6	14	1	46.49	2.97
6	14	1	45.30	3.98
6	14	1	51.34	3.19
6	14	1	48.45	3.57
-7	-14	-1	40.15	3.57
-7	-14	-1	46.06	5.37
-7	-14	-1	46.42	3.89

7	14	1	42.18	3.51
7	14	1	44.25	4.00
-8	-14	-1	59.07	4.53
-8	-14	-1	65.60	6.00
-8	-14	-1	57.39	4.68
8	14	1	64.76	4.35
8	14	1	61.34	4.82
-9	-14	-1	50.74	5.50
-9	-14	-1	41.69	4.18
-9	-14	-1	44.25	4.33
9	14	1	50.16	4.56
9	14	1	51.41	4.02
-10	-14	-1	35.30	4.44
-10	-14	-1	43.34	5.48
-10	-14	-1	35.48	4.38
10	14	1	33.81	3.80
10	14	1	37.77	4.66
-11	-14	-1	24.45	4.27
-11	-14	-1	17.60	2.90
-11	-14	-1	24.57	4.04
11	14	1	20.65	2.20
11	14	1	23.26	3.74
11	14	1	26.42	5.11
-12	-14	-1	40.69	5.18
-12	-14	-1	38.28	5.36
-12	-14	-1	40.86	5.88
12	14	1	38.42	2.96
12	14	1	36.05	6.30
-1	-15	-1	25.55	5.25
1	15	1	28.25	2.67
-2	-15	-1	47.48	5.95
2	15	1	58.16	3.60
2	15	1	55.20	4.10
-3	-15	-1	41.88	5.59
3	15	1	46.56	3.68
3	15	1	49.19	3.28
-4	-15	-1	56.67	5.96
4	15	1	61.31	3.83
4	15	1	62.28	4.27
-5	-15	-1	46.89	5.84
5	15	1	48.71	3.36
5	15	1	47.48	3.84
-6	-15	-1	49.75	5.68
6	15	1	42.87	3.19

6	15	1	44.98	3.83
6	15	1	50.58	3.33
-7	-15	-1	42.72	5.83
7	15	1	38.68	5.17
7	15	1	40.98	3.11
7	15	1	34.58	2.71
7	15	1	39.96	3.62
-8	-15	-1	48.95	5.95
8	15	1	45.28	3.10
8	15	1	49.34	3.43
8	15	1	47.01	4.07
-9	-15	-1	24.59	4.18
9	15	1	29.03	3.78
9	15	1	33.17	2.84
9	15	1	28.46	2.51
-10	-15	-1	34.29	5.80
10	15	1	31.22	4.06
10	15	1	31.71	2.76
-11	-15	-1	22.73	4.23
-11	-15	-1	24.19	4.38
11	15	1	28.68	4.32
11	15	1	23.20	2.74
-5	-16	-1	39.55	6.15
5	16	1	48.77	3.48
5	16	1	47.42	4.03
-6	-16	-1	44.41	6.21
6	16	1	47.26	3.55
-7	-16	-1	43.62	6.15
7	16	1	44.37	3.53
7	16	1	44.65	4.29
-8	-16	-1	29.14	6.09
8	16	1	28.61	4.01
8	16	1	29.34	3.07
8	15	-2	-2.22	2.31
8	15	-2	0.45	1.31
8	15	-2	0.05	1.19
-8	-15	2	2.26	3.51
7	15	-2	1.06	2.17
7	15	-2	0.41	1.08
7	15	-2	-2.19	1.21
-7	-15	2	-3.46	3.27
6	15	-2	-0.97	1.98
6	15	-2	1.00	1.25
-6	-15	2	0.92	3.30

5	15	-2	-2.00	1.97
5	15	-2	0.20	1.21
-5	-15	2	2.18	3.41
4	15	-2	1.09	1.94
4	15	-2	-0.25	1.19
-4	-15	2	1.11	3.33
3	15	-2	1.42	1.20
3	15	-2	-0.17	1.86
-3	-15	2	-3.10	3.42
11	14	-2	-1.03	2.61
11	14	-2	2.68	3.38
-11	-14	2	6.79	3.80
-11	-14	2	1.53	2.81
-11	-14	2	1.35	2.50
10	14	-2	0.12	1.15
10	14	-2	0.68	2.31
10	14	-2	0.08	2.99
-10	-14	2	0.63	2.39
-10	-14	2	-0.49	2.58
-10	-14	2	-1.18	3.57
9	14	-2	1.50	1.05
9	14	-2	-0.19	2.11
9	14	-2	6.39	2.67
9	14	-2	2.77	0.99
-9	-14	2	3.49	3.30
-9	-14	2	2.71	2.45
-9	-14	2	1.12	2.26
8	14	-2	-0.29	2.01
8	14	-2	0.36	0.99
8	14	-2	5.39	2.60
-8	-14	2	0.45	2.06
-8	-14	2	1.42	2.21
-8	-14	2	3.23	3.19
7	14	-2	-1.69	1.86
7	14	-2	0.47	1.05
7	14	-2	-0.09	1.06
7	14	-2	2.73	2.16
-7	-14	2	0.51	2.23
-7	-14	2	-1.10	1.93
-7	-14	2	6.17	3.24
6	14	-2	-0.20	0.99
6	14	-2	1.13	1.96
6	14	-2	-0.17	1.77
6	14	-2	1.73	0.90

-6	-14	2	-0.49	2.98
-6	-14	2	-2.19	1.72
5	14	-2	-0.16	1.80
5	14	-2	1.64	0.94
5	14	-2	-0.90	1.73
5	14	-2	-0.27	1.01
-5	-14	2	1.24	3.05
-5	-14	2	-3.11	1.79
4	14	-2	-0.88	0.97
4	14	-2	-1.04	1.95
4	14	-2	-0.06	0.93
4	14	-2	0.91	1.70
-4	-14	2	-1.13	2.90
3	14	-2	-0.46	1.04
3	14	-2	0.37	1.67
-3	-14	2	-1.97	2.94
2	14	-2	-0.94	1.69
2	14	-2	-0.16	1.06
-2	-14	2	-0.10	2.87
1	14	-2	2.47	1.84
1	14	-2	0.50	1.07
-1	-14	2	-0.71	2.94
0	14	-2	0.10	1.14
0	-14	2	-4.68	2.95
12	13	-2	0.19	2.65
12	13	-2	-1.11	3.30
-12	-13	2	2.65	3.02
-12	-13	2	0.03	2.66
-12	-13	2	3.10	3.65
11	13	-2	3.07	2.97
11	13	-2	1.86	2.23
-11	-13	2	-2.97	3.36
-11	-13	2	0.88	2.48
-11	-13	2	-1.44	2.41
10	13	-2	0.29	2.00
10	13	-2	-0.27	0.97
10	13	-2	-3.58	2.54
-10	-13	2	5.50	3.17
-10	-13	2	0.45	2.20
-10	-13	2	-0.94	2.09
9	13	-2	2.02	0.90
9	13	-2	1.81	0.96
9	13	-2	3.49	2.25
-9	-13	2	1.68	1.92

-9	-13	2	0.80	1.97
-9	-13	2	4.82	3.02
8	13	-2	-0.70	1.98
8	13	-2	1.70	0.88
8	13	-2	1.62	0.86
-8	-13	2	2.00	1.88
-8	-13	2	-2.01	2.82
-8	-13	2	-0.79	1.80
7	13	-2	1.11	1.76
7	13	-2	2.87	1.63
7	13	-2	0.40	0.80
7	13	-2	0.86	0.83
-7	-13	2	1.86	1.72
-7	-13	2	1.82	2.83
-7	-13	2	4.53	1.73
6	13	-2	1.16	1.50
6	13	-2	0.92	0.70
6	13	-2	0.47	0.83
6	13	-2	3.68	1.75
-6	-13	2	4.21	2.80
-6	-13	2	2.18	1.70
-6	-13	2	-0.46	1.65
5	13	-2	1.82	1.49
5	13	-2	3.24	0.93
5	13	-2	3.28	3.93
5	13	-2	3.11	1.69
-5	-13	2	2.28	1.60
-5	-13	2	4.57	2.70
-5	-13	2	3.99	1.74
4	13	-2	1.17	0.86
4	13	-2	-0.73	1.41
4	13	-2	-0.19	1.49
4	13	-2	0.13	0.80
-4	-13	2	-3.04	2.43
-4	-13	2	-0.30	1.50
-4	-13	2	1.63	2.65
3	13	-2	-0.28	0.87
3	13	-2	-0.08	1.45
3	13	-2	-0.14	0.81
3	13	-2	-0.11	1.45
-3	-13	2	-0.33	2.58
-3	-13	2	-1.64	1.41
2	13	-2	0.84	1.45
2	13	-2	-0.01	0.82

2	13	-2	0.32	0.90
2	13	-2	-1.50	1.44
-2	-13	2	-2.81	1.89
-2	-13	2	-2.87	2.54
1	13	-2	-0.58	1.56
1	13	-2	-0.68	0.94
-1	-13	2	2.15	2.71
0	13	-2	-0.39	0.95
0	13	-2	-0.77	1.63
0	-13	2	-0.01	2.80
-1	13	-2	-1.59	1.00
1	-13	2	-1.53	2.93
-2	13	-2	-0.90	1.06
13	12	-2	-1.73	2.43
-13	-12	2	3.99	3.72
-13	-12	2	0.37	3.06
12	12	-2	1.56	3.04
12	12	-2	5.12	2.24
-12	-12	2	1.40	2.82
-12	-12	2	2.54	3.36
-12	-12	2	2.27	2.43
11	12	-2	-4.16	2.64
11	12	-2	-0.19	1.90
-11	-12	2	-3.21	3.13
-11	-12	2	-1.22	2.19
-11	-12	2	-0.04	2.14
10	12	-2	1.32	2.35
10	12	-2	1.13	1.77
-10	-12	2	-0.54	3.03
-10	-12	2	1.03	2.07
-10	-12	2	0.26	1.90
9	12	-2	1.14	1.63
9	12	-2	0.15	1.96
9	12	-2	0.66	0.72
9	12	-2	4.09	3.45
-9	-12	2	-1.28	1.69
-9	-12	2	0.57	2.70
-9	-12	2	-0.77	1.70
8	12	-2	4.17	2.99
8	12	-2	1.45	0.74
8	12	-2	1.37	0.77
8	12	-2	1.45	4.09
8	12	-2	1.80	1.50
-8	-12	2	3.97	1.64

-8	-12	2	2.95	2.62
-8	-12	2	1.66	1.60
7	12	-2	-1.41	1.70
7	12	-2	-0.47	0.65
7	12	-2	0.80	1.37
7	12	-2	-0.52	0.68
-7	-12	2	0.20	1.50
-7	-12	2	-2.35	2.44
-7	-12	2	-1.86	1.47
6	12	-2	5.87	0.91
6	12	-2	7.36	1.73
6	12	-2	9.10	1.53
-6	-12	2	5.51	1.52
-6	-12	2	7.15	1.63
-6	-12	2	10.39	2.61
5	12	-2	1.98	0.70
5	12	-2	1.93	1.28
5	12	-2	2.14	1.45
-5	-12	2	3.89	2.44
-5	-12	2	1.21	1.40
-5	-12	2	3.02	1.44
4	12	-2	3.00	1.41
4	12	-2	2.94	1.26
4	12	-2	2.17	0.74
4	12	-2	1.85	0.70
-4	-12	2	0.34	2.36
-4	-12	2	1.14	1.33
-4	-12	2	3.09	1.71
3	12	-2	-1.02	1.23
3	12	-2	0.37	0.74
3	12	-2	0.61	1.34
-3	-12	2	-1.45	2.33
-3	-12	2	-0.24	1.24
-3	-12	2	-2.36	1.33
2	12	-2	-0.63	1.20
2	12	-2	0.03	1.29
2	12	-2	-0.51	0.72
-2	-12	2	0.82	1.19
-2	-12	2	-1.30	2.29
1	12	-2	0.90	1.34
1	12	-2	-0.49	1.25
1	12	-2	-1.08	0.75
1	12	-2	-0.87	0.72
-1	-12	2	-0.76	2.36

0	12	-2	0.16	0.84
0	12	-2	2.99	2.65
0	12	-2	1.33	1.55
0	12	-2	-0.53	0.82
0	12	-2	0.12	1.46
0	-12	2	-1.56	2.44
-1	12	-2	-0.39	0.89
-1	12	-2	1.73	1.81
1	-12	2	-0.06	2.50
-2	12	-2	-0.83	0.93
-3	12	-2	0.25	0.93
-4	12	-2	-0.33	1.06
-14	-11	2	4.47	4.13
-14	-11	2	-2.43	2.24
-14	-11	2	1.17	3.43
13	11	-2	-2.61	3.61
13	11	-2	0.73	2.15
-13	-11	2	1.76	2.91
-13	-11	2	0.68	2.47
-13	-11	2	-0.28	3.46
12	11	-2	4.51	2.88
12	11	-2	0.03	1.93
-12	-11	2	0.43	3.17
-12	-11	2	-2.21	2.09
-12	-11	2	-0.49	2.43
11	11	-2	-2.47	2.40
11	11	-2	-1.80	1.68
-11	-11	2	1.27	2.90
-11	-11	2	-1.28	2.11
-11	-11	2	0.07	1.84
10	11	-2	0.87	1.55
10	11	-2	-1.18	2.11
10	11	-2	2.48	3.64
-10	-11	2	3.08	1.76
-10	-11	2	0.92	2.79
-10	-11	2	-0.19	1.90
9	11	-2	4.22	1.46
9	11	-2	5.54	1.99
-9	-11	2	4.01	1.67
-9	-11	2	4.05	1.80
-9	-11	2	7.37	2.53
8	11	-2	-0.02	1.25
8	11	-2	0.84	0.63
8	11	-2	1.29	1.75

-8	-11	2	1.36	1.48
-8	-11	2	-1.09	1.45
-8	-11	2	0.47	2.37
7	11	-2	0.90	0.59
7	11	-2	1.50	1.22
7	11	-2	1.05	0.62
7	11	-2	0.93	1.58
-7	-11	2	1.54	2.22
-7	-11	2	1.86	1.38
-7	-11	2	-0.05	1.25
6	11	-2	7.88	1.24
6	11	-2	6.03	1.59
6	11	-2	7.06	1.10
-6	-11	2	7.83	2.29
-6	-11	2	5.05	1.49
-6	-11	2	5.92	1.48
5	11	-2	5.53	1.24
5	11	-2	4.66	1.43
5	11	-2	4.87	0.68
5	11	-2	5.19	0.75
-5	-11	2	5.75	1.40
-5	-11	2	7.21	2.25
-5	-11	2	3.21	1.40
4	11	-2	-0.04	0.55
4	11	-2	-0.79	1.26
4	11	-2	-1.74	1.04
4	11	-2	-0.58	0.53
-4	-11	2	-2.60	2.01
-4	-11	2	-1.28	1.18
-4	-11	2	-2.03	1.25
3	11	-2	1.16	1.15
3	11	-2	1.15	0.58
3	11	-2	-0.05	1.12
3	11	-2	0.22	0.59
-3	-11	2	-1.03	1.34
-3	-11	2	0.30	2.09
-3	-11	2	0.78	1.14
2	11	-2	1.45	0.66
2	11	-2	1.37	0.67
2	11	-2	1.29	1.22
2	11	-2	-0.98	1.16
-2	-11	2	1.19	2.04
-2	-11	2	0.28	1.07
1	11	-2	-0.08	0.65

1	11	-2	-1.19	1.10
1	11	-2	-0.19	1.19
-1	-11	2	-1.57	1.00
-1	-11	2	-0.95	2.08
0	11	-2	-1.87	1.26
0	11	-2	-0.73	0.66
0	11	-2	0.60	1.18
0	-11	2	-0.41	2.18
-1	11	-2	0.24	0.74
-1	11	-2	1.49	2.66
1	-11	2	-0.44	2.13
-2	11	-2	-1.17	0.80
-2	11	-2	2.34	2.52
2	-11	2	-0.13	2.29
-3	11	-2	0.88	0.89
-4	11	-2	0.37	0.95
-5	11	-2	-0.17	0.98
-14	-10	2	-1.38	3.34
-14	-10	2	5.37	3.69
13	10	-2	1.56	3.24
13	10	-2	-1.19	1.85
-13	-10	2	1.51	2.24
-13	-10	2	-1.96	3.37
-13	-10	2	-1.65	2.73
12	10	-2	2.29	2.61
12	10	-2	2.18	1.73
-12	-10	2	-0.93	1.95
-12	-10	2	-3.27	2.31
-12	-10	2	0.44	3.12
11	10	-2	-0.51	1.53
11	10	-2	-1.64	2.22
11	10	-2	2.01	3.60
-11	-10	2	0.09	2.72
-11	-10	2	1.65	1.70
-11	-10	2	-0.92	2.01
10	10	-2	0.35	3.29
10	10	-2	0.00	1.96
10	10	-2	-0.34	1.41
-10	-10	2	-0.95	1.57
-10	-10	2	-1.33	1.75
-10	-10	2	0.64	2.40
9	10	-2	4.37	1.81
9	10	-2	1.41	1.31
-9	-10	2	2.24	2.28

-9	-10	2	2.51	1.84
-9	-10	2	-1.05	1.60
8	10	-2	1.52	1.60
8	10	-2	1.82	0.56
8	10	-2	0.78	1.16
8	10	-2	1.83	0.57
-8	-10	2	3.95	2.14
-8	-10	2	-1.77	1.39
7	10	-2	2.90	1.14
7	10	-2	2.41	0.58
7	10	-2	2.02	1.47
7	10	-2	1.34	0.56
-7	-10	2	2.84	1.97
-7	-10	2	3.73	1.32
-7	-10	2	0.35	1.35
6	10	-2	1.25	3.50
6	10	-2	0.52	1.27
6	10	-2	-0.91	1.02
6	10	-2	0.02	0.47
6	10	-2	0.44	0.51
-6	-10	2	0.30	1.12
5	10	-2	-1.52	1.20
5	10	-2	1.11	2.12
5	10	-2	0.57	0.51
5	10	-2	0.48	0.46
-5	-10	2	0.13	1.87
-5	-10	2	-0.89	1.05
4	10	-2	1.51	0.51
4	10	-2	2.05	0.55
4	10	-2	2.73	2.08
4	10	-2	0.98	1.11
4	10	-2	0.95	1.00
-4	-10	2	4.21	1.94
-4	-10	2	-1.54	1.11
-4	-10	2	1.07	1.09
3	10	-2	-0.43	0.93
3	10	-2	-0.09	0.48
3	10	-2	0.39	0.51
3	10	-2	-0.35	1.10
-3	-10	2	-1.15	1.51
-3	-10	2	-0.92	1.01
-3	-10	2	-0.53	1.74
2	10	-2	-0.02	0.93
2	10	-2	0.34	0.52

2	10	-2	0.34	0.54
2	10	-2	0.38	1.08
-2	-10	2	0.65	0.97
-2	-10	2	1.53	3.01
1	10	-2	9.09	1.78
1	10	-2	10.83	1.45
1	10	-2	11.80	1.28
-1	-10	2	12.20	2.71
-1	-10	2	9.64	1.51
0	10	-2	-0.19	0.56
0	10	-2	1.52	1.10
0	10	-2	0.67	1.21
0	-10	2	1.13	2.02
-1	10	-2	1.21	0.66
-1	10	-2	0.05	1.16
1	-10	2	-0.82	1.83
-2	10	-2	0.36	0.71
-2	10	-2	0.36	1.24
2	-10	2	-1.76	1.99
-3	10	-2	-0.10	0.77
-4	10	-2	0.05	0.81
-4	10	-2	1.28	2.97
-5	10	-2	-0.14	0.87
-6	10	-2	-0.03	0.95
-7	10	-2	-0.74	1.02
-15	-9	2	-0.02	3.43
-15	-9	2	-4.96	4.09
14	9	-2	1.01	1.95
-14	-9	2	-2.33	2.01
-14	-9	2	-2.49	2.82
-14	-9	2	-1.48	3.69
13	9	-2	-1.44	1.68
13	9	-2	-1.62	3.05
-13	-9	2	1.85	3.22
-13	-9	2	0.42	2.56
-13	-9	2	-2.10	2.05
12	9	-2	-0.33	2.51
12	9	-2	2.04	3.71
12	9	-2	-0.05	1.48
-12	-9	2	0.53	2.93
-12	-9	2	1.11	2.21
-12	-9	2	0.68	1.79
11	9	-2	3.03	2.16
11	9	-2	1.96	1.42

11	9	-2	3.64	3.32
-11	-9	2	1.94	2.55
-11	-9	2	1.33	1.61
-11	-9	2	-0.10	1.97
10	9	-2	-0.02	1.22
10	9	-2	0.56	1.90
-10	-9	2	0.12	1.45
-10	-9	2	-0.41	2.27
-10	-9	2	-1.48	1.69
9	9	-2	0.35	1.59
9	9	-2	-0.47	2.60
9	9	-2	0.06	1.09
-9	-9	2	1.40	1.22
-9	-9	2	1.02	2.01
-9	-9	2	0.93	1.52
8	9	-2	1.13	1.08
8	9	-2	1.39	1.44
8	9	-2	1.90	2.33
-8	-9	2	-2.27	1.38
-8	-9	2	2.45	2.40
-8	-9	2	0.38	1.20
7	9	-2	-0.84	1.26
7	9	-2	0.18	0.43
7	9	-2	0.94	2.14
7	9	-2	0.49	0.47
7	9	-2	-1.03	0.95
-7	-9	2	1.24	1.69
-7	-9	2	0.09	1.12
6	9	-2	0.05	0.43
6	9	-2	-1.27	0.89
6	9	-2	0.01	1.82
6	9	-2	0.03	0.40
-6	-9	2	-0.98	1.03
-6	-9	2	-1.24	1.57
5	9	-2	2.09	0.46
5	9	-2	3.29	1.72
5	9	-2	0.65	1.15
5	9	-2	2.15	0.49
5	9	-2	1.82	0.91
-5	-9	2	3.26	1.53
-5	-9	2	1.32	1.08
4	9	-2	0.97	0.46
4	9	-2	1.45	0.89
4	9	-2	0.67	0.42

4	9	-2	1.40	1.61
4	9	-2	0.22	1.01
-4	-9	2	0.65	1.61
-4	-9	2	-1.96	1.01
-4	-9	2	0.36	1.04
3	9	-2	1.80	0.52
3	9	-2	0.50	0.99
3	9	-2	1.29	1.56
3	9	-2	1.88	0.91
3	9	-2	2.58	0.51
-3	-9	2	3.84	1.68
-3	-9	2	0.77	0.91
-3	-9	2	-0.52	1.15
2	9	-2	1.57	1.53
2	9	-2	0.66	0.45
2	9	-2	-0.19	0.91
2	9	-2	0.33	0.82
-2	-9	2	0.81	1.61
-2	-9	2	0.98	0.87
1	9	-2	0.15	0.91
1	9	-2	1.07	0.52
1	9	-2	1.64	1.57
1	9	-2	-0.13	0.94
-1	-9	2	-1.09	1.59
-1	-9	2	1.28	1.56
-1	-9	2	0.29	0.84
0	9	-2	8.27	1.88
0	9	-2	5.59	1.17
0	9	-2	8.18	1.09
0	9	-2	6.74	1.08
0	-9	2	7.86	1.86
0	-9	2	6.87	1.41
-1	9	-2	-1.02	0.96
-1	9	-2	-0.60	0.54
-1	9	-2	-0.46	1.00
1	-9	2	-0.74	1.99
-2	9	-2	1.41	0.63
-2	9	-2	0.79	1.23
2	-9	2	0.06	1.66
-3	9	-2	2.10	0.78
-4	9	-2	-0.33	0.70
-5	9	-2	1.59	2.99
-5	9	-2	1.48	0.83
-6	9	-2	-1.06	0.86

-7	9	-2	0.56	0.95
-8	9	-2	-1.96	1.00
-8	9	-2	-1.05	3.72
-15	-8	2	-3.91	3.23
-15	-8	2	-0.45	4.22
14	8	-2	0.41	1.67
-14	-8	2	-0.81	2.41
-14	-8	2	3.19	3.58
-14	-8	2	-3.85	2.75
13	8	-2	-1.09	2.74
13	8	-2	-2.59	1.99
-13	-8	2	-2.72	1.91
-13	-8	2	-0.98	3.30
-13	-8	2	-4.25	2.32
12	8	-2	1.40	1.42
12	8	-2	0.50	2.32
12	8	-2	0.14	3.23
-12	-8	2	2.49	1.75
-12	-8	2	2.98	2.90
-12	-8	2	-2.13	2.03
11	8	-2	0.27	1.32
11	8	-2	-2.09	2.05
-11	-8	2	2.67	2.50
-11	-8	2	-0.28	1.48
-11	-8	2	-2.45	1.82
10	8	-2	3.17	1.85
10	8	-2	6.65	2.65
10	8	-2	7.45	1.35
-10	-8	2	4.23	1.69
-10	-8	2	6.24	1.49
-10	-8	2	8.09	2.16
9	8	-2	-1.85	1.43
9	8	-2	-0.92	1.03
-9	-8	2	-0.16	1.14
-9	-8	2	-1.28	1.86
-9	-8	2	-2.38	1.40
8	8	-2	12.81	1.95
8	8	-2	11.57	1.69
8	8	-2	16.35	2.49
-8	-8	2	15.38	2.51
-8	-8	2	11.08	1.68
-8	-8	2	12.27	2.06
7	8	-2	0.76	1.82
7	8	-2	-0.67	0.87

7	8	-2	-1.64	1.12
-7	-8	2	-0.93	0.98
-7	-8	2	0.58	1.53
6	8	-2	2.30	0.43
6	8	-2	4.61	1.77
6	8	-2	2.58	1.12
6	8	-2	2.70	0.45
6	8	-2	3.05	0.86
-6	-8	2	3.69	1.48
-6	-8	2	3.38	1.00
-6	-8	2	1.72	1.15
5	8	-2	-0.57	0.74
5	8	-2	-0.46	1.47
5	8	-2	0.07	0.33
5	8	-2	-0.41	0.36
-5	-8	2	-0.92	0.85
-5	-8	2	-0.17	1.33
4	8	-2	7.31	0.82
4	8	-2	7.23	1.15
4	8	-2	9.02	1.60
4	8	-2	7.84	1.00
4	8	-2	9.30	0.90
-4	-8	2	5.31	1.09
-4	-8	2	7.63	1.08
-4	-8	2	9.63	1.57
3	8	-2	7.49	0.98
3	8	-2	7.08	0.84
3	8	-2	8.17	0.86
3	8	-2	5.99	1.08
3	8	-2	9.42	1.60
-3	-8	2	8.03	1.55
-3	-8	2	5.34	1.07
-3	-8	2	7.78	1.04
2	8	-2	4.54	0.87
2	8	-2	3.60	0.53
2	8	-2	3.37	0.97
2	8	-2	5.37	1.45
-2	-8	2	5.50	1.47
-2	-8	2	3.20	0.95
-2	-8	2	3.94	1.08
1	8	-2	-0.47	0.76
1	8	-2	-0.16	0.77
1	8	-2	1.54	1.36
1	8	-2	0.28	0.40

-1	-8	2	0.75	1.33
-1	-8	2	-0.67	0.70
0	8	-2	5.84	0.95
0	8	-2	7.79	1.60
0	8	-2	7.35	1.05
0	8	-2	6.58	0.97
0	-8	2	6.26	0.89
0	-8	2	8.24	1.65
-1	8	-2	0.00	0.47
-1	8	-2	-0.18	0.86
-1	8	-2	-0.71	0.87
-1	8	-2	0.70	1.43
1	-8	2	0.25	1.51
-2	8	-2	1.26	1.58
-2	8	-2	-0.08	0.49
2	-8	2	-0.68	1.36
-3	8	-2	0.07	0.90
-4	8	-2	1.06	0.68
-5	8	-2	0.89	0.73
-6	8	-2	0.16	0.80
-7	8	-2	0.71	0.82
-8	8	-2	-0.39	0.93
-8	8	-2	-1.95	3.46
-9	8	-2	0.23	4.02
-9	8	-2	0.75	1.04
-15	-7	2	0.33	3.36
-15	-7	2	1.91	4.02
-14	-7	2	-1.11	2.84
-14	-7	2	0.65	1.97
-14	-7	2	-2.23	3.53
-14	-7	2	-0.09	2.07
-14	-7	2	-3.42	3.04
13	7	-2	1.36	3.62
13	7	-2	-2.43	2.46
-13	-7	2	-2.11	1.76
-13	-7	2	-2.99	2.48
-13	-7	2	0.77	3.11
-13	-7	2	-2.20	2.33
12	7	-2	-1.63	2.11
12	7	-2	5.26	3.35
-12	-7	2	-1.17	2.79
-12	-7	2	-2.38	2.06
-12	-7	2	-1.94	1.52
11	7	-2	-0.45	1.34

11	7	-2	-1.04	2.03
-11	-7	2	-0.77	1.36
-11	-7	2	1.99	2.30
-11	-7	2	-2.26	1.76
10	7	-2	0.23	1.68
10	7	-2	0.61	2.46
10	7	-2	1.61	1.10
-10	-7	2	-0.21	1.55
-10	-7	2	2.02	1.28
9	7	-2	-1.45	2.13
9	7	-2	-1.47	1.38
9	7	-2	-0.91	0.91
-9	-7	2	-2.32	1.28
-9	-7	2	-0.11	1.06
-9	-7	2	-0.04	1.70
8	7	-2	-0.65	1.25
8	7	-2	-2.65	1.85
8	7	-2	-0.63	0.83
-8	-7	2	-0.01	1.54
-8	-7	2	-1.22	0.91
-8	-7	2	-1.67	1.11
7	7	-2	0.70	1.13
7	7	-2	2.80	0.85
7	7	-2	2.25	1.78
-7	-7	2	2.47	0.96
-7	-7	2	2.24	1.36
-7	-7	2	0.84	1.15
6	7	-2	0.51	0.69
6	7	-2	-0.29	0.98
-6	-7	2	0.43	1.23
-6	-7	2	1.02	0.83
5	7	-2	-0.07	0.29
5	7	-2	-1.07	1.29
5	7	-2	-1.04	0.84
5	7	-2	-0.41	0.29
5	7	-2	-0.19	0.62
-5	-7	2	0.14	1.13
-5	-7	2	0.43	0.76
4	7	-2	3.49	1.29
4	7	-2	1.43	0.33
4	7	-2	1.73	0.35
4	7	-2	0.53	0.83
4	7	-2	1.34	0.64
-4	-7	2	0.94	1.15

-4	-7	2	1.80	0.76
3	7	-2	1.13	0.34
3	7	-2	1.08	0.34
3	7	-2	2.35	1.26
3	7	-2	-1.02	0.73
-3	-7	2	0.67	1.17
-3	-7	2	0.35	0.69
-3	-7	2	-0.83	0.76
2	7	-2	-0.08	0.30
2	7	-2	-1.15	0.69
2	7	-2	-0.08	1.11
2	7	-2	0.42	0.66
-2	-7	2	-0.08	0.63
-2	-7	2	0.28	1.19
-2	-7	2	-1.99	1.03
1	7	-2	-0.29	0.62
1	7	-2	-1.28	0.73
1	7	-2	0.05	0.32
1	7	-2	-0.56	1.17
-1	-7	2	-0.12	1.22
-1	-7	2	-0.90	0.58
-1	-7	2	-0.07	0.62
0	7	-2	1.62	0.44
0	7	-2	1.57	0.75
0	7	-2	2.21	1.45
0	7	-2	2.13	0.75
0	-7	2	1.30	0.64
0	-7	2	1.27	0.97
0	-7	2	3.67	1.28
-1	7	-2	-0.95	0.74
-1	7	-2	-0.22	0.39
-1	7	-2	0.19	0.74
-1	7	-2	1.68	1.25
1	-7	2	-0.82	0.58
1	-7	2	0.97	1.21
-2	7	-2	5.35	0.67
-2	7	-2	5.29	1.44
-2	7	-2	7.11	1.55
2	-7	2	5.47	1.37
-3	7	-2	1.05	0.54
-3	7	-2	2.58	1.47
-4	7	-2	8.44	1.18
-4	7	-2	10.78	2.01
-5	7	-2	-0.25	0.59

-6	7	-2	4.16	0.97
-6	7	-2	4.15	0.80
-7	7	-2	-0.41	0.71
-8	7	-2	1.03	1.15
-8	7	-2	0.28	3.21
-8	7	-2	1.14	0.86
-9	7	-2	-1.90	3.61
-9	7	-2	-0.99	0.91
-10	7	-2	1.10	1.10
-10	7	-2	-2.37	4.38
-15	-6	2	3.17	3.27
-15	-6	2	0.07	3.63
-15	-6	2	4.40	4.21
14	6	-2	-1.14	1.62
-14	-6	2	1.76	2.84
-14	-6	2	-3.71	3.60
-14	-6	2	-3.31	3.02
-14	-6	2	-2.09	1.79
13	6	-2	-1.27	2.33
13	6	-2	0.71	3.67
-13	-6	2	0.20	1.67
-13	-6	2	0.08	1.58
-13	-6	2	-0.13	2.36
-13	-6	2	-0.14	2.50
-13	-6	2	-1.50	3.19
12	6	-2	-2.80	1.94
-12	-6	2	0.37	2.67
11	6	-2	5.01	1.24
11	6	-2	2.75	1.93
11	6	-2	10.20	2.84
-11	-6	2	4.02	1.42
-11	-6	2	3.07	1.88
10	6	-2	-0.01	1.69
10	6	-2	1.73	2.34
-10	-6	2	2.26	1.98
-10	-6	2	-0.41	1.49
-10	-6	2	1.14	1.20
9	6	-2	0.90	1.01
9	6	-2	-0.32	1.32
9	6	-2	-1.35	1.97
-9	-6	2	-1.87	1.27
-9	-6	2	0.97	1.62
-9	-6	2	-1.11	1.03
8	6	-2	6.61	1.84

8	6	-2	6.42	0.97
8	6	-2	4.39	1.30
-8	-6	2	6.39	1.57
-8	-6	2	2.93	1.23
-8	-6	2	5.30	1.04
7	6	-2	14.27	1.98
7	6	-2	13.28	1.54
-7	-6	2	9.34	1.86
-7	-6	2	12.39	1.66
-7	-6	2	14.81	1.99
6	6	-2	-0.54	0.65
6	6	-2	-1.34	1.27
-6	-6	2	-0.13	0.70
-6	-6	2	-1.12	1.05
5	6	-2	-0.83	0.78
5	6	-2	0.21	1.17
5	6	-2	-0.04	0.59
-5	-6	2	0.23	0.99
-5	-6	2	-0.15	0.68
4	6	-2	7.47	1.26
4	6	-2	5.39	0.60
4	6	-2	5.59	1.07
4	6	-2	6.67	0.64
4	6	-2	5.84	0.91
-4	-6	2	5.80	0.82
-4	-6	2	7.85	1.20
-4	-6	2	5.28	0.90
3	6	-2	46.55	2.50
3	6	-2	44.53	2.80
3	6	-2	50.61	2.72
3	6	-2	51.36	3.14
-3	-6	2	48.87	3.02
-3	-6	2	50.81	2.75
-3	-6	2	46.44	2.76
2	6	-2	5.38	1.15
2	6	-2	6.71	0.65
2	6	-2	5.28	0.79
2	6	-2	5.47	1.05
-2	-6	2	5.18	0.81
-2	-6	2	5.88	1.22
-2	-6	2	5.28	0.75
1	6	-2	7.50	0.71
1	6	-2	7.14	1.23
1	6	-2	7.27	0.85

1	6	-2	6.27	1.05
-1	-6	2	6.19	1.26
-1	-6	2	6.90	1.06
0	6	-2	3.81	0.71
0	6	-2	3.07	0.43
0	6	-2	2.84	0.72
0	6	-2	4.34	1.15
0	-6	2	3.70	1.12
0	-6	2	3.16	0.62
-1	6	-2	3.69	0.50
-1	6	-2	5.29	1.24
-1	6	-2	4.10	0.73
-1	6	-2	4.78	0.76
1	-6	2	4.85	1.24
1	-6	2	4.48	0.66
1	-6	2	4.45	0.96
-2	6	-2	3.56	0.52
-2	6	-2	5.90	1.32
2	-6	2	4.22	1.13
-3	6	-2	0.50	1.21
-3	6	-2	0.42	0.42
-4	6	-2	2.06	1.44
-4	6	-2	1.12	0.50
-5	6	-2	2.04	1.56
-6	6	-2	1.16	1.08
-6	6	-2	0.49	0.80
-7	6	-2	-0.55	0.61
-8	6	-2	2.16	1.06
-8	6	-2	3.31	0.81
-8	6	-2	1.12	2.99
8	-6	2	1.39	1.40
-9	6	-2	-1.59	3.48
-9	6	-2	1.42	0.89
-9	6	-2	-0.35	1.23
9	-6	2	-0.74	1.63
-10	6	-2	0.29	0.98
-10	6	-2	0.54	3.98
-11	6	-2	8.50	4.75
-15	-5	2	2.08	4.21
-15	-5	2	-5.10	3.65
-15	-5	2	1.11	3.13
-14	-5	2	-1.91	1.80
-14	-5	2	-0.85	2.78
-14	-5	2	2.00	3.82

-14	-5	2	-1.90	3.25
13	5	-2	-0.88	3.35
13	5	-2	-0.81	1.53
-13	-5	2	-2.38	3.29
-13	-5	2	-1.26	2.39
-13	-5	2	-2.46	2.59
-13	-5	2	-2.13	1.41
-13	-5	2	-1.86	1.55
-12	-5	2	-1.43	2.72
-12	-5	2	-3.58	1.99
-12	-5	2	-1.62	1.37
11	5	-2	1.29	1.66
11	5	-2	0.39	2.63
-11	-5	2	1.42	2.34
-11	-5	2	0.33	1.21
-11	-5	2	0.49	1.08
-11	-5	2	-2.03	2.62
10	5	-2	3.39	1.62
10	5	-2	8.04	2.42
-10	-5	2	3.16	1.58
-10	-5	2	4.97	2.09
-10	-5	2	4.71	1.19
9	5	-2	-0.81	0.85
9	5	-2	-0.79	1.87
9	5	-2	1.30	1.31
-9	-5	2	-1.15	1.59
-9	-5	2	0.46	0.93
-9	-5	2	-1.28	1.23
8	5	-2	-0.57	1.20
8	5	-2	-1.45	1.09
8	5	-2	-1.25	1.68
-8	-5	2	0.01	0.83
-8	-5	2	-1.05	1.29
7	5	-2	-0.67	1.40
7	5	-2	0.29	0.96
7	5	-2	-0.11	0.70
-7	-5	2	0.26	1.15
-7	-5	2	-1.63	0.87
-7	-5	2	-0.39	0.73
6	5	-2	0.53	1.19
6	5	-2	-0.37	0.56
-6	-5	2	0.74	0.98
-6	-5	2	-0.27	0.63
5	5	-2	6.37	1.07

5	5	-2	8.03	1.28
5	5	-2	5.54	0.95
-5	-5	2	7.57	1.18
-5	-5	2	7.46	1.11
4	5	-2	1.39	0.98
4	5	-2	0.82	0.24
4	5	-2	1.31	0.53
4	5	-2	1.12	0.67
-4	-5	2	2.22	0.88
-4	-5	2	1.28	0.58
-4	-5	2	-0.06	0.64
3	5	-2	7.14	0.59
3	5	-2	8.01	1.14
3	5	-2	5.62	1.12
3	5	-2	7.49	0.95
-3	-5	2	6.37	1.30
-3	-5	2	7.22	1.01
-3	-5	2	6.45	0.78
2	5	-2	24.35	1.42
2	5	-2	28.01	1.65
2	5	-2	24.79	1.70
2	5	-2	25.66	2.02
-2	-5	2	25.90	1.96
-2	-5	2	25.85	1.70
-2	-5	2	26.96	1.65
1	5	-2	-0.13	0.85
1	5	-2	0.45	0.24
1	5	-2	-0.19	0.49
1	5	-2	0.51	0.49
-1	-5	2	-0.05	0.53
-1	-5	2	0.24	0.83
-1	-5	2	0.77	0.46
0	5	-2	2.25	0.57
0	5	-2	0.98	0.29
0	5	-2	2.31	0.93
0	5	-2	1.75	0.53
0	-5	2	1.52	0.49
0	-5	2	1.01	0.88
-1	5	-2	20.59	1.42
-1	5	-2	20.03	1.20
-1	5	-2	18.35	1.39
-1	5	-2	20.97	1.82
1	-5	2	18.53	1.67
1	-5	2	16.39	1.85

1	-5	2	17.77	1.30
-2	5	-2	9.07	1.22
-2	5	-2	7.35	0.79
-2	5	-2	8.54	1.56
2	-5	2	7.08	1.42
-3	5	-2	13.80	1.04
-3	5	-2	15.91	1.81
-3	5	-2	11.66	1.18
-3	5	-2	9.45	1.65
3	-5	2	12.64	1.10
-4	5	-2	7.90	0.92
-4	5	-2	10.05	1.49
4	-5	2	7.52	1.04
-5	5	-2	0.66	0.48
-5	5	-2	0.26	1.32
5	-5	2	0.12	0.53
5	-5	2	-1.05	0.72
-6	5	-2	6.56	1.69
-6	5	-2	3.36	0.60
-6	5	-2	3.35	0.82
6	-5	2	2.67	0.96
-7	5	-2	-0.32	0.55
-7	5	-2	-0.30	2.20
-7	5	-2	-0.65	0.79
7	-5	2	-0.76	1.00
-8	5	-2	-0.44	0.91
-8	5	-2	-1.64	2.54
-8	5	-2	0.44	0.65
8	-5	2	-0.94	1.19
-9	5	-2	9.09	3.23
-9	5	-2	7.52	1.31
-9	5	-2	11.02	1.47
9	-5	2	7.45	1.70
-10	5	-2	6.63	3.82
-11	5	-2	13.24	4.72
-15	-4	2	-4.78	3.86
-15	-4	2	-1.26	4.19
-14	-4	2	-2.41	3.80
-14	-4	2	1.76	3.45
13	4	-2	2.29	3.30
-13	-4	2	-1.78	3.22
-13	-4	2	-2.43	2.35
-13	-4	2	-0.93	2.74
-13	-4	2	-2.40	1.45

-12	-4	2	0.41	2.01
-12	-4	2	-0.13	2.38
-12	-4	2	-0.75	2.81
11	4	-2	-1.11	0.93
11	4	-2	-4.12	2.45
-11	-4	2	-3.11	2.43
-11	-4	2	-2.87	2.71
-11	-4	2	-1.09	1.02
-11	-4	2	0.79	1.15
10	4	-2	0.48	2.18
10	4	-2	0.94	1.39
-10	-4	2	-0.99	1.94
-10	-4	2	1.05	1.02
-10	-4	2	-0.65	1.36
-10	-4	2	1.52	1.56
9	4	-2	-0.95	1.11
9	4	-2	-1.58	1.82
9	4	-2	-2.27	1.22
-9	-4	2	-0.82	0.85
-9	-4	2	-1.11	1.57
-9	-4	2	-1.52	1.29
8	4	-2	-1.28	1.06
8	4	-2	-5.22	2.93
-8	-4	2	-0.23	1.29
-8	-4	2	-1.01	0.75
7	4	-2	0.99	0.64
7	4	-2	3.09	1.38
7	4	-2	0.75	0.92
-7	-4	2	1.08	0.69
-7	-4	2	-0.14	0.89
-7	-4	2	2.57	1.15
-7	-4	2	0.69	0.96
6	4	-2	-0.73	1.11
6	4	-2	-1.25	0.78
-6	-4	2	-0.80	0.56
-6	-4	2	-0.36	0.92
5	4	-2	3.26	1.04
5	4	-2	2.85	0.60
5	4	-2	1.11	0.76
-5	-4	2	2.79	0.62
-5	-4	2	1.27	0.69
-5	-4	2	3.03	0.85
4	4	-2	5.83	0.79
4	4	-2	8.67	1.43

4	4	-2	7.48	0.89
-4	-4	2	8.10	1.19
-4	-4	2	5.62	0.78
-4	-4	2	6.57	0.97
3	4	-2	4.97	0.77
3	4	-2	3.35	0.85
3	4	-2	4.13	0.62
-3	-4	2	4.17	0.62
-3	-4	2	3.65	0.73
-3	-4	2	4.84	0.84
2	4	-2	10.42	0.66
2	4	-2	11.05	1.02
2	4	-2	11.20	0.96
2	4	-2	7.94	1.35
-2	-4	2	9.46	1.02
-2	-4	2	7.98	1.22
-2	-4	2	10.50	0.93
1	4	-2	0.28	0.38
1	4	-2	0.37	0.19
-1	-4	2	-0.87	0.83
-1	-4	2	0.29	0.37
0	4	-2	0.55	0.72
0	4	-2	0.41	0.23
0	4	-2	0.72	0.44
0	4	-2	0.49	0.45
0	-4	2	0.40	0.25
0	-4	2	1.01	0.40
0	-4	2	0.51	0.69
-1	4	-2	25.66	1.42
-1	4	-2	28.75	1.57
-1	4	-2	25.94	1.56
-1	4	-2	24.87	1.82
1	-4	2	23.03	2.00
1	-4	2	20.69	1.81
1	-4	2	26.65	1.50
1	-4	2	25.59	1.46
-2	4	-2	3.59	0.68
-2	4	-2	5.30	0.94
-2	4	-2	4.27	0.61
2	-4	2	5.49	0.93
2	-4	2	2.85	0.44
-3	4	-2	11.62	0.89
-3	4	-2	9.79	1.06
-3	4	-2	12.60	1.47

3	-4	2	10.82	0.95
-4	4	-2	0.37	0.35
-4	4	-2	1.23	0.95
4	-4	2	0.56	0.40
-5	4	-2	0.49	0.40
-5	4	-2	0.15	1.17
5	-4	2	-0.62	0.64
5	-4	2	0.83	0.54
-6	4	-2	0.28	0.44
-6	4	-2	1.23	1.33
-6	4	-2	0.21	0.64
6	-4	2	0.29	0.52
-7	4	-2	0.27	0.51
-7	4	-2	-0.68	0.74
-7	4	-2	-0.07	1.91
-7	4	-2	0.08	1.45
7	-4	2	0.14	0.61
-8	4	-2	-0.35	0.86
-8	4	-2	0.77	2.19
-8	4	-2	0.66	0.59
8	-4	2	0.44	0.71
-9	4	-2	-0.34	2.77
-9	4	-2	-1.15	0.99
-9	4	-2	0.26	0.69
9	-4	2	-1.14	1.32
-10	4	-2	8.33	3.51
-10	4	-2	2.20	0.85
-10	4	-2	2.97	1.25
-11	4	-2	4.90	4.09
-12	4	-2	5.70	5.07
-15	-3	2	-0.73	4.19
-15	-3	2	6.56	4.18
-14	-3	2	0.08	3.49
-14	-3	2	1.31	3.75
13	3	-2	0.61	3.31
-13	-3	2	-2.25	2.93
-13	-3	2	0.14	1.77
-13	-3	2	-3.49	2.23
-13	-3	2	-0.75	3.23
-13	-3	2	-0.80	1.50
-12	-3	2	-1.20	2.56
-12	-3	2	-0.51	1.89
-12	-3	2	-1.60	2.78
11	3	-2	-3.80	2.37

-11	-3	2	1.98	2.03
-11	-3	2	-1.99	2.67
-11	-3	2	-1.52	1.10
-11	-3	2	-0.64	1.59
-11	-3	2	-0.45	1.33
10	3	-2	-1.77	2.10
-10	-3	2	-0.88	0.96
-10	-3	2	-1.13	1.68
-10	-3	2	-2.38	1.94
9	3	-2	-0.24	0.71
9	3	-2	-1.39	1.71
-9	-3	2	0.80	1.69
-9	-3	2	-0.97	1.15
-9	-3	2	-0.41	0.87
-9	-3	2	-0.27	1.41
8	3	-2	-0.10	1.65
-8	-3	2	0.24	0.94
-8	-3	2	0.12	1.37
-8	-3	2	1.35	0.75
-8	-3	2	0.08	1.16
7	3	-2	-0.45	0.83
7	3	-2	-1.91	1.27
-7	-3	2	-0.97	1.11
-7	-3	2	-0.38	0.92
-7	-3	2	-0.19	0.61
-7	-3	2	-1.30	0.76
6	3	-2	5.63	1.24
6	3	-2	4.60	0.84
-6	-3	2	3.01	0.81
-6	-3	2	6.98	1.09
-6	-3	2	4.30	0.66
5	3	-2	2.10	0.96
5	3	-2	1.09	0.66
-5	-3	2	0.75	0.62
-5	-3	2	2.62	0.69
-5	-3	2	2.61	0.82
-5	-3	2	1.91	0.52
4	3	-2	0.62	0.61
4	3	-2	0.21	0.51
4	3	-2	-0.51	0.76
-4	-3	2	-0.12	0.47
-4	-3	2	0.41	0.41
-4	-3	2	0.07	0.63
3	3	-2	11.75	0.96

3	3	-2	14.38	1.21
3	3	-2	10.97	1.06
-3	-3	2	13.56	1.11
-3	-3	2	9.50	1.03
-3	-3	2	11.35	0.95
2	3	-2	22.63	1.52
2	3	-2	22.23	1.30
2	3	-2	22.09	1.34
2	3	-2	20.24	1.12
-2	-3	2	21.46	1.29
-2	-3	2	21.13	1.33
-2	-3	2	21.63	1.43
1	3	-2	-0.05	0.34
1	3	-2	-0.65	0.53
1	3	-2	0.12	0.33
1	3	-2	0.07	0.15
-1	-3	2	0.01	0.56
-1	-3	2	-0.14	0.41
-1	-3	2	0.13	0.17
-1	-3	2	0.39	0.31
0	3	-2	32.64	1.69
0	3	-2	31.09	1.60
0	3	-2	31.11	1.70
0	-3	2	30.76	1.62
0	-3	2	29.09	1.88
0	-3	2	30.56	1.68
-1	3	-2	6.99	0.79
-1	3	-2	6.10	0.53
-1	3	-2	6.11	0.79
-1	3	-2	6.19	0.98
1	-3	2	6.39	0.68
1	-3	2	6.00	0.55
1	-3	2	5.36	1.00
-2	3	-2	0.81	0.58
-2	3	-2	0.98	0.26
-2	3	-2	1.63	0.65
-2	3	-2	-0.36	0.57
2	-3	2	0.73	0.26
2	-3	2	0.71	0.44
2	-3	2	1.14	0.62
2	-3	2	1.43	0.45
-3	3	-2	3.71	0.54
-3	3	-2	2.87	0.46
-3	3	-2	4.56	0.86

3	-3	2	2.66	0.50
3	-3	2	3.52	0.60
-4	3	-2	16.14	1.58
-4	3	-2	17.58	1.04
-4	3	-2	13.22	1.22
4	-3	2	13.57	1.32
4	-3	2	16.36	1.09
-5	3	-2	0.19	0.32
-5	3	-2	0.96	0.95
-5	3	-2	0.00	0.50
5	-3	2	-0.73	0.57
5	-3	2	0.37	0.37
-6	3	-2	6.94	0.83
-6	3	-2	7.34	1.37
-6	3	-2	7.51	1.62
6	-3	2	6.56	0.96
-7	3	-2	0.94	0.47
-7	3	-2	2.25	1.39
-7	3	-2	-0.86	1.69
-7	3	-2	0.43	0.72
7	-3	2	0.09	0.84
7	-3	2	0.47	0.53
-8	3	-2	15.00	1.91
-8	3	-2	18.88	2.92
-8	3	-2	16.87	1.46
-8	3	-2	19.99	3.19
8	-3	2	14.15	2.12
8	-3	2	15.62	1.54
-9	3	-2	2.56	2.46
-9	3	-2	-0.01	0.61
-9	3	-2	-0.23	0.97
9	-3	2	0.21	0.74
9	-3	2	0.53	1.21
-10	3	-2	-1.14	1.08
-10	3	-2	-0.27	0.84
-10	3	-2	1.99	3.00
10	-3	2	0.83	0.92
-11	3	-2	0.08	0.87
-11	3	-2	-2.64	3.29
-11	3	-2	1.48	3.73
-11	3	-2	-1.63	1.28
-12	3	-2	1.75	1.08
-12	3	-2	2.47	4.73
-13	3	-2	4.06	5.53

-13	3	-2	-2.78	1.26
-15	-2	2	3.79	4.56
-15	-2	2	2.14	4.74
-14	-2	2	-4.85	3.95
-14	-2	2	-1.76	3.87
13	2	-2	-0.47	3.08
-13	-2	2	-0.37	1.55
-13	-2	2	-0.72	3.31
-13	-2	2	-1.95	3.49
-12	-2	2	-0.94	2.71
-12	-2	2	-2.36	2.84
11	2	-2	-2.46	2.32
-11	-2	2	1.17	2.55
-11	-2	2	-1.24	2.21
10	2	-2	-0.68	2.06
-10	-2	2	-0.12	1.86
-10	-2	2	-1.37	0.95
-10	-2	2	-0.61	2.04
9	2	-2	0.12	1.71
-9	-2	2	-1.06	1.35
-9	-2	2	-1.18	0.82
-9	-2	2	-0.34	1.53
8	2	-2	-1.48	1.52
-8	-2	2	-0.30	1.50
-8	-2	2	0.57	1.27
7	2	-2	-0.23	1.26
7	2	-2	-1.28	0.77
-7	-2	2	-0.43	1.16
-7	-2	2	-1.28	0.99
-7	-2	2	-0.33	0.59
6	2	-2	4.68	1.20
-6	-2	2	5.13	0.96
-6	-2	2	5.34	1.11
-6	-2	2	3.11	0.76
-6	-2	2	3.99	0.64
5	2	-2	23.44	1.62
5	2	-2	21.70	1.86
-5	-2	2	21.76	1.80
-5	-2	2	22.34	1.43
-5	-2	2	21.54	1.71
-5	-2	2	22.54	1.57
4	2	-2	6.03	0.93
4	2	-2	6.52	1.13
-4	-2	2	4.60	0.72

-4	-2	2	4.26	0.69
-4	-2	2	6.44	0.97
-4	-2	2	4.41	0.84
3	2	-2	0.09	0.20
3	2	-2	0.07	0.46
3	2	-2	0.61	0.56
3	2	-2	0.03	0.37
-3	-2	2	0.44	0.49
-3	-2	2	0.18	0.38
-3	-2	2	0.22	0.30
2	2	-2	0.36	0.34
2	2	-2	1.16	0.49
2	2	-2	0.26	0.46
-2	-2	2	0.64	0.30
-2	-2	2	0.90	0.46
-2	-2	2	0.08	0.34
1	2	-2	155.16	6.84
1	2	-2	135.53	6.89
1	2	-2	138.68	6.90
1	2	-2	135.51	6.77
-1	-2	2	127.56	6.77
-1	-2	2	150.66	6.83
-1	-2	2	139.90	6.89
-1	-2	2	137.29	6.83
0	2	-2	107.63	4.68
0	2	-2	98.63	4.62
0	2	-2	95.02	4.67
0	2	-2	90.12	4.74
0	-2	2	82.71	4.73
0	-2	2	95.28	4.67
0	-2	2	93.24	4.62
-1	2	-2	30.55	1.67
-1	2	-2	31.20	1.79
-1	2	-2	32.58	1.64
-1	2	-2	31.88	1.82
1	-2	2	32.45	1.65
1	-2	2	29.26	1.83
1	-2	2	35.59	1.76
-2	2	-2	0.63	0.19
-2	2	-2	0.57	0.42
-2	2	-2	0.40	0.57
-2	2	-2	1.29	0.50
2	-2	2	0.92	0.48
2	-2	2	0.88	0.39

2	-2	2	0.50	0.20
-3	2	-2	20.15	1.16
-3	2	-2	20.66	1.28
-3	2	-2	22.95	1.30
-3	2	-2	20.49	1.48
-3	2	-2	20.09	1.61
3	-2	2	19.56	1.18
3	-2	2	20.18	1.32
-4	2	-2	10.59	1.23
-4	2	-2	11.56	0.76
-4	2	-2	8.73	0.97
4	-2	2	9.40	1.06
4	-2	2	10.76	0.79
-5	2	-2	0.85	1.06
-5	2	-2	-0.39	0.45
-5	2	-2	0.40	0.29
5	-2	2	-0.69	0.52
5	-2	2	0.15	0.30
-6	2	-2	-0.59	0.53
-6	2	-2	-0.27	0.32
-6	2	-2	-0.14	1.28
6	-2	2	-0.31	0.61
6	-2	2	0.10	0.37
-7	2	-2	0.66	0.43
-7	2	-2	3.61	1.55
-7	2	-2	0.26	0.66
7	-2	2	-0.07	0.78
7	-2	2	0.56	0.45
-8	2	-2	12.98	2.16
-8	2	-2	9.36	1.67
-8	2	-2	9.12	1.04
8	-2	2	7.69	1.21
8	-2	2	8.98	1.18
-9	2	-2	13.29	1.89
-9	2	-2	14.89	1.34
9	-2	2	15.86	1.51
-10	2	-2	3.65	2.72
-10	2	-2	-0.23	0.69
-10	2	-2	0.92	0.83
10	-2	2	0.56	0.78
-11	2	-2	-1.92	1.32
-11	2	-2	0.02	1.17
-11	2	-2	2.81	3.40
11	-2	2	0.66	1.00

-12	2	-2	-0.10	4.13
-12	2	-2	0.63	0.97
-13	2	-2	6.50	4.74
-14	-1	2	-3.26	4.05
-14	-1	2	0.45	4.08
-13	-1	2	2.60	3.51
-13	-1	2	-3.73	3.56
-12	-1	2	0.78	2.96
11	1	-2	0.71	2.23
-11	-1	2	-0.71	2.52
-11	-1	2	1.78	2.56
10	1	-2	-0.77	2.02
-10	-1	2	-0.73	2.12
-10	-1	2	-1.54	1.46
-10	-1	2	-2.68	2.10
9	1	-2	-2.83	1.71
-9	-1	2	-0.99	1.77
8	1	-2	-0.29	1.70
-8	-1	2	-0.48	0.68
-8	-1	2	1.13	1.50
7	1	-2	0.54	1.27
-7	-1	2	1.49	1.23
-7	-1	2	2.29	1.27
6	1	-2	6.51	1.24
-6	-1	2	5.96	1.14
-6	-1	2	7.71	1.21
5	1	-2	35.46	2.39
-5	-1	2	39.31	2.43
-5	-1	2	36.47	2.35
-5	-1	2	36.59	2.03
4	1	-2	14.48	1.37
-4	-1	2	15.77	1.38
-4	-1	2	15.78	1.02
-4	-1	2	14.66	1.30
-4	-1	2	16.00	1.12
3	1	-2	61.17	3.14
3	1	-2	62.55	2.98
3	1	-2	57.17	3.05
-3	-1	2	55.92	3.07
-3	-1	2	56.36	2.93
-3	-1	2	58.98	2.95
-3	-1	2	55.76	3.02
2	1	-2	35.77	2.01
2	1	-2	38.25	1.92

-2	-1	2	36.05	1.99
-2	-1	2	34.40	1.94
-2	-1	2	38.77	1.91
-2	-1	2	36.86	1.89
1	1	-2	67.28	3.13
-1	-1	2	57.35	3.12
-1	-1	2	62.62	3.18
-1	-1	2	65.76	3.13
0	1	-2	10.70	0.72
0	1	-2	9.90	0.69
0	1	-2	8.77	0.53
0	-1	2	11.49	0.78
0	-1	2	9.40	0.55
0	-1	2	9.13	0.67
-1	1	-2	124.58	6.65
-1	1	-2	137.39	6.63
-1	1	-2	153.73	6.71
-1	1	-2	130.63	6.69
-1	1	-2	155.30	6.80
1	-1	2	124.03	6.66
1	-1	2	142.36	6.64
1	-1	2	146.73	6.78
1	-1	2	119.96	6.69
-2	1	-2	2.16	0.33
-2	1	-2	1.46	0.53
-2	1	-2	2.22	0.31
-2	1	-2	1.74	0.30
2	-1	2	1.89	0.30
2	-1	2	1.11	0.59
2	-1	2	2.41	0.34
-3	1	-2	24.42	1.40
-3	1	-2	26.65	1.32
-3	1	-2	26.94	1.55
-3	1	-2	22.54	1.42
3	-1	2	27.50	1.73
3	-1	2	26.87	1.35
3	-1	2	23.36	1.45
3	-1	2	23.23	1.39
-4	1	-2	1.04	0.38
-4	1	-2	2.60	0.83
-4	1	-2	1.61	0.27
4	-1	2	0.84	0.40
4	-1	2	1.69	0.29
-5	1	-2	16.38	1.71

-5	1	-2	13.12	1.28
-5	1	-2	16.04	1.28
-5	1	-2	18.02	0.98
5	-1	2	13.19	1.33
5	-1	2	16.15	1.01
-6	1	-2	1.16	0.57
-6	1	-2	1.43	1.13
-6	1	-2	1.18	0.35
6	-1	2	0.64	0.60
6	-1	2	1.10	0.36
-7	1	-2	13.82	2.13
-7	1	-2	7.95	1.46
-7	1	-2	10.46	0.91
7	-1	2	11.24	0.96
7	-1	2	9.47	1.50
-8	1	-2	-0.73	0.75
-8	1	-2	1.12	1.61
-8	1	-2	-0.03	0.47
8	-1	2	0.51	0.80
8	-1	2	0.93	0.49
-9	1	-2	27.76	1.84
-9	1	-2	33.42	3.39
-9	1	-2	28.19	2.39
9	-1	2	26.95	1.86
-10	1	-2	1.13	2.39
-10	1	-2	-0.37	0.66
10	-1	2	0.04	0.67
-11	1	-2	12.35	3.24
-12	1	-2	0.67	1.34
-12	1	-2	6.88	3.62
12	-1	2	1.86	1.08
-13	1	-2	12.92	4.47
-14	1	-2	5.46	5.22
-14	0	2	-2.52	4.35
-14	0	2	0.38	4.21
-13	0	2	-2.95	3.75
-13	0	2	6.04	3.89
-12	0	2	2.52	3.10
-12	0	2	-2.01	3.33
-11	0	2	-1.60	2.58
-10	0	2	-0.95	2.24
-9	0	2	0.42	0.87
-9	0	2	-1.06	1.86
8	0	-2	1.19	2.57

-8	0	2	0.17	1.50
-8	0	2	-1.21	0.73
7	0	-2	11.07	1.94
-7	0	2	6.76	1.31
-7	0	2	9.11	1.51
6	0	-2	7.68	1.31
-6	0	2	8.99	1.62
5	0	-2	37.17	2.37
-5	0	2	34.53	2.33
4	0	-2	5.54	1.14
-4	0	2	3.06	0.78
3	0	-2	6.09	0.92
-3	0	2	5.05	0.98
-3	0	2	6.38	0.90
2	0	-2	3.20	0.53
-2	0	2	3.62	0.74
1	0	-2	17.30	1.14
1	0	-2	20.35	1.04
-1	0	2	19.92	1.06
-1	0	2	21.05	1.04
0	0	-2	5.42	0.50
0	0	-2	5.92	0.51
0	0	2	5.60	0.62
0	0	2	4.39	0.48
0	0	2	5.23	0.32
-1	0	-2	4.10	0.36
-1	0	-2	4.47	0.31
-1	0	-2	4.21	0.42
-1	0	-2	3.20	0.66
1	0	2	4.05	0.35
1	0	2	4.77	0.32
1	0	2	4.17	0.57
-2	0	-2	1.85	0.28
-2	0	-2	1.68	0.47
-2	0	-2	1.15	0.34
-2	0	-2	1.11	0.27
-2	0	-2	0.96	0.23
2	0	2	1.34	0.36
2	0	2	1.56	0.19
2	0	2	0.96	0.24
-3	0	-2	17.70	1.11
-3	0	-2	16.66	1.04
-3	0	-2	18.08	0.94
-3	0	-2	17.14	1.28

-3	0	-2	15.62	1.03
3	0	2	17.35	1.11
3	0	2	16.55	1.07
3	0	2	17.92	0.94
-4	0	-2	24.25	1.27
-4	0	-2	24.78	1.71
-4	0	-2	24.04	1.45
-4	0	-2	21.91	1.41
-4	0	-2	24.18	1.38
4	0	2	23.00	1.44
4	0	2	24.14	1.45
4	0	2	23.21	1.27
-5	0	-2	3.28	0.49
-5	0	-2	2.87	0.59
-5	0	-2	2.89	0.51
-5	0	-2	3.77	0.96
-5	0	-2	2.34	0.49
5	0	2	2.32	0.52
5	0	2	2.97	0.48
5	0	2	2.66	0.51
-6	0	-2	1.26	0.67
-6	0	-2	0.33	0.48
-6	0	-2	0.53	0.32
-6	0	-2	1.40	1.04
6	0	2	0.65	0.30
6	0	2	0.20	0.54
-7	0	-2	-0.52	0.79
-7	0	-2	-0.81	0.56
-7	0	-2	1.31	1.29
-7	0	-2	-0.47	0.36
7	0	2	0.04	0.34
7	0	2	-0.19	0.65
-8	0	-2	15.05	2.45
-8	0	-2	14.14	1.60
-8	0	-2	13.41	1.15
8	0	2	13.48	1.11
8	0	2	13.74	1.71
-9	0	-2	3.80	1.99
-9	0	-2	2.18	0.65
-9	0	-2	2.65	1.07
-9	0	-2	1.40	0.86
9	0	2	2.36	0.56
-10	0	-2	4.33	0.80
-10	0	-2	6.75	4.14

10	0	2	4.45	0.72
-11	0	-2	2.18	2.72
-11	0	-2	-0.45	0.82
11	0	2	-0.22	0.69
-12	0	-2	4.98	3.22
-12	0	-2	1.22	1.00
12	0	2	1.10	0.94
-13	0	-2	3.49	3.86
13	0	2	0.59	1.14
-14	0	-2	8.56	4.61
-14	1	2	5.00	4.66
-14	1	2	0.09	4.61
-13	1	2	2.21	3.96
-13	1	2	-5.45	4.14
-12	1	2	5.69	3.42
-11	1	2	-3.05	2.69
-10	1	2	0.22	0.88
-9	1	2	0.44	0.94
-9	1	2	1.50	1.88
-9	1	2	0.25	0.53
-8	1	2	-0.37	0.39
-8	1	2	0.09	1.59
-7	1	2	0.64	0.34
-7	1	2	2.26	1.36
-7	1	2	0.46	0.80
-6	1	2	10.27	0.73
-6	1	2	12.54	1.77
-6	1	2	7.40	1.25
-5	1	2	3.44	0.61
-5	1	2	4.64	0.48
-3	1	2	28.84	1.69
-3	1	2	27.10	1.43
-2	1	2	16.93	1.30
-2	1	2	17.34	0.89
1	-1	-2	10.63	0.58
-1	1	2	10.68	0.57
0	-1	-2	83.34	3.79
0	1	2	72.52	3.77
-1	-1	-2	0.27	0.16
-1	-1	-2	0.43	0.10
-1	-1	-2	0.13	0.12
1	1	2	0.40	0.20
1	1	2	0.22	0.17
1	1	2	0.54	0.10

-2	-1	-2	56.28	3.05
-2	-1	-2	65.66	3.01
-2	-1	-2	63.49	3.08
-2	-1	-2	63.29	3.12
2	1	2	66.50	3.01
2	1	2	57.40	3.08
2	1	2	55.40	3.05
2	1	2	61.15	3.03
2	1	2	62.70	3.08
-3	-1	-2	2.31	0.38
-3	-1	-2	2.73	0.44
-3	-1	-2	3.98	0.69
-3	-1	-2	3.34	0.37
3	1	2	3.41	0.35
3	1	2	3.46	0.60
3	1	2	2.90	0.43
3	1	2	2.36	0.51
-4	-1	-2	4.59	0.72
-4	-1	-2	4.85	0.65
-4	-1	-2	5.33	1.10
-4	-1	-2	5.58	0.50
-4	-1	-2	4.82	0.81
4	1	2	5.84	0.78
4	1	2	5.49	0.45
4	1	2	4.75	0.70
4	1	2	5.57	1.00
-5	-1	-2	9.28	0.66
-5	-1	-2	9.46	1.41
-5	-1	-2	7.87	0.89
-5	-1	-2	7.15	0.92
-5	-1	-2	7.11	1.08
5	1	2	9.07	0.62
5	1	2	8.29	1.26
5	1	2	7.15	0.93
5	1	2	7.42	1.01
-6	-1	-2	70.29	3.75
-6	-1	-2	75.65	3.53
-6	-1	-2	65.98	3.67
-6	-1	-2	74.25	4.01
-6	-1	-2	66.22	3.66
6	1	2	67.31	3.69
6	1	2	71.68	3.50
6	1	2	73.29	3.91
6	1	2	65.87	3.70

-7	-1	-2	13.79	2.04
-7	-1	-2	12.19	1.37
-7	-1	-2	14.20	1.00
-7	-1	-2	12.77	1.35
-7	-1	-2	13.06	1.62
7	1	2	13.99	0.92
7	1	2	14.96	1.81
7	1	2	10.78	1.36
7	1	2	13.42	1.43
7	1	2	11.88	1.40
-8	-1	-2	3.48	0.61
-8	-1	-2	5.18	0.90
-8	-1	-2	6.06	1.59
-8	-1	-2	4.72	1.09
8	1	2	4.31	0.74
-9	-1	-2	-0.10	0.58
-9	-1	-2	0.91	1.74
-9	-1	-2	0.06	0.81
9	1	2	0.89	0.46
-10	-1	-2	8.17	1.65
-10	-1	-2	7.23	1.17
-10	-1	-2	11.64	2.29
-10	-1	-2	5.64	0.88
10	1	2	8.35	1.11
-11	-1	-2	8.49	2.51
-11	-1	-2	4.29	1.48
-11	-1	-2	2.33	0.84
11	1	2	3.51	0.73
-12	-1	-2	12.50	3.20
12	1	2	10.39	1.57
-13	-1	-2	2.55	1.02
-13	-1	-2	3.44	1.83
13	1	2	3.37	1.05
-14	-1	-2	4.30	1.50
-14	-1	-2	5.87	4.49
14	1	2	4.13	1.31
-15	-1	-2	5.66	5.25
-15	-1	-2	2.96	1.29
-13	2	2	1.51	4.31
-12	2	2	-0.94	3.58
-12	2	2	-1.38	0.98
-11	2	2	2.80	1.25
-11	2	2	6.35	3.09
-11	2	2	1.70	0.84

-10	2	2	1.89	1.07
-10	2	2	3.34	2.48
-10	2	2	2.07	0.67
-9	2	2	-0.59	0.97
-9	2	2	5.03	2.17
-8	2	2	1.20	0.47
-8	2	2	0.27	0.86
-7	2	2	2.11	0.80
-6	2	2	0.36	0.31
-6	2	2	1.48	1.14
-6	2	2	-0.43	0.61
-5	2	2	19.91	1.16
-4	2	2	1.42	0.25
-3	2	2	2.99	0.45
-2	2	2	0.27	0.15
1	-2	-2	0.54	0.15
-1	2	2	0.67	0.16
0	-2	-2	135.57	6.40
0	2	2	128.93	6.39
-1	-2	-2	0.29	0.22
-1	-2	-2	0.59	0.13
1	2	2	0.31	0.26
1	2	2	0.43	0.12
-2	-2	-2	61.15	3.37
-2	-2	-2	67.55	3.29
-2	-2	-2	74.36	3.41
2	2	2	61.74	3.37
2	2	2	70.99	3.39
2	2	2	70.18	3.29
2	2	2	65.91	3.36
-3	-2	-2	0.77	0.45
3	2	2	0.28	0.42
3	2	2	0.71	0.43
-4	-2	-2	3.34	0.71
-4	-2	-2	2.71	0.36
-4	-2	-2	2.88	0.53
4	2	2	2.46	0.51
4	2	2	2.72	0.46
4	2	2	2.91	0.39
4	2	2	3.15	0.73
-5	-2	-2	6.69	1.25
-5	-2	-2	5.85	0.51
5	2	2	6.02	0.52
5	2	2	4.39	0.71

5	2	2	6.60	0.94
5	2	2	5.49	0.83
-6	-2	-2	9.42	1.58
-6	-2	-2	6.15	0.63
-6	-2	-2	4.31	0.85
-6	-2	-2	7.49	0.99
6	2	2	6.15	0.60
6	2	2	4.99	0.83
6	2	2	7.70	1.50
6	2	2	8.18	1.00
-7	-2	-2	13.34	1.76
-7	-2	-2	19.63	2.08
7	2	2	18.12	1.07
7	2	2	19.05	1.93
7	2	2	17.11	1.65
-8	-2	-2	1.30	0.46
-8	-2	-2	1.70	0.69
-8	-2	-2	3.92	1.46
-8	-2	-2	0.64	1.05
8	2	2	2.35	0.40
8	2	2	4.31	1.38
8	2	2	1.51	0.92
-9	-2	-2	6.78	1.49
-9	-2	-2	9.58	1.84
-9	-2	-2	4.90	1.37
9	2	2	9.13	0.87
9	2	2	6.44	1.15
9	2	2	10.60	1.79
9	2	2	7.73	1.07
-10	-2	-2	0.07	0.63
-10	-2	-2	0.91	1.89
-10	-2	-2	-2.22	1.41
-10	-2	-2	1.40	1.07
-10	-2	-2	-1.42	0.84
10	2	2	0.01	1.80
10	2	2	0.34	0.48
-11	-2	-2	6.22	1.38
-11	-2	-2	4.64	0.83
-11	-2	-2	9.52	2.55
-11	-2	-2	3.27	1.15
11	2	2	4.18	0.71
-12	-2	-2	4.04	2.94
-12	-2	-2	0.63	2.22
-12	-2	-2	2.80	0.84

12	2	2	1.02	0.70
-13	-2	-2	4.44	1.99
-13	-2	-2	2.98	1.44
-13	-2	-2	12.10	3.69
-13	-2	-2	4.66	0.97
13	2	2	3.88	0.94
-14	-2	-2	6.78	4.05
-14	-2	-2	1.00	0.98
14	2	2	0.60	1.10
-15	-2	-2	4.78	4.99
15	2	2	1.74	1.32
-13	3	2	8.40	4.68
-12	3	2	3.52	4.05
-11	3	2	8.99	3.45
-11	3	2	2.99	0.89
-11	3	2	0.83	1.36
-10	3	2	1.74	1.13
-10	3	2	1.78	0.73
-10	3	2	-3.14	2.76
-9	3	2	5.34	1.13
-9	3	2	5.53	0.75
-8	3	2	3.71	0.59
-8	3	2	2.93	1.00
-7	3	2	17.38	1.84
-7	3	2	17.79	1.25
-6	3	2	9.88	0.88
-5	3	2	27.26	1.90
-5	3	2	28.50	1.57
-4	3	2	5.77	0.62
-3	3	2	9.33	0.73
-2	3	2	8.12	0.59
-2	3	2	10.21	1.00
1	-3	-2	33.59	1.68
-1	3	2	27.06	1.54
0	-3	-2	16.04	0.86
0	-3	-2	16.16	1.05
0	3	2	13.93	1.09
0	3	2	15.05	0.85
-1	-3	-2	204.57	9.98
-1	-3	-2	230.24	10.11
-1	-3	-2	207.46	10.07
-1	-3	-2	192.34	9.99
1	3	2	199.46	10.02
1	3	2	205.20	10.01

1	3	2	197.77	9.98
1	3	2	216.67	10.05
-2	-3	-2	7.38	0.78
-2	-3	-2	10.41	0.58
2	3	2	8.22	0.89
2	3	2	10.43	0.57
2	3	2	8.09	0.80
-3	-3	-2	26.87	1.59
-3	-3	-2	23.53	1.33
-3	-3	-2	25.79	1.51
-3	-3	-2	29.62	1.63
3	3	2	24.31	1.33
3	3	2	27.54	1.57
3	3	2	26.42	1.58
3	3	2	25.94	1.53
-4	-3	-2	47.62	2.65
-4	-3	-2	44.73	2.57
-4	-3	-2	51.50	2.40
-4	-3	-2	47.25	2.63
4	3	2	46.20	2.60
4	3	2	46.27	2.65
4	3	2	53.69	2.41
4	3	2	48.89	2.63
-5	-3	-2	0.64	0.68
-5	-3	-2	0.81	0.56
5	3	2	1.19	0.66
5	3	2	1.06	0.58
-6	-3	-2	2.67	0.66
-6	-3	-2	2.92	0.82
-6	-3	-2	4.91	0.50
-6	-3	-2	5.24	1.06
6	3	2	3.32	1.05
6	3	2	4.68	1.12
6	3	2	4.63	0.55
6	3	2	3.14	0.77
-7	-3	-2	43.84	2.82
-7	-3	-2	52.14	2.52
-7	-3	-2	48.41	3.00
-7	-3	-2	50.77	3.17
-7	-3	-2	46.81	2.76
7	3	2	53.25	2.54
7	3	2	49.09	2.95
7	3	2	50.34	3.13
-8	-3	-2	3.54	0.72

-8	-3	-2	4.34	0.66
-8	-3	-2	2.66	0.81
-8	-3	-2	2.26	1.06
-8	-3	-2	4.13	1.43
8	3	2	3.19	1.00
8	3	2	4.21	0.61
8	3	2	4.83	1.40
-9	-3	-2	13.55	2.30
-9	-3	-2	19.06	2.61
-9	-3	-2	16.85	2.10
9	3	2	19.42	1.19
9	3	2	18.19	2.05
9	3	2	21.17	2.49
-10	-3	-2	3.85	1.61
-10	-3	-2	8.42	0.96
-10	-3	-2	4.95	1.05
-10	-3	-2	7.49	2.09
10	3	2	9.28	1.96
10	3	2	6.79	0.86
10	3	2	6.32	1.15
-11	-3	-2	1.85	0.64
-11	-3	-2	1.66	1.00
-11	-3	-2	0.64	1.80
11	3	2	1.15	0.54
-12	-3	-2	6.72	2.92
-12	-3	-2	6.11	1.53
-12	-3	-2	0.92	2.21
-12	-3	-2	4.25	0.76
12	3	2	3.74	0.74
-13	-3	-2	8.42	1.35
-13	-3	-2	5.35	2.79
-13	-3	-2	12.32	3.39
-13	-3	-2	7.88	1.80
-13	-3	-2	5.85	1.43
13	3	2	4.55	0.91
-14	-3	-2	11.54	3.92
-14	-3	-2	4.60	2.24
-14	-3	-2	3.30	3.53
14	3	2	5.53	1.06
-15	-3	-2	11.67	4.51
-12	4	2	4.98	4.49
-11	4	2	-1.64	0.99
-11	4	2	-1.66	3.71
-10	4	2	7.94	3.16

-10	4	2	1.21	1.26
-10	4	2	2.31	0.81
-10	4	2	1.90	2.32
-9	4	2	-1.26	0.99
-9	4	2	-0.50	0.62
-9	4	2	0.24	1.96
-9	4	2	1.61	2.44
-8	4	2	0.28	0.54
-8	4	2	-0.41	0.93
-8	4	2	0.87	1.80
-7	4	2	5.82	1.78
-7	4	2	2.16	0.86
-7	4	2	3.48	0.59
-7	4	2	6.16	1.73
-6	4	2	7.73	0.93
-6	4	2	12.19	2.15
-5	4	2	9.12	1.87
-5	4	2	6.87	0.80
-4	4	2	6.32	0.74
-4	4	2	9.27	1.57
-3	4	2	3.46	0.89
-2	4	2	5.21	0.54
1	-4	-2	103.16	5.11
-1	4	2	94.89	5.17
-1	4	2	107.41	5.00
0	4	2	3.64	0.44
0	4	2	5.21	0.88
-1	-4	-2	18.80	1.02
-1	-4	-2	18.26	1.36
1	4	2	18.77	1.28
1	4	2	18.20	1.01
1	4	2	17.90	1.11
-2	-4	-2	0.73	0.19
-2	-4	-2	1.69	0.56
-2	-4	-2	0.23	0.40
2	4	2	1.55	0.52
2	4	2	0.05	0.36
2	4	2	0.94	0.20
-3	-4	-2	2.48	0.35
-3	-4	-2	3.18	0.62
3	4	2	2.38	0.35
3	4	2	1.41	0.46
3	4	2	2.99	0.60
-4	-4	-2	52.78	3.01

-4	-4	-2	59.11	3.06
-4	-4	-2	61.04	3.11
-4	-4	-2	51.43	2.80
4	4	2	61.82	3.08
4	4	2	50.93	2.99
4	4	2	54.36	2.80
4	4	2	62.49	3.10
-5	-4	-2	7.11	1.21
-5	-4	-2	6.63	0.50
-5	-4	-2	4.56	0.75
5	4	2	4.51	0.85
5	4	2	5.02	0.71
5	4	2	7.80	1.25
5	4	2	6.59	0.51
-6	-4	-2	1.73	0.75
6	4	2	0.89	0.87
6	4	2	2.65	0.72
-7	-4	-2	0.56	1.09
-7	-4	-2	0.28	0.27
7	4	2	-0.69	0.85
7	4	2	-0.79	1.07
7	4	2	0.04	0.31
7	4	2	-0.73	0.72
-8	-4	-2	11.77	1.61
-8	-4	-2	11.00	1.46
-8	-4	-2	16.99	2.20
8	4	2	15.73	0.98
8	4	2	17.89	2.20
8	4	2	9.91	1.85
-9	-4	-2	-1.54	1.23
-9	-4	-2	-0.69	0.83
-9	-4	-2	1.48	1.63
-9	-4	-2	-0.08	0.37
9	4	2	1.13	1.55
9	4	2	-0.73	0.86
9	4	2	-0.51	0.36
-10	-4	-2	2.66	0.90
-10	-4	-2	3.17	1.06
-10	-4	-2	3.24	0.53
-10	-4	-2	5.95	2.03
-10	-4	-2	1.70	1.47
10	4	2	3.97	0.53
10	4	2	4.70	1.35
10	4	2	6.36	1.90

-11	-4	-2	-0.79	0.94
-11	-4	-2	-0.09	0.53
-11	-4	-2	-1.71	1.63
11	4	2	0.42	1.52
11	4	2	-0.17	2.84
11	4	2	-0.54	0.44
11	4	2	-0.55	1.43
-12	-4	-2	2.14	2.20
-12	-4	-2	4.89	1.20
-12	-4	-2	7.94	2.90
-12	-4	-2	4.33	0.68
12	4	2	5.47	1.35
12	4	2	1.48	1.64
-13	-4	-2	3.37	1.53
-13	-4	-2	7.42	3.45
-13	-4	-2	-1.37	2.71
13	4	2	3.09	0.87
13	4	2	0.16	1.45
-14	-4	-2	4.39	1.61
-14	-4	-2	8.77	4.00
-14	-4	-2	3.02	3.23
-15	-4	-2	0.66	3.76
-15	-4	-2	10.37	4.48
-12	5	2	1.94	1.20
-12	5	2	12.56	4.95
-11	5	2	6.22	4.02
-10	5	2	-0.18	2.56
-10	5	2	4.30	3.38
-10	5	2	-0.18	0.78
-10	5	2	0.07	1.38
-9	5	2	1.63	0.76
-9	5	2	2.96	2.24
-9	5	2	1.63	1.14
-8	5	2	5.14	0.80
-8	5	2	9.04	2.21
-8	5	2	10.73	2.39
-7	5	2	0.99	0.57
-7	5	2	3.41	1.91
-7	5	2	0.20	1.80
-6	5	2	9.03	1.10
-6	5	2	11.24	1.91
-6	5	2	12.48	2.43
-5	5	2	10.54	1.01
-4	5	2	24.12	2.24

-4	5	2	23.06	1.47
-3	5	2	1.88	1.01
-3	5	2	1.20	0.37
-2	5	2	24.93	1.96
-2	5	2	23.82	1.39
1	-5	-2	29.81	1.82
-1	5	2	25.46	1.46
-1	5	2	24.53	1.89
0	-5	-2	71.24	3.65
0	5	2	64.12	3.65
0	5	2	68.97	3.41
-1	-5	-2	5.53	0.87
1	5	2	4.69	0.51
1	5	2	5.99	0.83
-2	-5	-2	6.25	0.89
-2	-5	-2	5.84	0.54
-2	-5	-2	4.67	0.82
2	5	2	3.89	0.79
2	5	2	5.40	0.50
2	5	2	6.70	0.74
2	5	2	5.39	1.01
-3	-5	-2	54.66	2.80
-3	-5	-2	52.78	3.06
-3	-5	-2	56.63	2.96
-3	-5	-2	54.14	3.01
3	5	2	58.80	3.03
3	5	2	56.37	2.79
3	5	2	56.33	2.95
3	5	2	56.42	3.04
-4	-5	-2	12.72	1.35
-4	-5	-2	14.18	0.84
-4	-5	-2	13.35	1.24
4	5	2	14.86	1.40
4	5	2	10.79	1.35
4	5	2	12.63	1.20
4	5	2	15.24	0.84
-5	-5	-2	63.37	3.80
-5	-5	-2	71.25	3.74
-5	-5	-2	65.05	3.68
-5	-5	-2	69.70	3.42
5	5	2	66.28	3.79
5	5	2	72.78	3.41
5	5	2	65.94	3.68
5	5	2	75.09	3.78

-6	-5	-2	6.41	0.94
-6	-5	-2	9.10	0.69
-6	-5	-2	12.29	1.52
6	5	2	12.18	1.60
6	5	2	9.15	0.67
-7	-5	-2	16.94	1.80
-7	-5	-2	21.53	2.02
-7	-5	-2	23.32	1.24
7	5	2	22.94	2.12
7	5	2	18.93	1.79
7	5	2	23.95	1.25
-8	-5	-2	33.50	2.60
-8	-5	-2	26.79	2.24
8	5	2	29.79	1.69
8	5	2	34.28	2.67
8	5	2	29.04	2.28
-9	-5	-2	3.85	1.61
-9	-5	-2	2.55	0.42
-9	-5	-2	-0.90	1.33
-9	-5	-2	1.49	0.94
9	5	2	2.64	0.46
9	5	2	5.18	1.69
9	5	2	0.97	1.24
9	5	2	0.66	1.06
-10	-5	-2	0.59	0.99
-10	-5	-2	-1.44	1.48
-10	-5	-2	0.87	0.42
-10	-5	-2	0.57	1.82
10	5	2	0.97	1.90
10	5	2	0.54	1.34
10	5	2	0.49	0.42
-11	-5	-2	4.84	1.90
-11	-5	-2	6.09	1.27
11	5	2	7.91	0.89
11	5	2	4.29	1.71
11	5	2	7.37	1.33
11	5	2	6.75	2.23
-12	-5	-2	5.22	2.58
-12	-5	-2	3.52	3.36
12	5	2	1.99	1.81
-13	-5	-2	5.43	2.66
13	5	2	3.40	2.02
-14	-5	-2	1.31	3.72
-14	-5	-2	5.44	3.12

-14	-5	-2	4.95	1.60
-15	-5	-2	2.41	4.18
-16	-5	-2	5.73	4.87
-16	-5	-2	6.76	5.40
-11	6	2	11.41	4.36
-10	6	2	5.02	3.75
-9	6	2	0.72	1.31
-9	6	2	1.85	0.85
-9	6	2	8.61	3.15
-9	6	2	3.32	2.79
-8	6	2	2.68	2.44
-8	6	2	0.94	2.37
-8	6	2	-0.70	0.96
-7	6	2	12.00	2.43
-6	6	2	4.39	0.70
-6	6	2	4.33	2.00
-5	6	2	21.52	1.50
-5	6	2	20.38	2.72
-4	6	2	3.64	0.58
-4	6	2	6.99	2.23
-3	6	2	7.49	0.93
-3	6	2	9.02	1.45
-2	6	2	2.13	1.06
-2	6	2	0.72	0.35
-2	6	2	0.52	0.37
-1	6	2	10.19	0.81
-1	6	2	8.49	1.55
0	-6	-2	6.82	1.29
0	6	2	3.58	0.60
0	6	2	4.56	0.98
-1	-6	-2	19.20	1.74
1	6	2	19.75	1.66
1	6	2	19.30	1.14
1	6	2	18.34	1.44
-2	-6	-2	8.45	1.45
-2	-6	-2	7.02	1.00
-2	-6	-2	9.23	0.70
2	6	2	9.14	1.29
2	6	2	7.58	0.67
2	6	2	7.81	0.93
2	6	2	7.90	1.15
-3	-6	-2	36.43	2.22
-3	-6	-2	39.62	2.44
-3	-6	-2	37.81	2.01

3	6	2	37.93	2.21
3	6	2	42.22	2.38
3	6	2	38.53	1.99
3	6	2	33.70	2.35
-4	-6	-2	1.84	0.86
-4	-6	-2	1.71	0.32
-4	-6	-2	0.82	0.62
4	6	2	2.13	0.85
4	6	2	0.21	0.55
4	6	2	1.29	0.29
-5	-6	-2	2.20	0.74
5	6	2	0.08	0.87
5	6	2	2.00	0.71
-6	-6	-2	6.80	0.98
-6	-6	-2	9.30	1.59
-6	-6	-2	8.31	0.71
6	6	2	6.42	0.93
6	6	2	10.11	1.65
6	6	2	9.36	0.69
-7	-6	-2	25.77	1.43
-7	-6	-2	26.52	2.20
-7	-6	-2	21.15	1.96
7	6	2	20.66	2.27
7	6	2	28.01	2.35
7	6	2	23.32	1.98
7	6	2	27.63	1.40
-8	-6	-2	12.20	1.71
-8	-6	-2	7.89	1.48
8	6	2	11.83	1.71
8	6	2	10.00	1.43
-9	-6	-2	36.46	2.95
-9	-6	-2	25.74	2.50
9	6	2	32.81	1.84
9	6	2	31.33	2.52
9	6	2	38.59	3.08
-10	-6	-2	2.79	1.79
-10	-6	-2	2.18	1.10
10	6	2	3.41	1.99
10	6	2	2.16	1.09
10	6	2	2.75	0.51
-11	-6	-2	16.65	2.34
-11	-6	-2	20.23	2.35
11	6	2	16.78	2.97
11	6	2	21.10	2.59

11	6	2	17.95	3.57
-12	-6	-2	-2.51	2.24
-12	-6	-2	3.64	2.57
-12	-6	-2	0.63	1.34
12	6	2	-2.03	1.82
-13	-6	-2	3.89	2.65
13	6	2	5.12	2.19
-14	-6	-2	1.98	1.51
-14	-6	-2	6.23	3.66
-14	-6	-2	-3.63	3.02
14	6	2	-0.18	2.30
-15	-6	-2	5.11	3.91
-15	-6	-2	8.24	4.25
-16	-6	-2	7.90	4.69
-16	-6	-2	3.66	4.16
-16	-6	-2	6.56	4.98
-10	7	2	4.50	3.86
-10	7	2	0.22	1.03
-10	7	2	1.29	1.23
-9	7	2	4.85	1.08
-9	7	2	6.01	1.07
-9	7	2	8.34	3.38
-8	7	2	4.02	0.92
-8	7	2	10.54	2.87
-8	7	2	4.80	2.81
-7	7	2	9.80	2.61
-7	7	2	6.87	0.96
-6	7	2	20.16	3.47
-6	7	2	16.10	1.57
-5	7	2	28.29	3.38
-5	7	2	24.96	1.79
-4	7	2	8.95	1.93
-4	7	2	7.35	1.07
-3	7	2	11.96	1.14
-3	7	2	12.48	1.12
-3	7	2	13.12	2.26
-2	7	2	25.74	2.44
-2	7	2	25.39	1.64
1	-7	-2	9.28	1.56
-1	7	2	6.46	0.79
-1	7	2	7.29	1.32
0	-7	-2	5.31	1.10
0	7	2	4.10	0.50
0	7	2	3.59	1.07

-1	-7	-2	13.43	1.72
1	7	2	12.34	1.66
1	7	2	12.63	0.93
1	7	2	11.98	1.06
-2	-7	-2	47.76	3.17
2	7	2	48.05	2.74
2	7	2	50.93	2.86
2	7	2	54.01	3.06
2	7	2	51.23	2.61
-3	-7	-2	47.93	2.69
-3	-7	-2	46.30	3.02
3	7	2	50.14	2.69
3	7	2	46.62	2.77
3	7	2	49.61	2.49
3	7	2	47.10	2.94
-4	-7	-2	25.91	1.92
-4	-7	-2	28.31	1.59
-4	-7	-2	29.70	2.23
4	7	2	25.65	2.07
4	7	2	31.68	2.17
4	7	2	28.32	1.89
4	7	2	28.65	1.57
-5	-7	-2	4.02	1.11
-5	-7	-2	1.62	0.37
-5	-7	-2	-0.05	0.81
-5	-7	-2	2.01	0.73
5	7	2	2.72	1.09
5	7	2	0.47	0.83
5	7	2	2.13	0.35
5	7	2	1.76	0.67
-6	-7	-2	10.06	1.38
-6	-7	-2	6.16	0.98
-6	-7	-2	8.44	0.77
6	7	2	5.24	1.12
6	7	2	10.58	1.43
6	7	2	6.74	0.97
6	7	2	8.82	0.72
-7	-7	-2	15.51	1.06
-7	-7	-2	10.34	1.70
-7	-7	-2	16.45	2.04
7	7	2	16.32	1.01
7	7	2	16.20	2.23
-8	-7	-2	43.83	3.08
-8	-7	-2	37.31	2.81

-8	-7	-2	43.33	2.26
8	7	2	45.95	3.27
8	7	2	42.20	2.83
8	7	2	37.86	3.14
8	7	2	45.65	2.25
-9	-7	-2	19.51	2.48
-9	-7	-2	11.02	1.73
-9	-7	-2	15.12	1.98
-9	-7	-2	16.81	1.13
9	7	2	13.49	2.44
9	7	2	15.65	2.76
9	7	2	16.49	1.12
9	7	2	14.51	2.00
-10	-7	-2	26.38	2.98
-10	-7	-2	25.16	1.48
-10	-7	-2	18.52	2.37
10	7	2	20.24	2.41
10	7	2	22.52	1.49
10	7	2	27.37	3.26
-11	-7	-2	26.23	2.64
-11	-7	-2	19.73	2.52
11	7	2	22.61	3.33
11	7	2	25.38	2.60
-12	-7	-2	15.34	2.54
-12	-7	-2	16.97	2.57
12	7	2	18.95	2.55
12	7	2	16.23	2.34
-13	-7	-2	0.35	2.52
-13	-7	-2	4.34	1.59
-13	-7	-2	9.85	3.32
13	7	2	3.16	1.87
13	7	2	0.28	2.11
-14	-7	-2	6.09	1.65
-14	-7	-2	6.46	1.94
-14	-7	-2	5.20	3.00
-14	-7	-2	13.43	3.90
14	7	2	3.58	2.60
14	7	2	8.18	2.20
-15	-7	-2	2.24	3.71
-15	-7	-2	11.07	4.27
-16	-7	-2	2.73	4.68
-16	-7	-2	10.84	4.76
-16	-7	-2	4.98	4.11
-9	8	2	2.78	3.59

-9	8	2	0.90	1.06
-8	8	2	5.20	1.08
-8	8	2	5.45	1.20
-8	8	2	9.30	3.23
-7	8	2	5.52	2.66
-7	8	2	5.66	2.93
-6	8	2	19.67	3.94
-6	8	2	13.89	1.65
-6	8	2	11.68	1.62
-5	8	2	2.18	2.26
-5	8	2	0.33	0.65
-5	8	2	0.68	0.66
-4	8	2	6.79	2.10
-4	8	2	3.14	0.66
-4	8	2	2.74	0.67
-3	8	2	1.02	0.51
-3	8	2	0.44	0.52
-3	8	2	1.93	1.66
2	-8	-2	4.50	1.23
-2	8	2	2.93	0.59
-2	8	2	3.17	0.56
-2	8	2	3.59	1.49
1	-8	-2	5.65	1.25
-1	8	2	3.68	0.60
-1	8	2	5.15	0.85
0	-8	-2	20.89	2.22
0	8	2	20.54	2.20
-1	-8	-2	21.43	2.22
1	8	2	20.36	2.11
1	8	2	19.88	1.32
-2	-8	-2	18.12	2.18
2	8	2	20.17	1.71
2	8	2	18.14	1.50
2	8	2	20.72	1.99
2	8	2	19.90	1.25
-3	-8	-2	1.38	0.67
-3	-8	-2	2.80	1.27
3	8	2	2.00	0.38
3	8	2	1.47	0.76
3	8	2	3.27	1.10
3	8	2	2.10	0.62
-4	-8	-2	27.30	1.94
-4	-8	-2	26.12	2.24
-4	-8	-2	25.88	2.38

4	8	2	32.25	2.31
4	8	2	26.46	1.92
4	8	2	28.14	1.57
4	8	2	23.82	2.14
-5	-8	-2	8.28	1.41
-5	-8	-2	5.04	0.93
-5	-8	-2	6.34	0.80
5	8	2	4.55	1.08
5	8	2	7.44	1.38
5	8	2	7.19	0.74
5	8	2	5.05	0.92
-6	-8	-2	23.46	1.40
-6	-8	-2	18.56	1.94
-6	-8	-2	23.26	2.28
6	8	2	18.24	1.95
6	8	2	24.98	2.42
6	8	2	23.15	1.35
-7	-8	-2	1.73	0.44
-7	-8	-2	2.21	1.39
-7	-8	-2	0.64	0.92
7	8	2	1.03	0.86
7	8	2	-1.00	1.07
7	8	2	1.18	1.49
7	8	2	1.52	0.39
-8	-8	-2	18.25	1.24
-8	-8	-2	14.86	2.37
8	8	2	12.29	2.04
8	8	2	18.32	1.17
8	8	2	21.91	2.69
-9	-8	-2	0.23	0.45
-9	-8	-2	-0.48	1.54
-9	-8	-2	-0.16	1.05
9	8	2	-1.57	1.38
9	8	2	0.62	0.42
9	8	2	-0.85	0.98
9	8	2	1.21	1.85
-10	-8	-2	9.98	2.07
-10	-8	-2	7.47	1.02
-10	-8	-2	7.20	1.42
10	8	2	4.56	1.80
10	8	2	8.54	2.32
10	8	2	8.48	0.97
10	8	2	5.94	1.33
-11	-8	-2	2.30	1.44

-11	-8	-2	3.04	0.59
-11	-8	-2	-1.51	1.84
-11	-8	-2	5.75	2.25
11	8	2	2.00	1.26
11	8	2	0.79	2.27
11	8	2	1.90	1.87
-12	-8	-2	15.73	2.58
-12	-8	-2	25.22	3.96
-12	-8	-2	20.03	2.77
12	8	2	14.73	2.52
12	8	2	24.29	2.76
-13	-8	-2	0.34	2.51
-13	-8	-2	5.01	1.41
-13	-8	-2	3.27	1.67
-13	-8	-2	8.07	3.15
13	8	2	6.65	3.32
13	8	2	0.53	2.28
13	8	2	4.15	1.70
-14	-8	-2	0.97	2.94
-14	-8	-2	11.15	3.77
-14	-8	-2	5.19	1.91
14	8	2	2.50	2.81
14	8	2	6.08	1.99
-15	-8	-2	4.51	4.16
-15	-8	-2	-0.18	3.69
-15	-8	-2	3.92	1.75
-16	-8	-2	8.09	4.07
-16	-8	-2	4.42	4.66
-8	9	2	4.24	3.41
-8	9	2	2.38	1.11
-7	9	2	15.68	2.10
-7	9	2	12.11	1.93
-7	9	2	16.17	3.29
-6	9	2	14.22	1.87
-6	9	2	14.80	1.85
-6	9	2	23.51	4.64
-5	9	2	24.82	2.00
-5	9	2	21.42	1.97
-5	9	2	26.27	4.13
-4	9	2	17.71	1.66
-4	9	2	17.47	1.63
-4	9	2	17.08	2.75
-3	9	2	9.79	2.20
-3	9	2	9.79	1.32

-3	9	2	12.29	2.07
2	-9	-2	7.79	1.58
-2	9	2	8.20	1.98
-2	9	2	7.21	1.15
-2	9	2	7.29	1.16
1	-9	-2	8.46	1.53
-1	9	2	8.96	1.77
-1	9	2	6.24	1.12
-1	9	2	8.31	1.07
0	-9	-2	33.00	2.84
0	9	2	35.03	2.00
0	9	2	31.49	2.89
-1	-9	-2	21.66	2.52
1	9	2	20.87	2.42
1	9	2	21.58	1.46
1	9	2	24.19	2.01
-2	-9	-2	21.61	2.60
-2	-9	-2	23.86	1.84
2	9	2	23.67	1.48
2	9	2	23.99	3.32
2	9	2	22.06	1.97
-3	-9	-2	57.93	3.81
-3	-9	-2	63.38	4.07
3	9	2	62.25	3.56
3	9	2	63.61	3.42
-4	-9	-2	25.02	2.56
-4	-9	-2	23.37	2.02
4	9	2	23.35	2.11
4	9	2	25.76	1.49
4	9	2	22.59	1.88
-5	-9	-2	40.81	3.14
-5	-9	-2	38.77	2.63
-5	-9	-2	36.44	2.91
5	9	2	37.04	2.82
5	9	2	41.51	2.62
5	9	2	48.33	3.11
-6	-9	-2	11.15	1.51
-6	-9	-2	15.45	2.35
-6	-9	-2	16.93	1.19
-6	-9	-2	12.18	1.81
6	9	2	16.54	2.41
6	9	2	16.64	1.12
6	9	2	14.25	1.79
-7	-9	-2	34.16	1.93

-7	-9	-2	35.96	2.97
-7	-9	-2	27.27	2.54
7	9	2	29.14	2.55
7	9	2	36.17	1.89
-8	-9	-2	14.69	1.20
-8	-9	-2	15.31	2.58
-8	-9	-2	10.88	2.01
8	9	2	15.04	2.20
8	9	2	11.44	2.06
8	9	2	15.63	1.13
-9	-9	-2	18.16	2.82
-9	-9	-2	13.12	2.26
-9	-9	-2	18.66	1.36
9	9	2	20.30	1.30
9	9	2	16.45	2.22
9	9	2	21.39	3.44
9	9	2	12.98	2.00
-10	-9	-2	6.75	2.64
-10	-9	-2	4.66	1.40
-10	-9	-2	4.48	0.66
-10	-9	-2	1.27	1.63
10	9	2	5.00	2.34
10	9	2	5.88	0.94
10	9	2	3.67	1.30
10	9	2	3.23	2.05
-11	-9	-2	23.86	3.55
-11	-9	-2	25.45	1.66
-11	-9	-2	25.16	2.89
11	9	2	23.27	2.69
11	9	2	18.30	3.62
-12	-9	-2	19.70	3.84
-12	-9	-2	11.36	1.86
-12	-9	-2	6.16	2.34
12	9	2	10.82	1.74
12	9	2	5.43	2.38
-13	-9	-2	19.18	3.15
-13	-9	-2	27.35	4.49
13	9	2	18.00	3.12
13	9	2	25.05	5.10
-14	-9	-2	12.03	3.64
-14	-9	-2	4.05	2.00
-14	-9	-2	2.65	3.03
14	9	2	5.88	2.99
14	9	2	3.94	1.98

-15	-9	-2	4.37	3.96
-15	-9	-2	2.38	1.89
-15	-9	-2	0.02	3.58
-7	10	2	6.11	1.25
-6	10	2	2.70	1.05
-5	10	2	19.59	2.01
-5	10	2	17.96	2.01
-5	10	2	16.80	3.55
-4	10	2	5.68	0.95
-4	10	2	5.59	0.93
-4	10	2	6.71	2.70
-3	10	2	21.46	1.85
-3	10	2	23.67	1.86
-3	10	2	23.21	3.84
2	-10	-2	0.03	1.67
-2	10	2	0.69	0.61
-2	10	2	2.36	2.02
-2	10	2	0.65	0.47
1	-10	-2	11.24	1.86
-1	10	2	8.86	2.03
0	-10	-2	7.93	1.84
0	10	2	6.31	0.84
0	10	2	6.32	1.06
0	10	2	8.89	1.87
-1	-10	-2	31.34	3.03
1	10	2	27.29	2.95
1	10	2	28.75	1.84
-2	-10	-2	4.95	1.77
2	10	2	3.42	1.57
2	10	2	2.96	0.56
2	10	2	3.80	1.00
2	10	2	3.76	0.77
-3	-10	-2	21.35	1.98
-3	-10	-2	26.87	2.08
-3	-10	-2	24.04	2.94
3	10	2	22.36	1.53
3	10	2	25.14	2.16
3	10	2	21.32	1.85
3	10	2	24.13	2.70
-4	-10	-2	8.44	1.15
-4	-10	-2	11.79	1.96
4	10	2	9.77	0.98
4	10	2	10.89	2.27
4	10	2	9.80	1.91

4	10	2	9.02	1.10
-5	-10	-2	10.43	1.83
-5	-10	-2	5.88	1.58
-5	-10	-2	7.79	1.16
5	10	2	9.94	0.97
5	10	2	11.93	1.83
5	10	2	7.20	1.32
5	10	2	8.06	1.16
-6	-10	-2	1.16	1.18
-6	-10	-2	2.69	1.03
-6	-10	-2	4.28	1.83
6	10	2	2.92	0.55
6	10	2	5.32	1.71
6	10	2	2.61	1.01
6	10	2	1.11	1.17
-7	-10	-2	6.84	1.36
-7	-10	-2	4.01	1.45
7	10	2	9.50	2.09
7	10	2	7.04	1.24
-8	-10	-2	7.02	1.13
-8	-10	-2	8.90	2.03
8	10	2	8.98	1.19
8	10	2	6.65	1.29
8	10	2	9.17	2.24
8	10	2	5.22	1.56
-9	-10	-2	6.70	1.56
-9	-10	-2	9.55	1.19
9	10	2	11.16	1.14
9	10	2	7.17	1.78
9	10	2	8.50	1.40
9	10	2	10.79	2.49
-10	-10	-2	0.46	1.42
-10	-10	-2	2.87	2.13
10	10	2	1.81	0.61
10	10	2	2.40	2.35
10	10	2	0.69	1.25
-11	-10	-2	25.56	3.77
-11	-10	-2	28.68	1.85
-11	-10	-2	26.82	3.20
11	10	2	24.75	3.01
11	10	2	26.73	1.85
11	10	2	23.62	4.01
-12	-10	-2	2.92	1.78
-12	-10	-2	2.44	2.26

12	10	2	2.96	1.61
12	10	2	0.49	2.41
12	10	2	6.11	3.16
-13	-10	-2	4.85	1.97
-13	-10	-2	9.05	3.33
13	10	2	7.07	1.90
13	10	2	-0.50	2.71
-14	-10	-2	5.46	2.17
-14	-10	-2	3.35	3.63
-14	-10	-2	1.84	3.10
14	10	2	1.50	2.19
14	10	2	1.01	3.13
-15	-10	-2	2.17	1.86
-15	-10	-2	6.75	4.19
-15	-10	-2	2.61	3.70
-6	11	2	16.11	2.24
-5	11	2	7.18	1.17
-4	11	2	22.17	3.01
-4	11	2	19.76	2.06
-4	11	2	16.39	3.53
-3	11	2	15.32	1.79
-3	11	2	15.29	1.80
-3	11	2	14.36	2.95
-2	11	2	36.27	4.17
-2	11	2	39.71	2.57
1	-11	-2	31.86	3.37
-1	11	2	31.30	3.75
-1	11	2	31.27	2.20
0	-11	-2	45.10	3.85
0	11	2	43.76	3.95
0	11	2	43.72	2.70
-1	-11	-2	37.49	5.59
1	11	2	47.10	2.84
1	11	2	53.25	2.89
1	11	2	45.00	3.85
-2	-11	-2	30.29	3.44
2	11	2	32.43	2.03
2	11	2	31.30	2.62
2	11	2	34.00	3.35
2	11	2	33.39	2.27
-3	-11	-2	17.70	3.02
3	11	2	20.77	2.14
3	11	2	20.69	1.82
3	11	2	18.64	1.48

3	11	2	24.14	3.00
-4	-11	-2	29.93	2.39
-4	-11	-2	29.73	3.41
4	11	2	28.60	2.22
4	11	2	29.45	1.87
4	11	2	28.60	2.58
4	11	2	33.75	3.17
-5	-11	-2	18.48	3.13
-5	-11	-2	17.54	2.09
-5	-11	-2	16.35	2.43
5	11	2	16.56	1.35
5	11	2	15.74	2.25
5	11	2	18.47	2.87
5	11	2	17.12	1.93
-6	-11	-2	34.79	3.53
-6	-11	-2	34.77	2.73
-6	-11	-2	32.92	2.92
6	11	2	34.43	2.03
6	11	2	34.93	3.49
6	11	2	31.02	2.90
6	11	2	35.89	2.68
-7	-11	-2	21.83	2.41
-7	-11	-2	20.91	3.89
-7	-11	-2	18.17	2.55
7	11	2	23.61	3.46
7	11	2	16.88	2.61
7	11	2	21.66	1.52
7	11	2	22.48	2.36
-8	-11	-2	36.22	3.66
-8	-11	-2	26.60	3.17
-8	-11	-2	31.55	2.97
8	11	2	40.47	3.98
8	11	2	27.47	3.24
8	11	2	38.78	2.09
8	11	2	33.52	2.93
-9	-11	-2	20.64	3.04
-9	-11	-2	25.12	3.53
-9	-11	-2	20.46	2.77
9	11	2	24.94	3.29
9	11	2	26.83	1.70
9	11	2	23.82	2.75
9	11	2	27.59	3.93
-10	-11	-2	34.09	3.72
-10	-11	-2	44.32	4.12

-10	-11	-2	37.66	3.43
10	11	2	36.07	3.32
10	11	2	34.99	4.43
10	11	2	39.65	2.28
-11	-11	-2	12.28	2.09
-11	-11	-2	23.35	4.06
-11	-11	-2	18.73	1.58
11	11	2	14.45	2.85
11	11	2	15.25	3.23
11	11	2	12.60	2.61
11	11	2	18.06	1.54
-12	-11	-2	9.33	2.56
-12	-11	-2	10.56	2.08
12	11	2	11.47	3.55
12	11	2	11.95	2.03
12	11	2	8.01	2.79
-13	-11	-2	3.56	2.16
-13	-11	-2	9.81	3.34
13	11	2	1.88	2.89
13	11	2	7.44	2.09
-14	-11	-2	11.85	2.51
-14	-11	-2	8.23	3.29
-14	-11	-2	9.72	3.62
14	11	2	10.34	3.56
14	11	2	12.53	2.44
-15	-11	-2	4.14	3.84
-15	-11	-2	10.62	4.26
-5	12	2	1.73	1.13
-4	12	2	4.33	1.09
-3	12	2	2.49	0.93
-2	12	2	17.13	1.90
-2	12	2	22.05	4.42
-2	12	2	18.36	1.97
1	-12	-2	14.44	2.49
-1	12	2	11.60	2.69
-1	12	2	14.01	1.72
0	-12	-2	22.42	3.55
0	12	2	20.97	1.89
0	12	2	19.89	3.64
-1	-12	-2	6.07	2.28
1	12	2	2.88	2.07
1	12	2	4.03	0.84
-2	-12	-2	25.57	3.53
2	12	2	17.82	3.31

2	12	2	20.24	2.02
2	12	2	22.04	1.85
-3	-12	-2	19.97	2.10
-3	-12	-2	19.07	3.58
3	12	2	17.41	1.51
3	12	2	18.52	3.26
3	12	2	18.63	2.22
3	12	2	17.69	1.95
-4	-12	-2	16.50	3.35
4	12	2	19.03	3.11
4	12	2	13.87	2.03
4	12	2	14.77	1.36
4	12	2	16.10	2.25
-5	-12	-2	20.72	2.77
-5	-12	-2	20.01	2.32
5	12	2	18.63	1.52
5	12	2	20.96	2.50
5	12	2	18.41	3.23
5	12	2	21.91	2.13
-6	-12	-2	39.11	4.14
-6	-12	-2	43.00	3.38
-6	-12	-2	43.22	3.16
6	12	2	39.81	3.32
6	12	2	41.11	2.43
6	12	2	40.87	3.96
6	12	2	43.79	3.02
-7	-12	-2	18.26	2.82
-7	-12	-2	19.95	2.58
-7	-12	-2	21.16	3.62
7	12	2	21.70	2.85
7	12	2	22.52	2.42
7	12	2	22.02	1.64
7	12	2	22.18	3.71
-8	-12	-2	9.42	1.73
-8	-12	-2	10.90	2.68
-8	-12	-2	7.03	1.62
8	12	2	11.51	1.30
8	12	2	8.63	1.52
8	12	2	9.30	2.63
8	12	2	6.99	1.78
-9	-12	-2	21.16	3.82
-9	-12	-2	16.22	3.00
-9	-12	-2	14.49	2.86
9	12	2	22.11	1.69

9	12	2	24.18	3.32
9	12	2	24.56	4.30
9	12	2	16.26	2.73
-10	-12	-2	26.02	3.49
-10	-12	-2	29.41	4.22
-10	-12	-2	25.23	3.36
10	12	2	29.05	2.02
10	12	2	35.67	4.80
10	12	2	27.20	3.19
-11	-12	-2	16.55	3.19
-11	-12	-2	11.72	2.24
-11	-12	-2	9.28	2.32
11	12	2	10.93	2.73
11	12	2	16.15	1.67
11	12	2	13.93	3.62
11	12	2	12.97	2.02
-12	-12	-2	15.79	3.36
-12	-12	-2	6.98	2.22
-12	-12	-2	8.68	2.62
12	12	2	7.30	3.02
12	12	2	11.07	2.12
12	12	2	12.80	1.67
-13	-12	-2	3.85	3.55
-13	-12	-2	1.24	2.40
-13	-12	-2	9.32	3.76
13	12	2	2.89	2.08
13	12	2	2.13	3.18
-14	-12	-2	4.39	2.59
-14	-12	-2	7.03	3.87
-14	-12	-2	1.40	3.27
14	12	2	1.50	3.80
14	12	2	0.79	2.36
-4	13	2	4.79	1.23
-3	13	2	12.93	2.12
-2	13	2	8.36	1.25
1	-13	-2	20.16	4.15
-1	13	2	18.41	2.03
0	-13	-2	17.55	2.78
0	13	2	16.84	3.04
0	13	2	15.81	1.91
0	13	2	14.86	2.63
-1	-13	-2	22.25	4.11
1	13	2	18.70	1.86
1	13	2	22.38	2.01

1	13	2	22.44	3.05
-2	-13	-2	9.53	2.59
2	13	2	10.37	2.53
2	13	2	10.04	1.40
2	13	2	9.33	1.61
-3	-13	-2	18.53	3.81
3	13	2	17.52	2.81
3	13	2	16.59	1.60
3	13	2	16.93	2.52
3	13	2	18.11	1.87
-4	-13	-2	14.27	2.24
-4	-13	-2	15.86	2.81
4	13	2	13.04	2.45
4	13	2	13.26	2.08
4	13	2	14.92	2.54
-5	-13	-2	20.77	4.02
-5	-13	-2	20.45	2.49
5	13	2	22.40	2.35
5	13	2	22.04	2.80
5	13	2	17.12	3.57
-6	-13	-2	34.05	4.30
-6	-13	-2	28.04	2.85
6	13	2	30.06	3.92
6	13	2	28.26	3.11
6	13	2	27.36	2.68
-7	-13	-2	29.81	4.12
-7	-13	-2	24.53	2.92
-7	-13	-2	21.58	2.84
7	13	2	23.71	1.84
7	13	2	23.55	2.66
7	13	2	24.74	4.03
7	13	2	24.43	3.16
-8	-13	-2	10.09	1.83
-8	-13	-2	16.20	3.06
-8	-13	-2	8.80	1.82
8	13	2	11.19	1.46
8	13	2	9.67	2.03
8	13	2	15.58	3.10
-9	-13	-2	21.59	3.39
-9	-13	-2	24.40	4.47
-9	-13	-2	18.46	3.13
9	13	2	24.05	1.91
9	13	2	17.41	2.84
9	13	2	22.44	3.48

9	13	2	27.22	4.86
-10	-13	-2	22.68	4.41
-10	-13	-2	13.50	2.26
-10	-13	-2	12.14	2.36
10	13	2	16.90	3.99
10	13	2	16.03	1.72
10	13	2	12.50	1.97
-11	-13	-2	20.87	3.50
-11	-13	-2	19.06	3.69
-11	-13	-2	20.15	3.83
11	13	2	19.53	1.91
11	13	2	14.02	2.93
11	13	2	14.06	2.20
-12	-13	-2	11.52	3.60
-12	-13	-2	10.71	2.68
-12	-13	-2	5.97	2.43
12	13	2	8.24	2.15
12	13	2	6.25	3.16
-13	-13	-2	11.28	3.36
-13	-13	-2	14.62	3.09
-13	-13	-2	20.32	3.85
13	13	2	16.76	2.66
13	13	2	15.53	3.83
-14	-13	-2	6.40	4.11
-14	-13	-2	3.90	3.44
14	13	2	3.59	2.41
14	13	2	-1.17	3.86
-2	14	2	6.58	1.27
1	-14	-2	13.39	3.24
-1	14	2	8.27	1.23
0	-14	-2	8.73	2.91
0	14	2	5.75	1.13
-1	-14	-2	16.84	3.26
1	14	2	18.30	2.08
-2	-14	-2	22.39	4.50
2	14	2	17.42	2.13
2	14	2	19.51	2.49
-3	-14	-2	24.12	4.31
3	14	2	23.39	3.02
3	14	2	19.58	1.91
3	14	2	25.52	2.22
3	14	2	26.70	4.54
-4	-14	-2	14.24	2.98
-4	-14	-2	13.36	2.51

4	14	2	13.71	2.86
4	14	2	12.50	1.57
4	14	2	12.19	1.88
4	14	2	14.02	1.62
4	14	2	12.05	1.91
-5	-14	-2	16.33	2.78
-5	-14	-2	21.50	4.38
-5	-14	-2	19.95	2.75
5	14	2	19.47	2.96
5	14	2	16.96	1.76
5	14	2	16.81	2.50
5	14	2	18.01	3.09
-6	-14	-2	19.28	3.33
-6	-14	-2	17.55	2.88
6	14	2	16.42	1.70
6	14	2	13.86	2.08
6	14	2	19.04	3.16
6	14	2	16.79	2.56
-7	-14	-2	16.64	3.03
-7	-14	-2	17.52	3.27
-7	-14	-2	22.96	4.72
7	14	2	20.63	3.30
7	14	2	18.98	2.77
7	14	2	19.15	3.50
-8	-14	-2	27.79	4.77
-8	-14	-2	19.15	3.27
-8	-14	-2	22.16	3.31
8	14	2	22.41	3.43
8	14	2	18.76	2.97
-9	-14	-2	17.39	3.42
-9	-14	-2	23.39	3.75
-9	-14	-2	21.79	3.59
9	14	2	21.90	3.17
9	14	2	20.51	3.70
-10	-14	-2	14.49	3.62
-10	-14	-2	8.18	2.42
-10	-14	-2	12.40	2.40
10	14	2	8.92	1.95
10	14	2	6.95	3.02
-11	-14	-2	7.12	2.64
-11	-14	-2	9.77	2.65
-11	-14	-2	10.13	3.61
11	14	2	6.35	2.03
11	14	2	3.63	3.02

-12	-14	-2	15.12	3.97
-12	-14	-2	8.21	2.80
-12	-14	-2	13.40	3.03
12	14	2	10.76	2.88
12	14	2	7.13	3.61
-13	-14	-2	12.39	4.12
-13	-14	-2	6.82	3.20
-13	-14	-2	5.30	3.23
13	14	2	7.51	1.51
13	14	2	6.78	4.18
0	-15	-2	14.73	3.65
0	15	2	17.56	2.36
-1	-15	-2	6.67	3.23
1	15	2	9.28	1.33
-2	-15	-2	27.29	5.23
2	15	2	31.11	2.65
-3	-15	-2	15.95	3.51
3	15	2	20.20	2.79
3	15	2	15.87	2.21
-4	-15	-2	24.24	4.89
4	15	2	23.39	2.96
4	15	2	28.72	2.55
4	15	2	25.29	4.10
-5	-15	-2	20.61	3.57
5	15	2	20.00	3.24
5	15	2	15.65	2.82
5	15	2	16.66	2.41
-6	-15	-2	32.23	5.08
6	15	2	24.33	3.38
6	15	2	18.97	2.11
6	15	2	22.58	2.48
6	15	2	21.82	2.94
-7	-15	-2	8.84	3.47
7	15	2	13.51	2.23
7	15	2	11.89	1.82
7	15	2	11.28	1.85
-8	-15	-2	27.02	5.38
8	15	2	25.40	3.83
8	15	2	19.73	3.33
8	15	2	21.44	2.18
9	15	2	12.22	2.22
9	15	2	12.53	2.76
-10	-15	-2	27.25	5.39
-10	-15	-2	10.75	2.39

10	15	2	15.84	2.41
10	15	2	20.54	2.24
-11	-15	-2	14.10	2.89
-11	-15	-2	10.09	4.08
11	15	2	11.14	2.40
11	15	2	8.77	1.38
-4	-16	-2	9.98	3.69
4	16	2	10.62	1.94
4	16	2	9.19	1.42
-5	-16	-2	18.85	3.85
5	16	2	21.23	2.64
5	16	2	22.61	3.22
-6	-16	-2	18.52	3.90
6	16	2	24.17	2.69
6	16	2	18.62	3.34
-7	-16	-2	16.30	3.90
7	16	2	11.93	2.14
7	16	2	15.92	2.62
-8	-16	-2	6.32	3.88
8	16	2	6.61	1.98
8	16	2	6.47	1.46
-9	-16	-2	8.69	3.88
9	16	2	7.15	2.20
10	14	-3	10.70	5.03
10	14	-3	7.19	2.79
-10	-14	3	7.30	2.76
-10	-14	3	12.05	3.76
-10	-14	3	5.08	2.85
9	14	-3	9.11	2.54
9	14	-3	5.63	3.35
9	14	-3	7.81	1.24
-9	-14	3	8.94	2.62
-9	-14	3	10.21	2.62
-9	-14	3	10.95	3.68
8	14	-3	14.53	3.19
8	14	-3	13.10	2.45
8	14	-3	14.07	2.15
-8	-14	3	15.28	3.57
-8	-14	3	13.61	3.47
-8	-14	3	14.87	2.70
7	14	-3	16.63	2.04
7	14	-3	18.48	3.53
7	14	-3	16.40	2.23
-7	-14	3	18.43	3.59

6	14	-3	11.47	1.93
6	14	-3	12.47	1.48
6	14	-3	12.70	2.21
-6	-14	3	15.31	3.40
5	14	-3	12.04	2.14
5	14	-3	16.87	2.29
5	14	-3	14.32	2.00
-5	-14	3	23.73	4.90
4	14	-3	19.57	2.42
4	14	-3	19.72	3.24
-4	-14	3	19.05	3.48
3	14	-3	16.88	2.36
3	14	-3	13.93	2.28
-3	-14	3	19.72	3.43
2	14	-3	20.47	2.47
2	14	-3	20.84	3.61
-2	-14	3	21.37	3.73
1	14	-3	12.21	2.50
1	14	-3	16.39	2.29
-1	-14	3	18.10	3.76
11	13	-3	11.44	2.62
11	13	-3	7.68	3.23
-11	-13	3	6.42	2.70
-11	-13	3	11.12	2.95
-11	-13	3	7.11	3.53
10	13	-3	5.98	2.81
10	13	-3	1.04	2.21
-10	-13	3	2.57	2.32
-10	-13	3	4.18	3.30
-10	-13	3	3.60	2.48
9	13	-3	17.53	1.94
9	13	-3	17.36	1.89
9	13	-3	9.63	4.07
9	13	-3	9.92	2.70
-9	-13	3	15.58	3.43
-9	-13	3	13.34	2.48
-9	-13	3	15.67	2.46
8	13	-3	5.99	2.11
8	13	-3	9.91	1.72
8	13	-3	6.88	2.36
8	13	-3	11.43	1.74
-8	-13	3	9.68	2.24
-8	-13	3	10.20	3.13
-8	-13	3	18.64	3.57

7	13	-3	12.51	2.11
7	13	-3	16.51	1.78
7	13	-3	19.12	3.70
7	13	-3	18.55	1.88
-7	-13	3	11.74	2.23
-7	-13	3	16.95	3.28
-7	-13	3	14.00	2.27
6	13	-3	4.65	0.99
6	13	-3	6.00	1.71
6	13	-3	8.36	2.02
-6	-13	3	6.65	1.97
-6	-13	3	9.03	3.01
-6	-13	3	6.83	1.92
5	13	-3	7.24	1.94
5	13	-3	9.53	1.19
5	13	-3	9.79	1.83
-5	-13	3	8.25	1.92
-5	-13	3	6.92	1.98
-5	-13	3	10.66	2.95
4	13	-3	5.98	1.63
4	13	-3	3.10	1.70
4	13	-3	5.40	1.03
4	13	-3	3.36	0.94
-4	-13	3	5.25	2.76
-4	-13	3	3.45	2.10
-4	-13	3	3.23	1.76
3	13	-3	12.54	1.87
3	13	-3	8.49	1.24
3	13	-3	16.02	2.91
3	13	-3	8.92	1.28
-3	-13	3	13.23	2.99
2	13	-3	11.04	1.86
2	13	-3	10.03	1.89
2	13	-3	8.57	1.36
2	13	-3	11.58	1.97
-2	-13	3	11.06	2.99
1	13	-3	17.15	3.27
1	13	-3	11.26	2.07
1	13	-3	9.92	1.34
-1	-13	3	11.84	3.24
0	13	-3	4.79	2.29
0	13	-3	5.18	1.18
0	-13	3	1.88	2.98
-1	13	-3	3.05	1.07

1	-13	3	4.24	3.13
-2	13	-3	3.67	1.14
12	12	-3	7.84	4.73
12	12	-3	12.63	2.65
-12	-12	3	11.16	3.55
-12	-12	3	9.09	3.01
-12	-12	3	11.87	2.78
11	12	-3	7.06	2.35
11	12	-3	6.94	2.88
-11	-12	3	6.62	2.62
-11	-12	3	7.05	3.40
-11	-12	3	6.39	2.35
10	12	-3	14.86	2.81
10	12	-3	17.26	3.35
-10	-12	3	15.07	2.51
-10	-12	3	14.93	2.47
-10	-12	3	17.27	3.49
9	12	-3	7.03	2.32
9	12	-3	10.47	2.04
-9	-12	3	13.37	3.17
-9	-12	3	9.42	2.05
-9	-12	3	7.64	2.06
8	12	-3	15.25	2.05
8	12	-3	26.16	5.23
8	12	-3	19.82	3.41
8	12	-3	16.12	1.72
8	12	-3	17.10	1.67
-8	-12	3	19.75	4.08
-8	-12	3	13.74	2.14
-8	-12	3	19.16	3.23
7	12	-3	12.68	3.37
7	12	-3	10.13	2.06
7	12	-3	11.75	1.46
7	12	-3	13.82	1.56
7	12	-3	12.46	1.83
-7	-12	3	12.87	2.88
-7	-12	3	10.16	1.97
-7	-12	3	9.15	1.90
6	12	-3	32.64	3.58
6	12	-3	31.70	2.16
6	12	-3	33.05	4.58
6	12	-3	26.26	2.02
6	12	-3	29.26	3.39
-6	-12	3	28.50	3.07

-6	-12	3	26.87	3.22
-6	-12	3	31.60	4.29
5	12	-3	27.38	3.02
5	12	-3	23.89	2.00
5	12	-3	28.13	3.26
5	12	-3	35.59	4.40
5	12	-3	28.54	2.90
-5	-12	3	30.46	4.24
-5	-12	3	30.06	2.99
-5	-12	3	28.59	3.08
4	12	-3	23.16	1.94
4	12	-3	20.57	3.87
4	12	-3	24.52	2.98
4	12	-3	22.33	2.65
-4	-12	3	21.23	2.84
-4	-12	3	23.09	4.01
-4	-12	3	19.73	2.74
3	12	-3	12.06	4.06
3	12	-3	13.63	1.66
3	12	-3	11.66	1.81
3	12	-3	12.24	2.38
-3	-12	3	11.61	1.74
-3	-12	3	8.59	2.37
-3	-12	3	13.58	2.79
2	12	-3	14.13	2.92
2	12	-3	14.57	1.79
2	12	-3	15.95	2.81
2	12	-3	16.77	1.80
2	12	-3	14.54	2.48
-2	-12	3	11.19	1.72
-2	-12	3	17.58	2.88
1	12	-3	9.31	1.71
1	12	-3	8.89	1.14
1	12	-3	10.70	1.78
1	12	-3	9.42	2.74
1	12	-3	8.00	1.14
-1	-12	3	9.37	2.77
0	12	-3	16.80	1.96
0	12	-3	17.91	2.97
0	-12	3	16.33	3.07
-1	12	-3	10.73	1.88
-1	12	-3	16.16	3.04
1	-12	3	13.06	2.92
-2	12	-3	10.30	2.55

-2	12	-3	15.54	1.96
-2	12	-3	13.53	2.45
-3	12	-3	2.26	1.02
13	11	-3	1.56	2.50
-13	-11	3	9.09	3.08
-13	-11	3	6.57	3.60
12	11	-3	5.13	3.09
12	11	-3	7.30	2.52
-12	-11	3	8.00	2.76
-12	-11	3	11.92	3.61
-12	-11	3	9.60	2.38
11	11	-3	8.80	2.74
11	11	-3	8.26	2.09
-11	-11	3	8.25	2.10
-11	-11	3	6.96	2.51
-11	-11	3	10.18	3.25
10	11	-3	20.24	3.04
10	11	-3	18.39	4.09
10	11	-3	11.55	2.59
-10	-11	3	20.77	3.33
-10	-11	3	19.29	3.26
-10	-11	3	14.25	2.40
9	11	-3	13.57	3.79
9	11	-3	22.07	3.60
9	11	-3	14.76	2.75
-9	-11	3	17.09	3.05
-9	-11	3	12.36	2.10
-9	-11	3	17.74	3.01
8	11	-3	8.48	1.28
8	11	-3	8.68	3.15
8	11	-3	8.44	1.94
8	11	-3	8.22	1.68
-8	-11	3	9.84	2.65
-8	-11	3	6.76	1.68
-8	-11	3	6.66	1.74
7	11	-3	4.82	1.46
7	11	-3	6.99	1.21
7	11	-3	6.07	1.79
7	11	-3	3.96	0.71
-7	-11	3	5.96	2.35
-7	-11	3	6.52	1.60
-7	-11	3	6.01	1.66
6	11	-3	17.50	1.51
6	11	-3	13.84	1.37

6	11	-3	13.44	2.35
6	11	-3	18.10	2.84
-6	-11	3	15.04	2.46
-6	-11	3	15.13	2.63
-6	-11	3	17.54	2.67
5	11	-3	9.51	1.23
5	11	-3	7.13	1.63
5	11	-3	9.96	1.50
5	11	-3	10.48	1.36
-5	-11	3	8.82	1.56
-5	-11	3	10.36	1.64
-5	-11	3	11.78	2.60
4	11	-3	8.65	1.30
4	11	-3	6.42	0.84
4	11	-3	6.92	1.60
4	11	-3	7.57	1.39
-4	-11	3	9.94	2.36
-4	-11	3	9.25	1.54
-4	-11	3	6.29	1.47
3	11	-3	6.44	0.87
3	11	-3	8.34	1.50
3	11	-3	3.98	2.26
-3	-11	3	9.33	2.51
-3	-11	3	9.89	1.78
-3	-11	3	7.29	1.43
2	11	-3	14.77	2.48
2	11	-3	13.72	2.15
2	11	-3	10.69	1.42
2	11	-3	13.17	1.52
-2	-11	3	16.45	2.66
-2	-11	3	11.83	2.20
1	11	-3	5.94	1.37
1	11	-3	4.89	0.87
1	11	-3	4.20	1.50
-1	-11	3	6.67	2.35
-1	-11	3	4.74	1.72
0	11	-3	6.63	1.56
0	11	-3	7.83	1.03
0	11	-3	9.75	1.61
0	-11	3	12.82	2.67
-1	11	-3	11.54	1.74
-1	11	-3	16.22	2.68
1	-11	3	15.40	2.74
-2	11	-3	6.91	2.20

-2	11	-3	10.15	1.68
2	-11	3	13.99	3.00
-3	11	-3	7.48	1.10
-4	11	-3	2.20	0.98
-5	11	-3	2.02	1.07
-14	-10	3	8.02	3.40
-14	-10	3	11.93	4.14
-13	-10	3	11.79	2.68
-13	-10	3	19.20	3.88
-13	-10	3	12.09	3.11
12	10	-3	8.25	2.03
12	10	-3	7.14	2.78
-12	-10	3	7.39	3.38
-12	-10	3	8.23	2.64
-12	-10	3	7.88	2.20
11	10	-3	13.26	2.67
11	10	-3	20.04	2.90
11	10	-3	17.64	3.94
-11	-10	3	18.39	3.27
-11	-10	3	23.55	4.24
-11	-10	3	13.84	2.94
10	10	-3	24.47	3.89
10	10	-3	29.01	5.03
10	10	-3	22.51	2.95
-10	-10	3	21.43	3.41
-10	-10	3	24.29	3.16
-10	-10	3	28.37	4.08
9	10	-3	25.96	3.52
9	10	-3	28.96	2.81
-9	-10	3	20.54	3.22
-9	-10	3	29.59	3.92
8	10	-3	19.75	2.46
8	10	-3	18.52	3.17
-8	-10	3	15.02	2.69
-8	-10	3	18.19	2.74
-8	-10	3	24.95	3.47
7	10	-3	33.23	4.34
7	10	-3	38.26	3.44
7	10	-3	35.18	2.12
7	10	-3	36.95	2.16
7	10	-3	36.29	2.92
-7	-10	3	38.98	3.09
-7	-10	3	40.00	3.70
-7	-10	3	34.06	3.02

6	10	-3	19.89	1.41
6	10	-3	16.90	2.64
6	10	-3	22.79	3.66
6	10	-3	14.46	1.43
6	10	-3	15.93	2.22
-6	-10	3	18.37	2.92
-6	-10	3	13.80	2.33
-6	-10	3	17.45	2.42
5	10	-3	19.69	1.52
5	10	-3	19.51	2.65
5	10	-3	21.57	2.31
5	10	-3	26.76	3.44
5	10	-3	26.50	1.63
-5	-10	3	21.46	2.51
-5	-10	3	19.75	2.42
-5	-10	3	23.21	4.02
4	10	-3	6.49	1.10
4	10	-3	4.39	1.18
4	10	-3	6.26	1.05
4	10	-3	8.70	2.09
4	10	-3	4.11	1.41
-4	-10	3	4.55	1.30
-4	-10	3	3.61	1.35
3	10	-3	21.31	2.26
3	10	-3	25.15	2.56
3	10	-3	21.74	1.67
3	10	-3	29.96	3.66
3	10	-3	23.46	1.70
-3	-10	3	21.99	2.37
-3	-10	3	26.17	2.50
-3	-10	3	22.08	4.61
2	10	-3	8.61	1.44
2	10	-3	7.27	1.18
2	10	-3	7.67	1.24
-2	-10	3	6.49	1.23
-2	-10	3	8.33	1.45
-2	-10	3	9.17	2.12
1	10	-3	29.89	2.88
1	10	-3	30.44	2.62
-1	-10	3	29.56	3.40
-1	-10	3	24.81	2.40
0	10	-3	28.43	2.65
0	10	-3	30.06	2.07
0	10	-3	30.25	2.71

0	-10	3	30.56	3.68
-1	10	-3	25.35	1.92
-1	10	-3	22.16	2.77
-1	10	-3	26.87	2.58
1	-10	3	19.38	3.44
-2	10	-3	9.06	1.54
-2	10	-3	13.75	2.69
2	-10	3	10.95	2.42
-3	10	-3	21.56	1.93
-4	10	-3	8.80	1.17
-5	10	-3	7.53	1.12
-6	10	-3	2.89	1.00
-14	-9	3	2.03	3.01
-14	-9	3	6.40	3.73
13	9	-3	3.89	1.84
-13	-9	3	13.43	3.51
-13	-9	3	7.29	2.33
-13	-9	3	6.02	2.88
12	9	-3	5.67	2.69
12	9	-3	12.63	4.27
12	9	-3	7.34	1.94
-12	-9	3	8.08	2.10
-12	-9	3	7.58	2.52
-12	-9	3	9.68	3.16
11	9	-3	12.32	3.64
11	9	-3	16.29	2.55
11	9	-3	9.14	2.43
-11	-9	3	12.39	2.77
-11	-9	3	15.69	3.02
-11	-9	3	10.43	1.95
10	9	-3	8.83	2.14
10	9	-3	12.66	2.31
-10	-9	3	11.22	2.59
-10	-9	3	8.98	2.05
-10	-9	3	9.32	1.75
9	9	-3	1.25	3.36
9	9	-3	6.91	1.37
9	9	-3	6.47	1.81
-9	-9	3	2.71	1.62
-9	-9	3	5.07	1.50
-9	-9	3	9.52	2.27
8	9	-3	4.78	1.84
8	9	-3	4.72	1.26
8	9	-3	4.69	2.62

-8	-9	3	5.16	1.45
-8	-9	3	6.07	2.66
-8	-9	3	3.17	1.46
7	9	-3	12.62	1.07
7	9	-3	11.51	2.00
7	9	-3	10.06	1.70
7	9	-3	17.42	3.56
-7	-9	3	9.30	1.58
-7	-9	3	16.31	2.71
-7	-9	3	15.82	2.23
6	9	-3	13.47	2.38
6	9	-3	13.77	2.36
6	9	-3	13.89	1.93
6	9	-3	13.10	1.11
6	9	-3	12.33	1.08
-6	-9	3	11.44	1.49
-6	-9	3	14.63	2.59
-6	-9	3	14.30	2.13
5	9	-3	3.09	1.06
5	9	-3	3.23	0.53
5	9	-3	2.97	1.25
5	9	-3	5.44	1.94
-5	-9	3	3.04	1.22
-5	-9	3	5.17	1.69
4	9	-3	17.44	2.88
4	9	-3	12.31	1.14
4	9	-3	11.17	2.21
4	9	-3	12.07	1.83
4	9	-3	13.59	1.17
-4	-9	3	13.12	2.59
-4	-9	3	13.40	2.02
-4	-9	3	11.51	1.90
3	9	-3	20.73	1.44
3	9	-3	24.34	3.06
3	9	-3	19.44	2.26
3	9	-3	18.03	2.01
-3	-9	3	20.48	2.18
-3	-9	3	14.62	1.98
-3	-9	3	22.73	2.82
2	9	-3	8.29	1.10
2	9	-3	8.07	1.17
2	9	-3	8.30	1.33
2	9	-3	10.83	1.89
-2	-9	3	8.12	1.18

-2	-9	3	10.79	1.92
1	9	-3	3.47	0.65
1	9	-3	4.01	1.09
1	9	-3	5.81	1.75
1	9	-3	3.76	1.12
-1	-9	3	3.82	1.71
0	9	-3	9.75	1.18
0	9	-3	9.22	1.26
0	9	-3	8.72	1.30
0	9	-3	10.59	1.96
0	-9	3	7.47	1.16
0	-9	3	12.04	2.07
0	-9	3	7.54	1.60
-1	9	-3	7.22	1.28
1	-9	3	7.51	1.46
1	-9	3	7.23	1.97
-2	9	-3	0.61	0.66
-2	9	-3	-0.59	1.24
-2	9	-3	-0.18	1.35
2	-9	3	2.04	1.84
-3	9	-3	2.98	1.60
-3	9	-3	2.72	0.76
-4	9	-3	7.30	1.00
-5	9	-3	6.88	1.06
-5	9	-3	6.37	2.78
-6	9	-3	3.16	0.96
-7	9	-3	2.38	0.99
-8	9	-3	8.11	4.11
-15	-8	3	5.47	3.40
-15	-8	3	0.69	4.37
-14	-8	3	3.19	3.91
-14	-8	3	3.03	3.04
-14	-8	3	2.89	3.17
13	8	-3	9.17	3.60
13	8	-3	10.48	2.51
-13	-8	3	8.52	2.19
-13	-8	3	12.09	2.85
-13	-8	3	16.08	3.61
12	8	-3	16.08	3.90
12	8	-3	13.40	2.87
12	8	-3	20.25	2.74
-12	-8	3	16.61	3.18
-12	-8	3	23.32	4.33
-12	-8	3	19.58	3.77

11	8	-3	8.21	2.26
11	8	-3	9.79	1.58
-11	-8	3	7.45	1.81
-11	-8	3	13.68	2.77
10	8	-3	13.17	2.19
10	8	-3	15.68	2.16
-10	-8	3	14.11	2.17
-10	-8	3	18.56	3.35
-10	-8	3	16.74	2.62
9	8	-3	1.74	1.07
9	8	-3	-0.11	2.53
9	8	-3	1.03	1.58
-9	-8	3	-0.62	1.51
-9	-8	3	1.54	1.27
8	8	-3	16.24	2.06
8	8	-3	15.13	2.28
-8	-8	3	21.54	2.33
-8	-8	3	13.52	2.43
7	8	-3	17.00	2.01
7	8	-3	17.52	2.54
7	8	-3	21.43	3.20
-7	-8	3	15.69	2.17
-7	-8	3	13.06	2.34
-7	-8	3	19.82	2.63
6	8	-3	33.84	2.93
6	8	-3	41.22	3.55
6	8	-3	34.74	1.97
6	8	-3	37.26	2.58
-6	-8	3	41.06	3.10
-6	-8	3	29.96	2.74
-6	-8	3	37.73	2.72
5	8	-3	19.44	2.29
5	8	-3	25.81	2.93
5	8	-3	22.30	1.38
5	8	-3	21.59	1.36
5	8	-3	22.10	2.01
-5	-8	3	24.35	2.13
-5	-8	3	24.75	2.55
-5	-8	3	17.54	2.09
4	8	-3	30.67	2.59
4	8	-3	35.88	3.06
4	8	-3	30.91	1.86
4	8	-3	34.87	1.89
4	8	-3	33.34	2.35

-4	-8	3	35.24	2.93
-4	-8	3	32.32	2.40
-4	-8	3	32.95	2.44
3	8	-3	30.14	1.73
3	8	-3	26.38	2.32
3	8	-3	28.90	2.14
3	8	-3	30.47	2.95
-3	-8	3	28.87	2.86
-3	-8	3	28.50	2.18
-3	-8	3	28.78	2.24
2	8	-3	66.63	4.09
2	8	-3	60.90	3.64
2	8	-3	62.51	3.55
2	8	-3	65.03	3.27
-2	-8	3	63.44	3.62
-2	-8	3	62.62	4.08
-2	-8	3	52.17	4.10
1	8	-3	15.31	2.42
1	8	-3	13.79	1.68
1	8	-3	13.45	1.75
1	8	-3	13.62	1.14
-1	-8	3	11.92	2.42
-1	-8	3	10.66	1.30
-1	-8	3	10.30	1.56
0	8	-3	78.10	4.67
0	8	-3	76.03	3.89
0	8	-3	74.49	4.13
0	8	-3	71.64	4.15
0	-8	3	72.27	5.45
0	-8	3	71.08	4.06
0	-8	3	77.11	4.69
-1	8	-3	10.49	1.34
-1	8	-3	12.45	1.88
-1	8	-3	11.88	1.18
-1	8	-3	9.68	1.85
1	-8	3	10.30	1.85
1	-8	3	7.85	1.09
-2	8	-3	14.59	2.23
-2	8	-3	19.38	2.75
-2	8	-3	15.95	1.44
2	-8	3	15.80	2.60
-3	8	-3	0.22	1.34
-3	8	-3	1.78	0.64
-4	8	-3	14.26	1.49

-5	8	-3	6.30	0.94
-6	8	-3	18.73	1.79
-6	8	-3	16.20	4.07
-7	8	-3	6.32	1.06
-8	8	-3	3.70	1.03
-8	8	-3	9.17	3.63
8	-8	3	2.79	1.97
-9	8	-3	-0.56	1.12
-9	8	-3	3.51	4.18
9	-8	3	0.97	2.12
-15	-7	3	7.79	4.48
-15	-7	3	2.74	3.11
-14	-7	3	3.70	3.93
-14	-7	3	3.35	2.85
13	7	-3	6.43	4.68
13	7	-3	2.29	2.75
-13	-7	3	2.19	1.94
-13	-7	3	3.01	2.54
-13	-7	3	7.76	3.43
12	7	-3	12.43	3.51
12	7	-3	9.78	1.76
12	7	-3	4.72	2.50
-12	-7	3	14.38	3.01
-12	-7	3	9.92	1.91
-12	-7	3	8.91	2.33
11	7	-3	10.79	1.67
11	7	-3	5.87	2.24
-11	-7	3	12.85	2.63
-11	-7	3	9.02	1.73
10	7	-3	8.85	2.82
10	7	-3	7.45	1.93
10	7	-3	8.15	1.29
-10	-7	3	7.94	1.53
-10	-7	3	10.13	2.52
-10	-7	3	5.71	1.75
9	7	-3	-0.92	1.47
9	7	-3	1.69	1.03
9	7	-3	-0.20	2.23
-9	-7	3	-1.56	1.41
-9	-7	3	1.68	1.14
-9	-7	3	0.99	1.80
8	7	-3	8.05	1.14
8	7	-3	6.10	2.03
8	7	-3	8.03	2.14

-8	-7	3	8.17	1.26
-8	-7	3	5.38	1.43
-8	-7	3	8.81	1.83
7	7	-3	13.85	2.29
7	7	-3	12.70	1.69
7	7	-3	12.45	2.15
-7	-7	3	10.31	2.05
-7	-7	3	11.89	1.89
-7	-7	3	12.98	2.27
6	7	-3	3.74	1.71
6	7	-3	3.03	1.10
6	7	-3	3.01	0.86
-6	-7	3	3.39	0.95
-6	-7	3	2.57	1.07
-6	-7	3	2.95	1.35
5	7	-3	13.41	0.92
5	7	-3	13.64	1.56
5	7	-3	10.49	1.84
5	7	-3	12.22	0.90
-5	-7	3	14.80	2.01
-5	-7	3	9.46	1.75
-5	-7	3	11.89	1.64
4	7	-3	18.76	2.34
4	7	-3	14.14	1.05
4	7	-3	14.23	1.85
4	7	-3	16.84	1.58
4	7	-3	16.55	1.06
-4	-7	3	14.19	1.70
-4	-7	3	17.67	2.06
-4	-7	3	17.05	1.68
3	7	-3	8.36	1.36
3	7	-3	8.28	1.11
3	7	-3	9.54	0.84
3	7	-3	12.02	2.15
-3	-7	3	8.00	1.49
-3	-7	3	7.14	1.36
-3	-7	3	10.78	1.57
2	7	-3	6.44	1.05
2	7	-3	10.72	1.98
2	7	-3	7.46	0.97
2	7	-3	7.13	0.82
-2	-7	3	9.57	1.47
-2	-7	3	7.58	1.08
-2	-7	3	6.72	1.30

1	7	-3	16.39	1.72
1	7	-3	18.04	1.67
1	7	-3	18.89	1.24
-1	-7	3	16.49	1.61
-1	-7	3	17.96	2.06
-1	-7	3	20.07	2.27
0	7	-3	9.07	1.09
0	7	-3	9.78	0.97
0	7	-3	10.07	2.06
0	7	-3	8.49	1.11
0	-7	3	11.39	1.61
-1	7	-3	9.53	1.55
-1	7	-3	9.21	0.99
-1	7	-3	6.49	1.11
1	-7	3	8.93	1.60
1	-7	3	7.77	1.28
-2	7	-3	1.46	1.13
-2	7	-3	1.85	0.54
-2	7	-3	1.10	0.97
-2	7	-3	3.98	1.49
2	-7	3	3.46	1.40
-3	7	-3	-0.42	1.50
-3	7	-3	0.17	1.20
-3	7	-3	3.09	1.59
-3	7	-3	1.78	0.55
-4	7	-3	-1.69	1.68
-4	7	-3	0.24	0.95
-5	7	-3	0.36	0.61
-7	7	-3	2.25	1.05
-7	7	-3	3.16	0.85
-8	7	-3	2.51	3.29
-8	7	-3	1.21	0.87
-9	7	-3	0.91	3.74
-9	7	-3	2.40	1.01
-10	7	-3	3.26	4.54
-15	-6	3	9.62	4.34
-15	-6	3	8.95	3.13
-15	-6	3	2.88	3.72
-14	-6	3	0.15	2.23
-14	-6	3	4.62	2.82
-14	-6	3	4.32	3.90
-14	-6	3	5.14	3.25
13	6	-3	14.30	3.92
13	6	-3	4.93	2.65

13	6	-3	7.54	1.68
-13	-6	3	9.22	2.78
-13	-6	3	9.14	1.99
-13	-6	3	9.03	2.48
-13	-6	3	13.97	3.40
-13	-6	3	6.67	1.82
12	6	-3	2.72	2.13
12	6	-3	5.60	3.62
12	6	-3	1.92	1.65
-12	-6	3	1.07	2.04
-12	-6	3	7.47	2.83
-12	-6	3	4.27	1.67
11	6	-3	19.78	3.52
11	6	-3	12.00	3.46
-11	-6	3	23.77	2.72
-11	-6	3	18.32	2.84
10	6	-3	9.60	1.95
10	6	-3	14.11	2.72
-10	-6	3	17.28	3.21
-10	-6	3	10.43	1.83
-10	-6	3	12.24	2.34
9	6	-3	17.38	2.14
9	6	-3	18.42	3.43
9	6	-3	17.49	2.70
-9	-6	3	14.52	1.80
-9	-6	3	16.84	2.17
-9	-6	3	20.31	2.77
8	6	-3	21.92	3.01
8	6	-3	17.96	1.85
8	6	-3	13.74	2.24
-8	-6	3	13.74	1.70
-8	-6	3	17.93	2.01
7	6	-3	43.23	3.21
7	6	-3	48.27	3.64
7	6	-3	47.88	2.87
-7	-6	3	49.24	3.25
-7	-6	3	46.13	2.99
-7	-6	3	44.61	3.10
6	6	-3	6.72	0.94
6	6	-3	3.96	1.12
6	6	-3	7.08	1.60
-6	-6	3	8.17	1.37
-6	-6	3	6.24	1.00
5	6	-3	9.35	1.33

5	6	-3	10.71	1.61
5	6	-3	8.46	1.63
-5	-6	3	8.77	1.43
-5	-6	3	8.30	1.12
-5	-6	3	10.61	1.70
4	6	-3	3.96	0.94
4	6	-3	4.97	0.59
4	6	-3	5.86	0.60
4	6	-3	6.07	1.29
4	6	-3	5.54	0.81
-4	-6	3	3.46	0.90
-4	-6	3	6.55	1.18
-4	-6	3	5.10	0.84
3	6	-3	50.72	2.65
3	6	-3	53.14	2.92
3	6	-3	50.97	3.00
3	6	-3	53.87	3.34
-3	-6	3	50.32	2.92
-3	-6	3	52.68	3.20
-3	-6	3	50.39	2.94
2	6	-3	41.17	2.44
2	6	-3	41.90	2.19
2	6	-3	45.64	2.87
2	6	-3	36.64	2.52
-2	-6	3	39.42	2.45
-2	-6	3	40.22	2.51
-2	-6	3	45.23	2.87
1	6	-3	66.00	3.27
1	6	-3	65.27	3.47
1	6	-3	66.10	3.80
1	6	-3	56.97	3.47
-1	-6	3	64.81	3.44
-1	-6	3	60.21	3.81
-1	-6	3	65.80	3.78
0	6	-3	11.48	1.38
0	6	-3	9.25	1.41
0	6	-3	12.20	1.88
0	6	-3	14.13	0.97
0	-6	3	10.11	1.25
0	-6	3	11.28	1.84
-1	6	-3	26.51	2.37
-1	6	-3	25.83	1.89
-1	6	-3	27.12	1.57
1	-6	3	22.01	2.50

1	-6	3	24.76	1.76
1	-6	3	25.57	2.37
-2	6	-3	12.39	1.03
-2	6	-3	8.60	1.68
-2	6	-3	12.58	2.37
-2	6	-3	8.02	1.51
2	-6	3	10.30	1.84
2	-6	3	10.31	1.63
-3	6	-3	49.62	2.57
-3	6	-3	39.58	2.88
-3	6	-3	49.36	3.24
-4	6	-3	9.33	1.67
-4	6	-3	6.97	1.05
-5	6	-3	25.97	1.79
-5	6	-3	28.89	3.03
-6	6	-3	7.90	1.64
-6	6	-3	8.15	1.19
-7	6	-3	17.12	1.53
-7	6	-3	13.77	2.03
7	-6	3	14.65	2.40
-8	6	-3	0.77	0.76
-8	6	-3	2.70	3.11
-8	6	-3	0.89	1.06
-9	6	-3	4.82	1.65
-9	6	-3	2.26	3.55
-9	6	-3	6.27	1.03
9	-6	3	3.74	1.72
-10	6	-3	0.43	1.00
-10	6	-3	4.81	4.27
10	-6	3	2.27	2.01
-15	-5	3	12.56	4.55
-15	-5	3	2.38	3.55
-15	-5	3	2.58	2.97
-14	-5	3	2.57	2.02
-14	-5	3	9.11	4.07
-14	-5	3	1.88	2.79
-14	-5	3	3.14	3.25
13	5	-3	9.50	3.87
-13	-5	3	5.22	1.79
-13	-5	3	10.52	3.59
-13	-5	3	6.44	1.80
-13	-5	3	7.98	2.46
-13	-5	3	5.49	2.68
12	5	-3	3.88	1.34

-12	-5	3	7.18	3.02
-12	-5	3	4.18	2.11
-12	-5	3	3.53	2.29
-12	-5	3	4.90	1.58
11	5	-3	8.72	2.80
-11	-5	3	5.76	1.46
-11	-5	3	11.63	2.52
-11	-5	3	6.77	1.90
10	5	-3	10.30	1.88
10	5	-3	14.02	2.64
-10	-5	3	10.28	1.48
-10	-5	3	9.76	1.71
-10	-5	3	13.00	2.28
-10	-5	3	12.69	2.76
9	5	-3	8.87	1.64
9	5	-3	8.04	1.57
9	5	-3	12.51	2.31
-9	-5	3	9.97	1.86
-9	-5	3	12.83	2.47
-9	-5	3	8.37	1.53
-9	-5	3	14.48	2.60
8	5	-3	16.96	2.51
8	5	-3	20.17	2.90
-8	-5	3	17.53	1.95
-8	-5	3	21.54	2.40
7	5	-3	5.93	1.24
7	5	-3	8.27	1.50
-7	-5	3	7.91	1.05
-7	-5	3	11.26	1.87
-7	-5	3	4.80	1.18
6	5	-3	2.45	1.06
6	5	-3	4.94	0.78
6	5	-3	5.78	1.42
-6	-5	3	4.90	0.89
-6	-5	3	6.66	1.23
5	5	-3	-0.08	0.60
5	5	-3	1.07	1.18
5	5	-3	-1.50	0.79
-5	-5	3	-0.34	0.64
-5	-5	3	0.74	0.95
4	5	-3	-0.21	1.03
4	5	-3	0.07	0.70
4	5	-3	0.68	0.58
-4	-5	3	0.36	0.66

-4	-5	3	0.20	0.61
-4	-5	3	-0.35	0.83
3	5	-3	0.68	0.58
3	5	-3	-0.20	0.68
3	5	-3	1.47	0.26
3	5	-3	1.91	1.01
-3	-5	3	1.21	0.58
-3	-5	3	-0.66	0.67
-3	-5	3	0.97	0.87
2	5	-3	10.23	1.58
2	5	-3	9.98	0.73
2	5	-3	9.13	1.11
-2	-5	3	10.12	1.52
-2	-5	3	9.53	1.10
-2	-5	3	8.19	0.89
1	5	-3	0.02	0.60
1	5	-3	0.81	0.95
1	5	-3	0.62	0.27
-1	-5	3	0.34	0.55
-1	-5	3	-0.87	0.68
-1	-5	3	0.64	0.91
0	5	-3	1.93	0.98
0	5	-3	1.57	0.65
0	5	-3	2.16	0.36
0	-5	3	1.71	0.60
0	-5	3	0.34	0.61
0	-5	3	1.25	0.94
-1	5	-3	0.85	0.95
-1	5	-3	-0.43	0.69
-1	5	-3	0.17	0.32
1	-5	3	0.94	0.99
1	-5	3	0.28	0.55
1	-5	3	0.05	0.37
-2	5	-3	2.68	1.01
-2	5	-3	1.46	0.86
-2	5	-3	2.33	0.43
2	-5	3	2.01	0.61
2	-5	3	2.04	0.98
2	-5	3	1.59	0.46
-3	5	-3	1.35	1.05
-3	5	-3	-0.17	0.99
-3	5	-3	0.63	0.39
3	-5	3	-0.08	0.52
-4	5	-3	5.63	0.86

-4	5	-3	5.52	1.33
-4	5	-3	6.55	1.36
-4	5	-3	2.47	1.58
4	-5	3	4.60	0.67
-5	5	-3	3.44	0.58
-5	5	-3	4.05	1.36
-5	5	-3	2.11	0.71
5	-5	3	2.61	0.64
5	-5	3	2.35	0.84
-6	5	-3	12.65	2.42
-6	5	-3	12.30	1.17
-6	5	-3	8.32	1.65
6	-5	3	10.19	1.39
-7	5	-3	1.78	2.37
-7	5	-3	2.24	0.92
-7	5	-3	1.24	0.74
7	-5	3	2.17	0.78
-8	5	-3	0.90	1.02
-8	5	-3	4.88	2.87
-8	5	-3	1.29	0.74
8	-5	3	0.02	1.30
-9	5	-3	-0.57	0.75
-9	5	-3	-1.42	3.04
-9	5	-3	0.07	1.09
9	-5	3	-1.09	1.51
-10	5	-3	-1.26	3.82
-10	5	-3	1.43	0.93
10	-5	3	1.80	1.90
-11	5	-3	8.22	4.49
-15	-4	3	4.30	4.40
-15	-4	3	0.84	3.83
-14	-4	3	5.01	3.36
-14	-4	3	6.59	2.44
-14	-4	3	11.21	4.01
-14	-4	3	6.30	2.63
13	4	-3	3.23	3.65
-13	-4	3	4.22	2.77
-13	-4	3	2.71	2.33
-13	-4	3	2.09	1.66
-13	-4	3	7.54	3.35
-12	-4	3	22.39	4.14
-12	-4	3	19.99	2.81
-12	-4	3	18.65	3.38
-12	-4	3	28.69	4.35

11	4	-3	2.84	1.47
11	4	-3	5.24	2.63
-11	-4	3	9.45	2.59
-11	-4	3	5.37	1.99
-11	-4	3	5.62	1.33
-11	-4	3	2.83	1.73
10	4	-3	6.26	1.62
10	4	-3	5.77	2.41
-10	-4	3	4.47	1.50
-10	-4	3	10.39	2.17
-10	-4	3	6.76	1.27
-10	-4	3	8.09	1.69
9	4	-3	6.44	2.09
9	4	-3	1.27	1.38
-9	-4	3	1.81	1.40
-9	-4	3	3.64	1.76
-9	-4	3	3.99	1.03
8	4	-3	25.55	4.80
8	4	-3	24.85	2.48
-8	-4	3	29.23	2.66
-8	-4	3	25.31	2.08
-8	-4	3	21.06	2.33
-8	-4	3	29.44	2.53
7	4	-3	25.92	2.35
7	4	-3	31.31	2.75
7	4	-3	27.28	1.91
-7	-4	3	32.09	2.40
-7	-4	3	27.21	2.08
6	4	-3	24.25	2.32
6	4	-3	17.94	1.95
-6	-4	3	21.68	1.71
-6	-4	3	25.09	1.95
-6	-4	3	16.90	1.82
5	4	-3	42.77	2.41
5	4	-3	42.49	2.78
5	4	-3	35.73	2.58
-5	-4	3	41.69	2.43
-5	-4	3	45.50	2.55
-5	-4	3	35.86	2.48
4	4	-3	66.71	3.42
4	4	-3	64.70	3.53
4	4	-3	62.35	3.67
-4	-4	3	66.65	3.54
-4	-4	3	64.11	3.44

-4	-4	3	64.34	3.46
3	4	-3	2.95	0.66
3	4	-3	4.53	0.97
3	4	-3	4.58	0.62
-3	-4	3	2.98	0.64
-3	-4	3	3.44	0.57
-3	-4	3	4.12	0.84
2	4	-3	58.67	2.88
2	4	-3	59.78	3.29
2	4	-3	55.45	3.07
2	4	-3	58.93	3.05
-2	-4	3	58.26	3.21
-2	-4	3	55.88	3.04
-2	-4	3	56.79	3.04
1	4	-3	0.53	0.50
1	4	-3	0.74	0.23
1	4	-3	0.36	0.79
1	4	-3	0.56	0.54
-1	-4	3	0.69	0.26
-1	-4	3	1.04	0.56
-1	-4	3	-0.14	0.77
-1	-4	3	1.13	0.46
0	4	-3	18.25	1.31
0	4	-3	16.22	1.31
0	4	-3	17.29	1.61
0	-4	3	16.26	1.57
0	-4	3	17.99	1.09
0	-4	3	18.31	1.25
0	-4	3	15.49	1.47
-1	4	-3	20.83	1.22
-1	4	-3	18.09	1.50
-1	4	-3	21.73	1.75
-1	4	-3	21.16	1.53
1	-4	3	22.37	1.43
1	-4	3	21.11	1.27
1	-4	3	19.23	1.76
-2	4	-3	50.30	3.00
-2	4	-3	54.73	2.63
-2	4	-3	50.04	2.94
2	-4	3	51.02	2.67
2	-4	3	41.85	2.94
2	-4	3	53.95	2.75
-3	4	-3	0.47	0.89
-3	4	-3	1.04	0.37

-3	4	-3	-0.82	0.82
-3	4	-3	0.12	0.95
3	-4	3	1.02	0.38
-4	4	-3	1.06	0.54
-4	4	-3	1.34	1.02
-4	4	-3	2.29	1.08
-4	4	-3	2.00	0.42
-4	4	-3	3.26	1.09
4	-4	3	1.58	0.44
4	-4	3	1.32	0.61
-5	4	-3	16.87	1.97
-5	4	-3	15.46	1.13
5	-4	3	14.33	1.24
5	-4	3	12.37	1.61
-6	4	-3	15.81	2.17
-6	4	-3	11.11	1.57
-6	4	-3	13.92	1.16
6	-4	3	9.88	1.76
6	-4	3	12.51	1.29
-7	4	-3	-0.17	0.52
-7	4	-3	-0.66	2.04
-7	4	-3	-0.49	0.78
-7	4	-3	-0.72	1.49
7	-4	3	-0.07	0.63
7	-4	3	-0.98	0.90
-8	4	-3	0.93	2.53
-8	4	-3	3.47	0.99
8	-4	3	3.75	1.21
8	-4	3	4.59	0.87
-9	4	-3	2.40	1.06
-9	4	-3	2.04	3.06
-9	4	-3	3.07	0.78
9	-4	3	3.22	1.47
-10	4	-3	0.03	0.83
-10	4	-3	-0.63	1.20
-10	4	-3	6.53	3.67
-10	4	-3	1.32	2.28
10	-4	3	1.46	1.13
10	-4	3	1.08	1.67
-11	4	-3	-2.49	4.36
-11	4	-3	-0.88	0.94
-12	4	-3	5.82	5.24
-15	-3	3	4.57	4.26
-15	-3	3	11.59	4.57

-14	-3	3	9.04	3.95
-14	-3	3	2.26	3.54
13	3	-3	8.96	3.60
-13	-3	3	9.46	3.45
-13	-3	3	6.78	3.16
-13	-3	3	4.46	1.75
-12	-3	3	10.13	3.02
-12	-3	3	6.25	1.52
-12	-3	3	6.69	2.55
11	3	-3	6.34	2.65
-11	-3	3	7.63	2.64
-11	-3	3	5.55	2.10
10	3	-3	1.09	2.22
-10	-3	3	-1.38	1.63
-10	-3	3	1.15	1.99
9	3	-3	3.12	1.95
-9	-3	3	3.56	1.47
-9	-3	3	0.83	1.26
-9	-3	3	6.27	1.82
8	3	-3	10.14	2.17
8	3	-3	7.97	1.25
-8	-3	3	10.74	1.73
-8	-3	3	5.90	1.18
-8	-3	3	7.48	1.46
-8	-3	3	7.89	1.34
7	3	-3	4.44	1.06
7	3	-3	7.52	1.53
7	3	-3	5.38	0.83
-7	-3	3	5.81	0.85
-7	-3	3	8.35	1.77
-7	-3	3	8.19	1.35
-7	-3	3	3.91	0.96
6	3	-3	2.99	0.89
-6	-3	3	5.89	1.11
-6	-3	3	5.25	0.95
-6	-3	3	3.81	0.72
5	3	-3	27.12	1.99
5	3	-3	30.46	2.25
5	3	-3	28.76	2.26
-5	-3	3	27.10	1.94
-5	-3	3	28.04	1.80
-5	-3	3	25.41	1.89
-5	-3	3	30.80	2.03
4	3	-3	13.89	1.39

4	3	-3	16.84	1.63
4	3	-3	17.76	1.56
-4	-3	3	16.07	1.25
-4	-3	3	16.51	1.41
3	3	-3	71.72	3.71
3	3	-3	75.72	3.68
3	3	-3	71.11	3.79
3	3	-3	69.27	3.79
-3	-3	3	70.24	3.66
-3	-3	3	71.88	3.67
-3	-3	3	70.64	3.73
2	3	-3	14.80	1.06
2	3	-3	12.19	1.33
2	3	-3	15.42	0.77
2	3	-3	12.36	1.09
-2	-3	3	12.27	0.79
-2	-3	3	12.56	1.15
-2	-3	3	14.37	1.02
-2	-3	3	12.75	1.08
-2	-3	3	13.01	1.18
1	3	-3	56.29	2.71
1	3	-3	55.70	2.84
1	3	-3	54.39	2.99
1	3	-3	57.58	2.85
-1	-3	3	51.94	2.96
-1	-3	3	54.40	2.83
-1	-3	3	52.26	2.83
-1	-3	3	52.97	2.73
0	3	-3	8.30	0.92
0	3	-3	10.21	0.65
0	3	-3	8.16	1.16
0	3	-3	8.64	0.96
0	-3	3	8.55	1.13
0	-3	3	10.34	0.68
0	-3	3	10.11	0.89
-1	3	-3	76.50	4.10
-1	3	-3	87.13	3.99
-1	3	-3	91.58	4.17
-1	3	-3	74.94	4.17
1	-3	3	83.42	4.01
1	-3	3	73.48	4.02
1	-3	3	68.72	4.19
1	-3	3	87.28	4.10
-2	3	-3	6.10	0.87

-2	3	-3	7.99	1.31
-2	3	-3	9.11	0.67
-2	3	-3	8.40	1.18
-2	3	-3	6.77	0.83
2	-3	3	10.44	1.06
2	-3	3	7.04	1.18
2	-3	3	9.06	0.72
-3	3	-3	9.87	0.98
-3	3	-3	11.22	1.30
-3	3	-3	10.08	0.76
-3	3	-3	7.38	1.17
-3	3	-3	7.09	1.39
3	-3	3	8.70	0.81
3	-3	3	11.74	1.27
3	-3	3	8.75	1.04
3	-3	3	9.85	1.00
-4	3	-3	0.24	0.30
-4	3	-3	0.87	0.86
-4	3	-3	-0.29	0.46
4	-3	3	0.42	0.34
4	-3	3	-0.68	0.49
-5	3	-3	3.57	1.03
-5	3	-3	4.15	0.68
-5	3	-3	4.71	0.70
-5	3	-3	6.98	1.16
-5	3	-3	4.97	1.32
5	-3	3	4.11	0.76
5	-3	3	5.26	0.80
-6	3	-3	2.48	1.71
-6	3	-3	1.49	0.64
-6	3	-3	4.02	1.21
-6	3	-3	2.46	0.48
6	-3	3	2.93	0.57
6	-3	3	1.54	0.79
-7	3	-3	1.11	0.51
-7	3	-3	0.72	1.96
-7	3	-3	-0.03	0.72
-7	3	-3	2.40	1.37
7	-3	3	-0.55	0.90
7	-3	3	1.05	0.54
-8	3	-3	2.97	0.62
-8	3	-3	4.88	2.67
-8	3	-3	1.17	0.89
8	-3	3	2.49	0.68

8	-3	3	1.51	1.10
-9	3	-3	-0.09	0.94
-9	3	-3	0.68	2.65
-9	3	-3	0.23	0.75
9	-3	3	0.23	0.73
9	-3	3	-1.74	1.20
-10	3	-3	-0.94	3.24
-10	3	-3	0.01	0.73
-10	3	-3	0.79	1.10
10	-3	3	-0.70	0.90
10	-3	3	1.85	1.49
-11	3	-3	1.25	0.93
-11	3	-3	2.73	4.08
11	-3	3	0.56	1.19
-12	3	-3	-0.07	1.06
-12	3	-3	5.72	4.93
12	-3	3	-0.29	1.41
-13	3	-3	13.18	5.99
-14	-2	3	2.39	3.69
-14	-2	3	4.23	4.06
13	2	-3	9.19	3.39
-13	-2	3	10.59	3.64
-13	-2	3	5.61	3.38
-12	-2	3	2.30	3.03
-12	-2	3	2.89	2.64
11	2	-3	14.48	2.67
-11	-2	3	11.15	2.43
-11	-2	3	16.94	2.94
10	2	-3	6.59	2.23
-10	-2	3	4.62	1.14
-10	-2	3	8.24	2.29
-10	-2	3	6.51	1.98
9	2	-3	21.09	2.79
-9	-2	3	14.11	2.01
-9	-2	3	16.46	2.83
-9	-2	3	20.53	2.88
8	2	-3	13.03	1.97
-8	-2	3	13.33	1.71
-8	-2	3	14.01	2.31
-8	-2	3	10.12	1.31
-8	-2	3	13.56	2.38
7	2	-3	25.25	2.44
-7	-2	3	24.74	2.30
-7	-2	3	26.18	2.26

-7	-2	3	19.48	1.92
6	2	-3	29.83	2.33
6	2	-3	25.06	1.98
-6	-2	3	28.63	2.13
-6	-2	3	23.27	1.91
-6	-2	3	28.53	1.81
-6	-2	3	31.71	2.22
5	2	-3	35.36	2.29
5	2	-3	40.96	2.50
-5	-2	3	33.28	2.20
-5	-2	3	37.80	2.15
-5	-2	3	39.56	2.33
-5	-2	3	37.99	2.40
4	2	-3	33.55	2.16
4	2	-3	33.56	2.01
4	2	-3	34.01	2.22
-4	-2	3	34.12	1.95
-4	-2	3	34.46	2.03
-4	-2	3	32.96	2.11
-4	-2	3	33.60	1.90
3	2	-3	18.43	1.36
3	2	-3	14.72	1.19
-3	-2	3	16.96	1.22
-3	-2	3	13.48	1.17
-3	-2	3	17.49	1.12
-3	-2	3	17.51	1.27
2	2	-3	125.35	5.99
2	2	-3	122.84	6.09
2	2	-3	116.57	6.04
-2	-2	3	118.29	5.98
-2	-2	3	126.18	5.98
-2	-2	3	119.90	6.04
1	2	-3	22.39	1.63
1	2	-3	26.72	1.30
1	2	-3	24.00	1.59
1	2	-3	27.35	1.49
1	2	-3	26.62	1.49
-1	-2	3	27.51	1.47
-1	-2	3	26.59	1.32
-1	-2	3	25.25	1.46
-1	-2	3	24.01	1.57
-1	-2	3	24.75	1.56
0	2	-3	25.22	1.45
0	2	-3	24.89	1.27

0	2	-3	22.80	1.41
0	2	-3	25.61	1.48
0	-2	3	26.98	1.47
0	-2	3	22.19	1.53
0	-2	3	25.64	1.30
0	-2	3	20.74	1.39
0	-2	3	26.08	1.54
-1	2	-3	3.91	0.62
-1	2	-3	4.59	0.57
-1	2	-3	5.58	0.44
-1	2	-3	6.18	0.86
-1	2	-3	3.91	0.65
1	-2	3	4.62	0.56
1	-2	3	5.86	0.49
1	-2	3	4.51	0.85
1	-2	3	5.92	0.86
-2	2	-3	43.48	2.41
-2	2	-3	43.17	2.52
-2	2	-3	48.36	2.35
-2	2	-3	48.11	2.73
-2	2	-3	47.59	2.55
2	-2	3	49.35	2.63
2	-2	3	49.62	2.38
2	-2	3	41.41	2.41
-3	2	-3	42.69	2.46
-3	2	-3	42.06	2.33
-3	2	-3	40.82	2.66
-3	2	-3	45.70	2.22
-3	2	-3	42.35	2.35
3	-2	3	44.96	2.63
3	-2	3	46.29	2.26
3	-2	3	40.14	2.34
-4	2	-3	12.38	1.55
-4	2	-3	9.56	1.10
-4	2	-3	15.23	1.41
-4	2	-3	12.68	0.85
-4	2	-3	13.53	1.15
4	-2	3	10.10	1.19
4	-2	3	13.42	0.92
4	-2	3	11.14	1.66
-5	2	-3	49.31	2.72
-5	2	-3	46.64	2.35
-5	2	-3	42.36	2.51
-5	2	-3	43.69	2.51

5	-2	3	44.14	2.39
5	-2	3	42.12	2.60
-6	2	-3	2.50	0.66
-6	2	-3	2.52	1.48
-6	2	-3	2.60	0.44
6	-2	3	2.57	0.49
6	-2	3	2.13	0.73
-7	2	-3	26.70	2.03
-7	2	-3	28.54	2.92
-7	2	-3	26.06	1.98
-7	2	-3	27.72	1.63
7	-2	3	24.20	1.94
7	-2	3	26.90	2.18
-8	2	-3	3.91	2.00
-8	2	-3	2.97	1.13
-8	2	-3	1.93	0.55
-8	2	-3	0.31	0.81
8	-2	3	-0.28	0.99
8	-2	3	1.35	0.57
-9	2	-3	5.38	2.90
-9	2	-3	-0.18	0.68
-9	2	-3	0.47	0.60
9	-2	3	0.10	0.62
9	-2	3	0.06	1.36
-10	2	-3	2.36	0.81
-10	2	-3	1.20	2.81
10	-2	3	2.72	1.42
10	-2	3	2.07	0.82
-11	2	-3	8.65	3.67
-11	2	-3	2.58	1.29
-11	2	-3	2.80	0.90
-12	2	-3	-0.16	1.08
-12	2	-3	1.40	4.29
12	-2	3	3.71	1.31
-13	2	-3	10.59	4.82
13	-2	3	0.98	1.51
-14	-1	3	5.61	4.46
-14	-1	3	3.52	3.83
-13	-1	3	6.27	3.42
-13	-1	3	5.05	3.65
-12	-1	3	8.54	2.99
-12	-1	3	9.72	3.15
11	1	-3	3.50	2.37
-11	-1	3	5.40	2.47

-11	-1	3	6.51	2.60
10	1	-3	5.60	2.16
-10	-1	3	4.73	2.26
9	1	-3	0.95	1.72
-9	-1	3	-0.07	0.84
-9	-1	3	-1.03	1.57
-9	-1	3	1.12	1.80
8	1	-3	14.80	2.95
-8	-1	3	10.81	1.62
-8	-1	3	11.29	2.37
7	1	-3	5.75	1.44
-7	-1	3	4.85	1.35
-7	-1	3	5.00	1.21
6	1	-3	39.95	2.74
-6	-1	3	39.70	2.63
-6	-1	3	39.50	2.75
5	1	-3	13.46	1.65
-5	-1	3	13.50	1.10
-5	-1	3	13.19	1.54
-5	-1	3	14.03	1.57
4	1	-3	3.09	0.82
4	1	-3	3.32	0.85
-4	-1	3	3.11	0.66
-4	-1	3	3.34	0.72
-4	-1	3	2.72	0.43
3	1	-3	1.42	0.44
3	1	-3	2.33	0.60
-3	-1	3	2.13	0.56
-3	-1	3	2.48	0.53
-3	-1	3	1.80	0.41
-3	-1	3	2.69	0.38
2	1	-3	95.15	4.90
2	1	-3	100.32	4.81
-2	-1	3	94.85	4.80
-2	-1	3	104.98	4.80
-2	-1	3	91.59	4.86
1	1	-3	11.73	0.85
1	1	-3	10.29	1.04
1	1	-3	11.72	0.62
-1	-1	3	11.68	0.81
-1	-1	3	11.58	0.63
-1	-1	3	9.88	0.85
-1	-1	3	10.95	0.96
0	1	-3	20.90	1.34

0	1	-3	21.71	1.32
0	1	-3	23.25	1.46
0	1	-3	21.46	1.11
0	-1	3	22.28	1.39
0	-1	3	19.49	1.30
0	-1	3	22.40	1.13
0	-1	3	21.89	1.39
-1	1	-3	0.38	0.23
-1	1	-3	-0.54	0.59
-1	1	-3	-0.53	0.44
1	-1	3	-0.42	0.61
1	-1	3	0.52	0.37
1	-1	3	0.22	0.24
1	-1	3	0.44	0.18
1	-1	3	-0.59	0.60
-2	1	-3	12.01	1.29
-2	1	-3	14.26	0.79
-2	1	-3	13.10	1.00
-2	1	-3	14.07	0.96
-2	1	-3	13.70	0.91
2	-1	3	15.26	1.35
2	-1	3	14.63	0.82
2	-1	3	13.05	0.90
-3	1	-3	48.01	2.57
-3	1	-3	46.48	2.29
-3	1	-3	43.62	2.37
-3	1	-3	43.36	2.38
3	-1	3	47.88	2.68
3	-1	3	45.38	2.31
3	-1	3	41.99	2.38
-4	1	-3	9.17	0.95
-4	1	-3	9.71	1.20
-4	1	-3	9.86	0.93
-4	1	-3	9.02	1.44
-4	1	-3	10.99	0.71
4	-1	3	10.06	0.74
4	-1	3	9.61	1.00
4	-1	3	8.84	1.07
-5	1	-3	1.30	0.47
-5	1	-3	1.97	0.54
-5	1	-3	1.96	0.34
-5	1	-3	2.29	0.70
-5	1	-3	1.93	1.09
5	-1	3	0.96	0.88

5	-1	3	0.96	0.53
5	-1	3	2.16	0.38
-6	1	-3	11.70	0.92
-6	1	-3	12.66	1.28
-6	1	-3	12.68	1.44
-6	1	-3	11.16	1.99
6	-1	3	11.78	0.96
-7	1	-3	1.55	0.87
-7	1	-3	0.71	0.41
-7	1	-3	0.87	0.76
-7	1	-3	-0.02	0.64
-7	1	-3	1.63	1.51
7	-1	3	-0.32	0.73
7	-1	3	0.39	0.42
-8	1	-3	3.94	1.87
-8	1	-3	6.05	1.11
-8	1	-3	3.78	0.89
-8	1	-3	5.40	0.93
8	-1	3	3.38	1.01
-9	1	-3	11.73	2.37
-9	1	-3	11.23	1.20
-9	1	-3	11.28	1.93
-9	1	-3	8.60	1.84
9	-1	3	10.37	1.22
9	-1	3	8.15	1.35
-10	1	-3	1.86	0.73
-10	1	-3	1.67	2.71
10	-1	3	0.17	1.28
10	-1	3	1.80	0.69
-11	1	-3	7.01	3.27
-11	1	-3	2.28	0.96
11	-1	3	2.49	0.91
-12	1	-3	1.65	0.95
-12	1	-3	-0.60	1.87
-12	1	-3	6.56	3.77
12	-1	3	-0.05	0.96
-13	1	-3	4.37	4.48
13	-1	3	1.88	1.36
-14	1	-3	2.91	5.26
-14	1	-3	-1.20	1.36
14	-1	3	1.73	1.63
-14	0	3	6.95	4.53
-14	0	3	1.51	4.22
-13	0	3	6.84	4.05

-13	0	3	4.26	3.74
-12	0	3	13.52	3.47
-12	0	3	6.02	1.75
-12	0	3	6.43	3.34
-11	0	3	10.18	2.83
-11	0	3	16.66	2.97
-10	0	3	17.00	2.62
-10	0	3	15.69	2.58
9	0	-3	-0.27	1.77
-9	0	3	2.14	1.94
-9	0	3	-0.16	1.76
-9	0	3	0.66	0.95
-8	0	3	8.20	1.55
-8	0	3	6.49	1.70
-8	0	3	8.58	1.74
7	0	-3	11.47	2.13
-7	0	3	9.83	2.02
-7	0	3	10.57	2.23
6	0	-3	33.07	2.37
-6	0	3	30.09	2.31
-6	0	3	26.62	2.52
5	0	-3	38.06	2.41
-5	0	3	36.67	2.33
-5	0	3	32.42	2.50
4	0	-3	25.16	1.77
-4	0	3	25.06	1.69
-4	0	3	24.30	1.82
3	0	-3	16.47	1.52
-3	0	3	18.63	1.35
2	0	-3	35.71	2.09
-2	0	3	39.04	1.92
-2	0	3	36.79	2.06
1	0	-3	14.84	1.13
1	0	-3	16.84	0.96
1	0	-3	13.39	0.79
-1	0	3	16.48	0.94
-1	0	3	15.28	1.06
-1	0	3	14.86	1.04
0	0	-3	48.79	2.55
0	0	-3	49.18	2.53
0	0	-3	50.27	2.41
0	0	3	49.56	2.54
0	0	3	50.23	2.66
0	0	3	47.75	2.54

0	0	3	46.93	2.41
-1	0	-3	6.67	0.96
-1	0	-3	7.49	0.47
-1	0	-3	6.64	0.65
1	0	3	7.53	0.73
1	0	3	7.03	0.97
1	0	3	7.28	0.49
1	0	3	5.39	0.57
-2	0	-3	106.04	5.73
-2	0	-3	108.83	5.73
-2	0	-3	122.81	5.71
-2	0	-3	134.86	5.84
2	0	3	108.79	5.76
2	0	3	124.81	5.72
2	0	3	128.59	5.84
2	0	3	104.96	5.74
-3	0	-3	88.40	4.20
-3	0	-3	83.99	4.37
-3	0	-3	84.52	4.26
-3	0	-3	79.28	4.24
-3	0	-3	90.63	4.33
3	0	3	86.94	4.21
3	0	3	88.72	4.33
3	0	3	81.42	4.26
-4	0	-3	15.32	1.07
-4	0	-3	15.83	1.20
-4	0	-3	16.56	1.46
-4	0	-3	16.34	0.92
-4	0	-3	12.79	1.06
4	0	3	15.29	1.22
4	0	3	16.12	0.92
4	0	3	13.09	1.11
-5	0	-3	55.66	2.56
-5	0	-3	49.74	3.01
-5	0	-3	48.06	2.75
-5	0	-3	48.69	2.67
-5	0	-3	46.81	2.68
5	0	3	51.08	2.56
5	0	3	47.52	2.73
5	0	3	50.93	2.77
-6	0	-3	23.16	1.29
-6	0	-3	21.47	1.53
-6	0	-3	25.24	1.70
-6	0	-3	23.93	2.17

-6	0	-3	18.79	1.54
6	0	3	21.45	1.56
6	0	3	19.99	1.29
6	0	3	21.92	1.69
6	0	3	19.01	1.66
-7	0	-3	2.99	1.40
-7	0	-3	2.43	0.46
-7	0	-3	2.71	0.73
-7	0	-3	2.87	0.86
-7	0	-3	1.97	0.66
7	0	3	1.97	0.73
7	0	3	2.97	0.75
7	0	3	2.78	0.45
-8	0	-3	5.20	1.76
-8	0	-3	4.49	0.62
-8	0	-3	2.55	0.82
-8	0	-3	5.73	0.93
8	0	3	3.03	0.92
8	0	3	4.76	1.08
8	0	3	3.98	0.59
-9	0	-3	1.85	1.26
-9	0	-3	-1.52	1.93
-9	0	-3	-0.05	0.62
-9	0	-3	0.54	1.16
9	0	3	0.01	0.64
9	0	3	-0.74	0.97
-10	0	-3	3.50	0.78
10	0	3	2.70	0.69
-11	0	-3	5.38	1.98
-11	0	-3	3.50	1.51
-11	0	-3	7.03	2.87
-11	0	-3	3.62	0.93
11	0	3	3.20	0.82
-12	0	-3	3.54	3.41
-12	0	-3	-0.99	1.02
-12	0	-3	0.64	2.35
12	0	3	1.23	0.93
-13	0	-3	9.32	4.12
-13	0	-3	-0.36	1.07
-14	0	-3	-0.71	1.33
-14	0	-3	6.46	4.63
14	0	3	0.59	1.40
-14	1	3	10.27	4.88
-14	1	3	-0.61	4.40

-13	1	3	15.52	4.29
-13	1	3	6.52	3.92
-12	1	3	2.98	3.46
-12	1	3	1.94	3.40
-11	1	3	12.74	3.07
-11	1	3	7.46	3.00
-10	1	3	-0.06	0.83
-10	1	3	-1.50	2.37
-9	1	3	4.76	1.11
-9	1	3	5.78	2.10
-8	1	3	2.93	1.68
-8	1	3	2.86	0.92
-7	1	3	25.37	2.64
-7	1	3	26.98	2.52
-6	1	3	-1.71	1.17
-6	1	3	-0.06	1.10
5	-1	-3	0.96	1.01
-5	1	3	0.63	0.93
-5	1	3	2.20	0.97
-4	1	3	-0.18	0.72
-3	1	3	10.52	1.18
2	-1	-3	45.51	2.51
2	-1	-3	44.14	2.22
-2	1	3	43.41	2.43
-2	1	3	46.70	2.21
1	-1	-3	51.25	2.69
1	-1	-3	52.13	2.54
-1	1	3	52.04	2.53
0	-1	-3	44.00	2.01
0	-1	-3	36.58	1.92
0	1	3	38.00	2.03
0	1	3	35.29	1.92
0	1	3	41.36	2.01
-1	-1	-3	1.24	0.26
1	1	3	1.23	0.28
1	1	3	0.90	0.16
1	1	3	0.96	0.31
-2	-1	-3	0.40	0.38
-2	-1	-3	0.48	0.28
-2	-1	-3	0.85	0.19
2	1	3	0.49	0.34
2	1	3	0.57	0.28
2	1	3	0.74	0.20
-3	-1	-3	29.08	1.60

-3	-1	-3	29.22	1.46
-3	-1	-3	27.69	1.53
-3	-1	-3	27.66	1.73
-3	-1	-3	26.12	1.50
3	1	3	28.49	1.59
3	1	3	26.16	1.52
3	1	3	26.51	1.62
3	1	3	28.65	1.45
-4	-1	-3	0.51	0.73
-4	-1	-3	0.55	0.25
-4	-1	-3	0.53	0.44
-4	-1	-3	0.24	0.29
-4	-1	-3	0.84	0.36
4	1	3	0.00	0.44
4	1	3	0.61	0.24
4	1	3	0.13	0.32
4	1	3	0.67	0.64
-5	-1	-3	1.79	0.43
-5	-1	-3	0.83	0.98
-5	-1	-3	2.35	0.50
-5	-1	-3	1.54	0.58
-5	-1	-3	2.09	0.35
5	1	3	1.97	0.82
5	1	3	1.87	0.60
5	1	3	2.07	0.32
5	1	3	1.86	0.46
-6	-1	-3	28.87	1.84
-6	-1	-3	33.06	1.68
-6	-1	-3	28.88	1.87
-6	-1	-3	28.43	2.41
-6	-1	-3	29.37	2.00
6	1	3	28.36	2.12
6	1	3	28.78	1.93
6	1	3	31.82	1.64
6	1	3	29.52	1.95
-7	-1	-3	0.63	0.81
-7	-1	-3	0.18	0.63
-7	-1	-3	-0.21	0.40
-7	-1	-3	-0.88	0.54
-7	-1	-3	-0.40	1.34
7	1	3	0.32	0.70
7	1	3	-0.17	1.08
7	1	3	-0.80	0.62
7	1	3	0.80	0.73

7	1	3	-0.07	0.31
-8	-1	-3	0.35	0.54
-8	-1	-3	1.21	0.77
-8	-1	-3	1.41	1.00
-8	-1	-3	1.63	1.62
-8	-1	-3	-0.17	1.02
8	1	3	0.91	0.42
8	1	3	0.77	0.82
8	1	3	0.41	0.78
-9	-1	-3	-1.16	0.84
-9	-1	-3	2.11	0.92
-9	-1	-3	1.32	1.89
-9	-1	-3	0.08	0.62
-9	-1	-3	2.99	1.30
9	1	3	-0.30	0.95
9	1	3	0.39	0.47
-10	-1	-3	3.68	1.11
-10	-1	-3	3.63	1.52
-10	-1	-3	1.27	0.78
-10	-1	-3	1.34	1.01
-10	-1	-3	3.37	2.23
10	1	3	2.58	0.66
-11	-1	-3	3.72	2.66
-11	-1	-3	4.01	1.83
-12	-1	-3	0.32	0.94
-12	-1	-3	-2.64	2.21
-12	-1	-3	-0.81	1.45
-12	-1	-3	-0.64	1.70
-12	-1	-3	3.89	3.16
12	1	3	-0.39	0.82
-13	-1	-3	-0.75	1.19
-13	-1	-3	6.29	3.87
13	1	3	-0.18	0.98
-14	-1	-3	-1.03	1.16
-14	-1	-3	5.60	4.50
-15	-1	-3	7.69	5.33
-15	-1	-3	-0.27	1.38
15	1	3	0.60	1.45
-13	2	3	11.18	4.45
-13	2	3	5.24	3.87
-12	2	3	1.00	3.48
-12	2	3	5.42	3.66
-11	2	3	2.66	0.86
-11	2	3	3.97	3.37

-11	2	3	7.12	3.13
-11	2	3	2.24	1.35
-10	2	3	3.62	2.53
-10	2	3	1.76	1.14
-9	2	3	17.39	1.37
-9	2	3	16.68	2.16
-9	2	3	15.99	3.22
-8	2	3	16.79	1.21
-8	2	3	14.53	1.92
-8	2	3	16.34	2.80
-7	2	3	23.84	2.04
-7	2	3	26.37	1.51
-7	2	3	26.28	2.63
-6	2	3	10.22	0.80
-6	2	3	10.08	1.79
-6	2	3	8.91	1.35
-5	2	3	5.67	1.03
-5	2	3	5.05	0.58
-4	2	3	22.55	1.23
-4	2	3	23.77	1.27
-4	2	3	22.13	1.69
-3	2	3	53.15	2.65
-2	2	3	37.06	1.86
1	-2	-3	14.36	0.81
-1	2	3	14.76	0.80
0	-2	-3	108.60	5.20
0	2	3	105.43	5.19
-1	-2	-3	33.75	1.78
-1	-2	-3	35.36	1.74
1	2	3	35.24	1.73
1	2	3	33.13	1.79
1	2	3	35.94	1.84
-2	-2	-3	0.64	0.17
-2	-2	-3	0.83	0.29
-2	-2	-3	0.37	0.38
2	2	3	0.60	0.32
2	2	3	0.44	0.35
2	2	3	0.66	0.17
-3	-2	-3	27.83	1.72
-3	-2	-3	30.12	1.84
-3	-2	-3	31.88	1.81
-3	-2	-3	33.65	1.62
3	2	3	33.37	1.62
3	2	3	33.00	1.80

-4	-2	-3	81.52	3.89
-4	-2	-3	75.94	3.98
-4	-2	-3	75.05	4.10
-4	-2	-3	80.17	4.04
4	2	3	83.28	3.90
4	2	3	83.67	4.03
4	2	3	77.08	4.01
4	2	3	73.72	4.05
-5	-2	-3	51.31	2.70
-5	-2	-3	49.77	2.88
-5	-2	-3	46.50	2.60
-5	-2	-3	46.98	2.44
5	2	3	48.47	2.77
5	2	3	50.76	2.67
5	2	3	48.80	2.44
5	2	3	42.92	2.94
5	2	3	45.27	2.57
-6	-2	-3	3.23	0.77
-6	-2	-3	2.82	0.41
-6	-2	-3	3.70	0.65
-6	-2	-3	3.27	1.13
6	2	3	3.35	0.56
6	2	3	4.28	1.06
6	2	3	3.79	0.75
-7	-2	-3	0.00	0.36
-7	-2	-3	-0.63	0.52
-7	-2	-3	-1.98	1.20
-7	-2	-3	0.27	0.83
-7	-2	-3	-0.29	0.63
7	2	3	-0.62	0.58
7	2	3	0.12	0.32
7	2	3	0.00	0.51
7	2	3	-0.18	0.74
7	2	3	0.65	1.09
-8	-2	-3	1.28	0.77
-8	-2	-3	1.87	1.49
-8	-2	-3	1.07	0.48
-8	-2	-3	0.30	0.67
-8	-2	-3	1.28	1.01
8	2	3	0.72	0.73
8	2	3	1.69	0.39
8	2	3	1.63	0.91
8	2	3	1.77	1.34
-9	-2	-3	-0.83	0.74

-9	-2	-3	0.19	1.78
-9	-2	-3	0.59	0.85
-9	-2	-3	-0.25	1.18
-9	-2	-3	-0.68	0.55
9	2	3	0.12	1.51
9	2	3	-0.22	0.39
9	2	3	0.25	0.89
9	2	3	-0.24	0.99
-10	-2	-3	5.62	2.18
-10	-2	-3	8.63	1.64
-10	-2	-3	5.39	1.12
-10	-2	-3	3.73	0.79
10	2	3	6.77	1.87
10	2	3	5.13	1.28
10	2	3	6.72	0.99
-11	-2	-3	0.03	0.77
-11	-2	-3	4.46	2.51
-11	-2	-3	2.24	1.20
11	2	3	0.59	0.64
-12	-2	-3	-0.75	0.86
-12	-2	-3	-0.26	3.00
12	2	3	-0.74	0.72
-13	-2	-3	0.17	2.02
-13	-2	-3	-1.24	0.91
-13	-2	-3	-1.53	1.45
-13	-2	-3	2.45	3.59
13	2	3	-0.06	0.86
-14	-2	-3	0.35	1.33
-14	-2	-3	5.54	4.36
14	2	3	0.18	1.10
-15	-2	-3	10.88	4.98
-13	3	3	0.96	4.11
-13	3	3	-1.67	1.90
-13	3	3	8.64	4.60
-12	3	3	3.52	1.12
-12	3	3	7.13	3.83
-12	3	3	4.87	0.83
-12	3	3	10.58	4.03
-11	3	3	0.54	1.37
-11	3	3	1.42	3.41
-11	3	3	-0.55	0.84
-11	3	3	0.39	0.64
-10	3	3	6.80	1.33
-10	3	3	8.65	1.32

-10	3	3	9.59	3.02
-9	3	3	0.13	1.04
-9	3	3	-0.82	0.56
-8	3	3	46.86	3.84
-8	3	3	45.45	2.51
-8	3	3	44.05	3.08
-7	3	3	1.49	0.84
-7	3	3	3.87	1.58
-7	3	3	1.18	0.49
-6	3	3	15.70	2.06
-6	3	3	13.38	1.56
-6	3	3	14.34	1.05
-5	3	3	4.05	1.06
-5	3	3	3.86	0.64
-4	3	3	6.35	0.63
-4	3	3	7.38	1.27
-4	3	3	7.85	0.68
-3	3	3	18.42	1.06
-2	3	3	20.17	1.12
1	-3	-3	9.23	0.62
-1	3	3	8.78	0.59
0	-3	-3	33.32	1.68
0	3	3	32.49	1.67
-1	-3	-3	35.57	1.70
-1	-3	-3	31.34	1.74
1	3	3	31.66	1.79
1	3	3	34.95	1.70
-2	-3	-3	3.68	0.37
-2	-3	-3	3.17	0.59
2	3	3	4.08	0.61
2	3	3	3.59	0.36
2	3	3	3.80	0.48
-3	-3	-3	0.44	0.48
-3	-3	-3	0.47	0.16
-3	-3	-3	0.24	0.51
3	3	3	-0.05	0.49
3	3	3	0.48	0.17
3	3	3	0.79	0.42
3	3	3	0.91	0.36
-4	-3	-3	20.05	1.50
-4	-3	-3	21.89	1.47
-4	-3	-3	21.58	1.11
4	3	3	21.16	1.43
4	3	3	21.85	1.12

4	3	3	18.65	1.36
4	3	3	18.02	1.50
-5	-3	-3	10.73	0.69
-5	-3	-3	8.42	1.21
-5	-3	-3	10.75	1.39
5	3	3	9.82	1.20
5	3	3	10.80	0.72
5	3	3	7.93	1.35
5	3	3	10.28	1.15
-6	-3	-3	7.12	1.38
-6	-3	-3	8.45	1.56
-6	-3	-3	8.47	0.67
6	3	3	8.20	0.70
6	3	3	7.75	1.31
6	3	3	8.62	1.21
6	3	3	7.14	1.28
-7	-3	-3	2.67	0.62
-7	-3	-3	4.18	1.28
-7	-3	-3	2.55	0.97
7	3	3	5.27	1.22
7	3	3	2.88	0.91
7	3	3	2.60	1.36
7	3	3	2.55	0.64
-8	-3	-3	-0.42	1.02
-8	-3	-3	0.04	0.38
-8	-3	-3	1.08	1.46
8	3	3	-0.62	0.93
8	3	3	-0.12	0.34
8	3	3	-0.02	0.79
8	3	3	0.04	1.29
-9	-3	-3	-0.61	0.77
-9	-3	-3	-1.10	1.25
-9	-3	-3	0.65	0.48
-9	-3	-3	-1.33	1.70
-9	-3	-3	-0.39	0.89
9	3	3	-0.01	1.05
9	3	3	0.30	1.49
9	3	3	0.18	0.40
-10	-3	-3	1.88	2.09
-10	-3	-3	2.03	1.10
-10	-3	-3	4.07	1.55
-10	-3	-3	2.07	0.63
10	3	3	5.01	1.86
10	3	3	2.15	1.53

10	3	3	2.17	0.51
10	3	3	4.20	1.24
-11	-3	-3	-1.16	0.96
-11	-3	-3	-1.13	1.73
-11	-3	-3	0.57	1.17
-11	-3	-3	0.24	0.66
-11	-3	-3	-0.14	4.02
11	3	3	-0.73	1.16
11	3	3	0.03	1.88
11	3	3	-0.72	0.55
-12	-3	-3	2.12	2.20
-12	-3	-3	2.45	2.85
-12	-3	-3	2.62	0.78
-12	-3	-3	1.73	1.37
12	3	3	1.09	0.67
12	3	3	-1.30	1.53
-13	-3	-3	-1.26	1.29
-13	-3	-3	-0.88	2.71
-13	-3	-3	-0.39	1.53
-13	-3	-3	0.09	0.85
-13	-3	-3	-0.39	3.48
13	3	3	0.47	0.80
-14	-3	-3	3.36	3.86
-14	-3	-3	1.94	0.92
-14	-3	-3	1.69	3.32
14	3	3	0.43	1.09
-15	-3	-3	0.35	1.34
-15	-3	-3	-1.23	4.39
-15	-3	-3	3.76	3.90
15	3	3	0.00	1.17
-12	4	3	0.72	0.91
-12	4	3	1.29	1.15
-12	4	3	2.94	4.36
-12	4	3	1.20	3.87
-11	4	3	2.67	4.36
-11	4	3	3.23	1.04
-11	4	3	4.53	0.84
-11	4	3	5.79	3.78
-10	4	3	2.92	0.81
-10	4	3	2.29	0.71
-10	4	3	1.48	1.25
-10	4	3	3.39	3.12
-9	4	3	1.69	2.40
-9	4	3	1.13	0.66

-8	4	3	6.21	1.17
-8	4	3	8.99	1.17
-8	4	3	10.41	2.23
-7	4	3	27.59	2.28
-7	4	3	27.00	2.99
-7	4	3	29.23	1.78
-6	4	3	16.72	1.23
-6	4	3	16.19	2.29
-5	4	3	8.37	0.87
-5	4	3	9.05	1.44
-5	4	3	9.30	1.76
-5	4	3	7.12	0.79
-4	4	3	14.31	1.72
-4	4	3	12.70	0.95
-4	4	3	12.06	0.90
-3	4	3	6.29	1.01
-3	4	3	4.68	0.58
-2	4	3	38.75	2.01
-1	4	3	0.21	0.21
0	-4	-3	29.57	1.54
0	4	3	28.79	1.53
-1	-4	-3	36.42	1.86
1	4	3	36.71	1.85
1	4	3	35.56	1.98
-2	-4	-3	0.18	0.18
2	4	3	0.03	0.41
2	4	3	0.25	0.33
2	4	3	0.00	0.17
-3	-4	-3	3.88	0.51
-3	-4	-3	4.34	0.43
-3	-4	-3	4.26	0.80
-3	-4	-3	4.66	0.90
3	4	3	3.64	0.52
3	4	3	5.16	0.66
3	4	3	4.44	0.41
3	4	3	3.79	0.58
-4	-4	-3	10.45	1.18
-4	-4	-3	11.03	1.02
-4	-4	-3	9.68	1.13
-4	-4	-3	11.18	0.69
4	4	3	10.79	0.68
4	4	3	9.77	1.21
4	4	3	10.10	1.14
4	4	3	11.24	1.08

-5	-4	-3	4.92	0.68
-5	-4	-3	5.32	0.50
-5	-4	-3	4.74	0.84
-5	-4	-3	6.05	0.93
5	4	3	4.86	0.77
5	4	3	6.54	0.97
5	4	3	5.65	0.51
5	4	3	4.91	0.71
-6	-4	-3	2.22	0.97
-6	-4	-3	1.42	0.81
-6	-4	-3	1.85	0.63
-6	-4	-3	1.71	0.30
6	4	3	1.29	1.00
6	4	3	1.86	0.32
6	4	3	1.22	0.62
6	4	3	1.47	0.75
-7	-4	-3	0.87	0.70
-7	-4	-3	1.52	0.32
-7	-4	-3	0.07	1.16
-7	-4	-3	0.27	0.88
7	4	3	1.04	0.83
7	4	3	1.49	0.37
7	4	3	1.22	1.19
7	4	3	0.90	0.74
-8	-4	-3	3.68	0.61
-8	-4	-3	3.66	1.49
-8	-4	-3	0.08	1.08
8	4	3	1.57	1.03
8	4	3	0.70	0.89
8	4	3	2.10	0.42
8	4	3	2.18	1.39
-9	-4	-3	7.37	1.47
-9	-4	-3	9.89	0.91
-9	-4	-3	10.30	1.93
9	4	3	9.60	0.90
9	4	3	11.12	2.27
9	4	3	5.04	1.97
-10	-4	-3	-0.15	1.45
-10	-4	-3	-2.78	1.95
-10	-4	-3	0.40	0.50
10	4	3	0.12	1.78
10	4	3	-0.69	1.26
10	4	3	-0.57	0.99
10	4	3	-0.18	0.45

-11	-4	-3	2.54	0.62
-11	-4	-3	2.11	1.78
11	4	3	3.39	2.07
11	4	3	2.05	0.57
11	4	3	1.00	1.45
-12	-4	-3	-1.42	1.06
-12	-4	-3	-2.12	2.18
-12	-4	-3	-0.08	2.87
-12	-4	-3	0.31	1.40
-12	-4	-3	0.09	0.65
12	4	3	0.07	0.95
12	4	3	-2.47	1.58
12	4	3	1.13	1.32
12	4	3	0.63	2.50
-13	-4	-3	2.55	0.77
-13	-4	-3	0.17	1.48
-13	-4	-3	-1.39	2.64
-13	-4	-3	1.51	1.58
-13	-4	-3	5.98	3.53
13	4	3	1.01	0.72
13	4	3	-1.68	2.58
13	4	3	0.39	1.58
-14	-4	-3	1.82	1.72
-14	-4	-3	1.06	0.83
-14	-4	-3	-1.24	1.51
-14	-4	-3	-0.46	3.11
-14	-4	-3	4.13	4.04
14	4	3	-0.74	0.87
-15	-4	-3	1.97	4.42
-15	-4	-3	0.59	0.88
15	4	3	-0.51	1.05
-12	5	3	0.40	1.09
-12	5	3	4.54	4.77
-12	5	3	-1.89	1.21
-11	5	3	3.51	0.97
-11	5	3	2.57	1.08
-10	5	3	2.27	0.95
-10	5	3	3.31	2.72
-10	5	3	12.56	3.56
-10	5	3	4.03	0.88
-10	5	3	4.47	1.53
-9	5	3	7.18	1.39
-9	5	3	15.99	3.03
-9	5	3	10.46	2.40

-9	5	3	12.45	1.44
-9	5	3	10.64	1.44
-8	5	3	0.17	0.63
-8	5	3	-0.15	0.59
-8	5	3	1.05	2.29
-7	5	3	7.17	1.04
-7	5	3	5.26	1.92
-7	5	3	5.77	0.76
-7	5	3	8.59	2.00
-6	5	3	1.27	1.53
-6	5	3	0.69	0.50
-6	5	3	0.53	1.59
-5	5	3	12.45	2.05
-5	5	3	12.42	1.05
-5	5	3	12.46	1.10
-4	5	3	2.62	0.46
-4	5	3	2.75	0.45
-4	5	3	3.91	1.22
-3	5	3	33.84	1.82
-3	5	3	32.20	2.39
-2	5	3	6.97	0.69
-2	5	3	7.42	1.42
-1	5	3	3.84	0.85
-1	5	3	3.31	0.53
0	-5	-3	7.73	1.03
0	5	3	5.84	1.09
0	5	3	5.36	0.57
-1	-5	-3	32.10	1.73
-1	-5	-3	33.29	2.02
1	5	3	32.16	1.71
1	5	3	32.65	1.96
-2	-5	-3	5.49	0.81
-2	-5	-3	3.77	0.72
-2	-5	-3	4.76	0.52
2	5	3	6.50	0.78
2	5	3	4.39	0.50
2	5	3	4.94	0.59
2	5	3	4.77	0.63
-3	-5	-3	5.18	1.04
-3	-5	-3	5.10	0.52
-3	-5	-3	4.87	0.61
3	5	3	4.63	0.90
3	5	3	4.78	0.72
3	5	3	6.16	0.99

3	5	3	5.32	0.51
-4	-5	-3	13.92	0.84
-4	-5	-3	12.80	1.18
-4	-5	-3	12.66	1.34
-4	-5	-3	11.42	1.45
4	5	3	13.85	0.82
4	5	3	12.33	1.20
4	5	3	13.27	1.30
4	5	3	12.31	1.31
-5	-5	-3	-0.31	0.55
-5	-5	-3	0.61	0.26
-5	-5	-3	0.88	0.77
5	5	3	-0.26	0.68
5	5	3	0.22	0.82
5	5	3	0.38	0.24
5	5	3	-0.29	0.55
-6	-5	-3	0.11	0.81
-6	-5	-3	0.50	0.93
-6	-5	-3	1.30	0.29
-6	-5	-3	0.20	0.63
6	5	3	1.17	0.80
6	5	3	1.36	0.66
6	5	3	1.45	1.00
6	5	3	1.30	0.28
-7	-5	-3	-0.01	0.70
-7	-5	-3	0.61	0.31
-7	-5	-3	-0.13	0.93
-7	-5	-3	-0.69	1.02
7	5	3	0.65	0.32
7	5	3	0.18	0.69
7	5	3	0.23	1.14
7	5	3	0.56	0.88
-8	-5	-3	-0.14	1.09
-8	-5	-3	2.26	1.34
-8	-5	-3	-0.01	0.79
8	5	3	0.34	1.37
8	5	3	0.94	1.01
8	5	3	0.37	0.79
-9	-5	-3	13.01	2.37
-9	-5	-3	8.07	1.20
-9	-5	-3	8.48	1.53
-9	-5	-3	11.23	0.90
9	5	3	8.81	1.44
9	5	3	12.44	1.89

9	5	3	9.30	1.30
9	5	3	10.89	0.96
-10	-5	-3	0.33	0.45
-10	-5	-3	-2.78	1.89
10	5	3	-0.19	0.45
10	5	3	-2.32	1.82
10	5	3	-1.25	1.29
-11	-5	-3	0.51	0.53
-11	-5	-3	1.40	3.23
11	5	3	-0.03	0.48
11	5	3	-2.74	1.54
11	5	3	-1.41	1.67
11	5	3	1.30	2.11
-12	-5	-3	2.40	0.63
-12	-5	-3	3.32	2.75
12	5	3	1.33	0.58
12	5	3	1.54	1.24
12	5	3	2.38	2.06
12	5	3	-2.36	1.72
-13	-5	-3	0.96	0.68
-13	-5	-3	2.11	3.22
13	5	3	-0.04	0.62
13	5	3	4.18	2.53
13	5	3	-1.42	1.87
-14	-5	-3	-1.96	3.88
-14	-5	-3	-0.46	3.16
-14	-5	-3	0.69	1.77
-14	-5	-3	-1.84	1.48
-14	-5	-3	-0.13	0.74
14	5	3	-1.63	2.64
14	5	3	-0.48	0.79
14	5	3	-1.72	1.75
-15	-5	-3	1.55	3.94
-15	-5	-3	1.32	4.29
15	5	3	0.12	0.99
-16	-5	-3	-1.28	4.54
-16	-5	-3	1.12	4.90
-11	6	3	2.54	1.15
-11	6	3	12.70	4.46
-10	6	3	4.78	1.00
-10	6	3	4.96	1.06
-10	6	3	9.55	3.68
-9	6	3	2.00	2.73
-9	6	3	6.35	0.96

-9	6	3	8.65	3.08
-9	6	3	4.24	0.92
-8	6	3	11.65	2.63
-8	6	3	11.14	1.49
-8	6	3	13.20	1.68
-8	6	3	9.64	2.68
-7	6	3	1.67	0.70
-7	6	3	0.45	2.17
-7	6	3	1.94	0.66
-6	6	3	1.28	1.74
-6	6	3	1.05	0.57
-6	6	3	-0.07	0.52
-6	6	3	0.49	1.89
-5	6	3	0.94	0.49
-5	6	3	0.47	0.47
-5	6	3	0.53	1.60
-4	6	3	6.94	0.95
-4	6	3	8.19	1.84
-4	6	3	6.22	1.08
-3	6	3	13.27	1.02
-3	6	3	11.15	1.98
-3	6	3	13.06	1.08
-2	6	3	22.72	1.35
-2	6	3	20.86	2.04
1	-6	-3	54.16	3.04
-1	6	3	57.37	2.82
-1	6	3	51.38	3.18
0	-6	-3	33.26	2.03
0	6	3	27.45	2.06
0	6	3	28.99	1.65
-1	-6	-3	3.75	0.88
-1	-6	-3	2.99	0.40
1	6	3	3.30	0.84
1	6	3	2.66	0.37
-2	-6	-3	0.78	0.46
-2	-6	-3	1.31	0.86
-2	-6	-3	0.95	0.30
2	6	3	0.98	0.26
2	6	3	0.76	0.45
2	6	3	0.78	0.71
-3	-6	-3	2.10	0.83
-3	-6	-3	0.46	0.44
-3	-6	-3	0.90	0.58
-3	-6	-3	1.70	0.33

3	6	3	0.64	0.69
3	6	3	0.58	0.54
3	6	3	2.68	0.75
3	6	3	1.45	0.29
-4	-6	-3	57.26	3.25
-4	-6	-3	55.46	3.12
-4	-6	-3	58.17	2.92
4	6	3	57.25	3.23
4	6	3	57.27	2.90
4	6	3	58.28	3.23
4	6	3	58.50	3.14
-5	-6	-3	50.44	2.48
-5	-6	-3	46.69	2.77
-5	-6	-3	45.27	2.92
-5	-6	-3	47.49	2.88
5	6	3	51.22	2.92
5	6	3	45.16	2.89
5	6	3	49.56	2.46
5	6	3	47.86	2.77
-6	-6	-3	17.07	1.61
-6	-6	-3	17.94	1.78
-6	-6	-3	13.52	1.84
-6	-6	-3	18.60	1.10
6	6	3	17.80	1.58
6	6	3	15.08	1.78
6	6	3	19.02	1.07
6	6	3	18.39	1.93
-7	-6	-3	0.04	0.32
-7	-6	-3	-0.96	0.73
-7	-6	-3	-0.09	1.08
7	6	3	0.14	0.30
7	6	3	-1.30	0.91
7	6	3	-0.93	1.11
7	6	3	-0.75	0.72
-8	-6	-3	10.78	1.68
-8	-6	-3	11.30	2.11
-8	-6	-3	13.91	0.98
-8	-6	-3	13.69	2.14
8	6	3	12.58	1.73
8	6	3	15.95	2.49
8	6	3	12.38	2.02
8	6	3	14.65	0.99
-9	-6	-3	-0.32	1.48
-9	-6	-3	0.23	0.95

9	6	3	0.16	0.98
9	6	3	1.79	1.63
9	6	3	-0.75	1.21
9	6	3	0.99	0.43
-10	-6	-3	2.91	0.50
-10	-6	-3	-0.35	1.60
-10	-6	-3	0.55	1.85
-10	-6	-3	1.48	1.08
10	6	3	2.02	1.44
10	6	3	1.50	1.13
10	6	3	0.89	2.05
-11	-6	-3	1.01	1.92
-11	-6	-3	3.89	1.31
11	6	3	6.50	0.94
11	6	3	3.92	1.35
11	6	3	7.60	2.36
11	6	3	3.67	1.71
-12	-6	-3	2.37	0.63
-12	-6	-3	5.07	2.82
-12	-6	-3	0.93	2.12
12	6	3	0.13	1.86
12	6	3	1.60	0.56
12	6	3	0.21	1.55
12	6	3	4.15	2.17
-13	-6	-3	0.45	3.18
13	6	3	1.43	2.05
13	6	3	5.17	2.46
13	6	3	-2.07	1.60
13	6	3	-1.27	1.23
-14	-6	-3	-3.49	3.04
-14	-6	-3	0.58	3.81
-14	-6	-3	-0.63	1.50
14	6	3	0.21	0.73
-15	-6	-3	-1.87	3.76
-15	-6	-3	0.23	4.19
15	6	3	0.82	1.84
-16	-6	-3	2.71	4.92
-16	-6	-3	-4.33	4.48
-10	7	3	2.26	1.15
-10	7	3	2.66	1.08
-10	7	3	9.53	3.87
-9	7	3	3.99	3.23
-9	7	3	0.52	0.95
-9	7	3	2.41	0.98

-8	7	3	8.67	2.87
-8	7	3	9.80	2.77
-8	7	3	6.69	1.04
-8	7	3	9.38	1.59
-7	7	3	3.67	2.35
-7	7	3	2.75	2.47
-6	7	3	2.15	0.70
-6	7	3	1.28	0.66
-6	7	3	0.89	2.22
-5	7	3	1.03	0.56
-5	7	3	0.70	0.55
-5	7	3	0.49	1.91
-4	7	3	3.60	1.72
-4	7	3	1.90	0.54
-4	7	3	0.87	0.49
-3	7	3	2.54	0.53
-3	7	3	2.65	0.52
-2	7	3	10.59	1.53
-2	7	3	10.64	1.20
1	-7	-3	1.09	0.85
-1	7	3	0.70	1.05
0	-7	-3	13.44	1.63
0	7	3	11.49	0.94
0	7	3	12.52	1.63
-1	-7	-3	0.83	0.94
1	7	3	0.20	0.30
1	7	3	0.80	0.90
-2	-7	-3	8.08	1.62
-2	-7	-3	9.86	0.84
2	7	3	9.54	1.40
2	7	3	9.34	0.76
2	7	3	7.91	1.04
2	7	3	6.81	1.32
-3	-7	-3	19.87	1.24
-3	-7	-3	19.86	1.49
-3	-7	-3	18.53	1.89
3	7	3	19.83	1.19
3	7	3	20.47	1.50
3	7	3	16.92	1.66
3	7	3	20.94	1.74
-4	-7	-3	1.51	0.72
-4	-7	-3	2.45	0.41
-4	-7	-3	3.66	1.06
4	7	3	2.47	0.37

4	7	3	4.33	0.95
4	7	3	1.79	0.67
4	7	3	1.08	0.81
-5	-7	-3	11.43	1.44
-5	-7	-3	11.76	1.31
-5	-7	-3	14.87	1.74
-5	-7	-3	13.81	0.97
5	7	3	12.93	1.73
5	7	3	11.99	1.64
5	7	3	13.86	1.44
5	7	3	14.42	0.94
-6	-7	-3	0.16	1.08
-6	-7	-3	0.14	0.33
-6	-7	-3	-0.89	0.69
6	7	3	0.09	0.31
6	7	3	-0.18	1.08
6	7	3	-0.48	0.68
-7	-7	-3	-0.55	0.79
-7	-7	-3	0.09	0.35
-7	-7	-3	0.11	1.17
7	7	3	-0.62	0.78
7	7	3	0.34	1.27
7	7	3	0.22	0.33
-8	-7	-3	0.57	1.32
-8	-7	-3	0.31	0.40
8	7	3	0.54	0.37
8	7	3	-0.58	0.86
8	7	3	-0.33	1.55
-9	-7	-3	1.23	0.49
-9	-7	-3	-2.44	1.34
-9	-7	-3	0.74	1.50
-9	-7	-3	0.32	0.99
9	7	3	0.91	0.44
9	7	3	1.79	1.66
9	7	3	-0.35	0.97
9	7	3	0.58	1.31
-10	-7	-3	0.70	0.50
-10	-7	-3	-0.30	1.78
-10	-7	-3	-1.75	1.02
-10	-7	-3	-2.51	1.58
10	7	3	0.62	0.49
10	7	3	-1.20	1.49
10	7	3	2.16	2.06
10	7	3	-1.08	1.07

-11	-7	-3	1.71	3.29
-11	-7	-3	0.49	1.24
-11	-7	-3	-2.28	1.84
-11	-7	-3	2.68	0.60
11	7	3	1.11	1.26
11	7	3	3.31	2.39
11	7	3	1.49	1.69
-12	-7	-3	0.17	0.61
-12	-7	-3	-1.11	1.02
-12	-7	-3	-0.89	1.35
-12	-7	-3	-0.06	2.70
12	7	3	4.07	2.48
12	7	3	-2.53	1.30
-13	-7	-3	-0.90	1.23
-13	-7	-3	-1.97	3.15
13	7	3	1.10	2.10
13	7	3	1.90	2.80
13	7	3	-1.20	1.60
-14	-7	-3	-1.28	1.52
-14	-7	-3	-3.35	3.69
14	7	3	-4.28	2.46
-15	-7	-3	-2.34	4.17
-15	-7	-3	-0.29	3.72
-15	-7	-3	-2.22	2.12
15	7	3	0.56	2.20
-16	-7	-3	-3.72	4.76
-16	-7	-3	-0.34	4.39
-9	8	3	6.29	3.51
-9	8	3	4.10	1.62
-9	8	3	3.05	1.14
-8	8	3	4.28	2.92
-8	8	3	1.89	1.00
-8	8	3	-0.01	0.95
-7	8	3	5.20	0.96
-7	8	3	7.22	3.01
-7	8	3	4.99	1.01
-7	8	3	7.11	2.68
-6	8	3	3.83	2.60
-6	8	3	2.57	0.80
-6	8	3	3.05	0.84
-5	8	3	16.91	1.60
-5	8	3	19.99	3.59
-4	8	3	2.31	0.62
-4	8	3	2.44	2.00

-4	8	3	2.75	0.65
-3	8	3	11.08	1.21
-3	8	3	10.34	1.16
-3	8	3	10.41	1.93
-2	8	3	1.53	0.49
-2	8	3	0.92	1.42
-2	8	3	1.06	0.55
1	-8	-3	15.03	1.82
-1	8	3	13.82	1.12
-1	8	3	12.84	2.06
0	-8	-3	8.66	1.75
0	8	3	5.22	0.98
0	8	3	7.42	1.33
-1	-8	-3	9.68	1.40
1	8	3	6.85	0.81
1	8	3	8.96	1.35
-2	-8	-3	0.98	1.09
2	8	3	0.24	0.92
2	8	3	-0.71	0.72
2	8	3	0.59	0.34
-3	-8	-3	9.57	1.88
-3	-8	-3	7.67	1.22
-3	-8	-3	10.58	1.31
-3	-8	-3	9.68	0.90
3	8	3	7.53	1.17
3	8	3	8.94	0.82
3	8	3	10.55	1.59
3	8	3	9.66	1.06
-4	-8	-3	0.55	1.12
-4	-8	-3	0.67	0.38
-4	-8	-3	-0.45	0.65
4	8	3	-0.06	0.63
4	8	3	0.66	0.34
4	8	3	0.22	0.96
-5	-8	-3	7.50	1.03
-5	-8	-3	10.20	0.92
-5	-8	-3	10.91	1.87
5	8	3	10.38	0.84
5	8	3	7.93	1.17
5	8	3	8.11	1.04
5	8	3	11.17	1.76
-6	-8	-3	-1.14	0.78
-6	-8	-3	0.46	0.40
-6	-8	-3	1.38	1.27

6	8	3	2.11	1.21
6	8	3	1.30	0.39
-7	-8	-3	7.37	0.87
-7	-8	-3	5.19	1.06
-7	-8	-3	7.06	1.55
7	8	3	7.97	0.79
7	8	3	5.41	0.99
-8	-8	-3	3.58	1.49
-8	-8	-3	0.84	1.03
-8	-8	-3	3.16	0.52
8	8	3	4.42	0.76
8	8	3	0.41	1.32
8	8	3	4.04	1.66
8	8	3	1.76	0.96
-9	-8	-3	0.53	1.07
-9	-8	-3	1.15	0.51
-9	-8	-3	-0.95	1.39
-9	-8	-3	1.64	1.71
9	8	3	-0.41	1.38
9	8	3	0.45	1.02
9	8	3	-2.24	1.80
9	8	3	1.17	0.46
-10	-8	-3	0.42	0.54
-10	-8	-3	-0.02	1.12
-10	-8	-3	0.72	1.88
-10	-8	-3	-1.73	1.56
10	8	3	1.16	0.54
10	8	3	0.38	1.12
10	8	3	-0.65	2.04
-11	-8	-3	7.98	1.12
-11	-8	-3	4.37	1.40
-11	-8	-3	9.02	2.30
-11	-8	-3	4.31	1.90
11	8	3	3.36	1.85
11	8	3	5.05	0.72
11	8	3	4.85	1.38
11	8	3	6.83	2.51
-12	-8	-3	2.37	0.68
-12	-8	-3	1.58	1.43
-12	-8	-3	-3.30	2.11
-12	-8	-3	2.20	2.60
12	8	3	1.43	2.68
12	8	3	2.07	0.68
12	8	3	-1.64	2.04

12	8	3	1.67	1.37
-13	-8	-3	1.00	1.27
-13	-8	-3	4.18	3.21
-13	-8	-3	-0.69	2.63
-13	-8	-3	0.14	1.58
13	8	3	-0.67	1.58
13	8	3	0.68	3.06
13	8	3	1.44	2.32
-14	-8	-3	-0.95	3.66
14	8	3	1.10	2.76
14	8	3	-2.16	1.76
-15	-8	-3	-3.46	4.03
-15	-8	-3	3.28	3.86
-15	-8	-3	-0.20	1.76
15	8	3	1.01	2.35
-16	-8	-3	-2.43	4.86
-16	-8	-3	-1.23	4.24
-8	9	3	1.71	1.11
-8	9	3	3.87	3.20
-7	9	3	7.74	2.86
-7	9	3	3.79	1.08
-7	9	3	9.91	4.96
-7	9	3	4.28	1.09
-6	9	3	0.91	0.84
-6	9	3	3.13	2.96
-6	9	3	2.23	0.90
-5	9	3	6.74	2.59
-5	9	3	8.65	1.49
-5	9	3	6.30	0.94
-4	9	3	1.80	0.68
-4	9	3	1.87	0.71
-4	9	3	3.02	2.24
-3	9	3	1.94	1.90
-3	9	3	3.20	0.66
-2	9	3	1.66	0.59
-2	9	3	1.35	1.71
-2	9	3	0.88	0.54
1	-9	-3	6.21	1.35
-1	9	3	4.88	0.66
-1	9	3	4.07	1.55
0	-9	-3	2.07	1.26
0	9	3	0.09	0.43
0	9	3	0.56	1.24
-1	-9	-3	2.23	1.28

-2	-9	-3	0.00	1.30
2	9	3	-0.46	0.80
2	9	3	-0.60	0.36
2	9	3	0.04	1.09
-3	-9	-3	2.90	1.42
-3	-9	-3	2.78	0.87
-3	-9	-3	1.13	0.91
3	9	3	2.53	0.71
3	9	3	2.22	0.94
3	9	3	3.96	1.24
-4	-9	-3	2.70	1.37
-4	-9	-3	1.30	1.10
-4	-9	-3	0.57	0.71
4	9	3	1.58	0.42
4	9	3	2.79	1.18
4	9	3	1.38	0.72
4	9	3	0.02	0.91
-5	-9	-3	-0.95	0.78
-5	-9	-3	-0.31	1.35
-5	-9	-3	0.22	0.44
5	9	3	0.42	0.39
5	9	3	0.17	0.75
-6	-9	-3	0.78	0.45
-6	-9	-3	-0.40	0.84
-6	-9	-3	1.77	1.37
6	9	3	-0.61	0.82
6	9	3	-1.06	1.35
6	9	3	0.10	0.40
-7	-9	-3	-1.04	1.38
-7	-9	-3	0.06	0.47
7	9	3	1.54	1.51
7	9	3	0.72	0.43
7	9	3	-1.51	0.90
-8	-9	-3	1.53	0.52
-8	-9	-3	-0.76	1.05
-8	-9	-3	1.22	1.64
8	9	3	0.71	1.68
-9	-9	-3	1.21	0.56
-9	-9	-3	-0.05	1.14
-9	-9	-3	1.16	1.70
9	9	3	1.81	2.01
9	9	3	1.87	0.53
9	9	3	-1.43	1.50
9	9	3	-0.38	1.06

-10	-9	-3	-2.05	1.57
-10	-9	-3	-0.77	1.26
-10	-9	-3	-0.43	0.57
10	9	3	-1.92	2.17
10	9	3	-0.07	0.53
10	9	3	-2.14	1.12
-11	-9	-3	1.78	2.27
-11	-9	-3	0.60	0.63
-11	-9	-3	-0.26	1.34
11	9	3	-2.66	2.34
11	9	3	-1.78	1.24
11	9	3	0.35	0.62
-12	-9	-3	-1.36	1.43
-12	-9	-3	-3.85	2.15
-12	-9	-3	1.64	2.52
-12	-9	-3	0.78	0.65
12	9	3	0.28	2.12
12	9	3	2.08	2.90
12	9	3	-0.68	1.32
12	9	3	-1.54	1.39
-13	-9	-3	0.58	3.06
-13	-9	-3	-2.71	1.60
-13	-9	-3	-0.21	1.30
13	9	3	-2.27	2.36
13	9	3	3.06	3.40
13	9	3	-1.32	1.61
-14	-9	-3	-4.61	2.93
-14	-9	-3	-1.16	1.84
-14	-9	-3	1.25	3.56
14	9	3	-0.14	2.77
14	9	3	-1.77	1.83
-15	-9	-3	0.72	1.79
-15	-9	-3	-3.20	3.59
-15	-9	-3	3.19	4.13
15	9	3	-0.13	2.13
-7	10	3	-0.75	1.07
-7	10	3	0.78	3.03
-6	10	3	7.84	1.20
-5	10	3	0.42	0.86
-5	10	3	-0.14	0.85
-4	10	3	9.77	1.57
-4	10	3	10.00	1.50
-4	10	3	7.36	2.61
-3	10	3	-0.11	2.29

-3	10	3	1.66	0.68
-3	10	3	1.48	0.71
-2	10	3	7.88	1.27
-2	10	3	9.49	1.35
-2	10	3	10.59	2.21
-1	10	3	0.31	0.43
-1	10	3	0.81	1.69
0	-10	-3	1.91	1.56
0	10	3	2.15	1.55
0	10	3	2.08	0.58
-1	-10	-3	2.73	1.49
1	10	3	2.30	0.53
1	10	3	1.29	1.38
2	10	3	8.26	1.03
2	10	3	11.78	1.67
2	10	3	9.29	2.02
2	10	3	8.87	1.35
-3	-10	-3	6.60	1.66
-3	-10	-3	4.40	0.87
3	10	3	3.98	0.85
3	10	3	5.01	1.47
3	10	3	3.92	0.58
3	10	3	4.45	1.06
-4	-10	-3	15.00	1.75
-4	-10	-3	15.32	2.59
4	10	3	13.57	1.91
4	10	3	16.34	1.20
4	10	3	18.49	2.20
4	10	3	13.29	1.63
-5	-10	-3	-0.85	1.19
-5	-10	-3	2.44	1.54
-5	-10	-3	0.85	0.89
5	10	3	1.02	0.85
5	10	3	1.79	1.43
5	10	3	-0.04	1.03
-6	-10	-3	4.55	0.66
-6	-10	-3	6.25	1.79
-6	-10	-3	4.72	1.06
-6	-10	-3	2.94	1.25
6	10	3	3.97	0.58
6	10	3	3.35	1.17
6	10	3	4.77	1.03
6	10	3	5.68	1.70
-7	-10	-3	2.02	0.59

-7	-10	-3	0.33	1.07
-7	-10	-3	3.34	1.72
7	10	3	1.27	1.24
7	10	3	1.04	1.02
7	10	3	4.18	1.78
7	10	3	2.98	0.54
-8	-10	-3	1.39	1.76
-8	-10	-3	0.31	0.56
-8	-10	-3	-2.30	1.49
8	10	3	-0.59	1.91
8	10	3	-0.59	0.46
8	10	3	-1.50	1.36
-9	-10	-3	2.22	0.66
-9	-10	-3	-0.45	1.50
-9	-10	-3	2.29	2.55
9	10	3	0.55	2.13
9	10	3	2.42	0.57
9	10	3	1.43	1.21
9	10	3	0.21	1.62
-10	-10	-3	2.85	1.74
-10	-10	-3	6.00	2.24
-10	-10	-3	3.94	1.80
-10	-10	-3	4.85	0.77
10	10	3	5.20	2.23
10	10	3	4.80	1.40
10	10	3	6.31	1.07
10	10	3	6.64	2.49
-11	-10	-3	1.61	0.68
-11	-10	-3	-0.10	2.29
-11	-10	-3	-2.68	1.51
11	10	3	-0.13	2.67
11	10	3	-2.03	1.36
11	10	3	-0.10	0.64
-12	-10	-3	0.15	2.74
-12	-10	-3	-0.09	0.72
-12	-10	-3	-2.25	1.54
12	10	3	-0.32	0.72
12	10	3	-1.85	1.53
-13	-10	-3	-2.32	1.63
-13	-10	-3	1.60	3.02
-13	-10	-3	1.17	0.79
-13	-10	-3	-4.13	3.01
13	10	3	-0.47	1.68
13	10	3	6.53	4.87

13	10	3	-2.07	2.52
-14	-10	-3	4.10	3.60
-14	-10	-3	-0.76	3.05
-14	-10	-3	-1.09	1.96
-14	-10	-3	1.86	1.39
14	10	3	-3.71	2.81
14	10	3	-3.19	1.96
-15	-10	-3	-0.12	3.64
-15	-10	-3	0.11	4.06
-15	-10	-3	-1.07	1.77
-6	11	3	0.49	1.13
-5	11	3	1.57	1.02
-4	11	3	1.52	0.90
-4	11	3	1.44	0.89
-4	11	3	6.76	3.25
-3	11	3	-0.24	2.50
-3	11	3	1.70	0.85
-3	11	3	2.09	0.81
-2	11	3	2.16	0.78
-2	11	3	0.88	0.66
-2	11	3	4.14	2.24
1	-11	-3	4.05	1.72
-1	11	3	3.53	0.80
-1	11	3	4.82	2.13
0	11	3	6.13	0.91
0	11	3	7.56	1.94
-1	-11	-3	1.79	1.64
1	11	3	1.54	1.66
-2	-11	-3	3.95	1.72
2	11	3	3.19	0.84
2	11	3	5.87	1.70
2	11	3	3.74	1.23
2	11	3	3.44	0.61
-3	-11	-3	6.88	1.89
3	11	3	5.99	0.93
3	11	3	5.38	0.70
3	11	3	6.43	1.22
3	11	3	6.17	1.75
-4	-11	-3	11.40	2.14
-4	-11	-3	8.80	1.35
4	11	3	15.12	2.48
4	11	3	10.00	1.15
4	11	3	9.38	1.65
4	11	3	10.47	1.37

-5	-11	-3	15.40	2.95
-5	-11	-3	13.54	1.96
5	11	3	15.08	2.62
5	11	3	13.46	1.94
5	11	3	13.02	2.13
5	11	3	15.40	1.26
-6	-11	-3	-0.82	1.02
-6	-11	-3	0.48	1.76
6	11	3	0.54	1.75
6	11	3	-1.23	0.95
6	11	3	-1.54	1.11
6	11	3	0.87	0.52
-7	-11	-3	-0.76	1.12
-7	-11	-3	-0.91	1.89
7	11	3	-1.69	1.02
7	11	3	-0.88	1.22
7	11	3	-0.21	0.52
7	11	3	-0.52	1.92
-8	-11	-3	-1.66	1.19
-8	-11	-3	0.06	0.63
8	11	3	1.88	2.14
8	11	3	-2.09	1.48
8	11	3	0.97	0.58
8	11	3	-1.63	1.14
-9	-11	-3	-0.99	1.34
-9	-11	-3	0.47	2.08
-9	-11	-3	-2.81	1.54
-9	-11	-3	0.00	0.64
9	11	3	-2.08	1.73
9	11	3	0.56	0.60
9	11	3	-1.04	1.20
9	11	3	2.33	2.42
-10	-11	-3	-1.96	1.45
-10	-11	-3	0.52	0.70
-10	-11	-3	0.13	2.29
10	11	3	0.00	2.68
10	11	3	0.43	0.65
10	11	3	-1.91	2.07
10	11	3	-2.14	1.25
-11	-11	-3	-1.05	2.48
-11	-11	-3	-2.22	1.62
-11	-11	-3	-3.61	2.01
-11	-11	-3	0.52	0.75
11	11	3	0.02	2.84

11	11	3	0.73	0.69
11	11	3	-1.19	1.46
11	11	3	-2.96	2.20
-12	-11	-3	-0.95	2.83
-12	-11	-3	-3.47	2.28
-12	-11	-3	-0.38	0.77
-12	-11	-3	-0.85	1.65
12	11	3	3.00	3.40
12	11	3	-3.75	2.34
12	11	3	1.15	0.80
12	11	3	-0.74	1.61
-13	-11	-3	-2.95	2.49
-13	-11	-3	0.42	0.86
-13	-11	-3	0.43	3.19
-13	-11	-3	-1.74	1.75
13	11	3	-0.32	0.89
13	11	3	-2.95	2.73
13	11	3	-3.05	1.84
-14	-11	-3	1.07	2.06
-14	-11	-3	2.01	3.74
-14	-11	-3	-4.53	2.93
14	11	3	-3.84	3.08
14	11	3	-0.49	2.21
-15	-11	-3	1.33	1.95
-15	-11	-3	1.18	4.30
-15	-11	-3	0.32	3.72
-5	12	3	2.24	1.14
-4	12	3	1.33	0.97
-3	12	3	0.26	3.54
-3	12	3	1.39	0.90
-2	12	3	0.31	0.73
-2	12	3	3.68	2.64
-2	12	3	1.20	0.84
1	-12	-3	-0.01	1.94
-1	12	3	1.56	0.81
-1	12	3	0.09	1.02
-1	12	3	1.56	2.28
0	-12	-3	0.66	1.88
0	12	3	1.52	0.76
0	12	3	1.39	2.12
-1	-12	-3	0.47	1.93
1	12	3	1.76	1.84
1	12	3	0.53	0.73
-2	-12	-3	4.02	1.97

2	12	3	1.93	1.89
2	12	3	2.56	1.32
-3	-12	-3	2.24	2.15
3	12	3	3.48	1.97
3	12	3	2.56	0.61
3	12	3	2.01	0.86
-4	-12	-3	-0.11	1.03
-4	-12	-3	1.46	2.57
-4	-12	-3	0.57	1.00
4	12	3	0.67	0.54
4	12	3	0.34	1.14
4	12	3	1.83	1.77
4	12	3	1.37	1.16
-5	-12	-3	-0.01	2.31
-5	-12	-3	0.99	1.06
5	12	3	0.46	0.98
5	12	3	1.01	0.54
5	12	3	1.28	1.22
5	12	3	0.72	1.85
-6	-12	-3	1.73	1.16
-6	-12	-3	-0.27	2.17
-6	-12	-3	1.64	2.11
6	12	3	0.43	1.28
6	12	3	0.67	1.08
6	12	3	1.41	1.99
6	12	3	0.52	0.56
-7	-12	-3	-1.01	1.22
-7	-12	-3	-0.56	2.17
-7	-12	-3	-1.58	1.28
7	12	3	0.23	1.15
7	12	3	0.15	1.34
7	12	3	0.11	0.58
7	12	3	-0.22	2.20
-8	-12	-3	-0.68	1.27
-8	-12	-3	-1.12	2.36
8	12	3	-1.59	1.24
8	12	3	0.23	2.26
8	12	3	0.44	0.64
8	12	3	-0.90	1.50
-9	-12	-3	-0.96	1.59
-9	-12	-3	1.13	2.45
-9	-12	-3	-0.69	1.47
9	12	3	0.53	2.59
9	12	3	-1.01	1.77

9	12	3	0.19	0.64
9	12	3	-2.47	1.33
-10	-12	-3	0.93	2.50
-10	-12	-3	-0.72	1.66
10	12	3	1.72	0.74
10	12	3	-1.75	2.08
10	12	3	-1.95	1.44
10	12	3	2.22	2.97
-11	-12	-3	1.51	2.78
-11	-12	-3	-3.41	1.98
-11	-12	-3	0.10	0.78
-11	-12	-3	-2.62	1.79
11	12	3	-3.52	2.26
11	12	3	-2.53	3.38
11	12	3	-0.21	0.79
-12	-12	-3	-0.78	1.98
-12	-12	-3	-3.84	2.37
-12	-12	-3	1.14	0.85
-12	-12	-3	1.71	3.19
12	12	3	-0.15	1.01
12	12	3	-1.88	1.75
12	12	3	-4.24	2.64
-13	-12	-3	-2.47	2.69
-13	-12	-3	0.27	3.39
-13	-12	-3	-0.98	2.10
-13	-12	-3	-0.06	0.88
13	12	3	-2.55	2.98
13	12	3	-2.41	2.04
13	12	3	-0.46	0.98
-14	-12	-3	2.17	3.78
-14	-12	-3	1.00	3.31
-14	-12	-3	2.36	2.41
14	12	3	0.39	5.47
14	12	3	-0.40	2.29
-4	13	3	1.04	1.13
-3	13	3	0.92	0.94
-2	13	3	0.55	0.93
1	-13	-3	-0.94	2.23
-1	13	3	3.42	2.90
-1	13	3	0.82	0.87
0	-13	-3	1.65	2.14
0	13	3	1.38	2.46
0	13	3	-0.12	0.80
0	13	3	1.33	0.80

-1	-13	-3	0.97	2.26
1	13	3	-1.08	0.78
1	13	3	0.45	2.23
1	13	3	-0.15	0.69
-2	-13	-3	-1.75	2.10
2	13	3	0.98	2.14
2	13	3	0.13	0.62
2	13	3	-0.36	0.91
2	13	3	0.33	0.82
-3	-13	-3	3.25	2.27
3	13	3	-1.78	2.07
3	13	3	0.12	0.61
-4	-13	-3	-0.42	2.16
4	13	3	0.02	0.96
4	13	3	1.44	1.96
4	13	3	0.66	1.31
-5	-13	-3	2.03	1.33
-5	-13	-3	1.44	1.12
-5	-13	-3	-0.38	2.31
5	13	3	1.83	1.37
5	13	3	0.61	2.10
5	13	3	1.86	1.06
-6	-13	-3	-0.01	1.43
-6	-13	-3	1.78	2.48
-6	-13	-3	2.00	1.25
6	13	3	1.05	1.44
6	13	3	1.64	2.20
6	13	3	0.85	1.12
6	13	3	0.36	0.56
-7	-13	-3	2.35	2.51
-7	-13	-3	0.21	1.35
-7	-13	-3	-0.92	1.52
7	13	3	1.37	2.37
7	13	3	1.88	1.27
7	13	3	2.03	1.52
7	13	3	1.44	0.69
-8	-13	-3	-0.64	1.62
-8	-13	-3	-1.22	1.45
-8	-13	-3	-0.06	2.59
8	13	3	1.44	2.58
8	13	3	-0.29	1.64
8	13	3	0.08	0.72
-9	-13	-3	-1.91	1.74
-9	-13	-3	-1.82	1.62

-9	-13	-3	1.02	2.85
9	13	3	1.39	2.91
9	13	3	-0.78	0.77
9	13	3	-2.26	1.48
9	13	3	-1.76	1.85
-10	-13	-3	-0.68	2.93
-10	-13	-3	-1.79	2.00
-10	-13	-3	-0.74	1.82
10	13	3	0.06	3.24
10	13	3	-0.73	0.83
10	13	3	-2.37	2.20
10	13	3	-1.12	1.63
-11	-13	-3	-2.61	1.99
-11	-13	-3	-2.73	2.15
-11	-13	-3	1.72	3.19
11	13	3	-0.98	1.75
11	13	3	1.26	0.92
11	13	3	-2.06	2.46
-12	-13	-3	-2.18	2.49
-12	-13	-3	4.56	3.29
-12	-13	-3	-4.25	2.26
12	13	3	0.64	2.96
12	13	3	-1.20	1.89
12	13	3	0.12	0.97
-13	-13	-3	-0.40	2.34
-13	-13	-3	4.05	3.56
-13	-13	-3	-4.70	2.91
13	13	3	-3.26	3.36
13	13	3	-1.14	2.22
13	13	3	2.33	1.11
-14	-13	-3	-0.11	3.98
-14	-13	-3	-0.75	2.62
-14	-13	-3	-4.98	3.44
14	13	3	-2.65	2.47
14	13	3	-2.51	1.31
14	13	3	-4.59	3.81
-2	14	3	1.24	1.10
-1	14	3	-0.56	0.93
0	-14	-3	-0.33	2.57
0	14	3	-0.63	0.91
-1	-14	-3	0.56	2.63
1	14	3	1.30	0.93
-2	-14	-3	1.70	2.54
2	14	3	0.62	1.53

2	14	3	1.91	2.47
2	14	3	0.65	0.92
-3	-14	-3	-1.07	2.44
3	14	3	0.12	0.66
3	14	3	-0.83	2.56
3	14	3	-0.17	0.90
-4	-14	-3	2.90	2.61
4	14	3	3.55	2.42
4	14	3	0.10	1.47
4	14	3	0.55	0.73
4	14	3	1.47	1.17
-5	-14	-3	0.25	1.37
-5	-14	-3	0.05	2.58
5	14	3	0.67	0.69
5	14	3	0.94	1.50
5	14	3	-0.43	1.16
5	14	3	-1.40	2.42
-6	-14	-3	-0.30	1.41
-6	-14	-3	-0.03	2.76
6	14	3	-0.06	1.48
6	14	3	-0.23	1.27
6	14	3	0.42	0.74
6	14	3	2.86	2.67
-7	-14	-3	-0.12	1.46
-7	-14	-3	0.46	2.97
-7	-14	-3	0.20	2.03
7	14	3	1.56	2.90
7	14	3	-0.59	0.83
7	14	3	-0.30	1.62
7	14	3	-1.79	1.34
-8	-14	-3	1.44	1.72
-8	-14	-3	1.47	2.87
-8	-14	-3	-0.32	1.62
8	14	3	3.40	3.10
8	14	3	-1.45	1.75
8	14	3	-1.29	1.42
-9	-14	-3	-0.02	3.19
-9	-14	-3	-0.68	1.77
9	14	3	-0.19	2.09
9	14	3	-2.95	1.56
-10	-14	-3	-1.16	1.92
-10	-14	-3	5.97	3.44
-10	-14	-3	-0.34	2.19
10	14	3	-1.44	1.72

10	14	3	-0.89	2.76
-11	-14	-3	-0.93	2.24
-11	-14	-3	1.79	3.51
-11	-14	-3	-1.62	2.38
11	14	3	-3.33	2.89
11	14	3	-3.42	1.80
-12	-14	-3	-2.65	2.50
-12	-14	-3	4.64	3.76
-12	-14	-3	0.80	2.75
12	14	3	-0.42	2.16
12	14	3	-1.70	3.35
-13	-14	-3	5.03	3.98
-13	-14	-3	-1.42	3.01
-13	-14	-3	-0.09	2.67
13	14	3	1.01	3.81
0	-15	-3	-1.44	3.02
0	15	3	-0.31	1.05
-1	-15	-3	1.74	2.91
1	15	3	0.73	1.02
-2	-15	-3	0.77	2.95
2	15	3	-0.83	0.98
-3	-15	-3	0.55	2.89
3	15	3	0.09	1.01
-4	-15	-3	-0.43	2.92
4	15	3	0.23	1.05
4	15	3	-0.61	1.73
-5	-15	-3	1.95	3.04
5	15	3	-0.24	1.38
5	15	3	0.00	1.65
-6	-15	-3	2.24	1.45
-6	-15	-3	1.91	3.10
6	15	3	0.85	1.02
6	15	3	0.39	1.65
6	15	3	-0.89	1.44
-7	-15	-3	2.12	1.55
-7	-15	-3	-1.77	3.16
7	15	3	0.12	0.86
7	15	3	2.42	1.81
7	15	3	0.00	1.49
-8	-15	-3	1.23	1.46
-8	-15	-3	1.19	3.35
8	15	3	1.58	0.98
8	15	3	0.49	1.95
8	15	3	-0.53	1.66

-9	-15	-3	4.86	1.70
-9	-15	-3	6.83	3.58
9	15	3	-1.45	1.00
9	15	3	0.49	2.25
9	15	3	0.49	1.75
-10	-15	-3	2.09	2.04
-10	-15	-3	-0.55	3.79
10	15	3	-0.51	1.03
10	15	3	-1.38	1.88
-11	-15	-3	4.45	3.82
-11	-15	-3	3.47	2.66
11	15	3	0.38	2.11
-12	-15	-3	1.86	4.10
12	15	3	0.64	2.43
12	15	3	1.34	1.27
-3	-16	-3	0.99	3.32
3	16	3	0.95	1.18
-4	-16	-3	-3.15	3.08
4	16	3	0.14	1.56
4	16	3	0.28	1.09
-5	-16	-3	-2.40	3.14
5	16	3	0.15	1.54
5	16	3	-1.13	1.17
-6	-16	-3	2.36	3.18
6	16	3	0.15	1.59
6	16	3	0.65	1.25
-7	-16	-3	0.60	3.36
7	16	3	-1.10	1.68
-8	-16	-3	-1.65	3.70
8	16	3	-0.66	1.78
-9	-16	-3	-0.07	4.09
9	16	3	-2.13	1.92
8	14	-4	35.17	4.35
8	14	-4	36.15	2.82
-8	-14	4	31.18	5.58
7	14	-4	29.00	2.59
7	14	-4	32.57	2.73
7	14	-4	36.87	4.16
-7	-14	4	29.96	5.34
6	14	-4	23.91	2.54
6	14	-4	33.47	2.73
6	14	-4	33.07	3.89
-6	-14	4	30.20	5.04
5	14	-4	30.18	3.76

5	14	-4	31.52	2.76
5	14	-4	24.66	2.65
-5	-14	4	32.73	5.15
4	14	-4	37.63	4.01
4	14	-4	44.11	3.13
-4	-14	4	37.83	5.27
3	14	-4	30.19	3.97
3	14	-4	35.96	2.89
-3	-14	4	35.94	5.26
2	14	-4	38.27	4.68
2	14	-4	41.30	3.08
-2	-14	4	41.61	5.74
10	13	-4	16.04	3.48
10	13	-4	22.07	4.22
-10	-13	4	14.82	2.89
-10	-13	4	18.43	3.74
-10	-13	4	24.43	4.53
9	13	-4	36.74	2.62
9	13	-4	40.86	5.26
-9	-13	4	38.63	5.29
-9	-13	4	37.66	4.59
-9	-13	4	37.28	4.46
8	13	-4	32.07	2.74
8	13	-4	36.03	2.63
8	13	-4	47.15	4.91
8	13	-4	36.24	3.95
-8	-13	4	42.10	4.21
-8	-13	4	32.79	4.21
-8	-13	4	36.89	5.20
7	13	-4	49.21	4.76
7	13	-4	45.73	4.03
7	13	-4	47.11	2.99
-7	-13	4	44.57	4.34
-7	-13	4	49.08	5.37
-7	-13	4	41.05	4.50
6	13	-4	33.97	4.18
6	13	-4	33.72	2.57
6	13	-4	34.96	3.61
-6	-13	4	35.64	4.10
-6	-13	4	35.25	3.69
-6	-13	4	29.55	4.78
5	13	-4	46.02	3.79
5	13	-4	40.10	2.89
5	13	-4	42.74	4.32

5	13	-4	48.00	3.00
-5	-13	4	43.09	4.11
-5	-13	4	49.01	5.11
-5	-13	4	42.57	3.89
4	13	-4	37.37	2.79
4	13	-4	43.32	4.06
4	13	-4	41.33	2.90
4	13	-4	42.29	3.68
-4	-13	4	40.91	4.99
3	13	-4	51.83	4.39
3	13	-4	46.22	4.03
3	13	-4	56.83	3.37
3	13	-4	48.25	3.35
-3	-13	4	57.06	5.37
2	13	-4	40.43	4.13
2	13	-4	44.59	3.01
-2	-13	4	39.88	5.30
1	13	-4	52.25	4.80
1	13	-4	49.96	3.37
-1	-13	4	55.71	5.65
0	13	-4	25.38	2.42
0	-13	4	26.31	4.81
-1	13	-4	33.64	4.91
-1	13	-4	30.14	2.63
1	-13	4	25.34	5.31
12	12	-4	21.88	3.81
-12	-12	4	28.43	5.67
-12	-12	4	26.18	4.90
11	12	-4	17.46	3.52
11	12	-4	27.36	4.56
-11	-12	4	27.34	4.48
-11	-12	4	27.92	5.25
-11	-12	4	23.23	4.30
10	12	-4	31.56	4.90
10	12	-4	26.96	3.93
-10	-12	4	25.99	3.99
-10	-12	4	30.15	4.10
-10	-12	4	30.33	4.98
9	12	-4	36.36	3.90
9	12	-4	35.91	4.58
-9	-12	4	30.93	3.86
-9	-12	4	35.29	4.87
-9	-12	4	34.57	4.02
8	12	-4	37.18	2.56

8	12	-4	39.08	2.58
8	12	-4	46.77	4.48
8	12	-4	42.83	3.68
-8	-12	4	36.15	3.99
-8	-12	4	45.75	4.94
-8	-12	4	37.53	3.77
7	12	-4	40.81	6.07
7	12	-4	38.41	3.50
7	12	-4	39.38	2.54
7	12	-4	44.74	4.36
7	12	-4	38.81	2.54
-7	-12	4	34.80	3.84
-7	-12	4	42.99	4.82
-7	-12	4	40.06	3.77
6	12	-4	48.26	4.28
6	12	-4	48.88	5.29
6	12	-4	52.19	3.04
6	12	-4	44.23	2.94
-6	-12	4	50.96	3.95
-6	-12	4	54.95	4.99
-6	-12	4	53.07	4.05
5	12	-4	49.36	5.23
5	12	-4	54.02	4.23
5	12	-4	53.93	3.10
5	12	-4	54.13	3.74
5	12	-4	48.09	3.03
-5	-12	4	56.94	3.91
-5	-12	4	44.03	3.97
-5	-12	4	47.30	4.87
4	12	-4	47.51	3.95
4	12	-4	46.63	3.47
4	12	-4	44.35	2.83
4	12	-4	40.81	4.88
-4	-12	4	50.32	4.78
-4	-12	4	36.98	3.65
-4	-12	4	46.82	3.81
3	12	-4	40.53	3.40
3	12	-4	46.26	2.80
3	12	-4	44.37	3.85
3	12	-4	43.84	4.92
-3	-12	4	41.17	4.58
-3	-12	4	35.99	3.53
-3	-12	4	44.05	4.65
2	12	-4	42.68	3.57

2	12	-4	49.75	3.95
2	12	-4	47.66	2.91
2	12	-4	40.05	2.95
-2	-12	4	40.06	3.59
-2	-12	4	46.96	4.74
1	12	-4	42.37	3.76
1	12	-4	42.07	2.79
1	12	-4	38.14	3.92
-1	-12	4	42.92	4.86
0	12	-4	51.07	3.35
0	12	-4	51.76	4.81
0	12	-4	61.56	4.30
0	-12	4	53.63	5.17
-1	12	-4	31.58	4.26
-1	12	-4	38.23	2.69
1	-12	4	40.13	4.83
-2	12	-4	36.02	2.71
-3	12	-4	22.11	2.26
-13	-11	4	12.87	3.39
-13	-11	4	16.66	4.38
12	11	-4	13.77	3.55
12	11	-4	18.92	3.24
-12	-11	4	15.70	2.95
-12	-11	4	16.29	2.79
-12	-11	4	20.29	3.88
11	11	-4	17.79	2.73
11	11	-4	11.39	3.02
-11	-11	4	20.78	3.82
-11	-11	4	14.45	2.64
-11	-11	4	22.33	3.63
10	11	-4	32.70	3.62
10	11	-4	38.01	4.70
-10	-11	4	33.86	3.89
-10	-11	4	36.56	3.84
-10	-11	4	36.76	4.85
9	11	-4	31.32	5.84
9	11	-4	34.36	3.49
9	11	-4	40.92	4.19
-9	-11	4	34.48	4.71
-9	-11	4	34.26	3.64
-9	-11	4	33.54	3.65
8	11	-4	42.76	4.15
8	11	-4	43.29	5.41
8	11	-4	38.08	3.46

-8	-11	4	39.81	3.65
-8	-11	4	35.62	3.66
-8	-11	4	40.60	4.48
7	11	-4	33.56	4.87
7	11	-4	33.77	2.17
7	11	-4	30.89	2.12
7	11	-4	33.59	3.07
-7	-11	4	33.55	3.35
-7	-11	4	38.11	4.18
-7	-11	4	35.62	3.22
6	11	-4	54.13	4.22
6	11	-4	49.74	3.04
6	11	-4	54.85	3.75
6	11	-4	58.02	5.12
6	11	-4	56.97	3.11
-6	-11	4	57.25	3.87
-6	-11	4	56.56	4.83
-6	-11	4	53.63	3.99
5	11	-4	26.53	4.65
5	11	-4	28.99	2.79
5	11	-4	33.23	3.26
5	11	-4	29.92	2.05
5	11	-4	27.40	1.97
-5	-11	4	30.13	3.07
-5	-11	4	31.58	2.94
-5	-11	4	29.91	4.01
4	11	-4	34.34	2.87
4	11	-4	31.80	3.23
4	11	-4	30.40	2.12
4	11	-4	30.20	2.10
4	11	-4	26.81	4.93
-4	-11	4	28.45	3.92
-4	-11	4	29.46	2.94
-4	-11	4	30.32	2.92
3	11	-4	27.53	3.01
3	11	-4	27.29	3.02
3	11	-4	23.15	1.94
3	11	-4	24.02	1.93
3	11	-4	26.64	3.83
-3	-11	4	28.69	3.84
-3	-11	4	23.00	2.68
-3	-11	4	24.54	2.99
2	11	-4	43.02	3.27
2	11	-4	46.36	3.58

2	11	-4	44.34	4.29
2	11	-4	46.60	2.73
-2	-11	4	44.05	4.28
-2	-11	4	39.99	3.24
-2	-11	4	39.54	4.63
1	11	-4	32.31	3.16
1	11	-4	30.90	2.19
1	11	-4	31.03	3.03
1	11	-4	29.40	3.87
-1	-11	4	29.15	4.09
-1	-11	4	26.89	2.69
0	11	-4	41.71	3.72
0	11	-4	38.44	2.66
0	11	-4	39.97	4.53
0	11	-4	44.29	3.61
0	-11	4	42.16	3.88
0	-11	4	42.66	4.54
-1	11	-4	51.92	3.85
-1	11	-4	40.01	4.06
-1	11	-4	48.47	2.96
1	-11	4	46.40	4.54
-2	11	-4	49.48	3.04
-2	11	-4	43.20	4.35
-3	11	-4	37.79	3.87
-3	11	-4	29.54	2.50
-4	11	-4	21.67	2.11
-13	-10	4	26.89	4.80
-13	-10	4	21.70	4.22
12	10	-4	14.52	3.35
-12	-10	4	12.13	2.52
-12	-10	4	17.29	2.95
11	10	-4	28.20	4.55
11	10	-4	30.52	3.63
-11	-10	4	25.99	4.04
-11	-10	4	35.57	4.77
-11	-10	4	30.33	3.70
10	10	-4	43.30	5.75
10	10	-4	38.68	3.56
10	10	-4	38.54	4.39
-10	-10	4	41.73	4.66
-10	-10	4	33.98	3.77
9	10	-4	39.96	4.15
9	10	-4	45.05	5.33
9	10	-4	43.50	3.44

-9	-10	4	38.34	3.59
-9	-10	4	34.70	4.04
-9	-10	4	47.16	4.43
8	10	-4	36.63	3.23
8	10	-4	44.13	3.94
-8	-10	4	42.70	4.20
-8	-10	4	38.86	3.46
-8	-10	4	40.01	3.43
7	10	-4	72.94	4.29
7	10	-4	63.06	3.61
-7	-10	4	68.34	4.46
-7	-10	4	71.11	4.39
-7	-10	4	71.29	4.98
6	10	-4	52.35	2.89
6	10	-4	54.47	3.99
6	10	-4	45.09	3.34
6	10	-4	55.83	3.51
-6	-10	4	51.23	3.58
-6	-10	4	53.92	4.28
-6	-10	4	51.26	3.67
5	10	-4	74.04	3.83
5	10	-4	73.58	4.32
5	10	-4	74.14	4.69
5	10	-4	72.99	5.25
5	10	-4	66.41	3.79
-5	-10	4	72.88	4.48
-5	-10	4	71.22	5.04
-5	-10	4	75.52	4.38
4	10	-4	43.38	4.18
4	10	-4	44.34	3.14
4	10	-4	40.76	2.54
4	10	-4	43.65	3.49
4	10	-4	46.25	2.57
-4	-10	4	44.35	4.01
-4	-10	4	43.88	3.26
-4	-10	4	46.31	3.22
3	10	-4	61.43	4.02
3	10	-4	54.72	4.50
3	10	-4	59.09	3.73
3	10	-4	60.28	3.27
-3	-10	4	63.11	4.54
-3	-10	4	54.86	3.76
-3	-10	4	62.29	3.78
2	10	-4	44.34	2.59

2	10	-4	46.60	3.37
2	10	-4	43.23	3.08
-2	-10	4	46.75	3.95
-2	-10	4	42.12	4.00
-2	-10	4	39.78	3.08
1	10	-4	45.40	2.65
1	10	-4	48.93	3.37
1	10	-4	33.31	4.42
-1	-10	4	39.83	3.05
-1	-10	4	49.63	4.13
0	10	-4	61.00	4.02
0	10	-4	58.94	3.36
0	10	-4	62.93	3.99
0	-10	4	50.62	3.64
0	-10	4	63.26	4.81
0	-10	4	61.32	4.33
-1	10	-4	73.15	4.53
-1	10	-4	83.06	6.01
-1	10	-4	64.62	4.72
-1	10	-4	72.31	3.97
1	-10	4	71.33	5.13
-2	10	-4	41.72	2.61
-2	10	-4	43.32	4.24
-2	10	-4	32.44	3.71
-2	10	-4	42.39	3.37
2	-10	4	36.36	4.23
-3	10	-4	57.92	6.02
-3	10	-4	45.70	3.04
-4	10	-4	22.35	3.95
-4	10	-4	22.28	2.01
-5	10	-4	32.86	2.50
-6	10	-4	17.24	2.10
-14	-9	4	9.52	3.22
-14	-9	4	12.01	4.19
-13	-9	4	27.31	5.42
-13	-9	4	16.41	3.25
12	9	-4	23.99	3.09
-12	-9	4	24.62	4.04
-12	-9	4	25.21	4.94
-12	-9	4	20.49	3.67
11	9	-4	36.07	5.96
11	9	-4	37.10	4.43
11	9	-4	36.05	3.29
-11	-9	4	39.85	3.93

-11	-9	4	34.47	3.65
-11	-9	4	35.12	4.70
10	9	-4	38.72	3.23
10	9	-4	38.95	4.16
10	9	-4	33.34	5.58
-10	-9	4	40.01	3.84
-10	-9	4	37.67	3.51
-10	-9	4	41.50	4.37
9	9	-4	26.51	2.64
9	9	-4	31.25	3.61
-9	-9	4	28.63	3.09
-9	-9	4	24.42	2.95
-9	-9	4	26.90	3.72
8	9	-4	30.51	2.70
8	9	-4	26.86	3.32
8	9	-4	28.14	4.27
-8	-9	4	31.94	2.94
-8	-9	4	31.90	3.57
-8	-9	4	29.13	2.95
7	9	-4	56.07	3.62
7	9	-4	54.27	4.86
7	9	-4	61.44	4.36
-7	-9	4	53.32	3.74
-7	-9	4	55.66	3.77
6	9	-4	45.72	3.40
6	9	-4	42.76	4.10
6	9	-4	40.25	2.36
6	9	-4	43.27	2.99
6	9	-4	37.49	2.33
-6	-9	4	47.04	3.11
-6	-9	4	44.94	3.68
-6	-9	4	43.72	3.36
5	9	-4	52.62	2.78
5	9	-4	51.09	3.25
5	9	-4	53.55	4.21
5	9	-4	45.96	2.76
5	9	-4	52.40	3.64
-5	-9	4	46.67	3.46
-5	-9	4	55.08	3.36
-5	-9	4	55.40	3.96
4	9	-4	57.09	3.75
4	9	-4	56.66	3.05
4	9	-4	57.04	3.50
4	9	-4	56.06	4.26

-4	-9	4	54.67	4.19
-4	-9	4	54.95	3.54
-4	-9	4	59.85	3.55
3	9	-4	102.60	5.56
3	9	-4	101.71	5.72
3	9	-4	107.54	6.13
3	9	-4	100.81	5.23
-3	-9	4	98.26	5.57
-3	-9	4	105.34	6.12
-3	-9	4	106.03	5.55
2	9	-4	53.86	3.44
2	9	-4	51.62	2.87
2	9	-4	50.48	3.31
2	9	-4	50.51	3.95
-2	-9	4	56.11	3.49
-2	-9	4	52.27	4.05
-2	-9	4	47.74	3.26
1	9	-4	54.27	3.43
1	9	-4	51.28	3.91
1	9	-4	52.03	3.27
1	9	-4	51.45	2.84
-1	-9	4	50.13	4.06
-1	-9	4	48.01	3.70
-1	-9	4	46.41	3.19
0	9	-4	31.69	2.68
0	9	-4	30.03	3.32
0	9	-4	34.89	2.66
0	9	-4	28.96	2.02
0	-9	4	27.53	4.09
0	-9	4	26.02	2.37
-1	9	-4	59.67	4.86
-1	9	-4	59.87	4.51
-1	9	-4	71.30	4.00
-1	9	-4	68.08	3.51
1	-9	4	64.43	4.52
1	-9	4	57.35	3.76
-2	9	-4	37.27	2.91
-2	9	-4	35.89	2.21
-2	9	-4	25.67	3.21
2	-9	4	30.84	3.65
-3	9	-4	39.58	2.50
-3	9	-4	37.86	3.28
-4	9	-4	42.44	4.24
-4	9	-4	36.09	2.45

-4	9	-4	31.88	3.82
-5	9	-4	36.80	2.55
-6	9	-4	27.29	2.25
-7	9	-4	8.38	2.25
-7	9	-4	11.91	1.87
-14	-8	4	10.11	3.38
-14	-8	4	15.64	4.25
-14	-8	4	14.06	3.17
-13	-8	4	24.15	4.41
-13	-8	4	27.55	5.32
-13	-8	4	26.71	3.84
12	8	-4	32.82	5.86
12	8	-4	33.02	4.73
12	8	-4	37.08	3.78
-12	-8	4	34.62	4.13
-12	-8	4	31.78	3.78
-12	-8	4	38.00	5.02
11	8	-4	36.93	3.10
11	8	-4	33.07	4.14
11	8	-4	34.68	5.26
-11	-8	4	33.84	3.75
-11	-8	4	34.84	3.52
-11	-8	4	37.48	4.43
10	8	-4	32.71	2.80
10	8	-4	32.47	3.82
-10	-8	4	31.78	3.35
-10	-8	4	31.85	3.96
-10	-8	4	32.75	3.17
9	8	-4	18.68	3.14
9	8	-4	17.78	2.22
9	8	-4	14.40	3.08
-9	-8	4	17.96	2.75
-9	-8	4	17.21	3.10
-9	-8	4	15.76	2.54
8	8	-4	36.41	3.37
8	8	-4	36.43	4.07
8	8	-4	33.66	2.68
-8	-8	4	38.00	3.13
-8	-8	4	38.14	3.48
-8	-8	4	35.87	2.95
7	8	-4	59.57	3.60
7	8	-4	61.15	4.69
7	8	-4	57.35	4.07
-7	-8	4	57.26	3.73

-7	-8	4	54.55	3.75
-7	-8	4	62.04	4.11
6	8	-4	98.71	5.30
6	8	-4	98.95	6.07
6	8	-4	96.79	5.57
-6	-8	4	95.14	5.38
-6	-8	4	100.73	5.72
-6	-8	4	99.34	5.38
5	8	-4	67.06	4.04
5	8	-4	64.85	3.76
5	8	-4	63.95	4.48
5	8	-4	62.53	3.30
5	8	-4	56.17	3.28
-5	-8	4	66.75	4.21
-5	-8	4	61.15	3.80
-5	-8	4	71.95	3.86
4	8	-4	115.24	6.11
4	8	-4	116.46	6.28
4	8	-4	117.74	6.60
4	8	-4	114.56	5.83
-4	-8	4	119.01	6.54
-4	-8	4	122.97	6.17
-4	-8	4	113.39	6.16
3	8	-4	44.06	2.86
3	8	-4	44.46	2.45
3	8	-4	48.62	3.09
3	8	-4	43.22	3.56
-3	-8	4	41.41	3.49
-3	-8	4	50.00	2.95
-3	-8	4	38.27	3.58
2	8	-4	120.95	6.73
2	8	-4	120.93	6.44
2	8	-4	123.73	6.37
2	8	-4	121.00	6.10
-2	-8	4	129.58	6.82
-2	-8	4	123.78	6.35
-2	-8	4	114.66	6.33
1	8	-4	55.01	3.79
1	8	-4	53.03	3.35
1	8	-4	52.54	3.27
1	8	-4	53.93	2.88
-1	-8	4	49.83	3.19
-1	-8	4	53.21	3.88
0	8	-4	104.42	5.94

0	8	-4	103.21	5.57
0	8	-4	104.31	5.27
0	8	-4	105.41	5.59
0	-8	4	98.05	5.46
0	-8	4	104.97	5.98
-1	8	-4	79.11	4.72
-1	8	-4	70.23	4.23
-1	8	-4	74.79	3.83
-1	8	-4	70.79	4.32
1	-8	4	73.26	4.66
1	-8	4	66.34	4.04
-2	8	-4	69.23	4.04
-2	8	-4	61.43	4.31
-2	8	-4	68.66	4.54
-3	8	-4	23.09	2.56
-3	8	-4	27.84	1.86
-3	8	-4	22.95	2.86
-4	8	-4	25.07	1.74
-4	8	-4	18.26	3.17
-4	8	-4	14.91	2.29
-5	8	-4	26.93	2.02
-6	8	-4	70.17	7.03
-6	8	-4	51.19	3.28
-7	8	-4	31.61	2.98
-7	8	-4	30.81	2.42
7	-8	4	37.33	3.57
-8	8	-4	20.73	2.17
-8	8	-4	18.77	4.12
8	-8	4	26.24	3.76
-14	-7	4	9.78	4.17
-13	-7	4	12.87	2.23
-13	-7	4	13.84	2.93
-13	-7	4	14.69	2.74
-13	-7	4	7.82	3.69
12	7	-4	34.35	5.53
12	7	-4	25.48	3.40
12	7	-4	28.62	4.55
-12	-7	4	34.41	3.90
-12	-7	4	32.50	3.55
-12	-7	4	37.72	4.78
11	7	-4	28.78	3.89
11	7	-4	27.30	3.02
-11	-7	4	35.51	4.24
-11	-7	4	28.88	3.24

-11	-7	4	31.48	3.55
10	7	-4	67.44	3.94
10	7	-4	58.46	4.70
10	7	-4	55.75	5.39
-10	-7	4	72.29	4.80
-10	-7	4	64.02	4.43
-10	-7	4	62.87	4.19
9	7	-4	18.11	3.05
9	7	-4	17.02	2.85
9	7	-4	18.95	2.11
-9	-7	4	16.11	2.61
-9	-7	4	20.49	3.07
-9	-7	4	16.48	2.40
8	7	-4	28.85	2.36
8	7	-4	30.75	2.93
8	7	-4	26.03	3.63
-8	-7	4	29.76	2.62
-8	-7	4	30.62	2.72
-8	-7	4	28.40	3.06
7	7	-4	64.19	4.06
7	7	-4	63.41	3.66
7	7	-4	62.21	4.61
-7	-7	4	60.98	3.82
-7	-7	4	63.64	4.13
-7	-7	4	61.64	3.78
6	7	-4	53.80	3.26
6	7	-4	55.07	3.59
6	7	-4	54.61	4.14
-6	-7	4	58.51	3.42
-6	-7	4	54.99	3.71
-6	-7	4	52.58	3.35
5	7	-4	67.70	3.77
5	7	-4	63.43	4.36
5	7	-4	66.09	3.98
-5	-7	4	71.71	3.84
-5	-7	4	65.04	4.13
-5	-7	4	66.05	3.81
4	7	-4	78.83	5.59
4	7	-4	83.82	4.30
4	7	-4	86.34	4.59
4	7	-4	88.04	4.74
-4	-7	4	84.12	4.63
-4	-7	4	92.69	4.64
-4	-7	4	85.17	4.93

3	7	-4	53.55	2.86
3	7	-4	54.02	3.21
3	7	-4	54.13	3.32
-3	-7	4	54.21	3.65
-3	-7	4	51.44	3.20
-3	-7	4	62.02	3.25
2	7	-4	47.60	2.57
2	7	-4	49.08	2.93
2	7	-4	47.95	3.01
-2	-7	4	47.97	2.90
-2	-7	4	48.39	2.88
1	7	-4	68.02	4.17
1	7	-4	66.68	3.83
1	7	-4	66.62	3.51
1	7	-4	70.58	3.83
-1	-7	4	71.91	3.94
-1	-7	4	65.76	4.19
-1	-7	4	65.16	3.78
0	7	-4	71.84	3.64
0	7	-4	70.98	3.97
0	7	-4	74.93	4.32
0	7	-4	73.57	3.98
0	-7	4	70.12	4.32
0	-7	4	67.15	3.85
0	-7	4	60.55	4.05
-1	7	-4	37.30	3.15
-1	7	-4	40.10	2.22
-1	7	-4	39.24	2.66
-1	7	-4	40.48	2.79
1	-7	4	35.91	2.49
1	-7	4	37.21	3.19
-2	7	-4	81.33	3.96
-2	7	-4	71.86	4.46
-2	7	-4	82.81	4.70
-2	7	-4	73.43	4.28
2	-7	4	70.12	4.60
2	-7	4	72.54	4.12
-3	7	-4	35.87	3.16
-3	7	-4	38.57	2.09
-3	7	-4	26.75	2.61
-3	7	-4	29.90	2.94
-4	7	-4	43.05	2.46
-4	7	-4	33.96	4.14
-4	7	-4	44.92	3.58

-5	7	-4	16.66	1.52
-6	7	-4	41.09	2.58
-7	7	-4	31.36	2.25
-7	7	-4	31.22	4.17
-7	7	-4	31.31	2.73
-8	7	-4	25.25	2.17
-8	7	-4	25.21	4.10
-9	7	-4	20.95	2.19
-9	7	-4	18.46	4.41
9	-7	4	26.32	3.94
-14	-6	4	12.75	3.09
-14	-6	4	13.64	3.44
-14	-6	4	17.72	4.30
13	6	-4	14.49	4.71
-13	-6	4	16.28	2.96
-13	-6	4	12.66	2.14
-13	-6	4	14.89	3.55
-13	-6	4	15.11	2.80
12	6	-4	17.24	2.47
12	6	-4	10.16	3.84
-12	-6	4	22.27	4.44
-12	-6	4	14.69	2.43
-12	-6	4	17.79	3.02
11	6	-4	41.12	4.30
-11	-6	4	48.79	3.71
-11	-6	4	53.34	4.53
-11	-6	4	45.90	3.98
10	6	-4	40.16	3.85
10	6	-4	41.25	3.05
10	6	-4	44.26	4.56
-10	-6	4	45.11	3.30
-10	-6	4	41.74	3.55
9	6	-4	41.41	3.58
9	6	-4	41.57	4.28
9	6	-4	41.95	2.96
-9	-6	4	44.70	3.65
-9	-6	4	42.51	3.30
-9	-6	4	42.18	3.10
8	6	-4	18.31	1.94
8	6	-4	17.49	2.57
8	6	-4	20.13	3.27
-8	-6	4	21.15	2.67
-8	-6	4	21.00	2.14
-8	-6	4	17.27	2.34

7	6	-4	75.26	4.12
7	6	-4	70.01	4.81
7	6	-4	77.64	4.47
-7	-6	4	71.54	4.22
-7	-6	4	72.60	4.48
-7	-6	4	79.77	4.28
6	6	-4	55.99	3.28
6	6	-4	60.68	3.58
-6	-6	4	54.15	3.36
-6	-6	4	58.93	3.41
-6	-6	4	55.32	3.63
5	6	-4	59.55	3.37
5	6	-4	57.44	3.92
5	6	-4	60.35	3.60
-5	-6	4	61.17	3.45
-5	-6	4	57.60	3.42
-5	-6	4	61.06	3.65
4	6	-4	49.13	3.08
4	6	-4	52.84	3.45
4	6	-4	50.02	2.92
4	6	-4	49.58	2.59
-4	-6	4	49.18	3.21
-4	-6	4	52.63	3.00
-4	-6	4	49.70	2.95
3	6	-4	68.70	3.50
3	6	-4	68.84	3.86
3	6	-4	68.94	3.78
3	6	-4	70.73	4.17
-3	-6	4	66.02	3.77
-3	-6	4	68.21	4.08
-3	-6	4	74.63	3.80
2	6	-4	71.90	3.87
2	6	-4	72.85	3.61
2	6	-4	72.33	4.18
2	6	-4	67.86	3.92
-2	-6	4	71.78	4.20
-2	-6	4	72.71	3.86
-2	-6	4	68.72	3.85
1	6	-4	107.94	5.27
1	6	-4	107.35	5.48
1	6	-4	105.90	5.72
1	6	-4	105.66	5.50
-1	-6	4	108.15	5.76
-1	-6	4	99.73	5.43

-1	-6	4	104.84	5.50
0	6	-4	29.81	2.49
0	6	-4	31.39	1.67
0	6	-4	25.43	2.09
0	6	-4	27.57	2.09
0	-6	4	27.04	1.98
0	-6	4	27.40	2.40
-1	6	-4	82.30	4.09
-1	6	-4	78.93	4.42
-1	6	-4	79.06	4.34
1	-6	4	78.55	4.59
1	-6	4	80.75	4.64
-2	6	-4	44.65	3.25
-2	6	-4	42.03	3.10
-2	6	-4	52.10	3.42
-2	6	-4	57.62	2.75
2	-6	4	53.15	2.94
2	-6	4	50.23	2.84
2	-6	4	54.90	3.20
2	-6	4	47.68	3.34
-3	6	-4	81.55	5.19
-3	6	-4	106.04	5.53
-3	6	-4	92.79	5.44
-3	6	-4	108.00	4.97
3	-6	4	90.73	5.04
3	-6	4	103.31	5.20
-4	6	-4	24.39	1.56
-4	6	-4	17.43	2.81
-4	6	-4	24.19	2.75
4	-6	4	20.64	1.78
-5	6	-4	42.70	2.92
-6	6	-4	31.31	2.40
-6	6	-4	29.15	2.02
6	-6	4	31.63	2.64
-7	6	-4	33.59	2.27
-7	6	-4	35.79	2.67
7	-6	4	38.11	3.09
7	-6	4	28.72	2.50
-8	6	-4	21.64	1.94
-8	6	-4	22.61	2.46
-8	6	-4	22.68	3.80
-8	6	-4	19.19	3.83
8	-6	4	26.12	3.09
-9	6	-4	25.68	4.38

-9	6	-4	29.70	2.30
-10	6	-4	18.47	4.72
-10	6	-4	12.60	2.01
10	-6	4	19.95	3.83
-15	-5	4	13.38	4.01
-15	-5	4	14.39	3.27
-15	-5	4	8.27	4.75
-14	-5	4	11.63	3.39
-14	-5	4	7.40	4.00
-14	-5	4	6.44	2.74
13	5	-4	14.15	4.24
-13	-5	4	16.28	3.14
-13	-5	4	18.13	3.19
-13	-5	4	24.31	3.95
-13	-5	4	18.46	3.41
-13	-5	4	22.93	3.97
12	5	-4	15.01	4.53
-12	-5	4	23.37	4.22
-12	-5	4	24.11	4.55
-12	-5	4	21.14	3.38
-12	-5	4	22.59	2.94
-11	-5	4	37.72	4.03
-11	-5	4	30.98	3.33
-11	-5	4	28.04	2.98
-11	-5	4	35.71	3.96
10	5	-4	33.11	3.49
10	5	-4	34.22	4.19
-10	-5	4	32.68	3.17
-10	-5	4	40.34	3.64
-10	-5	4	36.90	2.90
-10	-5	4	36.60	4.03
9	5	-4	56.87	4.05
9	5	-4	59.63	4.59
-9	-5	4	56.87	3.68
-9	-5	4	60.21	4.03
-9	-5	4	56.23	3.88
-9	-5	4	65.30	4.22
8	5	-4	47.35	3.95
8	5	-4	49.16	3.47
-8	-5	4	48.42	3.16
-8	-5	4	51.06	3.42
-8	-5	4	52.89	3.56
-8	-5	4	46.65	3.26
7	5	-4	64.07	3.63

7	5	-4	58.34	5.89
7	5	-4	63.69	3.95
7	5	-4	53.09	5.21
-7	-5	4	62.69	3.73
-7	-5	4	68.92	3.96
-7	-5	4	66.93	3.69
-7	-5	4	66.24	3.86
6	5	-4	63.77	3.75
6	5	-4	59.85	4.02
6	5	-4	63.51	3.48
-6	-5	4	61.99	3.54
-6	-5	4	64.02	3.74
-6	-5	4	62.17	3.60
5	5	-4	79.35	4.80
5	5	-4	81.56	4.38
5	5	-4	87.28	4.55
-5	-5	4	81.56	4.57
-5	-5	4	87.95	4.45
-5	-5	4	80.68	4.42
4	5	-4	74.14	4.01
4	5	-4	76.78	4.15
4	5	-4	75.31	4.35
-4	-5	4	74.17	4.04
-4	-5	4	80.14	4.08
-4	-5	4	73.15	4.18
3	5	-4	74.14	4.22
3	5	-4	76.05	4.00
3	5	-4	74.55	3.92
3	5	-4	73.99	3.69
-3	-5	4	72.08	4.10
-3	-5	4	70.91	3.92
-3	-5	4	77.05	3.93
2	5	-4	87.16	4.26
2	5	-4	83.99	4.49
2	5	-4	87.71	4.70
2	5	-4	87.63	4.48
-2	-5	4	81.60	4.66
-2	-5	4	82.82	4.45
-2	-5	4	88.51	4.47
1	5	-4	135.27	6.71
1	5	-4	139.02	6.87
1	5	-4	134.43	6.52
1	5	-4	138.34	6.70
-1	-5	4	122.69	6.55

-1	-5	4	128.06	6.65
-1	-5	4	135.43	6.69
-1	-5	4	127.56	6.86
0	5	-4	70.02	3.91
0	5	-4	71.82	4.10
0	5	-4	72.55	3.92
0	5	-4	73.53	3.68
0	-5	4	73.11	4.13
0	-5	4	73.33	3.86
0	-5	4	72.58	3.74
0	-5	4	74.54	4.24
-1	5	-4	64.81	3.78
-1	5	-4	70.96	3.32
-1	5	-4	66.37	3.66
-1	5	-4	55.74	3.55
1	-5	4	63.47	3.38
1	-5	4	67.33	3.52
1	-5	4	62.31	3.80
-2	5	-4	66.15	3.99
-2	5	-4	71.40	4.08
-2	5	-4	74.74	3.62
2	-5	4	75.61	3.82
2	-5	4	69.86	3.69
2	-5	4	63.08	4.01
-3	5	-4	55.93	4.31
-3	5	-4	59.16	3.96
-3	5	-4	69.16	3.37
3	-5	4	62.92	3.46
3	-5	4	66.45	3.63
3	-5	4	70.72	3.61
-4	5	-4	56.29	3.82
-4	5	-4	69.54	4.10
-4	5	-4	65.38	3.53
-4	5	-4	65.48	3.95
-4	5	-4	65.25	3.35
4	-5	4	60.93	3.46
-5	5	-4	71.44	5.06
-5	5	-4	71.41	3.85
-5	5	-4	73.26	4.33
-5	5	-4	69.79	3.65
5	-5	4	62.81	3.76
5	-5	4	68.70	3.95
-6	5	-4	41.82	3.56
-6	5	-4	48.24	3.57

-6	5	-4	50.44	2.86
-6	5	-4	43.81	4.62
-6	5	-4	48.41	2.98
6	-5	4	49.96	3.15
6	-5	4	47.11	2.85
-7	5	-4	30.28	2.40
-7	5	-4	29.80	2.00
7	-5	4	29.17	2.70
-8	5	-4	22.02	4.51
-8	5	-4	22.74	2.34
-8	5	-4	23.18	1.79
8	-5	4	21.35	2.77
-9	5	-4	21.98	2.61
-9	5	-4	21.99	1.95
-9	5	-4	22.51	4.04
-9	5	-4	22.65	4.07
9	-5	4	19.32	2.38
9	-5	4	28.70	3.33
-10	5	-4	23.45	2.14
-10	5	-4	24.64	4.66
10	-5	4	28.72	3.80
-11	5	-4	13.94	2.07
-11	5	-4	20.96	4.99
11	-5	4	13.26	2.49
-15	-4	4	6.46	3.92
-15	-4	4	13.93	4.79
-15	-4	4	11.60	3.04
-14	-4	4	15.56	3.63
13	4	-4	3.21	4.02
-13	-4	4	9.43	2.46
-13	-4	4	15.04	3.80
-13	-4	4	9.61	2.93
-12	-4	4	34.05	3.29
-12	-4	4	42.69	4.90
-12	-4	4	35.45	3.42
-12	-4	4	37.08	4.74
11	4	-4	12.12	3.08
-11	-4	4	17.86	2.81
-11	-4	4	18.24	3.46
-11	-4	4	15.64	2.49
-11	-4	4	20.83	3.74
10	4	-4	7.80	2.61
-10	-4	4	6.74	1.67
-10	-4	4	11.22	2.56

-10	-4	4	12.46	2.36
-10	-4	4	9.39	1.41
9	4	-4	2.38	2.21
9	4	-4	0.47	1.43
-9	-4	4	2.15	1.33
-9	-4	4	4.56	1.87
8	4	-4	44.40	3.31
8	4	-4	45.39	3.74
-8	-4	4	44.29	3.12
-8	-4	4	49.07	3.31
-8	-4	4	45.94	2.97
-8	-4	4	50.49	3.40
7	4	-4	39.84	2.81
7	4	-4	44.49	3.27
7	4	-4	33.51	2.37
-7	-4	4	39.24	2.57
-7	-4	4	46.07	2.89
-7	-4	4	35.74	2.65
6	4	-4	55.70	3.67
6	4	-4	56.12	3.65
6	4	-4	53.90	3.38
-6	-4	4	52.50	3.21
-6	-4	4	58.77	3.36
-6	-4	4	56.63	3.18
5	4	-4	87.64	4.76
5	4	-4	88.06	4.94
5	4	-4	88.49	4.87
5	4	-4	89.28	4.63
-5	-4	4	86.75	4.63
-5	-4	4	93.49	4.76
-5	-4	4	87.21	4.67
4	4	-4	98.26	5.40
4	4	-4	106.15	5.24
4	4	-4	91.03	5.39
4	4	-4	107.26	5.15
-4	-4	4	106.11	5.17
-4	-4	4	98.58	5.26
-4	-4	4	98.77	5.16
3	4	-4	15.05	1.33
3	4	-4	13.02	1.50
3	4	-4	12.24	1.71
-3	-4	4	14.72	1.39
-3	-4	4	12.50	1.45
-3	-4	4	14.20	1.46

-3	-4	4	15.35	1.29
2	4	-4	80.96	3.88
2	4	-4	78.60	4.08
2	4	-4	77.59	4.10
2	4	-4	83.37	4.30
-2	-4	4	79.27	4.06
-2	-4	4	78.59	4.20
-2	-4	4	73.55	3.90
-2	-4	4	77.64	4.13
-2	-4	4	76.81	4.06
1	4	-4	44.18	2.75
1	4	-4	45.49	2.56
1	4	-4	44.44	2.57
1	4	-4	46.01	2.31
-1	-4	4	44.15	2.35
-1	-4	4	45.30	2.52
-1	-4	4	42.35	2.72
-1	-4	4	47.59	2.57
0	4	-4	149.72	7.59
0	4	-4	149.06	7.48
0	4	-4	158.76	7.51
0	4	-4	156.37	7.34
0	4	-4	135.92	7.43
0	-4	4	155.95	7.54
0	-4	4	157.40	7.49
0	-4	4	152.13	7.38
0	-4	4	143.87	7.59
0	-4	4	141.85	7.43
-1	4	-4	46.60	2.26
-1	4	-4	44.39	2.64
-1	4	-4	36.79	2.37
-1	4	-4	43.37	2.71
1	-4	4	46.58	2.51
1	-4	4	39.45	2.65
1	-4	4	45.92	2.32
1	-4	4	38.59	2.37
1	-4	4	43.66	2.73
-2	4	-4	150.91	7.67
-2	4	-4	136.49	7.57
-2	4	-4	154.99	7.70
-2	4	-4	161.72	7.43
2	-4	4	131.55	7.65
2	-4	4	168.63	7.60
2	-4	4	153.03	7.47

-3	4	-4	61.20	3.41
-3	4	-4	67.90	3.87
-3	4	-4	61.91	3.66
-3	4	-4	67.16	3.28
-3	4	-4	61.31	3.64
3	-4	4	70.93	3.56
3	-4	4	58.31	3.32
3	-4	4	60.81	3.56
-4	4	-4	67.94	3.56
-4	4	-4	67.53	3.39
-4	4	-4	66.07	3.84
-4	4	-4	70.84	4.05
-4	4	-4	60.55	4.32
4	-4	4	65.31	3.59
4	-4	4	59.61	3.55
-5	4	-4	56.74	3.49
-5	4	-4	58.19	2.92
-5	4	-4	52.56	3.64
-5	4	-4	56.12	3.11
-5	4	-4	50.56	3.51
5	-4	4	52.85	3.00
5	-4	4	54.99	3.20
-6	4	-4	56.12	3.61
-6	4	-4	52.25	2.82
-6	4	-4	40.79	4.06
-6	4	-4	52.51	3.65
-6	4	-4	54.96	3.09
6	-4	4	48.21	2.92
6	-4	4	53.76	3.23
-7	4	-4	3.96	2.59
-7	4	-4	8.53	1.84
-7	4	-4	3.21	1.51
-7	4	-4	9.05	1.12
-7	4	-4	6.73	1.03
7	-4	4	8.15	1.34
7	-4	4	6.14	1.19
-8	4	-4	30.30	2.39
-8	4	-4	28.49	1.88
-8	4	-4	27.36	4.32
8	-4	4	27.82	2.70
8	-4	4	22.13	2.05
-9	4	-4	22.97	2.43
-9	4	-4	21.21	3.77
-9	4	-4	19.63	1.77

9	-4	4	21.93	3.00
-10	4	-4	17.73	1.83
-10	4	-4	22.02	3.08
-10	4	-4	24.26	4.45
10	-4	4	21.10	3.42
10	-4	4	13.64	2.34
-11	4	-4	11.36	1.82
-11	4	-4	12.35	4.82
11	-4	4	9.81	2.23
-12	4	-4	20.94	2.36
-12	4	-4	29.18	6.05
-14	-3	4	9.08	3.64
-14	-3	4	11.49	4.25
13	3	-4	5.39	3.88
-13	-3	4	14.75	3.89
-13	-3	4	14.35	3.35
-12	-3	4	38.16	4.89
-12	-3	4	30.77	3.20
-12	-3	4	35.69	4.88
11	3	-4	14.15	2.91
-11	-3	4	13.38	2.34
-11	-3	4	22.24	3.70
10	3	-4	12.01	2.62
-10	-3	4	17.88	3.20
9	3	-4	18.17	3.28
-9	-3	4	20.71	2.70
-9	-3	4	16.78	2.04
-9	-3	4	20.87	2.83
-8	-3	4	30.27	2.74
-8	-3	4	22.48	2.23
-8	-3	4	28.53	2.63
-8	-3	4	26.29	2.12
7	3	-4	28.43	2.43
7	3	-4	28.92	1.93
7	3	-4	27.45	2.74
-7	-3	4	28.14	2.06
-7	-3	4	29.92	2.50
-7	-3	4	24.99	2.16
-7	-3	4	29.35	2.47
6	3	-4	68.85	4.06
6	3	-4	60.76	4.23
6	3	-4	68.05	3.82
-6	-3	4	66.89	3.69
-6	-3	4	69.81	3.89

-6	-3	4	66.27	3.64
-6	-3	4	70.39	3.82
5	3	-4	135.99	6.94
5	3	-4	128.26	6.92
5	3	-4	135.99	6.78
-5	-3	4	129.80	6.76
-5	-3	4	136.01	6.82
-5	-3	4	135.64	6.70
-5	-3	4	132.00	6.69
4	3	-4	94.33	4.84
4	3	-4	88.86	4.97
-4	-3	4	95.35	4.77
-4	-3	4	97.57	4.79
-4	-3	4	91.96	4.86
-4	-3	4	92.25	4.84
3	3	-4	82.52	4.18
3	3	-4	83.73	4.14
3	3	-4	73.99	4.26
-3	-3	4	79.70	4.12
-3	-3	4	82.02	4.13
-3	-3	4	80.03	4.22
2	3	-4	5.04	0.42
2	3	-4	4.05	0.65
2	3	-4	3.09	0.89
-2	-3	4	3.74	0.80
-2	-3	4	4.77	0.47
-2	-3	4	3.74	0.66
-2	-3	4	3.56	0.69
-2	-3	4	4.37	0.62
1	3	-4	29.22	2.02
1	3	-4	31.57	2.04
1	3	-4	32.32	1.63
1	3	-4	32.35	1.91
1	3	-4	31.23	1.91
-1	-3	4	31.98	1.66
-1	-3	4	33.84	1.88
-1	-3	4	27.95	1.91
-1	-3	4	31.98	1.89
0	3	-4	9.40	1.22
0	3	-4	11.58	1.10
0	3	-4	11.10	1.23
0	3	-4	13.28	0.79
0	3	-4	11.73	1.32
0	-3	4	10.64	1.30

0	-3	4	11.67	1.06
0	-3	4	14.27	1.17
0	-3	4	14.00	0.84
0	-3	4	12.62	1.41
-1	3	-4	107.27	5.83
-1	3	-4	112.53	5.97
-1	3	-4	126.18	6.01
-1	3	-4	106.11	5.91
-1	3	-4	122.99	5.78
1	-3	4	124.83	5.82
1	-3	4	130.95	5.97
1	-3	4	110.67	5.85
-2	3	-4	47.03	2.64
-2	3	-4	49.24	3.02
-2	3	-4	47.81	2.89
-2	3	-4	53.50	2.55
-2	3	-4	49.45	2.84
2	-3	4	55.84	2.89
2	-3	4	42.47	2.82
2	-3	4	52.18	2.60
2	-3	4	46.70	2.66
-3	3	-4	78.19	4.27
-3	3	-4	72.15	3.87
-3	3	-4	77.20	3.76
-3	3	-4	74.84	4.06
-3	3	-4	70.26	4.00
3	-3	4	76.68	3.81
3	-3	4	79.80	4.10
3	-3	4	68.58	3.88
-4	3	-4	38.78	2.55
-4	3	-4	39.02	2.83
-4	3	-4	41.89	2.53
-4	3	-4	38.71	2.31
-4	3	-4	41.65	2.11
4	-3	4	35.96	2.36
4	-3	4	35.87	2.27
4	-3	4	39.27	2.17
-5	3	-4	39.07	2.04
-5	3	-4	34.53	2.43
-5	3	-4	37.95	2.76
-5	3	-4	35.27	2.82
5	-3	4	35.36	2.37
-6	3	-4	38.20	2.91
-6	3	-4	39.46	2.84

-6	3	-4	39.99	2.18
-6	3	-4	37.42	2.56
-6	3	-4	40.25	2.46
6	-3	4	36.00	2.24
6	-3	4	38.74	2.60
-7	3	-4	25.45	2.57
-7	3	-4	33.20	1.87
-7	3	-4	30.06	2.29
-7	3	-4	25.99	3.64
7	-3	4	29.15	1.97
7	-3	4	33.51	2.50
-8	3	-4	16.38	1.96
-8	3	-4	17.64	1.39
-8	3	-4	15.30	2.60
8	-3	4	13.98	1.53
-9	3	-4	20.33	2.14
-9	3	-4	21.50	4.40
-9	3	-4	20.73	1.63
9	-3	4	20.96	2.76
9	-3	4	15.67	1.81
-10	3	-4	10.70	3.71
-10	3	-4	10.95	2.24
-10	3	-4	9.69	2.77
-10	3	-4	11.53	1.52
10	-3	4	7.84	1.79
-11	3	-4	23.48	4.80
-11	3	-4	20.53	2.02
11	-3	4	21.13	2.52
11	-3	4	28.05	3.75
-12	3	-4	8.32	1.31
-12	3	-4	19.48	5.32
-14	-2	4	7.59	4.24
-14	-2	4	10.79	3.68
-13	-2	4	20.85	4.07
-13	-2	4	28.30	5.55
-12	-2	4	28.21	4.78
-12	-2	4	25.15	4.74
11	2	-4	20.25	3.78
-11	-2	4	27.80	3.96
-11	-2	4	28.88	3.96
10	2	-4	7.01	2.48
-10	-2	4	13.48	2.42
-10	-2	4	10.74	2.46
9	2	-4	52.80	4.15

-9	-2	4	54.03	4.03
-9	-2	4	62.32	4.09
8	2	-4	18.03	3.43
-8	-2	4	24.50	2.56
-8	-2	4	25.68	2.66
7	2	-4	41.55	3.17
-7	-2	4	45.18	2.98
-7	-2	4	38.37	2.59
-7	-2	4	43.33	2.99
6	2	-4	69.62	3.90
6	2	-4	66.79	4.14
-6	-2	4	71.93	3.96
-6	-2	4	74.06	4.05
-6	-2	4	69.89	3.80
-6	-2	4	72.53	3.77
5	2	-4	91.15	4.99
5	2	-4	92.55	4.84
5	2	-4	95.72	5.12
-5	-2	4	92.03	4.74
-5	-2	4	98.03	4.95
-5	-2	4	88.79	4.75
4	2	-4	131.42	6.67
4	2	-4	131.05	6.70
4	2	-4	132.10	6.57
-4	-2	4	127.32	6.50
-4	-2	4	131.68	6.62
-4	-2	4	132.08	6.52
3	2	-4	140.12	6.79
3	2	-4	139.92	6.92
-3	-2	4	140.08	6.76
-3	-2	4	130.37	6.82
-3	-2	4	133.40	6.75
2	2	-4	72.37	3.56
2	2	-4	69.75	3.54
-2	-2	4	67.82	3.49
-2	-2	4	68.31	3.51
-2	-2	4	64.01	3.59
1	2	-4	50.00	2.58
1	2	-4	40.46	2.68
1	2	-4	46.33	2.34
1	2	-4	49.64	2.55
-1	-2	4	50.71	2.55
-1	-2	4	51.08	2.38
-1	-2	4	42.85	2.60

-1	-2	4	46.21	2.52
0	2	-4	50.74	2.82
0	2	-4	49.72	2.81
0	2	-4	51.64	2.63
0	2	-4	56.37	2.89
0	-2	4	58.76	2.87
0	-2	4	46.74	2.82
0	-2	4	55.53	2.88
0	-2	4	49.24	2.80
0	-2	4	57.62	2.67
-1	2	-4	111.32	5.42
-1	2	-4	110.56	5.58
-1	2	-4	117.25	5.68
-1	2	-4	100.95	5.48
-1	2	-4	106.89	5.56
1	-2	4	104.77	5.50
1	-2	4	113.49	5.58
1	-2	4	119.27	5.46
1	-2	4	113.09	5.64
-2	2	-4	46.62	2.22
-2	2	-4	40.31	2.42
-2	2	-4	46.67	2.74
-2	2	-4	38.60	2.31
-2	2	-4	45.33	2.54
2	-2	4	47.72	2.28
2	-2	4	43.22	2.44
2	-2	4	39.51	2.33
2	-2	4	41.60	2.63
-3	2	-4	149.53	7.14
-3	2	-4	146.04	7.26
-3	2	-4	146.20	7.46
-3	2	-4	144.08	7.22
-3	2	-4	142.68	7.28
3	-2	4	150.77	7.18
3	-2	4	135.07	7.21
3	-2	4	153.69	7.48
-4	2	-4	57.43	2.85
-4	2	-4	54.15	3.34
-4	2	-4	53.02	2.98
-4	2	-4	52.86	3.11
-4	2	-4	55.75	3.00
4	-2	4	52.78	3.03
4	-2	4	62.61	3.42
4	-2	4	56.61	2.90

-5	2	-4	99.49	4.88
-5	2	-4	105.81	5.28
-5	2	-4	98.60	5.16
-5	2	-4	98.77	5.03
-5	2	-4	94.78	5.01
5	-2	4	96.08	5.07
5	-2	4	106.28	5.40
5	-2	4	87.51	4.97
5	-2	4	94.86	4.93
-6	2	-4	28.55	1.59
-6	2	-4	30.20	2.31
-6	2	-4	25.76	2.83
-6	2	-4	24.11	1.96
-6	2	-4	25.62	1.93
6	-2	4	23.85	1.86
6	-2	4	26.97	2.03
-7	2	-4	56.41	3.31
-7	2	-4	56.24	3.29
-7	2	-4	58.05	3.54
-7	2	-4	58.68	4.20
-7	2	-4	57.84	3.02
7	-2	4	57.47	3.46
7	-2	4	54.69	3.09
-8	2	-4	36.34	2.56
-8	2	-4	37.02	2.83
-8	2	-4	37.02	3.91
-8	2	-4	38.18	2.15
8	-2	4	34.46	2.78
8	-2	4	33.50	2.23
-9	2	-4	27.29	1.91
-9	2	-4	30.62	4.12
-9	2	-4	32.96	2.83
9	-2	4	27.40	2.83
9	-2	4	27.36	2.02
-10	2	-4	7.54	3.31
-10	2	-4	9.34	1.37
10	-2	4	6.27	1.03
10	-2	4	8.25	1.66
-11	2	-4	19.92	1.90
-11	2	-4	19.04	2.60
-11	2	-4	27.82	5.67
11	-2	4	20.33	3.36
-12	2	-4	5.90	4.67
-12	2	-4	6.50	1.17

12	-2	4	5.27	1.37
-13	2	-4	18.84	5.43
-13	2	-4	8.87	1.46
-14	-1	4	11.95	3.98
-14	-1	4	7.88	4.60
-13	-1	4	9.07	3.26
-12	-1	4	33.50	5.19
-12	-1	4	36.36	5.47
11	1	-4	15.29	2.91
-11	-1	4	24.92	4.15
-11	-1	4	24.81	4.06
10	1	-4	21.98	3.57
-10	-1	4	28.61	3.71
-10	-1	4	30.64	3.59
9	1	-4	23.74	3.03
-9	-1	4	26.57	3.21
8	1	-4	44.39	4.70
-8	-1	4	57.37	3.78
-8	-1	4	56.32	3.83
7	1	-4	26.80	2.73
-7	-1	4	33.31	2.62
-7	-1	4	31.01	2.64
6	1	-4	74.82	4.31
-6	-1	4	75.79	4.27
-6	-1	4	75.83	4.24
-5	-1	4	111.84	5.64
-5	-1	4	113.72	5.86
4	1	-4	35.09	2.30
4	1	-4	30.23	1.94
-4	-1	4	27.65	1.85
-4	-1	4	35.09	2.12
-4	-1	4	32.32	1.83
3	1	-4	86.06	4.28
3	1	-4	85.52	4.43
-3	-1	4	82.98	4.24
-3	-1	4	86.60	4.25
-3	-1	4	82.13	4.36
2	1	-4	113.49	5.37
2	1	-4	108.55	5.49
-2	-1	4	108.21	5.33
-2	-1	4	108.22	5.36
-2	-1	4	102.18	5.42
1	1	-4	1.16	0.53
1	1	-4	-0.31	0.58

1	1	-4	1.98	0.28
-1	-1	4	1.99	0.45
-1	-1	4	0.54	0.49
0	1	-4	125.06	5.88
0	1	-4	115.54	5.64
0	1	-4	112.29	5.77
0	1	-4	113.26	5.78
0	-1	4	107.18	5.85
0	-1	4	117.92	5.66
0	-1	4	106.74	5.76
0	-1	4	130.72	5.81
-1	1	-4	27.06	1.65
-1	1	-4	24.45	1.44
-1	1	-4	26.68	1.34
1	-1	4	23.85	1.46
1	-1	4	24.88	1.60
1	-1	4	24.39	1.82
1	-1	4	27.57	1.38
-2	1	-4	134.23	6.80
-2	1	-4	142.06	6.98
-2	1	-4	138.07	6.72
-2	1	-4	131.03	6.77
-2	1	-4	144.08	6.82
2	-1	4	135.77	6.79
2	-1	4	143.03	6.75
2	-1	4	136.67	6.93
-3	1	-4	7.89	1.01
-3	1	-4	9.06	1.03
-3	1	-4	9.68	0.66
-3	1	-4	7.97	0.96
3	-1	4	8.41	1.09
3	-1	4	9.04	0.72
-4	1	-4	63.82	3.33
-4	1	-4	60.62	3.34
-4	1	-4	67.76	3.56
-4	1	-4	65.37	3.22
4	-1	4	59.83	3.37
4	-1	4	66.86	3.69
4	-1	4	63.28	3.25
-5	1	-4	81.23	4.14
-5	1	-4	74.96	4.32
-5	1	-4	71.39	3.91
-5	1	-4	71.56	3.95
-5	1	-4	81.03	3.81

5	-1	4	78.76	4.28
5	-1	4	75.73	3.84
5	-1	4	71.73	4.00
-6	1	-4	86.44	4.36
-6	1	-4	83.46	4.04
-6	1	-4	77.30	4.18
-6	1	-4	76.88	4.20
-6	1	-4	74.50	4.68
6	-1	4	86.92	4.57
6	-1	4	79.81	4.09
6	-1	4	79.77	4.30
6	-1	4	75.76	4.21
-7	1	-4	19.61	1.99
-7	1	-4	17.79	2.70
-7	1	-4	20.07	1.67
-7	1	-4	18.23	1.70
-7	1	-4	21.31	1.31
7	-1	4	13.68	2.27
7	-1	4	16.97	1.55
7	-1	4	16.91	1.83
-8	1	-4	40.65	2.60
-8	1	-4	40.05	2.64
-8	1	-4	41.45	2.25
-8	1	-4	34.89	3.77
8	-1	4	36.83	2.29
8	-1	4	38.53	2.82
-9	1	-4	20.57	2.46
-9	1	-4	19.65	1.55
-9	1	-4	18.61	3.56
9	-1	4	18.41	2.84
9	-1	4	18.88	1.59
-10	1	-4	20.86	4.27
-10	1	-4	19.33	2.45
-10	1	-4	22.17	1.74
10	-1	4	18.17	1.76
10	-1	4	20.48	2.81
-11	1	-4	18.77	1.85
-11	1	-4	18.82	3.85
11	-1	4	23.01	3.18
11	-1	4	19.76	1.95
-12	1	-4	29.17	5.98
-12	1	-4	22.73	2.17
-13	1	-4	4.64	1.33
-13	1	-4	8.47	4.78

13	-1	4	6.53	1.45
-14	0	4	4.95	4.35
-14	0	4	12.88	4.87
-13	0	4	8.01	3.76
-13	0	4	10.15	4.14
-12	0	4	20.14	3.74
-12	0	4	14.03	3.30
-12	0	4	15.65	3.16
-11	0	4	14.78	3.16
-11	0	4	11.24	2.75
-10	0	4	30.18	3.76
-10	0	4	23.75	3.93
9	0	-4	13.69	2.24
-9	0	4	14.49	3.12
-9	0	4	16.87	2.72
-9	0	4	15.79	2.00
-8	0	4	48.84	3.57
-8	0	4	43.46	3.66
7	0	-4	15.40	2.40
-7	0	4	19.40	2.32
-7	0	4	21.00	2.39
6	0	-4	89.47	5.01
-6	0	4	92.59	5.06
-6	0	4	92.84	4.98
-5	0	4	52.57	3.18
-4	0	4	71.51	3.86
3	0	-4	36.88	2.32
-3	0	4	37.79	2.22
2	0	-4	56.46	3.20
2	0	-4	62.45	3.03
-2	0	4	54.69	3.12
-2	0	4	59.17	3.00
-2	0	4	65.74	3.07
1	0	-4	14.78	1.03
1	0	-4	14.39	1.26
1	0	-4	14.92	0.81
-1	0	4	14.63	1.12
-1	0	4	15.54	0.81
-1	0	4	16.29	1.06
-1	0	4	11.64	1.18
0	0	-4	159.11	7.73
0	0	-4	153.43	7.79
0	0	-4	157.25	7.70
0	0	-4	159.39	7.59

0	0	4	160.32	7.83
0	0	4	153.59	7.59
0	0	4	153.03	7.71
0	0	4	157.73	7.69
-1	0	-4	192.71	9.12
-1	0	-4	187.13	9.05
-1	0	-4	192.98	9.25
-1	0	-4	170.54	9.09
1	0	4	193.48	9.14
1	0	4	179.29	9.05
1	0	4	179.01	9.11
1	0	4	201.23	9.28
-2	0	-4	3.54	0.65
-2	0	-4	4.36	0.42
-2	0	-4	4.51	0.65
2	0	4	4.48	0.46
2	0	4	2.88	0.54
2	0	4	4.26	0.75
2	0	4	5.56	0.70
-3	0	-4	76.15	3.88
-3	0	-4	84.31	4.03
-3	0	-4	73.43	3.88
-3	0	-4	82.87	3.83
-3	0	-4	78.15	4.01
3	0	4	76.54	3.83
3	0	4	83.01	4.04
3	0	4	71.83	3.88
3	0	4	68.44	4.18
-4	0	-4	145.07	7.39
-4	0	-4	150.82	7.15
-4	0	-4	141.49	7.22
-4	0	-4	139.87	7.20
-4	0	-4	154.94	7.32
4	0	4	150.96	7.32
4	0	4	141.20	7.24
4	0	4	147.05	7.17
-5	0	-4	40.94	2.40
-5	0	-4	46.58	2.57
-5	0	-4	42.01	2.41
-5	0	-4	41.79	2.83
-5	0	-4	46.02	2.27
5	0	4	45.07	2.60
5	0	4	42.10	2.46
5	0	4	41.00	2.27

-6	0	-4	109.20	5.58
-6	0	-4	109.93	6.00
-6	0	-4	113.28	5.49
-6	0	-4	113.08	5.71
-6	0	-4	105.83	5.59
6	0	4	106.08	5.49
6	0	4	110.69	5.66
6	0	4	115.79	5.74
-7	0	-4	16.71	1.79
-7	0	-4	18.20	1.55
-7	0	-4	15.34	2.27
-7	0	-4	16.81	1.21
-7	0	-4	12.76	2.40
7	0	4	16.47	1.85
7	0	4	14.92	1.71
7	0	4	18.80	1.64
7	0	4	16.55	1.21
-8	0	-4	57.61	4.15
-8	0	-4	55.64	3.38
-8	0	-4	63.90	3.16
-8	0	-4	60.29	3.57
-8	0	-4	56.76	3.35
8	0	4	58.20	3.41
8	0	4	57.80	3.53
-9	0	-4	23.73	2.40
-9	0	-4	21.48	1.59
-9	0	-4	18.92	2.08
-9	0	-4	22.26	2.07
-9	0	-4	19.91	2.80
9	0	4	19.77	1.58
9	0	4	19.89	2.32
-10	0	-4	44.52	2.54
-10	0	-4	39.20	4.75
-10	0	-4	39.94	2.98
-10	0	-4	41.68	4.95
10	0	4	41.30	3.28
10	0	4	39.28	2.51
-11	0	-4	23.97	1.97
-11	0	-4	24.18	3.31
-11	0	-4	24.58	4.59
11	0	4	23.82	3.08
11	0	4	20.46	1.92
-12	0	-4	20.44	4.13
-12	0	-4	12.65	1.88

-12	0	-4	13.26	2.86
-13	0	-4	6.70	1.31
-13	0	-4	4.76	4.39
-14	0	-4	6.30	1.50
-14	0	-4	9.53	5.20
14	0	4	7.33	1.64
-13	1	4	24.97	4.65
-13	1	4	14.02	3.96
-12	1	4	7.62	3.46
-11	1	4	21.71	3.43
-11	1	4	16.16	2.57
-11	1	4	15.07	3.09
-9	1	4	26.88	3.44
-9	1	4	25.15	2.45
-9	1	4	21.75	4.02
-8	1	4	31.09	3.45
-8	1	4	33.97	3.20
-8	1	4	34.55	2.49
-7	1	4	77.27	4.46
-7	1	4	72.59	4.74
6	-1	-4	22.73	2.25
-6	1	4	26.81	2.25
-6	1	4	25.56	2.43
5	-1	-4	54.08	3.19
-5	1	4	52.36	3.12
-5	1	4	53.50	3.27
-4	1	4	14.57	1.54
3	-1	-4	44.51	2.77
-3	1	4	45.86	2.58
2	-1	-4	43.67	2.15
-2	1	4	43.16	2.39
1	-1	-4	5.71	0.91
1	-1	-4	5.13	0.38
-1	1	4	4.96	0.37
-1	1	4	4.35	0.70
0	-1	-4	992.37	46.41
0	1	4	984.54	46.45
0	1	4	918.55	46.40
-1	-1	-4	718.37	34.72
-1	-1	-4	704.28	34.67
1	1	4	725.20	34.69
1	1	4	735.57	34.72
-2	-1	-4	13.18	1.07
-2	-1	-4	13.20	0.76

-2	-1	-4	11.91	0.88
2	1	4	11.17	0.88
2	1	4	13.28	0.76
2	1	4	12.10	0.99
-3	-1	-4	61.72	3.24
-3	-1	-4	66.09	3.03
-3	-1	-4	58.47	3.06
-3	-1	-4	65.01	3.17
-3	-1	-4	60.57	3.08
3	1	4	61.46	3.14
3	1	4	55.92	3.06
3	1	4	55.56	3.09
3	1	4	59.73	3.02
-4	-1	-4	12.63	1.12
-4	-1	-4	11.26	0.98
-4	-1	-4	11.68	1.40
-4	-1	-4	12.73	0.80
4	1	4	8.89	0.95
4	1	4	9.95	1.14
4	1	4	13.23	1.13
4	1	4	11.62	0.79
-5	-1	-4	15.49	1.26
-5	-1	-4	18.11	1.48
-5	-1	-4	15.57	1.81
-5	-1	-4	18.21	1.32
-5	-1	-4	20.86	1.14
5	1	4	20.02	1.12
5	1	4	18.96	1.45
5	1	4	16.18	1.33
-6	-1	-4	12.98	1.97
-6	-1	-4	13.03	1.23
-6	-1	-4	13.70	0.99
-6	-1	-4	13.29	1.45
-6	-1	-4	9.83	1.18
6	1	4	15.03	1.43
6	1	4	9.81	1.29
6	1	4	13.52	0.94
-7	-1	-4	56.18	3.25
-7	-1	-4	55.52	3.28
-7	-1	-4	64.17	3.13
-7	-1	-4	63.93	3.46
-7	-1	-4	58.69	3.90
7	1	4	63.23	3.39
7	1	4	57.23	3.38

7	1	4	57.51	3.09
-8	-1	-4	16.05	2.73
-8	-1	-4	15.71	1.58
-8	-1	-4	13.98	1.23
-8	-1	-4	10.89	1.56
8	1	4	10.64	1.74
8	1	4	12.92	1.11
8	1	4	15.57	1.92
8	1	4	13.94	1.97
-9	-1	-4	51.84	3.28
-9	-1	-4	47.64	2.90
-9	-1	-4	47.60	2.63
-9	-1	-4	41.92	4.07
-9	-1	-4	42.06	2.96
9	1	4	40.51	2.73
9	1	4	43.29	3.15
9	1	4	47.47	3.08
-10	-1	-4	13.60	2.71
-10	-1	-4	14.19	2.01
-10	-1	-4	12.23	1.48
-10	-1	-4	13.01	2.00
10	1	4	11.53	2.24
10	1	4	11.10	1.33
-11	-1	-4	23.36	4.23
-11	-1	-4	28.20	3.31
-11	-1	-4	24.92	2.02
-11	-1	-4	25.44	2.57
11	1	4	22.25	1.89
-12	-1	-4	9.35	3.60
-12	-1	-4	8.47	1.72
-12	-1	-4	6.04	1.43
-12	-1	-4	6.36	1.20
12	1	4	6.84	1.03
-13	-1	-4	13.13	2.07
-13	-1	-4	12.88	2.56
-13	-1	-4	23.46	4.55
-14	-1	-4	14.49	4.81
14	1	4	8.05	1.54
-13	2	4	10.38	3.78
-13	2	4	9.28	4.69
-12	2	4	5.16	3.47
-12	2	4	9.20	3.84
-11	2	4	7.76	1.67
-11	2	4	11.51	3.23

-11	2	4	13.93	3.48
-10	2	4	11.15	2.83
-10	2	4	6.17	1.36
-10	2	4	5.67	2.79
-9	2	4	34.27	3.66
-9	2	4	30.30	1.80
-9	2	4	30.71	4.67
-9	2	4	33.35	2.73
-8	2	4	11.23	2.07
-8	2	4	11.97	0.96
-8	2	4	12.95	2.15
-8	2	4	12.28	1.95
-7	2	4	35.70	2.48
-7	2	4	34.76	1.90
-7	2	4	38.16	3.08
-7	2	4	33.56	3.34
-6	2	4	7.06	0.69
-6	2	4	8.01	0.73
-5	2	4	44.04	2.72
-5	2	4	42.05	2.19
-5	2	4	42.33	2.22
-4	2	4	4.61	0.50
-3	2	4	24.64	1.30
2	-2	-4	12.78	0.77
-2	2	4	12.91	0.75
1	-2	-4	35.97	1.80
-1	2	4	35.22	1.79
0	-2	-4	71.38	3.55
0	-2	-4	71.95	3.52
0	2	4	72.81	3.57
0	2	4	71.33	3.52
-1	-2	-4	288.18	13.49
-1	-2	-4	271.44	13.50
1	2	4	281.51	13.54
1	2	4	288.30	13.48
1	2	4	269.01	13.52
-2	-2	-4	27.48	1.50
-2	-2	-4	23.52	1.94
-2	-2	-4	30.28	1.43
2	2	4	26.84	1.57
2	2	4	26.44	1.51
2	2	4	27.53	1.41
-3	-2	-4	13.26	0.77
-3	-2	-4	11.48	0.93

-3	-2	-4	13.17	1.08
3	2	4	13.60	0.77
3	2	4	12.39	1.06
3	2	4	11.51	0.98
-4	-2	-4	68.95	3.71
-4	-2	-4	69.62	3.47
-4	-2	-4	67.07	3.55
-4	-2	-4	72.10	3.65
4	2	4	73.06	3.48
4	2	4	72.48	3.63
4	2	4	63.84	3.63
-5	-2	-4	54.83	2.86
-5	-2	-4	50.12	2.61
-5	-2	-4	49.91	2.73
-5	-2	-4	51.56	3.05
-5	-2	-4	47.86	2.67
5	2	4	47.58	2.89
5	2	4	50.41	2.60
5	2	4	54.13	2.85
-6	-2	-4	13.37	0.95
-6	-2	-4	13.72	1.49
-6	-2	-4	12.90	1.90
-6	-2	-4	11.73	1.16
-6	-2	-4	13.54	1.24
6	2	4	11.82	1.65
6	2	4	13.82	1.47
6	2	4	12.88	0.93
-7	-2	-4	15.16	2.35
-7	-2	-4	18.90	1.55
-7	-2	-4	19.47	1.26
-7	-2	-4	19.81	1.85
7	2	4	20.96	1.71
7	2	4	16.94	2.02
7	2	4	17.97	1.64
7	2	4	20.02	1.76
7	2	4	19.02	1.22
-8	-2	-4	11.28	1.57
-8	-2	-4	14.03	1.92
-8	-2	-4	13.91	1.16
-8	-2	-4	13.96	2.04
-8	-2	-4	14.32	1.56
8	2	4	13.91	1.07
8	2	4	14.20	1.75
8	2	4	13.74	1.95

-9	-2	-4	29.41	3.33
-9	-2	-4	29.18	2.29
-9	-2	-4	32.74	2.27
9	2	4	30.90	2.45
9	2	4	32.11	2.44
9	2	4	28.75	1.82
9	2	4	28.50	2.90
-10	-2	-4	26.28	3.74
-10	-2	-4	28.32	2.49
-10	-2	-4	36.49	3.05
-10	-2	-4	32.45	2.48
-10	-2	-4	29.02	2.07
10	2	4	28.96	2.69
10	2	4	30.95	1.94
10	2	4	30.52	2.67
10	2	4	34.40	2.59
-11	-2	-4	23.44	2.46
-11	-2	-4	25.42	4.87
-11	-2	-4	26.06	2.40
-11	-2	-4	18.88	1.88
-11	-2	-4	23.38	4.07
11	2	4	21.69	1.73
-12	-2	-4	4.99	1.46
-12	-2	-4	8.84	3.29
-12	-2	-4	8.33	2.28
-12	-2	-4	3.15	0.97
12	2	4	3.51	0.90
-13	-2	-4	4.09	1.30
-13	-2	-4	3.74	1.54
-13	-2	-4	6.91	4.11
-13	-2	-4	7.61	2.77
-13	-2	-4	4.49	1.74
13	2	4	3.63	1.02
-14	-2	-4	7.75	4.77
-14	-2	-4	4.65	2.18
-14	-2	-4	4.92	1.25
-15	-2	-4	11.96	5.52
-15	-2	-4	3.76	1.45
15	2	4	3.59	1.44
-13	3	4	9.58	5.06
-13	3	4	4.19	4.11
-12	3	4	10.95	3.69
-11	3	4	4.23	1.77
-11	3	4	4.20	3.05

-11	3	4	9.70	3.59
-11	3	4	4.06	1.01
-10	3	4	24.50	2.79
-10	3	4	24.61	4.36
-10	3	4	24.05	1.82
-10	3	4	20.89	3.24
-9	3	4	11.07	2.23
-9	3	4	6.95	2.45
-9	3	4	11.53	1.25
-9	3	4	10.55	3.38
-9	3	4	9.99	1.07
-8	3	4	72.98	4.93
-8	3	4	72.83	5.26
-8	3	4	70.69	4.20
-8	3	4	73.33	3.71
-8	3	4	66.87	3.63
-7	3	4	47.06	3.56
-7	3	4	39.32	4.06
-7	3	4	50.02	2.55
-7	3	4	51.42	3.05
-7	3	4	44.24	2.48
-6	3	4	62.48	3.78
-6	3	4	60.41	4.26
-6	3	4	62.18	3.17
-6	3	4	61.01	3.13
-5	3	4	32.03	1.75
-5	3	4	29.89	1.69
-5	3	4	31.26	2.34
-4	3	4	39.99	2.39
-4	3	4	36.89	2.01
-3	3	4	19.62	1.12
2	-3	-4	72.81	3.58
-2	3	4	71.33	3.56
1	-3	-4	123.52	6.11
-1	3	4	127.00	6.10
0	-3	-4	154.49	7.48
0	3	4	153.54	7.47
-1	-3	-4	16.00	1.01
-1	-3	-4	17.30	1.16
-1	-3	-4	18.23	0.95
1	3	4	16.45	1.05
1	3	4	17.18	0.94
1	3	4	17.42	1.13
-2	-3	-4	63.29	3.35

-2	-3	-4	70.45	3.30
2	3	4	65.85	3.41
2	3	4	63.42	3.38
2	3	4	70.21	3.29
-3	-3	-4	75.36	3.70
-3	-3	-4	73.44	3.79
-3	-3	-4	78.88	3.89
3	3	4	75.80	3.85
3	3	4	72.56	3.70
-4	-3	-4	91.48	5.01
-4	-3	-4	106.52	4.92
-4	-3	-4	99.79	5.11
-4	-3	-4	106.15	5.11
4	3	4	102.86	5.08
4	3	4	104.68	4.92
4	3	4	91.17	5.09
4	3	4	99.27	5.05
-5	-3	-4	42.21	2.12
-5	-3	-4	39.04	2.53
-5	-3	-4	41.17	2.44
-5	-3	-4	39.93	2.32
5	3	4	43.34	2.14
5	3	4	40.05	2.33
5	3	4	43.15	2.42
5	3	4	38.41	2.50
-6	-3	-4	96.17	5.22
-6	-3	-4	107.13	5.12
-6	-3	-4	98.24	4.82
-6	-3	-4	89.92	4.98
6	3	4	93.30	5.13
6	3	4	96.48	4.83
6	3	4	94.24	5.02
6	3	4	102.78	5.06
-7	-3	-4	30.90	2.73
-7	-3	-4	30.15	2.15
-7	-3	-4	32.69	1.82
-7	-3	-4	36.68	2.39
7	3	4	30.45	2.51
7	3	4	31.56	2.30
7	3	4	32.50	1.82
7	3	4	36.79	2.30
-8	-3	-4	25.14	1.55
-8	-3	-4	21.93	1.88
-8	-3	-4	26.54	2.29

-8	-3	-4	24.41	1.97
-8	-3	-4	21.98	2.76
8	3	4	26.94	2.17
8	3	4	24.55	2.47
8	3	4	26.70	1.52
-9	-3	-4	11.97	2.15
-9	-3	-4	9.39	1.63
-9	-3	-4	9.65	1.10
-9	-3	-4	10.41	2.09
9	3	4	9.35	1.81
9	3	4	10.48	1.80
9	3	4	8.43	1.74
9	3	4	8.11	0.99
-10	-3	-4	60.31	3.83
-10	-3	-4	47.28	2.90
-10	-3	-4	54.24	4.41
-10	-3	-4	48.20	3.28
10	3	4	47.19	2.85
10	3	4	58.45	3.53
10	3	4	52.17	4.01
-11	-3	-4	13.74	2.15
-11	-3	-4	9.89	2.07
-11	-3	-4	16.09	2.95
-11	-3	-4	11.94	4.10
-11	-3	-4	12.17	1.48
11	3	4	11.48	2.33
11	3	4	12.49	2.32
11	3	4	9.90	1.67
11	3	4	10.89	1.26
-12	-3	-4	24.06	4.56
-12	-3	-4	23.78	2.60
-12	-3	-4	26.52	3.68
-12	-3	-4	20.52	1.85
12	3	4	16.17	3.26
12	3	4	17.80	1.70
12	3	4	17.17	2.78
-13	-3	-4	7.99	1.62
-13	-3	-4	7.08	1.69
-13	-3	-4	13.03	2.78
-13	-3	-4	6.40	1.09
-13	-3	-4	11.15	3.87
13	3	4	9.04	1.92
13	3	4	6.66	1.06
-14	-3	-4	8.67	1.56

-14	-3	-4	9.59	1.99
-14	-3	-4	15.96	4.52
14	3	4	7.67	1.25
-15	-3	-4	3.92	1.24
-15	-3	-4	5.47	4.91
-15	-3	-4	6.43	3.94
-12	4	4	5.43	0.94
-12	4	4	4.44	3.66
-12	4	4	2.74	1.80
-12	4	4	8.00	4.52
-11	4	4	7.89	1.31
-11	4	4	4.51	3.23
-11	4	4	9.76	3.40
-11	4	4	9.89	3.90
-11	4	4	5.39	1.08
-10	4	4	14.25	3.21
-10	4	4	16.04	2.68
-10	4	4	22.63	3.65
-10	4	4	18.78	1.75
-10	4	4	19.73	1.54
-9	4	4	0.45	2.38
-9	4	4	1.57	1.15
-9	4	4	1.09	0.56
-9	4	4	1.38	0.67
-9	4	4	3.12	2.65
-8	4	4	30.40	3.53
-8	4	4	28.43	1.94
-8	4	4	29.18	2.61
-8	4	4	30.85	2.06
-7	4	4	30.17	1.85
-7	4	4	32.89	1.95
-7	4	4	31.60	3.26
-7	4	4	32.30	2.47
-6	4	4	44.13	3.22
-6	4	4	42.24	2.32
-6	4	4	42.66	2.37
-5	4	4	34.19	1.89
-5	4	4	34.43	2.65
-5	4	4	30.80	1.81
-5	4	4	32.55	2.49
-4	4	4	53.02	3.03
-4	4	4	49.02	2.62
-3	4	4	8.54	0.74
-2	4	4	37.47	1.96

1	-4	-4	19.08	1.11
-1	4	4	19.40	1.09
0	-4	-4	42.23	2.10
0	4	4	39.16	2.08
-1	-4	-4	31.02	1.64
1	4	4	31.40	1.63
-2	-4	-4	20.31	1.32
-2	-4	-4	24.15	1.23
2	4	4	23.13	1.38
2	4	4	22.41	1.43
2	4	4	22.18	1.21
-3	-4	-4	43.23	2.45
-3	-4	-4	48.14	2.62
-3	-4	-4	46.89	2.33
3	4	4	45.01	2.51
3	4	4	45.30	2.47
3	4	4	47.37	2.32
-4	-4	-4	1.02	0.69
-4	-4	-4	1.89	0.51
-4	-4	-4	1.34	0.64
-4	-4	-4	1.70	0.27
4	4	4	1.57	0.52
4	4	4	1.28	0.26
4	4	4	1.82	0.58
-5	-4	-4	43.46	2.55
-5	-4	-4	41.18	2.53
-5	-4	-4	43.58	2.14
-5	-4	-4	37.19	2.39
5	4	4	38.71	2.53
5	4	4	41.96	2.13
5	4	4	40.67	2.42
5	4	4	44.68	2.50
-6	-4	-4	49.39	3.17
-6	-4	-4	47.82	2.97
-6	-4	-4	54.33	2.67
-6	-4	-4	57.83	3.12
6	4	4	53.80	2.98
6	4	4	44.37	3.16
6	4	4	55.31	2.69
6	4	4	55.41	3.05
-7	-4	-4	41.56	2.72
-7	-4	-4	46.98	2.35
-7	-4	-4	43.92	3.09
-7	-4	-4	45.15	2.89

7	4	4	40.27	2.99
7	4	4	45.60	2.74
7	4	4	46.64	2.83
7	4	4	46.53	2.38
-8	-4	-4	34.03	1.88
-8	-4	-4	35.53	2.98
-8	-4	-4	35.30	2.66
-8	-4	-4	28.18	2.36
8	4	4	33.94	2.48
8	4	4	37.79	2.53
8	4	4	32.06	2.75
8	4	4	32.68	1.89
-9	-4	-4	32.97	2.61
-9	-4	-4	34.54	3.32
-9	-4	-4	39.87	2.07
-9	-4	-4	37.13	2.97
9	4	4	35.22	2.80
9	4	4	34.18	3.13
9	4	4	37.95	2.07
-10	-4	-4	20.97	1.49
-10	-4	-4	27.76	2.76
-10	-4	-4	15.43	2.26
-10	-4	-4	20.63	3.39
10	4	4	19.89	2.86
10	4	4	15.52	2.97
10	4	4	25.42	2.43
10	4	4	20.39	1.45
-11	-4	-4	17.79	1.42
-11	-4	-4	15.29	2.09
-11	-4	-4	18.53	3.00
-11	-4	-4	14.07	2.32
11	4	4	13.24	2.41
11	4	4	14.09	1.41
11	4	4	17.37	2.53
-12	-4	-4	7.51	2.25
-12	-4	-4	5.19	3.03
-12	-4	-4	4.35	1.41
-12	-4	-4	4.87	0.83
-12	-4	-4	3.20	1.20
12	4	4	2.49	2.42
12	4	4	3.53	0.71
12	4	4	3.73	1.71
-13	-4	-4	18.04	3.77
-13	-4	-4	12.91	1.56

-13	-4	-4	11.55	1.79
-13	-4	-4	14.44	2.92
-13	-4	-4	9.28	1.71
13	4	4	11.11	1.56
13	4	4	7.24	1.99
13	4	4	9.20	2.93
13	4	4	12.23	1.98
-14	-4	-4	11.14	4.34
-14	-4	-4	4.39	1.78
-14	-4	-4	5.35	1.01
-14	-4	-4	11.29	3.30
14	4	4	2.42	1.04
-15	-4	-4	4.98	1.15
-15	-4	-4	4.17	3.84
-15	-4	-4	7.85	4.78
15	4	4	1.62	1.12
-11	5	4	16.04	2.04
-11	5	4	15.72	3.69
-11	5	4	23.31	4.55
-11	5	4	15.72	1.73
-10	5	4	8.80	1.20
-10	5	4	6.27	3.39
-10	5	4	15.37	3.66
-10	5	4	11.40	1.43
-10	5	4	6.56	1.60
-10	5	4	6.75	2.79
-9	5	4	32.00	4.40
-9	5	4	28.02	1.95
-9	5	4	28.69	2.82
-9	5	4	20.43	3.68
-9	5	4	29.30	2.05
-8	5	4	7.27	1.12
-8	5	4	4.42	1.19
-8	5	4	5.62	2.18
-8	5	4	6.05	0.85
-7	5	4	35.22	2.14
-7	5	4	33.96	2.05
-7	5	4	32.84	3.50
-7	5	4	34.46	3.45
-6	5	4	12.75	1.18
-6	5	4	14.43	1.24
-6	5	4	12.77	2.42
-6	5	4	14.07	2.59
-5	5	4	52.28	3.58

-5	5	4	54.22	3.45
-5	5	4	52.88	2.84
-5	5	4	51.55	2.78
-4	5	4	21.24	2.17
-4	5	4	21.06	1.38
-4	5	4	20.25	1.30
-3	5	4	33.03	1.75
-3	5	4	29.08	2.32
-2	5	4	65.67	3.33
-1	5	4	28.41	1.56
0	-5	-4	51.41	2.63
0	5	4	51.12	2.61
-1	-5	-4	32.20	1.71
1	5	4	31.22	1.68
-2	-5	-4	14.56	1.08
-2	-5	-4	15.40	0.93
2	5	4	14.00	1.21
2	5	4	15.69	1.14
2	5	4	14.92	0.92
-3	-5	-4	21.95	1.59
-3	-5	-4	23.17	1.26
3	5	4	22.56	1.49
3	5	4	21.54	1.57
3	5	4	22.32	1.24
-4	-5	-4	14.17	1.41
-4	-5	-4	14.71	1.55
-4	-5	-4	15.09	0.92
4	5	4	14.78	0.91
4	5	4	14.71	1.37
4	5	4	13.92	1.26
-5	-5	-4	42.96	2.68
-5	-5	-4	48.26	2.73
-5	-5	-4	43.68	2.30
5	5	4	42.17	2.69
5	5	4	47.26	2.68
5	5	4	45.72	2.30
5	5	4	44.36	2.59
-6	-5	-4	20.46	1.98
-6	-5	-4	25.09	1.95
-6	-5	-4	22.00	1.28
6	5	4	22.27	1.29
6	5	4	23.95	1.90
6	5	4	21.42	1.75
-7	-5	-4	50.08	3.02

-7	-5	-4	44.36	3.08
-7	-5	-4	46.20	2.39
7	5	4	43.62	2.83
7	5	4	47.88	2.42
7	5	4	47.68	2.93
7	5	4	41.52	3.08
-8	-5	-4	11.83	2.09
-8	-5	-4	16.69	1.07
-8	-5	-4	15.07	2.27
8	5	4	15.37	1.79
8	5	4	14.82	2.01
8	5	4	15.85	1.13
8	5	4	12.06	2.33
-9	-5	-4	45.78	2.51
-9	-5	-4	49.72	3.63
-9	-5	-4	46.87	3.41
9	5	4	45.13	3.09
9	5	4	49.07	3.29
9	5	4	47.75	3.61
9	5	4	46.95	2.57
-10	-5	-4	3.30	2.21
-10	-5	-4	6.21	0.96
-10	-5	-4	6.57	1.68
10	5	4	3.00	2.06
10	5	4	5.97	0.97
10	5	4	5.10	1.30
10	5	4	7.26	1.49
-11	-5	-4	11.14	1.14
-11	-5	-4	10.51	3.76
-11	-5	-4	9.17	2.05
-11	-5	-4	7.26	1.54
-11	-5	-4	7.04	1.19
11	5	4	10.14	2.37
11	5	4	9.73	1.68
11	5	4	9.50	1.79
11	5	4	9.24	1.28
-12	-5	-4	-0.08	3.19
-12	-5	-4	-1.45	2.86
-12	-5	-4	1.34	1.43
-12	-5	-4	2.31	0.68
-12	-5	-4	0.98	1.10
12	5	4	-0.78	1.56
12	5	4	2.21	2.43
12	5	4	0.99	0.64

12	5	4	-0.52	1.75
-13	-5	-4	15.82	3.71
-13	-5	-4	8.12	1.86
-13	-5	-4	13.31	1.43
-13	-5	-4	15.31	2.93
13	5	4	11.02	2.95
13	5	4	10.06	1.41
13	5	4	8.45	2.12
-14	-5	-4	5.96	1.79
-14	-5	-4	4.46	0.89
-14	-5	-4	3.35	4.19
-14	-5	-4	8.72	3.37
-14	-5	-4	2.31	1.70
14	5	4	4.22	1.02
14	5	4	2.71	1.97
14	5	4	2.55	2.72
-15	-5	-4	5.05	1.12
-15	-5	-4	7.07	4.75
-15	-5	-4	8.32	3.96
15	5	4	5.11	1.17
-11	6	4	10.02	3.86
-11	6	4	6.08	1.11
-11	6	4	6.45	1.28
-10	6	4	12.10	1.63
-10	6	4	12.01	3.73
-10	6	4	7.26	1.18
-10	6	4	11.48	3.95
-9	6	4	12.25	2.90
-9	6	4	14.80	1.73
-9	6	4	20.13	3.45
-9	6	4	15.73	1.67
-8	6	4	20.40	1.68
-8	6	4	14.54	2.80
-8	6	4	22.68	1.83
-8	6	4	22.48	3.85
-7	6	4	9.49	2.45
-7	6	4	9.07	1.26
-7	6	4	10.71	1.38
-7	6	4	9.47	2.94
-6	6	4	2.86	1.92
-6	6	4	2.85	0.57
-6	6	4	2.69	1.81
-6	6	4	2.57	0.63
-5	6	4	13.99	1.27

-5	6	4	13.51	1.17
-5	6	4	13.74	2.03
-4	6	4	33.63	1.94
-4	6	4	30.46	2.83
-4	6	4	35.13	2.01
-3	6	4	46.84	3.20
-3	6	4	48.27	2.55
-2	6	4	46.65	2.37
-2	6	4	41.45	2.87
-1	6	4	121.74	6.55
-1	6	4	134.09	6.31
0	-6	-4	40.28	2.37
0	-6	-4	38.65	2.11
0	6	4	38.63	2.07
-1	-6	-4	49.17	2.53
1	6	4	46.85	2.73
1	6	4	49.05	2.50
-2	-6	-4	50.87	2.77
-2	-6	-4	51.42	2.59
2	6	4	50.71	2.71
2	6	4	49.43	2.78
2	6	4	49.09	2.78
2	6	4	48.27	2.56
-3	-6	-4	6.68	0.69
3	6	4	6.20	1.04
3	6	4	7.08	0.65
3	6	4	5.63	1.14
3	6	4	7.91	0.88
-4	-6	-4	65.01	3.47
-4	-6	-4	66.50	3.30
-4	-6	-4	63.47	3.59
4	6	4	66.03	3.28
4	6	4	64.79	3.56
4	6	4	67.25	3.50
4	6	4	62.77	3.56
-5	-6	-4	21.06	1.86
-5	-6	-4	20.80	1.25
-5	-6	-4	20.47	1.65
-5	-6	-4	21.89	1.86
5	6	4	20.94	1.69
5	6	4	22.39	1.82
5	6	4	21.12	1.81
5	6	4	21.11	1.24
-6	-6	-4	73.20	4.26

-6	-6	-4	79.81	4.15
-6	-6	-4	82.05	4.30
-6	-6	-4	74.51	3.87
6	6	4	76.16	4.17
6	6	4	77.85	3.86
6	6	4	74.33	4.29
6	6	4	79.81	4.25
-7	-6	-4	7.08	1.33
-7	-6	-4	5.37	0.91
-7	-6	-4	6.60	0.72
-7	-6	-4	7.10	1.22
7	6	4	5.53	0.97
7	6	4	7.25	1.13
7	6	4	6.96	0.71
7	6	4	5.64	1.38
-8	-6	-4	34.36	2.79
-8	-6	-4	35.70	2.74
-8	-6	-4	33.53	1.86
-8	-6	-4	33.13	2.47
8	6	4	30.25	2.86
8	6	4	33.04	1.87
8	6	4	32.39	2.42
8	6	4	35.16	2.67
-9	-6	-4	20.73	1.31
-9	-6	-4	15.23	2.60
-9	-6	-4	22.77	2.57
9	6	4	19.26	2.17
9	6	4	15.52	2.79
9	6	4	21.25	2.44
9	6	4	19.43	1.35
-10	-6	-4	6.17	1.78
-10	-6	-4	6.84	2.14
-10	-6	-4	9.12	0.98
10	6	4	7.96	1.37
10	6	4	7.63	1.59
10	6	4	2.93	2.19
10	6	4	8.19	1.06
-11	-6	-4	0.67	0.55
-11	-6	-4	-1.80	1.85
-11	-6	-4	-0.69	3.11
11	6	4	-2.01	2.24
11	6	4	0.45	0.57
11	6	4	-0.73	1.26
11	6	4	-0.91	1.64

-12	-6	-4	12.67	2.17
-12	-6	-4	17.56	1.35
-12	-6	-4	18.73	3.20
-12	-6	-4	12.00	2.53
12	6	4	12.53	2.16
12	6	4	14.21	1.35
12	6	4	14.34	1.95
12	6	4	14.46	2.74
-13	-6	-4	7.43	2.70
-13	-6	-4	4.07	0.77
-13	-6	-4	11.96	3.42
-13	-6	-4	2.34	1.83
13	6	4	4.28	0.77
13	6	4	3.35	2.03
13	6	4	1.43	2.85
-14	-6	-4	3.46	0.83
-14	-6	-4	3.79	1.90
-14	-6	-4	0.17	1.54
-14	-6	-4	4.03	3.27
-14	-6	-4	8.57	4.02
14	6	4	1.03	2.32
14	6	4	1.72	1.83
-15	-6	-4	3.28	0.89
-15	-6	-4	6.53	3.88
-15	-6	-4	10.85	4.62
15	6	4	1.25	0.99
-16	-6	-4	11.07	4.66
-16	-6	-4	10.50	5.21
-10	7	4	9.29	1.38
-10	7	4	12.66	4.05
-10	7	4	15.34	2.13
-10	7	4	17.08	4.33
-9	7	4	8.19	1.20
-9	7	4	11.29	1.72
-9	7	4	15.44	3.44
-9	7	4	2.99	5.07
-8	7	4	27.11	4.33
-8	7	4	20.52	3.40
-8	7	4	27.14	2.02
-8	7	4	26.41	2.14
-7	7	4	12.70	2.81
-7	7	4	9.99	1.40
-7	7	4	10.32	2.46
-7	7	4	8.59	1.45

-6	7	4	31.23	3.46
-6	7	4	27.55	3.78
-6	7	4	30.60	2.00
-6	7	4	30.20	2.08
-5	7	4	20.21	1.58
-5	7	4	18.90	3.10
-5	7	4	18.20	1.45
-4	7	4	44.21	3.60
-4	7	4	45.06	2.53
-4	7	4	46.53	2.61
-3	7	4	12.66	1.10
-3	7	4	11.68	2.27
-2	7	4	33.69	2.17
-2	7	4	36.74	2.69
-1	7	4	28.99	2.39
-1	7	4	34.01	1.82
0	-7	-4	81.79	4.27
0	7	4	75.19	4.29
0	7	4	79.65	3.99
-1	-7	-4	31.98	1.81
-1	-7	-4	32.84	2.26
1	7	4	32.44	1.76
1	7	4	29.86	2.17
-2	-7	-4	90.27	4.48
-2	-7	-4	90.66	4.82
2	7	4	87.17	4.63
2	7	4	88.78	4.69
2	7	4	88.57	4.45
-3	-7	-4	49.52	3.02
-3	-7	-4	51.99	2.61
-3	-7	-4	47.46	2.70
3	7	4	51.33	2.57
3	7	4	47.57	2.86
3	7	4	49.17	2.77
3	7	4	48.23	2.87
-4	-7	-4	23.55	1.99
-4	-7	-4	23.11	1.35
-4	-7	-4	21.17	1.63
4	7	4	20.57	1.67
4	7	4	20.66	1.82
4	7	4	19.73	1.81
4	7	4	23.41	1.31
-5	-7	-4	99.16	4.93
-5	-7	-4	95.68	5.28

-5	-7	-4	101.29	5.29
-5	-7	-4	97.03	5.12
5	7	4	96.68	5.24
5	7	4	98.62	5.14
5	7	4	100.86	5.23
5	7	4	101.12	4.90
-6	-7	-4	46.45	3.02
-6	-7	-4	45.98	2.43
-6	-7	-4	44.07	2.99
-6	-7	-4	44.81	2.77
6	7	4	45.20	2.79
6	7	4	49.59	2.96
6	7	4	45.16	2.40
6	7	4	47.24	3.02
-7	-7	-4	8.64	1.51
-7	-7	-4	9.65	0.91
-7	-7	-4	10.74	1.53
-7	-7	-4	8.58	1.33
7	7	4	8.38	1.57
7	7	4	10.29	0.86
7	7	4	10.38	1.59
7	7	4	9.09	1.28
-8	-7	-4	20.38	2.38
-8	-7	-4	18.37	1.93
-8	-7	-4	19.47	1.31
-8	-7	-4	17.98	2.38
8	7	4	22.38	2.27
8	7	4	19.11	1.26
8	7	4	19.51	1.99
-9	-7	-4	7.24	1.61
-9	-7	-4	9.51	1.82
-9	-7	-4	6.01	1.74
-9	-7	-4	7.64	0.92
9	7	4	6.08	1.18
9	7	4	5.06	1.89
9	7	4	7.27	0.92
9	7	4	7.35	1.41
-10	-7	-4	35.98	2.87
-10	-7	-4	38.24	2.13
-10	-7	-4	36.95	3.47
-10	-7	-4	39.50	3.43
10	7	4	32.68	2.90
10	7	4	39.00	4.06
10	7	4	38.26	2.17

10	7	4	34.76	3.83
-11	-7	-4	5.70	2.01
11	7	4	5.82	2.45
11	7	4	6.24	0.89
11	7	4	5.80	1.82
11	7	4	6.47	1.43
-12	-7	-4	10.91	2.13
-12	-7	-4	16.89	1.40
-12	-7	-4	18.06	3.13
-12	-7	-4	15.47	2.64
12	7	4	14.66	2.34
12	7	4	17.57	2.82
12	7	4	15.05	1.40
12	7	4	15.50	2.91
-13	-7	-4	0.95	1.29
-13	-7	-4	4.25	0.78
-13	-7	-4	0.30	2.64
-13	-7	-4	-1.60	3.28
13	7	4	3.59	2.19
13	7	4	4.37	3.10
13	7	4	2.76	1.65
13	7	4	2.54	0.72
-14	-7	-4	0.19	3.98
-14	-7	-4	0.71	2.09
-14	-7	-4	3.74	0.86
-14	-7	-4	2.87	1.65
-14	-7	-4	3.16	3.09
14	7	4	0.39	2.08
14	7	4	1.93	0.81
14	7	4	-0.01	2.54
-15	-7	-4	-0.11	1.82
-15	-7	-4	-0.27	3.72
-15	-7	-4	-1.18	4.39
15	7	4	-1.57	2.36
15	7	4	1.41	0.90
-16	-7	-4	0.91	5.18
-16	-7	-4	8.76	4.55
-9	8	4	7.33	1.19
-9	8	4	10.08	3.78
-9	8	4	7.40	1.26
-8	8	4	8.85	3.04
-8	8	4	6.34	1.03
-8	8	4	6.45	1.16
-8	8	4	3.49	3.54

-7	8	4	10.55	2.82
-7	8	4	12.15	1.61
-7	8	4	9.31	3.17
-7	8	4	10.90	1.78
-6	8	4	16.81	1.64
-6	8	4	20.81	3.42
-6	8	4	21.45	4.09
-6	8	4	15.68	1.73
-5	8	4	34.62	3.99
-5	8	4	34.07	2.19
-4	8	4	18.83	1.55
-4	8	4	17.87	1.48
-4	8	4	17.55	2.44
-3	8	4	14.16	1.34
-3	8	4	13.11	2.54
-3	8	4	13.44	1.23
-2	8	4	4.17	0.59
-1	8	4	19.38	1.29
-1	8	4	16.88	2.12
0	-8	-4	17.31	1.85
0	8	4	13.32	1.18
0	8	4	14.34	1.85
-1	-8	-4	19.77	1.97
1	8	4	18.18	1.21
1	8	4	16.99	1.84
-2	-8	-4	13.22	1.05
-2	-8	-4	12.76	1.91
2	8	4	12.80	1.59
2	8	4	12.81	0.97
2	8	4	9.41	1.55
-3	-8	-4	22.19	1.84
-3	-8	-4	22.57	2.26
-3	-8	-4	23.59	1.45
-3	-8	-4	22.60	1.63
3	8	4	22.26	1.82
3	8	4	24.04	1.71
3	8	4	22.43	1.40
3	8	4	23.68	1.94
-4	-8	-4	4.96	1.29
-4	-8	-4	5.33	0.79
-4	-8	-4	5.76	0.79
4	8	4	4.30	0.81
4	8	4	6.27	1.13
4	8	4	3.98	0.71

4	8	4	4.26	0.96
-5	-8	-4	31.73	2.46
-5	-8	-4	29.34	2.11
-5	-8	-4	30.86	1.78
5	8	4	28.69	2.35
5	8	4	32.11	2.39
5	8	4	30.77	1.73
5	8	4	28.42	2.17
-6	-8	-4	13.34	1.03
-6	-8	-4	9.40	1.37
-6	-8	-4	9.77	1.55
-6	-8	-4	10.33	1.98
6	8	4	11.01	1.36
6	8	4	12.49	0.93
6	8	4	12.38	2.00
6	8	4	10.56	1.60
-7	-8	-4	18.34	1.31
-7	-8	-4	18.50	1.85
-7	-8	-4	15.88	2.19
-7	-8	-4	18.44	2.35
7	8	4	19.61	1.26
7	8	4	18.82	2.72
7	8	4	21.76	2.26
7	8	4	20.79	1.96
-8	-8	-4	3.27	0.56
-8	-8	-4	4.15	1.56
-8	-8	-4	3.40	1.01
-8	-8	-4	2.30	1.42
8	8	4	3.65	1.30
8	8	4	3.81	1.05
8	8	4	4.35	1.65
8	8	4	3.36	0.52
-9	-8	-4	8.11	1.85
-9	-8	-4	8.11	1.03
-9	-8	-4	9.63	1.88
-9	-8	-4	8.88	1.61
9	8	4	9.58	1.57
9	8	4	7.10	1.30
9	8	4	8.53	0.97
9	8	4	8.08	2.04
-10	-8	-4	7.95	1.36
-10	-8	-4	10.13	1.81
-10	-8	-4	11.55	2.09
-10	-8	-4	9.52	1.16

10	8	4	9.13	1.46
10	8	4	8.50	1.10
10	8	4	9.69	2.39
-11	-8	-4	51.28	4.22
-11	-8	-4	47.08	4.06
-11	-8	-4	43.43	3.43
-11	-8	-4	47.45	2.64
11	8	4	40.44	2.87
11	8	4	47.28	4.47
11	8	4	48.60	4.03
11	8	4	46.40	3.50
-12	-8	-4	8.59	2.84
-12	-8	-4	6.80	2.32
12	8	4	5.67	2.15
12	8	4	1.82	2.90
12	8	4	5.88	0.84
12	8	4	5.46	1.57
-13	-8	-4	9.65	1.54
-13	-8	-4	7.48	3.81
-13	-8	-4	13.34	3.48
13	8	4	9.73	2.57
13	8	4	9.21	1.37
13	8	4	10.37	3.49
13	8	4	11.80	1.89
-14	-8	-4	-1.78	3.94
-14	-8	-4	2.16	0.86
-14	-8	-4	-3.36	3.13
14	8	4	-1.36	2.60
14	8	4	1.50	0.78
14	8	4	1.06	1.96
-15	-8	-4	1.39	1.78
-15	-8	-4	1.32	4.48
-15	-8	-4	3.03	3.88
15	8	4	-0.27	1.93
15	8	4	2.05	0.87
-16	-8	-4	4.00	4.59
-8	9	4	6.34	3.36
-8	9	4	6.05	1.27
-7	9	4	16.96	3.87
-7	9	4	22.72	2.24
-7	9	4	19.00	2.14
-7	9	4	21.95	4.17
-6	9	4	15.40	1.74
-6	9	4	13.59	3.26

-6	9	4	13.25	1.81
-5	9	4	43.40	4.62
-5	9	4	43.20	2.77
-5	9	4	43.24	2.69
-4	9	4	20.58	3.56
-4	9	4	21.85	1.75
-4	9	4	23.56	1.83
-3	9	4	30.14	3.43
-3	9	4	33.54	2.04
-3	9	4	30.17	2.47
-2	9	4	14.14	1.32
-2	9	4	14.86	2.05
1	-9	-4	23.00	2.22
-1	9	4	23.96	1.59
-1	9	4	23.26	2.51
0	-9	-4	16.84	2.17
0	9	4	14.97	1.22
-1	-9	-4	42.67	3.11
1	9	4	42.59	2.37
1	9	4	43.55	2.99
-2	-9	-4	30.04	2.65
2	9	4	31.03	2.41
2	9	4	26.49	2.47
2	9	4	29.67	1.74
-3	-9	-4	37.62	2.77
-3	-9	-4	48.77	2.49
-3	-9	-4	44.08	3.25
3	9	4	40.97	2.80
3	9	4	45.96	2.65
3	9	4	46.20	2.95
3	9	4	46.78	2.41
-4	-9	-4	12.22	2.17
-4	-9	-4	12.74	1.10
4	9	4	11.18	1.48
4	9	4	10.12	1.26
4	9	4	11.06	1.79
-5	-9	-4	58.61	2.88
-5	-9	-4	52.22	3.22
-5	-9	-4	54.70	3.59
-5	-9	-4	51.03	3.45
5	9	4	55.55	3.17
5	9	4	48.31	3.30
5	9	4	57.37	3.42
5	9	4	46.19	2.95

-6	-9	-4	12.16	1.73
-6	-9	-4	16.11	2.32
-6	-9	-4	15.82	1.19
-6	-9	-4	10.27	1.48
6	9	4	13.21	1.78
6	9	4	12.89	1.14
6	9	4	11.29	1.48
-7	-9	-4	13.22	2.37
-7	-9	-4	12.41	1.80
-7	-9	-4	13.50	1.17
-7	-9	-4	8.57	1.58
7	9	4	12.36	2.42
7	9	4	12.89	1.19
7	9	4	11.75	1.58
7	9	4	12.35	1.86
-8	-9	-4	9.52	1.10
-8	-9	-4	4.77	1.58
-8	-9	-4	11.61	1.96
-8	-9	-4	8.40	1.27
8	9	4	9.04	1.57
8	9	4	7.64	1.28
8	9	4	11.97	2.00
8	9	4	7.53	0.99
-9	-9	-4	10.73	2.01
-9	-9	-4	8.16	1.71
-9	-9	-4	9.17	1.39
-9	-9	-4	11.81	1.20
9	9	4	10.64	1.76
9	9	4	11.05	1.48
9	9	4	10.92	2.24
9	9	4	9.72	1.67
-10	-9	-4	3.66	1.75
-10	-9	-4	1.75	2.30
-10	-9	-4	3.32	0.71
-10	-9	-4	3.10	1.19
10	9	4	3.60	1.26
10	9	4	3.41	0.65
10	9	4	2.25	1.71
10	9	4	0.75	2.26
-11	-9	-4	21.79	3.50
-11	-9	-4	23.25	3.60
-11	-9	-4	23.54	1.78
-11	-9	-4	23.13	2.72
11	9	4	25.83	1.73

11	9	4	22.79	2.82
11	9	4	20.74	4.03
11	9	4	24.81	3.48
-12	-9	-4	5.07	2.29
-12	-9	-4	5.44	2.80
-12	-9	-4	4.63	1.57
-12	-9	-4	4.97	0.85
12	9	4	6.16	2.18
12	9	4	5.47	2.94
12	9	4	4.85	0.83
12	9	4	4.45	1.57
-13	-9	-4	7.03	3.62
-13	-9	-4	12.95	3.38
-13	-9	-4	10.33	1.50
13	9	4	8.94	3.74
13	9	4	10.17	1.49
13	9	4	9.20	2.58
13	9	4	8.39	1.97
-14	-9	-4	4.67	3.07
-14	-9	-4	4.37	0.94
-14	-9	-4	9.13	3.81
14	9	4	3.52	0.87
14	9	4	5.77	2.80
14	9	4	3.10	2.02
-15	-9	-4	11.13	4.45
-15	-9	-4	8.41	4.12
-7	10	4	5.41	3.30
-7	10	4	3.24	1.20
-6	10	4	13.07	2.25
-6	10	4	13.51	2.02
-6	10	4	10.72	3.84
-5	10	4	3.27	3.02
-5	10	4	5.40	0.98
-5	10	4	5.82	1.09
-4	10	4	11.21	2.74
-4	10	4	13.30	1.71
-4	10	4	13.80	1.58
-3	10	4	5.25	2.33
-3	10	4	3.94	0.86
-3	10	4	5.47	0.83
-2	10	4	12.71	1.48
-2	10	4	11.77	2.17
-2	10	4	13.09	1.38
1	-10	-4	4.45	1.35

-1	10	4	3.45	1.64
-1	10	4	3.54	0.64
0	-10	-4	5.73	1.53
0	10	4	4.46	0.67
0	10	4	4.97	1.50
-1	-10	-4	1.78	1.38
1	10	4	0.44	0.46
1	10	4	0.89	1.26
-2	-10	-4	17.09	2.43
2	10	4	13.01	1.42
2	10	4	12.51	1.14
2	10	4	9.83	2.05
2	10	4	14.12	1.96
-3	-10	-4	17.99	2.55
3	10	4	15.44	2.13
3	10	4	15.93	1.57
3	10	4	15.61	1.23
3	10	4	11.64	1.88
-4	-10	-4	40.10	2.59
-4	-10	-4	40.01	3.33
-4	-10	-4	40.01	2.78
4	10	4	37.76	2.74
4	10	4	42.22	2.96
4	10	4	42.71	2.63
4	10	4	40.40	2.26
-5	-10	-4	13.01	1.22
-5	-10	-4	10.55	1.69
-5	-10	-4	12.52	2.50
5	10	4	11.25	1.38
5	10	4	12.43	1.10
5	10	4	10.33	1.68
5	10	4	13.47	2.17
-6	-10	-4	14.55	2.62
-6	-10	-4	17.54	1.34
-6	-10	-4	12.90	1.86
-6	-10	-4	12.79	1.68
6	10	4	14.18	2.45
6	10	4	16.16	1.23
6	10	4	15.84	1.91
6	10	4	14.17	2.17
-7	-10	-4	-0.82	0.98
-7	-10	-4	0.01	0.55
-7	-10	-4	1.54	1.67
-7	-10	-4	-2.20	1.23

7	10	4	-0.25	0.48
7	10	4	-0.26	1.65
7	10	4	0.00	0.96
7	10	4	-1.03	1.22
-8	-10	-4	5.29	1.93
-8	-10	-4	2.53	1.46
-8	-10	-4	4.45	0.73
-8	-10	-4	5.13	1.17
8	10	4	4.53	1.18
8	10	4	5.32	1.99
8	10	4	5.24	1.53
8	10	4	5.73	0.97
-9	-10	-4	-2.01	1.51
-9	-10	-4	0.87	1.31
-9	-10	-4	1.52	0.66
-9	-10	-4	2.53	1.98
9	10	4	-1.41	1.59
9	10	4	1.47	0.57
9	10	4	1.51	1.09
9	10	4	1.70	2.14
-10	-10	-4	10.18	1.94
-10	-10	-4	9.98	1.35
-10	-10	-4	11.50	2.43
10	10	4	9.25	1.96
10	10	4	8.36	2.56
10	10	4	9.57	1.20
10	10	4	9.86	1.55
-11	-10	-4	-3.26	2.37
-11	-10	-4	0.31	0.74
-11	-10	-4	-2.22	1.94
11	10	4	-2.85	1.98
11	10	4	0.17	1.36
11	10	4	0.89	2.65
11	10	4	-0.07	0.67
-12	-10	-4	3.04	0.82
-12	-10	-4	1.40	2.32
-12	-10	-4	4.45	2.84
12	10	4	2.30	1.71
12	10	4	2.76	0.83
12	10	4	0.78	2.26
12	10	4	-1.66	2.93
-13	-10	-4	0.73	0.85
-13	-10	-4	2.20	3.24
-13	-10	-4	0.28	2.39

-13	-10	-4	-2.07	2.57
13	10	4	-0.93	3.62
13	10	4	0.07	0.85
13	10	4	1.08	1.78
13	10	4	-0.76	2.54
-14	-10	-4	2.85	3.23
-14	-10	-4	0.59	1.49
-14	-10	-4	1.94	3.68
14	10	4	1.42	0.93
14	10	4	3.47	3.00
14	10	4	1.03	2.11
-15	-10	-4	-1.05	1.82
-15	-10	-4	-3.31	3.85
-15	-10	-4	-1.42	4.09
15	10	4	-0.72	0.90
15	10	4	4.14	2.64
-6	11	4	12.21	1.50
-5	11	4	14.18	2.08
-4	11	4	28.55	2.37
-4	11	4	28.07	2.30
-4	11	4	28.98	4.87
-3	11	4	14.03	1.69
-3	11	4	15.78	1.83
-3	11	4	15.43	2.78
-2	11	4	24.97	1.94
-2	11	4	25.76	2.08
-2	11	4	26.16	3.55
1	-11	-4	16.93	3.21
-1	11	4	18.46	1.76
-1	11	4	15.87	3.05
0	-11	-4	44.55	3.43
0	11	4	40.87	3.57
-1	-11	-4	5.26	1.65
1	11	4	3.40	1.65
-2	-11	-4	8.70	1.86
2	11	4	10.46	1.77
2	11	4	7.39	1.09
-3	-11	-4	22.20	3.02
3	11	4	19.45	1.50
3	11	4	18.52	2.54
3	11	4	21.60	2.19
-4	-11	-4	53.65	3.47
-4	-11	-4	56.08	4.24
-4	-11	-4	55.50	3.33

4	11	4	57.53	3.33
4	11	4	55.42	3.73
4	11	4	54.32	3.48
4	11	4	56.83	3.02
-5	-11	-4	31.69	2.56
-5	-11	-4	37.91	3.50
5	11	4	33.97	2.04
5	11	4	36.04	3.07
5	11	4	33.82	2.51
5	11	4	31.28	2.63
-6	-11	-4	19.08	2.96
-6	-11	-4	18.70	2.14
-6	-11	-4	13.71	2.23
-6	-11	-4	18.66	1.54
6	11	4	17.11	1.38
6	11	4	21.29	2.78
6	11	4	16.34	2.09
6	11	4	15.45	2.27
-7	-11	-4	8.10	2.13
-7	-11	-4	5.18	1.42
-7	-11	-4	6.17	0.84
-7	-11	-4	6.55	1.27
7	11	4	7.55	1.07
7	11	4	8.60	2.06
7	11	4	6.72	1.50
7	11	4	6.38	1.26
-8	-11	-4	12.46	1.42
-8	-11	-4	9.88	1.51
-8	-11	-4	14.12	2.60
8	11	4	8.81	1.44
8	11	4	9.38	1.79
8	11	4	11.98	1.20
8	11	4	12.92	2.33
-9	-11	-4	6.28	1.45
-9	-11	-4	9.41	2.31
-9	-11	-4	10.18	1.39
-9	-11	-4	3.73	1.70
9	11	4	7.06	1.92
9	11	4	6.61	1.41
9	11	4	9.36	1.20
9	11	4	11.20	2.56
-10	-11	-4	6.26	1.96
-10	-11	-4	10.17	1.48
-10	-11	-4	8.36	1.64

-10	-11	-4	13.47	2.72
10	11	4	7.74	1.62
10	11	4	8.97	1.32
10	11	4	12.92	2.86
10	11	4	9.22	2.18
-11	-11	-4	1.11	2.04
-11	-11	-4	7.28	2.72
-11	-11	-4	3.83	1.59
-11	-11	-4	5.15	0.91
11	11	4	5.25	0.85
11	11	4	6.07	3.00
11	11	4	5.49	1.63
11	11	4	4.40	2.21
-12	-11	-4	6.37	3.00
-12	-11	-4	1.66	2.31
-12	-11	-4	3.85	1.66
-12	-11	-4	5.40	0.93
12	11	4	5.41	0.95
12	11	4	6.42	3.42
12	11	4	3.94	2.52
12	11	4	3.52	1.76
-13	-11	-4	3.11	3.38
-13	-11	-4	2.72	0.94
-13	-11	-4	-0.18	2.60
-13	-11	-4	2.54	1.79
13	11	4	3.53	1.92
13	11	4	-0.53	2.68
13	11	4	2.58	1.38
-14	-11	-4	-1.01	3.03
-14	-11	-4	-0.36	2.13
-14	-11	-4	0.32	0.97
-14	-11	-4	-3.35	3.74
14	11	4	0.27	0.98
14	11	4	1.41	2.98
14	11	4	-3.36	2.09
-15	-11	-4	-3.01	4.34
-15	-11	-4	1.36	3.76
-5	12	4	14.45	2.29
-4	12	4	3.32	1.08
-3	12	4	17.97	2.12
-3	12	4	15.58	3.24
-2	12	4	5.95	0.91
-2	12	4	5.50	1.05
-2	12	4	3.29	2.48

1	-12	-4	22.64	3.25
-1	12	4	24.60	2.09
-1	12	4	19.26	3.53
-1	12	4	21.23	1.79
0	-12	-4	15.17	2.84
0	12	4	15.82	2.47
0	12	4	14.37	1.78
-1	-12	-4	19.14	3.07
1	12	4	18.85	3.00
-2	-12	-4	1.84	2.52
2	12	4	3.79	1.75
3	12	4	10.81	1.99
3	12	4	8.11	1.82
3	12	4	7.26	1.12
-4	-12	-4	5.29	2.68
-4	-12	-4	5.17	1.17
4	12	4	6.09	1.71
4	12	4	4.59	0.70
4	12	4	4.64	1.18
4	12	4	5.18	1.13
-5	-12	-4	15.91	3.38
-5	-12	-4	13.93	1.97
-5	-12	-4	16.60	2.77
5	12	4	14.95	2.11
5	12	4	13.65	2.71
5	12	4	13.78	1.33
5	12	4	15.43	2.46
-6	-12	-4	1.56	1.14
-6	-12	-4	0.99	1.37
6	12	4	1.84	0.60
6	12	4	0.87	1.02
6	12	4	0.63	1.90
6	12	4	1.86	1.27
-7	-12	-4	5.51	2.35
-7	-12	-4	2.16	1.24
-7	-12	-4	1.12	1.38
7	12	4	3.15	1.44
7	12	4	2.66	1.17
7	12	4	2.90	0.68
7	12	4	3.23	2.16
-8	-12	-4	4.40	1.36
-8	-12	-4	4.74	0.86
-8	-12	-4	3.11	1.60
-8	-12	-4	5.46	2.42

8	12	4	4.34	1.33
8	12	4	3.41	1.65
8	12	4	5.23	2.32
8	12	4	4.07	0.76
-9	-12	-4	6.82	0.98
-9	-12	-4	7.43	1.63
-9	-12	-4	10.94	2.69
9	12	4	9.05	1.33
9	12	4	7.98	1.60
9	12	4	7.82	1.99
9	12	4	10.45	2.74
-10	-12	-4	-0.32	1.89
-10	-12	-4	2.78	2.57
-10	-12	-4	3.26	0.88
-10	-12	-4	1.82	1.53
10	12	4	1.87	2.19
10	12	4	2.71	2.81
10	12	4	3.93	0.80
10	12	4	3.70	1.52
-11	-12	-4	7.44	1.04
-11	-12	-4	11.39	2.99
-11	-12	-4	6.58	1.76
-11	-12	-4	5.85	2.18
11	12	4	6.90	1.85
11	12	4	5.46	2.37
11	12	4	8.04	1.02
11	12	4	9.10	3.32
-12	-12	-4	0.53	0.89
-12	-12	-4	-1.44	1.76
-12	-12	-4	-0.82	2.33
-12	-12	-4	1.33	3.19
12	12	4	1.01	2.61
12	12	4	-1.13	1.78
12	12	4	0.70	0.93
-13	-12	-4	1.60	1.03
-13	-12	-4	1.36	2.67
-13	-12	-4	1.87	3.44
-13	-12	-4	4.73	2.03
13	12	4	4.03	2.99
13	12	4	3.31	2.09
13	12	4	2.58	1.07
-14	-12	-4	-1.01	2.17
-14	-12	-4	0.57	3.22
-14	-12	-4	4.80	3.84

14	12	4	-3.14	3.16
14	12	4	-0.73	2.28
14	12	4	-1.28	1.05
-4	13	4	13.34	2.28
-3	13	4	6.99	1.23
-2	13	4	11.79	2.01
1	-13	-4	19.24	3.64
-1	13	4	13.34	3.01
-1	13	4	14.62	2.04
0	-13	-4	25.59	3.49
0	13	4	22.40	2.13
0	13	4	17.48	2.71
0	13	4	19.92	1.87
-1	-13	-4	27.59	3.77
1	13	4	31.45	3.68
-2	-13	-4	22.32	3.50
2	13	4	20.55	2.04
2	13	4	18.93	3.15
-3	-13	-4	18.84	3.49
3	13	4	16.45	3.09
3	13	4	19.65	1.64
3	13	4	16.61	3.11
3	13	4	21.50	2.13
-4	-13	-4	5.45	2.29
4	13	4	6.31	2.01
4	13	4	4.68	1.13
4	13	4	3.98	1.35
-5	-13	-4	2.08	1.09
-5	-13	-4	1.74	2.43
-5	-13	-4	0.03	1.23
5	13	4	4.40	2.06
5	13	4	3.67	1.15
5	13	4	3.86	1.34
-6	-13	-4	3.68	2.56
-6	-13	-4	4.43	1.26
6	13	4	3.88	1.42
6	13	4	3.33	1.26
6	13	4	4.16	2.15
-7	-13	-4	7.14	1.44
-7	-13	-4	11.54	2.70
-7	-13	-4	7.72	1.85
7	13	4	6.34	1.66
7	13	4	10.47	2.59
7	13	4	8.44	1.51

-8	-13	-4	6.54	1.77
-8	-13	-4	8.09	1.54
8	13	4	10.72	2.58
8	13	4	7.63	1.83
8	13	4	5.86	0.88
8	13	4	7.81	1.86
-9	-13	-4	5.18	1.88
-9	-13	-4	7.78	1.67
-9	-13	-4	11.10	3.06
9	13	4	4.67	2.00
9	13	4	7.81	2.68
9	13	4	6.89	0.92
9	13	4	5.71	2.92
-10	-13	-4	7.46	1.82
-10	-13	-4	11.45	3.21
-10	-13	-4	4.58	2.11
10	13	4	3.89	2.25
10	13	4	8.53	3.37
10	13	4	7.61	1.00
10	13	4	6.03	1.68
-11	-13	-4	4.26	2.28
-11	-13	-4	3.81	1.88
-11	-13	-4	8.04	3.23
-11	-13	-4	5.49	1.02
11	13	4	5.50	1.83
11	13	4	5.21	2.48
11	13	4	4.59	1.02
-12	-13	-4	0.72	2.54
-12	-13	-4	2.19	3.41
-12	-13	-4	3.80	1.03
-12	-13	-4	4.64	2.05
12	13	4	2.89	1.94
12	13	4	2.17	2.79
12	13	4	2.69	1.00
-13	-13	-4	0.98	2.21
-13	-13	-4	2.12	3.67
-13	-13	-4	-0.94	2.94
-13	-13	-4	0.97	1.06
13	13	4	0.60	2.20
13	13	4	-0.05	1.03
13	13	4	0.99	3.26
-14	-13	-4	-0.55	3.43
-14	-13	-4	3.07	4.30
-14	-13	-4	1.35	1.10

-14	-13	-4	2.84	2.56
14	13	4	-1.31	1.10
14	13	4	-4.17	3.66
14	13	4	1.45	2.60
-2	14	4	6.97	1.30
-1	14	4	1.46	1.01
0	-14	-4	8.10	2.65
0	14	4	8.20	1.25
-1	-14	-4	1.19	2.40
1	14	4	4.15	1.05
1	14	4	4.63	2.74
-2	-14	-4	13.37	2.69
2	14	4	8.57	2.66
-3	-14	-4	5.11	2.59
3	14	4	4.27	0.85
3	14	4	5.41	2.35
3	14	4	4.13	1.80
-4	-14	-4	17.92	3.01
4	14	4	13.83	2.27
4	14	4	14.41	2.49
4	14	4	14.81	1.65
4	14	4	15.26	2.58
-5	-14	-4	1.21	2.57
5	14	4	3.78	2.22
5	14	4	2.59	1.38
5	14	4	2.72	0.77
5	14	4	4.13	1.26
-6	-14	-4	3.53	2.43
-6	-14	-4	2.15	2.78
-6	-14	-4	2.47	1.34
6	14	4	4.24	1.51
6	14	4	4.35	1.34
6	14	4	3.63	0.82
6	14	4	3.37	2.41
-7	-14	-4	-0.11	1.95
-7	-14	-4	-3.00	2.84
-7	-14	-4	0.75	1.37
7	14	4	-0.76	2.65
7	14	4	-0.11	0.80
7	14	4	1.82	1.53
7	14	4	0.47	1.38
-8	-14	-4	-0.66	1.67
-8	-14	-4	3.61	2.98
-8	-14	-4	2.09	1.49

8	14	4	2.33	2.98
8	14	4	1.89	0.94
8	14	4	3.28	1.79
8	14	4	0.96	1.50
-9	-14	-4	1.61	3.11
-9	-14	-4	2.37	1.62
-9	-14	-4	0.90	1.85
9	14	4	2.47	3.29
9	14	4	0.85	1.65
9	14	4	-1.15	2.12
9	14	4	2.31	1.32
-10	-14	-4	1.13	2.09
-10	-14	-4	3.77	3.64
-10	-14	-4	1.69	1.87
10	14	4	3.93	2.48
10	14	4	3.75	1.87
-11	-14	-4	-0.95	2.38
-11	-14	-4	0.94	3.56
-11	-14	-4	-1.80	1.98
11	14	4	2.38	2.79
11	14	4	0.05	1.91
-12	-14	-4	1.19	2.71
-12	-14	-4	3.36	2.42
-12	-14	-4	4.33	3.72
12	14	4	3.33	2.26
12	14	4	5.51	3.31
-13	-14	-4	-1.68	2.36
-13	-14	-4	-2.67	3.91
-13	-14	-4	-3.05	2.98
13	14	4	3.87	3.62
13	14	4	-0.32	2.62
13	14	4	0.21	1.60
0	-15	-4	5.79	3.06
0	15	4	4.73	1.20
-1	-15	-4	13.13	3.07
1	15	4	11.83	1.51
-2	-15	-4	12.02	3.16
2	15	4	10.51	2.02
2	15	4	12.33	1.50
-3	-15	-4	10.76	3.07
3	15	4	11.17	1.94
3	15	4	13.18	2.20
-4	-15	-4	15.41	3.35
4	15	4	12.70	2.04

-5	-15	-4	15.45	3.43
5	15	4	16.66	2.69
5	15	4	14.44	2.85
-6	-15	-4	7.87	3.23
6	15	4	8.15	1.76
6	15	4	7.82	1.63
-7	-15	-4	6.62	3.49
7	15	4	4.75	1.01
7	15	4	5.02	1.82
7	15	4	5.84	1.63
-8	-15	-4	5.15	1.84
-8	-15	-4	5.08	3.62
8	15	4	4.29	1.73
8	15	4	5.12	1.05
8	15	4	4.60	2.05
-9	-15	-4	6.06	3.68
-9	-15	-4	3.28	1.74
9	15	4	6.05	1.17
9	15	4	3.94	1.89
9	15	4	2.25	2.39
-10	-15	-4	4.00	2.05
-10	-15	-4	7.44	3.86
10	15	4	3.01	1.14
10	15	4	2.54	3.13
10	15	4	2.63	2.06
-11	-15	-4	11.02	3.96
-11	-15	-4	8.81	2.48
11	15	4	4.65	1.25
11	15	4	2.34	2.20
-12	-15	-4	2.27	4.22
12	15	4	1.06	1.30
12	15	4	0.20	2.40
-3	-16	-4	2.36	3.22
3	16	4	5.17	1.35
-4	-16	-4	-0.33	3.22
4	16	4	1.01	1.20
4	16	4	-0.11	1.43
-5	-16	-4	1.40	3.39
5	16	4	4.46	1.55
-6	-16	-4	0.55	3.39
6	16	4	-0.14	1.56
-7	-16	-4	3.85	3.55
7	16	4	4.01	1.81
-8	-16	-4	3.33	3.82

8	16	4	1.10	1.82
-9	-16	-4	6.21	3.90
9	16	4	3.14	1.95
-10	-16	-4	2.73	4.17
10	16	4	-0.15	2.12
9	13	-5	19.99	3.18
9	13	-5	27.51	5.30
-9	-13	5	26.78	4.32
-9	-13	5	25.45	4.11
-9	-13	5	21.87	3.19
8	13	-5	27.59	5.16
8	13	-5	26.56	4.00
8	13	-5	25.50	2.30
-8	-13	5	23.01	3.11
-8	-13	5	31.24	5.12
-8	-13	5	20.70	4.86
7	13	-5	31.24	3.96
7	13	-5	36.79	4.90
7	13	-5	32.27	2.42
-7	-13	5	28.84	4.04
-7	-13	5	29.41	5.18
-7	-13	5	32.11	4.49
6	13	-5	19.00	2.11
6	13	-5	21.14	3.49
6	13	-5	27.78	4.62
-6	-13	5	20.32	3.51
-6	-13	5	22.78	3.61
-6	-13	5	20.63	4.00
5	13	-5	36.52	4.41
5	13	-5	26.18	3.56
5	13	-5	27.89	2.38
5	13	-5	26.93	2.43
-5	-13	5	27.60	4.98
4	13	-5	26.86	2.57
4	13	-5	29.55	3.62
4	13	-5	31.41	2.46
-4	-13	5	27.84	4.87
3	13	-5	40.44	2.88
3	13	-5	35.89	4.20
-3	-13	5	42.76	4.96
2	13	-5	29.44	4.36
2	13	-5	31.09	2.60
-2	-13	5	29.10	4.85
1	13	-5	30.46	4.90

1	13	-5	31.22	2.61
-1	-13	5	30.66	5.07
0	13	-5	14.56	3.13
0	13	-5	18.18	2.24
0	-13	5	17.16	3.57
11	12	-5	25.15	4.18
11	12	-5	30.48	5.77
-11	-12	5	17.40	3.24
-11	-12	5	21.52	4.28
-11	-12	5	18.70	3.18
10	12	-5	14.68	2.72
10	12	-5	15.71	3.24
-10	-12	5	27.19	5.22
-10	-12	5	14.68	2.73
9	12	-5	27.31	3.98
9	12	-5	29.47	4.77
-9	-12	5	23.82	4.08
-9	-12	5	27.13	4.02
-9	-12	5	27.73	5.04
8	12	-5	32.68	3.64
8	12	-5	38.35	4.70
-8	-12	5	26.96	4.93
-8	-12	5	30.42	3.96
-8	-12	5	31.24	3.92
7	12	-5	26.60	2.24
7	12	-5	30.21	2.16
7	12	-5	36.08	4.27
7	12	-5	35.39	4.04
-7	-12	5	28.32	3.79
-7	-12	5	29.06	4.75
-7	-12	5	33.67	3.64
6	12	-5	26.75	2.16
6	12	-5	29.20	3.52
-6	-12	5	25.78	3.43
-6	-12	5	28.27	3.61
-6	-12	5	26.41	4.58
5	12	-5	32.20	3.85
5	12	-5	27.25	2.12
5	12	-5	25.88	3.07
-5	-12	5	25.49	3.48
-5	-12	5	27.82	3.49
-5	-12	5	25.69	4.47
4	12	-5	29.98	3.78
4	12	-5	25.25	2.11

4	12	-5	27.80	3.09
-4	-12	5	20.65	3.49
-4	-12	5	23.31	3.29
-4	-12	5	24.50	4.45
3	12	-5	41.61	4.00
3	12	-5	34.88	2.64
3	12	-5	36.73	2.48
3	12	-5	32.91	3.41
-3	-12	5	24.40	4.13
-3	-12	5	34.78	4.62
-3	-12	5	31.62	3.51
2	12	-5	30.43	3.72
2	12	-5	35.46	3.91
2	12	-5	30.84	2.44
-2	-12	5	33.11	4.62
1	12	-5	29.30	4.22
1	12	-5	27.31	2.42
1	12	-5	36.83	3.81
-1	-12	5	30.82	4.54
0	12	-5	27.83	2.35
0	12	-5	26.30	4.43
0	-12	5	26.11	4.58
-1	12	-5	27.42	4.49
-1	12	-5	26.54	2.32
1	-12	5	23.44	4.63
-2	12	-5	23.93	4.43
-2	12	-5	19.24	2.15
-12	-11	5	19.75	3.23
-12	-11	5	16.29	4.08
11	11	-5	13.37	3.32
11	11	-5	21.08	3.69
-11	-11	5	17.67	2.92
-11	-11	5	21.31	3.82
-11	-11	5	15.71	2.69
10	11	-5	35.41	3.75
10	11	-5	28.23	4.77
-10	-11	5	24.27	4.29
-10	-11	5	32.17	4.00
-10	-11	5	24.77	5.10
9	11	-5	26.54	4.25
9	11	-5	29.80	3.41
-9	-11	5	30.05	4.70
-9	-11	5	25.35	3.60
-9	-11	5	22.64	3.65

8	11	-5	56.22	3.97
8	11	-5	47.61	5.85
8	11	-5	51.03	4.73
-8	-11	5	46.94	4.05
-8	-11	5	50.89	5.01
-8	-11	5	47.25	4.14
7	11	-5	39.25	5.41
7	11	-5	39.23	2.57
7	11	-5	48.71	4.22
7	11	-5	46.51	3.62
-7	-11	5	46.96	3.74
-7	-11	5	40.81	3.83
-7	-11	5	44.13	4.72
6	11	-5	47.74	5.07
6	11	-5	50.71	3.64
6	11	-5	45.48	2.78
6	11	-5	47.56	5.56
6	11	-5	44.47	2.77
-6	-11	5	51.73	3.85
-6	-11	5	49.38	4.76
-6	-11	5	47.21	3.89
5	11	-5	31.97	2.14
5	11	-5	31.39	3.61
5	11	-5	31.06	2.18
5	11	-5	31.75	3.02
5	11	-5	34.47	4.36
-5	-11	5	30.86	3.19
-5	-11	5	29.12	3.13
-5	-11	5	32.62	4.21
4	11	-5	40.99	3.73
4	11	-5	33.66	2.42
4	11	-5	30.70	4.35
4	11	-5	38.94	4.30
-4	-11	5	37.85	3.36
-4	-11	5	33.04	3.26
-4	-11	5	37.83	4.26
3	11	-5	31.17	3.39
3	11	-5	27.07	2.86
3	11	-5	26.15	2.01
3	11	-5	28.59	4.07
-3	-11	5	27.62	2.86
-3	-11	5	25.36	3.21
-3	-11	5	21.98	4.01
2	11	-5	58.81	3.25

2	11	-5	56.67	4.06
2	11	-5	55.78	4.86
2	11	-5	58.70	4.32
-2	-11	5	61.04	4.90
-2	-11	5	46.40	4.93
-2	-11	5	52.44	3.88
1	11	-5	49.57	4.00
1	11	-5	52.98	4.02
1	11	-5	44.06	4.74
1	11	-5	43.37	2.87
-1	-11	5	47.47	4.62
-1	-11	5	43.46	3.52
0	11	-5	48.14	4.53
0	11	-5	65.14	4.41
0	11	-5	53.23	3.32
0	11	-5	65.08	5.71
0	-11	5	51.47	4.50
0	-11	5	57.40	4.96
-1	11	-5	46.40	3.03
-1	11	-5	43.97	4.65
-1	11	-5	57.17	4.05
1	-11	5	46.14	4.81
1	-11	5	44.70	4.10
-2	11	-5	59.90	4.41
-2	11	-5	52.57	3.36
-3	11	-5	24.15	2.22
-3	11	-5	26.36	4.48
-4	11	-5	22.79	2.27
-13	-10	5	8.45	3.13
-13	-10	5	6.14	4.06
-12	-10	5	12.87	3.16
-12	-10	5	15.30	3.88
-12	-10	5	14.11	2.86
11	10	-5	16.64	3.12
11	10	-5	27.13	3.45
-11	-10	5	24.72	3.86
-11	-10	5	18.93	2.75
10	10	-5	30.96	3.28
10	10	-5	21.46	4.17
10	10	-5	28.10	4.39
-10	-10	5	32.04	4.82
-10	-10	5	18.42	3.43
-10	-10	5	23.11	3.31
9	10	-5	40.90	4.31

9	10	-5	41.39	3.48
9	10	-5	48.79	5.60
-9	-10	5	37.97	3.80
-9	-10	5	34.92	4.60
-9	-10	5	37.95	3.61
8	10	-5	37.97	4.16
8	10	-5	42.08	3.24
8	10	-5	37.50	4.96
-8	-10	5	40.07	4.36
-8	-10	5	34.87	3.49
-8	-10	5	36.25	3.46
7	10	-5	42.83	4.03
7	10	-5	44.38	3.33
-7	-10	5	43.04	4.27
-7	-10	5	42.89	3.58
-7	-10	5	41.93	3.46
6	10	-5	18.70	1.59
6	10	-5	20.41	1.59
6	10	-5	24.94	3.23
6	10	-5	23.76	2.55
-6	-10	5	16.90	3.58
-6	-10	5	24.28	2.77
-6	-10	5	23.97	2.74
5	10	-5	31.10	2.08
5	10	-5	35.97	3.44
5	10	-5	33.35	2.06
5	10	-5	33.90	2.86
-5	-10	5	33.38	2.97
-5	-10	5	30.29	3.90
-5	-10	5	32.62	3.01
4	10	-5	50.30	2.83
4	10	-5	50.71	3.85
4	10	-5	53.79	3.47
4	10	-5	44.35	2.84
-4	-10	5	49.30	3.59
-4	-10	5	52.21	4.33
-4	-10	5	51.18	3.48
3	10	-5	43.34	2.54
3	10	-5	43.97	3.58
3	10	-5	45.27	3.19
-3	-10	5	40.03	3.21
-3	-10	5	39.84	3.48
-3	-10	5	44.93	4.17
2	10	-5	25.16	2.94

2	10	-5	28.71	3.07
2	10	-5	26.34	2.48
-2	-10	5	22.87	3.04
-2	-10	5	27.55	2.64
-2	-10	5	24.37	3.58
1	10	-5	18.92	2.72
1	10	-5	17.76	1.62
-1	-10	5	20.63	3.46
-1	-10	5	16.77	3.00
-1	-10	5	15.42	2.32
0	10	-5	41.50	3.31
0	10	-5	36.00	3.92
0	10	-5	33.05	3.48
0	10	-5	34.33	2.32
0	-10	5	38.42	4.08
0	-10	5	31.64	2.85
-1	10	-5	66.00	5.00
-1	10	-5	53.31	4.61
-1	10	-5	62.97	3.54
-1	10	-5	67.61	4.33
1	-10	5	67.51	4.96
1	-10	5	67.77	4.35
1	-10	5	53.77	3.91
-2	10	-5	43.49	2.74
-2	10	-5	40.01	3.99
-2	10	-5	44.62	3.68
-2	10	-5	43.26	4.50
2	-10	5	43.19	4.39
-3	10	-5	40.48	3.79
-3	10	-5	39.25	2.67
-4	10	-5	20.23	2.06
-4	10	-5	25.91	4.01
-5	10	-5	22.32	2.19
-5	10	-5	29.08	4.08
-13	-9	5	17.82	4.09
-13	-9	5	13.56	3.31
12	9	-5	13.53	4.51
-12	-9	5	17.14	2.83
-12	-9	5	19.34	3.77
-12	-9	5	18.48	3.84
11	9	-5	26.38	4.44
11	9	-5	27.08	3.23
11	9	-5	31.91	5.99
-11	-9	5	28.24	4.79

-11	-9	5	27.75	3.53
-11	-9	5	25.59	3.69
10	9	-5	34.83	5.59
10	9	-5	33.38	4.22
10	9	-5	34.62	3.22
-10	-9	5	32.43	3.43
-10	-9	5	33.33	4.52
9	9	-5	35.37	3.90
9	9	-5	33.40	3.01
-9	-9	5	32.44	3.35
-9	-9	5	35.46	4.21
-9	-9	5	32.66	3.24
8	9	-5	37.94	2.98
8	9	-5	38.40	3.83
-8	-9	5	31.97	3.21
-8	-9	5	35.73	3.26
-8	-9	5	34.32	3.86
7	9	-5	72.67	4.75
7	9	-5	71.25	4.25
7	9	-5	67.49	5.50
-7	-9	5	66.90	4.45
-7	-9	5	75.29	4.93
-7	-9	5	68.33	4.37
6	9	-5	34.46	2.24
6	9	-5	42.97	3.52
6	9	-5	40.63	4.25
6	9	-5	42.36	2.98
-6	-9	5	45.03	3.12
-6	-9	5	36.94	5.22
-6	-9	5	41.88	4.23
5	9	-5	67.01	3.52
5	9	-5	62.60	3.53
5	9	-5	70.30	4.05
5	9	-5	69.89	4.91
5	9	-5	69.39	4.43
-5	-9	5	65.99	4.12
-5	-9	5	67.53	4.13
4	9	-5	81.03	5.26
4	9	-5	83.15	4.65
4	9	-5	81.56	4.89
4	9	-5	78.88	4.15
-4	-9	5	75.89	4.65
-4	-9	5	80.06	5.64
-4	-9	5	82.37	4.64

3	9	-5	133.49	6.71
3	9	-5	139.42	7.07
3	9	-5	133.97	7.24
3	9	-5	137.78	7.62
-3	-9	5	135.11	7.06
-3	-9	5	126.06	7.07
-3	-9	5	132.56	7.91
2	9	-5	54.38	3.43
2	9	-5	54.63	3.64
2	9	-5	50.96	4.06
2	9	-5	50.22	2.83
-2	-9	5	46.18	3.34
-2	-9	5	49.34	3.54
1	9	-5	57.80	3.18
1	9	-5	58.31	4.29
1	9	-5	61.03	3.88
1	9	-5	61.57	3.82
-1	-9	5	55.84	3.63
-1	-9	5	56.32	4.34
-1	-9	5	55.41	4.38
0	9	-5	38.89	2.94
0	9	-5	32.80	2.15
0	9	-5	33.04	3.47
0	9	-5	28.71	3.39
0	-9	5	32.42	2.67
0	-9	5	33.31	3.56
-1	9	-5	77.09	4.03
-1	9	-5	77.85	4.63
-1	9	-5	67.08	4.74
1	-9	5	77.31	4.87
1	-9	5	73.30	5.01
-2	9	-5	46.39	3.88
-2	9	-5	46.74	5.01
-2	9	-5	49.11	2.86
-2	9	-5	53.93	3.67
2	-9	5	45.35	3.22
2	-9	5	49.22	4.23
-3	9	-5	45.41	4.27
-3	9	-5	41.92	3.58
-3	9	-5	45.37	2.74
-4	9	-5	43.16	4.57
-4	9	-5	40.77	4.16
-4	9	-5	38.50	2.63
-5	9	-5	42.74	2.80

-6	9	-5	24.84	2.23
-6	9	-5	22.12	3.81
-7	9	-5	14.80	2.63
-7	9	-5	21.60	2.24
-14	-8	5	9.66	3.13
-14	-8	5	10.61	4.13
-13	-8	5	19.68	4.00
-13	-8	5	12.86	2.85
12	8	-5	29.32	5.42
12	8	-5	24.32	3.53
-12	-8	5	28.95	4.03
-12	-8	5	31.97	5.07
-12	-8	5	24.57	3.74
11	8	-5	24.78	4.21
11	8	-5	22.04	4.17
11	8	-5	22.46	2.90
-11	-8	5	29.64	3.67
-11	-8	5	30.68	4.46
-11	-8	5	27.49	3.31
10	8	-5	29.59	3.86
10	8	-5	29.45	2.84
-10	-8	5	25.91	4.10
-10	-8	5	27.58	3.13
9	8	-5	38.94	3.79
9	8	-5	33.39	2.90
-9	-8	5	35.88	3.93
-9	-8	5	35.84	3.17
-9	-8	5	38.23	3.28
8	8	-5	31.52	2.63
8	8	-5	33.14	3.46
8	8	-5	25.70	4.12
-8	-8	5	30.27	2.87
-8	-8	5	32.03	2.91
7	8	-5	41.18	3.40
7	8	-5	40.43	2.79
7	8	-5	34.61	3.99
-7	-8	5	37.11	3.00
-7	-8	5	36.10	2.95
-7	-8	5	38.94	3.51
6	8	-5	63.54	3.80
6	8	-5	65.24	4.19
6	8	-5	62.00	4.78
-6	-8	5	65.44	3.93
-6	-8	5	61.63	4.32

-6	-8	5	61.70	3.89
5	8	-5	37.03	2.30
5	8	-5	44.08	2.94
5	8	-5	42.01	2.31
5	8	-5	44.93	3.28
5	8	-5	41.44	3.85
-5	-8	5	40.01	3.46
-5	-8	5	46.87	3.03
-5	-8	5	42.93	2.98
4	8	-5	110.96	5.88
4	8	-5	110.28	6.06
4	8	-5	106.22	5.50
4	8	-5	111.42	6.36
-4	-8	5	107.99	5.92
-4	-8	5	114.73	5.89
-4	-8	5	109.92	6.30
3	8	-5	75.60	3.85
3	8	-5	77.71	4.28
3	8	-5	76.68	4.85
3	8	-5	74.77	4.43
-3	-8	5	72.52	4.77
-3	-8	5	78.23	4.25
-3	-8	5	65.60	4.36
2	8	-5	104.24	5.46
2	8	-5	107.24	5.90
2	8	-5	111.91	5.84
2	8	-5	103.46	6.17
-2	-8	5	108.26	5.79
-2	-8	5	116.19	6.29
-2	-8	5	109.19	5.85
1	8	-5	45.44	2.55
1	8	-5	41.39	3.58
1	8	-5	47.72	3.16
1	8	-5	47.12	3.14
-1	-8	5	47.11	3.41
-1	-8	5	45.01	2.99
-1	-8	5	44.51	3.59
0	8	-5	37.84	2.94
0	8	-5	37.14	2.15
0	8	-5	38.80	2.87
0	8	-5	34.36	3.28
0	-8	5	33.57	2.62
0	-8	5	34.76	3.72
0	-8	5	34.21	3.32

-1	8	-5	54.57	2.96
-1	8	-5	49.82	3.64
-1	8	-5	49.84	3.93
-1	8	-5	54.47	3.53
1	-8	5	50.27	3.28
1	-8	5	53.77	4.80
-2	8	-5	40.19	3.62
-2	8	-5	40.55	2.42
-2	8	-5	38.94	3.07
2	-8	5	47.34	3.12
2	-8	5	37.28	2.83
2	-8	5	38.46	3.58
-3	8	-5	29.51	1.89
-3	8	-5	21.94	3.12
-3	8	-5	21.59	2.63
-4	8	-5	23.08	3.45
-4	8	-5	25.25	3.56
-4	8	-5	27.98	1.97
-5	8	-5	26.18	2.05
-5	8	-5	25.92	4.08
-6	8	-5	35.40	2.52
-7	8	-5	23.85	2.13
-7	8	-5	16.90	2.64
-8	8	-5	13.11	2.60
-8	8	-5	20.92	2.16
-8	8	-5	18.90	4.36
8	-8	5	23.13	3.70
-14	-7	5	18.06	3.11
-14	-7	5	17.28	3.47
-14	-7	5	21.48	4.53
-13	-7	5	16.40	3.95
-13	-7	5	16.56	2.83
-13	-7	5	16.99	2.88
12	7	-5	38.39	6.13
12	7	-5	35.10	4.92
-12	-7	5	39.89	5.20
-12	-7	5	44.47	4.79
-12	-7	5	40.28	3.92
-12	-7	5	46.84	4.24
11	7	-5	35.72	4.25
11	7	-5	28.04	5.62
-11	-7	5	37.46	4.64
-11	-7	5	39.45	4.08
-11	-7	5	35.09	3.44

-11	-7	5	39.20	3.75
10	7	-5	65.93	4.16
10	7	-5	68.01	4.95
-10	-7	5	65.20	5.12
-10	-7	5	65.90	4.36
-10	-7	5	65.75	4.81
9	7	-5	33.34	3.60
9	7	-5	32.11	4.33
9	7	-5	33.18	2.72
-9	-7	5	34.03	3.14
-9	-7	5	33.05	2.97
8	7	-5	37.43	4.16
8	7	-5	40.12	2.86
8	7	-5	46.07	3.57
-8	-7	5	38.64	3.12
-8	-7	5	39.36	3.54
-8	-7	5	41.37	3.07
7	7	-5	59.32	4.84
7	7	-5	66.41	3.84
7	7	-5	69.16	4.29
-7	-7	5	66.96	4.38
-7	-7	5	64.29	3.96
-7	-7	5	68.88	4.00
6	7	-5	97.77	6.02
6	7	-5	100.98	5.30
6	7	-5	98.50	5.60
-6	-7	5	103.96	5.39
-6	-7	5	101.22	5.66
-6	-7	5	92.40	5.36
5	7	-5	97.20	5.92
5	7	-5	101.68	5.59
5	7	-5	101.64	5.35
-5	-7	5	97.66	5.40
-5	-7	5	99.41	5.68
-5	-7	5	105.04	5.39
4	7	-5	136.82	6.93
4	7	-5	143.67	7.38
4	7	-5	140.68	7.61
4	7	-5	145.10	7.23
-4	-7	5	133.99	7.24
-4	-7	5	137.07	7.50
-4	-7	5	148.57	7.23
3	7	-5	96.89	5.07
3	7	-5	92.28	4.72

3	7	-5	90.10	5.45
3	7	-5	97.27	5.18
-3	-7	5	91.34	5.04
-3	-7	5	91.20	5.43
-3	-7	5	98.86	5.02
2	7	-5	143.93	7.07
2	7	-5	136.93	7.33
2	7	-5	134.32	6.77
2	7	-5	136.08	7.10
-2	-7	5	133.01	7.35
-2	-7	5	135.99	7.03
-2	-7	5	136.69	7.02
1	7	-5	104.72	5.60
1	7	-5	114.66	5.61
1	7	-5	103.59	5.24
1	7	-5	104.62	5.85
-1	-7	5	100.89	5.88
-1	-7	5	106.02	5.63
-1	-7	5	93.96	5.96
0	7	-5	168.77	8.51
0	7	-5	165.48	8.98
0	7	-5	179.65	8.84
0	7	-5	168.79	8.79
0	-7	5	168.69	8.72
0	-7	5	169.42	9.01
0	-7	5	188.22	9.19
-1	7	-5	88.36	5.21
-1	7	-5	88.71	5.04
-1	7	-5	89.24	4.55
-1	7	-5	85.20	4.88
1	-7	5	92.59	4.82
1	-7	5	85.70	5.19
-2	7	-5	101.09	5.93
-2	7	-5	103.62	6.08
-2	7	-5	119.45	6.30
-2	7	-5	114.71	5.63
2	-7	5	112.41	6.24
2	-7	5	113.28	5.87
2	-7	5	111.06	6.24
-3	7	-5	36.19	3.41
-3	7	-5	32.93	2.96
-3	7	-5	43.78	3.51
-3	7	-5	46.45	2.64
3	-7	5	35.23	2.55

3	-7	5	47.57	3.00
-4	7	-5	71.28	4.51
-4	7	-5	75.51	3.57
-4	7	-5	54.74	4.02
-4	7	-5	55.64	4.49
-5	7	-5	52.09	3.25
-5	7	-5	49.69	2.96
-6	7	-5	68.00	5.16
-6	7	-5	65.65	3.73
-6	7	-5	66.40	4.02
-7	7	-5	44.54	3.00
7	-7	5	51.97	3.84
-8	7	-5	35.55	2.62
-8	7	-5	35.07	5.52
8	-7	5	39.75	3.80
-9	7	-5	20.67	2.21
-9	7	-5	21.31	4.72
-14	-6	5	9.16	4.24
-14	-6	5	12.98	2.72
-14	-6	5	7.01	3.31
-13	-6	5	13.09	3.14
-13	-6	5	22.21	3.73
-13	-6	5	19.09	3.33
-13	-6	5	13.04	3.87
12	6	-5	21.79	4.46
12	6	-5	22.70	4.47
12	6	-5	21.50	3.28
-12	-6	5	31.25	3.72
-12	-6	5	27.21	4.77
-12	-6	5	27.17	3.37
-12	-6	5	30.17	4.39
11	6	-5	46.65	4.36
-11	-6	5	51.33	3.94
-11	-6	5	44.49	4.79
-11	-6	5	47.34	3.77
10	6	-5	54.10	3.68
10	6	-5	56.89	4.42
10	6	-5	55.97	5.23
-10	-6	5	61.23	4.85
-10	-6	5	56.04	3.85
9	6	-5	48.64	4.81
9	6	-5	51.82	3.47
9	6	-5	55.15	4.10
-9	-6	5	56.95	3.73

-9	-6	5	52.44	4.16
-9	-6	5	54.76	3.61
8	6	-5	23.96	2.17
8	6	-5	31.43	2.89
8	6	-5	21.05	2.91
-8	-6	5	25.22	2.34
-8	-6	5	18.99	2.98
-8	-6	5	26.23	2.56
7	6	-5	44.24	4.01
7	6	-5	55.28	3.56
7	6	-5	50.86	3.09
-7	-6	5	48.82	3.17
-7	-6	5	53.55	3.30
-7	-6	5	46.56	3.55
6	6	-5	57.76	3.43
6	6	-5	49.75	3.06
6	6	-5	42.33	3.81
-6	-6	5	47.03	3.46
-6	-6	5	55.45	3.19
-6	-6	5	50.17	3.13
5	6	-5	50.07	3.20
5	6	-5	44.57	3.86
5	6	-5	47.63	2.91
-5	-6	5	52.16	3.02
-5	-6	5	46.46	3.26
-5	-6	5	46.45	2.96
4	6	-5	52.48	2.99
4	6	-5	48.16	2.57
4	6	-5	50.98	3.17
4	6	-5	47.32	3.50
-4	-6	5	47.24	3.28
-4	-6	5	53.75	3.04
-4	-6	5	48.99	2.98
3	6	-5	76.89	4.60
3	6	-5	79.99	4.23
3	6	-5	80.08	4.33
3	6	-5	77.65	3.89
-3	-6	5	83.53	4.22
-3	-6	5	69.70	4.48
-3	-6	5	73.90	4.20
2	6	-5	51.83	2.71
2	6	-5	54.39	3.12
2	6	-5	54.75	3.15
2	6	-5	49.63	3.45

-2	-6	5	53.81	3.07
-2	-6	5	48.38	3.42
-2	-6	5	50.09	3.03
1	6	-5	101.19	5.42
1	6	-5	99.18	5.61
1	6	-5	101.43	5.12
1	6	-5	108.79	5.43
-1	-6	5	100.69	5.35
-1	-6	5	102.14	5.63
-1	-6	5	103.49	5.41
0	6	-5	34.83	2.44
0	6	-5	28.83	2.33
0	6	-5	27.24	3.65
0	6	-5	31.98	1.82
0	-6	5	30.86	1.96
0	-6	5	31.30	2.70
0	-6	5	32.96	2.23
0	-6	5	31.13	2.61
-1	6	-5	78.87	4.45
-1	6	-5	78.53	3.97
-1	6	-5	69.93	4.28
-1	6	-5	79.74	4.55
1	-6	5	77.96	4.29
1	-6	5	80.44	4.59
1	-6	5	74.00	4.10
1	-6	5	80.04	4.49
-2	6	-5	53.46	3.72
-2	6	-5	63.05	3.93
-2	6	-5	70.39	3.36
-2	6	-5	65.28	4.06
2	-6	5	61.43	3.95
2	-6	5	65.48	3.51
-3	6	-5	56.03	2.83
-3	6	-5	42.47	3.26
-3	6	-5	49.34	3.66
-3	6	-5	56.64	3.68
3	-6	5	51.12	3.00
-4	6	-5	30.57	3.17
-4	6	-5	35.79	2.18
-4	6	-5	33.10	3.09
-5	6	-5	50.26	3.80
-5	6	-5	46.97	3.88
-5	6	-5	46.83	2.75
-5	6	-5	49.26	3.04

-6	6	-5	50.96	3.28
-6	6	-5	47.84	4.04
-6	6	-5	51.32	2.98
6	-6	5	55.63	3.54
-7	6	-5	40.89	4.25
-7	6	-5	48.62	2.89
-7	6	-5	49.50	3.26
7	-6	5	40.96	3.11
7	-6	5	55.68	3.64
-8	6	-5	20.83	4.06
-8	6	-5	25.12	2.11
-8	6	-5	24.81	2.65
8	-6	5	29.59	3.17
8	-6	5	27.89	3.05
-9	6	-5	26.96	4.71
-9	6	-5	27.77	2.38
9	-6	5	37.01	3.84
-10	6	-5	18.71	2.18
-10	6	-5	15.74	5.03
10	-6	5	24.38	4.00
-14	-5	5	9.13	3.35
-14	-5	5	11.04	4.22
-14	-5	5	10.24	2.60
13	5	-5	15.23	5.05
-13	-5	5	27.76	3.50
-13	-5	5	33.77	5.27
-13	-5	5	25.65	3.83
-13	-5	5	27.53	5.31
-12	-5	5	26.99	4.74
-12	-5	5	25.55	3.19
-12	-5	5	25.09	4.52
-12	-5	5	24.71	3.38
11	5	-5	45.73	4.24
-11	-5	5	43.67	4.50
-11	-5	5	45.88	3.55
-11	-5	5	47.25	4.65
-11	-5	5	45.36	3.72
10	5	-5	35.49	4.48
10	5	-5	40.83	3.76
-10	-5	5	35.71	3.94
-10	-5	5	36.68	5.22
-10	-5	5	38.43	3.72
-10	-5	5	42.76	3.09
9	5	-5	81.40	5.85

9	5	-5	88.59	5.35
-9	-5	5	91.30	5.05
-9	-5	5	90.41	5.34
-9	-5	5	91.74	5.47
-9	-5	5	85.48	4.99
8	5	-5	63.27	4.88
8	5	-5	72.08	4.40
-8	-5	5	69.43	4.08
-8	-5	5	72.59	4.48
-8	-5	5	73.19	4.16
-8	-5	5	70.94	4.39
7	5	-5	88.30	6.54
7	5	-5	100.06	5.38
7	5	-5	112.57	5.70
-7	-5	5	102.63	5.63
-7	-5	5	96.91	5.42
-7	-5	5	101.19	5.69
-7	-5	5	109.84	5.46
6	5	-5	73.51	4.03
6	5	-5	58.73	4.31
6	5	-5	67.54	3.71
-6	-5	5	65.13	3.78
-6	-5	5	66.45	4.02
-6	-5	5	70.50	3.81
5	5	-5	112.71	5.83
5	5	-5	102.83	6.20
5	5	-5	121.33	6.01
-5	-5	5	124.08	5.88
-5	-5	5	109.44	5.85
-5	-5	5	108.47	6.00
4	5	-5	117.45	5.92
4	5	-5	114.69	6.20
4	5	-5	116.57	6.02
-4	-5	5	110.91	6.07
-4	-5	5	122.12	5.93
-4	-5	5	111.02	5.93
3	5	-5	99.92	4.88
3	5	-5	94.39	5.12
3	5	-5	92.83	4.59
3	5	-5	78.91	5.14
3	5	-5	97.58	4.94
-3	-5	5	91.82	5.03
-3	-5	5	91.84	4.85
-3	-5	5	99.12	4.85

-3	-5	5	89.50	4.94
2	5	-5	113.29	5.55
2	5	-5	120.65	5.81
2	5	-5	112.69	6.02
2	5	-5	115.84	5.82
-2	-5	5	100.81	5.59
-2	-5	5	107.66	5.98
-2	-5	5	113.19	5.77
-2	-5	5	116.88	5.74
1	5	-5	158.03	7.61
1	5	-5	150.63	7.61
1	5	-5	149.19	7.75
1	5	-5	150.05	7.38
-1	-5	5	147.01	7.44
-1	-5	5	153.43	7.58
-1	-5	5	146.61	7.56
-1	-5	5	149.12	7.74
0	5	-5	106.64	5.32
0	5	-5	96.28	5.24
0	5	-5	101.37	5.00
0	5	-5	99.99	5.41
0	5	-5	89.37	5.19
0	-5	5	97.24	5.38
0	-5	5	104.19	5.36
0	-5	5	107.27	5.25
0	-5	5	100.52	5.08
-1	5	-5	170.04	8.54
-1	5	-5	155.44	8.28
-1	5	-5	173.95	8.48
-1	5	-5	172.59	8.19
-1	5	-5	146.33	8.37
1	-5	5	191.37	8.44
1	-5	5	162.42	8.53
1	-5	5	165.86	8.27
1	-5	5	161.96	8.31
-2	5	-5	69.71	4.13
-2	5	-5	72.94	4.21
-2	5	-5	74.85	3.62
-2	5	-5	60.88	3.92
2	-5	5	65.78	4.07
2	-5	5	76.07	4.28
2	-5	5	70.29	3.76
-3	5	-5	123.20	5.85
-3	5	-5	107.19	6.08

-3	5	-5	116.08	5.97
-3	5	-5	111.06	6.33
-3	5	-5	120.16	6.27
3	-5	5	110.65	5.98
3	-5	5	117.38	5.95
3	-5	5	126.94	6.11
-4	5	-5	86.30	4.88
-4	5	-5	82.66	4.76
-4	5	-5	83.15	4.16
-4	5	-5	84.16	4.35
-4	5	-5	74.34	4.61
4	-5	5	78.49	4.43
4	-5	5	74.89	4.27
-5	5	-5	111.13	6.11
-5	5	-5	109.12	6.17
-5	5	-5	117.67	5.75
-5	5	-5	112.82	5.55
-5	5	-5	94.60	5.97
5	-5	5	112.75	5.81
5	-5	5	105.31	5.71
-6	5	-5	72.63	4.58
-6	5	-5	58.51	4.44
-6	5	-5	72.71	3.73
-6	5	-5	72.82	3.99
-7	5	-5	66.06	3.78
-7	5	-5	71.24	4.09
7	-5	5	73.28	4.34
-8	5	-5	29.26	3.86
-8	5	-5	36.64	3.70
-8	5	-5	31.17	5.00
-8	5	-5	35.26	2.38
-8	5	-5	39.71	2.83
8	-5	5	34.31	2.66
8	-5	5	40.47	3.33
-9	5	-5	34.65	3.69
-9	5	-5	29.41	4.72
-9	5	-5	34.63	2.42
9	-5	5	32.62	2.83
9	-5	5	37.99	3.60
-10	5	-5	30.78	2.47
-10	5	-5	31.46	5.25
10	-5	5	36.74	4.07
-11	5	-5	22.59	2.34
-11	5	-5	17.38	5.53

11	-5	5	28.87	4.32
-14	-4	5	19.81	4.70
-14	-4	5	17.73	3.68
-14	-4	5	20.58	3.74
-13	-4	5	15.20	3.32
-13	-4	5	19.51	3.96
-13	-4	5	23.06	3.40
-12	-4	5	39.85	5.04
-12	-4	5	36.34	3.59
-12	-4	5	35.75	5.09
-12	-4	5	38.84	3.64
-11	-4	5	20.95	3.91
-11	-4	5	22.27	2.84
-11	-4	5	16.68	3.08
-11	-4	5	21.23	2.70
10	4	-5	15.26	1.92
10	4	-5	8.43	2.92
-10	-4	5	17.68	3.12
-10	-4	5	14.77	2.25
-10	-4	5	14.69	2.59
-10	-4	5	13.61	2.44
-9	-4	5	29.13	2.59
-9	-4	5	31.01	3.06
-9	-4	5	28.41	3.29
-9	-4	5	28.57	2.47
8	4	-5	74.32	4.44
8	4	-5	61.72	4.90
-8	-4	5	78.19	4.55
-8	-4	5	76.76	4.20
-8	-4	5	73.51	4.57
-8	-4	5	73.04	4.14
7	4	-5	102.26	5.59
7	4	-5	98.04	5.92
-7	-4	5	108.73	5.44
-7	-4	5	103.88	5.64
-7	-4	5	101.79	5.64
-7	-4	5	98.69	5.39
6	4	-5	138.52	6.89
6	4	-5	125.82	7.11
6	4	-5	125.81	7.08
-6	-4	5	135.94	6.74
-6	-4	5	137.47	6.88
-6	-4	5	128.33	6.73
-6	-4	5	131.71	6.91

5	4	-5	79.52	5.09
5	4	-5	85.22	5.10
5	4	-5	94.40	4.77
5	4	-5	99.72	4.92
-5	-4	5	98.78	4.78
-5	-4	5	88.45	4.75
-5	-4	5	88.97	4.90
4	4	-5	173.87	9.75
4	4	-5	201.35	9.58
4	4	-5	203.79	9.66
-4	-4	5	178.69	9.64
-4	-4	5	213.17	9.58
-4	-4	5	188.94	9.58
3	4	-5	97.82	4.84
3	4	-5	90.89	5.03
3	4	-5	91.56	4.56
3	4	-5	97.92	4.88
3	4	-5	87.17	5.12
-3	-4	5	92.19	4.81
-3	-4	5	98.29	4.80
-3	-4	5	88.23	4.92
2	4	-5	86.79	4.71
2	4	-5	90.94	4.56
2	4	-5	84.90	4.27
2	4	-5	91.35	4.53
-2	-4	5	84.53	4.62
-2	-4	5	81.81	4.31
-2	-4	5	86.76	4.50
-2	-4	5	88.08	4.46
-2	-4	5	83.86	4.56
1	4	-5	123.85	6.71
1	4	-5	135.97	6.67
1	4	-5	126.98	6.76
1	4	-5	131.28	6.45
-1	-4	5	142.66	6.67
-1	-4	5	134.02	6.50
-1	-4	5	133.50	6.63
-1	-4	5	124.91	6.74
0	4	-5	165.11	8.76
0	4	-5	189.59	8.84
0	4	-5	171.01	8.85
0	4	-5	179.75	8.59
0	-4	5	168.48	8.84
0	-4	5	164.76	8.73

0	-4	5	176.32	8.65
0	-4	5	193.11	8.82
0	-4	5	177.12	8.81
-1	4	-5	94.15	4.56
-1	4	-5	76.97	4.78
-1	4	-5	88.78	4.89
-1	4	-5	92.11	4.91
-1	4	-5	83.57	4.67
1	-4	5	90.11	4.91
1	-4	5	96.53	4.64
1	-4	5	101.61	4.85
1	-4	5	97.67	4.89
-2	4	-5	178.02	8.42
-2	4	-5	170.40	8.60
-2	4	-5	171.88	8.69
-2	4	-5	163.97	8.50
-2	4	-5	171.12	8.74
2	-4	5	152.70	8.65
2	-4	5	180.44	8.64
2	-4	5	190.72	8.67
2	-4	5	166.50	8.53
2	-4	5	170.60	8.48
-3	4	-5	81.46	4.48
-3	4	-5	73.46	4.24
-3	4	-5	76.05	3.85
-3	4	-5	72.20	4.26
3	-4	5	79.60	4.24
3	-4	5	71.02	3.99
-4	4	-5	99.87	4.90
-4	4	-5	102.33	5.06
-4	4	-5	92.81	5.36
-4	4	-5	101.54	5.49
-4	4	-5	94.06	5.29
4	-4	5	95.69	5.09
4	-4	5	95.71	5.14
4	-4	5	94.60	4.98
-5	4	-5	45.36	3.66
-5	4	-5	51.54	3.38
-5	4	-5	54.00	2.82
-5	4	-5	54.17	3.04
5	-4	5	48.20	2.89
5	-4	5	54.68	3.13
-6	4	-5	59.73	3.22
-6	4	-5	56.12	2.92

-6	4	-5	45.04	3.71
-6	4	-5	53.68	3.67
-6	4	-5	48.93	3.84
6	-4	5	57.02	3.33
6	-4	5	48.53	3.03
-7	4	-5	21.79	1.65
-7	4	-5	20.39	3.31
-7	4	-5	23.35	2.04
-7	4	-5	21.33	2.85
7	-4	5	24.38	2.32
7	-4	5	20.04	1.83
-8	4	-5	42.23	2.48
-8	4	-5	37.00	4.72
-8	4	-5	38.95	3.60
-8	4	-5	42.99	2.88
-8	4	-5	31.76	3.64
8	-4	5	44.50	3.27
-9	4	-5	29.79	2.62
-9	4	-5	25.42	1.99
-9	4	-5	23.28	3.73
-9	4	-5	27.46	5.01
9	-4	5	20.96	2.30
9	-4	5	29.35	3.11
-10	4	-5	36.61	6.14
-10	4	-5	33.74	2.50
10	-4	5	43.25	3.97
10	-4	5	32.21	2.92
-11	4	-5	17.23	2.09
-11	4	-5	26.07	5.41
11	-4	5	22.95	3.89
-14	-3	5	17.83	3.80
-14	-3	5	21.69	3.60
-14	-3	5	20.54	4.64
-13	-3	5	25.94	3.71
-13	-3	5	18.54	4.01
-13	-3	5	25.60	5.42
-12	-3	5	48.45	5.36
-12	-3	5	36.44	3.69
-12	-3	5	41.87	5.18
-12	-3	5	44.26	3.69
11	3	-5	9.24	3.07
-11	-3	5	11.15	2.31
-11	-3	5	13.32	2.99
-10	-3	5	33.54	3.64

-10	-3	5	28.32	3.76
-10	-3	5	29.62	2.82
-10	-3	5	34.17	2.89
-9	-3	5	42.73	2.99
-9	-3	5	46.38	4.01
-9	-3	5	46.66	3.68
-9	-3	5	41.02	3.08
-8	-3	5	82.52	4.57
-8	-3	5	87.73	4.60
-8	-3	5	80.69	5.01
-8	-3	5	84.30	5.05
7	3	-5	79.34	5.11
7	3	-5	87.17	4.73
-7	-3	5	88.07	4.56
-7	-3	5	86.80	4.85
-7	-3	5	83.47	4.82
-7	-3	5	84.86	4.55
6	3	-5	93.88	4.92
6	3	-5	90.26	5.29
-6	-3	5	90.24	5.00
-6	-3	5	89.89	4.76
-6	-3	5	89.56	4.93
-6	-3	5	92.50	4.79
5	3	-5	161.24	8.80
5	3	-5	169.96	8.86
5	3	-5	185.08	8.69
-5	-3	5	167.49	8.58
-5	-3	5	170.50	8.73
-5	-3	5	182.40	8.59
4	3	-5	168.28	8.77
4	3	-5	179.58	8.59
-4	-3	5	180.65	8.51
-4	-3	5	163.07	8.63
-4	-3	5	168.30	8.52
3	3	-5	105.62	5.13
3	3	-5	104.01	5.15
3	3	-5	92.07	5.39
-3	-3	5	89.89	5.20
-3	-3	5	101.53	5.10
-3	-3	5	106.71	5.09
2	3	-5	100.89	5.89
2	3	-5	110.02	5.50
2	3	-5	123.51	5.74
2	3	-5	121.21	5.73

-2	-3	5	117.94	5.72
-2	-3	5	107.25	5.79
-2	-3	5	118.63	5.66
-2	-3	5	106.41	5.74
-2	-3	5	109.53	5.53
1	3	-5	102.90	5.28
1	3	-5	107.58	5.56
1	3	-5	114.54	5.53
1	3	-5	110.57	5.50
1	3	-5	97.76	5.59
-1	-3	5	110.45	5.34
-1	-3	5	112.76	5.51
-1	-3	5	107.89	5.47
0	3	-5	149.80	8.17
0	3	-5	166.99	8.02
0	3	-5	175.81	8.26
0	-3	5	178.58	8.24
0	-3	5	154.37	8.22
0	-3	5	149.89	8.16
0	-3	5	174.68	8.08
0	-3	5	167.78	8.22
-1	3	-5	179.78	9.03
-1	3	-5	180.38	8.87
-1	3	-5	192.36	9.14
-1	3	-5	175.68	9.07
-1	3	-5	168.24	8.95
1	-3	5	189.90	8.93
1	-3	5	167.15	9.06
1	-3	5	172.47	8.98
1	-3	5	198.53	9.12
1	-3	5	197.00	9.07
-2	3	-5	203.60	10.57
-2	3	-5	221.12	10.82
-2	3	-5	228.16	10.69
-2	3	-5	215.23	10.69
-2	3	-5	217.37	10.50
2	-3	5	227.06	10.57
2	-3	5	188.25	10.67
2	-3	5	222.63	11.07
2	-3	5	211.88	10.61
2	-3	5	222.57	10.77
-3	3	-5	171.04	8.13
-3	3	-5	159.33	8.23
-3	3	-5	173.09	8.36

-3	3	-5	156.60	8.33
-3	3	-5	182.65	8.55
3	-3	5	165.58	8.44
3	-3	5	167.76	8.20
3	-3	5	152.64	8.23
-4	3	-5	85.64	4.58
-4	3	-5	91.88	4.86
-4	3	-5	86.72	4.41
-4	3	-5	87.12	4.25
-4	3	-5	80.70	4.61
4	-3	5	80.92	4.36
4	-3	5	79.85	4.72
4	-3	5	80.38	4.42
-5	3	-5	88.43	4.33
-5	3	-5	87.42	4.50
-5	3	-5	82.58	4.94
-5	3	-5	81.47	4.66
5	-3	5	86.78	4.56
5	-3	5	84.04	4.41
-6	3	-5	106.80	5.89
-6	3	-5	105.15	5.71
-6	3	-5	106.84	5.43
-6	3	-5	109.26	5.26
-6	3	-5	94.03	5.51
6	-3	5	106.72	5.54
6	-3	5	99.00	5.33
-7	3	-5	90.46	5.29
-7	3	-5	81.23	5.78
-7	3	-5	95.50	4.85
-7	3	-5	94.03	5.20
-7	3	-5	88.05	4.58
7	-3	5	78.66	4.67
7	-3	5	95.59	5.00
-8	3	-5	58.51	3.50
-8	3	-5	66.22	4.32
-8	3	-5	61.56	5.11
-8	3	-5	65.33	5.11
-8	3	-5	63.83	4.27
8	-3	5	70.93	4.06
8	-3	5	57.63	3.54
-9	3	-5	36.48	3.50
-9	3	-5	39.48	4.95
-9	3	-5	34.57	2.36
9	-3	5	42.18	3.32

-10	3	-5	24.22	4.30
-10	3	-5	26.14	2.06
-10	3	-5	28.02	2.84
-10	3	-5	24.90	3.69
10	-3	5	25.60	2.42
10	-3	5	30.02	3.46
-11	3	-5	43.03	6.61
-11	3	-5	41.33	2.70
11	-3	5	33.21	3.11
11	-3	5	43.78	4.25
-12	3	-5	26.71	2.62
-12	3	-5	40.84	7.49
12	-3	5	36.79	4.46
-14	-2	5	2.49	3.57
-14	-2	5	6.16	2.36
-14	-2	5	10.94	4.47
13	2	-5	11.73	4.08
-13	-2	5	17.74	3.46
-13	-2	5	23.32	4.11
12	2	-5	11.59	4.45
-12	-2	5	25.51	3.70
-12	-2	5	17.09	3.06
-12	-2	5	19.61	3.07
11	2	-5	13.70	3.06
-11	-2	5	19.68	3.13
-11	-2	5	20.77	3.91
-10	-2	5	15.06	2.71
-10	-2	5	16.61	3.27
9	2	-5	62.27	4.90
-9	-2	5	71.81	4.16
-9	-2	5	77.66	4.86
-9	-2	5	71.15	4.78
-8	-2	5	48.79	3.06
-8	-2	5	52.93	3.65
-8	-2	5	45.61	3.66
7	2	-5	45.63	3.88
-7	-2	5	58.92	3.20
-7	-2	5	57.44	3.66
-7	-2	5	57.91	3.62
-7	-2	5	58.38	3.21
6	2	-5	60.66	3.49
6	2	-5	57.52	4.07
6	2	-5	55.35	3.80
-6	-2	5	61.26	3.32

-6	-2	5	63.17	3.67
-6	-2	5	60.39	3.57
-6	-2	5	63.65	3.34
5	2	-5	104.40	5.39
5	2	-5	103.99	5.57
5	2	-5	100.83	5.68
-5	-2	5	103.21	5.29
-5	-2	5	105.97	5.48
-5	-2	5	103.39	5.28
4	2	-5	83.22	4.85
4	2	-5	93.61	4.63
-4	-2	5	87.87	4.53
-4	-2	5	93.33	4.55
-4	-2	5	84.99	4.71
3	2	-5	105.84	5.71
3	2	-5	112.10	5.48
-3	-2	5	100.74	5.54
-3	-2	5	114.15	5.43
2	2	-5	74.54	3.71
2	2	-5	67.68	3.45
2	2	-5	74.86	3.73
2	2	-5	62.35	3.93
-2	-2	5	71.30	3.48
-2	-2	5	74.25	3.69
-2	-2	5	72.13	3.63
-2	-2	5	64.58	3.74
1	2	-5	226.91	10.88
1	2	-5	211.04	10.99
1	2	-5	212.05	10.72
1	2	-5	233.31	10.93
-1	-2	5	226.74	10.92
-1	-2	5	210.60	10.92
-1	-2	5	229.65	10.77
-1	-2	5	225.54	10.86
0	2	-5	71.37	3.67
0	2	-5	61.87	3.59
0	2	-5	62.09	3.56
0	2	-5	70.30	3.34
0	-2	5	75.21	3.40
0	-2	5	57.98	3.55
0	-2	5	71.19	3.64
0	-2	5	68.88	3.66
-1	2	-5	204.76	9.40
-1	2	-5	166.38	9.30

-1	2	-5	197.12	9.51
1	-2	5	180.59	9.34
1	-2	5	208.17	9.29
1	-2	5	168.91	9.46
1	-2	5	204.50	9.38
-2	2	-5	48.04	2.88
-2	2	-5	54.17	3.03
-2	2	-5	48.79	2.78
-2	2	-5	58.95	3.25
-2	2	-5	53.17	2.65
2	-2	5	51.07	3.17
2	-2	5	50.01	2.79
2	-2	5	54.17	2.72
2	-2	5	51.57	2.99
-3	2	-5	152.35	7.54
-3	2	-5	142.83	7.51
-3	2	-5	149.77	7.35
-3	2	-5	149.11	7.45
-3	2	-5	158.43	7.73
3	-2	5	141.99	7.45
3	-2	5	152.69	7.41
3	-2	5	169.30	7.78
3	-2	5	136.70	7.55
-4	2	-5	68.07	3.83
-4	2	-5	72.37	3.90
-4	2	-5	66.20	4.15
-4	2	-5	73.91	3.78
-4	2	-5	73.41	3.63
4	-2	5	73.79	3.71
4	-2	5	68.21	3.78
4	-2	5	77.83	4.26
-5	2	-5	95.60	4.64
-5	2	-5	95.98	5.17
-5	2	-5	86.02	4.79
-5	2	-5	91.83	4.93
-5	2	-5	89.62	4.81
5	-2	5	89.66	4.71
5	-2	5	101.84	5.26
5	-2	5	89.65	4.82
-6	2	-5	65.52	3.38
-6	2	-5	68.58	3.91
-6	2	-5	63.84	4.28
-6	2	-5	67.15	4.00
-6	2	-5	69.47	3.59

-6	2	-5	60.94	3.62
6	-2	5	64.20	4.13
6	-2	5	68.55	3.68
6	-2	5	63.75	3.46
6	-2	5	58.10	3.57
-7	2	-5	112.23	5.94
-7	2	-5	103.78	5.36
-7	2	-5	109.54	5.59
-7	2	-5	111.58	5.85
-7	2	-5	99.96	5.57
-7	2	-5	102.06	6.36
7	-2	5	109.72	5.68
7	-2	5	100.79	5.44
-8	2	-5	67.97	3.46
-8	2	-5	56.60	3.79
-8	2	-5	64.06	4.17
-8	2	-5	71.78	4.24
-8	2	-5	60.70	4.98
8	-2	5	66.84	4.18
8	-2	5	62.51	3.55
-9	2	-5	39.34	2.73
-9	2	-5	40.87	3.73
-9	2	-5	52.04	4.76
9	-2	5	42.50	2.69
-10	2	-5	15.60	2.45
-10	2	-5	13.49	1.61
-10	2	-5	16.82	3.64
10	-2	5	16.83	2.90
-11	2	-5	34.90	2.50
-11	2	-5	39.21	3.95
-11	2	-5	44.90	6.17
-11	2	-5	32.13	4.22
11	-2	5	30.43	2.76
-12	2	-5	15.81	5.30
-12	2	-5	13.49	2.01
12	-2	5	21.17	3.85
12	-2	5	14.92	2.51
-13	2	-5	26.82	6.36
-13	2	-5	20.05	2.46
-14	-1	5	17.73	4.89
-14	-1	5	13.84	3.94
-13	-1	5	19.01	3.66
-13	-1	5	18.70	4.25
-12	-1	5	30.66	5.14

-12	-1	5	27.77	4.35
-12	-1	5	31.89	5.07
-11	-1	5	33.43	4.64
-11	-1	5	37.00	4.47
10	1	-5	30.46	4.02
-10	-1	5	36.08	4.18
-10	-1	5	43.36	4.05
9	1	-5	64.16	4.88
-9	-1	5	79.35	4.98
-9	-1	5	80.04	5.08
-8	-1	5	84.87	5.18
-8	-1	5	85.81	5.11
-7	-1	5	68.09	4.20
-7	-1	5	70.38	4.19
6	1	-5	82.92	5.00
-6	-1	5	90.51	4.59
-6	-1	5	94.23	4.96
5	1	-5	140.17	7.43
-5	-1	5	138.18	7.22
-5	-1	5	145.47	7.00
-5	-1	5	139.40	7.00
4	1	-5	134.56	7.16
-4	-1	5	135.67	7.04
-4	-1	5	147.22	6.88
-4	-1	5	137.83	6.87
3	1	-5	160.85	7.48
3	1	-5	145.46	7.65
-3	-1	5	145.12	7.55
-3	-1	5	153.16	7.43
2	1	-5	166.18	8.43
2	1	-5	178.00	8.28
-2	-1	5	158.84	8.34
-2	-1	5	169.22	8.30
-2	-1	5	169.00	8.23
1	1	-5	59.70	3.38
1	1	-5	56.71	3.24
1	1	-5	65.33	3.03
-1	-1	5	63.17	3.29
-1	-1	5	60.25	3.04
-1	-1	5	58.81	3.31
-1	-1	5	63.61	3.23
0	1	-5	190.17	9.07
0	1	-5	182.54	8.92
0	1	-5	191.32	9.20

0	1	-5	182.32	9.09
0	-1	5	202.33	9.10
0	-1	5	177.54	9.08
0	-1	5	184.14	8.95
0	-1	5	163.37	9.15
-1	1	-5	88.67	4.42
-1	1	-5	103.75	4.81
-1	1	-5	95.36	4.63
-1	1	-5	78.84	4.52
1	-1	5	94.95	4.81
1	-1	5	82.06	4.54
1	-1	5	90.23	4.46
1	-1	5	87.72	4.59
-2	1	-5	172.01	9.30
-2	1	-5	188.62	9.23
-2	1	-5	197.15	9.52
-2	1	-5	201.89	9.38
2	-1	5	197.16	9.28
2	-1	5	179.19	9.32
2	-1	5	196.24	9.37
-3	1	-5	130.97	6.66
-3	1	-5	126.76	6.64
-3	1	-5	130.51	6.89
-3	1	-5	139.77	6.57
3	-1	5	154.09	7.00
3	-1	5	132.45	6.71
3	-1	5	137.51	6.61
3	-1	5	121.84	6.65
-4	1	-5	107.19	5.16
-4	1	-5	102.63	5.34
-4	1	-5	105.79	5.27
-4	1	-5	103.50	5.27
-4	1	-5	112.16	5.54
4	-1	5	99.61	5.28
4	-1	5	88.32	5.26
4	-1	5	117.02	5.63
4	-1	5	103.48	5.20
-5	1	-5	124.81	6.60
-5	1	-5	140.63	6.85
-5	1	-5	134.35	6.49
-5	1	-5	125.86	6.92
-5	1	-5	131.32	6.61
5	-1	5	129.19	6.53
5	-1	5	128.27	6.64

5	-1	5	138.91	6.96
-6	1	-5	108.81	5.38
-6	1	-5	114.44	5.79
-6	1	-5	103.74	5.52
-6	1	-5	103.00	6.00
6	-1	5	108.83	5.60
6	-1	5	111.03	5.92
6	-1	5	104.97	5.44
-7	1	-5	102.78	5.04
-7	1	-5	96.03	5.84
-7	1	-5	91.28	5.19
-7	1	-5	103.39	5.24
-7	1	-5	102.21	5.48
7	-1	5	105.49	5.33
7	-1	5	96.41	5.10
-8	1	-5	128.35	6.10
-8	1	-5	115.83	6.27
-8	1	-5	114.34	7.06
-8	1	-5	124.41	6.57
-8	1	-5	127.54	6.34
8	-1	5	128.40	6.46
8	-1	5	120.72	6.15
8	-1	5	106.27	6.73
-9	1	-5	76.56	8.44
-9	1	-5	91.65	5.27
-9	1	-5	104.24	5.03
-9	1	-5	99.09	6.07
9	-1	5	105.74	5.70
9	-1	5	101.34	5.32
9	-1	5	93.09	5.09
-10	1	-5	42.63	3.19
-10	1	-5	44.39	5.09
-10	1	-5	44.40	2.80
-10	1	-5	45.82	3.76
10	-1	5	43.48	2.80
10	-1	5	48.60	3.66
-11	1	-5	25.13	3.53
-11	1	-5	32.49	5.51
-11	1	-5	29.83	3.67
-11	1	-5	33.20	2.32
11	-1	5	38.01	3.62
-12	1	-5	39.14	2.85
-12	1	-5	45.84	6.67
12	-1	5	34.99	3.04

12	-1	5	49.00	4.33
-13	1	-5	20.28	5.34
-13	1	-5	15.61	2.32
13	-1	5	17.74	3.01
-13	0	5	19.38	4.52
-13	0	5	10.66	3.77
-12	0	5	33.38	5.32
-12	0	5	26.44	5.46
-11	0	5	14.51	3.20
-11	0	5	11.37	2.77
-10	0	5	47.79	4.52
-10	0	5	46.81	4.59
9	0	-5	22.10	3.24
-9	0	5	29.76	3.50
8	0	-5	68.12	6.42
-8	0	5	85.84	5.01
-8	0	5	81.86	4.99
-7	0	5	42.81	3.09
-7	0	5	34.65	3.06
-6	0	5	63.49	3.85
-5	0	5	45.50	2.94
4	0	-5	79.57	4.40
-4	0	5	76.66	4.21
3	0	-5	65.49	3.66
-3	0	5	63.57	3.54
-3	0	5	67.80	3.35
2	0	-5	122.47	5.60
2	0	-5	113.06	5.76
2	0	-5	96.34	5.72
-2	0	5	118.15	5.65
-2	0	5	108.17	5.67
-2	0	5	116.63	5.57
1	0	-5	163.86	8.16
1	0	-5	161.69	7.93
1	0	-5	171.14	8.04
-1	0	5	165.32	7.93
-1	0	5	158.84	8.09
-1	0	5	145.50	8.13
-1	0	5	179.59	8.06
0	0	-5	464.88	24.58
0	0	-5	488.54	24.26
0	0	-5	550.39	24.37
0	0	-5	490.93	24.41
0	0	5	490.05	24.39

0	0	5	507.02	24.36
0	0	5	477.39	24.27
0	0	5	564.65	24.52
-1	0	-5	335.84	16.41
-1	0	-5	357.18	16.30
-1	0	-5	308.24	16.27
-1	0	-5	337.80	16.19
1	0	5	367.81	16.46
1	0	5	306.39	16.27
1	0	5	331.03	16.29
1	0	5	341.40	16.21
-2	0	-5	89.66	4.62
-2	0	-5	81.92	4.60
-2	0	-5	98.24	4.84
-2	0	-5	100.05	4.55
2	0	5	90.30	4.56
2	0	5	86.75	4.62
2	0	5	101.24	4.88
2	0	5	91.15	4.66
-3	0	-5	14.59	1.15
-3	0	-5	15.22	0.88
-3	0	-5	12.98	1.04
-3	0	-5	13.62	1.48
3	0	5	13.10	1.18
3	0	5	14.19	0.92
3	0	5	12.20	1.53
3	0	5	13.20	1.04
-4	0	-5	123.74	5.70
-4	0	-5	125.06	5.99
-4	0	-5	110.31	6.00
-4	0	-5	114.39	5.78
-4	0	-5	116.37	5.78
4	0	5	101.70	5.80
4	0	5	111.16	5.78
4	0	5	120.99	5.99
4	0	5	116.97	5.72
-5	0	-5	42.30	2.96
-5	0	-5	46.34	2.76
-5	0	-5	48.76	2.34
-5	0	-5	43.77	2.47
-5	0	-5	45.29	2.50
5	0	5	37.80	2.50
5	0	5	41.88	2.34
5	0	5	43.76	2.78

5	0	5	44.83	2.52
-6	0	-5	114.16	5.44
-6	0	-5	110.33	5.56
-6	0	-5	104.26	5.54
-6	0	-5	110.67	5.74
-6	0	-5	105.65	5.95
6	0	5	111.12	5.78
6	0	5	105.74	5.45
6	0	5	109.86	5.62
-7	0	-5	35.65	1.93
-7	0	-5	31.39	3.03
-7	0	-5	30.88	2.22
-7	0	-5	32.97	2.16
-7	0	-5	30.59	2.53
7	0	5	31.82	2.10
7	0	5	34.73	2.29
-8	0	-5	101.98	5.24
-8	0	-5	96.65	5.43
-8	0	-5	94.37	5.94
-8	0	-5	100.91	5.02
-8	0	-5	94.65	5.21
8	0	5	101.60	5.27
8	0	5	92.72	5.04
8	0	5	104.83	5.39
-9	0	-5	30.84	4.03
-9	0	-5	42.23	3.17
-9	0	-5	39.01	2.38
-9	0	-5	39.37	2.76
-9	0	-5	40.38	2.76
9	0	5	38.24	2.42
9	0	5	43.36	2.99
-10	0	-5	49.83	3.41
-10	0	-5	51.69	3.98
-10	0	-5	55.21	3.07
-10	0	-5	47.58	4.93
10	0	5	56.34	3.81
10	0	5	51.45	3.05
-11	0	-5	32.86	2.40
-11	0	-5	30.21	2.92
-11	0	-5	37.19	5.04
-11	0	-5	31.26	3.61
11	0	5	38.90	3.46
11	0	5	31.52	2.42
-12	0	-5	35.78	2.61

-12	0	-5	35.52	5.81
-12	0	-5	30.74	4.13
-12	0	-5	32.54	3.35
12	0	5	40.97	3.99
-13	0	-5	10.09	2.16
-13	0	-5	15.17	4.88
13	0	5	9.04	1.46
-14	0	-5	15.38	2.52
-14	0	-5	21.59	5.86
-13	1	5	20.41	4.02
-13	1	5	21.69	4.75
-12	1	5	31.39	5.66
-12	1	5	26.81	5.53
-11	1	5	32.07	5.22
-11	1	5	27.54	4.82
-11	1	5	24.49	3.18
-10	1	5	20.47	2.45
-9	1	5	58.96	4.68
-9	1	5	57.87	3.79
-9	1	5	62.21	4.98
-8	1	5	71.50	4.95
-8	1	5	83.55	5.00
-7	1	5	124.82	7.08
-7	1	5	140.64	7.23
-6	1	5	97.12	5.37
-5	1	5	63.96	3.72
-4	1	5	125.18	6.42
3	-1	-5	83.25	4.48
-3	1	5	82.57	4.35
2	-1	-5	90.35	4.76
2	-1	-5	97.34	4.48
-2	1	5	94.20	4.47
-2	1	5	85.01	4.67
1	-1	-5	157.07	8.14
1	-1	-5	159.26	8.34
-1	1	5	161.18	8.14
-1	1	5	175.54	8.26
-1	1	5	189.73	8.40
0	-1	-5	815.83	37.44
0	1	5	769.23	37.44
0	1	5	724.42	37.47
0	1	5	800.40	37.53
-1	-1	-5	904.23	45.03
-1	-1	-5	945.78	45.09

-1	-1	-5	925.85	45.23
1	1	5	1000.00	45.13
1	1	5	863.53	45.07
1	1	5	979.24	45.08
-2	-1	-5	244.15	10.81
-2	-1	-5	216.78	10.68
-2	-1	-5	226.73	10.65
-2	-1	-5	201.72	10.67
2	1	5	212.59	10.70
2	1	5	206.92	10.68
2	1	5	222.96	10.64
2	1	5	225.60	10.77
-3	-1	-5	144.36	7.49
-3	-1	-5	162.37	7.47
-3	-1	-5	171.38	7.65
-3	-1	-5	149.04	7.51
3	1	5	144.19	7.52
3	1	5	156.42	7.59
3	1	5	156.09	7.47
3	1	5	140.31	7.50
-4	-1	-5	68.52	3.58
-4	-1	-5	77.18	3.53
-4	-1	-5	66.36	3.60
-4	-1	-5	76.67	3.75
4	1	5	71.47	3.72
4	1	5	67.03	3.59
4	1	5	69.55	3.52
4	1	5	62.90	3.63
-5	-1	-5	162.09	7.46
-5	-1	-5	151.39	7.52
-5	-1	-5	153.23	7.76
-5	-1	-5	150.18	7.53
-5	-1	-5	152.49	7.64
5	1	5	152.50	7.46
5	1	5	150.69	7.62
5	1	5	143.32	7.57
5	1	5	150.55	7.54
-6	-1	-5	108.62	5.82
-6	-1	-5	110.12	5.58
-6	-1	-5	113.29	5.33
-6	-1	-5	103.15	5.41
-6	-1	-5	105.95	5.42
6	1	5	103.15	5.32
6	1	5	107.22	5.55

6	1	5	101.55	5.45
-7	-1	-5	144.98	6.80
-7	-1	-5	136.60	6.90
-7	-1	-5	133.31	6.90
-7	-1	-5	133.79	7.03
-7	-1	-5	137.75	7.37
7	1	5	134.12	6.79
7	1	5	137.48	7.03
7	1	5	136.15	6.97
-8	-1	-5	143.38	7.95
-8	-1	-5	155.85	7.25
-8	-1	-5	142.18	7.36
-8	-1	-5	138.76	7.52
-8	-1	-5	140.15	7.36
8	1	5	144.96	7.49
8	1	5	150.34	7.48
-9	-1	-5	134.32	7.89
-9	-1	-5	137.08	7.33
-9	-1	-5	130.95	7.10
-9	-1	-5	141.33	7.15
-9	-1	-5	148.07	6.96
9	1	5	134.53	7.27
9	1	5	142.54	7.27
9	1	5	135.50	6.93
9	1	5	137.42	7.34
-10	-1	-5	45.44	3.06
-10	-1	-5	46.40	2.72
-10	-1	-5	47.47	3.08
-10	-1	-5	40.31	5.93
-10	-1	-5	46.66	3.53
10	1	5	38.14	3.37
10	1	5	42.48	2.64
-11	-1	-5	48.10	3.38
-11	-1	-5	46.66	4.03
-11	-1	-5	47.19	5.05
-11	-1	-5	53.06	2.99
11	1	5	53.48	3.82
11	1	5	46.92	2.95
11	1	5	38.90	3.67
-12	-1	-5	32.00	2.50
-12	-1	-5	37.53	5.34
-12	-1	-5	37.15	4.06
-12	-1	-5	34.94	3.18
12	1	5	26.86	2.42

-13	-1	-5	44.23	6.38
-13	-1	-5	34.85	3.58
-13	-1	-5	35.46	2.86
-14	-1	-5	19.73	2.58
14	1	5	24.89	2.77
-13	2	5	11.10	3.91
-13	2	5	16.01	5.12
-12	2	5	5.79	3.39
-12	2	5	14.74	4.12
-11	2	5	22.46	3.15
-11	2	5	24.17	5.02
-11	2	5	21.23	3.82
-10	2	5	23.02	4.15
-10	2	5	24.69	4.66
-10	2	5	21.81	2.81
-9	2	5	44.09	3.41
-9	2	5	48.96	4.33
-9	2	5	46.84	4.25
-8	2	5	27.75	2.46
-8	2	5	21.28	3.26
-8	2	5	30.08	3.34
-7	2	5	83.93	5.18
-7	2	5	92.25	5.38
-6	2	5	16.94	2.26
-5	2	5	97.01	5.27
-4	2	5	54.49	2.78
-4	2	5	56.63	3.25
3	-2	-5	103.25	5.04
-3	2	5	103.84	5.03
-3	2	5	101.81	5.30
2	-2	-5	30.63	1.61
-2	2	5	31.16	1.59
1	-2	-5	118.18	5.73
-1	2	5	117.22	5.72
0	-2	-5	64.42	3.15
0	-2	-5	59.21	3.02
0	2	5	60.62	3.01
0	2	5	61.15	3.12
0	2	5	59.61	3.14
-1	-2	-5	304.17	14.72
-1	-2	-5	303.24	14.69
1	2	5	307.42	14.68
1	2	5	301.17	14.76
1	2	5	306.03	14.74

-2	-2	-5	198.79	9.35
-2	-2	-5	179.32	9.38
-2	-2	-5	215.48	9.82
2	2	5	190.75	9.43
2	2	5	180.82	9.40
2	2	5	192.95	9.34
-3	-2	-5	108.06	5.21
-3	-2	-5	102.57	5.04
-3	-2	-5	97.64	5.10
3	2	5	109.87	5.05
3	2	5	101.22	5.17
3	2	5	96.13	5.13
-4	-2	-5	79.50	3.80
-4	-2	-5	76.14	3.88
-4	-2	-5	77.97	4.00
4	2	5	76.71	3.96
4	2	5	73.83	3.92
4	2	5	76.77	3.79
4	2	5	70.54	3.84
-5	-2	-5	63.66	3.39
-5	-2	-5	66.57	3.78
-5	-2	-5	68.11	3.56
-5	-2	-5	68.15	3.32
-5	-2	-5	62.70	3.44
5	2	5	63.12	3.41
5	2	5	65.36	3.32
5	2	5	63.30	3.55
5	2	5	67.00	3.53
-6	-2	-5	84.64	4.32
-6	-2	-5	83.56	4.51
-6	-2	-5	82.38	4.36
-6	-2	-5	85.84	4.78
-6	-2	-5	88.95	4.25
6	2	5	84.79	4.48
6	2	5	86.59	4.24
6	2	5	74.68	4.55
-7	-2	-5	95.84	4.71
-7	-2	-5	96.35	5.36
-7	-2	-5	90.09	4.82
-7	-2	-5	92.80	5.00
-7	-2	-5	86.83	4.78
7	2	5	93.13	4.89
7	2	5	90.11	5.03
7	2	5	95.59	4.95

7	2	5	98.57	5.16
7	2	5	89.66	4.68
-8	-2	-5	37.42	2.47
-8	-2	-5	42.39	2.81
-8	-2	-5	34.67	2.71
-8	-2	-5	39.38	3.40
-8	-2	-5	36.87	2.21
8	2	5	36.47	2.17
8	2	5	37.95	2.59
8	2	5	38.86	2.66
8	2	5	37.86	2.78
-9	-2	-5	102.00	5.16
-9	-2	-5	98.81	5.32
-9	-2	-5	101.08	5.32
-9	-2	-5	99.80	5.58
-9	-2	-5	96.08	6.12
9	2	5	96.91	5.11
9	2	5	99.90	5.48
9	2	5	106.02	5.49
-10	-2	-5	32.92	2.62
-10	-2	-5	29.26	3.96
-10	-2	-5	35.23	2.56
-10	-2	-5	35.38	2.25
-10	-2	-5	31.25	3.13
10	2	5	30.28	2.91
10	2	5	35.54	2.88
10	2	5	33.08	2.10
-11	-2	-5	50.82	3.52
-11	-2	-5	55.42	5.08
-11	-2	-5	47.89	3.15
-11	-2	-5	49.85	4.75
11	2	5	50.32	3.07
11	2	5	59.64	3.85
11	2	5	58.02	3.78
-12	-2	-5	32.51	3.98
-12	-2	-5	32.91	2.95
-12	-2	-5	33.02	3.01
-12	-2	-5	40.09	5.10
-12	-2	-5	25.08	2.54
12	2	5	34.55	3.28
12	2	5	28.01	2.30
12	2	5	24.94	3.30
-13	-2	-5	24.00	3.21
-13	-2	-5	19.67	2.35

-13	-2	-5	28.53	3.17
-13	-2	-5	34.44	4.42
-14	-2	-5	19.50	4.99
-14	-2	-5	19.06	3.53
14	2	5	14.96	2.42
-15	-2	-5	8.37	1.65
-15	-2	-5	15.94	4.07
-15	-2	-5	16.55	5.99
15	2	5	9.04	1.65
-12	3	5	19.11	4.71
-12	3	5	13.65	3.67
-11	3	5	20.29	3.96
-11	3	5	16.35	3.37
-10	3	5	28.97	3.12
-10	3	5	33.54	5.06
-10	3	5	30.06	4.59
-9	3	5	26.53	4.22
-9	3	5	34.43	4.03
-9	3	5	29.91	2.86
-8	3	5	62.39	4.78
-8	3	5	62.91	4.66
-8	3	5	60.66	3.90
-7	3	5	97.47	4.89
-7	3	5	99.61	5.31
-7	3	5	95.78	5.75
-7	3	5	101.14	5.90
-7	3	5	92.97	4.85
-6	3	5	104.40	5.26
-6	3	5	100.55	5.80
-6	3	5	108.46	5.22
-6	3	5	108.09	6.00
-5	3	5	54.06	2.77
-5	3	5	53.99	2.82
-4	3	5	144.15	7.04
-3	3	5	154.02	7.50
2	-3	-5	106.63	5.22
-2	3	5	105.97	5.20
1	-3	-5	135.39	6.61
-1	3	5	135.73	6.60
0	-3	-5	136.86	6.65
0	3	5	136.24	6.64
-1	-3	-5	61.54	3.26
-1	-3	-5	69.48	3.24
-1	-3	-5	63.60	3.47

1	3	5	66.37	3.34
1	3	5	68.10	3.23
1	3	5	61.30	3.28
-2	-3	-5	73.66	3.79
-2	-3	-5	79.59	3.73
2	3	5	74.42	3.85
2	3	5	76.86	3.72
2	3	5	72.67	3.81
-3	-3	-5	228.87	11.63
-3	-3	-5	237.73	11.58
-3	-3	-5	249.25	11.72
3	3	5	248.57	11.58
3	3	5	229.94	11.67
3	3	5	241.10	11.69
-4	-3	-5	137.22	7.33
-4	-3	-5	155.16	7.26
-4	-3	-5	150.74	7.42
4	3	5	151.39	7.41
-5	-3	-5	87.88	4.37
-5	-3	-5	89.34	4.60
-5	-3	-5	82.89	4.48
5	3	5	91.48	4.60
5	3	5	90.44	4.38
5	3	5	85.01	4.61
-6	-3	-5	253.22	12.00
-6	-3	-5	234.81	11.79
-6	-3	-5	233.97	11.89
-6	-3	-5	244.54	12.10
6	3	5	227.56	12.00
6	3	5	244.21	11.80
6	3	5	247.90	11.94
6	3	5	254.53	11.98
-7	-3	-5	92.55	4.89
-7	-3	-5	95.71	5.34
-7	-3	-5	90.90	4.72
-7	-3	-5	96.03	5.07
7	3	5	96.65	4.98
7	3	5	91.39	4.72
7	3	5	88.74	5.12
7	3	5	99.89	5.03
-8	-3	-5	84.62	4.74
-8	-3	-5	85.06	5.09
-8	-3	-5	81.36	4.51
-8	-3	-5	81.63	4.31

-8	-3	-5	80.41	4.44
8	3	5	83.76	4.85
8	3	5	86.22	4.30
8	3	5	86.52	4.68
8	3	5	88.81	4.68
-9	-3	-5	64.75	3.80
-9	-3	-5	67.64	4.63
-9	-3	-5	72.18	4.13
-9	-3	-5	68.08	3.73
-9	-3	-5	60.47	3.53
9	3	5	72.32	4.00
9	3	5	62.40	4.29
9	3	5	61.72	3.60
-10	-3	-5	99.60	5.64
-10	-3	-5	106.59	5.89
-10	-3	-5	115.07	6.23
-10	-3	-5	116.35	6.75
-10	-3	-5	114.81	5.88
10	3	5	98.83	5.99
10	3	5	119.54	6.04
10	3	5	114.07	6.05
10	3	5	110.60	6.37
10	3	5	107.52	5.60
-11	-3	-5	38.21	2.65
-11	-3	-5	47.69	3.75
-11	-3	-5	44.66	3.05
-11	-3	-5	43.14	3.08
-11	-3	-5	47.21	4.95
11	3	5	39.34	3.79
11	3	5	38.88	3.33
11	3	5	39.82	2.52
-12	-3	-5	59.37	4.67
-12	-3	-5	46.69	3.27
-12	-3	-5	57.74	3.79
-12	-3	-5	65.85	5.63
12	3	5	58.32	3.23
12	3	5	48.14	3.96
-13	-3	-5	44.16	4.66
-13	-3	-5	37.94	3.52
-13	-3	-5	34.13	3.28
-13	-3	-5	40.91	5.80
-13	-3	-5	27.75	3.14
13	3	5	33.81	2.58
13	3	5	30.69	3.68

-14	-3	-5	36.74	3.70
-14	-3	-5	45.69	5.57
-14	-3	-5	31.46	2.72
-15	-3	-5	20.80	5.57
-15	-3	-5	14.94	2.44
-15	-3	-5	15.69	3.97
15	3	5	16.37	2.53
-12	4	5	14.24	3.82
-12	4	5	18.18	5.02
-11	4	5	13.78	4.03
-11	4	5	3.08	2.87
-11	4	5	8.70	3.30
-10	4	5	29.38	1.95
-10	4	5	26.77	5.05
-10	4	5	32.47	2.20
-10	4	5	29.64	5.34
-10	4	5	31.62	3.31
-9	4	5	6.75	2.90
-9	4	5	7.34	2.50
-9	4	5	5.02	1.39
-9	4	5	4.96	0.82
-9	4	5	6.49	1.07
-8	4	5	43.64	4.10
-8	4	5	41.12	3.07
-8	4	5	37.26	2.27
-8	4	5	40.77	2.39
-7	4	5	47.15	3.70
-7	4	5	41.75	2.43
-7	4	5	46.08	2.51
-6	4	5	91.93	4.62
-6	4	5	90.41	5.29
-6	4	5	93.67	4.69
-5	4	5	34.25	2.71
-5	4	5	34.29	1.89
-5	4	5	34.32	1.98
-4	4	5	89.67	4.47
-3	4	5	97.53	4.82
2	-4	-5	126.49	6.29
-2	4	5	129.46	6.27
1	-4	-5	83.03	4.07
-1	4	5	80.34	4.05
0	-4	-5	149.30	7.20
0	4	5	145.41	7.18
-1	-4	-5	123.54	6.09

1	4	5	125.27	6.08
-2	-4	-5	147.00	6.86
-2	-4	-5	132.24	6.89
2	4	5	139.92	6.96
2	4	5	142.98	6.85
-3	-4	-5	113.54	5.93
-3	-4	-5	121.87	5.86
-3	-4	-5	116.29	6.07
3	4	5	121.93	6.01
3	4	5	124.69	5.86
-4	-4	-5	95.05	5.05
-4	-4	-5	100.00	4.94
-4	-4	-5	104.05	5.18
4	4	5	102.27	4.94
4	4	5	102.67	5.14
4	4	5	97.95	5.08
-5	-4	-5	111.15	6.08
-5	-4	-5	128.56	6.14
-5	-4	-5	122.97	5.86
-5	-4	-5	115.44	5.99
5	4	5	107.47	6.08
5	4	5	128.42	6.09
5	4	5	119.22	6.03
5	4	5	119.85	5.86
-6	-4	-5	185.38	9.65
-6	-4	-5	199.10	9.60
-6	-4	-5	193.90	9.36
-6	-4	-5	187.85	9.50
6	4	5	192.59	9.37
6	4	5	203.04	9.58
6	4	5	179.09	9.64
6	4	5	194.18	9.54
-7	-4	-5	108.54	5.61
-7	-4	-5	100.24	5.44
-7	-4	-5	102.95	5.77
-7	-4	-5	107.42	5.25
7	4	5	107.07	5.51
7	4	5	104.58	5.26
7	4	5	99.16	5.65
7	4	5	112.16	5.56
-8	-4	-5	60.27	3.94
-8	-4	-5	54.72	3.37
-8	-4	-5	57.86	3.03
-8	-4	-5	62.60	3.65

8	4	5	64.21	3.54
8	4	5	55.26	3.04
8	4	5	51.67	3.80
8	4	5	56.67	3.42
-9	-4	-5	60.85	3.18
-9	-4	-5	59.91	3.89
-9	-4	-5	56.69	4.27
-9	-4	-5	60.68	3.57
9	4	5	60.89	3.19
9	4	5	51.99	4.01
9	4	5	65.30	3.77
-10	-4	-5	68.26	5.01
-10	-4	-5	70.48	4.07
-10	-4	-5	77.08	4.47
-10	-4	-5	63.10	3.63
10	4	5	63.72	3.63
10	4	5	55.94	4.61
10	4	5	74.36	4.24
10	4	5	74.04	4.31
-11	-4	-5	30.68	2.71
-11	-4	-5	28.85	2.49
-11	-4	-5	26.88	1.98
-11	-4	-5	34.17	3.38
11	4	5	30.19	3.00
11	4	5	27.13	1.93
11	4	5	20.56	3.68
11	4	5	29.94	3.17
-12	-4	-5	28.63	2.12
-12	-4	-5	34.63	3.79
-12	-4	-5	29.02	4.69
-12	-4	-5	35.98	2.95
12	4	5	23.80	3.98
12	4	5	25.74	3.18
12	4	5	26.77	2.05
-13	-4	-5	42.54	5.56
-13	-4	-5	36.06	2.50
-13	-4	-5	37.69	4.70
-13	-4	-5	39.90	3.42
-13	-4	-5	39.31	3.44
13	4	5	33.41	2.52
13	4	5	35.28	4.75
13	4	5	32.30	3.63
13	4	5	28.83	4.53
-14	-4	-5	35.46	5.25

-14	-4	-5	27.06	4.95
-14	-4	-5	21.87	2.23
-14	-4	-5	24.59	3.31
-15	-4	-5	12.82	2.17
-15	-4	-5	22.01	5.10
-15	-4	-5	12.32	4.08
-11	5	5	18.20	3.73
-11	5	5	21.11	4.74
-11	5	5	19.35	2.25
-11	5	5	23.13	1.94
-10	5	5	18.76	1.93
-10	5	5	17.59	3.29
-10	5	5	18.58	1.69
-10	5	5	24.11	4.23
-9	5	5	31.15	4.90
-9	5	5	36.10	4.71
-9	5	5	29.01	2.13
-9	5	5	27.98	2.96
-9	5	5	28.12	1.94
-8	5	5	14.82	2.33
-8	5	5	9.87	2.36
-8	5	5	17.20	3.73
-8	5	5	15.72	1.53
-8	5	5	14.56	1.33
-7	5	5	55.34	2.93
-7	5	5	49.76	2.82
-7	5	5	54.12	4.19
-7	5	5	46.05	4.00
-7	5	5	56.51	4.34
-6	5	5	42.96	2.31
-6	5	5	42.19	2.40
-6	5	5	35.78	3.38
-6	5	5	42.53	3.43
-5	5	5	84.83	4.40
-5	5	5	86.01	4.33
-5	5	5	86.03	4.91
-4	5	5	69.00	3.52
-3	5	5	60.66	3.11
-2	5	5	177.09	8.64
1	-5	-5	173.95	8.45
-1	5	5	171.59	8.43
0	-5	-5	150.41	7.25
0	5	5	145.11	7.22
-1	-5	-5	157.28	7.71

1	5	5	158.22	7.69
-2	-5	-5	118.08	5.73
-2	-5	-5	116.82	5.61
-2	-5	-5	107.65	5.63
2	5	5	115.87	5.59
2	5	5	111.57	5.71
2	5	5	113.24	5.68
-3	-5	-5	133.62	6.29
-3	-5	-5	120.88	6.35
3	5	5	127.71	6.40
3	5	5	134.19	6.28
3	5	5	123.99	6.42
-4	-5	-5	62.12	3.50
-4	-5	-5	68.55	3.69
-4	-5	-5	67.03	3.34
4	5	5	65.13	3.59
4	5	5	69.24	3.33
4	5	5	65.95	3.54
-5	-5	-5	246.88	11.56
-5	-5	-5	231.94	11.47
-5	-5	-5	227.87	11.32
5	5	5	235.74	11.32
5	5	5	235.24	11.49
5	5	5	243.84	11.53
5	5	5	212.05	11.51
-6	-5	-5	147.64	8.16
-6	-5	-5	158.29	8.08
-6	-5	-5	162.69	7.87
-6	-5	-5	178.88	8.20
6	5	5	160.07	7.87
6	5	5	167.75	8.10
6	5	5	141.20	8.17
6	5	5	172.02	8.15
-7	-5	-5	154.90	7.50
-7	-5	-5	146.26	7.88
-7	-5	-5	161.95	7.88
-7	-5	-5	148.60	7.74
7	5	5	157.93	7.77
7	5	5	156.13	7.81
7	5	5	153.50	7.52
7	5	5	144.16	7.92
-8	-5	-5	55.73	3.95
-8	-5	-5	65.18	3.83
-8	-5	-5	61.08	3.60

8	5	5	65.77	3.68
8	5	5	65.37	3.72
8	5	5	55.22	4.01
8	5	5	60.08	3.21
-9	-5	-5	100.33	5.69
-9	-5	-5	104.84	6.15
-9	-5	-5	114.85	5.96
-9	-5	-5	104.81	5.31
9	5	5	102.19	5.34
9	5	5	98.42	6.06
9	5	5	114.86	5.85
9	5	5	107.35	5.75
-10	-5	-5	33.86	2.05
-10	-5	-5	34.23	2.74
-10	-5	-5	34.36	3.22
-10	-5	-5	28.61	3.83
10	5	5	37.30	2.87
10	5	5	37.89	2.94
10	5	5	29.35	2.58
-11	-5	-5	51.27	2.84
-11	-5	-5	50.98	3.30
-11	-5	-5	51.14	3.60
-11	-5	-5	49.17	4.11
-11	-5	-5	53.47	6.51
11	5	5	53.20	3.79
11	5	5	49.96	2.85
11	5	5	39.64	4.30
11	5	5	55.84	3.80
-12	-5	-5	16.79	3.54
-12	-5	-5	19.85	1.67
-12	-5	-5	20.81	3.65
-12	-5	-5	22.02	2.70
-12	-5	-5	20.15	2.51
12	5	5	17.92	3.05
12	5	5	17.89	3.08
12	5	5	15.88	1.65
-13	-5	-5	57.18	5.05
-13	-5	-5	46.98	2.83
-13	-5	-5	50.62	3.85
13	5	5	39.88	4.10
13	5	5	40.04	5.05
13	5	5	46.86	2.88
13	5	5	45.69	4.26
-14	-5	-5	28.32	3.49

-14	-5	-5	26.08	3.38
-14	-5	-5	29.55	4.90
-14	-5	-5	25.60	2.46
-14	-5	-5	36.17	5.35
14	5	5	20.00	2.20
-15	-5	-5	27.79	5.40
-15	-5	-5	38.80	6.31
-15	-5	-5	25.39	2.33
-11	6	5	19.50	2.40
-11	6	5	24.39	5.13
-11	6	5	20.51	2.13
-10	6	5	14.71	1.70
-10	6	5	10.57	3.33
-10	6	5	22.11	4.29
-10	6	5	11.80	1.95
-9	6	5	26.90	4.20
-9	6	5	27.36	5.11
-9	6	5	35.03	4.91
-9	6	5	30.45	2.07
-9	6	5	23.71	2.58
-8	6	5	20.29	1.66
-8	6	5	19.37	1.82
-8	6	5	22.11	4.37
-8	6	5	19.28	3.04
-7	6	5	23.43	3.80
-7	6	5	21.83	3.53
-7	6	5	27.35	1.86
-7	6	5	26.47	1.74
-7	6	5	18.26	2.82
-6	6	5	1.97	2.01
-6	6	5	6.24	1.87
-6	6	5	6.05	1.97
-6	6	5	6.62	1.04
-6	6	5	7.97	1.20
-5	6	5	41.42	3.29
-5	6	5	35.65	3.33
-5	6	5	41.20	2.30
-5	6	5	43.01	2.40
-4	6	5	51.34	2.74
-3	6	5	58.38	3.04
-2	6	5	40.84	2.20
-1	6	5	117.74	5.82
0	-6	-5	67.61	3.38
0	6	5	63.97	3.33

-1	-6	-5	93.19	4.55
1	6	5	88.46	4.51
-2	-6	-5	136.94	6.49
2	6	5	130.95	6.57
2	6	5	131.15	6.47
2	6	5	127.77	6.60
-3	-6	-5	74.97	4.03
-3	-6	-5	78.47	3.94
3	6	5	77.84	4.13
3	6	5	80.87	4.09
3	6	5	79.24	3.92
-4	-6	-5	87.38	4.71
-4	-6	-5	81.38	4.45
-4	-6	-5	88.15	4.31
4	6	5	82.22	4.51
4	6	5	88.58	4.49
4	6	5	87.25	4.54
4	6	5	87.85	4.29
-5	-6	-5	69.95	3.67
-5	-6	-5	81.05	4.10
5	6	5	67.12	4.02
5	6	5	74.82	3.99
5	6	5	70.82	3.65
5	6	5	73.60	3.93
-6	-6	-5	144.43	7.09
-6	-6	-5	137.64	6.73
-6	-6	-5	128.21	7.04
6	6	5	140.08	7.02
6	6	5	139.07	6.72
6	6	5	127.68	7.05
6	6	5	141.03	6.99
-7	-6	-5	73.07	3.87
-7	-6	-5	85.54	4.43
-7	-6	-5	71.40	4.41
7	6	5	81.37	4.28
7	6	5	69.43	4.47
7	6	5	74.45	3.87
7	6	5	82.71	4.35
-8	-6	-5	106.81	5.71
-8	-6	-5	103.72	5.16
-8	-6	-5	95.58	5.72
8	6	5	107.33	5.56
8	6	5	98.68	5.81
8	6	5	105.42	5.67

8	6	5	105.25	5.18
-9	-6	-5	86.72	4.85
-9	-6	-5	79.02	4.96
-9	-6	-5	77.67	4.07
9	6	5	82.62	4.61
9	6	5	69.22	4.99
9	6	5	77.80	4.11
9	6	5	86.62	4.73
-10	-6	-5	29.22	1.85
-10	-6	-5	28.59	3.53
-10	-6	-5	29.29	2.75
-10	-6	-5	35.74	3.25
10	6	5	33.81	2.78
10	6	5	33.58	2.97
10	6	5	28.07	2.59
10	6	5	22.96	3.53
-11	-6	-5	11.92	1.72
-11	-6	-5	15.94	3.85
-11	-6	-5	16.57	3.14
-11	-6	-5	12.33	1.30
-11	-6	-5	12.24	1.91
11	6	5	18.26	2.89
11	6	5	10.34	1.30
11	6	5	9.67	2.65
11	6	5	11.59	1.70
-12	-6	-5	25.49	3.00
-12	-6	-5	25.79	1.81
-12	-6	-5	25.06	4.38
-12	-6	-5	24.24	3.81
-12	-6	-5	24.69	2.53
12	6	5	20.30	4.01
12	6	5	21.16	3.31
12	6	5	22.81	1.80
12	6	5	23.24	2.98
-13	-6	-5	35.70	3.57
-13	-6	-5	43.56	5.26
-13	-6	-5	33.15	2.33
-13	-6	-5	45.02	4.73
13	6	5	32.42	2.39
13	6	5	39.28	4.05
13	6	5	39.80	3.93
13	6	5	25.21	4.70
-14	-6	-5	23.94	1.97
-14	-6	-5	26.14	3.45

-14	-6	-5	25.23	3.28
-14	-6	-5	30.51	5.88
-14	-6	-5	30.56	5.25
14	6	5	22.62	3.94
14	6	5	19.45	2.08
14	6	5	20.94	3.66
-15	-6	-5	14.46	1.87
-15	-6	-5	24.04	4.28
-15	-6	-5	21.93	5.06
-10	7	5	16.59	2.32
-10	7	5	26.14	4.74
-10	7	5	18.67	2.08
-10	7	5	15.97	3.75
-9	7	5	23.77	3.93
-9	7	5	24.56	2.13
-9	7	5	25.35	5.31
-9	7	5	26.66	2.30
-8	7	5	31.26	4.64
-8	7	5	39.25	2.61
-8	7	5	39.18	5.17
-8	7	5	33.54	4.74
-8	7	5	38.54	2.45
-7	7	5	18.25	3.13
-7	7	5	19.21	3.66
-7	7	5	23.10	4.12
-7	7	5	22.03	1.71
-6	7	5	68.55	3.55
-6	7	5	61.56	4.92
-6	7	5	68.74	3.65
-6	7	5	62.38	4.65
-6	7	5	65.00	4.86
-5	7	5	36.61	3.74
-5	7	5	42.13	2.37
-5	7	5	41.72	2.49
-4	7	5	96.86	4.94
-4	7	5	95.33	4.85
-4	7	5	94.04	5.60
-3	7	5	59.68	3.15
-1	7	5	107.55	5.36
0	7	5	117.09	5.81
-1	-7	-5	75.11	3.83
1	7	5	74.70	3.79
-2	-7	-5	162.02	8.11
2	7	5	169.91	8.19

2	7	5	162.96	8.08
-3	-7	-5	75.67	3.82
-3	-7	-5	73.75	4.14
-3	-7	-5	76.30	4.07
3	7	5	74.47	3.99
3	7	5	75.63	3.79
3	7	5	74.68	4.04
-4	-7	-5	79.87	4.03
-4	-7	-5	76.42	4.35
4	7	5	81.27	4.31
4	7	5	81.09	4.24
4	7	5	79.55	4.01
-5	-7	-5	79.92	4.02
-5	-7	-5	84.24	4.49
-5	-7	-5	74.89	4.41
5	7	5	80.45	4.36
5	7	5	76.63	4.37
5	7	5	79.85	4.00
5	7	5	80.83	4.29
-6	-7	-5	89.00	4.67
-6	-7	-5	101.08	5.11
-6	-7	-5	88.19	5.08
6	7	5	96.33	4.98
6	7	5	91.80	4.65
6	7	5	88.84	5.10
6	7	5	97.43	5.06
-7	-7	-5	44.77	3.23
-7	-7	-5	48.58	2.60
-7	-7	-5	52.60	3.27
7	7	5	52.14	3.19
7	7	5	44.91	3.28
7	7	5	52.42	3.09
7	7	5	47.94	2.58
-8	-7	-5	105.11	6.04
-8	-7	-5	124.50	6.11
-8	-7	-5	101.31	5.47
8	7	5	115.90	6.00
8	7	5	112.22	5.89
8	7	5	102.06	5.47
-9	-7	-5	49.64	3.69
-9	-7	-5	55.44	3.68
-9	-7	-5	46.03	2.59
9	7	5	43.60	2.60
9	7	5	48.52	3.23

9	7	5	53.48	3.49
9	7	5	40.72	3.80
-10	-7	-5	98.11	5.86
-10	-7	-5	101.99	5.77
-10	-7	-5	97.48	4.85
10	7	5	84.12	4.90
10	7	5	101.44	5.44
10	7	5	89.75	6.04
-11	-7	-5	43.05	3.93
-11	-7	-5	38.32	2.83
-11	-7	-5	41.02	2.38
11	7	5	36.48	4.32
11	7	5	39.68	2.43
11	7	5	44.89	3.72
11	7	5	43.50	3.32
-12	-7	-5	54.72	3.73
-12	-7	-5	54.67	3.14
-12	-7	-5	65.10	5.25
-12	-7	-5	66.16	4.93
-12	-7	-5	52.56	4.20
12	7	5	58.02	4.58
12	7	5	51.89	3.16
12	7	5	58.46	4.18
12	7	5	56.83	5.14
-13	-7	-5	20.18	3.79
-13	-7	-5	17.69	1.67
-13	-7	-5	24.94	4.50
-13	-7	-5	18.75	3.21
-13	-7	-5	17.25	2.66
13	7	5	10.20	3.31
13	7	5	16.66	1.72
13	7	5	20.36	3.25
13	7	5	14.41	2.52
-14	-7	-5	26.08	2.11
-14	-7	-5	31.20	3.79
-14	-7	-5	34.45	5.83
-14	-7	-5	34.56	5.40
-14	-7	-5	30.02	3.40
14	7	5	24.73	4.34
14	7	5	23.82	2.22
14	7	5	35.43	3.92
-15	-7	-5	15.66	4.75
-15	-7	-5	8.86	1.41
-15	-7	-5	6.34	2.46

-15	-7	-5	16.16	3.99
15	7	5	7.80	1.15
-9	8	5	19.56	2.16
-9	8	5	18.63	2.31
-9	8	5	18.78	4.04
-9	8	5	12.57	3.59
-8	8	5	25.48	2.15
-8	8	5	21.53	3.69
-8	8	5	24.63	2.34
-8	8	5	25.87	5.14
-7	8	5	20.97	4.44
-7	8	5	19.20	1.83
-7	8	5	16.08	3.33
-7	8	5	16.03	3.06
-7	8	5	22.89	2.00
-6	8	5	33.12	4.54
-6	8	5	37.06	2.46
-6	8	5	40.34	4.64
-6	8	5	37.58	2.35
-6	8	5	32.65	3.88
-5	8	5	50.29	2.82
-5	8	5	48.75	3.15
-5	8	5	48.57	4.42
-5	8	5	47.66	4.60
-4	8	5	22.48	3.27
-4	8	5	26.13	1.74
-4	8	5	26.60	1.89
-3	8	5	38.08	3.33
-3	8	5	44.19	2.39
-2	8	5	63.91	4.18
-2	8	5	73.39	3.61
1	-8	-5	49.37	3.04
-1	8	5	48.84	3.21
-1	8	5	51.46	2.69
0	-8	-5	64.80	3.67
0	8	5	62.38	3.29
-1	-8	-5	58.38	3.00
1	8	5	53.93	3.28
-2	-8	-5	57.31	3.04
2	8	5	57.82	3.00
-3	-8	-5	86.71	4.29
-3	-8	-5	83.56	4.68
3	8	5	84.74	4.51
3	8	5	77.54	4.46

3	8	5	85.49	4.23
3	8	5	85.07	4.43
-4	-8	-5	50.24	2.65
-4	-8	-5	46.48	3.22
4	8	5	49.34	2.61
4	8	5	45.64	3.01
4	8	5	51.61	3.02
4	8	5	52.56	2.91
-5	-8	-5	68.59	3.54
-5	-8	-5	64.29	4.00
5	8	5	70.34	3.50
5	8	5	66.70	3.93
5	8	5	68.18	3.82
5	8	5	71.47	3.95
-6	-8	-5	34.33	2.81
-6	-8	-5	34.63	2.05
-6	-8	-5	37.63	2.75
6	8	5	38.63	2.70
6	8	5	38.77	2.53
6	8	5	34.96	2.72
6	8	5	36.06	2.02
-7	-8	-5	58.89	4.07
-7	-8	-5	70.68	4.09
-7	-8	-5	65.62	3.46
7	8	5	71.64	4.05
7	8	5	69.47	3.89
7	8	5	66.13	3.43
7	8	5	63.24	4.17
-8	-8	-5	77.03	4.43
-8	-8	-5	70.15	3.70
-8	-8	-5	67.64	4.40
8	8	5	69.25	4.51
8	8	5	75.82	4.39
8	8	5	65.14	3.74
8	8	5	75.11	4.19
-9	-8	-5	37.50	3.21
-9	-8	-5	33.17	3.23
-9	-8	-5	33.76	2.04
9	8	5	35.69	2.77
9	8	5	28.23	2.12
9	8	5	35.24	3.36
9	8	5	36.75	3.08
-10	-8	-5	30.01	3.30
-10	-8	-5	35.04	3.37

-10	-8	-5	23.78	1.78
10	8	5	25.45	1.79
10	8	5	22.99	3.64
10	8	5	28.02	2.71
10	8	5	32.57	3.60
-11	-8	-5	79.75	5.26
-11	-8	-5	71.02	3.93
-11	-8	-5	80.98	5.64
11	8	5	72.61	5.57
11	8	5	71.10	3.95
11	8	5	74.39	4.71
11	8	5	75.76	5.16
-12	-8	-5	37.58	2.44
-12	-8	-5	47.03	4.48
-12	-8	-5	35.33	2.96
-12	-8	-5	45.08	4.70
12	8	5	42.98	4.28
12	8	5	36.18	4.76
12	8	5	38.03	2.48
12	8	5	42.71	3.62
-13	-8	-5	27.08	5.08
-13	-8	-5	25.80	1.99
-13	-8	-5	29.70	2.91
13	8	5	24.09	2.03
13	8	5	28.00	4.22
13	8	5	32.23	3.52
13	8	5	21.59	3.80
-14	-8	-5	21.73	3.89
-14	-8	-5	21.31	1.91
-14	-8	-5	20.24	4.66
-14	-8	-5	21.17	3.68
14	8	5	26.10	3.75
14	8	5	16.65	3.02
14	8	5	19.04	1.93
-15	-8	-5	11.17	2.11
-15	-8	-5	7.41	3.87
-15	-8	-5	12.23	4.89
15	8	5	10.91	1.86
-8	9	5	14.76	3.45
-8	9	5	20.36	2.39
-8	9	5	16.94	3.80
-7	9	5	37.84	2.66
-7	9	5	31.65	4.59
-7	9	5	31.62	5.31

-7	9	5	23.19	4.21
-7	9	5	31.61	2.47
-6	9	5	24.18	4.76
-6	9	5	29.45	2.22
-6	9	5	31.75	2.37
-6	9	5	28.67	3.99
-5	9	5	67.45	5.50
-5	9	5	67.61	3.76
-5	9	5	70.00	3.89
-4	9	5	17.35	3.50
-4	9	5	27.12	1.84
-4	9	5	25.14	1.97
-3	9	5	49.67	4.00
-3	9	5	51.42	3.35
-3	9	5	55.29	2.94
-2	9	5	50.84	2.73
-2	9	5	46.07	3.57
1	-9	-5	82.92	4.75
-1	9	5	84.76	4.91
-1	9	5	87.78	4.39
0	-9	-5	65.36	3.77
0	9	5	64.80	3.31
0	9	5	57.86	3.76
-1	-9	-5	132.88	6.85
1	9	5	128.16	6.80
1	9	5	129.92	6.49
-2	-9	-5	80.60	4.09
-2	-9	-5	80.20	4.54
2	9	5	79.39	4.02
2	9	5	74.63	4.38
-3	-9	-5	141.93	6.83
-3	-9	-5	137.16	7.25
3	9	5	136.95	6.78
3	9	5	134.43	7.03
3	9	5	129.28	7.04
3	9	5	140.56	6.95
-4	-9	-5	77.83	4.46
-4	-9	-5	74.34	3.74
-4	-9	-5	69.28	4.29
4	9	5	65.76	4.07
4	9	5	72.70	3.95
4	9	5	71.62	3.68
4	9	5	69.64	4.07
-5	-9	-5	140.89	7.79

-5	-9	-5	144.78	7.66
-5	-9	-5	153.03	7.38
5	9	5	151.22	7.67
5	9	5	152.91	7.59
5	9	5	146.38	7.70
5	9	5	149.59	7.33
-6	-9	-5	91.82	5.11
-6	-9	-5	82.28	5.09
-6	-9	-5	84.29	5.13
-6	-9	-5	91.95	4.57
6	9	5	85.81	5.09
6	9	5	93.20	5.02
6	9	5	92.11	4.88
6	9	5	90.85	4.52
-7	-9	-5	106.10	5.32
-7	-9	-5	101.69	5.91
-7	-9	-5	106.47	5.62
-7	-9	-5	107.48	5.83
7	9	5	104.86	5.27
7	9	5	104.71	5.67
7	9	5	108.96	5.83
7	9	5	99.88	5.91
-8	-9	-5	83.82	4.77
-8	-9	-5	85.08	5.02
-8	-9	-5	81.40	5.10
-8	-9	-5	81.98	4.35
8	9	5	90.06	5.04
8	9	5	84.80	5.20
8	9	5	81.39	4.32
8	9	5	89.39	4.83
-9	-9	-5	63.88	4.59
-9	-9	-5	74.03	4.25
-9	-9	-5	74.12	4.58
-9	-9	-5	69.12	3.70
9	9	5	67.35	4.88
9	9	5	67.76	3.64
9	9	5	70.12	4.32
9	9	5	73.06	4.53
-10	-9	-5	11.32	1.32
-10	-9	-5	11.19	2.00
-10	-9	-5	9.64	2.32
10	9	5	10.56	1.82
10	9	5	9.32	1.18
10	9	5	10.73	1.62

10	9	5	8.98	2.40
-11	-9	-5	61.62	3.61
-11	-9	-5	70.07	5.03
-11	-9	-5	72.48	4.97
11	9	5	60.14	5.32
11	9	5	66.92	4.44
11	9	5	76.06	4.87
11	9	5	63.22	3.60
-12	-9	-5	46.15	4.30
-12	-9	-5	44.57	4.63
-12	-9	-5	36.59	2.44
-12	-9	-5	34.60	2.99
12	9	5	35.23	2.45
12	9	5	41.44	3.59
12	9	5	36.57	4.28
12	9	5	38.05	4.87
-13	-9	-5	45.86	3.71
-13	-9	-5	49.51	2.95
-13	-9	-5	56.05	5.20
-13	-9	-5	54.20	5.51
13	9	5	52.21	5.15
13	9	5	49.73	4.34
13	9	5	42.52	5.84
13	9	5	47.01	3.00
-14	-9	-5	23.09	4.33
-14	-9	-5	26.84	5.19
-14	-9	-5	20.62	1.96
14	9	5	16.67	1.96
14	9	5	25.97	3.91
14	9	5	13.97	3.11
-15	-9	-5	22.04	4.27
-15	-9	-5	23.06	2.71
-15	-9	-5	24.66	4.89
-15	-9	-5	22.61	3.55
15	9	5	16.28	2.04
-7	10	5	10.95	3.34
-7	10	5	9.73	1.42
-7	10	5	5.78	3.38
-6	10	5	16.89	3.26
-6	10	5	20.67	2.04
-6	10	5	13.83	3.78
-6	10	5	21.21	2.16
-6	10	5	23.43	4.78
-5	10	5	31.97	2.51

-5	10	5	33.14	4.68
-5	10	5	28.51	4.73
-5	10	5	32.66	2.32
-4	10	5	20.81	3.90
-4	10	5	23.72	1.87
-4	10	5	23.34	2.07
-3	10	5	15.12	2.46
-3	10	5	15.77	1.53
-3	10	5	16.57	1.72
-2	10	5	27.20	3.14
-2	10	5	29.35	1.91
1	-10	-5	30.56	2.72
-1	10	5	30.51	2.94
-1	10	5	30.47	1.94
0	-10	-5	44.44	3.14
0	10	5	43.45	2.49
0	10	5	43.93	3.23
-1	-10	-5	41.54	3.01
1	10	5	37.60	2.91
1	10	5	39.16	2.27
-2	-10	-5	28.61	2.71
2	10	5	24.45	2.38
2	10	5	27.84	1.71
-3	-10	-5	39.82	3.24
3	10	5	37.25	2.23
3	10	5	41.16	2.54
3	10	5	40.66	2.75
-4	-10	-5	57.60	3.72
-4	-10	-5	62.84	4.19
-4	-10	-5	62.12	3.56
-4	-10	-5	68.68	3.43
4	10	5	64.38	3.79
4	10	5	61.74	3.33
4	10	5	66.53	3.64
4	10	5	61.02	4.41
-5	-10	-5	49.31	3.06
-5	-10	-5	51.54	2.81
-5	-10	-5	48.49	3.69
5	10	5	50.93	2.73
5	10	5	50.68	3.39
5	10	5	50.33	3.31
5	10	5	49.57	3.14
-6	-10	-5	56.65	4.08
-6	-10	-5	51.35	4.02

-6	-10	-5	61.05	3.52
-6	-10	-5	60.35	3.22
6	10	5	57.45	3.92
6	10	5	61.85	3.60
6	10	5	60.73	3.14
6	10	5	60.86	3.78
-7	-10	-5	51.43	3.22
-7	-10	-5	51.71	3.85
-7	-10	-5	51.15	2.82
-7	-10	-5	45.98	3.55
7	10	5	51.51	3.35
7	10	5	51.88	3.58
7	10	5	51.04	3.74
7	10	5	47.05	2.72
-8	-10	-5	65.57	3.47
-8	-10	-5	61.61	4.44
-8	-10	-5	63.22	3.88
-8	-10	-5	66.22	4.24
8	10	5	63.02	4.24
8	10	5	66.17	4.00
8	10	5	63.04	3.37
8	10	5	62.33	4.52
-9	-10	-5	28.54	3.12
-9	-10	-5	28.83	1.94
-9	-10	-5	31.01	2.63
-9	-10	-5	26.37	3.37
9	10	5	26.57	3.54
9	10	5	25.98	1.80
9	10	5	27.09	3.17
9	10	5	27.84	2.75
-10	-10	-5	9.98	1.57
-10	-10	-5	12.61	2.81
-10	-10	-5	11.39	1.43
-10	-10	-5	11.14	2.03
10	10	5	12.85	2.51
10	10	5	14.19	2.05
10	10	5	9.24	2.65
10	10	5	9.42	1.28
-11	-10	-5	19.31	3.05
-11	-10	-5	20.46	1.79
-11	-10	-5	23.54	3.60
-11	-10	-5	24.15	2.89
11	10	5	25.70	3.01
11	10	5	19.47	1.72

11	10	5	22.13	3.53
11	10	5	20.06	3.15
-12	-10	-5	27.01	2.14
-12	-10	-5	33.08	4.14
-12	-10	-5	27.96	4.36
12	10	5	33.82	3.43
12	10	5	28.32	2.15
12	10	5	34.02	4.30
12	10	5	22.63	4.73
-13	-10	-5	15.41	1.84
-13	-10	-5	18.14	3.67
-13	-10	-5	23.79	4.52
13	10	5	16.92	2.92
13	10	5	20.05	3.52
13	10	5	17.17	1.92
13	10	5	15.13	3.79
-14	-10	-5	31.03	5.42
-14	-10	-5	26.28	3.37
-14	-10	-5	34.01	5.61
-14	-10	-5	27.13	2.47
14	10	5	31.20	5.48
14	10	5	27.51	2.36
14	10	5	34.99	4.29
-15	-10	-5	13.73	4.60
-15	-10	-5	7.33	1.32
-15	-10	-5	13.50	4.11
-15	-10	-5	7.09	2.05
15	10	5	3.91	1.08
-6	11	5	23.79	2.54
-6	11	5	17.09	3.51
-5	11	5	16.08	2.13
-4	11	5	43.35	2.88
-4	11	5	44.01	4.97
-4	11	5	46.46	3.05
-3	11	5	25.70	1.95
-3	11	5	18.78	3.76
-3	11	5	26.28	2.21
-2	11	5	46.65	2.70
-2	11	5	41.56	3.95
-2	11	5	45.29	2.92
-1	11	5	22.24	3.04
-1	11	5	24.95	1.76
-1	-11	-5	37.15	3.07
1	11	5	31.51	3.00

-2	-11	-5	33.54	3.14
2	11	5	30.90	2.80
-3	-11	-5	41.36	3.49
3	11	5	41.55	2.37
3	11	5	36.41	3.06
3	11	5	44.11	2.66
-4	-11	-5	162.76	8.76
4	11	5	160.50	8.36
4	11	5	166.18	8.28
4	11	5	164.03	8.07
4	11	5	159.09	8.47
-5	-11	-5	60.58	3.58
-5	-11	-5	64.57	3.37
-5	-11	-5	58.73	4.36
5	11	5	59.28	3.94
5	11	5	59.94	3.63
5	11	5	61.44	3.26
5	11	5	59.30	3.80
-6	-11	-5	56.14	3.42
-6	-11	-5	54.14	4.20
-6	-11	-5	51.36	4.07
-6	-11	-5	57.34	3.12
6	11	5	55.06	3.73
6	11	5	56.95	3.52
6	11	5	57.25	3.03
6	11	5	55.53	3.96
-7	-11	-5	38.25	3.75
-7	-11	-5	37.90	3.26
-7	-11	-5	41.11	2.91
-7	-11	-5	45.75	2.54
7	11	5	41.96	3.05
7	11	5	44.30	3.31
7	11	5	39.55	3.61
7	11	5	41.78	2.41
-8	-11	-5	67.30	4.67
-8	-11	-5	63.64	3.98
-8	-11	-5	58.11	4.22
-8	-11	-5	64.75	3.55
8	11	5	63.91	4.43
8	11	5	65.53	4.06
8	11	5	65.08	3.44
8	11	5	65.24	4.60
-9	-11	-5	19.57	2.48
-9	-11	-5	21.97	1.73

-9	-11	-5	18.50	3.84
-9	-11	-5	16.61	2.91
9	11	5	16.78	3.13
9	11	5	20.23	2.59
9	11	5	19.12	1.55
9	11	5	19.24	3.54
-10	-11	-5	27.66	3.50
-10	-11	-5	28.87	2.88
-10	-11	-5	27.98	4.00
-10	-11	-5	28.00	2.11
10	11	5	33.79	3.72
10	11	5	28.72	1.99
10	11	5	22.41	4.03
10	11	5	33.05	3.06
-11	-11	-5	28.29	2.18
-11	-11	-5	29.82	3.12
-11	-11	-5	29.86	4.26
-11	-11	-5	29.55	3.78
11	11	5	32.01	3.25
11	11	5	36.28	4.00
11	11	5	24.62	3.59
11	11	5	29.36	2.10
-12	-11	-5	29.84	4.59
-12	-11	-5	32.78	3.42
-12	-11	-5	29.80	2.21
-12	-11	-5	30.86	4.25
12	11	5	25.75	3.95
12	11	5	28.12	3.49
12	11	5	26.75	2.20
12	11	5	30.45	4.35
-13	-11	-5	14.95	2.28
-13	-11	-5	13.18	2.96
-13	-11	-5	10.91	3.73
13	11	5	14.25	1.94
13	11	5	19.29	3.52
13	11	5	15.29	2.96
-14	-11	-5	13.53	3.46
-14	-11	-5	16.79	4.21
-14	-11	-5	16.37	2.07
14	11	5	14.57	2.58
14	11	5	13.01	3.42
14	11	5	13.39	2.04
-15	-11	-5	6.42	3.98
-15	-11	-5	6.48	1.22

-15	-11	-5	6.96	4.48
-5	12	5	20.61	2.48
-4	12	5	9.34	1.34
-3	12	5	22.57	2.32
-3	12	5	23.30	4.40
-2	12	5	21.31	1.89
-2	12	5	23.55	2.17
-2	12	5	19.50	2.89
1	-12	-5	36.65	3.45
-1	12	5	33.52	2.30
-1	12	5	32.64	3.65
-1	12	5	35.79	2.61
0	-12	-5	43.66	3.52
0	12	5	43.58	3.79
-1	-12	-5	56.70	4.92
1	12	5	46.38	3.83
-2	-12	-5	15.53	2.40
2	12	5	14.44	2.02
-3	-12	-5	18.76	3.01
3	12	5	16.40	2.35
3	12	5	17.04	1.89
3	12	5	14.64	2.53
-4	-12	-5	15.39	2.26
-4	-12	-5	11.21	2.04
4	12	5	15.87	2.13
4	12	5	13.27	1.32
4	12	5	13.16	2.44
4	12	5	14.76	1.86
-5	-12	-5	37.70	2.90
-5	-12	-5	34.22	3.79
5	12	5	38.23	2.26
5	12	5	36.71	3.29
5	12	5	38.23	2.95
-6	-12	-5	27.80	3.59
-6	-12	-5	27.52	2.35
6	12	5	28.17	2.51
6	12	5	25.58	3.16
6	12	5	27.61	2.74
6	12	5	25.42	1.81
-7	-12	-5	33.09	3.45
-7	-12	-5	39.80	2.95
7	12	5	43.51	3.42
7	12	5	41.48	3.84
7	12	5	40.49	2.42

7	12	5	43.17	3.11
-8	-12	-5	28.49	3.34
-8	-12	-5	35.32	2.90
-8	-12	-5	38.45	4.08
-8	-12	-5	34.72	3.05
8	12	5	38.51	2.25
8	12	5	37.12	3.96
8	12	5	38.27	3.51
8	12	5	39.57	3.10
-9	-12	-5	32.24	4.14
-9	-12	-5	28.41	3.21
-9	-12	-5	30.74	2.86
9	12	5	30.03	3.52
9	12	5	27.52	2.01
9	12	5	30.02	4.02
9	12	5	28.22	2.99
-10	-12	-5	18.50	3.12
-10	-12	-5	12.97	2.16
-10	-12	-5	15.67	2.66
10	12	5	21.47	3.49
10	12	5	17.74	1.70
10	12	5	18.84	3.22
10	12	5	16.40	2.87
-11	-12	-5	35.95	3.96
-11	-12	-5	31.30	3.36
-11	-12	-5	31.74	4.68
-11	-12	-5	33.54	3.08
11	12	5	30.96	2.29
11	12	5	27.10	4.82
11	12	5	32.32	3.49
11	12	5	32.26	4.14
-12	-12	-5	17.49	3.24
-12	-12	-5	16.29	3.55
-12	-12	-5	16.90	1.91
-12	-12	-5	13.50	2.74
12	12	5	13.98	1.90
12	12	5	16.47	2.90
12	12	5	15.06	3.73
12	12	5	21.24	3.48
-13	-12	-5	15.91	3.12
-13	-12	-5	20.47	4.05
-13	-12	-5	16.38	2.03
13	12	5	15.12	3.09
13	12	5	14.89	2.49

-14	-12	-5	14.82	3.94
-14	-12	-5	12.19	2.12
-14	-12	-5	15.30	4.13
14	12	5	11.32	2.64
14	12	5	8.70	3.47
-4	13	5	10.12	1.52
-3	13	5	25.22	2.57
-2	13	5	19.54	2.28
-2	13	5	18.45	3.43
-1	13	5	43.73	4.53
-1	13	5	43.55	3.09
0	-13	-5	31.82	3.58
0	13	5	32.98	2.38
0	13	5	37.10	2.72
0	13	5	34.10	3.82
-1	-13	-5	52.64	4.21
1	13	5	46.67	4.06
-2	-13	-5	23.63	3.43
2	13	5	24.09	3.14
-3	-13	-5	43.87	4.09
3	13	5	43.81	2.58
3	13	5	38.95	3.59
-4	-13	-5	18.46	3.46
4	13	5	17.84	2.84
4	13	5	19.10	2.24
4	13	5	18.43	2.44
-5	-13	-5	25.78	3.65
5	13	5	22.12	2.54
5	13	5	21.47	2.37
5	13	5	18.75	3.03
-6	-13	-5	27.95	4.05
-6	-13	-5	27.44	2.44
6	13	5	26.96	3.48
6	13	5	28.34	2.63
6	13	5	26.16	2.88
-7	-13	-5	37.34	4.31
-7	-13	-5	38.62	2.97
7	13	5	42.30	3.22
7	13	5	35.95	3.49
7	13	5	39.57	4.10
-8	-13	-5	35.04	4.38
-8	-13	-5	30.32	2.81
-8	-13	-5	23.15	3.21
-8	-13	-5	33.21	2.31

8	13	5	29.10	3.44
8	13	5	29.54	3.88
8	13	5	31.58	3.00
-9	-13	-5	44.23	2.72
-9	-13	-5	39.20	4.79
-9	-13	-5	35.09	3.66
-9	-13	-5	38.44	3.30
9	13	5	38.57	4.16
9	13	5	41.39	4.58
-10	-13	-5	27.80	3.73
-10	-13	-5	29.41	4.93
-10	-13	-5	26.12	3.08
-10	-13	-5	27.06	2.30
10	13	5	31.02	4.04
10	13	5	30.65	4.03
10	13	5	33.09	4.87
-11	-13	-5	28.08	2.35
-11	-13	-5	27.29	3.41
-11	-13	-5	26.94	4.20
-11	-13	-5	26.76	4.86
11	13	5	28.47	3.48
11	13	5	27.49	4.22
11	13	5	25.91	5.14
-12	-13	-5	14.38	2.03
-12	-13	-5	12.32	2.92
-12	-13	-5	20.18	3.78
-12	-13	-5	11.35	2.16
12	13	5	13.72	2.29
12	13	5	11.98	3.04
-13	-13	-5	11.81	2.51
-13	-13	-5	13.59	3.98
-13	-13	-5	16.32	2.11
13	13	5	10.89	2.45
13	13	5	5.72	3.36
-14	-13	-5	19.01	5.69
-14	-13	-5	16.94	2.33
-14	-13	-5	14.16	4.46
14	13	5	11.01	2.69
14	13	5	8.56	3.74
-2	14	5	25.49	2.65
-1	14	5	13.70	2.25
0	-14	-5	26.06	4.01
0	14	5	27.30	2.63
0	14	5	32.26	6.27

-1	-14	-5	20.50	3.70
1	14	5	18.17	3.81
-2	-14	-5	29.17	4.06
2	14	5	28.96	3.78
-3	-14	-5	19.62	3.86
3	14	5	23.34	1.87
3	14	5	20.51	3.30
3	14	5	24.55	2.81
-4	-14	-5	28.71	4.10
4	14	5	28.34	3.10
4	14	5	24.85	3.37
4	14	5	24.29	1.98
-5	-14	-5	14.34	2.89
-5	-14	-5	9.45	1.58
5	14	5	10.82	1.53
5	14	5	14.57	2.37
5	14	5	15.78	2.51
5	14	5	14.65	2.51
-6	-14	-5	24.30	4.35
-6	-14	-5	22.49	2.64
-6	-14	-5	20.62	2.75
6	14	5	22.78	2.79
6	14	5	23.77	1.87
6	14	5	22.95	3.63
6	14	5	23.79	2.93
-7	-14	-5	15.74	3.24
-7	-14	-5	8.45	1.75
-7	-14	-5	13.43	2.37
7	14	5	14.01	1.83
7	14	5	12.22	1.62
7	14	5	15.93	2.83
7	14	5	9.81	1.89
-8	-14	-5	17.71	3.47
-8	-14	-5	21.00	2.70
-8	-14	-5	24.84	3.59
8	14	5	20.71	3.40
8	14	5	21.68	3.06
8	14	5	19.09	1.92
8	14	5	21.12	3.25
-9	-14	-5	25.91	3.75
-9	-14	-5	16.10	2.45
-9	-14	-5	18.47	2.88
9	14	5	21.07	3.24
9	14	5	19.00	3.63

9	14	5	21.24	1.93
9	14	5	23.03	3.90
-10	-14	-5	17.46	3.13
-10	-14	-5	26.70	5.21
-10	-14	-5	17.28	2.73
10	14	5	23.31	4.28
10	14	5	24.38	3.46
10	14	5	25.09	2.12
-11	-14	-5	6.64	2.64
-11	-14	-5	9.16	2.09
-11	-14	-5	14.90	2.11
-11	-14	-5	10.17	3.89
11	14	5	13.51	2.96
11	14	5	9.96	2.18
11	14	5	14.07	1.96
-12	-14	-5	14.18	2.43
-12	-14	-5	14.03	2.97
12	14	5	16.21	2.17
12	14	5	18.49	3.47
12	14	5	15.32	2.56
-13	-14	-5	6.29	2.61
-13	-14	-5	12.85	4.05
-13	-14	-5	13.49	2.31
-13	-14	-5	6.38	3.22
13	14	5	10.59	2.71
13	14	5	11.16	1.49
13	14	5	14.58	3.77
0	-15	-5	17.96	3.20
0	15	5	15.45	2.49
-1	-15	-5	20.51	3.19
1	15	5	21.00	2.49
1	15	5	20.44	3.01
-2	-15	-5	33.14	4.75
2	15	5	35.33	2.94
2	15	5	30.94	3.27
-3	-15	-5	26.52	4.56
3	15	5	26.20	3.19
-4	-15	-5	35.61	4.87
4	15	5	45.40	6.04
4	15	5	28.52	4.22
-5	-15	-5	23.89	4.72
5	15	5	19.98	2.83
5	15	5	21.99	4.42
5	15	5	19.91	2.97

-6	-15	-5	23.22	4.73
6	15	5	20.77	3.06
6	15	5	26.75	4.97
6	15	5	22.07	3.01
-7	-15	-5	15.83	3.58
7	15	5	18.20	2.72
7	15	5	16.83	3.00
7	15	5	11.21	2.10
-8	-15	-5	22.68	4.04
8	15	5	21.62	3.28
8	15	5	14.88	2.40
8	15	5	19.06	2.12
-9	-15	-5	24.90	4.14
9	15	5	23.50	3.73
9	15	5	21.77	4.01
9	15	5	22.09	2.16
-10	-15	-5	20.64	4.29
10	15	5	19.20	3.87
10	15	5	18.85	2.12
10	15	5	17.48	2.61
-11	-15	-5	8.86	2.36
-11	-15	-5	9.75	4.29
11	15	5	8.37	2.37
11	15	5	8.04	1.25
-12	-15	-5	14.09	4.54
12	15	5	9.14	1.41
12	15	5	10.41	2.65
-3	-16	-5	30.17	5.00
3	16	5	27.74	3.00
-4	-16	-5	9.11	3.28
-5	-16	-5	31.91	5.38
-6	-16	-5	12.75	3.45
6	16	5	7.70	1.82
-7	-16	-5	22.22	3.96
7	16	5	14.95	2.16
-8	-16	-5	13.37	4.01
8	16	5	7.65	2.02
-9	-16	-5	16.98	4.37
9	16	5	14.25	2.36
-10	-16	-5	8.25	4.19
10	16	5	5.64	2.30
8	13	-6	1.51	2.51
8	13	-6	0.28	1.25
-8	-13	6	-0.85	3.52

-8	-13	6	-0.16	2.73
-8	-13	6	-0.64	2.77
7	13	-6	0.29	0.90
7	13	-6	0.87	2.39
-7	-13	6	2.09	2.64
-7	-13	6	0.23	3.36
6	13	-6	0.28	2.26
6	13	-6	2.39	1.18
6	13	-6	-0.14	0.92
-6	-13	6	1.97	3.16
-6	-13	6	-3.45	2.58
5	13	-6	0.25	0.96
5	13	-6	0.21	2.23
5	13	-6	0.79	1.15
-5	-13	6	0.91	3.03
4	13	-6	0.66	2.46
4	13	-6	0.67	1.03
4	13	-6	2.11	3.49
4	13	-6	0.84	1.33
-4	-13	6	0.90	3.00
3	13	-6	4.43	1.09
3	13	-6	4.49	2.66
-3	-13	6	5.40	2.97
2	13	-6	3.39	1.12
2	13	-6	4.31	3.13
-2	-13	6	8.60	3.29
10	12	-6	-4.98	2.87
10	12	-6	4.26	3.57
-10	-12	6	-1.08	2.56
-10	-12	6	0.76	2.89
-10	-12	6	3.40	3.51
9	12	-6	2.53	3.22
9	12	-6	0.45	3.64
9	12	-6	1.96	2.41
-9	-12	6	-1.62	3.31
-9	-12	6	1.89	2.60
-9	-12	6	2.13	2.51
8	12	-6	2.48	2.95
8	12	-6	-0.82	2.12
-8	-12	6	-2.42	2.47
-8	-12	6	2.49	2.28
-8	-12	6	-1.50	3.30
7	12	-6	-0.06	0.93
7	12	-6	-0.65	0.75

7	12	-6	2.77	2.72
7	12	-6	1.99	3.12
-7	-12	6	1.47	2.23
-7	-12	6	-0.88	2.33
-7	-12	6	-0.82	3.07
6	12	-6	-0.79	0.78
6	12	-6	2.11	2.53
6	12	-6	-0.35	1.86
6	12	-6	-0.41	0.94
-6	-12	6	-3.09	2.86
-6	-12	6	-2.00	2.22
-6	-12	6	2.36	2.20
5	12	-6	-0.17	1.86
5	12	-6	-0.59	0.79
-5	-12	6	1.53	2.90
-5	-12	6	-2.03	2.27
-5	-12	6	1.65	2.00
4	12	-6	-0.56	1.04
4	12	-6	-0.17	1.97
4	12	-6	-1.09	0.78
4	12	-6	3.33	2.24
-4	-12	6	-1.12	2.32
-4	-12	6	-3.93	2.71
-4	-12	6	0.39	2.03
3	12	-6	-0.52	2.12
3	12	-6	7.31	2.42
3	12	-6	2.55	0.93
3	12	-6	3.30	1.15
-3	-12	6	-2.13	2.33
-3	-12	6	4.89	2.72
2	12	-6	2.10	0.91
2	12	-6	-0.11	2.47
2	12	-6	4.55	2.62
-2	-12	6	0.43	2.70
-2	-12	6	0.02	2.41
1	12	-6	1.69	0.96
1	12	-6	0.65	2.64
-1	-12	6	3.91	2.93
0	12	-6	0.97	0.96
0	12	-6	-0.58	2.79
0	-12	6	-3.00	2.63
-1	12	-6	2.57	1.00
-1	12	-6	3.24	2.83
1	-12	6	4.16	3.06

11	11	-6	2.94	2.62
-11	-11	6	3.68	3.71
-11	-11	6	-0.27	2.88
10	11	-6	9.26	3.07
10	11	-6	3.44	2.46
-10	-11	6	7.13	2.52
-10	-11	6	2.06	3.35
-10	-11	6	1.62	2.45
9	11	-6	4.87	3.00
9	11	-6	3.10	2.10
-9	-11	6	3.93	2.38
-9	-11	6	4.93	2.23
-9	-11	6	4.96	3.13
8	11	-6	5.16	2.02
8	11	-6	8.10	2.73
-8	-11	6	7.87	2.28
-8	-11	6	9.07	2.98
-8	-11	6	10.47	2.19
7	11	-6	5.21	3.42
7	11	-6	4.12	2.40
7	11	-6	3.99	0.87
7	11	-6	4.65	1.87
-7	-11	6	5.27	2.00
-7	-11	6	6.07	2.90
-7	-11	6	2.29	2.10
6	11	-6	7.88	2.20
6	11	-6	2.44	2.62
6	11	-6	3.13	0.71
6	11	-6	3.12	1.67
6	11	-6	1.59	3.09
6	11	-6	4.75	0.90
-6	-11	6	4.82	1.98
-6	-11	6	4.25	1.88
-6	-11	6	2.16	2.77
5	11	-6	0.55	0.83
5	11	-6	0.64	1.64
5	11	-6	0.29	0.67
5	11	-6	-1.04	2.46
5	11	-6	1.95	2.81
5	11	-6	-0.82	2.33
-5	-11	6	0.65	1.66
-5	-11	6	-0.86	1.83
-5	-11	6	-2.95	2.56
4	11	-6	4.15	1.97

4	11	-6	2.63	1.91
4	11	-6	1.26	0.93
4	11	-6	1.54	0.73
4	11	-6	3.54	2.69
-4	-11	6	1.52	2.66
-4	-11	6	1.67	1.92
-4	-11	6	1.87	1.60
3	11	-6	3.25	2.60
3	11	-6	1.64	1.67
3	11	-6	0.45	0.72
3	11	-6	1.15	2.25
3	11	-6	3.45	2.06
-3	-11	6	1.22	1.62
-3	-11	6	0.68	1.96
-3	-11	6	-0.15	2.43
2	11	-6	5.90	2.01
2	11	-6	6.93	2.05
2	11	-6	5.37	2.54
2	11	-6	4.77	0.86
-2	-11	6	2.94	2.19
-2	-11	6	5.53	2.50
-2	-11	6	5.53	1.69
1	11	-6	7.54	2.12
1	11	-6	5.29	0.95
1	11	-6	7.56	2.37
-1	-11	6	4.40	2.70
-1	-11	6	6.68	2.68
0	11	-6	11.06	1.66
0	11	-6	11.50	2.24
0	-11	6	11.00	2.81
-1	11	-6	2.96	0.92
-1	11	-6	6.91	2.28
-1	11	-6	4.22	2.62
1	-11	6	5.21	2.71
-2	11	-6	8.03	1.15
-2	11	-6	10.08	2.76
-3	11	-6	5.95	2.65
-3	11	-6	0.79	0.99
-12	-10	6	1.66	3.78
-12	-10	6	-1.63	2.76
11	10	-6	-1.24	2.48
11	10	-6	1.03	3.15
-11	-10	6	4.25	3.56
-11	-10	6	0.01	2.97

-11	-10	6	0.78	2.60
10	10	-6	-0.16	1.98
10	10	-6	3.53	2.82
-10	-10	6	-1.66	2.16
-10	-10	6	4.79	3.14
-10	-10	6	1.33	2.06
9	10	-6	5.91	1.91
9	10	-6	3.66	3.79
9	10	-6	4.00	2.69
-9	-10	6	0.83	2.01
-9	-10	6	3.51	2.93
-9	-10	6	7.26	1.87
8	10	-6	5.63	1.71
8	10	-6	6.12	2.41
8	10	-6	5.93	3.33
-8	-10	6	5.05	2.67
-8	-10	6	2.73	1.99
-8	-10	6	5.40	1.81
7	10	-6	5.19	1.64
7	10	-6	2.05	2.32
7	10	-6	2.80	3.14
7	10	-6	6.10	2.19
-7	-10	6	6.42	1.78
-7	-10	6	5.29	2.57
-7	-10	6	3.57	1.84
6	10	-6	0.34	1.90
6	10	-6	1.36	0.59
6	10	-6	1.27	0.67
6	10	-6	2.80	1.52
6	10	-6	0.51	2.26
-6	-10	6	1.58	2.40
-6	-10	6	0.76	1.70
-6	-10	6	1.82	1.60
5	10	-6	0.69	1.40
5	10	-6	0.20	1.73
5	10	-6	0.72	0.72
5	10	-6	0.28	0.58
-5	-10	6	-0.23	1.46
-5	-10	6	-0.54	1.60
-5	-10	6	0.39	2.35
4	10	-6	9.24	1.89
4	10	-6	8.06	1.60
4	10	-6	7.94	1.21
4	10	-6	4.18	2.09

-4	-10	6	8.06	1.79
-4	-10	6	7.94	1.65
-4	-10	6	6.34	2.40
3	10	-6	8.37	1.95
3	10	-6	9.70	1.68
3	10	-6	8.09	1.33
-3	-10	6	8.19	2.42
-3	-10	6	9.02	1.65
-3	-10	6	6.09	1.82
2	10	-6	2.91	0.72
2	10	-6	1.93	1.72
2	10	-6	1.00	2.71
2	10	-6	0.99	2.96
-2	-10	6	0.81	1.85
-2	-10	6	3.34	2.22
-2	-10	6	3.55	1.45
1	10	-6	1.97	1.81
1	10	-6	0.72	0.68
1	10	-6	0.41	2.26
1	10	-6	1.78	1.76
-1	-10	6	1.04	1.37
-1	-10	6	0.24	2.09
-1	-10	6	0.89	2.15
0	10	-6	3.88	1.74
0	10	-6	4.75	2.26
0	10	-6	4.53	2.05
0	10	-6	2.88	0.76
0	-10	6	2.44	1.47
0	-10	6	0.81	1.65
0	-10	6	2.39	2.31
-1	10	-6	6.83	2.45
-1	10	-6	4.40	0.90
-1	10	-6	7.41	1.83
-1	10	-6	6.67	2.19
1	-10	6	7.22	1.58
1	-10	6	6.70	2.41
-2	10	-6	5.35	1.91
-2	10	-6	4.89	2.41
-2	10	-6	4.93	0.96
2	-10	6	5.61	1.85
-3	10	-6	4.07	0.97
-3	10	-6	5.61	2.51
-3	10	-6	5.01	1.99
-4	10	-6	2.16	0.97

-4	10	-6	5.08	2.46
-5	10	-6	3.19	2.46
-5	10	-6	0.50	1.00
-13	-9	6	3.42	3.98
-13	-9	6	-1.11	2.99
-13	-9	6	-2.74	2.96
12	9	-6	2.85	2.21
-12	-9	6	1.60	3.53
-12	-9	6	-0.04	2.52
11	9	-6	5.87	1.95
11	9	-6	5.14	2.96
-11	-9	6	4.33	2.09
-11	-9	6	0.39	2.24
-11	-9	6	3.08	3.24
10	9	-6	5.74	2.51
10	9	-6	3.45	4.09
10	9	-6	2.10	1.69
-10	-9	6	3.06	1.84
-10	-9	6	-0.09	2.00
-10	-9	6	1.04	3.02
9	9	-6	5.13	3.44
9	9	-6	8.99	1.70
9	9	-6	7.33	2.45
-9	-9	6	10.14	2.80
-9	-9	6	6.34	1.89
-9	-9	6	9.27	1.88
8	9	-6	4.74	2.15
8	9	-6	3.47	1.50
-8	-9	6	2.43	2.52
-8	-9	6	2.76	1.57
-8	-9	6	0.08	1.65
7	9	-6	8.92	1.55
7	9	-6	8.67	2.06
-7	-9	6	9.82	2.38
-7	-9	6	7.37	1.77
6	9	-6	5.44	1.82
6	9	-6	6.55	1.42
6	9	-6	3.32	2.44
-6	-9	6	3.63	1.56
-6	-9	6	4.15	1.45
-6	-9	6	4.96	2.15
5	9	-6	21.30	3.04
5	9	-6	18.85	3.49
5	9	-6	18.00	1.42

5	9	-6	21.07	2.39
-5	-9	6	19.73	2.52
-5	-9	6	19.10	3.18
-5	-9	6	19.05	2.49
4	9	-6	23.52	3.01
4	9	-6	18.84	1.47
4	9	-6	23.69	3.35
4	9	-6	20.42	2.42
-4	-9	6	19.03	2.56
-4	-9	6	19.00	2.49
-4	-9	6	17.76	3.26
3	9	-6	47.01	4.00
3	9	-6	48.00	2.66
3	9	-6	47.76	3.38
3	9	-6	48.78	3.72
-3	-9	6	47.98	4.06
-3	-9	6	46.80	3.40
-3	-9	6	42.74	3.59
2	9	-6	20.83	2.84
2	9	-6	19.14	1.50
2	9	-6	19.98	3.04
2	9	-6	19.04	2.66
-2	-9	6	19.01	3.14
-2	-9	6	14.97	2.82
-2	-9	6	19.68	2.42
1	9	-6	14.67	2.73
1	9	-6	16.83	2.70
1	9	-6	10.41	2.29
-1	-9	6	11.88	2.30
-1	-9	6	16.33	3.01
-1	-9	6	12.00	1.97
0	9	-6	4.68	1.61
0	9	-6	3.67	2.28
0	9	-6	4.65	0.75
0	-9	6	5.29	2.09
0	-9	6	5.81	2.31
0	-9	6	4.90	1.35
-1	9	-6	18.39	3.17
-1	9	-6	17.92	1.60
-1	9	-6	18.83	2.90
1	-9	6	17.63	3.14
1	-9	6	17.71	2.38
-2	9	-6	6.99	2.03
-2	9	-6	6.66	0.94

-2	9	-6	8.00	2.25
-2	9	-6	8.27	1.83
2	-9	6	7.25	2.27
2	-9	6	6.96	1.66
-3	9	-6	14.37	1.64
-3	9	-6	14.62	2.41
-3	9	-6	12.40	2.65
-3	9	-6	8.36	1.88
-4	9	-6	8.22	1.12
-4	9	-6	7.13	1.87
-4	9	-6	8.09	2.33
-5	9	-6	13.33	1.84
-6	9	-6	6.47	2.26
-6	9	-6	3.09	1.08
-13	-8	6	-0.70	2.71
-13	-8	6	-0.05	2.88
-13	-8	6	-1.89	3.87
12	8	-6	-0.60	1.78
-12	-8	6	0.98	2.47
-12	-8	6	2.82	2.16
-12	-8	6	2.56	3.48
11	8	-6	-0.12	2.72
11	8	-6	0.82	1.64
11	8	-6	1.93	4.25
-11	-8	6	1.89	1.92
-11	-8	6	0.62	2.25
-11	-8	6	1.51	3.10
-11	-8	6	-0.27	2.08
10	8	-6	2.03	1.51
10	8	-6	-1.20	3.77
10	8	-6	1.99	2.24
-10	-8	6	0.61	2.75
-10	-8	6	1.49	1.67
-10	-8	6	0.01	1.77
9	8	-6	7.24	2.23
9	8	-6	8.52	1.49
-9	-8	6	8.74	1.70
-9	-8	6	4.16	1.73
-9	-8	6	6.53	2.54
8	8	-6	3.60	1.99
8	8	-6	4.44	1.34
8	8	-6	3.38	2.67
-8	-8	6	1.79	2.31
-8	-8	6	6.22	1.49

-8	-8	6	1.78	1.55
7	8	-6	2.18	2.40
7	8	-6	0.23	1.69
7	8	-6	1.34	1.19
-7	-8	6	1.42	1.29
-7	-8	6	-1.54	1.41
6	8	-6	2.79	2.29
6	8	-6	3.37	1.21
6	8	-6	3.11	1.65
-6	-8	6	1.08	1.35
-6	-8	6	3.97	1.87
-6	-8	6	2.04	1.24
5	8	-6	5.09	1.27
5	8	-6	4.29	2.07
5	8	-6	5.17	0.87
5	8	-6	5.56	1.53
-5	-8	6	6.62	1.90
-5	-8	6	4.12	1.30
-5	-8	6	6.05	1.44
4	8	-6	12.26	2.15
4	8	-6	9.88	1.68
4	8	-6	11.66	1.08
4	8	-6	13.80	2.03
-4	-8	6	13.38	2.79
-4	-8	6	10.69	2.07
3	8	-6	15.77	1.27
3	8	-6	16.61	2.52
3	8	-6	15.78	2.91
3	8	-6	19.40	2.22
-3	-8	6	14.12	2.10
-3	-8	6	15.53	2.32
-3	-8	6	14.61	2.77
2	8	-6	14.32	2.66
2	8	-6	17.36	2.41
2	8	-6	17.27	2.30
2	8	-6	14.00	1.26
-2	-8	6	16.42	2.17
-2	-8	6	14.60	2.05
-2	-8	6	13.08	1.72
1	8	-6	14.29	1.28
1	8	-6	17.35	2.48
1	8	-6	15.17	2.61
1	8	-6	15.02	2.41
-1	-8	6	15.70	2.14

-1	-8	6	16.53	2.13
-1	-8	6	13.86	1.80
0	8	-6	-0.17	0.51
0	8	-6	-0.45	1.38
0	8	-6	0.36	1.45
0	8	-6	0.12	1.68
0	-8	6	-0.14	2.02
0	-8	6	-0.87	1.53
0	-8	6	0.68	1.12
-1	8	-6	-0.07	0.57
-1	8	-6	-0.93	1.72
-1	8	-6	1.69	1.57
-1	8	-6	-0.42	1.36
1	-8	6	1.44	1.15
1	-8	6	-0.66	1.24
1	-8	6	0.87	1.73
-2	8	-6	5.07	1.72
-2	8	-6	3.60	0.73
-2	8	-6	3.26	1.46
2	-8	6	5.42	1.96
2	-8	6	2.64	1.79
2	-8	6	3.66	1.23
-3	8	-6	2.13	1.51
-3	8	-6	4.87	0.80
-3	8	-6	6.76	1.94
3	-8	6	4.57	1.43
-4	8	-6	7.85	1.25
-4	8	-6	13.09	2.48
-4	8	-6	8.82	2.09
-4	8	-6	9.31	1.50
-5	8	-6	8.27	1.13
-5	8	-6	8.64	3.27
-6	8	-6	11.79	2.25
-6	8	-6	7.34	1.13
-6	8	-6	1.94	3.65
6	-8	6	7.79	1.60
-7	8	-6	1.87	2.27
-7	8	-6	3.49	1.04
-13	-7	6	2.15	2.58
-13	-7	6	-1.55	3.65
-13	-7	6	3.22	2.88
12	7	-6	13.90	4.31
-12	-7	6	6.44	2.47
-12	-7	6	13.11	2.73

-12	-7	6	14.94	3.50
-12	-7	6	9.52	2.17
11	7	-6	3.07	3.80
11	7	-6	0.72	2.50
-11	-7	6	4.07	2.28
-11	-7	6	6.07	1.89
-11	-7	6	5.74	2.90
-11	-7	6	3.88	2.10
10	7	-6	7.71	1.60
10	7	-6	9.26	2.24
-10	-7	6	8.08	1.73
-10	-7	6	5.94	1.88
-10	-7	6	6.40	2.77
9	7	-6	12.41	2.12
9	7	-6	11.63	2.12
9	7	-6	10.04	2.98
-9	-7	6	7.18	1.75
-9	-7	6	8.31	2.47
-9	-7	6	14.67	2.45
8	7	-6	7.84	1.90
8	7	-6	4.45	2.50
8	7	-6	8.80	1.35
-8	-7	6	7.97	1.58
-8	-7	6	11.92	2.24
-8	-7	6	8.77	2.23
7	7	-6	5.93	1.25
7	7	-6	5.62	1.67
7	7	-6	5.85	2.39
-7	-7	6	4.84	1.39
-7	-7	6	6.29	1.99
-7	-7	6	7.25	1.31
6	7	-6	24.21	3.38
6	7	-6	20.16	3.23
6	7	-6	26.42	2.26
-6	-7	6	24.02	2.38
-6	-7	6	23.85	2.89
-6	-7	6	22.58	2.34
5	7	-6	20.23	2.57
5	7	-6	15.05	2.96
5	7	-6	19.11	2.04
-5	-7	6	18.74	2.02
-5	-7	6	15.84	3.11
-5	-7	6	17.62	2.21
4	7	-6	42.22	2.36

4	7	-6	46.66	3.55
4	7	-6	47.39	2.96
4	7	-6	41.18	3.22
-4	-7	6	43.63	2.97
-4	-7	6	42.37	3.34
-4	-7	6	42.64	2.95
3	7	-6	7.51	1.29
3	7	-6	8.49	1.42
3	7	-6	8.60	0.91
3	7	-6	8.34	1.78
-3	-7	6	7.10	1.28
-3	-7	6	6.76	1.70
-3	-7	6	6.47	1.19
2	7	-6	32.24	2.75
2	7	-6	39.02	2.69
2	7	-6	35.12	1.98
2	7	-6	32.87	2.98
-2	-7	6	35.62	3.28
-2	-7	6	31.63	2.68
-2	-7	6	33.13	3.02
1	7	-6	17.59	2.52
1	7	-6	23.13	2.37
1	7	-6	18.40	2.29
1	7	-6	17.21	1.34
-1	-7	6	16.37	2.42
-1	-7	6	18.00	2.50
-1	-7	6	21.54	2.05
0	7	-6	46.74	3.40
0	7	-6	47.88	3.53
0	7	-6	42.16	3.25
0	7	-6	46.90	2.62
0	-7	6	53.31	3.14
0	-7	6	48.14	3.49
0	-7	6	47.57	3.58
-1	7	-6	16.68	1.32
-1	7	-6	15.97	2.58
-1	7	-6	18.21	2.52
-1	7	-6	11.67	2.18
1	-7	6	18.71	2.09
1	-7	6	14.64	1.67
1	-7	6	18.94	3.24
1	-7	6	16.14	2.50
-2	7	-6	20.77	2.72
-2	7	-6	19.06	2.28

-2	7	-6	22.91	2.85
2	-7	6	23.32	2.80
2	-7	6	27.18	2.33
2	-7	6	23.82	2.01
-3	7	-6	7.06	1.20
-3	7	-6	8.68	1.92
3	-7	6	6.65	1.40
3	-7	6	6.93	1.07
3	-7	6	6.78	1.31
-4	7	-6	7.42	1.59
-4	7	-6	11.25	1.99
-5	7	-6	13.48	1.58
-5	7	-6	13.44	2.41
-5	7	-6	15.90	3.05
5	-7	6	12.64	2.28
-6	7	-6	26.18	3.97
-6	7	-6	24.78	2.55
-6	7	-6	26.03	2.10
-6	7	-6	27.57	3.53
6	-7	6	23.64	2.59
6	-7	6	30.49	2.94
-7	7	-6	15.86	1.88
-7	7	-6	14.38	2.53
7	-7	6	19.48	3.03
-8	7	-6	13.42	1.93
-8	7	-6	16.87	3.96
8	-7	6	16.40	3.44
-14	-6	6	-0.35	3.25
-14	-6	6	3.83	4.28
-14	-6	6	3.37	2.78
-13	-6	6	1.88	2.50
-13	-6	6	-0.35	3.67
-13	-6	6	1.65	2.87
12	6	-6	4.74	2.04
12	6	-6	2.29	4.44
-12	-6	6	8.46	3.32
-12	-6	6	7.16	1.96
-12	-6	6	6.46	2.27
-12	-6	6	7.20	2.60
-11	-6	6	10.06	2.28
-11	-6	6	9.31	3.08
-11	-6	6	10.46	2.16
-11	-6	6	9.47	1.85
10	6	-6	23.92	4.39

10	6	-6	24.49	3.59
10	6	-6	21.38	2.46
-10	-6	6	18.57	3.59
-10	-6	6	27.50	3.85
-10	-6	6	21.72	2.92
-10	-6	6	25.55	2.88
9	6	-6	19.26	3.13
9	6	-6	22.70	3.26
9	6	-6	18.81	2.45
-9	-6	6	17.82	2.61
-9	-6	6	25.47	2.55
-9	-6	6	16.50	2.94
-9	-6	6	25.83	3.39
8	6	-6	10.40	1.37
8	6	-6	9.70	2.58
8	6	-6	11.96	1.92
8	6	-6	9.68	2.18
-8	-6	6	13.05	2.11
-8	-6	6	9.13	1.67
-8	-6	6	10.44	2.15
-8	-6	6	8.02	1.60
7	6	-6	6.10	1.53
7	6	-6	3.98	2.09
7	6	-6	3.56	1.77
7	6	-6	6.29	1.14
-7	-6	6	3.52	1.37
-7	-6	6	4.60	1.29
-7	-6	6	7.12	1.23
-7	-6	6	5.61	1.87
6	6	-6	14.54	2.12
6	6	-6	9.32	1.83
6	6	-6	12.29	2.09
6	6	-6	14.03	1.78
-6	-6	6	14.30	1.86
-6	-6	6	12.82	1.96
-6	-6	6	13.31	1.89
5	6	-6	20.55	2.36
5	6	-6	20.92	1.90
5	6	-6	13.38	2.83
-5	-6	6	17.45	1.93
-5	-6	6	20.75	2.06
-5	-6	6	16.72	2.39
4	6	-6	20.54	2.18
4	6	-6	17.55	1.86

4	6	-6	13.28	1.96
-4	-6	6	21.17	1.97
-4	-6	6	14.16	2.26
-4	-6	6	19.31	1.88
3	6	-6	34.14	1.96
3	6	-6	36.45	2.68
3	6	-6	39.44	3.15
3	6	-6	36.95	2.54
-3	-6	6	36.44	2.52
-3	-6	6	33.84	2.89
-3	-6	6	34.70	2.48
2	6	-6	9.89	1.84
2	6	-6	9.35	0.91
2	6	-6	9.72	1.75
2	6	-6	11.07	1.79
-2	-6	6	9.32	1.59
-2	-6	6	11.18	1.63
-2	-6	6	10.83	1.81
1	6	-6	24.55	2.23
1	6	-6	20.54	1.41
1	6	-6	20.38	2.20
-1	-6	6	20.61	2.40
-1	-6	6	21.50	1.61
-1	-6	6	22.27	2.26
-1	-6	6	25.06	2.01
0	6	-6	9.91	1.87
0	6	-6	10.68	1.05
0	6	-6	15.51	2.07
0	6	-6	12.44	2.09
0	-6	6	12.30	2.12
0	-6	6	11.46	2.07
0	-6	6	13.04	1.32
-1	6	-6	9.47	1.05
-1	6	-6	9.08	1.48
-1	6	-6	10.11	1.58
-1	6	-6	6.78	1.29
1	-6	6	15.05	2.12
1	-6	6	11.69	1.75
1	-6	6	10.80	1.62
1	-6	6	9.80	1.35
-2	6	-6	15.84	2.38
-2	6	-6	14.11	1.24
-2	6	-6	11.28	1.53
-2	6	-6	15.17	1.63

-2	6	-6	14.34	2.33
2	-6	6	14.90	1.56
2	-6	6	13.86	2.07
2	-6	6	17.59	1.97
2	-6	6	12.17	2.34
-3	6	-6	6.98	1.69
-3	6	-6	6.27	1.06
-3	6	-6	7.35	1.61
-3	6	-6	4.15	1.30
3	-6	6	5.92	0.96
-4	6	-6	11.72	1.61
-4	6	-6	16.23	2.64
-4	6	-6	18.12	2.80
-4	6	-6	14.78	1.38
4	-6	6	13.97	2.19
4	-6	6	16.07	1.89
-5	6	-6	10.38	1.87
-5	6	-6	9.62	1.32
-5	6	-6	11.00	1.81
-5	6	-6	7.50	1.54
-5	6	-6	3.83	2.56
5	-6	6	13.40	2.02
-6	6	-6	20.07	2.19
-6	6	-6	18.52	3.08
-6	6	-6	22.63	3.22
-6	6	-6	20.72	1.77
-7	6	-6	21.86	3.77
-7	6	-6	16.96	2.27
-7	6	-6	15.73	1.77
-7	6	-6	13.75	2.41
7	-6	6	17.59	2.19
7	-6	6	21.66	2.73
-8	6	-6	9.66	3.71
-8	6	-6	7.97	1.13
8	-6	6	7.58	1.81
-9	6	-6	6.47	1.18
-9	6	-6	8.32	4.13
9	-6	6	8.58	2.04
-14	-5	6	2.41	2.49
-14	-5	6	-0.21	3.34
-14	-5	6	0.57	4.19
-13	-5	6	2.80	2.53
-13	-5	6	2.61	3.64
-13	-5	6	1.41	2.86

12	5	-6	1.11	4.14
-12	-5	6	4.47	2.02
-12	-5	6	2.95	2.50
-12	-5	6	-0.91	3.12
-12	-5	6	3.90	1.83
11	5	-6	8.29	1.59
11	5	-6	13.78	2.37
-11	-5	6	10.46	2.40
-11	-5	6	11.59	1.81
-11	-5	6	12.40	2.08
-11	-5	6	13.14	3.00
10	5	-6	0.98	1.21
10	5	-6	1.54	2.85
10	5	-6	1.59	1.88
-10	-5	6	1.79	1.85
-10	-5	6	1.32	1.34
-10	-5	6	-0.32	1.53
9	5	-6	22.86	3.13
9	5	-6	19.62	3.82
-9	-5	6	21.18	3.42
-9	-5	6	22.56	3.27
-9	-5	6	25.29	2.44
-9	-5	6	21.26	2.52
8	5	-6	15.47	2.80
8	5	-6	11.78	2.16
8	5	-6	16.01	2.63
-8	-5	6	16.34	2.17
-8	-5	6	16.94	2.07
-8	-5	6	13.94	2.86
-8	-5	6	13.65	2.56
7	5	-6	11.15	2.28
7	5	-6	12.17	1.89
7	5	-6	14.78	2.43
7	5	-6	9.50	1.95
-7	-5	6	12.62	1.91
-7	-5	6	11.67	1.84
-7	-5	6	11.18	1.92
-7	-5	6	11.32	2.30
6	5	-6	5.39	1.63
6	5	-6	8.96	2.15
-6	-5	6	11.40	1.64
-6	-5	6	8.85	1.63
-6	-5	6	8.05	1.21
-6	-5	6	8.12	1.35

5	5	-6	32.00	2.54
5	5	-6	24.53	2.91
5	5	-6	33.15	2.21
-5	-5	6	30.14	2.23
-5	-5	6	27.81	2.49
-5	-5	6	29.57	2.30
4	5	-6	32.74	2.27
4	5	-6	33.91	2.47
4	5	-6	33.08	2.77
-4	-5	6	31.53	2.27
-4	-5	6	34.01	2.32
-4	-5	6	31.29	2.53
3	5	-6	54.27	3.06
3	5	-6	49.82	3.13
3	5	-6	52.79	3.36
-3	-5	6	50.15	3.00
-3	-5	6	50.58	2.98
-3	-5	6	51.32	2.98
-3	-5	6	45.95	3.08
-3	-5	6	48.04	3.23
2	5	-6	23.46	2.06
2	5	-6	23.43	1.37
2	5	-6	19.50	2.35
2	5	-6	27.00	2.04
-2	-5	6	22.08	1.95
-2	-5	6	24.29	1.88
-2	-5	6	22.60	1.50
1	5	-6	32.65	1.78
1	5	-6	31.40	2.36
1	5	-6	34.73	2.40
1	5	-6	29.81	2.54
-1	-5	6	30.71	2.49
-1	-5	6	32.53	2.37
-1	-5	6	31.39	1.92
0	5	-6	33.57	2.45
0	5	-6	30.90	1.75
0	5	-6	25.99	2.28
0	5	-6	30.51	2.46
0	-5	6	32.43	2.44
0	-5	6	31.09	1.92
0	-5	6	29.19	2.38
0	-5	6	25.88	2.11
0	-5	6	37.08	2.32
-1	5	-6	76.99	4.44

-1	5	-6	76.77	3.96
-1	5	-6	77.73	4.46
1	-5	6	80.19	4.37
1	-5	6	86.64	4.41
1	-5	6	76.08	4.18
1	-5	6	70.94	4.40
-2	5	-6	28.79	2.42
-2	5	-6	31.15	2.14
-2	5	-6	32.61	1.86
-2	5	-6	32.62	2.73
-2	5	-6	29.58	2.78
2	-5	6	31.13	2.05
2	-5	6	31.52	2.18
2	-5	6	34.89	2.46
2	-5	6	31.93	2.57
-3	5	-6	40.83	2.97
-3	5	-6	46.15	2.77
-3	5	-6	46.00	2.52
-3	5	-6	46.68	3.20
-3	5	-6	43.03	3.34
3	-5	6	43.31	2.67
3	-5	6	49.95	3.09
-4	5	-6	10.61	1.49
-4	5	-6	13.51	2.49
-4	5	-6	12.04	1.18
-4	5	-6	12.71	2.36
-4	5	-6	13.26	1.61
4	-5	6	10.33	1.57
4	-5	6	10.10	1.42
4	-5	6	10.69	2.08
4	-5	6	13.03	1.67
-5	5	-6	33.62	2.19
-5	5	-6	37.10	2.48
-5	5	-6	38.22	3.13
-5	5	-6	34.60	3.24
-5	5	-6	31.08	2.95
5	-5	6	36.62	2.64
5	-5	6	32.17	2.63
-6	5	-6	5.27	0.80
-6	5	-6	4.58	1.81
-6	5	-6	3.91	1.76
-6	5	-6	5.76	1.02
6	-5	6	6.49	1.17
-7	5	-6	10.34	1.94

-7	5	-6	12.60	1.49
-7	5	-6	6.75	1.83
7	-5	6	9.81	1.50
7	-5	6	13.11	2.36
-8	5	-6	10.60	2.67
-8	5	-6	12.83	2.24
-8	5	-6	10.63	1.59
-8	5	-6	11.99	3.53
8	-5	6	9.31	1.36
8	-5	6	14.82	2.79
-9	5	-6	14.65	1.83
-9	5	-6	11.50	4.06
9	-5	6	16.25	3.24
-10	5	-6	12.60	4.60
-10	5	-6	6.08	1.23
10	-5	6	8.12	2.12
-14	-4	6	1.89	4.33
-14	-4	6	4.97	3.53
-14	-4	6	6.91	2.59
13	4	-6	2.64	6.28
-13	-4	6	5.12	3.65
-13	-4	6	3.28	2.26
-13	-4	6	1.36	3.04
12	4	-6	2.41	4.06
-12	-4	6	4.13	2.61
-12	-4	6	8.20	1.96
-12	-4	6	8.22	3.26
-11	-4	6	8.15	2.35
-11	-4	6	13.18	2.55
-11	-4	6	10.31	2.86
-11	-4	6	9.89	1.82
10	4	-6	-1.90	2.79
-10	-4	6	1.32	2.47
-10	-4	6	3.82	1.35
-10	-4	6	2.10	1.54
-10	-4	6	4.87	1.88
9	4	-6	12.15	1.82
9	4	-6	10.51	2.65
-9	-4	6	13.27	2.33
-9	-4	6	9.87	2.29
-9	-4	6	14.99	2.08
8	4	-6	27.09	2.91
8	4	-6	20.42	3.45
-8	-4	6	27.80	2.42

-8	-4	6	31.22	2.86
-8	-4	6	28.73	2.32
-8	-4	6	24.87	3.02
7	4	-6	33.96	2.78
7	4	-6	31.44	3.37
7	4	-6	21.28	4.46
-7	-4	6	26.94	2.77
-7	-4	6	30.48	2.35
-7	-4	6	29.21	2.81
-7	-4	6	29.80	2.28
6	4	-6	18.04	2.74
-6	-4	6	20.50	2.23
-6	-4	6	22.77	1.88
-6	-4	6	21.14	1.99
-6	-4	6	18.97	2.26
5	4	-6	34.30	2.35
5	4	-6	29.34	2.82
5	4	-6	35.82	2.56
-5	-4	6	33.08	2.27
-5	-4	6	31.29	2.50
-5	-4	6	32.05	2.56
-5	-4	6	35.48	2.29
4	4	-6	46.84	2.84
4	4	-6	39.52	3.06
4	4	-6	44.42	2.68
-4	-4	6	43.83	2.66
-4	-4	6	45.44	2.67
3	4	-6	36.92	2.39
3	4	-6	33.68	2.57
3	4	-6	34.34	1.83
3	4	-6	40.17	2.37
-3	-4	6	37.61	2.27
-3	-4	6	33.43	1.90
-3	-4	6	31.07	2.38
-3	-4	6	31.10	2.43
-3	-4	6	38.05	2.24
2	4	-6	9.25	1.29
2	4	-6	13.56	1.57
2	4	-6	10.01	1.40
2	4	-6	11.58	0.82
-2	-4	6	8.51	1.48
-2	-4	6	13.16	0.94
-2	-4	6	9.61	1.65
-2	-4	6	14.85	1.48

1	4	-6	13.37	0.91
1	4	-6	10.57	1.71
1	4	-6	13.08	1.54
1	4	-6	16.62	1.66
-1	-4	6	14.43	1.58
-1	-4	6	12.40	1.05
-1	-4	6	10.80	1.64
0	4	-6	38.53	2.51
0	4	-6	30.39	2.37
0	4	-6	35.71	2.35
0	4	-6	35.79	1.96
0	4	-6	35.61	2.49
0	-4	6	37.53	2.09
0	-4	6	36.22	2.45
0	-4	6	41.88	2.44
0	-4	6	36.62	2.52
-1	4	-6	53.12	3.14
-1	4	-6	56.50	3.41
-1	4	-6	56.61	3.46
-1	4	-6	50.89	3.28
-1	4	-6	58.19	2.94
1	-4	6	60.02	3.37
1	-4	6	54.22	3.17
1	-4	6	63.54	3.31
1	-4	6	58.52	3.06
-2	4	-6	60.05	3.60
-2	4	-6	60.49	3.52
-2	4	-6	59.34	3.38
-2	4	-6	60.50	3.16
-2	4	-6	62.76	3.79
2	-4	6	54.89	3.54
2	-4	6	61.10	3.33
2	-4	6	62.73	3.40
2	-4	6	62.78	3.64
2	-4	6	65.78	3.63
-3	4	-6	27.15	2.37
-3	4	-6	26.30	1.61
-3	4	-6	23.58	2.30
-3	4	-6	27.38	1.90
-3	4	-6	27.93	2.60
3	-4	6	26.81	1.77
3	-4	6	23.99	2.29
3	-4	6	27.34	1.90
-4	4	-6	42.02	3.07

-4	4	-6	44.16	3.04
-4	4	-6	46.81	2.72
-4	4	-6	46.11	3.31
-4	4	-6	44.79	2.45
4	-4	6	41.88	3.13
4	-4	6	43.80	2.72
4	-4	6	42.96	2.59
-5	4	-6	15.04	1.62
-5	4	-6	9.11	1.56
-5	4	-6	11.00	1.80
-5	4	-6	12.90	1.18
-5	4	-6	11.49	2.18
5	-4	6	15.20	1.73
5	-4	6	11.96	1.39
5	-4	6	9.85	1.32
-6	4	-6	9.71	1.88
-6	4	-6	9.14	1.40
-6	4	-6	9.07	1.66
-6	4	-6	13.06	2.32
6	-4	6	13.73	1.85
-7	4	-6	-2.74	1.53
-7	4	-6	0.92	2.41
-7	4	-6	1.33	0.67
-7	4	-6	1.66	0.87
-7	4	-6	0.41	1.71
-7	4	-6	0.38	1.70
7	-4	6	-0.52	1.24
7	-4	6	2.34	1.14
-8	4	-6	6.62	3.04
-8	4	-6	3.03	2.86
-8	4	-6	3.12	2.02
-8	4	-6	4.84	1.09
-8	4	-6	5.87	0.91
8	-4	6	3.93	1.00
8	-4	6	5.38	1.42
-9	4	-6	10.07	2.60
-9	4	-6	11.80	1.62
-9	4	-6	13.38	3.72
-9	4	-6	8.48	2.34
-9	4	-6	12.26	2.22
9	-4	6	7.79	1.31
9	-4	6	13.64	2.88
-10	4	-6	13.31	1.89
-10	4	-6	20.30	4.60

-11	4	-6	5.94	1.21
-11	4	-6	12.19	4.94
11	-4	6	7.53	2.16
-14	-3	6	6.28	3.66
-14	-3	6	12.60	4.79
13	3	-6	6.11	4.54
-13	-3	6	6.71	3.89
-13	-3	6	7.06	2.04
-13	-3	6	6.41	3.16
12	3	-6	10.43	4.04
-12	-3	6	16.76	3.59
-12	-3	6	10.22	2.98
11	3	-6	6.32	3.27
-11	-3	6	6.38	2.37
-11	-3	6	7.10	1.67
-11	-3	6	7.71	1.60
-11	-3	6	5.71	2.88
10	3	-6	4.71	2.89
-10	-3	6	11.15	1.68
-10	-3	6	11.11	2.67
-10	-3	6	8.99	2.21
-10	-3	6	13.72	2.21
9	3	-6	0.81	2.53
-9	-3	6	6.70	1.28
-9	-3	6	5.34	2.23
-9	-3	6	7.16	2.18
8	3	-6	59.02	3.70
-8	-3	6	51.45	3.87
-8	-3	6	50.82	3.31
-8	-3	6	58.49	3.36
-8	-3	6	53.61	3.86
7	3	-6	41.89	3.51
7	3	-6	37.40	2.82
-7	-3	6	32.80	2.40
-7	-3	6	31.13	2.94
-7	-3	6	35.48	2.46
6	3	-6	54.24	3.91
6	3	-6	46.37	3.60
6	3	-6	43.92	3.72
-6	-3	6	56.83	3.06
-6	-3	6	53.85	3.01
-6	-3	6	52.01	3.40
5	3	-6	31.45	2.92
5	3	-6	37.78	2.55

-5	-3	6	38.81	2.30
-5	-3	6	33.36	2.58
-5	-3	6	36.26	2.28
4	3	-6	48.36	2.80
4	3	-6	37.46	3.31
4	3	-6	49.29	2.83
-4	-3	6	51.33	2.70
-4	-3	6	40.88	2.94
-4	-3	6	46.55	2.67
3	3	-6	29.14	2.06
3	3	-6	29.68	2.04
-3	-3	6	29.07	1.95
-3	-3	6	29.16	1.92
2	3	-6	97.67	4.89
2	3	-6	96.19	4.87
2	3	-6	88.45	4.56
-2	-3	6	92.47	4.63
-2	-3	6	91.34	4.78
-2	-3	6	97.12	4.87
-2	-3	6	88.17	4.91
1	3	-6	53.09	3.11
1	3	-6	53.23	2.78
1	3	-6	51.01	3.26
1	3	-6	61.39	3.21
-1	-3	6	58.12	3.14
-1	-3	6	50.75	3.13
-1	-3	6	58.05	2.88
-1	-3	6	55.47	3.10
0	3	-6	67.51	3.94
0	3	-6	70.00	3.97
0	3	-6	74.20	3.99
0	3	-6	81.28	4.07
0	-3	6	80.96	3.78
0	-3	6	77.39	4.02
0	-3	6	75.91	4.04
0	-3	6	70.12	3.94
0	-3	6	64.55	3.91
-1	3	-6	27.40	2.06
-1	3	-6	31.47	2.31
-1	3	-6	30.02	1.64
-1	3	-6	28.34	1.92
1	-3	6	27.34	2.22
1	-3	6	28.53	1.94
1	-3	6	32.53	1.79

1	-3	6	32.33	2.08
-2	3	-6	66.56	3.78
-2	3	-6	72.16	3.82
-2	3	-6	69.31	3.47
-2	3	-6	64.85	3.62
-2	3	-6	68.78	4.01
2	-3	6	71.99	3.83
2	-3	6	61.02	3.93
2	-3	6	72.80	3.59
2	-3	6	67.60	3.66
-3	3	-6	85.14	4.58
-3	3	-6	78.44	4.16
-3	3	-6	81.64	4.37
-3	3	-6	80.72	4.01
-3	3	-6	75.44	4.33
3	-3	6	79.12	4.84
3	-3	6	76.87	4.18
3	-3	6	79.02	4.11
3	-3	6	77.21	4.52
-4	3	-6	65.00	3.68
-4	3	-6	71.23	3.97
-4	3	-6	60.81	3.71
-4	3	-6	64.12	3.25
-4	3	-6	65.78	3.45
4	-3	6	63.15	3.47
4	-3	6	61.30	3.37
4	-3	6	55.68	4.40
4	-3	6	54.84	3.52
-5	3	-6	28.00	2.91
-5	3	-6	27.50	2.55
-5	3	-6	36.47	2.17
-5	3	-6	33.32	2.52
-5	3	-6	31.79	1.88
5	-3	6	30.77	2.02
5	-3	6	33.38	2.28
5	-3	6	27.61	2.85
-6	3	-6	47.11	2.60
-6	3	-6	48.49	2.91
-6	3	-6	42.93	3.23
-6	3	-6	49.47	3.32
-6	3	-6	42.26	3.66
6	-3	6	45.57	2.98
6	-3	6	45.87	2.76
6	-3	6	51.40	3.05

-7	3	-6	20.78	1.90
-7	3	-6	16.02	2.23
-7	3	-6	22.97	2.68
-7	3	-6	20.85	3.45
7	-3	6	19.66	1.74
-8	3	-6	17.08	1.50
-8	3	-6	10.92	2.13
-8	3	-6	14.32	3.01
8	-3	6	18.83	2.40
-9	3	-6	11.91	2.35
-9	3	-6	5.98	1.68
-9	3	-6	10.06	1.45
-9	3	-6	10.05	3.40
-9	3	-6	9.35	2.11
-9	3	-6	9.51	1.82
9	-3	6	8.02	1.16
9	-3	6	14.18	2.68
-10	3	-6	13.13	1.72
-10	3	-6	9.19	2.39
-10	3	-6	14.23	3.92
-10	3	-6	12.64	2.53
10	-3	6	18.02	3.17
10	-3	6	8.69	1.43
-11	3	-6	18.32	4.97
-11	3	-6	11.32	1.93
-11	3	-6	13.60	2.66
11	-3	6	12.67	2.33
-12	3	-6	7.63	1.36
-12	3	-6	21.17	6.02
12	-3	6	10.45	2.50
-14	-2	6	6.67	3.73
-14	-2	6	2.77	4.66
13	2	-6	6.99	4.78
-13	-2	6	7.92	3.34
-13	-2	6	-1.21	3.88
-13	-2	6	5.73	1.91
-13	-2	6	5.74	1.81
12	2	-6	9.73	3.90
-12	-2	6	6.13	3.53
-12	-2	6	9.15	1.90
-12	-2	6	8.47	2.97
11	2	-6	9.64	3.27
-11	-2	6	14.61	2.73
-11	-2	6	18.64	2.53

-11	-2	6	19.42	3.24
-11	-2	6	18.12	2.37
10	2	-6	8.42	2.97
-10	-2	6	13.11	2.77
-10	-2	6	17.68	2.14
-10	-2	6	13.97	2.26
9	2	-6	17.26	2.76
-9	-2	6	18.87	3.87
-9	-2	6	21.07	3.34
-8	-2	6	19.50	2.78
-8	-2	6	24.10	2.06
-8	-2	6	23.15	3.00
-7	-2	6	41.71	3.24
-7	-2	6	43.92	2.78
-7	-2	6	49.05	2.80
6	2	-6	26.77	2.86
-6	-2	6	29.32	2.01
-6	-2	6	27.29	1.95
-6	-2	6	26.34	2.43
5	2	-6	91.82	4.53
5	2	-6	74.88	4.86
-5	-2	6	82.00	4.35
-5	-2	6	91.36	4.36
-5	-2	6	75.59	4.60
4	2	-6	56.67	2.97
4	2	-6	42.85	3.36
-4	-2	6	42.81	3.05
-4	-2	6	53.65	2.84
-4	-2	6	56.55	2.84
3	2	-6	40.97	2.79
3	2	-6	51.15	2.68
3	2	-6	41.36	3.11
-3	-2	6	39.67	2.79
-3	-2	6	49.33	2.64
-3	-2	6	50.60	2.57
2	2	-6	42.19	2.37
2	2	-6	34.98	1.98
2	2	-6	42.85	2.50
-2	-2	6	36.66	2.04
-2	-2	6	39.65	2.29
-2	-2	6	39.43	2.36
1	2	-6	68.04	3.48
1	2	-6	62.63	3.10
1	2	-6	56.42	3.61

1	2	-6	61.59	3.38
-1	-2	6	62.40	3.45
-1	-2	6	64.86	3.17
-1	-2	6	62.95	3.39
-1	-2	6	58.90	3.43
0	2	-6	11.41	1.43
0	2	-6	14.43	0.87
0	2	-6	15.66	1.66
0	-2	6	14.84	1.52
0	-2	6	14.96	1.01
0	-2	6	10.97	1.56
-1	2	-6	106.33	5.07
-1	2	-6	97.38	4.82
-1	2	-6	87.64	4.97
-1	2	-6	103.65	5.23
1	-2	6	106.33	5.05
1	-2	6	89.95	4.99
1	-2	6	88.90	5.19
1	-2	6	100.69	4.90
-2	2	-6	18.27	1.59
-2	2	-6	18.79	1.39
-2	2	-6	19.43	1.14
-2	2	-6	21.21	2.02
-2	2	-6	16.29	1.76
2	-2	6	17.28	1.66
2	-2	6	20.32	1.40
2	-2	6	15.88	1.97
2	-2	6	18.44	1.26
-3	2	-6	44.57	2.48
-3	2	-6	41.76	2.74
-3	2	-6	42.60	2.60
-3	2	-6	44.85	2.99
-3	2	-6	41.78	2.30
3	-2	6	44.14	2.50
3	-2	6	43.24	2.41
3	-2	6	47.39	3.06
3	-2	6	43.21	2.71
-4	2	-6	42.07	2.76
-4	2	-6	42.86	2.35
-4	2	-6	43.92	2.70
-4	2	-6	45.59	2.57
-4	2	-6	43.22	3.09
4	-2	6	43.85	2.55
4	-2	6	43.05	3.27

4	-2	6	44.42	2.47
4	-2	6	48.78	3.20
-5	2	-6	31.72	2.87
-5	2	-6	31.44	2.34
-5	2	-6	36.97	2.24
-5	2	-6	35.76	1.97
-5	2	-6	36.40	2.47
5	-2	6	33.37	2.09
5	-2	6	35.86	2.28
5	-2	6	34.37	3.48
-6	2	-6	26.03	2.24
-6	2	-6	16.94	2.61
-6	2	-6	22.45	2.85
-6	2	-6	18.55	2.02
-6	2	-6	25.44	1.57
-6	2	-6	25.63	1.88
6	-2	6	23.72	1.70
6	-2	6	19.71	2.76
6	-2	6	26.69	2.03
-7	2	-6	69.44	4.50
-7	2	-6	65.50	3.90
-7	2	-6	70.01	3.84
-7	2	-6	63.89	4.82
-7	2	-6	74.04	4.18
-7	2	-6	68.68	3.59
7	-2	6	70.26	3.97
7	-2	6	65.83	3.90
7	-2	6	67.61	3.72
7	-2	6	62.85	4.52
-8	2	-6	25.72	4.27
-8	2	-6	22.17	2.49
-8	2	-6	34.34	2.90
-8	2	-6	27.60	2.41
-8	2	-6	26.43	3.56
8	-2	6	27.32	2.09
8	-2	6	31.16	2.57
-9	2	-6	30.84	4.51
-9	2	-6	28.31	1.97
-9	2	-6	20.93	2.65
-9	2	-6	26.65	3.41
9	-2	6	33.97	2.95
-10	2	-6	5.99	1.00
-10	2	-6	7.58	2.18
-10	2	-6	3.99	2.05

-10	2	-6	7.01	3.50
10	-2	6	5.80	1.12
10	-2	6	7.77	1.63
-11	2	-6	20.87	4.64
-11	2	-6	13.90	2.73
-11	2	-6	16.06	1.91
11	-2	6	22.58	3.54
11	-2	6	15.23	2.36
-12	2	-6	4.42	1.18
-12	2	-6	7.90	5.21
12	-2	6	7.37	2.17
-13	-1	6	-0.07	4.01
-13	-1	6	2.80	3.31
12	1	-6	1.30	3.36
-12	-1	6	4.31	2.87
-12	-1	6	7.29	3.53
-11	-1	6	27.22	4.46
-11	-1	6	25.98	4.50
10	1	-6	16.79	3.07
-10	-1	6	18.35	3.82
-10	-1	6	21.14	3.75
9	1	-6	17.97	3.32
-9	-1	6	25.35	3.54
-9	-1	6	27.08	2.14
-9	-1	6	25.11	3.49
-8	-1	6	9.13	1.68
-8	-1	6	11.58	1.60
-8	-1	6	8.73	2.09
-7	-1	6	32.05	2.92
-6	-1	6	29.49	2.51
5	1	-6	49.80	3.69
-5	-1	6	50.67	3.34
-5	-1	6	62.27	3.01
-5	-1	6	55.25	3.00
4	1	-6	116.65	5.61
4	1	-6	99.75	5.88
-4	-1	6	115.97	5.52
-4	-1	6	101.13	5.73
-4	-1	6	114.32	5.55
3	1	-6	64.63	3.22
3	1	-6	50.49	3.60
-3	-1	6	51.89	3.33
-3	-1	6	64.56	3.22
-3	-1	6	63.78	3.13

2	1	-6	106.92	5.16
2	1	-6	108.87	4.96
2	1	-6	95.72	5.39
-2	-1	6	95.37	5.23
-2	-1	6	102.33	5.11
-2	-1	6	98.55	5.19
1	1	-6	109.90	5.14
1	1	-6	92.50	5.13
1	1	-6	95.08	4.95
1	1	-6	105.33	4.76
-1	-1	6	98.96	5.04
-1	-1	6	85.28	5.04
-1	-1	6	93.21	4.78
-1	-1	6	95.63	4.97
0	1	-6	13.90	1.41
0	1	-6	14.62	1.67
0	1	-6	15.19	0.92
0	-1	6	17.58	1.41
0	-1	6	14.44	0.98
-1	1	-6	73.49	3.61
-1	1	-6	67.13	3.79
-1	1	-6	75.62	3.89
-1	1	-6	78.13	4.07
1	-1	6	73.10	3.87
1	-1	6	66.92	3.80
1	-1	6	79.92	4.12
1	-1	6	68.51	3.66
-2	1	-6	84.71	4.69
-2	1	-6	96.79	5.00
-2	1	-6	94.74	4.83
-2	1	-6	95.05	4.59
2	-1	6	90.90	4.73
2	-1	6	95.36	4.81
2	-1	6	91.89	4.64
-3	1	-6	106.32	5.50
-3	1	-6	101.82	5.52
-3	1	-6	113.31	5.41
-3	1	-6	107.75	5.57
-3	1	-6	107.55	5.80
3	-1	6	109.73	5.46
3	-1	6	123.73	5.91
3	-1	6	108.94	5.60
3	-1	6	106.59	5.53
-4	1	-6	96.56	4.78

-4	1	-6	92.16	4.92
-4	1	-6	93.28	5.23
-4	1	-6	97.40	4.91
4	-1	6	90.81	4.91
4	-1	6	96.69	4.99
4	-1	6	108.70	5.34
4	-1	6	92.71	4.84
-5	1	-6	87.05	4.30
-5	1	-6	85.06	4.55
-5	1	-6	88.82	4.46
-5	1	-6	79.64	4.83
-5	1	-6	86.02	4.81
-5	1	-6	86.07	4.48
5	-1	6	92.81	4.92
5	-1	6	83.96	4.48
5	-1	6	76.71	4.65
5	-1	6	85.75	4.37
-6	1	-6	40.97	2.69
-6	1	-6	37.90	3.19
-6	1	-6	37.24	2.47
-6	1	-6	38.26	2.15
-6	1	-6	40.16	2.40
-6	1	-6	40.32	2.97
6	-1	6	35.41	2.24
6	-1	6	38.53	2.47
-7	1	-6	38.60	2.18
-7	1	-6	33.76	2.56
-7	1	-6	33.36	3.05
-7	1	-6	37.72	3.47
-7	1	-6	41.58	2.43
7	-1	6	35.23	2.27
7	-1	6	27.96	3.25
7	-1	6	41.94	2.60
-8	1	-6	54.17	3.49
-8	1	-6	63.77	3.78
-8	1	-6	59.39	3.98
-8	1	-6	50.21	4.52
-8	1	-6	59.23	3.17
8	-1	6	63.99	3.64
8	-1	6	57.00	3.29
8	-1	6	52.00	4.15
-9	1	-6	66.15	4.18
-9	1	-6	73.73	6.43
9	-1	6	79.03	4.48

9	-1	6	67.69	3.95
-10	1	-6	36.45	4.83
-10	1	-6	30.31	2.13
-10	1	-6	25.07	2.75
-10	1	-6	28.23	3.45
10	-1	6	32.69	3.16
-11	1	-6	12.68	2.70
-11	1	-6	17.28	4.27
-11	1	-6	8.94	1.76
-11	1	-6	11.36	1.74
-11	1	-6	6.51	2.36
11	-1	6	14.98	3.18
11	-1	6	10.74	1.39
-12	1	-6	14.75	2.80
-12	1	-6	19.70	5.10
-12	1	-6	14.12	2.08
12	-1	6	15.59	2.41
12	-1	6	12.42	2.25
-13	1	-6	7.45	1.41
-13	1	-6	15.55	5.47
13	-1	6	12.28	2.52
-13	0	6	4.79	3.42
-13	0	6	6.30	4.48
-12	0	6	13.73	3.96
-12	0	6	11.13	3.20
-11	0	6	5.23	3.27
-11	0	6	3.86	1.57
-11	0	6	6.51	2.60
-10	0	6	31.43	4.39
-10	0	6	31.16	4.20
9	0	-6	8.65	2.28
-9	0	6	13.35	2.64
-9	0	6	14.70	3.06
8	0	-6	31.93	5.24
-8	0	6	46.15	3.56
-8	0	6	42.93	3.72
-7	0	6	21.89	2.73
-6	0	6	14.27	2.18
5	0	-6	25.13	2.57
-5	0	6	21.52	2.16
4	0	-6	58.30	3.67
-4	0	6	59.50	3.43
3	0	-6	47.72	3.20
-3	0	6	48.05	3.00

-3	0	6	55.02	2.77
-3	0	6	56.63	2.86
2	0	-6	90.73	4.93
2	0	-6	89.99	4.50
2	0	-6	101.43	4.68
-2	0	6	92.95	4.65
-2	0	6	88.74	4.77
-2	0	6	85.93	4.75
-2	0	6	93.27	4.50
1	0	-6	53.80	3.06
1	0	-6	60.48	2.85
1	0	-6	51.94	3.29
-1	0	6	61.02	3.11
-1	0	6	56.67	3.25
-1	0	6	54.10	3.12
-1	0	6	59.95	2.86
0	0	-6	84.26	4.70
0	0	-6	89.90	4.43
0	0	-6	100.15	5.20
0	0	-6	89.25	4.63
0	0	6	88.75	4.44
0	0	6	99.13	4.87
0	0	6	84.79	4.62
0	0	6	85.64	4.60
-1	0	-6	74.75	3.86
-1	0	-6	79.28	4.03
-1	0	-6	68.11	3.82
-1	0	-6	76.78	3.63
1	0	6	63.75	3.78
1	0	6	80.19	4.09
1	0	6	71.76	3.83
1	0	6	70.42	3.64
-2	0	-6	116.84	5.67
-2	0	-6	115.63	5.79
-2	0	-6	113.62	5.99
-2	0	-6	109.42	5.75
2	0	6	120.42	6.04
2	0	6	114.66	5.78
2	0	6	117.33	5.84
-3	0	-6	11.75	0.81
-3	0	-6	11.34	1.02
-3	0	-6	9.16	1.19
-3	0	-6	9.30	1.14
3	0	6	8.87	1.21

3	0	6	8.28	1.22
3	0	6	11.05	0.86
3	0	6	10.76	1.02
-4	0	-6	63.71	3.40
-4	0	-6	65.95	3.37
-4	0	-6	66.63	3.26
-4	0	-6	59.64	3.66
-4	0	-6	68.37	3.71
4	0	6	61.82	3.29
4	0	6	63.92	3.37
4	0	6	60.18	3.43
4	0	6	64.51	3.73
-5	0	-6	15.82	1.93
-5	0	-6	14.14	1.48
-5	0	-6	15.10	2.03
-5	0	-6	18.25	1.35
-5	0	-6	17.28	1.12
5	0	6	14.38	1.55
5	0	6	17.74	1.39
5	0	6	14.44	1.97
5	0	6	15.20	1.16
-6	0	-6	37.95	2.90
-6	0	-6	35.54	2.70
-6	0	-6	32.52	2.31
-6	0	-6	33.65	2.95
-6	0	-6	38.84	2.05
6	0	6	32.04	2.69
6	0	6	34.17	2.80
6	0	6	37.31	2.30
6	0	6	40.18	2.15
-7	0	-6	18.77	2.77
-7	0	-6	15.91	1.83
-7	0	-6	21.90	1.70
-7	0	-6	17.50	2.31
-7	0	-6	20.69	1.41
7	0	6	18.44	1.45
7	0	6	17.09	2.43
7	0	6	21.47	1.83
-8	0	-6	43.04	3.03
-8	0	-6	52.25	4.06
-8	0	-6	54.62	3.01
-8	0	-6	49.64	2.71
-8	0	-6	43.90	3.45
8	0	6	38.42	3.58

8	0	6	43.86	2.75
8	0	6	53.69	3.15
-9	0	-6	28.25	3.79
-9	0	-6	28.96	2.65
-9	0	-6	22.92	1.80
-9	0	-6	23.28	2.26
-9	0	-6	20.63	2.78
9	0	6	26.03	2.37
9	0	6	19.97	1.84
9	0	6	18.56	2.99
-10	0	-6	26.55	3.30
-10	0	-6	30.83	4.47
-10	0	-6	23.62	2.32
-10	0	-6	26.82	2.56
10	0	6	30.02	2.77
10	0	6	32.81	2.94
10	0	6	26.77	2.18
-11	0	-6	11.22	3.70
-11	0	-6	11.57	1.69
-11	0	-6	9.46	1.63
-11	0	-6	10.11	2.25
11	0	6	10.36	1.87
-12	0	-6	29.73	4.84
-12	0	-6	23.50	2.25
-12	0	-6	21.89	2.94
-12	0	-6	18.81	2.96
12	0	6	21.37	2.49
12	0	6	29.85	3.69
-13	0	-6	7.41	3.02
-13	0	-6	9.60	1.48
-13	0	-6	11.87	4.98
13	0	6	6.24	1.46
13	0	6	8.51	2.38
-14	0	-6	10.76	1.67
-14	0	-6	18.32	6.25
-13	1	6	7.58	4.99
-13	1	6	10.34	3.71
-12	1	6	14.93	3.39
-12	1	6	17.26	4.20
-11	1	6	11.56	3.43
-11	1	6	17.38	3.10
-11	1	6	13.25	2.98
-10	1	6	26.33	2.93
-10	1	6	28.77	4.20

-9	1	6	20.84	3.82
-9	1	6	26.24	3.44
-8	1	6	23.03	3.06
-7	1	6	35.00	3.19
-6	1	6	46.24	3.28
-5	1	6	24.00	2.26
4	-1	-6	99.39	5.32
-4	1	6	91.81	5.07
3	-1	-6	43.62	2.90
-3	1	6	44.88	2.68
2	-1	-6	74.43	3.68
2	-1	-6	72.76	4.10
-2	1	6	71.20	3.94
-2	1	6	80.29	3.68
1	-1	-6	48.75	2.92
1	-1	-6	47.32	2.49
1	-1	-6	51.07	2.68
1	-1	-6	45.25	2.95
-1	1	6	49.24	2.48
-1	1	6	55.41	2.71
0	-1	-6	220.64	10.85
0	-1	-6	240.81	10.97
0	-1	-6	213.24	11.04
0	1	6	216.80	10.84
0	1	6	218.72	11.01
0	1	6	224.23	10.97
0	1	6	234.82	11.11
-1	-1	-6	220.15	10.97
-1	-1	-6	211.79	11.34
-1	-1	-6	228.99	10.89
1	1	6	237.95	10.89
1	1	6	219.71	11.00
1	1	6	228.76	11.11
-2	-1	-6	67.00	3.66
-2	-1	-6	70.85	3.36
-2	-1	-6	62.82	3.45
2	1	6	69.36	3.36
2	1	6	66.29	3.64
2	1	6	64.40	3.51
-3	-1	-6	64.42	3.43
-3	-1	-6	73.78	3.37
-3	-1	-6	70.41	3.72
-3	-1	-6	63.76	3.48
3	1	6	69.41	3.38

3	1	6	63.33	3.51
3	1	6	61.81	3.63
-4	-1	-6	71.12	3.66
-4	-1	-6	70.36	3.72
-4	-1	-6	74.52	3.94
-4	-1	-6	79.39	3.60
4	1	6	71.23	3.60
4	1	6	67.56	3.65
4	1	6	68.76	3.88
4	1	6	65.15	3.72
-5	-1	-6	57.25	2.78
-5	-1	-6	51.25	2.93
-5	-1	-6	54.94	3.18
5	1	6	51.68	2.97
5	1	6	51.88	2.89
5	1	6	52.15	2.78
5	1	6	51.50	3.14
-6	-1	-6	42.28	3.12
-6	-1	-6	43.76	2.29
-6	-1	-6	43.04	2.44
-6	-1	-6	43.43	2.82
-6	-1	-6	40.68	2.52
6	1	6	40.37	2.78
6	1	6	42.46	2.49
6	1	6	40.16	2.63
6	1	6	39.29	2.31
-7	-1	-6	45.70	3.49
-7	-1	-6	41.07	2.99
-7	-1	-6	44.92	2.43
-7	-1	-6	44.28	2.72
-7	-1	-6	39.58	2.68
7	1	6	46.42	2.72
7	1	6	42.37	2.98
-8	-1	-6	75.57	4.87
-8	-1	-6	69.49	4.01
-8	-1	-6	76.49	3.80
-8	-1	-6	68.73	4.30
-8	-1	-6	73.99	3.98
8	1	6	75.73	4.11
8	1	6	65.03	4.06
8	1	6	68.71	4.29
8	1	6	69.76	3.79
-9	-1	-6	53.00	3.40
-9	-1	-6	55.61	4.63

-9	-1	-6	58.05	3.11
-9	-1	-6	53.08	3.85
-9	-1	-6	56.87	3.37
9	1	6	57.97	3.59
9	1	6	49.65	3.78
9	1	6	52.69	3.09
-10	-1	-6	36.72	2.35
-10	-1	-6	38.06	2.76
-10	-1	-6	35.18	3.60
-10	-1	-6	33.41	2.79
10	1	6	32.20	2.35
10	1	6	40.26	3.04
10	1	6	38.67	3.18
10	1	6	27.21	3.30
-11	-1	-6	17.13	3.38
-11	-1	-6	21.43	2.64
-11	-1	-6	29.58	4.70
-11	-1	-6	25.42	2.05
11	1	6	25.40	3.08
11	1	6	20.23	2.05
11	1	6	26.14	2.88
11	1	6	19.02	3.18
-12	-1	-6	20.74	2.85
-12	-1	-6	19.46	2.77
-12	-1	-6	15.55	2.76
-12	-1	-6	20.02	4.26
-12	-1	-6	19.77	2.14
-13	-1	-6	20.24	2.45
-13	-1	-6	21.96	4.92
13	1	6	25.71	2.59
-14	-1	-6	14.94	2.53
-14	-1	-6	24.47	5.63
-14	-1	-6	13.05	3.39
-12	2	6	11.78	3.48
-12	2	6	13.15	4.51
-11	2	6	24.85	4.18
-11	2	6	29.39	5.17
-10	2	6	25.49	4.64
-10	2	6	21.30	3.02
-10	2	6	20.21	3.46
-9	2	6	46.46	4.20
-9	2	6	40.60	3.33
-8	2	6	18.30	3.08
-8	2	6	12.95	2.44

-7	2	6	65.00	4.37
-5	2	6	92.17	5.09
-4	2	6	73.31	4.11
3	-2	-6	127.32	6.32
-3	2	6	135.18	6.30
-3	2	6	125.83	6.58
2	-2	-6	78.41	3.93
-2	2	6	80.72	3.91
1	-2	-6	191.44	9.69
-1	2	6	197.04	9.79
-1	2	6	210.15	9.93
-1	2	6	202.17	9.69
0	-2	-6	68.87	3.66
0	-2	-6	68.94	3.55
0	-2	-6	68.96	3.40
0	2	6	70.05	3.62
0	2	6	68.20	3.39
0	2	6	65.78	3.54
-1	-2	-6	216.83	10.38
-1	-2	-6	205.77	10.42
1	2	6	213.65	10.37
1	2	6	224.10	10.48
1	2	6	210.49	10.50
-2	-2	-6	142.76	6.84
-2	-2	-6	130.34	6.68
-2	-2	-6	138.96	6.63
2	2	6	130.95	6.75
2	2	6	129.00	6.71
2	2	6	140.57	6.62
-3	-2	-6	159.26	7.75
-3	-2	-6	157.51	7.81
-3	-2	-6	167.25	7.94
3	2	6	166.90	7.76
3	2	6	144.85	7.82
3	2	6	156.34	7.88
-4	-2	-6	123.95	6.26
-4	-2	-6	119.79	6.12
-4	-2	-6	132.24	6.06
4	2	6	117.59	6.20
4	2	6	116.27	6.15
4	2	6	125.08	6.05
-5	-2	-6	70.82	3.97
-5	-2	-6	69.23	3.80
-5	-2	-6	76.54	3.67

5	2	6	70.80	3.92
5	2	6	68.88	3.83
5	2	6	74.73	3.66
-6	-2	-6	48.54	3.23
-6	-2	-6	44.52	2.50
-6	-2	-6	38.48	2.79
-6	-2	-6	44.72	2.62
-6	-2	-6	46.63	2.41
6	2	6	41.09	2.77
6	2	6	45.97	2.57
6	2	6	43.40	2.39
-7	-2	-6	89.50	4.95
-7	-2	-6	87.35	4.75
-7	-2	-6	92.97	5.31
-7	-2	-6	90.30	4.69
-7	-2	-6	96.32	4.61
7	2	6	88.51	4.89
7	2	6	86.46	4.58
7	2	6	89.33	4.76
-8	-2	-6	22.04	2.01
-8	-2	-6	20.79	1.60
-8	-2	-6	21.75	2.36
-8	-2	-6	22.95	1.83
-8	-2	-6	25.19	3.07
8	2	6	18.52	1.54
8	2	6	24.81	2.03
8	2	6	20.25	2.27
-9	-2	-6	58.95	4.53
-9	-2	-6	55.14	3.49
-9	-2	-6	51.54	3.76
-9	-2	-6	60.91	3.19
9	2	6	61.20	3.60
9	2	6	51.87	3.70
9	2	6	58.74	3.72
9	2	6	52.70	3.13
-10	-2	-6	9.94	2.81
-10	-2	-6	10.77	1.78
-10	-2	-6	12.71	1.87
-10	-2	-6	8.06	1.10
-10	-2	-6	12.80	2.13
10	2	6	10.44	1.71
10	2	6	8.90	1.39
10	2	6	15.29	2.40
-11	-2	-6	30.65	2.38

-11	-2	-6	34.17	2.88
-11	-2	-6	32.30	4.63
-11	-2	-6	30.12	3.55
11	2	6	28.96	2.27
11	2	6	37.95	4.32
11	2	6	24.39	3.21
11	2	6	35.91	3.22
-12	-2	-6	20.95	3.86
-12	-2	-6	34.39	5.18
-12	-2	-6	23.07	2.90
-12	-2	-6	26.37	2.81
-12	-2	-6	26.04	2.32
12	2	6	27.01	3.29
12	2	6	33.68	3.44
-13	-2	-6	25.61	2.57
-13	-2	-6	23.09	4.47
-13	-2	-6	30.73	3.24
13	2	6	31.22	3.77
-14	-2	-6	7.31	1.55
-14	-2	-6	16.65	5.41
-14	-2	-6	14.62	3.46
14	2	6	9.78	1.61
-12	3	6	9.98	4.63
-12	3	6	8.29	3.36
-11	3	6	25.96	5.25
-11	3	6	17.32	4.16
-10	3	6	27.24	3.36
-10	3	6	30.92	5.06
-10	3	6	26.23	4.78
-9	3	6	37.62	3.23
-9	3	6	28.49	4.28
-9	3	6	38.83	4.42
-8	3	6	15.86	2.35
-8	3	6	16.32	2.60
-7	3	6	37.93	3.48
-6	3	6	39.57	2.27
-6	3	6	44.42	3.30
-5	3	6	65.71	3.30
-5	3	6	65.03	3.97
-4	3	6	106.54	5.24
3	-3	-6	89.69	4.51
-3	3	6	92.16	4.49
2	-3	-6	39.54	2.06
-2	3	6	39.33	2.03

1	-3	-6	74.35	3.68
-1	3	6	73.58	3.66
0	-3	-6	37.56	1.93
0	3	6	36.46	2.06
0	3	6	37.21	1.91
-1	-3	-6	98.72	4.61
-1	-3	-6	90.43	4.64
1	3	6	99.11	4.60
1	3	6	88.65	4.71
1	3	6	89.96	4.68
-2	-3	-6	51.17	2.71
-2	-3	-6	52.70	2.63
2	3	6	54.76	2.62
2	3	6	50.13	2.79
2	3	6	49.33	2.74
-3	-3	-6	56.32	2.68
-3	-3	-6	50.24	2.80
-3	-3	-6	52.85	2.99
3	3	6	53.40	2.67
3	3	6	52.86	2.91
3	3	6	49.38	2.84
-4	-3	-6	164.48	8.29
-4	-3	-6	173.19	8.40
-4	-3	-6	164.81	8.21
4	3	6	172.40	8.37
4	3	6	171.07	8.21
4	3	6	162.28	8.33
-5	-3	-6	120.00	6.14
-5	-3	-6	115.24	6.01
-5	-3	-6	116.67	5.90
5	3	6	122.97	5.91
5	3	6	122.02	6.12
-6	-3	-6	92.11	5.05
-6	-3	-6	93.18	4.75
-6	-3	-6	89.13	4.89
-6	-3	-6	98.70	5.19
6	3	6	96.58	5.03
6	3	6	98.13	4.76
-7	-3	-6	120.61	6.27
-7	-3	-6	112.18	5.84
-7	-3	-6	108.73	5.68
-7	-3	-6	108.02	6.00
7	3	6	117.17	5.96
7	3	6	113.21	5.69

7	3	6	115.91	5.90
7	3	6	113.57	6.05
-8	-3	-6	79.32	4.70
-8	-3	-6	81.84	4.28
-8	-3	-6	80.43	4.47
-8	-3	-6	89.39	5.15
8	3	6	84.29	4.57
8	3	6	84.37	4.65
8	3	6	80.56	4.79
8	3	6	72.35	4.59
8	3	6	89.78	4.52
-9	-3	-6	35.41	3.04
-9	-3	-6	41.67	2.47
-9	-3	-6	41.97	3.71
-9	-3	-6	33.60	2.26
-9	-3	-6	35.48	2.63
9	3	6	36.69	3.14
9	3	6	36.51	2.89
9	3	6	37.33	2.96
9	3	6	32.04	2.19
-10	-3	-6	62.12	3.84
-10	-3	-6	72.28	4.98
-10	-3	-6	59.33	4.30
-10	-3	-6	67.05	3.74
-10	-3	-6	60.72	3.54
10	3	6	58.55	4.09
10	3	6	62.87	3.46
-11	-3	-6	34.16	2.64
-11	-3	-6	24.78	2.26
-11	-3	-6	28.90	3.61
-11	-3	-6	33.78	4.45
-11	-3	-6	30.55	2.77
11	3	6	27.90	2.12
11	3	6	24.61	3.05
11	3	6	34.68	3.00
-12	-3	-6	27.25	4.05
-12	-3	-6	30.47	3.28
-12	-3	-6	41.41	3.28
12	3	6	41.63	3.69
12	3	6	35.80	2.55
-13	-3	-6	26.37	3.15
-13	-3	-6	18.60	2.29
-13	-3	-6	20.49	3.21
-13	-3	-6	27.26	3.08

13	3	6	27.02	3.61
13	3	6	19.26	3.54
-14	-3	-6	30.23	4.43
-14	-3	-6	35.72	6.58
-14	-3	-6	27.47	5.24
14	3	6	22.36	2.73
-15	-3	-6	20.92	5.97
-15	-3	-6	7.51	1.61
-15	-3	-6	4.83	3.85
15	3	6	6.47	1.52
-12	4	6	15.25	5.30
-12	4	6	9.15	3.71
-11	4	6	14.36	4.42
-11	4	6	11.81	3.32
-10	4	6	28.32	5.12
-10	4	6	24.64	5.09
-10	4	6	23.13	3.31
-10	4	6	25.43	2.07
-9	4	6	13.61	2.75
-9	4	6	11.11	3.31
-9	4	6	15.06	1.57
-8	4	6	25.54	3.97
-8	4	6	27.68	1.90
-8	4	6	27.79	1.72
-8	4	6	28.81	3.70
-7	4	6	32.62	1.94
-7	4	6	29.55	1.80
-6	4	6	102.94	5.20
-6	4	6	102.63	5.12
-5	4	6	45.47	2.41
-4	4	6	75.43	3.79
-3	4	6	48.57	2.51
2	-4	-6	142.93	7.09
-2	4	6	146.35	7.07
1	-4	-6	128.76	6.34
-1	4	6	129.30	6.31
0	-4	-6	186.63	9.09
0	4	6	186.67	9.07
-1	-4	-6	71.89	3.44
1	4	6	65.78	3.57
1	4	6	64.99	3.51
1	4	6	71.44	3.43
-2	-4	-6	76.17	3.67
-2	-4	-6	68.90	3.73

2	4	6	79.78	3.67
2	4	6	71.33	3.83
2	4	6	71.23	3.79
-3	-4	-6	72.26	3.55
-3	-4	-6	69.94	3.65
-3	-4	-6	62.55	3.89
3	4	6	77.76	3.54
3	4	6	71.29	3.71
3	4	6	69.86	3.75
-4	-4	-6	176.62	9.18
-4	-4	-6	187.56	9.30
-4	-4	-6	194.28	9.11
4	4	6	186.34	9.26
4	4	6	189.07	9.10
-5	-4	-6	158.38	7.93
-5	-4	-6	158.55	7.68
-5	-4	-6	149.57	7.78
5	4	6	159.47	7.68
5	4	6	157.66	7.89
-6	-4	-6	122.15	6.45
-6	-4	-6	126.36	6.60
-6	-4	-6	128.05	6.66
-6	-4	-6	129.14	6.32
6	4	6	129.54	6.57
6	4	6	128.09	6.31
6	4	6	131.52	6.53
6	4	6	126.50	6.61
-7	-4	-6	73.24	4.15
-7	-4	-6	72.71	3.70
-7	-4	-6	72.72	4.31
-7	-4	-6	70.76	3.92
7	4	6	72.17	3.70
7	4	6	67.59	4.21
7	4	6	73.02	4.09
7	4	6	72.47	4.01
-8	-4	-6	61.87	3.60
-8	-4	-6	66.58	4.22
-8	-4	-6	60.64	3.89
-8	-4	-6	60.73	3.29
8	4	6	61.36	3.30
8	4	6	63.62	3.72
8	4	6	58.17	3.99
8	4	6	66.54	3.79
-9	-4	-6	44.08	2.59

-9	-4	-6	45.50	3.38
-9	-4	-6	49.63	3.87
-9	-4	-6	48.11	3.01
9	4	6	43.16	3.56
9	4	6	43.63	2.58
9	4	6	49.80	3.16
9	4	6	40.48	4.25
-10	-4	-6	68.25	4.92
-10	-4	-6	61.79	3.84
-10	-4	-6	57.80	3.43
-10	-4	-6	62.75	4.24
10	4	6	60.16	3.41
10	4	6	55.87	4.54
10	4	6	66.01	4.08
10	4	6	70.70	4.11
-11	-4	-6	18.05	2.37
-11	-4	-6	10.47	1.59
-11	-4	-6	11.40	2.11
11	4	6	11.88	1.50
11	4	6	16.28	2.68
11	4	6	13.78	2.49
11	4	6	17.32	2.91
-12	-4	-6	25.65	2.22
-12	-4	-6	30.17	3.85
-12	-4	-6	34.89	5.03
-12	-4	-6	32.84	3.03
12	4	6	27.10	2.20
12	4	6	25.21	3.29
12	4	6	37.01	3.58
12	4	6	26.45	3.89
-13	-4	-6	25.01	2.19
-13	-4	-6	31.30	3.17
-13	-4	-6	24.95	4.38
-13	-4	-6	31.24	5.51
13	4	6	19.86	2.16
13	4	6	19.23	3.48
-14	-4	-6	29.94	5.21
-14	-4	-6	26.01	2.58
-14	-4	-6	34.99	3.61
-14	-4	-6	40.16	6.51
-15	-4	-6	8.65	3.88
-15	-4	-6	7.58	1.51
-15	-4	-6	16.97	5.42
15	4	6	7.70	1.52

-11	5	6	15.53	3.56
-11	5	6	17.42	4.72
-11	5	6	16.55	1.79
-10	5	6	14.80	1.94
-10	5	6	20.18	1.67
-10	5	6	24.57	5.15
-10	5	6	18.63	4.23
-9	5	6	25.18	4.73
-9	5	6	23.82	1.71
-9	5	6	22.71	3.70
-9	5	6	21.98	2.99
-9	5	6	23.52	2.01
-8	5	6	20.95	3.68
-8	5	6	23.36	3.73
-8	5	6	21.38	1.73
-8	5	6	19.88	1.51
-8	5	6	15.92	2.54
-7	5	6	17.67	1.47
-7	5	6	18.75	3.19
-7	5	6	13.19	2.21
-7	5	6	15.51	2.58
-7	5	6	15.70	1.37
-6	5	6	32.66	1.92
-6	5	6	32.77	2.05
-5	5	6	75.51	3.85
-4	5	6	78.71	3.98
-3	5	6	66.34	3.38
2	-5	-6	77.54	3.92
-2	5	6	77.42	3.89
1	-5	-6	175.57	8.56
-1	5	6	174.14	8.53
0	-5	-6	132.19	6.38
0	5	6	126.97	6.35
-1	-5	-6	81.54	4.13
1	5	6	83.46	4.11
-2	-5	-6	65.21	3.45
-2	-5	-6	66.70	3.39
-2	-5	-6	65.16	3.59
2	5	6	65.02	3.56
2	5	6	73.46	3.39
-3	-5	-6	50.13	2.84
-3	-5	-6	54.98	2.74
3	5	6	55.60	2.73
3	5	6	53.02	2.99

-4	-5	-6	72.13	3.96
-4	-5	-6	70.49	3.61
-4	-5	-6	70.20	3.74
4	5	6	72.15	3.88
4	5	6	71.14	3.59
4	5	6	73.78	3.82
-5	-5	-6	150.26	7.78
-5	-5	-6	158.17	7.65
-5	-5	-6	156.29	7.92
5	5	6	158.02	7.84
5	5	6	157.96	7.65
5	5	6	156.74	7.88
-6	-5	-6	110.06	5.36
-6	-5	-6	111.59	5.72
-6	-5	-6	102.90	5.68
-6	-5	-6	103.89	5.56
6	5	6	108.90	5.36
6	5	6	106.45	5.61
6	5	6	111.48	5.68
-7	-5	-6	115.87	6.50
-7	-5	-6	124.68	6.10
-7	-5	-6	120.21	6.32
-7	-5	-6	125.50	6.49
7	5	6	121.78	6.55
7	5	6	123.00	6.11
7	5	6	127.97	6.39
7	5	6	123.96	6.42
-8	-5	-6	84.09	4.69
-8	-5	-6	80.36	4.86
-8	-5	-6	79.68	4.13
-8	-5	-6	80.75	4.47
8	5	6	81.69	4.58
8	5	6	75.43	4.82
8	5	6	76.63	4.14
8	5	6	87.67	4.55
-9	-5	-6	87.19	5.22
-9	-5	-6	81.52	4.24
-9	-5	-6	80.91	4.91
-9	-5	-6	84.17	4.65
9	5	6	75.42	4.26
9	5	6	86.20	4.76
9	5	6	86.60	4.81
9	5	6	77.51	5.02
-10	-5	-6	20.10	3.57

-10	-5	-6	24.60	2.37
-10	-5	-6	19.58	1.57
-10	-5	-6	20.91	2.86
10	5	6	17.27	1.54
10	5	6	20.97	2.63
10	5	6	15.00	2.50
10	5	6	22.77	2.54
-11	-5	-6	39.07	2.74
-11	-5	-6	36.93	2.36
-11	-5	-6	41.39	3.12
-11	-5	-6	34.10	3.68
11	5	6	32.11	2.29
11	5	6	40.59	3.17
11	5	6	36.26	3.41
11	5	6	32.95	3.90
-12	-5	-6	19.56	2.35
-12	-5	-6	18.80	3.65
-12	-5	-6	15.69	3.86
-12	-5	-6	15.02	1.74
-12	-5	-6	23.03	2.80
12	5	6	14.14	1.74
12	5	6	18.99	3.17
12	5	6	16.74	3.17
12	5	6	11.22	2.93
-13	-5	-6	44.14	3.42
-13	-5	-6	39.51	4.85
-13	-5	-6	51.58	6.02
-13	-5	-6	45.67	3.75
13	5	6	40.14	2.81
13	5	6	35.74	4.05
13	5	6	40.73	4.94
-14	-5	-6	22.09	2.26
-14	-5	-6	26.35	5.10
-14	-5	-6	24.53	5.28
-14	-5	-6	26.44	3.50
-14	-5	-6	27.67	3.35
-15	-5	-6	20.93	4.14
-15	-5	-6	36.41	7.05
-15	-5	-6	21.03	2.34
15	5	6	14.56	2.52
-10	6	6	10.93	3.23
-10	6	6	12.78	1.64
-10	6	6	11.47	4.41
-9	6	6	34.89	5.16

-9	6	6	31.53	5.32
-9	6	6	30.65	3.26
-9	6	6	27.28	2.07
-8	6	6	21.49	1.84
-8	6	6	18.95	4.07
-8	6	6	19.53	3.22
-8	6	6	13.52	2.77
-8	6	6	18.53	1.57
-7	6	6	27.20	1.78
-7	6	6	28.95	1.97
-7	6	6	22.84	3.79
-7	6	6	19.10	3.53
-6	6	6	15.21	1.30
-6	6	6	18.03	3.02
-6	6	6	17.53	1.51
-6	6	6	13.18	2.29
-5	6	6	61.37	3.33
-5	6	6	60.84	3.22
-4	6	6	60.00	3.15
-3	6	6	71.71	3.67
-2	6	6	69.71	3.55
1	-6	-6	185.63	8.95
-1	6	6	179.02	8.91
0	-6	-6	214.27	10.37
0	6	6	210.35	10.34
-1	-6	-6	193.02	9.44
1	6	6	193.28	9.42
-2	-6	-6	44.58	2.56
-2	-6	-6	48.76	2.79
-2	-6	-6	49.12	2.51
2	6	6	50.49	2.50
2	6	6	48.02	2.68
2	6	6	46.18	2.77
-3	-6	-6	124.88	6.49
-3	-6	-6	128.56	6.44
3	6	6	137.05	6.43
3	6	6	129.47	6.61
3	6	6	133.28	6.57
-4	-6	-6	129.25	6.18
-4	-6	-6	119.39	6.28
-4	-6	-6	122.68	6.46
4	6	6	123.28	6.39
4	6	6	129.52	6.16
4	6	6	126.11	6.36

-5	-6	-6	132.20	7.06
-5	-6	-6	145.37	7.24
-5	-6	-6	139.72	6.91
5	6	6	139.52	7.16
5	6	6	140.41	7.11
5	6	6	145.17	6.90
-6	-6	-6	150.35	7.39
-6	-6	-6	140.68	7.22
-6	-6	-6	141.79	7.01
-6	-6	-6	130.28	7.30
6	6	6	147.05	7.26
6	6	6	139.31	6.99
6	6	6	147.05	7.31
-7	-6	-6	117.03	5.95
-7	-6	-6	102.42	5.93
-7	-6	-6	109.20	5.47
-7	-6	-6	109.99	5.77
7	6	6	104.80	5.46
7	6	6	114.93	5.81
7	6	6	114.19	5.86
7	6	6	105.74	5.97
-8	-6	-6	93.97	5.30
-8	-6	-6	92.88	4.72
-8	-6	-6	94.30	5.11
-8	-6	-6	91.17	5.33
8	6	6	98.52	5.16
8	6	6	89.13	4.75
8	6	6	94.88	5.48
8	6	6	92.64	5.20
-9	-6	-6	102.02	5.62
-9	-6	-6	97.55	5.74
-9	-6	-6	93.07	4.92
-9	-6	-6	98.25	5.38
9	6	6	95.93	5.76
9	6	6	88.78	4.95
9	6	6	102.78	5.44
9	6	6	101.27	5.51
-10	-6	-6	24.42	3.11
-10	-6	-6	28.84	3.50
-10	-6	-6	28.60	2.73
-10	-6	-6	29.66	1.83
10	6	6	26.50	2.88
10	6	6	21.92	3.42
10	6	6	30.42	2.75

10	6	6	25.23	1.84
-11	-6	-6	29.56	2.75
-11	-6	-6	22.87	3.39
-11	-6	-6	25.78	1.83
-11	-6	-6	25.41	4.25
11	6	6	27.26	2.87
11	6	6	18.30	3.66
11	6	6	21.39	1.80
11	6	6	24.11	3.04
-12	-6	-6	22.41	1.79
-12	-6	-6	21.94	2.40
-12	-6	-6	21.13	4.37
-12	-6	-6	19.04	3.71
-12	-6	-6	23.86	2.91
12	6	6	18.28	3.36
12	6	6	17.84	1.75
12	6	6	22.58	3.13
12	6	6	14.89	2.98
-13	-6	-6	42.96	4.81
-13	-6	-6	38.62	2.81
-13	-6	-6	46.79	3.84
-13	-6	-6	54.68	5.64
13	6	6	37.98	4.16
13	6	6	38.31	2.74
13	6	6	46.27	4.00
13	6	6	29.06	4.80
-14	-6	-6	34.64	3.84
-14	-6	-6	32.90	5.21
-14	-6	-6	28.10	2.40
-14	-6	-6	33.96	3.40
-14	-6	-6	36.51	6.15
14	6	6	23.30	4.24
14	6	6	35.43	4.63
-15	-6	-6	22.96	2.35
-15	-6	-6	23.12	4.23
-15	-6	-6	28.79	5.30
-10	7	6	11.15	1.48
-10	7	6	10.57	1.85
-10	7	6	19.89	4.79
-10	7	6	13.64	3.54
-9	7	6	27.27	5.43
-9	7	6	13.09	3.59
-9	7	6	17.65	2.22
-9	7	6	21.19	1.89

-9	7	6	16.81	3.32
-8	7	6	29.11	4.80
-8	7	6	27.06	1.97
-8	7	6	23.51	4.60
-8	7	6	29.83	2.25
-8	7	6	18.84	3.36
-7	7	6	25.71	4.01
-7	7	6	22.01	2.23
-7	7	6	18.80	3.04
-7	7	6	19.32	3.03
-7	7	6	21.69	1.67
-6	7	6	41.25	4.11
-6	7	6	40.18	3.98
-6	7	6	42.60	2.48
-6	7	6	45.74	4.16
-6	7	6	41.49	2.63
-5	7	6	29.84	2.09
-5	7	6	26.23	3.36
-5	7	6	31.16	1.91
-5	7	6	33.79	3.21
-4	7	6	74.62	3.87
-3	7	6	54.31	2.89
-2	7	6	93.72	4.86
-1	7	6	94.33	4.74
0	-7	-6	74.23	3.77
0	7	6	72.01	3.72
-1	-7	-6	58.27	3.01
1	7	6	56.08	2.96
-2	-7	-6	140.58	6.78
2	7	6	142.81	6.75
2	7	6	126.76	7.08
-3	-7	-6	97.81	4.96
-3	-7	-6	94.91	4.71
-3	-7	-6	87.47	4.77
3	7	6	94.61	4.68
3	7	6	90.60	4.92
3	7	6	96.58	4.87
-4	-7	-6	121.08	6.10
-4	-7	-6	118.45	6.22
4	7	6	124.24	6.08
4	7	6	128.32	6.30
4	7	6	122.65	6.33
-5	-7	-6	40.05	2.24
-5	-7	-6	42.60	2.93

-5	-7	-6	39.84	2.55
5	7	6	40.39	2.22
5	7	6	43.62	2.65
5	7	6	41.81	2.73
-6	-7	-6	118.03	6.05
-6	-7	-6	111.25	5.88
-6	-7	-6	108.18	6.01
-6	-7	-6	112.43	5.63
6	7	6	115.32	5.99
6	7	6	117.62	5.62
6	7	6	104.62	5.95
6	7	6	116.56	5.95
-7	-7	-6	76.79	4.45
-7	-7	-6	78.44	4.25
-7	-7	-6	73.95	3.86
-7	-7	-6	70.95	4.40
7	7	6	78.03	4.38
7	7	6	73.20	4.42
7	7	6	73.67	3.84
7	7	6	79.25	4.29
-8	-7	-6	116.57	6.54
-8	-7	-6	119.00	6.37
-8	-7	-6	126.66	6.60
-8	-7	-6	112.19	5.97
8	7	6	127.08	6.51
8	7	6	124.23	6.39
8	7	6	114.02	6.88
-9	-7	-6	72.42	3.89
-9	-7	-6	81.08	4.77
-9	-7	-6	73.05	4.80
-9	-7	-6	74.85	4.48
9	7	6	73.47	4.86
9	7	6	70.43	3.90
9	7	6	80.60	4.52
9	7	6	76.89	4.62
-10	-7	-6	92.25	5.81
-10	-7	-6	98.32	5.67
-10	-7	-6	89.29	4.77
-10	-7	-6	96.59	5.41
10	7	6	95.35	5.93
10	7	6	94.93	5.39
10	7	6	88.46	4.80
-11	-7	-6	38.67	3.91
-11	-7	-6	42.63	3.48

-11	-7	-6	38.71	2.40
11	7	6	42.28	3.46
11	7	6	41.59	3.79
11	7	6	37.55	2.45
11	7	6	39.21	4.27
-12	-7	-6	69.42	4.63
-12	-7	-6	64.81	3.62
-12	-7	-6	74.77	5.63
-12	-7	-6	67.61	5.20
-12	-7	-6	68.02	4.10
12	7	6	60.81	5.31
12	7	6	68.54	4.56
12	7	6	60.45	3.64
12	7	6	63.74	4.96
-13	-7	-6	26.09	4.69
-13	-7	-6	26.62	3.32
-13	-7	-6	32.84	5.16
-13	-7	-6	22.01	1.99
-13	-7	-6	16.08	2.66
13	7	6	14.47	3.42
13	7	6	29.59	3.55
13	7	6	21.91	2.01
13	7	6	25.38	3.82
-14	-7	-6	32.97	2.52
-14	-7	-6	43.42	6.23
-14	-7	-6	38.34	5.65
-14	-7	-6	37.71	3.68
-14	-7	-6	41.02	4.05
14	7	6	38.54	4.32
14	7	6	34.77	4.71
-15	-7	-6	17.37	4.08
-15	-7	-6	25.11	5.32
-15	-7	-6	15.78	2.03
-9	8	6	14.72	2.39
-9	8	6	23.62	4.37
-9	8	6	17.18	3.44
-9	8	6	18.62	2.15
-8	8	6	34.05	2.61
-8	8	6	31.51	2.29
-8	8	6	23.68	4.14
-8	8	6	34.36	5.29
-8	8	6	26.29	5.13
-7	8	6	16.24	3.21
-7	8	6	20.31	1.76

-7	8	6	14.85	3.25
-7	8	6	22.55	2.02
-7	8	6	21.62	4.43
-6	8	6	43.75	4.41
-6	8	6	40.69	4.23
-6	8	6	45.96	2.85
-6	8	6	39.99	4.66
-6	8	6	46.94	2.65
-5	8	6	54.70	2.96
-5	8	6	54.42	4.17
-5	8	6	50.39	4.49
-5	8	6	49.89	3.33
-4	8	6	39.10	2.49
-4	8	6	39.06	2.30
-3	8	6	32.65	3.19
-3	8	6	37.01	2.09
-2	8	6	86.11	4.40
-1	8	6	74.89	3.86
0	8	6	79.93	4.07
-1	-8	-6	55.70	2.92
1	8	6	52.99	2.87
-2	-8	-6	105.11	5.22
2	8	6	101.53	5.47
-3	-8	-6	79.44	4.26
-3	-8	-6	77.54	4.01
3	8	6	77.93	4.19
3	8	6	77.04	4.29
-4	-8	-6	107.50	5.13
-4	-8	-6	97.45	5.25
4	8	6	90.80	5.14
4	8	6	107.59	5.35
4	8	6	104.41	5.40
-5	-8	-6	84.00	5.07
-5	-8	-6	89.84	4.36
-5	-8	-6	86.15	4.59
5	8	6	89.60	4.66
5	8	6	75.13	4.54
5	8	6	88.08	4.74
-6	-8	-6	80.07	4.08
-6	-8	-6	77.40	4.59
-6	-8	-6	82.50	4.61
-6	-8	-6	77.07	4.39
6	8	6	82.93	4.44
6	8	6	78.52	4.53

6	8	6	78.58	4.54
-7	-8	-6	75.56	3.97
-7	-8	-6	71.80	4.55
-7	-8	-6	81.78	4.61
7	8	6	75.50	3.96
7	8	6	74.25	4.55
7	8	6	81.63	4.43
7	8	6	79.04	4.54
-8	-8	-6	87.31	5.22
-8	-8	-6	87.89	4.55
-8	-8	-6	96.98	5.26
8	8	6	78.18	5.44
8	8	6	93.57	5.05
8	8	6	92.91	5.17
8	8	6	86.97	4.52
-9	-8	-6	43.63	2.51
-9	-8	-6	40.08	3.63
-9	-8	-6	45.55	3.58
9	8	6	47.38	3.70
9	8	6	40.44	2.46
9	8	6	47.95	3.55
9	8	6	48.36	3.26
-10	-8	-6	51.14	4.06
-10	-8	-6	52.45	4.04
-10	-8	-6	47.97	2.80
-10	-8	-6	50.00	3.67
10	8	6	50.47	4.18
10	8	6	50.03	4.31
10	8	6	52.13	3.63
10	8	6	46.89	2.78
-11	-8	-6	65.36	5.04
-11	-8	-6	65.15	3.64
-11	-8	-6	72.86	4.61
-11	-8	-6	77.12	6.20
11	8	6	63.24	3.67
11	8	6	67.15	4.52
11	8	6	72.03	5.32
11	8	6	62.06	4.82
-12	-8	-6	63.72	5.16
-12	-8	-6	57.26	3.42
-12	-8	-6	73.06	5.50
-12	-8	-6	57.95	3.83
-12	-8	-6	63.38	4.55
12	8	6	55.00	3.49

12	8	6	68.20	4.52
12	8	6	62.46	5.47
12	8	6	66.45	5.00
-13	-8	-6	31.90	3.58
-13	-8	-6	29.64	2.91
-13	-8	-6	27.65	5.15
-13	-8	-6	25.06	2.08
13	8	6	28.97	3.62
13	8	6	18.22	2.85
13	8	6	20.52	3.62
13	8	6	18.94	2.07
-14	-8	-6	27.45	3.85
-14	-8	-6	28.05	5.20
-14	-8	-6	23.76	2.14
-14	-8	-6	25.82	4.78
14	8	6	19.52	2.19
14	8	6	27.28	4.09
14	8	6	25.79	4.42
-15	-8	-6	18.42	5.08
-15	-8	-6	17.20	2.05
-15	-8	-6	14.02	4.22
-15	-8	-6	17.63	3.47
-8	9	6	16.22	3.44
-8	9	6	16.53	3.96
-8	9	6	19.52	2.87
-8	9	6	18.44	2.46
-7	9	6	27.78	5.54
-7	9	6	38.83	2.80
-7	9	6	35.95	2.54
-7	9	6	35.72	4.89
-7	9	6	42.17	5.32
-6	9	6	24.74	4.67
-6	9	6	34.89	2.47
-6	9	6	23.38	4.13
-6	9	6	27.39	5.09
-6	9	6	32.35	2.19
-5	9	6	51.95	3.20
-5	9	6	50.83	2.98
-5	9	6	54.96	4.52
-5	9	6	49.84	4.68
-4	9	6	22.56	1.71
-4	9	6	20.19	3.42
-4	9	6	22.84	1.97
-3	9	6	35.95	3.27

-3	9	6	36.56	2.20
-2	9	6	34.14	2.06
-1	9	6	104.31	5.29
0	9	6	64.78	3.41
1	9	6	126.64	6.31
-2	-9	-6	55.35	2.99
2	9	6	54.98	2.92
-3	-9	-6	112.96	5.60
-3	-9	-6	109.11	5.99
3	9	6	112.00	5.86
3	9	6	109.77	5.54
3	9	6	109.48	5.74
-4	-9	-6	55.86	3.63
-4	-9	-6	61.23	3.46
-4	-9	-6	54.52	3.23
-4	-9	-6	58.25	3.08
4	9	6	58.03	3.49
4	9	6	51.83	3.41
4	9	6	57.84	3.01
4	9	6	59.02	3.34
-5	-9	-6	112.22	6.27
-5	-9	-6	114.87	5.84
5	9	6	115.69	6.18
5	9	6	115.98	5.78
5	9	6	118.12	6.19
5	9	6	118.55	6.09
-6	-9	-6	101.28	5.49
-6	-9	-6	92.75	5.42
-6	-9	-6	95.05	4.90
6	9	6	97.98	5.35
6	9	6	95.85	5.22
6	9	6	94.23	5.37
6	9	6	95.80	4.85
-7	-9	-6	108.50	5.96
-7	-9	-6	100.82	5.97
-7	-9	-6	106.93	5.39
7	9	6	109.85	5.77
7	9	6	101.02	5.32
7	9	6	110.72	5.92
-8	-9	-6	67.90	4.43
-8	-9	-6	69.34	4.47
-8	-9	-6	66.98	3.61
8	9	6	70.64	4.15
8	9	6	72.51	4.37

8	9	6	63.75	3.55
8	9	6	67.79	4.44
-9	-9	-6	59.67	4.28
-9	-9	-6	65.55	4.35
-9	-9	-6	60.00	3.27
9	9	6	60.05	4.41
9	9	6	64.41	4.23
9	9	6	60.10	3.95
9	9	6	53.92	3.20
-10	-9	-6	14.07	2.50
-10	-9	-6	16.86	3.24
-10	-9	-6	17.32	1.55
10	9	6	19.74	2.63
10	9	6	15.15	1.41
10	9	6	19.81	3.05
10	9	6	14.32	2.61
-11	-9	-6	57.83	4.74
-11	-9	-6	55.87	3.25
-11	-9	-6	58.75	4.77
11	9	6	60.77	4.21
11	9	6	61.32	4.74
11	9	6	57.32	5.10
11	9	6	55.10	3.25
-12	-9	-6	44.76	2.81
-12	-9	-6	44.56	4.90
-12	-9	-6	54.49	4.11
-12	-9	-6	44.82	4.70
12	9	6	42.12	2.86
12	9	6	53.24	5.30
12	9	6	47.25	4.07
12	9	6	46.52	4.62
-13	-9	-6	67.10	5.80
-13	-9	-6	67.39	4.42
-13	-9	-6	65.06	4.06
-13	-9	-6	70.25	6.10
-13	-9	-6	72.87	5.08
13	9	6	64.42	6.27
13	9	6	71.28	5.08
13	9	6	64.49	3.84
13	9	6	61.76	5.65
-14	-9	-6	33.75	4.11
-14	-9	-6	22.21	2.20
-14	-9	-6	21.40	3.74
-14	-9	-6	33.28	5.90

14	9	6	32.35	4.20
14	9	6	28.17	4.92
-15	-9	-6	22.64	4.27
-15	-9	-6	22.53	3.55
-15	-9	-6	22.07	2.22
-7	10	6	26.32	2.60
-7	10	6	26.54	5.55
-7	10	6	18.06	3.80
-6	10	6	30.81	2.40
-6	10	6	24.76	4.59
-6	10	6	36.33	5.49
-6	10	6	33.58	2.65
-6	10	6	32.71	4.97
-5	10	6	53.61	3.25
-5	10	6	56.36	5.09
-5	10	6	56.37	3.48
-4	10	6	49.86	4.52
-4	10	6	54.06	3.09
-4	10	6	55.47	3.34
-3	10	6	42.78	2.71
-3	10	6	38.21	2.40
-3	10	6	37.70	3.73
-2	10	6	34.98	2.15
-2	10	6	34.19	3.20
-1	10	6	48.34	2.72
0	10	6	83.93	4.20
0	10	6	76.82	4.64
1	10	6	126.99	6.31
1	10	6	124.59	6.89
-2	-10	-6	87.65	4.83
2	10	6	73.91	4.90
2	10	6	84.77	4.25
-3	-10	-6	149.47	7.14
3	10	6	148.79	7.26
3	10	6	141.57	7.06
3	10	6	128.08	7.33
-4	-10	-6	117.46	5.70
-4	-10	-6	105.37	5.94
4	10	6	110.65	5.62
4	10	6	111.81	6.00
4	10	6	113.10	5.89
-5	-10	-6	132.56	7.36
-5	-10	-6	140.79	6.86
5	10	6	137.75	7.19

5	10	6	133.40	6.78
5	10	6	136.60	7.08
-6	-10	-6	106.32	6.25
-6	-10	-6	115.78	5.64
6	10	6	112.12	6.06
6	10	6	102.23	6.06
6	10	6	115.57	5.94
6	10	6	109.86	5.56
-7	-10	-6	124.25	6.97
-7	-10	-6	126.82	6.88
-7	-10	-6	125.72	6.37
7	10	6	125.18	6.92
7	10	6	127.39	6.75
7	10	6	124.11	6.31
7	10	6	129.65	6.89
-8	-10	-6	79.60	4.72
-8	-10	-6	69.59	4.86
-8	-10	-6	75.63	3.99
8	10	6	68.91	3.89
8	10	6	79.26	4.55
8	10	6	76.12	4.95
8	10	6	76.58	4.74
-9	-10	-6	56.63	4.09
-9	-10	-6	53.00	3.05
-9	-10	-6	55.57	4.20
9	10	6	50.09	2.97
9	10	6	52.56	4.28
9	10	6	59.73	3.84
9	10	6	55.85	4.08
-10	-10	-6	24.21	1.88
-10	-10	-6	29.57	3.46
10	10	6	23.82	3.76
10	10	6	24.69	2.94
10	10	6	26.38	3.38
10	10	6	23.09	1.77
-11	-10	-6	42.00	2.70
-11	-10	-6	44.86	4.32
-11	-10	-6	40.42	4.37
11	10	6	41.06	4.66
11	10	6	40.75	2.63
11	10	6	48.32	3.77
11	10	6	48.74	4.29
-12	-10	-6	53.43	5.16
-12	-10	-6	50.21	3.33

-12	-10	-6	57.82	5.09
12	10	6	59.33	5.15
12	10	6	48.50	3.22
12	10	6	62.43	4.40
12	10	6	57.64	5.54
-13	-10	-6	41.01	4.88
-13	-10	-6	30.97	2.64
-13	-10	-6	36.68	5.23
13	10	6	33.53	2.65
13	10	6	42.69	5.82
13	10	6	44.26	5.13
13	10	6	45.79	4.24
-14	-10	-6	36.78	5.63
-14	-10	-6	40.11	6.02
-14	-10	-6	47.18	4.64
-14	-10	-6	35.69	2.69
-14	-10	-6	34.52	3.57
14	10	6	43.32	4.72
14	10	6	30.74	5.55
-15	-10	-6	16.73	2.22
-15	-10	-6	18.42	3.45
-15	-10	-6	20.91	4.80
-15	-10	-6	19.15	4.35
-6	11	6	22.92	3.80
-6	11	6	30.87	5.51
-6	11	6	27.23	2.74
-5	11	6	15.11	3.54
-5	11	6	22.88	2.40
-5	11	6	18.62	3.78
-4	11	6	44.23	2.86
-4	11	6	39.78	4.64
-4	11	6	50.32	3.16
-3	11	6	32.12	2.25
-3	11	6	37.21	2.61
-3	11	6	29.55	3.72
-2	11	6	42.76	2.58
-2	11	6	43.05	3.71
-1	11	6	20.65	1.55
-1	11	6	16.07	2.74
0	-11	-6	61.49	3.81
0	11	6	56.42	3.97
-1	-11	-6	38.23	3.11
1	11	6	37.52	2.97
-2	-11	-6	55.97	3.78

2	11	6	52.88	3.52
-3	-11	-6	43.88	3.35
3	11	6	35.81	2.88
-4	-11	-6	171.50	8.97
-4	-11	-6	170.26	8.44
4	11	6	172.99	8.69
4	11	6	169.08	8.58
4	11	6	169.51	8.36
4	11	6	157.76	8.69
-5	-11	-6	58.21	3.18
-5	-11	-6	52.63	3.66
-5	-11	-6	57.38	4.15
5	11	6	57.33	3.52
5	11	6	55.87	3.07
5	11	6	59.61	3.76
5	11	6	54.45	3.75
-6	-11	-6	85.50	4.42
-6	-11	-6	78.55	5.26
6	11	6	83.95	4.78
6	11	6	84.42	5.04
6	11	6	83.56	4.33
6	11	6	84.34	4.95
-7	-11	-6	42.88	3.95
-7	-11	-6	48.13	2.76
-7	-11	-6	46.95	3.62
7	11	6	43.47	3.78
7	11	6	49.75	3.57
7	11	6	46.59	2.63
7	11	6	48.51	3.36
-8	-11	-6	75.76	4.12
-8	-11	-6	78.94	5.14
-8	-11	-6	75.85	4.89
8	11	6	74.33	4.01
8	11	6	76.01	4.66
8	11	6	76.07	5.15
8	11	6	80.27	4.96
-9	-11	-6	44.94	2.80
-9	-11	-6	46.31	3.91
9	11	6	46.38	4.32
9	11	6	46.81	3.57
9	11	6	45.64	2.69
9	11	6	51.06	3.95
-10	-11	-6	41.79	4.33
-10	-11	-6	42.06	2.78

-10	-11	-6	49.61	4.09
10	11	6	48.77	3.73
10	11	6	47.88	4.12
10	11	6	40.32	4.43
10	11	6	43.91	2.65
-11	-11	-6	48.15	4.42
-11	-11	-6	47.94	4.71
11	11	6	51.66	4.00
11	11	6	49.67	4.44
11	11	6	43.06	4.95
11	11	6	44.15	2.85
-12	-11	-6	51.81	3.38
-12	-11	-6	57.46	5.40
-12	-11	-6	60.40	5.21
12	11	6	53.01	3.41
12	11	6	58.12	5.79
12	11	6	66.16	5.32
12	11	6	64.67	4.57
-13	-11	-6	23.39	2.16
-13	-11	-6	21.56	3.98
-13	-11	-6	29.17	4.89
13	11	6	28.18	4.94
13	11	6	25.79	3.97
-14	-11	-6	30.99	6.12
-14	-11	-6	34.05	5.86
-14	-11	-6	32.39	2.57
14	11	6	31.74	4.47
14	11	6	34.55	5.71
-15	-11	-6	11.88	4.26
-15	-11	-6	13.79	2.19
-15	-11	-6	9.82	4.72
-5	12	6	23.92	2.67
-4	12	6	30.98	2.66
-4	12	6	19.54	4.28
-3	12	6	34.82	4.30
-3	12	6	36.77	2.86
-2	12	6	43.14	2.82
-2	12	6	43.65	4.21
-2	12	6	50.80	3.20
-1	12	6	56.53	4.34
-1	12	6	59.38	3.31
0	-12	-6	66.76	4.12
0	12	6	50.75	3.31
0	12	6	66.60	4.26

-1	-12	-6	65.34	4.78
1	12	6	72.50	4.48
-2	-12	-6	58.44	4.05
2	12	6	56.97	3.94
-3	-12	-6	48.80	3.73
3	12	6	41.73	3.33
-4	-12	-6	47.99	3.86
4	12	6	47.79	3.33
4	12	6	41.49	3.44
4	12	6	50.18	3.10
-5	-12	-6	57.17	3.84
-5	-12	-6	65.84	3.45
-5	-12	-6	63.94	4.55
5	12	6	57.41	3.30
5	12	6	62.81	3.85
5	12	6	55.90	4.08
5	12	6	62.76	3.90
-6	-12	-6	72.36	3.83
-6	-12	-6	70.73	4.89
6	12	6	70.09	4.41
6	12	6	65.77	4.64
6	12	6	67.33	3.70
-7	-12	-6	81.38	4.15
-7	-12	-6	71.08	5.38
7	12	6	75.73	4.01
7	12	6	71.01	4.98
7	12	6	76.07	4.84
7	12	6	76.20	4.55
-8	-12	-6	62.86	6.03
-8	-12	-6	66.49	4.56
-8	-12	-6	71.87	3.89
8	12	6	74.13	4.43
8	12	6	68.26	3.73
8	12	6	70.15	4.74
8	12	6	69.90	5.09
-9	-12	-6	57.21	4.28
-9	-12	-6	61.06	3.50
-9	-12	-6	63.85	5.04
9	12	6	64.46	4.60
9	12	6	61.88	4.16
9	12	6	63.51	5.00
9	12	6	54.85	3.32
-10	-12	-6	69.01	3.74
-10	-12	-6	66.89	4.76

-10	-12	-6	64.48	5.36
10	12	6	64.15	5.32
10	12	6	56.06	3.58
10	12	6	68.93	4.51
10	12	6	66.14	4.94
-11	-12	-6	64.98	5.01
-11	-12	-6	57.99	5.40
-11	-12	-6	58.02	3.52
11	12	6	60.71	4.49
11	12	6	57.03	5.17
11	12	6	57.99	5.61
-12	-12	-6	47.87	5.39
-12	-12	-6	50.84	5.01
-12	-12	-6	46.85	3.02
12	12	6	47.17	4.31
12	12	6	39.00	5.68
12	12	6	43.32	5.07
-13	-12	-6	40.90	5.69
-13	-12	-6	40.43	5.46
-13	-12	-6	37.23	2.81
13	12	6	38.64	5.60
13	12	6	36.83	4.44
-14	-12	-6	21.24	5.67
-14	-12	-6	23.33	2.41
14	12	6	18.28	3.11
14	12	6	18.63	3.86
14	12	6	24.14	4.59
-3	13	6	25.19	2.60
-2	13	6	27.94	2.59
-2	13	6	22.58	4.23
-1	13	6	55.99	4.86
-1	13	6	66.60	3.94
0	-13	-6	49.79	3.88
0	13	6	38.65	3.92
0	13	6	43.52	3.43
-1	-13	-6	54.36	4.05
1	13	6	51.74	3.06
1	13	6	50.17	4.00
-2	-13	-6	27.21	3.34
2	13	6	27.08	2.68
2	13	6	28.90	3.16
-3	-13	-6	60.83	4.46
3	13	6	59.92	3.82
3	13	6	51.35	4.00

-4	-13	-6	33.72	3.54
4	13	6	31.35	2.02
4	13	6	26.31	3.06
4	13	6	29.42	3.27
-5	-13	-6	45.64	3.97
5	13	6	32.83	3.48
5	13	6	40.92	2.96
5	13	6	37.28	3.19
-6	-13	-6	39.20	4.19
-6	-13	-6	39.15	3.30
6	13	6	40.97	3.08
6	13	6	32.66	3.67
6	13	6	37.54	3.23
-7	-13	-6	57.76	4.86
-7	-13	-6	63.46	3.46
7	13	6	54.93	4.56
7	13	6	58.66	3.96
7	13	6	53.35	4.23
-8	-13	-6	25.66	2.78
-8	-13	-6	21.69	3.17
-8	-13	-6	27.53	2.14
-8	-13	-6	20.52	4.24
8	13	6	19.09	3.77
8	13	6	24.93	2.93
8	13	6	27.55	3.44
-9	-13	-6	52.00	3.08
-9	-13	-6	51.53	5.11
-9	-13	-6	45.22	3.73
-9	-13	-6	42.25	4.02
9	13	6	48.71	4.78
9	13	6	50.54	3.79
9	13	6	47.42	4.29
-10	-13	-6	42.74	4.36
-10	-13	-6	50.27	3.16
-10	-13	-6	51.15	5.44
10	13	6	56.45	5.40
10	13	6	48.33	4.64
-11	-13	-6	49.66	5.49
-11	-13	-6	45.81	4.63
-11	-13	-6	53.49	3.22
11	13	6	43.28	5.55
11	13	6	45.51	5.84
-12	-13	-6	27.85	2.48
-12	-13	-6	25.76	4.54

-12	-13	-6	24.43	4.02
12	13	6	19.93	2.33
12	13	6	26.04	4.86
12	13	6	32.17	3.83
-13	-13	-6	24.39	4.38
-13	-13	-6	31.04	5.21
-13	-13	-6	29.28	2.62
13	13	6	22.32	2.51
13	13	6	40.30	5.69
13	13	6	28.05	4.34
-14	-13	-6	16.38	4.53
-14	-13	-6	21.45	2.51
-14	-13	-6	16.77	5.22
14	13	6	15.67	2.46
14	13	6	11.88	4.04
14	13	6	13.56	2.99
-2	14	6	31.65	2.96
-1	14	6	31.26	2.83
0	-14	-6	50.28	4.44
0	14	6	47.27	3.43
0	14	6	43.25	4.58
-1	-14	-6	48.77	4.14
1	14	6	42.30	4.10
-2	-14	-6	52.63	4.43
2	14	6	47.85	4.11
2	14	6	45.62	3.56
-3	-14	-6	53.06	4.53
3	14	6	51.68	3.82
3	14	6	47.96	4.05
3	14	6	53.13	3.73
-4	-14	-6	67.05	5.01
4	14	6	57.96	3.43
4	14	6	56.57	4.85
4	14	6	55.17	4.40
4	14	6	67.59	3.97
-5	-14	-6	51.40	4.56
5	14	6	36.78	3.93
5	14	6	44.28	2.70
5	14	6	43.20	3.51
-6	-14	-6	72.09	5.35
6	14	6	58.93	3.50
6	14	6	65.05	4.17
6	14	6	55.40	4.76
6	14	6	60.04	4.28

-7	-14	-6	65.63	5.46
7	14	6	54.87	4.98
7	14	6	58.14	4.39
7	14	6	61.93	4.19
7	14	6	58.16	3.41
-8	-14	-6	55.95	4.05
-8	-14	-6	56.12	5.40
8	14	6	53.24	3.27
8	14	6	56.02	4.53
8	14	6	52.74	4.94
8	14	6	59.51	4.16
-9	-14	-6	34.13	4.09
-9	-14	-6	45.30	5.48
-9	-14	-6	45.13	3.05
-9	-14	-6	45.25	3.86
9	14	6	44.76	2.86
9	14	6	45.14	5.16
9	14	6	52.37	4.02
9	14	6	46.70	4.39
-10	-14	-6	58.25	5.90
-10	-14	-6	52.37	3.39
-10	-14	-6	50.95	4.30
-10	-14	-6	41.12	4.54
10	14	6	51.04	4.90
10	14	6	53.09	4.36
10	14	6	53.12	3.22
10	14	6	60.06	6.10
-11	-14	-6	38.00	5.92
-11	-14	-6	34.79	4.49
-11	-14	-6	34.99	2.75
11	14	6	34.29	4.08
11	14	6	31.45	2.63
-12	-14	-6	45.97	6.13
-12	-14	-6	34.25	4.99
-12	-14	-6	37.53	3.05
12	14	6	37.07	2.87
12	14	6	43.88	5.64
12	14	6	38.97	4.48
-13	-14	-6	34.00	6.06
-13	-14	-6	39.36	5.49
-13	-14	-6	34.10	2.99
13	14	6	32.45	4.83
13	14	6	29.45	2.89
13	14	6	33.62	6.13

0	15	6	29.50	3.00
-1	-15	-6	21.53	3.15
1	15	6	26.82	2.75
-2	-15	-6	48.19	4.63
2	15	6	44.00	3.69
-3	-15	-6	39.06	4.47
3	15	6	33.38	4.22
3	15	6	38.71	3.53
-4	-15	-6	58.37	5.06
4	15	6	48.65	4.06
4	15	6	42.95	4.42
-5	-15	-6	37.90	4.86
5	15	6	29.38	4.09
5	15	6	33.56	3.23
5	15	6	33.31	3.42
-6	-15	-6	46.84	5.16
6	15	6	48.41	3.80
6	15	6	40.49	4.70
6	15	6	42.30	3.79
-7	-15	-6	34.54	5.20
7	15	6	30.27	3.63
7	15	6	27.56	4.62
7	15	6	35.11	3.58
-8	-15	-6	49.49	5.77
8	15	6	48.38	4.13
8	15	6	46.07	4.41
-9	-15	-6	46.64	5.95
9	15	6	42.70	4.31
9	15	6	47.17	4.86
-10	-15	-6	46.72	6.18
10	15	6	41.04	4.41
-11	-15	-6	30.12	6.18
11	15	6	24.31	4.26
-12	-15	-6	35.39	3.04
-12	-15	-6	29.47	4.89
12	15	6	29.87	4.77
-3	-16	-6	38.57	5.13
-4	-16	-6	30.55	4.92
-5	-16	-6	48.31	5.42
5	16	6	41.62	3.70
-6	-16	-6	25.74	5.27
6	16	6	25.51	3.33
-7	-16	-6	39.86	5.69
7	16	6	38.03	3.92

-8	-16	-6	25.50	4.44
8	16	6	28.56	3.87
-9	-16	-6	38.40	6.46
9	16	6	39.44	4.46
-10	-16	-6	35.57	6.38
10	16	6	26.28	4.48
8	12	-7	8.82	3.58
8	12	-7	4.18	4.02
8	12	-7	1.79	3.16
-8	-12	7	4.42	2.68
-8	-12	7	7.16	3.59
-8	-12	7	0.69	2.74
7	12	-7	-0.72	4.64
7	12	-7	6.52	3.32
7	12	-7	0.34	0.89
7	12	-7	-2.30	2.39
7	12	-7	2.10	1.04
-7	-12	7	2.74	2.47
-7	-12	7	-1.70	2.66
-7	-12	7	3.38	3.25
6	12	-7	2.32	0.84
6	12	-7	1.06	2.30
6	12	-7	2.73	3.88
6	12	-7	9.68	3.21
6	12	-7	1.15	0.96
-6	-12	7	4.38	2.49
-6	-12	7	1.49	2.59
-6	-12	7	6.80	3.15
5	12	-7	2.30	3.26
5	12	-7	4.71	2.89
5	12	-7	3.93	2.32
5	12	-7	2.32	0.86
-5	-12	7	1.93	2.99
-5	-12	7	0.65	2.72
-5	-12	7	4.74	2.40
4	12	-7	1.80	3.29
4	12	-7	0.97	1.21
4	12	-7	2.50	0.89
-4	-12	7	1.98	2.98
3	12	-7	2.21	2.66
3	12	-7	-1.03	3.02
3	12	-7	0.11	0.88
-3	-12	7	-4.78	2.57
-3	-12	7	0.17	2.84

2	12	-7	-0.79	3.02
2	12	-7	0.82	0.95
-2	-12	7	0.83	3.03
1	12	-7	0.56	3.15
1	12	-7	0.29	0.96
-1	-12	7	-1.80	2.88
0	12	-7	1.09	1.00
0	12	-7	3.47	3.06
0	-12	7	3.81	2.99
10	11	-7	6.08	3.90
10	11	-7	0.65	2.41
-10	-11	7	0.18	2.78
-10	-11	7	2.61	3.09
-10	-11	7	5.65	3.54
9	11	-7	1.09	2.24
9	11	-7	5.16	3.31
9	11	-7	-0.44	4.29
-9	-11	7	5.59	3.33
-9	-11	7	0.19	2.92
-9	-11	7	2.94	2.49
8	11	-7	1.22	2.15
8	11	-7	2.65	3.50
8	11	-7	6.39	3.06
-8	-11	7	3.77	3.23
-8	-11	7	-2.74	2.42
-8	-11	7	1.85	2.28
7	11	-7	1.93	3.10
7	11	-7	3.19	2.81
7	11	-7	3.90	2.03
-7	-11	7	3.18	2.18
-7	-11	7	0.64	2.31
-7	-11	7	4.57	3.13
6	11	-7	2.39	1.94
6	11	-7	4.26	0.96
6	11	-7	3.84	2.90
6	11	-7	2.23	0.71
6	11	-7	5.28	2.58
-6	-11	7	3.66	2.21
-6	-11	7	4.53	2.07
-6	-11	7	2.89	2.82
5	11	-7	6.29	3.40
5	11	-7	8.92	3.26
5	11	-7	11.86	1.83
5	11	-7	11.33	2.69

5	11	-7	16.09	3.71
5	11	-7	12.57	1.91
-5	-11	7	13.42	2.96
-5	-11	7	8.89	2.18
-5	-11	7	10.17	2.48
4	11	-7	10.05	3.81
4	11	-7	3.52	0.79
4	11	-7	6.83	2.15
4	11	-7	6.71	3.79
4	11	-7	3.53	2.83
-4	-11	7	1.35	2.28
-4	-11	7	6.79	2.68
3	11	-7	2.44	0.81
3	11	-7	5.90	2.42
3	11	-7	3.34	2.14
3	11	-7	3.15	2.42
-3	-11	7	0.32	1.92
-3	-11	7	5.12	2.57
-3	-11	7	0.39	2.34
2	11	-7	2.98	2.36
2	11	-7	2.67	2.30
2	11	-7	0.63	0.77
-2	-11	7	1.06	1.87
-2	-11	7	1.77	2.63
-2	-11	7	0.57	2.32
1	11	-7	0.12	0.82
1	11	-7	1.51	2.32
1	11	-7	-3.02	2.71
-1	-11	7	0.06	2.65
0	11	-7	-1.23	0.83
0	11	-7	-1.59	2.58
0	-11	7	-0.17	2.52
0	-11	7	-1.34	2.04
-1	11	-7	-0.54	2.84
-1	11	-7	0.45	0.95
1	-11	7	1.96	2.81
-2	11	-7	0.89	2.95
-2	11	-7	0.82	0.93
-11	-10	7	2.66	3.59
-11	-10	7	-1.41	2.73
10	10	-7	1.30	3.21
10	10	-7	0.93	2.20
-10	-10	7	4.53	2.30
-10	-10	7	4.11	3.32

-10	-10	7	2.24	2.52
9	10	-7	9.22	3.02
9	10	-7	7.81	2.20
9	10	-7	6.88	3.25
-9	-10	7	7.59	2.25
-9	-10	7	6.50	3.04
-9	-10	7	9.11	2.73
8	10	-7	4.09	2.71
8	10	-7	2.15	1.80
8	10	-7	6.23	3.73
8	10	-7	2.06	2.89
-8	-10	7	2.53	1.92
-8	-10	7	1.78	2.10
-8	-10	7	6.25	2.80
7	10	-7	4.29	3.37
7	10	-7	2.78	2.43
7	10	-7	-0.70	2.62
7	10	-7	0.00	1.76
-7	-10	7	-1.22	2.03
-7	-10	7	1.62	1.75
-7	-10	7	2.55	2.75
6	10	-7	1.76	0.77
6	10	-7	3.33	2.25
6	10	-7	3.62	3.04
6	10	-7	1.77	0.60
6	10	-7	2.08	2.56
6	10	-7	2.88	1.75
-6	-10	7	5.05	2.70
-6	-10	7	2.26	1.91
-6	-10	7	3.29	1.83
5	10	-7	8.67	1.88
5	10	-7	12.34	2.89
5	10	-7	7.28	1.20
5	10	-7	10.99	2.27
5	10	-7	6.79	2.56
5	10	-7	7.29	1.01
-5	-10	7	10.49	2.63
-5	-10	7	7.06	1.90
-5	-10	7	8.58	2.08
4	10	-7	2.79	0.67
4	10	-7	3.63	2.39
4	10	-7	4.48	2.52
4	10	-7	5.24	2.13
4	10	-7	4.62	1.71

-4	-10	7	1.36	1.94
-4	-10	7	3.30	2.40
-4	-10	7	3.74	1.67
3	10	-7	-0.29	2.17
3	10	-7	3.16	2.04
3	10	-7	1.38	2.39
3	10	-7	1.66	1.97
3	10	-7	1.81	0.71
-3	-10	7	2.19	2.32
-3	-10	7	0.66	1.93
-3	-10	7	2.68	1.71
2	10	-7	3.81	1.80
2	10	-7	4.41	2.03
2	10	-7	3.40	2.38
2	10	-7	2.64	0.74
2	10	-7	2.51	2.05
-2	-10	7	1.58	2.04
-2	-10	7	2.94	1.69
-2	-10	7	2.87	2.21
1	10	-7	1.44	2.44
1	10	-7	2.00	0.75
1	10	-7	3.16	2.04
1	10	-7	-1.43	2.31
-1	-10	7	0.87	2.35
-1	-10	7	1.96	1.66
-1	-10	7	0.24	1.94
0	10	-7	0.65	0.76
0	10	-7	0.22	2.02
0	10	-7	-1.46	2.32
0	10	-7	2.33	2.34
0	-10	7	-0.98	3.33
0	-10	7	-0.63	2.24
0	-10	7	-0.71	1.71
-1	10	-7	2.24	2.55
-1	10	-7	3.72	2.03
-1	10	-7	3.27	0.89
1	-10	7	6.12	2.48
-2	10	-7	0.54	2.53
-2	10	-7	1.11	2.20
-2	10	-7	0.02	0.87
2	-10	7	-1.22	1.62
-3	10	-7	0.83	2.68
-3	10	-7	1.94	0.96
-4	10	-7	0.70	2.48

-4	10	-7	-1.69	0.95
-12	-9	7	3.71	3.87
-12	-9	7	-0.69	2.85
11	9	-7	-2.89	1.80
-11	-9	7	2.62	3.37
-11	-9	7	-0.18	2.52
-11	-9	7	3.24	2.68
10	9	-7	2.79	4.08
10	9	-7	2.28	1.93
10	9	-7	2.75	2.85
-10	-9	7	0.59	2.23
-10	-9	7	4.17	2.09
-10	-9	7	0.54	2.97
9	9	-7	1.57	2.90
9	9	-7	4.58	2.56
9	9	-7	1.98	1.74
9	9	-7	-3.25	3.45
-9	-9	7	3.20	2.93
-9	-9	7	0.87	1.84
-9	-9	7	0.72	2.27
8	9	-7	3.09	1.66
8	9	-7	5.96	3.52
8	9	-7	2.34	2.33
8	9	-7	4.08	2.62
-8	-9	7	4.23	2.60
-8	-9	7	0.99	1.89
7	9	-7	4.98	2.20
7	9	-7	2.54	1.53
7	9	-7	1.79	2.40
-7	-9	7	3.49	1.82
-7	-9	7	5.83	2.37
-7	-9	7	4.65	1.66
6	9	-7	5.00	1.57
6	9	-7	5.69	2.13
6	9	-7	6.99	2.43
-6	-9	7	7.32	2.36
-6	-9	7	4.81	1.79
-6	-9	7	4.77	1.64
5	9	-7	2.03	1.87
5	9	-7	0.56	0.52
5	9	-7	-0.40	2.24
5	9	-7	-1.11	1.46
-5	-9	7	-1.45	2.12
-5	-9	7	2.42	1.46

-5	-9	7	-1.84	1.59
4	9	-7	0.57	1.85
4	9	-7	0.05	0.54
4	9	-7	-0.54	1.45
4	9	-7	0.81	2.01
4	9	-7	-0.41	2.60
-4	-9	7	0.18	2.05
-4	-9	7	0.60	1.46
-4	-9	7	-1.95	1.61
3	9	-7	-1.68	1.65
3	9	-7	-1.07	1.74
3	9	-7	1.61	1.79
3	9	-7	0.24	2.47
3	9	-7	0.31	0.54
-3	-9	7	-0.32	2.00
-3	-9	7	1.00	1.48
2	9	-7	0.25	1.72
2	9	-7	-0.48	1.78
2	9	-7	0.79	0.64
-2	-9	7	-0.81	1.49
-2	-9	7	2.44	1.97
-2	-9	7	-1.95	1.69
1	9	-7	0.72	1.80
1	9	-7	0.58	2.13
1	9	-7	1.66	0.64
-1	-9	7	0.48	1.82
-1	-9	7	0.57	1.47
-1	-9	7	2.54	2.05
0	9	-7	8.00	2.19
0	9	-7	9.02	2.07
0	9	-7	13.06	1.39
0	-9	7	14.00	2.38
0	-9	7	10.75	2.04
0	-9	7	10.43	1.77
-1	9	-7	4.54	2.15
-1	9	-7	2.32	0.75
-1	9	-7	1.90	2.06
-1	9	-7	-0.34	1.76
1	-9	7	3.35	1.59
1	-9	7	-0.97	1.60
1	-9	7	0.30	2.01
-2	9	-7	4.47	2.18
-2	9	-7	3.76	2.42
-2	9	-7	1.28	1.90

-2	9	-7	2.58	0.79
2	-9	7	3.40	1.61
-3	9	-7	-0.61	0.82
-3	9	-7	0.54	2.31
-3	9	-7	-1.74	1.82
3	-9	7	0.17	1.72
-4	9	-7	2.61	2.35
-4	9	-7	0.96	1.44
-4	9	-7	1.06	0.93
-4	9	-7	-1.05	2.56
-5	9	-7	0.40	2.28
-5	9	-7	-1.64	0.91
-12	-8	7	7.70	3.62
-12	-8	7	1.84	2.75
-12	-8	7	0.93	2.71
11	8	-7	2.27	4.15
11	8	-7	-2.74	1.64
-11	-8	7	-0.15	2.01
-11	-8	7	2.45	3.20
-11	-8	7	-0.67	2.31
10	8	-7	-0.19	2.52
10	8	-7	4.15	1.71
10	8	-7	7.90	3.84
-10	-8	7	5.45	1.90
-10	-8	7	2.34	2.83
-10	-8	7	2.52	2.06
-10	-8	7	2.04	2.28
9	8	-7	0.14	1.47
9	8	-7	0.45	2.36
-9	-8	7	1.94	1.67
-9	-8	7	1.59	2.02
-9	-8	7	-0.27	2.66
8	8	-7	3.82	2.24
8	8	-7	1.16	1.46
-8	-8	7	-1.72	1.68
-8	-8	7	0.67	2.31
-8	-8	7	-0.12	1.46
7	8	-7	3.13	2.54
7	8	-7	1.11	1.95
7	8	-7	1.94	1.34
-7	-8	7	2.23	2.12
-7	-8	7	0.81	1.59
-7	-8	7	1.87	1.42
6	8	-7	5.87	2.41

6	8	-7	2.68	1.39
6	8	-7	4.68	1.91
6	8	-7	0.64	2.16
-6	-8	7	2.71	1.38
-6	-8	7	3.16	1.60
5	8	-7	-0.23	0.46
5	8	-7	0.37	1.29
5	8	-7	0.33	1.91
5	8	-7	0.83	2.09
5	8	-7	-0.97	2.00
-5	-8	7	-0.48	1.94
-5	-8	7	-2.06	1.42
4	8	-7	-0.31	1.81
4	8	-7	0.26	0.48
4	8	-7	-0.52	1.33
4	8	-7	-1.05	1.61
4	8	-7	2.16	1.93
-4	-8	7	1.06	1.92
-4	-8	7	0.46	1.25
-4	-8	7	0.95	1.43
3	8	-7	-0.14	0.47
3	8	-7	-0.38	1.57
3	8	-7	1.16	1.83
3	8	-7	-1.59	1.43
-3	-8	7	-0.86	1.26
-3	-8	7	-0.51	1.74
-3	-8	7	-0.59	1.52
2	8	-7	1.46	1.55
2	8	-7	0.74	0.54
2	8	-7	0.09	1.55
2	8	-7	1.79	1.79
-2	-8	7	0.47	1.62
-2	-8	7	2.26	1.78
-2	-8	7	0.48	1.31
1	8	-7	3.57	1.77
1	8	-7	1.51	1.56
1	8	-7	2.50	0.61
1	8	-7	0.06	1.72
-1	-8	7	0.76	1.56
-1	-8	7	1.27	1.35
0	8	-7	2.51	0.84
0	8	-7	-1.07	1.54
0	8	-7	3.32	1.79
0	8	-7	-1.13	1.74

0	-8	7	2.51	1.43
0	-8	7	1.63	1.41
0	-8	7	2.86	1.83
-1	8	-7	4.77	0.73
-1	8	-7	1.10	1.63
-1	8	-7	1.22	2.33
1	-8	7	4.69	1.49
1	-8	7	3.66	2.15
-2	8	-7	-0.91	1.86
-2	8	-7	-0.98	0.64
2	-8	7	0.86	1.37
2	-8	7	-0.58	2.10
-3	8	-7	-1.83	1.58
-3	8	-7	-0.43	0.73
-3	8	-7	3.94	1.99
-3	8	-7	3.10	2.09
3	-8	7	0.03	1.53
3	-8	7	1.62	1.43
-4	8	-7	3.42	2.41
-4	8	-7	1.39	2.02
4	-8	7	2.43	1.79
-5	8	-7	0.21	1.31
-5	8	-7	1.54	2.15
-5	8	-7	-0.66	0.83
5	-8	7	-0.74	1.42
-6	8	-7	-1.21	2.16
-6	8	-7	-0.15	0.95
6	-8	7	1.25	1.61
-7	8	-7	-2.02	1.03
-7	8	-7	-0.59	2.44
7	-8	7	1.44	1.82
-13	-7	7	-0.71	4.04
-13	-7	7	-5.03	2.80
-13	-7	7	-0.70	2.87
-12	-7	7	-2.21	2.55
-12	-7	7	2.46	3.38
11	7	-7	3.50	3.34
11	7	-7	9.08	3.89
11	7	-7	-2.15	2.16
-11	-7	7	2.31	3.11
-11	-7	7	2.26	1.91
-11	-7	7	-0.11	2.19
-11	-7	7	1.97	2.30
10	7	-7	1.44	1.69

10	7	-7	2.85	2.32
-10	-7	7	1.73	1.86
-10	-7	7	-0.59	1.99
-10	-7	7	1.87	2.70
-10	-7	7	3.67	1.73
9	7	-7	3.25	2.19
9	7	-7	1.44	3.30
9	7	-7	0.96	1.36
-9	-7	7	-1.63	1.79
-9	-7	7	0.84	1.83
-9	-7	7	0.61	1.49
-9	-7	7	1.99	2.35
8	7	-7	0.63	1.23
8	7	-7	2.21	1.99
8	7	-7	-0.22	2.58
-8	-7	7	1.56	1.35
-8	-7	7	-0.72	1.48
7	7	-7	8.41	1.47
7	7	-7	10.27	2.49
7	7	-7	8.43	2.03
-7	-7	7	11.65	2.20
-7	-7	7	8.10	1.55
-7	-7	7	7.88	1.66
6	7	-7	1.33	1.19
6	7	-7	2.60	1.68
6	7	-7	1.98	2.15
-6	-7	7	3.18	1.22
-6	-7	7	2.33	1.37
-6	-7	7	2.21	1.81
5	7	-7	11.13	1.52
5	7	-7	7.89	2.29
5	7	-7	12.30	2.20
-5	-7	7	12.62	2.21
-5	-7	7	10.51	1.52
-5	-7	7	13.35	2.06
4	7	-7	-0.58	1.42
4	7	-7	-0.78	1.21
4	7	-7	2.29	1.74
4	7	-7	1.10	0.46
-4	-7	7	0.68	1.74
-4	-7	7	0.61	1.28
-4	-7	7	0.70	1.11
3	7	-7	7.44	1.53
3	7	-7	9.90	1.90

3	7	-7	8.09	0.97
3	7	-7	8.73	1.60
-3	-7	7	8.65	1.50
-3	-7	7	7.28	1.43
2	7	-7	4.69	1.65
2	7	-7	4.35	1.49
2	7	-7	3.63	0.57
2	7	-7	1.85	1.42
-2	-7	7	3.47	1.43
-2	-7	7	4.60	1.26
-2	-7	7	5.33	1.71
1	7	-7	4.00	1.50
1	7	-7	6.94	1.01
1	7	-7	8.15	1.65
1	7	-7	8.04	1.66
-1	-7	7	8.65	1.42
-1	-7	7	8.31	1.70
-1	-7	7	7.18	1.54
0	7	-7	0.04	1.43
0	7	-7	1.97	1.59
0	7	-7	2.56	1.61
0	7	-7	2.09	0.54
0	-7	7	2.46	0.85
0	-7	7	3.13	1.35
0	-7	7	2.05	1.62
0	-7	7	3.68	1.31
-1	7	-7	7.58	1.11
-1	7	-7	8.35	1.82
-1	7	-7	4.42	1.74
1	-7	7	6.65	1.47
1	-7	7	7.34	1.71
1	-7	7	7.69	1.51
1	-7	7	5.38	1.03
-2	7	-7	0.59	1.62
-2	7	-7	0.13	1.66
-2	7	-7	-0.02	0.57
2	-7	7	1.35	1.48
2	-7	7	0.32	1.32
2	-7	7	-0.37	0.86
-3	7	-7	-1.21	0.93
-3	7	-7	2.11	1.77
-3	7	-7	-1.00	0.60
3	-7	7	0.03	1.34
3	-7	7	-1.05	0.87

3	-7	7	-0.99	0.94
-4	7	-7	-2.11	1.47
-4	7	-7	-0.89	0.69
-4	7	-7	0.71	1.75
4	-7	7	-0.16	0.90
4	-7	7	0.39	1.44
-5	7	-7	-0.03	1.05
-5	7	-7	1.39	0.81
-5	7	-7	-0.58	1.48
-5	7	-7	2.19	1.90
-5	7	-7	5.28	2.32
5	-7	7	0.49	1.01
5	-7	7	0.46	1.21
-6	7	-7	-0.06	1.17
-6	7	-7	-2.18	1.60
-6	7	-7	-1.13	0.84
6	-7	7	-0.81	1.34
-7	7	-7	-2.25	2.27
7	-7	7	0.75	1.61
-8	7	-7	-2.03	1.02
-8	7	-7	-3.01	2.39
-8	7	-7	4.21	3.84
8	-7	7	-0.21	1.80
-13	-6	7	2.22	3.81
-13	-6	7	-2.42	2.54
-13	-6	7	-1.42	2.72
12	6	-7	0.34	1.44
12	6	-7	1.73	4.38
-12	-6	7	-2.48	2.51
-12	-6	7	-1.10	3.23
-12	-6	7	2.12	2.03
11	6	-7	1.22	2.65
11	6	-7	3.23	3.97
11	6	-7	-0.83	2.26
-11	-6	7	3.29	2.99
-11	-6	7	0.12	2.18
-11	-6	7	-2.37	1.98
-11	-6	7	1.07	1.72
10	6	-7	-1.63	1.27
10	6	-7	-0.78	2.16
-10	-6	7	-2.67	1.86
-10	-6	7	1.90	1.56
-10	-6	7	-2.97	1.68
-10	-6	7	-0.82	2.47

9	6	-7	-0.80	1.93
9	6	-7	3.37	2.93
9	6	-7	-0.60	1.55
-9	-6	7	-1.52	1.58
-9	-6	7	0.41	1.34
8	6	-7	0.83	1.83
8	6	-7	-1.13	2.22
8	6	-7	-0.93	2.46
8	6	-7	-0.82	1.12
-8	-6	7	-2.57	1.47
-8	-6	7	-1.41	2.04
-8	-6	7	-0.70	1.23
7	6	-7	9.18	2.32
7	6	-7	10.18	1.89
7	6	-7	9.82	1.40
-7	-6	7	10.80	1.70
-7	-6	7	9.87	1.47
-7	-6	7	9.18	1.54
6	6	-7	-0.44	1.04
6	6	-7	1.41	1.91
6	6	-7	0.29	1.48
-6	-6	7	0.56	1.09
-6	-6	7	-0.18	1.14
-6	-6	7	-0.94	1.24
-6	-6	7	0.16	1.61
5	6	-7	-1.09	1.05
5	6	-7	-0.51	1.69
-5	-6	7	-0.86	1.01
-5	-6	7	-0.16	1.11
-5	-6	7	0.39	1.50
-5	-6	7	-2.02	1.15
4	6	-7	0.15	1.57
4	6	-7	-2.36	1.28
4	6	-7	-1.92	1.11
4	6	-7	-1.61	1.47
-4	-6	7	-0.74	1.02
3	6	-7	-0.40	1.46
3	6	-7	-1.55	1.16
3	6	-7	-0.18	0.41
3	6	-7	-1.97	1.13
-3	-6	7	-0.03	0.85
-3	-6	7	0.71	1.47
-3	-6	7	-0.57	0.97
-3	-6	7	-1.10	1.14

2	6	-7	0.42	1.29
2	6	-7	2.64	0.49
2	6	-7	0.47	1.25
2	6	-7	3.22	1.52
-2	-6	7	1.88	0.62
-2	-6	7	1.52	1.24
-2	-6	7	2.65	1.09
-2	-6	7	2.25	1.41
-2	-6	7	1.95	0.90
1	6	-7	2.40	0.52
1	6	-7	4.18	1.12
1	6	-7	3.89	1.35
1	6	-7	0.65	1.24
1	6	-7	3.65	1.33
-1	-6	7	3.06	0.83
-1	-6	7	4.01	1.15
-1	-6	7	2.38	1.38
-1	-6	7	3.81	1.35
0	6	-7	-0.61	0.44
0	6	-7	-0.78	1.31
0	6	-7	-0.32	1.36
0	6	-7	0.53	0.90
0	-6	7	-0.04	1.28
0	-6	7	-0.50	1.07
0	-6	7	-0.31	0.62
0	-6	7	-0.60	0.76
0	-6	7	0.00	1.18
-1	6	-7	-1.07	1.40
-1	6	-7	0.25	0.51
-1	6	-7	0.66	1.37
-1	6	-7	0.79	0.87
1	-6	7	0.32	0.70
1	-6	7	1.37	1.11
1	-6	7	0.99	1.11
1	-6	7	0.67	0.81
-2	6	-7	-0.52	1.27
-2	6	-7	2.46	1.53
-2	6	-7	2.00	1.57
-2	6	-7	0.44	0.54
2	-6	7	0.61	0.74
2	-6	7	1.17	1.39
2	-6	7	0.03	1.43
2	-6	7	1.36	1.25
-3	6	-7	-1.22	1.25

-3	6	-7	0.50	0.83
-3	6	-7	-0.08	1.68
-3	6	-7	0.04	0.60
-3	6	-7	1.02	1.52
3	-6	7	0.05	1.06
3	-6	7	0.28	0.76
3	-6	7	0.77	1.37
-4	6	-7	1.64	0.66
-4	6	-7	0.38	0.94
-4	6	-7	3.22	2.27
-4	6	-7	3.18	1.81
-4	6	-7	-0.77	1.34
4	-6	7	-0.73	0.67
4	-6	7	0.69	1.46
4	-6	7	0.97	0.99
-5	6	-7	2.24	1.70
-5	6	-7	0.24	0.95
-5	6	-7	-0.35	1.41
-5	6	-7	0.53	0.70
5	-6	7	1.43	1.11
5	-6	7	1.64	1.28
-6	6	-7	1.41	1.92
-6	6	-7	-1.22	1.45
-6	6	-7	-0.36	0.77
-6	6	-7	2.74	2.12
-6	6	-7	0.19	0.99
6	-6	7	1.05	1.24
-7	6	-7	-1.48	0.84
-7	6	-7	-1.54	2.14
-7	6	-7	-2.05	2.56
-7	6	-7	0.23	1.17
-7	6	-7	-1.07	1.61
7	-6	7	1.93	1.46
-8	6	-7	3.39	3.58
-8	6	-7	-1.77	2.36
8	-6	7	-0.31	1.68
-9	6	-7	0.26	4.00
-9	6	-7	-1.38	1.02
-9	6	-7	-1.02	2.59
9	-6	7	-2.51	1.87
-13	-5	7	-2.94	2.37
-13	-5	7	-0.19	2.94
-13	-5	7	-3.31	3.77
12	5	-7	2.71	4.19

-12	-5	7	1.52	3.21
-12	-5	7	-0.28	1.83
-12	-5	7	-0.68	2.53
-12	-5	7	-2.78	2.08
11	5	-7	0.06	1.43
-11	-5	7	-1.63	2.18
-11	-5	7	-0.67	1.56
-11	-5	7	-3.43	1.83
-11	-5	7	-1.43	2.79
10	5	-7	9.41	3.27
10	5	-7	8.08	2.16
-10	-5	7	7.91	2.15
-10	-5	7	10.70	2.67
-10	-5	7	5.78	1.77
-10	-5	7	8.67	1.66
9	5	-7	-1.99	1.80
9	5	-7	-0.83	2.64
-9	-5	7	1.72	2.16
-9	-5	7	1.26	1.29
-9	-5	7	-1.77	1.44
-9	-5	7	0.83	1.60
8	5	-7	2.28	1.73
8	5	-7	2.35	2.36
8	5	-7	0.23	2.16
8	5	-7	1.59	1.20
-8	-5	7	-1.16	1.28
-8	-5	7	2.24	1.20
-8	-5	7	3.05	1.98
7	5	-7	6.23	1.63
7	5	-7	3.79	1.28
7	5	-7	8.46	2.21
7	5	-7	4.57	1.95
-7	-5	7	4.82	1.43
-7	-5	7	5.85	1.22
-7	-5	7	5.52	1.77
-7	-5	7	3.46	1.32
6	5	-7	3.02	2.09
6	5	-7	3.98	1.12
6	5	-7	4.90	1.46
-6	-5	7	4.30	1.60
-6	-5	7	1.75	1.19
-6	-5	7	4.39	1.29
-6	-5	7	4.48	1.13
5	5	-7	-1.77	1.56

5	5	-7	-0.99	1.00
5	5	-7	-1.50	1.53
-5	-5	7	0.14	0.92
-5	-5	7	-1.32	1.01
-5	-5	7	-1.17	1.05
4	5	-7	-0.21	1.13
4	5	-7	0.80	1.42
4	5	-7	0.63	0.99
-4	-5	7	0.66	0.94
-4	-5	7	-0.26	0.98
3	5	-7	0.35	1.06
3	5	-7	-0.81	1.29
3	5	-7	-0.27	1.04
3	5	-7	0.89	0.37
-3	-5	7	1.24	0.45
-3	-5	7	1.58	1.03
-3	-5	7	2.38	0.94
2	5	-7	20.70	2.23
2	5	-7	20.84	2.11
2	5	-7	16.45	1.20
2	5	-7	16.96	2.10
-2	-5	7	18.13	2.08
-2	-5	7	20.89	1.94
-2	-5	7	17.77	1.40
-2	-5	7	18.01	2.12
1	5	-7	9.34	1.87
1	5	-7	6.24	1.24
1	5	-7	7.02	1.34
1	5	-7	7.71	1.37
1	5	-7	7.82	0.85
-1	-5	7	9.67	1.72
-1	-5	7	7.65	1.29
-1	-5	7	7.55	1.01
-1	-5	7	7.41	1.09
0	5	-7	5.93	1.25
0	5	-7	9.67	1.45
0	5	-7	8.60	1.44
0	5	-7	8.32	0.92
0	-5	7	10.09	1.82
0	-5	7	9.18	1.44
0	-5	7	7.53	1.06
0	-5	7	8.52	1.27
-1	5	-7	-0.44	0.43
-1	5	-7	0.27	1.26

-1	5	-7	0.22	0.75
1	-5	7	-0.14	0.57
1	-5	7	0.74	0.95
1	-5	7	0.21	0.69
-2	5	-7	0.16	0.47
-2	5	-7	-0.75	0.72
-2	5	-7	-0.68	1.09
-2	5	-7	0.55	1.29
-2	5	-7	-0.24	1.33
2	-5	7	-0.71	1.20
2	-5	7	0.39	1.12
2	-5	7	0.54	0.64
2	-5	7	-1.29	1.10
-3	5	-7	0.51	1.36
-3	5	-7	-2.07	1.10
-3	5	-7	0.43	0.50
-3	5	-7	0.00	0.78
-3	5	-7	1.97	1.40
3	-5	7	0.56	0.73
3	-5	7	0.25	1.39
3	-5	7	0.40	1.25
3	-5	7	0.25	0.66
-4	5	-7	0.02	0.82
-4	5	-7	0.43	1.39
-4	5	-7	0.08	1.23
-4	5	-7	0.86	1.46
4	-5	7	0.15	0.82
4	-5	7	-0.07	1.31
4	-5	7	0.16	0.70
4	-5	7	-0.30	1.79
-5	5	-7	-0.22	0.60
-5	5	-7	0.02	0.86
-5	5	-7	0.21	1.69
-5	5	-7	-1.14	1.32
5	-5	7	-0.79	1.16
5	-5	7	-0.80	0.92
5	-5	7	-2.19	1.40
-6	5	-7	5.87	1.91
-6	5	-7	4.64	1.06
-6	5	-7	3.02	1.52
6	-5	7	1.54	1.50
6	-5	7	4.66	1.27
-7	5	-7	-0.63	1.57
-7	5	-7	2.57	1.99

-7	5	-7	1.74	1.01
-7	5	-7	1.90	2.11
-7	5	-7	-0.42	0.77
7	-5	7	1.58	1.29
-8	5	-7	-0.50	2.43
-8	5	-7	-1.84	0.87
-8	5	-7	-2.02	2.22
-8	5	-7	-3.33	1.74
-8	5	-7	2.49	3.21
8	-5	7	0.38	1.43
-9	5	-7	0.39	0.97
-9	5	-7	2.10	3.91
9	-5	7	1.18	1.80
-10	5	-7	-1.33	1.02
-10	5	-7	0.60	2.71
-10	5	-7	-0.33	4.50
10	-5	7	-2.18	1.95
-13	-4	7	-2.62	2.04
-13	-4	7	-1.82	3.77
-13	-4	7	0.78	2.94
12	4	-7	3.34	3.85
-12	-4	7	1.89	2.01
-12	-4	7	-1.71	2.02
-12	-4	7	2.34	3.16
-12	-4	7	0.37	2.60
-11	-4	7	0.77	1.61
-11	-4	7	-0.29	1.75
-11	-4	7	-1.29	2.23
-11	-4	7	-2.98	2.69
10	4	-7	-0.21	3.14
-10	-4	7	4.02	1.41
-10	-4	7	1.18	1.98
-10	-4	7	0.96	1.53
9	4	-7	-0.27	2.55
-9	-4	7	-0.18	1.13
-9	-4	7	-0.74	1.33
-9	-4	7	-2.37	2.02
-9	-4	7	0.42	1.66
8	4	-7	-2.11	2.19
8	4	-7	-1.26	2.08
8	4	-7	-0.99	1.60
-8	-4	7	2.24	1.81
-8	-4	7	-2.01	1.18
7	4	-7	-2.31	1.82

7	4	-7	0.63	0.89
7	4	-7	-0.60	1.42
-7	-4	7	-0.99	1.21
-7	-4	7	0.82	0.94
-7	-4	7	0.51	1.56
6	4	-7	2.64	1.58
6	4	-7	2.16	1.33
-6	-4	7	1.71	1.19
-6	-4	7	1.35	1.07
-6	-4	7	2.60	0.97
5	4	-7	-1.45	1.40
5	4	-7	-1.02	1.05
-5	-4	7	0.11	0.87
-5	-4	7	-1.06	0.98
-5	-4	7	-0.94	1.00
4	4	-7	-0.28	0.94
4	4	-7	-0.64	1.39
4	4	-7	0.77	1.11
-4	-4	7	1.44	0.90
-4	-4	7	0.99	0.87
-4	-4	7	1.13	0.93
3	4	-7	4.58	1.06
3	4	-7	4.56	1.10
-3	-4	7	4.92	0.76
-3	-4	7	4.40	0.95
-3	-4	7	6.09	1.02
-3	-4	7	3.93	1.02
2	4	-7	6.07	0.70
2	4	-7	5.13	1.12
2	4	-7	6.48	1.33
2	4	-7	9.56	1.73
-2	-4	7	7.03	1.49
-2	-4	7	6.86	0.87
-2	-4	7	7.66	1.13
-2	-4	7	8.90	1.56
1	4	-7	13.48	1.92
1	4	-7	13.90	1.85
1	4	-7	8.71	1.24
1	4	-7	12.45	0.91
-1	-4	7	11.38	1.81
-1	-4	7	12.34	1.72
-1	-4	7	12.73	1.10
-1	-4	7	9.68	1.55
0	4	-7	-0.49	0.97

0	4	-7	-0.46	0.35
0	4	-7	-1.61	0.92
0	4	-7	0.42	1.04
0	-4	7	-0.46	0.92
0	-4	7	-0.83	0.97
0	-4	7	-0.16	0.48
0	-4	7	-0.66	0.95
0	-4	7	-0.50	0.66
-1	4	-7	-0.32	1.16
-1	4	-7	1.46	1.06
-1	4	-7	0.55	0.41
-1	4	-7	-1.06	0.96
-1	4	-7	0.84	0.68
1	-4	7	-1.05	0.99
1	-4	7	-0.06	0.61
1	-4	7	-0.19	0.51
1	-4	7	1.01	0.90
-2	4	-7	0.62	1.16
-2	4	-7	0.53	0.62
-2	4	-7	1.58	1.22
-2	4	-7	-1.09	0.94
2	-4	7	-1.62	1.12
2	-4	7	0.57	0.93
2	-4	7	0.26	0.63
-3	4	-7	1.38	1.27
-3	4	-7	1.59	1.52
-3	4	-7	-1.23	1.07
-3	4	-7	-0.10	0.46
-3	4	-7	0.33	0.65
3	-4	7	1.24	1.10
3	-4	7	0.63	0.66
3	-4	7	-1.22	1.23
3	-4	7	-0.91	0.57
-4	4	-7	2.11	1.33
-4	4	-7	1.42	0.77
-4	4	-7	1.52	1.29
-4	4	-7	0.78	0.55
-4	4	-7	0.90	1.19
4	-4	7	-0.74	1.33
4	-4	7	1.34	0.75
4	-4	7	1.82	1.15
4	-4	7	1.20	0.67
-5	4	-7	-0.01	0.72
-5	4	-7	0.24	0.79

-5	4	-7	0.59	1.40
-5	4	-7	0.80	1.31
-5	4	-7	0.22	1.43
5	-4	7	1.02	0.86
5	-4	7	0.33	1.44
-6	4	-7	-0.53	1.56
-6	4	-7	-1.80	2.61
-6	4	-7	0.08	0.61
6	-4	7	-0.18	0.97
6	-4	7	-0.91	1.09
-7	4	-7	-0.50	1.86
-7	4	-7	-0.43	1.56
-7	4	-7	1.51	2.55
-7	4	-7	1.59	0.77
-7	4	-7	2.44	0.94
7	-4	7	1.68	1.17
-8	4	-7	-0.42	0.99
-8	4	-7	-2.32	2.03
-8	4	-7	0.49	2.88
-8	4	-7	2.38	2.06
-8	4	-7	-1.32	0.79
-8	4	-7	-1.22	1.69
8	-4	7	0.46	0.93
8	-4	7	0.44	1.34
-9	4	-7	-0.14	1.97
-9	4	-7	-2.06	2.38
-9	4	-7	-0.51	0.88
-9	4	-7	2.17	3.50
-9	4	-7	2.16	3.01
9	-4	7	-2.21	1.59
-10	4	-7	2.09	4.32
-10	4	-7	0.66	1.00
-10	4	-7	2.27	2.65
10	-4	7	0.81	1.88
-11	4	-7	1.14	1.18
-11	4	-7	6.42	5.21
11	-4	7	0.51	2.16
-13	-3	7	-1.65	3.01
-13	-3	7	-0.46	3.97
-13	-3	7	0.72	1.92
12	3	-7	6.73	3.85
-12	-3	7	-3.85	2.67
-12	-3	7	-1.12	1.81
-12	-3	7	-1.24	3.20

11	3	-7	6.56	3.88
-11	-3	7	1.96	2.80
-11	-3	7	-0.11	2.43
-11	-3	7	1.84	1.85
-11	-3	7	0.73	1.65
10	3	-7	3.55	2.93
-10	-3	7	0.64	1.93
-10	-3	7	-0.43	1.45
-10	-3	7	-2.69	2.49
-10	-3	7	-2.63	1.41
9	3	-7	0.99	2.50
-9	-3	7	2.90	2.13
-9	-3	7	4.03	1.24
-9	-3	7	2.43	1.70
-9	-3	7	3.00	1.34
8	3	-7	0.10	1.01
8	3	-7	0.25	1.42
-8	-3	7	-1.11	1.13
-8	-3	7	-0.09	2.03
7	3	-7	3.75	1.38
7	3	-7	1.77	1.00
7	3	-7	4.40	1.87
-7	-3	7	1.96	1.11
-7	-3	7	2.08	1.37
-7	-3	7	4.34	1.01
6	3	-7	0.08	1.46
6	3	-7	-1.45	1.15
-6	-3	7	-1.00	1.06
-6	-3	7	-0.17	0.82
5	3	-7	1.42	1.02
5	3	-7	1.26	1.34
-5	-3	7	2.68	1.04
-5	-3	7	2.98	0.94
-5	-3	7	3.49	0.89
4	3	-7	-0.38	1.02
4	3	-7	0.16	1.00
-4	-3	7	-0.45	0.87
-4	-3	7	0.42	0.90
-4	-3	7	2.40	0.83
3	3	-7	1.74	1.23
3	3	-7	3.41	0.98
3	3	-7	4.89	1.02
-3	-3	7	4.02	0.92
-3	-3	7	3.71	0.92

-3	-3	7	4.94	0.93
2	3	-7	0.99	0.98
2	3	-7	0.72	0.30
2	3	-7	-0.54	0.83
2	3	-7	0.20	0.98
-2	-3	7	-0.08	0.70
-2	-3	7	0.74	0.83
-2	-3	7	1.56	0.88
-2	-3	7	0.54	0.39
1	3	-7	1.68	0.95
1	3	-7	0.76	0.32
1	3	-7	0.89	0.88
1	3	-7	-0.54	0.83
-1	-3	7	0.92	0.87
-1	-3	7	0.47	0.67
-1	-3	7	0.49	0.42
-1	-3	7	0.67	0.89
0	3	-7	0.61	0.85
0	3	-7	1.75	0.73
0	3	-7	1.62	1.02
0	3	-7	2.20	0.39
0	-3	7	1.31	0.50
0	-3	7	1.85	0.87
0	-3	7	2.16	1.02
-1	3	-7	3.95	0.73
-1	3	-7	3.17	1.12
-1	3	-7	4.44	0.94
-1	3	-7	4.15	0.66
1	-3	7	4.52	0.90
1	-3	7	3.49	0.61
1	-3	7	3.28	1.08
1	-3	7	3.74	0.74
-2	3	-7	12.21	1.72
-2	3	-7	13.14	2.02
-2	3	-7	9.55	0.91
-2	3	-7	10.43	1.62
2	-3	7	12.99	1.64
2	-3	7	11.45	1.37
2	-3	7	8.73	1.40
2	-3	7	9.12	1.14
-3	3	-7	2.16	1.06
-3	3	-7	0.50	0.60
-3	3	-7	1.14	0.43
-3	3	-7	2.04	1.06

-3	3	-7	3.50	1.15
3	-3	7	0.49	1.22
3	-3	7	0.35	0.53
3	-3	7	1.15	0.94
3	-3	7	0.76	0.63
-4	3	-7	6.06	1.23
-4	3	-7	7.13	1.34
-4	3	-7	10.40	1.97
-4	3	-7	6.33	0.91
-4	3	-7	7.47	1.40
4	-3	7	4.56	1.46
4	-3	7	5.02	0.78
4	-3	7	6.75	1.36
4	-3	7	5.56	1.50
-5	3	-7	1.56	0.53
-5	3	-7	2.46	1.41
-5	3	-7	1.98	1.24
-5	3	-7	1.14	0.73
-5	3	-7	-0.70	1.19
5	-3	7	1.30	0.95
5	-3	7	1.21	0.68
5	-3	7	-0.47	1.41
5	-3	7	1.39	0.77
-6	3	-7	-0.32	1.29
-6	3	-7	-0.31	0.61
-6	3	-7	1.29	1.39
-6	3	-7	-1.11	1.51
-6	3	-7	-0.06	0.75
6	-3	7	0.64	0.91
6	-3	7	-2.71	1.53
-7	3	-7	4.96	1.66
-7	3	-7	2.42	0.68
-7	3	-7	0.24	1.38
7	-3	7	1.54	1.07
7	-3	7	3.33	1.14
-8	3	-7	-2.98	1.94
-8	3	-7	-0.13	0.71
-8	3	-7	-1.60	1.47
-8	3	-7	-0.08	0.72
-8	3	-7	0.34	2.71
8	-3	7	-0.08	1.17
8	-3	7	-0.73	1.21
-9	3	-7	0.62	1.07
-9	3	-7	0.11	3.18

-9	3	-7	-0.57	1.61
-9	3	-7	-0.53	0.79
-9	3	-7	-0.83	2.17
-9	3	-7	1.43	2.09
9	-3	7	-0.92	1.43
9	-3	7	-1.21	0.91
-10	3	-7	-0.42	2.61
-10	3	-7	-0.28	0.94
-10	3	-7	3.36	3.93
10	-3	7	0.38	1.64
-11	3	-7	4.00	4.80
-11	3	-7	-1.84	1.02
11	-3	7	-0.94	1.91
-13	-2	7	-1.01	1.87
-13	-2	7	-0.84	3.98
-13	-2	7	-1.58	3.10
12	2	-7	3.96	3.72
-12	-2	7	-1.51	3.36
-12	-2	7	-1.32	2.76
-12	-2	7	-1.08	1.71
11	2	-7	2.27	3.22
-11	-2	7	0.30	2.93
-11	-2	7	-1.29	2.40
-11	-2	7	-2.79	1.57
10	2	-7	1.32	2.76
-10	-2	7	-2.16	1.95
-10	-2	7	0.65	2.55
-9	-2	7	-2.25	1.63
-9	-2	7	-1.25	2.14
-8	-2	7	0.16	1.06
-8	-2	7	-0.62	1.10
-8	-2	7	-2.71	1.64
-7	-2	7	0.18	0.89
-7	-2	7	-0.45	1.20
6	2	-7	-1.54	1.51
6	2	-7	-0.68	0.92
6	2	-7	0.23	1.06
-6	-2	7	-0.82	1.12
-6	-2	7	-1.19	0.85
-6	-2	7	0.25	0.81
5	2	-7	0.28	1.28
-5	-2	7	0.32	0.83
-5	-2	7	0.28	0.93
-5	-2	7	1.59	0.77

4	2	-7	0.28	0.87
4	2	-7	0.34	0.70
-4	-2	7	-1.38	0.81
-4	-2	7	0.89	0.72
-4	-2	7	-0.82	0.74
3	2	-7	-1.68	1.16
3	2	-7	-0.70	1.48
3	2	-7	-0.74	0.82
-3	-2	7	0.64	0.80
-3	-2	7	-1.20	0.75
-3	-2	7	0.14	0.77
2	2	-7	3.21	0.51
2	2	-7	3.35	0.88
2	2	-7	3.87	1.01
2	2	-7	1.92	1.09
-2	-2	7	3.26	0.82
-2	-2	7	5.36	0.87
-2	-2	7	3.17	0.45
-2	-2	7	3.19	0.87
1	2	-7	17.29	1.77
1	2	-7	15.10	1.61
1	2	-7	16.76	1.00
1	2	-7	17.95	1.88
-1	-2	7	15.69	1.13
-1	-2	7	12.69	1.72
-1	-2	7	16.55	1.55
-1	-2	7	17.04	1.68
0	2	-7	0.10	0.92
0	2	-7	-0.16	0.27
0	2	-7	-0.20	0.67
0	2	-7	-0.98	0.73
0	-2	7	0.06	0.37
0	-2	7	-0.86	0.91
0	-2	7	0.51	0.73
-1	2	-7	4.21	0.45
-1	2	-7	3.32	0.72
-1	2	-7	5.32	1.06
-1	2	-7	4.47	0.91
1	-2	7	2.84	1.07
1	-2	7	5.01	0.85
1	-2	7	3.39	0.70
1	-2	7	3.86	0.55
-2	2	-7	0.48	0.98
-2	2	-7	-0.09	0.52

-2	2	-7	-0.24	0.32
-2	2	-7	0.28	0.85
2	-2	7	-0.19	0.42
2	-2	7	0.03	0.53
2	-2	7	0.22	0.80
2	-2	7	0.09	1.05
-3	2	-7	-0.37	0.35
-3	2	-7	0.85	1.03
-3	2	-7	-0.15	0.89
-3	2	-7	0.98	0.84
-3	2	-7	-0.05	0.54
3	-2	7	-1.18	1.13
3	-2	7	0.40	0.53
3	-2	7	0.19	0.75
3	-2	7	-0.20	0.46
-4	2	-7	1.42	0.94
-4	2	-7	0.52	0.43
-4	2	-7	1.10	0.60
-4	2	-7	1.87	1.14
-4	2	-7	0.16	0.97
4	-2	7	0.93	0.92
4	-2	7	0.59	1.33
4	-2	7	0.71	0.52
4	-2	7	0.89	0.57
-5	2	-7	2.47	1.36
-5	2	-7	7.18	1.33
-5	2	-7	3.48	0.57
-5	2	-7	4.40	1.07
-5	2	-7	4.57	0.80
5	-2	7	3.82	0.70
5	-2	7	4.71	0.80
5	-2	7	4.54	1.57
-6	2	-7	-0.36	0.50
-6	2	-7	1.12	1.76
-6	2	-7	-0.80	1.02
-6	2	-7	0.31	0.69
-6	2	-7	1.48	1.24
-6	2	-7	-1.09	1.40
6	-2	7	0.53	0.84
6	-2	7	0.22	0.75
6	-2	7	-1.03	1.51
6	-2	7	-0.07	0.62
-7	2	-7	4.85	2.15
-7	2	-7	3.98	0.76

-7	2	-7	7.20	1.58
-7	2	-7	2.68	1.66
-7	2	-7	4.88	1.18
7	-2	7	4.53	0.86
7	-2	7	4.76	1.77
-8	2	-7	2.72	1.63
-8	2	-7	3.02	4.18
-8	2	-7	-0.82	2.34
-8	2	-7	0.13	0.67
-8	2	-7	-0.25	1.21
8	-2	7	0.25	0.73
8	-2	7	2.48	1.16
8	-2	7	-0.15	1.85
8	-2	7	0.34	1.08
-9	2	-7	3.06	0.83
-9	2	-7	1.24	1.34
-9	2	-7	1.53	2.06
-9	2	-7	5.48	3.11
9	-2	7	-0.95	1.91
9	-2	7	4.96	1.40
-10	2	-7	0.41	1.08
-10	2	-7	3.61	3.64
-10	2	-7	-0.53	2.28
-10	2	-7	-1.46	2.10
-10	2	-7	-1.46	1.46
10	-2	7	0.26	1.51
10	-2	7	-0.43	1.06
-11	2	-7	-1.56	0.95
-11	2	-7	-3.04	1.71
-11	2	-7	-4.26	4.40
-11	2	-7	-4.39	2.61
11	-2	7	-0.67	1.76
-12	2	-7	5.30	5.27
-12	2	-7	-1.58	2.84
-12	2	-7	-1.19	1.15
12	-2	7	1.27	2.09
-13	-1	7	-0.92	4.20
-13	-1	7	3.10	3.40
-12	-1	7	-2.45	3.54
-12	-1	7	1.80	3.11
11	1	-7	1.41	3.31
-11	-1	7	0.74	2.59
-11	-1	7	-1.34	3.02
-10	-1	7	-0.73	2.11

-9	-1	7	-1.97	1.70
-8	-1	7	4.32	2.06
-7	-1	7	-1.09	1.25
6	1	-7	-0.01	1.51
-6	-1	7	-0.31	1.09
-6	-1	7	-1.22	0.85
-6	-1	7	1.73	0.85
5	1	-7	0.14	1.41
-5	-1	7	2.43	0.79
-5	-1	7	0.56	0.97
4	1	-7	1.17	0.86
4	1	-7	-0.89	1.13
-4	-1	7	1.48	0.68
-4	-1	7	-0.72	0.83
3	1	-7	4.84	1.27
3	1	-7	8.43	1.37
-3	-1	7	6.18	1.01
-3	-1	7	9.75	1.27
2	1	-7	2.92	0.78
2	1	-7	2.87	0.50
2	1	-7	2.39	0.87
-2	-1	7	3.84	0.77
-2	-1	7	1.54	0.75
1	1	-7	3.81	0.52
1	1	-7	2.99	1.04
1	1	-7	2.43	0.92
1	1	-7	2.93	0.76
-1	-1	7	2.49	0.73
-1	-1	7	3.68	0.62
-1	-1	7	1.03	0.90
-1	-1	7	3.81	0.82
0	1	-7	7.35	1.27
0	1	-7	6.66	1.41
0	1	-7	9.42	1.59
0	1	-7	7.87	0.64
0	-1	7	6.47	0.74
0	-1	7	5.02	0.80
0	-1	7	6.53	1.14
0	-1	7	8.65	1.26
-1	1	-7	2.30	0.79
-1	1	-7	1.85	0.35
-1	1	-7	2.12	0.64
-1	1	-7	2.71	0.95
1	-1	7	2.22	0.42

1	-1	7	2.09	1.05
1	-1	7	1.21	0.59
1	-1	7	2.54	0.73
-2	1	-7	0.92	0.33
-2	1	-7	0.69	0.80
-2	1	-7	1.42	0.92
-2	1	-7	0.79	0.50
2	-1	7	-0.28	1.04
2	-1	7	0.60	0.71
2	-1	7	1.02	0.39
2	-1	7	0.58	0.50
-3	1	-7	1.25	0.38
-3	1	-7	2.03	1.01
-3	1	-7	1.65	0.79
-3	1	-7	1.60	0.55
3	-1	7	1.61	0.75
3	-1	7	1.54	1.11
3	-1	7	2.01	0.56
3	-1	7	1.93	0.47
-4	1	-7	2.81	0.60
-4	1	-7	1.66	1.08
-4	1	-7	2.00	0.79
-4	1	-7	2.00	0.44
4	-1	7	1.73	0.50
4	-1	7	2.20	0.57
4	-1	7	1.31	1.22
4	-1	7	2.04	0.76
-5	1	-7	-0.15	1.35
-5	1	-7	0.93	0.81
-5	1	-7	0.64	0.43
-5	1	-7	1.57	0.94
-5	1	-7	-0.56	1.12
-5	1	-7	0.91	0.59
5	-1	7	0.37	0.58
5	-1	7	-0.36	1.28
5	-1	7	1.02	0.87
5	-1	7	0.03	0.48
-6	1	-7	-0.17	1.31
-6	1	-7	0.30	0.88
-6	1	-7	-0.15	0.63
-6	1	-7	-0.09	0.48
-6	1	-7	1.25	1.61
6	-1	7	-0.51	0.53
6	-1	7	0.27	0.71

6	-1	7	-1.56	1.38
-7	1	-7	-2.45	1.41
-7	1	-7	0.09	1.89
-7	1	-7	-1.30	0.97
-7	1	-7	1.20	1.24
-7	1	-7	-0.53	0.70
-7	1	-7	-0.56	0.55
7	-1	7	-0.19	0.81
7	-1	7	-1.73	1.50
7	-1	7	0.12	0.83
-8	1	-7	2.13	1.49
-8	1	-7	3.09	2.32
-8	1	-7	0.35	0.84
-8	1	-7	0.05	1.08
-8	1	-7	-0.87	1.58
8	-1	7	-0.04	0.73
8	-1	7	0.79	1.16
8	-1	7	-0.69	1.68
-9	1	-7	4.99	1.38
-9	1	-7	12.09	2.89
-9	1	-7	10.42	1.96
-9	1	-7	7.75	2.05
-9	1	-7	8.00	1.35
9	-1	7	8.00	1.42
9	-1	7	3.75	1.96
-10	1	-7	0.85	2.08
-10	1	-7	0.90	0.86
-10	1	-7	0.74	1.36
-10	1	-7	1.68	3.31
10	-1	7	0.84	1.23
10	-1	7	2.11	1.42
10	-1	7	0.52	1.99
10	-1	7	1.38	1.51
-11	1	-7	-2.74	2.33
-11	1	-7	-1.54	1.49
-11	1	-7	-0.92	0.98
-11	1	-7	-0.32	2.31
-11	1	-7	6.16	4.05
11	-1	7	-2.48	1.12
11	-1	7	-0.46	1.71
-12	1	-7	6.21	5.06
-12	1	-7	-1.24	1.10
-12	1	-7	0.00	2.75
-12	1	-7	-0.38	2.28

12	-1	7	-1.05	2.06
-13	1	-7	0.68	1.32
-13	1	-7	6.74	5.76
-13	1	-7	-4.49	3.11
13	-1	7	-0.63	2.33
-13	0	7	-0.55	4.39
-13	0	7	0.99	3.62
-12	0	7	-3.70	3.03
-12	0	7	2.29	3.69
-11	0	7	0.14	3.08
-11	0	7	-3.63	2.57
-10	0	7	-1.48	2.18
-10	0	7	-0.97	2.85
-9	0	7	-1.36	1.85
-5	0	7	0.27	0.98
-5	0	7	1.73	0.79
4	0	-7	5.13	1.28
-4	0	7	7.99	1.09
3	0	-7	23.12	1.66
3	0	-7	15.80	2.23
-3	0	7	17.40	1.83
-3	0	7	24.74	1.59
-3	0	7	21.62	1.70
2	0	-7	3.68	0.51
2	0	-7	4.30	0.74
2	0	-7	1.68	1.11
-2	0	7	4.23	0.78
-2	0	7	5.08	0.54
-2	0	7	3.08	0.91
1	0	-7	0.19	0.63
1	0	-7	0.23	0.27
1	0	-7	0.51	0.66
1	0	-7	-0.93	0.86
-1	0	7	-0.07	0.59
-1	0	7	-1.32	0.84
-1	0	7	1.41	0.65
-1	0	7	0.39	0.27
0	0	-7	13.96	1.42
0	0	-7	11.19	1.63
0	0	-7	13.68	0.92
0	0	-7	18.36	1.82
0	0	7	14.88	1.80
0	0	7	15.05	0.95
0	0	7	12.87	1.37

0	0	7	16.23	1.38
-1	0	-7	5.58	0.82
-1	0	-7	4.24	0.74
-1	0	-7	6.01	0.97
-1	0	-7	5.49	0.60
1	0	7	5.24	0.80
1	0	7	6.09	1.11
1	0	7	5.50	0.64
1	0	7	3.59	0.68
-2	0	-7	19.18	1.94
-2	0	-7	20.68	1.19
-2	0	-7	19.59	1.64
-2	0	-7	19.89	1.45
2	0	7	17.49	1.61
2	0	7	20.62	1.47
2	0	7	20.18	1.24
2	0	7	18.00	1.98
-3	0	-7	9.66	1.23
-3	0	-7	7.94	1.20
-3	0	-7	10.54	1.10
-3	0	-7	10.08	0.83
3	0	7	10.42	0.91
3	0	7	9.67	1.78
3	0	7	10.01	1.35
3	0	7	9.92	1.08
-4	0	-7	14.32	1.92
-4	0	-7	17.11	1.45
-4	0	-7	16.62	1.33
-4	0	-7	15.19	1.08
4	0	7	13.40	1.14
4	0	7	15.72	1.51
4	0	7	12.78	1.97
4	0	7	15.78	1.31
-5	0	-7	-0.31	0.71
-5	0	-7	0.06	0.41
-5	0	-7	0.19	0.53
-5	0	-7	-1.02	1.03
5	0	7	0.25	0.48
5	0	7	0.52	0.52
5	0	7	-2.02	1.12
5	0	7	0.20	0.66
-6	0	-7	2.60	1.58
-6	0	-7	1.20	0.83
-6	0	-7	1.90	0.52

-6	0	-7	1.78	0.63
-6	0	-7	0.07	1.19
6	0	7	0.76	1.36
6	0	7	2.84	0.60
6	0	7	2.75	0.95
6	0	7	2.23	0.70
-7	0	-7	0.57	1.33
-7	0	-7	1.09	0.59
-7	0	-7	2.31	1.80
-7	0	-7	1.44	0.70
-7	0	-7	0.81	0.94
7	0	7	1.43	1.40
7	0	7	0.87	0.63
7	0	7	1.28	0.82
-8	0	-7	2.99	0.72
-8	0	-7	3.86	2.26
-8	0	-7	3.96	0.87
-8	0	-7	2.95	1.10
-8	0	-7	4.14	1.59
8	0	7	2.06	1.70
8	0	7	3.15	0.79
8	0	7	3.74	1.03
-9	0	-7	3.25	1.68
-9	0	-7	2.05	1.17
-9	0	-7	-0.11	1.19
-9	0	-7	3.43	2.57
9	0	7	1.17	1.82
9	0	7	3.00	1.19
9	0	7	1.99	0.84
-10	0	-7	6.93	2.08
-10	0	-7	13.23	3.29
-10	0	-7	7.55	1.04
-10	0	-7	8.30	1.49
10	0	7	9.12	1.64
10	0	7	7.81	1.55
-11	0	-7	4.08	3.72
-11	0	-7	-1.00	0.92
-11	0	-7	-2.73	2.21
-11	0	-7	-0.01	1.21
11	0	7	0.66	1.04
11	0	7	-1.35	1.57
11	0	7	0.14	1.65
-12	0	-7	-2.32	2.58
-12	0	-7	-2.11	1.56

-12	0	-7	-0.33	1.08
-12	0	-7	4.65	4.54
12	0	7	0.81	1.90
12	0	7	0.75	1.31
-13	0	-7	0.36	3.07
-13	0	-7	-0.14	1.30
-13	0	-7	5.45	5.22
13	0	7	1.17	2.14
-13	1	7	-2.44	4.77
-13	1	7	-3.93	3.49
-12	1	7	-2.59	4.24
-12	1	7	-0.38	3.16
-11	1	7	-1.60	3.33
-11	1	7	2.03	2.80
-10	1	7	-1.42	2.28
-9	1	7	-1.16	1.95
-7	1	7	3.41	1.51
-5	1	7	7.36	1.24
4	-1	-7	-0.13	1.15
-4	1	7	-0.85	0.86
3	-1	-7	4.02	1.08
-3	1	7	2.97	0.84
2	-1	-7	0.45	0.26
2	-1	-7	0.36	0.61
-2	1	7	0.45	0.24
-2	1	7	0.89	0.67
-2	1	7	-1.03	0.85
1	-1	-7	0.42	0.28
1	-1	-7	0.79	0.63
-1	1	7	1.21	0.28
-1	1	7	-0.18	0.91
-1	1	7	1.67	0.59
0	-1	-7	2.82	0.38
0	-1	-7	3.29	0.69
0	-1	-7	2.54	0.81
0	1	7	2.99	0.51
0	1	7	3.32	0.69
0	1	7	2.18	0.90
-1	-1	-7	21.68	1.20
-1	-1	-7	20.70	1.57
-1	-1	-7	20.03	1.91
1	1	7	19.46	1.57
1	1	7	21.67	1.19
1	1	7	17.74	1.50

1	1	7	19.51	1.86
-2	-1	-7	0.28	0.31
-2	-1	-7	-0.89	0.78
-2	-1	-7	0.16	0.55
-2	-1	-7	0.03	0.48
2	1	7	0.39	0.31
2	1	7	0.32	0.45
2	1	7	-0.31	0.83
2	1	7	0.22	0.58
-3	-1	-7	15.70	1.24
-3	-1	-7	15.09	1.75
-3	-1	-7	15.34	1.34
-3	-1	-7	16.34	1.04
3	1	7	15.94	1.21
3	1	7	15.47	1.05
3	1	7	14.49	1.70
3	1	7	14.68	1.40
-4	-1	-7	10.07	1.70
-4	-1	-7	11.09	1.12
-4	-1	-7	11.47	0.89
-4	-1	-7	11.50	1.30
4	1	7	10.90	1.09
4	1	7	12.02	0.94
4	1	7	10.33	1.62
4	1	7	10.41	1.33
-5	-1	-7	-1.42	0.90
-5	-1	-7	0.02	0.50
-5	-1	-7	-0.25	0.38
-5	-1	-7	0.22	0.65
5	1	7	0.27	0.48
5	1	7	0.08	0.65
5	1	7	-0.74	0.90
5	1	7	0.17	0.39
-6	-1	-7	-0.32	0.42
-6	-1	-7	0.16	0.65
-6	-1	-7	-1.79	1.44
6	1	7	-0.53	0.57
6	1	7	-0.34	0.77
6	1	7	-0.83	0.72
-7	-1	-7	-0.80	0.49
-7	-1	-7	0.02	0.61
-7	-1	-7	-0.60	0.84
-7	-1	-7	0.28	1.21
-7	-1	-7	2.89	1.76

7	1	7	0.23	0.71
7	1	7	-0.10	1.39
7	1	7	-0.18	0.50
7	1	7	1.17	0.98
-8	-1	-7	2.03	2.03
-8	-1	-7	-1.01	0.60
-8	-1	-7	-1.03	0.97
-8	-1	-7	-1.09	1.29
-8	-1	-7	0.54	0.72
8	1	7	0.62	0.87
8	1	7	-1.89	1.36
-9	-1	-7	0.10	0.82
-9	-1	-7	-0.54	0.69
-9	-1	-7	1.97	2.41
-9	-1	-7	-2.20	1.46
-9	-1	-7	-1.32	1.03
9	1	7	-1.25	1.55
9	1	7	-0.53	1.10
9	1	7	-2.10	1.17
9	1	7	-1.15	0.65
-10	-1	-7	5.09	3.11
-10	-1	-7	3.45	1.29
-10	-1	-7	3.57	0.95
10	1	7	3.99	1.36
10	1	7	2.77	1.80
10	1	7	3.28	0.91
-11	-1	-7	1.75	3.47
-11	-1	-7	-0.70	0.92
-11	-1	-7	1.24	1.42
-11	-1	-7	0.19	2.08
-11	-1	-7	-0.93	1.15
11	1	7	-0.04	1.50
11	1	7	-1.53	1.57
-12	-1	-7	0.66	1.65
-12	-1	-7	-1.88	1.52
-12	-1	-7	-1.37	1.06
-12	-1	-7	-2.43	2.46
-12	-1	-7	7.09	4.10
12	1	7	-1.13	1.83
12	1	7	-2.07	1.13
12	1	7	-0.26	1.79
-13	-1	-7	6.31	5.05
-13	-1	-7	-0.64	2.45
-13	-1	-7	-2.01	3.01

-13	-1	-7	-0.86	1.24
13	1	7	0.21	1.31
13	1	7	-0.03	2.07
-14	-1	-7	0.82	1.46
-14	-1	-7	1.80	5.83
-12	2	7	4.44	4.16
-12	2	7	-1.56	3.09
-11	2	7	4.32	2.93
-10	2	7	-2.06	2.39
-10	2	7	-1.31	1.52
-10	2	7	-1.92	2.99
-9	2	7	3.37	2.07
-8	2	7	-0.79	1.66
-7	2	7	1.83	1.55
-6	2	7	-1.32	1.32
-5	2	7	16.57	2.21
4	-2	-7	6.26	1.45
-4	2	7	4.62	1.07
3	-2	-7	0.13	0.27
-3	2	7	0.38	0.25
2	-2	-7	9.08	0.70
-2	2	7	9.12	0.67
1	-2	-7	9.55	0.71
-1	2	7	9.43	1.18
-1	2	7	9.56	0.67
-1	2	7	7.90	1.09
0	-2	-7	1.16	0.61
0	-2	-7	0.87	0.29
0	2	7	0.54	0.25
0	2	7	-0.45	0.73
0	2	7	0.61	0.58
-1	-2	-7	3.31	0.53
-1	-2	-7	3.07	0.60
1	2	7	2.51	0.74
1	2	7	3.12	0.64
1	2	7	2.89	0.50
-2	-2	-7	8.92	0.76
-2	-2	-7	10.58	1.54
-2	-2	-7	9.12	1.05
2	2	7	7.81	1.30
2	2	7	9.30	1.15
2	2	7	10.54	0.75
-3	-2	-7	5.37	0.72
-3	-2	-7	6.02	0.66

-3	-2	-7	5.80	0.95
3	2	7	5.04	0.86
3	2	7	5.04	0.74
3	2	7	6.66	0.67
-4	-2	-7	0.42	0.82
-4	-2	-7	0.64	0.38
-4	-2	-7	0.61	0.59
4	2	7	1.08	0.60
4	2	7	0.68	0.78
4	2	7	1.26	0.37
-5	-2	-7	0.89	0.67
-5	-2	-7	0.88	0.92
-5	-2	-7	2.01	0.45
5	2	7	1.40	0.88
5	2	7	1.49	0.72
5	2	7	1.66	0.41
5	2	7	2.08	0.49
-6	-2	-7	4.91	1.15
-6	-2	-7	5.40	1.11
-6	-2	-7	6.53	0.85
-6	-2	-7	7.58	1.61
-6	-2	-7	4.82	0.89
6	2	7	5.51	1.17
6	2	7	4.78	1.10
6	2	7	5.63	0.93
6	2	7	6.12	0.84
-7	-2	-7	33.68	2.78
-7	-2	-7	34.75	2.47
-7	-2	-7	39.97	3.33
-7	-2	-7	39.21	2.14
7	2	7	35.05	2.12
7	2	7	34.07	2.74
7	2	7	38.63	2.42
-8	-2	-7	3.45	0.71
-8	-2	-7	4.51	0.82
-8	-2	-7	2.79	1.36
-8	-2	-7	4.59	2.09
-8	-2	-7	4.50	1.04
8	2	7	4.28	0.93
8	2	7	4.28	1.33
8	2	7	3.97	0.72
-9	-2	-7	13.14	2.61
-9	-2	-7	9.91	1.33
-9	-2	-7	10.47	1.37

-9	-2	-7	10.17	1.74
-9	-2	-7	7.83	1.69
9	2	7	9.77	1.65
9	2	7	8.42	1.30
9	2	7	8.90	1.52
9	2	7	9.64	2.01
-10	-2	-7	-0.76	0.81
-10	-2	-7	-0.22	1.65
-10	-2	-7	3.90	2.83
-10	-2	-7	0.86	1.22
-10	-2	-7	1.62	0.97
10	2	7	0.91	1.58
10	2	7	-0.03	1.29
10	2	7	-0.72	1.42
10	2	7	-0.74	0.74
-11	-2	-7	2.95	1.47
-11	-2	-7	5.37	1.06
-11	-2	-7	1.69	2.01
11	2	7	2.94	0.94
11	2	7	2.93	1.80
11	2	7	5.21	1.53
-12	-2	-7	1.57	1.58
-12	-2	-7	2.11	3.87
-12	-2	-7	1.05	1.37
-12	-2	-7	-1.55	2.42
-12	-2	-7	0.10	1.09
12	2	7	2.12	1.75
12	2	7	0.33	2.06
12	2	7	0.10	1.89
-13	-2	-7	5.89	1.77
-13	-2	-7	2.32	1.32
-13	-2	-7	3.19	4.72
-13	-2	-7	5.18	2.92
13	2	7	1.74	1.93
13	2	7	5.14	1.39
13	2	7	7.79	2.23
-14	-2	-7	2.59	3.34
-14	-2	-7	7.12	5.43
-14	-2	-7	-0.81	1.43
-12	3	7	2.40	4.82
-12	3	7	-3.78	3.35
-11	3	7	2.02	3.90
-11	3	7	-0.90	2.89
-10	3	7	-1.27	3.22

-10	3	7	-1.90	2.54
-10	3	7	-0.95	1.64
-9	3	7	5.27	2.37
-9	3	7	3.33	1.45
-8	3	7	0.05	1.82
-7	3	7	-0.79	1.50
-4	3	7	1.24	0.30
3	-3	-7	8.55	0.77
-3	3	7	7.63	0.68
2	-3	-7	15.98	1.04
-2	3	7	16.53	1.00
1	-3	-7	19.53	1.17
-1	3	7	20.39	1.15
0	-3	-7	9.31	0.70
0	3	7	8.64	1.11
0	3	7	8.08	0.68
-1	-3	-7	0.50	0.46
-1	-3	-7	0.31	0.27
1	3	7	0.01	0.60
1	3	7	-0.40	0.49
1	3	7	0.40	0.25
-2	-3	-7	5.84	0.96
-2	-3	-7	7.26	0.67
-2	-3	-7	5.67	0.85
2	3	7	6.60	0.81
2	3	7	6.43	1.01
2	3	7	7.63	0.62
-3	-3	-7	0.38	0.32
-3	-3	-7	-1.35	0.79
-3	-3	-7	0.02	0.53
3	3	7	0.46	0.67
3	3	7	-0.24	0.55
-4	-3	-7	1.56	0.62
-4	-3	-7	2.01	0.40
-4	-3	-7	1.07	0.84
4	3	7	1.69	0.65
4	3	7	1.44	0.78
4	3	7	1.92	0.39
-5	-3	-7	0.40	0.40
-5	-3	-7	-0.83	0.63
-5	-3	-7	-0.67	0.84
5	3	7	0.78	0.37
5	3	7	0.02	0.80
5	3	7	0.39	0.65

-6	-3	-7	-0.17	0.72
-6	-3	-7	0.82	0.44
-6	-3	-7	-1.18	0.96
6	3	7	0.47	0.43
-7	-3	-7	9.84	1.06
-7	-3	-7	7.58	1.32
-7	-3	-7	12.55	2.50
-7	-3	-7	12.50	1.59
7	3	7	8.98	1.29
-8	-3	-7	1.29	0.64
-8	-3	-7	1.46	1.27
-8	-3	-7	2.34	1.93
-8	-3	-7	1.39	0.97
8	3	7	1.74	0.57
8	3	7	0.19	1.20
8	3	7	1.62	1.00
-9	-3	-7	-1.82	2.20
-9	-3	-7	-1.28	1.05
-9	-3	-7	-1.14	0.72
9	3	7	0.28	0.97
9	3	7	-2.18	1.27
9	3	7	0.71	1.12
9	3	7	-0.43	0.59
-10	-3	-7	0.19	0.92
-10	-3	-7	-0.97	0.79
-10	-3	-7	1.64	1.21
-10	-3	-7	-1.17	2.49
-10	-3	-7	-1.93	1.67
10	3	7	-0.41	0.69
10	3	7	-0.46	1.51
10	3	7	-0.74	1.42
10	3	7	0.74	1.10
-11	-3	-7	-0.45	0.97
-11	-3	-7	1.14	3.06
-11	-3	-7	0.82	2.87
-11	-3	-7	2.14	1.41
11	3	7	0.89	0.85
11	3	7	0.80	1.72
11	3	7	2.29	1.41
-12	-3	-7	0.28	1.24
-12	-3	-7	-0.20	1.07
-12	-3	-7	1.90	1.59
-12	-3	-7	4.61	3.58
-12	-3	-7	-1.49	2.26

12	3	7	-1.59	1.90
12	3	7	3.33	1.66
12	3	7	-2.95	1.59
-13	-3	-7	0.67	1.71
-13	-3	-7	-1.06	2.78
-13	-3	-7	0.66	1.54
-13	-3	-7	7.61	4.39
13	3	7	2.12	1.97
13	3	7	2.07	2.62
13	3	7	-0.73	1.23
-14	-3	-7	4.71	5.39
-14	-3	-7	-2.01	1.33
-14	-3	-7	0.37	3.30
14	3	7	-0.15	1.42
-11	4	7	-0.36	4.24
-11	4	7	0.54	3.10
-11	4	7	-1.82	2.72
-10	4	7	6.17	3.82
-10	4	7	1.96	2.80
-9	4	7	2.78	2.41
-9	4	7	2.11	1.49
-8	4	7	1.76	1.92
-7	4	7	0.73	0.55
-7	4	7	-0.56	0.43
-6	4	7	22.84	1.72
-6	4	7	28.17	1.58
-5	4	7	5.23	0.77
-4	4	7	25.85	1.50
3	-4	-7	2.51	0.44
-3	4	7	2.41	0.38
2	-4	-7	14.62	1.06
-2	4	7	15.89	0.99
1	-4	-7	17.76	1.11
-1	4	7	17.02	1.07
0	-4	-7	52.13	2.71
0	4	7	53.33	2.69
-1	-4	-7	3.40	0.73
-1	-4	-7	4.31	0.59
1	4	7	3.42	0.68
1	4	7	4.02	0.62
1	4	7	4.29	0.56
-2	-4	-7	24.04	1.30
-2	-4	-7	20.73	1.46
2	4	7	23.53	1.28

2	4	7	21.08	1.53
2	4	7	20.98	1.62
-3	-4	-7	25.41	1.75
-3	-4	-7	29.84	1.54
-3	-4	-7	23.72	2.72
3	4	7	30.34	1.54
3	4	7	26.94	1.82
3	4	7	25.78	1.91
-4	-4	-7	18.81	1.85
-4	-4	-7	18.68	1.58
-4	-4	-7	23.86	1.31
4	4	7	22.54	1.28
4	4	7	20.96	1.79
4	4	7	21.94	1.67
-5	-4	-7	12.22	1.74
-5	-4	-7	13.29	1.41
-5	-4	-7	15.85	1.04
5	4	7	12.72	1.67
5	4	7	15.70	1.03
-6	-4	-7	3.77	1.10
-6	-4	-7	5.67	0.80
-6	-4	-7	4.83	0.85
6	4	7	5.71	0.80
6	4	7	5.76	1.06
-7	-4	-7	5.65	0.91
-7	-4	-7	6.07	0.95
-7	-4	-7	4.58	1.23
-7	-4	-7	7.31	1.63
7	4	7	6.88	1.36
7	4	7	4.92	0.95
7	4	7	6.50	1.05
7	4	7	5.77	1.17
-8	-4	-7	24.58	2.19
-8	-4	-7	19.60	2.60
-8	-4	-7	33.52	3.04
-8	-4	-7	25.72	1.72
8	4	7	26.07	2.47
8	4	7	28.88	2.67
8	4	7	24.95	2.31
8	4	7	28.61	1.71
-9	-4	-7	8.11	2.11
-9	-4	-7	2.07	1.50
-9	-4	-7	3.24	0.75
-9	-4	-7	3.68	1.09

9	4	7	7.09	1.76
9	4	7	4.21	1.22
9	4	7	3.87	1.40
9	4	7	3.51	0.68
-10	-4	-7	23.78	3.69
-10	-4	-7	13.40	1.58
-10	-4	-7	17.08	2.25
10	4	7	16.84	2.62
10	4	7	20.62	2.93
10	4	7	18.40	2.43
10	4	7	16.37	1.53
-11	-4	-7	0.72	3.38
-11	-4	-7	-0.47	1.26
-11	-4	-7	-1.05	1.04
-11	-4	-7	-1.73	1.84
-11	-4	-7	-1.75	0.85
11	4	7	0.59	1.65
11	4	7	-0.38	0.76
11	4	7	0.82	1.46
-12	-4	-7	4.71	1.62
-12	-4	-7	10.93	3.66
-12	-4	-7	1.51	1.04
-12	-4	-7	1.45	2.27
12	4	7	2.18	0.98
12	4	7	2.32	1.87
12	4	7	2.76	2.31
-13	-4	-7	-3.51	2.63
-13	-4	-7	4.97	4.21
-13	-4	-7	3.11	1.78
-13	-4	-7	0.54	1.12
13	4	7	0.93	2.09
13	4	7	-1.34	2.12
-14	-4	-7	9.28	4.92
-14	-4	-7	7.07	2.48
-14	-4	-7	8.54	3.37
-14	-4	-7	2.16	1.31
14	4	7	5.24	1.34
-15	-4	-7	-1.77	1.45
-15	-4	-7	-2.34	3.81
-15	-4	-7	-5.22	5.61
15	4	7	0.75	1.60
-11	5	7	-2.56	3.20
-11	5	7	0.16	1.16
-11	5	7	0.49	4.58

-10	5	7	0.98	4.04
-10	5	7	-0.54	0.98
-10	5	7	1.79	2.96
-10	5	7	1.10	0.74
-10	5	7	-0.71	2.62
-9	5	7	0.28	0.62
-9	5	7	-0.97	2.56
-9	5	7	-3.36	2.30
-9	5	7	-0.23	0.86
-9	5	7	-0.89	3.33
-8	5	7	3.33	0.64
-8	5	7	2.87	2.28
-8	5	7	3.08	0.80
-7	5	7	1.16	0.66
-7	5	7	0.55	0.76
-6	5	7	-0.18	0.57
-6	5	7	-0.02	0.46
-5	5	7	1.43	0.47
-4	5	7	2.86	0.47
-3	5	7	1.81	0.39
2	-5	-7	0.64	0.38
-2	5	7	0.11	0.32
1	-5	-7	4.22	0.72
-1	5	7	4.27	0.64
0	-5	-7	17.84	1.15
0	5	7	17.15	1.10
-1	-5	-7	0.74	0.33
1	5	7	-0.81	0.53
1	5	7	0.65	0.31
-2	-5	-7	8.64	0.77
-2	-5	-7	7.43	0.96
-2	-5	-7	6.55	0.97
2	5	7	6.92	1.26
2	5	7	8.80	0.73
2	5	7	9.29	1.15
-3	-5	-7	9.41	0.75
-3	-5	-7	6.66	0.79
3	5	7	6.23	0.93
3	5	7	9.16	0.74
-4	-5	-7	7.36	1.20
-4	-5	-7	10.25	0.82
-4	-5	-7	7.12	1.21
4	5	7	8.75	1.07
4	5	7	10.72	0.79

-5	-5	-7	9.99	0.83
-5	-5	-7	6.82	1.26
-5	-5	-7	7.79	1.19
5	5	7	8.10	1.00
5	5	7	8.31	1.12
5	5	7	10.65	0.81
-6	-5	-7	9.65	1.32
-6	-5	-7	9.02	1.39
-6	-5	-7	12.41	0.96
6	5	7	10.12	1.73
6	5	7	12.25	0.95
6	5	7	10.56	1.55
-7	-5	-7	9.61	0.97
-7	-5	-7	8.09	1.48
-7	-5	-7	7.55	1.36
7	5	7	9.64	1.81
7	5	7	9.07	1.40
7	5	7	9.06	1.16
-8	-5	-7	3.87	1.75
-8	-5	-7	1.75	1.31
-8	-5	-7	3.60	0.63
-8	-5	-7	3.03	0.98
8	5	7	3.23	0.59
8	5	7	3.61	1.23
8	5	7	2.68	1.06
8	5	7	2.76	1.58
-9	-5	-7	2.16	0.65
-9	-5	-7	0.14	1.42
-9	-5	-7	2.32	1.06
-9	-5	-7	3.94	2.02
9	5	7	1.66	1.16
9	5	7	1.00	1.38
9	5	7	1.73	0.62
9	5	7	2.89	1.72
-10	-5	-7	-0.48	0.72
-10	-5	-7	2.64	2.43
-10	-5	-7	-0.33	1.11
-10	-5	-7	2.27	1.65
10	5	7	-2.07	1.90
10	5	7	0.13	1.46
10	5	7	-0.01	0.64
10	5	7	1.49	1.25
-11	-5	-7	1.99	1.05
-11	-5	-7	1.96	1.35

-11	-5	-7	0.87	0.85
-11	-5	-7	-0.27	1.87
11	5	7	1.00	2.26
11	5	7	0.46	0.74
11	5	7	-0.63	1.69
11	5	7	1.60	1.53
-12	-5	-7	0.57	1.10
-12	-5	-7	-1.41	1.22
-12	-5	-7	2.60	1.51
-12	-5	-7	5.10	3.49
12	5	7	1.32	1.88
12	5	7	2.09	0.95
12	5	7	0.66	2.53
12	5	7	0.37	1.64
-13	-5	-7	0.34	2.64
-13	-5	-7	2.52	1.13
-13	-5	-7	0.64	1.44
-13	-5	-7	8.34	4.27
-13	-5	-7	3.83	1.78
13	5	7	1.37	2.87
13	5	7	1.80	2.04
13	5	7	4.89	2.27
-14	-5	-7	0.83	1.20
-14	-5	-7	4.56	5.08
-14	-5	-7	-1.62	3.18
-14	-5	-7	3.04	1.96
14	5	7	-1.74	1.13
14	5	7	3.85	2.46
-15	-5	-7	2.46	3.87
-15	-5	-7	2.87	5.42
-15	-5	-7	3.12	1.37
15	5	7	0.65	1.40
-10	6	7	4.48	4.42
-10	6	7	-1.66	1.09
-10	6	7	0.50	3.10
-10	6	7	0.63	0.87
-9	6	7	2.12	0.77
-9	6	7	2.59	2.76
-9	6	7	-2.74	2.70
-9	6	7	2.48	1.02
-8	6	7	2.54	0.82
-8	6	7	-0.13	2.39
-8	6	7	1.14	0.63
-8	6	7	-0.73	2.39

-7	6	7	6.90	2.10
-7	6	7	6.13	0.91
-7	6	7	7.22	1.16
-6	6	7	1.61	0.55
-6	6	7	2.83	0.74
-5	6	7	19.76	1.42
-3	6	7	17.90	1.26
2	-6	-7	2.03	0.47
-2	6	7	1.88	0.43
1	-6	-7	36.70	2.06
-1	6	7	36.48	2.01
0	-6	-7	29.94	1.74
0	6	7	30.45	1.70
-1	-6	-7	30.81	1.77
1	6	7	31.27	1.73
-2	-6	-7	1.56	0.38
2	6	7	-0.39	0.76
2	6	7	1.47	0.35
-3	-6	-7	27.60	1.82
-3	-6	-7	29.42	1.66
3	6	7	25.66	2.06
3	6	7	30.95	1.64
3	6	7	29.47	1.97
-4	-6	-7	10.08	1.24
-4	-6	-7	12.84	0.92
-4	-6	-7	8.06	1.23
4	6	7	9.54	1.42
4	6	7	9.92	1.16
4	6	7	12.53	0.90
-5	-6	-7	56.35	3.51
-5	-6	-7	59.44	3.00
-5	-6	-7	56.26	3.18
5	6	7	54.59	3.38
5	6	7	60.91	2.99
5	6	7	57.55	3.31
-6	-6	-7	24.06	2.37
-6	-6	-7	24.80	1.91
-6	-6	-7	26.75	1.54
6	6	7	22.94	2.19
6	6	7	26.21	1.52
6	6	7	24.68	2.06
-7	-6	-7	19.37	2.34
-7	-6	-7	24.50	1.41
-7	-6	-7	20.51	1.92

7	6	7	16.67	1.88
7	6	7	21.58	2.06
7	6	7	19.99	2.17
-8	-6	-7	12.31	2.36
-8	-6	-7	9.29	1.00
-8	-6	-7	7.79	1.19
8	6	7	5.71	1.39
8	6	7	9.28	1.03
8	6	7	8.28	1.32
8	6	7	9.72	1.78
-9	-6	-7	16.26	1.33
-9	-6	-7	11.52	1.86
-9	-6	-7	18.52	2.79
-9	-6	-7	14.77	2.10
9	6	7	14.88	1.33
9	6	7	18.40	2.71
9	6	7	11.88	1.71
9	6	7	14.93	2.25
-10	-6	-7	2.95	2.18
-10	-6	-7	1.24	0.70
-10	-6	-7	0.47	1.23
-10	-6	-7	-0.05	1.67
10	6	7	1.46	2.00
10	6	7	-1.53	1.27
10	6	7	0.49	0.66
10	6	7	0.37	1.50
-11	-6	-7	7.44	2.09
-11	-6	-7	9.55	1.65
-11	-6	-7	12.06	1.47
-11	-6	-7	10.94	1.90
11	6	7	8.32	1.96
11	6	7	11.07	1.75
11	6	7	9.24	1.37
11	6	7	8.77	2.44
-12	-6	-7	-2.27	2.16
-12	-6	-7	2.82	1.64
-12	-6	-7	4.54	1.26
-12	-6	-7	7.07	3.17
-12	-6	-7	4.65	1.44
12	6	7	1.85	2.01
12	6	7	3.50	2.56
12	6	7	3.08	1.72
12	6	7	2.32	0.89
-13	-6	-7	3.80	2.66

-13	-6	-7	8.44	3.95
-13	-6	-7	8.86	1.90
-13	-6	-7	7.81	1.17
13	6	7	3.15	2.29
13	6	7	5.96	2.07
13	6	7	11.08	3.02
-14	-6	-7	2.43	1.18
-14	-6	-7	0.32	3.15
-14	-6	-7	3.47	4.58
-14	-6	-7	2.11	1.73
-14	-6	-7	0.90	1.97
14	6	7	3.96	2.24
14	6	7	0.46	2.96
-15	-6	-7	10.51	5.19
-15	-6	-7	6.36	3.96
-15	-6	-7	5.54	1.36
15	6	7	3.73	1.29
-9	7	7	1.49	0.82
-9	7	7	-1.51	2.90
-9	7	7	0.59	1.11
-9	7	7	4.14	4.21
-9	7	7	-1.82	3.31
-8	7	7	0.30	2.84
-8	7	7	1.11	0.92
-8	7	7	0.77	0.70
-8	7	7	-0.10	2.52
-7	7	7	2.80	0.98
-7	7	7	2.78	0.68
-7	7	7	1.53	2.19
-6	7	7	7.49	2.03
-6	7	7	7.34	1.17
-6	7	7	9.67	1.50
-5	7	7	6.63	1.09
-5	7	7	8.25	1.42
-4	7	7	17.02	1.32
-3	7	7	4.54	0.59
-2	7	7	7.15	0.90
1	-7	-7	1.82	0.51
0	-7	-7	1.28	0.46
0	7	7	1.05	0.40
-1	-7	-7	2.31	0.48
1	7	7	1.59	0.41
-2	-7	-7	25.04	1.57
2	7	7	25.71	1.51

-3	-7	-7	31.03	1.79
-3	-7	-7	30.39	2.34
3	7	7	28.64	2.25
3	7	7	30.10	2.12
3	7	7	32.98	1.75
-4	-7	-7	20.04	1.32
-4	-7	-7	18.64	1.55
4	7	7	17.64	1.89
4	7	7	19.41	1.76
4	7	7	22.56	1.30
-5	-7	-7	0.81	1.23
-5	-7	-7	2.82	0.48
-5	-7	-7	2.36	0.72
5	7	7	2.71	0.51
5	7	7	1.98	0.84
5	7	7	1.51	0.95
-6	-7	-7	6.95	0.98
-6	-7	-7	6.08	1.30
-6	-7	-7	8.46	0.88
6	7	7	5.09	0.94
6	7	7	6.72	1.07
6	7	7	6.16	1.18
-7	-7	-7	10.56	2.14
-7	-7	-7	9.37	1.54
-7	-7	-7	11.63	1.04
-7	-7	-7	12.52	1.71
7	7	7	13.06	1.89
7	7	7	9.66	1.42
7	7	7	11.79	1.03
-8	-7	-7	18.76	2.07
-8	-7	-7	12.95	1.83
-8	-7	-7	18.92	1.30
8	7	7	14.98	2.23
8	7	7	16.76	1.26
8	7	7	13.30	1.72
8	7	7	17.21	2.60
-9	-7	-7	20.10	2.31
-9	-7	-7	16.25	2.86
-9	-7	-7	19.19	1.42
-9	-7	-7	13.66	2.74
9	7	7	16.24	1.41
9	7	7	20.67	3.01
9	7	7	18.64	2.38
9	7	7	16.00	2.59

-10	-7	-7	24.13	3.29
-10	-7	-7	20.67	2.56
-10	-7	-7	21.67	1.59
10	7	7	19.65	2.64
10	7	7	16.17	2.65
10	7	7	19.27	1.60
10	7	7	19.76	3.39
-11	-7	-7	9.39	1.69
-11	-7	-7	8.38	1.35
11	7	7	5.35	1.70
11	7	7	9.15	2.55
11	7	7	4.85	1.91
11	7	7	8.60	1.38
-12	-7	-7	20.84	3.13
-12	-7	-7	22.72	2.33
-12	-7	-7	23.14	4.37
-12	-7	-7	19.50	2.40
12	7	7	18.43	3.22
12	7	7	24.77	4.01
-13	-7	-7	4.89	3.69
-13	-7	-7	1.18	1.79
-13	-7	-7	-0.49	2.76
-13	-7	-7	0.99	0.92
-13	-7	-7	0.49	1.38
13	7	7	0.86	1.89
13	7	7	0.61	0.91
13	7	7	5.30	2.99
13	7	7	2.29	2.22
-14	-7	-7	5.17	1.72
-14	-7	-7	2.58	3.24
-14	-7	-7	4.29	1.13
-14	-7	-7	5.04	2.14
-14	-7	-7	4.91	4.50
14	7	7	5.52	2.36
14	7	7	1.50	2.77
-15	-7	-7	2.19	5.11
-15	-7	-7	0.19	1.20
-15	-7	-7	-2.82	3.81
15	7	7	2.78	1.25
-9	8	7	1.44	1.32
-9	8	7	4.63	4.32
-9	8	7	1.30	3.13
-9	8	7	3.04	1.00
-8	8	7	3.62	2.89

-8	8	7	2.59	3.35
-8	8	7	11.56	3.75
-8	8	7	5.72	0.92
-8	8	7	6.62	1.22
-7	8	7	1.16	2.38
-7	8	7	1.74	0.75
-7	8	7	2.75	0.93
-7	8	7	1.99	2.97
-7	8	7	-2.81	2.79
-6	8	7	14.38	2.77
-6	8	7	16.17	1.80
-6	8	7	15.11	1.52
-5	8	7	13.30	1.75
-5	8	7	14.32	1.40
-4	8	7	27.65	1.83
-3	8	7	13.51	1.24
-2	8	7	22.75	1.53
-1	8	7	13.86	1.16
0	-8	-7	36.93	2.19
0	8	7	38.07	2.12
-1	-8	-7	16.14	1.27
1	8	7	15.72	1.17
-2	-8	-7	86.41	4.41
2	8	7	85.74	4.36
-3	-8	-7	34.45	2.63
-3	-8	-7	39.67	2.19
3	8	7	38.43	2.50
3	8	7	41.10	2.15
-4	-8	-7	63.01	3.29
4	8	7	63.50	3.25
4	8	7	64.29	3.60
4	8	7	59.72	3.68
-5	-8	-7	10.88	2.19
-5	-8	-7	14.75	2.05
-5	-8	-7	16.37	1.19
5	8	7	16.35	1.14
5	8	7	14.66	1.84
5	8	7	10.61	1.99
-6	-8	-7	15.42	2.49
-6	-8	-7	20.49	1.36
-6	-8	-7	18.93	1.87
6	8	7	19.52	1.31
6	8	7	15.53	2.22
6	8	7	19.90	2.06

-7	-8	-7	9.51	1.62
-7	-8	-7	9.67	1.02
-7	-8	-7	4.52	1.53
-7	-8	-7	9.52	1.81
7	8	7	8.68	1.62
7	8	7	6.11	1.44
7	8	7	8.01	0.94
7	8	7	5.87	1.24
-8	-8	-7	21.88	2.75
-8	-8	-7	22.20	2.32
-8	-8	-7	20.40	2.78
-8	-8	-7	22.07	1.52
8	8	7	21.42	1.48
8	8	7	20.48	2.60
8	8	7	20.00	2.40
-9	-8	-7	16.51	1.38
-9	-8	-7	16.87	2.36
-9	-8	-7	13.91	2.82
-9	-8	-7	11.59	1.98
9	8	7	15.52	2.97
9	8	7	14.11	2.45
9	8	7	14.13	1.28
9	8	7	12.80	1.83
-10	-8	-7	32.73	3.08
-10	-8	-7	30.72	3.55
-10	-8	-7	30.97	2.02
-10	-8	-7	30.26	3.45
10	8	7	32.19	3.87
10	8	7	28.47	2.01
10	8	7	32.86	3.67
10	8	7	33.31	3.07
-11	-8	-7	15.73	2.62
-11	-8	-7	24.59	3.14
-11	-8	-7	24.12	4.55
-11	-8	-7	23.70	1.77
11	8	7	20.80	1.77
11	8	7	24.28	4.02
11	8	7	20.72	3.05
11	8	7	19.08	3.42
-12	-8	-7	33.27	4.40
-12	-8	-7	41.12	4.83
-12	-8	-7	38.80	3.07
-12	-8	-7	45.20	3.37
-12	-8	-7	40.30	3.69

12	8	7	34.08	4.08
12	8	7	36.12	2.57
12	8	7	40.13	3.69
12	8	7	44.57	4.62
-13	-8	-7	3.91	1.91
-13	-8	-7	8.35	3.67
-13	-8	-7	-1.65	3.91
-13	-8	-7	4.20	1.00
-13	-8	-7	3.38	1.48
13	8	7	1.79	0.97
13	8	7	1.65	2.11
13	8	7	7.19	2.98
13	8	7	1.21	2.41
-14	-8	-7	10.48	4.53
-14	-8	-7	7.00	3.48
-14	-8	-7	6.23	1.18
-14	-8	-7	7.59	2.22
14	8	7	8.67	2.54
14	8	7	6.40	2.87
-15	-8	-7	14.08	5.05
-15	-8	-7	3.80	1.24
-15	-8	-7	6.32	1.86
-15	-8	-7	4.42	4.12
15	8	7	1.74	1.30
-8	9	7	0.44	3.04
-8	9	7	4.87	3.91
-8	9	7	1.64	1.12
-8	9	7	0.95	1.27
-7	9	7	4.78	1.18
-7	9	7	3.57	0.91
-7	9	7	3.22	3.31
-7	9	7	0.90	3.20
-7	9	7	6.99	2.75
-6	9	7	2.90	2.25
-6	9	7	3.87	0.81
-6	9	7	4.06	1.05
-5	9	7	9.34	2.44
-5	9	7	6.80	1.95
-5	9	7	6.67	1.07
-5	9	7	6.56	0.87
-4	9	7	0.16	0.60
-3	9	7	5.36	0.73
-2	9	7	-0.24	0.49
-1	9	7	5.95	1.00

0	9	7	2.75	0.55
-1	-9	-7	22.17	1.57
1	9	7	19.70	1.41
-2	-9	-7	6.59	1.03
2	9	7	6.53	0.90
-3	-9	-7	17.82	1.38
3	9	7	17.55	2.11
3	9	7	16.36	1.26
-4	-9	-7	11.58	1.13
-4	-9	-7	10.25	1.40
-4	-9	-7	9.79	1.87
4	9	7	9.35	1.66
4	9	7	10.30	1.93
4	9	7	10.38	1.03
-5	-9	-7	14.88	1.75
-5	-9	-7	16.56	1.34
5	9	7	15.22	1.97
5	9	7	16.77	1.25
5	9	7	16.96	2.18
-6	-9	-7	9.65	1.74
-6	-9	-7	9.21	1.72
-6	-9	-7	10.69	1.14
6	9	7	9.82	1.03
6	9	7	9.06	1.40
6	9	7	9.66	1.36
-7	-9	-7	7.41	1.09
-7	-9	-7	3.99	1.60
-7	-9	-7	6.98	1.20
7	9	7	7.30	1.01
7	9	7	7.73	1.47
7	9	7	7.14	1.33
7	9	7	8.59	1.95
-8	-9	-7	4.71	0.74
-8	-9	-7	4.34	1.65
-8	-9	-7	5.26	1.27
-8	-9	-7	7.52	1.84
8	9	7	5.66	1.81
8	9	7	3.66	1.50
8	9	7	3.52	1.25
8	9	7	6.00	0.99
-9	-9	-7	20.12	1.58
-9	-9	-7	18.21	3.02
-9	-9	-7	20.24	3.10
-9	-9	-7	19.81	3.07

9	9	7	18.25	2.54
9	9	7	16.52	2.97
9	9	7	20.17	3.15
9	9	7	18.85	1.48
-10	-9	-7	1.00	1.83
-10	-9	-7	2.01	2.19
-10	-9	-7	1.86	0.74
10	9	7	2.40	1.42
10	9	7	1.14	0.66
10	9	7	-0.43	2.27
10	9	7	1.08	1.73
-11	-9	-7	5.61	2.55
-11	-9	-7	5.35	2.21
-11	-9	-7	5.09	0.88
11	9	7	3.66	1.64
11	9	7	4.49	0.86
11	9	7	3.69	2.03
11	9	7	7.18	2.73
-12	-9	-7	10.27	2.66
-12	-9	-7	16.61	3.27
-12	-9	-7	10.32	1.81
12	9	7	7.64	2.38
12	9	7	10.72	2.12
12	9	7	9.16	1.62
12	9	7	13.00	3.03
-13	-9	-7	16.43	3.22
-13	-9	-7	17.80	2.78
-13	-9	-7	19.21	1.95
-13	-9	-7	27.87	5.09
13	9	7	17.66	2.53
13	9	7	16.23	2.98
-14	-9	-7	3.59	1.12
-14	-9	-7	2.88	3.38
-14	-9	-7	10.69	4.43
14	9	7	1.46	2.44
14	9	7	5.05	2.95
-15	-9	-7	5.02	1.28
-15	-9	-7	4.20	4.12
-15	-9	-7	14.34	5.05
-15	-9	-7	5.86	1.92
15	9	7	3.48	1.23
-7	10	7	6.01	3.18
-7	10	7	5.81	1.32
-7	10	7	9.08	3.71

-6	10	7	5.30	0.95
-6	10	7	4.43	2.61
-6	10	7	6.53	1.27
-6	10	7	4.55	3.08
-6	10	7	9.15	3.22
-5	10	7	21.01	3.86
-5	10	7	22.04	4.00
-5	10	7	17.99	1.81
-5	10	7	20.32	2.24
-4	10	7	18.97	1.70
-4	10	7	19.81	2.18
-4	10	7	19.22	3.32
-3	10	7	16.30	1.54
-2	10	7	9.83	1.26
-1	10	7	11.84	1.26
0	10	7	11.26	1.19
1	10	7	64.46	3.43
-2	-10	-7	33.03	2.11
2	10	7	33.82	2.01
-3	-10	-7	102.03	5.19
3	10	7	97.02	5.96
3	10	7	100.54	5.12
3	10	7	103.19	5.37
-4	-10	-7	42.27	2.46
4	10	7	43.91	3.05
4	10	7	40.34	2.35
4	10	7	39.13	2.80
-5	-10	-7	63.33	3.33
-5	-10	-7	56.83	3.57
-5	-10	-7	60.70	3.99
5	10	7	62.33	3.71
5	10	7	63.72	3.87
5	10	7	58.66	3.24
-6	-10	-7	21.46	2.19
-6	-10	-7	23.05	2.76
-6	-10	-7	23.03	1.66
6	10	7	23.20	1.56
6	10	7	21.08	2.33
6	10	7	22.68	2.60
-7	-10	-7	36.48	2.34
-7	-10	-7	40.55	3.34
-7	-10	-7	42.01	3.40
-7	-10	-7	39.38	2.90
7	10	7	36.11	2.24

7	10	7	38.83	3.01
7	10	7	39.58	3.23
-8	-10	-7	17.95	2.40
-8	-10	-7	15.90	2.96
-8	-10	-7	16.79	1.47
-8	-10	-7	14.32	2.76
8	10	7	18.76	2.88
8	10	7	16.01	2.80
8	10	7	16.05	1.38
8	10	7	16.05	2.45
-9	-10	-7	37.01	3.65
-9	-10	-7	32.64	3.55
-9	-10	-7	36.24	2.28
-9	-10	-7	37.61	3.21
9	10	7	36.04	3.54
9	10	7	38.28	3.75
9	10	7	34.29	2.17
9	10	7	33.61	3.20
-10	-10	-7	6.27	0.96
-10	-10	-7	8.47	4.15
-10	-10	-7	7.83	1.67
-10	-10	-7	4.37	2.08
10	10	7	6.22	2.02
10	10	7	5.41	2.44
10	10	7	6.02	0.84
10	10	7	7.14	1.72
-11	-10	-7	9.60	2.47
-11	-10	-7	14.18	2.81
-11	-10	-7	14.57	2.25
-11	-10	-7	11.31	2.00
11	10	7	12.38	1.94
11	10	7	14.51	2.94
11	10	7	11.74	2.42
11	10	7	10.03	1.53
-12	-10	-7	21.67	3.38
-12	-10	-7	19.18	3.37
-12	-10	-7	12.23	2.81
12	10	7	11.58	1.74
12	10	7	16.43	2.32
12	10	7	17.10	2.76
-13	-10	-7	23.14	3.69
-13	-10	-7	14.37	3.06
-13	-10	-7	13.25	3.69
13	10	7	18.73	3.89

13	10	7	10.60	2.93
13	10	7	14.15	2.43
-14	-10	-7	6.01	1.22
-14	-10	-7	1.29	4.29
-14	-10	-7	5.84	3.39
-14	-10	-7	4.86	1.74
-14	-10	-7	7.97	2.66
14	10	7	4.24	3.11
14	10	7	7.37	2.54
-15	-10	-7	3.38	4.06
-15	-10	-7	7.87	4.76
-15	-10	-7	5.93	1.32
-15	-10	-7	2.82	1.98
15	10	7	2.69	1.22
-6	11	7	4.94	1.34
-6	11	7	5.02	3.02
-6	11	7	2.14	3.46
-5	11	7	6.36	1.27
-5	11	7	5.92	3.18
-4	11	7	16.26	2.13
-4	11	7	13.21	2.73
-4	11	7	13.24	1.70
-3	11	7	8.84	1.46
-3	11	7	7.96	1.31
-2	11	7	13.71	1.47
-2	11	7	14.39	2.73
-1	11	7	3.17	0.67
0	-11	-7	17.96	2.15
0	11	7	14.34	2.21
0	11	7	11.64	1.31
1	11	7	0.53	0.40
-3	-11	-7	2.21	0.77
3	11	7	2.57	1.31
4	11	7	34.37	2.71
4	11	7	39.06	3.10
-5	-11	-7	6.03	1.06
-5	-11	-7	9.01	1.87
-5	-11	-7	5.55	1.29
5	11	7	5.05	0.73
5	11	7	6.37	1.18
5	11	7	6.98	1.41
-6	-11	-7	10.52	1.97
-6	-11	-7	10.07	1.57
-6	-11	-7	12.15	2.10

6	11	7	9.95	1.19
6	11	7	11.06	2.08
6	11	7	11.97	2.33
6	11	7	13.99	2.43
-7	-11	-7	13.87	2.36
-7	-11	-7	11.99	2.16
-7	-11	-7	10.47	1.69
-7	-11	-7	11.30	1.93
7	11	7	16.55	2.59
7	11	7	10.39	2.02
7	11	7	11.01	1.24
7	11	7	10.61	1.53
-8	-11	-7	23.51	3.26
-8	-11	-7	15.79	2.94
8	11	7	21.55	3.24
8	11	7	16.66	2.62
8	11	7	19.51	2.97
8	11	7	16.79	1.48
-9	-11	-7	15.87	2.09
-9	-11	-7	14.93	2.69
-9	-11	-7	12.59	2.07
9	11	7	15.33	2.78
9	11	7	15.26	1.47
9	11	7	16.63	3.39
9	11	7	20.37	3.25
-10	-11	-7	12.87	2.64
-10	-11	-7	11.63	1.55
-10	-11	-7	8.46	2.12
10	11	7	9.47	1.39
10	11	7	10.26	2.66
10	11	7	12.02	2.10
10	11	7	9.83	1.88
-11	-11	-7	8.74	2.79
-11	-11	-7	5.50	0.97
-11	-11	-7	7.35	1.97
-11	-11	-7	5.31	2.32
11	11	7	5.21	2.26
11	11	7	5.37	2.85
11	11	7	3.37	0.85
11	11	7	5.57	1.92
-12	-11	-7	17.53	3.02
-12	-11	-7	26.64	3.73
-12	-11	-7	21.76	2.05
-12	-11	-7	21.79	4.56

12	11	7	26.30	4.98
12	11	7	16.32	2.96
12	11	7	19.53	3.64
-13	-11	-7	4.22	1.16
-13	-11	-7	9.39	2.44
-13	-11	-7	3.26	3.05
-13	-11	-7	8.31	3.64
13	11	7	12.56	4.87
13	11	7	4.41	2.28
13	11	7	8.06	3.08
-14	-11	-7	6.65	4.15
-14	-11	-7	11.41	2.72
-14	-11	-7	10.38	4.21
-14	-11	-7	9.12	1.37
14	11	7	12.51	2.79
14	11	7	7.28	1.36
14	11	7	10.32	3.34
-15	-11	-7	5.37	4.23
-15	-11	-7	2.92	4.77
-15	-11	-7	6.29	1.41
-5	12	7	4.01	1.39
-4	12	7	19.87	2.51
-3	12	7	12.58	1.54
-3	12	7	10.83	2.56
-2	12	7	27.29	3.47
-2	12	7	27.29	2.16
-1	12	7	28.58	2.11
0	12	7	28.50	2.92
0	12	7	27.63	1.93
2	12	7	53.98	3.66
-3	-12	-7	26.32	2.89
3	12	7	19.61	2.43
-4	-12	-7	38.14	2.51
-4	-12	-7	41.78	3.44
4	12	7	38.83	3.26
4	12	7	36.73	3.07
4	12	7	40.51	2.85
-5	-12	-7	18.39	2.08
-5	-12	-7	19.13	3.06
-5	-12	-7	19.98	1.76
5	12	7	20.09	2.57
5	12	7	17.01	2.69
5	12	7	20.95	2.30
-6	-12	-7	48.07	2.87

-6	-12	-7	51.38	3.98
-6	-12	-7	45.25	3.24
6	12	7	48.81	3.62
6	12	7	45.20	3.77
6	12	7	43.78	3.42
-7	-12	-7	43.61	3.96
-7	-12	-7	39.99	2.56
-7	-12	-7	35.74	3.92
7	12	7	39.05	3.69
7	12	7	38.12	3.42
-8	-12	-7	45.23	2.75
-8	-12	-7	38.63	3.58
8	12	7	46.04	3.79
8	12	7	45.33	4.11
8	12	7	40.74	3.52
-9	-12	-7	32.28	3.51
-9	-12	-7	35.82	2.45
-9	-12	-7	37.91	4.24
9	12	7	32.53	3.32
9	12	7	33.52	3.70
9	12	7	35.65	4.15
-10	-12	-7	43.63	4.25
-10	-12	-7	45.85	4.68
-10	-12	-7	42.89	2.83
10	12	7	42.87	3.89
10	12	7	43.77	4.50
10	12	7	44.25	4.27
-11	-12	-7	16.70	2.81
-11	-12	-7	24.69	2.06
-11	-12	-7	22.07	4.46
11	12	7	15.15	3.50
11	12	7	16.64	3.36
11	12	7	23.04	3.52
-12	-12	-7	21.56	3.70
-12	-12	-7	13.26	3.02
-12	-12	-7	17.54	2.85
-12	-12	-7	20.97	2.11
12	12	7	23.07	3.79
12	12	7	13.46	2.94
12	12	7	14.27	2.21
12	12	7	26.99	5.39
-13	-12	-7	13.21	3.93
-13	-12	-7	5.90	3.34
-13	-12	-7	11.48	2.67

13	12	7	10.71	3.24
13	12	7	7.81	2.53
13	12	7	8.86	1.34
-14	-12	-7	8.55	2.90
-14	-12	-7	8.42	1.39
-14	-12	-7	9.56	4.15
14	12	7	4.95	2.72
14	12	7	7.97	1.36
14	12	7	4.33	3.56
-3	13	7	10.69	1.54
-2	13	7	6.67	2.60
-2	13	7	9.06	1.45
-1	13	7	28.22	3.66
0	-13	-7	19.43	2.98
0	13	7	14.10	2.94
-1	-13	-7	23.12	2.84
1	13	7	18.73	2.77
1	13	7	18.46	1.68
-2	-13	-7	11.39	2.00
2	13	7	10.51	1.89
-3	-13	-7	22.38	3.61
3	13	7	22.88	2.64
4	13	7	3.34	1.63
4	13	7	2.93	2.03
5	13	7	15.28	1.52
5	13	7	15.64	2.33
5	13	7	18.43	2.82
5	13	7	16.56	2.69
-6	-13	-7	13.26	1.87
-6	-13	-7	19.52	3.71
-6	-13	-7	15.80	1.84
6	13	7	16.58	2.42
6	13	7	16.66	1.59
6	13	7	17.83	3.13
6	13	7	18.01	2.79
-7	-13	-7	42.59	4.22
-7	-13	-7	40.78	2.69
7	13	7	39.68	2.64
7	13	7	41.39	3.69
7	13	7	40.21	3.84
7	13	7	38.76	3.37
-8	-13	-7	18.40	1.92
-8	-13	-7	19.36	3.98
-8	-13	-7	11.99	2.18

8	13	7	12.70	2.06
8	13	7	12.38	1.95
8	13	7	18.55	2.74
8	13	7	17.25	2.74
-9	-13	-7	19.64	3.25
-9	-13	-7	17.85	3.25
-9	-13	-7	17.84	1.99
9	13	7	15.20	1.81
9	13	7	22.02	4.08
9	13	7	12.92	2.28
9	13	7	21.61	3.01
-10	-13	-7	20.91	3.58
-10	-13	-7	25.43	2.18
-10	-13	-7	22.78	3.55
10	13	7	20.31	3.24
10	13	7	15.35	3.27
10	13	7	13.91	2.55
10	13	7	19.35	1.93
-11	-13	-7	32.37	2.53
-11	-13	-7	27.54	4.23
-11	-13	-7	36.76	5.08
11	13	7	26.74	2.31
11	13	7	27.81	5.24
11	13	7	25.24	3.86
-12	-13	-7	17.43	3.82
-12	-13	-7	11.91	2.98
12	13	7	15.49	3.01
12	13	7	11.12	1.97
-13	-13	-7	15.90	2.30
-13	-13	-7	14.77	3.06
-13	-13	-7	9.99	3.41
-13	-13	-7	15.72	4.23
13	13	7	8.98	3.77
13	13	7	12.12	3.50
13	13	7	13.41	2.23
-14	-13	-7	5.21	1.42
-14	-13	-7	6.09	3.86
-14	-13	-7	2.08	4.26
-14	-13	-7	7.40	3.27
14	13	7	5.88	1.44
14	13	7	4.27	4.07
14	13	7	5.88	2.87
-2	14	7	15.87	2.67
-1	14	7	15.35	4.25

-1	14	7	15.40	2.53
0	14	7	21.45	3.69
-1	-14	-7	23.91	3.38
1	14	7	24.80	3.38
-2	-14	-7	26.30	3.48
2	14	7	22.25	3.17
-3	-14	-7	21.76	3.44
3	14	7	18.63	2.90
3	14	7	20.76	2.93
-4	-14	-7	32.19	3.78
4	14	7	22.71	3.10
4	14	7	22.97	3.35
4	14	7	26.80	2.06
-5	-14	-7	31.58	3.87
5	14	7	27.63	2.05
5	14	7	29.33	3.18
5	14	7	26.36	3.30
5	14	7	26.56	2.80
-6	-14	-7	32.14	2.91
-6	-14	-7	38.64	4.22
-6	-14	-7	29.67	3.22
6	14	7	34.83	3.40
6	14	7	33.86	3.16
6	14	7	31.29	3.80
6	14	7	33.30	2.32
-7	-14	-7	45.50	4.75
-7	-14	-7	41.73	2.91
7	14	7	42.52	3.88
7	14	7	40.89	2.71
7	14	7	45.89	4.26
7	14	7	40.97	3.60
-8	-14	-7	26.94	2.40
-8	-14	-7	21.86	3.98
-8	-14	-7	35.35	4.52
8	14	7	27.34	3.23
8	14	7	29.66	3.64
8	14	7	27.62	2.15
8	14	7	22.36	4.06
-9	-14	-7	24.03	2.28
-9	-14	-7	22.24	3.81
-9	-14	-7	17.14	3.34
9	14	7	24.23	4.46
9	14	7	21.12	2.06
9	14	7	25.94	3.74

9	14	7	19.86	3.35
-10	-14	-7	14.74	2.61
-10	-14	-7	22.62	2.32
-10	-14	-7	25.16	5.22
10	14	7	22.00	4.26
10	14	7	18.60	3.53
10	14	7	21.31	2.09
10	14	7	19.32	3.78
-11	-14	-7	21.39	2.36
-11	-14	-7	22.42	4.26
11	14	7	19.59	2.13
11	14	7	15.03	2.48
-12	-14	-7	10.85	3.01
-12	-14	-7	21.72	2.46
-12	-14	-7	19.41	4.28
12	14	7	13.11	2.59
12	14	7	15.65	3.32
12	14	7	19.33	2.22
-13	-14	-7	10.40	3.23
-13	-14	-7	15.76	4.42
-13	-14	-7	11.04	3.28
13	14	7	11.86	2.83
0	15	7	16.95	2.82
-1	-15	-7	13.68	2.73
-2	-15	-7	32.72	3.98
2	15	7	28.84	3.82
2	15	7	31.30	3.36
-3	-15	-7	17.64	2.89
3	15	7	16.26	3.43
3	15	7	22.12	3.08
-4	-15	-7	24.86	4.03
4	15	7	18.43	3.53
4	15	7	24.01	3.14
-5	-15	-7	17.19	2.99
5	15	7	10.49	2.54
5	15	7	10.47	2.21
5	15	7	11.59	1.78
-6	-15	-7	31.59	4.52
6	15	7	21.60	4.09
6	15	7	27.43	3.44
6	15	7	27.65	3.29
-7	-15	-7	14.09	3.26
7	15	7	12.60	2.04
7	15	7	16.55	3.17

7	15	7	18.62	3.28
-8	-15	-7	27.60	5.09
8	15	7	20.95	4.43
8	15	7	22.63	3.46
8	15	7	27.82	3.89
-9	-15	-7	26.91	5.27
9	15	7	14.75	2.31
-10	-15	-7	23.22	2.62
-10	-15	-7	24.20	4.33
10	15	7	24.75	4.01
-11	-15	-7	12.17	4.43
-11	-15	-7	9.63	1.47
11	15	7	10.85	2.51
-12	-15	-7	19.86	4.71
-12	-15	-7	18.17	2.58
12	15	7	14.16	2.94
-3	-16	-7	30.65	4.74
-4	-16	-7	25.06	4.60
-5	-16	-7	36.39	5.11
-6	-16	-7	26.70	5.03
6	16	7	29.19	3.47
-7	-16	-7	39.13	5.48
7	16	7	34.39	3.91
-8	-16	-7	19.74	4.13
8	16	7	21.66	3.76
-9	-16	-7	34.93	6.06
9	16	7	22.27	4.18
-10	-16	-7	22.12	4.52
10	16	7	16.76	2.73
6	12	-8	11.03	3.06
6	12	-8	5.58	1.00
-6	-12	8	11.63	3.42
-6	-12	8	5.88	3.49
-6	-12	8	11.00	3.12
5	12	-8	1.15	3.97
5	12	-8	6.26	2.92
5	12	-8	2.58	0.94
-5	-12	8	2.56	3.61
-5	-12	8	6.71	3.31
4	12	-8	5.65	1.08
4	12	-8	7.54	4.16
4	12	-8	8.99	1.58
4	12	-8	8.43	3.46
-4	-12	8	3.87	3.12

3	12	-8	5.57	3.26
-3	-12	8	6.53	3.27
9	11	-8	8.27	2.71
9	11	-8	13.23	4.06
-9	-11	8	9.69	3.68
-9	-11	8	5.29	3.02
-9	-11	8	5.99	3.56
8	11	-8	7.99	3.72
8	11	-8	10.32	2.76
-8	-11	8	12.79	3.62
-8	-11	8	10.35	2.91
7	11	-8	5.00	3.81
7	11	-8	6.63	3.40
7	11	-8	9.90	2.58
-7	-11	8	8.73	2.57
-7	-11	8	12.23	3.41
-7	-11	8	4.88	2.68
6	11	-8	15.87	3.36
6	11	-8	9.40	4.19
6	11	-8	8.63	1.54
6	11	-8	12.55	2.81
-6	-11	8	14.83	3.28
-6	-11	8	9.99	2.82
-6	-11	8	10.04	2.46
5	11	-8	35.59	5.19
5	11	-8	23.45	4.11
5	11	-8	25.65	4.18
-5	-11	8	22.58	4.09
-5	-11	8	18.23	3.08
-5	-11	8	25.37	4.70
4	11	-8	12.80	2.92
4	11	-8	15.75	3.42
4	11	-8	13.15	1.64
-4	-11	8	13.36	2.67
-4	-11	8	8.42	2.91
-4	-11	8	16.83	3.15
3	11	-8	14.92	3.18
3	11	-8	9.86	1.63
3	11	-8	11.28	2.87
-3	-11	8	12.35	3.02
-3	-11	8	11.56	2.47
-3	-11	8	11.44	2.82
2	11	-8	9.81	2.69
2	11	-8	11.12	1.72

2	11	-8	12.91	3.22
2	11	-8	6.32	3.01
-2	-11	8	13.37	3.01
1	11	-8	6.57	1.06
1	11	-8	6.25	3.05
-1	-11	8	6.40	3.04
0	11	-8	5.59	1.13
0	11	-8	8.32	3.32
0	-11	8	8.36	2.88
-1	11	-8	6.54	1.15
-1	11	-8	7.79	3.24
1	-11	8	7.60	3.00
-10	-10	8	9.78	2.93
-10	-10	8	10.93	3.71
9	10	-8	14.46	3.66
9	10	-8	16.01	2.67
-9	-10	8	19.03	3.65
-9	-10	8	17.37	2.95
-9	-10	8	18.71	2.98
8	10	-8	6.73	2.36
8	10	-8	11.16	3.26
8	10	-8	7.48	3.72
-8	-10	8	13.32	3.24
-8	-10	8	9.44	2.42
7	10	-8	8.83	3.00
7	10	-8	7.67	2.14
7	10	-8	4.55	3.59
7	10	-8	4.58	3.07
-7	-10	8	3.51	2.29
-7	-10	8	4.84	2.14
-7	-10	8	8.92	3.06
6	10	-8	6.63	1.03
6	10	-8	7.79	2.17
6	10	-8	8.26	3.58
6	10	-8	9.93	2.80
6	10	-8	2.86	3.07
-6	-10	8	6.39	2.38
-6	-10	8	6.20	2.20
-6	-10	8	7.69	2.96
5	10	-8	11.64	2.32
5	10	-8	10.34	3.11
5	10	-8	9.61	2.62
5	10	-8	11.89	2.92
5	10	-8	10.27	1.32

-5	-10	8	11.89	2.27
-5	-10	8	13.12	2.90
-5	-10	8	9.06	2.48
4	10	-8	9.62	2.99
4	10	-8	9.63	2.50
4	10	-8	6.86	2.25
4	10	-8	9.07	2.60
-4	-10	8	5.06	2.14
-4	-10	8	7.85	2.66
-4	-10	8	5.10	2.40
3	10	-8	6.04	2.26
3	10	-8	5.64	0.88
3	10	-8	8.25	3.31
3	10	-8	9.93	2.87
3	10	-8	5.81	2.98
-3	-10	8	5.12	2.54
-3	-10	8	5.82	2.46
-3	-10	8	6.46	1.99
2	10	-8	5.09	2.88
2	10	-8	2.41	2.49
2	10	-8	5.32	2.40
2	10	-8	4.60	0.87
2	10	-8	7.02	2.11
-2	-10	8	8.48	2.52
-2	-10	8	5.43	2.40
-2	-10	8	5.03	2.04
1	10	-8	7.14	2.69
1	10	-8	4.69	2.39
1	10	-8	5.79	0.93
1	10	-8	4.96	2.85
-1	-10	8	6.66	2.23
-1	-10	8	8.17	2.71
-1	-10	8	5.08	2.05
0	10	-8	0.05	2.31
0	10	-8	0.12	2.74
0	10	-8	2.11	0.89
0	-10	8	0.99	2.31
0	-10	8	-1.28	1.75
-1	10	-8	8.32	1.13
-1	10	-8	7.96	2.98
1	-10	8	11.10	2.73
-2	10	-8	4.59	1.07
-2	10	-8	4.34	2.90
-3	10	-8	9.66	2.98

-3	10	-8	7.69	1.22
-11	-9	8	10.11	3.92
-11	-9	8	11.14	3.04
-10	-9	8	21.16	3.96
-10	-9	8	14.74	2.87
-10	-9	8	24.40	5.02
9	9	-8	14.12	3.24
9	9	-8	15.39	3.77
9	9	-8	17.81	3.27
9	9	-8	13.29	4.05
-9	-9	8	17.70	3.41
-9	-9	8	20.52	3.77
-9	-9	8	17.60	2.64
8	9	-8	16.01	3.08
8	9	-8	14.01	2.25
8	9	-8	15.93	3.74
8	9	-8	14.68	3.29
-8	-9	8	14.73	2.32
-8	-9	8	21.56	4.29
7	9	-8	26.14	4.53
7	9	-8	14.93	3.12
7	9	-8	20.07	3.04
7	9	-8	20.21	3.69
-7	-9	8	22.99	3.32
-7	-9	8	26.97	4.04
-7	-9	8	20.84	3.24
6	9	-8	24.76	3.16
6	9	-8	27.75	4.88
6	9	-8	30.13	4.38
-6	-9	8	33.48	4.12
-6	-9	8	24.00	3.39
-6	-9	8	27.43	3.31
5	9	-8	19.22	1.49
5	9	-8	14.67	2.86
5	9	-8	19.36	3.11
5	9	-8	22.75	3.87
-5	-9	8	19.12	3.29
-5	-9	8	23.32	3.73
-5	-9	8	16.73	3.10
4	9	-8	14.13	2.49
4	9	-8	12.34	2.23
4	9	-8	14.45	2.50
4	9	-8	14.11	1.32
-4	-9	8	13.39	2.26

-4	-9	8	16.68	3.38
-4	-9	8	11.96	2.10
3	9	-8	6.07	2.08
3	9	-8	6.88	1.20
3	9	-8	6.85	2.08
3	9	-8	8.42	2.18
-3	-9	8	7.62	2.14
-3	-9	8	6.56	1.90
-3	-9	8	7.82	2.23
2	9	-8	14.61	1.45
2	9	-8	11.69	2.47
2	9	-8	14.47	2.37
-2	-9	8	13.14	2.40
-2	-9	8	15.94	2.45
-2	-9	8	15.93	2.17
1	9	-8	13.75	1.46
1	9	-8	10.11	3.19
1	9	-8	10.61	2.34
1	9	-8	12.72	2.88
-1	-9	8	13.23	2.11
-1	-9	8	9.41	2.03
-1	-9	8	14.74	2.64
0	9	-8	19.06	1.68
0	9	-8	20.81	3.67
0	9	-8	13.48	2.57
0	9	-8	13.37	2.43
0	-9	8	21.89	3.39
0	-9	8	16.23	3.18
0	-9	8	18.35	3.02
-1	9	-8	12.16	2.62
-1	9	-8	11.78	2.75
-1	9	-8	8.35	2.32
-1	9	-8	14.63	1.60
1	-9	8	19.89	3.60
1	-9	8	10.51	2.28
1	-9	8	11.63	2.25
-2	9	-8	13.84	3.52
-2	9	-8	7.79	2.66
-2	9	-8	7.93	2.35
-2	9	-8	9.38	1.64
-3	9	-8	6.43	2.66
-3	9	-8	4.53	3.35
-3	9	-8	4.65	0.99
-4	9	-8	12.05	2.68

-4	9	-8	6.55	1.13
-5	9	-8	5.13	2.55
-5	9	-8	2.19	1.08
-12	-8	8	14.21	4.06
-12	-8	8	11.15	3.07
-12	-8	8	8.91	3.06
-11	-8	8	9.05	3.52
-11	-8	8	5.25	2.58
10	8	-8	20.54	4.37
10	8	-8	11.93	2.48
10	8	-8	9.67	3.34
-10	-8	8	15.18	2.67
-10	-8	8	13.24	2.44
-10	-8	8	19.33	3.57
-10	-8	8	17.10	2.57
9	8	-8	9.42	2.76
9	8	-8	5.36	1.83
9	8	-8	7.49	3.74
9	8	-8	5.88	3.43
-9	-8	8	6.55	2.12
-9	-8	8	5.77	2.17
-9	-8	8	10.64	2.92
-9	-8	8	6.85	1.97
8	8	-8	7.96	2.61
8	8	-8	5.49	1.80
8	8	-8	3.03	2.94
-8	-8	8	5.82	1.83
-8	-8	8	5.95	2.61
7	8	-8	7.79	1.73
7	8	-8	7.45	2.38
7	8	-8	6.38	2.58
-7	-8	8	10.38	2.45
-7	-8	8	9.39	1.87
-7	-8	8	9.97	1.87
6	8	-8	15.21	2.73
6	8	-8	22.88	4.07
6	8	-8	13.54	2.51
6	8	-8	18.38	2.75
-6	-8	8	17.60	2.84
-6	-8	8	12.72	3.20
-6	-8	8	22.20	3.35
-6	-8	8	19.07	2.84
5	8	-8	7.13	2.39
5	8	-8	5.13	2.04

5	8	-8	4.96	1.72
5	8	-8	8.29	2.46
-5	-8	8	6.52	1.81
-5	-8	8	7.02	2.74
-5	-8	8	5.65	1.69
4	8	-8	16.17	1.30
4	8	-8	19.29	3.30
4	8	-8	13.52	2.04
4	8	-8	11.57	2.37
4	8	-8	19.59	3.67
-4	-8	8	15.81	2.96
-4	-8	8	14.92	2.70
3	8	-8	21.08	3.38
3	8	-8	17.04	3.33
3	8	-8	22.06	3.21
3	8	-8	16.14	1.39
3	8	-8	16.93	3.02
-3	-8	8	14.18	2.16
-3	-8	8	17.07	2.76
2	8	-8	19.63	3.22
2	8	-8	17.55	3.19
2	8	-8	17.07	3.20
2	8	-8	19.83	1.47
-2	-8	8	16.69	3.13
-2	-8	8	16.98	2.23
-2	-8	8	18.83	2.86
1	8	-8	6.99	1.98
1	8	-8	1.94	1.98
1	8	-8	3.03	1.84
-1	-8	8	4.96	1.74
-1	-8	8	5.19	1.77
-1	-8	8	7.39	1.98
0	8	-8	9.59	1.25
0	8	-8	3.22	2.17
0	-8	8	7.55	1.86
0	-8	8	6.41	1.70
0	-8	8	8.86	2.02
-1	8	-8	7.65	1.31
-1	8	-8	3.41	2.21
1	-8	8	6.43	1.76
1	-8	8	5.68	1.70
1	-8	8	9.02	2.05
-2	8	-8	2.98	1.94
-2	8	-8	10.69	2.43

-2	8	-8	6.96	2.34
-2	8	-8	8.55	1.60
-2	8	-8	6.88	0.97
2	-8	8	10.02	1.89
2	-8	8	8.89	2.45
-3	8	-8	-1.27	1.82
-3	8	-8	0.43	2.13
-3	8	-8	-0.25	0.79
-3	8	-8	-0.34	2.43
3	-8	8	1.71	1.82
3	-8	8	1.92	1.23
-4	8	-8	1.71	1.99
-4	8	-8	6.20	1.57
-4	8	-8	6.07	1.03
-4	8	-8	7.72	2.38
4	-8	8	7.98	1.68
4	-8	8	4.56	1.77
-5	8	-8	3.98	2.36
-5	8	-8	2.00	0.99
5	-8	8	-2.32	2.15
-6	8	-8	9.40	2.52
-6	8	-8	5.06	1.19
6	-8	8	8.05	1.89
-12	-7	8	14.68	4.13
-12	-7	8	11.44	2.72
-12	-7	8	9.56	2.93
11	7	-8	7.80	4.32
-11	-7	8	15.30	2.59
-11	-7	8	18.89	3.55
-11	-7	8	12.49	2.49
-11	-7	8	15.71	2.83
10	7	-8	8.00	2.34
10	7	-8	17.81	4.03
10	7	-8	11.56	2.97
-10	-7	8	10.76	2.22
-10	-7	8	14.14	2.53
-10	-7	8	12.05	2.22
-10	-7	8	14.90	3.12
9	7	-8	6.27	1.68
9	7	-8	10.21	2.66
-9	-7	8	10.36	1.90
-9	-7	8	8.22	2.03
-9	-7	8	13.47	2.76
8	7	-8	4.51	2.34

8	7	-8	1.71	2.75
8	7	-8	1.90	1.48
-8	-7	8	5.00	2.40
-8	-7	8	3.54	1.72
-8	-7	8	4.34	1.61
7	7	-8	15.47	2.49
7	7	-8	20.44	3.77
7	7	-8	20.39	3.79
-7	-7	8	20.32	2.92
-7	-7	8	20.84	4.29
-7	-7	8	20.89	2.73
-7	-7	8	19.73	2.78
6	7	-8	28.81	3.64
6	7	-8	29.29	3.69
6	7	-8	22.28	2.67
-6	-7	8	25.84	2.86
-6	-7	8	25.34	2.70
-6	-7	8	28.77	3.25
-6	-7	8	22.36	2.84
5	7	-8	32.61	4.24
5	7	-8	33.36	3.50
5	7	-8	30.78	3.57
5	7	-8	28.23	2.96
-5	-7	8	24.08	2.90
-5	-7	8	28.73	3.31
-5	-7	8	32.48	2.87
4	7	-8	23.04	3.97
4	7	-8	28.03	3.26
4	7	-8	24.35	2.84
4	7	-8	26.42	1.63
4	7	-8	26.33	3.67
-4	-7	8	26.62	2.77
-4	-7	8	25.35	3.16
-4	-7	8	24.61	2.84
3	7	-8	29.23	3.06
3	7	-8	26.99	3.12
3	7	-8	27.81	3.00
3	7	-8	26.82	1.73
-3	-7	8	25.57	3.43
-3	-7	8	26.42	3.06
-3	-7	8	27.00	3.05
2	7	-8	20.42	1.46
2	7	-8	17.14	2.81
2	7	-8	16.20	2.89

2	7	-8	22.41	2.94
-2	-7	8	20.74	2.58
-2	-7	8	20.01	2.21
-2	-7	8	19.49	2.87
-2	-7	8	19.78	2.82
1	7	-8	31.81	1.91
1	7	-8	27.32	3.26
1	7	-8	30.77	3.00
-1	-7	8	29.99	2.35
-1	-7	8	27.76	3.09
-1	-7	8	33.03	3.02
-1	-7	8	32.87	3.01
-1	-7	8	28.82	2.71
0	7	-8	34.80	2.97
0	7	-8	32.82	1.97
0	7	-8	33.71	3.13
0	7	-8	24.76	3.35
0	7	-8	23.50	3.13
0	-7	8	30.16	2.48
0	-7	8	32.99	3.10
0	-7	8	30.39	2.97
0	-7	8	33.28	3.20
-1	7	-8	35.21	3.28
-1	7	-8	30.58	3.53
-1	7	-8	33.72	2.04
-1	7	-8	31.07	2.97
-1	7	-8	25.07	3.12
1	-7	8	30.82	2.61
1	-7	8	35.12	3.20
1	-7	8	32.27	2.98
-2	7	-8	9.94	2.06
-2	7	-8	7.49	1.82
-2	7	-8	11.16	2.30
-2	7	-8	10.49	1.35
2	-7	8	8.88	1.35
2	-7	8	10.54	2.09
2	-7	8	9.61	1.81
2	-7	8	10.68	1.86
-3	7	-8	8.76	2.13
-3	7	-8	5.42	1.80
-3	7	-8	10.35	1.41
3	-7	8	10.37	1.45
3	-7	8	8.30	1.87
3	-7	8	7.71	1.75

3	-7	8	8.12	1.37
-4	7	-8	0.85	1.69
-4	7	-8	1.68	2.26
-4	7	-8	4.47	2.00
-4	7	-8	1.28	0.82
4	-7	8	1.27	1.16
4	-7	8	1.58	1.31
4	-7	8	0.31	1.76
-5	7	-8	5.13	1.35
-5	7	-8	10.44	2.70
-5	7	-8	4.48	1.77
-5	7	-8	7.05	2.13
-5	7	-8	5.82	1.03
5	-7	8	7.50	1.88
5	-7	8	8.85	1.59
-6	7	-8	4.35	2.18
-6	7	-8	5.85	1.10
6	-7	8	8.17	1.79
-7	7	-8	10.96	2.63
-7	7	-8	7.32	1.19
12	6	-8	16.41	5.79
-12	-6	8	6.51	2.65
-12	-6	8	8.94	3.69
-12	-6	8	4.93	2.40
11	6	-8	20.91	4.14
-11	-6	8	18.43	3.36
-11	-6	8	12.36	2.66
-11	-6	8	16.30	3.31
-11	-6	8	17.05	3.31
10	6	-8	11.02	2.80
-10	-6	8	16.95	2.97
-10	-6	8	9.46	2.34
-10	-6	8	9.96	2.11
-10	-6	8	12.37	2.05
9	6	-8	11.91	3.11
9	6	-8	10.40	2.57
9	6	-8	11.52	2.46
9	6	-8	16.26	3.30
-9	-6	8	9.16	1.93
-9	-6	8	15.19	2.74
-9	-6	8	12.16	2.17
-9	-6	8	14.61	2.68
8	6	-8	3.21	2.56
8	6	-8	3.91	2.10

8	6	-8	2.03	1.35
-8	-6	8	2.39	1.74
-8	-6	8	1.00	1.42
-8	-6	8	5.40	2.22
7	6	-8	26.64	3.50
7	6	-8	19.33	2.57
7	6	-8	23.99	3.56
-7	-6	8	26.49	3.14
-7	-6	8	24.67	2.65
-7	-6	8	21.52	2.67
-7	-6	8	24.60	2.95
6	6	-8	11.59	2.20
6	6	-8	12.07	2.05
6	6	-8	10.47	2.34
-6	-6	8	13.86	2.32
-6	-6	8	14.11	2.32
-6	-6	8	12.70	2.12
5	6	-8	7.25	1.49
5	6	-8	8.67	2.16
5	6	-8	8.53	1.81
-5	-6	8	9.49	1.51
-5	-6	8	8.36	1.49
4	6	-8	11.26	1.04
4	6	-8	12.05	2.10
4	6	-8	14.13	2.02
4	6	-8	10.43	1.69
-4	-6	8	13.26	2.53
-4	-6	8	12.68	2.29
-4	-6	8	13.12	2.04
-4	-6	8	12.31	2.20
3	6	-8	26.24	2.73
3	6	-8	27.74	3.20
3	6	-8	19.18	2.81
3	6	-8	23.04	2.72
3	6	-8	23.14	1.49
-3	-6	8	20.15	2.65
-3	-6	8	23.09	2.47
-3	-6	8	24.80	2.27
2	6	-8	8.04	1.50
2	6	-8	8.03	0.96
2	6	-8	5.08	1.70
-2	-6	8	5.26	1.57
-2	-6	8	6.08	1.39
-2	-6	8	7.93	1.61

-2	-6	8	6.95	1.12
-2	-6	8	7.60	1.46
1	6	-8	23.19	2.57
1	6	-8	25.63	2.65
1	6	-8	16.15	2.73
1	6	-8	21.60	1.50
-1	-6	8	19.38	2.73
-1	-6	8	22.73	1.87
-1	-6	8	22.56	2.45
-1	-6	8	20.41	2.16
-1	-6	8	20.36	2.96
0	6	-8	9.11	1.62
0	6	-8	6.23	1.02
0	6	-8	2.60	1.51
0	6	-8	4.27	1.72
0	-6	8	6.11	1.49
0	-6	8	7.07	1.62
0	-6	8	6.23	0.97
0	-6	8	5.88	1.39
-1	6	-8	6.41	1.81
-1	6	-8	10.86	1.80
-1	6	-8	10.58	2.09
-1	6	-8	11.20	1.16
1	-6	8	9.21	1.47
1	-6	8	10.85	1.78
1	-6	8	9.00	1.89
1	-6	8	8.71	1.16
-2	6	-8	6.87	1.85
-2	6	-8	2.65	1.53
-2	6	-8	5.30	1.70
-2	6	-8	4.88	1.17
-2	6	-8	7.32	0.98
2	-6	8	6.03	1.37
2	-6	8	5.38	1.05
2	-6	8	3.06	1.64
2	-6	8	4.75	1.72
-3	6	-8	11.77	2.00
-3	6	-8	8.78	1.29
-3	6	-8	9.69	1.69
-3	6	-8	8.83	1.29
-3	6	-8	8.18	1.97
3	-6	8	9.63	2.60
3	-6	8	8.52	1.91
3	-6	8	8.99	1.25

3	-6	8	11.33	2.02
-4	6	-8	2.90	0.75
-4	6	-8	1.42	1.09
-4	6	-8	2.55	1.73
-4	6	-8	0.29	1.53
4	-6	8	-0.18	1.51
4	-6	8	1.19	1.12
4	-6	8	0.89	1.70
4	-6	8	2.37	0.98
-5	6	-8	-0.76	1.06
-5	6	-8	1.14	0.80
-5	6	-8	2.41	1.80
5	-6	8	0.55	1.70
5	-6	8	-0.73	1.52
5	-6	8	0.54	0.99
5	-6	8	-0.01	1.24
-6	6	-8	4.86	2.10
-6	6	-8	0.92	0.87
-6	6	-8	1.65	1.15
-6	6	-8	-0.98	1.54
-6	6	-8	3.12	2.49
6	-6	8	2.06	1.43
6	-6	8	-0.09	1.90
-7	6	-8	5.46	1.09
-7	6	-8	3.09	2.42
7	-6	8	8.71	1.77
-8	6	-8	1.53	1.02
-8	6	-8	-0.50	3.67
-8	6	-8	2.96	2.63
8	-6	8	2.77	1.79
-13	-5	8	3.68	2.64
-13	-5	8	7.18	4.16
-13	-5	8	5.69	3.10
12	5	-8	7.39	4.45
12	5	-8	2.43	2.12
-12	-5	8	11.75	3.60
-12	-5	8	6.59	2.44
-12	-5	8	7.56	2.91
11	5	-8	12.88	5.39
-11	-5	8	7.80	2.07
-11	-5	8	12.95	3.17
-11	-5	8	11.37	2.02
-11	-5	8	10.21	2.59
-10	-5	8	21.87	3.02

-10	-5	8	30.87	4.01
-10	-5	8	18.75	3.01
-10	-5	8	25.71	3.80
9	5	-8	16.55	3.18
-9	-5	8	23.38	3.76
-9	-5	8	19.05	3.31
-9	-5	8	17.63	2.66
-9	-5	8	14.10	2.68
8	5	-8	13.75	2.19
8	5	-8	7.05	2.68
8	5	-8	7.59	1.64
-8	-5	8	11.92	1.90
-8	-5	8	10.56	1.59
7	5	-8	17.38	2.61
7	5	-8	22.11	3.21
7	5	-8	22.00	4.04
-7	-5	8	25.25	2.87
-7	-5	8	22.25	2.42
-7	-5	8	20.17	2.47
6	5	-8	25.49	3.21
6	5	-8	28.44	3.82
6	5	-8	22.98	2.53
-6	-5	8	27.21	2.76
-6	-5	8	26.07	2.53
-6	-5	8	26.76	2.60
5	5	-8	10.08	1.72
5	5	-8	8.25	1.51
5	5	-8	9.67	2.12
-5	-5	8	10.32	1.45
-5	-5	8	11.07	2.01
4	5	-8	11.01	1.98
4	5	-8	10.30	1.58
-4	-5	8	11.48	2.04
-4	-5	8	12.32	1.99
-4	-5	8	11.54	1.50
3	5	-8	17.55	1.20
3	5	-8	14.63	2.43
3	5	-8	21.05	2.88
3	5	-8	16.47	2.37
-3	-5	8	18.00	1.43
-3	-5	8	17.75	2.04
-3	-5	8	19.24	2.16
-3	-5	8	17.45	2.35
2	5	-8	31.47	2.88

2	5	-8	25.62	2.85
2	5	-8	37.27	3.02
2	5	-8	34.94	1.88
-2	-5	8	35.39	2.54
-2	-5	8	27.96	2.78
-2	-5	8	32.89	2.70
-2	-5	8	34.16	2.09
1	5	-8	22.20	2.43
1	5	-8	16.20	2.55
1	5	-8	22.13	2.22
1	5	-8	21.25	1.38
-1	-5	8	19.86	2.03
-1	-5	8	17.24	2.30
-1	-5	8	15.36	2.41
-1	-5	8	21.57	1.65
0	5	-8	30.47	2.56
0	5	-8	33.28	1.85
0	5	-8	27.82	2.90
0	-5	8	27.83	2.42
0	-5	8	32.38	2.59
0	-5	8	32.55	2.64
0	-5	8	26.97	2.76
0	-5	8	32.26	2.11
-1	5	-8	17.27	2.21
-1	5	-8	15.65	1.25
-1	5	-8	15.03	2.08
-1	5	-8	11.78	1.85
-1	5	-8	12.31	1.67
1	-5	8	13.52	1.84
1	-5	8	14.51	2.35
1	-5	8	16.54	2.12
1	-5	8	16.74	2.10
1	-5	8	16.11	1.58
-2	5	-8	17.44	2.30
-2	5	-8	21.64	2.43
-2	5	-8	17.13	2.09
-2	5	-8	15.33	2.73
2	-5	8	20.04	2.28
2	-5	8	14.74	2.50
2	-5	8	17.00	1.75
2	-5	8	19.18	2.06
-3	5	-8	17.32	2.86
-3	5	-8	18.53	2.63
-3	5	-8	20.61	2.35

-3	5	-8	12.76	1.70
-3	5	-8	17.65	2.06
3	-5	8	13.83	1.96
3	-5	8	18.24	2.52
3	-5	8	19.05	1.80
-4	5	-8	19.82	1.61
-4	5	-8	21.80	2.78
-4	5	-8	26.13	2.89
-4	5	-8	19.23	2.20
-4	5	-8	20.69	2.49
4	-5	8	20.70	3.09
4	-5	8	19.32	1.94
4	-5	8	20.00	2.21
-5	5	-8	9.63	1.97
-5	5	-8	10.75	1.36
-5	5	-8	7.83	1.65
-5	5	-8	11.29	1.92
-5	5	-8	11.40	2.03
5	-5	8	9.39	1.39
-6	5	-8	18.45	1.77
-6	5	-8	25.98	3.41
-6	5	-8	19.10	2.31
6	-5	8	19.80	2.88
6	-5	8	21.53	2.65
-7	5	-8	6.69	1.30
-7	5	-8	5.95	2.51
-7	5	-8	7.10	1.04
-7	5	-8	3.50	1.70
-7	5	-8	9.02	2.95
-7	5	-8	7.40	2.28
7	-5	8	5.07	1.41
7	-5	8	12.21	2.62
7	-5	8	2.04	1.87
-8	5	-8	5.57	3.56
-8	5	-8	3.17	1.86
-8	5	-8	4.25	2.63
-8	5	-8	4.06	1.03
8	-5	8	5.13	1.71
-9	5	-8	1.89	1.02
-9	5	-8	0.24	2.79
-9	5	-8	3.03	3.92
9	-5	8	2.88	1.88
-13	-4	8	1.78	3.09
-13	-4	8	4.73	4.09

-13	-4	8	3.15	2.20
12	4	-8	6.24	4.11
-12	-4	8	13.79	3.62
-12	-4	8	8.64	2.89
-12	-4	8	7.79	2.29
-11	-4	8	8.24	2.51
-11	-4	8	9.95	1.92
-11	-4	8	10.86	3.12
-11	-4	8	6.52	1.97
10	4	-8	24.17	4.77
-10	-4	8	14.40	2.73
-10	-4	8	19.20	2.73
-10	-4	8	14.60	2.54
-10	-4	8	20.89	4.18
9	4	-8	7.32	1.98
-9	-4	8	8.44	1.93
-9	-4	8	9.35	1.57
-9	-4	8	6.84	1.66
8	4	-8	17.19	3.22
8	4	-8	15.85	2.81
-8	-4	8	19.71	2.36
-8	-4	8	23.95	2.98
-8	-4	8	16.21	2.45
7	4	-8	11.93	2.27
7	4	-8	10.18	1.91
-7	-4	8	11.07	1.50
-7	-4	8	11.43	2.10
6	4	-8	29.48	3.05
6	4	-8	25.50	3.62
-6	-4	8	26.18	2.41
-6	-4	8	25.45	2.76
-6	-4	8	27.47	2.43
5	4	-8	4.08	1.50
5	4	-8	4.90	1.44
5	4	-8	7.62	1.85
-5	-4	8	7.70	1.30
-5	-4	8	7.87	1.29
-5	-4	8	7.84	1.29
4	4	-8	15.95	2.99
4	4	-8	14.69	2.14
-4	-4	8	13.00	2.05
-4	-4	8	14.01	2.00
-4	-4	8	14.95	1.94
3	4	-8	2.89	1.44

3	4	-8	3.06	0.42
3	4	-8	1.71	1.25
3	4	-8	0.40	1.19
-3	-4	8	2.89	1.17
-3	-4	8	2.94	0.53
-3	-4	8	4.26	1.11
-3	-4	8	3.38	0.95
2	4	-8	9.05	0.85
2	4	-8	9.21	1.44
2	4	-8	8.05	1.36
2	4	-8	12.00	2.18
-2	-4	8	8.63	1.64
-2	-4	8	9.50	1.08
-2	-4	8	9.29	1.31
-2	-4	8	10.41	1.87
1	4	-8	12.16	2.17
1	4	-8	15.34	1.09
1	4	-8	17.96	2.15
-1	-4	8	13.63	2.05
-1	-4	8	14.20	1.31
-1	-4	8	13.55	1.76
-1	-4	8	15.38	2.05
0	4	-8	5.61	0.81
0	4	-8	5.27	1.01
0	4	-8	1.27	1.14
0	4	-8	3.50	1.30
0	-4	8	4.28	0.71
0	-4	8	3.95	1.08
0	-4	8	3.76	0.89
0	-4	8	2.18	1.25
-1	4	-8	1.11	1.34
-1	4	-8	1.28	0.85
-1	4	-8	-0.49	1.08
-1	4	-8	1.01	0.46
1	-4	8	1.17	0.97
1	-4	8	0.97	0.62
1	-4	8	-1.12	1.21
1	-4	8	0.44	0.76
-2	4	-8	1.38	1.13
-2	4	-8	2.92	0.57
-2	4	-8	2.78	1.38
-2	4	-8	4.43	1.83
-2	4	-8	2.40	0.85
2	-4	8	2.61	0.74

2	-4	8	2.61	0.89
2	-4	8	2.44	1.09
-3	4	-8	0.77	1.19
-3	4	-8	1.00	0.83
-3	4	-8	0.82	1.34
-3	4	-8	2.12	0.56
-3	4	-8	3.68	1.35
3	-4	8	1.55	0.72
3	-4	8	-0.25	1.40
3	-4	8	1.92	1.13
3	-4	8	1.37	0.84
-4	4	-8	4.98	1.53
-4	4	-8	2.20	0.93
-4	4	-8	4.20	0.74
-4	4	-8	4.09	1.33
-4	4	-8	5.50	1.49
4	-4	8	2.72	1.58
4	-4	8	3.23	0.95
4	-4	8	2.22	0.80
4	-4	8	2.57	1.35
-5	4	-8	2.89	1.43
-5	4	-8	5.06	1.69
-5	4	-8	3.32	0.70
-5	4	-8	7.22	1.66
-5	4	-8	3.63	0.99
5	-4	8	3.70	1.37
5	-4	8	1.87	1.65
5	-4	8	2.87	1.01
-6	4	-8	11.36	1.78
-6	4	-8	13.02	1.97
-6	4	-8	12.44	1.44
-6	4	-8	13.06	2.15
-6	4	-8	14.13	2.69
6	-4	8	12.89	2.23
-7	4	-8	4.24	1.66
-7	4	-8	2.98	1.07
-7	4	-8	4.95	2.56
-7	4	-8	3.44	2.03
-7	4	-8	3.79	0.87
7	-4	8	4.46	1.53
7	-4	8	4.27	1.35
7	-4	8	2.01	1.78
-8	4	-8	3.08	2.39
-8	4	-8	2.19	3.09

-8	4	-8	1.33	2.38
-8	4	-8	0.77	1.74
-8	4	-8	0.97	1.18
-8	4	-8	0.76	0.87
8	-4	8	2.66	1.46
8	-4	8	0.75	1.04
8	-4	8	-0.06	1.88
-9	4	-8	0.67	2.74
-9	4	-8	-1.14	1.98
-9	4	-8	2.16	3.66
-9	4	-8	1.27	0.95
9	-4	8	1.58	1.68
-10	4	-8	0.92	4.47
-10	4	-8	-0.45	2.95
-10	4	-8	0.79	1.04
10	-4	8	-0.61	1.90
-13	-3	8	6.80	4.20
-13	-3	8	6.01	2.41
-13	-3	8	3.01	3.28
12	3	-8	13.62	4.08
-12	-3	8	15.14	3.54
-12	-3	8	10.62	3.11
-12	-3	8	12.23	2.19
-11	-3	8	23.93	4.26
-11	-3	8	23.87	3.54
-11	-3	8	20.92	2.99
-11	-3	8	22.42	4.50
-10	-3	8	3.71	2.11
-10	-3	8	2.35	1.60
-9	-3	8	5.01	1.80
-9	-3	8	5.84	1.40
-9	-3	8	1.91	1.50
-8	-3	8	7.79	1.71
-8	-3	8	8.43	1.38
-8	-3	8	8.53	1.55
7	3	-8	23.08	3.90
7	3	-8	28.02	2.99
-7	-3	8	27.55	3.31
-7	-3	8	25.06	2.35
-7	-3	8	26.27	2.35
6	3	-8	14.50	2.63
6	3	-8	13.46	2.15
-6	-3	8	14.84	2.32
-6	-3	8	17.68	1.95

-6	-3	8	15.47	2.04
5	3	-8	24.89	2.61
5	3	-8	32.48	3.34
5	3	-8	31.98	2.82
-5	-3	8	31.54	2.70
-5	-3	8	31.84	2.40
-5	-3	8	30.47	2.37
4	3	-8	43.62	3.37
4	3	-8	53.58	3.87
-4	-3	8	47.57	3.13
-4	-3	8	48.82	3.07
-4	-3	8	50.60	3.21
3	3	-8	39.68	2.88
3	3	-8	39.20	3.33
3	3	-8	40.03	2.83
-3	-3	8	36.93	2.19
-3	-3	8	40.59	2.77
-3	-3	8	41.55	2.81
-3	-3	8	42.68	2.73
2	3	-8	26.73	2.42
2	3	-8	31.08	1.70
2	3	-8	28.82	2.56
2	3	-8	34.41	2.76
-2	-3	8	31.57	1.86
-2	-3	8	29.93	2.46
-2	-3	8	31.66	2.43
-2	-3	8	34.20	2.30
1	3	-8	25.18	2.36
1	3	-8	19.82	2.16
1	3	-8	27.65	1.47
1	3	-8	25.58	2.36
-1	-3	8	26.23	2.07
-1	-3	8	24.26	2.25
0	3	-8	15.46	1.02
0	3	-8	15.46	1.80
0	3	-8	13.89	2.09
0	3	-8	11.09	1.80
0	-3	8	10.40	1.51
0	-3	8	12.60	1.61
0	-3	8	16.73	1.84
0	-3	8	14.39	1.29
-1	3	-8	29.71	2.31
-1	3	-8	26.80	2.59
-1	3	-8	29.06	2.20

-1	3	-8	30.04	1.67
1	-3	8	30.05	1.90
1	-3	8	30.85	2.25
1	-3	8	26.73	2.16
1	-3	8	24.39	2.52
-2	3	-8	28.79	2.17
-2	3	-8	31.55	2.44
-2	3	-8	31.41	2.73
-2	3	-8	29.40	1.74
2	-3	8	27.80	2.67
2	-3	8	31.50	2.38
2	-3	8	30.13	2.19
2	-3	8	28.74	1.98
-3	3	-8	26.60	1.71
-3	3	-8	28.82	2.46
-3	3	-8	26.87	2.11
-3	3	-8	28.79	2.36
-3	3	-8	31.93	2.72
3	-3	8	27.72	2.19
3	-3	8	27.61	1.96
3	-3	8	28.28	2.40
-4	3	-8	0.04	1.20
-4	3	-8	1.56	0.55
-4	3	-8	1.18	0.81
-4	3	-8	1.30	1.35
-4	3	-8	2.90	1.32
4	-3	8	1.10	0.68
4	-3	8	-0.38	1.40
4	-3	8	1.50	1.14
4	-3	8	1.67	0.81
-5	3	-8	14.50	1.86
-5	3	-8	17.90	1.40
-5	3	-8	21.24	2.78
-5	3	-8	17.39	2.21
-5	3	-8	18.31	2.39
5	-3	8	13.34	1.59
5	-3	8	18.00	2.84
5	-3	8	18.17	1.97
5	-3	8	17.28	2.79
-6	3	-8	5.68	1.55
-6	3	-8	6.15	1.80
-6	3	-8	5.88	1.61
-6	3	-8	6.36	0.83
6	-3	8	4.36	1.81

6	-3	8	4.05	1.25
6	-3	8	5.80	1.17
-7	3	-8	5.27	1.97
-7	3	-8	3.88	1.61
-7	3	-8	5.16	0.84
-7	3	-8	4.57	1.76
-7	3	-8	6.26	2.49
7	-3	8	1.23	1.81
7	-3	8	4.84	1.26
-8	3	-8	-0.31	0.84
-8	3	-8	-0.33	0.76
-8	3	-8	-2.15	2.12
-8	3	-8	-1.22	1.65
-8	3	-8	-1.06	2.70
8	-3	8	0.25	1.29
8	-3	8	-0.49	1.85
8	-3	8	-0.63	0.88
-9	3	-8	6.94	1.07
-9	3	-8	6.88	2.47
-9	3	-8	7.67	3.45
-9	3	-8	4.37	2.58
-9	3	-8	2.58	1.81
9	-3	8	3.15	1.21
9	-3	8	6.01	1.66
-10	3	-8	-1.51	2.03
-10	3	-8	0.07	0.95
-10	3	-8	0.48	2.86
-10	3	-8	1.73	4.06
10	-3	8	1.00	1.80
-11	3	-8	2.79	3.06
-11	3	-8	5.37	5.08
-11	3	-8	3.44	1.12
11	-3	8	3.39	2.06
-13	-2	8	11.02	4.52
-13	-2	8	4.99	3.33
-13	-2	8	2.74	2.20
12	2	-8	0.32	3.71
-12	-2	8	6.80	1.98
-12	-2	8	13.20	3.74
-12	-2	8	7.35	3.04
-11	-2	8	15.83	3.19
-11	-2	8	9.95	2.77
-11	-2	8	9.89	1.95
-10	-2	8	2.89	2.16

-9	-2	8	6.55	1.48
-9	-2	8	7.36	1.93
-9	-2	8	7.43	1.62
-8	-2	8	5.85	1.62
7	2	-8	10.75	2.32
-7	-2	8	11.45	1.86
-7	-2	8	11.81	1.87
-7	-2	8	12.40	2.55
6	2	-8	4.89	1.28
6	2	-8	5.64	1.40
6	2	-8	4.44	1.81
-6	-2	8	7.31	1.12
-6	-2	8	8.22	1.21
-6	-2	8	7.73	1.33
5	2	-8	50.11	3.79
5	2	-8	47.14	3.28
-5	-2	8	47.68	3.26
-5	-2	8	49.76	2.98
-5	-2	8	46.33	2.92
4	2	-8	24.61	2.93
-4	-2	8	26.26	2.12
-4	-2	8	24.47	2.27
-4	-2	8	26.59	2.09
3	2	-8	46.98	3.83
3	2	-8	54.61	4.05
3	2	-8	45.73	3.09
-3	-2	8	48.01	3.03
-3	-2	8	47.19	2.95
-3	-2	8	45.89	3.05
2	2	-8	21.33	1.19
2	2	-8	18.82	2.79
2	2	-8	17.69	1.99
2	2	-8	17.99	2.15
-2	-2	8	22.97	2.00
-2	-2	8	19.33	1.32
-2	-2	8	20.79	1.92
1	2	-8	17.57	1.10
1	2	-8	14.09	1.83
1	2	-8	21.10	2.10
1	2	-8	14.48	2.04
-1	-2	8	19.81	1.75
-1	-2	8	17.39	1.25
-1	-2	8	19.21	1.90
0	2	-8	14.89	1.75

0	2	-8	17.27	1.07
0	2	-8	16.09	1.82
0	2	-8	17.18	2.09
0	-2	8	14.90	1.66
0	-2	8	14.97	1.21
0	-2	8	18.78	1.71
0	-2	8	12.14	1.99
-1	2	-8	18.26	2.11
-1	2	-8	12.34	2.00
-1	2	-8	18.81	1.80
-1	2	-8	17.27	1.09
1	-2	8	19.98	1.77
1	-2	8	15.40	2.10
1	-2	8	13.23	1.63
1	-2	8	15.37	1.27
-2	2	-8	28.52	2.26
-2	2	-8	28.48	2.10
-2	2	-8	27.42	1.65
-2	2	-8	33.62	2.50
2	-2	8	28.20	2.06
2	-2	8	25.94	1.81
2	-2	8	31.30	2.25
2	-2	8	28.11	2.58
-3	2	-8	23.15	2.16
-3	2	-8	20.87	1.81
-3	2	-8	22.26	1.42
-3	2	-8	25.05	2.42
3	-2	8	23.07	2.11
3	-2	8	21.24	1.87
3	-2	8	26.85	2.50
3	-2	8	19.74	1.59
-4	2	-8	14.34	1.64
-4	2	-8	15.72	1.21
-4	2	-8	14.40	1.85
-4	2	-8	16.23	2.12
-4	2	-8	18.69	2.43
4	-2	8	16.04	1.95
4	-2	8	17.90	2.49
4	-2	8	13.96	1.41
4	-2	8	16.50	1.65
-5	2	-8	2.85	1.32
-5	2	-8	2.38	0.79
-5	2	-8	2.64	0.56
-5	2	-8	2.43	1.22

-5	2	-8	2.46	1.48
5	-2	8	2.31	0.83
5	-2	8	3.23	1.56
5	-2	8	1.06	1.11
5	-2	8	2.16	0.70
-6	2	-8	8.43	1.17
-6	2	-8	7.42	1.80
-6	2	-8	10.60	2.01
-6	2	-8	11.08	2.11
-6	2	-8	10.06	2.07
6	-2	8	9.03	2.40
6	-2	8	8.81	1.85
6	-2	8	8.71	1.81
-7	2	-8	1.97	0.67
-7	2	-8	1.53	2.15
-7	2	-8	2.82	1.32
-7	2	-8	1.93	1.55
-7	2	-8	1.03	1.77
7	-2	8	2.16	1.06
7	-2	8	2.28	1.85
7	-2	8	-0.44	1.08
-8	2	-8	0.26	1.74
-8	2	-8	1.53	1.38
-8	2	-8	1.17	2.51
-8	2	-8	0.40	0.71
-8	2	-8	0.55	2.00
8	-2	8	-1.01	1.14
-9	2	-8	-1.46	2.28
-9	2	-8	2.86	3.22
-9	2	-8	-1.04	0.77
-9	2	-8	-0.25	1.49
9	-2	8	0.27	1.36
9	-2	8	-0.08	0.95
-10	2	-8	0.04	2.61
-10	2	-8	2.37	3.69
-10	2	-8	0.22	0.90
-10	2	-8	-1.27	1.55
10	-2	8	0.27	1.33
10	-2	8	0.76	1.66
-11	2	-8	-2.21	1.02
-11	2	-8	4.34	4.65
-11	2	-8	-2.00	2.94
11	-2	8	-0.37	1.86
-12	2	-8	5.43	5.49

-12	2	-8	-2.15	3.27
-12	2	-8	-0.39	1.18
12	-2	8	-0.61	2.20
-13	-1	8	4.05	3.61
-13	-1	8	7.42	4.58
-12	-1	8	12.47	3.32
-12	-1	8	22.36	4.20
-11	-1	8	8.22	2.82
-10	-1	8	9.06	2.44
-9	-1	8	6.74	2.01
-8	-1	8	20.57	3.05
-8	-1	8	21.03	2.17
-7	-1	8	8.79	1.37
-7	-1	8	5.48	1.51
6	1	-8	40.00	3.89
-6	-1	8	43.19	2.68
-6	-1	8	36.70	3.14
5	1	-8	24.26	2.93
-5	-1	8	24.03	2.37
-5	-1	8	25.34	1.94
-5	-1	8	26.12	2.04
4	1	-8	44.84	3.25
4	1	-8	34.71	2.75
4	1	-8	37.84	3.42
-4	-1	8	42.67	2.82
-4	-1	8	43.37	2.58
3	1	-8	15.04	2.59
3	1	-8	12.68	1.75
-3	-1	8	16.26	1.67
-3	-1	8	14.33	1.77
-3	-1	8	14.11	1.71
2	1	-8	64.66	3.98
2	1	-8	57.63	3.59
2	1	-8	67.13	3.19
-2	-1	8	60.04	3.58
-2	-1	8	66.89	3.64
-2	-1	8	62.96	3.22
1	1	-8	34.74	1.73
1	1	-8	30.75	2.59
1	1	-8	29.04	2.24
1	1	-8	33.37	2.56
-1	-1	8	27.47	2.45
-1	-1	8	30.46	2.24
-1	-1	8	33.75	2.24

-1	-1	8	33.07	1.79
0	1	-8	13.17	1.93
0	1	-8	14.30	1.61
0	1	-8	16.66	1.02
0	1	-8	13.82	1.85
0	-1	8	17.47	1.59
0	-1	8	15.12	1.61
0	-1	8	16.34	1.13
0	-1	8	13.75	1.98
-1	1	-8	19.67	1.78
-1	1	-8	17.17	1.72
-1	1	-8	17.97	1.14
-1	1	-8	20.73	2.01
1	-1	8	17.40	1.23
1	-1	8	18.51	2.11
1	-1	8	18.65	1.74
1	-1	8	15.67	1.60
-2	1	-8	21.25	1.93
-2	1	-8	23.20	2.22
-2	1	-8	23.20	1.34
-2	1	-8	21.25	1.74
2	-1	8	20.43	1.74
2	-1	8	22.48	1.91
2	-1	8	21.50	1.44
2	-1	8	22.51	2.32
-3	1	-8	20.06	1.97
-3	1	-8	23.69	2.25
-3	1	-8	21.53	1.32
3	-1	8	21.43	1.90
3	-1	8	18.52	1.43
3	-1	8	21.12	1.70
3	-1	8	20.98	2.36
-4	1	-8	27.66	1.69
-4	1	-8	28.47	2.02
-4	1	-8	31.10	2.71
-4	1	-8	27.70	2.26
4	-1	8	28.11	2.02
4	-1	8	27.29	1.82
4	-1	8	32.85	2.71
4	-1	8	29.27	2.30
-5	1	-8	17.38	1.30
-5	1	-8	17.54	2.52
-5	1	-8	18.06	1.90
-5	1	-8	19.40	1.88

-5	1	-8	18.07	1.69
5	-1	8	22.14	2.53
5	-1	8	15.96	1.46
5	-1	8	17.78	1.72
5	-1	8	19.25	1.95
-6	1	-8	35.10	2.49
-6	1	-8	34.01	2.35
-6	1	-8	34.75	2.66
-6	1	-8	31.39	3.18
-6	1	-8	31.59	1.99
-6	1	-8	31.46	3.17
6	-1	8	30.87	2.15
6	-1	8	35.45	2.68
6	-1	8	32.69	2.42
6	-1	8	35.25	3.15
-7	1	-8	7.15	1.22
-7	1	-8	6.86	1.86
-7	1	-8	5.52	0.82
-7	1	-8	5.92	2.17
7	-1	8	6.45	1.81
7	-1	8	4.94	1.43
7	-1	8	4.13	0.86
-8	1	-8	18.67	1.65
-8	1	-8	20.89	2.33
-8	1	-8	19.63	2.98
-8	1	-8	24.20	2.61
-8	1	-8	18.08	3.27
8	-1	8	21.01	2.42
8	-1	8	19.93	3.26
-9	1	-8	-2.88	2.06
-9	1	-8	0.41	0.79
-9	1	-8	-0.39	1.65
-9	1	-8	1.70	2.83
-9	1	-8	0.29	1.33
9	-1	8	0.24	1.27
9	-1	8	0.33	2.01
-10	1	-8	2.39	2.39
-10	1	-8	-0.86	1.49
-10	1	-8	3.20	2.11
-10	1	-8	0.67	1.57
-10	1	-8	2.16	0.96
-10	1	-8	3.28	3.36
10	-1	8	0.64	1.04
10	-1	8	-0.18	1.55

-11	1	-8	3.77	3.04
-11	1	-8	4.69	2.84
-11	1	-8	5.30	4.25
11	-1	8	0.91	1.78
-12	1	-8	5.58	1.28
-12	1	-8	5.02	3.24
-12	1	-8	9.70	5.23
12	-1	8	7.18	2.23
-12	0	8	4.56	3.25
-12	0	8	11.95	4.19
-11	0	8	6.54	2.85
-10	0	8	7.34	2.59
-9	0	8	3.15	2.04
-8	0	8	7.12	1.81
-7	0	8	6.53	1.55
6	0	-8	9.66	2.14
-6	0	8	12.59	2.27
5	0	-8	49.41	3.89
-5	0	8	54.95	2.93
-5	0	8	47.79	3.38
4	0	-8	14.09	2.99
-4	0	8	13.33	1.91
-4	0	8	17.10	1.61
3	0	-8	5.38	1.11
3	0	-8	5.24	0.97
3	0	-8	7.30	1.34
-3	0	8	6.38	1.02
-3	0	8	4.14	1.03
-3	0	8	7.38	1.42
2	0	-8	39.18	2.02
2	0	-8	39.00	2.97
2	0	-8	38.47	2.44
-2	0	8	40.06	2.02
-2	0	8	40.19	2.47
-2	0	8	36.47	2.51
-2	0	8	34.06	2.63
1	0	-8	3.49	1.02
1	0	-8	3.35	0.41
1	0	-8	1.90	0.83
1	0	-8	2.29	1.03
-1	0	8	2.62	0.79
-1	0	8	1.65	1.07
-1	0	8	3.38	0.79
0	0	-8	2.44	0.40

0	0	-8	3.61	1.08
0	0	-8	2.76	0.89
0	0	-8	1.91	0.84
0	0	8	2.84	0.77
0	0	8	1.89	1.09
0	0	8	2.29	0.67
-1	0	-8	16.81	1.63
-1	0	-8	16.99	1.07
-1	0	-8	18.95	1.89
-1	0	-8	19.44	1.67
1	0	8	15.82	2.04
1	0	8	15.84	1.12
1	0	8	16.84	1.64
1	0	8	13.48	1.50
-2	0	-8	15.14	0.98
-2	0	-8	13.47	1.43
-2	0	-8	14.51	1.92
-2	0	-8	12.23	1.61
2	0	8	14.38	1.59
2	0	8	11.09	1.40
2	0	8	14.43	1.04
2	0	8	14.27	1.98
-3	0	-8	7.30	1.22
-3	0	-8	7.72	0.80
-3	0	-8	7.22	1.27
-3	0	-8	7.26	1.40
3	0	8	8.82	0.89
3	0	8	7.37	1.32
3	0	8	7.80	1.48
3	0	8	7.27	1.17
-4	0	-8	3.53	0.83
-4	0	-8	3.26	0.49
-4	0	-8	2.22	1.13
-4	0	-8	2.99	0.75
4	0	8	3.20	1.25
4	0	8	3.69	0.71
4	0	8	2.45	0.69
4	0	8	4.29	0.88
-5	0	-8	12.16	1.59
-5	0	-8	13.78	1.44
-5	0	-8	12.89	1.06
-5	0	-8	11.24	1.63
5	0	8	11.25	1.60
5	0	8	11.43	1.15

5	0	8	12.73	1.70
5	0	8	12.37	1.44
-6	0	-8	9.35	1.52
-6	0	-8	12.26	1.70
-6	0	-8	11.05	1.11
-6	0	-8	13.55	1.77
-6	0	-8	13.23	2.50
6	0	8	9.84	1.21
6	0	8	12.20	1.77
6	0	8	9.96	1.61
6	0	8	8.12	2.44
-7	0	-8	7.99	1.10
-7	0	-8	6.68	1.01
-7	0	-8	8.24	1.77
-7	0	-8	9.67	1.78
-7	0	-8	10.78	2.00
-7	0	-8	3.94	2.07
7	0	8	8.08	1.32
7	0	8	6.90	1.68
7	0	8	6.91	0.93
7	0	8	8.20	1.70
-8	0	-8	-1.78	2.16
-8	0	-8	0.90	1.12
-8	0	-8	-0.07	1.68
-8	0	-8	-1.14	0.81
8	0	8	-1.17	1.76
8	0	8	-0.66	2.74
8	0	8	0.26	0.71
-9	0	-8	0.31	0.73
-9	0	-8	0.72	1.25
-9	0	-8	-0.21	2.79
9	0	8	-0.07	0.83
9	0	8	0.62	1.14
9	0	8	2.00	1.99
-10	0	-8	1.64	3.13
-10	0	-8	-2.51	2.13
-10	0	-8	-0.97	0.82
-10	0	-8	0.13	1.40
10	0	8	-1.21	1.41
10	0	8	-1.92	1.06
10	0	8	1.92	2.11
-11	0	-8	2.64	1.05
-11	0	-8	2.40	4.09
-11	0	-8	6.37	2.70

-11	0	-8	3.24	1.57
-11	0	-8	3.83	1.61
11	0	8	3.57	1.74
11	0	8	4.82	1.81
11	0	8	5.71	1.28
11	0	8	5.09	2.41
-12	0	-8	6.39	4.90
-12	0	-8	1.99	1.19
-12	0	-8	2.13	3.00
-12	0	-8	2.57	1.83
12	0	8	4.03	2.01
-13	0	-8	-1.38	1.28
-13	0	-8	-2.76	3.27
-13	0	-8	6.40	5.33
13	0	8	1.05	2.31
-12	1	8	5.10	3.30
-11	1	8	13.51	3.18
-10	1	8	10.12	2.74
-9	1	8	15.72	2.47
-8	1	8	2.56	1.73
-7	1	8	23.46	2.87
-6	1	8	8.80	1.51
-5	1	8	49.77	3.36
-4	1	8	24.50	2.22
3	-1	-8	17.10	2.27
-3	1	8	15.63	1.82
-3	1	8	14.10	1.82
2	-1	-8	9.97	1.59
2	-1	-8	5.71	0.93
2	-1	-8	8.36	0.72
2	-1	-8	6.60	1.27
-2	1	8	8.93	1.33
-2	1	8	9.98	1.26
1	-1	-8	14.74	2.03
1	-1	-8	16.47	1.08
1	-1	-8	17.47	1.56
-1	1	8	17.83	1.06
-1	1	8	15.86	1.63
-1	1	8	18.62	1.99
-1	1	8	19.19	1.53
0	-1	-8	5.76	0.89
0	-1	-8	8.53	1.61
0	-1	-8	4.43	0.99
0	-1	-8	6.62	0.71

0	1	8	7.32	1.32
0	1	8	6.68	1.15
0	1	8	6.20	1.23
0	1	8	7.80	0.69
-1	-1	-8	43.38	2.29
-1	-1	-8	43.07	2.96
-1	-1	-8	45.87	2.65
-1	-1	-8	43.83	2.69
1	1	8	41.43	2.90
1	1	8	43.82	2.63
1	1	8	43.77	2.30
-2	-1	-8	3.15	0.74
-2	-1	-8	3.10	0.99
-2	-1	-8	2.79	0.45
2	1	8	3.56	0.80
2	1	8	3.83	0.65
2	1	8	2.58	1.01
-3	-1	-8	32.15	2.57
-3	-1	-8	33.40	2.12
-3	-1	-8	37.80	1.92
-3	-1	-8	34.00	2.16
3	1	8	36.45	1.94
3	1	8	35.02	2.29
3	1	8	34.09	2.14
3	1	8	33.23	2.58
-4	-1	-8	41.42	2.49
-4	-1	-8	42.82	2.55
-4	-1	-8	44.50	2.30
-4	-1	-8	42.82	2.99
4	1	8	42.12	2.64
4	1	8	43.78	2.33
4	1	8	41.45	2.92
4	1	8	40.99	2.49
-5	-1	-8	35.83	2.29
-5	-1	-8	35.93	1.98
-5	-1	-8	36.28	2.77
5	1	8	34.41	2.12
5	1	8	32.70	2.19
5	1	8	34.33	2.34
5	1	8	34.62	2.72
-6	-1	-8	17.63	2.40
-6	-1	-8	17.46	1.59
-6	-1	-8	14.43	2.56
-6	-1	-8	17.32	1.78

-6	-1	-8	17.00	1.26
6	1	8	14.73	2.27
6	1	8	15.33	1.31
6	1	8	17.06	1.65
6	1	8	17.67	1.86
-7	-1	-8	26.44	2.03
-7	-1	-8	26.32	3.20
-7	-1	-8	29.20	2.21
-7	-1	-8	27.84	2.86
-7	-1	-8	27.91	1.77
7	1	8	26.56	2.95
7	1	8	26.59	2.34
7	1	8	25.30	1.82
7	1	8	27.77	2.20
-8	-1	-8	5.19	1.12
-8	-1	-8	4.73	0.94
-8	-1	-8	4.25	0.75
-8	-1	-8	6.40	2.34
-8	-1	-8	4.58	1.66
8	1	8	4.01	1.02
8	1	8	4.54	0.84
8	1	8	3.48	1.60
-9	-1	-8	6.64	1.33
-9	-1	-8	6.46	1.09
-9	-1	-8	7.37	1.84
-9	-1	-8	4.57	2.71
-9	-1	-8	6.07	0.96
9	1	8	6.57	1.27
9	1	8	4.96	0.91
9	1	8	7.39	1.83
-10	-1	-8	1.05	0.85
-10	-1	-8	1.39	1.31
-10	-1	-8	0.42	2.14
-10	-1	-8	0.26	3.02
10	1	8	-0.04	1.36
10	1	8	0.41	1.42
10	1	8	1.06	1.89
-11	-1	-8	6.97	1.40
-11	-1	-8	7.73	2.40
-11	-1	-8	8.10	1.17
-11	-1	-8	7.16	1.61
-11	-1	-8	10.07	3.73
11	1	8	7.25	1.24
11	1	8	7.50	1.75

11	1	8	7.04	2.26
-12	-1	-8	2.68	4.24
-12	-1	-8	1.76	1.15
-12	-1	-8	2.97	2.79
-12	-1	-8	-0.02	1.64
12	1	8	-0.06	1.83
12	1	8	0.82	1.30
12	1	8	0.49	1.86
-13	-1	-8	5.78	5.17
-13	-1	-8	2.00	1.30
-13	-1	-8	2.59	3.19
-12	2	8	2.93	3.40
-11	2	8	-0.01	2.94
-10	2	8	2.82	2.72
-10	2	8	0.49	1.73
-9	2	8	1.21	2.23
-8	2	8	13.69	2.20
-7	2	8	9.85	1.82
-6	2	8	8.45	1.55
-5	2	8	6.09	1.30
-4	2	8	4.37	1.14
3	-2	-8	16.33	1.06
3	-2	-8	15.75	2.30
-3	2	8	16.99	1.02
2	-2	-8	10.80	0.86
-2	2	8	11.58	0.82
-2	2	8	13.19	1.30
1	-2	-8	1.78	0.88
1	-2	-8	3.82	0.60
-1	2	8	4.28	0.54
-1	2	8	2.95	1.05
-1	2	8	2.22	0.74
-1	2	8	3.73	0.78
0	-2	-8	11.87	1.33
0	-2	-8	9.83	0.82
0	2	8	8.78	1.18
0	2	8	9.74	1.33
0	2	8	10.27	0.77
-1	-2	-8	62.06	4.00
-1	-2	-8	76.21	3.69
-1	-2	-8	74.24	3.88
1	2	8	76.06	3.93
1	2	8	71.69	3.67
1	2	8	76.33	3.91

1	2	8	74.94	4.07
-2	-2	-8	0.87	0.98
-2	-2	-8	0.32	0.34
-2	-2	-8	0.75	0.58
2	2	8	-0.02	0.66
2	2	8	0.38	0.32
2	2	8	0.92	0.83
-3	-2	-8	1.66	0.97
-3	-2	-8	2.85	0.46
-3	-2	-8	2.67	0.67
3	2	8	2.26	0.73
3	2	8	2.40	0.89
3	2	8	2.72	0.42
-4	-2	-8	39.55	2.12
-4	-2	-8	40.93	2.73
-4	-2	-8	38.04	2.35
4	2	8	37.00	2.10
4	2	8	38.57	2.27
4	2	8	38.66	2.41
4	2	8	37.26	2.59
-5	-2	-8	3.52	0.80
-5	-2	-8	2.90	0.67
-5	-2	-8	3.64	0.55
-5	-2	-8	4.10	1.19
5	2	8	4.21	0.84
5	2	8	4.32	1.06
5	2	8	4.97	0.80
-6	-2	-8	0.01	0.44
-6	-2	-8	-0.50	0.78
-6	-2	-8	-0.83	1.13
6	2	8	-0.26	1.04
6	2	8	-0.49	0.58
6	2	8	-0.29	0.79
6	2	8	0.26	0.45
-7	-2	-8	0.02	1.27
-7	-2	-8	-0.39	0.87
-7	-2	-8	0.21	0.67
-7	-2	-8	-2.37	1.89
-7	-2	-8	0.59	0.54
7	2	8	0.29	0.70
7	2	8	0.14	1.20
7	2	8	0.42	0.97
7	2	8	0.62	0.52
-8	-2	-8	1.30	0.97

-8	-2	-8	1.21	2.25
-8	-2	-8	0.60	1.46
-8	-2	-8	1.13	0.66
-8	-2	-8	1.67	0.77
8	2	8	1.90	1.38
8	2	8	0.88	0.63
8	2	8	0.60	0.88
-9	-2	-8	-1.03	0.70
-9	-2	-8	-0.58	0.82
-9	-2	-8	-1.47	1.60
-9	-2	-8	-1.23	1.07
-9	-2	-8	1.39	2.47
9	2	8	-1.20	0.64
9	2	8	-1.02	1.50
9	2	8	0.16	1.16
9	2	8	-0.96	1.02
-10	-2	-8	-0.82	0.88
-10	-2	-8	-0.10	0.83
-10	-2	-8	0.12	1.23
-10	-2	-8	0.16	1.83
-10	-2	-8	2.11	5.20
10	2	8	0.36	0.80
10	2	8	-0.83	1.24
10	2	8	0.22	1.71
10	2	8	1.88	1.50
-11	-2	-8	1.25	1.43
-11	-2	-8	0.41	1.17
-11	-2	-8	1.88	3.38
-11	-2	-8	1.17	0.98
-11	-2	-8	0.75	2.14
11	2	8	0.69	1.45
11	2	8	0.06	1.74
11	2	8	-0.80	1.90
-12	-2	-8	2.31	4.25
-12	-2	-8	-0.64	1.10
-12	-2	-8	-1.06	1.57
-12	-2	-8	1.48	1.32
-12	-2	-8	-0.09	2.56
12	2	8	0.68	1.74
12	2	8	1.24	1.15
12	2	8	-2.07	2.29
-13	-2	-8	2.06	4.96
-13	-2	-8	-1.45	1.98
-13	-2	-8	-0.47	1.23

-13	-2	-8	-2.58	3.07
13	2	8	-1.69	1.35
13	2	8	1.25	1.95
13	2	8	-1.03	1.95
-14	-2	-8	-4.88	3.48
-14	-2	-8	1.68	5.60
-14	-2	-8	-0.03	1.48
14	2	8	-0.52	2.28
-11	3	8	3.89	4.18
-11	3	8	4.25	3.12
-10	3	8	8.13	2.95
-9	3	8	5.07	1.57
-9	3	8	3.48	2.44
-8	3	8	19.01	3.57
-6	3	8	20.23	2.77
-5	3	8	2.14	1.31
4	-3	-8	37.93	2.09
-4	3	8	39.06	2.03
3	-3	-8	6.00	0.74
-3	3	8	5.09	0.64
2	-3	-8	63.18	3.25
-2	3	8	65.00	3.23
1	-3	-8	12.67	0.90
-1	3	8	11.93	0.85
0	-3	-8	70.89	3.74
0	-3	-8	73.17	3.88
0	-3	-8	72.91	3.60
0	3	8	69.29	3.79
0	3	8	69.08	3.90
0	3	8	72.10	3.57
-1	-3	-8	60.49	3.36
-1	-3	-8	66.08	3.26
1	3	8	63.51	3.46
1	3	8	67.20	3.23
1	3	8	61.71	3.51
-2	-3	-8	20.69	1.29
-2	-3	-8	20.60	1.52
-2	-3	-8	20.68	1.87
2	3	8	22.53	1.65
2	3	8	19.86	1.72
2	3	8	21.53	1.26
-3	-3	-8	4.50	1.00
-3	-3	-8	3.35	0.69
-3	-3	-8	3.81	0.49

3	3	8	5.20	0.66
3	3	8	2.61	0.69
3	3	8	3.43	0.84
-4	-3	-8	24.87	2.08
-4	-3	-8	22.27	1.42
-4	-3	-8	23.45	1.69
4	3	8	21.01	1.92
4	3	8	24.07	1.42
4	3	8	22.57	1.79
-5	-3	-8	41.61	2.29
-5	-3	-8	43.79	2.85
-5	-3	-8	40.85	2.52
5	3	8	41.81	2.74
5	3	8	41.86	2.62
5	3	8	41.39	2.28
-6	-3	-8	18.84	1.32
-6	-3	-8	17.77	1.72
-6	-3	-8	19.31	2.15
6	3	8	17.93	2.04
6	3	8	18.40	1.82
6	3	8	19.55	1.35
-7	-3	-8	1.31	1.23
-7	-3	-8	1.05	0.54
-7	-3	-8	1.11	0.91
7	3	8	2.22	0.55
7	3	8	0.26	1.13
-8	-3	-8	5.78	2.24
-8	-3	-8	6.84	1.13
-8	-3	-8	6.93	1.16
-8	-3	-8	5.21	1.49
8	3	8	7.95	1.08
8	3	8	7.47	2.17
-9	-3	-8	1.33	1.52
-9	-3	-8	0.52	2.44
-9	-3	-8	-0.12	0.70
-9	-3	-8	0.92	1.08
-9	-3	-8	0.08	0.80
9	3	8	0.08	1.01
9	3	8	-0.49	1.37
9	3	8	1.32	0.66
9	3	8	-0.74	1.10
-10	-3	-8	6.75	1.13
-10	-3	-8	7.36	1.89
-10	-3	-8	5.13	2.89

-10	-3	-8	6.53	1.38
10	3	8	5.87	1.71
10	3	8	7.44	1.44
10	3	8	7.07	0.97
-11	-3	-8	2.88	1.49
-11	-3	-8	3.11	1.04
-11	-3	-8	1.88	3.28
-11	-3	-8	0.80	3.13
11	3	8	3.63	1.75
11	3	8	2.52	1.47
11	3	8	3.13	1.86
-12	-3	-8	6.26	4.06
-12	-3	-8	2.44	1.31
-12	-3	-8	0.44	1.58
-12	-3	-8	1.46	1.13
-12	-3	-8	-0.34	2.49
12	3	8	0.11	2.06
12	3	8	5.38	3.23
12	3	8	2.83	1.70
12	3	8	1.55	1.30
-13	-3	-8	-3.02	2.89
-13	-3	-8	-2.36	4.59
-13	-3	-8	-1.87	1.79
13	3	8	-0.12	1.28
13	3	8	0.53	1.96
-14	-3	-8	0.30	5.50
-14	-3	-8	0.88	1.47
-14	-3	-8	-0.79	3.43
-11	4	8	1.88	3.17
-11	4	8	1.70	2.81
-10	4	8	3.94	2.80
-9	4	8	2.73	1.70
-9	4	8	2.53	2.52
-8	4	8	7.02	2.08
-6	4	8	4.06	0.55
-5	4	8	10.34	0.95
-4	4	8	7.11	0.79
3	-4	-8	9.91	0.93
-3	4	8	9.00	0.81
2	-4	-8	16.86	1.13
-2	4	8	15.00	1.05
1	-4	-8	48.10	2.50
-1	4	8	46.61	2.46
0	-4	-8	49.47	2.58

0	4	8	51.01	2.55
0	4	8	47.16	2.75
-1	-4	-8	5.06	0.94
-1	-4	-8	4.64	0.67
-1	-4	-8	3.52	0.84
1	4	8	5.85	1.08
1	4	8	4.45	0.62
1	4	8	4.27	0.78
-2	-4	-8	1.53	0.39
-2	-4	-8	0.87	0.54
2	4	8	0.68	0.68
2	4	8	1.12	0.60
2	4	8	1.15	0.34
-3	-4	-8	8.42	1.14
-3	-4	-8	8.37	0.79
3	4	8	6.18	0.94
3	4	8	7.58	1.24
3	4	8	8.67	0.75
-4	-4	-8	12.03	0.97
-4	-4	-8	10.43	1.72
-4	-4	-8	10.47	1.32
4	4	8	13.11	1.43
4	4	8	11.76	0.93
4	4	8	11.62	1.56
-5	-4	-8	16.26	1.97
-5	-4	-8	16.08	1.20
-5	-4	-8	16.79	1.58
5	4	8	17.44	1.70
5	4	8	18.71	1.20
5	4	8	17.34	1.81
-6	-4	-8	16.96	1.66
-6	-4	-8	15.86	1.24
-6	-4	-8	17.18	2.05
6	4	8	17.87	1.42
6	4	8	18.58	1.94
-7	-4	-8	6.77	1.01
-7	-4	-8	6.71	1.32
-7	-4	-8	6.01	0.98
7	4	8	7.30	1.24
7	4	8	7.32	0.97
-8	-4	-8	0.76	1.30
-8	-4	-8	0.32	0.96
-8	-4	-8	-0.17	0.58
8	4	8	0.56	1.38

8	4	8	0.25	0.52
8	4	8	0.40	0.98
8	4	8	0.02	1.22
-9	-4	-8	1.18	0.71
-9	-4	-8	0.27	2.25
-9	-4	-8	1.74	1.50
-9	-4	-8	1.08	1.10
9	4	8	0.82	1.37
9	4	8	2.23	0.64
9	4	8	2.38	1.16
-10	-4	-8	0.19	1.23
-10	-4	-8	-1.55	1.67
-10	-4	-8	-1.43	2.64
10	4	8	-0.57	1.55
10	4	8	-1.90	1.32
10	4	8	-0.98	0.65
-11	-4	-8	3.14	2.08
-11	-4	-8	5.06	3.28
-11	-4	-8	2.84	0.99
-11	-4	-8	4.75	1.48
-11	-4	-8	2.68	1.14
11	4	8	3.09	0.92
11	4	8	1.59	1.80
11	4	8	5.38	1.57
-12	-4	-8	-0.23	1.62
-12	-4	-8	-0.35	3.77
-12	-4	-8	-0.53	2.29
-12	-4	-8	-0.98	1.60
-12	-4	-8	-0.86	1.03
12	4	8	-0.39	1.63
12	4	8	-2.81	1.93
12	4	8	0.86	1.85
-13	-4	-8	0.49	1.19
-13	-4	-8	1.15	4.51
-13	-4	-8	0.34	1.81
-13	-4	-8	-0.05	2.81
-13	-4	-8	1.48	1.50
13	4	8	1.12	1.15
13	4	8	-1.73	4.11
13	4	8	-0.42	1.86
13	4	8	-2.35	2.32
-14	-4	-8	-0.22	5.19
-14	-4	-8	-0.54	1.33
-14	-4	-8	-0.55	3.36

14	4	8	-1.33	1.33
-10	5	8	2.32	3.19
-10	5	8	4.81	2.95
-9	5	8	7.88	2.71
-9	5	8	5.05	1.85
-9	5	8	7.13	1.10
-9	5	8	3.48	2.33
-8	5	8	3.74	2.19
-8	5	8	2.16	0.62
-8	5	8	2.50	0.78
-7	5	8	24.24	2.13
-7	5	8	21.69	1.80
-7	5	8	22.07	3.28
-6	5	8	13.11	1.20
-5	5	8	17.84	1.29
-4	5	8	11.22	1.01
3	-5	-8	19.74	1.38
-3	5	8	20.98	1.31
2	-5	-8	5.11	0.83
-2	5	8	4.18	0.72
1	-5	-8	21.81	1.39
-1	5	8	21.77	1.32
0	-5	-8	3.59	0.49
0	5	8	2.78	0.43
-1	-5	-8	61.28	3.12
1	5	8	61.60	3.09
1	5	8	58.65	3.27
-2	-5	-8	4.49	0.68
-2	-5	-8	3.32	0.62
-2	-5	-8	2.65	0.88
2	5	8	3.08	0.79
2	5	8	2.89	0.70
2	5	8	4.97	0.66
-3	-5	-8	16.77	1.13
-3	-5	-8	14.02	1.38
3	5	8	16.87	1.66
3	5	8	17.60	1.10
3	5	8	17.05	1.58
-4	-5	-8	1.28	0.66
-4	-5	-8	1.91	0.45
-4	-5	-8	1.23	1.02
4	5	8	0.82	0.83
4	5	8	1.42	0.40
4	5	8	2.02	0.77

-5	-5	-8	-0.06	0.67
-5	-5	-8	-1.03	0.95
-5	-5	-8	-0.01	0.38
5	5	8	-0.57	0.89
5	5	8	0.07	0.38
-6	-5	-8	9.43	0.94
-6	-5	-8	8.45	1.45
-6	-5	-8	8.10	1.30
6	5	8	10.28	0.97
6	5	8	8.92	1.24
-7	-5	-8	13.19	1.15
-7	-5	-8	11.77	1.57
-7	-5	-8	13.62	1.70
7	5	8	13.15	2.02
7	5	8	11.70	1.84
7	5	8	13.81	1.14
-8	-5	-8	3.87	1.05
-8	-5	-8	3.95	0.68
-8	-5	-8	2.79	1.42
8	5	8	4.97	1.15
8	5	8	4.49	0.66
8	5	8	5.22	1.35
8	5	8	4.99	1.60
-9	-5	-8	12.00	1.34
-9	-5	-8	10.42	2.00
-9	-5	-8	11.91	2.42
-9	-5	-8	8.05	1.75
9	5	8	13.55	1.28
9	5	8	11.50	2.17
9	5	8	13.83	2.00
-10	-5	-8	4.35	1.80
-10	-5	-8	5.63	1.35
-10	-5	-8	10.13	2.72
-10	-5	-8	5.70	0.94
10	5	8	6.44	1.47
10	5	8	5.60	1.69
10	5	8	8.99	2.20
10	5	8	7.61	1.23
-11	-5	-8	1.63	1.12
-11	-5	-8	1.45	1.02
-11	-5	-8	-1.75	5.04
-11	-5	-8	-0.46	1.38
-11	-5	-8	-0.67	1.96
11	5	8	2.30	1.44

11	5	8	1.14	2.23
11	5	8	-0.08	1.73
11	5	8	1.42	0.79
-12	-5	-8	-1.49	3.05
-12	-5	-8	-0.49	1.58
-12	-5	-8	-2.80	3.65
-12	-5	-8	-0.85	0.99
12	5	8	-2.02	1.95
12	5	8	2.24	2.45
12	5	8	-2.28	1.74
-13	-5	-8	-0.11	4.42
-13	-5	-8	-1.09	1.85
-13	-5	-8	1.56	1.50
-13	-5	-8	0.27	1.18
-13	-5	-8	-0.42	2.78
13	5	8	-2.01	2.23
13	5	8	-1.04	1.96
13	5	8	0.25	1.10
-14	-5	-8	-2.81	2.65
-14	-5	-8	-1.93	5.35
-14	-5	-8	-0.11	1.32
-14	-5	-8	3.14	3.34
14	5	8	1.06	2.48
14	5	8	-0.55	1.25
-15	-5	-8	-0.02	1.46
-15	-5	-8	-1.57	5.92
-15	-5	-8	2.59	3.98
-10	6	8	5.18	3.24
-10	6	8	5.35	1.11
-10	6	8	1.19	3.27
-10	6	8	2.09	1.26
-10	6	8	10.18	5.01
-9	6	8	4.23	2.82
-9	6	8	6.29	1.18
-9	6	8	0.68	2.75
-9	6	8	4.78	0.87
-8	6	8	3.43	2.37
-8	6	8	3.46	2.48
-8	6	8	4.45	0.77
-8	6	8	4.87	0.95
-7	6	8	2.88	2.03
-7	6	8	3.47	0.84
-7	6	8	2.60	0.67
-6	6	8	5.94	0.89

-6	6	8	6.24	0.75
-5	6	8	0.38	0.52
-4	6	8	10.70	1.16
-3	6	8	4.69	0.64
2	-6	-8	31.73	1.85
-2	6	8	29.11	1.76
1	-6	-8	7.34	0.95
-1	6	8	6.34	0.83
0	-6	-8	24.76	1.53
0	6	8	24.17	1.48
-1	-6	-8	4.71	0.80
1	6	8	5.18	0.74
-2	-6	-8	18.69	1.22
-2	-6	-8	15.97	1.73
-2	-6	-8	15.86	1.37
2	6	8	15.77	1.71
2	6	8	19.44	1.18
-3	-6	-8	6.99	1.17
-3	-6	-8	7.93	0.84
3	6	8	7.15	0.80
3	6	8	6.42	0.99
-4	-6	-8	10.44	1.84
-4	-6	-8	12.72	1.39
-4	-6	-8	14.34	1.04
4	6	8	12.90	1.67
4	6	8	14.09	1.01
-5	-6	-8	9.11	0.89
-5	-6	-8	5.93	1.27
-5	-6	-8	7.32	0.93
5	6	8	7.90	1.13
5	6	8	7.51	1.05
-6	-6	-8	17.33	2.26
-6	-6	-8	19.88	1.33
-6	-6	-8	19.25	1.74
6	6	8	19.04	1.29
6	6	8	18.99	2.07
6	6	8	20.71	1.95
-7	-6	-8	3.35	0.88
-7	-6	-8	2.91	0.55
-7	-6	-8	0.50	1.25
7	6	8	2.66	1.16
7	6	8	1.89	0.97
7	6	8	3.02	0.53
-8	-6	-8	3.45	1.44

-8	-6	-8	4.33	1.04
-8	-6	-8	4.82	0.70
8	6	8	4.61	1.18
8	6	8	4.83	0.67
8	6	8	5.23	1.32
-9	-6	-8	2.23	0.69
-9	-6	-8	1.16	1.10
-9	-6	-8	0.62	1.59
9	6	8	1.88	0.64
9	6	8	0.52	1.20
9	6	8	2.01	1.42
9	6	8	2.51	1.74
-10	-6	-8	8.71	1.47
-10	-6	-8	12.84	1.42
-10	-6	-8	8.18	1.96
-10	-6	-8	12.90	2.59
10	6	8	11.91	1.35
10	6	8	9.16	1.85
10	6	8	13.01	2.42
10	6	8	13.04	2.28
-11	-6	-8	-1.29	1.05
-11	-6	-8	-0.67	1.43
-11	-6	-8	-1.64	1.39
11	6	8	-1.92	2.24
11	6	8	0.33	0.73
11	6	8	0.29	1.57
-12	-6	-8	-0.79	1.23
-12	-6	-8	-0.64	0.93
-12	-6	-8	0.84	3.40
-12	-6	-8	-2.21	1.53
-12	-6	-8	-1.01	2.30
12	6	8	-0.59	0.81
12	6	8	-1.58	2.00
12	6	8	-1.43	1.72
12	6	8	2.42	2.62
-13	-6	-8	2.27	4.08
-13	-6	-8	-1.69	1.74
-13	-6	-8	-0.03	1.08
-13	-6	-8	-0.98	1.71
13	6	8	-0.70	2.12
13	6	8	2.24	2.94
13	6	8	1.66	2.02
-14	-6	-8	2.87	5.04
-14	-6	-8	1.72	1.26

-14	-6	-8	-0.31	1.74
-14	-6	-8	0.10	3.27
-14	-6	-8	1.98	2.09
14	6	8	-0.84	2.12
14	6	8	0.52	1.15
-15	-6	-8	1.68	3.85
-15	-6	-8	0.41	1.38
-15	-6	-8	3.98	5.48
15	6	8	-1.78	1.53
-9	7	8	5.91	3.63
-9	7	8	3.82	1.25
-9	7	8	4.35	0.95
-9	7	8	3.77	2.99
-8	7	8	13.81	2.92
-8	7	8	11.84	1.93
-8	7	8	9.70	2.80
-8	7	8	13.22	1.62
-7	7	8	6.54	2.25
-7	7	8	7.80	1.36
-7	7	8	7.35	1.19
-6	7	8	0.97	0.79
-6	7	8	1.10	0.62
-5	7	8	-0.75	0.55
-4	7	8	7.99	1.10
-3	7	8	13.73	1.19
-2	7	8	25.30	1.62
1	-7	-8	31.44	1.88
-1	7	8	29.94	1.80
0	-7	-8	25.50	1.65
0	7	8	25.13	1.57
-1	-7	-8	7.53	0.94
1	7	8	6.44	1.08
-2	-7	-8	13.53	1.11
2	7	8	13.91	1.43
-3	-7	-8	0.62	0.40
-3	-7	-8	-0.64	0.97
3	7	8	0.20	0.71
3	7	8	-1.09	0.87
3	7	8	0.48	0.46
-4	-7	-8	6.55	0.86
-4	-7	-8	5.74	0.86
4	7	8	6.99	0.99
4	7	8	6.97	0.82
4	7	8	5.56	1.10

-5	-7	-8	28.76	1.67
-5	-7	-8	22.23	2.76
-5	-7	-8	26.49	1.95
5	7	8	28.11	2.18
5	7	8	28.32	1.63
5	7	8	26.51	2.31
-6	-7	-8	32.46	1.85
-6	-7	-8	27.25	2.64
-6	-7	-8	31.18	2.18
6	7	8	30.81	2.53
6	7	8	31.61	1.81
6	7	8	29.58	2.40
-7	-7	-8	6.91	1.07
-7	-7	-8	6.31	1.47
-7	-7	-8	7.22	0.99
7	7	8	7.32	1.32
7	7	8	7.70	0.92
7	7	8	7.76	1.22
-8	-7	-8	3.17	1.05
-8	-7	-8	3.31	0.64
-8	-7	-8	2.54	1.45
8	7	8	3.02	1.33
8	7	8	2.81	0.61
8	7	8	2.02	1.21
-9	-7	-8	9.27	1.17
-9	-7	-8	6.74	1.71
-9	-7	-8	6.52	1.28
9	7	8	6.96	1.56
9	7	8	7.06	1.42
9	7	8	6.76	1.16
-10	-7	-8	5.48	2.26
-10	-7	-8	4.43	0.79
-10	-7	-8	4.38	1.85
-10	-7	-8	5.18	1.38
10	7	8	3.26	1.48
10	7	8	4.91	2.22
10	7	8	3.94	1.75
10	7	8	3.52	0.74
-11	-7	-8	0.05	1.39
-11	-7	-8	-1.38	1.46
11	7	8	-1.32	1.47
11	7	8	0.24	0.75
11	7	8	-0.57	1.77
11	7	8	0.57	2.34

-12	-7	-8	1.48	0.87
-12	-7	-8	-0.69	3.20
-12	-7	-8	-0.96	1.59
-12	-7	-8	-2.69	2.26
-12	-7	-8	-1.05	1.25
12	7	8	-1.86	1.72
12	7	8	-0.94	0.76
12	7	8	-0.02	2.10
12	7	8	2.59	2.62
-13	-7	-8	4.36	2.70
-13	-7	-8	7.53	4.08
-13	-7	-8	1.59	1.84
-13	-7	-8	4.01	1.05
13	7	8	3.43	2.05
13	7	8	1.12	2.95
13	7	8	-0.13	2.31
-14	-7	-8	-0.26	2.03
-14	-7	-8	5.13	4.91
-14	-7	-8	0.65	1.10
-14	-7	-8	2.27	3.32
-14	-7	-8	-0.03	1.77
14	7	8	1.12	2.24
14	7	8	-2.10	2.70
14	7	8	-1.67	1.11
-15	-7	-8	2.36	4.13
-15	-7	-8	0.29	1.24
-15	-7	-8	5.25	5.63
15	7	8	0.38	1.26
-8	8	8	0.60	1.17
-8	8	8	1.80	0.88
-8	8	8	6.36	6.02
-8	8	8	-0.36	2.77
-7	8	8	5.13	1.13
-7	8	8	4.74	0.87
-7	8	8	4.08	2.53
-6	8	8	0.57	0.87
-6	8	8	1.05	1.89
-6	8	8	0.16	0.67
-5	8	8	2.24	0.66
-4	8	8	-0.38	0.55
-3	8	8	8.30	1.14
-2	8	8	-0.45	0.46
1	-8	-8	10.76	1.22
-1	8	8	11.06	1.11

0	-8	-8	5.26	0.70
0	8	8	4.67	0.62
-1	-8	-8	17.55	1.36
1	8	8	16.05	1.25
-2	-8	-8	0.38	0.47
2	8	8	0.06	0.41
-3	-8	-8	6.01	0.93
3	8	8	3.63	1.11
3	8	8	5.98	0.99
3	8	8	5.91	0.84
-4	-8	-8	2.18	1.43
-4	-8	-8	3.94	0.78
-4	-8	-8	4.79	0.62
4	8	8	3.98	0.56
4	8	8	5.39	1.01
4	8	8	3.59	1.05
-5	-8	-8	9.72	1.03
-5	-8	-8	7.58	1.38
-5	-8	-8	6.22	1.47
5	8	8	9.17	1.22
5	8	8	7.89	1.29
5	8	8	9.54	0.96
-6	-8	-8	11.00	1.10
-6	-8	-8	10.08	1.75
-6	-8	-8	8.41	1.58
6	8	8	9.38	1.40
6	8	8	10.44	1.02
6	8	8	9.27	1.31
-7	-8	-8	6.04	1.00
-7	-8	-8	2.71	1.48
7	8	8	4.00	0.60
7	8	8	4.60	1.32
7	8	8	4.66	1.16
-8	-8	-8	1.19	1.49
-8	-8	-8	1.80	1.65
-8	-8	-8	2.05	0.60
8	8	8	2.08	1.40
8	8	8	1.46	1.23
8	8	8	1.67	0.57
-9	-8	-8	-0.75	1.63
-9	-8	-8	0.30	0.61
-9	-8	-8	0.53	1.31
-9	-8	-8	-0.66	1.92
9	8	8	-0.64	1.30

9	8	8	-1.23	1.49
9	8	8	-1.47	1.96
9	8	8	0.80	0.60
-10	-8	-8	-0.45	1.85
-10	-8	-8	0.56	0.67
-10	-8	-8	0.13	1.41
-10	-8	-8	0.51	2.24
10	8	8	-0.41	2.29
10	8	8	-0.35	1.40
10	8	8	-0.98	0.63
10	8	8	-0.33	2.24
-11	-8	-8	1.66	1.61
-11	-8	-8	3.82	0.99
-11	-8	-8	1.77	2.11
11	8	8	1.50	0.75
11	8	8	-0.73	1.91
11	8	8	-0.14	1.66
11	8	8	1.11	2.39
-12	-8	-8	1.66	0.89
-12	-8	-8	1.42	1.74
-12	-8	-8	-1.82	2.39
-12	-8	-8	2.15	3.15
12	8	8	1.06	0.85
12	8	8	0.96	1.82
12	8	8	0.62	2.15
12	8	8	4.94	2.68
-13	-8	-8	3.09	1.02
-13	-8	-8	1.46	1.52
-13	-8	-8	0.93	1.98
-13	-8	-8	3.91	3.79
13	8	8	1.51	2.45
13	8	8	1.36	2.09
13	8	8	3.39	3.09
-14	-8	-8	0.55	1.10
-14	-8	-8	-1.10	1.79
-14	-8	-8	-1.94	2.14
-14	-8	-8	-1.12	4.56
-14	-8	-8	-1.26	3.27
14	8	8	-0.78	2.37
14	8	8	-1.83	1.06
14	8	8	-1.40	2.86
-15	-8	-8	3.31	5.15
-15	-8	-8	-2.28	3.95
-15	-8	-8	0.08	1.28

15	8	8	-1.51	1.30
-7	9	8	2.86	2.65
-7	9	8	2.80	1.19
-7	9	8	1.92	0.92
-6	9	8	2.86	1.11
-6	9	8	3.16	2.26
-6	9	8	3.67	0.87
-5	9	8	5.88	1.09
-5	9	8	5.56	0.84
-4	9	8	14.72	1.50
-3	9	8	4.46	0.71
-2	9	8	7.54	1.15
-1	9	8	4.06	0.62
0	-9	-8	8.60	1.18
0	9	8	8.37	1.07
-1	-9	-8	5.99	0.76
1	9	8	5.89	0.95
-2	-9	-8	5.99	0.76
2	9	8	5.96	0.70
-3	-9	-8	10.59	1.18
3	9	8	7.52	1.45
3	9	8	10.63	1.28
3	9	8	10.28	1.06
-4	-9	-8	-0.64	1.16
4	9	8	-0.40	1.12
4	9	8	-0.04	0.98
4	9	8	2.01	0.51
-5	-9	-8	4.27	0.64
-5	-9	-8	3.17	0.89
5	9	8	1.42	1.24
5	9	8	4.55	0.61
5	9	8	2.96	1.08
-6	-9	-8	9.41	2.08
-6	-9	-8	10.77	1.72
-6	-9	-8	13.16	1.22
6	9	8	12.47	1.15
6	9	8	9.87	1.58
6	9	8	12.61	1.48
-7	-9	-8	1.67	1.54
-7	-9	-8	3.77	1.10
-7	-9	-8	4.48	0.70
7	9	8	2.00	1.40
7	9	8	5.17	0.66
7	9	8	3.20	1.24

-8	-9	-8	0.91	0.62
-8	-9	-8	0.60	1.10
-8	-9	-8	-2.56	1.49
8	9	8	-0.90	1.39
8	9	8	0.81	0.55
8	9	8	-0.99	1.25
-9	-9	-8	-0.99	1.22
-9	-9	-8	-0.59	0.65
-9	-9	-8	0.30	1.98
9	9	8	-1.13	1.53
9	9	8	-1.60	1.97
9	9	8	-1.22	1.34
9	9	8	-0.64	0.58
-10	-9	-8	1.68	1.94
-10	-9	-8	1.02	1.45
-10	-9	-8	2.09	2.23
10	9	8	0.86	1.55
10	9	8	0.42	0.68
10	9	8	1.39	2.36
10	9	8	-1.75	1.73
-11	-9	-8	5.41	2.32
-11	-9	-8	8.16	2.78
-11	-9	-8	8.67	1.80
-11	-9	-8	10.10	1.49
11	9	8	6.61	2.78
11	9	8	7.09	2.14
11	9	8	6.59	0.97
11	9	8	6.67	1.84
-12	-9	-8	1.84	3.05
-12	-9	-8	-1.73	2.57
-12	-9	-8	3.63	0.97
-12	-9	-8	2.99	1.79
12	9	8	0.81	1.89
12	9	8	-0.28	2.31
12	9	8	3.72	2.91
-13	-9	-8	-0.55	2.87
-13	-9	-8	-0.08	1.95
-13	-9	-8	1.61	1.02
-13	-9	-8	0.98	3.74
13	9	8	-1.93	2.58
13	9	8	9.69	3.47
13	9	8	-1.60	2.19
-14	-9	-8	0.35	2.24
-14	-9	-8	-2.14	3.35

-14	-9	-8	1.99	1.12
-14	-9	-8	3.37	4.52
14	9	8	-0.88	2.91
14	9	8	-1.05	1.07
14	9	8	-3.73	2.46
-15	-9	-8	4.61	4.24
-15	-9	-8	-2.05	5.02
-15	-9	-8	0.33	2.10
-15	-9	-8	1.19	1.26
15	9	8	-0.13	1.27
-7	10	8	3.36	4.03
-7	10	8	1.54	1.30
-7	10	8	2.05	3.08
-6	10	8	4.48	2.58
-6	10	8	3.56	1.21
-6	10	8	4.19	1.03
-5	10	8	2.66	1.12
-5	10	8	2.17	0.85
-4	10	8	6.28	0.89
-3	10	8	0.90	0.63
-2	10	8	4.72	0.74
-1	10	8	-1.05	0.52
0	10	8	4.29	0.67
-1	-10	-8	-0.59	0.92
1	10	8	-0.65	0.49
-2	-10	-8	9.10	1.25
2	10	8	8.62	1.06
-3	-10	-8	1.70	0.64
3	10	8	1.38	0.52
3	10	8	1.40	1.36
3	10	8	1.20	0.95
-4	-10	-8	-1.62	1.18
-4	-10	-8	1.37	0.63
4	10	8	-1.34	1.17
4	10	8	0.29	1.00
4	10	8	1.91	0.56
-5	-10	-8	-0.47	0.56
-5	-10	-8	-1.05	0.83
5	10	8	-1.76	1.18
5	10	8	-1.23	1.02
5	10	8	-0.27	0.52
-6	-10	-8	8.43	1.24
-6	-10	-8	7.54	1.16
-6	-10	-8	5.92	1.63

6	10	8	9.07	1.10
6	10	8	8.31	1.41
6	10	8	7.78	1.53
-7	-10	-8	-0.63	1.02
-7	-10	-8	-3.00	1.58
-7	-10	-8	0.12	0.60
7	10	8	-0.49	0.55
7	10	8	-1.73	1.36
7	10	8	-0.97	1.23
-8	-10	-8	0.36	0.76
-8	-10	-8	0.93	1.88
-8	-10	-8	-0.11	1.19
8	10	8	0.10	1.29
8	10	8	0.11	0.56
8	10	8	-1.73	1.76
-9	-10	-8	-1.04	1.80
-9	-10	-8	0.95	1.16
-9	-10	-8	0.85	2.08
-9	-10	-8	0.66	1.42
9	10	8	0.13	1.76
9	10	8	-0.23	1.40
9	10	8	0.38	2.14
9	10	8	0.56	0.61
-10	-10	-8	0.95	2.41
-10	-10	-8	-1.54	2.04
-10	-10	-8	-1.80	1.56
-10	-10	-8	0.61	0.81
10	10	8	0.50	1.87
10	10	8	-0.55	1.48
10	10	8	0.73	0.69
10	10	8	-1.36	2.42
-11	-10	-8	1.40	2.56
-11	-10	-8	0.32	0.86
-11	-10	-8	-1.53	2.25
-11	-10	-8	0.88	1.68
11	10	8	-0.64	0.83
11	10	8	-0.22	1.68
11	10	8	-2.83	2.06
11	10	8	-0.94	2.78
-12	-10	-8	1.14	1.85
-12	-10	-8	2.46	0.96
-12	-10	-8	-1.98	2.58
-12	-10	-8	0.86	3.11
12	10	8	-2.00	1.88

12	10	8	4.69	3.19
12	10	8	-0.13	2.46
-13	-10	-8	1.04	2.97
-13	-10	-8	2.89	1.12
-13	-10	-8	-3.21	3.56
-13	-10	-8	0.06	2.08
13	10	8	-0.43	2.15
13	10	8	-0.43	2.75
13	10	8	1.06	3.67
13	10	8	0.65	1.09
-14	-10	-8	-0.45	2.37
-14	-10	-8	-1.91	4.33
-14	-10	-8	1.94	1.17
-14	-10	-8	2.33	3.50
14	10	8	-2.44	3.00
14	10	8	-0.72	2.53
14	10	8	-0.05	1.11
-15	-10	-8	1.43	1.28
-15	-10	-8	-4.72	4.06
-15	-10	-8	2.03	4.96
-15	-10	-8	-1.00	2.05
15	10	8	-1.02	1.22
-5	11	8	1.95	1.25
-4	11	8	0.76	1.11
-4	11	8	0.85	0.85
-3	11	8	2.86	0.79
-2	11	8	2.95	0.72
-1	11	8	4.78	0.75
0	11	8	1.82	0.62
1	11	8	0.59	0.55
-2	-11	-8	0.37	0.64
2	11	8	1.24	0.62
-3	-11	-8	12.13	1.43
3	11	8	12.95	2.41
-4	-11	-8	11.69	1.44
4	11	8	9.96	1.58
-5	-11	-8	9.34	1.72
-5	-11	-8	10.02	1.39
5	11	8	9.02	1.54
5	11	8	9.83	1.64
-6	-11	-8	7.08	0.92
-6	-11	-8	6.87	1.21
6	11	8	6.81	1.15
6	11	8	6.60	1.52

6	11	8	7.36	1.36
-7	-11	-8	-0.06	1.13
-7	-11	-8	-1.38	2.03
-7	-11	-8	0.12	0.68
7	11	8	-1.83	1.18
7	11	8	-0.48	0.58
7	11	8	-1.37	1.77
7	11	8	-0.57	1.46
-8	-11	-8	-2.66	1.67
-8	-11	-8	0.79	1.98
-8	-11	-8	-0.73	0.70
-8	-11	-8	-0.33	1.26
8	11	8	-0.71	1.62
8	11	8	-0.19	0.62
8	11	8	-0.82	1.35
-9	-11	-8	0.53	0.75
-9	-11	-8	-1.61	1.44
-9	-11	-8	1.34	2.23
-9	-11	-8	-3.46	1.77
9	11	8	-0.87	1.49
9	11	8	0.18	0.60
9	11	8	-1.14	1.80
9	11	8	-0.91	2.25
-10	-11	-8	0.93	2.56
-10	-11	-8	-1.22	2.01
-10	-11	-8	0.99	1.65
-10	-11	-8	1.27	0.85
10	11	8	-0.97	1.67
10	11	8	-2.78	1.98
10	11	8	2.56	2.43
-11	-11	-8	1.90	0.97
-11	-11	-8	-3.87	2.35
-11	-11	-8	-0.39	2.80
-11	-11	-8	2.03	1.83
11	11	8	2.06	1.90
11	11	8	0.20	2.30
11	11	8	1.94	2.86
-12	-11	-8	-2.82	2.96
-12	-11	-8	-1.03	2.00
-12	-11	-8	0.59	1.03
-12	-11	-8	-4.84	2.61
12	11	8	1.40	2.56
12	11	8	-0.97	2.09
12	11	8	1.37	1.08

12	11	8	-0.88	3.22
-13	-11	-8	0.55	1.09
-13	-11	-8	3.95	3.61
-13	-11	-8	-2.77	3.11
-13	-11	-8	1.01	2.33
13	11	8	0.51	2.90
13	11	8	-0.58	4.26
13	11	8	-0.93	2.27
13	11	8	1.06	1.06
-14	-11	-8	1.23	4.49
-14	-11	-8	-0.35	2.60
-14	-11	-8	1.32	1.24
-14	-11	-8	-1.94	4.19
14	11	8	-3.92	3.28
14	11	8	-2.35	2.65
14	11	8	-0.30	1.28
-4	12	8	0.05	1.27
-3	12	8	3.95	1.29
-3	12	8	3.67	2.21
-2	12	8	-1.12	1.26
-1	12	8	0.49	0.71
0	12	8	0.99	0.65
1	12	8	2.85	0.66
-3	-12	-8	6.46	0.96
-4	-12	-8	4.15	0.89
4	12	8	3.63	1.17
-5	-12	-8	23.52	1.97
5	12	8	24.73	2.83
5	12	8	22.98	2.57
-6	-12	-8	-0.01	1.33
-6	-12	-8	4.36	1.98
-6	-12	-8	1.31	0.81
-6	-12	-8	0.76	1.10
6	12	8	2.72	1.47
6	12	8	0.54	1.22
-7	-12	-8	0.61	0.81
-7	-12	-8	0.38	1.60
-7	-12	-8	2.32	1.23
7	12	8	1.15	1.54
7	12	8	0.09	1.33
7	12	8	0.90	1.98
-8	-12	-8	-0.99	1.75
-8	-12	-8	1.95	2.44
-8	-12	-8	1.28	0.85

-8	-12	-8	-1.00	1.36
8	12	8	2.92	2.22
8	12	8	-0.22	1.70
-9	-12	-8	0.61	1.54
-9	-12	-8	1.15	0.89
-9	-12	-8	1.60	2.52
-9	-12	-8	-0.70	1.93
9	12	8	-0.21	1.80
9	12	8	-0.59	2.32
9	12	8	-2.13	2.62
-10	-12	-8	1.20	1.67
-10	-12	-8	0.72	0.93
-10	-12	-8	-2.67	2.16
-10	-12	-8	0.79	2.68
10	12	8	0.25	1.32
10	12	8	-0.54	1.73
10	12	8	1.48	2.71
-11	-12	-8	1.20	2.92
-11	-12	-8	-1.15	2.44
-11	-12	-8	3.51	1.99
-11	-12	-8	1.02	0.99
11	12	8	1.60	0.95
11	12	8	-2.44	1.88
11	12	8	2.33	2.94
-12	-12	-8	4.41	3.44
-12	-12	-8	0.11	2.78
-12	-12	-8	1.09	1.05
-12	-12	-8	0.65	2.32
12	12	8	-2.57	2.11
12	12	8	-0.99	0.98
12	12	8	1.19	3.60
12	12	8	-3.34	2.63
-13	-12	-8	0.85	3.84
-13	-12	-8	0.40	1.15
-13	-12	-8	0.18	2.44
-13	-12	-8	-1.92	3.25
13	12	8	-2.05	3.14
13	12	8	0.40	1.16
13	12	8	0.41	2.44
-14	-12	-8	1.42	4.15
-14	-12	-8	-1.33	2.86
-14	-12	-8	2.00	5.27
-14	-12	-8	1.72	1.24
14	12	8	1.96	3.58

14	12	8	-2.45	2.67
14	12	8	1.19	1.28
-3	13	8	-0.72	1.26
-1	-13	-8	0.31	1.55
1	13	8	-0.52	0.72
2	13	8	0.89	0.67
-3	-13	-8	2.95	2.35
4	13	8	3.01	1.79
-5	-13	-8	-0.26	0.80
-5	-13	-8	1.53	1.88
5	13	8	-0.87	0.64
5	13	8	-0.57	1.51
5	13	8	0.17	1.16
-6	-13	-8	0.70	1.15
-6	-13	-8	-0.03	0.79
6	13	8	-1.41	1.23
6	13	8	0.21	0.69
6	13	8	0.61	1.53
-7	-13	-8	2.35	1.35
-7	-13	-8	1.04	0.84
7	13	8	0.45	0.72
7	13	8	0.54	1.42
7	13	8	1.75	1.71
-8	-13	-8	-0.47	0.88
-8	-13	-8	1.65	1.42
-8	-13	-8	3.48	2.62
-8	-13	-8	3.12	2.67
8	13	8	1.38	0.74
8	13	8	0.78	1.72
8	13	8	-0.86	1.54
8	13	8	-0.33	2.35
-9	-13	-8	0.48	1.83
-9	-13	-8	-0.10	1.56
-9	-13	-8	3.73	2.85
-9	-13	-8	2.15	1.00
9	13	8	1.89	1.94
9	13	8	0.22	1.67
9	13	8	1.43	2.69
9	13	8	0.73	0.83
-10	-13	-8	-1.68	1.75
-10	-13	-8	1.07	1.06
-10	-13	-8	2.51	3.07
-10	-13	-8	-2.22	2.09
10	13	8	-2.63	2.75

10	13	8	0.52	0.89
10	13	8	0.54	2.12
10	13	8	-1.04	1.86
-11	-13	-8	-0.31	2.52
-11	-13	-8	1.14	2.07
-11	-13	-8	0.60	1.08
-11	-13	-8	-1.10	3.21
11	13	8	-1.17	2.05
11	13	8	-2.31	3.27
11	13	8	-0.64	0.96
-12	-13	-8	1.82	3.55
-12	-13	-8	0.38	1.18
-12	-13	-8	0.59	2.32
-12	-13	-8	-2.75	2.78
12	13	8	-0.14	2.91
12	13	8	0.39	1.07
12	13	8	-0.73	2.23
-13	-13	-8	1.51	1.25
-13	-13	-8	1.68	2.63
-13	-13	-8	-0.76	4.12
-13	-13	-8	-0.98	3.26
13	13	8	0.67	2.51
13	13	8	1.66	1.48
13	13	8	-5.35	3.39
-1	14	8	1.21	2.27
0	14	8	1.72	1.91
-1	-14	-8	1.90	1.83
-2	-14	-8	1.47	1.86
2	14	8	0.89	1.77
3	14	8	0.74	1.47
4	14	8	-2.17	1.80
4	14	8	-0.19	1.59
-5	-14	-8	0.96	2.05
5	14	8	1.39	1.30
5	14	8	1.46	1.62
5	14	8	0.66	1.26
5	14	8	1.36	1.97
-6	-14	-8	1.40	0.93
6	14	8	-0.96	1.30
6	14	8	1.68	1.64
6	14	8	0.50	0.80
6	14	8	-1.61	2.11
-7	-14	-8	1.89	1.75
-7	-14	-8	1.53	0.95

-7	-14	-8	4.81	2.63
7	14	8	2.48	0.82
7	14	8	0.56	1.77
7	14	8	1.89	1.55
7	14	8	3.33	2.34
-8	-14	-8	-0.23	1.51
-8	-14	-8	0.48	1.90
-8	-14	-8	1.66	2.79
-8	-14	-8	-0.48	0.94
8	14	8	-0.24	1.62
8	14	8	-0.01	0.82
8	14	8	2.27	2.60
8	14	8	-1.30	1.86
-9	-14	-8	1.90	1.03
-9	-14	-8	-0.42	1.70
-9	-14	-8	-1.37	1.90
-9	-14	-8	2.58	3.19
9	14	8	0.18	2.90
9	14	8	-1.61	1.69
9	14	8	-1.12	0.87
9	14	8	-1.79	2.06
-10	-14	-8	2.37	2.13
-10	-14	-8	-0.73	1.08
-10	-14	-8	-2.08	1.91
-10	-14	-8	-0.25	3.53
10	14	8	0.78	2.36
10	14	8	-1.96	1.86
10	14	8	-0.63	1.22
10	14	8	-1.31	3.37
-11	-14	-8	1.60	2.28
-11	-14	-8	3.70	3.89
-11	-14	-8	0.88	1.21
-11	-14	-8	0.29	2.31
11	14	8	0.29	3.17
11	14	8	-1.06	2.08
-12	-14	-8	2.48	4.03
-12	-14	-8	1.99	1.34
-12	-14	-8	0.08	2.63
-12	-14	-8	0.15	2.74
12	14	8	0.81	4.14
12	14	8	0.01	2.32
-13	-14	-8	1.26	1.34
-13	-14	-8	-0.64	4.23
-13	-14	-8	5.89	4.52

13	14	8	-3.50	2.71
-1	-15	-8	0.74	2.46
1	15	8	-1.53	2.72
-2	-15	-8	1.97	2.18
-3	-15	-8	0.40	2.30
3	15	8	-2.28	1.71
3	15	8	-0.14	1.99
-4	-15	-8	1.15	2.26
4	15	8	0.14	1.78
4	15	8	-0.78	2.03
-5	-15	-8	5.48	2.50
5	15	8	5.61	2.32
5	15	8	0.59	2.95
-6	-15	-8	2.21	2.55
6	15	8	1.23	1.82
6	15	8	-0.44	2.52
6	15	8	-0.78	1.49
-7	-15	-8	-1.19	2.72
7	15	8	1.53	2.59
7	15	8	-0.64	2.04
7	15	8	-1.23	1.63
-8	-15	-8	1.41	1.09
-8	-15	-8	1.79	3.25
8	15	8	0.86	3.01
8	15	8	0.31	1.78
-9	-15	-8	1.32	2.57
-9	-15	-8	2.26	3.36
-9	-15	-8	-0.18	1.12
9	15	8	1.72	3.68
9	15	8	-0.22	1.89
-10	-15	-8	2.49	3.88
-10	-15	-8	-1.01	1.15
10	15	8	-1.82	2.11
-11	-15	-8	0.63	1.30
-11	-15	-8	-1.41	4.24
11	15	8	-2.17	2.27
-4	-16	-8	0.97	2.66
-5	-16	-8	0.41	2.76
-6	-16	-8	3.93	2.95
6	16	8	1.03	1.66
-7	-16	-8	2.79	3.00
7	16	8	-0.78	1.80
-8	-16	-8	2.88	3.35
8	16	8	-0.64	1.90

-9	-16	-8	3.23	3.98
9	16	8	1.53	2.16
7	11	-9	15.61	3.34
7	11	-9	18.33	4.25
-7	-11	9	12.37	3.86
-7	-11	9	28.93	5.07
-7	-11	9	22.19	4.04
6	11	-9	17.77	1.93
6	11	-9	18.88	2.37
6	11	-9	18.07	4.75
-6	-11	9	25.88	4.94
-6	-11	9	27.65	5.11
-6	-11	9	15.91	3.41
5	11	-9	20.12	3.39
5	11	-9	18.20	1.86
-5	-11	9	25.10	4.89
-5	-11	9	16.86	3.25
-5	-11	9	21.45	3.35
4	11	-9	12.77	3.51
4	11	-9	20.78	1.91
4	11	-9	22.32	4.67
-4	-11	9	13.37	3.39
-4	-11	9	19.73	3.51
-4	-11	9	20.63	3.24
3	11	-9	12.97	3.40
3	11	-9	16.42	1.86
-3	-11	9	17.28	3.52
2	11	-9	17.09	2.00
2	11	-9	11.81	3.54
-2	-11	9	24.58	4.74
1	11	-9	16.03	3.81
1	11	-9	14.11	2.00
-1	-11	9	14.50	3.26
-9	-10	9	16.68	3.17
-9	-10	9	13.41	3.85
8	10	-9	14.18	5.00
8	10	-9	16.50	2.96
-8	-10	9	14.99	3.04
-8	-10	9	16.27	3.67
-8	-10	9	19.15	3.06
7	10	-9	23.36	4.27
7	10	-9	30.45	5.81
-7	-10	9	29.90	4.54
-7	-10	9	23.29	3.86

-7	-10	9	21.02	3.51
6	10	-9	19.06	3.05
6	10	-9	32.97	5.68
6	10	-9	23.85	2.21
-6	-10	9	24.01	4.28
-6	-10	9	17.14	3.09
-6	-10	9	23.38	4.69
5	10	-9	24.89	5.09
5	10	-9	20.87	3.06
5	10	-9	16.39	3.83
5	10	-9	17.44	1.99
-5	-10	9	27.43	4.51
-5	-10	9	23.20	4.15
-5	-10	9	16.87	3.13
4	10	-9	13.45	3.32
4	10	-9	13.33	1.55
4	10	-9	10.78	3.18
4	10	-9	17.89	3.41
4	10	-9	12.14	4.08
-4	-10	9	12.30	2.90
-4	-10	9	13.86	2.91
-4	-10	9	13.85	2.68
3	10	-9	16.09	3.04
3	10	-9	18.01	3.23
3	10	-9	12.32	3.01
3	10	-9	10.38	3.22
3	10	-9	15.21	1.70
-3	-10	9	15.06	2.70
-3	-10	9	16.98	2.79
-3	-10	9	13.84	3.13
2	10	-9	15.15	3.23
2	10	-9	15.55	1.74
2	10	-9	20.17	4.16
-2	-10	9	20.82	3.94
-2	-10	9	13.49	2.79
-2	-10	9	13.31	2.66
1	10	-9	15.19	3.23
1	10	-9	22.22	1.96
1	10	-9	19.04	3.23
-1	-10	9	25.19	4.29
0	10	-9	11.28	3.22
0	10	-9	12.18	1.81
0	-10	9	11.97	2.87
-1	10	-9	24.16	2.25

-1	10	-9	33.91	5.44
-2	10	-9	22.31	3.72
-2	10	-9	21.83	2.23
-10	-9	9	17.73	3.10
-10	-9	9	12.10	3.77
9	9	-9	17.03	2.83
-9	-9	9	25.62	4.17
-9	-9	9	20.80	2.99
-9	-9	9	26.28	5.06
-9	-9	9	22.15	4.29
8	9	-9	13.42	2.65
8	9	-9	16.96	3.44
8	9	-9	16.08	3.94
-8	-9	9	15.59	3.32
-8	-9	9	17.90	2.77
-8	-9	9	20.19	3.68
7	9	-9	28.63	3.92
7	9	-9	29.72	5.10
7	9	-9	30.55	5.32
-7	-9	9	24.37	4.27
-7	-9	9	26.16	4.56
-7	-9	9	27.09	3.98
6	9	-9	17.83	3.55
6	9	-9	14.39	3.28
6	9	-9	16.19	3.08
6	9	-9	20.90	3.61
-6	-9	9	22.09	3.69
-6	-9	9	24.97	4.31
-6	-9	9	17.43	2.66
5	9	-9	16.32	2.65
5	9	-9	14.51	2.98
5	9	-9	17.70	1.52
5	9	-9	23.40	4.44
5	9	-9	18.82	3.33
-5	-9	9	15.48	2.64
-5	-9	9	16.11	2.48
-5	-9	9	21.26	3.91
4	9	-9	22.13	4.19
4	9	-9	30.12	4.77
4	9	-9	27.21	4.34
4	9	-9	25.20	1.82
4	9	-9	27.47	4.38
-4	-9	9	19.79	2.83
-4	-9	9	27.25	3.94

-4	-9	9	23.81	3.73
3	9	-9	16.37	2.76
3	9	-9	25.61	4.38
3	9	-9	19.66	3.90
3	9	-9	20.65	1.71
3	9	-9	22.79	4.07
-3	-9	9	19.66	2.66
-3	-9	9	23.50	4.11
-3	-9	9	21.94	3.65
2	9	-9	18.53	1.70
2	9	-9	19.58	3.80
2	9	-9	23.25	3.91
2	9	-9	13.27	3.38
2	9	-9	19.06	3.06
-2	-9	9	17.53	2.64
-2	-9	9	19.51	3.53
-2	-9	9	21.42	3.58
1	9	-9	7.62	2.58
1	9	-9	13.47	2.75
1	9	-9	16.78	1.61
1	9	-9	10.85	2.53
-1	-9	9	13.25	2.25
-1	-9	9	13.30	2.44
-1	-9	9	16.05	2.76
0	9	-9	16.71	1.76
0	9	-9	23.82	4.14
0	9	-9	11.60	2.90
0	-9	9	17.86	3.15
0	-9	9	18.44	2.76
0	-9	9	12.73	2.60
-1	9	-9	36.86	4.68
-1	9	-9	31.84	4.93
-1	9	-9	30.10	2.35
1	-9	9	32.65	4.27
-2	9	-9	27.92	4.83
-2	9	-9	24.79	2.22
-3	9	-9	27.14	2.35
-3	9	-9	31.37	4.82
-4	9	-9	14.43	2.08
-4	9	-9	16.51	2.98
-11	-8	9	12.41	2.81
-11	-8	9	11.55	4.22
10	8	-9	30.85	6.37
-10	-8	9	26.96	5.03

-10	-8	9	22.72	4.20
9	8	-9	15.10	3.48
9	8	-9	14.34	4.14
9	8	-9	12.59	2.32
-9	-8	9	13.41	2.54
-9	-8	9	13.26	3.24
-9	-8	9	13.16	2.44
8	8	-9	17.21	3.30
8	8	-9	8.49	3.50
8	8	-9	16.02	3.12
8	8	-9	11.23	3.80
-8	-8	9	13.92	2.37
-8	-8	9	16.35	3.03
-8	-8	9	16.88	3.54
-8	-8	9	13.36	2.34
7	8	-9	14.07	3.22
7	8	-9	20.90	3.26
-7	-8	9	17.60	2.65
-7	-8	9	18.73	3.23
-7	-8	9	13.71	3.31
-7	-8	9	17.09	2.88
6	8	-9	38.38	3.73
6	8	-9	40.89	4.62
6	8	-9	35.31	5.21
-6	-8	9	34.30	3.69
-6	-8	9	35.15	4.16
-6	-8	9	33.11	3.76
5	8	-9	22.66	4.10
5	8	-9	18.65	3.20
5	8	-9	21.57	4.50
-5	-8	9	17.81	3.61
-5	-8	9	19.50	3.39
-5	-8	9	18.79	3.21
4	8	-9	32.60	2.07
4	8	-9	35.43	3.76
4	8	-9	36.53	4.34
4	8	-9	34.66	3.94
4	8	-9	34.41	4.12
-4	-8	9	32.93	3.69
-4	-8	9	30.46	3.55
-4	-8	9	31.71	3.83
3	8	-9	28.80	4.09
3	8	-9	34.12	3.84
3	8	-9	45.19	4.11

3	8	-9	36.61	4.16
3	8	-9	37.23	2.26
-3	-8	9	40.25	3.75
-3	-8	9	35.70	4.02
-3	-8	9	37.95	3.76
2	8	-9	30.13	2.54
2	8	-9	33.23	4.08
2	8	-9	26.99	4.19
2	8	-9	28.30	3.94
2	8	-9	35.37	3.62
-2	-8	9	30.31	3.77
-2	-8	9	31.94	3.46
-2	-8	9	30.65	3.61
-2	-8	9	36.13	3.07
1	8	-9	9.76	2.23
1	8	-9	15.88	1.48
1	8	-9	14.55	2.48
-1	-8	9	14.68	2.26
-1	-8	9	13.12	2.10
-1	-8	9	18.15	3.17
0	8	-9	17.14	1.56
0	8	-9	8.85	3.85
0	8	-9	11.98	2.30
0	8	-9	15.91	3.07
0	-8	9	15.49	3.03
0	-8	9	12.07	1.92
0	-8	9	14.05	2.32
-1	8	-9	14.89	2.74
-1	8	-9	16.40	3.05
-1	8	-9	16.05	1.67
-1	8	-9	15.17	2.78
-1	8	-9	15.10	2.42
1	-8	9	16.35	2.48
1	-8	9	19.21	3.32
1	-8	9	16.01	2.53
1	-8	9	16.62	2.82
1	-8	9	16.67	3.03
-2	8	-9	40.47	4.28
-2	8	-9	30.29	3.82
-2	8	-9	32.04	2.35
-2	8	-9	29.40	4.43
2	-8	9	33.13	4.06
2	-8	9	36.00	3.35
2	-8	9	36.68	3.80

2	-8	9	30.90	3.24
-3	8	-9	9.08	2.54
-3	8	-9	6.30	1.04
-3	8	-9	6.40	2.78
-3	8	-9	3.39	2.10
3	-8	9	6.41	1.68
3	-8	9	1.56	1.84
-4	8	-9	18.39	2.87
-4	8	-9	17.17	1.99
-5	8	-9	9.29	2.64
-5	8	-9	8.58	1.22
5	-8	9	9.40	2.00
11	7	-9	14.25	5.49
-11	-7	9	17.14	3.17
-11	-7	9	24.51	3.96
-11	-7	9	26.23	5.39
10	7	-9	17.40	4.14
-10	-7	9	19.78	3.66
-10	-7	9	24.42	3.77
-10	-7	9	25.28	4.43
9	7	-9	15.16	3.41
9	7	-9	19.24	4.08
-9	-7	9	20.37	3.40
-9	-7	9	18.74	3.44
-9	-7	9	22.60	3.82
-9	-7	9	21.14	4.27
8	7	-9	11.17	2.03
8	7	-9	13.68	2.81
8	7	-9	10.70	3.19
-8	-7	9	11.02	2.72
-8	-7	9	12.04	2.13
-8	-7	9	11.84	2.17
-8	-7	9	10.81	2.06
7	7	-9	17.60	3.04
7	7	-9	15.33	2.84
7	7	-9	21.49	4.07
7	7	-9	14.64	3.08
-7	-7	9	13.68	2.76
-7	-7	9	14.32	2.27
-7	-7	9	16.90	3.03
-7	-7	9	21.70	3.09
6	7	-9	40.19	4.31
6	7	-9	34.90	5.01
6	7	-9	42.80	3.63

6	7	-9	46.78	4.50
-6	-7	9	39.85	3.69
-6	-7	9	45.68	3.56
-6	-7	9	51.42	5.02
5	7	-9	37.17	4.68
5	7	-9	35.13	4.06
5	7	-9	36.71	4.19
5	7	-9	34.82	3.45
-5	-7	9	34.07	3.48
-5	-7	9	37.65	3.15
-5	-7	9	39.74	3.77
-5	-7	9	32.32	3.42
4	7	-9	42.50	3.78
4	7	-9	40.92	2.38
4	7	-9	41.76	3.90
4	7	-9	46.63	4.48
4	7	-9	39.07	4.04
-4	-7	9	41.14	3.76
-4	-7	9	43.91	3.74
3	7	-9	34.70	3.90
3	7	-9	38.69	2.29
3	7	-9	42.18	4.00
3	7	-9	39.19	3.68
3	7	-9	41.29	3.71
-3	-7	9	38.59	3.51
-3	-7	9	40.76	3.10
-3	-7	9	39.80	3.60
-3	-7	9	35.81	3.73
2	7	-9	36.45	3.77
2	7	-9	41.41	3.66
2	7	-9	32.95	3.68
2	7	-9	37.91	2.26
2	7	-9	36.52	3.60
-2	-7	9	40.37	3.04
-2	-7	9	36.50	3.74
-2	-7	9	36.04	3.52
1	7	-9	27.54	3.50
1	7	-9	37.97	3.37
1	7	-9	32.61	2.06
1	7	-9	27.89	3.63
1	7	-9	30.41	3.28
-1	-7	9	32.51	3.26
-1	-7	9	34.72	2.92
-1	-7	9	34.66	3.23

-1	-7	9	31.34	3.40
-1	-7	9	30.35	2.66
0	7	-9	21.46	3.28
0	7	-9	21.48	3.55
0	7	-9	29.11	3.04
0	7	-9	30.87	3.27
0	-7	9	26.05	3.36
0	-7	9	26.71	2.91
0	-7	9	26.19	2.51
-1	7	-9	38.46	2.32
-1	7	-9	33.06	3.91
-1	7	-9	31.46	3.51
-1	7	-9	37.97	3.33
-1	7	-9	36.99	3.61
1	-7	9	34.05	3.26
1	-7	9	35.94	3.76
1	-7	9	39.73	3.22
1	-7	9	35.57	2.97
1	-7	9	41.01	3.38
-2	7	-9	31.35	3.15
-2	7	-9	26.00	3.82
-2	7	-9	26.44	3.36
2	-7	9	31.71	3.21
2	-7	9	25.15	3.54
2	-7	9	34.63	2.98
2	-7	9	30.67	2.85
-3	7	-9	18.84	3.67
-3	7	-9	21.04	1.81
-3	7	-9	13.93	2.27
-3	7	-9	24.39	2.85
-3	7	-9	18.18	3.77
3	-7	9	19.19	2.70
3	-7	9	21.74	2.80
3	-7	9	15.55	2.44
3	-7	9	17.88	2.63
-4	7	-9	9.96	2.30
-4	7	-9	10.18	1.65
-4	7	-9	7.71	2.07
-4	7	-9	15.65	2.96
-4	7	-9	7.75	1.63
4	-7	9	10.80	1.73
4	-7	9	6.13	2.26
-5	7	-9	10.12	2.10
-5	7	-9	12.11	1.80

-5	7	-9	11.29	2.44
5	-7	9	17.55	3.16
-6	7	-9	15.15	2.75
-6	7	-9	13.87	1.96
6	-7	9	17.79	3.48
-7	7	-9	17.35	2.22
-7	7	-9	23.06	4.43
-12	-6	9	13.21	4.18
-12	-6	9	9.76	2.93
-12	-6	9	8.03	2.94
11	6	-9	17.47	4.64
-11	-6	9	27.78	4.74
-11	-6	9	25.50	3.86
-11	-6	9	27.82	5.09
-10	-6	9	22.24	3.56
-10	-6	9	24.25	4.33
-10	-6	9	24.47	3.52
9	6	-9	27.93	4.83
9	6	-9	38.15	6.42
-9	-6	9	30.72	4.21
-9	-6	9	28.89	3.37
-9	-6	9	30.17	3.48
-9	-6	9	32.52	4.08
8	6	-9	6.92	1.81
8	6	-9	5.15	3.03
8	6	-9	9.18	2.46
-8	-6	9	10.11	1.91
-8	-6	9	7.84	1.96
-8	-6	9	7.54	1.82
7	6	-9	23.95	3.02
7	6	-9	31.06	4.05
7	6	-9	20.26	3.80
-7	-6	9	25.09	3.18
-7	-6	9	27.64	3.07
-7	-6	9	27.99	3.21
6	6	-9	26.24	4.55
6	6	-9	26.39	3.01
6	6	-9	36.27	3.90
-6	-6	9	34.99	3.01
-6	-6	9	28.43	3.11
-6	-6	9	31.11	3.17
5	6	-9	47.29	4.94
5	6	-9	51.76	3.79
5	6	-9	52.29	4.33

-5	-6	9	49.67	3.65
-5	-6	9	51.60	3.70
-5	-6	9	51.17	3.82
4	6	-9	42.67	3.55
4	6	-9	42.55	4.41
4	6	-9	45.00	3.81
-4	-6	9	41.31	3.41
-4	-6	9	41.78	3.17
-4	-6	9	41.97	3.44
3	6	-9	49.15	4.10
3	6	-9	43.81	4.08
3	6	-9	45.17	2.50
3	6	-9	49.68	3.69
-3	-6	9	43.02	3.54
-3	-6	9	45.94	3.56
-3	-6	9	41.45	2.83
2	6	-9	25.09	1.62
2	6	-9	26.20	3.09
2	6	-9	27.54	3.11
2	6	-9	23.59	2.92
2	6	-9	17.14	3.00
-2	-6	9	22.57	3.04
-2	-6	9	22.37	2.79
-2	-6	9	24.06	2.01
1	6	-9	42.13	3.26
1	6	-9	37.47	3.44
1	6	-9	42.31	2.36
1	6	-9	42.90	3.68
-1	-6	9	40.13	2.78
-1	-6	9	39.86	3.35
-1	-6	9	38.62	3.38
-1	-6	9	42.93	3.28
-1	-6	9	35.01	3.81
0	6	-9	24.73	3.30
0	6	-9	27.95	2.89
0	6	-9	27.52	1.74
0	6	-9	22.05	2.93
0	6	-9	27.11	2.85
0	-6	9	21.58	2.48
0	-6	9	24.55	2.27
0	-6	9	19.84	3.02
0	-6	9	24.47	2.81
0	-6	9	24.87	2.70
-1	6	-9	37.78	3.27

-1	6	-9	30.07	3.56
-1	6	-9	37.38	2.60
-1	6	-9	33.95	3.08
-1	6	-9	35.53	3.21
1	-6	9	32.35	3.42
1	-6	9	32.31	2.69
1	-6	9	33.71	2.92
1	-6	9	35.57	3.04
1	-6	9	34.88	2.99
-2	6	-9	22.56	3.41
-2	6	-9	28.52	2.86
-2	6	-9	26.84	3.16
-2	6	-9	24.44	2.92
-2	6	-9	28.53	1.91
2	-6	9	27.92	2.46
2	-6	9	27.01	2.93
2	-6	9	28.34	2.79
2	-6	9	21.73	3.38
-3	6	-9	32.62	3.40
-3	6	-9	30.41	2.95
-3	6	-9	28.31	3.15
-3	6	-9	33.11	2.15
3	-6	9	29.75	2.67
3	-6	9	33.92	3.27
3	-6	9	32.28	2.94
-4	6	-9	10.96	1.54
-4	6	-9	11.30	1.91
-4	6	-9	14.20	1.57
-4	6	-9	13.08	2.18
4	-6	9	13.03	2.59
4	-6	9	13.57	2.27
-5	6	-9	13.42	1.67
-5	6	-9	10.49	2.68
-5	6	-9	9.50	1.93
-5	6	-9	12.94	2.49
-5	6	-9	19.40	3.38
5	-6	9	9.83	1.76
5	-6	9	15.10	2.81
5	-6	9	7.50	2.32
-6	6	-9	10.25	2.83
-6	6	-9	6.40	1.06
-6	6	-9	7.33	2.32
6	-6	9	7.53	1.73
6	-6	9	2.70	1.85

-7	6	-9	14.79	1.98
-7	6	-9	17.16	2.82
-8	6	-9	5.25	3.82
-8	6	-9	4.96	1.19
-8	6	-9	6.25	2.92
-12	-5	9	9.42	2.86
-12	-5	9	10.98	2.60
-11	-5	9	20.66	3.54
-11	-5	9	23.06	4.51
-10	-5	9	26.22	3.34
-10	-5	9	26.99	3.37
-10	-5	9	26.50	4.19
9	5	-9	33.58	6.21
9	5	-9	40.26	4.69
-9	-5	9	34.59	3.45
-9	-5	9	34.12	4.07
-9	-5	9	34.40	3.38
8	5	-9	12.22	3.14
8	5	-9	22.25	3.86
-8	-5	9	16.85	2.69
-8	-5	9	17.04	2.79
-8	-5	9	19.71	3.34
7	5	-9	15.39	2.86
7	5	-9	22.35	3.63
7	5	-9	16.57	2.89
-7	-5	9	24.06	3.51
-7	-5	9	23.29	3.02
-7	-5	9	20.25	2.70
6	5	-9	45.76	4.07
6	5	-9	38.78	3.40
6	5	-9	42.54	4.75
-6	-5	9	38.25	3.40
-6	-5	9	48.67	3.19
-6	-5	9	43.52	3.46
5	5	-9	25.60	3.26
5	5	-9	23.11	4.10
5	5	-9	25.13	2.84
-5	-5	9	22.41	2.62
-5	-5	9	24.06	3.25
-5	-5	9	23.96	2.71
4	5	-9	26.73	3.14
4	5	-9	25.36	3.86
4	5	-9	23.32	2.90
-4	-5	9	29.67	2.86

-4	-5	9	25.75	2.73
-4	-5	9	27.44	2.89
3	5	-9	31.48	1.82
3	5	-9	33.23	3.05
3	5	-9	35.67	3.60
3	5	-9	30.01	3.83
-3	-5	9	28.74	2.89
-3	-5	9	32.81	2.62
-3	-5	9	34.66	2.95
-3	-5	9	26.53	2.07
2	5	-9	27.70	2.94
2	5	-9	18.24	2.84
2	5	-9	28.93	3.04
2	5	-9	26.79	1.61
-2	-5	9	20.54	2.90
-2	-5	9	24.80	1.90
-2	-5	9	26.68	2.37
1	5	-9	46.47	3.45
1	5	-9	34.74	3.55
1	5	-9	49.42	2.41
-1	-5	9	41.06	3.28
-1	-5	9	45.34	3.02
-1	-5	9	42.55	2.67
-1	-5	9	32.71	3.38
0	5	-9	28.64	2.82
0	5	-9	25.52	3.11
0	5	-9	31.23	2.72
0	5	-9	33.39	1.88
0	5	-9	25.34	2.84
0	-5	9	24.47	4.11
0	-5	9	31.64	2.67
0	-5	9	27.09	2.54
0	-5	9	30.26	2.20
-1	5	-9	27.97	1.68
-1	5	-9	16.28	3.12
-1	5	-9	19.51	2.68
-1	5	-9	27.42	2.62
1	-5	9	25.47	2.54
1	-5	9	22.37	2.08
-2	5	-9	34.69	2.59
-2	5	-9	40.36	2.99
-2	5	-9	40.36	3.12
-2	5	-9	32.95	3.48
-2	5	-9	38.10	3.16

2	-5	9	37.73	2.64
2	-5	9	36.32	3.02
2	-5	9	28.02	3.38
2	-5	9	39.90	2.92
-3	5	-9	35.66	3.26
-3	5	-9	33.44	2.11
-3	5	-9	31.97	2.95
-3	5	-9	32.06	3.28
-3	5	-9	31.44	2.73
3	-5	9	30.51	2.50
3	-5	9	29.95	3.00
3	-5	9	33.93	2.77
-4	5	-9	26.03	2.87
-4	5	-9	30.08	4.26
-4	5	-9	27.64	2.60
-4	5	-9	29.95	2.00
-4	5	-9	23.57	3.28
4	-5	9	25.75	2.33
4	-5	9	32.43	2.71
4	-5	9	25.32	3.27
-5	5	-9	20.25	1.77
-5	5	-9	14.03	2.05
-5	5	-9	21.14	2.45
-5	5	-9	20.67	3.21
5	-5	9	21.92	2.66
5	-5	9	18.41	2.12
5	-5	9	20.95	3.29
-6	5	-9	27.82	3.65
-6	5	-9	22.45	2.65
-6	5	-9	22.11	1.95
-6	5	-9	18.46	2.98
-6	5	-9	23.45	3.62
6	-5	9	20.05	2.35
-7	5	-9	14.64	2.99
-7	5	-9	15.90	3.34
-7	5	-9	16.13	2.70
-7	5	-9	16.14	1.86
-8	5	-9	12.38	2.99
-8	5	-9	18.31	3.83
-8	5	-9	14.44	1.99
-9	5	-9	6.38	1.23
-9	5	-9	4.27	3.17
-9	5	-9	6.59	4.10
12	4	-9	15.55	4.69

-12	-4	9	19.83	3.31
-12	-4	9	15.65	3.24
-11	-4	9	12.81	3.53
-11	-4	9	9.38	2.68
-11	-4	9	16.61	3.15
-10	-4	9	25.37	4.07
-10	-4	9	24.98	3.14
-10	-4	9	25.79	3.05
9	4	-9	20.09	3.78
-9	-4	9	19.20	2.76
-9	-4	9	20.18	2.84
-9	-4	9	21.56	3.53
8	4	-9	38.75	3.95
8	4	-9	30.50	5.10
-8	-4	9	39.44	3.63
-8	-4	9	34.83	3.14
-8	-4	9	36.01	3.08
7	4	-9	32.24	3.67
7	4	-9	30.97	4.53
-7	-4	9	29.37	2.84
-7	-4	9	32.77	3.29
6	4	-9	42.58	4.51
6	4	-9	39.66	5.08
6	4	-9	44.35	3.80
-6	-4	9	38.87	3.22
-6	-4	9	40.80	3.36
-6	-4	9	45.21	3.06
5	4	-9	34.06	3.39
5	4	-9	42.75	4.23
-5	-4	9	37.78	2.90
-5	-4	9	31.86	2.93
-5	-4	9	33.45	3.05
4	4	-9	57.07	3.90
4	4	-9	52.10	3.78
4	4	-9	54.31	4.57
-4	-4	9	55.15	3.66
-4	-4	9	59.53	3.63
-4	-4	9	55.16	3.69
3	4	-9	33.56	3.05
3	4	-9	38.78	3.65
3	4	-9	30.48	3.91
3	4	-9	35.34	1.95
-3	-4	9	39.80	2.73
-3	-4	9	33.37	2.13

-3	-4	9	36.87	2.90
2	4	-9	38.19	3.23
2	4	-9	42.35	2.28
2	4	-9	40.98	3.27
2	4	-9	43.97	3.48
-2	-4	9	44.18	2.93
-2	-4	9	47.02	3.25
-2	-4	9	39.53	2.50
1	4	-9	52.19	3.69
1	4	-9	56.14	3.93
1	4	-9	59.65	2.98
1	4	-9	60.92	3.82
-1	-4	9	55.32	3.19
-1	-4	9	51.85	3.64
-1	-4	9	57.99	3.50
0	4	-9	68.98	4.16
0	4	-9	61.89	4.38
0	4	-9	70.10	3.52
0	4	-9	65.42	4.11
0	-4	9	67.05	3.74
0	-4	9	68.01	4.03
0	-4	9	70.93	4.02
-1	4	-9	36.52	2.00
-1	4	-9	33.28	2.76
-1	4	-9	31.89	3.20
-1	4	-9	33.20	2.81
1	-4	9	33.38	2.72
1	-4	9	32.60	2.61
1	-4	9	26.33	3.05
1	-4	9	36.05	2.31
-2	4	-9	45.69	2.53
-2	4	-9	46.71	3.24
-2	4	-9	45.14	3.14
-2	4	-9	43.21	3.57
2	-4	9	38.74	3.52
2	-4	9	44.33	3.12
2	-4	9	46.23	2.85
2	-4	9	48.11	3.18
-3	4	-9	37.06	3.23
-3	4	-9	35.42	2.14
-3	4	-9	36.04	2.90
-3	4	-9	34.13	3.06
-3	4	-9	34.21	2.72
3	-4	9	33.22	2.45

3	-4	9	28.50	3.27
3	-4	9	35.83	2.93
-4	4	-9	54.37	3.58
-4	4	-9	58.35	3.84
-4	4	-9	64.58	4.10
-4	4	-9	53.17	3.13
-4	4	-9	58.20	3.94
4	-4	9	59.84	3.67
4	-4	9	58.56	3.84
4	-4	9	53.62	4.17
4	-4	9	51.84	3.42
-5	4	-9	39.74	3.57
-5	4	-9	28.48	3.15
-5	4	-9	34.39	2.72
-5	4	-9	36.79	3.32
-5	4	-9	35.06	2.24
5	-4	9	38.42	2.98
5	-4	9	29.66	3.60
5	-4	9	33.88	3.42
-6	4	-9	41.04	2.94
-6	4	-9	35.37	3.47
-6	4	-9	37.12	2.43
-6	4	-9	41.16	3.88
6	-4	9	30.33	3.47
6	-4	9	43.57	3.26
6	-4	9	30.35	3.67
-7	4	-9	16.77	2.61
-7	4	-9	19.74	1.82
-7	4	-9	18.36	3.22
-7	4	-9	19.56	3.92
-7	4	-9	14.62	2.17
-7	4	-9	22.63	2.47
7	-4	9	17.61	2.24
-8	4	-9	15.62	2.86
-8	4	-9	9.08	2.82
-8	4	-9	13.81	3.54
-8	4	-9	16.01	1.85
-8	4	-9	9.54	2.11
8	-4	9	21.34	3.08
-9	4	-9	2.23	3.01
-9	4	-9	6.02	1.14
-9	4	-9	2.25	3.92
9	-4	9	9.55	1.98
-10	4	-9	7.72	1.26

-10	4	-9	10.54	4.79
-10	4	-9	6.37	3.46
10	-4	9	9.26	2.27
-12	-3	9	12.39	2.23
-12	-3	9	20.41	4.02
-11	-3	9	21.25	4.56
-11	-3	9	20.80	3.13
-11	-3	9	23.47	3.87
-10	-3	9	1.71	2.32
-10	-3	9	2.72	1.72
-9	-3	9	5.18	1.52
-9	-3	9	5.45	1.64
-9	-3	9	5.45	2.00
-8	-3	9	14.80	2.40
-8	-3	9	13.68	2.46
-8	-3	9	16.26	3.01
7	3	-9	22.96	3.20
-7	-3	9	22.27	2.43
-7	-3	9	22.59	2.91
6	3	-9	43.96	3.65
-6	-3	9	42.32	3.12
-6	-3	9	41.76	2.96
5	3	-9	23.21	2.95
5	3	-9	22.97	3.56
-5	-3	9	27.33	2.42
-5	-3	9	23.60	2.38
-5	-3	9	25.04	2.61
4	3	-9	52.45	3.61
4	3	-9	45.14	3.71
4	3	-9	57.03	4.37
-4	-3	9	55.02	3.40
-4	-3	9	49.69	3.47
-4	-3	9	51.70	3.46
3	3	-9	20.91	2.51
3	3	-9	21.37	3.06
3	3	-9	26.50	3.17
3	3	-9	24.80	1.41
-3	-3	9	26.40	2.34
-3	-3	9	27.82	2.44
-3	-3	9	19.80	2.46
2	3	-9	2.51	1.42
2	3	-9	4.44	1.42
-2	-3	9	4.16	0.67
-2	-3	9	2.31	1.28

-2	-3	9	4.36	0.97
-2	-3	9	1.73	1.29
1	3	-9	11.29	1.66
1	3	-9	12.37	2.08
1	3	-9	15.84	1.08
1	3	-9	16.15	2.26
-1	-3	9	14.05	1.85
-1	-3	9	16.62	2.09
-1	-3	9	13.61	1.36
0	3	-9	22.30	1.42
0	3	-9	20.41	2.26
0	3	-9	25.62	2.32
0	3	-9	20.79	2.60
0	-3	9	24.44	2.15
0	-3	9	21.34	2.06
0	-3	9	22.37	1.70
-1	3	-9	33.39	2.52
-1	3	-9	34.62	1.87
-1	3	-9	33.07	2.58
-1	3	-9	31.35	2.90
1	-3	9	28.38	2.45
1	-3	9	25.22	2.79
1	-3	9	31.87	2.11
1	-3	9	34.12	2.52
-2	3	-9	32.68	1.89
-2	3	-9	29.60	2.47
-2	3	-9	35.18	2.88
-2	3	-9	32.65	2.59
2	-3	9	30.58	2.15
2	-3	9	30.15	2.44
2	-3	9	33.10	2.57
2	-3	9	28.45	2.88
-3	3	-9	41.25	2.42
-3	3	-9	41.30	2.92
-3	3	-9	46.34	3.38
-3	3	-9	44.72	3.16
3	-3	9	44.23	3.11
3	-3	9	41.69	2.71
3	-3	9	43.36	3.00
3	-3	9	44.20	3.42
-4	3	-9	20.38	2.39
-4	3	-9	19.52	2.48
-4	3	-9	17.66	1.46
-4	3	-9	25.52	2.75

4	-3	9	19.80	2.44
4	-3	9	17.72	2.81
4	-3	9	17.65	1.79
4	-3	9	16.89	2.69
-5	3	-9	34.26	2.29
-5	3	-9	39.76	3.21
-5	3	-9	40.49	3.52
-5	3	-9	33.93	3.11
5	-3	9	40.70	3.57
5	-3	9	39.82	2.91
5	-3	9	38.35	3.16
-6	3	-9	20.56	2.90
-6	3	-9	22.50	1.70
-6	3	-9	20.90	3.09
-6	3	-9	20.45	2.78
-6	3	-9	23.82	3.33
6	-3	9	21.86	3.60
6	-3	9	16.50	2.37
-7	3	-9	12.68	2.08
-7	3	-9	16.45	1.66
-7	3	-9	15.86	2.93
-7	3	-9	13.84	2.42
7	-3	9	17.42	2.55
-8	3	-9	6.05	2.59
-8	3	-9	8.43	3.13
-8	3	-9	7.02	2.00
-8	3	-9	7.67	1.31
-8	3	-9	9.09	1.57
-8	3	-9	9.66	2.50
8	-3	9	4.47	2.25
8	-3	9	11.19	2.66
8	-3	9	14.76	2.76
8	-3	9	9.14	1.32
-9	3	-9	16.09	3.90
-9	3	-9	19.24	3.80
-9	3	-9	17.78	1.87
-9	3	-9	11.32	2.30
-9	3	-9	13.31	3.14
-10	3	-9	6.23	3.36
-10	3	-9	6.41	4.14
-10	3	-9	6.26	1.15
10	-3	9	8.33	2.04
-12	-2	9	11.57	2.23
-12	-2	9	11.77	3.13

-11	-2	9	22.98	4.54
-11	-2	9	15.59	2.93
-10	-2	9	5.50	2.37
-9	-2	9	18.15	3.58
-9	-2	9	20.70	3.42
-8	-2	9	21.16	3.11
-8	-2	9	20.70	2.44
-8	-2	9	18.48	2.46
-7	-2	9	32.74	2.75
-7	-2	9	34.81	3.19
6	2	-9	18.93	2.81
6	2	-9	15.00	3.26
-6	-2	9	16.76	2.73
-6	-2	9	23.87	2.15
-6	-2	9	21.96	2.21
5	2	-9	72.76	5.21
5	2	-9	80.27	4.66
-5	-2	9	76.85	4.42
-5	-2	9	75.05	4.59
-5	-2	9	78.14	4.35
4	2	-9	58.91	4.46
4	2	-9	60.13	3.81
-4	-2	9	59.20	3.75
-4	-2	9	62.05	3.61
-4	-2	9	54.74	3.67
3	2	-9	92.88	5.37
3	2	-9	78.54	5.05
3	2	-9	84.86	5.10
3	2	-9	87.08	4.23
-3	-2	9	89.68	4.80
-3	-2	9	88.24	4.73
-3	-2	9	73.07	4.83
2	2	-9	24.68	2.83
2	2	-9	26.25	1.41
2	2	-9	19.71	2.32
2	2	-9	20.83	2.47
-2	-2	9	25.23	2.33
-2	-2	9	23.79	1.56
-2	-2	9	24.22	2.16
1	2	-9	12.72	1.72
1	2	-9	18.22	2.28
1	2	-9	13.11	1.49
1	2	-9	18.68	1.10
-1	-2	9	11.12	2.17

-1	-2	9	14.77	1.95
-1	-2	9	16.27	1.81
-1	-2	9	18.14	1.28
0	2	-9	24.84	2.54
0	2	-9	25.94	2.25
0	2	-9	27.71	2.40
0	2	-9	26.34	1.52
0	-2	9	23.88	2.10
0	-2	9	23.78	2.49
0	-2	9	26.34	1.71
0	-2	9	27.65	2.15
-1	2	-9	31.44	2.46
-1	2	-9	34.92	2.47
-1	2	-9	34.99	2.75
-1	2	-9	33.58	1.87
1	-2	9	38.96	2.48
1	-2	9	31.80	2.76
1	-2	9	31.26	2.04
1	-2	9	27.84	2.37
-2	2	-9	43.67	2.38
-2	2	-9	48.08	2.97
-2	2	-9	41.85	2.85
-2	2	-9	47.33	3.19
2	-2	9	41.07	2.85
2	-2	9	43.05	2.91
2	-2	9	46.19	3.28
2	-2	9	40.48	2.54
-3	2	-9	47.89	3.23
-3	2	-9	42.03	2.35
-3	2	-9	40.09	2.75
-3	2	-9	42.05	3.02
3	-2	9	45.28	3.31
3	-2	9	40.76	2.83
3	-2	9	43.15	2.97
3	-2	9	40.11	2.53
-4	2	-9	21.81	2.63
-4	2	-9	19.36	1.37
-4	2	-9	16.92	1.93
-4	2	-9	18.25	2.34
4	-2	9	18.49	2.29
4	-2	9	18.28	1.64
4	-2	9	16.59	1.94
4	-2	9	18.08	2.90
-5	2	-9	16.65	2.80

-5	2	-9	18.22	2.16
-5	2	-9	18.59	1.40
-5	2	-9	16.85	2.44
5	-2	9	16.88	2.30
5	-2	9	17.99	2.84
5	-2	9	16.87	2.00
5	-2	9	13.65	1.63
-6	2	-9	17.27	3.06
-6	2	-9	15.51	2.63
-6	2	-9	17.85	1.47
-6	2	-9	17.55	2.98
-6	2	-9	15.43	2.35
6	-2	9	16.74	3.04
6	-2	9	18.56	2.23
6	-2	9	20.10	2.56
-7	2	-9	24.37	1.83
-7	2	-9	22.83	3.73
-7	2	-9	23.39	2.82
-7	2	-9	22.35	3.51
-7	2	-9	21.89	2.91
7	-2	9	29.00	2.67
7	-2	9	30.90	5.01
7	-2	9	20.55	3.40
-8	2	-9	10.43	1.50
-8	2	-9	9.84	1.84
-8	2	-9	7.93	2.47
-8	2	-9	8.13	2.95
8	-2	9	7.78	2.22
8	-2	9	13.12	2.55
-9	2	-9	8.71	2.80
-9	2	-9	11.87	1.66
-9	2	-9	7.82	1.89
-9	2	-9	10.37	3.48
-9	2	-9	11.51	2.54
-9	2	-9	12.56	2.43
9	-2	9	9.79	1.44
-10	2	-9	4.51	3.65
-10	2	-9	3.72	1.05
-10	2	-9	7.50	3.23
-10	2	-9	3.17	1.90
-10	2	-9	5.25	4.03
10	-2	9	7.09	1.86
-11	2	-9	7.07	4.65
-11	2	-9	6.43	1.23

-11	2	-9	5.41	3.48
11	-2	9	7.29	2.16
-12	-1	9	17.09	3.55
-11	-1	9	11.14	3.02
-10	-1	9	9.29	2.57
-9	-1	9	17.18	3.59
-8	-1	9	35.04	3.74
-8	-1	9	36.26	3.01
-8	-1	9	40.24	2.89
-7	-1	9	19.84	2.85
6	1	-9	30.26	4.08
-6	-1	9	31.34	2.39
-6	-1	9	30.59	2.87
-6	-1	9	30.49	2.49
5	1	-9	35.61	3.66
-5	-1	9	35.63	2.71
-5	-1	9	34.35	2.92
-5	-1	9	39.58	2.66
4	1	-9	67.47	4.60
4	1	-9	69.11	4.07
-4	-1	9	70.21	3.89
-4	-1	9	66.93	4.11
-4	-1	9	63.82	3.98
3	1	-9	40.03	2.82
3	1	-9	39.17	3.40
-3	-1	9	37.23	2.67
-3	-1	9	30.28	2.72
-3	-1	9	37.92	2.67
2	1	-9	62.08	3.95
2	1	-9	59.53	2.95
2	1	-9	50.58	3.48
-2	-1	9	57.13	3.00
-2	-1	9	54.23	3.48
-2	-1	9	62.21	3.50
1	1	-9	43.46	3.02
1	1	-9	34.21	2.90
1	1	-9	38.82	2.01
1	1	-9	33.30	2.62
-1	-1	9	37.20	2.53
-1	-1	9	38.49	2.10
-1	-1	9	33.88	2.84
-1	-1	9	36.62	2.54
0	1	-9	33.32	1.77
0	1	-9	32.57	2.97

0	1	-9	31.43	2.61
0	1	-9	27.25	2.33
0	-1	9	32.75	2.68
0	-1	9	31.71	2.29
0	-1	9	31.58	1.85
0	-1	9	31.26	2.31
-1	1	-9	32.69	1.84
-1	1	-9	31.77	2.42
-1	1	-9	35.71	2.44
-1	1	-9	36.18	2.67
1	-1	9	32.55	2.37
1	-1	9	34.27	2.77
1	-1	9	30.38	2.34
1	-1	9	33.51	1.96
-2	1	-9	42.32	2.68
-2	1	-9	36.54	2.56
-2	1	-9	37.08	2.09
-2	1	-9	45.37	2.91
2	-1	9	39.10	2.99
2	-1	9	33.25	2.53
2	-1	9	32.64	2.23
2	-1	9	42.46	2.68
-3	1	-9	38.87	2.87
-3	1	-9	32.03	1.95
-3	1	-9	33.89	2.36
-3	1	-9	35.52	2.63
3	-1	9	35.48	2.40
3	-1	9	38.71	2.96
3	-1	9	29.73	2.08
3	-1	9	36.17	2.57
-4	1	-9	45.08	2.86
-4	1	-9	46.78	3.15
-4	1	-9	49.88	3.47
-4	1	-9	43.86	2.52
4	-1	9	48.19	2.95
4	-1	9	41.64	2.63
4	-1	9	46.86	3.12
4	-1	9	46.83	3.47
-5	1	-9	14.22	1.24
-5	1	-9	15.19	2.61
-5	1	-9	14.54	2.12
-5	1	-9	14.93	1.78
5	-1	9	13.63	1.44
5	-1	9	14.51	1.76

5	-1	9	13.34	2.18
5	-1	9	15.13	2.14
-6	1	-9	37.64	2.95
-6	1	-9	36.17	3.57
-6	1	-9	42.45	2.91
-6	1	-9	44.69	3.27
-6	1	-9	33.16	3.47
-6	1	-9	38.67	2.44
6	-1	9	41.40	2.86
6	-1	9	44.12	3.01
6	-1	9	42.74	3.55
-7	1	-9	11.05	1.73
-7	1	-9	8.78	2.08
-7	1	-9	9.84	2.38
-7	1	-9	11.78	2.21
-7	1	-9	11.51	1.35
7	-1	9	8.32	2.04
7	-1	9	9.76	1.60
7	-1	9	11.37	2.10
-8	1	-9	23.11	4.95
-8	1	-9	18.47	4.79
-8	1	-9	28.24	2.09
-8	1	-9	30.50	2.80
8	-1	9	26.45	3.67
8	-1	9	35.39	2.88
-9	1	-9	10.84	3.20
-9	1	-9	14.56	2.56
-9	1	-9	10.75	2.67
-9	1	-9	11.35	1.61
9	-1	9	13.26	2.73
9	-1	9	8.71	2.38
9	-1	9	7.43	1.26
-10	1	-9	8.45	1.15
-10	1	-9	10.97	2.51
-10	1	-9	10.28	3.08
-10	1	-9	8.46	3.82
-10	1	-9	7.83	1.78
10	-1	9	7.51	2.51
10	-1	9	11.86	1.92
10	-1	9	7.78	1.42
-11	1	-9	5.45	4.58
-11	1	-9	3.14	2.25
-11	1	-9	3.57	1.10
-11	1	-9	2.86	3.28

11	-1	9	4.00	2.00
11	-1	9	0.43	1.82
-12	1	-9	12.84	3.79
-12	1	-9	17.39	5.64
-12	1	-9	13.61	2.11
12	-1	9	11.98	2.45
-12	0	9	13.13	3.59
-11	0	9	12.32	3.12
-10	0	9	21.66	4.58
-9	0	9	17.15	3.73
-8	0	9	44.41	3.98
-7	0	9	33.80	2.48
-7	0	9	31.48	3.13
6	0	-9	44.19	4.45
-6	0	9	46.44	3.46
5	0	-9	46.23	4.19
-5	0	9	53.55	3.14
-5	0	9	50.35	3.46
4	0	-9	54.78	3.98
4	0	-9	48.22	3.35
-4	0	9	52.14	3.43
-4	0	9	48.06	3.29
-4	0	9	58.05	3.18
3	0	-9	34.43	2.48
3	0	-9	35.37	2.65
3	0	-9	35.49	3.25
-3	0	9	34.12	2.61
-3	0	9	35.41	2.47
-3	0	9	28.11	2.56
2	0	-9	69.40	4.04
-2	0	9	68.55	4.06
-2	0	9	70.56	3.61
-2	0	9	75.41	3.99
-2	0	9	74.12	4.21
1	0	-9	20.49	2.59
1	0	-9	19.32	2.08
1	0	-9	26.68	1.47
1	0	-9	26.89	2.57
-1	0	9	27.33	1.49
-1	0	9	21.94	2.44
-1	0	9	28.57	2.06
-1	0	9	23.15	2.01
0	0	-9	21.30	2.47
0	0	-9	26.97	1.48

0	0	-9	26.74	2.32
0	0	9	22.62	2.46
0	0	9	24.78	1.50
0	0	9	23.67	2.05
0	0	9	26.23	2.01
-1	0	-9	50.06	3.16
-1	0	-9	51.85	2.68
-1	0	-9	52.24	3.10
-1	0	-9	57.00	3.38
1	0	9	48.07	2.72
1	0	9	49.23	3.06
1	0	9	51.57	3.10
1	0	9	51.76	3.44
-2	0	-9	59.06	3.52
-2	0	-9	53.61	3.23
-2	0	-9	55.96	2.82
-2	0	-9	50.50	3.47
2	0	9	48.51	3.16
2	0	9	53.76	3.56
2	0	9	55.46	3.26
2	0	9	54.85	2.88
-3	0	-9	31.42	1.78
-3	0	-9	30.64	2.33
-3	0	-9	34.86	2.74
-3	0	-9	27.53	2.18
3	0	9	33.26	2.72
3	0	9	34.01	2.40
3	0	9	27.59	2.20
-4	0	-9	23.79	1.50
-4	0	-9	22.79	2.06
-4	0	-9	23.24	2.52
-4	0	-9	23.72	1.93
4	0	9	22.76	1.94
4	0	9	23.37	2.53
4	0	9	24.38	1.60
4	0	9	24.20	2.18
-5	0	-9	32.11	1.93
-5	0	-9	32.84	2.34
-5	0	-9	31.02	3.03
-5	0	-9	34.39	2.41
5	0	9	29.88	2.02
5	0	9	32.23	3.03
5	0	9	33.67	2.34
5	0	9	34.22	2.57

-6	0	-9	48.67	2.68
-6	0	-9	48.86	3.11
-6	0	-9	48.67	3.02
-6	0	-9	49.94	3.85
6	0	9	49.25	3.06
6	0	9	52.43	3.30
6	0	9	41.48	2.77
6	0	9	47.21	3.75
-7	0	-9	30.86	3.47
-7	0	-9	23.87	3.51
-7	0	-9	32.64	2.59
-7	0	-9	32.60	2.42
-7	0	-9	34.10	2.62
7	0	9	30.92	3.12
7	0	9	31.55	3.34
7	0	9	34.13	2.74
-8	0	-9	22.21	2.58
-8	0	-9	20.36	2.40
-8	0	-9	17.79	3.60
-8	0	-9	18.46	1.65
-8	0	-9	17.06	2.51
8	0	9	21.72	2.40
8	0	9	17.30	3.26
8	0	9	20.54	2.63
-9	0	-9	21.40	3.01
-9	0	-9	20.12	2.66
-9	0	-9	20.43	1.86
-9	0	-9	20.60	3.12
-9	0	-9	23.83	2.91
-9	0	-9	20.30	3.79
9	0	9	22.09	3.57
9	0	9	24.48	2.76
-10	0	-9	8.47	1.76
-10	0	-9	8.70	3.62
-10	0	-9	10.91	2.35
-10	0	-9	8.01	2.70
-10	0	-9	11.26	1.64
10	0	9	9.94	1.40
10	0	9	10.06	1.78
10	0	9	9.60	2.42
-11	0	-9	7.15	4.36
-11	0	-9	7.19	2.72
-11	0	-9	9.41	3.15
-11	0	-9	6.31	1.78

-11	0	-9	7.00	1.20
11	0	9	6.12	2.58
11	0	9	6.98	1.94
-12	0	-9	7.41	5.01
-12	0	-9	11.92	1.47
-12	0	-9	13.03	3.56
12	0	9	11.99	2.25
-12	1	9	8.20	3.36
-11	1	9	10.32	3.14
-10	1	9	11.31	2.98
-9	1	9	28.98	4.07
-8	1	9	12.63	2.15
-7	1	9	38.48	3.42
-6	1	9	16.61	2.58
5	-1	-9	40.83	3.87
-5	1	9	40.85	3.14
-4	1	9	32.15	2.36
-4	1	9	31.65	2.63
3	-1	-9	39.23	2.57
3	-1	-9	40.33	3.33
-3	1	9	39.79	2.57
-3	1	9	38.73	2.05
-3	1	9	39.80	2.77
-3	1	9	34.41	2.80
2	-1	-9	24.08	2.63
2	-1	-9	20.76	1.35
2	-1	-9	20.75	2.11
-2	1	9	22.39	1.84
-2	1	9	20.82	2.00
-2	1	9	23.99	2.30
-2	1	9	23.63	1.32
1	-1	-9	38.18	2.99
1	-1	-9	36.84	2.06
1	-1	-9	36.32	2.47
-1	1	9	41.18	2.45
-1	1	9	42.13	2.88
-1	1	9	35.80	2.54
-1	1	9	37.75	2.04
0	-1	-9	54.13	3.26
0	-1	-9	50.42	2.61
0	-1	-9	48.88	3.00
0	-1	-9	47.85	3.31
0	1	9	50.83	2.95
0	1	9	49.83	2.59

0	1	9	48.52	3.00
0	1	9	44.67	3.34
-1	-1	-9	142.56	6.91
-1	-1	-9	140.04	7.38
-1	-1	-9	141.36	7.13
1	1	9	136.09	7.35
1	1	9	143.23	7.17
1	1	9	137.77	6.91
1	1	9	140.31	7.15
-2	-1	-9	32.76	2.33
-2	-1	-9	34.83	1.85
-2	-1	-9	33.46	2.68
-2	-1	-9	30.24	2.35
2	1	9	30.60	2.64
2	1	9	32.73	2.33
2	1	9	32.54	1.86
-3	-1	-9	14.84	1.66
-3	-1	-9	15.67	2.18
-3	-1	-9	19.54	1.18
-3	-1	-9	17.86	1.69
3	1	9	16.22	1.81
3	1	9	16.40	2.22
3	1	9	14.22	1.60
-4	-1	-9	24.90	1.49
-4	-1	-9	23.08	1.88
-4	-1	-9	23.06	2.51
4	1	9	24.40	2.07
4	1	9	21.57	2.45
4	1	9	21.50	1.86
4	1	9	24.42	1.53
-5	-1	-9	38.45	2.52
-5	-1	-9	38.99	2.44
-5	-1	-9	39.17	2.15
-5	-1	-9	36.97	3.11
5	1	9	40.13	2.66
5	1	9	35.84	2.19
5	1	9	33.64	3.04
5	1	9	37.56	2.43
-6	-1	-9	23.51	2.04
-6	-1	-9	21.43	1.88
-6	-1	-9	21.20	1.50
-6	-1	-9	23.11	2.79
6	1	9	21.88	2.16
6	1	9	16.18	2.66

6	1	9	22.66	1.97
6	1	9	19.93	1.58
-7	-1	-9	26.57	3.49
-7	-1	-9	34.31	2.51
-7	-1	-9	30.29	2.33
-7	-1	-9	30.55	3.32
7	1	9	33.27	2.45
7	1	9	30.89	2.65
7	1	9	32.37	3.13
7	1	9	28.33	2.05
-8	-1	-9	5.38	1.01
-8	-1	-9	2.18	2.51
-8	-1	-9	6.68	1.30
-8	-1	-9	5.93	0.90
-8	-1	-9	6.69	1.95
8	1	9	6.56	1.36
8	1	9	5.31	0.93
8	1	9	5.47	1.88
-9	-1	-9	26.24	3.58
-9	-1	-9	16.68	3.33
-9	-1	-9	25.90	2.57
9	1	9	21.14	3.47
9	1	9	26.67	2.68
-10	-1	-9	13.03	2.59
-10	-1	-9	10.59	1.70
-10	-1	-9	12.19	2.17
-10	-1	-9	14.91	1.65
-10	-1	-9	10.46	3.48
10	1	9	9.71	1.31
10	1	9	10.86	2.38
10	1	9	11.58	2.36
10	1	9	10.33	1.73
-11	-1	-9	18.78	3.11
-11	-1	-9	19.15	2.08
-11	-1	-9	18.54	2.87
-11	-1	-9	20.62	2.58
-11	-1	-9	15.67	4.18
11	1	9	19.87	3.24
11	1	9	15.50	2.34
11	1	9	21.47	4.18
-12	-1	-9	5.51	1.88
-12	-1	-9	2.28	4.49
-12	-1	-9	7.32	3.18
-12	-1	-9	6.82	1.28

12	1	9	7.16	1.97
-13	-1	-9	9.70	3.70
-13	-1	-9	9.89	5.44
-13	-1	-9	9.73	1.53
13	1	9	10.53	2.39
-11	2	9	10.70	3.15
-10	2	9	11.40	2.92
-9	2	9	23.07	4.33
-8	2	9	21.18	3.58
-7	2	9	54.66	4.08
-6	2	9	43.23	3.45
4	-2	-9	21.34	3.02
3	-2	-9	43.28	2.35
-3	2	9	45.39	2.31
2	-2	-9	26.62	1.59
-2	2	9	26.72	1.55
-2	2	9	31.00	2.00
1	-2	-9	44.98	2.66
1	-2	-9	45.07	2.36
1	-2	-9	45.64	3.22
1	-2	-9	37.58	2.89
-1	2	9	46.24	2.72
-1	2	9	48.25	3.11
-1	2	9	42.10	2.79
-1	2	9	44.88	2.32
0	-2	-9	100.42	5.59
0	-2	-9	108.53	5.28
0	-2	-9	100.06	5.11
0	2	9	107.48	5.35
0	2	9	103.39	5.36
0	2	9	97.85	5.53
0	2	9	99.42	5.09
-1	-2	-9	278.43	13.61
-1	-2	-9	261.54	13.82
-1	-2	-9	297.06	13.50
1	2	9	275.53	13.79
1	2	9	276.31	13.48
1	2	9	276.46	13.67
-2	-2	-9	159.53	7.19
-2	-2	-9	150.89	7.62
-2	-2	-9	141.11	7.32
2	2	9	143.00	7.53
2	2	9	129.27	7.35
2	2	9	147.70	7.17

2	2	9	148.08	7.42
-3	-2	-9	32.18	2.15
-3	-2	-9	34.39	1.89
-3	-2	-9	32.37	2.67
3	2	9	33.83	1.89
3	2	9	31.46	2.55
3	2	9	33.57	2.31
-4	-2	-9	24.70	2.43
-4	-2	-9	24.52	1.89
-4	-2	-9	25.56	1.52
4	2	9	21.83	1.81
4	2	9	24.42	2.27
4	2	9	24.50	2.02
-5	-2	-9	31.58	1.76
-5	-2	-9	27.07	2.10
-5	-2	-9	28.63	2.70
5	2	9	26.61	2.53
5	2	9	29.61	1.76
5	2	9	27.38	2.25
-6	-2	-9	28.33	2.19
-6	-2	-9	27.60	1.73
-6	-2	-9	28.67	2.84
6	2	9	27.89	2.30
6	2	9	24.57	1.74
6	2	9	27.41	2.65
-7	-2	-9	28.68	1.82
-7	-2	-9	29.84	2.31
-7	-2	-9	31.67	2.97
-7	-2	-9	28.39	2.17
7	2	9	27.36	2.50
7	2	9	26.08	1.85
7	2	9	24.26	3.07
7	2	9	27.63	2.26
-8	-2	-9	32.06	3.32
-8	-2	-9	33.62	2.54
-8	-2	-9	32.13	2.04
-8	-2	-9	30.24	2.34
8	2	9	30.83	3.09
8	2	9	26.83	2.06
8	2	9	32.18	2.54
-9	-2	-9	31.50	2.13
-9	-2	-9	29.84	2.42
-9	-2	-9	33.28	2.71
-9	-2	-9	23.67	4.22

-9	-2	-9	35.65	3.57
9	2	9	30.35	2.77
9	2	9	26.72	2.16
9	2	9	33.47	3.43
-10	-2	-9	10.25	4.92
-10	-2	-9	15.38	3.08
-10	-2	-9	16.42	2.45
-10	-2	-9	15.45	1.71
10	2	9	11.90	2.23
10	2	9	15.48	2.59
10	2	9	13.44	2.62
-11	-2	-9	8.33	2.59
-11	-2	-9	6.24	1.15
-11	-2	-9	6.66	1.36
-11	-2	-9	7.98	1.66
-11	-2	-9	5.72	3.80
11	2	9	8.49	1.79
11	2	9	7.47	2.10
11	2	9	6.69	1.19
11	2	9	7.56	2.28
-12	-2	-9	7.88	1.32
-12	-2	-9	6.80	1.85
-12	-2	-9	7.68	3.02
-12	-2	-9	10.72	4.61
12	2	9	10.26	3.07
12	2	9	6.55	1.38
12	2	9	6.01	2.23
12	2	9	8.95	2.00
-13	-2	-9	6.28	5.38
-13	-2	-9	5.03	3.50
-13	-2	-9	2.97	1.39
13	2	9	6.30	2.21
-11	3	9	4.43	3.25
-10	3	9	8.79	2.97
-9	3	9	10.23	2.72
-8	3	9	30.15	3.95
-7	3	9	20.86	3.22
4	-3	-9	24.26	1.52
3	-3	-9	12.54	1.03
-3	3	9	9.02	1.89
-3	3	9	13.34	0.93
2	-3	-9	23.09	1.42
-2	3	9	23.10	1.36
1	-3	-9	9.52	0.86

-1	3	9	8.28	1.46
-1	3	9	8.83	0.79
-1	3	9	6.40	1.37
0	-3	-9	19.06	1.30
0	-3	-9	21.04	1.99
0	-3	-9	22.67	1.68
0	3	9	19.40	1.24
0	3	9	18.71	1.75
0	3	9	19.03	2.07
-1	-3	-9	38.69	2.18
-1	-3	-9	39.36	2.43
1	3	9	41.07	2.52
1	3	9	39.36	2.14
1	3	9	39.85	2.65
-2	-3	-9	58.74	3.31
-2	-3	-9	62.30	3.19
-2	-3	-9	60.82	4.18
2	3	9	59.23	3.56
2	3	9	63.79	3.47
2	3	9	62.65	3.16
-3	-3	-9	12.17	1.97
-3	-3	-9	12.18	1.04
-3	-3	-9	14.77	1.45
3	3	9	13.66	1.03
3	3	9	11.61	1.76
3	3	9	14.59	1.59
-4	-3	-9	35.00	2.03
-4	-3	-9	37.45	2.77
-4	-3	-9	38.20	2.31
4	3	9	36.23	2.42
4	3	9	35.13	2.58
4	3	9	33.28	2.15
-5	-3	-9	8.32	0.97
-5	-3	-9	10.70	1.42
-5	-3	-9	10.35	1.48
5	3	9	9.72	1.64
5	3	9	8.97	0.99
5	3	9	10.55	1.87
-6	-3	-9	31.42	2.80
-6	-3	-9	33.89	2.30
-6	-3	-9	30.79	1.91
6	3	9	30.69	1.89
6	3	9	31.84	2.67
6	3	9	31.20	2.48

-7	-3	-9	14.08	1.81
-7	-3	-9	14.54	1.28
-7	-3	-9	16.40	2.59
7	3	9	12.85	1.26
7	3	9	14.65	2.33
7	3	9	13.80	2.02
-8	-3	-9	10.95	1.85
-8	-3	-9	9.50	1.28
-8	-3	-9	11.40	1.89
8	3	9	11.91	1.86
8	3	9	9.10	1.22
8	3	9	9.95	1.75
-9	-3	-9	4.05	0.99
-9	-3	-9	5.71	1.25
-9	-3	-9	3.69	1.31
-9	-3	-9	2.88	2.77
-9	-3	-9	4.23	1.85
9	3	9	6.20	1.36
9	3	9	3.80	0.82
9	3	9	6.31	1.69
-10	-3	-9	29.82	2.20
-10	-3	-9	29.56	2.72
-10	-3	-9	23.32	4.56
-10	-3	-9	31.68	3.65
10	3	9	32.46	3.00
10	3	9	29.69	2.95
10	3	9	28.87	3.46
-11	-3	-9	9.99	1.92
-11	-3	-9	14.73	2.60
-11	-3	-9	14.76	2.56
-11	-3	-9	8.19	3.67
-11	-3	-9	12.44	1.83
11	3	9	10.88	1.79
11	3	9	7.96	1.56
11	3	9	9.76	2.17
11	3	9	15.05	2.80
-12	-3	-9	9.58	4.52
-12	-3	-9	10.67	2.91
-12	-3	-9	14.09	2.55
-12	-3	-9	12.67	2.06
-12	-3	-9	9.55	1.87
12	3	9	8.98	1.41
12	3	9	8.22	2.47
12	3	9	10.02	2.33

12	3	9	11.82	2.01
-13	-3	-9	5.94	5.14
-13	-3	-9	2.27	3.23
-13	-3	-9	-0.17	2.04
-13	-3	-9	0.25	1.28
13	3	9	0.05	2.48
13	3	9	0.75	2.08
-14	-3	-9	9.32	6.13
-14	-3	-9	5.93	3.84
-14	-3	-9	5.14	1.60
14	3	9	4.71	2.40
-10	4	9	8.56	2.58
-10	4	9	11.13	3.18
-9	4	9	10.99	2.86
-9	4	9	10.53	2.02
-8	4	9	19.37	3.96
-7	4	9	55.42	4.35
-5	4	9	38.04	2.09
4	-4	-9	46.88	2.61
-4	4	9	48.53	2.52
3	-4	-9	45.56	2.50
2	-4	-9	58.74	3.00
-2	4	9	55.08	2.92
1	-4	-9	50.77	2.75
-1	4	9	53.43	2.71
0	-4	-9	96.92	4.84
0	4	9	91.92	5.15
0	4	9	100.47	4.81
-1	-4	-9	36.24	2.47
-1	-4	-9	32.58	2.14
-1	-4	-9	36.32	1.97
1	4	9	35.31	2.39
1	4	9	34.38	2.28
1	4	9	34.85	1.92
-2	-4	-9	25.86	1.56
-2	-4	-9	25.27	1.76
2	4	9	26.43	1.51
2	4	9	24.88	1.90
2	4	9	26.03	2.03
-3	-4	-9	73.42	4.57
-3	-4	-9	76.30	3.73
-3	-4	-9	69.85	3.85
3	4	9	72.67	3.69
3	4	9	73.38	3.97

3	4	9	69.20	4.02
-4	-4	-9	49.96	2.77
-4	-4	-9	54.92	3.30
-4	-4	-9	54.03	2.98
4	4	9	53.51	3.12
4	4	9	47.15	2.93
4	4	9	53.87	3.20
-5	-4	-9	31.44	2.54
-5	-4	-9	27.83	1.79
-5	-4	-9	31.68	2.12
5	4	9	31.15	2.29
5	4	9	29.85	2.39
5	4	9	29.83	1.79
-6	-4	-9	45.51	2.79
-6	-4	-9	48.47	3.25
-6	-4	-9	42.75	2.50
6	4	9	43.75	2.48
6	4	9	46.67	3.06
6	4	9	43.52	2.94
-7	-4	-9	60.85	3.89
-7	-4	-9	55.97	3.15
-7	-4	-9	59.49	3.46
7	4	9	60.24	3.73
7	4	9	54.56	3.13
-8	-4	-9	19.51	2.08
-8	-4	-9	15.97	1.46
-8	-4	-9	18.80	2.70
8	4	9	18.79	2.48
8	4	9	16.51	1.42
-9	-4	-9	20.42	2.29
-9	-4	-9	17.91	1.78
-9	-4	-9	20.58	3.03
-9	-4	-9	20.07	3.79
9	4	9	19.74	2.75
9	4	9	19.83	1.59
9	4	9	19.35	2.42
-10	-4	-9	21.73	1.88
-10	-4	-9	19.62	3.39
-10	-4	-9	22.63	2.55
-10	-4	-9	23.71	3.25
10	4	9	18.70	1.74
10	4	9	23.98	2.66
10	4	9	21.09	3.16
-11	-4	-9	22.42	2.13

-11	-4	-9	25.81	3.78
-11	-4	-9	22.81	2.83
-11	-4	-9	23.49	4.85
11	4	9	26.12	3.12
11	4	9	25.30	3.45
-12	-4	-9	9.52	2.65
-12	-4	-9	8.26	1.81
-12	-4	-9	9.73	4.24
-12	-4	-9	7.27	1.32
-12	-4	-9	9.13	1.53
12	4	9	10.81	2.07
12	4	9	9.57	2.30
12	4	9	10.16	1.94
12	4	9	8.63	1.29
-13	-4	-9	9.10	2.04
-13	-4	-9	11.42	3.21
-13	-4	-9	10.25	1.49
-13	-4	-9	13.20	4.89
13	4	9	9.88	1.44
13	4	9	5.15	2.29
-14	-4	-9	3.33	3.69
-14	-4	-9	8.56	5.63
-14	-4	-9	3.49	1.50
14	4	9	4.86	2.36
-10	5	9	5.19	3.16
-10	5	9	0.72	2.68
-9	5	9	10.33	2.92
-8	5	9	3.19	2.38
-8	5	9	4.27	0.90
-7	5	9	36.91	4.16
-7	5	9	31.87	2.38
-7	5	9	39.49	2.27
-6	5	9	25.75	1.81
-5	5	9	28.51	1.75
-4	5	9	14.87	1.17
3	-5	-9	45.70	2.53
-3	5	9	45.57	2.45
2	-5	-9	15.38	1.24
-2	5	9	16.21	1.14
1	-5	-9	29.59	1.79
-1	5	9	29.93	1.71
0	-5	-9	3.93	0.55
0	5	9	3.82	0.50
-1	-5	-9	50.65	2.67

1	5	9	46.92	2.88
1	5	9	51.68	2.63
-2	-5	-9	25.45	1.70
-2	-5	-9	23.01	2.07
-2	-5	-9	24.56	1.53
2	5	9	25.13	1.49
2	5	9	24.98	2.02
2	5	9	25.24	1.90
-3	-5	-9	22.53	1.70
-3	-5	-9	21.49	1.43
3	5	9	21.77	1.97
3	5	9	24.01	1.41
3	5	9	22.20	1.86
-4	-5	-9	8.87	1.38
-4	-5	-9	8.80	0.96
-4	-5	-9	10.88	1.38
4	5	9	10.40	1.59
4	5	9	9.41	1.23
4	5	9	9.70	1.66
-5	-5	-9	13.91	1.54
-5	-5	-9	11.93	1.08
-5	-5	-9	13.69	2.00
5	5	9	9.43	1.28
5	5	9	13.99	1.86
-6	-5	-9	15.71	1.30
-6	-5	-9	17.59	1.77
-6	-5	-9	16.43	2.24
6	5	9	18.77	2.07
6	5	9	16.76	1.28
-7	-5	-9	37.78	2.41
-7	-5	-9	46.56	2.83
-7	-5	-9	45.50	3.26
7	5	9	44.23	3.06
7	5	9	40.38	2.42
-8	-5	-9	14.25	2.60
-8	-5	-9	12.60	1.91
-8	-5	-9	9.85	1.27
8	5	9	12.71	1.28
8	5	9	12.26	2.33
8	5	9	14.89	2.12
-9	-5	-9	20.46	2.39
-9	-5	-9	16.45	3.51
-9	-5	-9	20.61	2.95
-9	-5	-9	20.71	1.74

9	5	9	26.04	2.52
9	5	9	22.89	1.67
9	5	9	22.99	3.18
-10	-5	-9	10.62	1.60
-10	-5	-9	11.70	2.11
-10	-5	-9	10.63	2.96
-10	-5	-9	13.72	2.37
10	5	9	10.96	1.91
10	5	9	10.85	1.41
10	5	9	11.63	1.74
10	5	9	13.22	2.32
-11	-5	-9	12.54	4.10
-11	-5	-9	16.66	2.67
-11	-5	-9	14.86	2.46
-11	-5	-9	16.57	2.26
-11	-5	-9	14.14	1.87
11	5	9	13.59	2.22
11	5	9	19.70	2.79
-12	-5	-9	-3.56	3.96
-12	-5	-9	1.03	1.66
-12	-5	-9	-0.98	2.53
-12	-5	-9	0.72	1.13
12	5	9	1.34	1.06
12	5	9	1.10	2.10
12	5	9	2.68	1.80
-13	-5	-9	4.44	1.95
-13	-5	-9	1.86	4.71
-13	-5	-9	3.78	3.05
-13	-5	-9	4.39	1.37
-13	-5	-9	6.74	1.73
13	5	9	6.85	2.66
13	5	9	7.32	2.27
13	5	9	6.02	1.32
-14	-5	-9	-3.20	5.59
-14	-5	-9	3.99	3.53
-14	-5	-9	2.70	1.52
-9	6	9	16.76	2.32
-9	6	9	14.96	3.27
-9	6	9	22.41	1.87
-8	6	9	10.38	1.76
-8	6	9	6.50	2.67
-7	6	9	17.28	1.47
-7	6	9	11.15	2.40
-7	6	9	13.95	1.80

-6	6	9	29.60	1.94
-5	6	9	25.04	1.68
-4	6	9	57.45	3.06
-3	6	9	40.35	2.29
2	-6	-9	53.97	2.95
1	-6	-9	68.22	3.43
-1	6	9	61.44	3.47
0	-6	-9	51.94	2.68
0	6	9	45.62	2.69
-1	-6	-9	39.22	2.13
1	6	9	36.85	2.39
1	6	9	35.94	2.20
-2	-6	-9	54.20	2.89
2	6	9	53.20	3.23
2	6	9	55.38	3.18
-3	-6	-9	34.73	2.01
3	6	9	33.54	2.48
3	6	9	37.29	2.00
3	6	9	34.31	2.41
-4	-6	-9	25.41	2.50
-4	-6	-9	28.54	1.72
-4	-6	-9	29.71	2.03
4	6	9	30.13	1.69
4	6	9	27.66	2.31
-5	-6	-9	20.20	2.41
-5	-6	-9	21.36	1.87
-5	-6	-9	23.33	1.46
5	6	9	22.47	1.42
5	6	9	19.59	2.15
-6	-6	-9	31.20	2.83
-6	-6	-9	32.05	1.97
-6	-6	-9	35.69	2.34
6	6	9	32.01	1.94
6	6	9	33.62	2.53
6	6	9	32.33	2.65
-7	-6	-9	13.33	1.30
-7	-6	-9	14.59	2.40
-7	-6	-9	15.81	1.87
7	6	9	15.15	1.29
7	6	9	16.08	2.21
7	6	9	18.03	2.11
-8	-6	-9	21.23	2.74
-8	-6	-9	22.86	2.23
-8	-6	-9	22.38	1.63

8	6	9	21.32	2.41
8	6	9	22.14	1.61
8	6	9	20.59	2.55
-9	-6	-9	17.23	1.56
-9	-6	-9	14.51	2.04
-9	-6	-9	18.81	2.30
9	6	9	17.76	2.65
9	6	9	17.41	1.50
9	6	9	17.05	2.43
-10	-6	-9	26.06	2.09
-10	-6	-9	26.02	3.46
-10	-6	-9	28.60	2.78
10	6	9	26.05	1.95
10	6	9	30.94	2.96
10	6	9	21.98	3.49
10	6	9	30.63	3.12
-11	-6	-9	2.61	2.15
-11	-6	-9	4.43	1.04
-11	-6	-9	2.07	1.57
11	6	9	2.44	2.51
11	6	9	5.36	1.67
11	6	9	5.33	1.98
-12	-6	-9	4.24	2.75
-12	-6	-9	3.94	1.13
-12	-6	-9	2.70	3.62
-12	-6	-9	4.89	1.78
12	6	9	2.03	1.88
12	6	9	2.38	2.06
12	6	9	7.57	2.67
-13	-6	-9	3.39	1.27
-13	-6	-9	2.60	1.58
-13	-6	-9	3.09	2.83
-13	-6	-9	0.38	4.41
-13	-6	-9	1.23	1.92
13	6	9	2.51	2.24
13	6	9	3.79	2.39
13	6	9	0.70	1.14
-14	-6	-9	8.54	1.57
-14	-6	-9	5.18	3.55
-14	-6	-9	11.94	5.32
14	6	9	10.39	1.73
-9	7	9	9.37	1.48
-9	7	9	8.52	3.33
-9	7	9	12.77	1.79

-9	7	9	10.08	3.24
-8	7	9	25.78	2.05
-8	7	9	23.86	4.99
-8	7	9	22.26	2.37
-7	7	9	13.02	2.61
-7	7	9	14.21	2.14
-7	7	9	18.10	1.63
-6	7	9	11.71	1.41
-6	7	9	8.66	2.14
-6	7	9	13.56	1.73
-5	7	9	5.48	0.77
-4	7	9	28.72	1.87
-3	7	9	22.55	1.56
2	-7	-9	37.19	2.23
-2	7	9	36.45	2.13
1	-7	-9	39.26	2.33
-1	7	9	39.74	2.23
0	-7	-9	77.27	4.02
0	7	9	77.19	3.95
-1	-7	-9	17.99	1.42
1	7	9	20.67	1.34
-2	-7	-9	64.47	3.37
2	7	9	61.15	3.86
2	7	9	65.45	3.32
-3	-7	-9	35.52	2.22
-3	-7	-9	32.44	2.58
-3	-7	-9	36.80	2.06
3	7	9	35.38	2.54
3	7	9	36.27	2.02
-4	-7	-9	37.06	2.14
-4	-7	-9	36.95	2.37
4	7	9	38.58	2.12
4	7	9	36.70	2.58
4	7	9	36.67	2.65
-5	-7	-9	26.85	2.27
-5	-7	-9	24.19	3.14
-5	-7	-9	29.67	1.78
5	7	9	29.34	1.74
5	7	9	30.78	2.44
5	7	9	29.61	2.33
-6	-7	-9	51.65	2.87
-6	-7	-9	51.08	3.55
6	7	9	52.27	2.84
6	7	9	55.83	3.34

6	7	9	52.12	3.43
-7	-7	-9	8.28	1.33
-7	-7	-9	8.28	1.54
-7	-7	-9	9.38	1.11
7	7	9	11.54	2.15
7	7	9	7.79	1.06
7	7	9	9.12	1.33
-8	-7	-9	21.77	2.80
-8	-7	-9	20.00	2.17
-8	-7	-9	20.89	1.56
8	7	9	19.42	1.52
8	7	9	18.38	2.41
8	7	9	21.77	2.59
-9	-7	-9	20.99	2.35
-9	-7	-9	18.49	1.60
-9	-7	-9	19.35	2.99
9	7	9	22.41	2.78
9	7	9	20.37	2.55
9	7	9	17.50	1.57
-10	-7	-9	10.40	2.55
-10	-7	-9	13.22	2.40
-10	-7	-9	8.07	2.08
-10	-7	-9	11.64	1.50
10	7	9	13.63	2.62
10	7	9	11.20	1.41
10	7	9	10.10	2.40
10	7	9	10.15	1.98
-11	-7	-9	1.10	0.87
-11	-7	-9	-0.30	1.51
-11	-7	-9	-1.44	2.08
11	7	9	-2.09	2.47
11	7	9	-1.04	1.63
11	7	9	-0.14	1.98
-12	-7	-9	2.89	1.75
-12	-7	-9	4.75	1.34
-12	-7	-9	2.22	3.44
-12	-7	-9	6.27	1.13
-12	-7	-9	0.18	2.40
12	7	9	2.44	1.92
12	7	9	5.87	2.84
12	7	9	4.12	2.22
-13	-7	-9	1.68	4.21
-13	-7	-9	5.93	1.24
-13	-7	-9	3.29	1.98

-13	-7	-9	1.77	2.94
13	7	9	3.92	3.20
13	7	9	2.95	2.45
13	7	9	2.14	1.08
13	7	9	7.46	2.28
-14	-7	-9	6.21	2.46
-14	-7	-9	7.05	1.41
-14	-7	-9	8.25	5.10
-14	-7	-9	9.24	3.57
-14	-7	-9	5.68	1.89
14	7	9	1.80	2.40
-15	-7	-9	3.82	1.42
-15	-7	-9	0.38	4.16
-15	-7	-9	0.76	5.67
-8	8	9	15.25	3.27
-8	8	9	21.44	2.11
-8	8	9	19.62	2.51
-7	8	9	19.89	1.87
-7	8	9	13.38	2.78
-7	8	9	19.57	2.25
-6	8	9	19.94	3.72
-6	8	9	20.63	1.79
-6	8	9	18.33	2.11
-5	8	9	36.11	2.29
-4	8	9	23.78	1.76
-3	8	9	49.88	2.80
-2	8	9	15.96	1.37
1	-8	-9	47.26	2.68
-1	8	9	44.19	2.55
0	-8	-9	41.59	2.46
0	8	9	41.85	2.36
-1	-8	-9	79.72	4.08
1	8	9	75.42	3.99
-2	-8	-9	40.68	2.37
2	8	9	39.71	2.27
2	8	9	40.87	2.95
-3	-8	-9	74.49	3.90
-3	-8	-9	68.20	4.26
3	8	9	77.34	3.86
3	8	9	76.47	4.16
3	8	9	76.48	4.27
-4	-8	-9	24.30	1.88
-4	-8	-9	19.80	2.40
-4	-8	-9	24.54	1.62

4	8	9	23.74	1.56
4	8	9	25.19	2.11
4	8	9	23.96	2.27
-5	-8	-9	65.38	3.50
-5	-8	-9	63.88	3.68
5	8	9	66.04	3.46
5	8	9	64.91	3.95
5	8	9	69.24	3.89
-6	-8	-9	41.14	2.69
-6	-8	-9	39.30	3.41
-6	-8	-9	41.51	2.39
6	8	9	41.63	3.07
6	8	9	39.77	2.34
6	8	9	45.21	2.99
-7	-8	-9	28.73	1.87
-7	-8	-9	26.52	2.96
-7	-8	-9	29.24	2.32
7	8	9	29.98	2.62
7	8	9	29.65	2.75
7	8	9	29.40	1.86
-8	-8	-9	14.68	1.99
-8	-8	-9	17.14	1.44
-8	-8	-9	16.22	2.09
8	8	9	15.89	2.56
8	8	9	18.01	2.40
8	8	9	13.86	1.34
-9	-8	-9	32.20	2.76
-9	-8	-9	30.79	2.69
-9	-8	-9	29.09	3.32
9	8	9	34.69	3.20
9	8	9	32.47	3.04
9	8	9	30.48	2.05
-10	-8	-9	9.40	2.17
-10	-8	-9	15.24	2.53
-10	-8	-9	12.43	2.62
-10	-8	-9	15.91	1.53
10	8	9	10.21	2.52
10	8	9	12.13	1.82
10	8	9	12.74	1.44
-11	-8	-9	24.55	3.90
-11	-8	-9	31.47	2.25
-11	-8	-9	31.78	3.51
11	8	9	31.47	3.69
11	8	9	28.75	3.45

11	8	9	31.58	4.07
-12	-8	-9	7.23	2.66
-12	-8	-9	7.92	1.17
-12	-8	-9	9.45	3.45
-12	-8	-9	6.21	2.27
12	8	9	5.55	2.10
12	8	9	8.47	2.44
12	8	9	9.28	2.91
-13	-8	-9	7.46	4.25
-13	-8	-9	9.09	1.29
-13	-8	-9	8.90	2.41
-13	-8	-9	9.42	4.05
13	8	9	6.08	1.19
13	8	9	5.41	3.32
13	8	9	11.23	2.42
13	8	9	6.04	2.74
-14	-8	-9	3.59	3.48
-14	-8	-9	0.74	4.77
-14	-8	-9	1.99	1.81
-14	-8	-9	4.36	2.44
-14	-8	-9	4.46	1.31
14	8	9	1.85	1.18
14	8	9	2.74	2.63
14	8	9	1.53	3.03
-15	-8	-9	9.47	4.27
-15	-8	-9	-5.75	5.38
-15	-8	-9	6.87	1.48
-7	9	9	13.66	2.15
-7	9	9	16.05	2.42
-7	9	9	8.05	3.00
-6	9	9	11.78	2.58
-6	9	9	12.73	1.71
-6	9	9	9.91	1.40
-5	9	9	36.05	2.47
-5	9	9	40.65	2.88
-4	9	9	24.29	1.84
-3	9	9	14.03	1.42
-2	9	9	14.69	1.42
-1	9	9	15.04	1.36
0	-9	-9	10.22	1.29
0	9	9	9.63	1.16
-1	-9	-9	31.75	2.03
1	9	9	30.21	1.92
-2	-9	-9	12.15	1.30

2	9	9	11.33	1.17
-3	-9	-9	26.28	1.76
3	9	9	24.26	2.20
3	9	9	26.11	1.68
-4	-9	-9	9.41	1.49
-4	-9	-9	9.57	1.15
-4	-9	-9	6.36	1.41
4	9	9	9.18	1.08
4	9	9	8.46	1.39
4	9	9	9.29	1.31
-5	-9	-9	14.08	1.70
-5	-9	-9	13.95	1.33
5	9	9	12.36	2.22
5	9	9	15.01	1.27
5	9	9	14.88	2.11
-6	-9	-9	11.84	1.26
-6	-9	-9	10.90	1.72
6	9	9	13.01	2.11
6	9	9	10.24	1.55
6	9	9	10.50	1.20
-7	-9	-9	15.98	1.90
-7	-9	-9	12.72	1.88
-7	-9	-9	13.97	1.37
7	9	9	13.43	2.52
7	9	9	12.03	1.27
7	9	9	16.41	2.33
-8	-9	-9	12.92	2.24
-8	-9	-9	17.45	2.17
-8	-9	-9	13.52	1.98
8	9	9	14.41	2.47
8	9	9	12.45	1.36
8	9	9	14.40	2.67
-9	-9	-9	22.24	2.59
-9	-9	-9	20.61	1.74
-9	-9	-9	19.93	3.18
9	9	9	22.66	2.85
9	9	9	21.95	3.09
9	9	9	17.76	1.60
-10	-9	-9	-0.16	2.30
-10	-9	-9	4.88	1.54
-10	-9	-9	3.99	0.85
-10	-9	-9	2.79	2.09
10	9	9	2.94	1.66
10	9	9	1.23	2.51

10	9	9	2.70	1.91
-11	-9	-9	21.07	3.54
-11	-9	-9	24.66	2.05
-11	-9	-9	25.19	3.91
-11	-9	-9	24.57	3.18
11	9	9	24.19	4.24
11	9	9	28.27	3.50
11	9	9	23.86	3.65
-12	-9	-9	25.24	3.43
-12	-9	-9	23.55	4.33
-12	-9	-9	22.84	4.65
-12	-9	-9	25.84	2.13
12	9	9	16.19	2.84
12	9	9	28.55	4.66
12	9	9	22.54	3.70
12	9	9	18.06	2.23
-13	-9	-9	9.99	3.12
-13	-9	-9	8.76	2.19
-13	-9	-9	11.24	4.04
13	9	9	6.32	2.68
13	9	9	13.10	3.56
13	9	9	8.10	2.43
13	9	9	6.37	1.19
-14	-9	-9	-1.19	4.54
-14	-9	-9	0.96	2.39
-14	-9	-9	0.19	3.49
14	9	9	4.45	2.70
14	9	9	2.37	3.12
14	9	9	1.99	1.20
-15	-9	-9	3.33	2.09
-15	-9	-9	7.25	4.38
-15	-9	-9	2.11	5.44
-15	-9	-9	5.09	1.44
-6	10	9	14.15	3.04
-6	10	9	19.13	2.68
-6	10	9	20.23	2.62
-5	10	9	21.34	2.07
-5	10	9	24.15	2.58
-4	10	9	39.09	2.52
-3	10	9	13.12	1.49
-2	10	9	44.33	2.63
-1	10	9	22.00	1.67
0	10	9	28.57	1.90
-1	-10	-9	23.59	1.78

1	10	9	21.70	1.64
-2	-10	-9	54.83	3.86
2	10	9	63.66	3.25
3	10	9	28.23	2.84
-4	-10	-9	43.67	2.83
4	10	9	47.37	3.36
4	10	9	48.39	3.23
-5	-10	-9	23.97	2.80
-5	-10	-9	27.75	2.17
5	10	9	29.93	2.76
5	10	9	29.52	2.65
-6	-10	-9	38.75	2.73
6	10	9	41.09	3.13
6	10	9	40.81	3.25
6	10	9	39.36	2.37
-7	-10	-9	8.80	1.97
-7	-10	-9	11.27	1.40
-7	-10	-9	11.67	1.96
7	10	9	13.40	2.57
7	10	9	11.33	1.64
7	10	9	10.95	1.30
-8	-10	-9	28.94	2.01
-8	-10	-9	30.64	2.67
-8	-10	-9	23.97	3.33
8	10	9	31.17	3.07
8	10	9	27.24	3.21
8	10	9	24.52	1.85
-9	-10	-9	17.34	3.29
-9	-10	-9	19.68	2.70
-9	-10	-9	17.44	1.71
9	10	9	18.09	3.17
9	10	9	20.69	2.98
-10	-10	-9	11.52	1.82
-10	-10	-9	8.92	1.56
-10	-10	-9	11.35	2.54
-10	-10	-9	6.79	2.24
10	10	9	8.18	2.67
10	10	9	10.05	2.19
10	10	9	8.55	1.89
-11	-10	-9	7.66	2.78
-11	-10	-9	5.88	1.07
-11	-10	-9	3.83	2.39
-11	-10	-9	5.59	1.88
11	10	9	4.34	2.22

11	10	9	4.70	1.92
11	10	9	4.41	3.00
-12	-10	-9	23.80	3.71
-12	-10	-9	24.21	2.15
-12	-10	-9	16.47	3.10
-12	-10	-9	19.01	3.60
12	10	9	25.47	4.28
12	10	9	22.19	3.79
12	10	9	21.29	2.13
12	10	9	25.75	4.99
-13	-10	-9	2.86	3.84
-13	-10	-9	3.83	3.11
-13	-10	-9	5.02	2.28
-13	-10	-9	5.71	1.25
13	10	9	-0.16	2.78
13	10	9	5.21	1.20
13	10	9	3.30	2.45
13	10	9	7.50	3.77
-14	-10	-9	6.94	2.68
-14	-10	-9	5.89	4.62
-14	-10	-9	9.97	1.45
-14	-10	-9	8.59	3.78
14	10	9	5.00	3.75
14	10	9	6.60	2.76
14	10	9	6.20	1.37
-5	11	9	11.16	1.60
-4	11	9	13.61	1.69
-3	11	9	10.42	1.61
-2	11	9	12.26	1.48
-1	11	9	16.75	1.56
0	11	9	12.81	1.40
1	11	9	5.55	0.78
-2	-11	-9	9.82	1.47
-3	-11	-9	8.13	0.98
3	11	9	7.52	1.30
3	11	9	5.80	1.47
-4	-11	-9	33.34	2.29
4	11	9	33.79	3.13
-5	-11	-9	19.84	1.76
-5	-11	-9	15.42	2.55
5	11	9	18.87	2.67
5	11	9	20.64	2.52
-6	-11	-9	20.59	2.12
-6	-11	-9	19.07	1.75

6	11	9	21.66	2.89
-7	-11	-9	5.73	0.94
-7	-11	-9	6.57	1.27
7	11	9	6.83	1.72
7	11	9	7.02	1.61
-8	-11	-9	7.22	1.96
-8	-11	-9	10.67	1.53
-8	-11	-9	12.75	2.33
8	11	9	11.46	1.83
8	11	9	12.05	1.97
-9	-11	-9	4.48	2.02
-9	-11	-9	7.06	1.04
-9	-11	-9	7.68	1.65
-9	-11	-9	9.31	2.42
9	11	9	5.52	2.45
9	11	9	7.50	1.95
9	11	9	9.10	1.81
-10	-11	-9	3.81	1.70
-10	-11	-9	3.53	2.25
-10	-11	-9	4.93	1.02
-10	-11	-9	4.21	3.16
10	11	9	3.38	1.79
10	11	9	6.22	2.65
10	11	9	3.80	1.38
10	11	9	4.27	2.08
-11	-11	-9	4.97	1.93
-11	-11	-9	3.82	1.06
-11	-11	-9	2.41	2.90
-11	-11	-9	4.94	2.58
11	11	9	3.14	0.99
11	11	9	2.23	1.92
11	11	9	5.05	2.43
11	11	9	0.98	3.07
-12	-11	-9	1.78	2.91
-12	-11	-9	5.34	2.15
-12	-11	-9	9.94	3.30
-12	-11	-9	6.51	1.21
12	11	9	4.06	1.11
12	11	9	4.61	2.27
12	11	9	6.26	2.77
12	11	9	5.92	3.60
-13	-11	-9	4.70	3.81
-13	-11	-9	4.82	1.28
-13	-11	-9	4.56	2.41

-13	-11	-9	-3.72	3.30
13	11	9	2.44	1.15
13	11	9	2.89	3.05
13	11	9	1.86	2.54
13	11	9	9.34	4.17
-14	-11	-9	2.90	5.21
-14	-11	-9	4.93	1.41
-14	-11	-9	3.12	2.75
-14	-11	-9	0.51	4.50
14	11	9	1.37	2.88
14	11	9	1.03	3.40
-4	12	9	20.81	2.93
-1	12	9	31.52	2.21
0	12	9	26.27	1.99
1	12	9	38.56	2.41
2	12	9	4.41	0.75
-3	-12	-9	25.67	2.09
-4	-12	-9	36.23	2.54
4	12	9	41.58	3.06
-5	-12	-9	78.04	4.28
5	12	9	78.81	4.73
5	12	9	75.11	4.86
-6	-12	-9	20.92	1.89
-6	-12	-9	16.21	2.75
6	12	9	18.88	2.73
6	12	9	18.17	2.89
-7	-12	-9	20.70	2.33
-7	-12	-9	23.14	1.97
7	12	9	19.81	1.89
7	12	9	18.37	2.91
7	12	9	17.14	3.13
-8	-12	-9	16.68	1.80
-8	-12	-9	16.62	2.46
-8	-12	-9	10.98	2.35
8	12	9	18.89	3.01
8	12	9	13.63	1.60
8	12	9	12.95	2.20
-9	-12	-9	10.20	2.30
-9	-12	-9	16.85	1.85
-9	-12	-9	16.28	2.73
9	12	9	16.98	2.71
9	12	9	12.30	2.10
9	12	9	13.42	2.37
9	12	9	15.38	1.60

-10	-12	-9	4.49	1.04
-10	-12	-9	1.74	2.68
-10	-12	-9	2.37	2.31
-10	-12	-9	3.28	1.71
10	12	9	3.42	2.18
10	12	9	2.15	2.53
10	12	9	3.77	0.91
10	12	9	2.43	2.79
-11	-12	-9	8.71	2.83
-11	-12	-9	16.90	2.03
-11	-12	-9	17.23	3.35
-11	-12	-9	13.16	2.24
11	12	9	24.83	4.74
11	12	9	13.85	1.88
-12	-12	-9	4.76	3.13
-12	-12	-9	7.36	1.35
-12	-12	-9	4.49	3.58
-12	-12	-9	7.83	2.34
12	12	9	11.27	1.93
12	12	9	6.28	3.81
12	12	9	12.03	2.99
12	12	9	6.90	2.86
-13	-12	-9	7.08	2.55
-13	-12	-9	4.83	3.56
-13	-12	-9	7.90	4.00
-13	-12	-9	4.74	1.38
13	12	9	3.14	2.69
13	12	9	11.57	3.44
-14	-12	-9	2.94	4.33
-14	-12	-9	3.63	1.44
-14	-12	-9	1.59	2.88
-14	-12	-9	5.43	5.05
14	12	9	-0.13	2.93
14	12	9	-1.26	3.75
-3	13	9	9.91	1.69
2	13	9	16.01	1.64
3	13	9	5.72	0.86
-4	-13	-9	13.31	1.81
-5	-13	-9	15.50	1.83
5	13	9	14.11	1.79
5	13	9	15.21	2.56
5	13	9	15.97	2.90
-6	-13	-9	13.53	1.85
6	13	9	13.20	1.92

6	13	9	11.77	1.54
6	13	9	14.81	2.70
-7	-13	-9	7.39	1.41
-7	-13	-9	7.49	1.14
7	13	9	8.89	1.90
7	13	9	9.69	1.75
7	13	9	7.57	0.99
-8	-13	-9	8.07	3.13
-8	-13	-9	11.12	1.90
-8	-13	-9	9.47	1.63
8	13	9	9.07	1.57
8	13	9	10.77	1.96
8	13	9	12.72	2.71
8	13	9	10.24	2.09
-9	-13	-9	11.50	1.92
-9	-13	-9	8.32	1.73
-9	-13	-9	7.96	2.17
-9	-13	-9	11.61	2.99
9	13	9	7.69	1.11
9	13	9	8.26	2.80
9	13	9	10.69	2.33
9	13	9	10.05	2.11
-10	-13	-9	7.19	3.38
-10	-13	-9	1.77	2.43
-10	-13	-9	4.03	1.15
-10	-13	-9	4.68	1.83
10	13	9	3.02	3.12
10	13	9	4.26	2.06
10	13	9	0.79	2.35
10	13	9	3.75	0.98
-11	-13	-9	11.28	3.48
-11	-13	-9	4.48	1.22
-11	-13	-9	7.34	2.12
-11	-13	-9	3.67	2.74
11	13	9	4.86	1.27
11	13	9	7.82	3.42
11	13	9	6.70	3.83
11	13	9	7.20	2.29
-12	-13	-9	6.65	1.36
-12	-13	-9	0.58	3.08
-12	-13	-9	5.90	2.54
-12	-13	-9	8.52	3.69
12	13	9	10.03	5.07
12	13	9	5.88	2.53

12	13	9	8.98	3.14
-13	-13	-9	1.17	4.27
-13	-13	-9	4.36	3.47
-13	-13	-9	0.23	1.32
-13	-13	-9	3.27	2.70
13	13	9	2.28	3.59
13	13	9	1.50	2.69
4	14	9	26.61	3.35
-5	-14	-9	5.42	1.07
5	14	9	5.18	1.52
5	14	9	3.16	2.75
-6	-14	-9	15.82	2.04
6	14	9	13.69	2.11
6	14	9	13.11	1.89
-7	-14	-9	7.18	1.89
-7	-14	-9	10.10	1.29
7	14	9	10.39	2.64
7	14	9	9.24	1.89
7	14	9	10.44	2.00
-8	-14	-9	18.13	3.07
-8	-14	-9	18.09	2.13
8	14	9	12.83	2.28
8	14	9	12.72	2.27
-9	-14	-9	9.49	1.38
-9	-14	-9	14.20	3.35
-9	-14	-9	5.29	2.10
9	14	9	14.83	3.39
9	14	9	10.94	2.30
9	14	9	8.63	2.38
-10	-14	-9	20.19	4.05
-10	-14	-9	20.86	2.44
10	14	9	16.07	2.61
10	14	9	18.85	3.59
10	14	9	14.65	2.82
-11	-14	-9	7.67	1.41
-11	-14	-9	9.60	4.01
11	14	9	7.63	2.53
11	14	9	7.06	4.97
-12	-14	-9	16.29	4.31
-12	-14	-9	8.44	1.54
12	14	9	8.30	2.85
-3	-15	-9	14.38	2.51
4	15	9	13.56	2.57
4	15	9	11.77	2.42

5	15	9	12.90	2.94
5	15	9	14.95	2.87
6	15	9	6.98	1.75
6	15	9	4.34	2.58
-7	-15	-9	8.88	2.78
-7	-15	-9	7.90	1.30
7	15	9	9.06	2.79
7	15	9	6.33	1.89
-8	-15	-9	0.93	1.13
-8	-15	-9	1.17	3.14
8	15	9	3.57	1.94
8	15	9	2.00	3.03
-9	-15	-9	8.84	3.53
-9	-15	-9	4.47	1.27
9	15	9	5.92	2.19
9	15	9	6.58	3.53
-10	-15	-9	6.56	4.00
-10	-15	-9	6.35	1.39
10	15	9	5.63	2.38
-11	-15	-9	10.45	4.39
-11	-15	-9	7.26	1.52
11	15	9	5.98	2.67
-4	-16	-9	12.52	2.85
-5	-16	-9	20.10	3.20
-6	-16	-9	13.58	3.12
6	16	9	10.92	1.98
-7	-16	-9	15.89	3.42
7	16	9	12.15	2.10
-8	-16	-9	9.02	3.36
8	16	9	7.05	2.18
7	10	-10	25.53	4.89
7	10	-10	39.87	7.24
-7	-10	10	19.21	3.63
-7	-10	10	33.31	5.28
6	10	-10	17.46	3.43
6	10	-10	18.97	4.14
-6	-10	10	21.30	3.48
-6	-10	10	15.43	3.46
-6	-10	10	28.36	4.96
5	10	-10	15.82	1.78
5	10	-10	16.32	3.82
-5	-10	10	18.18	3.33
-5	-10	10	14.19	3.54
-5	-10	10	17.00	3.47

4	10	-10	12.52	3.69
4	10	-10	9.78	1.15
4	10	-10	9.44	3.75
-4	-10	10	6.63	2.87
-4	-10	10	13.47	3.33
-4	-10	10	12.94	3.52
3	10	-10	14.68	1.76
3	10	-10	10.18	3.50
3	10	-10	13.33	3.49
-3	-10	10	12.25	3.02
-3	-10	10	12.74	3.22
-3	-10	10	15.97	3.24
2	10	-10	14.61	1.85
2	10	-10	14.83	3.72
-2	-10	10	9.14	2.59
-2	-10	10	17.06	3.23
1	10	-10	33.35	5.82
1	10	-10	23.20	2.20
0	10	-10	22.57	2.24
0	10	-10	30.74	5.76
0	-10	10	24.63	4.39
-9	-9	10	17.11	3.79
-9	-9	10	14.37	3.20
8	9	-10	18.76	3.42
-8	-9	10	18.71	3.73
-8	-9	10	27.71	5.37
-8	-9	10	13.73	3.12
-8	-9	10	20.86	3.85
7	9	-10	25.32	4.34
7	9	-10	16.49	3.85
7	9	-10	18.63	3.74
-7	-9	10	24.52	4.96
-7	-9	10	16.85	3.15
-7	-9	10	28.16	4.68
6	9	-10	10.47	3.83
6	9	-10	23.07	4.42
6	9	-10	17.47	3.52
-6	-9	10	21.00	3.34
-6	-9	10	14.89	3.07
-6	-9	10	16.86	2.97
5	9	-10	13.10	3.36
5	9	-10	12.46	3.71
5	9	-10	16.23	1.58
5	9	-10	19.00	3.16

-5	-9	10	15.22	2.99
-5	-9	10	17.09	3.16
-5	-9	10	19.79	3.27
-5	-9	10	12.03	3.14
4	9	-10	15.11	3.27
4	9	-10	23.16	4.72
4	9	-10	15.49	3.16
4	9	-10	27.47	5.22
-4	-9	10	18.83	3.21
-4	-9	10	30.75	4.34
-4	-9	10	23.67	3.19
-4	-9	10	21.12	3.18
3	9	-10	36.03	4.93
3	9	-10	34.51	2.36
3	9	-10	28.78	4.97
3	9	-10	38.70	5.11
3	9	-10	33.52	4.58
-3	-9	10	37.42	4.14
-3	-9	10	38.90	4.73
-3	-9	10	33.96	4.52
2	9	-10	16.49	3.01
2	9	-10	29.93	5.68
2	9	-10	26.32	4.36
2	9	-10	20.79	1.86
2	9	-10	18.96	3.56
-2	-9	10	22.37	4.12
-2	-9	10	15.77	2.76
-2	-9	10	17.31	2.93
1	9	-10	14.73	3.13
1	9	-10	17.18	1.80
1	9	-10	13.70	3.22
1	9	-10	9.69	3.35
-1	-9	10	14.59	2.32
-1	-9	10	18.88	3.82
-1	-9	10	13.61	2.82
0	9	-10	12.68	2.91
0	9	-10	15.09	1.83
0	9	-10	17.29	3.38
0	-9	10	17.33	3.39
0	-9	10	10.25	2.11
0	-9	10	15.27	2.82
-1	9	-10	31.80	2.52
-1	9	-10	40.49	5.53
-2	9	-10	23.67	2.34

-2	9	-10	31.22	5.17
2	-9	10	31.38	3.77
-10	-8	10	13.29	3.87
-10	-8	10	16.03	3.00
-10	-8	10	14.48	2.83
9	8	-10	12.73	4.25
-9	-8	10	13.09	3.62
-9	-8	10	16.59	2.74
-9	-8	10	14.63	2.87
8	8	-10	13.32	3.73
8	8	-10	15.96	2.78
-8	-8	10	17.52	3.23
-8	-8	10	16.41	2.73
-8	-8	10	21.73	3.80
7	8	-10	28.22	5.48
7	8	-10	20.39	4.03
7	8	-10	20.29	3.93
-7	-8	10	20.22	2.85
-7	-8	10	25.06	4.44
-7	-8	10	27.46	4.07
-7	-8	10	24.44	3.52
6	8	-10	30.73	5.17
6	8	-10	26.34	4.21
6	8	-10	27.57	5.80
-6	-8	10	20.24	3.95
-6	-8	10	30.47	3.34
-6	-8	10	31.96	4.03
-6	-8	10	31.53	4.42
5	8	-10	27.99	5.47
5	8	-10	29.75	4.82
5	8	-10	23.20	4.08
-5	-8	10	29.21	3.97
-5	-8	10	29.91	3.96
-5	-8	10	28.98	3.23
-5	-8	10	20.29	4.09
4	8	-10	37.80	2.40
4	8	-10	43.75	4.77
4	8	-10	38.99	5.08
4	8	-10	37.04	4.35
-4	-8	10	41.48	4.45
-4	-8	10	42.46	3.52
-4	-8	10	37.30	4.19
3	8	-10	41.62	2.82
3	8	-10	44.34	4.62

3	8	-10	47.92	4.83
3	8	-10	57.52	4.91
-3	-8	10	50.34	4.51
-3	-8	10	48.10	4.29
-3	-8	10	44.59	4.63
2	8	-10	57.14	5.22
2	8	-10	52.29	5.05
2	8	-10	63.04	3.39
2	8	-10	63.84	5.09
-2	-8	10	63.17	4.80
-2	-8	10	54.65	4.75
-2	-8	10	57.94	5.00
-2	-8	10	66.63	4.34
1	8	-10	33.62	2.29
1	8	-10	38.56	4.10
1	8	-10	35.73	4.85
-1	-8	10	27.99	3.91
-1	-8	10	26.94	4.11
-1	-8	10	35.39	3.44
-1	-8	10	36.27	3.63
0	8	-10	30.32	4.00
0	8	-10	27.97	4.03
0	8	-10	28.40	3.94
0	8	-10	29.38	4.28
0	8	-10	30.63	2.13
0	-8	10	26.23	4.10
0	-8	10	26.30	3.68
0	-8	10	27.02	3.37
0	-8	10	29.00	3.39
-1	8	-10	24.81	4.18
-1	8	-10	28.02	3.86
-1	8	-10	27.46	2.19
-1	8	-10	29.64	4.35
-1	8	-10	29.81	4.46
1	-8	10	31.07	3.39
1	-8	10	25.56	3.47
1	-8	10	25.33	4.25
1	-8	10	30.77	3.30
-2	8	-10	47.82	4.44
-2	8	-10	43.89	4.91
-2	8	-10	40.40	2.90
-2	8	-10	45.35	5.01
2	-8	10	50.30	4.09
-3	8	-10	24.39	4.53

-3	8	-10	21.91	2.16
3	-8	10	25.06	3.61
-4	8	-10	25.02	2.32
-4	8	-10	27.62	4.44
-10	-7	10	14.45	2.81
-10	-7	10	15.78	3.74
-10	-7	10	13.11	2.73
9	7	-10	14.50	4.06
-9	-7	10	12.20	2.53
-9	-7	10	9.11	2.54
-9	-7	10	15.07	2.68
8	7	-10	12.81	2.48
8	7	-10	15.53	3.49
-8	-7	10	19.31	3.49
-8	-7	10	24.85	3.81
-8	-7	10	19.75	3.77
7	7	-10	7.18	3.26
7	7	-10	8.91	2.91
7	7	-10	1.44	3.06
7	7	-10	4.72	2.19
-7	-7	10	7.62	2.14
-7	-7	10	7.84	2.20
-7	-7	10	8.32	1.89
6	7	-10	20.39	3.55
-6	-7	10	24.38	3.11
-6	-7	10	20.04	3.46
-6	-7	10	27.79	3.56
-6	-7	10	18.82	3.67
5	7	-10	24.50	4.65
5	7	-10	15.51	2.77
5	7	-10	22.31	3.45
-5	-7	10	17.02	2.52
4	7	-10	32.20	2.09
4	7	-10	33.41	4.19
4	7	-10	32.80	4.24
4	7	-10	34.63	4.60
4	7	-10	30.17	3.86
-4	-7	10	36.87	3.71
-4	-7	10	38.94	3.20
-4	-7	10	33.90	3.94
3	7	-10	41.64	4.10
3	7	-10	34.67	2.30
3	7	-10	34.73	4.08
3	7	-10	41.70	4.47

-3	-7	10	42.86	4.44
-3	-7	10	38.42	4.01
-3	-7	10	37.49	3.87
2	7	-10	39.86	4.03
2	7	-10	41.82	2.48
2	7	-10	44.63	3.96
2	7	-10	41.78	4.15
2	7	-10	42.81	3.93
-2	-7	10	36.52	3.97
-2	-7	10	38.63	2.97
-2	-7	10	39.70	3.82
1	7	-10	38.11	3.84
1	7	-10	40.09	2.42
1	7	-10	39.78	3.70
1	7	-10	37.00	4.15
-1	-7	10	37.60	3.04
-1	-7	10	38.72	3.29
-1	-7	10	33.95	3.85
-1	-7	10	38.52	3.59
0	7	-10	27.29	3.87
0	7	-10	26.41	3.41
0	7	-10	20.69	3.27
0	7	-10	24.39	3.24
0	7	-10	23.05	1.82
0	-7	10	22.89	2.89
0	-7	10	22.50	2.95
0	-7	10	19.36	3.13
0	-7	10	22.66	2.69
0	-7	10	17.61	3.47
-1	7	-10	19.60	3.08
-1	7	-10	14.78	1.63
-1	7	-10	13.37	2.24
1	-7	10	14.91	2.22
1	-7	10	16.26	2.60
1	-7	10	12.04	2.42
1	-7	10	15.04	2.87
1	-7	10	16.90	2.81
-2	7	-10	28.74	2.23
-2	7	-10	29.93	3.61
-2	7	-10	26.92	4.01
-2	7	-10	31.46	3.55
2	-7	10	32.94	3.26
2	-7	10	32.60	3.24
2	-7	10	30.90	3.39

-3	7	-10	15.15	2.34
-3	7	-10	18.07	1.91
-3	7	-10	16.86	2.65
-3	7	-10	24.00	3.94
3	-7	10	22.08	3.17
3	-7	10	15.59	2.78
3	-7	10	18.25	3.25
-4	7	-10	20.70	2.02
-4	7	-10	22.67	4.11
-4	7	-10	18.11	3.34
4	-7	10	19.76	3.34
-5	7	-10	16.01	2.71
-5	7	-10	15.89	2.01
-6	7	-10	18.78	2.24
-6	7	-10	26.26	4.53
-11	-6	10	30.77	5.07
-11	-6	10	25.01	4.31
-10	-6	10	20.35	3.73
-9	-6	10	29.06	3.90
-9	-6	10	25.47	3.66
-9	-6	10	33.45	4.30
8	6	-10	11.59	3.47
8	6	-10	14.23	3.09
8	6	-10	13.07	2.42
-8	-6	10	15.86	2.34
7	6	-10	27.21	3.62
7	6	-10	33.27	4.71
-7	-6	10	34.07	3.73
-7	-6	10	34.31	3.49
-7	-6	10	34.36	3.65
6	6	-10	38.96	4.42
6	6	-10	25.15	5.07
6	6	-10	29.73	3.44
-6	-6	10	31.74	3.59
-6	-6	10	31.92	3.33
-6	-6	10	29.45	3.40
5	6	-10	65.96	5.53
5	6	-10	60.94	4.92
5	6	-10	55.14	4.42
-5	-6	10	59.04	4.35
-5	-6	10	58.15	4.47
-5	-6	10	61.67	4.14
4	6	-10	47.83	4.74
4	6	-10	47.33	3.86

4	6	-10	46.67	4.23
4	6	-10	38.80	2.45
-4	-6	10	45.15	3.89
-4	-6	10	49.45	3.45
-4	-6	10	41.72	3.85
3	6	-10	52.63	4.16
3	6	-10	44.39	4.20
3	6	-10	56.49	4.42
3	6	-10	51.04	2.77
-3	-6	10	43.12	3.10
-3	-6	10	57.55	3.60
-3	-6	10	49.34	4.07
-3	-6	10	45.82	5.09
2	6	-10	47.75	3.97
2	6	-10	41.42	3.89
2	6	-10	46.75	2.58
2	6	-10	48.71	3.98
-2	-6	10	39.33	3.74
-2	-6	10	40.01	3.00
-2	-6	10	47.75	3.31
-2	-6	10	43.41	3.76
1	6	-10	70.31	4.48
1	6	-10	64.48	3.46
1	6	-10	64.93	5.59
1	6	-10	64.79	4.69
-1	-6	10	71.02	4.17
-1	-6	10	57.39	3.90
-1	-6	10	60.83	4.27
-1	-6	10	55.75	4.57
0	6	-10	63.49	4.42
0	6	-10	65.66	4.41
0	6	-10	63.49	4.80
0	6	-10	67.44	4.46
0	-6	10	54.36	4.70
0	-6	10	64.04	4.14
0	-6	10	68.83	4.24
0	-6	10	62.72	4.01
-1	6	-10	62.19	4.18
-1	6	-10	58.01	4.56
-1	6	-10	58.88	4.08
-1	6	-10	58.52	3.23
-1	6	-10	58.02	4.26
1	-6	10	56.36	3.77
1	-6	10	58.82	3.95

1	-6	10	59.54	4.04
1	-6	10	45.12	4.43
1	-6	10	56.35	3.98
-2	6	-10	44.86	2.64
-2	6	-10	45.28	3.65
-2	6	-10	40.12	3.58
-2	6	-10	41.71	3.89
-2	6	-10	42.12	4.19
2	-6	10	45.03	3.51
2	-6	10	46.11	3.56
2	-6	10	31.65	4.17
2	-6	10	40.79	3.22
-3	6	-10	47.68	3.79
-3	6	-10	44.44	4.15
-3	6	-10	49.88	3.83
-3	6	-10	45.94	2.87
3	-6	10	38.94	3.44
3	-6	10	50.46	3.76
3	-6	10	51.36	3.88
-4	6	-10	20.79	4.04
-4	6	-10	19.78	1.90
-4	6	-10	21.67	2.96
-4	6	-10	21.89	3.64
-4	6	-10	17.73	3.00
4	-6	10	13.25	2.75
4	-6	10	26.36	3.06
4	-6	10	17.09	2.71
4	-6	10	18.10	3.53
-5	6	-10	32.02	4.03
-5	6	-10	28.88	3.41
-5	6	-10	28.89	2.36
-5	6	-10	30.93	4.73
5	-6	10	37.49	3.63
-6	6	-10	25.30	2.35
-6	6	-10	27.94	4.40
6	-6	10	36.44	3.84
-7	6	-10	40.59	4.89
-7	6	-10	33.23	5.05
-7	6	-10	33.20	2.70
-11	-5	10	20.49	4.05
-11	-5	10	22.12	4.75
-10	-5	10	27.87	4.29
-10	-5	10	24.38	3.64
-10	-5	10	22.47	4.40

-9	-5	10	43.04	4.64
-9	-5	10	36.81	3.75
-9	-5	10	37.84	3.85
8	5	-10	12.83	3.70
8	5	-10	29.14	4.55
-8	-5	10	21.44	3.18
-8	-5	10	19.52	3.60
-8	-5	10	20.94	3.03
7	5	-10	26.28	4.07
7	5	-10	10.55	2.46
-7	-5	10	19.88	3.07
-7	-5	10	15.28	3.10
-7	-5	10	18.19	2.88
6	5	-10	16.71	2.86
6	5	-10	12.91	2.83
6	5	-10	28.89	3.88
-6	-5	10	22.24	3.09
-6	-5	10	19.82	3.05
-6	-5	10	19.92	2.98
5	5	-10	25.47	3.51
5	5	-10	17.88	2.99
5	5	-10	19.95	4.32
-5	-5	10	19.69	2.91
-5	-5	10	24.93	3.04
-5	-5	10	20.62	2.77
4	5	-10	18.58	2.86
4	5	-10	11.55	2.43
4	5	-10	11.49	2.15
-4	-5	10	16.18	2.76
-4	-5	10	11.90	2.32
-4	-5	10	14.42	2.79
3	5	-10	26.27	3.18
3	5	-10	28.83	3.67
3	5	-10	25.17	1.66
3	5	-10	27.69	3.18
-3	-5	10	29.01	3.13
-3	-5	10	29.84	2.60
-3	-5	10	24.77	3.01
-3	-5	10	21.46	2.04
2	5	-10	20.08	1.48
2	5	-10	23.43	3.16
2	5	-10	22.18	2.94
2	5	-10	21.92	3.04
-2	-5	10	23.06	2.35

-2	-5	10	23.15	2.82
-2	-5	10	15.45	2.87
-2	-5	10	18.28	1.87
1	5	-10	35.44	3.26
1	5	-10	32.61	3.54
1	5	-10	34.20	3.46
1	5	-10	34.01	2.04
-1	-5	10	33.53	2.74
-1	-5	10	28.79	2.40
-1	-5	10	32.25	2.92
0	5	-10	17.57	2.69
0	5	-10	13.07	2.58
0	5	-10	17.34	1.41
0	5	-10	18.72	3.08
0	-5	10	13.84	1.90
0	-5	10	13.50	2.12
0	-5	10	17.48	2.35
0	-5	10	15.89	2.27
-1	5	-10	24.33	2.67
-1	5	-10	17.08	1.95
-1	5	-10	23.12	2.73
-1	5	-10	21.09	3.17
1	-5	10	18.13	2.13
1	-5	10	22.26	2.51
1	-5	10	23.55	2.62
1	-5	10	18.61	2.42
-2	5	-10	25.37	3.25
-2	5	-10	27.14	2.84
-2	5	-10	22.23	1.77
-2	5	-10	26.16	2.75
2	-5	10	19.22	2.32
2	-5	10	27.45	2.75
2	-5	10	25.86	2.74
2	-5	10	17.47	3.28
-3	5	-10	29.09	2.79
-3	5	-10	25.34	3.29
-3	5	-10	30.31	3.24
-3	5	-10	25.62	1.93
-3	5	-10	27.24	2.89
3	-5	10	27.17	2.94
3	-5	10	20.88	2.43
3	-5	10	22.19	3.40
3	-5	10	27.88	2.86
-4	5	-10	16.94	2.79

-4	5	-10	21.92	1.77
-4	5	-10	24.80	3.33
-4	5	-10	17.85	3.39
4	-5	10	20.21	3.05
4	-5	10	18.29	2.28
4	-5	10	15.07	2.36
-5	5	-10	16.61	2.46
-5	5	-10	21.96	2.71
-5	5	-10	21.77	1.90
-5	5	-10	19.44	2.90
5	-5	10	22.14	2.44
5	-5	10	15.89	2.50
5	-5	10	22.50	3.68
5	-5	10	26.46	2.99
-6	5	-10	21.89	3.30
-6	5	-10	31.71	4.08
-6	5	-10	27.93	2.24
-6	5	-10	34.40	4.22
6	-5	10	23.18	3.96
-7	5	-10	25.45	3.83
-7	5	-10	28.80	4.42
-7	5	-10	29.32	2.37
-8	5	-10	19.54	2.21
-8	5	-10	13.10	4.05
-8	5	-10	15.58	3.32
-11	-4	10	22.15	4.49
-11	-4	10	14.22	2.51
-10	-4	10	27.52	4.31
-10	-4	10	22.20	3.38
-10	-4	10	26.55	3.43
-9	-4	10	24.83	3.15
-9	-4	10	23.42	3.87
-9	-4	10	22.61	3.15
8	4	-10	53.91	6.47
-8	-4	10	60.24	4.77
-8	-4	10	62.72	4.05
-8	-4	10	60.19	4.11
-8	-4	10	57.80	4.28
7	4	-10	47.25	4.49
7	4	-10	41.44	5.37
-7	-4	10	45.09	3.95
-7	-4	10	42.72	3.71
-7	-4	10	49.08	3.46
6	4	-10	41.45	5.04

6	4	-10	61.02	4.44
6	4	-10	42.46	4.96
-6	-4	10	54.72	3.95
-6	-4	10	50.55	4.23
-6	-4	10	53.25	3.87
5	4	-10	60.05	4.28
5	4	-10	46.32	4.92
5	4	-10	47.59	3.77
-5	-4	10	54.07	3.63
-5	-4	10	50.45	3.79
-5	-4	10	50.70	3.84
4	4	-10	62.57	4.26
4	4	-10	53.63	4.14
4	4	-10	60.18	4.99
-4	-4	10	60.64	3.97
-4	-4	10	63.77	3.93
-4	-4	10	52.84	4.07
3	4	-10	54.35	3.83
3	4	-10	58.15	4.44
3	4	-10	51.88	3.83
3	4	-10	52.47	2.72
-3	-4	10	42.98	3.74
-3	-4	10	45.04	2.90
-3	-4	10	58.75	3.81
2	4	-10	73.00	4.46
2	4	-10	70.65	3.61
2	4	-10	73.07	4.56
2	4	-10	72.32	4.81
-2	-4	10	62.05	3.82
-2	-4	10	71.58	4.35
1	4	-10	51.37	3.90
1	4	-10	55.85	3.94
1	4	-10	54.15	2.84
1	4	-10	51.57	3.90
-1	-4	10	49.90	3.11
-1	-4	10	49.17	4.37
-1	-4	10	56.53	3.55
0	4	-10	77.25	4.76
0	4	-10	76.38	4.92
0	4	-10	82.05	4.00
0	4	-10	81.61	4.61
0	-4	10	75.21	4.25
0	-4	10	67.68	4.83
0	-4	10	76.69	4.50

-1	4	-10	57.05	3.67
-1	4	-10	53.34	3.68
-1	4	-10	60.98	4.02
-1	4	-10	59.17	2.96
1	-4	10	50.24	3.56
1	-4	10	46.44	3.93
1	-4	10	46.96	3.86
1	-4	10	55.28	3.30
-2	4	-10	57.74	3.06
-2	4	-10	58.93	3.73
-2	4	-10	49.24	3.64
-2	4	-10	55.10	3.99
2	-4	10	58.48	3.69
2	-4	10	51.24	4.04
2	-4	10	51.56	3.67
2	-4	10	51.02	3.32
-3	4	-10	37.91	2.31
-3	4	-10	39.15	3.08
-3	4	-10	38.55	3.39
3	-4	10	36.23	3.57
3	-4	10	37.76	3.09
3	-4	10	34.92	2.70
3	-4	10	37.64	3.13
-4	4	-10	42.87	3.42
-4	4	-10	44.89	3.82
-4	4	-10	43.82	2.63
-4	4	-10	44.78	3.70
4	-4	10	39.34	2.99
4	-4	10	51.61	3.40
4	-4	10	38.11	3.95
4	-4	10	43.59	3.62
-5	4	-10	39.52	3.78
-5	4	-10	38.91	2.49
-5	4	-10	43.47	3.07
5	-4	10	46.34	3.40
5	-4	10	29.15	3.80
5	-4	10	37.72	3.58
5	-4	10	36.72	2.88
-6	4	-10	46.06	3.29
-6	4	-10	43.04	2.65
-6	4	-10	44.44	4.13
-6	4	-10	33.59	3.64
6	-4	10	35.16	4.01
6	-4	10	35.50	3.04

6	-4	10	41.46	4.99
-7	4	-10	15.98	3.29
-7	4	-10	20.96	2.84
-7	4	-10	15.19	2.30
7	-4	10	14.19	2.66
-8	4	-10	24.16	2.23
-8	4	-10	22.59	3.36
-8	4	-10	24.45	3.93
-9	4	-10	12.64	4.31
-9	4	-10	10.68	3.48
-9	4	-10	15.19	2.04
-12	-3	10	7.23	3.20
-12	-3	10	8.86	2.54
-11	-3	10	11.16	2.78
-11	-3	10	11.08	2.44
-10	-3	10	10.59	2.03
-10	-3	10	8.41	2.14
-10	-3	10	6.11	2.39
-9	-3	10	7.62	2.17
-9	-3	10	6.94	1.87
-9	-3	10	8.56	1.80
-8	-3	10	30.36	2.91
-8	-3	10	29.36	3.63
-8	-3	10	24.87	2.98
7	3	-10	46.63	4.18
-7	-3	10	51.24	3.50
-7	-3	10	43.75	3.56
-7	-3	10	46.23	3.99
6	3	-10	55.05	4.28
-6	-3	10	51.60	3.77
-6	-3	10	52.46	3.94
5	3	-10	48.72	3.85
-5	-3	10	48.94	3.38
-5	-3	10	41.08	3.45
-5	-3	10	43.53	3.43
4	3	-10	76.03	4.53
4	3	-10	63.37	4.55
-4	-3	10	76.63	4.33
-4	-3	10	59.94	4.39
3	3	-10	50.84	2.67
3	3	-10	48.69	3.69
3	3	-10	52.77	4.30
3	3	-10	50.07	3.67
-3	-3	10	51.59	3.55

-3	-3	10	53.14	3.47
2	3	-10	37.46	3.30
2	3	-10	40.91	3.60
2	3	-10	41.18	2.15
2	3	-10	37.56	3.09
-2	-3	10	39.33	3.13
-2	-3	10	43.22	2.88
-2	-3	10	34.09	2.39
1	3	-10	58.59	3.91
1	3	-10	57.15	3.91
1	3	-10	51.92	2.87
1	3	-10	51.64	3.84
-1	-3	10	56.99	3.50
-1	-3	10	51.10	3.11
-1	-3	10	55.12	3.44
0	3	-10	60.98	4.19
0	3	-10	67.64	4.09
0	3	-10	63.45	3.29
0	3	-10	68.69	3.97
0	-3	10	59.98	3.50
0	-3	10	60.47	3.80
0	-3	10	53.74	4.12
0	-3	10	64.72	3.81
-1	3	-10	73.30	4.21
-1	3	-10	68.49	4.47
-1	3	-10	68.42	3.64
-1	3	-10	69.78	4.25
1	-3	10	68.69	4.16
1	-3	10	69.46	4.18
1	-3	10	69.40	3.88
1	-3	10	71.25	4.53
-2	3	-10	82.04	4.25
-2	3	-10	82.56	4.97
-2	3	-10	85.55	4.73
2	-3	10	82.43	4.52
2	-3	10	78.97	4.93
2	-3	10	82.28	4.72
-3	3	-10	54.45	3.06
-3	3	-10	55.57	3.93
-3	3	-10	53.74	3.71
3	-3	10	50.05	3.32
3	-3	10	58.79	4.06
3	-3	10	61.47	3.86
3	-3	10	53.17	3.72

-4	3	-10	40.09	2.43
-4	3	-10	42.11	3.49
-4	3	-10	39.73	3.28
4	-3	10	44.48	3.11
4	-3	10	37.78	3.65
4	-3	10	37.89	2.79
4	-3	10	43.56	3.30
-5	3	-10	29.41	3.13
-5	3	-10	33.31	2.12
-5	3	-10	35.11	3.44
5	-3	10	31.56	3.23
5	-3	10	28.85	2.43
5	-3	10	30.16	3.60
-6	3	-10	18.83	3.18
-6	3	-10	23.87	3.26
-6	3	-10	16.39	3.36
-6	3	-10	25.64	1.86
6	-3	10	20.63	3.47
6	-3	10	21.03	3.24
-7	3	-10	19.93	1.81
-7	3	-10	18.80	2.70
-7	3	-10	13.65	2.25
-7	3	-10	16.64	3.12
7	-3	10	20.43	4.12
7	-3	10	15.61	2.27
7	-3	10	15.43	2.47
-8	3	-10	6.59	2.14
-8	3	-10	10.60	2.94
-8	3	-10	13.44	1.76
-8	3	-10	13.91	2.72
-8	3	-10	7.17	3.22
8	-3	10	7.92	2.67
-9	3	-10	18.02	4.03
-9	3	-10	17.04	2.01
-9	3	-10	14.95	3.51
-10	3	-10	13.91	4.42
-10	3	-10	12.90	2.05
-12	-2	10	7.70	2.48
-12	-2	10	8.68	3.27
-11	-2	10	12.82	3.12
-11	-2	10	16.42	3.20
-10	-2	10	7.01	2.46
-10	-2	10	9.11	2.00
-9	-2	10	17.71	3.80

-9	-2	10	19.32	2.77
-8	-2	10	26.17	3.01
-8	-2	10	25.52	3.53
-8	-2	10	32.65	2.83
-7	-2	10	56.51	3.57
-7	-2	10	50.31	4.06
-7	-2	10	50.35	3.66
6	2	-10	29.03	3.05
-6	-2	10	38.03	2.95
-6	-2	10	35.80	3.29
5	2	-10	68.38	4.34
-5	-2	10	65.66	3.94
-5	-2	10	55.45	4.04
4	2	-10	59.33	3.91
4	2	-10	58.10	4.66
-4	-2	10	58.64	3.73
-4	-2	10	45.75	3.75
-4	-2	10	57.11	3.66
3	2	-10	73.43	5.04
3	2	-10	62.41	4.52
3	2	-10	70.59	4.33
-3	-2	10	71.20	4.22
-3	-2	10	71.82	4.24
2	2	-10	52.74	3.97
2	2	-10	54.96	2.75
2	2	-10	49.16	3.65
2	2	-10	55.44	3.50
-2	-2	10	50.44	2.87
-2	-2	10	55.31	3.36
-2	-2	10	43.39	3.63
-2	-2	10	56.08	3.47
1	2	-10	83.05	4.09
1	2	-10	84.34	4.90
1	2	-10	78.59	5.05
1	2	-10	79.23	4.84
-1	-2	10	79.86	4.52
-1	-2	10	81.51	4.23
-1	-2	10	73.38	4.84
-1	-2	10	86.24	4.61
0	2	-10	70.56	3.64
0	2	-10	74.13	4.16
0	2	-10	70.95	4.37
0	2	-10	68.05	4.35
0	-2	10	72.26	4.09

0	-2	10	71.98	4.41
0	-2	10	70.14	4.12
0	-2	10	69.78	3.81
-1	2	-10	93.72	5.32
-1	2	-10	100.43	5.46
-1	2	-10	92.55	4.81
-1	2	-10	99.18	5.22
1	-2	10	98.20	5.52
1	-2	10	95.18	4.97
1	-2	10	90.90	5.20
1	-2	10	94.47	5.26
-2	2	-10	84.60	4.84
-2	2	-10	80.31	4.17
-2	2	-10	78.86	4.67
-2	2	-10	85.66	4.65
2	-2	10	84.03	4.63
2	-2	10	72.56	4.62
2	-2	10	79.65	4.34
2	-2	10	85.39	4.94
-3	2	-10	109.48	5.69
-3	2	-10	114.62	6.13
-3	2	-10	112.17	6.08
-3	2	-10	117.69	6.31
3	-2	10	114.25	6.14
3	-2	10	108.47	5.86
3	-2	10	115.16	6.15
3	-2	10	112.44	6.46
-4	2	-10	72.32	4.52
-4	2	-10	71.17	3.92
-4	2	-10	79.73	4.74
4	-2	10	66.77	4.08
4	-2	10	75.15	4.53
4	-2	10	83.47	4.88
-5	2	-10	57.69	4.18
-5	2	-10	62.54	3.46
-5	2	-10	66.83	4.46
5	-2	10	66.03	3.99
5	-2	10	62.02	4.16
5	-2	10	68.54	4.58
-6	2	-10	39.29	3.79
-6	2	-10	38.49	3.51
-6	2	-10	41.46	2.48
-6	2	-10	39.91	3.33
-6	2	-10	34.32	3.66

6	-2	10	47.93	3.59
6	-2	10	39.00	3.91
-7	2	-10	58.99	4.15
-7	2	-10	48.81	4.29
-7	2	-10	55.75	3.23
-7	2	-10	44.51	5.52
7	-2	10	67.25	4.47
-8	2	-10	22.95	3.50
-8	2	-10	31.20	2.78
-8	2	-10	25.18	2.11
-8	2	-10	21.40	4.37
-8	2	-10	19.77	3.14
8	-2	10	25.98	2.48
-9	2	-10	22.44	3.85
-9	2	-10	21.76	3.56
-9	2	-10	20.01	3.57
-9	2	-10	25.33	4.02
-9	2	-10	23.87	2.15
-10	2	-10	8.88	4.44
-10	2	-10	10.00	3.73
-10	2	-10	12.23	1.91
-11	2	-10	18.24	2.28
-11	2	-10	21.73	5.05
-11	2	-10	20.90	4.38
11	-2	10	27.88	4.20
-11	-1	10	11.62	3.03
-10	-1	10	14.22	2.74
-9	-1	10	18.99	3.84
-9	-1	10	18.83	2.57
-8	-1	10	26.24	3.58
-8	-1	10	27.04	3.04
-8	-1	10	30.79	2.64
-7	-1	10	40.17	3.02
-7	-1	10	36.22	3.53
-7	-1	10	37.38	3.74
-6	-1	10	27.48	2.64
-6	-1	10	27.64	3.01
-6	-1	10	29.97	3.18
5	1	-10	28.86	5.59
5	1	-10	32.29	3.06
-5	-1	10	31.29	2.66
-5	-1	10	32.61	3.36
-5	-1	10	26.89	2.72
4	1	-10	40.31	4.00

4	1	-10	39.68	3.16
-4	-1	10	32.34	3.00
-4	-1	10	38.59	3.03
-4	-1	10	41.34	2.98
3	1	-10	24.66	2.67
3	1	-10	31.89	3.51
-3	-1	10	28.08	2.56
-3	-1	10	30.65	2.59
2	1	-10	24.63	3.62
2	1	-10	24.54	1.49
2	1	-10	27.16	3.12
2	1	-10	20.98	2.40
-2	-1	10	25.19	2.33
-2	-1	10	19.27	2.56
-2	-1	10	26.96	2.33
-2	-1	10	27.39	1.59
1	1	-10	35.38	2.99
1	1	-10	40.18	2.13
1	1	-10	43.25	3.22
-1	-1	10	37.16	3.04
-1	-1	10	39.06	2.74
-1	-1	10	37.67	2.68
-1	-1	10	37.79	2.21
0	1	-10	31.23	2.52
0	1	-10	33.36	1.83
0	1	-10	32.08	2.80
0	1	-10	33.79	2.75
0	-1	10	29.99	2.44
0	-1	10	31.54	2.49
0	-1	10	33.63	1.96
0	-1	10	31.57	2.85
-1	1	-10	21.49	1.40
-1	1	-10	22.06	2.26
-1	1	-10	23.01	2.41
-1	1	-10	22.88	2.18
1	-1	10	21.40	2.56
1	-1	10	23.58	2.24
1	-1	10	21.80	2.17
1	-1	10	18.80	2.08
-2	1	-10	41.40	3.02
-2	1	-10	35.99	2.11
-2	1	-10	37.91	2.75
-2	1	-10	40.43	2.78
2	-1	10	36.88	2.26

2	-1	10	38.60	3.13
2	-1	10	34.10	2.71
2	-1	10	38.32	2.77
-3	1	-10	25.52	1.67
-3	1	-10	28.08	2.29
-3	1	-10	25.58	2.55
-3	1	-10	28.77	2.78
3	-1	10	26.84	2.27
3	-1	10	26.05	1.85
3	-1	10	26.23	2.83
3	-1	10	25.52	2.47
-4	1	-10	60.95	3.99
-4	1	-10	52.13	3.02
-4	1	-10	57.36	3.76
-4	1	-10	55.28	3.45
4	-1	10	56.72	4.04
4	-1	10	56.54	3.69
4	-1	10	52.39	3.16
4	-1	10	61.23	3.54
-5	1	-10	30.37	3.40
-5	1	-10	28.32	2.88
-5	1	-10	27.32	2.07
-5	1	-10	33.31	3.18
5	-1	10	32.59	2.80
5	-1	10	33.50	3.26
5	-1	10	28.59	2.11
5	-1	10	29.71	3.29
-6	1	-10	29.48	3.10
-6	1	-10	34.50	2.15
-6	1	-10	28.71	3.53
6	-1	10	36.05	3.09
6	-1	10	36.08	3.62
6	-1	10	38.72	2.83
-7	1	-10	30.52	3.94
-7	1	-10	34.91	2.27
-7	1	-10	33.61	3.82
-7	1	-10	33.98	3.22
-7	1	-10	34.94	3.12
7	-1	10	40.02	3.36
7	-1	10	33.46	3.85
-8	1	-10	56.63	5.31
-8	1	-10	55.79	3.30
-8	1	-10	59.57	4.32
-8	1	-10	50.15	5.51

-8	1	-10	57.95	4.12
8	-1	10	64.99	4.51
8	-1	10	53.34	4.76
-9	1	-10	34.34	2.87
-9	1	-10	28.00	3.30
-9	1	-10	25.19	4.77
-9	1	-10	23.55	3.49
-9	1	-10	28.74	2.22
9	-1	10	23.15	4.17
9	-1	10	31.66	2.70
-10	1	-10	19.14	2.03
-10	1	-10	20.10	4.07
-10	1	-10	17.54	4.20
-10	1	-10	17.71	3.22
-10	1	-10	21.09	3.74
-11	1	-10	11.25	4.60
-11	1	-10	4.84	3.77
-11	1	-10	7.33	1.25
11	-1	10	11.22	2.18
-11	0	10	14.21	3.14
-10	0	10	31.34	4.70
-9	0	10	23.28	3.94
-8	0	10	42.52	4.10
-7	0	10	33.22	3.42
6	0	-10	59.43	5.28
-6	0	10	58.67	4.02
-6	0	10	57.09	3.64
5	0	-10	30.44	4.02
-5	0	10	35.88	3.01
4	0	-10	83.66	5.34
4	0	-10	75.04	4.51
-4	0	10	65.41	4.54
-4	0	10	85.47	4.66
-4	0	10	79.62	4.45
3	0	-10	52.05	3.46
3	0	-10	53.16	2.78
3	0	-10	53.45	4.30
-3	0	10	57.40	3.37
-3	0	10	52.92	3.52
-3	0	10	49.95	3.50
2	0	-10	95.69	5.76
2	0	-10	100.00	4.80
2	0	-10	93.39	5.18
-2	0	10	96.36	5.28

-2	0	10	93.74	5.12
-2	0	10	92.04	4.79
-2	0	10	100.06	5.41
1	0	-10	83.93	4.13
1	0	-10	69.90	4.95
1	0	-10	84.14	5.00
-1	0	10	82.31	4.15
-1	0	10	86.73	4.81
-1	0	10	78.85	4.46
-1	0	10	84.42	4.55
0	0	-10	86.59	4.20
0	0	-10	82.55	4.85
0	0	-10	79.77	4.78
0	0	-10	83.63	4.56
0	0	10	83.92	4.89
0	0	10	80.48	4.59
0	0	10	84.27	4.56
-1	0	-10	58.90	3.85
-1	0	-10	63.05	3.97
-1	0	-10	66.09	3.27
-1	0	-10	66.25	3.71
1	0	10	58.96	4.03
1	0	10	63.22	3.32
1	0	10	64.23	3.72
1	0	10	62.23	3.68
-2	0	-10	104.78	5.42
-2	0	-10	100.45	5.06
-2	0	-10	96.82	5.45
-2	0	-10	109.96	5.67
2	0	10	104.41	5.47
2	0	10	98.02	5.16
2	0	10	93.39	5.42
2	0	10	100.33	5.72
-3	0	-10	59.09	3.81
-3	0	-10	57.76	2.96
-3	0	-10	56.19	3.49
-3	0	-10	54.26	3.41
3	0	10	56.80	3.51
3	0	10	49.77	3.39
3	0	10	53.86	3.81
3	0	10	58.65	3.06
-4	0	-10	64.46	4.28
-4	0	-10	66.65	3.82
-4	0	-10	65.65	3.45

-4	0	-10	64.18	3.97
4	0	10	63.45	4.28
4	0	10	62.45	3.53
4	0	10	67.20	4.02
4	0	10	71.38	3.91
-5	0	-10	67.60	3.62
-5	0	-10	68.80	4.15
-5	0	-10	66.31	4.56
-5	0	-10	71.41	3.99
5	0	10	64.96	3.73
5	0	10	66.03	4.55
5	0	10	71.51	4.02
5	0	10	74.30	4.23
-6	0	-10	69.77	4.94
-6	0	-10	71.04	4.30
-6	0	-10	78.03	4.20
6	0	10	75.98	4.44
6	0	10	71.55	4.82
6	0	10	70.48	4.77
-7	0	-10	25.76	3.66
-7	0	-10	27.95	2.40
-7	0	-10	32.11	2.03
-7	0	-10	32.57	2.78
7	0	10	33.77	3.00
7	0	10	27.31	3.53
-8	0	-10	50.89	4.73
-8	0	-10	50.95	4.77
-8	0	-10	54.56	3.75
-8	0	-10	53.78	3.11
8	0	10	47.75	4.54
8	0	10	62.02	4.06
-9	0	-10	55.42	3.30
-9	0	-10	55.79	3.97
-9	0	-10	52.21	4.15
-9	0	-10	60.71	5.33
-9	0	-10	60.21	3.73
-9	0	-10	50.81	5.32
9	0	10	53.32	3.53
9	0	10	67.18	4.14
9	0	10	47.69	4.70
-10	0	-10	43.03	2.86
-10	0	-10	40.32	3.66
-10	0	-10	48.77	5.37
-10	0	-10	44.27	5.49

10	0	10	31.19	4.83
10	0	10	42.91	3.75
10	0	10	52.83	3.96
-11	0	-10	17.08	2.95
-11	0	-10	17.95	4.62
-11	0	-10	11.61	3.68
-11	0	-10	12.11	2.30
-11	0	-10	18.23	2.00
11	0	10	14.90	2.33
-12	0	-10	25.61	5.69
-12	0	-10	19.26	2.39
-12	0	-10	23.50	4.40
12	0	10	28.11	4.10
-11	1	10	10.11	3.12
-10	1	10	13.01	2.84
-9	1	10	28.03	4.15
-8	1	10	18.35	3.47
-7	1	10	39.37	3.83
6	-1	-10	21.31	4.30
-6	1	10	27.98	2.94
5	-1	-10	18.04	3.77
-5	1	10	23.82	2.65
4	-1	-10	21.65	3.31
-4	1	10	21.09	2.47
-4	1	10	22.02	2.18
3	-1	-10	37.04	3.45
-3	1	10	35.90	2.49
-3	1	10	34.84	2.77
-3	1	10	30.05	2.83
2	-1	-10	11.79	1.02
2	-1	-10	10.53	1.76
-2	1	10	10.59	1.89
-2	1	10	11.47	0.96
-2	1	10	11.93	1.59
-2	1	10	14.05	1.65
1	-1	-10	44.48	2.39
1	-1	-10	44.63	2.84
1	-1	-10	42.31	3.41
-1	1	10	45.01	2.81
-1	1	10	45.92	3.22
-1	1	10	42.10	2.36
0	-1	-10	51.02	3.79
0	-1	-10	50.81	3.22
0	-1	-10	44.33	4.69

0	-1	-10	49.35	2.62
0	1	10	42.67	3.46
0	1	10	53.24	3.05
0	1	10	48.97	3.09
0	1	10	47.48	2.60
-1	-1	-10	104.84	6.90
-1	-1	-10	128.14	6.59
-1	-1	-10	126.01	6.32
-1	-1	-10	128.38	6.03
1	1	10	114.57	6.58
1	1	10	118.04	6.03
1	1	10	122.92	6.32
1	1	10	124.68	6.35
-2	-1	-10	48.43	3.07
-2	-1	-10	49.59	2.53
-2	-1	-10	51.54	3.41
-2	-1	-10	48.41	3.05
2	1	10	48.40	3.08
2	1	10	44.52	2.95
2	1	10	39.26	2.69
2	1	10	44.28	3.37
-3	-1	-10	17.36	1.93
-3	-1	-10	19.76	2.04
-3	-1	-10	21.02	1.31
-3	-1	-10	17.65	2.44
3	1	10	14.56	1.82
3	1	10	19.14	2.09
3	1	10	14.98	2.47
3	1	10	18.77	1.35
-4	-1	-10	19.47	1.31
-4	-1	-10	15.31	2.60
-4	-1	-10	17.47	1.83
-4	-1	-10	18.12	1.94
4	1	10	18.29	1.39
4	1	10	18.15	1.85
4	1	10	19.18	2.11
4	1	10	13.94	2.54
-5	-1	-10	29.69	1.87
-5	-1	-10	26.64	3.14
-5	-1	-10	30.90	2.42
-5	-1	-10	34.95	2.33
5	1	10	33.04	2.64
5	1	10	25.60	3.08
5	1	10	32.76	2.34

5	1	10	29.48	1.96
-6	-1	-10	17.95	3.00
-6	-1	-10	17.49	1.46
-6	-1	-10	18.64	2.18
-6	-1	-10	21.11	1.99
6	1	10	17.47	3.07
6	1	10	19.28	2.38
6	1	10	22.22	1.99
6	1	10	17.26	1.57
-7	-1	-10	34.63	2.77
-7	-1	-10	28.32	3.66
-7	-1	-10	37.08	2.56
-7	-1	-10	34.41	2.17
7	1	10	29.88	3.56
7	1	10	34.58	2.95
-8	-1	-10	16.85	2.67
-8	-1	-10	19.98	2.52
-8	-1	-10	21.58	3.12
-8	-1	-10	21.59	1.71
8	1	10	22.77	2.77
8	1	10	18.30	3.55
-9	-1	-10	53.98	4.00
-9	-1	-10	47.95	3.01
-9	-1	-10	48.62	3.65
-9	-1	-10	45.99	5.61
-9	-1	-10	53.13	5.00
9	1	10	46.02	3.16
9	1	10	44.89	4.60
9	1	10	60.27	3.73
-10	-1	-10	24.20	4.59
-10	-1	-10	26.57	3.02
-10	-1	-10	27.28	2.72
-10	-1	-10	25.70	2.18
-10	-1	-10	19.58	4.13
10	1	10	31.92	3.19
10	1	10	23.05	2.46
10	1	10	26.29	4.20
-11	-1	-10	22.37	3.59
-11	-1	-10	16.99	2.07
-11	-1	-10	21.97	3.88
-11	-1	-10	15.73	3.10
-11	-1	-10	12.17	4.43
11	1	10	15.44	3.15
11	1	10	23.34	3.47

-12	-1	-10	16.25	5.12
-12	-1	-10	8.57	1.39
-12	-1	-10	7.62	3.74
-11	2	10	16.25	3.46
-10	2	10	11.11	2.96
-9	2	10	32.00	4.34
-8	2	10	23.45	3.66
-7	2	10	54.61	4.21
-6	2	10	51.07	3.95
5	-2	-10	51.84	4.57
3	-2	-10	78.31	3.96
-3	2	10	75.80	3.91
-3	2	10	76.68	4.52
-3	2	10	80.18	4.23
2	-2	-10	50.71	2.85
-2	2	10	62.89	3.69
-2	2	10	53.13	2.83
-2	2	10	53.63	3.21
1	-2	-10	61.06	4.03
1	-2	-10	68.47	3.52
1	-2	-10	68.68	3.80
-1	2	10	67.42	3.93
-1	2	10	74.48	4.22
-1	2	10	68.44	3.48
-1	2	10	68.62	3.83
0	-2	-10	132.71	7.07
0	-2	-10	130.99	6.54
0	2	10	126.23	6.98
0	2	10	132.12	6.52
0	2	10	137.01	6.80
-1	-2	-10	284.72	13.83
-1	-2	-10	288.22	13.68
-1	-2	-10	288.30	15.00
1	2	10	277.45	13.67
1	2	10	272.31	14.04
1	2	10	276.43	13.89
-2	-2	-10	171.74	9.29
-2	-2	-10	173.88	9.05
-2	-2	-10	197.39	8.86
2	2	10	186.11	8.86
2	2	10	170.47	9.27
2	2	10	179.07	9.12
-3	-2	-10	98.92	4.63
-3	-2	-10	89.98	4.86

-3	-2	-10	88.20	5.28
3	2	10	92.34	5.02
3	2	10	82.02	5.19
3	2	10	93.50	4.63
-4	-2	-10	42.97	2.19
-4	-2	-10	39.42	2.56
-4	-2	-10	35.78	3.16
4	2	10	39.34	2.77
4	2	10	37.06	2.55
4	2	10	33.30	3.04
4	2	10	38.00	2.19
-5	-2	-10	58.45	3.49
-5	-2	-10	60.00	3.54
-5	-2	-10	65.14	3.23
-5	-2	-10	59.04	4.11
5	2	10	57.27	3.99
5	2	10	62.31	3.71
5	2	10	60.06	3.50
5	2	10	59.33	3.22
-6	-2	-10	37.52	2.18
-6	-2	-10	33.52	3.45
-6	-2	-10	38.17	2.54
-6	-2	-10	37.67	2.64
6	2	10	31.95	2.18
6	2	10	34.40	3.18
6	2	10	37.41	2.53
6	2	10	39.17	2.89
-7	-2	-10	62.25	4.43
-7	-2	-10	62.18	3.74
-7	-2	-10	57.58	3.38
-7	-2	-10	63.83	3.59
7	2	10	60.08	3.91
7	2	10	61.05	3.65
7	2	10	56.64	4.73
-8	-2	-10	54.73	3.20
-8	-2	-10	58.07	3.70
-8	-2	-10	59.16	3.53
-8	-2	-10	60.58	4.52
8	2	10	55.84	3.89
8	2	10	56.66	4.33
-9	-2	-10	65.46	5.19
-9	-2	-10	56.86	5.44
-9	-2	-10	65.10	4.19
-9	-2	-10	67.13	3.71

9	2	10	66.32	4.92
9	2	10	73.98	4.23
-10	-2	-10	35.53	2.48
-10	-2	-10	39.99	2.91
-10	-2	-10	32.86	3.19
-10	-2	-10	25.90	4.62
-10	-2	-10	23.92	4.90
10	2	10	36.85	3.48
10	2	10	35.48	4.13
10	2	10	39.72	3.32
10	2	10	33.39	2.59
-11	-2	-10	26.02	4.64
-11	-2	-10	14.00	2.11
-11	-2	-10	22.17	2.64
-11	-2	-10	20.35	2.10
-11	-2	-10	21.33	4.35
11	2	10	15.93	2.27
11	2	10	21.52	3.25
11	2	10	14.93	2.77
11	2	10	26.07	3.27
-12	-2	-10	16.31	3.21
-12	-2	-10	21.77	2.38
-12	-2	-10	21.73	5.06
-12	-2	-10	17.93	3.71
12	2	10	24.72	3.67
-13	-2	-10	21.41	5.76
-13	-2	-10	21.57	2.64
-13	-2	-10	18.02	4.28
13	2	10	30.38	4.14
-10	3	10	12.81	3.11
-9	3	10	11.95	2.84
-8	3	10	18.54	3.78
-7	3	10	19.33	3.34
-6	3	10	38.46	3.42
4	-3	-10	41.83	2.26
4	-3	-10	34.87	3.91
3	-3	-10	37.22	2.09
-3	3	10	34.83	1.97
2	-3	-10	38.52	2.22
-2	3	10	41.50	2.18
1	-3	-10	25.75	1.61
-1	3	10	26.56	2.14
-1	3	10	26.21	1.55
0	-3	-10	24.97	2.44

0	-3	-10	28.40	2.02
0	-3	-10	25.89	1.65
0	3	10	27.27	2.15
0	3	10	26.59	1.60
0	3	10	27.73	2.53
-1	-3	-10	49.49	2.70
1	3	10	46.38	3.32
1	3	10	51.33	3.11
1	3	10	51.97	2.67
-2	-3	-10	104.60	5.37
-2	-3	-10	105.97	5.19
2	3	10	99.16	5.17
2	3	10	98.48	5.61
2	3	10	105.99	5.49
-3	-3	-10	28.32	2.11
-3	-3	-10	31.68	1.78
-3	-3	-10	29.13	2.66
3	3	10	27.33	2.33
3	3	10	28.17	2.55
3	3	10	28.17	1.79
-4	-3	-10	76.36	4.05
-4	-3	-10	75.78	3.82
-4	-3	-10	72.72	4.52
4	3	10	74.72	4.21
4	3	10	71.06	3.79
4	3	10	70.30	4.34
-5	-3	-10	29.37	2.92
-5	-3	-10	28.44	1.80
-5	-3	-10	30.96	2.26
5	3	10	28.47	2.70
5	3	10	29.60	2.46
5	3	10	27.78	1.80
-6	-3	-10	28.41	1.79
-6	-3	-10	28.02	3.07
-6	-3	-10	28.01	2.32
6	3	10	27.09	2.79
6	3	10	26.47	2.54
6	3	10	26.53	1.78
-7	-3	-10	18.61	1.54
-7	-3	-10	22.39	3.11
-7	-3	-10	20.65	2.16
7	3	10	17.89	1.54
7	3	10	20.58	2.77
7	3	10	22.01	2.43

-8	-3	-10	24.35	1.98
-8	-3	-10	25.89	2.20
-8	-3	-10	25.30	2.44
-8	-3	-10	23.20	3.38
8	3	10	20.17	1.82
8	3	10	27.84	2.33
8	3	10	25.14	3.06
-9	-3	-10	20.29	3.59
-9	-3	-10	18.20	2.15
-9	-3	-10	18.00	1.79
-9	-3	-10	20.70	2.48
9	3	10	20.55	2.44
9	3	10	21.89	3.28
-10	-3	-10	37.53	2.54
-10	-3	-10	35.66	4.23
-10	-3	-10	34.16	3.15
-10	-3	-10	26.35	5.63
10	3	10	36.34	3.05
10	3	10	41.61	3.29
10	3	10	34.26	3.99
10	3	10	39.60	3.32
-11	-3	-10	18.42	2.06
-11	-3	-10	12.40	4.06
-11	-3	-10	17.59	2.83
-11	-3	-10	19.60	2.50
-11	-3	-10	13.97	2.86
11	3	10	21.39	3.10
11	3	10	15.11	2.11
11	3	10	14.05	2.61
11	3	10	19.59	3.12
-12	-3	-10	21.55	2.33
-12	-3	-10	15.12	3.39
-12	-3	-10	22.80	3.25
-12	-3	-10	19.52	5.02
12	3	10	26.96	3.65
12	3	10	26.11	3.58
12	3	10	15.08	2.99
-13	-3	-10	9.59	1.58
-13	-3	-10	11.11	5.50
-13	-3	-10	13.89	3.93
-10	4	10	14.84	3.32
-9	4	10	15.95	2.98
-8	4	10	21.81	3.98
-7	4	10	33.06	3.76

-5	4	10	35.14	1.99
4	-4	-10	65.90	3.46
-4	4	10	64.94	3.35
3	-4	-10	62.31	3.30
-3	4	10	62.36	3.21
2	-4	-10	69.04	3.54
-2	4	10	66.75	3.46
1	-4	-10	63.34	3.35
-1	4	10	64.86	3.30
0	-4	-10	89.85	4.42
0	4	10	78.07	4.88
0	4	10	85.89	4.69
0	4	10	92.33	4.39
-1	-4	-10	66.92	3.49
-1	-4	-10	65.03	3.69
1	4	10	67.05	3.44
1	4	10	66.81	3.93
1	4	10	68.47	3.80
-2	-4	-10	82.32	4.17
2	4	10	82.75	4.47
2	4	10	78.98	4.25
2	4	10	80.14	4.53
-3	-4	-10	68.64	3.54
-3	-4	-10	61.09	3.34
-3	-4	-10	62.13	4.11
3	4	10	60.71	3.78
3	4	10	62.39	3.32
3	4	10	65.90	3.69
-4	-4	-10	98.81	5.09
-4	-4	-10	103.04	5.60
-4	-4	-10	101.88	5.25
4	4	10	102.23	5.41
4	4	10	96.48	5.06
4	4	10	100.33	5.48
-5	-4	-10	88.90	4.65
-5	-4	-10	87.15	5.06
-5	-4	-10	83.24	4.43
5	4	10	85.16	4.90
5	4	10	90.14	4.85
5	4	10	83.12	4.42
-6	-4	-10	80.57	4.32
-6	-4	-10	86.29	4.85
-6	-4	-10	76.58	4.07
6	4	10	79.01	4.62

6	4	10	69.98	4.03
6	4	10	75.99	4.54
-7	-4	-10	84.26	4.95
-7	-4	-10	79.61	4.37
-7	-4	-10	74.90	4.08
7	4	10	79.28	4.71
7	4	10	70.92	4.05
-8	-4	-10	67.93	4.03
-8	-4	-10	72.74	4.72
8	4	10	64.79	3.68
8	4	10	71.31	5.86
-9	-4	-10	29.16	2.12
-9	-4	-10	29.10	3.61
-9	-4	-10	27.69	2.69
9	4	10	31.15	2.93
9	4	10	33.27	3.34
-10	-4	-10	44.21	3.44
-10	-4	-10	44.38	4.40
-10	-4	-10	40.21	5.04
-10	-4	-10	49.41	2.95
10	4	10	52.53	3.70
10	4	10	48.44	4.15
-11	-4	-10	35.10	3.14
-11	-4	-10	25.28	5.20
-11	-4	-10	31.43	2.43
-11	-4	-10	22.80	4.28
11	4	10	35.95	3.42
11	4	10	28.27	2.40
11	4	10	29.60	3.89
-12	-4	-10	21.42	3.18
-12	-4	-10	23.64	3.05
-12	-4	-10	21.23	2.34
-12	-4	-10	27.80	4.72
-12	-4	-10	18.37	4.64
12	4	10	22.53	4.12
12	4	10	25.52	3.47
12	4	10	29.67	3.62
12	4	10	17.54	2.35
-13	-4	-10	22.78	5.50
-13	-4	-10	22.16	3.84
-13	-4	-10	25.24	4.82
-13	-4	-10	22.53	2.59
-14	-4	-10	16.93	2.75
-14	-4	-10	19.42	6.08

-14	-4	-10	19.30	4.33
-9	5	10	7.97	2.91
-8	5	10	4.28	2.46
-7	5	10	21.01	1.98
-7	5	10	25.32	3.59
-7	5	10	22.44	1.58
-6	5	10	33.47	2.60
-5	5	10	42.36	2.53
-4	5	10	19.83	1.38
3	-5	-10	34.43	2.09
-3	5	10	34.61	1.97
2	-5	-10	37.04	2.14
-2	5	10	34.76	2.02
1	-5	-10	59.87	3.21
-1	5	10	60.87	3.15
0	-5	-10	34.96	2.00
0	5	10	33.44	1.97
0	5	10	32.77	2.73
-1	-5	-10	52.84	2.68
-1	-5	-10	46.46	2.84
1	5	10	52.77	2.99
1	5	10	43.83	2.89
1	5	10	47.89	3.15
-2	-5	-10	18.75	2.34
-2	-5	-10	24.11	1.81
-2	-5	-10	25.29	1.61
2	5	10	26.15	2.06
2	5	10	26.79	1.58
2	5	10	25.54	2.21
-3	-5	-10	33.82	2.00
-3	-5	-10	28.51	2.66
3	5	10	34.10	2.56
3	5	10	35.80	1.95
3	5	10	35.67	2.48
-4	-5	-10	25.08	2.72
-4	-5	-10	24.56	2.32
-4	-5	-10	23.81	1.64
4	5	10	24.84	2.31
4	5	10	27.28	2.23
4	5	10	24.89	1.59
-5	-5	-10	73.27	4.34
-5	-5	-10	74.75	3.91
-5	-5	-10	60.84	3.64
5	5	10	71.27	4.16

5	5	10	71.85	4.11
5	5	10	67.59	3.64
-6	-5	-10	45.28	2.79
-6	-5	-10	54.39	3.62
-6	-5	-10	53.68	3.11
6	5	10	52.43	3.43
6	5	10	48.03	2.78
-7	-5	-10	74.92	4.50
-7	-5	-10	77.31	4.01
-7	-5	-10	61.69	3.66
7	5	10	62.93	3.65
7	5	10	68.93	4.30
-8	-5	-10	18.33	1.92
-8	-5	-10	24.98	2.31
-8	-5	-10	20.90	3.05
8	5	10	24.60	2.76
8	5	10	22.91	2.54
8	5	10	18.36	1.65
-9	-5	-10	36.98	2.92
-9	-5	-10	33.81	3.61
-9	-5	-10	31.94	2.32
9	5	10	39.19	3.15
9	5	10	31.96	2.23
9	5	10	38.40	3.32
-10	-5	-10	22.32	3.48
-10	-5	-10	22.17	2.12
-10	-5	-10	25.21	3.66
-10	-5	-10	27.80	2.83
10	5	10	32.41	2.98
10	5	10	27.88	3.37
-11	-5	-10	29.07	4.02
-11	-5	-10	22.75	4.15
-11	-5	-10	25.78	2.33
-11	-5	-10	27.39	3.00
11	5	10	23.96	2.15
11	5	10	32.16	3.36
11	5	10	29.07	3.68
-12	-5	-10	7.34	4.35
-12	-5	-10	12.80	2.76
-12	-5	-10	7.57	2.76
-12	-5	-10	7.53	1.40
-12	-5	-10	9.48	1.96
12	5	10	7.37	1.33
12	5	10	10.47	2.10

12	5	10	10.51	2.42
-13	-5	-10	13.31	1.74
-13	-5	-10	20.23	3.60
-13	-5	-10	17.89	5.27
-13	-5	-10	13.39	2.32
13	5	10	15.28	2.94
13	5	10	12.75	2.46
-14	-5	-10	9.93	4.12
-14	-5	-10	8.95	1.78
-14	-5	-10	9.64	6.00
-9	6	10	15.62	3.27
-9	6	10	16.32	2.38
-8	6	10	12.57	1.99
-8	6	10	11.46	2.75
-8	6	10	12.75	1.53
-7	6	10	16.06	1.44
-7	6	10	9.80	2.49
-7	6	10	11.45	1.84
-6	6	10	20.88	1.61
-5	6	10	38.49	2.26
-4	6	10	47.72	2.62
3	-6	-10	39.84	2.35
-3	6	10	38.80	2.22
2	-6	-10	32.09	2.03
-2	6	10	31.89	1.89
1	-6	-10	99.10	5.05
-1	6	10	98.67	4.98
0	-6	-10	100.71	5.07
0	6	10	98.30	5.00
-1	-6	-10	80.39	4.06
1	6	10	74.32	4.30
1	6	10	79.99	4.01
-2	-6	-10	73.44	3.79
2	6	10	71.04	4.14
2	6	10	75.03	4.08
2	6	10	71.82	3.74
-3	-6	-10	56.18	3.06
-3	-6	-10	56.67	3.22
3	6	10	58.94	3.48
3	6	10	57.89	3.45
3	6	10	55.75	3.01
-4	-6	-10	53.79	3.97
-4	-6	-10	62.97	3.45
-4	-6	-10	60.20	3.25

4	6	10	61.66	3.68
4	6	10	61.47	3.69
4	6	10	64.01	3.23
-5	-6	-10	69.11	4.40
-5	-6	-10	74.77	4.07
-5	-6	-10	68.64	3.77
5	6	10	72.33	3.74
5	6	10	73.76	4.22
-6	-6	-10	72.49	3.97
-6	-6	-10	79.09	4.65
6	6	10	72.99	3.94
6	6	10	78.81	4.46
-7	-6	-10	85.49	4.91
-7	-6	-10	84.88	5.05
-7	-6	-10	76.97	4.35
7	6	10	89.74	4.84
7	6	10	85.34	4.87
7	6	10	78.88	4.33
-8	-6	-10	80.10	4.40
-8	-6	-10	64.64	4.16
-8	-6	-10	79.58	4.87
8	6	10	78.81	4.61
8	6	10	68.46	3.97
8	6	10	79.31	4.57
-9	-6	-10	59.13	4.35
-9	-6	-10	60.41	3.76
-9	-6	-10	48.96	3.23
9	6	10	50.70	3.19
9	6	10	64.05	5.61
9	6	10	65.63	3.95
-10	-6	-10	44.18	3.33
-10	-6	-10	35.44	2.63
-10	-6	-10	37.24	3.99
10	6	10	44.53	3.56
10	6	10	42.01	3.72
-11	-6	-10	14.51	1.96
-11	-6	-10	13.82	5.34
-11	-6	-10	19.95	3.73
-11	-6	-10	17.87	2.82
11	6	10	14.41	2.01
11	6	10	16.29	2.76
11	6	10	17.57	3.35
-12	-6	-10	9.89	1.41
-12	-6	-10	6.80	1.93

-12	-6	-10	13.57	4.34
-12	-6	-10	8.53	3.85
12	6	10	10.38	2.19
12	6	10	8.83	1.26
12	6	10	9.45	2.46
-13	-6	-10	10.97	2.28
-13	-6	-10	13.61	4.83
-13	-6	-10	12.00	3.41
-13	-6	-10	14.99	3.06
-13	-6	-10	9.87	1.57
13	6	10	13.96	2.62
13	6	10	18.52	2.50
13	6	10	10.43	2.82
-14	-6	-10	23.45	5.69
-14	-6	-10	34.61	5.93
-14	-6	-10	24.47	2.90
-8	7	10	21.02	2.45
-8	7	10	23.59	2.01
-8	7	10	18.15	3.16
-7	7	10	14.52	1.65
-6	7	10	7.54	2.15
-6	7	10	10.81	1.40
-5	7	10	8.15	1.30
-4	7	10	31.44	2.02
-3	7	10	19.56	1.51
2	-7	-10	27.34	1.90
-2	7	10	28.06	1.77
1	-7	-10	30.17	2.00
-1	7	10	30.13	1.85
0	-7	-10	75.87	3.99
0	7	10	75.14	3.90
-1	-7	-10	18.81	1.45
1	7	10	18.35	1.37
-2	-7	-10	27.13	1.76
2	7	10	25.27	2.41
2	7	10	28.07	1.70
-3	-7	-10	6.64	1.08
3	7	10	8.22	1.01
3	7	10	7.73	1.28
-4	-7	-10	35.41	2.33
-4	-7	-10	34.22	2.07
4	7	10	34.23	2.70
4	7	10	33.02	2.01
-5	-7	-10	10.70	1.18

-5	-7	-10	8.14	1.94
-5	-7	-10	11.78	1.67
5	7	10	12.17	2.12
5	7	10	10.33	1.13
-6	-7	-10	39.99	2.47
-6	-7	-10	44.83	2.84
-6	-7	-10	43.42	3.38
6	7	10	44.92	3.11
6	7	10	43.20	2.46
6	7	10	42.08	3.18
-7	-7	-10	22.48	1.81
-7	-7	-10	25.38	2.90
-7	-7	-10	29.43	2.32
7	7	10	28.98	2.74
7	7	10	23.35	1.77
7	7	10	29.99	2.57
-8	-7	-10	48.95	3.17
-8	-7	-10	47.44	3.69
8	7	10	40.74	2.64
8	7	10	46.13	3.35
8	7	10	44.81	3.53
-9	-7	-10	29.23	3.31
-9	-7	-10	23.42	1.93
-9	-7	-10	27.74	2.66
9	7	10	28.61	2.89
9	7	10	25.91	3.09
9	7	10	20.51	1.83
-10	-7	-10	31.46	3.74
-10	-7	-10	35.48	3.72
-10	-7	-10	27.99	2.24
10	7	10	28.22	3.45
10	7	10	34.76	3.27
-11	-7	-10	0.62	2.33
-11	-7	-10	3.89	1.08
11	7	10	6.07	1.86
11	7	10	4.25	2.08
11	7	10	4.15	1.26
11	7	10	2.74	2.60
-12	-7	-10	10.87	3.62
-12	-7	-10	12.43	2.83
-12	-7	-10	12.05	1.99
12	7	10	7.55	3.03
12	7	10	8.49	1.22
12	7	10	10.58	2.41

12	7	10	11.58	2.29
-13	-7	-10	12.20	3.19
-13	-7	-10	13.47	2.23
-13	-7	-10	11.45	1.90
-13	-7	-10	12.37	4.55
13	7	10	6.55	3.35
13	7	10	10.42	2.82
13	7	10	10.10	2.59
13	7	10	7.52	1.33
-14	-7	-10	13.13	2.81
-14	-7	-10	13.18	5.28
-14	-7	-10	19.99	3.89
-7	8	10	20.24	2.50
-7	8	10	24.16	4.64
-7	8	10	21.29	2.01
-6	8	10	20.08	2.34
-6	8	10	22.98	1.90
-6	8	10	23.07	3.80
-5	8	10	34.38	2.27
-4	8	10	37.08	2.31
-3	8	10	45.66	2.65
-2	8	10	52.19	2.90
1	-8	-10	89.07	4.65
-1	8	10	88.48	4.57
0	-8	-10	66.99	3.61
0	8	10	65.77	3.51
-1	-8	-10	75.61	3.97
1	8	10	73.82	3.89
-2	-8	-10	64.79	3.49
2	8	10	64.66	3.42
-3	-8	-10	78.41	4.09
3	8	10	75.65	4.48
3	8	10	78.82	4.03
3	8	10	77.20	4.37
-4	-8	-10	43.68	2.79
-4	-8	-10	47.17	2.57
-4	-8	-10	38.26	3.19
4	8	10	47.08	3.06
4	8	10	45.80	3.12
4	8	10	46.02	2.54
-5	-8	-10	50.48	2.84
-5	-8	-10	50.10	3.11
5	8	10	51.77	3.33
5	8	10	52.42	3.41

5	8	10	49.60	2.79
-6	-8	-10	30.11	3.23
-6	-8	-10	30.59	2.06
-6	-8	-10	33.78	2.44
6	8	10	34.56	2.83
6	8	10	31.91	2.01
6	8	10	32.87	2.70
-7	-8	-10	40.42	2.77
-7	-8	-10	43.00	3.55
-7	-8	-10	45.40	2.96
7	8	10	46.30	3.24
7	8	10	40.13	2.48
7	8	10	43.80	3.35
-8	-8	-10	45.75	3.09
-8	-8	-10	39.79	2.53
-8	-8	-10	41.02	3.64
8	8	10	44.88	3.35
8	8	10	36.42	2.46
8	8	10	42.69	3.45
-9	-8	-10	40.92	3.84
-9	-8	-10	38.73	2.60
-9	-8	-10	44.52	3.25
9	8	10	44.19	3.49
9	8	10	43.14	3.72
-10	-8	-10	32.32	2.35
-10	-8	-10	33.20	3.84
-10	-8	-10	33.50	3.18
10	8	10	31.77	4.62
10	8	10	37.61	3.42
-11	-8	-10	30.52	4.19
-11	-8	-10	33.06	2.45
-11	-8	-10	32.69	3.38
11	8	10	37.52	3.71
11	8	10	29.75	3.97
11	8	10	29.74	2.45
-12	-8	-10	22.20	4.38
-12	-8	-10	24.39	3.42
-12	-8	-10	24.48	3.88
-12	-8	-10	24.60	2.30
12	8	10	20.70	2.16
12	8	10	20.36	3.78
12	8	10	23.60	4.11
-13	-8	-10	17.80	4.44
-13	-8	-10	21.99	2.39

-13	-8	-10	15.41	4.42
-13	-8	-10	26.16	3.75
13	8	10	19.13	3.74
13	8	10	17.00	2.16
13	8	10	15.84	2.96
13	8	10	27.04	4.31
-14	-8	-10	16.28	3.93
-14	-8	-10	12.06	1.69
-14	-8	-10	7.74	5.10
-14	-8	-10	10.34	3.17
-14	-8	-10	11.31	2.26
14	8	10	12.04	3.44
14	8	10	13.62	3.03
-7	9	10	17.08	2.65
-7	9	10	15.67	3.05
-7	9	10	16.76	2.15
-6	9	10	10.31	1.53
-6	9	10	12.57	2.70
-5	9	10	34.25	2.38
-4	9	10	19.51	1.74
-3	9	10	20.46	1.70
-2	9	10	19.85	1.63
1	-9	-10	46.09	3.04
-1	9	10	51.44	2.78
0	-9	-10	28.87	2.80
0	9	10	28.35	1.87
-1	-9	-10	42.52	2.64
1	9	10	42.66	2.45
-2	-9	-10	23.18	1.73
2	9	10	21.34	1.59
-3	-9	-10	47.15	2.79
3	9	10	48.03	2.67
-4	-9	-10	24.59	2.04
-4	-9	-10	18.78	2.59
-4	-9	-10	22.61	1.71
4	9	10	26.22	2.34
4	9	10	23.32	2.51
-5	-9	-10	38.51	2.57
-5	-9	-10	36.19	2.62
5	9	10	39.71	3.10
5	9	10	39.93	2.94
-6	-9	-10	26.29	4.53
-6	-9	-10	21.55	2.17
6	9	10	23.92	2.74

6	9	10	24.54	2.54
-7	-9	-10	44.51	2.74
-7	-9	-10	48.83	3.17
-7	-9	-10	46.47	3.76
7	9	10	48.71	3.49
7	9	10	50.15	3.62
7	9	10	42.83	2.67
-8	-9	-10	24.66	1.92
-8	-9	-10	23.74	3.19
-8	-9	-10	27.62	2.57
8	9	10	27.93	2.93
8	9	10	25.54	3.11
-9	-9	-10	31.41	2.33
-9	-9	-10	35.97	3.08
-9	-9	-10	35.67	3.69
9	9	10	40.82	3.39
9	9	10	32.64	3.48
-10	-9	-10	5.29	1.59
-10	-9	-10	3.71	2.17
-10	-9	-10	3.34	0.94
10	9	10	4.53	2.13
10	9	10	3.10	1.68
-11	-9	-10	24.58	4.09
-11	-9	-10	22.98	3.80
-11	-9	-10	28.19	3.27
-11	-9	-10	27.56	2.22
11	9	10	28.34	3.68
11	9	10	23.17	4.33
11	9	10	26.19	3.85
11	9	10	23.21	2.11
-12	-9	-10	27.38	2.38
-12	-9	-10	28.92	4.65
-12	-9	-10	27.95	4.93
-12	-9	-10	25.83	3.54
12	9	10	24.46	4.29
12	9	10	26.91	4.06
12	9	10	21.17	2.28
12	9	10	22.74	4.68
-13	-9	-10	27.85	2.46
-13	-9	-10	22.85	3.95
-13	-9	-10	23.30	4.51
-13	-9	-10	30.38	5.20
13	9	10	29.99	4.46
13	9	10	15.12	3.88

13	9	10	19.78	2.33
13	9	10	15.21	3.22
-14	-9	-10	9.70	1.60
-14	-9	-10	11.96	2.71
-14	-9	-10	12.04	2.23
-14	-9	-10	10.45	4.84
-14	-9	-10	13.36	3.94
14	9	10	13.21	3.50
14	9	10	11.81	3.01
-6	10	10	22.72	4.91
-6	10	10	19.38	2.84
-5	10	10	28.94	2.39
-5	10	10	32.09	2.96
-4	10	10	36.76	2.48
-3	10	10	19.63	1.78
-2	10	10	36.69	2.33
-1	10	10	38.68	2.39
0	-10	-10	56.66	3.26
0	10	10	53.97	3.08
-1	-10	-10	47.63	2.82
1	10	10	44.65	2.67
-2	-10	-10	64.34	3.53
2	10	10	60.79	3.39
-3	-10	-10	44.68	2.71
-4	-10	-10	62.43	3.37
4	10	10	63.33	3.98
4	10	10	51.93	4.52
-5	-10	-10	47.43	3.68
-5	-10	-10	54.33	2.96
-5	-10	-10	48.34	3.12
5	10	10	53.24	3.60
5	10	10	48.45	3.87
-6	-10	-10	80.03	4.44
-6	-10	-10	79.01	4.64
6	10	10	90.53	5.01
6	10	10	85.13	5.07
-7	-10	-10	55.65	3.59
-7	-10	-10	52.01	4.42
-7	-10	-10	56.96	3.26
7	10	10	61.82	4.02
7	10	10	58.40	4.10
-8	-10	-10	64.41	4.11
-8	-10	-10	63.49	3.70
-8	-10	-10	62.21	4.83

8	10	10	70.03	4.59
8	10	10	72.69	4.52
-9	-10	-10	41.65	2.74
-9	-10	-10	43.16	3.43
-9	-10	-10	39.77	4.08
9	10	10	45.92	3.99
9	10	10	47.22	3.85
-10	-10	-10	24.14	2.97
-10	-10	-10	20.95	3.74
-10	-10	-10	20.80	2.00
10	10	10	28.03	3.53
10	10	10	19.72	1.88
10	10	10	21.75	3.41
-11	-10	-10	9.16	2.64
-11	-10	-10	11.44	3.01
-11	-10	-10	14.27	1.89
-11	-10	-10	13.74	2.07
11	10	10	14.11	3.20
11	10	10	10.65	1.78
11	10	10	8.71	2.42
11	10	10	12.70	2.29
-12	-10	-10	38.21	4.93
-12	-10	-10	43.63	4.08
-12	-10	-10	37.35	2.75
-12	-10	-10	29.34	5.13
12	10	10	32.09	2.69
12	10	10	35.58	5.30
12	10	10	35.10	4.45
12	10	10	32.23	4.74
-13	-10	-10	29.07	5.20
-13	-10	-10	25.25	2.54
-13	-10	-10	23.66	4.53
-13	-10	-10	22.16	4.13
13	10	10	29.19	5.63
13	10	10	19.30	3.36
13	10	10	30.11	4.60
-14	-10	-10	20.96	4.24
-14	-10	-10	27.10	2.82
-14	-10	-10	24.28	5.03
-14	-10	-10	28.11	4.68
14	10	10	20.93	3.87
14	10	10	31.65	5.02
-5	11	10	12.81	1.81
-3	11	10	13.97	1.73

-2	11	10	16.86	1.75
-1	11	10	10.01	1.42
0	11	10	31.45	2.12
-1	-11	-10	25.88	2.11
1	11	10	25.56	1.86
-2	-11	-10	27.55	2.06
2	11	10	26.22	1.87
-4	-11	-10	48.05	2.96
4	11	10	50.42	3.53
-5	-11	-10	30.54	2.15
5	11	10	28.50	2.95
5	11	10	28.90	3.01
-6	-11	-10	39.38	2.93
-6	-11	-10	41.71	2.70
6	11	10	46.46	3.48
6	11	10	44.42	3.52
-7	-11	-10	23.40	4.01
-7	-11	-10	30.17	2.18
-7	-11	-10	29.85	2.57
7	11	10	31.17	3.29
7	11	10	31.76	3.30
-8	-11	-10	30.72	3.67
-8	-11	-10	31.85	2.31
-8	-11	-10	34.75	2.81
8	11	10	26.74	2.40
8	11	10	34.11	3.56
-9	-11	-10	14.37	2.60
-9	-11	-10	13.29	2.28
-9	-11	-10	13.92	1.78
9	11	10	13.75	2.29
9	11	10	14.28	2.39
9	11	10	11.03	1.55
-10	-11	-10	8.99	2.48
-10	-11	-10	10.69	1.76
-10	-11	-10	7.77	1.82
10	11	10	8.66	2.30
10	11	10	7.31	1.05
10	11	10	8.95	2.04
-11	-11	-10	4.07	1.17
-11	-11	-10	4.95	2.71
-11	-11	-10	3.69	1.94
-11	-11	-10	5.21	2.97
11	11	10	7.31	2.48
11	11	10	2.65	1.07

11	11	10	-2.08	3.33
11	11	10	4.36	2.15
-12	-11	-10	17.57	3.30
-12	-11	-10	22.49	3.85
-12	-11	-10	17.81	2.22
-12	-11	-10	18.49	3.57
12	11	10	18.87	3.97
12	11	10	12.54	2.55
12	11	10	16.87	3.02
12	11	10	12.32	2.17
-13	-11	-10	10.56	1.57
-13	-11	-10	11.69	3.61
-13	-11	-10	9.95	2.59
-13	-11	-10	8.58	4.04
13	11	10	8.46	2.85
13	11	10	10.83	4.11
13	11	10	15.73	3.37
-14	-11	-10	10.91	3.01
-14	-11	-10	20.57	2.66
-14	-11	-10	15.66	4.94
-14	-11	-10	9.70	5.47
14	11	10	13.71	3.76
14	11	10	15.47	3.17
0	12	10	24.32	1.94
1	12	10	44.15	2.73
-2	-12	-10	15.55	1.85
2	12	10	16.49	1.58
3	12	10	14.31	1.41
-4	-12	-10	56.60	3.37
4	12	10	54.95	3.83
-5	-12	-10	80.55	4.44
5	12	10	76.58	4.96
5	12	10	83.55	4.94
-6	-12	-10	39.41	2.62
-6	-12	-10	30.16	3.48
6	12	10	40.65	3.43
6	12	10	40.47	3.44
-7	-12	-10	39.57	3.00
-7	-12	-10	42.74	2.77
7	12	10	45.61	3.76
7	12	10	42.62	3.71
7	12	10	38.69	2.58
-8	-12	-10	29.54	2.34
-8	-12	-10	26.61	4.06

-8	-12	-10	26.38	2.76
8	12	10	32.68	3.56
8	12	10	26.14	2.11
8	12	10	33.61	3.50
-9	-12	-10	31.28	3.13
-9	-12	-10	32.96	2.46
-9	-12	-10	28.14	4.00
9	12	10	37.80	3.77
9	12	10	29.09	2.27
9	12	10	31.78	3.88
-10	-12	-10	12.54	2.01
-10	-12	-10	11.97	2.66
-10	-12	-10	18.38	2.03
10	12	10	13.75	2.60
10	12	10	12.56	1.79
10	12	10	21.42	3.65
-11	-12	-10	28.61	3.97
-11	-12	-10	24.34	4.75
-11	-12	-10	23.14	2.40
-11	-12	-10	29.94	4.73
11	12	10	26.59	4.11
11	12	10	21.10	2.32
11	12	10	27.01	4.58
-12	-12	-10	25.22	4.13
-12	-12	-10	16.75	3.44
-12	-12	-10	20.48	2.47
-12	-12	-10	16.11	3.90
12	12	10	14.95	3.33
12	12	10	22.86	4.44
12	12	10	18.24	4.28
-13	-12	-10	18.09	3.81
-13	-12	-10	14.99	2.89
-13	-12	-10	20.34	4.26
-13	-12	-10	22.78	2.62
13	12	10	14.61	3.67
13	12	10	12.37	3.74
-3	-13	-10	12.65	1.88
3	13	10	13.32	2.02
-4	-13	-10	7.47	1.10
4	13	10	9.94	1.44
-5	-13	-10	18.54	2.03
5	13	10	18.30	1.67
5	13	10	17.31	3.07
-6	-13	-10	17.01	1.96

6	13	10	16.66	2.98
6	13	10	13.95	1.65
6	13	10	15.74	3.00
-7	-13	-10	23.12	2.11
-7	-13	-10	13.92	2.28
-7	-13	-10	17.42	2.42
7	13	10	18.15	3.24
7	13	10	18.19	1.80
7	13	10	21.70	3.34
-8	-13	-10	21.33	2.18
-8	-13	-10	19.71	2.67
8	13	10	19.28	1.96
8	13	10	20.42	3.49
8	13	10	19.68	3.46
-9	-13	-10	19.29	2.19
-9	-13	-10	13.73	2.56
-9	-13	-10	21.18	3.01
9	13	10	23.32	3.79
9	13	10	20.66	3.74
9	13	10	21.93	2.97
-10	-13	-10	21.29	3.50
-10	-13	-10	11.56	2.64
-10	-13	-10	18.13	2.23
10	13	10	14.28	2.49
10	13	10	14.90	2.71
-11	-13	-10	22.29	4.08
-11	-13	-10	18.40	2.39
-11	-13	-10	26.51	5.29
-11	-13	-10	13.47	3.02
11	13	10	24.11	4.45
11	13	10	12.91	3.64
11	13	10	15.81	4.35
-12	-13	-10	15.11	2.44
-12	-13	-10	13.41	2.85
-12	-13	-10	12.77	3.99
-12	-13	-10	11.77	3.32
12	13	10	16.32	4.57
12	13	10	15.53	2.99
12	13	10	16.47	3.69
-13	-13	-10	6.17	4.45
-13	-13	-10	9.29	1.58
-13	-13	-10	12.65	3.99
-13	-13	-10	7.00	2.94
13	13	10	7.99	3.16

-5	-14	-10	11.49	1.95
5	14	10	7.04	3.15
-6	-14	-10	19.02	2.09
6	14	10	13.58	2.24
6	14	10	20.50	3.25
-7	-14	-10	16.60	2.18
7	14	10	21.11	3.38
7	14	10	20.44	3.45
-8	-14	-10	22.07	2.33
8	14	10	21.40	3.81
8	14	10	23.75	3.73
-9	-14	-10	17.65	2.35
-9	-14	-10	20.64	3.57
9	14	10	16.18	2.54
9	14	10	12.74	2.71
-10	-14	-10	37.79	2.95
-10	-14	-10	41.18	5.67
10	14	10	37.76	4.73
10	14	10	26.80	5.60
-11	-14	-10	21.95	4.42
-11	-14	-10	19.70	2.57
11	14	10	17.57	4.34
11	14	10	15.50	2.95
-12	-14	-10	19.63	4.51
-12	-14	-10	13.51	1.75
12	14	10	13.20	3.26
-6	-15	-10	15.25	2.19
-7	-15	-10	17.99	2.26
7	15	10	14.26	2.25
-8	-15	-10	10.89	3.25
8	15	10	11.13	2.28
-9	-15	-10	20.47	2.43
9	15	10	12.72	2.62
-10	-15	-10	19.55	2.62
10	15	10	22.26	5.18
10	15	10	16.40	2.87
7	16	10	19.97	4.03
-7	-9	11	0.51	3.31
6	9	-11	4.51	4.04
6	9	-11	-1.20	5.03
6	9	-11	2.04	3.43
-6	-9	11	-3.08	2.99
-6	-9	11	-1.78	3.21
-6	-9	11	2.14	3.13

-6	-9	11	1.30	1.78
5	9	-11	3.03	3.58
5	9	-11	-1.90	3.21
5	9	-11	-0.89	1.05
5	9	-11	0.31	3.85
-5	-9	11	-3.91	3.35
-5	-9	11	-0.18	1.67
-5	-9	11	0.95	2.95
4	9	-11	1.67	3.28
4	9	-11	-0.06	3.22
4	9	-11	1.10	0.86
4	9	-11	1.39	3.33
-4	-9	11	-2.14	3.00
-4	-9	11	2.40	2.91
-4	-9	11	0.19	2.83
-4	-9	11	-0.68	1.67
3	9	-11	6.93	1.03
3	9	-11	6.07	4.73
3	9	-11	0.89	3.25
3	9	-11	6.30	3.10
-3	-9	11	5.51	2.83
-3	-9	11	5.06	2.99
-3	-9	11	6.05	2.85
-3	-9	11	5.81	1.84
2	9	-11	-0.28	3.78
2	9	-11	4.04	3.45
2	9	-11	1.81	0.94
2	9	-11	2.70	3.18
-2	-9	11	-0.06	2.70
-2	-9	11	-1.88	2.38
-2	-9	11	-0.79	3.04
-2	-9	11	0.54	1.79
1	9	-11	2.58	3.38
1	9	-11	0.64	0.98
-1	-9	11	0.77	1.89
-1	-9	11	-0.74	2.20
0	9	-11	0.39	1.02
0	9	-11	5.15	3.68
0	-9	11	1.11	1.98
0	-9	11	1.94	2.74
-1	9	-11	1.53	1.09
-1	9	-11	5.28	3.45
1	-9	11	2.20	1.96
8	8	-11	9.31	4.18

-8	-8	11	1.63	3.01
-8	-8	11	2.45	2.15
7	8	-11	12.03	4.55
7	8	-11	4.96	3.14
-7	-8	11	0.08	2.94
-7	-8	11	0.07	2.75
-7	-8	11	6.40	3.35
6	8	-11	2.89	2.90
6	8	-11	2.45	3.42
6	8	-11	3.51	3.29
-6	-8	11	-1.70	2.74
-6	-8	11	6.59	2.83
-6	-8	11	3.93	1.81
5	8	-11	1.06	2.90
5	8	-11	2.84	3.15
-5	-8	11	4.47	1.69
-5	-8	11	3.84	2.95
-5	-8	11	5.07	2.80
4	8	-11	10.21	1.42
4	8	-11	7.18	3.02
4	8	-11	9.16	3.02
-4	-8	11	8.60	2.90
-4	-8	11	10.04	2.81
3	8	-11	18.98	1.68
3	8	-11	14.96	3.22
3	8	-11	13.09	3.05
3	8	-11	13.05	4.35
-3	-8	11	15.53	3.07
-3	-8	11	13.80	2.80
2	8	-11	12.64	2.94
2	8	-11	16.31	3.20
2	8	-11	18.05	1.80
-2	-8	11	15.64	2.53
-2	-8	11	16.99	3.02
-2	-8	11	16.91	3.04
1	8	-11	9.35	1.12
1	8	-11	6.48	2.66
1	8	-11	8.61	3.79
1	8	-11	13.13	2.99
-1	-8	11	9.17	2.76
-1	-8	11	10.19	1.94
-1	-8	11	2.96	2.73
-1	-8	11	8.47	2.20
0	8	-11	4.98	3.09

0	8	-11	0.04	2.53
0	8	-11	3.16	1.00
0	8	-11	4.46	2.80
0	-8	11	5.71	2.93
0	-8	11	4.29	2.38
0	-8	11	4.76	1.72
0	-8	11	3.95	1.83
0	-8	11	3.01	2.01
-1	8	-11	3.50	3.00
-1	8	-11	1.76	2.91
-1	8	-11	0.81	1.00
1	-8	11	-0.90	1.90
1	-8	11	1.14	1.86
-2	8	-11	6.08	2.96
-2	8	-11	4.46	1.10
2	-8	11	3.74	1.92
-3	8	-11	5.50	1.17
-3	8	-11	9.18	3.02
3	-8	11	8.42	2.09
-9	-7	11	-0.12	2.75
-9	-7	11	-1.94	2.23
8	7	-11	2.86	2.39
-8	-7	11	4.50	2.67
-8	-7	11	8.08	2.26
7	7	-11	2.81	3.46
7	7	-11	3.52	2.57
-7	-7	11	1.37	1.83
-7	-7	11	4.31	2.62
-7	-7	11	-1.69	2.51
6	7	-11	4.61	2.94
6	7	-11	2.01	2.64
-6	-7	11	3.82	1.74
-6	-7	11	-2.37	2.50
5	7	-11	1.32	2.89
5	7	-11	0.67	2.53
5	7	-11	2.94	3.00
-5	-7	11	0.30	2.42
-5	-7	11	0.10	2.22
4	7	-11	12.69	2.63
4	7	-11	13.42	2.79
4	7	-11	13.19	1.39
4	7	-11	10.23	2.78
-4	-7	11	14.09	2.65
-4	-7	11	14.34	2.73

-4	-7	11	10.76	2.66
3	7	-11	12.92	2.65
3	7	-11	14.59	2.86
3	7	-11	12.34	1.50
3	7	-11	15.52	2.82
-3	-7	11	14.20	2.62
-3	-7	11	13.05	2.56
-3	-7	11	19.59	2.69
2	7	-11	11.04	2.33
2	7	-11	13.05	1.61
2	7	-11	10.63	2.54
2	7	-11	15.22	2.81
-2	-7	11	15.22	2.47
-2	-7	11	9.53	2.56
-2	-7	11	16.09	2.76
-2	-7	11	14.80	2.28
1	7	-11	12.89	1.59
1	7	-11	11.98	2.82
1	7	-11	12.42	2.43
1	7	-11	13.40	2.25
-1	-7	11	13.45	2.76
-1	-7	11	13.36	2.08
-1	-7	11	12.30	2.60
-1	-7	11	9.73	1.66
0	7	-11	13.64	2.87
0	7	-11	18.71	3.50
0	7	-11	12.68	2.45
0	7	-11	11.05	1.63
0	-7	11	12.33	1.87
0	-7	11	10.51	2.62
0	-7	11	11.00	1.73
0	-7	11	11.25	2.14
0	-7	11	10.85	1.95
-1	7	-11	1.86	2.73
-1	7	-11	2.49	2.51
-1	7	-11	-0.94	0.86
1	-7	11	-0.26	1.63
1	-7	11	0.65	1.67
1	-7	11	-0.49	2.36
1	-7	11	1.08	1.53
-2	7	-11	4.25	1.04
-2	7	-11	5.32	2.16
-2	7	-11	4.36	2.57
-2	7	-11	5.30	2.78

2	-7	11	4.63	1.87
2	-7	11	5.65	1.77
-3	7	-11	5.40	2.39
-3	7	-11	3.38	2.42
-3	7	-11	4.97	2.91
-3	7	-11	2.41	1.04
3	-7	11	4.17	1.80
3	-7	11	-1.63	1.65
-4	7	-11	2.48	1.04
-4	7	-11	3.66	2.50
4	-7	11	4.28	1.92
-5	7	-11	2.92	2.65
5	-7	11	4.12	2.19
-10	-6	11	4.32	2.74
-10	-6	11	2.94	2.60
-9	-6	11	6.61	2.42
-9	-6	11	4.21	2.49
8	6	-11	-1.17	2.43
-8	-6	11	7.90	2.54
-8	-6	11	3.94	2.42
7	6	-11	6.85	3.21
-7	-6	11	5.14	2.02
-7	-6	11	1.67	2.33
-7	-6	11	6.37	2.37
6	6	-11	3.04	2.27
6	6	-11	6.95	2.75
6	6	-11	2.07	3.21
-6	-6	11	6.78	1.73
-6	-6	11	6.58	2.28
-6	-6	11	3.79	2.32
5	6	-11	9.83	2.60
5	6	-11	14.10	3.29
5	6	-11	7.69	2.44
-5	-6	11	12.29	1.80
-5	-6	11	12.23	2.54
-5	-6	11	12.17	2.41
4	6	-11	8.47	1.14
4	6	-11	11.25	2.84
4	6	-11	12.09	2.36
4	6	-11	9.79	2.47
-4	-6	11	9.94	1.66
-4	-6	11	9.96	2.32
-4	-6	11	6.50	2.50
3	6	-11	15.69	1.39

3	6	-11	20.99	3.65
3	6	-11	13.67	2.53
3	6	-11	13.34	2.44
-3	-6	11	18.04	2.59
-3	-6	11	13.92	2.33
-3	-6	11	12.07	2.31
-3	-6	11	10.64	1.39
2	6	-11	6.84	0.87
2	6	-11	6.76	2.18
2	6	-11	9.82	2.30
2	6	-11	9.80	1.98
-2	-6	11	7.91	1.47
-2	-6	11	9.36	1.95
-2	-6	11	3.68	2.10
-2	-6	11	8.75	1.34
1	6	-11	10.52	2.17
1	6	-11	15.06	3.23
1	6	-11	9.45	2.41
-1	-6	11	10.24	1.60
-1	-6	11	5.58	2.24
-1	-6	11	15.41	2.69
-1	-6	11	6.98	1.39
0	6	-11	29.46	3.75
0	6	-11	22.95	1.80
0	6	-11	21.82	3.62
0	6	-11	27.15	3.48
0	-6	11	21.23	2.95
0	-6	11	17.53	2.62
0	-6	11	16.77	2.52
0	-6	11	20.67	2.86
-1	6	-11	18.65	3.24
-1	6	-11	19.59	3.12
-1	6	-11	19.01	3.67
-1	6	-11	15.78	1.70
1	-6	11	17.84	2.89
1	-6	11	14.81	2.57
1	-6	11	13.48	1.77
1	-6	11	19.68	2.83
-2	6	-11	8.94	1.77
-2	6	-11	2.11	2.87
-2	6	-11	8.68	1.09
-2	6	-11	9.32	3.65
-2	6	-11	6.76	1.90
2	-6	11	9.03	1.73

2	-6	11	7.42	2.40
2	-6	11	6.25	1.50
2	-6	11	9.12	1.75
-3	6	-11	13.75	1.76
-3	6	-11	18.30	3.00
-3	6	-11	12.84	2.33
3	-6	11	12.93	1.85
3	-6	11	11.23	2.63
3	-6	11	20.38	3.10
3	-6	11	14.96	3.08
-4	6	-11	8.02	2.25
-4	6	-11	6.16	2.00
-4	6	-11	6.70	1.11
-4	6	-11	7.38	2.87
4	-6	11	5.00	2.76
4	-6	11	7.30	1.83
4	-6	11	4.66	1.99
-5	6	-11	14.49	2.64
-5	6	-11	11.68	1.90
5	-6	11	11.34	2.16
-6	6	-11	9.74	1.34
-6	6	-11	12.48	2.81
6	-6	11	18.78	3.89
-10	-5	11	4.03	2.44
-10	-5	11	8.17	2.69
9	5	-11	7.87	2.97
-9	-5	11	9.48	2.47
-9	-5	11	14.13	3.01
-9	-5	11	13.36	2.56
8	5	-11	5.57	2.38
-8	-5	11	4.20	2.27
-8	-5	11	7.40	2.29
-8	-5	11	7.74	2.24
7	5	-11	5.70	2.89
7	5	-11	3.83	2.55
7	5	-11	0.82	3.13
-7	-5	11	5.71	2.16
-7	-5	11	2.84	1.93
-7	-5	11	3.12	2.16
6	5	-11	0.18	2.04
6	5	-11	-1.76	2.84
6	5	-11	0.86	2.34
-6	-5	11	1.67	2.00
-6	-5	11	2.05	1.65

-6	-5	11	-0.30	2.05
5	5	-11	11.87	2.50
5	5	-11	12.75	2.21
5	5	-11	11.42	2.78
-5	-5	11	14.39	2.37
-5	-5	11	10.70	2.28
-5	-5	11	16.38	2.81
4	5	-11	9.69	2.51
4	5	-11	8.71	2.14
4	5	-11	7.48	2.22
-4	-5	11	9.99	1.56
-4	-5	11	9.36	2.05
-4	-5	11	6.82	2.04
3	5	-11	31.24	3.88
3	5	-11	24.20	3.47
3	5	-11	29.27	3.37
3	5	-11	23.30	1.62
-3	-5	11	20.32	2.15
-3	-5	11	27.18	3.28
-3	-5	11	18.05	3.31
2	5	-11	17.28	3.17
2	5	-11	20.75	3.30
2	5	-11	18.42	1.47
2	5	-11	20.50	3.40
-2	-5	11	21.64	2.49
-2	-5	11	14.78	1.99
-2	-5	11	18.74	2.85
1	5	-11	13.38	1.41
1	5	-11	17.36	3.31
1	5	-11	16.29	2.86
1	5	-11	20.88	2.99
-1	-5	11	12.12	1.48
-1	-5	11	16.07	2.40
-1	-5	11	18.74	2.54
-1	-5	11	12.16	2.26
0	5	-11	8.36	2.18
0	5	-11	11.43	2.15
0	5	-11	9.39	1.73
0	-5	11	8.49	1.55
0	-5	11	8.57	1.55
0	-5	11	9.32	1.34
0	-5	11	8.25	2.18
-1	5	-11	17.04	1.58
-1	5	-11	15.76	2.78

-1	5	-11	17.58	3.24
-1	5	-11	17.23	2.81
1	-5	11	17.81	2.64
1	-5	11	12.49	2.19
1	-5	11	17.92	2.60
1	-5	11	13.94	2.39
-2	5	-11	18.17	3.16
-2	5	-11	14.61	2.71
-2	5	-11	19.34	2.81
-2	5	-11	15.27	1.60
2	-5	11	10.94	2.37
2	-5	11	15.84	2.70
2	-5	11	11.29	1.56
2	-5	11	17.16	2.61
-3	5	-11	14.28	2.69
-3	5	-11	15.28	2.49
-3	5	-11	18.28	3.34
-3	5	-11	13.70	1.66
-3	5	-11	14.17	2.89
3	-5	11	11.61	2.35
3	-5	11	14.92	2.82
3	-5	11	18.85	2.79
3	-5	11	10.17	1.58
-4	5	-11	3.92	1.77
-4	5	-11	6.34	0.99
-4	5	-11	7.19	2.12
-4	5	-11	8.54	2.02
4	-5	11	7.49	1.68
4	-5	11	4.40	1.32
4	-5	11	6.09	2.18
4	-5	11	6.16	1.78
-5	5	-11	9.49	1.98
-5	5	-11	12.01	2.50
-5	5	-11	14.28	3.03
-5	5	-11	11.21	1.78
5	-5	11	6.70	2.44
5	-5	11	16.58	3.18
5	-5	11	13.47	2.18
-6	5	-11	10.51	2.64
-6	5	-11	13.51	3.15
-6	5	-11	8.55	1.24
6	-5	11	11.53	2.08
-7	5	-11	7.49	3.39
-7	5	-11	5.01	2.67

-7	5	-11	5.71	1.20
7	-5	11	10.32	2.11
-11	-4	11	2.61	2.41
-11	-4	11	3.01	2.79
-10	-4	11	4.46	2.51
-10	-4	11	3.99	2.29
9	4	-11	2.66	2.11
-9	-4	11	6.01	2.03
-9	-4	11	3.46	2.20
-9	-4	11	3.26	2.34
8	4	-11	12.16	2.49
8	4	-11	19.96	3.74
-8	-4	11	13.74	2.37
-8	-4	11	18.04	3.83
-8	-4	11	19.46	3.40
7	4	-11	9.36	2.56
7	4	-11	2.57	3.30
7	4	-11	2.23	2.06
-7	-4	11	4.24	1.93
-7	-4	11	3.76	1.95
-7	-4	11	-1.14	1.93
6	4	-11	5.10	2.27
6	4	-11	3.24	3.07
6	4	-11	3.94	2.57
-6	-4	11	3.32	1.97
-6	-4	11	6.07	2.01
-6	-4	11	6.50	1.81
5	4	-11	5.63	1.88
5	4	-11	6.60	2.41
5	4	-11	7.55	2.21
-5	-4	11	9.47	2.43
-5	-4	11	8.69	2.01
-5	-4	11	10.35	1.74
4	4	-11	12.80	2.15
4	4	-11	16.07	3.08
-4	-4	11	18.32	2.55
-4	-4	11	15.75	2.86
-4	-4	11	9.74	2.06
3	4	-11	14.26	1.16
3	4	-11	10.64	2.06
3	4	-11	16.79	2.90
-3	-4	11	15.89	2.79
-3	-4	11	11.74	1.53
-3	-4	11	13.35	2.37

2	4	-11	10.10	1.09
2	4	-11	12.05	2.05
2	4	-11	11.47	1.89
-2	-4	11	13.33	2.47
-2	-4	11	9.79	1.56
-2	-4	11	10.87	1.49
-2	-4	11	8.21	1.93
1	4	-11	5.66	0.71
1	4	-11	7.13	1.84
1	4	-11	6.13	1.59
-1	-4	11	4.49	1.85
-1	-4	11	5.38	1.00
-1	-4	11	6.44	1.39
-1	-4	11	4.86	1.23
0	4	-11	14.46	2.17
0	4	-11	18.66	2.91
0	4	-11	16.16	1.40
0	-4	11	14.64	2.34
0	-4	11	17.00	2.35
0	-4	11	15.39	1.92
-1	4	-11	39.76	3.42
-1	4	-11	38.94	3.16
1	-4	11	33.62	2.63
1	-4	11	31.05	3.42
1	-4	11	34.29	2.94
1	-4	11	36.20	3.04
-2	4	-11	33.10	2.18
-2	4	-11	33.83	3.32
-2	4	-11	39.25	3.08
2	-4	11	35.32	2.96
2	-4	11	35.13	3.07
2	-4	11	31.27	2.64
2	-4	11	31.43	3.48
-3	4	-11	21.23	1.76
-3	4	-11	21.68	2.70
-3	4	-11	20.04	3.09
3	-4	11	20.06	3.40
3	-4	11	26.19	2.82
3	-4	11	25.01	2.88
3	-4	11	18.44	2.25
-4	4	-11	24.37	3.39
-4	4	-11	27.18	2.77
-4	4	-11	18.25	2.78
-4	4	-11	21.91	1.85

-4	4	-11	22.02	3.22
4	-4	11	19.95	3.42
4	-4	11	17.91	2.34
4	-4	11	20.35	3.01
4	-4	11	25.10	2.93
-5	4	-11	11.14	2.57
-5	4	-11	15.60	2.92
-5	4	-11	15.25	2.33
-5	4	-11	21.47	2.61
-5	4	-11	21.40	1.81
5	-4	11	14.63	2.56
5	-4	11	21.16	3.27
5	-4	11	16.65	2.40
-6	4	-11	17.25	3.53
-6	4	-11	17.83	1.88
-6	4	-11	19.35	3.72
6	-4	11	19.22	3.71
6	-4	11	11.19	2.74
-7	4	-11	2.48	2.97
-7	4	-11	3.57	1.05
-7	4	-11	5.00	3.10
-7	4	-11	3.94	2.51
-7	4	-11	0.35	2.41
7	-4	11	6.66	1.97
-8	4	-11	6.14	3.10
-8	4	-11	7.41	1.22
-8	4	-11	11.33	3.60
8	-4	11	11.43	2.27
-11	-3	11	1.91	2.55
-11	-3	11	2.78	2.80
-10	-3	11	4.65	2.51
-10	-3	11	4.07	2.35
-9	-3	11	-1.61	2.15
-9	-3	11	2.50	1.85
-9	-3	11	2.15	2.09
8	3	-11	9.54	4.09
8	3	-11	14.64	2.83
-8	-3	11	11.22	2.30
-8	-3	11	18.20	3.05
-8	-3	11	9.92	2.17
7	3	-11	22.24	3.96
-7	-3	11	16.55	3.30
-7	-3	11	12.05	2.17
-7	-3	11	17.74	3.04

6	3	-11	19.42	3.61
-6	-3	11	15.00	2.84
-6	-3	11	14.26	2.95
-6	-3	11	16.94	3.01
5	3	-11	12.17	2.18
5	3	-11	8.81	2.63
-5	-3	11	13.17	2.04
-5	-3	11	15.07	2.76
4	3	-11	18.96	3.03
4	3	-11	22.62	3.12
-4	-3	11	14.33	2.22
-4	-3	11	22.80	2.85
-4	-3	11	21.11	2.65
3	3	-11	14.58	2.86
3	3	-11	18.00	2.81
-3	-3	11	16.24	1.53
-3	-3	11	19.58	2.66
-3	-3	11	12.12	2.09
-3	-3	11	19.55	2.35
2	3	-11	23.05	2.86
2	3	-11	19.88	2.68
2	3	-11	20.02	1.39
-2	-3	11	18.81	1.74
-2	-3	11	23.07	2.49
-2	-3	11	18.03	2.31
1	3	-11	4.24	1.56
1	3	-11	3.34	0.61
1	3	-11	3.35	1.38
-1	-3	11	3.83	0.87
-1	-3	11	2.98	1.08
-1	-3	11	3.00	1.63
-1	-3	11	5.14	1.27
0	3	-11	13.87	2.60
0	3	-11	12.72	1.80
0	3	-11	12.08	1.21
0	-3	11	13.47	1.66
0	-3	11	14.36	2.14
0	-3	11	11.95	2.06
0	-3	11	13.90	2.63
-1	3	-11	14.54	2.34
-1	3	-11	11.93	1.19
-1	3	-11	13.78	2.56
1	-3	11	13.65	2.16
1	-3	11	8.19	1.40

1	-3	11	10.19	1.86
1	-3	11	11.16	1.69
-2	3	-11	24.45	2.57
-2	3	-11	24.55	2.83
-2	3	-11	21.28	1.74
2	-3	11	21.46	2.48
2	-3	11	23.89	2.51
2	-3	11	23.31	2.12
2	-3	11	26.60	2.94
-3	3	-11	18.05	2.79
-3	3	-11	18.19	1.57
-3	3	-11	17.28	2.52
3	-3	11	18.99	2.51
3	-3	11	20.30	2.93
3	-3	11	19.78	2.51
3	-3	11	15.09	2.01
-4	3	-11	22.33	3.13
-4	3	-11	25.97	1.87
-4	3	-11	22.17	2.86
4	-3	11	23.56	3.33
4	-3	11	26.62	2.76
4	-3	11	22.78	2.29
4	-3	11	27.76	2.97
-5	3	-11	16.62	1.59
-5	3	-11	10.25	1.97
-5	3	-11	18.54	3.10
5	-3	11	14.74	2.06
5	-3	11	13.45	2.00
5	-3	11	12.73	2.25
-6	3	-11	1.87	1.90
-6	3	-11	6.06	2.07
-6	3	-11	9.71	2.35
-6	3	-11	9.44	1.54
6	-3	11	4.51	1.21
6	-3	11	4.29	2.12
6	-3	11	6.17	1.98
-7	3	-11	1.75	2.77
-7	3	-11	3.41	0.98
-7	3	-11	-1.96	2.04
-7	3	-11	0.92	2.37
7	-3	11	3.29	2.19
7	-3	11	-3.01	2.27
-8	3	-11	-4.24	2.53
-8	3	-11	2.40	2.93

-8	3	-11	1.36	2.94
-8	3	-11	1.16	1.05
-8	3	-11	-0.06	3.19
8	-3	11	4.88	1.92
-9	3	-11	2.38	3.56
-9	3	-11	9.73	3.95
-9	3	-11	3.46	1.16
9	-3	11	7.10	2.10
-11	-2	11	6.46	3.02
-11	-2	11	5.68	2.51
-10	-2	11	5.90	2.61
-10	-2	11	2.62	2.11
-9	-2	11	10.09	2.37
8	2	-11	11.42	3.62
-8	-2	11	9.57	2.13
-8	-2	11	12.54	2.26
-7	-2	11	17.73	2.99
-7	-2	11	21.75	3.42
-7	-2	11	22.38	2.99
6	2	-11	34.82	3.66
-6	-2	11	26.93	3.08
-6	-2	11	29.06	3.40
-6	-2	11	28.52	3.17
5	2	-11	51.88	3.99
-5	-2	11	44.99	3.56
-5	-2	11	36.16	3.58
4	2	-11	30.41	3.19
4	2	-11	34.50	3.30
-4	-2	11	31.84	3.03
-4	-2	11	31.99	2.99
3	2	-11	43.47	3.58
3	2	-11	46.01	3.36
-3	-2	11	42.49	3.21
-3	-2	11	31.66	3.59
-3	-2	11	35.15	3.44
2	2	-11	30.46	1.85
2	2	-11	33.75	2.98
2	2	-11	35.92	2.89
-2	-2	11	31.28	3.10
-2	-2	11	31.33	2.67
-2	-2	11	31.49	2.06
1	2	-11	38.08	2.90
1	2	-11	38.73	2.09
1	2	-11	37.01	3.09

-1	-2	11	37.28	2.88
-1	-2	11	35.17	3.23
-1	-2	11	32.29	2.59
-1	-2	11	37.86	2.78
0	2	-11	31.64	2.71
0	2	-11	30.35	2.84
0	2	-11	29.80	1.86
0	-2	11	29.07	2.56
0	-2	11	32.43	2.53
0	-2	11	32.14	2.09
0	-2	11	30.22	3.01
-1	2	-11	43.01	2.53
-1	2	-11	48.48	3.38
-1	2	-11	45.09	3.37
1	-2	11	49.03	2.78
1	-2	11	44.47	4.01
1	-2	11	47.54	3.51
1	-2	11	42.79	3.17
-2	2	-11	23.37	2.42
-2	2	-11	19.58	1.51
-2	2	-11	20.41	2.64
2	-2	11	19.49	2.76
2	-2	11	24.94	2.41
2	-2	11	22.36	1.88
-3	2	-11	31.56	2.87
-3	2	-11	28.61	2.12
-3	2	-11	31.71	3.06
3	-2	11	32.06	2.30
3	-2	11	34.18	2.77
3	-2	11	31.79	2.83
3	-2	11	30.90	3.25
-4	2	-11	24.39	3.01
-4	2	-11	28.79	1.99
-4	2	-11	33.25	3.20
4	-2	11	32.40	2.98
4	-2	11	34.96	2.85
4	-2	11	32.58	3.35
4	-2	11	25.76	2.28
-5	2	-11	33.78	3.45
-5	2	-11	28.27	3.23
-5	2	-11	30.36	2.11
5	-2	11	29.06	3.16
5	-2	11	31.34	2.40
5	-2	11	36.46	2.96

-6	2	-11	9.13	2.43
-6	2	-11	8.50	1.96
-6	2	-11	10.88	1.50
-6	2	-11	11.74	2.17
6	-2	11	13.28	1.95
6	-2	11	8.96	2.23
6	-2	11	9.03	1.23
-7	2	-11	19.95	3.80
-7	2	-11	20.46	3.24
-7	2	-11	20.71	1.85
-7	2	-11	17.15	3.39
-7	2	-11	20.80	2.88
-7	2	-11	16.38	2.85
7	-2	11	14.21	2.59
7	-2	11	20.37	3.51
7	-2	11	16.40	2.31
-8	2	-11	11.05	1.73
-8	2	-11	9.41	3.17
-8	2	-11	6.58	2.27
-8	2	-11	6.45	2.89
8	-2	11	14.64	2.52
-9	2	-11	5.75	3.63
-9	2	-11	6.61	1.16
-9	2	-11	6.61	2.88
-9	2	-11	10.45	3.51
-9	2	-11	5.50	3.41
9	-2	11	4.18	3.26
-10	2	-11	3.26	1.16
-10	2	-11	6.68	4.12
-10	2	-11	0.02	4.01
10	-2	11	7.03	2.24
-11	-1	11	6.73	3.13
-10	-1	11	8.33	2.72
-9	-1	11	10.45	2.54
-8	-1	11	10.87	2.00
-8	-1	11	11.31	2.17
-7	-1	11	12.32	2.07
-6	-1	11	18.47	2.65
-6	-1	11	16.34	2.66
-6	-1	11	15.71	3.05
5	1	-11	30.49	2.97
-5	-1	11	33.96	3.53
-5	-1	11	30.68	3.09
-5	-1	11	37.38	3.22

4	1	-11	30.71	3.09
-4	-1	11	23.13	2.95
-4	-1	11	29.36	2.93
-4	-1	11	32.65	2.81
3	1	-11	33.33	2.07
3	1	-11	38.07	4.25
3	1	-11	41.98	3.14
-3	-1	11	38.45	3.11
-3	-1	11	34.16	3.21
-3	-1	11	38.28	2.91
2	1	-11	31.09	3.08
2	1	-11	29.70	3.60
2	1	-11	31.16	1.91
2	1	-11	36.75	2.82
-2	-1	11	33.67	2.69
-2	-1	11	32.49	1.99
-2	-1	11	30.55	2.99
-2	-1	11	39.36	2.65
1	1	-11	36.03	2.70
1	1	-11	34.30	1.98
1	1	-11	35.14	2.89
1	1	-11	35.26	3.42
-1	-1	11	34.43	2.68
-1	-1	11	36.40	3.02
-1	-1	11	34.39	2.59
-1	-1	11	33.05	2.09
0	1	-11	31.62	2.49
0	1	-11	28.18	2.96
0	1	-11	30.50	2.75
0	1	-11	28.95	1.78
0	-1	11	28.01	2.45
0	-1	11	32.06	2.11
0	-1	11	28.07	2.86
0	-1	11	29.83	2.44
-1	1	-11	44.42	3.23
-1	1	-11	42.78	2.40
-1	1	-11	43.14	3.75
-1	1	-11	41.33	3.29
1	-1	11	44.89	2.57
1	-1	11	41.83	3.00
1	-1	11	43.69	3.37
1	-1	11	45.15	3.00
-2	1	-11	28.24	1.82
-2	1	-11	29.95	2.66

-2	1	-11	31.05	2.83
-2	1	-11	30.48	2.61
2	-1	11	29.11	2.00
2	-1	11	28.99	2.93
2	-1	11	29.17	2.63
2	-1	11	27.85	2.53
-3	1	-11	21.74	1.57
-3	1	-11	24.39	2.73
-3	1	-11	20.29	2.51
3	-1	11	21.30	2.49
3	-1	11	19.35	2.83
3	-1	11	22.81	2.52
3	-1	11	23.69	1.82
-4	1	-11	36.42	3.25
-4	1	-11	46.51	3.49
4	-1	11	42.34	3.28
4	-1	11	39.91	2.60
4	-1	11	38.97	4.20
4	-1	11	45.15	3.18
-5	1	-11	32.87	3.32
-5	1	-11	36.78	2.29
-5	1	-11	36.83	3.46
5	-1	11	44.09	2.95
5	-1	11	41.70	3.25
5	-1	11	32.74	2.48
5	-1	11	33.84	3.67
-6	1	-11	13.43	2.04
-6	1	-11	16.93	1.54
-6	1	-11	15.73	2.36
6	-1	11	12.16	2.32
6	-1	11	18.95	2.92
-7	1	-11	16.16	3.17
-7	1	-11	20.21	1.76
-7	1	-11	20.95	3.68
7	-1	11	16.09	2.65
-8	1	-11	26.07	4.00
-8	1	-11	16.61	3.10
-8	1	-11	22.33	3.42
-8	1	-11	23.53	2.01
8	-1	11	22.71	2.34
8	-1	11	30.59	3.65
-9	1	-11	17.06	1.88
-9	1	-11	11.74	2.37
-9	1	-11	16.79	2.85

-9	1	-11	18.77	3.63
-9	1	-11	9.46	3.44
9	-1	11	15.67	2.68
9	-1	11	11.05	2.93
-10	1	-11	10.96	2.99
-10	1	-11	15.32	4.02
-10	1	-11	8.50	1.27
-10	1	-11	10.14	3.94
-11	1	-11	1.14	1.18
-11	1	-11	-2.53	4.26
-11	1	-11	7.81	4.77
11	-1	11	7.02	2.35
-11	0	11	5.93	3.17
-10	0	11	14.11	2.97
-9	0	11	12.78	2.55
-8	0	11	12.25	2.25
7	0	-11	8.71	3.10
-7	0	11	10.39	1.98
-6	0	11	23.17	4.15
-6	0	11	17.64	3.05
-6	0	11	20.38	2.66
5	0	-11	11.26	2.55
-5	0	11	17.67	2.40
-5	0	11	18.88	2.76
4	0	-11	55.63	4.66
-4	0	11	45.47	3.65
-4	0	11	53.36	3.45
3	0	-11	34.65	2.89
3	0	-11	34.50	4.07
-3	0	11	38.65	3.05
-3	0	11	41.42	2.80
-3	0	11	37.14	3.15
2	0	-11	61.78	3.19
2	0	-11	57.75	4.59
2	0	-11	63.37	3.76
-2	0	11	64.26	3.99
-2	0	11	58.66	3.61
-2	0	11	58.47	3.77
1	0	-11	26.03	3.10
1	0	-11	24.96	1.59
1	0	-11	23.27	3.16
1	0	-11	30.32	2.38
-1	0	11	25.67	1.65
-1	0	11	24.32	2.66

-1	0	11	23.90	2.29
-1	0	11	23.88	2.22
0	0	-11	31.21	2.83
0	0	-11	33.14	2.53
0	0	-11	33.95	1.91
0	0	-11	31.06	3.04
0	0	11	31.72	2.92
0	0	11	32.18	2.05
0	0	11	33.66	2.59
0	0	11	34.24	2.54
-1	0	-11	20.93	1.38
-1	0	-11	18.37	2.52
-1	0	-11	19.06	3.45
-1	0	-11	18.73	2.47
1	0	11	17.76	2.59
1	0	11	20.63	2.13
1	0	11	22.44	1.52
1	0	11	17.84	2.06
-2	0	-11	67.77	3.48
-2	0	-11	67.19	4.23
-2	0	-11	68.06	3.99
-2	0	-11	66.20	4.03
2	0	11	66.02	3.56
2	0	11	69.94	4.01
2	0	11	66.27	3.99
2	0	11	61.24	4.29
-3	0	-11	41.96	3.22
-3	0	-11	44.06	2.46
-3	0	-11	49.42	3.13
-3	0	-11	47.41	3.48
3	0	11	45.85	2.60
3	0	11	40.53	2.98
3	0	11	38.51	3.49
3	0	11	43.44	3.25
-4	0	-11	54.68	3.83
-4	0	-11	56.66	3.16
-4	0	-11	66.31	3.70
-4	0	-11	58.00	4.08
4	0	11	57.06	3.86
4	0	11	57.07	3.27
4	0	11	63.03	3.71
4	0	11	55.43	4.16
-5	0	-11	39.22	2.69
-5	0	-11	31.92	3.05

-5	0	-11	27.61	2.42
-5	0	-11	36.04	3.41
5	0	11	34.04	2.25
5	0	11	36.09	3.07
5	0	11	31.74	3.78
-6	0	-11	35.29	3.78
-6	0	-11	38.57	3.26
-6	0	-11	37.54	2.33
6	0	11	43.07	3.34
6	0	11	35.26	3.80
-7	0	-11	11.83	2.48
-7	0	-11	16.72	1.57
-7	0	-11	13.94	2.69
7	0	11	16.83	2.98
7	0	11	13.93	2.40
-8	0	-11	38.42	2.86
-8	0	-11	26.33	4.04
-8	0	-11	28.58	3.24
-8	0	-11	25.82	4.81
-8	0	-11	37.36	3.40
-8	0	-11	28.77	2.26
8	0	11	40.32	3.61
8	0	11	26.41	4.10
-9	0	-11	31.27	2.83
-9	0	-11	18.90	3.48
-9	0	-11	23.29	3.24
-9	0	-11	25.51	4.55
-9	0	-11	25.34	2.16
9	0	11	26.88	2.87
9	0	11	33.44	3.77
9	0	11	25.97	4.30
-10	0	-11	42.14	2.95
-10	0	-11	46.71	4.52
-10	0	-11	40.72	6.05
-10	0	-11	54.54	5.73
-10	0	-11	36.26	3.94
10	0	11	52.88	4.22
10	0	11	37.18	6.64
-11	0	-11	15.93	4.80
-11	0	-11	9.65	1.35
-11	0	-11	4.11	4.09
11	0	11	11.86	2.18
-10	1	11	7.14	2.84
-9	1	11	10.47	2.75

-8	1	11	17.52	3.65
-7	1	11	13.08	2.13
-6	1	11	10.70	1.83
5	-1	-11	14.64	2.61
-5	1	11	16.84	2.25
-5	1	11	15.46	2.62
4	-1	-11	26.04	3.81
-4	1	11	18.67	2.90
-4	1	11	26.40	2.42
-4	1	11	26.01	2.76
3	-1	-11	33.04	2.04
3	-1	-11	35.02	3.86
-3	1	11	39.48	2.92
-3	1	11	38.11	3.01
-3	1	11	39.29	2.56
-3	1	11	32.42	2.00
2	-1	-11	32.49	1.87
2	-1	-11	29.69	2.51
2	-1	-11	29.97	3.55
2	-1	-11	27.25	2.65
-2	1	11	33.58	2.80
-2	1	11	30.11	2.64
-2	1	11	30.54	2.38
-2	1	11	31.20	1.81
1	-1	-11	42.16	2.81
1	-1	-11	39.57	3.61
1	-1	-11	41.31	2.31
-1	1	11	39.82	2.27
-1	1	11	41.12	2.87
-1	1	11	40.49	2.79
-1	1	11	44.22	3.20
0	-1	-11	53.76	2.85
0	-1	-11	54.81	3.76
0	-1	-11	52.52	3.26
0	-1	-11	53.44	5.06
0	1	11	54.47	3.32
0	1	11	51.73	3.69
0	1	11	52.01	3.31
0	1	11	53.04	2.85
-1	-1	-11	70.43	3.60
-1	-1	-11	69.50	4.09
-1	-1	-11	69.32	4.34
1	1	11	71.58	4.02
1	1	11	72.53	3.74

1	1	11	67.74	4.08
1	1	11	62.81	4.36
-2	-1	-11	48.33	2.53
-2	-1	-11	45.32	3.48
-2	-1	-11	46.03	3.46
-2	-1	-11	48.24	3.20
2	1	11	47.43	2.57
2	1	11	47.40	3.06
2	1	11	39.85	3.46
2	1	11	42.88	3.14
-3	-1	-11	18.33	2.70
-3	-1	-11	20.35	2.40
-3	-1	-11	23.29	1.49
3	1	11	23.02	1.56
3	1	11	19.80	2.37
3	1	11	18.44	2.74
-4	-1	-11	33.77	2.95
-4	-1	-11	38.43	2.18
-4	-1	-11	42.38	2.78
-4	-1	-11	35.39	3.34
4	1	11	39.73	2.69
4	1	11	28.68	3.29
4	1	11	36.71	2.97
4	1	11	36.28	2.26
-5	-1	-11	28.02	2.65
-5	-1	-11	31.47	1.94
-5	-1	-11	23.40	3.29
-5	-1	-11	35.28	2.47
5	1	11	23.01	3.25
5	1	11	30.06	2.87
5	1	11	28.71	2.00
5	1	11	37.26	2.54
-6	-1	-11	12.84	2.24
-6	-1	-11	12.79	1.64
-6	-1	-11	18.59	1.42
6	1	11	13.36	2.16
6	1	11	16.92	2.57
-7	-1	-11	21.85	3.73
-7	-1	-11	29.57	2.04
-7	-1	-11	30.38	2.87
7	1	11	34.79	3.12
7	1	11	36.57	2.73
7	1	11	22.18	3.72
-8	-1	-11	18.84	2.66

-8	-1	-11	18.68	1.73
-8	-1	-11	14.57	2.79
8	1	11	14.46	2.64
8	1	11	20.80	3.04
-9	-1	-11	20.50	2.00
-9	-1	-11	25.69	2.55
-9	-1	-11	20.75	2.95
-9	-1	-11	14.39	4.81
9	1	11	27.67	3.28
9	1	11	20.97	2.20
-10	-1	-11	11.68	3.43
-10	-1	-11	19.20	4.02
-10	-1	-11	14.70	1.92
-10	-1	-11	15.02	2.13
10	1	11	11.36	2.82
-11	-1	-11	12.51	1.93
-11	-1	-11	9.35	4.56
-11	-1	-11	9.02	4.02
-11	-1	-11	10.04	2.30
-11	-1	-11	13.93	2.94
-12	-1	-11	3.52	1.29
-12	-1	-11	10.10	5.16
-12	-1	-11	-0.76	4.28
12	1	11	6.53	2.22
-10	2	11	6.60	2.87
-9	2	11	20.50	4.24
-8	2	11	12.27	2.44
-7	2	11	19.95	3.34
-6	2	11	9.99	1.85
5	-2	-11	14.88	2.65
-5	2	11	18.96	2.75
4	-2	-11	14.82	2.36
3	-2	-11	56.89	2.96
-3	2	11	57.14	3.31
-3	2	11	52.41	2.88
2	-2	-11	18.79	1.41
-2	2	11	20.62	2.03
-2	2	11	20.10	1.34
-2	2	11	20.66	2.33
-2	2	11	22.12	2.59
1	-2	-11	58.60	3.46
1	-2	-11	51.27	3.73
1	-2	-11	57.48	3.08
-1	2	11	58.43	3.03

-1	2	11	65.15	3.89
-1	2	11	57.31	3.56
-1	2	11	57.78	3.50
0	-2	-11	36.41	3.27
0	-2	-11	37.43	2.20
0	-2	-11	39.27	2.58
0	2	11	37.08	2.14
0	2	11	40.34	2.72
0	2	11	36.13	3.09
0	2	11	39.87	2.68
-1	-2	-11	116.32	6.32
-1	-2	-11	122.72	5.82
-1	-2	-11	120.70	7.54
-1	-2	-11	114.87	6.06
1	2	11	119.20	6.12
1	2	11	119.27	6.01
1	2	11	105.23	6.35
1	2	11	109.20	6.13
-2	-2	-11	87.72	4.21
-2	-2	-11	83.01	4.71
-2	-2	-11	78.14	4.93
2	2	11	71.60	4.86
2	2	11	80.02	4.67
2	2	11	86.48	4.20
-3	-2	-11	57.80	3.65
-3	-2	-11	57.27	3.53
-3	-2	-11	61.46	3.06
-3	-2	-11	53.58	3.97
3	2	11	58.85	3.07
3	2	11	58.00	3.64
3	2	11	53.27	3.94
3	2	11	53.77	3.47
-4	-2	-11	30.58	3.28
-4	-2	-11	36.17	2.68
-4	-2	-11	38.98	2.12
4	2	11	36.24	2.90
4	2	11	35.71	2.58
4	2	11	37.50	2.13
4	2	11	29.52	3.22
-5	-2	-11	73.30	4.28
-5	-2	-11	67.62	4.85
-5	-2	-11	76.10	3.89
-5	-2	-11	81.20	4.24
5	2	11	81.56	4.54

5	2	11	72.87	3.92
5	2	11	64.22	4.77
-6	-2	-11	13.12	2.17
-6	-2	-11	14.07	2.12
-6	-2	-11	11.59	1.99
6	2	11	14.43	2.45
6	2	11	11.31	2.07
-7	-2	-11	38.60	2.37
-7	-2	-11	43.69	2.81
-7	-2	-11	37.98	3.01
-7	-2	-11	28.75	3.99
7	2	11	39.53	3.32
7	2	11	32.88	3.73
-8	-2	-11	34.11	3.02
-8	-2	-11	32.89	4.09
-8	-2	-11	32.25	2.32
8	2	11	42.05	2.97
8	2	11	27.53	3.91
8	2	11	38.36	3.36
-9	-2	-11	30.14	4.42
-9	-2	-11	31.16	2.22
-9	-2	-11	30.15	3.02
-9	-2	-11	24.77	4.35
9	2	11	29.24	2.34
9	2	11	25.01	4.04
-10	-2	-11	24.09	3.04
-10	-2	-11	22.13	2.11
-10	-2	-11	20.89	3.43
-10	-2	-11	26.55	2.83
-10	-2	-11	27.09	4.88
10	2	11	20.01	2.28
10	2	11	29.24	3.21
10	2	11	15.94	2.95
-11	-2	-11	21.85	2.26
-11	-2	-11	20.34	3.86
-11	-2	-11	24.90	3.34
11	2	11	19.64	3.74
11	2	11	16.78	3.33
-12	-2	-11	12.52	4.08
-12	-2	-11	8.63	1.47
-12	-2	-11	19.77	5.09
12	2	11	10.91	2.31
-10	3	11	7.49	3.14
-9	3	11	15.33	2.98

-8	3	11	19.54	4.11
-7	3	11	11.33	2.15
-6	3	11	25.67	3.26
-5	3	11	33.64	3.58
4	-3	-11	56.40	3.05
3	-3	-11	29.31	1.83
-3	3	11	28.63	1.70
2	-3	-11	31.24	1.92
-2	3	11	33.58	2.41
-2	3	11	32.06	1.86
1	-3	-11	28.14	1.80
-1	3	11	24.40	2.90
-1	3	11	31.66	1.76
-1	3	11	29.94	2.44
0	-3	-11	49.93	2.71
0	-3	-11	47.85	3.01
0	3	11	48.70	3.50
0	3	11	49.59	2.68
0	3	11	50.31	3.16
-1	-3	-11	27.34	2.23
-1	-3	-11	28.50	1.76
1	3	11	27.14	2.36
1	3	11	24.67	2.68
1	3	11	27.17	1.94
-2	-3	-11	72.57	3.72
-2	-3	-11	65.21	4.87
-2	-3	-11	70.93	4.04
2	3	11	66.60	4.37
2	3	11	71.92	3.68
2	3	11	75.05	4.15
-3	-3	-11	41.42	2.11
-3	-3	-11	31.29	3.16
-3	-3	-11	26.19	3.44
3	3	11	34.94	2.78
3	3	11	33.73	3.02
3	3	11	36.60	2.08
-4	-3	-11	22.14	2.94
-4	-3	-11	26.03	1.71
-4	-3	-11	26.31	2.27
4	3	11	27.55	2.45
4	3	11	21.89	2.76
4	3	11	25.99	1.71
-5	-3	-11	34.87	2.66
-5	-3	-11	30.15	3.43

-5	-3	-11	37.84	2.12
5	3	11	33.97	3.16
5	3	11	34.28	2.12
5	3	11	34.95	2.97
-6	-3	-11	41.26	2.97
-6	-3	-11	40.64	3.82
-6	-3	-11	43.99	2.45
6	3	11	35.05	3.43
6	3	11	43.72	3.28
-7	-3	-11	37.64	3.87
-7	-3	-11	40.92	2.97
-7	-3	-11	38.00	2.37
7	3	11	34.44	3.94
7	3	11	41.49	3.29
-8	-3	-11	30.32	4.11
-8	-3	-11	33.92	3.01
-8	-3	-11	41.22	2.78
-8	-3	-11	36.44	2.35
8	3	11	40.55	3.31
8	3	11	32.53	3.70
-9	-3	-11	22.12	1.88
-9	-3	-11	21.15	2.89
-9	-3	-11	20.67	2.68
9	3	11	17.45	2.54
9	3	11	18.24	1.97
-10	-3	-11	24.70	2.55
-10	-3	-11	14.63	3.16
-10	-3	-11	17.12	2.89
-10	-3	-11	23.61	2.04
10	3	11	19.77	2.08
10	3	11	20.36	3.77
10	3	11	20.95	3.24
-11	-3	-11	16.88	1.99
-11	-3	-11	11.15	2.03
-11	-3	-11	12.35	3.25
-11	-3	-11	11.93	4.12
11	3	11	11.18	2.18
11	3	11	12.18	2.73
-12	-3	-11	22.30	5.23
-12	-3	-11	18.05	3.54
-12	-3	-11	23.50	4.00
-12	-3	-11	21.87	2.44
12	3	11	30.88	3.82
-13	-3	-11	10.10	4.40

-13	-3	-11	9.14	1.62
-13	-3	-11	13.46	5.63
-9	4	11	13.61	3.06
-8	4	11	20.49	4.22
-7	4	11	6.78	2.14
-5	4	11	19.65	1.37
4	-4	-11	44.35	2.52
-4	4	11	43.09	2.38
3	-4	-11	52.16	2.85
-3	4	11	51.26	2.74
2	-4	-11	47.83	2.62
-2	4	11	45.98	2.52
1	-4	-11	46.54	2.51
-1	4	11	40.83	3.01
-1	4	11	45.71	2.48
0	-4	-11	59.08	4.02
0	-4	-11	71.58	3.43
0	4	11	61.12	3.83
-1	-4	-11	18.02	1.90
-1	-4	-11	16.26	1.36
-1	-4	-11	16.54	2.35
1	4	11	16.78	1.27
1	4	11	16.29	2.30
1	4	11	16.32	2.00
-2	-4	-11	42.88	2.45
-2	-4	-11	41.68	2.78
2	4	11	44.14	2.95
2	4	11	44.30	2.39
2	4	11	39.89	3.11
-3	-4	-11	37.51	2.50
-3	-4	-11	36.21	3.41
-3	-4	-11	35.74	2.19
3	4	11	38.69	2.16
3	4	11	35.60	2.95
3	4	11	38.56	2.76
-4	-4	-11	67.19	3.51
-4	-4	-11	59.73	4.02
-4	-4	-11	65.07	4.36
4	4	11	69.60	4.04
4	4	11	63.98	4.11
4	4	11	67.12	3.48
-5	-4	-11	49.44	2.66
-5	-4	-11	44.47	3.69
-5	-4	-11	44.39	3.71

5	4	11	45.21	2.63
5	4	11	44.27	3.46
5	4	11	51.81	3.33
-6	-4	-11	74.06	4.86
-6	-4	-11	76.67	4.22
-6	-4	-11	66.92	3.78
6	4	11	70.78	4.54
6	4	11	64.53	3.76
6	4	11	76.04	4.46
-7	-4	-11	37.17	3.86
-7	-4	-11	34.01	2.59
-7	-4	-11	42.18	2.96
7	4	11	49.03	3.30
7	4	11	39.73	3.48
-8	-4	-11	52.25	3.42
-8	-4	-11	51.65	4.37
-8	-4	-11	48.52	2.89
8	4	11	43.18	4.08
-9	-4	-11	13.18	2.56
-9	-4	-11	17.26	1.78
-9	-4	-11	20.12	2.55
9	4	11	17.32	3.34
-10	-4	-11	28.52	2.33
-10	-4	-11	28.55	3.03
-10	-4	-11	29.59	4.25
-10	-4	-11	30.00	4.59
10	4	11	31.81	3.34
10	4	11	27.02	2.25
10	4	11	31.10	3.85
10	4	11	35.75	3.11
-11	-4	-11	20.89	2.22
-11	-4	-11	23.16	3.11
-11	-4	-11	25.67	2.88
-11	-4	-11	23.68	4.01
-11	-4	-11	16.68	3.74
11	4	11	26.14	3.44
11	4	11	26.51	3.29
11	4	11	19.74	2.20
11	4	11	20.23	3.96
-12	-4	-11	21.17	4.70
-12	-4	-11	19.84	3.43
-12	-4	-11	17.21	3.71
-12	-4	-11	22.49	2.39
12	4	11	12.87	3.10

12	4	11	24.10	3.85
-13	-4	-11	16.18	2.51
-13	-4	-11	14.13	4.32
-9	5	11	12.01	3.11
-8	5	11	11.10	2.77
-7	5	11	9.79	2.40
4	-5	-11	36.60	2.25
-4	5	11	34.70	2.07
3	-5	-11	50.15	2.74
-3	5	11	44.85	2.60
2	-5	-11	34.31	2.07
-2	5	11	32.08	1.95
1	-5	-11	48.24	2.59
-1	5	11	47.72	2.49
-1	5	11	39.81	2.98
0	-5	-11	69.37	3.58
0	5	11	59.90	4.80
0	5	11	70.97	3.52
-1	-5	-11	63.18	3.23
-1	-5	-11	52.51	3.80
1	5	11	63.43	3.18
1	5	11	58.31	3.81
1	5	11	60.80	3.64
-2	-5	-11	20.24	1.46
-2	-5	-11	17.48	1.80
-2	-5	-11	14.89	2.62
2	5	11	18.66	1.37
2	5	11	19.87	2.07
2	5	11	21.30	2.28
-3	-5	-11	37.90	2.30
-3	-5	-11	38.35	2.58
3	5	11	40.55	2.25
3	5	11	36.27	2.97
3	5	11	42.80	2.88
-4	-5	-11	17.95	1.54
-4	-5	-11	19.25	2.95
4	5	11	20.16	1.49
4	5	11	24.08	2.35
4	5	11	22.58	2.46
-5	-5	-11	54.57	3.21
-5	-5	-11	45.78	3.85
-5	-5	-11	47.64	2.84
5	5	11	50.81	2.83
5	5	11	54.13	3.52

5	5	11	50.72	3.55
-6	-5	-11	51.29	3.62
-6	-5	-11	53.66	3.01
-6	-5	-11	52.31	4.03
6	5	11	53.67	3.73
6	5	11	60.14	3.70
6	5	11	50.80	2.98
-7	-5	-11	61.81	4.30
-7	-5	-11	48.69	3.71
7	5	11	59.15	3.97
-8	-5	-11	37.60	3.65
-8	-5	-11	34.29	3.74
-8	-5	-11	34.06	2.33
8	5	11	39.45	3.42
-9	-5	-11	39.04	3.27
-9	-5	-11	36.73	4.07
-9	-5	-11	35.47	2.57
9	5	11	43.28	3.49
9	5	11	44.27	3.74
-10	-5	-11	23.32	3.90
-10	-5	-11	21.50	2.12
-10	-5	-11	26.37	2.87
10	5	11	27.73	3.56
10	5	11	20.16	1.93
-11	-5	-11	24.96	2.99
-11	-5	-11	21.33	3.93
-11	-5	-11	21.95	2.27
-11	-5	-11	19.13	3.13
11	5	11	24.70	3.46
11	5	11	20.73	2.09
11	5	11	21.75	3.87
-12	-5	-11	8.90	1.53
-12	-5	-11	9.92	2.09
-12	-5	-11	17.13	4.54
-12	-5	-11	9.57	3.34
12	5	11	10.01	2.71
12	5	11	13.21	2.38
-13	-5	-11	22.51	5.41
-13	-5	-11	16.37	4.03
-13	-5	-11	15.67	2.60
-8	6	11	7.04	2.87
-7	6	11	8.12	1.20
-7	6	11	11.49	1.36
-7	6	11	9.23	2.56

-6	6	11	7.77	1.22
-5	6	11	26.11	1.76
-4	6	11	36.86	2.17
3	-6	-11	42.26	2.45
-3	6	11	38.94	2.30
2	-6	-11	43.07	2.50
-2	6	11	40.14	2.32
1	-6	-11	83.07	4.27
-1	6	11	79.47	4.16
0	-6	-11	92.75	4.70
0	6	11	89.37	4.63
-1	-6	-11	99.63	4.92
1	6	11	98.34	4.85
1	6	11	88.28	5.20
-2	-6	-11	29.44	2.88
-2	-6	-11	36.18	2.41
-2	-6	-11	39.74	2.23
2	6	11	39.91	2.78
2	6	11	39.50	2.14
2	6	11	34.28	2.85
-3	-6	-11	47.48	2.96
-3	-6	-11	47.51	2.72
3	6	11	48.73	3.29
3	6	11	49.04	2.67
3	6	11	48.60	3.26
-4	-6	-11	37.95	3.86
-4	-6	-11	52.17	3.04
-4	-6	-11	49.34	2.75
4	6	11	50.14	3.36
4	6	11	51.85	3.32
4	6	11	46.27	2.67
-5	-6	-11	68.96	3.92
-5	-6	-11	79.64	4.16
-5	-6	-11	73.52	4.72
5	6	11	70.19	3.89
5	6	11	73.29	4.46
5	6	11	79.97	4.46
-6	-6	-11	66.17	3.63
-6	-6	-11	55.72	3.29
-6	-6	-11	61.02	4.22
6	6	11	60.20	3.96
6	6	11	57.09	3.41
-7	-6	-11	79.20	5.14
-7	-6	-11	83.95	4.62

7	6	11	80.76	4.96
-8	-6	-11	31.11	2.34
-8	-6	-11	34.04	3.68
-8	-6	-11	39.95	2.89
8	6	11	35.26	3.38
8	6	11	41.16	3.27
-9	-6	-11	41.58	4.05
-9	-6	-11	35.68	2.59
-9	-6	-11	38.98	3.21
9	6	11	46.72	3.49
9	6	11	36.71	4.00
-10	-6	-11	15.98	2.60
-10	-6	-11	18.65	2.98
-10	-6	-11	12.80	1.88
10	6	11	19.91	2.92
10	6	11	14.17	2.29
10	6	11	15.98	1.71
-11	-6	-11	9.78	1.41
-11	-6	-11	7.70	2.75
-11	-6	-11	12.08	2.63
11	6	11	14.69	2.23
11	6	11	12.42	2.42
11	6	11	12.07	1.73
-12	-6	-11	9.53	4.04
-12	-6	-11	15.41	2.90
-12	-6	-11	3.05	3.92
-12	-6	-11	8.21	1.47
-12	-6	-11	13.51	3.06
12	6	11	8.15	1.50
12	6	11	11.71	2.33
12	6	11	10.16	2.68
-13	-6	-11	17.05	4.97
-13	-6	-11	22.08	3.88
-13	-6	-11	12.11	3.78
13	6	11	17.93	3.16
13	6	11	16.14	2.85
-14	-6	-11	12.01	1.93
-14	-6	-11	17.37	5.80
-14	-6	-11	16.93	4.47
-8	7	11	10.12	1.45
-8	7	11	11.99	3.08
-7	7	11	11.48	1.57
-7	7	11	7.73	1.36
-7	7	11	10.07	2.67

-6	7	11	6.49	2.15
-6	7	11	9.87	1.39
-5	7	11	8.12	1.31
-4	7	11	33.96	2.14
-3	7	11	50.19	2.81
2	-7	-11	49.43	2.83
-2	7	11	47.13	2.69
1	-7	-11	40.92	2.47
-1	7	11	39.46	2.31
0	-7	-11	102.88	5.18
0	7	11	97.86	5.08
-1	-7	-11	57.23	3.11
1	7	11	54.70	3.54
1	7	11	56.35	3.04
-2	-7	-11	73.35	3.85
2	7	11	74.31	3.79
2	7	11	69.20	4.21
2	7	11	72.08	4.33
-3	-7	-11	46.73	2.63
-3	-7	-11	45.67	2.85
3	7	11	49.32	3.16
3	7	11	40.38	3.20
3	7	11	46.64	2.57
-4	-7	-11	74.15	4.08
-4	-7	-11	72.05	3.89
4	7	11	75.54	3.86
4	7	11	71.35	4.35
-5	-7	-11	41.33	2.41
-5	-7	-11	32.25	4.03
-5	-7	-11	42.83	2.74
5	7	11	39.31	2.34
5	7	11	40.75	3.06
-6	-7	-11	88.99	5.32
-6	-7	-11	75.56	4.87
-6	-7	-11	96.07	4.86
6	7	11	90.81	5.13
-7	-7	-11	48.97	3.16
-7	-7	-11	59.99	3.60
-7	-7	-11	53.96	4.14
7	7	11	61.23	3.88
7	7	11	58.67	3.89
-8	-7	-11	55.17	3.43
-8	-7	-11	56.04	4.51
-8	-7	-11	66.88	3.91

8	7	11	65.45	4.16
8	7	11	62.30	4.19
-9	-7	-11	46.72	3.28
-9	-7	-11	34.75	2.62
-9	-7	-11	41.50	4.05
9	7	11	37.87	3.63
9	7	11	45.26	3.52
-10	-7	-11	39.66	3.35
-10	-7	-11	33.60	2.56
-10	-7	-11	35.28	4.20
10	7	11	34.53	2.48
10	7	11	39.41	3.67
10	7	11	38.77	3.77
-11	-7	-11	7.77	1.33
-11	-7	-11	9.32	1.84
-11	-7	-11	8.55	2.68
11	7	11	8.08	1.13
11	7	11	9.00	2.35
-12	-7	-11	27.82	5.12
-12	-7	-11	17.69	3.27
-12	-7	-11	18.49	2.36
-12	-7	-11	23.48	3.45
12	7	11	24.75	3.96
12	7	11	18.05	2.15
12	7	11	16.64	2.85
-13	-7	-11	11.88	1.76
-13	-7	-11	19.82	3.82
-13	-7	-11	16.25	3.69
-13	-7	-11	18.22	4.72
-13	-7	-11	21.59	3.42
13	7	11	10.26	2.98
13	7	11	18.07	2.91
-14	-7	-11	9.64	4.36
-14	-7	-11	29.42	5.70
-14	-7	-11	18.97	2.91
-7	8	11	6.06	1.43
-7	8	11	5.76	2.83
-6	8	11	20.54	1.89
-6	8	11	19.09	4.08
-5	8	11	16.93	1.69
-4	8	11	23.72	1.83
-3	8	11	22.56	1.72
2	-8	-11	33.13	2.42
-2	8	11	32.17	2.08

1	-8	-11	41.10	2.52
-1	8	11	38.92	2.35
0	-8	-11	36.98	2.33
0	8	11	35.56	2.17
-1	-8	-11	30.30	2.02
1	8	11	29.49	1.91
-2	-8	-11	46.90	2.68
2	8	11	46.09	2.60
2	8	11	43.28	3.65
-3	-8	-11	52.87	3.04
3	8	11	56.47	2.97
3	8	11	51.91	3.61
-4	-8	-11	65.11	3.45
-4	-8	-11	60.87	3.61
4	8	11	62.58	3.88
4	8	11	61.41	3.97
4	8	11	61.33	3.34
-5	-8	-11	41.91	2.81
5	8	11	45.59	3.14
5	8	11	37.72	3.23
-6	-8	-11	43.14	4.10
-6	-8	-11	55.58	3.36
-6	-8	-11	52.50	3.02
6	8	11	50.87	3.75
6	8	11	58.42	3.70
-7	-8	-11	57.68	3.35
-7	-8	-11	62.56	3.74
-7	-8	-11	53.15	4.37
7	8	11	66.00	4.02
7	8	11	57.64	4.14
-8	-8	-11	50.94	4.26
-8	-8	-11	57.08	3.67
-8	-8	-11	51.20	3.12
8	8	11	54.00	4.09
8	8	11	55.90	3.90
-9	-8	-11	39.88	2.81
-9	-8	-11	47.37	4.09
-9	-8	-11	45.65	3.44
9	8	11	46.15	3.94
9	8	11	47.94	3.72
-10	-8	-11	52.01	3.34
-10	-8	-11	56.17	4.71
-10	-8	-11	59.88	4.04
10	8	11	54.01	4.39

10	8	11	63.76	4.34
10	8	11	48.73	3.23
-11	-8	-11	34.90	2.70
-11	-8	-11	39.80	3.61
-11	-8	-11	33.94	4.46
11	8	11	35.26	4.04
11	8	11	30.77	2.54
11	8	11	40.12	3.97
-12	-8	-11	37.31	5.18
-12	-8	-11	35.64	3.92
-12	-8	-11	31.37	4.87
-12	-8	-11	31.68	2.75
12	8	11	39.81	4.32
12	8	11	31.23	2.69
12	8	11	37.95	4.54
-13	-8	-11	20.47	4.04
-13	-8	-11	21.52	4.02
-13	-8	-11	27.85	3.56
-13	-8	-11	20.38	2.63
-13	-8	-11	22.40	4.50
13	8	11	20.16	3.10
13	8	11	18.26	3.14
-14	-8	-11	22.27	3.92
-14	-8	-11	12.88	5.16
-14	-8	-11	23.21	4.35
-6	9	11	14.30	1.89
-6	9	11	12.25	2.83
-6	9	11	10.61	1.65
-5	9	11	34.33	2.42
-4	9	11	24.55	2.00
-3	9	11	33.40	2.23
-2	9	11	27.55	1.97
1	-9	-11	45.32	2.75
-1	9	11	41.92	2.57
0	-9	-11	28.14	2.07
0	9	11	26.68	1.85
-1	-9	-11	36.47	2.32
1	9	11	32.55	2.13
-2	-9	-11	15.71	1.56
2	9	11	15.60	1.42
-3	-9	-11	55.39	3.05
3	9	11	50.16	3.87
3	9	11	52.44	3.61
3	9	11	52.29	2.94

-4	-9	-11	16.81	2.75
-4	-9	-11	26.98	1.82
4	9	11	21.08	3.69
4	9	11	22.81	2.64
-5	-9	-11	36.31	2.74
-5	-9	-11	40.35	2.42
5	9	11	39.81	3.10
5	9	11	39.28	3.19
-6	-9	-11	13.36	2.06
-6	-9	-11	15.83	1.55
-6	-9	-11	12.59	2.67
6	9	11	15.01	2.48
6	9	11	17.02	2.64
-7	-9	-11	32.85	3.54
-7	-9	-11	34.05	2.37
-7	-9	-11	37.09	2.87
7	9	11	38.10	3.38
7	9	11	42.33	3.30
-8	-9	-11	19.36	2.44
-8	-9	-11	15.58	1.72
-8	-9	-11	13.81	2.19
8	9	11	17.10	2.82
8	9	11	18.80	3.02
-9	-9	-11	35.35	4.00
-9	-9	-11	43.72	3.30
-9	-9	-11	35.99	2.58
9	9	11	42.99	3.64
9	9	11	40.56	3.86
9	9	11	33.76	2.55
-10	-9	-11	23.39	3.97
-10	-9	-11	24.74	3.00
-10	-9	-11	18.77	2.00
10	9	11	21.53	3.41
10	9	11	17.72	1.90
-11	-9	-11	39.20	2.81
-11	-9	-11	44.34	3.86
-11	-9	-11	38.06	4.64
11	9	11	33.69	2.69
11	9	11	42.47	4.20
11	9	11	37.17	4.38
-12	-9	-11	39.07	5.23
-12	-9	-11	40.03	5.26
-12	-9	-11	44.58	4.25
-12	-9	-11	39.81	2.96

12	9	11	41.78	3.76
12	9	11	30.55	5.15
12	9	11	39.91	4.81
12	9	11	42.37	4.62
-13	-9	-11	37.67	3.08
-13	-9	-11	37.73	6.60
-13	-9	-11	40.69	4.59
-13	-9	-11	32.47	6.09
13	9	11	39.12	5.02
13	9	11	30.94	5.00
-14	-9	-11	13.50	2.38
-14	-9	-11	10.75	5.05
-14	-9	-11	10.38	4.26
-14	-9	-11	12.11	1.83
14	9	11	16.61	3.60
14	9	11	5.34	3.51
-5	10	11	27.80	2.36
-4	10	11	22.28	2.10
-3	10	11	19.68	1.84
-2	10	11	18.71	1.73
-1	10	11	28.10	2.00
0	-10	-11	42.40	2.65
0	10	11	38.17	2.43
-1	-10	-11	62.44	3.52
1	10	11	59.29	3.34
-2	-10	-11	66.43	3.66
2	10	11	62.51	3.50
-3	-10	-11	41.78	2.59
3	10	11	41.14	2.44
3	10	11	38.76	3.10
-4	-10	-11	54.50	3.14
4	10	11	54.93	3.67
4	10	11	50.48	3.76
-5	-10	-11	46.35	2.74
-5	-10	-11	43.01	2.98
-5	-10	-11	38.62	3.62
5	10	11	51.19	3.44
5	10	11	44.26	3.46
-6	-10	-11	65.17	3.54
-6	-10	-11	60.77	3.79
6	10	11	64.09	4.27
6	10	11	57.75	4.55
-7	-10	-11	55.46	3.62
-7	-10	-11	53.06	4.55

-7	-10	-11	55.61	3.25
7	10	11	57.91	4.15
-8	-10	-11	35.64	2.94
-8	-10	-11	29.21	3.77
-8	-10	-11	30.33	2.31
8	10	11	30.35	2.21
8	10	11	33.22	3.53
8	10	11	37.49	3.42
-9	-10	-11	40.17	4.06
-9	-10	-11	38.28	2.63
-9	-10	-11	42.86	3.39
9	10	11	32.70	2.47
9	10	11	38.79	3.93
9	10	11	42.78	3.84
-10	-10	-11	20.02	2.07
-10	-10	-11	18.19	2.76
-10	-10	-11	18.02	3.01
10	10	11	26.47	3.54
10	10	11	20.23	3.52
10	10	11	17.85	1.94
-11	-10	-11	8.93	2.79
-11	-10	-11	11.72	1.98
-11	-10	-11	9.15	2.09
11	10	11	10.77	2.29
11	10	11	6.66	1.15
11	10	11	13.45	2.52
-12	-10	-11	38.19	4.20
-12	-10	-11	33.41	2.85
-12	-10	-11	35.44	5.19
12	10	11	35.16	5.04
12	10	11	39.07	4.68
-13	-10	-11	39.57	3.30
-13	-10	-11	44.36	6.04
-13	-10	-11	50.92	4.86
-13	-10	-11	44.44	5.97
13	10	11	35.46	5.99
13	10	11	41.34	5.74
13	10	11	47.91	5.32
-2	11	11	23.13	2.02
-1	11	11	15.46	1.69
0	11	11	28.04	2.04
-1	-11	-11	39.01	2.61
1	11	11	36.32	2.37
-2	-11	-11	65.86	3.66

2	11	11	60.42	3.47
-3	-11	-11	44.45	2.84
3	11	11	43.50	2.63
-4	-11	-11	87.74	4.59
4	11	11	79.15	5.41
-5	-11	-11	55.71	3.27
5	11	11	55.83	3.98
5	11	11	53.34	4.01
-6	-11	-11	57.56	3.21
-6	-11	-11	52.57	3.41
-6	-11	-11	41.94	4.18
6	11	11	57.55	4.02
6	11	11	53.78	3.99
-7	-11	-11	33.04	2.34
-7	-11	-11	25.39	3.67
-7	-11	-11	30.26	2.70
7	11	11	32.06	3.41
7	11	11	33.58	3.46
-8	-11	-11	44.90	2.91
-8	-11	-11	38.15	4.25
-8	-11	-11	45.27	3.35
8	11	11	41.22	2.73
8	11	11	49.58	4.13
8	11	11	50.44	4.03
-9	-11	-11	26.44	2.26
-9	-11	-11	25.29	2.94
-9	-11	-11	27.05	3.90
9	11	11	20.83	2.04
9	11	11	33.51	3.63
9	11	11	25.35	3.80
-10	-11	-11	35.50	3.37
-10	-11	-11	27.73	4.39
-10	-11	-11	30.05	2.43
10	11	11	29.53	4.25
10	11	11	30.72	4.08
10	11	11	24.12	2.26
-11	-11	-11	9.89	1.38
-11	-11	-11	8.58	2.16
-11	-11	-11	6.00	3.03
11	11	11	9.66	2.68
-12	-11	-11	27.88	5.09
-12	-11	-11	32.18	4.23
-12	-11	-11	30.79	2.83
-12	-11	-11	36.54	5.43

12	11	11	30.74	4.77
12	11	11	34.38	5.00
-13	-11	-11	20.68	4.47
-13	-11	-11	30.13	2.87
-13	-11	-11	32.28	5.86
-13	-11	-11	27.51	4.59
13	11	11	35.03	6.13
13	11	11	19.23	3.68
13	11	11	25.87	5.07
1	12	11	28.65	2.14
-2	-12	-11	37.84	2.61
2	12	11	34.12	2.36
-3	-12	-11	26.60	2.25
3	12	11	26.55	1.99
-4	-12	-11	50.41	3.08
4	12	11	44.06	2.84
4	12	11	49.76	3.71
4	12	11	49.57	4.21
-5	-12	-11	62.90	3.74
5	12	11	66.77	4.44
5	12	11	61.68	4.45
-6	-12	-11	27.19	2.61
-6	-12	-11	31.50	2.39
6	12	11	32.15	3.37
6	12	11	31.19	3.43
-7	-12	-11	32.57	2.93
-7	-12	-11	37.17	2.61
7	12	11	44.74	3.78
7	12	11	31.35	2.40
7	12	11	36.98	3.66
-8	-12	-11	27.40	2.87
-8	-12	-11	31.38	2.40
-8	-12	-11	23.28	4.00
8	12	11	34.80	3.82
8	12	11	27.15	2.17
8	12	11	35.16	3.80
-9	-12	-11	32.92	3.26
-9	-12	-11	38.17	2.63
-9	-12	-11	26.18	4.28
9	12	11	29.62	4.10
9	12	11	35.61	2.66
9	12	11	35.81	4.09
-10	-12	-11	24.86	3.24
-10	-12	-11	16.99	3.03

-10	-12	-11	23.07	2.31
10	12	11	25.33	3.97
10	12	11	15.00	2.81
-11	-12	-11	23.09	2.44
-11	-12	-11	26.63	3.70
-11	-12	-11	15.03	3.36
11	12	11	17.08	3.71
11	12	11	27.49	4.35
-12	-12	-11	22.37	3.80
-12	-12	-11	27.95	2.86
-12	-12	-11	32.64	4.51
-12	-12	-11	24.81	4.20
12	12	11	31.03	5.30
12	12	11	31.51	4.90
-13	-12	-11	29.05	4.92
-13	-12	-11	25.76	4.55
-13	-12	-11	22.49	4.23
-13	-12	-11	33.17	3.01
13	12	11	32.55	5.39
13	12	11	20.23	4.62
-3	-13	-11	37.06	2.77
-4	-13	-11	22.13	2.16
-5	-13	-11	42.66	2.97
5	13	11	43.14	3.70
-6	-13	-11	32.75	2.62
6	13	11	32.45	3.50
6	13	11	33.83	3.56
-7	-13	-11	48.37	3.21
7	13	11	49.90	4.14
7	13	11	46.42	4.24
-8	-13	-11	36.27	2.88
8	13	11	42.64	4.17
8	13	11	41.37	4.27
-9	-13	-11	30.27	4.48
-9	-13	-11	42.05	2.97
9	13	11	43.84	4.55
9	13	11	44.44	4.48
-10	-13	-11	36.36	2.87
10	13	11	28.42	4.64
10	13	11	41.77	4.61
-11	-13	-11	37.67	3.07
11	13	11	42.62	5.04
-12	-13	-11	22.75	4.18
-12	-13	-11	24.85	2.76

12	13	11	27.41	5.19
12	13	11	25.63	4.90
-4	-14	-11	24.64	2.42
-5	-14	-11	12.96	2.04
5	14	11	7.33	2.49
-6	-14	-11	25.09	2.44
6	14	11	27.59	3.43
-7	-14	-11	32.82	2.72
7	14	11	35.82	4.67
7	14	11	29.05	3.95
-8	-14	-11	33.27	2.82
8	14	11	37.60	4.34
-9	-14	-11	24.20	2.51
9	14	11	23.88	4.30
-10	-14	-11	34.91	3.00
10	14	11	34.75	5.01
-11	-14	-11	22.88	2.75
11	14	11	28.46	5.17
-5	-15	-11	26.31	2.61
5	15	11	29.18	3.43
-6	-15	-11	30.04	2.69
6	15	11	26.90	3.63
-7	-15	-11	33.86	2.79
7	15	11	33.91	4.07
-8	-15	-11	36.25	2.90
8	15	11	34.80	4.49
-9	-15	-11	24.61	2.66
9	15	11	21.45	3.03
6	8	-12	3.60	3.76
-6	-8	12	2.95	3.49
-6	-8	12	3.78	1.86
5	8	-12	4.18	3.80
5	8	-12	2.08	3.36
-5	-8	12	1.92	1.72
-5	-8	12	0.78	3.28
-5	-8	12	-1.31	3.23
4	8	-12	4.76	3.29
4	8	-12	1.32	0.83
4	8	-12	1.55	3.43
-4	-8	12	0.84	1.63
-4	-8	12	-5.00	3.03
-4	-8	12	-1.02	3.17
3	8	-12	0.97	3.33
3	8	-12	0.13	3.38

3	8	-12	-0.71	0.80
3	8	-12	1.04	3.28
-3	-8	12	-1.19	2.69
-3	-8	12	0.05	1.62
2	8	-12	-0.29	0.88
2	8	-12	4.49	3.22
2	8	-12	5.21	4.55
-2	-8	12	-0.50	1.68
-2	-8	12	-0.55	3.01
-2	-8	12	-2.01	2.29
1	8	-12	0.59	0.97
1	8	-12	-1.37	3.35
1	8	-12	2.26	3.45
1	8	-12	1.49	2.91
-1	-8	12	-3.75	2.22
-1	-8	12	-0.10	1.79
-1	-8	12	-2.61	3.61
-1	-8	12	-1.83	2.61
0	8	-12	3.13	3.29
0	8	-12	6.72	3.54
0	8	-12	3.02	1.04
0	-8	12	0.05	2.18
0	-8	12	3.45	1.96
-1	8	-12	-0.24	3.38
-1	8	-12	1.95	1.09
1	-8	12	3.40	2.06
-8	-7	12	-0.28	2.14
-8	-7	12	-1.26	3.04
-7	-7	12	-4.59	3.08
-7	-7	12	1.12	1.80
6	7	-12	0.55	3.08
6	7	-12	3.34	3.69
-6	-7	12	1.57	1.74
-6	-7	12	0.79	2.92
-6	-7	12	-1.48	2.98
5	7	-12	1.41	3.00
5	7	-12	-2.57	3.15
-5	-7	12	2.92	1.66
-5	-7	12	-3.69	2.76
-5	-7	12	-4.53	2.77
4	7	-12	1.80	3.10
4	7	-12	-0.67	0.68
4	7	-12	1.01	2.85
-4	-7	12	-0.25	3.02

-4	-7	12	0.54	1.55
3	7	-12	4.49	3.01
3	7	-12	1.76	2.72
3	7	-12	1.19	0.85
-3	-7	12	3.41	1.57
-3	-7	12	-1.95	2.25
-3	-7	12	-2.42	2.67
2	7	-12	0.17	3.08
2	7	-12	-0.94	2.58
2	7	-12	-0.23	0.80
-2	-7	12	0.78	1.52
-2	-7	12	1.69	1.46
-2	-7	12	-1.61	2.65
-2	-7	12	1.31	2.12
1	7	-12	0.13	0.82
1	7	-12	1.45	2.87
1	7	-12	0.30	2.51
-1	-7	12	0.70	1.97
-1	-7	12	0.71	1.59
-1	-7	12	-1.90	2.59
-1	-7	12	-1.86	1.46
0	7	-12	-2.00	2.47
0	7	-12	1.40	4.10
0	7	-12	0.91	0.94
0	-7	12	-0.24	2.63
0	-7	12	-1.53	1.61
0	-7	12	-0.21	1.63
0	-7	12	-0.28	2.04
-1	7	-12	6.28	2.86
-1	7	-12	4.44	2.82
-1	7	-12	4.52	2.59
-1	7	-12	4.30	1.06
1	-7	12	3.52	2.94
1	-7	12	4.67	2.08
1	-7	12	4.31	1.91
-2	7	-12	6.87	2.77
-2	7	-12	0.18	2.99
-2	7	-12	-0.38	1.01
2	-7	12	-2.13	1.79
2	-7	12	-0.90	1.81
-3	7	-12	-1.49	1.02
-3	7	-12	0.91	2.72
3	-7	12	-0.46	1.87
-9	-6	12	-4.46	2.94

-9	-6	12	1.73	2.47
-8	-6	12	0.64	2.15
7	6	-12	6.60	2.50
-7	-6	12	4.17	2.81
-7	-6	12	-1.24	2.66
-7	-6	12	0.50	1.97
6	6	-12	2.34	2.68
6	6	-12	5.69	3.23
-6	-6	12	0.35	2.62
-6	-6	12	-0.99	2.68
-6	-6	12	3.78	1.73
5	6	-12	4.36	2.85
5	6	-12	-0.19	2.61
-5	-6	12	1.21	1.59
-5	-6	12	-2.83	2.55
-5	-6	12	-1.14	2.55
4	6	-12	-1.30	2.62
4	6	-12	2.27	2.60
-4	-6	12	-4.04	2.75
-4	-6	12	-1.14	1.46
3	6	-12	-1.13	2.54
3	6	-12	-0.22	0.65
3	6	-12	0.93	2.51
-3	-6	12	1.85	1.44
-3	-6	12	1.16	2.34
-3	-6	12	1.18	2.04
-3	-6	12	0.55	1.16
2	6	-12	4.40	2.62
2	6	-12	0.48	2.61
2	6	-12	0.47	2.31
-2	-6	12	2.76	1.28
-2	-6	12	1.51	1.93
-2	-6	12	2.12	1.42
-2	-6	12	0.66	2.35
1	6	-12	1.82	0.81
1	6	-12	3.35	2.21
1	6	-12	2.91	2.61
1	6	-12	3.59	2.56
-1	-6	12	-1.45	2.29
-1	-6	12	3.09	1.53
-1	-6	12	2.40	1.32
-1	-6	12	1.89	1.77
0	6	-12	1.05	2.44
0	6	-12	0.28	0.81

0	6	-12	1.84	2.10
0	-6	12	2.84	1.72
0	-6	12	-0.57	2.41
0	-6	12	1.00	1.55
0	-6	12	1.73	1.33
-1	6	-12	2.11	0.90
-1	6	-12	4.68	2.56
-1	6	-12	1.99	2.00
1	-6	12	3.85	1.47
1	-6	12	-0.62	2.34
1	-6	12	0.50	1.84
1	-6	12	1.23	1.67
-2	6	-12	0.91	2.27
-2	6	-12	-0.14	2.27
-2	6	-12	0.81	0.91
2	-6	12	2.63	2.57
2	-6	12	1.96	1.83
2	-6	12	1.13	1.56
2	-6	12	0.45	1.69
-3	6	-12	2.74	2.38
-3	6	-12	1.16	2.79
-3	6	-12	-0.61	2.62
-3	6	-12	-0.60	0.96
3	-6	12	-0.44	1.84
3	-6	12	-2.97	1.75
3	-6	12	-1.45	2.71
-4	6	-12	3.70	3.09
-4	6	-12	-0.53	0.98
-4	6	-12	-1.22	2.42
4	-6	12	-1.59	1.79
-5	6	-12	0.30	2.71
-5	6	-12	-0.26	1.10
5	-6	12	-1.43	2.07
-9	-5	12	-4.50	2.61
-9	-5	12	-2.86	2.22
8	5	-12	2.33	2.64
-8	-5	12	-2.22	2.10
-8	-5	12	-3.32	2.57
7	5	-12	1.77	2.76
-7	-5	12	-1.87	1.87
-7	-5	12	-2.56	2.49
-7	-5	12	-1.46	2.53
6	5	-12	0.79	2.49
6	5	-12	7.26	3.14

-6	-5	12	4.24	1.79
-6	-5	12	0.34	2.50
-6	-5	12	0.34	2.47
5	5	-12	-3.06	2.45
5	5	-12	1.17	2.33
5	5	-12	-1.30	3.37
-5	-5	12	-3.40	2.31
-5	-5	12	0.08	1.47
4	5	-12	0.93	2.64
4	5	-12	-2.28	2.22
4	5	-12	0.34	0.55
4	5	-12	0.09	2.33
-4	-5	12	-2.63	2.18
-4	-5	12	-2.66	2.81
-4	-5	12	-0.56	1.37
3	5	-12	0.22	2.22
3	5	-12	-1.76	2.24
3	5	-12	-0.74	0.60
3	5	-12	-1.35	2.06
-3	-5	12	-1.57	1.82
-3	-5	12	-0.15	0.92
-3	-5	12	-0.60	1.30
-3	-5	12	-3.81	2.04
2	5	-12	1.05	1.98
2	5	-12	-1.09	1.96
2	5	-12	-0.37	0.59
2	5	-12	-0.25	2.27
-2	-5	12	0.18	1.01
-2	-5	12	-0.44	1.21
-2	-5	12	-0.28	1.66
-2	-5	12	-3.28	2.04
1	5	-12	1.09	1.96
1	5	-12	0.82	2.23
1	5	-12	-1.62	1.18
1	5	-12	2.47	1.86
-1	-5	12	-0.06	1.27
-1	-5	12	0.85	1.51
-1	-5	12	-0.40	2.08
-1	-5	12	-0.51	1.01
0	5	-12	-0.23	0.72
0	5	-12	1.73	2.12
0	5	-12	0.73	1.77
0	5	-12	0.59	1.80
0	-5	12	-0.10	1.10

0	-5	12	0.57	1.38
0	-5	12	-1.25	2.13
0	-5	12	1.64	1.57
-1	5	-12	2.52	2.07
-1	5	-12	1.38	1.68
-1	5	-12	-0.12	0.76
-1	5	-12	0.23	1.73
1	-5	12	0.22	1.15
1	-5	12	-2.72	2.12
1	-5	12	0.24	1.51
1	-5	12	-0.25	1.42
-2	5	-12	0.49	1.97
-2	5	-12	-3.41	1.96
-2	5	-12	-0.64	0.79
-2	5	-12	1.97	2.03
2	-5	12	-1.57	2.11
2	-5	12	0.53	1.53
2	-5	12	-1.65	1.15
2	-5	12	-1.24	1.50
-3	5	-12	-1.20	0.84
-3	5	-12	1.04	2.13
-3	5	-12	1.46	2.05
3	-5	12	-1.49	1.62
3	-5	12	-1.52	1.25
3	-5	12	-2.90	2.20
3	-5	12	0.61	1.62
-4	5	-12	2.87	2.21
-4	5	-12	-0.34	1.85
-4	5	-12	-1.17	0.88
4	-5	12	-1.95	2.46
4	-5	12	-1.44	1.59
4	-5	12	-0.28	1.86
-5	5	-12	0.31	0.98
-5	5	-12	4.48	3.01
-5	5	-12	1.03	2.36
-5	5	-12	-2.90	2.91
5	-5	12	-1.54	1.85
5	-5	12	-1.66	1.87
-6	5	-12	-0.61	2.94
-6	5	-12	-0.07	2.58
-6	5	-12	-1.14	0.98
6	-5	12	-2.18	2.01
-10	-4	12	-3.52	2.53
-10	-4	12	-2.91	2.62

-9	-4	12	-4.88	2.49
-9	-4	12	-1.21	2.30
-8	-4	12	-0.87	2.15
-8	-4	12	-1.58	2.28
-7	-4	12	-2.76	2.24
-7	-4	12	1.85	1.95
-7	-4	12	-4.34	2.31
6	4	-12	3.91	3.24
6	4	-12	-0.83	2.72
6	4	-12	-0.11	3.04
-6	-4	12	-1.70	2.23
-6	-4	12	0.99	1.77
-6	-4	12	0.47	2.18
5	4	-12	0.00	2.05
5	4	-12	-1.12	2.29
5	4	-12	2.37	2.74
-5	-4	12	-0.95	2.15
-5	-4	12	0.17	1.50
4	4	-12	0.04	2.32
4	4	-12	-2.58	2.05
-4	-4	12	-0.47	1.32
3	4	-12	1.21	1.94
3	4	-12	-0.82	1.97
3	4	-12	0.04	0.52
3	4	-12	-1.07	1.91
-3	-4	12	0.03	1.23
-3	-4	12	-0.23	0.83
-3	-4	12	-2.89	1.98
-3	-4	12	-0.08	1.67
2	4	-12	-0.21	1.79
2	4	-12	2.98	1.96
2	4	-12	2.14	0.63
2	4	-12	1.73	1.66
-2	-4	12	0.74	0.89
-2	-4	12	0.63	1.95
-2	-4	12	2.32	1.22
-2	-4	12	1.50	1.54
1	4	-12	3.17	1.75
1	4	-12	2.54	0.65
1	4	-12	2.07	1.96
-1	-4	12	3.40	1.22
-1	-4	12	3.21	1.01
-1	-4	12	3.24	1.42
-1	-4	12	-0.69	2.48

0	4	-12	3.92	1.93
0	4	-12	1.32	1.63
0	4	-12	1.65	1.14
0	4	-12	0.60	1.33
0	-4	12	2.09	1.05
0	-4	12	3.36	1.29
0	-4	12	3.66	1.44
0	-4	12	0.50	1.89
-1	4	-12	0.84	1.37
-1	4	-12	-0.01	0.68
-1	4	-12	0.13	1.76
-1	4	-12	-0.95	1.53
1	-4	12	-2.24	1.87
1	-4	12	1.29	1.35
1	-4	12	0.71	1.05
1	-4	12	-0.70	1.24
-2	4	-12	0.54	1.80
-2	4	-12	2.05	1.47
-2	4	-12	-0.63	1.74
-2	4	-12	-0.04	0.73
2	-4	12	-0.34	1.81
2	-4	12	0.22	1.44
2	-4	12	-1.59	1.35
2	-4	12	-0.97	1.02
-3	4	-12	1.32	1.55
-3	4	-12	0.97	1.78
-3	4	-12	-1.52	0.77
-3	4	-12	-1.21	1.82
3	-4	12	2.35	1.45
3	-4	12	-2.38	1.93
3	-4	12	-0.28	1.50
-4	4	-12	-1.53	2.61
-4	4	-12	-0.91	0.84
-4	4	-12	-2.27	1.63
4	-4	12	-1.85	1.52
4	-4	12	0.17	2.13
4	-4	12	-0.82	1.63
4	-4	12	-1.39	1.14
-5	4	-12	-2.72	1.71
-5	4	-12	1.36	2.03
-5	4	-12	-1.26	0.90
5	-4	12	-0.63	1.58
5	-4	12	1.12	1.90
5	-4	12	1.18	2.29

-6	4	-12	-0.86	2.19
-6	4	-12	-1.10	0.94
6	-4	12	-0.16	1.84
6	-4	12	1.24	2.02
-7	4	-12	6.59	3.22
-7	4	-12	2.71	2.73
-7	4	-12	5.21	4.30
-7	4	-12	1.68	1.08
7	-4	12	3.63	2.08
-10	-3	12	-2.42	2.57
-10	-3	12	-1.77	2.40
-9	-3	12	-0.50	2.42
-9	-3	12	0.96	2.32
-8	-3	12	0.43	1.99
-8	-3	12	-1.50	2.01
-8	-3	12	-4.53	2.35
7	3	-12	-3.54	2.20
-7	-3	12	-1.44	2.19
-7	-3	12	-1.83	1.85
-7	-3	12	-1.86	2.01
6	3	-12	-0.74	2.98
6	3	-12	-0.36	2.44
-6	-3	12	0.17	1.65
-6	-3	12	-0.69	2.05
5	3	-12	-0.40	3.20
-5	-3	12	-1.31	1.49
-5	-3	12	-2.00	1.93
4	3	-12	0.72	1.95
-4	-3	12	-3.31	1.96
-4	-3	12	1.13	1.34
3	3	-12	-3.37	1.76
3	3	-12	0.48	1.86
3	3	-12	1.10	0.48
-3	-3	12	0.38	1.16
-3	-3	12	0.10	1.49
-3	-3	12	-1.82	1.92
-3	-3	12	1.54	0.75
2	3	-12	1.77	1.71
2	3	-12	-0.28	1.67
2	3	-12	2.92	0.59
-2	-3	12	2.33	1.42
-2	-3	12	3.09	1.19
-2	-3	12	1.56	1.90
-2	-3	12	3.52	0.87

1	3	-12	-0.50	0.53
1	3	-12	-0.52	1.58
1	3	-12	-1.08	1.49
-1	-3	12	-1.49	1.72
-1	-3	12	-1.09	1.03
-1	-3	12	0.29	1.21
-1	-3	12	0.13	0.86
0	3	-12	-1.65	1.39
0	3	-12	0.79	0.59
0	3	-12	0.92	1.63
0	-3	12	-1.31	1.71
0	-3	12	1.22	1.29
0	-3	12	0.79	0.94
0	-3	12	1.13	1.10
-1	3	-12	-0.85	1.55
-1	3	-12	1.21	1.41
-1	3	-12	0.80	1.23
1	-3	12	0.24	1.15
1	-3	12	1.75	0.99
1	-3	12	2.51	1.97
1	-3	12	2.51	1.27
-2	3	-12	0.40	1.64
-2	3	-12	2.50	1.60
-2	3	-12	1.27	0.69
2	-3	12	1.90	1.27
2	-3	12	-0.02	0.99
2	-3	12	1.13	1.25
2	-3	12	2.71	1.70
-3	3	-12	2.61	1.62
-3	3	-12	-0.84	1.71
-3	3	-12	-0.04	0.69
3	-3	12	-0.34	0.99
3	-3	12	0.00	1.34
3	-3	12	2.27	1.38
3	-3	12	-0.21	1.83
-4	3	-12	2.55	0.81
-4	3	-12	1.34	1.57
-4	3	-12	3.24	1.73
4	-3	12	3.32	1.97
4	-3	12	0.64	1.43
4	-3	12	2.32	1.14
4	-3	12	3.54	1.56
-5	3	-12	0.37	0.82
-5	3	-12	2.18	1.64

-5	3	-12	-1.08	1.70
5	-3	12	0.22	1.71
5	-3	12	0.01	2.03
5	-3	12	-0.83	1.44
5	-3	12	-1.17	1.12
-6	3	-12	-0.67	0.89
-6	3	-12	0.53	2.09
6	-3	12	-1.04	1.93
6	-3	12	-0.79	1.62
6	-3	12	-2.75	2.10
-7	3	-12	1.10	0.99
-7	3	-12	1.10	2.72
-7	3	-12	1.23	2.48
-7	3	-12	-1.08	2.13
7	-3	12	2.65	1.92
7	-3	12	-0.02	2.17
-8	3	-12	0.23	0.99
-8	3	-12	3.08	3.52
-8	3	-12	-0.34	3.01
-8	3	-12	1.63	3.34
8	-3	12	2.01	1.99
-10	-2	12	-1.94	2.59
-10	-2	12	-1.15	2.64
-9	-2	12	-1.47	2.36
-8	-2	12	-2.81	2.26
-8	-2	12	-3.28	2.05
7	2	-12	2.31	2.50
7	2	-12	-4.62	3.98
-7	-2	12	-1.82	1.82
-7	-2	12	-0.21	1.88
6	2	-12	1.02	1.89
-6	-2	12	-1.31	1.81
-6	-2	12	-3.10	1.92
-6	-2	12	-0.09	1.69
-5	-2	12	-2.06	1.78
-5	-2	12	0.09	1.50
4	2	-12	0.86	1.83
4	2	-12	-0.09	1.78
-4	-2	12	2.56	1.43
-4	-2	12	-0.46	2.29
-4	-2	12	-1.53	1.92
3	2	-12	-1.07	1.67
3	2	-12	2.20	0.52
3	2	-12	1.28	1.81

-3	-2	12	1.27	1.19
-3	-2	12	1.80	1.44
-3	-2	12	-1.02	1.83
2	2	-12	2.51	1.55
2	2	-12	2.96	0.58
2	2	-12	3.15	1.59
-2	-2	12	3.60	1.21
-2	-2	12	4.80	1.31
-2	-2	12	3.49	1.20
-2	-2	12	-0.66	1.74
1	2	-12	5.04	1.51
1	2	-12	4.31	0.64
1	2	-12	3.95	1.46
-1	-2	12	5.57	1.28
-1	-2	12	4.26	1.72
-1	-2	12	3.76	1.14
-1	-2	12	4.35	0.81
0	2	-12	1.54	1.37
0	2	-12	2.31	0.59
0	2	-12	1.04	1.39
0	-2	12	2.55	0.83
0	-2	12	1.69	1.20
0	-2	12	2.48	1.60
0	-2	12	0.81	1.02
-1	2	-12	-0.78	1.23
-1	2	-12	0.24	0.57
-1	2	-12	1.44	1.45
1	-2	12	-0.52	1.07
1	-2	12	0.40	1.48
1	-2	12	0.86	1.19
1	-2	12	0.29	0.78
-2	2	-12	-0.30	1.00
-2	2	-12	0.35	1.32
2	-2	12	1.59	1.22
2	-2	12	-0.03	0.83
2	-2	12	-0.68	1.11
-3	2	-12	-0.34	1.51
-3	2	-12	0.35	0.64
-3	2	-12	1.14	1.95
3	-2	12	0.22	0.87
3	-2	12	0.42	1.68
3	-2	12	-0.64	1.18
3	-2	12	1.32	1.29
-4	2	-12	-0.32	1.52

-4	2	-12	1.25	1.58
4	-2	12	-0.81	1.35
4	-2	12	0.11	1.82
4	-2	12	-0.55	1.26
4	-2	12	0.18	0.86
-5	2	-12	-0.72	0.72
-5	2	-12	0.17	1.73
-5	2	-12	-1.56	1.64
5	-2	12	-1.18	0.93
5	-2	12	-0.47	1.91
5	-2	12	-0.03	1.58
5	-2	12	0.22	1.35
-6	2	-12	-1.58	0.78
-6	2	-12	-0.13	1.81
-6	2	-12	-0.05	1.24
6	-2	12	2.06	2.09
6	-2	12	0.11	1.07
6	-2	12	-1.43	1.43
6	-2	12	-1.57	1.67
-7	2	-12	-0.77	2.38
-7	2	-12	-0.91	0.87
7	-2	12	1.73	2.27
7	-2	12	-0.90	1.63
7	-2	12	-2.15	1.97
-8	2	-12	-3.10	2.25
-8	2	-12	-0.77	3.03
-8	2	-12	-0.42	0.95
-8	2	-12	-2.07	2.80
8	-2	12	0.34	2.16
8	-2	12	-1.99	1.82
-9	2	-12	-2.53	3.35
-9	2	-12	0.90	3.55
9	-2	12	1.71	2.07
-10	-1	12	-1.51	2.66
-9	-1	12	-1.41	2.42
-8	-1	12	-1.83	2.06
7	1	-12	-0.07	3.30
-7	-1	12	-1.26	1.89
-7	-1	12	-0.76	1.79
-6	-1	12	-1.35	1.74
-6	-1	12	-0.52	1.73
-6	-1	12	-2.41	1.80
5	1	-12	0.65	1.82
5	1	-12	0.82	1.52

-5	-1	12	-1.62	1.67
-5	-1	12	-2.40	1.72
-5	-1	12	1.24	1.54
4	1	-12	-0.12	1.93
4	1	-12	-1.21	1.70
-4	-1	12	-1.30	1.44
-4	-1	12	-1.24	1.34
-4	-1	12	-3.12	1.66
3	1	-12	-2.05	1.55
3	1	-12	0.75	1.40
3	1	-12	1.11	0.55
-3	-1	12	1.33	1.18
-3	-1	12	1.76	0.58
-3	-1	12	-1.12	1.64
-3	-1	12	1.02	1.27
2	1	-12	0.96	1.52
2	1	-12	-0.60	1.35
2	1	-12	-0.59	0.47
-2	-1	12	0.02	0.54
-2	-1	12	-0.48	1.53
-2	-1	12	0.68	1.13
-2	-1	12	-0.20	1.03
1	1	-12	-0.46	1.25
1	1	-12	-0.11	0.47
1	1	-12	1.04	1.31
-1	-1	12	-0.55	0.99
-1	-1	12	0.46	1.11
-1	-1	12	0.25	0.58
-1	-1	12	-0.33	1.49
0	1	-12	0.99	1.19
0	1	-12	0.44	0.53
0	1	-12	2.17	1.32
0	-1	12	1.13	0.65
0	-1	12	0.08	1.01
0	-1	12	0.93	1.08
0	-1	12	1.32	1.49
-1	1	-12	1.53	1.17
-1	1	-12	3.30	1.34
-1	1	-12	1.55	0.56
1	-1	12	1.36	0.70
1	-1	12	1.73	1.10
1	-1	12	0.08	1.01
1	-1	12	2.18	1.48
-2	1	-12	-0.56	1.19

-2	1	-12	-0.37	0.53
-2	1	-12	-0.29	1.34
2	-1	12	0.22	0.69
2	-1	12	-1.16	1.04
2	-1	12	0.65	1.12
2	-1	12	-1.50	1.46
-3	1	-12	0.57	1.46
-3	1	-12	0.23	0.79
3	-1	12	-0.79	0.70
3	-1	12	0.17	1.88
3	-1	12	-0.55	1.05
3	-1	12	0.19	1.24
-4	1	-12	-2.85	1.47
-4	1	-12	-1.65	1.53
4	-1	12	-1.24	1.18
4	-1	12	-1.06	0.72
4	-1	12	-0.27	1.72
4	-1	12	0.20	1.34
-5	1	-12	0.63	1.60
-5	1	-12	4.02	1.77
-5	1	-12	0.78	0.70
5	-1	12	1.70	1.92
5	-1	12	2.11	1.51
5	-1	12	2.15	1.30
5	-1	12	0.74	0.82
-6	1	-12	0.20	0.76
-6	1	-12	0.18	1.97
-6	1	-12	-1.26	1.74
-6	1	-12	-1.11	1.74
6	-1	12	-1.22	2.04
6	-1	12	-0.33	1.30
6	-1	12	-0.27	1.64
6	-1	12	0.74	0.87
-7	1	-12	1.31	1.26
-7	1	-12	0.42	1.93
-7	1	-12	-0.01	0.85
7	-1	12	-0.58	1.42
7	-1	12	-0.43	1.84
7	-1	12	-0.17	2.20
7	-1	12	-1.14	0.91
-8	1	-12	-1.35	2.66
-8	1	-12	-0.83	0.89
-8	1	-12	-1.57	2.10
8	-1	12	1.11	1.64

8	-1	12	1.09	2.03
8	-1	12	-0.35	2.46
-9	1	-12	-1.05	3.31
-9	1	-12	-1.47	2.58
-9	1	-12	-0.74	1.00
-9	1	-12	0.57	2.54
-9	1	-12	-2.08	3.32
9	-1	12	3.45	2.23
9	-1	12	0.39	1.83
-10	1	-12	-0.34	1.07
-10	1	-12	-3.58	3.92
-10	1	-12	5.06	4.16
10	-1	12	-0.11	2.10
-10	0	12	-2.29	2.75
-9	0	12	-1.72	2.37
-8	0	12	-1.25	2.07
7	0	-12	-3.77	3.47
-7	0	12	-0.22	1.88
6	0	-12	1.89	2.90
-5	0	12	-0.80	1.50
-5	0	12	0.83	1.50
-4	0	12	1.31	1.29
-4	0	12	0.02	1.34
-4	0	12	-0.61	1.61
3	0	-12	1.96	1.44
3	0	-12	-1.53	2.12
3	0	-12	1.38	1.27
3	0	-12	0.83	0.56
-3	0	12	1.09	1.54
-3	0	12	0.66	1.21
-3	0	12	1.65	1.21
2	0	-12	0.34	1.91
2	0	-12	0.00	1.31
2	0	-12	-0.01	1.09
-2	0	12	1.66	1.50
-2	0	12	1.71	1.06
1	0	-12	3.02	1.46
1	0	-12	2.37	1.22
1	0	-12	2.70	0.59
1	0	-12	2.84	1.68
-1	0	12	3.61	1.29
-1	0	12	2.22	1.50
-1	0	12	2.21	1.13
0	0	-12	0.28	1.23

0	0	-12	-0.15	0.50
0	0	-12	0.14	1.11
0	0	-12	-1.08	1.37
0	0	12	0.36	1.04
0	0	12	0.79	0.56
0	0	12	0.04	1.38
0	0	12	-0.80	0.92
-1	0	-12	1.12	1.30
-1	0	-12	3.52	1.33
-1	0	-12	0.79	0.54
-1	0	-12	1.90	1.12
1	0	12	1.90	0.62
1	0	12	0.36	0.97
1	0	12	0.54	1.44
1	0	12	1.69	1.12
-2	0	-12	1.34	1.22
-2	0	-12	1.11	1.35
-2	0	-12	-0.12	1.16
-2	0	-12	-0.23	0.53
2	0	12	1.27	0.65
2	0	12	1.79	1.13
2	0	12	-0.43	1.51
2	0	12	-0.45	1.48
-3	0	-12	0.06	1.44
-3	0	-12	-0.46	0.54
-3	0	-12	-1.10	1.54
-3	0	-12	-0.16	1.13
3	0	12	-0.41	1.18
3	0	12	-0.88	1.58
3	0	12	0.56	0.66
3	0	12	-0.85	1.35
-4	0	-12	-0.77	1.66
-4	0	-12	0.64	1.58
-4	0	-12	0.41	0.58
-4	0	-12	0.92	1.15
4	0	12	-0.31	1.29
4	0	12	-0.46	0.68
4	0	12	-0.09	1.77
4	0	12	-0.69	1.02
-5	0	-12	-0.97	1.69
-5	0	-12	-1.74	1.54
-5	0	-12	-1.19	0.65
5	0	12	-1.25	1.80
5	0	12	-0.97	1.09

5	0	12	-0.34	0.73
5	0	12	-1.08	1.33
-6	0	-12	-0.54	1.92
-6	0	-12	-0.04	0.71
-6	0	-12	-1.40	1.62
6	0	12	0.05	1.21
6	0	12	0.04	0.75
6	0	12	-0.62	2.00
6	0	12	0.68	1.54
-7	0	-12	-0.90	0.76
-7	0	-12	-2.58	2.16
-7	0	-12	-0.86	1.74
-7	0	-12	-0.15	1.17
7	0	12	-0.24	1.34
7	0	12	0.05	2.21
7	0	12	-0.37	0.83
7	0	12	-0.97	1.74
-8	0	-12	-1.35	1.82
-8	0	-12	-0.25	2.95
-8	0	-12	-0.03	0.86
-8	0	-12	-0.87	1.26
8	0	12	1.47	1.15
8	0	12	-0.02	1.44
8	0	12	0.36	2.36
8	0	12	1.75	1.95
-9	0	-12	-4.00	3.18
-9	0	-12	-1.19	0.94
-9	0	-12	0.71	3.10
-9	0	-12	-2.64	1.99
9	0	12	0.31	2.82
9	0	12	0.58	2.11
9	0	12	0.91	1.73
-10	0	-12	8.05	2.90
-10	0	-12	11.39	4.19
-10	0	-12	-0.36	3.99
10	0	12	8.56	2.07
-11	0	-12	-4.76	4.47
-11	0	-12	-0.43	1.14
-11	0	-12	1.60	4.60
11	0	12	0.31	2.10
-10	1	12	-0.41	2.81
-9	1	12	-1.42	2.41
-8	1	12	-1.65	2.20
-7	1	12	-0.85	1.94

6	-1	-12	1.38	2.92
-6	1	12	-1.45	1.67
-5	1	12	-1.52	1.49
4	-1	-12	-1.10	2.19
-4	1	12	-0.60	1.37
-4	1	12	-1.89	1.54
3	-1	-12	7.29	1.03
3	-1	-12	6.14	1.37
3	-1	-12	3.79	2.20
-3	1	12	8.34	1.66
-3	1	12	6.27	1.73
2	-1	-12	8.98	1.42
2	-1	-12	8.12	1.45
2	-1	-12	6.49	2.10
2	-1	-12	10.93	1.13
-2	1	12	7.39	1.39
-2	1	12	11.74	1.70
-2	1	12	10.43	1.06
-2	1	12	13.10	1.90
1	-1	-12	1.86	0.60
1	-1	-12	1.61	1.14
1	-1	-12	2.32	1.14
1	-1	-12	2.82	1.74
-1	1	12	2.02	1.02
-1	1	12	2.24	1.44
-1	1	12	3.59	1.10
-1	1	12	1.53	0.72
0	-1	-12	14.98	2.03
0	-1	-12	17.99	1.32
0	-1	-12	12.48	1.95
0	1	12	17.71	2.04
0	1	12	16.53	1.30
0	1	12	14.42	2.05
0	1	12	14.22	2.50
-1	-1	-12	3.73	1.41
-1	-1	-12	5.28	1.42
-1	-1	-12	2.82	1.16
-1	-1	-12	5.12	0.68
1	1	12	2.71	1.49
1	1	12	4.65	0.67
1	1	12	3.89	1.03
1	1	12	3.86	1.16
-2	-1	-12	6.05	1.27
-2	-1	-12	5.06	1.33

-2	-1	-12	5.82	0.99
-2	-1	-12	6.83	1.51
2	1	12	4.54	1.12
2	1	12	6.46	1.09
2	1	12	5.35	1.30
2	1	12	4.97	1.65
-3	-1	-12	1.81	0.59
-3	-1	-12	2.04	1.46
-3	-1	-12	1.24	1.28
-3	-1	-12	2.18	1.14
3	1	12	2.17	1.25
3	1	12	1.05	1.60
3	1	12	0.51	1.00
3	1	12	2.19	0.64
-4	-1	-12	4.77	1.76
-4	-1	-12	6.83	1.26
-4	-1	-12	4.27	2.56
-4	-1	-12	4.02	0.92
4	1	12	3.71	1.49
4	1	12	5.49	1.40
4	1	12	5.54	0.79
4	1	12	3.34	1.73
-5	-1	-12	-0.24	1.77
-5	-1	-12	2.61	1.11
-5	-1	-12	0.78	0.66
-5	-1	-12	0.70	1.55
5	1	12	-0.12	1.97
5	1	12	2.36	1.09
5	1	12	0.50	1.38
5	1	12	1.76	0.74
-6	-1	-12	0.53	0.68
-6	-1	-12	-1.20	1.46
-6	-1	-12	-2.19	1.89
6	1	12	-0.34	0.57
6	1	12	-0.51	1.45
6	1	12	-1.08	1.87
6	1	12	0.90	1.08
-7	-1	-12	-0.91	0.73
-7	-1	-12	-0.71	2.11
-7	-1	-12	-1.03	1.45
7	1	12	-0.80	1.17
7	1	12	-1.04	1.57
7	1	12	-1.41	2.07
-8	-1	-12	-0.25	1.58

-8	-1	-12	-0.47	0.83
-8	-1	-12	-2.94	2.51
-8	-1	-12	-0.08	1.18
8	1	12	0.36	1.41
8	1	12	2.94	1.94
8	1	12	-2.82	2.27
-9	-1	-12	-2.48	2.89
-9	-1	-12	0.02	1.41
-9	-1	-12	-0.81	0.94
9	1	12	0.50	1.99
9	1	12	0.27	1.56
9	1	12	-0.19	2.47
-10	-1	-12	-3.62	3.52
-10	-1	-12	2.43	1.08
-10	-1	-12	1.24	2.00
10	1	12	3.24	2.99
10	1	12	3.17	1.85
-11	-1	-12	-0.83	1.04
-11	-1	-12	1.40	2.63
-11	-1	-12	0.20	4.44
-11	-1	-12	0.26	4.29
11	1	12	0.87	1.96
-10	2	12	-0.78	3.09
-9	2	12	-1.72	2.62
-8	2	12	-0.95	2.24
-7	2	12	-0.19	1.89
-6	2	12	-1.27	1.68
-5	2	12	-1.82	1.57
4	-2	-12	-0.37	2.29
-4	2	12	2.13	1.05
-4	2	12	1.32	1.45
3	-2	-12	1.79	0.59
-3	2	12	2.14	0.50
-3	2	12	3.09	1.53
2	-2	-12	-0.39	0.50
2	-2	-12	1.27	1.25
2	-2	-12	-1.64	1.91
-2	2	12	-0.95	1.10
-2	2	12	0.36	0.48
-2	2	12	0.42	1.01
-2	2	12	-1.42	1.33
1	-2	-12	3.19	0.66
1	-2	-12	3.40	1.37
1	-2	-12	4.11	1.81

1	-2	-12	2.49	1.09
-1	2	12	5.45	1.08
-1	2	12	3.67	1.06
-1	2	12	4.35	1.51
-1	2	12	4.15	1.07
0	-2	-12	0.09	1.23
0	-2	-12	-1.54	1.50
0	-2	-12	-0.80	0.54
0	-2	-12	-0.78	0.99
0	2	12	0.05	1.04
0	2	12	-0.61	0.90
0	2	12	-2.41	1.34
0	2	12	-0.50	0.47
-1	-2	-12	2.52	1.68
-1	-2	-12	3.65	0.64
-1	-2	-12	3.23	1.10
1	2	12	3.42	0.61
1	2	12	4.69	1.14
1	2	12	2.42	1.48
-2	-2	-12	-0.55	1.21
-2	-2	-12	-0.61	1.42
-2	-2	-12	-0.60	1.11
-2	-2	-12	0.00	0.56
2	2	12	-1.12	1.43
2	2	12	0.11	1.11
2	2	12	0.62	0.55
-3	-2	-12	3.65	1.16
-3	-2	-12	2.21	0.62
-3	-2	-12	2.11	1.24
-3	-2	-12	0.75	1.51
3	2	12	0.38	1.54
3	2	12	2.72	0.62
3	2	12	2.71	1.24
-4	-2	-12	-1.95	1.27
-4	-2	-12	-0.38	1.56
-4	-2	-12	1.83	1.08
-4	-2	-12	0.82	0.59
4	2	12	-0.34	1.25
4	2	12	0.53	0.59
4	2	12	-1.99	1.58
4	2	12	-0.18	0.91
-5	-2	-12	-1.68	1.73
-5	-2	-12	-0.10	1.01
-5	-2	-12	-0.70	0.65

5	2	12	0.45	0.62
5	2	12	-0.48	0.95
5	2	12	0.37	1.31
5	2	12	-0.90	1.71
-6	-2	-12	-2.64	1.92
-6	-2	-12	-0.66	0.63
-6	-2	-12	-2.03	1.38
-6	-2	-12	-0.20	1.06
6	2	12	-0.48	1.41
-7	-2	-12	-0.12	0.68
-7	-2	-12	-2.50	2.19
-7	-2	-12	-0.85	1.39
7	2	12	0.31	1.56
7	2	12	0.35	1.10
7	2	12	0.30	2.08
-8	-2	-12	-2.99	2.40
-8	-2	-12	-0.90	0.78
-8	-2	-12	-0.98	1.43
8	2	12	1.24	1.68
8	2	12	0.98	2.25
8	2	12	0.19	1.26
-9	-2	-12	-1.40	0.87
-9	-2	-12	-2.38	2.75
-9	-2	-12	-0.20	1.27
-9	-2	-12	0.08	1.63
9	2	12	2.17	1.49
9	2	12	0.17	0.92
9	2	12	1.29	2.52
-10	-2	-12	-1.82	1.78
-10	-2	-12	-0.67	0.97
-10	-2	-12	1.18	3.27
10	2	12	3.89	2.83
10	2	12	0.65	1.68
-11	-2	-12	4.03	2.60
-11	-2	-12	-1.79	2.21
-11	-2	-12	2.73	4.32
-11	-2	-12	-4.20	4.08
-11	-2	-12	0.08	1.07
11	2	12	-1.63	2.16
11	2	12	1.57	1.95
-12	-2	-12	-0.09	1.21
-12	-2	-12	6.40	5.17
-12	-2	-12	-0.53	4.77
12	2	12	1.15	2.18

-9	3	12	1.25	2.85
-8	3	12	0.35	2.43
-7	3	12	0.22	2.06
-6	3	12	-0.48	1.76
-5	3	12	3.62	1.66
4	-3	-12	2.17	2.45
4	-3	-12	6.16	0.76
-4	3	12	4.50	0.63
3	-3	-12	6.56	1.12
-3	3	12	5.67	0.95
2	-3	-12	6.78	1.07
-2	3	12	7.47	1.84
-2	3	12	5.25	0.72
1	-3	-12	10.47	1.18
1	-3	-12	9.02	1.79
-1	3	12	9.83	1.65
-1	3	12	10.60	1.91
-1	3	12	12.29	1.22
0	-3	-12	18.49	1.47
0	-3	-12	16.31	1.97
0	3	12	20.35	2.19
0	3	12	17.79	2.61
0	3	12	19.43	1.35
-1	-3	-12	3.50	0.67
-1	-3	-12	2.11	1.08
-1	-3	-12	-0.34	1.22
1	3	12	1.14	1.40
1	3	12	3.47	0.58
1	3	12	2.64	1.18
-2	-3	-12	14.52	1.31
-2	-3	-12	10.60	1.98
-2	-3	-12	11.25	1.43
2	3	12	13.09	1.19
2	3	12	12.01	2.09
2	3	12	10.93	1.74
-3	-3	-12	33.89	3.51
-3	-3	-12	40.14	3.00
-3	-3	-12	46.19	2.43
3	3	12	38.49	3.49
3	3	12	46.25	2.44
3	3	12	39.65	3.15
-4	-3	-12	-0.53	1.59
-4	-3	-12	0.49	0.62
-4	-3	-12	0.14	1.09

4	3	12	0.69	1.23
4	3	12	1.40	0.62
4	3	12	-0.95	1.54
-5	-3	-12	1.81	1.17
-5	-3	-12	-0.83	1.75
5	3	12	2.70	0.65
5	3	12	0.94	1.34
5	3	12	1.39	1.72
-6	-3	-12	3.74	1.99
-6	-3	-12	4.50	1.84
-6	-3	-12	5.48	0.80
6	3	12	6.01	1.90
6	3	12	7.04	1.56
-7	-3	-12	14.66	1.51
-7	-3	-12	14.04	2.44
-7	-3	-12	12.79	2.16
7	3	12	16.55	2.42
7	3	12	16.38	2.87
-8	-3	-12	2.69	2.39
-8	-3	-12	6.40	1.65
-8	-3	-12	9.05	1.07
8	3	12	10.06	2.26
8	3	12	9.74	1.84
-9	-3	-12	0.95	2.70
-9	-3	-12	3.08	0.97
-9	-3	-12	1.71	1.55
9	3	12	1.75	2.33
9	3	12	2.32	0.97
-10	-3	-12	3.51	1.74
-10	-3	-12	1.31	1.03
-10	-3	-12	5.76	3.75
-10	-3	-12	2.69	1.47
10	3	12	5.00	2.62
10	3	12	4.22	1.65
-11	-3	-12	0.72	3.57
-11	-3	-12	0.74	1.08
-11	-3	-12	3.43	4.11
-11	-3	-12	1.04	1.94
11	3	12	1.85	2.36
11	3	12	2.96	3.00
11	3	12	2.81	1.85
-12	-3	-12	4.54	4.94
-12	-3	-12	0.21	1.20
-12	-3	-12	-4.41	4.23

12	3	12	2.15	2.09
-9	4	12	-0.47	2.86
-8	4	12	-0.06	2.54
-7	4	12	-0.10	2.09
4	-4	-12	2.57	0.71
-4	4	12	2.61	0.79
3	-4	-12	1.88	0.66
-3	4	12	1.17	0.70
2	-4	-12	1.72	0.63
-2	4	12	1.73	0.54
1	-4	-12	1.26	0.58
-1	4	12	1.05	0.50
0	-4	-12	1.26	0.62
0	-4	-12	0.96	0.96
0	4	12	0.56	1.41
0	4	12	1.19	1.08
0	4	12	2.18	0.55
-1	-4	-12	3.83	0.70
-1	-4	-12	4.08	1.08
1	4	12	5.15	0.65
1	4	12	2.76	1.45
1	4	12	3.37	1.22
-2	-4	-12	1.74	0.66
-2	-4	-12	0.14	1.39
-2	-4	-12	0.61	1.10
2	4	12	1.70	0.57
2	4	12	0.92	1.18
2	4	12	-2.15	1.39
-3	-4	-12	-1.49	1.72
-3	-4	-12	0.04	0.61
-3	-4	-12	0.40	1.04
3	4	12	-0.37	1.42
3	4	12	-0.36	1.17
3	4	12	0.80	0.57
-4	-4	-12	6.19	1.20
-4	-4	-12	6.40	1.85
-4	-4	-12	6.45	0.83
4	4	12	4.96	1.61
4	4	12	8.00	1.13
4	4	12	7.50	1.48
-5	-4	-12	-2.83	1.66
-5	-4	-12	-0.85	0.86
-5	-4	-12	-0.87	1.05
5	4	12	0.78	0.60

5	4	12	0.57	1.31
5	4	12	-1.12	1.52
-6	-4	-12	-1.20	1.14
-6	-4	-12	-0.03	0.64
6	4	12	0.08	1.34
6	4	12	-0.46	0.60
6	4	12	-2.07	1.55
-7	-4	-12	-1.74	1.23
-7	-4	-12	-1.58	1.96
-7	-4	-12	0.50	0.72
7	4	12	0.10	1.75
7	4	12	1.20	1.48
-8	-4	-12	7.33	1.88
-8	-4	-12	4.29	2.33
-8	-4	-12	7.87	1.09
8	4	12	7.31	2.07
-9	-4	-12	-1.89	2.70
-9	-4	-12	0.84	0.91
-9	-4	-12	-2.71	2.57
9	4	12	0.61	2.15
9	4	12	-0.51	0.82
-10	-4	-12	1.96	1.02
-10	-4	-12	8.14	3.28
-10	-4	-12	4.09	1.50
-10	-4	-12	2.53	2.84
-10	-4	-12	1.17	2.82
10	4	12	3.30	2.07
10	4	12	2.26	2.43
10	4	12	2.75	0.97
-11	-4	-12	0.80	2.20
-11	-4	-12	2.99	3.70
-11	-4	-12	1.03	1.50
-11	-4	-12	-0.29	1.12
-11	-4	-12	-3.04	3.28
11	4	12	0.87	2.80
11	4	12	1.60	1.72
11	4	12	-1.11	2.20
-12	-4	-12	-3.83	3.86
-12	-4	-12	-0.16	1.26
-12	-4	-12	4.57	4.69
-12	-4	-12	-0.78	2.45
12	4	12	1.90	2.37
12	4	12	1.39	2.03
12	4	12	-1.27	4.36

-13	-4	-12	-2.24	1.40
-13	-4	-12	4.11	5.33
-13	-4	-12	-2.33	4.80
13	4	12	2.91	2.31
-8	5	12	1.00	2.72
-7	5	12	-1.45	2.15
-5	5	12	7.27	1.08
4	-5	-12	6.11	0.85
-4	5	12	7.02	1.02
3	-5	-12	8.75	1.36
-3	5	12	10.08	1.09
2	-5	-12	7.27	1.25
-2	5	12	6.79	1.00
1	-5	-12	10.58	1.30
-1	5	12	10.63	1.12
0	-5	-12	10.87	1.24
0	5	12	7.01	1.38
0	5	12	7.02	2.06
0	5	12	10.04	1.10
-1	-5	-12	13.52	2.58
-1	-5	-12	21.66	1.54
1	5	12	17.10	2.24
1	5	12	20.07	1.40
1	5	12	15.11	2.57
-2	-5	-12	10.47	2.20
-2	-5	-12	15.35	2.05
-2	-5	-12	16.69	1.42
2	5	12	14.81	2.20
2	5	12	15.18	2.48
2	5	12	17.86	1.35
-3	-5	-12	21.88	1.69
-3	-5	-12	22.92	2.20
-3	-5	-12	16.74	3.02
3	5	12	26.45	2.54
3	5	12	26.06	1.67
3	5	12	20.45	2.69
-4	-5	-12	10.97	1.29
-4	-5	-12	9.52	1.97
-4	-5	-12	10.10	1.30
4	5	12	11.31	1.61
4	5	12	9.71	1.64
-5	-5	-12	8.99	2.02
-5	-5	-12	10.59	1.41
-5	-5	-12	12.55	1.81

5	5	12	8.63	1.75
5	5	12	14.63	2.44
5	5	12	11.52	1.28
-6	-5	-12	7.63	1.27
-6	-5	-12	7.96	1.35
-6	-5	-12	5.79	2.00
6	5	12	6.84	1.73
6	5	12	9.49	1.70
-7	-5	-12	3.08	0.81
-7	-5	-12	3.97	1.26
-7	-5	-12	0.48	2.04
7	5	12	6.63	1.57
7	5	12	3.70	1.76
-8	-5	-12	14.54	1.61
-8	-5	-12	11.07	2.41
-8	-5	-12	15.11	2.37
-9	-5	-12	8.58	1.16
-9	-5	-12	10.05	1.66
-9	-5	-12	7.38	2.56
9	5	12	9.81	2.25
9	5	12	11.26	1.57
-10	-5	-12	-0.20	2.75
-10	-5	-12	3.10	1.13
-10	-5	-12	4.49	1.64
10	5	12	3.39	1.96
10	5	12	3.44	0.96
10	5	12	2.89	2.27
-11	-5	-12	2.00	3.72
-11	-5	-12	3.90	1.86
-11	-5	-12	1.39	1.20
-11	-5	-12	3.20	1.59
11	5	12	4.65	2.25
11	5	12	-0.18	2.53
-12	-5	-12	1.86	3.72
-12	-5	-12	-0.32	4.34
-12	-5	-12	1.25	2.10
-12	-5	-12	1.11	1.31
12	5	12	2.15	2.47
12	5	12	-0.91	2.96
-13	-5	-12	6.50	5.35
-13	-5	-12	1.54	1.48
-13	-5	-12	-4.23	4.35
13	5	12	4.50	2.40
-8	6	12	1.75	2.87

-7	6	12	0.35	2.47
-6	6	12	-0.34	2.04
-6	6	12	-0.22	0.62
-5	6	12	1.00	0.61
-4	6	12	1.97	0.58
3	-6	-12	4.48	0.83
-3	6	12	2.80	0.62
2	-6	-12	1.00	0.72
-2	6	12	1.49	0.57
1	-6	-12	2.50	0.75
-1	6	12	3.41	0.60
0	-6	-12	1.96	0.66
0	6	12	2.02	0.58
1	6	12	2.15	0.58
1	6	12	0.68	1.14
1	6	12	-1.08	1.46
-2	-6	-12	-0.33	0.58
2	6	12	-0.28	1.10
2	6	12	-2.27	1.32
2	6	12	0.61	0.57
-3	-6	-12	7.29	1.15
-3	-6	-12	5.24	1.11
3	6	12	6.58	1.37
3	6	12	5.75	1.49
3	6	12	7.25	1.08
-4	-6	-12	-0.47	1.99
-4	-6	-12	1.19	1.05
-4	-6	-12	0.87	0.66
4	6	12	1.19	1.35
4	6	12	1.30	1.29
4	6	12	0.55	0.59
-5	-6	-12	18.63	3.12
-5	-6	-12	23.72	2.30
5	6	12	21.12	1.68
5	6	12	23.89	2.82
5	6	12	29.30	2.74
-6	-6	-12	3.34	0.76
-6	-6	-12	3.17	1.20
-6	-6	-12	1.04	1.78
6	6	12	3.44	1.58
-7	-6	-12	2.79	0.83
-7	-6	-12	3.52	1.29
-7	-6	-12	0.99	1.95
7	6	12	2.53	1.69

-8	-6	-12	0.16	0.83
-8	-6	-12	-2.53	2.08
-8	-6	-12	1.21	1.34
8	6	12	0.69	1.79
-9	-6	-12	3.54	1.03
-9	-6	-12	3.36	1.48
-9	-6	-12	2.52	2.36
9	6	12	4.91	0.89
9	6	12	5.20	1.82
9	6	12	3.27	1.91
-10	-6	-12	-1.72	2.52
-10	-6	-12	-2.04	1.49
10	6	12	0.55	2.12
10	6	12	1.44	1.83
10	6	12	-0.05	0.87
-11	-6	-12	0.42	1.17
-11	-6	-12	2.53	1.78
-11	-6	-12	-3.16	2.90
11	6	12	1.70	2.36
11	6	12	-0.15	2.00
-12	-6	-12	-4.07	3.38
-12	-6	-12	4.46	4.09
-12	-6	-12	-0.06	1.99
-12	-6	-12	-0.79	1.83
-12	-6	-12	-1.42	1.26
12	6	12	3.22	2.40
12	6	12	1.43	2.66
-13	-6	-12	5.95	4.84
-13	-6	-12	-2.41	4.06
-13	-6	-12	-0.79	1.45
13	6	12	-3.14	3.35
-7	7	12	1.74	2.65
-7	7	12	3.00	1.28
-7	7	12	3.61	0.91
-6	7	12	4.33	0.84
-6	7	12	4.15	2.43
-5	7	12	6.92	0.88
3	-7	-12	12.35	1.62
-3	7	12	11.49	1.34
2	-7	-12	9.09	1.50
-2	7	12	8.29	1.19
1	-7	-12	8.44	1.40
-1	7	12	8.91	1.19
0	-7	-12	20.02	1.73

0	7	12	19.79	1.53
-1	-7	-12	12.26	1.38
1	7	12	11.68	1.23
-2	-7	-12	18.56	1.57
2	7	12	13.13	2.65
2	7	12	17.90	1.43
2	7	12	20.35	3.10
-3	-7	-12	3.71	1.54
-3	-7	-12	6.36	0.86
3	7	12	7.30	1.13
3	7	12	6.50	1.44
3	7	12	8.19	1.44
-4	-7	-12	14.38	2.01
4	7	12	16.90	1.42
4	7	12	15.03	2.52
4	7	12	17.94	2.40
-5	-7	-12	6.49	1.19
-5	-7	-12	5.37	2.40
-5	-7	-12	6.53	0.87
5	7	12	4.62	1.52
-6	-7	-12	7.52	1.94
-6	-7	-12	8.77	1.36
-6	-7	-12	7.89	1.33
6	7	12	7.68	1.70
-7	-7	-12	4.65	0.90
-7	-7	-12	2.83	1.92
-7	-7	-12	4.86	1.34
7	7	12	2.76	1.52
7	7	12	6.14	1.73
-8	-7	-12	10.95	2.24
-8	-7	-12	14.83	2.36
-8	-7	-12	11.38	1.65
8	7	12	14.30	1.93
8	7	12	13.43	2.05
-9	-7	-12	6.36	1.11
-9	-7	-12	7.64	1.63
-9	-7	-12	6.84	2.39
9	7	12	10.05	1.50
9	7	12	7.53	1.86
-10	-7	-12	1.56	1.06
-10	-7	-12	2.60	2.48
-10	-7	-12	2.98	1.68
10	7	12	4.35	2.13
10	7	12	3.18	1.85

10	7	12	3.88	0.98
-11	-7	-12	-1.02	1.15
-11	-7	-12	-1.09	1.80
-11	-7	-12	-1.17	2.87
11	7	12	0.53	2.16
11	7	12	2.04	2.33
11	7	12	3.16	1.60
-12	-7	-12	4.10	1.44
-12	-7	-12	6.50	2.17
-12	-7	-12	7.21	3.83
-12	-7	-12	4.27	3.86
-12	-7	-12	6.02	1.88
12	7	12	2.99	2.72
12	7	12	4.95	2.53
-13	-7	-12	2.84	3.97
-13	-7	-12	3.11	1.52
-13	-7	-12	1.60	2.48
-13	-7	-12	2.38	2.09
-13	-7	-12	2.98	4.52
13	7	12	3.64	3.12
13	7	12	3.96	2.75
-6	8	12	5.06	2.65
-6	8	12	5.57	0.98
-5	8	12	11.47	1.59
-4	8	12	14.81	1.63
-3	8	12	9.41	1.39
2	-8	-12	12.34	1.65
-2	8	12	11.33	1.38
1	-8	-12	4.27	0.95
-1	8	12	4.71	0.76
0	-8	-12	9.80	1.35
0	8	12	11.32	1.28
-1	-8	-12	6.10	1.33
1	8	12	6.93	1.19
-2	-8	-12	20.20	1.86
2	8	12	14.65	2.37
2	8	12	20.35	1.55
-3	-8	-12	19.95	1.69
3	8	12	16.21	2.65
3	8	12	21.44	1.58
-4	-8	-12	34.69	2.24
-4	-8	-12	32.72	2.60
4	8	12	32.94	3.03
4	8	12	34.67	2.13

-5	-8	-12	4.59	1.17
-5	-8	-12	0.78	1.51
-5	-8	-12	4.84	0.83
5	8	12	3.53	1.44
-6	-8	-12	5.37	0.85
-6	-8	-12	0.96	1.93
-6	-8	-12	3.25	1.19
6	8	12	4.02	1.46
6	8	12	2.57	1.49
-7	-8	-12	-1.62	1.85
-7	-8	-12	0.73	1.21
-7	-8	-12	1.47	0.79
7	8	12	0.28	1.54
7	8	12	1.24	1.47
-8	-8	-12	9.34	1.58
-8	-8	-12	8.27	2.12
-8	-8	-12	9.85	1.57
8	8	12	8.03	1.88
8	8	12	9.82	1.81
-9	-8	-12	7.14	1.60
-9	-8	-12	5.77	1.10
-9	-8	-12	6.21	2.28
9	8	12	8.36	1.79
9	8	12	7.95	1.96
9	8	12	5.30	0.95
-10	-8	-12	8.43	1.29
-10	-8	-12	9.41	1.83
-10	-8	-12	8.72	2.61
10	8	12	9.36	2.17
10	8	12	11.41	2.17
10	8	12	10.53	1.70
-11	-8	-12	5.06	1.28
-11	-8	-12	5.08	1.99
-11	-8	-12	6.65	2.89
11	8	12	6.52	2.20
11	8	12	6.29	2.42
-12	-8	-12	8.94	2.27
-12	-8	-12	5.50	3.32
-12	-8	-12	10.56	1.55
12	8	12	6.55	2.80
12	8	12	10.92	2.56
-13	-8	-12	-1.22	2.47
-13	-8	-12	1.92	3.81
-13	-8	-12	1.21	1.44

-13	-8	-12	-0.90	4.22
-13	-8	-12	5.38	2.16
13	8	12	0.46	2.76
13	8	12	-2.38	2.92
-6	9	12	3.89	1.10
-6	9	12	2.39	1.58
-6	9	12	5.91	2.88
-5	9	12	10.26	1.80
-4	9	12	12.41	1.68
-3	9	12	9.86	1.50
-2	9	12	6.64	0.89
1	-9	-12	13.90	1.73
-1	9	12	16.16	1.53
0	-9	-12	10.76	1.59
0	9	12	7.63	1.29
-1	-9	-12	21.54	1.90
1	9	12	20.62	1.67
-2	-9	-12	23.82	1.95
2	9	12	21.72	1.72
-3	-9	-12	50.66	2.84
3	9	12	43.61	3.65
3	9	12	44.49	2.70
-4	-9	-12	34.09	2.18
4	9	12	31.20	3.02
4	9	12	27.64	2.91
4	9	12	28.87	1.99
-5	-9	-12	47.78	3.16
-5	-9	-12	48.89	2.86
5	9	12	45.56	3.62
5	9	12	46.78	3.53
-6	-9	-12	1.26	2.19
-6	-9	-12	5.28	1.25
-6	-9	-12	6.81	0.93
6	9	12	3.12	1.64
-7	-9	-12	10.32	1.50
-7	-9	-12	13.56	1.60
-7	-9	-12	6.12	2.01
7	9	12	11.35	1.87
7	9	12	9.99	1.93
-8	-9	-12	12.90	2.31
-8	-9	-12	14.77	2.53
-8	-9	-12	16.48	1.76
8	9	12	14.68	2.01
8	9	12	14.38	2.17

-9	-9	-12	28.87	3.06
-9	-9	-12	22.85	3.98
-9	-9	-12	29.74	2.26
9	9	12	27.40	3.62
9	9	12	23.97	2.12
9	9	12	29.08	3.48
-10	-9	-12	10.51	2.72
-10	-9	-12	12.12	1.98
-10	-9	-12	14.66	1.93
10	9	12	10.45	2.83
10	9	12	11.85	2.15
10	9	12	11.86	1.77
-11	-9	-12	21.30	2.24
-11	-9	-12	11.02	3.06
-11	-9	-12	17.34	3.41
11	9	12	22.11	3.92
11	9	12	13.59	2.70
-12	-9	-12	12.49	2.49
-12	-9	-12	12.86	3.60
-12	-9	-12	18.72	2.36
12	9	12	7.57	2.84
12	9	12	16.07	2.84
-13	-9	-12	13.95	6.08
-13	-9	-12	16.10	2.62
-13	-9	-12	10.61	2.73
13	9	12	9.22	3.17
13	9	12	4.03	4.06
13	9	12	8.09	3.04
-4	10	12	1.83	0.92
-3	10	12	2.15	0.90
-2	10	12	0.30	0.77
1	-10	-12	3.19	0.93
-1	10	12	3.10	0.79
0	-10	-12	2.81	0.87
0	10	12	1.75	0.74
-1	-10	-12	7.09	1.09
1	10	12	9.96	1.36
-2	-10	-12	12.82	1.69
2	10	12	10.32	1.39
-3	-10	-12	13.17	1.67
3	10	12	12.12	1.42
-4	-10	-12	11.59	1.57
4	10	12	9.68	1.32
4	10	12	6.94	1.76

4	10	12	8.96	1.65
-5	-10	-12	14.23	1.68
-5	-10	-12	9.26	1.95
5	10	12	11.43	1.89
5	10	12	13.42	1.83
-6	-10	-12	6.02	0.99
-6	-10	-12	7.09	1.30
6	10	12	8.09	1.68
6	10	12	5.39	1.79
-7	-10	-12	10.15	1.61
-7	-10	-12	6.21	2.67
-7	-10	-12	7.44	1.44
7	10	12	9.35	1.97
7	10	12	9.88	1.84
-8	-10	-12	5.63	1.48
-8	-10	-12	5.80	1.05
-8	-10	-12	1.54	2.13
8	10	12	3.84	1.79
8	10	12	4.27	0.88
8	10	12	4.82	2.01
-9	-10	-12	10.71	2.47
-9	-10	-12	17.60	1.91
-9	-10	-12	15.66	2.76
9	10	12	12.91	2.65
9	10	12	12.74	1.74
9	10	12	13.54	2.38
-10	-10	-12	4.45	1.14
-10	-10	-12	2.41	1.79
-10	-10	-12	5.14	2.63
10	10	12	3.62	1.32
10	10	12	2.26	2.63
-11	-10	-12	2.15	1.98
-11	-10	-12	1.80	3.01
-11	-10	-12	3.34	1.20
11	10	12	0.24	2.52
11	10	12	-0.34	2.42
-12	-10	-12	3.69	3.44
-12	-10	-12	5.14	2.35
-12	-10	-12	3.97	1.35
12	10	12	-2.06	2.89
12	10	12	1.05	2.70
-13	-10	-12	8.60	2.79
-13	-10	-12	10.06	4.00
13	10	12	10.06	3.42

13	10	12	14.65	3.19
13	10	12	11.53	4.18
-1	11	12	6.85	1.01
0	-11	-12	11.47	1.32
0	11	12	12.00	1.60
-1	-11	-12	12.36	1.80
1	11	12	11.90	1.57
-2	-11	-12	33.59	2.47
2	11	12	30.45	2.20
-3	-11	-12	27.86	2.25
3	11	12	25.83	1.99
-4	-11	-12	50.96	3.10
4	11	12	45.15	4.32
4	11	12	48.19	2.90
4	11	12	50.72	3.76
-5	-11	-12	28.78	2.26
5	11	12	23.35	3.14
5	11	12	28.50	3.17
-6	-11	-12	23.39	2.09
6	11	12	23.78	3.17
6	11	12	26.28	3.19
-7	-11	-12	6.01	2.12
-7	-11	-12	14.42	1.83
-7	-11	-12	10.61	1.56
7	11	12	11.97	1.74
7	11	12	11.64	1.99
7	11	12	11.24	2.07
-8	-11	-12	35.39	2.47
-8	-11	-12	30.42	3.02
-8	-11	-12	27.11	4.30
8	11	12	33.79	3.79
8	11	12	32.89	3.71
8	11	12	27.58	2.27
-9	-11	-12	14.22	2.80
-9	-11	-12	12.88	2.69
-9	-11	-12	17.28	2.04
9	11	12	11.98	2.33
9	11	12	19.75	3.61
-10	-11	-12	19.18	3.25
-10	-11	-12	22.28	2.33
-10	-11	-12	16.30	3.08
10	11	12	23.33	4.09
10	11	12	21.13	4.08
-11	-11	-12	5.69	2.18

-11	-11	-12	7.64	3.14
-11	-11	-12	7.04	1.34
11	11	12	3.98	2.74
11	11	12	5.88	2.52
-12	-11	-12	10.32	2.61
-12	-11	-12	10.18	3.50
12	11	12	7.77	3.28
12	11	12	13.86	3.02
-13	-11	-12	15.37	2.62
-13	-11	-12	4.78	3.94
-13	-11	-12	9.65	2.95
13	11	12	3.88	3.69
13	11	12	12.03	3.23
-1	-12	-12	6.69	1.23
-2	-12	-12	17.10	2.06
-3	-12	-12	7.82	1.19
3	12	12	7.23	1.28
-4	-12	-12	12.98	1.90
4	12	12	8.36	1.14
-5	-12	-12	14.50	1.91
5	12	12	12.04	1.75
5	12	12	10.07	1.96
-6	-12	-12	5.85	1.98
-6	-12	-12	7.06	1.14
6	12	12	6.85	1.89
6	12	12	6.89	1.83
6	12	12	7.70	1.32
-7	-12	-12	8.02	1.56
-7	-12	-12	12.30	1.92
7	12	12	9.74	2.14
7	12	12	11.67	2.18
-8	-12	-12	18.54	2.76
-8	-12	-12	20.88	2.14
8	12	12	16.95	2.54
8	12	12	22.14	3.78
-9	-12	-12	16.55	2.11
-9	-12	-12	16.19	2.98
-9	-12	-12	10.42	2.67
9	12	12	15.04	2.56
9	12	12	9.95	2.52
-10	-12	-12	10.14	2.87
-10	-12	-12	7.49	2.10
-10	-12	-12	7.17	1.27
10	12	12	3.53	2.64

10	12	12	9.05	2.62
-11	-12	-12	5.91	3.13
-11	-12	-12	2.49	2.26
-11	-12	-12	6.27	1.41
11	12	12	0.23	2.95
11	12	12	5.80	2.68
-12	-12	-12	9.81	3.64
-12	-12	-12	9.08	1.60
-12	-12	-12	6.79	2.99
12	12	12	10.62	3.06
-2	-13	-12	20.04	2.30
-3	-13	-12	21.71	2.27
-4	-13	-12	6.53	1.13
4	13	12	4.06	1.86
-5	-13	-12	13.85	2.00
5	13	12	10.58	2.58
5	13	12	10.69	1.96
-6	-13	-12	9.68	1.29
6	13	12	12.15	2.12
6	13	12	7.52	2.07
-7	-13	-12	25.38	2.43
7	13	12	29.41	3.87
7	13	12	24.08	3.55
-8	-13	-12	19.04	2.28
8	13	12	20.22	3.92
8	13	12	20.22	3.74
-9	-13	-12	21.40	2.35
9	13	12	16.12	2.80
-10	-13	-12	16.19	2.29
10	13	12	14.73	2.90
-11	-13	-12	23.29	2.65
11	13	12	26.96	5.08
-4	-14	-12	9.33	1.33
-5	-14	-12	6.73	1.23
-6	-14	-12	16.95	2.27
6	14	12	13.93	2.25
-7	-14	-12	20.58	2.42
7	14	12	20.42	3.87
-8	-14	-12	22.02	2.44
8	14	12	23.67	4.36
-9	-14	-12	8.83	1.40
9	14	12	7.96	2.58
-10	-14	-12	8.20	1.49
10	14	12	14.43	2.96

-5	-15	-12	16.85	2.42
-6	-15	-12	23.28	2.60
6	15	12	24.15	3.77
-7	-15	-12	20.86	2.58
7	15	12	27.82	4.03
-8	-15	-12	23.05	2.70
8	15	12	28.38	4.47
-6	-7	13	7.30	3.55
-6	-7	13	9.70	2.13
5	7	-13	15.85	3.94
-5	-7	13	6.07	3.33
-5	-7	13	9.38	1.94
4	7	-13	8.73	3.80
4	7	-13	12.23	3.52
-4	-7	13	2.11	3.36
-4	-7	13	6.22	2.89
-4	-7	13	6.44	1.70
3	7	-13	12.45	1.67
3	7	-13	15.03	3.62
3	7	-13	9.70	3.18
-3	-7	13	9.57	3.49
-3	-7	13	15.84	3.04
2	7	-13	9.81	3.51
2	7	-13	8.29	3.05
2	7	-13	7.74	1.09
-2	-7	13	9.73	1.87
-2	-7	13	3.78	3.30
-2	-7	13	5.75	2.39
-2	-7	13	5.50	1.79
1	7	-13	7.39	1.13
1	7	-13	7.62	3.01
1	7	-13	6.61	4.53
-1	-7	13	7.27	1.84
-1	-7	13	2.31	2.34
-1	-7	13	4.60	3.12
-1	-7	13	3.77	1.96
0	7	-13	3.90	3.17
0	7	-13	2.38	1.04
0	7	-13	1.21	2.92
0	-7	13	1.85	2.28
0	-7	13	2.77	1.84
0	-7	13	1.79	3.34
-1	7	-13	3.77	1.12
-1	7	-13	6.12	3.27

1	-7	13	6.28	2.20
-7	-6	13	8.15	2.15
-7	-6	13	9.43	3.42
-6	-6	13	20.19	3.41
-6	-6	13	13.45	3.36
5	6	-13	23.17	4.83
5	6	-13	32.84	4.82
-5	-6	13	29.75	3.59
-5	-6	13	26.14	4.76
4	6	-13	15.00	3.27
4	6	-13	13.35	1.42
4	6	-13	13.95	3.33
-4	-6	13	19.46	3.15
-4	-6	13	11.41	2.71
-4	-6	13	10.38	3.22
3	6	-13	18.53	3.39
3	6	-13	16.55	3.14
-3	-6	13	14.70	3.25
-3	-6	13	19.20	2.77
2	6	-13	15.86	2.94
2	6	-13	18.14	3.30
2	6	-13	15.83	1.71
-2	-6	13	21.33	3.06
-2	-6	13	15.84	2.46
-2	-6	13	22.25	2.91
-2	-6	13	12.75	3.08
1	6	-13	18.09	3.28
1	6	-13	22.51	4.11
1	6	-13	18.75	1.88
-1	-6	13	21.32	3.01
-1	-6	13	16.37	2.47
-1	-6	13	17.24	3.19
0	6	-13	17.50	2.81
0	6	-13	17.31	3.13
0	6	-13	18.57	1.92
0	-6	13	18.46	3.17
0	-6	13	18.45	3.57
0	-6	13	16.48	3.20
0	-6	13	23.67	3.41
-1	6	-13	26.23	3.95
-1	6	-13	20.91	2.09
-1	6	-13	15.30	3.51
1	-6	13	19.61	3.58
1	-6	13	19.03	3.16

1	-6	13	18.36	3.32
1	-6	13	20.50	3.60
-2	6	-13	13.53	2.96
-2	6	-13	15.47	2.63
-2	6	-13	17.79	2.05
2	-6	13	14.37	2.52
2	-6	13	18.89	3.72
2	-6	13	10.37	3.02
-3	6	-13	11.08	1.93
-3	6	-13	11.64	2.95
3	-6	13	7.59	2.23
-8	-5	13	4.96	3.11
-8	-5	13	6.96	2.44
-7	-5	13	2.41	2.99
-7	-5	13	4.23	2.00
-6	-5	13	8.57	2.89
-6	-5	13	0.93	3.07
5	5	-13	4.60	3.01
5	5	-13	7.65	2.78
-5	-5	13	8.89	1.84
-5	-5	13	7.15	2.78
-5	-5	13	6.92	2.90
4	5	-13	13.24	2.86
4	5	-13	12.17	2.91
-4	-5	13	12.00	1.87
-4	-5	13	9.70	2.52
-4	-5	13	8.97	2.86
3	5	-13	10.73	1.29
3	5	-13	13.52	2.66
3	5	-13	12.88	2.88
-3	-5	13	9.12	1.39
-3	-5	13	9.26	2.84
-3	-5	13	15.97	2.80
2	5	-13	8.96	2.71
2	5	-13	10.80	2.51
-2	-5	13	13.65	2.70
-2	-5	13	11.77	2.05
-2	-5	13	14.86	2.29
-2	-5	13	9.02	2.70
1	5	-13	9.63	2.31
1	5	-13	9.40	1.44
1	5	-13	9.72	2.63
-1	-5	13	8.81	2.56
-1	-5	13	9.72	2.10

-1	-5	13	12.87	2.79
-1	-5	13	9.13	1.44
0	5	-13	8.28	2.52
0	5	-13	11.52	1.60
0	5	-13	9.37	2.26
0	-5	13	10.98	1.81
0	-5	13	14.81	2.48
0	-5	13	9.48	1.96
0	-5	13	6.01	2.68
-1	5	-13	8.36	1.08
-1	5	-13	8.55	2.14
-1	5	-13	8.26	2.41
1	-5	13	6.74	1.89
1	-5	13	6.46	1.76
1	-5	13	8.29	2.76
1	-5	13	6.96	1.46
-2	5	-13	7.82	2.19
-2	5	-13	13.01	1.75
-2	5	-13	12.72	3.63
2	-5	13	9.04	2.08
2	-5	13	6.73	2.71
2	-5	13	12.54	2.13
2	-5	13	18.10	2.75
-3	5	-13	5.85	2.36
-3	5	-13	5.63	1.13
-3	5	-13	4.19	2.14
3	-5	13	3.70	2.64
3	-5	13	3.89	1.98
3	-5	13	5.62	2.05
-4	5	-13	5.87	2.54
-4	5	-13	6.12	1.17
4	-5	13	6.42	2.05
4	-5	13	1.42	1.90
-5	5	-13	3.57	2.71
-5	5	-13	1.89	1.13
5	-5	13	-0.42	2.13
-8	-4	13	9.66	3.23
-7	-4	13	8.59	2.24
-7	-4	13	6.46	2.86
-6	-4	13	33.75	3.74
-6	-4	13	23.33	3.42
-6	-4	13	26.89	4.38
5	4	-13	14.77	3.07
5	4	-13	14.74	2.65

-5	-4	13	14.79	2.78
-5	-4	13	10.02	2.90
4	4	-13	11.24	2.61
4	4	-13	11.06	2.59
-4	-4	13	10.64	2.73
-4	-4	13	12.50	2.37
-4	-4	13	17.73	2.94
3	4	-13	15.33	2.48
3	4	-13	12.84	2.53
3	4	-13	14.20	3.02
3	4	-13	14.93	1.38
-3	-4	13	14.63	3.09
-3	-4	13	17.36	1.94
-3	-4	13	19.85	2.85
2	4	-13	17.67	1.49
2	4	-13	15.70	2.57
2	4	-13	23.56	3.87
2	4	-13	17.75	3.43
-2	-4	13	20.46	2.15
-2	-4	13	16.09	2.69
-2	-4	13	21.29	2.74
-2	-4	13	16.20	2.94
1	4	-13	20.14	3.33
1	4	-13	16.11	2.60
1	4	-13	23.05	3.66
-1	-4	13	22.13	3.03
-1	-4	13	22.28	2.32
-1	-4	13	18.62	2.76
-1	-4	13	22.72	2.79
0	4	-13	28.86	3.64
0	4	-13	28.57	3.32
0	4	-13	26.38	1.92
0	4	-13	25.03	3.63
0	-4	13	31.35	3.11
0	-4	13	26.37	2.55
0	-4	13	18.84	2.83
0	-4	13	22.87	3.05
-1	4	-13	10.76	2.02
-1	4	-13	14.43	1.58
-1	4	-13	12.67	2.25
1	-4	13	11.40	2.37
1	-4	13	15.32	2.85
1	-4	13	14.15	2.23
1	-4	13	12.18	1.91

-2	4	-13	15.63	1.68
-2	4	-13	10.55	2.30
-2	4	-13	15.61	2.99
2	-4	13	11.76	2.52
2	-4	13	18.48	2.41
2	-4	13	15.81	2.92
2	-4	13	15.60	3.10
-3	4	-13	13.90	2.44
-3	4	-13	14.24	1.78
-3	4	-13	16.28	3.04
3	-4	13	12.58	2.57
3	-4	13	17.70	3.33
3	-4	13	11.69	1.66
3	-4	13	18.70	3.20
-4	4	-13	13.51	1.84
-4	4	-13	11.20	2.33
-4	4	-13	10.60	2.19
4	-4	13	10.06	2.09
4	-4	13	6.81	2.53
4	-4	13	12.17	2.21
-5	4	-13	10.25	2.54
-5	4	-13	10.61	1.85
-5	4	-13	6.92	3.29
5	-4	13	9.31	3.20
5	-4	13	8.70	2.36
5	-4	13	10.40	2.13
-6	4	-13	7.53	1.25
-6	4	-13	8.22	3.11
-6	4	-13	11.65	2.75
6	-4	13	10.94	2.36
-9	-3	13	2.76	2.73
-9	-3	13	6.40	2.51
-8	-3	13	6.86	2.21
-8	-3	13	3.19	2.84
7	3	-13	4.97	2.06
-7	-3	13	5.77	2.54
-7	-3	13	5.71	2.69
-6	-3	13	2.47	2.45
-6	-3	13	2.46	2.34
5	3	-13	7.09	2.73
-5	-3	13	6.43	2.52
-5	-3	13	9.74	2.35
4	3	-13	4.67	2.29
4	3	-13	3.43	2.15

4	3	-13	5.24	2.69
-4	-3	13	6.52	1.54
-4	-3	13	6.45	1.97
-4	-3	13	1.28	2.41
3	3	-13	13.13	2.30
3	3	-13	12.70	1.17
3	3	-13	10.36	2.24
-3	-3	13	10.79	2.11
-3	-3	13	10.61	2.57
2	3	-13	7.53	1.96
2	3	-13	6.91	1.99
2	3	-13	7.69	1.13
-2	-3	13	8.31	1.51
-2	-3	13	5.08	2.25
-2	-3	13	11.16	1.67
-2	-3	13	8.25	1.64
1	3	-13	16.16	3.04
1	3	-13	14.04	2.07
1	3	-13	15.12	1.38
-1	-3	13	10.97	2.30
-1	-3	13	13.50	2.63
-1	-3	13	18.35	2.46
-1	-3	13	19.10	2.01
0	3	-13	12.80	2.12
0	3	-13	10.15	1.89
0	3	-13	15.23	1.83
0	-3	13	10.97	1.74
0	-3	13	17.23	2.54
0	-3	13	15.03	1.99
-1	3	-13	6.60	1.77
-1	3	-13	5.21	1.89
-1	3	-13	6.76	0.89
1	-3	13	7.11	1.58
1	-3	13	6.96	1.21
1	-3	13	6.03	2.01
1	-3	13	6.11	1.53
-2	3	-13	5.83	1.68
-2	3	-13	4.82	0.88
-2	3	-13	4.27	2.51
-2	3	-13	4.70	1.85
2	-3	13	4.24	1.58
2	-3	13	2.64	1.98
2	-3	13	4.98	1.55
2	-3	13	3.74	1.18

-3	3	-13	7.86	1.78
-3	3	-13	8.49	1.88
-3	3	-13	7.03	1.00
-3	3	-13	6.44	2.03
3	-3	13	7.26	2.15
3	-3	13	4.90	1.73
3	-3	13	8.77	1.72
3	-3	13	13.13	2.11
-4	3	-13	2.74	1.75
-4	3	-13	5.36	0.97
-4	3	-13	4.42	2.04
4	-3	13	7.40	1.90
4	-3	13	5.29	1.26
4	-3	13	5.52	2.18
4	-3	13	3.63	1.79
-5	3	-13	6.79	1.10
-5	3	-13	3.95	2.76
-5	3	-13	8.45	2.24
5	-3	13	7.97	2.16
5	-3	13	2.99	2.36
5	-3	13	7.20	1.93
-6	3	-13	8.55	2.57
-6	3	-13	1.47	2.72
-6	3	-13	3.65	1.06
6	-3	13	3.78	2.03
6	-3	13	3.05	2.48
6	-3	13	7.42	2.45
-7	3	-13	6.93	2.93
-7	3	-13	8.31	3.33
-7	3	-13	6.39	1.24
7	-3	13	9.87	2.33
-9	-2	13	5.02	2.70
-9	-2	13	-0.51	2.64
-8	-2	13	5.14	2.60
-8	-2	13	7.09	2.38
-7	-2	13	8.55	2.17
-7	-2	13	5.14	2.25
-7	-2	13	2.51	2.47
6	2	-13	9.13	2.13
-6	-2	13	7.54	2.36
-6	-2	13	7.68	2.33
5	2	-13	11.17	2.64
5	2	-13	10.47	3.11
-5	-2	13	10.91	2.17

-5	-2	13	10.59	2.53
4	2	-13	12.33	2.38
4	2	-13	13.25	2.37
-4	-2	13	18.55	2.69
-4	-2	13	18.48	2.94
3	2	-13	25.12	3.41
3	2	-13	24.26	3.38
-3	-2	13	21.89	3.72
-3	-2	13	33.25	3.01
-3	-2	13	27.49	2.93
2	2	-13	10.20	1.97
2	2	-13	13.14	1.19
2	2	-13	11.65	1.95
-2	-2	13	14.34	1.61
-2	-2	13	12.20	2.36
-2	-2	13	15.80	2.50
-2	-2	13	11.32	2.24
1	2	-13	10.01	1.15
1	2	-13	9.70	1.75
1	2	-13	8.58	1.77
-1	-2	13	8.91	2.10
-1	-2	13	8.32	1.57
-1	-2	13	10.80	2.27
-1	-2	13	12.29	1.62
0	2	-13	8.33	1.83
0	2	-13	12.03	1.25
0	2	-13	10.80	1.75
0	-2	13	10.43	1.69
0	-2	13	10.93	1.63
0	-2	13	10.84	1.99
0	-2	13	12.44	2.29
-1	2	-13	19.01	1.65
-1	2	-13	16.69	2.64
-1	2	-13	17.13	2.87
1	-2	13	18.01	2.99
1	-2	13	18.43	1.95
1	-2	13	16.54	2.47
1	-2	13	20.37	2.60
-2	2	-13	19.79	2.71
-2	2	-13	18.53	1.62
-2	2	-13	17.76	2.89
2	-2	13	20.55	2.74
2	-2	13	17.64	2.98
2	-2	13	16.83	2.66

2	-2	13	21.27	2.03
-3	2	-13	21.96	3.09
-3	2	-13	20.72	1.77
-3	2	-13	19.67	2.82
3	-2	13	17.21	3.16
3	-2	13	22.37	3.03
3	-2	13	24.32	2.91
3	-2	13	22.55	2.15
-4	2	-13	11.87	1.56
-4	2	-13	11.08	1.90
-4	2	-13	12.51	2.17
-4	2	-13	11.87	2.13
4	-2	13	11.32	1.94
4	-2	13	18.42	2.94
4	-2	13	10.06	2.18
4	-2	13	11.27	1.95
-5	2	-13	7.52	1.06
-5	2	-13	3.53	2.48
-5	2	-13	8.01	2.09
5	-2	13	8.88	2.01
5	-2	13	8.36	1.90
5	-2	13	4.37	2.22
5	-2	13	7.05	1.27
-6	2	-13	13.81	2.57
-6	2	-13	10.77	1.75
-6	2	-13	8.93	2.97
6	-2	13	13.76	2.42
6	-2	13	12.54	2.10
6	-2	13	11.64	2.49
-7	2	-13	0.50	2.43
-7	2	-13	5.88	1.12
-7	2	-13	5.55	2.81
7	-2	13	6.03	2.13
7	-2	13	5.66	2.67
7	-2	13	9.49	2.58
-8	2	-13	4.18	3.19
-8	2	-13	2.85	1.12
-8	2	-13	4.00	3.19
8	-2	13	4.00	2.17
-9	-1	13	3.02	2.68
-8	-1	13	7.02	2.44
-8	-1	13	3.91	2.44
-7	-1	13	3.43	2.28
-7	-1	13	6.71	2.20

-7	-1	13	9.26	2.20
6	1	-13	3.77	4.44
-6	-1	13	4.87	2.09
-6	-1	13	3.28	2.04
5	1	-13	6.42	1.81
5	1	-13	13.00	3.03
-5	-1	13	9.90	1.91
-5	-1	13	8.03	2.24
-5	-1	13	14.71	3.04
4	1	-13	24.41	3.47
4	1	-13	18.77	3.68
-4	-1	13	27.58	3.13
-4	-1	13	23.18	2.92
-4	-1	13	17.76	3.50
3	1	-13	15.19	2.98
3	1	-13	16.76	1.38
-3	-1	13	15.71	2.52
-3	-1	13	14.42	2.33
-3	-1	13	16.55	1.53
-3	-1	13	20.26	2.67
2	1	-13	21.75	2.95
2	1	-13	25.06	1.64
-2	-1	13	19.98	3.24
-2	-1	13	27.38	2.72
-2	-1	13	26.57	1.82
-2	-1	13	23.56	2.85
1	1	-13	7.87	1.71
1	1	-13	10.96	1.12
1	1	-13	8.20	1.65
-1	-1	13	8.79	1.46
-1	-1	13	8.82	1.43
-1	-1	13	11.21	1.37
-1	-1	13	8.57	1.89
0	1	-13	9.57	1.63
0	1	-13	9.97	1.66
0	1	-13	12.44	1.17
0	-1	13	9.43	1.42
0	-1	13	13.30	1.50
0	-1	13	10.69	1.56
0	-1	13	10.56	1.86
-1	1	-13	2.80	1.43
-1	1	-13	2.17	1.50
-1	1	-13	3.19	0.70
1	-1	13	4.89	0.86

1	-1	13	2.96	1.62
1	-1	13	5.38	1.32
1	-1	13	3.16	1.23
-2	1	-13	7.97	1.56
-2	1	-13	10.71	1.22
-2	1	-13	8.82	1.77
2	-1	13	8.59	1.52
2	-1	13	10.23	1.57
2	-1	13	8.26	1.49
2	-1	13	8.45	1.92
-3	1	-13	10.33	1.70
-3	1	-13	8.61	1.85
-3	1	-13	9.33	1.27
3	-1	13	10.07	1.65
3	-1	13	8.54	1.55
3	-1	13	12.17	1.60
3	-1	13	11.68	2.03
-4	1	-13	11.85	1.90
-4	1	-13	14.72	1.51
-4	1	-13	17.40	3.20
-4	1	-13	15.68	2.95
4	-1	13	12.43	2.21
4	-1	13	11.85	1.80
4	-1	13	17.76	2.74
4	-1	13	10.77	1.73
-5	1	-13	0.89	1.83
-5	1	-13	4.18	1.95
-5	1	-13	0.65	1.51
-5	1	-13	2.57	0.83
5	-1	13	2.58	2.02
5	-1	13	1.65	1.73
5	-1	13	1.79	0.97
5	-1	13	1.34	1.51
-6	1	-13	6.85	2.28
-6	1	-13	8.40	1.09
6	-1	13	5.75	1.72
6	-1	13	9.28	2.12
6	-1	13	6.23	1.27
6	-1	13	6.23	2.36
-7	1	-13	5.65	2.73
-7	1	-13	4.01	1.05
-7	1	-13	3.38	2.65
7	-1	13	6.36	1.82
7	-1	13	9.19	2.39

7	-1	13	3.24	2.48
-8	1	-13	2.25	1.03
-8	1	-13	1.10	2.52
-8	1	-13	1.02	3.22
8	-1	13	-0.33	2.68
8	-1	13	4.21	2.50
8	-1	13	3.68	1.98
-9	1	-13	-0.13	2.97
-9	1	-13	-0.02	1.07
-9	1	-13	0.41	3.84
9	-1	13	-0.92	2.18
-9	0	13	8.47	2.71
-8	0	13	12.29	2.64
-8	0	13	10.37	2.27
-7	0	13	7.98	2.11
-7	0	13	13.55	2.32
6	0	-13	4.06	3.60
5	0	-13	17.59	3.31
-5	0	13	20.27	2.84
-5	0	13	27.21	3.27
4	0	-13	38.01	4.78
-4	0	13	28.44	3.51
-4	0	13	27.59	2.91
-4	0	13	34.15	3.32
3	0	-13	25.12	3.04
3	0	-13	22.50	2.85
-3	0	13	26.64	1.77
-3	0	13	27.82	3.27
-3	0	13	29.93	3.02
-3	0	13	28.81	2.63
2	0	-13	30.78	1.94
2	0	-13	26.74	2.95
-2	0	13	28.85	1.98
-2	0	13	34.03	2.87
-2	0	13	31.63	3.25
1	0	-13	30.44	2.92
1	0	-13	38.06	2.11
1	0	-13	30.87	3.31
-1	0	13	32.11	2.83
-1	0	13	36.90	2.96
-1	0	13	36.42	3.26
-1	0	13	38.00	2.17
0	0	-13	16.13	2.35
0	0	-13	17.59	1.39

0	0	-13	15.82	2.56
0	0	13	16.31	2.27
0	0	13	17.00	2.25
0	0	13	18.72	1.54
0	0	13	15.25	2.71
-1	0	-13	17.90	2.62
-1	0	-13	20.53	1.47
-1	0	-13	18.34	2.43
1	0	13	19.74	2.85
1	0	13	19.93	2.44
1	0	13	20.29	1.68
1	0	13	19.21	2.37
-2	0	-13	26.82	2.90
-2	0	-13	21.41	1.96
-2	0	-13	22.72	2.71
2	0	13	26.77	2.67
2	0	13	25.81	1.91
2	0	13	25.45	3.10
2	0	13	27.52	2.66
-3	0	-13	19.15	1.56
-3	0	-13	20.85	2.89
-3	0	-13	16.99	2.72
3	0	13	19.75	1.75
3	0	13	20.84	2.66
3	0	13	16.72	3.16
3	0	13	21.12	2.62
-4	0	-13	11.98	1.87
-4	0	-13	18.87	2.94
-4	0	-13	16.20	1.46
4	0	13	16.43	2.70
4	0	13	12.48	2.60
4	0	13	14.02	1.63
4	0	13	11.41	2.32
-5	0	-13	13.64	1.47
-5	0	-13	16.68	3.16
-5	0	-13	14.48	2.71
-5	0	-13	12.70	2.01
5	0	13	15.24	2.70
5	0	13	16.22	2.90
5	0	13	10.41	2.32
5	0	13	13.24	1.69
-6	0	-13	9.34	2.50
-6	0	-13	10.85	1.71
-6	0	-13	11.41	1.50

-6	0	-13	11.49	2.44
6	0	13	12.67	2.16
6	0	13	10.63	2.43
6	0	13	9.27	1.74
6	0	13	10.09	1.73
-7	0	-13	16.43	2.84
-7	0	-13	17.42	3.74
-7	0	-13	17.68	1.81
-7	0	-13	19.65	3.24
7	0	13	14.73	2.81
7	0	13	24.26	3.24
-8	0	-13	2.27	2.50
-8	0	-13	6.53	1.06
-8	0	-13	6.95	3.11
8	0	13	11.48	2.51
8	0	13	7.97	2.81
8	0	13	9.51	1.95
-9	0	-13	2.09	3.75
-9	0	-13	1.02	2.58
-9	0	-13	3.62	1.06
9	0	13	8.14	4.81
9	0	13	4.50	2.54
9	0	13	7.87	2.09
-10	0	-13	-0.12	4.38
-10	0	-13	-0.42	1.05
10	0	13	1.05	2.19
-9	1	13	5.25	2.68
-8	1	13	3.91	2.35
-7	1	13	7.76	2.38
6	-1	-13	4.20	3.65
-6	1	13	6.61	2.01
5	-1	-13	11.77	3.07
-5	1	13	20.83	3.15
-5	1	13	17.32	2.49
-4	1	13	14.03	2.26
-4	1	13	10.44	1.99
-4	1	13	16.59	2.87
3	-1	-13	8.84	1.16
3	-1	-13	6.97	1.54
3	-1	-13	6.69	1.63
-3	1	13	10.05	1.79
-3	1	13	9.26	1.12
-3	1	13	8.77	1.44
-3	1	13	9.11	1.61

2	-1	-13	4.32	1.41
2	-1	-13	4.18	0.73
2	-1	-13	3.73	1.43
2	-1	-13	2.40	2.10
-2	1	13	4.54	1.61
-2	1	13	4.30	0.72
-2	1	13	4.72	1.32
-2	1	13	2.89	1.25
1	-1	-13	14.63	1.39
1	-1	-13	18.98	3.45
1	-1	-13	14.75	2.30
-1	1	13	14.49	2.58
-1	1	13	17.39	2.19
-1	1	13	16.07	2.22
-1	1	13	14.85	1.33
0	-1	-13	2.84	1.65
0	-1	-13	-0.09	1.23
0	-1	-13	0.67	0.58
0	-1	-13	1.83	1.61
0	1	13	-0.36	1.54
0	1	13	0.53	1.02
0	1	13	1.38	0.61
0	1	13	1.82	1.17
-1	-1	-13	8.14	1.61
-1	-1	-13	6.84	1.35
-1	-1	-13	8.30	1.11
-1	-1	-13	6.29	1.57
1	1	13	6.14	1.20
1	1	13	8.83	1.24
1	1	13	6.59	1.77
1	1	13	7.02	1.37
-2	-1	-13	5.47	1.44
-2	-1	-13	3.32	0.65
-2	-1	-13	6.41	1.57
-2	-1	-13	5.30	1.37
2	1	13	4.36	1.34
2	1	13	3.49	1.16
2	1	13	2.85	1.72
2	1	13	2.85	0.73
-3	-1	-13	6.43	1.02
-3	-1	-13	7.00	1.43
-3	-1	-13	7.22	1.55
-3	-1	-13	7.50	1.76
3	1	13	4.50	1.82

3	1	13	7.76	1.49
3	1	13	5.99	0.85
3	1	13	4.26	1.26
-4	-1	-13	2.51	1.55
-4	-1	-13	2.09	0.66
-4	-1	-13	3.97	1.81
4	1	13	0.99	1.20
4	1	13	2.67	0.75
4	1	13	1.77	1.90
4	1	13	1.82	1.41
-5	-1	-13	5.79	0.83
-5	-1	-13	5.88	1.74
-5	-1	-13	5.60	2.00
5	1	13	6.18	0.91
5	1	13	7.06	1.76
5	1	13	5.42	2.11
5	1	13	6.30	1.45
-6	-1	-13	6.30	1.51
-6	-1	-13	5.13	2.21
-6	-1	-13	4.25	1.83
-6	-1	-13	6.48	0.93
6	1	13	5.71	1.46
6	1	13	4.55	2.21
6	1	13	5.20	0.93
-7	-1	-13	11.15	1.57
-7	-1	-13	10.18	2.39
-7	-1	-13	10.64	2.71
-7	-1	-13	10.80	1.67
7	1	13	13.97	2.32
7	1	13	7.87	2.50
7	1	13	9.91	1.71
7	1	13	10.44	1.76
-8	-1	-13	1.16	0.90
-8	-1	-13	-1.95	3.11
-8	-1	-13	1.86	1.68
8	1	13	1.68	1.62
8	1	13	-0.47	2.62
8	1	13	6.63	2.18
-9	-1	-13	0.06	2.40
-9	-1	-13	1.19	1.00
-9	-1	-13	-1.71	3.29
9	1	13	3.20	2.93
9	1	13	2.51	2.46
9	1	13	3.82	1.78

-10	-1	-13	-0.85	1.00
-10	-1	-13	-7.07	4.11
-10	-1	-13	-1.04	3.98
10	1	13	2.19	1.94
-9	2	13	3.98	2.81
-8	2	13	6.85	2.55
-7	2	13	6.68	2.31
6	-2	-13	15.54	3.80
-6	2	13	12.62	2.30
5	-2	-13	2.18	3.16
-4	2	13	28.39	2.75
-4	2	13	35.60	3.35
3	-2	-13	26.23	1.80
-3	2	13	25.71	2.51
-3	2	13	26.88	2.91
2	-2	-13	28.27	1.88
-2	2	13	28.73	2.57
-2	2	13	27.22	1.82
-2	2	13	31.52	2.98
-2	2	13	28.30	2.77
1	-2	-13	18.45	3.63
1	-2	-13	16.08	2.58
1	-2	-13	16.84	2.27
1	-2	-13	19.23	1.53
-1	2	13	19.09	1.42
-1	2	13	19.81	2.68
0	-2	-13	18.47	3.22
0	-2	-13	17.80	2.26
0	-2	-13	20.11	1.55
0	2	13	20.75	2.71
0	2	13	16.92	2.79
0	2	13	19.90	1.49
0	2	13	19.37	2.29
-1	-2	-13	19.22	3.15
-1	-2	-13	20.92	1.59
-1	-2	-13	20.62	2.92
-1	-2	-13	20.84	2.32
1	2	13	16.56	2.93
1	2	13	20.77	1.58
1	2	13	21.61	2.47
-2	-2	-13	39.16	2.28
-2	-2	-13	40.43	3.06
-2	-2	-13	39.46	3.43
2	2	13	39.24	3.14

2	2	13	37.77	2.32
2	2	13	39.55	3.04
2	2	13	32.78	3.48
-3	-2	-13	15.93	1.84
-3	-2	-13	18.03	2.89
-3	-2	-13	20.48	2.60
3	2	13	18.16	3.00
3	2	13	18.39	1.53
3	2	13	19.34	2.55
-4	-2	-13	26.54	3.35
-4	-2	-13	29.84	1.86
-4	-2	-13	26.98	2.99
-4	-2	-13	28.38	2.87
4	2	13	27.01	3.34
4	2	13	31.52	3.00
4	2	13	27.79	1.96
4	2	13	22.67	2.71
-5	-2	-13	18.09	2.62
-5	-2	-13	24.43	1.74
-5	-2	-13	25.22	3.50
-5	-2	-13	25.84	2.94
5	2	13	30.29	3.06
5	2	13	24.38	2.65
5	2	13	20.83	3.50
5	2	13	22.97	1.83
-6	-2	-13	18.94	2.73
-6	-2	-13	17.64	1.55
-6	-2	-13	14.07	2.56
6	2	13	17.07	1.67
6	2	13	14.01	2.41
6	2	13	21.10	3.20
6	2	13	15.75	2.48
-7	-2	-13	8.77	1.42
-7	-2	-13	7.78	1.73
-7	-2	-13	4.28	2.49
-7	-2	-13	8.33	1.60
7	2	13	10.81	2.03
7	2	13	8.91	1.58
7	2	13	9.41	2.39
-8	-2	-13	7.36	1.03
-8	-2	-13	9.27	1.97
-8	-2	-13	6.74	2.90
-8	-2	-13	6.39	1.56
8	2	13	10.51	1.79

8	2	13	9.60	2.66
8	2	13	6.84	1.11
8	2	13	10.64	2.30
-9	-2	-13	1.92	0.98
-9	-2	-13	-0.66	2.57
9	2	13	3.80	2.85
9	2	13	2.78	2.35
9	2	13	4.13	1.71
-10	-2	-13	2.91	3.91
-10	-2	-13	3.95	1.09
10	2	13	11.32	3.39
10	2	13	5.69	1.99
-11	-2	-13	3.04	4.86
-11	-2	-13	1.94	4.71
-11	-2	-13	2.03	1.11
11	2	13	4.89	2.19
-8	3	13	6.59	2.58
-7	3	13	6.83	2.37
-6	3	13	10.87	2.26
5	-3	-13	1.95	3.19
4	-3	-13	3.09	0.78
-4	3	13	2.09	0.70
3	-3	-13	5.60	0.83
-3	3	13	4.70	0.75
-3	3	13	5.73	1.23
2	-3	-13	9.84	1.29
-2	3	13	8.90	1.72
-2	3	13	9.45	1.14
-2	3	13	13.09	2.09
1	-3	-13	0.55	1.45
1	-3	-13	0.57	0.62
1	-3	-13	1.77	2.01
-1	3	13	1.82	1.22
-1	3	13	-0.76	1.48
-1	3	13	0.66	0.56
0	-3	-13	11.62	1.97
0	-3	-13	9.31	1.72
0	-3	-13	10.53	1.34
0	3	13	11.21	1.83
0	3	13	12.75	1.21
0	3	13	15.60	2.21
-1	-3	-13	24.70	2.39
-1	-3	-13	24.57	1.75
1	3	13	21.69	3.01

1	3	13	22.02	1.65
1	3	13	22.79	2.52
-2	-3	-13	26.38	1.82
-2	-3	-13	23.01	3.52
-2	-3	-13	28.51	2.64
2	3	13	26.19	2.73
2	3	13	23.64	3.17
2	3	13	26.07	1.81
-3	-3	-13	-0.43	0.62
-3	-3	-13	-1.50	1.28
-3	-3	-13	0.42	1.66
3	3	13	0.50	0.61
3	3	13	0.92	1.25
3	3	13	-0.19	1.65
-4	-3	-13	4.08	1.81
-4	-3	-13	4.72	1.45
-4	-3	-13	3.89	0.75
4	3	13	3.70	1.85
4	3	13	4.44	0.75
4	3	13	3.62	1.42
-5	-3	-13	7.68	1.25
-5	-3	-13	5.23	2.05
-5	-3	-13	8.59	1.54
5	3	13	10.51	1.78
5	3	13	9.74	1.30
5	3	13	7.98	2.07
-6	-3	-13	11.74	1.39
-6	-3	-13	8.67	2.42
-6	-3	-13	9.88	1.63
6	3	13	8.56	1.42
6	3	13	8.98	2.25
6	3	13	12.83	1.97
-7	-3	-13	0.45	2.43
-7	-3	-13	1.81	0.76
-7	-3	-13	1.49	1.47
7	3	13	2.79	2.20
7	3	13	1.83	1.30
7	3	13	3.28	1.83
-8	-3	-13	0.68	0.80
-8	-3	-13	-2.60	2.62
-8	-3	-13	3.23	1.51
8	3	13	2.41	1.41
8	3	13	4.14	1.96
8	3	13	3.26	2.41

8	3	13	0.74	1.36
-9	-3	-13	-2.60	3.03
-9	-3	-13	0.63	0.93
-9	-3	-13	-0.14	1.70
9	3	13	2.44	1.55
9	3	13	1.53	2.66
-10	-3	-13	1.41	1.93
-10	-3	-13	0.11	0.99
-10	-3	-13	1.98	3.66
10	3	13	3.47	1.78
10	3	13	1.22	2.98
-11	-3	-13	1.54	4.08
-11	-3	-13	-0.07	2.54
-11	-3	-13	0.43	1.09
-11	-3	-13	2.24	4.19
11	3	13	-0.85	2.55
11	3	13	2.93	3.88
11	3	13	2.74	2.02
-8	4	13	2.25	2.61
-7	4	13	9.51	2.46
4	-4	-13	3.62	0.86
-4	4	13	2.67	0.62
3	-4	-13	13.82	1.51
-3	4	13	11.34	1.23
2	-4	-13	13.27	1.44
-2	4	13	12.76	2.19
-2	4	13	12.28	1.25
1	-4	-13	34.75	2.15
-1	4	13	35.59	2.82
-1	4	13	29.63	3.43
-1	4	13	31.80	2.03
0	-4	-13	7.48	0.93
0	-4	-13	5.76	1.67
0	-4	-13	8.76	1.21
0	4	13	8.33	1.71
0	4	13	9.82	1.16
-1	-4	-13	21.17	3.15
-1	-4	-13	26.94	2.47
-1	-4	-13	27.81	1.94
1	4	13	27.26	3.12
1	4	13	30.67	2.76
1	4	13	28.72	1.84
-2	-4	-13	14.97	1.46
-2	-4	-13	14.24	2.28

2	4	13	15.12	2.42
2	4	13	13.86	2.04
2	4	13	15.71	1.38
-3	-4	-13	14.09	2.32
-3	-4	-13	14.57	2.36
-3	-4	-13	15.59	1.51
3	4	13	15.54	3.05
3	4	13	16.01	2.53
3	4	13	15.64	1.40
-4	-4	-13	14.54	1.48
-4	-4	-13	9.04	2.18
-4	-4	-13	12.04	2.22
4	4	13	14.35	2.56
4	4	13	13.91	1.39
4	4	13	11.89	2.05
-5	-4	-13	19.70	1.64
-5	-4	-13	17.67	2.51
-5	-4	-13	21.13	2.47
5	4	13	20.03	1.66
5	4	13	20.19	3.23
5	4	13	25.09	2.97
-6	-4	-13	9.63	2.29
-6	-4	-13	11.35	1.59
-6	-4	-13	11.87	1.44
6	4	13	13.75	2.00
6	4	13	12.05	2.20
-7	-4	-13	9.76	2.54
-7	-4	-13	13.62	2.47
-7	-4	-13	12.17	1.53
7	4	13	14.84	2.18
7	4	13	15.32	2.34
-8	-4	-13	-1.23	2.56
-8	-4	-13	1.17	0.84
-8	-4	-13	1.31	1.52
8	4	13	2.44	1.92
8	4	13	2.46	2.20
8	4	13	2.65	1.35
-9	-4	-13	1.08	2.95
-9	-4	-13	1.81	1.61
-9	-4	-13	1.61	0.94
9	4	13	2.98	2.43
9	4	13	2.56	0.94
9	4	13	3.95	1.49
-10	-4	-13	2.08	1.06

-10	-4	-13	2.06	1.77
-10	-4	-13	2.11	3.34
-10	-4	-13	2.40	1.75
10	4	13	3.17	2.72
10	4	13	3.06	1.70
-11	-4	-13	3.84	3.87
-11	-4	-13	7.20	2.12
-11	-4	-13	7.11	3.95
-11	-4	-13	6.54	1.29
11	4	13	7.94	2.02
11	4	13	6.30	2.72
11	4	13	5.39	3.09
-12	-4	-13	3.76	4.86
-12	-4	-13	1.07	1.27
-12	-4	-13	0.08	4.72
12	4	13	1.86	2.26
-8	5	13	1.96	2.83
-7	5	13	7.97	2.56
-6	5	13	10.04	2.24
-5	5	13	4.92	0.71
4	-5	-13	3.37	0.87
-4	5	13	2.69	0.66
3	-5	-13	9.52	1.54
-3	5	13	10.04	1.24
2	-5	-13	6.20	0.89
-2	5	13	3.95	0.69
1	-5	-13	4.22	0.82
-1	5	13	4.65	0.69
0	-5	-13	10.58	1.33
0	5	13	7.37	1.45
0	5	13	8.37	1.18
0	5	13	6.28	1.79
-1	-5	-13	7.63	1.25
-1	-5	-13	7.81	0.95
-1	-5	-13	6.21	1.70
1	5	13	8.25	1.23
1	5	13	9.12	1.80
-2	-5	-13	0.38	0.68
-2	-5	-13	1.20	1.21
-2	-5	-13	-1.09	2.48
2	5	13	1.39	0.64
2	5	13	1.62	1.60
2	5	13	1.58	1.30
-3	-5	-13	0.74	0.91

-3	-5	-13	-0.41	1.84
-3	-5	-13	2.51	1.21
3	5	13	1.99	1.65
3	5	13	1.98	0.66
3	5	13	1.96	1.35
-4	-5	-13	0.81	0.72
-4	-5	-13	0.58	1.14
-4	-5	-13	-1.84	1.77
4	5	13	0.64	1.37
4	5	13	1.42	1.62
4	5	13	-0.82	1.00
4	5	13	0.61	0.64
-5	-5	-13	1.84	0.77
-5	-5	-13	1.13	1.94
-5	-5	-13	2.76	1.22
5	5	13	3.61	1.54
5	5	13	1.76	1.69
5	5	13	2.78	0.71
-6	-5	-13	1.34	0.79
-6	-5	-13	3.18	1.35
-6	-5	-13	-0.51	2.07
6	5	13	1.31	1.56
6	5	13	0.95	1.76
-7	-5	-13	9.44	2.41
-7	-5	-13	9.42	1.61
-7	-5	-13	11.16	1.50
7	5	13	10.21	2.08
7	5	13	11.10	1.96
-8	-5	-13	0.55	0.83
-8	-5	-13	0.98	2.42
-8	-5	-13	1.54	1.46
8	5	13	2.24	2.02
-9	-5	-13	-1.94	2.68
-9	-5	-13	0.04	0.91
-9	-5	-13	-2.04	1.55
9	5	13	1.14	0.83
9	5	13	-0.91	2.17
-10	-5	-13	0.52	3.17
-10	-5	-13	-2.19	1.70
10	5	13	2.51	2.46
10	5	13	2.35	1.61
10	5	13	-1.17	2.14
-11	-5	-13	1.06	1.71
-11	-5	-13	0.40	1.99

-11	-5	-13	1.30	5.22
-11	-5	-13	2.68	1.19
11	5	13	0.07	2.37
11	5	13	-0.30	2.77
11	5	13	3.75	1.93
-12	-5	-13	5.34	4.51
-12	-5	-13	5.57	4.30
12	5	13	4.75	2.75
12	5	13	3.03	2.14
12	5	13	-0.72	3.27
-7	6	13	4.77	2.61
-6	6	13	19.60	3.98
-6	6	13	12.53	1.53
-5	6	13	5.23	0.79
4	-6	-13	4.09	0.95
-4	6	13	3.61	0.71
3	-6	-13	6.61	1.00
-3	6	13	7.34	1.19
2	-6	-13	13.17	1.64
-2	6	13	12.79	1.32
1	-6	-13	4.28	0.91
-1	6	13	5.22	0.75
0	-6	-13	8.23	1.40
0	6	13	7.25	2.00
0	6	13	8.92	1.22
0	6	13	7.07	1.46
-1	-6	-13	5.33	0.86
1	6	13	5.85	1.48
1	6	13	5.90	0.77
1	6	13	5.28	1.74
-2	-6	-13	13.49	1.82
-2	-6	-13	11.45	1.88
-2	-6	-13	14.68	2.21
2	6	13	15.53	2.81
2	6	13	17.10	2.99
2	6	13	18.48	1.44
-3	-6	-13	3.97	1.02
-3	-6	-13	4.15	1.20
3	6	13	5.23	1.50
3	6	13	3.39	1.60
3	6	13	4.21	0.76
-4	-6	-13	10.12	1.41
-4	-6	-13	11.92	1.46
-4	-6	-13	9.15	3.06

4	6	13	16.92	2.65
4	6	13	12.38	1.86
4	6	13	11.99	1.42
-5	-6	-13	1.63	1.77
-5	-6	-13	1.08	0.78
-5	-6	-13	2.69	1.24
5	6	13	1.99	0.69
5	6	13	2.77	1.57
5	6	13	3.02	1.63
-6	-6	-13	5.87	1.38
-6	-6	-13	4.15	2.00
-6	-6	-13	7.19	1.00
6	6	13	7.68	1.83
6	6	13	5.77	1.80
-7	-6	-13	3.32	0.89
-7	-6	-13	3.46	2.22
-7	-6	-13	3.27	1.40
7	6	13	3.98	1.84
-8	-6	-13	6.37	1.06
-8	-6	-13	7.37	1.63
-8	-6	-13	7.27	2.63
8	6	13	6.96	2.04
-9	-6	-13	1.62	0.97
-9	-6	-13	2.26	2.58
-9	-6	-13	-0.63	1.56
9	6	13	-0.15	1.92
9	6	13	0.13	2.07
9	6	13	1.68	0.84
-10	-6	-13	7.77	1.87
-10	-6	-13	7.92	1.30
-10	-6	-13	8.38	3.10
10	6	13	6.12	2.22
10	6	13	4.63	2.45
-11	-6	-13	2.70	1.84
-11	-6	-13	1.83	2.00
-11	-6	-13	1.18	1.17
-11	-6	-13	-0.62	3.52
11	6	13	0.19	2.71
11	6	13	1.92	2.50
-12	-6	-13	1.23	1.33
-12	-6	-13	-0.98	2.26
-12	-6	-13	-3.40	3.89
-12	-6	-13	3.77	4.05
12	6	13	0.43	2.71

12	6	13	2.06	6.16
-6	7	13	3.23	0.87
-6	7	13	3.25	2.40
-5	7	13	1.23	0.76
-4	7	13	3.46	0.75
3	-7	-13	9.49	1.70
-3	7	13	10.28	1.37
-2	7	13	10.74	1.33
-1	7	13	9.45	1.25
0	7	13	3.42	0.70
-1	-7	-13	0.25	0.89
1	7	13	1.01	0.64
-2	-7	-13	5.10	0.89
2	7	13	4.78	0.78
2	7	13	5.38	1.54
-3	-7	-13	5.40	0.88
-3	-7	-13	3.17	1.73
-3	-7	-13	3.75	1.13
3	7	13	4.09	1.56
3	7	13	5.99	1.60
-4	-7	-13	0.19	1.12
-4	-7	-13	0.69	0.74
4	7	13	1.28	0.65
4	7	13	-0.66	1.52
-5	-7	-13	-0.16	0.72
-5	-7	-13	-1.03	2.48
-5	-7	-13	-0.04	1.15
5	7	13	-2.18	1.53
5	7	13	0.18	0.65
-6	-7	-13	1.59	2.03
-6	-7	-13	1.91	0.81
-6	-7	-13	1.89	1.28
6	7	13	1.17	1.63
-7	-7	-13	0.49	0.84
-7	-7	-13	-0.62	1.31
-7	-7	-13	-0.07	1.96
7	7	13	-1.02	1.68
-8	-7	-13	0.10	1.39
-8	-7	-13	-0.84	0.91
-8	-7	-13	-2.16	2.26
8	7	13	-1.43	1.78
8	7	13	-1.21	1.69
-9	-7	-13	0.49	0.97
-9	-7	-13	2.09	1.57

-9	-7	-13	0.53	2.58
9	7	13	2.27	1.93
9	7	13	1.31	2.01
9	7	13	1.70	0.87
-10	-7	-13	2.11	1.14
-10	-7	-13	1.71	1.75
-10	-7	-13	1.07	2.94
10	7	13	2.20	2.15
10	7	13	6.54	2.33
-11	-7	-13	1.73	3.41
-11	-7	-13	1.14	1.96
-11	-7	-13	0.90	1.18
11	7	13	-1.20	2.28
11	7	13	4.57	2.59
-12	-7	-13	-0.72	1.32
-12	-7	-13	-2.55	6.22
-12	-7	-13	-0.43	2.13
-12	-7	-13	-2.09	2.24
12	7	13	-0.92	2.65
12	7	13	-1.27	2.84
-6	8	13	0.72	2.64
-6	8	13	0.75	0.94
-5	8	13	-0.14	0.82
-4	8	13	-0.69	0.78
-3	8	13	4.07	0.85
2	-8	-13	6.69	1.05
-2	8	13	5.70	0.84
1	-8	-13	18.35	1.92
-1	8	13	15.12	1.57
0	-8	-13	20.76	1.90
0	8	13	17.55	1.62
-1	-8	-13	28.05	2.10
1	8	13	23.27	1.81
-2	-8	-13	6.15	1.01
2	8	13	7.14	1.24
-3	-8	-13	12.32	1.98
-3	-8	-13	18.86	1.74
3	8	13	15.55	2.79
3	8	13	17.92	1.53
-4	-8	-13	4.26	1.22
-4	-8	-13	5.12	0.93
4	8	13	4.21	0.78
4	8	13	3.75	1.53
-5	-8	-13	21.07	2.29

-5	-8	-13	19.46	1.77
5	8	13	17.25	2.83
5	8	13	14.29	1.50
-6	-8	-13	17.76	2.30
-6	-8	-13	18.25	1.77
-6	-8	-13	11.84	2.47
6	8	13	16.59	2.93
-7	-8	-13	14.47	2.35
-7	-8	-13	9.39	2.37
-7	-8	-13	13.87	1.67
7	8	13	12.46	2.05
7	8	13	10.81	2.02
-8	-8	-13	-2.38	1.34
-8	-8	-13	-3.52	2.19
-8	-8	-13	0.52	0.93
8	8	13	-2.50	1.82
-9	-8	-13	3.15	1.56
-9	-8	-13	4.46	1.08
-9	-8	-13	3.15	2.51
9	8	13	2.60	1.92
9	8	13	1.66	2.08
9	8	13	2.30	0.91
-10	-8	-13	-1.99	2.67
-10	-8	-13	-0.61	1.80
-10	-8	-13	-0.29	1.15
10	8	13	-0.39	2.21
10	8	13	0.62	2.06
-11	-8	-13	1.35	1.96
-11	-8	-13	0.23	1.23
-11	-8	-13	-2.04	3.25
11	8	13	1.40	2.36
11	8	13	-0.31	2.49
-12	-8	-13	-2.56	2.23
-12	-8	-13	0.36	3.74
-12	-8	-13	-2.08	1.31
12	8	13	-3.81	2.64
12	8	13	-5.31	2.81
-5	9	13	0.56	1.02
-4	9	13	-0.09	0.93
-3	9	13	0.76	0.84
2	-9	-13	0.38	0.99
-2	9	13	-0.73	0.76
1	-9	-13	0.58	0.94
-1	9	13	0.54	0.76

0	-9	-13	1.81	0.93
0	9	13	0.82	0.71
-1	-9	-13	1.91	0.86
1	9	13	1.04	0.70
-2	-9	-13	2.84	0.95
2	9	13	2.18	0.73
-3	-9	-13	2.13	0.86
3	9	13	1.88	0.70
3	9	13	-1.84	1.58
-4	-9	-13	1.36	0.87
4	9	13	-0.31	1.46
4	9	13	-0.72	1.54
4	9	13	1.70	0.71
-5	-9	-13	-0.71	1.11
-5	-9	-13	-1.69	2.47
-5	-9	-13	-0.89	0.83
5	9	13	-1.84	1.43
5	9	13	-0.41	1.58
5	9	13	0.20	0.66
-6	-9	-13	6.10	0.99
-6	-9	-13	3.37	1.25
-6	-9	-13	5.80	2.36
6	9	13	5.20	1.74
6	9	13	4.56	1.66
-7	-9	-13	0.78	1.28
-7	-9	-13	0.34	2.04
-7	-9	-13	2.17	0.91
7	9	13	1.50	1.65
7	9	13	0.16	1.73
-8	-9	-13	-0.02	1.41
-8	-9	-13	0.62	0.93
8	9	13	-1.61	0.84
8	9	13	-0.94	1.76
8	9	13	-2.40	1.85
-9	-9	-13	-2.10	1.56
-9	-9	-13	0.64	0.99
-9	-9	-13	-2.85	2.47
9	9	13	-2.52	1.94
9	9	13	-0.16	1.16
-10	-9	-13	0.53	1.09
-10	-9	-13	-2.08	2.90
-10	-9	-13	0.10	1.80
10	9	13	-0.68	2.16
10	9	13	0.21	2.80

-11	-9	-13	-2.26	2.08
-11	-9	-13	-1.46	1.16
-11	-9	-13	-2.44	3.21
11	9	13	-2.13	2.54
11	9	13	-0.27	2.42
-12	-9	-13	-3.67	3.64
-12	-9	-13	-0.55	2.34
-12	-9	-13	1.36	1.38
12	9	13	-0.51	2.74
12	9	13	-0.80	3.04
-3	10	13	1.42	0.97
-2	10	13	2.06	0.92
1	-10	-13	0.53	1.03
-1	10	13	-0.27	0.83
0	-10	-13	1.47	0.99
0	10	13	-0.04	0.78
-1	-10	-13	0.96	0.96
1	10	13	-1.12	0.70
-2	-10	-13	4.02	1.02
2	10	13	4.06	0.82
-3	-10	-13	0.17	0.92
3	10	13	0.06	1.40
3	10	13	0.09	0.73
3	10	13	0.30	1.99
-4	-10	-13	1.53	1.83
-4	-10	-13	3.63	0.96
4	10	13	2.01	0.74
4	10	13	0.22	1.61
4	10	13	2.70	1.55
-5	-10	-13	-2.01	1.86
-5	-10	-13	0.21	0.89
5	10	13	-2.45	1.56
5	10	13	-0.38	0.69
5	10	13	-0.84	1.48
-6	-10	-13	3.01	1.28
-6	-10	-13	5.74	1.03
6	10	13	3.37	1.73
6	10	13	4.32	1.78
6	10	13	4.62	0.95
-7	-10	-13	1.06	1.29
-7	-10	-13	0.95	3.36
-7	-10	-13	1.53	0.94
7	10	13	1.49	0.92
7	10	13	0.33	1.77

7	10	13	0.57	1.81
-8	-10	-13	0.97	1.52
-8	-10	-13	3.54	1.03
-8	-10	-13	2.39	2.35
8	10	13	1.93	1.05
8	10	13	2.37	1.98
8	10	13	1.65	1.88
-9	-10	-13	1.29	1.09
-9	-10	-13	-1.75	2.54
-9	-10	-13	-1.73	1.62
9	10	13	-2.37	2.08
9	10	13	1.18	1.93
-10	-10	-13	-2.46	1.89
-10	-10	-13	0.39	2.92
-10	-10	-13	2.70	1.15
10	10	13	0.12	2.16
-11	-10	-13	-2.29	2.01
-11	-10	-13	0.10	1.22
-11	-10	-13	-1.37	3.17
11	10	13	1.46	2.48
11	10	13	-1.49	2.63
-12	-10	-13	2.72	2.63
-12	-10	-13	8.89	3.72
12	10	13	3.73	2.96
12	10	13	1.48	3.14
0	-11	-13	0.36	1.03
-1	-11	-13	0.06	0.93
1	11	13	0.57	0.84
-2	-11	-13	1.01	1.00
2	11	13	-0.17	0.79
-3	-11	-13	2.00	0.97
3	11	13	-0.76	0.76
-4	-11	-13	1.22	0.93
4	11	13	-0.02	0.76
-5	-11	-13	1.40	1.02
5	11	13	-0.26	1.61
5	11	13	1.26	0.76
5	11	13	0.18	1.68
-6	-11	-13	-1.20	2.00
-6	-11	-13	0.45	0.93
-6	-11	-13	-0.62	1.27
6	11	13	-1.62	1.75
6	11	13	-0.18	1.72
6	11	13	0.13	0.92

-7	-11	-13	1.02	1.37
-7	-11	-13	0.22	1.01
-7	-11	-13	1.24	2.32
7	11	13	0.19	1.93
7	11	13	-2.22	1.76
-8	-11	-13	-1.30	2.62
-8	-11	-13	-0.99	1.59
-8	-11	-13	1.74	1.05
8	11	13	-1.28	2.08
8	11	13	-0.34	1.91
-9	-11	-13	-3.90	2.55
-9	-11	-13	2.52	1.09
-9	-11	-13	-1.41	1.80
9	11	13	-0.50	2.28
9	11	13	-0.64	2.13
-10	-11	-13	-2.46	2.87
-10	-11	-13	1.57	1.15
-10	-11	-13	-3.28	2.00
10	11	13	-0.86	2.53
10	11	13	0.73	2.36
-11	-11	-13	-1.94	3.43
-11	-11	-13	-1.12	2.26
-11	-11	-13	1.63	1.25
11	11	13	-0.47	2.70
11	11	13	0.01	2.84
-12	-11	-13	-4.81	2.58
-12	-11	-13	1.71	1.43
-12	-11	-13	1.19	3.81
12	11	13	-0.61	2.98
-1	-12	-13	-1.16	1.04
-2	-12	-13	0.21	1.02
-3	-12	-13	0.80	1.05
-4	-12	-13	1.02	1.02
-5	-12	-13	1.38	1.02
5	12	13	0.93	1.81
5	12	13	1.68	1.83
-6	-12	-13	1.63	1.07
6	12	13	0.42	1.84
6	12	13	2.93	1.86
-7	-12	-13	0.82	1.03
-7	-12	-13	1.88	2.40
7	12	13	-0.73	1.94
7	12	13	0.75	2.00
-8	-12	-13	0.31	1.10

-8	-12	-13	1.86	3.07
8	12	13	-3.53	2.11
8	12	13	-2.59	2.09
-9	-12	-13	3.01	2.25
-9	-12	-13	1.94	1.20
9	12	13	-3.36	2.44
9	12	13	-1.68	2.43
-10	-12	-13	1.73	2.58
-10	-12	-13	1.62	1.25
10	12	13	-2.78	2.55
-11	-12	-13	1.23	1.28
11	12	13	-2.66	2.91
-2	-13	-13	-0.30	1.11
-3	-13	-13	1.44	1.08
-4	-13	-13	2.03	1.15
-5	-13	-13	5.62	1.20
5	13	13	3.16	1.90
5	13	13	1.83	2.49
-6	-13	-13	2.32	1.16
6	13	13	1.34	2.04
-7	-13	-13	0.05	1.15
-7	-13	-13	4.76	2.26
7	13	13	0.77	2.17
-8	-13	-13	1.94	1.19
8	13	13	-1.37	2.35
-9	-13	-13	0.63	1.14
9	13	13	-0.96	2.56
-10	-13	-13	0.24	1.21
10	13	13	-3.56	2.76
-3	-14	-13	-1.45	1.13
-4	-14	-13	0.27	1.16
-5	-14	-13	-0.18	1.09
-6	-14	-13	0.96	1.11
6	14	13	-0.59	1.85
-7	-14	-13	1.41	1.19
7	14	13	-3.47	2.09
-8	-14	-13	0.15	1.23
8	14	13	-0.32	2.37
-9	-14	-13	0.57	1.25
9	14	13	-2.72	2.55
-5	-6	14	14.66	2.29
-5	-6	14	10.65	3.25
4	6	-14	13.95	2.22
-4	-6	14	9.15	2.90

-4	-6	14	16.38	3.33
3	6	-14	14.24	3.50
3	6	-14	17.48	1.75
3	6	-14	15.51	3.37
-3	-6	14	16.79	2.82
-3	-6	14	14.70	3.75
-3	-6	14	17.90	3.29
-3	-6	14	15.00	1.97
2	6	-14	11.13	1.81
-2	-6	14	6.10	2.83
-2	-6	14	8.66	1.92
-2	-6	14	9.27	1.81
-2	-6	14	8.01	3.34
1	6	-14	11.06	2.95
1	6	-14	19.26	2.01
1	6	-14	11.18	3.28
-1	-6	14	15.85	2.15
-1	-6	14	16.02	3.38
-1	-6	14	13.33	3.46
-1	-6	14	13.14	2.58
0	6	-14	15.52	4.53
0	6	-14	8.46	1.27
0	-6	14	9.58	2.53
0	-6	14	10.84	2.15
-1	6	-14	9.09	3.20
-1	6	-14	11.85	1.99
1	-6	14	10.56	3.38
1	-6	14	8.24	2.35
1	-6	14	11.56	2.33
-6	-5	14	10.25	3.19
-6	-5	14	12.52	2.20
-5	-5	14	22.38	3.59
-5	-5	14	24.73	4.48
4	5	-14	12.76	3.04
-4	-5	14	12.53	2.65
-4	-5	14	20.09	3.29
-4	-5	14	19.65	4.27
3	5	-14	26.48	4.71
3	5	-14	29.33	4.73
-3	-5	14	21.13	2.82
-3	-5	14	14.77	3.34
-3	-5	14	20.37	3.18
-3	-5	14	19.64	3.75
2	5	-14	14.95	2.88

2	5	-14	23.16	4.47
2	5	-14	17.44	1.70
-2	-5	14	15.05	3.23
-2	-5	14	16.28	2.68
-2	-5	14	21.85	3.13
-2	-5	14	13.26	2.55
1	5	-14	16.71	1.87
1	5	-14	19.34	3.07
1	5	-14	14.90	2.70
-1	-5	14	9.88	3.04
-1	-5	14	13.43	2.37
-1	-5	14	18.15	3.16
-1	-5	14	19.17	2.76
0	5	-14	19.80	3.89
0	5	-14	14.48	3.04
0	5	-14	21.94	2.02
0	-5	14	15.85	3.06
0	-5	14	15.35	2.46
0	-5	14	23.12	2.94
0	-5	14	19.61	3.27
-1	5	-14	7.69	2.76
-1	5	-14	13.21	1.89
-1	5	-14	12.68	2.56
1	-5	14	17.21	3.36
1	-5	14	9.46	3.14
1	-5	14	11.76	2.24
1	-5	14	14.66	2.42
-2	5	-14	11.26	2.61
-2	5	-14	15.21	2.03
-2	5	-14	13.49	2.89
2	-5	14	20.14	3.76
2	-5	14	13.38	2.37
2	-5	14	10.62	3.13
-3	5	-14	14.70	2.06
-3	5	-14	13.02	3.03
3	-5	14	11.55	2.91
3	-5	14	11.74	2.42
-7	-4	14	5.34	2.23
-7	-4	14	7.94	3.19
-6	-4	14	20.96	3.56
-6	-4	14	15.92	3.19
-5	-4	14	13.56	2.10
-5	-4	14	12.14	2.76
4	4	-14	17.58	3.92

4	4	-14	13.31	2.65
-4	-4	14	16.70	3.17
-4	-4	14	13.52	3.15
-4	-4	14	12.81	2.50
3	4	-14	13.49	2.71
3	4	-14	10.73	1.33
3	4	-14	11.27	2.64
-3	-4	14	13.66	2.87
-3	-4	14	12.22	2.28
-3	-4	14	16.34	2.15
-3	-4	14	9.38	3.04
2	4	-14	15.88	1.90
2	4	-14	15.57	2.77
2	4	-14	11.82	2.54
-2	-4	14	15.71	2.78
-2	-4	14	16.56	2.23
-2	-4	14	9.25	2.89
-2	-4	14	9.95	2.09
1	4	-14	12.90	2.35
1	4	-14	11.82	1.53
1	4	-14	10.20	2.57
-1	-4	14	16.03	2.31
-1	-4	14	12.87	2.16
-1	-4	14	9.70	2.75
-1	-4	14	17.73	2.85
0	4	-14	25.72	3.53
0	4	-14	21.97	1.92
0	4	-14	16.00	2.70
0	-4	14	23.20	2.60
0	-4	14	14.49	2.92
0	-4	14	21.07	3.24
0	-4	14	24.88	3.17
-1	4	-14	9.38	2.26
-1	4	-14	11.32	1.73
-1	4	-14	9.83	2.52
1	-4	14	10.84	1.60
1	-4	14	10.17	2.57
1	-4	14	11.55	2.10
1	-4	14	15.24	3.06
-2	4	-14	12.60	1.84
-2	4	-14	11.29	2.27
-2	4	-14	12.45	2.52
2	-4	14	12.28	1.85
2	-4	14	13.16	2.20

2	-4	14	11.77	2.58
2	-4	14	16.51	3.45
-3	4	-14	9.89	3.64
-3	4	-14	10.87	1.89
-3	4	-14	14.17	2.34
3	-4	14	10.67	2.31
3	-4	14	9.09	2.68
3	-4	14	13.29	2.25
-4	4	-14	15.72	2.05
-4	4	-14	12.63	2.77
-4	4	-14	10.89	2.58
4	-4	14	15.38	2.41
4	-4	14	21.53	3.97
-5	4	-14	8.19	2.93
-5	4	-14	10.58	1.41
5	-4	14	12.25	2.50
-8	-3	14	9.13	3.14
-7	-3	14	30.93	4.23
-7	-3	14	25.04	4.64
-6	-3	14	23.48	3.65
-6	-3	14	15.86	2.97
-5	-3	14	30.56	3.57
-5	-3	14	23.62	3.43
-5	-3	14	28.93	4.02
4	3	-14	12.31	2.46
4	3	-14	15.10	2.93
-4	-3	14	16.26	3.07
-4	-3	14	21.44	3.39
3	3	-14	22.05	3.83
3	3	-14	24.74	3.55
3	3	-14	19.93	1.63
-3	-3	14	21.32	2.11
-3	-3	14	28.13	3.17
-3	-3	14	22.34	3.29
-3	-3	14	20.53	3.10
2	3	-14	18.31	1.55
2	3	-14	18.70	3.45
2	3	-14	20.47	3.44
-2	-3	14	16.75	2.88
-2	-3	14	18.88	2.97
-2	-3	14	22.48	2.13
-2	-3	14	23.15	2.98
1	3	-14	22.18	3.45
1	3	-14	22.07	1.78

1	3	-14	23.99	3.32
-1	-3	14	19.26	3.48
-1	-3	14	25.17	2.32
-1	-3	14	24.78	2.93
-1	-3	14	15.43	3.25
0	3	-14	24.39	1.89
0	3	-14	19.90	3.63
0	3	-14	20.34	3.29
0	-3	14	22.48	3.08
0	-3	14	27.97	3.12
0	-3	14	21.04	3.55
0	-3	14	30.96	2.52
-1	3	-14	16.26	3.08
-1	3	-14	20.70	1.83
-1	3	-14	20.51	3.49
1	-3	14	26.41	2.47
1	-3	14	24.71	3.18
1	-3	14	15.39	2.51
1	-3	14	19.48	3.04
-2	3	-14	11.45	1.68
-2	3	-14	10.72	2.28
-2	3	-14	9.78	2.04
2	-3	14	11.23	1.90
2	-3	14	10.01	2.44
2	-3	14	14.62	2.34
2	-3	14	17.55	3.02
-3	3	-14	19.28	1.91
-3	3	-14	14.16	2.23
-3	3	-14	13.84	2.44
3	-3	14	19.32	3.36
3	-3	14	11.50	2.71
3	-3	14	18.47	2.63
3	-3	14	19.25	3.52
-4	3	-14	13.93	1.88
-4	3	-14	10.28	2.21
4	-3	14	12.29	2.25
4	-3	14	9.58	2.56
4	-3	14	11.94	2.32
-5	3	-14	12.84	2.43
-5	3	-14	13.78	2.67
-5	3	-14	17.04	2.05
5	-3	14	16.67	2.76
5	-3	14	19.87	3.89
5	-3	14	13.57	2.93

-6	3	-14	11.79	2.01
-6	3	-14	10.95	2.96
6	-3	14	13.18	2.58
-8	-2	14	0.67	2.77
-8	-2	14	7.69	2.46
-7	-2	14	8.88	2.72
-6	-2	14	9.43	3.40
-6	-2	14	15.58	2.61
-5	-2	14	21.16	3.55
-5	-2	14	28.39	3.50
-5	-2	14	20.48	3.08
4	2	-14	21.48	3.72
4	2	-14	23.85	4.12
-4	-2	14	27.49	3.36
-4	-2	14	26.83	4.28
-4	-2	14	34.71	3.39
3	2	-14	38.43	3.94
3	2	-14	32.57	3.79
-3	-2	14	41.67	3.56
-3	-2	14	35.73	3.45
-3	-2	14	34.41	4.30
-3	-2	14	38.52	2.49
2	2	-14	12.59	2.66
2	2	-14	21.73	3.18
2	2	-14	12.02	2.18
2	2	-14	16.75	1.42
-2	-2	14	18.09	2.80
-2	-2	14	14.65	2.77
-2	-2	14	17.71	1.81
-2	-2	14	15.30	2.53
1	2	-14	7.78	1.91
1	2	-14	10.09	1.25
1	2	-14	9.33	2.34
1	2	-14	9.03	1.89
-1	-2	14	10.42	2.24
-1	-2	14	10.56	1.64
-1	-2	14	14.01	1.81
0	2	-14	16.15	3.02
0	2	-14	12.19	2.31
0	2	-14	11.20	1.92
0	2	-14	12.68	1.39
0	-2	14	11.99	1.78
0	-2	14	14.70	2.61
0	-2	14	15.76	1.92

0	-2	14	11.92	2.19
-1	2	-14	22.48	1.81
-1	2	-14	21.55	3.33
-1	2	-14	21.32	3.43
-1	2	-14	24.86	3.02
1	-2	14	26.03	2.24
1	-2	14	19.42	2.90
1	-2	14	25.28	3.02
1	-2	14	17.83	3.34
-2	2	-14	26.94	2.03
-2	2	-14	29.52	4.60
-2	2	-14	26.58	3.43
-2	2	-14	26.63	3.16
2	-2	14	29.88	2.46
2	-2	14	26.40	3.40
2	-2	14	31.23	3.30
2	-2	14	26.68	3.15
-3	2	-14	19.38	3.13
-3	2	-14	19.11	3.41
-3	2	-14	23.41	1.92
3	-2	14	16.74	3.18
3	-2	14	20.65	3.47
3	-2	14	24.01	2.37
3	-2	14	25.91	3.34
-4	2	-14	9.64	2.22
-4	2	-14	9.50	1.18
-4	2	-14	7.28	2.05
4	-2	14	10.79	1.75
4	-2	14	11.51	2.07
4	-2	14	12.75	2.07
4	-2	14	9.98	2.40
-5	2	-14	14.48	3.74
-5	2	-14	14.00	1.87
-5	2	-14	8.56	2.28
5	-2	14	10.57	2.13
5	-2	14	8.20	2.43
5	-2	14	14.93	2.42
-6	2	-14	12.19	1.91
-6	2	-14	11.39	2.77
-6	2	-14	6.46	2.48
6	-2	14	12.07	2.32
6	-2	14	7.70	2.78
6	-2	14	12.89	2.76
-7	2	-14	12.19	3.27

-7	2	-14	10.87	1.41
7	-2	14	10.46	2.49
-8	-1	14	10.03	2.58
-8	-1	14	3.83	2.56
-7	-1	14	8.66	2.40
-7	-1	14	12.33	2.39
-6	-1	14	12.56	2.30
-6	-1	14	7.81	2.46
-5	-1	14	18.36	3.43
-5	-1	14	15.74	3.09
-5	-1	14	15.97	2.67
4	1	-14	35.29	6.69
4	1	-14	31.05	4.00
4	1	-14	24.06	3.91
-4	-1	14	29.16	3.27
-4	-1	14	33.74	4.08
-4	-1	14	36.19	3.44
3	1	-14	29.05	1.96
3	1	-14	29.24	3.56
3	1	-14	21.87	3.18
3	1	-14	34.76	5.07
-3	-1	14	31.99	2.05
-3	-1	14	29.17	3.73
-3	-1	14	35.71	3.24
-3	-1	14	28.35	3.11
2	1	-14	34.56	3.29
2	1	-14	30.72	4.62
2	1	-14	29.72	1.97
2	1	-14	29.31	3.46
-2	-1	14	30.60	2.14
-2	-1	14	33.57	3.16
-2	-1	14	28.41	3.68
-2	-1	14	28.46	3.05
1	1	-14	16.94	2.77
1	1	-14	23.64	3.73
1	1	-14	16.65	1.46
1	1	-14	14.39	2.87
-1	-1	14	14.18	2.68
-1	-1	14	15.46	2.28
-1	-1	14	20.03	1.77
-1	-1	14	19.30	2.63
0	1	-14	24.00	2.16
0	1	-14	27.27	3.76
0	1	-14	22.10	3.04

0	1	-14	22.35	2.93
0	-1	14	26.42	2.80
0	-1	14	29.68	2.88
0	-1	14	26.28	2.06
0	-1	14	25.03	3.21
-1	1	-14	20.35	2.75
-1	1	-14	20.03	2.96
-1	1	-14	24.28	3.37
-1	1	-14	19.32	1.57
1	-1	14	19.20	2.74
1	-1	14	18.65	2.65
1	-1	14	15.52	2.25
1	-1	14	20.12	1.89
-2	1	-14	20.86	1.78
-2	1	-14	22.96	2.87
-2	1	-14	19.91	3.46
-2	1	-14	20.97	3.17
2	-1	14	27.43	2.07
2	-1	14	21.63	2.92
2	-1	14	23.27	2.90
-3	1	-14	21.75	3.13
-3	1	-14	24.01	1.89
-3	1	-14	23.26	3.29
-3	1	-14	24.13	3.52
3	-1	14	22.01	3.32
3	-1	14	26.47	3.14
3	-1	14	23.96	3.08
3	-1	14	25.48	2.18
-4	1	-14	24.13	2.01
-4	1	-14	30.83	4.08
-4	1	-14	19.78	2.46
-4	1	-14	26.37	3.49
4	-1	14	26.14	3.38
4	-1	14	26.60	3.66
4	-1	14	28.86	3.47
4	-1	14	31.08	2.40
-5	1	-14	9.21	2.32
-5	1	-14	3.65	2.08
-5	1	-14	7.93	1.07
5	-1	14	4.81	1.86
5	-1	14	5.61	2.32
5	-1	14	9.05	2.12
-6	1	-14	11.58	1.74
-6	1	-14	14.90	3.80

-6	1	-14	9.29	2.42
6	-1	14	11.87	2.57
6	-1	14	8.05	2.61
6	-1	14	10.42	2.12
-7	1	-14	3.72	2.56
-7	1	-14	7.50	2.89
-7	1	-14	9.01	1.27
7	-1	14	10.10	2.87
7	-1	14	2.92	2.88
7	-1	14	9.18	2.20
-8	1	-14	8.66	1.30
-8	1	-14	9.50	3.72
8	-1	14	10.96	2.49
-8	0	14	24.83	4.47
-7	0	14	19.99	3.34
-6	0	14	23.71	3.62
-6	0	14	16.20	3.13
-6	0	14	14.41	2.69
-5	0	14	14.86	2.83
-5	0	14	17.13	3.38
4	0	-14	21.97	3.70
4	0	-14	19.13	3.45
-4	0	14	25.80	3.58
-4	0	14	27.97	3.27
-4	0	14	25.44	2.91
3	0	-14	28.12	3.31
3	0	-14	30.60	5.21
3	0	-14	26.17	1.87
3	0	-14	19.41	3.03
-3	0	14	34.17	3.14
-3	0	14	25.93	3.48
-3	0	14	25.98	2.88
2	0	-14	25.78	1.81
2	0	-14	25.39	3.03
2	0	-14	30.03	4.37
-2	0	14	21.18	2.72
-2	0	14	25.52	1.85
-2	0	14	28.68	2.93
-2	0	14	25.23	3.28
1	0	-14	18.37	3.23
1	0	-14	25.60	2.85
1	0	-14	28.47	1.82
1	0	-14	30.24	3.98
-1	0	14	26.48	1.93

-1	0	14	23.69	2.75
-1	0	14	30.12	2.93
-1	0	14	24.90	3.25
0	0	-14	20.30	2.62
0	0	-14	18.96	1.51
0	0	-14	16.17	2.86
0	0	-14	24.11	3.60
0	0	14	17.58	2.57
0	0	14	19.24	2.52
0	0	14	18.98	1.70
0	0	14	15.53	2.21
-1	0	-14	16.24	2.62
-1	0	-14	12.33	1.91
-1	0	-14	15.53	1.58
-1	0	-14	18.55	3.26
1	0	14	17.67	2.54
1	0	14	15.40	2.46
1	0	14	13.57	2.09
1	0	14	18.62	1.68
-2	0	-14	25.24	2.88
-2	0	-14	25.49	1.81
-2	0	-14	27.65	3.11
-2	0	-14	25.71	3.34
2	0	14	23.89	2.04
2	0	14	26.44	2.91
2	0	14	28.57	2.94
2	0	14	21.65	3.22
-3	0	-14	20.53	3.05
-3	0	-14	24.37	3.31
-3	0	-14	26.91	3.28
-3	0	-14	19.93	1.75
3	0	14	15.86	3.74
3	0	14	22.27	1.99
3	0	14	24.70	3.00
3	0	14	29.81	3.01
-4	0	-14	23.19	3.28
-4	0	-14	21.92	1.81
-4	0	-14	21.11	3.30
-4	0	-14	20.83	3.31
4	0	14	21.25	2.01
4	0	14	23.05	3.09
4	0	14	18.15	3.49
4	0	14	22.08	3.15
-5	0	-14	18.43	3.45

-5	0	-14	17.73	1.79
-5	0	-14	20.66	3.28
-5	0	-14	23.06	3.49
5	0	14	18.76	3.11
5	0	14	20.02	2.08
5	0	14	15.95	2.66
5	0	14	23.00	3.45
-6	0	-14	15.41	1.77
-6	0	-14	17.93	2.73
-6	0	-14	12.31	2.38
6	0	14	22.08	3.71
6	0	14	10.36	2.64
6	0	14	16.76	3.30
-7	0	-14	13.46	2.63
-7	0	-14	16.96	1.84
7	0	14	19.20	3.71
7	0	14	10.10	2.94
7	0	14	16.42	2.88
-8	0	-14	12.49	1.87
-8	0	-14	11.41	2.81
-8	0	-14	13.47	3.54
8	0	14	16.51	3.09
-9	0	-14	12.28	1.97
-9	0	-14	12.71	4.33
-8	1	14	10.87	2.72
-6	1	14	16.73	2.75
-6	1	14	15.14	2.65
5	-1	-14	22.71	4.56
-5	1	14	32.14	3.05
-5	1	14	36.26	3.79
4	-1	-14	24.41	5.29
-4	1	14	19.90	2.71
-4	1	14	20.03	3.27
3	-1	-14	30.97	2.09
3	-1	-14	40.10	5.00
-3	1	14	30.88	2.92
-3	1	14	31.65	3.38
-3	1	14	32.49	3.23
2	-1	-14	20.47	1.73
2	-1	-14	25.51	4.80
2	-1	-14	22.82	2.77
-2	1	14	21.68	3.02
-2	1	14	25.28	2.76
-2	1	14	24.70	2.62

1	-1	-14	30.42	1.98
1	-1	-14	27.77	2.77
-1	1	14	26.63	2.76
-1	1	14	34.78	2.85
-1	1	14	28.12	1.98
-1	1	14	25.70	3.23
0	-1	-14	15.28	2.47
0	-1	-14	15.99	3.07
0	-1	-14	21.77	1.59
0	1	14	19.86	2.42
0	1	14	16.30	2.94
0	1	14	19.17	1.65
-1	-1	-14	26.24	3.10
-1	-1	-14	22.60	1.94
-1	-1	-14	22.84	2.71
1	1	14	25.36	1.93
1	1	14	30.28	2.65
1	1	14	21.80	3.12
1	1	14	24.66	2.73
-2	-1	-14	23.77	1.80
-2	-1	-14	25.77	3.08
-2	-1	-14	24.97	2.89
2	1	14	25.88	2.86
2	1	14	26.16	1.98
2	1	14	28.29	2.81
2	1	14	23.90	3.37
-3	-1	-14	21.98	3.03
-3	-1	-14	25.31	1.81
-3	-1	-14	28.52	3.27
3	1	14	26.20	2.01
3	1	14	23.88	3.51
3	1	14	27.71	2.85
3	1	14	27.80	3.04
-4	-1	-14	11.53	1.39
-4	-1	-14	11.56	2.23
-4	-1	-14	8.82	1.91
-4	-1	-14	10.35	1.81
4	1	14	9.28	2.98
4	1	14	15.99	2.85
4	1	14	12.89	1.62
4	1	14	8.89	1.66
-5	-1	-14	31.89	3.36
-5	-1	-14	25.54	1.96
-5	-1	-14	27.30	3.56

-5	-1	-14	29.12	3.77
5	1	14	31.96	3.53
5	1	14	27.13	3.22
5	1	14	24.08	2.12
5	1	14	21.80	3.84
-6	-1	-14	20.22	3.21
-6	-1	-14	17.38	2.76
-6	-1	-14	17.70	3.47
-6	-1	-14	18.01	1.71
6	1	14	25.99	3.71
6	1	14	14.03	2.77
6	1	14	16.81	3.07
6	1	14	13.77	1.92
-7	-1	-14	20.55	1.90
-7	-1	-14	15.30	2.53
-7	-1	-14	15.78	3.04
7	1	14	23.82	4.08
7	1	14	15.75	3.02
7	1	14	24.64	3.56
-8	-1	-14	2.65	4.11
-8	-1	-14	4.11	2.39
-8	-1	-14	6.77	1.11
8	1	14	3.56	2.90
8	1	14	6.59	1.95
8	1	14	8.31	2.77
-9	-1	-14	12.18	2.87
-9	-1	-14	13.77	1.87
-9	-1	-14	8.57	4.08
9	1	14	11.93	3.40
-10	-1	-14	7.91	1.35
-10	-1	-14	8.72	4.83
10	1	14	12.57	2.55
-8	2	14	11.49	2.89
-7	2	14	10.38	2.24
-6	2	14	32.68	4.34
-5	2	14	24.09	3.60
4	-2	-14	14.20	3.34
-4	2	14	23.77	3.20
-4	2	14	17.32	2.51
-4	2	14	16.11	3.42
3	-2	-14	15.99	3.13
3	-2	-14	16.32	1.61
-3	2	14	20.10	3.06
-3	2	14	17.38	2.47

-3	2	14	17.05	1.45
2	-2	-14	15.66	2.59
2	-2	-14	17.09	1.56
-2	2	14	15.80	2.80
-2	2	14	20.54	2.42
-2	2	14	14.50	1.47
-2	2	14	17.63	2.63
1	-2	-14	14.68	2.36
1	-2	-14	18.73	1.61
-1	2	14	17.34	2.46
-1	2	14	17.97	2.43
-1	2	14	16.79	2.78
-1	2	14	17.50	1.55
0	-2	-14	17.63	2.44
0	-2	-14	19.80	1.59
0	2	14	14.01	2.10
0	2	14	17.54	1.56
0	2	14	18.78	2.38
0	2	14	14.91	2.42
-1	-2	-14	38.98	3.16
-1	-2	-14	34.54	3.81
-1	-2	-14	43.26	2.46
1	2	14	42.15	3.04
1	2	14	33.71	3.70
1	2	14	42.91	3.48
1	2	14	38.15	2.44
-2	-2	-14	42.68	3.76
-2	-2	-14	40.53	2.44
-2	-2	-14	41.37	3.28
2	2	14	35.56	3.76
2	2	14	38.64	3.35
2	2	14	36.69	2.43
-3	-2	-14	15.06	2.88
-3	-2	-14	17.61	3.05
-3	-2	-14	15.39	1.48
3	2	14	15.51	2.71
3	2	14	13.86	2.26
3	2	14	16.60	1.59
3	2	14	15.90	2.49
-4	-2	-14	11.73	1.91
-4	-2	-14	12.78	1.41
-4	-2	-14	14.13	2.22
4	2	14	13.87	1.98
4	2	14	9.73	1.64

4	2	14	12.70	1.51
4	2	14	12.25	2.29
-5	-2	-14	9.83	1.36
-5	-2	-14	10.42	2.44
-5	-2	-14	8.39	1.96
5	2	14	10.80	1.52
5	2	14	8.21	1.65
5	2	14	11.65	2.02
-6	-2	-14	10.64	2.17
-6	-2	-14	13.12	1.54
-6	-2	-14	16.57	2.94
-6	-2	-14	9.47	2.61
6	2	14	15.56	2.74
6	2	14	12.43	1.70
6	2	14	13.81	2.52
6	2	14	18.65	3.48
-7	-2	-14	15.15	1.69
-7	-2	-14	11.52	3.04
-7	-2	-14	12.86	2.25
-7	-2	-14	17.80	3.09
7	2	14	13.43	2.72
7	2	14	16.74	2.61
7	2	14	16.53	3.15
-8	-2	-14	11.30	1.63
-8	-2	-14	3.51	3.22
-8	-2	-14	9.00	2.17
8	2	14	13.04	2.04
8	2	14	9.54	2.92
-9	-2	-14	8.22	2.35
-9	-2	-14	12.01	1.78
-9	-2	-14	6.08	3.80
9	2	14	12.02	3.00
9	2	14	9.08	3.17
-10	-2	-14	9.32	4.63
-10	-2	-14	15.49	2.00
-7	3	14	24.43	4.47
-6	3	14	43.88	4.39
-5	3	14	29.86	3.84
4	-3	-14	27.53	1.98
4	-3	-14	19.31	3.71
-4	3	14	21.18	1.61
3	-3	-14	22.49	1.88
-3	3	14	24.83	3.09
-3	3	14	20.46	1.59

2	-3	-14	35.36	2.24
-2	3	14	32.44	2.09
-2	3	14	32.40	2.92
-2	3	14	33.81	3.28
1	-3	-14	14.29	2.32
1	-3	-14	16.66	1.61
-1	3	14	14.70	2.82
-1	3	14	21.24	2.47
-1	3	14	17.44	2.48
-1	3	14	15.32	1.45
0	-3	-14	45.89	2.68
0	-3	-14	43.45	3.17
0	3	14	43.40	3.76
0	3	14	47.81	3.43
0	3	14	41.97	2.60
-1	-3	-14	67.50	3.45
-1	-3	-14	61.97	3.89
-1	-3	-14	67.08	4.78
1	3	14	58.90	3.38
1	3	14	56.31	5.16
1	3	14	52.92	4.42
-2	-3	-14	94.40	6.30
-2	-3	-14	103.17	5.74
-2	-3	-14	99.58	5.48
2	3	14	103.87	5.26
2	3	14	107.74	5.90
2	3	14	99.93	6.20
-3	-3	-14	27.63	3.07
-3	-3	-14	28.27	3.65
-3	-3	-14	30.09	2.02
3	3	14	32.17	3.16
3	3	14	29.29	2.82
3	3	14	27.04	2.03
3	3	14	26.31	3.54
-4	-3	-14	12.07	2.31
-4	-3	-14	17.06	1.54
-4	-3	-14	17.71	2.98
4	3	14	17.51	1.58
4	3	14	12.04	2.46
4	3	14	15.36	2.37
4	3	14	18.02	2.91
-5	-3	-14	10.35	1.43
-5	-3	-14	12.09	1.92
-5	-3	-14	8.26	2.35

5	3	14	13.55	2.09
5	3	14	8.47	1.61
5	3	14	11.20	1.46
5	3	14	13.76	2.30
-6	-3	-14	12.51	1.97
-6	-3	-14	7.75	2.75
-6	-3	-14	13.24	1.53
6	3	14	18.03	3.47
6	3	14	12.59	1.57
6	3	14	10.46	1.68
6	3	14	10.65	3.54
-7	-3	-14	8.23	1.09
-7	-3	-14	6.83	1.84
-7	-3	-14	5.69	2.87
7	3	14	10.15	2.34
7	3	14	6.02	1.62
7	3	14	5.43	0.98
7	3	14	10.30	2.57
-8	-3	-14	13.86	2.16
-8	-3	-14	15.35	1.80
-8	-3	-14	13.55	3.43
-8	-3	-14	18.18	3.11
8	3	14	21.07	2.98
8	3	14	21.80	3.21
8	3	14	13.98	3.00
-9	-3	-14	5.64	3.64
-9	-3	-14	6.86	2.09
-9	-3	-14	7.69	1.19
9	3	14	7.02	3.10
9	3	14	10.75	2.03
9	3	14	12.21	2.93
-10	-3	-14	13.81	1.92
-10	-3	-14	14.17	4.45
-10	-3	-14	10.68	2.42
10	3	14	14.42	3.53
-11	-3	-14	8.53	4.59
-11	-3	-14	5.15	5.21
-11	-3	-14	5.62	1.22
11	3	14	7.29	2.46
-7	4	14	15.26	2.83
-6	4	14	30.77	4.38
4	-4	-14	23.13	2.08
-4	4	14	23.26	3.38
3	-4	-14	22.56	2.00

2	-4	-14	18.43	1.79
-2	4	14	19.70	1.56
1	-4	-14	28.34	2.01
-1	4	14	25.72	1.85
0	-4	-14	14.23	1.59
0	4	14	17.43	2.60
0	4	14	13.84	1.45
0	4	14	13.94	2.06
-1	-4	-14	21.65	3.60
-1	-4	-14	28.94	2.68
-1	-4	-14	30.63	2.29
1	4	14	34.35	3.43
1	4	14	29.54	2.04
1	4	14	34.89	3.01
-2	-4	-14	52.82	3.04
-2	-4	-14	47.90	3.48
2	4	14	52.17	4.16
2	4	14	42.07	2.79
-3	-4	-14	31.55	4.10
-3	-4	-14	34.80	3.30
-3	-4	-14	39.77	2.41
3	4	14	39.48	4.27
3	4	14	39.50	3.42
3	4	14	38.82	3.88
3	4	14	36.06	2.38
-4	-4	-14	30.49	2.14
-4	-4	-14	30.07	3.13
-4	-4	-14	23.78	3.94
4	4	14	32.72	2.15
4	4	14	35.57	3.40
4	4	14	31.54	3.81
-5	-4	-14	25.57	1.99
-5	-4	-14	24.66	3.93
-5	-4	-14	27.83	3.04
5	4	14	26.01	1.97
5	4	14	23.50	2.81
5	4	14	33.50	3.55
5	4	14	34.08	3.95
-6	-4	-14	9.76	1.77
-6	-4	-14	8.08	2.54
-6	-4	-14	11.32	1.47
6	4	14	12.52	2.16
6	4	14	12.17	1.49
6	4	14	19.51	3.63

6	4	14	8.56	1.49
-7	-4	-14	17.54	2.84
-7	-4	-14	13.52	1.68
-7	-4	-14	11.60	2.96
7	4	14	21.98	3.87
7	4	14	16.85	2.69
7	4	14	13.05	1.67
7	4	14	18.81	2.61
-8	-4	-14	10.73	1.87
-8	-4	-14	7.95	1.20
-8	-4	-14	7.81	3.19
8	4	14	14.25	2.54
8	4	14	10.62	2.67
8	4	14	14.06	2.83
-9	-4	-14	7.76	1.15
-9	-4	-14	5.60	3.65
-9	-4	-14	7.14	2.00
9	4	14	12.62	2.97
9	4	14	10.13	1.88
-10	-4	-14	9.38	2.28
-10	-4	-14	7.35	1.25
10	4	14	11.54	3.28
-11	-4	-14	11.03	4.84
-11	-4	-14	10.95	4.23
-11	-4	-14	10.38	1.48
11	4	14	10.17	3.67
11	4	14	10.05	3.21
-7	5	14	25.05	4.94
-6	5	14	37.31	4.49
-5	5	14	29.51	1.98
4	-5	-14	27.67	2.18
-4	5	14	20.33	1.71
3	-5	-14	40.39	2.63
-3	5	14	36.81	2.33
2	-5	-14	21.89	1.95
-2	5	14	19.75	1.63
1	-5	-14	34.35	2.32
-1	5	14	30.42	3.10
-1	5	14	33.47	2.13
0	-5	-14	29.26	2.27
0	5	14	31.86	3.54
0	5	14	29.83	3.09
0	5	14	29.55	2.02
-1	-5	-14	24.32	3.51

-1	-5	-14	34.00	2.71
1	5	14	30.96	2.14
1	5	14	37.86	3.60
1	5	14	35.75	3.27
-2	-5	-14	26.16	2.07
-2	-5	-14	27.84	2.73
2	5	14	31.49	3.09
2	5	14	31.39	3.54
2	5	14	23.67	1.94
-3	-5	-14	26.21	3.86
-3	-5	-14	32.99	2.24
-3	-5	-14	31.08	3.03
3	5	14	31.72	2.14
3	5	14	32.74	3.76
-4	-5	-14	21.93	1.85
-4	-5	-14	19.10	3.75
-4	-5	-14	22.15	2.72
4	5	14	23.48	3.16
4	5	14	25.21	3.50
4	5	14	20.22	1.75
4	5	14	15.00	2.46
-5	-5	-14	34.06	4.27
-5	-5	-14	34.45	3.08
-5	-5	-14	36.27	2.37
5	5	14	30.44	3.04
5	5	14	36.36	2.34
5	5	14	37.75	3.88
-6	-5	-14	19.24	2.66
-6	-5	-14	18.75	1.77
-6	-5	-14	18.60	2.82
6	5	14	16.37	2.91
6	5	14	22.72	3.48
6	5	14	15.07	1.70
6	5	14	22.19	3.54
-7	-5	-14	31.52	2.29
-7	-5	-14	33.68	4.65
-7	-5	-14	32.30	3.11
7	5	14	31.51	3.02
7	5	14	26.18	2.20
7	5	14	39.16	4.07
-8	-5	-14	22.76	3.45
-8	-5	-14	25.94	3.11
-8	-5	-14	23.92	2.07
8	5	14	22.32	3.90

8	5	14	26.20	3.02
8	5	14	22.52	2.10
-9	-5	-14	9.42	2.05
-9	-5	-14	14.28	1.82
-9	-5	-14	6.31	3.35
9	5	14	16.58	3.10
9	5	14	13.32	2.78
-10	-5	-14	13.05	3.82
-10	-5	-14	9.79	1.32
-10	-5	-14	7.55	2.15
10	5	14	8.17	2.83
10	5	14	10.96	2.14
-11	-5	-14	13.05	4.45
-11	-5	-14	20.59	3.87
-11	-5	-14	16.27	2.16
11	5	14	17.41	3.24
11	5	14	14.90	3.43
-6	6	14	19.40	1.82
-6	6	14	25.96	4.56
-5	6	14	27.76	2.01
4	-6	-14	17.94	2.51
-4	6	14	19.10	1.66
3	-6	-14	18.09	2.48
-3	6	14	17.64	1.61
2	-6	-14	27.41	3.12
-2	6	14	25.24	1.88
1	-6	-14	21.89	2.84
-1	6	14	17.44	1.59
0	-6	-14	23.40	2.10
0	6	14	22.49	1.75
-1	-6	-14	17.82	1.80
1	6	14	17.70	1.63
1	6	14	20.60	3.51
1	6	14	20.87	2.91
-2	-6	-14	28.85	2.67
-2	-6	-14	25.61	2.09
2	6	14	24.86	1.96
2	6	14	28.58	3.25
2	6	14	29.59	3.50
-3	-6	-14	16.79	2.58
-3	-6	-14	15.99	1.80
3	6	14	19.48	3.10
3	6	14	24.04	3.29
3	6	14	15.13	1.63

-4	-6	-14	21.12	2.51
-4	-6	-14	18.62	4.16
-4	-6	-14	18.17	1.86
4	6	14	25.91	3.35
4	6	14	18.05	2.39
4	6	14	18.91	3.26
4	6	14	19.37	1.73
-5	-6	-14	12.18	1.69
-5	-6	-14	14.52	2.47
-5	-6	-14	15.42	2.40
5	6	14	11.46	2.23
5	6	14	14.51	2.20
5	6	14	13.74	1.59
-6	-6	-14	15.23	1.72
-6	-6	-14	18.29	2.57
-6	-6	-14	12.39	2.55
6	6	14	10.10	1.54
6	6	14	12.78	2.22
-7	-6	-14	16.55	2.68
-7	-6	-14	11.52	1.78
-7	-6	-14	16.92	2.92
7	6	14	13.61	1.69
7	6	14	17.85	3.40
-8	-6	-14	22.21	2.94
-8	-6	-14	17.88	1.96
-8	-6	-14	28.34	4.75
8	6	14	16.12	3.51
8	6	14	18.60	1.94
-9	-6	-14	7.89	1.26
-9	-6	-14	7.66	1.90
-9	-6	-14	9.11	3.23
9	6	14	10.71	2.53
-10	-6	-14	18.21	3.88
-10	-6	-14	20.29	2.27
-10	-6	-14	18.61	3.53
10	6	14	27.54	4.60
10	6	14	19.21	3.18
10	6	14	27.29	3.44
-11	-6	-14	8.70	1.41
-11	-6	-14	9.97	2.51
-11	-6	-14	10.28	2.40
-11	-6	-14	9.30	4.62
11	6	14	9.42	2.91
11	6	14	6.05	3.03

-12	-6	-14	3.19	1.43
-12	-6	-14	7.17	4.22
-12	-6	-14	-4.07	4.74
12	6	14	5.03	3.55
12	6	14	4.93	2.41
12	6	14	8.12	3.54
-5	7	14	20.42	4.26
-5	7	14	15.15	1.71
-4	7	14	25.42	1.94
3	-7	-14	23.51	2.19
-3	7	14	19.11	1.74
2	-7	-14	21.85	2.16
-2	7	14	20.46	1.73
1	-7	-14	30.53	2.34
-1	7	14	26.64	1.97
0	-7	-14	51.50	3.02
0	7	14	43.01	3.82
0	7	14	45.81	2.79
-1	-7	-14	24.65	2.09
1	7	14	26.31	4.99
1	7	14	23.47	1.85
-2	-7	-14	36.54	2.44
2	7	14	37.06	3.58
2	7	14	30.73	2.23
2	7	14	38.24	3.76
-3	-7	-14	28.98	2.75
-3	-7	-14	20.13	3.53
-3	-7	-14	27.48	2.20
3	7	14	32.94	3.47
3	7	14	27.86	3.39
3	7	14	30.38	2.04
-4	-7	-14	24.95	2.61
-4	-7	-14	25.95	2.13
4	7	14	30.96	3.47
4	7	14	26.12	3.43
4	7	14	24.20	1.97
-5	-7	-14	15.13	1.85
-5	-7	-14	16.27	3.52
-5	-7	-14	19.56	2.48
5	7	14	18.59	1.74
5	7	14	24.85	3.35
5	7	14	17.35	3.31
-6	-7	-14	19.27	1.93
-6	-7	-14	17.08	2.72

-6	-7	-14	22.69	2.64
6	7	14	18.31	1.77
6	7	14	21.05	3.43
-7	-7	-14	11.96	1.87
-7	-7	-14	14.40	2.72
-7	-7	-14	18.89	2.63
7	7	14	15.88	2.35
7	7	14	13.18	1.73
-8	-7	-14	19.98	3.11
-8	-7	-14	17.53	2.06
-8	-7	-14	20.05	2.94
8	7	14	23.11	3.63
8	7	14	20.20	2.10
-9	-7	-14	16.51	3.41
-9	-7	-14	14.98	2.02
-9	-7	-14	16.24	3.09
9	7	14	16.18	2.53
9	7	14	10.64	2.44
-10	-7	-14	10.73	2.20
-10	-7	-14	11.89	3.68
-10	-7	-14	14.27	2.08
10	7	14	16.59	2.74
10	7	14	15.13	2.88
-11	-7	-14	6.90	1.40
-11	-7	-14	7.31	2.29
-11	-7	-14	5.67	4.02
11	7	14	9.20	2.82
11	7	14	9.05	3.08
-12	-7	-14	-1.45	4.82
-12	-7	-14	2.13	1.44
-12	-7	-14	2.99	2.52
12	7	14	6.38	3.18
12	7	14	0.61	3.29
-5	8	14	10.60	1.77
-5	8	14	8.84	2.69
-4	8	14	12.99	1.73
3	-8	-14	24.94	2.44
-3	8	14	22.04	1.94
2	-8	-14	15.97	2.15
-2	8	14	12.03	1.61
1	-8	-14	24.15	2.34
-1	8	14	25.03	1.90
0	-8	-14	38.80	2.65
0	8	14	31.74	2.27

-1	-8	-14	40.17	2.64
1	8	14	32.44	2.30
-2	-8	-14	16.15	1.90
2	8	14	13.76	1.56
-3	-8	-14	25.46	2.13
3	8	14	22.73	1.87
3	8	14	23.56	3.38
-4	-8	-14	15.59	2.30
-4	-8	-14	15.79	1.86
-4	-8	-14	12.59	2.31
4	8	14	16.30	1.63
4	8	14	20.09	3.19
-5	-8	-14	25.59	2.20
-5	-8	-14	26.16	2.76
5	8	14	30.49	3.42
5	8	14	24.93	2.02
-6	-8	-14	22.70	2.59
-6	-8	-14	18.00	1.99
-6	-8	-14	14.51	2.86
6	8	14	18.12	1.84
-7	-8	-14	20.16	2.95
-7	-8	-14	22.68	2.78
-7	-8	-14	16.50	2.04
7	8	14	18.08	1.90
7	8	14	25.03	3.57
7	8	14	25.35	3.39
-8	-8	-14	7.99	2.73
-8	-8	-14	8.99	1.71
-8	-8	-14	6.37	1.23
8	8	14	9.12	2.16
8	8	14	11.79	2.20
-9	-8	-14	18.59	3.27
-9	-8	-14	23.32	3.16
-9	-8	-14	16.10	2.16
-10	-8	-14	9.69	3.45
-10	-8	-14	8.85	2.03
-10	-8	-14	6.79	1.42
10	8	14	8.84	2.60
10	8	14	10.23	3.75
-11	-8	-14	6.35	1.41
-11	-8	-14	7.80	2.29
-11	-8	-14	3.63	3.82
11	8	14	9.25	3.01
11	8	14	8.66	2.79

-12	-8	-14	-1.47	2.49
-12	-8	-14	0.71	4.39
-12	-8	-14	1.00	1.44
12	8	14	2.91	3.04
12	8	14	2.53	3.19
-4	9	14	23.24	2.20
-3	9	14	25.95	2.17
2	-9	-14	23.79	2.54
-2	9	14	23.29	2.04
1	-9	-14	35.73	2.69
-1	9	14	30.08	2.29
0	-9	-14	35.96	2.68
0	9	14	31.67	2.30
-1	-9	-14	42.07	2.92
1	9	14	42.21	2.62
-2	-9	-14	40.58	2.77
2	9	14	35.09	2.42
-3	-9	-14	38.83	2.74
3	9	14	38.63	3.86
3	9	14	35.74	2.43
-4	-9	-14	24.20	2.55
-4	-9	-14	23.75	2.26
4	9	14	24.68	2.01
4	9	14	27.74	3.26
-5	-9	-14	16.59	2.62
-5	-9	-14	20.68	2.59
-5	-9	-14	19.54	2.09
5	9	14	22.29	3.17
5	9	14	25.24	3.48
5	9	14	21.88	1.87
-6	-9	-14	20.12	2.55
-6	-9	-14	12.98	2.61
-6	-9	-14	17.27	2.04
6	9	14	16.12	3.16
6	9	14	20.09	3.45
6	9	14	16.14	1.77
-7	-9	-14	18.19	2.83
-7	-9	-14	18.68	2.64
-7	-9	-14	17.81	2.09
7	9	14	17.45	2.20
7	9	14	18.32	3.33
7	9	14	27.08	3.67
-8	-9	-14	17.56	2.83
-8	-9	-14	15.05	2.84

-8	-9	-14	15.99	2.09
8	9	14	21.36	3.74
8	9	14	15.01	2.45
-9	-9	-14	12.43	2.07
-9	-9	-14	14.89	3.12
-9	-9	-14	17.52	3.10
9	9	14	12.51	2.52
9	9	14	13.09	2.57
-10	-9	-14	10.79	2.08
-10	-9	-14	8.77	3.32
-10	-9	-14	11.08	1.54
10	9	14	9.84	2.62
10	9	14	11.40	2.69
-11	-9	-14	14.45	3.84
-11	-9	-14	10.32	1.53
-11	-9	-14	10.64	2.35
11	9	14	14.19	2.97
11	9	14	12.51	3.12
-12	-9	-14	6.69	2.67
-12	-9	-14	10.13	1.68
-12	-9	-14	8.77	4.19
12	9	14	6.70	3.36
12	9	14	7.98	3.35
1	-10	-14	20.46	2.46
-1	10	14	17.25	1.98
0	-10	-14	25.29	2.44
0	10	14	20.67	2.03
-1	-10	-14	18.75	2.21
1	10	14	14.34	1.79
-2	-10	-14	30.05	2.46
2	10	14	25.31	2.09
2	10	14	26.75	3.21
-3	-10	-14	19.65	2.24
3	10	14	15.05	2.76
3	10	14	19.71	1.84
-4	-10	-14	25.98	2.33
4	10	14	26.42	3.30
4	10	14	24.38	2.05
4	10	14	24.48	3.29
-5	-10	-14	13.86	1.95
5	10	14	8.68	1.95
5	10	14	13.95	2.10
5	10	14	11.03	1.67
-6	-10	-14	19.00	2.16

-6	-10	-14	20.78	2.61
6	10	14	16.04	2.30
6	10	14	20.82	3.47
-7	-10	-14	12.54	1.49
-7	-10	-14	15.88	2.64
7	10	14	12.18	2.22
7	10	14	13.49	2.29
-8	-10	-14	17.12	2.15
-8	-10	-14	16.35	2.94
-8	-10	-14	11.70	2.86
8	10	14	15.56	2.57
8	10	14	16.37	2.48
-9	-10	-14	8.03	2.96
-9	-10	-14	7.20	1.38
-9	-10	-14	8.47	1.95
9	10	14	10.59	2.53
9	10	14	9.04	2.46
-10	-10	-14	7.06	2.27
-10	-10	-14	7.68	1.42
-10	-10	-14	5.91	3.32
10	10	14	5.88	2.72
10	10	14	11.30	2.68
-11	-10	-14	3.34	1.34
-11	-10	-14	4.54	3.62
-11	-10	-14	4.24	2.40
11	10	14	7.71	3.49
11	10	14	6.41	2.93
0	-11	-14	27.00	2.73
-1	-11	-14	20.99	2.49
-2	-11	-14	28.56	2.68
-3	-11	-14	25.82	2.54
-4	-11	-14	24.89	2.47
4	11	14	22.89	3.40
-5	-11	-14	27.77	2.56
5	11	14	31.65	3.72
-6	-11	-14	22.93	2.31
-6	-11	-14	14.86	2.57
6	11	14	26.60	3.70
6	11	14	20.72	3.28
-7	-11	-14	20.08	2.29
7	11	14	23.67	3.89
7	11	14	17.04	2.45
-8	-11	-14	16.71	2.26
8	11	14	13.78	2.50

8	11	14	14.90	2.61
-9	-11	-14	13.93	2.35
9	11	14	18.21	3.02
9	11	14	25.42	4.40
-10	-11	-14	3.11	2.99
-10	-11	-14	8.74	1.44
10	11	14	9.86	2.83
-11	-11	-14	5.14	1.50
11	11	14	5.48	3.02
-1	-12	-14	22.19	2.64
-2	-12	-14	7.60	1.37
-3	-12	-14	11.54	1.53
4	12	14	10.49	2.33
-5	-12	-14	31.08	2.75
5	12	14	33.79	3.90
5	12	14	29.02	3.72
-6	-12	-14	21.27	2.50
6	12	14	26.57	3.98
-7	-12	-14	24.63	2.57
7	12	14	28.61	4.39
-8	-12	-14	9.37	1.49
8	12	14	8.16	2.53
-9	-12	-14	10.00	1.45
9	12	14	5.46	2.67
-10	-12	-14	4.81	1.43
10	12	14	1.82	2.86
-2	-13	-14	16.85	2.51
-3	-13	-14	6.37	1.32
-4	-13	-14	21.29	2.53
-5	-13	-14	41.88	3.21
5	13	14	41.24	4.34
-6	-13	-14	34.95	2.89
6	13	14	31.84	4.39
-7	-13	-14	16.25	2.39
7	13	14	18.40	2.83
-8	-13	-14	14.78	2.50
8	13	14	18.12	3.00
-9	-13	-14	11.12	1.59
9	13	14	14.55	3.13
-5	-14	-14	8.99	1.51
5	14	14	6.18	2.11
-6	-14	-14	9.10	1.49
6	14	14	8.73	2.38
3	5	-15	11.12	1.55

-3	-5	15	6.60	1.81
-3	-5	15	7.99	2.62
-3	-5	15	12.80	2.33
2	5	-15	16.72	1.92
2	5	-15	16.39	4.29
-2	-5	15	14.00	2.74
-2	-5	15	16.23	3.31
-2	-5	15	13.61	1.90
1	5	-15	14.34	1.91
1	5	-15	14.47	3.02
-1	-5	15	13.34	3.64
-1	-5	15	10.11	2.55
-1	-5	15	12.57	1.92
-1	-5	15	10.91	2.05
0	5	-15	25.43	4.52
0	5	-15	20.64	2.22
0	5	-15	15.07	3.17
0	-5	15	17.80	3.51
0	-5	15	16.06	3.42
-5	-4	15	4.97	1.97
-5	-4	15	6.67	2.71
-4	-4	15	6.72	1.91
-4	-4	15	7.46	2.46
3	4	-15	2.74	2.75
3	4	-15	5.80	0.97
-3	-4	15	5.16	1.74
-3	-4	15	7.49	1.37
-3	-4	15	4.05	2.27
2	4	-15	12.95	1.58
2	4	-15	9.42	2.80
2	4	-15	15.02	3.36
-2	-4	15	10.22	2.39
-2	-4	15	16.93	3.26
-2	-4	15	14.27	2.97
-2	-4	15	17.15	2.47
1	4	-15	10.24	2.64
1	4	-15	11.20	2.61
1	4	-15	8.19	1.14
-1	-4	15	9.68	2.28
-1	-4	15	8.96	1.55
-1	-4	15	8.95	1.81
-1	-4	15	9.51	3.14
0	4	-15	9.24	1.18
0	4	-15	9.46	2.70

0	4	-15	6.82	2.38
0	-4	15	9.07	1.67
0	-4	15	9.42	1.89
0	-4	15	6.86	3.07
-1	4	-15	4.82	2.71
-1	4	-15	5.16	2.33
-1	4	-15	6.46	1.14
1	-4	15	2.75	2.68
1	-4	15	5.53	1.90
1	-4	15	5.10	2.17
-2	4	-15	11.10	1.35
-2	4	-15	8.52	2.93
-2	4	-15	4.24	2.35
2	-4	15	7.92	2.30
2	-4	15	4.41	2.80
2	-4	15	8.43	2.15
-3	4	-15	5.13	1.22
-3	4	-15	3.19	2.80
3	-4	15	4.80	2.30
3	-4	15	5.12	2.17
3	-4	15	3.22	3.09
-6	-3	15	11.35	2.75
-6	-3	15	9.91	2.22
-5	-3	15	20.61	3.89
-5	-3	15	10.88	2.08
-4	-3	15	19.69	3.37
-4	-3	15	20.11	3.72
3	3	-15	13.82	1.52
-3	-3	15	17.74	3.46
-3	-3	15	16.39	2.20
-3	-3	15	18.46	3.22
-3	-3	15	17.68	3.32
2	3	-15	14.75	1.55
2	3	-15	14.30	2.59
2	3	-15	13.43	2.51
-2	-3	15	14.32	2.19
-2	-3	15	18.31	3.04
-2	-3	15	14.31	2.30
-2	-3	15	15.21	3.07
1	3	-15	16.60	1.64
1	3	-15	13.55	2.66
1	3	-15	22.75	3.70
-1	-3	15	17.27	3.26
-1	-3	15	17.95	2.33

-1	-3	15	11.93	2.94
-1	-3	15	19.17	3.06
0	3	-15	18.13	3.41
0	3	-15	19.71	3.89
0	3	-15	20.50	1.91
0	-3	15	15.23	2.59
0	-3	15	17.35	2.83
0	-3	15	23.76	3.28
0	-3	15	25.67	2.54
-1	3	-15	25.46	4.08
-1	3	-15	22.95	3.71
-1	3	-15	28.54	2.23
1	-3	15	22.03	4.24
1	-3	15	33.33	2.93
1	-3	15	25.06	3.59
1	-3	15	32.19	3.56
-2	3	-15	22.76	3.61
-2	3	-15	17.17	2.87
-2	3	-15	23.71	2.11
2	-3	15	22.62	3.60
2	-3	15	21.49	4.13
2	-3	15	27.79	3.54
-3	3	-15	25.37	3.79
-3	3	-15	22.93	4.23
-3	3	-15	22.15	2.29
3	-3	15	30.76	4.01
3	-3	15	24.02	4.03
-4	3	-15	10.14	2.56
-4	3	-15	10.17	2.72
-4	3	-15	14.38	2.07
4	-3	15	8.99	2.96
4	-3	15	13.28	2.52
4	-3	15	14.22	2.46
-6	-2	15	9.95	2.27
-6	-2	15	9.27	2.49
-5	-2	15	12.34	2.40
-5	-2	15	20.34	3.44
-4	-2	15	16.43	3.34
-4	-2	15	18.81	3.38
3	2	-15	15.18	1.52
-3	-2	15	19.33	3.18
-3	-2	15	12.48	2.21
-3	-2	15	16.46	3.12
2	2	-15	6.79	2.11

2	2	-15	10.03	2.27
2	2	-15	6.42	1.06
-2	-2	15	5.55	2.70
-2	-2	15	10.49	1.99
-2	-2	15	10.37	1.71
1	2	-15	10.02	2.17
1	2	-15	11.08	1.41
1	2	-15	9.71	2.12
-1	-2	15	7.20	2.47
-1	-2	15	10.19	1.66
-1	-2	15	12.00	1.95
-1	-2	15	9.75	1.86
0	2	-15	10.66	1.46
0	2	-15	9.34	2.28
0	2	-15	8.94	2.07
0	-2	15	15.45	2.83
0	-2	15	8.37	1.88
0	-2	15	14.19	2.04
-1	2	-15	17.71	1.82
-1	2	-15	18.38	3.15
-1	2	-15	19.54	3.59
1	-2	15	21.85	3.31
1	-2	15	14.97	2.74
1	-2	15	25.01	3.17
1	-2	15	25.29	2.37
-2	2	-15	19.43	1.99
-2	2	-15	22.55	3.67
-2	2	-15	20.28	3.27
2	-2	15	22.70	3.26
2	-2	15	18.76	3.70
2	-2	15	22.08	2.53
2	-2	15	25.44	3.39
-3	2	-15	18.24	2.67
-3	2	-15	16.16	2.38
3	-2	15	23.02	3.55
3	-2	15	16.39	2.68
3	-2	15	16.81	2.42
-4	2	-15	11.97	1.86
-4	2	-15	8.43	2.46
-4	2	-15	8.19	2.26
4	-2	15	12.18	2.55
4	-2	15	11.93	2.35
4	-2	15	13.41	2.36
-5	2	-15	8.92	2.56

-5	2	-15	11.57	2.79
-5	2	-15	14.23	1.97
5	-2	15	12.14	2.78
5	-2	15	16.26	2.77
5	-2	15	11.86	2.36
-6	2	-15	6.77	3.09
-6	2	-15	7.15	1.30
6	-2	15	7.01	2.41
-7	-1	15	12.42	2.52
-7	-1	15	9.02	2.44
-6	-1	15	20.90	3.87
-6	-1	15	13.10	2.38
-5	-1	15	22.40	3.62
-5	-1	15	21.23	3.42
-4	-1	15	28.67	3.45
-4	-1	15	24.66	3.35
3	1	-15	22.47	1.81
-3	-1	15	22.11	3.22
-3	-1	15	24.39	3.17
-3	-1	15	20.67	3.22
2	1	-15	22.17	1.79
2	1	-15	28.65	3.48
2	1	-15	22.05	3.40
-2	-1	15	26.00	3.16
-2	-1	15	22.29	3.11
-2	-1	15	23.61	2.03
-2	-1	15	24.00	3.79
1	1	-15	17.65	1.67
1	1	-15	16.86	3.02
1	1	-15	12.01	2.18
-1	-1	15	13.59	2.51
-1	-1	15	12.87	1.96
-1	-1	15	18.76	2.81
-1	-1	15	16.61	1.82
0	1	-15	20.19	3.25
0	1	-15	21.26	2.97
0	1	-15	21.66	1.77
0	-1	15	25.50	3.06
0	-1	15	24.86	2.93
0	-1	15	25.35	2.14
0	-1	15	18.47	3.36
-1	1	-15	22.39	3.00
-1	1	-15	21.84	1.76
-1	1	-15	21.12	3.32

1	-1	15	15.82	2.53
1	-1	15	22.84	2.11
1	-1	15	22.13	3.05
-2	1	-15	22.28	1.85
-2	1	-15	22.47	3.22
-2	1	-15	22.03	3.53
2	-1	15	24.56	2.24
2	-1	15	27.46	3.20
2	-1	15	18.34	3.41
2	-1	15	21.38	3.15
-3	1	-15	18.77	3.47
-3	1	-15	22.23	1.95
-3	1	-15	21.13	3.30
3	-1	15	19.86	2.36
3	-1	15	20.27	3.57
3	-1	15	24.31	3.29
3	-1	15	24.31	3.33
-4	1	-15	29.98	2.35
-4	1	-15	30.31	3.98
-4	1	-15	22.22	3.75
4	-1	15	29.19	3.92
4	-1	15	28.91	4.06
4	-1	15	39.22	3.89
-5	1	-15	12.54	2.62
-5	1	-15	21.84	4.07
-5	1	-15	20.39	2.02
5	-1	15	20.02	3.78
5	-1	15	16.54	2.82
5	-1	15	23.79	4.13
-6	1	-15	15.12	1.94
-6	1	-15	14.99	3.13
6	-1	15	17.75	2.92
6	-1	15	13.49	2.35
6	-1	15	8.06	2.97
-7	1	-15	9.78	1.34
-7	1	-15	11.59	3.43
7	-1	15	11.24	2.53
-7	0	15	9.33	2.52
-7	0	15	7.08	2.25
-6	0	15	10.21	2.20
-6	0	15	8.49	1.97
-5	0	15	11.41	2.20
-5	0	15	9.41	1.97
-4	0	15	17.88	3.31

-4	0	15	15.59	2.77
-4	0	15	12.46	2.03
3	0	-15	9.69	1.49
3	0	-15	9.03	1.96
-3	0	15	10.70	1.46
-3	0	15	10.38	2.26
-3	0	15	10.38	1.83
-3	0	15	17.01	2.96
2	0	-15	16.77	3.05
2	0	-15	13.01	1.51
2	0	-15	17.46	3.58
-2	0	15	17.28	2.92
-2	0	15	10.83	1.78
-2	0	15	13.68	2.29
-2	0	15	13.13	1.57
1	0	-15	13.23	2.21
1	0	-15	13.82	2.77
1	0	-15	12.13	1.88
1	0	-15	10.98	1.83
-1	0	15	12.37	1.82
-1	0	15	14.04	1.62
-1	0	15	14.46	2.67
-1	0	15	11.06	2.40
0	0	-15	12.60	1.42
0	0	-15	13.42	2.45
0	0	-15	10.95	1.85
0	0	-15	9.54	1.99
0	0	15	14.70	2.70
0	0	15	14.77	1.66
0	0	15	10.39	2.13
0	0	15	13.38	2.48
-1	0	-15	10.73	1.39
-1	0	-15	9.13	1.92
-1	0	-15	9.82	1.72
-1	0	-15	10.29	2.17
1	0	15	8.67	1.10
1	0	15	9.38	1.64
1	0	15	7.65	2.04
1	0	15	6.35	1.99
-2	0	-15	9.93	1.83
-2	0	-15	11.22	2.09
-2	0	-15	9.86	2.08
-2	0	-15	9.79	1.41
2	0	15	11.30	1.76

2	0	15	10.51	1.87
2	0	15	8.65	2.11
2	0	15	11.39	1.75
-3	0	-15	10.02	2.65
-3	0	-15	4.32	1.89
-3	0	-15	6.01	0.95
-3	0	-15	4.74	2.17
3	0	15	3.07	2.05
3	0	15	8.17	1.86
3	0	15	7.28	1.09
3	0	15	7.08	1.74
-4	0	-15	12.62	1.58
-4	0	-15	11.82	2.45
-4	0	-15	8.35	2.15
4	0	15	11.62	2.10
4	0	15	12.82	2.22
4	0	15	11.48	1.97
4	0	15	10.11	2.42
-5	0	-15	12.34	1.72
-5	0	-15	10.02	2.59
-5	0	-15	8.11	2.40
5	0	15	14.14	2.39
5	0	15	11.03	2.69
5	0	15	13.01	2.14
-6	0	-15	10.77	1.80
-6	0	-15	8.03	2.58
-6	0	-15	9.54	2.83
6	0	15	14.62	2.72
6	0	15	10.08	2.82
6	0	15	13.37	2.28
-7	0	-15	10.99	3.27
-7	0	-15	6.60	1.14
-7	0	-15	2.04	2.71
7	0	15	9.73	2.99
7	0	15	5.00	3.10
7	0	15	8.65	2.18
-8	0	-15	14.13	2.14
-8	0	-15	12.67	4.03
8	0	15	23.12	4.54
-7	1	15	8.64	1.98
-7	1	15	12.39	2.66
-6	1	15	17.25	3.78
-5	1	15	15.71	2.97
-5	1	15	22.09	3.77

-4	1	15	19.11	3.33
-4	1	15	20.23	2.92
-4	1	15	13.92	2.72
3	-1	-15	30.21	6.25
3	-1	-15	30.69	2.23
-3	1	15	37.89	3.50
-3	1	15	34.64	3.64
-3	1	15	32.81	3.21
2	-1	-15	23.02	1.79
2	-1	-15	17.81	2.93
2	-1	-15	18.24	3.21
-2	1	15	20.26	3.43
-2	1	15	24.26	2.81
-2	1	15	25.23	3.02
-2	1	15	21.06	1.77
1	-1	-15	32.58	4.78
1	-1	-15	27.85	2.03
1	-1	-15	27.35	2.99
-1	1	15	26.97	2.99
-1	1	15	26.68	3.30
-1	1	15	27.75	2.04
-1	1	15	30.65	3.07
0	-1	-15	24.10	4.10
0	-1	-15	19.12	3.43
0	-1	-15	23.04	2.83
0	-1	-15	24.13	1.94
0	1	15	22.15	1.90
0	1	15	24.69	2.88
0	1	15	23.67	3.25
0	1	15	26.67	2.84
-1	-1	-15	23.58	2.94
-1	-1	-15	30.36	3.92
-1	-1	-15	25.84	1.91
-1	-1	-15	26.34	3.39
1	1	15	27.93	2.88
1	1	15	24.09	2.98
1	1	15	21.78	3.37
1	1	15	27.43	2.05
-2	-1	-15	31.31	3.86
-2	-1	-15	26.83	3.43
-2	-1	-15	29.25	3.12
-2	-1	-15	26.34	2.02
2	1	15	33.48	3.22
2	1	15	28.84	2.21

2	1	15	29.94	3.64
-3	-1	-15	29.30	2.12
-3	-1	-15	35.88	3.95
-3	-1	-15	28.40	3.44
-3	-1	-15	30.05	3.63
3	1	15	29.87	2.32
3	1	15	35.31	3.35
3	1	15	28.31	3.87
3	1	15	30.61	3.39
-4	-1	-15	11.99	2.41
-4	-1	-15	8.71	2.14
-4	-1	-15	12.91	1.53
-4	-1	-15	17.25	3.48
4	1	15	12.47	1.81
4	1	15	11.58	2.06
4	1	15	10.80	2.43
4	1	15	15.52	2.98
-5	-1	-15	28.15	4.12
-5	-1	-15	27.60	2.14
-5	-1	-15	23.90	3.85
5	1	15	31.78	2.77
5	1	15	31.97	3.71
5	1	15	32.41	3.99
5	1	15	24.18	4.31
-6	-1	-15	14.94	2.97
-6	-1	-15	14.37	1.73
-6	-1	-15	12.54	2.58
6	1	15	11.39	2.95
6	1	15	23.27	4.10
6	1	15	13.01	2.18
-7	-1	-15	13.29	3.32
-7	-1	-15	15.28	2.86
-7	-1	-15	16.43	1.86
7	1	15	14.60	3.20
7	1	15	19.21	3.29
-8	-1	-15	8.06	2.98
-8	-1	-15	10.59	1.84
-8	-1	-15	9.76	3.84
8	1	15	10.40	2.29
8	1	15	7.81	3.61
8	1	15	16.50	3.29
-7	2	15	9.55	2.61
-6	2	15	12.38	2.48
-5	2	15	9.43	2.25

-4	2	15	10.73	2.10
3	-2	-15	5.37	3.21
3	-2	-15	11.33	1.58
-3	2	15	8.50	1.37
-3	2	15	10.66	1.96
-3	2	15	15.19	2.54
2	-2	-15	8.35	2.96
2	-2	-15	7.33	0.95
2	-2	-15	7.44	1.82
-2	2	15	6.90	1.87
-2	2	15	7.05	1.60
-2	2	15	5.86	0.85
1	-2	-15	9.60	1.68
1	-2	-15	10.13	2.65
1	-2	-15	11.85	1.52
-1	2	15	9.05	1.89
-1	2	15	13.63	2.53
-1	2	15	13.11	2.56
-1	2	15	9.83	1.44
0	-2	-15	20.58	2.27
0	-2	-15	21.31	2.63
0	-2	-15	21.65	4.04
0	2	15	25.41	2.67
0	2	15	16.35	2.34
0	2	15	19.12	1.77
0	2	15	22.34	2.80
-1	-2	-15	42.06	2.57
-1	-2	-15	33.41	4.07
-1	-2	-15	35.76	3.30
-1	-2	-15	48.91	4.44
1	2	15	41.11	2.59
1	2	15	41.13	3.50
1	2	15	38.94	3.92
1	2	15	45.43	3.29
-2	-2	-15	31.56	3.21
-2	-2	-15	40.71	3.87
-2	-2	-15	29.51	3.67
-2	-2	-15	32.66	2.20
2	2	15	33.37	3.41
2	2	15	26.05	3.80
-3	-2	-15	15.55	2.40
-3	-2	-15	16.23	1.60
-3	-2	-15	20.45	3.35
-3	-2	-15	13.41	2.03

3	2	15	19.71	2.71
3	2	15	13.41	1.72
3	2	15	13.32	2.43
3	2	15	18.18	3.37
-4	-2	-15	5.30	2.29
-4	-2	-15	4.86	1.96
-4	-2	-15	7.56	1.01
-4	-2	-15	10.84	2.05
4	2	15	7.36	1.66
4	2	15	10.41	1.60
4	2	15	8.18	1.97
4	2	15	7.85	2.33
-5	-2	-15	11.10	1.55
-5	-2	-15	10.82	2.26
-5	-2	-15	13.01	2.13
-5	-2	-15	8.39	2.55
5	2	15	9.55	2.57
5	2	15	8.81	1.87
5	2	15	12.31	1.75
5	2	15	12.46	2.22
-6	-2	-15	8.04	2.82
-6	-2	-15	8.70	2.07
-6	-2	-15	8.34	2.34
-6	-2	-15	9.01	1.54
6	2	15	5.51	2.71
6	2	15	6.50	1.84
6	2	15	9.42	2.47
-7	-2	-15	9.09	3.32
-7	-2	-15	6.19	2.54
-7	-2	-15	11.50	1.65
7	2	15	8.60	3.04
7	2	15	13.62	2.83
7	2	15	10.58	2.11
-8	-2	-15	10.19	1.24
-8	-2	-15	4.63	2.66
-8	-2	-15	8.36	3.89
8	2	15	10.98	3.27
8	2	15	10.65	3.12
8	2	15	11.13	2.27
-9	-2	-15	4.60	4.18
-9	-2	-15	11.29	1.89
-9	-2	-15	10.57	3.19
9	2	15	16.55	3.61
9	2	15	10.40	3.80

9	2	15	13.20	2.38
-6	3	15	23.04	4.29
-5	3	15	17.94	3.72
4	-3	-15	17.69	2.05
4	-3	-15	9.89	4.68
-4	3	15	21.38	3.42
3	-3	-15	12.79	3.64
3	-3	-15	19.13	1.89
-3	3	15	21.15	3.25
-3	3	15	19.24	2.68
-3	3	15	16.27	1.59
2	-3	-15	19.70	1.82
-2	3	15	15.40	1.64
-2	3	15	23.89	2.95
-2	3	15	20.23	2.67
-2	3	15	18.29	3.02
1	-3	-15	9.58	1.55
1	-3	-15	7.53	3.02
-1	3	15	13.72	2.54
-1	3	15	14.35	2.63
-1	3	15	11.81	1.96
-1	3	15	7.51	0.97
0	-3	-15	25.39	2.77
0	-3	-15	27.96	2.32
0	3	15	25.68	2.00
0	3	15	31.31	3.39
0	3	15	30.99	3.09
-1	-3	-15	43.20	2.57
-1	-3	-15	38.28	3.15
1	3	15	37.39	2.52
1	3	15	41.05	3.50
1	3	15	34.42	3.92
-2	-3	-15	81.46	5.04
-2	-3	-15	87.98	4.48
-2	-3	-15	72.90	5.69
2	3	15	77.65	5.65
2	3	15	94.18	5.10
2	3	15	84.98	5.23
2	3	15	78.82	4.45
-3	-3	-15	35.34	4.12
-3	-3	-15	38.37	3.64
-3	-3	-15	46.60	2.62
3	3	15	35.61	2.58
3	3	15	48.84	3.48

3	3	15	37.90	4.20
3	3	15	37.17	4.97
-4	-3	-15	18.50	2.78
-4	-3	-15	18.07	3.31
-4	-3	-15	23.53	1.84
4	3	15	22.85	2.91
4	3	15	27.47	3.45
4	3	15	19.23	1.87
4	3	15	23.06	3.88
-5	-3	-15	18.05	3.42
-5	-3	-15	16.06	2.88
-5	-3	-15	18.19	1.76
-5	-3	-15	21.77	3.46
5	3	15	21.61	3.68
5	3	15	18.21	2.72
5	3	15	19.01	1.88
5	3	15	14.13	3.00
-6	-3	-15	15.42	3.09
-6	-3	-15	18.15	1.82
-6	-3	-15	15.50	2.49
6	3	15	23.16	4.02
6	3	15	21.89	3.11
6	3	15	19.94	2.56
6	3	15	25.49	4.20
-7	-3	-15	6.86	1.08
-7	-3	-15	8.25	2.28
-7	-3	-15	7.05	3.22
-7	-3	-15	9.37	2.04
7	3	15	9.40	2.88
7	3	15	7.96	1.82
7	3	15	9.95	2.74
-8	-3	-15	21.00	2.05
-8	-3	-15	24.41	3.86
-8	-3	-15	15.73	4.00
8	3	15	31.11	5.08
-9	-3	-15	13.29	1.89
-9	-3	-15	11.06	2.67
9	3	15	21.42	3.62
9	3	15	20.01	3.61
-10	-3	-15	10.85	5.20
-10	-3	-15	14.30	2.13
10	3	15	21.95	4.53
-6	4	15	14.38	2.76
-5	4	15	23.42	3.91

4	-4	-15	9.86	4.21
-4	4	15	14.43	1.48
3	-4	-15	14.35	1.88
-3	4	15	10.73	1.43
2	-4	-15	13.84	1.79
-2	4	15	13.80	2.70
-2	4	15	13.79	1.49
1	-4	-15	5.16	1.66
-1	4	15	6.23	0.86
-1	4	15	6.08	1.85
-1	4	15	3.87	1.60
0	-4	-15	6.58	2.06
0	-4	-15	9.23	1.60
0	4	15	9.73	2.00
0	4	15	9.59	1.74
0	4	15	9.36	1.42
-1	-4	-15	8.02	2.37
-1	-4	-15	9.21	1.62
-1	-4	-15	9.51	1.53
1	4	15	12.00	2.18
1	4	15	8.92	1.47
1	4	15	12.26	1.86
-2	-4	-15	21.36	1.88
-2	-4	-15	20.05	2.73
-2	-4	-15	12.65	2.55
2	4	15	23.00	3.12
2	4	15	14.79	1.72
2	4	15	19.18	3.59
-3	-4	-15	19.78	2.94
-3	-4	-15	12.38	2.66
-3	-4	-15	20.87	1.87
3	4	15	18.92	3.21
3	4	15	22.62	2.67
3	4	15	17.03	1.78
3	4	15	28.78	3.88
-4	-4	-15	8.18	1.95
-4	-4	-15	10.35	1.56
-4	-4	-15	6.18	2.47
4	4	15	8.79	1.56
4	4	15	9.34	2.75
4	4	15	10.13	1.55
4	4	15	10.92	2.39
-5	-4	-15	9.96	1.59
-5	-4	-15	9.98	2.02

-5	-4	-15	7.06	2.61
5	4	15	8.09	2.44
5	4	15	8.53	1.09
5	4	15	11.01	2.41
5	4	15	8.60	1.66
-6	-4	-15	6.28	2.93
-6	-4	-15	12.93	1.63
-6	-4	-15	10.90	2.10
6	4	15	11.53	1.65
6	4	15	10.80	3.39
6	4	15	9.56	1.68
6	4	15	14.42	2.58
-7	-4	-15	9.49	3.24
-7	-4	-15	10.67	2.15
-7	-4	-15	11.45	1.70
7	4	15	9.44	2.79
7	4	15	16.36	3.06
7	4	15	14.72	2.74
-8	-4	-15	5.56	3.51
-8	-4	-15	5.99	1.09
-8	-4	-15	6.03	2.15
8	4	15	7.73	2.99
8	4	15	11.74	3.02
8	4	15	7.89	1.97
-9	-4	-15	5.98	1.17
-9	-4	-15	6.20	4.22
-9	-4	-15	0.82	2.20
9	4	15	9.16	2.01
9	4	15	12.50	3.33
-10	-4	-15	7.05	1.31
-10	-4	-15	3.62	4.90
-10	-4	-15	3.98	2.47
10	4	15	10.05	3.70
10	4	15	10.86	2.36
-6	5	15	25.00	4.52
-5	5	15	33.43	4.45
-4	5	15	16.41	1.64
3	-5	-15	31.51	2.97
-3	5	15	28.20	2.07
2	-5	-15	31.87	2.97
-2	5	15	25.26	1.89
-2	5	15	17.26	3.04
1	-5	-15	36.20	2.47
-1	5	15	32.26	2.19

-1	5	15	29.90	3.36
0	-5	-15	32.24	2.32
0	5	15	32.11	3.90
0	5	15	31.07	3.41
0	5	15	28.24	2.11
-1	-5	-15	30.98	2.86
-1	-5	-15	31.84	2.37
1	5	15	35.43	3.80
1	5	15	35.25	3.47
1	5	15	29.85	2.20
-2	-5	-15	16.53	2.64
-2	-5	-15	8.70	3.71
-2	-5	-15	13.88	1.81
2	5	15	19.98	3.17
2	5	15	13.14	1.59
2	5	15	15.37	2.40
-3	-5	-15	29.87	2.27
-3	-5	-15	28.53	3.21
-3	-5	-15	20.87	3.22
3	5	15	27.95	2.12
3	5	15	33.53	4.10
3	5	15	33.51	3.65
3	5	15	32.06	2.96
-4	-5	-15	27.17	2.09
-4	-5	-15	17.95	2.95
-4	-5	-15	23.29	3.20
4	5	15	26.23	4.09
4	5	15	29.82	3.75
4	5	15	23.62	1.98
4	5	15	21.08	2.76
-5	-5	-15	35.52	2.43
-5	-5	-15	24.79	4.49
-5	-5	-15	34.90	3.49
5	5	15	41.50	4.42
5	5	15	32.74	2.41
5	5	15	29.03	3.15
5	5	15	37.56	4.16
-6	-5	-15	27.20	4.85
-6	-5	-15	25.26	3.24
-6	-5	-15	25.23	2.17
6	5	15	31.16	4.27
6	5	15	27.23	2.97
6	5	15	28.15	2.20
-7	-5	-15	39.43	3.73

-7	-5	-15	26.75	5.39
-7	-5	-15	36.62	2.64
7	5	15	46.40	4.74
7	5	15	37.94	3.51
7	5	15	38.65	5.94
-8	-5	-15	10.78	3.80
-8	-5	-15	20.05	3.31
-8	-5	-15	17.30	1.96
8	5	15	23.24	3.39
8	5	15	23.76	4.56
8	5	15	23.62	4.57
-9	-5	-15	12.56	1.94
-9	-5	-15	10.40	3.96
-9	-5	-15	11.97	2.33
9	5	15	20.55	3.63
-10	-5	-15	8.17	1.30
-10	-5	-15	8.72	4.48
-10	-5	-15	8.96	2.49
-5	6	15	20.86	1.91
-5	6	15	25.73	4.29
-4	6	15	13.07	1.58
3	-6	-15	18.46	2.20
-3	6	15	15.69	1.63
2	-6	-15	19.07	2.10
-2	6	15	14.05	1.62
1	-6	-15	18.93	2.00
-1	6	15	14.59	1.63
0	-6	-15	25.40	2.17
0	6	15	16.45	3.51
-1	-6	-15	26.55	2.23
1	6	15	29.39	3.89
1	6	15	25.03	1.98
1	6	15	24.25	3.47
-2	-6	-15	17.21	2.66
-2	-6	-15	13.36	1.42
2	6	15	17.43	2.27
2	6	15	11.59	1.58
2	6	15	22.49	3.80
-3	-6	-15	9.63	1.21
-3	-6	-15	7.01	1.70
3	6	15	11.67	2.23
3	6	15	11.60	2.43
3	6	15	7.97	1.05
-4	-6	-15	10.31	1.82

-4	-6	-15	10.38	1.76
4	6	15	9.03	1.09
4	6	15	12.47	2.28
4	6	15	10.69	2.36
-5	-6	-15	8.58	2.66
-5	-6	-15	9.52	1.24
-5	-6	-15	7.63	1.72
5	6	15	9.62	1.64
5	6	15	8.29	1.66
5	6	15	12.16	2.41
5	6	15	15.02	2.49
-6	-6	-15	18.14	3.10
-6	-6	-15	23.25	2.96
-6	-6	-15	23.38	2.10
6	6	15	25.90	3.97
6	6	15	20.27	2.70
6	6	15	31.04	4.12
6	6	15	17.99	1.99
-7	-6	-15	24.94	2.33
-7	-6	-15	24.16	3.60
-7	-6	-15	28.18	3.25
7	6	15	31.58	4.24
7	6	15	36.72	5.71
7	6	15	31.69	3.13
-8	-6	-15	22.25	2.20
-8	-6	-15	26.38	3.43
-8	-6	-15	17.72	3.81
8	6	15	26.83	4.34
8	6	15	32.06	3.42
-9	-6	-15	10.96	1.42
-9	-6	-15	10.29	4.01
-9	-6	-15	9.65	2.23
9	6	15	12.14	3.02
9	6	15	12.36	2.06
-10	-6	-15	10.03	2.41
-10	-6	-15	9.67	4.30
-10	-6	-15	13.25	2.08
10	6	15	9.81	3.36
10	6	15	9.07	3.25
-11	-6	-15	10.23	1.48
-11	-6	-15	6.04	6.77
-11	-6	-15	7.83	2.57
11	6	15	9.06	3.59
11	6	15	4.94	3.27

-4	7	15	25.35	2.07
3	-7	-15	24.49	2.49
-3	7	15	20.18	1.90
2	-7	-15	28.49	2.48
-2	7	15	20.91	1.95
1	-7	-15	31.96	2.54
-1	7	15	24.85	2.06
0	7	15	41.70	4.07
0	7	15	44.12	2.68
-1	-7	-15	43.92	2.90
1	7	15	38.03	2.57
-2	-7	-15	32.62	2.45
2	7	15	28.58	3.89
2	7	15	29.74	2.22
2	7	15	34.56	4.16
-3	-7	-15	16.08	1.94
3	7	15	14.25	1.72
3	7	15	22.49	3.71
3	7	15	16.37	2.49
-4	-7	-15	23.98	3.00
-4	-7	-15	23.23	2.21
4	7	15	25.43	3.73
4	7	15	30.42	3.96
4	7	15	22.22	1.98
-5	-7	-15	20.06	3.87
-5	-7	-15	19.36	2.10
-5	-7	-15	21.30	2.80
5	7	15	18.35	1.93
5	7	15	21.42	3.65
-6	-7	-15	26.59	2.32
-6	-7	-15	29.99	3.09
-6	-7	-15	21.98	3.27
6	7	15	25.98	3.90
6	7	15	26.22	2.26
-7	-7	-15	28.91	3.21
-7	-7	-15	23.69	2.37
-7	-7	-15	23.92	3.49
7	7	15	29.40	4.04
-8	-7	-15	29.87	3.48
-8	-7	-15	29.68	5.41
-8	-7	-15	28.82	2.43
8	7	15	26.46	4.61
-9	-7	-15	16.75	2.15
-9	-7	-15	10.97	2.17

-9	-7	-15	12.31	3.78
9	7	15	15.02	2.93
9	7	15	15.77	3.09
-10	-7	-15	25.14	3.82
-10	-7	-15	22.35	2.36
-10	-7	-15	12.53	4.43
10	7	15	18.94	3.40
-11	-7	-15	5.17	1.40
-11	-7	-15	7.03	2.58
-11	-7	-15	3.92	4.66
11	7	15	11.40	4.07
11	7	15	8.31	3.44
-4	8	15	4.92	1.01
-3	8	15	14.57	1.79
-2	8	15	16.66	1.77
-1	8	15	11.74	1.63
0	-8	-15	34.03	2.65
0	8	15	28.36	2.22
-1	-8	-15	34.77	2.65
1	8	15	38.25	4.47
1	8	15	29.11	2.31
-2	-8	-15	19.02	2.19
2	8	15	17.57	1.77
-3	-8	-15	18.87	2.93
-3	-8	-15	17.81	2.12
3	8	15	22.35	3.69
3	8	15	15.31	2.40
3	8	15	16.52	1.78
-4	-8	-15	9.13	1.31
-4	-8	-15	10.37	1.72
-4	-8	-15	10.35	2.42
4	8	15	9.54	1.68
4	8	15	12.91	2.28
-5	-8	-15	9.75	1.75
-5	-8	-15	10.90	1.92
5	8	15	10.20	1.75
5	8	15	14.14	2.33
-6	-8	-15	11.42	1.40
-6	-8	-15	9.07	3.10
-6	-8	-15	11.39	1.84
6	8	15	13.56	2.41
-7	-8	-15	23.14	3.08
-7	-8	-15	16.48	3.19
-7	-8	-15	16.15	2.16

7	8	15	16.88	2.65
-8	-8	-15	10.38	1.99
-8	-8	-15	10.24	1.43
-8	-8	-15	13.29	3.32
8	8	15	10.23	2.62
8	8	15	14.69	2.75
-9	-8	-15	13.67	3.79
-9	-8	-15	16.22	2.30
-9	-8	-15	18.38	3.48
9	8	15	16.93	2.92
9	8	15	26.58	4.50
-10	-8	-15	6.58	4.06
-10	-8	-15	6.75	1.40
-10	-8	-15	4.06	3.87
10	8	15	10.90	3.00
10	8	15	6.25	3.12
-11	-8	-15	4.90	1.43
-11	-8	-15	2.55	2.53
-11	-8	-15	0.12	4.41
11	8	15	3.46	3.52
11	8	15	8.19	3.28
-3	9	15	21.69	2.34
2	-9	-15	17.61	2.62
-2	9	15	16.14	1.91
1	-9	-15	38.28	2.96
-1	9	15	27.78	2.35
0	-9	-15	27.85	2.60
0	9	15	21.53	2.06
-1	-9	-15	36.74	2.84
1	9	15	31.28	2.39
-2	-9	-15	27.07	2.43
2	9	15	19.57	2.01
-3	-9	-15	31.41	2.59
3	9	15	27.37	2.24
-4	-9	-15	30.71	2.56
4	9	15	29.33	2.44
4	9	15	27.84	3.77
-5	-9	-15	20.85	2.31
5	9	15	20.33	3.63
-6	-9	-15	16.80	2.19
-6	-9	-15	19.13	3.21
-6	-9	-15	10.14	2.92
6	9	15	19.86	3.65
6	9	15	17.62	2.66

-7	-9	-15	26.01	2.56
-7	-9	-15	24.87	4.67
-7	-9	-15	31.82	3.49
7	9	15	34.52	4.42
7	9	15	34.86	4.07
-8	-9	-15	18.31	3.27
-8	-9	-15	12.57	2.25
-8	-9	-15	17.16	3.21
8	9	15	25.38	4.33
8	9	15	19.94	3.90
-9	-9	-15	11.75	2.30
-9	-9	-15	13.52	3.47
-9	-9	-15	10.80	1.55
9	9	15	18.11	2.98
-10	-9	-15	15.90	3.97
-10	-9	-15	13.01	2.50
-10	-9	-15	10.90	1.62
10	9	15	14.11	3.84
10	9	15	17.08	3.15
-11	-9	-15	9.84	1.62
-11	-9	-15	13.01	3.08
-11	-9	-15	10.90	6.43
11	9	15	19.44	3.57
1	-10	-15	23.37	2.76
0	-10	-15	26.11	2.80
-1	-10	-15	20.78	2.58
-2	-10	-15	19.41	2.50
-3	-10	-15	15.81	2.35
3	10	15	13.31	2.21
-4	-10	-15	20.84	2.38
4	10	15	20.65	3.63
4	10	15	16.07	2.53
-5	-10	-15	13.28	2.21
5	10	15	13.38	2.40
5	10	15	12.54	2.22
-6	-10	-15	15.86	2.26
6	10	15	19.13	3.53
6	10	15	17.39	2.60
-7	-10	-15	9.91	1.46
7	10	15	12.22	2.40
7	10	15	16.01	2.68
-8	-10	-15	16.95	2.36
8	10	15	18.91	3.19
8	10	15	15.87	2.90

-9	-10	-15	9.39	1.46
-9	-10	-15	1.50	3.01
9	10	15	15.01	2.96
-10	-10	-15	7.11	1.48
10	10	15	14.05	3.17
0	-11	-15	32.77	3.06
-1	-11	-15	29.78	2.90
-2	-11	-15	41.71	3.27
-3	-11	-15	22.17	2.56
-4	-11	-15	24.38	2.60
4	11	15	21.82	3.73
4	11	15	18.05	3.39
-5	-11	-15	25.51	2.61
5	11	15	24.08	4.03
-6	-11	-15	34.57	2.97
6	11	15	35.58	4.49
-7	-11	-15	34.72	2.96
7	11	15	37.52	4.70
-8	-11	-15	22.95	2.62
8	11	15	33.80	4.82
-9	-11	-15	19.53	2.65
9	11	15	27.80	4.99
-10	-11	-15	15.36	2.63
10	11	15	28.15	5.35
-1	-12	-15	21.56	2.74
-2	-12	-15	21.65	2.71
-3	-12	-15	17.95	2.54
-4	-12	-15	15.76	2.46
4	12	15	12.65	2.24
-5	-12	-15	30.70	2.80
5	12	15	28.07	4.12
-6	-12	-15	18.35	2.53
6	12	15	17.05	2.72
-7	-12	-15	18.20	2.51
7	12	15	24.59	4.62
-8	-12	-15	9.21	1.60
8	12	15	12.58	2.88
-9	-12	-15	10.95	1.69
9	12	15	12.59	3.07
-4	-13	-15	23.24	2.88
-5	-13	-15	38.81	3.28
-6	-13	-15	30.30	2.99
-7	-13	-15	18.76	2.69
7	13	15	19.72	3.07

3	3	-16	4.93	0.89
-3	-3	16	4.51	2.49
-3	-3	16	5.43	1.81
2	3	-16	4.66	0.93
-2	-3	16	4.35	2.28
-2	-3	16	4.02	1.69
-2	-3	16	4.37	1.33
1	3	-16	1.88	0.89
-1	-3	16	2.92	2.21
-1	-3	16	1.84	1.29
-1	-3	16	1.78	1.67
0	3	-16	7.01	2.67
0	3	-16	3.22	0.97
0	3	-16	6.06	3.77
0	-3	16	2.59	1.41
0	-3	16	2.84	1.77
0	-3	16	4.23	2.15
-1	3	-16	8.16	2.62
-1	3	-16	6.09	1.17
-1	3	-16	5.92	2.47
1	-3	16	4.22	3.07
1	-3	16	7.07	2.25
1	-3	16	6.62	1.92
-2	3	-16	8.23	2.49
-2	3	-16	10.83	2.82
-2	3	-16	12.38	2.08
2	-3	16	11.20	2.21
2	-3	16	7.16	3.00
-4	-2	16	6.05	2.01
-4	-2	16	7.86	2.29
3	2	-16	8.59	1.40
-3	-2	16	5.24	2.20
-3	-2	16	6.75	1.24
-3	-2	16	8.37	1.96
2	2	-16	8.56	1.49
-2	-2	16	8.66	1.31
-2	-2	16	5.46	1.74
-2	-2	16	7.70	2.16
1	2	-16	6.39	0.97
-1	-2	16	6.54	1.69
-1	-2	16	7.54	2.01
-1	-2	16	6.01	1.26
0	2	-16	4.14	2.49
0	2	-16	4.78	2.36

0	2	-16	3.35	0.91
0	-2	16	3.61	2.01
0	-2	16	1.07	1.33
0	-2	16	3.11	1.62
0	-2	16	2.54	2.63
-1	2	-16	3.50	2.15
-1	2	-16	9.90	1.65
-1	2	-16	8.49	2.44
1	-2	16	6.44	1.81
1	-2	16	9.32	2.09
1	-2	16	5.51	2.73
1	-2	16	5.52	1.37
-2	2	-16	5.14	2.45
-2	2	-16	5.44	2.16
-2	2	-16	4.86	1.09
2	-2	16	6.16	1.94
2	-2	16	4.27	2.62
2	-2	16	3.39	2.17
-3	2	-16	8.24	2.64
-3	2	-16	5.57	1.21
-3	2	-16	6.23	2.33
3	-2	16	8.63	2.18
3	-2	16	7.67	2.78
3	-2	16	8.57	2.41
-4	2	-16	3.53	2.46
-4	2	-16	5.91	1.21
-4	2	-16	7.05	4.10
4	-2	16	0.32	2.80
4	-2	16	4.85	2.24
4	-2	16	4.16	2.39
-5	-1	16	9.40	2.10
-5	-1	16	8.53	2.25
-4	-1	16	7.97	1.95
-4	-1	16	8.81	2.23
3	1	-16	11.46	1.60
-3	-1	16	10.71	2.17
-3	-1	16	9.21	1.88
-3	-1	16	11.26	1.88
2	1	-16	12.78	1.63
-2	-1	16	16.87	3.04
-2	-1	16	12.15	2.17
-2	-1	16	12.49	1.98
1	1	-16	7.59	0.98
-1	-1	16	7.46	1.18

-1	-1	16	8.67	2.44
-1	-1	16	9.02	1.70
-1	-1	16	7.38	1.88
0	1	-16	7.62	2.12
0	1	-16	10.34	1.54
0	1	-16	8.77	2.24
0	-1	16	7.51	2.02
0	-1	16	7.50	1.25
0	-1	16	9.28	1.79
0	-1	16	9.04	2.44
-1	1	-16	10.01	1.61
-1	1	-16	9.84	2.41
-1	1	-16	9.69	2.18
1	-1	16	11.54	1.92
1	-1	16	9.87	2.40
1	-1	16	10.72	1.42
1	-1	16	12.87	2.06
-2	1	-16	14.25	1.70
-2	1	-16	11.62	2.43
-2	1	-16	12.24	2.12
2	-1	16	16.18	3.17
2	-1	16	10.58	2.13
2	-1	16	10.59	1.54
2	-1	16	8.81	2.66
-3	1	-16	9.90	1.71
-3	1	-16	8.48	2.56
-3	1	-16	6.71	2.21
3	-1	16	10.44	2.03
3	-1	16	8.30	2.69
-4	1	-16	6.88	2.78
-4	1	-16	6.41	2.34
-4	1	-16	11.21	1.85
4	-1	16	11.10	2.48
4	-1	16	19.27	3.70
4	-1	16	13.33	2.72
-5	1	-16	8.52	2.67
-5	1	-16	12.47	1.99
-5	1	-16	16.67	3.72
5	-1	16	10.80	2.95
5	-1	16	14.03	2.49
5	-1	16	17.43	2.85
-5	0	16	4.42	2.00
-5	0	16	6.23	2.08
-4	0	16	11.17	2.05

-4	0	16	10.27	1.95
3	0	-16	6.44	0.95
-3	0	16	7.44	1.01
-3	0	16	6.62	1.95
-3	0	16	7.55	1.79
2	0	-16	10.90	1.81
-2	0	16	12.92	1.73
-2	0	16	10.24	1.97
-2	0	16	14.12	2.97
-2	0	16	10.15	2.48
1	0	-16	2.46	1.98
1	0	-16	7.37	0.98
-1	0	16	7.75	1.61
-1	0	16	5.80	1.02
-1	0	16	6.21	2.15
-1	0	16	7.55	1.82
0	0	-16	6.32	2.04
0	0	-16	5.35	1.88
0	0	-16	5.63	0.93
0	0	16	5.28	1.57
0	0	16	7.35	1.78
0	0	16	5.88	1.05
0	0	16	5.93	2.37
-1	0	-16	6.99	0.98
-1	0	-16	4.61	1.87
-1	0	-16	8.87	2.13
1	0	16	5.91	1.12
1	0	16	5.43	2.23
1	0	16	6.52	1.92
1	0	16	4.78	1.57
-2	0	-16	10.89	1.61
-2	0	-16	8.79	2.35
-2	0	-16	7.75	2.03
2	0	16	11.26	2.61
2	0	16	10.31	1.80
2	0	16	9.38	1.32
2	0	16	9.51	2.01
-3	0	-16	4.64	1.97
-3	0	-16	5.77	0.99
-3	0	-16	3.08	2.39
3	0	16	5.90	2.39
3	0	16	7.19	2.32
3	0	16	5.17	1.74
-4	0	-16	11.24	2.63

-4	0	-16	12.02	1.76
4	0	16	12.51	2.48
4	0	16	13.06	2.71
4	0	16	18.21	3.57
-5	0	-16	7.94	1.17
-5	0	-16	8.14	2.86
5	0	16	11.41	2.66
5	0	16	9.94	2.90
-6	0	-16	11.21	4.48
-6	0	-16	4.27	2.76
-6	0	-16	9.29	1.24
6	0	16	9.37	2.29
6	0	16	4.07	3.05
6	0	16	14.72	2.95
-5	1	16	4.70	2.05
-4	1	16	7.58	1.87
-4	1	16	7.14	1.96
-3	1	16	18.38	2.99
-3	1	16	11.62	1.65
-3	1	16	18.33	3.26
-2	1	16	15.63	2.89
-2	1	16	10.28	2.17
-2	1	16	12.35	1.88
-2	1	16	9.69	1.60
1	-1	-16	12.33	1.62
-1	1	16	9.83	2.16
-1	1	16	10.24	1.87
-1	1	16	8.72	1.07
-1	1	16	8.85	1.68
0	-1	-16	2.61	2.17
0	-1	-16	5.90	0.93
0	-1	-16	5.11	1.86
0	1	16	5.34	1.49
0	1	16	5.54	1.74
0	1	16	5.34	2.27
0	1	16	5.41	1.00
-1	-1	-16	6.87	1.02
-1	-1	-16	5.13	1.79
-1	-1	-16	2.02	2.02
1	1	16	5.54	1.03
1	1	16	5.83	1.84
1	1	16	6.24	2.17
1	1	16	7.59	1.61
-2	-1	-16	5.94	1.86

-2	-1	-16	7.62	2.24
-2	-1	-16	8.54	1.50
2	1	16	8.91	1.76
2	1	16	12.58	2.33
2	1	16	8.21	1.14
2	1	16	9.35	1.96
-3	-1	-16	9.87	2.15
-3	-1	-16	12.37	1.67
-3	-1	-16	11.90	2.57
3	1	16	13.54	2.21
3	1	16	16.04	3.15
3	1	16	15.42	3.71
3	1	16	15.38	2.00
-4	-1	-16	9.57	1.67
-4	-1	-16	13.36	2.71
-4	-1	-16	5.07	2.25
4	1	16	10.55	2.66
4	1	16	16.88	3.21
-5	-1	-16	9.97	2.55
-5	-1	-16	15.79	1.80
-5	-1	-16	13.58	2.91
5	1	16	14.18	3.03
5	1	16	21.78	4.06
-6	-1	-16	10.09	3.16
-6	-1	-16	10.19	1.23
-6	-1	-16	5.20	2.69
6	1	16	10.88	2.25
6	1	16	7.32	3.03
6	1	16	14.06	2.97
-7	-1	-16	6.57	2.92
-7	-1	-16	8.64	1.26
7	1	16	11.48	2.49
7	1	16	7.37	3.43
7	1	16	15.14	3.33
-5	2	16	5.98	2.21
-4	2	16	6.01	1.70
-4	2	16	4.47	1.95
3	-2	-16	14.39	1.76
-3	2	16	11.63	1.59
-3	2	16	11.35	1.88
-3	2	16	12.68	1.98
-3	2	16	6.12	2.51
-2	2	16	15.48	2.85
-2	2	16	16.15	3.10

-2	2	16	11.29	1.64
-2	2	16	12.38	2.09
1	-2	-16	9.91	1.62
-1	2	16	10.67	1.78
-1	2	16	8.91	1.98
-1	2	16	7.61	1.03
-1	2	16	9.53	1.71
0	-2	-16	11.69	1.67
0	-2	-16	11.72	2.21
0	-2	-16	10.43	2.80
0	2	16	12.32	2.18
0	2	16	13.46	1.72
0	2	16	14.49	2.71
0	2	16	10.24	1.91
-1	-2	-16	19.25	2.96
-1	-2	-16	26.07	4.21
-1	-2	-16	19.30	1.88
-1	-2	-16	16.35	2.55
1	2	16	20.25	3.15
1	2	16	18.30	3.58
1	2	16	20.08	1.93
-2	-2	-16	13.74	1.61
-2	-2	-16	10.78	2.32
-2	-2	-16	8.18	2.30
-2	-2	-16	8.68	1.88
2	2	16	9.31	1.98
2	2	16	7.93	2.37
2	2	16	9.95	1.75
2	2	16	13.69	2.80
-3	-2	-16	10.63	1.60
-3	-2	-16	10.23	2.44
-3	-2	-16	12.20	2.46
-3	-2	-16	7.66	2.02
3	2	16	10.86	2.51
3	2	16	10.43	1.82
3	2	16	10.70	2.05
3	2	16	9.04	1.22
-4	-2	-16	6.78	1.01
-4	-2	-16	6.17	2.64
-4	-2	-16	1.62	2.13
4	2	16	6.49	1.46
4	2	16	8.74	2.14
4	2	16	6.07	1.75
-5	-2	-16	13.72	1.71

-5	-2	-16	8.25	2.44
-5	-2	-16	11.80	2.96
5	2	16	9.99	2.95
5	2	16	12.09	2.10
-6	-2	-16	5.76	3.12
-6	-2	-16	4.00	1.02
-6	-2	-16	0.93	2.46
6	2	16	5.49	2.64
6	2	16	4.18	1.92
6	2	16	3.87	3.06
-7	-2	-16	6.42	3.55
-7	-2	-16	3.59	2.81
-7	-2	-16	6.20	1.11
7	2	16	8.04	3.32
7	2	16	5.50	2.11
7	2	16	9.85	3.25
-8	-2	-16	-1.04	5.04
-8	-2	-16	4.46	3.09
-8	-2	-16	2.95	1.07
8	2	16	7.33	2.32
8	2	16	6.00	3.52
8	2	16	5.94	3.64
-5	3	16	4.80	2.25
4	-3	-16	6.16	1.19
-4	3	16	3.75	2.06
3	-3	-16	9.50	1.36
-3	3	16	10.10	1.57
-3	3	16	7.83	2.50
-3	3	16	9.82	1.72
2	-3	-16	13.03	1.92
-2	3	16	11.86	1.68
-2	3	16	17.04	3.03
-2	3	16	16.80	2.94
1	-3	-16	6.90	2.10
-1	3	16	10.41	1.61
-1	3	16	9.98	1.87
-1	3	16	9.93	2.15
0	-3	-16	21.08	1.92
0	3	16	19.91	3.30
0	3	16	18.52	3.11
0	3	16	16.03	1.86
-1	-3	-16	22.05	1.99
-1	-3	-16	16.42	2.77
-1	-3	-16	16.98	2.89

1	3	16	19.92	3.16
1	3	16	18.10	1.86
1	3	16	20.22	3.53
-2	-3	-16	32.43	4.72
-2	-3	-16	38.52	2.42
-2	-3	-16	28.80	3.36
-2	-3	-16	26.85	4.22
2	3	16	38.19	3.73
2	3	16	38.38	3.39
2	3	16	31.20	2.40
2	3	16	29.13	4.16
-3	-3	-16	18.36	3.37
-3	-3	-16	20.52	3.96
-3	-3	-16	26.41	2.03
3	3	16	16.38	2.91
3	3	16	23.18	3.11
3	3	16	20.14	2.06
3	3	16	23.29	3.56
-4	-3	-16	14.66	2.48
-4	-3	-16	15.78	1.76
-4	-3	-16	14.32	2.83
4	3	16	18.83	3.00
4	3	16	11.64	1.87
4	3	16	14.84	2.84
4	3	16	19.06	3.63
-5	-3	-16	10.92	3.14
-5	-3	-16	11.55	1.68
-5	-3	-16	7.66	2.37
5	3	16	9.83	3.53
5	3	16	13.84	2.48
5	3	16	9.50	1.90
-6	-3	-16	13.46	3.36
-6	-3	-16	11.96	2.61
-6	-3	-16	17.12	1.78
6	3	16	14.43	3.11
6	3	16	15.79	3.43
-7	-3	-16	0.04	3.52
-7	-3	-16	5.50	1.09
-7	-3	-16	3.51	2.60
7	3	16	7.75	3.28
7	3	16	11.18	3.00
7	3	16	7.45	2.02
-8	-3	-16	10.71	1.88
-8	-3	-16	6.55	2.83

-8	-3	-16	11.80	4.11
8	3	16	17.96	3.66
8	3	16	18.86	4.08
8	3	16	15.75	3.68
-5	4	16	5.02	2.46
4	-4	-16	8.90	1.43
-4	4	16	9.42	1.49
-4	4	16	7.33	2.28
3	-4	-16	10.21	1.53
-3	4	16	9.24	1.50
2	-4	-16	14.86	1.95
-2	4	16	12.84	1.61
-2	4	16	11.31	2.74
1	-4	-16	8.88	1.16
-1	4	16	9.08	2.03
-1	4	16	6.88	0.98
-1	4	16	8.91	1.88
0	-4	-16	10.82	2.38
0	-4	-16	18.03	1.91
0	4	16	20.87	3.40
0	4	16	13.75	1.76
0	4	16	18.29	3.12
-1	-4	-16	9.14	1.21
-1	-4	-16	4.91	2.48
1	4	16	9.01	1.57
1	4	16	11.53	2.07
1	4	16	7.98	2.24
-2	-4	-16	10.68	1.84
-2	-4	-16	10.27	2.63
-2	-4	-16	14.20	1.81
2	4	16	13.40	2.18
2	4	16	9.95	1.69
2	4	16	12.74	2.53
2	4	16	16.74	2.66
-3	-4	-16	27.17	2.21
-3	-4	-16	16.81	3.38
3	4	16	27.77	3.03
3	4	16	27.24	4.26
3	4	16	23.51	2.14
3	4	16	29.27	3.80
-4	-4	-16	17.55	1.87
-4	-4	-16	12.61	2.22
-4	-4	-16	13.44	2.84
4	4	16	14.76	2.91

4	4	16	14.88	1.90
4	4	16	17.06	2.55
4	4	16	17.44	2.97
-5	-4	-16	4.81	2.22
-5	-4	-16	4.54	2.93
-5	-4	-16	8.43	1.18
5	4	16	7.61	2.59
5	4	16	6.82	1.84
5	4	16	8.94	2.82
-6	-4	-16	10.87	2.51
-6	-4	-16	10.51	3.46
-6	-4	-16	13.42	1.77
6	4	16	12.12	1.97
6	4	16	14.90	3.10
6	4	16	17.65	2.89
-7	-4	-16	8.76	1.21
-7	-4	-16	9.70	2.54
-7	-4	-16	9.86	3.70
7	4	16	10.41	2.01
7	4	16	15.62	3.24
-8	-4	-16	8.65	1.24
-8	-4	-16	4.12	4.00
-8	-4	-16	8.84	2.57
8	4	16	17.11	3.79
8	4	16	11.06	2.24
-9	-4	-16	4.78	1.17
-9	-4	-16	-0.11	4.53
-9	-4	-16	4.40	2.64
9	4	16	7.05	2.45
-4	5	16	4.23	0.93
3	-5	-16	16.82	2.23
-3	5	16	11.26	1.65
2	-5	-16	12.74	2.01
-2	5	16	9.18	1.54
-2	5	16	6.83	1.83
1	-5	-16	9.58	1.30
-1	5	16	9.53	1.53
-1	5	16	8.66	1.92
-1	5	16	10.34	2.95
0	-5	-16	11.15	1.85
0	-5	-16	1.97	2.30
0	5	16	7.34	1.02
0	5	16	8.31	1.99
-1	-5	-16	6.71	1.16

-1	-5	-16	3.52	2.33
1	5	16	10.26	2.30
1	5	16	5.56	0.97
1	5	16	7.42	2.00
-2	-5	-16	2.14	1.00
-2	-5	-16	0.40	3.04
-2	-5	-16	3.25	1.74
2	5	16	4.39	2.24
2	5	16	3.35	0.91
2	5	16	3.88	1.98
2	5	16	1.06	1.36
-3	-5	-16	9.02	1.91
-3	-5	-16	9.56	1.25
-3	-5	-16	4.86	3.04
3	5	16	7.49	1.14
3	5	16	9.80	1.69
3	5	16	13.42	2.34
3	5	16	9.55	2.55
-4	-5	-16	8.40	2.81
-4	-5	-16	12.92	1.83
-4	-5	-16	7.42	2.11
4	5	16	14.62	2.60
4	5	16	9.04	1.18
4	5	16	10.78	2.71
4	5	16	9.97	1.71
-5	-5	-16	18.21	1.99
-5	-5	-16	13.10	2.39
-5	-5	-16	14.74	3.04
5	5	16	24.35	4.62
5	5	16	19.14	2.85
5	5	16	14.35	3.04
-6	-5	-16	11.55	2.28
-6	-5	-16	8.81	3.34
-6	-5	-16	11.35	1.84
6	5	16	12.35	3.01
6	5	16	11.46	3.06
6	5	16	9.49	1.82
-7	-5	-16	14.85	2.50
-7	-5	-16	12.82	3.85
-7	-5	-16	19.55	2.07
7	5	16	23.19	3.48
7	5	16	18.63	3.35
7	5	16	26.30	4.91
-8	-5	-16	14.24	1.95

-8	-5	-16	10.67	2.52
-8	-5	-16	14.49	4.09
8	5	16	19.41	3.93
8	5	16	15.05	3.52
8	5	16	18.06	3.74
-9	-5	-16	12.98	1.98
-9	-5	-16	10.57	2.58
-9	-5	-16	11.36	4.51
9	5	16	11.12	3.73
4	-6	-16	15.60	2.54
-4	6	16	11.25	1.73
-4	6	16	9.52	2.48
3	-6	-16	15.04	2.44
-3	6	16	10.88	1.68
2	-6	-16	18.23	2.29
-2	6	16	11.66	1.69
1	-6	-16	17.31	2.22
-1	6	16	15.83	1.76
0	-6	-16	17.57	2.16
0	6	16	15.65	1.73
0	6	16	11.42	2.22
-1	-6	-16	21.45	2.18
1	6	16	22.21	3.69
1	6	16	19.97	3.89
1	6	16	20.19	1.89
-2	-6	-16	12.25	1.97
2	6	16	12.89	2.40
2	6	16	15.68	2.59
2	6	16	11.03	1.71
-3	-6	-16	10.58	1.34
-3	-6	-16	11.06	2.36
3	6	16	16.43	2.61
3	6	16	14.93	2.67
3	6	16	9.31	1.66
3	6	16	8.88	1.19
-4	-6	-16	3.12	1.96
-4	-6	-16	8.50	1.24
4	6	16	4.77	1.04
4	6	16	8.76	2.47
4	6	16	9.55	2.70
4	6	16	5.54	1.53
-5	-6	-16	10.19	1.33
-5	-6	-16	8.31	2.22
-5	-6	-16	5.36	2.96

5	6	16	13.31	2.86
5	6	16	13.14	2.86
5	6	16	7.47	1.71
-6	-6	-16	18.60	3.42
-6	-6	-16	14.18	3.44
-6	-6	-16	16.76	2.07
6	6	16	16.92	3.01
6	6	16	19.99	3.11
-7	-6	-16	13.16	1.96
-7	-6	-16	5.00	3.53
-7	-6	-16	11.50	2.30
7	6	16	16.58	3.12
-8	-6	-16	9.23	3.95
-8	-6	-16	11.76	2.34
-8	-6	-16	12.40	1.99
8	6	16	18.37	3.45
-9	-6	-16	13.90	2.14
-9	-6	-16	20.27	3.92
-9	-6	-16	10.44	4.38
9	6	16	18.57	3.73
9	6	16	24.05	3.95
3	-7	-16	16.13	2.55
-3	7	16	9.56	2.28
-3	7	16	9.95	1.70
-2	7	16	10.77	1.68
1	-7	-16	13.77	2.23
-1	7	16	7.69	2.11
-1	7	16	9.23	1.59
0	-7	-16	24.36	2.41
0	7	16	16.27	2.52
0	7	16	20.43	1.94
-1	-7	-16	17.67	2.28
1	7	16	15.83	3.14
1	7	16	17.54	2.63
1	7	16	14.80	1.80
-2	-7	-16	19.37	2.21
2	7	16	17.34	2.79
2	7	16	18.15	2.71
2	7	16	15.69	1.95
-3	-7	-16	10.82	1.39
-3	-7	-16	6.02	2.67
3	7	16	11.26	2.57
3	7	16	11.59	2.66
3	7	16	8.23	1.13

-4	-7	-16	13.15	2.02
4	7	16	17.69	2.97
4	7	16	10.49	1.71
4	7	16	17.68	2.70
4	7	16	14.91	2.72
-5	-7	-16	11.24	4.51
-5	-7	-16	12.89	2.03
-5	-7	-16	14.37	2.45
5	7	16	8.07	1.64
-6	-7	-16	23.34	2.35
-6	-7	-16	24.40	3.57
-6	-7	-16	20.71	3.63
6	7	16	26.59	4.55
6	7	16	23.92	3.09
6	7	16	35.58	4.94
-7	-7	-16	13.92	2.14
-7	-7	-16	13.55	2.29
-7	-7	-16	13.05	3.55
7	7	16	14.63	3.00
7	7	16	18.90	3.08
-8	-7	-16	18.83	2.22
-8	-7	-16	15.45	4.10
-8	-7	-16	22.29	3.73
8	7	16	15.41	3.19
-9	-7	-16	6.42	4.12
-9	-7	-16	12.06	2.11
-9	-7	-16	9.80	2.57
9	7	16	11.53	3.45
9	7	16	14.94	3.66
-2	8	16	13.59	1.96
1	-8	-16	17.21	2.50
-1	8	16	8.08	2.14
-1	8	16	11.87	1.83
0	-8	-16	25.47	2.63
0	8	16	21.22	3.97
0	8	16	19.95	2.05
-1	-8	-16	20.69	2.42
1	8	16	17.62	2.65
1	8	16	17.67	1.92
-2	-8	-16	17.54	2.33
2	8	16	14.50	2.94
2	8	16	14.66	1.95
2	8	16	13.35	2.77
-3	-8	-16	19.92	2.36

3	8	16	24.76	4.24
3	8	16	20.58	2.99
-4	-8	-16	14.80	2.28
4	8	16	21.11	4.06
4	8	16	22.38	3.08
-5	-8	-16	13.23	2.20
5	8	16	15.02	2.69
-6	-8	-16	20.35	2.31
6	8	16	19.01	2.88
-7	-8	-16	18.24	2.33
7	8	16	24.31	4.29
-8	-8	-16	5.86	3.38
-8	-8	-16	12.64	2.14
8	8	16	20.16	4.42
8	8	16	15.53	3.22
-9	-8	-16	13.85	2.31
-9	-8	-16	17.40	4.83
9	8	16	21.27	3.65
1	-9	-16	17.76	2.76
0	-9	-16	9.16	1.54
-1	-9	-16	15.67	2.54
-2	-9	-16	9.76	1.52
2	9	16	10.05	3.21
-3	-9	-16	15.70	2.47
-4	-9	-16	18.45	2.42
4	9	16	16.30	2.82
-5	-9	-16	14.65	1.71
5	9	16	19.83	3.18
-6	-9	-16	4.16	1.28
-6	-9	-16	-0.39	2.70
-7	-9	-16	9.70	1.50
7	9	16	13.65	3.10
-8	-9	-16	5.59	1.33
8	9	16	11.85	3.12
-9	-9	-16	13.67	2.40
9	9	16	17.74	3.51
0	-10	-16	19.38	2.83
-1	-10	-16	16.16	2.70
-2	-10	-16	17.06	2.62
3	10	16	10.89	2.50
-4	-10	-16	18.37	2.59
-5	-10	-16	15.45	2.48
5	10	16	15.22	2.78
-6	-10	-16	10.36	1.50

6	10	16	13.41	2.85
-7	-10	-16	5.55	1.38
7	10	16	5.47	2.88
-8	-10	-16	8.55	1.52
8	10	16	11.10	3.15
-9	-10	-16	6.45	1.47
9	10	16	7.05	3.35
-1	-11	-16	9.57	1.64
-2	-11	-16	25.65	2.95
-3	-11	-16	17.87	2.65
-4	-11	-16	18.38	2.61
4	11	16	13.31	2.50
-5	-11	-16	19.05	2.53
5	11	16	14.23	2.79
-6	-11	-16	23.66	2.67
6	11	16	17.47	2.98
-7	-11	-16	11.70	1.68
7	11	16	12.39	3.05
-8	-11	-16	11.07	1.57
8	11	16	13.70	3.23
-4	-12	-16	30.02	3.11
-5	-12	-16	34.96	3.22
5	12	16	29.76	4.72
-6	-12	-16	20.49	2.78
6	12	16	17.51	2.92
2	1	-17	-1.11	0.89
-2	-1	17	-1.13	1.11
-2	-1	17	-2.26	1.59
-2	-1	17	0.84	2.09
1	1	-17	0.03	0.94
-1	-1	17	-0.14	1.16
-1	-1	17	-1.69	2.05
0	1	-17	-0.90	0.89
0	-1	17	-1.75	1.58
0	-1	17	-0.22	1.29
0	-1	17	1.25	2.01
-1	1	-17	-1.02	0.98
1	-1	17	-0.25	1.67
1	-1	17	-0.96	2.14
-2	1	-17	-0.68	0.98
2	-1	17	-1.96	1.75
2	-1	17	0.00	2.25
3	0	-17	0.30	0.92
-3	0	17	-0.12	1.95

-3	0	17	-0.17	1.76
2	0	-17	1.45	0.94
-2	0	17	-1.77	1.59
-2	0	17	0.84	1.95
-2	0	17	-0.29	0.98
1	0	-17	-0.02	0.91
-1	0	17	-1.30	1.43
-1	0	17	-0.01	1.02
-1	0	17	-1.14	1.88
0	0	-17	-0.44	0.90
0	0	17	0.50	1.33
0	0	17	-0.93	1.48
0	0	17	2.34	1.99
-1	0	-17	-0.61	0.89
1	0	17	-0.56	1.56
1	0	17	-0.54	2.03
1	0	17	0.79	1.11
-2	0	-17	0.93	2.44
-2	0	-17	-0.45	0.91
2	0	17	2.70	2.05
2	0	17	-0.89	1.60
-3	0	-17	-1.40	2.53
-3	0	-17	0.19	0.98
3	0	17	-0.79	1.74
3	0	17	-2.41	2.17
-4	0	-17	-4.12	2.53
-4	0	-17	-0.71	0.97
-4	0	-17	2.58	2.76
4	0	17	-4.43	3.59
4	0	17	1.36	2.46
4	0	17	2.20	2.07
3	-1	-17	0.77	1.02
-3	1	17	-2.12	1.81
-3	1	17	-2.02	1.73
2	-1	-17	0.21	0.88
-2	1	17	1.45	1.81
-2	1	17	-0.45	0.90
-2	1	17	0.31	1.62
1	-1	-17	0.55	0.95
-1	1	17	-0.12	1.78
-1	1	17	0.46	1.51
-1	1	17	-1.61	0.88
0	-1	-17	-0.75	0.90
0	1	17	-0.78	0.96

0	1	17	0.03	1.85
0	1	17	-1.57	1.46
-1	-1	-17	-1.39	0.91
-1	-1	-17	-2.45	2.07
1	1	17	1.24	2.01
1	1	17	-0.77	1.00
1	1	17	-0.36	1.55
-2	-1	-17	0.13	0.92
-2	-1	-17	-0.15	2.10
2	1	17	-1.41	1.57
2	1	17	0.45	1.93
-3	-1	-17	-1.64	0.90
-3	-1	-17	-1.11	2.42
3	1	17	1.70	2.72
3	1	17	-0.02	1.65
3	1	17	0.80	2.14
-4	-1	-17	0.17	2.60
-4	-1	-17	-0.55	0.97
4	1	17	-1.94	1.75
4	1	17	4.57	2.99
4	1	17	1.64	2.35
-5	-1	-17	-2.16	2.82
-5	-1	-17	-0.73	0.97
5	1	17	-0.76	2.01
5	1	17	-0.49	2.62
5	1	17	3.17	3.10
3	-2	-17	0.90	1.06
-3	2	17	-0.62	1.73
-3	2	17	-0.15	1.68
2	-2	-17	2.38	0.97
-2	2	17	2.00	1.68
-2	2	17	3.22	1.72
1	-2	-17	1.88	0.95
-1	2	17	2.70	1.86
-1	2	17	0.90	1.55
-1	2	17	2.19	0.90
0	-2	-17	0.75	2.71
0	-2	-17	0.88	0.96
0	2	17	0.65	1.43
0	2	17	1.84	1.81
0	2	17	1.02	0.93
-1	-2	-17	5.77	1.06
1	2	17	6.17	2.08
1	2	17	3.65	1.58

1	2	17	1.61	1.06
-2	-2	-17	-1.42	2.01
-2	-2	-17	1.07	0.95
2	2	17	5.92	2.74
2	2	17	-0.87	1.05
2	2	17	-0.18	1.56
2	2	17	2.12	2.04
-3	-2	-17	2.18	0.99
-3	-2	-17	1.79	2.42
3	2	17	3.50	2.12
3	2	17	3.03	3.05
3	2	17	0.86	1.69
-4	-2	-17	-2.74	2.52
-4	-2	-17	0.64	0.94
-4	-2	-17	-0.97	2.66
4	2	17	3.60	2.34
4	2	17	2.63	2.76
4	2	17	2.06	1.81
-5	-2	-17	0.85	2.96
-5	-2	-17	2.19	1.01
-5	-2	-17	-3.83	2.52
5	2	17	1.95	1.86
5	2	17	5.86	2.56
5	2	17	0.61	3.14
-6	-2	-17	-0.78	0.96
-6	-2	-17	-3.65	2.79
-6	-2	-17	-4.29	3.23
6	2	17	-0.67	2.89
6	2	17	-2.34	3.66
6	2	17	0.56	1.94
3	-3	-17	0.65	1.10
-3	3	17	-0.60	1.78
-3	3	17	0.11	0.88
2	-3	-17	-0.72	1.02
-2	3	17	-0.60	1.72
-2	3	17	-0.84	1.67
1	-3	-17	-0.16	0.94
-1	3	17	-1.02	0.88
-1	3	17	1.36	1.76
-1	3	17	-0.10	1.57
0	-3	-17	1.93	0.97
0	-3	-17	-1.02	2.22
0	3	17	-0.97	0.88
0	3	17	2.25	1.88

0	3	17	0.43	1.45
-1	-3	-17	0.49	2.07
-1	-3	-17	0.02	0.97
1	3	17	3.83	3.02
1	3	17	1.69	1.86
1	3	17	0.29	0.97
1	3	17	0.39	1.47
-2	-3	-17	1.08	1.02
-2	-3	-17	-2.55	2.14
2	3	17	0.70	2.00
2	3	17	0.73	1.57
2	3	17	-1.39	2.48
2	3	17	-1.14	0.95
-3	-3	-17	0.63	1.01
-3	-3	-17	-1.57	2.19
3	3	17	-0.78	1.56
3	3	17	4.49	2.13
3	3	17	-0.45	2.68
-4	-3	-17	1.36	1.00
-4	-3	-17	0.94	2.80
4	3	17	-0.81	2.97
4	3	17	2.82	2.35
4	3	17	1.25	1.71
-5	-3	-17	-3.70	2.72
-5	-3	-17	0.41	3.08
-5	-3	-17	-0.22	0.99
5	3	17	0.77	3.04
5	3	17	0.13	2.59
5	3	17	0.18	1.80
-6	-3	-17	-0.60	3.32
-6	-3	-17	-3.67	2.73
-6	-3	-17	-1.22	0.93
6	3	17	-0.72	2.88
6	3	17	0.55	1.94
6	3	17	2.65	3.56
-7	-3	-17	-1.60	3.75
-7	-3	-17	-0.92	2.97
-7	-3	-17	0.28	1.03
7	3	17	-1.36	2.12
7	3	17	2.13	3.24
7	3	17	-2.05	5.10
3	-4	-17	2.83	1.27
-3	4	17	1.02	0.92
-3	4	17	1.47	1.88

2	-4	-17	3.88	1.17
-2	4	17	2.41	1.73
-2	4	17	0.35	0.91
1	-4	-17	3.13	1.07
-1	4	17	1.70	1.81
-1	4	17	1.39	0.91
0	-4	-17	7.18	1.16
0	4	17	5.12	1.62
0	4	17	3.76	1.96
0	4	17	5.27	1.01
-1	-4	-17	2.06	1.02
1	4	17	0.76	3.33
1	4	17	0.66	0.98
1	4	17	0.56	1.48
1	4	17	1.10	1.93
-2	-4	-17	-3.91	2.34
-2	-4	-17	1.32	1.04
2	4	17	3.61	2.10
2	4	17	1.42	0.96
2	4	17	-0.83	1.51
2	4	17	1.96	2.73
-3	-4	-17	6.60	1.17
-3	-4	-17	0.18	2.37
3	4	17	9.96	2.91
3	4	17	5.68	1.67
3	4	17	9.43	2.43
-4	-4	-17	8.77	1.26
4	4	17	7.93	3.03
4	4	17	9.46	1.87
4	4	17	10.40	2.65
-5	-4	-17	-0.21	3.02
-5	-4	-17	-0.39	1.00
5	4	17	-0.40	2.63
5	4	17	-0.18	1.82
-6	-4	-17	-0.79	3.50
-6	-4	-17	1.97	3.24
-6	-4	-17	1.69	1.02
6	4	17	6.51	3.76
6	4	17	-0.29	1.85
6	4	17	1.10	2.88
-7	-4	-17	0.77	1.06
-7	-4	-17	-1.21	3.05
-7	-4	-17	2.87	3.87
7	4	17	4.78	3.35

7	4	17	-0.74	2.13
3	-5	-17	3.40	1.34
-3	5	17	0.14	1.99
-3	5	17	1.84	0.94
2	-5	-17	0.19	1.21
-2	5	17	0.59	1.73
-2	5	17	1.33	0.90
1	-5	-17	-1.32	1.13
-1	5	17	-0.98	1.81
-1	5	17	0.84	0.91
0	-5	-17	-3.82	2.60
0	-5	-17	0.38	1.10
0	5	17	1.70	1.99
0	5	17	0.93	0.93
0	5	17	-0.98	1.43
-1	-5	-17	-3.00	2.25
-1	-5	-17	0.56	1.09
1	5	17	-0.13	1.42
1	5	17	0.99	0.97
1	5	17	1.09	2.10
-2	-5	-17	-1.91	1.01
2	5	17	-1.04	1.45
2	5	17	0.81	2.07
2	5	17	-1.31	3.20
2	5	17	0.48	0.98
-3	-5	-17	-0.01	1.04
-3	-5	-17	-2.45	2.96
3	5	17	6.14	3.05
3	5	17	2.95	2.37
3	5	17	1.13	1.53
-4	-5	-17	1.73	1.13
-4	-5	-17	2.49	2.50
4	5	17	2.22	1.60
4	5	17	1.93	2.65
4	5	17	3.59	3.20
-5	-5	-17	0.39	1.06
-5	-5	-17	-0.51	2.74
5	5	17	1.79	2.85
5	5	17	0.75	1.73
5	5	17	3.02	3.65
-6	-5	-17	3.17	3.18
-6	-5	-17	-0.09	1.01
6	5	17	-0.27	1.80
6	5	17	2.02	3.01

-7	-5	-17	-1.74	1.02
-7	-5	-17	2.51	4.06
7	5	17	-0.01	1.96
7	5	17	0.40	3.29
-8	-5	-17	1.53	1.09
8	5	17	-0.31	2.21
8	5	17	2.65	3.76
2	-6	-17	3.40	1.35
-2	6	17	0.39	0.93
-2	6	17	0.41	1.78
-2	6	17	-0.08	1.83
1	-6	-17	4.65	1.30
-1	6	17	1.78	1.94
-1	6	17	1.56	1.17
0	-6	-17	1.07	1.22
0	6	17	1.60	0.96
0	6	17	0.34	1.99
-1	-6	-17	5.19	1.24
-1	-6	-17	-1.90	2.68
1	6	17	3.92	2.25
1	6	17	3.14	0.97
1	6	17	2.44	1.50
-2	-6	-17	4.93	1.21
2	6	17	5.45	1.47
2	6	17	2.80	1.58
2	6	17	6.96	2.49
-3	-6	-17	9.46	1.36
3	6	17	14.46	2.83
3	6	17	7.57	1.74
-4	-6	-17	3.03	1.16
4	6	17	3.31	1.61
4	6	17	3.64	2.70
-5	-6	-17	5.98	2.87
-5	-6	-17	7.00	1.24
5	6	17	7.24	2.98
5	6	17	5.07	1.88
-6	-6	-17	4.79	1.21
-6	-6	-17	3.15	3.30
6	6	17	3.36	1.85
6	6	17	9.99	3.35
-7	-6	-17	2.12	1.17
7	6	17	0.43	1.90
7	6	17	3.26	3.37
-8	-6	-17	2.03	1.12

8	6	17	1.62	2.07
2	-7	-17	1.22	1.49
-2	7	17	-0.34	1.03
-2	7	17	0.69	1.98
1	-7	-17	2.83	1.34
-1	7	17	0.11	0.97
-1	7	17	-0.22	2.08
0	-7	-17	3.61	1.31
0	7	17	3.33	1.14
0	7	17	0.87	2.15
-1	-7	-17	2.18	1.27
1	7	17	-1.14	1.36
1	7	17	0.14	2.34
-2	-7	-17	1.24	3.68
-2	-7	-17	0.20	1.23
2	7	17	-0.30	2.48
2	7	17	-0.25	1.44
-3	-7	-17	0.68	1.19
-3	-7	-17	-1.09	3.31
3	7	17	-0.28	1.45
3	7	17	-0.18	2.60
-4	-7	-17	2.44	1.22
4	7	17	0.28	1.47
4	7	17	2.18	3.01
-5	-7	-17	1.44	1.16
5	7	17	1.98	2.94
5	7	17	-0.05	1.64
-6	-7	-17	4.94	1.24
6	7	17	6.63	3.33
6	7	17	1.57	1.70
-7	-7	-17	1.60	1.17
7	7	17	0.11	1.83
-8	-7	-17	-0.77	1.16
8	7	17	3.52	2.14
1	-8	-17	2.88	1.51
-1	8	17	1.72	2.26
0	-8	-17	8.16	1.51
0	8	17	4.32	2.46
-1	-8	-17	7.58	1.49
1	8	17	6.68	2.57
-2	-8	-17	9.48	1.52
2	8	17	6.30	2.74
-3	-8	-17	6.31	1.36
3	8	17	4.79	2.88

-4	-8	-17	7.63	1.43
4	8	17	10.54	3.07
-5	-8	-17	7.85	1.39
-6	-8	-17	4.43	1.27
-7	-8	-17	0.93	1.20
7	8	17	1.94	1.94
-8	-8	-17	4.06	1.27
-1	-9	-17	3.50	1.49
-2	-9	-17	3.21	1.41
-3	-9	-17	6.09	1.48
-4	-9	-17	5.89	1.45
-5	-9	-17	5.98	1.36
-6	-9	-17	0.56	1.28
-7	-9	-17	0.53	1.25
7	9	17	-3.97	3.24
-2	-10	-17	4.73	1.54
-3	-10	-17	17.89	2.81
4	10	17	3.95	2.80
-5	-10	-17	17.11	2.70
5	10	17	9.91	3.12
-6	-10	-17	8.28	1.54
6	10	17	6.17	3.11
0	-2	-18	4.07	1.10
0	2	18	1.74	1.66
0	2	18	0.96	1.19
0	2	18	2.96	2.17
-1	-2	-18	2.23	1.12
1	2	18	1.11	1.62
1	2	18	3.40	2.17
-2	-2	-18	4.46	1.14
2	2	18	2.90	1.75
2	2	18	4.80	2.40
-3	-2	-18	2.70	1.11
3	2	18	2.79	1.89
3	2	18	3.49	2.47
0	-3	-18	3.38	1.13
0	3	18	3.46	2.06
0	3	18	1.01	1.14
0	3	18	3.48	1.70
-1	-3	-18	4.43	1.17
1	3	18	3.55	2.23
1	3	18	3.78	1.73
-2	-3	-18	13.32	2.00
2	3	18	7.80	1.95

2	3	18	10.01	2.45
-3	-3	-18	6.04	1.20
3	3	18	3.69	1.91
3	3	18	6.18	2.60
-4	-3	-18	4.96	1.17
4	3	18	6.52	2.06
4	3	18	7.92	2.82
0	-4	-18	2.85	1.15
0	4	18	1.29	1.64
0	4	18	6.15	2.19
0	4	18	-0.06	1.12
-1	-4	-18	6.98	1.27
1	4	18	3.92	2.22
1	4	18	4.99	1.75
-2	-4	-18	8.93	1.31
2	4	18	9.79	2.50
2	4	18	7.50	1.94
-3	-4	-18	4.66	1.21
3	4	18	6.92	2.54
3	4	18	4.81	1.89
-4	-4	-18	1.10	1.14
4	4	18	-0.08	2.77
4	4	18	0.00	1.84
-5	-4	-18	3.37	1.13
5	4	18	2.02	2.07
5	4	18	7.49	3.32
0	-5	-18	2.34	1.28
0	5	18	4.45	2.16
0	5	18	4.08	1.71
-1	-5	-18	5.10	1.27
1	5	18	6.21	2.40
1	5	18	1.77	1.63
-2	-5	-18	5.52	1.25
2	5	18	8.25	2.50
2	5	18	5.26	1.76
-3	-5	-18	4.80	1.21
3	5	18	4.99	1.79
3	5	18	5.24	2.71
-4	-5	-18	2.98	1.19
4	5	18	0.75	1.78
4	5	18	4.54	2.97
-5	-5	-18	4.32	1.20
5	5	18	4.36	3.25
5	5	18	0.97	1.90

```
   0  -6 -18    3.52    1.42
   0   6  18    4.52    2.29
   0   6  18    2.43    1.58
  -1  -6 -18    1.64    1.28
   1   6  18    2.79    2.42
   1   6  18    0.93    1.63
  -2  -6 -18    1.85    1.26
   2   6  18    3.22    1.71
   2   6  18    3.94    2.70
  -3  -6 -18    1.29    1.21
   3   6  18    5.73    2.87
   3   6  18    1.09    1.67
  -4  -6 -18    0.51    1.20
   4   6  18    2.73    2.94
   4   6  18   -0.19    1.72
  -5  -6 -18    0.27    1.16
   5   6  18   -1.36    1.87
   5   6  18   -1.51    3.33
  -1  -7 -18   -0.84    1.41
   1   7  18    0.70    1.61
   1   7  18    2.28    2.62
  -2  -7 -18    2.12    1.36
   2   7  18    0.16    2.72
   2   7  18    0.89    1.66
  -3  -7 -18    2.53    1.32
   3   7  18    0.82    2.95
   3   7  18    3.50    1.71
  -4  -7 -18    0.38    1.23
   4   7  18    2.75    3.30
   4   7  18    0.47    1.70
  -5  -7 -18    0.90    1.24
   5   7  18    3.24    3.37
   5   7  18   -1.20    1.83
  -3  -8 -18    1.78    1.38
   3   8  18    2.23    3.04
  -4  -8 -18    3.58    1.36
   4   8  18    2.42    3.23
   4   8  18    1.43    1.75
   0   0   0    0.00    0.00
;
_shelx_hkl_checksum   70330
```
