# Supplementary material for: Structure-property relationships of photofunctional diiridium(II) complexes with tetracationic charge and an unsupported Ir–Ir bond
Source: Commun Chem. 2022 Nov 23;5:159. doi: 10.1038/s42004-022-00775-4 (PMC9814866; doi:10.1038/s42004-022-00775-4)
Supplement: Supplementary file 8 — Supplementary Data 5 [file 42004_2022_775_MOESM8_ESM.pdf]

```
data_[Ir(nBu-bzimpy)(PPh3)(CO)2]2[OTf]4

_audit_creation_method            'SHELXL-2014/7'
_shelx_SHELXL_version_number      '2014/7'
_chemical_name_systematic         ?
_chemical_name_common             ?
_chemical_melting_point           ?
_chemical_formula_moiety            'C48 H44 Ir N3 O P, 2(C F3 O3 S),
2(C2 H3 N)'
_chemical_formula_sum
 'C54 H50 F6 Ir N5 O7 P S2'
_chemical_formula_weight          1282.28

loop_
 _atom_type_symbol
 _atom_type_description
 _atom_type_scat_dispersion_real
 _atom_type_scat_dispersion_imag
 _atom_type_scat_source
 'C'  'C'   0.0033   0.0016
 'International Tables Vol C Tables 4.2.6.8 and 6.1.1.4'
 'H'  'H'   0.0000   0.0000
 'International Tables Vol C Tables 4.2.6.8 and 6.1.1.4'
 'N'  'N'   0.0061   0.0033
 'International Tables Vol C Tables 4.2.6.8 and 6.1.1.4'
 'O'  'O'   0.0106   0.0060
 'International Tables Vol C Tables 4.2.6.8 and 6.1.1.4'
 'F'  'F'   0.0171   0.0103
 'International Tables Vol C Tables 4.2.6.8 and 6.1.1.4'
 'P'  'P'   0.1023   0.0942
 'International Tables Vol C Tables 4.2.6.8 and 6.1.1.4'
 'S'  'S'   0.1246   0.1234
 'International Tables Vol C Tables 4.2.6.8 and 6.1.1.4'
 'Ir'  'Ir'  -1.4442   7.9887
 'International Tables Vol C Tables 4.2.6.8 and 6.1.1.4'

_space_group_crystal_system       triclinic
_space_group_IT_number            2
_space_group_name_H-M_alt         'P -1'
_space_group_name_Hall            '-P 1'

_shelx_space_group_comment
;
```

```
The symmetry employed for this shelxl refinement is uniquely defined
by the following loop, which should always be used as a source of
symmetry information in preference to the above space-group names.
They are only intended as comments.
;

loop_
 _space_group_symop_operation_xyz
 'x, y, z'
 '-x, -y, -z'

_cell_length_a                    14.0846(12)
_cell_length_b                    14.6810(12)
_cell_length_c                    15.1434(13)
_cell_angle_alpha                 99.697(3)
_cell_angle_beta                  111.760(3)
_cell_angle_gamma                 108.904(3)
_cell_volume                      2598.8(4)
_cell_formula_units_Z             2
_cell_measurement_temperature     150(2)
_cell_measurement_reflns_used     9306
_cell_measurement_theta_min       2.45
_cell_measurement_theta_max       27.45

_exptl_crystal_description        stick
_exptl_crystal_colour             brown
_exptl_crystal_density_meas       ?
_exptl_crystal_density_method     ?
_exptl_crystal_density_diffrn     1.639
_exptl_crystal_F_000              1286
_exptl_transmission_factor_min    ?
_exptl_transmission_factor_max    ?
_exptl_crystal_size_max           0.210
_exptl_crystal_size_mid           0.190
_exptl_crystal_size_min           0.170
_exptl_absorpt_coefficient_mu     2.760
_shelx_estimated_absorpt_T_min    0.595
_shelx_estimated_absorpt_T_max    0.651
_exptl_absorpt_correction_type    multi-scan
_exptl_absorpt_correction_T_min   0.5501
_exptl_absorpt_correction_T_max   0.7455
_exptl_absorpt_process_details
'SADABS-2014/5 (Bruker,2014/5) was used for absorption correction.
wR2(int) was 0.1483 before and 0.0761 after correction. The Ratio of
```

```
minimum to maximum transmission is 0.7738. The \l/2 correction factor
is Not present.'
_exptl_absorpt_special_details    ?
_diffrn_ambient_temperature       150(2)
_diffrn_radiation_wavelength      0.70999
_diffrn_radiation_type            MoK\a
_diffrn_source                    ?
_diffrn_measurement_device_type   'Bruker APEX-II CCD'
_diffrn_measurement_method        '\f and \w scans'
_diffrn_detector_area_resol_mean  ?
_diffrn_reflns_number             55676
_diffrn_reflns_av_unetI/netI      0.0419
_diffrn_reflns_av_R_equivalents   0.0460
_diffrn_reflns_limit_h_min        -18
_diffrn_reflns_limit_h_max        18
_diffrn_reflns_limit_k_min        -19
_diffrn_reflns_limit_k_max        19
_diffrn_reflns_limit_l_min        -19
_diffrn_reflns_limit_l_max        19
_diffrn_reflns_theta_min          2.446
_diffrn_reflns_theta_max          27.497
_diffrn_reflns_theta_full         25.214
_diffrn_measured_fraction_theta_max   0.996
_diffrn_measured_fraction_theta_full  0.999
_diffrn_reflns_Laue_measured_fraction_max    0.996
_diffrn_reflns_Laue_measured_fraction_full   0.999
_diffrn_reflns_point_group_measured_fraction_max   0.996
_diffrn_reflns_point_group_measured_fraction_full  0.999
_reflns_number_total              11940
_reflns_number_gt                 10616
_reflns_threshold_expression      'I > 2\s(I)'
_reflns_Friedel_coverage          0.000
_reflns_Friedel_fraction_max      .
_reflns_Friedel_fraction_full     .

_reflns_special_details
;
 Reflections were merged by SHELXL according to the crystal
 class for the calculation of statistics and refinement.

 _reflns_Friedel_fraction is defined as the number of unique
 Friedel pairs measured divided by the number that would be
 possible theoretically, ignoring centric projections and
 systematic absences.
```

```
;

_computing_data_collection        'Bruker APEX2'
_computing_cell_refinement        'Bruker SAINT'
_computing_data_reduction         'Bruker SAINT'
_computing_structure_solution     'SHELXS-97 (Sheldrick 2008)'
_computing_structure_refinement 'SHELXL-2014/7 (Sheldrick, 2014)'
_computing_molecular_graphics     'Bruker SHELXTL'
_computing_publication_material   'Bruker SHELXTL'
_refine_special_details           ?
_refine_ls_structure_factor_coef  Fsqd
_refine_ls_matrix_type            full
_refine_ls_weighting_scheme       calc
_refine_ls_weighting_details
'w=1/[\s^2^(Fo^2^)+(0.0304P)^2^+5.3985P] where P=(Fo^2^+2Fc^2^)/3'
_atom_sites_solution_primary      ?
_atom_sites_solution_secondary    ?
_atom_sites_solution_hydrogens    geom
_refine_ls_hydrogen_treatment     constr
_refine_ls_extinction_method      none
_refine_ls_extinction_coef        .
_refine_ls_number_reflns          11940
_refine_ls_number_parameters      708
_refine_ls_number_restraints      7
_refine_ls_R_factor_all           0.0411
_refine_ls_R_factor_gt            0.0321
_refine_ls_wR_factor_ref          0.0747
_refine_ls_wR_factor_gt           0.0717
_refine_ls_goodness_of_fit_ref    1.059
_refine_ls_restrained_S_all       1.059
_refine_ls_shift/su_max           0.002
_refine_ls_shift/su_mean          0.000

loop_
 _atom_site_label
 _atom_site_type_symbol
 _atom_site_fract_x
 _atom_site_fract_y
 _atom_site_fract_z
 _atom_site_U_iso_or_equiv
 _atom_site_adp_type
 _atom_site_occupancy
 _atom_site_site_symmetry_order
 _atom_site_calc_flag
```

_atom_site_refinement_flags_posn
_atom_site_refinement_flags_adp
_atom_site_refinement_flags_occupancy
_atom_site_disorder_assembly
_atom_site_disorder_group
Ir1 Ir 0.01040(2) 0.51618(2) 0.10336(2) 0.01354(4) Uani 1 1 d . . . . .
S1 S -0.23545(9) 0.01665(8) -0.03785(8) 0.0318(2) Uani 1 1 d . . . . .
S2 S 0.31360(11) 1.01983(9) 0.58065(10) 0.0449(3) Uani 1 1 d . . . . .
P1 P 0.01740(8) 0.53765(7) 0.26975(7) 0.01821(18) Uani 1 1 d . . . . .
F1 F -0.3773(3) 0.1021(3) -0.0859(3) 0.0720(10) Uani 1 1 d . . . . .
F2 F -0.3211(4) 0.0942(3) 0.0635(3) 0.0844(11) Uani 1 1 d . . . . .
F3 F -0.4403(3) -0.0365(3) -0.0584(3) 0.0899(13) Uani 1 1 d . . . . .
F4 F 0.3740(5) 0.9726(4) 0.4439(3) 0.134(2) Uani 1 1 d . . . . .
F5 F 0.4421(3) 1.1286(4) 0.5179(3) 0.1092(17) Uani 1 1 d . . . . .
F6 F 0.2740(3) 1.0554(3) 0.4094(3) 0.0763(10) Uani 1 1 d . . . . .
O1 O -0.1940(2) 0.31547(19) 0.00917(19) 0.0265(6) Uani 1 1 d . . . . .
O2 O -0.1478(3) 0.1148(3) -0.0005(3) 0.0630(10) Uani 1 1 d . . . . .
O3 O -0.2231(3) -0.0468(3) 0.0234(3) 0.0606(11) Uani 1 1 d . . . . .
O4 O -0.2794(4) -0.0320(3) -0.1447(3) 0.0727(13) Uani 1 1 d . . . . .
O5 O 0.4049(3) 1.0071(4) 0.6479(3) 0.0709(12) Uani 1 1 d . . . . .
O6 O 0.2766(9) 1.0948(6) 0.6014(7) 0.057(3) Uani 0.677(19) 1 d . . P A
1
O6' O 0.370(2) 1.1379(10) 0.6454(10) 0.067(7) Uani 0.323(19) 1 d . . P
A 2
O7 O 0.2156(7) 0.9179(6) 0.5185(9) 0.082(3) Uani 0.677(19) 1 d . . P A
1
O7' O 0.2131(12) 0.971(2) 0.5629(15) 0.090(10) Uani 0.323(19) 1 d . . P
A 2
N1 N 0.1438(2) 0.4765(2) 0.1492(2) 0.0169(6) Uani 1 1 d . . . . .
N2 N 0.1446(2) 0.6538(2) 0.1621(2) 0.0152(6) Uani 1 1 d . . . . .
N3 N -0.0670(2) 0.6116(2) 0.0802(2) 0.0152(6) Uani 1 1 d . . . . .
N4 N -0.4082(4) 0.4118(4) 0.2460(4) 0.0572(12) Uani 1 1 d . . . . .
N5 N 0.5165(4) 1.2779(4) 0.8881(4) 0.0567(12) Uani 1 1 d . . . . .
C1 C 0.1623(3) 0.3900(3) 0.1547(3) 0.0202(7) Uani 1 1 d . . . . .
C2 C 0.0871(3) 0.2877(3) 0.1172(3) 0.0220(7) Uani 1 1 d . . . . .
H2 H 0.0079 0.2662 0.0817 0.026 Uiso 1 1 calc R U . . .
C3 C 0.1342(4) 0.2188(3) 0.1345(3) 0.0269(8) Uani 1 1 d . . . . .
H3 H 0.0857 0.1482 0.1107 0.032 Uiso 1 1 calc R U . . .
C4 C 0.2510(4) 0.2499(3) 0.1861(3) 0.0298(9) Uani 1 1 d . . . . .
H4 H 0.2794 0.2001 0.1967 0.036 Uiso 1 1 calc R U . . .
C5 C 0.3256(4) 0.3511(3) 0.2216(3) 0.0284(9) Uani 1 1 d . . . . .
H5 H 0.4048 0.3721 0.2558 0.034 Uiso 1 1 calc R U . . .
C6 C 0.2790(3) 0.4214(3) 0.2050(3) 0.0217(7) Uani 1 1 d . U . . .
C7 C 0.3295(2) 0.5252(2) 0.2283(2) 0.0098(6) Uani 1 1 d . U . . .

C8 C 0.2463(3) 0.5563(3) 0.1944(2) 0.0183(7) Uani 1 1 d . . . . .
C9 C 0.2507(3) 0.6583(3) 0.2031(2) 0.0179(7) Uani 1 1 d . . . . .
C10 C 0.3430(3) 0.7524(3) 0.2462(3) 0.0207(7) Uani 1 1 d . . . . .
H10 H 0.4176 0.7569 0.2741 0.025 Uiso 1 1 calc R U . . .
C11 C 0.3242(3) 0.8394(3) 0.2476(3) 0.0231(8) Uani 1 1 d . . . . .
H11 H 0.3866 0.9041 0.2761 0.028 Uiso 1 1 calc R U . . .
C12 C 0.2151(3) 0.8334(3) 0.2080(3) 0.0213(7) Uani 1 1 d . . . . .
H12 H 0.2030 0.8936 0.2107 0.026 Uiso 1 1 calc R U . . .
C13 C 0.1251(3) 0.7389(3) 0.1648(2) 0.0161(7) Uani 1 1 d . . . . .
C14 C 0.0033(3) 0.7109(3) 0.1177(2) 0.0166(7) Uani 1 1 d . . . . .
C15 C -0.0547(2) 0.7693(2) 0.1074(2) 0.0088(6) Uani 1 1 d . U . . .
C16 C -0.1681(3) 0.7037(3) 0.0623(3) 0.0195(7) Uani 1 1 d . . . . .
C17 C -0.2636(3) 0.7233(3) 0.0366(3) 0.0246(8) Uani 1 1 d . . . . .
H17 H -0.2586 0.7907 0.0474 0.029 Uiso 1 1 calc R U . . .
C18 C -0.3660(3) 0.6392(3) -0.0054(3) 0.0272(8) Uani 1 1 d . . . . .
H18 H -0.4331 0.6493 -0.0231 0.033 Uiso 1 1 calc R U . . .
C19 C -0.3741(3) 0.5393(3) -0.0227(3) 0.0247(8) Uani 1 1 d . . . . .
H19 H -0.4463 0.4837 -0.0520 0.030 Uiso 1 1 calc R U . . .
C20 C -0.2794(3) 0.5200(3) 0.0018(2) 0.0205(7) Uani 1 1 d . . . . .
H20 H -0.2849 0.4525 -0.0103 0.025 Uiso 1 1 calc R U . . .
C21 C -0.1754(3) 0.6042(3) 0.0452(2) 0.0169(7) Uani 1 1 d . . . . .
C22 C 0.4533(3) 0.5837(3) 0.2762(3) 0.0234(8) Uani 1 1 d . . . . .
H22A H 0.4714 0.6461 0.2570 0.028 Uiso 1 1 calc R U . . .
H22B H 0.4868 0.5422 0.2512 0.028 Uiso 1 1 calc R U . . .
C23 C 0.5050(3) 0.6137(3) 0.3903(3) 0.0277(8) Uani 1 1 d . . . . .
H23A H 0.4783 0.6618 0.4160 0.033 Uiso 1 1 calc R U . . .
H23B H 0.4796 0.5522 0.4093 0.033 Uiso 1 1 calc R U . . .
C24 C 0.6329(3) 0.6631(3) 0.4390(3) 0.0316(9) Uani 1 1 d . . . . .
H24A H 0.6585 0.7271 0.4238 0.038 Uiso 1 1 calc R U . . .
H24B H 0.6595 0.6170 0.4098 0.038 Uiso 1 1 calc R U . . .
C25 C 0.6849(4) 0.6865(4) 0.5524(3) 0.0455(12) Uani 1 1 d . . . . .
H25A H 0.6595 0.6233 0.5677 0.068 Uiso 1 1 calc R U . . .
H25B H 0.6615 0.7346 0.5819 0.068 Uiso 1 1 calc R U . . .
H25C H 0.7673 0.7167 0.5808 0.068 Uiso 1 1 calc R U . . .
C26 C -0.0141(3) 0.8810(3) 0.1375(3) 0.0222(7) Uani 1 1 d . . . . .
H26A H -0.0677 0.8992 0.0881 0.027 Uiso 1 1 calc R U . . .
H26B H 0.0600 0.9118 0.1381 0.027 Uiso 1 1 calc R U . . .
C27 C -0.0028(3) 0.9229(3) 0.2417(3) 0.0276(8) Uani 1 1 d . . . . .
H27A H -0.0745 0.8854 0.2429 0.033 Uiso 1 1 calc R U . . .
H27B H 0.0571 0.9112 0.2917 0.033 Uiso 1 1 calc R U . . .
C28 C 0.0255(4) 1.0361(3) 0.2715(3) 0.0335(9) Uani 1 1 d . . . . .
H28A H -0.0306 1.0490 0.2185 0.040 Uiso 1 1 calc R U . . .
H28B H 0.1006 1.0745 0.2766 0.040 Uiso 1 1 calc R U . . .
C29 C 0.0260(4) 1.0744(4) 0.3720(3) 0.0444(12) Uani 1 1 d . . . . .

H29A H -0.0488 1.0377 0.3665 0.067 Uiso 1 1 calc R U . . .
H29B H 0.0450 1.1476 0.3892 0.067 Uiso 1 1 calc R U . . .
H29C H 0.0821 1.0625 0.4246 0.067 Uiso 1 1 calc R U . . .
C30 C -0.0487(3) 0.6187(3) 0.2988(3) 0.0210(7) Uani 1 1 d . . . . .
C31 C 0.0083(4) 0.7144(3) 0.3733(3) 0.0285(8) Uani 1 1 d . . . . .
H31 H 0.0878 0.7421 0.4125 0.034 Uiso 1 1 calc R U . . .
C32 C -0.0509(4) 0.7692(3) 0.3903(3) 0.0361(10) Uani 1 1 d . . . . .
H32 H -0.0115 0.8342 0.4410 0.043 Uiso 1 1 calc R U . . .
C33 C -0.1664(4) 0.7299(4) 0.3340(4) 0.0398(11) Uani 1 1 d . . . . .
H33 H -0.2060 0.7684 0.3453 0.048 Uiso 1 1 calc R U . . .
C34 C -0.2241(4) 0.6351(4) 0.2617(3) 0.0363(10) Uani 1 1 d . . . . .
H34 H -0.3037 0.6076 0.2236 0.044 Uiso 1 1 calc R U . . .
C35 C -0.1649(3) 0.5792(3) 0.2445(3) 0.0286(9) Uani 1 1 d . . . . .
H35 H -0.2051 0.5135 0.1949 0.034 Uiso 1 1 calc R U . . .
C36 C -0.0532(3) 0.4219(3) 0.2913(3) 0.0211(7) Uani 1 1 d . . . . .
C37 C -0.0908(3) 0.4279(3) 0.3646(3) 0.0272(8) Uani 1 1 d . . . . .
H37 H -0.0878 0.4907 0.3974 0.033 Uiso 1 1 calc R U . . .
C38 C -0.1328(4) 0.3415(3) 0.3892(3) 0.0334(9) Uani 1 1 d . . . . .
H38 H -0.1585 0.3455 0.4389 0.040 Uiso 1 1 calc R U . . .
C39 C -0.1374(4) 0.2499(3) 0.3424(3) 0.0346(10) Uani 1 1 d . . . . .
H39 H -0.1656 0.1915 0.3602 0.042 Uiso 1 1 calc R U . . .
C40 C -0.1008(4) 0.2433(3) 0.2696(3) 0.0331(9) Uani 1 1 d . . . . .
H40 H -0.1044 0.1802 0.2368 0.040 Uiso 1 1 calc R U . . .
C41 C -0.0586(3) 0.3294(3) 0.2444(3) 0.0271(8) Uani 1 1 d . . . . .
H41 H -0.0332 0.3248 0.1945 0.033 Uiso 1 1 calc R U . . .
C42 C 0.1607(3) 0.5906(3) 0.3720(3) 0.0226(8) Uani 1 1 d . . . . .
C43 C 0.1989(4) 0.5292(3) 0.4242(3) 0.0299(9) Uani 1 1 d . . . . .
H43 H 0.1526 0.4583 0.4024 0.036 Uiso 1 1 calc R U . . .
C44 C 0.3037(4) 0.5714(4) 0.5072(3) 0.0365(10) Uani 1 1 d . . . . .
H44 H 0.3293 0.5291 0.5418 0.044 Uiso 1 1 calc R U . . .
C45 C 0.3709(4) 0.6736(4) 0.5400(3) 0.0370(10) Uani 1 1 d . . . . .
H45 H 0.4414 0.7025 0.5988 0.044 Uiso 1 1 calc R U . . .
C46 C 0.3364(3) 0.7350(3) 0.4877(3) 0.0317(9) Uani 1 1 d . . . . .
H46 H 0.3838 0.8057 0.5099 0.038 Uiso 1 1 calc R U . . .
C47 C 0.2331(3) 0.6938(3) 0.4033(3) 0.0264(8) Uani 1 1 d . . . . .
H47 H 0.2110 0.7358 0.3662 0.032 Uiso 1 1 calc R U . . .
C48 C -0.1159(3) 0.3903(3) 0.0432(2) 0.0183(7) Uani 1 1 d . . . . .
C49 C -0.3507(4) 0.0438(4) -0.0312(4) 0.0476(12) Uani 1 1 d . . . . .
C50 C 0.3483(4) 1.0406(4) 0.4814(4) 0.0481(12) Uani 1 1 d . . . . .
C51 C -0.3976(4) 0.3492(4) 0.2063(4) 0.0521(13) Uani 1 1 d . . . . .
C52 C -0.3781(7) 0.2703(6) 0.1517(6) 0.116(3) Uani 1 1 d . . . . .
H52A H -0.4068 0.2656 0.0807 0.173 Uiso 1 1 calc R U . . .
H52B H -0.4174 0.2046 0.1572 0.173 Uiso 1 1 calc R U . . .
H52C H -0.2971 0.2881 0.1808 0.173 Uiso 1 1 calc R U . . .

```
C53 C 0.4462(5) 1.2006(4) 0.8638(4) 0.0429(11) Uani 1 1 d . . . . .
C54 C 0.3557(5) 1.1012(4) 0.8336(4) 0.0514(13) Uani 1 1 d . . . . .
H54A H 0.2897 1.0926 0.7739 0.077 Uiso 1 1 calc R U . . .
H54B H 0.3359 1.0956 0.8886 0.077 Uiso 1 1 calc R U . . .
H54C H 0.3804 1.0481 0.8178 0.077 Uiso 1 1 calc R U . . .

loop_
 _atom_site_aniso_label
 _atom_site_aniso_U_11
 _atom_site_aniso_U_22
 _atom_site_aniso_U_33
 _atom_site_aniso_U_23
 _atom_site_aniso_U_13
 _atom_site_aniso_U_12
Ir1 0.01944(7) 0.01089(7) 0.01579(7) 0.00533(5) 0.01129(5) 0.00850(5)
S1 0.0397(6) 0.0248(5) 0.0491(6) 0.0200(5) 0.0296(5) 0.0198(4)
S2 0.0457(7) 0.0408(7) 0.0668(8) 0.0277(6) 0.0356(6) 0.0232(6)
P1 0.0254(5) 0.0186(5) 0.0185(4) 0.0080(4) 0.0145(4) 0.0122(4)
F1 0.076(2) 0.092(3) 0.096(3) 0.063(2) 0.050(2) 0.064(2)
F2 0.118(3) 0.108(3) 0.081(2) 0.039(2) 0.069(2) 0.077(3)
F3 0.0435(19) 0.075(3) 0.154(4) 0.050(3) 0.050(2) 0.0171(18)
F4 0.240(6) 0.180(5) 0.073(3) 0.050(3) 0.090(3) 0.171(5)
F5 0.058(2) 0.149(4) 0.086(3) 0.047(3) 0.037(2) -0.006(2)
F6 0.062(2) 0.107(3) 0.064(2) 0.045(2) 0.0202(17) 0.042(2)
O1 0.0314(15) 0.0177(14) 0.0279(14) 0.0049(11) 0.0168(12) 0.0050(12)
O2 0.044(2) 0.047(2) 0.079(3) 0.021(2) 0.0231(19) 0.0029(17)
O3 0.081(3) 0.074(3) 0.088(3) 0.064(2) 0.059(2) 0.060(2)
O4 0.144(4) 0.043(2) 0.064(2) 0.0252(19) 0.066(3) 0.052(3)
O5 0.053(2) 0.114(4) 0.055(2) 0.037(2) 0.0244(19) 0.043(2)
O6 0.094(8) 0.060(5) 0.075(5) 0.040(4) 0.066(6) 0.059(6)
O6' 0.106(19) 0.041(8) 0.047(8) 0.006(6) 0.035(9) 0.025(9)
O7 0.061(4) 0.039(4) 0.110(8) 0.016(4) 0.029(5) -0.005(3)
O7' 0.025(6) 0.14(2) 0.071(12) 0.054(13) 0.014(7) -0.006(10)
N1 0.0238(15) 0.0166(15) 0.0186(14) 0.0080(12) 0.0130(12) 0.0133(12)
N2 0.0195(14) 0.0149(14) 0.0157(13) 0.0063(11) 0.0112(11) 0.0082(11)
N3 0.0208(14) 0.0127(14) 0.0171(13) 0.0052(11) 0.0111(11) 0.0098(11)
N4 0.048(3) 0.065(3) 0.065(3) 0.020(3) 0.029(2) 0.029(2)
N5 0.070(3) 0.043(3) 0.072(3) 0.018(2) 0.045(3) 0.026(2)
C1 0.0306(19) 0.0221(19) 0.0206(17) 0.0114(15) 0.0174(15) 0.0169(16)
C2 0.033(2) 0.0203(19) 0.0216(17) 0.0074(15) 0.0180(16) 0.0148(16)
C3 0.048(2) 0.0186(19) 0.0288(19) 0.0119(16) 0.0248(18) 0.0200(17)
C4 0.048(2) 0.030(2) 0.032(2) 0.0169(18) 0.0244(19) 0.030(2)
C5 0.040(2) 0.036(2) 0.029(2) 0.0182(18) 0.0212(18) 0.0282(19)
C6 0.0305(19) 0.0263(19) 0.0194(17) 0.0096(15) 0.0157(15) 0.0189(16)
```

C7 0.0124(14) 0.0136(15) 0.0101(13) 0.0052(12) 0.0074(12) 0.0100(12)
C8 0.0215(17) 0.0210(18) 0.0173(16) 0.0069(14) 0.0119(14) 0.0108(14)
C9 0.0228(17) 0.0209(18) 0.0165(16) 0.0072(14) 0.0117(14) 0.0132(14)
C10 0.0206(17) 0.0224(19) 0.0205(17) 0.0064(14) 0.0109(14) 0.0095(15)
C11 0.0266(19) 0.0177(18) 0.0238(18) 0.0043(15) 0.0131(15) 0.0076(15)
C12 0.031(2) 0.0150(18) 0.0216(17) 0.0053(14) 0.0141(15) 0.0116(15)
C13 0.0252(18) 0.0140(16) 0.0155(15) 0.0061(13) 0.0127(14) 0.0112(14)
C14 0.0244(17) 0.0163(17) 0.0161(15) 0.0069(13) 0.0131(14) 0.0114(14)
C15 0.0145(14) 0.0061(14) 0.0115(13) 0.0035(11) 0.0081(11) 0.0083(11)
C16 0.0272(19) 0.0204(18) 0.0198(16) 0.0081(14) 0.0151(15) 0.0145(15)
C17 0.032(2) 0.029(2) 0.0263(18) 0.0115(16) 0.0172(16) 0.0218(17)
C18 0.026(2) 0.038(2) 0.0279(19) 0.0104(17) 0.0159(16) 0.0219(18)
C19 0.0232(19) 0.031(2) 0.0230(18) 0.0071(16) 0.0136(15) 0.0120(16)
C20 0.0258(18) 0.0218(19) 0.0189(16) 0.0064(14) 0.0132(14) 0.0125(15)
C21 0.0215(17) 0.0218(18) 0.0167(15) 0.0078(14) 0.0135(14) 0.0136(14)
C22 0.0214(18) 0.027(2) 0.0274(19) 0.0095(16) 0.0134(15) 0.0135(15)
C23 0.029(2) 0.031(2) 0.0272(19) 0.0097(17) 0.0150(16) 0.0137(17)
C24 0.029(2) 0.029(2) 0.035(2) 0.0121(18) 0.0128(17) 0.0105(17)
C25 0.040(3) 0.040(3) 0.035(2) 0.009(2) 0.004(2) 0.009(2)
C26 0.031(2) 0.0156(18) 0.0272(18) 0.0080(15) 0.0158(16) 0.0148(15)
C27 0.038(2) 0.022(2) 0.0258(19) 0.0060(16) 0.0151(17) 0.0155(17)
C28 0.044(2) 0.024(2) 0.033(2) 0.0047(17) 0.0167(19) 0.0193(19)
C29 0.060(3) 0.040(3) 0.036(2) 0.001(2) 0.021(2) 0.031(2)
C30 0.034(2) 0.0214(19) 0.0217(17) 0.0112(15) 0.0206(16) 0.0168(16)
C31 0.041(2) 0.026(2) 0.031(2) 0.0117(17) 0.0250(18) 0.0165(18)
C32 0.062(3) 0.026(2) 0.043(2) 0.0142(19) 0.039(2) 0.026(2)
C33 0.066(3) 0.043(3) 0.053(3) 0.029(2) 0.049(3) 0.041(2)
C34 0.043(2) 0.051(3) 0.042(2) 0.025(2) 0.031(2) 0.032(2)
C35 0.040(2) 0.032(2) 0.0259(19) 0.0137(17) 0.0205(17) 0.0208(19)
C36 0.0294(19) 0.0212(19) 0.0207(17) 0.0112(15) 0.0157(15) 0.0130(15)
C37 0.040(2) 0.022(2) 0.0269(19) 0.0098(16) 0.0220(17) 0.0132(17)
C38 0.049(3) 0.035(2) 0.029(2) 0.0151(18) 0.0285(19) 0.018(2)
C39 0.051(3) 0.027(2) 0.040(2) 0.0221(19) 0.029(2) 0.017(2)
C40 0.054(3) 0.024(2) 0.040(2) 0.0177(18) 0.032(2) 0.023(2)
C41 0.043(2) 0.026(2) 0.0289(19) 0.0131(17) 0.0256(18) 0.0204(18)
C42 0.033(2) 0.027(2) 0.0166(16) 0.0075(15) 0.0164(15) 0.0168(16)
C43 0.039(2) 0.031(2) 0.0260(19) 0.0100(17) 0.0149(17) 0.0220(18)
C44 0.043(2) 0.048(3) 0.028(2) 0.017(2) 0.0134(19) 0.031(2)
C45 0.031(2) 0.052(3) 0.026(2) 0.009(2) 0.0103(17) 0.020(2)
C46 0.028(2) 0.034(2) 0.027(2) 0.0057(17) 0.0151(17) 0.0066(17)
C47 0.032(2) 0.033(2) 0.0210(18) 0.0120(16) 0.0164(16) 0.0147(17)
C48 0.0252(18) 0.0195(19) 0.0176(16) 0.0085(14) 0.0135(14) 0.0127(16)
C49 0.048(3) 0.043(3) 0.064(3) 0.027(3) 0.029(3) 0.024(2)
C50 0.047(3) 0.051(3) 0.035(2) 0.000(2) 0.014(2) 0.023(2)

```
C51 0.036(3) 0.047(3) 0.053(3) 0.010(3) 0.001(2) 0.018(2)
C52 0.108(6) 0.093(6) 0.100(6) -0.010(5) 0.001(5) 0.065(5)
C53 0.063(3) 0.044(3) 0.053(3) 0.026(2) 0.044(3) 0.034(3)
C54 0.067(3) 0.047(3) 0.060(3) 0.026(3) 0.045(3) 0.023(3)

_geom_special_details
;
 All esds (except the esd in the dihedral angle between two l.s. planes)
 are estimated using the full covariance matrix.  The cell esds are
taken
 into account individually in the estimation of esds in distances,
angles
 and torsion angles; correlations between esds in cell parameters are
only
 used when they are defined by crystal symmetry.  An approximate
(isotropic)
 treatment of cell esds is used for estimating esds involving l.s.
planes.
;

loop_
 _geom_bond_atom_site_label_1
 _geom_bond_atom_site_label_2
 _geom_bond_distance
 _geom_bond_site_symmetry_2
 _geom_bond_publ_flag
Ir1 C48 1.874(4) . ?
Ir1 N2 2.029(3) . ?
Ir1 N3 2.037(3) . ?
Ir1 N1 2.057(3) . ?
Ir1 P1 2.4467(9) . ?
Ir1 Ir1 2.9731(4) 2_565 ?
S1 O2 1.406(4) . ?
S1 O3 1.426(3) . ?
S1 O4 1.446(4) . ?
S1 C49 1.826(5) . ?
S2 O7' 1.260(14) . ?
S2 O6 1.399(5) . ?
S2 O5 1.405(4) . ?
S2 O7 1.502(8) . ?
S2 O6' 1.598(13) . ?
S2 C50 1.787(5) . ?
P1 C36 1.821(4) . ?
P1 C30 1.822(4) . ?
```

P1 C42 1.826(4) . ?
F1 C49 1.332(6) . ?
F2 C49 1.333(6) . ?
F3 C49 1.280(6) . ?
F4 C50 1.283(6) . ?
F5 C50 1.353(6) . ?
F6 C50 1.308(6) . ?
O1 C48 1.135(4) . ?
N1 C8 1.352(4) . ?
N1 C1 1.385(4) . ?
N2 C13 1.362(4) . ?
N2 C9 1.363(4) . ?
N3 C14 1.340(4) . ?
N3 C21 1.377(4) . ?
N4 C51 1.094(7) . ?
N5 C53 1.125(6) . ?
C1 C2 1.392(5) . ?
C1 C6 1.400(5) . ?
C2 C3 1.388(5) . ?
C3 C4 1.401(6) . ?
C4 C5 1.378(6) . ?
C5 C6 1.400(5) . ?
C6 C7 1.372(5) . ?
C7 C8 1.351(4) . ?
C7 C22 1.478(4) . ?
C8 C9 1.460(5) . ?
C9 C10 1.388(5) . ?
C10 C11 1.384(5) . ?
C11 C12 1.392(5) . ?
C12 C13 1.378(5) . ?
C13 C14 1.468(5) . ?
C14 C15 1.353(4) . ?
C15 C16 1.385(5) . ?
C15 C26 1.473(4) . ?
C16 C17 1.395(5) . ?
C16 C21 1.402(5) . ?
C17 C18 1.382(5) . ?
C18 C19 1.404(5) . ?
C19 C20 1.382(5) . ?
C20 C21 1.395(5) . ?
C22 C23 1.521(5) . ?
C23 C24 1.518(5) . ?
C24 C25 1.521(6) . ?
C26 C27 1.523(5) . ?

```
C27 C28 1.519(5) . ?
C28 C29 1.528(6) . ?
C30 C35 1.385(5) . ?
C30 C31 1.399(5) . ?
C31 C32 1.389(6) . ?
C32 C33 1.380(7) . ?
C33 C34 1.375(7) . ?
C34 C35 1.403(5) . ?
C36 C41 1.386(5) . ?
C36 C37 1.398(5) . ?
C37 C38 1.392(5) . ?
C38 C39 1.380(6) . ?
C39 C40 1.381(6) . ?
C40 C41 1.393(5) . ?
C42 C47 1.399(5) . ?
C42 C43 1.403(5) . ?
C43 C44 1.383(6) . ?
C44 C45 1.370(6) . ?
C45 C46 1.384(6) . ?
C46 C47 1.381(5) . ?
C51 C52 1.467(9) . ?
C53 C54 1.449(7) . ?

loop_
 _geom_angle_atom_site_label_1
 _geom_angle_atom_site_label_2
 _geom_angle_atom_site_label_3
 _geom_angle
 _geom_angle_site_symmetry_1
 _geom_angle_site_symmetry_3
 _geom_angle_publ_flag
C48 Ir1 N2 177.23(13) . . ?
C48 Ir1 N3 100.08(13) . . ?
N2 Ir1 N3 78.44(11) . . ?
C48 Ir1 N1 103.36(13) . . ?
N2 Ir1 N1 78.06(11) . . ?
N3 Ir1 N1 156.50(11) . . ?
C48 Ir1 P1 93.58(10) . . ?
N2 Ir1 P1 88.78(8) . . ?
N3 Ir1 P1 90.31(8) . . ?
N1 Ir1 P1 89.91(8) . . ?
C48 Ir1 Ir1 83.90(10) . 2_565 ?
N2 Ir1 Ir1 93.71(7) . 2_565 ?
N3 Ir1 Ir1 88.84(7) . 2_565 ?
```

N1 Ir1 Ir1 91.96(7) . 2_565 ?
P1 Ir1 Ir1 177.15(2) . 2_565 ?
O2 S1 O3 117.0(3) . . ?
O2 S1 O4 113.2(2) . . ?
O3 S1 O4 116.3(2) . . ?
O2 S1 C49 102.2(2) . . ?
O3 S1 C49 102.6(2) . . ?
O4 S1 C49 102.4(3) . . ?
O7' S2 O5 123.1(10) . . ?
O6 S2 O5 126.8(5) . . ?
O6 S2 O7 110.9(5) . . ?
O5 S2 O7 109.8(4) . . ?
O7' S2 O6' 115.6(13) . . ?
O5 S2 O6' 90.2(8) . . ?
O7' S2 C50 121.1(8) . . ?
O6 S2 C50 103.8(3) . . ?
O5 S2 C50 104.4(2) . . ?
O7 S2 C50 95.9(5) . . ?
O6' S2 C50 95.1(6) . . ?
C36 P1 C30 103.00(16) . . ?
C36 P1 C42 102.77(17) . . ?
C30 P1 C42 107.11(17) . . ?
C36 P1 Ir1 116.18(12) . . ?
C30 P1 Ir1 113.44(11) . . ?
C42 P1 Ir1 113.19(11) . . ?
C8 N1 C1 106.8(3) . . ?
C8 N1 Ir1 114.3(2) . . ?
C1 N1 Ir1 138.7(2) . . ?
C13 N2 C9 122.3(3) . . ?
C13 N2 Ir1 118.5(2) . . ?
C9 N2 Ir1 119.1(2) . . ?
C14 N3 C21 107.7(3) . . ?
C14 N3 Ir1 114.6(2) . . ?
C21 N3 Ir1 137.2(2) . . ?
N1 C1 C2 130.9(3) . . ?
N1 C1 C6 107.3(3) . . ?
C2 C1 C6 121.7(3) . . ?
C3 C2 C1 116.3(4) . . ?
C2 C3 C4 122.1(4) . . ?
C5 C4 C3 121.6(3) . . ?
C4 C5 C6 116.8(4) . . ?
C7 C6 C1 107.7(3) . . ?
C7 C6 C5 130.9(4) . . ?
C1 C6 C5 121.4(4) . . ?

C8 C7 C6 107.2(3) . . ?
C8 C7 C22 130.2(3) . . ?
C6 C7 C22 122.5(3) . . ?
C7 C8 N1 111.0(3) . . ?
C7 C8 C9 130.9(3) . . ?
N1 C8 C9 118.0(3) . . ?
N2 C9 C10 119.5(3) . . ?
N2 C9 C8 110.5(3) . . ?
C10 C9 C8 130.0(3) . . ?
C11 C10 C9 118.7(3) . . ?
C10 C11 C12 121.0(3) . . ?
C13 C12 C11 119.1(3) . . ?
N2 C13 C12 119.4(3) . . ?
N2 C13 C14 110.3(3) . . ?
C12 C13 C14 130.3(3) . . ?
N3 C14 C15 110.9(3) . . ?
N3 C14 C13 118.0(3) . . ?
C15 C14 C13 131.0(3) . . ?
C14 C15 C16 107.0(3) . . ?
C14 C15 C26 130.4(3) . . ?
C16 C15 C26 122.6(3) . . ?
C15 C16 C17 130.9(3) . . ?
C15 C16 C21 107.1(3) . . ?
C17 C16 C21 122.0(3) . . ?
C18 C17 C16 116.3(3) . . ?
C17 C18 C19 122.1(3) . . ?
C20 C19 C18 121.5(3) . . ?
C19 C20 C21 117.1(3) . . ?
N3 C21 C20 131.6(3) . . ?
N3 C21 C16 107.3(3) . . ?
C20 C21 C16 121.1(3) . . ?
C7 C22 C23 111.8(3) . . ?
C24 C23 C22 111.8(3) . . ?
C23 C24 C25 111.9(4) . . ?
C15 C26 C27 110.2(3) . . ?
C28 C27 C26 112.5(3) . . ?
C27 C28 C29 111.5(4) . . ?
C35 C30 C31 118.6(3) . . ?
C35 C30 P1 116.3(3) . . ?
C31 C30 P1 125.0(3) . . ?
C32 C31 C30 120.3(4) . . ?
C33 C32 C31 120.5(4) . . ?
C34 C33 C32 120.0(4) . . ?
C33 C34 C35 119.8(4) . . ?

C30 C35 C34 120.8(4) . . ?
C41 C36 C37 119.1(3) . . ?
C41 C36 P1 120.8(3) . . ?
C37 C36 P1 119.7(3) . . ?
C38 C37 C36 119.6(4) . . ?
C39 C38 C37 120.8(4) . . ?
C38 C39 C40 119.8(4) . . ?
C39 C40 C41 119.8(4) . . ?
C36 C41 C40 120.9(3) . . ?
C47 C42 C43 118.3(4) . . ?
C47 C42 P1 121.2(3) . . ?
C43 C42 P1 120.3(3) . . ?
C44 C43 C42 120.3(4) . . ?
C45 C44 C43 120.5(4) . . ?
C44 C45 C46 120.1(4) . . ?
C47 C46 C45 120.2(4) . . ?
C46 C47 C42 120.4(4) . . ?
O1 C48 Ir1 177.3(3) . . ?
F3 C49 F1 108.4(4) . . ?
F3 C49 F2 105.6(5) . . ?
F1 C49 F2 106.8(4) . . ?
F3 C49 S1 113.3(4) . . ?
F1 C49 S1 112.2(4) . . ?
F2 C49 S1 110.1(4) . . ?
F4 C50 F6 110.1(4) . . ?
F4 C50 F5 104.3(5) . . ?
F6 C50 F5 102.9(5) . . ?
F4 C50 S2 113.9(4) . . ?
F6 C50 S2 115.0(4) . . ?
F5 C50 S2 109.5(3) . . ?
N4 C51 C52 176.2(7) . . ?
N5 C53 C54 179.4(5) . . ?

loop_
 _geom_torsion_atom_site_label_1
 _geom_torsion_atom_site_label_2
 _geom_torsion_atom_site_label_3
 _geom_torsion_atom_site_label_4
 _geom_torsion
 _geom_torsion_site_symmetry_1
 _geom_torsion_site_symmetry_2
 _geom_torsion_site_symmetry_3
 _geom_torsion_site_symmetry_4
 _geom_torsion_publ_flag

C8 N1 C1 C2 -177.4(3) . . . . ?
Ir1 N1 C1 C2 8.4(6) . . . . ?
C8 N1 C1 C6 0.3(4) . . . . ?
Ir1 N1 C1 C6 -173.9(2) . . . . ?
N1 C1 C2 C3 178.8(3) . . . . ?
C6 C1 C2 C3 1.4(5) . . . . ?
C1 C2 C3 C4 -0.6(5) . . . . ?
C2 C3 C4 C5 -0.6(6) . . . . ?
C3 C4 C5 C6 0.8(5) . . . . ?
N1 C1 C6 C7 -0.6(4) . . . . ?
C2 C1 C6 C7 177.3(3) . . . . ?
N1 C1 C6 C5 -179.1(3) . . . . ?
C2 C1 C6 C5 -1.2(5) . . . . ?
C4 C5 C6 C7 -178.1(3) . . . . ?
C4 C5 C6 C1 0.0(5) . . . . ?
C1 C6 C7 C8 0.8(4) . . . . ?
C5 C6 C7 C8 179.1(4) . . . . ?
C1 C6 C7 C22 -176.0(3) . . . . ?
C5 C6 C7 C22 2.3(6) . . . . ?
C6 C7 C8 N1 -0.6(4) . . . . ?
C22 C7 C8 N1 175.8(3) . . . . ?
C6 C7 C8 C9 176.4(3) . . . . ?
C22 C7 C8 C9 -7.2(6) . . . . ?
C1 N1 C8 C7 0.2(4) . . . . ?
Ir1 N1 C8 C7 176.0(2) . . . . ?
C1 N1 C8 C9 -177.2(3) . . . . ?
Ir1 N1 C8 C9 -1.5(4) . . . . ?
C13 N2 C9 C10 -2.3(5) . . . . ?
Ir1 N2 C9 C10 -178.1(2) . . . . ?
C13 N2 C9 C8 176.8(3) . . . . ?
Ir1 N2 C9 C8 1.0(3) . . . . ?
C7 C8 C9 N2 -176.6(3) . . . . ?
N1 C8 C9 N2 0.3(4) . . . . ?
C7 C8 C9 C10 2.4(6) . . . . ?
N1 C8 C9 C10 179.3(3) . . . . ?
N2 C9 C10 C11 1.1(5) . . . . ?
C8 C9 C10 C11 -177.8(3) . . . . ?
C9 C10 C11 C12 0.8(5) . . . . ?
C10 C11 C12 C13 -1.5(5) . . . . ?
C9 N2 C13 C12 1.6(5) . . . . ?
Ir1 N2 C13 C12 177.4(2) . . . . ?
C9 N2 C13 C14 -178.3(3) . . . . ?
Ir1 N2 C13 C14 -2.5(3) . . . . ?
C11 C12 C13 N2 0.3(5) . . . . ?

C11 C12 C13 C14 -179.8(3) . . . . ?
C21 N3 C14 C15 -0.9(4) . . . . ?
Ir1 N3 C14 C15 -174.4(2) . . . . ?
C21 N3 C14 C13 176.9(3) . . . . ?
Ir1 N3 C14 C13 3.4(4) . . . . ?
N2 C13 C14 N3 -0.6(4) . . . . ?
C12 C13 C14 N3 179.4(3) . . . . ?
N2 C13 C14 C15 176.6(3) . . . . ?
C12 C13 C14 C15 -3.3(6) . . . . ?
N3 C14 C15 C16 1.0(4) . . . . ?
C13 C14 C15 C16 -176.4(3) . . . . ?
N3 C14 C15 C26 179.6(3) . . . . ?
C13 C14 C15 C26 2.3(6) . . . . ?
C14 C15 C16 C17 178.5(4) . . . . ?
C26 C15 C16 C17 -0.3(6) . . . . ?
C14 C15 C16 C21 -0.6(4) . . . . ?
C26 C15 C16 C21 -179.4(3) . . . . ?
C15 C16 C17 C18 -178.1(3) . . . . ?
C21 C16 C17 C18 0.9(5) . . . . ?
C16 C17 C18 C19 -0.9(5) . . . . ?
C17 C18 C19 C20 0.3(6) . . . . ?
C18 C19 C20 C21 0.4(5) . . . . ?
C14 N3 C21 C20 -178.1(3) . . . . ?
Ir1 N3 C21 C20 -6.9(6) . . . . ?
C14 N3 C21 C16 0.5(3) . . . . ?
Ir1 N3 C21 C16 171.7(2) . . . . ?
C19 C20 C21 N3 178.1(3) . . . . ?
C19 C20 C21 C16 -0.4(5) . . . . ?
C15 C16 C21 N3 0.1(4) . . . . ?
C17 C16 C21 N3 -179.1(3) . . . . ?
C15 C16 C21 C20 178.9(3) . . . . ?
C17 C16 C21 C20 -0.3(5) . . . . ?
C8 C7 C22 C23 97.6(4) . . . . ?
C6 C7 C22 C23 -86.4(4) . . . . ?
C7 C22 C23 C24 173.5(3) . . . . ?
C22 C23 C24 C25 -176.1(4) . . . . ?
C14 C15 C26 C27 -93.3(4) . . . . ?
C16 C15 C26 C27 85.2(4) . . . . ?
C15 C26 C27 C28 -173.3(3) . . . . ?
C26 C27 C28 C29 174.9(4) . . . . ?
C36 P1 C30 C35 -57.0(3) . . . . ?
C42 P1 C30 C35 -165.0(3) . . . . ?
Ir1 P1 C30 C35 69.4(3) . . . . ?
C36 P1 C30 C31 120.4(3) . . . . ?

C42 P1 C30 C31 12.5(4) . . . . ?
Ir1 P1 C30 C31 -113.1(3) . . . . ?
C35 C30 C31 C32 -1.5(5) . . . . ?
P1 C30 C31 C32 -178.9(3) . . . . ?
C30 C31 C32 C33 0.1(6) . . . . ?
C31 C32 C33 C34 1.1(6) . . . . ?
C32 C33 C34 C35 -0.9(6) . . . . ?
C31 C30 C35 C34 1.8(5) . . . . ?
P1 C30 C35 C34 179.4(3) . . . . ?
C33 C34 C35 C30 -0.6(6) . . . . ?
C30 P1 C36 C41 156.2(3) . . . . ?
C42 P1 C36 C41 -92.5(3) . . . . ?
Ir1 P1 C36 C41 31.6(4) . . . . ?
C30 P1 C36 C37 -31.3(3) . . . . ?
C42 P1 C36 C37 79.9(3) . . . . ?
Ir1 P1 C36 C37 -155.9(3) . . . . ?
C41 C36 C37 C38 0.1(6) . . . . ?
P1 C36 C37 C38 -172.5(3) . . . . ?
C36 C37 C38 C39 0.2(6) . . . . ?
C37 C38 C39 C40 -0.5(7) . . . . ?
C38 C39 C40 C41 0.5(7) . . . . ?
C37 C36 C41 C40 0.0(6) . . . . ?
P1 C36 C41 C40 172.5(3) . . . . ?
C39 C40 C41 C36 -0.3(7) . . . . ?
C36 P1 C42 C47 -159.3(3) . . . . ?
C30 P1 C42 C47 -51.2(3) . . . . ?
Ir1 P1 C42 C47 74.6(3) . . . . ?
C36 P1 C42 C43 17.6(3) . . . . ?
C30 P1 C42 C43 125.8(3) . . . . ?
Ir1 P1 C42 C43 -108.5(3) . . . . ?
C47 C42 C43 C44 2.8(6) . . . . ?
P1 C42 C43 C44 -174.2(3) . . . . ?
C42 C43 C44 C45 0.6(6) . . . . ?
C43 C44 C45 C46 -2.7(6) . . . . ?
C44 C45 C46 C47 1.2(6) . . . . ?
C45 C46 C47 C42 2.3(6) . . . . ?
C43 C42 C47 C46 -4.3(5) . . . . ?
P1 C42 C47 C46 172.8(3) . . . . ?
O2 S1 C49 F3 -177.4(4) . . . . ?
O3 S1 C49 F3 -55.8(5) . . . . ?
O4 S1 C49 F3 65.2(4) . . . . ?
O2 S1 C49 F1 59.4(4) . . . . ?
O3 S1 C49 F1 -179.0(4) . . . . ?
O4 S1 C49 F1 -58.1(4) . . . . ?

```
O2 S1 C49 F2 -59.5(4) . . . . ?
O3 S1 C49 F2 62.1(4) . . . . ?
O4 S1 C49 F2 -176.9(3) . . . . ?
O7' S2 C50 F4 -92.3(19) . . . . ?
O6 S2 C50 F4 -173.4(6) . . . . ?
O5 S2 C50 F4 52.1(5) . . . . ?
O7 S2 C50 F4 -60.2(6) . . . . ?
O6' S2 C50 F4 143.6(10) . . . . ?
O7' S2 C50 F6 36.1(19) . . . . ?
O6 S2 C50 F6 -45.0(6) . . . . ?
O5 S2 C50 F6 -179.5(4) . . . . ?
O7 S2 C50 F6 68.2(5) . . . . ?
O6' S2 C50 F6 -88.0(10) . . . . ?
O7' S2 C50 F5 151.3(18) . . . . ?
O6 S2 C50 F5 70.2(6) . . . . ?
O5 S2 C50 F5 -64.2(5) . . . . ?
O7 S2 C50 F5 -176.5(5) . . . . ?
O6' S2 C50 F5 27.3(10) . . . . ?

_refine_diff_density_max    1.361
_refine_diff_density_min   -0.797
_refine_diff_density_rms    0.115

_shelx_res_file
;

    z.res created by SHELXL-2014/7


TITL z in P-1
CELL 0.71 14.0846 14.681 15.1434 99.6968 111.7595 108.9035
ZERR 2 0.0012 0.0012 0.0013 0.0027 0.0026 0.0026
LATT 1
SFAC C H N O F P S Ir
UNIT 108 100 10 14 12 2 4 2

L.S. 20
PLAN  1
TEMP -123.15
fmap 2
htab
size 0.21 0.19 0.17
conf
isor 0.005 C15
```

```
delu 0.005 C6 C7
ACTA
REM <olex2.extras>
REM <HklSrc "%.\\z.hkl">
REM </olex2.extras>

WGHT    0.030400    5.398500
FVAR       0.07747   0.67749
IR1   8    0.010401    0.516179    0.103357    11.00000    0.01944
0.01089 =
         0.01579    0.00533    0.01129    0.00850
S1    7   -0.235451    0.016650   -0.037851    11.00000    0.03970
0.02478 =
         0.04912    0.02000    0.02955    0.01983
S2    7    0.313603    1.019827    0.580653    11.00000    0.04570
0.04080 =
         0.06684    0.02772    0.03562    0.02317
P1    6    0.017397    0.537645    0.269746    11.00000    0.02535
0.01855 =
         0.01853    0.00796    0.01447    0.01219
F1    5   -0.377292    0.102110   -0.085893    11.00000    0.07598
0.09221 =
         0.09641    0.06262    0.04959    0.06427
F2    5   -0.321103    0.094212    0.063517    11.00000    0.11792
0.10757 =
         0.08062    0.03900    0.06904    0.07730
F3    5   -0.440253   -0.036502   -0.058425    11.00000    0.04352
0.07529 =
         0.15433    0.05025    0.05001    0.01711
F4    5    0.373977    0.972606    0.443889    11.00000    0.24018
0.18038 =
         0.07285    0.05019    0.08983    0.17081
F5    5    0.442065    1.128553    0.517859    11.00000    0.05811
0.14861 =
         0.08640    0.04691    0.03746   -0.00588
F6    5    0.274016    1.055373    0.409397    11.00000    0.06166
0.10721 =
         0.06394    0.04493    0.02016    0.04166
O1    4   -0.193974    0.315466    0.009175    11.00000    0.03139
0.01771 =
         0.02791    0.00485    0.01677    0.00503
O2    4   -0.147761    0.114796   -0.000531    11.00000    0.04384
0.04724 =
         0.07903    0.02072    0.02306    0.00290
```

```
O3     4    -0.223136    -0.046780     0.023356     11.00000     0.08058
0.07379 =
         0.08781    0.06362    0.05925    0.05982
O4     4    -0.279444    -0.031985    -0.144685     11.00000     0.14423
0.04289 =
         0.06371    0.02520    0.06604    0.05196
O5     4     0.404870     1.007101     0.647856     11.00000     0.05339
0.11422 =
         0.05487    0.03673    0.02439    0.04264
PART   1
O6     4     0.276585     1.094768     0.601372     21.00000     0.09415
0.06001 =
         0.07545    0.04017    0.06600    0.05863
PART   2
O6'    4     0.370343     1.137904     0.645446    -21.00000     0.10564
0.04085 =
         0.04698    0.00578    0.03453    0.02533
PART   1
O7     4     0.215580     0.917856     0.518542     21.00000     0.06111
0.03905 =
         0.10982    0.01593    0.02910   -0.00529
PART   2
O7'    4     0.213099     0.970727     0.562870    -21.00000     0.02465
0.13970 =
         0.07051    0.05419    0.01394   -0.00555
PART   0
N1     3     0.143808     0.476453     0.149215     11.00000     0.02383
0.01657 =
         0.01862    0.00795    0.01301    0.01335
N2     3     0.144600     0.653777     0.162130     11.00000     0.01950
0.01494 =
         0.01567    0.00632    0.01117    0.00820
N3     3    -0.067024     0.611648     0.080222     11.00000     0.02079
0.01275 =
         0.01708    0.00516    0.01112    0.00980
N4     3    -0.408236     0.411804     0.246003     11.00000     0.04821
0.06479 =
         0.06521    0.01955    0.02863    0.02918
N5     3     0.516523     1.277936     0.888120     11.00000     0.07008
0.04266 =
         0.07167    0.01826    0.04489    0.02608
C1     1     0.162274     0.390004     0.154705     11.00000     0.03062
0.02212 =
         0.02056    0.01139    0.01744    0.01687
```

```
C2     1     0.087124     0.287673     0.117217     11.00000     0.03290
0.02032 =
         0.02159    0.00741    0.01796    0.01483
AFIX  43
H2    2    0.007944    0.266152    0.081720    11.00000   -1.20000
AFIX   0
C3     1     0.134198     0.218767     0.134534     11.00000     0.04769
0.01863 =
         0.02878    0.01187    0.02477    0.02005
AFIX  43
H3    2    0.085697    0.148216    0.110688    11.00000   -1.20000
AFIX   0
C4     1     0.250974     0.249928     0.186063     11.00000     0.04820
0.03018 =
         0.03153    0.01691    0.02436    0.02978
AFIX  43
H4    2    0.279403    0.200126    0.196723    11.00000   -1.20000
AFIX   0
C5     1     0.325556     0.351085     0.221644     11.00000     0.03962
0.03563 =
         0.02939    0.01819    0.02122    0.02815
AFIX  43
H5    2    0.404761    0.372132    0.255801    11.00000   -1.20000
AFIX   0
C6     1     0.279026     0.421353     0.205048     11.00000     0.03049
0.02634 =
         0.01939    0.00964    0.01566    0.01889
C7     1     0.329528     0.525156     0.228280     11.00000     0.01241
0.01356 =
         0.01012    0.00522    0.00745    0.00998
C8     1     0.246303     0.556280     0.194435     11.00000     0.02148
0.02101 =
         0.01731    0.00688    0.01187    0.01080
C9     1     0.250718     0.658327     0.203122     11.00000     0.02277
0.02090 =
         0.01649    0.00717    0.01169    0.01316
C10    1     0.342954     0.752358     0.246166     11.00000     0.02062
0.02238 =
         0.02054    0.00644    0.01088    0.00946
AFIX  43
H10   2    0.417552    0.756937    0.274081    11.00000   -1.20000
AFIX   0
C11    1     0.324150     0.839373     0.247638     11.00000     0.02660
0.01774 =
```

```
         0.02380    0.00426    0.01311    0.00757
AFIX  43
H11   2    0.386598    0.904145    0.276137    11.00000   -1.20000
AFIX   0
C12   1     0.215115     0.833385     0.207985     11.00000     0.03079
0.01500 =
         0.02161    0.00534    0.01412    0.01155
AFIX  43
H12   2    0.202951    0.893557    0.210686    11.00000   -1.20000
AFIX   0
C13   1     0.125068     0.738925     0.164757     11.00000     0.02518
0.01403 =
         0.01548    0.00608    0.01269    0.01119
C14   1     0.003323     0.710920     0.117696     11.00000     0.02437
0.01631 =
         0.01609    0.00693    0.01311    0.01138
C15   1    -0.054659     0.769327     0.107403     11.00000     0.01450
0.00611 =
         0.01153    0.00352    0.00806    0.00833
C16   1    -0.168069     0.703662     0.062341     11.00000     0.02723
0.02044 =
         0.01975    0.00808    0.01509    0.01453
C17   1    -0.263612     0.723294     0.036615     11.00000     0.03179
0.02852 =
         0.02629    0.01149    0.01720    0.02183
AFIX  43
H17   2   -0.258578    0.790699    0.047352    11.00000   -1.20000
AFIX   0
C18   1    -0.366012     0.639194    -0.005369     11.00000     0.02634
0.03790 =
         0.02789    0.01035    0.01589    0.02193
AFIX  43
H18   2   -0.433073    0.649299   -0.023052    11.00000   -1.20000
AFIX   0
C19   1    -0.374132     0.539292    -0.022734     11.00000     0.02315
0.03074 =
         0.02301    0.00712    0.01357    0.01203
AFIX  43
H19   2   -0.446331    0.483724   -0.052027    11.00000   -1.20000
AFIX   0
C20   1    -0.279421     0.520041     0.001849     11.00000     0.02584
0.02176 =
         0.01893    0.00637    0.01320    0.01250
AFIX  43
```

```
H20   2   -0.284930    0.452452   -0.010272    11.00000   -1.20000
AFIX   0
C21    1    -0.175392     0.604202     0.045236     11.00000     0.02149
0.02177 =
         0.01666    0.00776    0.01351    0.01361
C22    1     0.453292     0.583750     0.276226     11.00000     0.02144
0.02678 =
         0.02742    0.00950    0.01343    0.01347
AFIX  23
H22A  2    0.471431    0.646063    0.257016    11.00000   -1.20000
H22B  2    0.486781    0.542163    0.251195    11.00000   -1.20000
AFIX   0
C23    1     0.505018     0.613679     0.390319     11.00000     0.02875
0.03054 =
         0.02716    0.00966    0.01504    0.01374
AFIX  23
H23A  2    0.478256    0.661762    0.415987    11.00000   -1.20000
H23B  2    0.479561    0.552197    0.409306    11.00000   -1.20000
AFIX   0
C24    1     0.632907     0.663114     0.438952     11.00000     0.02854
0.02869 =
         0.03514    0.01208    0.01281    0.01055
AFIX  23
H24A  2    0.658467    0.727149    0.423836    11.00000   -1.20000
H24B  2    0.659453    0.616959    0.409764    11.00000   -1.20000
AFIX   0
C25    1     0.684910     0.686520     0.552426     11.00000     0.04021
0.04009 =
         0.03503    0.00864    0.00439    0.00898
AFIX 137
H25A  2    0.659480    0.623344    0.567697    11.00000   -1.50000
H25B  2    0.661474    0.734574    0.581934    11.00000   -1.50000
H25C  2    0.767291    0.716725    0.580780    11.00000   -1.50000
AFIX   0
C26    1    -0.014069     0.881001     0.137469     11.00000     0.03112
0.01559 =
         0.02718    0.00797    0.01580    0.01480
AFIX  23
H26A  2   -0.067681    0.899201    0.088092    11.00000   -1.20000
H26B  2    0.060039    0.911834    0.138055    11.00000   -1.20000
AFIX   0
C27    1    -0.002830     0.922947     0.241687     11.00000     0.03771
0.02178 =
         0.02582    0.00599    0.01514    0.01546
```

```
AFIX  23
H27A  2   -0.074547    0.885444    0.242865    11.00000   -1.20000
H27B  2    0.057050    0.911188    0.291741    11.00000   -1.20000
AFIX   0
C28    1     0.025528     1.036143     0.271461     11.00000     0.04438
0.02440 =
         0.03300    0.00474    0.01667    0.01929
AFIX  23
H28A  2   -0.030586    1.048963    0.218528    11.00000   -1.20000
H28B  2    0.100635    1.074481    0.276617    11.00000   -1.20000
AFIX   0
C29    1     0.025993     1.074411     0.371963     11.00000     0.06002
0.03997 =
         0.03633    0.00074    0.02119    0.03144
AFIX 137
H29A  2   -0.048826    1.037738    0.366538    11.00000   -1.50000
H29B  2    0.045024    1.147559    0.389184    11.00000   -1.50000
H29C  2    0.082079    1.062483    0.424633    11.00000   -1.50000
AFIX   0
C30    1    -0.048734     0.618678     0.298778     11.00000     0.03399
0.02139 =
         0.02171    0.01118    0.02061    0.01684
C31    1     0.008299     0.714411     0.373342     11.00000     0.04078
0.02559 =
         0.03094    0.01172    0.02497    0.01654
AFIX  43
H31   2    0.087806    0.742088    0.412531    11.00000   -1.20000
AFIX   0
C32    1    -0.050860     0.769166     0.390309     11.00000     0.06197
0.02630 =
         0.04279    0.01421    0.03922    0.02574
AFIX  43
H32   2   -0.011485    0.834156    0.441045    11.00000   -1.20000
AFIX   0
C33    1    -0.166376     0.729900     0.334048     11.00000     0.06646
0.04253 =
         0.05290    0.02853    0.04871    0.04089
AFIX  43
H33   2   -0.206040    0.768360    0.345299    11.00000   -1.20000
AFIX   0
C34    1    -0.224055     0.635133     0.261721     11.00000     0.04320
0.05057 =
         0.04199    0.02457    0.03138    0.03247
AFIX  43
```

```
H34   2   -0.303696    0.607596    0.223559    11.00000   -1.20000
AFIX   0
C35    1    -0.164913     0.579225     0.244455     11.00000     0.03995
0.03213 =
         0.02588    0.01371    0.02050    0.02082
AFIX  43
H35   2   -0.205075    0.513481    0.194876    11.00000   -1.20000
AFIX   0
C36    1    -0.053189     0.421901     0.291307     11.00000     0.02937
0.02123 =
         0.02074    0.01120    0.01568    0.01298
C37    1    -0.090843     0.427944     0.364626     11.00000     0.04045
0.02181 =
         0.02689    0.00983    0.02202    0.01319
AFIX  43
H37   2   -0.087823    0.490713    0.397430    11.00000   -1.20000
AFIX   0
C38    1    -0.132758     0.341463     0.389242     11.00000     0.04947
0.03479 =
         0.02858    0.01514    0.02851    0.01767
AFIX  43
H38   2   -0.158529    0.345543    0.438914    11.00000   -1.20000
AFIX   0
C39    1    -0.137361     0.249875     0.342421     11.00000     0.05104
0.02669 =
         0.04021    0.02212    0.02928    0.01733
AFIX  43
H39   2   -0.165606    0.191471    0.360224    11.00000   -1.20000
AFIX   0
C40    1    -0.100802     0.243285     0.269585     11.00000     0.05436
0.02414 =
         0.03962    0.01771    0.03169    0.02280
AFIX  43
H40   2   -0.104420    0.180224    0.236755    11.00000   -1.20000
AFIX   0
C41    1    -0.058610     0.329447     0.244449     11.00000     0.04291
0.02575 =
         0.02886    0.01314    0.02556    0.02041
AFIX  43
H41   2   -0.033231    0.324751    0.194540    11.00000   -1.20000
AFIX   0
C42    1     0.160675     0.590625     0.371950     11.00000     0.03325
0.02667 =
         0.01658    0.00747    0.01639    0.01675
```

```
C43   1    0.198892    0.529195    0.424182    11.00000    0.03893
0.03147 =
         0.02596   0.01001   0.01494   0.02197
AFIX  43
H43   2   0.152586   0.458301   0.402426   11.00000   -1.20000
AFIX   0
C44   1    0.303686    0.571398    0.507206    11.00000    0.04306
0.04798 =
         0.02756   0.01715   0.01336   0.03066
AFIX  43
H44   2   0.329251   0.529144   0.541750   11.00000   -1.20000
AFIX   0
C45   1    0.370894    0.673647    0.540009    11.00000    0.03098
0.05202 =
         0.02565   0.00859   0.01027   0.02003
AFIX  43
H45   2   0.441361   0.702498   0.598798   11.00000   -1.20000
AFIX   0
C46   1    0.336363    0.735015    0.487691    11.00000    0.02827
0.03433 =
         0.02741   0.00575   0.01508   0.00660
AFIX  43
H46   2   0.383838   0.805670   0.509902   11.00000   -1.20000
AFIX   0
C47   1    0.233059    0.693820    0.403264    11.00000    0.03178
0.03298 =
         0.02097   0.01202   0.01639   0.01467
AFIX  43
H47   2   0.211003   0.735804   0.366217   11.00000   -1.20000
AFIX   0
C48   1   -0.115936    0.390324    0.043160    11.00000    0.02521
0.01954 =
         0.01763   0.00849   0.01350   0.01271
C49   1   -0.350745    0.043848   -0.031214    11.00000    0.04819
0.04281 =
         0.06370   0.02724   0.02883   0.02424
C50   1    0.348268    1.040628    0.481387    11.00000    0.04748
0.05142 =
         0.03547   0.00005   0.01360   0.02268
C51   1   -0.397596    0.349248    0.206329    11.00000    0.03558
0.04693 =
         0.05252   0.00960   0.00084   0.01835
C52   1   -0.378097    0.270344    0.151722    11.00000    0.10800
0.09346 =
```

```
         0.09984   -0.00956    0.00075    0.06498
AFIX 137
H52A  2   -0.406845    0.265606    0.080656    11.00000   -1.50000
H52B  2   -0.417367    0.204588    0.157246    11.00000   -1.50000
H52C  2   -0.297056    0.288139    0.180792    11.00000   -1.50000
AFIX   0
C53   1     0.446183     1.200574     0.863804     11.00000     0.06287
0.04412 =
         0.05310    0.02576    0.04367    0.03406
C54   1     0.355724     1.101163     0.833595     11.00000     0.06670
0.04685 =
         0.05957    0.02647    0.04510    0.02251
AFIX 137
H54A  2    0.289689    1.092629    0.773931    11.00000   -1.50000
H54B  2    0.335887    1.095564    0.888635    11.00000   -1.50000
H54C  2    0.380433    1.048078    0.817792    11.00000   -1.50000

AFIX   0
HKLF 4

REM  z in P-1
REM R1 =  0.0321 for   10616 Fo > 4sig(Fo)  and  0.0411 for all   11940
data
REM    708 parameters refined using      7 restraints

END

WGHT      0.0274      5.5053

REM Instructions for potential hydrogen bonds
HTAB C2 O2
EQIV $1 -x+1, -y+2, -z+1
HTAB C10 O6'_$1
EQIV $2 -x, -y+1, -z
HTAB C12 O4_$2
HTAB C31 O7
EQIV $3 -x-1, -y, -z
HTAB C52 F3_$3
HTAB C54 O6
EQIV $4 -x, -y+1, -z+1
HTAB C54 O3_$4

REM Highest difference peak  1.361,  deepest hole -0.797,  1-sigma
level  0.115
```

```
Q1    1   0.2746  0.9287  0.4210  11.00000  0.05    1.36
;
_shelx_res_checksum   20071

_shelx_hkl_file
;
  -2   0   0   15.73    0.56
  -2   0   0   15.94    0.63
  -2   0   0   17.86    0.65
  -2   0   0   16.97    0.55
   2   0   0   15.94    0.56
   2   0   0   16.30    0.62
   2   0   0   17.66    0.65
   2   0   0   16.97    0.55
  -3   0   0  304.49    9.30
  -3   0   0  326.07    9.45
  -3   0   0  301.72    9.33
  -3   0   0  316.59    9.44
   3   0   0  307.30    9.40
   3   0   0  309.76    9.33
   3   0   0  329.65    9.48
   3   0   0  309.87    9.30
  -4   0   0   80.00    2.56
  -4   0   0   80.82    2.53
  -4   0   0   85.93    2.72
  -4   0   0   78.87    2.70
   4   0   0   83.25    2.76
   4   0   0   80.61    2.57
   4   0   0   84.19    2.66
   4   0   0   83.69    2.54
  -5   0   0  134.14    4.25
  -5   0   0  130.33    4.42
  -5   0   0  145.71    4.46
  -5   0   0  138.32    4.22
   5   0   0  143.38    4.22
   5   0   0  134.94    4.35
   5   0   0  138.10    4.27
   5   0   0  142.44    4.52
  -6   0   0  123.77    3.88
  -6   0   0  116.12    3.60
   6   0   0  115.21    3.95
   6   0   0  116.91    3.74
   6   0   0  113.95    3.75
   6   0   0  105.80    3.85
```

6	0	0	120.67	3.68
6	0	0	118.06	3.60
-7	0	0	62.35	2.11
-7	0	0	69.13	2.45
7	0	0	65.72	2.29
7	0	0	62.16	2.52
7	0	0	64.90	2.28
7	0	0	68.45	2.13
7	0	0	62.07	2.29
7	0	0	63.79	2.20
-8	0	0	54.46	2.21
-8	0	0	52.12	1.83
8	0	0	52.03	2.05
8	0	0	54.61	2.37
8	0	0	54.91	1.95
8	0	0	55.53	1.85
8	0	0	53.45	1.99
-9	0	0	44.21	1.56
-9	0	0	43.78	2.00
9	0	0	40.87	1.79
9	0	0	43.19	1.56
9	0	0	40.14	1.68
9	0	0	43.97	1.71
9	0	0	41.39	2.16
9	0	0	43.48	1.78
-10	0	0	9.93	0.70
10	0	0	9.27	0.82
10	0	0	10.83	1.44
10	0	0	10.33	0.99
10	0	0	9.23	0.95
-11	0	0	11.71	0.80
11	0	0	11.49	0.93
11	0	0	9.85	1.69
11	0	0	11.55	1.16
11	0	0	9.82	1.10
-12	0	0	16.04	1.01
12	0	0	15.27	1.08
12	0	0	16.47	2.06
12	0	0	13.92	1.40
12	0	0	15.14	1.41
-13	0	0	15.00	1.15
13	0	0	14.81	1.14
13	0	0	18.33	2.44
13	0	0	15.48	1.61

-14	0	0	5.82	0.82
14	0	0	5.38	1.97
14	0	0	5.93	1.20
-15	0	0	3.03	0.86
15	0	0	2.31	1.17
15	0	0	2.11	2.10
15	-1	0	7.26	2.21
15	-1	0	8.32	1.46
-15	1	0	4.42	0.85
14	-1	0	7.33	1.85
14	-1	0	4.55	1.04
-14	1	0	3.18	0.72
13	-1	0	8.80	1.78
13	-1	0	10.59	0.92
13	-1	0	10.24	1.42
-13	1	0	9.90	0.95
12	-1	0	12.55	1.29
12	-1	0	12.30	0.87
12	-1	0	10.95	1.88
12	-1	0	11.64	1.14
-12	1	0	12.45	0.89
11	-1	0	44.23	2.60
11	-1	0	44.48	1.72
11	-1	0	47.04	1.86
-11	1	0	44.09	1.70
10	-1	0	47.71	1.78
10	-1	0	45.53	2.43
10	-1	0	44.35	1.68
10	-1	0	43.30	1.95
-10	1	0	47.01	1.67
-10	1	0	44.46	2.14
9	-1	0	16.51	1.00
9	-1	0	15.74	0.79
9	-1	0	13.04	1.33
9	-1	0	16.75	0.92
9	-1	0	15.59	0.93
9	-1	0	15.12	0.82
-9	1	0	16.72	1.17
-9	1	0	15.98	0.78
8	-1	0	29.36	1.31
8	-1	0	28.82	1.19
8	-1	0	30.33	1.12
8	-1	0	27.98	1.60
8	-1	0	29.55	1.17

8	-1	0	27.79	1.20
-8	1	0	28.49	1.09
-8	1	0	28.20	1.41
7	-1	0	38.06	1.38
7	-1	0	39.21	1.42
7	-1	0	39.22	1.54
7	-1	0	37.11	1.42
7	-1	0	38.10	1.78
7	-1	0	40.42	1.36
-7	1	0	37.48	1.34
-7	1	0	40.59	1.64
6	-1	0	133.21	4.35
6	-1	0	131.63	3.99
6	-1	0	134.80	3.97
6	-1	0	128.17	4.11
6	-1	0	126.70	4.01
-6	1	0	131.04	4.20
-6	1	0	120.28	4.09
-6	1	0	123.81	3.95
5	-1	0	16.08	0.60
5	-1	0	14.98	0.63
5	-1	0	15.87	0.87
5	-1	0	14.70	0.61
5	-1	0	14.69	0.70
-5	1	0	14.91	0.59
-5	1	0	14.79	0.87
-5	1	0	14.72	0.71
4	-1	0	23.59	1.03
4	-1	0	22.95	0.81
4	-1	0	23.32	0.79
4	-1	0	21.45	0.87
4	-1	0	22.94	0.78
-4	1	0	22.66	0.78
-4	1	0	22.06	0.94
-4	1	0	22.13	0.85
3	-1	0	38.60	1.24
3	-1	0	39.16	1.29
3	-1	0	36.64	1.20
3	-1	0	39.54	1.41
3	-1	0	38.51	1.23
-3	1	0	40.33	1.37
-3	1	0	39.53	1.25
-3	1	0	38.15	1.22
2	-1	0	226.45	6.80

2	-1	0	220.34	6.70
2	-1	0	217.42	6.66
-2	1	0	218.29	6.66
-2	1	0	226.56	6.70
-2	1	0	226.98	6.64
-2	1	0	233.41	6.79
1	1	0	252.79	7.56
1	1	0	253.03	7.41
1	1	0	254.46	7.44
1	1	0	241.65	7.55
-2	-1	0	168.89	4.98
-2	-1	0	164.92	5.01
-2	-1	0	164.25	5.17
2	1	0	162.32	4.97
2	1	0	168.20	5.02
2	1	0	167.51	5.16
2	1	0	167.39	5.18
2	1	0	172.51	5.11
-3	-1	0	9.38	0.62
-3	-1	0	10.00	0.39
-3	-1	0	9.96	0.45
3	1	0	9.81	0.60
3	1	0	9.39	0.65
3	1	0	10.73	0.45
3	1	0	10.23	0.39
-4	-1	0	80.94	2.49
-4	-1	0	79.53	2.54
4	1	0	80.98	2.71
4	1	0	83.37	2.85
4	1	0	81.25	2.66
4	1	0	78.95	2.76
4	1	0	84.73	2.49
4	1	0	75.33	2.58
-5	-1	0	94.03	3.08
-5	-1	0	96.70	3.03
5	1	0	94.13	3.43
5	1	0	97.55	3.30
5	1	0	101.55	3.12
5	1	0	102.97	3.04
5	1	0	97.39	3.22
5	1	0	99.00	3.31
-6	-1	0	90.43	2.98
-6	-1	0	92.51	2.92
6	1	0	92.22	3.42

6	1	0	94.81	3.27
6	1	0	95.71	3.03
6	1	0	91.86	3.20
6	1	0	93.86	2.93
6	1	0	94.78	3.15
-7	-1	0	62.79	2.11
7	1	0	63.37	2.23
7	1	0	66.87	2.37
7	1	0	66.40	2.73
7	1	0	64.38	2.12
7	1	0	62.41	2.40
7	1	0	66.76	2.56
-8	-1	0	53.44	1.86
8	1	0	54.38	2.00
8	1	0	56.83	1.88
8	1	0	53.31	2.57
8	1	0	54.71	2.38
8	1	0	55.37	2.10
8	1	0	54.58	2.16
8	1	0	54.59	2.14
-9	-1	0	3.83	0.52
9	1	0	3.73	0.85
9	1	0	4.10	0.68
9	1	0	4.03	0.61
9	1	0	4.76	0.83
9	1	0	4.12	1.02
9	1	0	4.31	0.73
9	1	0	4.31	0.55
-10	-1	0	4.90	0.62
10	1	0	6.00	1.05
10	1	0	5.84	0.93
10	1	0	5.73	0.90
10	1	0	5.22	1.05
-11	-1	0	9.80	0.85
11	1	0	14.30	1.37
11	1	0	12.09	1.18
11	1	0	13.62	1.14
-12	-1	0	11.29	0.95
12	1	0	11.91	1.17
12	1	0	12.02	1.36
12	1	0	10.18	1.64
12	1	0	9.12	1.57
-13	-1	0	3.08	0.69
13	1	0	3.32	0.80

13	1	0	4.53	1.09
13	1	0	3.70	1.58
-14	-1	0	4.12	0.84
14	1	0	3.58	1.77
14	1	0	5.57	1.23
15	1	0	0.38	2.01
16	-2	0	7.61	2.41
-16	2	0	3.91	0.91
15	-2	0	0.32	0.97
15	-2	0	-0.03	1.93
-15	2	0	1.56	0.72
14	-2	0	8.13	1.94
14	-2	0	9.62	1.32
-14	2	0	9.87	1.05
13	-2	0	26.21	1.74
13	-2	0	25.59	2.58
13	-2	0	21.39	1.06
-13	2	0	23.83	1.25
12	-2	0	27.12	1.29
12	-2	0	27.12	1.63
12	-2	0	22.21	1.11
-12	2	0	28.16	1.24
11	-2	0	26.30	1.22
11	-2	0	25.86	1.49
11	-2	0	23.27	1.10
11	-2	0	25.80	2.11
-11	2	0	28.00	1.15
10	-2	0	63.00	2.96
10	-2	0	56.75	2.25
10	-2	0	64.55	2.22
10	-2	0	66.41	2.51
10	-2	0	64.15	2.22
-10	2	0	65.54	2.20
-10	2	0	65.24	2.61
9	-2	0	61.70	2.07
9	-2	0	56.37	1.99
9	-2	0	62.08	2.16
9	-2	0	59.94	2.30
9	-2	0	59.62	2.07
9	-2	0	62.18	2.73
-9	2	0	61.79	2.05
-9	2	0	59.81	2.38
8	-2	0	58.48	2.58
8	-2	0	59.69	1.99

8	-2	0	61.69	2.06
8	-2	0	66.88	2.07
8	-2	0	60.80	2.25
8	-2	0	61.16	2.12
-8	2	0	62.37	2.35
-8	2	0	60.01	2.04
7	-2	0	87.06	2.67
7	-2	0	87.16	2.72
7	-2	0	77.26	2.59
7	-2	0	81.24	2.82
7	-2	0	84.00	3.14
-7	2	0	85.95	2.93
-7	2	0	80.94	2.73
-7	2	0	82.56	2.65
6	-2	0	32.88	1.14
6	-2	0	31.10	1.47
6	-2	0	28.14	1.02
6	-2	0	31.84	1.09
6	-2	0	30.87	1.22
-6	2	0	31.19	1.33
-6	2	0	30.60	1.14
-6	2	0	30.56	1.08
5	-2	0	298.95	9.46
5	-2	0	309.05	9.45
5	-2	0	296.43	9.26
5	-2	0	312.44	9.69
5	-2	0	319.65	9.35
-5	2	0	306.91	9.33
-5	2	0	330.32	9.60
-5	2	0	324.98	9.34
4	-2	0	197.43	5.76
4	-2	0	183.97	5.69
4	-2	0	189.76	5.77
4	-2	0	193.89	6.07
4	-2	0	191.38	5.85
-4	2	0	196.16	5.72
-4	2	0	187.99	5.75
-4	2	0	192.65	5.99
3	-2	0	409.76	12.74
3	-2	0	454.53	13.06
3	-2	0	414.97	12.78
3	-2	0	430.10	12.85
-3	2	0	429.22	12.78
-3	2	0	430.69	12.83

-3	2	0	448.87	13.01
2	-2	0	41.40	1.34
2	-2	0	41.05	1.29
2	-2	0	41.07	1.52
2	-2	0	42.41	1.32
-2	2	0	41.21	1.29
-2	2	0	43.10	1.31
-2	2	0	42.95	1.34
-2	2	0	41.82	1.51
1	-2	0	63.83	2.05
1	-2	0	66.50	1.97
-1	2	0	67.89	2.01
-1	2	0	64.65	2.05
-1	2	0	66.07	2.20
-1	2	0	64.93	1.97
0	-2	0	287.73	8.91
0	-2	0	286.64	8.72
0	-2	0	285.87	8.81
0	2	0	306.90	8.73
0	2	0	307.15	8.82
0	2	0	287.28	8.92
-1	-2	0	39.59	1.49
-1	-2	0	41.69	1.30
1	2	0	43.34	1.26
1	2	0	38.76	1.50
1	2	0	41.67	1.25
1	2	0	40.76	1.43
1	2	0	42.53	1.32
-2	-2	0	38.36	1.44
-2	-2	0	37.27	1.22
-2	-2	0	36.51	1.17
2	2	0	36.41	1.36
2	2	0	39.07	1.24
2	2	0	36.94	1.46
2	2	0	37.88	1.17
-3	-2	0	103.53	3.18
-3	-2	0	103.51	3.23
3	2	0	104.12	3.38
3	2	0	105.14	3.40
3	2	0	106.86	3.57
3	2	0	97.92	3.49
3	2	0	109.22	3.17
3	2	0	106.66	3.25
-4	-2	0	146.02	4.49

-4	-2	0	145.69	4.43
4	2	0	147.17	4.68
4	2	0	150.09	4.53
4	2	0	150.71	4.43
4	2	0	141.85	4.89
4	2	0	147.51	4.68
4	2	0	141.47	4.77
-5	-2	0	18.17	0.77
-5	-2	0	18.00	0.71
5	2	0	19.91	0.83
5	2	0	18.62	1.01
5	2	0	18.81	0.71
5	2	0	17.42	1.07
5	2	0	19.23	1.00
5	2	0	17.59	1.27
-6	-2	0	62.06	2.04
-6	-2	0	63.55	2.11
6	2	0	64.77	2.34
6	2	0	65.38	2.17
6	2	0	63.44	2.43
6	2	0	52.94	2.59
6	2	0	66.73	2.04
6	2	0	61.51	2.37
-7	-2	0	52.76	1.87
-7	-2	0	53.76	1.80
7	2	0	53.65	2.22
7	2	0	55.14	2.22
7	2	0	48.18	2.51
7	2	0	54.11	2.14
7	2	0	54.43	1.80
7	2	0	52.71	1.94
-8	-2	0	12.08	0.68
8	2	0	10.77	1.16
8	2	0	13.76	1.10
8	2	0	10.25	1.63
8	2	0	12.81	0.69
8	2	0	12.99	1.14
8	2	0	13.77	0.89
8	2	0	11.38	0.88
-9	-2	0	13.14	0.76
9	2	0	15.57	1.28
9	2	0	13.14	1.21
9	2	0	15.20	1.95
9	2	0	13.44	1.04

9	2	0	13.44	1.39
9	2	0	13.94	0.81
-10	-2	0	6.07	0.68
10	2	0	7.21	1.25
10	2	0	6.62	1.15
10	2	0	7.71	1.17
-11	-2	0	3.07	0.96
11	2	0	3.95	0.85
11	2	0	2.86	1.13
11	2	0	5.14	1.00
11	2	0	3.11	0.78
-12	-2	0	0.87	0.57
12	2	0	2.16	1.18
12	2	0	2.36	0.82
12	2	0	1.26	0.82
-13	-2	0	4.40	0.81
13	2	0	3.56	0.90
13	2	0	6.83	1.17
13	2	0	2.26	1.47
-14	-2	0	1.80	0.89
14	2	0	3.54	1.24
14	2	0	2.26	1.91
16	-3	0	7.55	2.48
16	-3	0	3.47	1.26
-16	3	0	4.66	0.89
15	-3	0	10.23	2.36
15	-3	0	7.93	1.28
-15	3	0	10.10	1.14
14	-3	0	16.03	1.69
14	-3	0	24.96	2.92
-14	3	0	16.69	1.17
13	-3	0	16.81	2.37
13	-3	0	14.46	1.45
-13	3	0	14.69	1.01
12	-3	0	9.41	1.14
12	-3	0	10.00	0.70
12	-3	0	9.00	1.63
12	-3	0	9.76	0.53
-12	3	0	8.39	0.76
11	-3	0	41.40	1.91
11	-3	0	42.31	1.37
11	-3	0	39.45	2.73
11	-3	0	38.70	1.47
-11	3	0	41.20	1.56

10	-3	0	48.61	2.00
10	-3	0	44.76	1.60
10	-3	0	46.55	2.49
10	-3	0	47.66	1.52
10	-3	0	47.18	1.81
10	-3	0	45.80	1.70
-10	3	0	45.62	2.08
-10	3	0	46.66	1.69
9	-3	0	50.13	1.83
9	-3	0	47.42	1.98
9	-3	0	46.66	2.39
9	-3	0	53.09	1.60
9	-3	0	49.82	1.67
9	-3	0	50.69	1.76
-9	3	0	50.16	2.09
-9	3	0	49.45	1.74
8	-3	0	47.32	2.21
8	-3	0	46.28	1.85
8	-3	0	49.63	1.65
8	-3	0	47.80	1.70
8	-3	0	47.89	1.49
-8	3	0	45.03	1.94
-8	3	0	47.00	1.63
7	-3	0	94.61	2.79
7	-3	0	84.13	2.63
7	-3	0	83.54	3.26
7	-3	0	90.09	2.81
7	-3	0	84.60	2.94
-7	3	0	86.53	3.08
-7	3	0	85.44	2.75
-7	3	0	85.36	2.73
6	-3	0	128.02	4.16
6	-3	0	133.76	4.05
6	-3	0	134.77	4.03
6	-3	0	120.30	4.44
-6	3	0	128.75	3.96
-6	3	0	138.39	4.36
-6	3	0	129.20	4.01
5	-3	0	90.00	3.14
5	-3	0	87.57	2.83
5	-3	0	89.93	2.73
5	-3	0	86.63	2.75
-5	3	0	88.25	2.63
-5	3	0	86.08	2.71

-5	3	0	81.46	3.17
4	-3	0	100.65	3.01
4	-3	0	96.25	2.98
4	-3	0	98.70	3.11
4	-3	0	98.46	3.40
-4	3	0	97.75	3.31
-4	3	0	99.08	3.08
-4	3	0	95.12	2.94
-4	3	0	98.51	3.00
3	-3	0	91.22	2.89
3	-3	0	92.84	2.82
3	-3	0	92.44	2.85
3	-3	0	92.19	3.18
-3	3	0	97.56	3.16
-3	3	0	90.97	2.81
-3	3	0	92.13	2.88
-3	3	0	92.12	2.84
2	-3	0	365.57	10.86
2	-3	0	351.28	10.59
2	-3	0	344.52	10.49
2	-3	0	356.15	10.55
-2	3	0	346.16	10.49
-2	3	0	362.74	10.84
-2	3	0	349.84	10.58
-2	3	0	356.44	10.54
1	-3	0	124.32	4.03
1	-3	0	137.62	4.13
1	-3	0	127.29	4.31
1	-3	0	132.87	3.97
-1	3	0	131.29	4.32
-1	3	0	135.62	3.97
-1	3	0	139.02	4.04
-1	3	0	137.05	4.13
0	-3	0	15.39	0.48
0	-3	0	15.57	0.84
0	-3	0	14.12	0.55
0	-3	0	15.67	0.70
0	3	0	14.37	0.57
0	3	0	14.29	0.48
0	3	0	14.31	0.69
-1	-3	0	163.29	4.86
-1	-3	0	141.61	5.30
1	3	0	175.55	4.90
1	3	0	161.04	4.80

1	3	0	165.66	5.05
1	3	0	159.49	4.80
-2	-3	0	120.84	3.82
2	3	0	116.68	3.89
2	3	0	114.22	3.70
2	3	0	106.04	3.81
2	3	0	112.73	3.42
2	3	0	113.38	3.52
-3	-3	0	66.76	2.11
-3	-3	0	65.27	2.41
3	3	0	69.60	2.04
3	3	0	72.25	2.17
3	3	0	57.01	2.45
3	3	0	63.60	2.45
-4	-3	0	61.29	2.03
-4	-3	0	63.61	2.09
4	3	0	65.57	2.64
4	3	0	66.85	2.15
4	3	0	63.05	2.29
4	3	0	59.03	2.45
4	3	0	67.44	2.37
4	3	0	67.76	2.02
-5	-3	0	20.91	0.88
-5	-3	0	21.62	0.82
5	3	0	20.71	1.52
5	3	0	21.79	0.96
5	3	0	21.34	1.10
5	3	0	22.73	0.81
5	3	0	23.65	1.19
5	3	0	20.22	1.24
-6	-3	0	32.57	1.24
-6	-3	0	32.90	1.18
6	3	0	34.61	1.17
6	3	0	31.24	1.51
6	3	0	33.18	1.67
6	3	0	33.76	1.34
6	3	0	32.64	1.56
6	3	0	26.19	1.91
-7	-3	0	48.22	1.69
-7	-3	0	50.59	1.77
7	3	0	49.70	2.20
7	3	0	51.93	1.69
7	3	0	48.55	1.87
7	3	0	49.13	2.11

7	3	0	48.75	2.09
7	3	0	42.34	2.52
-8	-3	0	13.64	0.81
-8	-3	0	13.92	0.73
8	3	0	13.32	0.98
8	3	0	16.22	1.28
8	3	0	13.06	0.73
8	3	0	13.37	1.24
8	3	0	14.37	1.30
8	3	0	13.78	1.95
-9	-3	0	13.10	0.79
9	3	0	12.37	0.81
9	3	0	12.03	1.38
9	3	0	13.65	1.44
9	3	0	11.98	1.03
9	3	0	13.01	1.35
-10	-3	0	6.69	0.75
10	3	0	10.38	1.49
10	3	0	6.79	1.03
10	3	0	7.03	1.04
-11	-3	0	9.47	0.91
11	3	0	9.32	1.25
11	3	0	11.70	1.47
11	3	0	6.96	1.27
11	3	0	12.34	1.86
-12	-3	0	3.48	0.72
12	3	0	2.67	0.98
12	3	0	2.68	0.92
12	3	0	1.65	1.34
-13	-3	0	0.08	0.78
13	3	0	0.05	0.95
13	3	0	-0.10	0.99
13	3	0	-1.30	1.46
-14	-3	0	0.79	0.97
14	3	0	0.08	1.79
14	3	0	1.63	1.22
16	-4	0	5.11	1.29
16	-4	0	8.26	2.46
15	-4	0	16.97	3.15
15	-4	0	15.39	1.89
-15	4	0	16.30	1.24
14	-4	0	15.58	2.70
14	-4	0	17.46	1.73
-14	4	0	17.95	1.16

13	-4	0	32.20	1.93
13	-4	0	35.39	2.85
-13	4	0	33.05	1.48
12	-4	0	50.24	2.99
12	-4	0	47.35	2.22
-12	4	0	49.86	1.85
11	-4	0	29.98	2.24
11	-4	0	29.43	1.57
-11	4	0	30.18	1.24
10	-4	0	66.73	3.17
10	-4	0	71.19	2.45
10	-4	0	69.63	2.36
10	-4	0	69.32	2.63
-10	4	0	66.08	2.73
-10	4	0	68.05	2.32
9	-4	0	65.36	2.19
9	-4	0	59.07	2.80
9	-4	0	60.62	2.35
9	-4	0	63.08	2.13
-9	4	0	63.41	2.52
-9	4	0	61.28	2.10
8	-4	0	75.06	2.46
8	-4	0	76.57	2.42
8	-4	0	67.79	2.99
8	-4	0	74.59	2.62
-8	4	0	70.87	2.39
-8	4	0	75.03	2.81
7	-4	0	138.54	4.14
7	-4	0	136.19	4.12
7	-4	0	125.26	4.26
7	-4	0	129.51	4.70
-7	4	0	130.52	4.09
-7	4	0	133.00	4.50
6	-4	0	35.23	1.72
6	-4	0	35.04	1.36
6	-4	0	35.58	1.24
6	-4	0	36.62	1.25
-6	4	0	36.18	1.62
-6	4	0	34.56	1.21
-6	4	0	35.35	1.12
5	-4	0	136.64	4.68
5	-4	0	134.22	4.14
5	-4	0	132.88	4.28
5	-4	0	141.76	4.17

-5	4	0	137.03	4.58
-5	4	0	133.78	4.15
-5	4	0	136.30	4.09
-5	4	0	137.30	4.25
4	-4	0	304.79	8.95
4	-4	0	285.13	9.10
4	-4	0	306.72	9.42
4	-4	0	290.15	8.91
-4	4	0	310.43	9.35
-4	4	0	305.93	8.99
-4	4	0	286.39	8.90
-4	4	0	298.12	8.92
3	-4	0	142.55	4.29
3	-4	0	135.09	4.19
3	-4	0	138.34	4.30
-3	4	0	138.78	4.19
-3	4	0	141.80	4.26
-3	4	0	136.54	4.28
-3	4	0	140.54	4.64
2	-4	0	18.34	0.61
2	-4	0	17.50	0.78
2	-4	0	16.37	1.19
2	-4	0	17.82	0.70
-2	4	0	18.36	0.78
-2	4	0	17.94	1.04
-2	4	0	18.29	0.61
-2	4	0	19.00	0.69
1	-4	0	151.33	4.62
1	-4	0	150.71	4.88
1	-4	0	131.57	5.23
1	-4	0	162.95	4.72
-1	4	0	165.13	5.13
-1	4	0	156.07	4.62
-1	4	0	161.39	4.72
-1	4	0	159.01	4.85
0	-4	0	174.25	5.30
0	-4	0	167.60	5.21
0	4	0	184.26	5.76
0	4	0	178.55	5.34
0	4	0	181.02	5.66
0	4	0	167.65	5.22
-1	-4	0	67.24	2.12
-1	-4	0	65.95	2.50
1	4	0	65.15	2.49

1	4	0	70.72	2.18
1	4	0	72.02	2.62
1	4	0	62.76	2.34
1	4	0	66.64	2.04
1	4	0	67.88	2.05
-2	-4	0	76.21	2.81
-2	-4	0	75.77	2.41
2	4	0	76.60	2.92
2	4	0	74.19	2.83
2	4	0	80.87	2.48
2	4	0	77.22	2.33
2	4	0	75.45	2.68
2	4	0	74.96	2.33
-3	-4	0	97.35	3.46
-3	-4	0	95.87	3.08
3	4	0	102.17	3.17
3	4	0	97.49	3.00
3	4	0	100.85	3.67
3	4	0	92.66	3.51
3	4	0	100.22	3.00
3	4	0	99.12	3.38
-4	-4	0	36.73	1.66
-4	-4	0	32.80	1.18
-4	-4	0	34.23	1.24
4	4	0	35.42	1.34
4	4	0	33.05	1.90
4	4	0	34.57	1.56
4	4	0	34.77	1.72
-5	-4	0	13.72	0.69
-5	-4	0	14.76	0.64
5	4	0	15.23	0.82
5	4	0	14.38	1.50
5	4	0	15.18	0.62
5	4	0	13.91	1.16
5	4	0	14.71	1.06
5	4	0	13.09	0.94
-6	-4	0	32.43	1.19
-6	-4	0	31.95	1.26
6	4	0	33.38	1.78
6	4	0	31.03	1.54
6	4	0	31.61	1.64
6	4	0	33.93	1.39
6	4	0	33.15	1.17
6	4	0	30.61	2.19

-7	-4	0	8.75	0.61
-7	-4	0	7.84	0.65
7	4	0	9.40	1.25
7	4	0	10.08	0.83
7	4	0	9.50	1.16
7	4	0	11.05	0.60
7	4	0	9.96	1.52
7	4	0	8.64	1.03
-8	-4	0	16.86	0.85
-8	-4	0	17.06	0.93
8	4	0	15.35	1.45
8	4	0	15.17	1.51
8	4	0	16.20	1.50
8	4	0	16.65	0.85
-9	-4	0	26.19	1.18
9	4	0	28.13	1.91
9	4	0	23.50	1.84
9	4	0	24.70	1.17
9	4	0	25.48	1.81
9	4	0	29.05	1.49
-10	-4	0	2.57	0.54
10	4	0	1.40	0.99
10	4	0	1.63	0.93
10	4	0	1.09	0.66
10	4	0	4.49	1.45
-11	-4	0	1.75	0.61
11	4	0	-0.18	0.79
11	4	0	-0.65	1.13
11	4	0	2.68	1.00
-12	-4	0	0.83	0.73
12	4	0	0.69	0.90
12	4	0	-0.35	1.37
12	4	0	0.42	1.28
-13	-4	0	0.15	0.91
13	4	0	1.22	1.11
13	4	0	1.20	1.11
13	4	0	4.49	1.83
16	-5	0	4.46	2.53
16	-5	0	6.33	1.39
-16	5	0	4.83	0.82
15	-5	0	23.80	2.08
15	-5	0	26.54	3.37
-15	5	0	24.30	1.41
14	-5	0	27.32	3.11

14	-5	0	31.41	2.06
-14	5	0	31.68	1.49
13	-5	0	32.75	1.93
13	-5	0	33.50	2.90
-13	5	0	32.24	1.45
12	-5	0	35.08	2.25
12	-5	0	35.42	2.65
-12	5	0	35.48	1.44
11	-5	0	75.96	3.72
11	-5	0	84.83	3.09
-11	5	0	79.84	2.70
10	-5	0	76.70	2.69
10	-5	0	83.08	2.77
10	-5	0	78.47	3.58
10	-5	0	78.36	2.96
-10	5	0	81.37	3.17
-10	5	0	83.37	2.67
9	-5	0	74.19	2.48
9	-5	0	71.17	2.67
9	-5	0	73.67	3.24
9	-5	0	75.90	2.45
-9	5	0	68.85	2.40
-9	5	0	73.46	2.89
8	-5	0	52.05	1.75
8	-5	0	50.08	1.94
8	-5	0	49.52	2.44
8	-5	0	50.66	1.76
-8	5	0	47.53	1.71
-8	5	0	51.76	2.22
7	-5	0	97.04	3.89
7	-5	0	98.64	3.40
7	-5	0	105.10	3.24
7	-5	0	106.64	3.26
-7	5	0	105.73	3.73
-7	5	0	100.92	3.21
-7	5	0	102.39	3.09
6	-5	0	246.34	7.76
6	-5	0	256.97	7.61
6	-5	0	256.32	7.59
6	-5	0	249.45	8.26
-6	5	0	260.42	7.71
-6	5	0	247.44	7.52
-6	5	0	251.24	7.59
-6	5	0	248.36	8.10

5	-5	0	93.24	3.60
5	-5	0	97.31	3.01
5	-5	0	99.05	3.06
5	-5	0	94.17	3.13
-5	5	0	94.80	3.01
-5	5	0	103.99	3.56
-5	5	0	93.96	2.99
-5	5	0	99.25	3.10
4	-5	0	144.12	4.90
4	-5	0	146.42	4.41
4	-5	0	140.42	4.28
4	-5	0	125.40	4.48
-4	5	0	142.60	4.27
-4	5	0	143.79	4.38
-4	5	0	144.09	4.36
-4	5	0	141.89	4.87
3	-5	0	280.63	8.50
3	-5	0	276.55	8.98
3	-5	0	282.65	8.57
3	-5	0	280.17	8.39
-3	5	0	277.84	8.46
-3	5	0	282.34	8.38
-3	5	0	283.87	8.55
-3	5	0	286.01	8.97
2	-5	0	41.71	1.31
2	-5	0	41.91	1.56
2	-5	0	39.80	1.42
2	-5	0	42.94	1.89
-2	5	0	38.44	1.83
-2	5	0	42.26	1.30
-2	5	0	42.32	1.54
-2	5	0	41.89	1.40
1	-5	0	77.75	2.37
1	-5	0	81.15	2.69
1	-5	0	82.15	2.97
1	-5	0	78.75	2.49
-1	5	0	76.68	2.96
-1	5	0	77.49	2.37
-1	5	0	76.66	2.66
-1	5	0	75.72	2.50
0	-5	0	95.34	2.86
0	-5	0	92.65	3.43
0	-5	0	98.77	2.97
0	5	0	97.96	2.87

0	5	0	86.13	3.33
0	5	0	96.22	3.40
0	5	0	97.53	3.03
-1	-5	0	123.96	4.24
-1	-5	0	103.01	3.78
1	5	0	122.06	3.65
1	5	0	122.68	4.23
1	5	0	127.51	4.36
1	5	0	125.42	3.65
1	5	0	124.97	3.81
-2	-5	0	89.62	2.85
-2	-5	0	92.50	3.34
2	5	0	94.39	3.52
2	5	0	89.14	3.36
2	5	0	90.16	2.93
2	5	0	87.57	3.16
2	5	0	88.32	2.75
2	5	0	91.70	2.76
-3	-5	0	45.75	1.58
-3	-5	0	44.78	2.03
3	5	0	47.57	1.68
3	5	0	44.69	1.48
3	5	0	46.13	1.94
3	5	0	47.17	1.47
3	5	0	48.50	2.31
3	5	0	45.30	2.10
-4	-5	0	27.08	1.07
-4	-5	0	28.23	1.55
4	5	0	27.23	1.47
4	5	0	30.02	1.92
4	5	0	27.52	0.97
4	5	0	28.40	0.97
4	5	0	28.20	1.19
4	5	0	27.17	1.61
-5	-5	0	40.42	1.52
5	5	0	42.60	2.13
5	5	0	43.69	1.42
5	5	0	42.49	1.66
5	5	0	41.23	2.46
5	5	0	41.82	1.95
5	5	0	42.37	1.83
5	5	0	41.75	1.42
-6	-5	0	22.21	0.99
6	5	0	22.41	0.88

6	5	0	21.42	1.45
6	5	0	18.45	2.08
6	5	0	21.15	1.33
6	5	0	22.56	1.16
6	5	0	21.78	1.59
-7	-5	0	13.12	0.80
-7	-5	0	10.84	0.74
7	5	0	14.64	1.20
7	5	0	13.01	0.71
7	5	0	14.95	1.53
7	5	0	15.20	1.38
7	5	0	11.64	2.18
7	5	0	12.71	1.04
-8	-5	0	34.91	1.41
-8	-5	0	34.89	1.45
8	5	0	33.96	2.17
8	5	0	33.84	1.90
8	5	0	34.79	2.07
8	5	0	33.91	1.35
-9	-5	0	6.08	0.79
9	5	0	7.64	1.24
9	5	0	5.97	0.87
9	5	0	5.57	1.10
9	5	0	5.37	0.72
10	5	0	1.71	0.79
10	5	0	2.36	0.97
10	5	0	1.49	1.05
11	5	0	6.49	1.07
11	5	0	5.75	1.39
11	5	0	5.51	0.84
11	5	0	3.62	1.16
12	5	0	0.76	0.61
12	5	0	-0.98	1.17
12	5	0	1.02	0.99
12	5	0	-0.49	1.55
16	-6	0	18.14	2.13
16	-6	0	12.68	2.71
-16	6	0	17.41	1.31
15	-6	0	9.73	2.17
15	-6	0	8.06	1.30
14	-6	0	16.37	2.75
14	-6	0	19.58	1.76
-14	6	0	17.42	1.14
13	-6	0	32.10	2.88

13	-6	0	36.87	2.02
-13	6	0	37.18	1.55
12	-6	0	23.45	2.35
12	-6	0	26.46	1.91
-12	6	0	25.52	1.16
11	-6	0	25.44	1.49
11	-6	0	24.52	2.16
-11	6	0	25.28	1.12
10	-6	0	87.61	3.08
10	-6	0	82.68	2.82
10	-6	0	80.03	2.76
10	-6	0	81.96	3.73
-10	6	0	82.14	2.73
-10	6	0	81.57	3.32
9	-6	0	83.15	3.00
9	-6	0	83.20	3.62
9	-6	0	85.30	2.75
9	-6	0	86.85	2.78
-9	6	0	79.81	3.26
-9	6	0	80.78	2.71
8	-6	0	53.30	2.69
8	-6	0	56.05	1.78
8	-6	0	55.00	1.91
8	-6	0	58.70	1.93
8	-6	0	54.34	2.12
-8	6	0	56.94	1.74
-8	6	0	57.09	2.48
-8	6	0	55.16	1.88
7	-6	0	135.13	4.16
7	-6	0	132.64	4.37
7	-6	0	137.43	4.20
7	-6	0	131.36	4.96
-7	6	0	136.54	4.78
-7	6	0	132.03	4.17
-7	6	0	131.41	4.09
-7	6	0	141.87	4.31
6	-6	0	107.91	3.22
6	-6	0	102.48	3.26
6	-6	0	94.78	3.90
6	-6	0	101.08	3.37
-6	6	0	102.88	3.20
-6	6	0	104.85	3.31
-6	6	0	103.95	3.83
-6	6	0	100.58	3.19

5	-6	0	216.89	6.70
5	-6	0	213.72	7.27
5	-6	0	214.94	6.67
5	-6	0	221.21	6.56
-5	6	0	214.59	7.18
-5	6	0	221.07	6.64
-5	6	0	212.38	6.54
-5	6	0	220.90	6.64
4	-6	0	140.30	4.41
4	-6	0	140.69	4.98
4	-6	0	142.50	4.27
4	-6	0	141.80	4.47
-4	6	0	139.66	4.44
-4	6	0	138.76	4.35
-4	6	0	136.43	4.26
-4	6	0	143.50	4.94
3	-6	0	89.27	3.50
3	-6	0	88.00	2.95
3	-6	0	94.01	3.08
3	-6	0	93.76	2.82
-3	6	0	98.19	2.92
-3	6	0	95.64	3.52
-3	6	0	80.61	2.84
-3	6	0	95.21	3.07
2	-6	0	20.99	0.70
2	-6	0	18.95	1.30
2	-6	0	20.45	0.85
2	-6	0	19.05	0.98
-2	6	0	18.07	1.31
-2	6	0	20.73	0.81
-2	6	0	19.08	0.68
1	-6	0	81.48	2.46
1	-6	0	75.97	3.11
1	-6	0	79.16	2.58
-1	6	0	83.77	2.46
-1	6	0	77.94	3.01
-1	6	0	84.11	2.84
-1	6	0	78.87	2.62
-1	6	0	84.43	3.22
0	-6	0	21.64	0.69
0	-6	0	18.87	1.29
0	-6	0	20.98	0.88
0	6	0	20.92	1.27
0	6	0	19.50	0.89

0	6	0	21.15	1.11
0	6	0	20.37	1.43
0	6	0	21.28	0.71
-1	-6	0	0.83	0.23
-1	-6	0	0.77	0.50
1	6	0	1.35	0.19
1	6	0	0.91	0.31
1	6	0	1.49	0.21
1	6	0	2.25	0.72
1	6	0	1.68	0.55
1	6	0	1.22	0.38
-2	-6	0	15.05	0.66
-2	-6	0	13.37	1.15
2	6	0	14.32	0.53
2	6	0	17.55	1.46
2	6	0	15.47	1.06
2	6	0	12.25	1.15
2	6	0	12.78	0.57
2	6	0	14.44	0.78
-3	-6	0	26.88	1.60
-3	-6	0	27.69	1.05
3	6	0	27.96	1.21
3	6	0	26.81	0.95
3	6	0	27.09	1.92
3	6	0	26.92	1.48
3	6	0	26.93	1.66
3	6	0	28.25	0.94
-4	-6	0	10.17	1.15
-4	-6	0	11.20	0.62
4	6	0	11.35	0.51
4	6	0	9.76	1.62
4	6	0	10.29	1.23
4	6	0	10.60	0.52
4	6	0	11.55	0.82
4	6	0	10.77	1.09
-5	-6	0	17.17	1.41
-5	-6	0	15.99	0.83
5	6	0	15.82	1.50
5	6	0	16.39	0.70
5	6	0	15.66	1.36
5	6	0	16.66	1.03
5	6	0	16.40	0.71
5	6	0	16.90	2.03
-6	-6	0	4.43	0.57

6	6	0	5.75	0.88
6	6	0	5.12	0.49
6	6	0	5.49	0.49
6	6	0	5.26	1.36
6	6	0	6.19	0.97
6	6	0	5.38	0.96
6	6	0	5.17	1.04
-7	-6	0	0.21	0.37
7	6	0	0.08	0.81
7	6	0	0.37	0.78
7	6	0	-0.26	0.31
7	6	0	1.08	0.35
7	6	0	-0.26	0.61
7	6	0	2.05	1.09
-8	-6	0	4.16	0.69
8	6	0	3.65	1.08
8	6	0	4.33	0.57
8	6	0	2.47	1.52
8	6	0	3.91	0.49
9	6	0	5.75	0.89
9	6	0	5.91	0.73
9	6	0	4.91	1.43
9	6	0	4.65	1.08
10	6	0	0.98	1.18
10	6	0	0.72	0.51
10	6	0	-0.53	1.05
10	6	0	1.56	0.84
11	6	0	0.50	0.95
11	6	0	1.04	0.62
11	6	0	-1.43	1.29
11	6	0	-0.59	1.23
12	6	0	-0.45	1.61
12	6	0	1.47	1.08
12	6	0	-0.74	0.68
12	6	0	1.37	1.42
16	-7	0	15.24	2.79
16	-7	0	14.87	2.07
-16	7	0	12.19	1.20
15	-7	0	28.41	2.21
15	-7	0	20.56	3.33
-15	7	0	25.82	1.44
14	-7	0	31.27	2.13
14	-7	0	32.89	3.28
-14	7	0	32.06	1.51

13	-7	0	21.25	2.72
13	-7	0	21.22	1.67
-13	7	0	25.19	1.21
12	-7	0	16.30	1.33
12	-7	0	13.19	2.15
-12	7	0	16.82	0.93
11	-7	0	25.38	2.32
11	-7	0	24.50	1.48
-11	7	0	25.01	1.11
10	-7	0	22.25	1.07
10	-7	0	21.77	1.02
10	-7	0	18.61	1.97
10	-7	0	20.36	1.27
-10	7	0	22.09	0.96
-10	7	0	19.75	1.64
9	-7	0	65.49	2.18
9	-7	0	65.04	2.17
9	-7	0	56.45	3.06
9	-7	0	63.80	2.41
-9	7	0	62.83	2.13
-9	7	0	62.18	2.81
8	-7	0	67.28	2.48
8	-7	0	68.92	2.27
8	-7	0	64.97	3.16
-8	7	0	67.90	2.38
-8	7	0	70.22	2.14
-8	7	0	65.76	2.90
-8	7	0	67.97	2.24
7	-7	0	64.50	2.14
7	-7	0	65.01	2.19
7	-7	0	64.55	2.33
7	-7	0	58.73	2.93
-7	7	0	66.20	2.24
-7	7	0	70.28	2.86
-7	7	0	63.76	2.11
-7	7	0	62.88	2.10
6	-7	0	207.62	6.39
6	-7	0	203.30	7.07
6	-7	0	213.20	6.21
6	-7	0	200.90	6.33
-6	7	0	204.61	6.30
-6	7	0	199.19	6.17
-6	7	0	207.72	6.28
-6	7	0	193.04	6.90

5	-7	0	143.01	5.36
5	-7	0	148.26	4.71
5	-7	0	155.33	4.55
5	-7	0	149.32	4.75
-5	7	0	145.17	4.52
-5	7	0	149.71	5.31
-5	7	0	144.59	4.70
-5	7	0	151.75	4.64
4	-7	0	35.71	2.00
4	-7	0	37.53	1.26
4	-7	0	37.16	1.52
4	-7	0	38.13	1.45
-4	7	0	38.26	1.25
-4	7	0	35.81	1.98
-4	7	0	38.99	1.36
3	-7	0	41.27	1.67
3	-7	0	37.94	1.55
3	-7	0	41.43	1.33
3	-7	0	38.52	2.08
-3	7	0	41.74	2.12
-3	7	0	41.73	1.44
-3	7	0	41.96	1.64
-3	7	0	40.29	1.33
2	-7	0	135.95	5.08
2	-7	0	138.54	4.42
2	-7	0	143.64	4.28
-2	7	0	147.29	4.42
-2	7	0	135.51	4.85
-2	7	0	140.19	4.65
-2	7	0	149.17	5.16
-2	7	0	142.20	4.27
1	-7	0	69.11	2.14
1	-7	0	67.41	2.92
-1	7	0	66.33	2.80
-1	7	0	69.96	2.35
-1	7	0	71.78	3.05
-1	7	0	68.01	2.58
-1	7	0	73.39	2.14
0	-7	0	22.21	0.87
0	-7	0	20.55	0.69
0	-7	0	17.89	1.40
0	7	0	20.94	1.19
0	7	0	19.55	1.58
0	7	0	21.48	0.98

0	7	0	20.11	1.37
0	7	0	19.90	0.71
-1	-7	0	33.98	1.21
-1	-7	0	32.29	1.82
1	7	0	33.33	1.08
1	7	0	34.00	1.10
1	7	0	30.40	1.79
1	7	0	34.01	2.07
1	7	0	35.41	1.64
1	7	0	31.22	1.47
-2	-7	0	11.09	0.58
-2	-7	0	10.37	1.18
2	7	0	10.55	1.22
2	7	0	11.29	1.51
2	7	0	10.22	0.51
2	7	0	10.16	0.44
2	7	0	12.23	1.04
2	7	0	10.19	0.75
-3	-7	0	43.73	2.17
-3	-7	0	42.10	1.50
3	7	0	40.41	1.39
3	7	0	42.12	2.00
3	7	0	43.63	1.70
3	7	0	42.85	1.36
3	7	0	40.38	2.49
3	7	0	37.77	2.19
-4	-7	0	7.81	0.57
-4	-7	0	9.40	1.27
4	7	0	7.36	0.48
4	7	0	7.61	1.32
4	7	0	6.09	1.33
4	7	0	8.11	0.45
4	7	0	6.34	0.83
4	7	0	8.86	1.15
-5	-7	0	-0.44	0.66
-5	-7	0	0.18	0.32
5	7	0	0.93	0.27
5	7	0	0.64	0.26
5	7	0	0.34	0.65
5	7	0	0.01	0.51
5	7	0	0.84	1.17
5	7	0	-0.02	0.69
-6	-7	0	0.66	0.35
6	7	0	0.59	0.31

6	7	0	0.72	0.86
6	7	0	-0.42	0.62
6	7	0	0.63	0.31
6	7	0	0.46	0.65
-7	-7	0	2.80	0.50
7	7	0	1.42	0.42
7	7	0	2.17	0.41
7	7	0	2.03	1.01
-8	-7	0	3.17	0.61
8	7	0	3.02	0.98
8	7	0	2.85	1.13
8	7	0	3.58	0.55
8	7	0	3.71	0.54
9	7	0	0.94	1.11
9	7	0	-0.18	0.49
9	7	0	2.35	0.78
9	7	0	0.26	0.96
10	7	0	0.74	0.87
10	7	0	0.18	1.24
10	7	0	2.25	1.25
11	7	0	-0.34	0.67
11	7	0	-0.25	1.04
11	7	0	-0.04	1.46
11	7	0	-1.66	1.36
16	-8	0	22.90	2.32
16	-8	0	30.00	4.03
-16	8	0	23.76	1.48
15	-8	0	23.24	2.11
15	-8	0	22.03	3.45
-15	8	0	20.14	1.29
14	-8	0	27.84	3.21
14	-8	0	22.73	1.90
-14	8	0	22.50	1.27
13	-8	0	46.13	2.31
13	-8	0	44.54	3.37
-13	8	0	43.02	1.77
12	-8	0	48.71	2.22
-12	8	0	44.11	1.75
11	-8	0	13.70	1.21
11	-8	0	14.46	2.08
-11	8	0	13.77	0.81
10	-8	0	25.68	1.18
10	-8	0	24.86	2.25
10	-8	0	24.64	1.14

10	-8	0	25.88	1.43
-10	8	0	26.25	1.10
-10	8	0	27.20	1.94
9	-8	0	28.72	1.16
9	-8	0	27.48	1.14
9	-8	0	26.01	1.36
9	-8	0	25.48	2.16
-9	8	0	26.23	0.95
-9	8	0	26.27	1.22
-9	8	0	27.64	1.09
-9	8	0	28.47	1.95
8	-8	0	104.99	3.64
8	-8	0	106.76	4.46
8	-8	0	105.60	3.41
-8	8	0	105.38	3.38
-8	8	0	109.25	3.53
-8	8	0	109.72	3.36
-8	8	0	104.34	4.18
7	-8	0	77.75	3.55
7	-8	0	81.84	2.61
7	-8	0	79.70	2.82
-7	8	0	82.84	2.67
-7	8	0	80.55	2.71
-7	8	0	77.77	2.57
-7	8	0	81.44	3.45
6	-8	0	47.78	1.85
6	-8	0	50.07	2.60
6	-8	0	50.53	1.66
6	-8	0	48.82	1.85
-6	8	0	47.07	1.62
-6	8	0	49.96	1.75
-6	8	0	47.44	2.48
5	-8	0	29.81	1.98
5	-8	0	31.90	1.42
5	-8	0	30.96	1.32
5	-8	0	32.45	1.13
-5	8	0	32.20	1.38
-5	8	0	32.06	1.11
-5	8	0	32.09	1.25
-5	8	0	34.50	2.04
4	-8	0	18.27	1.04
4	-8	0	20.51	1.59
4	-8	0	17.41	0.71
4	-8	0	18.70	0.95

-4	8	0	18.40	0.83
-4	8	0	16.12	1.52
-4	8	0	19.62	1.03
-4	8	0	18.41	0.69
3	-8	0	28.88	0.99
3	-8	0	26.62	1.79
3	-8	0	29.99	1.23
-3	8	0	28.66	1.13
-3	8	0	27.76	1.86
-3	8	0	27.47	0.98
-3	8	0	30.06	1.62
-3	8	0	30.37	1.39
2	-8	0	57.60	2.74
2	-8	0	59.16	2.06
2	-8	0	57.89	1.87
-2	8	0	56.60	2.54
-2	8	0	60.47	1.87
-2	8	0	64.34	2.89
-2	8	0	61.00	2.06
-2	8	0	60.59	2.34
1	-8	0	61.27	1.91
1	-8	0	60.73	2.83
1	-8	0	57.85	2.07
-1	8	0	62.98	2.44
-1	8	0	64.70	2.18
-1	8	0	60.91	2.69
-1	8	0	61.83	2.94
-1	8	0	61.89	1.91
0	-8	0	55.05	1.84
0	-8	0	54.10	1.69
0	-8	0	51.75	2.60
0	8	0	54.24	2.55
0	8	0	57.23	2.84
0	8	0	53.24	2.00
0	8	0	53.93	2.27
0	8	0	55.31	1.71
-1	-8	0	11.91	0.62
-1	-8	0	10.35	1.29
1	8	0	11.01	0.46
1	8	0	11.64	0.84
1	8	0	9.51	1.07
1	8	0	10.23	1.27
1	8	0	11.67	0.51
1	8	0	10.25	1.62

-2	-8	0	14.80	1.45
-2	-8	0	13.12	0.67
2	8	0	12.20	0.59
2	8	0	11.75	0.91
2	8	0	12.64	0.52
2	8	0	10.48	1.15
2	8	0	13.62	1.80
2	8	0	10.99	1.40
-3	-8	0	11.73	1.42
-3	-8	0	11.80	0.67
3	8	0	10.48	0.57
3	8	0	13.64	0.98
3	8	0	11.94	1.48
3	8	0	9.27	1.26
3	8	0	11.42	1.73
-4	-8	0	1.95	0.39
-4	-8	0	1.77	0.88
4	8	0	2.12	0.28
4	8	0	4.39	1.44
4	8	0	2.04	0.99
4	8	0	2.57	0.94
4	8	0	2.23	0.31
4	8	0	2.54	0.62
-5	-8	0	1.81	0.41
-5	-8	0	1.27	0.88
5	8	0	2.81	0.39
5	8	0	3.22	0.69
5	8	0	2.20	0.33
5	8	0	1.42	0.91
-6	-8	0	5.54	0.69
6	8	0	4.27	1.11
6	8	0	6.39	1.16
6	8	0	5.55	0.59
6	8	0	4.50	0.60
-7	-8	0	0.18	0.47
7	8	0	0.50	0.44
7	8	0	0.25	0.41
7	8	0	0.83	1.05
7	8	0	1.59	0.89
-8	-8	0	0.15	0.54
8	8	0	0.64	0.85
8	8	0	0.39	0.49
8	8	0	-1.24	0.90
8	8	0	-1.28	1.08

9	8	0	0.95	0.57
9	8	0	1.97	1.17
9	8	0	0.44	1.35
9	8	0	0.35	0.87
10	8	0	0.24	0.65
10	8	0	-0.70	1.01
10	8	0	1.76	1.38
10	8	0	1.14	1.50
16	-9	0	13.78	2.11
16	-9	0	8.12	2.64
-16	9	0	10.41	1.18
15	-9	0	29.48	3.76
15	-9	0	29.93	2.29
-15	9	0	31.34	1.55
14	-9	0	22.18	3.18
14	-9	0	24.03	1.93
-14	9	0	24.79	1.31
13	-9	0	28.53	3.03
13	-9	0	27.89	1.86
-13	9	0	28.15	1.33
12	-9	0	58.94	3.69
12	-9	0	59.05	2.59
-12	9	0	59.10	2.14
11	-9	0	46.75	3.17
11	-9	0	52.47	2.25
11	-9	0	51.14	1.99
-11	9	0	49.59	1.84
10	-9	0	58.79	2.08
10	-9	0	56.01	2.36
10	-9	0	57.06	3.33
10	-9	0	60.68	2.09
-10	9	0	56.22	1.84
-10	9	0	57.01	2.01
-10	9	0	55.50	2.08
9	-9	0	67.31	2.59
9	-9	0	68.43	2.32
9	-9	0	71.66	3.55
-9	9	0	66.13	2.21
-9	9	0	68.11	2.27
-9	9	0	66.49	3.21
-9	9	0	64.41	2.51
-9	9	0	67.38	2.42
8	-9	0	33.10	1.52
8	-9	0	33.44	2.38

8	-9	0	34.47	1.29
-8	9	0	31.19	1.37
-8	9	0	30.98	1.56
-8	9	0	32.55	1.23
-8	9	0	32.24	2.21
-8	9	0	34.57	1.34
7	-9	0	34.58	1.52
7	-9	0	34.08	2.31
7	-9	0	36.36	1.30
-7	9	0	32.81	1.67
-7	9	0	36.21	1.44
-7	9	0	32.71	2.24
-7	9	0	36.27	1.41
-7	9	0	33.21	1.25
6	-9	0	56.80	2.95
6	-9	0	54.52	2.11
6	-9	0	60.58	1.93
-6	9	0	56.75	2.86
-6	9	0	58.05	2.14
-6	9	0	56.22	2.01
-6	9	0	58.19	1.89
5	-9	0	19.13	1.75
5	-9	0	19.27	0.79
5	-9	0	19.62	1.03
-5	9	0	19.47	1.11
-5	9	0	19.43	1.34
-5	9	0	19.90	1.77
-5	9	0	20.17	0.77
4	-9	0	44.84	1.81
4	-9	0	50.05	2.64
-4	9	0	50.36	2.01
-4	9	0	51.08	1.75
-4	9	0	49.36	1.59
-4	9	0	49.80	2.62
-4	9	0	47.31	2.24
3	-9	0	17.52	0.93
3	-9	0	15.48	0.66
3	-9	0	17.11	1.62
-3	9	0	15.84	0.65
-3	9	0	15.70	1.36
-3	9	0	16.62	1.70
-3	9	0	18.37	0.85
-3	9	0	16.34	1.14
2	-9	0	65.07	3.12

2	-9	0	68.41	2.12
2	-9	0	66.93	2.30
-2	9	0	68.52	2.68
-2	9	0	66.11	2.92
-2	9	0	65.99	3.23
-2	9	0	71.62	2.35
1	-9	0	20.85	0.89
1	-9	0	18.22	1.67
1	-9	0	20.36	0.72
-1	9	0	21.15	0.73
-1	9	0	20.88	1.06
-1	9	0	21.38	1.89
-1	9	0	20.58	1.35
-1	9	0	18.52	1.54
0	-9	0	14.51	0.53
0	-9	0	15.01	0.72
0	-9	0	13.59	1.57
0	9	0	15.07	1.80
0	9	0	13.39	0.56
0	9	0	12.23	0.93
0	9	0	12.74	1.20
0	9	0	13.90	1.47
-1	-9	0	20.44	1.75
-1	-9	0	22.82	0.95
1	9	0	23.38	1.25
1	9	0	22.62	0.78
1	9	0	21.26	1.78
1	9	0	22.60	1.50
1	9	0	19.22	2.09
-2	-9	0	6.60	0.56
-2	-9	0	6.56	1.07
2	9	0	8.28	1.69
2	9	0	6.99	0.40
2	9	0	7.23	0.92
2	9	0	8.06	1.37
2	9	0	6.93	0.49
2	9	0	8.16	1.18
-3	-9	0	4.81	1.06
-3	-9	0	5.71	0.56
3	9	0	6.07	1.23
3	9	0	6.37	0.84
3	9	0	4.81	0.46
3	9	0	5.45	0.42
-4	-9	0	7.13	1.22

-4	-9	0	6.28	0.64
4	9	0	7.86	0.95
4	9	0	7.67	0.57
4	9	0	7.38	0.50
4	9	0	8.38	1.43
-5	-9	0	3.86	0.63
-5	-9	0	3.12	1.19
5	9	0	3.47	0.48
5	9	0	3.38	0.52
5	9	0	2.95	1.18
5	9	0	3.98	0.92
-6	-9	0	4.58	0.72
6	9	0	3.90	0.50
6	9	0	3.39	0.50
6	9	0	5.23	1.39
6	9	0	4.56	0.98
-7	-9	0	0.38	0.52
7	9	0	-0.02	0.92
7	9	0	1.25	0.48
7	9	0	1.23	1.25
8	9	0	-0.40	0.55
8	9	0	-0.78	0.96
8	9	0	-0.15	1.37
9	9	0	0.64	0.98
9	9	0	1.02	1.48
9	9	0	0.37	0.65
9	9	0	-1.18	1.23
10	9	0	-0.27	1.58
10	9	0	-0.62	1.05
10	9	0	-1.63	1.36
16	-10	0	28.03	2.45
16	-10	0	16.63	3.03
-16	10	0	23.23	1.45
15	-10	0	16.59	1.98
15	-10	0	13.21	2.56
-15	10	0	14.88	1.18
14	-10	0	11.66	2.37
14	-10	0	16.30	1.79
-14	10	0	14.56	1.07
13	-10	0	44.40	2.33
13	-10	0	40.43	3.61
-13	10	0	44.76	1.78
12	-10	0	45.56	3.45
12	-10	0	47.07	2.30

-12	10	0	49.13	1.83
11	-10	0	62.79	2.61
11	-10	0	59.75	2.27
-11	10	0	61.66	2.16
-11	10	0	59.98	2.16
-11	10	0	60.79	1.95
10	-10	0	90.67	3.06
10	-10	0	86.94	4.41
10	-10	0	95.28	3.42
-10	10	0	87.48	3.22
-10	10	0	97.92	2.95
-10	10	0	89.50	3.01
9	-10	0	12.05	0.79
9	-10	0	11.69	2.07
9	-10	0	11.65	1.08
-9	10	0	11.90	1.09
-9	10	0	11.04	0.88
-9	10	0	11.29	0.70
-9	10	0	11.97	0.76
8	-10	0	18.33	0.88
8	-10	0	16.03	2.00
8	-10	0	16.58	1.13
-8	10	0	17.26	0.81
-8	10	0	18.87	1.01
-8	10	0	17.02	1.89
-8	10	0	16.80	0.96
-8	10	0	17.50	1.28
7	-10	0	93.80	4.31
7	-10	0	101.45	3.13
7	-10	0	94.40	3.36
-7	10	0	94.35	3.60
-7	10	0	98.76	3.08
-7	10	0	90.31	4.20
-7	10	0	96.73	3.22
-7	10	0	102.82	3.36
6	-10	0	25.61	1.31
6	-10	0	26.71	2.14
6	-10	0	27.48	1.06
-6	10	0	27.32	1.16
-6	10	0	28.16	2.18
-6	10	0	25.69	1.63
-6	10	0	28.37	1.40
-6	10	0	26.72	1.01
5	-10	0	25.94	0.98

5	-10	0	26.16	1.25
5	-10	0	25.97	2.09
-5	10	0	25.59	1.10
-5	10	0	21.83	1.59
-5	10	0	24.63	2.14
-5	10	0	25.50	0.94
-5	10	0	25.77	1.40
4	-10	0	63.11	2.29
4	-10	0	64.08	2.07
4	-10	0	61.41	3.21
-4	10	0	64.89	2.56
-4	10	0	67.81	3.32
-4	10	0	59.87	2.80
-4	10	0	65.87	2.25
-4	10	0	65.42	2.05
3	-10	0	10.95	0.78
3	-10	0	12.60	0.57
3	-10	0	12.10	1.61
-3	10	0	12.39	0.78
-3	10	0	13.19	1.36
-3	10	0	10.25	1.09
-3	10	0	11.66	0.53
-3	10	0	11.48	1.73
2	-10	0	6.55	1.18
2	-10	0	5.63	0.57
2	-10	0	6.08	0.38
-2	10	0	5.42	0.69
-2	10	0	5.93	0.38
-2	10	0	7.78	1.30
-2	10	0	6.54	1.28
-2	10	0	6.50	1.05
1	-10	0	12.27	1.64
1	-10	0	12.02	0.70
1	-10	0	11.44	0.49
-1	10	0	12.91	1.28
-1	10	0	9.57	1.45
-1	10	0	12.05	1.73
-1	10	0	12.84	0.90
0	-10	0	11.07	1.58
0	-10	0	10.79	0.65
0	10	0	10.75	0.96
0	10	0	10.66	1.29
0	10	0	9.72	0.48
0	10	0	11.60	1.79

0	10	0	10.07	1.44
-1	-10	0	17.22	0.82
-1	-10	0	16.03	1.77
1	10	0	14.95	1.70
1	10	0	15.71	1.20
1	10	0	16.98	0.63
1	10	0	15.51	0.69
1	10	0	15.63	2.30
-2	-10	0	12.55	1.76
-2	-10	0	15.42	0.82
2	10	0	16.11	1.28
2	10	0	17.53	0.64
2	10	0	15.69	0.70
2	10	0	13.55	1.81
-3	-10	0	-0.26	0.32
-3	-10	0	0.34	0.96
3	10	0	0.74	0.24
3	10	0	0.74	1.10
3	10	0	0.59	0.31
3	10	0	-0.17	0.70
-4	-10	0	0.58	1.03
-4	-10	0	0.54	0.42
4	10	0	2.62	1.24
4	10	0	0.39	0.84
4	10	0	0.77	0.36
4	10	0	0.81	0.30
-5	-10	0	3.42	1.23
-5	-10	0	2.96	0.53
5	10	0	3.03	1.30
5	10	0	3.64	0.49
5	10	0	2.90	0.43
-6	-10	0	1.24	0.55
6	10	0	0.94	0.48
6	10	0	-0.50	1.19
6	10	0	0.61	0.93
-7	-10	0	1.07	0.61
7	10	0	0.27	1.06
7	10	0	-0.37	0.53
7	10	0	2.28	1.48
8	10	0	0.19	0.94
8	10	0	0.14	1.20
8	10	0	-0.49	1.38
9	10	0	-0.68	1.01
9	10	0	-1.05	1.40

9	10	0	-0.30	1.62
16	-11	0	7.09	1.60
16	-11	0	7.90	2.83
-16	11	0	11.91	1.21
15	-11	0	23.12	3.77
15	-11	0	18.57	2.17
-15	11	0	19.15	1.31
14	-11	0	28.08	3.64
14	-11	0	25.11	2.14
-14	11	0	27.50	1.40
13	-11	0	18.30	1.75
13	-11	0	22.15	3.27
-13	11	0	21.35	1.17
12	-11	0	32.06	3.26
12	-11	0	31.80	1.89
-12	11	0	28.89	1.35
11	-11	0	35.81	3.21
11	-11	0	42.04	2.03
11	-11	0	36.61	1.64
-11	11	0	41.00	1.44
-11	11	0	40.86	1.57
-11	11	0	39.88	1.80
10	-11	0	23.48	1.51
10	-11	0	22.35	3.04
-10	11	0	26.57	1.13
-10	11	0	22.56	1.07
-10	11	0	22.06	1.48
9	-11	0	30.63	2.65
9	-11	0	29.54	1.29
9	-11	0	30.66	1.61
-9	11	0	30.60	1.69
-9	11	0	29.55	1.39
-9	11	0	30.29	1.21
-9	11	0	28.48	1.38
8	-11	0	5.49	0.61
8	-11	0	5.42	1.42
8	-11	0	4.80	0.72
-8	11	0	3.57	0.50
-8	11	0	4.25	0.68
-8	11	0	4.24	0.63
-8	11	0	5.12	0.85
7	-11	0	14.51	0.77
7	-11	0	12.08	1.03
7	-11	0	14.52	1.97

-7	11	0	14.24	1.11
-7	11	0	13.05	0.86
-7	11	0	12.91	1.33
-7	11	0	12.88	0.69
6	-11	0	49.03	2.99
6	-11	0	48.77	2.00
6	-11	0	50.78	1.73
-6	11	0	50.36	2.15
-6	11	0	47.00	2.42
-6	11	0	49.34	2.96
-6	11	0	51.39	1.85
-6	11	0	51.40	1.69
5	-11	0	28.86	2.29
5	-11	0	26.42	1.33
5	-11	0	26.47	1.06
-5	11	0	27.25	1.84
-5	11	0	26.68	1.58
-5	11	0	28.80	1.02
-5	11	0	27.15	2.38
-5	11	0	27.76	1.20
4	-11	0	4.80	1.20
4	-11	0	5.01	0.69
4	-11	0	4.01	0.44
-4	11	0	4.71	0.63
-4	11	0	6.02	0.87
-4	11	0	5.01	1.26
-4	11	0	5.97	1.04
-4	11	0	5.34	0.40
3	-11	0	0.87	1.03
3	-11	0	1.53	0.28
3	-11	0	1.22	0.40
-3	11	0	1.44	1.13
-3	11	0	0.89	0.49
-3	11	0	1.07	0.22
-3	11	0	1.21	0.64
-3	11	0	1.96	0.92
2	-11	0	9.65	0.48
2	-11	0	10.13	0.69
2	-11	0	9.42	1.66
-2	11	0	7.06	1.44
-2	11	0	11.63	1.56
-2	11	0	10.27	1.29
-2	11	0	9.70	0.50
-2	11	0	9.93	0.86

1	-11	0	1.81	0.36
1	-11	0	1.77	1.02
1	-11	0	2.11	0.23
-1	11	0	2.18	0.30
-1	11	0	1.51	0.55
-1	11	0	2.92	0.97
0	-11	0	5.58	0.57
0	-11	0	7.86	1.43
0	11	0	5.60	0.39
0	11	0	5.70	0.97
0	11	0	5.81	1.17
-1	-11	0	17.40	0.89
-1	-11	0	16.61	1.93
1	11	0	20.37	1.40
1	11	0	20.72	2.02
1	11	0	18.15	0.68
1	11	0	17.11	0.76
-2	-11	0	6.24	0.65
2	11	0	8.21	1.33
2	11	0	7.85	1.06
2	11	0	6.42	0.44
-3	-11	0	2.80	1.21
-3	-11	0	3.60	0.49
3	11	0	2.58	0.39
3	11	0	2.21	0.98
3	11	0	0.03	1.12
3	11	0	3.96	0.44
-4	-11	0	1.05	1.29
-4	-11	0	0.52	0.47
4	11	0	1.05	0.40
4	11	0	1.66	1.19
4	11	0	0.43	1.01
-5	-11	0	0.55	0.53
-5	-11	0	1.43	1.37
5	11	0	0.86	0.45
5	11	0	2.61	1.35
-6	-11	0	1.04	0.61
6	11	0	2.46	1.07
6	11	0	2.95	1.52
-7	-11	0	0.18	0.68
7	11	0	2.04	1.28
7	11	0	1.00	1.53
8	11	0	2.77	1.12
8	11	0	3.29	1.44

8	11	0	4.47	1.77
16	-12	0	17.48	2.58
16	-12	0	16.41	3.53
-16	12	0	17.07	1.37
15	-12	0	20.33	2.31
15	-12	0	23.49	3.98
-15	12	0	20.56	1.34
14	-12	0	11.61	2.59
14	-12	0	11.27	1.89
-14	12	0	12.00	1.07
13	-12	0	27.67	3.51
13	-12	0	25.19	1.96
-13	12	0	26.97	1.33
12	-12	0	47.48	3.77
12	-12	0	42.67	2.32
-12	12	0	42.95	1.93
-12	12	0	44.46	1.75
11	-12	0	18.12	1.15
11	-12	0	20.27	2.89
11	-12	0	20.48	1.59
-11	12	0	19.33	1.03
-11	12	0	20.46	1.44
-11	12	0	20.90	1.02
10	-12	0	23.72	2.85
10	-12	0	25.27	1.59
-10	12	0	25.62	1.14
-10	12	0	28.26	1.31
-10	12	0	23.25	1.33
-10	12	0	20.12	1.61
9	-12	0	6.64	1.06
9	-12	0	6.78	0.77
-9	12	0	6.78	0.63
-9	12	0	6.07	0.95
-9	12	0	6.47	0.73
-9	12	0	6.93	0.96
8	-12	0	10.16	0.76
8	-12	0	9.81	1.88
8	-12	0	9.10	1.08
-8	12	0	9.01	0.65
-8	12	0	9.83	1.11
-8	12	0	9.31	0.85
-8	12	0	9.20	1.35
7	-12	0	36.24	2.81
7	-12	0	35.83	1.40

7	-12	0	34.36	1.70
-7	12	0	34.69	2.09
-7	12	0	38.04	1.34
-7	12	0	36.10	1.49
-7	12	0	36.79	1.83
6	-12	0	5.71	0.86
6	-12	0	5.66	0.59
6	-12	0	7.75	1.54
-6	12	0	5.46	1.05
-6	12	0	5.85	0.48
-6	12	0	6.12	0.93
-6	12	0	5.16	0.67
5	-12	0	4.71	0.55
5	-12	0	1.58	1.04
5	-12	0	5.32	0.79
-5	12	0	1.89	0.91
-5	12	0	3.07	0.34
-5	12	0	3.17	0.49
-5	12	0	3.73	0.83
4	-12	0	9.13	1.69
4	-12	0	8.67	0.58
4	-12	0	9.78	0.83
-4	12	0	8.34	0.50
-4	12	0	5.74	0.98
-4	12	0	8.84	1.29
-4	12	0	8.03	0.78
3	-12	0	3.10	0.50
3	-12	0	3.56	1.30
-3	12	0	4.00	0.58
-3	12	0	1.68	0.93
-3	12	0	3.73	0.37
2	-12	0	1.82	1.19
2	-12	0	2.49	0.34
2	-12	0	1.34	0.39
-2	12	0	1.62	0.56
-2	12	0	2.60	1.01
-2	12	0	1.72	0.29
1	-12	0	1.66	1.24
1	-12	0	1.34	0.39
1	-12	0	1.58	0.23
-1	12	0	2.06	1.05
-1	12	0	0.67	0.61
0	-12	0	4.99	0.60
0	12	0	5.65	0.88

0	12	0	2.90	1.09
-1	-12	0	2.17	1.19
-1	-12	0	3.12	0.48
1	12	0	2.31	1.15
1	12	0	3.22	0.91
-2	-12	0	0.44	0.40
-2	-12	0	2.98	1.25
2	12	0	-0.20	0.91
2	12	0	1.33	1.26
-3	-12	0	-0.43	1.24
-3	-12	0	0.04	0.45
3	12	0	0.99	1.32
3	12	0	-0.52	1.06
-4	-12	0	1.19	0.57
-4	-12	0	-0.34	1.33
4	12	0	1.44	0.64
4	12	0	-1.83	1.38
-5	-12	0	0.74	1.52
-5	-12	0	-0.43	0.58
5	12	0	2.33	1.48
-6	-12	0	0.91	0.70
6	12	0	0.54	1.16
6	12	0	-2.80	1.51
7	12	0	1.45	1.39
7	12	0	1.97	1.07
7	12	0	-0.60	1.82
16	-13	0	7.20	2.91
16	-13	0	8.26	1.78
-16	13	0	9.89	1.25
15	-13	0	14.46	2.87
15	-13	0	14.44	2.33
-15	13	0	15.05	1.27
14	-13	0	15.28	2.89
14	-13	0	20.00	2.16
-14	13	0	18.79	1.24
13	-13	0	18.39	3.05
13	-13	0	19.81	1.90
-13	13	0	16.74	1.14
12	-13	0	10.61	2.27
12	-13	0	13.81	1.64
-12	13	0	12.82	1.33
-12	13	0	14.59	0.97
11	-13	0	23.67	1.51
11	-13	0	25.31	1.79

11	-13	0	21.40	3.14
-11	13	0	23.18	1.75
-11	13	0	27.18	1.32
10	-13	0	18.65	1.53
10	-13	0	16.22	2.85
-10	13	0	16.23	1.68
-10	13	0	17.57	1.22
-10	13	0	17.38	0.96
-10	13	0	21.99	1.32
9	-13	0	23.03	1.18
9	-13	0	22.79	1.57
9	-13	0	21.13	2.78
-9	13	0	23.07	1.27
-9	13	0	23.82	1.50
-9	13	0	23.68	1.84
-9	13	0	23.55	1.06
8	-13	0	7.17	1.85
8	-13	0	8.22	1.15
8	-13	0	9.34	0.78
-8	13	0	8.01	0.88
-8	13	0	9.21	0.65
-8	13	0	8.01	1.22
-8	13	0	6.73	1.22
7	-13	0	16.76	1.23
7	-13	0	17.14	0.93
7	-13	0	16.39	2.41
-7	13	0	15.12	1.40
-7	13	0	16.58	0.81
-7	13	0	16.11	1.01
-7	13	0	15.71	1.75
6	-13	0	25.39	1.44
6	-13	0	27.37	1.14
-6	13	0	23.75	2.03
-6	13	0	24.04	1.21
-6	13	0	26.42	1.01
-6	13	0	26.40	1.79
5	-13	0	5.26	0.85
5	-13	0	6.62	0.63
-5	13	0	6.66	1.23
-5	13	0	8.01	0.77
-5	13	0	5.85	0.50
4	-13	0	-0.13	0.42
4	-13	0	0.72	0.32
-4	13	0	0.48	0.48

-4	13	0	1.36	1.01
-4	13	0	0.48	0.25
3	-13	0	8.13	1.62
3	-13	0	8.60	0.73
3	-13	0	8.20	0.57
-3	13	0	10.94	0.95
-3	13	0	8.08	1.33
-3	13	0	9.34	0.51
2	-13	0	8.12	1.66
2	-13	0	5.24	0.42
2	-13	0	4.28	0.63
-2	13	0	5.21	1.63
-2	13	0	6.21	0.97
1	-13	0	3.52	0.50
1	-13	0	1.12	1.41
-1	13	0	1.71	0.77
-1	13	0	1.99	1.10
0	-13	0	-0.47	1.14
0	-13	0	1.75	0.43
0	13	0	1.30	0.92
0	13	0	3.48	1.38
-1	-13	0	2.21	1.43
-1	-13	0	3.76	0.55
1	13	0	4.44	1.05
1	13	0	1.34	1.25
-2	-13	0	-0.12	0.46
-2	-13	0	-0.78	1.38
2	13	0	1.88	1.43
-3	-13	0	-0.40	0.54
-3	-13	0	-1.16	1.41
3	13	0	-0.90	1.43
-4	-13	0	-0.13	1.56
-4	-13	0	0.65	0.61
4	13	0	1.07	1.47
-5	-13	0	1.70	0.73
-5	-13	0	0.54	1.75
5	13	0	2.02	1.64
-6	-13	0	2.08	0.86
6	13	0	1.90	1.39
6	13	0	1.32	1.95
15	-14	0	6.57	1.68
15	-14	0	7.79	3.16
-15	14	0	7.74	0.91
14	-14	0	8.31	2.86

14	-14	0	6.62	1.51
-14	14	0	7.14	1.05
13	-14	0	19.30	3.58
13	-14	0	14.23	2.02
-13	14	0	17.06	1.19
12	-14	0	10.03	2.45
12	-14	0	15.86	1.83
-12	14	0	10.76	1.59
-12	14	0	11.08	0.96
11	-14	0	16.54	2.71
11	-14	0	11.95	1.73
-11	14	0	13.67	1.78
-11	14	0	14.36	0.94
10	-14	0	13.34	2.59
10	-14	0	12.20	1.56
-10	14	0	10.84	1.16
-10	14	0	10.65	0.83
-10	14	0	12.15	1.33
-10	14	0	14.24	1.76
9	-14	0	6.10	0.86
9	-14	0	8.89	1.09
9	-14	0	5.59	1.90
-9	14	0	8.16	0.72
-9	14	0	10.13	1.02
-9	14	0	8.86	1.31
-9	14	0	7.56	1.30
8	-14	0	18.18	2.83
8	-14	0	16.75	1.47
8	-14	0	19.37	1.09
-8	14	0	17.86	0.92
-8	14	0	19.94	2.04
-8	14	0	18.10	1.17
-8	14	0	18.39	1.61
7	-14	0	32.41	1.38
7	-14	0	35.29	3.18
7	-14	0	31.05	1.72
-7	14	0	32.10	2.39
-7	14	0	34.59	1.50
-7	14	0	32.11	1.27
6	-14	0	1.99	1.47
6	-14	0	2.29	0.70
6	-14	0	2.62	0.49
-6	14	0	1.86	0.37
-6	14	0	3.05	0.57

-6	14	0	3.18	1.16
5	-14	0	3.66	1.59
5	-14	0	1.77	0.43
5	-14	0	1.06	0.60
-5	14	0	0.90	1.05
-5	14	0	1.56	0.32
-5	14	0	2.03	0.55
4	-14	0	17.41	0.84
4	-14	0	17.98	1.08
4	-14	0	24.75	2.75
-4	14	0	17.60	0.77
-4	14	0	14.85	2.21
-4	14	0	18.83	1.18
3	-14	0	15.86	2.65
3	-14	0	13.29	0.67
3	-14	0	11.66	0.87
-3	14	0	13.18	1.16
-3	14	0	11.68	2.08
-3	14	0	12.72	0.61
2	-14	0	3.71	0.53
2	-14	0	1.27	1.50
-2	14	0	4.46	1.31
-2	14	0	4.63	0.82
1	-14	0	-1.61	1.41
1	-14	0	0.16	0.42
-1	14	0	1.40	1.22
-1	14	0	1.80	1.06
0	-14	0	1.33	0.48
0	-14	0	1.66	1.72
0	14	0	0.75	1.40
-1	-14	0	-0.83	1.41
-1	-14	0	0.55	0.49
1	14	0	1.32	1.38
-2	-14	0	-0.15	0.51
-2	-14	0	0.76	1.61
2	14	0	0.21	1.40
-3	-14	0	4.42	0.73
-3	-14	0	2.88	1.81
3	14	0	2.76	1.70
-4	-14	0	-2.61	1.66
4	14	0	-0.10	1.73
15	-15	0	7.59	3.25
14	-15	0	17.00	2.51
14	-15	0	12.38	2.99

-14	15	0	18.68	1.30
13	-15	0	24.40	3.99
13	-15	0	21.18	2.33
-13	15	0	18.25	1.25
12	-15	0	3.71	1.28
12	-15	0	1.56	2.24
-12	15	0	1.83	0.62
11	-15	0	4.82	2.18
11	-15	0	5.75	1.31
-11	15	0	3.28	1.21
-11	15	0	4.87	0.61
10	-15	0	9.38	2.18
10	-15	0	11.06	1.69
-10	15	0	16.08	2.04
-10	15	0	12.89	0.90
9	-15	0	13.27	1.60
9	-15	0	13.31	2.64
-9	15	0	11.85	2.08
-9	15	0	13.64	0.87
8	-15	0	1.80	0.88
8	-15	0	1.21	0.56
8	-15	0	1.06	1.71
-8	15	0	2.09	0.44
-8	15	0	0.43	1.15
7	-15	0	3.21	0.85
7	-15	0	1.44	0.51
7	-15	0	2.72	1.86
-7	15	0	1.74	0.42
-7	15	0	1.19	1.15
6	-15	0	8.10	2.01
6	-15	0	6.82	0.89
6	-15	0	7.64	0.78
-6	15	0	6.45	0.61
-6	15	0	7.18	1.49
5	-15	0	9.24	0.73
5	-15	0	8.59	0.99
5	-15	0	7.68	2.09
-5	15	0	11.14	1.85
-5	15	0	8.37	0.60
4	-15	0	0.98	0.54
4	-15	0	0.40	1.67
-4	15	0	0.65	0.29
-4	15	0	0.23	1.16
3	-15	0	2.70	0.57

3	-15	0	3.77	1.82
-3	15	0	2.92	1.48
2	-15	0	1.57	1.77
-2	15	0	0.51	1.39
1	-15	0	1.20	0.50
1	-15	0	-2.05	1.62
-1	15	0	-0.79	1.37
0	-15	0	-0.40	0.48
0	-15	0	1.56	1.81
0	15	0	-0.96	1.40
-1	-15	0	-2.01	1.70
-1	-15	0	0.29	0.59
1	15	0	-2.02	1.60
-2	-15	0	-1.47	1.70
2	15	0	2.21	1.69
-3	-15	0	2.52	1.98
3	15	0	1.11	1.84
14	-16	0	10.41	1.84
14	-16	0	7.49	3.16
-14	16	0	8.26	1.15
13	-16	0	10.22	2.91
13	-16	0	10.25	1.77
-13	16	0	7.36	1.09
12	-16	0	4.82	2.41
12	-16	0	7.10	1.49
-12	16	0	6.93	1.01
11	-16	0	6.87	1.42
11	-16	0	10.44	2.78
-11	16	0	6.39	0.92
-11	16	0	5.41	1.49
10	-16	0	4.76	1.23
10	-16	0	3.39	2.19
-10	16	0	2.45	1.43
-10	16	0	4.58	0.61
9	-16	0	1.72	1.05
9	-16	0	-0.54	1.91
-9	16	0	1.84	0.47
-9	16	0	4.05	1.55
8	-16	0	2.33	2.18
8	-16	0	5.55	1.09
-8	16	0	4.30	0.54
-8	16	0	3.38	1.36
7	-16	0	5.83	2.11
7	-16	0	4.89	0.93

-7	16	0	3.63	1.49
-7	16	0	5.07	0.53
6	-16	0	-0.47	0.66
6	-16	0	-2.74	1.79
-6	16	0	0.51	0.37
-6	16	0	-1.69	1.39
5	-16	0	-0.02	0.62
5	-16	0	-2.61	1.81
-5	16	0	-0.18	1.33
-5	16	0	0.99	0.44
4	-16	0	0.62	1.85
4	-16	0	0.97	0.55
-4	16	0	-0.04	1.53
3	-16	0	0.25	1.87
-3	16	0	2.43	1.53
2	-16	0	-0.52	1.82
-2	16	0	0.13	1.54
1	-16	0	-0.15	1.83
-1	16	0	1.30	1.55
0	-16	0	5.92	2.24
0	16	0	4.71	1.87
-1	-16	0	-2.22	2.01
1	16	0	6.53	1.91
-2	-16	0	-0.78	2.13
13	-17	0	6.94	4.46
-13	17	0	3.95	0.79
12	-17	0	1.57	2.41
-12	17	0	4.00	0.70
11	-17	0	1.40	2.53
-11	17	0	1.44	0.58
-11	17	0	0.71	1.52
10	-17	0	-1.28	2.09
-10	17	0	0.83	1.60
-10	17	0	2.90	0.62
9	-17	0	0.86	2.38
-9	17	0	1.92	0.53
-9	17	0	1.25	1.55
8	-17	0	0.55	2.09
-8	17	0	0.73	1.58
-8	17	0	0.76	0.56
7	-17	0	-1.61	2.02
-7	17	0	-0.63	1.45
6	-17	0	1.28	2.03
-6	17	0	1.43	1.61

5	-17	0	0.93	2.00
-5	17	0	-2.00	1.61
4	-17	0	2.01	2.08
-4	17	0	-1.76	1.56
3	-17	0	0.62	2.07
-3	17	0	-0.07	1.64
2	-17	0	-0.18	2.12
-2	17	0	0.97	1.66
1	-17	0	0.76	2.08
-1	17	0	1.30	1.96
0	-17	0	-0.39	2.37
11	-18	0	-1.40	4.91
10	-18	0	-0.25	2.57
-10	18	0	2.00	1.84
9	-18	0	0.03	2.56
-9	18	0	3.64	1.95
8	-18	0	-0.68	2.50
-8	18	0	-1.40	1.75
7	-18	0	-1.56	2.34
-7	18	0	0.09	1.82
6	-18	0	-0.18	2.36
-6	18	0	-1.39	1.82
5	-18	0	1.27	2.32
-5	18	0	0.80	1.89
4	-18	0	1.94	2.38
-4	18	0	0.29	1.80
3	-18	0	-0.93	2.45
-3	18	0	-2.10	1.92
-2	18	-1	-1.38	2.04
2	-18	1	3.29	2.51
-3	18	-1	8.21	2.18
3	-18	1	9.99	2.68
-4	18	-1	5.61	2.10
4	-18	1	4.60	2.44
-5	18	-1	2.89	1.95
5	-18	1	4.04	2.51
-6	18	-1	9.53	2.05
6	-18	1	10.64	2.77
-7	18	-1	11.73	2.25
7	-18	1	15.71	3.22
-8	18	-1	12.86	2.28
8	-18	1	16.71	3.07
-9	18	-1	18.37	2.52
9	-18	1	23.71	4.26

-10	18	-1	11.65	1.22
-10	18	-1	10.54	2.20
10	-18	1	6.22	2.98
-11	18	-1	10.55	1.13
0	17	-1	-0.50	1.84
0	-17	1	-0.47	2.11
-1	17	-1	7.08	2.01
1	-17	1	6.85	2.35
-2	17	-1	7.40	1.94
2	-17	1	5.76	2.33
-3	17	-1	1.81	1.72
3	-17	1	3.51	2.19
-4	17	-1	10.01	2.04
4	-17	1	10.00	2.42
-5	17	-1	17.81	2.91
5	-17	1	14.09	2.74
-6	17	-1	10.10	1.99
6	-17	1	14.58	2.69
-7	17	-1	25.28	1.19
-7	17	-1	26.33	3.09
7	-17	1	30.11	3.95
-8	17	-1	8.83	1.94
-8	17	-1	8.53	0.86
8	-17	1	8.38	2.57
-9	17	-1	23.47	1.22
-9	17	-1	22.76	2.87
9	-17	1	23.92	3.51
-10	17	-1	24.38	2.98
-10	17	-1	26.22	1.32
10	-17	1	32.88	4.30
-11	17	-1	8.61	1.81
-11	17	-1	8.58	1.03
11	-17	1	10.99	2.89
-12	17	-1	9.48	1.11
12	-17	1	10.81	3.07
2	16	-1	0.64	1.86
-2	-16	1	1.96	2.14
1	16	-1	4.37	1.89
-1	-16	1	5.52	2.17
0	16	-1	0.16	1.64
0	-16	1	0.86	1.95
-1	16	-1	4.22	1.59
1	-16	1	7.16	2.05
-2	16	-1	11.30	2.01

2	-16	1	20.01	3.35
-3	16	-1	20.92	2.89
3	-16	1	22.11	3.42
-4	16	-1	14.99	0.79
-4	16	-1	12.78	1.98
4	-16	1	15.20	2.66
-5	16	-1	25.29	1.08
-5	16	-1	26.39	2.91
5	-16	1	27.59	3.57
5	-16	1	26.18	1.42
-6	16	-1	29.83	1.22
-6	16	-1	31.33	2.93
6	-16	1	23.35	3.48
6	-16	1	29.28	1.43
-7	16	-1	23.64	2.69
-7	16	-1	27.45	1.16
7	-16	1	20.43	3.33
7	-16	1	24.23	1.51
-8	16	-1	24.47	2.59
-8	16	-1	21.51	1.09
8	-16	1	17.73	1.59
8	-16	1	22.40	3.59
-9	16	-1	29.02	1.32
-9	16	-1	32.53	2.80
9	-16	1	29.21	3.74
9	-16	1	24.48	1.94
-10	16	-1	21.75	2.58
-10	16	-1	18.75	1.12
10	-16	1	16.21	1.90
10	-16	1	23.95	3.79
-11	16	-1	24.89	1.32
-11	16	-1	22.29	2.57
11	-16	1	28.03	4.05
11	-16	1	25.19	2.21
-12	16	-1	23.89	1.37
12	-16	1	24.44	4.09
12	-16	1	22.77	2.38
-13	16	-1	16.27	1.26
13	-16	1	14.45	2.90
4	15	-1	1.51	2.52
-4	-15	1	1.10	2.09
3	15	-1	0.40	1.78
-3	-15	1	3.57	1.96
2	15	-1	0.95	1.68

-2	-15	1	3.43	1.92
1	15	-1	4.91	1.85
-1	-15	1	6.21	2.09
0	15	-1	8.22	1.84
0	-15	1	10.01	2.17
0	-15	1	11.32	0.79
-1	15	-1	9.97	1.82
1	-15	1	14.25	2.28
1	-15	1	9.89	0.72
-2	15	-1	12.86	0.67
-2	15	-1	10.16	1.84
2	-15	1	13.48	0.78
2	-15	1	16.49	3.00
-3	15	-1	13.44	2.04
-3	15	-1	13.90	0.72
3	-15	1	12.48	2.21
3	-15	1	14.86	0.82
-4	15	-1	27.38	1.07
-4	15	-1	26.73	2.62
4	-15	1	26.41	1.14
4	-15	1	24.85	1.16
4	-15	1	32.94	3.23
-5	15	-1	43.16	1.55
-5	15	-1	44.36	2.97
5	-15	1	42.01	1.67
5	-15	1	43.18	3.65
5	-15	1	43.18	1.71
-6	15	-1	48.28	1.70
6	-15	1	47.72	3.77
6	-15	1	47.13	1.79
6	-15	1	46.02	1.93
-7	15	-1	34.61	1.35
-7	15	-1	34.79	2.57
7	-15	1	31.85	1.48
7	-15	1	32.58	3.47
7	-15	1	35.17	1.75
-8	15	-1	25.43	2.33
-8	15	-1	26.11	1.16
8	-15	1	25.49	3.22
8	-15	1	23.60	1.30
8	-15	1	24.57	1.66
-9	15	-1	33.93	2.68
-9	15	-1	36.18	1.48
9	-15	1	37.73	3.79

9	-15	1	36.21	2.06
-10	15	-1	39.47	2.71
10	-15	1	38.37	4.00
10	-15	1	38.93	2.27
-11	15	-1	30.13	1.41
-11	15	-1	29.41	2.40
11	-15	1	32.54	3.94
11	-15	1	29.73	2.19
-12	15	-1	14.32	1.11
12	-15	1	15.19	2.87
12	-15	1	17.01	2.02
-13	15	-1	44.74	1.88
13	-15	1	40.83	2.75
13	-15	1	38.25	4.44
-14	15	-1	30.66	1.58
14	-15	1	17.85	3.36
14	-15	1	30.51	2.72
5	14	-1	4.91	1.96
-5	-14	1	3.52	1.99
4	14	-1	1.95	1.71
-4	-14	1	5.13	1.83
3	14	-1	5.18	1.81
-3	-14	1	2.42	1.75
-3	-14	1	0.45	0.56
2	14	-1	11.53	2.19
-2	-14	1	9.20	0.83
-2	-14	1	8.05	1.83
1	14	-1	14.67	2.58
-1	-14	1	12.10	0.83
-1	-14	1	12.91	2.14
0	14	-1	1.72	1.32
0	-14	1	6.82	0.64
0	-14	1	6.37	1.78
-1	14	-1	7.63	0.56
-1	14	-1	6.38	1.02
-1	14	-1	5.50	1.50
1	-14	1	8.13	0.54
1	-14	1	7.27	1.88
1	-14	1	7.20	0.62
-2	14	-1	16.27	2.32
-2	14	-1	24.57	1.38
-2	14	-1	22.33	0.91
2	-14	1	23.46	0.92
2	-14	1	22.88	0.99

2	-14	1	19.20	2.61
-3	14	-1	23.21	2.33
-3	14	-1	26.30	1.01
3	-14	1	27.17	1.06
3	-14	1	25.66	1.09
3	-14	1	23.57	2.77
-4	14	-1	15.61	0.77
-4	14	-1	15.45	2.72
4	-14	1	15.25	0.91
4	-14	1	18.37	2.65
4	-14	1	17.16	0.87
-5	14	-1	19.33	2.12
-5	14	-1	17.12	0.83
-5	14	-1	17.15	0.95
5	-14	1	20.02	2.69
5	-14	1	17.48	1.08
5	-14	1	16.96	0.95
-6	14	-1	65.52	2.20
-6	14	-1	65.58	2.27
-6	14	-1	66.38	3.38
6	-14	1	63.38	2.43
6	-14	1	66.88	4.04
6	-14	1	65.36	2.28
-7	14	-1	42.72	1.54
-7	14	-1	42.51	2.67
-7	14	-1	43.87	1.63
7	-14	1	38.68	3.34
7	-14	1	39.26	1.86
7	-14	1	38.47	1.63
-8	14	-1	56.23	2.98
-8	14	-1	55.45	2.49
-8	14	-1	55.45	2.07
-8	14	-1	57.83	1.98
8	-14	1	52.60	2.34
8	-14	1	55.89	3.91
8	-14	1	54.25	2.07
-9	14	-1	48.63	2.72
-9	14	-1	46.60	1.79
-9	14	-1	48.14	2.21
9	-14	1	49.79	2.29
9	-14	1	47.72	3.84
9	-14	1	48.27	1.78
-10	14	-1	17.03	1.00
-10	14	-1	18.24	1.35

-10	14	-1	16.71	1.90
10	-14	1	16.39	1.06
10	-14	1	17.31	1.62
10	-14	1	17.55	3.17
-11	14	-1	40.61	1.68
-11	14	-1	41.51	2.49
11	-14	1	44.32	3.97
11	-14	1	42.60	2.33
11	-14	1	42.19	1.75
-12	14	-1	43.39	1.77
-12	14	-1	39.77	2.34
12	-14	1	45.44	2.49
12	-14	1	36.90	4.14
-13	14	-1	19.08	1.22
13	-14	1	14.25	2.75
13	-14	1	18.29	2.09
-14	14	-1	19.09	1.32
14	-14	1	22.79	4.06
14	-14	1	22.16	2.37
-15	14	-1	20.69	1.45
15	-14	1	19.06	3.44
15	-14	1	18.43	2.69
6	13	-1	1.47	1.36
6	13	-1	3.25	1.91
-6	-13	1	3.52	1.90
5	13	-1	5.03	1.88
-5	-13	1	3.61	1.67
4	13	-1	6.36	1.74
-4	-13	1	6.05	0.88
-4	-13	1	5.95	1.74
3	13	-1	21.05	2.79
3	13	-1	17.29	0.90
-3	-13	1	15.00	2.55
-3	-13	1	18.67	1.04
2	13	-1	14.02	2.10
2	13	-1	7.89	1.70
-2	-13	1	13.65	0.84
-2	-13	1	12.32	2.00
1	13	-1	2.41	1.30
1	13	-1	3.51	0.46
1	13	-1	3.13	0.39
1	13	-1	2.10	0.91
-1	-13	1	4.13	1.48
-1	-13	1	2.74	0.46

0	13	-1	25.63	2.56
0	13	-1	22.39	0.93
0	13	-1	23.75	0.94
0	13	-1	23.35	1.47
0	-13	1	24.62	1.00
0	-13	1	22.41	2.53
-1	13	-1	25.46	2.27
-1	13	-1	24.76	0.96
-1	13	-1	25.45	1.32
1	-13	1	24.00	1.01
1	-13	1	24.30	2.56
1	-13	1	24.03	0.93
-2	13	-1	18.39	1.35
-2	13	-1	23.12	2.23
-2	13	-1	21.97	0.86
2	-13	1	21.11	0.93
2	-13	1	22.57	0.88
-3	13	-1	35.40	2.46
-3	13	-1	35.74	1.29
-3	13	-1	36.94	1.45
3	-13	1	35.22	3.34
3	-13	1	35.83	1.37
3	-13	1	37.45	1.33
-4	13	-1	47.54	1.67
-4	13	-1	46.49	2.77
-4	13	-1	46.83	1.56
4	-13	1	39.23	1.66
4	-13	1	46.98	3.27
4	-13	1	44.99	1.69
-5	13	-1	31.65	1.41
-5	13	-1	31.58	1.91
-5	13	-1	35.01	1.23
-5	13	-1	32.41	2.26
5	-13	1	31.78	1.43
5	-13	1	32.92	3.12
5	-13	1	31.70	1.30
-6	13	-1	58.27	3.00
-6	13	-1	58.95	2.59
-6	13	-1	61.05	2.06
-6	13	-1	60.19	1.99
6	-13	1	52.17	2.21
6	-13	1	60.08	2.07
-7	13	-1	37.53	1.49
-7	13	-1	38.00	1.41

-7	13	-1	35.17	1.90
-7	13	-1	35.79	2.31
7	-13	1	35.95	2.98
7	-13	1	38.18	1.75
7	-13	1	38.48	1.50
-8	13	-1	29.05	1.29
-8	13	-1	27.23	1.61
-8	13	-1	25.75	2.02
-8	13	-1	28.40	1.18
8	-13	1	28.70	1.57
8	-13	1	28.97	2.88
8	-13	1	26.70	1.29
-9	13	-1	42.15	1.63
-9	13	-1	40.70	2.36
-9	13	-1	43.66	1.96
9	-13	1	45.09	2.06
9	-13	1	40.76	3.38
9	-13	1	43.35	1.64
-10	13	-1	73.48	2.59
-10	13	-1	68.03	2.41
-10	13	-1	64.28	3.03
10	-13	1	68.61	4.35
10	-13	1	69.35	2.84
10	-13	1	68.67	2.42
-11	13	-1	28.75	2.00
-11	13	-1	33.16	1.49
-11	13	-1	34.49	1.42
11	-13	1	31.59	1.97
11	-13	1	30.87	1.49
11	-13	1	31.82	3.57
-12	13	-1	14.85	1.52
-12	13	-1	15.41	1.05
12	-13	1	15.78	1.67
12	-13	1	18.40	3.30
-13	13	-1	32.55	1.52
13	-13	1	34.04	4.00
13	-13	1	29.59	2.18
-14	13	-1	38.48	1.72
14	-13	1	34.21	2.53
14	-13	1	32.62	4.22
-15	13	-1	18.48	1.35
15	-13	1	17.77	2.45
15	-13	1	19.80	3.40
7	12	-1	1.45	1.72

7	12	-1	0.87	1.31
7	12	-1	1.60	0.98
6	12	-1	1.93	1.54
6	12	-1	2.81	1.01
-6	-12	1	3.69	1.69
5	12	-1	8.55	0.86
5	12	-1	8.86	1.95
-5	-12	1	9.32	0.94
-5	-12	1	6.13	1.65
4	12	-1	13.45	0.85
4	12	-1	17.23	1.90
4	12	-1	16.46	2.58
-4	-12	1	13.94	2.34
-4	-12	1	13.81	0.94
3	12	-1	8.66	0.70
3	12	-1	6.28	1.56
3	12	-1	8.58	1.22
3	12	-1	7.91	0.59
-3	-12	1	8.27	0.73
-3	-12	1	12.26	1.78
2	12	-1	23.08	0.99
2	12	-1	26.06	1.68
2	12	-1	23.87	0.93
2	12	-1	25.42	2.53
-2	-12	1	22.02	2.34
-2	-12	1	24.16	1.05
1	12	-1	39.19	1.88
1	12	-1	39.16	1.37
1	12	-1	39.28	1.35
1	12	-1	34.98	2.70
-1	-12	1	40.43	1.44
0	12	-1	11.81	1.04
0	12	-1	8.81	0.54
0	12	-1	7.26	1.44
0	-12	1	9.53	1.57
0	-12	1	8.87	0.58
-1	12	-1	15.43	1.85
-1	12	-1	13.60	0.65
1	-12	1	14.69	0.63
1	-12	1	12.19	1.80
1	-12	1	13.06	0.70
-2	12	-1	49.79	1.65
-2	12	-1	48.17	2.81
-2	12	-1	49.43	1.83

2	-12	1	51.16	1.65
2	-12	1	47.08	3.02
2	-12	1	49.48	1.71
-3	12	-1	107.41	4.20
-3	12	-1	117.32	3.65
-3	12	-1	116.61	3.53
-3	12	-1	109.94	4.59
3	-12	1	107.63	3.61
3	-12	1	119.75	5.09
3	-12	1	116.73	3.56
-4	12	-1	92.55	3.53
-4	12	-1	92.62	2.86
-4	12	-1	94.91	2.95
-4	12	-1	83.54	3.81
4	-12	1	90.08	4.33
4	-12	1	91.88	2.90
4	-12	1	85.13	3.00
-5	12	-1	48.95	2.24
-5	12	-1	48.50	1.67
-5	12	-1	44.91	2.56
5	-12	1	52.75	3.17
5	-12	1	48.37	1.89
5	-12	1	49.93	1.74
-6	12	-1	45.26	1.59
-6	12	-1	43.99	2.08
-6	12	-1	44.43	2.41
-6	12	-1	45.60	1.66
6	-12	1	44.45	1.84
6	-12	1	48.58	1.67
6	-12	1	42.10	2.96
-7	12	-1	28.58	1.60
-7	12	-1	28.29	1.94
-7	12	-1	30.54	1.26
-7	12	-1	29.57	1.17
7	-12	1	31.39	2.60
7	-12	1	27.84	1.24
7	-12	1	31.27	1.51
-8	12	-1	35.80	1.70
-8	12	-1	38.00	2.09
-8	12	-1	35.55	1.38
-8	12	-1	34.44	1.48
8	-12	1	35.87	1.46
8	-12	1	37.07	1.72
8	-12	1	34.36	2.88

-9	12	-1	32.93	2.00
-9	12	-1	39.89	1.68
-9	12	-1	36.96	1.45
9	-12	1	36.10	3.07
9	-12	1	35.67	1.79
9	-12	1	36.70	1.47
9	-12	1	41.36	1.74
-10	12	-1	42.77	1.66
-10	12	-1	40.75	1.59
-10	12	-1	37.46	2.11
10	-12	1	38.68	3.33
10	-12	1	43.05	2.01
10	-12	1	39.11	1.62
-11	12	-1	36.99	2.00
-11	12	-1	41.22	1.53
-11	12	-1	40.60	1.60
11	-12	1	36.75	1.69
11	-12	1	39.33	3.55
11	-12	1	42.30	2.08
-12	12	-1	65.03	2.34
-12	12	-1	60.72	2.49
12	-12	1	65.68	2.85
12	-12	1	60.88	4.34
-13	12	-1	15.60	1.10
13	-12	1	14.30	2.52
13	-12	1	16.77	1.79
-14	12	-1	9.91	1.04
14	-12	1	9.86	2.48
14	-12	1	12.43	1.94
-15	12	-1	29.64	1.60
15	-12	1	31.58	4.28
15	-12	1	28.98	2.61
-16	12	-1	12.10	1.35
16	-12	1	15.05	3.24
16	-12	1	12.22	1.88
8	11	-1	3.75	1.78
8	11	-1	0.75	1.00
8	11	-1	2.36	1.37
7	11	-1	8.11	1.11
7	11	-1	6.40	1.74
7	11	-1	3.86	1.30
7	11	-1	6.56	1.04
6	11	-1	7.99	0.89
6	11	-1	8.19	1.83

-6	-11	1	9.14	1.72
-6	-11	1	7.89	0.93
5	11	-1	9.92	1.71
5	11	-1	10.28	1.75
5	11	-1	9.71	0.82
-5	-11	1	9.87	1.77
-5	-11	1	11.33	0.88
4	11	-1	13.69	0.83
4	11	-1	10.09	1.52
4	11	-1	12.34	0.74
4	11	-1	19.91	2.30
-4	-11	1	12.94	1.98
-4	-11	1	15.75	0.87
3	11	-1	27.14	1.11
3	11	-1	30.27	1.71
3	11	-1	23.16	2.38
3	11	-1	28.00	1.04
-3	-11	1	25.78	3.52
-3	-11	1	27.40	1.17
2	11	-1	33.94	1.74
2	11	-1	33.30	1.17
2	11	-1	33.78	1.25
-2	-11	1	33.68	1.28
-2	-11	1	32.45	2.40
1	11	-1	39.35	1.38
1	11	-1	40.88	1.37
1	11	-1	40.52	1.79
1	11	-1	40.37	2.58
-1	-11	1	36.48	2.54
-1	-11	1	40.83	1.44
0	11	-1	53.13	2.03
0	11	-1	54.33	1.71
0	11	-1	48.30	2.82
0	-11	1	47.65	2.92
0	-11	1	54.26	1.77
0	-11	1	49.42	1.84
-1	11	-1	78.77	2.43
-1	11	-1	68.18	3.83
-1	11	-1	76.46	3.55
-1	11	-1	74.98	3.15
-1	11	-1	83.74	2.68
1	-11	1	74.21	3.67
1	-11	1	72.84	2.44
1	-11	1	79.95	2.48

-2	11	-1	64.17	3.22
-2	11	-1	72.64	2.42
-2	11	-1	69.52	3.72
-2	11	-1	69.15	2.93
-2	11	-1	72.15	2.27
2	-11	1	74.06	2.36
2	-11	1	69.55	3.54
2	-11	1	74.62	2.28
-3	11	-1	72.62	3.72
-3	11	-1	73.04	2.31
-3	11	-1	71.46	2.91
-3	11	-1	75.21	2.42
-3	11	-1	69.19	3.24
3	-11	1	78.65	3.68
3	-11	1	68.39	2.42
3	-11	1	72.00	2.34
-4	11	-1	118.52	5.15
-4	11	-1	120.76	4.38
-4	11	-1	125.65	3.95
-4	11	-1	124.56	3.88
4	-11	1	130.70	4.50
4	-11	1	122.20	3.90
4	-11	1	136.41	4.06
4	-11	1	120.94	5.17
-5	11	-1	75.66	3.71
-5	11	-1	69.53	2.49
-5	11	-1	75.64	2.85
-5	11	-1	75.85	2.41
5	-11	1	76.50	2.44
5	-11	1	78.10	2.97
5	-11	1	76.26	2.62
5	-11	1	65.24	3.59
-6	11	-1	75.07	2.50
-6	11	-1	69.72	3.59
-6	11	-1	74.99	2.44
-6	11	-1	74.19	2.80
6	-11	1	76.50	2.68
6	-11	1	76.26	2.92
6	-11	1	77.90	2.48
6	-11	1	70.41	3.70
-7	11	-1	50.14	2.06
-7	11	-1	50.83	1.77
-7	11	-1	51.02	1.84
7	-11	1	55.31	1.83

7	-11	1	49.20	2.16
7	-11	1	49.21	2.02
7	-11	1	48.17	3.09
-8	11	-1	10.76	0.68
-8	11	-1	10.00	0.91
8	-11	1	9.96	1.04
8	-11	1	12.76	1.90
8	-11	1	11.10	1.00
8	-11	1	10.21	0.76
-9	11	-1	47.57	1.72
-9	11	-1	48.07	1.81
9	-11	1	47.18	1.73
9	-11	1	46.19	2.05
9	-11	1	46.34	1.77
9	-11	1	44.41	3.18
-10	11	-1	100.17	3.79
-10	11	-1	109.05	3.45
-10	11	-1	103.85	3.47
10	-11	1	107.84	3.51
10	-11	1	106.98	5.31
10	-11	1	110.18	3.86
-11	11	-1	32.89	1.71
-11	11	-1	33.51	1.60
-11	11	-1	38.16	1.36
11	-11	1	35.20	1.64
11	-11	1	35.46	1.90
11	-11	1	32.14	3.12
-12	11	-1	16.08	1.00
-12	11	-1	12.96	0.70
12	-11	1	15.63	2.88
12	-11	1	15.51	1.49
-13	11	-1	21.81	1.24
13	-11	1	23.62	1.85
13	-11	1	21.51	3.28
-14	11	-1	16.45	1.16
14	-11	1	20.93	3.56
14	-11	1	15.42	1.87
-15	11	-1	11.94	1.16
15	-11	1	13.63	1.72
15	-11	1	16.60	2.97
-16	11	-1	8.42	1.23
16	-11	1	7.05	1.55
16	-11	1	6.40	2.79
9	10	-1	1.11	1.70

9	10	-1	2.64	1.42
9	10	-1	3.90	1.13
8	10	-1	3.75	0.70
8	10	-1	3.24	1.25
8	10	-1	4.67	0.98
8	10	-1	5.13	1.76
7	10	-1	8.11	1.75
7	10	-1	7.81	1.14
7	10	-1	9.98	1.74
7	10	-1	7.69	0.93
6	10	-1	18.36	1.07
6	10	-1	19.54	0.99
6	10	-1	20.65	2.49
-6	-10	1	22.01	2.27
-6	-10	1	21.02	1.11
5	10	-1	17.41	0.85
5	10	-1	15.59	0.91
5	10	-1	17.79	1.50
5	10	-1	16.35	2.21
-5	-10	1	17.56	0.95
-5	-10	1	16.90	2.01
4	10	-1	20.82	2.19
4	10	-1	18.59	0.92
4	10	-1	20.72	1.49
4	10	-1	19.71	0.84
-4	-10	1	18.24	1.89
-4	-10	1	20.50	0.96
3	10	-1	19.53	2.05
3	10	-1	22.05	0.83
3	10	-1	20.61	1.37
3	10	-1	21.20	0.90
-3	-10	1	21.05	1.95
-3	-10	1	19.80	0.94
2	10	-1	55.72	2.19
2	10	-1	55.17	2.99
2	10	-1	54.85	1.78
2	10	-1	58.55	3.63
2	10	-1	53.81	1.82
-2	-10	1	52.29	2.85
-2	-10	1	55.18	1.87
1	10	-1	98.60	3.25
1	10	-1	92.39	4.01
1	10	-1	91.39	4.38
1	10	-1	87.55	3.62

1	10	-1	94.67	2.92
-1	-10	1	88.86	3.98
-1	-10	1	96.22	2.97
0	10	-1	104.42	3.64
0	10	-1	107.03	4.44
0	10	-1	111.03	4.79
0	10	-1	114.81	3.41
0	10	-1	108.91	4.10
0	-10	1	111.79	3.45
0	-10	1	107.57	4.51
0	-10	1	115.41	3.38
-1	10	-1	87.03	2.82
-1	10	-1	86.57	3.63
-1	10	-1	82.35	3.90
-1	10	-1	84.16	2.62
-1	10	-1	82.46	3.25
1	-10	1	84.39	2.68
1	-10	1	82.73	2.61
1	-10	1	79.67	3.73
-2	10	-1	63.10	2.59
-2	10	-1	69.31	3.34
-2	10	-1	64.60	2.03
-2	10	-1	64.37	2.15
2	-10	1	59.07	2.23
2	-10	1	62.15	3.17
-3	10	-1	90.07	4.06
-3	10	-1	99.41	3.00
-3	10	-1	93.50	2.91
-3	10	-1	88.59	3.39
3	-10	1	96.65	3.18
3	-10	1	90.85	4.05
3	-10	1	93.92	3.48
3	-10	1	90.96	2.93
-4	10	-1	104.43	3.25
-4	10	-1	105.53	3.31
-4	10	-1	106.53	3.70
-4	10	-1	106.19	4.40
4	-10	1	103.61	3.42
4	-10	1	102.50	3.27
4	-10	1	103.82	3.74
4	-10	1	100.58	4.42
-5	10	-1	77.29	3.66
-5	10	-1	81.20	2.64
-5	10	-1	82.22	2.96

-5	10	-1	81.03	2.59
5	-10	1	83.99	2.62
5	-10	1	81.50	3.03
5	-10	1	80.50	2.78
5	-10	1	78.81	3.78
-6	10	-1	61.49	2.13
-6	10	-1	64.29	2.37
-6	10	-1	64.06	3.17
-6	10	-1	62.74	2.07
6	-10	1	63.61	3.25
6	-10	1	59.65	2.27
6	-10	1	62.68	2.46
6	-10	1	63.48	2.11
-7	10	-1	72.73	2.52
-7	10	-1	73.45	2.44
-7	10	-1	69.84	3.50
-7	10	-1	75.63	2.66
7	-10	1	75.46	3.69
7	-10	1	74.03	2.48
7	-10	1	78.92	2.70
7	-10	1	75.55	2.77
-8	10	-1	62.18	2.25
-8	10	-1	56.95	3.19
-8	10	-1	63.40	2.15
8	-10	1	64.91	2.39
8	-10	1	59.23	3.41
8	-10	1	65.27	2.21
8	-10	1	66.56	2.16
8	-10	1	65.31	2.42
-9	10	-1	58.61	2.09
-9	10	-1	62.22	2.10
9	-10	1	65.38	3.55
9	-10	1	61.20	2.12
9	-10	1	60.28	2.15
9	-10	1	59.32	2.41
9	-10	1	57.83	2.25
-10	10	-1	62.21	2.21
-10	10	-1	66.24	2.11
10	-10	1	63.74	3.67
10	-10	1	62.55	2.26
10	-10	1	61.09	2.55
-11	10	-1	44.76	1.56
11	-10	1	47.46	3.29
11	-10	1	47.45	2.16

-12	10	-1	40.02	1.60
12	-10	1	37.54	3.32
12	-10	1	37.48	2.03
-13	10	-1	18.05	1.11
13	-10	1	14.29	2.38
13	-10	1	20.17	1.71
-14	10	-1	12.08	1.08
14	-10	1	10.34	1.70
14	-10	1	10.90	2.38
-15	10	-1	19.53	1.28
15	-10	1	15.53	2.97
15	-10	1	15.84	2.09
-16	10	-1	8.03	1.18
16	-10	1	4.54	2.63
16	-10	1	9.42	1.61
10	9	-1	5.98	1.24
10	9	-1	7.27	1.05
10	9	-1	5.66	1.53
10	9	-1	6.89	1.80
9	9	-1	2.67	1.00
9	9	-1	4.99	1.54
9	9	-1	4.34	0.75
9	9	-1	3.82	1.33
8	9	-1	6.06	0.93
8	9	-1	7.51	1.30
8	9	-1	3.16	1.39
8	9	-1	6.08	0.70
7	9	-1	14.01	1.22
7	9	-1	14.94	0.92
7	9	-1	13.60	1.92
7	9	-1	13.53	0.94
7	9	-1	12.79	1.65
6	9	-1	16.91	0.95
6	9	-1	15.28	1.47
6	9	-1	17.81	0.89
6	9	-1	16.92	2.20
-6	-9	1	18.36	0.98
-6	-9	1	19.28	1.91
5	9	-1	11.30	0.65
5	9	-1	11.04	0.72
5	9	-1	11.24	1.82
5	9	-1	11.02	1.20
-5	-9	1	11.50	0.75
-5	-9	1	8.52	1.59

4	9	-1	35.25	2.42
4	9	-1	33.47	1.28
4	9	-1	34.88	1.67
4	9	-1	34.29	1.21
-4	-9	1	35.42	1.34
-4	-9	1	33.58	2.21
3	9	-1	83.30	3.87
3	9	-1	91.12	2.72
3	9	-1	87.80	3.08
3	9	-1	88.66	4.23
3	9	-1	83.66	2.76
3	9	-1	93.01	4.24
-3	-9	1	82.02	3.65
-3	-9	1	86.68	2.82
2	9	-1	39.92	1.50
2	9	-1	38.86	2.67
2	9	-1	45.72	1.74
2	9	-1	41.19	2.41
2	9	-1	43.25	2.12
2	9	-1	39.55	1.43
-2	-9	1	41.06	2.37
-2	-9	1	44.04	1.47
1	9	-1	50.96	2.31
1	9	-1	44.97	2.80
1	9	-1	51.08	1.69
1	9	-1	55.23	2.70
-1	-9	1	53.68	1.72
-1	-9	1	50.01	2.62
0	9	-1	85.45	3.29
0	9	-1	88.86	2.92
0	9	-1	82.21	3.84
0	9	-1	84.21	3.60
0	9	-1	87.03	2.71
0	-9	1	90.20	3.73
0	-9	1	89.56	2.69
0	-9	1	87.17	2.76
-1	9	-1	70.44	3.47
-1	9	-1	79.32	2.45
-1	9	-1	75.23	2.96
-1	9	-1	80.77	2.61
1	-9	1	81.70	3.05
1	-9	1	80.83	3.50
1	-9	1	78.82	2.45
-2	9	-1	87.38	3.25

-2	9	-1	92.22	2.79
-2	9	-1	87.45	2.87
-2	9	-1	89.06	3.84
2	-9	1	90.73	2.87
2	-9	1	92.94	2.81
2	-9	1	90.19	3.31
2	-9	1	86.63	3.80
-3	9	-1	72.41	2.24
-3	9	-1	75.09	2.68
-3	9	-1	68.91	3.24
-3	9	-1	73.79	2.30
3	-9	1	68.24	2.26
3	-9	1	71.21	3.27
3	-9	1	69.43	2.69
3	-9	1	66.22	2.36
-4	9	-1	42.65	1.41
-4	9	-1	42.12	2.38
-4	9	-1	40.44	1.75
-4	9	-1	42.44	1.45
4	-9	1	39.72	1.58
4	-9	1	42.62	1.43
4	-9	1	42.05	1.82
4	-9	1	39.79	2.36
-5	9	-1	50.73	1.67
-5	9	-1	47.52	2.60
-5	9	-1	49.35	1.72
-5	9	-1	51.00	1.95
5	-9	1	48.25	2.68
5	-9	1	50.41	2.04
5	-9	1	49.87	1.69
5	-9	1	51.16	1.84
-6	9	-1	50.48	1.93
-6	9	-1	51.08	1.73
-6	9	-1	51.23	2.68
-6	9	-1	51.53	1.79
6	-9	1	48.54	2.76
6	-9	1	51.82	1.76
6	-9	1	52.53	2.03
6	-9	1	50.67	1.92
-7	9	-1	29.35	1.23
-7	9	-1	28.72	1.20
-7	9	-1	28.49	1.11
-7	9	-1	31.80	2.10
7	-9	1	30.09	1.16

7	-9	1	29.08	1.38
7	-9	1	27.93	1.33
7	-9	1	28.75	2.19
-8	9	-1	22.11	0.94
-8	9	-1	20.14	1.85
8	-9	1	23.04	0.97
8	-9	1	21.45	1.18
8	-9	1	21.86	1.14
8	-9	1	21.28	2.05
8	-9	1	22.06	1.00
-9	9	-1	59.16	3.36
-9	9	-1	60.67	2.10
-9	9	-1	60.24	2.02
9	-9	1	61.36	2.16
9	-9	1	64.96	3.43
9	-9	1	63.28	2.15
9	-9	1	61.74	2.22
9	-9	1	58.36	2.37
-10	9	-1	68.97	2.25
-10	9	-1	69.56	2.41
10	-9	1	71.22	2.73
10	-9	1	73.34	2.44
10	-9	1	66.25	3.69
10	-9	1	71.78	2.48
-11	9	-1	10.93	0.76
11	-9	1	4.22	2.26
11	-9	1	10.70	1.13
-12	9	-1	9.63	0.82
12	-9	1	11.79	1.29
12	-9	1	14.22	2.43
-13	9	-1	20.54	1.18
13	-9	1	20.54	2.90
13	-9	1	22.08	1.72
-14	9	-1	21.77	1.29
14	-9	1	22.00	3.31
14	-9	1	21.53	1.94
-15	9	-1	6.08	0.81
15	-9	1	7.43	2.25
15	-9	1	9.83	1.54
-16	9	-1	1.87	0.76
16	-9	1	5.63	2.62
16	-9	1	2.53	1.30
11	8	-1	7.60	1.75
11	8	-1	7.32	1.71

11	8	-1	6.56	0.90
11	8	-1	8.44	1.31
10	8	-1	5.43	1.13
10	8	-1	6.08	1.68
10	8	-1	8.14	1.53
10	8	-1	6.77	1.04
9	8	-1	9.74	0.95
9	8	-1	10.35	1.35
9	8	-1	7.03	1.59
9	8	-1	8.50	1.43
8	8	-1	14.92	2.21
8	8	-1	20.68	1.05
8	8	-1	18.29	1.07
8	8	-1	16.30	1.71
8	8	-1	18.56	1.69
7	8	-1	14.67	0.87
7	8	-1	13.16	1.74
7	8	-1	13.50	0.84
-7	-8	1	15.32	0.94
6	8	-1	15.50	1.26
6	8	-1	14.34	0.76
6	8	-1	13.74	0.80
6	8	-1	12.98	1.89
-6	-8	1	12.02	1.65
-6	-8	1	14.81	0.86
5	8	-1	23.77	0.95
5	8	-1	24.48	1.37
5	8	-1	23.51	2.04
5	8	-1	24.49	1.00
5	8	-1	25.48	3.05
-5	-8	1	23.91	1.06
-5	-8	1	24.98	1.82
4	8	-1	51.80	3.08
4	8	-1	51.25	2.73
4	8	-1	50.33	1.73
4	8	-1	44.40	2.38
4	8	-1	53.14	1.69
4	8	-1	53.07	2.02
-4	-8	1	50.14	2.55
-4	-8	1	51.94	1.78
3	8	-1	77.48	3.62
3	8	-1	78.48	2.36
3	8	-1	74.51	2.40
3	8	-1	71.98	2.62

3	8	-1	72.73	3.32
3	8	-1	71.93	3.00
-3	-8	1	75.40	2.44
-3	-8	1	76.45	3.23
2	8	-1	49.37	1.82
2	8	-1	47.09	2.47
2	8	-1	46.87	1.59
2	8	-1	50.71	2.72
2	8	-1	49.12	2.18
2	8	-1	47.97	1.56
-2	-8	1	47.30	2.39
-2	-8	1	46.70	1.62
1	8	-1	96.19	3.88
1	8	-1	97.03	3.57
1	8	-1	97.99	3.22
1	8	-1	98.37	3.03
1	8	-1	92.90	4.04
-1	-8	1	94.71	3.85
-1	-8	1	102.54	3.00
-1	-8	1	100.70	3.07
0	8	-1	130.60	4.09
0	8	-1	133.00	4.95
0	8	-1	129.82	4.43
0	8	-1	132.22	3.93
0	-8	1	126.63	4.45
0	-8	1	118.02	4.74
0	-8	1	128.79	3.90
-1	8	-1	145.34	4.88
-1	8	-1	140.49	5.35
-1	8	-1	146.16	4.42
-1	8	-1	152.41	4.54
1	-8	1	145.99	4.92
1	-8	1	147.38	4.42
1	-8	1	133.16	5.25
1	-8	1	152.87	4.47
-2	8	-1	94.32	2.97
-2	8	-1	94.58	3.37
-2	8	-1	91.61	3.85
-2	8	-1	97.57	3.03
2	-8	1	93.68	2.98
2	-8	1	97.86	3.42
2	-8	1	99.03	3.05
2	-8	1	102.35	3.92
-3	8	-1	168.40	5.94

-3	8	-1	165.27	5.37
-3	8	-1	172.11	5.07
-3	8	-1	162.56	5.02
3	-8	1	169.09	5.14
3	-8	1	169.58	5.05
3	-8	1	151.18	5.88
3	-8	1	166.12	5.42
-4	8	-1	133.43	5.22
-4	8	-1	142.34	4.68
-4	8	-1	147.48	4.46
-4	8	-1	144.64	4.41
4	-8	1	150.05	4.43
4	-8	1	147.41	5.38
4	-8	1	143.96	4.74
-5	8	-1	137.20	4.22
-5	8	-1	133.00	4.37
-5	8	-1	132.42	4.17
-5	8	-1	135.36	5.02
5	-8	1	139.37	4.32
5	-8	1	134.74	5.13
5	-8	1	138.89	4.19
5	-8	1	131.50	4.43
-6	8	-1	31.28	1.14
-6	8	-1	30.15	1.20
6	-8	1	29.72	2.02
6	-8	1	30.81	1.31
6	-8	1	32.52	1.17
6	-8	1	32.63	1.38
-7	8	-1	65.91	2.20
-7	8	-1	69.21	2.25
-7	8	-1	66.99	2.99
7	-8	1	65.18	3.19
7	-8	1	65.37	2.36
7	-8	1	67.81	2.24
7	-8	1	65.47	2.39
7	-8	1	68.84	2.21
-8	8	-1	129.76	4.20
-8	8	-1	131.93	4.98
-8	8	-1	138.61	4.16
8	-8	1	134.64	4.30
8	-8	1	140.69	4.23
8	-8	1	136.75	4.24
8	-8	1	131.27	4.42
8	-8	1	126.42	5.23

-9	8	-1	21.04	1.80
-9	8	-1	22.89	0.86
-9	8	-1	23.13	1.00
9	-8	1	21.87	2.06
9	-8	1	22.54	1.04
9	-8	1	23.64	1.06
9	-8	1	23.97	1.07
9	-8	1	23.15	1.27
-10	8	-1	13.07	0.61
-10	8	-1	13.99	0.77
10	-8	1	13.29	0.91
10	-8	1	12.74	0.70
10	-8	1	13.75	1.10
10	-8	1	13.00	1.96
-11	8	-1	43.42	1.63
11	-8	1	40.10	2.01
11	-8	1	42.00	2.94
-12	8	-1	19.19	1.02
12	-8	1	16.02	2.35
12	-8	1	17.69	1.73
-13	8	-1	17.11	1.07
13	-8	1	17.68	2.73
13	-8	1	18.53	1.65
-14	8	-1	19.31	1.21
14	-8	1	19.58	1.89
14	-8	1	20.08	3.23
-15	8	-1	7.43	1.05
15	-8	1	3.21	2.26
15	-8	1	4.30	1.24
-16	8	-1	7.69	1.18
16	-8	1	6.07	1.44
16	-8	1	8.34	2.71
12	7	-1	5.18	1.29
12	7	-1	6.30	0.87
12	7	-1	4.36	1.75
12	7	-1	6.65	1.76
11	7	-1	7.37	1.25
11	7	-1	7.61	1.67
11	7	-1	7.12	1.06
11	7	-1	9.79	1.71
10	7	-1	11.14	1.45
10	7	-1	11.23	1.75
10	7	-1	8.86	1.49
9	7	-1	25.39	2.32

9	7	-1	25.82	1.23
9	7	-1	24.80	1.59
9	7	-1	23.93	2.03
8	7	-1	23.06	1.09
8	7	-1	24.33	1.42
8	7	-1	21.72	1.11
8	7	-1	22.92	1.77
8	7	-1	22.38	2.16
7	7	-1	28.49	1.61
7	7	-1	32.14	1.26
7	7	-1	32.96	1.25
7	7	-1	31.45	2.31
7	7	-1	32.34	1.86
-7	-7	1	32.23	1.35
6	7	-1	23.85	1.33
6	7	-1	23.99	1.00
6	7	-1	25.40	0.99
6	7	-1	20.80	2.48
6	7	-1	25.06	1.52
6	7	-1	23.41	1.94
6	7	-1	23.59	1.75
-6	-7	1	24.02	1.07
5	7	-1	15.72	2.02
5	7	-1	16.26	0.77
5	7	-1	16.65	1.65
5	7	-1	16.82	1.05
5	7	-1	18.01	0.75
5	7	-1	18.52	1.48
-5	-7	1	16.65	1.48
-5	-7	1	17.96	0.84
4	7	-1	23.18	0.86
4	7	-1	24.67	1.15
4	7	-1	20.81	1.44
4	7	-1	22.81	0.88
4	7	-1	20.62	1.95
4	7	-1	20.75	1.71
-4	-7	1	22.46	0.94
-4	-7	1	22.81	1.60
3	7	-1	125.49	4.42
3	7	-1	124.11	4.87
3	7	-1	127.48	3.87
3	7	-1	128.68	4.74
3	7	-1	131.96	4.08
3	7	-1	112.55	3.89

-3	-7	1	129.12	3.92
-3	-7	1	128.15	4.60
2	7	-1	45.49	1.67
2	7	-1	47.85	2.44
2	7	-1	42.19	2.20
2	7	-1	45.10	1.45
2	7	-1	44.36	1.97
2	7	-1	43.03	1.47
-2	-7	1	44.00	1.49
-2	-7	1	44.50	2.16
1	7	-1	37.25	1.29
1	7	-1	40.61	1.45
1	7	-1	37.89	1.74
1	7	-1	41.41	2.20
1	7	-1	36.90	1.95
-1	-7	1	37.53	1.98
-1	-7	1	39.01	1.25
-1	-7	1	40.09	1.32
0	7	-1	82.99	3.32
0	7	-1	76.61	2.56
0	7	-1	77.64	2.86
0	-7	1	78.86	2.42
0	-7	1	80.38	2.90
0	-7	1	79.81	2.47
0	-7	1	75.00	3.16
-1	7	-1	52.97	2.05
-1	7	-1	51.52	2.44
-1	7	-1	53.47	1.75
1	-7	1	50.89	1.67
1	-7	1	49.75	2.05
1	-7	1	55.48	1.71
1	-7	1	52.39	2.42
-2	7	-1	77.96	2.42
-2	7	-1	79.21	2.75
-2	7	-1	79.21	2.45
-2	7	-1	78.93	3.19
2	-7	1	79.46	2.43
2	-7	1	75.74	2.48
2	-7	1	73.12	3.15
2	-7	1	77.35	2.77
-3	7	-1	69.19	2.89
-3	7	-1	68.42	2.40
-3	7	-1	63.85	2.11
-3	7	-1	63.84	2.14

3	-7	1	65.78	2.43
3	-7	1	66.64	2.89
3	-7	1	71.14	2.14
3	-7	1	69.45	2.22
-4	7	-1	82.34	2.52
-4	7	-1	83.24	2.56
-4	7	-1	77.60	2.85
-4	7	-1	79.62	3.25
4	-7	1	82.16	2.53
4	-7	1	80.13	2.77
4	-7	1	73.18	3.25
-5	7	-1	145.07	5.10
-5	7	-1	144.37	4.42
-5	7	-1	154.39	4.51
-5	7	-1	139.89	4.36
5	-7	1	123.17	5.09
5	-7	1	147.90	4.39
5	-7	1	141.30	4.56
5	-7	1	139.10	4.49
-6	7	-1	122.53	3.75
-6	7	-1	120.39	4.39
-6	7	-1	113.17	3.66
-6	7	-1	116.47	3.71
6	-7	1	119.90	3.82
6	-7	1	115.54	3.82
6	-7	1	118.49	3.69
6	-7	1	115.80	4.53
-7	7	-1	94.73	3.79
-7	7	-1	99.12	3.14
-7	7	-1	96.47	3.09
7	-7	1	99.51	3.33
7	-7	1	93.57	4.01
7	-7	1	104.51	3.15
7	-7	1	102.03	3.17
-8	7	-1	11.20	0.51
-8	7	-1	12.01	1.31
-8	7	-1	11.10	0.63
8	-7	1	12.74	0.67
8	-7	1	11.76	0.69
8	-7	1	11.81	0.68
8	-7	1	10.54	0.84
8	-7	1	11.63	1.50
-9	7	-1	64.09	2.90
-9	7	-1	68.64	2.15

-9	7	-1	66.75	2.28
9	-7	1	65.63	2.22
9	-7	1	67.02	2.55
9	-7	1	70.08	2.35
9	-7	1	69.94	2.34
9	-7	1	71.12	3.33
-10	7	-1	47.93	1.76
-10	7	-1	54.08	3.43
10	-7	1	46.08	2.82
10	-7	1	47.92	1.86
10	-7	1	48.33	2.07
-11	7	-1	6.42	0.63
11	-7	1	6.51	0.97
11	-7	1	5.35	1.42
-12	7	-1	19.44	1.03
12	-7	1	16.89	2.34
-13	7	-1	23.08	1.23
13	-7	1	25.41	2.88
13	-7	1	24.51	1.74
-14	7	-1	4.70	0.70
14	-7	1	6.65	1.17
14	-7	1	6.75	2.04
-15	7	-1	3.59	0.70
15	-7	1	1.57	1.10
15	-7	1	6.40	2.19
-16	7	-1	2.32	0.76
16	-7	1	1.13	2.37
16	-7	1	0.31	1.24
12	6	-1	7.03	0.86
12	6	-1	6.81	1.32
12	6	-1	12.32	1.95
12	6	-1	6.55	1.55
11	6	-1	12.91	1.03
11	6	-1	10.25	1.68
11	6	-1	12.18	1.56
11	6	-1	14.92	2.17
10	6	-1	24.46	1.65
10	6	-1	17.93	2.67
10	6	-1	20.61	2.03
10	6	-1	22.56	1.16
9	6	-1	29.37	1.97
9	6	-1	31.21	2.30
9	6	-1	32.32	1.33
8	6	-1	31.48	1.28

8	6	-1	30.73	1.88
8	6	-1	33.70	2.26
8	6	-1	30.73	1.58
8	6	-1	32.65	1.28
7	6	-1	38.57	1.98
7	6	-1	42.18	1.82
7	6	-1	42.80	1.52
7	6	-1	42.82	1.51
7	6	-1	42.19	2.44
7	6	-1	40.18	2.24
7	6	-1	41.66	3.12
-7	-6	1	42.62	1.61
6	6	-1	63.61	2.09
6	6	-1	61.88	2.54
6	6	-1	64.20	2.70
6	6	-1	63.57	2.96
6	6	-1	60.26	3.29
6	6	-1	63.52	2.33
6	6	-1	63.99	2.08
-6	-6	1	63.07	2.17
5	6	-1	81.15	2.74
5	6	-1	75.93	3.32
5	6	-1	76.88	3.57
5	6	-1	79.35	3.09
5	6	-1	79.24	2.53
5	6	-1	82.29	2.52
-5	-6	1	79.28	3.17
-5	-6	1	79.39	2.60
4	6	-1	200.31	6.59
4	6	-1	197.92	6.86
4	6	-1	207.42	6.25
4	6	-1	195.26	6.06
4	6	-1	209.30	7.04
4	6	-1	197.05	6.05
-4	-6	1	194.68	6.12
-4	-6	1	203.42	6.71
3	6	-1	40.97	1.48
3	6	-1	38.30	1.32
3	6	-1	40.14	1.31
3	6	-1	40.63	2.16
3	6	-1	38.01	2.00
3	6	-1	38.29	1.78
-3	-6	1	40.79	1.37
-3	-6	1	40.05	1.94

2	6	-1	91.19	3.42
2	6	-1	81.91	3.00
2	6	-1	81.05	2.56
2	6	-1	82.01	2.55
2	6	-1	76.63	3.20
2	6	-1	85.93	2.70
-2	-6	1	82.91	2.59
-2	-6	1	78.48	3.15
1	6	-1	191.73	5.64
1	6	-1	190.06	5.94
1	6	-1	180.45	6.27
1	6	-1	176.75	5.53
-1	-6	1	188.29	5.55
-1	-6	1	186.17	5.50
-1	-6	1	176.48	6.13
-1	-6	1	179.46	5.94
0	6	-1	175.25	5.27
0	6	-1	170.50	5.53
0	6	-1	172.77	5.18
0	6	-1	170.32	5.88
0	-6	1	169.12	5.82
0	-6	1	174.65	5.57
0	-6	1	172.99	5.19
0	-6	1	171.36	5.16
-1	6	-1	103.50	3.52
-1	6	-1	100.72	3.86
-1	6	-1	108.91	3.22
-1	6	-1	111.52	3.28
1	-6	1	104.11	3.56
1	-6	1	104.66	3.22
1	-6	1	102.64	3.87
1	-6	1	108.38	3.25
-2	6	-1	44.49	1.36
-2	6	-1	39.39	1.95
-2	6	-1	40.31	1.61
2	-6	1	41.57	1.99
2	-6	1	40.42	1.34
2	-6	1	41.36	1.40
2	-6	1	40.86	1.62
-3	6	-1	132.44	4.23
-3	6	-1	134.43	4.06
-3	6	-1	129.89	4.66
-3	6	-1	131.04	4.04
3	-6	1	129.48	4.27

3	-6	1	139.81	4.12
3	-6	1	138.40	4.05
3	-6	1	124.78	4.69
-4	6	-1	91.67	2.82
-4	6	-1	91.35	2.85
-4	6	-1	98.99	3.49
-4	6	-1	90.40	2.95
4	-6	1	87.91	3.48
4	-6	1	89.52	3.00
4	-6	1	85.29	2.89
-5	6	-1	129.47	4.13
-5	6	-1	137.09	4.20
-5	6	-1	141.50	4.78
-5	6	-1	133.43	4.19
5	-6	1	132.49	4.15
5	-6	1	135.31	4.25
5	-6	1	135.18	4.40
-6	6	-1	75.35	2.48
-6	6	-1	76.04	2.46
-6	6	-1	79.54	3.08
6	-6	1	79.54	2.49
6	-6	1	79.55	2.51
6	-6	1	75.94	3.21
6	-6	1	79.80	2.64
6	-6	1	75.75	2.55
-7	6	-1	102.97	3.35
-7	6	-1	111.29	3.28
-7	6	-1	102.58	3.91
7	-6	1	103.30	4.13
7	-6	1	105.77	3.38
7	-6	1	104.57	3.37
7	-6	1	113.09	3.39
7	-6	1	106.75	3.53
-8	6	-1	73.71	2.43
-8	6	-1	73.77	2.32
-8	6	-1	71.99	2.97
8	-6	1	74.16	2.46
8	-6	1	78.18	2.49
8	-6	1	74.86	2.65
8	-6	1	74.52	2.39
8	-6	1	69.71	3.24
-9	6	-1	50.81	1.78
-9	6	-1	50.87	1.69
-9	6	-1	48.81	2.33

9	-6	1	52.91	1.84
9	-6	1	52.19	1.87
9	-6	1	52.60	2.06
9	-6	1	47.04	2.66
-10	6	-1	13.48	0.77
-10	6	-1	13.47	1.34
10	-6	1	13.27	1.05
10	-6	1	14.64	0.91
10	-6	1	13.06	1.71
-11	6	-1	29.83	1.29
11	-6	1	30.97	2.45
11	-6	1	32.90	1.65
-12	6	-1	23.05	1.13
12	-6	1	23.74	2.49
12	-6	1	22.96	1.54
-13	6	-1	2.62	0.52
13	-6	1	2.93	1.64
13	-6	1	1.98	0.87
-14	6	-1	4.59	0.70
14	-6	1	4.75	1.95
14	-6	1	6.49	1.16
-15	6	-1	1.74	0.68
15	-6	1	1.88	2.02
15	-6	1	3.30	1.17
-16	6	-1	1.46	0.73
16	-6	1	-2.24	2.22
16	-6	1	1.81	1.33
13	5	-1	7.16	1.10
13	5	-1	5.30	1.41
13	5	-1	5.41	1.31
13	5	-1	7.38	1.95
12	5	-1	10.79	1.70
12	5	-1	13.02	1.11
12	5	-1	12.55	2.08
12	5	-1	15.70	2.48
11	5	-1	21.46	2.09
11	5	-1	17.94	2.13
11	5	-1	18.80	1.62
11	5	-1	18.24	1.08
10	5	-1	30.34	2.15
10	5	-1	31.64	2.28
10	5	-1	33.09	1.37
10	5	-1	33.79	1.79
9	5	-1	11.24	1.64

9	5	-1	11.21	1.62
9	5	-1	12.11	0.79
9	5	-1	12.14	0.86
9	5	-1	11.34	1.11
9	5	-1	11.13	1.43
8	5	-1	26.35	1.13
8	5	-1	27.69	1.14
8	5	-1	27.37	1.56
8	5	-1	27.14	1.65
8	5	-1	27.04	1.97
8	5	-1	29.23	1.44
8	5	-1	28.26	1.80
7	5	-1	58.05	2.15
7	5	-1	56.22	1.91
7	5	-1	56.47	2.69
7	5	-1	57.25	2.48
7	5	-1	56.74	1.91
7	5	-1	56.83	2.37
7	5	-1	53.11	3.10
-7	-5	1	57.08	2.01
6	5	-1	123.10	4.36
6	5	-1	123.24	4.46
6	5	-1	128.62	4.72
6	5	-1	128.85	3.96
6	5	-1	130.45	3.96
6	5	-1	128.26	4.98
6	5	-1	133.51	4.17
-6	-5	1	126.55	4.05
5	5	-1	82.68	2.50
5	5	-1	83.10	2.68
5	5	-1	78.42	2.50
5	5	-1	76.47	3.38
5	5	-1	80.18	2.99
5	5	-1	74.02	3.16
-5	-5	1	79.76	3.06
-5	-5	1	77.05	2.57
4	5	-1	37.09	1.39
4	5	-1	35.49	1.86
4	5	-1	36.64	1.23
4	5	-1	36.25	1.66
4	5	-1	36.33	2.03
4	5	-1	36.73	1.24
-4	-5	1	36.99	1.29
-4	-5	1	39.01	1.78

3	5	-1	210.05	7.29
3	5	-1	232.56	7.41
3	5	-1	213.69	6.88
3	5	-1	218.61	6.67
3	5	-1	224.80	6.67
3	5	-1	239.15	7.12
-3	-5	1	219.66	7.19
-3	-5	1	220.97	6.72
2	5	-1	213.72	6.59
2	5	-1	202.43	6.20
2	5	-1	215.92	6.30
2	5	-1	215.10	6.88
-2	-5	1	201.75	6.72
-2	-5	1	199.42	6.57
-2	-5	1	200.29	6.23
1	5	-1	134.38	4.07
1	5	-1	129.33	4.39
1	5	-1	133.13	4.14
-1	-5	1	138.13	4.08
-1	-5	1	135.34	4.05
-1	-5	1	137.78	4.61
-1	-5	1	136.11	4.43
0	5	-1	123.40	3.63
0	5	-1	121.66	3.95
0	5	-1	123.39	4.21
0	5	-1	105.33	3.66
0	-5	1	117.08	4.14
0	-5	1	121.76	3.94
0	-5	1	126.52	3.63
0	-5	1	119.73	3.61
-1	5	-1	25.75	1.11
-1	5	-1	26.80	0.90
-1	5	-1	26.16	0.88
-1	5	-1	26.68	1.39
1	-5	1	26.41	0.90
1	-5	1	26.78	0.88
1	-5	1	26.54	1.15
1	-5	1	26.62	1.39
-2	5	-1	126.44	3.95
-2	5	-1	129.83	4.13
-2	5	-1	135.58	4.50
-2	5	-1	128.33	3.94
2	-5	1	128.73	3.94
2	-5	1	132.34	3.98

2	-5	1	130.59	4.16
2	-5	1	132.20	4.52
-3	5	-1	87.86	3.46
-3	5	-1	91.85	2.79
-3	5	-1	94.79	2.82
-3	5	-1	95.70	2.93
3	-5	1	76.14	3.26
3	-5	1	90.42	2.84
3	-5	1	95.01	2.80
3	-5	1	91.00	2.96
-4	5	-1	48.22	1.56
-4	5	-1	47.05	1.99
-4	5	-1	48.44	1.55
-4	5	-1	48.14	1.51
4	-5	1	41.74	2.02
4	-5	1	44.03	1.66
4	-5	1	45.62	1.60
4	-5	1	44.23	1.52
-5	5	-1	36.74	1.31
-5	5	-1	38.06	1.31
-5	5	-1	41.78	1.83
5	-5	1	40.65	1.33
5	-5	1	38.98	1.43
5	-5	1	41.65	1.35
5	-5	1	38.76	1.38
5	-5	1	38.81	1.89
-6	5	-1	173.35	5.05
-6	5	-1	163.76	5.11
-6	5	-1	168.01	5.60
6	-5	1	167.60	5.12
6	-5	1	160.33	5.76
6	-5	1	172.70	5.13
6	-5	1	162.76	5.24
6	-5	1	168.73	5.13
-7	5	-1	52.65	1.69
-7	5	-1	51.10	1.77
-7	5	-1	51.95	2.24
7	-5	1	55.15	1.82
7	-5	1	49.85	2.42
7	-5	1	52.13	1.74
7	-5	1	55.90	1.81
7	-5	1	53.87	1.96
-8	5	-1	21.42	0.83
-8	5	-1	18.93	1.30

-8	5	-1	19.41	0.86
8	-5	1	21.15	0.81
8	-5	1	21.07	0.90
8	-5	1	21.14	0.95
8	-5	1	21.80	1.10
8	-5	1	21.58	1.60
-9	5	-1	32.37	1.22
-9	5	-1	30.69	1.19
-9	5	-1	31.04	1.70
9	-5	1	31.00	1.10
9	-5	1	33.10	1.49
9	-5	1	28.50	2.01
9	-5	1	30.13	1.24
9	-5	1	32.11	1.31
-10	5	-1	31.37	1.81
-10	5	-1	31.78	1.30
10	-5	1	35.14	1.44
10	-5	1	35.18	2.30
10	-5	1	32.31	1.59
-11	5	-1	8.39	0.67
11	-5	1	10.43	1.04
11	-5	1	6.20	1.30
-12	5	-1	22.23	1.12
12	-5	1	21.30	2.35
12	-5	1	23.41	1.55
-13	5	-1	8.76	0.87
13	-5	1	8.00	1.34
13	-5	1	8.55	1.86
-14	5	-1	3.33	0.68
14	-5	1	5.76	1.92
14	-5	1	3.30	1.00
-15	5	-1	4.27	0.74
15	-5	1	4.89	1.19
15	-5	1	3.97	2.17
-16	5	-1	-0.59	0.73
16	-5	1	0.84	2.52
16	-5	1	-2.83	1.13
13	4	-1	9.21	1.07
13	4	-1	6.17	1.82
13	4	-1	10.89	1.76
12	4	-1	12.02	2.19
12	4	-1	11.67	1.81
12	4	-1	12.28	1.58
-12	-4	1	11.38	1.23

11	4	-1	22.61	1.64
11	4	-1	22.66	2.12
11	4	-1	25.14	2.02
-11	-4	1	23.92	1.31
10	4	-1	14.78	1.70
10	4	-1	13.42	1.71
10	4	-1	14.38	1.26
10	4	-1	15.22	1.66
-10	-4	1	15.86	0.99
9	4	-1	47.10	2.34
9	4	-1	46.29	2.04
9	4	-1	47.81	2.35
9	4	-1	48.25	2.45
9	4	-1	46.91	1.74
-9	-4	1	49.86	1.79
8	4	-1	42.09	1.50
8	4	-1	37.54	1.95
8	4	-1	41.87	1.77
8	4	-1	40.24	2.18
8	4	-1	41.96	1.74
8	4	-1	40.77	2.01
8	4	-1	42.26	2.80
-8	-4	1	41.77	1.54
7	4	-1	53.38	1.81
7	4	-1	53.87	2.45
7	4	-1	53.38	2.02
7	4	-1	52.56	2.21
7	4	-1	55.87	2.30
7	4	-1	53.87	1.98
7	4	-1	50.98	2.78
-7	-4	1	52.44	1.85
6	4	-1	40.66	1.54
6	4	-1	42.47	1.82
6	4	-1	36.70	1.95
6	4	-1	39.63	1.71
6	4	-1	41.36	2.28
6	4	-1	41.83	1.38
-6	-4	1	37.86	1.44
-6	-4	1	38.35	1.41
5	4	-1	62.96	2.42
5	4	-1	66.20	2.17
5	4	-1	62.30	2.61
5	4	-1	64.45	2.03
5	4	-1	62.98	2.78

5	4	-1	63.26	2.35
5	4	-1	62.72	2.03
-5	-4	1	63.05	2.10
4	4	-1	289.84	9.52
4	4	-1	311.46	9.09
4	4	-1	307.37	9.35
4	4	-1	312.75	9.65
4	4	-1	285.87	8.96
-4	-4	1	293.38	9.02
-4	-4	1	301.93	9.40
3	4	-1	77.80	2.98
3	4	-1	79.68	2.43
3	4	-1	83.36	2.52
3	4	-1	77.61	2.76
3	4	-1	75.02	2.93
3	4	-1	80.57	2.43
-3	-4	1	76.73	2.85
-3	-4	1	78.03	2.47
2	4	-1	106.21	3.67
2	4	-1	105.56	3.15
2	4	-1	103.92	3.15
2	4	-1	88.26	3.55
2	4	-1	109.56	3.22
-2	-4	1	102.86	3.56
-2	-4	1	107.57	3.17
-2	-4	1	104.49	3.46
1	4	-1	202.01	5.97
1	4	-1	202.46	6.41
1	4	-1	192.73	5.93
1	4	-1	203.37	6.21
-1	-4	1	195.37	5.93
-1	-4	1	198.49	6.36
-1	-4	1	200.71	6.22
-1	-4	1	194.55	5.92
0	4	-1	156.77	4.65
0	4	-1	158.68	4.86
0	4	-1	154.26	5.07
0	4	-1	150.42	4.63
0	-4	1	150.92	4.62
0	-4	1	151.69	4.61
0	-4	1	158.85	4.89
-1	4	-1	3.37	0.21
-1	4	-1	3.38	0.36
-1	4	-1	3.62	0.21

-1	4	-1	3.43	0.57
1	-4	1	3.70	0.22
1	-4	1	3.16	0.57
1	-4	1	4.12	0.23
1	-4	1	3.62	0.39
-2	4	-1	167.85	5.06
-2	4	-1	163.54	4.94
-2	4	-1	158.61	4.93
-2	4	-1	168.65	5.37
2	-4	1	171.61	5.46
2	-4	1	160.82	4.96
2	-4	1	163.97	4.94
2	-4	1	161.55	5.07
-3	4	-1	50.92	1.60
-3	4	-1	46.21	1.57
-3	4	-1	51.61	1.63
3	-4	1	48.74	1.58
3	-4	1	50.08	1.62
3	-4	1	49.57	1.66
3	-4	1	49.77	2.03
-4	4	-1	133.90	4.11
-4	4	-1	133.01	4.10
-4	4	-1	133.52	4.72
4	-4	1	138.38	4.60
4	-4	1	133.57	4.14
4	-4	1	132.73	4.11
4	-4	1	137.68	4.18
4	-4	1	136.12	4.10
-5	4	-1	58.27	1.80
-5	4	-1	61.66	2.27
-5	4	-1	57.90	1.86
5	-4	1	57.14	1.96
5	-4	1	59.91	1.86
5	-4	1	61.21	1.88
5	-4	1	48.03	1.92
5	-4	1	57.58	2.37
-6	4	-1	13.49	0.60
-6	4	-1	14.27	0.55
-6	4	-1	13.99	0.97
6	-4	1	13.16	0.72
6	-4	1	13.92	0.62
6	-4	1	13.26	0.56
6	-4	1	12.69	1.07
6	-4	1	14.30	0.64

-7	4	-1	18.31	1.13
-7	4	-1	17.30	0.71
-7	4	-1	16.81	0.73
7	-4	1	15.39	0.64
7	-4	1	17.85	0.75
7	-4	1	17.97	0.79
7	-4	1	15.85	1.28
7	-4	1	15.59	0.87
-8	4	-1	16.25	0.76
-8	4	-1	18.01	1.18
-8	4	-1	17.13	0.82
8	-4	1	16.79	0.79
8	-4	1	16.77	0.66
8	-4	1	17.39	0.85
8	-4	1	18.63	0.99
8	-4	1	14.94	1.37
-9	4	-1	18.56	0.85
-9	4	-1	18.91	1.30
-9	4	-1	19.71	0.96
9	-4	1	18.04	0.72
9	-4	1	17.56	1.08
9	-4	1	18.92	0.97
9	-4	1	19.70	1.64
9	-4	1	18.46	0.88
-10	4	-1	3.56	0.55
-10	4	-1	3.25	0.42
-10	4	-1	4.09	0.75
10	-4	1	3.50	1.01
10	-4	1	3.33	0.37
10	-4	1	1.96	0.50
10	-4	1	3.62	0.66
-11	4	-1	7.85	0.68
11	-4	1	11.20	1.77
11	-4	1	6.32	0.65
11	-4	1	8.30	1.02
11	-4	1	7.59	0.49
-12	4	-1	3.78	0.53
12	-4	1	3.57	0.43
12	-4	1	3.81	1.29
12	-4	1	1.92	0.70
-13	4	-1	2.64	0.53
13	-4	1	2.52	1.51
13	-4	1	1.00	0.79
-14	4	-1	2.33	0.63

14	-4	1	0.31	0.93
14	-4	1	5.54	1.83
-15	4	-1	0.56	0.63
15	-4	1	-0.16	1.03
15	-4	1	2.48	2.01
-16	4	-1	-0.77	0.74
16	-4	1	0.03	1.37
16	-4	1	-1.62	2.27
14	3	-1	9.51	1.45
14	3	-1	7.38	2.12
-14	-3	1	10.26	1.52
13	3	-1	17.38	1.76
13	3	-1	12.28	1.73
13	3	-1	14.71	2.46
-13	-3	1	15.66	1.35
12	3	-1	10.33	1.60
12	3	-1	11.36	1.51
12	3	-1	14.49	1.62
-12	-3	1	11.66	1.11
11	3	-1	26.63	1.91
11	3	-1	27.83	1.92
11	3	-1	26.56	1.66
11	3	-1	27.48	2.15
-11	-3	1	27.98	1.63
10	3	-1	30.75	1.80
10	3	-1	29.61	1.85
10	3	-1	28.13	1.61
10	3	-1	27.51	1.99
-10	-3	1	29.45	1.28
9	3	-1	20.26	2.18
9	3	-1	18.94	1.44
9	3	-1	19.75	1.52
9	3	-1	17.66	1.17
9	3	-1	18.34	0.93
9	3	-1	17.69	1.47
-9	-3	1	18.56	0.94
8	3	-1	65.46	2.10
8	3	-1	61.57	2.55
8	3	-1	62.34	2.34
8	3	-1	61.93	2.65
8	3	-1	58.26	3.06
8	3	-1	62.14	2.26
8	3	-1	63.66	2.50
-8	-3	1	60.23	2.11

7	3	-1	85.62	2.85
7	3	-1	77.97	3.49
7	3	-1	88.41	2.71
7	3	-1	84.70	3.25
7	3	-1	84.03	3.08
7	3	-1	85.01	2.91
7	3	-1	87.26	3.09
-7	-3	1	82.01	2.73
6	3	-1	184.82	5.58
6	3	-1	183.36	5.46
6	3	-1	178.41	5.77
6	3	-1	176.98	6.16
6	3	-1	181.67	5.80
6	3	-1	183.62	5.98
-6	-3	1	170.28	5.47
-6	-3	1	174.07	5.51
5	3	-1	37.58	1.87
5	3	-1	37.80	1.57
5	3	-1	38.05	1.26
5	3	-1	36.83	1.73
5	3	-1	38.57	1.37
5	3	-1	38.69	1.54
-5	-3	1	37.31	1.32
-5	-3	1	36.31	1.28
4	3	-1	25.75	1.08
4	3	-1	29.40	1.47
4	3	-1	27.08	1.35
4	3	-1	26.29	1.37
4	3	-1	28.21	0.94
-4	-3	1	27.69	0.99
-4	-3	1	29.12	1.31
-4	-3	1	26.94	0.96
3	3	-1	241.20	7.29
3	3	-1	222.76	7.02
3	3	-1	246.70	7.08
3	3	-1	238.69	7.45
3	3	-1	227.45	7.42
3	3	-1	233.82	7.02
-3	-3	1	231.31	7.05
-3	-3	1	237.22	7.34
2	3	-1	114.68	3.50
2	3	-1	120.40	3.88
2	3	-1	114.34	3.49
2	3	-1	120.37	3.54

2	3	-1	110.53	3.72
-2	-3	1	116.48	3.74
-2	-3	1	115.93	3.52
1	3	-1	71.13	2.27
1	3	-1	77.96	2.28
1	3	-1	75.90	2.47
1	3	-1	76.00	2.61
-1	-3	1	74.00	2.26
-1	-3	1	73.86	2.26
-1	-3	1	75.80	2.48
-1	-3	1	73.22	2.57
0	3	-1	186.39	5.65
0	3	-1	203.93	6.00
0	3	-1	182.36	5.64
0	3	-1	194.09	5.82
0	-3	1	179.55	5.65
0	-3	1	188.39	5.82
0	-3	1	194.52	5.97
-1	3	-1	46.19	1.42
-1	3	-1	45.32	1.53
-1	3	-1	48.34	1.75
-1	3	-1	47.24	1.40
1	-3	1	44.51	1.54
1	-3	1	45.11	1.42
1	-3	1	44.11	1.72
-2	3	-1	51.85	1.70
-2	3	-1	53.59	1.75
-2	3	-1	56.02	2.01
2	-3	1	55.82	1.72
2	-3	1	59.64	2.06
2	-3	1	53.54	1.70
2	-3	1	54.29	1.77
-3	3	-1	158.78	4.85
-3	3	-1	175.22	5.19
3	-3	1	163.33	4.86
3	-3	1	147.32	5.23
3	-3	1	160.77	4.85
3	-3	1	160.92	4.88
3	-3	1	163.27	4.90
-4	3	-1	61.61	1.93
-4	3	-1	64.50	2.25
-4	3	-1	60.59	1.89
4	-3	1	62.27	2.01
4	-3	1	59.97	1.93

4	-3	1	58.90	1.91
4	-3	1	62.76	1.94
4	-3	1	58.74	2.29
-5	3	-1	57.07	2.10
-5	3	-1	53.47	1.79
-5	3	-1	57.25	1.77
5	-3	1	56.14	2.23
5	-3	1	54.02	1.89
5	-3	1	55.60	1.80
5	-3	1	55.17	1.82
5	-3	1	55.92	1.75
-6	3	-1	28.17	1.03
-6	3	-1	29.72	1.35
-6	3	-1	27.45	1.04
6	-3	1	26.74	1.47
6	-3	1	28.90	1.07
6	-3	1	28.75	0.96
6	-3	1	28.98	1.05
6	-3	1	28.83	1.16
-7	3	-1	12.97	0.94
-7	3	-1	12.16	0.61
-7	3	-1	13.25	0.71
7	-3	1	14.12	0.56
7	-3	1	13.79	0.69
7	-3	1	12.28	1.08
7	-3	1	12.94	0.77
7	-3	1	14.28	0.65
-8	3	-1	40.38	1.57
-8	3	-1	40.12	1.44
-8	3	-1	39.54	1.77
8	-3	1	40.12	1.65
8	-3	1	42.82	1.47
8	-3	1	37.89	1.36
8	-3	1	42.42	2.13
8	-3	1	40.98	1.53
-9	3	-1	34.16	1.69
-9	3	-1	33.97	1.30
-9	3	-1	33.79	1.48
9	-3	1	32.34	1.23
9	-3	1	35.20	1.33
9	-3	1	34.43	2.08
9	-3	1	35.78	1.56
9	-3	1	34.22	1.42
-10	3	-1	16.34	1.25

-10	3	-1	14.90	0.80
10	-3	1	14.73	0.86
10	-3	1	14.06	0.78
10	-3	1	16.73	1.12
10	-3	1	15.16	1.65
-11	3	-1	0.52	0.34
11	-3	1	0.70	0.46
11	-3	1	1.04	1.00
11	-3	1	0.58	0.36
11	-3	1	0.79	0.61
-12	3	-1	4.89	0.73
12	-3	1	6.35	1.50
12	-3	1	5.17	0.65
12	-3	1	5.41	0.85
12	-3	1	4.82	0.88
-13	3	-1	8.17	0.89
13	-3	1	9.82	1.40
13	-3	1	8.01	1.77
13	-3	1	8.16	0.71
-14	3	-1	0.84	0.62
14	-3	1	2.76	1.71
14	-3	1	0.06	0.89
-15	3	-1	-0.03	0.67
15	-3	1	1.60	2.17
15	-3	1	-0.67	1.01
14	2	-1	23.40	2.04
14	2	-1	20.13	2.91
-14	-2	1	20.32	1.53
13	2	-1	14.38	2.36
13	2	-1	18.79	1.76
13	2	-1	16.17	1.50
-13	-2	1	17.84	1.30
12	2	-1	26.48	1.76
12	2	-1	23.53	2.19
12	2	-1	23.65	1.71
12	2	-1	27.12	1.85
-12	-2	1	26.13	1.34
11	2	-1	18.35	1.56
11	2	-1	19.79	1.41
11	2	-1	20.90	1.53
11	2	-1	17.79	1.81
-11	-2	1	17.51	1.07
10	2	-1	5.85	0.86
10	2	-1	4.00	0.78

10	2	-1	5.36	0.87
10	2	-1	4.65	1.03
-10	-2	1	4.65	0.64
9	2	-1	72.89	2.77
9	2	-1	72.50	2.66
9	2	-1	79.73	2.98
9	2	-1	76.56	3.07
9	2	-1	69.61	3.37
9	2	-1	77.08	2.51
9	2	-1	77.67	2.86
-9	-2	1	75.06	2.53
8	2	-1	127.65	4.28
8	2	-1	131.27	4.50
8	2	-1	129.47	4.47
8	2	-1	130.31	4.18
8	2	-1	129.75	4.34
8	2	-1	136.35	4.06
8	2	-1	113.16	4.73
-8	-2	1	132.98	4.07
7	2	-1	77.53	2.80
7	2	-1	79.56	2.89
7	2	-1	79.88	2.74
7	2	-1	78.53	2.94
7	2	-1	74.04	3.18
7	2	-1	82.38	2.54
7	2	-1	82.03	2.66
-7	-2	1	76.46	2.55
6	2	-1	63.60	2.48
6	2	-1	62.53	2.24
6	2	-1	66.20	2.35
6	2	-1	68.60	2.19
6	2	-1	68.43	2.40
6	2	-1	67.44	2.09
6	2	-1	57.30	2.60
-6	-2	1	63.32	2.10
5	2	-1	104.95	3.39
5	2	-1	107.03	3.23
5	2	-1	105.09	3.15
5	2	-1	96.42	3.60
5	2	-1	102.87	3.40
5	2	-1	100.01	3.51
-5	-2	1	99.51	3.19
-5	-2	1	100.11	3.16
4	2	-1	179.59	5.95

4	2	-1	187.93	5.77
4	2	-1	190.87	5.63
4	2	-1	190.78	5.57
4	2	-1	186.62	5.78
4	2	-1	180.35	5.91
-4	-2	1	181.90	5.60
-4	-2	1	183.27	5.58
3	2	-1	84.49	2.76
3	2	-1	81.96	2.43
3	2	-1	80.97	2.62
3	2	-1	81.38	2.47
-3	-2	1	78.33	2.45
-3	-2	1	75.30	2.49
-3	-2	1	77.47	2.62
-3	-2	1	75.23	2.78
2	2	-1	79.88	2.43
2	2	-1	83.99	2.68
2	2	-1	81.50	2.42
2	2	-1	79.21	2.58
2	2	-1	78.68	2.41
-2	-2	1	76.22	2.58
-2	-2	1	78.51	2.43
-2	-2	1	79.93	2.62
1	2	-1	344.80	10.22
1	2	-1	340.09	10.14
1	2	-1	334.10	10.00
-1	-2	1	338.24	10.19
-1	-2	1	331.55	10.14
0	2	-1	67.22	2.11
0	2	-1	67.24	2.02
0	2	-1	74.39	2.23
0	-2	1	67.00	2.02
0	-2	1	58.04	2.10
-1	2	-1	13.53	0.63
-1	2	-1	12.77	0.43
-1	2	-1	13.28	0.48
1	-2	1	10.89	0.59
1	-2	1	12.51	0.41
1	-2	1	13.05	0.44
1	-2	1	12.36	0.48
-2	2	-1	35.98	1.16
-2	2	-1	37.98	1.37
-2	2	-1	35.94	1.15
-2	2	-1	36.37	1.16

2	-2	1	35.36	1.16
2	-2	1	38.09	1.40
2	-2	1	37.13	1.16
2	-2	1	38.44	1.18
-3	2	-1	1.06	0.14
-3	2	-1	1.44	0.19
-3	2	-1	1.32	0.29
-3	2	-1	1.17	0.15
3	-2	1	1.05	0.37
3	-2	1	1.20	0.16
3	-2	1	0.90	0.14
3	-2	1	0.86	0.13
-4	2	-1	116.29	3.64
-4	2	-1	111.38	3.40
-4	2	-1	110.93	3.41
-4	2	-1	107.16	3.41
4	-2	1	114.88	3.41
4	-2	1	108.96	3.42
4	-2	1	108.83	3.37
-5	2	-1	57.44	2.08
-5	2	-1	57.67	1.84
-5	2	-1	59.08	1.90
5	-2	1	59.29	2.24
5	-2	1	58.76	1.88
5	-2	1	57.80	1.85
5	-2	1	52.09	1.81
5	-2	1	55.85	1.94
-6	2	-1	29.59	1.08
-6	2	-1	30.42	1.21
-6	2	-1	30.85	1.35
6	-2	1	30.28	1.22
6	-2	1	30.56	1.14
6	-2	1	31.80	1.55
6	-2	1	28.86	1.07
6	-2	1	31.56	1.10
-7	2	-1	6.64	0.73
-7	2	-1	6.97	0.66
-7	2	-1	6.37	0.44
7	-2	1	6.67	0.45
7	-2	1	6.67	0.89
7	-2	1	7.00	0.60
7	-2	1	7.09	0.47
7	-2	1	7.29	0.54
-8	2	-1	13.35	0.94

-8	2	-1	13.49	0.67
-8	2	-1	14.09	0.99
8	-2	1	13.12	0.77
8	-2	1	12.60	0.68
8	-2	1	14.76	1.28
8	-2	1	12.80	0.85
8	-2	1	13.51	0.71
-9	2	-1	6.77	0.53
-9	2	-1	5.08	0.73
-9	2	-1	7.50	0.94
9	-2	1	6.84	0.76
9	-2	1	5.75	0.57
9	-2	1	7.90	1.26
9	-2	1	6.37	0.58
9	-2	1	4.02	0.67
-10	2	-1	1.42	0.36
-10	2	-1	3.07	1.23
10	-2	1	1.89	0.39
10	-2	1	0.74	0.91
10	-2	1	1.40	0.52
-11	2	-1	6.45	0.67
11	-2	1	4.89	0.66
11	-2	1	6.66	0.98
11	-2	1	6.56	0.75
11	-2	1	4.92	1.26
-12	2	-1	0.72	0.44
12	-2	1	1.64	1.36
12	-2	1	0.03	0.62
12	-2	1	0.76	0.68
12	-2	1	1.55	0.45
-13	2	-1	2.73	0.63
13	-2	1	4.02	1.56
13	-2	1	4.43	0.95
13	-2	1	2.70	0.51
-14	2	-1	-0.14	0.63
14	-2	1	-2.10	0.88
14	-2	1	0.99	1.72
-15	2	-1	1.29	0.72
15	-2	1	-0.37	1.09
15	-2	1	-2.10	2.12
15	1	-1	11.05	2.33
-15	-1	1	16.95	1.52
14	1	-1	22.00	1.94
14	1	-1	21.70	2.87

-14	-1	1	19.71	1.44
13	1	-1	24.68	2.52
13	1	-1	23.69	1.48
13	1	-1	26.26	1.85
-13	-1	1	22.80	1.37
12	1	-1	29.18	1.79
12	1	-1	26.15	2.29
12	1	-1	26.00	1.55
12	1	-1	28.16	1.73
-12	-1	1	28.49	1.35
11	1	-1	28.18	1.60
11	1	-1	29.94	1.56
11	1	-1	27.17	2.07
11	1	-1	28.88	1.59
-11	-1	1	27.57	1.27
10	1	-1	39.08	1.77
10	1	-1	38.40	1.78
10	1	-1	38.69	1.78
10	1	-1	41.02	2.18
10	1	-1	37.65	2.42
-10	-1	1	38.50	1.50
9	1	-1	50.33	2.02
9	1	-1	48.60	1.97
9	1	-1	48.72	1.96
9	1	-1	43.89	2.41
9	1	-1	46.80	1.84
9	1	-1	49.37	1.72
9	1	-1	49.33	2.28
-9	-1	1	47.30	1.72
8	1	-1	44.48	2.01
8	1	-1	46.50	1.58
8	1	-1	43.45	1.87
8	1	-1	45.91	1.79
8	1	-1	43.77	1.78
8	1	-1	46.37	1.68
8	1	-1	43.44	2.20
-8	-1	1	44.09	1.58
7	1	-1	93.69	3.50
7	1	-1	91.69	3.16
7	1	-1	93.85	3.06
7	1	-1	94.44	3.27
7	1	-1	97.70	2.99
7	1	-1	96.16	3.36
7	1	-1	93.23	3.17

-7	-1	1	94.25	2.99
6	1	-1	93.25	3.06
6	1	-1	97.07	2.90
6	1	-1	92.23	3.18
6	1	-1	84.74	3.29
6	1	-1	93.37	3.07
6	1	-1	90.41	3.19
6	1	-1	96.30	2.97
-6	-1	1	92.10	2.90
5	1	-1	68.64	3.02
5	1	-1	83.78	2.57
5	1	-1	84.95	2.62
5	1	-1	80.83	2.69
5	1	-1	85.31	2.80
5	1	-1	85.58	2.73
-5	-1	1	85.94	2.58
4	1	-1	141.48	4.90
4	1	-1	155.19	4.79
4	1	-1	149.71	4.88
4	1	-1	159.32	4.66
4	1	-1	154.27	4.76
4	1	-1	157.82	4.69
4	1	-1	163.89	4.85
-4	-1	1	152.38	4.66
3	1	-1	112.06	3.41
3	1	-1	103.39	3.52
3	1	-1	112.46	3.34
3	1	-1	116.82	3.46
3	1	-1	109.36	3.45
3	1	-1	110.04	3.36
-3	-1	1	108.57	3.45
-3	-1	1	111.15	3.35
2	1	-1	118.20	3.53
2	1	-1	115.38	3.46
2	1	-1	116.61	3.50
2	1	-1	117.11	3.55
2	1	-1	112.22	3.47
2	1	-1	119.14	3.60
-2	-1	1	113.70	3.55
-2	-1	1	111.65	3.47
1	1	-1	169.79	5.18
1	1	-1	181.02	5.20
-1	-1	1	170.70	5.11
-1	-1	1	167.74	5.13

-1	1	-1	39.67	1.27
-1	1	-1	40.44	1.18
1	-1	1	32.01	1.17
1	-1	1	38.60	1.17
1	-1	1	39.28	1.17
-2	1	-1	65.30	1.99
-2	1	-1	66.11	2.10
-2	1	-1	63.88	1.97
-2	1	-1	64.28	1.98
2	-1	1	66.50	2.14
2	-1	1	64.46	1.98
2	-1	1	64.28	1.99
2	-1	1	61.71	1.97
-3	1	-1	139.33	4.21
-3	1	-1	121.54	4.11
-3	1	-1	126.41	4.05
-3	1	-1	138.40	4.11
3	-1	1	134.94	4.08
3	-1	1	145.42	4.08
3	-1	1	133.52	4.05
-4	1	-1	81.00	2.59
-4	1	-1	76.13	2.50
-4	1	-1	80.09	2.66
-4	1	-1	76.26	2.47
4	-1	1	86.55	2.52
4	-1	1	80.41	2.76
4	-1	1	79.48	2.48
4	-1	1	79.28	2.52
-5	1	-1	63.67	2.22
-5	1	-1	59.32	2.02
-5	1	-1	60.57	1.98
-5	1	-1	62.18	2.16
5	-1	1	64.08	2.03
5	-1	1	61.98	2.05
5	-1	1	63.95	1.99
5	-1	1	62.23	2.34
-6	1	-1	89.99	3.06
-6	1	-1	84.39	2.78
-6	1	-1	86.17	3.01
-6	1	-1	86.18	2.83
6	-1	1	89.29	2.87
6	-1	1	93.70	2.81
6	-1	1	89.98	3.24
-7	1	-1	13.27	0.69

-7	1	-1	13.57	0.94
-7	1	-1	13.89	0.64
-7	1	-1	13.57	0.96
7	-1	1	13.42	0.74
7	-1	1	14.00	0.67
7	-1	1	13.27	1.11
7	-1	1	13.42	0.72
-8	1	-1	5.73	0.91
-8	1	-1	5.97	0.47
-8	1	-1	4.50	0.68
8	-1	1	5.80	0.53
8	-1	1	5.76	0.65
8	-1	1	6.04	0.50
8	-1	1	5.86	1.01
8	-1	1	6.12	0.61
-9	1	-1	0.71	0.59
-9	1	-1	0.91	0.30
9	-1	1	1.22	0.35
9	-1	1	0.55	0.42
9	-1	1	1.05	0.69
9	-1	1	0.73	0.40
9	-1	1	1.13	0.35
9	-1	1	1.08	0.45
-10	1	-1	2.00	0.38
10	-1	1	1.93	0.58
10	-1	1	1.78	0.55
10	-1	1	1.66	0.93
10	-1	1	2.60	0.44
-11	1	-1	6.93	0.73
11	-1	1	6.69	0.77
11	-1	1	7.91	1.04
11	-1	1	6.64	0.88
11	-1	1	6.49	1.58
-12	1	-1	0.65	0.45
12	-1	1	0.52	0.73
12	-1	1	-0.06	0.46
12	-1	1	0.25	0.71
12	-1	1	2.51	1.29
-13	1	-1	-0.77	0.53
13	-1	1	-0.16	0.53
13	-1	1	-0.81	1.48
13	-1	1	-0.21	0.85
-14	1	-1	-0.05	0.63
14	-1	1	-0.74	0.98

14	-1	1	1.71	1.72
-15	1	-1	-0.96	0.78
15	-1	1	1.75	2.09
15	-1	1	2.07	1.21
15	0	-1	18.73	1.98
15	0	-1	19.00	3.21
-15	0	1	17.12	1.45
14	0	-1	25.79	1.94
14	0	-1	18.40	2.78
-14	0	1	24.63	1.46
13	0	-1	36.01	2.70
13	0	-1	30.34	1.96
13	0	-1	29.94	1.48
-13	0	1	31.06	1.51
12	0	-1	32.53	2.45
12	0	-1	36.41	1.59
12	0	-1	37.31	1.95
12	0	-1	38.26	1.75
-12	0	1	34.11	1.51
11	0	-1	40.77	1.75
11	0	-1	37.14	1.98
11	0	-1	39.99	2.41
11	0	-1	40.55	1.67
-11	0	1	41.54	1.59
10	0	-1	36.82	1.44
10	0	-1	37.16	1.74
10	0	-1	38.73	2.14
10	0	-1	38.14	1.59
10	0	-1	36.27	1.59
-10	0	1	36.71	1.44
-10	0	1	36.62	1.91
9	0	-1	82.97	3.27
9	0	-1	68.63	2.95
9	0	-1	83.77	2.81
9	0	-1	82.79	2.86
9	0	-1	88.87	2.73
9	0	-1	83.55	2.98
-9	0	1	86.00	3.10
-9	0	1	84.96	2.72
8	0	-1	82.03	2.58
8	0	-1	79.50	3.02
8	0	-1	79.52	2.75
8	0	-1	78.70	2.79
8	0	-1	78.68	2.69

8	0	-1	77.38	2.65
-8	0	1	76.73	2.57
-8	0	1	83.70	2.91
7	0	-1	68.40	2.50
7	0	-1	72.43	2.39
7	0	-1	70.93	2.69
7	0	-1	77.05	2.34
7	0	-1	69.40	2.43
-7	0	1	70.39	2.33
-7	0	1	78.04	2.62
6	0	-1	14.29	0.89
6	0	-1	14.25	0.70
6	0	-1	13.48	0.64
6	0	-1	13.91	0.73
6	0	-1	14.59	0.76
6	0	-1	14.41	0.60
-6	0	1	14.97	0.82
-6	0	1	13.43	0.60
5	0	-1	119.58	3.81
5	0	-1	120.06	3.78
5	0	-1	126.36	3.98
5	0	-1	123.31	3.78
5	0	-1	120.21	3.87
5	0	-1	120.60	3.88
-5	0	1	129.37	3.94
-5	0	1	121.22	3.75
4	0	-1	81.53	2.50
4	0	-1	80.37	2.60
4	0	-1	81.47	2.53
4	0	-1	78.11	2.55
4	0	-1	76.95	2.61
4	0	-1	84.98	2.67
-4	0	1	80.79	2.50
-4	0	1	86.32	2.65
3	0	-1	16.08	0.54
3	0	-1	15.25	0.64
3	0	-1	15.38	0.58
3	0	-1	15.60	0.62
-3	0	1	15.24	0.54
-3	0	1	14.89	0.58
-3	0	1	15.27	0.60
-3	0	1	16.32	0.64
2	0	-1	39.14	1.21
2	0	-1	37.96	1.26

2	0	-1	40.40	1.26
2	0	-1	39.55	1.22
-2	0	1	40.54	1.25
-2	0	1	38.68	1.21
-2	0	1	37.96	1.25
-1	0	-1	61.55	1.99
-1	0	-1	68.38	2.02
1	0	1	62.16	1.96
1	0	1	62.25	1.93
1	0	1	65.51	1.99
-2	0	-1	48.77	1.53
-2	0	-1	52.63	1.67
-2	0	-1	47.36	1.56
-2	0	-1	47.98	1.65
2	0	1	52.16	1.69
2	0	1	46.90	1.61
2	0	1	49.91	1.53
2	0	1	50.96	1.58
-3	0	-1	64.95	2.18
-3	0	-1	69.11	2.19
-3	0	-1	62.80	2.05
-3	0	-1	62.35	2.00
3	0	1	65.71	2.01
3	0	1	64.69	2.22
3	0	1	65.41	2.07
3	0	1	64.34	2.12
-4	0	-1	31.67	1.30
-4	0	-1	31.93	1.07
-4	0	-1	32.64	1.13
4	0	1	29.75	1.20
4	0	1	33.68	1.08
4	0	1	33.02	1.35
4	0	1	32.40	1.20
-5	0	-1	43.85	1.77
-5	0	-1	43.59	1.55
-5	0	-1	43.97	1.50
-5	0	-1	48.55	1.78
5	0	1	47.91	1.67
5	0	1	45.88	1.85
5	0	1	46.61	1.51
5	0	1	46.04	1.59
-6	0	-1	20.70	0.88
-6	0	-1	20.31	1.19
-6	0	-1	22.42	1.16

-6	0	-1	21.64	0.84
6	0	1	22.50	1.27
6	0	1	22.21	0.95
6	0	1	23.73	0.86
6	0	1	21.12	1.00
-7	0	-1	7.09	0.52
-7	0	-1	8.93	0.86
-7	0	-1	6.81	0.46
-7	0	-1	6.93	0.94
7	0	1	7.12	0.50
7	0	1	6.82	0.59
7	0	1	5.91	0.88
7	0	1	6.41	0.63
-8	0	-1	21.05	0.90
-8	0	-1	21.31	1.33
-8	0	-1	19.84	0.97
8	0	1	22.58	1.20
8	0	1	20.45	1.05
8	0	1	21.70	0.93
8	0	1	21.44	1.06
8	0	1	21.64	1.48
-9	0	-1	1.63	0.68
-9	0	-1	2.11	0.37
9	0	1	2.88	0.61
9	0	1	1.25	0.46
9	0	1	2.48	0.47
9	0	1	1.79	0.40
9	0	1	2.38	0.77
-10	0	-1	2.02	0.41
10	0	1	2.08	0.51
10	0	1	1.78	0.89
10	0	1	1.67	0.58
10	0	1	2.86	0.69
11	0	1	2.29	0.58
11	0	1	4.25	0.85
11	0	1	2.21	0.70
11	0	1	2.06	1.07
-12	0	-1	0.33	0.49
12	0	1	-0.49	0.71
12	0	1	1.97	1.31
12	0	1	-0.32	0.54
-13	0	-1	0.60	0.60
13	0	1	1.15	1.48
13	0	1	1.34	0.87

13	0	1	0.08	0.61
-14	0	-1	-0.82	0.66
14	0	1	-0.05	1.76
14	0	1	-0.35	0.97
16	-1	-1	11.09	1.94
16	-1	-1	19.55	3.49
-16	1	1	15.51	1.50
15	-1	-1	18.07	3.12
15	-1	-1	15.02	1.93
-15	1	1	19.61	1.43
14	-1	-1	16.43	1.72
14	-1	-1	13.86	2.55
-14	1	1	15.38	1.16
13	-1	-1	23.76	2.44
13	-1	-1	20.22	1.65
13	-1	-1	20.48	1.09
-13	1	1	21.44	1.19
12	-1	-1	20.75	1.07
12	-1	-1	21.75	1.48
12	-1	-1	18.61	2.06
-12	1	1	24.40	1.14
11	-1	-1	52.79	1.99
11	-1	-1	55.06	2.79
11	-1	-1	53.19	2.29
11	-1	-1	56.01	1.94
-11	1	1	54.89	1.96
-11	1	1	51.09	2.42
10	-1	-1	93.85	3.45
10	-1	-1	103.73	3.17
10	-1	-1	94.34	3.29
10	-1	-1	94.66	3.82
10	-1	-1	96.76	3.16
10	-1	-1	95.84	3.19
-10	1	1	96.28	3.15
-10	1	1	96.37	3.53
9	-1	-1	24.12	1.28
9	-1	-1	25.22	1.13
9	-1	-1	26.05	1.62
9	-1	-1	26.15	1.05
9	-1	-1	25.68	1.09
-9	1	1	25.59	1.05
-9	1	1	26.23	1.38
8	-1	-1	37.44	1.81
8	-1	-1	39.70	1.37

8	-1	-1	38.50	1.38
8	-1	-1	37.13	1.38
8	-1	-1	37.69	1.42
8	-1	-1	36.13	1.56
-8	1	1	36.94	1.35
-8	1	1	38.58	1.63
7	-1	-1	178.26	5.54
7	-1	-1	187.11	5.59
7	-1	-1	175.60	5.91
7	-1	-1	179.15	5.54
7	-1	-1	175.36	5.71
7	-1	-1	183.58	5.53
-7	1	1	189.18	5.77
-7	1	1	180.27	5.53
6	-1	-1	5.45	0.35
6	-1	-1	4.89	0.62
6	-1	-1	5.13	0.38
6	-1	-1	5.22	0.36
6	-1	-1	5.83	0.37
6	-1	-1	4.47	0.45
-6	1	1	5.05	0.53
-6	1	1	4.96	0.33
5	-1	-1	3.57	0.26
5	-1	-1	3.61	0.51
5	-1	-1	3.24	0.37
5	-1	-1	3.86	0.28
-5	1	1	4.17	0.44
-5	1	1	3.48	0.26
4	-1	-1	5.10	0.38
4	-1	-1	5.22	0.27
4	-1	-1	5.27	0.25
4	-1	-1	5.16	0.45
4	-1	-1	5.46	0.28
-4	1	1	5.72	0.35
-4	1	1	5.51	0.28
-4	1	1	5.67	0.42
3	-1	-1	80.80	2.62
3	-1	-1	82.14	2.55
3	-1	-1	80.59	2.46
-3	1	1	83.18	2.60
-3	1	1	80.23	2.46
-3	1	1	78.94	2.53
2	-1	-1	12.84	0.43
2	-1	-1	12.77	0.49

2	-1	-1	13.12	0.56
2	-1	-1	12.72	0.42
-2	1	1	13.33	0.50
-2	1	1	12.74	0.43
-2	1	1	12.34	0.54
0	-1	-1	4.04	0.28
-1	-1	-1	49.76	1.68
-1	-1	-1	47.88	1.68
-1	-1	-1	48.57	1.56
-1	-1	-1	51.35	1.49
1	1	1	50.06	1.67
1	1	1	49.74	1.49
1	1	1	47.36	1.70
1	1	1	49.74	1.57
-2	-1	-1	97.44	3.08
-2	-1	-1	101.71	3.26
-2	-1	-1	99.83	3.01
2	1	1	98.64	3.28
2	1	1	99.48	3.01
2	1	1	102.94	3.20
2	1	1	100.65	3.10
2	1	1	100.44	3.22
-3	-1	-1	92.07	2.80
-3	-1	-1	91.77	3.08
-3	-1	-1	90.51	2.85
3	1	1	84.80	3.15
3	1	1	90.91	3.01
3	1	1	94.03	2.88
3	1	1	92.25	3.13
3	1	1	95.22	2.79
-4	-1	-1	0.71	0.33
-4	-1	-1	0.79	0.18
-4	-1	-1	0.86	0.15
4	1	1	0.49	0.38
4	1	1	0.89	0.16
4	1	1	0.73	0.21
4	1	1	0.73	0.32
4	1	1	0.80	0.28
-5	-1	-1	10.49	0.48
-5	-1	-1	9.30	0.54
-5	-1	-1	9.84	0.87
5	1	1	10.71	0.61
5	1	1	11.01	0.74
5	1	1	11.54	0.99

5	1	1	11.09	0.49
5	1	1	10.70	0.79
-6	-1	-1	9.49	0.57
-6	-1	-1	10.81	1.02
-6	-1	-1	10.16	0.51
6	1	1	9.66	0.52
6	1	1	9.64	0.79
6	1	1	10.26	1.09
6	1	1	10.02	0.86
6	1	1	10.07	0.65
-7	-1	-1	6.62	0.47
-7	-1	-1	6.45	0.54
7	1	1	6.41	0.92
7	1	1	6.10	0.73
7	1	1	7.21	0.63
7	1	1	6.07	0.79
7	1	1	4.30	0.88
7	1	1	7.14	0.49
-8	-1	-1	0.46	0.26
-8	-1	-1	0.03	0.27
8	1	1	0.18	0.43
8	1	1	0.10	0.60
8	1	1	-1.17	0.78
8	1	1	0.22	0.29
8	1	1	0.22	0.49
8	1	1	0.10	0.44
-9	-1	-1	2.16	0.36
9	1	1	1.65	0.40
9	1	1	1.98	0.69
9	1	1	2.03	0.50
9	1	1	1.59	0.50
9	1	1	2.08	0.83
-10	-1	-1	-0.48	0.31
10	1	1	0.04	0.53
10	1	1	-0.96	0.70
10	1	1	0.27	0.84
10	1	1	0.72	0.60
-11	-1	-1	0.21	0.43
11	1	1	-0.38	0.56
11	1	1	0.45	1.07
11	1	1	0.95	0.85
11	1	1	-0.41	0.65
-12	-1	-1	-0.24	0.52
12	1	1	0.71	0.66

12	1	1	0.73	0.77
12	1	1	0.58	1.23
-13	-1	-1	1.11	0.66
13	1	1	2.11	0.77
13	1	1	1.20	1.53
13	1	1	0.72	0.91
-14	-1	-1	0.23	0.80
14	1	1	-0.69	1.84
14	1	1	0.49	1.11
16	-2	-1	10.79	2.61
16	-2	-1	12.70	2.05
-16	2	1	8.82	1.35
15	-2	-1	14.75	2.45
15	-2	-1	12.62	1.82
-15	2	1	10.70	1.18
14	-2	-1	24.23	1.88
14	-2	-1	25.89	2.82
-14	2	1	23.04	1.34
13	-2	-1	21.93	1.05
13	-2	-1	28.02	2.53
13	-2	-1	24.72	1.70
-13	2	1	29.56	1.31
12	-2	-1	37.02	1.92
12	-2	-1	38.32	1.43
12	-2	-1	38.74	2.58
-12	2	1	37.15	1.53
11	-2	-1	42.91	2.42
11	-2	-1	40.50	1.86
11	-2	-1	42.19	1.47
11	-2	-1	33.73	1.37
-11	2	1	36.65	1.95
-11	2	1	43.62	1.56
10	-2	-1	34.84	1.34
10	-2	-1	37.71	1.73
10	-2	-1	41.29	1.42
10	-2	-1	38.62	2.19
10	-2	-1	40.08	1.58
10	-2	-1	41.91	1.51
-10	2	1	40.13	1.85
-10	2	1	40.53	1.48
9	-2	-1	91.70	3.53
9	-2	-1	86.43	2.81
9	-2	-1	90.78	2.93
9	-2	-1	90.70	3.19

9	-2	-1	90.15	2.85
9	-2	-1	92.34	3.01
-9	2	1	91.36	2.93
-9	2	1	90.02	3.22
8	-2	-1	78.86	2.52
8	-2	-1	79.12	2.49
8	-2	-1	80.03	2.82
8	-2	-1	78.15	3.08
8	-2	-1	81.00	2.66
8	-2	-1	84.46	2.61
-8	2	1	82.57	2.87
-8	2	1	80.15	2.59
7	-2	-1	39.66	1.79
7	-2	-1	41.81	1.59
7	-2	-1	42.71	1.41
7	-2	-1	40.74	1.31
7	-2	-1	41.21	1.44
7	-2	-1	39.07	1.28
-7	2	1	39.60	1.64
-7	2	1	39.88	1.38
6	-2	-1	51.50	1.63
6	-2	-1	47.73	1.77
6	-2	-1	50.78	2.00
6	-2	-1	50.08	1.66
-6	2	1	48.94	2.03
-6	2	1	48.82	1.62
5	-2	-1	177.75	5.52
5	-2	-1	184.82	5.85
5	-2	-1	182.08	5.65
-5	2	1	177.87	5.51
-5	2	1	187.01	5.63
-5	2	1	187.33	5.75
4	-2	-1	2.11	0.19
4	-2	-1	2.28	0.31
4	-2	-1	2.27	0.41
-4	2	1	2.26	0.20
-4	2	1	2.98	0.28
-4	2	1	2.35	0.38
3	-2	-1	197.16	6.00
3	-2	-1	191.75	5.90
3	-2	-1	197.07	6.16
-3	2	1	195.33	5.88
-3	2	1	210.08	6.15
-3	2	1	191.89	5.89

-3	2	1	201.85	5.99
2	-2	-1	67.38	2.14
2	-2	-1	67.75	2.29
2	-2	-1	66.36	2.13
2	-2	-1	67.86	2.03
-2	2	1	69.05	2.29
-2	2	1	68.09	2.13
-2	2	1	66.73	2.03
-2	2	1	66.80	2.12
1	-2	-1	0.95	0.18
1	-2	-1	1.17	0.06
1	-2	-1	1.23	0.18
1	-2	-1	1.12	0.23
-1	2	1	1.19	0.17
-1	2	1	1.20	0.19
-1	2	1	0.82	0.22
-1	2	1	1.12	0.06
0	-2	-1	51.13	1.73
0	-2	-1	49.16	1.70
0	-2	-1	46.99	1.55
0	2	1	49.70	1.57
0	2	1	44.49	1.72
0	2	1	46.23	1.67
-1	-2	-1	50.37	1.81
-1	-2	-1	48.11	1.78
-1	-2	-1	48.26	1.48
-1	-2	-1	47.94	1.58
1	2	1	49.99	1.49
1	2	1	48.17	1.77
1	2	1	51.08	1.62
-2	-2	-1	2.36	0.39
-2	-2	-1	3.55	0.29
-2	-2	-1	3.13	0.18
-2	-2	-1	2.60	0.41
2	2	1	3.16	0.18
2	2	1	3.58	0.53
2	2	1	3.21	0.49
2	2	1	3.56	0.30
-3	-2	-1	65.46	2.07
-3	-2	-1	66.64	2.40
3	2	1	68.46	1.99
3	2	1	64.05	2.23
3	2	1	59.07	2.19
3	2	1	65.34	2.33

3	2	1	57.61	2.62
-4	-2	-1	3.20	0.26
-4	-2	-1	2.89	0.63
-4	-2	-1	3.20	0.32
4	2	1	2.80	0.59
4	2	1	3.14	0.50
4	2	1	3.66	0.28
4	2	1	3.57	0.41
4	2	1	4.20	0.60
-5	-2	-1	3.53	0.31
-5	-2	-1	3.35	0.37
-5	-2	-1	2.63	0.62
5	2	1	2.92	0.30
5	2	1	3.14	0.52
5	2	1	3.81	0.48
5	2	1	2.58	0.50
5	2	1	2.88	0.70
-6	-2	-1	0.17	0.23
-6	-2	-1	0.47	0.20
6	2	1	1.15	0.74
6	2	1	0.75	0.33
6	2	1	0.52	0.41
6	2	1	0.32	0.41
6	2	1	0.18	0.21
6	2	1	-0.08	0.39
-7	-2	-1	0.25	0.28
-7	-2	-1	0.12	0.23
7	2	1	0.65	0.38
7	2	1	-1.25	0.79
7	2	1	0.11	0.46
7	2	1	-1.07	0.45
7	2	1	-0.55	0.46
7	2	1	0.40	0.24
-8	-2	-1	1.25	0.32
-8	-2	-1	1.41	0.36
8	2	1	1.69	1.07
8	2	1	1.52	0.60
8	2	1	0.85	0.48
8	2	1	1.20	0.64
8	2	1	1.14	0.61
8	2	1	1.14	0.33
-9	-2	-1	0.04	0.33
-9	-2	-1	0.58	0.34
9	2	1	-0.16	0.73

9	2	1	-0.37	0.37
9	2	1	-0.19	0.71
9	2	1	0.40	0.61
-10	-2	-1	0.97	0.41
10	2	1	0.33	0.86
10	2	1	-0.15	0.61
10	2	1	-0.10	0.55
10	2	1	0.53	0.94
-11	-2	-1	2.18	0.53
11	2	1	3.73	1.25
11	2	1	2.44	0.83
11	2	1	1.80	0.77
11	2	1	2.56	1.05
-12	-2	-1	0.44	0.58
12	2	1	1.23	0.80
12	2	1	0.09	1.25
12	2	1	1.72	0.87
-13	-2	-1	0.67	0.71
13	2	1	0.25	1.58
13	2	1	0.31	0.80
13	2	1	-0.46	0.91
-14	-2	-1	0.44	0.90
14	2	1	2.40	1.92
14	2	1	0.37	1.22
16	-3	-1	12.26	1.99
16	-3	-1	12.88	2.62
-16	3	1	12.36	1.27
15	-3	-1	17.01	1.90
15	-3	-1	22.25	3.10
-15	3	1	18.77	1.29
14	-3	-1	16.47	1.65
14	-3	-1	15.33	2.54
-14	3	1	18.44	1.14
13	-3	-1	22.15	2.81
13	-3	-1	26.73	1.69
-13	3	1	23.44	1.22
12	-3	-1	12.26	1.20
12	-3	-1	11.68	1.86
-12	3	1	12.16	0.97
11	-3	-1	18.51	1.85
11	-3	-1	20.81	1.31
-11	3	1	21.96	1.00
-11	3	1	22.56	1.50
10	-3	-1	73.71	3.29

10	-3	-1	76.85	2.59
10	-3	-1	78.70	2.88
10	-3	-1	78.52	2.68
-10	3	1	76.63	2.57
-10	3	1	78.90	2.94
9	-3	-1	31.12	1.44
9	-3	-1	29.87	1.79
9	-3	-1	32.77	1.21
9	-3	-1	30.90	1.26
-9	3	1	32.01	1.53
-9	3	1	30.75	1.19
8	-3	-1	47.42	1.61
8	-3	-1	45.90	1.82
8	-3	-1	47.61	1.66
8	-3	-1	45.21	2.12
-8	3	1	46.29	1.59
-8	3	1	46.04	1.91
7	-3	-1	79.13	3.09
7	-3	-1	84.75	2.67
7	-3	-1	81.63	2.81
7	-3	-1	85.22	2.63
-7	3	1	79.44	2.61
-7	3	1	84.76	2.95
6	-3	-1	91.78	3.40
6	-3	-1	97.58	2.96
6	-3	-1	90.47	3.11
-6	3	1	92.96	2.94
-6	3	1	98.07	3.08
-6	3	1	97.50	3.29
5	-3	-1	4.94	0.69
5	-3	-1	5.41	0.45
5	-3	-1	5.83	0.33
-5	3	1	6.14	0.32
-5	3	1	6.15	0.43
-5	3	1	5.50	0.61
4	-3	-1	51.47	1.78
4	-3	-1	52.34	1.63
-4	3	1	53.34	1.97
-4	3	1	51.37	1.61
-4	3	1	53.92	1.74
-4	3	1	48.40	1.66
3	-3	-1	233.06	6.98
3	-3	-1	234.56	7.24
3	-3	-1	228.92	6.85

-3	3	1	237.74	6.97
-3	3	1	223.66	6.85
-3	3	1	220.54	6.95
-3	3	1	227.24	6.91
-3	3	1	236.38	7.22
2	-3	-1	39.06	1.37
2	-3	-1	40.00	1.62
2	-3	-1	41.76	1.23
-2	3	1	40.47	1.62
-2	3	1	40.98	1.23
-2	3	1	40.08	1.36
-2	3	1	38.42	1.43
1	-3	-1	43.99	1.54
1	-3	-1	46.37	1.70
1	-3	-1	45.67	1.78
-1	3	1	45.41	1.67
-1	3	1	47.16	1.56
-1	3	1	49.47	1.82
0	-3	-1	21.34	0.81
0	-3	-1	21.52	1.03
0	-3	-1	20.35	1.19
0	3	1	24.02	0.86
0	3	1	20.61	1.11
-1	-3	-1	1.09	0.35
-1	-3	-1	1.13	0.37
-1	-3	-1	2.13	0.25
-1	-3	-1	1.37	0.12
1	3	1	1.33	0.41
1	3	1	1.23	0.11
1	3	1	1.11	0.35
1	3	1	2.05	0.28
-2	-3	-1	43.80	1.49
-2	-3	-1	44.84	1.79
-2	-3	-1	42.16	1.84
-2	-3	-1	44.63	1.38
2	3	1	45.96	1.38
2	3	1	42.54	1.78
2	3	1	46.19	1.55
-3	-3	-1	49.24	1.67
-3	-3	-1	45.68	1.61
-3	-3	-1	50.98	1.99
-3	-3	-1	50.96	2.10
3	3	1	52.11	2.18
3	3	1	49.99	1.57

3	3	1	52.17	1.75
3	3	1	47.06	1.99
-4	-3	-1	3.63	0.29
-4	-3	-1	3.83	0.38
-4	-3	-1	5.81	0.88
4	3	1	5.07	0.61
4	3	1	3.78	0.60
4	3	1	4.17	0.30
4	3	1	4.31	0.78
4	3	1	4.90	0.48
-5	-3	-1	0.59	0.23
-5	-3	-1	0.37	0.18
5	3	1	0.99	0.33
5	3	1	0.34	0.43
5	3	1	0.66	0.38
5	3	1	1.32	0.71
5	3	1	0.57	0.18
5	3	1	0.45	0.41
-6	-3	-1	4.60	0.49
-6	-3	-1	4.28	0.40
6	3	1	5.11	0.87
6	3	1	4.25	0.64
6	3	1	4.74	0.79
6	3	1	4.52	0.41
6	3	1	5.07	0.87
6	3	1	4.24	1.00
-7	-3	-1	2.97	0.44
-7	-3	-1	3.25	0.37
7	3	1	1.88	0.60
7	3	1	3.24	0.56
7	3	1	3.99	0.46
7	3	1	2.77	1.11
7	3	1	4.53	0.78
7	3	1	2.76	0.68
-8	-3	-1	-0.37	0.28
-8	-3	-1	-0.20	0.32
8	3	1	-0.35	0.60
8	3	1	-0.53	0.31
8	3	1	0.35	0.68
8	3	1	0.27	0.62
-9	-3	-1	1.85	0.41
-9	-3	-1	2.94	0.61
9	3	1	2.40	0.90
9	3	1	2.09	0.76

9	3	1	1.67	0.84
9	3	1	1.57	0.43
-10	-3	-1	1.59	0.47
10	3	1	0.55	0.99
10	3	1	1.58	1.03
10	3	1	1.49	0.67
10	3	1	3.03	0.85
-11	-3	-1	1.94	0.56
11	3	1	2.24	0.85
11	3	1	0.03	1.18
11	3	1	1.98	0.88
-12	-3	-1	2.89	0.74
12	3	1	1.08	0.94
12	3	1	1.70	0.93
12	3	1	1.49	1.48
-13	-3	-1	2.04	0.91
13	3	1	0.65	1.71
13	3	1	3.11	1.40
13	3	1	2.53	1.11
16	-4	-1	14.87	2.74
16	-4	-1	12.93	1.95
-16	4	1	14.11	1.28
15	-4	-1	17.79	2.63
15	-4	-1	20.02	1.88
-15	4	1	19.79	1.27
14	-4	-1	32.29	2.03
14	-4	-1	25.59	2.86
13	-4	-1	27.28	1.76
-13	4	1	27.73	1.29
12	-4	-1	40.45	2.69
11	-4	-1	35.65	1.77
11	-4	-1	36.38	2.34
-11	4	1	35.41	1.87
-11	4	1	34.67	1.39
10	-4	-1	49.77	2.57
10	-4	-1	52.27	1.81
10	-4	-1	48.87	2.07
10	-4	-1	49.29	1.86
-10	4	1	49.20	1.77
-10	4	1	51.03	2.21
9	-4	-1	60.58	2.32
9	-4	-1	62.59	2.08
9	-4	-1	60.36	2.78
9	-4	-1	62.13	2.13

-9	4	1	59.77	2.45
-9	4	1	61.36	2.06
8	-4	-1	83.73	3.48
8	-4	-1	97.76	2.91
8	-4	-1	88.81	3.12
8	-4	-1	93.96	2.94
-8	4	1	91.11	3.29
-8	4	1	86.95	2.88
7	-4	-1	31.33	1.11
7	-4	-1	29.84	1.64
7	-4	-1	29.40	1.28
-7	4	1	31.50	1.24
-7	4	1	28.77	1.08
-7	4	1	32.25	1.51
6	-4	-1	22.10	1.00
6	-4	-1	23.95	0.84
6	-4	-1	21.95	1.33
-6	4	1	21.74	0.82
-6	4	1	21.94	1.21
-6	4	1	22.55	0.96
5	-4	-1	100.15	3.74
5	-4	-1	107.40	3.42
5	-4	-1	109.30	3.26
-5	4	1	108.49	3.38
-5	4	1	107.81	3.22
-5	4	1	107.47	3.74
-5	4	1	100.88	3.27
-5	4	1	104.99	3.24
4	-4	-1	103.37	3.66
4	-4	-1	106.16	3.16
4	-4	-1	106.47	3.31
-4	4	1	96.00	3.18
-4	4	1	106.62	3.62
-4	4	1	100.18	3.27
-4	4	1	103.00	3.28
-4	4	1	106.17	3.22
3	-4	-1	4.11	0.37
3	-4	-1	4.64	0.23
3	-4	-1	4.21	0.66
-3	4	1	4.87	0.38
-3	4	1	4.55	0.22
-3	4	1	4.24	0.43
-3	4	1	4.15	0.62
-3	4	1	4.75	0.36

2	-4	-1	40.46	1.22
2	-4	-1	36.36	1.38
2	-4	-1	39.35	1.72
-2	4	1	38.74	1.37
-2	4	1	39.11	1.53
-2	4	1	42.53	1.74
-2	4	1	39.11	1.21
-2	4	1	40.42	1.44
1	-4	-1	1.97	0.48
1	-4	-1	2.53	0.32
-1	4	1	1.62	0.36
-1	4	1	2.58	0.33
-1	4	1	1.58	0.29
-1	4	1	2.80	0.55
0	-4	-1	9.96	0.79
0	-4	-1	8.69	0.48
0	4	1	9.10	0.52
0	4	1	7.83	0.59
0	4	1	7.79	0.68
0	4	1	7.71	0.80
-1	-4	-1	22.45	1.23
-1	-4	-1	23.18	0.89
1	4	1	23.01	1.21
1	4	1	23.96	0.73
1	4	1	24.26	0.96
1	4	1	22.93	1.13
1	4	1	22.11	0.73
1	4	1	23.68	1.38
-2	-4	-1	3.50	0.60
-2	-4	-1	3.81	0.25
-2	-4	-1	3.85	0.36
2	4	1	4.80	0.87
2	4	1	3.84	0.57
2	4	1	4.51	0.67
2	4	1	4.85	0.23
-3	-4	-1	1.58	0.25
-3	-4	-1	1.50	0.20
-3	-4	-1	1.75	0.52
3	4	1	1.50	0.21
3	4	1	0.91	0.45
3	4	1	0.99	0.39
3	4	1	2.40	0.69
3	4	1	1.61	0.32
-4	-4	-1	18.21	0.81

-4	-4	-1	16.55	0.69
4	4	1	19.28	1.55
4	4	1	19.43	1.24
4	4	1	19.35	1.15
4	4	1	19.80	1.05
4	4	1	20.55	0.95
4	4	1	15.12	0.86
-5	-4	-1	1.72	0.25
-5	-4	-1	1.62	0.31
5	4	1	1.89	0.59
5	4	1	1.53	0.44
5	4	1	1.59	0.55
5	4	1	1.84	0.26
5	4	1	1.67	0.48
5	4	1	1.19	0.91
-6	-4	-1	9.89	0.57
-6	-4	-1	9.48	0.66
6	4	1	10.61	1.18
6	4	1	10.56	1.00
6	4	1	9.20	0.87
6	4	1	8.96	1.09
6	4	1	9.77	0.56
6	4	1	10.44	1.75
-7	-4	-1	2.62	0.42
-7	-4	-1	2.01	0.36
7	4	1	2.51	1.30
7	4	1	2.49	0.68
7	4	1	2.77	0.76
7	4	1	1.65	0.73
7	4	1	2.30	0.36
7	4	1	1.42	0.54
-8	-4	-1	5.14	0.67
-8	-4	-1	5.63	0.62
8	4	1	6.24	1.02
8	4	1	5.42	0.95
8	4	1	5.11	0.64
8	4	1	7.93	1.33
-9	-4	-1	3.10	0.50
-9	-4	-1	2.92	0.55
9	4	1	2.38	0.90
9	4	1	3.79	1.09
9	4	1	2.72	0.95
9	4	1	2.32	0.52
-10	-4	-1	3.71	0.61

10	4	1	3.93	1.01
10	4	1	5.85	0.93
10	4	1	3.90	1.17
-11	-4	-1	-0.01	0.62
11	4	1	0.33	0.95
11	4	1	1.60	0.83
11	4	1	0.47	1.24
-12	-4	-1	2.99	0.84
12	4	1	3.37	1.60
12	4	1	2.48	1.03
12	4	1	3.90	1.14
-13	-4	-1	1.65	0.97
13	4	1	1.55	1.85
13	4	1	1.52	1.19
17	-5	-1	7.32	2.46
17	-5	-1	2.10	2.27
-17	5	1	6.25	1.30
16	-5	-1	4.86	2.30
16	-5	-1	7.23	1.26
-16	5	1	7.56	1.10
15	-5	-1	16.00	1.85
15	-5	-1	12.68	2.37
-15	5	1	18.01	1.20
14	-5	-1	29.03	2.94
14	-5	-1	26.08	1.92
-14	5	1	25.75	1.33
13	-5	-1	39.10	2.07
-13	5	1	36.66	1.57
12	-5	-1	9.72	1.14
12	-5	-1	9.87	1.60
-12	5	1	11.09	1.01
11	-5	-1	30.84	1.64
11	-5	-1	30.00	2.24
-11	5	1	31.91	1.87
-11	5	1	32.12	1.28
10	-5	-1	70.16	2.42
10	-5	-1	69.83	2.37
10	-5	-1	68.41	2.66
10	-5	-1	66.56	3.19
-10	5	1	69.78	2.33
-10	5	1	70.84	2.85
9	-5	-1	10.65	0.63
9	-5	-1	11.09	0.67
9	-5	-1	9.70	0.84

9	-5	-1	10.23	1.38
-9	5	1	10.60	1.08
-9	5	1	10.63	0.59
8	-5	-1	45.09	1.50
8	-5	-1	40.53	2.15
8	-5	-1	40.82	1.70
-8	5	1	41.18	1.46
-8	5	1	43.25	1.63
-8	5	1	40.89	1.95
7	-5	-1	61.40	2.22
7	-5	-1	63.46	2.03
7	-5	-1	59.93	2.66
-7	5	1	61.28	2.51
-7	5	1	58.47	1.99
-7	5	1	64.68	2.17
6	-5	-1	71.26	2.23
6	-5	-1	68.52	2.84
6	-5	-1	68.61	2.41
-6	5	1	75.11	2.78
-6	5	1	70.18	2.18
-6	5	1	66.22	2.25
-6	5	1	70.84	2.36
-6	5	1	68.00	2.21
5	-5	-1	16.28	0.60
5	-5	-1	13.04	1.11
5	-5	-1	15.21	0.77
-5	5	1	15.67	1.11
-5	5	1	15.84	0.73
-5	5	1	14.10	0.57
-5	5	1	14.65	0.75
-5	5	1	14.62	0.65
4	-5	-1	52.63	2.20
4	-5	-1	50.84	1.77
4	-5	-1	51.84	1.58
-4	5	1	53.54	1.73
-4	5	1	48.14	1.81
-4	5	1	46.93	1.61
-4	5	1	50.44	1.72
-4	5	1	40.61	2.73
3	-5	-1	80.39	2.45
3	-5	-1	82.60	3.08
3	-5	-1	80.36	2.67
-3	5	1	80.49	2.60
-3	5	1	75.48	3.07

-3	5	1	82.23	2.44
-3	5	1	77.31	2.96
-3	5	1	83.14	2.68
2	-5	-1	13.86	0.71
2	-5	-1	15.38	0.50
2	-5	-1	13.97	1.04
-2	5	1	14.11	0.78
-2	5	1	15.14	0.70
-2	5	1	14.79	0.49
-2	5	1	14.42	0.92
-2	5	1	14.59	1.10
1	-5	-1	2.19	0.36
1	-5	-1	1.74	0.45
-1	5	1	3.44	0.40
-1	5	1	3.15	0.52
-1	5	1	1.93	0.44
-1	5	1	1.45	0.58
0	-5	-1	20.41	1.25
0	-5	-1	20.83	0.86
0	5	1	20.13	0.91
0	5	1	20.62	1.04
0	5	1	21.37	1.36
0	5	1	18.93	1.15
-1	-5	-1	17.89	1.16
-1	-5	-1	17.66	0.78
1	5	1	19.85	1.37
1	5	1	18.03	0.60
1	5	1	17.92	0.59
1	5	1	18.60	0.89
1	5	1	18.42	1.16
-2	-5	-1	3.55	0.39
-2	-5	-1	2.50	0.58
2	5	1	2.87	0.50
2	5	1	3.05	0.57
2	5	1	4.41	0.88
2	5	1	3.69	0.23
2	5	1	3.38	0.22
2	5	1	3.13	0.48
-3	-5	-1	0.38	0.23
-3	-5	-1	0.89	0.50
3	5	1	0.48	0.15
3	5	1	0.50	0.33
3	5	1	0.42	0.49
3	5	1	0.55	0.17

3	5	1	1.30	0.72
3	5	1	0.68	0.45
-4	-5	-1	8.65	0.58
-4	-5	-1	7.73	0.47
-4	-5	-1	8.26	1.06
4	5	1	12.80	1.51
4	5	1	9.23	0.46
4	5	1	9.57	0.76
4	5	1	7.53	1.02
4	5	1	8.75	1.00
-5	-5	-1	2.56	0.39
-5	-5	-1	2.80	0.46
5	5	1	2.51	0.51
5	5	1	1.98	0.58
5	5	1	2.98	1.13
5	5	1	3.01	0.76
5	5	1	2.31	0.30
5	5	1	3.44	0.70
-6	-5	-1	0.18	0.32
-6	-5	-1	0.28	0.27
6	5	1	0.16	0.50
6	5	1	0.92	0.50
6	5	1	0.48	0.66
6	5	1	1.40	1.23
6	5	1	0.59	0.25
6	5	1	-0.03	0.69
-7	-5	-1	0.49	0.40
-7	-5	-1	-0.38	0.33
7	5	1	-0.51	0.68
7	5	1	-0.52	0.31
7	5	1	0.09	0.60
7	5	1	-0.79	0.79
-8	-5	-1	0.72	0.42
-8	-5	-1	0.28	0.37
8	5	1	0.30	0.41
8	5	1	0.42	0.85
-9	-5	-1	3.76	0.64
-9	-5	-1	4.86	0.63
9	5	1	3.29	1.04
9	5	1	5.34	1.13
-10	-5	-1	2.74	0.66
10	5	1	2.66	1.22
10	5	1	2.46	0.85
10	5	1	1.92	1.09

-11	-5	-1	0.03	0.75
11	5	1	-0.46	1.11
11	5	1	0.90	0.90
11	5	1	0.42	1.38
12	5	1	1.24	1.09
12	5	1	0.40	0.67
12	5	1	-2.15	1.15
12	5	1	0.79	1.61
17	-6	-1	9.87	1.75
17	-6	-1	9.82	2.83
-17	6	1	11.07	1.39
16	-6	-1	9.48	1.47
16	-6	-1	12.49	2.70
-16	6	1	13.20	1.16
15	-6	-1	10.61	2.20
15	-6	-1	10.09	1.44
-15	6	1	11.77	1.08
14	-6	-1	24.64	1.89
14	-6	-1	26.00	2.90
-14	6	1	25.15	1.30
13	-6	-1	18.15	1.53
-13	6	1	16.77	1.01
12	-6	-1	15.34	1.31
12	-6	-1	14.18	2.04
-12	6	1	14.92	1.02
11	-6	-1	10.90	1.71
11	-6	-1	9.53	1.03
-11	6	1	10.89	1.33
-11	6	1	9.24	0.68
10	-6	-1	15.92	0.84
10	-6	-1	15.25	1.71
10	-6	-1	14.71	0.85
10	-6	-1	15.80	1.10
-10	6	1	14.37	1.36
-10	6	1	16.16	0.79
9	-6	-1	17.02	0.79
9	-6	-1	14.73	1.01
9	-6	-1	14.26	1.57
-9	6	1	15.79	1.33
-9	6	1	15.02	0.74
-9	6	1	16.77	0.95
8	-6	-1	9.98	0.80
8	-6	-1	11.63	0.60
8	-6	-1	10.70	1.29

-8	6	1	9.47	1.11
-8	6	1	10.93	0.74
-8	6	1	10.15	0.55
7	-6	-1	27.09	1.24
7	-6	-1	27.47	1.02
7	-6	-1	28.98	1.75
-7	6	1	27.53	1.17
-7	6	1	26.04	1.06
-7	6	1	26.74	0.95
-7	6	1	26.54	0.99
-7	6	1	27.88	1.60
6	-6	-1	3.62	0.50
6	-6	-1	3.80	0.32
6	-6	-1	3.71	0.82
-6	6	1	2.94	0.51
-6	6	1	4.13	0.45
-6	6	1	2.99	0.66
-6	6	1	3.25	0.29
-6	6	1	2.93	0.37
5	-6	-1	6.83	0.95
5	-6	-1	6.09	0.55
5	-6	-1	6.64	0.38
-5	6	1	7.66	1.03
-5	6	1	7.20	0.51
-5	6	1	6.37	0.35
-5	6	1	6.61	0.82
-5	6	1	6.81	0.60
4	-6	-1	17.02	0.64
4	-6	-1	18.24	0.89
4	-6	-1	15.58	1.25
-4	6	1	17.23	1.03
-4	6	1	17.61	0.80
-4	6	1	18.30	0.87
-4	6	1	17.01	1.29
-4	6	1	17.50	0.63
3	-6	-1	3.02	0.21
3	-6	-1	3.00	0.48
3	-6	-1	2.39	0.59
-3	6	1	3.11	0.38
-3	6	1	3.31	0.69
-3	6	1	2.56	0.20
-3	6	1	3.53	0.61
-3	6	1	2.94	0.50
2	-6	-1	15.27	1.15

2	-6	-1	15.02	0.80
2	-6	-1	14.31	0.52
-2	6	1	14.78	1.04
-2	6	1	15.38	0.51
-2	6	1	14.52	0.75
-2	6	1	15.63	0.90
-2	6	1	13.63	1.20
1	-6	-1	17.78	1.30
1	-6	-1	22.13	0.95
-1	6	1	23.03	1.29
-1	6	1	23.44	1.51
-1	6	1	21.51	0.98
-1	6	1	23.20	1.16
0	-6	-1	0.82	0.50
0	-6	-1	0.98	0.25
0	6	1	0.07	0.34
0	6	1	0.33	0.57
0	6	1	1.15	0.33
0	6	1	0.77	0.48
-1	-6	-1	2.31	0.33
-1	-6	-1	2.04	0.61
1	6	1	1.30	0.43
1	6	1	1.07	0.44
1	6	1	1.92	0.19
1	6	1	2.06	0.43
1	6	1	1.88	0.71
1	6	1	1.92	0.16
-2	-6	-1	5.91	0.87
-2	-6	-1	6.13	0.48
2	6	1	5.79	0.86
2	6	1	7.58	0.67
2	6	1	6.84	1.27
2	6	1	6.19	0.33
2	6	1	6.45	0.31
2	6	1	6.28	0.99
-3	-6	-1	13.94	0.72
-3	-6	-1	12.98	1.22
3	6	1	13.77	0.58
3	6	1	14.25	1.16
3	6	1	14.64	0.56
3	6	1	11.80	1.23
3	6	1	14.27	1.62
3	6	1	15.19	0.91
-4	-6	-1	4.87	0.86

-4	-6	-1	4.68	0.51
4	6	1	5.58	0.78
4	6	1	5.94	1.04
4	6	1	3.49	0.77
4	6	1	5.94	0.39
4	6	1	5.81	1.25
4	6	1	4.93	0.38
-5	-6	-1	1.47	0.36
5	6	1	0.85	0.72
5	6	1	1.57	0.29
5	6	1	0.10	0.67
5	6	1	1.96	1.32
5	6	1	1.27	0.28
5	6	1	0.99	0.55
5	6	1	0.86	0.52
-6	-6	-1	6.92	0.69
6	6	1	6.94	1.04
6	6	1	6.26	0.52
6	6	1	6.60	0.85
6	6	1	8.23	1.11
6	6	1	6.84	0.55
6	6	1	4.91	1.00
-7	-6	-1	10.77	0.85
7	6	1	11.95	1.60
7	6	1	11.48	0.74
7	6	1	11.40	0.69
7	6	1	9.79	1.32
-8	-6	-1	4.89	0.78
8	6	1	5.44	0.59
8	6	1	3.65	0.95
8	6	1	5.02	0.66
8	6	1	3.95	1.11
-9	-6	-1	1.43	0.60
9	6	1	2.35	1.08
9	6	1	1.47	1.00
9	6	1	2.14	0.50
10	6	1	2.41	1.26
10	6	1	4.27	0.98
10	6	1	4.38	0.95
10	6	1	4.36	1.35
11	6	1	2.95	1.50
11	6	1	3.19	1.12
11	6	1	1.29	0.62
11	6	1	3.81	1.39

17	-7	-1	15.81	2.30
17	-7	-1	16.97	3.13
-17	7	1	15.61	1.45
16	-7	-1	14.39	2.07
16	-7	-1	11.56	2.64
-16	7	1	15.93	1.24
15	-7	-1	17.85	3.17
15	-7	-1	13.00	1.85
-15	7	1	17.21	1.17
14	-7	-1	23.86	2.90
14	-7	-1	19.74	1.74
-14	7	1	21.24	1.21
13	-7	-1	37.92	2.13
13	-7	-1	40.41	3.14
-13	7	1	42.51	1.65
12	-7	-1	8.07	1.66
12	-7	-1	8.17	1.13
-12	7	1	6.14	0.80
11	-7	-1	5.60	0.93
11	-7	-1	6.01	1.31
11	-7	-1	5.01	0.73
-11	7	1	4.39	0.98
-11	7	1	6.40	0.58
10	-7	-1	15.77	1.82
10	-7	-1	17.17	1.18
10	-7	-1	17.00	0.89
10	-7	-1	18.01	0.92
-10	7	1	17.05	0.83
-10	7	1	18.36	1.60
9	-7	-1	3.59	1.00
9	-7	-1	3.10	0.56
9	-7	-1	3.85	0.47
-9	7	1	4.54	0.95
-9	7	1	3.39	0.54
-9	7	1	3.14	0.42
8	-7	-1	7.90	0.54
8	-7	-1	7.93	0.78
8	-7	-1	7.76	1.35
-8	7	1	8.51	0.44
-8	7	1	8.52	0.50
-8	7	1	9.26	0.70
-8	7	1	9.57	1.24
-8	7	1	8.20	0.61
7	-7	-1	52.17	2.64

7	-7	-1	52.83	2.04
7	-7	-1	55.16	1.81
-7	7	1	54.31	1.78
-7	7	1	52.35	1.98
-7	7	1	51.55	1.82
-7	7	1	54.07	1.95
-7	7	1	58.28	2.59
6	-7	-1	41.35	2.22
6	-7	-1	41.81	1.42
6	-7	-1	40.64	1.64
-6	7	1	41.55	1.56
-6	7	1	40.96	1.73
-6	7	1	41.81	1.39
-6	7	1	41.14	1.54
-6	7	1	45.02	2.21
5	-7	-1	0.87	0.34
5	-7	-1	1.44	0.69
5	-7	-1	1.12	0.23
-5	7	1	1.24	0.19
-5	7	1	0.97	0.41
-5	7	1	0.83	0.32
-5	7	1	0.90	0.34
-5	7	1	1.97	0.72
4	-7	-1	3.16	0.71
4	-7	-1	2.86	0.44
4	-7	-1	2.74	0.26
-4	7	1	3.11	0.26
-4	7	1	3.61	0.45
-4	7	1	1.57	0.69
-4	7	1	3.10	0.54
-4	7	1	2.44	0.55
3	-7	-1	16.61	0.59
3	-7	-1	13.39	1.30
3	-7	-1	15.48	0.87
-3	7	1	15.81	0.57
-3	7	1	16.42	0.95
-3	7	1	15.83	1.10
-3	7	1	18.58	1.44
-3	7	1	16.67	0.81
2	-7	-1	44.57	1.61
2	-7	-1	41.75	1.34
2	-7	-1	39.84	2.11
-2	7	1	42.87	1.32
-2	7	1	41.92	2.20

-2	7	1	43.26	1.79
-2	7	1	40.03	1.94
-2	7	1	45.96	1.63
1	-7	-1	1.93	0.63
1	-7	-1	4.31	0.48
-1	7	1	2.76	0.60
-1	7	1	5.11	0.76
-1	7	1	3.43	0.76
-1	7	1	3.62	0.55
0	-7	-1	5.87	0.49
0	-7	-1	3.89	0.78
0	7	1	4.94	0.88
0	7	1	6.23	0.66
0	7	1	6.18	0.77
0	7	1	5.67	1.08
-1	-7	-1	2.09	0.72
-1	-7	-1	1.65	0.30
1	7	1	1.35	0.18
1	7	1	1.43	0.44
1	7	1	0.89	0.55
1	7	1	0.89	0.50
1	7	1	1.27	0.17
1	7	1	0.35	0.84
-2	-7	-1	5.17	0.51
-2	-7	-1	6.24	1.13
2	7	1	5.85	0.31
2	7	1	5.06	0.34
2	7	1	6.24	0.77
2	7	1	6.03	0.95
2	7	1	6.79	1.25
2	7	1	5.18	0.81
-3	-7	-1	3.02	0.47
-3	-7	-1	5.11	0.94
3	7	1	5.33	0.86
3	7	1	4.85	0.64
3	7	1	4.24	1.20
3	7	1	3.74	0.38
3	7	1	3.61	0.31
3	7	1	2.64	0.76
-4	-7	-1	13.89	1.43
-4	-7	-1	14.96	0.82
4	7	1	12.99	2.15
4	7	1	12.91	1.50
4	7	1	13.15	1.45

4	7	1	14.23	0.65
4	7	1	15.38	1.12
4	7	1	16.32	0.65
-5	-7	-1	1.90	0.84
-5	-7	-1	0.44	0.36
5	7	1	0.53	0.73
5	7	1	0.78	0.27
5	7	1	0.98	0.29
5	7	1	1.38	0.63
-6	-7	-1	0.82	0.41
6	7	1	1.14	0.36
6	7	1	1.58	0.72
6	7	1	1.08	0.35
6	7	1	-0.14	0.83
-7	-7	-1	5.09	0.77
7	7	1	5.40	0.55
7	7	1	4.54	0.94
7	7	1	4.20	0.52
-8	-7	-1	8.18	0.93
8	7	1	7.80	1.42
8	7	1	7.00	0.79
8	7	1	8.84	0.88
8	7	1	8.71	1.60
-9	-7	-1	2.96	0.74
9	7	1	4.21	1.16
9	7	1	3.57	1.31
9	7	1	3.73	0.62
10	7	1	3.63	1.43
10	7	1	2.71	1.00
10	7	1	4.37	0.84
10	7	1	5.49	1.60
11	7	1	1.35	1.59
11	7	1	0.18	1.32
11	7	1	0.41	1.09
17	-8	-1	7.92	2.86
17	-8	-1	8.22	1.59
-17	8	1	8.17	1.26
16	-8	-1	24.74	3.79
16	-8	-1	21.93	2.25
-16	8	1	18.46	1.36
15	-8	-1	9.52	2.27
15	-8	-1	12.38	1.75
-15	8	1	11.97	1.07
14	-8	-1	13.66	2.36

14	-8	-1	13.62	1.60
-14	8	1	13.84	1.03
13	-8	-1	34.34	3.04
13	-8	-1	38.06	2.09
-13	8	1	37.95	1.55
12	-8	-1	20.44	2.37
12	-8	-1	21.38	1.48
-12	8	1	18.76	1.38
11	-8	-1	1.06	0.45
11	-8	-1	0.73	0.57
11	-8	-1	2.27	1.25
-11	8	1	1.23	0.38
10	-8	-1	2.67	0.42
10	-8	-1	2.07	1.13
10	-8	-1	1.88	0.58
-10	8	1	3.12	0.55
-10	8	1	3.14	0.97
-10	8	1	2.33	0.36
9	-8	-1	12.87	1.00
9	-8	-1	9.60	1.64
9	-8	-1	12.99	0.71
-9	8	1	8.74	1.44
-9	8	1	12.33	0.85
-9	8	1	12.19	0.77
-9	8	1	12.16	0.66
-9	8	1	11.86	0.55
8	-8	-1	38.52	1.68
8	-8	-1	35.69	2.34
8	-8	-1	40.90	1.41
-8	8	1	37.73	1.56
-8	8	1	37.64	1.37
-8	8	1	41.76	2.32
-8	8	1	38.67	1.41
7	-8	-1	19.38	1.73
7	-8	-1	18.27	1.07
7	-8	-1	20.21	0.82
-7	8	1	18.95	1.13
-7	8	1	19.32	0.94
-7	8	1	19.35	0.78
-7	8	1	18.82	0.97
-7	8	1	19.59	1.68
6	-8	-1	16.77	0.97
6	-8	-1	14.45	1.51
6	-8	-1	18.39	0.72

-6	8	1	15.48	1.05
-6	8	1	17.85	1.58
-6	8	1	17.50	0.69
-6	8	1	17.06	0.87
-6	8	1	16.10	1.11
5	-8	-1	0.79	0.68
5	-8	-1	0.04	0.34
5	-8	-1	0.34	0.34
-5	8	1	0.48	0.17
-5	8	1	0.48	0.35
-5	8	1	0.41	0.45
-5	8	1	0.70	0.31
-5	8	1	-0.06	0.72
4	-8	-1	1.58	0.70
4	-8	-1	0.03	0.33
4	-8	-1	0.54	0.17
-4	8	1	0.44	0.37
-4	8	1	0.68	0.48
-4	8	1	0.83	0.76
-4	8	1	0.57	0.17
-4	8	1	0.89	0.41
3	-8	-1	1.34	0.19
3	-8	-1	0.76	0.64
3	-8	-1	1.65	0.41
-3	8	1	0.14	0.72
-3	8	1	1.40	0.37
-3	8	1	1.24	0.54
-3	8	1	1.02	0.44
-3	8	1	1.52	0.17
2	-8	-1	1.29	0.69
2	-8	-1	0.57	0.32
2	-8	-1	0.35	0.13
-2	8	1	-0.20	0.50
-2	8	1	0.36	0.33
-2	8	1	0.37	0.11
-2	8	1	0.36	0.42
-2	8	1	0.36	0.68
1	-8	-1	0.79	0.62
1	-8	-1	0.81	0.32
-1	8	1	0.74	0.82
-1	8	1	0.55	0.48
-1	8	1	0.68	0.63
-1	8	1	0.44	0.38
0	-8	-1	4.71	0.48

0	-8	-1	4.79	0.95
0	8	1	4.91	0.72
0	8	1	3.69	0.74
0	8	1	5.04	0.64
0	8	1	3.06	0.91
-1	-8	-1	3.83	0.87
-1	-8	-1	3.58	0.47
1	8	1	3.42	1.12
1	8	1	3.52	0.24
1	8	1	4.11	0.76
1	8	1	4.49	0.65
1	8	1	3.44	0.76
-2	-8	-1	5.57	0.55
-2	-8	-1	6.32	1.12
2	8	1	7.39	0.91
2	8	1	4.78	0.85
2	8	1	5.55	0.39
2	8	1	6.16	1.01
2	8	1	8.27	1.41
2	8	1	6.34	0.34
-3	-8	-1	6.67	0.62
-3	-8	-1	7.75	1.30
3	8	1	6.74	0.46
3	8	1	6.16	0.40
3	8	1	4.19	0.96
3	8	1	6.83	0.98
3	8	1	6.00	0.96
3	8	1	6.07	1.35
-4	-8	-1	1.49	0.86
-4	-8	-1	1.32	0.38
4	8	1	1.40	0.30
4	8	1	0.92	0.78
4	8	1	0.37	0.66
4	8	1	1.31	0.26
-5	-8	-1	2.02	0.46
-5	-8	-1	2.34	0.99
5	8	1	1.35	0.91
5	8	1	2.16	0.33
5	8	1	2.03	0.72
5	8	1	2.23	0.36
-6	-8	-1	2.70	0.56
6	8	1	3.10	0.87
6	8	1	2.32	0.43
6	8	1	1.41	1.04

6	8	1	1.53	0.41
-7	-8	-1	5.53	0.85
7	8	1	3.70	0.55
7	8	1	5.27	0.72
7	8	1	4.05	1.04
7	8	1	3.92	1.31
-8	-8	-1	3.98	0.73
8	8	1	4.30	1.19
8	8	1	1.59	1.25
8	8	1	3.19	0.57
-9	-8	-1	3.01	0.74
9	8	1	0.57	1.34
9	8	1	2.68	1.16
9	8	1	2.99	0.63
10	8	1	-0.10	1.40
10	8	1	1.34	1.47
10	8	1	1.84	1.09
17	-9	-1	6.03	1.55
17	-9	-1	6.29	2.85
-17	9	1	3.33	0.82
16	-9	-1	5.83	2.41
16	-9	-1	9.18	1.57
-16	9	1	10.18	1.10
15	-9	-1	12.34	2.34
15	-9	-1	11.27	1.82
-15	9	1	10.31	1.07
14	-9	-1	21.99	3.09
14	-9	-1	21.96	1.84
-14	9	1	21.90	1.21
13	-9	-1	21.43	2.85
13	-9	-1	23.91	1.77
-13	9	1	25.59	1.22
12	-9	-1	4.30	0.85
12	-9	-1	2.80	1.68
11	-9	-1	27.25	2.53
11	-9	-1	27.70	1.60
11	-9	-1	26.45	1.25
-11	9	1	24.24	1.13
10	-9	-1	27.70	2.39
10	-9	-1	25.40	1.18
10	-9	-1	27.99	1.54
-10	9	1	25.74	1.17
-10	9	1	28.76	2.53
-10	9	1	27.36	0.97

-10	9	1	27.36	1.34
-10	9	1	25.48	1.11
9	-9	-1	3.35	0.45
9	-9	-1	2.09	1.10
9	-9	-1	2.67	0.59
-9	9	1	2.85	0.52
-9	9	1	2.94	0.58
-9	9	1	2.05	1.14
-9	9	1	2.96	0.38
-9	9	1	2.58	0.36
8	-9	-1	6.85	1.32
8	-9	-1	7.32	0.55
8	-9	-1	5.96	0.80
-8	9	1	5.29	0.82
-8	9	1	4.56	0.72
-8	9	1	7.62	1.33
-8	9	1	5.86	0.69
-8	9	1	5.66	0.47
7	-9	-1	42.77	1.47
7	-9	-1	43.42	1.78
7	-9	-1	37.21	2.47
-7	9	1	44.89	2.51
-7	9	1	38.91	1.84
-7	9	1	41.55	1.63
-7	9	1	38.83	1.42
-7	9	1	43.04	1.68
6	-9	-1	1.93	0.48
6	-9	-1	2.40	0.29
6	-9	-1	1.65	0.82
-6	9	1	2.06	0.26
-6	9	1	2.20	0.98
-6	9	1	1.50	0.53
-6	9	1	1.71	0.40
-6	9	1	2.04	0.47
5	-9	-1	5.15	1.07
5	-9	-1	4.12	0.36
5	-9	-1	4.03	0.65
-5	9	1	5.33	0.94
-5	9	1	6.30	0.79
-5	9	1	4.23	0.57
-5	9	1	3.84	0.97
-5	9	1	4.06	0.34
4	-9	-1	3.69	0.33
4	-9	-1	1.69	0.76

4	-9	-1	2.93	0.53
-4	9	1	2.72	0.30
-4	9	1	4.64	0.77
-4	9	1	2.64	0.69
-4	9	1	2.36	0.96
-4	9	1	3.13	0.48
3	-9	-1	0.23	0.16
3	-9	-1	-0.81	0.65
3	-9	-1	-0.17	0.37
-3	9	1	0.11	0.14
-3	9	1	-0.17	0.45
-3	9	1	-0.97	0.81
-3	9	1	0.16	0.33
-3	9	1	-0.47	0.55
2	-9	-1	1.91	0.81
2	-9	-1	2.45	0.42
-2	9	1	2.98	0.70
-2	9	1	2.70	0.94
-2	9	1	1.45	0.73
-2	9	1	2.85	0.50
1	-9	-1	2.00	0.87
1	-9	-1	1.50	0.34
-1	9	1	1.30	0.77
-1	9	1	0.77	0.55
-1	9	1	1.46	0.49
-1	9	1	0.90	0.94
0	-9	-1	0.94	0.31
0	-9	-1	-0.43	0.71
0	9	1	1.27	0.62
0	9	1	1.63	1.05
0	9	1	1.14	0.64
0	9	1	0.62	0.53
-1	-9	-1	5.19	0.58
-1	-9	-1	5.61	1.05
1	9	1	7.43	0.94
1	9	1	5.68	0.93
1	9	1	6.70	1.14
1	9	1	7.66	1.59
-2	-9	-1	0.47	0.33
-2	-9	-1	0.73	0.80
2	9	1	0.49	0.18
2	9	1	0.21	0.57
2	9	1	0.46	0.78
-3	-9	-1	7.95	1.47

-3	-9	-1	9.72	0.72
3	9	1	9.72	1.61
3	9	1	9.72	1.23
3	9	1	9.55	0.49
3	9	1	9.04	0.55
-4	-9	-1	8.91	0.76
4	9	1	9.49	0.54
4	9	1	8.76	1.40
4	9	1	7.63	0.60
-5	-9	-1	4.33	0.73
-5	-9	-1	5.48	1.28
5	9	1	5.48	1.32
5	9	1	5.36	0.56
5	9	1	4.92	0.60
-6	-9	-1	4.73	0.80
6	9	1	3.45	1.24
6	9	1	4.56	0.54
6	9	1	2.32	0.94
-7	-9	-1	3.53	0.69
7	9	1	2.81	1.33
7	9	1	1.76	1.05
7	9	1	1.76	0.54
-8	-9	-1	3.03	0.76
8	9	1	3.99	1.25
8	9	1	3.51	1.51
8	9	1	1.79	0.64
9	9	1	4.10	1.45
9	9	1	2.43	1.11
9	9	1	2.66	1.64
17	-10	-1	3.86	2.71
17	-10	-1	5.22	1.63
-17	10	1	3.52	0.88
16	-10	-1	7.50	2.62
16	-10	-1	7.06	1.56
-16	10	1	5.01	0.77
15	-10	-1	6.85	1.33
15	-10	-1	5.80	2.16
-15	10	1	7.77	0.99
14	-10	-1	7.69	1.26
14	-10	-1	6.29	2.24
-14	10	1	7.81	0.92
13	-10	-1	10.25	2.23
13	-10	-1	8.39	1.20
-13	10	1	8.71	0.84

12	-10	-1	13.35	1.37
12	-10	-1	12.54	2.06
-12	10	1	10.45	1.23
11	-10	-1	29.93	1.73
11	-10	-1	26.24	2.67
-11	10	1	29.40	1.03
-11	10	1	27.99	1.21
-11	10	1	28.94	1.24
-11	10	1	29.58	1.54
10	-10	-1	1.57	1.17
10	-10	-1	-0.11	0.58
10	-10	-1	-0.27	0.40
-10	10	1	-0.15	0.46
-10	10	1	0.26	0.32
-10	10	1	0.13	0.50
-10	10	1	-0.17	0.30
9	-10	-1	4.36	0.60
9	-10	-1	3.76	1.29
9	-10	-1	5.22	0.78
-9	10	1	5.38	0.52
-9	10	1	3.79	0.66
-9	10	1	5.11	0.72
-9	10	1	4.80	0.76
8	-10	-1	8.24	1.49
8	-10	-1	7.05	0.60
8	-10	-1	8.27	0.92
-8	10	1	6.88	0.52
-8	10	1	7.52	0.99
-8	10	1	6.62	0.78
-8	10	1	6.51	1.33
-8	10	1	7.06	0.75
7	-10	-1	0.92	0.29
7	-10	-1	1.35	0.54
7	-10	-1	2.24	1.00
-7	10	1	2.26	1.08
-7	10	1	1.69	0.66
-7	10	1	2.00	0.52
-7	10	1	1.10	0.28
-7	10	1	1.38	0.43
6	-10	-1	2.71	0.39
6	-10	-1	2.07	0.53
6	-10	-1	1.88	1.04
-6	10	1	2.63	0.60
-6	10	1	0.25	1.00

-6	10	1	2.80	0.68
-6	10	1	2.86	0.47
-6	10	1	2.49	0.29
5	-10	-1	1.86	0.28
5	-10	-1	1.42	0.48
5	-10	-1	0.55	0.80
-5	10	1	1.47	0.41
-5	10	1	1.15	0.57
-5	10	1	1.14	0.64
-5	10	1	1.83	0.25
-5	10	1	2.90	1.14
4	-10	-1	5.10	0.76
4	-10	-1	6.40	1.26
4	-10	-1	7.66	0.44
-4	10	1	8.51	0.74
-4	10	1	6.30	1.31
-4	10	1	7.37	0.41
-4	10	1	7.01	1.21
-4	10	1	7.08	0.98
3	-10	-1	1.44	0.89
3	-10	-1	1.68	0.23
3	-10	-1	1.35	0.45
-3	10	1	4.41	1.20
-3	10	1	1.74	0.24
-3	10	1	2.27	0.47
-3	10	1	2.60	0.78
-3	10	1	1.71	0.65
2	-10	-1	-0.22	0.82
2	-10	-1	-0.32	0.34
-2	10	1	1.44	0.62
-2	10	1	0.45	1.12
-2	10	1	-0.29	0.62
-2	10	1	-0.58	0.44
1	-10	-1	18.18	0.91
1	-10	-1	16.76	1.82
-1	10	1	12.93	2.11
-1	10	1	18.22	1.17
-1	10	1	14.42	1.44
-1	10	1	18.09	1.62
0	-10	-1	0.71	0.35
0	-10	-1	0.98	0.83
0	10	1	0.87	0.72
0	10	1	0.82	1.31
0	10	1	2.08	0.91

0	10	1	1.27	0.64
-1	-10	-1	5.52	0.61
-1	-10	-1	4.93	1.19
1	10	1	5.37	1.16
1	10	1	4.83	0.86
-2	-10	-1	13.87	1.81
-2	-10	-1	13.01	0.83
2	10	1	13.95	1.76
2	10	1	12.15	1.36
-3	-10	-1	18.77	1.95
-3	-10	-1	21.17	1.06
3	10	1	18.37	2.02
3	10	1	20.72	1.59
-4	-10	-1	5.32	1.30
-4	-10	-1	6.52	0.77
4	10	1	6.33	0.51
4	10	1	4.73	0.57
4	10	1	5.04	1.16
4	10	1	5.76	1.35
-5	-10	-1	3.06	1.31
-5	-10	-1	3.32	0.62
5	10	1	4.34	0.63
5	10	1	5.43	1.40
-6	-10	-1	1.81	0.61
6	10	1	1.06	1.02
6	10	1	1.86	1.29
6	10	1	2.09	0.54
-7	-10	-1	1.59	0.71
7	10	1	3.14	1.24
7	10	1	1.61	1.40
-8	-10	-1	8.31	1.29
8	10	1	3.17	1.65
8	10	1	5.07	1.47
16	-11	-1	2.07	2.48
16	-11	-1	1.64	1.36
-16	11	1	1.29	0.71
15	-11	-1	3.06	2.33
15	-11	-1	6.42	1.35
-15	11	1	6.03	0.75
14	-11	-1	11.92	2.60
14	-11	-1	13.53	1.85
-14	11	1	15.89	1.09
13	-11	-1	4.20	1.00
13	-11	-1	5.29	2.01

-13	11	1	3.81	0.61
12	-11	-1	1.54	0.83
12	-11	-1	2.46	1.73
-12	11	1	2.16	0.50
11	-11	-1	6.59	1.78
11	-11	-1	8.48	1.23
-11	11	1	6.63	0.69
-11	11	1	6.27	0.98
-11	11	1	7.39	1.02
-11	11	1	6.53	0.61
10	-11	-1	14.14	1.26
10	-11	-1	11.17	0.84
10	-11	-1	12.98	2.32
-10	11	1	11.16	1.02
-10	11	1	12.69	0.89
-10	11	1	11.85	1.14
-10	11	1	11.53	0.75
9	-11	-1	0.87	1.25
9	-11	-1	4.27	0.62
9	-11	-1	2.82	0.68
-9	11	1	3.23	0.41
-9	11	1	3.26	0.56
-9	11	1	3.45	0.76
-9	11	1	3.36	0.58
8	-11	-1	0.93	0.36
8	-11	-1	1.10	0.58
8	-11	-1	0.74	1.13
-8	11	1	0.81	0.31
-8	11	1	0.99	0.47
-8	11	1	1.39	0.57
-8	11	1	0.92	0.61
7	-11	-1	6.08	0.79
7	-11	-1	5.18	1.42
7	-11	-1	5.29	0.55
-7	11	1	5.00	0.47
-7	11	1	5.52	0.72
-7	11	1	7.73	1.03
-7	11	1	6.73	1.17
6	-11	-1	0.60	1.00
6	-11	-1	0.32	0.27
6	-11	-1	-0.32	0.49
-6	11	1	0.57	0.43
-6	11	1	-0.14	1.16
-6	11	1	-0.24	0.56

-6	11	1	-0.04	0.70
-6	11	1	0.14	0.22
5	-11	-1	-0.31	0.25
5	-11	-1	0.68	1.00
5	-11	-1	0.03	0.46
-5	11	1	0.36	1.17
-5	11	1	0.43	0.43
-5	11	1	-0.07	0.61
-5	11	1	-0.01	0.71
-5	11	1	0.25	0.21
4	-11	-1	-0.44	0.42
4	-11	-1	0.23	0.94
-4	11	1	-2.00	1.10
-4	11	1	-1.38	0.65
-4	11	1	0.30	0.20
-4	11	1	0.34	0.65
-4	11	1	0.67	0.47
3	-11	-1	26.04	0.91
3	-11	-1	26.31	1.26
3	-11	-1	24.35	2.16
-3	11	1	26.63	1.33
-3	11	1	23.13	2.73
-3	11	1	25.25	0.91
-3	11	1	31.03	1.82
-3	11	1	24.70	1.91
2	-11	-1	15.80	1.90
2	-11	-1	16.19	0.92
-2	11	1	16.04	1.17
-2	11	1	15.78	1.67
-2	11	1	16.04	1.51
1	-11	-1	0.84	0.97
1	-11	-1	0.40	0.36
-1	11	1	-0.12	0.61
-1	11	1	-1.27	0.74
0	-11	-1	9.67	0.75
0	-11	-1	8.51	1.45
0	11	1	9.68	1.66
0	11	1	8.30	1.18
-1	-11	-1	3.68	0.52
1	11	1	3.40	0.92
1	11	1	2.27	1.09
-2	-11	-1	2.61	0.50
-2	-11	-1	1.55	1.12
2	11	1	1.82	1.12

2	11	1	2.89	0.99
-3	-11	-1	4.85	0.75
-3	-11	-1	5.74	1.37
3	11	1	5.02	1.20
3	11	1	4.66	1.37
-4	-11	-1	7.35	1.59
-4	-11	-1	7.91	0.88
4	11	1	7.68	1.58
-5	-11	-1	1.27	1.41
-5	-11	-1	3.27	0.82
5	11	1	2.10	1.46
-6	-11	-1	1.03	0.67
6	11	1	-1.15	1.07
6	11	1	0.15	1.50
-7	-11	-1	2.60	0.84
7	11	1	5.16	1.42
7	11	1	1.58	1.71
16	-12	-1	9.28	1.79
16	-12	-1	8.10	2.98
-16	12	1	9.88	1.20
15	-12	-1	6.54	1.45
15	-12	-1	6.61	2.41
14	-12	-1	0.84	2.04
14	-12	-1	1.17	1.06
-14	12	1	-0.73	0.53
13	-12	-1	1.75	1.00
13	-12	-1	2.48	2.05
-13	12	1	2.38	0.57
12	-12	-1	16.72	1.65
12	-12	-1	19.20	3.02
-12	12	1	17.76	1.01
-12	12	1	17.69	1.24
-12	12	1	18.24	1.13
11	-12	-1	1.43	1.61
11	-12	-1	1.82	0.83
-11	12	1	1.22	0.48
-11	12	1	1.72	0.66
-11	12	1	2.12	0.45
10	-12	-1	2.31	1.37
10	-12	-1	0.89	0.44
10	-12	-1	1.97	0.76
-10	12	1	1.91	0.41
-10	12	1	1.93	0.61
-10	12	1	1.91	0.79

-10	12	1	2.41	0.59
9	-12	-1	1.90	0.72
9	-12	-1	2.61	0.47
-9	12	1	2.57	0.79
-9	12	1	3.64	0.64
-9	12	1	3.51	0.45
-9	12	1	3.37	0.70
8	-12	-1	0.60	0.65
8	-12	-1	1.69	0.42
8	-12	-1	1.79	1.36
-8	12	1	0.06	0.63
-8	12	1	0.84	0.33
-8	12	1	1.28	0.73
-8	12	1	0.41	0.50
7	-12	-1	3.30	0.70
7	-12	-1	1.96	0.41
7	-12	-1	2.01	1.26
-7	12	1	2.12	0.53
-7	12	1	3.27	0.81
-7	12	1	2.46	0.34
-7	12	1	2.54	0.82
6	-12	-1	-0.02	0.31
6	-12	-1	0.70	1.07
6	-12	-1	-0.63	0.54
-6	12	1	0.40	0.72
-6	12	1	0.25	0.44
-6	12	1	0.47	0.25
-6	12	1	0.73	0.77
5	-12	-1	0.31	0.51
5	-12	-1	0.33	0.30
5	-12	-1	1.44	1.11
-5	12	1	0.16	0.48
-5	12	1	0.38	0.80
-5	12	1	-0.92	0.75
-5	12	1	0.29	0.24
4	-12	-1	0.53	1.05
4	-12	-1	0.19	0.49
4	-12	-1	-0.31	0.24
-4	12	1	0.96	0.77
-4	12	1	0.66	0.81
-4	12	1	0.67	0.53
-4	12	1	0.22	0.22
3	-12	-1	8.91	1.60
3	-12	-1	8.01	0.80

-3	12	1	6.13	0.92
-3	12	1	7.72	1.28
2	-12	-1	14.72	0.90
2	-12	-1	15.34	2.04
-2	12	1	13.91	1.22
-2	12	1	12.43	1.79
1	-12	-1	9.51	0.80
1	-12	-1	9.41	2.52
-1	12	1	12.25	1.28
0	-12	-1	7.46	1.58
0	-12	-1	8.15	0.77
0	12	1	8.57	1.41
0	12	1	6.16	1.30
-1	-12	-1	1.38	0.48
-1	-12	-1	-0.15	1.17
1	12	1	1.44	1.10
1	12	1	0.57	0.94
-2	-12	-1	3.88	0.59
-2	-12	-1	6.27	1.54
2	12	1	4.40	1.29
2	12	1	5.59	1.23
-3	-12	-1	11.14	1.81
-3	-12	-1	10.65	1.00
-4	-12	-1	3.97	1.67
-4	-12	-1	2.44	0.65
4	12	1	1.79	1.49
-5	-12	-1	5.75	1.75
-5	-12	-1	4.64	0.79
5	12	1	7.47	1.88
-6	-12	-1	4.09	0.92
6	12	1	4.71	1.38
6	12	1	1.12	1.61
16	-13	-1	3.80	2.83
16	-13	-1	1.72	1.52
-16	13	1	1.21	0.72
15	-13	-1	1.67	2.50
15	-13	-1	2.13	1.39
-15	13	1	1.58	0.66
14	-13	-1	6.87	2.61
14	-13	-1	8.68	1.50
-14	13	1	7.23	1.00
13	-13	-1	1.76	1.07
13	-13	-1	-1.41	1.98
-13	13	1	-0.33	0.53

12	-13	-1	0.61	1.96
12	-13	-1	0.17	0.90
-12	13	1	-0.45	0.68
-12	13	1	-0.29	0.45
11	-13	-1	0.44	1.61
11	-13	-1	-0.12	0.87
-11	13	1	2.36	0.63
-11	13	1	2.05	0.84
-11	13	1	1.98	0.48
10	-13	-1	3.43	0.92
10	-13	-1	3.26	0.66
10	-13	-1	5.08	1.81
-10	13	1	2.48	0.45
-10	13	1	3.52	0.74
-10	13	1	3.76	0.93
9	-13	-1	0.21	0.43
9	-13	-1	2.12	1.65
9	-13	-1	-0.54	0.70
-9	13	1	0.55	0.35
-9	13	1	-0.63	0.79
-9	13	1	0.33	0.59
-9	13	1	0.66	0.72
8	-13	-1	0.09	0.43
8	-13	-1	-0.01	1.45
8	-13	-1	0.59	0.66
-8	13	1	0.76	0.83
-8	13	1	0.12	0.53
-8	13	1	0.69	0.34
-8	13	1	0.79	0.74
7	-13	-1	0.90	0.41
7	-13	-1	0.94	0.64
7	-13	-1	3.03	1.33
-7	13	1	0.77	0.31
-7	13	1	0.64	0.90
-7	13	1	0.74	0.55
-7	13	1	1.56	0.84
6	-13	-1	4.07	0.60
6	-13	-1	2.77	1.32
6	-13	-1	4.84	0.74
-6	13	1	3.83	0.93
-6	13	1	4.14	1.03
-6	13	1	4.44	0.67
-6	13	1	3.52	0.45
5	-13	-1	2.15	1.37

5	-13	-1	2.09	0.36
5	-13	-1	0.92	0.58
-5	13	1	1.89	0.30
-5	13	1	1.09	0.60
-5	13	1	2.18	1.03
4	-13	-1	4.38	0.66
4	-13	-1	5.81	0.48
4	-13	-1	5.35	1.57
-4	13	1	6.56	0.81
-4	13	1	5.26	1.27
-4	13	1	4.87	0.44
3	-13	-1	9.45	1.77
3	-13	-1	5.04	0.65
-3	13	1	3.29	1.46
-3	13	1	5.50	0.81
2	-13	-1	0.76	0.47
2	-13	-1	0.13	1.34
-2	13	1	0.19	0.96
-2	13	1	0.07	0.75
1	-13	-1	1.19	0.47
1	-13	-1	0.38	1.28
-1	13	1	0.58	0.88
-1	13	1	0.86	1.11
0	-13	-1	4.79	1.54
0	-13	-1	5.76	0.79
0	13	1	5.67	1.54
0	13	1	6.43	1.19
-1	-13	-1	8.14	0.88
-1	-13	-1	6.92	1.65
1	13	1	7.36	1.65
1	13	1	9.10	1.34
-2	-13	-1	1.85	1.47
-2	-13	-1	3.53	0.61
2	13	1	5.11	1.69
-3	-13	-1	4.97	1.68
-3	-13	-1	5.64	0.76
3	13	1	2.95	1.51
-4	-13	-1	2.97	1.67
-4	-13	-1	3.26	0.77
4	13	1	4.19	1.74
-5	-13	-1	8.24	2.05
-5	-13	-1	8.10	1.28
5	13	1	6.52	1.98
15	-14	-1	-0.33	2.64

15	-14	-1	7.43	1.64
-15	14	1	3.47	0.77
14	-14	-1	-1.66	2.33
14	-14	-1	1.96	1.32
-14	14	1	1.24	0.64
13	-14	-1	0.69	1.14
13	-14	-1	-0.40	2.26
-13	14	1	-0.62	0.54
12	-14	-1	1.93	1.13
12	-14	-1	0.79	2.09
-12	14	1	2.21	0.56
-12	14	1	2.99	0.95
11	-14	-1	-0.80	1.02
11	-14	-1	3.22	1.84
-11	14	1	2.11	0.98
-11	14	1	0.45	0.45
10	-14	-1	1.93	1.76
10	-14	-1	1.00	0.94
-10	14	1	2.18	0.77
-10	14	1	0.32	0.92
-10	14	1	1.04	0.80
-10	14	1	1.56	0.45
9	-14	-1	-0.64	0.50
9	-14	-1	1.62	1.59
9	-14	-1	-1.29	0.78
-9	14	1	-2.51	0.89
-9	14	1	0.76	0.40
-9	14	1	0.05	0.67
-9	14	1	1.04	0.85
8	-14	-1	0.46	1.58
8	-14	-1	0.35	0.77
8	-14	-1	-0.36	0.45
-8	14	1	0.20	0.35
-8	14	1	0.26	0.97
-8	14	1	-0.24	0.85
-8	14	1	-0.16	0.59
7	-14	-1	5.34	0.93
7	-14	-1	5.31	1.72
7	-14	-1	6.05	0.73
-7	14	1	3.79	1.14
-7	14	1	4.81	0.74
-7	14	1	3.96	0.53
6	-14	-1	3.38	1.73
6	-14	-1	3.53	0.77

6	-14	-1	3.68	0.47
-6	14	1	3.39	1.15
-6	14	1	5.20	0.78
-6	14	1	3.47	0.48
5	-14	-1	6.35	1.78
5	-14	-1	4.56	0.54
5	-14	-1	4.22	0.74
-5	14	1	5.93	0.84
-5	14	1	3.56	0.46
-5	14	1	5.19	1.84
4	-14	-1	0.11	0.56
4	-14	-1	1.05	1.41
-4	14	1	0.26	1.06
-4	14	1	-0.33	0.67
3	-14	-1	3.42	1.65
3	-14	-1	2.60	0.60
-3	14	1	1.95	0.82
-3	14	1	2.15	1.18
2	-14	-1	2.45	0.58
2	-14	-1	2.85	1.66
-2	14	1	2.98	0.93
-2	14	1	0.53	1.21
1	-14	-1	16.31	1.07
1	-14	-1	11.72	2.01
-1	14	1	19.90	2.47
0	-14	-1	3.26	1.67
0	-14	-1	5.07	0.71
0	14	1	7.58	1.82
-1	-14	-1	2.87	0.66
-1	-14	-1	1.61	1.69
1	14	1	2.22	1.59
-2	-14	-1	8.38	1.04
-2	-14	-1	10.35	2.10
2	14	1	6.69	1.78
-3	-14	-1	3.65	1.80
3	14	1	8.85	1.97
-4	-14	-1	3.63	0.89
-4	-14	-1	1.54	1.87
4	14	1	1.33	1.82
15	-15	-1	0.78	2.74
15	-15	-1	0.43	1.64
-15	15	1	0.57	0.74
14	-15	-1	14.91	3.18
14	-15	-1	11.05	1.74

-14	15	1	11.59	1.20
13	-15	-1	1.88	1.41
13	-15	-1	2.18	2.36
-13	15	1	-0.41	0.58
12	-15	-1	0.57	1.36
12	-15	-1	-1.47	1.98
-12	15	1	1.86	1.12
-12	15	1	1.99	0.58
11	-15	-1	-2.15	1.98
11	-15	-1	0.52	1.13
-11	15	1	0.80	0.48
-11	15	1	0.50	1.04
10	-15	-1	-1.15	1.84
10	-15	-1	0.63	1.04
-10	15	1	2.79	1.15
-10	15	1	0.49	1.22
-10	15	1	0.08	0.46
9	-15	-1	0.38	1.80
9	-15	-1	0.43	0.94
9	-15	-1	-0.22	0.58
-9	15	1	0.67	1.21
-9	15	1	0.63	0.41
8	-15	-1	1.39	0.94
8	-15	-1	2.60	1.85
8	-15	-1	1.88	0.54
-8	15	1	1.97	0.43
-8	15	1	0.77	1.19
7	-15	-1	6.35	2.05
7	-15	-1	5.58	0.99
7	-15	-1	6.14	0.66
-7	15	1	3.80	0.56
6	-15	-1	1.92	1.80
6	-15	-1	2.07	0.78
-6	15	1	-0.75	1.03
5	-15	-1	1.46	0.70
5	-15	-1	2.18	1.70
-5	15	1	0.23	1.20
4	-15	-1	6.13	0.80
4	-15	-1	6.21	1.85
-4	15	1	2.25	1.37
3	-15	-1	6.24	0.94
3	-15	-1	5.68	1.99
-3	15	1	6.65	1.63
2	-15	-1	10.37	2.29

2	-15	-1	9.93	0.98
-2	15	1	7.66	1.76
1	-15	-1	6.86	0.97
1	-15	-1	8.41	2.11
-1	15	1	7.74	1.86
0	-15	-1	9.48	2.23
0	-15	-1	6.80	0.97
0	15	1	6.27	1.77
-1	-15	-1	8.63	1.05
-1	-15	-1	9.53	2.29
1	15	1	7.10	1.90
-2	-15	-1	0.66	1.94
2	15	1	3.76	1.85
14	-16	-1	-0.86	1.67
14	-16	-1	2.07	2.75
-14	16	1	0.30	0.70
13	-16	-1	2.22	2.53
13	-16	-1	-0.77	1.44
-13	16	1	-0.18	0.65
12	-16	-1	-2.44	2.27
12	-16	-1	-1.92	2.43
-12	16	1	1.20	0.60
11	-16	-1	2.80	1.32
11	-16	-1	-0.22	2.27
-11	16	1	0.50	1.01
-11	16	1	1.37	1.25
10	-16	-1	2.17	1.17
10	-16	-1	1.94	2.09
-10	16	1	2.74	0.57
-10	16	1	2.74	1.32
9	-16	-1	2.87	2.00
9	-16	-1	1.48	1.08
-9	16	1	1.03	2.33
-9	16	1	1.18	0.47
8	-16	-1	1.60	0.98
8	-16	-1	-1.66	1.89
-8	16	1	0.78	0.42
-8	16	1	1.85	1.31
7	-16	-1	1.79	2.06
7	-16	-1	2.66	0.94
-7	16	1	2.10	0.41
-7	16	1	0.41	1.29
6	-16	-1	3.36	2.00
6	-16	-1	1.32	0.80

-6	16	1	1.14	1.44
5	-16	-1	6.92	0.91
5	-16	-1	7.86	2.24
-5	16	1	4.60	1.55
4	-16	-1	5.00	0.79
4	-16	-1	4.67	1.98
-4	16	1	3.30	1.55
3	-16	-1	4.38	0.74
3	-16	-1	4.12	2.10
-3	16	1	3.20	1.68
2	-16	-1	9.25	2.27
-2	16	1	12.77	2.17
1	-16	-1	12.39	2.32
-1	16	1	14.19	2.37
0	-16	-1	4.41	2.25
0	16	1	4.28	1.95
-1	-16	-1	1.93	2.18
1	16	1	-0.94	2.39
13	-17	-1	1.89	2.91
-13	17	1	-0.80	0.67
12	-17	-1	3.31	2.55
11	-17	-1	-0.56	2.33
-11	17	1	0.33	0.66
10	-17	-1	-0.50	2.11
-10	17	1	-0.31	0.62
-10	17	1	1.22	1.50
9	-17	-1	5.43	2.49
-9	17	1	3.26	1.59
8	-17	-1	4.38	2.38
-8	17	1	1.70	1.61
7	-17	-1	5.82	2.47
-7	17	1	6.30	1.84
6	-17	-1	2.65	2.15
-6	17	1	6.13	1.86
5	-17	-1	-0.57	1.99
-5	17	1	3.05	1.78
4	-17	-1	1.07	2.14
-4	17	1	3.03	1.73
3	-17	-1	16.48	2.89
-3	17	1	14.28	2.34
2	-17	-1	4.59	2.37
-2	17	1	3.47	1.97
1	-17	-1	5.12	2.69
-1	17	1	2.04	1.93

11	-18	-1	2.97	4.66
10	-18	-1	5.85	2.91
-10	18	1	3.96	1.80
9	-18	-1	2.56	2.61
-9	18	1	3.94	1.91
8	-18	-1	3.15	2.48
-8	18	1	1.68	1.79
7	-18	-1	1.27	2.37
-7	18	1	0.65	1.77
6	-18	-1	4.15	2.37
-6	18	1	1.79	1.81
5	-18	-1	11.32	2.77
-5	18	1	8.55	2.15
4	-18	-1	10.23	3.86
-4	18	1	6.98	2.15
-6	19	-2	12.23	2.50
-7	19	-2	14.20	2.65
1	-18	2	16.12	3.23
-2	18	-2	23.02	3.62
2	-18	2	18.39	3.01
-3	18	-2	28.25	3.70
3	-18	2	37.98	4.51
-4	18	-2	23.14	3.46
4	-18	2	18.86	3.09
-5	18	-2	13.79	2.46
5	-18	2	9.93	2.65
-6	18	-2	20.17	3.42
6	-18	2	23.11	4.03
-7	18	-2	36.09	3.65
7	-18	2	23.78	4.24
-8	18	-2	35.37	1.83
-8	18	-2	35.60	3.72
8	-18	2	34.63	4.52
-9	18	-2	25.56	3.54
-9	18	-2	23.99	1.32
9	-18	2	26.85	4.35
-10	18	-2	19.51	1.27
-10	18	-2	24.45	3.46
10	-18	2	12.82	3.12
-11	18	-2	28.71	1.53
1	17	-2	14.14	3.75
-1	-17	2	13.96	2.81
0	17	-2	26.23	3.58
0	-17	2	18.88	3.04

-1	17	-2	24.48	3.37
1	-17	2	26.55	3.79
-2	17	-2	28.63	3.39
2	-17	2	24.09	3.25
-3	17	-2	15.30	2.37
3	-17	2	18.60	2.90
-4	17	-2	17.85	3.04
4	-17	2	20.29	2.91
-5	17	-2	39.60	1.51
-5	17	-2	33.26	3.33
5	-17	2	39.11	4.10
-6	17	-2	43.36	1.63
-6	17	-2	39.34	3.45
6	-17	2	40.52	4.23
-7	17	-2	19.18	1.10
-7	17	-2	19.04	2.94
7	-17	2	19.54	3.24
-8	17	-2	20.77	1.16
-8	17	-2	17.94	2.90
8	-17	2	26.09	3.83
-9	17	-2	39.64	3.34
-9	17	-2	41.78	1.74
9	-17	2	47.70	4.52
-10	17	-2	35.58	3.26
-10	17	-2	32.07	1.54
10	-17	2	24.26	4.16
-11	17	-2	13.39	1.18
-11	17	-2	18.32	3.18
11	-17	2	13.26	3.38
-12	17	-2	14.71	1.23
12	-17	2	8.25	3.26
-3	-16	2	11.99	2.52
2	16	-2	19.22	3.38
-2	-16	2	15.25	2.58
1	16	-2	18.81	3.19
-1	-16	2	26.38	3.54
0	16	-2	23.12	3.36
0	-16	2	28.88	3.55
-1	16	-2	24.93	3.13
1	-16	2	25.18	3.58
-2	16	-2	30.62	3.22
-2	16	-2	32.66	1.41
2	-16	2	31.52	3.66
-3	16	-2	43.24	1.51

-3	16	-2	38.22	3.31
3	-16	2	34.08	3.70
-4	16	-2	39.13	1.46
-4	16	-2	37.41	3.19
4	-16	2	36.58	3.69
-5	16	-2	28.44	1.23
-5	16	-2	28.44	2.86
5	-16	2	32.76	3.63
-6	16	-2	38.03	3.12
-6	16	-2	41.79	1.60
6	-16	2	48.22	2.37
6	-16	2	35.74	3.78
-7	16	-2	44.82	1.75
-7	16	-2	41.86	3.22
7	-16	2	48.71	2.18
7	-16	2	50.44	4.15
-8	16	-2	28.88	2.89
-8	16	-2	28.50	1.32
8	-16	2	33.01	3.81
8	-16	2	30.81	1.73
-9	16	-2	24.72	1.28
-9	16	-2	24.56	2.71
9	-16	2	25.27	3.50
9	-16	2	29.24	1.83
-10	16	-2	32.16	2.85
-10	16	-2	29.92	1.45
10	-16	2	34.74	4.20
10	-16	2	29.46	2.06
-11	16	-2	17.37	2.58
-11	16	-2	16.99	1.21
11	-16	2	24.86	4.13
11	-16	2	20.34	2.04
-12	16	-2	19.17	1.30
12	-16	2	17.77	3.27
12	-16	2	20.60	2.20
-13	16	-2	12.00	1.22
13	-16	2	16.59	2.65
13	-16	2	14.79	3.21
4	15	-2	24.78	3.60
-4	-15	2	28.03	3.53
3	15	-2	18.54	3.26
-3	-15	2	19.29	3.20
2	15	-2	21.20	3.25
-2	-15	2	21.38	3.19

1	15	-2	24.87	3.08
-1	-15	2	29.05	3.33
0	15	-2	22.32	1.01
0	15	-2	30.70	3.02
0	-15	2	22.72	3.09
-1	15	-2	32.40	1.25
-1	15	-2	37.08	3.18
1	-15	2	29.13	3.35
-2	15	-2	49.36	1.80
-2	15	-2	49.30	3.41
2	-15	2	57.98	2.00
2	-15	2	51.25	3.79
-3	15	-2	22.61	2.63
-3	15	-2	24.33	1.07
3	-15	2	23.73	3.09
3	-15	2	28.12	1.25
-4	15	-2	42.40	1.53
-4	15	-2	40.56	3.04
4	-15	2	41.39	1.59
4	-15	2	41.11	3.55
-5	15	-2	37.00	1.38
-5	15	-2	34.12	2.70
5	-15	2	34.29	1.47
5	-15	2	30.09	3.29
-6	15	-2	33.44	4.51
-6	15	-2	41.82	1.53
6	-15	2	37.95	1.64
6	-15	2	45.54	3.70
6	-15	2	39.00	1.63
-7	15	-2	23.11	2.32
-7	15	-2	24.04	1.09
7	-15	2	21.25	1.38
7	-15	2	23.96	3.15
7	-15	2	22.62	1.27
-8	15	-2	29.95	1.28
-8	15	-2	27.16	2.45
8	-15	2	28.40	1.69
8	-15	2	26.87	3.44
-9	15	-2	32.28	1.46
-9	15	-2	30.99	2.64
9	-15	2	36.04	3.76
9	-15	2	34.21	1.92
-10	15	-2	24.36	2.40
-10	15	-2	23.91	1.22

10	-15	2	23.99	3.70
10	-15	2	20.13	1.83
-11	15	-2	10.86	1.59
-11	15	-2	10.85	1.00
11	-15	2	11.00	1.74
11	-15	2	12.65	2.85
-12	15	-2	13.46	1.11
12	-15	2	12.90	2.79
12	-15	2	13.98	1.93
-13	15	-2	14.32	1.22
13	-15	2	11.84	2.97
13	-15	2	16.92	2.21
-14	15	-2	12.40	1.26
14	-15	2	14.91	2.49
14	-15	2	15.01	3.39
5	14	-2	19.04	3.38
-5	-14	2	18.71	2.53
4	14	-2	21.45	3.19
-4	-14	2	22.18	3.09
3	14	-2	31.96	3.26
-3	-14	2	33.13	3.25
2	14	-2	41.27	1.55
2	14	-2	41.97	3.47
-2	-14	2	42.07	1.54
-2	-14	2	43.09	3.38
1	14	-2	39.97	1.44
1	14	-2	33.22	3.15
-1	-14	2	38.56	3.14
-1	-14	2	40.05	1.41
0	14	-2	33.60	2.90
0	14	-2	34.58	1.30
0	-14	2	35.11	1.24
0	-14	2	33.94	3.12
-1	14	-2	35.25	1.34
-1	14	-2	37.37	2.82
1	-14	2	38.77	3.16
1	-14	2	36.98	1.33
-2	14	-2	36.48	2.75
-2	14	-2	38.11	1.40
2	-14	2	35.01	3.11
2	-14	2	40.80	1.42
-3	14	-2	56.31	1.91
-3	14	-2	47.61	3.14
3	-14	2	57.41	1.95

3	-14	2	62.43	3.78
-4	14	-2	57.28	3.23
-4	14	-2	61.26	2.01
4	-14	2	58.46	2.06
4	-14	2	54.29	3.72
-5	14	-2	6.99	0.57
-5	14	-2	6.75	1.40
-5	14	-2	8.06	0.64
5	-14	2	7.28	0.84
5	-14	2	6.05	1.63
5	-14	2	6.53	0.77
-6	14	-2	25.42	1.09
-6	14	-2	23.20	2.19
6	-14	2	22.47	2.78
6	-14	2	25.10	1.22
6	-14	2	24.52	1.29
-7	14	-2	77.73	3.33
-7	14	-2	72.25	2.43
7	-14	2	63.53	2.50
7	-14	2	75.74	2.68
7	-14	2	69.26	4.27
7	-14	2	70.44	2.33
-8	14	-2	46.12	2.19
-8	14	-2	45.93	1.72
8	-14	2	44.89	3.62
8	-14	2	46.70	1.66
8	-14	2	43.02	2.05
-9	14	-2	31.38	1.36
-9	14	-2	31.63	2.33
-9	14	-2	31.33	1.72
9	-14	2	21.66	3.17
9	-14	2	32.39	1.34
9	-14	2	32.52	1.81
-10	14	-2	21.43	1.34
-10	14	-2	18.97	1.08
-10	14	-2	16.35	2.00
10	-14	2	21.90	1.62
10	-14	2	18.58	1.13
10	-14	2	19.36	3.29
-11	14	-2	13.66	1.03
-11	14	-2	13.46	1.95
11	-14	2	16.97	1.49
11	-14	2	16.17	1.65
11	-14	2	15.25	2.81

-12	14	-2	18.48	1.19
12	-14	2	22.15	3.72
12	-14	2	17.32	1.88
-13	14	-2	5.97	0.78
13	-14	2	4.73	2.43
13	-14	2	9.44	1.41
-14	14	-2	7.29	0.88
14	-14	2	5.91	2.71
14	-14	2	6.76	1.55
7	13	-2	15.12	2.29
7	13	-2	7.06	2.11
6	13	-2	7.49	2.00
-6	-13	2	12.65	2.21
5	13	-2	19.14	1.37
5	13	-2	16.51	2.51
-5	-13	2	16.69	2.29
4	13	-2	33.89	1.64
4	13	-2	38.83	3.44
4	13	-2	35.38	1.47
-4	-13	2	33.25	3.06
3	13	-2	49.50	3.49
3	13	-2	46.42	1.74
-3	-13	2	49.17	1.75
-3	-13	2	48.50	3.37
2	13	-2	42.56	2.20
2	13	-2	44.58	1.57
2	13	-2	44.51	1.61
2	13	-2	41.63	3.24
-2	-13	2	44.89	3.13
-2	-13	2	44.43	1.58
1	13	-2	47.62	1.65
1	13	-2	43.77	2.01
1	13	-2	48.98	3.17
1	13	-2	47.43	1.66
-1	-13	2	48.06	1.61
-1	-13	2	45.16	3.11
0	13	-2	25.28	2.44
0	13	-2	29.89	1.39
0	13	-2	27.71	1.10
0	-13	2	29.60	1.09
0	-13	2	27.66	2.65
0	-13	2	28.02	1.04
-1	13	-2	36.34	1.41
-1	13	-2	35.42	1.30

-1	13	-2	27.33	2.60
1	-13	2	37.25	1.30
1	-13	2	36.90	1.28
-2	13	-2	46.94	2.93
-2	13	-2	53.37	1.77
2	-13	2	46.88	3.24
2	-13	2	55.19	1.79
-3	13	-2	42.89	1.49
-3	13	-2	39.50	2.58
3	-13	2	42.07	1.54
3	-13	2	42.74	3.05
-4	13	-2	11.30	0.62
-4	13	-2	12.34	0.68
4	-13	2	12.65	1.99
4	-13	2	13.18	0.80
4	-13	2	10.87	1.45
-5	13	-2	59.60	1.99
-5	13	-2	57.18	1.92
-5	13	-2	56.15	2.53
5	-13	2	57.35	2.04
5	-13	2	57.78	2.10
5	-13	2	61.67	2.73
5	-13	2	54.27	3.58
-6	13	-2	59.04	2.01
-6	13	-2	58.54	2.55
6	-13	2	55.98	2.17
6	-13	2	58.22	2.08
6	-13	2	58.10	2.66
-7	13	-2	7.29	1.13
-7	13	-2	8.12	0.66
7	-13	2	8.17	0.80
7	-13	2	7.44	0.60
7	-13	2	6.67	0.90
7	-13	2	8.07	1.08
7	-13	2	9.69	1.80
-8	13	-2	44.38	1.73
-8	13	-2	47.35	2.09
8	-13	2	49.22	1.70
8	-13	2	46.61	2.27
8	-13	2	46.69	3.44
8	-13	2	46.47	2.03
8	-13	2	49.08	2.02
-9	13	-2	69.94	2.61
-9	13	-2	66.46	2.38

9	-13	2	67.41	2.84
9	-13	2	68.90	2.37
9	-13	2	68.02	4.18
9	-13	2	69.24	2.74
-10	13	-2	17.49	1.06
-10	13	-2	18.07	1.15
10	-13	2	18.01	1.09
10	-13	2	15.31	1.44
10	-13	2	17.89	2.96
-11	13	-2	13.59	0.98
-11	13	-2	13.29	1.70
11	-13	2	14.89	2.61
11	-13	2	13.73	1.11
11	-13	2	14.16	1.54
-12	13	-2	9.53	0.93
-12	13	-2	10.27	1.57
12	-13	2	10.17	1.58
12	-13	2	9.00	2.31
-13	13	-2	11.36	1.09
13	-13	2	12.43	1.81
13	-13	2	10.83	2.54
-14	13	-2	19.76	1.32
14	-13	2	20.76	2.29
14	-13	2	24.85	3.96
-15	13	-2	4.90	0.81
15	-13	2	2.70	2.66
15	-13	2	1.48	1.48
8	12	-2	4.14	1.52
8	12	-2	7.65	1.30
8	12	-2	1.93	2.02
7	12	-2	13.15	2.11
7	12	-2	12.42	2.27
7	12	-2	14.57	1.55
6	12	-2	24.85	1.60
6	12	-2	23.90	1.32
6	12	-2	23.52	3.18
-6	-12	2	26.51	2.79
5	12	-2	31.91	3.08
5	12	-2	26.58	1.51
5	12	-2	27.31	1.32
-5	-12	2	29.02	2.73
4	12	-2	44.08	2.28
4	12	-2	42.84	1.65
4	12	-2	40.70	3.29

-4	-12	2	46.77	3.03
3	12	-2	53.51	3.45
3	12	-2	53.33	1.81
3	12	-2	53.02	2.40
3	12	-2	53.06	1.89
-3	-12	2	52.70	3.18
-3	-12	2	53.59	1.86
2	12	-2	44.36	1.53
2	12	-2	43.33	1.57
2	12	-2	44.34	2.00
2	12	-2	41.29	2.95
-2	-12	2	43.55	1.53
1	12	-2	61.23	2.06
1	12	-2	59.59	3.45
1	12	-2	61.51	2.35
1	12	-2	62.10	2.06
-1	-12	2	62.84	2.02
-1	-12	2	62.12	3.42
0	12	-2	15.00	0.74
0	12	-2	16.65	1.01
0	12	-2	15.52	2.00
0	-12	2	16.00	2.08
0	-12	2	16.48	0.73
0	-12	2	16.12	0.69
-1	12	-2	23.11	0.99
-1	12	-2	21.72	0.89
-1	12	-2	20.53	1.89
1	-12	2	22.75	0.90
1	-12	2	17.40	2.15
-2	12	-2	48.45	1.65
2	-12	2	50.34	1.67
2	-12	2	45.34	3.01
2	-12	2	46.90	2.39
-3	12	-2	126.02	3.83
-3	12	-2	120.59	4.47
-3	12	-2	123.51	3.88
3	-12	2	118.94	5.27
3	-12	2	126.64	3.85
3	-12	2	127.40	3.91
3	-12	2	124.92	4.58
-4	12	-2	56.03	1.89
-4	12	-2	58.64	2.51
-4	12	-2	58.40	1.94
4	-12	2	60.42	2.63

4	-12	2	55.07	3.32
4	-12	2	57.01	1.98
-5	12	-2	27.06	1.05
-5	12	-2	25.75	1.56
-5	12	-2	25.49	1.01
5	-12	2	27.39	1.14
5	-12	2	23.64	2.33
5	-12	2	25.77	1.22
5	-12	2	24.67	1.69
-6	12	-2	39.49	1.86
-6	12	-2	38.53	1.44
6	-12	2	41.76	2.03
6	-12	2	38.30	1.50
6	-12	2	39.62	1.64
6	-12	2	33.30	2.73
-7	12	-2	44.70	1.93
-7	12	-2	41.98	1.56
7	-12	2	37.52	2.92
7	-12	2	42.30	1.50
7	-12	2	43.55	2.08
7	-12	2	42.56	1.63
7	-12	2	41.34	1.78
-8	12	-2	10.77	1.00
-8	12	-2	9.06	0.70
8	-12	2	9.25	0.70
8	-12	2	10.16	0.97
8	-12	2	8.21	0.80
8	-12	2	12.21	2.63
8	-12	2	8.33	1.14
-9	12	-2	15.00	1.02
-9	12	-2	14.40	0.86
9	-12	2	14.31	1.25
9	-12	2	15.72	2.43
9	-12	2	14.08	0.90
9	-12	2	13.14	1.15
-10	12	-2	28.45	1.26
-10	12	-2	21.82	1.58
10	-12	2	25.11	1.26
10	-12	2	22.75	2.86
10	-12	2	27.04	1.54
10	-12	2	26.23	1.61
-11	12	-2	15.70	0.89
-11	12	-2	14.22	0.99
11	-12	2	14.96	1.46

11	-12	2	15.11	1.34
11	-12	2	16.53	2.89
-12	12	-2	10.69	1.41
-12	12	-2	12.30	0.98
12	-12	2	14.63	1.55
12	-12	2	12.68	2.44
-13	12	-2	6.60	0.94
13	-12	2	5.57	2.19
13	-12	2	5.35	1.20
-14	12	-2	0.47	0.64
14	-12	2	1.09	1.20
14	-12	2	-2.31	1.95
-15	12	-2	1.45	0.69
15	-12	2	1.57	2.63
15	-12	2	1.03	1.39
9	11	-2	9.75	2.13
9	11	-2	7.63	1.30
9	11	-2	7.46	1.61
8	11	-2	18.37	1.65
8	11	-2	16.60	2.40
8	11	-2	15.60	2.19
8	11	-2	16.12	1.58
7	11	-2	25.46	1.64
7	11	-2	26.58	2.21
7	11	-2	25.68	3.07
7	11	-2	23.09	1.33
6	11	-2	19.55	1.15
6	11	-2	20.04	1.36
6	11	-2	19.39	2.80
-6	-11	2	23.71	2.43
5	11	-2	37.94	1.60
5	11	-2	41.97	1.78
5	11	-2	40.51	3.07
5	11	-2	43.14	2.20
5	11	-2	39.74	1.51
-5	-11	2	41.81	2.80
4	11	-2	51.31	2.39
4	11	-2	54.55	1.94
4	11	-2	54.58	3.39
4	11	-2	56.83	1.86
-4	-11	2	56.29	1.93
-4	-11	2	51.60	3.20
3	11	-2	46.77	1.62
3	11	-2	47.20	3.01

3	11	-2	45.71	1.69
3	11	-2	49.32	2.08
-3	-11	2	46.46	2.80
-3	-11	2	47.89	1.66
2	11	-2	28.88	1.14
2	11	-2	26.47	2.30
2	11	-2	28.02	1.47
2	11	-2	30.46	1.11
-2	-11	2	29.43	2.29
-2	-11	2	28.45	1.08
1	11	-2	19.06	1.23
1	11	-2	14.78	1.85
1	11	-2	18.36	0.82
-1	-11	2	17.81	1.97
-1	-11	2	18.63	0.76
-1	-11	2	20.08	0.78
0	11	-2	31.09	1.35
0	11	-2	33.60	2.70
0	11	-2	33.45	1.20
0	11	-2	32.09	2.24
0	-11	2	34.70	1.17
0	-11	2	31.51	2.34
0	-11	2	33.46	1.15
-1	11	-2	73.54	2.37
-1	11	-2	68.29	2.96
-1	11	-2	71.27	2.32
-1	11	-2	75.03	3.84
1	-11	2	73.14	2.26
1	-11	2	64.34	3.45
1	-11	2	75.42	2.32
1	-11	2	73.98	3.05
-2	11	-2	97.95	3.74
-2	11	-2	96.67	4.51
-2	11	-2	102.22	3.15
-2	11	-2	99.64	3.17
2	-11	2	104.18	3.86
2	-11	2	103.12	3.12
2	-11	2	101.70	3.18
2	-11	2	100.36	4.41
-3	11	-2	67.35	3.44
-3	11	-2	63.36	2.66
-3	11	-2	67.95	2.16
3	-11	2	66.14	2.15
3	-11	2	65.13	3.38

3	-11	2	65.98	2.18
3	-11	2	64.36	2.75
-4	11	-2	41.46	1.43
-4	11	-2	39.52	1.87
-4	11	-2	40.91	1.38
-4	11	-2	39.00	2.72
4	-11	2	44.60	1.48
4	-11	2	41.74	2.01
4	-11	2	40.77	2.67
4	-11	2	32.84	1.59
-5	11	-2	122.32	4.06
-5	11	-2	111.14	4.84
-5	11	-2	117.81	3.65
-5	11	-2	115.19	3.62
5	-11	2	117.42	4.18
5	-11	2	104.87	4.86
5	-11	2	116.14	3.78
5	-11	2	119.44	3.69
-6	11	-2	60.54	2.10
-6	11	-2	63.51	2.43
-6	11	-2	64.56	3.34
6	-11	2	63.45	2.58
6	-11	2	62.82	2.15
6	-11	2	61.56	3.41
6	-11	2	61.34	2.27
-7	11	-2	3.60	0.51
-7	11	-2	4.59	0.80
7	-11	2	4.42	0.62
7	-11	2	5.56	1.32
7	-11	2	3.93	0.49
7	-11	2	4.42	0.63
7	-11	2	2.75	0.69
-8	11	-2	15.08	0.98
-8	11	-2	13.98	0.80
8	-11	2	15.89	0.83
8	-11	2	16.17	1.07
8	-11	2	15.46	0.93
8	-11	2	12.98	1.19
8	-11	2	16.50	2.17
-9	11	-2	57.72	2.03
-9	11	-2	52.08	1.95
9	-11	2	54.41	1.96
9	-11	2	51.13	3.44
9	-11	2	53.49	2.23

9	-11	2	54.21	2.24
-10	11	-2	22.21	1.09
-10	11	-2	20.77	1.02
10	-11	2	22.12	1.44
10	-11	2	22.04	1.14
10	-11	2	22.48	1.33
10	-11	2	20.24	2.61
-11	11	-2	14.93	0.95
-11	11	-2	17.54	0.84
11	-11	2	13.70	2.29
11	-11	2	14.20	1.12
11	-11	2	14.68	1.36
-12	11	-2	2.51	0.53
12	-11	2	4.74	1.80
12	-11	2	4.04	0.93
-13	11	-2	3.91	0.63
13	-11	2	0.39	1.82
13	-11	2	2.68	0.96
-14	11	-2	2.39	0.65
14	-11	2	1.08	1.13
14	-11	2	2.98	2.05
-15	11	-2	-0.77	0.59
15	-11	2	-0.17	1.31
15	-11	2	5.22	2.49
10	10	-2	10.38	2.08
10	10	-2	15.82	2.31
10	10	-2	13.69	1.94
9	10	-2	17.13	2.80
9	10	-2	12.72	1.43
9	10	-2	16.56	1.76
9	10	-2	16.69	2.25
8	10	-2	18.56	1.30
8	10	-2	20.94	1.58
8	10	-2	19.55	2.07
8	10	-2	20.12	2.82
7	10	-2	24.61	2.76
7	10	-2	25.64	1.50
7	10	-2	24.63	2.02
7	10	-2	23.16	1.28
6	10	-2	41.21	3.05
6	10	-2	41.10	1.54
6	10	-2	39.53	2.14
6	10	-2	40.86	1.64
6	10	-2	41.76	1.84

-6	-10	2	43.50	2.70
5	10	-2	30.86	1.66
5	10	-2	29.87	1.15
5	10	-2	28.84	1.25
5	10	-2	27.39	2.52
-5	-10	2	25.38	2.21
4	10	-2	55.24	2.22
4	10	-2	56.06	1.82
4	10	-2	51.86	1.87
4	10	-2	51.85	3.22
-4	-10	2	52.67	1.86
-4	-10	2	54.40	2.88
3	10	-2	26.20	3.11
3	10	-2	22.92	1.31
3	10	-2	21.73	1.00
3	10	-2	24.31	0.94
3	10	-2	21.26	2.04
-3	-10	2	24.77	0.96
-3	-10	2	23.09	1.94
2	10	-2	19.65	0.85
2	10	-2	18.34	1.93
2	10	-2	23.38	2.43
2	10	-2	19.07	1.13
2	10	-2	20.14	0.83
-2	-10	2	16.53	1.77
-2	-10	2	19.86	0.81
1	10	-2	54.05	2.01
1	10	-2	55.60	1.82
1	10	-2	52.15	2.77
1	10	-2	53.82	3.14
1	10	-2	52.16	2.44
-1	-10	2	54.59	2.87
-1	-10	2	55.60	1.77
-1	-10	2	54.72	1.76
0	10	-2	100.53	3.21
0	10	-2	107.30	3.32
0	10	-2	100.65	3.80
0	10	-2	98.79	4.43
0	-10	2	101.85	3.87
0	-10	2	105.17	3.18
0	-10	2	95.28	4.23
0	-10	2	106.95	3.18
-1	10	-2	54.62	2.36
-1	10	-2	53.24	1.81

-1	10	-2	56.20	2.99
-1	10	-2	54.00	1.79
1	-10	2	55.17	2.87
1	-10	2	54.06	1.73
1	-10	2	54.81	2.41
1	-10	2	54.18	1.78
-2	10	-2	40.22	1.31
-2	10	-2	36.20	1.80
-2	10	-2	41.17	2.51
-2	10	-2	37.76	1.34
2	-10	2	40.36	2.45
2	-10	2	36.61	1.87
2	-10	2	39.11	1.36
-3	10	-2	97.67	3.03
-3	10	-2	95.27	2.98
-3	10	-2	94.14	3.45
-3	10	-2	89.33	4.05
3	-10	2	98.03	3.05
3	-10	2	99.55	3.57
3	-10	2	97.09	4.17
3	-10	2	97.28	3.05
-4	10	-2	54.25	1.75
-4	10	-2	54.90	2.17
-4	10	-2	53.82	1.79
-4	10	-2	50.57	2.81
4	-10	2	53.94	2.26
4	-10	2	51.41	1.88
4	-10	2	52.73	2.89
4	-10	2	54.18	1.82
-5	10	-2	17.19	1.84
-5	10	-2	18.50	0.79
-5	10	-2	19.61	1.12
-5	10	-2	17.81	0.79
5	-10	2	18.47	0.84
5	-10	2	17.09	1.21
5	-10	2	16.35	1.80
5	-10	2	19.12	0.98
-6	10	-2	15.37	1.88
-6	10	-2	20.21	0.89
-6	10	-2	22.45	1.15
6	-10	2	21.31	0.94
6	-10	2	21.59	1.30
6	-10	2	21.68	0.84
6	-10	2	18.83	1.95

6	-10	2	21.08	1.06
-7	10	-2	61.68	3.15
-7	10	-2	60.68	2.26
-7	10	-2	62.86	2.12
7	-10	2	62.47	2.43
7	-10	2	63.64	2.16
7	-10	2	64.76	2.08
7	-10	2	60.90	2.29
7	-10	2	60.45	3.29
-8	10	-2	10.05	0.66
-8	10	-2	8.97	0.71
8	-10	2	10.53	1.81
8	-10	2	7.79	0.94
8	-10	2	10.28	0.75
8	-10	2	9.16	0.89
8	-10	2	10.89	0.70
-9	10	-2	20.56	0.92
-9	10	-2	17.94	0.93
9	-10	2	19.81	1.02
9	-10	2	17.56	1.17
9	-10	2	20.64	2.26
9	-10	2	18.08	1.17
-10	10	-2	27.91	1.21
-10	10	-2	26.55	1.16
10	-10	2	27.81	1.30
10	-10	2	25.77	1.51
10	-10	2	28.07	1.37
-11	10	-2	-0.19	0.33
-11	10	-2	1.05	0.41
11	-10	2	0.33	1.32
11	-10	2	0.90	0.66
11	-10	2	0.66	0.56
-12	10	-2	6.42	0.77
12	-10	2	4.12	1.78
-13	10	-2	2.22	0.56
13	-10	2	3.48	0.98
13	-10	2	2.28	1.85
-14	10	-2	-0.11	0.57
14	-10	2	0.63	1.90
14	-10	2	-0.62	1.00
-15	10	-2	-0.39	0.61
15	-10	2	2.07	2.31
15	-10	2	2.27	1.16
-16	10	-2	-0.87	1.06

16	-10	2	0.81	1.35
16	-10	2	-2.89	2.58
10	9	-2	11.32	1.72
10	9	-2	8.58	1.56
10	9	-2	8.19	1.18
10	9	-2	11.44	1.94
9	9	-2	14.00	2.06
9	9	-2	13.48	1.52
9	9	-2	17.72	1.99
9	9	-2	16.33	1.19
8	9	-2	32.19	1.48
8	9	-2	34.03	2.20
8	9	-2	31.85	1.76
8	9	-2	30.10	2.91
7	9	-2	42.89	2.34
7	9	-2	41.64	2.95
7	9	-2	44.33	1.94
7	9	-2	41.98	1.68
7	9	-2	43.33	1.62
6	9	-2	21.60	1.40
6	9	-2	19.95	1.19
6	9	-2	19.80	1.00
6	9	-2	18.07	0.91
-6	-9	2	17.75	1.87
5	9	-2	21.48	1.03
5	9	-2	27.74	2.26
5	9	-2	23.94	0.97
5	9	-2	22.14	1.35
-5	-9	2	22.92	1.01
-5	-9	2	24.44	1.97
4	9	-2	51.50	2.94
4	9	-2	57.88	1.85
4	9	-2	55.05	3.37
4	9	-2	54.63	1.90
4	9	-2	56.25	2.14
-4	-9	2	56.45	2.74
-4	-9	2	54.97	1.89
3	9	-2	48.78	2.67
3	9	-2	44.01	2.23
3	9	-2	44.51	2.86
3	9	-2	48.10	1.58
3	9	-2	44.05	1.61
3	9	-2	46.30	1.82
-3	-9	2	48.59	2.51

-3	-9	2	47.21	1.59
2	9	-2	38.46	1.36
2	9	-2	40.35	2.33
2	9	-2	41.03	2.62
2	9	-2	37.29	1.95
2	9	-2	39.71	1.38
-2	-9	2	38.94	2.25
-2	-9	2	38.75	1.33
1	9	-2	72.18	3.43
1	9	-2	69.66	2.28
1	9	-2	66.95	2.40
1	9	-2	69.58	2.83
-1	-9	2	75.08	2.23
-1	-9	2	66.67	3.15
-1	-9	2	72.55	2.23
-1	-9	2	71.76	2.89
0	9	-2	35.00	1.71
0	9	-2	35.48	2.27
0	9	-2	34.46	1.21
0	9	-2	33.53	1.28
0	-9	2	33.38	1.74
0	-9	2	34.69	1.13
0	-9	2	32.62	2.09
0	-9	2	34.49	1.17
-1	9	-2	103.66	3.14
-1	9	-2	97.89	4.16
-1	9	-2	99.82	3.62
-1	9	-2	101.55	3.15
1	-9	2	97.94	4.09
1	-9	2	100.83	3.08
1	-9	2	104.37	3.71
1	-9	2	100.43	3.14
-2	9	-2	72.57	3.36
-2	9	-2	76.19	2.35
-2	9	-2	74.74	2.81
-2	9	-2	74.54	2.40
2	-9	2	75.74	2.41
2	-9	2	75.66	2.36
2	-9	2	77.44	3.39
2	-9	2	75.99	2.89
-3	9	-2	14.59	0.63
-3	9	-2	14.07	0.58
-3	9	-2	16.33	1.60
-3	9	-2	15.24	0.98

3	-9	2	13.74	1.53
3	-9	2	13.20	0.67
3	-9	2	13.79	0.65
3	-9	2	13.58	1.02
-4	9	-2	49.73	1.99
-4	9	-2	49.37	1.66
-4	9	-2	49.23	1.69
-4	9	-2	50.11	2.61
4	-9	2	51.34	1.80
4	-9	2	54.70	2.75
4	-9	2	51.46	1.71
4	-9	2	49.01	2.06
-5	9	-2	35.99	2.21
-5	9	-2	36.27	1.52
-5	9	-2	37.03	1.29
5	-9	2	35.30	1.62
5	-9	2	37.35	2.29
5	-9	2	34.77	1.40
-6	9	-2	22.73	1.10
-6	9	-2	22.53	0.93
-6	9	-2	22.92	1.81
6	-9	2	23.04	0.97
6	-9	2	23.80	0.90
6	-9	2	23.07	1.24
6	-9	2	21.00	1.92
6	-9	2	24.53	1.10
-7	9	-2	4.48	0.52
-7	9	-2	4.22	0.46
-7	9	-2	3.36	0.89
7	-9	2	3.19	1.03
7	-9	2	4.34	0.50
7	-9	2	4.57	0.48
7	-9	2	4.05	0.59
7	-9	2	4.12	0.61
-8	9	-2	35.71	2.22
-8	9	-2	36.43	1.39
-8	9	-2	37.57	1.39
8	-9	2	36.19	1.59
8	-9	2	37.89	1.45
8	-9	2	33.50	2.45
8	-9	2	39.63	1.42
8	-9	2	36.24	1.59
-9	9	-2	21.14	1.01
-9	9	-2	20.75	1.23

9	-9	2	21.49	1.10
9	-9	2	22.80	2.21
9	-9	2	22.62	1.17
9	-9	2	23.45	1.29
9	-9	2	24.83	1.10
-10	9	-2	2.11	0.42
-10	9	-2	2.44	0.36
10	-9	2	1.44	0.58
10	-9	2	1.24	1.20
10	-9	2	2.34	0.56
10	-9	2	1.83	0.53
-11	9	-2	2.87	0.46
-11	9	-2	3.51	0.57
11	-9	2	2.86	0.71
11	-9	2	2.82	0.53
11	-9	2	2.30	1.31
-12	9	-2	2.80	0.52
12	-9	2	4.00	1.12
12	-9	2	1.16	1.42
-13	9	-2	-0.58	0.48
13	-9	2	-1.56	1.54
13	-9	2	0.74	0.82
-14	9	-2	2.35	0.61
14	-9	2	-1.42	1.91
14	-9	2	0.84	0.98
-15	9	-2	0.20	0.62
15	-9	2	-0.18	1.13
15	-9	2	-1.99	2.15
-16	9	-2	0.20	0.89
16	-9	2	3.16	1.41
16	-9	2	6.45	2.75
11	8	-2	14.63	2.39
11	8	-2	11.13	1.87
11	8	-2	13.08	2.03
11	8	-2	15.38	1.26
10	8	-2	16.06	2.54
10	8	-2	18.28	1.68
10	8	-2	17.54	1.20
10	8	-2	18.98	2.07
9	8	-2	37.53	2.86
9	8	-2	35.12	2.30
9	8	-2	35.15	1.88
9	8	-2	35.72	1.54
8	8	-2	31.68	1.40

8	8	-2	35.05	1.44
8	8	-2	36.90	2.73
8	8	-2	33.58	1.73
8	8	-2	35.05	2.08
7	8	-2	39.43	1.48
7	8	-2	36.20	1.85
7	8	-2	39.31	1.76
7	8	-2	39.59	2.11
7	8	-2	38.46	2.66
7	8	-2	41.99	1.54
6	8	-2	35.87	1.39
6	8	-2	35.02	1.67
6	8	-2	40.45	2.56
6	8	-2	35.27	1.57
6	8	-2	35.63	1.33
-6	-8	2	35.92	2.20
5	8	-2	33.57	1.99
5	8	-2	35.06	1.26
5	8	-2	36.04	1.54
5	8	-2	35.74	1.31
5	8	-2	33.03	2.31
5	8	-2	35.39	2.69
-5	-8	2	34.82	1.32
-5	-8	2	34.41	2.08
4	8	-2	58.30	1.86
4	8	-2	54.84	2.86
4	8	-2	57.84	2.09
4	8	-2	56.37	1.90
4	8	-2	58.82	3.14
4	8	-2	54.16	2.51
-4	-8	2	54.85	2.65
-4	-8	2	55.66	1.89
3	8	-2	55.20	1.77
3	8	-2	52.44	2.34
3	8	-2	58.99	1.96
3	8	-2	49.97	1.79
3	8	-2	53.20	2.71
3	8	-2	52.54	2.89
-3	-8	2	52.37	2.54
-3	-8	2	53.56	1.78
2	8	-2	77.02	2.96
2	8	-2	80.74	2.56
2	8	-2	80.81	3.49
2	8	-2	76.29	2.44

-2	-8	2	71.17	3.16
-2	-8	2	76.02	2.40
-2	-8	2	70.80	2.94
-2	-8	2	75.86	2.38
1	8	-2	92.62	3.02
1	8	-2	93.71	3.41
1	8	-2	102.53	3.97
1	8	-2	93.06	2.93
-1	-8	2	84.32	3.66
-1	-8	2	95.60	2.88
-1	-8	2	94.13	3.47
0	8	-2	89.94	3.69
0	8	-2	88.42	3.22
0	8	-2	89.09	2.77
0	8	-2	85.42	2.81
0	-8	2	90.78	2.75
0	-8	2	87.56	3.59
0	-8	2	89.73	2.71
0	-8	2	87.29	3.26
-1	8	-2	23.37	0.84
-1	8	-2	24.18	1.75
-1	8	-2	24.87	1.27
1	-8	2	22.18	1.26
1	-8	2	23.25	0.79
1	-8	2	22.44	1.62
1	-8	2	24.10	0.95
-2	8	-2	89.72	3.48
-2	8	-2	81.32	2.47
-2	8	-2	79.97	2.88
-2	8	-2	79.27	2.53
2	-8	2	78.30	2.54
2	-8	2	80.75	2.94
2	-8	2	74.87	3.34
-3	8	-2	76.99	3.29
-3	8	-2	81.69	2.48
-3	8	-2	79.41	2.43
-3	8	-2	78.79	2.77
3	-8	2	75.17	2.50
3	-8	2	76.48	3.34
3	-8	2	80.68	2.49
3	-8	2	75.23	2.81
-4	8	-2	26.08	1.74
-4	8	-2	26.51	0.94
-4	8	-2	25.32	1.17

-4	8	-2	25.73	0.96
4	-8	2	26.46	1.05
4	-8	2	26.11	0.97
4	-8	2	25.26	1.24
4	-8	2	21.68	1.69
-5	8	-2	21.29	0.84
-5	8	-2	21.44	1.00
-5	8	-2	19.84	1.60
5	-8	2	21.12	0.80
5	-8	2	20.59	0.95
5	-8	2	22.87	1.68
5	-8	2	21.77	0.87
5	-8	2	20.35	1.12
-6	8	-2	20.79	0.92
-6	8	-2	18.42	1.53
-6	8	-2	20.16	0.83
6	-8	2	17.74	1.69
6	-8	2	20.93	0.82
6	-8	2	19.96	0.87
6	-8	2	19.97	1.08
6	-8	2	19.86	0.96
-7	8	-2	49.67	1.72
-7	8	-2	48.21	2.46
7	-8	2	48.65	1.75
7	-8	2	49.59	1.89
7	-8	2	50.89	1.72
7	-8	2	50.31	1.89
-8	8	-2	2.07	0.79
-8	8	-2	2.07	0.36
-8	8	-2	2.55	0.36
8	-8	2	2.03	0.40
8	-8	2	1.97	0.46
8	-8	2	2.77	0.49
8	-8	2	2.61	0.93
8	-8	2	3.16	0.52
-9	8	-2	1.55	0.32
-9	8	-2	1.32	0.28
-9	8	-2	2.58	1.01
9	-8	2	0.38	0.35
9	-8	2	0.90	0.45
9	-8	2	1.26	0.48
9	-8	2	1.68	0.43
9	-8	2	1.24	1.06
-10	8	-2	0.97	0.35

-10	8	-2	0.77	0.32
10	-8	2	0.43	0.49
10	-8	2	0.72	0.54
10	-8	2	2.70	1.13
-11	8	-2	2.84	0.47
-11	8	-2	2.77	0.47
11	-8	2	2.83	1.57
11	-8	2	3.41	0.45
11	-8	2	3.34	0.72
-12	8	-2	3.16	0.54
12	-8	2	2.11	0.76
12	-8	2	2.15	1.43
-13	8	-2	0.75	0.51
13	-8	2	-0.68	1.62
13	-8	2	1.01	0.89
-14	8	-2	0.32	0.55
14	-8	2	1.81	1.01
14	-8	2	1.67	2.02
-15	8	-2	0.27	0.64
15	-8	2	1.05	1.18
15	-8	2	-2.79	2.23
-16	8	-2	0.84	0.84
16	-8	2	-1.62	1.26
16	-8	2	-0.33	2.61
12	7	-2	9.83	1.48
12	7	-2	12.44	1.22
12	7	-2	12.62	1.98
12	7	-2	9.88	1.86
11	7	-2	23.77	2.36
11	7	-2	15.78	2.61
11	7	-2	19.60	1.83
11	7	-2	21.41	1.28
10	7	-2	21.99	2.39
10	7	-2	25.62	1.69
10	7	-2	20.26	2.02
9	7	-2	30.69	1.35
9	7	-2	30.80	2.04
9	7	-2	28.81	1.91
8	7	-2	49.92	2.91
8	7	-2	49.97	2.08
8	7	-2	45.56	1.76
8	7	-2	50.06	1.79
8	7	-2	47.99	2.35
7	7	-2	41.72	2.63

7	7	-2	43.84	1.87
7	7	-2	44.24	1.57
7	7	-2	44.33	1.60
7	7	-2	43.35	1.84
7	7	-2	42.92	2.13
6	7	-2	29.01	1.32
6	7	-2	26.58	1.77
6	7	-2	23.16	2.38
6	7	-2	28.83	1.35
6	7	-2	28.25	1.08
6	7	-2	26.40	1.10
6	7	-2	30.86	2.15
-6	-7	2	29.71	1.86
5	7	-2	19.92	1.98
5	7	-2	21.96	1.05
5	7	-2	20.10	1.75
5	7	-2	19.38	0.86
5	7	-2	20.68	0.83
5	7	-2	18.69	1.40
-5	-7	2	18.86	1.50
-5	-7	2	20.17	0.87
4	7	-2	64.94	2.20
4	7	-2	62.85	2.06
4	7	-2	63.77	2.94
4	7	-2	61.82	2.57
4	7	-2	63.24	2.04
4	7	-2	63.35	3.05
-4	-7	2	61.43	2.72
-4	-7	2	61.85	2.06
3	7	-2	26.25	0.98
3	7	-2	23.89	1.18
3	7	-2	25.83	0.95
3	7	-2	28.72	1.93
3	7	-2	26.30	1.44
-3	-7	2	26.37	1.46
-3	-7	2	27.67	1.64
-3	-7	2	25.97	0.95
2	7	-2	49.34	2.49
2	7	-2	50.88	2.09
2	7	-2	48.57	1.65
2	7	-2	53.97	1.74
-2	-7	2	51.01	2.32
-2	-7	2	49.62	1.62
-2	-7	2	47.26	2.10

1	7	-2	66.09	2.04
1	7	-2	61.72	2.43
1	7	-2	61.33	2.08
1	7	-2	66.56	2.86
-1	-7	2	63.45	1.97
-1	-7	2	65.08	1.99
-1	-7	2	63.12	2.70
-1	-7	2	59.89	2.45
0	7	-2	28.46	1.35
0	7	-2	31.43	1.76
0	7	-2	28.03	1.00
0	7	-2	28.27	1.00
0	-7	2	28.29	1.38
0	-7	2	25.93	1.61
0	-7	2	27.89	0.91
0	-7	2	28.53	0.98
-1	7	-2	23.64	0.87
-1	7	-2	24.57	1.19
-1	7	-2	24.25	1.57
1	-7	2	23.49	1.21
1	-7	2	24.34	0.87
1	-7	2	23.60	1.53
-2	7	-2	25.29	0.92
-2	7	-2	25.17	1.18
-2	7	-2	27.10	1.62
-2	7	-2	26.45	0.85
2	-7	2	24.88	1.62
2	-7	2	24.84	1.21
2	-7	2	26.22	0.94
-3	7	-2	24.22	0.89
-3	7	-2	26.80	1.59
-3	7	-2	24.75	0.85
-3	7	-2	23.22	1.09
3	-7	2	23.94	0.90
3	-7	2	24.01	1.17
3	-7	2	22.88	1.58
3	-7	2	24.95	0.92
-4	7	-2	35.76	1.86
-4	7	-2	29.56	1.39
-4	7	-2	32.52	1.14
4	-7	2	32.20	1.47
4	-7	2	28.82	1.81
4	-7	2	33.13	1.38
4	-7	2	33.63	1.15

4	-7	2	32.41	1.08
-5	7	-2	67.26	2.87
-5	7	-2	64.43	2.22
-5	7	-2	75.00	2.33
5	-7	2	66.73	3.01
5	-7	2	69.12	2.19
5	-7	2	70.19	2.31
5	-7	2	69.36	2.25
5	-7	2	66.46	2.40
-6	7	-2	21.86	0.88
-6	7	-2	20.92	1.46
-6	7	-2	18.87	0.83
6	-7	2	21.33	0.98
6	-7	2	19.25	1.60
6	-7	2	21.81	1.03
6	-7	2	20.75	0.84
6	-7	2	22.94	0.89
-7	7	-2	5.18	0.45
-7	7	-2	4.99	0.40
-7	7	-2	6.08	1.01
7	-7	2	4.69	0.58
7	-7	2	3.32	0.87
7	-7	2	4.87	0.48
7	-7	2	5.17	0.47
7	-7	2	4.95	0.65
-8	7	-2	2.15	0.33
-8	7	-2	2.80	0.34
-8	7	-2	2.77	0.75
8	-7	2	2.78	0.45
8	-7	2	2.26	0.49
8	-7	2	2.43	0.88
8	-7	2	3.21	0.43
8	-7	2	3.23	0.40
-9	7	-2	1.95	0.31
-9	7	-2	2.27	0.37
-9	7	-2	3.62	0.86
9	-7	2	2.24	0.46
9	-7	2	2.62	0.98
9	-7	2	2.40	0.52
9	-7	2	2.20	0.38
-10	7	-2	1.08	0.36
-10	7	-2	0.33	0.30
10	-7	2	0.10	0.50
10	-7	2	-0.08	0.46

10	-7	2	0.46	0.36
10	-7	2	-0.28	1.00
-11	7	-2	6.73	0.78
-11	7	-2	6.32	0.70
11	-7	2	7.23	1.04
11	-7	2	7.01	0.62
-12	7	-2	0.50	0.42
12	-7	2	-0.69	0.67
12	-7	2	2.39	1.39
-13	7	-2	1.23	0.51
13	-7	2	0.77	1.65
13	-7	2	1.64	0.83
-14	7	-2	-0.77	0.51
14	-7	2	1.30	1.01
14	-7	2	2.08	2.07
-15	7	-2	0.43	0.63
15	-7	2	2.47	2.28
15	-7	2	0.49	1.09
-16	7	-2	1.39	0.86
16	-7	2	4.84	1.50
16	-7	2	6.12	2.69
13	6	-2	13.51	1.99
13	6	-2	11.34	2.07
13	6	-2	14.19	1.91
13	6	-2	17.31	1.31
12	6	-2	14.16	2.38
12	6	-2	14.32	1.78
12	6	-2	16.93	2.46
12	6	-2	11.21	1.15
11	6	-2	24.05	2.24
11	6	-2	21.27	1.75
11	6	-2	18.28	2.31
11	6	-2	24.29	1.24
10	6	-2	40.36	2.00
10	6	-2	38.45	2.55
10	6	-2	38.42	1.57
10	6	-2	36.27	2.30
9	6	-2	41.02	1.54
9	6	-2	37.27	2.10
9	6	-2	39.62	1.87
9	6	-2	35.83	2.48
8	6	-2	43.11	1.86
8	6	-2	41.44	1.87
8	6	-2	42.82	2.29

8	6	-2	44.32	1.59
8	6	-2	46.42	2.60
8	6	-2	40.05	2.10
8	6	-2	43.41	1.59
7	6	-2	52.37	2.40
7	6	-2	51.08	1.77
7	6	-2	50.16	2.01
7	6	-2	53.30	3.11
7	6	-2	50.78	1.78
7	6	-2	49.64	2.67
7	6	-2	52.60	2.00
7	6	-2	51.52	2.26
6	6	-2	62.86	2.60
6	6	-2	59.86	2.89
6	6	-2	63.66	2.04
6	6	-2	61.29	3.12
6	6	-2	61.33	2.23
6	6	-2	59.08	2.43
6	6	-2	63.34	2.23
6	6	-2	61.59	2.05
5	6	-2	65.51	2.16
5	6	-2	61.49	1.99
5	6	-2	59.69	2.92
5	6	-2	57.92	2.47
5	6	-2	59.53	1.99
5	6	-2	59.55	2.80
-5	-6	2	59.93	2.03
-5	-6	2	62.74	2.57
4	6	-2	65.49	3.00
4	6	-2	75.70	2.31
4	6	-2	72.54	2.42
4	6	-2	71.27	3.13
4	6	-2	72.71	2.78
4	6	-2	71.68	2.31
-4	-6	2	73.88	2.89
-4	-6	2	74.67	2.33
3	6	-2	51.97	1.67
3	6	-2	48.31	1.66
3	6	-2	54.01	2.48
3	6	-2	51.16	2.09
3	6	-2	54.36	1.75
-3	-6	2	50.75	1.66
-3	-6	2	50.01	2.23
-3	-6	2	49.46	2.10

2	6	-2	43.64	1.81
2	6	-2	43.82	1.46
2	6	-2	42.69	2.11
2	6	-2	42.35	1.43
-2	-6	2	43.32	1.40
-2	-6	2	42.71	1.83
-2	-6	2	42.78	1.39
-2	-6	2	43.61	1.98
1	6	-2	55.18	2.09
1	6	-2	51.55	1.74
1	6	-2	61.16	2.45
1	6	-2	54.58	1.75
-1	-6	2	54.41	1.71
-1	-6	2	54.34	1.68
-1	-6	2	52.15	2.09
-1	-6	2	51.42	2.28
0	6	-2	122.51	4.34
0	6	-2	120.11	3.70
0	6	-2	122.70	4.01
0	-6	2	121.46	4.32
0	-6	2	119.51	3.62
0	-6	2	120.79	4.05
0	-6	2	121.66	3.69
-1	6	-2	1.75	0.25
-1	6	-2	2.69	0.58
-1	6	-2	1.63	0.35
1	-6	2	1.84	0.56
1	-6	2	1.69	0.38
1	-6	2	2.00	0.24
-2	6	-2	96.13	2.92
-2	6	-2	96.94	3.52
-2	6	-2	95.67	2.85
-2	6	-2	83.35	3.22
2	-6	2	96.57	2.92
2	-6	2	95.97	3.18
2	-6	2	99.52	2.90
2	-6	2	87.93	3.52
-3	6	-2	36.11	1.73
-3	6	-2	32.93	1.30
-3	6	-2	33.98	1.15
3	-6	2	33.37	1.35
3	-6	2	33.26	1.16
3	-6	2	32.56	1.72
3	-6	2	33.80	1.08

-4	6	-2	2.52	0.27
-4	6	-2	1.93	0.62
-4	6	-2	2.76	0.36
4	-6	2	2.98	0.25
4	-6	2	3.30	0.67
4	-6	2	2.86	0.43
4	-6	2	2.20	0.29
4	-6	2	2.74	0.49
-5	6	-2	14.72	1.14
-5	6	-2	14.49	0.65
-5	6	-2	14.53	0.63
5	-6	2	12.79	1.21
5	-6	2	14.23	0.78
5	-6	2	14.33	0.62
5	-6	2	15.40	0.65
5	-6	2	15.03	0.72
-6	6	-2	13.96	0.63
-6	6	-2	14.01	0.66
-6	6	-2	14.96	1.18
6	-6	2	16.13	1.34
6	-6	2	15.51	0.81
6	-6	2	14.96	0.70
6	-6	2	15.73	0.69
6	-6	2	15.78	0.79
-7	6	-2	12.28	0.62
-7	6	-2	11.59	1.15
-7	6	-2	12.35	0.56
7	-6	2	12.29	0.66
7	-6	2	13.29	1.39
7	-6	2	12.15	0.78
7	-6	2	11.07	0.64
-8	6	-2	7.64	0.48
-8	6	-2	6.59	1.06
-8	6	-2	7.61	0.54
8	-6	2	8.01	0.57
8	-6	2	7.05	0.72
8	-6	2	7.66	0.61
8	-6	2	7.10	0.66
8	-6	2	8.53	1.32
-9	6	-2	0.16	0.68
-9	6	-2	0.18	0.28
-9	6	-2	0.24	0.25
9	-6	2	0.04	0.32
9	-6	2	0.64	0.85

9	-6	2	0.03	0.40
9	-6	2	0.16	0.35
9	-6	2	-0.10	0.28
-10	6	-2	0.23	0.29
-10	6	-2	0.67	0.42
10	-6	2	1.42	1.08
10	-6	2	-0.22	0.45
10	-6	2	0.71	0.50
10	-6	2	0.47	0.31
-11	6	-2	2.71	0.57
-11	6	-2	1.40	0.58
11	-6	2	1.13	1.30
11	-6	2	1.88	0.65
11	-6	2	2.19	0.37
-12	6	-2	-0.48	0.38
12	-6	2	-0.52	1.27
12	-6	2	-0.85	0.64
-13	6	-2	-0.08	0.48
13	-6	2	1.09	1.62
13	-6	2	-1.05	0.74
-14	6	-2	1.13	0.58
14	-6	2	-1.03	1.88
14	-6	2	0.78	0.97
-15	6	-2	2.32	0.67
15	-6	2	1.26	1.10
15	-6	2	2.62	2.27
-16	6	-2	0.91	0.93
16	-6	2	0.26	1.39
16	-6	2	3.02	2.59
13	5	-2	5.22	1.76
13	5	-2	5.55	1.44
13	5	-2	7.46	1.09
13	5	-2	4.66	1.24
12	5	-2	16.08	2.71
12	5	-2	19.60	1.76
12	5	-2	21.93	2.49
12	5	-2	19.70	1.19
11	5	-2	25.70	1.24
11	5	-2	26.41	2.18
11	5	-2	24.49	2.19
11	5	-2	25.63	1.79
10	5	-2	17.41	1.66
10	5	-2	16.33	1.87
10	5	-2	15.52	0.93

10	5	-2	17.84	1.40
9	5	-2	25.32	1.80
9	5	-2	22.50	1.60
9	5	-2	23.44	1.39
9	5	-2	20.23	1.90
9	5	-2	22.80	1.06
9	5	-2	22.84	1.05
8	5	-2	29.45	1.47
8	5	-2	31.60	1.22
8	5	-2	30.86	2.62
8	5	-2	32.95	1.51
8	5	-2	32.34	1.82
8	5	-2	29.25	1.71
8	5	-2	33.33	2.07
8	5	-2	30.96	1.23
7	5	-2	26.19	1.27
7	5	-2	24.49	1.43
7	5	-2	27.72	1.07
7	5	-2	30.24	2.19
7	5	-2	26.98	1.57
7	5	-2	27.51	1.23
7	5	-2	26.46	1.05
7	5	-2	26.61	1.86
6	5	-2	20.74	1.19
6	5	-2	21.77	1.59
6	5	-2	19.76	1.32
6	5	-2	21.61	1.00
6	5	-2	20.82	0.84
6	5	-2	20.85	1.79
6	5	-2	20.51	0.84
6	5	-2	21.16	1.02
5	5	-2	44.90	1.56
5	5	-2	42.77	1.44
5	5	-2	42.17	1.57
5	5	-2	38.87	2.05
5	5	-2	44.26	2.24
5	5	-2	45.15	1.90
5	5	-2	40.45	1.43
-5	-5	2	42.25	1.92
4	5	-2	48.67	1.97
4	5	-2	46.17	1.56
4	5	-2	49.19	1.65
4	5	-2	46.52	1.57
4	5	-2	51.37	2.28

-4	-5	2	47.11	1.59
-4	-5	2	46.34	1.96
-4	-5	2	49.14	2.04
3	5	-2	110.73	3.85
3	5	-2	105.46	3.25
3	5	-2	104.94	3.56
3	5	-2	99.36	3.19
3	5	-2	104.35	3.21
-3	-5	2	99.76	3.57
-3	-5	2	102.03	3.20
-3	-5	2	105.95	3.67
2	5	-2	61.64	3.51
2	5	-2	75.82	2.32
2	5	-2	72.28	2.61
2	5	-2	76.65	2.31
-2	-5	2	70.95	2.63
-2	-5	2	77.87	2.28
-2	-5	2	73.41	2.76
-2	-5	2	74.92	2.27
1	5	-2	6.57	0.81
1	5	-2	7.56	0.62
1	5	-2	7.37	0.35
-1	-5	2	7.69	0.30
-1	-5	2	8.27	0.65
-1	-5	2	6.92	0.78
-1	-5	2	8.11	0.35
0	5	-2	80.10	2.74
0	5	-2	81.06	2.51
0	5	-2	78.54	2.98
0	-5	2	77.78	2.75
0	-5	2	84.01	3.02
0	-5	2	82.56	2.49
-1	5	-2	53.61	2.17
-1	5	-2	54.86	1.88
1	-5	2	51.85	1.90
1	-5	2	51.73	1.68
1	-5	2	52.38	2.19
-2	5	-2	12.50	0.64
-2	5	-2	13.19	0.96
-2	5	-2	12.44	0.52
2	-5	2	12.52	0.43
2	-5	2	13.09	0.52
2	-5	2	13.31	0.71
2	-5	2	12.71	0.49

2	-5	2	11.18	0.94
-3	5	-2	3.14	0.25
-3	5	-2	2.86	0.62
-3	5	-2	3.01	0.33
3	-5	2	3.06	0.28
3	-5	2	2.48	0.54
3	-5	2	2.90	0.21
3	-5	2	2.67	0.46
3	-5	2	2.75	0.26
-4	5	-2	33.49	1.15
-4	5	-2	32.07	1.17
-4	5	-2	36.55	1.62
4	-5	2	34.20	1.13
4	-5	2	34.86	1.17
4	-5	2	31.55	1.34
4	-5	2	34.21	1.21
4	-5	2	29.97	1.64
-5	5	-2	17.87	1.15
-5	5	-2	17.43	0.72
-5	5	-2	17.16	0.69
5	-5	2	19.16	0.75
5	-5	2	18.97	1.30
5	-5	2	17.94	0.82
5	-5	2	18.33	0.79
-6	5	-2	6.05	0.81
-6	5	-2	5.24	0.34
-6	5	-2	5.54	0.39
6	-5	2	5.37	0.43
6	-5	2	5.83	0.43
6	-5	2	4.67	0.41
6	-5	2	5.93	0.93
6	-5	2	4.90	0.49
-7	5	-2	2.59	0.34
-7	5	-2	3.19	0.67
-7	5	-2	2.90	0.36
7	-5	2	2.65	0.78
7	-5	2	2.79	0.36
7	-5	2	2.85	0.38
7	-5	2	2.79	0.42
7	-5	2	2.40	0.41
-8	5	-2	2.90	0.39
-8	5	-2	2.93	0.45
-8	5	-2	2.74	0.67
8	-5	2	3.25	0.40

8	-5	2	1.71	0.73
8	-5	2	2.41	0.38
8	-5	2	2.45	0.46
8	-5	2	3.47	0.52
-9	5	-2	1.57	0.31
-9	5	-2	2.26	0.78
-9	5	-2	1.92	0.41
9	-5	2	1.39	0.84
9	-5	2	1.95	0.43
9	-5	2	1.63	0.38
9	-5	2	1.47	0.37
9	-5	2	1.76	0.52
-10	5	-2	1.16	0.33
-10	5	-2	0.74	0.51
10	-5	2	1.22	1.00
10	-5	2	1.32	0.54
-11	5	-2	-0.03	0.61
-11	5	-2	0.02	0.65
11	-5	2	0.59	1.13
11	-5	2	-0.18	0.53
-12	5	-2	2.97	0.54
12	-5	2	3.57	0.88
12	-5	2	3.16	1.54
12	-5	2	3.41	0.53
-13	5	-2	0.56	0.51
13	-5	2	0.55	0.81
13	-5	2	1.32	1.61
-14	5	-2	0.07	0.58
14	-5	2	1.65	1.95
14	-5	2	1.17	1.00
-15	5	-2	-0.69	0.63
15	-5	2	2.76	2.21
15	-5	2	-1.03	1.03
14	4	-2	10.61	1.19
14	4	-2	8.61	1.46
14	4	-2	8.76	1.99
13	4	-2	24.80	2.71
13	4	-2	27.71	2.08
13	4	-2	25.72	2.22
13	4	-2	26.88	1.41
12	4	-2	15.11	1.97
12	4	-2	14.98	2.14
12	4	-2	17.69	1.10
12	4	-2	20.00	1.70

11	4	-2	17.28	1.84
11	4	-2	19.73	1.98
11	4	-2	16.31	1.82
11	4	-2	17.79	1.49
11	4	-2	20.32	1.05
10	4	-2	49.82	1.82
10	4	-2	52.12	2.41
10	4	-2	48.49	2.55
10	4	-2	51.49	2.22
10	4	-2	49.10	2.48
9	4	-2	35.39	2.82
9	4	-2	39.15	1.97
9	4	-2	39.30	1.47
9	4	-2	38.39	1.98
9	4	-2	35.56	2.18
9	4	-2	38.55	1.76
8	4	-2	5.91	0.71
8	4	-2	6.35	0.93
8	4	-2	5.26	0.99
8	4	-2	6.55	1.02
8	4	-2	6.19	0.55
8	4	-2	5.17	1.27
8	4	-2	5.30	0.75
8	4	-2	6.29	0.52
7	4	-2	25.69	1.42
7	4	-2	25.52	1.62
7	4	-2	24.91	1.11
7	4	-2	25.02	0.98
7	4	-2	23.22	1.16
7	4	-2	23.55	0.96
7	4	-2	23.48	1.31
7	4	-2	26.21	1.91
6	4	-2	16.64	0.99
6	4	-2	15.30	1.29
6	4	-2	16.66	0.80
6	4	-2	16.04	0.68
6	4	-2	15.65	0.69
6	4	-2	16.51	1.46
6	4	-2	15.86	1.09
6	4	-2	15.80	0.85
5	4	-2	42.56	2.02
5	4	-2	39.49	1.92
5	4	-2	40.61	1.39
5	4	-2	41.93	1.52

5	4	-2	41.85	1.75
5	4	-2	41.59	1.39
5	4	-2	42.79	1.48
4	4	-2	122.18	3.68
4	4	-2	121.95	3.77
4	4	-2	115.62	3.83
4	4	-2	119.87	4.22
4	4	-2	122.94	4.01
4	4	-2	120.77	3.68
-4	-4	2	115.91	4.00
-4	-4	2	121.74	4.06
3	4	-2	140.43	4.19
3	4	-2	139.06	4.48
3	4	-2	138.01	4.18
3	4	-2	139.11	4.66
3	4	-2	141.13	4.21
-3	-4	2	134.35	4.53
-3	-4	2	131.33	4.48
2	4	-2	18.09	0.64
2	4	-2	18.98	1.09
2	4	-2	18.48	0.89
-2	-4	2	17.58	0.63
-2	-4	2	17.96	0.62
-2	-4	2	16.72	0.89
-2	-4	2	17.28	1.02
1	4	-2	58.29	1.74
1	4	-2	59.11	2.18
1	4	-2	56.90	1.99
1	4	-2	56.09	1.78
-1	-4	2	58.04	2.03
-1	-4	2	57.68	1.76
-1	-4	2	49.30	2.27
0	4	-2	53.44	1.84
0	4	-2	52.02	2.03
0	4	-2	53.98	1.67
0	-4	2	53.83	1.87
0	-4	2	49.71	2.03
0	-4	2	53.58	1.66
-1	4	-2	10.38	0.42
-1	4	-2	10.89	0.76
-1	4	-2	10.51	0.54
1	-4	2	9.01	0.56
1	-4	2	8.32	0.88
1	-4	2	10.23	0.42

-2	4	-2	0.60	0.32
-2	4	-2	0.70	0.17
-2	4	-2	0.79	0.14
2	-4	2	0.63	0.18
2	-4	2	0.71	0.12
2	-4	2	0.41	0.13
2	-4	2	0.70	0.12
2	-4	2	2.00	0.45
-3	4	-2	35.34	1.18
-3	4	-2	37.34	1.21
-3	4	-2	33.84	1.51
3	-4	2	35.99	1.20
3	-4	2	34.74	1.15
3	-4	2	35.40	1.60
3	-4	2	33.75	1.24
3	-4	2	35.93	1.19
-4	4	-2	50.19	1.92
-4	4	-2	51.23	1.54
-4	4	-2	43.17	1.53
4	-4	2	47.15	1.54
4	-4	2	43.82	1.99
4	-4	2	47.46	1.58
4	-4	2	46.66	1.60
4	-4	2	46.12	1.55
-5	4	-2	6.55	0.38
-5	4	-2	6.49	0.71
-5	4	-2	6.41	0.36
5	-4	2	5.99	0.40
5	-4	2	6.20	0.80
5	-4	2	6.77	0.39
5	-4	2	5.94	0.47
5	-4	2	6.54	0.40
-6	4	-2	11.21	0.89
-6	4	-2	10.39	0.52
-6	4	-2	9.95	0.52
6	-4	2	10.44	0.57
6	-4	2	10.79	0.65
6	-4	2	11.12	0.56
6	-4	2	10.21	0.51
6	-4	2	10.08	1.01
-7	4	-2	5.83	0.79
-7	4	-2	5.01	0.41
-7	4	-2	6.00	0.50
7	-4	2	6.67	0.48

7	-4	2	6.49	0.41
7	-4	2	5.11	0.50
7	-4	2	5.89	0.94
7	-4	2	5.42	0.57
-8	4	-2	1.79	0.39
-8	4	-2	0.25	0.55
-8	4	-2	0.72	0.24
8	-4	2	1.26	0.37
8	-4	2	0.91	0.34
8	-4	2	1.07	0.32
8	-4	2	1.06	0.73
-9	4	-2	0.53	0.26
-9	4	-2	0.72	0.60
-9	4	-2	0.68	0.43
9	-4	2	0.19	0.37
9	-4	2	1.12	0.30
9	-4	2	-0.03	0.38
9	-4	2	0.71	0.34
9	-4	2	0.81	0.80
-10	4	-2	4.63	0.56
-10	4	-2	4.28	0.69
10	-4	2	5.23	1.22
10	-4	2	4.96	0.83
10	-4	2	5.89	0.57
-11	4	-2	3.15	0.75
-11	4	-2	4.68	0.85
11	-4	2	3.30	1.43
11	-4	2	4.65	0.59
-12	4	-2	0.48	0.41
12	-4	2	0.29	0.37
12	-4	2	0.12	0.64
12	-4	2	-0.20	1.27
12	-4	2	0.36	0.59
-13	4	-2	0.58	0.50
13	-4	2	2.24	1.67
13	-4	2	1.72	0.86
-14	4	-2	2.16	0.69
14	-4	2	4.74	1.19
14	-4	2	1.20	1.93
-15	4	-2	2.50	0.72
15	-4	2	5.24	2.22
15	-4	2	1.82	1.18
14	3	-2	12.90	2.07
14	3	-2	14.07	1.93

13	3	-2	14.08	2.31
13	3	-2	18.50	1.77
13	3	-2	16.00	1.70
-13	-3	2	16.92	1.36
12	3	-2	23.58	1.70
12	3	-2	24.07	2.17
12	3	-2	21.63	1.83
12	3	-2	22.64	1.83
-12	-3	2	20.88	1.30
11	3	-2	24.27	1.83
11	3	-2	24.10	1.96
11	3	-2	24.46	1.59
11	3	-2	26.19	1.73
-11	-3	2	23.73	1.53
10	3	-2	15.76	2.37
10	3	-2	15.29	1.35
10	3	-2	14.97	1.46
10	3	-2	14.41	1.18
10	3	-2	14.52	1.52
-10	-3	2	14.65	0.92
9	3	-2	14.39	0.77
9	3	-2	14.59	1.20
9	3	-2	11.99	1.35
9	3	-2	12.80	1.93
9	3	-2	11.27	0.90
9	3	-2	13.24	1.00
9	3	-2	12.45	1.26
-9	-3	2	14.24	0.81
8	3	-2	42.28	1.82
8	3	-2	38.06	2.31
8	3	-2	39.38	1.83
8	3	-2	39.29	1.66
8	3	-2	37.30	1.99
8	3	-2	41.82	1.58
8	3	-2	39.88	1.43
-8	-3	2	38.31	1.47
7	3	-2	31.48	1.73
7	3	-2	33.95	1.33
7	3	-2	34.79	1.55
7	3	-2	34.30	1.53
7	3	-2	33.67	1.99
7	3	-2	33.44	1.41
7	3	-2	35.22	1.23
-7	-3	2	32.95	1.26

6	3	-2	19.35	1.07
6	3	-2	19.46	0.93
6	3	-2	21.73	1.41
6	3	-2	19.07	1.25
6	3	-2	19.91	0.86
6	3	-2	19.35	1.01
6	3	-2	19.19	0.77
-6	-3	2	17.52	0.80
5	3	-2	103.73	3.19
5	3	-2	101.64	3.35
5	3	-2	102.62	3.14
5	3	-2	103.32	3.64
5	3	-2	101.52	3.27
5	3	-2	100.71	3.13
5	3	-2	93.76	3.57
5	3	-2	102.19	3.41
4	3	-2	23.48	0.82
4	3	-2	24.11	0.87
4	3	-2	24.38	0.86
4	3	-2	23.83	1.24
4	3	-2	23.20	0.92
4	3	-2	22.66	1.07
-4	-3	2	23.06	1.08
-4	-3	2	23.15	1.11
3	3	-2	85.14	2.65
3	3	-2	84.83	2.59
3	3	-2	87.02	2.61
3	3	-2	80.96	2.92
3	3	-2	85.20	2.60
3	3	-2	86.03	2.82
-3	-3	2	80.76	2.83
2	3	-2	77.12	2.35
2	3	-2	75.42	2.54
2	3	-2	77.48	2.66
2	3	-2	76.80	2.33
2	3	-2	78.49	2.37
-2	-3	2	71.22	2.58
-2	-3	2	76.94	2.34
-2	-3	2	78.19	2.61
1	3	-2	54.94	1.66
1	3	-2	53.05	1.68
1	3	-2	55.03	1.63
1	3	-2	54.09	1.95
1	3	-2	55.76	1.85

-1	-3	2	51.46	1.92
-1	-3	2	54.39	1.67
-1	-3	2	53.52	1.85
0	3	-2	3.26	0.45
0	3	-2	3.08	0.21
0	3	-2	3.18	0.31
0	-3	2	2.94	0.32
0	-3	2	3.62	0.22
0	-3	2	3.15	0.45
-1	3	-2	247.43	7.51
-1	3	-2	245.56	7.77
-1	3	-2	271.08	7.60
-1	3	-2	237.48	7.46
1	-3	2	242.84	7.47
1	-3	2	250.20	7.61
1	-3	2	270.14	7.84
-2	3	-2	109.51	3.48
-2	3	-2	106.63	3.42
-2	3	-2	130.56	3.77
-2	3	-2	108.00	3.46
2	-3	2	110.59	3.43
2	-3	2	113.95	3.46
2	-3	2	118.05	3.52
-3	3	-2	24.46	0.84
-3	3	-2	24.02	0.83
-3	3	-2	23.97	0.85
-3	3	-2	25.18	1.12
3	-3	2	26.29	1.21
3	-3	2	23.57	0.85
3	-3	2	23.39	0.84
3	-3	2	24.07	0.86
-4	3	-2	20.77	0.75
-4	3	-2	21.86	1.07
-4	3	-2	20.86	0.76
-4	3	-2	19.38	0.75
4	-3	2	21.61	0.77
4	-3	2	19.96	0.77
4	-3	2	20.47	0.75
4	-3	2	18.33	1.10
-5	3	-2	0.51	0.18
-5	3	-2	0.72	0.32
-5	3	-2	0.50	0.18
-5	3	-2	0.49	0.16
5	-3	2	0.53	0.16

5	-3	2	0.59	0.15
5	-3	2	0.51	0.19
5	-3	2	0.76	0.42
-6	3	-2	22.07	0.87
-6	3	-2	23.13	1.20
-6	3	-2	21.85	0.90
-6	3	-2	22.96	0.95
6	-3	2	23.75	0.90
6	-3	2	20.96	0.85
6	-3	2	23.62	0.94
-7	3	-2	0.51	0.23
-7	3	-2	0.31	0.42
-7	3	-2	0.63	0.34
7	-3	2	0.88	0.31
7	-3	2	0.99	0.25
7	-3	2	0.98	0.32
7	-3	2	1.05	0.26
7	-3	2	1.80	0.63
-8	3	-2	1.28	0.43
-8	3	-2	0.41	0.49
-8	3	-2	0.53	0.25
8	-3	2	0.89	0.34
8	-3	2	0.22	0.60
8	-3	2	0.16	0.25
8	-3	2	0.19	0.26
8	-3	2	0.44	0.37
-9	3	-2	6.09	0.97
-9	3	-2	5.49	0.93
-9	3	-2	5.02	0.52
9	-3	2	5.59	0.73
9	-3	2	4.10	0.50
9	-3	2	6.29	0.76
9	-3	2	6.68	1.20
9	-3	2	4.85	0.59
-10	3	-2	-0.23	0.76
-10	3	-2	0.58	0.32
10	-3	2	0.84	0.95
10	-3	2	-0.04	0.47
10	-3	2	0.59	0.32
-11	3	-2	-0.04	0.47
11	-3	2	0.27	1.77
11	-3	2	0.36	0.57
11	-3	2	0.03	0.38
-12	3	-2	2.81	0.54

12	-3	2	2.61	0.48
12	-3	2	4.07	0.75
12	-3	2	2.45	1.36
12	-3	2	2.54	0.78
-13	3	-2	3.13	0.63
13	-3	2	2.23	1.62
13	-3	2	1.38	0.86
13	-3	2	2.54	0.54
-14	3	-2	0.59	0.63
14	-3	2	2.33	1.01
14	-3	2	3.44	1.90
-15	3	-2	0.81	0.72
15	-3	2	3.79	2.15
15	-3	2	1.98	1.18
15	2	-2	8.45	1.50
15	2	-2	9.39	2.23
-15	-2	2	10.09	1.49
14	2	-2	18.75	1.94
14	2	-2	22.19	2.80
-14	-2	2	22.52	1.52
13	2	-2	26.18	1.78
13	2	-2	25.36	2.44
13	2	-2	24.28	1.84
-13	-2	2	23.63	1.42
12	2	-2	11.42	1.34
12	2	-2	10.00	1.41
12	2	-2	8.83	1.36
-12	-2	2	9.57	0.96
11	2	-2	9.26	1.17
11	2	-2	7.19	1.06
11	2	-2	6.82	1.21
11	2	-2	7.42	1.23
-11	-2	2	6.71	0.76
10	2	-2	19.59	1.29
10	2	-2	18.76	1.26
10	2	-2	18.98	1.54
10	2	-2	18.49	1.39
10	2	-2	15.13	2.06
-10	-2	2	18.95	0.97
9	2	-2	49.75	2.21
9	2	-2	49.17	1.89
9	2	-2	50.72	1.75
9	2	-2	49.68	2.03
9	2	-2	49.68	2.04

9	2	-2	48.80	2.58
9	2	-2	49.33	2.23
-9	-2	2	48.83	1.78
8	2	-2	30.40	1.26
8	2	-2	29.92	1.88
8	2	-2	33.18	1.57
8	2	-2	29.51	1.38
8	2	-2	30.06	1.58
8	2	-2	31.99	1.16
8	2	-2	32.13	1.44
-8	-2	2	28.07	1.17
7	2	-2	27.85	1.01
7	2	-2	25.14	1.59
7	2	-2	26.06	1.45
7	2	-2	27.19	1.09
7	2	-2	25.69	1.18
7	2	-2	27.90	1.31
7	2	-2	27.18	1.25
-7	-2	2	26.21	1.03
6	2	-2	21.45	1.21
6	2	-2	21.78	0.84
6	2	-2	22.95	0.99
6	2	-2	23.25	1.07
6	2	-2	23.21	0.90
6	2	-2	19.91	1.17
6	2	-2	21.27	1.31
-6	-2	2	22.29	0.87
5	2	-2	10.36	0.47
5	2	-2	9.56	0.49
5	2	-2	9.99	0.88
5	2	-2	10.26	0.59
5	2	-2	10.39	0.67
5	2	-2	10.57	0.64
-5	-2	2	9.18	0.48
-5	-2	2	10.36	0.69
4	2	-2	28.12	1.05
4	2	-2	28.65	0.95
4	2	-2	29.67	1.26
4	2	-2	27.68	1.12
4	2	-2	27.71	0.97
-4	-2	2	27.05	0.97
-4	-2	2	27.01	1.18
3	2	-2	36.25	1.32
3	2	-2	36.36	1.23

3	2	-2	35.91	1.17
3	2	-2	35.98	1.39
3	2	-2	36.35	1.18
-3	-2	2	36.41	1.32
-3	-2	2	35.78	1.34
2	2	-2	99.17	3.16
2	2	-2	99.65	3.06
2	2	-2	99.98	3.03
2	2	-2	100.10	3.21
-2	-2	2	102.70	3.18
-2	-2	2	98.49	3.18
-2	-2	2	100.32	3.05
-2	-2	2	101.32	3.03
1	2	-2	23.57	0.80
1	2	-2	25.25	0.96
1	2	-2	23.90	0.90
1	2	-2	25.34	0.81
-1	-2	2	24.86	0.95
-1	-2	2	25.23	0.80
-1	-2	2	24.83	0.79
-1	-2	2	24.57	0.91
0	2	-2	45.80	1.46
0	2	-2	45.28	1.54
0	2	-2	46.89	1.38
0	2	-2	42.89	1.40
0	-2	2	44.20	1.40
0	-2	2	45.11	1.39
0	-2	2	42.83	1.46
0	-2	2	45.89	1.56
-1	2	-2	4.84	0.39
-1	2	-2	5.58	0.27
-1	2	-2	4.76	0.24
-1	2	-2	5.01	0.22
1	-2	2	4.93	0.41
1	-2	2	5.19	0.28
1	-2	2	4.94	0.23
1	-2	2	5.01	0.24
-2	2	-2	4.14	0.40
-2	2	-2	3.71	0.22
-2	2	-2	4.12	0.23
-2	2	-2	3.72	0.24
2	-2	2	3.89	0.24
2	-2	2	3.89	0.43
2	-2	2	3.67	0.23

2	-2	2	3.86	0.24
-3	2	-2	3.48	0.27
-3	2	-2	3.44	0.24
-3	2	-2	2.86	0.40
-3	2	-2	3.79	0.26
3	-2	2	3.61	0.25
3	-2	2	3.38	0.24
3	-2	2	2.95	0.49
3	-2	2	3.94	0.27
-4	2	-2	6.98	0.41
-4	2	-2	6.20	0.36
-4	2	-2	6.60	0.34
-4	2	-2	6.80	0.57
4	-2	2	6.42	0.44
4	-2	2	5.87	0.41
4	-2	2	5.76	0.62
4	-2	2	6.19	0.36
-5	2	-2	23.11	0.96
-5	2	-2	22.43	0.88
-5	2	-2	22.07	0.84
-5	2	-2	24.36	1.11
5	-2	2	24.43	0.87
5	-2	2	22.78	0.91
5	-2	2	20.88	0.86
-6	2	-2	25.57	1.11
-6	2	-2	23.17	0.92
-6	2	-2	25.17	1.20
-6	2	-2	23.08	0.96
6	-2	2	25.43	0.94
6	-2	2	24.50	1.39
6	-2	2	23.58	0.97
6	-2	2	24.85	1.02
-7	2	-2	0.51	0.41
-7	2	-2	0.47	0.38
-7	2	-2	-0.02	0.20
-7	2	-2	0.20	0.23
7	-2	2	-0.09	0.22
7	-2	2	0.46	0.28
7	-2	2	0.26	0.25
7	-2	2	-0.14	0.48
-8	2	-2	5.49	0.87
-8	2	-2	6.18	0.50
8	-2	2	6.32	0.52
8	-2	2	6.30	1.14

8	-2	2	6.82	0.71
8	-2	2	6.98	0.57
8	-2	2	6.77	0.68
-9	2	-2	1.58	0.65
-9	2	-2	0.33	0.28
-9	2	-2	-0.94	0.58
9	-2	2	0.21	0.40
9	-2	2	0.75	0.35
9	-2	2	0.09	0.44
9	-2	2	-0.41	0.31
9	-2	2	-0.20	0.70
-10	2	-2	2.14	0.38
-10	2	-2	3.86	0.89
10	-2	2	2.96	1.06
10	-2	2	2.17	0.59
10	-2	2	2.73	0.45
-11	2	-2	2.16	0.45
11	-2	2	1.15	0.60
11	-2	2	1.53	0.47
11	-2	2	1.39	1.14
11	-2	2	2.68	0.70
-12	2	-2	0.56	0.48
12	-2	2	0.71	0.70
12	-2	2	0.11	0.46
12	-2	2	0.17	1.28
-13	2	-2	1.57	0.58
13	-2	2	1.97	1.67
13	-2	2	1.03	0.58
13	-2	2	1.75	0.93
-14	2	-2	4.16	0.74
14	-2	2	6.24	2.02
14	-2	2	4.87	1.17
-15	2	-2	1.25	0.77
15	-2	2	0.98	2.26
15	-2	2	0.26	1.26
15	1	-2	17.19	3.15
15	1	-2	16.02	1.95
-15	-1	2	20.83	1.52
14	1	-2	15.57	1.71
14	1	-2	18.20	2.65
-14	-1	2	15.31	1.29
13	1	-2	8.25	1.16
13	1	-2	11.89	2.07
13	1	-2	10.72	1.45

-13	-1	2	11.33	1.05
12	1	-2	16.23	1.39
12	1	-2	15.82	1.87
12	1	-2	14.68	1.28
12	1	-2	18.20	1.31
-12	-1	2	15.73	1.04
11	1	-2	28.48	1.60
11	1	-2	28.03	1.49
11	1	-2	27.90	1.56
11	1	-2	28.43	2.48
11	1	-2	28.02	1.97
-11	-1	2	29.86	1.28
10	1	-2	32.11	1.60
10	1	-2	34.29	1.52
10	1	-2	29.59	2.09
10	1	-2	30.60	1.87
10	1	-2	31.39	1.30
10	1	-2	32.06	1.58
-10	-1	2	33.31	1.31
9	1	-2	30.32	1.31
9	1	-2	30.10	1.68
9	1	-2	31.19	1.55
9	1	-2	27.65	1.85
9	1	-2	32.51	1.50
9	1	-2	31.85	1.22
9	1	-2	33.80	1.41
-9	-1	2	32.36	1.25
8	1	-2	46.17	2.16
8	1	-2	46.34	1.94
8	1	-2	47.20	1.69
8	1	-2	47.08	1.62
8	1	-2	47.20	2.00
8	1	-2	46.62	1.85
-8	-1	2	46.76	1.64
7	1	-2	20.13	1.02
7	1	-2	20.66	1.30
7	1	-2	23.75	1.17
7	1	-2	22.28	1.02
7	1	-2	21.97	0.91
7	1	-2	21.34	1.19
7	1	-2	22.81	0.86
-7	-1	2	20.56	0.86
6	1	-2	39.07	1.67
6	1	-2	42.06	1.39

6	1	-2	40.52	1.54
6	1	-2	43.49	1.63
6	1	-2	43.21	1.44
6	1	-2	37.06	1.75
6	1	-2	41.67	1.52
-6	-1	2	41.46	1.40
5	1	-2	20.97	0.81
5	1	-2	18.18	1.03
5	1	-2	21.13	0.91
5	1	-2	22.43	0.78
5	1	-2	23.06	0.97
5	1	-2	21.09	0.89
-5	-1	2	22.04	0.92
-5	-1	2	21.71	0.80
4	1	-2	56.12	1.86
4	1	-2	52.19	1.94
4	1	-2	56.55	1.76
4	1	-2	55.55	1.87
4	1	-2	56.89	1.87
-4	-1	2	57.80	1.84
-4	-1	2	55.39	1.87
-4	-1	2	55.93	1.77
3	1	-2	105.00	3.32
3	1	-2	109.31	3.26
3	1	-2	104.72	3.21
3	1	-2	107.47	3.28
3	1	-2	104.80	3.28
-3	-1	2	105.06	3.30
-3	-1	2	103.36	3.22
-3	-1	2	106.17	3.26
2	1	-2	113.29	3.37
2	1	-2	111.58	3.36
2	1	-2	114.01	3.36
2	1	-2	110.35	3.30
-2	-1	2	113.34	3.35
-2	-1	2	113.04	3.30
-2	-1	2	94.07	3.32
-2	-1	2	109.93	3.38
1	1	-2	67.91	2.01
1	1	-2	68.23	2.03
1	1	-2	64.16	2.02
1	1	-2	59.12	1.98
-1	-1	2	66.37	1.98
-1	-1	2	65.67	2.01

0	1	-2	41.37	1.35
0	1	-2	47.11	1.34
0	1	-2	41.84	1.33
0	-1	2	41.27	1.34
0	-1	2	38.91	1.33
-1	1	-2	41.04	1.37
-1	1	-2	42.41	1.35
-1	1	-2	45.33	1.45
-1	1	-2	44.15	1.39
1	-1	2	44.77	1.39
1	-1	2	43.30	1.38
1	-1	2	42.35	1.47
1	-1	2	43.15	1.35
-2	1	-2	28.60	0.98
-2	1	-2	30.16	1.04
-2	1	-2	28.44	1.01
-2	1	-2	30.91	1.12
2	-1	2	29.52	0.98
2	-1	2	31.53	1.19
2	-1	2	29.93	1.03
2	-1	2	31.86	1.03
-3	1	-2	14.08	0.55
-3	1	-2	14.74	0.71
-3	1	-2	13.79	0.58
-3	1	-2	15.26	0.66
3	-1	2	14.51	0.60
3	-1	2	14.75	0.61
3	-1	2	14.66	0.55
3	-1	2	14.52	0.81
-4	1	-2	20.32	0.80
-4	1	-2	19.89	0.76
-4	1	-2	21.62	0.97
-4	1	-2	21.30	0.92
4	-1	2	20.80	1.08
4	-1	2	22.33	0.85
4	-1	2	21.12	0.84
-5	1	-2	5.62	0.58
-5	1	-2	4.12	0.32
-5	1	-2	4.02	0.55
-5	1	-2	4.38	0.38
5	-1	2	4.89	0.34
5	-1	2	4.97	0.43
5	-1	2	5.27	0.43
5	-1	2	6.17	0.73

-6	1	-2	3.87	0.64
-6	1	-2	4.22	0.60
-6	1	-2	2.73	0.32
-6	1	-2	3.51	0.38
6	-1	2	2.42	0.55
6	-1	2	4.18	0.44
6	-1	2	2.82	0.45
6	-1	2	3.84	0.37
-7	1	-2	0.48	0.25
-7	1	-2	-0.26	0.41
-7	1	-2	-0.28	0.46
-7	1	-2	0.44	0.22
7	-1	2	0.38	0.28
7	-1	2	-0.33	0.22
7	-1	2	-0.32	0.27
7	-1	2	0.38	0.56
-8	1	-2	1.72	0.31
-8	1	-2	1.84	0.36
-8	1	-2	1.04	0.55
-8	1	-2	1.54	0.65
8	-1	2	2.07	0.38
8	-1	2	1.77	0.32
8	-1	2	0.75	0.39
8	-1	2	2.03	0.76
-9	1	-2	21.14	0.93
-9	1	-2	19.10	1.68
-9	1	-2	21.59	1.59
9	-1	2	17.53	1.63
9	-1	2	17.92	1.16
9	-1	2	21.13	1.02
9	-1	2	20.12	0.97
-10	1	-2	-0.29	0.32
10	-1	2	0.59	0.60
10	-1	2	1.14	0.60
10	-1	2	0.09	0.41
10	-1	2	-0.27	0.85
-11	1	-2	-0.17	0.39
11	-1	2	0.05	0.44
11	-1	2	-0.32	1.10
11	-1	2	-0.32	0.59
11	-1	2	-1.09	0.76
-12	1	-2	8.64	0.91
12	-1	2	9.62	0.99
12	-1	2	12.78	2.28

12	-1	2	12.33	1.38
-13	1	-2	6.80	0.97
13	-1	2	4.23	0.99
13	-1	2	3.34	1.80
13	-1	2	5.03	0.73
-14	1	-2	2.35	0.77
14	-1	2	0.09	1.00
14	-1	2	4.23	1.98
16	0	-2	13.68	2.64
16	0	-2	18.32	2.31
-16	0	2	13.30	1.53
15	0	-2	7.32	1.36
15	0	-2	4.00	1.95
-15	0	2	5.25	0.87
14	0	-2	19.01	2.63
14	0	-2	20.09	1.86
-14	0	2	23.72	1.39
13	0	-2	14.60	2.10
13	0	-2	15.64	1.06
13	0	-2	16.44	1.53
-13	0	2	15.79	1.08
12	0	-2	2.23	1.01
12	0	-2	3.86	0.63
12	0	-2	3.50	0.60
12	0	-2	3.61	0.82
-12	0	2	2.67	0.50
11	0	-2	27.20	1.33
11	0	-2	27.50	1.93
11	0	-2	27.55	1.30
11	0	-2	24.64	2.07
11	0	-2	27.23	1.53
-11	0	2	26.54	1.20
10	0	-2	28.66	1.30
10	0	-2	29.82	1.21
10	0	-2	28.83	1.47
10	0	-2	29.00	1.34
10	0	-2	27.11	1.31
10	0	-2	29.06	1.78
-10	0	2	28.44	1.19
-10	0	2	31.07	1.63
9	0	-2	35.01	1.82
9	0	-2	36.42	1.43
9	0	-2	36.11	1.35
9	0	-2	36.09	1.57

9	0	-2	36.72	1.45
9	0	-2	36.88	1.62
-9	0	2	40.13	1.73
-9	0	2	34.48	1.36
8	0	-2	19.45	0.89
8	0	-2	18.04	1.05
8	0	-2	18.99	0.88
8	0	-2	19.43	1.19
8	0	-2	17.85	1.01
8	0	-2	19.74	0.82
-8	0	2	18.51	0.81
-8	0	2	20.40	1.11
7	0	-2	0.09	0.23
7	0	-2	0.07	0.18
7	0	-2	0.38	0.33
7	0	-2	0.77	0.36
7	0	-2	0.26	0.27
7	0	-2	0.79	0.23
-7	0	2	0.50	0.32
-7	0	2	0.38	0.21
6	0	-2	2.14	0.29
6	0	-2	1.74	0.31
6	0	-2	2.39	0.27
6	0	-2	1.49	0.26
6	0	-2	1.53	0.34
6	0	-2	1.94	0.38
-6	0	2	1.87	0.26
-6	0	2	2.57	0.38
5	0	-2	7.73	0.41
5	0	-2	7.59	0.49
-5	0	2	6.57	0.53
-5	0	2	6.86	0.42
-5	0	2	6.94	0.37
-5	0	2	7.55	0.49
4	0	-2	4.36	0.27
4	0	-2	4.26	0.35
4	0	-2	3.90	0.36
4	0	-2	4.24	0.24
-4	0	2	3.96	0.30
-4	0	2	4.04	0.26
-4	0	2	4.03	0.32
-4	0	2	4.51	0.33
3	0	-2	23.35	0.88
3	0	-2	23.06	0.84

3	0	-2	23.73	0.80
3	0	-2	23.91	0.79
-3	0	2	25.53	0.83
-3	0	2	24.36	0.87
-3	0	2	23.15	0.81
-3	0	2	23.40	0.79
2	0	-2	13.57	0.46
2	0	-2	13.69	0.48
2	0	-2	13.31	0.47
2	0	-2	12.90	0.53
2	0	-2	13.56	0.47
-2	0	2	13.43	0.48
-2	0	2	13.22	0.53
-2	0	2	13.96	0.46
1	0	-2	11.00	0.39
1	0	-2	10.12	0.44
1	0	-2	11.76	0.41
-1	0	2	10.59	0.40
-1	0	2	10.99	0.45
-1	0	2	10.80	0.37
0	0	-2	42.41	1.29
0	0	-2	38.55	1.30
0	0	-2	40.21	1.29
0	0	2	38.10	1.27
0	0	2	40.42	1.31
0	0	2	38.79	1.22
-1	0	-2	145.28	4.45
-1	0	-2	141.01	4.49
-1	0	-2	158.83	4.52
-1	0	-2	144.01	4.39
1	0	2	137.23	4.46
1	0	2	155.17	4.53
1	0	2	146.36	4.39
-2	0	-2	71.99	2.33
-2	0	-2	69.88	2.17
-2	0	-2	74.53	2.35
2	0	2	69.88	2.17
2	0	2	70.47	2.25
2	0	2	67.77	2.27
-3	0	-2	7.81	0.53
-3	0	-2	6.38	0.52
-3	0	-2	6.34	0.38
-3	0	-2	6.47	0.33
3	0	2	7.44	0.43

3	0	2	7.36	0.56
3	0	2	6.27	0.32
-4	0	-2	2.04	0.39
-4	0	-2	1.37	0.22
-4	0	-2	1.63	0.24
-4	0	-2	1.43	0.34
4	0	2	1.76	0.31
4	0	2	1.97	0.42
4	0	2	1.57	0.27
-5	0	-2	1.33	0.39
-5	0	-2	0.89	0.18
-5	0	-2	0.96	0.22
-5	0	-2	1.19	0.43
5	0	2	0.56	0.38
5	0	2	0.69	0.27
5	0	2	0.60	0.25
5	0	2	0.61	0.18
-6	0	-2	1.88	0.51
-6	0	-2	1.72	0.30
-6	0	-2	1.46	0.23
-6	0	-2	1.67	0.47
6	0	2	1.77	0.36
6	0	2	1.78	0.35
6	0	2	2.07	0.29
6	0	2	1.82	0.56
-7	0	-2	3.39	0.63
-7	0	-2	2.26	0.36
-7	0	-2	2.05	0.67
-7	0	-2	2.16	0.32
7	0	2	2.52	0.43
7	0	2	3.97	0.57
7	0	2	3.11	0.74
7	0	2	2.90	0.42
-8	0	-2	4.42	0.89
-8	0	-2	2.55	0.40
-8	0	-2	3.05	0.45
-8	0	-2	2.46	0.72
8	0	2	2.04	0.76
8	0	2	3.38	0.42
8	0	2	2.99	0.53
8	0	2	3.57	0.52
-9	0	-2	7.82	0.61
-9	0	-2	8.07	1.13
-9	0	-2	6.22	0.66

9	0	2	4.49	0.95
9	0	2	6.01	0.75
9	0	2	7.70	0.66
-10	0	-2	6.52	0.68
10	0	2	6.65	0.97
10	0	2	5.61	1.93
10	0	2	6.19	0.83
10	0	2	6.91	1.23
-11	0	-2	1.40	0.47
11	0	2	1.11	0.57
11	0	2	-0.12	1.14
11	0	2	0.99	0.69
11	0	2	1.09	0.92
-12	0	-2	2.79	0.58
12	0	2	3.57	1.57
12	0	2	3.16	0.68
12	0	2	1.33	0.80
-13	0	-2	3.74	0.73
13	0	2	1.05	1.58
13	0	2	2.21	0.80
13	0	2	4.17	1.04
-14	0	-2	2.22	0.80
14	0	2	2.57	1.17
14	0	2	4.43	2.02
16	-1	-2	8.13	1.42
16	-1	-2	13.42	2.51
-16	1	2	10.72	1.40
15	-1	-2	14.44	1.86
15	-1	-2	13.44	2.29
-15	1	2	15.05	1.28
14	-1	-2	13.63	2.39
14	-1	-2	13.12	1.57
-14	1	2	13.11	1.12
13	-1	-2	8.60	1.33
13	-1	-2	7.34	0.78
13	-1	-2	8.26	1.82
13	-1	-2	9.99	0.88
-13	1	2	7.35	0.86
12	-1	-2	17.86	1.94
12	-1	-2	16.82	1.32
12	-1	-2	17.04	0.94
12	-1	-2	17.51	0.97
-12	1	2	14.81	1.03
11	-1	-2	47.76	1.77

11	-1	-2	45.74	1.69
11	-1	-2	47.04	2.50
11	-1	-2	43.86	2.05
-11	1	2	47.91	1.75
-11	1	2	47.23	2.17
10	-1	-2	44.43	2.24
10	-1	-2	43.09	1.72
10	-1	-2	46.80	1.62
10	-1	-2	45.64	1.68
10	-1	-2	45.33	1.65
10	-1	-2	44.03	1.92
-10	1	2	44.18	1.64
-10	1	2	46.58	1.99
9	-1	-2	20.67	0.88
9	-1	-2	20.40	0.90
9	-1	-2	20.48	0.86
9	-1	-2	17.88	1.33
9	-1	-2	18.93	1.10
9	-1	-2	20.67	0.94
-9	1	2	18.43	0.87
-9	1	2	21.83	1.18
8	-1	-2	22.60	1.32
8	-1	-2	22.34	1.12
8	-1	-2	22.88	0.97
8	-1	-2	24.43	0.92
8	-1	-2	22.94	0.95
8	-1	-2	23.64	0.89
-8	1	2	21.87	0.92
-8	1	2	24.21	1.16
7	-1	-2	16.07	0.64
7	-1	-2	15.64	0.85
7	-1	-2	15.10	1.00
7	-1	-2	14.96	0.71
7	-1	-2	16.45	0.68
7	-1	-2	16.70	0.73
-7	1	2	16.93	0.88
-7	1	2	15.87	0.68
6	-1	-2	30.56	1.13
6	-1	-2	32.04	1.05
6	-1	-2	31.01	1.25
6	-1	-2	32.41	1.10
6	-1	-2	31.76	1.38
-6	1	2	30.82	1.10
-6	1	2	33.10	1.23

-6	1	2	32.03	1.26
5	-1	-2	0.48	0.09
5	-1	-2	0.12	0.24
5	-1	-2	0.33	0.19
5	-1	-2	0.39	0.12
-5	1	2	0.45	0.12
-5	1	2	0.51	0.20
-5	1	2	0.34	0.13
-5	1	2	0.72	0.24
4	-1	-2	10.21	0.58
4	-1	-2	10.75	0.36
4	-1	-2	10.17	0.41
-4	1	2	10.06	0.43
-4	1	2	10.79	0.52
-4	1	2	10.27	0.54
3	-1	-2	4.77	0.23
3	-1	-2	4.99	0.22
3	-1	-2	4.98	0.35
3	-1	-2	4.30	0.38
-3	1	2	4.93	0.24
-3	1	2	4.61	0.35
-3	1	2	5.31	0.21
-3	1	2	4.79	0.33
2	-1	-2	75.07	2.40
2	-1	-2	73.75	2.38
2	-1	-2	77.64	2.31
2	-1	-2	73.06	2.26
-2	1	2	74.16	2.29
-2	1	2	73.65	2.37
-2	1	2	73.87	2.26
-2	1	2	78.04	2.40
1	-1	-2	2.19	0.26
1	-1	-2	1.46	0.09
1	-1	-2	2.10	0.21
1	-1	-2	1.51	0.21
-1	1	2	1.89	0.18
-1	1	2	1.82	0.09
-1	1	2	1.80	0.27
-1	1	2	1.85	0.23
0	-1	-2	89.02	2.82
0	-1	-2	85.50	2.69
0	-1	-2	94.91	2.87
0	-1	-2	90.16	2.88
0	1	2	91.25	2.84

0	1	2	90.48	2.91
0	1	2	88.72	2.69
0	1	2	91.29	2.84
-1	-1	-2	11.09	0.50
-1	-1	-2	11.25	0.40
-1	-1	-2	11.21	0.62
1	1	2	11.30	0.40
1	1	2	11.88	0.68
1	1	2	11.10	0.53
1	1	2	11.29	0.58
1	1	2	12.01	0.57
-2	-1	-2	6.41	0.39
-2	-1	-2	6.66	0.54
-2	-1	-2	6.94	0.29
2	1	2	6.71	0.42
2	1	2	7.20	0.51
2	1	2	7.61	0.65
2	1	2	6.50	0.29
2	1	2	6.42	0.56
-3	-1	-2	5.48	0.38
-3	-1	-2	6.23	0.31
-3	-1	-2	5.72	0.60
3	1	2	5.47	0.67
3	1	2	5.98	0.43
3	1	2	5.67	0.30
3	1	2	5.90	0.51
3	1	2	4.86	0.52
-4	-1	-2	17.37	0.65
-4	-1	-2	14.42	1.52
-4	-1	-2	17.76	0.75
4	1	2	17.61	0.81
4	1	2	20.29	1.17
4	1	2	16.87	0.89
4	1	2	16.59	0.95
-5	-1	-2	0.19	0.15
-5	-1	-2	0.03	0.40
-5	-1	-2	0.23	0.18
5	1	2	0.07	0.25
5	1	2	0.43	0.17
5	1	2	0.32	0.53
5	1	2	0.57	0.33
5	1	2	0.12	0.35
-6	-1	-2	0.95	0.27
-6	-1	-2	0.58	0.57

-6	-1	-2	0.96	0.23
6	1	2	1.23	0.65
6	1	2	1.30	0.26
6	1	2	0.83	0.35
6	1	2	0.90	0.38
6	1	2	1.84	0.54
-7	-1	-2	7.13	0.50
-7	-1	-2	8.26	1.26
-7	-1	-2	6.34	0.58
7	1	2	6.89	0.78
7	1	2	5.76	0.71
7	1	2	6.61	1.02
7	1	2	6.74	0.52
7	1	2	5.50	1.02
-8	-1	-2	7.12	0.56
-8	-1	-2	7.05	0.64
-8	-1	-2	8.41	1.14
8	1	2	6.15	0.59
8	1	2	6.44	0.84
8	1	2	8.01	1.20
8	1	2	8.71	1.18
8	1	2	6.65	1.22
-9	-1	-2	22.77	1.02
-9	-1	-2	22.79	1.14
9	1	2	19.40	1.73
9	1	2	26.13	1.36
9	1	2	22.67	1.54
9	1	2	20.42	1.05
-10	-1	-2	0.19	0.37
10	1	2	-0.17	0.93
10	1	2	-0.59	0.51
10	1	2	0.50	0.84
10	1	2	-0.56	0.53
-11	-1	-2	1.53	0.52
11	1	2	2.98	1.10
11	1	2	1.86	1.25
11	1	2	1.63	0.80
11	1	2	1.06	0.63
-12	-1	-2	8.76	1.00
12	1	2	7.27	1.58
12	1	2	9.58	1.44
12	1	2	8.54	1.18
-13	-1	-2	5.43	0.83
13	1	2	5.15	1.74

13	1	2	4.42	1.10
-14	-1	-2	1.38	0.87
14	1	2	2.26	1.64
14	1	2	2.72	1.97
16	-2	-2	4.34	2.19
16	-2	-2	5.33	1.37
-16	2	2	4.12	0.85
15	-2	-2	4.41	1.86
15	-2	-2	1.69	1.02
-15	2	2	2.21	0.69
14	-2	-2	18.42	2.46
14	-2	-2	20.96	1.74
-14	2	2	19.84	1.24
13	-2	-2	14.21	2.04
13	-2	-2	16.52	1.47
-13	2	2	15.09	0.99
12	-2	-2	5.88	1.25
12	-2	-2	5.50	1.13
-12	2	2	5.08	1.19
11	-2	-2	9.08	0.99
11	-2	-2	8.99	1.49
-11	2	2	9.06	0.69
-11	2	2	9.60	1.14
10	-2	-2	34.83	1.41
10	-2	-2	33.95	1.60
10	-2	-2	32.03	1.95
10	-2	-2	34.18	1.33
-10	2	2	35.97	1.64
-10	2	2	33.60	1.31
9	-2	-2	2.89	0.36
9	-2	-2	2.99	0.40
9	-2	-2	2.42	0.49
9	-2	-2	2.16	0.68
-9	2	2	2.68	0.39
-9	2	2	2.71	0.55
8	-2	-2	20.51	0.81
8	-2	-2	18.09	1.22
8	-2	-2	18.73	1.01
8	-2	-2	18.88	0.85
-8	2	2	18.31	0.79
-8	2	2	19.70	1.04
7	-2	-2	4.25	0.34
7	-2	-2	4.12	0.72
7	-2	-2	4.35	0.51

-7	2	2	5.07	0.58
-7	2	2	4.65	0.48
-7	2	2	3.97	0.33
6	-2	-2	0.30	0.34
6	-2	-2	0.25	0.14
6	-2	-2	0.31	0.23
-6	2	2	0.49	0.15
-6	2	2	0.24	0.27
-6	2	2	0.09	0.21
5	-2	-2	76.49	2.37
5	-2	-2	73.76	2.53
-5	2	2	75.50	2.37
-5	2	2	77.31	2.51
-5	2	2	78.49	2.61
4	-2	-2	5.71	0.43
4	-2	-2	5.72	0.27
4	-2	-2	5.79	0.28
4	-2	-2	6.45	0.55
-4	2	2	6.37	0.52
-4	2	2	6.12	0.40
3	-2	-2	17.55	0.60
3	-2	-2	18.58	0.68
3	-2	-2	18.08	0.76
3	-2	-2	17.31	0.87
-3	2	2	18.68	0.75
-3	2	2	17.87	0.64
-3	2	2	19.26	0.88
-3	2	2	17.90	0.61
2	-2	-2	58.17	1.79
2	-2	-2	61.01	1.95
2	-2	-2	58.61	2.07
2	-2	-2	59.93	1.97
-2	2	2	60.02	1.96
-2	2	2	58.90	1.80
-2	2	2	59.59	1.92
-2	2	2	60.41	2.08
1	-2	-2	1.97	0.28
1	-2	-2	1.98	0.32
1	-2	-2	2.17	0.32
-1	2	2	1.98	0.29
-1	2	2	2.05	0.34
-1	2	2	2.08	0.29
0	-2	-2	53.20	1.91
0	-2	-2	54.50	1.93

0	-2	-2	52.80	1.78
0	2	2	53.61	1.97
0	2	2	53.86	1.88
0	2	2	54.72	1.81
-1	-2	-2	18.23	0.74
-1	-2	-2	17.66	0.59
-1	-2	-2	19.36	0.94
-1	-2	-2	17.87	0.93
1	2	2	18.68	0.77
1	2	2	18.21	0.60
1	2	2	18.78	0.99
1	2	2	18.25	0.89
-2	-2	-2	1.46	0.15
-2	-2	-2	0.98	0.34
-2	-2	-2	1.54	0.26
2	2	2	2.10	0.32
2	2	2	1.45	0.16
2	2	2	1.55	0.33
2	2	2	1.67	0.50
-3	-2	-2	1.86	0.30
-3	-2	-2	2.55	0.23
-3	-2	-2	1.64	0.45
3	2	2	2.92	0.45
3	2	2	2.77	0.38
3	2	2	2.25	0.22
3	2	2	2.28	0.43
3	2	2	2.87	0.58
-4	-2	-2	2.72	0.26
-4	-2	-2	2.64	0.58
-4	-2	-2	2.03	0.30
4	2	2	3.31	0.55
4	2	2	1.86	0.42
4	2	2	2.93	0.46
4	2	2	1.53	0.56
-5	-2	-2	8.46	0.44
-5	-2	-2	9.89	1.07
-5	-2	-2	8.49	0.55
5	2	2	8.48	1.17
5	2	2	8.40	0.46
5	2	2	7.39	0.78
5	2	2	7.96	0.66
5	2	2	8.80	0.79
-6	-2	-2	4.10	0.40
-6	-2	-2	3.99	0.48

-6	-2	-2	4.42	0.86
6	2	2	3.90	0.63
6	2	2	4.67	0.79
6	2	2	5.43	1.02
6	2	2	4.50	0.41
6	2	2	3.41	0.50
-7	-2	-2	24.21	1.87
-7	-2	-2	26.66	1.05
-7	-2	-2	26.23	1.16
7	2	2	26.95	1.07
7	2	2	28.39	2.08
7	2	2	26.24	1.58
7	2	2	26.85	1.35
7	2	2	26.98	1.45
-8	-2	-2	-0.06	0.28
-8	-2	-2	0.83	0.35
8	2	2	-0.11	0.32
8	2	2	1.24	0.68
8	2	2	-0.17	0.53
8	2	2	0.81	0.69
-9	-2	-2	0.29	0.55
-9	-2	-2	0.36	0.34
9	2	2	0.42	0.41
9	2	2	-0.29	0.81
9	2	2	0.62	0.91
9	2	2	0.42	0.59
-10	-2	-2	10.68	0.84
-10	-2	-2	11.65	0.97
10	2	2	10.78	1.22
10	2	2	12.92	1.78
10	2	2	9.52	1.71
-11	-2	-2	6.23	0.86
11	2	2	7.96	1.31
11	2	2	6.39	0.94
11	2	2	4.58	1.41
-12	-2	-2	4.69	0.78
12	2	2	7.01	1.00
12	2	2	5.41	1.60
12	2	2	5.14	1.02
-13	-2	-2	5.23	0.90
13	2	2	4.67	1.20
13	2	2	3.03	1.77
17	-3	-2	-4.37	2.24
-17	3	2	2.86	1.02

16	-3	-2	1.56	2.05
16	-3	-2	2.26	1.14
-16	3	2	3.79	0.78
15	-3	-2	14.31	2.25
15	-3	-2	19.41	1.86
-15	3	2	20.15	1.29
14	-3	-2	8.56	1.68
14	-3	-2	10.29	1.44
-14	3	2	8.41	0.92
13	-3	-2	5.79	0.94
13	-3	-2	5.38	1.35
-13	3	2	5.32	0.75
12	-3	-2	7.93	1.37
12	-3	-2	5.26	0.82
-12	3	2	5.27	0.91
-12	3	2	7.37	0.68
11	-3	-2	10.92	1.53
11	-3	-2	11.90	1.05
-11	3	2	11.87	0.73
-11	3	2	11.94	1.17
10	-3	-2	9.74	0.70
10	-3	-2	9.44	1.33
10	-3	-2	8.48	0.88
10	-3	-2	9.07	0.63
-10	3	2	8.96	0.60
-10	3	2	9.48	0.96
9	-3	-2	0.92	0.27
9	-3	-2	1.13	0.29
9	-3	-2	1.05	0.40
9	-3	-2	1.32	0.63
-9	3	2	1.06	0.48
-9	3	2	0.84	0.25
8	-3	-2	5.53	0.61
8	-3	-2	5.00	0.90
8	-3	-2	5.79	0.44
-8	3	2	6.24	0.58
-8	3	2	5.74	0.73
-8	3	2	4.79	0.41
7	-3	-2	46.96	1.80
7	-3	-2	47.89	2.08
7	-3	-2	48.89	1.60
-7	3	2	49.86	1.76
-7	3	2	46.16	1.58
-7	3	2	46.08	1.89

6	-3	-2	12.76	0.54
6	-3	-2	11.56	0.92
6	-3	-2	12.59	0.71
-6	3	2	12.33	0.53
-6	3	2	13.98	0.87
-6	3	2	13.77	0.69
5	-3	-2	40.46	1.52
5	-3	-2	40.55	1.76
5	-3	-2	42.18	1.35
-5	3	2	42.22	1.50
-5	3	2	40.82	1.34
-5	3	2	43.20	1.68
4	-3	-2	23.29	0.96
4	-3	-2	23.94	0.88
4	-3	-2	20.47	1.12
4	-3	-2	22.32	0.77
-4	3	2	24.62	1.13
-4	3	2	23.94	0.81
-4	3	2	22.20	0.77
-4	3	2	22.46	0.92
3	-3	-2	33.65	1.26
3	-3	-2	33.93	1.07
3	-3	-2	34.94	1.47
3	-3	-2	33.90	1.26
-3	3	2	33.94	1.07
-3	3	2	35.53	1.47
-3	3	2	33.28	1.21
-3	3	2	34.80	1.24
2	-3	-2	100.76	3.32
2	-3	-2	97.15	3.17
2	-3	-2	96.61	3.11
2	-3	-2	95.51	2.89
-2	3	2	94.59	2.89
-2	3	2	96.09	3.10
-2	3	2	100.07	3.33
-2	3	2	96.03	3.13
1	-3	-2	4.38	0.40
1	-3	-2	4.04	0.65
1	-3	-2	5.46	0.51
-1	3	2	5.42	0.42
-1	3	2	4.74	0.75
-1	3	2	5.06	0.61
0	-3	-2	40.52	1.61
0	-3	-2	45.58	1.79

0	3	2	44.56	1.83
0	3	2	44.37	1.57
0	3	2	45.55	1.71
-1	-3	-2	14.19	0.45
-1	-3	-2	13.36	0.89
-1	-3	-2	12.77	0.86
-1	-3	-2	13.03	0.63
1	3	2	13.97	0.45
1	3	2	14.08	0.68
1	3	2	12.85	1.11
1	3	2	12.17	0.80
-2	-3	-2	3.30	0.36
-2	-3	-2	2.82	0.20
-2	-3	-2	2.63	0.55
-2	-3	-2	4.03	0.62
2	3	2	2.78	0.43
2	3	2	2.79	0.62
2	3	2	2.55	0.19
2	3	2	2.06	0.45
-3	-3	-2	15.29	1.14
-3	-3	-2	15.14	0.70
-3	-3	-2	13.69	0.55
3	3	2	14.19	0.54
3	3	2	15.57	0.79
3	3	2	13.46	0.94
3	3	2	13.60	1.20
3	3	2	13.62	0.84
-4	-3	-2	17.72	1.30
-4	-3	-2	15.05	0.60
-4	-3	-2	14.61	0.73
4	3	2	13.48	1.35
4	3	2	15.30	0.96
4	3	2	15.58	1.06
4	3	2	15.96	0.87
-5	-3	-2	5.46	0.38
-5	-3	-2	5.41	0.51
-5	-3	-2	7.92	1.18
5	3	2	4.78	0.73
5	3	2	5.22	0.67
5	3	2	6.00	0.83
5	3	2	5.55	0.41
5	3	2	7.02	1.09
-6	-3	-2	8.21	0.63
-6	-3	-2	7.20	0.48

-6	-3	-2	7.67	1.15
6	3	2	7.95	0.90
6	3	2	7.75	0.82
6	3	2	6.83	1.21
6	3	2	7.61	0.52
6	3	2	8.07	1.01
-7	-3	-2	1.50	0.38
-7	-3	-2	0.81	0.30
7	3	2	-0.01	1.08
7	3	2	1.21	0.59
7	3	2	0.54	0.57
7	3	2	1.12	0.63
7	3	2	1.41	0.33
7	3	2	1.51	0.56
-8	-3	-2	13.31	0.75
-8	-3	-2	13.37	0.88
8	3	2	12.37	1.48
8	3	2	14.68	1.35
8	3	2	13.02	0.81
8	3	2	14.66	1.42
-9	-3	-2	20.10	1.01
-9	-3	-2	21.60	1.17
9	3	2	21.64	1.58
9	3	2	17.86	1.86
9	3	2	20.92	1.81
-10	-3	-2	6.10	0.77
-10	-3	-2	8.38	0.93
10	3	2	8.21	1.38
10	3	2	6.10	1.36
-11	-3	-2	9.66	1.01
11	3	2	11.78	2.18
11	3	2	10.91	1.44
11	3	2	9.81	1.22
-12	-3	-2	9.56	1.23
12	3	2	5.74	1.10
12	3	2	9.69	1.35
12	3	2	7.45	1.73
-13	-3	-2	4.81	1.01
13	3	2	5.38	1.99
13	3	2	4.76	1.25
17	-4	-2	4.50	2.52
17	-4	-2	5.70	1.47
-17	4	2	5.01	0.89
16	-4	-2	4.63	2.12

16	-4	-2	4.77	1.18
-16	4	2	1.88	0.69
15	-4	-2	5.71	1.98
15	-4	-2	12.44	1.70
-15	4	2	7.47	1.02
14	-4	-2	12.24	1.50
14	-4	-2	13.06	2.42
-14	4	2	10.40	0.94
13	-4	-2	12.83	1.37
13	-4	-2	14.14	2.20
-13	4	2	15.14	0.92
12	-4	-2	2.51	1.21
12	-4	-2	3.45	0.71
-12	4	2	4.49	0.59
-12	4	2	2.17	0.77
11	-4	-2	0.71	0.53
11	-4	-2	1.29	0.98
-11	4	2	2.97	0.76
-11	4	2	0.38	0.31
10	-4	-2	6.13	0.83
10	-4	-2	7.59	0.58
10	-4	-2	6.18	1.10
10	-4	-2	7.29	0.64
-10	4	2	5.88	0.97
-10	4	2	6.38	0.55
9	-4	-2	0.33	0.37
9	-4	-2	0.98	0.65
9	-4	-2	0.38	0.24
-9	4	2	0.62	0.48
-9	4	2	0.25	0.22
-9	4	2	0.92	0.36
8	-4	-2	39.57	1.38
8	-4	-2	40.02	1.99
8	-4	-2	38.58	1.62
-8	4	2	37.74	1.36
-8	4	2	40.57	1.78
-8	4	2	39.97	1.56
7	-4	-2	5.40	0.58
7	-4	-2	6.22	0.88
7	-4	-2	5.50	0.39
-7	4	2	6.56	0.56
-7	4	2	6.08	0.76
-7	4	2	5.76	0.38
6	-4	-2	19.74	0.75

6	-4	-2	20.21	1.28
6	-4	-2	19.46	0.94
-6	4	2	21.15	1.20
-6	4	2	20.40	0.91
-6	4	2	19.26	0.74
5	-4	-2	10.19	0.63
5	-4	-2	10.84	0.91
5	-4	-2	11.38	0.46
-5	4	2	12.36	0.91
-5	4	2	11.08	0.51
-5	4	2	10.83	0.45
-5	4	2	10.15	0.69
-5	4	2	10.57	0.50
4	-4	-2	44.12	1.62
4	-4	-2	46.00	1.96
4	-4	-2	44.16	1.63
4	-4	-2	45.02	1.42
-4	4	2	43.71	1.41
-4	4	2	44.55	1.57
-4	4	2	44.87	1.60
-4	4	2	47.06	1.92
3	-4	-2	0.54	0.09
3	-4	-2	0.38	0.26
3	-4	-2	0.90	0.28
3	-4	-2	0.84	0.42
-3	4	2	0.39	0.09
-3	4	2	1.15	0.44
-3	4	2	0.48	0.22
-3	4	2	0.44	0.21
2	-4	-2	0.97	0.33
2	-4	-2	1.50	0.46
-2	4	2	1.37	0.28
-2	4	2	1.08	0.30
-2	4	2	1.40	0.44
1	-4	-2	0.11	0.35
1	-4	-2	0.35	0.33
1	-4	-2	0.40	0.25
-1	4	2	-0.06	0.20
-1	4	2	-0.29	0.36
-1	4	2	0.13	0.25
0	-4	-2	5.19	0.44
0	-4	-2	5.03	0.68
0	-4	-2	4.22	0.67
0	4	2	5.72	0.94

0	4	2	5.28	0.62
0	4	2	4.88	0.49
-1	-4	-2	7.71	0.50
-1	-4	-2	7.43	0.29
-1	-4	-2	8.35	0.85
-1	-4	-2	7.69	0.79
1	4	2	8.85	0.98
1	4	2	7.49	0.29
1	4	2	7.06	0.71
-2	-4	-2	36.78	1.37
-2	-4	-2	35.57	1.71
-2	-4	-2	36.56	1.20
-2	-4	-2	38.91	1.86
2	4	2	37.98	1.20
2	4	2	38.36	1.48
2	4	2	43.47	2.02
2	4	2	36.76	1.71
-3	-4	-2	9.36	0.44
-3	-4	-2	9.41	0.60
-3	-4	-2	11.04	1.14
-3	-4	-2	10.56	0.93
3	4	2	10.30	0.92
3	4	2	8.89	1.23
3	4	2	9.60	0.43
3	4	2	11.05	0.75
-4	-4	-2	2.25	0.73
-4	-4	-2	2.61	0.44
-4	-4	-2	2.80	0.29
4	4	2	3.08	0.36
4	4	2	4.33	0.98
4	4	2	2.72	0.51
4	4	2	2.14	0.56
4	4	2	3.74	0.54
-5	-4	-2	2.23	0.37
-5	-4	-2	2.17	0.33
5	4	2	2.69	1.03
5	4	2	1.85	0.56
5	4	2	2.09	0.28
5	4	2	2.16	0.50
5	4	2	2.77	0.64
5	4	2	2.98	0.71
-6	-4	-2	0.01	0.30
-6	-4	-2	0.41	0.26
6	4	2	0.79	0.56

6	4	2	0.47	0.27
6	4	2	1.12	0.63
6	4	2	0.44	0.51
6	4	2	0.27	1.09
6	4	2	2.08	0.69
-7	-4	-2	6.15	0.56
-7	-4	-2	6.87	0.70
7	4	2	4.98	0.83
7	4	2	7.13	1.04
7	4	2	6.64	0.61
7	4	2	7.27	1.06
-8	-4	-2	7.06	0.66
8	4	2	9.58	0.74
8	4	2	9.02	1.31
8	4	2	8.94	1.65
8	4	2	8.92	1.35
-9	-4	-2	7.40	0.77
-9	-4	-2	7.51	0.91
9	4	2	6.34	1.21
9	4	2	9.23	1.45
-10	-4	-2	10.30	1.07
-10	-4	-2	10.01	0.99
10	4	2	11.43	2.02
10	4	2	8.78	1.56
-11	-4	-2	9.54	1.11
11	4	2	6.31	1.09
11	4	2	9.56	1.69
11	4	2	6.64	1.61
-12	-4	-2	3.64	0.94
12	4	2	2.89	1.69
12	4	2	3.43	1.17
12	4	2	4.14	1.21
17	-5	-2	0.74	1.33
17	-5	-2	4.50	2.51
-17	5	2	1.53	0.81
16	-5	-2	2.72	1.13
16	-5	-2	3.48	2.25
-16	5	2	3.62	0.75
15	-5	-2	12.29	1.70
15	-5	-2	10.78	2.23
-15	5	2	10.94	1.05
14	-5	-2	8.92	1.41
14	-5	-2	10.14	1.92
-14	5	2	8.43	0.88

13	-5	-2	2.13	1.27
13	-5	-2	4.70	0.96
-13	5	2	3.04	0.51
12	-5	-2	3.71	1.21
12	-5	-2	2.26	0.70
-12	5	2	2.07	0.39
-12	5	2	2.46	0.89
11	-5	-2	8.35	0.91
11	-5	-2	7.93	0.97
11	-5	-2	5.35	1.20
-11	5	2	8.28	1.08
-11	5	2	6.12	0.60
10	-5	-2	1.92	0.34
10	-5	-2	1.48	0.89
10	-5	-2	0.87	0.48
-10	5	2	0.95	0.29
-10	5	2	1.90	0.67
9	-5	-2	0.47	0.37
9	-5	-2	2.15	0.73
9	-5	-2	1.31	0.29
-9	5	2	0.77	0.24
-9	5	2	0.96	0.54
-9	5	2	1.63	0.40
8	-5	-2	0.59	0.61
8	-5	-2	0.48	0.21
8	-5	-2	-0.11	0.30
-8	5	2	0.53	0.50
-8	5	2	-0.44	0.29
-8	5	2	0.28	0.20
7	-5	-2	13.29	1.19
7	-5	-2	12.62	0.80
7	-5	-2	11.77	0.58
-7	5	2	12.30	0.56
-7	5	2	13.58	1.09
-7	5	2	12.82	0.75
6	-5	-2	3.87	0.71
6	-5	-2	3.89	0.31
6	-5	-2	3.41	0.48
-6	5	2	3.02	0.45
-6	5	2	3.49	0.29
-6	5	2	4.46	0.71
-6	5	2	3.95	0.34
-6	5	2	3.99	0.37
5	-5	-2	0.16	0.27

5	-5	-2	0.59	0.51
5	-5	-2	0.39	0.14
-5	5	2	0.05	0.48
-5	5	2	0.28	0.21
-5	5	2	0.55	0.25
-5	5	2	0.30	0.14
-5	5	2	0.62	0.29
4	-5	-2	22.54	1.38
4	-5	-2	24.61	0.81
4	-5	-2	23.62	1.05
-4	5	2	24.11	1.02
-4	5	2	23.70	1.00
-4	5	2	24.02	0.80
3	-5	-2	0.64	0.33
3	-5	-2	0.22	0.09
3	-5	-2	0.65	0.43
3	-5	-2	0.45	0.28
-3	5	2	0.43	0.24
-3	5	2	0.42	0.09
-3	5	2	0.44	0.32
2	-5	-2	16.74	1.04
2	-5	-2	14.85	0.82
2	-5	-2	14.62	1.09
-2	5	2	15.55	0.79
-2	5	2	14.77	0.92
-2	5	2	14.40	1.13
1	-5	-2	39.00	1.48
1	-5	-2	37.80	1.76
1	-5	-2	38.48	1.83
-1	5	2	37.90	1.69
-1	5	2	41.19	1.93
-1	5	2	40.46	1.52
0	-5	-2	2.23	0.56
0	-5	-2	2.23	0.33
0	-5	-2	2.27	0.58
0	5	2	2.34	0.48
0	5	2	3.56	0.73
0	5	2	1.78	0.41
-1	-5	-2	1.73	0.52
-1	-5	-2	1.67	0.58
-1	-5	-2	2.07	0.33
1	5	2	1.84	0.63
1	5	2	2.04	0.40
1	5	2	1.69	0.44

-2	-5	-2	30.45	1.18
-2	-5	-2	28.32	1.56
-2	-5	-2	29.86	1.73
2	5	2	31.37	1.89
2	5	2	28.47	0.96
2	5	2	28.66	1.54
2	5	2	30.25	0.96
2	5	2	30.18	1.31
-3	-5	-2	31.99	1.13
-3	-5	-2	33.98	1.31
-3	-5	-2	31.60	1.73
-3	-5	-2	33.95	1.96
3	5	2	36.06	1.48
3	5	2	31.95	1.72
3	5	2	33.97	1.11
3	5	2	35.25	2.14
-4	-5	-2	6.24	0.57
-4	-5	-2	7.15	0.43
4	5	2	7.00	0.99
4	5	2	5.77	0.79
4	5	2	6.78	0.41
4	5	2	7.22	1.02
4	5	2	6.48	1.20
4	5	2	5.89	0.81
-5	-5	-2	5.59	0.45
-5	-5	-2	4.99	0.57
5	5	2	4.99	1.33
5	5	2	6.41	0.87
5	5	2	6.35	0.93
5	5	2	5.64	0.44
5	5	2	5.70	0.92
5	5	2	5.91	0.90
-6	-5	-2	6.97	0.55
-6	-5	-2	6.15	0.66
6	5	2	6.42	1.06
6	5	2	6.24	1.05
6	5	2	7.85	1.04
6	5	2	8.24	1.14
6	5	2	7.40	0.56
-7	-5	-2	1.48	0.43
-7	-5	-2	1.98	0.39
7	5	2	0.86	0.76
7	5	2	1.33	0.38
7	5	2	1.32	0.79

-8	-5	-2	14.68	0.91
-8	-5	-2	14.53	1.03
8	5	2	16.46	0.92
8	5	2	14.33	1.65
8	5	2	16.01	1.59
-9	-5	-2	12.21	0.99
-9	-5	-2	14.81	1.12
9	5	2	14.20	1.81
9	5	2	12.74	1.94
-10	-5	-2	5.08	0.79
-10	-5	-2	6.57	1.38
10	5	2	3.92	1.16
10	5	2	6.66	1.40
-11	-5	-2	2.97	0.86
11	5	2	3.32	1.59
11	5	2	2.09	1.20
11	5	2	1.89	1.02
17	-6	-2	8.86	1.49
17	-6	-2	14.97	2.93
-17	6	2	10.12	1.26
16	-6	-2	4.70	1.30
16	-6	-2	3.28	2.15
-16	6	2	4.48	0.77
15	-6	-2	2.99	1.03
15	-6	-2	1.60	1.81
-15	6	2	2.80	0.63
14	-6	-2	9.32	1.89
14	-6	-2	10.69	1.46
-14	6	2	9.58	0.90
13	-6	-2	2.74	1.40
13	-6	-2	2.25	0.73
-13	6	2	2.82	0.51
12	-6	-2	1.73	1.20
12	-6	-2	1.50	0.61
-12	6	2	0.59	0.35
-12	6	2	3.03	1.47
11	-6	-2	9.26	1.72
11	-6	-2	9.23	1.02
11	-6	-2	7.79	0.73
-11	6	2	7.21	1.29
-11	6	2	8.13	0.63
10	-6	-2	0.44	0.45
10	-6	-2	0.45	0.31
10	-6	-2	0.82	0.86

-10	6	2	0.62	0.41
-10	6	2	0.34	0.26
-10	6	2	-0.22	0.64
9	-6	-2	0.41	0.40
9	-6	-2	0.04	0.25
9	-6	-2	0.18	0.72
-9	6	2	0.15	0.23
-9	6	2	0.42	0.36
-9	6	2	0.22	0.62
8	-6	-2	2.84	0.38
8	-6	-2	2.86	0.87
8	-6	-2	2.16	0.46
-8	6	2	2.51	0.33
-8	6	2	2.64	0.74
-8	6	2	3.58	0.49
7	-6	-2	4.76	0.39
7	-6	-2	6.26	1.09
7	-6	-2	3.97	0.60
-7	6	2	5.74	0.57
-7	6	2	4.36	0.45
-7	6	2	4.71	0.35
-7	6	2	4.95	0.41
-7	6	2	5.06	0.83
6	-6	-2	13.45	0.56
6	-6	-2	11.79	0.78
6	-6	-2	13.05	1.22
-6	6	2	12.09	0.74
-6	6	2	13.55	0.75
-6	6	2	12.94	0.67
-6	6	2	12.39	0.54
-6	6	2	13.40	1.23
5	-6	-2	0.30	0.30
5	-6	-2	0.08	0.13
5	-6	-2	0.08	0.55
-5	6	2	0.27	0.54
-5	6	2	0.30	0.25
-5	6	2	0.62	0.27
-5	6	2	0.19	0.35
-5	6	2	0.12	0.14
4	-6	-2	3.05	0.63
4	-6	-2	4.73	0.26
4	-6	-2	4.24	0.53
-4	6	2	5.58	0.96
-4	6	2	3.89	0.48

-4	6	2	3.94	0.25
-4	6	2	4.40	0.55
-4	6	2	4.08	0.61
3	-6	-2	5.63	0.57
3	-6	-2	6.35	0.28
3	-6	-2	5.54	0.88
-3	6	2	5.90	0.62
-3	6	2	5.32	0.55
-3	6	2	6.80	0.74
-3	6	2	6.66	0.26
-3	6	2	8.40	1.06
2	-6	-2	12.02	1.11
2	-6	-2	13.38	0.77
-2	6	2	14.56	1.27
-2	6	2	14.60	0.82
-2	6	2	14.57	0.90
-2	6	2	14.40	0.98
1	-6	-2	1.73	0.59
1	-6	-2	1.79	0.35
-1	6	2	2.36	0.48
-1	6	2	1.78	0.47
-1	6	2	2.74	0.79
0	-6	-2	1.64	0.33
0	-6	-2	1.84	0.55
0	6	2	1.27	0.40
0	6	2	1.53	0.70
0	6	2	1.51	0.48
0	6	2	1.30	0.39
-1	-6	-2	14.31	1.20
-1	-6	-2	12.12	0.70
1	6	2	14.20	1.46
1	6	2	13.16	0.87
1	6	2	10.90	1.08
-2	-6	-2	7.77	1.06
-2	-6	-2	8.64	0.62
2	6	2	9.05	0.94
2	6	2	8.91	0.39
2	6	2	10.43	1.49
2	6	2	9.51	1.05
2	6	2	10.52	0.85
2	6	2	9.50	0.39
-3	-6	-2	21.11	0.98
-3	-6	-2	20.65	1.49
3	6	2	19.66	1.46

3	6	2	20.84	0.74
3	6	2	20.64	0.76
3	6	2	21.10	1.20
3	6	2	19.92	1.96
3	6	2	19.89	1.38
-4	-6	-2	9.15	0.68
-4	-6	-2	8.92	1.22
4	6	2	9.19	0.48
4	6	2	7.73	1.54
4	6	2	8.86	0.95
4	6	2	9.38	1.23
4	6	2	8.53	0.49
4	6	2	9.80	1.23
-5	-6	-2	1.19	0.40
5	6	2	-0.25	0.63
5	6	2	0.38	0.25
5	6	2	-0.62	0.60
5	6	2	-0.43	0.73
5	6	2	0.66	0.59
-6	-6	-2	23.88	1.16
6	6	2	22.77	0.96
6	6	2	20.28	1.52
6	6	2	20.66	1.80
-7	-6	-2	19.85	1.00
7	6	2	18.42	0.96
7	6	2	18.79	1.61
7	6	2	20.54	1.83
-8	-6	-2	8.46	0.94
8	6	2	8.28	1.32
8	6	2	8.20	0.87
8	6	2	5.79	1.11
-9	-6	-2	9.17	1.09
9	6	2	9.07	0.88
9	6	2	13.28	1.89
9	6	2	6.04	1.30
-10	-6	-2	10.69	1.27
10	6	2	7.65	1.56
10	6	2	12.29	2.14
10	6	2	7.60	1.15
11	6	2	3.07	0.71
11	6	2	2.97	1.35
11	6	2	3.69	1.18
11	6	2	1.09	1.67
17	-7	-2	5.86	1.38

17	-7	-2	8.47	2.76
-17	7	2	2.60	0.81
16	-7	-2	5.58	1.27
16	-7	-2	8.52	2.45
-16	7	2	4.17	0.78
15	-7	-2	10.39	2.19
15	-7	-2	5.80	1.16
-15	7	2	7.55	0.94
14	-7	-2	5.52	1.05
14	-7	-2	6.08	1.80
-14	7	2	5.10	0.80
13	-7	-2	0.48	0.68
13	-7	-2	1.18	1.33
-13	7	2	0.03	0.41
12	-7	-2	0.25	1.20
12	-7	-2	0.24	0.54
-12	7	2	0.24	0.35
11	-7	-2	7.59	0.99
11	-7	-2	5.55	1.31
-11	7	2	9.38	1.45
-11	7	2	6.86	0.89
-11	7	2	6.50	0.60
10	-7	-2	0.45	0.54
10	-7	-2	0.18	0.88
10	-7	-2	1.08	0.33
-10	7	2	0.95	0.46
-10	7	2	0.83	0.30
-10	7	2	0.90	0.76
9	-7	-2	1.12	0.86
9	-7	-2	1.25	0.29
9	-7	-2	0.71	0.45
-9	7	2	0.45	0.24
-9	7	2	0.36	0.37
-9	7	2	0.71	0.74
8	-7	-2	0.74	0.41
8	-7	-2	0.84	0.77
8	-7	-2	0.25	0.22
-8	7	2	0.41	0.27
-8	7	2	0.40	0.34
-8	7	2	0.03	0.21
-8	7	2	0.66	0.75
-8	7	2	0.17	0.19
7	-7	-2	5.32	0.68
7	-7	-2	5.46	0.40

7	-7	-2	5.07	0.98
-7	7	2	5.94	1.04
-7	7	2	5.11	0.51
-7	7	2	5.97	0.61
-7	7	2	5.56	0.38
6	-7	-2	0.13	0.17
6	-7	-2	0.04	0.34
6	-7	-2	-0.40	0.58
-6	7	2	-0.38	0.27
-6	7	2	0.12	0.16
-6	7	2	-1.29	0.58
-6	7	2	0.03	0.36
-6	7	2	-0.06	0.29
5	-7	-2	12.50	1.30
5	-7	-2	10.53	0.81
5	-7	-2	12.36	0.51
-5	7	2	9.55	1.17
-5	7	2	11.06	0.89
-5	7	2	11.28	0.73
-5	7	2	11.61	0.49
-5	7	2	14.22	1.39
4	-7	-2	75.20	2.29
4	-7	-2	74.43	3.16
4	-7	-2	69.55	2.60
-4	7	2	75.00	3.18
-4	7	2	73.76	2.27
-4	7	2	76.13	2.56
-4	7	2	76.35	2.66
-4	7	2	71.15	2.76
3	-7	-2	0.83	0.38
3	-7	-2	0.02	0.11
3	-7	-2	0.03	0.52
-3	7	2	0.35	0.39
-3	7	2	-0.15	0.63
-3	7	2	-0.49	0.34
-3	7	2	0.06	0.35
-3	7	2	0.13	0.09
2	-7	-2	5.46	0.91
2	-7	-2	5.79	0.62
-2	7	2	5.66	0.86
-2	7	2	6.58	1.18
-2	7	2	6.25	0.77
-2	7	2	5.66	0.65
1	-7	-2	31.03	1.30

1	-7	-2	32.46	1.83
-1	7	2	28.20	1.65
-1	7	2	32.74	2.01
-1	7	2	30.41	1.54
-1	7	2	34.16	1.44
0	-7	-2	16.16	0.83
0	-7	-2	14.91	1.32
0	7	2	14.84	1.22
0	7	2	15.32	1.60
0	7	2	16.54	1.03
0	7	2	15.58	1.15
-1	-7	-2	3.36	0.74
-1	-7	-2	3.28	0.49
1	7	2	3.38	0.96
1	7	2	2.45	0.64
1	7	2	2.75	0.55
1	7	2	2.57	0.63
-2	-7	-2	14.97	0.82
-2	-7	-2	16.52	1.43
2	7	2	14.81	1.86
2	7	2	15.31	1.29
2	7	2	14.78	1.09
2	7	2	14.61	0.56
2	7	2	15.03	0.55
2	7	2	14.72	1.34
-3	-7	-2	7.90	1.26
-3	-7	-2	7.52	0.66
3	7	2	7.24	1.02
3	7	2	7.19	1.23
3	7	2	6.32	1.41
3	7	2	8.49	0.46
3	7	2	9.10	0.99
3	7	2	7.67	0.42
-4	-7	-2	6.21	0.67
4	7	2	6.18	0.45
4	7	2	6.39	1.06
4	7	2	6.76	0.44
4	7	2	5.41	1.01
-5	-7	-2	7.68	0.76
5	7	2	6.74	1.10
5	7	2	7.79	1.17
5	7	2	8.91	0.56
5	7	2	7.36	0.55
5	7	2	9.74	1.51

-6	-7	-2	17.05	1.03
6	7	2	17.04	0.85
6	7	2	15.11	2.14
6	7	2	16.40	0.82
6	7	2	16.49	1.55
-7	-7	-2	11.20	1.00
7	7	2	12.47	1.81
7	7	2	13.87	0.86
7	7	2	11.40	1.56
7	7	2	11.12	0.81
-8	-7	-2	15.37	1.17
8	7	2	16.62	1.91
8	7	2	13.88	2.05
8	7	2	14.30	0.97
-9	-7	-2	13.91	1.30
9	7	2	15.46	2.08
9	7	2	16.60	2.24
9	7	2	16.12	1.08
10	7	2	7.91	1.26
10	7	2	3.24	1.46
10	7	2	4.69	1.50
17	-8	-2	2.75	1.46
17	-8	-2	4.10	2.63
-17	8	2	4.30	0.82
16	-8	-2	7.14	2.33
16	-8	-2	4.53	1.24
-16	8	2	5.20	0.77
15	-8	-2	0.57	1.87
15	-8	-2	2.19	1.08
-15	8	2	1.44	0.59
14	-8	-2	3.08	1.71
14	-8	-2	2.65	0.94
-14	8	2	2.35	0.57
13	-8	-2	0.49	0.72
-13	8	2	0.69	0.43
12	-8	-2	-0.70	1.13
12	-8	-2	-0.15	0.64
-12	8	2	-0.08	0.33
11	-8	-2	-0.35	1.06
11	-8	-2	0.10	0.56
-11	8	2	0.19	0.31
-11	8	2	1.72	0.95
-11	8	2	0.05	0.52
10	-8	-2	3.17	0.42

10	-8	-2	2.46	1.14
10	-8	-2	3.59	0.68
-10	8	2	3.39	0.48
-10	8	2	3.91	0.59
-10	8	2	2.81	1.01
9	-8	-2	1.41	0.89
9	-8	-2	2.57	0.38
9	-8	-2	2.08	0.55
-9	8	2	2.41	0.40
-9	8	2	2.45	0.51
-9	8	2	2.57	0.95
-9	8	2	2.18	0.38
-9	8	2	2.30	0.33
8	-8	-2	-0.69	0.76
8	-8	-2	0.08	0.42
8	-8	-2	-0.26	0.23
-8	8	2	-0.10	0.20
-8	8	2	0.33	0.42
-8	8	2	-0.26	0.28
-8	8	2	0.58	0.37
-8	8	2	-1.67	0.71
7	-8	-2	3.49	0.96
7	-8	-2	2.99	0.33
7	-8	-2	2.83	0.56
-7	8	2	3.63	0.59
-7	8	2	1.71	0.89
-7	8	2	3.45	0.49
-7	8	2	2.21	0.33
-7	8	2	2.56	0.50
6	-8	-2	25.06	1.28
6	-8	-2	26.42	0.95
6	-8	-2	23.54	1.84
-6	8	2	26.62	0.93
-6	8	2	25.87	1.19
-6	8	2	25.12	1.23
-6	8	2	25.57	1.37
-6	8	2	28.30	1.93
5	-8	-2	7.62	1.26
5	-8	-2	8.79	0.44
5	-8	-2	8.89	0.78
-5	8	2	8.99	1.41
-5	8	2	8.62	0.69
-5	8	2	8.62	0.89
-5	8	2	8.85	0.42

-5	8	2	8.89	0.80
4	-8	-2	3.68	0.53
4	-8	-2	3.33	0.27
4	-8	-2	4.44	0.96
-4	8	2	3.46	0.25
-4	8	2	3.63	0.66
-4	8	2	2.53	0.88
-4	8	2	3.70	0.61
-4	8	2	3.86	0.57
3	-8	-2	16.45	1.51
3	-8	-2	16.57	0.99
-3	8	2	17.31	0.99
-3	8	2	19.67	1.18
-3	8	2	20.38	1.72
-3	8	2	18.10	1.28
2	-8	-2	4.06	0.88
2	-8	-2	4.24	0.54
-2	8	2	3.42	0.91
-2	8	2	4.47	0.62
-2	8	2	2.94	0.68
-2	8	2	3.86	0.65
1	-8	-2	0.67	0.35
1	-8	-2	0.64	0.63
-1	8	2	0.99	0.53
-1	8	2	0.69	0.44
-1	8	2	1.03	0.86
-1	8	2	-0.04	0.54
0	-8	-2	19.70	0.97
0	-8	-2	20.71	1.64
0	8	2	19.14	1.36
0	8	2	19.50	1.90
0	8	2	20.35	1.53
0	8	2	17.81	1.21
-1	-8	-2	14.64	1.51
-1	-8	-2	17.99	0.91
1	8	2	17.99	1.22
1	8	2	19.45	1.51
1	8	2	14.50	1.93
1	8	2	16.76	1.37
-2	-8	-2	9.42	0.74
-2	-8	-2	9.88	1.34
2	8	2	10.62	1.06
2	8	2	6.75	1.37
2	8	2	11.44	1.35

2	8	2	8.45	1.28
-3	-8	-2	16.44	0.92
-3	-8	-2	14.25	1.60
3	8	2	15.67	0.62
3	8	2	14.57	1.33
3	8	2	15.65	1.66
3	8	2	15.21	0.67
-4	-8	-2	1.83	0.46
-4	-8	-2	2.39	0.93
4	8	2	1.63	0.27
4	8	2	4.01	0.90
4	8	2	0.66	0.79
4	8	2	1.55	0.32
-5	-8	-2	8.46	0.82
5	8	2	6.23	1.23
5	8	2	6.26	0.56
5	8	2	7.74	1.25
5	8	2	7.54	0.59
-6	-8	-2	11.06	1.05
6	8	2	10.38	0.75
6	8	2	11.69	1.63
6	8	2	11.34	0.76
-7	-8	-2	19.82	1.26
7	8	2	19.59	2.19
7	8	2	17.23	0.99
7	8	2	15.66	1.83
-8	-8	-2	8.92	1.16
8	8	2	6.83	0.88
8	8	2	6.02	1.39
8	8	2	7.34	1.49
-9	-8	-2	4.14	0.86
9	8	2	4.67	1.41
9	8	2	8.98	1.71
17	-9	-2	0.44	1.38
17	-9	-2	2.57	2.49
-17	9	2	0.14	0.70
16	-9	-2	2.47	2.15
16	-9	-2	-0.24	1.19
-16	9	2	1.12	0.62
15	-9	-2	3.79	1.23
15	-9	-2	3.72	2.14
-15	9	2	3.52	0.65
14	-9	-2	-1.14	0.89
14	-9	-2	-0.32	1.62

-14	9	2	-0.29	0.46
13	-9	-2	-1.14	1.39
13	-9	-2	-1.20	0.76
-13	9	2	0.74	0.42
12	-9	-2	2.10	1.48
12	-9	-2	0.45	0.68
-12	9	2	0.07	0.36
11	-9	-2	1.83	0.68
11	-9	-2	1.56	1.28
-11	9	2	-0.21	0.56
-11	9	2	0.85	0.37
10	-9	-2	0.29	0.52
10	-9	-2	-1.30	1.02
10	-9	-2	0.20	0.34
-10	9	2	-1.25	0.91
-10	9	2	0.62	0.36
-10	9	2	-0.37	0.46
-10	9	2	0.20	0.25
-10	9	2	-0.42	0.30
9	-9	-2	2.34	0.61
9	-9	-2	2.29	0.38
9	-9	-2	1.16	1.04
-9	9	2	2.05	1.07
-9	9	2	2.28	0.35
-9	9	2	2.55	0.53
-9	9	2	2.94	0.43
-9	9	2	2.23	0.52
8	-9	-2	7.30	0.90
8	-9	-2	10.48	1.65
8	-9	-2	8.24	0.57
-8	9	2	11.06	1.49
-8	9	2	7.60	0.85
-8	9	2	6.75	0.50
-8	9	2	8.00	0.76
-8	9	2	7.77	0.76
7	-9	-2	-0.16	0.24
7	-9	-2	-0.91	0.80
7	-9	-2	-0.18	0.42
-7	9	2	-0.17	0.42
-7	9	2	0.00	0.38
-7	9	2	0.05	0.39
-7	9	2	0.00	0.87
-7	9	2	-0.14	0.19
6	-9	-2	0.93	0.88

6	-9	-2	1.65	0.50
6	-9	-2	1.43	0.25
-6	9	2	0.62	0.90
-6	9	2	0.95	0.22
-6	9	2	1.87	0.52
-6	9	2	0.82	0.53
-6	9	2	1.24	0.44
5	-9	-2	-0.58	0.40
5	-9	-2	0.02	0.74
5	-9	-2	0.43	0.18
-5	9	2	-0.63	0.82
-5	9	2	0.08	0.17
-5	9	2	0.69	0.41
-5	9	2	0.44	0.47
-5	9	2	-0.58	0.49
4	-9	-2	17.89	1.71
4	-9	-2	19.08	0.70
4	-9	-2	18.26	1.10
-4	9	2	21.52	1.92
-4	9	2	19.50	0.68
-4	9	2	20.74	1.09
-4	9	2	18.11	1.39
-4	9	2	19.82	1.26
3	-9	-2	2.52	0.89
3	-9	-2	2.75	0.50
-3	9	2	1.38	0.86
-3	9	2	1.58	0.62
-3	9	2	2.26	0.59
-3	9	2	2.89	0.55
2	-9	-2	1.76	0.87
2	-9	-2	0.60	0.38
-2	9	2	0.52	0.90
-2	9	2	1.44	0.51
-2	9	2	0.94	0.61
-2	9	2	2.12	0.64
1	-9	-2	52.16	1.98
1	-9	-2	54.56	2.76
-1	9	2	54.25	2.19
-1	9	2	53.58	2.61
-1	9	2	52.05	3.02
-1	9	2	53.62	2.42
0	-9	-2	5.14	1.02
0	-9	-2	7.55	0.67
0	9	2	9.99	1.70

0	9	2	5.78	0.92
0	9	2	7.97	1.29
0	9	2	7.93	1.01
-1	-9	-2	4.36	0.55
-1	-9	-2	4.79	1.10
1	9	2	3.63	1.88
1	9	2	4.39	0.78
1	9	2	3.49	0.87
-2	-9	-2	12.43	1.68
-2	-9	-2	16.29	0.92
2	9	2	14.36	1.64
2	9	2	16.63	1.33
-3	-9	-2	8.54	0.78
-3	-9	-2	8.49	1.63
3	9	2	11.26	1.37
3	9	2	9.59	1.44
-4	-9	-2	8.44	0.85
-4	-9	-2	8.51	1.34
4	9	2	8.51	0.64
4	9	2	9.44	0.60
4	9	2	10.20	1.71
-5	-9	-2	25.14	1.30
5	9	2	21.35	2.26
5	9	2	23.09	1.02
5	9	2	26.74	1.03
5	9	2	24.02	1.87
-6	-9	-2	15.48	1.16
6	9	2	12.00	0.85
6	9	2	13.83	1.81
6	9	2	17.57	2.26
-7	-9	-2	12.51	1.19
7	9	2	13.26	1.90
7	9	2	12.73	2.23
-8	-9	-2	9.87	1.35
8	9	2	10.88	1.81
8	9	2	11.87	2.12
-9	-9	-2	11.50	1.48
9	9	2	10.53	1.78
9	9	2	10.48	1.83
17	-10	-2	1.17	1.39
17	-10	-2	-2.72	2.51
-17	10	2	-0.33	0.70
16	-10	-2	-1.01	2.23
16	-10	-2	1.73	1.26

-16	10	2	1.33	0.65
15	-10	-2	0.65	2.00
15	-10	-2	0.98	1.08
-15	10	2	-0.78	0.51
14	-10	-2	3.30	1.98
14	-10	-2	0.91	1.00
-14	10	2	0.34	0.50
13	-10	-2	-2.09	1.45
13	-10	-2	1.89	0.87
-13	10	2	-0.11	0.41
12	-10	-2	1.59	0.73
-12	10	2	1.29	0.42
11	-10	-2	-0.19	0.62
11	-10	-2	3.18	1.42
-11	10	2	0.64	0.27
-11	10	2	0.31	0.35
-11	10	2	-0.52	0.70
-11	10	2	1.03	0.37
10	-10	-2	1.41	0.65
10	-10	-2	0.24	1.21
10	-10	-2	0.61	0.39
-10	10	2	0.89	0.38
-10	10	2	1.08	0.36
-10	10	2	1.39	0.45
-10	10	2	0.53	0.52
9	-10	-2	6.16	0.99
9	-10	-2	8.49	1.59
9	-10	-2	5.95	0.61
-9	10	2	9.05	0.86
-9	10	2	6.18	0.54
-9	10	2	8.68	1.47
-9	10	2	6.51	0.77
-9	10	2	6.44	0.89
8	-10	-2	-0.80	0.99
8	-10	-2	0.25	0.54
8	-10	-2	0.36	0.29
-8	10	2	0.36	0.25
-8	10	2	1.09	1.16
-8	10	2	-0.30	0.46
-8	10	2	-0.23	0.43
-8	10	2	-0.26	0.55
7	-10	-2	4.08	1.25
7	-10	-2	3.73	0.67
7	-10	-2	3.81	0.44

-7	10	2	3.85	0.68
-7	10	2	5.80	1.36
-7	10	2	3.07	0.69
-7	10	2	2.87	0.38
-7	10	2	3.68	0.56
6	-10	-2	4.16	1.24
6	-10	-2	5.69	0.75
6	-10	-2	4.56	0.41
-6	10	2	4.17	1.19
-6	10	2	4.20	0.78
-6	10	2	4.82	0.74
-6	10	2	3.98	0.37
-6	10	2	5.06	0.73
5	-10	-2	2.21	0.99
5	-10	-2	2.10	0.53
5	-10	-2	1.31	0.23
-5	10	2	1.08	0.62
-5	10	2	1.19	0.57
-5	10	2	3.12	1.12
-5	10	2	1.43	0.48
-5	10	2	1.04	0.22
4	-10	-2	3.68	1.09
4	-10	-2	2.70	0.31
4	-10	-2	3.71	0.58
-4	10	2	2.87	0.26
-4	10	2	2.64	0.67
-4	10	2	3.62	0.84
-4	10	2	3.55	0.59
-4	10	2	7.03	1.40
3	-10	-2	10.62	0.86
3	-10	-2	10.62	1.56
-3	10	2	8.33	1.17
-3	10	2	7.18	1.30
-3	10	2	10.42	0.97
-3	10	2	7.82	1.39
2	-10	-2	30.51	2.22
2	-10	-2	31.86	1.41
-2	10	2	33.88	1.61
-2	10	2	27.85	2.52
-2	10	2	28.25	1.82
-2	10	2	32.04	2.02
1	-10	-2	14.79	1.73
1	-10	-2	15.22	0.90
-1	10	2	15.36	2.60

-1	10	2	11.57	1.39
-1	10	2	11.96	1.19
-1	10	2	14.04	1.52
0	-10	-2	2.54	0.99
0	-10	-2	3.17	0.52
0	10	2	1.46	0.80
0	10	2	3.40	0.79
-1	-10	-2	1.89	1.05
-1	-10	-2	1.03	0.43
1	10	2	1.06	0.82
1	10	2	0.83	0.76
-2	-10	-2	19.04	2.01
-2	-10	-2	17.64	1.05
2	10	2	16.12	1.56
2	10	2	19.19	1.88
-3	-10	-2	33.53	2.45
-3	-10	-2	31.83	1.43
3	10	2	24.74	2.01
3	10	2	33.69	2.46
-4	-10	-2	17.25	2.14
-4	-10	-2	14.63	1.05
4	10	2	15.97	2.07
-5	-10	-2	18.30	1.20
5	10	2	15.75	0.85
5	10	2	17.75	2.37
-6	-10	-2	13.11	1.23
6	10	2	16.09	2.45
6	10	2	13.84	1.93
-7	-10	-2	17.66	1.42
7	10	2	17.87	2.67
7	10	2	13.05	2.11
-8	-10	-2	6.47	1.06
8	10	2	8.47	1.62
8	10	2	7.18	1.88
17	-11	-2	5.06	2.76
17	-11	-2	-0.35	1.36
-17	11	2	0.73	0.74
16	-11	-2	1.07	1.35
16	-11	-2	-4.92	2.26
-16	11	2	0.00	0.64
15	-11	-2	-0.69	1.12
15	-11	-2	-0.16	2.06
-15	11	2	0.67	0.62
14	-11	-2	-2.15	1.82

14	-11	-2	-0.44	0.93
-14	11	2	0.05	0.53
13	-11	-2	2.21	1.74
13	-11	-2	1.66	0.93
-13	11	2	1.53	0.50
12	-11	-2	0.74	0.81
12	-11	-2	1.78	1.67
-12	11	2	0.70	0.42
11	-11	-2	4.29	0.89
-11	11	2	4.86	0.82
-11	11	2	5.21	0.66
-11	11	2	4.84	0.66
10	-11	-2	0.86	0.42
10	-11	-2	1.03	0.69
10	-11	-2	1.18	1.32
-10	11	2	1.63	0.58
-10	11	2	1.07	0.51
-10	11	2	1.43	0.38
-10	11	2	1.61	0.57
9	-11	-2	1.16	1.21
9	-11	-2	1.11	0.39
9	-11	-2	1.28	0.65
-9	11	2	0.85	0.53
-9	11	2	1.14	0.61
-9	11	2	1.16	0.60
-9	11	2	2.15	0.36
8	-11	-2	8.96	0.67
8	-11	-2	7.60	1.09
8	-11	-2	10.57	1.77
-8	11	2	9.63	0.92
-8	11	2	9.72	0.58
-8	11	2	9.50	1.20
-8	11	2	8.27	1.08
7	-11	-2	13.39	1.14
7	-11	-2	15.51	2.07
7	-11	-2	11.02	0.65
-7	11	2	11.61	0.59
-7	11	2	11.98	0.95
-7	11	2	10.42	1.30
-7	11	2	11.58	1.23
6	-11	-2	0.47	0.26
6	-11	-2	1.22	1.02
6	-11	-2	-0.19	0.53
-6	11	2	0.90	0.69

-6	11	2	0.73	0.46
-6	11	2	0.43	0.65
-6	11	2	-0.54	1.52
-6	11	2	0.61	0.22
5	-11	-2	9.42	1.01
5	-11	-2	10.97	0.56
5	-11	-2	11.56	1.89
-5	11	2	10.93	0.51
-5	11	2	9.89	1.99
-5	11	2	10.75	1.32
-5	11	2	10.94	0.95
-5	11	2	8.52	1.37
4	-11	-2	26.58	2.31
4	-11	-2	25.07	1.33
-4	11	2	25.85	1.85
-4	11	2	22.17	1.66
-4	11	2	23.87	1.34
3	-11	-2	13.84	1.01
3	-11	-2	12.96	1.89
-3	11	2	16.34	1.19
-3	11	2	14.49	1.45
-3	11	2	15.05	1.59
2	-11	-2	18.15	2.00
2	-11	-2	17.63	1.06
-2	11	2	18.10	1.35
-2	11	2	18.81	1.79
1	-11	-2	7.35	1.43
1	-11	-2	6.45	0.76
-1	11	2	6.65	1.17
-1	11	2	6.46	0.98
0	-11	-2	5.14	0.72
0	11	2	5.67	0.97
-1	-11	-2	10.79	0.89
-1	-11	-2	7.47	1.39
1	11	2	12.01	1.56
1	11	2	10.56	1.77
-2	-11	-2	21.91	1.21
-2	-11	-2	26.82	2.34
2	11	2	19.87	1.92
2	11	2	20.94	2.27
-3	-11	-2	27.66	2.55
-3	-11	-2	26.88	1.37
3	11	2	27.93	2.57
-4	-11	-2	19.87	2.41

4	11	2	16.80	2.40
-5	-11	-2	21.19	1.40
5	11	2	21.74	2.71
-6	-11	-2	13.43	1.35
6	11	2	10.39	1.89
6	11	2	11.48	2.08
-7	-11	-2	3.62	0.99
7	11	2	1.73	1.42
7	11	2	5.50	1.84
16	-12	-2	-2.61	2.44
16	-12	-2	2.30	1.39
-16	12	2	1.10	0.68
15	-12	-2	3.72	1.37
15	-12	-2	2.93	2.38
-15	12	2	1.12	0.66
14	-12	-2	3.12	2.00
14	-12	-2	1.18	1.08
-14	12	2	1.82	0.58
13	-12	-2	0.92	0.99
13	-12	-2	3.66	2.04
-13	12	2	1.52	0.51
12	-12	-2	0.90	0.84
12	-12	-2	-1.38	1.68
-12	12	2	-0.13	0.46
-12	12	2	-0.09	0.50
-12	12	2	0.41	0.41
11	-12	-2	1.75	0.86
11	-12	-2	2.95	1.63
-11	12	2	0.97	0.44
-11	12	2	2.32	0.57
-11	12	2	1.41	0.65
10	-12	-2	5.58	1.66
10	-12	-2	4.57	0.58
10	-12	-2	5.88	0.92
-10	12	2	4.82	0.84
-10	12	2	3.89	0.58
-10	12	2	6.32	1.04
-10	12	2	4.65	0.74
9	-12	-2	4.51	2.67
9	-12	-2	1.83	0.43
9	-12	-2	0.09	0.71
-9	12	2	1.02	0.67
-9	12	2	1.95	0.58
-9	12	2	0.52	0.32

-9	12	2	0.82	0.72
8	-12	-2	1.73	1.27
8	-12	-2	0.29	0.36
8	-12	-2	0.27	0.65
-8	12	2	1.17	0.75
-8	12	2	1.59	0.56
-8	12	2	1.97	0.78
-8	12	2	0.71	0.30
7	-12	-2	14.95	1.27
7	-12	-2	17.84	2.26
7	-12	-2	17.08	0.82
-7	12	2	17.23	1.52
-7	12	2	15.95	1.16
-7	12	2	17.43	1.58
-7	12	2	16.46	0.73
6	-12	-2	8.47	1.54
6	-12	-2	7.35	0.57
6	-12	-2	8.84	1.09
-6	12	2	9.32	0.51
-6	12	2	10.21	1.00
-6	12	2	8.84	1.36
-6	12	2	13.26	1.57
5	-12	-2	4.58	1.33
5	-12	-2	6.29	0.48
5	-12	-2	6.70	0.97
-5	12	2	6.33	0.44
-5	12	2	6.98	1.06
-5	12	2	6.72	0.96
-5	12	2	5.67	1.08
4	-12	-2	12.60	1.07
4	-12	-2	11.67	2.02
-4	12	2	11.60	1.67
-4	12	2	13.13	1.16
3	-12	-2	30.12	2.55
3	-12	-2	28.46	1.42
-3	12	2	27.88	1.62
-3	12	2	27.45	2.13
2	-12	-2	6.75	1.52
2	-12	-2	7.85	0.86
-2	12	2	6.51	1.04
-2	12	2	8.50	1.55
1	-12	-2	10.83	0.90
1	-12	-2	12.56	2.05
-1	12	2	9.89	1.57

-1	12	2	10.73	1.48
0	-12	-2	24.75	1.28
0	-12	-2	25.64	2.52
0	12	2	22.53	1.87
0	12	2	29.45	2.39
-1	-12	-2	14.65	1.03
-1	-12	-2	11.92	2.23
1	12	2	10.42	1.45
1	12	2	11.77	2.08
-2	-12	-2	17.89	2.52
-2	-12	-2	21.18	1.25
2	12	2	21.77	2.47
-3	-12	-2	24.21	1.40
-3	-12	-2	27.65	2.80
3	12	2	23.04	2.71
-4	-12	-2	5.53	1.70
-4	-12	-2	7.50	1.12
4	12	2	6.45	1.68
-5	-12	-2	10.14	1.30
5	12	2	7.77	1.40
5	12	2	9.26	1.94
-6	-12	-2	11.03	1.51
6	12	2	9.36	1.94
6	12	2	15.80	2.40
16	-13	-2	3.16	2.77
16	-13	-2	1.49	1.55
-16	13	2	0.92	0.71
15	-13	-2	0.07	1.41
15	-13	-2	-2.61	2.31
-15	13	2	-0.08	0.62
14	-13	-2	0.82	2.18
14	-13	-2	1.02	1.18
-14	13	2	0.74	0.59
13	-13	-2	0.07	1.94
13	-13	-2	1.39	1.09
-13	13	2	0.44	0.52
12	-13	-2	-0.27	0.94
12	-13	-2	-0.80	1.81
-12	13	2	-1.23	0.63
11	-13	-2	3.01	1.02
11	-13	-2	1.28	1.73
-11	13	2	3.26	0.83
-11	13	2	2.23	0.48
-11	13	2	2.96	0.72

10	-13	-2	5.78	1.08
10	-13	-2	5.58	1.79
10	-13	-2	4.70	0.64
-10	13	2	5.67	0.84
-10	13	2	6.00	0.87
-10	13	2	3.48	0.95
-10	13	2	5.00	0.63
9	-13	-2	0.28	0.43
9	-13	-2	1.39	0.81
9	-13	-2	-0.55	1.42
-9	13	2	0.74	0.75
-9	13	2	0.02	0.33
-9	13	2	0.73	0.83
-9	13	2	0.98	0.64
8	-13	-2	8.88	1.28
8	-13	-2	9.24	0.73
8	-13	-2	9.13	1.93
-8	13	2	8.59	0.61
-8	13	2	8.08	1.06
-8	13	2	8.78	1.43
-8	13	2	9.17	1.48
7	-13	-2	10.93	1.27
7	-13	-2	11.73	0.75
7	-13	-2	12.32	2.02
-7	13	2	12.30	1.54
-7	13	2	12.38	1.14
-7	13	2	11.73	0.64
-7	13	2	13.83	1.69
6	-13	-2	1.72	0.36
6	-13	-2	1.09	0.70
6	-13	-2	1.84	1.37
-6	13	2	1.91	0.31
-6	13	2	2.58	0.71
-6	13	2	0.93	0.87
5	-13	-2	12.21	1.16
5	-13	-2	16.84	2.40
-5	13	2	11.15	1.77
-5	13	2	11.03	1.21
4	-13	-2	16.85	1.19
4	-13	-2	14.94	2.41
-4	13	2	17.07	1.93
-4	13	2	15.28	1.36
3	-13	-2	11.40	0.99
3	-13	-2	7.16	1.63

-3	13	2	7.70	1.33
-3	13	2	12.97	1.94
2	-13	-2	7.67	0.91
2	-13	-2	7.34	1.71
-2	13	2	8.01	1.16
-2	13	2	8.31	1.56
1	-13	-2	16.65	2.49
1	-13	-2	16.64	1.09
-1	13	2	14.13	2.22
-1	13	2	17.07	1.81
0	-13	-2	18.79	1.20
0	-13	-2	17.04	2.62
0	13	2	18.49	2.39
0	13	2	21.12	2.10
-1	-13	-2	28.71	1.49
-1	-13	-2	28.80	2.92
1	13	2	27.97	2.78
-2	-13	-2	23.91	1.39
-2	-13	-2	22.45	2.82
2	13	2	23.50	2.77
-3	-13	-2	6.33	1.85
-3	-13	-2	7.33	1.14
3	13	2	7.66	1.80
-4	-13	-2	8.56	2.05
-4	-13	-2	8.37	1.04
4	13	2	6.37	1.86
16	-14	-2	1.22	2.82
15	-14	-2	-1.72	2.52
15	-14	-2	-0.50	1.47
-15	14	2	-0.44	0.64
14	-14	-2	-1.78	2.19
14	-14	-2	-0.96	1.32
-14	14	2	0.93	0.62
13	-14	-2	0.91	2.10
13	-14	-2	-0.41	1.17
-13	14	2	1.23	0.56
12	-14	-2	-1.34	1.91
-12	14	2	0.78	0.49
-12	14	2	0.15	0.80
11	-14	-2	3.58	1.94
11	-14	-2	3.20	1.12
-11	14	2	1.03	0.95
-11	14	2	1.20	0.47
10	-14	-2	12.09	1.01

10	-14	-2	11.28	2.46
10	-14	-2	13.89	1.68
-10	14	2	13.16	1.38
-10	14	2	10.87	0.80
-10	14	2	9.96	1.68
-10	14	2	11.24	1.51
9	-14	-2	23.61	3.02
9	-14	-2	18.56	1.05
9	-14	-2	22.82	1.73
-9	14	2	17.59	1.41
-9	14	2	18.90	0.92
-9	14	2	18.67	1.85
-9	14	2	15.40	1.75
8	-14	-2	10.06	2.22
8	-14	-2	9.97	0.82
8	-14	-2	9.59	1.18
-8	14	2	10.31	1.70
-8	14	2	10.87	1.22
-8	14	2	10.15	0.67
7	-14	-2	5.96	1.02
7	-14	-2	4.78	1.76
7	-14	-2	7.06	0.71
-7	14	2	5.66	1.22
-7	14	2	6.60	0.54
-7	14	2	5.89	0.85
6	-14	-2	20.65	2.92
6	-14	-2	18.61	1.44
6	-14	-2	19.07	0.89
-6	14	2	19.46	1.47
-6	14	2	20.32	0.81
-6	14	2	21.83	2.08
5	-14	-2	18.24	1.36
5	-14	-2	16.55	2.79
-5	14	2	19.70	2.16
-5	14	2	19.82	1.52
4	-14	-2	5.25	0.83
4	-14	-2	6.47	1.72
-4	14	2	5.60	1.39
-4	14	2	6.50	1.03
3	-14	-2	7.94	1.00
3	-14	-2	12.52	2.09
-3	14	2	10.45	1.52
-3	14	2	8.08	1.47
2	-14	-2	12.35	1.10

2	-14	-2	17.32	2.65
-2	14	2	13.42	2.30
-2	14	2	14.33	1.78
1	-14	-2	18.48	1.22
1	-14	-2	15.54	2.41
-1	14	2	14.81	2.46
0	-14	-2	22.18	2.99
0	-14	-2	22.75	1.37
0	14	2	20.61	2.72
-1	-14	-2	10.58	1.17
-1	-14	-2	9.28	1.99
1	14	2	9.75	1.99
-2	-14	-2	19.77	2.66
-2	-14	-2	17.68	1.35
2	14	2	21.29	2.93
-3	-14	-2	16.56	1.45
-3	-14	-2	16.55	2.45
3	14	2	16.63	2.46
15	-15	-2	1.32	1.73
15	-15	-2	0.03	2.84
-15	15	2	-0.39	0.66
14	-15	-2	1.12	2.50
14	-15	-2	1.77	1.61
-14	15	2	-0.02	0.65
13	-15	-2	-1.51	1.36
13	-15	-2	0.59	2.30
-13	15	2	-0.69	0.58
12	-15	-2	2.55	1.35
12	-15	-2	-0.35	2.31
-12	15	2	3.16	0.62
-12	15	2	0.78	1.02
11	-15	-2	4.23	1.28
11	-15	-2	6.48	2.21
-11	15	2	4.17	0.60
-11	15	2	1.67	1.11
10	-15	-2	3.47	1.19
10	-15	-2	3.61	2.10
-10	15	2	2.59	1.25
-10	15	2	3.16	0.51
9	-15	-2	11.22	2.36
9	-15	-2	11.99	1.65
-9	15	2	10.08	0.75
-9	15	2	10.42	1.50
8	-15	-2	21.50	1.83

8	-15	-2	24.67	3.38
-8	15	2	21.51	0.95
-8	15	2	18.34	2.14
7	-15	-2	11.25	1.40
7	-15	-2	10.46	2.31
-7	15	2	9.05	1.51
-7	15	2	7.59	0.59
6	-15	-2	6.65	2.02
6	-15	-2	5.02	0.96
-6	15	2	5.04	1.41
5	-15	-2	7.90	1.23
5	-15	-2	11.18	2.29
-5	15	2	9.86	1.74
4	-15	-2	21.63	3.20
4	-15	-2	20.21	1.41
-4	15	2	22.33	2.56
3	-15	-2	17.54	1.28
3	-15	-2	11.61	2.39
-3	15	2	14.61	2.00
2	-15	-2	13.44	1.18
2	-15	-2	6.95	1.96
-2	15	2	17.17	2.55
1	-15	-2	16.79	1.29
1	-15	-2	19.38	3.18
-1	15	2	13.20	2.10
0	-15	-2	12.83	2.29
0	-15	-2	15.71	1.26
0	15	2	13.43	2.11
-1	-15	-2	12.76	2.51
-1	-15	-2	11.32	1.82
1	15	2	13.59	2.11
-2	-15	-2	12.15	2.64
2	15	2	10.79	3.07
14	-16	-2	5.91	2.98
14	-16	-2	3.50	1.83
-14	16	2	3.40	0.77
13	-16	-2	5.63	2.57
13	-16	-2	1.79	1.70
-13	16	2	0.63	0.65
12	-16	-2	2.27	1.51
12	-16	-2	2.79	2.47
-12	16	2	3.26	0.62
11	-16	-2	9.18	2.45
11	-16	-2	12.94	2.11

-11	16	2	10.09	0.92
-11	16	2	11.19	1.56
10	-16	-2	18.56	2.86
10	-16	-2	12.23	1.59
-10	16	2	15.49	0.94
-10	16	2	14.31	2.27
9	-16	-2	5.65	2.32
9	-16	-2	10.58	1.45
-9	16	2	8.14	1.59
-9	16	2	8.01	0.75
8	-16	-2	7.09	1.24
8	-16	-2	8.67	2.36
-8	16	2	5.42	1.52
7	-16	-2	7.27	1.15
7	-16	-2	8.83	2.38
-7	16	2	8.61	1.72
6	-16	-2	15.89	1.52
6	-16	-2	17.24	2.76
-6	16	2	14.12	2.51
5	-16	-2	19.32	3.37
5	-16	-2	15.66	1.43
-5	16	2	19.55	2.63
4	-16	-2	7.75	1.24
4	-16	-2	13.50	2.64
-4	16	2	8.12	1.88
3	-16	-2	20.08	3.34
3	-16	-2	18.79	1.39
-3	16	2	21.14	2.89
2	-16	-2	21.09	3.38
2	-16	-2	17.94	1.96
-2	16	2	16.42	2.29
1	-16	-2	22.56	3.63
-1	16	2	21.89	3.23
0	-16	-2	9.13	2.47
0	16	2	11.25	2.12
13	-17	-2	3.21	3.03
13	-17	-2	-0.20	1.97
12	-17	-2	3.89	2.65
12	-17	-2	7.40	1.84
11	-17	-2	14.98	3.13
-11	17	2	12.24	1.84
10	-17	-2	5.63	1.49
10	-17	-2	7.29	2.70
-10	17	2	5.75	1.69

9	-17	-2	6.01	2.39
9	-17	-2	3.61	1.41
-9	17	2	3.81	1.59
8	-17	-2	12.56	2.17
8	-17	-2	11.28	2.59
-8	17	2	12.89	2.00
7	-17	-2	10.40	2.66
-7	17	2	19.87	2.86
6	-17	-2	5.90	2.31
-6	17	2	5.55	1.70
5	-17	-2	5.88	2.32
-5	17	2	11.40	2.03
4	-17	-2	22.26	3.84
-4	17	2	22.99	3.15
3	-17	-2	23.67	3.78
-3	17	2	28.77	3.43
2	-17	-2	18.67	3.26
-2	17	2	10.78	2.22
10	-18	-2	22.89	3.98
-10	18	2	18.77	2.39
9	-18	-2	4.14	2.72
-9	18	2	9.67	2.10
8	-18	-2	0.42	2.66
-8	18	2	3.30	1.86
7	-18	-2	1.19	2.63
-7	18	2	3.10	1.92
6	-18	-2	8.01	3.07
-6	18	2	10.49	2.27
-5	18	2	21.07	3.39
-5	19	-3	9.87	2.46
5	-19	3	10.76	3.27
-6	19	-3	28.84	3.91
6	-19	3	34.39	5.39
-7	19	-3	11.80	2.56
-1	18	-3	16.45	2.81
1	-18	3	20.63	3.29
-2	18	-3	28.82	3.78
2	-18	3	33.40	4.44
-3	18	-3	21.92	3.52
3	-18	3	23.29	3.94
-4	18	-3	13.87	2.47
4	-18	3	13.89	2.80
-5	18	-3	11.01	2.24
5	-18	3	11.37	2.79

-6	18	-3	20.67	3.33
-6	18	-3	18.42	1.64
6	-18	3	24.27	4.10
-7	18	-3	19.49	3.28
7	-18	3	15.93	2.83
-8	18	-3	17.17	1.14
-8	18	-3	14.47	2.43
8	-18	3	14.91	3.01
-9	18	-3	4.69	2.15
-9	18	-3	8.57	1.06
9	-18	3	3.22	2.61
-10	18	-3	4.22	2.05
-10	18	-3	6.96	0.83
10	-18	3	3.01	2.88
1	17	-3	12.00	2.45
-1	-17	3	10.29	2.53
0	17	-3	16.57	2.58
0	-17	3	20.94	3.69
-1	17	-3	35.73	3.65
1	-17	3	36.16	4.10
-2	17	-3	21.08	3.18
2	-17	3	26.61	3.80
-3	17	-3	10.74	2.23
3	-17	3	12.86	2.74
-4	17	-3	14.39	2.19
-4	17	-3	15.81	0.97
4	-17	3	16.59	2.91
-5	17	-3	23.54	1.15
-5	17	-3	20.69	3.09
5	-17	3	23.21	3.64
-6	17	-3	11.24	0.92
-6	17	-3	8.41	1.97
6	-17	3	11.44	2.64
-7	17	-3	9.01	1.91
-7	17	-3	5.88	0.86
7	-17	3	5.76	2.27
-8	17	-3	7.77	1.94
-8	17	-3	9.45	0.97
8	-17	3	6.42	2.47
-9	17	-3	13.81	1.13
-9	17	-3	16.47	2.26
9	-17	3	15.78	3.16
-10	17	-3	7.86	1.92
-10	17	-3	7.07	1.06

10	-17	3	6.97	2.53
-11	17	-3	0.66	0.66
11	-17	3	-0.56	2.67
-12	17	-3	2.80	0.73
12	-17	3	-5.02	4.65
3	16	-3	6.99	2.19
-3	-16	3	10.98	2.35
2	16	-3	22.89	3.40
-2	-16	3	19.73	3.51
1	16	-3	37.43	3.64
-1	-16	3	34.33	3.74
0	16	-3	16.09	2.37
0	-16	3	25.41	3.19
-1	16	-3	32.99	3.29
-1	16	-3	28.16	1.22
1	-16	3	25.60	3.50
-2	16	-3	30.13	3.13
-2	16	-3	38.54	1.43
2	-16	3	31.83	3.59
-3	16	-3	34.63	3.22
-3	16	-3	35.67	1.43
3	-16	3	37.07	3.68
-4	16	-3	9.22	1.79
-4	16	-3	11.23	0.82
4	-16	3	9.25	2.34
-5	16	-3	18.26	2.66
-5	16	-3	16.57	0.96
5	-16	3	23.91	3.34
5	-16	3	16.34	1.10
-6	16	-3	18.36	1.03
-6	16	-3	14.90	2.47
6	-16	3	22.57	3.39
-7	16	-3	10.76	1.86
-7	16	-3	11.36	0.90
7	-16	3	14.59	2.53
-8	16	-3	18.47	1.58
-8	16	-3	19.24	2.57
8	-16	3	20.74	3.62
-9	16	-3	3.61	1.67
-9	16	-3	8.27	0.90
9	-16	3	10.38	2.46
-10	16	-3	1.92	0.61
-10	16	-3	2.91	1.57
10	-16	3	2.43	2.31

-11	16	-3	2.77	0.68
-11	16	-3	1.12	1.61
11	-16	3	3.99	2.37
-12	16	-3	3.10	0.74
12	-16	3	0.26	2.67
-13	16	-3	-0.61	0.67
13	-16	3	0.35	3.56
5	15	-3	17.93	4.68
-5	-15	3	12.24	2.44
4	15	-3	11.89	2.30
-4	-15	3	13.02	2.50
3	15	-3	26.71	3.49
-3	-15	3	27.99	3.35
2	15	-3	13.19	2.23
-2	-15	3	20.62	3.12
1	15	-3	18.40	2.93
1	15	-3	20.35	1.00
-1	-15	3	16.19	2.99
0	15	-3	33.52	3.09
0	15	-3	32.37	1.32
0	-15	3	32.72	3.37
-1	15	-3	19.03	0.96
-1	15	-3	15.69	2.63
1	-15	3	15.99	2.91
1	-15	3	20.05	1.13
-2	15	-3	20.56	1.01
-2	15	-3	15.39	2.46
2	-15	3	22.22	3.02
2	-15	3	22.70	1.03
-3	15	-3	18.97	2.44
-3	15	-3	21.69	1.04
3	-15	3	23.33	1.10
3	-15	3	24.42	3.04
-4	15	-3	24.18	1.10
4	-15	3	25.32	1.21
4	-15	3	21.98	3.01
-5	15	-3	26.19	1.16
5	-15	3	20.17	2.99
5	-15	3	24.25	1.30
5	-15	3	29.33	2.14
-6	15	-3	16.11	0.92
6	-15	3	15.92	2.10
6	-15	3	16.34	3.00
6	-15	3	13.70	1.06

-7	15	-3	15.17	0.94
7	-15	3	18.94	3.04
7	-15	3	11.39	1.72
7	-15	3	16.17	1.17
-8	15	-3	5.65	0.75
8	-15	3	6.60	0.90
8	-15	3	3.41	1.91
8	-15	3	7.13	1.24
-9	15	-3	5.20	0.64
9	-15	3	6.64	1.20
9	-15	3	4.41	2.14
9	-15	3	3.31	0.96
-10	15	-3	5.64	0.70
10	-15	3	-0.19	2.06
10	-15	3	6.79	1.09
-11	15	-3	0.54	0.57
-11	15	-3	1.14	1.40
11	-15	3	-1.62	1.04
11	-15	3	-1.93	2.21
-12	15	-3	-0.55	0.59
12	-15	3	0.33	1.15
12	-15	3	1.30	2.45
-13	15	-3	1.40	0.68
13	-15	3	-2.44	2.57
13	-15	3	2.01	1.32
6	14	-3	17.02	2.63
-6	-14	3	20.03	3.30
5	14	-3	26.48	3.45
-5	-14	3	22.94	3.21
4	14	-3	17.19	2.42
-4	-14	3	19.74	2.93
3	14	-3	23.00	1.17
3	14	-3	17.63	3.01
-3	-14	3	26.90	3.01
2	14	-3	32.17	1.51
2	14	-3	26.57	3.01
2	14	-3	30.83	1.32
-2	-14	3	29.23	3.00
1	14	-3	30.37	2.93
1	14	-3	30.20	1.27
-1	-14	3	32.06	2.99
0	14	-3	14.74	2.36
0	14	-3	16.64	0.86
0	-14	3	15.33	0.83

0	-14	3	13.98	2.54
-1	14	-3	9.20	0.70
-1	14	-3	12.79	1.91
1	-14	3	10.53	0.71
1	-14	3	7.53	1.71
-2	14	-3	17.98	0.90
2	-14	3	15.19	2.56
2	-14	3	18.88	0.94
-3	14	-3	37.53	1.41
3	-14	3	36.21	1.44
3	-14	3	32.42	3.00
3	-14	3	36.58	2.70
-4	14	-3	11.17	0.72
4	-14	3	11.20	1.95
4	-14	3	11.03	1.55
4	-14	3	12.40	0.89
-5	14	-3	17.00	0.91
5	-14	3	18.49	1.76
5	-14	3	21.61	2.65
5	-14	3	18.35	1.05
-6	14	-3	9.11	0.72
6	-14	3	8.78	1.95
6	-14	3	8.40	0.54
6	-14	3	8.51	1.51
6	-14	3	10.28	0.92
6	-14	3	8.42	0.98
-7	14	-3	7.63	0.73
7	-14	3	7.92	1.19
7	-14	3	5.85	0.98
7	-14	3	4.06	1.74
7	-14	3	8.27	0.61
-8	14	-3	11.11	1.24
-8	14	-3	7.10	0.77
8	-14	3	8.26	0.74
8	-14	3	9.97	1.16
8	-14	3	9.49	1.20
8	-14	3	9.66	2.10
-9	14	-3	3.22	0.53
-9	14	-3	3.51	0.75
9	-14	3	2.69	0.96
9	-14	3	2.53	1.76
9	-14	3	2.65	0.62
9	-14	3	3.64	0.85
-10	14	-3	0.35	0.63

-10	14	-3	0.04	0.47
10	-14	3	0.06	0.85
10	-14	3	0.98	0.84
10	-14	3	-1.63	1.80
10	-14	3	0.85	0.77
-11	14	-3	4.88	0.67
11	-14	3	2.36	1.01
11	-14	3	2.76	2.20
11	-14	3	3.35	0.98
-12	14	-3	2.25	0.63
12	-14	3	1.90	2.19
12	-14	3	2.08	1.07
-13	14	-3	2.05	0.69
13	-14	3	2.61	2.59
13	-14	3	0.66	1.19
-14	14	-3	-0.08	0.65
14	-14	3	0.02	2.52
14	-14	3	0.62	1.41
7	13	-3	18.01	2.72
6	13	-3	9.33	2.27
-6	-13	3	12.05	2.04
5	13	-3	14.12	1.23
5	13	-3	10.02	2.05
5	13	-3	14.40	1.08
-5	-13	3	18.28	2.73
4	13	-3	28.08	3.08
4	13	-3	26.35	1.38
4	13	-3	27.00	1.30
-4	-13	3	25.81	2.70
3	13	-3	29.90	1.33
3	13	-3	30.71	1.35
3	13	-3	33.18	3.05
-3	-13	3	32.06	2.83
2	13	-3	13.29	0.84
2	13	-3	10.32	1.81
2	13	-3	16.32	1.21
2	13	-3	14.13	0.87
2	13	-3	13.64	0.86
-2	-13	3	14.14	2.01
1	13	-3	15.36	2.21
1	13	-3	18.67	1.10
1	13	-3	17.86	0.92
-1	-13	3	19.60	0.87
-1	-13	3	16.29	2.26

0	13	-3	21.91	0.96
0	13	-3	14.04	2.09
0	-13	3	22.95	0.95
0	-13	3	25.96	3.77
-1	13	-3	43.74	1.60
1	-13	3	45.88	1.60
1	-13	3	44.77	3.09
-2	13	-3	14.29	0.75
2	-13	3	12.50	1.52
2	-13	3	12.52	2.15
2	-13	3	13.62	0.80
-3	13	-3	0.64	0.32
3	-13	3	1.18	1.18
3	-13	3	1.03	0.76
3	-13	3	0.15	0.40
-4	13	-3	6.88	0.60
4	-13	3	6.69	1.59
4	-13	3	7.74	1.12
4	-13	3	6.51	0.72
-5	13	-3	12.25	0.73
-5	13	-3	9.30	1.23
5	-13	3	11.78	0.84
5	-13	3	13.31	1.47
5	-13	3	12.50	2.20
5	-13	3	11.64	0.53
-6	13	-3	1.47	0.74
-6	13	-3	1.12	0.36
6	-13	3	0.44	0.77
6	-13	3	-0.09	0.47
6	-13	3	0.89	0.29
6	-13	3	0.14	0.48
-7	13	-3	1.23	0.39
-7	13	-3	1.48	0.67
7	-13	3	0.64	0.74
7	-13	3	0.30	0.48
7	-13	3	-2.70	1.16
7	-13	3	0.70	0.34
7	-13	3	0.64	0.52
-8	13	-3	1.01	0.59
-8	13	-3	0.29	0.40
8	-13	3	1.50	0.83
8	-13	3	0.98	0.42
8	-13	3	1.08	0.59
8	-13	3	0.62	1.39

-9	13	-3	5.99	0.93
-9	13	-3	5.07	0.70
9	-13	3	6.27	0.79
9	-13	3	6.68	1.12
9	-13	3	5.32	0.96
9	-13	3	3.99	1.81
-10	13	-3	2.27	0.58
-10	13	-3	3.39	0.55
10	-13	3	1.50	0.58
10	-13	3	2.77	0.85
10	-13	3	3.66	1.81
10	-13	3	3.90	0.93
-11	13	-3	-0.20	0.50
11	-13	3	1.55	0.80
11	-13	3	-0.24	0.83
11	-13	3	3.60	1.95
-12	13	-3	0.02	0.53
12	-13	3	2.06	2.08
12	-13	3	0.39	0.98
-13	13	-3	0.56	0.60
13	-13	3	1.23	2.10
13	-13	3	-0.85	1.07
-14	13	-3	0.69	0.65
14	-13	3	0.00	2.51
14	-13	3	1.20	1.29
8	12	-3	9.44	2.21
8	12	-3	17.90	2.23
8	12	-3	13.44	2.27
7	12	-3	15.31	2.32
7	12	-3	13.99	1.47
7	12	-3	15.23	1.27
-7	-12	3	16.15	2.78
6	12	-3	15.76	1.33
6	12	-3	16.01	1.17
6	12	-3	13.82	2.12
-6	-12	3	17.07	2.48
5	12	-3	32.77	1.58
5	12	-3	30.61	1.45
5	12	-3	30.48	3.14
-5	-12	3	36.85	2.78
4	12	-3	27.47	1.13
4	12	-3	22.85	2.76
4	12	-3	24.17	1.25
4	12	-3	25.03	1.22

4	12	-3	25.46	1.69
-4	-12	3	24.41	2.43
3	12	-3	7.99	1.71
3	12	-3	7.23	0.74
3	12	-3	5.42	0.71
3	12	-3	5.85	0.90
3	12	-3	6.62	0.68
-3	-12	3	8.58	2.43
2	12	-3	43.77	1.60
2	12	-3	44.77	1.64
2	12	-3	45.49	3.03
2	12	-3	45.47	1.63
-2	-12	3	43.75	2.83
1	12	-3	20.88	0.98
1	12	-3	23.18	1.13
-1	-12	3	21.88	2.23
-1	-12	3	23.51	0.95
0	12	-3	7.36	0.58
0	-12	3	5.21	0.96
0	-12	3	6.78	0.56
0	-12	3	6.93	1.42
-1	12	-3	49.73	1.72
1	-12	3	51.69	1.72
1	-12	3	46.82	3.05
1	-12	3	48.93	2.46
-2	12	-3	31.03	1.15
2	-12	3	26.38	2.38
2	-12	3	28.71	1.18
2	-12	3	30.55	1.88
-3	12	-3	1.89	0.69
-3	12	-3	2.17	0.36
3	-12	3	1.39	0.39
3	-12	3	1.44	1.15
3	-12	3	2.51	0.75
-4	12	-3	4.26	0.81
-4	12	-3	4.53	0.51
4	-12	3	3.88	0.60
4	-12	3	2.92	1.21
4	-12	3	4.35	0.86
-5	12	-3	-0.36	0.57
-5	12	-3	0.45	0.31
5	-12	3	1.06	1.12
5	-12	3	0.57	0.22
5	-12	3	1.07	0.40

5	-12	3	0.54	0.70
-6	12	-3	0.78	0.33
-6	12	-3	-0.26	0.57
6	-12	3	0.68	0.67
6	-12	3	0.20	1.11
6	-12	3	0.94	0.46
6	-12	3	0.45	0.41
6	-12	3	0.94	0.28
-7	12	-3	1.41	0.38
-7	12	-3	1.92	0.59
7	-12	3	1.53	1.21
7	-12	3	0.81	0.48
7	-12	3	1.28	0.47
7	-12	3	1.71	0.36
7	-12	3	0.99	0.67
-8	12	-3	0.42	0.39
-8	12	-3	0.57	0.49
8	-12	3	0.99	0.39
8	-12	3	0.79	0.52
8	-12	3	0.76	1.43
8	-12	3	0.84	0.70
-9	12	-3	0.44	0.40
-9	12	-3	0.25	0.47
9	-12	3	0.37	0.67
9	-12	3	0.25	1.41
9	-12	3	0.42	0.46
9	-12	3	0.89	0.60
-10	12	-3	0.64	0.45
-10	12	-3	0.24	0.43
10	-12	3	0.81	0.69
10	-12	3	0.20	0.52
10	-12	3	-0.95	0.66
10	-12	3	-0.36	1.45
-11	12	-3	0.60	0.48
-11	12	-3	1.06	0.51
11	-12	3	0.77	0.80
11	-12	3	1.66	1.86
11	-12	3	-0.15	0.68
-12	12	-3	-0.35	0.51
12	-12	3	-0.30	1.82
-13	12	-3	2.30	0.63
13	-12	3	2.28	2.05
13	-12	3	2.46	1.08
-14	12	-3	1.09	0.63

14	-12	3	0.05	1.18
14	-12	3	-3.27	2.00
-15	12	-3	0.70	0.73
15	-12	3	-1.09	1.28
15	-12	3	-1.39	2.47
9	11	-3	15.42	2.20
9	11	-3	12.88	2.34
9	11	-3	15.50	1.80
8	11	-3	18.39	2.71
8	11	-3	24.35	3.13
8	11	-3	14.48	1.28
8	11	-3	15.99	2.13
7	11	-3	16.93	2.83
7	11	-3	16.98	1.19
7	11	-3	15.47	1.41
-7	-11	3	17.28	2.49
6	11	-3	25.13	2.82
6	11	-3	20.90	1.20
6	11	-3	21.65	1.07
6	11	-3	20.04	1.69
6	11	-3	20.99	1.35
-6	-11	3	23.62	2.38
5	11	-3	23.11	2.62
5	11	-3	20.89	1.14
5	11	-3	23.25	1.04
5	11	-3	20.93	1.56
5	11	-3	22.05	1.26
-5	-11	3	23.13	2.22
4	11	-3	44.64	3.05
4	11	-3	42.81	1.69
4	11	-3	41.45	1.99
4	11	-3	45.10	1.74
4	11	-3	45.04	1.61
-4	-11	3	50.28	2.84
3	11	-3	15.19	0.85
3	11	-3	15.70	0.82
3	11	-3	15.97	0.87
3	11	-3	16.40	1.12
-3	-11	3	14.39	1.92
2	11	-3	4.38	0.58
2	11	-3	4.94	0.54
2	11	-3	2.58	1.05
2	11	-3	4.83	0.66
-2	-11	3	4.90	0.50

-2	-11	3	3.89	1.11
1	11	-3	10.58	0.79
1	11	-3	12.10	2.19
1	11	-3	10.84	0.67
-1	-11	3	11.51	0.61
-1	-11	3	11.70	1.74
-1	-11	3	12.06	1.34
0	11	-3	60.24	3.10
0	11	-3	55.17	3.26
0	11	-3	52.66	1.83
0	-11	3	53.32	2.54
0	-11	3	54.53	1.81
0	-11	3	49.22	2.94
-1	11	-3	25.05	1.62
-1	11	-3	24.70	2.36
-1	11	-3	27.32	1.07
1	-11	3	29.11	1.73
1	-11	3	29.30	1.09
1	-11	3	30.48	2.30
-2	11	-3	13.76	1.19
-2	11	-3	15.38	2.02
-2	11	-3	14.42	0.72
2	-11	3	14.25	0.74
2	-11	3	15.00	1.33
2	-11	3	16.97	1.89
-3	11	-3	4.02	0.45
-3	11	-3	4.26	1.25
-3	11	-3	3.33	0.66
3	-11	3	4.20	0.52
3	-11	3	4.23	0.76
3	-11	3	5.17	1.12
-4	11	-3	7.22	0.53
-4	11	-3	7.25	1.36
-4	11	-3	9.43	0.96
4	-11	3	7.16	0.36
4	-11	3	6.15	1.31
4	-11	3	6.39	1.05
4	-11	3	6.82	0.62
-5	11	-3	17.59	1.99
-5	11	-3	14.29	0.76
-5	11	-3	15.36	1.10
5	-11	3	14.61	1.29
5	-11	3	14.80	0.83
5	-11	3	14.02	0.89

5	-11	3	15.26	1.90
5	-11	3	14.46	0.62
-6	11	-3	20.64	3.20
-6	11	-3	20.85	0.94
-6	11	-3	20.96	1.24
6	-11	3	19.02	2.17
6	-11	3	21.33	1.07
6	-11	3	22.35	1.03
6	-11	3	19.65	1.40
-7	11	-3	0.36	0.32
-7	11	-3	0.37	0.44
7	-11	3	0.65	0.39
7	-11	3	-0.07	0.41
7	-11	3	0.34	0.57
7	-11	3	0.69	1.12
7	-11	3	0.61	0.33
-8	11	-3	-0.31	0.40
-8	11	-3	0.14	0.34
8	-11	3	-0.19	0.55
8	-11	3	-0.22	1.11
8	-11	3	0.02	0.38
8	-11	3	-0.03	0.50
-9	11	-3	12.30	0.80
-9	11	-3	12.06	0.84
9	-11	3	11.09	1.04
9	-11	3	13.26	1.21
9	-11	3	11.03	0.86
9	-11	3	9.86	2.01
-10	11	-3	2.54	0.47
-10	11	-3	2.70	0.47
10	-11	3	2.64	1.57
10	-11	3	1.56	0.68
10	-11	3	2.35	0.69
10	-11	3	2.70	0.60
-11	11	-3	-0.24	0.40
-11	11	-3	0.14	0.42
11	-11	3	-0.07	1.63
11	-11	3	-0.38	0.69
11	-11	3	0.62	0.60
-12	11	-3	0.87	0.49
12	-11	3	0.51	1.71
12	-11	3	0.20	0.61
12	-11	3	-0.23	0.92
-13	11	-3	0.80	0.58

13	-11	3	1.55	1.00
13	-11	3	-0.51	1.84
-14	11	-3	0.03	0.61
14	-11	3	1.53	2.25
14	-11	3	2.12	1.17
-15	11	-3	-0.86	0.68
15	-11	3	-1.51	2.47
15	-11	3	2.33	1.38
10	10	-3	15.80	1.91
10	10	-3	15.75	1.45
10	10	-3	17.85	2.52
10	10	-3	14.15	2.31
9	10	-3	19.18	2.14
9	10	-3	14.38	2.37
9	10	-3	19.27	1.73
9	10	-3	16.43	1.59
8	10	-3	14.39	1.39
8	10	-3	13.86	1.14
8	10	-3	18.28	2.73
8	10	-3	9.79	1.79
7	10	-3	19.76	1.17
7	10	-3	19.71	1.08
7	10	-3	23.55	2.72
7	10	-3	19.48	1.37
7	10	-3	19.20	1.70
6	10	-3	25.37	1.13
6	10	-3	26.84	2.60
6	10	-3	25.06	1.23
6	10	-3	24.64	1.60
6	10	-3	26.55	1.39
5	10	-3	21.15	1.10
5	10	-3	24.67	1.41
5	10	-3	24.30	1.03
5	10	-3	22.44	2.40
5	10	-3	24.75	1.22
-5	-10	3	24.80	2.07
4	10	-3	21.21	2.23
4	10	-3	24.09	1.29
4	10	-3	22.82	1.10
4	10	-3	22.45	1.06
4	10	-3	26.36	1.01
-4	-10	3	22.52	2.00
3	10	-3	1.74	1.37
3	10	-3	0.58	0.32

3	10	-3	1.09	0.35
3	10	-3	0.89	0.88
3	10	-3	0.26	0.34
3	10	-3	1.00	0.50
-3	-10	3	0.91	0.89
2	10	-3	13.74	2.04
2	10	-3	12.24	0.72
2	10	-3	14.02	0.88
2	10	-3	17.59	1.86
-2	-10	3	12.86	0.64
-2	-10	3	12.35	1.61
-2	-10	3	13.27	1.34
1	10	-3	38.87	1.50
1	10	-3	42.33	1.46
1	10	-3	40.72	2.68
1	10	-3	38.79	2.03
-1	-10	3	41.33	1.40
-1	-10	3	44.44	2.50
-1	-10	3	39.98	2.05
0	10	-3	5.18	0.49
0	10	-3	5.38	0.81
0	10	-3	8.52	1.39
0	-10	3	5.83	1.02
0	-10	3	3.78	0.99
0	-10	3	6.39	0.45
-1	10	-3	2.62	0.66
-1	10	-3	3.57	0.41
-1	10	-3	1.92	1.03
1	-10	3	2.95	0.40
1	-10	3	2.60	0.96
1	-10	3	3.09	0.70
-2	10	-3	3.60	1.02
-2	10	-3	2.92	0.38
-2	10	-3	2.14	0.58
2	-10	3	2.72	0.64
2	-10	3	1.12	0.82
2	-10	3	2.51	0.36
-3	10	-3	0.89	0.26
-3	10	-3	-0.41	0.85
-3	10	-3	0.68	0.44
3	-10	3	0.65	0.28
3	-10	3	0.57	0.49
3	-10	3	0.94	0.86
-4	10	-3	3.41	0.40

-4	10	-3	2.97	1.08
-4	10	-3	4.57	0.76
4	-10	3	3.96	0.51
4	-10	3	3.04	0.62
4	-10	3	1.82	0.91
4	-10	3	4.12	0.49
4	-10	3	3.87	0.28
-5	10	-3	0.22	0.42
-5	10	-3	-0.50	0.87
-5	10	-3	-0.15	0.23
5	-10	3	-0.03	0.91
5	-10	3	0.27	0.47
5	-10	3	0.52	0.21
5	-10	3	0.51	0.39
5	-10	3	0.32	0.31
-6	10	-3	4.29	0.57
-6	10	-3	5.39	1.22
-6	10	-3	3.83	0.45
6	-10	3	4.46	0.62
6	-10	3	4.35	0.71
6	-10	3	4.27	0.42
6	-10	3	4.30	1.21
6	-10	3	3.71	0.54
-7	10	-3	5.62	0.65
-7	10	-3	5.75	1.13
-7	10	-3	4.71	0.53
7	-10	3	4.24	0.66
7	-10	3	5.92	0.62
7	-10	3	4.99	0.74
7	-10	3	4.66	0.52
7	-10	3	4.22	1.11
-8	10	-3	2.15	0.38
-8	10	-3	2.10	0.42
8	-10	3	1.90	0.45
8	-10	3	2.92	1.21
8	-10	3	2.86	0.65
8	-10	3	1.69	0.43
8	-10	3	1.73	0.51
-9	10	-3	12.22	0.77
-9	10	-3	12.24	0.74
9	-10	3	10.11	0.85
9	-10	3	8.48	1.62
9	-10	3	11.37	1.00
9	-10	3	10.44	1.04

-10	10	-3	2.93	0.66
-10	10	-3	3.67	0.59
10	-10	3	3.50	0.72
10	-10	3	2.76	0.60
10	-10	3	4.46	0.69
10	-10	3	3.34	1.42
-11	10	-3	0.37	0.41
-11	10	-3	0.18	0.41
11	-10	3	-0.23	0.61
11	-10	3	-0.44	0.57
11	-10	3	0.85	1.47
-12	10	-3	0.14	0.46
12	-10	3	0.58	0.61
12	-10	3	3.08	1.83
12	-10	3	-0.11	0.75
-13	10	-3	3.84	0.67
13	-10	3	5.25	2.23
13	-10	3	4.59	1.07
-14	10	-3	1.64	0.63
14	-10	3	0.09	2.17
14	-10	3	1.99	1.19
-15	10	-3	0.24	0.68
15	-10	3	0.70	2.52
15	-10	3	0.53	1.31
11	9	-3	21.81	3.09
11	9	-3	14.10	1.42
11	9	-3	12.58	1.96
11	9	-3	15.37	2.39
10	9	-3	10.20	1.94
10	9	-3	8.08	1.49
10	9	-3	12.56	1.71
10	9	-3	10.03	1.17
9	9	-3	13.57	1.45
9	9	-3	16.95	2.64
9	9	-3	12.45	1.10
9	9	-3	13.13	1.85
8	9	-3	17.80	2.46
8	9	-3	15.56	1.07
8	9	-3	15.61	1.35
8	9	-3	15.47	1.69
7	9	-3	25.86	1.77
7	9	-3	23.44	1.13
7	9	-3	24.18	2.48
7	9	-3	23.80	1.20

7	9	-3	24.94	1.41
7	9	-3	27.19	1.60
6	9	-3	13.88	2.05
6	9	-3	16.67	0.87
6	9	-3	16.95	1.08
6	9	-3	17.15	0.95
6	9	-3	16.86	1.20
-6	-9	3	17.80	1.74
5	9	-3	10.56	0.75
5	9	-3	11.08	0.95
5	9	-3	11.00	1.84
5	9	-3	11.01	0.67
5	9	-3	8.61	1.59
5	9	-3	10.68	0.82
-5	-9	3	9.74	1.53
4	9	-3	34.96	2.63
4	9	-3	36.46	1.39
4	9	-3	33.48	1.48
4	9	-3	33.72	1.34
4	9	-3	35.42	1.30
4	9	-3	34.19	2.39
-4	-9	3	38.56	2.21
3	9	-3	44.03	2.16
3	9	-3	53.52	2.87
3	9	-3	44.75	1.59
3	9	-3	47.48	1.58
3	9	-3	39.95	1.74
3	9	-3	42.08	1.59
-3	-9	3	45.03	2.40
-3	-9	3	45.56	2.21
2	9	-3	3.92	0.59
2	9	-3	6.34	0.98
2	9	-3	5.54	0.49
2	9	-3	5.43	1.21
-2	-9	3	5.10	0.95
-2	-9	3	4.96	0.79
-2	-9	3	5.51	0.41
1	9	-3	20.33	1.35
1	9	-3	21.41	0.93
1	9	-3	23.35	0.92
1	9	-3	24.16	1.96
-1	-9	3	22.41	0.86
-1	-9	3	23.74	1.47
-1	-9	3	23.36	1.79

0	9	-3	25.71	1.93
0	9	-3	23.39	1.34
0	9	-3	23.18	0.92
0	-9	3	23.74	0.90
0	-9	3	21.16	1.76
0	-9	3	23.70	1.41
-1	9	-3	6.81	0.83
-1	9	-3	6.91	0.44
-1	9	-3	8.17	1.39
1	-9	3	4.83	0.71
1	-9	3	5.58	1.10
1	-9	3	5.22	0.44
-2	9	-3	19.48	1.10
-2	9	-3	18.27	0.75
-2	9	-3	17.02	1.65
2	-9	3	17.68	0.76
2	-9	3	15.54	1.13
2	-9	3	15.65	1.57
-3	9	-3	2.59	0.31
-3	9	-3	2.57	0.88
-3	9	-3	2.82	0.51
3	-9	3	2.61	0.33
3	-9	3	1.95	0.49
3	-9	3	1.85	0.81
3	-9	3	2.51	0.20
-4	9	-3	1.45	0.39
-4	9	-3	1.57	0.29
-4	9	-3	2.43	0.83
4	-9	3	1.26	0.48
4	-9	3	1.49	0.85
4	-9	3	1.74	0.24
4	-9	3	2.07	0.33
4	-9	3	1.72	0.33
-5	9	-3	0.17	0.34
-5	9	-3	0.19	0.77
-5	9	-3	0.03	0.21
5	-9	3	0.17	0.75
5	-9	3	0.02	0.24
5	-9	3	0.04	0.32
5	-9	3	0.18	0.19
5	-9	3	-0.42	0.38
-6	9	-3	2.03	0.80
-6	9	-3	2.12	0.40
-6	9	-3	1.79	0.30

6	-9	3	3.27	0.47
6	-9	3	2.55	0.37
6	-9	3	2.44	0.99
6	-9	3	2.40	0.52
6	-9	3	2.93	0.44
-7	9	-3	2.27	0.35
-7	9	-3	0.96	0.84
-7	9	-3	2.45	0.39
7	-9	3	3.44	1.07
7	-9	3	2.48	0.55
7	-9	3	2.33	0.48
7	-9	3	2.94	0.44
7	-9	3	2.90	0.39
-8	9	-3	2.92	0.39
-8	9	-3	3.58	1.27
-8	9	-3	2.40	0.38
8	-9	3	2.94	0.46
8	-9	3	2.35	0.43
8	-9	3	2.17	0.51
8	-9	3	2.97	1.12
8	-9	3	2.67	0.54
-9	9	-3	0.21	0.30
-9	9	-3	0.35	0.29
9	-9	3	0.06	0.43
9	-9	3	-0.20	0.95
9	-9	3	0.90	0.48
9	-9	3	0.34	0.43
-10	9	-3	-0.30	0.38
10	-9	3	-0.68	0.49
10	-9	3	0.57	0.55
10	-9	3	0.12	0.49
-11	9	-3	1.69	0.50
-11	9	-3	3.80	0.53
11	-9	3	3.41	1.47
11	-9	3	3.32	0.62
11	-9	3	2.85	0.72
-12	9	-3	6.86	0.81
12	-9	3	5.63	1.75
12	-9	3	9.21	1.32
-13	9	-3	0.95	0.56
13	-9	3	1.56	1.90
13	-9	3	1.14	0.91
-14	9	-3	2.75	0.70
14	-9	3	5.78	1.26

14	-9	3	3.43	2.15
-15	9	-3	4.60	0.83
15	-9	3	2.76	2.36
15	-9	3	3.07	1.33
12	8	-3	5.49	1.35
12	8	-3	4.68	0.88
12	8	-3	2.06	1.57
12	8	-3	5.05	1.87
11	8	-3	16.91	2.27
11	8	-3	11.80	2.00
11	8	-3	13.38	1.77
11	8	-3	13.76	1.27
10	8	-3	7.73	0.98
10	8	-3	6.95	1.31
10	8	-3	9.42	1.17
10	8	-3	8.43	1.75
9	8	-3	12.77	0.97
9	8	-3	11.04	1.29
9	8	-3	16.39	1.69
9	8	-3	13.01	1.92
8	8	-3	9.83	0.84
8	8	-3	9.04	0.82
8	8	-3	9.93	1.44
8	8	-3	8.83	1.10
7	8	-3	2.80	0.62
7	8	-3	2.79	0.42
7	8	-3	1.41	0.44
7	8	-3	2.27	0.79
7	8	-3	2.68	1.24
7	8	-3	1.98	0.61
6	8	-3	7.12	0.86
6	8	-3	6.11	1.25
6	8	-3	7.05	0.60
6	8	-3	10.12	1.76
6	8	-3	6.90	0.65
6	8	-3	6.65	0.78
-6	-8	3	8.31	1.37
5	8	-3	17.87	1.49
5	8	-3	18.85	0.90
5	8	-3	20.15	0.84
5	8	-3	18.22	2.15
5	8	-3	16.82	1.84
5	8	-3	18.54	0.98
5	8	-3	18.99	1.02

-5	-8	3	21.18	1.63
4	8	-3	2.67	0.38
4	8	-3	1.76	0.68
4	8	-3	2.99	0.36
4	8	-3	2.80	1.12
4	8	-3	2.38	0.38
4	8	-3	3.55	0.56
-4	-8	3	3.27	0.85
-4	-8	3	2.55	0.67
3	8	-3	1.68	0.33
3	8	-3	1.50	0.31
3	8	-3	1.84	0.59
3	8	-3	2.91	1.01
3	8	-3	1.73	0.35
3	8	-3	1.24	0.28
-3	-8	3	0.80	0.53
-3	-8	3	1.27	0.72
2	8	-3	13.08	1.52
2	8	-3	12.42	0.64
2	8	-3	11.62	1.01
2	8	-3	11.11	0.60
2	8	-3	11.04	0.55
-2	-8	3	11.99	1.07
-2	-8	3	12.23	0.54
-2	-8	3	10.35	1.27
1	8	-3	4.41	0.76
1	8	-3	4.55	0.94
1	8	-3	4.63	0.40
1	8	-3	5.05	0.41
1	8	-3	4.07	0.29
-1	-8	3	4.03	0.34
-1	-8	3	4.86	0.90
-1	-8	3	3.74	0.64
0	8	-3	0.73	0.44
0	8	-3	0.17	0.68
0	8	-3	1.01	0.24
0	-8	3	1.00	0.22
0	-8	3	0.19	0.39
0	-8	3	0.75	0.59
-1	8	-3	-0.50	0.63
-1	8	-3	0.40	0.39
-1	8	-3	0.45	0.21
1	-8	3	0.91	0.63
1	-8	3	0.03	0.30

1	-8	3	0.23	0.19
-2	8	-3	3.00	0.49
-2	8	-3	2.06	0.29
-2	8	-3	2.67	0.77
2	-8	3	1.79	0.27
2	-8	3	1.76	0.46
2	-8	3	1.76	0.69
-3	8	-3	6.87	0.66
-3	8	-3	6.52	0.42
-3	8	-3	6.93	1.18
3	-8	3	5.82	0.70
3	-8	3	6.06	1.12
3	-8	3	6.73	0.31
3	-8	3	7.51	0.47
3	-8	3	6.28	0.42
-4	8	-3	0.98	0.34
-4	8	-3	1.07	0.23
-4	8	-3	0.34	0.65
4	-8	3	1.20	0.28
4	-8	3	0.58	0.35
4	-8	3	1.05	0.27
4	-8	3	1.04	0.18
4	-8	3	0.85	0.70
-5	8	-3	6.22	0.55
-5	8	-3	6.66	1.18
-5	8	-3	5.25	0.42
5	-8	3	7.39	1.29
5	-8	3	5.00	0.47
5	-8	3	5.65	0.68
5	-8	3	5.77	0.51
5	-8	3	5.20	0.39
-6	8	-3	22.46	0.99
-6	8	-3	22.38	1.65
-6	8	-3	22.98	0.95
6	-8	3	23.47	1.04
6	-8	3	23.18	0.98
6	-8	3	22.86	1.17
6	-8	3	23.36	0.92
6	-8	3	24.15	1.85
-7	8	-3	2.31	0.39
-7	8	-3	1.90	0.73
-7	8	-3	1.97	0.33
7	-8	3	2.20	0.48
7	-8	3	2.34	0.38

7	-8	3	2.16	0.46
7	-8	3	4.59	1.08
7	-8	3	2.12	0.36
-8	8	-3	1.01	0.31
-8	8	-3	0.97	0.28
-8	8	-3	-0.38	0.69
8	-8	3	0.88	0.43
8	-8	3	-0.21	0.31
8	-8	3	1.37	0.94
8	-8	3	0.11	0.36
8	-8	3	0.28	0.42
-9	8	-3	12.01	0.76
9	-8	3	13.89	0.90
9	-8	3	12.59	0.98
9	-8	3	11.72	0.90
9	-8	3	12.16	1.83
-10	8	-3	1.70	0.39
-10	8	-3	2.01	0.43
10	-8	3	1.30	1.15
10	-8	3	1.16	0.48
10	-8	3	0.48	0.49
10	-8	3	1.35	0.59
-11	8	-3	1.39	0.43
-11	8	-3	1.37	0.53
11	-8	3	1.19	1.27
11	-8	3	1.41	0.56
11	-8	3	2.50	0.69
-12	8	-3	13.80	1.12
-12	8	-3	10.84	0.92
12	-8	3	8.94	1.98
12	-8	3	14.58	1.39
-13	8	-3	3.25	0.63
13	-8	3	4.83	1.02
13	-8	3	0.33	1.83
-14	8	-3	8.02	1.04
14	-8	3	10.11	2.58
14	-8	3	8.56	1.40
-15	8	-3	4.53	0.80
15	-8	3	5.44	1.31
15	-8	3	5.89	2.50
12	7	-3	7.16	1.72
12	7	-3	8.56	1.77
12	7	-3	7.85	1.14
12	7	-3	7.80	1.37

11	7	-3	15.60	1.64
11	7	-3	14.99	2.07
11	7	-3	14.42	1.13
11	7	-3	17.96	2.49
10	7	-3	14.23	1.43
10	7	-3	16.19	1.73
10	7	-3	12.84	2.21
9	7	-3	18.65	1.39
9	7	-3	18.26	1.64
9	7	-3	19.02	1.03
9	7	-3	18.31	2.20
8	7	-3	18.75	1.00
8	7	-3	18.24	1.28
8	7	-3	20.42	1.28
8	7	-3	23.25	2.23
8	7	-3	21.45	1.02
8	7	-3	22.52	1.57
7	7	-3	12.36	2.22
7	7	-3	14.49	1.50
7	7	-3	12.38	0.96
7	7	-3	13.90	1.25
7	7	-3	11.75	0.96
7	7	-3	12.57	0.72
7	7	-3	11.73	1.80
7	7	-3	12.27	0.76
6	7	-3	6.67	0.59
6	7	-3	8.18	0.73
6	7	-3	8.18	0.76
6	7	-3	7.65	1.20
6	7	-3	8.95	1.47
6	7	-3	7.11	0.55
6	7	-3	6.56	1.24
-6	-7	3	9.10	1.21
5	7	-3	5.59	0.82
5	7	-3	6.27	1.20
5	7	-3	5.83	0.49
5	7	-3	5.54	0.49
5	7	-3	5.76	1.05
5	7	-3	6.12	0.62
5	7	-3	6.39	0.61
-5	-7	3	5.97	1.10
4	7	-3	15.32	0.76
4	7	-3	15.17	0.77
4	7	-3	15.99	1.19

4	7	-3	12.89	1.60
4	7	-3	14.34	0.71
4	7	-3	14.57	0.69
-4	-7	3	16.52	1.34
-4	-7	3	15.47	1.18
3	7	-3	32.16	1.62
3	7	-3	33.20	1.20
3	7	-3	32.99	1.22
3	7	-3	32.50	1.21
3	7	-3	34.73	2.04
-3	-7	3	35.22	1.15
-3	-7	3	31.61	1.66
-3	-7	3	33.95	1.80
2	7	-3	2.65	0.35
2	7	-3	2.80	0.31
2	7	-3	2.82	0.81
2	7	-3	2.76	0.50
2	7	-3	2.83	0.36
-2	-7	3	2.90	0.28
-2	-7	3	2.40	0.53
-2	-7	3	2.72	0.71
1	7	-3	4.05	0.37
1	7	-3	4.58	0.82
1	7	-3	4.64	0.66
-1	-7	3	4.41	0.33
-1	-7	3	4.82	0.68
-1	-7	3	3.82	0.72
0	7	-3	2.95	0.33
0	7	-3	3.08	0.47
0	7	-3	4.70	0.86
0	-7	3	3.73	0.63
0	-7	3	2.91	0.68
0	-7	3	2.62	0.30
-1	7	-3	0.20	0.30
-1	7	-3	0.23	0.16
-1	7	-3	-0.06	0.55
1	-7	3	0.31	0.53
1	-7	3	0.14	0.34
1	-7	3	0.05	0.17
-2	7	-3	1.48	0.21
-2	7	-3	1.69	0.38
-2	7	-3	2.43	0.66
2	-7	3	1.80	0.26
2	-7	3	1.38	0.39

2	-7	3	1.95	0.66
-3	7	-3	11.06	0.54
-3	7	-3	11.77	1.19
-3	7	-3	13.27	0.75
3	-7	3	10.97	0.79
3	-7	3	11.82	0.46
3	-7	3	12.47	0.59
3	-7	3	12.93	1.23
3	-7	3	11.96	0.54
-4	7	-3	6.02	1.01
-4	7	-3	5.51	0.39
-4	7	-3	5.41	0.50
4	-7	3	6.08	0.64
4	-7	3	6.77	1.05
4	-7	3	6.15	0.35
4	-7	3	5.37	0.52
4	-7	3	6.21	0.44
-5	7	-3	1.07	0.23
-5	7	-3	1.08	0.60
-5	7	-3	1.10	0.27
5	-7	3	0.17	0.58
5	-7	3	1.57	0.26
5	-7	3	1.17	0.24
5	-7	3	1.35	0.30
5	-7	3	1.53	0.38
-6	7	-3	3.47	0.39
-6	7	-3	2.50	0.42
-6	7	-3	1.41	0.64
6	-7	3	3.38	0.89
6	-7	3	3.37	0.40
6	-7	3	2.95	0.45
6	-7	3	3.40	0.43
6	-7	3	3.72	0.48
-7	7	-3	1.50	0.29
-7	7	-3	3.26	0.74
7	-7	3	1.95	0.83
7	-7	3	1.80	0.41
7	-7	3	1.35	0.36
7	-7	3	1.22	0.33
7	-7	3	1.49	0.33
-8	7	-3	14.12	0.75
-8	7	-3	11.76	1.33
-8	7	-3	13.65	0.73
8	-7	3	15.02	0.82

8	-7	3	14.99	0.84
8	-7	3	15.39	0.86
8	-7	3	14.42	1.60
8	-7	3	15.70	0.96
-9	7	-3	0.15	0.30
-9	7	-3	0.04	0.31
9	-7	3	0.60	0.45
9	-7	3	-0.08	0.40
9	-7	3	0.37	0.35
9	-7	3	-0.26	0.89
-10	7	-3	-0.37	0.42
-10	7	-3	0.60	0.35
10	-7	3	0.63	1.09
10	-7	3	0.99	0.58
10	-7	3	0.41	0.41
-11	7	-3	8.79	0.75
-11	7	-3	9.14	1.03
11	-7	3	11.14	1.88
11	-7	3	10.48	1.12
11	-7	3	9.33	0.88
-12	7	-3	13.46	0.93
-12	7	-3	12.85	1.25
12	-7	3	12.72	1.14
12	-7	3	9.14	2.06
12	-7	3	11.73	1.36
-13	7	-3	2.97	0.61
13	-7	3	3.24	1.02
13	-7	3	4.90	2.05
-14	7	-3	5.17	0.77
14	-7	3	7.33	2.14
14	-7	3	5.55	1.29
-15	7	-3	4.29	0.79
15	-7	3	5.48	1.39
15	-7	3	3.72	2.32
13	6	-3	7.37	1.12
13	6	-3	3.04	1.24
13	6	-3	4.99	1.65
13	6	-3	0.70	1.63
12	6	-3	7.37	1.64
12	6	-3	6.62	1.00
12	6	-3	8.59	1.64
12	6	-3	5.57	1.16
11	6	-3	10.20	1.79
11	6	-3	12.76	0.97

11	6	-3	11.68	2.14
11	6	-3	13.40	1.51
10	6	-3	6.58	1.44
10	6	-3	8.20	1.45
10	6	-3	9.02	0.80
9	6	-3	25.44	1.11
9	6	-3	23.03	1.64
9	6	-3	19.72	2.11
9	6	-3	22.79	1.45
8	6	-3	14.29	0.80
8	6	-3	16.18	1.54
8	6	-3	14.88	1.05
8	6	-3	13.69	1.27
8	6	-3	12.79	1.08
8	6	-3	14.80	2.29
8	6	-3	13.97	0.79
8	6	-3	13.80	1.79
7	6	-3	4.63	0.51
7	6	-3	5.05	0.75
7	6	-3	4.50	1.26
7	6	-3	6.67	0.54
7	6	-3	5.73	0.70
7	6	-3	6.88	0.96
7	6	-3	7.39	1.18
7	6	-3	6.46	1.13
6	6	-3	4.08	0.58
6	6	-3	4.43	1.12
6	6	-3	4.22	0.60
6	6	-3	4.57	0.45
6	6	-3	3.82	0.98
6	6	-3	4.28	0.45
6	6	-3	4.86	0.76
-6	-6	3	4.01	0.80
5	6	-3	46.44	2.45
5	6	-3	49.69	1.65
5	6	-3	49.49	1.77
5	6	-3	47.82	2.10
5	6	-3	49.43	1.73
5	6	-3	48.84	1.66
-5	-6	3	49.11	2.15
-5	-6	3	48.85	2.16
4	6	-3	49.05	2.36
4	6	-3	47.88	2.00
4	6	-3	48.11	1.65

4	6	-3	48.23	1.58
4	6	-3	48.68	1.59
4	6	-3	41.83	1.67
-4	-6	3	47.14	2.04
-4	-6	3	45.08	2.08
3	6	-3	2.71	0.52
3	6	-3	3.72	0.34
3	6	-3	2.61	0.34
3	6	-3	2.23	0.70
3	6	-3	3.64	0.38
-3	-6	3	3.58	0.64
-3	-6	3	3.57	0.29
-3	-6	3	1.92	0.44
2	6	-3	0.33	0.18
2	6	-3	-0.22	0.49
2	6	-3	0.16	0.15
2	6	-3	0.33	0.16
2	6	-3	0.31	0.36
-2	-6	3	-0.17	0.30
-2	-6	3	0.31	0.48
-2	-6	3	0.23	0.14
1	6	-3	0.48	0.17
1	6	-3	1.40	0.53
1	6	-3	0.85	0.35
-1	-6	3	0.59	0.17
-1	-6	3	0.92	0.49
-1	-6	3	0.14	0.30
0	6	-3	0.34	0.27
0	6	-3	1.32	0.68
0	6	-3	0.47	0.16
0	-6	3	0.54	0.49
0	-6	3	0.53	0.29
0	-6	3	0.76	0.18
-1	6	-3	3.30	0.46
-1	6	-3	4.39	0.75
-1	6	-3	4.09	0.30
1	-6	3	4.31	0.32
1	-6	3	3.26	0.51
1	-6	3	3.07	0.63
-2	6	-3	1.34	0.28
-2	6	-3	1.43	0.21
-2	6	-3	1.78	0.59
2	-6	3	1.35	0.19
2	-6	3	1.65	0.37

2	-6	3	0.44	0.48
2	-6	3	1.39	0.15
2	-6	3	1.37	0.21
-3	6	-3	2.07	0.55
-3	6	-3	1.94	0.24
3	-6	3	1.38	0.53
3	-6	3	1.32	0.30
3	-6	3	1.82	0.28
3	-6	3	1.81	0.27
3	-6	3	1.76	0.20
-4	6	-3	2.92	0.57
-4	6	-3	3.87	0.38
-4	6	-3	3.25	0.32
4	-6	3	4.57	0.86
4	-6	3	4.33	0.47
4	-6	3	3.47	0.30
4	-6	3	3.41	0.33
4	-6	3	2.96	0.39
-5	6	-3	0.16	0.48
-5	6	-3	0.50	0.20
-5	6	-3	1.15	0.22
5	-6	3	0.82	0.29
5	-6	3	1.12	0.28
5	-6	3	1.24	0.62
5	-6	3	1.11	0.24
5	-6	3	1.18	0.24
-6	6	-3	6.40	0.45
-6	6	-3	6.87	0.96
-6	6	-3	6.48	0.44
6	-6	3	6.48	0.55
6	-6	3	5.90	0.55
6	-6	3	5.78	1.00
6	-6	3	6.30	0.48
6	-6	3	6.98	0.49
-7	6	-3	0.52	0.52
-7	6	-3	0.79	0.26
-7	6	-3	0.89	0.26
7	-6	3	0.74	0.27
7	-6	3	0.86	0.32
7	-6	3	1.37	0.75
7	-6	3	0.89	0.31
7	-6	3	1.10	0.36
-8	6	-3	1.87	0.33
-8	6	-3	2.88	0.70

-8	6	-3	1.92	0.35
8	-6	3	1.25	0.87
8	-6	3	2.10	0.39
8	-6	3	2.27	0.48
8	-6	3	1.87	0.43
8	-6	3	1.66	0.37
-9	6	-3	6.64	0.70
-9	6	-3	6.99	0.60
9	-6	3	7.16	0.62
9	-6	3	7.47	1.36
9	-6	3	6.43	0.76
9	-6	3	5.48	0.73
-10	6	-3	2.09	0.38
-10	6	-3	1.07	0.53
10	-6	3	0.84	1.04
10	-6	3	2.30	0.63
10	-6	3	1.98	0.41
-11	6	-3	0.38	0.39
-11	6	-3	0.04	0.64
11	-6	3	0.00	1.17
11	-6	3	0.32	0.40
-12	6	-3	11.05	1.32
-12	6	-3	9.59	0.88
12	-6	3	9.14	0.84
12	-6	3	8.55	1.28
12	-6	3	8.69	1.85
-13	6	-3	7.90	0.92
13	-6	3	6.98	2.07
13	-6	3	8.96	1.24
-14	6	-3	0.60	0.61
14	-6	3	1.64	2.09
14	-6	3	2.19	1.04
-15	6	-3	1.56	0.74
15	-6	3	7.23	2.68
15	-6	3	2.30	1.26
14	5	-3	3.22	0.79
14	5	-3	5.71	1.76
13	5	-3	5.50	1.53
13	5	-3	7.86	1.03
13	5	-3	7.93	1.28
13	5	-3	8.55	1.78
12	5	-3	6.16	0.89
12	5	-3	7.96	1.47
12	5	-3	6.41	1.41

12	5	-3	7.54	1.22
11	5	-3	9.33	2.17
11	5	-3	14.71	1.38
11	5	-3	11.85	0.89
11	5	-3	13.12	1.99
10	5	-3	19.18	1.60
10	5	-3	17.74	0.97
10	5	-3	17.50	1.91
10	5	-3	19.26	1.39
10	5	-3	17.14	1.68
9	5	-3	1.23	0.40
9	5	-3	0.86	1.38
9	5	-3	1.34	0.59
9	5	-3	1.05	0.78
9	5	-3	1.27	0.68
9	5	-3	0.74	0.85
9	5	-3	0.98	0.36
9	5	-3	0.91	0.89
8	5	-3	2.73	0.51
8	5	-3	3.25	0.94
8	5	-3	4.07	1.22
8	5	-3	2.40	0.38
8	5	-3	2.63	0.39
8	5	-3	2.51	0.58
8	5	-3	3.29	0.70
8	5	-3	3.05	0.75
7	5	-3	33.08	1.24
7	5	-3	36.13	2.25
7	5	-3	33.28	2.05
7	5	-3	33.38	1.46
7	5	-3	33.87	1.58
7	5	-3	34.20	1.37
7	5	-3	33.65	1.25
7	5	-3	33.22	1.71
6	5	-3	5.63	0.60
6	5	-3	5.68	0.73
6	5	-3	5.67	1.00
6	5	-3	6.49	0.47
6	5	-3	5.72	0.45
6	5	-3	7.13	0.90
6	5	-3	6.67	0.55
6	5	-3	5.39	0.90
5	5	-3	8.59	0.87
5	5	-3	9.73	0.55

5	5	-3	8.35	0.49
5	5	-3	8.88	0.48
5	5	-3	8.95	1.18
5	5	-3	9.40	0.60
-5	-5	3	7.71	0.87
-5	-5	3	9.12	0.87
4	5	-3	25.60	0.95
4	5	-3	23.90	0.92
4	5	-3	24.96	1.00
4	5	-3	25.88	1.52
4	5	-3	26.10	1.29
4	5	-3	25.75	0.94
-4	-5	3	27.53	1.39
-4	-5	3	24.95	1.30
3	5	-3	2.77	0.49
3	5	-3	2.41	0.32
3	5	-3	2.17	0.29
3	5	-3	2.51	0.26
3	5	-3	1.31	1.05
-3	-5	3	1.43	0.39
-3	-5	3	2.71	0.54
-3	-5	3	2.59	0.26
2	5	-3	17.55	0.66
2	5	-3	17.49	0.93
2	5	-3	17.33	0.68
2	5	-3	17.51	0.62
2	5	-3	19.57	1.18
-2	-5	3	16.67	0.94
-2	-5	3	17.39	0.65
-2	-5	3	15.96	1.06
1	5	-3	7.73	0.41
1	5	-3	7.71	0.81
1	5	-3	7.49	0.59
-1	-5	3	8.56	0.39
-1	-5	3	7.42	0.63
-1	-5	3	7.86	0.84
0	5	-3	2.40	0.49
0	5	-3	1.44	0.20
0	-5	3	1.10	0.27
0	-5	3	1.68	0.24
0	-5	3	1.99	0.53
-1	5	-3	4.40	0.69
-1	5	-3	4.29	0.42
-1	5	-3	4.65	0.31

1	-5	3	4.39	0.31
1	-5	3	4.07	0.75
1	-5	3	4.13	0.46
-2	5	-3	20.59	0.92
-2	5	-3	23.57	0.84
-2	5	-3	23.12	1.24
2	-5	3	23.64	0.78
2	-5	3	22.96	0.76
2	-5	3	22.26	0.85
2	-5	3	22.80	1.28
2	-5	3	23.54	1.00
-3	5	-3	12.13	0.55
-3	5	-3	13.81	0.95
-3	5	-3	14.25	0.62
3	-5	3	12.09	0.51
3	-5	3	12.38	0.55
3	-5	3	12.06	1.02
3	-5	3	12.88	0.56
3	-5	3	11.77	0.67
-4	5	-3	0.83	0.19
-4	5	-3	0.80	0.19
-4	5	-3	1.15	0.46
4	-5	3	0.70	0.19
4	-5	3	1.00	0.48
4	-5	3	0.91	0.22
4	-5	3	0.61	0.18
4	-5	3	0.63	0.25
-5	5	-3	19.12	0.75
-5	5	-3	17.49	0.75
-5	5	-3	17.66	1.18
5	-5	3	19.58	0.78
5	-5	3	17.74	0.82
5	-5	3	17.98	0.83
5	-5	3	18.03	0.77
5	-5	3	18.04	1.31
-6	5	-3	3.91	0.69
-6	5	-3	3.83	0.38
-6	5	-3	2.84	0.35
6	-5	3	2.95	0.42
6	-5	3	3.23	0.43
6	-5	3	3.43	0.79
6	-5	3	3.59	0.40
6	-5	3	4.08	0.48
-7	5	-3	13.32	0.69

-7	5	-3	12.83	1.10
-7	5	-3	15.47	0.73
7	-5	3	14.38	0.77
7	-5	3	14.51	0.73
7	-5	3	14.26	0.85
7	-5	3	11.66	1.36
7	-5	3	14.15	0.72
-8	5	-3	9.11	0.70
-8	5	-3	8.07	0.57
-8	5	-3	9.10	1.06
8	-5	3	7.87	0.70
8	-5	3	8.28	0.62
8	-5	3	8.51	0.59
8	-5	3	7.99	1.35
8	-5	3	8.39	0.77
-9	5	-3	2.48	0.39
-9	5	-3	3.42	0.59
-9	5	-3	3.56	0.96
9	-5	3	2.75	0.57
9	-5	3	3.58	0.49
9	-5	3	3.21	0.56
9	-5	3	2.52	1.06
-10	5	-3	4.68	0.60
10	-5	3	3.75	1.13
10	-5	3	5.50	0.84
10	-5	3	5.52	0.59
-11	5	-3	22.14	1.57
-11	5	-3	22.81	1.11
11	-5	3	20.65	1.46
11	-5	3	19.71	2.39
11	-5	3	22.31	1.05
-12	5	-3	8.79	1.48
-12	5	-3	13.47	0.94
12	-5	3	9.93	1.35
12	-5	3	10.37	1.92
12	-5	3	13.67	0.87
-13	5	-3	7.65	0.93
13	-5	3	10.19	1.47
13	-5	3	6.54	1.96
-14	5	-3	5.21	0.75
14	-5	3	5.36	2.15
14	-5	3	7.02	1.27
-15	5	-3	2.31	0.77
15	-5	3	3.94	1.25

15	-5	3	0.54	2.36
14	4	-3	11.33	2.00
14	4	-3	11.58	1.17
14	4	-3	14.68	2.01
13	4	-3	5.94	1.33
13	4	-3	7.09	0.94
13	4	-3	6.75	1.55
13	4	-3	5.88	1.12
12	4	-3	8.53	1.29
12	4	-3	10.44	0.90
12	4	-3	9.87	1.41
12	4	-3	8.25	1.44
12	4	-3	10.17	1.41
11	4	-3	16.45	1.37
11	4	-3	14.72	1.72
11	4	-3	13.36	1.55
11	4	-3	15.61	1.84
11	4	-3	11.23	1.00
10	4	-3	6.66	1.04
10	4	-3	8.71	0.69
10	4	-3	8.17	1.21
10	4	-3	7.92	1.28
10	4	-3	8.60	1.33
10	4	-3	10.65	1.76
9	4	-3	4.50	0.53
9	4	-3	5.44	0.76
9	4	-3	4.92	1.03
9	4	-3	5.67	1.04
9	4	-3	4.99	0.85
9	4	-3	7.29	1.30
9	4	-3	5.57	0.87
9	4	-3	4.62	0.60
8	4	-3	1.03	0.29
8	4	-3	1.10	0.93
8	4	-3	0.78	0.52
8	4	-3	0.88	0.43
8	4	-3	0.48	0.28
8	4	-3	0.53	0.69
8	4	-3	1.08	0.39
8	4	-3	0.40	0.54
7	4	-3	5.88	0.47
7	4	-3	6.02	0.91
7	4	-3	5.63	0.56
7	4	-3	6.54	0.48

7	4	-3	6.42	0.85
7	4	-3	5.81	0.66
7	4	-3	7.51	1.10
7	4	-3	6.25	0.77
6	4	-3	2.70	0.35
6	4	-3	2.58	0.32
6	4	-3	3.26	0.57
6	4	-3	2.15	0.33
6	4	-3	2.30	0.44
6	4	-3	2.82	0.43
6	4	-3	2.53	0.73
-6	-4	3	2.14	0.49
5	4	-3	15.06	0.95
5	4	-3	14.37	0.76
5	4	-3	16.10	0.66
5	4	-3	14.99	0.64
5	4	-3	15.31	0.68
5	4	-3	15.71	1.19
-5	-4	3	14.70	0.97
-5	-4	3	17.31	1.02
4	4	-3	17.98	0.80
4	4	-3	19.33	1.17
4	4	-3	18.25	0.72
4	4	-3	17.65	0.97
4	4	-3	17.86	0.70
4	4	-3	17.04	0.69
-4	-4	3	18.22	1.01
-4	-4	3	19.21	1.01
3	4	-3	6.62	0.32
3	4	-3	7.04	0.76
3	4	-3	6.60	0.35
3	4	-3	5.86	0.40
3	4	-3	6.89	0.58
-3	-4	3	6.46	0.71
-3	-4	3	5.80	0.61
-3	-4	3	6.86	0.34
2	4	-3	0.94	0.36
2	4	-3	0.88	0.12
2	4	-3	0.86	0.17
2	4	-3	0.95	0.29
2	4	-3	0.89	0.15
-2	-4	3	0.64	0.34
-2	-4	3	0.83	0.14
-2	-4	3	0.91	0.28

1	4	-3	1.02	0.09
1	4	-3	0.96	0.23
1	4	-3	1.58	0.38
1	4	-3	1.59	0.22
-1	-4	3	1.67	0.19
-1	-4	3	0.94	0.27
-1	-4	3	1.73	0.39
0	4	-3	3.87	0.28
0	4	-3	3.83	0.55
0	4	-3	3.95	0.37
0	-4	3	3.17	0.57
0	-4	3	3.67	0.26
0	-4	3	3.07	0.40
-1	4	-3	8.32	0.31
-1	4	-3	8.19	0.68
-1	4	-3	9.43	0.50
-1	4	-3	8.21	0.40
1	-4	3	8.96	0.35
1	-4	3	10.11	0.55
1	-4	3	9.11	0.41
-2	4	-3	0.11	0.12
-2	4	-3	-0.03	0.30
-2	4	-3	-0.07	0.12
-2	4	-3	-0.05	0.08
2	-4	3	-0.25	0.16
2	-4	3	0.04	0.13
2	-4	3	0.56	0.36
2	-4	3	0.08	0.10
-3	4	-3	0.70	0.16
-3	4	-3	0.54	0.15
3	-4	3	0.74	0.21
3	-4	3	0.76	0.16
3	-4	3	0.61	0.15
3	-4	3	0.86	0.44
-4	4	-3	0.38	0.15
-4	4	-3	0.62	0.17
-4	4	-3	0.53	0.33
-4	4	-3	0.58	0.17
4	-4	3	0.59	0.19
4	-4	3	0.33	0.39
4	-4	3	0.57	0.17
4	-4	3	0.36	0.19
-5	4	-3	0.36	0.17
-5	4	-3	0.14	0.16

-5	4	-3	-0.23	0.33
-5	4	-3	0.07	0.17
5	-4	3	0.42	0.19
5	-4	3	0.21	0.21
5	-4	3	0.23	0.45
5	-4	3	0.21	0.20
-6	4	-3	28.86	1.14
-6	4	-3	32.94	1.19
-6	4	-3	31.79	1.51
-6	4	-3	30.51	1.14
6	-4	3	28.98	1.72
6	-4	3	31.01	1.16
6	-4	3	30.43	1.16
6	-4	3	31.05	1.20
-7	4	-3	0.54	0.32
-7	4	-3	0.48	0.22
-7	4	-3	0.92	0.50
7	-4	3	0.57	0.25
7	-4	3	0.43	0.63
7	-4	3	0.57	0.25
7	-4	3	0.51	0.31
-8	4	-3	0.34	0.44
-8	4	-3	0.53	0.28
-8	4	-3	1.64	0.56
8	-4	3	0.47	0.73
8	-4	3	1.68	0.42
8	-4	3	0.39	0.28
8	-4	3	0.58	0.39
8	-4	3	0.79	0.27
-9	4	-3	11.64	1.05
-9	4	-3	10.91	0.69
9	-4	3	10.82	0.70
9	-4	3	12.40	1.66
9	-4	3	10.43	0.88
9	-4	3	10.63	0.89
9	-4	3	12.45	0.96
-10	4	-3	6.16	0.62
-10	4	-3	6.54	0.89
10	-4	3	6.55	0.65
10	-4	3	7.02	0.90
10	-4	3	5.67	1.41
-11	4	-3	6.17	0.72
-11	4	-3	6.73	1.05
11	-4	3	6.38	1.05

11	-4	3	4.83	1.51
11	-4	3	6.54	0.71
-12	4	-3	12.21	1.69
-12	4	-3	10.16	0.90
12	-4	3	9.37	1.90
12	-4	3	11.23	1.33
12	-4	3	11.87	0.89
-13	4	-3	8.02	0.99
13	-4	3	7.47	2.03
13	-4	3	7.65	1.27
-14	4	-3	8.91	1.13
14	-4	3	4.38	2.34
14	-4	3	7.45	1.30
-15	4	-3	2.89	0.80
15	-4	3	3.89	1.37
15	-4	3	3.62	2.53
15	3	-3	3.63	0.72
15	3	-3	2.41	1.85
15	3	-3	5.17	1.37
14	3	-3	4.40	1.19
14	3	-3	6.88	1.01
14	3	-3	7.37	1.74
13	3	-3	6.80	1.52
13	3	-3	6.98	1.24
13	3	-3	4.34	0.64
13	3	-3	3.71	1.03
12	3	-3	6.41	1.04
12	3	-3	5.29	1.07
12	3	-3	6.20	1.21
12	3	-3	4.47	0.94
11	3	-3	14.37	1.62
11	3	-3	14.78	1.35
11	3	-3	16.42	1.64
-11	-3	3	14.62	1.04
10	3	-3	5.29	0.98
10	3	-3	7.82	0.99
10	3	-3	7.64	1.09
10	3	-3	6.80	1.26
10	3	-3	7.85	1.48
-10	-3	3	5.12	0.72
9	3	-3	2.33	0.38
9	3	-3	2.78	0.49
9	3	-3	2.24	1.05
9	3	-3	2.88	0.81

9	3	-3	2.67	0.60
9	3	-3	2.58	0.60
9	3	-3	2.94	0.72
-9	-3	3	2.08	0.42
8	3	-3	10.74	0.95
8	3	-3	9.86	1.47
8	3	-3	11.45	0.85
8	3	-3	10.57	0.62
8	3	-3	11.13	1.23
8	3	-3	11.42	0.72
8	3	-3	11.78	0.97
7	3	-3	1.18	0.69
7	3	-3	0.52	0.23
7	3	-3	0.86	0.22
7	3	-3	-0.14	0.51
7	3	-3	1.00	0.34
7	3	-3	0.87	0.28
7	3	-3	0.91	0.42
7	3	-3	0.80	0.38
6	3	-3	15.93	0.68
6	3	-3	15.88	0.93
6	3	-3	16.15	0.72
6	3	-3	16.24	0.66
6	3	-3	16.51	1.19
6	3	-3	14.99	0.81
6	3	-3	15.80	0.88
-6	-3	3	15.77	0.95
5	3	-3	7.32	0.55
5	3	-3	6.49	0.37
5	3	-3	7.09	0.64
5	3	-3	7.05	0.42
5	3	-3	7.30	0.41
5	3	-3	5.95	0.78
5	3	-3	6.85	0.52
-5	-3	3	6.03	0.65
4	3	-3	31.67	1.15
4	3	-3	30.28	1.04
4	3	-3	30.10	1.07
4	3	-3	30.61	1.04
4	3	-3	30.51	1.25
4	3	-3	29.08	1.36
-4	-3	3	31.06	1.29
-4	-3	3	29.42	1.38
3	3	-3	8.92	0.37

3	3	-3	9.09	0.57
3	3	-3	9.02	0.46
3	3	-3	8.79	0.40
3	3	-3	8.71	0.67
-3	-3	3	8.94	0.39
-3	-3	3	9.34	0.62
-3	-3	3	9.59	0.63
2	3	-3	0.25	0.12
2	3	-3	0.28	0.16
2	3	-3	0.36	0.13
2	3	-3	0.06	0.24
-2	-3	3	0.21	0.22
-2	-3	3	0.29	0.10
-2	-3	3	0.23	0.23
-2	-3	3	0.35	0.11
1	3	-3	66.50	2.11
1	3	-3	60.51	1.96
1	3	-3	62.62	2.19
-1	-3	3	62.51	1.97
-1	-3	3	66.74	2.21
-1	-3	3	61.56	1.94
0	3	-3	57.91	1.90
0	3	-3	58.16	1.81
0	3	-3	56.99	2.01
0	3	-3	55.81	1.76
0	-3	3	57.49	1.79
0	-3	3	57.45	1.81
0	-3	3	56.94	1.92
0	-3	3	59.13	2.06
-1	3	-3	16.32	0.81
-1	3	-3	15.08	0.55
-1	3	-3	15.53	0.67
-1	3	-3	16.70	0.65
1	-3	3	15.03	0.58
1	-3	3	15.96	0.60
1	-3	3	16.86	0.68
1	-3	3	15.52	0.86
-2	3	-3	25.62	0.90
-2	3	-3	25.67	1.13
-2	3	-3	29.83	0.95
-2	3	-3	25.33	0.88
2	-3	3	26.25	0.96
2	-3	3	25.17	0.90
2	-3	3	24.79	0.90

2	-3	3	25.53	1.22
-3	3	-3	7.41	0.39
-3	3	-3	7.71	0.62
-3	3	-3	7.81	0.39
-3	3	-3	6.96	0.37
3	-3	3	7.45	0.41
3	-3	3	7.03	0.67
-4	3	-3	49.68	1.65
-4	3	-3	50.50	1.67
-4	3	-3	46.57	1.63
-4	3	-3	48.80	1.89
4	-3	3	49.80	1.67
4	-3	3	50.33	1.65
4	-3	3	48.39	2.04
4	-3	3	52.91	1.74
-5	3	-3	15.29	0.95
-5	3	-3	15.19	0.66
-5	3	-3	17.13	0.75
-5	3	-3	15.76	0.69
5	-3	3	15.47	1.12
5	-3	3	15.29	0.74
5	-3	3	15.60	0.68
5	-3	3	15.87	0.71
-6	3	-3	9.93	0.85
-6	3	-3	10.30	0.57
-6	3	-3	10.14	0.54
-6	3	-3	10.19	0.67
6	-3	3	12.06	0.67
6	-3	3	10.06	1.05
6	-3	3	9.21	0.57
6	-3	3	10.26	0.56
-7	3	-3	12.36	0.64
-7	3	-3	11.59	0.94
-7	3	-3	12.76	0.69
7	-3	3	12.63	0.77
7	-3	3	13.25	1.29
7	-3	3	9.79	0.63
7	-3	3	12.51	0.67
-8	3	-3	15.42	1.06
-8	3	-3	14.22	1.11
-8	3	-3	14.12	0.79
-8	3	-3	13.16	0.71
8	-3	3	13.12	0.89
8	-3	3	12.94	0.76

8	-3	3	14.62	1.49
-9	3	-3	19.94	0.97
-9	3	-3	22.40	1.43
9	-3	3	19.05	1.90
9	-3	3	21.31	1.22
9	-3	3	21.36	1.10
-10	3	-3	5.00	0.94
-10	3	-3	2.88	0.43
10	-3	3	1.85	0.43
10	-3	3	4.76	1.36
10	-3	3	2.66	0.62
-11	3	-3	17.72	1.01
-11	3	-3	17.78	1.84
11	-3	3	16.29	1.02
11	-3	3	18.38	1.37
11	-3	3	19.68	2.28
-12	3	-3	8.82	0.89
12	-3	3	6.90	0.88
12	-3	3	10.45	1.33
12	-3	3	7.57	1.83
-13	3	-3	6.23	0.97
13	-3	3	7.12	2.05
13	-3	3	6.03	1.13
-14	3	-3	3.01	0.77
14	-3	3	2.82	1.12
14	-3	3	2.08	2.03
15	-3	3	1.33	2.54
15	2	-3	5.47	1.93
15	2	-3	5.06	1.22
14	2	-3	7.15	1.69
14	2	-3	6.87	1.23
-14	-2	3	4.77	0.91
13	2	-3	2.06	0.87
13	2	-3	1.35	0.96
13	2	-3	0.40	1.15
-13	-2	3	2.04	0.63
12	2	-3	12.61	1.45
12	2	-3	10.36	1.69
12	2	-3	12.51	1.32
12	2	-3	12.30	1.28
-12	-2	3	12.15	0.98
11	2	-3	0.60	0.82
11	2	-3	1.32	0.59
11	2	-3	0.44	0.58

11	2	-3	-2.00	1.19
11	2	-3	0.67	0.69
-11	-2	3	0.37	0.41
10	2	-3	1.05	0.51
10	2	-3	0.31	0.69
10	2	-3	1.42	0.52
10	2	-3	1.26	1.03
10	2	-3	0.79	0.50
10	2	-3	1.48	0.72
-10	-2	3	1.20	0.39
9	2	-3	28.60	1.34
9	2	-3	25.18	1.57
9	2	-3	27.74	1.20
9	2	-3	27.87	1.88
9	2	-3	26.12	1.50
9	2	-3	27.12	1.38
9	2	-3	26.93	1.10
-9	-2	3	26.91	1.15
8	2	-3	12.10	0.85
8	2	-3	13.18	0.64
8	2	-3	10.78	0.84
8	2	-3	11.90	1.11
8	2	-3	12.53	0.71
8	2	-3	13.05	1.28
8	2	-3	10.40	0.94
-8	-2	3	12.21	0.68
7	2	-3	0.92	0.26
7	2	-3	0.59	0.54
7	2	-3	0.66	0.30
7	2	-3	0.27	0.48
7	2	-3	0.54	0.33
7	2	-3	0.89	0.38
7	2	-3	0.56	0.20
-7	-2	3	0.77	0.25
6	2	-3	2.57	0.65
6	2	-3	1.63	0.33
6	2	-3	2.11	0.30
6	2	-3	2.11	0.38
6	2	-3	1.95	0.32
6	2	-3	2.79	0.47
-6	-2	3	1.66	0.35
-6	-2	3	2.40	0.34
5	2	-3	0.10	0.17
5	2	-3	0.36	0.26

5	2	-3	0.54	0.36
5	2	-3	0.35	0.22
5	2	-3	0.46	0.15
5	2	-3	0.43	0.25
5	2	-3	0.39	0.14
-5	-2	3	0.36	0.29
4	2	-3	2.06	0.21
4	2	-3	2.59	0.35
4	2	-3	2.60	0.31
4	2	-3	2.21	0.37
4	2	-3	1.87	0.28
4	2	-3	2.62	0.25
-4	-2	3	2.07	0.34
-4	-2	3	1.95	0.28
3	2	-3	19.00	0.67
3	2	-3	18.57	0.74
3	2	-3	20.21	0.84
3	2	-3	18.85	0.79
-3	-2	3	19.01	0.66
-3	-2	3	17.51	0.71
-3	-2	3	18.88	0.83
-3	-2	3	19.41	0.77
2	2	-3	8.50	0.36
2	2	-3	8.60	0.40
2	2	-3	8.00	0.44
2	2	-3	8.42	0.47
-2	-2	3	8.42	0.39
-2	-2	3	8.18	0.35
-2	-2	3	8.10	0.47
-2	-2	3	8.45	0.45
1	2	-3	2.41	0.25
1	2	-3	2.20	0.29
1	2	-3	2.41	0.20
1	2	-3	2.21	0.22
-1	-2	3	2.34	0.19
-1	-2	3	2.17	0.31
-1	-2	3	2.66	0.22
-1	-2	3	2.45	0.27
0	2	-3	23.91	0.83
0	2	-3	25.11	0.93
0	2	-3	25.28	0.87
0	-2	3	22.83	0.96
0	-2	3	24.61	0.86
0	-2	3	24.38	0.82

0	-2	3	23.51	0.88
-1	2	-3	87.19	2.52
-1	2	-3	74.29	2.47
-1	2	-3	79.92	2.61
-1	2	-3	81.33	2.47
1	-2	3	82.02	2.52
1	-2	3	79.52	2.47
1	-2	3	77.05	2.50
1	-2	3	79.21	2.66
-2	2	-3	26.43	0.97
-2	2	-3	29.66	1.01
-2	2	-3	29.21	1.13
-2	2	-3	28.32	0.96
2	-2	3	28.44	1.23
2	-2	3	29.37	1.01
2	-2	3	28.12	1.00
2	-2	3	27.64	0.96
-3	2	-3	9.13	0.44
-3	2	-3	8.34	0.60
-3	2	-3	9.02	0.48
-3	2	-3	8.43	0.41
3	-2	3	8.71	0.69
3	-2	3	8.90	0.42
3	-2	3	9.47	0.48
-4	2	-3	1.02	0.18
-4	2	-3	1.00	0.21
-4	2	-3	1.39	0.25
-4	2	-3	1.12	0.31
4	-2	3	0.98	0.19
4	-2	3	0.73	0.21
4	-2	3	1.07	0.23
4	-2	3	0.65	0.33
-5	2	-3	6.39	0.64
-5	2	-3	6.80	0.40
-5	2	-3	6.47	0.57
-5	2	-3	6.02	0.44
5	-2	3	6.74	0.83
5	-2	3	6.06	0.46
5	-2	3	6.36	0.41
5	-2	3	6.19	0.50
-6	2	-3	43.95	1.77
-6	2	-3	42.22	1.48
-6	2	-3	43.38	1.54
-6	2	-3	36.14	2.12

6	-2	3	43.60	1.60
6	-2	3	43.19	2.07
6	-2	3	42.33	1.54
6	-2	3	44.32	1.51
-7	2	-3	11.85	0.95
-7	2	-3	12.33	0.99
-7	2	-3	10.94	0.67
-7	2	-3	11.32	0.62
7	-2	3	12.35	0.72
7	-2	3	10.16	1.21
7	-2	3	11.95	0.78
7	-2	3	12.14	0.65
-8	2	-3	23.65	1.45
-8	2	-3	25.01	1.52
-8	2	-3	25.85	1.05
-8	2	-3	24.17	1.11
8	-2	3	25.26	1.83
8	-2	3	25.38	1.08
8	-2	3	26.88	1.14
8	-2	3	23.82	1.24
-9	2	-3	42.31	1.68
-9	2	-3	43.08	2.17
-9	2	-3	42.12	1.58
9	-2	3	44.28	1.81
9	-2	3	41.44	1.66
9	-2	3	43.00	2.51
-10	2	-3	6.18	1.14
-10	2	-3	3.81	0.59
10	-2	3	4.39	0.74
10	-2	3	4.58	0.61
-11	2	-3	3.92	0.55
-11	2	-3	5.13	1.26
11	-2	3	4.83	0.80
11	-2	3	4.97	0.84
11	-2	3	3.62	1.33
-12	2	-3	7.93	0.89
12	-2	3	6.80	1.75
12	-2	3	7.80	1.31
12	-2	3	7.23	0.95
-13	2	-3	3.03	0.69
13	-2	3	5.86	1.94
13	-2	3	4.96	1.04
-14	2	-3	2.62	0.76
14	-2	3	2.93	1.18

14	-2	3	-0.97	2.00
16	1	-3	6.38	2.23
16	1	-3	4.60	1.43
-16	-1	3	3.78	1.01
15	1	-3	2.88	1.15
15	1	-3	4.23	1.87
-15	-1	3	3.55	0.83
14	1	-3	5.14	1.10
14	1	-3	4.61	1.63
-14	-1	3	3.19	0.78
13	1	-3	3.10	1.38
13	1	-3	1.45	0.72
13	1	-3	1.42	0.83
-13	-1	3	2.05	0.59
12	1	-3	-0.27	0.62
12	1	-3	0.64	0.95
12	1	-3	0.41	0.65
12	1	-3	0.11	0.54
-12	-1	3	0.42	0.46
11	1	-3	23.66	1.42
11	1	-3	22.51	1.75
11	1	-3	25.19	1.44
11	1	-3	24.78	1.26
11	1	-3	20.67	2.02
-11	-1	3	22.05	1.12
10	1	-3	9.81	0.69
10	1	-3	10.33	1.05
10	1	-3	10.98	0.85
10	1	-3	10.38	0.80
10	1	-3	9.32	0.95
10	1	-3	11.24	1.49
10	1	-3	10.32	1.20
-10	-1	3	9.29	0.69
9	1	-3	5.56	0.58
9	1	-3	6.30	0.99
9	1	-3	6.52	0.67
9	1	-3	5.22	0.51
9	1	-3	5.66	0.93
9	1	-3	6.09	0.76
9	1	-3	5.98	0.90
-9	-1	3	4.93	0.53
8	1	-3	1.91	0.37
8	1	-3	2.23	0.43
8	1	-3	1.60	0.26

8	1	-3	0.12	0.59
8	1	-3	1.56	0.49
8	1	-3	1.29	0.31
8	1	-3	0.73	0.45
-8	-1	3	1.50	0.30
7	1	-3	0.83	0.21
7	1	-3	0.89	0.29
7	1	-3	1.22	0.51
7	1	-3	0.52	0.37
7	1	-3	0.81	0.24
7	1	-3	1.21	0.34
-7	-1	3	0.71	0.33
-7	-1	3	0.94	0.24
6	1	-3	11.99	0.52
6	1	-3	10.52	0.66
6	1	-3	11.34	0.55
6	1	-3	10.36	0.81
6	1	-3	12.15	0.74
6	1	-3	11.25	0.62
-6	-1	3	10.22	0.80
-6	-1	3	10.80	0.54
5	1	-3	0.86	0.31
5	1	-3	0.32	0.21
5	1	-3	0.17	0.13
5	1	-3	0.57	0.19
5	1	-3	0.24	0.22
-5	-1	3	0.31	0.15
-5	-1	3	0.58	0.24
-5	-1	3	0.40	0.22
4	1	-3	2.00	0.25
4	1	-3	1.69	0.28
4	1	-3	1.89	0.28
4	1	-3	2.01	0.22
4	1	-3	1.60	0.19
4	1	-3	1.26	0.21
-4	-1	3	1.84	0.27
-4	-1	3	1.54	0.25
3	1	-3	4.63	0.31
3	1	-3	5.10	0.25
3	1	-3	4.77	0.31
3	1	-3	4.94	0.35
-3	-1	3	4.94	0.33
-3	-1	3	5.06	0.25
-3	-1	3	5.13	0.34

2	1	-3	32.24	1.10
2	1	-3	31.69	1.07
2	1	-3	31.30	1.02
-2	-1	3	32.30	1.09
-2	-1	3	30.41	1.08
-2	-1	3	31.18	1.01
1	1	-3	2.57	0.25
1	1	-3	2.81	0.18
1	1	-3	2.74	0.21
1	1	-3	2.79	0.19
-1	-1	3	2.82	0.19
-1	-1	3	2.44	0.26
-1	-1	3	2.54	0.21
0	1	-3	0.45	0.14
0	1	-3	0.28	0.10
0	1	-3	0.56	0.15
0	1	-3	0.39	0.12
0	-1	3	0.27	0.10
0	-1	3	0.48	0.16
0	-1	3	0.34	0.10
0	-1	3	0.37	0.15
-1	1	-3	5.52	0.28
-1	1	-3	5.71	0.34
-1	1	-3	6.76	0.40
-1	1	-3	6.11	0.35
1	-1	3	5.56	0.44
1	-1	3	5.83	0.37
1	-1	3	6.12	0.34
1	-1	3	5.79	0.29
-2	1	-3	21.39	0.86
-2	1	-3	19.44	0.76
-2	1	-3	19.56	0.72
-2	1	-3	20.34	0.80
2	-1	3	19.66	0.78
2	-1	3	18.79	0.83
2	-1	3	19.53	0.70
2	-1	3	20.01	0.96
-3	1	-3	1.11	0.25
-3	1	-3	1.40	0.32
-3	1	-3	1.07	0.17
-3	1	-3	0.96	0.18
3	-1	3	0.98	0.21
3	-1	3	0.89	0.16
3	-1	3	1.40	0.37

-4	1	-3	5.50	0.40
-4	1	-3	5.91	0.36
-4	1	-3	5.45	0.55
-4	1	-3	5.97	0.58
4	-1	3	5.63	0.69
4	-1	3	6.33	0.37
4	-1	3	5.92	0.47
4	-1	3	6.26	0.45
-5	1	-3	4.20	0.60
-5	1	-3	4.35	0.40
-5	1	-3	4.10	0.34
5	-1	3	4.11	0.36
5	-1	3	5.05	0.45
5	-1	3	4.72	0.50
5	-1	3	4.14	0.74
-6	1	-3	45.85	1.89
-6	1	-3	43.16	1.52
-6	1	-3	43.76	1.84
-6	1	-3	42.10	1.59
6	-1	3	43.66	1.67
6	-1	3	43.18	2.08
6	-1	3	47.14	1.65
6	-1	3	44.73	1.54
-7	1	-3	44.40	1.95
-7	1	-3	39.21	1.45
-7	1	-3	40.24	1.85
-7	1	-3	40.30	1.55
7	-1	3	41.20	1.58
7	-1	3	41.26	2.11
7	-1	3	40.92	1.49
7	-1	3	41.78	1.65
-8	1	-3	30.79	1.20
-8	1	-3	29.28	1.64
-8	1	-3	33.90	1.83
-8	1	-3	30.80	1.29
8	-1	3	31.31	1.24
8	-1	3	29.29	1.41
8	-1	3	28.93	1.92
8	-1	3	30.48	1.32
-9	1	-3	23.65	1.82
-9	1	-3	19.91	0.97
-9	1	-3	19.93	1.06
9	-1	3	21.07	1.86
9	-1	3	22.34	1.12

9	-1	3	20.67	1.27
-10	1	-3	11.47	1.90
-10	1	-3	10.47	0.78
10	-1	3	11.04	1.09
10	-1	3	11.97	1.76
10	-1	3	11.30	0.92
-11	1	-3	7.88	0.80
11	-1	3	6.63	1.11
11	-1	3	6.63	1.49
11	-1	3	8.33	0.96
11	-1	3	8.64	1.19
-12	1	-3	7.61	0.92
12	-1	3	5.88	1.71
12	-1	3	6.31	0.97
12	-1	3	5.19	1.01
-13	1	-3	12.15	1.15
13	-1	3	10.89	1.65
13	-1	3	12.05	2.35
-14	1	-3	2.69	0.83
14	-1	3	6.28	2.29
14	-1	3	4.02	1.26
16	0	-3	2.29	2.10
16	0	-3	2.22	1.30
-16	0	3	1.98	0.88
15	0	-3	1.89	1.68
15	0	-3	4.09	1.11
-15	0	3	2.31	0.77
14	0	-3	2.43	1.44
14	0	-3	4.25	1.04
-14	0	3	3.64	0.70
13	0	-3	1.54	0.80
13	0	-3	0.11	0.54
13	0	-3	0.25	0.53
13	0	-3	-0.30	1.10
-13	0	3	0.60	0.51
12	0	-3	2.73	0.55
12	0	-3	0.54	0.98
12	0	-3	2.18	0.58
12	0	-3	0.39	0.59
12	0	-3	0.90	1.16
-12	0	3	1.58	0.48
11	0	-3	4.04	0.66
11	0	-3	2.31	0.60
11	0	-3	3.91	0.61

11	0	-3	4.10	0.95
-11	0	3	2.85	0.77
-11	0	3	2.48	0.42
10	0	-3	0.47	0.38
10	0	-3	0.76	0.47
10	0	-3	-0.40	0.44
10	0	-3	0.88	0.67
10	0	-3	0.65	0.32
10	0	-3	0.51	0.34
-10	0	3	0.87	0.35
-10	0	3	0.94	0.58
9	0	-3	0.89	0.30
9	0	-3	0.26	0.47
9	0	-3	0.43	0.53
9	0	-3	0.57	0.26
9	0	-3	0.68	0.31
9	0	-3	0.22	0.39
-9	0	3	0.65	0.27
-9	0	3	0.24	0.48
8	0	-3	2.30	0.49
8	0	-3	2.24	0.56
8	0	-3	1.86	0.42
8	0	-3	1.79	0.31
8	0	-3	1.86	0.28
8	0	-3	2.20	0.38
-8	0	3	2.58	0.47
-8	0	3	1.44	0.27
7	0	-3	26.13	0.99
7	0	-3	27.64	0.97
7	0	-3	25.10	1.22
7	0	-3	26.65	1.18
-7	0	3	25.28	0.98
-7	0	3	27.30	1.12
-7	0	3	24.37	1.03
6	0	-3	0.53	0.17
6	0	-3	0.66	0.17
6	0	-3	0.18	0.29
6	0	-3	0.40	0.24
-6	0	3	0.35	0.17
-6	0	3	0.49	0.22
-6	0	3	0.66	0.21
-6	0	3	0.80	0.28
5	0	-3	14.23	0.71
5	0	-3	14.10	0.58

5	0	-3	14.77	0.70
5	0	-3	14.03	0.63
-5	0	3	14.74	0.59
-5	0	3	14.12	0.69
-5	0	3	14.27	0.63
-5	0	3	15.82	0.66
4	0	-3	27.93	0.92
4	0	-3	25.57	0.98
4	0	-3	26.76	0.92
4	0	-3	27.40	1.06
-4	0	3	26.18	0.93
-4	0	3	26.23	0.95
-4	0	3	28.68	0.95
-4	0	3	28.08	1.03
3	0	-3	18.28	0.68
3	0	-3	18.92	0.65
3	0	-3	18.79	0.66
3	0	-3	18.64	0.77
3	0	-3	18.76	0.66
-3	0	3	19.38	0.69
-3	0	3	19.05	0.64
-3	0	3	19.06	0.76
2	0	-3	2.68	0.20
2	0	-3	2.33	0.28
2	0	-3	2.42	0.17
-2	0	3	2.56	0.28
-2	0	3	2.55	0.16
-2	0	3	2.45	0.20
1	0	-3	0.12	0.10
1	0	-3	0.33	0.16
1	0	-3	0.24	0.08
-1	0	3	0.48	0.14
-1	0	3	0.16	0.15
-1	0	3	0.12	0.08
-1	0	3	0.25	0.09
0	0	-3	23.25	0.85
0	0	-3	21.70	0.74
0	0	-3	21.66	0.85
0	0	3	22.31	0.74
0	0	3	21.30	0.82
0	0	3	23.28	0.88
0	0	3	22.63	0.85
-1	0	-3	7.63	0.46
-1	0	-3	7.01	0.45

-1	0	-3	6.72	0.31
1	0	3	6.83	0.31
1	0	3	7.81	0.43
1	0	3	7.36	0.48
1	0	3	7.58	0.44
-2	0	-3	27.89	1.08
-2	0	-3	22.74	0.90
-2	0	-3	26.23	1.07
-2	0	-3	25.45	0.96
2	0	3	25.01	1.11
2	0	3	27.37	1.01
2	0	3	26.40	0.88
2	0	3	26.72	1.01
-3	0	-3	69.71	2.47
-3	0	-3	73.88	2.47
-3	0	-3	67.83	2.29
-3	0	-3	67.87	2.20
3	0	3	71.85	2.35
3	0	3	69.98	2.51
3	0	3	73.00	2.36
3	0	3	71.26	2.22
-4	0	-3	0.62	0.45
-4	0	-3	0.46	0.19
-4	0	-3	0.55	0.16
-4	0	-3	0.60	0.36
4	0	3	0.51	0.16
4	0	3	0.89	0.36
4	0	3	0.67	0.26
4	0	3	1.18	0.28
-5	0	-3	17.30	1.01
-5	0	-3	16.21	1.06
-5	0	-3	15.76	0.66
-5	0	-3	15.34	0.75
5	0	3	16.49	0.85
5	0	3	15.66	0.83
5	0	3	15.40	0.68
5	0	3	14.53	1.08
-6	0	-3	39.72	1.87
-6	0	-3	38.38	1.48
-6	0	-3	40.42	1.78
-6	0	-3	37.77	1.37
6	0	3	40.61	1.60
6	0	3	40.10	1.94
6	0	3	38.38	1.55

6	0	3	39.93	1.40
-7	0	-3	7.56	0.97
-7	0	-3	7.00	0.60
-7	0	-3	6.83	1.17
-7	0	-3	7.20	0.50
7	0	3	7.30	1.10
7	0	3	6.51	0.54
7	0	3	7.97	0.72
7	0	3	7.30	0.76
-8	0	-3	23.13	1.10
-8	0	-3	23.24	1.00
-8	0	-3	25.95	1.86
8	0	3	22.73	1.19
8	0	3	23.05	1.03
8	0	3	21.53	1.73
-9	0	-3	26.36	2.15
-9	0	-3	24.86	1.27
-9	0	-3	25.89	1.14
9	0	3	28.86	1.36
9	0	3	27.70	2.06
-10	0	-3	9.30	0.87
-10	0	-3	8.91	0.76
-10	0	-3	11.93	2.01
10	0	3	7.47	1.32
10	0	3	9.47	0.95
-11	0	-3	10.15	0.87
11	0	3	7.55	1.26
11	0	3	10.97	1.78
11	0	3	8.39	1.67
11	0	3	11.19	1.07
-12	0	-3	8.63	0.99
12	0	3	9.16	1.14
12	0	3	12.82	1.52
12	0	3	8.24	1.88
-13	0	-3	9.32	1.15
13	0	3	5.01	1.85
13	0	3	9.80	1.36
14	0	3	10.74	2.51
16	-1	-3	5.40	2.15
16	-1	-3	2.54	1.18
-16	1	3	1.92	0.79
15	-1	-3	1.51	1.03
15	-1	-3	2.33	1.64
-15	1	3	1.75	0.72

14	-1	-3	3.69	1.01
14	-1	-3	3.91	1.43
-14	1	3	3.50	0.67
13	-1	-3	0.45	0.70
13	-1	-3	-0.19	0.33
13	-1	-3	-0.13	1.05
-13	1	3	0.65	0.43
12	-1	-3	2.92	1.07
12	-1	-3	2.04	0.37
-12	1	3	2.36	0.85
11	-1	-3	13.67	0.70
11	-1	-3	13.42	1.48
11	-1	-3	12.91	1.12
11	-1	-3	12.88	0.78
-11	1	3	14.24	0.83
-11	1	3	14.41	1.22
10	-1	-3	14.12	1.04
10	-1	-3	14.29	1.30
10	-1	-3	14.54	0.67
10	-1	-3	12.21	0.78
10	-1	-3	12.92	0.82
10	-1	-3	13.49	0.75
-10	1	3	14.36	0.76
-10	1	3	15.31	1.10
9	-1	-3	0.20	0.19
9	-1	-3	0.43	0.29
9	-1	-3	0.04	0.22
9	-1	-3	-0.09	0.35
9	-1	-3	0.47	0.56
9	-1	-3	0.79	0.28
-9	1	3	0.41	0.40
-9	1	3	0.34	0.25
8	-1	-3	0.70	0.21
8	-1	-3	0.28	0.16
8	-1	-3	0.52	0.28
8	-1	-3	0.67	0.35
8	-1	-3	-0.05	0.40
-8	1	3	-0.24	0.27
-8	1	3	0.22	0.19
-8	1	3	0.77	0.35
7	-1	-3	11.39	0.46
7	-1	-3	10.42	0.80
7	-1	-3	10.87	0.73
7	-1	-3	11.17	0.53

-7	1	3	11.16	0.68
-7	1	3	12.42	0.71
-7	1	3	10.44	0.53
-7	1	3	10.26	0.53
6	-1	-3	1.78	0.24
6	-1	-3	1.35	0.40
6	-1	-3	1.10	0.17
6	-1	-3	0.95	0.28
-6	1	3	1.18	0.19
-6	1	3	1.25	0.20
-6	1	3	1.25	0.30
-6	1	3	1.24	0.26
5	-1	-3	14.82	0.57
5	-1	-3	15.18	0.78
5	-1	-3	14.29	0.51
5	-1	-3	14.16	0.72
-5	1	3	14.74	0.70
-5	1	3	14.06	0.67
-5	1	3	14.53	0.57
-5	1	3	14.23	0.56
4	-1	-3	35.93	1.14
4	-1	-3	34.83	1.31
4	-1	-3	35.16	1.15
-4	1	3	35.22	1.28
-4	1	3	35.29	1.14
-4	1	3	33.90	1.16
-4	1	3	36.72	1.27
3	-1	-3	3.89	0.25
3	-1	-3	4.08	0.22
3	-1	-3	3.73	0.36
3	-1	-3	3.80	0.21
3	-1	-3	3.61	0.37
-3	1	3	3.95	0.36
-3	1	3	3.63	0.35
-3	1	3	3.76	0.22
2	-1	-3	1.51	0.23
2	-1	-3	1.27	0.23
2	-1	-3	1.07	0.12
2	-1	-3	1.14	0.23
-2	1	3	1.44	0.24
-2	1	3	1.33	0.19
-2	1	3	1.12	0.12
-2	1	3	1.38	0.25
1	-1	-3	28.72	1.08

1	-1	-3	28.20	1.05
1	-1	-3	28.34	0.90
1	-1	-3	28.75	1.09
-1	1	3	27.96	1.01
-1	1	3	28.68	0.90
-1	1	3	28.63	1.12
-1	1	3	29.17	1.10
0	-1	-3	64.49	2.17
0	-1	-3	62.97	1.95
0	-1	-3	62.60	2.14
0	1	3	65.10	1.95
0	1	3	62.02	2.12
0	1	3	64.91	2.14
0	1	3	67.00	2.24
-1	-1	-3	24.26	1.06
-1	-1	-3	24.83	0.81
-1	-1	-3	24.94	0.96
1	1	3	25.14	0.81
1	1	3	24.56	1.13
1	1	3	25.95	1.01
1	1	3	23.59	0.98
1	1	3	25.23	1.02
-2	-1	-3	69.43	2.37
-2	-1	-3	73.99	2.57
-2	-1	-3	69.41	2.24
2	1	3	76.35	2.47
2	1	3	73.24	2.25
2	1	3	75.89	2.51
2	1	3	72.21	2.74
-3	-1	-3	21.57	0.79
-3	-1	-3	21.45	0.89
-3	-1	-3	21.74	1.16
3	1	3	23.95	1.04
3	1	3	24.87	1.27
3	1	3	22.32	0.80
3	1	3	21.06	1.06
3	1	3	22.11	0.98
-4	-1	-3	32.34	1.13
-4	-1	-3	31.61	1.56
-4	-1	-3	31.58	1.24
4	1	3	35.64	1.69
4	1	3	33.29	1.14
4	1	3	33.33	1.38
4	1	3	32.02	1.48

4	1	3	34.90	1.34
-5	-1	-3	23.94	1.43
-5	-1	-3	23.00	0.89
-5	-1	-3	22.83	1.00
5	1	3	25.06	1.18
5	1	3	23.76	1.13
5	1	3	26.58	1.56
5	1	3	24.58	1.34
5	1	3	24.39	0.92
-6	-1	-3	11.11	1.25
-6	-1	-3	9.26	0.65
-6	-1	-3	10.01	0.53
6	1	3	9.94	0.78
6	1	3	10.12	1.29
6	1	3	9.44	1.02
6	1	3	9.73	0.57
6	1	3	10.18	0.82
-7	-1	-3	56.34	2.56
-7	-1	-3	48.97	1.73
-7	-1	-3	48.08	1.86
7	1	3	46.47	2.32
7	1	3	50.79	2.60
7	1	3	50.19	1.76
7	1	3	52.51	2.04
7	1	3	50.29	2.01
-8	-1	-3	28.68	2.16
-8	-1	-3	28.85	1.30
-8	-1	-3	27.66	1.16
8	1	3	30.42	1.49
8	1	3	29.78	1.21
8	1	3	30.54	1.96
-9	-1	-3	1.79	0.46
-9	-1	-3	2.03	0.42
-9	-1	-3	2.03	1.17
9	1	3	2.11	0.60
9	1	3	1.36	1.01
-10	-1	-3	15.40	1.10
-10	-1	-3	16.27	0.93
10	1	3	16.15	1.26
10	1	3	14.49	1.78
10	1	3	16.77	1.99
-11	-1	-3	9.28	0.90
11	1	3	7.90	1.29
11	1	3	5.24	1.45

11	1	3	9.74	1.15
11	1	3	7.92	1.53
-12	-1	-3	14.22	1.16
12	1	3	15.81	2.57
12	1	3	13.85	1.65
12	1	3	11.36	1.31
-13	-1	-3	12.66	1.32
13	1	3	14.89	2.40
13	1	3	11.30	1.83
17	-2	-3	0.56	2.26
-17	2	3	0.62	0.94
16	-2	-3	1.08	1.98
16	-2	-3	-0.77	1.09
-16	2	3	2.14	0.79
15	-2	-3	0.74	1.53
15	-2	-3	-0.03	0.91
-15	2	3	0.21	0.62
14	-2	-3	1.25	0.88
-14	2	3	0.71	0.54
13	-2	-3	0.48	0.69
13	-2	-3	-1.82	1.06
-13	2	3	0.41	0.45
12	-2	-3	-0.42	0.94
12	-2	-3	1.50	0.66
-12	2	3	0.43	0.36
-12	2	3	1.15	0.73
11	-2	-3	0.27	0.82
11	-2	-3	-0.16	0.48
-11	2	3	-0.07	0.31
-11	2	3	-0.19	0.59
10	-2	-3	5.10	0.58
10	-2	-3	5.05	0.93
10	-2	-3	6.08	0.54
10	-2	-3	5.17	0.77
-10	2	3	5.56	0.53
-10	2	3	7.44	0.86
9	-2	-3	6.37	1.00
9	-2	-3	5.77	0.46
9	-2	-3	5.24	0.70
-9	2	3	5.23	0.64
-9	2	3	6.41	0.73
-9	2	3	5.80	0.47
8	-2	-3	11.86	1.03
8	-2	-3	13.22	0.85

8	-2	-3	13.47	0.62
-8	2	3	13.12	0.79
-8	2	3	13.04	0.63
-8	2	3	13.83	0.85
7	-2	-3	9.58	0.85
7	-2	-3	9.71	0.48
7	-2	-3	7.90	0.64
-7	2	3	9.73	0.65
-7	2	3	8.41	0.68
-7	2	3	9.42	0.48
6	-2	-3	6.16	0.74
6	-2	-3	6.33	0.35
6	-2	-3	5.56	0.28
6	-2	-3	6.13	0.52
-6	2	3	5.97	0.29
-6	2	3	6.44	0.50
-6	2	3	5.99	0.36
-6	2	3	5.96	0.56
5	-2	-3	0.48	0.20
5	-2	-3	0.24	0.30
5	-2	-3	0.58	0.13
5	-2	-3	0.66	0.14
-5	2	3	0.49	0.22
-5	2	3	0.58	0.30
-5	2	3	0.46	0.14
-5	2	3	0.62	0.10
4	-2	-3	72.30	2.52
4	-2	-3	72.98	2.30
4	-2	-3	72.58	2.45
4	-2	-3	70.99	2.24
-4	2	3	74.99	2.43
-4	2	3	72.20	2.24
-4	2	3	74.26	2.49
3	-2	-3	19.89	0.93
3	-2	-3	21.16	0.80
3	-2	-3	19.36	0.67
-3	2	3	20.48	0.86
-3	2	3	19.25	0.68
-3	2	3	20.95	0.96
-3	2	3	20.36	0.74
2	-2	-3	0.79	0.22
2	-2	-3	1.14	0.11
2	-2	-3	1.00	0.13
2	-2	-3	1.49	0.25

2	-2	-3	0.79	0.27
-2	2	3	0.92	0.24
-2	2	3	1.37	0.21
-2	2	3	1.23	0.31
1	-2	-3	29.60	0.91
1	-2	-3	27.76	1.16
1	-2	-3	28.67	1.21
-1	2	3	29.51	0.90
-1	2	3	29.41	1.15
-1	2	3	30.55	1.28
0	-2	-3	3.83	0.18
0	-2	-3	4.35	0.54
0	-2	-3	4.10	0.41
0	-2	-3	3.80	0.48
0	2	3	3.73	0.18
0	2	3	3.36	0.42
0	2	3	4.14	0.42
0	2	3	3.87	0.57
-1	-2	-3	42.47	1.38
-1	-2	-3	43.86	1.56
-1	-2	-3	43.03	1.75
-1	-2	-3	47.47	1.76
1	2	3	45.10	1.68
1	2	3	46.20	1.87
1	2	3	43.95	1.38
1	2	3	44.97	1.62
-2	-2	-3	37.98	1.19
-2	-2	-3	38.93	1.64
-2	-2	-3	37.73	1.36
2	2	3	35.94	1.46
2	2	3	39.32	1.74
2	2	3	36.52	1.42
2	2	3	34.59	1.49
2	2	3	36.80	1.20
-3	-2	-3	10.59	0.61
-3	-2	-3	11.78	0.48
-3	-2	-3	10.96	0.96
3	2	3	11.03	0.48
3	2	3	12.32	0.79
3	2	3	10.72	1.08
3	2	3	12.26	0.73
3	2	3	11.34	0.77
-4	-2	-3	11.48	0.51
-4	-2	-3	11.58	0.65

-4	-2	-3	12.14	1.10
4	2	3	11.09	0.51
4	2	3	11.13	0.84
4	2	3	9.17	0.80
4	2	3	13.78	1.23
4	2	3	11.44	0.77
-5	-2	-3	34.06	1.31
-5	-2	-3	31.46	1.15
-5	-2	-3	32.83	1.82
5	2	3	29.00	1.88
5	2	3	33.23	1.54
5	2	3	32.24	1.57
5	2	3	34.37	1.18
5	2	3	34.92	1.45
-6	-2	-3	63.09	2.30
-6	-2	-3	67.44	2.94
-6	-2	-3	65.47	2.15
6	2	3	62.16	2.99
6	2	3	71.17	2.58
6	2	3	65.22	2.45
6	2	3	66.58	2.17
6	2	3	64.76	2.67
-7	-2	-3	21.77	1.05
-7	-2	-3	20.18	0.89
-7	-2	-3	17.70	1.89
7	2	3	18.96	1.48
7	2	3	22.30	1.33
7	2	3	20.05	0.92
-8	-2	-3	10.16	1.51
-8	-2	-3	9.79	0.66
-8	-2	-3	8.91	0.78
8	2	3	9.25	0.71
8	2	3	8.96	1.38
8	2	3	9.35	1.08
-9	-2	-3	7.99	0.73
-9	-2	-3	9.96	0.90
9	2	3	9.98	1.63
9	2	3	8.94	1.12
9	2	3	10.48	1.65
-10	-2	-3	14.52	1.07
-10	-2	-3	14.93	1.11
10	2	3	14.69	1.41
10	2	3	10.70	1.90
-11	-2	-3	10.31	1.02

11	2	3	15.25	2.28
11	2	3	10.20	1.34
11	2	3	10.01	1.44
-12	-2	-3	15.36	1.27
12	2	3	15.78	1.74
12	2	3	11.19	1.99
-13	-2	-3	5.86	0.97
13	2	3	1.31	1.87
13	2	3	4.63	1.47
17	-3	-3	0.90	1.26
17	-3	-3	1.16	2.37
-17	3	3	1.82	0.87
16	-3	-3	2.33	2.08
16	-3	-3	1.81	1.11
-16	3	3	0.89	0.70
15	-3	-3	2.62	1.65
15	-3	-3	2.38	1.01
-15	3	3	2.31	0.68
14	-3	-3	0.25	0.74
14	-3	-3	-0.29	1.25
-14	3	3	-0.52	0.45
13	-3	-3	0.27	0.67
13	-3	-3	0.52	1.16
-13	3	3	0.99	0.42
12	-3	-3	0.86	0.61
12	-3	-3	-0.50	0.97
-12	3	3	1.09	0.71
-12	3	3	0.50	0.36
11	-3	-3	2.48	0.61
11	-3	-3	3.15	0.97
-11	3	3	2.37	0.69
-11	3	3	3.47	0.51
10	-3	-3	4.57	0.50
10	-3	-3	3.31	0.57
10	-3	-3	4.16	0.55
10	-3	-3	5.96	1.00
-10	3	3	5.75	0.88
-10	3	3	4.60	0.48
9	-3	-3	-0.11	0.59
9	-3	-3	0.36	0.24
9	-3	-3	0.60	0.34
-9	3	3	1.07	0.49
-9	3	3	1.28	0.42
-9	3	3	0.92	0.24

8	-3	-3	1.05	0.35
8	-3	-3	1.30	0.59
8	-3	-3	1.36	0.25
-8	3	3	1.05	0.34
-8	3	3	1.79	0.46
-8	3	3	1.09	0.22
7	-3	-3	6.76	0.62
7	-3	-3	6.83	0.40
7	-3	-3	6.19	0.85
-7	3	3	6.47	0.56
-7	3	3	6.52	0.40
6	-3	-3	13.21	0.78
6	-3	-3	13.11	1.02
6	-3	-3	15.03	0.58
6	-3	-3	13.77	0.57
-6	3	3	15.14	0.91
-6	3	3	14.97	0.50
-6	3	3	14.76	0.76
-6	3	3	14.12	0.59
5	-3	-3	241.82	7.57
5	-3	-3	259.51	7.75
5	-3	-3	252.94	7.50
5	-3	-3	246.62	8.16
-5	3	3	241.99	7.49
-5	3	3	265.12	7.88
-5	3	3	248.84	7.51
4	-3	-3	8.00	0.72
4	-3	-3	7.78	0.55
4	-3	-3	7.23	0.47
4	-3	-3	7.44	0.32
-4	3	3	7.38	0.70
-4	3	3	7.87	0.40
-4	3	3	7.53	0.51
-4	3	3	7.26	0.33
3	-3	-3	6.81	0.66
3	-3	-3	7.01	0.55
3	-3	-3	7.14	0.51
3	-3	-3	7.12	0.28
-3	3	3	7.13	0.50
-3	3	3	7.26	0.45
-3	3	3	7.49	0.69
-3	3	3	6.99	0.29
2	-3	-3	2.73	0.42
2	-3	-3	2.54	0.13

2	-3	-3	2.76	0.42
2	-3	-3	2.69	0.13
-2	3	3	2.94	0.40
-2	3	3	2.15	0.43
-2	3	3	2.46	0.40
1	-3	-3	7.21	0.62
1	-3	-3	7.92	0.57
1	-3	-3	7.54	0.73
-1	3	3	7.87	0.57
-1	3	3	7.07	0.57
-1	3	3	6.44	0.73
0	-3	-3	25.49	1.05
0	-3	-3	26.06	1.25
0	3	3	25.98	1.35
0	3	3	25.14	1.17
0	3	3	26.35	1.11
-1	-3	-3	12.68	0.88
-1	-3	-3	12.73	0.44
-1	-3	-3	12.18	0.93
-1	-3	-3	13.31	0.67
1	3	3	13.08	0.74
1	3	3	12.50	0.82
1	3	3	12.28	0.45
1	3	3	13.99	1.04
-2	-3	-3	26.68	1.35
-2	-3	-3	25.89	1.07
-2	-3	-3	26.72	1.45
-2	-3	-3	26.37	0.87
2	3	3	26.89	1.17
2	3	3	24.64	1.53
2	3	3	27.13	1.30
2	3	3	26.80	0.88
-3	-3	-3	29.95	1.65
-3	-3	-3	28.85	1.17
-3	-3	-3	28.83	0.99
3	3	3	28.69	1.43
3	3	3	28.87	1.74
3	3	3	31.73	1.33
3	3	3	29.00	1.34
3	3	3	29.32	1.00
-4	-3	-3	23.82	1.05
-4	-3	-3	24.03	0.87
-4	-3	-3	28.21	1.67
4	3	3	25.46	1.22

4	3	3	21.98	1.73
4	3	3	22.51	1.24
4	3	3	24.82	0.89
4	3	3	24.03	1.34
-5	-3	-3	8.80	1.29
-5	-3	-3	10.54	0.70
-5	-3	-3	9.06	0.51
5	3	3	10.40	0.53
5	3	3	9.39	0.93
5	3	3	9.56	0.99
5	3	3	8.57	1.49
5	3	3	9.73	0.86
-6	-3	-3	10.17	1.61
-6	-3	-3	8.82	0.70
-6	-3	-3	9.63	0.55
6	3	3	9.01	1.03
6	3	3	9.80	1.05
6	3	3	10.00	0.60
6	3	3	7.69	1.33
6	3	3	12.07	1.03
-7	-3	-3	15.92	0.94
-7	-3	-3	14.36	1.99
-7	-3	-3	15.94	0.78
7	3	3	17.67	0.84
7	3	3	14.31	1.35
7	3	3	16.89	1.33
-8	-3	-3	17.72	0.89
-8	-3	-3	15.70	1.04
8	3	3	16.31	1.56
8	3	3	15.81	0.93
8	3	3	18.27	1.74
8	3	3	19.57	1.46
-9	-3	-3	20.73	1.04
-9	-3	-3	20.50	1.22
9	3	3	20.88	1.59
9	3	3	19.74	1.96
-10	-3	-3	9.97	1.06
10	3	3	8.84	1.61
10	3	3	11.37	1.41
-11	-3	-3	7.81	1.07
11	3	3	9.22	1.79
11	3	3	8.08	1.18
11	3	3	6.12	1.11
-12	-3	-3	14.95	1.36

12	3	3	8.91	1.90
12	3	3	13.37	1.72
12	3	3	10.54	1.77
17	-4	-3	3.63	2.29
17	-4	-3	-0.13	1.15
-17	4	3	0.21	0.75
16	-4	-3	1.80	1.07
16	-4	-3	1.80	1.91
-16	4	3	1.82	0.68
15	-4	-3	1.34	0.94
15	-4	-3	0.00	1.67
-15	4	3	-0.82	0.47
14	-4	-3	0.15	0.72
14	-4	-3	1.14	1.36
-14	4	3	-0.86	0.43
13	-4	-3	2.52	1.41
13	-4	-3	2.38	0.72
-13	4	3	2.54	0.48
12	-4	-3	3.94	0.77
12	-4	-3	4.02	1.20
-12	4	3	4.03	0.58
-12	4	3	4.15	0.85
11	-4	-3	0.82	0.36
11	-4	-3	1.19	0.56
11	-4	-3	1.65	0.98
11	-4	-3	1.23	0.50
-11	4	3	1.32	0.67
-11	4	3	0.53	0.31
10	-4	-3	1.97	0.83
10	-4	-3	1.86	0.35
10	-4	-3	1.31	0.47
-10	4	3	1.29	0.58
-10	4	3	1.63	0.30
-10	4	3	1.93	0.47
9	-4	-3	5.95	0.96
9	-4	-3	4.89	0.45
9	-4	-3	5.59	0.69
-9	4	3	4.26	0.42
-9	4	3	5.53	0.64
-9	4	3	4.77	0.81
8	-4	-3	-0.21	0.34
8	-4	-3	0.77	0.60
8	-4	-3	0.40	0.20
-8	4	3	0.19	0.43

-8	4	3	1.02	0.32
-8	4	3	0.18	0.18
7	-4	-3	21.73	0.80
7	-4	-3	21.04	1.37
7	-4	-3	19.71	1.05
-7	4	3	21.30	1.00
-7	4	3	19.54	0.78
-7	4	3	19.96	1.20
6	-4	-3	41.85	1.34
6	-4	-3	39.76	1.43
6	-4	-3	41.66	1.89
6	-4	-3	38.56	1.58
-6	4	3	37.71	1.84
-6	4	3	41.06	1.35
-6	4	3	40.69	1.31
-6	4	3	42.64	1.57
5	-4	-3	5.89	0.50
5	-4	-3	5.75	0.55
5	-4	-3	5.93	0.31
5	-4	-3	5.77	0.81
-5	4	3	6.36	0.40
-5	4	3	5.92	0.78
-5	4	3	6.29	0.33
-5	4	3	6.11	0.52
4	-4	-3	35.24	1.13
4	-4	-3	36.54	1.69
4	-4	-3	34.64	1.40
4	-4	-3	34.27	1.40
-4	4	3	35.91	1.65
-4	4	3	35.80	1.33
-4	4	3	35.32	1.13
-4	4	3	35.19	1.37
3	-4	-3	1.55	0.32
3	-4	-3	1.46	0.35
3	-4	-3	1.10	0.13
3	-4	-3	1.06	0.45
-3	4	3	1.32	0.31
-3	4	3	0.82	0.27
-3	4	3	1.06	0.43
-3	4	3	1.12	0.13
2	-4	-3	8.62	0.63
2	-4	-3	8.47	0.80
2	-4	-3	9.71	0.74
-2	4	3	9.36	0.64

-2	4	3	9.37	0.88
-2	4	3	8.64	0.64
1	-4	-3	21.95	1.23
1	-4	-3	22.41	1.19
1	-4	-3	22.32	1.01
-1	4	3	21.55	1.53
-1	4	3	23.06	1.12
-1	4	3	23.21	1.05
0	-4	-3	12.77	0.94
0	-4	-3	12.12	0.71
0	-4	-3	13.32	0.98
0	4	3	13.87	1.13
0	4	3	11.77	0.84
0	4	3	13.30	0.79
-1	-4	-3	49.69	2.21
-1	-4	-3	52.27	1.84
-1	-4	-3	51.02	1.57
-1	-4	-3	52.22	2.16
1	4	3	54.32	2.38
1	4	3	52.99	2.11
1	4	3	49.15	1.92
-2	-4	-3	106.74	3.51
-2	-4	-3	107.71	4.00
-2	-4	-3	109.26	3.27
-2	-4	-3	105.89	3.84
2	4	3	106.39	3.82
2	4	3	115.04	3.65
2	4	3	107.64	4.13
2	4	3	107.33	3.28
-3	-4	-3	39.75	1.31
-3	-4	-3	38.92	2.09
-3	-4	-3	39.35	1.52
3	4	3	44.07	1.71
3	4	3	35.78	2.22
3	4	3	40.10	1.72
3	4	3	41.21	1.87
3	4	3	40.89	1.32
-4	-4	-3	4.28	0.34
-4	-4	-3	3.93	0.51
-4	-4	-3	3.43	0.85
4	4	3	4.77	1.10
4	4	3	5.41	0.77
4	4	3	4.25	0.35
4	4	3	5.65	0.78

4	4	3	4.00	0.68
-5	-4	-3	18.39	0.76
-5	-4	-3	16.67	1.78
-5	-4	-3	20.09	0.98
5	4	3	18.48	1.20
5	4	3	14.96	2.04
5	4	3	17.84	1.35
5	4	3	19.92	0.79
5	4	3	18.00	1.24
-6	-4	-3	11.74	1.92
-6	-4	-3	13.02	0.65
-6	-4	-3	11.70	0.84
6	4	3	12.70	1.29
6	4	3	12.27	1.21
6	4	3	13.11	0.69
-7	-4	-3	12.31	0.89
-7	-4	-3	10.61	0.69
7	4	3	12.33	1.61
7	4	3	13.18	0.77
7	4	3	11.50	1.34
7	4	3	10.98	1.48
-8	-4	-3	11.65	0.79
-8	-4	-3	12.55	0.97
8	4	3	11.48	1.53
8	4	3	11.42	0.88
8	4	3	11.12	1.43
-9	-4	-3	14.68	0.99
-9	-4	-3	17.32	1.18
9	4	3	14.89	1.68
9	4	3	15.06	1.91
-10	-4	-3	10.29	1.07
-10	-4	-3	12.40	1.20
10	4	3	12.06	1.65
10	4	3	14.43	2.17
-11	-4	-3	13.21	1.29
11	4	3	11.62	1.66
11	4	3	8.08	1.34
11	4	3	9.34	1.84
12	4	3	7.70	1.46
12	4	3	3.71	1.90
12	4	3	5.34	1.28
17	-5	-3	0.47	1.23
17	-5	-3	-1.16	2.24
-17	5	3	-0.02	0.73

16	-5	-3	1.67	1.94
16	-5	-3	-0.96	1.04
-16	5	3	1.47	0.67
15	-5	-3	1.65	1.59
15	-5	-3	-0.32	0.88
-15	5	3	1.26	0.56
14	-5	-3	0.43	0.75
14	-5	-3	2.95	1.45
-14	5	3	0.54	0.48
13	-5	-3	2.71	0.77
13	-5	-3	2.59	1.32
-13	5	3	2.06	0.46
12	-5	-3	0.39	1.09
12	-5	-3	1.29	0.60
-12	5	3	2.06	0.40
-12	5	3	1.91	0.79
11	-5	-3	4.50	0.58
11	-5	-3	5.45	1.14
11	-5	-3	5.36	0.86
-11	5	3	4.66	0.54
-11	5	3	4.90	0.84
-11	5	3	4.25	0.65
10	-5	-3	3.92	0.50
10	-5	-3	4.54	0.69
10	-5	-3	5.44	1.09
-10	5	3	5.95	0.50
-10	5	3	4.42	0.71
-10	5	3	5.93	0.86
9	-5	-3	11.19	0.61
9	-5	-3	10.01	1.33
9	-5	-3	10.24	0.90
-9	5	3	13.10	1.13
-9	5	3	11.94	0.60
-9	5	3	11.46	0.83
8	-5	-3	21.71	1.54
8	-5	-3	21.74	0.85
8	-5	-3	22.03	1.15
-8	5	3	19.00	0.81
-8	5	3	20.48	1.34
-8	5	3	20.94	1.05
7	-5	-3	2.28	0.45
7	-5	-3	3.06	0.72
7	-5	-3	2.59	0.30
-7	5	3	2.33	0.25

-7	5	3	3.11	0.68
-7	5	3	2.52	0.42
-7	5	3	2.41	0.29
-7	5	3	2.04	0.27
6	-5	-3	2.82	0.44
6	-5	-3	1.63	0.59
6	-5	-3	2.05	0.26
-6	5	3	1.97	0.34
-6	5	3	2.80	0.65
-6	5	3	2.67	0.42
-6	5	3	2.45	0.26
5	-5	-3	5.81	0.92
5	-5	-3	5.97	0.62
5	-5	-3	5.80	0.63
5	-5	-3	6.39	0.33
-5	5	3	6.29	0.33
-5	5	3	6.28	0.57
-5	5	3	6.34	0.88
4	-5	-3	3.79	0.22
4	-5	-3	2.66	0.62
4	-5	-3	3.70	0.55
4	-5	-3	3.12	0.46
-4	5	3	3.32	0.53
-4	5	3	4.19	0.72
-4	5	3	3.63	0.22
-4	5	3	3.61	0.49
3	-5	-3	4.96	0.83
3	-5	-3	5.55	0.23
3	-5	-3	5.25	0.56
3	-5	-3	5.03	0.79
-3	5	3	4.92	0.86
-3	5	3	4.53	0.57
-3	5	3	5.22	0.23
2	-5	-3	17.59	1.19
2	-5	-3	17.05	1.11
2	-5	-3	17.67	0.92
-2	5	3	17.20	1.01
-2	5	3	19.16	1.31
1	-5	-3	50.54	1.85
1	-5	-3	49.19	2.14
1	-5	-3	47.14	2.13
-1	5	3	51.96	1.91
-1	5	3	49.66	2.32
-1	5	3	49.59	2.03

0	-5	-3	66.26	2.71
0	-5	-3	65.25	2.34
0	-5	-3	68.72	2.76
0	5	3	68.96	2.92
0	5	3	68.61	2.45
0	5	3	68.66	2.63
-1	-5	-3	53.05	2.37
-1	-5	-3	56.67	2.01
-1	-5	-3	57.68	2.52
1	5	3	60.27	2.17
1	5	3	55.07	2.32
1	5	3	56.66	2.67
-2	-5	-3	49.69	2.22
-2	-5	-3	49.24	1.53
-2	-5	-3	47.15	2.36
-2	-5	-3	49.27	1.80
2	5	3	48.72	1.53
2	5	3	48.53	2.17
2	5	3	46.92	2.54
2	5	3	49.39	1.96
-3	-5	-3	46.23	1.77
-3	-5	-3	47.28	1.54
-3	-5	-3	47.15	2.44
-3	-5	-3	47.20	2.23
3	5	3	49.44	1.97
3	5	3	48.61	1.53
3	5	3	45.26	2.18
3	5	3	49.71	2.67
-4	-5	-3	58.03	2.10
-4	-5	-3	56.33	2.89
-4	-5	-3	56.89	1.86
4	5	3	58.96	1.86
4	5	3	58.53	2.38
4	5	3	59.91	2.33
4	5	3	59.28	2.56
4	5	3	50.67	3.07
-5	-5	-3	31.08	2.41
-5	-5	-3	33.39	1.24
-5	-5	-3	35.51	1.47
5	5	3	35.42	1.97
5	5	3	35.84	1.82
5	5	3	34.82	1.76
5	5	3	36.68	1.26
-6	-5	-3	17.45	0.82

-6	-5	-3	17.35	1.01
6	5	3	17.18	1.41
6	5	3	16.34	1.51
6	5	3	17.02	0.82
-7	-5	-3	10.43	0.91
-7	-5	-3	11.58	0.78
7	5	3	10.61	1.35
7	5	3	13.45	1.55
7	5	3	13.57	0.80
-8	-5	-3	19.05	1.18
-8	-5	-3	15.57	0.96
8	5	3	12.63	2.15
8	5	3	15.98	1.03
8	5	3	18.93	1.81
-9	-5	-3	18.67	1.17
-9	-5	-3	21.00	1.33
9	5	3	17.71	2.04
9	5	3	20.57	1.90
-10	-5	-3	12.99	1.31
-10	-5	-3	11.30	1.20
10	5	3	10.11	1.74
10	5	3	15.60	2.00
-11	-5	-3	9.74	1.41
11	5	3	4.25	1.20
11	5	3	5.02	1.86
11	5	3	6.93	1.38
17	-6	-3	-0.21	1.29
17	-6	-3	-0.17	2.35
-17	6	3	-0.61	0.74
16	-6	-3	-0.71	2.03
16	-6	-3	-1.68	0.96
-16	6	3	1.76	0.63
15	-6	-3	-0.31	0.93
15	-6	-3	1.42	1.69
-15	6	3	-0.04	0.52
14	-6	-3	1.40	1.68
14	-6	-3	1.60	0.81
-14	6	3	1.52	0.48
13	-6	-3	1.30	1.32
13	-6	-3	1.23	0.65
-13	6	3	1.07	0.43
12	-6	-3	0.97	0.57
12	-6	-3	1.59	1.09
-12	6	3	1.23	0.88

-12	6	3	0.20	0.32
11	-6	-3	10.59	0.74
11	-6	-3	10.84	1.07
11	-6	-3	13.88	1.77
-11	6	3	11.53	1.00
-11	6	3	11.76	1.36
-11	6	3	12.51	0.71
10	-6	-3	1.17	0.32
10	-6	-3	0.76	0.48
10	-6	-3	0.49	0.85
-10	6	3	2.14	0.71
-10	6	3	1.49	0.46
-10	6	3	1.06	0.29
9	-6	-3	4.31	0.66
9	-6	-3	3.28	0.44
9	-6	-3	3.94	0.96
-9	6	3	3.90	0.42
-9	6	3	3.77	0.58
-9	6	3	3.69	0.85
8	-6	-3	22.68	1.21
8	-6	-3	21.13	0.85
8	-6	-3	20.67	1.62
-8	6	3	21.20	0.82
-8	6	3	20.52	0.77
-8	6	3	20.06	0.83
-8	6	3	21.54	1.08
-8	6	3	20.53	1.51
7	-6	-3	47.46	1.94
7	-6	-3	50.91	1.62
7	-6	-3	45.86	2.36
-7	6	3	47.95	1.60
-7	6	3	49.28	1.71
-7	6	3	49.57	1.70
-7	6	3	48.95	1.87
-7	6	3	47.98	2.31
6	-6	-3	6.67	0.68
6	-6	-3	6.82	1.01
6	-6	-3	6.59	0.39
-6	6	3	7.28	0.63
-6	6	3	7.61	0.63
-6	6	3	7.22	1.08
-6	6	3	6.94	0.58
-6	6	3	6.74	0.38
5	-6	-3	0.81	0.16

5	-6	-3	0.75	0.36
5	-6	-3	0.59	0.56
-5	6	3	0.71	0.32
-5	6	3	0.89	0.66
-5	6	3	0.58	0.16
-5	6	3	0.53	0.30
4	-6	-3	41.48	2.08
4	-6	-3	42.11	1.85
4	-6	-3	44.52	1.34
4	-6	-3	42.66	1.72
-4	6	3	42.10	1.65
-4	6	3	41.96	1.33
-4	6	3	43.34	2.13
-4	6	3	40.41	1.73
3	-6	-3	1.80	0.42
3	-6	-3	1.09	0.54
3	-6	-3	1.85	0.58
-3	6	3	2.23	0.49
-3	6	3	1.47	0.66
-3	6	3	1.51	0.41
2	-6	-3	20.98	1.05
2	-6	-3	21.73	1.38
2	-6	-3	19.56	1.39
-2	6	3	23.28	1.12
-2	6	3	20.71	1.22
-2	6	3	20.25	1.55
1	-6	-3	84.78	2.90
1	-6	-3	85.19	3.33
1	-6	-3	85.25	3.34
-1	6	3	84.43	3.18
-1	6	3	84.91	2.99
-1	6	3	86.49	3.53
0	-6	-3	25.97	1.71
0	-6	-3	27.51	1.22
0	-6	-3	27.88	1.66
0	6	3	29.57	1.93
0	6	3	27.79	1.54
0	6	3	30.81	1.39
-1	-6	-3	32.99	1.99
-1	-6	-3	35.31	1.41
-1	-6	-3	34.82	1.87
1	6	3	34.97	1.58
1	6	3	35.24	2.19
1	6	3	34.05	1.78

-2	-6	-3	84.46	3.44
-2	-6	-3	89.25	2.94
-2	-6	-3	85.07	3.60
2	6	3	92.18	3.89
2	6	3	83.46	3.13
2	6	3	84.46	3.39
-3	-6	-3	20.57	1.04
-3	-6	-3	20.72	1.56
-3	-6	-3	24.45	1.91
3	6	3	21.38	1.28
3	6	3	19.99	1.52
3	6	3	21.79	2.16
3	6	3	20.78	0.77
-4	-6	-3	18.25	0.77
-4	-6	-3	19.10	1.01
4	6	3	17.63	1.30
4	6	3	18.76	0.74
4	6	3	17.03	1.51
4	6	3	17.10	1.53
4	6	3	18.40	1.36
-5	-6	-3	60.13	2.00
-5	-6	-3	58.66	2.22
5	6	3	62.10	1.98
5	6	3	56.20	2.56
5	6	3	60.71	2.83
-6	-6	-3	32.66	1.49
-6	-6	-3	31.98	1.28
6	6	3	35.57	1.27
6	6	3	31.92	1.91
6	6	3	30.24	2.08
-7	-6	-3	32.04	1.36
-7	-6	-3	30.84	1.55
7	6	3	33.82	1.35
7	6	3	33.48	2.22
7	6	3	32.58	2.11
-8	-6	-3	21.94	1.36
-8	-6	-3	22.14	1.21
8	6	3	22.67	2.05
8	6	3	20.49	1.96
-9	-6	-3	13.53	1.29
-9	-6	-3	13.80	1.24
9	6	3	11.68	1.97
9	6	3	14.16	2.08
-10	-6	-3	8.64	1.33

10	6	3	13.58	2.37
10	6	3	8.71	1.04
10	6	3	11.15	1.59
17	-7	-3	2.56	1.32
17	-7	-3	2.05	2.41
-17	7	3	1.94	0.73
16	-7	-3	1.10	1.10
16	-7	-3	-0.50	2.00
-16	7	3	0.58	0.60
15	-7	-3	1.63	1.00
15	-7	-3	1.87	1.79
-15	7	3	0.82	0.52
14	-7	-3	0.14	0.85
14	-7	-3	-1.47	2.40
-14	7	3	-0.10	0.43
13	-7	-3	7.20	1.67
13	-7	-3	6.96	1.03
-13	7	3	7.44	0.73
12	-7	-3	1.35	1.21
12	-7	-3	0.94	0.72
-12	7	3	2.18	0.91
-12	7	3	1.04	0.37
11	-7	-3	2.20	1.14
11	-7	-3	1.52	0.64
11	-7	-3	1.16	0.39
-11	7	3	1.49	0.95
-11	7	3	1.26	0.53
-11	7	3	1.21	0.34
10	-7	-3	1.17	0.51
10	-7	-3	1.93	1.00
10	-7	-3	1.30	0.32
-10	7	3	1.02	0.45
-10	7	3	1.88	0.86
-10	7	3	1.18	0.31
9	-7	-3	40.06	1.43
9	-7	-3	41.87	2.45
9	-7	-3	41.44	1.83
-9	7	3	39.60	2.17
-9	7	3	39.47	1.68
-9	7	3	39.65	1.35
-9	7	3	39.37	1.41
-9	7	3	38.23	1.32
8	-7	-3	10.28	0.90
8	-7	-3	10.64	0.56

8	-7	-3	8.96	1.36
-8	7	3	9.45	0.63
-8	7	3	10.21	0.63
-8	7	3	10.06	0.54
-8	7	3	10.17	1.33
-8	7	3	10.31	0.81
7	-7	-3	6.62	0.42
7	-7	-3	6.43	0.74
7	-7	-3	7.18	1.13
-7	7	3	6.56	0.40
-7	7	3	7.00	1.07
-7	7	3	5.94	0.68
-7	7	3	6.37	0.62
-7	7	3	6.30	0.66
6	-7	-3	39.40	1.75
6	-7	-3	39.93	2.22
-6	7	3	40.54	1.94
-6	7	3	40.14	1.69
-6	7	3	44.92	1.70
-6	7	3	41.51	1.36
-6	7	3	45.44	2.28
5	-7	-3	64.85	2.39
5	-7	-3	60.47	2.83
5	-7	-3	65.11	1.99
-5	7	3	62.47	2.89
-5	7	3	63.96	1.98
-5	7	3	63.06	2.30
-5	7	3	61.12	2.40
-5	7	3	66.29	2.36
4	-7	-3	15.15	0.54
4	-7	-3	14.21	0.94
4	-7	-3	14.75	1.37
-4	7	3	15.25	0.90
-4	7	3	16.45	1.00
-4	7	3	16.12	1.50
-4	7	3	14.84	1.03
-4	7	3	14.74	0.53
3	-7	-3	31.26	1.82
3	-7	-3	32.93	1.41
-3	7	3	28.35	1.53
-3	7	3	30.62	1.95
-3	7	3	30.03	1.38
2	-7	-3	26.72	1.26
2	-7	-3	27.30	1.68

2	-7	-3	27.19	1.74
-2	7	3	25.19	1.48
-2	7	3	29.37	1.36
-2	7	3	28.82	1.94
1	-7	-3	52.67	1.95
1	-7	-3	50.99	2.45
1	-7	-3	50.46	2.43
-1	7	3	49.28	2.27
-1	7	3	53.62	2.09
-1	7	3	48.66	2.66
0	-7	-3	38.30	1.54
0	-7	-3	36.57	2.13
0	-7	-3	39.29	2.09
0	7	3	37.09	2.35
0	7	3	36.89	1.93
0	7	3	38.13	1.72
-1	-7	-3	40.44	1.64
-1	-7	-3	42.41	2.20
1	7	3	42.48	2.61
1	7	3	42.62	1.87
1	7	3	41.35	2.10
1	7	3	41.58	2.01
-2	-7	-3	15.36	0.90
-2	-7	-3	14.18	1.41
2	7	3	14.95	1.20
2	7	3	16.83	2.12
2	7	3	14.85	1.38
2	7	3	16.89	1.37
-3	-7	-3	11.61	1.43
-3	-7	-3	13.45	0.87
3	7	3	11.81	1.39
3	7	3	12.60	1.94
3	7	3	13.20	0.59
3	7	3	13.68	1.26
3	7	3	12.28	0.54
-4	-7	-3	25.90	1.95
-4	-7	-3	26.70	1.27
4	7	3	25.99	1.88
4	7	3	25.90	0.97
4	7	3	25.54	1.70
4	7	3	26.44	0.97
-5	-7	-3	34.75	1.51
5	7	3	29.16	2.19
5	7	3	33.83	1.22

5	7	3	34.44	1.23
5	7	3	30.86	1.90
-6	-7	-3	20.65	1.21
6	7	3	19.32	0.95
6	7	3	19.54	1.73
6	7	3	19.91	1.93
6	7	3	19.54	0.93
-7	-7	-3	24.74	1.43
7	7	3	27.02	2.08
7	7	3	24.18	1.14
7	7	3	23.58	2.22
-8	-7	-3	17.02	1.31
8	7	3	11.94	1.84
8	7	3	14.92	1.00
8	7	3	17.83	2.14
-9	-7	-3	14.56	1.37
9	7	3	11.28	1.73
9	7	3	10.94	1.69
-10	-7	-3	8.22	1.13
10	7	3	6.48	1.79
10	7	3	7.46	1.61
17	-8	-3	-1.30	2.28
17	-8	-3	0.68	1.32
-17	8	3	0.54	0.73
16	-8	-3	-0.13	1.19
16	-8	-3	-1.60	2.00
-16	8	3	-0.95	0.56
15	-8	-3	0.47	1.70
15	-8	-3	-0.91	1.01
-15	8	3	0.42	0.54
14	-8	-3	-0.79	0.80
14	-8	-3	2.36	1.57
-14	8	3	-0.14	0.45
13	-8	-3	-0.69	1.31
13	-8	-3	0.85	0.76
-13	8	3	0.18	0.41
12	-8	-3	-0.61	1.23
12	-8	-3	0.42	0.64
-12	8	3	-0.33	0.35
11	-8	-3	9.77	0.74
11	-8	-3	9.91	1.63
11	-8	-3	11.27	1.13
-11	8	3	10.68	0.70
-11	8	3	11.68	1.71

-11	8	3	9.85	1.04
10	-8	-3	3.71	0.74
10	-8	-3	3.40	1.21
10	-8	-3	3.84	0.54
-10	8	3	3.86	0.63
-10	8	3	3.61	0.41
-10	8	3	3.24	0.96
9	-8	-3	5.37	0.86
9	-8	-3	6.10	0.53
9	-8	-3	5.21	1.16
-9	8	3	6.45	0.57
-9	8	3	5.70	0.75
-9	8	3	5.71	0.49
-9	8	3	9.04	1.54
8	-8	-3	4.52	0.45
8	-8	-3	3.67	1.08
8	-8	-3	4.44	0.68
-8	8	3	5.11	1.09
-8	8	3	4.79	0.42
-8	8	3	6.02	0.73
-8	8	3	5.27	0.66
-8	8	3	5.16	0.66
7	-8	-3	14.36	0.62
7	-8	-3	14.38	1.03
7	-8	-3	12.66	1.56
-7	8	3	13.42	0.94
-7	8	3	11.16	0.88
-7	8	3	12.72	0.90
-7	8	3	13.22	0.59
-7	8	3	13.35	1.58
6	-8	-3	8.46	1.37
6	-8	-3	10.67	0.92
-6	8	3	10.98	0.83
-6	8	3	10.36	0.86
-6	8	3	7.85	1.44
-6	8	3	9.75	0.46
-6	8	3	7.12	0.83
5	-8	-3	47.85	2.55
5	-8	-3	51.02	1.57
5	-8	-3	46.90	1.99
-5	8	3	49.35	1.56
-5	8	3	48.26	1.92
-5	8	3	51.07	2.03
-5	8	3	48.50	2.60

-5	8	3	47.02	2.07
4	-8	-3	53.74	2.15
4	-8	-3	53.38	2.71
-4	8	3	54.47	2.82
-4	8	3	57.19	2.26
-4	8	3	57.59	2.14
-4	8	3	54.43	1.70
-4	8	3	55.04	2.35
3	-8	-3	20.79	1.16
3	-8	-3	22.13	1.71
-3	8	3	19.67	1.82
-3	8	3	22.47	1.22
-3	8	3	22.20	1.32
-3	8	3	21.00	1.40
2	-8	-3	45.75	1.80
2	-8	-3	43.79	2.36
-2	8	3	45.79	1.92
-2	8	3	41.88	2.54
-2	8	3	41.77	2.10
-2	8	3	44.54	2.01
1	-8	-3	61.37	2.93
1	-8	-3	63.36	2.33
-1	8	3	65.71	2.50
-1	8	3	64.93	3.23
-1	8	3	63.51	2.63
-1	8	3	59.33	2.74
0	-8	-3	13.38	0.87
0	-8	-3	13.45	1.44
0	8	3	11.97	1.14
0	8	3	13.76	1.87
0	8	3	13.52	1.22
0	8	3	10.89	1.27
-1	-8	-3	21.11	1.70
-1	-8	-3	22.84	1.12
1	8	3	19.94	1.58
1	8	3	17.10	2.22
1	8	3	21.64	1.41
-2	-8	-3	61.51	2.93
-2	-8	-3	62.49	2.24
2	8	3	56.34	2.84
2	8	3	59.67	2.54
-3	-8	-3	22.14	1.88
-3	-8	-3	25.68	1.24
3	8	3	22.10	1.69

3	8	3	24.82	1.90
-4	-8	-3	7.20	1.30
-4	-8	-3	6.87	0.81
4	8	3	6.09	1.19
4	8	3	6.85	0.51
5	8	3	19.44	1.72
5	8	3	19.85	0.86
5	8	3	18.25	1.95
5	8	3	18.13	0.86
-6	-8	-3	18.29	1.20
6	8	3	17.16	1.78
6	8	3	14.73	0.86
6	8	3	15.45	2.02
-7	-8	-3	14.81	1.25
7	8	3	9.16	0.87
7	8	3	11.98	1.72
7	8	3	13.44	1.91
-8	-8	-3	8.86	1.26
8	8	3	5.01	1.35
8	8	3	8.55	1.56
-9	-8	-3	14.61	1.52
9	8	3	13.17	2.39
9	8	3	14.05	1.93
17	-9	-3	-1.52	1.36
17	-9	-3	-2.23	2.36
-17	9	3	0.65	0.69
16	-9	-3	3.01	1.33
16	-9	-3	-0.13	2.10
-16	9	3	1.17	0.64
15	-9	-3	0.24	0.99
15	-9	-3	-0.94	1.82
-15	9	3	-0.88	0.48
14	-9	-3	-0.30	0.87
14	-9	-3	-1.18	1.59
-14	9	3	-0.30	0.48
13	-9	-3	5.15	0.94
13	-9	-3	3.74	2.66
-13	9	3	4.80	0.70
12	-9	-3	4.02	1.51
12	-9	-3	5.33	0.95
-12	9	3	4.58	0.64
11	-9	-3	4.68	1.42
11	-9	-3	4.27	0.83
11	-9	-3	3.15	0.46

-11	9	3	3.73	0.57
10	-9	-3	16.29	1.29
10	-9	-3	15.99	0.84
10	-9	-3	17.80	2.12
-10	9	3	14.97	0.78
-10	9	3	15.46	1.09
-10	9	3	13.12	1.98
-10	9	3	13.76	0.79
9	-9	-3	22.94	2.14
9	-9	-3	23.28	0.96
9	-9	-3	21.74	1.38
-9	9	3	18.16	1.18
-9	9	3	21.85	1.12
-9	9	3	21.89	1.10
-9	9	3	22.51	2.09
-9	9	3	22.26	0.91
8	-9	-3	4.89	1.18
8	-9	-3	5.31	0.82
8	-9	-3	5.78	0.49
-8	9	3	6.25	1.28
-8	9	3	5.38	0.46
-8	9	3	5.97	0.78
-8	9	3	4.44	0.76
-8	9	3	5.65	0.78
7	-9	-3	6.82	0.46
7	-9	-3	5.08	1.16
7	-9	-3	6.93	0.90
-7	9	3	8.51	1.38
-7	9	3	7.45	0.85
-7	9	3	6.47	0.44
-7	9	3	5.45	0.83
-7	9	3	6.46	0.79
6	-9	-3	44.84	2.60
6	-9	-3	42.38	1.91
-6	9	3	44.86	2.65
-6	9	3	43.88	1.82
-6	9	3	43.03	1.99
-6	9	3	44.68	1.94
-6	9	3	44.25	1.44
5	-9	-3	14.18	0.56
5	-9	-3	13.86	1.05
5	-9	-3	13.93	1.66
-5	9	3	14.52	1.01
-5	9	3	14.15	1.77

-5	9	3	13.97	1.13
-5	9	3	12.15	1.13
4	-9	-3	13.17	1.62
4	-9	-3	17.34	1.10
-4	9	3	17.10	1.87
-4	9	3	16.13	1.08
-4	9	3	13.42	1.27
-4	9	3	15.37	1.22
3	-9	-3	52.32	2.79
3	-9	-3	53.44	2.14
-3	9	3	54.18	2.49
-3	9	3	54.87	3.05
-3	9	3	54.02	2.36
-3	9	3	56.27	2.23
2	-9	-3	41.18	2.39
2	-9	-3	41.20	1.70
-2	9	3	37.99	2.64
-2	9	3	42.21	1.89
-2	9	3	39.75	2.14
-2	9	3	39.44	2.03
1	-9	-3	40.90	1.68
1	-9	-3	40.57	2.36
-1	9	3	38.96	2.19
-1	9	3	38.48	2.08
-1	9	3	42.00	1.92
-1	9	3	39.67	2.83
0	-9	-3	39.01	1.63
0	-9	-3	37.65	2.36
0	9	3	40.64	1.95
0	9	3	43.40	3.77
0	9	3	34.90	2.17
-1	-9	-3	34.38	1.47
-1	-9	-3	32.09	2.22
1	9	3	33.24	1.86
1	9	3	30.97	2.07
-2	-9	-3	27.21	2.08
-2	-9	-3	29.45	1.35
2	9	3	26.08	1.82
2	9	3	26.30	2.00
-3	-9	-3	22.98	1.24
-3	-9	-3	21.05	2.00
3	9	3	24.07	2.05
-4	-9	-3	26.70	2.30
-4	-9	-3	27.62	1.38

4	9	3	27.70	2.28
-5	-9	-3	26.89	1.43
5	9	3	25.95	2.03
5	9	3	26.12	2.35
-6	-9	-3	17.26	1.30
6	9	3	16.21	2.21
6	9	3	16.38	1.96
-7	-9	-3	11.94	1.30
7	9	3	11.06	1.78
7	9	3	12.34	2.04
-8	-9	-3	12.11	1.44
8	9	3	8.63	1.75
8	9	3	6.21	1.51
17	-10	-3	0.82	2.68
17	-10	-3	-1.24	1.25
-17	10	3	0.10	0.69
16	-10	-3	1.49	1.28
16	-10	-3	2.52	2.37
-16	10	3	1.23	0.63
15	-10	-3	4.20	1.16
15	-10	-3	-0.49	1.93
-15	10	3	0.90	0.55
14	-10	-3	5.91	1.17
14	-10	-3	6.96	2.12
-14	10	3	6.87	0.86
13	-10	-3	0.67	1.58
13	-10	-3	0.66	0.80
-13	10	3	-0.05	0.43
12	-10	-3	7.92	1.30
12	-10	-3	13.82	2.20
-12	10	3	10.76	0.79
11	-10	-3	45.47	1.73
11	-10	-3	44.57	2.22
11	-10	-3	41.06	3.15
-11	10	3	46.04	1.68
-11	10	3	47.00	1.64
10	-10	-3	9.97	1.17
10	-10	-3	7.79	0.70
10	-10	-3	9.34	1.76
-10	10	3	8.42	0.81
-10	10	3	8.69	0.63
-10	10	3	6.40	1.00
9	-10	-3	9.03	0.65
9	-10	-3	7.16	1.07

9	-10	-3	8.86	1.59
-9	10	3	9.27	0.92
-9	10	3	9.05	0.94
-9	10	3	8.93	0.59
-9	10	3	8.50	1.60
-9	10	3	7.12	0.91
8	-10	-3	22.78	1.44
8	-10	-3	21.46	2.23
8	-10	-3	23.48	0.98
-8	10	3	24.95	1.38
-8	10	3	23.38	0.92
-8	10	3	23.34	1.29
-8	10	3	24.06	2.32
-8	10	3	22.00	1.35
7	-10	-3	41.07	1.98
7	-10	-3	45.50	1.51
7	-10	-3	40.87	2.75
-7	10	3	44.67	1.47
-7	10	3	43.85	1.99
-7	10	3	44.04	2.85
-7	10	3	44.24	2.06
-7	10	3	42.76	1.84
6	-10	-3	27.00	2.21
6	-10	-3	23.78	1.43
6	-10	-3	25.64	0.98
-6	10	3	25.77	1.56
-6	10	3	26.34	1.37
-6	10	3	27.09	1.62
-6	10	3	24.22	2.36
-6	10	3	27.43	0.93
5	-10	-3	39.56	2.55
5	-10	-3	40.96	1.25
5	-10	-3	35.46	1.76
-5	10	3	38.34	2.70
-5	10	3	39.16	1.71
-5	10	3	32.09	1.26
-5	10	3	38.33	2.00
-5	10	3	39.08	1.93
4	-10	-3	26.66	2.15
4	-10	-3	25.41	1.40
-4	10	3	24.19	2.45
-4	10	3	29.52	1.72
-4	10	3	29.94	1.80
-4	10	3	27.24	1.44

3	-10	-3	13.01	1.76
3	-10	-3	15.57	1.06
-3	10	3	13.11	1.19
-3	10	3	16.83	1.51
-3	10	3	14.48	1.42
-3	10	3	16.25	2.15
2	-10	-3	64.79	2.47
2	-10	-3	63.02	3.28
-2	10	3	65.47	3.01
-2	10	3	61.16	2.85
-2	10	3	74.35	2.73
1	-10	-3	53.62	2.08
1	-10	-3	56.60	2.94
-1	10	3	51.22	2.71
-1	10	3	50.58	2.34
0	-10	-3	6.37	1.32
0	-10	-3	9.35	0.85
0	10	3	9.39	1.35
0	10	3	6.47	1.14
-1	-10	-3	8.30	1.75
-1	-10	-3	11.08	0.91
1	10	3	9.69	1.51
1	10	3	10.38	1.82
-2	-10	-3	40.12	1.75
-2	-10	-3	43.04	2.71
2	10	3	40.10	2.61
2	10	3	42.48	2.73
-3	-10	-3	17.44	1.17
-3	-10	-3	19.74	2.21
3	10	3	19.37	2.11
-4	-10	-3	19.64	1.24
-4	-10	-3	18.94	2.37
4	10	3	17.44	2.27
-5	-10	-3	18.61	1.35
5	10	3	19.74	2.01
5	10	3	17.89	2.37
-6	-10	-3	17.45	1.46
6	10	3	21.82	2.54
6	10	3	16.70	2.08
-7	-10	-3	13.38	1.47
7	10	3	15.01	2.63
7	10	3	8.01	1.59
17	-11	-3	-2.50	1.39
17	-11	-3	3.90	2.73

-17	11	3	1.19	0.76
16	-11	-3	3.12	1.39
16	-11	-3	3.03	2.40
-16	11	3	3.69	0.75
15	-11	-3	0.86	2.18
15	-11	-3	2.33	1.21
-15	11	3	-0.05	0.57
14	-11	-3	4.40	2.12
14	-11	-3	2.48	1.07
-14	11	3	3.35	0.59
13	-11	-3	17.60	2.91
13	-11	-3	11.69	1.66
-13	11	3	13.71	0.94
12	-11	-3	26.20	1.80
12	-11	-3	24.94	2.94
-12	11	3	23.45	1.14
-12	11	3	22.76	0.98
11	-11	-3	23.81	3.26
11	-11	-3	21.52	1.12
11	-11	-3	24.81	1.71
-11	11	3	23.46	1.21
-11	11	3	24.10	1.08
-11	11	3	22.56	1.23
10	-11	-3	5.33	0.98
10	-11	-3	5.59	1.59
10	-11	-3	5.16	0.67
-10	11	3	4.63	0.57
-10	11	3	4.34	0.73
-10	11	3	6.25	0.90
-10	11	3	4.79	0.72
9	-11	-3	11.68	1.29
9	-11	-3	11.77	0.77
9	-11	-3	9.39	1.71
-9	11	3	12.47	1.17
-9	11	3	12.76	0.69
-9	11	3	12.58	1.10
-9	11	3	12.92	1.19
8	-11	-3	34.20	1.24
8	-11	-3	28.55	2.61
8	-11	-3	32.71	1.78
-8	11	3	32.23	1.58
-8	11	3	31.55	1.18
-8	11	3	28.60	1.73
-8	11	3	35.35	1.78

7	-11	-3	43.47	1.46
7	-11	-3	40.23	2.87
7	-11	-3	42.13	2.03
-7	11	3	42.22	1.41
-7	11	3	41.82	1.85
-7	11	3	39.41	2.12
-7	11	3	40.35	2.07
6	-11	-3	63.06	3.55
6	-11	-3	66.21	2.12
6	-11	-3	65.98	2.67
-6	11	3	65.36	2.56
-6	11	3	67.40	2.92
-6	11	3	67.35	2.10
-6	11	3	68.59	2.85
5	-11	-3	65.62	2.62
5	-11	-3	66.59	3.56
-5	11	3	64.71	2.85
-5	11	3	69.01	2.60
-5	11	3	64.70	2.98
4	-11	-3	66.88	3.60
4	-11	-3	69.85	2.67
-4	11	3	70.27	3.14
-4	11	3	64.88	2.96
-4	11	3	71.77	2.72
3	-11	-3	66.33	3.59
3	-11	-3	72.91	2.70
-3	11	3	70.87	2.86
-3	11	3	75.06	3.32
2	-11	-3	26.27	1.37
2	-11	-3	24.72	2.28
-2	11	3	23.81	1.92
-2	11	3	27.12	1.73
1	-11	-3	13.97	1.03
-1	11	3	13.00	1.50
-1	11	3	12.37	2.03
0	-11	-3	54.89	3.13
0	-11	-3	54.75	2.13
0	11	3	55.74	2.60
0	11	3	49.28	2.93
-1	-11	-3	45.45	1.87
-1	-11	-3	43.73	2.86
1	11	3	41.98	2.48
1	11	3	47.38	2.86
-2	-11	-3	28.35	2.50

-2	-11	-3	25.42	1.39
2	11	3	25.62	2.46
-3	-11	-3	24.95	2.52
-3	-11	-3	20.23	1.72
3	11	3	25.76	2.49
-4	-11	-3	29.31	2.82
-4	-11	-3	25.94	1.52
4	11	3	27.63	2.77
-5	-11	-3	18.72	1.44
5	11	3	16.98	2.63
5	11	3	14.85	2.06
-6	-11	-3	13.09	1.50
6	11	3	15.56	2.26
6	11	3	15.26	2.69
17	-12	-3	4.20	1.78
17	-12	-3	0.45	2.71
-17	12	3	4.06	0.83
16	-12	-3	4.26	1.49
16	-12	-3	3.70	2.61
-16	12	3	3.80	0.76
15	-12	-3	2.31	2.27
15	-12	-3	3.95	1.34
-15	12	3	3.12	0.68
14	-12	-3	10.84	2.51
14	-12	-3	12.99	1.92
-14	12	3	14.20	1.04
13	-12	-3	0.56	1.73
13	-12	-3	0.28	0.97
12	-12	-3	11.71	1.76
12	-12	-3	15.88	2.83
-12	12	3	12.47	0.95
-12	12	3	12.27	0.86
-12	12	3	8.94	1.08
11	-12	-3	18.93	1.69
11	-12	-3	18.05	1.09
11	-12	-3	20.66	2.86
-11	12	3	19.10	1.30
-11	12	3	20.38	1.27
-11	12	3	18.81	0.97
10	-12	-3	13.21	0.90
10	-12	-3	13.84	2.49
10	-12	-3	16.26	1.53
-10	12	3	11.58	1.24
-10	12	3	14.11	0.80

-10	12	3	14.20	1.26
-10	12	3	13.52	1.28
9	-12	-3	8.95	0.73
9	-12	-3	8.91	1.34
-9	12	3	9.78	1.12
-9	12	3	7.26	1.24
-9	12	3	8.78	1.30
-9	12	3	8.39	0.63
8	-12	-3	5.33	1.54
8	-12	-3	8.19	0.63
8	-12	-3	5.69	0.94
-8	12	3	7.54	0.87
-8	12	3	8.77	1.35
-8	12	3	7.63	0.54
-8	12	3	5.43	0.95
7	-12	-3	9.99	0.66
7	-12	-3	11.16	1.88
7	-12	-3	10.99	1.24
-7	12	3	12.10	1.52
-7	12	3	12.72	1.17
-7	12	3	12.22	0.59
-7	12	3	13.83	1.46
6	-12	-3	22.56	0.89
6	-12	-3	23.40	1.52
6	-12	-3	20.88	2.49
-6	12	3	22.67	1.81
-6	12	3	22.31	1.45
-6	12	3	21.97	1.83
-6	12	3	23.25	0.85
5	-12	-3	62.25	3.57
5	-12	-3	64.07	2.53
-5	12	3	59.71	2.51
-5	12	3	59.83	2.97
4	-12	-3	55.18	2.22
4	-12	-3	48.53	4.03
-4	12	3	50.44	2.76
-4	12	3	54.16	2.35
3	-12	-3	13.39	1.13
3	-12	-3	14.61	2.15
-3	12	3	12.20	1.71
-3	12	3	16.60	1.49
2	-12	-3	17.72	2.26
2	-12	-3	17.56	1.16
-2	12	3	15.25	1.64

-2	12	3	16.43	1.93
1	-12	-3	42.40	1.81
1	-12	-3	38.59	2.92
-1	12	3	41.39	2.76
-1	12	3	44.03	2.39
0	-12	-3	26.22	2.54
0	-12	-3	25.81	1.39
0	12	3	22.37	2.31
0	12	3	21.86	2.06
-1	-12	-3	27.51	2.70
-1	-12	-3	27.12	1.46
1	12	3	29.19	2.54
-2	-12	-3	36.20	1.92
-2	-12	-3	37.58	3.05
2	12	3	33.09	2.94
-3	-12	-3	19.57	1.40
-3	-12	-3	21.83	2.71
3	12	3	19.67	2.61
-4	-12	-3	15.86	2.21
-4	-12	-3	13.25	1.39
4	12	3	11.73	2.11
-5	-12	-3	12.98	1.53
5	12	3	12.09	2.03
5	12	3	15.55	2.25
16	-13	-3	16.00	3.16
16	-13	-3	13.36	2.01
-16	13	3	16.97	1.33
15	-13	-3	3.94	2.54
15	-13	-3	3.63	1.39
-15	13	3	2.67	0.71
14	-13	-3	6.40	2.40
14	-13	-3	6.23	1.39
-14	13	3	4.39	0.70
13	-13	-3	11.52	1.63
13	-13	-3	14.63	2.65
-13	13	3	11.20	0.95
12	-13	-3	20.06	2.52
12	-13	-3	22.20	3.31
-12	13	3	19.47	1.38
-12	13	3	21.45	1.10
11	-13	-3	26.68	2.02
11	-13	-3	22.83	3.19
11	-13	-3	30.41	1.52
-11	13	3	25.57	1.18

-11	13	3	24.88	1.67
-11	13	3	26.61	1.62
10	-13	-3	34.65	3.26
10	-13	-3	34.77	1.43
10	-13	-3	32.60	2.11
-10	13	3	34.60	1.34
-10	13	3	32.86	1.93
-10	13	3	32.38	1.95
9	-13	-3	15.87	2.65
9	-13	-3	14.15	1.55
9	-13	-3	11.77	0.83
-9	13	3	12.28	1.49
-9	13	3	14.73	1.56
-9	13	3	10.47	0.72
-9	13	3	13.94	1.33
8	-13	-3	31.29	1.92
8	-13	-3	31.08	1.26
8	-13	-3	33.10	3.12
-8	13	3	31.40	2.08
-8	13	3	32.42	2.14
-8	13	3	31.69	1.18
-8	13	3	31.76	1.72
7	-13	-3	26.90	2.80
7	-13	-3	19.05	0.90
7	-13	-3	16.87	1.57
-7	13	3	21.12	0.83
-7	13	3	20.63	1.49
-7	13	3	18.84	1.84
6	-13	-3	12.67	2.06
6	-13	-3	11.31	1.27
-6	13	3	7.97	1.27
-6	13	3	10.61	1.66
5	-13	-3	29.41	2.91
5	-13	-3	31.62	1.73
-5	13	3	31.64	1.83
-5	13	3	29.85	2.26
4	-13	-3	44.73	3.24
4	-13	-3	39.48	1.90
-4	13	3	41.07	2.59
-4	13	3	40.36	2.11
3	-13	-3	40.39	3.17
3	-13	-3	36.59	1.77
-3	13	3	38.55	2.67
-3	13	3	38.41	2.19

2	-13	-3	19.75	2.59
2	-13	-3	18.03	1.28
-2	13	3	21.44	1.96
-2	13	3	22.27	2.30
1	-13	-3	20.74	1.35
1	-13	-3	23.43	2.79
-1	13	3	20.46	2.16
-1	13	3	21.94	2.44
0	-13	-3	42.23	1.90
0	-13	-3	45.11	3.32
0	13	3	40.55	3.07
-1	-13	-3	27.81	3.03
-1	-13	-3	33.02	1.69
1	13	3	36.14	3.01
-2	-13	-3	19.57	1.42
-2	-13	-3	20.83	2.90
2	13	3	17.94	2.77
-3	-13	-3	19.10	1.50
-3	-13	-3	15.64	2.29
3	13	3	23.13	2.90
-4	-13	-3	12.02	1.51
-4	-13	-3	11.05	2.26
4	13	3	6.94	1.90
16	-14	-3	4.65	2.88
16	-14	-3	1.67	1.75
-16	14	3	0.99	0.74
15	-14	-3	9.49	1.83
15	-14	-3	7.50	2.97
-15	14	3	5.74	0.84
14	-14	-3	11.22	2.67
14	-14	-3	14.16	2.38
-14	14	3	13.33	1.12
13	-14	-3	5.74	1.55
13	-14	-3	4.02	2.37
-13	14	3	5.49	0.67
12	-14	-3	10.27	2.63
12	-14	-3	12.68	1.93
-12	14	3	12.51	1.45
11	-14	-3	17.27	1.92
11	-14	-3	18.03	2.79
-11	14	3	17.83	1.70
-11	14	3	16.49	0.99
10	-14	-3	29.43	1.32
10	-14	-3	26.71	2.11

10	-14	-3	32.78	3.46
-10	14	3	27.89	1.20
-10	14	3	25.53	2.02
-10	14	3	29.14	2.07
9	-14	-3	23.13	1.90
9	-14	-3	21.04	3.11
9	-14	-3	24.92	1.14
-9	14	3	23.93	1.98
-9	14	3	22.61	1.64
-9	14	3	23.35	1.04
-9	14	3	27.32	2.40
8	-14	-3	21.00	1.76
8	-14	-3	19.04	2.98
8	-14	-3	19.57	0.98
-8	14	3	20.00	0.86
-8	14	3	17.62	1.58
-8	14	3	15.97	1.91
7	-14	-3	19.06	1.66
7	-14	-3	21.24	0.92
7	-14	-3	23.11	3.11
-7	14	3	22.35	2.10
-7	14	3	22.51	1.61
-7	14	3	19.55	0.85
6	-14	-3	34.40	3.19
6	-14	-3	28.43	1.82
-6	14	3	34.58	2.45
-6	14	3	30.70	1.89
5	-14	-3	34.68	3.26
5	-14	-3	33.48	1.87
-5	14	3	37.20	2.61
-5	14	3	36.86	2.05
4	-14	-3	20.22	2.98
4	-14	-3	21.67	1.53
-4	14	3	25.57	1.87
-4	14	3	25.37	2.42
3	-14	-3	11.68	2.17
3	-14	-3	16.39	1.30
-3	14	3	14.26	2.30
-3	14	3	17.81	1.95
2	-14	-3	30.71	1.64
2	-14	-3	28.05	3.14
-2	14	3	34.73	2.80
-2	14	3	31.19	3.27
1	-14	-3	31.21	1.63

1	-14	-3	27.85	3.16
-1	14	3	28.05	2.80
0	-14	-3	23.96	3.21
0	-14	-3	26.74	1.56
0	14	3	25.07	2.86
-1	-14	-3	18.59	1.47
-1	-14	-3	18.02	2.62
1	14	3	23.49	2.92
-2	-14	-3	18.75	1.60
-2	-14	-3	24.46	3.29
2	14	3	22.58	3.19
15	-15	-3	8.33	3.03
15	-15	-3	9.01	2.04
-15	15	3	9.35	1.18
14	-15	-3	5.92	1.75
14	-15	-3	0.92	2.60
-14	15	3	3.34	0.76
13	-15	-3	14.64	2.86
13	-15	-3	20.27	2.50
-13	15	3	16.94	1.14
12	-15	-3	25.62	3.86
12	-15	-3	22.65	2.38
-12	15	3	25.38	1.90
-12	15	3	17.71	1.13
11	-15	-3	22.49	2.68
11	-15	-3	19.82	3.10
-11	15	3	19.46	1.07
-11	15	3	20.47	2.01
10	-15	-3	24.21	3.57
10	-15	-3	21.18	2.09
-10	15	3	24.04	2.22
-10	15	3	24.84	1.10
9	-15	-3	13.52	1.96
9	-15	-3	14.30	2.49
-9	15	3	17.74	2.03
-9	15	3	14.09	0.81
8	-15	-3	40.75	2.35
8	-15	-3	41.77	3.82
-8	15	3	39.22	1.43
-8	15	3	42.74	2.79
7	-15	-3	45.37	3.94
7	-15	-3	50.11	2.47
-7	15	3	51.58	3.07
6	-15	-3	30.98	1.90

6	-15	-3	26.09	3.37
-6	15	3	35.37	2.76
5	-15	-3	27.76	1.74
5	-15	-3	23.76	3.27
-5	15	3	26.03	2.62
4	-15	-3	42.25	2.06
4	-15	-3	47.29	3.90
-4	15	3	44.27	3.22
3	-15	-3	34.35	3.52
3	-15	-3	33.63	1.80
-3	15	3	32.67	3.05
2	-15	-3	16.64	1.40
2	-15	-3	17.70	3.12
-2	15	3	14.91	2.57
1	-15	-3	18.85	1.48
1	-15	-3	17.34	3.11
-1	15	3	26.76	2.99
0	-15	-3	23.59	1.62
0	-15	-3	19.60	3.20
0	15	3	30.07	3.26
-1	-15	-3	11.46	2.37
-1	-15	-3	9.01	1.42
1	15	3	6.04	1.93
14	-16	-3	13.06	2.16
14	-16	-3	13.73	3.23
-14	16	3	13.43	1.18
13	-16	-3	7.91	1.86
13	-16	-3	7.58	2.70
-13	16	3	7.49	0.80
12	-16	-3	13.81	2.87
12	-16	-3	14.16	1.96
-12	16	3	13.10	1.03
11	-16	-3	29.20	4.28
11	-16	-3	35.60	2.72
-11	16	3	37.27	2.62
-11	16	3	37.65	1.53
10	-16	-3	21.72	2.30
10	-16	-3	30.93	3.99
-10	16	3	19.90	2.51
9	-16	-3	20.92	3.62
9	-16	-3	19.61	2.13
-9	16	3	20.44	2.42
8	-16	-3	28.93	3.86
8	-16	-3	29.83	2.21

-8	16	3	28.18	2.69
7	-16	-3	30.73	2.14
7	-16	-3	32.96	3.81
-7	16	3	30.09	2.77
6	-16	-3	28.54	1.94
6	-16	-3	28.32	3.73
-6	16	3	24.36	2.87
5	-16	-3	45.57	4.14
5	-16	-3	41.08	2.14
-5	16	3	41.55	3.36
4	-16	-3	14.70	1.51
4	-16	-3	21.56	3.08
-4	16	3	20.56	2.84
3	-16	-3	27.21	1.74
3	-16	-3	31.22	3.68
-3	16	3	32.90	3.21
2	-16	-3	31.35	1.91
2	-16	-3	29.31	3.81
-2	16	3	31.61	3.32
1	-16	-3	22.98	3.09
-1	16	3	13.35	2.20
12	-17	-3	19.54	3.42
12	-17	-3	17.29	2.81
11	-17	-3	12.55	3.11
11	-17	-3	23.73	2.67
-11	17	3	24.53	2.64
10	-17	-3	21.53	3.97
-10	17	3	17.96	2.72
9	-17	-3	23.17	2.23
9	-17	-3	23.76	3.94
-9	17	3	20.44	2.87
8	-17	-3	25.91	2.20
8	-17	-3	24.02	3.91
-8	17	3	32.06	3.09
7	-17	-3	27.83	3.54
7	-17	-3	23.02	2.54
-7	17	3	27.23	3.02
6	-17	-3	10.25	2.68
-6	17	3	11.94	2.07
5	-17	-3	10.88	2.65
-5	17	3	6.20	1.85
4	-17	-3	23.34	3.88
-4	17	3	19.65	3.09
3	-17	-3	22.01	4.25

-3	17	3	29.17	3.47
9	-18	-3	20.59	3.85
-9	18	3	18.41	3.22
8	-18	-3	28.80	5.13
-8	18	3	30.08	3.44
-7	18	3	14.19	2.25
-5	19	-4	4.83	2.10
5	-19	4	0.45	2.78
-6	19	-4	1.32	2.32
6	-19	4	2.54	3.10
7	-19	4	3.38	4.03
-1	18	-4	4.80	2.16
1	-18	4	4.70	2.59
-2	18	-4	1.26	2.04
2	-18	4	-0.29	2.18
-3	18	-4	7.53	2.12
3	-18	4	6.74	2.58
-4	18	-4	2.21	1.94
4	-18	4	-0.17	2.27
-5	18	-4	2.07	1.85
-5	18	-4	0.40	0.55
5	-18	4	2.46	2.33
-6	18	-4	1.27	0.58
-6	18	-4	2.03	1.95
6	-18	4	-1.30	2.25
-7	18	-4	3.43	2.06
-7	18	-4	6.26	0.97
7	-18	4	4.31	2.48
-8	18	-4	-1.34	1.89
-8	18	-4	0.97	0.65
8	-18	4	3.47	2.47
-9	18	-4	4.24	2.19
-9	18	-4	0.25	0.65
9	-18	4	0.28	2.61
-10	18	-4	-0.53	2.04
-10	18	-4	0.66	0.71
10	-18	4	0.19	4.03
-2	-17	4	9.86	2.63
1	17	-4	11.96	2.33
-1	-17	4	20.19	3.70
0	17	-4	5.04	2.08
0	-17	4	5.42	2.37
-1	17	-4	8.39	2.06
1	-17	4	4.69	2.39

-2	17	-4	3.16	0.58
-2	17	-4	1.44	1.80
2	-17	4	1.81	2.15
-3	17	-4	1.40	1.77
3	-17	4	2.24	2.13
-4	17	-4	6.52	1.97
-4	17	-4	6.85	0.87
4	-17	4	4.66	2.33
-5	17	-4	7.83	0.88
-5	17	-4	7.52	2.09
5	-17	4	7.19	2.45
-6	17	-4	0.02	0.59
6	-17	4	0.74	2.15
-7	17	-4	-0.36	0.54
7	-17	4	1.15	2.15
-8	17	-4	2.93	0.64
8	-17	4	1.17	2.30
-9	17	-4	0.46	0.63
9	-17	4	0.85	2.39
-10	17	-4	0.60	0.66
-10	17	-4	2.20	1.82
10	-17	4	-3.09	2.39
-11	17	-4	-0.48	0.64
11	-17	4	0.84	2.60
3	16	-4	2.71	2.06
-3	-16	4	1.95	2.11
2	16	-4	6.55	1.90
-2	-16	4	8.36	2.29
1	16	-4	8.86	2.03
-1	-16	4	9.70	2.46
0	16	-4	6.70	0.82
0	16	-4	5.44	1.99
0	-16	4	6.32	2.16
-1	16	-4	2.64	1.72
-1	16	-4	1.78	0.50
1	-16	4	2.40	2.00
-2	16	-4	3.71	1.52
-2	16	-4	3.05	0.54
2	-16	4	0.41	1.83
-3	16	-4	8.93	0.80
3	-16	4	8.11	2.23
-4	16	-4	0.74	0.47
4	-16	4	0.35	1.09
4	-16	4	1.17	1.79

-5	16	-4	0.21	0.45
5	-16	4	2.90	1.81
5	-16	4	0.58	1.12
-6	16	-4	3.06	0.54
6	-16	4	0.49	1.80
6	-16	4	1.54	1.08
-7	16	-4	2.02	0.59
7	-16	4	1.38	2.11
7	-16	4	1.25	1.08
-8	16	-4	-0.28	0.49
8	-16	4	-1.19	2.02
8	-16	4	-1.57	1.03
-9	16	-4	-0.83	0.52
9	-16	4	-0.04	1.27
9	-16	4	0.50	1.96
-10	16	-4	-0.11	0.57
10	-16	4	-3.98	2.07
10	-16	4	0.10	1.12
-11	16	-4	0.13	0.64
11	-16	4	2.66	2.51
-12	16	-4	2.66	0.75
12	-16	4	0.79	2.54
5	15	-4	1.85	2.22
-5	-15	4	4.63	2.13
4	15	-4	7.27	2.22
-4	-15	4	8.18	2.15
3	15	-4	3.40	1.80
-3	-15	4	-0.69	1.81
2	15	-4	3.76	0.60
2	15	-4	4.90	1.92
-2	-15	4	5.58	1.92
1	15	-4	2.22	1.60
-1	-15	4	0.65	1.79
0	15	-4	4.59	0.56
0	15	-4	2.02	1.43
0	-15	4	2.74	1.74
-1	15	-4	2.48	0.49
1	-15	4	1.70	1.68
1	-15	4	1.24	0.47
-2	15	-4	0.01	0.39
2	-15	4	-0.16	1.59
2	-15	4	-1.79	0.92
2	-15	4	0.05	0.46
-3	15	-4	3.64	0.52

3	-15	4	1.99	1.63
3	-15	4	4.77	1.19
3	-15	4	3.47	0.59
-4	15	-4	1.14	0.43
4	-15	4	0.94	0.92
4	-15	4	0.42	0.51
4	-15	4	4.19	1.85
-5	15	-4	6.45	0.73
5	-15	4	6.20	1.17
5	-15	4	6.62	2.01
-6	15	-4	1.11	0.45
6	-15	4	-1.60	1.58
-7	15	-4	-0.47	0.41
7	-15	4	0.48	0.96
7	-15	4	0.58	1.77
-8	15	-4	0.19	0.45
8	-15	4	0.51	1.03
8	-15	4	1.38	0.73
8	-15	4	-0.09	1.76
-9	15	-4	0.15	0.51
9	-15	4	-0.97	1.84
9	-15	4	0.20	0.81
9	-15	4	-0.53	1.06
-10	15	-4	-0.78	0.51
10	-15	4	0.49	1.09
10	-15	4	-4.08	1.97
10	-15	4	0.33	0.90
-11	15	-4	3.82	0.69
11	-15	4	1.85	1.11
11	-15	4	1.10	1.07
11	-15	4	2.67	2.33
-12	15	-4	8.29	1.12
12	-15	4	8.93	1.42
12	-15	4	8.79	2.76
-13	15	-4	2.04	0.75
13	-15	4	-1.15	2.71
13	-15	4	4.20	1.44
6	14	-4	8.15	2.39
-6	-14	4	2.14	1.99
5	14	-4	2.47	1.94
-5	-14	4	4.68	1.88
4	14	-4	4.07	1.81
4	14	-4	6.21	0.94
-4	-14	4	3.76	1.75

3	14	-4	2.77	0.57
3	14	-4	2.03	1.61
-3	-14	4	4.46	1.78
2	14	-4	5.47	1.72
2	14	-4	7.76	0.84
-2	-14	4	8.38	1.87
1	14	-4	3.84	1.44
1	14	-4	4.63	0.57
-1	-14	4	4.67	0.52
-1	-14	4	4.45	1.64
0	14	-4	0.07	0.39
0	-14	4	0.38	0.91
0	-14	4	0.73	0.41
0	-14	4	-0.75	1.32
-1	14	-4	6.56	0.69
1	-14	4	5.89	1.13
1	-14	4	8.42	0.72
1	-14	4	6.40	1.72
-2	14	-4	6.84	0.65
2	-14	4	6.21	0.75
2	-14	4	2.79	1.48
2	-14	4	7.07	1.26
-3	14	-4	3.72	0.49
3	-14	4	2.81	0.52
3	-14	4	-0.03	1.41
3	-14	4	1.40	1.19
-4	14	-4	1.00	0.39
4	-14	4	-0.11	0.85
4	-14	4	1.04	0.51
4	-14	4	2.49	1.51
-5	14	-4	2.03	0.45
5	-14	4	2.18	1.00
5	-14	4	2.01	1.53
5	-14	4	2.14	0.52
-6	14	-4	0.42	0.40
6	-14	4	0.35	0.84
6	-14	4	0.29	1.38
-7	14	-4	0.48	0.41
7	-14	4	0.59	0.60
7	-14	4	0.86	0.90
7	-14	4	0.78	1.45
-8	14	-4	1.86	0.59
-8	14	-4	1.50	0.70
8	-14	4	0.27	0.88

8	-14	4	0.65	0.45
8	-14	4	1.74	0.70
8	-14	4	0.56	1.56
-9	14	-4	0.70	0.68
-9	14	-4	2.04	0.50
9	-14	4	3.20	0.82
9	-14	4	-0.79	1.78
9	-14	4	4.10	1.04
-10	14	-4	1.62	0.59
10	-14	4	3.29	1.03
10	-14	4	-1.47	1.90
10	-14	4	2.29	0.89
-11	14	-4	3.04	0.62
11	-14	4	2.68	2.14
11	-14	4	1.73	0.99
11	-14	4	3.52	1.07
-12	14	-4	1.72	0.63
12	-14	4	-1.20	2.02
-13	14	-4	0.87	0.67
13	-14	4	3.32	1.34
13	-14	4	1.37	2.60
-14	14	-4	1.37	0.75
14	-14	4	0.13	1.48
7	13	-4	6.93	2.16
-7	-13	4	4.98	1.95
6	13	-4	2.15	1.95
6	13	-4	1.95	0.70
-6	-13	4	0.05	1.66
5	13	-4	2.65	1.75
5	13	-4	5.03	0.98
5	13	-4	3.80	0.65
-5	-13	4	4.70	1.64
4	13	-4	6.44	0.72
4	13	-4	6.51	0.89
4	13	-4	6.28	1.90
-4	-13	4	7.89	1.77
3	13	-4	5.29	0.73
3	13	-4	6.07	1.71
3	13	-4	5.80	0.64
3	13	-4	6.39	0.79
-3	-13	4	8.84	1.68
2	13	-4	5.88	0.79
2	13	-4	6.16	1.49
2	13	-4	6.27	0.62

-2	-13	4	5.28	0.63
-2	-13	4	5.45	1.47
1	13	-4	0.56	0.42
-1	-13	4	-0.76	0.34
-1	-13	4	-0.01	1.57
-1	-13	4	0.06	0.87
0	13	-4	0.10	0.36
0	-13	4	0.79	0.39
0	-13	4	-0.93	1.10
0	-13	4	0.03	0.74
-1	13	-4	3.77	0.56
1	-13	4	4.11	0.62
1	-13	4	3.32	1.43
1	-13	4	5.42	1.46
-2	13	-4	-0.12	0.32
2	-13	4	0.83	0.40
2	-13	4	0.83	1.19
2	-13	4	-0.25	0.72
-3	13	-4	5.09	0.60
3	-13	4	5.64	0.59
3	-13	4	5.82	1.50
3	-13	4	3.99	0.97
-4	13	-4	2.75	0.44
4	-13	4	3.36	0.94
4	-13	4	0.14	1.27
4	-13	4	3.96	0.53
-5	13	-4	2.91	0.86
-5	13	-4	1.90	0.42
5	-13	4	1.03	1.25
5	-13	4	1.69	0.54
5	-13	4	1.66	0.82
-6	13	-4	6.52	0.66
-6	13	-4	5.06	0.81
6	-13	4	6.63	1.04
6	-13	4	6.73	1.80
6	-13	4	6.60	0.53
6	-13	4	6.92	0.95
6	-13	4	7.05	0.89
-7	13	-4	6.41	0.71
-7	13	-4	8.78	1.12
7	-13	4	6.48	1.80
7	-13	4	8.01	0.64
7	-13	4	7.76	1.36
7	-13	4	7.08	0.89

-8	13	-4	4.05	0.68
-8	13	-4	4.82	0.68
8	-13	4	3.85	1.68
8	-13	4	4.14	0.70
8	-13	4	3.45	0.92
8	-13	4	4.67	0.76
-9	13	-4	6.04	0.96
-9	13	-4	6.73	0.75
9	-13	4	3.85	1.78
9	-13	4	5.96	1.05
9	-13	4	5.53	0.84
9	-13	4	5.27	0.87
-10	13	-4	8.24	0.88
-10	13	-4	7.58	0.92
10	-13	4	9.10	1.46
10	-13	4	5.17	1.93
10	-13	4	6.12	1.01
10	-13	4	7.62	1.06
-11	13	-4	9.77	1.00
11	-13	4	11.40	1.49
11	-13	4	11.77	1.56
11	-13	4	13.77	2.71
-12	13	-4	5.50	0.76
12	-13	4	1.91	1.21
12	-13	4	3.77	2.24
-13	13	-4	1.36	0.66
13	-13	4	2.18	1.20
13	-13	4	0.41	2.24
-14	13	-4	4.76	0.80
14	-13	4	3.88	2.68
14	-13	4	6.20	1.49
8	12	-4	3.04	1.98
8	12	-4	1.92	1.34
8	12	-4	0.69	0.81
7	12	-4	7.61	1.27
7	12	-4	3.96	0.78
7	12	-4	7.06	2.07
-7	-12	4	4.51	1.73
6	12	-4	9.12	1.06
6	12	-4	7.88	1.15
6	12	-4	10.12	2.07
-6	-12	4	9.92	1.81
5	12	-4	2.54	0.64
5	12	-4	3.76	0.63

5	12	-4	4.71	1.76
5	12	-4	3.18	0.53
-5	-12	4	3.28	1.38
4	12	-4	3.71	0.57
4	12	-4	3.65	0.84
4	12	-4	3.51	0.57
4	12	-4	2.95	0.51
4	12	-4	2.82	1.42
-4	-12	4	0.88	1.35
3	12	-4	0.71	0.43
3	12	-4	0.45	0.41
3	12	-4	1.01	1.15
3	12	-4	0.79	0.44
-3	-12	4	0.52	1.16
2	12	-4	0.77	0.46
2	12	-4	0.97	0.42
-2	-12	4	1.37	0.37
-2	-12	4	0.68	1.18
-2	-12	4	0.93	0.79
1	12	-4	3.34	0.46
-1	-12	4	2.85	0.41
-1	-12	4	2.14	1.18
-1	-12	4	3.24	0.96
0	12	-4	11.24	0.69
0	-12	4	10.36	1.89
0	-12	4	7.60	1.35
0	-12	4	9.28	0.66
-1	12	-4	1.18	0.35
-1	12	-4	1.03	1.74
1	-12	4	-0.16	1.03
1	-12	4	0.92	0.37
1	-12	4	0.60	0.70
-2	12	-4	6.52	1.68
-2	12	-4	3.67	0.51
2	-12	4	4.41	0.90
2	-12	4	4.10	1.31
2	-12	4	4.43	0.60
-3	12	-4	12.96	0.75
-3	12	-4	11.77	1.19
3	-12	4	13.62	0.83
3	-12	4	12.60	1.33
3	-12	4	14.07	2.02
-4	12	-4	-0.25	0.59
-4	12	-4	0.30	0.31

4	-12	4	-1.26	1.02
4	-12	4	0.43	0.39
4	-12	4	-0.46	0.61
-5	12	-4	10.39	1.11
-5	12	-4	9.93	0.70
5	-12	4	10.36	1.30
5	-12	4	11.41	0.74
5	-12	4	10.70	1.74
5	-12	4	11.46	0.82
5	-12	4	10.06	0.52
-6	12	-4	-0.01	0.54
-6	12	-4	-0.14	0.31
6	-12	4	0.09	0.41
6	-12	4	-0.82	0.43
6	-12	4	0.71	0.68
6	-12	4	-0.14	1.13
6	-12	4	-0.13	0.28
-7	12	-4	0.03	0.36
-7	12	-4	0.22	0.52
7	-12	4	-0.24	0.66
7	-12	4	0.09	0.45
7	-12	4	-0.84	1.37
7	-12	4	0.16	0.36
-8	12	-4	13.09	0.86
-8	12	-4	14.36	1.03
8	-12	4	14.83	2.41
8	-12	4	11.83	1.02
8	-12	4	13.52	0.87
8	-12	4	9.34	1.29
-9	12	-4	2.22	0.46
-9	12	-4	2.04	0.54
9	-12	4	1.73	0.80
9	-12	4	0.91	0.61
9	-12	4	0.58	0.53
9	-12	4	2.90	1.45
-10	12	-4	3.45	0.55
-10	12	-4	2.78	0.54
10	-12	4	3.64	0.87
10	-12	4	2.53	0.76
10	-12	4	1.09	1.61
10	-12	4	2.78	0.67
-11	12	-4	3.20	0.61
11	-12	4	0.40	1.73
11	-12	4	2.19	0.82

11	-12	4	1.81	0.84
-12	12	-4	1.34	0.58
12	-12	4	0.33	0.85
12	-12	4	-1.64	1.98
-13	12	-4	3.82	0.69
13	-12	4	2.07	1.14
13	-12	4	4.57	2.34
-14	12	-4	9.65	1.18
14	-12	4	6.13	2.66
14	-12	4	9.34	1.58
9	11	-4	4.85	0.83
9	11	-4	1.61	1.01
9	11	-4	1.78	1.36
9	11	-4	1.78	1.95
8	11	-4	3.07	0.77
8	11	-4	1.93	0.92
8	11	-4	1.61	1.76
8	11	-4	2.54	1.18
7	11	-4	6.31	0.90
7	11	-4	5.50	1.82
7	11	-4	6.26	0.81
-7	-11	4	5.81	1.47
6	11	-4	5.25	0.60
6	11	-4	4.71	1.52
6	11	-4	5.09	0.69
6	11	-4	6.11	0.98
6	11	-4	6.68	1.01
-6	-11	4	4.55	1.35
5	11	-4	11.39	0.92
5	11	-4	8.58	1.62
5	11	-4	10.90	0.81
5	11	-4	10.35	1.20
5	11	-4	11.34	0.95
-5	-11	4	12.27	1.68
4	11	-4	1.23	1.19
4	11	-4	2.51	0.49
4	11	-4	2.35	0.50
4	11	-4	3.05	0.70
4	11	-4	1.67	0.44
-4	-11	4	1.94	1.03
3	11	-4	0.60	0.37
3	11	-4	0.21	0.37
3	11	-4	1.21	0.51
3	11	-4	0.22	0.39

-3	-11	4	-0.01	0.72
-3	-11	4	-1.11	0.95
2	11	-4	-0.24	0.40
2	11	-4	-2.35	1.26
2	11	-4	0.35	0.35
-2	-11	4	-0.75	0.59
-2	-11	4	1.11	1.01
-2	-11	4	-0.39	0.27
1	11	-4	0.84	1.32
1	11	-4	0.47	0.35
-1	-11	4	-0.34	0.90
-1	-11	4	0.45	0.29
-1	-11	4	0.53	0.70
0	11	-4	4.84	1.38
0	11	-4	4.74	0.54
0	11	-4	4.78	1.00
0	-11	4	3.51	0.82
0	-11	4	4.07	1.16
0	-11	4	3.70	0.50
-1	11	-4	11.28	1.90
-1	11	-4	9.79	1.16
-1	11	-4	9.99	0.63
1	-11	4	10.88	1.22
1	-11	4	9.03	0.63
1	-11	4	6.80	1.33
-2	11	-4	3.09	1.13
-2	11	-4	2.02	0.64
-2	11	-4	3.31	0.40
2	-11	4	2.80	1.10
2	-11	4	2.87	0.43
2	-11	4	2.46	0.73
-3	11	-4	9.89	0.64
-3	11	-4	11.31	1.07
-3	11	-4	11.50	1.87
3	-11	4	9.87	1.17
3	-11	4	10.29	0.71
3	-11	4	11.45	1.74
-4	11	-4	5.35	1.35
-4	11	-4	5.24	0.73
-4	11	-4	3.93	0.50
4	-11	4	4.42	0.79
4	-11	4	3.87	0.49
4	-11	4	2.79	1.09
-5	11	-4	2.65	0.58

-5	11	-4	1.31	1.11
-5	11	-4	2.44	0.39
5	-11	4	1.61	0.42
5	-11	4	1.60	0.65
5	-11	4	2.27	0.48
5	-11	4	1.88	1.09
5	-11	4	2.11	0.35
-6	11	-4	12.36	0.97
-6	11	-4	10.74	0.70
6	-11	4	7.96	1.59
6	-11	4	10.64	0.79
6	-11	4	9.50	0.82
6	-11	4	10.28	1.23
-7	11	-4	10.47	0.74
-7	11	-4	11.87	0.93
7	-11	4	13.35	0.99
7	-11	4	11.85	0.90
7	-11	4	11.68	0.73
7	-11	4	12.51	1.26
7	-11	4	12.19	2.08
-8	11	-4	2.65	0.51
-8	11	-4	2.59	0.46
8	-11	4	3.35	0.51
8	-11	4	2.32	0.56
8	-11	4	3.21	0.75
8	-11	4	3.75	1.38
-9	11	-4	8.88	0.80
9	-11	4	9.65	1.02
9	-11	4	7.84	0.98
9	-11	4	7.75	1.83
9	-11	4	8.92	0.87
-10	11	-4	12.02	0.90
-10	11	-4	12.08	0.88
10	-11	4	11.34	1.29
10	-11	4	11.59	1.21
10	-11	4	8.94	1.93
10	-11	4	11.17	1.09
-11	11	-4	5.55	0.68
-11	11	-4	6.14	0.98
11	-11	4	8.96	1.21
11	-11	4	1.98	1.71
11	-11	4	5.77	0.99
-12	11	-4	12.68	1.06
12	-11	4	9.13	1.54

12	-11	4	12.82	2.54
-13	11	-4	7.11	0.98
13	-11	4	3.60	1.19
13	-11	4	4.46	2.41
-14	11	-4	2.31	0.68
14	-11	4	1.69	1.23
14	-11	4	3.20	2.32
15	-11	4	9.66	5.15
15	-11	4	3.77	1.50
10	10	-4	4.60	1.41
10	10	-4	4.39	1.99
10	10	-4	3.65	0.88
10	10	-4	3.71	1.11
9	10	-4	3.28	1.10
9	10	-4	4.00	1.83
9	10	-4	1.70	0.76
9	10	-4	2.95	1.22
8	10	-4	0.82	0.62
8	10	-4	0.57	0.76
8	10	-4	-1.61	0.88
8	10	-4	1.91	1.60
7	10	-4	3.95	1.58
7	10	-4	4.82	0.79
7	10	-4	3.83	0.67
7	10	-4	3.42	0.88
-7	-10	4	4.49	1.44
6	10	-4	2.37	0.54
6	10	-4	2.98	0.73
6	10	-4	1.93	0.64
6	10	-4	1.81	0.46
6	10	-4	0.74	1.24
-6	-10	4	2.30	1.10
5	10	-4	5.24	0.80
5	10	-4	7.40	0.66
5	10	-4	5.68	0.92
5	10	-4	7.23	0.72
-5	-10	4	9.97	1.64
4	10	-4	2.06	0.52
4	10	-4	2.05	0.44
4	10	-4	1.23	0.39
4	10	-4	1.25	1.47
4	10	-4	1.98	0.45
-4	-10	4	1.51	0.95
-4	-10	4	1.54	0.79

3	10	-4	7.15	0.73
3	10	-4	6.70	0.66
3	10	-4	8.18	1.64
3	10	-4	7.00	0.61
-3	-10	4	7.68	1.26
-3	-10	4	7.97	1.28
-3	-10	4	8.37	0.56
2	10	-4	-0.35	0.29
2	10	-4	-1.87	1.02
2	10	-4	0.18	0.61
2	10	-4	-0.17	0.23
-2	-10	4	0.31	0.25
-2	-10	4	0.19	0.56
-2	-10	4	0.06	0.85
1	10	-4	16.06	1.88
1	10	-4	17.17	1.33
1	10	-4	17.80	0.84
-1	-10	4	16.58	1.36
-1	-10	4	18.22	1.75
-1	-10	4	17.43	0.77
0	10	-4	3.81	0.69
0	10	-4	7.18	1.29
0	10	-4	3.87	0.48
0	-10	4	2.90	0.68
0	-10	4	4.54	1.06
0	-10	4	4.08	0.47
-1	10	-4	3.95	1.08
-1	10	-4	3.39	0.45
-1	10	-4	3.50	0.65
1	-10	4	3.82	1.06
1	-10	4	3.96	0.78
1	-10	4	4.00	0.46
-2	10	-4	-0.05	0.22
-2	10	-4	-0.58	0.89
-2	10	-4	0.40	0.44
2	-10	4	1.20	0.94
2	-10	4	-0.46	0.48
2	-10	4	0.76	0.29
-3	10	-4	2.59	0.53
-3	10	-4	3.31	1.03
-3	10	-4	2.10	0.32
3	-10	4	2.15	0.96
3	-10	4	1.77	0.57
3	-10	4	2.17	0.36

-4	10	-4	9.15	0.86
-4	10	-4	7.58	1.23
-4	10	-4	8.56	0.58
4	-10	4	8.65	0.63
4	-10	4	9.34	1.02
4	-10	4	7.79	1.37
4	-10	4	8.70	0.42
4	-10	4	9.37	0.57
-5	10	-4	35.25	1.31
-5	10	-4	31.23	2.26
-5	10	-4	36.03	1.53
5	-10	4	33.62	1.34
5	-10	4	32.31	1.18
5	-10	4	35.55	1.35
5	-10	4	31.13	2.36
5	-10	4	34.28	1.70
-6	10	-4	10.16	1.59
-6	10	-4	10.90	0.67
-6	10	-4	10.90	0.82
6	-10	4	10.27	0.75
6	-10	4	13.71	1.86
6	-10	4	11.94	1.09
6	-10	4	10.04	0.72
6	-10	4	10.76	0.62
-7	10	-4	2.51	0.38
-7	10	-4	2.29	0.46
7	-10	4	2.14	0.48
7	-10	4	2.55	0.41
7	-10	4	2.24	0.45
7	-10	4	1.96	0.59
7	-10	4	2.86	1.16
-8	10	-4	13.36	0.83
-8	10	-4	13.06	0.79
8	-10	4	11.63	0.84
8	-10	4	11.63	0.96
8	-10	4	13.90	1.14
8	-10	4	13.86	1.99
-9	10	-4	41.41	1.69
-9	10	-4	46.45	1.69
9	-10	4	41.89	3.84
9	-10	4	44.49	1.93
9	-10	4	43.60	1.97
9	-10	4	43.26	1.77
-10	10	-4	15.63	0.99

-10	10	-4	17.13	0.99
10	-10	4	17.72	1.27
10	-10	4	15.15	2.44
10	-10	4	16.31	1.14
10	-10	4	15.17	1.28
-11	10	-4	13.28	1.00
-11	10	-4	16.17	1.03
11	-10	4	13.62	1.37
11	-10	4	12.82	2.26
11	-10	4	14.26	1.24
-12	10	-4	4.45	0.65
12	-10	4	4.88	1.94
12	-10	4	6.39	1.13
12	-10	4	6.90	1.13
-13	10	-4	3.98	0.69
13	-10	4	3.37	1.05
13	-10	4	3.45	2.08
-14	10	-4	8.94	1.11
14	-10	4	5.62	2.48
14	-10	4	9.76	1.47
-15	10	-4	0.40	0.87
15	-10	4	2.04	2.98
15	-10	4	6.89	1.45
11	9	-4	2.49	1.41
11	9	-4	3.67	0.83
11	9	-4	3.84	1.25
11	9	-4	2.92	1.85
10	9	-4	1.04	1.65
10	9	-4	0.82	0.65
10	9	-4	1.13	0.93
10	9	-4	1.38	1.13
9	9	-4	1.92	1.53
9	9	-4	4.49	1.09
9	9	-4	3.09	0.87
8	9	-4	4.65	0.83
8	9	-4	3.20	0.62
8	9	-4	3.90	1.06
8	9	-4	2.54	0.95
8	9	-4	2.87	1.50
7	9	-4	-0.16	0.56
7	9	-4	-0.64	1.12
7	9	-4	-0.36	0.62
7	9	-4	-0.25	0.43
7	9	-4	0.34	0.41

-7	-9	4	-0.27	0.91
6	9	-4	0.70	0.37
6	9	-4	0.46	0.41
6	9	-4	0.82	0.55
6	9	-4	-0.30	0.46
6	9	-4	1.10	1.35
6	9	-4	-0.56	1.01
-6	-9	4	-0.19	0.86
5	9	-4	7.10	0.76
5	9	-4	8.64	1.59
5	9	-4	6.54	0.72
5	9	-4	5.70	0.66
5	9	-4	7.46	0.64
-5	-9	4	7.85	1.28
-5	-9	4	6.52	1.14
4	9	-4	-0.12	0.36
4	9	-4	-0.09	0.29
4	9	-4	0.37	0.77
4	9	-4	0.32	0.33
4	9	-4	-1.29	1.09
4	9	-4	0.34	0.34
-4	-9	4	1.02	0.69
-4	-9	4	-0.10	0.72
3	9	-4	4.02	0.81
3	9	-4	3.49	0.52
3	9	-4	3.99	1.26
3	9	-4	3.75	0.52
3	9	-4	3.37	0.54
-3	-9	4	3.84	0.91
-3	-9	4	3.28	0.78
-3	-9	4	4.36	0.43
2	9	-4	2.75	0.33
2	9	-4	3.49	0.48
2	9	-4	3.08	0.63
2	9	-4	3.00	0.37
2	9	-4	2.89	1.02
-2	-9	4	2.91	0.38
-2	-9	4	1.55	0.54
-2	-9	4	2.12	0.85
1	9	-4	10.36	1.52
1	9	-4	8.74	0.95
1	9	-4	8.31	0.58
-1	-9	4	8.84	1.31
-1	-9	4	9.09	0.52

-1	-9	4	8.85	1.01
0	9	-4	7.19	0.85
0	9	-4	5.70	0.49
0	9	-4	5.74	1.11
0	-9	4	5.48	0.45
0	-9	4	4.91	0.86
0	-9	4	5.18	1.02
-1	9	-4	7.22	0.84
-1	9	-4	8.13	0.51
-1	9	-4	7.98	1.25
1	-9	4	8.23	0.54
1	-9	4	7.65	1.30
1	-9	4	8.24	0.91
-2	9	-4	5.27	0.73
-2	9	-4	5.39	1.06
-2	9	-4	5.04	0.43
2	-9	4	4.76	0.98
2	-9	4	5.08	0.47
2	-9	4	6.30	0.83
-3	9	-4	3.62	0.56
-3	9	-4	3.88	0.99
-3	9	-4	3.57	0.38
3	-9	4	2.94	0.44
3	-9	4	4.09	1.01
3	-9	4	4.15	0.73
-4	9	-4	-0.43	0.73
-4	9	-4	0.30	0.34
-4	9	-4	0.17	0.21
4	-9	4	0.96	0.82
4	-9	4	0.16	0.42
4	-9	4	0.13	0.27
4	-9	4	0.26	0.18
4	-9	4	-0.01	0.26
-5	9	-4	13.29	0.83
-5	9	-4	13.80	1.49
-5	9	-4	11.93	0.67
5	-9	4	12.46	0.71
5	-9	4	10.38	1.54
5	-9	4	12.97	1.02
5	-9	4	12.00	0.60
5	-9	4	11.99	0.71
-6	9	-4	16.14	1.60
-6	9	-4	17.55	0.91
-6	9	-4	16.88	0.82

6	-9	4	16.82	1.15
6	-9	4	13.87	1.80
6	-9	4	18.70	0.89
6	-9	4	17.92	0.80
6	-9	4	17.86	0.91
-7	9	-4	19.51	0.92
-7	9	-4	20.50	0.95
-7	9	-4	17.38	1.65
7	-9	4	17.35	1.95
7	-9	4	17.78	1.02
7	-9	4	19.82	0.98
7	-9	4	18.99	1.20
7	-9	4	19.39	0.93
-8	9	-4	5.38	0.60
-8	9	-4	6.57	0.61
8	-9	4	3.15	1.19
8	-9	4	6.78	0.73
8	-9	4	6.28	0.90
8	-9	4	6.19	0.80
-9	9	-4	3.15	0.47
-9	9	-4	4.34	0.63
9	-9	4	3.11	0.64
9	-9	4	2.75	1.24
9	-9	4	2.92	0.57
9	-9	4	4.73	0.68
-10	9	-4	23.01	1.13
-10	9	-4	20.92	1.13
10	-9	4	22.62	1.32
10	-9	4	22.69	1.53
10	-9	4	21.45	1.40
10	-9	4	23.49	2.58
-11	9	-4	27.26	1.39
-11	9	-4	27.95	1.33
11	-9	4	25.13	1.61
11	-9	4	28.32	1.47
11	-9	4	27.50	3.01
-12	9	-4	16.12	1.07
12	-9	4	14.54	1.20
12	-9	4	12.76	1.47
12	-9	4	15.00	2.56
-13	9	-4	7.90	0.99
13	-9	4	11.82	2.56
13	-9	4	9.59	1.34
-14	9	-4	5.00	0.77

14	-9	4	6.45	1.32
14	-9	4	3.93	2.37
-15	9	-4	3.15	0.93
15	-9	4	4.32	1.48
15	-9	4	4.37	2.83
12	8	-4	0.45	1.53
12	8	-4	-1.12	1.12
12	8	-4	0.72	0.76
12	8	-4	-0.18	1.65
11	8	-4	3.41	1.33
11	8	-4	1.40	1.65
11	8	-4	0.69	0.99
11	8	-4	2.06	0.69
10	8	-4	4.57	0.70
10	8	-4	3.33	1.51
10	8	-4	3.22	1.08
10	8	-4	4.52	0.98
9	8	-4	3.42	0.98
9	8	-4	4.22	0.84
9	8	-4	1.73	1.33
9	8	-4	3.55	0.60
8	8	-4	-0.61	0.76
8	8	-4	-0.36	0.42
8	8	-4	0.42	1.22
8	8	-4	-0.08	0.59
8	8	-4	-0.96	0.65
7	8	-4	1.53	1.44
7	8	-4	0.12	0.49
7	8	-4	1.01	0.39
7	8	-4	0.51	0.38
7	8	-4	0.92	0.55
7	8	-4	-0.95	1.00
6	8	-4	0.73	1.56
6	8	-4	0.29	0.41
6	8	-4	-0.46	1.23
6	8	-4	0.35	0.35
6	8	-4	-0.85	0.41
6	8	-4	0.68	0.31
-6	-8	4	0.60	0.73
5	8	-4	0.09	0.66
5	8	-4	-0.03	0.30
5	8	-4	0.38	0.36
5	8	-4	0.11	0.34
5	8	-4	1.83	1.08

5	8	-4	0.24	0.30
-5	-8	4	-0.26	0.60
-5	-8	4	0.13	0.66
4	8	-4	0.06	0.52
4	8	-4	0.66	0.31
4	8	-4	0.46	0.32
4	8	-4	0.42	0.29
4	8	-4	0.98	0.94
4	8	-4	0.37	0.29
-4	-8	4	1.49	0.71
-4	-8	4	-0.13	0.52
3	8	-4	9.88	0.57
3	8	-4	10.58	0.71
3	8	-4	10.99	1.01
3	8	-4	11.35	1.55
3	8	-4	9.34	0.61
-3	-8	4	10.20	0.55
-3	-8	4	10.79	1.12
-3	-8	4	10.34	1.20
2	8	-4	5.14	0.48
2	8	-4	7.81	1.20
2	8	-4	5.68	0.38
2	8	-4	5.47	0.41
2	8	-4	5.04	0.81
-2	-8	4	6.85	0.42
-2	-8	4	5.76	0.94
-2	-8	4	5.60	0.86
1	8	-4	0.86	0.71
1	8	-4	-0.05	0.38
1	8	-4	0.65	0.24
-1	-8	4	0.53	0.21
-1	-8	4	0.70	0.66
-1	-8	4	0.79	0.42
0	8	-4	25.61	0.99
0	8	-4	25.73	1.33
0	8	-4	26.66	1.78
0	-8	4	25.50	1.70
0	-8	4	24.17	1.38
0	-8	4	26.56	0.98
-1	8	-4	22.92	1.62
-1	8	-4	22.58	0.86
-1	8	-4	19.25	1.12
1	-8	4	20.79	1.20
1	-8	4	20.84	0.86

1	-8	4	19.97	1.57
-2	8	-4	20.49	1.03
-2	8	-4	17.02	1.46
-2	8	-4	16.86	0.75
2	-8	4	16.21	1.05
2	-8	4	17.99	1.49
2	-8	4	17.54	0.78
-3	8	-4	7.38	0.46
-3	8	-4	6.36	0.64
-3	8	-4	7.50	1.22
3	-8	4	7.16	0.77
3	-8	4	6.62	0.49
3	-8	4	6.46	0.34
3	-8	4	7.01	1.14
3	-8	4	6.15	0.41
-4	8	-4	31.55	1.92
-4	8	-4	30.41	1.19
-4	8	-4	34.24	1.35
4	-8	4	34.10	2.08
4	-8	4	30.59	1.27
4	-8	4	31.63	1.11
4	-8	4	31.44	1.47
4	-8	4	34.53	1.24
-5	8	-4	29.34	1.83
-5	8	-4	29.89	1.23
-5	8	-4	30.86	1.16
5	-8	4	29.76	2.01
5	-8	4	31.83	1.20
5	-8	4	31.99	1.20
5	-8	4	24.44	1.17
5	-8	4	31.32	1.41
-6	8	-4	18.15	0.83
-6	8	-4	18.59	1.49
-6	8	-4	17.53	0.86
6	-8	4	18.50	1.06
6	-8	4	17.45	0.90
6	-8	4	14.08	1.62
6	-8	4	18.85	0.83
6	-8	4	17.33	0.86
-7	8	-4	4.94	0.98
-7	8	-4	3.85	0.48
-7	8	-4	5.57	0.51
7	-8	4	4.16	0.57
7	-8	4	4.64	0.61

7	-8	4	3.95	0.70
7	-8	4	4.04	0.57
7	-8	4	2.99	0.97
-8	8	-4	4.00	0.54
-8	8	-4	4.46	0.53
8	-8	4	4.81	0.69
8	-8	4	4.49	0.64
8	-8	4	4.57	0.60
8	-8	4	3.13	1.19
-9	8	-4	0.96	0.40
-9	8	-4	-0.09	0.32
9	-8	4	0.91	0.51
9	-8	4	0.38	0.49
9	-8	4	1.65	1.10
9	-8	4	0.89	0.44
-10	8	-4	10.33	0.79
-10	8	-4	11.19	0.93
10	-8	4	8.57	1.61
10	-8	4	11.29	1.10
10	-8	4	10.79	0.92
-11	8	-4	21.69	1.31
-11	8	-4	19.81	1.12
11	-8	4	18.12	2.67
11	-8	4	20.08	1.20
-12	8	-4	8.93	0.90
12	-8	4	6.26	1.99
12	-8	4	9.11	1.17
12	-8	4	7.34	1.01
-13	8	-4	13.35	1.08
13	-8	4	12.60	1.68
13	-8	4	11.06	2.34
-14	8	-4	17.63	1.27
14	-8	4	17.34	2.01
14	-8	4	12.08	2.64
-15	8	-4	6.43	0.93
15	-8	4	8.48	1.54
15	-8	4	9.81	2.97
13	7	-4	3.30	1.28
13	7	-4	2.49	0.74
13	7	-4	0.90	1.65
13	7	-4	0.85	1.66
12	7	-4	6.60	1.25
12	7	-4	7.96	1.10
12	7	-4	9.34	1.85

12	7	-4	9.06	1.71
11	7	-4	2.70	0.98
11	7	-4	3.59	1.18
11	7	-4	4.13	0.66
11	7	-4	2.67	1.42
10	7	-4	5.65	1.28
10	7	-4	5.16	0.67
9	7	-4	6.55	1.11
9	7	-4	8.44	0.77
9	7	-4	8.62	1.37
9	7	-4	10.44	1.63
8	7	-4	11.70	1.93
8	7	-4	12.82	1.03
8	7	-4	12.75	1.26
8	7	-4	13.20	0.84
8	7	-4	12.26	0.80
8	7	-4	16.38	2.46
8	7	-4	13.76	1.10
7	7	-4	0.61	0.93
7	7	-4	0.36	0.59
7	7	-4	0.27	0.46
7	7	-4	0.38	0.69
7	7	-4	0.32	0.32
7	7	-4	-0.01	1.08
7	7	-4	0.90	0.47
7	7	-4	0.97	0.36
6	7	-4	5.77	1.24
6	7	-4	4.54	0.79
6	7	-4	4.29	0.51
6	7	-4	4.18	0.52
6	7	-4	4.88	0.67
6	7	-4	5.29	0.61
-6	-7	4	4.64	0.85
-6	-7	4	5.60	0.91
5	7	-4	0.41	0.28
5	7	-4	0.94	0.32
5	7	-4	0.33	0.28
5	7	-4	1.46	0.98
5	7	-4	0.69	0.35
5	7	-4	0.80	0.52
-5	-7	4	1.06	0.62
-5	-7	4	0.28	0.52
4	7	-4	-0.52	0.44
4	7	-4	0.80	0.27

4	7	-4	1.02	0.30
4	7	-4	1.26	0.29
4	7	-4	1.31	0.77
4	7	-4	0.88	0.28
-4	-7	4	0.36	0.57
-4	-7	4	0.65	0.51
3	7	-4	10.91	0.60
3	7	-4	11.89	1.34
3	7	-4	11.44	0.97
3	7	-4	10.57	0.60
3	7	-4	11.63	0.54
-3	-7	4	11.94	1.13
-3	-7	4	11.45	1.01
-3	-7	4	12.19	0.55
2	7	-4	0.32	0.22
2	7	-4	0.21	0.16
2	7	-4	0.35	0.16
2	7	-4	0.57	0.62
2	7	-4	0.47	0.38
-2	-7	4	-0.06	0.37
-2	-7	4	0.28	0.50
-2	-7	4	0.29	0.17
1	7	-4	5.36	0.90
1	7	-4	4.65	0.39
1	7	-4	5.16	0.67
-1	-7	4	4.15	0.36
-1	-7	4	6.51	0.93
-1	-7	4	4.52	0.68
0	7	-4	10.23	0.55
0	7	-4	14.37	1.23
0	7	-4	11.27	0.80
0	-7	4	10.06	1.06
0	-7	4	11.80	0.87
0	-7	4	11.80	0.56
-1	7	-4	0.34	0.56
-1	7	-4	0.44	0.19
-1	7	-4	0.47	0.29
1	-7	4	-0.08	0.49
1	-7	4	0.45	0.33
1	-7	4	0.46	0.20
-2	7	-4	7.56	0.63
-2	7	-4	7.14	1.04
-2	7	-4	7.25	0.44
2	-7	4	6.98	0.47

2	-7	4	8.02	1.08
2	-7	4	8.19	0.71
-3	7	-4	15.10	0.68
-3	7	-4	15.21	1.29
-3	7	-4	14.99	0.81
3	-7	4	16.22	0.61
3	-7	4	15.57	0.64
3	-7	4	13.51	1.30
3	-7	4	15.32	0.91
3	-7	4	16.23	0.71
-4	7	-4	12.73	0.63
-4	7	-4	13.58	0.72
-4	7	-4	11.49	1.17
4	-7	4	13.55	0.67
4	-7	4	14.44	1.36
4	-7	4	14.03	0.67
4	-7	4	12.18	0.81
4	-7	4	13.11	0.59
-5	7	-4	19.30	0.85
-5	7	-4	20.02	1.40
-5	7	-4	17.90	0.80
5	-7	4	18.59	0.83
5	-7	4	17.57	0.96
5	-7	4	17.76	0.78
5	-7	4	17.38	0.84
5	-7	4	16.94	1.53
-6	7	-4	23.30	0.96
-6	7	-4	21.28	1.47
-6	7	-4	21.35	0.95
6	-7	4	24.98	0.99
6	-7	4	21.78	1.08
6	-7	4	22.09	0.98
6	-7	4	24.24	1.80
6	-7	4	23.84	1.04
-7	7	-4	13.61	0.70
-7	7	-4	12.94	1.26
-7	7	-4	12.43	0.70
7	-7	4	12.62	0.82
7	-7	4	10.66	1.51
7	-7	4	13.47	0.84
7	-7	4	12.44	0.73
7	-7	4	12.57	0.79
-8	7	-4	6.60	1.18
-8	7	-4	5.66	0.56

-8	7	-4	5.81	0.58
8	-7	4	5.51	0.66
8	-7	4	4.57	0.60
8	-7	4	5.75	0.68
8	-7	4	5.32	1.25
8	-7	4	5.13	0.72
-9	7	-4	5.39	0.72
-9	7	-4	5.56	0.59
9	-7	4	5.76	0.70
9	-7	4	5.81	0.73
9	-7	4	4.21	0.67
9	-7	4	8.41	1.52
-10	7	-4	0.92	0.38
-10	7	-4	1.15	0.58
10	-7	4	3.08	1.36
10	-7	4	1.97	0.62
10	-7	4	1.85	0.47
-11	7	-4	11.07	1.24
-11	7	-4	12.04	0.88
11	-7	4	12.33	1.24
11	-7	4	13.25	2.09
11	-7	4	12.54	0.96
-12	7	-4	16.37	1.07
12	-7	4	15.10	1.52
12	-7	4	15.12	1.08
12	-7	4	10.30	2.15
-13	7	-4	9.03	1.01
13	-7	4	7.31	2.18
13	-7	4	8.82	1.31
-14	7	-4	3.43	0.73
14	-7	4	6.21	2.44
14	-7	4	5.32	1.30
-15	7	-4	13.16	1.36
15	-7	4	8.47	2.95
15	-7	4	13.20	2.18
13	6	-4	0.10	1.03
13	6	-4	1.71	1.48
13	6	-4	0.96	1.53
13	6	-4	0.76	0.66
12	6	-4	-0.63	1.21
12	6	-4	0.38	0.93
12	6	-4	-0.36	0.55
12	6	-4	0.17	1.33
11	6	-4	1.23	0.50

11	6	-4	1.96	1.24
11	6	-4	1.58	0.86
10	6	-4	0.50	0.69
10	6	-4	-0.72	1.05
10	6	-4	0.35	0.79
10	6	-4	0.45	0.42
9	6	-4	1.38	1.05
9	6	-4	0.92	0.81
9	6	-4	0.08	0.36
9	6	-4	-0.06	0.61
9	6	-4	-1.19	0.67
9	6	-4	-0.41	1.46
9	6	-4	-0.53	0.58
8	6	-4	1.38	0.62
8	6	-4	0.35	0.46
8	6	-4	0.61	0.35
8	6	-4	1.15	1.21
8	6	-4	0.38	0.69
8	6	-4	0.35	0.36
8	6	-4	0.72	0.97
8	6	-4	0.12	0.48
7	6	-4	-0.13	0.58
7	6	-4	0.57	0.57
7	6	-4	0.60	0.44
7	6	-4	0.69	0.93
7	6	-4	1.14	0.32
7	6	-4	1.37	0.39
7	6	-4	0.83	0.31
7	6	-4	-0.29	0.78
6	6	-4	0.08	0.29
6	6	-4	0.21	0.83
6	6	-4	0.51	0.26
6	6	-4	0.37	0.36
6	6	-4	0.21	0.25
6	6	-4	0.73	0.53
-6	-6	4	0.40	0.53
-6	-6	4	0.30	0.50
5	6	-4	2.61	0.39
5	6	-4	2.94	0.42
5	6	-4	2.66	0.58
5	6	-4	2.92	0.86
5	6	-4	2.62	0.33
5	6	-4	2.85	0.40
-5	-6	4	2.35	0.59

-5	-6	4	2.56	0.61
4	6	-4	0.75	0.44
4	6	-4	0.83	0.24
4	6	-4	0.73	0.65
4	6	-4	0.61	0.28
4	6	-4	0.65	0.21
-4	-6	4	0.77	0.45
-4	-6	4	0.29	0.47
3	6	-4	0.33	0.16
3	6	-4	0.35	0.21
3	6	-4	-0.18	0.35
3	6	-4	0.08	0.58
3	6	-4	0.02	0.17
-3	-6	4	0.45	0.39
-3	-6	4	0.03	0.16
-3	-6	4	0.66	0.52
2	6	-4	0.16	0.19
2	6	-4	0.43	0.58
2	6	-4	0.23	0.31
2	6	-4	0.18	0.12
2	6	-4	0.26	0.15
-2	-6	4	0.29	0.34
-2	-6	4	0.05	0.14
-2	-6	4	0.41	0.47
1	6	-4	-0.11	0.56
1	6	-4	0.28	0.27
1	6	-4	0.22	0.19
-1	-6	4	0.49	0.33
-1	-6	4	0.03	0.16
-1	-6	4	0.29	0.46
0	6	-4	6.52	0.42
0	6	-4	6.27	1.32
0	6	-4	6.61	0.59
0	-6	4	5.72	0.86
0	-6	4	5.79	0.61
0	-6	4	6.55	0.41
-1	6	-4	45.26	1.59
-1	6	-4	49.23	1.78
-1	6	-4	47.98	2.09
1	-6	4	46.87	1.83
1	-6	4	50.95	2.19
1	-6	4	47.46	1.60
-2	6	-4	3.65	0.31
-2	6	-4	3.24	0.42

-2	6	-4	2.88	0.60
2	-6	4	3.08	0.49
2	-6	4	3.40	0.26
2	-6	4	3.21	0.35
2	-6	4	3.14	0.65
2	-6	4	2.83	0.24
-3	6	-4	12.97	0.60
-3	6	-4	12.33	1.03
-3	6	-4	11.88	0.66
3	-6	4	13.52	0.78
3	-6	4	12.19	0.54
3	-6	4	12.95	0.62
3	-6	4	13.02	1.17
3	-6	4	12.58	0.56
-4	6	-4	53.09	2.30
-4	6	-4	59.44	1.89
-4	6	-4	51.72	1.82
4	-6	4	53.99	1.80
4	-6	4	51.27	1.95
4	-6	4	55.79	1.84
4	-6	4	53.98	2.47
4	-6	4	55.18	1.83
-5	6	-4	72.28	2.45
-5	6	-4	78.44	2.47
-5	6	-4	78.95	2.97
5	-6	4	74.74	2.46
5	-6	4	69.87	3.14
5	-6	4	75.41	2.50
5	-6	4	72.72	2.56
5	-6	4	76.83	2.45
-6	6	-4	2.41	0.39
-6	6	-4	3.19	0.69
-6	6	-4	2.23	0.31
6	-6	4	2.70	0.36
6	-6	4	2.45	0.82
6	-6	4	2.45	0.40
6	-6	4	2.59	0.38
6	-6	4	2.38	0.40
-7	6	-4	8.47	1.06
-7	6	-4	7.73	0.58
-7	6	-4	8.04	0.56
7	-6	4	8.65	0.65
7	-6	4	8.18	0.70
7	-6	4	8.40	0.67

7	-6	4	7.45	1.36
7	-6	4	8.51	0.62
-8	6	-4	21.18	0.95
-8	6	-4	22.32	1.04
-8	6	-4	21.82	1.48
8	-6	4	22.17	1.16
8	-6	4	19.83	1.09
8	-6	4	18.47	0.98
8	-6	4	19.93	0.98
8	-6	4	19.08	1.84
-9	6	-4	28.20	1.25
-9	6	-4	33.54	1.45
9	-6	4	28.71	1.48
9	-6	4	30.26	1.46
9	-6	4	29.22	2.34
9	-6	4	31.44	1.30
-10	6	-4	8.26	0.82
-10	6	-4	8.72	1.10
10	-6	4	8.67	0.77
10	-6	4	10.01	1.03
10	-6	4	5.36	1.55
-11	6	-4	12.65	0.89
-11	6	-4	13.29	1.44
11	-6	4	13.35	1.27
11	-6	4	12.43	0.92
11	-6	4	14.10	2.37
-12	6	-4	14.40	1.05
12	-6	4	16.92	1.53
12	-6	4	10.39	2.04
12	-6	4	16.11	1.01
-13	6	-4	10.44	1.03
13	-6	4	8.04	1.27
13	-6	4	5.22	2.15
-14	6	-4	10.25	1.16
14	-6	4	9.79	1.41
14	-6	4	13.12	2.71
-15	6	-4	6.02	0.94
15	-6	4	4.61	2.97
15	-6	4	5.28	1.40
14	5	-4	2.33	1.23
14	5	-4	3.93	1.71
14	5	-4	0.95	0.71
13	5	-4	1.11	1.47
13	5	-4	0.23	0.58

13	5	-4	1.87	1.03
13	5	-4	0.28	1.29
12	5	-4	3.47	0.96
12	5	-4	1.87	1.24
12	5	-4	3.87	1.28
12	5	-4	4.07	0.63
11	5	-4	3.71	0.57
11	5	-4	5.96	1.31
11	5	-4	4.14	1.25
11	5	-4	2.36	0.97
11	5	-4	3.65	0.82
10	5	-4	5.52	1.57
10	5	-4	7.05	1.27
10	5	-4	6.20	0.68
10	5	-4	5.48	0.85
10	5	-4	6.00	1.02
10	5	-4	5.65	1.22
9	5	-4	-2.06	1.19
9	5	-4	-1.16	0.67
9	5	-4	-0.31	0.59
9	5	-4	1.59	0.50
9	5	-4	0.33	0.34
9	5	-4	0.29	0.37
9	5	-4	1.12	0.59
9	5	-4	-1.30	0.84
8	5	-4	0.46	0.57
8	5	-4	0.15	0.51
8	5	-4	1.45	0.86
8	5	-4	0.55	0.47
8	5	-4	0.48	0.30
8	5	-4	1.95	1.04
8	5	-4	0.00	0.38
8	5	-4	0.01	0.30
7	5	-4	3.53	0.54
7	5	-4	3.88	0.54
7	5	-4	2.97	0.43
7	5	-4	4.33	0.47
7	5	-4	3.57	0.65
7	5	-4	4.03	0.61
7	5	-4	3.12	1.01
-7	-5	4	2.68	0.64
6	5	-4	2.67	0.46
6	5	-4	2.71	0.43
6	5	-4	3.95	0.87

6	5	-4	2.85	0.34
6	5	-4	2.67	0.36
6	5	-4	2.81	0.50
6	5	-4	3.58	0.58
-6	-5	4	2.37	0.58
5	5	-4	1.87	0.47
5	5	-4	1.96	0.27
5	5	-4	1.95	0.29
5	5	-4	1.84	0.27
5	5	-4	3.03	0.71
5	5	-4	2.32	0.37
-5	-5	4	2.23	0.52
-5	-5	4	1.74	0.49
4	5	-4	0.81	0.38
4	5	-4	1.52	0.22
4	5	-4	1.21	0.23
4	5	-4	1.33	0.31
-4	-5	4	1.28	0.47
-4	-5	4	1.50	0.21
3	5	-4	4.98	0.36
3	5	-4	4.59	0.28
3	5	-4	4.98	0.60
3	5	-4	4.52	0.81
3	5	-4	5.12	0.37
-3	-5	4	3.95	0.31
-3	-5	4	4.09	0.74
-3	-5	4	3.31	0.58
2	5	-4	18.44	0.95
2	5	-4	18.70	1.14
2	5	-4	18.16	0.69
2	5	-4	19.02	0.62
2	5	-4	17.82	0.74
-2	-5	4	18.49	1.13
-2	-5	4	19.50	0.71
-2	-5	4	17.62	0.97
1	5	-4	59.45	1.93
1	5	-4	60.73	2.33
1	5	-4	60.49	2.12
-1	-5	4	57.13	2.33
-1	-5	4	59.48	2.17
0	5	-4	18.25	1.09
0	5	-4	18.25	0.86
0	5	-4	18.02	0.71
0	-5	4	17.89	1.17

0	-5	4	17.93	0.91
-1	5	-4	3.14	0.38
-1	5	-4	3.46	0.30
-1	5	-4	3.44	0.18
-1	5	-4	3.00	0.56
1	-5	4	3.27	0.27
1	-5	4	3.66	0.59
1	-5	4	2.98	0.44
1	-5	4	3.36	0.31
-2	5	-4	96.03	3.09
-2	5	-4	98.08	3.00
-2	5	-4	102.43	3.54
2	-5	4	101.43	3.10
2	-5	4	94.34	3.57
2	-5	4	98.47	3.05
2	-5	4	100.86	3.25
-3	5	-4	150.10	4.70
-3	5	-4	161.22	5.12
-3	5	-4	156.56	4.66
3	-5	4	153.91	4.68
3	-5	4	147.14	5.23
3	-5	4	147.07	4.81
3	-5	4	155.86	4.71
-4	5	-4	4.46	0.72
-4	5	-4	3.25	0.33
-4	5	-4	3.22	0.30
4	-5	4	3.34	0.36
4	-5	4	2.85	0.67
4	-5	4	2.85	0.40
4	-5	4	3.52	0.45
-5	5	-4	33.97	1.28
-5	5	-4	35.22	1.38
-5	5	-4	35.84	1.27
-5	5	-4	38.74	1.71
5	-5	4	35.87	1.32
5	-5	4	32.81	1.87
5	-5	4	36.74	1.36
5	-5	4	36.44	1.31
-6	5	-4	80.90	3.06
-6	5	-4	81.32	2.68
-6	5	-4	81.38	2.64
-6	5	-4	80.52	2.65
6	-5	4	83.13	2.70
6	-5	4	74.83	3.33

6	-5	4	81.29	2.67
6	-5	4	84.51	2.71
-7	5	-4	5.57	0.51
-7	5	-4	5.28	0.96
-7	5	-4	6.26	0.55
-7	5	-4	7.49	0.62
7	-5	4	6.54	0.57
7	-5	4	8.84	1.29
7	-5	4	6.01	0.54
7	-5	4	5.83	0.64
-8	5	-4	32.91	1.81
-8	5	-4	37.46	1.41
-8	5	-4	36.87	1.55
8	-5	4	36.25	1.59
8	-5	4	38.45	1.56
8	-5	4	35.57	1.43
8	-5	4	38.19	1.45
8	-5	4	35.24	2.29
-9	5	-4	40.87	1.78
-9	5	-4	35.66	1.49
9	-5	4	37.19	1.52
9	-5	4	38.83	1.77
9	-5	4	48.01	2.36
9	-5	4	38.09	2.56
-10	5	-4	7.91	1.23
10	-5	4	8.03	1.68
10	-5	4	7.56	0.99
10	-5	4	7.00	0.74
-11	5	-4	23.35	1.19
-11	5	-4	23.39	1.82
11	-5	4	24.78	2.77
11	-5	4	22.98	1.55
11	-5	4	26.07	1.24
-12	5	-4	20.23	1.20
12	-5	4	21.74	3.06
12	-5	4	23.27	1.70
12	-5	4	21.58	1.20
-13	5	-4	13.20	1.15
13	-5	4	12.26	2.57
13	-5	4	13.28	1.75
-14	5	-4	3.80	0.75
14	-5	4	7.41	1.35
14	-5	4	3.61	2.23
14	4	-4	4.66	1.29

14	4	-4	3.23	0.70
14	4	-4	5.57	1.73
13	4	-4	0.11	1.17
13	4	-4	1.14	0.92
13	4	-4	-0.09	0.55
13	4	-4	-0.73	1.09
12	4	-4	0.95	0.93
12	4	-4	1.70	0.51
12	4	-4	1.77	0.89
12	4	-4	1.67	1.08
12	4	-4	0.51	1.03
11	4	-4	0.30	0.98
11	4	-4	1.43	0.93
11	4	-4	0.88	0.50
11	4	-4	-0.03	0.76
10	4	-4	0.13	0.83
10	4	-4	0.25	1.20
10	4	-4	1.36	0.68
10	4	-4	0.57	0.34
10	4	-4	0.41	0.69
10	4	-4	0.46	0.62
9	4	-4	1.32	1.04
9	4	-4	2.60	0.49
9	4	-4	2.92	0.92
9	4	-4	3.02	0.63
9	4	-4	2.78	0.69
9	4	-4	3.13	0.67
9	4	-4	3.28	0.41
9	4	-4	2.94	0.42
8	4	-4	0.15	0.48
8	4	-4	0.59	0.26
8	4	-4	1.25	0.70
8	4	-4	0.40	0.79
8	4	-4	0.14	0.28
8	4	-4	-0.05	0.31
8	4	-4	-0.10	0.42
8	4	-4	-0.24	0.40
7	4	-4	0.39	0.23
7	4	-4	0.73	0.69
7	4	-4	0.66	0.29
7	4	-4	0.34	0.24
7	4	-4	0.48	0.36
7	4	-4	-0.68	0.39
7	4	-4	0.36	0.40

-7	-4	4	0.46	0.47
6	4	-4	0.58	0.24
6	4	-4	1.02	0.39
6	4	-4	0.94	0.40
6	4	-4	1.07	0.32
6	4	-4	1.10	0.62
6	4	-4	1.23	0.24
6	4	-4	0.65	0.22
-6	-4	4	0.50	0.41
5	4	-4	5.78	0.52
5	4	-4	5.54	0.85
5	4	-4	5.68	0.38
5	4	-4	5.36	0.38
5	4	-4	5.85	0.42
5	4	-4	4.64	0.63
-5	-4	4	4.80	0.66
-5	-4	4	5.63	0.67
4	4	-4	0.81	0.17
4	4	-4	0.87	0.33
4	4	-4	0.64	0.17
4	4	-4	1.34	0.47
4	4	-4	0.70	0.26
-4	-4	4	0.71	0.31
-4	-4	4	1.41	0.45
-4	-4	4	0.63	0.17
3	4	-4	17.94	0.87
3	4	-4	17.12	0.71
3	4	-4	16.64	0.67
3	4	-4	17.43	1.01
-3	-4	4	17.22	0.66
-3	-4	4	15.19	0.88
-3	-4	4	17.41	0.65
-3	-4	4	15.78	0.92
2	4	-4	1.19	0.19
2	4	-4	0.63	0.28
2	4	-4	1.36	0.41
2	4	-4	0.98	0.18
-2	-4	4	1.09	0.31
-2	-4	4	1.18	0.17
-2	-4	4	1.22	0.19
-2	-4	4	1.24	0.37
1	4	-4	22.76	1.03
1	4	-4	26.45	0.87
1	4	-4	24.76	0.90

1	4	-4	27.19	1.19
-1	-4	4	24.36	0.88
-1	-4	4	24.64	1.20
-1	-4	4	24.82	0.84
-1	-4	4	24.90	1.07
0	4	-4	114.21	3.75
0	4	-4	111.38	3.47
0	4	-4	122.93	3.61
0	4	-4	109.32	3.40
0	-4	4	108.86	3.79
0	-4	4	109.21	3.47
0	-4	4	114.48	3.64
0	-4	4	111.92	3.45
-1	4	-4	43.45	1.45
-1	4	-4	43.27	1.38
-1	4	-4	44.78	1.53
-1	4	-4	46.46	1.74
1	-4	4	43.79	1.82
1	-4	4	43.26	1.44
1	-4	4	41.87	1.46
1	-4	4	43.07	1.57
-2	4	-4	68.05	2.37
-2	4	-4	71.46	2.13
-2	4	-4	62.08	2.01
2	-4	4	59.92	2.14
2	-4	4	62.06	2.05
2	-4	4	61.70	2.44
-3	4	-4	1.23	0.20
-3	4	-4	1.54	0.21
-3	4	-4	1.90	0.29
-3	4	-4	1.41	0.36
3	-4	4	1.04	0.46
3	-4	4	1.14	0.23
3	-4	4	1.62	0.23
3	-4	4	1.45	0.25
-4	4	-4	55.79	2.12
-4	4	-4	52.83	1.80
-4	4	-4	50.94	1.78
-4	4	-4	55.06	1.83
4	-4	4	54.22	1.81
4	-4	4	56.18	1.86
4	-4	4	52.92	1.83
4	-4	4	54.39	2.32
-5	4	-4	83.59	2.98

-5	4	-4	80.40	2.63
-5	4	-4	79.12	2.64
5	-4	4	76.29	3.20
5	-4	4	84.21	2.71
5	-4	4	87.54	2.71
5	-4	4	81.89	2.65
-6	4	-4	22.32	0.93
-6	4	-4	22.01	0.94
-6	4	-4	23.28	1.04
-6	4	-4	22.17	1.28
6	-4	4	22.68	1.59
6	-4	4	23.60	1.04
6	-4	4	23.36	0.99
6	-4	4	21.76	0.94
-7	4	-4	37.08	1.53
-7	4	-4	38.23	1.42
-7	4	-4	36.55	1.37
-7	4	-4	35.95	1.74
7	-4	4	37.47	1.50
7	-4	4	36.54	1.40
7	-4	4	36.19	1.42
-8	4	-4	19.01	1.31
-8	4	-4	19.46	1.18
-8	4	-4	18.31	0.94
-8	4	-4	20.04	0.90
8	-4	4	19.09	1.74
8	-4	4	18.12	0.93
8	-4	4	18.59	1.11
8	-4	4	18.33	0.94
-9	4	-4	9.93	1.20
-9	4	-4	8.95	0.67
9	-4	4	8.29	0.73
9	-4	4	9.96	1.69
9	-4	4	9.25	0.93
-10	4	-4	18.15	1.59
10	-4	4	14.80	2.06
10	-4	4	17.73	1.04
-11	4	-4	10.28	0.83
-11	4	-4	13.28	1.75
11	-4	4	9.87	1.23
11	-4	4	8.00	1.76
11	-4	4	8.96	0.89
-12	4	-4	9.12	0.93
12	-4	4	9.58	1.40

12	-4	4	10.04	0.99
12	-4	4	7.28	1.88
-13	4	-4	15.97	1.21
13	-4	4	18.87	1.78
-14	4	-4	11.24	1.23
14	-4	4	13.02	2.70
14	-4	4	12.28	1.94
15	3	-4	0.59	1.18
15	3	-4	0.85	0.66
15	3	-4	4.24	1.91
14	3	-4	1.40	0.63
14	3	-4	-0.51	1.46
14	3	-4	0.57	1.31
14	3	-4	1.04	1.02
13	3	-4	0.83	1.05
13	3	-4	1.23	0.50
13	3	-4	0.72	0.86
13	3	-4	1.45	1.36
12	3	-4	2.06	0.81
12	3	-4	2.68	0.51
12	3	-4	3.23	1.05
12	3	-4	2.62	1.01
12	3	-4	4.40	0.84
11	3	-4	1.78	0.68
11	3	-4	0.84	1.35
11	3	-4	2.03	0.95
11	3	-4	1.52	0.41
11	3	-4	1.01	0.84
11	3	-4	1.70	0.68
10	3	-4	2.43	0.85
10	3	-4	0.22	0.53
10	3	-4	1.31	0.74
10	3	-4	1.69	1.12
10	3	-4	1.20	0.34
10	3	-4	0.41	0.54
9	3	-4	2.65	0.95
9	3	-4	3.49	0.82
9	3	-4	2.90	0.38
9	3	-4	2.87	0.61
9	3	-4	2.37	0.58
9	3	-4	3.15	0.67
9	3	-4	2.51	0.35
9	3	-4	4.05	0.60
8	3	-4	0.46	0.44

8	3	-4	0.40	0.26
8	3	-4	0.63	0.42
8	3	-4	0.85	0.74
8	3	-4	0.28	0.40
8	3	-4	0.77	0.31
8	3	-4	0.98	0.25
-8	-3	4	0.54	0.44
7	3	-4	1.55	0.41
7	3	-4	0.90	0.38
7	3	-4	1.28	0.24
7	3	-4	2.38	0.64
7	3	-4	1.09	0.37
7	3	-4	1.17	0.26
7	3	-4	1.30	0.29
-7	-3	4	1.08	0.42
6	3	-4	0.38	0.20
6	3	-4	0.51	0.28
6	3	-4	0.82	0.30
6	3	-4	0.12	0.44
6	3	-4	0.72	0.20
6	3	-4	0.42	0.30
6	3	-4	0.46	0.20
-6	-3	4	0.44	0.35
5	3	-4	41.47	1.33
5	3	-4	38.27	1.44
5	3	-4	39.24	1.63
5	3	-4	39.19	1.30
5	3	-4	32.80	1.34
5	3	-4	38.78	1.43
5	3	-4	39.74	1.51
-5	-3	4	36.83	1.57
4	3	-4	16.71	0.65
4	3	-4	17.17	0.77
4	3	-4	13.52	0.89
4	3	-4	16.74	0.92
-4	-3	4	16.70	0.70
-4	-3	4	17.41	0.88
-4	-3	4	17.76	0.81
-4	-3	4	17.01	0.64
3	3	-4	2.48	0.46
3	3	-4	2.33	0.35
3	3	-4	2.35	0.24
3	3	-4	2.64	0.31
-3	-3	4	2.00	0.34

-3	-3	4	2.46	0.40
-3	-3	4	2.39	0.26
-3	-3	4	1.90	0.22
2	3	-4	0.67	0.18
2	3	-4	0.84	0.28
2	3	-4	0.60	0.14
2	3	-4	0.71	0.20
-2	-3	4	0.49	0.20
-2	-3	4	0.68	0.15
-2	-3	4	1.13	0.17
-2	-3	4	1.15	0.28
1	3	-4	23.46	0.84
1	3	-4	23.09	0.98
1	3	-4	24.47	0.92
1	3	-4	23.02	0.84
-1	-3	4	23.18	0.96
-1	-3	4	22.06	1.01
-1	-3	4	23.37	0.84
-1	-3	4	22.65	0.82
0	3	-4	38.37	1.44
0	3	-4	37.61	1.28
0	3	-4	38.02	1.34
0	3	-4	38.62	1.27
0	-3	4	40.13	1.39
0	-3	4	37.81	1.28
0	-3	4	39.23	1.51
0	-3	4	39.07	1.32
-1	3	-4	22.77	0.82
-1	3	-4	25.32	1.03
-1	3	-4	21.77	0.81
-1	3	-4	22.82	0.87
1	-3	4	23.06	0.91
1	-3	4	22.78	0.83
1	-3	4	22.22	0.86
-2	3	-4	71.05	2.29
-2	3	-4	70.94	2.33
-2	3	-4	73.68	2.51
-2	3	-4	72.18	2.30
2	-3	4	71.44	2.33
2	-3	4	75.49	2.64
2	-3	4	71.98	2.36
-3	3	-4	96.69	3.25
-3	3	-4	94.13	3.01
-3	3	-4	92.37	3.01

-3	3	-4	98.72	3.07
3	-3	4	97.44	3.07
3	-3	4	98.53	3.44
3	-3	4	93.94	3.01
3	-3	4	99.71	3.09
-4	3	-4	43.32	1.51
-4	3	-4	44.10	1.50
-4	3	-4	46.31	1.78
-4	3	-4	42.17	1.57
4	-3	4	47.16	1.52
4	-3	4	44.83	1.59
4	-3	4	42.27	1.55
4	-3	4	44.56	1.98
-5	3	-4	45.06	1.67
-5	3	-4	43.90	1.58
-5	3	-4	45.60	1.83
-5	3	-4	45.53	1.55
5	-3	4	42.75	1.61
5	-3	4	46.43	1.57
5	-3	4	46.58	1.66
-6	3	-4	40.96	1.46
-6	3	-4	41.19	1.50
-6	3	-4	40.77	1.77
-6	3	-4	39.97	1.63
6	-3	4	43.70	1.49
6	-3	4	37.26	1.51
6	-3	4	42.69	1.60
-7	3	-4	15.69	0.82
-7	3	-4	15.56	0.76
-7	3	-4	16.21	1.12
-7	3	-4	16.47	1.06
7	-3	4	14.40	1.49
7	-3	4	15.83	0.79
7	-3	4	17.65	0.97
7	-3	4	13.23	0.81
-8	3	-4	29.29	1.63
-8	3	-4	32.45	1.67
-8	3	-4	30.48	1.24
-8	3	-4	32.20	1.33
8	-3	4	33.08	1.48
8	-3	4	32.14	2.18
8	-3	4	30.16	1.29
-9	3	-4	24.74	1.07
-9	3	-4	27.12	1.70

-9	3	-4	24.12	1.16
9	-3	4	20.03	1.13
9	-3	4	26.61	2.19
-10	3	-4	9.38	1.58
10	-3	4	6.85	0.99
10	-3	4	4.55	1.36
10	-3	4	5.08	0.77
-11	3	-4	11.87	0.89
-11	3	-4	14.27	1.99
11	-3	4	9.51	0.97
11	-3	4	11.31	1.29
11	-3	4	12.78	2.02
-12	3	-4	21.93	1.25
12	-3	4	24.40	1.75
12	-3	4	19.81	1.30
12	-3	4	20.81	2.96
-13	3	-4	12.01	1.15
13	-3	4	10.67	2.34
13	-3	4	11.62	1.69
-14	3	-4	4.04	0.81
14	-3	4	4.15	2.39
14	-3	4	4.16	1.21
15	2	-4	-0.16	1.11
15	2	-4	-1.31	1.67
15	2	-4	0.32	0.60
14	2	-4	3.99	0.67
14	2	-4	4.68	1.49
14	2	-4	5.23	1.14
14	2	-4	4.43	1.07
13	2	-4	4.23	0.98
13	2	-4	3.56	0.98
13	2	-4	2.41	1.18
12	2	-4	2.29	0.97
12	2	-4	1.61	0.69
12	2	-4	1.76	0.68
12	2	-4	1.08	1.72
12	2	-4	0.75	0.79
-12	-2	4	0.95	0.52
11	2	-4	0.56	1.16
11	2	-4	0.62	0.73
11	2	-4	0.04	0.75
11	2	-4	1.07	0.62
11	2	-4	0.29	0.53
-11	-2	4	0.81	0.45

10	2	-4	1.86	0.72
10	2	-4	0.16	0.41
10	2	-4	1.11	0.50
10	2	-4	0.58	0.48
10	2	-4	0.57	0.41
10	2	-4	0.40	0.96
10	2	-4	1.68	0.69
-10	-2	4	0.67	0.40
9	2	-4	1.67	0.61
9	2	-4	1.26	0.63
9	2	-4	1.41	0.47
9	2	-4	2.53	0.42
9	2	-4	2.17	0.34
9	2	-4	2.00	0.83
9	2	-4	2.27	0.45
-9	-2	4	2.26	0.41
8	2	-4	9.32	0.56
8	2	-4	9.10	0.73
8	2	-4	9.47	0.79
8	2	-4	10.57	0.85
8	2	-4	8.86	1.15
8	2	-4	9.72	0.62
8	2	-4	9.32	0.55
-8	-2	4	8.97	0.80
7	2	-4	0.74	0.20
7	2	-4	0.47	0.30
7	2	-4	0.01	0.29
7	2	-4	0.61	0.35
7	2	-4	0.52	0.21
7	2	-4	0.12	0.21
7	2	-4	1.03	0.56
-7	-2	4	0.47	0.35
6	2	-4	3.73	0.47
6	2	-4	4.18	0.50
6	2	-4	4.06	0.67
6	2	-4	4.20	0.33
6	2	-4	3.92	0.36
6	2	-4	3.61	0.48
-6	-2	4	4.00	0.43
-6	-2	4	2.99	0.45
5	2	-4	12.82	0.60
5	2	-4	12.44	0.67
5	2	-4	12.80	0.76
5	2	-4	13.28	0.57

5	2	-4	11.66	0.53
5	2	-4	12.32	0.66
-5	-2	4	11.11	0.72
4	2	-4	1.36	0.23
4	2	-4	1.21	0.18
4	2	-4	1.50	0.26
4	2	-4	1.21	0.29
4	2	-4	1.33	0.25
4	2	-4	1.05	0.25
-4	-2	4	1.27	0.23
-4	-2	4	1.22	0.29
3	2	-4	72.96	2.27
3	2	-4	69.66	2.29
3	2	-4	70.46	2.20
3	2	-4	68.77	2.29
-3	-2	4	69.35	2.26
-3	-2	4	71.14	2.21
-3	-2	4	69.66	2.19
-3	-2	4	70.28	2.34
2	2	-4	8.51	0.40
2	2	-4	9.27	0.44
2	2	-4	9.20	0.46
2	2	-4	8.71	0.48
-2	-2	4	8.76	0.51
-2	-2	4	9.58	0.46
-2	-2	4	8.58	0.38
-2	-2	4	7.52	0.44
1	2	-4	33.03	1.20
1	2	-4	34.64	1.20
1	2	-4	35.71	1.18
1	2	-4	36.10	1.20
-1	-2	4	33.18	1.23
-1	-2	4	34.32	1.13
-1	-2	4	34.13	1.20
-1	-2	4	32.43	1.31
0	2	-4	8.42	0.42
0	2	-4	9.89	0.50
0	2	-4	8.98	0.46
0	2	-4	9.72	0.45
0	-2	4	9.46	0.48
0	-2	4	8.98	0.50
0	-2	4	8.29	0.54
0	-2	4	9.24	0.41
-1	2	-4	146.04	4.46

-1	2	-4	153.06	4.53
-1	2	-4	143.21	4.49
-1	2	-4	145.98	4.58
1	-2	4	144.66	4.53
1	-2	4	146.34	4.54
1	-2	4	148.91	4.46
-2	2	-4	36.03	1.35
-2	2	-4	33.14	1.21
-2	2	-4	36.03	1.27
-2	2	-4	33.27	1.18
2	-2	4	36.89	1.26
2	-2	4	34.83	1.18
2	-2	4	34.72	1.27
2	-2	4	36.05	1.49
-3	2	-4	4.15	0.41
-3	2	-4	3.86	0.35
-3	2	-4	4.61	0.49
-3	2	-4	3.94	0.30
3	-2	4	4.54	0.63
3	-2	4	4.96	0.43
3	-2	4	3.65	0.36
3	-2	4	4.20	0.32
-4	2	-4	26.58	1.21
-4	2	-4	25.77	0.97
-4	2	-4	24.95	1.01
4	-2	4	26.11	1.05
4	-2	4	27.34	1.43
4	-2	4	26.64	1.09
4	-2	4	27.47	0.98
-5	2	-4	16.93	0.95
-5	2	-4	16.49	0.78
-5	2	-4	16.61	0.99
-5	2	-4	16.50	0.72
5	-2	4	17.66	0.75
5	-2	4	18.05	0.83
5	-2	4	17.36	0.89
5	-2	4	17.39	1.25
-6	2	-4	12.24	0.70
-6	2	-4	11.78	0.62
-6	2	-4	12.85	0.96
-6	2	-4	12.04	0.92
6	-2	4	12.01	0.80
6	-2	4	12.77	0.72
6	-2	4	11.13	1.21

6	-2	4	13.24	0.67
-7	2	-4	26.19	1.07
-7	2	-4	27.61	1.49
-7	2	-4	27.04	1.15
-7	2	-4	26.40	1.44
7	-2	4	28.02	1.19
7	-2	4	26.82	1.10
7	-2	4	24.65	1.26
-8	2	-4	35.65	1.49
-8	2	-4	37.13	1.96
-8	2	-4	38.72	1.41
8	-2	4	35.96	2.32
8	-2	4	37.66	1.53
8	-2	4	35.36	1.44
-9	2	-4	35.72	1.43
-9	2	-4	37.89	2.21
-9	2	-4	36.42	1.54
9	-2	4	37.84	1.57
9	-2	4	35.90	2.47
-10	2	-4	40.45	1.70
-10	2	-4	38.83	2.47
10	-2	4	39.69	1.70
10	-2	4	38.83	2.80
-11	2	-4	13.47	2.21
-11	2	-4	14.14	0.96
11	-2	4	15.29	1.11
11	-2	4	12.55	2.06
11	-2	4	13.49	1.37
-12	2	-4	12.35	1.08
12	-2	4	13.77	1.18
12	-2	4	11.02	2.34
12	-2	4	15.72	1.58
-13	2	-4	7.94	1.11
13	-2	4	10.71	2.19
13	-2	4	9.82	1.38
-14	2	-4	14.97	1.38
14	-2	4	14.34	2.37
14	-2	4	11.22	2.70
16	1	-4	0.69	1.29
16	1	-4	-2.84	1.91
16	1	-4	0.65	0.70
15	1	-4	1.33	1.06
15	1	-4	-1.70	1.59
-15	-1	4	1.35	0.81

14	1	-4	-0.16	1.34
14	1	-4	0.79	0.92
14	1	-4	1.36	0.75
-14	-1	4	0.83	0.65
13	1	-4	-0.17	1.10
13	1	-4	1.68	0.81
13	1	-4	1.15	0.76
13	1	-4	1.60	0.83
-13	-1	4	0.71	0.57
12	1	-4	-0.56	0.67
12	1	-4	-0.18	0.58
12	1	-4	-0.17	0.50
12	1	-4	0.58	0.93
12	1	-4	-0.74	1.17
-12	-1	4	0.58	0.45
11	1	-4	3.70	0.87
11	1	-4	4.43	1.15
11	1	-4	5.23	0.81
11	1	-4	3.38	0.73
11	1	-4	4.60	0.60
-11	-1	4	4.77	0.66
10	1	-4	3.45	0.73
10	1	-4	3.81	0.68
10	1	-4	2.63	0.40
10	1	-4	4.45	0.92
10	1	-4	3.45	0.50
10	1	-4	3.54	0.50
10	1	-4	3.31	0.74
-10	-1	4	3.13	0.44
9	1	-4	25.12	1.44
9	1	-4	26.52	1.41
9	1	-4	26.46	1.07
9	1	-4	30.61	1.63
9	1	-4	25.34	1.12
9	1	-4	25.65	1.34
-9	-1	4	26.30	1.12
8	1	-4	14.14	0.75
8	1	-4	11.92	1.04
8	1	-4	13.25	0.70
8	1	-4	13.03	0.64
8	1	-4	12.77	0.91
8	1	-4	14.14	0.90
-8	-1	4	13.18	0.70
-8	-1	4	12.44	0.81

7	1	-4	22.51	1.18
7	1	-4	22.39	0.95
7	1	-4	22.08	1.06
7	1	-4	21.22	1.07
7	1	-4	23.08	0.88
-7	-1	4	22.54	0.92
-7	-1	4	21.85	1.06
-7	-1	4	22.78	1.02
6	1	-4	33.12	1.22
6	1	-4	34.09	1.16
6	1	-4	32.94	1.34
6	1	-4	33.17	1.14
6	1	-4	31.81	1.27
6	1	-4	32.37	1.32
-6	-1	4	33.96	1.33
-6	-1	4	31.91	1.27
5	1	-4	40.92	1.37
5	1	-4	39.95	1.41
5	1	-4	39.18	1.46
5	1	-4	38.99	1.30
5	1	-4	40.00	1.32
5	1	-4	38.61	1.34
-5	-1	4	34.95	1.49
4	1	-4	59.28	1.86
4	1	-4	58.57	1.84
4	1	-4	58.89	1.87
4	1	-4	59.14	2.00
4	1	-4	56.78	1.82
4	1	-4	59.06	1.90
-4	-1	4	57.25	1.95
-4	-1	4	56.72	1.95
3	1	-4	2.86	0.23
3	1	-4	2.74	0.33
3	1	-4	2.87	0.26
-3	-1	4	2.76	0.31
-3	-1	4	2.63	0.21
-3	-1	4	2.53	0.28
2	1	-4	191.72	5.57
2	1	-4	179.34	5.63
2	1	-4	173.74	5.56
-2	-1	4	190.29	5.63
-2	-1	4	179.81	5.58
-2	-1	4	185.98	5.50
1	1	-4	398.32	12.08

1	1	-4	408.07	11.96
1	1	-4	415.71	11.96
1	1	-4	409.38	12.01
-1	-1	4	403.91	11.95
-1	-1	4	367.47	12.07
-1	-1	4	401.44	12.00
-1	-1	4	413.06	12.09
0	1	-4	5.25	0.35
0	1	-4	4.80	0.30
0	1	-4	5.28	0.39
0	1	-4	5.38	0.34
0	-1	4	5.41	0.35
0	-1	4	4.88	0.29
0	-1	4	5.15	0.42
0	-1	4	5.10	0.40
-1	1	-4	13.33	0.53
-1	1	-4	12.94	0.62
-1	1	-4	13.89	0.63
-1	1	-4	14.26	0.65
1	-1	4	13.14	0.75
1	-1	4	13.64	0.53
1	-1	4	13.38	0.65
1	-1	4	12.57	0.60
-2	1	-4	31.13	1.23
-2	1	-4	29.68	1.11
-2	1	-4	29.76	1.04
-2	1	-4	31.78	1.20
2	-1	4	30.91	1.04
2	-1	4	30.98	1.14
2	-1	4	29.08	1.18
2	-1	4	30.71	1.33
-3	1	-4	1.07	0.18
-3	1	-4	1.00	0.32
-3	1	-4	1.34	0.24
3	-1	4	1.21	0.25
3	-1	4	1.69	0.30
3	-1	4	1.28	0.41
3	-1	4	1.09	0.19
-4	1	-4	14.37	0.71
-4	1	-4	14.65	0.89
-4	1	-4	14.60	0.63
-4	1	-4	15.03	0.89
4	-1	4	14.97	1.05
4	-1	4	14.31	0.64

4	-1	4	15.72	0.76
4	-1	4	16.74	0.82
-5	1	-4	25.16	1.24
-5	1	-4	23.87	1.02
-5	1	-4	23.58	0.93
-5	1	-4	25.02	1.27
5	-1	4	25.44	0.97
5	-1	4	24.95	1.14
5	-1	4	24.16	1.06
5	-1	4	25.18	1.47
-6	1	-4	38.32	1.46
-6	1	-4	37.32	1.71
-6	1	-4	38.21	1.36
-6	1	-4	38.65	1.80
6	-1	4	37.43	1.57
6	-1	4	35.31	1.96
6	-1	4	39.42	1.39
6	-1	4	37.89	1.50
-7	1	-4	7.90	1.05
-7	1	-4	9.08	0.67
-7	1	-4	9.32	1.18
-7	1	-4	9.95	0.59
7	-1	4	9.46	0.62
7	-1	4	7.32	0.88
7	-1	4	8.39	1.21
7	-1	4	9.25	0.73
-8	1	-4	61.27	2.22
-8	1	-4	61.73	2.10
-8	1	-4	59.67	2.79
8	-1	4	59.35	2.14
8	-1	4	63.86	2.28
8	-1	4	58.79	2.94
-9	1	-4	61.51	2.20
-9	1	-4	58.28	2.55
-9	1	-4	63.95	3.04
9	-1	4	68.43	2.40
-10	1	-4	10.18	1.18
-10	1	-4	8.71	1.49
-10	1	-4	11.03	0.95
10	-1	4	7.10	1.44
10	-1	4	10.28	0.98
-11	1	-4	23.06	2.60
-11	1	-4	22.55	1.19
11	-1	4	21.79	1.61

11	-1	4	20.93	1.34
11	-1	4	17.17	2.50
-12	1	-4	21.59	1.31
12	-1	4	20.58	1.78
12	-1	4	23.55	1.40
12	-1	4	21.28	2.87
-13	1	-4	9.12	1.17
13	-1	4	11.14	1.71
13	-1	4	10.00	2.29
16	0	-4	1.20	1.13
16	0	-4	0.17	1.96
-16	0	4	1.06	0.84
15	0	-4	1.27	0.98
15	0	-4	0.09	1.57
-15	0	4	-0.67	0.69
14	0	-4	2.72	1.35
14	0	-4	0.31	0.57
14	0	-4	0.74	0.89
-14	0	4	1.21	0.63
13	0	-4	2.97	0.65
13	0	-4	1.86	0.76
13	0	-4	2.67	1.18
-13	0	4	3.33	0.60
12	0	-4	0.65	0.45
12	0	-4	0.42	0.90
12	0	-4	1.64	0.57
12	0	-4	-0.76	0.98
-12	0	4	0.46	0.74
11	0	-4	0.71	0.53
11	0	-4	0.10	0.34
11	0	-4	-0.33	0.75
11	0	-4	0.77	0.53
-11	0	4	0.35	0.34
-11	0	4	0.63	0.64
10	0	-4	3.68	0.68
10	0	-4	3.23	0.59
10	0	-4	3.43	0.53
10	0	-4	4.27	0.62
10	0	-4	3.32	0.78
10	0	-4	3.33	0.51
-10	0	4	4.35	0.67
-10	0	4	2.94	0.42
9	0	-4	0.59	0.41
9	0	-4	0.55	0.31

9	0	-4	1.64	0.53
9	0	-4	0.53	0.51
9	0	-4	0.82	0.28
9	0	-4	1.23	0.27
-9	0	4	1.36	0.45
-9	0	4	0.61	0.28
8	0	-4	46.52	1.85
8	0	-4	46.04	1.59
8	0	-4	44.52	1.89
8	0	-4	46.05	1.59
-8	0	4	44.38	1.62
-8	0	4	49.54	1.76
-8	0	4	47.29	1.77
7	0	-4	46.63	1.56
7	0	-4	46.32	1.79
7	0	-4	46.64	1.57
7	0	-4	45.58	1.80
-7	0	4	47.13	1.72
-7	0	4	50.19	1.70
-7	0	4	44.75	1.65
-7	0	4	44.21	1.58
6	0	-4	11.95	0.54
6	0	-4	11.75	0.69
6	0	-4	11.04	0.69
6	0	-4	12.19	0.53
-6	0	4	11.03	0.60
-6	0	4	11.46	0.56
-6	0	4	12.02	0.68
-6	0	4	13.00	0.61
5	0	-4	2.32	0.24
5	0	-4	2.44	0.40
5	0	-4	2.44	0.35
5	0	-4	2.43	0.25
5	0	-4	2.04	0.23
-5	0	4	2.26	0.29
-5	0	4	2.31	0.28
4	0	-4	46.30	1.51
4	0	-4	46.87	1.53
4	0	-4	46.75	1.56
4	0	-4	47.28	1.70
4	0	-4	48.32	1.54
-4	0	4	47.84	1.57
-4	0	4	48.34	1.50
-4	0	4	48.05	1.66

3	0	-4	7.27	0.36
-3	0	4	6.24	0.30
-3	0	4	6.49	0.45
-3	0	4	6.45	0.36
2	0	-4	531.97	15.54
2	0	-4	516.02	15.46
2	0	-4	527.44	15.65
-2	0	4	506.08	15.45
-2	0	4	533.91	15.52
-2	0	4	507.88	15.52
-2	0	4	529.34	15.64
1	0	-4	409.85	12.54
1	0	-4	421.22	12.61
1	0	-4	409.14	12.43
-1	0	4	432.78	12.50
-1	0	4	419.08	12.55
-1	0	4	405.97	12.43
-1	0	4	435.15	12.63
0	0	-4	29.02	1.09
0	0	-4	28.93	1.11
0	0	-4	27.50	0.95
0	0	4	28.82	1.13
0	0	4	28.96	0.95
0	0	4	29.41	1.10
0	0	4	30.47	1.15
0	0	4	26.77	1.05
-1	0	-4	41.22	1.39
-1	0	-4	36.01	1.31
-1	0	-4	30.52	1.21
-1	0	-4	36.72	1.38
1	0	4	37.32	1.41
1	0	4	36.71	1.36
1	0	4	35.71	1.18
1	0	4	36.50	1.31
-2	0	-4	36.43	1.38
-2	0	-4	34.38	1.26
-2	0	-4	33.48	1.14
2	0	4	31.48	1.27
2	0	4	34.35	1.43
2	0	4	35.12	1.33
2	0	4	34.08	1.14
-3	0	-4	61.77	2.12
-3	0	-4	54.28	1.92
-3	0	-4	54.68	1.81

-3	0	-4	57.78	2.13
3	0	4	56.77	1.98
3	0	4	56.11	2.16
3	0	4	53.38	1.88
3	0	4	57.44	2.01
-4	0	-4	33.89	1.18
-4	0	-4	35.06	1.51
-4	0	-4	35.02	1.58
-4	0	-4	32.91	1.29
4	0	4	33.41	1.35
4	0	4	33.34	1.59
4	0	4	34.37	1.39
4	0	4	35.05	1.20
-5	0	-4	29.28	1.19
-5	0	-4	29.88	1.54
-5	0	-4	30.32	1.46
-5	0	-4	28.21	1.07
5	0	4	29.77	1.26
5	0	4	27.20	1.57
5	0	4	30.36	1.10
5	0	4	30.22	1.31
-6	0	-4	54.56	1.90
-6	0	-4	57.85	2.33
-6	0	-4	55.73	2.03
-6	0	-4	57.81	2.47
6	0	4	59.07	2.18
6	0	4	59.49	1.94
6	0	4	50.63	2.48
6	0	4	58.90	2.12
-7	0	-4	58.58	2.12
-7	0	-4	59.11	2.70
-7	0	-4	58.13	1.98
7	0	4	57.85	2.01
7	0	4	54.13	2.25
7	0	4	57.89	2.71
7	0	4	57.87	2.76
7	0	4	61.34	2.21
-8	0	-4	28.87	1.31
-8	0	-4	27.81	2.10
-8	0	-4	29.86	1.18
8	0	4	29.49	2.00
8	0	4	28.04	1.40
8	0	4	28.90	1.24
-9	0	-4	14.78	0.99

-9	0	-4	14.52	0.84
-9	0	-4	15.98	2.09
9	0	4	15.90	1.08
9	0	4	12.63	1.71
-10	0	-4	23.54	1.33
-10	0	-4	31.12	2.69
-10	0	-4	29.60	1.44
10	0	4	29.09	1.51
10	0	4	23.50	2.39
-11	0	-4	24.24	1.29
11	0	4	21.60	2.62
11	0	4	24.55	1.47
-12	0	-4	15.58	1.21
12	0	4	16.43	1.74
12	0	4	15.11	2.78
12	0	4	18.31	1.42
-13	0	-4	9.91	1.30
13	0	4	11.85	1.87
13	0	4	9.23	2.32
17	-1	-4	0.33	2.26
17	-1	-4	-0.90	2.22
-17	1	4	1.56	1.00
16	-1	-4	2.12	1.08
16	-1	-4	-0.09	1.90
-16	1	4	0.98	0.79
15	-1	-4	-0.67	1.00
15	-1	-4	2.61	1.55
-15	1	4	0.33	0.66
14	-1	-4	4.90	1.41
14	-1	-4	4.60	1.05
-14	1	4	7.88	0.97
13	-1	-4	4.30	0.91
13	-1	-4	4.07	1.19
13	-1	-4	3.96	0.47
13	-1	-4	4.45	0.46
-13	1	4	3.75	0.56
-13	1	4	4.64	0.93
12	-1	-4	3.63	0.79
12	-1	-4	4.25	0.43
12	-1	-4	3.67	1.07
12	-1	-4	4.29	0.62
-12	1	4	4.83	0.88
-12	1	4	4.19	0.56
11	-1	-4	0.61	0.23

11	-1	-4	0.31	0.54
11	-1	-4	1.17	0.40
11	-1	-4	0.12	0.77
-11	1	4	1.23	0.38
-11	1	4	1.40	0.65
10	-1	-4	5.02	0.65
10	-1	-4	4.98	0.59
10	-1	-4	6.02	0.40
10	-1	-4	5.00	0.53
10	-1	-4	4.23	0.65
10	-1	-4	5.42	0.88
-10	1	4	5.28	0.54
-10	1	4	6.11	0.80
9	-1	-4	18.70	0.71
9	-1	-4	18.99	0.81
9	-1	-4	18.28	1.23
9	-1	-4	17.92	1.08
9	-1	-4	17.32	0.93
-9	1	4	17.20	0.84
-9	1	4	18.15	1.01
-9	1	4	20.55	1.03
8	-1	-4	19.12	0.68
8	-1	-4	18.70	1.03
8	-1	-4	18.97	0.77
8	-1	-4	17.56	1.11
-8	1	4	17.35	0.78
-8	1	4	17.80	1.05
-8	1	4	17.68	0.79
-8	1	4	18.14	0.95
7	-1	-4	8.19	0.37
7	-1	-4	8.63	0.45
7	-1	-4	7.29	0.64
7	-1	-4	7.39	0.80
-7	1	4	8.06	0.61
-7	1	4	8.53	0.59
-7	1	4	7.55	0.46
-7	1	4	7.14	0.46
6	-1	-4	17.04	0.88
6	-1	-4	17.26	0.91
6	-1	-4	17.46	0.63
6	-1	-4	17.29	0.68
-6	1	4	17.17	0.69
-6	1	4	16.86	0.69
-6	1	4	17.37	0.84

-6	1	4	16.99	0.78
5	-1	-4	68.51	2.48
5	-1	-4	80.87	2.41
5	-1	-4	76.52	2.62
5	-1	-4	78.42	2.59
-5	1	4	76.78	2.43
-5	1	4	79.18	2.51
-5	1	4	79.49	2.42
4	-1	-4	17.90	0.85
4	-1	-4	17.46	0.65
4	-1	-4	18.44	0.66
4	-1	-4	18.18	0.69
4	-1	-4	18.70	0.81
-4	1	4	18.19	0.77
-4	1	4	19.01	0.84
-4	1	4	17.61	0.67
3	-1	-4	18.68	0.73
3	-1	-4	18.03	0.63
3	-1	-4	17.84	0.87
3	-1	-4	17.94	0.77
-3	1	4	18.59	0.70
-3	1	4	18.72	0.63
-3	1	4	18.32	0.81
-3	1	4	18.99	0.83
2	-1	-4	123.67	3.93
2	-1	-4	123.20	3.84
2	-1	-4	122.01	3.70
2	-1	-4	125.42	3.85
-2	1	4	126.17	3.92
-2	1	4	123.49	3.91
-2	1	4	121.15	3.69
-2	1	4	116.68	3.86
1	-1	-4	191.51	5.96
1	-1	-4	191.90	5.93
1	-1	-4	201.11	5.95
1	-1	-4	190.65	5.74
-1	1	4	199.44	5.99
-1	1	4	182.54	5.87
-1	1	4	192.29	5.99
-1	1	4	187.34	5.73
0	-1	-4	43.72	1.60
0	-1	-4	40.75	1.34
0	-1	-4	42.16	1.55
0	1	4	42.49	1.51

0	1	4	42.02	1.58
0	1	4	44.37	1.68
0	1	4	42.39	1.35
0	1	4	43.56	1.53
-1	-1	-4	82.38	2.57
-1	-1	-4	80.35	2.74
1	1	4	87.42	2.81
1	1	4	85.90	3.00
1	1	4	82.71	2.57
1	1	4	85.57	2.83
1	1	4	82.98	2.77
-2	-1	-4	34.56	1.15
-2	-1	-4	36.22	1.51
-2	-1	-4	31.38	1.29
2	1	4	34.54	1.37
2	1	4	34.89	1.38
2	1	4	34.02	1.15
2	1	4	35.89	1.43
2	1	4	36.39	1.62
-3	-1	-4	42.29	1.81
-3	-1	-4	40.19	1.51
-3	-1	-4	40.45	1.36
3	1	4	42.24	1.63
3	1	4	45.66	1.93
3	1	4	39.56	1.59
3	1	4	40.53	1.70
-4	-1	-4	73.71	2.57
-4	-1	-4	75.01	2.42
-4	-1	-4	79.36	2.96
4	1	4	79.63	2.45
4	1	4	79.85	2.71
4	1	4	73.85	2.84
4	1	4	79.07	3.04
4	1	4	74.41	2.68
-5	-1	-4	55.82	2.45
-5	-1	-4	55.30	2.01
-5	-1	-4	55.73	1.85
5	1	4	55.75	2.14
5	1	4	56.46	2.14
5	1	4	59.43	1.89
5	1	4	54.53	2.34
5	1	4	57.48	2.55
-6	-1	-4	61.19	2.04
-6	-1	-4	66.82	2.81

-6	-1	-4	60.74	2.19
6	1	4	61.54	2.07
6	1	4	63.74	2.86
6	1	4	58.98	2.35
6	1	4	62.22	2.64
6	1	4	58.23	2.31
-7	-1	-4	27.62	1.12
-7	-1	-4	29.89	1.30
-7	-1	-4	29.10	2.08
7	1	4	27.61	1.43
7	1	4	29.11	1.17
7	1	4	26.71	1.83
-8	-1	-4	36.63	2.39
-8	-1	-4	27.85	1.58
-8	-1	-4	29.15	1.22
8	1	4	29.66	1.52
8	1	4	31.11	1.30
8	1	4	31.43	2.14
-9	-1	-4	14.53	2.42
-9	-1	-4	14.33	0.86
-9	-1	-4	15.53	1.03
9	1	4	15.43	1.16
9	1	4	11.38	1.80
-10	-1	-4	10.26	0.86
-10	-1	-4	10.81	1.05
10	1	4	9.43	1.16
10	1	4	12.77	2.10
-11	-1	-4	18.99	1.18
-11	-1	-4	18.90	1.34
11	1	4	12.52	2.01
11	1	4	18.17	1.44
-12	-1	-4	21.29	1.39
12	1	4	22.42	1.91
12	1	4	17.03	2.74
13	1	4	6.59	2.24
17	-2	-4	-2.82	1.19
17	-2	-4	0.10	2.27
-17	2	4	1.17	0.89
16	-2	-4	-3.44	1.77
16	-2	-4	1.09	1.13
-16	2	4	0.36	0.72
15	-2	-4	4.82	1.15
15	-2	-4	7.12	1.79
-15	2	4	4.81	0.77

14	-2	-4	-0.60	1.24
14	-2	-4	0.01	0.79
-14	2	4	0.82	0.56
13	-2	-4	0.96	0.68
13	-2	-4	1.42	1.13
-13	2	4	0.26	0.43
-13	2	4	1.06	0.80
12	-2	-4	10.31	1.13
12	-2	-4	9.52	1.61
-12	2	4	12.16	1.23
-12	2	4	9.79	0.75
11	-2	-4	13.71	1.12
11	-2	-4	13.31	1.47
-11	2	4	13.11	0.80
-11	2	4	13.84	1.10
10	-2	-4	7.47	0.56
10	-2	-4	7.42	0.88
10	-2	-4	6.47	1.13
-10	2	4	7.27	0.76
-10	2	4	9.05	0.86
-10	2	4	8.17	0.58
9	-2	-4	10.81	1.12
9	-2	-4	11.78	0.61
9	-2	-4	9.83	0.85
-9	2	4	11.67	0.83
-9	2	4	12.13	0.81
-9	2	4	11.06	0.63
8	-2	-4	0.83	0.14
8	-2	-4	0.69	0.21
8	-2	-4	1.08	0.35
8	-2	-4	0.56	0.50
-8	2	4	0.92	0.31
-8	2	4	0.90	0.23
-8	2	4	0.55	0.19
-8	2	4	0.03	0.35
7	-2	-4	35.19	1.66
7	-2	-4	39.29	1.27
7	-2	-4	35.14	1.51
7	-2	-4	32.75	1.19
-7	2	4	38.15	1.47
-7	2	4	36.46	1.27
-7	2	4	38.53	1.47
-7	2	4	37.07	1.23
6	-2	-4	69.99	2.38

6	-2	-4	75.90	2.64
6	-2	-4	74.43	2.72
6	-2	-4	80.03	2.40
-6	2	4	77.56	2.37
-6	2	4	80.23	2.62
-6	2	4	75.23	2.41
5	-2	-4	2.30	0.23
5	-2	-4	2.52	0.44
5	-2	-4	1.52	0.31
5	-2	-4	2.88	0.29
-5	2	4	2.49	0.40
-5	2	4	1.83	0.32
-5	2	4	2.14	0.24
-5	2	4	2.40	0.21
4	-2	-4	35.33	1.23
4	-2	-4	33.84	1.35
4	-2	-4	33.50	1.37
4	-2	-4	33.28	1.12
4	-2	-4	33.48	1.11
-4	2	4	34.78	1.33
-4	2	4	33.59	1.37
-4	2	4	34.49	1.16
3	-2	-4	109.71	3.59
3	-2	-4	113.69	3.51
3	-2	-4	110.81	3.59
3	-2	-4	109.42	3.31
3	-2	-4	108.67	3.33
-3	2	4	112.94	3.64
-3	2	4	111.35	3.57
-3	2	4	102.67	3.43
2	-2	-4	29.45	1.23
2	-2	-4	29.96	1.23
2	-2	-4	28.64	1.18
2	-2	-4	29.14	0.94
-2	2	4	29.26	0.93
-2	2	4	29.54	1.22
-2	2	4	30.37	1.12
-2	2	4	30.34	1.30
1	-2	-4	44.61	1.65
1	-2	-4	43.15	1.60
1	-2	-4	42.51	1.33
1	-2	-4	42.16	1.66
-1	2	4	42.38	1.55
-1	2	4	43.62	1.63

-1	2	4	42.02	1.33
-1	2	4	43.23	1.74
0	-2	-4	42.39	1.91
0	-2	-4	44.23	1.73
0	-2	-4	44.06	1.38
0	-2	-4	42.84	1.62
0	2	4	46.85	1.87
0	2	4	45.42	1.39
0	2	4	46.74	1.66
0	2	4	41.64	1.90
-1	-2	-4	28.39	1.15
-1	-2	-4	29.07	0.94
-1	-2	-4	29.23	1.36
1	2	4	28.24	1.20
1	2	4	26.60	1.21
1	2	4	29.81	1.49
1	2	4	29.09	0.95
1	2	4	29.54	1.25
-2	-2	-4	47.09	1.50
-2	-2	-4	46.19	1.71
-2	-2	-4	45.60	1.98
2	2	4	46.96	1.82
2	2	4	48.63	2.12
2	2	4	48.73	1.84
2	2	4	47.06	1.51
2	2	4	47.04	1.80
-3	-2	-4	68.23	2.77
-3	-2	-4	68.38	2.39
-3	-2	-4	70.30	2.21
3	2	4	70.35	2.58
3	2	4	70.67	2.53
3	2	4	73.55	2.58
3	2	4	71.82	2.23
-4	-2	-4	27.47	1.17
-4	-2	-4	27.52	1.64
-4	-2	-4	27.59	0.99
4	2	4	28.35	1.01
4	2	4	28.57	1.33
4	2	4	26.39	1.39
4	2	4	28.27	1.35
4	2	4	26.73	1.77
-5	-2	-4	54.57	1.81
-5	-2	-4	53.16	1.99
-5	-2	-4	52.92	2.53

5	2	4	51.03	2.28
5	2	4	57.19	2.19
5	2	4	55.38	2.21
5	2	4	55.76	2.69
5	2	4	58.54	1.85
-6	-2	-4	43.45	1.53
-6	-2	-4	43.49	2.45
-6	-2	-4	44.83	1.72
6	2	4	43.37	2.53
6	2	4	42.62	2.11
6	2	4	45.69	1.94
6	2	4	43.92	1.91
6	2	4	45.46	1.58
-7	-2	-4	41.07	1.70
-7	-2	-4	41.64	2.63
-7	-2	-4	41.60	1.50
7	2	4	42.73	1.56
7	2	4	41.76	1.93
7	2	4	41.00	2.18
-8	-2	-4	4.34	0.73
-8	-2	-4	6.20	1.40
-8	-2	-4	4.73	0.60
8	2	4	2.60	0.97
8	2	4	4.01	0.54
8	2	4	5.47	0.84
-9	-2	-4	14.41	1.06
-9	-2	-4	14.94	0.88
9	2	4	11.04	1.88
9	2	4	15.50	1.34
-10	-2	-4	28.65	1.53
-10	-2	-4	26.54	1.32
10	2	4	26.21	2.48
10	2	4	28.87	1.73
-11	-2	-4	12.35	1.10
-11	-2	-4	13.35	1.28
11	2	4	12.18	1.42
11	2	4	8.95	1.79
-12	-2	-4	11.32	1.30
12	2	4	12.31	1.52
12	2	4	6.73	1.24
12	2	4	7.43	1.85
17	-3	-4	2.02	1.22
17	-3	-4	4.72	2.39
-17	3	4	2.66	0.87

16	-3	-4	0.41	1.09
16	-3	-4	1.10	1.88
-16	3	4	0.43	0.71
15	-3	-4	0.49	0.93
15	-3	-4	1.79	1.59
-15	3	4	0.45	0.56
14	-3	-4	6.51	1.06
14	-3	-4	8.71	1.72
-14	3	4	6.07	0.85
13	-3	-4	0.68	1.14
13	-3	-4	1.83	0.75
-13	3	4	2.28	0.80
-13	3	4	0.62	0.42
12	-3	-4	0.69	1.01
12	-3	-4	1.64	0.64
-12	3	4	0.68	0.36
-12	3	4	0.87	0.67
11	-3	-4	5.46	1.09
11	-3	-4	5.66	0.89
11	-3	-4	5.26	0.60
-11	3	4	7.59	0.95
-11	3	4	5.78	0.58
-11	3	4	7.07	0.87
10	-3	-4	9.21	1.28
10	-3	-4	8.14	0.57
10	-3	-4	7.02	0.85
-10	3	4	7.99	0.57
-10	3	4	8.44	0.92
-10	3	4	7.15	0.77
9	-3	-4	8.95	0.81
9	-3	-4	7.68	0.51
9	-3	-4	6.11	1.05
-9	3	4	9.56	0.83
-9	3	4	7.58	0.52
-9	3	4	7.96	0.73
8	-3	-4	2.33	0.34
8	-3	-4	2.58	0.65
8	-3	-4	2.24	0.47
-8	3	4	2.93	0.52
-8	3	4	2.59	0.45
-8	3	4	2.10	0.32
7	-3	-4	7.09	0.41
7	-3	-4	6.58	0.40
7	-3	-4	6.60	0.64

7	-3	-4	7.57	0.89
-7	3	4	7.10	0.42
-7	3	4	6.75	0.62
-7	3	4	6.58	0.32
-7	3	4	6.70	0.71
6	-3	-4	40.97	1.81
6	-3	-4	40.68	1.34
6	-3	-4	40.36	1.61
6	-3	-4	40.46	1.41
-6	3	4	38.97	1.35
-6	3	4	41.93	1.58
-6	3	4	40.62	1.31
-6	3	4	38.90	1.67
5	-3	-4	95.10	2.89
5	-3	-4	93.69	3.03
5	-3	-4	91.50	3.17
-5	3	4	90.39	2.90
-5	3	4	92.52	2.93
-5	3	4	98.44	3.15
-5	3	4	97.33	3.28
4	-3	-4	43.72	1.55
4	-3	-4	41.21	1.76
4	-3	-4	41.83	1.32
4	-3	-4	42.04	1.63
-4	3	4	38.07	1.51
-4	3	4	43.46	1.75
3	-3	-4	60.15	2.18
3	-3	-4	60.12	1.86
3	-3	-4	60.75	2.16
3	-3	-4	60.21	1.86
-3	3	4	61.70	2.07
-3	3	4	60.02	2.16
-3	3	4	63.21	2.33
2	-3	-4	82.75	2.46
2	-3	-4	80.01	2.89
2	-3	-4	84.31	2.84
2	-3	-4	76.80	2.77
-2	3	4	84.24	2.84
-2	3	4	79.63	2.96
-2	3	4	82.64	2.73
1	-3	-4	133.28	4.37
1	-3	-4	133.00	4.49
1	-3	-4	137.11	4.49
1	-3	-4	131.36	4.04

-1	3	4	137.24	4.39
-1	3	4	133.03	4.03
-1	3	4	139.32	4.44
-1	3	4	139.74	4.63
0	-3	-4	52.21	2.13
0	-3	-4	53.92	1.67
0	-3	-4	57.54	2.20
0	-3	-4	54.63	1.96
0	3	4	54.13	2.29
0	3	4	55.61	2.04
0	3	4	54.90	2.06
0	3	4	55.57	1.68
-1	-3	-4	104.05	3.70
-1	-3	-4	105.72	3.62
-1	-3	-4	102.24	3.39
-1	-3	-4	103.29	3.12
1	3	4	104.30	3.49
1	3	4	102.09	3.13
1	3	4	103.12	3.83
1	3	4	102.50	3.54
-2	-3	-4	61.56	2.19
-2	-3	-4	61.15	2.57
2	3	4	64.58	2.75
2	3	4	61.77	2.30
2	3	4	65.93	2.32
2	3	4	62.21	1.95
2	3	4	60.22	2.37
-3	-3	-4	35.32	1.88
-3	-3	-4	34.84	1.15
-3	-3	-4	33.46	1.38
3	3	4	33.48	1.54
3	3	4	33.81	1.59
3	3	4	34.96	1.54
3	3	4	35.00	1.17
3	3	4	36.06	2.02
-4	-3	-4	36.74	1.27
-4	-3	-4	35.90	2.06
-4	-3	-4	39.71	1.51
4	3	4	37.69	1.69
4	3	4	36.57	1.71
4	3	4	37.38	2.27
4	3	4	37.30	1.75
4	3	4	38.98	1.30
-5	-3	-4	76.27	2.47

-5	-3	-4	82.69	3.45
-5	-3	-4	77.68	2.70
5	3	4	74.22	2.89
5	3	4	75.02	2.98
5	3	4	80.57	3.00
5	3	4	74.29	3.58
5	3	4	80.40	2.51
-6	-3	-4	15.77	0.73
-6	-3	-4	15.65	0.94
-6	-3	-4	14.86	1.86
6	3	4	14.85	1.25
6	3	4	14.08	1.28
6	3	4	15.08	0.77
-7	-3	-4	17.36	2.21
-7	-3	-4	17.04	0.83
-7	-3	-4	16.99	1.16
7	3	4	18.16	0.91
7	3	4	18.27	1.40
-8	-3	-4	39.32	1.72
-8	-3	-4	40.29	1.50
8	3	4	36.92	2.31
8	3	4	38.36	2.02
8	3	4	38.00	1.56
-9	-3	-4	22.59	1.11
-9	-3	-4	22.55	1.36
9	3	4	21.55	2.20
9	3	4	20.95	1.63
-10	-3	-4	11.85	1.22
-10	-3	-4	14.04	1.04
10	3	4	10.05	1.73
10	3	4	13.64	1.53
-11	-3	-4	11.23	1.30
-11	-3	-4	11.08	1.18
11	3	4	8.40	1.76
11	3	4	12.96	1.67
12	3	4	1.33	2.03
17	-4	-4	0.47	2.31
17	-4	-4	0.79	1.17
-17	4	4	-0.19	0.74
16	-4	-4	3.43	2.03
16	-4	-4	4.98	1.26
-16	4	4	7.15	1.10
15	-4	-4	7.90	1.30
15	-4	-4	4.81	1.85

-15	4	4	8.58	0.99
14	-4	-4	1.09	0.81
14	-4	-4	1.67	1.39
-14	4	4	1.67	0.55
13	-4	-4	11.76	2.01
13	-4	-4	10.09	1.25
-13	4	4	11.52	1.35
-13	4	4	10.52	0.82
12	-4	-4	16.12	1.29
12	-4	-4	17.96	1.95
-12	4	4	19.42	1.45
-12	4	4	18.96	0.94
11	-4	-4	11.49	1.06
11	-4	-4	12.10	0.74
11	-4	-4	12.96	1.62
-11	4	4	11.66	0.97
-11	4	4	11.92	0.73
-11	4	4	12.43	1.17
10	-4	-4	0.16	0.44
10	-4	-4	-0.20	0.74
10	-4	-4	0.99	0.29
-10	4	4	0.67	0.27
-10	4	4	1.60	0.58
-10	4	4	0.73	0.42
9	-4	-4	22.63	0.91
9	-4	-4	21.94	1.24
9	-4	-4	21.42	1.57
-9	4	4	23.04	0.93
-9	4	4	21.92	1.13
-9	4	4	21.94	1.30
8	-4	-4	24.44	1.55
8	-4	-4	25.02	1.29
8	-4	-4	26.52	0.97
-8	4	4	27.61	1.40
-8	4	4	25.18	1.19
-8	4	4	24.71	0.97
7	-4	-4	44.64	1.50
7	-4	-4	43.63	1.58
7	-4	-4	45.14	2.08
7	-4	-4	45.77	1.82
-7	4	4	44.38	1.51
-7	4	4	47.45	1.78
-7	4	4	46.35	2.00
-7	4	4	44.62	1.44

6	-4	-4	71.85	2.80
6	-4	-4	70.44	2.40
6	-4	-4	72.83	2.57
6	-4	-4	70.25	2.24
-6	4	4	73.34	2.74
-6	4	4	69.69	2.26
-6	4	4	72.61	2.51
-6	4	4	70.15	2.25
5	-4	-4	17.28	0.96
5	-4	-4	18.26	0.65
5	-4	-4	17.22	0.89
5	-4	-4	17.76	1.21
-5	4	4	18.06	0.92
-5	4	4	18.99	1.20
-5	4	4	18.23	0.68
4	-4	-4	20.81	1.01
4	-4	-4	18.97	0.66
4	-4	-4	19.07	1.19
4	-4	-4	19.28	1.01
-4	4	4	19.67	0.96
-4	4	4	19.88	0.89
-4	4	4	19.13	1.21
3	-4	-4	36.88	1.68
3	-4	-4	34.40	1.49
3	-4	-4	35.39	1.13
3	-4	-4	37.50	1.13
3	-4	-4	34.22	1.55
-3	4	4	38.04	1.45
-3	4	4	37.81	1.50
-3	4	4	36.97	1.74
2	-4	-4	141.51	4.70
2	-4	-4	137.77	4.61
2	-4	-4	135.86	4.48
-2	4	4	137.40	4.50
-2	4	4	139.19	4.85
-2	4	4	136.47	4.50
1	-4	-4	87.15	3.18
1	-4	-4	86.74	3.19
1	-4	-4	88.12	2.98
-1	4	4	89.46	3.36
-1	4	4	88.28	3.03
-1	4	4	83.27	3.04
0	-4	-4	105.50	3.85
0	-4	-4	104.93	3.54

0	-4	-4	105.84	3.80
0	4	4	113.93	4.05
0	4	4	109.22	3.64
0	4	4	103.96	3.69
-1	-4	-4	78.76	2.43
-1	-4	-4	78.97	3.14
-1	-4	-4	80.99	2.75
-1	-4	-4	80.08	3.05
1	4	4	83.49	3.35
1	4	4	80.87	2.97
1	4	4	80.04	2.45
1	4	4	81.38	2.88
-2	-4	-4	93.33	3.20
-2	-4	-4	96.30	3.56
-2	-4	-4	93.57	3.69
-2	-4	-4	97.15	2.94
2	4	4	97.07	3.36
2	4	4	96.77	3.88
2	4	4	94.48	2.93
2	4	4	98.97	3.50
-3	-4	-4	44.27	1.69
-3	-4	-4	44.38	1.42
-3	-4	-4	44.32	2.30
3	4	4	40.80	1.95
3	4	4	45.34	1.89
3	4	4	44.76	1.44
3	4	4	43.33	1.88
3	4	4	43.19	2.46
-4	-4	-4	51.50	1.70
-4	-4	-4	53.26	2.67
-4	-4	-4	51.22	1.95
4	4	4	49.28	2.17
4	4	4	54.24	2.31
4	4	4	54.15	1.72
4	4	4	52.13	2.17
4	4	4	51.20	2.91
-5	-4	-4	48.80	1.66
-5	-4	-4	49.75	1.92
-5	-4	-4	50.50	2.83
5	4	4	47.32	2.22
5	4	4	51.98	1.70
5	4	4	49.79	2.29
5	4	4	48.69	2.20
-6	-4	-4	34.39	2.75

-6	-4	-4	28.63	1.13
-6	-4	-4	30.12	1.38
6	4	4	30.52	1.18
6	4	4	30.88	1.80
6	4	4	28.12	1.72
-7	-4	-4	56.55	2.05
-7	-4	-4	61.45	2.31
7	4	4	60.94	2.10
7	4	4	59.42	2.79
7	4	4	62.09	2.74
-8	-4	-4	12.56	0.84
-8	-4	-4	11.80	1.05
8	4	4	14.33	1.99
8	4	4	12.49	0.93
8	4	4	9.50	1.65
-9	-4	-4	19.72	1.35
-9	-4	-4	17.49	1.08
9	4	4	21.13	1.77
9	4	4	17.17	2.11
-10	-4	-4	23.21	1.54
-10	-4	-4	22.51	1.35
10	4	4	21.77	2.51
10	4	4	23.50	2.02
-11	-4	-4	5.23	0.93
-11	-4	-4	8.45	1.35
11	4	4	7.40	1.91
11	4	4	12.78	1.85
17	-5	-4	10.32	2.62
17	-5	-4	6.86	1.43
16	-5	-4	-2.28	1.84
16	-5	-4	0.62	0.99
-16	5	4	0.62	0.63
15	-5	-4	-1.32	1.59
15	-5	-4	0.81	0.87
-15	5	4	0.48	0.56
14	-5	-4	17.00	1.59
14	-5	-4	16.88	2.46
-14	5	4	16.68	1.07
13	-5	-4	18.82	1.57
13	-5	-4	25.01	2.38
-13	5	4	26.48	1.75
-13	5	4	24.18	1.15
12	-5	-4	13.17	1.24
12	-5	-4	12.17	1.87

-12	5	4	10.52	1.33
-12	5	4	11.48	0.78
11	-5	-4	27.66	1.57
11	-5	-4	28.66	1.19
11	-5	-4	29.66	2.14
-11	5	4	28.82	1.45
-11	5	4	29.13	1.71
-11	5	4	27.89	1.16
10	-5	-4	28.89	1.13
10	-5	-4	28.97	1.98
10	-5	-4	28.56	1.52
-10	5	4	29.79	1.66
-10	5	4	27.98	1.13
-10	5	4	27.12	1.37
9	-5	-4	29.44	1.13
9	-5	-4	30.19	1.91
9	-5	-4	30.47	1.49
-9	5	4	29.04	1.12
-9	5	4	29.64	1.38
-9	5	4	31.52	1.67
8	-5	-4	102.74	3.17
8	-5	-4	98.05	3.51
8	-5	-4	102.76	3.89
-8	5	4	99.04	3.69
-8	5	4	102.91	3.44
-8	5	4	99.71	3.16
-8	5	4	99.04	3.06
7	-5	-4	73.92	2.33
7	-5	-4	71.88	2.51
7	-5	-4	70.74	2.67
7	-5	-4	70.65	2.99
-7	5	4	76.16	2.62
-7	5	4	72.96	2.33
-7	5	4	73.99	2.34
-7	5	4	76.15	2.93
6	-5	-4	15.94	1.30
6	-5	-4	16.08	0.92
6	-5	-4	16.19	0.97
-6	5	4	16.32	1.25
-6	5	4	16.46	0.78
-6	5	4	15.75	0.63
-6	5	4	15.91	0.92
5	-5	-4	103.46	3.93
5	-5	-4	105.16	3.64

5	-5	-4	106.83	3.27
5	-5	-4	106.44	3.65
-5	5	4	112.70	3.98
-5	5	4	110.39	3.51
-5	5	4	109.24	3.60
-5	5	4	106.34	3.28
4	-5	-4	98.31	3.00
4	-5	-4	96.75	3.40
4	-5	-4	99.89	3.69
-4	5	4	99.52	3.34
-4	5	4	104.99	3.38
-4	5	4	99.24	3.01
-4	5	4	96.15	3.69
3	-5	-4	14.54	1.11
3	-5	-4	13.77	1.00
3	-5	-4	13.91	0.89
-3	5	4	15.02	0.92
-3	5	4	15.36	1.24
-3	5	4	16.56	0.92
2	-5	-4	54.55	2.37
2	-5	-4	55.32	2.09
2	-5	-4	57.04	2.33
-2	5	4	59.85	2.56
-2	5	4	54.86	2.15
-2	5	4	55.50	2.18
1	-5	-4	70.10	2.51
1	-5	-4	67.39	2.79
1	-5	-4	69.47	2.82
-1	5	4	71.60	2.59
-1	5	4	73.44	3.02
-1	5	4	71.49	2.68
0	-5	-4	82.57	3.21
0	-5	-4	77.44	3.23
0	-5	-4	79.61	2.86
0	5	4	89.00	3.01
0	5	4	83.91	3.48
0	5	4	86.53	3.12
-1	-5	-4	73.06	3.02
-1	-5	-4	79.45	3.20
-1	-5	-4	76.06	2.66
1	5	4	77.53	2.82
1	5	4	73.53	2.94
1	5	4	80.23	3.40
-2	-5	-4	42.26	1.30

-2	-5	-4	37.60	2.17
-2	-5	-4	40.06	1.62
-2	-5	-4	42.72	2.05
2	5	4	41.07	2.43
2	5	4	40.39	1.82
2	5	4	40.83	1.96
2	5	4	40.68	1.31
-3	-5	-4	83.32	3.61
-3	-5	-4	84.40	2.88
-3	-5	-4	85.21	2.58
3	5	4	83.07	3.83
3	5	4	86.72	3.12
3	5	4	79.33	3.23
3	5	4	83.59	2.59
3	5	4	78.96	3.08
-4	-5	-4	89.06	4.04
-4	-5	-4	91.85	2.91
-4	-5	-4	95.30	3.21
4	5	4	97.80	2.93
4	5	4	91.79	3.46
4	5	4	92.54	3.62
4	5	4	93.32	3.48
-5	-5	-4	51.97	1.98
-5	-5	-4	51.32	1.71
5	5	4	48.18	2.28
5	5	4	50.78	2.46
5	5	4	51.04	1.72
-6	-5	-4	27.27	1.10
6	5	4	26.07	1.77
6	5	4	27.78	1.86
6	5	4	28.41	1.13
-7	-5	-4	23.64	1.08
-7	-5	-4	24.02	1.33
7	5	4	24.08	2.20
7	5	4	23.52	1.10
7	5	4	19.64	1.77
-8	-5	-4	32.21	1.39
-8	-5	-4	32.43	1.65
8	5	4	31.58	1.45
8	5	4	34.18	2.16
8	5	4	29.06	2.26
-9	-5	-4	21.75	1.47
-9	-5	-4	20.51	1.25
9	5	4	20.39	1.99

9	5	4	17.35	2.20
-10	-5	-4	10.21	1.32
-10	-5	-4	5.41	0.88
10	5	4	8.27	1.77
10	5	4	7.42	1.34
17	-6	-4	-0.33	2.22
17	-6	-4	-2.35	1.17
-17	6	4	-0.59	0.71
16	-6	-4	3.72	1.22
16	-6	-4	4.69	2.08
-16	6	4	3.08	0.69
15	-6	-4	7.13	1.98
15	-6	-4	5.24	1.20
-15	6	4	4.73	0.69
14	-6	-4	6.42	1.71
14	-6	-4	2.72	0.88
-14	6	4	4.94	0.63
13	-6	-4	9.18	1.27
13	-6	-4	11.44	1.84
-13	6	4	9.28	1.45
-13	6	4	10.04	0.79
12	-6	-4	15.49	1.31
12	-6	-4	17.85	2.12
-12	6	4	15.62	1.53
-12	6	4	16.20	0.88
11	-6	-4	42.76	1.98
11	-6	-4	39.30	2.56
11	-6	-4	40.61	1.53
-11	6	4	39.15	1.81
-11	6	4	39.10	1.50
-11	6	4	41.15	2.17
10	-6	-4	24.06	1.94
10	-6	-4	25.05	1.40
10	-6	-4	25.41	1.03
-10	6	4	23.70	1.00
-10	6	4	22.46	1.28
-10	6	4	24.28	1.62
9	-6	-4	43.10	2.37
9	-6	-4	43.56	1.51
9	-6	-4	42.83	1.90
-9	6	4	43.64	1.79
-9	6	4	42.67	1.45
-9	6	4	42.38	1.37
-9	6	4	42.71	1.51

-9	6	4	44.30	2.17
8	-6	-4	55.64	1.80
8	-6	-4	54.65	2.65
8	-6	-4	51.36	2.16
-8	6	4	55.61	1.87
-8	6	4	53.68	2.48
-8	6	4	54.36	2.10
-8	6	4	51.64	1.79
-8	6	4	54.93	1.78
7	-6	-4	126.29	4.30
7	-6	-4	124.20	4.71
7	-6	-4	128.34	3.90
-7	6	4	126.39	4.62
-7	6	4	124.97	3.89
-7	6	4	129.04	4.02
-7	6	4	126.47	4.20
6	-6	-4	85.68	3.49
6	-6	-4	86.85	3.12
6	-6	-4	84.19	2.73
6	-6	-4	87.46	3.15
-6	6	4	89.18	3.50
-6	6	4	85.69	2.72
-6	6	4	93.93	3.08
-6	6	4	89.30	2.96
5	-6	-4	89.55	2.69
5	-6	-4	85.05	3.45
5	-6	-4	85.64	3.11
5	-6	-4	88.25	3.19
-5	6	4	89.21	3.04
-5	6	4	87.40	3.49
-5	6	4	87.99	2.74
-5	6	4	87.77	3.05
4	-6	-4	10.62	0.84
4	-6	-4	10.82	0.40
4	-6	-4	10.19	1.14
4	-6	-4	10.37	0.99
-4	6	4	10.15	0.81
-4	6	4	9.59	1.22
-4	6	4	11.37	0.82
3	-6	-4	62.69	2.69
3	-6	-4	58.95	2.33
3	-6	-4	63.45	2.59
-3	6	4	66.59	2.39
-3	6	4	61.99	2.42

-3	6	4	61.92	2.81
2	-6	-4	62.24	2.39
2	-6	-4	66.78	2.78
2	-6	-4	66.41	2.73
-2	6	4	61.33	2.52
-2	6	4	67.69	2.97
-2	6	4	64.56	2.46
1	-6	-4	18.48	1.03
1	-6	-4	17.63	1.39
1	-6	-4	19.19	1.44
-1	6	4	19.24	1.25
-1	6	4	21.31	1.69
-1	6	4	21.45	1.21
0	-6	-4	30.42	1.75
0	-6	-4	29.70	1.82
0	-6	-4	28.02	1.30
0	6	4	27.12	1.47
0	6	4	32.06	2.11
0	6	4	28.57	1.57
-1	-6	-4	57.77	2.76
-1	-6	-4	59.82	2.62
-1	-6	-4	59.92	2.16
1	6	4	56.92	2.32
1	6	4	54.77	2.95
1	6	4	56.67	2.51
-2	-6	-4	89.07	2.98
-2	-6	-4	85.61	3.44
-2	-6	-4	82.72	3.67
2	6	4	86.61	3.40
2	6	4	85.62	3.20
2	6	4	81.67	3.92
-3	-6	-4	44.60	2.30
-3	-6	-4	43.92	1.75
-3	-6	-4	44.03	1.45
-3	-6	-4	40.74	2.59
3	6	4	42.52	1.42
3	6	4	42.35	3.69
3	6	4	43.03	2.18
3	6	4	44.45	2.06
-4	-6	-4	33.62	1.52
-4	-6	-4	36.13	1.23
4	6	4	31.78	1.99
4	6	4	33.01	1.81
4	6	4	35.39	1.20

-5	-6	-4	18.12	1.09
-5	-6	-4	18.17	0.83
5	6	4	17.84	1.59
5	6	4	17.68	0.81
5	6	4	15.90	1.40
-6	-6	-4	38.20	1.72
-6	-6	-4	38.49	1.46
6	6	4	37.16	2.19
6	6	4	38.42	1.44
6	6	4	36.48	2.26
-7	-6	-4	39.88	1.81
-7	-6	-4	38.57	1.55
7	6	4	35.75	2.32
7	6	4	39.26	2.40
-8	-6	-4	18.57	1.20
-8	-6	-4	18.71	1.36
8	6	4	17.01	2.04
8	6	4	19.20	2.00
-9	-6	-4	13.22	1.25
-9	-6	-4	11.06	1.32
9	6	4	15.29	2.05
9	6	4	10.35	1.75
-10	-6	-4	12.93	1.49
10	6	4	7.31	1.85
10	6	4	9.05	1.53
18	-7	-4	3.95	2.76
17	-7	-4	2.36	2.40
17	-7	-4	2.81	1.37
-17	7	4	3.98	0.74
16	-7	-4	2.66	1.20
16	-7	-4	6.19	2.25
-16	7	4	2.47	0.67
15	-7	-4	13.23	1.76
15	-7	-4	11.36	2.09
-15	7	4	11.80	1.02
14	-7	-4	11.16	2.06
14	-7	-4	15.92	1.65
-14	7	4	15.79	1.01
13	-7	-4	8.72	1.83
13	-7	-4	6.86	1.03
-13	7	4	6.54	0.73
12	-7	-4	7.34	1.59
12	-7	-4	6.34	1.08
-12	7	4	6.93	0.65

-12	7	4	6.65	1.19
11	-7	-4	6.38	0.65
11	-7	-4	6.76	1.38
11	-7	-4	6.46	1.01
-11	7	4	7.69	1.20
-11	7	4	6.91	0.59
-11	7	4	4.76	0.82
10	-7	-4	36.14	1.35
10	-7	-4	36.43	2.38
10	-7	-4	34.62	1.77
-10	7	4	36.72	2.15
-10	7	4	34.77	1.63
-10	7	4	33.85	1.32
9	-7	-4	51.37	1.72
9	-7	-4	48.01	2.70
9	-7	-4	47.84	2.14
-9	7	4	49.52	1.75
-9	7	4	49.73	1.71
-9	7	4	49.24	2.01
-9	7	4	51.88	2.55
-9	7	4	51.33	1.67
8	-7	-4	34.27	1.23
8	-7	-4	35.98	2.16
8	-7	-4	33.15	1.65
-8	7	4	35.21	2.11
-8	7	4	33.04	1.21
-8	7	4	34.13	1.54
-8	7	4	34.72	1.35
-8	7	4	34.94	1.39
7	-7	-4	135.16	5.13
7	-7	-4	139.07	4.65
7	-7	-4	138.62	4.19
-7	7	4	140.20	5.08
-7	7	4	139.16	4.53
-7	7	4	132.84	4.72
-7	7	4	136.35	4.41
-7	7	4	131.34	4.18
6	-7	-4	44.30	2.36
6	-7	-4	45.61	1.50
6	-7	-4	45.69	1.94
-6	7	4	45.28	1.86
-6	7	4	51.25	2.47
-6	7	4	45.02	1.50
-6	7	4	47.86	1.86

5	-7	-4	27.72	1.58
5	-7	-4	26.96	1.79
5	-7	-4	27.35	1.41
5	-7	-4	28.24	0.94
-5	7	4	31.16	1.93
-5	7	4	28.97	1.38
-5	7	4	25.93	0.95
-5	7	4	28.97	1.36
4	-7	-4	84.42	3.57
4	-7	-4	91.23	3.46
4	-7	-4	87.13	3.19
-4	7	4	89.75	3.26
-4	7	4	94.40	3.19
-4	7	4	89.13	3.74
3	-7	-4	51.34	2.40
3	-7	-4	50.42	2.48
3	-7	-4	53.49	2.08
-3	7	4	55.69	2.13
-3	7	4	47.87	2.63
-3	7	4	50.56	2.20
2	-7	-4	60.69	2.29
2	-7	-4	61.82	2.74
2	-7	-4	59.02	2.74
-2	7	4	59.67	2.51
-2	7	4	59.80	2.39
-2	7	4	57.96	2.96
1	-7	-4	65.17	2.89
1	-7	-4	66.29	2.40
1	-7	-4	62.35	2.91
-1	7	4	61.51	3.15
-1	7	4	67.45	2.57
-1	7	4	63.76	2.68
0	-7	-4	42.49	2.25
0	-7	-4	42.65	2.39
0	-7	-4	43.39	1.76
0	7	4	43.65	2.13
0	7	4	42.56	2.67
0	7	4	44.31	1.98
-1	-7	-4	60.52	2.75
-1	-7	-4	58.76	2.18
-1	-7	-4	52.44	2.90
1	7	4	54.27	2.58
1	7	4	60.98	2.45
1	7	4	58.64	3.28

-2	-7	-4	38.87	1.61
-2	-7	-4	37.74	2.93
-2	-7	-4	37.03	2.17
2	7	4	36.55	2.08
2	7	4	36.11	1.89
-3	-7	-4	20.78	1.74
-3	-7	-4	20.11	1.14
3	7	4	21.11	1.67
3	7	4	20.56	1.60
-4	-7	-4	18.90	1.12
4	7	4	17.35	0.77
4	7	4	18.41	0.77
4	7	4	20.21	1.80
4	7	4	19.21	1.48
-5	-7	-4	13.17	1.05
5	7	4	14.88	0.76
5	7	4	17.34	1.74
5	7	4	15.52	0.76
5	7	4	15.52	1.55
-6	-7	-4	23.06	1.38
6	7	4	22.17	2.04
6	7	4	23.11	1.92
6	7	4	22.53	1.01
-7	-7	-4	20.08	1.40
7	7	4	19.00	2.11
7	7	4	18.51	1.01
7	7	4	18.52	2.02
-8	-7	-4	14.01	1.39
8	7	4	14.24	2.03
8	7	4	14.10	2.13
-9	-7	-4	5.07	1.00
9	7	4	5.28	1.56
9	7	4	8.49	1.55
17	-8	-4	2.12	1.38
17	-8	-4	3.70	2.47
-17	8	4	2.45	0.71
16	-8	-4	16.52	2.14
16	-8	-4	18.37	2.91
-16	8	4	18.73	1.25
15	-8	-4	16.57	2.93
15	-8	-4	8.38	1.35
-15	8	4	12.14	1.03
14	-8	-4	13.74	1.63
14	-8	-4	9.66	3.04

-14	8	4	13.58	0.97
13	-8	-4	16.87	2.50
13	-8	-4	16.56	1.55
-13	8	4	18.03	1.10
12	-8	-4	8.72	1.24
12	-8	-4	11.89	2.13
-12	8	4	7.08	1.28
-12	8	4	9.15	0.72
11	-8	-4	12.22	0.84
11	-8	-4	15.92	1.33
11	-8	-4	13.76	2.11
-11	8	4	12.77	1.83
-11	8	4	15.02	0.79
10	-8	-4	61.79	3.28
10	-8	-4	56.16	2.49
10	-8	-4	58.82	2.05
-10	8	4	60.30	1.95
-10	8	4	59.38	2.36
-10	8	4	59.07	2.03
-10	8	4	60.10	2.03
-10	8	4	63.30	3.03
9	-8	-4	20.88	1.34
9	-8	-4	21.72	1.97
9	-8	-4	20.81	0.90
-9	8	4	20.82	0.87
-9	8	4	20.93	0.98
-9	8	4	19.45	1.92
-9	8	4	20.39	1.05
-9	8	4	21.36	1.21
8	-8	-4	31.25	1.59
8	-8	-4	29.48	1.10
8	-8	-4	26.31	2.09
-8	8	4	32.28	2.13
-8	8	4	29.07	1.08
-8	8	4	28.15	1.36
-8	8	4	30.48	1.43
-8	8	4	28.72	1.30
7	-8	-4	43.06	2.48
7	-8	-4	41.47	1.92
7	-8	-4	44.28	1.45
-7	8	4	46.15	2.51
-7	8	4	43.16	1.79
-7	8	4	42.16	1.83
-7	8	4	42.12	1.44

-7	8	4	43.63	1.81
6	-8	-4	71.76	2.84
6	-8	-4	75.66	2.35
6	-8	-4	74.80	3.41
-6	8	4	73.37	2.80
-6	8	4	78.50	2.80
-6	8	4	75.76	2.34
-6	8	4	73.76	3.41
-6	8	4	76.55	2.76
5	-8	-4	39.65	2.36
5	-8	-4	40.48	1.85
5	-8	-4	42.51	1.35
-5	8	4	41.86	1.35
-5	8	4	41.61	2.47
-5	8	4	44.47	1.88
-5	8	4	43.05	1.93
-5	8	4	43.22	1.81
4	-8	-4	41.32	2.39
4	-8	-4	40.64	2.21
4	-8	-4	45.15	1.93
-4	8	4	48.64	2.63
-4	8	4	44.98	2.06
-4	8	4	47.98	1.95
3	-8	-4	116.39	3.97
3	-8	-4	114.00	4.39
3	-8	-4	114.20	4.52
-3	8	4	121.92	4.05
-3	8	4	109.15	4.68
-3	8	4	116.06	4.21
2	-8	-4	101.08	4.14
2	-8	-4	99.43	4.10
2	-8	-4	100.61	3.57
-2	8	4	109.17	3.74
-2	8	4	102.61	3.90
-2	8	4	106.47	4.47
1	-8	-4	59.62	2.96
1	-8	-4	61.97	2.96
1	-8	-4	63.74	2.39
-1	8	4	63.08	2.77
-1	8	4	61.39	3.32
-1	8	4	65.92	2.60
0	-8	-4	43.99	2.64
0	-8	-4	46.16	1.86
0	-8	-4	44.88	2.45

0	8	4	46.81	2.13
0	8	4	46.39	2.30
0	8	4	43.61	2.93
-1	-8	-4	52.16	2.05
-1	-8	-4	50.18	2.67
1	8	4	50.11	2.55
1	8	4	53.80	2.40
-2	-8	-4	74.12	3.33
-2	-8	-4	73.21	2.65
2	8	4	73.18	3.23
2	8	4	67.73	2.97
-3	-8	-4	26.68	1.38
-3	-8	-4	27.27	2.06
3	8	4	27.15	2.19
-4	-8	-4	19.13	1.66
4	8	4	22.54	1.96
4	8	4	19.48	1.64
-5	-8	-4	28.82	1.49
5	8	4	25.61	1.97
5	8	4	30.14	2.26
5	8	4	24.12	1.03
-6	-8	-4	24.67	1.44
6	8	4	22.45	2.19
6	8	4	17.67	0.99
6	8	4	23.92	2.07
-7	-8	-4	15.27	1.39
7	8	4	14.28	2.02
7	8	4	15.59	2.27
-8	-8	-4	10.00	1.40
8	8	4	8.56	1.59
17	-9	-4	12.37	3.06
17	-9	-4	15.04	2.26
-17	9	4	10.04	1.20
16	-9	-4	10.83	2.71
16	-9	-4	5.83	1.40
-16	9	4	7.09	1.05
15	-9	-4	3.77	1.97
15	-9	-4	4.82	1.14
-15	9	4	5.06	0.68
14	-9	-4	21.71	1.89
14	-9	-4	20.82	2.89
-14	9	4	19.64	1.14
13	-9	-4	13.04	2.09
13	-9	-4	11.95	1.48

-13	9	4	9.77	0.96
12	-9	-4	61.39	3.69
12	-9	-4	61.30	2.76
-12	9	4	62.73	2.18
11	-9	-4	48.81	2.33
11	-9	-4	47.26	1.80
11	-9	-4	48.96	3.17
-11	9	4	49.49	1.69
-11	9	4	48.24	2.85
-11	9	4	49.58	1.77
-11	9	4	49.76	1.62
10	-9	-4	20.55	2.26
10	-9	-4	24.70	1.06
10	-9	-4	22.54	1.53
-10	9	4	25.73	1.17
-10	9	4	23.04	1.40
-10	9	4	24.09	2.28
-10	9	4	24.59	1.07
-10	9	4	24.83	1.02
9	-9	-4	37.99	1.94
9	-9	-4	40.45	2.71
9	-9	-4	40.93	1.44
-9	9	4	40.83	2.66
-9	9	4	38.98	1.61
-9	9	4	39.02	1.41
-9	9	4	40.99	1.69
-9	9	4	37.14	1.77
8	-9	-4	78.82	3.79
8	-9	-4	79.69	2.58
8	-9	-4	78.78	3.10
-8	9	4	77.94	3.71
-8	9	4	78.84	2.95
-8	9	4	85.01	2.96
-8	9	4	83.05	2.93
-8	9	4	80.82	2.57
7	-9	-4	37.77	1.82
7	-9	-4	34.10	2.36
7	-9	-4	35.95	1.26
-7	9	4	36.30	1.25
-7	9	4	37.74	1.74
-7	9	4	36.14	2.50
-7	9	4	36.25	1.68
-7	9	4	36.54	1.71
6	-9	-4	66.52	2.04

6	-9	-4	61.19	3.19
6	-9	-4	61.54	2.57
-6	9	4	60.12	2.57
-6	9	4	64.20	2.48
-6	9	4	69.50	2.62
-6	9	4	64.15	2.03
-6	9	4	67.24	3.35
5	-9	-4	80.54	2.46
5	-9	-4	80.35	3.04
5	-9	-4	79.72	3.66
-5	9	4	86.86	3.85
-5	9	4	78.10	3.07
-5	9	4	79.17	3.15
-5	9	4	78.02	2.95
4	-9	-4	85.04	3.14
4	-9	-4	83.48	3.76
-4	9	4	86.67	4.00
-4	9	4	83.09	3.35
-4	9	4	84.25	3.26
-4	9	4	87.68	3.16
3	-9	-4	61.96	3.07
3	-9	-4	59.21	2.42
-3	9	4	59.85	2.61
-3	9	4	59.91	2.52
-3	9	4	63.65	2.76
-3	9	4	66.38	3.44
2	-9	-4	60.45	2.97
2	-9	-4	60.23	2.30
-2	9	4	53.05	3.31
-2	9	4	57.74	2.48
-2	9	4	59.58	2.69
-2	9	4	53.75	2.56
1	-9	-4	77.45	2.88
1	-9	-4	79.16	3.61
-1	9	4	93.50	4.07
-1	9	4	79.10	3.15
-1	9	4	74.89	3.37
0	-9	-4	39.57	1.71
0	-9	-4	40.67	2.46
0	9	4	37.27	2.07
0	9	4	40.78	2.27
-1	-9	-4	32.00	2.26
-1	-9	-4	36.91	1.58
1	9	4	31.40	2.01

1	9	4	31.93	2.13
-2	-9	-4	32.94	1.56
-2	-9	-4	31.30	2.33
2	9	4	33.60	2.24
-3	-9	-4	14.33	1.08
-3	-9	-4	12.27	1.89
3	9	4	14.13	1.85
-4	-9	-4	35.50	1.73
4	9	4	35.65	2.25
4	9	4	40.06	2.59
-5	-9	-4	20.45	1.37
5	9	4	18.12	2.19
5	9	4	18.06	1.96
-6	-9	-4	4.51	0.87
6	9	4	2.41	1.27
6	9	4	2.66	1.20
-7	-9	-4	5.31	0.97
7	9	4	7.04	1.59
7	9	4	5.94	1.53
17	-10	-4	9.44	2.91
17	-10	-4	5.65	1.51
-17	10	4	5.37	0.83
16	-10	-4	8.39	1.50
16	-10	-4	10.89	2.65
-16	10	4	8.86	1.07
15	-10	-4	4.80	1.31
15	-10	-4	6.18	2.29
-15	10	4	4.84	0.70
14	-10	-4	15.94	2.59
14	-10	-4	17.26	1.85
-14	10	4	14.49	1.03
13	-10	-4	21.74	1.83
13	-10	-4	22.19	2.98
-13	10	4	20.64	1.22
12	-10	-4	47.73	3.39
12	-10	-4	48.18	2.44
-12	10	4	48.39	1.79
11	-10	-4	60.44	2.23
11	-10	-4	64.16	2.82
11	-10	-4	62.41	3.73
-11	10	4	64.24	2.20
-11	10	4	64.23	2.26
-11	10	4	65.26	2.20
10	-10	-4	24.08	1.65

10	-10	-4	19.99	2.43
10	-10	-4	25.34	1.07
-10	10	4	24.55	1.21
-10	10	4	20.52	1.37
-10	10	4	22.79	1.01
-10	10	4	24.46	1.29
9	-10	-4	36.99	2.69
9	-10	-4	33.42	1.27
9	-10	-4	31.46	1.83
-9	10	4	32.25	1.56
-9	10	4	32.29	1.22
-9	10	4	32.70	2.57
-9	10	4	32.51	1.66
-9	10	4	30.80	1.61
8	-10	-4	57.28	3.29
8	-10	-4	64.33	2.06
8	-10	-4	59.51	2.60
-8	10	4	63.99	2.03
-8	10	4	65.46	2.56
-8	10	4	58.89	3.38
-8	10	4	62.70	2.47
-8	10	4	61.37	2.49
7	-10	-4	65.42	3.40
7	-10	-4	63.40	2.03
7	-10	-4	61.92	2.61
-7	10	4	60.46	3.46
-7	10	4	61.47	2.59
-7	10	4	61.47	2.48
-7	10	4	64.26	2.01
-7	10	4	61.72	2.60
6	-10	-4	114.32	4.95
6	-10	-4	120.58	4.23
6	-10	-4	120.93	3.63
-6	10	4	112.56	5.04
-6	10	4	121.68	3.62
-6	10	4	117.42	4.28
-6	10	4	121.07	4.34
-6	10	4	118.60	4.12
5	-10	-4	77.15	3.78
5	-10	-4	84.96	3.12
-5	10	4	79.11	3.22
-5	10	4	79.41	3.03
-5	10	4	82.80	4.04
-5	10	4	79.38	3.27

4	-10	-4	55.24	2.27
4	-10	-4	55.30	3.01
-4	10	4	52.40	2.50
-4	10	4	53.13	2.32
-4	10	4	48.66	3.21
-4	10	4	55.28	2.57
3	-10	-4	108.03	4.59
3	-10	-4	110.42	3.79
-3	10	4	108.69	4.23
-3	10	4	99.75	4.05
-3	10	4	110.21	3.94
2	-10	-4	59.85	3.17
2	-10	-4	62.29	2.39
-2	10	4	56.46	2.61
-2	10	4	63.11	2.92
1	-10	-4	42.32	1.85
1	-10	-4	42.32	2.69
-1	10	4	43.40	2.48
-1	10	4	42.58	2.22
0	-10	-4	57.39	3.57
0	-10	-4	60.69	2.30
0	10	4	58.47	2.77
0	10	4	54.57	3.32
-1	-10	-4	29.01	1.47
-1	-10	-4	27.80	2.32
1	10	4	28.15	2.17
-2	-10	-4	37.74	2.68
-2	-10	-4	38.16	1.75
2	10	4	38.04	2.61
-3	-10	-4	27.81	1.49
-3	-10	-4	22.93	2.46
3	10	4	26.85	2.41
-4	-10	-4	12.55	1.25
4	10	4	17.29	1.89
4	10	4	14.64	2.17
-5	-10	-4	8.74	1.25
5	10	4	8.02	1.48
5	10	4	7.07	1.59
-6	-10	-4	9.50	1.38
6	10	4	7.24	1.58
6	10	4	5.97	1.38
17	-11	-4	11.80	3.18
17	-11	-4	17.20	2.53
-17	11	4	12.31	1.31

16	-11	-4	8.63	2.66
16	-11	-4	6.16	1.51
-16	11	4	6.25	0.85
15	-11	-4	13.18	2.69
15	-11	-4	13.16	2.02
-15	11	4	11.72	1.09
14	-11	-4	23.48	3.03
14	-11	-4	25.78	2.10
-14	11	4	24.46	1.28
13	-11	-4	38.02	2.29
13	-11	-4	38.85	3.48
-13	11	4	35.45	1.57
12	-11	-4	48.89	3.58
12	-11	-4	49.74	3.55
-12	11	4	48.81	1.82
-12	11	4	49.50	1.81
-12	11	4	48.31	1.76
11	-11	-4	40.15	2.21
11	-11	-4	35.35	3.17
11	-11	-4	39.62	1.56
-11	11	4	40.36	1.64
-11	11	4	37.23	1.48
-11	11	4	37.76	1.70
10	-11	-4	6.23	0.68
10	-11	-4	5.90	1.68
10	-11	-4	7.01	1.07
-10	11	4	6.44	1.05
-10	11	4	6.67	1.10
-10	11	4	6.59	0.93
-10	11	4	6.43	0.59
9	-11	-4	30.27	1.19
9	-11	-4	27.13	2.64
9	-11	-4	26.87	1.76
-9	11	4	28.93	1.63
-9	11	4	29.34	1.61
-9	11	4	31.20	1.76
-9	11	4	28.79	1.13
8	-11	-4	77.47	3.99
8	-11	-4	72.26	3.08
8	-11	-4	79.66	2.49
-8	11	4	74.97	3.05
-8	11	4	78.23	2.45
-8	11	4	74.78	2.91
-8	11	4	75.04	3.05

7	-11	-4	42.81	2.95
7	-11	-4	40.65	1.38
7	-11	-4	35.06	1.95
-7	11	4	40.59	2.11
-7	11	4	39.72	1.91
-7	11	4	40.06	1.34
-7	11	4	38.82	2.05
6	-11	-4	67.03	3.62
6	-11	-4	64.79	2.09
6	-11	-4	65.24	2.75
-6	11	4	68.16	2.95
-6	11	4	66.77	2.66
-6	11	4	66.59	2.91
5	-11	-4	86.29	4.05
5	-11	-4	76.24	3.13
-5	11	4	84.75	3.17
-5	11	4	81.21	3.41
-5	11	4	85.04	3.49
4	-11	-4	76.81	2.88
4	-11	-4	71.61	3.72
-4	11	4	70.14	2.95
-4	11	4	72.89	3.25
3	-11	-4	27.70	1.55
3	-11	-4	27.14	2.38
-3	11	4	30.45	1.87
-3	11	4	29.29	2.02
2	-11	-4	16.59	1.19
-2	11	4	15.65	1.86
-2	11	4	15.45	1.64
1	-11	-4	48.54	2.06
1	-11	-4	49.59	3.07
-1	11	4	50.90	2.79
-1	11	4	45.96	2.54
0	-11	-4	33.86	1.68
0	-11	-4	32.81	2.66
0	11	4	36.28	2.48
-1	-11	-4	41.81	2.88
-1	-11	-4	40.64	1.85
1	11	4	42.59	2.78
-2	-11	-4	23.09	1.42
-2	-11	-4	21.55	2.49
2	11	4	24.72	2.39
-3	-11	-4	13.03	2.03
-3	-11	-4	14.76	1.29

3	11	4	15.35	2.28
-4	-11	-4	8.00	1.26
4	11	4	11.95	1.85
4	11	4	6.78	1.31
-5	-11	-4	8.32	1.41
5	11	4	4.72	1.55
5	11	4	7.27	1.44
17	-12	-4	18.65	3.36
17	-12	-4	11.41	1.98
-17	12	4	9.56	1.29
16	-12	-4	17.99	2.48
16	-12	-4	18.66	3.32
-16	12	4	16.17	1.31
15	-12	-4	16.42	2.24
15	-12	-4	16.56	2.87
-15	12	4	18.38	1.24
14	-12	-4	33.58	2.44
14	-12	-4	32.85	3.83
-14	12	4	30.12	1.47
13	-12	-4	33.57	2.36
13	-12	-4	36.50	3.69
-13	12	4	36.25	1.51
12	-12	-4	22.54	2.00
12	-12	-4	24.51	3.16
-12	12	4	25.41	1.53
-12	12	4	25.10	1.25
-12	12	4	23.26	1.15
11	-12	-4	26.57	1.30
11	-12	-4	30.78	3.17
11	-12	-4	24.58	1.96
-11	12	4	29.61	1.64
-11	12	4	28.20	1.54
-11	12	4	28.43	1.20
10	-12	-4	31.38	1.37
10	-12	-4	33.60	3.07
10	-12	-4	32.36	2.08
-10	12	4	35.44	1.92
-10	12	4	33.95	1.30
-10	12	4	34.76	1.80
9	-12	-4	23.17	1.71
9	-12	-4	21.25	1.01
-9	12	4	23.56	1.72
-9	12	4	20.19	1.55
-9	12	4	22.01	0.94

-9	12	4	21.70	1.49
8	-12	-4	36.40	3.51
8	-12	-4	35.92	1.33
8	-12	-4	37.60	2.04
-8	12	4	35.24	1.28
-8	12	4	33.94	2.00
-8	12	4	37.22	2.12
-8	12	4	35.04	1.88
7	-12	-4	41.57	3.36
7	-12	-4	44.28	2.26
-7	12	4	45.63	2.15
-7	12	4	53.47	2.51
-7	12	4	42.81	2.35
-7	12	4	45.14	1.51
6	-12	-4	41.87	4.54
6	-12	-4	42.09	2.10
-6	12	4	42.21	2.07
-6	12	4	40.76	2.35
5	-12	-4	46.17	2.20
5	-12	-4	47.77	4.08
-5	12	4	49.62	2.63
-5	12	4	49.67	2.29
4	-12	-4	16.74	1.33
4	-12	-4	17.60	2.34
-4	12	4	19.54	1.61
-4	12	4	18.06	1.81
3	-12	-4	19.19	1.36
3	-12	-4	17.98	2.34
-3	12	4	19.66	1.95
-3	12	4	17.72	1.75
2	-12	-4	22.11	2.44
2	-12	-4	20.79	1.35
-2	12	4	20.27	2.10
-2	12	4	19.60	1.91
1	-12	-4	29.23	1.55
1	-12	-4	24.56	2.63
-1	12	4	28.19	2.37
0	-12	-4	18.91	2.47
0	-12	-4	20.32	1.33
0	12	4	16.37	2.26
-1	-12	-4	24.39	2.70
-1	-12	-4	19.28	1.61
1	12	4	23.26	2.49
-2	-12	-4	16.06	2.61

-2	-12	-4	18.10	1.41
2	12	4	21.99	2.48
-3	-12	-4	14.66	1.41
-3	-12	-4	13.32	2.08
3	12	4	19.34	2.58
-4	-12	-4	2.88	0.93
4	12	4	2.96	1.32
4	12	4	2.64	1.57
16	-13	-4	33.24	4.65
16	-13	-4	27.25	2.89
-16	13	4	24.20	1.54
15	-13	-4	19.35	2.52
15	-13	-4	23.24	3.44
-15	13	4	20.17	1.32
14	-13	-4	21.72	2.33
14	-13	-4	17.39	2.78
-14	13	4	19.55	1.22
13	-13	-4	33.26	2.51
13	-13	-4	33.79	3.88
-13	13	4	35.94	1.51
12	-13	-4	49.77	2.77
12	-13	-4	59.13	4.18
-12	13	4	49.64	1.90
-12	13	4	51.27	2.16
11	-13	-4	54.87	2.10
11	-13	-4	57.66	4.12
11	-13	-4	56.71	2.85
-11	13	4	55.75	2.47
-11	13	4	57.36	2.51
-11	13	4	57.18	2.01
10	-13	-4	28.12	3.23
10	-13	-4	27.89	2.03
10	-13	-4	29.25	1.28
-10	13	4	31.71	1.96
-10	13	4	27.81	1.20
-10	13	4	30.43	1.85
9	-13	-4	56.29	2.69
9	-13	-4	53.82	1.93
9	-13	-4	51.26	3.79
-9	13	4	56.89	2.78
-9	13	4	55.29	2.64
-9	13	4	56.08	1.88
8	-13	-4	92.60	3.82
8	-13	-4	95.66	3.10

8	-13	-4	97.76	4.97
-8	13	4	93.22	3.63
-8	13	4	98.18	3.94
-8	13	4	113.20	4.51
-8	13	4	97.83	3.07
7	-13	-4	31.18	3.07
7	-13	-4	37.09	2.03
7	-13	-4	31.59	1.18
-7	13	4	31.40	2.20
-7	13	4	32.16	1.96
6	-13	-4	13.03	1.44
6	-13	-4	16.94	2.56
-6	13	4	12.86	1.72
-6	13	4	14.30	1.61
5	-13	-4	41.05	2.12
5	-13	-4	45.66	3.42
-5	13	4	42.61	2.33
-5	13	4	46.58	2.73
4	-13	-4	41.98	3.22
4	-13	-4	36.16	1.90
-4	13	4	39.92	2.62
-4	13	4	33.40	2.21
3	-13	-4	18.80	2.62
3	-13	-4	16.50	1.36
-3	13	4	19.53	2.00
-3	13	4	17.87	2.16
2	-13	-4	24.68	1.55
2	-13	-4	28.29	2.82
-2	13	4	25.15	2.43
1	-13	-4	13.89	2.16
1	-13	-4	11.26	1.26
-1	13	4	13.43	2.21
0	-13	-4	9.47	1.90
0	-13	-4	12.91	1.60
0	13	4	15.11	2.41
-1	-13	-4	23.92	1.59
-1	-13	-4	20.15	2.91
1	13	4	28.70	2.86
-2	-13	-4	5.48	0.97
-2	-13	-4	4.81	1.87
2	13	4	5.37	1.59
-3	-13	-4	5.67	1.05
-3	-13	-4	5.06	1.92
3	13	4	3.97	1.60

16	-14	-4	15.44	3.31
16	-14	-4	10.94	2.11
-16	14	4	10.06	1.21
15	-14	-4	29.66	2.97
15	-14	-4	30.95	4.58
-15	14	4	27.05	1.53
14	-14	-4	17.79	3.09
14	-14	-4	21.70	2.62
-14	14	4	21.57	1.30
13	-14	-4	22.89	2.41
13	-14	-4	25.57	3.82
-13	14	4	20.14	1.21
12	-14	-4	29.47	2.37
12	-14	-4	26.83	3.64
-12	14	4	27.91	1.30
-12	14	4	25.87	1.78
11	-14	-4	32.39	3.63
11	-14	-4	45.13	3.99
-11	14	4	43.77	1.62
-11	14	4	42.98	2.28
10	-14	-4	44.40	2.55
10	-14	-4	46.78	3.97
-10	14	4	45.51	2.45
-10	14	4	43.57	1.63
-10	14	4	43.65	2.50
9	-14	-4	48.26	2.66
9	-14	-4	56.81	4.03
-9	14	4	52.37	1.75
-9	14	4	45.69	2.65
8	-14	-4	40.95	3.62
8	-14	-4	39.24	2.35
-8	14	4	39.99	1.40
-8	14	4	40.24	2.49
7	-14	-4	31.65	3.38
7	-14	-4	32.69	2.08
-7	14	4	34.81	2.53
-7	14	4	35.99	2.43
6	-14	-4	40.19	2.22
6	-14	-4	41.49	3.52
-6	14	4	41.11	2.69
-6	14	4	39.93	2.36
5	-14	-4	25.31	1.76
5	-14	-4	24.84	3.15
-5	14	4	28.35	2.44

-5	14	4	28.21	2.49
4	-14	-4	21.23	3.03
4	-14	-4	22.47	1.67
-4	14	4	26.84	2.51
3	-14	-4	22.91	1.57
3	-14	-4	25.67	3.01
-3	14	4	21.35	2.45
2	-14	-4	20.57	1.52
2	-14	-4	21.65	3.11
-2	14	4	17.30	2.62
1	-14	-4	19.49	1.75
1	-14	-4	18.92	3.09
-1	14	4	21.38	2.70
0	-14	-4	16.35	2.96
0	-14	-4	13.70	1.45
0	14	4	14.93	2.66
-1	-14	-4	5.46	1.04
-1	-14	-4	11.56	2.12
1	14	4	5.84	1.80
-2	-14	-4	6.15	1.09
-2	-14	-4	11.75	3.83
15	-15	-4	30.34	3.25
15	-15	-4	26.66	4.80
-15	15	4	38.28	1.75
14	-15	-4	27.24	2.93
14	-15	-4	21.49	3.53
-14	15	4	21.55	1.34
13	-15	-4	23.47	2.66
13	-15	-4	27.00	4.20
-13	15	4	23.34	1.28
12	-15	-4	51.05	4.43
12	-15	-4	43.75	2.95
-12	15	4	40.48	1.68
-12	15	4	41.22	2.33
11	-15	-4	38.78	4.13
11	-15	-4	43.38	2.77
-11	15	4	40.63	2.48
-11	15	4	40.67	1.60
10	-15	-4	22.88	3.59
10	-15	-4	24.02	2.93
-10	15	4	18.00	0.99
-10	15	4	20.81	2.06
9	-15	-4	28.18	2.37
9	-15	-4	36.67	3.80

-9	15	4	36.07	2.89
8	-15	-4	50.15	4.02
8	-15	-4	44.63	2.54
-8	15	4	44.67	2.92
7	-15	-4	25.91	2.05
7	-15	-4	30.92	3.64
-7	15	4	35.35	2.65
6	-15	-4	18.82	2.78
6	-15	-4	16.59	1.71
-6	15	4	16.12	2.33
5	-15	-4	15.90	1.64
5	-15	-4	17.85	3.27
-5	15	4	19.60	2.56
4	-15	-4	28.42	3.39
4	-15	-4	27.91	1.77
-4	15	4	24.21	2.81
3	-15	-4	15.64	2.55
3	-15	-4	15.06	1.51
-3	15	4	19.43	2.66
2	-15	-4	10.58	1.44
2	-15	-4	12.89	2.47
-2	15	4	12.05	2.17
1	-15	-4	11.05	2.35
1	-15	-4	8.21	1.12
-1	15	4	12.06	2.12
0	-15	-4	8.62	1.14
0	-15	-4	2.08	2.06
0	15	4	7.32	1.99
14	-16	-4	27.76	4.78
14	-16	-4	26.09	3.26
-14	16	4	25.37	1.49
13	-16	-4	20.58	3.03
13	-16	-4	23.06	3.61
12	-16	-4	18.01	3.08
12	-16	-4	22.64	2.72
11	-16	-4	30.26	4.20
11	-16	-4	23.09	2.60
10	-16	-4	34.60	4.30
10	-16	-4	31.33	2.71
-10	16	4	32.44	2.60
9	-16	-4	31.57	4.11
-9	16	4	39.16	2.86
8	-16	-4	18.87	2.06
8	-16	-4	10.16	2.53

-8	16	4	16.42	2.51
7	-16	-4	25.43	2.05
7	-16	-4	19.31	3.62
-7	16	4	29.43	2.79
6	-16	-4	22.17	3.67
6	-16	-4	25.96	1.96
-6	16	4	22.85	2.86
5	-16	-4	17.29	1.68
5	-16	-4	15.67	2.75
-5	16	4	15.33	2.71
4	-16	-4	10.64	2.36
4	-16	-4	10.16	2.25
-4	16	4	13.64	2.03
3	-16	-4	14.78	1.66
3	-16	-4	20.00	3.63
-3	16	4	16.79	2.90
2	-16	-4	4.93	2.39
2	-16	-4	8.17	1.17
-2	16	4	6.27	1.94
12	-17	-4	17.67	3.28
12	-17	-4	17.92	2.27
11	-17	-4	18.79	2.24
11	-17	-4	13.54	3.08
-11	17	4	18.97	2.57
10	-17	-4	10.37	1.74
10	-17	-4	1.16	2.53
-10	17	4	7.04	1.71
9	-17	-4	20.19	3.20
9	-17	-4	20.71	2.38
-9	17	4	21.18	2.77
8	-17	-4	27.39	2.38
8	-17	-4	32.81	4.14
-8	17	4	25.38	3.05
7	-17	-4	7.77	2.60
7	-17	-4	8.37	1.62
-7	17	4	7.73	1.86
6	-17	-4	6.41	2.52
-6	17	4	3.60	1.76
5	-17	-4	6.77	2.67
-5	17	4	10.26	2.14
-4	17	4	7.17	1.95
5	-19	5	1.37	2.65
0	-18	5	2.82	2.63
-1	18	-5	-0.47	1.94

1	-18	5	4.85	2.43
-2	18	-5	0.53	2.04
2	-18	5	-0.67	2.37
-3	18	-5	-2.29	1.87
3	-18	5	-2.29	2.35
-4	18	-5	1.44	1.94
-4	18	-5	0.30	0.59
4	-18	5	3.27	2.58
-5	18	-5	0.41	0.57
5	-18	5	-3.17	2.26
-6	18	-5	1.71	0.62
6	-18	5	0.31	1.41
6	-18	5	-2.41	2.20
7	-18	5	1.88	2.51
7	-18	5	1.41	1.49
-8	18	-5	1.85	0.71
8	-18	5	5.89	2.76
8	-18	5	2.10	1.55
-9	18	-5	2.67	0.74
9	-18	5	2.96	1.55
9	-18	5	-0.78	2.85
2	17	-5	-3.93	3.27
-2	-17	5	-1.90	2.26
1	17	-5	-0.16	1.91
-1	-17	5	3.23	2.24
0	17	-5	1.00	1.74
0	-17	5	0.63	2.20
-1	17	-5	-0.25	1.76
-1	17	-5	0.00	0.57
1	-17	5	-2.79	2.02
-2	17	-5	0.23	0.53
-2	17	-5	2.43	1.72
2	-17	5	1.55	2.20
-3	17	-5	1.31	0.54
3	-17	5	2.04	2.17
3	-17	5	-0.72	1.26
-4	17	-5	0.50	0.55
4	-17	5	-1.62	1.20
4	-17	5	0.05	2.23
-5	17	-5	1.37	0.57
5	-17	5	-2.11	2.05
5	-17	5	1.79	1.33
-6	17	-5	1.31	0.58
6	-17	5	0.19	1.29

6	-17	5	1.06	2.20
-7	17	-5	1.82	0.65
7	-17	5	2.63	1.36
7	-17	5	0.02	2.17
-8	17	-5	3.41	0.71
8	-17	5	6.21	1.55
8	-17	5	5.20	2.48
-9	17	-5	1.67	0.66
9	-17	5	5.70	2.52
9	-17	5	-0.11	1.30
-10	17	-5	3.49	0.76
10	-17	5	4.71	1.47
10	-17	5	8.57	2.83
11	-17	5	-2.78	4.67
-4	-16	5	-0.39	2.19
3	16	-5	0.90	1.92
-3	-16	5	3.10	2.16
2	16	-5	3.25	1.73
-2	-16	5	2.16	1.97
1	16	-5	1.04	0.52
1	16	-5	2.10	1.84
-1	-16	5	0.79	1.98
0	16	-5	2.18	0.55
0	16	-5	1.28	1.56
0	-16	5	1.09	1.97
-1	16	-5	2.16	0.53
1	-16	5	1.21	2.00
1	-16	5	3.81	1.38
-2	16	-5	1.56	0.53
2	-16	5	0.44	1.87
2	-16	5	3.64	1.31
-3	16	-5	8.62	0.88
3	-16	5	10.92	2.37
3	-16	5	8.93	1.58
-4	16	-5	5.91	0.80
4	-16	5	4.78	2.07
4	-16	5	7.63	1.45
-5	16	-5	6.18	0.82
5	-16	5	6.93	2.12
5	-16	5	6.31	1.41
-6	16	-5	1.08	0.51
6	-16	5	2.06	2.08
6	-16	5	0.62	1.15
-7	16	-5	4.14	0.63

7	-16	5	3.00	1.20
7	-16	5	4.69	2.20
-8	16	-5	11.28	1.04
8	-16	5	10.93	2.73
8	-16	5	13.18	1.71
-9	16	-5	7.52	1.02
9	-16	5	8.04	1.52
9	-16	5	9.10	2.75
-10	16	-5	3.88	0.73
10	-16	5	2.28	2.25
10	-16	5	3.94	1.38
-11	16	-5	2.38	0.74
11	-16	5	-1.09	2.63
-12	16	-5	6.06	0.88
12	-16	5	6.45	4.08
5	15	-5	0.29	1.92
-5	-15	5	1.54	2.01
4	15	-5	3.69	1.89
4	15	-5	3.82	1.20
-4	-15	5	3.76	2.15
3	15	-5	0.77	1.70
3	15	-5	0.69	0.60
-3	-15	5	3.30	1.91
2	15	-5	-0.18	1.66
2	15	-5	0.99	0.55
-2	-15	5	3.09	1.84
1	15	-5	0.03	0.50
-1	-15	5	-1.60	1.08
-1	-15	5	-2.29	1.62
0	15	-5	0.11	0.45
0	-15	5	0.12	0.47
0	-15	5	0.91	1.13
0	-15	5	0.31	1.65
-1	15	-5	2.93	0.54
1	-15	5	6.28	0.78
1	-15	5	3.79	1.27
1	-15	5	5.31	1.91
-2	15	-5	3.60	0.54
2	-15	5	5.89	1.98
2	-15	5	5.12	0.63
2	-15	5	3.62	1.20
-3	15	-5	5.94	0.75
3	-15	5	6.79	2.01
3	-15	5	5.32	1.27

-4	15	-5	5.76	0.77
4	-15	5	6.66	1.25
4	-15	5	7.25	1.96
-5	15	-5	5.23	0.73
5	-15	5	5.15	1.91
5	-15	5	8.02	1.35
-6	15	-5	6.75	0.93
6	-15	5	11.03	1.48
6	-15	5	15.62	2.64
-7	15	-5	16.28	1.04
7	-15	5	18.43	2.15
7	-15	5	12.70	2.34
-8	15	-5	7.33	0.89
8	-15	5	7.52	0.92
8	-15	5	8.65	2.37
8	-15	5	9.45	1.40
-9	15	-5	2.69	0.61
9	-15	5	4.19	2.23
9	-15	5	4.90	1.27
9	-15	5	3.67	0.87
-10	15	-5	5.11	0.74
10	-15	5	6.99	1.37
10	-15	5	5.02	1.07
10	-15	5	4.48	2.32
-11	15	-5	8.10	1.09
11	-15	5	9.97	1.35
11	-15	5	5.87	2.74
-12	15	-5	10.07	1.21
12	-15	5	7.35	2.85
6	14	-5	-0.16	1.89
-6	-14	5	-0.89	1.81
5	14	-5	2.43	0.72
5	14	-5	5.72	2.07
-5	-14	5	3.42	1.91
4	14	-5	0.32	0.61
4	14	-5	2.02	1.61
-4	-14	5	2.63	1.64
3	14	-5	-0.17	0.53
3	14	-5	-2.45	1.45
-3	-14	5	0.30	1.48
2	14	-5	2.35	0.55
-2	-14	5	1.52	0.49
-2	-14	5	-1.29	1.01
-2	-14	5	1.25	1.53

1	14	-5	0.48	0.48
-1	-14	5	1.55	1.03
-1	-14	5	1.19	1.49
-1	-14	5	1.13	0.46
0	14	-5	10.27	0.84
0	-14	5	11.53	1.77
0	-14	5	10.51	0.81
0	-14	5	9.10	1.81
-1	14	-5	5.45	0.68
1	-14	5	4.07	0.56
1	-14	5	3.89	1.12
1	-14	5	6.62	1.76
-2	14	-5	3.76	0.51
2	-14	5	2.19	0.54
2	-14	5	3.92	1.11
2	-14	5	0.59	1.28
-3	14	-5	1.06	0.40
3	-14	5	0.19	0.96
3	-14	5	-0.39	0.44
3	-14	5	0.23	1.30
-4	14	-5	9.61	0.80
4	-14	5	10.63	1.70
4	-14	5	7.90	1.77
-5	14	-5	26.57	1.23
5	-14	5	27.61	2.03
5	-14	5	24.09	2.95
-6	14	-5	10.38	0.84
6	-14	5	10.60	1.63
6	-14	5	8.48	1.99
-7	14	-5	2.35	0.60
7	-14	5	3.32	1.04
7	-14	5	8.22	2.19
7	-14	5	2.92	0.65
7	-14	5	3.22	0.71
-8	14	-5	4.19	0.60
8	-14	5	5.84	0.83
8	-14	5	2.96	1.08
8	-14	5	5.03	2.09
-9	14	-5	11.91	1.41
-9	14	-5	8.45	0.95
9	-14	5	10.53	1.32
9	-14	5	7.71	2.28
9	-14	5	9.67	1.71
-10	14	-5	6.55	0.95

10	-14	5	6.09	2.26
10	-14	5	8.72	1.27
10	-14	5	8.75	1.41
-11	14	-5	2.38	0.67
11	-14	5	2.65	1.06
11	-14	5	4.93	1.14
11	-14	5	2.05	2.36
-12	14	-5	9.08	1.13
12	-14	5	9.27	1.43
12	-14	5	9.39	2.53
-13	14	-5	12.67	1.27
13	-14	5	3.89	2.86
13	-14	5	10.64	1.57
8	13	-5	2.46	2.21
8	13	-5	1.15	1.30
7	13	-5	0.67	0.78
7	13	-5	4.07	2.05
-7	-13	5	0.80	1.75
6	13	-5	-0.63	1.71
-6	-13	5	0.53	1.59
5	13	-5	0.31	1.71
5	13	-5	0.44	0.63
-5	-13	5	1.42	1.47
4	13	-5	0.97	1.43
4	13	-5	0.78	0.53
4	13	-5	-1.21	0.52
4	13	-5	0.53	0.52
-4	-13	5	0.84	1.37
3	13	-5	1.17	0.52
3	13	-5	1.10	0.58
-3	-13	5	0.01	0.95
-3	-13	5	1.87	1.32
2	13	-5	5.20	0.78
-2	-13	5	4.99	1.86
-2	-13	5	4.52	0.65
-2	-13	5	6.44	1.31
1	13	-5	0.56	0.45
-1	-13	5	-0.21	0.37
-1	-13	5	0.45	0.88
-1	-13	5	0.91	1.21
0	13	-5	0.57	0.39
0	-13	5	0.42	0.80
0	-13	5	0.29	1.24
0	-13	5	0.72	0.39

-1	13	-5	3.18	0.47
1	-13	5	4.95	1.36
1	-13	5	4.49	0.53
-2	13	-5	12.39	0.81
2	-13	5	13.30	1.55
2	-13	5	13.44	0.87
2	-13	5	11.68	1.87
-3	13	-5	13.24	0.82
3	-13	5	11.45	0.92
3	-13	5	13.02	1.53
3	-13	5	13.40	2.21
-4	13	-5	13.83	0.85
4	-13	5	12.07	0.90
4	-13	5	13.33	1.56
4	-13	5	12.45	2.21
-5	13	-5	8.61	0.75
5	-13	5	8.30	2.78
5	-13	5	9.61	1.46
-6	13	-5	5.24	0.81
-6	13	-5	4.67	0.68
6	-13	5	6.08	1.05
6	-13	5	6.18	0.71
6	-13	5	5.72	0.58
6	-13	5	6.79	1.65
-7	13	-5	9.74	0.82
-7	13	-5	10.63	1.10
7	-13	5	11.27	1.52
7	-13	5	13.53	0.99
7	-13	5	11.95	0.79
7	-13	5	12.84	2.25
-8	13	-5	11.12	0.85
-8	13	-5	10.50	1.13
8	-13	5	10.77	1.54
8	-13	5	10.88	0.90
8	-13	5	8.13	1.06
8	-13	5	7.60	1.98
-9	13	-5	9.71	0.92
-9	13	-5	9.33	1.04
9	-13	5	11.11	1.05
9	-13	5	11.02	1.26
9	-13	5	12.73	2.24
9	-13	5	13.66	1.60
-10	13	-5	17.68	1.14
10	-13	5	19.74	2.94

10	-13	5	14.12	1.70
10	-13	5	18.71	1.36
10	-13	5	17.49	1.49
-11	13	-5	14.18	1.13
11	-13	5	14.68	1.68
11	-13	5	16.45	2.89
11	-13	5	13.39	1.57
-12	13	-5	13.08	1.17
12	-13	5	10.84	2.82
12	-13	5	12.75	1.79
-13	13	-5	9.11	1.12
13	-13	5	9.30	1.50
13	-13	5	7.90	2.92
9	12	-5	-1.52	0.87
9	12	-5	-1.05	2.01
9	12	-5	-1.63	1.32
8	12	-5	1.42	0.78
8	12	-5	1.95	1.98
8	12	-5	0.21	1.23
7	12	-5	-0.20	0.74
7	12	-5	0.61	1.69
7	12	-5	-0.43	0.70
-7	-12	5	0.54	1.45
6	12	-5	0.14	0.64
6	12	-5	-0.11	1.53
6	12	-5	-0.71	0.57
-6	-12	5	-0.63	1.34
5	12	-5	1.71	0.57
5	12	-5	0.22	0.53
5	12	-5	1.35	1.46
5	12	-5	0.94	0.57
-5	-12	5	0.52	1.25
4	12	-5	4.78	0.59
4	12	-5	4.08	0.61
4	12	-5	4.99	0.62
4	12	-5	5.82	0.77
-4	-12	5	4.31	1.14
-4	-12	5	4.37	1.27
3	12	-5	7.98	0.82
-3	-12	5	7.96	1.22
-3	-12	5	9.55	1.57
-3	-12	5	7.01	0.68
2	12	-5	2.90	0.50
-2	-12	5	2.54	0.42

-2	-12	5	0.49	0.84
-2	-12	5	1.50	1.16
1	12	-5	0.70	0.40
-1	-12	5	0.13	0.80
-1	-12	5	-0.19	0.33
-1	-12	5	1.87	1.16
0	12	-5	1.76	1.48
0	12	-5	1.43	0.41
0	-12	5	1.57	0.38
0	-12	5	1.18	1.07
0	-12	5	0.71	0.66
-1	12	-5	0.69	1.31
-1	12	-5	0.31	0.36
1	-12	5	0.90	0.37
1	-12	5	0.51	0.74
1	-12	5	1.61	1.09
-2	12	-5	12.58	2.20
-2	12	-5	11.62	0.74
2	-12	5	12.53	0.83
2	-12	5	10.94	1.39
2	-12	5	12.84	1.92
-3	12	-5	5.17	0.58
-3	12	-5	4.53	0.75
3	-12	5	4.11	0.87
3	-12	5	5.09	1.50
3	-12	5	5.95	0.70
-4	12	-5	4.71	0.60
-4	12	-5	5.91	1.05
4	-12	5	5.95	0.99
4	-12	5	5.34	0.72
4	-12	5	5.23	1.50
-5	12	-5	20.25	0.99
-5	12	-5	20.09	1.36
5	-12	5	19.07	2.25
5	-12	5	18.02	1.07
5	-12	5	20.09	1.63
-6	12	-5	1.19	0.40
6	-12	5	1.54	1.32
6	-12	5	0.99	0.79
6	-12	5	1.43	0.36
6	-12	5	1.23	0.42
-7	12	-5	1.31	0.40
-7	12	-5	1.73	0.56
7	-12	5	-0.45	1.41

7	-12	5	1.49	0.45
7	-12	5	0.78	0.78
7	-12	5	2.87	0.54
-8	12	-5	20.62	1.22
-8	12	-5	19.30	1.13
8	-12	5	22.37	1.14
8	-12	5	19.78	2.63
8	-12	5	20.16	1.62
8	-12	5	19.59	1.22
-9	12	-5	23.24	1.26
-9	12	-5	22.15	1.20
9	-12	5	23.80	1.73
9	-12	5	23.00	1.42
9	-12	5	23.49	1.30
9	-12	5	25.87	3.16
-10	12	-5	24.92	1.29
-10	12	-5	25.25	1.31
10	-12	5	24.84	1.78
10	-12	5	23.11	1.48
10	-12	5	21.21	3.16
10	-12	5	25.17	1.60
-11	12	-5	8.65	0.98
11	-12	5	8.83	1.41
11	-12	5	10.57	1.50
11	-12	5	9.76	2.26
-12	12	-5	8.90	1.02
12	-12	5	5.99	1.14
12	-12	5	8.33	2.43
-13	12	-5	18.10	1.27
13	-12	5	14.71	2.94
13	-12	5	17.66	2.16
-14	12	-5	5.74	0.87
14	-12	5	5.43	1.56
14	-12	5	1.95	2.96
10	11	-5	1.86	0.94
10	11	-5	0.89	1.43
10	11	-5	0.08	2.03
9	11	-5	0.67	0.76
9	11	-5	-0.75	1.19
9	11	-5	0.14	0.92
9	11	-5	0.80	1.83
8	11	-5	2.12	1.79
8	11	-5	0.54	0.69
8	11	-5	-0.36	0.78

8	11	-5	1.62	1.11
7	11	-5	-2.44	1.45
7	11	-5	0.56	0.67
7	11	-5	-0.38	0.54
-7	-11	5	-0.21	1.26
6	11	-5	4.38	0.83
6	11	-5	5.59	1.52
6	11	-5	3.15	0.64
6	11	-5	3.77	0.67
-6	-11	5	3.38	1.28
5	11	-5	0.96	0.51
5	11	-5	1.60	0.66
5	11	-5	2.00	0.54
5	11	-5	0.23	0.50
-5	-11	5	1.00	1.09
-5	-11	5	3.36	1.05
4	11	-5	0.42	0.43
4	11	-5	0.35	0.39
4	11	-5	-0.29	0.43
4	11	-5	0.19	0.64
-4	-11	5	0.08	0.76
-4	-11	5	0.10	1.03
3	11	-5	14.75	0.89
3	11	-5	15.14	0.77
3	11	-5	16.90	0.94
3	11	-5	15.22	2.44
-3	-11	5	15.25	1.89
-3	-11	5	16.20	1.64
-3	-11	5	17.50	0.83
2	11	-5	35.68	1.45
2	11	-5	32.65	2.73
-2	-11	5	36.16	2.12
-2	-11	5	35.71	2.47
-2	-11	5	39.29	1.38
1	11	-5	9.68	0.72
1	11	-5	7.02	1.48
-1	-11	5	8.83	0.65
-1	-11	5	9.27	1.47
-1	-11	5	9.72	1.26
0	11	-5	3.84	1.27
0	11	-5	2.60	0.42
0	-11	5	2.65	1.10
0	-11	5	3.60	0.43
0	-11	5	2.55	0.74

-1	11	-5	1.55	0.59
-1	11	-5	1.23	1.08
-1	11	-5	0.82	0.32
1	-11	5	1.04	0.64
1	-11	5	1.09	0.37
1	-11	5	0.83	0.90
-2	11	-5	31.32	2.34
-2	11	-5	28.90	1.16
-2	11	-5	28.37	1.59
2	-11	5	26.86	1.19
2	-11	5	27.75	2.27
2	-11	5	26.64	1.70
-3	11	-5	30.93	1.22
-3	11	-5	29.43	1.57
-3	11	-5	27.66	2.33
3	-11	5	31.19	1.80
3	-11	5	29.77	1.26
3	-11	5	31.10	2.39
-4	11	-5	27.67	2.32
-4	11	-5	29.44	1.22
-4	11	-5	32.28	1.55
4	-11	5	27.81	1.27
4	-11	5	30.54	2.40
4	-11	5	30.05	1.74
-5	11	-5	17.69	2.50
-5	11	-5	18.46	0.93
-5	11	-5	20.37	1.21
5	-11	5	16.13	1.00
5	-11	5	18.37	1.43
5	-11	5	19.00	0.94
5	-11	5	19.27	2.21
5	-11	5	18.01	0.79
-6	11	-5	9.39	0.72
-6	11	-5	10.16	0.91
6	-11	5	9.23	0.76
6	-11	5	9.94	1.24
6	-11	5	10.17	0.68
6	-11	5	10.77	1.91
6	-11	5	9.83	0.91
-7	11	-5	29.00	1.37
-7	11	-5	26.52	1.21
7	-11	5	25.67	1.62
7	-11	5	27.61	1.31
7	-11	5	28.61	1.22

7	-11	5	25.56	2.62
-8	11	-5	27.33	1.30
-8	11	-5	30.72	1.38
8	-11	5	28.83	1.45
8	-11	5	27.19	2.75
8	-11	5	27.37	1.70
8	-11	5	30.02	1.36
-9	11	-5	37.30	1.56
-9	11	-5	34.89	1.52
9	-11	5	34.03	3.11
9	-11	5	36.96	1.64
9	-11	5	34.74	1.92
9	-11	5	36.52	1.76
-10	11	-5	25.59	1.31
-10	11	-5	27.07	1.34
10	-11	5	24.96	1.47
10	-11	5	26.33	3.08
10	-11	5	28.64	1.71
10	-11	5	24.82	1.60
-11	11	-5	31.42	1.51
11	-11	5	32.29	1.84
11	-11	5	33.06	1.88
11	-11	5	29.41	3.54
-12	11	-5	12.60	1.11
12	-11	5	11.47	2.45
12	-11	5	15.77	1.68
-13	11	-5	8.67	1.10
13	-11	5	15.72	1.92
13	-11	5	9.75	2.94
-14	11	-5	9.93	1.23
14	-11	5	7.90	2.88
14	-11	5	12.35	1.62
11	10	-5	1.13	1.38
11	10	-5	0.14	1.22
11	10	-5	-0.55	1.97
11	10	-5	-0.92	0.88
10	10	-5	2.19	1.90
10	10	-5	-0.96	0.76
10	10	-5	0.55	1.28
10	10	-5	1.37	1.02
9	10	-5	-0.42	1.05
9	10	-5	0.07	0.82
9	10	-5	0.09	1.63
9	10	-5	-0.32	0.65

8	10	-5	3.26	1.65
8	10	-5	5.03	0.73
8	10	-5	3.29	0.82
8	10	-5	2.90	1.03
7	10	-5	3.96	0.67
7	10	-5	3.82	0.75
7	10	-5	3.29	0.76
7	10	-5	1.76	1.38
-7	-10	5	3.60	1.18
6	10	-5	0.34	0.57
6	10	-5	0.04	0.51
6	10	-5	0.04	0.45
6	10	-5	0.58	0.43
6	10	-5	-0.65	1.11
-6	-10	5	1.43	1.04
5	10	-5	1.16	0.42
5	10	-5	0.74	1.48
5	10	-5	0.07	0.42
5	10	-5	0.71	0.47
5	10	-5	0.02	0.43
-5	-10	5	0.30	0.89
-5	-10	5	-0.78	0.82
4	10	-5	10.72	0.76
4	10	-5	13.75	1.89
4	10	-5	12.20	0.83
4	10	-5	12.02	0.83
-4	-10	5	11.31	1.65
-4	-10	5	13.50	1.47
3	10	-5	10.34	0.70
3	10	-5	10.07	0.62
3	10	-5	9.54	0.77
3	10	-5	13.77	2.03
-3	-10	5	11.41	0.67
-3	-10	5	9.85	1.52
-3	-10	5	11.47	1.29
2	10	-5	2.12	1.22
2	10	-5	3.28	0.43
2	10	-5	3.18	0.73
-2	-10	5	2.96	1.00
-2	-10	5	2.69	0.36
-2	-10	5	2.18	0.68
1	10	-5	8.18	1.43
1	10	-5	6.46	1.04
1	10	-5	6.77	0.61

-1	-10	5	8.26	0.56
-1	-10	5	6.46	1.06
-1	-10	5	7.40	1.23
0	10	-5	23.15	1.00
0	10	-5	23.20	1.99
0	10	-5	22.15	1.39
0	-10	5	23.19	1.51
0	-10	5	22.85	0.97
0	-10	5	19.53	1.86
-1	10	-5	6.62	0.92
-1	10	-5	6.86	0.56
-1	10	-5	8.14	1.37
1	-10	5	6.22	0.90
1	-10	5	7.54	0.57
1	-10	5	5.77	1.16
-2	10	-5	8.72	1.55
-2	10	-5	7.97	0.57
-2	10	-5	8.12	0.91
2	-10	5	7.49	0.60
2	-10	5	7.19	0.98
2	-10	5	8.07	1.35
-3	10	-5	9.46	0.61
-3	10	-5	8.18	0.86
-3	10	-5	10.59	1.51
3	-10	5	8.89	0.66
3	-10	5	8.18	1.34
3	-10	5	8.74	1.02
-4	10	-5	4.33	0.73
-4	10	-5	6.43	1.23
-4	10	-5	4.75	0.51
4	-10	5	5.78	1.28
4	-10	5	3.78	0.58
4	-10	5	5.06	0.49
4	-10	5	6.01	0.96
4	-10	5	5.48	0.41
-5	10	-5	46.03	1.68
-5	10	-5	45.60	2.61
-5	10	-5	48.24	1.86
5	-10	5	46.61	1.58
5	-10	5	43.57	1.69
5	-10	5	39.20	2.69
5	-10	5	48.50	2.12
5	-10	5	46.34	1.66
-6	10	-5	20.08	1.90

-6	10	-5	16.74	0.88
-6	10	-5	19.30	1.04
6	-10	5	13.43	1.95
6	-10	5	17.68	0.84
6	-10	5	18.35	1.29
6	-10	5	15.75	0.95
6	-10	5	16.22	0.91
-7	10	-5	1.91	0.41
-7	10	-5	2.55	0.48
7	-10	5	1.48	0.61
7	-10	5	1.71	0.47
7	-10	5	3.66	1.26
7	-10	5	1.82	0.45
-8	10	-5	37.91	1.62
-8	10	-5	43.34	1.67
8	-10	5	40.34	1.95
8	-10	5	43.22	1.71
8	-10	5	42.11	1.77
8	-10	5	45.63	3.08
-9	10	-5	58.21	2.10
-9	10	-5	57.48	2.10
9	-10	5	51.23	2.34
9	-10	5	58.32	2.19
9	-10	5	56.24	2.28
-10	10	-5	16.85	1.06
-10	10	-5	16.82	1.12
10	-10	5	20.94	1.38
10	-10	5	17.58	2.45
10	-10	5	19.60	1.41
-11	10	-5	22.02	1.25
11	-10	5	22.40	1.51
11	-10	5	18.01	2.98
-12	10	-5	14.27	1.12
12	-10	5	10.24	2.38
12	-10	5	14.02	1.59
-13	10	-5	17.26	1.28
13	-10	5	16.34	1.92
13	-10	5	17.09	2.97
-14	10	-5	11.58	1.23
14	-10	5	10.35	1.61
14	-10	5	6.45	2.69
11	9	-5	0.25	0.74
11	9	-5	0.07	1.05
11	9	-5	0.27	1.29

11	9	-5	2.31	1.83
10	9	-5	1.20	1.64
10	9	-5	-0.02	0.61
10	9	-5	-1.39	0.86
10	9	-5	-0.70	1.06
9	9	-5	0.15	0.57
9	9	-5	0.07	0.70
9	9	-5	-0.23	1.44
8	9	-5	2.50	1.39
8	9	-5	1.22	0.84
8	9	-5	1.17	0.53
8	9	-5	0.62	0.65
8	9	-5	0.33	0.70
7	9	-5	0.85	1.57
7	9	-5	0.65	0.59
7	9	-5	0.34	0.54
7	9	-5	0.95	0.49
7	9	-5	0.67	0.50
-7	-9	5	1.25	0.98
6	9	-5	4.54	0.59
6	9	-5	4.48	0.75
6	9	-5	3.33	1.48
6	9	-5	5.15	0.67
6	9	-5	5.14	0.59
-6	-9	5	5.44	1.08
-6	-9	5	4.77	0.97
5	9	-5	14.84	0.87
5	9	-5	15.40	2.15
5	9	-5	15.33	0.90
5	9	-5	16.59	0.88
5	9	-5	17.42	0.92
-5	-9	5	15.54	1.53
-5	-9	5	16.02	1.51
4	9	-5	0.46	0.34
4	9	-5	-0.25	1.11
4	9	-5	0.31	0.68
4	9	-5	0.16	0.33
4	9	-5	0.18	0.30
-4	-9	5	0.56	0.81
-4	-9	5	0.05	0.64
-4	-9	5	0.15	0.28
3	9	-5	5.99	1.22
3	9	-5	3.68	0.55
3	9	-5	4.22	0.46

3	9	-5	4.90	0.47
3	9	-5	3.93	0.72
-3	-9	5	4.53	0.96
-3	-9	5	4.46	0.47
-3	-9	5	4.27	0.83
2	9	-5	56.43	2.85
2	9	-5	58.15	1.96
2	9	-5	54.97	2.36
-2	-9	5	53.63	2.69
-2	-9	5	58.71	1.90
-2	-9	5	55.04	2.47
1	9	-5	50.52	1.74
1	9	-5	46.93	2.10
1	9	-5	48.51	2.61
-1	-9	5	47.22	2.21
-1	-9	5	51.05	1.69
-1	-9	5	50.58	2.53
0	9	-5	11.45	0.97
0	9	-5	11.19	0.64
0	9	-5	9.45	1.47
0	-9	5	10.35	0.61
0	-9	5	11.82	1.41
0	-9	5	10.83	1.04
-1	9	-5	32.47	1.23
-1	9	-5	32.06	1.54
-1	9	-5	35.39	2.12
1	-9	5	30.31	1.64
1	-9	5	33.41	1.25
1	-9	5	30.70	2.07
-2	9	-5	43.50	1.81
-2	9	-5	45.38	2.40
-2	9	-5	43.48	1.55
2	-9	5	40.69	2.41
2	-9	5	44.31	1.96
2	-9	5	43.18	1.57
-3	9	-5	13.25	1.48
-3	9	-5	11.68	0.86
-3	9	-5	11.45	0.66
3	-9	5	12.16	1.01
3	-9	5	11.75	0.70
3	-9	5	12.12	0.55
3	-9	5	8.81	1.47
-4	9	-5	29.25	1.88
-4	9	-5	25.64	1.07

-4	9	-5	28.15	1.25
4	-9	5	25.39	1.20
4	-9	5	24.65	1.40
4	-9	5	26.09	1.11
4	-9	5	26.87	0.98
4	-9	5	25.08	2.03
-5	9	-5	11.93	0.81
-5	9	-5	11.06	0.69
-5	9	-5	12.70	1.50
5	-9	5	11.29	1.63
5	-9	5	11.96	0.70
5	-9	5	12.96	1.01
5	-9	5	13.02	0.77
-6	9	-5	20.29	0.97
-6	9	-5	20.64	1.75
-6	9	-5	20.96	1.04
6	-9	5	20.78	1.03
6	-9	5	20.78	2.01
6	-9	5	22.98	1.04
6	-9	5	21.75	0.98
6	-9	5	20.81	1.27
-7	9	-5	49.79	1.83
-7	9	-5	47.93	1.80
7	-9	5	51.43	2.08
7	-9	5	47.79	2.99
7	-9	5	45.93	1.87
7	-9	5	50.64	1.83
-8	9	-5	20.25	1.02
-8	9	-5	19.51	0.98
8	-9	5	17.93	1.13
8	-9	5	19.01	1.24
8	-9	5	20.07	1.13
8	-9	5	17.58	2.15
-9	9	-5	14.33	0.95
-9	9	-5	12.38	0.86
9	-9	5	12.24	1.08
9	-9	5	11.75	1.09
9	-9	5	13.42	2.19
9	-9	5	12.41	1.10
-10	9	-5	20.31	1.08
-10	9	-5	17.45	1.17
10	-9	5	17.36	1.34
10	-9	5	19.36	2.70
10	-9	5	18.78	1.29

-11	9	-5	30.04	1.65
-11	9	-5	30.70	1.47
11	-9	5	32.39	3.33
11	-9	5	29.75	1.80
11	-9	5	30.65	1.63
-12	9	-5	22.36	1.37
12	-9	5	27.38	1.91
12	-9	5	22.90	3.59
12	-9	5	28.40	1.71
-13	9	-5	14.18	1.17
13	-9	5	10.17	2.73
13	-9	5	14.15	1.83
-14	9	-5	11.53	1.21
14	-9	5	6.82	2.83
14	-9	5	14.57	2.04
12	8	-5	-0.53	1.42
12	8	-5	-3.43	1.74
12	8	-5	-1.04	0.70
12	8	-5	0.42	1.12
11	8	-5	2.07	1.66
11	8	-5	1.80	1.18
11	8	-5	-0.13	0.97
11	8	-5	1.10	0.65
10	8	-5	1.15	1.44
10	8	-5	0.26	0.88
10	8	-5	-0.37	0.53
9	8	-5	-0.11	1.28
9	8	-5	0.49	0.82
9	8	-5	0.67	0.48
9	8	-5	-0.36	0.67
8	8	-5	1.44	1.21
8	8	-5	-0.10	0.55
8	8	-5	-0.24	1.52
8	8	-5	1.24	0.59
8	8	-5	-0.81	0.41
8	8	-5	-0.26	0.70
7	8	-5	0.61	0.40
7	8	-5	0.08	0.46
7	8	-5	0.58	0.40
7	8	-5	0.96	0.52
7	8	-5	2.17	1.32
-7	-8	5	1.26	0.82
-7	-8	5	1.50	0.76
6	8	-5	7.90	0.70

6	8	-5	8.50	0.71
6	8	-5	9.48	1.53
6	8	-5	7.01	0.78
6	8	-5	8.05	1.50
6	8	-5	7.29	0.67
-6	-8	5	9.10	1.28
-6	-8	5	9.44	1.31
5	8	-5	5.26	0.63
5	8	-5	7.27	0.62
5	8	-5	6.18	0.58
5	8	-5	8.04	0.64
5	8	-5	5.35	0.84
5	8	-5	9.68	1.66
-5	-8	5	7.65	1.15
-5	-8	5	7.15	1.05
4	8	-5	21.36	0.88
4	8	-5	22.79	1.42
4	8	-5	20.55	0.96
4	8	-5	21.13	0.95
4	8	-5	21.14	1.89
-4	-8	5	20.39	1.51
-4	-8	5	22.48	0.89
-4	-8	5	21.21	1.46
3	8	-5	15.49	0.65
3	8	-5	15.31	0.71
3	8	-5	14.17	1.13
3	8	-5	16.12	0.78
3	8	-5	17.60	1.62
-3	-8	5	15.26	1.40
-3	-8	5	16.05	0.70
-3	-8	5	15.76	1.23
2	8	-5	5.17	0.49
2	8	-5	5.00	0.98
2	8	-5	4.93	0.79
-2	-8	5	5.23	0.42
-2	-8	5	5.07	0.77
-2	-8	5	6.45	1.12
1	8	-5	22.88	1.69
1	8	-5	22.25	0.95
1	8	-5	23.69	1.26
-1	-8	5	23.02	0.91
-1	-8	5	23.38	1.63
-1	-8	5	21.78	1.32
0	8	-5	73.61	2.34

0	8	-5	71.07	2.64
0	8	-5	73.21	3.05
0	-8	5	66.41	3.03
0	-8	5	68.50	2.72
0	-8	5	72.42	2.33
-1	8	-5	50.76	2.36
-1	8	-5	46.00	1.62
-1	8	-5	46.34	1.88
1	-8	5	46.53	1.62
1	-8	5	43.10	2.34
1	-8	5	47.17	1.98
-2	8	-5	2.51	0.36
-2	8	-5	2.12	0.43
-2	8	-5	1.51	0.73
2	-8	5	2.11	0.32
2	-8	5	2.38	0.55
2	-8	5	2.27	0.82
-3	8	-5	59.56	2.05
-3	8	-5	64.61	2.23
-3	8	-5	64.27	2.77
3	-8	5	61.21	1.96
3	-8	5	59.73	2.34
3	-8	5	61.40	2.90
3	-8	5	57.76	2.05
3	-8	5	58.45	1.94
-4	8	-5	54.93	1.96
-4	8	-5	51.15	1.84
-4	8	-5	60.15	2.58
4	-8	5	52.13	1.82
4	-8	5	52.71	1.79
4	-8	5	55.81	2.13
4	-8	5	51.84	1.85
4	-8	5	48.86	2.66
-5	8	-5	29.79	1.20
-5	8	-5	32.11	1.28
-5	8	-5	28.15	1.79
5	-8	5	30.66	1.21
5	-8	5	27.57	2.07
5	-8	5	30.96	1.45
5	-8	5	31.54	1.23
5	-8	5	30.43	1.17
-6	8	-5	75.30	2.60
-6	8	-5	71.09	3.19
-6	8	-5	86.13	2.66

6	-8	5	79.83	2.61
6	-8	5	77.12	2.65
6	-8	5	75.35	2.62
6	-8	5	79.26	2.82
6	-8	5	76.57	3.60
-7	8	-5	7.50	0.62
-7	8	-5	6.92	1.25
-7	8	-5	10.05	0.69
7	-8	5	9.85	1.54
7	-8	5	7.65	0.82
7	-8	5	9.37	0.75
7	-8	5	7.09	0.69
7	-8	5	8.50	0.77
-8	8	-5	18.62	1.03
-8	8	-5	20.22	0.99
8	-8	5	19.91	1.17
8	-8	5	21.54	2.16
8	-8	5	20.09	1.14
8	-8	5	19.40	1.13
-9	8	-5	39.63	1.63
9	-8	5	37.01	1.70
9	-8	5	32.81	1.65
9	-8	5	35.56	1.63
-10	8	-5	15.73	1.16
-10	8	-5	16.39	0.97
10	-8	5	14.59	1.11
10	-8	5	8.67	1.90
10	-8	5	13.81	1.22
-11	8	-5	20.87	1.57
-11	8	-5	25.92	1.28
11	-8	5	23.18	1.38
11	-8	5	24.85	3.12
11	-8	5	24.75	1.64
-12	8	-5	27.34	1.39
12	-8	5	24.65	1.46
12	-8	5	25.88	1.85
12	-8	5	28.61	3.55
-13	8	-5	18.93	1.33
13	-8	5	20.19	1.94
13	-8	5	24.98	3.80
-14	8	-5	14.53	1.32
14	-8	5	14.57	2.05
14	-8	5	25.86	4.38
13	7	-5	1.57	1.64

13	7	-5	-0.65	1.62
13	7	-5	0.97	1.22
13	7	-5	1.29	0.73
12	7	-5	1.65	1.59
12	7	-5	-2.61	0.97
12	7	-5	-0.71	1.23
12	7	-5	0.35	0.62
11	7	-5	-0.99	1.34
11	7	-5	-0.37	0.80
11	7	-5	1.16	0.58
11	7	-5	0.34	1.14
10	7	-5	-0.10	1.25
10	7	-5	1.01	0.74
10	7	-5	1.43	0.81
10	7	-5	0.98	0.47
9	7	-5	4.63	0.79
9	7	-5	4.89	0.92
9	7	-5	4.90	1.36
9	7	-5	2.42	0.68
9	7	-5	4.75	0.59
9	7	-5	2.09	1.53
8	7	-5	2.17	0.45
8	7	-5	0.69	0.50
8	7	-5	1.79	1.25
8	7	-5	1.99	0.50
8	7	-5	1.23	1.09
8	7	-5	1.77	0.59
8	7	-5	0.96	0.66
7	7	-5	1.21	0.49
7	7	-5	0.69	0.35
7	7	-5	-0.01	1.09
7	7	-5	0.40	0.57
7	7	-5	0.13	0.68
7	7	-5	0.45	0.41
7	7	-5	0.24	0.33
-7	-7	5	0.23	0.67
6	7	-5	13.45	0.74
6	7	-5	11.58	0.87
6	7	-5	14.94	0.77
6	7	-5	10.97	1.18
6	7	-5	13.21	0.75
6	7	-5	15.38	1.75
-6	-7	5	13.80	1.29
-6	-7	5	13.93	1.26

5	7	-5	30.41	1.14
5	7	-5	30.48	1.20
5	7	-5	28.56	1.26
5	7	-5	31.52	1.61
5	7	-5	28.26	1.97
5	7	-5	30.48	1.20
-5	-7	5	31.54	1.72
-5	-7	5	29.65	1.68
4	7	-5	5.46	0.83
4	7	-5	3.82	0.93
4	7	-5	4.77	0.39
4	7	-5	4.82	0.54
4	7	-5	4.13	0.46
-4	-7	5	3.90	0.68
-4	-7	5	4.85	0.81
-4	-7	5	5.13	0.41
3	7	-5	3.25	0.42
3	7	-5	2.89	0.39
3	7	-5	2.25	0.51
3	7	-5	3.04	0.29
3	7	-5	2.76	0.79
-3	-7	5	2.77	0.71
-3	-7	5	2.86	0.60
-3	-7	5	2.55	0.34
2	7	-5	32.96	1.80
2	7	-5	31.17	1.19
2	7	-5	31.60	1.47
-2	-7	5	33.27	1.15
-2	-7	5	29.32	1.69
-2	-7	5	30.52	1.53
1	7	-5	88.41	3.11
1	7	-5	84.73	2.83
1	7	-5	98.84	3.48
-1	-7	5	88.45	2.81
-1	-7	5	87.96	3.42
-1	-7	5	82.94	3.16
0	7	-5	9.62	0.75
0	7	-5	7.91	1.05
0	7	-5	9.26	0.53
0	-7	5	8.70	0.80
0	-7	5	9.40	1.10
0	-7	5	9.47	0.54
-1	7	-5	11.01	0.58
-1	7	-5	11.74	1.16

-1	7	-5	12.44	0.79
1	-7	5	11.26	0.60
1	-7	5	11.19	0.84
1	-7	5	11.59	1.18
-2	7	-5	57.23	1.91
-2	7	-5	58.25	2.07
-2	7	-5	58.19	2.49
2	-7	5	56.38	1.83
2	-7	5	56.76	1.79
2	-7	5	56.36	2.17
2	-7	5	58.00	1.93
2	-7	5	53.09	2.55
-3	7	-5	48.06	1.67
-3	7	-5	42.25	1.53
-3	7	-5	44.93	2.09
3	-7	5	41.71	2.22
3	-7	5	45.75	1.78
3	-7	5	45.43	1.49
3	-7	5	37.57	1.64
-4	7	-5	66.39	2.75
-4	7	-5	65.53	2.20
-4	7	-5	68.64	2.27
4	-7	5	60.90	2.94
4	-7	5	66.57	2.17
4	-7	5	66.53	2.17
4	-7	5	68.04	2.22
4	-7	5	60.87	2.37
-5	7	-5	63.44	2.16
-5	7	-5	65.91	2.20
-5	7	-5	60.39	2.67
5	-7	5	65.10	2.34
5	-7	5	64.76	2.18
5	-7	5	64.07	2.16
5	-7	5	63.74	2.18
5	-7	5	61.62	2.98
-6	7	-5	10.49	1.18
-6	7	-5	10.93	0.67
-6	7	-5	8.96	0.62
6	-7	5	9.63	0.66
6	-7	5	10.79	0.72
6	-7	5	10.15	0.72
6	-7	5	8.90	1.42
6	-7	5	10.25	0.79
-7	7	-5	59.90	2.11

-7	7	-5	61.73	2.15
-7	7	-5	59.35	2.67
7	-7	5	57.39	3.07
7	-7	5	63.58	2.21
7	-7	5	62.48	2.26
7	-7	5	60.06	2.24
7	-7	5	59.39	2.14
-8	7	-5	42.67	1.69
-8	7	-5	48.31	1.79
8	-7	5	46.11	1.85
8	-7	5	45.60	2.86
8	-7	5	43.63	1.82
8	-7	5	46.92	1.89
-9	7	-5	10.36	1.26
-9	7	-5	8.36	0.75
9	-7	5	7.99	1.72
9	-7	5	13.01	1.03
9	-7	5	11.44	1.08
9	-7	5	10.31	0.91
-10	7	-5	11.92	0.85
-10	7	-5	12.30	1.25
10	-7	5	7.12	1.85
10	-7	5	10.30	1.00
10	-7	5	12.46	1.13
-11	7	-5	25.53	1.31
-11	7	-5	24.92	1.78
11	-7	5	26.93	1.67
11	-7	5	26.58	3.20
11	-7	5	24.65	1.37
-12	7	-5	19.00	1.20
12	-7	5	22.31	1.76
12	-7	5	23.90	3.41
12	-7	5	18.98	1.30
-13	7	-5	14.95	1.18
13	-7	5	15.08	2.89
13	-7	5	13.61	1.81
-14	7	-5	11.51	1.26
14	-7	5	13.23	3.08
14	-7	5	11.48	1.59
14	6	-5	0.82	1.21
14	6	-5	0.03	0.70
14	6	-5	3.45	1.77
13	6	-5	-0.68	1.43
13	6	-5	-0.24	0.61

13	6	-5	-1.12	1.41
13	6	-5	1.60	1.13
12	6	-5	2.16	0.61
12	6	-5	1.74	1.17
12	6	-5	0.51	0.96
11	6	-5	2.06	0.80
11	6	-5	3.56	0.59
11	6	-5	3.65	1.04
11	6	-5	1.13	1.26
10	6	-5	2.47	0.86
10	6	-5	1.72	1.49
10	6	-5	2.04	1.17
10	6	-5	1.35	0.47
10	6	-5	0.36	0.66
9	6	-5	0.20	1.01
9	6	-5	0.01	0.58
9	6	-5	0.60	0.56
9	6	-5	0.35	1.26
9	6	-5	-0.15	0.65
9	6	-5	0.62	0.38
9	6	-5	-0.41	0.73
8	6	-5	16.54	0.89
8	6	-5	14.75	1.33
8	6	-5	16.19	1.14
8	6	-5	18.46	0.88
8	6	-5	15.92	0.95
8	6	-5	17.32	1.21
8	6	-5	17.92	1.99
-8	-6	5	16.78	1.41
7	6	-5	22.03	0.96
7	6	-5	21.68	0.96
7	6	-5	21.66	1.16
7	6	-5	21.04	1.84
7	6	-5	15.95	1.31
7	6	-5	20.87	1.23
7	6	-5	22.22	0.99
-7	-6	5	23.06	1.50
6	6	-5	11.71	0.66
6	6	-5	12.00	0.82
6	6	-5	13.39	1.49
6	6	-5	9.27	1.02
6	6	-5	11.69	0.67
6	6	-5	11.52	0.69
-6	-6	5	14.37	1.08

-6	-6	5	13.16	1.14
5	6	-5	8.83	0.56
5	6	-5	9.83	0.52
5	6	-5	9.13	0.67
5	6	-5	7.77	1.09
5	6	-5	9.00	0.60
5	6	-5	10.37	0.93
-5	-6	5	8.49	0.93
-5	-6	5	7.73	0.96
4	6	-5	48.24	1.72
4	6	-5	47.88	1.99
4	6	-5	49.11	1.68
4	6	-5	48.86	1.59
4	6	-5	48.03	2.22
-4	-6	5	47.35	2.07
-4	-6	5	47.82	2.07
-4	-6	5	49.98	1.63
3	6	-5	38.94	1.40
3	6	-5	40.31	1.68
3	6	-5	39.96	1.38
3	6	-5	40.04	1.28
-3	-6	5	40.86	1.36
-3	-6	5	39.55	1.75
-3	-6	5	37.19	1.78
2	6	-5	24.53	0.78
2	6	-5	25.31	1.19
2	6	-5	25.38	1.44
2	6	-5	23.93	0.94
2	6	-5	22.89	0.87
-2	-6	5	23.29	1.23
-2	-6	5	21.43	1.34
-2	-6	5	25.64	0.91
1	6	-5	57.55	2.32
1	6	-5	55.77	1.85
1	6	-5	55.82	2.06
-1	-6	5	54.50	1.82
-1	-6	5	54.89	2.33
-1	-6	5	53.03	2.11
0	6	-5	13.21	0.76
0	6	-5	12.62	0.60
0	-6	5	12.34	0.60
0	-6	5	12.86	0.83
0	-6	5	10.99	1.06
-1	6	-5	87.22	2.83

-1	6	-5	88.01	2.69
-1	6	-5	90.52	3.29
-1	6	-5	87.76	2.97
1	-6	5	84.23	3.05
1	-6	5	91.01	2.78
1	-6	5	90.38	2.84
1	-6	5	94.25	3.44
-2	6	-5	30.06	1.12
-2	6	-5	31.11	1.23
-2	6	-5	32.87	1.58
-2	6	-5	29.96	1.01
2	-6	5	28.15	1.65
2	-6	5	29.65	1.31
2	-6	5	30.19	1.09
2	-6	5	32.04	1.15
-3	6	-5	76.97	2.68
-3	6	-5	83.31	2.60
-3	6	-5	82.47	2.76
-3	6	-5	82.03	3.12
3	-6	5	86.04	2.87
3	-6	5	89.12	3.38
3	-6	5	83.35	2.67
3	-6	5	85.21	2.70
-4	6	-5	114.47	4.01
-4	6	-5	110.36	3.57
-4	6	-5	111.20	3.46
-4	6	-5	108.17	3.53
4	-6	5	112.02	3.68
4	-6	5	109.17	4.22
4	-6	5	111.39	3.54
4	-6	5	114.51	3.55
-5	6	-5	89.66	2.94
-5	6	-5	95.23	2.98
-5	6	-5	89.44	3.40
-5	6	-5	90.60	2.89
5	-6	5	91.27	3.06
5	-6	5	85.32	3.69
5	-6	5	89.99	2.95
5	-6	5	91.99	2.97
-6	6	-5	67.46	2.78
-6	6	-5	68.68	2.32
-6	6	-5	70.81	2.36
-6	6	-5	68.57	2.29
6	-6	5	72.40	2.39

6	-6	5	66.52	2.41
6	-6	5	69.27	2.34
6	-6	5	64.82	3.14
-7	6	-5	43.03	1.70
-7	6	-5	44.19	1.63
-7	6	-5	45.34	2.13
7	-6	5	43.63	1.67
7	-6	5	48.14	1.77
7	-6	5	43.78	1.72
7	-6	5	41.13	2.53
-8	6	-5	82.95	2.84
-8	6	-5	79.19	2.71
8	-6	5	80.13	2.79
8	-6	5	79.18	3.81
8	-6	5	79.40	2.90
8	-6	5	80.48	2.87
-9	6	-5	32.51	1.63
-9	6	-5	31.59	1.35
9	-6	5	29.31	2.59
9	-6	5	32.76	1.61
9	-6	5	32.65	1.43
-10	6	-5	35.71	1.94
-10	6	-5	37.16	1.55
10	-6	5	38.47	1.63
10	-6	5	39.01	3.00
-11	6	-5	29.81	1.98
-11	6	-5	28.02	1.35
11	-6	5	27.28	3.08
11	-6	5	30.51	1.45
11	-6	5	25.83	1.69
-12	6	-5	17.88	1.19
12	-6	5	20.01	1.26
12	-6	5	19.40	3.17
12	-6	5	19.88	1.69
-13	6	-5	23.40	1.38
13	-6	5	23.30	2.03
13	-6	5	25.93	3.74
-14	6	-5	9.89	1.22
14	-6	5	5.13	2.55
14	-6	5	14.32	2.02
14	5	-5	-0.03	1.18
14	5	-5	-0.92	1.47
14	5	-5	0.07	0.68
13	5	-5	2.01	1.37

13	5	-5	1.21	1.02
13	5	-5	1.73	1.32
13	5	-5	2.06	0.59
12	5	-5	2.71	1.12
12	5	-5	3.35	0.61
12	5	-5	4.24	1.04
12	5	-5	4.73	1.42
11	5	-5	2.30	1.97
11	5	-5	1.64	0.91
11	5	-5	1.76	1.13
11	5	-5	1.90	0.83
11	5	-5	0.90	0.45
10	5	-5	12.40	1.23
10	5	-5	12.36	2.19
10	5	-5	12.11	1.81
10	5	-5	11.13	1.31
10	5	-5	12.12	0.81
10	5	-5	13.12	1.30
9	5	-5	13.81	1.29
9	5	-5	10.85	1.74
9	5	-5	17.36	1.22
9	5	-5	18.23	1.00
9	5	-5	16.85	2.01
9	5	-5	14.72	0.83
9	5	-5	14.63	1.18
8	5	-5	1.28	0.57
8	5	-5	1.59	0.53
8	5	-5	1.95	0.39
8	5	-5	1.46	0.34
8	5	-5	2.01	0.36
8	5	-5	1.62	0.55
8	5	-5	2.39	0.96
-8	-5	5	1.13	0.61
7	5	-5	5.75	0.85
7	5	-5	5.46	1.00
7	5	-5	6.31	0.75
7	5	-5	6.55	0.52
7	5	-5	6.56	0.73
7	5	-5	6.91	0.54
7	5	-5	5.25	0.54
-7	-5	5	5.22	0.75
6	5	-5	28.44	1.12
6	5	-5	27.95	1.25
6	5	-5	27.84	1.09

6	5	-5	29.45	1.09
6	5	-5	28.79	1.42
6	5	-5	27.22	1.66
-6	-5	5	28.83	1.51
-6	-5	5	28.51	1.39
5	5	-5	46.69	1.55
5	5	-5	45.80	1.88
5	5	-5	48.59	1.63
5	5	-5	45.38	1.58
5	5	-5	46.74	1.70
-5	-5	5	43.25	1.94
-5	-5	5	46.49	1.88
4	5	-5	22.47	0.82
4	5	-5	22.11	1.34
4	5	-5	24.50	1.18
4	5	-5	23.94	0.99
4	5	-5	22.30	0.90
-4	-5	5	23.14	0.87
-4	-5	5	22.50	1.21
-4	-5	5	22.87	1.24
3	5	-5	49.09	1.75
3	5	-5	54.89	1.99
3	5	-5	54.30	2.14
3	5	-5	51.82	1.75
-3	-5	5	54.40	2.10
-3	-5	5	51.47	2.04
-3	-5	5	50.55	1.69
-3	-5	5	52.73	1.71
2	5	-5	65.29	2.46
2	5	-5	62.57	2.10
2	5	-5	64.26	2.08
2	5	-5	68.19	2.32
-2	-5	5	66.04	2.08
-2	-5	5	66.12	2.03
-2	-5	5	60.99	2.49
-2	-5	5	64.23	2.36
1	5	-5	1.77	0.47
1	5	-5	0.60	0.25
1	5	-5	1.69	0.24
1	5	-5	1.41	0.20
-1	-5	5	1.41	0.52
-1	-5	5	1.49	0.22
-1	-5	5	1.29	0.21
-1	-5	5	1.23	0.35

0	5	-5	17.21	1.02
0	5	-5	16.04	0.58
0	5	-5	16.25	0.79
0	5	-5	15.81	0.68
0	-5	5	15.06	1.10
0	-5	5	15.95	0.65
0	-5	5	16.93	0.69
0	-5	5	15.32	0.84
-1	5	-5	85.37	2.63
-1	5	-5	81.89	3.07
-1	5	-5	84.61	2.82
-1	5	-5	86.87	2.72
1	-5	5	91.32	2.72
1	-5	5	87.01	2.73
1	-5	5	82.22	3.16
1	-5	5	84.36	2.89
-2	5	-5	146.31	4.62
-2	5	-5	159.48	4.71
-2	5	-5	152.02	4.98
-2	5	-5	143.71	4.56
2	-5	5	142.66	5.11
2	-5	5	151.71	4.63
2	-5	5	148.73	4.62
2	-5	5	154.63	4.78
-3	5	-5	24.53	0.92
-3	5	-5	28.48	1.04
-3	5	-5	23.94	0.96
-3	5	-5	24.88	1.32
3	-5	5	24.26	1.48
3	-5	5	25.17	0.99
3	-5	5	25.34	1.00
3	-5	5	25.22	1.08
-4	5	-5	65.72	2.24
-4	5	-5	69.34	2.62
-4	5	-5	70.14	2.29
-4	5	-5	66.50	2.21
4	-5	5	69.86	2.28
4	-5	5	68.44	2.25
4	-5	5	63.98	2.83
4	-5	5	69.07	2.35
-5	5	-5	53.44	1.90
-5	5	-5	55.25	1.89
-5	5	-5	56.30	2.31
-5	5	-5	60.05	1.98

5	-5	5	56.80	1.99
5	-5	5	55.56	1.92
5	-5	5	56.57	2.63
5	-5	5	54.50	2.00
-6	5	-5	31.24	1.22
-6	5	-5	29.75	1.29
-6	5	-5	30.02	1.62
-6	5	-5	31.27	1.22
6	-5	5	32.79	1.34
6	-5	5	30.91	1.30
6	-5	5	31.93	1.24
6	-5	5	34.48	2.05
-7	5	-5	45.23	2.22
-7	5	-5	53.54	1.86
-7	5	-5	50.60	1.95
-7	5	-5	50.35	1.82
7	-5	5	51.99	1.87
7	-5	5	54.39	1.93
7	-5	5	53.28	1.98
7	-5	5	48.57	2.72
-8	5	-5	64.47	2.31
-8	5	-5	71.35	2.56
-8	5	-5	67.88	2.36
8	-5	5	66.54	2.41
8	-5	5	66.30	2.51
8	-5	5	64.41	3.39
-9	5	-5	23.23	1.51
-9	5	-5	22.94	1.09
9	-5	5	23.96	1.34
9	-5	5	22.18	2.38
9	-5	5	22.15	1.19
-10	5	-5	37.11	2.11
-10	5	-5	38.25	1.57
10	-5	5	38.67	3.07
10	-5	5	37.16	1.67
10	-5	5	39.69	1.89
-11	5	-5	36.51	2.28
-11	5	-5	32.55	1.48
11	-5	5	28.60	3.13
11	-5	5	33.72	1.90
11	-5	5	33.97	1.60
-12	5	-5	15.70	1.12
12	-5	5	11.74	2.36
12	-5	5	15.79	1.60

12	-5	5	15.96	1.21
-13	5	-5	16.38	1.26
13	-5	5	17.18	1.83
13	-5	5	12.51	2.71
-14	5	-5	8.50	1.21
14	-5	5	6.08	2.41
14	-5	5	7.30	1.48
15	4	-5	0.22	1.25
15	4	-5	4.58	1.84
15	4	-5	0.96	0.70
14	4	-5	0.64	0.62
14	4	-5	-1.75	1.00
14	4	-5	-0.48	1.43
14	4	-5	-0.62	1.25
13	4	-5	0.39	0.92
13	4	-5	2.47	0.59
13	4	-5	4.31	1.29
12	4	-5	3.38	0.57
12	4	-5	2.63	0.89
12	4	-5	3.03	1.09
12	4	-5	5.17	1.29
12	4	-5	4.69	1.00
11	4	-5	13.72	1.35
11	4	-5	14.99	2.90
11	4	-5	14.03	0.93
11	4	-5	15.20	1.35
11	4	-5	16.06	1.86
11	4	-5	13.00	1.47
10	4	-5	6.36	1.07
10	4	-5	8.38	0.68
10	4	-5	9.16	1.47
10	4	-5	8.98	1.27
10	4	-5	6.81	1.14
10	4	-5	7.68	1.05
9	4	-5	1.48	0.36
9	4	-5	2.15	0.98
9	4	-5	1.94	0.61
9	4	-5	1.42	0.43
9	4	-5	2.07	0.36
9	4	-5	2.01	0.61
9	4	-5	3.18	0.64
-9	-4	5	2.23	0.63
8	4	-5	35.93	1.60
8	4	-5	35.73	1.34

8	4	-5	35.15	1.33
8	4	-5	35.27	1.38
8	4	-5	34.90	2.01
8	4	-5	34.56	1.54
8	4	-5	38.11	1.65
-8	-4	5	34.36	1.72
7	4	-5	15.57	0.96
7	4	-5	16.11	0.92
7	4	-5	14.10	1.27
7	4	-5	14.79	1.01
7	4	-5	16.50	0.77
7	4	-5	16.66	0.74
7	4	-5	16.67	0.77
-7	-4	5	15.33	1.11
6	4	-5	11.48	0.58
6	4	-5	10.23	0.61
6	4	-5	11.59	0.84
6	4	-5	11.13	0.74
6	4	-5	11.36	0.57
6	4	-5	10.96	0.70
6	4	-5	10.53	1.05
-6	-4	5	10.50	0.90
5	4	-5	31.28	1.29
5	4	-5	31.86	1.39
5	4	-5	32.49	1.54
5	4	-5	32.02	1.17
5	4	-5	33.92	1.22
-5	-4	5	34.54	1.40
-5	-4	5	31.36	1.46
4	4	-5	89.32	2.95
4	4	-5	88.96	2.85
4	4	-5	91.05	3.15
4	4	-5	93.86	3.07
-4	-4	5	91.12	2.87
-4	-4	5	89.69	3.03
-4	-4	5	92.31	2.83
-4	-4	5	87.54	3.12
3	4	-5	7.55	0.56
3	4	-5	6.56	0.47
3	4	-5	6.34	0.66
3	4	-5	7.25	0.41
-3	-4	5	7.12	0.39
-3	-4	5	6.81	0.63
-3	-4	5	7.10	0.41

-3	-4	5	6.73	0.60
2	4	-5	41.06	1.43
2	4	-5	45.45	1.58
2	4	-5	44.12	1.65
2	4	-5	41.92	1.42
-2	-4	5	42.12	1.40
-2	-4	5	42.51	1.40
-2	-4	5	39.34	1.64
-2	-4	5	38.42	1.59
1	4	-5	19.40	0.75
1	4	-5	18.56	0.75
1	4	-5	19.24	0.85
1	4	-5	18.82	0.96
-1	-4	5	19.24	1.04
-1	-4	5	18.28	0.90
-1	-4	5	18.87	0.74
-1	-4	5	19.18	0.74
0	4	-5	39.25	1.43
0	4	-5	36.46	1.34
0	4	-5	38.80	1.32
0	4	-5	41.36	1.58
0	-4	5	40.93	1.35
0	-4	5	39.00	1.38
0	-4	5	42.29	1.51
-1	4	-5	141.12	4.04
-1	4	-5	121.67	3.90
-1	4	-5	129.74	4.19
-1	4	-5	121.61	3.93
1	-4	5	134.62	4.34
1	-4	5	119.79	4.05
1	-4	5	124.63	3.98
-2	4	-5	114.63	3.59
-2	4	-5	111.94	3.61
-2	4	-5	119.08	3.89
-2	4	-5	119.35	3.68
2	-4	5	116.78	3.73
2	-4	5	114.31	4.04
2	-4	5	115.84	3.66
2	-4	5	117.34	3.62
-3	4	-5	106.16	3.53
-3	4	-5	108.35	3.30
-3	4	-5	99.00	3.21
-3	4	-5	99.69	3.23
3	-4	5	103.34	3.24

3	-4	5	107.38	3.35
3	-4	5	99.13	3.38
3	-4	5	98.33	3.70
-4	4	-5	19.53	0.83
-4	4	-5	20.84	1.15
-4	4	-5	19.28	0.83
-4	4	-5	20.78	0.91
4	-4	5	19.76	0.92
4	-4	5	20.06	0.84
4	-4	5	22.48	1.41
4	-4	5	20.37	0.92
-5	4	-5	68.10	2.28
-5	4	-5	68.47	2.29
-5	4	-5	71.29	2.38
-5	4	-5	66.18	2.60
5	-4	5	67.97	2.29
5	-4	5	71.17	2.38
5	-4	5	67.79	2.95
5	-4	5	68.79	2.38
-6	4	-5	86.10	2.83
-6	4	-5	87.54	2.99
-6	4	-5	84.30	3.16
-6	4	-5	88.22	2.87
6	-4	5	88.90	2.94
6	-4	5	85.67	3.63
6	-4	5	87.19	2.85
6	-4	5	86.30	2.97
-7	4	-5	53.50	1.99
-7	4	-5	45.26	2.09
-7	4	-5	47.10	1.75
-7	4	-5	48.04	1.71
7	-4	5	46.73	2.58
7	-4	5	43.82	1.79
7	-4	5	46.45	1.73
7	-4	5	47.85	1.90
-8	4	-5	77.68	2.57
-8	4	-5	80.86	2.93
-8	4	-5	77.51	2.64
8	-4	5	76.19	3.64
8	-4	5	63.13	2.62
8	-4	5	79.90	2.83
-9	4	-5	27.78	1.22
-9	4	-5	31.01	1.83
-9	4	-5	28.49	1.31

9	-4	5	24.30	1.31
-10	4	-5	24.22	1.16
-10	4	-5	25.93	1.95
10	-4	5	25.77	1.53
10	-4	5	19.19	1.24
10	-4	5	23.37	2.57
-11	4	-5	12.49	2.06
-11	4	-5	17.38	1.04
11	-4	5	12.81	1.13
11	-4	5	11.76	2.14
11	-4	5	16.83	1.46
-12	4	-5	17.93	1.21
12	-4	5	17.41	1.33
12	-4	5	20.23	3.22
12	-4	5	19.98	1.76
-13	4	-5	11.01	1.16
13	-4	5	12.59	2.61
13	-4	5	12.81	1.75
-14	4	-5	10.62	1.27
14	-4	5	9.41	2.86
14	-4	5	6.05	1.48
15	3	-5	1.47	1.24
15	3	-5	0.42	1.65
15	3	-5	1.71	0.70
14	3	-5	1.00	1.39
14	3	-5	0.44	1.06
14	3	-5	0.96	0.57
14	3	-5	1.14	1.00
13	3	-5	3.45	0.99
13	3	-5	3.87	1.25
13	3	-5	3.51	1.12
13	3	-5	2.57	0.56
12	3	-5	2.01	0.79
12	3	-5	3.15	0.53
12	3	-5	1.22	1.46
12	3	-5	5.48	1.17
12	3	-5	2.41	0.96
12	3	-5	5.04	0.85
11	3	-5	0.62	0.84
11	3	-5	0.68	0.61
11	3	-5	-0.75	0.61
11	3	-5	0.79	0.75
11	3	-5	0.82	0.38
11	3	-5	0.08	1.18

10	3	-5	17.45	1.62
10	3	-5	20.69	0.95
10	3	-5	19.51	1.31
10	3	-5	19.60	1.06
10	3	-5	18.31	1.37
10	3	-5	21.25	1.26
10	3	-5	17.31	1.86
9	3	-5	35.13	1.34
9	3	-5	36.34	1.60
9	3	-5	33.85	1.64
9	3	-5	34.97	1.36
9	3	-5	37.85	2.02
9	3	-5	35.15	1.67
9	3	-5	35.62	1.42
-9	-3	5	34.58	1.69
8	3	-5	26.56	1.28
8	3	-5	26.61	1.06
8	3	-5	25.30	1.24
8	3	-5	27.05	1.10
8	3	-5	25.93	1.55
8	3	-5	26.02	1.04
8	3	-5	25.64	1.31
-8	-3	5	26.44	1.38
7	3	-5	15.73	0.91
7	3	-5	15.46	0.83
7	3	-5	13.52	1.27
7	3	-5	14.98	0.69
7	3	-5	15.18	0.72
7	3	-5	14.88	0.71
7	3	-5	15.22	0.91
-7	-3	5	15.06	1.00
6	3	-5	19.33	1.09
6	3	-5	18.65	0.85
6	3	-5	17.10	0.73
6	3	-5	17.14	0.90
6	3	-5	19.11	0.79
6	3	-5	18.21	0.93
-6	-3	5	15.92	0.98
-6	-3	5	17.11	0.84
5	3	-5	63.42	2.05
5	3	-5	62.01	2.17
5	3	-5	64.60	2.20
5	3	-5	61.47	2.03
5	3	-5	58.98	2.24

5	3	-5	64.36	2.07
-5	-3	5	62.47	2.28
4	3	-5	6.23	0.51
4	3	-5	6.54	0.38
4	3	-5	6.61	0.58
4	3	-5	6.91	0.53
-4	-3	5	6.04	0.57
-4	-3	5	6.28	0.44
-4	-3	5	6.66	0.37
-4	-3	5	7.20	0.49
3	3	-5	144.60	4.56
3	3	-5	148.51	4.72
3	3	-5	147.56	4.48
3	3	-5	145.88	4.58
-3	-3	5	141.41	4.65
-3	-3	5	148.11	4.56
-3	-3	5	148.32	4.52
-3	-3	5	147.00	4.46
2	3	-5	40.91	1.41
2	3	-5	42.35	1.50
2	3	-5	43.24	1.49
-2	-3	5	42.23	1.45
-2	-3	5	42.98	1.55
-2	-3	5	41.70	1.56
1	3	-5	2.10	0.28
1	3	-5	1.30	0.27
1	3	-5	1.51	0.29
1	3	-5	2.02	0.25
-1	-3	5	1.88	0.37
-1	-3	5	2.03	0.32
-1	-3	5	1.49	0.25
-1	-3	5	1.83	0.31
0	3	-5	129.92	4.06
0	3	-5	116.18	4.08
0	3	-5	127.92	3.96
0	3	-5	130.44	3.93
0	-3	5	129.68	4.04
0	-3	5	131.68	4.18
0	-3	5	128.09	4.02
-1	3	-5	77.72	2.55
-1	3	-5	73.69	2.38
-1	3	-5	73.28	2.41
1	-3	5	78.33	2.49
1	-3	5	76.25	2.38

1	-3	5	73.80	2.47
1	-3	5	71.67	2.67
-2	3	-5	41.01	1.46
-2	3	-5	42.41	1.45
-2	3	-5	45.52	1.66
-2	3	-5	43.57	1.52
2	-3	5	41.74	1.54
2	-3	5	42.55	1.82
2	-3	5	45.46	1.46
2	-3	5	41.77	1.54
-3	3	-5	92.26	3.06
-3	3	-5	89.56	2.82
-3	3	-5	87.58	2.91
-3	3	-5	86.77	2.84
3	-3	5	89.14	2.92
3	-3	5	91.86	3.31
3	-3	5	90.76	2.83
3	-3	5	83.93	2.90
-4	3	-5	78.64	2.57
-4	3	-5	76.94	2.53
-4	3	-5	82.85	2.67
-4	3	-5	78.19	2.79
4	-3	5	69.42	2.59
4	-3	5	81.59	2.66
4	-3	5	76.03	3.07
4	-3	5	83.34	2.55
-5	3	-5	37.91	1.54
-5	3	-5	37.65	1.37
-5	3	-5	38.22	1.67
-5	3	-5	38.15	1.42
5	-3	5	37.88	2.03
5	-3	5	39.85	1.40
5	-3	5	35.87	1.46
5	-3	5	39.61	1.53
-6	3	-5	48.57	1.73
-6	3	-5	46.49	1.66
-6	3	-5	48.79	1.94
-6	3	-5	45.65	1.98
6	-3	5	47.69	1.68
6	-3	5	49.53	1.85
6	-3	5	43.50	1.75
-7	3	-5	77.89	2.54
-7	3	-5	71.15	2.90
-7	3	-5	77.59	2.62

-7	3	-5	82.06	2.91
7	-3	5	81.54	2.78
7	-3	5	67.88	2.63
7	-3	5	76.90	2.57
7	-3	5	72.76	3.40
-8	3	-5	60.81	2.54
-8	3	-5	59.19	2.09
-8	3	-5	56.17	1.99
8	-3	5	50.01	2.08
8	-3	5	55.09	3.00
-9	3	-5	18.15	1.76
-9	3	-5	18.50	1.32
-9	3	-5	20.82	0.98
9	-3	5	16.92	1.10
9	-3	5	22.47	2.30
-10	3	-5	11.46	0.85
-10	3	-5	11.29	0.96
-10	3	-5	12.72	1.95
10	-3	5	10.43	1.00
10	-3	5	9.07	1.79
-11	3	-5	22.89	1.20
-11	3	-5	22.97	2.37
11	-3	5	21.60	1.63
11	-3	5	17.73	2.78
11	-3	5	18.46	1.30
-12	3	-5	15.88	1.15
12	-3	5	13.41	2.42
12	-3	5	14.52	1.28
12	-3	5	15.36	1.65
-13	3	-5	3.36	0.77
13	-3	5	3.26	2.20
13	-3	5	2.65	1.14
16	2	-5	3.14	1.33
16	2	-5	3.66	1.93
16	2	-5	2.60	0.73
15	2	-5	4.11	1.81
15	2	-5	1.97	0.64
15	2	-5	2.96	1.16
14	2	-5	0.51	1.51
14	2	-5	2.01	0.55
14	2	-5	2.46	0.99
14	2	-5	2.75	1.00
13	2	-5	5.79	0.77
13	2	-5	5.27	0.98

13	2	-5	4.85	1.30
13	2	-5	6.50	1.12
12	2	-5	0.02	0.38
12	2	-5	2.21	0.91
12	2	-5	1.62	2.00
12	2	-5	0.84	0.89
12	2	-5	-0.17	0.57
12	2	-5	0.74	0.73
11	2	-5	5.05	0.83
11	2	-5	4.00	0.88
11	2	-5	3.35	0.73
11	2	-5	3.44	0.91
11	2	-5	3.99	0.56
11	2	-5	5.46	1.20
10	2	-5	16.13	1.03
10	2	-5	16.35	0.84
10	2	-5	17.71	1.23
10	2	-5	16.16	1.42
10	2	-5	18.39	1.62
10	2	-5	17.61	0.97
10	2	-5	19.83	1.24
10	2	-5	16.15	1.26
9	2	-5	13.92	1.29
9	2	-5	11.53	0.97
9	2	-5	11.27	0.96
9	2	-5	11.10	0.74
9	2	-5	11.80	0.84
9	2	-5	11.78	0.68
9	2	-5	12.10	0.66
-9	-2	5	10.87	0.93
8	2	-5	68.05	2.53
8	2	-5	63.77	2.14
8	2	-5	63.27	2.15
8	2	-5	66.46	2.42
8	2	-5	64.65	2.28
8	2	-5	65.23	2.19
8	2	-5	63.07	2.33
-8	-2	5	62.96	2.38
7	2	-5	68.09	2.35
7	2	-5	67.61	2.35
7	2	-5	68.10	2.45
7	2	-5	68.81	2.24
7	2	-5	67.94	2.22
7	2	-5	68.57	2.50

-7	-2	5	66.13	2.46
-7	-2	5	70.04	2.37
6	2	-5	37.02	1.29
6	2	-5	36.10	1.45
6	2	-5	37.64	1.46
6	2	-5	36.17	1.31
6	2	-5	34.48	1.25
-6	-2	5	38.07	1.40
-6	-2	5	34.27	1.46
5	2	-5	73.13	2.36
5	2	-5	73.14	2.46
5	2	-5	75.67	2.54
5	2	-5	74.11	2.47
5	2	-5	77.47	2.40
5	2	-5	74.09	2.34
-5	-2	5	73.28	2.54
4	2	-5	106.88	3.18
4	2	-5	103.58	3.26
4	2	-5	108.11	3.36
4	2	-5	104.11	3.40
-4	-2	5	106.17	3.25
-4	-2	5	110.33	3.37
-4	-2	5	98.09	3.40
3	2	-5	43.91	1.45
3	2	-5	44.28	1.53
-3	-2	5	43.62	1.54
-3	-2	5	43.72	1.58
2	2	-5	231.69	7.06
2	2	-5	234.76	7.10
2	2	-5	231.68	7.22
2	2	-5	236.49	7.17
-2	-2	5	241.20	7.09
-2	-2	5	233.40	7.21
-2	-2	5	235.60	7.14
-2	-2	5	243.88	7.21
1	2	-5	124.67	4.00
1	2	-5	122.79	3.89
1	2	-5	137.82	3.98
1	2	-5	128.08	3.91
-1	-2	5	126.62	4.00
-1	-2	5	120.88	3.98
-1	-2	5	127.85	3.89
-1	-2	5	128.89	4.02
0	2	-5	43.43	1.47

0	2	-5	38.70	1.57
0	2	-5	42.41	1.40
0	2	-5	43.40	1.47
0	-2	5	42.51	1.59
0	-2	5	44.01	1.55
0	-2	5	40.72	1.48
0	-2	5	43.23	1.40
-1	2	-5	83.05	2.73
-1	2	-5	83.52	2.61
-1	2	-5	80.01	2.67
1	-2	5	83.09	2.75
1	-2	5	85.01	2.89
1	-2	5	84.57	2.71
-2	2	-5	71.80	2.43
-2	2	-5	71.64	2.47
-2	2	-5	70.14	2.37
-2	2	-5	72.28	2.31
2	-2	5	73.83	2.45
2	-2	5	72.46	2.41
2	-2	5	74.17	2.32
2	-2	5	74.55	2.68
-3	2	-5	155.94	5.00
-3	2	-5	148.78	4.78
-3	2	-5	152.12	4.85
-3	2	-5	169.58	4.97
3	-2	5	156.68	4.93
3	-2	5	152.02	5.23
3	-2	5	154.44	4.89
3	-2	5	159.28	4.79
-4	2	-5	43.44	1.60
-4	2	-5	43.71	1.54
-4	2	-5	45.27	1.75
-4	2	-5	44.65	1.79
4	-2	5	47.26	1.56
4	-2	5	45.21	1.65
4	-2	5	47.09	1.72
4	-2	5	42.45	2.04
-5	2	-5	94.00	3.12
-5	2	-5	97.26	3.05
-5	2	-5	93.70	3.32
-5	2	-5	94.33	3.34
5	-2	5	98.83	3.25
5	-2	5	89.00	3.67
5	-2	5	97.25	3.07

5	-2	5	98.88	3.19
-6	2	-5	47.13	1.68
-6	2	-5	47.42	1.76
-6	2	-5	49.70	2.07
-6	2	-5	46.27	2.02
6	-2	5	52.39	1.84
6	-2	5	48.10	1.91
6	-2	5	45.10	2.39
6	-2	5	46.67	1.69
-7	2	-5	32.16	1.67
-7	2	-5	33.23	1.81
-7	2	-5	33.22	1.28
-7	2	-5	32.55	1.38
7	-2	5	34.01	1.57
7	-2	5	33.70	1.31
7	-2	5	35.72	1.45
7	-2	5	26.07	1.99
-8	2	-5	31.05	1.39
-8	2	-5	32.42	2.00
-8	2	-5	31.82	1.28
8	-2	5	30.43	1.43
8	-2	5	27.43	2.19
8	-2	5	38.15	1.66
-9	2	-5	45.82	1.83
-9	2	-5	45.61	1.70
-9	2	-5	43.28	2.53
9	-2	5	45.37	1.86
9	-2	5	41.28	2.83
-10	2	-5	20.26	1.22
-10	2	-5	19.77	1.07
-10	2	-5	21.45	2.33
10	-2	5	17.54	2.43
10	-2	5	20.26	1.24
-11	2	-5	7.99	2.15
-11	2	-5	10.63	0.93
11	-2	5	8.19	1.89
11	-2	5	12.03	1.13
11	-2	5	10.59	1.38
-12	2	-5	11.08	1.11
12	-2	5	12.10	1.66
12	-2	5	11.13	2.35
12	-2	5	12.21	1.22
-13	2	-5	2.88	0.76
13	-2	5	1.47	1.08

13	-2	5	2.18	2.12
16	1	-5	0.48	1.91
16	1	-5	-0.92	1.15
16	1	-5	0.34	0.65
15	1	-5	6.11	1.82
15	1	-5	6.58	0.96
15	1	-5	4.95	1.26
14	1	-5	5.84	1.51
14	1	-5	7.72	1.33
14	1	-5	6.78	0.85
14	1	-5	7.76	1.25
13	1	-5	8.70	1.41
13	1	-5	10.43	1.33
13	1	-5	9.58	1.41
13	1	-5	10.70	0.96
13	1	-5	13.16	2.21
-13	-1	5	10.24	1.03
12	1	-5	4.43	0.85
12	1	-5	3.61	0.55
12	1	-5	2.27	1.14
12	1	-5	3.43	0.97
-12	-1	5	2.32	0.55
11	1	-5	0.04	0.65
11	1	-5	1.30	0.90
11	1	-5	0.02	0.71
11	1	-5	0.92	0.39
11	1	-5	1.07	0.64
-11	-1	5	0.31	0.40
10	1	-5	37.48	1.88
10	1	-5	37.05	1.45
10	1	-5	41.05	1.53
10	1	-5	37.41	1.86
10	1	-5	39.07	1.55
10	1	-5	38.16	1.98
10	1	-5	40.31	1.83
-10	-1	5	40.88	1.55
9	1	-5	56.87	1.93
9	1	-5	56.52	2.24
9	1	-5	56.88	1.98
9	1	-5	55.69	1.92
9	1	-5	55.48	2.10
9	1	-5	58.92	2.32
9	1	-5	56.82	2.24
-9	-1	5	53.58	2.09

8	1	-5	47.28	1.60
8	1	-5	44.64	1.85
8	1	-5	47.19	1.66
8	1	-5	47.00	1.88
8	1	-5	45.46	1.81
8	1	-5	45.60	1.58
-8	-1	5	42.90	1.74
-8	-1	5	46.87	1.76
7	1	-5	34.96	1.48
7	1	-5	36.79	1.31
7	1	-5	36.04	1.45
7	1	-5	36.39	1.26
7	1	-5	32.53	1.23
-7	-1	5	32.55	1.40
-7	-1	5	36.92	1.42
6	1	-5	111.53	3.78
6	1	-5	116.03	3.59
6	1	-5	113.06	3.62
6	1	-5	117.52	3.63
6	1	-5	113.13	3.56
6	1	-5	117.43	3.66
-6	-1	5	114.95	3.72
-6	-1	5	116.93	3.72
5	1	-5	47.40	1.58
5	1	-5	48.90	1.59
5	1	-5	48.85	1.65
5	1	-5	49.01	1.79
5	1	-5	51.04	1.63
5	1	-5	49.47	1.60
-5	-1	5	45.18	1.85
-5	-1	5	48.41	1.72
4	1	-5	16.52	0.66
4	1	-5	18.12	0.73
4	1	-5	17.30	0.84
-4	-1	5	17.51	0.78
-4	-1	5	18.18	0.67
-4	-1	5	17.57	0.81
3	1	-5	333.98	10.01
3	1	-5	322.76	9.82
3	1	-5	342.05	9.89
-3	-1	5	328.05	9.81
-3	-1	5	331.49	9.97
-3	-1	5	316.09	9.91
2	1	-5	453.40	13.31

2	1	-5	434.82	13.24
2	1	-5	432.52	13.53
-2	-1	5	427.09	13.31
-2	-1	5	471.83	13.42
-2	-1	5	449.44	13.23
-2	-1	5	445.74	13.24
1	1	-5	341.25	10.39
1	1	-5	358.09	10.42
1	1	-5	353.08	10.55
1	1	-5	334.42	10.47
-1	-1	5	360.02	10.58
-1	-1	5	349.87	10.38
-1	-1	5	340.85	10.47
0	1	-5	71.38	2.24
0	1	-5	69.69	2.15
0	1	-5	68.97	2.29
0	-1	5	68.52	2.33
0	-1	5	67.17	2.14
0	-1	5	62.50	2.45
0	-1	5	68.04	2.24
-1	1	-5	70.60	2.35
-1	1	-5	66.48	2.16
-1	1	-5	65.70	2.27
-1	1	-5	73.35	2.32
1	-1	5	67.15	2.42
1	-1	5	66.33	2.27
1	-1	5	69.39	2.17
-2	1	-5	36.42	1.43
-2	1	-5	38.69	1.44
-2	1	-5	36.01	1.23
-2	1	-5	35.14	1.33
2	-1	5	35.18	1.35
2	-1	5	37.24	1.24
2	-1	5	36.94	1.42
2	-1	5	33.08	1.55
-3	1	-5	51.72	1.85
-3	1	-5	51.52	1.74
-3	1	-5	54.13	1.99
-3	1	-5	53.53	2.01
3	-1	5	54.36	1.89
3	-1	5	52.39	2.17
3	-1	5	54.39	1.76
3	-1	5	54.28	1.96
-4	1	-5	193.50	6.27

-4	1	-5	194.43	6.16
-4	1	-5	208.80	6.51
-4	1	-5	208.33	6.47
4	-1	5	206.19	6.33
4	-1	5	194.08	6.68
4	-1	5	206.87	6.39
4	-1	5	209.95	6.19
-5	1	-5	43.51	1.85
-5	1	-5	43.16	1.91
-5	1	-5	42.48	1.51
-5	1	-5	42.88	1.62
5	-1	5	40.38	2.08
5	-1	5	45.25	1.77
5	-1	5	45.42	1.69
5	-1	5	44.07	1.53
-6	1	-5	21.83	1.35
-6	1	-5	24.28	1.50
-6	1	-5	21.53	1.05
-6	1	-5	21.57	0.94
6	-1	5	22.44	1.24
6	-1	5	23.80	0.99
6	-1	5	25.19	1.15
6	-1	5	23.18	1.65
-7	1	-5	61.51	2.28
-7	1	-5	65.16	2.83
-7	1	-5	64.33	2.16
7	-1	5	64.70	2.19
7	-1	5	63.15	2.34
7	-1	5	61.05	2.97
-8	1	-5	11.84	0.74
-8	1	-5	12.26	0.87
-8	1	-5	10.04	1.70
8	-1	5	11.70	0.98
8	-1	5	12.27	0.94
8	-1	5	9.61	1.58
-9	1	-5	19.95	0.98
-9	1	-5	17.44	2.12
-9	1	-5	19.24	1.12
9	-1	5	17.72	1.18
9	-1	5	14.03	2.00
-10	1	-5	18.26	1.04
-10	1	-5	14.48	2.59
-10	1	-5	19.70	1.23
10	-1	5	19.25	1.25

10	-1	5	14.02	2.27
-11	1	-5	12.15	1.18
-11	1	-5	11.97	1.03
11	-1	5	13.70	1.22
11	-1	5	11.51	2.08
-12	1	-5	5.19	0.80
12	-1	5	9.01	2.06
12	-1	5	8.55	1.24
12	-1	5	6.57	1.17
-13	1	-5	5.20	0.88
13	-1	5	0.31	2.01
13	-1	5	4.61	1.29
16	0	-5	0.08	1.84
16	0	-5	-0.05	1.05
-16	0	5	1.84	0.87
15	0	-5	3.03	1.62
15	0	-5	3.70	1.09
-15	0	5	4.75	0.87
14	0	-5	5.76	0.97
14	0	-5	6.54	1.11
14	0	-5	6.36	1.52
-14	0	5	8.70	1.05
13	0	-5	9.15	1.50
13	0	-5	9.04	1.08
13	0	-5	9.73	1.45
13	0	-5	6.48	0.90
13	0	-5	8.07	1.27
-13	0	5	8.69	0.91
12	0	-5	5.57	0.82
12	0	-5	7.75	0.63
12	0	-5	8.30	1.44
12	0	-5	7.86	1.22
12	0	-5	7.53	1.10
11	0	-5	36.60	1.34
11	0	-5	36.05	1.79
11	0	-5	34.78	1.68
11	0	-5	33.23	1.94
-11	0	5	34.70	1.46
10	0	-5	12.47	1.04
10	0	-5	14.59	1.21
10	0	-5	13.63	0.70
10	0	-5	11.56	0.80
10	0	-5	14.44	0.76
-10	0	5	12.62	0.78

-10	0	5	14.87	1.01
9	0	-5	28.62	1.50
9	0	-5	30.82	1.14
9	0	-5	30.66	1.16
9	0	-5	28.50	1.46
-9	0	5	30.68	1.36
-9	0	5	30.02	1.22
-9	0	5	27.96	1.29
-9	0	5	34.25	1.36
8	0	-5	48.64	1.67
8	0	-5	47.57	1.93
8	0	-5	47.93	1.94
8	0	-5	50.07	1.65
-8	0	5	46.69	1.71
-8	0	5	47.66	1.79
-8	0	5	52.09	1.78
-8	0	5	48.16	1.85
7	0	-5	48.65	1.92
7	0	-5	49.64	1.66
7	0	-5	48.90	1.86
7	0	-5	51.05	1.68
7	0	-5	50.28	1.67
-7	0	5	49.72	1.85
-7	0	5	48.10	1.80
-7	0	5	52.99	1.73
6	0	-5	35.53	1.23
6	0	-5	36.98	1.26
6	0	-5	34.15	1.45
6	0	-5	36.71	1.25
6	0	-5	34.95	1.36
-6	0	5	37.85	1.25
-6	0	5	36.75	1.42
5	0	-5	68.41	2.39
5	0	-5	66.52	2.16
5	0	-5	68.23	2.21
5	0	-5	68.40	2.19
5	0	-5	68.80	2.19
-5	0	5	71.90	2.13
-5	0	5	70.82	2.35
-5	0	5	64.11	2.33
4	0	-5	42.45	1.60
4	0	-5	44.26	1.44
4	0	-5	41.60	1.39
-4	0	5	41.54	1.57

-4	0	5	42.73	1.38
-4	0	5	43.30	1.47
3	0	-5	91.54	3.06
3	0	-5	93.18	2.91
3	0	-5	91.43	2.83
-3	0	5	91.70	2.82
-3	0	5	92.75	2.87
-3	0	5	91.63	3.03
-3	0	5	91.21	2.91
2	0	-5	956.05	29.10
2	0	-5	990.45	29.21
-2	0	5	1000.00	29.21
-2	0	5	997.45	29.10
-2	0	5	957.94	28.97
1	0	-5	181.68	5.77
1	0	-5	177.72	5.70
-1	0	5	176.25	5.66
-1	0	5	192.10	5.67
-1	0	5	184.70	5.56
-1	0	5	188.48	5.74
-1	0	5	192.21	5.81
0	0	-5	7.04	0.37
0	0	-5	7.33	0.54
0	0	5	7.56	0.37
0	0	5	7.10	0.47
0	0	5	7.90	0.56
0	0	5	8.88	0.63
0	0	5	7.77	0.61
-1	0	-5	82.88	2.82
-1	0	-5	93.49	2.94
-1	0	-5	83.64	2.66
1	0	5	89.20	2.67
1	0	5	85.89	2.91
1	0	5	86.33	2.92
1	0	5	79.58	2.80
-2	0	-5	131.02	4.02
-2	0	-5	124.58	4.05
-2	0	-5	117.87	3.73
-2	0	-5	111.85	3.87
2	0	5	124.56	4.07
2	0	5	124.61	3.98
2	0	5	117.16	3.89
-3	0	-5	98.67	3.25
-3	0	-5	91.36	3.06

-3	0	-5	92.51	2.92
-3	0	-5	96.40	3.31
3	0	5	93.10	3.18
3	0	5	91.58	3.32
3	0	5	91.53	2.93
3	0	5	92.49	3.11
-4	0	-5	55.33	2.23
-4	0	-5	49.72	1.74
-4	0	-5	51.30	1.90
-4	0	-5	50.65	2.10
4	0	5	56.60	2.29
4	0	5	54.25	1.96
4	0	5	52.58	2.03
4	0	5	54.43	1.78
-5	0	-5	61.74	2.60
-5	0	-5	64.16	2.06
-5	0	-5	61.46	2.21
5	0	5	56.96	2.61
5	0	5	63.04	2.27
5	0	5	64.47	2.09
5	0	5	63.03	2.36
5	0	5	62.72	2.71
-6	0	-5	57.06	2.59
-6	0	-5	56.04	1.93
-6	0	-5	55.64	2.07
6	0	5	61.00	2.19
6	0	5	54.82	2.26
6	0	5	57.57	2.64
6	0	5	56.47	1.96
6	0	5	57.15	2.69
-7	0	-5	26.46	1.09
-7	0	-5	26.56	1.24
-7	0	-5	27.87	2.01
7	0	5	26.32	1.33
7	0	5	26.11	1.14
7	0	5	25.70	1.85
-8	0	-5	3.18	0.45
-8	0	-5	4.08	1.17
-8	0	-5	3.01	0.53
8	0	5	3.04	0.58
8	0	5	2.63	1.05
8	0	5	3.07	0.62
-9	0	-5	21.15	1.04
-9	0	-5	20.96	1.24

-9	0	-5	21.85	2.50
9	0	5	21.47	2.11
9	0	5	21.03	1.30
-10	0	-5	17.82	1.07
-10	0	-5	17.48	1.24
10	0	5	19.97	1.36
10	0	5	17.10	2.31
-11	0	-5	11.36	1.19
-11	0	-5	9.85	1.00
11	0	5	5.27	1.69
11	0	5	10.37	1.25
-12	0	-5	6.28	0.85
12	0	5	4.71	1.18
12	0	5	2.77	1.72
17	-1	-5	2.96	1.35
17	-1	-5	-0.29	2.17
-17	1	5	2.47	0.98
16	-1	-5	-0.59	1.07
16	-1	-5	0.46	1.89
-16	1	5	1.07	0.79
15	-1	-5	4.68	1.91
15	-1	-5	8.79	1.37
-15	1	5	6.90	0.90
14	-1	-5	14.05	2.16
14	-1	-5	12.11	1.54
-14	1	5	12.25	1.06
13	-1	-5	30.27	2.23
13	-1	-5	29.49	1.04
13	-1	-5	27.79	1.22
13	-1	-5	28.23	1.83
-13	1	5	29.07	1.76
-13	1	5	30.75	1.41
12	-1	-5	27.17	0.97
12	-1	-5	24.91	1.60
12	-1	-5	26.63	1.95
12	-1	-5	25.70	1.23
-12	1	5	26.21	1.24
-12	1	5	28.37	1.57
11	-1	-5	10.29	0.89
11	-1	-5	9.50	1.34
11	-1	-5	11.01	0.53
11	-1	-5	10.15	1.09
-11	1	5	11.37	1.03
-11	1	5	10.17	0.75

10	-1	-5	10.50	0.51
10	-1	-5	11.42	0.99
10	-1	-5	11.89	0.67
10	-1	-5	11.30	1.18
-10	1	5	12.01	0.88
-10	1	5	10.53	0.87
-10	1	5	10.46	0.68
-10	1	5	9.67	0.69
9	-1	-5	94.37	3.38
9	-1	-5	94.22	2.95
9	-1	-5	93.79	3.44
9	-1	-5	96.69	3.05
-9	1	5	92.12	3.07
-9	1	5	103.52	3.21
-9	1	5	92.30	3.09
-9	1	5	94.95	3.26
8	-1	-5	68.17	2.11
8	-1	-5	65.85	2.48
8	-1	-5	66.06	2.52
8	-1	-5	67.36	2.18
-8	1	5	65.08	2.38
-8	1	5	66.95	2.28
-8	1	5	64.43	2.21
-8	1	5	65.83	2.22
7	-1	-5	30.95	1.39
7	-1	-5	31.06	1.40
7	-1	-5	32.62	1.10
7	-1	-5	32.35	1.14
-7	1	5	32.75	1.22
-7	1	5	32.36	1.34
-7	1	5	30.80	1.17
-7	1	5	31.45	1.17
6	-1	-5	101.83	3.49
6	-1	-5	106.61	3.57
6	-1	-5	110.11	3.32
6	-1	-5	104.44	3.30
6	-1	-5	108.04	3.31
-6	1	5	105.18	3.34
-6	1	5	108.16	3.52
-6	1	5	109.18	3.38
5	-1	-5	35.54	1.19
5	-1	-5	36.90	1.22
5	-1	-5	34.80	1.16
5	-1	-5	35.07	1.33

5	-1	-5	33.53	1.41
-5	1	5	30.12	1.24
-5	1	5	35.84	1.26
-5	1	5	35.45	1.39
4	-1	-5	157.06	4.77
4	-1	-5	156.20	4.87
4	-1	-5	156.38	5.05
4	-1	-5	158.28	4.89
4	-1	-5	160.31	4.80
-4	1	5	162.47	4.91
-4	1	5	154.75	4.83
3	-1	-5	172.51	5.26
3	-1	-5	169.94	5.39
3	-1	-5	161.78	5.15
3	-1	-5	167.23	5.23
-3	1	5	173.32	5.37
-3	1	5	166.59	5.20
-3	1	5	171.52	5.10
-3	1	5	175.91	5.32
2	-1	-5	8.25	0.53
2	-1	-5	8.61	0.54
2	-1	-5	7.42	0.60
2	-1	-5	7.85	0.36
-2	1	5	7.27	0.45
-2	1	5	8.55	0.63
-2	1	5	7.43	0.34
-2	1	5	8.03	0.61
1	-1	-5	233.69	6.91
1	-1	-5	233.05	7.17
-1	1	5	228.55	6.90
-1	1	5	237.47	7.22
-1	1	5	221.83	7.06
0	-1	-5	77.17	2.41
0	-1	-5	73.16	2.63
0	-1	-5	78.76	2.72
0	1	5	77.79	2.70
0	1	5	77.97	2.41
0	1	5	80.06	2.62
0	1	5	78.44	2.60
0	1	5	81.81	2.81
-1	-1	-5	80.42	2.54
-1	-1	-5	81.51	2.91
-1	-1	-5	79.73	2.75
1	1	5	88.23	3.02

1	1	5	79.20	2.75
1	1	5	85.86	2.82
1	1	5	81.01	2.84
1	1	5	81.74	2.55
-2	-1	-5	94.19	3.23
-2	-1	-5	97.71	3.46
-2	-1	-5	95.29	3.03
2	1	5	106.50	3.58
2	1	5	96.28	3.36
2	1	5	98.90	3.33
2	1	5	99.28	3.04
2	1	5	98.60	3.27
-3	-1	-5	59.20	1.97
-3	-1	-5	61.57	2.47
-3	-1	-5	60.61	2.16
3	1	5	63.51	2.29
3	1	5	60.36	2.37
3	1	5	59.62	2.22
3	1	5	63.67	2.58
3	1	5	63.03	1.99
-4	-1	-5	71.68	2.36
-4	-1	-5	70.14	2.92
-4	-1	-5	72.31	2.54
4	1	5	76.19	2.65
4	1	5	75.74	2.85
4	1	5	73.20	2.71
4	1	5	74.79	2.38
4	1	5	76.72	3.04
-5	-1	-5	51.94	1.70
-5	-1	-5	48.81	1.88
-5	-1	-5	50.29	2.40
5	1	5	48.78	1.99
5	1	5	51.52	2.49
5	1	5	48.21	2.05
5	1	5	49.80	2.25
5	1	5	52.61	1.74
-6	-1	-5	25.87	1.21
-6	-1	-5	26.51	1.95
-6	-1	-5	25.53	1.03
6	1	5	25.87	1.33
6	1	5	23.09	1.66
6	1	5	27.80	1.09
-7	-1	-5	27.45	1.16
-7	-1	-5	31.90	2.25

-7	-1	-5	30.11	1.37
7	1	5	28.51	1.22
7	1	5	27.70	1.92
7	1	5	30.95	1.51
-8	-1	-5	6.82	0.64
-8	-1	-5	7.14	0.82
-8	-1	-5	10.67	1.55
8	1	5	4.84	1.17
8	1	5	7.13	0.98
8	1	5	8.27	0.96
-9	-1	-5	7.74	0.93
-9	-1	-5	6.90	0.73
9	1	5	6.04	1.41
9	1	5	6.00	1.05
-10	-1	-5	9.22	1.10
-10	-1	-5	8.54	0.91
10	1	5	5.72	1.49
10	1	5	9.81	1.19
-11	-1	-5	7.40	1.16
-11	-1	-5	7.09	1.04
11	1	5	8.03	1.28
11	1	5	3.32	1.53
-12	-1	-5	3.44	0.84
12	1	5	5.21	1.87
12	1	5	5.37	1.00
12	1	5	3.37	1.11
17	-2	-5	3.56	1.37
17	-2	-5	7.26	2.27
-17	2	5	2.01	0.90
16	-2	-5	5.97	2.03
16	-2	-5	11.91	1.90
-16	2	5	7.79	1.21
15	-2	-5	9.53	1.92
15	-2	-5	12.76	1.71
-15	2	5	13.62	1.18
14	-2	-5	16.31	2.29
14	-2	-5	15.15	1.60
-14	2	5	14.07	1.08
13	-2	-5	8.20	1.44
13	-2	-5	11.07	1.31
-13	2	5	9.28	1.25
-13	2	5	8.04	0.82
12	-2	-5	19.03	1.84
12	-2	-5	19.73	1.41

-12	2	5	19.17	1.01
-12	2	5	18.80	1.36
11	-2	-5	51.23	2.56
11	-2	-5	54.94	2.33
-11	2	5	56.11	2.42
-11	2	5	54.17	2.14
-11	2	5	52.62	1.94
10	-2	-5	32.33	1.81
10	-2	-5	29.98	1.06
10	-2	-5	31.38	1.21
10	-2	-5	31.56	1.57
-10	2	5	29.23	1.44
-10	2	5	30.59	1.24
-10	2	5	33.55	1.43
9	-2	-5	41.31	1.44
9	-2	-5	37.85	1.33
9	-2	-5	38.74	1.93
9	-2	-5	37.66	1.75
-9	2	5	41.07	1.68
-9	2	5	42.47	1.64
-9	2	5	40.86	1.41
-9	2	5	41.06	1.47
8	-2	-5	52.35	1.75
8	-2	-5	52.55	2.18
8	-2	-5	49.86	1.70
8	-2	-5	51.86	2.07
-8	2	5	50.71	1.78
-8	2	5	54.77	1.94
-8	2	5	52.56	1.74
-8	2	5	53.26	1.99
7	-2	-5	195.13	5.72
7	-2	-5	187.29	6.03
7	-2	-5	188.72	6.43
7	-2	-5	172.97	5.71
-7	2	5	182.95	5.74
-7	2	5	189.91	5.71
-7	2	5	194.96	5.91
-7	2	5	191.71	5.97
6	-2	-5	25.73	0.92
6	-2	-5	24.77	1.19
6	-2	-5	25.93	1.24
6	-2	-5	24.64	0.97
-6	2	5	25.48	1.12
-6	2	5	24.97	0.91

-6	2	5	26.52	1.16
5	-2	-5	77.11	2.47
5	-2	-5	79.77	2.45
5	-2	-5	78.35	2.75
5	-2	-5	79.35	2.73
5	-2	-5	78.26	2.58
-5	2	5	79.61	2.71
-5	2	5	79.92	2.71
4	-2	-5	123.76	4.18
4	-2	-5	126.71	3.88
4	-2	-5	133.75	4.08
4	-2	-5	128.41	3.89
4	-2	-5	123.22	4.13
-4	2	5	129.97	4.19
-4	2	5	128.92	4.16
-4	2	5	128.69	3.97
3	-2	-5	45.14	1.78
3	-2	-5	46.97	1.76
3	-2	-5	49.37	1.73
3	-2	-5	46.37	1.47
-3	2	5	44.87	1.79
-3	2	5	46.12	1.46
-3	2	5	47.11	1.83
-3	2	5	46.95	1.61
2	-2	-5	84.70	2.97
2	-2	-5	87.35	2.99
2	-2	-5	86.59	2.65
-2	2	5	87.00	2.64
-2	2	5	85.78	2.99
-2	2	5	86.51	2.84
-2	2	5	89.98	3.07
1	-2	-5	71.71	2.19
1	-2	-5	72.50	2.56
1	-2	-5	70.57	2.52
1	-2	-5	68.96	2.49
-1	2	5	75.45	2.69
-1	2	5	73.35	2.55
-1	2	5	70.64	2.18
-1	2	5	66.78	2.40
0	-2	-5	31.33	1.31
0	-2	-5	32.26	1.46
0	-2	-5	33.64	1.45
0	-2	-5	31.71	1.04
0	2	5	31.57	1.04

0	2	5	31.06	1.39
0	2	5	30.72	1.29
0	2	5	32.66	1.58
-1	-2	-5	145.86	4.80
-1	-2	-5	155.41	5.07
1	2	5	147.50	4.85
1	2	5	153.32	4.56
1	2	5	142.06	4.84
1	2	5	151.83	4.92
1	2	5	160.18	5.20
-2	-2	-5	120.42	4.17
-2	-2	-5	112.31	3.82
-2	-2	-5	112.67	3.58
2	2	5	119.12	3.60
2	2	5	126.07	4.32
2	2	5	114.47	3.94
2	2	5	120.18	3.94
2	2	5	115.19	3.93
-3	-2	-5	34.64	1.41
-3	-2	-5	37.67	1.85
-3	-2	-5	33.65	1.18
3	2	5	34.20	1.59
3	2	5	34.73	1.20
3	2	5	34.92	1.56
3	2	5	34.79	1.53
3	2	5	35.38	1.97
-4	-2	-5	40.53	1.59
-4	-2	-5	39.33	1.35
-4	-2	-5	38.28	2.06
4	2	5	38.73	1.82
4	2	5	37.92	1.72
4	2	5	41.66	1.77
4	2	5	40.99	1.38
-5	-2	-5	82.26	3.51
-5	-2	-5	84.08	2.89
-5	-2	-5	81.27	2.65
5	2	5	82.65	3.11
5	2	5	84.97	3.07
5	2	5	88.40	2.70
5	2	5	78.31	3.19
5	2	5	83.04	3.64
-6	-2	-5	38.17	1.60
-6	-2	-5	37.93	1.38
-6	-2	-5	42.51	2.52

6	2	5	38.16	1.43
6	2	5	38.22	1.81
6	2	5	35.14	1.98
-7	-2	-5	3.89	0.61
-7	-2	-5	3.54	1.21
-7	-2	-5	3.75	0.53
7	2	5	4.40	0.64
7	2	5	2.28	0.66
7	2	5	3.79	1.04
-8	-2	-5	12.26	1.01
-8	-2	-5	9.07	0.73
8	2	5	7.73	1.29
8	2	5	10.90	1.17
-9	-2	-5	11.40	0.88
-9	-2	-5	11.72	1.11
9	2	5	11.06	1.96
9	2	5	11.73	1.29
-10	-2	-5	4.31	0.64
-10	-2	-5	4.42	0.81
10	2	5	4.18	1.54
10	2	5	3.59	0.90
-11	-2	-5	5.66	0.85
-11	-2	-5	4.30	0.88
11	2	5	3.83	1.65
11	2	5	5.22	1.01
17	-3	-5	10.66	2.44
17	-3	-5	6.30	1.37
-17	3	5	9.13	1.29
16	-3	-5	19.34	3.01
16	-3	-5	13.76	1.90
-16	3	5	14.76	1.30
15	-3	-5	8.89	1.86
15	-3	-5	6.59	1.16
-15	3	5	7.50	1.01
14	-3	-5	17.26	2.31
14	-3	-5	19.52	1.66
-14	3	5	13.16	1.06
-14	3	5	15.58	1.79
13	-3	-5	41.30	2.13
13	-3	-5	41.45	2.65
-13	3	5	40.53	1.66
-13	3	5	37.63	1.94
12	-3	-5	32.65	2.21
12	-3	-5	32.15	1.75

-12	3	5	28.81	1.32
-12	3	5	31.01	1.64
11	-3	-5	43.28	1.62
11	-3	-5	41.94	2.35
11	-3	-5	44.84	2.05
-11	3	5	44.05	1.93
-11	3	5	43.65	1.64
-11	3	5	46.46	1.94
10	-3	-5	58.23	2.46
10	-3	-5	64.44	2.75
10	-3	-5	62.65	2.09
-10	3	5	60.11	2.11
-10	3	5	61.19	2.35
-10	3	5	63.19	2.36
9	-3	-5	78.31	2.88
9	-3	-5	75.69	3.05
9	-3	-5	76.00	2.50
-9	3	5	78.66	2.78
-9	3	5	77.28	2.41
-9	3	5	76.78	2.76
-9	3	5	75.21	2.52
8	-3	-5	94.42	3.45
8	-3	-5	94.38	2.96
8	-3	-5	95.03	2.95
8	-3	-5	94.08	3.30
-8	3	5	92.80	2.89
-8	3	5	87.82	3.39
-8	3	5	94.64	3.22
-8	3	5	91.53	2.96
7	-3	-5	62.32	2.03
7	-3	-5	60.38	1.94
7	-3	-5	58.09	2.43
7	-3	-5	60.83	2.28
-7	3	5	62.14	2.27
-7	3	5	59.37	1.92
-7	3	5	60.84	2.22
-7	3	5	56.28	1.96
6	-3	-5	84.21	3.01
6	-3	-5	82.32	2.54
6	-3	-5	79.45	2.87
6	-3	-5	83.51	2.70
-6	3	5	80.63	2.57
-6	3	5	73.08	2.60
-6	3	5	84.50	2.85

-6	3	5	82.49	2.90
5	-3	-5	161.42	4.92
5	-3	-5	158.86	5.27
5	-3	-5	165.67	5.15
5	-3	-5	159.73	4.91
-5	3	5	167.54	5.34
-5	3	5	163.10	5.01
-5	3	5	164.66	5.23
4	-3	-5	151.01	5.03
4	-3	-5	157.30	4.97
4	-3	-5	149.59	5.11
4	-3	-5	153.70	4.68
-4	3	5	156.69	5.13
-4	3	5	157.48	4.83
-4	3	5	160.81	5.01
3	-3	-5	60.28	2.29
3	-3	-5	62.63	1.94
3	-3	-5	63.96	1.94
3	-3	-5	62.33	2.37
-3	3	5	63.85	2.30
-3	3	5	62.27	2.15
-3	3	5	63.06	2.44
2	-3	-5	52.88	1.62
2	-3	-5	51.33	2.05
2	-3	-5	51.51	2.03
2	-3	-5	49.16	1.96
-2	3	5	52.50	2.01
-2	3	5	51.74	1.60
-2	3	5	52.14	1.90
-2	3	5	55.77	2.22
1	-3	-5	105.19	3.71
1	-3	-5	114.48	3.78
1	-3	-5	103.21	3.60
1	-3	-5	104.89	3.25
-1	3	5	109.64	3.67
-1	3	5	107.58	3.89
-1	3	5	107.18	3.25
-1	3	5	111.17	3.60
0	-3	-5	174.12	5.18
0	-3	-5	168.09	5.65
0	-3	-5	172.73	5.75
0	-3	-5	173.38	5.52
0	3	5	177.38	5.61
0	3	5	171.37	5.57

0	3	5	177.98	5.90
0	3	5	170.11	5.18
-1	-3	-5	88.55	3.39
-1	-3	-5	89.87	3.08
-1	-3	-5	89.41	2.77
1	3	5	92.95	3.21
1	3	5	91.21	3.20
1	3	5	93.58	3.56
1	3	5	90.20	2.78
-2	-3	-5	81.35	3.20
-2	-3	-5	80.98	2.49
-2	-3	-5	78.74	2.78
2	3	5	81.71	2.95
2	3	5	80.29	2.50
2	3	5	79.21	2.93
2	3	5	80.06	2.90
2	3	5	81.64	3.36
-3	-3	-5	45.03	1.77
-3	-3	-5	44.81	1.48
-3	-3	-5	43.83	2.26
3	3	5	46.35	1.51
3	3	5	44.76	1.93
3	3	5	46.32	2.44
3	3	5	46.38	1.94
3	3	5	45.96	1.96
-4	-3	-5	51.25	2.57
-4	-3	-5	49.94	1.64
-4	-3	-5	48.40	1.90
4	3	5	51.00	2.14
4	3	5	48.67	2.13
4	3	5	46.88	2.13
4	3	5	49.41	2.74
4	3	5	50.77	1.67
-5	-3	-5	22.57	1.14
-5	-3	-5	21.87	2.01
-5	-3	-5	21.73	0.88
5	3	5	22.22	0.92
5	3	5	21.21	1.40
5	3	5	21.21	1.39
-6	-3	-5	24.64	2.43
-6	-3	-5	28.87	1.35
-6	-3	-5	25.61	1.07
6	3	5	27.93	1.75
6	3	5	28.25	1.63

6	3	5	28.17	1.14
-7	-3	-5	20.02	1.21
-7	-3	-5	21.14	0.96
7	3	5	22.62	1.51
7	3	5	19.95	1.03
7	3	5	19.52	1.74
-8	-3	-5	6.74	0.72
-8	-3	-5	8.32	0.96
8	3	5	6.41	1.04
8	3	5	5.41	1.22
-9	-3	-5	8.14	1.10
-9	-3	-5	6.74	0.85
9	3	5	7.22	1.45
9	3	5	9.08	1.36
-10	-3	-5	3.47	0.81
-10	-3	-5	4.68	0.69
10	3	5	4.74	1.59
10	3	5	3.33	1.00
-11	-3	-5	1.03	0.83
-11	-3	-5	1.03	0.82
11	3	5	1.92	1.57
11	3	5	0.44	0.97
17	-4	-5	5.47	1.38
17	-4	-5	5.61	2.42
-17	4	5	9.38	1.25
16	-4	-5	16.74	2.97
16	-4	-5	12.01	1.88
-16	4	5	12.89	1.22
15	-4	-5	23.38	2.76
15	-4	-5	26.86	2.02
-15	4	5	24.91	1.42
14	-4	-5	33.79	2.05
14	-4	-5	30.35	2.68
-14	4	5	29.79	1.45
-14	4	5	30.61	2.11
13	-4	-5	20.57	2.15
13	-4	-5	16.14	1.46
-13	4	5	19.85	1.47
-13	4	5	18.13	1.03
12	-4	-5	71.81	3.41
12	-4	-5	71.63	2.97
-12	4	5	72.63	2.52
-12	4	5	74.34	2.88
11	-4	-5	84.60	3.18

11	-4	-5	77.77	2.69
11	-4	-5	82.70	3.53
-11	4	5	79.80	3.02
-11	4	5	82.70	3.08
-11	4	5	80.23	2.72
10	-4	-5	43.68	2.29
10	-4	-5	42.94	1.54
10	-4	-5	44.54	1.96
-10	4	5	42.31	1.92
-10	4	5	40.99	1.80
-10	4	5	42.75	1.56
9	-4	-5	66.80	2.20
9	-4	-5	65.75	2.59
9	-4	-5	66.59	2.85
-9	4	5	66.61	2.09
-9	4	5	68.30	2.49
-9	4	5	68.48	2.61
-9	4	5	66.13	2.22
8	-4	-5	90.86	3.45
8	-4	-5	89.67	2.81
8	-4	-5	88.50	3.19
8	-4	-5	84.60	2.87
-8	4	5	90.74	3.24
-8	4	5	86.39	2.82
-8	4	5	88.19	3.10
-8	4	5	89.84	2.74
7	-4	-5	116.03	3.78
7	-4	-5	115.62	3.99
7	-4	-5	118.16	3.62
7	-4	-5	114.76	4.19
-7	4	5	122.85	3.95
-7	4	5	118.16	3.62
-7	4	5	115.39	3.64
6	-4	-5	140.80	4.33
6	-4	-5	142.31	4.90
6	-4	-5	141.47	4.73
6	-4	-5	141.50	4.58
-6	4	5	136.98	4.36
-6	4	5	143.67	4.86
-6	4	5	151.17	4.69
-6	4	5	143.19	4.42
5	-4	-5	79.38	2.47
5	-4	-5	79.15	2.87
5	-4	-5	75.49	2.99

5	-4	-5	78.53	2.47
5	-4	-5	81.40	2.80
-5	4	5	80.45	2.82
-5	4	5	83.70	3.07
-5	4	5	83.59	2.64
4	-4	-5	74.72	2.66
4	-4	-5	69.70	2.75
4	-4	-5	73.00	2.23
4	-4	-5	69.84	2.64
4	-4	-5	72.11	2.23
-4	4	5	73.55	2.86
-4	4	5	71.86	2.47
-4	4	5	75.73	2.62
3	-4	-5	25.84	1.41
3	-4	-5	25.24	1.26
3	-4	-5	24.81	1.29
3	-4	-5	26.70	0.85
3	-4	-5	25.49	0.84
-3	4	5	25.47	1.52
-3	4	5	26.97	1.16
-3	4	5	26.32	1.27
2	-4	-5	160.32	5.38
2	-4	-5	159.36	5.21
2	-4	-5	158.54	5.35
-2	4	5	159.79	5.53
-2	4	5	165.41	5.27
-2	4	5	161.07	5.20
1	-4	-5	60.87	2.29
1	-4	-5	59.20	2.46
1	-4	-5	63.34	2.53
-1	4	5	65.91	2.73
-1	4	5	64.95	2.39
-1	4	5	60.31	2.33
0	-4	-5	91.95	3.52
0	-4	-5	93.23	3.45
0	-4	-5	92.68	3.21
0	4	5	96.46	3.73
0	4	5	92.82	2.83
0	4	5	95.27	3.33
0	4	5	93.92	3.33
-1	-4	-5	115.39	3.80
-1	-4	-5	113.23	4.08
-1	-4	-5	110.74	4.21
-1	-4	-5	111.20	3.44

1	4	5	117.10	3.96
1	4	5	112.12	3.45
1	4	5	117.17	4.00
1	4	5	117.29	4.43
-2	-4	-5	48.25	2.42
-2	-4	-5	50.39	1.58
-2	-4	-5	48.68	1.91
2	4	5	53.21	2.16
2	4	5	48.29	2.09
2	4	5	47.82	2.03
2	4	5	49.78	1.59
2	4	5	52.10	2.66
-3	-4	-5	20.47	0.76
-3	-4	-5	21.58	1.09
-3	-4	-5	18.31	1.74
3	4	5	19.68	1.97
3	4	5	20.79	1.27
3	4	5	21.19	1.34
3	4	5	20.39	1.33
-4	-4	-5	5.81	1.27
-4	-4	-5	7.96	0.47
-4	-4	-5	8.94	0.77
4	4	5	8.37	1.04
4	4	5	9.61	1.14
4	4	5	9.14	0.53
4	4	5	7.98	1.00
-5	-4	-5	15.19	0.74
-5	-4	-5	17.49	1.02
5	4	5	18.15	0.80
5	4	5	16.08	1.37
5	4	5	14.28	1.31
-6	-4	-5	44.03	1.60
-6	-4	-5	46.90	1.91
6	4	5	44.35	1.65
6	4	5	49.01	2.30
6	4	5	44.68	2.30
-7	-4	-5	12.43	1.08
-7	-4	-5	13.38	0.80
7	4	5	12.73	0.89
7	4	5	13.99	1.65
-8	-4	-5	4.62	0.60
-8	-4	-5	4.08	0.75
8	4	5	3.32	1.11
8	4	5	5.91	1.04

-9	-4	-5	3.68	0.64
-9	-4	-5	4.93	0.84
9	4	5	4.97	1.42
9	4	5	3.36	1.03
-10	-4	-5	1.77	0.82
-10	-4	-5	2.71	0.79
10	4	5	1.83	1.51
10	4	5	1.53	1.08
18	-5	-5	6.37	2.67
17	-5	-5	8.18	1.50
17	-5	-5	5.68	2.37
-17	5	5	5.80	0.90
16	-5	-5	19.80	2.08
16	-5	-5	22.11	3.12
-16	5	5	23.73	1.43
15	-5	-5	17.42	2.65
15	-5	-5	17.01	1.84
-15	5	5	18.19	1.19
14	-5	-5	22.98	2.58
14	-5	-5	21.68	1.80
-14	5	5	24.53	1.27
13	-5	-5	43.73	2.26
13	-5	-5	47.02	2.92
-13	5	5	43.17	1.75
-13	5	5	40.93	2.19
12	-5	-5	54.11	2.99
12	-5	-5	56.47	2.45
-12	5	5	52.20	2.40
-12	5	5	52.38	1.95
11	-5	-5	44.21	1.62
11	-5	-5	44.18	2.07
11	-5	-5	43.98	2.52
-11	5	5	44.21	1.63
-11	5	5	42.21	2.00
-11	5	5	44.32	2.11
10	-5	-5	82.94	3.58
10	-5	-5	86.85	2.79
10	-5	-5	82.51	3.22
-10	5	5	83.00	3.07
-10	5	5	86.61	2.80
-10	5	5	90.67	3.32
9	-5	-5	99.92	3.12
9	-5	-5	95.04	3.87
9	-5	-5	94.61	3.17

9	-5	-5	97.48	3.56
-9	5	5	98.71	3.43
-9	5	5	100.37	3.02
-9	5	5	102.61	3.67
-9	5	5	97.65	3.14
8	-5	-5	130.32	4.81
8	-5	-5	126.97	4.25
8	-5	-5	136.07	4.10
8	-5	-5	135.31	4.53
-8	5	5	136.02	4.43
-8	5	5	133.96	4.66
-8	5	5	127.88	4.10
-8	5	5	133.35	4.06
7	-5	-5	52.65	2.41
7	-5	-5	51.45	2.11
7	-5	-5	52.61	1.70
7	-5	-5	51.26	1.97
-7	5	5	51.33	1.71
-7	5	5	53.79	2.05
-7	5	5	51.44	2.30
-7	5	5	51.36	1.76
6	-5	-5	131.74	4.84
6	-5	-5	137.42	4.62
6	-5	-5	137.27	4.53
-6	5	5	132.84	4.20
-6	5	5	141.89	4.55
-6	5	5	142.43	4.35
-6	5	5	137.97	4.85
5	-5	-5	207.00	6.09
5	-5	-5	198.69	6.09
5	-5	-5	197.30	6.54
5	-5	-5	198.69	6.76
5	-5	-5	202.69	6.54
-5	5	5	210.24	6.84
-5	5	5	203.67	6.36
-5	5	5	209.39	6.50
4	-5	-5	50.99	2.32
4	-5	-5	53.81	1.69
4	-5	-5	56.12	1.69
4	-5	-5	55.91	2.23
4	-5	-5	53.00	2.15
-4	5	5	54.94	2.04
-4	5	5	53.15	2.46
-4	5	5	59.11	2.17

3	-5	-5	92.68	3.47
3	-5	-5	87.60	3.23
3	-5	-5	94.47	3.40
-3	5	5	92.04	3.21
-3	5	5	93.40	3.26
-3	5	5	91.18	3.60
2	-5	-5	49.11	2.20
2	-5	-5	49.41	2.22
2	-5	-5	50.12	1.99
-2	5	5	44.68	1.97
-2	5	5	50.35	2.45
-2	5	5	54.96	2.10
1	-5	-5	71.98	3.03
1	-5	-5	73.38	2.72
1	-5	-5	77.36	3.02
-1	5	5	80.27	2.87
-1	5	5	79.97	3.31
-1	5	5	74.45	2.84
0	-5	-5	92.79	3.20
0	-5	-5	94.26	3.53
0	-5	-5	87.06	3.57
0	5	5	90.71	3.38
0	5	5	93.08	3.85
0	5	5	94.34	3.35
-1	-5	-5	14.58	0.94
-1	-5	-5	15.56	1.30
-1	-5	-5	13.39	1.43
1	5	5	16.06	1.16
1	5	5	18.51	1.77
1	5	5	16.74	1.18
-2	-5	-5	23.26	1.58
-2	-5	-5	20.03	1.77
-2	-5	-5	23.43	1.17
2	5	5	21.29	1.39
2	5	5	22.06	1.39
2	5	5	21.88	0.79
2	5	5	21.50	2.10
-3	-5	-5	34.79	1.20
-3	-5	-5	35.68	1.55
-3	-5	-5	36.70	2.41
3	5	5	35.67	1.94
3	5	5	33.34	2.67
3	5	5	34.16	1.76
3	5	5	37.79	1.92

3	5	5	37.90	1.23
-4	-5	-5	22.41	1.19
-4	-5	-5	19.34	0.81
4	5	5	20.16	1.40
4	5	5	22.20	0.86
4	5	5	20.88	1.55
-5	-5	-5	1.45	0.32
-5	-5	-5	2.94	0.61
5	5	5	1.51	0.68
5	5	5	1.78	0.77
5	5	5	1.34	0.35
-6	-5	-5	17.23	1.16
-6	-5	-5	16.66	0.84
6	5	5	15.99	1.63
6	5	5	14.91	1.54
6	5	5	15.90	0.89
-7	-5	-5	8.35	0.78
-7	-5	-5	8.98	1.07
7	5	5	9.11	0.85
7	5	5	9.83	1.65
7	5	5	10.27	1.50
-8	-5	-5	4.66	0.84
-8	-5	-5	6.40	0.90
8	5	5	5.58	1.17
8	5	5	1.57	1.17
-9	-5	-5	2.54	0.78
-9	-5	-5	3.88	0.75
9	5	5	1.62	1.11
9	5	5	0.70	1.31
-10	-5	-5	0.95	0.88
10	5	5	1.95	1.16
10	5	5	1.50	1.71
18	-6	-5	11.75	2.91
18	-6	-5	11.19	2.45
-18	6	5	5.99	1.23
17	-6	-5	9.11	1.60
17	-6	-5	7.82	2.47
16	-6	-5	7.33	1.33
16	-6	-5	12.80	2.35
-16	6	5	11.42	1.11
15	-6	-5	31.80	2.19
15	-6	-5	30.30	3.09
-15	6	5	28.46	1.49
14	-6	-5	24.62	2.68

14	-6	-5	24.58	1.86
-14	6	5	25.63	1.27
13	-6	-5	0.71	1.23
13	-6	-5	0.86	0.71
-13	6	5	0.81	0.89
12	-6	-5	60.37	3.28
12	-6	-5	60.70	2.66
-12	6	5	61.07	2.16
-12	6	5	60.93	2.76
11	-6	-5	89.44	2.98
11	-6	-5	87.63	3.97
11	-6	-5	91.35	3.48
-11	6	5	91.29	3.59
-11	6	5	93.03	2.99
10	-6	-5	18.62	0.87
10	-6	-5	22.60	1.88
10	-6	-5	18.96	1.31
-10	6	5	18.32	0.85
-10	6	5	18.35	1.19
-10	6	5	16.81	1.52
9	-6	-5	82.59	3.14
9	-6	-5	84.28	3.59
9	-6	-5	83.23	2.68
-9	6	5	84.54	3.37
-9	6	5	83.66	2.61
-9	6	5	82.64	3.03
-9	6	5	82.17	2.69
8	-6	-5	94.82	3.54
8	-6	-5	98.98	3.96
8	-6	-5	97.41	3.09
8	-6	-5	97.13	3.37
-8	6	5	103.36	3.14
-8	6	5	97.52	3.44
-8	6	5	102.63	3.85
-8	6	5	95.21	3.10
7	-6	-5	135.12	4.78
7	-6	-5	143.64	4.31
7	-6	-5	145.02	5.17
7	-6	-5	137.12	4.73
-7	6	5	136.76	4.68
-7	6	5	138.06	4.32
-7	6	5	142.91	4.46
-7	6	5	150.33	5.13
6	-6	-5	60.92	2.78

6	-6	-5	64.19	2.00
6	-6	-5	61.83	2.47
6	-6	-5	59.46	2.45
-6	6	5	64.54	2.40
-6	6	5	62.34	2.01
-6	6	5	67.29	2.85
-6	6	5	66.10	2.28
5	-6	-5	50.40	2.38
5	-6	-5	49.21	2.09
5	-6	-5	49.37	2.16
5	-6	-5	49.96	1.58
-5	6	5	50.52	1.95
-5	6	5	50.74	2.47
-5	6	5	52.23	2.03
4	-6	-5	108.07	3.18
4	-6	-5	101.22	3.68
4	-6	-5	105.12	3.18
4	-6	-5	102.91	3.84
4	-6	-5	103.36	3.98
-4	6	5	105.02	3.65
-4	6	5	109.65	3.70
-4	6	5	108.98	4.15
3	-6	-5	75.19	2.83
3	-6	-5	76.13	3.08
3	-6	-5	75.29	3.14
-3	6	5	80.62	3.36
-3	6	5	76.09	2.87
-3	6	5	80.80	2.89
2	-6	-5	67.20	2.62
2	-6	-5	67.74	2.95
2	-6	-5	70.22	2.94
-2	6	5	72.44	2.75
-2	6	5	76.35	2.75
-2	6	5	69.14	3.20
1	-6	-5	22.66	1.22
1	-6	-5	21.49	1.60
1	-6	-5	22.13	1.56
-1	6	5	25.74	1.40
-1	6	5	22.68	1.37
-1	6	5	21.43	1.88
0	-6	-5	7.68	0.78
0	-6	-5	7.20	1.07
0	-6	-5	6.66	1.12
0	6	5	7.53	0.95

0	6	5	6.40	1.00
0	6	5	8.60	1.41
-1	-6	-5	45.91	2.32
-1	-6	-5	46.81	2.56
-1	-6	-5	45.61	1.87
1	6	5	50.69	2.88
1	6	5	43.23	2.08
1	6	5	46.06	2.19
-2	-6	-5	53.15	2.61
-2	-6	-5	55.46	2.91
-2	-6	-5	56.82	2.15
2	6	5	53.10	3.29
2	6	5	55.70	2.45
2	6	5	56.32	2.52
-3	-6	-5	2.22	0.29
-3	-6	-5	2.89	0.55
3	6	5	2.43	0.64
3	6	5	2.40	0.27
3	6	5	1.80	0.70
-4	-6	-5	5.52	0.80
-4	-6	-5	3.46	0.46
4	6	5	4.84	0.98
4	6	5	3.97	0.44
4	6	5	3.86	0.94
-5	-6	-5	7.43	0.64
-5	-6	-5	9.39	0.93
5	6	5	8.36	1.43
5	6	5	7.42	0.62
5	6	5	7.13	1.29
-6	-6	-5	2.61	0.49
-6	-6	-5	2.13	0.65
6	6	5	4.04	0.65
6	6	5	1.26	0.94
6	6	5	4.15	1.08
-7	-6	-5	6.85	0.88
-7	-6	-5	7.28	1.13
7	6	5	7.91	1.33
7	6	5	5.69	1.25
-8	-6	-5	0.99	0.66
-8	-6	-5	2.26	0.83
8	6	5	2.92	1.13
8	6	5	2.21	1.20
-9	-6	-5	1.40	0.82
9	6	5	1.63	1.23

9	6	5	3.06	1.50
18	-7	-5	12.37	3.08
18	-7	-5	8.74	2.08
-18	7	5	11.73	1.50
17	-7	-5	17.26	2.23
17	-7	-5	19.95	3.06
-17	7	5	18.79	1.39
16	-7	-5	17.83	3.30
16	-7	-5	22.09	2.18
-16	7	5	21.66	1.38
15	-7	-5	22.30	2.01
-15	7	5	24.12	1.32
14	-7	-5	37.25	2.24
14	-7	-5	36.99	3.14
-14	7	5	38.02	1.60
13	-7	-5	33.49	2.00
13	-7	-5	31.82	2.77
-13	7	5	31.36	2.11
12	-7	-5	20.66	2.32
12	-7	-5	20.55	1.53
-12	7	5	22.00	1.83
-12	7	5	22.22	1.05
11	-7	-5	25.55	1.14
11	-7	-5	26.56	2.30
11	-7	-5	26.61	1.67
-11	7	5	25.33	1.12
-11	7	5	29.02	1.99
10	-7	-5	98.09	4.20
10	-7	-5	95.51	3.61
10	-7	-5	95.11	3.10
-10	7	5	96.56	2.99
-10	7	5	98.10	3.11
-10	7	5	96.12	3.91
-10	7	5	94.88	3.46
-10	7	5	97.61	3.11
9	-7	-5	110.67	4.50
9	-7	-5	109.05	3.99
9	-7	-5	110.61	3.49
-9	7	5	107.27	3.84
-9	7	5	108.00	3.49
-9	7	5	112.07	3.63
-9	7	5	112.63	4.34
-9	7	5	114.99	3.51
8	-7	-5	103.68	3.22

8	-7	-5	100.14	4.18
8	-7	-5	102.05	3.73
-8	7	5	104.27	3.61
-8	7	5	102.16	3.35
-8	7	5	102.11	3.23
-8	7	5	105.78	4.12
7	-7	-5	42.90	1.43
7	-7	-5	43.31	1.96
7	-7	-5	44.77	2.38
7	-7	-5	40.81	1.93
-7	7	5	44.14	1.85
-7	7	5	42.23	2.35
-7	7	5	42.63	1.68
-7	7	5	41.90	1.44
6	-7	-5	63.60	2.55
6	-7	-5	62.00	2.61
6	-7	-5	65.06	2.01
6	-7	-5	62.45	2.91
-6	7	5	65.04	2.99
-6	7	5	65.26	2.46
-6	7	5	62.62	2.02
-6	7	5	62.98	2.38
5	-7	-5	48.22	2.42
5	-7	-5	44.86	2.03
5	-7	-5	47.19	1.49
5	-7	-5	45.63	2.20
5	-7	-5	48.55	1.48
-5	7	5	48.67	2.00
-5	7	5	47.63	2.56
-5	7	5	46.92	1.96
4	-7	-5	89.40	3.65
4	-7	-5	88.61	3.52
4	-7	-5	86.03	3.27
-4	7	5	90.76	3.30
-4	7	5	89.37	3.28
-4	7	5	96.26	3.90
3	-7	-5	64.96	3.04
3	-7	-5	71.39	2.69
3	-7	-5	69.68	3.03
-3	7	5	74.02	2.76
-3	7	5	73.12	2.82
-3	7	5	70.10	3.30
2	-7	-5	21.78	1.68
2	-7	-5	25.39	1.33

2	-7	-5	21.93	1.68
-2	7	5	23.81	1.44
-2	7	5	26.01	1.47
-2	7	5	23.54	2.03
1	-7	-5	32.80	2.12
1	-7	-5	35.52	1.59
1	-7	-5	33.46	2.04
-1	7	5	34.73	1.82
-1	7	5	34.46	2.45
-1	7	5	33.66	1.76
0	-7	-5	28.57	1.42
0	-7	-5	30.63	1.93
0	-7	-5	28.01	2.11
0	7	5	29.91	2.46
0	7	5	29.66	1.72
0	7	5	30.33	1.68
-1	-7	-5	37.66	2.24
-1	-7	-5	39.52	1.70
1	7	5	39.48	2.11
1	7	5	39.87	2.03
-2	-7	-5	3.47	0.58
-2	-7	-5	2.85	0.88
2	7	5	1.29	0.86
2	7	5	3.23	0.80
-3	-7	-5	3.49	0.60
3	7	5	4.46	1.08
3	7	5	4.26	1.10
-4	-7	-5	5.47	0.87
4	7	5	5.14	0.97
4	7	5	5.20	0.48
4	7	5	6.90	1.14
4	7	5	6.46	0.51
-5	-7	-5	3.47	0.70
5	7	5	4.84	1.06
5	7	5	3.01	1.03
-6	-7	-5	1.53	0.69
6	7	5	1.90	0.45
6	7	5	3.13	1.15
6	7	5	1.91	1.03
-7	-7	-5	2.20	0.76
7	7	5	1.84	1.16
7	7	5	0.10	1.06
-8	-7	-5	1.44	0.88
8	7	5	1.22	1.22

8	7	5	1.34	1.35
18	-8	-5	8.53	2.94
18	-8	-5	9.10	1.93
-18	8	5	9.73	1.38
17	-8	-5	26.07	3.98
17	-8	-5	18.64	2.33
-17	8	5	19.16	1.41
16	-8	-5	11.63	2.54
16	-8	-5	15.53	2.03
-16	8	5	16.17	1.24
15	-8	-5	43.48	3.58
15	-8	-5	44.98	2.65
-15	8	5	45.52	1.89
14	-8	-5	59.16	2.92
14	-8	-5	59.25	3.78
-14	8	5	60.79	2.24
13	-8	-5	15.59	2.44
13	-8	-5	17.33	1.60
-13	8	5	13.47	1.12
12	-8	-5	56.42	2.64
12	-8	-5	59.90	3.50
-12	8	5	59.35	3.02
-12	8	5	57.66	2.09
11	-8	-5	66.07	2.85
11	-8	-5	66.98	3.54
11	-8	-5	68.96	2.31
-11	8	5	67.99	3.22
-11	8	5	68.85	2.11
-11	8	5	65.93	2.21
-11	8	5	65.09	2.29
10	-8	-5	57.26	2.52
10	-8	-5	58.78	1.98
10	-8	-5	56.11	3.15
-10	8	5	56.53	1.94
-10	8	5	57.89	3.00
-10	8	5	58.02	2.08
-10	8	5	59.25	1.98
-10	8	5	56.03	2.34
9	-8	-5	50.91	2.28
9	-8	-5	52.80	1.73
9	-8	-5	51.97	2.88
-9	8	5	48.65	1.82
-9	8	5	48.63	2.77
-9	8	5	48.83	2.10

-9	8	5	49.52	1.72
-9	8	5	50.21	2.01
8	-8	-5	74.06	2.36
8	-8	-5	73.38	3.47
8	-8	-5	71.68	2.90
-8	8	5	75.47	2.70
-8	8	5	71.79	2.35
-8	8	5	72.64	2.60
-8	8	5	72.85	2.77
-8	8	5	73.33	3.40
7	-8	-5	61.75	2.56
7	-8	-5	63.29	2.01
7	-8	-5	60.47	3.09
-7	8	5	63.52	2.46
-7	8	5	63.87	2.38
-7	8	5	62.38	2.02
-7	8	5	61.74	3.11
6	-8	-5	44.34	2.21
6	-8	-5	41.07	2.46
6	-8	-5	42.43	2.00
6	-8	-5	43.01	1.40
-6	8	5	43.81	1.43
-6	8	5	43.56	2.54
-6	8	5	42.78	1.89
-6	8	5	43.03	1.89
5	-8	-5	85.61	3.26
5	-8	-5	84.79	3.51
5	-8	-5	90.82	2.65
5	-8	-5	87.28	3.74
-5	8	5	88.50	3.26
-5	8	5	88.73	3.21
-5	8	5	82.40	3.83
4	-8	-5	72.68	3.30
4	-8	-5	70.28	3.15
4	-8	-5	74.09	2.82
-4	8	5	69.61	2.83
-4	8	5	75.12	3.55
-4	8	5	74.28	2.91
3	-8	-5	39.94	2.35
3	-8	-5	40.79	1.85
3	-8	-5	37.43	2.27
-3	8	5	42.29	2.02
-3	8	5	40.57	2.66
-3	8	5	38.99	1.92

2	-8	-5	78.14	2.81
2	-8	-5	73.79	3.33
2	-8	-5	69.56	3.34
-2	8	5	71.03	2.94
-2	8	5	76.50	3.70
-2	8	5	74.36	3.07
1	-8	-5	40.38	1.79
1	-8	-5	38.64	2.44
1	-8	-5	39.99	2.35
-1	8	5	42.20	2.13
-1	8	5	41.73	2.04
-1	8	5	35.35	2.80
0	-8	-5	13.07	1.60
0	-8	-5	13.82	1.04
0	8	5	11.57	1.33
0	8	5	13.21	1.40
-1	-8	-5	26.35	1.41
-1	-8	-5	28.45	2.08
1	8	5	28.18	1.89
1	8	5	27.16	1.86
-2	-8	-5	16.61	1.13
-2	-8	-5	16.30	1.75
2	8	5	14.58	1.60
-3	-8	-5	3.78	1.22
3	8	5	2.25	0.83
-4	-8	-5	4.08	0.70
4	8	5	3.76	1.08
4	8	5	2.90	0.97
-5	-8	-5	5.10	0.80
5	8	5	3.49	1.11
5	8	5	5.45	1.33
-6	-8	-5	1.47	0.74
6	8	5	2.66	1.18
6	8	5	0.87	1.16
-7	-8	-5	2.18	0.82
7	8	5	2.01	1.35
7	8	5	-0.03	1.14
18	-9	-5	10.49	2.98
18	-9	-5	5.01	1.74
17	-9	-5	24.66	4.24
17	-9	-5	25.11	2.53
-17	9	5	25.39	1.55
16	-9	-5	27.10	3.69
16	-9	-5	27.85	2.37

-16	9	5	25.31	1.48
15	-9	-5	39.78	3.68
15	-9	-5	37.00	2.47
-15	9	5	35.76	1.66
14	-9	-5	42.99	2.51
-14	9	5	43.52	1.77
13	-9	-5	75.94	3.30
13	-9	-5	81.50	4.27
-13	9	5	71.34	2.73
12	-9	-5	60.31	2.74
12	-9	-5	56.26	3.58
-12	9	5	59.75	2.12
11	-9	-5	22.33	2.46
11	-9	-5	22.93	1.07
11	-9	-5	22.96	1.62
-11	9	5	22.54	2.29
-11	9	5	20.92	1.03
-11	9	5	22.50	0.94
-11	9	5	22.70	1.11
10	-9	-5	28.55	2.52
10	-9	-5	27.75	1.75
10	-9	-5	29.93	1.18
-10	9	5	27.33	1.40
-10	9	5	28.44	1.57
-10	9	5	33.49	2.60
-10	9	5	28.29	1.15
-10	9	5	27.80	1.22
9	-9	-5	57.15	2.59
9	-9	-5	58.85	3.29
9	-9	-5	60.67	2.01
-9	9	5	60.21	2.21
-9	9	5	59.29	1.99
-9	9	5	60.89	2.34
-9	9	5	55.51	3.17
-9	9	5	59.18	2.42
8	-9	-5	37.67	1.98
8	-9	-5	37.05	2.58
8	-9	-5	39.95	1.38
-8	9	5	39.00	2.66
-8	9	5	38.20	1.71
-8	9	5	39.97	1.86
-8	9	5	37.32	1.83
-8	9	5	40.38	1.37
7	-9	-5	20.73	1.45

7	-9	-5	20.55	0.82
7	-9	-5	20.49	2.03
-7	9	5	21.50	1.35
-7	9	5	20.74	2.18
-7	9	5	20.48	1.28
-7	9	5	21.12	0.83
-7	9	5	22.90	1.40
6	-9	-5	36.84	2.49
6	-9	-5	38.50	1.91
6	-9	-5	39.31	1.29
-6	9	5	38.75	1.84
-6	9	5	38.40	1.89
-6	9	5	39.05	1.91
-6	9	5	43.99	2.69
5	-9	-5	37.13	1.82
5	-9	-5	34.02	2.35
-5	9	5	35.43	1.77
-5	9	5	34.84	1.84
-5	9	5	36.02	2.59
4	-9	-5	32.40	2.15
4	-9	-5	30.49	2.23
4	-9	-5	29.96	1.63
-4	9	5	29.70	2.54
-4	9	5	32.02	1.80
-4	9	5	28.81	1.69
3	-9	-5	30.27	2.19
3	-9	-5	33.79	1.67
3	-9	-5	34.60	2.35
-3	9	5	31.85	2.69
-3	9	5	32.09	1.88
-3	9	5	34.35	1.84
2	-9	-5	13.07	1.08
2	-9	-5	12.56	1.73
-2	9	5	14.24	1.40
-2	9	5	10.09	1.30
1	-9	-5	12.28	1.70
1	-9	-5	13.48	1.10
-1	9	5	14.52	1.48
-1	9	5	15.38	1.49
0	-9	-5	34.38	2.34
0	-9	-5	34.24	1.63
0	9	5	31.26	2.05
0	9	5	34.00	2.13
-1	-9	-5	3.49	0.62

-1	-9	-5	2.39	1.13
1	9	5	1.89	0.92
-2	-9	-5	0.11	1.09
-2	-9	-5	-1.17	0.50
2	9	5	-0.35	0.82
-3	-9	-5	5.39	1.37
-3	-9	-5	5.77	0.81
3	9	5	5.45	1.20
-4	-9	-5	2.65	0.70
4	9	5	1.19	1.03
4	9	5	0.56	0.97
-5	-9	-5	0.13	0.72
5	9	5	0.28	1.14
5	9	5	-0.01	1.02
-6	-9	-5	0.98	0.81
6	9	5	1.22	1.20
6	9	5	-0.16	1.19
-7	-9	-5	0.29	0.88
7	9	5	0.74	2.73
7	9	5	1.24	1.33
17	-10	-5	14.64	3.16
17	-10	-5	18.69	2.46
-17	10	5	13.80	1.36
16	-10	-5	29.46	4.02
16	-10	-5	26.00	2.51
-16	10	5	30.20	1.56
15	-10	-5	53.63	3.05
15	-10	-5	52.95	4.27
-15	10	5	57.70	2.20
14	-10	-5	28.32	2.19
14	-10	-5	23.66	3.21
-14	10	5	30.91	1.40
13	-10	-5	57.42	2.75
-13	10	5	48.29	2.07
12	-10	-5	56.80	3.73
-12	10	5	59.01	2.15
-12	10	5	57.00	2.10
-12	10	5	68.15	2.26
11	-10	-5	32.91	2.02
11	-10	-5	33.75	1.38
11	-10	-5	32.61	2.92
-11	10	5	33.99	1.35
-11	10	5	35.22	1.59
-11	10	5	32.39	1.36

10	-10	-5	21.53	1.69
10	-10	-5	23.62	1.04
10	-10	-5	21.90	2.49
-10	10	5	23.58	1.01
-10	10	5	23.02	1.21
-10	10	5	21.05	1.40
9	-10	-5	48.30	2.41
9	-10	-5	47.63	3.09
9	-10	-5	51.88	1.77
-9	10	5	56.90	2.29
-9	10	5	51.85	1.74
-9	10	5	45.57	3.08
-9	10	5	51.54	2.09
-9	10	5	48.25	2.22
8	-10	-5	48.10	3.00
8	-10	-5	45.99	2.28
8	-10	-5	50.91	1.63
-8	10	5	46.20	1.60
-8	10	5	45.93	2.06
-8	10	5	47.13	3.09
-8	10	5	47.03	2.21
-8	10	5	44.29	2.11
7	-10	-5	47.52	2.25
7	-10	-5	46.08	2.89
-7	10	5	44.94	1.53
-7	10	5	49.27	2.26
-7	10	5	45.74	2.13
-7	10	5	42.71	2.08
-7	10	5	47.76	3.05
6	-10	-5	39.56	2.62
6	-10	-5	34.89	1.93
-6	10	5	39.58	2.06
-6	10	5	36.73	2.80
-6	10	5	35.54	1.87
-6	10	5	39.88	1.99
5	-10	-5	23.40	2.13
5	-10	-5	21.58	1.47
-5	10	5	23.71	1.64
-5	10	5	21.25	2.61
-5	10	5	23.55	1.73
-5	10	5	23.66	1.55
4	-10	-5	17.63	2.03
4	-10	-5	14.43	1.27
-4	10	5	17.85	1.48

-4	10	5	14.03	1.42
-4	10	5	13.31	1.50
3	-10	-5	35.18	1.73
3	-10	-5	29.97	2.36
-3	10	5	35.33	2.09
-3	10	5	32.70	1.95
2	-10	-5	26.01	1.49
2	-10	-5	27.71	2.28
-2	10	5	22.62	1.87
-2	10	5	27.14	1.91
1	-10	-5	25.21	1.42
1	-10	-5	25.62	2.38
-1	10	5	25.62	2.05
-1	10	5	24.85	1.92
0	-10	-5	14.94	1.16
0	-10	-5	15.88	1.98
0	10	5	12.13	1.71
-1	-10	-5	4.39	1.33
-1	-10	-5	4.05	0.70
1	10	5	1.28	0.97
-2	-10	-5	2.55	0.71
-2	-10	-5	2.46	1.35
2	10	5	2.18	1.10
-3	-10	-5	4.19	1.57
-3	-10	-5	2.38	0.78
3	10	5	2.25	1.19
-4	-10	-5	-0.35	0.73
4	10	5	2.23	1.06
4	10	5	0.91	1.14
-5	-10	-5	1.81	0.87
5	10	5	2.42	1.41
5	10	5	3.00	1.25
-6	-10	-5	1.10	0.95
6	10	5	1.07	1.66
6	10	5	-1.44	1.37
17	-11	-5	20.12	2.65
17	-11	-5	25.41	4.46
-17	11	5	25.14	1.57
16	-11	-5	30.03	4.19
16	-11	-5	23.81	2.56
-16	11	5	24.00	1.47
15	-11	-5	18.28	2.95
15	-11	-5	16.77	2.21
-15	11	5	22.73	1.30

14	-11	-5	28.81	2.28
14	-11	-5	31.09	3.51
-14	11	5	28.93	1.40
13	-11	-5	51.32	2.70
13	-11	-5	46.50	3.74
-13	11	5	44.96	1.94
12	-11	-5	21.62	1.86
12	-11	-5	17.46	3.21
-12	11	5	21.39	1.07
-12	11	5	20.07	1.23
-12	11	5	20.51	1.02
11	-11	-5	21.01	1.11
11	-11	-5	20.70	1.84
11	-11	-5	21.26	2.68
-11	11	5	23.60	1.49
-11	11	5	22.41	1.06
-11	11	5	24.66	1.23
10	-11	-5	11.35	0.77
10	-11	-5	10.16	1.85
10	-11	-5	8.58	1.46
-10	11	5	10.97	1.01
-10	11	5	8.84	1.33
-10	11	5	10.53	0.70
9	-11	-5	17.62	0.87
9	-11	-5	16.21	1.58
9	-11	-5	19.29	2.51
-9	11	5	20.63	1.58
-9	11	5	16.85	1.52
-9	11	5	20.03	1.33
-9	11	5	18.59	0.84
8	-11	-5	40.37	2.93
8	-11	-5	39.64	1.40
8	-11	-5	39.35	2.10
-8	11	5	37.03	1.99
-8	11	5	39.33	1.37
-8	11	5	38.33	1.98
-8	11	5	40.31	2.13
7	-11	-5	17.84	1.53
7	-11	-5	16.36	0.73
7	-11	-5	16.79	2.25
-7	11	5	17.66	0.74
-7	11	5	19.28	1.48
-7	11	5	18.29	1.48
-7	11	5	16.43	1.62

6	-11	-5	12.71	1.86
6	-11	-5	9.41	1.29
-6	11	5	16.33	1.63
-6	11	5	9.60	1.35
-6	11	5	11.54	1.31
5	-11	-5	4.23	0.82
5	-11	-5	3.85	1.29
-5	11	5	4.02	0.92
-5	11	5	6.14	0.96
4	-11	-5	2.91	0.79
4	-11	-5	3.55	1.30
-4	11	5	5.47	1.05
-4	11	5	3.43	0.88
3	-11	-5	6.16	1.03
3	-11	-5	5.05	1.56
-3	11	5	4.28	1.42
-3	11	5	9.96	1.58
2	-11	-5	3.97	1.35
2	-11	-5	1.19	0.66
-2	11	5	0.93	0.84
-2	11	5	2.29	1.44
1	-11	-5	7.82	1.62
1	-11	-5	6.88	0.89
-1	11	5	9.84	1.72
0	-11	-5	6.85	1.08
0	-11	-5	7.86	1.62
0	11	5	6.51	1.26
-1	-11	-5	1.34	0.71
-1	-11	-5	-1.43	1.24
1	11	5	1.46	1.17
-2	-11	-5	0.67	1.42
-2	-11	-5	0.76	0.71
2	11	5	-0.07	1.08
-3	-11	-5	1.69	1.53
-3	-11	-5	2.18	0.80
3	11	5	-0.24	1.18
-4	-11	-5	1.06	0.86
4	11	5	0.26	1.19
4	11	5	1.75	1.31
-5	-11	-5	-0.33	0.90
5	11	5	-0.23	1.83
5	11	5	-0.71	1.44
17	-12	-5	17.21	2.71
17	-12	-5	18.60	3.47

-17	12	5	19.38	1.47
16	-12	-5	24.45	2.70
16	-12	-5	16.07	3.09
-16	12	5	20.46	1.41
15	-12	-5	41.81	4.27
15	-12	-5	34.45	2.77
-15	12	5	36.98	1.71
14	-12	-5	38.95	3.87
14	-12	-5	38.01	2.68
-14	12	5	38.96	1.69
13	-12	-5	27.80	2.31
13	-12	-5	24.70	3.43
-13	12	5	22.54	1.44
-13	12	5	27.05	1.15
12	-12	-5	41.60	2.49
12	-12	-5	36.18	3.60
-12	12	5	42.23	1.64
-12	12	5	41.02	1.73
11	-12	-5	28.22	1.34
11	-12	-5	31.67	3.20
11	-12	-5	34.09	2.49
-11	12	5	31.35	1.63
-11	12	5	32.22	1.88
-11	12	5	32.06	1.32
10	-12	-5	21.95	1.09
10	-12	-5	22.24	2.80
10	-12	-5	25.78	1.91
-10	12	5	22.27	1.03
-10	12	5	20.65	1.47
-10	12	5	24.01	1.83
9	-12	-5	47.83	1.65
9	-12	-5	46.41	3.51
9	-12	-5	41.91	2.40
-9	12	5	46.90	1.62
-9	12	5	44.80	2.38
-9	12	5	44.32	2.22
8	-12	-5	24.08	1.81
8	-12	-5	25.05	0.97
8	-12	-5	21.10	3.26
-8	12	5	23.03	0.96
-8	12	5	25.69	2.18
-8	12	5	22.29	1.94
-8	12	5	23.49	1.69
7	-12	-5	17.45	2.83

7	-12	-5	13.69	1.51
-7	12	5	17.16	1.61
-7	12	5	14.29	1.51
6	-12	-5	50.96	2.43
6	-12	-5	55.49	3.42
-6	12	5	52.30	2.66
-6	12	5	48.09	2.42
5	-12	-5	17.72	1.51
5	-12	-5	19.82	2.50
-5	12	5	20.43	1.79
-5	12	5	16.95	1.68
4	-12	-5	1.18	0.75
4	-12	-5	0.50	1.24
-4	12	5	0.60	0.86
-4	12	5	0.83	1.05
3	-12	-5	6.38	1.71
3	-12	-5	5.18	0.90
-3	12	5	6.09	1.27
2	-12	-5	7.67	1.13
2	-12	-5	9.72	1.76
-2	12	5	7.00	1.33
1	-12	-5	2.24	1.50
1	-12	-5	0.36	0.65
-1	12	5	-0.80	1.02
0	-12	-5	-1.56	1.42
0	-12	-5	1.67	0.75
0	12	5	-0.68	1.23
-1	-12	-5	0.88	1.46
-1	-12	-5	1.04	0.81
1	12	5	-0.81	1.12
-2	-12	-5	-0.44	0.79
-2	-12	-5	1.19	1.59
2	12	5	1.56	1.40
-3	-12	-5	-1.56	1.57
-3	-12	-5	0.17	0.86
3	12	5	0.69	1.51
16	-13	-5	22.58	3.45
16	-13	-5	23.78	2.80
-16	13	5	25.54	1.54
15	-13	-5	10.16	1.84
15	-13	-5	12.48	2.85
-15	13	5	13.88	1.19
14	-13	-5	14.71	2.31
14	-13	-5	20.09	3.72

-14	13	5	15.58	1.14
13	-13	-5	23.05	3.69
13	-13	-5	29.31	2.42
-13	13	5	25.60	1.64
12	-13	-5	16.97	2.02
12	-13	-5	19.62	3.28
-12	13	5	17.36	1.33
-12	13	5	15.87	1.03
11	-13	-5	19.17	1.11
11	-13	-5	16.74	1.91
11	-13	-5	15.02	2.48
-11	13	5	18.99	1.49
-11	13	5	19.86	1.01
-11	13	5	17.76	1.73
10	-13	-5	17.89	3.01
10	-13	-5	19.25	1.89
10	-13	-5	19.43	1.01
-10	13	5	17.92	1.92
-10	13	5	19.12	1.58
-10	13	5	18.07	0.95
9	-13	-5	24.43	1.93
9	-13	-5	23.02	1.06
9	-13	-5	26.72	3.13
-9	13	5	24.20	2.08
-9	13	5	23.59	1.83
-9	13	5	24.54	1.03
8	-13	-5	17.39	0.94
8	-13	-5	18.63	1.81
8	-13	-5	17.78	2.44
-8	13	5	21.00	1.78
7	-13	-5	6.13	1.15
7	-13	-5	6.44	1.88
-7	13	5	6.75	1.14
6	-13	-5	11.55	2.06
6	-13	-5	12.76	1.55
-6	13	5	9.68	1.66
5	-13	-5	14.67	1.53
5	-13	-5	19.30	2.77
-5	13	5	16.76	1.94
4	-13	-5	20.31	1.56
4	-13	-5	21.96	2.77
-4	13	5	19.67	2.13
3	-13	-5	3.03	0.82
3	-13	-5	2.22	1.58

-3	13	5	3.20	1.17
2	-13	-5	3.10	1.65
2	-13	-5	-2.03	1.22
-2	13	5	-0.16	1.08
1	-13	-5	-0.13	1.47
1	-13	-5	0.66	0.75
-1	13	5	-0.31	1.26
0	-13	-5	0.93	1.66
0	-13	-5	1.50	0.82
0	13	5	-1.08	1.35
-1	-13	-5	0.73	1.62
-1	-13	-5	2.55	0.93
1	13	5	0.31	1.32
-2	-13	-5	-0.30	0.90
-2	-13	-5	-1.32	1.65
2	13	5	0.36	1.50
16	-14	-5	14.86	3.25
16	-14	-5	17.70	3.00
15	-14	-5	31.36	4.62
15	-14	-5	28.89	3.03
-15	14	5	33.94	1.68
14	-14	-5	17.90	3.25
14	-14	-5	16.07	2.09
-14	14	5	17.45	1.24
13	-14	-5	5.86	1.56
13	-14	-5	6.45	2.41
-13	14	5	4.37	1.11
12	-14	-5	13.25	2.23
12	-14	-5	11.87	2.46
-12	14	5	14.12	1.02
-12	14	5	13.15	1.50
11	-14	-5	19.52	2.17
11	-14	-5	19.66	3.40
-11	14	5	23.06	1.79
-11	14	5	19.30	1.07
10	-14	-5	24.50	2.21
10	-14	-5	23.83	3.43
-10	14	5	21.53	1.93
-10	14	5	21.03	1.03
9	-14	-5	22.26	2.05
9	-14	-5	20.41	3.30
-9	14	5	18.58	0.91
-9	14	5	19.42	1.88
8	-14	-5	4.89	2.05

8	-14	-5	6.12	1.23
-8	14	5	5.69	1.31
7	-14	-5	4.62	1.09
7	-14	-5	4.31	1.88
-7	14	5	4.63	1.22
6	-14	-5	13.35	1.64
6	-14	-5	16.69	2.59
-6	14	5	11.44	2.02
5	-14	-5	6.96	1.12
5	-14	-5	5.92	1.95
-5	14	5	4.25	1.24
4	-14	-5	-0.26	0.82
4	-14	-5	0.13	1.69
-4	14	5	-0.69	1.17
3	-14	-5	3.37	1.78
3	-14	-5	-0.05	0.84
-3	14	5	2.25	1.34
2	-14	-5	1.27	1.70
2	-14	-5	0.02	0.81
-2	14	5	-1.76	1.20
1	-14	-5	1.05	0.90
-1	14	5	1.14	1.52
0	-14	-5	1.53	0.97
0	-14	-5	-2.03	1.80
0	14	5	-1.03	1.49
-1	-14	-5	4.06	2.68
-1	-14	-5	1.20	1.04
1	14	5	1.39	2.95
15	-15	-5	10.88	3.08
15	-15	-5	9.50	2.12
-15	15	5	9.08	1.23
14	-15	-5	5.42	2.65
14	-15	-5	4.78	1.83
-14	15	5	8.13	1.10
13	-15	-5	9.55	2.90
13	-15	-5	12.21	2.00
-13	15	5	11.64	1.31
12	-15	-5	16.05	2.47
12	-15	-5	7.98	2.63
-12	15	5	9.83	1.63
-12	15	5	10.89	0.97
11	-15	-5	12.80	2.81
11	-15	-5	11.24	1.79
-11	15	5	9.93	1.77

10	-15	-5	5.24	2.28
10	-15	-5	7.80	1.58
9	-15	-5	4.65	2.17
9	-15	-5	4.63	1.35
-9	15	5	3.74	1.22
8	-15	-5	8.49	2.41
-8	15	5	10.57	1.59
7	-15	-5	13.64	2.50
-7	15	5	12.68	1.71
6	-15	-5	2.17	2.06
-6	15	5	2.68	1.30
5	-15	-5	2.29	1.10
5	-15	-5	0.60	1.78
-5	15	5	-0.56	1.29
4	-15	-5	3.65	1.92
4	-15	-5	1.69	0.98
-4	15	5	3.43	1.50
3	-15	-5	1.66	0.95
3	-15	-5	-2.06	1.82
-3	15	5	2.15	1.43
2	-15	-5	1.31	0.99
2	-15	-5	0.66	1.97
-2	15	5	0.51	1.44
1	-15	-5	-0.35	1.99
1	-15	-5	-0.15	1.02
-1	15	5	3.42	1.67
13	-16	-5	6.43	2.85
13	-16	-5	3.23	1.85
12	-16	-5	6.29	1.86
12	-16	-5	3.65	2.53
11	-16	-5	8.76	2.65
11	-16	-5	7.09	1.81
-11	16	5	5.73	1.39
10	-16	-5	1.56	1.53
10	-16	-5	4.08	2.36
-10	16	5	3.04	1.31
9	-16	-5	0.37	2.24
9	-16	-5	4.44	1.50
-9	16	5	2.18	1.36
8	-16	-5	-0.18	2.16
8	-16	-5	-2.28	1.22
-8	16	5	2.66	1.43
7	-16	-5	0.04	2.04
7	-16	-5	0.06	1.21

-7	16	5	0.21	1.41
6	-16	-5	4.41	2.19
6	-16	-5	1.59	1.17
-6	16	5	1.17	1.48
5	-16	-5	1.86	2.06
5	-16	-5	1.41	1.12
-5	16	5	1.07	1.49
4	-16	-5	2.31	2.10
4	-16	-5	-0.30	1.08
-4	16	5	0.81	1.61
3	-16	-5	1.76	1.11
3	-16	-5	3.72	2.31
-3	16	5	2.92	1.79
11	-17	-5	2.15	2.63
11	-17	-5	-0.05	1.80
-11	17	5	1.97	1.46
10	-17	-5	-0.05	1.58
10	-17	-5	-0.30	2.45
-10	17	5	-0.30	1.49
9	-17	-5	4.65	2.51
9	-17	-5	3.84	1.67
-9	17	5	1.27	1.58
8	-17	-5	3.04	1.40
8	-17	-5	-0.48	2.42
-8	17	5	2.62	1.57
7	-17	-5	0.71	2.45
7	-17	-5	-1.27	1.26
-7	17	5	1.36	1.68
6	-17	-5	-0.48	2.67
-6	17	5	1.94	1.72
0	18	-6	2.95	4.12
0	-18	6	3.68	2.72
-1	18	-6	7.49	2.36
1	-18	6	4.81	2.59
-2	18	-6	1.77	0.71
2	-18	6	-2.87	2.33
2	-18	6	1.28	1.50
-3	18	-6	2.11	0.64
3	-18	6	0.35	1.50
3	-18	6	-0.42	2.43
-4	18	-6	9.74	1.08
4	-18	6	10.20	1.95
4	-18	6	6.02	2.78
-5	18	-6	4.66	0.76

5	-18	6	5.46	2.70
5	-18	6	2.83	1.59
-6	18	-6	3.15	0.76
6	-18	6	1.76	1.49
6	-18	6	2.42	2.60
-7	18	-6	9.63	1.17
7	-18	6	11.68	2.02
7	-18	6	9.46	3.13
-8	18	-6	13.90	1.26
8	-18	6	8.46	2.98
8	-18	6	10.97	1.94
9	-18	6	4.21	4.26
2	17	-6	0.41	2.21
-2	-17	6	7.27	2.65
1	17	-6	5.89	2.15
-1	-17	6	10.08	2.69
0	17	-6	3.11	0.68
0	-17	6	2.25	2.32
-1	17	-6	2.98	0.75
1	-17	6	4.46	1.42
1	-17	6	5.68	2.38
-2	17	-6	8.31	1.41
2	-17	6	11.55	2.56
2	-17	6	9.94	1.65
-3	17	-6	14.55	1.11
3	-17	6	14.89	2.88
3	-17	6	14.27	2.46
-4	17	-6	7.65	0.98
4	-17	6	5.25	1.49
4	-17	6	6.88	2.43
-5	17	-6	14.74	1.15
5	-17	6	14.08	2.89
5	-17	6	20.53	2.50
-6	17	-6	14.61	1.15
6	-17	6	11.23	2.81
6	-17	6	14.96	2.00
-7	17	-6	11.12	1.12
7	-17	6	10.47	2.82
7	-17	6	14.56	2.42
-8	17	-6	11.42	1.18
8	-17	6	13.68	3.23
8	-17	6	18.81	2.60
-9	17	-6	12.57	1.23
9	-17	6	12.62	1.88

9	-17	6	8.16	2.83
-10	17	-6	10.32	1.23
10	-17	6	9.66	3.11
10	-17	6	11.08	2.09
4	16	-6	2.96	2.51
-4	-16	6	2.83	2.34
3	16	-6	1.35	1.89
-3	-16	6	5.69	2.29
2	16	-6	1.59	0.67
2	16	-6	1.24	1.81
-2	-16	6	1.10	2.08
1	16	-6	1.38	0.57
-1	-16	6	2.36	2.03
-1	-16	6	0.72	1.35
0	16	-6	4.11	0.64
0	-16	6	4.03	2.08
0	-16	6	5.06	1.45
-1	16	-6	11.54	1.00
1	-16	6	13.80	2.27
1	-16	6	12.02	2.49
-2	16	-6	3.66	0.63
2	-16	6	2.22	1.28
2	-16	6	0.74	2.01
-3	16	-6	10.28	0.95
3	-16	6	9.22	1.56
3	-16	6	6.10	2.32
-4	16	-6	26.27	1.36
4	-16	6	32.50	2.64
4	-16	6	23.61	3.50
-5	16	-6	20.14	1.17
5	-16	6	19.04	3.03
5	-16	6	18.95	2.34
-6	16	-6	6.57	0.93
6	-16	6	9.65	2.59
6	-16	6	12.95	2.09
-7	16	-6	13.40	1.10
7	-16	6	15.47	2.16
7	-16	6	17.42	2.92
-8	16	-6	26.87	1.45
8	-16	6	24.37	3.95
8	-16	6	29.90	2.49
-9	16	-6	18.18	1.29
9	-16	6	16.53	3.03
9	-16	6	18.96	2.33

-10	16	-6	13.81	1.27
10	-16	6	17.65	2.30
10	-16	6	13.30	2.79
-11	16	-6	25.33	1.50
11	-16	6	17.08	3.46
5	15	-6	1.55	2.03
-5	-15	6	1.17	2.08
4	15	-6	2.49	0.89
4	15	-6	2.06	1.93
-4	-15	6	5.33	2.06
3	15	-6	5.27	1.95
3	15	-6	4.44	0.74
-3	-15	6	3.41	1.99
2	15	-6	5.32	0.75
-2	-15	6	3.44	1.26
-2	-15	6	4.58	1.82
1	15	-6	3.22	0.63
-1	-15	6	3.48	1.84
-1	-15	6	3.07	1.29
0	15	-6	8.68	0.87
0	-15	6	10.33	1.64
0	-15	6	9.83	2.11
-1	15	-6	6.75	0.83
1	-15	6	5.45	1.37
1	-15	6	5.16	1.88
-2	15	-6	26.26	1.26
2	-15	6	25.50	3.12
2	-15	6	26.83	2.31
-3	15	-6	18.84	1.08
3	-15	6	20.06	2.12
3	-15	6	18.45	2.94
-4	15	-6	5.44	0.79
4	-15	6	5.17	2.00
4	-15	6	6.45	1.32
-5	15	-6	18.28	1.24
5	-15	6	18.43	3.07
5	-15	6	20.66	2.06
-6	15	-6	27.06	1.34
6	-15	6	27.87	2.23
6	-15	6	26.35	3.50
-7	15	-6	21.92	1.23
7	-15	6	19.78	3.41
7	-15	6	21.36	2.15
-8	15	-6	17.23	1.15

8	-15	6	16.52	3.01
-9	15	-6	18.71	1.22
9	-15	6	13.57	2.78
9	-15	6	20.69	2.19
-10	15	-6	21.33	1.34
10	-15	6	26.77	2.26
10	-15	6	13.63	3.04
-11	15	-6	17.80	1.33
11	-15	6	14.46	3.10
-12	15	-6	16.74	1.40
12	-15	6	14.28	3.44
7	14	-6	-0.31	2.09
-7	-14	6	0.70	2.01
6	14	-6	0.22	1.99
6	14	-6	1.10	0.79
-6	-14	6	3.33	1.97
5	14	-6	2.83	0.90
5	14	-6	3.35	1.91
-5	-14	6	0.09	1.73
4	14	-6	6.35	0.81
4	14	-6	9.11	1.98
-4	-14	6	12.98	2.14
3	14	-6	5.70	0.76
-3	-14	6	6.34	1.76
-3	-14	6	4.06	1.25
2	14	-6	4.57	0.69
-2	-14	6	5.11	1.70
1	14	-6	6.71	0.84
-1	-14	6	9.26	1.85
-1	-14	6	8.22	1.52
0	14	-6	25.51	1.23
0	-14	6	29.16	2.80
0	-14	6	20.62	2.16
-1	14	-6	26.76	1.25
1	-14	6	25.35	2.95
1	-14	6	27.90	2.15
-2	14	-6	13.48	0.92
2	-14	6	14.65	1.78
2	-14	6	14.95	2.31
-3	14	-6	4.08	0.57
3	-14	6	3.55	1.07
3	-14	6	2.09	1.45
-4	14	-6	43.46	1.61
4	-14	6	30.44	3.02

4	-14	6	36.24	3.25
-5	14	-6	49.50	1.92
5	-14	6	51.11	2.74
5	-14	6	48.69	3.65
-6	14	-6	21.95	1.20
6	-14	6	22.57	1.98
6	-14	6	27.44	3.17
-7	14	-6	55.01	2.15
7	-14	6	53.89	2.84
7	-14	6	56.88	4.10
7	-14	6	61.32	2.18
-8	14	-6	47.79	1.93
8	-14	6	46.17	2.64
8	-14	6	48.81	2.06
-9	14	-6	27.28	1.40
9	-14	6	24.60	2.16
9	-14	6	22.20	3.65
9	-14	6	28.17	1.68
-10	14	-6	25.54	1.39
10	-14	6	18.16	2.93
10	-14	6	23.79	2.16
10	-14	6	26.16	1.80
-11	14	-6	22.62	1.40
11	-14	6	21.44	1.91
11	-14	6	29.38	4.22
-12	14	-6	15.57	1.32
12	-14	6	14.00	3.21
12	-14	6	16.13	1.97
8	13	-6	1.01	1.26
8	13	-6	0.61	0.88
8	13	-6	1.20	2.16
7	13	-6	2.60	2.03
7	13	-6	2.66	0.85
-7	-13	6	7.14	1.88
6	13	-6	2.57	1.41
6	13	-6	5.11	2.06
-6	-13	6	5.31	1.76
5	13	-6	12.89	2.23
5	13	-6	9.56	1.07
-5	-13	6	9.76	1.83
4	13	-6	6.88	0.97
-4	-13	6	6.70	1.54
-4	-13	6	5.47	1.27
3	13	-6	8.02	0.92

-3	-13	6	10.27	2.17
-3	-13	6	7.50	1.30
2	13	-6	18.38	1.11
-2	-13	6	19.96	2.43
-2	-13	6	18.03	0.97
-2	-13	6	20.67	2.04
1	13	-6	7.98	0.84
-1	-13	6	11.95	2.20
-1	-13	6	9.45	0.77
-1	-13	6	10.48	1.68
0	13	-6	4.98	0.72
0	-13	6	3.45	1.00
0	-13	6	5.64	1.50
0	-13	6	6.17	0.73
-1	13	-6	20.21	1.03
1	-13	6	19.88	2.07
1	-13	6	19.52	1.05
1	-13	6	19.94	2.46
-2	13	-6	34.26	1.45
2	-13	6	34.03	2.16
2	-13	6	35.53	1.46
2	-13	6	36.90	2.86
-3	13	-6	30.39	1.29
3	-13	6	26.36	2.66
3	-13	6	27.96	1.99
-4	13	-6	21.35	1.07
4	-13	6	21.05	2.51
4	-13	6	19.17	1.76
-5	13	-6	25.48	1.22
5	-13	6	22.22	3.16
5	-13	6	26.92	1.94
-6	13	-6	23.63	1.20
6	-13	6	23.37	1.04
6	-13	6	19.60	2.78
6	-13	6	24.54	1.21
6	-13	6	25.64	1.95
-7	13	-6	37.45	1.56
7	-13	6	35.26	3.36
7	-13	6	35.73	2.23
7	-13	6	35.50	1.50
7	-13	6	38.18	1.62
-8	13	-6	36.58	1.76
-8	13	-6	32.65	1.55
8	-13	6	35.82	1.59

8	-13	6	36.72	2.22
8	-13	6	35.31	3.50
8	-13	6	35.87	1.70
-9	13	-6	17.08	1.11
-9	13	-6	18.56	1.26
9	-13	6	16.68	1.64
9	-13	6	18.80	1.41
9	-13	6	19.25	1.83
9	-13	6	19.47	3.30
-10	13	-6	17.50	1.19
10	-13	6	17.05	1.55
10	-13	6	19.23	1.87
10	-13	6	21.46	3.25
-11	13	-6	21.38	1.33
11	-13	6	21.99	1.84
11	-13	6	14.40	2.89
11	-13	6	25.57	2.01
-12	13	-6	15.10	1.25
12	-13	6	19.37	1.99
12	-13	6	14.07	2.98
-13	13	-6	11.18	1.30
13	-13	6	6.58	2.85
13	-13	6	16.32	2.24
9	12	-6	3.80	2.13
9	12	-6	2.52	1.34
9	12	-6	1.92	0.94
8	12	-6	5.89	1.41
8	12	-6	3.40	2.09
8	12	-6	4.93	0.92
7	12	-6	5.96	0.87
7	12	-6	6.04	1.33
-7	-12	6	4.33	1.59
6	12	-6	4.34	1.79
6	12	-6	5.43	0.78
6	12	-6	7.30	1.04
-6	-12	6	5.57	1.52
5	12	-6	15.51	1.07
5	12	-6	15.90	1.15
-5	-12	6	17.37	2.02
-5	-12	6	16.42	2.19
4	12	-6	14.77	1.05
4	12	-6	15.65	1.13
-4	-12	6	16.27	1.89
-4	-12	6	14.15	2.09

3	12	-6	46.55	1.83
-3	-12	6	46.48	2.62
-3	-12	6	44.39	1.69
-3	-12	6	46.46	2.91
2	12	-6	10.08	0.85
2	12	-6	11.82	2.44
-2	-12	6	12.23	1.73
-2	-12	6	9.00	1.45
-2	-12	6	9.29	0.74
1	12	-6	5.56	0.72
1	12	-6	5.45	1.60
-1	-12	6	5.58	1.34
-1	-12	6	6.35	0.66
0	12	-6	29.27	1.26
0	12	-6	25.79	2.64
0	-12	6	27.33	1.21
0	-12	6	33.46	2.55
0	-12	6	28.41	1.93
-1	12	-6	73.32	3.93
-1	12	-6	87.72	2.75
1	-12	6	80.40	2.71
1	-12	6	75.28	3.90
1	-12	6	81.50	3.42
-2	12	-6	38.99	1.54
-2	12	-6	41.13	2.86
2	-12	6	34.91	2.72
2	-12	6	39.96	1.58
2	-12	6	39.92	2.18
-3	12	-6	27.86	1.20
3	-12	6	25.75	1.27
3	-12	6	26.19	1.85
3	-12	6	30.99	2.51
-4	12	-6	72.47	2.50
-4	12	-6	69.42	2.83
4	-12	6	74.82	2.69
4	-12	6	71.48	3.09
4	-12	6	71.32	3.88
-5	12	-6	42.02	1.62
-5	12	-6	38.43	1.84
5	-12	6	41.23	2.20
5	-12	6	35.82	2.96
5	-12	6	43.63	1.61
5	-12	6	40.45	1.45
-6	12	-6	10.60	0.81

-6	12	-6	11.00	1.07
6	-12	6	10.98	0.85
6	-12	6	11.80	1.40
6	-12	6	12.73	2.25
-7	12	-6	23.76	1.39
-7	12	-6	24.45	1.38
7	-12	6	23.92	2.78
7	-12	6	26.69	1.30
7	-12	6	26.60	1.25
7	-12	6	24.48	1.76
-8	12	-6	37.58	1.72
-8	12	-6	37.88	1.62
8	-12	6	40.63	3.38
8	-12	6	37.16	2.12
8	-12	6	37.69	1.67
8	-12	6	41.28	1.77
-9	12	-6	34.96	1.61
-9	12	-6	35.88	1.56
9	-12	6	32.47	1.75
9	-12	6	36.00	2.06
9	-12	6	33.29	1.68
9	-12	6	32.50	3.38
-10	12	-6	30.56	1.51
10	-12	6	32.69	1.89
10	-12	6	30.26	2.00
10	-12	6	32.51	3.64
-11	12	-6	24.91	1.40
11	-12	6	26.02	3.09
11	-12	6	25.55	1.93
11	-12	6	19.44	3.55
-12	12	-6	11.06	1.13
12	-12	6	7.98	2.58
12	-12	6	11.58	1.80
-13	12	-6	15.99	1.36
13	-12	6	19.23	2.24
13	-12	6	20.04	3.46
10	11	-6	0.04	2.04
10	11	-6	3.01	0.93
10	11	-6	2.65	1.41
9	11	-6	1.62	1.92
9	11	-6	0.70	1.23
9	11	-6	-1.56	0.86
9	11	-6	2.06	0.86
8	11	-6	1.69	1.67

8	11	-6	2.48	0.83
8	11	-6	0.92	0.68
8	11	-6	1.58	0.97
7	11	-6	10.04	1.07
7	11	-6	11.31	1.20
7	11	-6	6.60	1.86
-7	-11	6	13.59	2.48
6	11	-6	8.66	1.19
6	11	-6	10.42	1.01
6	11	-6	8.34	0.98
-6	-11	6	10.60	1.54
-6	-11	6	12.94	1.83
5	11	-6	22.68	1.17
5	11	-6	23.25	1.35
5	11	-6	19.43	1.15
5	11	-6	20.70	1.19
-5	-11	6	21.91	1.99
-5	-11	6	21.84	2.14
4	11	-6	21.99	1.36
4	11	-6	18.52	0.98
4	11	-6	14.63	2.60
4	11	-6	19.08	1.08
-4	-11	6	14.95	1.93
-4	-11	6	17.61	1.76
-4	-11	6	17.90	0.95
3	11	-6	18.29	2.50
3	11	-6	21.30	1.12
-3	-11	6	21.75	2.05
-3	-11	6	18.90	1.76
-3	-11	6	22.52	0.99
2	11	-6	53.60	1.96
2	11	-6	52.50	3.22
-2	-11	6	55.33	1.88
-2	-11	6	50.24	2.56
-2	-11	6	52.26	2.91
1	11	-6	41.36	1.57
1	11	-6	38.28	2.75
-1	-11	6	40.65	1.51
-1	-11	6	39.45	2.59
-1	-11	6	40.94	2.19
0	11	-6	21.95	2.25
0	11	-6	24.25	1.08
0	-11	6	21.64	1.63
0	-11	6	24.05	2.16

0	-11	6	23.30	1.06
-1	11	-6	42.00	1.62
-1	11	-6	40.92	2.68
-1	11	-6	41.29	2.00
1	-11	6	42.48	1.62
1	-11	6	47.84	2.79
1	-11	6	45.51	2.21
-2	11	-6	80.32	2.69
-2	11	-6	81.52	3.06
-2	11	-6	79.06	3.71
2	-11	6	80.80	3.26
2	-11	6	79.83	2.71
2	-11	6	78.37	3.84
-3	11	-6	68.79	3.38
-3	11	-6	70.09	2.64
-3	11	-6	65.46	2.29
3	-11	6	63.56	2.31
3	-11	6	61.98	3.41
3	-11	6	66.77	2.84
-4	11	-6	31.95	1.32
-4	11	-6	33.48	1.60
-4	11	-6	29.93	2.37
4	-11	6	30.79	2.53
4	-11	6	32.45	1.84
-5	11	-6	41.46	1.60
-5	11	-6	42.65	1.80
5	-11	6	42.12	1.55
5	-11	6	39.82	2.07
5	-11	6	40.90	2.90
5	-11	6	40.01	1.49
-6	11	-6	26.22	1.31
-6	11	-6	22.25	1.12
6	-11	6	23.22	1.62
6	-11	6	23.17	1.12
6	-11	6	23.70	1.09
6	-11	6	23.30	2.37
-7	11	-6	29.81	1.42
-7	11	-6	28.14	1.30
7	-11	6	28.10	1.77
7	-11	6	29.79	1.36
7	-11	6	30.13	1.36
-8	11	-6	52.44	2.08
-8	11	-6	59.40	2.16
8	-11	6	56.66	2.51

8	-11	6	53.06	2.18
8	-11	6	56.66	2.16
8	-11	6	55.73	3.69
-9	11	-6	33.35	1.51
-9	11	-6	36.59	1.57
9	-11	6	33.09	1.91
9	-11	6	29.73	3.16
9	-11	6	33.90	1.67
9	-11	6	33.62	1.70
-10	11	-6	12.54	1.10
-10	11	-6	11.91	1.02
10	-11	6	14.08	1.49
10	-11	6	7.72	2.14
10	-11	6	12.97	1.31
-11	11	-6	27.78	1.45
11	-11	6	19.26	3.04
11	-11	6	26.83	1.79
11	-11	6	26.31	1.83
-12	11	-6	12.59	1.18
12	-11	6	15.91	1.78
12	-11	6	17.00	2.94
-13	11	-6	9.38	1.17
13	-11	6	6.23	1.36
13	-11	6	3.67	2.58
11	10	-6	0.12	0.82
11	10	-6	1.93	1.38
11	10	-6	-0.23	1.13
11	10	-6	3.35	2.03
10	10	-6	1.57	0.77
10	10	-6	-1.26	1.17
10	10	-6	0.41	0.99
10	10	-6	0.49	1.88
9	10	-6	2.76	1.75
9	10	-6	5.37	1.14
9	10	-6	4.57	1.00
9	10	-6	6.37	0.83
8	10	-6	10.75	1.93
8	10	-6	12.35	1.28
8	10	-6	10.37	1.52
7	10	-6	4.60	0.74
7	10	-6	3.32	0.63
7	10	-6	4.78	0.72
-7	-10	6	3.51	1.15
-7	-10	6	4.80	1.20

6	10	-6	11.88	1.94
6	10	-6	10.41	0.97
6	10	-6	11.56	0.94
6	10	-6	13.71	0.97
-6	-10	6	11.80	1.68
-6	-10	6	11.20	1.66
5	10	-6	26.90	1.27
5	10	-6	25.38	2.63
5	10	-6	24.85	1.23
5	10	-6	25.97	1.24
5	10	-6	27.64	1.17
-5	-10	6	23.51	2.11
-5	-10	6	27.34	2.04
4	10	-6	26.09	1.22
4	10	-6	22.57	2.41
4	10	-6	26.96	1.08
4	10	-6	24.17	1.17
-4	-10	6	27.04	1.10
-4	-10	6	26.60	1.84
-4	-10	6	27.14	1.98
3	10	-6	46.32	1.73
3	10	-6	41.39	2.79
-3	-10	6	46.43	2.55
-3	-10	6	45.20	2.33
-3	-10	6	46.53	1.63
2	10	-6	16.03	2.01
2	10	-6	19.93	1.01
-2	-10	6	21.97	0.94
-2	-10	6	20.70	1.86
-2	-10	6	20.61	1.52
1	10	-6	35.11	1.40
1	10	-6	34.09	2.40
1	10	-6	34.68	1.79
-1	-10	6	36.38	1.35
-1	-10	6	36.44	2.33
-1	-10	6	35.88	1.94
0	10	-6	74.78	2.48
0	10	-6	74.41	2.86
0	10	-6	71.21	3.38
0	-10	6	71.79	3.41
0	-10	6	72.54	3.00
0	-10	6	72.90	2.45
-1	10	-6	63.22	3.15
-1	10	-6	66.91	2.29

-1	10	-6	68.07	2.63
1	-10	6	70.97	3.32
1	-10	6	66.58	2.28
1	-10	6	65.87	2.78
-2	10	-6	39.31	1.46
-2	10	-6	38.83	1.76
-2	10	-6	34.91	2.32
2	-10	6	38.90	2.44
2	-10	6	38.30	1.48
2	-10	6	40.10	1.95
-3	10	-6	44.37	1.61
-3	10	-6	43.24	2.54
-3	10	-6	44.84	1.86
3	-10	6	43.38	2.06
3	-10	6	42.19	2.65
3	-10	6	41.17	1.61
-4	10	-6	54.42	2.15
-4	10	-6	52.46	2.79
-4	10	-6	55.55	1.96
4	-10	6	54.18	3.09
4	-10	6	56.30	2.39
4	-10	6	55.48	2.00
4	-10	6	55.65	1.86
-5	10	-6	45.34	1.67
-5	10	-6	40.81	2.47
-5	10	-6	46.93	1.83
5	-10	6	46.66	2.86
5	-10	6	46.91	1.63
5	-10	6	44.71	2.07
5	-10	6	36.82	1.79
-6	10	-6	38.18	1.62
-6	10	-6	37.89	1.52
6	-10	6	39.88	1.52
6	-10	6	38.17	2.78
6	-10	6	36.21	1.87
6	-10	6	39.37	1.54
-7	10	-6	56.94	2.03
-7	10	-6	54.05	2.06
7	-10	6	52.97	2.34
7	-10	6	53.92	3.36
7	-10	6	53.71	2.08
7	-10	6	55.78	2.07
-8	10	-6	73.41	2.57
-8	10	-6	70.95	2.59

8	-10	6	68.25	4.02
8	-10	6	75.47	2.88
8	-10	6	73.34	2.65
8	-10	6	74.21	2.69
-9	10	-6	28.17	1.36
-9	10	-6	27.63	1.43
9	-10	6	30.16	1.56
9	-10	6	26.60	2.99
9	-10	6	28.52	1.71
9	-10	6	30.12	1.60
-10	10	-6	23.48	1.41
-10	10	-6	22.80	1.28
10	-10	6	25.51	1.56
10	-10	6	24.65	1.59
10	-10	6	22.26	3.15
-11	10	-6	15.01	1.13
11	-10	6	15.47	1.46
11	-10	6	12.66	2.38
11	-10	6	15.64	1.54
-12	10	-6	9.56	1.10
12	-10	6	12.27	1.67
12	-10	6	11.40	2.83
-13	10	-6	18.11	1.29
13	-10	6	18.85	3.33
13	-10	6	13.40	2.04
14	-10	6	10.45	1.67
12	9	-6	1.62	0.84
12	9	-6	0.25	1.41
12	9	-6	2.69	1.98
12	9	-6	2.03	1.22
11	9	-6	3.41	0.82
11	9	-6	3.58	1.86
11	9	-6	0.29	1.21
11	9	-6	2.01	1.09
10	9	-6	8.15	1.12
10	9	-6	11.55	2.13
10	9	-6	12.39	1.52
10	9	-6	12.74	1.71
9	9	-6	12.62	1.38
9	9	-6	13.64	2.12
9	9	-6	12.62	1.08
9	9	-6	10.88	1.50
8	9	-6	9.91	2.54
8	9	-6	8.39	1.31

8	9	-6	9.16	1.06
8	9	-6	6.63	1.60
8	9	-6	8.79	0.92
8	9	-6	8.41	1.11
7	9	-6	15.73	1.04
7	9	-6	18.13	2.56
7	9	-6	15.51	1.16
7	9	-6	17.44	1.08
-7	-9	6	15.54	1.67
6	9	-6	21.12	1.17
6	9	-6	20.67	1.05
6	9	-6	23.82	2.43
6	9	-6	22.43	1.12
6	9	-6	21.20	1.06
-6	-9	6	21.38	1.72
-6	-9	6	19.03	1.72
5	9	-6	49.82	1.84
5	9	-6	48.26	1.84
5	9	-6	47.60	1.82
5	9	-6	49.46	3.00
5	9	-6	50.48	1.74
-5	-9	6	47.75	2.44
-5	-9	6	50.90	2.47
4	9	-6	37.80	1.45
4	9	-6	34.24	2.42
4	9	-6	34.27	1.40
4	9	-6	35.65	1.28
4	9	-6	37.56	2.20
-4	-9	6	37.33	2.10
-4	-9	6	37.19	2.04
-4	-9	6	33.83	1.34
3	9	-6	27.99	2.11
3	9	-6	30.50	1.24
3	9	-6	29.47	1.62
-3	-9	6	29.42	1.91
-3	-9	6	29.18	1.72
-3	-9	6	30.86	1.16
2	9	-6	59.72	2.42
2	9	-6	59.86	2.05
2	9	-6	57.80	2.90
-2	-9	6	56.98	2.55
-2	-9	6	58.96	1.97
-2	-9	6	55.60	2.72
1	9	-6	75.09	2.79

1	9	-6	75.00	3.26
1	9	-6	73.70	2.43
-1	-9	6	72.90	2.39
-1	-9	6	69.91	2.90
-1	-9	6	64.92	3.13
0	9	-6	57.00	2.78
0	9	-6	57.88	2.00
0	9	-6	57.93	2.32
0	-9	6	57.62	2.44
0	-9	6	57.10	1.98
0	-9	6	59.21	2.82
-1	9	-6	57.63	2.20
-1	9	-6	55.37	2.67
-1	9	-6	52.92	1.90
1	-9	6	55.90	2.79
1	-9	6	52.51	1.90
1	-9	6	53.68	2.32
-2	9	-6	95.15	3.29
-2	9	-6	93.37	3.80
-2	9	-6	95.35	3.06
2	-9	6	91.98	3.45
2	-9	6	93.26	3.07
2	-9	6	93.37	3.98
-3	9	-6	56.82	2.14
-3	9	-6	56.76	2.71
-3	9	-6	57.09	1.97
3	-9	6	57.06	2.33
3	-9	6	56.92	2.93
3	-9	6	54.79	1.83
3	-9	6	54.41	1.98
3	-9	6	55.95	1.86
-4	9	-6	42.23	2.35
-4	9	-6	45.37	1.65
-4	9	-6	45.75	1.78
4	-9	6	46.60	1.60
4	-9	6	42.27	1.67
4	-9	6	45.90	1.59
4	-9	6	43.06	2.63
4	-9	6	46.60	2.01
-5	9	-6	42.89	2.34
-5	9	-6	44.49	1.73
-5	9	-6	41.35	1.62
5	-9	6	43.56	1.61
5	-9	6	44.83	1.60

5	-9	6	41.57	2.64
5	-9	6	44.78	1.96
-6	9	-6	59.86	2.90
-6	9	-6	62.22	2.23
-6	9	-6	61.53	2.17
6	-9	6	57.98	3.27
6	-9	6	60.75	2.45
6	-9	6	61.76	2.19
6	-9	6	62.89	2.18
-7	9	-6	64.89	2.33
-7	9	-6	67.98	2.38
7	-9	6	64.52	2.58
7	-9	6	66.81	2.42
7	-9	6	66.65	2.41
7	-9	6	66.19	3.56
-8	9	-6	46.02	1.81
8	-9	6	43.71	1.97
8	-9	6	45.49	1.89
8	-9	6	43.17	1.87
-9	9	-6	13.87	0.94
-9	9	-6	12.71	1.05
9	-9	6	15.67	1.24
9	-9	6	13.33	2.40
9	-9	6	14.22	1.20
9	-9	6	11.53	1.13
-10	9	-6	17.51	1.36
-10	9	-6	21.23	1.17
10	-9	6	20.44	2.97
10	-9	6	20.81	1.47
10	-9	6	22.40	1.43
-11	9	-6	20.41	1.26
11	-9	6	23.86	1.54
11	-9	6	20.88	3.22
11	-9	6	21.32	1.65
-12	9	-6	26.92	1.47
12	-9	6	23.75	3.69
12	-9	6	28.01	2.03
-13	9	-6	10.94	1.12
13	-9	6	8.30	1.33
13	-9	6	5.33	2.59
14	-9	6	3.35	1.36
12	8	-6	5.24	1.34
12	8	-6	4.28	1.43
12	8	-6	4.74	1.96

12	8	-6	4.15	0.87
11	8	-6	5.65	0.79
11	8	-6	4.33	1.23
11	8	-6	5.20	1.09
11	8	-6	9.38	2.01
10	8	-6	12.42	1.01
10	8	-6	11.81	1.48
10	8	-6	11.03	1.42
10	8	-6	14.34	2.54
9	8	-6	16.02	1.36
9	8	-6	15.63	1.45
9	8	-6	17.58	2.38
9	8	-6	16.01	1.24
9	8	-6	12.17	2.08
8	8	-6	18.18	1.25
8	8	-6	18.34	1.05
8	8	-6	18.20	1.13
8	8	-6	18.90	1.44
8	8	-6	15.66	2.48
-8	-8	6	19.41	1.72
7	8	-6	1.86	0.49
7	8	-6	3.60	1.30
7	8	-6	2.27	0.46
7	8	-6	1.69	0.54
7	8	-6	1.80	0.47
-7	-8	6	1.24	0.79
-7	-8	6	1.85	0.84
6	8	-6	29.88	1.32
6	8	-6	33.75	2.14
6	8	-6	31.28	2.36
6	8	-6	31.14	1.39
6	8	-6	30.16	1.29
6	8	-6	29.72	1.23
-6	-8	6	29.40	1.86
-6	-8	6	34.53	1.87
5	8	-6	50.85	2.72
5	8	-6	50.35	1.71
5	8	-6	47.36	1.81
5	8	-6	50.57	1.85
5	8	-6	50.37	2.26
-5	-8	6	51.85	1.74
-5	-8	6	51.02	2.33
-5	-8	6	46.82	2.32
4	8	-6	29.59	1.60

4	8	-6	30.60	1.08
4	8	-6	30.40	1.23
4	8	-6	29.84	1.23
4	8	-6	29.78	2.01
-4	-8	6	28.17	1.70
-4	-8	6	30.25	1.14
-4	-8	6	29.25	1.76
3	8	-6	68.24	2.11
3	8	-6	66.01	2.20
3	8	-6	66.38	3.02
3	8	-6	68.24	2.31
3	8	-6	70.11	2.67
-3	-8	6	69.18	2.23
-3	-8	6	68.77	2.88
-3	-8	6	64.75	2.75
2	8	-6	43.55	2.21
2	8	-6	38.57	1.46
2	8	-6	38.33	1.76
-2	-8	6	40.32	1.90
-2	-8	6	38.83	1.41
-2	-8	6	38.74	2.06
1	8	-6	45.91	2.32
1	8	-6	47.67	1.98
1	8	-6	48.12	1.69
-1	-8	6	44.33	2.32
-1	-8	6	47.96	2.08
-1	-8	6	49.89	1.67
0	8	-6	105.69	3.42
0	8	-6	107.61	3.69
0	8	-6	111.35	4.10
0	-8	6	108.27	3.42
0	-8	6	103.83	4.13
0	-8	6	106.98	3.82
-1	8	-6	110.57	3.76
-1	8	-6	120.82	4.23
-1	8	-6	111.04	3.54
1	-8	6	103.86	4.26
1	-8	6	107.43	3.88
1	-8	6	112.00	3.54
-2	8	-6	46.99	2.31
-2	8	-6	46.81	1.68
-2	8	-6	48.69	1.85
2	-8	6	48.26	1.52
2	-8	6	47.25	1.99

2	-8	6	48.63	1.59
2	-8	6	42.94	2.40
-3	8	-6	98.65	3.24
-3	8	-6	95.12	3.81
-3	8	-6	111.13	3.41
3	-8	6	102.25	3.18
3	-8	6	100.83	3.53
3	-8	6	96.06	4.06
3	-8	6	95.95	3.24
-4	8	-6	132.20	3.93
-4	8	-6	116.63	3.80
-4	8	-6	115.94	4.39
4	-8	6	114.73	3.77
4	-8	6	122.25	4.08
4	-8	6	106.24	4.63
4	-8	6	117.80	3.74
4	-8	6	122.70	3.82
-5	8	-6	67.63	2.94
-5	8	-6	68.79	2.40
-5	8	-6	67.86	2.34
5	-8	6	69.64	2.34
5	-8	6	69.75	2.34
5	-8	6	65.39	2.56
5	-8	6	67.89	2.36
5	-8	6	71.52	3.36
-6	8	-6	51.85	1.89
-6	8	-6	50.60	2.51
-6	8	-6	56.61	1.96
6	-8	6	51.20	2.10
6	-8	6	47.64	2.85
6	-8	6	51.70	1.92
6	-8	6	54.23	1.95
6	-8	6	51.87	1.94
-7	8	-6	16.83	0.90
-7	8	-6	17.86	0.99
7	-8	6	17.83	1.09
7	-8	6	16.29	1.02
7	-8	6	20.09	2.14
7	-8	6	15.50	1.13
-8	8	-6	11.65	0.93
-8	8	-6	10.12	0.77
8	-8	6	10.52	1.86
8	-8	6	12.91	0.98
8	-8	6	9.00	1.03

8	-8	6	11.05	1.01
-9	8	-6	21.14	1.32
-9	8	-6	23.08	1.14
9	-8	6	19.32	2.51
9	-8	6	23.75	1.37
9	-8	6	20.57	1.33
-10	8	-6	33.42	1.50
-10	8	-6	33.95	1.80
10	-8	6	32.61	1.67
10	-8	6	32.62	3.22
-11	8	-6	12.32	1.04
11	-8	6	11.40	2.27
11	-8	6	10.41	1.25
11	-8	6	14.14	1.47
-12	8	-6	15.02	1.15
12	-8	6	9.37	2.36
12	-8	6	14.76	1.73
-13	8	-6	9.91	1.13
13	-8	6	7.31	2.65
13	-8	6	9.06	1.43
-14	8	-6	5.20	0.97
14	-8	6	6.88	1.54
13	7	-6	4.65	1.25
13	7	-6	3.04	1.80
13	7	-6	4.15	0.81
13	7	-6	4.43	1.56
12	7	-6	5.85	1.38
12	7	-6	7.27	1.27
12	7	-6	7.16	1.03
12	7	-6	5.05	1.79
11	7	-6	21.33	1.86
11	7	-6	20.21	1.25
11	7	-6	24.82	2.71
11	7	-6	21.14	1.71
10	7	-6	26.59	3.00
10	7	-6	29.71	2.64
10	7	-6	25.24	1.74
10	7	-6	25.74	1.70
10	7	-6	29.05	1.34
9	7	-6	12.56	1.86
9	7	-6	13.10	1.30
9	7	-6	12.22	1.06
9	7	-6	11.74	1.20
9	7	-6	12.47	0.88

-9	-7	6	12.42	1.55
8	7	-6	22.76	1.41
8	7	-6	25.48	1.19
8	7	-6	22.42	1.12
8	7	-6	23.93	1.36
8	7	-6	25.14	2.33
-8	-7	6	23.34	1.75
7	7	-6	32.16	1.84
7	7	-6	37.34	2.46
7	7	-6	36.26	1.41
7	7	-6	34.67	1.42
7	7	-6	36.70	1.40
7	7	-6	33.78	1.56
-7	-7	6	37.27	1.85
-7	-7	6	34.40	1.98
6	7	-6	42.74	2.38
6	7	-6	40.90	1.92
6	7	-6	42.02	1.47
6	7	-6	39.05	1.53
6	7	-6	39.95	1.64
6	7	-6	39.49	1.53
-6	-7	6	40.82	1.94
-6	-7	6	39.30	2.04
5	7	-6	45.65	1.77
5	7	-6	46.68	2.07
5	7	-6	48.52	1.61
5	7	-6	47.35	1.72
5	7	-6	51.35	2.47
-5	-7	6	48.33	1.65
-5	-7	6	47.56	2.12
-5	-7	6	46.66	2.18
4	7	-6	29.24	1.49
4	7	-6	29.56	1.79
4	7	-6	28.81	1.03
4	7	-6	29.59	1.20
4	7	-6	29.26	1.17
-4	-7	6	30.17	1.10
-4	-7	6	28.66	1.60
-4	-7	6	30.02	1.61
3	7	-6	32.36	1.26
3	7	-6	32.16	1.21
3	7	-6	34.19	1.09
3	7	-6	32.80	1.55
3	7	-6	33.68	1.84

-3	-7	6	34.68	1.21
-3	-7	6	33.12	1.74
-3	-7	6	32.40	1.65
2	7	-6	90.81	3.00
2	7	-6	92.56	3.52
2	7	-6	95.47	3.28
-2	-7	6	86.72	3.48
-2	-7	6	95.30	3.39
-2	-7	6	95.75	2.96
1	7	-6	100.63	3.38
1	7	-6	98.12	3.65
1	7	-6	93.73	3.13
-1	-7	6	95.50	3.48
-1	-7	6	100.96	3.12
-1	-7	6	97.70	3.71
0	7	-6	51.66	2.31
0	7	-6	52.25	1.81
0	7	-6	54.34	2.02
0	-7	6	52.62	1.81
0	-7	6	53.73	2.13
0	-7	6	53.44	2.41
-1	7	-6	75.90	2.92
-1	7	-6	70.19	2.39
-1	7	-6	76.27	2.57
1	-7	6	73.13	2.40
1	-7	6	75.57	2.32
1	-7	6	70.73	2.67
1	-7	6	64.69	2.97
-2	7	-6	169.61	5.30
-2	7	-6	151.31	4.73
-2	7	-6	143.88	4.57
-2	7	-6	146.17	4.84
2	-7	6	154.77	5.00
2	-7	6	147.30	5.41
2	-7	6	151.85	4.74
2	-7	6	149.66	4.69
-3	7	-6	100.42	3.34
-3	7	-6	102.95	3.84
-3	7	-6	107.17	3.45
-3	7	-6	102.34	3.22
3	-7	6	103.45	3.58
3	-7	6	108.48	4.15
3	-7	6	106.83	3.34
3	-7	6	104.33	3.36

-4	7	-6	67.30	2.24
-4	7	-6	62.20	2.70
-4	7	-6	64.03	2.13
-4	7	-6	71.14	2.31
4	-7	6	64.96	2.24
4	-7	6	65.67	2.24
4	-7	6	65.99	3.05
4	-7	6	64.93	2.43
-5	7	-6	62.76	2.70
-5	7	-6	65.89	2.22
-5	7	-6	67.75	2.27
-5	7	-6	61.78	2.12
5	-7	6	64.41	3.08
5	-7	6	64.86	2.23
5	-7	6	66.55	2.26
5	-7	6	61.21	2.38
-6	7	-6	45.30	1.65
-6	7	-6	47.27	1.70
-6	7	-6	48.03	1.77
-6	7	-6	44.07	2.23
6	-7	6	49.39	1.80
6	-7	6	47.55	1.88
6	-7	6	42.90	2.64
6	-7	6	43.62	1.72
-7	7	-6	48.02	1.89
-7	7	-6	48.38	1.80
7	-7	6	49.05	1.96
7	-7	6	47.06	2.90
7	-7	6	49.53	1.95
-8	7	-6	19.78	1.19
-8	7	-6	20.41	1.00
8	-7	6	18.70	1.16
8	-7	6	19.19	1.18
8	-7	6	16.29	2.18
8	-7	6	20.16	1.32
-9	7	-6	9.40	0.79
-9	7	-6	11.25	1.17
9	-7	6	8.45	1.02
9	-7	6	10.49	1.00
9	-7	6	12.92	2.01
-10	7	-6	23.60	1.65
-10	7	-6	22.98	1.22
10	-7	6	24.40	2.95
10	-7	6	23.40	1.53

10	-7	6	22.61	1.37
-11	7	-6	19.16	1.21
11	-7	6	21.87	1.41
11	-7	6	20.41	3.27
11	-7	6	18.24	1.58
-12	7	-6	12.99	1.10
12	-7	6	14.95	2.06
12	-7	6	13.58	1.65
12	-7	6	11.05	2.71
-13	7	-6	3.65	0.74
13	-7	6	6.33	2.62
13	-7	6	3.07	1.21
-14	7	-6	3.20	0.89
14	-7	6	1.92	1.31
14	6	-6	3.22	0.78
14	6	-6	2.79	1.31
14	6	-6	4.31	1.65
13	6	-6	10.14	1.88
13	6	-6	6.09	1.51
13	6	-6	11.25	1.13
13	6	-6	11.39	1.85
12	6	-6	14.27	1.11
12	6	-6	14.86	1.71
12	6	-6	16.07	2.54
12	6	-6	14.56	1.92
11	6	-6	10.93	0.92
11	6	-6	12.70	2.21
11	6	-6	10.33	1.53
11	6	-6	10.60	2.44
10	6	-6	12.63	2.11
10	6	-6	15.45	1.38
10	6	-6	15.22	2.37
10	6	-6	16.89	1.40
9	6	-6	37.99	1.74
9	6	-6	34.79	1.54
9	6	-6	30.86	2.52
9	6	-6	36.34	1.45
9	6	-6	38.12	1.80
9	6	-6	34.06	1.92
-9	-6	6	34.93	2.02
8	6	-6	68.61	2.74
8	6	-6	69.35	3.21
8	6	-6	64.68	2.32
8	6	-6	69.62	2.37

8	6	-6	66.88	2.59
8	6	-6	67.30	2.34
8	6	-6	68.75	2.55
-8	-6	6	69.39	2.90
7	6	-6	26.46	1.47
7	6	-6	25.86	1.93
7	6	-6	25.40	1.30
7	6	-6	26.43	1.09
7	6	-6	27.00	1.12
7	6	-6	25.19	1.29
7	6	-6	25.90	1.12
-7	-6	6	25.80	1.61
6	6	-6	85.44	2.85
6	6	-6	88.40	2.80
6	6	-6	89.85	3.20
6	6	-6	86.15	2.86
6	6	-6	84.97	3.47
6	6	-6	86.44	2.98
-6	-6	6	86.88	3.32
-6	-6	6	88.55	3.14
5	6	-6	31.93	1.57
5	6	-6	33.46	1.84
5	6	-6	32.33	1.37
5	6	-6	35.18	1.30
5	6	-6	32.44	1.20
-5	-6	6	34.85	1.63
-5	-6	6	36.04	1.24
-5	-6	6	34.65	1.71
4	6	-6	79.16	2.43
4	6	-6	76.89	2.55
4	6	-6	74.08	2.58
4	6	-6	77.60	3.07
4	6	-6	84.20	2.86
-4	-6	6	77.50	2.50
-4	-6	6	76.48	2.86
-4	-6	6	75.63	2.93
3	6	-6	81.74	2.84
3	6	-6	80.54	3.02
3	6	-6	79.26	2.59
3	6	-6	77.77	2.57
-3	-6	6	80.37	2.49
-3	-6	6	80.45	2.54
-3	-6	6	77.66	2.93
-3	-6	6	76.97	2.95

2	6	-6	95.04	3.12
2	6	-6	98.54	3.18
2	6	-6	100.52	3.40
2	6	-6	100.02	3.58
-2	-6	6	97.55	3.48
-2	-6	6	100.82	3.61
-2	-6	6	103.86	3.15
-2	-6	6	101.72	3.06
1	6	-6	55.39	2.17
1	6	-6	51.40	1.93
1	6	-6	51.20	1.76
1	6	-6	51.32	1.64
-1	-6	6	46.73	1.99
-1	-6	6	51.20	1.64
-1	-6	6	50.62	2.22
-1	-6	6	52.19	1.74
0	6	-6	85.86	3.32
0	6	-6	88.54	2.78
0	6	-6	93.15	2.93
0	6	-6	95.33	3.09
0	-6	6	89.35	2.86
0	-6	6	94.74	3.19
0	-6	6	87.06	3.41
0	-6	6	94.99	2.93
-1	6	-6	165.49	5.17
-1	6	-6	184.64	5.72
-1	6	-6	159.80	5.29
-1	6	-6	183.05	5.44
1	-6	6	170.84	5.86
1	-6	6	170.39	5.30
1	-6	6	167.96	5.29
1	-6	6	173.90	5.54
-2	6	-6	18.59	1.21
-2	6	-6	20.30	0.78
-2	6	-6	22.14	0.96
-2	6	-6	20.24	0.86
2	-6	6	20.10	1.41
2	-6	6	18.97	1.03
2	-6	6	21.25	0.90
2	-6	6	20.87	0.88
-3	6	-6	51.09	1.77
-3	6	-6	50.63	2.13
-3	6	-6	47.56	1.69
-3	6	-6	48.50	1.61

3	-6	6	47.35	1.86
3	-6	6	45.58	2.31
3	-6	6	47.97	1.74
-4	6	-6	102.56	3.24
-4	6	-6	103.00	3.31
-4	6	-6	101.17	3.72
-4	6	-6	109.97	3.38
4	-6	6	99.45	4.03
4	-6	6	99.24	3.30
4	-6	6	104.31	3.35
4	-6	6	102.27	3.46
-5	6	-6	70.77	2.42
-5	6	-6	72.02	2.85
-5	6	-6	71.59	2.38
-5	6	-6	73.69	2.49
5	-6	6	72.57	2.57
5	-6	6	68.14	3.21
5	-6	6	73.91	2.50
5	-6	6	72.28	2.44
-6	6	-6	72.10	2.91
-6	6	-6	72.78	2.45
-6	6	-6	76.28	2.57
-6	6	-6	71.60	2.47
6	-6	6	76.01	2.59
6	-6	6	71.52	2.60
6	-6	6	70.46	2.48
6	-6	6	72.36	3.41
-7	6	-6	30.22	1.25
-7	6	-6	29.49	1.40
-7	6	-6	28.28	1.23
7	-6	6	31.31	1.45
7	-6	6	29.38	1.38
7	-6	6	27.19	2.23
-8	6	-6	13.48	0.88
-8	6	-6	12.97	1.14
-8	6	-6	13.35	0.82
8	-6	6	12.89	1.11
8	-6	6	14.37	1.00
8	-6	6	12.98	1.97
-9	6	-6	39.20	1.60
-9	6	-6	38.20	1.98
9	-6	6	36.54	2.97
9	-6	6	40.71	1.78
9	-6	6	42.44	1.88

-10	6	-6	23.29	1.21
-10	6	-6	24.22	1.79
10	-6	6	24.62	1.54
10	-6	6	18.03	2.77
10	-6	6	23.38	1.39
-11	6	-6	7.98	0.90
11	-6	6	8.27	2.11
11	-6	6	8.37	1.34
11	-6	6	6.13	0.85
-12	6	-6	7.65	1.03
12	-6	6	8.24	2.33
12	-6	6	8.12	1.31
12	-6	6	9.45	1.56
-13	6	-6	6.03	0.80
13	-6	6	5.38	1.31
13	-6	6	3.30	2.51
14	5	-6	7.77	1.09
14	5	-6	3.55	1.60
14	5	-6	9.29	1.97
14	5	-6	8.28	1.43
13	5	-6	11.64	1.95
13	5	-6	12.11	1.77
13	5	-6	12.39	1.84
13	5	-6	12.40	1.03
12	5	-6	10.81	1.48
12	5	-6	11.00	0.91
12	5	-6	8.34	1.62
12	5	-6	10.54	1.70
11	5	-6	19.04	1.60
11	5	-6	24.36	2.64
11	5	-6	21.50	1.71
11	5	-6	22.19	1.60
11	5	-6	20.37	2.18
11	5	-6	22.61	1.12
10	5	-6	43.80	1.62
10	5	-6	41.61	2.01
10	5	-6	41.70	2.02
10	5	-6	41.26	1.96
10	5	-6	38.85	2.54
10	5	-6	35.31	2.64
9	5	-6	21.13	1.01
9	5	-6	22.29	1.92
9	5	-6	21.14	1.34
9	5	-6	21.54	1.08

9	5	-6	21.31	1.40
9	5	-6	20.28	1.26
-9	-5	6	21.08	1.52
8	5	-6	58.04	2.21
8	5	-6	52.95	2.09
8	5	-6	55.47	1.95
8	5	-6	54.48	2.25
8	5	-6	56.20	1.95
8	5	-6	54.43	2.61
8	5	-6	54.18	1.92
-8	-5	6	52.32	2.37
7	5	-6	64.81	2.23
7	5	-6	67.63	2.20
7	5	-6	65.85	2.42
7	5	-6	66.22	2.22
7	5	-6	62.82	2.32
7	5	-6	65.43	2.78
7	5	-6	65.10	2.51
-7	-5	6	66.57	2.67
6	5	-6	33.22	1.29
6	5	-6	33.70	1.41
6	5	-6	32.50	1.26
6	5	-6	33.65	1.22
6	5	-6	34.18	1.54
-6	-5	6	33.50	1.67
-6	-5	6	34.83	1.48
5	5	-6	120.13	3.80
5	5	-6	120.68	3.91
5	5	-6	124.76	4.06
5	5	-6	119.87	4.19
-5	-5	6	120.85	3.76
-5	-5	6	114.57	4.14
-5	-5	6	124.55	4.02
4	5	-6	164.54	5.16
4	5	-6	165.04	5.10
4	5	-6	169.16	5.33
4	5	-6	163.67	5.42
-4	-5	6	169.76	5.08
-4	-5	6	161.03	5.35
-4	-5	6	166.91	5.06
-4	-5	6	161.43	5.42
3	5	-6	84.88	2.77
3	5	-6	88.74	2.81
3	5	-6	89.68	2.97

3	5	-6	85.74	3.06
-3	-5	6	87.49	2.74
-3	-5	6	83.61	3.04
-3	-5	6	83.72	2.72
2	5	-6	133.68	4.16
2	5	-6	136.13	4.16
2	5	-6	135.96	4.43
2	5	-6	134.95	4.32
-2	-5	6	127.31	4.40
-2	-5	6	131.88	4.11
-2	-5	6	135.27	4.14
1	5	-6	77.90	2.49
1	5	-6	80.92	2.76
1	5	-6	76.98	2.62
1	5	-6	74.94	2.45
-1	-5	6	72.78	2.45
-1	-5	6	76.33	2.47
-1	-5	6	76.48	2.71
-1	-5	6	78.72	2.89
0	5	-6	35.83	1.35
0	5	-6	32.91	1.17
0	5	-6	36.25	1.52
0	5	-6	32.15	1.22
0	-5	6	32.93	1.41
0	-5	6	35.65	1.24
0	-5	6	33.06	1.25
0	-5	6	35.49	1.66
-1	5	-6	35.87	1.55
-1	5	-6	36.43	1.34
-1	5	-6	28.92	1.22
-1	5	-6	33.84	1.24
1	-5	6	35.50	1.31
1	-5	6	35.72	1.26
1	-5	6	37.47	1.44
1	-5	6	33.22	1.69
-2	5	-6	87.52	2.78
-2	5	-6	94.61	3.18
-2	5	-6	84.85	2.83
-2	5	-6	83.98	2.90
2	-5	6	87.86	2.99
2	-5	6	85.39	3.34
2	-5	6	91.60	2.85
2	-5	6	91.37	2.90
-3	5	-6	108.96	3.72

-3	5	-6	114.00	3.46
-3	5	-6	106.68	3.34
-3	5	-6	104.42	3.38
3	-5	6	108.71	3.39
3	-5	6	98.77	3.50
3	-5	6	103.42	3.97
3	-5	6	104.94	3.45
-4	5	-6	31.89	1.59
-4	5	-6	33.40	1.23
-4	5	-6	35.96	1.27
-4	5	-6	34.39	1.33
4	-5	6	32.99	1.27
4	-5	6	31.99	1.88
4	-5	6	33.36	1.35
4	-5	6	32.49	1.38
-5	5	-6	23.10	1.10
-5	5	-6	24.69	1.01
-5	5	-6	23.32	1.00
-5	5	-6	23.58	1.39
5	-5	6	26.58	1.80
5	-5	6	22.43	1.00
5	-5	6	22.65	1.13
5	-5	6	25.42	1.15
-6	5	-6	44.27	1.76
-6	5	-6	43.95	1.61
-6	5	-6	42.65	1.98
-6	5	-6	42.27	1.61
6	-5	6	44.98	2.54
6	-5	6	42.98	1.62
6	-5	6	44.26	1.75
6	-5	6	44.08	1.77
-7	5	-6	20.18	1.04
-7	5	-6	24.55	1.31
-7	5	-6	20.55	1.00
7	-5	6	22.33	1.17
7	-5	6	23.33	2.05
7	-5	6	21.23	1.16
7	-5	6	21.15	1.26
-8	5	-6	24.56	1.17
-8	5	-6	23.53	1.08
-8	5	-6	24.92	1.52
8	-5	6	19.79	2.16
8	-5	6	21.70	1.25
-9	5	-6	15.61	0.93

-9	5	-6	14.65	1.00
-9	5	-6	17.58	1.53
9	-5	6	11.97	2.24
9	-5	6	14.23	1.11
-10	5	-6	13.74	0.96
-10	5	-6	14.22	1.77
10	-5	6	13.34	1.18
10	-5	6	14.32	2.30
10	-5	6	13.33	1.29
-11	5	-6	18.02	1.11
11	-5	6	18.92	3.09
11	-5	6	15.59	1.32
11	-5	6	15.92	1.57
-12	5	-6	9.64	1.06
12	-5	6	9.39	2.47
12	-5	6	9.26	1.25
12	-5	6	11.47	1.63
-13	5	-6	5.47	0.83
13	-5	6	5.39	2.52
13	-5	6	3.19	1.11
15	4	-6	6.64	1.92
15	4	-6	5.26	0.83
15	4	-6	5.09	1.45
14	4	-6	5.14	1.56
14	4	-6	5.43	0.73
14	4	-6	5.51	1.25
14	4	-6	7.95	1.48
13	4	-6	18.07	1.74
13	4	-6	13.71	1.96
13	4	-6	14.99	1.07
13	4	-6	16.46	2.19
12	4	-6	17.60	1.06
12	4	-6	19.23	1.61
12	4	-6	18.84	2.73
12	4	-6	14.46	2.08
12	4	-6	17.10	1.50
12	4	-6	17.59	1.71
11	4	-6	38.08	1.52
11	4	-6	35.62	1.86
11	4	-6	35.32	2.57
11	4	-6	39.46	1.99
11	4	-6	38.25	1.99
11	4	-6	34.29	2.37
10	4	-6	21.24	2.01

10	4	-6	25.01	1.50
10	4	-6	23.86	1.21
10	4	-6	23.65	1.46
10	4	-6	24.63	1.43
10	4	-6	24.93	1.09
-10	-4	6	25.02	1.57
9	4	-6	73.24	2.71
9	4	-6	70.67	2.51
9	4	-6	78.26	2.79
9	4	-6	76.59	2.84
9	4	-6	72.00	3.13
9	4	-6	75.43	2.50
9	4	-6	75.18	2.55
-9	-4	6	73.71	2.94
8	4	-6	51.85	2.08
8	4	-6	53.00	1.95
8	4	-6	51.96	1.83
8	4	-6	48.27	2.30
8	4	-6	50.70	1.82
8	4	-6	53.13	2.07
8	4	-6	51.81	1.81
-8	-4	6	51.73	2.21
7	4	-6	52.19	1.79
7	4	-6	52.06	1.82
7	4	-6	50.58	1.79
7	4	-6	54.58	2.33
7	4	-6	52.88	2.02
7	4	-6	50.78	1.87
7	4	-6	52.30	2.03
-7	-4	6	51.48	2.15
6	4	-6	99.04	3.39
6	4	-6	104.33	3.45
6	4	-6	103.25	3.26
6	4	-6	98.94	3.22
6	4	-6	101.37	3.26
6	4	-6	101.86	3.55
-6	-4	6	102.78	3.29
-6	-4	6	99.38	3.55
5	4	-6	114.22	3.69
5	4	-6	106.99	3.41
5	4	-6	111.92	3.62
5	4	-6	107.55	3.54
-5	-4	6	108.30	3.38
-5	-4	6	103.13	3.70

-5	-4	6	101.15	3.55
-5	-4	6	112.93	3.51
4	4	-6	161.10	4.89
4	4	-6	154.28	4.89
4	4	-6	143.76	4.79
4	4	-6	149.48	4.70
-4	-4	6	153.44	4.67
-4	-4	6	155.25	4.73
-4	-4	6	153.48	4.81
-4	-4	6	149.06	4.96
3	4	-6	147.70	4.53
3	4	-6	150.72	4.53
3	4	-6	119.22	4.53
3	4	-6	140.03	4.37
-3	-4	6	143.74	4.34
-3	-4	6	140.43	4.52
-3	-4	6	141.59	4.60
-3	-4	6	146.54	4.39
2	4	-6	112.21	3.49
2	4	-6	111.69	3.50
2	4	-6	106.67	3.41
2	4	-6	105.36	3.36
-2	-4	6	106.38	3.37
-2	-4	6	104.19	3.55
-2	-4	6	99.68	3.55
-2	-4	6	108.94	3.34
1	4	-6	61.39	2.01
1	4	-6	61.58	2.10
1	4	-6	62.63	2.04
1	4	-6	62.37	2.16
-1	-4	6	60.69	2.28
-1	-4	6	59.84	1.99
-1	-4	6	58.88	2.16
-1	-4	6	59.95	2.05
0	4	-6	86.60	2.86
0	4	-6	88.39	2.77
0	4	-6	80.72	2.79
0	4	-6	93.26	2.98
0	-4	6	89.78	2.86
0	-4	6	85.42	2.92
0	-4	6	85.60	3.11
0	-4	6	87.51	2.77
-1	4	-6	144.92	4.49
-1	4	-6	141.85	4.41

-1	4	-6	137.89	4.39
1	-4	6	146.71	4.51
1	-4	6	137.25	4.53
1	-4	6	146.20	4.82
-2	4	-6	59.59	2.04
-2	4	-6	63.38	2.28
-2	4	-6	66.17	2.13
-2	4	-6	59.14	2.03
2	-4	6	63.81	2.05
2	-4	6	62.46	2.17
2	-4	6	57.13	2.45
-3	4	-6	63.71	2.24
-3	4	-6	67.30	2.44
-3	4	-6	66.50	2.16
-3	4	-6	62.19	2.15
3	-4	6	62.77	2.27
3	-4	6	66.04	2.72
3	-4	6	68.27	2.17
3	-4	6	64.20	2.27
-4	4	-6	55.79	1.96
-4	4	-6	60.32	2.08
-4	4	-6	56.02	2.24
-4	4	-6	58.28	1.96
4	-4	6	56.87	1.96
4	-4	6	59.83	2.10
4	-4	6	61.49	2.64
4	-4	6	52.86	2.05
-5	4	-6	29.42	1.27
-5	4	-6	28.53	1.09
-5	4	-6	26.43	1.10
-5	4	-6	26.40	1.39
5	-4	6	25.65	1.78
5	-4	6	23.95	1.18
5	-4	6	26.05	1.09
5	-4	6	25.87	1.24
-6	4	-6	15.08	1.15
-6	4	-6	16.24	0.81
-6	4	-6	15.63	0.85
-6	4	-6	18.82	1.10
6	-4	6	16.38	1.66
6	-4	6	17.39	0.86
6	-4	6	13.76	0.91
6	-4	6	18.91	1.08
-7	4	-6	20.62	0.95

-7	4	-6	22.87	1.37
-7	4	-6	21.36	1.01
7	-4	6	17.76	1.89
7	-4	6	16.11	1.08
7	-4	6	19.90	1.00
7	-4	6	21.27	1.28
-8	4	-6	9.82	0.71
-8	4	-6	10.09	1.30
-8	4	-6	9.05	0.77
8	-4	6	8.68	1.47
-9	4	-6	15.79	0.92
-9	4	-6	15.86	1.03
-9	4	-6	17.12	1.71
9	-4	6	16.66	2.27
9	-4	6	11.79	1.07
-10	4	-6	9.73	0.83
-10	4	-6	9.44	1.38
-10	4	-6	11.04	1.00
10	-4	6	7.54	1.83
10	-4	6	8.03	1.04
11	-4	6	6.80	1.13
11	-4	6	8.10	1.38
11	-4	6	10.12	2.27
-12	4	-6	2.96	0.70
12	-4	6	-0.10	2.03
12	-4	6	2.12	1.01
12	-4	6	2.87	0.77
-13	4	-6	3.96	0.79
13	-4	6	3.02	1.21
13	-4	6	1.94	2.36
15	3	-6	14.50	2.11
15	3	-6	10.40	1.95
15	3	-6	12.72	1.19
14	3	-6	14.81	2.30
14	3	-6	17.13	1.89
14	3	-6	18.85	1.19
14	3	-6	18.31	2.11
13	3	-6	12.69	2.36
13	3	-6	12.89	0.99
13	3	-6	15.34	1.89
13	3	-6	14.44	1.63
13	3	-6	13.81	1.90
12	3	-6	12.39	1.20
12	3	-6	12.87	0.87

12	3	-6	11.29	1.70
12	3	-6	11.30	1.79
12	3	-6	11.94	1.37
12	3	-6	11.51	1.53
11	3	-6	32.63	1.76
11	3	-6	28.03	1.41
11	3	-6	27.54	2.04
11	3	-6	30.28	1.77
11	3	-6	30.84	1.54
11	3	-6	30.95	2.20
10	3	-6	57.78	2.09
10	3	-6	56.15	2.61
10	3	-6	57.63	2.40
10	3	-6	57.85	2.23
10	3	-6	56.90	2.01
10	3	-6	57.79	2.34
-10	-3	6	53.61	2.36
9	3	-6	67.62	2.79
9	3	-6	68.89	2.37
9	3	-6	68.88	2.34
9	3	-6	66.81	2.52
9	3	-6	71.16	2.52
9	3	-6	69.61	2.33
9	3	-6	70.13	2.66
-9	-3	6	67.85	2.67
8	3	-6	53.50	1.98
8	3	-6	53.93	1.87
8	3	-6	52.69	1.84
8	3	-6	51.34	2.10
8	3	-6	53.47	1.86
8	3	-6	55.36	2.04
-8	-3	6	53.65	2.18
7	3	-6	52.03	2.04
7	3	-6	51.44	1.77
7	3	-6	49.90	1.90
7	3	-6	51.41	1.81
7	3	-6	50.47	1.74
7	3	-6	50.46	1.98
-7	-3	6	50.99	1.86
-7	-3	6	50.59	2.05
6	3	-6	50.97	1.94
6	3	-6	52.57	1.76
6	3	-6	52.17	1.88
6	3	-6	50.95	1.92

6	3	-6	50.07	1.72
6	3	-6	49.22	1.70
-6	-3	6	51.59	1.83
-6	-3	6	49.66	1.98
5	3	-6	172.27	5.23
5	3	-6	163.77	5.27
5	3	-6	167.74	5.25
5	3	-6	163.81	5.11
5	3	-6	164.23	5.05
-5	-3	6	169.15	5.23
-5	-3	6	165.26	5.35
-5	-3	6	167.42	5.09
4	3	-6	58.65	2.12
4	3	-6	59.28	1.99
4	3	-6	60.43	2.03
4	3	-6	63.20	2.12
-4	-3	6	60.90	1.96
-4	-3	6	63.97	2.08
-4	-3	6	60.86	1.97
-4	-3	6	59.33	2.18
3	3	-6	42.82	1.45
3	3	-6	41.48	1.44
3	3	-6	44.89	1.55
3	3	-6	40.61	1.57
-3	-3	6	44.11	1.53
-3	-3	6	41.57	1.42
-3	-3	6	42.25	1.49
-3	-3	6	41.57	1.61
2	3	-6	225.04	6.94
2	3	-6	232.81	6.96
-2	-3	6	218.12	7.08
-2	-3	6	232.31	6.92
-2	-3	6	232.94	7.07
-2	-3	6	234.05	7.03
1	3	-6	120.53	3.67
1	3	-6	114.04	3.63
1	3	-6	110.54	3.57
-1	-3	6	117.38	3.57
-1	-3	6	119.86	3.79
-1	-3	6	108.03	3.69
-1	-3	6	114.93	3.69
0	3	-6	59.46	2.01
0	3	-6	56.89	1.90
0	3	-6	62.03	1.99

0	3	-6	55.46	1.97
0	-3	6	57.76	1.90
0	-3	6	56.00	2.16
0	-3	6	60.45	2.03
0	-3	6	55.59	2.04
-1	3	-6	107.06	3.32
-1	3	-6	102.23	3.27
-1	3	-6	90.13	3.44
-1	3	-6	101.56	3.22
1	-3	6	98.39	3.33
1	-3	6	106.46	3.58
1	-3	6	108.07	3.24
1	-3	6	107.61	3.39
-2	3	-6	48.72	1.70
-2	3	-6	50.00	1.75
-2	3	-6	55.99	1.84
-2	3	-6	49.25	1.88
2	-3	6	51.83	1.71
2	-3	6	49.66	1.81
2	-3	6	48.07	2.09
2	-3	6	51.37	1.85
-3	3	-6	154.44	5.03
-3	3	-6	159.54	5.21
-3	3	-6	157.18	4.99
-3	3	-6	168.05	5.15
3	-3	6	165.64	5.16
3	-3	6	160.94	5.12
3	-3	6	162.47	5.54
3	-3	6	167.03	5.00
-4	3	-6	12.37	0.80
-4	3	-6	9.30	0.83
-4	3	-6	10.02	0.57
-4	3	-6	9.69	0.62
4	-3	6	10.62	1.16
4	-3	6	10.52	0.60
4	-3	6	9.82	0.69
4	-3	6	12.10	0.79
-5	3	-6	20.25	1.19
-5	3	-6	21.21	0.91
-5	3	-6	21.95	0.98
-5	3	-6	20.85	1.16
5	-3	6	20.86	1.59
5	-3	6	22.40	0.94
5	-3	6	20.73	1.14

5	-3	6	20.98	1.05
-6	3	-6	23.22	1.02
-6	3	-6	25.48	1.43
-6	3	-6	22.49	1.40
-6	3	-6	23.64	1.10
6	-3	6	23.78	1.17
6	-3	6	24.68	1.07
6	-3	6	25.41	1.90
6	-3	6	24.67	1.30
-7	3	-6	26.20	1.20
-7	3	-6	29.22	1.67
-7	3	-6	24.43	1.09
7	-3	6	24.19	1.27
7	-3	6	25.69	2.11
7	-3	6	27.34	1.57
7	-3	6	25.43	1.15
-8	3	-6	18.41	1.68
-8	3	-6	18.70	1.05
-8	3	-6	19.05	0.95
8	-3	6	17.80	1.12
8	-3	6	17.75	2.25
-9	3	-6	6.76	0.84
-9	3	-6	7.76	0.79
-9	3	-6	7.21	1.28
9	-3	6	6.91	0.89
9	-3	6	6.71	1.56
-10	3	-6	6.43	0.92
-10	3	-6	9.11	1.47
-10	3	-6	7.07	0.82
10	-3	6	6.78	1.01
10	-3	6	6.11	1.87
-11	3	-6	10.36	1.00
11	-3	6	8.68	2.02
11	-3	6	8.28	1.19
-12	3	-6	0.99	0.64
12	-3	6	-1.42	1.96
12	-3	6	1.25	0.78
12	-3	6	0.65	1.01
13	-3	6	2.07	2.39
13	-3	6	3.02	1.26
16	2	-6	14.81	1.29
16	2	-6	15.30	2.25
16	2	-6	17.60	3.08
15	2	-6	20.59	2.09

15	2	-6	22.78	2.75
15	2	-6	22.88	1.35
14	2	-6	18.94	2.29
14	2	-6	16.64	1.82
14	2	-6	17.87	1.14
14	2	-6	16.39	1.82
13	2	-6	19.13	1.72
13	2	-6	20.96	1.14
13	2	-6	21.73	1.89
13	2	-6	18.75	2.57
13	2	-6	25.60	1.55
13	2	-6	22.10	2.03
12	2	-6	23.78	1.11
12	2	-6	22.06	1.77
12	2	-6	21.41	1.80
12	2	-6	21.14	1.25
12	2	-6	19.68	2.09
11	2	-6	38.17	1.99
11	2	-6	39.49	2.28
11	2	-6	39.22	1.97
11	2	-6	35.59	2.09
11	2	-6	42.47	1.69
11	2	-6	40.64	1.54
10	2	-6	65.05	2.58
10	2	-6	68.16	2.27
10	2	-6	65.61	2.33
10	2	-6	63.58	2.76
10	2	-6	68.25	2.39
10	2	-6	66.92	2.65
-10	-2	6	64.35	2.52
9	2	-6	67.79	2.54
9	2	-6	67.62	2.33
9	2	-6	69.80	2.69
9	2	-6	71.81	2.44
9	2	-6	70.60	2.65
9	2	-6	69.02	2.31
-9	-2	6	68.14	2.58
-9	-2	6	67.68	2.49
8	2	-6	54.71	2.08
8	2	-6	52.00	1.95
8	2	-6	53.40	2.09
8	2	-6	51.34	1.79
8	2	-6	50.64	1.80
8	2	-6	54.17	1.91

-8	-2	6	52.27	2.05
-8	-2	6	50.58	1.95
7	2	-6	91.45	2.98
7	2	-6	91.21	3.21
7	2	-6	91.81	3.01
7	2	-6	98.48	3.08
7	2	-6	92.88	3.12
7	2	-6	91.98	2.96
-7	-2	6	89.33	3.18
-7	-2	6	97.14	3.13
6	2	-6	138.10	4.30
6	2	-6	139.33	4.39
6	2	-6	141.43	4.33
6	2	-6	137.95	4.37
6	2	-6	137.32	4.51
6	2	-6	138.04	4.26
-6	-2	6	140.37	4.44
-6	-2	6	139.10	4.50
5	2	-6	59.73	2.03
5	2	-6	58.86	1.94
5	2	-6	57.82	2.13
-5	-2	6	61.86	1.93
-5	-2	6	57.39	2.11
-5	-2	6	60.20	2.08
4	2	-6	76.71	2.69
4	2	-6	82.40	2.61
4	2	-6	77.41	2.52
-4	-2	6	79.81	2.50
-4	-2	6	79.20	2.68
-4	-2	6	81.10	2.66
3	2	-6	119.67	3.95
3	2	-6	126.53	3.87
3	2	-6	122.65	3.78
-3	-2	6	121.14	3.91
-3	-2	6	121.47	3.76
-3	-2	6	123.09	3.92
2	2	-6	29.14	1.09
2	2	-6	27.59	1.19
2	2	-6	26.54	1.01
2	2	-6	28.22	0.96
-2	-2	6	28.10	1.00
-2	-2	6	28.43	1.08
-2	-2	6	28.46	1.12
-2	-2	6	29.57	1.17

1	2	-6	100.50	3.18
1	2	-6	102.01	3.31
1	2	-6	98.99	3.17
1	2	-6	103.71	3.25
-1	-2	6	103.86	3.16
-1	-2	6	105.69	3.36
-1	-2	6	102.88	3.31
-1	-2	6	94.82	3.26
0	2	-6	109.11	3.54
0	2	-6	112.68	3.61
0	2	-6	121.86	3.66
0	2	-6	115.38	3.67
0	-2	6	117.02	3.74
0	-2	6	109.10	3.65
0	-2	6	113.70	3.54
0	-2	6	115.77	3.79
-1	2	-6	53.54	1.95
-1	2	-6	56.82	1.86
-1	2	-6	58.40	2.00
1	-2	6	55.91	2.17
1	-2	6	56.18	2.05
1	-2	6	56.96	1.98
1	-2	6	59.00	1.87
-2	2	-6	64.22	2.21
-2	2	-6	63.38	2.11
-2	2	-6	66.14	2.30
-2	2	-6	68.93	2.30
2	-2	6	64.56	2.24
2	-2	6	63.15	2.30
2	-2	6	66.44	2.12
2	-2	6	62.60	2.51
-3	2	-6	21.76	0.90
-3	2	-6	24.29	1.14
-3	2	-6	23.30	1.13
-3	2	-6	21.64	0.97
3	-2	6	22.78	1.12
3	-2	6	24.37	0.93
3	-2	6	23.41	1.39
3	-2	6	22.51	1.03
-4	2	-6	24.39	1.06
-4	2	-6	23.31	1.24
-4	2	-6	24.70	0.97
-4	2	-6	24.22	1.25
4	-2	6	24.92	1.11

4	-2	6	23.68	1.53
4	-2	6	24.27	0.98
4	-2	6	24.48	1.21
-5	2	-6	39.52	1.55
-5	2	-6	38.42	1.44
-5	2	-6	43.64	1.85
-5	2	-6	39.07	1.76
5	-2	6	41.56	1.61
5	-2	6	39.74	1.72
5	-2	6	38.59	2.14
5	-2	6	40.87	1.47
-6	2	-6	12.86	1.11
-6	2	-6	12.32	0.69
-6	2	-6	12.41	1.21
-6	2	-6	12.39	0.79
6	-2	6	11.08	1.05
6	-2	6	12.88	0.87
6	-2	6	11.18	1.42
6	-2	6	12.21	0.73
-7	2	-6	10.40	1.43
-7	2	-6	13.48	0.87
-7	2	-6	10.55	0.71
7	-2	6	12.56	0.81
7	-2	6	10.35	1.52
7	-2	6	12.85	0.93
-8	2	-6	11.42	1.73
-8	2	-6	10.38	0.76
8	-2	6	11.82	1.79
8	-2	6	12.61	0.98
-9	2	-6	14.55	1.05
-9	2	-6	12.21	1.00
-9	2	-6	13.05	2.08
9	-2	6	14.20	1.09
9	-2	6	14.67	2.19
-10	2	-6	8.11	1.62
-10	2	-6	7.48	1.00
-10	2	-6	6.53	0.83
10	-2	6	6.74	1.04
10	-2	6	9.00	1.90
-11	2	-6	-0.41	0.56
-11	2	-6	0.63	0.66
11	-2	6	-1.55	1.60
11	-2	6	-0.77	0.65
-12	2	-6	1.08	0.67

12	-2	6	0.07	0.74
12	-2	6	0.60	1.04
12	-2	6	1.87	1.95
16	1	-6	11.27	1.66
16	1	-6	9.69	1.12
16	1	-6	8.31	2.15
15	1	-6	26.64	2.17
15	1	-6	26.39	1.38
15	1	-6	24.82	2.69
14	1	-6	32.37	1.45
14	1	-6	30.74	2.11
14	1	-6	27.65	2.47
14	1	-6	30.57	1.86
13	1	-6	18.73	1.58
13	1	-6	18.53	1.05
13	1	-6	17.87	1.02
13	1	-6	17.15	1.90
13	1	-6	15.24	1.54
13	1	-6	17.95	2.15
12	1	-6	27.92	2.03
12	1	-6	27.83	1.21
12	1	-6	26.05	1.73
12	1	-6	25.11	1.72
12	1	-6	21.49	1.83
12	1	-6	30.00	1.25
11	1	-6	39.63	1.96
11	1	-6	40.04	1.55
11	1	-6	39.92	2.02
11	1	-6	39.04	1.51
11	1	-6	41.24	2.08
-11	-1	6	39.05	1.70
10	1	-6	56.21	2.03
10	1	-6	56.65	2.37
10	1	-6	57.14	2.43
10	1	-6	61.91	2.08
10	1	-6	59.32	2.03
10	1	-6	58.31	2.41
-10	-1	6	58.29	2.23
-10	-1	6	61.38	2.28
9	1	-6	83.30	2.71
9	1	-6	81.85	2.70
9	1	-6	83.15	2.96
9	1	-6	85.36	2.75
9	1	-6	82.69	3.04

-9	-1	6	81.01	2.90
-9	-1	6	81.52	2.89
8	1	-6	49.66	2.02
8	1	-6	52.50	1.78
8	1	-6	49.85	2.07
8	1	-6	53.01	1.96
8	1	-6	53.61	1.83
8	1	-6	52.32	1.79
-8	-1	6	51.16	1.96
-8	-1	6	53.85	1.98
7	1	-6	77.24	2.69
7	1	-6	75.16	2.42
7	1	-6	73.24	2.47
7	1	-6	71.35	2.47
7	1	-6	71.87	2.39
-7	-1	6	73.73	2.56
-7	-1	6	76.58	2.58
6	1	-6	48.76	1.87
6	1	-6	51.17	1.66
6	1	-6	44.84	1.77
6	1	-6	48.80	1.62
-6	-1	6	47.33	1.76
-6	-1	6	49.68	1.81
5	1	-6	164.51	5.05
5	1	-6	161.97	5.28
5	1	-6	164.33	5.13
-5	-1	6	171.82	5.24
-5	-1	6	163.83	5.18
-5	-1	6	167.44	5.04
4	1	-6	39.24	1.31
4	1	-6	34.73	1.45
4	1	-6	40.13	1.54
-4	-1	6	40.33	1.42
-4	-1	6	41.83	1.30
-4	-1	6	34.47	1.56
3	1	-6	48.23	1.61
3	1	-6	48.53	1.82
-3	-1	6	50.40	1.80
-3	-1	6	49.73	1.60
-3	-1	6	48.42	1.71
2	1	-6	538.00	16.25
2	1	-6	547.23	16.37
2	1	-6	529.07	16.15
-2	-1	6	540.05	16.19

-2	-1	6	558.61	16.37
-2	-1	6	560.21	16.19
-2	-1	6	533.75	16.15
-2	-1	6	538.48	16.26
1	1	-6	52.17	1.71
1	1	-6	49.90	1.89
1	1	-6	54.46	1.84
1	1	-6	55.75	1.78
-1	-1	6	50.65	1.81
-1	-1	6	51.51	1.71
-1	-1	6	53.29	1.85
-1	-1	6	52.87	1.95
0	1	-6	51.45	1.78
0	1	-6	51.00	1.82
0	1	-6	48.60	1.65
0	1	-6	49.91	1.83
0	-1	6	50.60	1.89
0	-1	6	50.56	1.90
0	-1	6	53.13	1.79
-1	1	-6	32.92	1.29
-1	1	-6	29.43	1.07
-1	1	-6	31.43	1.25
-1	1	-6	28.56	1.21
1	-1	6	29.67	1.07
1	-1	6	29.67	1.19
1	-1	6	30.63	1.32
1	-1	6	29.62	1.39
-2	1	-6	37.61	1.50
-2	1	-6	34.07	1.40
-2	1	-6	35.05	1.26
-2	1	-6	40.61	1.56
2	-1	6	36.35	1.28
2	-1	6	37.60	1.53
2	-1	6	35.75	1.40
2	-1	6	36.26	1.67
-3	1	-6	25.35	1.23
-3	1	-6	23.72	1.24
-3	1	-6	23.92	1.06
-3	1	-6	23.63	0.93
3	-1	6	23.96	1.09
3	-1	6	26.51	0.96
3	-1	6	21.08	1.34
3	-1	6	26.16	1.23
-4	1	-6	44.83	1.90

-4	1	-6	40.56	1.60
-4	1	-6	40.99	1.48
-4	1	-6	41.18	1.80
4	-1	6	43.28	1.66
4	-1	6	41.12	1.77
4	-1	6	45.27	1.51
4	-1	6	42.37	2.06
-5	1	-6	30.82	1.57
-5	1	-6	30.98	1.68
-5	1	-6	31.48	1.33
-5	1	-6	31.57	1.20
5	-1	6	30.76	1.87
5	-1	6	32.81	1.23
5	-1	6	31.44	1.52
5	-1	6	32.65	1.39
-6	1	-6	12.30	1.43
-6	1	-6	13.50	0.71
-6	1	-6	13.21	1.19
-6	1	-6	13.76	0.86
6	-1	6	12.73	1.47
6	-1	6	12.30	0.91
6	-1	6	12.11	0.76
6	-1	6	14.09	1.14
-7	1	-6	3.19	0.52
-7	1	-6	3.00	0.95
-7	1	-6	3.12	0.44
7	-1	6	2.78	0.48
7	-1	6	4.35	0.76
7	-1	6	1.80	0.86
-8	1	-6	3.14	0.46
-8	1	-6	2.78	0.56
-8	1	-6	3.01	1.15
8	-1	6	4.59	0.67
8	-1	6	3.79	1.18
-9	1	-6	6.40	0.95
-9	1	-6	5.96	0.90
-9	1	-6	6.75	1.43
9	-1	6	6.62	0.94
9	-1	6	3.42	1.26
-10	1	-6	0.16	0.58
-10	1	-6	0.91	0.50
10	-1	6	0.46	0.58
10	-1	6	-1.01	1.35
-11	1	-6	0.68	0.66

-11	1	-6	-0.34	0.57
11	-1	6	-0.05	1.52
11	-1	6	0.35	0.66
-12	1	-6	-0.53	0.70
12	-1	6	-0.16	0.92
12	-1	6	0.19	1.02
17	0	-6	7.06	2.42
17	0	-6	14.11	1.30
17	0	-6	10.11	1.65
16	0	-6	26.41	2.34
16	0	-6	31.26	1.54
16	0	-6	20.98	3.05
15	0	-6	24.82	2.75
15	0	-6	26.20	2.11
15	0	-6	25.08	1.36
14	0	-6	7.73	1.22
14	0	-6	6.12	1.53
14	0	-6	8.06	0.85
14	0	-6	7.53	1.10
14	0	-6	5.00	1.46
13	0	-6	46.21	2.13
13	0	-6	50.53	1.85
13	0	-6	48.35	2.64
13	0	-6	46.40	2.61
13	0	-6	47.05	2.38
12	0	-6	56.17	2.62
12	0	-6	51.52	2.27
12	0	-6	59.21	2.59
12	0	-6	56.94	2.46
12	0	-6	47.06	2.04
11	0	-6	59.27	2.46
11	0	-6	55.75	2.00
11	0	-6	55.00	2.52
11	0	-6	57.74	1.96
10	0	-6	60.40	2.53
10	0	-6	63.40	2.55
10	0	-6	60.04	2.15
10	0	-6	65.45	2.11
10	0	-6	64.77	2.15
-10	0	6	59.76	2.26
-10	0	6	67.72	2.30
-10	0	6	62.12	2.38
9	0	-6	47.25	1.62
9	0	-6	47.09	1.65

9	0	-6	45.82	2.00
9	0	-6	47.63	1.66
9	0	-6	45.48	1.96
-9	0	6	45.06	1.86
-9	0	6	45.10	1.78
-9	0	6	51.43	1.76
8	0	-6	49.49	1.76
8	0	-6	51.25	1.75
8	0	-6	52.97	1.75
8	0	-6	53.14	2.09
-8	0	6	49.33	1.87
-8	0	6	51.42	1.97
-8	0	6	54.89	1.80
7	0	-6	43.29	1.47
7	0	-6	41.69	1.45
7	0	-6	42.61	1.48
7	0	-6	41.29	1.60
7	0	-6	41.69	1.74
-7	0	6	43.21	1.68
-7	0	6	45.06	1.45
-7	0	6	40.81	1.56
6	0	-6	40.41	1.38
6	0	-6	36.29	1.60
6	0	-6	40.06	1.39
6	0	-6	39.85	1.35
-6	0	6	43.23	1.30
-6	0	6	37.70	1.44
-6	0	6	39.69	1.57
5	0	-6	5.61	0.41
5	0	-6	5.22	0.34
5	0	-6	6.15	0.58
5	0	-6	5.72	0.40
-5	0	6	5.97	0.55
-5	0	6	5.26	0.42
4	0	-6	143.14	4.38
4	0	-6	138.05	4.29
4	0	-6	141.92	4.58
-4	0	6	142.19	4.53
-4	0	6	141.15	4.28
-4	0	6	138.22	4.38
3	0	-6	342.35	10.58
3	0	-6	337.82	10.31
-3	0	6	365.65	10.30
-3	0	6	358.24	10.41

-3	0	6	325.84	10.40
-3	0	6	341.81	10.30
2	0	-6	223.66	6.76
2	0	-6	208.53	6.85
2	0	-6	218.92	6.60
-2	0	6	220.19	6.58
-2	0	6	216.71	6.71
-2	0	6	234.16	6.67
-2	0	6	207.07	7.03
-2	0	6	222.37	6.76
1	0	-6	55.52	1.78
1	0	-6	51.81	2.01
1	0	-6	55.67	1.96
-1	0	6	58.40	1.95
-1	0	6	53.61	1.89
-1	0	6	56.08	2.01
-1	0	6	55.00	1.77
-1	0	6	53.91	2.05
0	0	-6	101.86	3.42
0	0	-6	100.86	3.17
0	0	-6	98.17	3.38
0	0	6	102.41	3.17
0	0	6	105.62	3.48
0	0	6	107.47	3.48
0	0	6	103.82	3.39
0	0	6	100.54	3.32
-1	0	-6	46.63	1.79
-1	0	-6	46.55	1.60
-1	0	-6	46.99	1.90
1	0	6	50.94	1.92
1	0	6	50.92	1.69
1	0	6	46.93	1.76
1	0	6	52.26	1.91
1	0	6	49.62	1.96
-2	0	-6	34.85	1.56
-2	0	-6	32.44	1.18
-2	0	-6	32.09	1.36
2	0	6	34.33	1.37
2	0	6	37.28	1.67
2	0	6	34.49	1.20
2	0	6	33.85	1.55
2	0	6	34.38	1.50
-3	0	-6	28.94	1.25
-3	0	-6	28.19	1.08

-3	0	-6	32.82	1.56
3	0	6	28.65	1.53
3	0	6	27.71	1.26
3	0	6	30.75	1.43
3	0	6	34.42	1.69
3	0	6	31.79	1.11
-4	0	-6	40.41	1.61
-4	0	-6	38.76	1.42
-4	0	-6	41.34	1.95
4	0	6	40.80	1.45
4	0	6	41.54	2.06
4	0	6	41.21	1.99
4	0	6	43.38	1.80
4	0	6	41.19	1.65
-5	0	-6	17.53	1.50
-5	0	-6	20.09	0.86
-5	0	-6	19.79	1.02
5	0	6	19.94	1.52
5	0	6	19.15	1.31
5	0	6	21.12	1.11
5	0	6	17.72	1.63
5	0	6	20.42	0.90
-6	0	-6	21.51	0.94
-6	0	-6	22.26	1.12
-6	0	-6	21.37	1.83
6	0	6	20.79	1.66
6	0	6	22.37	1.20
6	0	6	21.76	1.00
-7	0	-6	0.44	0.45
-7	0	-6	0.27	1.02
-7	0	-6	0.05	0.31
7	0	6	1.38	0.50
7	0	6	0.73	1.16
7	0	6	0.45	0.41
-8	0	-6	-1.76	1.19
-8	0	-6	0.98	0.50
-8	0	-6	0.65	0.37
8	0	6	0.55	1.07
8	0	6	1.11	0.54
-9	0	-6	5.52	0.94
-9	0	-6	4.35	0.68
9	0	6	4.32	1.42
9	0	6	5.58	0.80
-10	0	-6	1.84	0.67

-10	0	-6	1.63	0.56
10	0	6	2.53	1.41
10	0	6	1.66	0.70
-11	0	-6	0.35	0.67
-11	0	-6	0.08	0.63
11	0	6	0.63	1.69
11	0	6	0.42	0.74
-12	0	-6	-0.07	0.75
12	0	6	-1.60	1.94
12	0	6	0.61	1.30
17	-1	-6	14.54	1.26
17	-1	-6	20.41	3.53
17	-1	-6	11.03	1.64
16	-1	-6	8.59	2.16
16	-1	-6	13.84	1.92
-16	1	6	10.90	1.35
15	-1	-6	23.62	2.02
15	-1	-6	23.02	2.73
-15	1	6	23.23	1.53
14	-1	-6	48.95	2.85
14	-1	-6	46.74	2.98
14	-1	-6	51.45	2.56
-14	1	6	50.23	2.08
13	-1	-6	41.11	1.39
13	-1	-6	40.35	1.72
13	-1	-6	41.36	2.17
13	-1	-6	38.39	2.45
-13	1	6	39.67	1.73
-13	1	6	42.92	2.00
12	-1	-6	82.85	2.64
12	-1	-6	82.47	3.30
12	-1	-6	81.08	3.49
-12	1	6	87.59	3.07
-12	1	6	82.99	2.91
11	-1	-6	43.69	2.21
11	-1	-6	43.59	2.07
11	-1	-6	42.34	1.48
-11	1	6	44.14	1.71
-11	1	6	47.30	1.84
-11	1	6	42.23	1.72
10	-1	-6	67.79	2.14
10	-1	-6	63.88	2.63
10	-1	-6	66.06	2.70
10	-1	-6	62.70	2.23

-10	1	6	70.66	2.37
-10	1	6	64.67	2.30
-10	1	6	65.65	2.48
-10	1	6	65.83	2.31
9	-1	-6	73.70	2.85
9	-1	-6	74.54	2.43
9	-1	-6	75.36	2.86
9	-1	-6	76.03	2.49
9	-1	-6	77.28	2.50
-9	1	6	75.37	2.74
-9	1	6	77.09	2.58
-9	1	6	79.21	2.59
8	-1	-6	45.64	1.50
8	-1	-6	43.72	1.85
8	-1	-6	42.69	1.51
8	-1	-6	43.42	1.53
-8	1	6	44.83	1.58
-8	1	6	43.83	1.77
-8	1	6	42.99	1.59
7	-1	-6	23.64	0.89
7	-1	-6	21.82	1.17
7	-1	-6	22.81	0.89
7	-1	-6	22.30	1.11
7	-1	-6	21.74	0.86
-7	1	6	23.70	1.13
-7	1	6	23.08	0.92
-7	1	6	21.69	0.94
6	-1	-6	34.53	1.17
6	-1	-6	32.65	1.46
6	-1	-6	33.89	1.19
6	-1	-6	31.97	1.32
6	-1	-6	35.24	1.23
-6	1	6	35.31	1.44
-6	1	6	33.54	1.23
-6	1	6	32.74	1.22
5	-1	-6	202.54	6.49
5	-1	-6	204.25	6.26
5	-1	-6	207.52	6.20
5	-1	-6	206.06	6.27
5	-1	-6	204.64	6.17
-5	1	6	209.31	6.44
-5	1	6	203.65	6.26
-5	1	6	196.43	6.23
4	-1	-6	17.49	0.81

4	-1	-6	19.18	0.70
4	-1	-6	18.13	1.00
4	-1	-6	19.38	0.85
-4	1	6	18.51	0.78
-4	1	6	18.73	0.70
-4	1	6	19.98	0.98
-4	1	6	19.50	0.91
3	-1	-6	19.52	0.88
3	-1	-6	20.21	0.95
3	-1	-6	20.63	1.05
3	-1	-6	20.66	0.76
-3	1	6	22.14	1.02
-3	1	6	20.84	1.04
-3	1	6	19.99	0.73
-3	1	6	20.89	0.86
2	-1	-6	46.72	1.51
2	-1	-6	44.81	1.78
2	-1	-6	47.10	1.74
-2	1	6	45.60	1.82
-2	1	6	45.53	1.48
-2	1	6	47.21	1.81
-2	1	6	46.40	1.64
1	-1	-6	44.09	1.43
1	-1	-6	42.72	1.71
1	-1	-6	46.12	1.75
-1	1	6	44.15	1.60
-1	1	6	41.42	1.59
-1	1	6	43.86	1.43
-1	1	6	43.68	1.80
-1	1	6	45.62	1.78
0	-1	-6	38.71	1.60
0	-1	-6	35.20	1.24
0	-1	-6	37.31	1.51
0	1	6	37.60	1.48
0	1	6	35.92	1.43
0	1	6	39.38	1.61
0	1	6	37.27	1.24
0	1	6	38.08	1.69
-1	-1	-6	47.61	1.83
-1	-1	-6	50.54	2.04
-1	-1	-6	48.57	1.60
1	1	6	48.87	1.95
1	1	6	50.51	1.91
1	1	6	49.74	1.60

1	1	6	53.41	2.14
1	1	6	43.86	1.79
-2	-1	-6	13.59	0.59
-2	-1	-6	12.67	1.05
-2	-1	-6	13.03	0.81
2	1	6	14.21	1.19
2	1	6	14.89	0.95
2	1	6	14.57	0.83
2	1	6	14.07	0.97
2	1	6	14.53	0.61
-3	-1	-6	1.11	0.35
-3	-1	-6	1.14	0.22
-3	-1	-6	0.36	0.57
3	1	6	1.57	0.38
3	1	6	0.92	0.49
3	1	6	1.35	0.66
3	1	6	1.63	0.26
3	1	6	1.49	0.50
-4	-1	-6	27.28	1.22
-4	-1	-6	26.12	1.01
-4	-1	-6	23.72	1.66
4	1	6	24.72	1.50
4	1	6	27.77	1.30
4	1	6	29.19	1.84
4	1	6	27.20	1.43
4	1	6	27.48	1.04
-5	-1	-6	5.23	0.48
-5	-1	-6	6.84	0.67
-5	-1	-6	4.64	0.97
5	1	6	5.94	1.06
5	1	6	5.58	0.84
5	1	6	5.23	0.76
5	1	6	6.62	1.22
5	1	6	6.19	0.54
-6	-1	-6	2.66	0.50
-6	-1	-6	3.43	0.47
-6	-1	-6	2.86	1.09
6	1	6	3.26	0.45
6	1	6	2.46	0.78
6	1	6	2.74	0.58
-7	-1	-6	0.96	1.33
-7	-1	-6	-0.39	0.32
-7	-1	-6	0.35	0.44
7	1	6	-0.80	0.83

7	1	6	0.33	0.50
7	1	6	0.36	0.43
-8	-1	-6	1.56	0.56
-8	-1	-6	2.05	0.44
8	1	6	3.31	1.19
8	1	6	2.25	0.66
-9	-1	-6	-0.72	0.54
-9	-1	-6	0.11	0.52
9	1	6	-0.58	0.59
9	1	6	-0.30	1.22
-10	-1	-6	-0.08	0.64
-10	-1	-6	-1.03	0.56
10	1	6	-3.04	1.28
10	1	6	0.31	0.71
-11	-1	-6	-1.71	0.66
-11	-1	-6	0.05	0.69
11	1	6	1.39	1.72
11	1	6	-1.20	0.74
17	-2	-6	9.55	2.44
17	-2	-6	9.53	1.69
-17	2	6	6.55	1.04
16	-2	-6	38.25	2.59
16	-2	-6	41.33	5.70
15	-2	-6	26.95	2.08
15	-2	-6	25.76	2.74
-15	2	6	27.47	1.53
14	-2	-6	16.39	1.63
14	-2	-6	18.06	2.26
-14	2	6	14.32	1.08
-14	2	6	10.53	1.51
13	-2	-6	35.22	2.43
13	-2	-6	35.88	2.04
-13	2	6	35.90	1.58
-13	2	6	37.33	1.82
12	-2	-6	81.27	3.25
12	-2	-6	78.94	3.47
-12	2	6	80.68	2.81
-12	2	6	83.46	2.98
11	-2	-6	73.21	2.45
11	-2	-6	78.49	3.23
11	-2	-6	73.79	3.03
-11	2	6	80.28	2.67
-11	2	6	81.94	2.79
-11	2	6	74.21	2.51

-11	2	6	79.24	3.18
10	-2	-6	40.06	1.88
10	-2	-6	42.10	1.49
10	-2	-6	39.26	2.03
10	-2	-6	40.07	1.41
-10	2	6	39.58	1.53
-10	2	6	40.69	1.48
-10	2	6	40.27	1.76
-10	2	6	41.02	1.64
9	-2	-6	52.05	2.13
9	-2	-6	48.61	1.70
9	-2	-6	49.16	1.73
9	-2	-6	48.78	2.19
-9	2	6	50.72	2.00
-9	2	6	53.06	1.91
-9	2	6	50.46	1.79
-9	2	6	50.65	1.75
8	-2	-6	63.14	2.07
8	-2	-6	65.62	2.51
8	-2	-6	61.83	2.42
8	-2	-6	62.64	2.06
8	-2	-6	61.08	2.09
-8	2	6	61.53	2.34
-8	2	6	64.02	2.22
7	-2	-6	116.60	3.60
7	-2	-6	113.96	3.58
7	-2	-6	108.77	3.66
7	-2	-6	118.47	3.93
7	-2	-6	115.03	3.95
-7	2	6	119.06	3.89
-7	2	6	116.57	3.76
-7	2	6	118.10	3.63
6	-2	-6	168.85	5.54
6	-2	-6	170.44	5.18
6	-2	-6	161.75	5.31
6	-2	-6	173.84	5.20
6	-2	-6	169.29	5.45
-6	2	6	174.34	5.50
-6	2	6	174.06	5.40
-6	2	6	172.57	5.23
5	-2	-6	106.77	3.33
5	-2	-6	105.55	3.54
5	-2	-6	107.46	3.30
5	-2	-6	104.69	3.48

5	-2	-6	105.06	3.65
-5	2	6	107.28	3.38
-5	2	6	109.91	3.61
-5	2	6	110.27	3.59
4	-2	-6	56.92	2.04
4	-2	-6	53.65	2.15
4	-2	-6	52.96	2.12
4	-2	-6	55.26	1.77
-4	2	6	56.36	1.90
-4	2	6	58.03	2.11
-4	2	6	56.97	2.13
3	-2	-6	73.57	2.57
3	-2	-6	72.89	2.71
3	-2	-6	73.42	2.27
3	-2	-6	70.62	2.61
-3	2	6	72.31	2.24
-3	2	6	74.45	2.63
-3	2	6	69.54	2.42
-3	2	6	74.39	2.67
2	-2	-6	53.77	2.01
2	-2	-6	55.30	2.06
2	-2	-6	46.13	1.74
2	-2	-6	52.96	2.04
-2	2	6	56.85	2.19
-2	2	6	55.33	2.10
-2	2	6	53.30	1.68
-2	2	6	54.29	1.90
1	-2	-6	9.24	0.79
1	-2	-6	7.05	0.54
1	-2	-6	8.91	0.72
1	-2	-6	8.25	0.78
-1	2	6	9.11	0.92
-1	2	6	9.00	0.40
-1	2	6	9.31	0.81
-1	2	6	8.74	0.60
0	-2	-6	38.12	1.74
0	-2	-6	37.21	1.56
0	-2	-6	37.81	1.26
0	2	6	38.82	1.26
0	2	6	38.66	1.68
0	2	6	39.77	1.87
0	2	6	36.40	1.51
0	2	6	39.23	1.54
-1	-2	-6	45.38	1.82

-1	-2	-6	45.95	1.54
-1	-2	-6	48.54	2.11
1	2	6	46.26	1.86
1	2	6	47.26	2.21
1	2	6	47.76	1.55
1	2	6	49.70	1.97
1	2	6	49.92	1.87
-2	-2	-6	10.41	0.49
-2	-2	-6	10.19	1.10
-2	-2	-6	9.94	0.74
2	2	6	9.92	0.84
2	2	6	8.98	0.93
2	2	6	11.71	1.29
2	2	6	10.28	0.79
2	2	6	10.84	0.52
-3	-2	-6	2.14	0.72
-3	-2	-6	2.00	0.30
-3	-2	-6	1.40	0.40
3	2	6	1.28	0.51
3	2	6	1.61	0.53
3	2	6	1.50	0.26
3	2	6	1.73	0.43
3	2	6	2.51	0.86
-4	-2	-6	9.61	1.46
-4	-2	-6	9.83	0.79
-4	-2	-6	10.68	0.56
4	2	6	10.53	1.12
4	2	6	10.23	0.92
4	2	6	10.55	1.06
4	2	6	10.50	0.60
4	2	6	9.57	1.66
-5	-2	-6	1.63	0.44
-5	-2	-6	-0.66	0.96
-5	-2	-6	1.58	0.31
5	2	6	2.49	0.59
5	2	6	1.01	0.34
5	2	6	1.02	0.62
-6	-2	-6	4.32	0.51
6	2	6	5.37	0.93
6	2	6	4.57	0.63
-7	-2	-6	2.70	0.43
-7	-2	-6	1.64	0.56
7	2	6	3.62	0.55
7	2	6	3.79	0.77

7	2	6	1.87	0.96
-8	-2	-6	0.07	0.57
-8	-2	-6	-0.35	0.40
8	2	6	0.89	1.11
-9	-2	-6	-0.36	0.59
-9	-2	-6	-0.02	0.53
9	2	6	0.38	0.70
9	2	6	-2.48	1.11
-10	-2	-6	-0.03	0.69
-10	-2	-6	0.13	0.61
10	2	6	0.17	0.84
10	2	6	-2.29	1.44
-11	-2	-6	0.77	0.81
-11	-2	-6	0.29	0.85
11	2	6	0.17	1.79
11	2	6	1.81	0.96
17	-3	-6	27.41	2.50
17	-3	-6	28.67	3.65
-17	3	6	25.32	1.68
16	-3	-6	18.04	2.02
-16	3	6	15.53	1.33
15	-3	-6	24.32	2.04
15	-3	-6	25.90	2.76
-15	3	6	27.05	1.47
14	-3	-6	30.68	2.69
14	-3	-6	35.89	2.20
-14	3	6	40.17	1.67
-14	3	6	34.17	1.95
13	-3	-6	17.59	1.54
13	-3	-6	12.97	1.95
-13	3	6	17.71	1.34
-13	3	6	18.06	1.04
12	-3	-6	63.63	2.77
12	-3	-6	67.64	3.14
-12	3	6	61.65	2.32
-12	3	6	69.52	2.57
11	-3	-6	38.82	1.92
11	-3	-6	36.52	2.18
11	-3	-6	36.75	1.45
-11	3	6	40.51	2.05
-11	3	6	37.31	1.49
-11	3	6	39.81	1.74
10	-3	-6	55.06	2.54
10	-3	-6	56.85	1.93

10	-3	-6	53.12	1.88
10	-3	-6	56.47	2.35
-10	3	6	58.98	2.19
-10	3	6	56.12	1.87
-10	3	6	56.19	1.97
-10	3	6	56.41	2.22
9	-3	-6	74.92	2.62
9	-3	-6	78.63	2.59
9	-3	-6	81.65	3.16
9	-3	-6	82.60	3.03
-9	3	6	78.69	2.64
-9	3	6	81.11	2.90
-9	3	6	83.80	2.90
-9	3	6	80.13	2.57
8	-3	-6	49.61	1.64
8	-3	-6	45.86	1.73
8	-3	-6	45.95	2.02
8	-3	-6	49.40	2.16
-8	3	6	49.33	1.95
-8	3	6	48.64	1.69
-8	3	6	51.17	1.95
7	-3	-6	17.69	0.71
7	-3	-6	17.83	0.72
7	-3	-6	17.25	1.27
7	-3	-6	15.71	1.05
7	-3	-6	17.47	0.89
-7	3	6	18.88	1.07
-7	3	6	17.05	0.73
-7	3	6	17.78	1.02
6	-3	-6	162.44	5.07
6	-3	-6	157.46	4.82
6	-3	-6	157.91	4.83
6	-3	-6	156.04	5.23
6	-3	-6	155.78	5.27
-6	3	6	164.63	5.23
-6	3	6	154.68	4.89
-6	3	6	160.90	5.17
5	-3	-6	70.37	2.64
5	-3	-6	71.21	2.24
5	-3	-6	69.27	2.62
5	-3	-6	70.08	2.22
-5	3	6	72.23	2.35
-5	3	6	69.34	2.57
-5	3	6	74.04	2.70

4	-3	-6	55.03	2.22
4	-3	-6	58.19	1.84
4	-3	-6	61.99	2.21
4	-3	-6	58.46	2.27
4	-3	-6	57.61	1.82
-4	3	6	57.85	2.21
-4	3	6	58.40	2.33
-4	3	6	55.24	1.99
3	-3	-6	15.24	0.58
3	-3	-6	15.30	0.97
3	-3	-6	15.76	1.00
3	-3	-6	14.12	0.95
-3	3	6	15.18	0.98
-3	3	6	14.78	0.55
-3	3	6	16.75	1.17
-3	3	6	14.68	0.77
2	-3	-6	1.97	0.51
2	-3	-6	1.53	0.49
2	-3	-6	1.31	0.40
2	-3	-6	1.40	0.22
-2	3	6	1.85	0.60
-2	3	6	1.71	0.35
-2	3	6	1.42	0.47
-2	3	6	1.30	0.16
1	-3	-6	15.23	1.06
1	-3	-6	17.34	1.15
1	-3	-6	17.44	0.62
1	-3	-6	16.85	0.98
-1	3	6	18.89	1.11
-1	3	6	16.87	1.31
-1	3	6	17.77	0.62
-1	3	6	16.68	0.92
0	-3	-6	5.70	0.32
0	-3	-6	5.71	0.81
0	-3	-6	4.21	0.70
0	-3	-6	5.72	0.65
0	3	6	5.98	0.33
0	3	6	7.15	0.80
0	3	6	3.78	0.79
0	3	6	5.56	0.63
-1	-3	-6	10.17	0.46
-1	-3	-6	8.71	0.75
-1	-3	-6	9.47	1.11
1	3	6	11.12	1.34

1	3	6	8.99	0.80
1	3	6	9.91	0.94
1	3	6	9.91	0.46
1	3	6	10.33	0.83
-2	-3	-6	6.76	1.12
-2	-3	-6	5.27	0.35
-2	-3	-6	4.91	0.64
2	3	6	3.90	0.43
2	3	6	5.78	0.74
2	3	6	4.21	0.73
2	3	6	5.41	1.03
2	3	6	4.66	0.71
-3	-3	-6	0.50	0.20
-3	-3	-6	0.05	0.34
-3	-3	-6	1.32	0.79
3	3	6	0.25	0.23
3	3	6	0.15	0.48
3	3	6	1.04	0.60
3	3	6	0.30	0.44
3	3	6	-1.14	0.82
-4	-3	-6	2.39	0.51
-4	-3	-6	2.72	1.01
-4	-3	-6	2.89	0.37
4	3	6	2.38	0.65
4	3	6	2.79	0.58
4	3	6	3.72	0.45
-5	-3	-6	6.27	0.50
-5	-3	-6	6.63	0.81
5	3	6	5.84	0.56
5	3	6	6.30	1.07
5	3	6	5.39	0.80
-6	-3	-6	0.21	0.44
-6	-3	-6	-0.12	0.31
6	3	6	-0.27	0.37
6	3	6	-0.20	0.71
6	3	6	-0.59	0.63
-7	-3	-6	-0.80	0.46
-7	-3	-6	0.14	0.37
7	3	6	0.75	0.91
7	3	6	-0.48	0.49
-8	-3	-6	0.07	0.44
-8	-3	-6	-0.26	0.58
8	3	6	0.18	0.71
8	3	6	0.06	1.11

-9	-3	-6	2.61	0.76
-9	-3	-6	2.54	0.65
9	3	6	3.70	0.94
9	3	6	3.95	1.41
-10	-3	-6	1.22	0.84
-10	-3	-6	2.28	0.77
10	3	6	2.98	1.69
10	3	6	2.48	1.00
18	-4	-6	8.66	2.69
17	-4	-6	16.84	2.24
17	-4	-6	18.98	3.47
-17	4	6	16.70	1.43
16	-4	-6	47.71	2.85
16	-4	-6	45.09	4.54
-16	4	6	48.25	2.10
15	-4	-6	37.76	3.15
15	-4	-6	38.20	2.43
-15	4	6	38.44	1.76
14	-4	-6	19.76	2.36
14	-4	-6	18.63	1.73
-14	4	6	19.91	1.17
-14	4	6	18.94	1.61
13	-4	-6	31.90	1.91
13	-4	-6	33.83	2.45
-13	4	6	30.58	1.75
-13	4	6	31.90	1.43
12	-4	-6	56.69	2.94
12	-4	-6	54.58	2.52
-12	4	6	58.91	2.41
-12	4	6	58.62	2.09
11	-4	-6	42.09	2.37
11	-4	-6	41.66	2.03
11	-4	-6	42.73	1.58
-11	4	6	42.67	1.61
-11	4	6	42.70	1.95
10	-4	-6	51.98	2.52
10	-4	-6	53.46	1.80
10	-4	-6	50.94	2.23
10	-4	-6	47.92	1.77
-10	4	6	51.30	2.11
-10	4	6	51.56	2.16
-10	4	6	52.94	1.84
-10	4	6	52.19	1.70
9	-4	-6	49.88	2.23

9	-4	-6	57.09	2.51
9	-4	-6	52.02	1.90
9	-4	-6	54.86	1.82
-9	4	6	54.50	2.21
-9	4	6	52.05	1.85
-9	4	6	53.46	2.15
-9	4	6	54.49	1.76
8	-4	-6	42.72	1.89
8	-4	-6	43.19	1.66
8	-4	-6	43.59	1.48
8	-4	-6	42.53	2.10
-8	4	6	44.26	1.47
-8	4	6	41.68	1.51
-8	4	6	42.71	1.93
-8	4	6	42.90	1.80
7	-4	-6	85.16	3.20
7	-4	-6	83.59	2.61
7	-4	-6	86.52	2.63
7	-4	-6	83.61	3.07
7	-4	-6	84.87	2.89
-7	4	6	78.47	3.34
-7	4	6	80.33	2.97
-7	4	6	82.43	2.67
6	-4	-6	125.84	4.32
6	-4	-6	125.85	3.86
6	-4	-6	123.94	4.40
6	-4	-6	128.23	3.86
-6	4	6	130.49	4.44
-6	4	6	125.97	4.24
-6	4	6	124.05	3.98
5	-4	-6	39.01	1.27
5	-4	-6	38.30	1.81
5	-4	-6	38.86	1.68
5	-4	-6	38.46	1.28
5	-4	-6	38.26	1.71
-5	4	6	40.98	1.68
-5	4	6	37.79	2.11
-5	4	6	39.33	1.47
4	-4	-6	15.08	0.57
4	-4	-6	14.11	0.99
4	-4	-6	15.53	1.14
4	-4	-6	15.50	1.03
-4	4	6	15.15	1.02
-4	4	6	15.75	1.23

-4	4	6	14.45	0.79
3	-4	-6	29.52	0.98
3	-4	-6	28.44	1.51
3	-4	-6	29.63	0.98
3	-4	-6	30.43	1.53
3	-4	-6	29.51	1.43
-3	4	6	29.00	1.29
-3	4	6	29.07	1.69
-3	4	6	31.10	1.47
2	-4	-6	39.92	1.89
2	-4	-6	39.72	1.28
2	-4	-6	40.76	1.87
2	-4	-6	40.99	1.74
-2	4	6	40.61	1.67
-2	4	6	41.59	1.27
-2	4	6	41.47	1.81
-2	4	6	40.91	2.09
1	-4	-6	35.94	1.60
1	-4	-6	35.61	1.84
1	-4	-6	36.48	1.78
-1	4	6	37.38	2.05
-1	4	6	35.81	1.69
-1	4	6	35.57	1.15
-1	4	6	38.45	1.62
0	-4	-6	4.65	0.66
0	-4	-6	4.87	0.28
0	-4	-6	3.72	0.74
0	-4	-6	5.07	0.77
0	4	6	4.59	0.69
0	4	6	4.73	0.72
0	4	6	4.50	0.29
0	4	6	3.71	0.88
-1	-4	-6	1.75	0.66
-1	-4	-6	2.36	0.51
1	4	6	2.44	0.60
1	4	6	3.19	0.57
1	4	6	3.51	0.97
1	4	6	2.64	0.29
-2	-4	-6	7.60	0.78
-2	-4	-6	7.91	1.41
-2	-4	-6	7.48	0.40
2	4	6	7.38	0.89
2	4	6	7.91	0.45
2	4	6	5.53	1.18

2	4	6	6.59	0.87
2	4	6	7.49	1.03
-3	-4	-6	0.39	0.20
-3	-4	-6	0.91	0.41
-3	-4	-6	1.39	0.98
3	4	6	0.16	0.49
3	4	6	-0.12	0.66
3	4	6	0.34	0.24
3	4	6	0.11	1.12
3	4	6	0.80	0.52
-4	-4	-6	0.10	0.24
-4	-4	-6	-0.17	0.39
4	4	6	-0.36	0.55
4	4	6	0.24	0.27
4	4	6	0.39	0.59
-5	-4	-6	1.44	0.30
-5	-4	-6	1.17	0.50
5	4	6	0.97	0.63
5	4	6	1.63	0.68
5	4	6	1.72	0.38
-6	-4	-6	1.52	0.37
-6	-4	-6	1.94	0.58
6	4	6	1.11	0.83
6	4	6	1.46	0.75
6	4	6	1.91	0.46
-7	-4	-6	1.18	0.67
-7	-4	-6	2.19	0.48
7	4	6	2.31	1.04
7	4	6	1.96	0.56
7	4	6	2.66	0.85
-8	-4	-6	4.18	0.62
-8	-4	-6	4.61	0.83
8	4	6	3.04	1.23
8	4	6	3.09	0.98
-9	-4	-6	1.96	0.73
-9	-4	-6	0.21	0.75
9	4	6	0.44	0.97
9	4	6	0.62	1.35
-10	-4	-6	-0.55	0.79
-10	-4	-6	1.48	0.95
10	4	6	0.47	1.04
10	4	6	3.84	2.49
18	-5	-6	12.76	2.17
18	-5	-6	13.89	2.94

-18	5	6	15.33	1.67
17	-5	-6	19.64	3.57
17	-5	-6	21.99	2.32
-17	5	6	22.43	1.53
16	-5	-6	17.45	2.05
-16	5	6	21.16	1.37
15	-5	-6	38.18	2.37
15	-5	-6	33.77	3.12
-15	5	6	35.56	1.67
14	-5	-6	28.52	1.96
14	-5	-6	27.66	2.65
-14	5	6	29.04	1.38
-14	5	6	26.40	1.89
13	-5	-6	35.47	2.04
13	-5	-6	33.35	2.56
-13	5	6	36.20	2.05
-13	5	6	36.73	1.99
12	-5	-6	33.63	2.39
12	-5	-6	33.24	1.86
-12	5	6	30.21	1.81
-12	5	6	32.56	1.37
11	-5	-6	19.34	0.96
11	-5	-6	22.76	1.48
11	-5	-6	20.45	1.86
-11	5	6	20.83	1.53
-11	5	6	20.24	0.97
10	-5	-6	27.50	1.98
10	-5	-6	26.90	1.19
10	-5	-6	27.29	1.56
10	-5	-6	28.59	1.12
-10	5	6	28.56	1.00
-10	5	6	27.14	1.65
-10	5	6	27.76	1.14
-10	5	6	28.23	1.46
9	-5	-6	57.78	2.68
9	-5	-6	57.97	1.90
9	-5	-6	53.35	2.06
9	-5	-6	58.94	2.40
-9	5	6	59.10	2.48
-9	5	6	51.71	1.97
-9	5	6	58.68	2.27
-9	5	6	57.48	1.94
8	-5	-6	46.51	1.89
8	-5	-6	46.33	2.33

8	-5	-6	47.66	2.10
8	-5	-6	49.88	1.63
-8	5	6	50.29	1.65
-8	5	6	46.17	1.66
-8	5	6	50.11	2.00
-8	5	6	49.64	2.25
7	-5	-6	32.68	1.14
7	-5	-6	32.76	1.63
7	-5	-6	31.27	1.69
7	-5	-6	33.44	1.83
-7	5	6	33.27	1.82
-7	5	6	32.61	1.54
-7	5	6	32.56	1.24
6	-5	-6	28.75	0.99
6	-5	-6	28.63	1.49
6	-5	-6	28.83	1.65
6	-5	-6	27.64	1.41
-6	5	6	29.36	1.42
-6	5	6	28.99	1.71
-6	5	6	28.55	1.18
5	-5	-6	43.42	1.93
5	-5	-6	43.73	1.43
5	-5	-6	46.39	1.44
5	-5	-6	46.30	1.96
5	-5	-6	41.47	2.07
-5	5	6	44.21	1.70
-5	5	6	45.19	2.21
-5	5	6	46.71	1.90
4	-5	-6	14.53	1.24
4	-5	-6	15.82	1.08
4	-5	-6	17.02	1.19
4	-5	-6	15.77	0.57
4	-5	-6	15.42	0.58
-4	5	6	14.49	1.35
-4	5	6	17.38	0.94
-4	5	6	16.68	1.11
3	-5	-6	5.45	0.95
3	-5	-6	5.29	0.76
3	-5	-6	5.12	0.28
3	-5	-6	5.35	0.81
3	-5	-6	4.95	0.26
-3	5	6	6.42	1.19
-3	5	6	5.56	0.82
-3	5	6	4.96	0.65

2	-5	-6	9.82	0.89
2	-5	-6	7.85	1.07
2	-5	-6	7.82	1.04
-2	5	6	11.05	1.00
-2	5	6	11.86	0.89
-2	5	6	10.49	1.38
1	-5	-6	29.14	1.66
1	-5	-6	27.91	1.40
1	-5	-6	28.75	1.76
-1	5	6	27.56	1.97
-1	5	6	28.56	1.54
-1	5	6	29.46	1.48
0	-5	-6	4.82	0.86
0	-5	-6	7.13	0.76
0	-5	-6	7.58	1.29
0	5	6	6.25	0.85
0	5	6	8.86	1.40
0	5	6	6.12	1.00
-1	-5	-6	1.27	0.43
-1	-5	-6	0.46	0.16
-1	-5	-6	0.35	0.65
-1	-5	-6	-0.38	0.71
1	5	6	-2.18	0.90
1	5	6	0.86	0.62
1	5	6	0.60	0.48
-2	-5	-6	0.32	0.21
-2	-5	-6	-0.43	0.86
-2	-5	-6	0.32	0.41
2	5	6	0.49	0.57
2	5	6	0.91	0.69
2	5	6	0.70	1.16
2	5	6	1.00	0.52
2	5	6	0.34	0.22
-3	-5	-6	4.44	0.40
-3	-5	-6	5.94	0.82
3	5	6	4.34	0.42
3	5	6	4.40	0.78
3	5	6	5.26	0.97
-4	-5	-6	0.63	0.51
4	5	6	-0.23	0.28
4	5	6	0.99	0.68
4	5	6	0.74	0.81
-5	-5	-6	0.45	0.32
-5	-5	-6	-0.16	0.51

5	5	6	0.23	0.37
5	5	6	1.47	0.76
5	5	6	-0.56	0.74
-6	-5	-6	2.05	0.65
-6	-5	-6	1.07	0.43
6	5	6	1.03	0.88
6	5	6	1.72	0.48
6	5	6	1.22	0.82
-7	-5	-6	1.25	0.68
-7	-5	-6	0.62	0.51
7	5	6	1.38	1.15
7	5	6	-0.01	0.86
-8	-5	-6	0.77	0.73
-8	-5	-6	0.19	0.62
8	5	6	0.75	1.23
8	5	6	-0.69	0.97
-9	-5	-6	0.48	0.84
9	5	6	1.76	1.52
9	5	6	0.23	1.02
18	-6	-6	14.23	3.00
18	-6	-6	9.86	1.93
-18	6	6	14.81	1.50
17	-6	-6	14.74	2.71
17	-6	-6	15.32	2.17
-17	6	6	12.45	1.31
16	-6	-6	42.48	2.68
16	-6	-6	39.28	3.61
-16	6	6	39.53	1.88
15	-6	-6	27.38	3.00
15	-6	-6	30.79	2.24
-15	6	6	28.00	1.49
14	-6	-6	10.96	1.54
14	-6	-6	13.90	2.43
-14	6	6	9.39	1.54
-14	6	6	7.95	0.90
13	-6	-6	29.49	1.87
13	-6	-6	30.77	2.56
-13	6	6	22.60	1.69
-13	6	6	31.01	1.92
12	-6	-6	20.22	1.55
12	-6	-6	21.23	2.18
-12	6	6	20.97	1.05
-12	6	6	19.63	1.69
11	-6	-6	25.81	1.65

11	-6	-6	25.71	1.14
11	-6	-6	25.23	2.16
-11	6	6	25.68	1.15
-11	6	6	29.57	1.84
10	-6	-6	19.53	1.84
10	-6	-6	19.59	1.10
10	-6	-6	18.59	1.35
10	-6	-6	21.35	0.91
-10	6	6	19.74	0.80
-10	6	6	18.48	1.27
-10	6	6	20.96	1.63
-10	6	6	21.47	0.94
9	-6	-6	44.06	1.50
9	-6	-6	42.08	2.38
9	-6	-6	41.18	1.80
9	-6	-6	43.03	2.02
-9	6	6	43.23	1.53
-9	6	6	42.56	1.88
-9	6	6	43.11	1.49
-9	6	6	41.68	2.25
8	-6	-6	41.47	1.98
8	-6	-6	41.93	2.30
8	-6	-6	39.46	1.97
8	-6	-6	42.86	1.44
-8	6	6	42.71	1.85
-8	6	6	40.55	1.51
-8	6	6	42.11	1.47
-8	6	6	43.25	2.23
7	-6	-6	45.59	2.06
7	-6	-6	44.80	1.53
7	-6	-6	44.73	2.36
7	-6	-6	47.65	2.03
-7	6	6	49.57	2.41
-7	6	6	46.47	1.71
-7	6	6	46.23	1.97
6	-6	-6	27.47	0.96
6	-6	-6	27.30	1.51
6	-6	-6	28.59	1.56
6	-6	-6	27.90	1.78
6	-6	-6	27.88	0.96
-6	6	6	27.21	1.45
-6	6	6	28.58	1.26
-6	6	6	30.13	1.88
5	-6	-6	7.28	1.16

5	-6	-6	7.19	1.03
5	-6	-6	7.76	0.37
5	-6	-6	8.00	0.92
-5	6	6	8.28	1.32
-5	6	6	8.22	0.91
-5	6	6	8.07	0.74
4	-6	-6	14.07	1.32
4	-6	-6	12.00	1.21
4	-6	-6	10.64	1.00
4	-6	-6	10.96	0.42
4	-6	-6	12.03	0.45
-4	6	6	10.07	1.43
-4	6	6	11.20	0.90
-4	6	6	10.44	1.04
3	-6	-6	27.19	1.68
3	-6	-6	23.56	1.61
3	-6	-6	23.69	1.37
-3	6	6	26.29	1.48
-3	6	6	25.55	1.92
-3	6	6	24.49	1.34
2	-6	-6	0.22	0.62
2	-6	-6	1.30	0.53
2	-6	-6	-0.38	0.61
-2	6	6	0.42	0.55
-2	6	6	1.26	0.90
-2	6	6	1.31	0.47
1	-6	-6	2.49	0.81
1	-6	-6	1.86	0.73
1	-6	-6	1.65	0.50
-1	6	6	2.33	0.68
-1	6	6	1.44	0.54
-1	6	6	0.10	0.87
0	-6	-6	0.16	0.79
0	-6	-6	0.45	0.73
0	-6	-6	0.52	0.43
0	6	6	1.42	0.68
0	6	6	1.07	0.57
0	6	6	-0.15	1.03
-1	-6	-6	0.82	1.19
-1	-6	-6	0.41	0.45
-1	-6	-6	-0.17	0.68
1	6	6	-0.01	0.50
1	6	6	0.86	0.74
1	6	6	0.72	1.14

-2	-6	-6	0.96	0.49
2	6	6	0.35	0.63
2	6	6	0.82	0.58
-3	-6	-6	0.14	0.46
-3	-6	-6	-0.07	0.24
3	6	6	1.42	0.73
3	6	6	0.43	0.27
-4	-6	-6	6.82	0.97
-4	-6	-6	7.64	0.56
4	6	6	9.40	1.38
4	6	6	7.76	0.58
4	6	6	8.67	1.26
-5	-6	-6	6.97	0.68
-5	-6	-6	9.80	1.07
5	6	6	9.48	1.49
5	6	6	8.90	1.53
5	6	6	9.05	0.79
-6	-6	-6	1.98	0.70
-6	-6	-6	3.48	0.55
6	6	6	2.94	1.02
6	6	6	4.03	1.05
-7	-6	-6	4.74	0.89
-7	-6	-6	3.65	0.67
7	6	6	5.16	1.30
7	6	6	5.09	1.16
-8	-6	-6	6.65	1.02
8	6	6	8.14	1.42
8	6	6	4.82	1.52
18	-7	-6	19.91	2.71
18	-7	-6	20.16	3.30
-18	7	6	25.82	1.71
17	-7	-6	26.76	2.50
17	-7	-6	25.23	3.94
-17	7	6	29.34	1.63
16	-7	-6	15.97	2.08
16	-7	-6	21.96	3.34
-16	7	6	18.11	1.32
15	-7	-6	29.27	3.14
15	-7	-6	35.90	2.41
-15	7	6	35.96	1.64
14	-7	-6	15.02	2.56
14	-7	-6	13.57	1.63
-14	7	6	13.80	1.02
13	-7	-6	8.71	1.77

13	-7	-6	6.03	1.02
-13	7	6	4.81	0.74
-13	7	6	6.47	1.25
12	-7	-6	8.88	1.63
12	-7	-6	9.28	1.28
-12	7	6	7.77	0.75
-12	7	6	9.92	1.63
11	-7	-6	11.14	1.89
11	-7	-6	11.80	0.78
11	-7	-6	10.50	1.23
-11	7	6	12.33	0.56
-11	7	6	12.58	0.76
-11	7	6	12.91	0.79
-11	7	6	14.49	1.71
10	-7	-6	35.30	2.39
10	-7	-6	33.82	1.29
10	-7	-6	33.13	1.83
-10	7	6	34.96	1.32
-10	7	6	32.99	1.22
-10	7	6	33.86	2.20
-10	7	6	31.31	1.69
9	-7	-6	69.71	3.31
9	-7	-6	67.39	2.85
9	-7	-6	65.89	2.72
9	-7	-6	73.94	2.29
-9	7	6	69.16	2.33
-9	7	6	70.59	3.20
-9	7	6	69.81	2.30
-9	7	6	68.02	2.69
8	-7	-6	40.36	1.98
8	-7	-6	39.60	2.37
8	-7	-6	40.16	1.94
8	-7	-6	41.67	1.40
-8	7	6	41.11	1.85
-8	7	6	40.40	1.43
-8	7	6	39.87	1.56
-8	7	6	40.16	2.38
7	-7	-6	2.14	0.31
7	-7	-6	1.31	0.56
7	-7	-6	3.51	0.72
7	-7	-6	1.24	0.76
-7	7	6	1.68	0.97
-7	7	6	4.10	0.70
-7	7	6	1.52	0.40

6	-7	-6	2.42	0.29
6	-7	-6	2.55	0.87
6	-7	-6	2.34	0.27
6	-7	-6	2.44	0.63
6	-7	-6	3.37	0.73
-6	7	6	2.22	0.59
-6	7	6	2.41	0.51
-6	7	6	2.24	0.98
5	-7	-6	9.94	1.36
5	-7	-6	10.14	0.42
5	-7	-6	8.72	1.24
5	-7	-6	10.24	0.42
5	-7	-6	10.85	1.08
-5	7	6	9.31	1.03
-5	7	6	9.10	1.52
-5	7	6	9.42	0.87
4	-7	-6	23.06	1.65
4	-7	-6	22.39	1.39
4	-7	-6	23.78	1.75
-4	7	6	24.38	1.45
-4	7	6	22.64	1.34
-4	7	6	20.81	1.95
3	-7	-6	11.53	1.35
3	-7	-6	10.20	1.38
3	-7	-6	10.34	1.01
-3	7	6	11.26	1.17
-3	7	6	10.44	1.01
-3	7	6	10.94	1.68
2	-7	-6	6.59	0.87
2	-7	-6	4.28	0.94
2	-7	-6	3.33	0.96
-2	7	6	6.24	1.28
-2	7	6	5.81	0.91
-2	7	6	5.80	0.88
1	-7	-6	4.14	0.69
1	-7	-6	2.61	0.98
1	-7	-6	3.89	0.94
-1	7	6	3.79	0.73
-1	7	6	4.03	1.35
-1	7	6	3.17	0.82
0	-7	-6	2.53	0.57
0	-7	-6	1.34	0.85
0	7	6	2.56	0.68
0	7	6	2.54	1.54

0	7	6	1.94	0.82
-1	-7	-6	-0.19	0.80
-1	-7	-6	-0.20	0.45
1	7	6	0.05	0.53
-2	-7	-6	7.77	0.94
-3	-7	-6	1.12	0.56
3	7	6	0.65	0.73
3	7	6	-0.39	0.66
-4	-7	-6	-0.75	0.54
4	7	6	-0.16	0.80
4	7	6	-0.39	0.81
-5	-7	-6	3.60	0.80
5	7	6	3.27	1.03
5	7	6	1.30	0.99
-6	-7	-6	5.20	0.92
6	7	6	4.42	1.20
6	7	6	5.83	1.24
-7	-7	-6	2.16	0.87
7	7	6	1.19	1.25
7	7	6	2.04	1.16
-8	-7	-6	2.62	0.98
8	7	6	2.87	1.32
18	-8	-6	6.32	1.86
18	-8	-6	4.05	2.88
-18	8	6	10.21	1.39
17	-8	-6	15.97	2.99
17	-8	-6	19.11	2.33
-17	8	6	21.01	1.48
16	-8	-6	28.13	2.38
16	-8	-6	33.67	3.60
-16	8	6	26.90	1.54
15	-8	-6	24.70	2.13
-15	8	6	23.58	1.35
14	-8	-6	13.42	1.73
14	-8	-6	18.44	2.64
-14	8	6	15.83	1.05
13	-8	-6	7.12	1.83
13	-8	-6	6.24	1.08
-13	8	6	8.12	1.71
-13	8	6	8.67	0.82
12	-8	-6	16.85	2.27
12	-8	-6	15.35	1.61
-12	8	6	16.98	2.07
-12	8	6	16.00	0.94

11	-8	-6	33.24	2.59
11	-8	-6	34.11	1.91
11	-8	-6	31.63	1.30
-11	8	6	32.67	1.31
-11	8	6	32.33	1.17
-11	8	6	28.76	2.32
-11	8	6	31.36	1.39
10	-8	-6	25.48	2.29
10	-8	-6	26.91	1.11
10	-8	-6	26.40	1.69
-10	8	6	26.62	1.38
-10	8	6	26.45	2.25
-10	8	6	25.96	1.11
-10	8	6	27.29	1.10
-10	8	6	27.64	1.82
9	-8	-6	25.14	1.01
9	-8	-6	23.93	1.60
9	-8	-6	26.78	2.24
-9	8	6	26.01	1.50
-9	8	6	26.36	2.17
-9	8	6	24.88	1.02
-9	8	6	23.95	1.12
8	-8	-6	6.99	1.29
8	-8	-6	7.03	0.49
8	-8	-6	7.88	1.13
8	-8	-6	8.18	1.26
-8	8	6	6.27	0.51
-8	8	6	7.02	0.72
-8	8	6	7.20	1.33
-8	8	6	6.24	0.84
7	-8	-6	6.22	1.22
7	-8	-6	5.59	0.88
7	-8	-6	6.35	0.42
7	-8	-6	7.59	1.25
-7	8	6	6.87	1.31
-7	8	6	6.23	0.76
-7	8	6	6.04	0.99
6	-8	-6	1.70	0.90
6	-8	-6	1.90	0.28
6	-8	-6	2.04	0.67
6	-8	-6	1.90	0.81
6	-8	-6	1.85	0.22
-6	8	6	1.23	0.59
-6	8	6	1.71	0.51

-6	8	6	1.81	1.06
5	-8	-6	5.50	1.11
5	-8	-6	5.99	1.11
5	-8	-6	5.40	0.87
-5	8	6	5.00	0.86
-5	8	6	6.85	1.01
-5	8	6	7.40	1.42
4	-8	-6	5.65	1.09
4	-8	-6	4.34	0.97
4	-8	-6	5.62	0.86
-4	8	6	5.29	0.94
-4	8	6	3.92	1.20
-4	8	6	7.54	1.10
3	-8	-6	4.85	0.81
3	-8	-6	6.56	1.22
3	-8	-6	4.65	1.07
-3	8	6	5.79	0.95
-3	8	6	6.53	1.51
-3	8	6	3.69	0.75
2	-8	-6	1.67	0.86
2	-8	-6	1.55	0.95
-2	8	6	-0.02	0.54
-2	8	6	2.12	1.25
-2	8	6	1.72	0.77
1	-8	-6	0.46	0.48
1	-8	-6	-0.09	0.74
-1	8	6	-0.44	0.57
-1	8	6	1.35	0.82
0	-8	-6	10.33	1.60
0	-8	-6	8.22	0.98
0	8	6	5.61	0.97
-1	-8	-6	8.82	1.39
-1	-8	-6	7.76	0.97
1	8	6	8.13	1.31
-2	-8	-6	0.10	0.65
-2	-8	-6	-0.48	0.98
2	8	6	1.26	0.79
-3	-8	-6	2.54	0.70
3	8	6	2.04	0.89
3	8	6	3.17	1.03
-4	-8	-6	4.66	0.79
4	8	6	3.26	1.06
4	8	6	3.29	1.03
-5	-8	-6	5.09	0.90

5	8	6	3.23	1.14
5	8	6	5.36	1.33
-6	-8	-6	3.86	0.92
6	8	6	4.07	1.29
6	8	6	1.39	1.24
-7	-8	-6	4.41	1.02
7	8	6	1.95	1.31
7	8	6	4.64	1.80
18	-9	-6	10.52	3.05
18	-9	-6	11.43	2.09
-18	9	6	6.09	1.01
17	-9	-6	16.02	2.28
17	-9	-6	14.69	3.05
-17	9	6	15.00	1.37
16	-9	-6	5.46	1.46
16	-9	-6	6.32	2.46
-16	9	6	7.51	1.08
15	-9	-6	16.77	2.05
15	-9	-6	18.95	3.11
-15	9	6	22.83	1.30
14	-9	-6	22.30	2.91
14	-9	-6	25.22	2.04
-14	9	6	24.23	1.27
13	-9	-6	24.23	2.83
13	-9	-6	25.94	1.98
-13	9	6	27.69	1.29
12	-9	-6	42.56	3.14
12	-9	-6	38.82	2.75
-12	9	6	44.17	1.67
-12	9	6	42.76	1.64
11	-9	-6	8.28	0.73
11	-9	-6	10.08	1.36
11	-9	-6	10.36	1.86
-11	9	6	8.39	0.63
-11	9	6	10.01	1.59
-11	9	6	9.89	0.73
-11	9	6	9.38	1.05
10	-9	-6	1.19	0.77
10	-9	-6	-0.69	1.10
10	-9	-6	1.16	0.38
-10	9	6	0.67	0.36
-10	9	6	0.94	0.37
-10	9	6	1.04	0.63
-10	9	6	2.06	1.37

9	-9	-6	34.95	1.30
9	-9	-6	36.16	1.96
9	-9	-6	33.94	2.62
-9	9	6	38.63	1.80
-9	9	6	34.69	2.65
-9	9	6	34.03	1.79
-9	9	6	34.94	1.53
-9	9	6	34.12	1.30
8	-9	-6	7.09	1.16
8	-9	-6	6.88	1.37
8	-9	-6	7.54	0.52
-8	9	6	6.72	0.86
-8	9	6	6.30	1.11
-8	9	6	7.12	1.13
-8	9	6	12.02	1.73
-8	9	6	6.64	0.53
7	-9	-6	0.80	0.98
7	-9	-6	2.22	0.86
7	-9	-6	1.85	0.23
7	-9	-6	1.34	0.28
7	-9	-6	2.08	0.74
-7	9	6	3.42	1.37
-7	9	6	1.91	0.68
-7	9	6	1.24	0.55
6	-9	-6	1.59	0.21
6	-9	-6	1.95	0.91
6	-9	-6	1.16	0.71
6	-9	-6	0.48	0.95
6	-9	-6	1.37	0.20
-6	9	6	3.06	1.28
-6	9	6	1.60	0.65
-6	9	6	0.87	0.52
5	-9	-6	1.26	0.97
5	-9	-6	0.82	0.64
5	-9	-6	0.92	0.89
-5	9	6	1.17	0.70
-5	9	6	0.52	0.55
-5	9	6	1.45	1.40
4	-9	-6	5.62	1.21
4	-9	-6	2.94	1.05
4	-9	-6	4.60	0.84
-4	9	6	5.94	1.40
-4	9	6	6.69	0.97
3	-9	-6	2.32	0.64

3	-9	-6	3.52	1.11
-3	9	6	1.15	0.71
-3	9	6	2.75	0.86
2	-9	-6	0.16	0.57
2	-9	-6	-0.64	0.87
-2	9	6	1.82	0.87
-2	9	6	0.23	0.61
1	-9	-6	0.24	0.58
1	-9	-6	0.72	0.96
-1	9	6	0.28	1.00
0	-9	-6	1.76	1.03
0	-9	-6	2.02	0.64
0	9	6	1.96	0.84
-1	-9	-6	10.98	1.63
1	9	6	8.82	1.36
-2	-9	-6	3.98	0.78
-2	-9	-6	3.54	1.36
2	9	6	3.39	1.13
-3	-9	-6	2.19	0.74
3	9	6	2.61	1.06
3	9	6	3.44	1.09
-4	-9	-6	0.92	0.74
4	9	6	3.36	1.13
4	9	6	0.65	1.02
-5	-9	-6	2.70	0.91
5	9	6	3.31	1.16
5	9	6	5.99	1.45
-6	-9	-6	5.39	1.06
6	9	6	3.65	1.46
6	9	6	2.26	1.44
17	-10	-6	11.67	2.92
17	-10	-6	7.00	1.67
-17	10	6	9.09	1.25
16	-10	-6	15.00	2.86
16	-10	-6	13.79	2.30
-16	10	6	16.36	1.29
15	-10	-6	7.93	1.44
15	-10	-6	9.80	2.32
-15	10	6	10.55	1.06
14	-10	-6	22.06	2.11
14	-10	-6	21.02	3.13
-14	10	6	21.72	1.24
13	-10	-6	30.79	3.07
13	-10	-6	29.12	2.17

-13	10	6	29.53	1.37
12	-10	-6	14.10	1.65
12	-10	-6	14.40	2.22
-12	10	6	12.79	0.89
-12	10	6	10.74	0.71
11	-10	-6	12.96	1.56
11	-10	-6	12.13	2.05
11	-10	-6	13.18	0.86
-11	10	6	13.85	0.87
-11	10	6	14.79	1.28
-11	10	6	14.14	0.84
10	-10	-6	7.63	1.60
10	-10	-6	6.80	0.64
10	-10	-6	4.55	1.03
-10	10	6	5.79	0.63
-10	10	6	5.52	0.75
-10	10	6	5.21	0.88
9	-10	-6	-0.20	1.22
9	-10	-6	0.25	0.76
9	-10	-6	0.38	0.33
-9	10	6	0.17	0.33
-9	10	6	1.53	1.66
-9	10	6	-0.04	0.48
-9	10	6	0.32	0.75
8	-10	-6	4.73	1.34
8	-10	-6	4.06	0.40
8	-10	-6	3.59	0.84
-8	10	6	4.12	0.89
-8	10	6	3.53	0.70
-8	10	6	5.86	1.65
-8	10	6	3.00	0.80
7	-10	-6	16.54	0.64
7	-10	-6	16.39	1.52
7	-10	-6	17.14	2.10
7	-10	-6	16.43	0.69
-7	10	6	16.29	1.49
-7	10	6	18.36	2.82
-7	10	6	16.29	1.28
-7	10	6	13.76	1.41
6	-10	-6	0.41	1.18
6	-10	-6	-0.41	0.67
-6	10	6	-0.77	0.54
-6	10	6	0.18	0.80
-6	10	6	-0.62	0.69

5	-10	-6	6.23	0.95
5	-10	-6	4.00	1.28
-5	10	6	4.30	0.97
-5	10	6	3.73	0.80
4	-10	-6	1.09	0.67
4	-10	-6	-0.01	0.99
-4	10	6	0.69	0.83
-4	10	6	-0.57	0.61
3	-10	-6	4.44	1.19
3	-10	-6	4.77	0.81
-3	10	6	4.47	1.09
-3	10	6	3.44	0.93
2	-10	-6	0.71	0.65
2	-10	-6	0.55	1.10
-2	10	6	1.12	0.82
1	-10	-6	-0.29	0.59
1	-10	-6	0.05	1.05
-1	10	6	1.00	0.86
0	-10	-6	1.02	1.16
0	10	6	1.05	0.92
-1	-10	-6	15.81	1.33
-1	-10	-6	15.99	2.28
1	10	6	15.34	1.94
-2	-10	-6	5.16	1.59
-2	-10	-6	4.79	0.86
2	10	6	6.06	1.27
-3	-10	-6	5.49	0.96
3	10	6	3.92	1.28
3	10	6	3.86	1.21
-4	-10	-6	7.16	1.02
4	10	6	4.06	1.19
4	10	6	6.42	1.40
-5	-10	-6	5.30	1.04
5	10	6	3.51	1.47
5	10	6	4.92	1.48
17	-11	-6	9.51	1.78
17	-11	-6	16.59	3.14
-17	11	6	11.95	1.35
16	-11	-6	3.72	2.54
16	-11	-6	4.73	1.55
-16	11	6	5.63	0.84
15	-11	-6	5.73	2.27
15	-11	-6	3.64	1.37
-15	11	6	6.17	0.82

14	-11	-6	10.60	2.34
14	-11	-6	11.02	1.62
-14	11	6	9.81	1.00
13	-11	-6	1.23	1.77
13	-11	-6	6.44	1.39
-13	11	6	4.27	0.64
-13	11	6	4.44	0.54
12	-11	-6	8.83	2.70
12	-11	-6	11.08	1.73
-12	11	6	12.45	0.90
-12	11	6	9.81	1.22
-12	11	6	11.89	0.86
11	-11	-6	3.49	1.63
11	-11	-6	3.88	0.53
-11	11	6	4.14	0.90
-11	11	6	4.39	0.63
-11	11	6	4.95	0.57
10	-11	-6	0.17	1.40
10	-11	-6	-0.33	0.38
10	-11	-6	0.89	0.86
-10	11	6	-0.15	0.51
-10	11	6	0.72	0.83
-10	11	6	0.24	0.38
9	-11	-6	0.47	1.36
9	-11	-6	2.84	0.43
9	-11	-6	2.99	0.88
-9	11	6	2.59	0.69
-9	11	6	2.70	0.43
-9	11	6	3.07	0.93
8	-11	-6	4.13	1.45
8	-11	-6	2.24	0.35
8	-11	-6	2.98	0.90
-8	11	6	2.62	0.75
-8	11	6	2.95	0.98
7	-11	-6	1.23	0.84
7	-11	-6	-1.09	1.19
-7	11	6	-0.60	0.86
-7	11	6	-0.09	0.64
6	-11	-6	-0.65	0.73
6	-11	-6	-0.33	1.14
-6	11	6	-0.39	1.05
-6	11	6	0.16	0.68
5	-11	-6	3.55	1.81
5	-11	-6	2.85	0.82

-5	11	6	2.77	0.84
4	-11	-6	8.20	1.24
4	-11	-6	8.30	1.64
3	-11	-6	4.32	1.41
3	-11	-6	4.78	0.87
-3	11	6	3.57	0.97
2	-11	-6	2.47	0.75
2	-11	-6	2.07	1.31
-2	11	6	1.66	0.94
1	-11	-6	0.69	0.86
1	-11	-6	3.05	1.41
-1	11	6	0.90	0.96
0	-11	-6	0.70	0.73
0	-11	-6	-1.47	1.24
0	11	6	1.80	1.10
-1	-11	-6	9.92	1.33
-1	-11	-6	9.36	1.77
1	11	6	8.92	1.58
-2	-11	-6	14.36	2.60
-2	-11	-6	8.52	1.40
2	11	6	13.98	2.17
-3	-11	-6	5.04	0.98
3	11	6	2.67	1.43
3	11	6	5.39	1.32
-4	-11	-6	6.12	1.15
4	11	6	6.39	1.56
4	11	6	2.87	1.41
17	-12	-6	3.39	2.87
17	-12	-6	2.89	1.69
-17	12	6	2.70	0.86
16	-12	-6	5.33	1.70
16	-12	-6	6.89	2.79
-16	12	6	8.98	1.23
15	-12	-6	6.00	1.64
15	-12	-6	7.76	2.52
-15	12	6	6.08	0.84
14	-12	-6	8.84	1.61
14	-12	-6	7.38	2.42
-14	12	6	8.70	1.02
13	-12	-6	5.59	2.10
13	-12	-6	4.54	1.36
-13	12	6	4.97	0.67
-13	12	6	5.14	0.72
12	-12	-6	2.80	1.78

12	-12	-6	-0.03	1.12
12	-12	-6	1.76	0.66
-12	12	6	0.99	0.55
-12	12	6	1.82	0.54
11	-12	-6	4.81	1.18
11	-12	-6	6.34	1.89
11	-12	-6	2.70	0.54
-11	12	6	3.69	0.53
-11	12	6	2.37	1.01
-11	12	6	3.34	0.71
10	-12	-6	10.34	0.80
10	-12	-6	9.82	1.60
10	-12	-6	13.38	2.29
-10	12	6	9.30	1.65
-10	12	6	9.44	0.77
-10	12	6	11.59	1.24
9	-12	-6	-0.57	1.41
9	-12	-6	1.25	0.92
9	-12	-6	0.67	0.37
-9	12	6	0.49	0.37
-9	12	6	0.63	0.71
-9	12	6	0.84	1.00
8	-12	-6	-1.84	1.31
8	-12	-6	0.94	0.25
8	-12	-6	-0.09	0.90
-8	12	6	1.91	0.80
7	-12	-6	-0.48	1.36
7	-12	-6	-0.27	0.82
-7	12	6	-0.95	0.72
6	-12	-6	-0.49	0.80
6	-12	-6	1.07	1.35
-6	12	6	-0.47	0.85
5	-12	-6	6.74	1.09
5	-12	-6	8.97	1.84
-5	12	6	5.02	1.11
4	-12	-6	13.44	2.07
4	-12	-6	11.99	1.42
-4	12	6	12.96	1.75
3	-12	-6	0.95	1.38
3	-12	-6	1.50	0.79
-3	12	6	1.38	0.97
2	-12	-6	3.75	0.83
2	-12	-6	4.88	1.65
-2	12	6	3.20	1.18

1	-12	-6	8.98	1.28
1	-12	-6	8.06	1.82
-1	12	6	5.72	1.35
0	-12	-6	3.46	1.67
0	-12	-6	4.41	0.92
0	12	6	2.23	1.27
-1	-12	-6	9.22	1.17
-1	-12	-6	11.45	2.07
1	12	6	12.94	1.84
-2	-12	-6	5.76	1.93
-2	-12	-6	2.31	1.03
2	12	6	3.95	1.41
-3	-12	-6	2.90	1.05
3	12	6	0.07	2.64
16	-13	-6	0.48	1.65
16	-13	-6	4.86	2.76
-16	13	6	2.58	0.80
15	-13	-6	4.26	2.57
15	-13	-6	1.41	1.58
-15	13	6	0.95	0.70
14	-13	-6	-0.25	2.10
14	-13	-6	1.66	1.42
-14	13	6	0.73	0.65
13	-13	-6	-0.47	1.99
13	-13	-6	5.07	1.52
-13	13	6	3.24	0.66
12	-13	-6	-0.05	1.27
12	-13	-6	-0.69	1.82
-12	13	6	-0.60	0.65
-12	13	6	0.41	0.51
11	-13	-6	-0.02	1.16
11	-13	-6	1.49	1.84
-11	13	6	-0.61	1.45
-11	13	6	0.26	0.76
-11	13	6	-0.58	0.46
10	-13	-6	-0.33	1.07
10	-13	-6	-1.09	1.71
-10	13	6	1.29	1.18
-10	13	6	1.20	0.78
-10	13	6	0.59	0.42
9	-13	-6	0.25	1.66
-9	13	6	0.92	0.78
8	-13	-6	2.22	1.67
8	-13	-6	0.46	1.02

-8	13	6	0.98	0.83
7	-13	-6	3.85	1.78
7	-13	-6	4.24	1.07
-7	13	6	4.54	1.14
6	-13	-6	3.34	1.71
6	-13	-6	1.34	0.98
-6	13	6	0.79	0.95
5	-13	-6	0.59	0.86
5	-13	-6	0.76	1.55
-5	13	6	-0.11	0.97
4	-13	-6	2.62	1.56
4	-13	-6	0.73	0.96
-4	13	6	0.31	1.11
3	-13	-6	10.94	2.15
3	-13	-6	8.06	1.36
-3	13	6	6.54	1.41
2	-13	-6	5.95	1.87
2	-13	-6	8.58	1.17
-2	13	6	7.43	1.52
1	-13	-6	5.92	1.09
1	-13	-6	6.92	1.94
-1	13	6	9.19	1.63
0	-13	-6	9.23	1.92
0	-13	-6	8.51	1.19
0	13	6	6.95	1.55
-1	-13	-6	9.80	2.18
-1	-13	-6	11.40	1.68
1	13	6	8.56	1.70
15	-14	-6	5.97	1.97
15	-14	-6	-0.90	2.57
-15	14	6	3.04	0.80
14	-14	-6	2.14	2.48
14	-14	-6	1.01	1.59
-14	14	6	1.17	0.69
13	-14	-6	-1.60	2.22
13	-14	-6	-0.55	1.43
-13	14	6	0.38	0.63
12	-14	-6	0.06	1.37
12	-14	-6	0.44	2.05
-12	14	6	0.17	0.79
-12	14	6	0.71	0.54
11	-14	-6	-0.78	1.28
11	-14	-6	-0.27	2.01
-11	14	6	-0.94	0.90

-11	14	6	-0.11	0.50
10	-14	-6	2.63	1.28
10	-14	-6	1.21	2.01
-10	14	6	2.30	1.04
9	-14	-6	6.46	1.40
9	-14	-6	10.52	2.26
-9	14	6	6.64	1.22
8	-14	-6	-0.74	1.81
8	-14	-6	2.96	1.19
-8	14	6	1.46	1.03
7	-14	-6	0.65	1.05
7	-14	-6	2.11	1.85
-7	14	6	0.47	1.09
6	-14	-6	-2.14	1.58
6	-14	-6	-1.46	0.93
-6	14	6	1.89	1.12
5	-14	-6	0.96	0.94
5	-14	-6	-2.18	1.67
-5	14	6	1.45	1.19
4	-14	-6	4.50	1.08
4	-14	-6	5.76	1.84
-4	14	6	8.58	1.61
3	-14	-6	3.05	1.89
3	-14	-6	4.80	1.09
-3	14	6	2.15	1.41
2	-14	-6	7.52	1.99
2	-14	-6	8.34	1.19
-2	14	6	6.44	1.64
1	-14	-6	3.34	1.89
1	-14	-6	4.62	1.15
-1	14	6	1.88	1.51
0	-14	-6	8.36	3.10
0	-14	-6	9.41	1.34
0	14	6	8.80	2.96
14	-15	-6	1.22	1.79
14	-15	-6	-0.03	2.65
13	-15	-6	2.54	1.74
13	-15	-6	1.83	2.54
12	-15	-6	2.04	1.61
12	-15	-6	1.47	2.32
-12	15	6	1.31	1.00
11	-15	-6	1.07	2.33
11	-15	-6	2.90	1.56
10	-15	-6	7.94	2.40

10	-15	-6	6.71	1.67
-10	15	6	7.21	1.32
9	-15	-6	0.42	1.35
9	-15	-6	3.79	2.13
-9	15	6	-0.27	1.14
8	-15	-6	1.96	2.04
8	-15	-6	-0.75	1.23
-8	15	6	-1.39	1.14
7	-15	-6	1.85	1.22
7	-15	-6	0.39	1.96
-7	15	6	1.03	1.25
6	-15	-6	4.84	2.11
6	-15	-6	6.01	1.35
-6	15	6	6.15	1.46
5	-15	-6	4.36	1.22
5	-15	-6	2.28	2.00
-5	15	6	4.64	1.50
4	-15	-6	6.41	2.05
4	-15	-6	4.62	1.19
-4	15	6	3.35	1.55
3	-15	-6	10.31	1.39
3	-15	-6	7.81	2.32
-3	15	6	6.44	1.80
2	-15	-6	9.12	2.46
2	-15	-6	10.62	1.40
-2	15	6	7.18	1.94
13	-16	-6	5.58	2.83
13	-16	-6	-1.86	1.91
12	-16	-6	3.46	2.57
12	-16	-6	6.24	1.95
11	-16	-6	0.68	1.73
11	-16	-6	1.60	2.51
-11	16	6	1.06	1.22
10	-16	-6	1.90	1.62
10	-16	-6	4.54	2.48
-10	16	6	0.03	1.25
9	-16	-6	9.61	2.70
9	-16	-6	7.87	1.75
-9	16	6	8.07	1.59
8	-16	-6	9.56	2.60
8	-16	-6	14.07	2.38
-8	16	6	10.16	1.75
7	-16	-6	6.20	1.50
7	-16	-6	4.24	2.45

-7	16	6	4.82	1.63
6	-16	-6	2.61	1.39
6	-16	-6	2.31	2.30
-6	16	6	3.03	1.50
5	-16	-6	12.47	2.62
5	-16	-6	9.51	1.51
-5	16	6	13.73	2.07
4	-16	-6	13.47	4.36
4	-16	-6	9.28	1.48
-4	16	6	4.57	1.75
0	-18	7	16.98	3.30
-1	18	-7	9.96	1.18
1	-18	7	9.01	1.97
1	-18	7	8.53	2.89
-2	18	-7	10.07	1.18
2	-18	7	18.80	3.24
2	-18	7	16.63	2.82
-3	18	-7	16.07	1.27
3	-18	7	18.34	3.22
3	-18	7	16.03	2.81
-4	18	-7	25.88	1.50
4	-18	7	24.22	2.93
4	-18	7	27.57	4.46
-5	18	-7	18.65	1.36
5	-18	7	22.25	3.51
5	-18	7	17.27	2.85
-6	18	-7	13.84	1.25
6	-18	7	13.63	2.98
6	-18	7	11.84	2.00
-7	18	-7	22.72	1.47
7	-18	7	22.92	2.92
7	-18	7	24.62	3.93
-8	18	-7	17.54	1.36
8	-18	7	16.27	3.64
2	17	-7	11.85	2.71
-2	-17	7	22.81	3.21
1	17	-7	18.45	1.30
-1	-17	7	22.39	4.02
-1	-17	7	17.69	2.72
0	17	-7	20.26	1.33
0	-17	7	24.40	2.67
0	-17	7	22.64	3.90
-1	17	-7	28.01	1.47
1	-17	7	31.35	2.86

1	-17	7	21.32	3.87
-2	17	-7	16.06	1.19
2	-17	7	16.05	2.54
2	-17	7	9.50	2.78
-3	17	-7	17.00	1.24
3	-17	7	17.74	2.87
3	-17	7	19.85	2.62
-4	17	-7	30.00	1.54
4	-17	7	23.21	4.03
4	-17	7	29.29	2.85
-5	17	-7	23.86	1.42
5	-17	7	24.79	3.95
5	-17	7	25.59	2.77
-6	17	-7	14.08	1.22
6	-17	7	18.49	3.30
6	-17	7	15.80	2.50
-7	17	-7	30.63	1.61
7	-17	7	29.56	2.86
7	-17	7	27.84	4.47
-8	17	-7	18.61	1.37
8	-17	7	20.64	3.45
8	-17	7	21.25	2.75
-9	17	-7	13.35	1.30
9	-17	7	14.79	3.23
9	-17	7	14.46	1.97
4	16	-7	9.65	2.58
-4	-16	7	13.95	2.72
3	16	-7	14.57	1.32
-3	-16	7	23.09	2.72
-3	-16	7	10.71	2.54
2	16	-7	11.72	1.17
-2	-16	7	10.56	1.84
-2	-16	7	11.32	2.45
1	16	-7	29.85	1.52
-1	-16	7	29.44	3.77
-1	-16	7	32.69	2.78
0	16	-7	32.79	1.60
0	-16	7	35.23	3.80
0	-16	7	36.51	2.85
-1	16	-7	26.68	1.41
1	-16	7	31.90	3.71
1	-16	7	33.59	2.75
-2	16	-7	24.97	1.32
2	-16	7	24.76	3.58

2	-16	7	25.23	2.53
-3	16	-7	27.57	1.40
3	-16	7	27.69	2.50
3	-16	7	25.59	3.59
-4	16	-7	35.87	1.66
4	-16	7	33.92	3.81
4	-16	7	40.33	2.84
-5	16	-7	32.32	1.58
5	-16	7	34.74	4.00
5	-16	7	33.77	2.65
-6	16	-7	22.72	1.36
6	-16	7	28.18	3.96
6	-16	7	26.30	2.51
-7	16	-7	23.07	1.35
7	-16	7	18.21	3.08
7	-16	7	19.44	2.46
-8	16	-7	24.40	1.45
8	-16	7	24.28	2.58
8	-16	7	29.82	4.19
-9	16	-7	13.53	1.26
9	-16	7	13.17	3.13
9	-16	7	20.93	2.47
-10	16	-7	7.92	1.18
10	-16	7	7.91	1.60
10	-16	7	8.29	3.05
5	15	-7	15.41	1.46
5	15	-7	16.13	2.77
-5	-15	7	17.86	2.59
4	15	-7	13.84	1.33
-4	-15	7	15.13	2.46
-4	-15	7	17.56	2.49
3	15	-7	21.99	1.46
-3	-15	7	24.39	3.26
-3	-15	7	25.85	2.57
2	15	-7	33.30	2.10
-2	-15	7	23.64	3.35
-2	-15	7	28.90	2.68
1	15	-7	16.52	1.15
-1	-15	7	20.04	3.10
-1	-15	7	16.33	2.30
0	15	-7	22.00	1.20
0	-15	7	17.93	2.23
0	-15	7	22.71	3.12
-1	15	-7	42.87	1.77

1	-15	7	42.03	2.82
1	-15	7	43.32	3.62
-2	15	-7	43.02	1.97
2	-15	7	45.28	3.72
2	-15	7	45.53	2.80
-3	15	-7	28.65	1.57
3	-15	7	31.17	3.41
3	-15	7	32.52	2.47
-4	15	-7	35.33	1.58
4	-15	7	37.03	2.58
4	-15	7	35.17	3.56
-5	15	-7	31.39	1.47
5	-15	7	32.61	2.44
5	-15	7	24.84	3.47
-6	15	-7	40.88	1.75
6	-15	7	35.51	3.81
6	-15	7	40.79	2.66
-7	15	-7	34.64	1.58
7	-15	7	29.49	3.76
7	-15	7	29.29	2.42
-8	15	-7	12.92	1.13
8	-15	7	17.74	2.16
8	-15	7	16.70	3.01
-9	15	-7	14.36	1.16
9	-15	7	12.11	1.64
9	-15	7	11.20	2.76
-10	15	-7	21.02	1.38
10	-15	7	18.79	3.34
-11	15	-7	21.31	1.46
11	-15	7	16.36	3.37
7	14	-7	10.67	1.50
7	14	-7	8.42	2.40
-7	-14	7	16.62	2.50
6	14	-7	9.78	1.36
6	14	-7	10.86	2.31
-6	-14	7	11.05	2.22
5	14	-7	32.82	1.76
-5	-14	7	30.30	3.25
-5	-14	7	29.77	2.81
-4	-14	7	45.89	3.43
-4	-14	7	41.26	2.96
3	14	-7	21.23	1.32
-3	-14	7	22.62	2.31
-3	-14	7	20.05	2.78

2	14	-7	19.31	1.21
-2	-14	7	17.96	2.70
-2	-14	7	20.09	2.20
1	14	-7	38.94	1.64
-1	-14	7	37.08	2.59
-1	-14	7	36.30	3.28
0	14	-7	35.32	1.52
0	-14	7	32.43	2.46
-1	14	-7	34.04	1.46
1	-14	7	27.95	4.53
1	-14	7	32.35	2.36
-2	14	-7	35.06	1.50
2	-14	7	37.12	3.21
2	-14	7	32.38	2.36
-3	14	-7	31.91	1.45
3	-14	7	35.37	2.33
3	-14	7	30.88	3.09
4	-14	7	35.59	2.36
4	-14	7	35.70	3.29
-5	14	-7	40.28	1.67
5	-14	7	34.56	3.39
5	-14	7	40.89	2.52
-6	14	-7	41.23	1.71
6	-14	7	36.86	3.62
-7	14	-7	27.43	1.39
7	-14	7	30.26	2.26
7	-14	7	27.59	1.43
7	-14	7	24.04	3.52
-8	14	-7	31.07	1.51
8	-14	7	29.38	1.61
8	-14	7	27.36	3.71
8	-14	7	30.75	2.31
-9	14	-7	26.87	1.45
9	-14	7	26.04	3.91
9	-14	7	28.44	2.30
9	-14	7	26.10	1.70
-10	14	-7	22.22	1.35
10	-14	7	21.05	1.73
10	-14	7	19.78	3.36
10	-14	7	19.60	2.19
11	-14	7	9.02	2.66
11	-14	7	9.26	1.36
-12	14	-7	16.81	1.44
12	-14	7	10.61	3.31

12	-14	7	20.87	2.17
8	13	-7	4.57	2.28
8	13	-7	5.88	1.06
7	13	-7	11.74	2.36
7	13	-7	10.68	1.42
-7	-13	7	17.36	2.76
6	13	-7	34.82	1.79
-6	-13	7	34.37	3.01
-6	-13	7	34.80	2.86
-5	-13	7	28.46	2.57
-5	-13	7	35.29	2.92
4	13	-7	15.91	1.18
-4	-13	7	17.15	2.46
-4	-13	7	15.86	2.07
3	13	-7	40.06	1.72
-3	-13	7	38.96	2.64
-3	-13	7	41.14	2.95
2	13	-7	27.23	1.36
-2	-13	7	24.12	2.56
-2	-13	7	27.82	2.22
1	13	-7	17.31	1.08
-1	-13	7	14.15	1.77
-1	-13	7	16.18	2.27
0	13	-7	49.01	1.87
0	-13	7	48.15	2.68
0	-13	7	44.90	3.16
-1	13	-7	55.24	1.99
1	-13	7	51.35	3.34
1	-13	7	48.11	2.69
-2	13	-7	17.95	1.02
2	-13	7	16.41	2.39
-3	13	-7	38.34	1.57
3	-13	7	40.95	2.35
3	-13	7	33.23	2.98
-4	13	-7	29.12	1.31
4	-13	7	21.66	3.46
4	-13	7	27.98	2.00
5	-13	7	4.64	1.05
5	-13	7	4.64	1.62
-6	13	-7	21.22	1.18
6	-13	7	20.57	1.83
6	-13	7	22.55	1.13
6	-13	7	22.91	2.90
6	-13	7	22.64	1.14

-7	13	-7	41.06	1.78
7	-13	7	47.13	2.49
7	-13	7	40.35	3.67
7	-13	7	45.75	1.79
7	-13	7	42.81	1.82
-8	13	-7	21.15	1.18
8	-13	7	16.17	2.60
8	-13	7	19.64	1.36
8	-13	7	18.88	1.87
-9	13	-7	12.67	1.07
9	-13	7	11.82	1.80
9	-13	7	14.66	1.37
9	-13	7	11.33	2.40
-10	13	-7	25.14	1.36
10	-13	7	19.16	2.05
10	-13	7	20.95	1.73
10	-13	7	22.90	3.84
-11	13	-7	10.64	1.13
11	-13	7	10.82	1.37
11	-13	7	6.46	2.65
-12	13	-7	6.72	0.93
12	-13	7	11.19	1.57
9	12	-7	10.81	2.52
9	12	-7	13.71	1.55
9	12	-7	10.07	1.52
8	12	-7	18.56	2.79
8	12	-7	18.80	1.54
7	12	-7	19.22	3.10
7	12	-7	21.69	1.46
-7	-12	7	22.72	2.50
6	12	-7	38.35	1.77
-6	-12	7	36.36	2.72
-6	-12	7	36.38	2.70
5	12	-7	29.01	1.49
-5	-12	7	28.51	2.38
-5	-12	7	33.50	2.55
4	12	-7	51.17	2.05
-4	-12	7	52.61	3.05
-4	-12	7	52.62	2.88
3	12	-7	35.94	3.30
3	12	-7	34.54	1.56
-3	-12	7	37.44	2.65
-3	-12	7	33.27	2.25
2	12	-7	5.02	0.63

2	12	-7	3.94	1.62
-2	-12	7	3.50	1.01
-2	-12	7	5.04	0.58
-2	-12	7	6.04	1.46
1	12	-7	71.56	2.45
1	12	-7	62.91	3.70
-1	-12	7	68.81	2.37
-1	-12	7	63.82	3.29
-1	-12	7	74.67	3.67
0	12	-7	120.18	3.84
0	12	-7	117.81	5.05
0	-12	7	112.84	3.79
0	-12	7	121.96	5.07
0	-12	7	113.07	4.53
-1	12	-7	67.40	3.68
-1	12	-7	76.59	2.53
1	-12	7	70.10	2.50
1	-12	7	72.38	3.72
1	-12	7	71.97	3.20
-2	12	-7	66.57	2.30
-2	12	-7	63.83	3.86
2	-12	7	60.69	3.53
2	-12	7	63.32	2.92
-3	12	-7	41.86	1.65
3	-12	7	41.51	2.26
3	-12	7	42.25	3.01
-4	12	-7	44.07	1.69
4	-12	7	42.44	3.04
4	-12	7	40.76	2.28
-5	12	-7	49.46	1.92
5	-12	7	51.45	3.39
5	-12	7	50.40	1.85
5	-12	7	52.49	1.83
5	-12	7	49.74	2.50
-6	12	-7	13.53	0.91
6	-12	7	12.30	0.98
6	-12	7	11.05	1.95
6	-12	7	13.00	1.50
6	-12	7	13.41	0.88
-7	12	-7	16.30	1.26
-7	12	-7	16.07	0.99
7	-12	7	12.84	2.18
7	-12	7	15.03	1.60
7	-12	7	17.17	1.10

7	-12	7	14.88	1.14
-8	12	-7	54.05	2.11
-8	12	-7	54.77	2.20
8	-12	7	55.27	2.64
8	-12	7	53.40	2.21
8	-12	7	52.08	3.93
8	-12	7	56.06	2.21
-9	12	-7	28.68	1.50
-9	12	-7	27.43	1.43
9	-12	7	34.75	3.67
9	-12	7	26.67	1.64
9	-12	7	30.18	2.01
9	-12	7	24.59	1.65
-10	12	-7	10.65	1.07
10	-12	7	10.56	1.41
10	-12	7	12.71	1.71
10	-12	7	15.43	2.79
-11	12	-7	1.17	0.63
11	-12	7	1.35	0.99
11	-12	7	2.27	1.06
11	-12	7	2.35	2.33
-12	12	-7	5.52	0.85
12	-12	7	7.51	1.40
12	-12	7	7.29	2.91
13	-12	7	2.66	1.45
10	11	-7	13.68	2.11
10	11	-7	14.19	2.67
10	11	-7	14.46	1.53
9	11	-7	16.76	1.96
9	11	-7	21.02	3.37
9	11	-7	15.70	1.44
9	11	-7	18.90	1.94
8	11	-7	14.14	1.27
8	11	-7	17.53	2.92
8	11	-7	15.92	1.44
7	11	-7	20.16	1.38
7	11	-7	18.51	1.28
-7	-11	7	18.68	2.20
-7	-11	7	20.14	2.14
6	11	-7	47.80	1.97
6	11	-7	48.90	1.99
-6	-11	7	49.41	2.87
-6	-11	7	50.17	2.86
5	11	-7	25.64	1.26

5	11	-7	25.05	2.97
5	11	-7	24.76	1.31
5	11	-7	25.99	1.35
-5	-11	7	29.20	2.22
-5	-11	7	26.91	2.14
4	11	-7	8.08	0.88
4	11	-7	9.73	1.82
-4	-11	7	9.71	1.72
-4	-11	7	7.97	1.26
3	11	-7	25.73	1.26
3	11	-7	27.73	2.62
-3	-11	7	26.00	2.21
-3	-11	7	24.54	1.11
2	11	-7	84.02	2.90
2	11	-7	86.99	4.03
-2	-11	7	87.14	2.84
-2	-11	7	88.49	3.61
-2	-11	7	78.44	3.79
1	11	-7	43.01	2.88
1	11	-7	46.77	1.80
-1	-11	7	48.04	1.74
-1	-11	7	44.49	2.40
-1	-11	7	52.44	2.85
0	11	-7	43.74	1.71
0	11	-7	43.00	2.78
0	-11	7	45.02	1.69
0	-11	7	47.54	2.30
0	-11	7	41.45	2.70
-1	11	-7	55.42	2.96
-1	11	-7	49.64	1.89
1	-11	7	51.01	1.90
1	-11	7	48.95	2.97
1	-11	7	52.65	2.49
-2	11	-7	65.80	2.71
-2	11	-7	59.69	3.13
-2	11	-7	58.70	2.13
2	-11	7	55.01	2.14
2	-11	7	60.76	3.31
2	-11	7	59.82	2.67
-3	11	-7	71.18	2.43
-3	11	-7	66.67	3.41
-3	11	-7	73.46	2.69
3	-11	7	69.68	2.94
3	-11	7	65.68	3.57

3	-11	7	67.44	2.46
-4	11	-7	58.26	2.18
-4	11	-7	77.42	4.72
-4	11	-7	56.79	2.39
4	-11	7	61.43	2.70
4	-11	7	61.80	2.04
4	-11	7	62.23	3.44
-5	11	-7	15.27	0.91
-5	11	-7	15.29	1.13
5	-11	7	15.59	0.93
5	-11	7	15.24	0.85
5	-11	7	15.00	1.42
5	-11	7	14.31	2.22
-6	11	-7	21.91	1.14
-6	11	-7	22.47	1.28
6	-11	7	22.77	1.11
6	-11	7	22.54	1.20
6	-11	7	21.37	1.59
6	-11	7	21.00	2.47
-7	11	-7	50.32	2.13
-7	11	-7	54.78	2.05
7	-11	7	52.28	2.09
7	-11	7	58.25	3.68
7	-11	7	55.29	2.13
7	-11	7	57.16	2.51
-8	11	-7	36.89	1.67
-8	11	-7	34.57	1.57
8	-11	7	36.56	2.00
8	-11	7	38.30	3.35
8	-11	7	34.49	1.70
8	-11	7	32.73	1.65
-9	11	-7	8.95	1.14
-9	11	-7	10.62	0.96
9	-11	7	9.34	2.10
9	-11	7	10.12	1.20
9	-11	7	8.28	1.29
9	-11	7	11.06	1.43
-10	11	-7	13.05	1.08
10	-11	7	13.49	1.56
10	-11	7	8.03	2.19
10	-11	7	13.50	1.40
-11	11	-7	4.83	0.72
11	-11	7	6.66	1.12
11	-11	7	5.57	1.09

11	-11	7	8.15	2.40
-12	11	-7	2.81	0.72
12	-11	7	2.52	1.17
12	-11	7	-3.04	2.21
-13	11	-7	1.06	0.90
13	-11	7	-1.33	3.02
13	-11	7	1.87	1.39
11	10	-7	13.33	1.96
11	10	-7	13.64	2.43
11	10	-7	15.19	2.13
11	10	-7	16.79	1.53
10	10	-7	21.54	2.06
10	10	-7	15.50	2.48
10	10	-7	24.01	1.91
10	10	-7	24.21	1.56
9	10	-7	29.20	1.54
9	10	-7	24.89	3.28
9	10	-7	26.13	1.96
9	10	-7	27.70	1.81
8	10	-7	49.22	2.42
8	10	-7	52.11	2.09
8	10	-7	49.39	2.23
-8	-10	7	56.66	3.03
7	10	-7	21.69	1.35
7	10	-7	18.62	1.21
7	10	-7	20.94	3.02
7	10	-7	21.01	1.22
-7	-10	7	20.64	1.93
-7	-10	7	21.57	2.02
6	10	-7	29.07	1.34
6	10	-7	32.17	1.48
6	10	-7	32.49	1.51
6	10	-7	35.92	2.91
-6	-10	7	33.08	2.22
-6	-10	7	31.74	2.15
5	10	-7	45.13	3.08
5	10	-7	48.07	1.94
5	10	-7	48.30	1.88
-5	-10	7	51.96	2.63
-5	-10	7	58.94	2.12
4	10	-7	71.41	3.55
4	10	-7	70.98	2.45
-4	-10	7	64.56	3.04
-4	-10	7	66.80	3.16

-4	-10	7	69.09	2.33
3	10	-7	52.70	1.97
3	10	-7	53.86	3.00
-3	-10	7	53.05	2.75
-3	-10	7	53.74	2.56
-3	-10	7	52.47	1.88
2	10	-7	76.27	2.60
2	10	-7	72.31	3.48
-2	-10	7	77.96	3.43
-2	-10	7	74.04	3.17
-2	-10	7	75.23	2.52
1	10	-7	65.71	2.60
1	10	-7	57.59	2.97
1	10	-7	60.24	2.15
-1	-10	7	60.67	2.10
-1	-10	7	59.45	3.03
-1	-10	7	58.23	2.67
0	10	-7	66.13	3.13
0	10	-7	61.31	2.56
0	10	-7	65.41	2.27
0	-10	7	64.17	2.24
0	-10	7	67.12	3.20
0	-10	7	65.53	2.79
-1	10	-7	49.45	2.70
-1	10	-7	48.41	2.11
-1	10	-7	50.60	1.82
1	-10	7	50.71	2.32
1	-10	7	49.23	1.82
1	-10	7	49.02	2.77
-2	10	-7	35.67	1.65
-2	10	-7	33.38	1.37
-2	10	-7	35.01	2.25
2	-10	7	34.11	2.38
2	-10	7	34.17	1.40
2	-10	7	35.02	1.86
-3	10	-7	29.10	1.46
-3	10	-7	29.03	2.07
-3	10	-7	32.36	1.29
3	-10	7	29.78	2.31
3	-10	7	31.94	1.73
3	-10	7	30.43	1.32
-4	10	-7	26.24	1.16
-4	10	-7	25.50	2.03
-4	10	-7	25.52	1.32

4	-10	7	28.06	1.62
4	-10	7	27.12	2.26
4	-10	7	25.68	1.13
4	-10	7	26.57	1.08
-5	10	-7	50.67	2.00
-5	10	-7	51.29	1.88
5	-10	7	48.66	3.08
5	-10	7	48.83	2.26
5	-10	7	53.64	1.90
5	-10	7	48.84	1.81
-6	10	-7	21.21	1.18
-6	10	-7	22.20	1.09
6	-10	7	20.80	2.30
6	-10	7	19.77	1.08
6	-10	7	24.11	1.21
6	-10	7	22.10	1.50
-7	10	-7	13.65	1.03
-7	10	-7	15.69	0.93
7	-10	7	14.00	1.02
7	-10	7	15.89	1.14
7	-10	7	15.30	1.33
-8	10	-7	34.98	1.50
-8	10	-7	32.20	1.57
8	-10	7	34.18	1.84
8	-10	7	37.87	3.12
8	-10	7	33.24	1.63
8	-10	7	34.01	1.69
-9	10	-7	8.38	1.09
-9	10	-7	9.15	0.91
9	-10	7	11.12	1.30
9	-10	7	11.02	2.05
9	-10	7	8.04	1.17
-10	10	-7	11.20	1.58
-10	10	-7	8.91	0.97
10	-10	7	8.95	2.34
10	-10	7	8.90	1.36
-11	10	-7	2.23	0.60
11	-10	7	2.21	0.96
11	-10	7	2.87	0.94
11	-10	7	4.24	2.30
-12	10	-7	1.40	0.66
12	-10	7	-0.05	2.32
12	-10	7	1.46	1.09
-13	10	-7	0.85	0.73

13	-10	7	1.59	1.30
13	-10	7	-1.50	2.68
12	9	-7	14.48	2.29
12	9	-7	11.32	1.39
12	9	-7	14.43	2.06
12	9	-7	15.96	2.54
11	9	-7	14.13	1.79
11	9	-7	12.43	1.31
11	9	-7	11.78	2.30
11	9	-7	14.10	1.89
10	9	-7	28.26	1.51
10	9	-7	22.93	1.84
10	9	-7	25.24	1.96
10	9	-7	24.28	3.19
9	9	-7	22.11	1.45
9	9	-7	23.73	1.75
9	9	-7	26.11	1.67
9	9	-7	26.67	1.39
-9	-9	7	23.21	2.25
8	9	-7	15.44	1.55
8	9	-7	16.94	1.16
8	9	-7	18.21	1.51
8	9	-7	21.95	2.89
8	9	-7	17.61	1.34
-8	-9	7	16.28	1.90
7	9	-7	25.33	1.27
7	9	-7	25.26	1.20
7	9	-7	22.74	2.62
7	9	-7	24.44	1.39
-7	-9	7	27.02	1.83
6	9	-7	66.96	2.34
6	9	-7	63.97	2.20
6	9	-7	62.47	2.37
6	9	-7	62.62	3.38
-6	-9	7	64.58	3.00
-6	-9	7	67.17	2.88
5	9	-7	67.00	2.31
5	9	-7	65.23	3.30
5	9	-7	67.01	2.35
-5	-9	7	65.20	2.94
-5	-9	7	64.50	2.24
-5	-9	7	63.97	2.87
4	9	-7	27.87	2.09
4	9	-7	25.22	0.93

4	9	-7	26.08	1.18
-4	-9	7	23.79	1.64
-4	-9	7	24.95	1.05
-4	-9	7	23.19	1.75
3	9	-7	67.84	2.39
3	9	-7	65.14	2.72
3	9	-7	72.70	3.21
-3	-9	7	69.43	2.31
-3	-9	7	67.65	2.91
-3	-9	7	71.41	3.07
2	9	-7	128.03	4.56
2	9	-7	135.32	4.97
2	9	-7	133.74	4.24
-2	-9	7	137.10	4.18
-2	-9	7	131.49	4.93
-2	-9	7	127.80	4.72
1	9	-7	71.18	2.50
1	9	-7	74.53	2.81
1	9	-7	78.60	3.28
-1	-9	7	72.16	2.46
-1	-9	7	73.55	2.98
-1	-9	7	73.73	3.25
0	9	-7	42.53	1.60
0	9	-7	45.35	2.36
0	9	-7	42.17	1.86
0	-9	7	41.22	2.39
0	-9	7	44.96	2.06
0	-9	7	42.47	1.58
-1	9	-7	37.52	1.63
-1	9	-7	34.34	1.39
-1	9	-7	38.10	2.13
1	-9	7	37.41	2.26
1	-9	7	36.34	1.41
1	-9	7	34.06	1.78
-2	9	-7	33.95	1.53
-2	9	-7	36.07	1.36
-2	9	-7	32.27	2.01
2	-9	7	32.15	1.36
2	-9	7	34.96	2.27
2	-9	7	36.11	1.74
-3	9	-7	38.16	1.46
-3	9	-7	36.03	2.11
-3	9	-7	38.36	1.60
3	-9	7	36.93	1.81

3	-9	7	39.39	1.42
3	-9	7	37.96	1.40
3	-9	7	37.69	2.39
3	-9	7	34.00	1.46
-4	9	-7	15.78	0.85
-4	9	-7	18.11	1.00
-4	9	-7	15.72	1.52
4	-9	7	14.88	1.85
4	-9	7	16.25	0.92
4	-9	7	14.81	0.77
4	-9	7	15.68	1.17
-5	9	-7	11.46	0.75
-5	9	-7	10.73	0.83
-5	9	-7	10.77	1.46
5	-9	7	10.66	0.84
5	-9	7	10.22	0.71
5	-9	7	11.90	1.07
5	-9	7	9.68	1.77
-6	9	-7	43.36	1.74
-6	9	-7	40.60	1.65
6	-9	7	42.95	1.75
6	-9	7	42.45	1.69
6	-9	7	44.62	1.97
6	-9	7	41.66	2.83
-7	9	-7	16.15	1.04
-7	9	-7	14.05	1.27
7	-9	7	17.18	1.06
7	-9	7	16.46	1.18
7	-9	7	17.52	1.27
7	-9	7	14.61	2.21
-8	9	-7	5.67	0.90
-8	9	-7	3.75	0.57
8	-9	7	4.31	0.79
8	-9	7	5.11	0.86
8	-9	7	5.14	0.80
8	-9	7	5.58	1.68
-9	9	-7	8.45	0.86
-9	9	-7	8.05	1.12
9	-9	7	14.88	2.69
9	-9	7	8.26	1.08
9	-9	7	8.93	1.19
-10	9	-7	7.90	1.30
-10	9	-7	3.83	0.62
10	-9	7	4.93	0.85

10	-9	7	4.79	0.88
10	-9	7	3.97	2.02
-11	9	-7	2.85	0.66
11	-9	7	6.83	2.33
11	-9	7	3.04	0.92
11	-9	7	2.57	0.87
-12	9	-7	0.78	0.69
12	-9	7	1.18	1.05
12	-9	7	0.02	2.21
-13	9	-7	0.89	0.71
13	-9	7	1.45	2.70
13	-9	7	1.24	1.27
13	8	-7	13.29	2.43
13	8	-7	15.33	1.42
13	8	-7	15.52	2.23
12	8	-7	19.65	2.08
12	8	-7	21.30	1.45
12	8	-7	19.58	3.29
11	8	-7	27.35	3.08
11	8	-7	25.87	1.95
11	8	-7	27.83	1.48
11	8	-7	25.91	2.02
10	8	-7	25.34	1.77
10	8	-7	22.92	3.84
10	8	-7	28.49	1.79
10	8	-7	27.78	1.41
10	8	-7	26.71	2.79
9	8	-7	20.79	1.52
9	8	-7	17.99	1.27
9	8	-7	19.37	1.17
9	8	-7	20.70	1.50
9	8	-7	23.84	2.78
-9	-8	7	19.63	2.31
8	8	-7	33.77	2.83
8	8	-7	38.74	1.89
8	8	-7	38.19	1.83
8	8	-7	38.58	1.60
8	8	-7	41.49	1.63
-8	-8	7	39.12	2.29
7	8	-7	36.55	1.51
7	8	-7	35.78	2.58
7	8	-7	35.07	1.42
7	8	-7	35.78	1.63
-7	-8	7	34.45	2.09

-7	-8	7	37.56	1.94
6	8	-7	11.65	1.85
6	8	-7	13.75	0.86
6	8	-7	13.50	0.89
6	8	-7	13.12	0.94
6	8	-7	14.11	0.74
-6	-8	7	12.93	1.40
-6	-8	7	14.61	1.34
5	8	-7	65.31	2.25
5	8	-7	63.56	2.61
5	8	-7	64.68	2.24
5	8	-7	58.89	2.94
-5	-8	7	63.78	2.67
-5	-8	7	60.11	2.72
-5	-8	7	64.60	2.15
4	8	-7	80.63	3.41
4	8	-7	82.72	3.11
4	8	-7	81.40	2.76
4	8	-7	80.57	2.56
-4	-8	7	83.24	2.68
-4	-8	7	82.52	3.28
-4	-8	7	83.60	3.26
3	8	-7	64.73	2.19
3	8	-7	63.09	2.48
3	8	-7	58.71	2.78
-3	-8	7	65.69	2.12
-3	-8	7	62.05	2.63
-3	-8	7	61.30	2.71
2	8	-7	52.91	2.14
2	8	-7	53.90	2.48
2	8	-7	53.04	1.87
-2	-8	7	49.71	1.80
-2	-8	7	53.04	2.47
-2	-8	7	51.49	2.28
1	8	-7	17.74	1.06
1	8	-7	18.60	1.48
1	8	-7	16.99	0.85
-1	-8	7	17.12	1.21
-1	-8	7	17.34	1.48
-1	-8	7	16.40	0.82
0	8	-7	72.38	2.47
0	8	-7	69.78	2.67
0	8	-7	78.72	3.08
0	-8	7	73.42	2.84

0	-8	7	75.22	3.19
0	-8	7	73.46	2.46
-1	8	-7	56.68	2.51
-1	8	-7	55.91	2.13
-1	8	-7	55.94	1.96
1	-8	7	59.92	2.74
1	-8	7	55.65	1.95
1	-8	7	51.73	2.26
-2	8	-7	21.64	0.77
-2	8	-7	21.99	1.10
-2	8	-7	22.04	1.55
-2	8	-7	21.65	0.96
2	-8	7	19.89	1.23
2	-8	7	22.35	0.99
2	-8	7	20.72	0.95
2	-8	7	25.42	1.82
-3	8	-7	42.43	2.15
-3	8	-7	45.16	1.74
-3	8	-7	42.00	1.61
-3	8	-7	43.28	1.46
3	-8	7	44.77	1.91
3	-8	7	44.64	2.49
3	-8	7	45.23	1.64
3	-8	7	44.12	1.65
-4	8	-7	22.21	1.61
-4	8	-7	23.43	1.03
-4	8	-7	23.30	1.11
-4	8	-7	23.29	0.90
4	-8	7	21.90	1.91
4	-8	7	23.71	1.09
4	-8	7	22.70	1.07
4	-8	7	20.74	1.27
-5	8	-7	59.98	2.11
-5	8	-7	59.81	2.00
-5	8	-7	61.81	2.19
-5	8	-7	56.66	2.67
5	-8	7	61.61	2.18
5	-8	7	60.71	2.38
5	-8	7	51.87	3.02
-6	8	-7	5.65	0.61
-6	8	-7	5.61	0.75
6	-8	7	5.91	1.33
6	-8	7	6.07	0.86
6	-8	7	6.90	0.92

-7	8	-7	0.59	0.36
-7	8	-7	0.28	0.50
7	-8	7	0.61	0.59
7	-8	7	1.36	0.55
7	-8	7	1.14	1.11
7	-8	7	0.34	0.47
-8	8	-7	4.84	0.72
-8	8	-7	5.40	0.68
8	-8	7	4.22	0.73
8	-8	7	3.84	0.65
8	-8	7	4.14	0.77
8	-8	7	4.33	1.52
-9	8	-7	8.98	1.24
-9	8	-7	8.87	0.87
9	-8	7	9.95	1.18
9	-8	7	11.61	1.14
9	-8	7	11.15	2.09
-10	8	-7	0.11	0.86
-10	8	-7	-0.02	0.49
10	-8	7	-0.47	0.66
10	-8	7	-0.33	0.71
10	-8	7	0.24	1.72
-11	8	-7	0.89	0.58
11	-8	7	-0.56	1.95
11	-8	7	1.22	0.76
11	-8	7	-0.09	0.85
-12	8	-7	0.08	0.63
12	-8	7	1.27	1.02
12	-8	7	-4.65	2.19
-13	8	-7	-0.25	0.68
13	-8	7	1.75	1.28
13	-8	7	1.58	2.69
13	7	-7	20.21	1.45
13	7	-7	21.05	2.23
13	7	-7	18.07	3.12
13	7	-7	19.94	2.33
12	7	-7	24.84	2.99
12	7	-7	27.96	1.48
12	7	-7	25.92	2.07
12	7	-7	24.25	2.19
11	7	-7	28.99	1.46
11	7	-7	24.40	2.78
11	7	-7	28.86	1.94
10	7	-7	56.99	2.50

10	7	-7	55.78	2.58
10	7	-7	62.83	3.64
10	7	-7	57.10	2.19
-10	-7	7	63.79	2.96
9	7	-7	51.55	1.98
9	7	-7	52.84	2.28
9	7	-7	58.34	2.44
9	7	-7	50.35	2.22
9	7	-7	48.50	3.06
-9	-7	7	48.60	2.59
8	7	-7	15.67	1.16
8	7	-7	14.71	0.91
8	7	-7	16.06	2.05
8	7	-7	14.52	0.91
8	7	-7	14.84	1.16
-8	-7	7	16.16	1.57
7	7	-7	42.20	1.53
7	7	-7	39.31	1.59
7	7	-7	39.78	1.59
7	7	-7	43.93	1.79
7	7	-7	43.04	2.44
7	7	-7	38.23	1.95
-7	-7	7	43.01	1.91
-7	-7	7	40.51	2.14
6	7	-7	123.06	3.86
6	7	-7	119.66	3.96
6	7	-7	119.85	3.97
6	7	-7	121.74	4.05
6	7	-7	127.73	4.33
6	7	-7	129.63	4.65
-6	-7	7	126.33	4.28
-6	-7	7	118.76	4.47
5	7	-7	32.62	1.91
5	7	-7	32.79	1.33
5	7	-7	33.38	1.40
5	7	-7	35.84	1.67
-5	-7	7	32.27	1.78
-5	-7	7	35.53	1.70
-5	-7	7	33.32	1.27
4	7	-7	23.02	0.83
4	7	-7	21.93	1.55
4	7	-7	23.00	1.27
4	7	-7	24.04	1.09
4	7	-7	23.25	1.02

-4	-7	7	20.77	1.40
-4	-7	7	22.73	0.93
-4	-7	7	23.34	1.43
3	7	-7	78.14	3.09
3	7	-7	77.90	2.62
3	7	-7	78.94	2.58
3	7	-7	81.17	2.88
-3	-7	7	78.36	2.56
-3	-7	7	79.74	3.07
-3	-7	7	76.74	3.01
2	7	-7	61.27	2.51
2	7	-7	59.69	1.93
2	7	-7	58.19	2.25
2	7	-7	56.67	2.02
-2	-7	7	57.51	2.52
-2	-7	7	61.33	2.00
-2	-7	7	57.71	2.38
-2	-7	7	58.96	1.86
1	7	-7	18.41	0.87
1	7	-7	20.40	1.36
1	7	-7	19.82	1.05
-1	-7	7	20.17	0.87
-1	-7	7	19.99	1.44
-1	-7	7	18.72	1.17
0	7	-7	8.80	0.40
0	7	-7	9.69	0.72
0	7	-7	8.50	0.58
0	7	-7	10.32	1.09
0	-7	7	7.62	1.11
0	-7	7	9.68	0.60
0	-7	7	9.08	0.51
0	-7	7	9.64	0.85
-1	7	-7	29.68	1.29
-1	7	-7	29.00	1.15
-1	7	-7	29.86	1.62
-1	7	-7	28.63	0.99
1	-7	7	29.30	1.42
1	-7	7	29.43	1.15
1	-7	7	29.63	1.16
1	-7	7	26.75	1.76
-2	7	-7	87.12	2.91
-2	7	-7	83.76	2.79
-2	7	-7	84.76	2.66
-2	7	-7	86.91	3.25

2	-7	7	84.87	2.81
2	-7	7	87.68	2.83
2	-7	7	80.87	3.48
2	-7	7	83.91	3.04
-3	7	-7	70.11	2.87
-3	7	-7	73.99	2.50
-3	7	-7	70.73	2.41
-3	7	-7	70.22	2.29
3	-7	7	73.48	2.42
3	-7	7	68.76	3.17
3	-7	7	71.42	2.58
3	-7	7	70.59	2.64
-4	7	-7	30.60	1.15
-4	7	-7	30.70	1.30
-4	7	-7	28.05	1.21
-4	7	-7	29.99	1.69
4	-7	7	32.70	1.35
4	-7	7	29.45	1.45
4	-7	7	30.36	1.25
4	-7	7	32.53	2.10
-5	7	-7	29.11	1.24
-5	7	-7	30.02	1.72
-5	7	-7	31.72	1.34
-5	7	-7	30.09	1.19
5	-7	7	31.46	1.48
5	-7	7	31.77	1.38
5	-7	7	28.99	1.28
5	-7	7	29.26	2.16
-6	7	-7	8.99	0.82
-6	7	-7	9.22	0.66
-6	7	-7	7.28	0.63
-6	7	-7	9.28	1.26
6	-7	7	7.39	0.87
6	-7	7	10.13	1.73
6	-7	7	7.76	0.89
-7	7	-7	7.43	0.70
-7	7	-7	8.74	0.93
-7	7	-7	7.78	0.68
7	-7	7	6.24	1.43
7	-7	7	8.23	1.02
7	-7	7	8.72	0.95
-8	7	-7	0.12	0.41
-8	7	-7	0.33	0.60
-8	7	-7	-0.22	0.36

8	-7	7	0.70	1.34
8	-7	7	0.32	0.51
-9	7	-7	6.47	0.98
-9	7	-7	5.66	0.76
9	-7	7	6.86	1.05
9	-7	7	8.78	2.01
9	-7	7	5.76	1.03
-10	7	-7	1.41	0.52
-10	7	-7	1.72	1.02
10	-7	7	3.06	0.86
10	-7	7	3.37	0.76
10	-7	7	2.02	1.78
-11	7	-7	-0.96	0.54
11	-7	7	-0.12	0.87
11	-7	7	1.14	0.73
11	-7	7	1.04	2.03
-12	7	-7	0.75	0.62
12	-7	7	1.76	1.01
12	-7	7	1.75	2.45
-13	7	-7	0.44	0.71
13	-7	7	-1.37	2.82
13	-7	7	1.50	1.22
14	6	-7	11.13	2.25
14	6	-7	14.32	2.14
14	6	-7	23.04	3.31
14	6	-7	12.13	1.26
13	6	-7	22.12	2.14
13	6	-7	20.89	2.91
13	6	-7	21.64	2.25
13	6	-7	25.04	1.41
12	6	-7	25.81	2.74
12	6	-7	27.12	1.42
12	6	-7	28.11	2.07
12	6	-7	27.79	1.99
11	6	-7	35.14	1.94
11	6	-7	22.29	2.88
11	6	-7	32.10	1.47
11	6	-7	34.02	1.95
11	6	-7	30.35	2.68
10	6	-7	26.50	1.66
10	6	-7	24.79	1.22
10	6	-7	27.62	2.53
10	6	-7	25.02	1.58
-10	-6	7	26.61	1.91

9	6	-7	38.86	1.54
9	6	-7	36.85	1.92
9	6	-7	37.47	1.72
9	6	-7	36.55	2.51
9	6	-7	40.25	1.87
9	6	-7	39.29	1.58
-9	-6	7	38.74	2.12
8	6	-7	99.58	3.48
8	6	-7	98.29	3.22
8	6	-7	98.60	3.58
8	6	-7	97.03	3.35
8	6	-7	102.46	3.97
8	6	-7	96.56	3.26
-8	-6	7	94.82	3.77
7	6	-7	52.45	1.80
7	6	-7	52.04	1.95
7	6	-7	52.65	2.19
7	6	-7	50.68	2.05
7	6	-7	52.50	1.86
7	6	-7	49.86	1.88
7	6	-7	52.52	2.51
-7	-6	7	50.34	2.36
6	6	-7	43.43	2.16
6	6	-7	46.59	1.58
6	6	-7	44.96	1.94
6	6	-7	44.85	1.67
6	6	-7	45.86	1.71
6	6	-7	48.04	1.82
-6	-6	7	45.03	1.88
-6	-6	7	45.28	2.10
5	6	-7	102.57	3.58
5	6	-7	100.52	3.31
5	6	-7	107.67	3.75
-5	-6	7	97.68	3.70
-5	-6	7	102.97	3.26
-5	-6	7	103.93	3.54
4	6	-7	66.36	2.64
4	6	-7	67.05	2.32
4	6	-7	68.06	2.27
4	6	-7	68.60	2.49
-4	-6	7	65.62	2.62
-4	-6	7	65.44	2.20
-4	-6	7	68.09	2.19
-4	-6	7	65.97	2.55

3	6	-7	56.08	1.96
3	6	-7	61.33	2.33
3	6	-7	55.01	1.97
3	6	-7	55.29	2.15
-3	-6	7	57.33	1.88
-3	-6	7	57.06	2.34
-3	-6	7	58.33	2.30
-3	-6	7	56.95	1.92
2	6	-7	78.89	2.85
2	6	-7	76.49	2.70
2	6	-7	75.72	2.50
2	6	-7	77.65	2.53
-2	-6	7	75.80	2.93
-2	-6	7	77.87	2.50
-2	-6	7	74.63	2.81
-2	-6	7	74.58	2.43
1	6	-7	43.63	1.51
1	6	-7	47.64	1.75
1	6	-7	42.87	1.58
1	6	-7	45.53	1.95
-1	-6	7	46.36	1.86
-1	-6	7	44.36	2.04
-1	-6	7	43.19	1.56
-1	-6	7	42.93	1.52
0	6	-7	24.37	1.33
0	6	-7	25.69	1.03
0	6	-7	27.60	1.17
0	6	-7	24.67	0.93
0	-6	7	25.66	1.05
0	-6	7	26.12	1.56
0	-6	7	26.24	1.04
0	-6	7	24.83	1.25
-1	6	-7	32.63	1.53
-1	6	-7	31.93	1.28
-1	6	-7	29.90	1.17
-1	6	-7	29.36	1.08
1	-6	7	30.42	1.71
1	-6	7	28.75	1.37
1	-6	7	30.57	1.25
1	-6	7	30.24	1.17
-2	6	-7	24.23	1.08
-2	6	-7	23.16	0.99
-2	6	-7	24.72	1.36
-2	6	-7	24.19	0.92

2	-6	7	23.93	1.01
2	-6	7	24.02	1.60
2	-6	7	24.57	1.11
2	-6	7	23.24	1.17
-3	6	-7	26.34	1.11
-3	6	-7	24.00	1.02
-3	6	-7	23.54	1.39
-3	6	-7	23.65	0.96
3	-6	7	25.53	1.14
3	-6	7	22.70	1.18
3	-6	7	25.59	1.05
3	-6	7	25.49	1.73
-4	6	-7	30.85	1.19
-4	6	-7	31.02	1.24
-4	6	-7	31.45	1.64
-4	6	-7	31.49	1.32
4	-6	7	30.70	1.25
4	-6	7	30.99	2.02
4	-6	7	31.61	1.39
4	-6	7	30.40	1.40
-5	6	-7	19.69	0.90
-5	6	-7	19.15	1.04
-5	6	-7	18.36	1.32
-5	6	-7	18.09	0.90
5	-6	7	20.07	1.82
5	-6	7	18.07	1.09
5	-6	7	19.38	1.09
5	-6	7	19.68	0.95
-6	6	-7	1.89	0.38
-6	6	-7	1.43	0.39
-6	6	-7	1.54	0.46
-6	6	-7	0.50	0.68
6	-6	7	0.79	0.65
6	-6	7	1.47	0.94
6	-6	7	0.77	0.55
6	-6	7	2.02	0.51
-7	6	-7	0.82	0.55
-7	6	-7	1.33	0.36
-7	6	-7	1.43	0.40
7	-6	7	1.03	0.65
7	-6	7	1.38	1.06
7	-6	7	1.08	0.52
-8	6	-7	2.14	0.45
-8	6	-7	1.96	0.50

-8	6	-7	2.57	0.74
8	-6	7	2.00	0.59
8	-6	7	1.32	1.22
-9	6	-7	3.23	0.61
-9	6	-7	2.66	0.53
-9	6	-7	4.34	0.94
9	-6	7	0.17	1.45
9	-6	7	3.74	0.73
-10	6	-7	0.49	0.50
-10	6	-7	-0.66	1.00
10	-6	7	0.45	1.65
10	-6	7	0.29	0.76
10	-6	7	0.02	0.65
-11	6	-7	0.78	0.61
11	-6	7	-2.54	1.83
11	-6	7	0.31	0.77
11	-6	7	0.58	0.88
-12	6	-7	0.11	0.64
12	-6	7	-1.13	0.96
12	-6	7	-0.50	2.26
-13	6	-7	-0.87	0.68
13	-6	7	-3.58	2.69
13	-6	7	-1.65	1.13
14	5	-7	24.55	2.28
14	5	-7	23.01	2.72
14	5	-7	19.45	1.37
13	5	-7	24.85	2.20
13	5	-7	28.39	2.12
13	5	-7	24.21	2.65
13	5	-7	25.79	1.38
12	5	-7	27.43	1.90
12	5	-7	26.44	1.31
12	5	-7	26.73	1.93
12	5	-7	21.97	3.21
12	5	-7	22.95	2.55
11	5	-7	26.17	1.24
11	5	-7	24.25	1.69
11	5	-7	25.40	1.71
11	5	-7	24.42	2.46
11	5	-7	24.76	1.66
-11	-5	7	25.51	1.84
10	5	-7	20.10	2.01
10	5	-7	17.69	0.98
10	5	-7	14.59	1.07

10	5	-7	17.42	1.36
10	5	-7	17.89	1.30
10	5	-7	19.93	1.42
-10	-5	7	18.16	1.56
9	5	-7	54.94	2.70
9	5	-7	54.68	2.16
9	5	-7	54.74	2.01
9	5	-7	57.34	2.35
9	5	-7	57.88	2.05
9	5	-7	57.51	2.34
-9	-5	7	56.66	2.52
8	5	-7	9.20	1.40
8	5	-7	13.05	1.03
8	5	-7	11.52	0.73
8	5	-7	10.92	0.71
8	5	-7	9.98	0.75
8	5	-7	10.38	0.82
8	5	-7	12.10	1.04
-8	-5	7	10.95	1.13
7	5	-7	45.48	2.58
7	5	-7	47.12	1.95
7	5	-7	48.01	1.92
7	5	-7	48.73	1.73
7	5	-7	45.00	1.72
7	5	-7	48.18	1.66
7	5	-7	48.06	1.70
-7	-5	7	44.44	2.11
6	5	-7	187.20	6.07
6	5	-7	191.51	6.16
6	5	-7	193.93	6.27
6	5	-7	186.00	5.93
-6	-5	7	194.31	5.96
-6	-5	7	185.38	6.30
-6	-5	7	204.55	6.04
5	5	-7	95.91	3.28
5	5	-7	98.67	3.43
5	5	-7	101.41	3.39
5	5	-7	99.57	3.17
-5	-5	7	95.84	3.50
-5	-5	7	98.41	3.20
-5	-5	7	98.29	3.12
4	5	-7	19.30	0.88
4	5	-7	20.34	1.14
4	5	-7	21.20	1.07

4	5	-7	20.84	1.02
-4	-5	7	20.61	1.17
-4	-5	7	21.54	0.89
-4	-5	7	21.53	1.12
-4	-5	7	19.76	0.83
3	5	-7	103.76	3.72
3	5	-7	115.28	3.74
3	5	-7	114.26	3.71
3	5	-7	114.08	3.56
-3	-5	7	110.92	3.78
-3	-5	7	111.32	3.51
-3	-5	7	112.09	3.83
-3	-5	7	113.60	3.54
2	5	-7	72.73	2.55
2	5	-7	70.92	2.36
2	5	-7	68.65	2.39
2	5	-7	75.35	2.51
-2	-5	7	72.69	2.34
-2	-5	7	68.90	2.59
-2	-5	7	72.50	2.36
1	5	-7	53.02	1.96
1	5	-7	50.70	1.75
1	5	-7	53.19	1.88
1	5	-7	51.60	1.76
-1	-5	7	53.00	1.74
-1	-5	7	47.57	1.78
-1	-5	7	50.58	1.96
-1	-5	7	47.33	2.12
0	5	-7	32.80	1.24
0	5	-7	34.04	1.35
0	5	-7	33.99	1.25
0	-5	7	34.53	1.68
0	-5	7	34.28	1.26
0	-5	7	34.21	1.44
0	-5	7	33.82	1.34
-1	5	-7	5.81	0.70
-1	5	-7	5.08	0.50
-1	5	-7	6.31	0.46
-1	5	-7	5.72	0.46
1	-5	7	6.06	0.60
1	-5	7	5.05	0.46
1	-5	7	5.61	0.88
1	-5	7	5.28	0.57
-2	5	-7	79.42	2.61

-2	5	-7	83.46	2.71
-2	5	-7	78.10	2.87
-2	5	-7	78.20	2.58
2	-5	7	79.41	2.73
2	-5	7	77.22	3.16
2	-5	7	80.91	2.62
2	-5	7	79.75	2.78
-3	5	-7	22.46	0.94
-3	5	-7	21.61	0.95
-3	5	-7	22.64	1.04
-3	5	-7	23.16	1.27
3	-5	7	23.59	1.10
3	-5	7	21.20	1.54
3	-5	7	21.90	1.10
3	-5	7	23.20	0.98
-4	5	-7	16.03	0.79
-4	5	-7	14.84	0.89
-4	5	-7	14.64	0.77
-4	5	-7	15.01	1.11
4	-5	7	15.25	0.80
4	-5	7	16.27	0.99
4	-5	7	15.98	1.46
4	-5	7	15.19	0.93
-5	5	-7	0.75	0.54
-5	5	-7	0.60	0.38
-5	5	-7	0.53	0.31
-5	5	-7	0.18	0.26
5	-5	7	0.16	0.31
5	-5	7	0.29	0.34
5	-5	7	0.77	0.48
5	-5	7	-0.63	0.75
-6	5	-7	0.31	0.59
-6	5	-7	0.22	0.30
-6	5	-7	0.71	0.36
-6	5	-7	0.84	0.48
6	-5	7	0.08	0.80
6	-5	7	0.29	0.35
6	-5	7	0.01	0.41
-7	5	-7	6.76	1.08
-7	5	-7	6.14	0.64
-7	5	-7	6.71	0.69
7	-5	7	8.33	0.91
7	-5	7	3.99	1.19
7	-5	7	7.02	1.05

-8	5	-7	-0.14	0.75
-8	5	-7	-0.41	0.40
8	-5	7	-0.50	1.16
8	-5	7	0.02	0.51
-9	5	-7	0.33	0.50
-9	5	-7	-0.38	0.42
-9	5	-7	1.46	0.89
9	-5	7	0.86	0.60
9	-5	7	-0.21	1.34
-10	5	-7	-0.31	0.55
-10	5	-7	0.61	0.52
-10	5	-7	1.34	1.24
10	-5	7	0.95	0.68
10	-5	7	1.38	1.65
-11	5	-7	0.16	0.56
11	-5	7	0.32	0.76
11	-5	7	3.93	2.03
11	-5	7	0.13	0.86
-12	5	-7	1.53	0.69
12	-5	7	0.51	1.04
12	-5	7	1.09	2.20
-13	5	-7	0.64	0.79
13	-5	7	1.66	4.63
13	-5	7	-0.02	1.19
15	4	-7	25.92	1.49
15	4	-7	24.43	2.44
15	4	-7	20.68	3.68
14	4	-7	19.54	2.28
14	4	-7	17.01	2.36
14	4	-7	18.26	2.03
14	4	-7	15.38	1.17
13	4	-7	25.78	1.99
13	4	-7	23.61	2.42
13	4	-7	21.69	2.05
13	4	-7	23.47	1.28
12	4	-7	33.85	1.50
12	4	-7	34.26	2.06
12	4	-7	37.93	2.07
12	4	-7	33.54	2.75
12	4	-7	29.54	2.49
12	4	-7	35.09	1.84
11	4	-7	37.94	2.51
11	4	-7	40.92	1.89
11	4	-7	39.91	1.99

11	4	-7	38.27	1.57
11	4	-7	40.42	2.07
10	4	-7	4.40	0.73
10	4	-7	3.66	0.57
10	4	-7	3.97	0.67
10	4	-7	3.92	0.76
10	4	-7	3.61	0.75
10	4	-7	3.47	1.04
-10	-4	7	2.88	0.75
9	4	-7	25.02	1.10
9	4	-7	25.43	1.34
9	4	-7	25.90	1.27
9	4	-7	24.36	1.45
9	4	-7	23.97	1.13
9	4	-7	27.21	1.71
-9	-4	7	24.21	1.55
8	4	-7	68.45	2.63
8	4	-7	68.12	2.39
8	4	-7	68.19	2.34
8	4	-7	72.06	2.86
8	4	-7	68.01	2.37
8	4	-7	71.75	2.56
-8	-4	7	68.37	2.74
7	4	-7	41.03	1.56
7	4	-7	43.70	1.73
7	4	-7	42.29	1.77
7	4	-7	41.65	1.56
7	4	-7	40.19	1.84
7	4	-7	42.63	1.54
-7	-4	7	43.68	1.64
-7	-4	7	42.32	1.87
6	4	-7	60.19	2.04
6	4	-7	58.99	2.24
6	4	-7	60.32	1.98
6	4	-7	61.96	2.22
6	4	-7	57.94	2.21
-6	-4	7	61.78	2.37
-6	-4	7	60.60	2.12
5	4	-7	78.18	2.71
5	4	-7	83.51	2.78
5	4	-7	77.14	2.74
5	4	-7	78.85	2.61
-5	-4	7	80.89	2.60
-5	-4	7	80.95	2.57

-5	-4	7	81.44	2.66
-5	-4	7	78.74	2.88
4	4	-7	58.63	2.08
4	4	-7	57.40	1.94
4	4	-7	54.10	2.06
4	4	-7	58.62	2.01
-4	-4	7	59.48	1.91
-4	-4	7	58.54	2.00
-4	-4	7	56.73	2.18
-4	-4	7	58.19	2.00
3	4	-7	5.28	0.51
3	4	-7	6.04	0.64
3	4	-7	6.24	0.54
3	4	-7	5.82	0.45
-3	-4	7	6.57	0.64
-3	-4	7	6.65	0.51
-3	-4	7	5.29	0.59
-3	-4	7	6.58	0.43
2	4	-7	41.89	1.51
2	4	-7	37.79	1.39
2	4	-7	37.96	1.51
2	4	-7	39.95	1.47
-2	-4	7	40.82	1.38
-2	-4	7	38.99	1.47
-2	-4	7	38.64	1.61
-2	-4	7	38.64	1.58
1	4	-7	30.05	1.23
1	4	-7	29.40	1.22
1	4	-7	31.11	1.21
1	4	-7	29.06	1.12
-1	-4	7	29.70	1.23
-1	-4	7	28.42	1.26
-1	-4	7	31.22	1.48
-1	-4	7	30.09	1.11
0	4	-7	20.07	0.89
0	4	-7	19.05	0.81
0	4	-7	18.42	0.87
0	4	-7	19.78	0.96
0	-4	7	18.95	0.95
0	-4	7	19.73	0.82
0	-4	7	20.88	0.98
0	-4	7	17.36	1.13
-1	4	-7	49.11	1.75
-1	4	-7	48.82	1.73

-1	4	-7	54.85	1.84
-1	4	-7	50.18	1.90
1	-4	7	49.26	2.15
1	-4	7	51.24	1.92
1	-4	7	51.35	1.75
1	-4	7	50.20	1.89
-2	4	-7	19.45	0.84
-2	4	-7	19.92	0.87
-2	4	-7	20.98	0.94
-2	4	-7	18.95	1.06
2	-4	7	19.23	0.97
2	-4	7	18.05	1.30
2	-4	7	19.43	0.86
2	-4	7	20.74	1.05
-3	4	-7	8.51	0.60
-3	4	-7	9.85	0.57
-3	4	-7	8.44	0.81
-3	4	-7	9.37	0.69
3	-4	7	8.81	0.77
3	-4	7	10.22	0.61
3	-4	7	10.26	1.14
3	-4	7	8.35	0.67
-4	4	-7	0.20	0.27
-4	4	-7	0.88	0.38
-4	4	-7	0.61	0.47
-4	4	-7	0.19	0.23
4	-4	7	0.16	0.30
4	-4	7	0.71	0.71
4	-4	7	0.06	0.42
4	-4	7	0.70	0.30
-5	4	-7	0.78	0.52
-5	4	-7	0.34	0.26
-5	4	-7	0.37	0.42
-5	4	-7	0.26	0.30
5	-4	7	0.28	0.32
5	-4	7	0.26	0.33
5	-4	7	0.72	0.76
5	-4	7	-0.12	0.47
-6	4	-7	2.22	0.39
-6	4	-7	2.54	0.66
-6	4	-7	3.46	0.65
-6	4	-7	3.04	0.47
6	-4	7	3.55	0.66
6	-4	7	1.89	0.95

6	-4	7	2.27	0.42
6	-4	7	2.36	0.51
-7	4	-7	-0.02	0.39
-7	4	-7	0.39	0.32
-7	4	-7	0.26	0.70
7	-4	7	-0.95	0.90
7	-4	7	0.18	0.46
-8	4	-7	0.40	0.40
-8	4	-7	0.20	0.47
-8	4	-7	0.75	0.84
8	-4	7	0.45	1.24
8	-4	7	0.14	0.48
-9	4	-7	1.61	1.09
-9	4	-7	0.19	0.42
-9	4	-7	0.98	0.53
9	-4	7	-0.69	1.38
9	-4	7	-0.10	0.59
-10	4	-7	3.54	0.61
-10	4	-7	3.03	1.33
-10	4	-7	3.98	0.71
10	-4	7	4.54	1.87
10	-4	7	3.45	0.76
-11	4	-7	2.36	0.63
11	-4	7	1.66	0.77
11	-4	7	1.44	1.98
-12	4	-7	0.69	0.66
12	-4	7	4.29	2.31
12	-4	7	1.03	1.05
16	3	-7	19.17	2.40
16	3	-7	20.38	1.44
15	3	-7	13.56	2.08
15	3	-7	13.04	2.02
15	3	-7	13.85	1.18
14	3	-7	15.16	2.14
14	3	-7	16.84	2.28
14	3	-7	17.50	1.16
14	3	-7	15.39	1.88
13	3	-7	21.86	1.20
13	3	-7	20.86	2.01
13	3	-7	23.29	1.89
13	3	-7	17.85	2.59
13	3	-7	22.63	2.15
12	3	-7	13.47	1.89
12	3	-7	14.24	1.89

12	3	-7	14.39	1.18
12	3	-7	14.72	1.58
12	3	-7	14.78	2.03
12	3	-7	13.65	0.92
11	3	-7	17.97	1.17
11	3	-7	17.25	1.40
11	3	-7	16.55	1.41
11	3	-7	15.17	1.72
-11	-3	7	17.96	1.39
10	3	-7	101.78	3.75
10	3	-7	102.30	3.35
10	3	-7	102.40	3.41
10	3	-7	106.91	3.54
10	3	-7	98.99	3.80
10	3	-7	100.39	3.57
-10	-3	7	106.37	3.74
9	3	-7	56.78	2.08
9	3	-7	54.36	2.26
9	3	-7	51.97	1.91
9	3	-7	53.94	1.91
9	3	-7	53.54	2.06
-9	-3	7	54.40	2.27
-9	-3	7	54.36	2.09
8	3	-7	18.99	0.99
8	3	-7	20.69	1.22
8	3	-7	20.04	1.04
8	3	-7	19.92	0.92
8	3	-7	19.67	0.88
8	3	-7	17.76	1.21
-8	-3	7	19.00	1.04
-8	-3	7	21.16	1.22
7	3	-7	58.15	2.04
7	3	-7	52.64	1.88
7	3	-7	54.00	2.07
7	3	-7	57.14	2.15
7	3	-7	56.13	1.92
7	3	-7	53.70	1.86
-7	-3	7	53.63	2.17
-7	-3	7	56.41	2.03
6	3	-7	45.64	1.69
6	3	-7	46.63	1.85
6	3	-7	46.95	1.64
6	3	-7	49.80	1.70
6	3	-7	49.49	1.78

-6	-3	7	46.30	1.75
-6	-3	7	46.02	1.88
5	3	-7	18.32	0.79
5	3	-7	20.15	0.91
5	3	-7	18.27	1.00
-5	-3	7	18.10	0.99
-5	-3	7	18.00	0.76
-5	-3	7	18.73	0.90
4	3	-7	14.35	0.67
4	3	-7	14.75	0.85
4	3	-7	16.01	0.78
-4	-3	7	15.32	0.65
-4	-3	7	14.60	0.84
-4	-3	7	13.24	0.88
3	3	-7	59.96	2.05
3	3	-7	65.55	2.15
3	3	-7	59.06	2.20
3	3	-7	60.72	1.96
-3	-3	7	64.38	2.03
-3	-3	7	60.08	2.09
-3	-3	7	62.97	2.17
-3	-3	7	63.57	2.23
2	3	-7	0.40	0.21
2	3	-7	0.54	0.20
2	3	-7	0.62	0.24
2	3	-7	0.67	0.32
-2	-3	7	0.80	0.21
-2	-3	7	0.38	0.33
-2	-3	7	0.70	0.33
-2	-3	7	0.33	0.21
1	3	-7	4.69	0.42
1	3	-7	4.83	0.38
1	3	-7	3.85	0.50
1	3	-7	5.17	0.44
-1	-3	7	3.79	0.58
-1	-3	7	4.40	0.46
-1	-3	7	4.89	0.39
0	3	-7	30.19	1.17
0	3	-7	28.58	1.08
0	3	-7	29.58	1.20
0	3	-7	31.34	1.19
0	-3	7	28.48	1.21
0	-3	7	28.31	1.43
0	-3	7	28.18	1.37

0	-3	7	28.44	1.08
-1	3	-7	7.75	0.57
-1	3	-7	9.57	0.63
-1	3	-7	8.22	0.64
-1	3	-7	9.09	0.51
1	-3	7	9.95	0.63
1	-3	7	9.68	0.90
1	-3	7	9.80	0.75
1	-3	7	9.02	0.54
-2	3	-7	1.14	0.31
-2	3	-7	0.36	0.26
-2	3	-7	1.13	0.35
-2	3	-7	0.44	0.20
2	-3	7	0.51	0.26
2	-3	7	0.24	0.47
2	-3	7	0.46	0.26
2	-3	7	0.33	0.39
-3	3	-7	0.86	0.41
-3	3	-7	0.96	0.29
-3	3	-7	0.66	0.25
-3	3	-7	0.18	0.33
3	-3	7	0.77	0.58
3	-3	7	0.85	0.42
3	-3	7	1.04	0.29
3	-3	7	0.49	0.28
-4	3	-7	0.37	0.30
-4	3	-7	0.22	0.22
-4	3	-7	-0.05	0.43
-4	3	-7	0.29	0.40
4	-3	7	-0.47	0.41
4	-3	7	0.21	0.60
4	-3	7	0.01	0.30
4	-3	7	-0.36	0.27
-5	3	-7	3.62	0.63
-5	3	-7	3.10	0.46
-5	3	-7	2.44	0.42
-5	3	-7	2.53	0.60
5	-3	7	1.45	0.75
5	-3	7	2.92	0.61
5	-3	7	3.51	0.61
5	-3	7	2.97	0.40
-6	3	-7	4.14	0.80
-6	3	-7	4.17	0.62
-6	3	-7	4.43	0.53

6	-3	7	5.89	1.21
6	-3	7	4.84	0.73
6	-3	7	4.63	0.71
6	-3	7	4.00	0.52
-7	3	-7	3.93	0.58
-7	3	-7	3.97	0.94
-7	3	-7	3.58	0.55
7	-3	7	4.09	0.64
7	-3	7	5.60	1.31
-8	3	-7	-0.26	0.36
-8	3	-7	-1.38	0.93
-8	3	-7	-0.28	0.43
8	-3	7	0.29	0.50
8	-3	7	-0.52	1.11
-9	3	-7	0.17	0.52
-9	3	-7	0.32	0.45
-9	3	-7	0.85	1.19
9	-3	7	0.37	0.55
9	-3	7	-1.38	1.31
-10	3	-7	2.46	0.56
-10	3	-7	1.66	0.64
10	-3	7	2.51	1.66
10	-3	7	1.64	0.71
-11	3	-7	0.41	0.63
-11	3	-7	-0.37	0.64
11	-3	7	-0.22	1.98
11	-3	7	0.89	0.77
-12	3	-7	1.77	0.73
12	-3	7	5.69	2.28
12	-3	7	-1.48	1.02
16	2	-7	17.24	2.32
16	2	-7	24.08	1.46
15	2	-7	8.61	1.76
15	2	-7	10.79	1.06
15	2	-7	9.33	1.42
14	2	-7	12.14	1.01
14	2	-7	13.03	1.84
14	2	-7	13.79	2.08
14	2	-7	14.27	1.76
13	2	-7	15.39	2.22
13	2	-7	14.10	1.82
13	2	-7	14.53	1.81
13	2	-7	16.80	1.16
13	2	-7	16.39	1.02

13	2	-7	19.16	1.67
12	2	-7	27.11	2.02
12	2	-7	32.45	1.45
12	2	-7	30.28	1.34
12	2	-7	31.99	1.94
12	2	-7	30.99	2.15
11	2	-7	68.81	2.39
11	2	-7	68.89	2.78
11	2	-7	71.12	2.48
11	2	-7	71.32	2.90
11	2	-7	68.11	2.86
-11	-2	7	64.88	2.66
10	2	-7	45.23	1.70
10	2	-7	41.28	1.58
10	2	-7	41.65	1.97
10	2	-7	41.36	1.87
10	2	-7	41.40	2.01
-10	-2	7	45.36	1.85
-10	-2	7	41.29	1.88
9	2	-7	36.82	1.36
9	2	-7	35.06	1.58
9	2	-7	36.95	1.67
9	2	-7	34.50	1.71
9	2	-7	36.12	1.39
9	2	-7	36.68	1.46
-9	-2	7	35.64	1.56
-9	-2	7	35.79	1.64
8	2	-7	65.56	2.21
8	2	-7	62.11	2.18
8	2	-7	61.86	2.25
8	2	-7	62.56	2.13
8	2	-7	61.63	2.10
8	2	-7	63.36	2.42
-8	-2	7	61.66	2.35
-8	-2	7	60.98	2.28
7	2	-7	47.33	1.72
7	2	-7	49.16	1.67
7	2	-7	46.78	1.65
7	2	-7	47.76	1.70
7	2	-7	46.57	1.93
7	2	-7	49.56	1.76
-7	-2	7	50.40	1.85
-7	-2	7	49.73	1.91
6	2	-7	25.00	0.99

6	2	-7	25.31	1.23
6	2	-7	27.71	1.09
6	2	-7	24.75	1.04
-6	-2	7	25.47	1.17
-6	-2	7	26.55	1.17
5	2	-7	77.86	2.51
5	2	-7	72.17	2.41
5	2	-7	72.03	2.63
-5	-2	7	77.48	2.59
-5	-2	7	75.55	2.40
-5	-2	7	73.67	2.58
4	2	-7	45.35	1.58
4	2	-7	44.49	1.72
4	2	-7	43.68	1.50
-4	-2	7	42.62	1.63
-4	-2	7	41.79	1.64
-4	-2	7	43.23	1.46
3	2	-7	1.58	0.40
3	2	-7	1.46	0.24
3	2	-7	1.53	0.26
-3	-2	7	1.28	0.22
-3	-2	7	1.96	0.32
-3	-2	7	1.82	0.29
2	2	-7	93.29	3.03
2	2	-7	94.11	2.99
2	2	-7	95.97	3.23
2	2	-7	99.24	3.14
-2	-2	7	98.93	3.14
-2	-2	7	98.09	3.02
-2	-2	7	94.59	3.16
1	2	-7	0.64	0.20
1	2	-7	0.41	0.32
1	2	-7	0.74	0.22
1	2	-7	0.47	0.22
-1	-2	7	0.68	0.34
-1	-2	7	0.47	0.20
-1	-2	7	0.73	0.44
-1	-2	7	0.71	0.20
0	2	-7	7.11	0.53
0	2	-7	7.49	0.57
0	2	-7	6.48	0.57
0	2	-7	6.76	0.43
0	-2	7	7.73	0.70
0	-2	7	7.52	0.71

0	-2	7	7.09	0.46
0	-2	7	6.58	0.53
-1	2	-7	3.60	0.47
-1	2	-7	3.45	0.50
-1	2	-7	2.78	0.42
-1	2	-7	3.35	0.33
1	-2	7	3.22	0.53
1	-2	7	3.54	0.38
1	-2	7	3.89	0.63
1	-2	7	2.56	0.41
-2	2	-7	0.69	0.36
-2	2	-7	0.51	0.20
-2	2	-7	0.70	0.32
-2	2	-7	0.97	0.31
2	-2	7	0.78	0.26
2	-2	7	0.60	0.41
2	-2	7	0.80	0.51
2	-2	7	1.35	0.27
-3	2	-7	-0.29	0.37
-3	2	-7	-0.10	0.36
-3	2	-7	0.20	0.25
-3	2	-7	0.17	0.21
3	-2	7	0.06	0.26
3	-2	7	-0.23	0.23
3	-2	7	0.52	0.42
3	-2	7	-0.44	0.48
-4	2	-7	1.36	0.38
-4	2	-7	2.40	0.55
-4	2	-7	1.71	0.50
-4	2	-7	1.97	0.32
4	-2	7	1.86	0.33
4	-2	7	2.09	0.43
4	-2	7	1.84	0.70
4	-2	7	2.83	0.57
-5	2	-7	1.69	0.32
-5	2	-7	0.70	0.36
-5	2	-7	2.13	0.67
-5	2	-7	1.48	0.61
5	-2	7	1.82	0.41
5	-2	7	0.92	0.69
5	-2	7	1.53	0.36
5	-2	7	0.84	0.60
-6	2	-7	0.73	0.33
-6	2	-7	-0.34	0.69

-6	2	-7	1.00	0.41
6	-2	7	0.78	0.42
6	-2	7	1.49	0.87
6	-2	7	0.57	0.38
-7	2	-7	13.22	0.80
-7	2	-7	11.82	0.92
-7	2	-7	10.56	1.65
7	-2	7	7.40	1.68
7	-2	7	14.61	1.03
-8	2	-7	1.59	1.09
-8	2	-7	1.46	0.43
-8	2	-7	0.61	0.48
8	-2	7	1.38	0.53
8	-2	7	1.19	1.24
-9	2	-7	-0.02	0.42
-9	2	-7	-0.27	0.52
9	-2	7	0.05	0.58
9	-2	7	0.40	1.33
-10	2	-7	1.10	0.66
-10	2	-7	2.13	0.59
10	-2	7	3.39	1.77
10	-2	7	1.77	0.71
-11	2	-7	3.66	0.74
-11	2	-7	4.24	0.79
11	-2	7	6.61	2.17
-12	2	-7	2.38	0.79
12	-2	7	3.29	1.19
12	-2	7	0.32	2.12
16	1	-7	16.84	3.87
16	1	-7	13.52	2.17
16	1	-7	15.84	1.22
15	1	-7	14.84	1.12
15	1	-7	13.50	1.97
15	1	-7	14.44	1.90
14	1	-7	19.39	1.13
14	1	-7	18.03	1.81
14	1	-7	17.10	1.70
14	1	-7	15.82	2.13
13	1	-7	43.20	1.68
13	1	-7	39.82	2.45
13	1	-7	38.02	2.50
13	1	-7	40.98	2.22
13	1	-7	41.99	2.29
13	1	-7	41.60	1.64

12	1	-7	48.50	1.80
12	1	-7	47.22	1.81
12	1	-7	49.06	2.40
12	1	-7	47.98	2.33
12	1	-7	46.55	2.40
-12	-1	7	48.54	2.02
11	1	-7	14.89	1.37
11	1	-7	14.86	0.84
11	1	-7	17.13	0.88
11	1	-7	16.12	1.33
11	1	-7	14.36	1.30
-11	-1	7	15.27	1.09
10	1	-7	46.88	2.42
10	1	-7	52.16	1.81
10	1	-7	49.29	2.19
10	1	-7	52.45	1.89
10	1	-7	47.64	2.09
10	1	-7	50.02	1.78
-10	-1	7	50.29	2.00
-10	-1	7	49.16	2.03
9	1	-7	24.18	1.35
9	1	-7	24.14	0.99
9	1	-7	23.74	1.22
9	1	-7	23.46	1.03
9	1	-7	23.38	1.28
9	1	-7	23.06	1.01
-9	-1	7	23.31	1.22
-9	-1	7	22.64	1.19
8	1	-7	21.71	1.23
8	1	-7	22.02	1.01
8	1	-7	23.88	0.96
8	1	-7	20.78	0.93
8	1	-7	20.94	1.05
8	1	-7	21.32	0.91
-8	-1	7	21.87	1.08
-8	-1	7	22.89	1.13
7	1	-7	45.01	1.87
7	1	-7	45.73	1.62
7	1	-7	45.50	1.58
7	1	-7	46.73	1.61
-7	-1	7	45.84	1.74
-7	-1	7	45.12	1.78
6	1	-7	47.17	1.90
6	1	-7	50.46	1.68

6	1	-7	47.44	1.67
6	1	-7	47.26	1.62
-6	-1	7	49.10	1.83
-6	-1	7	45.09	1.75
5	1	-7	5.66	0.63
5	1	-7	5.46	0.39
5	1	-7	5.99	0.44
-5	-1	7	5.39	0.58
-5	-1	7	6.31	0.39
-5	-1	7	4.66	0.46
4	1	-7	48.50	1.62
4	1	-7	49.56	1.89
4	1	-7	49.84	1.70
-4	-1	7	46.82	1.83
-4	-1	7	46.66	1.72
-4	-1	7	48.86	1.60
3	1	-7	55.10	1.83
3	1	-7	56.35	2.10
3	1	-7	57.37	1.94
-3	-1	7	56.38	1.81
-3	-1	7	55.58	1.85
-3	-1	7	57.53	2.08
-3	-1	7	52.55	1.93
-3	-1	7	55.23	1.82
2	1	-7	9.68	0.60
2	1	-7	9.92	0.50
2	1	-7	9.09	0.73
-2	-1	7	9.71	0.76
-2	-1	7	10.27	0.60
-2	-1	7	9.23	0.48
-2	-1	7	9.50	0.60
-2	-1	7	9.49	0.57
1	1	-7	0.74	0.27
1	1	-7	1.37	0.33
1	1	-7	0.59	0.20
-1	-1	7	0.86	0.21
-1	-1	7	0.73	0.22
-1	-1	7	0.61	0.38
-1	-1	7	1.16	0.39
-1	-1	7	1.06	0.34
0	1	-7	16.85	0.85
0	1	-7	16.57	0.88
0	1	-7	15.60	0.68
0	1	-7	16.00	0.88

0	-1	7	16.57	0.97
0	-1	7	16.32	0.69
0	-1	7	17.00	1.00
0	-1	7	16.28	0.81
-1	1	-7	2.84	0.45
-1	1	-7	3.37	0.50
-1	1	-7	3.32	0.50
-1	1	-7	2.61	0.32
1	-1	7	3.17	0.36
1	-1	7	3.19	0.50
1	-1	7	3.92	0.62
-2	1	-7	4.09	0.64
-2	1	-7	4.09	0.68
-2	1	-7	4.46	0.52
-2	1	-7	3.87	0.38
2	-1	7	4.36	0.79
2	-1	7	5.15	0.76
2	-1	7	3.61	0.47
2	-1	7	4.10	0.42
-3	1	-7	8.14	0.51
-3	1	-7	9.82	0.93
-3	1	-7	8.17	0.82
-3	1	-7	8.55	0.68
3	-1	7	7.78	1.03
3	-1	7	9.52	0.88
3	-1	7	9.58	0.57
3	-1	7	8.55	0.66
-4	1	-7	3.92	0.43
-4	1	-7	2.98	0.64
-4	1	-7	3.74	0.63
-4	1	-7	2.89	0.45
4	-1	7	4.52	0.71
4	-1	7	3.46	0.82
4	-1	7	3.04	0.47
4	-1	7	3.67	0.57
-5	1	-7	8.41	0.57
-5	1	-7	7.89	0.72
-5	1	-7	7.41	1.22
-5	1	-7	8.01	1.01
5	-1	7	7.14	0.73
5	-1	7	8.46	1.04
5	-1	7	5.79	0.97
5	-1	7	7.20	0.61
-6	1	-7	0.77	0.83

-6	1	-7	0.26	0.29
-6	1	-7	0.44	0.39
6	-1	7	0.15	0.39
6	-1	7	0.38	0.36
6	-1	7	-0.59	0.71
-7	1	-7	5.79	0.64
-7	1	-7	5.81	0.78
-7	1	-7	4.26	1.16
7	-1	7	8.11	1.34
7	-1	7	5.28	0.83
-8	1	-7	1.94	0.47
-8	1	-7	2.93	0.62
8	-1	7	1.99	1.25
8	-1	7	3.01	0.65
-9	1	-7	1.60	0.49
-9	1	-7	2.22	0.65
9	-1	7	0.66	1.21
9	-1	7	0.43	0.61
-10	1	-7	1.11	0.54
-10	1	-7	1.17	0.67
10	-1	7	-1.73	1.50
-11	1	-7	4.24	0.84
-11	1	-7	4.20	0.80
11	-1	7	4.54	0.88
11	-1	7	3.11	1.91
17	0	-7	15.94	1.31
17	0	-7	11.72	1.80
16	0	-7	20.63	1.29
16	0	-7	17.55	2.21
16	0	-7	18.43	2.99
15	0	-7	21.24	2.59
15	0	-7	21.80	2.11
15	0	-7	23.45	1.29
14	0	-7	10.47	1.58
14	0	-7	11.95	1.39
14	0	-7	11.40	1.63
14	0	-7	14.70	0.97
14	0	-7	11.28	2.05
13	0	-7	27.48	1.25
13	0	-7	26.37	1.74
13	0	-7	28.36	1.13
13	0	-7	25.72	1.98
13	0	-7	25.75	2.12
13	0	-7	27.45	1.86

12	0	-7	89.56	2.78
12	0	-7	77.33	3.07
12	0	-7	86.16	3.41
12	0	-7	86.13	3.46
12	0	-7	86.69	3.28
-12	0	7	83.23	2.99
11	0	-7	43.95	2.11
11	0	-7	45.17	1.63
11	0	-7	44.61	2.13
11	0	-7	43.23	1.95
11	0	-7	44.06	1.56
-11	0	7	42.27	1.79
10	0	-7	17.61	1.27
10	0	-7	17.25	0.78
10	0	-7	16.96	0.81
10	0	-7	15.95	1.24
10	0	-7	15.01	0.84
-10	0	7	17.26	1.00
-10	0	7	16.93	1.11
9	0	-7	26.33	1.44
9	0	-7	27.71	1.06
9	0	-7	27.05	1.07
9	0	-7	28.17	1.11
-9	0	7	26.62	1.30
-9	0	7	25.52	1.21
-9	0	7	26.61	1.08
8	0	-7	11.32	0.82
8	0	-7	11.26	0.59
8	0	-7	11.78	0.60
8	0	-7	11.23	0.94
8	0	-7	10.99	0.63
-8	0	7	11.23	0.57
-8	0	7	11.05	0.73
-8	0	7	11.60	0.85
7	0	-7	39.43	1.41
7	0	-7	39.28	1.72
7	0	-7	41.23	1.44
7	0	-7	41.60	1.44
-7	0	7	41.67	1.67
-7	0	7	39.20	1.53
6	0	-7	10.73	0.54
6	0	-7	11.29	0.60
6	0	-7	11.99	0.60
6	0	-7	10.58	0.82

-6	0	7	10.70	0.64
-6	0	7	11.05	0.80
5	0	-7	5.79	0.38
5	0	-7	5.37	0.66
5	0	-7	6.19	0.46
-5	0	7	5.69	0.36
-5	0	7	5.45	0.61
-5	0	7	5.62	0.46
4	0	-7	3.19	0.40
4	0	-7	3.55	0.57
4	0	-7	3.31	0.31
-4	0	7	3.99	0.30
-4	0	7	4.11	0.58
-4	0	7	3.61	0.36
3	0	-7	12.34	0.56
3	0	-7	12.15	0.68
3	0	-7	12.18	0.82
-3	0	7	11.58	0.52
-3	0	7	11.46	0.82
-3	0	7	11.97	0.73
-3	0	7	10.98	0.61
-3	0	7	11.89	0.56
2	0	-7	1.10	0.36
2	0	-7	1.20	0.20
-2	0	7	0.85	0.23
-2	0	7	0.98	0.39
-2	0	7	1.00	0.19
-2	0	7	1.43	0.39
1	0	-7	0.60	0.20
1	0	-7	0.37	0.32
1	0	-7	0.42	0.27
-1	0	7	0.68	0.37
-1	0	7	0.13	0.19
-1	0	7	0.60	0.19
-1	0	7	0.30	0.39
-1	0	7	0.62	0.28
0	0	-7	1.49	0.37
0	0	-7	1.46	0.36
0	0	-7	1.71	0.24
0	0	7	1.89	0.53
0	0	7	1.91	0.47
0	0	7	2.15	0.31
0	0	7	2.03	0.33
0	0	7	1.65	0.40

-1	0	-7	1.24	0.46
-1	0	-7	0.90	0.36
-1	0	-7	0.72	0.19
1	0	7	0.52	0.27
1	0	7	0.96	0.41
1	0	7	0.84	0.44
1	0	7	1.22	0.52
1	0	7	0.78	0.22
-2	0	-7	-0.40	0.40
-2	0	-7	0.01	0.32
-2	0	-7	0.10	0.20
2	0	7	0.54	0.30
2	0	7	0.39	0.23
2	0	7	0.38	0.49
2	0	7	-0.55	0.52
2	0	7	0.44	0.44
-3	0	-7	0.46	0.32
-3	0	-7	0.31	0.22
-3	0	-7	0.02	0.50
3	0	7	0.41	0.25
3	0	7	0.80	0.34
3	0	7	0.59	0.52
3	0	7	0.44	0.63
3	0	7	0.51	0.53
-4	0	-7	0.69	0.26
-4	0	-7	0.59	0.36
-4	0	-7	0.26	0.64
4	0	7	1.50	0.63
4	0	7	0.92	0.81
4	0	7	0.93	0.40
4	0	7	0.40	0.29
4	0	7	1.49	0.71
-5	0	-7	0.01	0.78
-5	0	-7	0.23	0.38
-5	0	-7	0.02	0.27
5	0	7	0.33	0.40
5	0	7	0.39	0.33
5	0	7	-0.42	0.68
-6	0	-7	6.99	0.62
-6	0	-7	7.81	0.80
-6	0	-7	9.99	1.73
6	0	7	6.31	0.69
6	0	7	7.64	1.25
6	0	7	8.10	0.91

-7	0	-7	12.03	0.98
-7	0	-7	10.71	0.76
7	0	7	10.26	1.70
7	0	7	12.56	1.08
-8	0	-7	6.42	0.72
-8	0	-7	7.00	0.97
8	0	7	5.72	1.35
8	0	7	7.55	1.02
-9	0	-7	2.93	0.56
-9	0	-7	2.65	0.71
9	0	7	2.10	0.83
9	0	7	3.40	1.48
-10	0	-7	4.17	0.69
-10	0	-7	1.80	0.75
10	0	7	4.70	1.77
10	0	7	3.91	0.85
-11	0	-7	8.70	1.34
-11	0	-7	6.36	0.89
11	0	7	5.43	0.99
11	0	7	6.52	2.08
17	-1	-7	11.54	1.77
17	-1	-7	14.80	1.24
17	-1	-7	16.48	2.65
16	-1	-7	14.29	2.04
16	-1	-7	12.18	2.14
16	-1	-7	9.43	1.07
15	-1	-7	15.60	2.41
15	-1	-7	18.55	1.13
15	-1	-7	11.92	1.54
14	-1	-7	13.85	1.33
14	-1	-7	17.11	1.03
14	-1	-7	15.52	1.95
14	-1	-7	15.52	2.11
14	-1	-7	14.62	1.71
13	-1	-7	25.80	1.21
13	-1	-7	24.81	1.82
13	-1	-7	24.82	1.62
13	-1	-7	27.04	1.03
13	-1	-7	26.25	2.12
-13	1	7	27.43	1.54
12	-1	-7	36.33	1.99
12	-1	-7	34.80	2.13
12	-1	-7	35.94	1.45
12	-1	-7	35.52	1.29

-12	1	7	35.84	1.52
-12	1	7	36.81	1.66
11	-1	-7	19.46	0.93
11	-1	-7	19.51	1.41
11	-1	-7	19.80	0.84
11	-1	-7	19.73	1.50
-11	1	7	18.75	1.04
-11	1	7	20.12	1.09
10	-1	-7	47.82	1.61
10	-1	-7	44.60	1.67
10	-1	-7	46.92	2.08
10	-1	-7	46.29	2.09
10	-1	-7	45.36	1.64
-10	1	7	44.87	1.95
-10	1	7	46.06	1.76
-10	1	7	47.32	1.76
9	-1	-7	25.82	0.99
9	-1	-7	23.67	1.01
9	-1	-7	23.99	0.99
9	-1	-7	23.02	1.49
9	-1	-7	24.66	1.38
-9	1	7	23.93	1.26
-9	1	7	24.31	1.09
-9	1	7	25.66	1.04
8	-1	-7	3.46	0.39
8	-1	-7	4.33	0.42
8	-1	-7	3.60	0.63
8	-1	-7	2.80	0.42
8	-1	-7	4.63	0.64
-8	1	7	4.07	0.44
-8	1	7	2.97	0.52
-8	1	7	3.01	0.46
7	-1	-7	37.03	1.30
7	-1	-7	36.45	1.65
7	-1	-7	37.97	1.33
7	-1	-7	36.25	1.45
-7	1	7	38.15	1.59
-7	1	7	38.28	1.31
-7	1	7	34.75	1.36
6	-1	-7	2.31	0.30
6	-1	-7	2.25	0.37
6	-1	-7	2.59	0.52
6	-1	-7	2.58	0.40
6	-1	-7	2.43	0.37

-6	1	7	2.61	0.37
-6	1	7	2.94	0.40
-6	1	7	2.81	0.49
5	-1	-7	4.62	0.65
5	-1	-7	4.22	0.43
5	-1	-7	5.46	0.49
5	-1	-7	4.88	0.34
-5	1	7	5.53	0.66
-5	1	7	4.99	0.55
-5	1	7	4.17	0.40
-5	1	7	4.79	0.33
4	-1	-7	9.84	0.50
4	-1	-7	10.78	0.67
4	-1	-7	9.62	0.60
4	-1	-7	10.19	0.82
-4	1	7	9.98	0.47
-4	1	7	11.71	0.83
-4	1	7	9.87	0.56
-4	1	7	11.03	0.76
3	-1	-7	-0.32	0.30
3	-1	-7	0.75	0.30
3	-1	-7	-0.13	0.15
-3	1	7	-0.41	0.35
-3	1	7	0.20	0.36
-3	1	7	-0.01	0.19
2	-1	-7	24.75	1.20
2	-1	-7	24.51	1.24
2	-1	-7	26.04	0.93
-2	1	7	26.03	1.07
-2	1	7	24.64	1.28
-2	1	7	25.56	1.31
-2	1	7	25.52	0.91
-2	1	7	25.99	1.06
1	-1	-7	11.40	0.54
1	-1	-7	12.69	0.90
1	-1	-7	12.12	0.85
-1	1	7	13.66	0.96
-1	1	7	12.46	0.74
-1	1	7	12.79	0.55
-1	1	7	12.51	0.71
-1	1	7	13.63	1.01
0	-1	-7	3.68	0.32
0	-1	-7	4.60	0.70
0	-1	-7	4.40	0.60

0	1	7	4.82	0.86
0	1	7	4.01	0.48
0	1	7	4.74	0.74
0	1	7	4.22	0.57
-1	-1	-7	1.29	0.49
-1	-1	-7	1.04	0.38
-1	-1	-7	0.68	0.20
1	1	7	1.12	0.49
1	1	7	1.12	0.24
1	1	7	1.93	0.63
1	1	7	0.79	0.37
1	1	7	0.78	0.32
-2	-1	-7	3.79	0.73
-2	-1	-7	3.17	0.51
-2	-1	-7	3.22	0.34
2	1	7	4.08	0.73
2	1	7	3.77	0.67
2	1	7	3.37	0.40
2	1	7	3.64	0.56
2	1	7	5.44	0.90
-3	-1	-7	1.93	0.46
-3	-1	-7	1.57	0.72
-3	-1	-7	1.10	0.27
3	1	7	1.62	0.56
3	1	7	1.53	0.41
3	1	7	2.21	0.32
3	1	7	1.43	0.79
3	1	7	0.49	0.56
-4	-1	-7	11.97	0.62
-4	-1	-7	11.85	0.87
-4	-1	-7	10.56	1.46
4	1	7	11.08	0.66
4	1	7	9.54	1.13
4	1	7	11.15	0.91
4	1	7	10.54	1.16
4	1	7	8.40	1.53
-5	-1	-7	15.82	0.79
-5	-1	-7	16.44	1.81
-5	-1	-7	16.18	1.02
5	1	7	16.56	1.10
5	1	7	16.69	0.84
5	1	7	14.34	1.40
-6	-1	-7	14.37	0.78
-6	-1	-7	14.25	1.02

6	1	7	13.20	1.10
6	1	7	10.63	1.52
6	1	7	14.76	0.87
-7	-1	-7	1.61	0.41
-7	-1	-7	1.47	0.56
7	1	7	1.46	0.99
7	1	7	1.79	0.63
-8	-1	-7	4.68	0.72
-8	-1	-7	3.76	0.74
8	1	7	4.04	1.34
8	1	7	4.73	0.86
-9	-1	-7	7.28	0.89
-9	-1	-7	7.03	1.12
9	1	7	7.90	1.26
9	1	7	5.18	1.50
-10	-1	-7	6.66	0.99
-10	-1	-7	8.26	1.11
10	1	7	11.43	1.49
10	1	7	5.10	1.70
-11	-1	-7	4.52	0.90
11	1	7	5.20	1.06
17	-2	-7	14.03	2.45
17	-2	-7	11.19	1.19
17	-2	-7	6.98	1.50
16	-2	-7	18.03	1.23
16	-2	-7	19.81	2.89
16	-2	-7	17.72	2.06
15	-2	-7	17.27	2.52
15	-2	-7	17.47	1.88
-15	2	7	16.21	1.63
14	-2	-7	6.80	1.18
14	-2	-7	7.78	1.51
-14	2	7	7.39	1.27
-14	2	7	6.82	0.99
13	-2	-7	5.28	0.98
13	-2	-7	7.87	1.38
-13	2	7	7.41	1.12
-13	2	7	9.35	0.88
12	-2	-7	28.34	1.96
12	-2	-7	25.43	1.68
12	-2	-7	23.99	1.05
-12	2	7	28.00	1.18
-12	2	7	28.64	1.42
11	-2	-7	19.24	1.55

11	-2	-7	17.48	0.84
11	-2	-7	17.47	0.87
11	-2	-7	19.33	1.40
-11	2	7	19.08	1.07
10	-2	-7	15.75	1.21
10	-2	-7	16.26	0.80
10	-2	-7	16.15	0.79
10	-2	-7	17.01	0.81
10	-2	-7	15.99	1.37
-10	2	7	16.08	0.88
-10	2	7	17.32	1.14
9	-2	-7	21.37	0.94
9	-2	-7	21.56	0.88
9	-2	-7	21.50	1.44
9	-2	-7	20.33	0.90
9	-2	-7	21.07	1.30
-9	2	7	21.65	1.03
-9	2	7	20.41	1.20
-9	2	7	19.70	0.93
8	-2	-7	13.33	1.12
8	-2	-7	13.46	1.01
8	-2	-7	13.39	0.65
8	-2	-7	14.10	0.76
8	-2	-7	13.55	0.66
-8	2	7	15.28	0.98
-8	2	7	12.47	0.79
-8	2	7	14.80	0.70
7	-2	-7	6.54	0.80
7	-2	-7	5.95	0.43
7	-2	-7	7.31	0.77
7	-2	-7	6.03	0.40
-7	2	7	5.49	0.45
-7	2	7	5.49	0.71
-7	2	7	5.25	0.62
6	-2	-7	0.57	0.30
6	-2	-7	0.22	0.20
6	-2	-7	0.25	0.37
6	-2	-7	0.56	0.39
6	-2	-7	0.06	0.15
-6	2	7	0.96	0.40
-6	2	7	0.50	0.39
-6	2	7	0.13	0.18
5	-2	-7	1.85	0.43
5	-2	-7	2.06	0.28

5	-2	-7	2.05	0.32
5	-2	-7	2.02	0.47
5	-2	-7	1.57	0.35
-5	2	7	1.70	0.27
-5	2	7	1.66	0.43
-5	2	7	1.65	0.47
4	-2	-7	0.36	0.17
4	-2	-7	0.13	0.37
4	-2	-7	0.45	0.32
-4	2	7	0.41	0.39
-4	2	7	0.44	0.20
-4	2	7	0.47	0.45
3	-2	-7	12.09	0.54
3	-2	-7	12.40	0.91
3	-2	-7	12.80	0.86
3	-2	-7	12.40	0.85
-3	2	7	11.81	0.91
-3	2	7	11.50	0.49
-3	2	7	11.79	0.68
-3	2	7	11.25	1.00
2	-2	-7	8.34	0.44
2	-2	-7	8.22	0.76
2	-2	-7	9.80	0.84
2	-2	-7	7.94	0.77
-2	2	7	7.17	0.95
-2	2	7	8.02	0.40
-2	2	7	9.29	0.85
-2	2	7	8.89	0.61
1	-2	-7	9.20	0.45
1	-2	-7	8.25	0.89
1	-2	-7	8.11	0.74
-1	2	7	8.65	0.67
-1	2	7	8.98	0.64
-1	2	7	8.67	0.89
-1	2	7	9.50	0.44
-1	2	7	8.76	1.05
0	-2	-7	10.29	0.81
0	-2	-7	11.04	1.00
0	-2	-7	8.66	0.45
0	2	7	10.07	0.48
0	2	7	9.53	0.76
0	2	7	10.79	1.19
0	2	7	9.32	0.71
0	2	7	9.82	0.95

-1	-2	-7	0.52	0.37
-1	-2	-7	0.84	0.51
-1	-2	-7	0.46	0.18
1	2	7	0.40	0.40
1	2	7	0.63	0.21
1	2	7	0.68	0.50
1	2	7	0.86	0.66
1	2	7	0.34	0.29
-2	-2	-7	2.95	0.49
-2	-2	-7	3.34	0.33
-2	-2	-7	3.94	0.74
2	2	7	2.51	0.63
2	2	7	2.90	0.83
2	2	7	2.70	0.61
2	2	7	2.89	0.50
2	2	7	2.93	0.39
-3	-2	-7	12.96	1.49
-3	-2	-7	12.61	0.90
-3	-2	-7	13.23	0.64
3	2	7	14.72	0.70
3	2	7	14.92	1.29
3	2	7	15.20	1.01
3	2	7	14.00	1.69
-4	-2	-7	19.11	0.82
-4	-2	-7	18.63	1.80
-4	-2	-7	19.54	1.12
4	2	7	19.61	1.42
4	2	7	19.19	1.19
4	2	7	18.00	0.87
-5	-2	-7	7.12	0.86
-5	-2	-7	6.32	0.54
5	2	7	7.42	0.98
5	2	7	5.50	1.00
5	2	7	6.45	0.63
-6	-2	-7	8.06	0.92
-6	-2	-7	8.47	0.65
6	2	7	8.53	1.06
6	2	7	9.06	1.46
6	2	7	8.38	0.76
-7	-2	-7	6.81	0.72
-7	-2	-7	6.40	0.82
7	2	7	6.45	0.92
7	2	7	6.66	0.87
7	2	7	5.29	1.22

-8	-2	-7	11.61	1.19
-8	-2	-7	12.34	0.91
8	2	7	12.44	2.09
8	2	7	9.23	1.29
-9	-2	-7	3.73	0.84
-9	-2	-7	6.61	0.97
9	2	7	4.88	0.97
9	2	7	3.83	1.64
-10	-2	-7	5.51	0.86
-10	-2	-7	7.16	1.02
10	2	7	6.38	1.13
10	2	7	5.58	1.88
17	-3	-7	23.78	3.34
17	-3	-7	17.79	2.29
-17	3	7	15.81	1.50
16	-3	-7	11.39	1.92
16	-3	-7	9.43	2.02
-16	3	7	10.35	1.26
15	-3	-7	6.75	1.79
15	-3	-7	6.87	1.24
-15	3	7	6.21	0.81
-15	3	7	8.27	1.49
14	-3	-7	18.38	1.77
14	-3	-7	20.57	2.28
-14	3	7	18.48	1.23
-14	3	7	17.98	1.46
13	-3	-7	24.48	1.76
13	-3	-7	25.39	2.18
-13	3	7	24.70	1.27
-13	3	7	25.93	1.51
12	-3	-7	0.13	0.96
12	-3	-7	1.19	0.66
-12	3	7	0.30	0.42
-12	3	7	1.15	0.58
11	-3	-7	1.19	0.37
11	-3	-7	1.91	0.39
11	-3	-7	1.35	0.62
11	-3	-7	2.48	0.96
-11	3	7	1.00	0.41
-11	3	7	2.43	0.39
-11	3	7	1.96	0.59
10	-3	-7	22.21	1.63
10	-3	-7	22.99	1.43
10	-3	-7	22.34	0.95

10	-3	-7	20.66	1.05
10	-3	-7	22.25	0.97
-10	3	7	23.97	1.22
-10	3	7	22.68	1.06
-10	3	7	22.58	1.31
9	-3	-7	14.08	1.12
9	-3	-7	14.77	0.71
9	-3	-7	13.23	0.85
9	-3	-7	14.44	0.69
9	-3	-7	13.80	1.28
-9	3	7	14.05	0.71
-9	3	7	15.67	0.98
-9	3	7	14.59	1.05
8	-3	-7	0.82	0.24
8	-3	-7	0.31	0.43
8	-3	-7	0.72	0.68
8	-3	-7	0.75	0.22
8	-3	-7	0.53	0.37
-8	3	7	0.83	0.45
-8	3	7	1.11	0.23
-8	3	7	0.63	0.48
7	-3	-7	9.48	0.50
7	-3	-7	7.47	0.71
7	-3	-7	8.60	0.47
7	-3	-7	7.25	0.86
7	-3	-7	8.07	1.01
-7	3	7	8.92	0.52
-7	3	7	8.29	0.86
-7	3	7	9.36	0.82
6	-3	-7	0.46	0.53
6	-3	-7	-0.18	0.17
6	-3	-7	0.62	0.36
6	-3	-7	0.17	0.16
6	-3	-7	-0.38	0.35
-6	3	7	0.34	0.52
-6	3	7	0.19	0.20
-6	3	7	0.40	0.39
5	-3	-7	-0.36	0.14
5	-3	-7	0.29	0.51
5	-3	-7	-0.32	0.17
5	-3	-7	0.22	0.39
5	-3	-7	-0.37	0.34
-5	3	7	-0.05	0.39
-5	3	7	0.12	0.20

-5	3	7	0.75	0.53
4	-3	-7	15.07	1.00
4	-3	-7	15.02	1.02
4	-3	-7	14.31	0.59
4	-3	-7	14.08	0.99
-4	3	7	13.89	0.75
-4	3	7	13.86	1.01
-4	3	7	13.85	1.14
3	-3	-7	-0.03	0.18
3	-3	-7	0.58	0.37
3	-3	-7	0.48	0.42
3	-3	-7	-0.08	0.41
-3	3	7	0.00	0.22
-3	3	7	-0.08	0.13
-3	3	7	-0.33	0.51
-3	3	7	-0.07	0.41
2	-3	-7	14.21	0.58
2	-3	-7	13.94	0.98
2	-3	-7	15.06	1.11
2	-3	-7	14.95	1.03
-2	3	7	13.01	0.54
-2	3	7	14.08	0.83
-2	3	7	12.94	1.24
-2	3	7	13.05	1.05
1	-3	-7	0.54	0.49
1	-3	-7	0.66	0.36
1	-3	-7	0.65	0.18
1	-3	-7	-0.19	0.42
-1	3	7	0.01	0.65
-1	3	7	0.34	0.16
-1	3	7	0.05	0.24
-1	3	7	-0.15	0.46
0	-3	-7	0.77	0.41
0	-3	-7	1.31	0.21
0	-3	-7	1.26	0.58
0	3	7	0.35	0.74
0	3	7	1.53	0.22
0	3	7	0.43	0.35
0	3	7	1.13	0.57
0	3	7	1.09	0.41
-1	-3	-7	33.83	1.50
-1	-3	-7	32.11	1.91
-1	-3	-7	31.76	1.10
1	3	7	32.01	1.13

1	3	7	32.55	1.51
1	3	7	32.21	2.04
1	3	7	31.93	1.66
1	3	7	31.64	1.51
-2	-3	-7	7.75	1.09
-2	-3	-7	10.17	0.85
-2	-3	-7	8.79	0.49
2	3	7	9.65	1.62
2	3	7	11.33	1.14
2	3	7	8.97	0.53
2	3	7	9.73	0.95
2	3	7	10.05	0.92
-3	-3	-7	0.08	0.21
-3	-3	-7	-0.15	0.40
-3	-3	-7	-0.52	0.81
3	3	7	0.77	0.50
3	3	7	-2.88	1.15
3	3	7	-0.11	0.54
3	3	7	0.07	0.28
-4	-3	-7	6.58	0.50
-4	-3	-7	6.02	0.82
4	3	7	5.56	0.96
4	3	7	6.32	0.59
4	3	7	6.78	1.03
-5	-3	-7	4.02	0.62
-5	-3	-7	2.59	0.38
5	3	7	3.42	0.75
5	3	7	2.01	0.82
5	3	7	3.14	0.46
-6	-3	-7	5.18	0.78
-6	-3	-7	5.79	0.62
6	3	7	4.90	0.75
6	3	7	5.61	0.87
6	3	7	6.08	1.21
-7	-3	-7	12.33	0.88
-7	-3	-7	14.65	1.25
7	3	7	13.49	1.07
7	3	7	13.90	1.40
7	3	7	12.56	1.87
-8	-3	-7	10.04	0.93
-8	-3	-7	8.86	1.21
8	3	7	8.09	1.60
8	3	7	8.99	1.20
-9	-3	-7	3.22	0.88

-9	-3	-7	4.00	0.71
9	3	7	2.22	1.44
9	3	7	4.69	1.08
18	-4	-7	9.15	3.40
18	-4	-7	6.66	2.95
-18	4	7	5.71	1.25
17	-4	-7	15.71	2.21
17	-4	-7	13.39	2.43
-17	4	7	9.96	1.35
16	-4	-7	15.69	2.92
16	-4	-7	17.45	2.06
-16	4	7	17.17	1.34
15	-4	-7	26.17	2.17
15	-4	-7	30.62	2.88
-15	4	7	31.52	1.59
-15	4	7	28.03	1.85
14	-4	-7	13.11	2.19
14	-4	-7	9.64	1.36
-14	4	7	9.96	1.36
-14	4	7	10.82	0.99
13	-4	-7	8.77	1.35
13	-4	-7	12.65	1.94
-13	4	7	10.80	1.28
-13	4	7	10.56	0.89
12	-4	-7	16.73	1.40
12	-4	-7	15.20	1.84
-12	4	7	19.77	1.36
-12	4	7	17.38	0.99
11	-4	-7	9.11	0.67
11	-4	-7	9.26	1.13
11	-4	-7	8.32	1.47
11	-4	-7	9.49	0.71
-11	4	7	9.13	0.59
-11	4	7	9.67	1.06
-11	4	7	7.81	0.70
10	-4	-7	3.48	0.49
10	-4	-7	4.06	1.00
10	-4	-7	3.64	0.70
10	-4	-7	3.33	0.52
-10	4	7	3.59	0.73
-10	4	7	3.35	0.45
-10	4	7	3.93	0.55
-10	4	7	2.91	0.60
9	-4	-7	9.90	1.04

9	-4	-7	8.50	1.25
9	-4	-7	9.25	0.55
9	-4	-7	10.01	0.84
9	-4	-7	9.43	0.59
-9	4	7	9.39	0.94
-9	4	7	12.07	1.04
-9	4	7	10.13	0.56
8	-4	-7	13.11	1.26
8	-4	-7	12.34	0.60
8	-4	-7	13.45	0.64
8	-4	-7	13.77	1.12
-8	4	7	13.39	1.32
-8	4	7	14.67	1.01
-8	4	7	13.55	0.64
7	-4	-7	2.68	0.58
7	-4	-7	3.75	0.72
7	-4	-7	3.70	0.58
7	-4	-7	3.37	0.33
7	-4	-7	3.40	0.36
-7	4	7	3.50	0.40
-7	4	7	3.82	0.66
6	-4	-7	0.09	0.50
6	-4	-7	0.04	0.15
6	-4	-7	0.61	0.42
6	-4	-7	-0.28	0.41
6	-4	-7	0.15	0.18
-6	4	7	-0.14	0.42
-6	4	7	0.03	0.21
-6	4	7	0.56	1.26
5	-4	-7	0.86	0.45
5	-4	-7	0.20	0.15
5	-4	-7	0.95	0.57
5	-4	-7	0.37	0.18
5	-4	-7	0.41	0.45
-5	4	7	0.23	0.24
-5	4	7	0.95	0.45
-5	4	7	0.35	0.60
4	-4	-7	10.36	1.00
4	-4	-7	10.82	0.49
4	-4	-7	10.64	0.96
4	-4	-7	10.09	0.47
4	-4	-7	10.55	1.00
-4	4	7	9.78	0.70
-4	4	7	9.13	0.94

-4	4	7	9.86	1.16
3	-4	-7	2.20	0.62
3	-4	-7	3.32	0.59
3	-4	-7	1.99	0.57
3	-4	-7	2.15	0.29
-3	4	7	3.03	0.52
-3	4	7	2.36	0.23
-3	4	7	2.02	0.73
-3	4	7	3.79	0.66
2	-4	-7	5.55	0.80
2	-4	-7	6.12	0.91
2	-4	-7	5.30	0.33
2	-4	-7	6.32	0.93
-2	4	7	6.74	0.91
-2	4	7	5.67	0.29
-2	4	7	5.38	0.66
-2	4	7	4.27	0.93
1	-4	-7	11.69	0.90
1	-4	-7	8.56	1.17
1	-4	-7	11.11	0.47
1	-4	-7	11.06	1.07
-1	4	7	11.40	1.09
-1	4	7	11.17	0.46
-1	4	7	12.27	0.89
-1	4	7	10.75	1.48
0	-4	-7	35.17	1.81
0	-4	-7	29.58	1.91
0	-4	-7	29.58	1.22
0	-4	-7	33.46	1.55
0	4	7	33.00	1.09
0	4	7	32.89	1.74
0	4	7	35.05	2.18
0	4	7	33.94	1.59
-1	-4	-7	13.15	1.49
-1	-4	-7	10.49	0.92
-1	-4	-7	10.18	0.48
1	4	7	9.24	0.92
1	4	7	10.44	0.98
1	4	7	8.99	1.20
1	4	7	11.03	0.52
1	4	7	10.47	1.69
-2	-4	-7	9.94	0.52
-2	-4	-7	10.80	1.68
-2	-4	-7	12.04	0.96

2	4	7	11.75	1.07
2	4	7	10.86	1.38
2	4	7	9.64	1.01
2	4	7	13.94	0.66
2	4	7	10.39	1.58
-3	-4	-7	9.23	0.50
-3	-4	-7	8.98	0.88
3	4	7	8.87	0.58
3	4	7	9.85	1.12
3	4	7	7.88	1.04
-4	-4	-7	1.24	0.60
-4	-4	-7	2.49	0.33
4	4	7	0.57	0.72
4	4	7	2.21	0.74
4	4	7	2.24	0.39
-5	-4	-7	1.21	0.58
-5	-4	-7	0.50	0.33
5	4	7	0.65	0.42
5	4	7	0.31	0.67
5	4	7	0.23	0.72
-6	-4	-7	5.41	0.85
-6	-4	-7	4.68	0.65
6	4	7	5.55	0.79
6	4	7	5.91	1.00
6	4	7	5.61	1.19
-7	-4	-7	9.88	1.26
-7	-4	-7	8.47	0.85
7	4	7	10.27	1.49
7	4	7	10.41	1.90
-8	-4	-7	7.30	0.96
-8	-4	-7	7.92	1.28
8	4	7	9.21	1.65
8	4	7	7.10	1.19
-9	-4	-7	3.87	0.94
-9	-4	-7	3.63	0.83
9	4	7	4.91	1.17
9	4	7	3.55	1.67
18	-5	-7	5.99	2.62
18	-5	-7	7.53	1.93
-18	5	7	4.29	1.05
17	-5	-7	11.92	2.39
17	-5	-7	5.73	1.41
-17	5	7	8.22	1.25
16	-5	-7	6.19	2.00

16	-5	-7	11.22	1.87
-16	5	7	9.59	1.18
15	-5	-7	3.47	1.08
15	-5	-7	5.65	1.74
-15	5	7	3.21	0.69
-15	5	7	3.31	1.05
14	-5	-7	0.22	1.42
14	-5	-7	4.04	1.07
-14	5	7	2.73	0.60
-14	5	7	4.43	0.97
13	-5	-7	17.99	2.18
13	-5	-7	18.77	1.61
-13	5	7	17.66	1.55
12	-5	-7	0.22	0.63
12	-5	-7	-1.30	0.97
-12	5	7	-0.97	0.36
-12	5	7	-0.95	0.72
11	-5	-7	-0.37	0.62
11	-5	-7	0.77	0.42
11	-5	-7	0.36	0.97
11	-5	-7	0.21	0.35
-11	5	7	0.00	0.71
-11	5	7	0.69	0.36
-11	5	7	0.30	0.25
10	-5	-7	7.30	1.03
10	-5	-7	6.46	0.57
10	-5	-7	6.91	0.80
10	-5	-7	7.21	1.19
-10	5	7	6.75	0.62
-10	5	7	5.66	0.91
-10	5	7	6.11	0.50
-10	5	7	8.15	1.19
9	-5	-7	7.62	0.97
9	-5	-7	6.47	1.01
9	-5	-7	8.75	0.55
9	-5	-7	8.36	1.21
-9	5	7	8.24	0.50
-9	5	7	9.34	1.18
-9	5	7	7.97	0.92
8	-5	-7	-0.18	0.22
8	-5	-7	-0.34	0.63
8	-5	-7	0.00	0.49
8	-5	-7	0.71	0.46
8	-5	-7	0.03	0.19

-8	5	7	1.26	0.69
-8	5	7	0.21	0.22
-8	5	7	0.27	0.49
7	-5	-7	0.62	0.49
7	-5	-7	-0.02	0.16
7	-5	-7	0.77	0.62
7	-5	-7	0.85	0.51
-7	5	7	-0.22	0.67
-7	5	7	0.03	0.23
-7	5	7	0.32	0.46
6	-5	-7	1.69	0.58
6	-5	-7	2.24	0.30
6	-5	-7	2.09	0.28
6	-5	-7	2.64	0.64
6	-5	-7	2.51	0.68
-6	5	7	2.36	0.57
-6	5	7	2.58	0.81
-6	5	7	2.16	0.38
5	-5	-7	1.18	0.64
5	-5	-7	0.43	0.52
5	-5	-7	1.14	0.20
5	-5	-7	0.11	0.55
5	-5	-7	1.13	0.19
-5	5	7	0.55	0.30
-5	5	7	1.75	0.54
-5	5	7	1.63	0.77
4	-5	-7	-0.29	0.14
4	-5	-7	0.18	0.18
4	-5	-7	0.41	0.56
4	-5	-7	-0.13	0.43
4	-5	-7	0.79	0.58
-4	5	7	-0.01	0.47
-4	5	7	0.09	0.31
-4	5	7	-0.59	0.68
3	-5	-7	7.87	1.06
3	-5	-7	9.45	1.15
3	-5	-7	8.04	0.91
-3	5	7	9.62	1.03
-3	5	7	7.49	0.76
-3	5	7	6.58	1.09
2	-5	-7	14.06	1.06
2	-5	-7	13.49	1.33
2	-5	-7	14.14	1.27
-2	5	7	14.13	1.20

-2	5	7	15.82	1.65
-2	5	7	13.13	1.00
1	-5	-7	1.62	0.63
1	-5	-7	1.63	0.82
1	-5	-7	1.35	0.51
-1	5	7	3.07	1.04
-1	5	7	1.46	0.51
-1	5	7	1.69	0.66
0	-5	-7	17.84	1.47
0	-5	-7	17.41	1.16
0	-5	-7	18.15	1.74
0	5	7	14.73	1.96
0	5	7	17.99	1.43
0	5	7	18.37	1.22
-1	-5	-7	38.95	1.40
-1	-5	-7	43.60	3.65
-1	-5	-7	41.36	1.82
1	5	7	41.86	1.91
1	5	7	41.23	2.13
1	5	7	42.90	1.36
1	5	7	40.89	2.77
1	5	7	43.43	2.01
-2	-5	-7	10.97	0.52
-2	-5	-7	11.96	0.99
2	5	7	9.99	1.09
2	5	7	11.89	0.58
2	5	7	12.33	1.18
-3	-5	-7	4.12	0.41
-3	-5	-7	4.43	0.81
3	5	7	4.65	0.49
3	5	7	4.70	0.88
-4	-5	-7	9.41	0.62
-4	-5	-7	10.09	1.07
4	5	7	11.23	0.71
4	5	7	11.66	1.33
-5	-5	-7	12.21	1.17
-5	-5	-7	13.68	0.77
5	5	7	15.68	1.57
5	5	7	12.17	0.84
5	5	7	13.43	1.55
-6	-5	-7	9.80	0.81
-6	-5	-7	11.23	1.23
6	5	7	9.75	1.56
-7	-5	-7	10.21	0.95

-7	-5	-7	8.45	1.06
7	5	7	10.41	1.67
7	5	7	12.71	2.07
-8	-5	-7	8.34	1.37
8	5	7	5.13	1.59
8	5	7	5.54	1.24
18	-6	-7	6.63	1.71
18	-6	-7	9.20	2.74
-18	6	7	7.31	1.04
17	-6	-7	5.64	1.34
17	-6	-7	4.99	2.26
-17	6	7	4.08	0.84
16	-6	-7	10.68	2.18
16	-6	-7	9.07	1.49
-16	6	7	9.49	1.14
15	-6	-7	10.09	2.08
15	-6	-7	8.25	1.33
-15	6	7	8.68	1.02
14	-6	-7	1.04	0.85
14	-6	-7	1.19	1.43
-14	6	7	-0.46	0.98
-14	6	7	0.75	0.51
13	-6	-7	0.83	1.23
13	-6	-7	0.32	0.75
-13	6	7	1.53	0.56
-13	6	7	1.80	0.93
12	-6	-7	6.39	1.05
12	-6	-7	9.65	1.54
-12	6	7	8.76	1.50
-12	6	7	7.76	0.77
11	-6	-7	0.65	0.37
11	-6	-7	0.78	0.51
11	-6	-7	1.21	0.71
11	-6	-7	-0.20	1.02
-11	6	7	3.76	0.97
-11	6	7	-0.14	0.34
10	-6	-7	3.11	0.65
10	-6	-7	3.38	0.52
10	-6	-7	3.60	0.80
10	-6	-7	2.12	1.02
-10	6	7	3.36	1.00
-10	6	7	2.02	0.62
-10	6	7	2.79	0.44
-10	6	7	2.86	0.38

9	-6	-7	2.17	0.61
9	-6	-7	1.59	0.91
9	-6	-7	1.25	0.59
9	-6	-7	1.42	0.31
-9	6	7	1.47	0.31
-9	6	7	1.77	0.64
-9	6	7	1.91	0.89
8	-6	-7	4.80	0.79
8	-6	-7	4.15	0.99
8	-6	-7	4.63	0.81
8	-6	-7	5.13	0.44
8	-6	-7	5.93	0.41
-8	6	7	6.05	1.14
-8	6	7	5.17	0.74
-8	6	7	5.05	0.52
7	-6	-7	5.05	0.36
7	-6	-7	4.76	0.39
7	-6	-7	7.13	1.03
7	-6	-7	5.28	0.96
7	-6	-7	4.50	0.79
-7	6	7	6.11	0.87
-7	6	7	5.54	0.58
-7	6	7	5.31	1.03
6	-6	-7	17.73	1.29
6	-6	-7	17.03	0.65
6	-6	-7	17.25	1.32
6	-6	-7	17.26	0.67
6	-6	-7	15.86	1.47
-6	6	7	17.61	1.68
-6	6	7	16.21	0.94
-6	6	7	16.09	1.19
5	-6	-7	11.67	0.48
5	-6	-7	12.47	1.34
5	-6	-7	11.54	0.52
5	-6	-7	10.63	1.21
5	-6	-7	13.05	1.13
-5	6	7	11.09	1.52
-5	6	7	9.44	1.08
-5	6	7	11.77	0.86
4	-6	-7	0.51	0.13
4	-6	-7	0.61	0.62
4	-6	-7	0.52	0.51
4	-6	-7	0.52	0.66
-4	6	7	0.36	0.40

-4	6	7	0.32	0.57
-4	6	7	-0.86	0.79
3	-6	-7	10.44	1.02
3	-6	-7	9.91	1.34
3	-6	-7	10.25	1.28
-3	6	7	12.69	1.69
-3	6	7	9.97	0.92
-3	6	7	8.95	1.12
2	-6	-7	7.91	1.15
2	-6	-7	7.16	0.92
2	-6	-7	8.44	1.23
-2	6	7	7.71	1.12
-2	6	7	7.23	1.46
-2	6	7	7.37	0.91
1	-6	-7	26.89	1.48
1	-6	-7	29.24	1.88
1	-6	-7	26.28	2.05
-1	6	7	26.98	1.73
-1	6	7	28.00	1.57
-1	6	7	29.07	2.39
0	-6	-7	30.48	1.93
0	-6	-7	29.50	1.52
0	6	7	27.10	1.84
0	6	7	33.25	2.60
0	6	7	29.83	1.68
-1	-6	-7	6.22	0.90
-1	-6	-7	7.96	1.40
1	6	7	8.02	1.12
-2	-6	-7	1.83	0.28
-2	-6	-7	1.38	0.58
2	6	7	0.11	0.91
-3	-6	-7	25.36	1.47
-3	-6	-7	25.59	0.95
3	6	7	24.09	1.69
3	6	7	25.93	1.78
-4	-6	-7	27.36	1.54
-4	-6	-7	22.26	0.98
4	6	7	23.78	1.83
4	6	7	23.37	1.83
-5	-6	-7	12.36	0.84
-5	-6	-7	13.23	1.28
5	6	7	14.03	1.70
5	6	7	15.56	1.74
-6	-6	-7	17.35	1.47

-6	-6	-7	18.45	1.07
6	6	7	17.44	1.88
6	6	7	17.60	2.00
-7	-6	-7	8.73	1.38
7	6	7	9.54	1.46
7	6	7	8.09	1.57
-8	-6	-7	3.87	1.08
8	6	7	7.44	1.37
18	-7	-7	7.54	2.83
18	-7	-7	5.19	1.60
-18	7	7	5.12	0.95
17	-7	-7	6.92	2.34
17	-7	-7	2.48	1.31
-17	7	7	5.52	0.91
16	-7	-7	6.86	1.44
-16	7	7	5.68	0.83
15	-7	-7	0.70	1.71
15	-7	-7	2.45	1.09
-15	7	7	1.34	0.64
14	-7	-7	1.80	1.05
14	-7	-7	2.64	1.58
-14	7	7	2.22	1.05
-14	7	7	2.51	0.59
13	-7	-7	3.47	1.49
13	-7	-7	2.05	0.92
-13	7	7	4.08	0.60
-13	7	7	4.52	1.10
12	-7	-7	-0.26	1.20
-12	7	7	0.77	0.40
-12	7	7	-1.11	1.00
-12	7	7	0.16	0.24
-12	7	7	-0.30	0.43
11	-7	-7	0.97	0.40
11	-7	-7	2.28	1.21
11	-7	-7	1.20	0.74
-11	7	7	1.76	1.06
-11	7	7	2.01	0.30
-11	7	7	1.07	0.42
10	-7	-7	0.74	0.67
10	-7	-7	0.87	0.62
10	-7	-7	-0.04	0.31
10	-7	-7	-0.39	1.05
-10	7	7	0.13	0.25
-10	7	7	0.18	0.35

-10	7	7	0.90	0.97
9	-7	-7	-0.16	0.83
9	-7	-7	-0.19	0.28
9	-7	-7	0.39	0.61
9	-7	-7	0.02	0.66
-9	7	7	1.86	1.06
-9	7	7	-0.24	0.27
-9	7	7	-1.23	0.54
8	-7	-7	0.47	0.61
8	-7	-7	0.51	0.25
8	-7	-7	-0.09	0.18
8	-7	-7	-0.97	0.77
8	-7	-7	-0.35	0.63
-8	7	7	0.12	0.98
-8	7	7	0.48	0.32
-8	7	7	-0.77	0.53
7	-7	-7	27.75	1.01
7	-7	-7	28.20	1.94
7	-7	-7	28.96	1.00
7	-7	-7	28.29	1.70
7	-7	-7	27.96	1.76
-7	7	7	28.07	1.31
-7	7	7	27.80	2.13
-7	7	7	30.00	1.60
6	-7	-7	15.21	1.49
6	-7	-7	19.10	1.68
6	-7	-7	18.58	1.36
6	-7	-7	17.49	0.68
6	-7	-7	17.85	0.68
-6	7	7	20.38	1.95
-6	7	7	17.80	1.28
-6	7	7	17.46	1.08
5	-7	-7	1.91	0.27
5	-7	-7	2.04	0.85
5	-7	-7	2.22	0.69
5	-7	-7	2.58	0.78
5	-7	-7	1.94	0.24
-5	7	7	2.58	0.73
-5	7	7	1.63	0.48
-5	7	7	3.94	1.08
4	-7	-7	1.73	0.78
4	-7	-7	0.68	0.59
4	-7	-7	0.53	0.76
-4	7	7	1.58	0.50

-4	7	7	0.33	0.64
-4	7	7	1.84	1.11
3	-7	-7	14.77	1.55
3	-7	-7	14.69	1.70
3	-7	-7	14.36	1.24
-3	7	7	17.40	2.04
-3	7	7	16.17	1.22
-3	7	7	17.26	1.40
2	-7	-7	14.30	1.73
2	-7	-7	11.67	1.07
2	-7	-7	10.36	1.42
-2	7	7	12.11	1.12
-2	7	7	10.28	1.36
-2	7	7	11.04	1.71
1	-7	-7	2.43	0.89
1	-7	-7	2.77	0.66
-1	7	7	2.37	0.67
-1	7	7	0.87	0.83
-1	7	7	5.62	1.68
0	-7	-7	8.20	1.01
0	-7	-7	7.94	1.21
0	7	7	10.89	1.22
-1	-7	-7	13.96	1.14
1	7	7	11.66	1.34
-2	-7	-7	4.12	0.72
2	7	7	3.90	0.91
2	7	7	4.64	0.98
-3	-7	-7	7.73	1.09
3	7	7	6.45	1.12
3	7	7	7.01	1.15
-4	-7	-7	7.16	1.16
-4	-7	-7	9.24	0.74
4	7	7	7.20	1.25
4	7	7	8.75	1.34
-5	-7	-7	13.89	1.43
5	7	7	11.56	1.83
5	7	7	13.30	1.80
-6	-7	-7	8.00	1.09
6	7	7	12.92	1.95
6	7	7	7.88	1.37
-7	-7	-7	5.98	1.10
7	7	7	6.61	1.86
7	7	7	5.75	1.41
18	-8	-7	7.42	2.78

18	-8	-7	3.65	1.65
-18	8	7	5.14	0.96
17	-8	-7	0.21	1.29
17	-8	-7	0.74	2.35
-17	8	7	0.56	0.73
16	-8	-7	4.84	2.19
16	-8	-7	6.29	1.35
-16	8	7	5.54	0.83
15	-8	-7	2.42	1.85
15	-8	-7	3.45	1.12
-15	8	7	3.18	0.66
14	-8	-7	-0.22	0.92
14	-8	-7	0.44	1.59
-14	8	7	1.01	0.56
13	-8	-7	3.16	1.51
13	-8	-7	1.53	0.92
-13	8	7	2.98	1.22
12	-8	-7	3.33	1.42
12	-8	-7	1.72	0.84
-12	8	7	5.03	1.30
-12	8	7	4.12	0.59
-12	8	7	3.30	0.55
-12	8	7	2.74	0.46
11	-8	-7	3.84	0.49
11	-8	-7	4.43	1.43
11	-8	-7	4.04	0.94
-11	8	7	3.98	1.30
-11	8	7	3.13	0.47
-11	8	7	3.47	0.51
-11	8	7	4.46	0.68
10	-8	-7	11.42	0.74
10	-8	-7	11.72	1.95
10	-8	-7	12.06	1.37
10	-8	-7	10.06	1.37
-10	8	7	11.18	1.66
-10	8	7	11.72	0.76
-10	8	7	11.07	0.73
9	-8	-7	-0.70	0.72
9	-8	-7	0.97	0.32
9	-8	-7	0.29	0.61
9	-8	-7	0.54	1.03
-9	8	7	-0.14	0.77
-9	8	7	3.15	1.25
-9	8	7	0.63	0.34

8	-8	-7	4.00	1.19
8	-8	-7	3.03	0.81
8	-8	-7	4.01	0.44
8	-8	-7	4.24	0.93
8	-8	-7	4.07	0.37
-8	8	7	4.59	0.81
-8	8	7	5.15	1.40
-8	8	7	4.18	0.58
7	-8	-7	3.32	0.77
7	-8	-7	5.76	0.36
7	-8	-7	7.06	1.11
7	-8	-7	3.96	0.49
7	-8	-7	3.54	1.10
-7	8	7	4.57	1.30
-7	8	7	5.04	0.82
-7	8	7	4.53	0.78
6	-8	-7	2.68	0.96
6	-8	-7	2.25	1.03
6	-8	-7	2.19	0.30
6	-8	-7	2.16	0.79
6	-8	-7	3.83	0.35
-6	8	7	2.25	0.74
-6	8	7	2.90	0.59
-6	8	7	5.62	1.42
5	-8	-7	37.69	1.94
5	-8	-7	31.97	2.21
5	-8	-7	34.73	2.30
-5	8	7	37.80	1.81
-5	8	7	36.27	1.97
-5	8	7	37.80	2.73
4	-8	-7	20.26	1.85
4	-8	-7	14.46	1.90
4	-8	-7	17.19	1.35
-4	8	7	21.37	2.36
-4	8	7	16.75	1.29
-4	8	7	17.74	1.53
3	-8	-7	3.97	0.99
3	-8	-7	5.92	0.98
-3	8	7	5.12	1.38
-3	8	7	6.29	1.01
-3	8	7	5.79	1.03
2	-8	-7	19.86	1.90
2	-8	-7	25.12	1.46
-2	8	7	21.38	1.53

1	-8	-7	22.62	1.40
1	-8	-7	21.16	1.92
-1	8	7	21.45	1.59
0	-8	-7	19.59	1.99
0	-8	-7	19.71	1.35
0	8	7	18.91	1.60
-1	-8	-7	19.55	1.38
-1	-8	-7	21.41	2.11
1	8	7	21.60	1.77
-2	-8	-7	4.96	0.81
2	8	7	5.18	1.01
2	8	7	3.92	1.00
-3	-8	-7	18.10	1.48
3	8	7	23.48	2.00
3	8	7	20.02	1.92
-4	-8	-7	15.26	1.45
4	8	7	12.80	1.90
4	8	7	14.88	1.81
-5	-8	-7	11.23	1.43
5	8	7	9.75	1.46
5	8	7	10.95	1.95
-6	-8	-7	5.32	1.02
6	8	7	3.17	1.44
6	8	7	4.47	1.35
18	-9	-7	1.86	1.86
18	-9	-7	0.50	2.77
-18	9	7	1.29	0.89
17	-9	-7	0.41	1.40
17	-9	-7	10.87	2.77
-17	9	7	1.26	0.80
16	-9	-7	-1.24	1.18
16	-9	-7	-2.62	1.92
-16	9	7	0.54	0.66
15	-9	-7	-0.39	1.08
15	-9	-7	2.33	2.29
-15	9	7	0.21	0.60
14	-9	-7	0.41	1.06
14	-9	-7	1.86	1.70
-14	9	7	1.59	0.57
13	-9	-7	1.08	0.96
13	-9	-7	3.23	1.69
-13	9	7	1.77	0.31
-13	9	7	2.48	0.56
12	-9	-7	2.65	0.97

12	-9	-7	4.60	1.71
-12	9	7	3.62	0.48
-12	9	7	5.47	1.81
-12	9	7	3.92	0.59
11	-9	-7	1.21	0.86
11	-9	-7	0.66	0.42
11	-9	-7	1.79	1.39
-11	9	7	0.55	0.40
-11	9	7	1.23	0.36
-11	9	7	0.22	0.70
-11	9	7	4.52	1.56
10	-9	-7	1.14	0.79
10	-9	-7	1.46	0.40
10	-9	-7	1.05	1.23
-10	9	7	1.44	0.71
-10	9	7	1.95	0.43
-10	9	7	0.93	1.41
-10	9	7	1.67	0.42
9	-9	-7	0.24	0.31
9	-9	-7	-0.19	0.72
9	-9	-7	-0.32	1.08
9	-9	-7	2.35	0.93
-9	9	7	0.17	1.38
-9	9	7	0.19	0.40
8	-9	-7	0.61	0.95
8	-9	-7	0.77	0.28
8	-9	-7	0.49	0.22
8	-9	-7	1.10	0.73
8	-9	-7	-0.81	1.02
-8	9	7	-0.01	0.46
-8	9	7	-0.25	1.32
7	-9	-7	0.85	0.95
7	-9	-7	1.32	1.06
7	-9	-7	0.76	0.74
7	-9	-7	0.73	0.20
7	-9	-7	0.71	0.24
-7	9	7	0.87	1.39
-7	9	7	1.16	0.72
-7	9	7	0.39	0.50
6	-9	-7	3.66	1.08
6	-9	-7	3.02	0.81
6	-9	-7	2.36	1.15
-6	9	7	2.98	1.50
-6	9	7	3.11	0.84

-6	9	7	2.49	0.63
5	-9	-7	-0.48	1.00
5	-9	-7	0.09	0.65
5	-9	-7	1.28	1.03
-5	9	7	0.00	0.52
-5	9	7	0.43	1.36
-5	9	7	0.25	0.83
4	-9	-7	1.50	0.72
4	-9	-7	2.55	1.10
-4	9	7	3.75	1.20
-4	9	7	2.85	0.77
3	-9	-7	34.45	1.84
3	-9	-7	30.66	2.32
-3	9	7	32.54	1.94
2	-9	-7	8.33	1.43
2	-9	-7	7.93	1.14
-2	9	7	10.00	1.59
1	-9	-7	0.81	1.10
1	-9	-7	0.62	0.64
-1	9	7	0.88	0.73
0	-9	-7	7.78	1.12
0	-9	-7	5.05	1.32
0	9	7	8.70	1.53
-1	-9	-7	23.10	1.53
-1	-9	-7	23.06	2.38
1	9	7	22.65	1.97
-2	-9	-7	17.24	1.44
2	9	7	18.16	1.89
2	9	7	16.92	1.76
-3	-9	-7	12.40	1.45
3	9	7	13.14	1.82
3	9	7	14.31	2.01
-4	-9	-7	12.93	1.48
4	9	7	13.17	2.07
4	9	7	13.27	1.93
-5	-9	-7	8.05	1.15
5	9	7	13.00	2.15
5	9	7	7.65	1.50
17	-10	-7	-0.10	1.44
17	-10	-7	-3.11	2.48
-17	10	7	-0.53	0.76
16	-10	-7	0.75	1.30
16	-10	-7	-0.93	2.25
-16	10	7	-0.94	0.69

15	-10	-7	-0.06	1.29
15	-10	-7	-2.00	1.87
-15	10	7	0.31	0.64
14	-10	-7	3.02	1.18
14	-10	-7	1.46	1.80
-14	10	7	1.60	0.60
13	-10	-7	6.28	1.31
13	-10	-7	10.88	2.10
-13	10	7	9.28	0.61
-13	10	7	8.82	0.90
12	-10	-7	2.07	1.55
12	-10	-7	2.69	0.54
12	-10	-7	0.17	0.93
-12	10	7	1.78	0.53
-12	10	7	1.84	0.42
-12	10	7	2.26	0.77
11	-10	-7	1.25	0.44
11	-10	-7	1.74	1.45
11	-10	-7	-0.42	0.82
-11	10	7	1.85	0.83
-11	10	7	0.90	0.43
-11	10	7	0.27	0.42
10	-10	-7	6.01	1.07
10	-10	-7	7.27	0.69
10	-10	-7	7.47	1.71
-10	10	7	7.62	0.71
-10	10	7	8.55	1.10
-10	10	7	7.92	0.87
9	-10	-7	13.54	0.64
9	-10	-7	13.16	1.52
9	-10	-7	12.33	0.73
9	-10	-7	15.48	2.26
-9	10	7	14.94	1.46
-9	10	7	11.52	1.04
8	-10	-7	23.83	1.83
8	-10	-7	26.39	0.95
8	-10	-7	26.85	1.00
8	-10	-7	25.20	2.52
-8	10	7	25.78	1.53
7	-10	-7	12.58	1.54
7	-10	-7	12.81	2.01
7	-10	-7	12.33	2.13
7	-10	-7	13.28	0.58
-7	10	7	12.61	1.21

6	-10	-7	5.24	1.06
6	-10	-7	5.58	1.45
-6	10	7	6.24	0.93
5	-10	-7	17.26	2.13
5	-10	-7	19.07	1.56
-5	10	7	19.33	1.56
4	-10	-7	33.91	1.94
4	-10	-7	33.87	2.59
-4	10	7	37.11	2.10
3	-10	-7	12.69	1.98
3	-10	-7	9.05	1.21
-3	10	7	8.75	1.35
2	-10	-7	11.45	1.97
2	-10	-7	8.38	1.35
-2	10	7	5.91	1.02
1	-10	-7	8.69	1.58
1	-10	-7	6.71	0.97
-1	10	7	4.42	1.13
0	-10	-7	18.26	1.49
0	-10	-7	20.62	2.37
0	10	7	17.82	1.96
-1	-10	-7	16.17	1.47
-1	-10	-7	14.04	2.45
1	10	7	15.24	1.99
-2	-10	-7	18.57	1.59
2	10	7	14.61	1.88
2	10	7	14.94	2.07
-3	-10	-7	14.47	1.56
3	10	7	10.97	1.98
3	10	7	17.56	2.19
-4	-10	-7	11.57	1.61
4	10	7	7.55	1.50
4	10	7	7.55	1.52
17	-11	-7	1.94	1.60
17	-11	-7	-3.33	2.47
-17	11	7	0.22	0.83
16	-11	-7	-1.86	2.34
16	-11	-7	-2.84	1.23
-16	11	7	1.54	0.76
15	-11	-7	2.41	1.39
15	-11	-7	4.89	2.37
-15	11	7	3.60	0.74
14	-11	-7	-0.01	1.21
14	-11	-7	1.80	1.91

-14	11	7	-1.20	0.58
13	-11	-7	4.37	1.30
13	-11	-7	2.92	1.89
-13	11	7	1.52	0.58
-13	11	7	2.17	0.43
12	-11	-7	1.66	1.06
12	-11	-7	2.23	2.04
12	-11	-7	2.03	0.54
-12	11	7	2.03	0.87
-12	11	7	2.67	0.52
-12	11	7	1.17	0.50
11	-11	-7	-1.09	0.85
11	-11	-7	-0.65	1.49
11	-11	-7	0.80	0.44
-11	11	7	-0.31	0.50
-11	11	7	0.57	0.44
-11	11	7	0.13	0.90
10	-11	-7	3.22	0.48
10	-11	-7	4.59	1.66
10	-11	-7	2.57	1.18
-10	11	7	2.36	1.00
-10	11	7	3.17	0.68
9	-11	-7	5.95	0.48
9	-11	-7	4.64	1.11
9	-11	-7	8.49	1.90
9	-11	-7	5.96	0.61
-9	11	7	7.30	1.06
-9	11	7	6.78	1.13
8	-11	-7	7.19	1.75
8	-11	-7	5.64	1.17
8	-11	-7	6.65	0.43
8	-11	-7	5.33	0.53
-8	11	7	6.01	1.14
-8	11	7	7.55	1.18
7	-11	-7	17.40	1.69
7	-11	-7	16.65	2.44
-7	11	7	17.60	1.49
6	-11	-7	35.38	2.12
-6	11	7	37.56	2.11
5	-11	-7	12.71	2.26
5	-11	-7	10.44	1.45
-5	11	7	14.15	1.50
4	-11	-7	16.68	2.30
4	-11	-7	14.50	1.46

-4	11	7	13.51	1.57
3	-11	-7	16.37	1.81
3	-11	-7	15.00	2.31
-3	11	7	16.68	1.78
2	-11	-7	16.12	1.47
2	-11	-7	16.78	2.38
-2	11	7	17.02	1.86
1	-11	-7	11.18	1.40
1	-11	-7	13.43	2.38
-1	11	7	12.85	1.87
0	-11	-7	16.77	2.60
0	-11	-7	17.90	1.61
0	11	7	20.99	2.18
-1	-11	-7	20.73	1.71
-1	-11	-7	16.44	2.68
1	11	7	19.50	2.36
-2	-11	-7	14.35	1.60
2	11	7	11.90	1.97
2	11	7	14.04	2.25
-3	-11	-7	6.52	1.16
3	11	7	7.49	1.58
3	11	7	7.00	1.56
16	-12	-7	0.42	2.55
16	-12	-7	1.72	1.61
-16	12	7	1.09	0.78
15	-12	-7	1.67	1.43
15	-12	-7	1.57	2.32
-15	12	7	0.00	0.70
14	-12	-7	2.42	2.14
14	-12	-7	0.24	1.29
-14	12	7	1.50	0.65
13	-12	-7	7.64	1.50
13	-12	-7	7.03	2.29
-13	12	7	7.13	0.84
-13	12	7	6.90	0.93
12	-12	-7	6.21	0.89
12	-12	-7	10.59	2.31
12	-12	-7	11.55	1.95
-12	12	7	9.79	0.88
-12	12	7	6.39	0.98
11	-12	-7	7.78	1.35
11	-12	-7	4.00	1.87
11	-12	-7	4.32	0.60
-11	12	7	4.21	1.14

-11	12	7	6.08	0.81
-11	12	7	4.84	0.79
10	-12	-7	3.58	1.78
10	-12	-7	1.79	0.36
10	-12	-7	0.57	1.07
10	-12	-7	1.51	0.44
-10	12	7	3.26	1.13
-10	12	7	2.21	0.73
9	-12	-7	6.86	0.60
9	-12	-7	5.88	1.74
9	-12	-7	8.81	1.38
9	-12	-7	5.15	0.48
-9	12	7	5.33	0.93
8	-12	-7	10.73	1.98
-8	12	7	10.53	1.41
7	-12	-7	2.49	1.60
6	-12	-7	15.24	2.26
-6	12	7	10.55	1.54
5	-12	-7	21.26	1.81
5	-12	-7	28.80	2.79
-5	12	7	23.15	1.97
4	-12	-7	15.08	2.53
4	-12	-7	13.45	1.53
-4	12	7	12.33	1.79
3	-12	-7	10.22	1.96
3	-12	-7	7.73	1.11
-3	12	7	6.27	1.28
2	-12	-7	16.09	1.59
2	-12	-7	16.61	2.69
-2	12	7	16.82	2.09
1	-12	-7	17.50	2.33
1	-12	-7	18.61	1.65
-1	12	7	20.05	2.20
0	-12	-7	16.13	1.72
0	-12	-7	18.81	2.80
0	12	7	16.31	2.24
-1	-12	-7	7.26	1.93
-1	-12	-7	6.03	1.18
1	12	7	7.14	1.60
-2	-12	-7	5.05	1.15
2	12	7	5.17	2.67
16	-13	-7	-0.81	1.75
16	-13	-7	1.78	2.68
-16	13	7	3.07	0.86

15	-13	-7	5.08	2.76
15	-13	-7	6.21	1.83
-15	13	7	3.05	0.79
14	-13	-7	1.27	1.58
14	-13	-7	0.47	2.21
-14	13	7	2.36	0.72
13	-13	-7	0.80	1.40
13	-13	-7	4.65	2.40
-13	13	7	1.15	0.64
12	-13	-7	8.71	2.31
12	-13	-7	8.70	1.56
-12	13	7	9.34	1.19
-12	13	7	10.50	0.97
11	-13	-7	13.10	1.81
11	-13	-7	9.18	2.18
-11	13	7	11.05	1.39
10	-13	-7	7.17	0.59
10	-13	-7	6.50	2.04
10	-13	-7	6.19	1.42
-10	13	7	8.11	1.14
9	-13	-7	8.04	1.97
9	-13	-7	8.14	1.46
-9	13	7	8.35	1.20
8	-13	-7	6.42	1.35
8	-13	-7	5.17	1.90
-8	13	7	7.23	1.14
7	-13	-7	10.03	2.15
7	-13	-7	6.86	1.27
-7	13	7	9.10	1.37
6	-13	-7	15.48	1.80
6	-13	-7	17.95	2.94
-6	13	7	16.30	1.87
5	-13	-7	9.01	1.30
5	-13	-7	9.16	2.05
-5	13	7	13.14	1.96
4	-13	-7	6.98	1.24
4	-13	-7	8.21	2.05
-4	13	7	6.89	1.40
3	-13	-7	21.96	1.82
3	-13	-7	18.43	2.93
-3	13	7	18.33	2.32
2	-13	-7	22.62	3.00
2	-13	-7	22.34	1.90
-2	13	7	26.95	2.50

1	-13	-7	13.45	1.71
1	-13	-7	13.18	2.22
-1	13	7	7.72	1.49
0	-13	-7	12.30	2.21
0	-13	-7	12.70	1.77
0	13	7	14.28	2.05
15	-14	-7	-1.28	1.74
15	-14	-7	2.36	2.66
-15	14	7	-0.87	0.75
14	-14	-7	4.22	1.76
14	-14	-7	2.05	2.56
-14	14	7	5.05	0.79
13	-14	-7	1.39	2.42
13	-14	-7	6.30	1.78
-13	14	7	6.97	1.01
12	-14	-7	2.50	2.27
12	-14	-7	1.67	1.54
-12	14	7	1.80	0.89
11	-14	-7	8.65	1.66
11	-14	-7	10.68	2.60
-11	14	7	9.55	1.58
10	-14	-7	6.83	1.64
10	-14	-7	7.88	2.32
-10	14	7	9.66	1.65
9	-14	-7	14.42	2.23
9	-14	-7	17.85	3.22
-9	14	7	11.31	1.79
8	-14	-7	11.24	1.61
8	-14	-7	11.81	2.43
-8	14	7	12.94	1.94
7	-14	-7	10.04	2.20
7	-14	-7	10.27	1.58
-7	14	7	8.61	1.42
6	-14	-7	10.26	1.45
6	-14	-7	7.12	2.19
-6	14	7	9.48	1.55
5	-14	-7	10.55	2.23
5	-14	-7	11.24	1.49
-5	14	7	10.29	1.65
4	-14	-7	8.55	1.35
4	-14	-7	9.54	2.08
-4	14	7	11.57	1.79
3	-14	-7	7.82	1.30
3	-14	-7	4.84	1.98

-3	14	7	6.49	1.46
2	-14	-7	10.65	2.24
2	-14	-7	12.28	1.87
-2	14	7	10.23	1.75
1	-14	-7	9.89	1.40
14	-15	-7	2.30	1.96
14	-15	-7	0.04	2.72
13	-15	-7	5.13	2.76
13	-15	-7	-0.40	1.74
12	-15	-7	11.69	2.77
12	-15	-7	10.84	2.02
-12	15	7	8.89	2.37
11	-15	-7	9.78	1.93
11	-15	-7	10.82	2.79
-11	15	7	10.07	1.42
10	-15	-7	10.54	2.52
10	-15	-7	7.46	1.67
-10	15	7	8.70	1.47
9	-15	-7	12.18	2.64
9	-15	-7	14.16	2.38
-9	15	7	12.84	2.06
8	-15	-7	11.24	1.67
8	-15	-7	9.37	2.58
-8	15	7	11.41	1.67
7	-15	-7	16.39	2.25
7	-15	-7	18.26	2.70
-7	15	7	17.54	2.27
6	-15	-7	28.20	2.40
6	-15	-7	21.63	3.58
-6	15	7	25.25	2.61
5	-15	-7	19.48	3.58
5	-15	-7	17.55	2.15
-5	15	7	18.42	2.56
4	-15	-7	14.96	2.66
4	-15	-7	12.14	1.98
-4	15	7	12.43	1.88
3	-15	-7	21.45	2.17
3	-15	-7	25.59	4.31
-3	15	7	19.48	2.88
12	-16	-7	10.61	2.21
12	-16	-7	9.40	2.97
11	-16	-7	4.54	1.90
11	-16	-7	-2.81	2.42
-11	16	7	0.39	1.33

10	-16	-7	6.40	2.65
10	-16	-7	4.91	1.79
-10	16	7	3.03	1.43
9	-16	-7	7.13	2.61
9	-16	-7	7.25	1.86
-9	16	7	8.55	1.66
8	-16	-7	6.05	1.67
8	-16	-7	2.21	2.54
-8	16	7	7.65	1.67
7	-16	-7	10.68	1.79
7	-16	-7	8.70	2.68
-7	16	7	9.24	1.74
6	-16	-7	4.81	1.51
6	-16	-7	6.02	2.67
-6	16	7	5.98	1.66
-1	18	-8	19.19	1.42
1	-18	8	22.96	3.08
1	-18	8	18.67	3.41
2	-18	8	8.80	1.93
2	-18	8	8.09	2.99
-3	18	-8	16.86	1.41
3	-18	8	25.98	2.97
3	-18	8	23.49	3.62
-4	18	-8	22.28	1.49
4	-18	8	28.56	4.53
4	-18	8	22.15	2.99
-5	18	-8	12.51	1.28
5	-18	8	21.92	3.00
5	-18	8	9.17	2.98
-6	18	-8	12.82	1.25
6	-18	8	13.52	3.16
6	-18	8	12.01	2.04
7	-18	8	20.40	3.78
2	17	-8	29.38	1.70
-2	-17	8	32.45	3.32
-2	-17	8	30.00	4.21
1	17	-8	29.47	1.61
-1	-17	8	32.60	4.34
-1	-17	8	28.85	3.11
0	17	-8	19.17	1.37
0	-17	8	19.22	2.66
0	-17	8	20.54	3.23
-1	17	-8	24.15	1.48
1	-17	8	26.95	4.12

1	-17	8	21.67	2.71
-2	17	-8	36.58	1.74
2	-17	8	33.91	4.24
2	-17	8	32.70	3.01
-3	17	-8	26.92	1.52
3	-17	8	28.82	4.20
3	-17	8	26.04	2.82
-4	17	-8	13.75	1.24
4	-17	8	18.63	2.63
4	-17	8	11.92	2.76
-5	17	-8	15.61	1.28
5	-17	8	15.69	2.61
5	-17	8	21.77	3.96
-6	17	-8	23.30	1.48
6	-17	8	24.39	3.64
6	-17	8	22.71	2.74
-7	17	-8	14.56	1.29
7	-17	8	15.09	2.58
7	-17	8	11.28	2.85
-8	17	-8	10.85	1.24
8	-17	8	11.32	3.13
8	-17	8	10.47	1.88
4	16	-8	19.74	1.58
-4	-16	8	20.52	3.03
-4	-16	8	16.87	2.91
3	16	-8	28.53	1.68
-3	-16	8	28.18	3.76
-3	-16	8	25.82	2.86
2	16	-8	26.13	1.57
-2	-16	8	25.43	3.67
-2	-16	8	30.26	2.93
1	16	-8	25.29	1.50
-1	-16	8	30.43	3.78
-1	-16	8	29.54	2.77
0	16	-8	29.06	1.53
0	-16	8	31.62	2.81
0	-16	8	31.22	3.82
-1	16	-8	37.21	1.73
1	-16	8	39.51	3.94
1	-16	8	37.39	2.87
-2	16	-8	19.91	1.27
2	-16	8	19.76	2.42
2	-16	8	20.43	3.57
-3	16	-8	25.00	1.37

3	-16	8	20.44	2.45
3	-16	8	21.59	3.06
-4	16	-8	36.14	1.72
4	-16	8	34.89	3.92
4	-16	8	39.99	2.86
-5	16	-8	11.69	1.12
5	-16	8	11.92	1.76
5	-16	8	8.99	2.40
-6	16	-8	13.25	1.20
6	-16	8	14.62	2.37
6	-16	8	14.30	2.92
-7	16	-8	16.66	1.26
7	-16	8	18.07	2.47
7	-16	8	13.59	2.81
-8	16	-8	8.91	0.98
8	-16	8	9.67	1.79
8	-16	8	10.95	2.89
-9	16	-8	2.83	0.76
9	-16	8	-1.36	2.63
9	-16	8	1.74	1.47
-10	16	-8	0.49	0.76
10	-16	8	0.93	3.18
6	15	-8	16.03	3.40
5	15	-8	16.12	1.54
-5	-15	8	20.66	2.82
-5	-15	8	23.06	2.83
4	15	-8	18.90	1.50
-4	-15	8	19.72	3.35
-4	-15	8	19.71	2.64
3	15	-8	25.32	1.52
-3	-15	8	22.25	2.58
-3	-15	8	22.83	3.25
2	15	-8	29.96	1.61
-2	-15	8	33.66	3.49
-2	-15	8	29.95	2.71
-1	-15	8	20.55	3.17
-1	-15	8	23.00	2.46
0	15	-8	14.74	1.10
0	-15	8	18.69	2.97
0	-15	8	15.66	2.21
-1	15	-8	21.62	1.24
1	-15	8	19.06	2.33
1	-15	8	24.83	3.15
-2	15	-8	42.33	1.81

2	-15	8	43.50	2.87
2	-15	8	43.21	3.73
-3	15	-8	14.77	1.10
3	-15	8	17.47	2.75
3	-15	8	19.35	2.18
-4	15	-8	14.85	1.10
4	-15	8	14.44	2.48
4	-15	8	16.62	2.14
-5	15	-8	27.09	1.43
5	-15	8	29.76	2.41
5	-15	8	25.88	3.52
-6	15	-8	19.30	1.26
6	-15	8	21.94	2.32
6	-15	8	18.57	2.90
-7	15	-8	9.16	1.04
7	-15	8	12.33	2.08
7	-15	8	12.10	2.83
-8	15	-8	6.66	0.87
8	-15	8	4.24	1.39
8	-15	8	6.29	2.61
-9	15	-8	3.37	0.73
9	-15	8	3.97	2.47
9	-15	8	3.75	1.45
10	-15	8	2.28	2.89
10	-15	8	3.65	1.47
7	14	-8	15.08	2.69
7	14	-8	8.51	1.16
-7	-14	8	14.01	2.36
6	14	-8	25.75	1.75
-6	-14	8	32.39	3.33
-6	-14	8	27.01	2.84
5	14	-8	21.27	1.57
-5	-14	8	25.63	3.09
-5	-14	8	27.37	2.74
4	14	-8	30.27	1.65
-4	-14	8	32.12	3.08
-4	-14	8	34.19	2.70
3	14	-8	28.20	1.50
-3	-14	8	25.99	2.94
-3	-14	8	28.98	2.57
2	14	-8	12.82	1.36
-2	-14	8	14.93	2.52
-2	-14	8	18.34	2.18
-1	-14	8	17.36	2.11

0	14	-8	19.64	1.44
0	-14	8	20.19	2.13
1	-14	8	15.79	1.92
-2	14	-8	4.22	0.64
2	-14	8	4.50	1.79
2	-14	8	4.54	1.26
-3	14	-8	16.49	1.06
3	-14	8	14.75	2.30
3	-14	8	16.67	1.92
-4	14	-8	18.92	1.12
4	-14	8	16.34	1.94
4	-14	8	18.54	2.86
-5	14	-8	3.89	0.62
5	-14	8	3.87	1.15
5	-14	8	1.64	1.78
-6	14	-8	4.98	0.70
6	-14	8	8.74	2.25
6	-14	8	6.67	1.24
-7	14	-8	6.40	0.92
7	-14	8	7.20	2.25
7	-14	8	6.47	1.04
7	-14	8	7.24	1.26
-8	14	-8	1.18	0.55
8	-14	8	2.53	1.11
8	-14	8	1.34	2.27
8	-14	8	1.69	0.78
-9	14	-8	0.13	0.59
9	-14	8	-1.08	0.84
9	-14	8	-1.15	2.34
9	-14	8	-0.09	1.15
-10	14	-8	-1.25	0.62
10	-14	8	0.28	2.50
10	-14	8	0.12	2.21
10	-14	8	1.84	1.24
-11	14	-8	0.37	0.68
11	-14	8	2.96	3.00
8	13	-8	15.21	2.58
8	13	-8	14.90	1.62
-8	-13	8	18.07	3.03
7	13	-8	15.88	1.53
-7	-13	8	17.75	2.63
-7	-13	8	17.93	2.78
6	13	-8	32.34	1.74
-6	-13	8	32.64	2.85

-6	-13	8	27.01	2.86
5	13	-8	25.20	1.51
-5	-13	8	26.42	3.25
-5	-13	8	28.72	2.63
4	13	-8	25.80	1.54
-4	-13	8	30.89	2.53
-4	-13	8	33.16	2.74
3	13	-8	28.22	1.44
-3	-13	8	24.92	2.24
-3	-13	8	28.37	2.68
2	13	-8	23.99	1.29
-2	-13	8	22.80	2.54
-2	-13	8	21.96	2.11
1	13	-8	40.21	1.69
-1	-13	8	36.49	2.94
-1	-13	8	37.83	2.47
0	13	-8	16.95	1.07
0	-13	8	15.32	2.40
0	-13	8	17.03	1.89
-1	13	-8	10.06	0.86
1	-13	8	8.30	1.79
1	-13	8	11.97	1.63
-2	13	-8	33.84	1.43
2	-13	8	27.71	2.77
2	-13	8	29.51	2.10
3	-13	8	16.35	2.46
3	-13	8	14.79	1.73
-4	13	-8	3.91	0.57
4	-13	8	3.76	1.86
-5	13	-8	8.43	0.88
5	-13	8	9.27	1.38
5	-13	8	10.20	2.01
-6	13	-8	0.65	0.49
6	-13	8	0.49	1.59
6	-13	8	1.05	0.52
6	-13	8	1.84	0.92
-7	13	-8	0.88	0.50
7	-13	8	0.03	1.70
7	-13	8	1.45	0.62
7	-13	8	1.56	0.99
-8	13	-8	5.29	0.72
8	-13	8	5.44	0.83
8	-13	8	5.52	2.13
8	-13	8	6.65	1.23

-9	13	-8	-0.16	0.58
9	-13	8	2.48	2.17
9	-13	8	-1.62	0.99
9	-13	8	0.61	0.82
-10	13	-8	0.18	0.61
10	-13	8	1.01	1.12
10	-13	8	-3.43	2.23
10	-13	8	1.07	1.00
-11	13	-8	2.71	0.78
11	-13	8	2.84	1.21
11	-13	8	0.94	2.68
9	12	-8	20.95	2.15
9	12	-8	24.90	1.79
9	12	-8	24.90	3.91
8	12	-8	31.76	1.84
-8	-12	8	31.46	2.89
-8	-12	8	30.73	2.85
7	12	-8	24.74	1.59
-7	-12	8	24.98	2.55
-7	-12	8	31.53	2.68
6	12	-8	32.31	1.64
-6	-12	8	29.55	2.54
-6	-12	8	31.63	2.62
-5	-12	8	22.80	2.36
-5	-12	8	21.91	2.14
4	12	-8	27.63	1.45
4	12	-8	29.93	3.17
-4	-12	8	29.28	2.24
-4	-12	8	31.16	2.52
3	12	-8	21.56	1.24
3	12	-8	20.32	2.83
-3	-12	8	22.81	1.95
-3	-12	8	24.49	2.32
2	12	-8	11.99	0.98
2	12	-8	15.43	2.58
-2	-12	8	13.47	1.62
-2	-12	8	11.61	2.02
1	12	-8	29.00	1.38
1	12	-8	30.34	2.70
-1	-12	8	29.34	2.52
-1	-12	8	29.46	2.09
0	12	-8	44.68	3.09
0	12	-8	52.50	1.94
0	-12	8	49.84	3.07

-1	12	-8	12.26	2.19
-1	12	-8	13.41	0.91
1	-12	8	14.43	2.13
1	-12	8	13.96	1.60
-2	12	-8	8.10	0.76
2	-12	8	6.52	1.52
2	-12	8	7.94	1.18
-3	12	-8	19.23	1.07
3	-12	8	21.47	1.72
3	-12	8	21.35	2.41
-4	12	-8	9.01	0.98
4	-12	8	10.45	1.48
4	-12	8	13.23	2.30
-5	12	-8	0.89	0.44
5	-12	8	0.84	0.83
5	-12	8	1.52	0.43
5	-12	8	1.92	0.57
-6	12	-8	-0.51	0.41
6	-12	8	-0.76	0.62
6	-12	8	0.43	0.47
6	-12	8	0.56	0.76
6	-12	8	-0.54	1.41
-7	12	-8	3.66	0.62
7	-12	8	3.84	1.84
7	-12	8	6.30	0.92
7	-12	8	4.76	1.04
7	-12	8	4.11	0.85
-8	12	-8	2.74	0.63
8	-12	8	4.93	2.06
8	-12	8	4.03	0.76
8	-12	8	4.02	1.04
8	-12	8	5.15	0.93
-9	12	-8	1.20	0.63
9	-12	8	3.75	2.06
9	-12	8	3.05	0.98
9	-12	8	2.53	0.86
-10	12	-8	0.32	0.61
10	-12	8	-1.10	1.05
10	-12	8	-2.67	1.94
10	-12	8	-0.06	1.03
-11	12	-8	0.07	0.64
11	-12	8	3.75	2.61
11	-12	8	0.63	1.11
-12	12	-8	0.41	0.78

12	-12	8	-0.59	2.94
12	-12	8	-1.55	1.21
10	11	-8	19.65	1.63
10	11	-8	16.66	2.05
10	11	-8	20.62	2.88
9	11	-8	14.75	2.54
9	11	-8	16.80	1.45
8	11	-8	36.93	1.97
8	11	-8	38.48	1.86
-8	-11	8	37.02	2.80
7	11	-8	39.56	1.85
7	11	-8	39.74	1.88
-7	-11	8	40.21	2.77
-7	-11	8	42.18	2.67
6	11	-8	37.40	3.40
6	11	-8	37.57	1.71
-6	-11	8	42.32	2.63
-6	-11	8	38.81	2.53
5	11	-8	15.21	2.09
5	11	-8	16.38	1.14
-5	-11	8	15.84	1.94
-5	-11	8	19.68	1.90
4	11	-8	5.12	1.57
4	11	-8	4.01	0.63
-4	-11	8	5.49	1.22
-4	-11	8	3.41	0.96
3	11	-8	22.43	2.43
3	11	-8	23.41	1.24
-3	-11	8	22.86	1.79
-3	-11	8	24.26	2.09
2	11	-8	66.12	2.38
2	11	-8	65.01	3.41
-2	-11	8	68.16	3.32
-2	-11	8	62.83	3.06
1	11	-8	9.76	0.83
1	11	-8	11.79	1.98
-1	-11	8	7.39	1.01
-1	-11	8	9.93	1.79
-1	-11	8	10.11	1.31
0	11	-8	9.70	1.54
0	11	-8	6.92	0.72
0	-11	8	5.74	1.32
0	-11	8	6.19	0.98
0	-11	8	7.29	0.98

-1	11	-8	10.40	1.86
-1	11	-8	14.47	0.90
1	-11	8	12.54	1.90
1	-11	8	14.97	1.40
-2	11	-8	8.89	0.74
-2	11	-8	10.34	1.90
2	-11	8	11.14	1.89
2	-11	8	7.48	1.27
-3	11	-8	10.31	1.48
-3	11	-8	8.54	0.75
3	-11	8	8.54	1.64
3	-11	8	9.10	1.32
-4	11	-8	0.88	0.39
4	-11	8	2.00	0.50
4	-11	8	1.58	1.31
4	-11	8	2.62	0.80
4	-11	8	1.62	0.40
5	-11	8	2.85	0.63
5	-11	8	1.30	1.28
5	-11	8	2.95	0.47
5	-11	8	3.60	0.86
-6	11	-8	1.00	0.44
6	-11	8	0.72	0.75
6	-11	8	1.17	0.48
6	-11	8	0.82	0.65
6	-11	8	0.53	1.56
-7	11	-8	0.20	0.44
7	-11	8	0.84	1.54
7	-11	8	2.52	0.76
7	-11	8	0.24	0.54
7	-11	8	0.92	0.79
-8	11	-8	0.16	0.49
8	-11	8	0.10	0.77
8	-11	8	-0.69	0.77
8	-11	8	-0.84	1.61
8	-11	8	0.05	0.63
-9	11	-8	-0.58	0.50
9	-11	8	-0.98	1.83
9	-11	8	-0.22	0.70
9	-11	8	0.89	0.85
-10	11	-8	0.44	0.57
10	-11	8	1.41	0.96
10	-11	8	0.01	2.19
10	-11	8	1.13	0.92

-11	11	-8	0.00	0.62
11	-11	8	0.91	1.16
11	-11	8	0.12	0.98
11	-11	8	-2.11	2.28
-12	11	-8	-0.29	0.72
12	-11	8	3.61	1.32
12	-11	8	0.53	2.64
11	10	-8	11.23	2.44
11	10	-8	11.84	1.43
11	10	-8	13.36	2.43
10	10	-8	15.43	1.36
10	10	-8	11.22	2.33
10	10	-8	13.50	1.74
10	10	-8	10.74	1.75
9	10	-8	41.45	1.91
9	10	-8	40.29	2.25
9	10	-8	40.19	2.19
-9	-10	8	44.01	2.90
8	10	-8	24.81	1.39
8	10	-8	24.96	1.61
8	10	-8	24.00	3.21
-8	-10	8	25.05	2.33
7	10	-8	18.67	2.77
7	10	-8	18.75	1.08
7	10	-8	19.50	1.28
7	10	-8	18.31	1.29
-7	-10	8	21.54	1.87
-7	-10	8	18.61	1.99
6	10	-8	35.98	2.92
6	10	-8	36.85	1.67
6	10	-8	38.97	1.68
6	10	-8	37.52	1.46
-6	-10	8	39.56	2.29
-6	-10	8	40.66	2.41
5	10	-8	8.76	0.64
5	10	-8	10.55	0.90
5	10	-8	9.46	0.99
5	10	-8	12.22	1.79
-5	-10	8	10.68	1.76
-5	-10	8	10.99	1.58
4	10	-8	18.02	1.05
4	10	-8	17.89	2.13
-4	-10	8	17.30	1.74
-4	-10	8	15.49	1.58

3	10	-8	58.14	2.13
3	10	-8	58.59	3.08
-3	-10	8	57.06	2.03
-3	-10	8	58.47	2.71
-3	-10	8	56.74	2.88
2	10	-8	5.93	1.28
2	10	-8	5.15	0.69
-2	-10	8	5.96	1.02
-2	-10	8	6.53	1.22
-2	-10	8	5.88	0.64
1	10	-8	5.87	0.68
1	10	-8	7.27	1.29
-1	-10	8	6.31	1.25
-1	-10	8	5.02	0.87
-1	-10	8	6.84	0.69
0	10	-8	27.34	2.04
0	10	-8	26.88	1.20
0	-10	8	25.00	2.09
0	-10	8	28.94	1.21
0	-10	8	25.06	1.68
-1	10	-8	4.47	0.65
-1	10	-8	6.75	0.65
-1	10	-8	6.42	1.16
1	-10	8	6.34	0.96
1	-10	8	6.13	0.68
1	-10	8	6.61	1.28
-2	10	-8	0.19	0.43
-2	10	-8	0.93	0.33
-2	10	-8	1.04	0.89
2	-10	8	-0.10	0.39
2	-10	8	0.65	0.99
2	-10	8	-0.02	0.55
-3	10	-8	5.88	0.83
-3	10	-8	4.99	0.61
-3	10	-8	6.85	1.20
3	-10	8	4.90	1.30
3	-10	8	4.26	0.67
3	-10	8	5.73	0.88
-4	10	-8	0.71	0.93
-4	10	-8	0.57	0.48
-4	10	-8	0.66	0.35
4	-10	8	0.35	0.31
4	-10	8	-1.86	0.99
4	-10	8	0.31	0.62

-5	10	-8	-0.16	0.33
-5	10	-8	-0.10	0.44
5	-10	8	0.28	0.55
5	-10	8	0.93	0.39
5	-10	8	-0.25	0.60
5	-10	8	1.65	1.10
-6	10	-8	1.04	0.52
-6	10	-8	1.78	0.44
6	-10	8	1.77	0.70
6	-10	8	1.43	0.70
6	-10	8	2.70	1.40
6	-10	8	2.39	0.48
-7	10	-8	2.19	0.61
-7	10	-8	2.51	0.52
7	-10	8	3.21	0.61
7	-10	8	3.49	0.80
7	-10	8	1.80	1.57
7	-10	8	3.98	0.78
-8	10	-8	6.15	1.09
-8	10	-8	8.13	0.86
8	-10	8	8.60	1.26
8	-10	8	10.04	1.35
8	-10	8	15.79	2.78
8	-10	8	7.93	1.04
-9	10	-8	0.68	0.54
-9	10	-8	2.04	0.75
9	-10	8	-0.09	1.85
9	-10	8	1.62	0.83
9	-10	8	2.51	0.75
-10	10	-8	0.02	0.56
10	-10	8	0.31	0.84
10	-10	8	-0.32	2.18
10	-10	8	1.18	0.88
-11	10	-8	3.51	0.72
11	-10	8	3.73	1.13
11	-10	8	2.35	0.97
11	-10	8	1.31	2.43
-12	10	-8	2.77	0.80
12	-10	8	4.23	1.33
12	-10	8	-0.11	2.75
12	9	-8	8.39	2.33
12	9	-8	10.78	1.55
12	9	-8	6.43	0.99
11	9	-8	22.28	1.53

11	9	-8	19.93	3.36
11	9	-8	24.38	1.99
11	9	-8	22.16	2.01
10	9	-8	17.14	1.74
10	9	-8	19.43	1.34
10	9	-8	20.38	1.75
-10	-9	8	22.83	2.31
9	9	-8	13.93	1.15
9	9	-8	16.39	3.27
9	9	-8	15.15	1.49
9	9	-8	12.68	1.40
-9	-9	8	15.28	2.07
8	9	-8	5.27	0.88
8	9	-8	8.06	1.86
8	9	-8	5.90	0.88
8	9	-8	6.06	0.88
8	9	-8	4.49	0.87
-8	-9	8	6.38	1.21
7	9	-8	25.93	1.20
7	9	-8	26.05	1.32
7	9	-8	27.94	1.46
7	9	-8	26.50	2.56
-7	-9	8	27.07	1.77
-7	-9	8	28.47	2.28
6	9	-8	9.80	0.68
6	9	-8	12.10	2.00
6	9	-8	11.88	0.90
6	9	-8	11.00	0.95
-6	-9	8	9.88	1.42
-6	-9	8	9.61	1.46
5	9	-8	16.06	1.93
5	9	-8	19.12	0.80
5	9	-8	17.32	1.07
5	9	-8	19.80	1.06
-5	-9	8	21.03	1.64
-5	-9	8	19.61	1.62
4	9	-8	8.52	0.73
4	9	-8	8.77	0.77
4	9	-8	10.02	1.68
4	9	-8	8.65	0.46
-4	-9	8	7.86	1.25
-4	-9	8	9.04	1.34
-4	-9	8	8.09	0.65
3	9	-8	9.91	1.31

3	9	-8	8.57	0.73
-3	-9	8	10.17	0.69
-3	-9	8	8.46	1.34
-3	-9	8	9.44	1.16
2	9	-8	20.67	1.74
2	9	-8	19.40	1.25
2	9	-8	20.23	1.00
-2	-9	8	19.65	1.42
-2	-9	8	20.39	1.72
-2	-9	8	18.01	0.91
1	9	-8	8.59	0.93
1	9	-8	9.29	1.44
1	9	-8	8.54	0.70
-1	-9	8	9.34	0.68
-1	-9	8	10.54	1.44
-1	-9	8	7.68	1.04
0	9	-8	5.73	1.02
0	9	-8	4.88	0.81
0	9	-8	5.32	0.61
0	-9	8	6.51	1.14
0	-9	8	4.66	0.84
0	-9	8	5.68	0.59
-1	9	-8	14.99	0.83
-1	9	-8	15.12	1.03
-1	9	-8	16.34	1.51
1	-9	8	14.01	1.17
1	-9	8	12.55	1.67
1	-9	8	15.20	0.87
-2	9	-8	9.55	0.86
-2	9	-8	9.58	0.69
-2	9	-8	11.87	1.41
2	-9	8	10.58	0.77
2	-9	8	9.59	0.71
2	-9	8	9.92	1.58
2	-9	8	11.07	1.09
-3	9	-8	1.58	0.39
-3	9	-8	1.91	0.85
-3	9	-8	1.48	0.26
-3	9	-8	1.56	0.44
3	-9	8	1.28	0.97
3	-9	8	0.72	0.54
3	-9	8	0.85	0.40
3	-9	8	1.58	0.47
-4	9	-8	2.23	0.90

-4	9	-8	1.11	0.37
-4	9	-8	1.17	0.45
4	-9	8	2.19	1.08
4	-9	8	2.00	0.56
4	-9	8	1.50	0.59
-5	9	-8	0.90	0.45
-5	9	-8	0.71	0.36
5	-9	8	1.46	0.62
5	-9	8	1.45	1.13
5	-9	8	2.07	0.61
-6	9	-8	2.17	0.52
6	-9	8	2.58	0.72
6	-9	8	1.85	0.51
6	-9	8	3.53	1.35
6	-9	8	2.61	0.72
-7	9	-8	-0.20	0.55
-7	9	-8	0.07	0.39
7	-9	8	0.16	0.59
7	-9	8	-0.50	0.47
7	-9	8	0.60	0.68
7	-9	8	-0.92	1.33
-8	9	-8	1.74	0.54
-8	9	-8	0.93	0.68
8	-9	8	1.99	0.72
8	-9	8	1.27	1.03
8	-9	8	0.35	1.51
8	-9	8	0.87	0.61
-9	9	-8	7.71	0.91
-9	9	-8	6.55	0.95
9	-9	8	7.60	1.16
9	-9	8	7.11	1.02
9	-9	8	6.70	1.99
-10	9	-8	7.32	0.98
10	-9	8	6.68	1.06
10	-9	8	6.32	1.08
10	-9	8	8.24	2.44
-11	9	-8	-1.30	0.59
11	-9	8	1.82	1.02
11	-9	8	0.69	0.87
11	-9	8	1.39	2.24
-12	9	-8	1.67	0.75
12	-9	8	9.45	2.98
12	-9	8	4.82	1.34
13	8	-8	17.85	2.34

13	8	-8	15.60	2.66
13	8	-8	19.01	1.47
12	8	-8	22.89	3.38
12	8	-8	23.13	1.48
12	8	-8	19.53	2.13
12	8	-8	22.67	2.10
11	8	-8	20.58	3.04
11	8	-8	23.23	1.83
11	8	-8	17.84	1.34
10	8	-8	20.50	1.70
10	8	-8	20.16	1.54
10	8	-8	18.31	1.26
-10	-8	8	23.21	2.17
9	8	-8	27.22	1.36
9	8	-8	25.80	1.34
9	8	-8	28.59	2.81
9	8	-8	24.65	1.67
9	8	-8	25.01	1.58
-9	-8	8	26.30	2.15
8	8	-8	10.72	0.86
8	8	-8	10.31	1.11
8	8	-8	8.23	1.15
8	8	-8	9.04	0.87
8	8	-8	11.98	1.74
-8	-8	8	12.12	1.60
7	8	-8	9.95	0.72
7	8	-8	9.29	0.83
7	8	-8	12.39	1.88
7	8	-8	10.12	1.02
-7	-8	8	9.43	1.40
-7	-8	8	9.81	1.19
6	8	-8	15.30	0.77
6	8	-8	16.68	1.75
6	8	-8	15.45	1.02
6	8	-8	16.71	0.97
-6	-8	8	15.07	1.51
-6	-8	8	16.69	1.35
5	8	-8	8.94	0.73
5	8	-8	7.66	0.79
5	8	-8	8.74	0.74
5	8	-8	7.85	1.04
5	8	-8	8.66	1.52
-5	-8	8	7.59	0.62
-5	-8	8	7.16	1.16

-5	-8	8	7.99	1.14
4	8	-8	27.04	1.51
4	8	-8	28.21	1.89
4	8	-8	28.17	1.00
4	8	-8	28.29	1.26
4	8	-8	34.36	1.80
-4	-8	8	31.14	1.76
-4	-8	8	28.77	1.70
-4	-8	8	28.32	1.16
3	8	-8	9.27	0.70
3	8	-8	12.22	1.37
3	8	-8	9.67	0.43
3	8	-8	10.18	0.96
3	8	-8	9.52	0.65
-3	-8	8	8.53	1.05
-3	-8	8	10.64	1.26
-3	-8	8	10.26	0.65
2	8	-8	1.30	0.37
2	8	-8	3.28	0.83
2	8	-8	1.96	0.48
-2	-8	8	1.26	0.71
-2	-8	8	1.04	0.54
-2	-8	8	1.59	0.34
1	8	-8	1.44	0.71
1	8	-8	0.65	0.37
1	8	-8	0.85	0.33
-1	-8	8	0.91	0.48
-1	-8	8	0.90	0.75
-1	-8	8	0.45	0.31
0	8	-8	10.25	1.22
0	8	-8	10.29	0.84
0	8	-8	10.65	0.69
0	-8	8	10.61	0.56
0	-8	8	10.32	0.99
0	-8	8	10.28	0.70
0	-8	8	9.96	1.39
-1	8	-8	17.26	0.67
-1	8	-8	19.30	1.05
-1	8	-8	19.77	1.46
-1	8	-8	17.64	0.88
1	-8	8	19.18	1.65
1	-8	8	16.94	0.86
1	-8	8	16.56	1.17
1	-8	8	16.97	0.89

-2	8	-8	0.42	0.37
-2	8	-8	-0.15	0.24
-2	8	-8	0.37	0.64
-2	8	-8	0.03	0.23
2	-8	8	-0.52	0.73
2	-8	8	-0.36	0.43
2	-8	8	0.06	0.40
2	-8	8	-0.39	0.32
-3	8	-8	0.05	0.35
-3	8	-8	-0.08	0.26
-3	8	-8	0.14	0.64
-3	8	-8	-0.08	0.24
3	-8	8	1.28	0.89
3	-8	8	0.66	0.46
3	-8	8	-0.11	0.41
3	-8	8	-0.25	0.38
-4	8	-8	1.21	0.36
-4	8	-8	1.09	0.73
-4	8	-8	1.84	0.34
-4	8	-8	1.44	0.44
4	-8	8	1.34	0.53
4	-8	8	2.28	0.99
4	-8	8	1.72	0.57
4	-8	8	0.88	0.48
-5	8	-8	0.19	0.32
-5	8	-8	0.08	0.74
-5	8	-8	0.64	0.32
-5	8	-8	0.36	0.42
5	-8	8	-0.43	0.98
5	-8	8	0.89	0.53
5	-8	8	0.54	0.57
-6	8	-8	5.13	0.84
-6	8	-8	3.70	0.50
-6	8	-8	4.28	0.60
6	-8	8	5.95	1.00
6	-8	8	6.10	1.52
6	-8	8	3.50	0.74
-7	8	-8	1.24	0.57
-7	8	-8	0.89	0.42
-7	8	-8	1.03	0.40
7	-8	8	1.23	0.62
7	-8	8	0.67	0.73
7	-8	8	0.50	2.24
-8	8	-8	-0.39	0.38

-8	8	-8	1.21	0.69
8	-8	8	0.28	0.62
8	-8	8	-0.95	1.42
-9	8	-8	0.98	0.50
-9	8	-8	2.22	0.93
9	-8	8	-0.67	0.63
9	-8	8	0.80	0.72
9	-8	8	0.44	1.77
-10	8	-8	2.39	0.65
10	-8	8	1.23	2.20
10	-8	8	4.30	0.91
10	-8	8	3.15	0.95
-11	8	-8	7.05	1.07
11	-8	8	6.82	1.09
11	-8	8	6.70	1.18
11	-8	8	6.76	2.69
-12	8	-8	5.39	0.86
12	-8	8	4.75	2.93
12	-8	8	6.01	1.36
13	7	-8	13.27	2.24
13	7	-8	12.46	2.11
13	7	-8	11.56	1.28
12	7	-8	19.11	1.90
12	7	-8	17.58	1.27
12	7	-8	17.43	2.91
12	7	-8	17.95	1.92
11	7	-8	25.64	1.85
11	7	-8	25.94	1.42
11	7	-8	29.47	1.97
-11	-7	8	36.06	2.36
10	7	-8	37.41	1.61
10	7	-8	35.52	1.86
10	7	-8	37.60	2.04
10	7	-8	36.60	2.98
-10	-7	8	37.69	2.33
9	7	-8	9.22	1.05
9	7	-8	8.74	0.89
9	7	-8	8.77	1.20
9	7	-8	12.77	2.13
9	7	-8	7.60	0.86
-9	-7	8	7.81	1.16
8	7	-8	12.17	0.84
8	7	-8	13.03	1.03
8	7	-8	11.17	1.15

8	7	-8	13.80	1.82
8	7	-8	11.93	0.88
-8	-7	8	11.93	1.48
7	7	-8	33.88	2.12
7	7	-8	30.60	1.75
7	7	-8	36.03	1.45
7	7	-8	36.13	1.33
7	7	-8	32.32	1.59
-7	-7	8	37.75	1.69
-7	-7	8	34.22	1.96
6	7	-8	14.89	1.20
6	7	-8	14.94	1.01
6	7	-8	15.25	0.88
6	7	-8	17.26	1.52
6	7	-8	16.97	0.76
-6	-7	8	15.43	1.13
-6	-7	8	13.42	0.79
-6	-7	8	16.43	1.38
5	7	-8	17.48	1.47
5	7	-8	14.33	0.77
5	7	-8	18.55	1.00
5	7	-8	16.10	1.14
5	7	-8	17.21	0.90
-5	-7	8	17.09	1.20
-5	-7	8	17.13	0.81
-5	-7	8	16.02	1.30
4	7	-8	5.39	0.75
4	7	-8	5.38	0.58
4	7	-8	5.50	0.78
-4	-7	8	6.15	0.86
-4	-7	8	5.75	0.99
-4	-7	8	5.44	0.46
-4	-7	8	4.91	0.50
3	7	-8	11.09	0.70
3	7	-8	11.45	0.88
3	7	-8	10.78	0.72
-3	-7	8	10.61	0.97
-3	-7	8	10.81	0.57
-3	-7	8	10.68	1.14
-3	-7	8	10.22	0.63
2	7	-8	8.54	0.59
2	7	-8	9.11	1.05
2	7	-8	8.44	0.74
2	7	-8	9.38	0.63

-2	-7	8	7.71	0.58
-2	-7	8	9.82	0.95
-2	-7	8	8.58	0.51
-2	-7	8	8.94	1.13
1	7	-8	-0.02	0.25
1	7	-8	0.05	0.23
1	7	-8	0.23	0.32
1	7	-8	0.39	0.53
-1	-7	8	0.11	0.38
-1	-7	8	-0.14	0.57
-1	-7	8	0.35	0.27
-1	-7	8	0.27	0.26
0	7	-8	-0.42	0.55
0	7	-8	-0.34	0.24
0	7	-8	0.25	0.21
0	7	-8	0.32	0.35
0	-7	8	0.01	0.29
0	-7	8	-0.13	0.36
0	-7	8	0.17	0.26
0	-7	8	-0.85	0.59
-1	7	-8	1.08	0.29
-1	7	-8	0.84	0.26
-1	7	-8	1.11	0.34
-1	7	-8	1.45	0.62
1	-7	8	1.37	0.47
1	-7	8	0.73	0.40
1	-7	8	1.11	0.32
1	-7	8	1.43	0.72
-2	7	-8	5.29	0.95
-2	7	-8	4.13	0.58
-2	7	-8	5.14	0.50
-2	7	-8	4.85	0.43
2	-7	8	4.61	0.56
2	-7	8	4.97	0.73
2	-7	8	5.27	1.03
-3	7	-8	1.60	0.34
-3	7	-8	1.56	0.31
-3	7	-8	3.17	0.56
-3	7	-8	2.56	0.70
3	-7	8	2.58	0.53
3	-7	8	1.54	0.50
3	-7	8	2.11	0.90
3	-7	8	1.89	0.38
-4	7	-8	1.20	0.32

-4	7	-8	0.96	0.65
-4	7	-8	1.91	0.44
-4	7	-8	0.51	0.30
4	-7	8	0.83	0.37
4	-7	8	0.64	0.90
4	-7	8	1.35	0.47
4	-7	8	0.99	0.52
-5	7	-8	1.20	0.72
-5	7	-8	1.83	0.39
-5	7	-8	2.60	0.50
-5	7	-8	2.44	0.41
5	-7	8	1.80	1.05
5	-7	8	2.91	0.60
5	-7	8	2.72	0.66
-6	7	-8	0.74	0.35
-6	7	-8	0.90	0.53
-6	7	-8	0.08	0.37
6	-7	8	-0.39	0.62
6	-7	8	0.11	0.48
6	-7	8	-0.82	0.98
-7	7	-8	1.27	0.40
-7	7	-8	0.79	0.43
-7	7	-8	0.84	0.60
7	-7	8	0.78	0.66
7	-7	8	1.06	0.57
-8	7	-8	-0.22	0.44
-8	7	-8	1.79	0.76
-8	7	-8	0.56	0.46
8	-7	8	1.24	1.43
8	-7	8	-0.28	0.58
-9	7	-8	3.36	0.64
-9	7	-8	3.97	0.63
-9	7	-8	4.49	1.06
9	-7	8	4.19	0.83
9	-7	8	2.74	1.71
-10	7	-8	7.21	0.95
10	-7	8	3.50	2.12
10	-7	8	5.68	1.06
10	-7	8	7.16	1.02
-11	7	-8	3.48	0.74
11	-7	8	3.09	2.38
11	-7	8	3.52	0.95
11	-7	8	3.81	1.08
-12	7	-8	2.28	0.69

12	-7	8	-2.46	2.48
12	-7	8	2.89	1.13
14	6	-8	11.35	1.23
14	6	-8	6.97	1.52
14	6	-8	11.55	2.08
13	6	-8	20.22	2.90
13	6	-8	25.88	2.25
13	6	-8	25.39	1.46
13	6	-8	26.06	2.15
12	6	-8	16.77	2.57
12	6	-8	17.85	1.15
12	6	-8	13.93	1.73
12	6	-8	12.26	1.70
11	6	-8	12.38	1.94
11	6	-8	10.79	1.47
11	6	-8	9.79	1.37
11	6	-8	11.26	0.96
-11	-6	8	8.44	1.32
10	6	-8	17.31	1.16
10	6	-8	17.67	2.28
10	6	-8	18.60	1.08
10	6	-8	19.03	1.51
10	6	-8	18.46	1.33
-10	-6	8	17.66	1.71
9	6	-8	21.84	1.46
9	6	-8	18.95	1.41
9	6	-8	20.18	1.08
9	6	-8	20.22	1.20
9	6	-8	20.78	1.94
9	6	-8	19.81	1.05
-9	-6	8	18.62	1.62
8	6	-8	6.91	0.70
8	6	-8	7.44	0.78
8	6	-8	8.30	1.02
8	6	-8	8.38	1.09
8	6	-8	8.74	1.46
8	6	-8	7.50	0.71
-8	-6	8	6.80	1.19
7	6	-8	16.00	0.92
7	6	-8	14.39	1.09
7	6	-8	15.24	0.88
7	6	-8	16.14	0.92
7	6	-8	17.55	0.81
7	6	-8	15.82	1.15

-7	-6	8	16.80	1.36
6	6	-8	20.66	0.97
6	6	-8	18.13	1.10
6	6	-8	18.63	1.40
6	6	-8	20.04	1.20
-6	-6	8	18.91	1.35
-6	-6	8	20.75	0.90
-6	-6	8	20.80	1.14
-6	-6	8	20.69	0.97
5	6	-8	7.08	0.60
5	6	-8	9.38	1.02
5	6	-8	6.52	0.76
5	6	-8	6.50	0.77
-5	-6	8	7.91	0.95
-5	-6	8	7.16	0.94
-5	-6	8	7.19	0.53
-5	-6	8	6.85	0.57
4	6	-8	3.23	0.64
4	6	-8	2.34	0.47
4	6	-8	1.78	0.36
4	6	-8	2.28	0.49
-4	-6	8	2.62	0.65
-4	-6	8	2.02	0.52
-4	-6	8	2.25	0.34
-4	-6	8	2.38	0.35
3	6	-8	0.72	0.32
3	6	-8	0.90	0.30
3	6	-8	1.93	0.45
3	6	-8	0.36	0.51
-3	-6	8	1.32	0.30
-3	-6	8	1.01	0.47
-3	-6	8	1.49	0.63
-3	-6	8	1.32	0.29
2	6	-8	4.94	0.62
2	6	-8	5.63	0.77
2	6	-8	5.52	0.51
-2	-6	8	6.98	0.51
-2	-6	8	6.93	1.00
-2	-6	8	6.13	0.73
-2	-6	8	4.81	0.49
1	6	-8	0.80	0.37
1	6	-8	0.27	0.28
1	6	-8	0.31	0.27
1	6	-8	0.54	0.54

-1	-6	8	0.06	0.32
-1	-6	8	1.12	0.58
-1	-6	8	0.57	0.25
-1	-6	8	0.33	0.36
0	6	-8	12.99	0.69
0	6	-8	11.17	0.93
0	6	-8	11.80	0.75
0	6	-8	11.17	0.65
0	-6	8	12.15	0.78
0	-6	8	11.76	0.67
0	-6	8	11.32	1.14
0	-6	8	9.16	0.84
-1	6	-8	10.44	0.70
-1	6	-8	9.57	0.61
-1	6	-8	10.12	0.93
-1	6	-8	9.05	0.57
1	-6	8	10.57	0.82
1	-6	8	8.95	1.19
1	-6	8	10.61	0.82
1	-6	8	9.14	0.63
-2	6	-8	3.63	0.65
-2	6	-8	2.86	0.39
-2	6	-8	3.15	0.43
-2	6	-8	3.99	0.54
2	-6	8	3.94	0.55
2	-6	8	3.32	0.42
2	-6	8	3.52	0.61
2	-6	8	3.71	0.82
-3	6	-8	1.05	0.29
-3	6	-8	1.00	0.30
-3	6	-8	0.04	0.53
-3	6	-8	0.29	0.31
3	-6	8	0.59	0.38
3	-6	8	-0.09	0.48
3	-6	8	0.37	0.80
3	-6	8	0.75	0.33
-4	6	-8	6.11	0.59
-4	6	-8	6.67	0.57
-4	6	-8	7.43	0.74
-4	6	-8	5.89	1.01
4	-6	8	7.34	0.89
4	-6	8	8.69	1.34
4	-6	8	6.74	0.66
4	-6	8	6.73	0.79

-5	6	-8	0.01	0.32
-5	6	-8	-0.25	0.28
-5	6	-8	-0.11	0.66
-5	6	-8	0.32	0.44
5	-6	8	-0.02	0.55
5	-6	8	0.76	0.88
5	-6	8	-0.32	0.69
5	-6	8	-0.17	0.41
-6	6	-8	0.21	0.39
-6	6	-8	1.29	0.38
-6	6	-8	0.11	0.54
6	-6	8	0.98	0.52
6	-6	8	-0.45	0.64
6	-6	8	1.58	1.13
-7	6	-8	24.37	1.50
-7	6	-8	23.87	1.19
7	-6	8	22.02	1.41
-8	6	-8	3.11	0.56
-8	6	-8	3.13	0.58
-8	6	-8	4.33	0.93
8	-6	8	3.51	0.76
8	-6	8	5.24	1.63
-9	6	-8	2.38	0.55
-9	6	-8	2.57	1.04
-9	6	-8	1.12	0.57
9	-6	8	2.11	1.72
9	-6	8	1.98	0.75
-10	6	-8	10.98	1.03
-10	6	-8	11.14	1.16
10	-6	8	7.44	2.21
10	-6	8	10.52	1.42
-11	6	-8	9.61	1.12
11	-6	8	3.93	2.27
11	-6	8	9.94	1.50
11	-6	8	9.91	1.32
-12	6	-8	0.53	0.68
12	-6	8	-1.09	2.47
12	-6	8	2.04	1.10
15	5	-8	10.26	1.27
15	5	-8	11.05	1.74
14	5	-8	10.10	1.84
14	5	-8	11.40	1.58
14	5	-8	13.42	1.19
13	5	-8	19.26	1.20

13	5	-8	14.92	1.93
13	5	-8	19.37	2.64
13	5	-8	18.20	2.00
12	5	-8	21.61	1.78
12	5	-8	22.22	1.21
12	5	-8	19.61	1.71
-12	-5	8	21.98	1.96
11	5	-8	9.19	1.36
11	5	-8	7.16	0.79
11	5	-8	8.19	1.24
11	5	-8	8.47	1.54
11	5	-8	7.52	1.22
-11	-5	8	8.23	1.47
10	5	-8	7.52	1.11
10	5	-8	7.91	0.84
10	5	-8	6.95	1.03
10	5	-8	6.91	1.01
10	5	-8	9.90	1.73
10	5	-8	4.25	0.77
-10	-5	8	7.23	1.08
9	5	-8	0.89	0.67
9	5	-8	1.47	0.56
9	5	-8	2.04	0.87
9	5	-8	0.36	0.45
9	5	-8	1.20	0.52
9	5	-8	1.51	0.41
-9	-5	8	1.21	0.67
8	5	-8	4.36	0.71
8	5	-8	3.85	0.55
8	5	-8	3.56	0.62
8	5	-8	4.15	0.65
8	5	-8	6.02	0.91
-8	-5	8	3.48	0.74
7	5	-8	3.25	0.47
7	5	-8	2.20	0.71
7	5	-8	3.04	0.45
7	5	-8	3.25	0.55
7	5	-8	2.48	0.41
7	5	-8	2.78	0.59
-7	-5	8	3.44	0.67
-7	-5	8	3.25	0.50
6	5	-8	11.19	0.86
6	5	-8	11.40	0.87
6	5	-8	9.75	0.94

6	5	-8	9.99	0.67
-6	-5	8	9.54	0.98
-6	-5	8	10.38	0.73
-6	-5	8	10.76	0.61
5	5	-8	5.03	0.50
5	5	-8	4.75	0.71
5	5	-8	5.18	0.74
5	5	-8	5.30	0.63
-5	-5	8	4.29	0.75
-5	-5	8	4.91	0.55
-5	-5	8	5.11	0.62
-5	-5	8	4.99	0.45
4	5	-8	7.49	0.67
4	5	-8	7.50	0.56
4	5	-8	7.17	0.71
4	5	-8	7.25	0.70
-4	-5	8	7.54	0.59
-4	-5	8	7.65	0.79
-4	-5	8	7.68	0.49
-4	-5	8	6.71	0.75
3	5	-8	0.66	0.39
3	5	-8	0.24	0.35
3	5	-8	0.19	0.27
3	5	-8	0.46	0.26
-3	-5	8	0.51	0.47
-3	-5	8	0.73	0.23
-3	-5	8	0.44	0.30
-3	-5	8	0.38	0.34
2	5	-8	7.72	0.69
2	5	-8	6.90	0.62
2	5	-8	7.05	0.52
2	5	-8	7.05	0.66
-2	-5	8	7.36	0.52
-2	-5	8	7.40	0.96
-2	-5	8	7.24	0.61
-2	-5	8	8.27	0.71
1	5	-8	4.01	0.49
1	5	-8	4.14	0.54
1	5	-8	3.56	0.60
1	5	-8	3.74	0.43
-1	-5	8	4.05	0.58
-1	-5	8	3.21	0.42
-1	-5	8	3.44	0.56
-1	-5	8	4.13	0.69

0	5	-8	4.39	0.50
0	5	-8	4.87	0.64
0	5	-8	3.66	0.43
0	5	-8	2.80	0.45
0	-5	8	4.15	0.46
0	-5	8	2.71	0.50
0	-5	8	4.89	0.79
0	-5	8	3.47	0.56
-1	5	-8	16.58	0.88
-1	5	-8	16.85	0.82
-1	5	-8	17.55	1.04
-1	5	-8	16.32	0.79
1	-5	8	16.91	0.81
1	-5	8	16.58	1.30
1	-5	8	17.15	1.01
1	-5	8	17.93	0.98
-2	5	-8	3.23	0.56
-2	5	-8	2.65	0.37
-2	5	-8	1.87	0.32
-2	5	-8	2.12	0.37
2	-5	8	2.72	0.54
2	-5	8	2.75	0.48
2	-5	8	2.40	0.70
2	-5	8	2.84	0.40
-3	5	-8	3.23	0.42
-3	5	-8	3.95	0.64
-3	5	-8	3.57	0.50
-3	5	-8	4.05	0.60
3	-5	8	3.69	0.53
3	-5	8	3.37	0.61
3	-5	8	3.86	0.57
3	-5	8	3.94	0.92
-4	5	-8	21.23	0.99
-4	5	-8	21.69	1.01
-4	5	-8	22.68	1.18
-4	5	-8	21.76	1.32
4	-5	8	22.96	1.04
4	-5	8	19.80	1.16
4	-5	8	20.04	1.78
4	-5	8	23.47	1.28
-5	5	-8	10.82	1.13
-5	5	-8	10.24	0.75
-5	5	-8	11.01	0.70
-5	5	-8	11.87	1.01

5	-5	8	10.35	0.79
5	-5	8	10.00	0.92
5	-5	8	10.23	1.10
5	-5	8	9.35	1.60
-6	5	-8	2.35	0.41
-6	5	-8	2.62	0.49
-6	5	-8	3.26	0.71
6	-5	8	2.67	1.13
6	-5	8	0.95	0.73
6	-5	8	2.67	0.59
-7	5	-8	4.59	0.84
-7	5	-8	3.70	0.58
-7	5	-8	3.50	0.51
7	-5	8	2.58	0.67
-8	5	-8	6.48	1.12
-8	5	-8	5.50	0.81
-8	5	-8	7.35	0.76
8	-5	8	4.12	1.52
8	-5	8	3.81	0.78
-9	5	-8	17.43	2.35
-9	5	-8	15.82	1.07
-9	5	-8	21.73	1.23
9	-5	8	20.72	2.89
9	-5	8	12.60	1.40
-10	5	-8	9.52	1.11
-10	5	-8	7.99	0.99
10	-5	8	7.08	1.04
10	-5	8	4.35	2.15
-11	5	-8	5.72	0.76
11	-5	8	4.21	0.99
11	-5	8	7.87	2.49
-12	5	-8	8.67	1.16
12	-5	8	4.48	1.27
15	4	-8	6.80	0.84
15	4	-8	6.46	1.50
14	4	-8	11.67	2.12
14	4	-8	14.26	1.13
14	4	-8	10.23	1.80
14	4	-8	15.53	2.04
13	4	-8	31.75	2.19
13	4	-8	31.74	2.64
13	4	-8	32.71	2.22
13	4	-8	28.93	1.48
12	4	-8	13.12	1.53

12	4	-8	13.71	0.95
12	4	-8	12.05	2.16
12	4	-8	11.11	1.54
12	4	-8	11.80	1.30
-12	-4	8	11.87	1.49
11	4	-8	0.88	0.43
11	4	-8	-0.66	1.00
11	4	-8	0.42	0.71
11	4	-8	0.82	0.59
11	4	-8	-0.46	0.75
-11	-4	8	0.26	0.73
10	4	-8	1.33	0.69
10	4	-8	1.69	0.61
10	4	-8	0.44	0.46
10	4	-8	1.55	0.88
10	4	-8	2.27	0.58
10	4	-8	1.74	0.42
-10	-4	8	1.06	0.67
9	4	-8	4.11	0.73
9	4	-8	3.78	0.58
9	4	-8	5.34	0.94
9	4	-8	5.08	0.80
9	4	-8	4.26	0.74
-9	-4	8	5.32	0.85
-9	-4	8	5.11	0.82
8	4	-8	2.86	0.70
8	4	-8	3.37	0.53
8	4	-8	2.89	0.63
8	4	-8	2.18	0.42
8	4	-8	2.12	0.38
-8	-4	8	3.52	0.69
-8	-4	8	3.30	0.55
7	4	-8	3.52	0.54
7	4	-8	4.10	0.65
7	4	-8	2.94	0.46
7	4	-8	3.22	0.57
7	4	-8	3.41	0.51
7	4	-8	3.13	0.41
-7	-4	8	3.77	0.53
-7	-4	8	2.64	0.59
6	4	-8	-0.31	0.40
6	4	-8	0.08	0.24
6	4	-8	0.22	0.31
-6	-4	8	-0.23	0.31

-6	-4	8	0.48	0.23
-6	-4	8	0.23	0.38
5	4	-8	3.59	0.44
5	4	-8	3.87	0.54
5	4	-8	3.77	0.56
-5	-4	8	3.83	0.64
-5	-4	8	3.72	0.39
-5	-4	8	3.57	0.53
4	4	-8	9.69	0.68
4	4	-8	9.67	0.79
4	4	-8	9.14	0.57
-4	-4	8	10.44	0.79
-4	-4	8	9.53	0.69
-4	-4	8	8.82	0.52
3	4	-8	1.19	0.27
3	4	-8	1.16	0.32
3	4	-8	1.15	0.37
3	4	-8	0.53	0.25
-3	-4	8	1.81	0.28
-3	-4	8	1.22	0.41
-3	-4	8	1.30	0.36
-3	-4	8	0.84	0.36
2	4	-8	62.96	2.12
2	4	-8	61.80	2.23
2	4	-8	65.62	2.23
2	4	-8	59.99	2.10
-2	-4	8	65.97	2.26
-2	-4	8	63.53	2.35
-2	-4	8	59.48	2.29
-2	-4	8	64.02	2.10
1	4	-8	12.89	0.68
1	4	-8	15.09	0.82
1	4	-8	14.27	0.77
1	4	-8	14.76	0.77
-1	-4	8	14.27	1.06
-1	-4	8	13.97	0.83
-1	-4	8	13.45	0.68
0	4	-8	0.21	0.22
0	4	-8	0.36	0.31
0	4	-8	0.04	0.26
0	4	-8	0.11	0.33
0	-4	8	0.32	0.24
0	-4	8	0.49	0.30
0	-4	8	0.84	0.42

0	-4	8	-0.17	0.45
-1	4	-8	-0.02	0.33
-1	4	-8	0.32	0.22
-1	4	-8	-0.23	0.24
-1	4	-8	0.38	0.28
1	-4	8	0.49	0.42
1	-4	8	0.14	0.25
1	-4	8	-0.28	0.47
1	-4	8	0.22	0.29
-2	4	-8	3.95	0.67
-2	4	-8	5.95	0.63
-2	4	-8	5.77	0.55
-2	4	-8	4.69	0.46
2	-4	8	6.53	0.80
2	-4	8	5.22	0.52
2	-4	8	5.53	0.63
2	-4	8	5.39	0.86
-3	4	-8	24.48	1.21
-3	4	-8	24.42	1.09
-3	4	-8	24.96	1.30
-3	4	-8	24.53	1.03
3	-4	8	22.77	1.20
3	-4	8	25.57	1.72
3	-4	8	24.74	1.06
3	-4	8	26.05	1.33
-4	4	-8	29.18	1.51
-4	4	-8	28.40	1.21
-4	4	-8	31.37	1.29
-4	4	-8	29.88	1.45
4	-4	8	30.16	2.03
4	-4	8	31.39	1.54
4	-4	8	30.88	1.42
4	-4	8	29.34	1.24
-5	4	-8	3.43	0.52
-5	4	-8	4.32	0.71
-5	4	-8	3.55	0.71
-5	4	-8	4.28	0.52
5	-4	8	3.15	0.48
5	-4	8	5.76	1.25
5	-4	8	3.48	0.55
5	-4	8	3.88	0.73
-6	4	-8	4.27	0.82
-6	4	-8	3.56	0.48
-6	4	-8	4.75	0.70

6	-4	8	4.11	1.27
6	-4	8	4.92	0.69
-7	4	-8	17.24	1.61
-7	4	-8	17.48	0.94
-7	4	-8	15.54	1.03
7	-4	8	13.45	2.09
7	-4	8	14.84	1.18
-8	4	-8	5.87	0.86
-8	4	-8	4.21	0.56
-8	4	-8	4.94	1.11
8	-4	8	5.17	0.79
8	-4	8	3.20	1.50
-9	4	-8	1.56	0.61
-9	4	-8	1.12	0.49
9	-4	8	3.33	1.72
9	-4	8	2.11	0.76
-10	4	-8	10.63	1.17
-10	4	-8	9.37	0.98
10	-4	8	6.67	1.03
10	-4	8	5.32	2.00
-11	4	-8	12.47	1.18
-11	4	-8	12.60	1.34
11	-4	8	10.27	1.37
11	-4	8	8.39	2.37
-12	4	-8	3.82	0.84
12	-4	8	2.60	3.34
12	-4	8	5.55	1.38
16	3	-8	4.26	1.50
16	3	-8	5.49	0.88
15	3	-8	14.66	2.15
15	3	-8	14.09	1.18
14	3	-8	16.29	2.11
14	3	-8	12.10	1.95
14	3	-8	13.32	2.17
14	3	-8	15.04	1.10
13	3	-8	0.99	0.91
13	3	-8	1.33	1.18
13	3	-8	2.26	1.13
13	3	-8	1.47	0.52
12	3	-8	1.09	0.44
12	3	-8	1.64	1.14
12	3	-8	1.65	0.59
12	3	-8	2.10	0.91
-12	-3	8	2.51	0.80

11	3	-8	4.20	0.91
11	3	-8	4.66	0.66
11	3	-8	3.05	0.49
11	3	-8	5.13	1.05
11	3	-8	4.67	0.81
-11	-3	8	4.73	0.79
10	3	-8	6.32	0.61
10	3	-8	7.34	0.94
10	3	-8	5.32	0.85
10	3	-8	6.30	0.81
-10	-3	8	6.25	1.05
-10	-3	8	3.49	0.71
9	3	-8	0.23	0.29
9	3	-8	0.64	0.57
9	3	-8	1.07	0.57
9	3	-8	0.58	0.61
9	3	-8	1.00	0.61
9	3	-8	0.72	0.40
-9	-3	8	1.75	0.57
-9	-3	8	0.54	0.48
8	3	-8	18.56	1.09
8	3	-8	18.44	0.87
8	3	-8	18.50	0.92
8	3	-8	18.13	0.87
8	3	-8	16.93	1.18
8	3	-8	18.17	1.00
-8	-3	8	19.20	1.19
-8	-3	8	21.07	1.09
7	3	-8	0.77	0.31
7	3	-8	0.54	0.27
7	3	-8	0.54	0.31
7	3	-8	-0.05	0.43
-7	-3	8	0.54	0.38
-7	-3	8	0.01	0.36
6	3	-8	1.28	0.46
6	3	-8	0.89	0.33
6	3	-8	0.72	0.26
-6	-3	8	0.74	0.24
-6	-3	8	0.67	0.34
-6	-3	8	0.59	0.36
5	3	-8	11.71	0.86
5	3	-8	11.43	0.63
5	3	-8	11.53	0.71
-5	-3	8	10.97	0.58

-5	-3	8	13.00	0.78
-5	-3	8	11.90	0.82
4	3	-8	0.43	0.25
4	3	-8	0.44	0.21
4	3	-8	0.44	0.38
-4	-3	8	0.58	0.20
-4	-3	8	0.47	0.29
-4	-3	8	0.52	0.31
3	3	-8	0.71	0.39
3	3	-8	0.01	0.19
3	3	-8	0.30	0.16
3	3	-8	-0.07	0.22
-3	-3	8	-0.12	0.24
-3	-3	8	0.12	0.19
-3	-3	8	0.20	0.32
-3	-3	8	0.30	0.32
2	3	-8	2.72	0.41
2	3	-8	2.12	0.44
2	3	-8	2.47	0.36
2	3	-8	2.17	0.33
-2	-3	8	1.83	0.32
-2	-3	8	2.42	0.31
-2	-3	8	2.67	0.48
-2	-3	8	2.99	0.51
1	3	-8	0.72	0.28
1	3	-8	1.27	0.38
1	3	-8	1.29	0.26
1	3	-8	0.63	0.24
-1	-3	8	0.71	0.39
-1	-3	8	0.58	0.25
-1	-3	8	0.98	0.25
-1	-3	8	1.17	0.49
0	3	-8	25.54	1.11
0	3	-8	27.93	1.17
0	3	-8	25.00	1.02
0	3	-8	25.86	1.17
0	-3	8	27.55	1.05
0	-3	8	24.30	1.16
0	-3	8	27.23	1.40
0	-3	8	28.48	1.33
-1	3	-8	-0.02	0.30
-1	3	-8	0.19	0.20
-1	3	-8	0.51	0.31
-1	3	-8	0.33	0.28

1	-3	8	0.04	0.25
1	-3	8	0.54	0.50
1	-3	8	0.56	0.29
1	-3	8	-0.09	0.40
-2	3	-8	8.83	0.56
-2	3	-8	8.37	0.75
-2	3	-8	8.40	0.74
-2	3	-8	7.27	0.63
2	-3	8	8.70	1.05
2	-3	8	9.01	0.59
2	-3	8	8.06	0.68
2	-3	8	9.34	0.87
-3	3	-8	33.44	1.50
-3	3	-8	30.93	1.48
-3	3	-8	31.85	1.24
-3	3	-8	30.40	1.33
3	-3	8	32.44	1.55
3	-3	8	31.45	1.89
3	-3	8	32.97	1.43
3	-3	8	32.68	1.26
-4	3	-8	7.91	0.94
-4	3	-8	9.41	0.63
-4	3	-8	8.15	0.93
-4	3	-8	8.79	0.72
4	-3	8	9.95	0.80
4	-3	8	9.24	1.03
4	-3	8	8.14	1.32
4	-3	8	8.70	0.69
-5	3	-8	14.51	1.29
-5	3	-8	12.97	0.77
-5	3	-8	13.31	1.18
-5	3	-8	15.03	0.89
5	-3	8	12.89	1.23
5	-3	8	15.40	1.69
5	-3	8	14.95	1.00
5	-3	8	14.39	0.87
-6	3	-8	17.23	0.92
-6	3	-8	19.83	1.57
-6	3	-8	17.95	1.04
6	-3	8	13.20	1.79
6	-3	8	17.60	1.16
-7	3	-8	8.01	0.72
-7	3	-8	6.13	1.05
-7	3	-8	6.67	0.95

7	-3	8	5.64	1.35
7	-3	8	7.22	0.95
-8	3	-8	9.05	0.97
-8	3	-8	7.84	0.81
8	-3	8	7.60	1.59
8	-3	8	9.44	1.09
-9	3	-8	8.59	1.06
-9	3	-8	8.13	0.88
9	-3	8	5.01	1.89
9	-3	8	7.44	1.16
-10	3	-8	8.16	1.10
-10	3	-8	6.48	0.97
10	-3	8	6.91	1.13
10	-3	8	7.97	2.14
-11	3	-8	4.59	0.83
-11	3	-8	3.99	0.78
11	-3	8	4.72	2.18
11	-3	8	6.26	1.04
16	2	-8	9.16	1.66
16	2	-8	7.27	1.13
15	2	-8	7.80	1.66
15	2	-8	6.31	0.77
15	2	-8	7.63	1.41
14	2	-8	5.61	1.25
14	2	-8	3.03	1.17
14	2	-8	3.71	1.32
14	2	-8	4.36	0.67
13	2	-8	3.66	0.61
13	2	-8	5.19	1.21
13	2	-8	3.09	0.57
13	2	-8	4.20	1.23
13	2	-8	2.94	1.01
12	2	-8	5.51	1.06
12	2	-8	5.32	0.99
12	2	-8	4.76	0.64
12	2	-8	5.53	1.07
12	2	-8	4.90	0.57
-12	-2	8	4.43	0.82
11	2	-8	6.25	1.15
11	2	-8	3.99	0.90
11	2	-8	3.06	0.50
11	2	-8	2.58	0.52
11	2	-8	2.84	0.83
-11	-2	8	2.50	0.68

10	2	-8	2.69	0.70
10	2	-8	1.00	0.63
10	2	-8	1.64	0.42
10	2	-8	2.17	0.71
10	2	-8	1.34	0.33
-10	-2	8	-0.14	0.58
-10	-2	8	1.24	0.54
9	2	-8	3.86	0.72
9	2	-8	3.38	0.43
9	2	-8	3.58	0.57
9	2	-8	3.97	0.56
9	2	-8	3.65	0.71
9	2	-8	4.01	0.62
-9	-2	8	4.55	0.81
-9	-2	8	3.90	0.64
8	2	-8	0.66	0.33
8	2	-8	1.10	0.27
8	2	-8	1.33	0.52
8	2	-8	1.01	0.35
-8	-2	8	0.73	0.44
-8	-2	8	1.18	0.44
7	2	-8	0.71	0.30
7	2	-8	0.94	0.27
7	2	-8	1.94	0.50
7	2	-8	1.40	0.31
-7	-2	8	1.49	0.41
-7	-2	8	0.69	0.38
6	2	-8	0.41	0.25
6	2	-8	0.39	0.41
6	2	-8	0.15	0.20
-6	-2	8	-0.44	0.32
-6	-2	8	0.69	0.31
-6	-2	8	0.47	0.21
5	2	-8	6.76	0.53
5	2	-8	6.79	0.47
5	2	-8	5.68	0.70
-5	-2	8	5.13	0.57
-5	-2	8	6.48	0.65
-5	-2	8	6.97	0.44
4	2	-8	0.31	0.19
4	2	-8	0.48	0.25
4	2	-8	0.05	0.33
-4	-2	8	0.20	0.18
-4	-2	8	0.78	0.35

-4	-2	8	0.23	0.23
3	2	-8	1.35	0.40
3	2	-8	1.50	0.26
3	2	-8	1.55	0.29
-3	-2	8	1.47	0.30
-3	-2	8	1.43	0.43
-3	-2	8	1.78	0.26
-3	-2	8	1.39	0.34
-3	-2	8	1.68	0.28
2	2	-8	2.11	0.29
2	2	-8	1.54	0.27
2	2	-8	1.72	0.32
2	2	-8	1.58	0.40
-2	-2	8	2.01	0.36
-2	-2	8	1.27	0.44
-2	-2	8	1.42	0.26
-2	-2	8	1.96	0.41
1	2	-8	1.00	0.29
1	2	-8	0.80	0.38
1	2	-8	0.51	0.22
1	2	-8	0.51	0.27
-1	-2	8	0.75	0.24
-1	-2	8	1.32	0.44
-1	-2	8	0.56	0.41
-1	-2	8	0.57	0.23
0	2	-8	5.36	0.55
0	2	-8	5.24	0.58
0	2	-8	5.08	0.62
0	2	-8	4.68	0.42
0	-2	8	5.41	0.53
0	-2	8	5.40	0.78
0	-2	8	6.50	0.46
0	-2	8	6.97	0.78
-1	2	-8	3.17	0.47
-1	2	-8	3.76	0.52
-1	2	-8	2.93	0.47
-1	2	-8	2.71	0.37
1	-2	8	3.83	0.71
1	-2	8	3.74	0.43
1	-2	8	4.16	0.63
1	-2	8	3.54	0.49
-2	2	-8	11.29	0.63
-2	2	-8	12.92	0.81
-2	2	-8	13.99	0.95

-2	2	-8	10.90	0.88
2	-2	8	12.06	0.67
2	-2	8	12.46	1.02
2	-2	8	11.79	1.14
2	-2	8	13.15	0.80
-3	2	-8	22.54	0.95
-3	2	-8	21.76	1.08
-3	2	-8	22.03	1.29
-3	2	-8	20.60	1.22
3	-2	8	22.39	1.10
3	-2	8	21.11	1.55
3	-2	8	21.50	0.96
3	-2	8	22.91	1.35
-4	2	-8	27.77	1.58
-4	2	-8	27.57	1.11
-4	2	-8	28.81	1.28
-4	2	-8	25.89	1.45
4	-2	8	25.77	1.13
4	-2	8	26.39	1.53
4	-2	8	28.37	1.32
4	-2	8	26.07	1.85
-5	2	-8	10.02	0.67
-5	2	-8	9.75	1.30
-5	2	-8	9.24	0.81
5	-2	8	9.53	0.75
5	-2	8	9.60	1.52
5	-2	8	10.18	0.88
-6	2	-8	13.02	1.61
-6	2	-8	12.59	0.80
-6	2	-8	14.17	0.99
6	-2	8	14.23	1.05
6	-2	8	13.25	1.75
-7	2	-8	39.55	1.75
-7	2	-8	39.02	1.56
7	-2	8	36.10	2.74
7	-2	8	40.40	1.82
-8	2	-8	17.89	1.24
-8	2	-8	17.40	1.04
8	-2	8	16.72	2.39
8	-2	8	18.37	1.28
-9	2	-8	3.44	0.62
-9	2	-8	4.37	0.78
9	-2	8	4.45	0.80
9	-2	8	1.41	1.67

-10	2	-8	13.75	1.12
-10	2	-8	11.12	1.25
10	-2	8	10.69	2.33
10	-2	8	10.32	1.36
-11	2	-8	9.55	1.21
-11	2	-8	8.41	1.33
11	-2	8	5.61	2.22
11	-2	8	8.09	1.08
16	1	-8	2.68	0.66
16	1	-8	3.55	1.34
15	1	-8	5.71	1.36
15	1	-8	7.08	1.72
15	1	-8	7.98	0.96
14	1	-8	0.55	1.03
14	1	-8	-0.46	1.21
14	1	-8	0.71	0.49
14	1	-8	0.40	0.97
13	1	-8	6.72	1.29
13	1	-8	5.76	1.07
13	1	-8	7.03	1.27
13	1	-8	6.32	0.76
13	1	-8	7.11	0.80
-13	-1	8	6.43	1.11
12	1	-8	5.15	0.67
12	1	-8	5.17	0.68
12	1	-8	6.38	1.05
12	1	-8	6.61	1.00
12	1	-8	4.68	1.01
-12	-1	8	5.10	0.75
11	1	-8	0.98	0.35
11	1	-8	1.32	0.77
11	1	-8	1.62	0.79
11	1	-8	0.54	0.65
11	1	-8	1.02	0.36
-11	-1	8	1.34	0.56
10	1	-8	0.52	0.66
10	1	-8	0.37	0.29
10	1	-8	0.31	0.30
10	1	-8	1.62	0.60
10	1	-8	0.58	0.51
-10	-1	8	0.73	0.45
-10	-1	8	1.32	0.57
9	1	-8	-0.78	0.45
9	1	-8	0.26	0.28

9	1	-8	-0.07	0.30
9	1	-8	0.13	0.40
9	1	-8	-0.14	0.22
-9	-1	8	-0.07	0.46
-9	-1	8	0.35	0.39
8	1	-8	0.14	0.25
8	1	-8	0.14	0.23
8	1	-8	0.08	0.43
8	1	-8	0.09	0.28
-8	-1	8	-0.13	0.31
-8	-1	8	-0.12	0.40
7	1	-8	-0.10	0.37
7	1	-8	0.15	0.22
7	1	-8	0.10	0.26
7	1	-8	-0.24	0.19
-7	-1	8	-0.08	0.29
-7	-1	8	0.51	0.37
6	1	-8	3.02	0.36
6	1	-8	2.43	0.52
6	1	-8	4.45	0.43
-6	-1	8	3.27	0.47
-6	-1	8	3.94	0.37
-6	-1	8	3.43	0.53
5	1	-8	4.58	0.40
5	1	-8	5.25	0.47
5	1	-8	4.50	0.68
-5	-1	8	4.20	0.37
-5	-1	8	4.33	0.48
-5	-1	8	4.64	0.65
4	1	-8	0.28	0.22
4	1	-8	-0.09	0.36
4	1	-8	0.58	0.20
-4	-1	8	0.31	0.35
-4	-1	8	0.24	0.18
-4	-1	8	0.30	0.19
3	1	-8	33.05	1.23
3	1	-8	36.23	1.38
3	1	-8	32.88	1.52
-3	-1	8	34.90	1.33
-3	-1	8	34.44	1.21
-3	-1	8	31.22	1.33
-3	-1	8	35.94	1.54
-3	-1	8	36.09	1.28
2	1	-8	0.69	0.22

2	1	-8	0.75	0.39
2	1	-8	0.12	0.23
-2	-1	8	0.51	0.22
-2	-1	8	0.37	0.41
-2	-1	8	0.98	0.39
-2	-1	8	0.48	0.21
1	1	-8	1.73	0.26
1	1	-8	1.94	0.42
1	1	-8	1.48	0.35
-1	-1	8	2.14	0.28
-1	-1	8	2.25	0.55
-1	-1	8	2.20	0.46
-1	-1	8	2.56	0.46
-1	-1	8	2.22	0.39
0	1	-8	27.21	1.26
0	1	-8	26.19	1.02
0	1	-8	26.53	1.27
0	-1	8	26.64	1.39
0	-1	8	25.42	1.15
0	-1	8	25.80	1.02
0	-1	8	27.67	1.32
0	-1	8	28.68	1.33
-1	1	-8	11.21	0.89
-1	1	-8	10.67	0.58
-1	1	-8	10.44	0.77
1	-1	8	11.87	0.98
1	-1	8	12.17	0.98
1	-1	8	10.77	0.72
1	-1	8	12.25	0.63
-2	1	-8	3.78	0.64
-2	1	-8	4.22	0.61
-2	1	-8	5.76	0.45
2	-1	8	6.01	0.96
2	-1	8	5.42	0.58
2	-1	8	5.38	0.88
2	-1	8	5.67	0.50
-3	1	-8	4.71	0.63
-3	1	-8	5.43	0.95
-3	1	-8	5.00	0.46
3	-1	8	4.67	0.52
3	-1	8	5.19	0.89
3	-1	8	4.50	0.60
3	-1	8	4.43	0.71
-4	1	-8	17.82	1.48

-4	1	-8	19.21	0.88
-4	1	-8	19.37	1.07
4	-1	8	22.18	1.62
4	-1	8	19.85	1.09
4	-1	8	19.85	0.93
4	-1	8	17.84	1.67
4	-1	8	17.00	1.35
-5	1	-8	14.14	0.77
-5	1	-8	9.72	1.55
-5	1	-8	13.60	0.95
5	-1	8	15.16	1.60
5	-1	8	13.79	0.85
5	-1	8	14.73	1.00
-6	1	-8	15.43	0.84
-6	1	-8	14.66	1.27
6	-1	8	18.20	2.62
6	-1	8	16.80	1.11
6	-1	8	12.57	0.96
-7	1	-8	20.62	1.06
-7	1	-8	21.82	1.30
7	-1	8	20.61	2.15
7	-1	8	19.80	1.28
-8	1	-8	12.65	0.89
-8	1	-8	11.62	1.13
8	-1	8	12.43	1.15
8	-1	8	6.81	1.60
-9	1	-8	9.73	0.97
-9	1	-8	9.88	1.16
9	-1	8	7.97	1.85
9	-1	8	10.67	1.22
-10	1	-8	12.73	1.32
-10	1	-8	10.11	1.12
10	-1	8	7.64	1.91
10	-1	8	14.00	1.43
-11	1	-8	3.19	1.08
-11	1	-8	4.10	0.81
11	-1	8	1.75	2.17
11	-1	8	1.76	0.93
17	0	-8	3.23	0.79
17	0	-8	1.87	1.50
16	0	-8	11.12	2.08
16	0	-8	7.57	1.53
16	0	-8	7.50	0.89
15	0	-8	0.18	1.09

15	0	-8	0.51	0.55
15	0	-8	0.87	1.43
14	0	-8	0.64	0.94
14	0	-8	3.67	1.33
14	0	-8	2.06	0.89
14	0	-8	1.21	0.52
13	0	-8	6.77	0.75
13	0	-8	7.67	0.64
13	0	-8	6.33	1.25
13	0	-8	7.12	1.12
13	0	-8	8.12	1.49
-13	0	8	6.49	0.98
12	0	-8	1.98	0.38
12	0	-8	2.07	0.43
12	0	-8	3.19	0.79
12	0	-8	2.51	0.93
12	0	-8	2.51	0.84
-12	0	8	2.82	0.58
11	0	-8	0.20	0.61
11	0	-8	0.76	0.75
11	0	-8	1.61	0.34
11	0	-8	0.95	0.31
-11	0	8	1.03	0.48
10	0	-8	-0.25	0.24
10	0	-8	0.52	0.27
10	0	-8	0.00	0.53
10	0	-8	0.43	0.34
10	0	-8	0.50	0.45
-10	0	8	0.62	0.54
-10	0	8	0.54	0.40
9	0	-8	8.73	0.61
9	0	-8	6.89	0.78
9	0	-8	7.92	0.55
9	0	-8	6.54	0.93
9	0	-8	7.55	0.56
9	0	-8	7.94	0.63
-9	0	8	8.09	0.85
-9	0	8	6.34	0.68
8	0	-8	9.55	0.61
8	0	-8	10.18	0.59
8	0	-8	10.93	0.97
8	0	-8	11.54	0.66
-8	0	8	10.26	0.72
-8	0	8	11.45	0.90

7	0	-8	0.51	0.27
7	0	-8	0.34	0.24
7	0	-8	0.28	0.21
7	0	-8	0.60	0.44
-7	0	8	0.44	0.38
-7	0	8	0.35	0.25
6	0	-8	13.73	0.72
6	0	-8	12.79	0.63
6	0	-8	12.78	0.97
-6	0	8	15.28	0.94
-6	0	8	13.28	0.73
5	0	-8	11.84	0.69
5	0	-8	10.11	0.87
5	0	-8	11.40	0.58
-5	0	8	12.31	0.88
-5	0	8	11.10	0.65
-5	0	8	12.34	0.56
4	0	-8	0.53	0.26
4	0	-8	0.62	0.37
4	0	-8	0.64	0.21
-4	0	8	0.76	0.36
-4	0	8	1.10	0.22
-4	0	8	0.63	0.17
-4	0	8	0.90	0.24
-4	0	8	1.33	0.45
3	0	-8	28.74	1.44
3	0	-8	30.48	1.13
3	0	-8	32.79	1.34
-3	0	8	31.88	1.49
-3	0	8	32.39	1.37
-3	0	8	33.60	1.20
-3	0	8	30.65	1.25
-3	0	8	29.87	1.10
2	0	-8	100.18	3.55
2	0	-8	100.84	3.23
2	0	-8	103.59	3.48
-2	0	8	107.43	3.38
-2	0	8	103.51	3.39
-2	0	8	101.62	3.50
-2	0	8	103.84	3.62
-2	0	8	103.07	3.22
1	0	-8	45.56	1.84
1	0	-8	45.11	1.54
1	0	-8	43.66	1.83

-1	0	8	44.83	1.90
-1	0	8	43.61	1.70
-1	0	8	45.59	1.54
-1	0	8	46.78	1.95
-1	0	8	48.53	1.78
0	0	-8	58.94	2.28
0	0	-8	60.87	2.00
0	0	-8	61.17	2.36
0	0	8	57.42	2.41
0	0	8	59.65	1.99
0	0	8	61.29	2.42
0	0	8	62.46	2.20
0	0	8	63.66	2.30
-1	0	-8	25.41	1.42
-1	0	-8	24.06	1.24
-1	0	-8	26.08	1.00
1	0	8	27.08	1.02
1	0	8	26.73	1.51
1	0	8	25.54	1.37
1	0	8	25.83	1.42
1	0	8	27.58	1.21
-2	0	-8	30.17	1.17
-2	0	-8	31.56	1.43
-2	0	-8	34.82	1.74
2	0	8	31.80	1.66
2	0	8	32.14	1.20
2	0	8	31.59	1.65
2	0	8	32.06	1.76
2	0	8	30.30	1.38
-3	0	-8	11.50	0.87
-3	0	-8	12.20	0.65
-3	0	-8	10.99	1.27
3	0	8	12.28	0.88
3	0	8	13.45	0.71
3	0	8	13.27	1.25
3	0	8	11.81	1.37
3	0	8	12.63	1.24
-4	0	-8	32.76	2.07
-4	0	-8	32.96	1.53
-4	0	-8	33.25	1.28
4	0	8	35.35	2.01
4	0	8	34.87	2.26
4	0	8	34.35	1.56
4	0	8	33.89	1.33

-5	0	-8	16.33	1.07
-5	0	-8	15.01	0.82
5	0	8	16.67	1.11
5	0	8	14.05	1.54
5	0	8	16.50	0.90
-6	0	-8	28.14	1.45
-6	0	-8	28.40	1.18
6	0	8	27.51	2.08
6	0	8	25.20	1.26
6	0	8	26.46	1.47
-7	0	-8	24.22	1.10
-7	0	-8	22.51	1.37
7	0	8	22.34	1.40
7	0	8	19.66	2.14
-8	0	-8	7.87	1.05
-8	0	-8	6.70	0.82
8	0	8	8.35	1.08
8	0	8	6.60	1.55
-9	0	-8	10.26	1.00
-9	0	-8	8.88	1.19
9	0	8	5.90	1.70
9	0	8	7.89	1.26
-10	0	-8	11.99	1.19
-10	0	-8	12.48	1.39
10	0	8	11.05	1.47
10	0	8	6.58	1.92
17	-1	-8	1.63	1.37
17	-1	-8	1.99	0.69
16	-1	-8	0.27	0.57
16	-1	-8	3.50	1.79
16	-1	-8	0.36	1.21
15	-1	-8	4.39	1.58
15	-1	-8	2.31	0.58
15	-1	-8	2.46	1.07
14	-1	-8	-0.95	1.20
14	-1	-8	1.05	0.92
14	-1	-8	-0.02	0.80
14	-1	-8	-0.83	1.04
14	-1	-8	0.25	0.46
13	-1	-8	-0.06	0.78
13	-1	-8	1.09	1.06
13	-1	-8	-0.54	0.35
13	-1	-8	-0.16	0.34
-13	1	8	-0.67	0.47

12	-1	-8	0.37	0.58
12	-1	-8	0.34	0.86
12	-1	-8	-0.05	0.66
-12	1	8	0.66	0.55
-12	1	8	-0.12	0.44
11	-1	-8	-0.08	0.28
11	-1	-8	0.04	0.29
11	-1	-8	-0.54	0.73
11	-1	-8	0.20	0.59
-11	1	8	-0.14	0.40
10	-1	-8	-0.44	0.31
10	-1	-8	0.31	0.26
10	-1	-8	0.31	0.29
10	-1	-8	-0.12	0.52
10	-1	-8	-0.68	0.69
-10	1	8	1.36	0.59
-10	1	8	-0.37	0.33
-10	1	8	0.19	0.33
9	-1	-8	0.07	0.28
9	-1	-8	0.15	0.22
9	-1	-8	0.40	0.28
9	-1	-8	-0.59	0.50
-9	1	8	0.80	0.47
-9	1	8	0.09	0.27
-9	1	8	-0.03	0.29
8	-1	-8	0.09	0.26
8	-1	-8	0.15	0.45
8	-1	-8	-0.08	0.25
8	-1	-8	0.04	0.20
-8	1	8	-0.24	0.23
-8	1	8	0.21	0.23
-8	1	8	0.40	0.42
7	-1	-8	13.79	1.06
7	-1	-8	14.04	0.71
7	-1	-8	14.79	0.79
7	-1	-8	14.05	0.66
-7	1	8	14.66	1.02
-7	1	8	13.85	0.74
6	-1	-8	14.63	0.71
6	-1	-8	16.67	0.85
6	-1	-8	16.02	0.74
6	-1	-8	15.85	0.69
6	-1	-8	16.23	1.08
-6	1	8	14.87	0.76

-6	1	8	15.27	0.79
-6	1	8	17.23	1.04
5	-1	-8	6.76	0.52
5	-1	-8	7.48	0.64
5	-1	-8	8.30	0.83
5	-1	-8	7.55	0.46
-5	1	8	7.42	0.53
-5	1	8	7.55	0.71
-5	1	8	7.41	0.43
-5	1	8	8.56	0.79
4	-1	-8	0.44	0.18
4	-1	-8	0.65	0.40
4	-1	-8	0.51	0.33
-4	1	8	0.63	0.18
-4	1	8	0.55	0.22
-4	1	8	0.25	0.41
-4	1	8	0.61	0.43
3	-1	-8	19.11	1.16
3	-1	-8	20.64	1.10
3	-1	-8	20.76	0.81
-3	1	8	21.08	0.78
-3	1	8	19.71	1.19
-3	1	8	20.71	0.92
-3	1	8	20.87	1.21
-3	1	8	19.55	0.93
2	-1	-8	10.79	0.84
2	-1	-8	9.19	0.84
2	-1	-8	9.68	0.52
-2	1	8	9.86	0.64
-2	1	8	10.06	0.50
-2	1	8	10.61	0.96
-2	1	8	9.91	0.98
-2	1	8	10.74	0.68
1	-1	-8	13.82	1.02
1	-1	-8	14.17	0.95
-1	1	8	13.53	1.12
-1	1	8	12.71	0.81
-1	1	8	14.05	0.61
-1	1	8	11.80	0.76
-1	1	8	14.03	1.08
0	-1	-8	22.65	1.24
0	-1	-8	24.79	0.92
0	-1	-8	23.85	1.40
0	1	8	23.30	1.14

0	1	8	26.13	1.54
0	1	8	24.59	1.22
0	1	8	23.21	1.40
0	1	8	25.62	0.94
-1	-1	-8	8.24	0.78
-1	-1	-8	7.00	1.02
-1	-1	-8	7.59	0.48
1	1	8	7.96	0.84
1	1	8	8.12	0.69
1	1	8	8.29	0.52
1	1	8	8.45	1.00
1	1	8	9.22	1.20
-2	-1	-8	19.40	1.09
-2	-1	-8	19.09	0.79
-2	-1	-8	17.86	1.45
2	1	8	19.76	0.85
2	1	8	17.06	1.22
2	1	8	19.49	1.62
2	1	8	18.44	1.05
2	1	8	17.98	1.33
-3	-1	-8	10.52	0.60
-3	-1	-8	10.59	0.90
-3	-1	-8	7.75	1.18
3	1	8	11.64	0.68
3	1	8	9.53	1.28
3	1	8	12.08	1.16
3	1	8	16.01	1.70
3	1	8	11.71	0.90
-4	-1	-8	15.32	2.25
-4	-1	-8	14.01	1.00
-4	-1	-8	14.02	0.74
4	1	8	14.41	1.11
4	1	8	15.38	0.83
4	1	8	13.44	1.38
-5	-1	-8	35.51	1.35
-5	-1	-8	34.73	1.64
5	1	8	34.99	1.70
5	1	8	34.55	1.42
-6	-1	-8	15.57	0.86
-6	-1	-8	17.04	1.20
6	1	8	14.10	1.80
6	1	8	14.62	1.21
6	1	8	16.77	1.00
-7	-1	-8	4.77	0.69

-7	-1	-8	4.13	0.76
7	1	8	4.63	1.33
7	1	8	4.55	0.82
-8	-1	-8	9.30	1.17
-8	-1	-8	6.98	0.85
8	1	8	7.94	1.58
8	1	8	9.93	1.26
-9	-1	-8	10.87	1.32
-9	-1	-8	14.23	1.11
9	1	8	12.31	1.50
9	1	8	9.66	1.83
-10	-1	-8	13.14	1.52
-10	-1	-8	11.18	1.29
10	1	8	13.67	1.57
10	1	8	6.47	1.88
17	-2	-8	2.87	0.70
17	-2	-8	2.40	1.30
16	-2	-8	3.78	1.26
16	-2	-8	5.17	1.79
15	-2	-8	0.37	0.48
15	-2	-8	-1.32	1.40
15	-2	-8	1.30	1.03
14	-2	-8	2.05	0.96
14	-2	-8	-0.59	0.39
14	-2	-8	0.52	1.20
-14	2	8	1.08	0.78
13	-2	-8	-0.35	0.73
13	-2	-8	0.25	0.34
13	-2	-8	0.24	1.06
13	-2	-8	0.46	0.39
-13	2	8	0.68	0.62
-13	2	8	1.29	0.82
12	-2	-8	4.63	0.64
12	-2	-8	5.11	0.59
12	-2	-8	3.76	1.05
12	-2	-8	3.94	0.82
-12	2	8	4.45	0.82
-12	2	8	4.23	0.75
11	-2	-8	2.07	0.36
11	-2	-8	2.72	0.88
11	-2	-8	1.51	0.66
11	-2	-8	1.99	0.42
-11	2	8	2.41	0.45
-11	2	8	2.17	0.51

10	-2	-8	-0.73	0.47
10	-2	-8	-0.05	0.72
10	-2	-8	-0.09	0.26
10	-2	-8	-0.01	0.30
-10	2	8	0.73	0.39
-10	2	8	1.07	0.56
-10	2	8	0.50	0.34
9	-2	-8	0.23	0.33
9	-2	-8	0.40	0.24
9	-2	-8	-0.12	0.25
9	-2	-8	-0.01	0.51
-9	2	8	0.12	0.49
-9	2	8	0.01	0.33
-9	2	8	0.03	0.26
8	-2	-8	19.13	0.82
8	-2	-8	18.51	0.78
8	-2	-8	16.59	1.21
8	-2	-8	16.39	1.07
8	-2	-8	18.32	0.95
-8	2	8	17.93	0.84
-8	2	8	17.87	1.14
-8	2	8	16.98	0.91
7	-2	-8	29.58	1.28
7	-2	-8	27.93	1.05
7	-2	-8	27.98	1.51
7	-2	-8	30.07	1.10
7	-2	-8	28.60	1.30
-7	2	8	28.22	1.13
-7	2	8	28.07	1.26
-7	2	8	26.67	1.41
6	-2	-8	19.41	1.24
6	-2	-8	21.35	0.84
6	-2	-8	20.27	1.07
6	-2	-8	18.59	1.01
6	-2	-8	19.57	0.79
-6	2	8	19.27	1.16
-6	2	8	19.30	0.88
-6	2	8	20.09	1.14
5	-2	-8	59.62	1.93
5	-2	-8	57.72	2.38
5	-2	-8	60.63	2.25
-5	2	8	57.33	1.90
-5	2	8	57.45	2.32
-5	2	8	58.73	2.05

-5	2	8	61.61	2.33
4	-2	-8	11.93	0.99
4	-2	-8	13.48	0.94
4	-2	-8	11.63	0.89
4	-2	-8	12.84	0.59
-4	2	8	13.98	1.10
-4	2	8	13.36	0.56
-4	2	8	12.91	0.72
-4	2	8	12.64	1.03
3	-2	-8	16.09	1.08
3	-2	-8	17.10	1.00
3	-2	-8	15.66	1.07
3	-2	-8	16.98	0.69
-3	2	8	16.49	1.18
-3	2	8	17.67	1.45
-3	2	8	16.59	0.66
-3	2	8	15.98	0.86
2	-2	-8	7.69	0.45
2	-2	-8	8.57	0.93
2	-2	-8	8.11	0.85
-2	2	8	7.74	0.65
-2	2	8	7.87	1.06
-2	2	8	7.34	0.94
-2	2	8	8.55	0.65
1	-2	-8	66.49	2.55
1	-2	-8	66.27	2.15
1	-2	-8	65.10	2.70
-1	2	8	72.59	2.86
-1	2	8	69.20	2.44
-1	2	8	67.44	2.67
-1	2	8	64.47	2.41
-1	2	8	67.28	2.21
0	-2	-8	7.73	0.81
0	-2	-8	6.39	1.01
0	-2	-8	7.14	0.43
0	2	8	7.20	0.45
0	2	8	6.22	0.66
0	2	8	5.68	0.82
0	2	8	7.29	1.04
0	2	8	6.65	0.74
-1	-2	-8	12.69	1.33
-1	-2	-8	13.47	0.62
-1	-2	-8	13.10	0.95
1	2	8	13.49	1.04

1	2	8	14.07	0.92
1	2	8	15.35	1.23
1	2	8	12.73	1.50
1	2	8	13.57	0.65
-2	-2	-8	17.40	1.64
-2	-2	-8	13.82	1.00
-2	-2	-8	13.99	0.66
2	2	8	15.00	1.13
2	2	8	12.12	1.25
2	2	8	14.91	0.72
2	2	8	12.48	1.71
2	2	8	15.34	1.00
-3	-2	-8	16.99	0.77
-3	-2	-8	16.42	1.08
3	2	8	15.91	1.11
3	2	8	18.56	1.35
3	2	8	16.79	0.81
-4	-2	-8	14.69	1.15
-4	-2	-8	16.21	0.78
4	2	8	16.28	0.87
4	2	8	15.86	1.42
4	2	8	17.32	1.18
-5	-2	-8	9.73	0.64
-5	-2	-8	7.17	0.97
5	2	8	7.71	1.01
5	2	8	6.68	0.77
5	2	8	6.33	1.12
-6	-2	-8	11.44	0.77
-6	-2	-8	11.90	1.14
6	2	8	10.98	1.71
6	2	8	10.22	0.95
6	2	8	12.99	1.23
-7	-2	-8	11.87	0.86
-7	-2	-8	10.73	1.21
7	2	8	12.31	1.34
7	2	8	11.05	2.05
-8	-2	-8	13.21	1.32
-8	-2	-8	15.11	1.04
8	2	8	10.68	1.85
8	2	8	13.85	1.51
-9	-2	-8	8.08	1.33
-9	-2	-8	8.18	1.14
9	2	8	7.23	1.12
9	2	8	7.87	1.81

17	-3	-8	3.99	2.12
17	-3	-8	4.59	1.41
16	-3	-8	-1.06	1.64
16	-3	-8	0.65	1.11
16	-3	-8	1.63	0.58
15	-3	-8	1.61	1.03
15	-3	-8	2.00	1.48
15	-3	-8	1.97	0.53
-15	3	8	2.46	1.02
14	-3	-8	2.76	1.02
14	-3	-8	3.64	1.41
14	-3	-8	2.81	0.55
-14	3	8	4.45	0.82
13	-3	-8	0.09	0.36
13	-3	-8	-0.14	1.08
13	-3	-8	-0.23	0.72
-13	3	8	-0.19	0.60
12	-3	-8	0.90	0.68
12	-3	-8	0.59	0.42
12	-3	-8	0.67	0.33
12	-3	-8	0.16	0.91
-12	3	8	0.13	0.64
-12	3	8	1.79	0.59
11	-3	-8	0.51	0.84
11	-3	-8	0.62	0.32
11	-3	-8	0.93	0.46
11	-3	-8	0.80	0.48
11	-3	-8	1.55	0.67
-11	3	8	1.41	0.55
-11	3	8	1.02	0.33
10	-3	-8	0.76	0.77
10	-3	-8	1.24	0.62
10	-3	-8	1.21	0.33
10	-3	-8	0.94	0.29
-10	3	8	0.89	0.30
-10	3	8	1.40	0.52
9	-3	-8	5.96	0.52
9	-3	-8	6.61	1.14
9	-3	-8	5.47	0.74
9	-3	-8	7.79	1.00
9	-3	-8	7.27	0.50
-9	3	8	6.60	0.52
-9	3	8	7.48	0.81
-9	3	8	5.66	0.86

8	-3	-8	12.08	1.15
8	-3	-8	10.60	0.87
8	-3	-8	11.72	0.58
8	-3	-8	12.06	0.63
8	-3	-8	10.58	1.07
-8	3	8	9.87	0.94
-8	3	8	11.80	0.99
-8	3	8	11.31	0.62
7	-3	-8	63.01	2.11
7	-3	-8	65.92	2.54
7	-3	-8	62.96	2.44
7	-3	-8	67.39	2.14
7	-3	-8	62.87	2.61
-7	3	8	67.56	2.22
-7	3	8	66.65	2.53
-7	3	8	63.70	2.52
6	-3	-8	0.73	0.24
6	-3	-8	0.48	0.20
6	-3	-8	0.57	0.57
6	-3	-8	0.26	0.45
6	-3	-8	0.06	0.43
-6	3	8	0.48	0.25
-6	3	8	0.74	0.43
-6	3	8	-0.01	0.57
5	-3	-8	1.34	0.49
5	-3	-8	1.52	0.25
5	-3	-8	1.24	0.51
5	-3	-8	1.32	0.26
5	-3	-8	1.87	0.53
-5	3	8	1.19	0.61
-5	3	8	1.23	0.30
-5	3	8	0.48	0.45
4	-3	-8	3.95	0.35
4	-3	-8	3.53	0.62
4	-3	-8	3.66	0.70
4	-3	-8	4.29	0.68
-4	3	8	3.58	0.49
-4	3	8	5.96	1.04
-4	3	8	2.85	0.61
3	-3	-8	60.54	2.42
3	-3	-8	58.60	2.50
3	-3	-8	61.18	2.36
3	-3	-8	63.48	1.95
-3	3	8	62.25	2.66

-3	3	8	57.29	2.19
-3	3	8	60.81	1.91
-3	3	8	57.29	2.44
2	-3	-8	35.46	1.21
2	-3	-8	35.05	1.69
2	-3	-8	34.35	1.83
2	-3	-8	33.53	1.68
-2	3	8	36.19	1.19
-2	3	8	36.66	1.80
-2	3	8	37.07	2.04
-2	3	8	37.12	1.52
1	-3	-8	9.23	0.90
1	-3	-8	10.85	1.15
1	-3	-8	9.10	0.47
-1	3	8	7.97	0.77
-1	3	8	9.15	0.78
-1	3	8	9.92	0.48
-1	3	8	11.65	1.10
-1	3	8	7.90	1.35
0	-3	-8	36.70	1.22
0	-3	-8	34.66	1.64
0	-3	-8	31.88	1.96
0	3	8	37.11	1.63
0	3	8	37.94	2.21
0	3	8	34.47	1.83
0	3	8	34.16	1.59
-1	-3	-8	38.90	1.33
-1	-3	-8	34.99	2.24
-1	-3	-8	39.26	1.74
1	3	8	40.36	1.36
1	3	8	41.96	1.78
1	3	8	38.61	1.79
1	3	8	37.12	2.01
1	3	8	41.35	2.44
-2	-3	-8	10.02	0.55
-2	-3	-8	9.79	0.94
2	3	8	9.42	1.05
2	3	8	11.73	1.00
2	3	8	12.02	1.88
2	3	8	10.43	0.62
-3	-3	-8	12.46	1.02
-3	-3	-8	12.94	0.65
3	3	8	13.09	0.73
3	3	8	13.26	1.21

3	3	8	13.60	1.12
-4	-3	-8	6.81	0.55
-4	-3	-8	6.94	0.94
4	3	8	6.54	1.02
4	3	8	5.09	0.90
4	3	8	6.85	0.68
-5	-3	-8	13.96	1.19
-5	-3	-8	14.12	0.78
5	3	8	14.36	1.60
5	3	8	13.31	0.91
5	3	8	15.41	1.34
-6	-3	-8	20.00	1.00
-6	-3	-8	19.28	1.39
6	3	8	19.87	1.59
6	3	8	18.08	1.95
6	3	8	18.45	1.16
-7	-3	-8	13.57	0.98
-7	-3	-8	14.18	1.34
7	3	8	15.61	2.15
7	3	8	16.23	1.54
-8	-3	-8	10.49	1.33
-8	-3	-8	8.03	1.03
8	3	8	9.20	1.51
8	3	8	7.84	1.73
-9	-3	-8	9.89	1.57
-9	-3	-8	11.59	1.24
9	3	8	8.11	1.23
9	3	8	5.84	2.07
18	-4	-8	3.73	2.28
18	-4	-8	-1.02	0.88
17	-4	-8	0.72	2.04
17	-4	-8	-1.23	1.19
17	-4	-8	-0.92	0.56
16	-4	-8	0.56	1.13
16	-4	-8	1.10	0.56
16	-4	-8	5.05	1.90
15	-4	-8	3.49	1.64
15	-4	-8	0.17	0.97
-15	4	8	2.70	0.87
14	-4	-8	0.34	0.89
14	-4	-8	-0.37	1.30
-14	4	8	1.86	0.81
13	-4	-8	3.66	1.24
13	-4	-8	1.13	0.81

13	-4	-8	1.99	0.45
-13	4	8	1.68	0.79
12	-4	-8	0.81	0.32
12	-4	-8	2.02	1.05
12	-4	-8	1.39	0.78
12	-4	-8	0.78	0.45
-12	4	8	1.02	0.69
-12	4	8	0.08	0.56
11	-4	-8	0.19	0.86
11	-4	-8	1.31	0.31
11	-4	-8	-0.02	0.57
11	-4	-8	0.59	0.35
11	-4	-8	0.85	0.48
-11	4	8	1.30	0.65
-11	4	8	0.60	0.32
10	-4	-8	2.16	0.87
10	-4	-8	3.71	0.72
10	-4	-8	2.92	0.44
10	-4	-8	2.99	0.41
-10	4	8	1.85	0.60
-10	4	8	3.91	0.44
-10	4	8	4.25	0.73
9	-4	-8	22.88	1.61
9	-4	-8	24.77	0.97
9	-4	-8	23.44	0.94
9	-4	-8	21.28	1.45
9	-4	-8	24.00	1.31
-9	4	8	23.13	0.96
-9	4	8	20.76	1.34
-9	4	8	21.53	1.42
8	-4	-8	17.68	0.75
8	-4	-8	19.00	1.21
8	-4	-8	18.75	1.36
8	-4	-8	18.64	0.80
8	-4	-8	19.02	1.32
-8	4	8	16.81	1.51
-8	4	8	15.94	1.19
-8	4	8	18.23	0.83
7	-4	-8	6.10	0.90
7	-4	-8	5.62	0.93
7	-4	-8	5.47	0.44
7	-4	-8	5.53	0.85
7	-4	-8	5.27	0.40
-7	4	8	6.69	0.84

-7	4	8	6.11	0.51
-7	4	8	5.70	0.99
6	-4	-8	21.15	1.39
6	-4	-8	22.62	0.86
6	-4	-8	20.92	1.40
6	-4	-8	22.28	1.37
6	-4	-8	21.23	0.83
-6	4	8	22.61	1.02
-6	4	8	23.06	1.33
-6	4	8	23.20	1.55
5	-4	-8	13.25	1.11
5	-4	-8	12.05	0.56
5	-4	-8	12.15	1.13
5	-4	-8	11.90	1.08
5	-4	-8	13.24	0.60
-5	4	8	11.19	1.31
-5	4	8	12.26	0.76
-5	4	8	11.05	1.07
4	-4	-8	13.98	1.16
4	-4	-8	13.13	1.11
4	-4	-8	14.28	0.60
4	-4	-8	13.45	1.21
-4	4	8	13.69	0.84
-4	4	8	13.11	1.42
-4	4	8	14.29	1.17
3	-4	-8	38.52	1.27
3	-4	-8	36.18	1.78
3	-4	-8	36.76	1.83
3	-4	-8	36.60	1.96
-3	4	8	36.39	1.85
-3	4	8	37.61	1.60
-3	4	8	37.91	1.22
-3	4	8	38.62	2.17
2	-4	-8	22.32	1.61
2	-4	-8	24.43	1.39
2	-4	-8	22.94	0.85
2	-4	-8	24.63	1.47
-2	4	8	24.96	1.51
-2	4	8	24.87	1.91
-2	4	8	24.25	1.24
-2	4	8	22.60	0.82
1	-4	-8	50.14	2.26
1	-4	-8	47.41	2.42
1	-4	-8	46.02	2.05

1	-4	-8	47.61	1.56
-1	4	8	48.52	2.64
-1	4	8	47.28	2.00
-1	4	8	50.51	2.24
-1	4	8	47.41	1.54
0	-4	-8	41.51	1.89
0	-4	-8	43.99	1.41
0	-4	-8	41.51	2.38
0	4	8	39.51	2.58
0	4	8	44.95	1.43
0	4	8	42.22	1.90
0	4	8	44.04	1.90
0	4	8	43.91	2.18
-1	-4	-8	25.54	1.38
-1	-4	-8	24.71	0.90
1	4	8	22.50	1.40
1	4	8	25.04	1.44
1	4	8	27.04	2.34
-2	-4	-8	13.51	1.11
-2	-4	-8	13.32	0.61
2	4	8	13.07	1.21
2	4	8	13.65	0.69
2	4	8	13.85	1.17
-3	-4	-8	34.36	1.24
-3	-4	-8	33.66	1.69
3	4	8	35.76	1.30
-4	-4	-8	7.77	1.05
-4	-4	-8	7.91	0.59
4	4	8	8.47	0.75
4	4	8	8.71	1.18
4	4	8	9.26	1.31
-5	-4	-8	12.32	1.19
-5	-4	-8	9.76	0.73
5	4	8	10.25	0.88
5	4	8	9.63	1.58
5	4	8	9.72	1.35
-6	-4	-8	12.07	1.33
-6	-4	-8	11.88	0.88
6	4	8	8.62	1.46
6	4	8	11.86	1.54
-7	-4	-8	8.48	0.94
-7	-4	-8	9.22	1.34
7	4	8	7.96	1.54
7	4	8	10.45	1.60

-8	-4	-8	10.86	1.45
8	4	8	11.83	1.71
8	4	8	5.61	1.61
18	-5	-8	-0.47	1.53
17	-5	-8	0.24	1.19
17	-5	-8	0.10	2.08
-17	5	8	-0.87	0.76
16	-5	-8	0.59	1.11
16	-5	-8	-0.10	1.83
-16	5	8	0.97	0.73
15	-5	-8	0.56	1.58
15	-5	-8	0.26	1.01
-15	5	8	2.57	0.68
-15	5	8	1.70	0.98
14	-5	-8	3.03	0.98
14	-5	-8	2.98	1.48
-14	5	8	2.23	0.60
-14	5	8	3.60	0.95
13	-5	-8	3.70	1.37
13	-5	-8	3.08	0.95
-13	5	8	4.86	0.93
-13	5	8	3.95	1.00
12	-5	-8	6.13	1.30
12	-5	-8	6.01	0.59
12	-5	-8	6.34	0.85
-12	5	8	6.61	1.22
11	-5	-8	10.74	0.69
11	-5	-8	11.96	1.00
11	-5	-8	10.74	1.65
11	-5	-8	12.39	0.79
11	-5	-8	10.75	1.27
-11	5	8	14.30	1.37
-11	5	8	11.42	0.64
10	-5	-8	18.22	0.89
10	-5	-8	18.16	1.43
10	-5	-8	17.76	1.67
10	-5	-8	20.31	1.27
10	-5	-8	17.79	0.82
-10	5	8	18.67	1.49
-10	5	8	18.52	0.83
9	-5	-8	1.26	0.66
9	-5	-8	1.86	0.34
9	-5	-8	1.54	0.81
9	-5	-8	1.63	0.61

9	-5	-8	1.71	0.30
-9	5	8	1.55	0.30
-9	5	8	1.77	0.83
-9	5	8	2.29	0.67
8	-5	-8	13.38	0.66
8	-5	-8	12.26	1.16
8	-5	-8	12.42	1.22
8	-5	-8	13.91	0.62
8	-5	-8	13.76	1.38
-8	5	8	13.35	0.72
-8	5	8	13.21	1.42
-8	5	8	12.75	1.11
7	-5	-8	27.22	1.64
7	-5	-8	23.87	1.53
7	-5	-8	26.38	0.96
7	-5	-8	25.39	1.55
7	-5	-8	24.41	0.93
-7	5	8	22.14	1.72
-7	5	8	26.00	1.13
-7	5	8	24.49	1.44
6	-5	-8	13.90	1.27
6	-5	-8	15.29	1.33
6	-5	-8	15.81	0.65
6	-5	-8	14.81	0.63
6	-5	-8	14.42	1.20
-6	5	8	14.42	1.54
-6	5	8	15.78	1.20
-6	5	8	15.28	0.87
5	-5	-8	15.72	0.65
5	-5	-8	16.67	1.37
5	-5	-8	16.48	1.28
5	-5	-8	15.20	1.37
5	-5	-8	16.25	0.66
-5	5	8	17.29	1.27
-5	5	8	15.95	0.95
-5	5	8	14.93	1.62
4	-5	-8	27.93	1.80
4	-5	-8	27.70	1.69
4	-5	-8	28.68	1.61
4	-5	-8	30.74	1.02
4	-5	-8	29.81	1.00
-4	5	8	27.75	1.35
-4	5	8	28.46	1.65
-4	5	8	27.35	1.99

3	-5	-8	43.96	1.48
3	-5	-8	44.00	2.07
3	-5	-8	45.93	2.22
3	-5	-8	51.02	2.42
-3	5	8	45.68	2.59
-3	5	8	47.54	1.95
-3	5	8	46.32	2.20
2	-5	-8	36.34	1.77
2	-5	-8	38.68	2.00
2	-5	-8	34.56	1.16
2	-5	-8	36.39	2.13
-2	5	8	35.28	1.69
-2	5	8	33.69	1.89
-2	5	8	37.42	2.43
1	-5	-8	35.03	2.28
1	-5	-8	42.47	1.90
1	-5	-8	38.95	2.11
-1	5	8	42.78	2.68
-1	5	8	38.99	1.83
-1	5	8	39.74	2.07
0	-5	-8	34.67	1.69
0	5	8	34.00	1.75
0	5	8	36.83	2.65
0	5	8	33.53	1.71
-1	-5	-8	38.51	1.28
-1	-5	-8	38.04	1.83
1	5	8	39.81	1.93
1	5	8	39.49	1.31
1	5	8	38.02	1.93
-2	-5	-8	41.45	2.33
-2	-5	-8	40.15	1.36
2	5	8	35.09	2.94
2	5	8	33.26	2.18
2	5	8	47.23	1.48
-3	-5	-8	31.61	1.16
-3	-5	-8	31.82	1.69
3	5	8	32.80	1.94
3	5	8	33.81	1.91
3	5	8	31.52	1.22
-4	-5	-8	24.19	1.50
-4	-5	-8	22.30	0.97
4	5	8	24.45	1.30
4	5	8	23.34	1.81
4	5	8	20.48	1.71

-5	-5	-8	20.65	1.51
-5	-5	-8	21.65	1.03
5	5	8	20.65	1.78
5	5	8	21.21	1.92
-6	-5	-8	17.75	1.04
-6	-5	-8	18.74	1.50
6	5	8	18.75	1.79
6	5	8	15.07	2.10
-7	-5	-8	12.65	1.48
7	5	8	10.23	1.46
7	5	8	17.37	2.30
18	-6	-8	1.57	1.56
-18	6	8	0.67	0.85
17	-6	-8	0.52	1.20
-17	6	8	-0.43	0.76
16	-6	-8	0.66	1.09
16	-6	-8	1.61	1.78
-16	6	8	0.27	0.70
15	-6	-8	5.01	1.82
15	-6	-8	3.37	1.12
-15	6	8	4.19	0.76
-15	6	8	5.99	1.15
14	-6	-8	-0.43	0.90
14	-6	-8	2.35	1.54
-14	6	8	0.96	0.58
-14	6	8	1.67	0.98
13	-6	-8	2.11	1.39
13	-6	-8	2.13	0.89
-13	6	8	3.29	0.98
-13	6	8	3.34	0.71
12	-6	-8	5.67	0.96
12	-6	-8	4.19	1.01
12	-6	-8	3.49	1.26
-12	6	8	5.82	0.59
-12	6	8	5.63	1.10
11	-6	-8	1.93	1.14
11	-6	-8	3.00	0.46
11	-6	-8	2.28	0.82
11	-6	-8	2.58	0.39
-11	6	8	2.83	0.97
-11	6	8	2.85	0.43
10	-6	-8	2.69	0.88
10	-6	-8	2.09	0.32
10	-6	-8	2.27	0.39

10	-6	-8	1.58	0.68
10	-6	-8	3.08	0.99
-10	6	8	2.92	1.02
-10	6	8	2.03	0.33
9	-6	-8	53.09	1.81
9	-6	-8	55.47	2.38
9	-6	-8	55.77	1.85
9	-6	-8	51.07	2.66
9	-6	-8	55.05	2.50
-9	6	8	54.10	2.69
-9	6	8	54.75	1.92
-9	6	8	52.19	2.36
8	-6	-8	11.48	0.58
8	-6	-8	13.40	1.28
8	-6	-8	10.30	1.19
8	-6	-8	12.48	0.63
8	-6	-8	11.04	1.50
-8	6	8	11.90	1.64
-8	6	8	12.19	1.20
-8	6	8	11.64	0.76
7	-6	-8	2.25	0.68
7	-6	-8	2.06	0.74
7	-6	-8	2.34	0.30
7	-6	-8	2.39	0.87
7	-6	-8	1.80	0.25
-7	6	8	3.13	0.78
-7	6	8	3.61	1.06
-7	6	8	2.09	0.40
6	-6	-8	51.90	2.36
6	-6	-8	50.03	2.50
6	-6	-8	52.48	1.68
6	-6	-8	51.37	2.46
-6	6	8	51.51	2.02
-6	6	8	52.31	2.77
-6	6	8	51.12	2.29
5	-6	-8	20.87	1.64
5	-6	-8	22.64	0.84
5	-6	-8	21.95	0.80
5	-6	-8	23.20	1.70
5	-6	-8	20.10	1.48
-5	6	8	22.48	1.51
-5	6	8	23.57	1.22
-5	6	8	22.22	2.02
4	-6	-8	13.98	0.56

4	-6	-8	14.16	0.54
4	-6	-8	12.04	1.19
4	-6	-8	12.91	1.53
4	-6	-8	13.49	1.46
-4	6	8	12.66	1.35
-4	6	8	14.43	1.04
-4	6	8	14.16	1.84
3	-6	-8	22.37	1.42
3	-6	-8	21.15	1.67
3	-6	-8	17.06	1.80
-3	6	8	23.73	2.17
-3	6	8	21.84	1.65
-3	6	8	22.80	1.31
2	-6	-8	13.80	1.50
2	-6	-8	10.15	1.70
2	-6	-8	12.90	1.13
-2	6	8	14.06	2.10
-2	6	8	13.06	1.10
-2	6	8	15.38	1.45
1	-6	-8	19.03	1.34
1	-6	-8	19.27	1.64
-1	6	8	20.00	1.35
-1	6	8	18.62	2.35
0	-6	-8	23.64	1.87
0	-6	-8	20.25	1.32
0	6	8	19.10	1.41
1	6	8	7.88	1.11
1	6	8	7.06	1.10
-2	-6	-8	15.97	1.28
2	6	8	15.81	0.70
2	6	8	16.69	1.52
2	6	8	15.72	1.50
-3	-6	-8	13.27	1.30
-3	-6	-8	15.84	0.72
3	6	8	14.93	1.57
3	6	8	14.85	1.54
-4	-6	-8	17.11	1.44
-4	-6	-8	17.58	0.86
4	6	8	13.86	1.76
4	6	8	15.60	1.70
-5	-6	-8	13.23	1.43
5	6	8	14.50	1.74
-6	-6	-8	8.65	1.41
6	6	8	11.41	1.80

6	6	8	6.61	1.43
-7	-6	-8	6.66	1.17
7	6	8	8.49	1.42
7	6	8	9.25	2.06
18	-7	-8	-1.29	1.57
18	-7	-8	2.38	2.89
-18	7	8	0.19	0.90
17	-7	-8	3.30	1.43
17	-7	-8	3.18	2.11
-17	7	8	0.80	0.79
16	-7	-8	3.88	1.26
16	-7	-8	5.93	2.07
-16	7	8	3.78	0.80
15	-7	-8	0.23	1.70
15	-7	-8	2.14	1.06
-15	7	8	2.53	0.67
14	-7	-8	1.97	0.96
14	-7	-8	0.29	1.54
-14	7	8	2.48	1.22
-14	7	8	0.83	0.55
13	-7	-8	0.83	1.34
13	-7	-8	0.13	0.85
-13	7	8	-0.04	0.50
-13	7	8	-1.47	1.09
12	-7	-8	-0.90	0.67
12	-7	-8	0.27	1.22
12	-7	-8	0.30	0.74
-12	7	8	-0.82	1.00
-12	7	8	0.27	0.28
11	-7	-8	17.70	2.08
11	-7	-8	18.32	0.94
11	-7	-8	17.55	1.41
11	-7	-8	19.24	0.88
11	-7	-8	19.03	1.64
-11	7	8	17.95	2.01
-11	7	8	18.10	0.84
10	-7	-8	5.71	0.59
10	-7	-8	4.57	0.86
10	-7	-8	7.42	1.39
10	-7	-8	5.64	0.94
10	-7	-8	5.66	0.49
-10	7	8	4.67	0.54
-10	7	8	4.97	1.29
9	-7	-8	2.31	0.80

9	-7	-8	1.95	0.36
9	-7	-8	2.15	1.05
9	-7	-8	0.08	0.72
9	-7	-8	1.65	0.29
-9	7	8	1.57	0.38
-9	7	8	-0.76	1.07
8	-7	-8	42.10	1.45
8	-7	-8	41.69	2.21
8	-7	-8	41.44	1.42
8	-7	-8	40.47	2.15
8	-7	-8	41.36	2.45
-8	7	8	41.12	1.68
-8	7	8	43.60	2.08
-8	7	8	44.37	2.68
7	-7	-8	72.15	2.24
7	-7	-8	70.49	2.24
7	-7	-8	68.83	3.09
7	-7	-8	66.55	2.94
7	-7	-8	70.34	3.23
-7	7	8	74.43	3.46
-7	7	8	71.26	2.60
-7	7	8	69.40	2.89
6	-7	-8	9.99	1.28
6	-7	-8	13.29	1.61
6	-7	-8	12.33	0.55
6	-7	-8	12.81	0.57
6	-7	-8	11.58	1.31
-6	7	8	13.25	2.00
-6	7	8	11.64	1.30
-6	7	8	13.62	0.99
5	-7	-8	12.75	1.55
5	-7	-8	14.08	0.54
5	-7	-8	15.69	1.72
5	-7	-8	14.34	1.33
-5	7	8	13.36	1.05
-5	7	8	15.93	2.07
-5	7	8	12.84	1.43
4	-7	-8	60.69	2.61
4	-7	-8	57.66	2.88
4	-7	-8	59.55	3.05
-4	7	8	59.55	2.72
-4	7	8	62.91	3.45
-4	7	8	63.25	2.51
3	-7	-8	30.32	1.69

3	-7	-8	26.29	1.98
-3	7	8	30.96	1.65
-3	7	8	29.79	2.69
-3	7	8	29.31	2.24
2	-7	-8	21.87	1.93
2	-7	-8	25.59	1.53
-2	7	8	19.77	3.25
-2	7	8	22.33	1.46
1	-7	-8	19.69	1.83
1	-7	-8	20.78	1.42
-1	7	8	22.36	1.50
0	7	8	19.93	1.53
-1	-7	-8	24.43	1.55
1	7	8	24.51	1.75
1	7	8	24.49	1.72
-2	-7	-8	15.38	1.35
2	7	8	17.32	1.65
2	7	8	14.03	1.60
-3	-7	-8	10.60	1.27
3	7	8	9.04	1.36
3	7	8	10.82	1.58
-4	-7	-8	13.20	1.46
4	7	8	16.00	1.88
4	7	8	13.35	1.85
-5	-7	-8	14.83	1.55
5	7	8	14.16	1.92
5	7	8	14.80	1.98
-6	-7	-8	10.49	1.57
6	7	8	12.19	2.03
6	7	8	7.32	1.53
18	-8	-8	1.30	2.16
18	-8	-8	3.36	2.78
-18	8	8	0.11	0.87
17	-8	-8	-0.06	1.28
17	-8	-8	3.22	2.27
-17	8	8	1.11	0.77
16	-8	-8	-1.20	1.20
16	-8	-8	-0.99	3.73
-16	8	8	0.64	0.67
15	-8	-8	1.88	1.79
15	-8	-8	1.69	1.20
-15	8	8	3.76	0.73
14	-8	-8	3.32	1.18
14	-8	-8	3.53	1.79

-14	8	8	2.78	0.64
13	-8	-8	3.16	1.07
13	-8	-8	1.70	1.44
-13	8	8	1.55	0.46
-13	8	8	1.54	1.38
-13	8	8	1.11	0.55
-13	8	8	2.04	0.55
12	-8	-8	6.45	1.14
12	-8	-8	7.48	1.63
-12	8	8	11.52	2.01
-12	8	8	6.16	0.56
11	-8	-8	4.92	0.95
11	-8	-8	4.70	1.42
11	-8	-8	5.17	0.68
11	-8	-8	4.33	0.54
-11	8	8	3.65	1.36
-11	8	8	3.66	0.52
10	-8	-8	6.85	0.53
10	-8	-8	9.09	1.47
10	-8	-8	6.98	0.66
10	-8	-8	6.19	1.45
10	-8	-8	6.77	1.05
-10	8	8	6.90	1.50
-10	8	8	6.49	0.67
9	-8	-8	30.60	1.16
9	-8	-8	27.30	1.86
9	-8	-8	28.13	2.34
9	-8	-8	27.65	2.02
9	-8	-8	29.17	1.09
-9	8	8	31.58	2.52
-9	8	8	29.80	1.34
8	-8	-8	25.18	0.97
8	-8	-8	25.11	0.93
8	-8	-8	25.43	2.15
8	-8	-8	22.56	1.92
8	-8	-8	24.08	1.75
-8	8	8	25.04	1.30
-8	8	8	23.22	2.41
7	-8	-8	2.97	1.12
7	-8	-8	2.63	0.35
7	-8	-8	3.22	1.00
7	-8	-8	3.89	0.91
-7	8	8	0.03	1.29
-7	8	8	3.29	0.61

6	-8	-8	25.75	2.15
6	-8	-8	25.02	1.74
6	-8	-8	25.75	2.15
-6	8	8	24.18	1.43
-6	8	8	29.76	2.62
5	-8	-8	17.19	1.49
5	-8	-8	17.10	1.98
5	-8	-8	17.81	1.85
-5	8	8	17.88	1.26
-5	8	8	22.01	2.61
4	-8	-8	10.69	1.65
4	-8	-8	10.56	1.29
-4	8	8	13.17	2.37
-4	8	8	10.61	1.15
3	-8	-8	25.70	2.09
3	-8	-8	23.76	1.60
-3	8	8	25.30	1.61
2	-8	-8	23.42	2.04
2	-8	-8	23.22	1.56
1	-8	-8	5.95	1.40
-1	8	8	7.15	1.24
0	-8	-8	21.00	2.13
0	-8	-8	17.30	1.42
0	8	8	22.50	1.75
-1	-8	-8	17.30	1.47
1	8	8	13.60	1.60
1	8	8	19.10	1.77
-2	-8	-8	6.40	0.99
2	8	8	4.48	1.09
2	8	8	5.91	1.16
-3	-8	-8	14.67	1.52
3	8	8	16.70	1.89
3	8	8	14.08	1.85
-4	-8	-8	15.14	1.59
4	8	8	14.61	2.05
4	8	8	16.74	2.04
5	8	8	5.72	1.29
5	8	8	3.90	1.42
17	-9	-8	-2.10	2.25
17	-9	-8	0.86	1.46
-17	9	8	0.61	0.80
16	-9	-8	1.75	1.29
16	-9	-8	1.78	2.13
-16	9	8	2.81	0.79

15	-9	-8	1.44	2.16
15	-9	-8	0.14	1.19
-15	9	8	0.00	0.62
14	-9	-8	7.58	1.44
14	-9	-8	3.51	1.86
-14	9	8	4.73	0.71
13	-9	-8	15.87	1.93
13	-9	-8	17.71	2.74
-13	9	8	14.93	0.78
-13	9	8	16.95	1.09
12	-9	-8	22.33	1.17
12	-9	-8	20.40	1.94
12	-9	-8	22.15	2.67
-12	9	8	22.26	1.01
-12	9	8	21.98	1.50
-12	9	8	21.46	2.58
11	-9	-8	18.31	0.89
11	-9	-8	19.16	2.48
11	-9	-8	19.12	1.83
11	-9	-8	20.88	1.02
-11	9	8	19.09	1.01
-11	9	8	18.81	2.55
10	-9	-8	29.29	2.57
10	-9	-8	21.96	2.14
10	-9	-8	22.63	2.05
10	-9	-8	26.10	1.10
10	-9	-8	24.21	0.99
-10	9	8	23.57	2.61
-10	9	8	25.74	1.25
9	-9	-8	13.98	1.91
9	-9	-8	16.07	0.81
9	-9	-8	15.84	0.70
9	-9	-8	15.76	1.60
9	-9	-8	14.54	2.13
-9	9	8	14.64	2.59
-9	9	8	15.89	1.05
8	-9	-8	33.33	2.47
8	-9	-8	35.30	2.11
8	-9	-8	36.86	2.65
8	-9	-8	35.96	1.29
8	-9	-8	36.33	1.24
-8	9	8	40.73	3.13
-8	9	8	37.30	1.73
7	-9	-8	44.72	1.44

7	-9	-8	44.27	2.79
7	-9	-8	42.30	1.43
7	-9	-8	44.54	2.34
7	-9	-8	41.20	2.78
-7	9	8	45.14	2.01
-7	9	8	39.45	3.16
6	-9	-8	11.05	1.89
6	-9	-8	10.33	1.41
-6	9	8	12.15	2.37
-6	9	8	11.12	1.16
5	-9	-8	14.57	1.45
5	-9	-8	18.89	2.04
-5	9	8	13.40	1.27
4	-9	-8	48.74	2.35
4	-9	-8	43.49	2.81
-4	9	8	51.42	2.37
3	-9	-8	37.30	2.91
3	-9	-8	41.61	2.10
-3	9	8	41.25	2.20
2	-9	-8	20.46	2.10
2	-9	-8	16.68	1.46
-2	9	8	17.09	1.61
1	-9	-8	3.34	0.83
1	-9	-8	3.12	1.24
-1	9	8	2.78	0.87
0	-9	-8	23.05	1.61
0	-9	-8	20.37	2.33
0	9	8	23.62	1.93
-1	-9	-8	15.02	1.51
1	9	8	14.06	1.83
-2	-9	-8	17.12	1.54
2	9	8	11.67	1.85
2	9	8	13.22	1.86
-3	-9	-8	21.03	1.77
3	9	8	21.85	2.15
3	9	8	17.49	2.10
-4	-9	-8	9.79	1.54
4	9	8	9.05	1.60
4	9	8	8.78	1.45
17	-10	-8	-0.38	1.57
17	-10	-8	3.60	2.47
-17	10	8	-0.73	0.83
16	-10	-8	2.63	2.29
16	-10	-8	-0.38	1.39

-16	10	8	2.38	0.79
15	-10	-8	4.74	2.19
15	-10	-8	-1.63	1.29
-15	10	8	1.96	0.71
14	-10	-8	9.85	1.57
14	-10	-8	12.23	3.91
-14	10	8	10.34	1.05
13	-10	-8	42.56	2.71
13	-10	-8	45.27	3.54
-13	10	8	45.56	1.83
-13	10	8	41.97	1.60
12	-10	-8	6.67	0.86
12	-10	-8	10.03	1.43
12	-10	-8	6.28	1.78
-12	10	8	9.66	0.79
-12	10	8	8.78	1.40
11	-10	-8	3.53	1.10
11	-10	-8	2.18	1.51
11	-10	-8	2.66	0.52
11	-10	-8	3.53	0.54
-11	10	8	5.24	1.01
-11	10	8	4.95	0.76
10	-10	-8	29.63	2.77
10	-10	-8	25.92	1.99
10	-10	-8	26.17	1.12
10	-10	-8	24.48	1.01
-10	10	8	23.00	1.79
-10	10	8	24.21	1.35
9	-10	-8	39.31	2.71
9	-10	-8	43.96	1.48
9	-10	-8	44.13	1.55
9	-10	-8	43.17	3.12
-9	10	8	44.21	1.97
8	-10	-8	24.26	1.89
8	-10	-8	23.99	0.89
8	-10	-8	19.48	2.40
8	-10	-8	24.55	2.46
-8	10	8	24.22	1.49
7	-10	-8	27.03	2.58
7	-10	-8	30.14	2.03
-7	10	8	30.26	1.72
6	-10	-8	40.84	2.26
6	-10	-8	39.59	2.86
-6	10	8	38.78	2.07

5	-10	-8	46.58	3.50
5	-10	-8	40.56	2.31
4	-10	-8	30.53	2.53
4	-10	-8	29.35	2.79
-4	10	8	24.79	1.85
3	-10	-8	11.74	1.42
3	-10	-8	12.49	2.13
-3	10	8	10.11	1.51
2	-10	-8	17.71	2.27
2	-10	-8	17.09	1.52
-2	10	8	15.21	1.75
1	-10	-8	12.70	2.28
1	-10	-8	10.80	1.45
-1	10	8	13.77	1.76
0	-10	-8	18.74	2.54
0	-10	-8	22.85	1.75
0	10	8	25.67	2.14
-1	-10	-8	18.96	1.65
1	10	8	19.99	2.12
-2	-10	-8	13.00	1.62
2	10	8	9.73	1.52
2	10	8	9.53	1.53
-3	-10	-8	13.08	1.68
3	10	8	12.05	2.17
3	10	8	11.93	2.13
17	-11	-8	2.89	1.71
17	-11	-8	-0.41	2.58
-17	11	8	2.19	0.91
16	-11	-8	4.09	2.64
16	-11	-8	4.57	1.66
-16	11	8	4.52	0.89
15	-11	-8	9.95	2.63
15	-11	-8	12.59	1.95
-15	11	8	13.93	1.30
14	-11	-8	3.95	2.09
14	-11	-8	7.50	1.58
-14	11	8	11.48	1.12
13	-11	-8	9.80	1.64
13	-11	-8	8.01	2.15
-13	11	8	10.45	0.84
12	-11	-8	10.80	2.16
12	-11	-8	6.21	1.40
12	-11	-8	4.96	0.67
-12	11	8	6.54	0.89

11	-11	-8	7.27	0.79
11	-11	-8	4.02	0.58
11	-11	-8	6.05	1.30
-11	11	8	6.15	1.18
-11	11	8	5.93	0.94
10	-11	-8	11.37	1.74
10	-11	-8	10.66	0.77
10	-11	-8	10.46	0.65
10	-11	-8	13.50	2.64
-10	11	8	11.81	1.14
-10	11	8	10.46	1.85
9	-11	-8	10.21	0.58
9	-11	-8	9.52	0.67
9	-11	-8	10.32	1.75
-9	11	8	11.20	1.22
8	-11	-8	17.17	1.87
-8	11	8	18.36	1.46
7	-11	-8	55.51	2.76
7	-11	-8	54.87	3.46
-7	11	8	51.48	2.57
6	-11	-8	31.14	2.11
6	-11	-8	28.95	2.77
-6	11	8	31.14	1.98
5	-11	-8	17.68	2.43
5	-11	-8	22.00	1.81
-5	11	8	22.66	1.83
4	-11	-8	8.42	1.76
4	-11	-8	6.06	1.05
-4	11	8	8.18	1.21
3	-11	-8	14.84	1.56
3	-11	-8	13.50	2.37
-3	11	8	10.84	1.73
2	-11	-8	20.08	2.58
2	-11	-8	20.15	1.68
-2	11	8	15.62	1.98
1	-11	-8	20.91	1.76
1	-11	-8	23.45	2.77
-1	11	8	19.72	2.09
0	-11	-8	17.49	1.71
0	-11	-8	13.80	2.07
0	11	8	18.56	2.18
-1	-11	-8	18.60	1.81
1	11	8	19.59	2.28
2	11	8	6.26	1.55

2	11	8	5.44	1.57
16	-12	-8	3.64	1.66
16	-12	-8	0.76	2.66
-16	12	8	2.41	0.85
15	-12	-8	8.16	2.66
15	-12	-8	5.59	1.72
-15	12	8	6.88	0.94
14	-12	-8	13.72	2.74
14	-12	-8	10.34	1.82
-14	12	8	12.05	1.22
13	-12	-8	24.04	2.43
13	-12	-8	21.55	3.53
-13	12	8	19.54	1.24
12	-12	-8	15.59	2.52
12	-12	-8	9.97	1.65
12	-12	-8	13.69	0.88
-12	12	8	12.25	1.15
11	-12	-8	2.76	0.44
11	-12	-8	4.80	1.35
11	-12	-8	6.14	1.97
-11	12	8	3.59	0.76
-11	12	8	1.36	1.12
10	-12	-8	15.48	0.76
10	-12	-8	15.57	2.01
10	-12	-8	13.68	2.26
-10	12	8	13.25	1.37
9	-12	-8	24.39	2.25
9	-12	-8	24.26	2.94
-9	12	8	24.76	1.70
8	-12	-8	4.82	1.78
8	-12	-8	8.21	1.34
-8	12	8	4.07	0.94
7	-12	-8	17.95	2.82
7	-12	-8	18.78	1.95
-7	12	8	19.32	1.73
6	-12	-8	25.47	2.85
6	-12	-8	26.65	2.10
-6	12	8	26.16	2.03
5	-12	-8	10.60	2.04
5	-12	-8	10.46	1.69
-5	12	8	12.64	1.77
4	-12	-8	6.65	1.25
4	-12	-8	6.79	1.76
-4	12	8	8.77	1.38

3	-12	-8	12.91	1.70
3	-12	-8	15.94	2.75
-3	12	8	15.62	2.02
2	-12	-8	16.07	2.28
2	-12	-8	15.26	1.73
-2	12	8	13.40	2.06
1	-12	-8	22.51	1.90
1	-12	-8	21.24	2.90
-1	12	8	17.99	2.28
0	-12	-8	21.26	1.92
0	-12	-8	18.13	2.96
0	12	8	20.77	2.39
-1	-12	-8	11.50	1.83
16	-13	-8	1.57	1.85
16	-13	-8	2.72	2.74
15	-13	-8	7.65	2.87
15	-13	-8	5.87	1.75
-15	13	8	6.73	0.96
14	-13	-8	12.47	2.81
14	-13	-8	11.70	1.95
13	-13	-8	15.08	2.55
13	-13	-8	13.03	0.97
13	-13	-8	18.62	2.86
12	-13	-8	18.68	1.03
12	-13	-8	21.94	2.53
12	-13	-8	20.24	3.01
-12	13	8	21.78	1.53
11	-13	-8	15.76	2.35
11	-13	-8	20.12	2.87
11	-13	-8	18.27	0.90
-11	13	8	17.43	1.58
10	-13	-8	29.05	3.45
10	-13	-8	19.76	2.44
9	-13	-8	17.38	3.14
-9	13	8	18.89	1.73
8	-13	-8	10.17	2.29
8	-13	-8	12.57	2.03
-8	13	8	8.74	1.25
7	-13	-8	24.59	2.26
7	-13	-8	30.44	3.26
-7	13	8	27.52	2.17
6	-13	-8	20.51	2.99
6	-13	-8	17.07	1.97
-6	13	8	19.40	2.05

5	-13	-8	12.15	2.34
5	-13	-8	11.52	1.45
-5	13	8	11.55	2.02
4	-13	-8	12.46	1.89
4	-13	-8	14.45	2.30
-4	13	8	10.76	1.60
3	-13	-8	19.66	1.97
3	-13	-8	20.36	3.12
-3	13	8	20.63	2.36
2	-13	-8	18.62	3.01
2	-13	-8	17.71	1.94
-2	13	8	16.42	2.41
1	-13	-8	19.28	2.01
1	-13	-8	21.45	3.27
-1	13	8	18.09	2.58
15	-14	-8	6.26	2.89
14	-14	-8	9.23	2.77
14	-14	-8	6.10	1.90
13	-14	-8	24.89	3.39
13	-14	-8	27.25	2.95
12	-14	-8	21.69	2.78
12	-14	-8	17.60	3.09
-12	14	8	17.88	1.68
11	-14	-8	11.46	1.95
11	-14	-8	4.62	2.35
-11	14	8	9.61	1.62
10	-14	-8	21.11	3.57
10	-14	-8	22.68	2.67
-10	14	8	23.01	1.98
9	-14	-8	36.35	3.82
9	-14	-8	26.05	2.59
-9	14	8	22.58	2.14
8	-14	-8	12.95	1.81
8	-14	-8	23.69	3.48
-8	14	8	17.28	2.12
7	-14	-8	8.55	2.22
7	-14	-8	10.20	1.67
-7	14	8	14.46	2.10
6	-14	-8	17.86	2.20
6	-14	-8	21.48	3.35
-6	14	8	22.86	2.35
5	-14	-8	14.15	2.13
5	-14	-8	17.19	2.59
-5	14	8	16.19	2.26

4	-14	-8	12.94	2.39
4	-14	-8	14.94	2.02
-4	14	8	13.69	2.28
3	-14	-8	24.03	3.48
3	-14	-8	19.28	2.11
-3	14	8	20.15	2.58
13	-15	-8	19.54	3.28
13	-15	-8	16.40	2.35
12	-15	-8	17.26	2.31
12	-15	-8	19.80	3.32
-12	15	8	16.28	1.88
11	-15	-8	26.97	4.17
11	-15	-8	19.46	2.97
-11	15	8	18.69	2.11
10	-15	-8	16.95	2.99
10	-15	-8	22.58	2.85
-10	15	8	23.62	2.31
9	-15	-8	16.50	2.05
9	-15	-8	14.73	2.91
-9	15	8	13.48	2.21
8	-15	-8	19.50	2.62
8	-15	-8	24.44	3.89
-8	15	8	22.50	2.41
7	-15	-8	15.90	2.81
7	-15	-8	17.25	1.98
-7	15	8	15.32	2.36
6	-15	-8	16.17	2.83
6	-15	-8	16.02	2.39
-6	15	8	16.35	2.41
5	-15	-8	14.87	2.71
5	-15	-8	15.74	2.25
-5	15	8	13.66	1.90
-2	18	-9	6.55	0.95
2	-18	9	9.11	3.06
2	-18	9	5.88	1.78
-3	18	-9	4.22	0.87
3	-18	9	4.99	2.91
3	-18	9	4.72	1.90
-4	18	-9	0.69	0.74
4	-18	9	2.95	2.87
4	-18	9	-1.25	1.58
-5	18	-9	1.90	0.78
5	-18	9	1.07	1.66
5	-18	9	4.54	2.89

2	17	-9	10.37	1.38
-2	-17	9	7.70	2.79
1	17	-9	2.32	0.78
-1	-17	9	2.44	2.55
-1	-17	9	3.16	1.66
0	17	-9	5.83	0.90
0	-17	9	6.18	1.75
0	-17	9	5.52	2.73
-1	17	-9	7.14	0.94
1	-17	9	6.27	2.60
1	-17	9	4.81	1.64
-2	17	-9	2.47	0.72
2	-17	9	1.11	2.34
2	-17	9	0.78	1.48
-3	17	-9	1.58	0.73
3	-17	9	-2.03	1.35
3	-17	9	2.65	2.35
-4	17	-9	2.26	0.77
4	-17	9	2.80	2.43
4	-17	9	2.35	1.52
-5	17	-9	0.69	0.72
5	-17	9	0.16	1.50
5	-17	9	3.33	2.64
-6	17	-9	2.01	0.82
6	-17	9	-1.20	2.58
6	-17	9	4.71	1.59
-7	17	-9	0.32	0.71
7	-17	9	1.79	2.71
7	-17	9	-0.19	1.50
4	16	-9	8.88	1.14
-4	-16	9	8.66	2.26
-4	-16	9	11.05	2.64
3	16	-9	4.33	0.93
-3	-16	9	6.64	2.53
-3	-16	9	2.28	1.58
2	16	-9	7.26	0.95
-2	-16	9	6.78	2.52
-2	-16	9	5.26	1.75
1	16	-9	7.65	1.19
-1	-16	9	9.17	2.44
-1	-16	9	7.52	1.63
0	16	-9	0.89	0.66
0	-16	9	1.99	1.38
0	-16	9	0.08	2.14

-1	16	-9	0.93	0.65
1	-16	9	3.14	1.44
1	-16	9	1.61	2.32
-2	16	-9	1.25	0.66
2	-16	9	0.15	2.03
2	-16	9	0.27	1.27
-3	16	-9	2.33	0.69
3	-16	9	2.91	1.39
3	-16	9	-0.83	2.08
-4	16	-9	2.32	0.69
4	-16	9	0.99	2.10
4	-16	9	1.84	1.37
-5	16	-9	2.06	0.70
5	-16	9	1.27	1.30
5	-16	9	-1.06	2.25
-6	16	-9	1.32	0.68
6	-16	9	1.35	2.30
6	-16	9	0.82	1.40
-7	16	-9	-0.47	0.67
7	-16	9	-0.54	1.34
7	-16	9	-2.40	2.53
-8	16	-9	-0.47	0.66
8	-16	9	-2.84	2.49
8	-16	9	-1.48	1.43
-9	16	-9	0.74	0.76
9	-16	9	1.95	1.57
9	-16	9	2.34	2.93
-6	-15	9	11.26	2.50
5	15	-9	2.26	0.93
-5	-15	9	4.86	1.72
-5	-15	9	3.30	2.17
4	15	-9	6.18	0.98
-4	-15	9	9.49	2.26
-4	-15	9	5.76	1.72
3	15	-9	10.53	1.36
-3	-15	9	11.27	2.31
-3	-15	9	12.32	1.96
2	15	-9	3.15	0.77
-2	-15	9	3.01	2.03
-2	-15	9	2.27	1.37
1	15	-9	1.76	0.66
-1	-15	9	0.72	1.22
-1	-15	9	3.39	1.90
0	15	-9	0.30	0.59

0	-15	9	0.90	1.21
0	-15	9	2.87	1.86
-1	15	-9	1.11	0.61
1	-15	9	2.47	1.24
1	-15	9	2.86	1.84
-2	15	-9	0.96	0.58
2	-15	9	0.89	1.21
2	-15	9	-2.00	1.74
-3	15	-9	1.22	0.61
3	-15	9	2.87	2.02
3	-15	9	1.32	1.23
-4	15	-9	0.51	0.62
4	-15	9	-2.75	1.88
4	-15	9	0.73	1.20
-5	15	-9	0.83	0.61
5	-15	9	0.86	2.22
5	-15	9	0.12	1.17
-6	15	-9	0.23	0.57
6	-15	9	-1.08	1.19
6	-15	9	1.83	2.17
-7	15	-9	0.56	0.63
7	-15	9	1.27	2.41
7	-15	9	0.80	1.31
-8	15	-9	0.44	0.71
8	-15	9	0.59	1.36
8	-15	9	1.53	2.52
-9	15	-9	0.94	0.68
9	-15	9	-0.89	1.37
9	-15	9	5.14	2.73
-10	15	-9	1.83	0.84
10	-15	9	1.60	3.05
7	14	-9	3.81	1.00
-7	-14	9	7.12	2.08
-7	-14	9	7.28	1.82
6	14	-9	4.94	1.01
-6	-14	9	6.79	1.70
-6	-14	9	4.02	1.95
5	14	-9	12.30	1.45
-5	-14	9	15.43	2.07
-5	-14	9	13.12	2.15
4	14	-9	9.34	1.26
-4	-14	9	15.07	2.72
-4	-14	9	11.46	1.75
3	14	-9	0.16	0.66

-3	-14	9	2.30	3.35
-3	-14	9	-0.50	1.18
2	14	-9	0.00	0.61
-2	-14	9	-0.92	1.04
-2	-14	9	-1.03	1.72
1	14	-9	1.14	0.61
-1	-14	9	3.16	1.87
-1	-14	9	0.25	1.02
0	14	-9	2.10	0.60
0	-14	9	2.00	1.09
-1	14	-9	0.69	0.53
1	-14	9	0.01	1.04
1	-14	9	0.94	1.94
-2	14	-9	0.05	0.51
2	-14	9	0.23	1.61
2	-14	9	0.12	0.99
-3	14	-9	0.30	0.52
3	-14	9	-1.47	1.56
3	-14	9	-0.47	1.02
-4	14	-9	3.28	0.63
4	-14	9	1.57	1.78
4	-14	9	2.48	1.22
-5	14	-9	-0.24	0.55
5	-14	9	1.26	1.85
5	-14	9	0.04	0.98
-6	14	-9	1.80	0.63
6	-14	9	0.23	1.08
6	-14	9	4.46	2.16
-7	14	-9	3.29	0.72
7	-14	9	1.11	2.14
7	-14	9	4.69	1.30
-8	14	-9	0.80	0.60
8	-14	9	1.86	1.19
8	-14	9	3.23	2.44
-9	14	-9	1.12	0.67
9	-14	9	2.78	2.54
9	-14	9	2.83	1.30
-10	14	-9	3.29	0.80
10	-14	9	2.53	2.95
10	-14	9	5.07	1.41
8	13	-9	4.06	1.01
-8	-13	9	5.31	1.84
-8	-13	9	4.59	1.68
7	13	-9	11.17	1.46

-7	-13	9	15.71	2.09
-7	-13	9	10.77	1.81
6	13	-9	9.94	1.28
-6	-13	9	11.98	2.28
-6	-13	9	8.13	1.72
5	13	-9	2.96	0.74
-5	-13	9	4.66	1.41
-5	-13	9	2.25	1.52
4	13	-9	2.88	0.71
-4	-13	9	3.51	1.54
-4	-13	9	4.41	1.38
3	13	-9	9.48	1.07
-3	-13	9	11.50	1.91
-3	-13	9	12.27	1.91
2	13	-9	4.20	0.69
-2	-13	9	3.29	1.21
-2	-13	9	5.23	1.65
1	13	-9	2.28	0.64
-1	-13	9	3.92	1.21
-1	-13	9	1.87	1.53
0	13	-9	0.60	0.51
0	-13	9	3.41	1.45
0	-13	9	2.03	1.04
-1	13	-9	0.58	0.46
1	-13	9	0.55	0.87
1	-13	9	-1.09	1.29
-2	13	-9	-0.03	0.45
2	-13	9	-0.48	0.85
2	-13	9	0.88	1.45
-3	13	-9	0.38	0.44
3	-13	9	-0.36	0.83
-4	13	-9	3.71	0.62
4	-13	9	5.54	1.82
4	-13	9	4.17	1.08
-5	13	-9	2.05	0.58
5	-13	9	1.36	1.75
5	-13	9	2.56	1.03
-6	13	-9	3.57	0.63
6	-13	9	3.62	2.08
6	-13	9	1.45	0.94
6	-13	9	2.89	0.60
-7	13	-9	1.94	0.61
7	-13	9	3.00	1.96
7	-13	9	3.35	1.05

7	-13	9	2.01	0.70
-8	13	-9	6.58	1.01
8	-13	9	8.42	1.29
8	-13	9	7.32	2.52
8	-13	9	10.84	1.45
-9	13	-9	4.32	0.76
9	-13	9	3.93	0.99
9	-13	9	4.80	1.26
9	-13	9	1.69	2.35
10	-13	9	1.92	1.24
10	-13	9	1.75	1.25
10	-13	9	2.69	2.57
-11	13	-9	0.86	0.80
11	-13	9	6.42	3.30
11	-13	9	3.82	1.31
9	12	-9	6.43	1.09
-9	-12	9	5.84	1.83
8	12	-9	9.09	1.39
-8	-12	9	12.19	1.86
-8	-12	9	11.35	1.79
7	12	-9	5.23	0.91
-7	-12	9	3.29	1.45
-7	-12	9	3.10	1.45
6	12	-9	1.82	0.72
-6	-12	9	1.39	1.28
-6	-12	9	-0.18	1.32
5	12	-9	0.02	1.82
5	12	-9	1.73	0.65
-5	-12	9	1.84	1.08
-5	-12	9	1.40	1.27
4	12	-9	2.32	1.76
4	12	-9	1.74	0.59
-4	-12	9	1.96	1.12
-4	-12	9	1.77	1.27
3	12	-9	0.62	0.83
3	12	-9	-0.22	1.53
-3	-12	9	-0.05	1.17
-3	-12	9	-1.44	0.88
2	12	-9	3.50	0.62
2	12	-9	2.47	1.60
-2	-12	9	4.20	1.30
-2	-12	9	6.11	1.17
1	12	-9	11.73	0.98
1	12	-9	13.73	2.38

-1	-12	9	11.00	1.59
-1	-12	9	12.91	1.78
0	12	-9	0.92	0.46
0	12	-9	-0.38	1.34
0	-12	9	0.84	0.81
0	-12	9	-0.71	1.14
-1	12	-9	0.84	0.44
-1	12	-9	2.11	1.44
1	-12	9	-0.12	0.77
1	-12	9	3.15	1.31
2	-12	9	2.23	1.18
2	-12	9	2.24	1.37
-3	12	-9	15.05	0.96
3	-12	9	14.17	2.35
3	-12	9	12.12	2.29
-4	12	-9	3.34	0.58
4	-12	9	4.81	1.67
4	-12	9	3.16	1.01
-5	12	-9	0.66	0.47
5	-12	9	-0.72	0.65
5	-12	9	0.26	0.44
5	-12	9	1.92	1.50
5	-12	9	0.02	0.77
-6	12	-9	11.15	0.97
6	-12	9	10.27	2.31
6	-12	9	9.92	1.49
6	-12	9	10.92	0.96
6	-12	9	9.28	1.32
-7	12	-9	7.68	0.94
7	-12	9	7.87	1.21
7	-12	9	11.77	2.21
7	-12	9	7.89	1.06
-8	12	-9	4.84	0.73
8	-12	9	7.17	0.97
8	-12	9	7.76	2.28
8	-12	9	7.37	1.28
-9	12	-9	3.35	0.71
9	-12	9	2.70	1.27
9	-12	9	3.57	0.96
9	-12	9	4.26	2.51
-10	12	-9	5.84	0.85
10	-12	9	6.76	1.20
10	-12	9	6.76	2.77
10	-12	9	8.47	2.30

-11	12	-9	3.53	0.81
11	-12	9	6.22	1.34
11	-12	9	1.01	2.79
10	11	-9	2.21	2.24
10	11	-9	3.59	0.94
9	11	-9	2.05	0.79
9	11	-9	0.23	1.00
-9	-11	9	1.66	1.43
8	11	-9	1.58	0.73
-8	-11	9	2.55	1.32
-8	-11	9	1.36	1.27
7	11	-9	10.67	1.18
7	11	-9	13.44	1.25
-7	-11	9	12.39	1.68
-7	-11	9	10.00	1.49
6	11	-9	6.78	1.01
6	11	-9	9.95	2.03
6	11	-9	6.43	0.82
-6	-11	9	8.04	1.42
-6	-11	9	8.28	1.40
5	11	-9	1.95	1.63
5	11	-9	1.59	0.78
-5	-11	9	0.48	1.11
-5	-11	9	2.85	1.00
4	11	-9	1.87	0.56
4	11	-9	0.78	1.39
-4	-11	9	1.33	0.88
-4	-11	9	0.55	1.09
3	11	-9	5.31	0.82
3	11	-9	4.03	1.41
-3	-11	9	3.92	1.24
-3	-11	9	5.22	1.14
2	11	-9	0.79	0.48
2	11	-9	0.61	1.19
-2	-11	9	-0.33	0.77
-2	-11	9	1.03	1.08
1	11	-9	1.83	1.20
1	11	-9	1.12	0.45
-1	-11	9	0.87	0.88
-1	-11	9	0.40	1.11
0	11	-9	0.40	0.40
0	11	-9	2.39	1.23
0	-11	9	-0.39	1.06
0	-11	9	0.23	0.67

-1	11	-9	9.07	0.79
-1	11	-9	9.21	1.35
1	-11	9	10.20	1.65
1	-11	9	8.60	1.35
-2	11	-9	1.84	0.44
-2	11	-9	5.67	1.29
2	-11	9	1.50	1.20
2	-11	9	2.62	0.80
-3	11	-9	7.31	0.76
3	-11	9	8.95	1.62
3	-11	9	6.55	0.70
3	-11	9	7.08	1.25
-4	11	-9	14.05	0.92
4	-11	9	14.26	1.48
4	-11	9	11.89	1.42
4	-11	9	13.92	0.81
4	-11	9	12.69	2.27
-5	11	-9	3.12	0.58
5	-11	9	4.80	0.81
5	-11	9	4.21	0.95
5	-11	9	1.46	1.58
5	-11	9	4.71	0.70
-6	11	-9	0.90	0.50
6	-11	9	0.07	0.75
6	-11	9	1.67	0.79
6	-11	9	1.60	0.55
6	-11	9	0.46	1.52
-7	11	-9	13.27	1.03
7	-11	9	10.60	1.47
7	-11	9	19.27	2.99
7	-11	9	13.65	1.14
7	-11	9	13.06	1.53
-8	11	-9	19.18	1.21
8	-11	9	17.40	1.75
8	-11	9	17.59	1.66
8	-11	9	18.21	1.41
8	-11	9	17.75	3.26
-9	11	-9	13.07	1.13
9	-11	9	9.34	1.40
9	-11	9	10.06	2.60
9	-11	9	11.67	1.74
-10	11	-9	5.06	0.81
10	-11	9	6.97	1.26
10	-11	9	8.21	2.77

10	-11	9	5.10	1.13
-11	11	-9	4.95	0.83
11	-11	9	4.69	2.95
11	-11	9	7.25	1.41
11	10	-9	5.72	2.24
11	10	-9	4.59	0.92
10	10	-9	0.66	0.99
10	10	-9	-0.24	0.73
10	10	-9	-0.10	0.98
-10	-10	9	2.27	1.39
9	10	-9	1.42	0.91
9	10	-9	-0.23	0.64
9	10	-9	0.98	0.90
-9	-10	9	1.17	1.25
8	10	-9	1.53	0.83
8	10	-9	2.61	0.71
-8	-10	9	2.34	1.19
7	10	-9	0.26	0.56
7	10	-9	-1.03	1.56
7	10	-9	0.37	0.73
7	10	-9	-0.06	0.65
-7	-10	9	1.44	0.94
-7	-10	9	1.31	1.00
6	10	-9	-0.45	0.62
6	10	-9	0.70	1.39
6	10	-9	0.38	0.38
-6	-10	9	-0.80	0.90
-6	-10	9	0.97	0.92
5	10	-9	2.23	0.56
5	10	-9	2.37	0.64
5	10	-9	2.15	0.33
5	10	-9	1.19	1.30
-5	-10	9	1.80	0.83
-5	-10	9	0.10	0.94
4	10	-9	0.59	1.14
4	10	-9	-0.24	0.46
-4	-10	9	0.01	0.91
3	10	-9	1.25	1.09
3	10	-9	1.55	0.48
-3	-10	9	1.79	0.73
-3	-10	9	1.72	1.00
2	10	-9	9.87	1.45
2	10	-9	8.86	0.80
-2	-10	9	9.87	1.24

-2	-10	9	11.05	1.62
1	10	-9	1.24	0.42
1	10	-9	1.08	1.04
-1	-10	9	1.68	0.67
-1	-10	9	0.84	0.98
0	10	-9	0.67	0.99
0	10	-9	0.57	0.38
0	-10	9	0.49	0.96
0	-10	9	0.32	0.60
-1	10	-9	3.39	0.52
-1	10	-9	2.32	0.97
1	-10	9	6.24	1.25
1	-10	9	3.32	0.75
-2	10	-9	1.29	1.02
-2	10	-9	0.82	0.39
2	-10	9	1.65	0.68
2	-10	9	0.56	1.02
-3	10	-9	26.24	1.24
-3	10	-9	25.67	2.03
3	-10	9	29.97	1.76
3	-10	9	26.01	2.36
3	-10	9	26.88	1.33
-4	10	-9	6.44	0.80
4	-10	9	7.79	0.64
4	-10	9	5.76	0.96
4	-10	9	7.86	1.02
4	-10	9	9.18	1.77
-5	10	-9	-0.34	0.39
5	-10	9	0.00	0.40
5	-10	9	1.46	0.73
5	-10	9	-1.31	1.20
5	-10	9	-0.30	0.66
-6	10	-9	8.46	0.83
6	-10	9	8.81	1.32
6	-10	9	6.67	0.86
6	-10	9	14.09	2.34
6	-10	9	9.67	1.29
-7	10	-9	27.80	1.37
7	-10	9	26.53	1.45
7	-10	9	30.18	1.86
7	-10	9	27.25	3.00
7	-10	9	25.40	1.73
-8	10	-9	9.41	0.96
8	-10	9	10.05	1.17

8	-10	9	8.85	2.22
8	-10	9	9.78	1.47
-9	10	-9	16.38	1.17
9	-10	9	13.47	1.47
9	-10	9	14.96	1.69
9	-10	9	15.35	2.82
-10	10	-9	16.29	1.29
10	-10	9	19.41	1.88
10	-10	9	18.96	1.76
10	-10	9	17.56	3.22
-11	10	-9	10.84	1.25
11	-10	9	8.43	1.41
11	-10	9	13.54	3.27
12	9	-9	1.21	2.07
12	9	-9	0.27	1.28
12	9	-9	0.68	0.83
11	9	-9	5.36	1.49
11	9	-9	5.78	0.92
-11	-9	9	6.91	1.66
10	9	-9	6.76	1.18
10	9	-9	4.38	0.83
10	9	-9	7.15	1.32
-10	-9	9	4.54	1.41
9	9	-9	1.44	0.64
9	9	-9	0.34	0.79
9	9	-9	0.07	0.75
-9	-9	9	1.00	1.13
8	9	-9	-0.29	0.71
8	9	-9	0.31	0.69
8	9	-9	0.08	0.51
8	9	-9	1.29	1.48
8	9	-9	-0.57	0.49
-8	-9	9	-0.17	0.89
7	9	-9	3.24	1.41
7	9	-9	3.22	0.51
7	9	-9	4.38	0.76
7	9	-9	3.92	0.81
-7	-9	9	4.21	1.03
-7	-9	9	4.43	0.93
6	9	-9	1.19	0.59
6	9	-9	1.00	1.17
6	9	-9	2.23	0.55
6	9	-9	1.18	0.37
-6	-9	9	0.86	0.80

-6	-9	9	1.29	0.95
5	9	-9	-0.13	0.26
5	9	-9	0.56	0.53
5	9	-9	0.40	0.42
5	9	-9	0.57	1.04
-5	-9	9	0.52	0.76
-5	-9	9	-0.37	0.68
4	9	-9	6.00	1.18
4	9	-9	6.00	0.70
4	9	-9	5.62	0.39
4	9	-9	5.68	0.73
-4	-9	9	6.30	1.04
-4	-9	9	5.26	0.86
3	9	-9	5.83	1.13
3	9	-9	3.33	0.51
-3	-9	9	3.61	0.80
-3	-9	9	5.11	0.76
-3	-9	9	5.94	1.08
2	9	-9	1.26	0.40
2	9	-9	1.10	0.90
-2	-9	9	1.88	0.39
-2	-9	9	1.33	0.65
-2	-9	9	0.73	0.84
1	9	-9	1.50	0.82
1	9	-9	2.00	0.43
-1	-9	9	0.58	0.59
-1	-9	9	1.55	0.40
-1	-9	9	0.66	0.89
0	9	-9	1.19	0.82
0	9	-9	2.57	0.43
0	9	-9	2.17	0.53
0	-9	9	2.22	0.99
0	-9	9	1.87	0.42
0	-9	9	2.12	0.64
-1	9	-9	5.77	0.81
-1	9	-9	5.22	0.63
-1	9	-9	7.02	1.30
-1	9	-9	4.62	0.36
1	-9	9	6.12	0.86
1	-9	9	4.79	1.17
1	-9	9	4.51	0.76
1	-9	9	5.38	0.61
-2	9	-9	1.60	0.82
-2	9	-9	2.60	0.45

-2	9	-9	2.89	0.39
-2	9	-9	3.19	0.57
2	-9	9	3.12	0.67
2	-9	9	4.06	1.17
2	-9	9	4.52	0.74
-3	9	-9	2.03	0.43
-3	9	-9	2.04	0.33
-3	9	-9	2.17	0.86
-3	9	-9	1.56	0.47
3	-9	9	2.12	1.08
3	-9	9	2.10	0.61
3	-9	9	2.01	0.66
-4	9	-9	16.66	1.07
-4	9	-9	14.29	0.76
-4	9	-9	16.01	0.93
-4	9	-9	15.71	1.60
4	-9	9	17.19	1.26
4	-9	9	16.39	1.35
4	-9	9	13.65	2.01
-5	9	-9	19.33	0.97
-5	9	-9	22.15	1.29
-5	9	-9	19.06	1.25
5	-9	9	17.72	2.29
5	-9	9	25.09	1.46
5	-9	9	22.42	1.51
-6	9	-9	0.72	0.44
-6	9	-9	0.44	0.39
6	-9	9	0.04	0.66
6	-9	9	0.23	0.74
-7	9	-9	4.02	0.58
7	-9	9	3.78	1.57
7	-9	9	5.61	1.01
7	-9	9	4.97	0.90
-8	9	-9	13.61	1.05
8	-9	9	13.86	2.31
8	-9	9	15.97	1.56
8	-9	9	13.72	1.25
-9	9	-9	19.59	1.28
9	-9	9	19.15	3.47
9	-9	9	20.24	1.74
9	-9	9	17.84	1.56
-10	9	-9	16.10	1.23
10	-9	9	13.40	1.67
10	-9	9	16.52	2.98

10	-9	9	12.68	1.74
-11	9	-9	4.02	0.79
11	-9	9	4.41	2.65
13	8	-9	3.08	0.89
13	8	-9	3.47	2.10
13	8	-9	1.50	1.45
12	8	-9	2.55	2.00
12	8	-9	3.63	1.29
12	8	-9	4.53	0.81
11	8	-9	4.27	1.15
11	8	-9	3.37	0.69
11	8	-9	5.83	1.07
-11	-8	9	4.78	1.41
10	8	-9	1.03	0.77
10	8	-9	1.23	0.59
10	8	-9	1.54	0.91
-10	-8	9	-0.61	1.06
9	8	-9	1.22	0.58
9	8	-9	1.85	0.80
9	8	-9	0.74	0.55
9	8	-9	0.83	1.45
9	8	-9	0.91	0.70
-9	-8	9	1.53	0.95
8	8	-9	5.56	0.95
8	8	-9	6.63	0.90
8	8	-9	3.89	1.33
8	8	-9	4.48	0.74
8	8	-9	5.37	0.81
-8	-8	9	4.36	1.12
7	8	-9	3.41	0.46
7	8	-9	4.33	0.59
7	8	-9	3.82	1.16
7	8	-9	2.97	0.69
-7	-8	9	2.48	0.87
-7	-8	9	3.27	0.74
6	8	-9	1.69	0.97
6	8	-9	1.08	0.43
6	8	-9	1.91	0.56
6	8	-9	2.29	0.35
-6	-8	9	1.67	0.79
-6	-8	9	1.10	0.66
5	8	-9	-0.57	0.48
5	8	-9	0.50	0.40
5	8	-9	-1.45	0.80

-5	-8	9	0.74	0.63
-5	-8	9	-0.39	0.57
4	8	-9	0.22	0.49
4	8	-9	1.19	0.78
4	8	-9	0.80	0.39
4	8	-9	-0.11	0.77
-4	-8	9	0.87	0.34
-4	-8	9	-0.05	0.28
-4	-8	9	0.25	0.66
-4	-8	9	0.57	0.61
3	8	-9	5.29	0.82
3	8	-9	4.56	0.62
3	8	-9	7.89	1.23
3	8	-9	4.48	0.63
-3	-8	9	6.02	0.47
-3	-8	9	6.93	1.20
-3	-8	9	5.75	0.61
-3	-8	9	7.55	0.98
2	8	-9	14.08	1.02
2	8	-9	14.45	0.84
2	8	-9	13.44	1.32
2	8	-9	13.58	0.69
-2	-8	9	12.50	0.64
-2	-8	9	12.80	0.80
-2	-8	9	14.97	1.42
-2	-8	9	13.49	1.16
1	8	-9	13.32	0.61
1	8	-9	13.14	1.28
1	8	-9	13.83	0.98
1	8	-9	12.39	0.78
-1	-8	9	12.90	1.12
-1	-8	9	13.21	1.44
-1	-8	9	12.36	0.68
-1	-8	9	14.23	0.81
0	8	-9	-0.15	0.63
0	8	-9	0.29	0.32
0	8	-9	0.27	0.28
0	8	-9	0.44	0.39
0	-8	9	0.30	0.37
0	-8	9	0.28	0.34
0	-8	9	0.06	0.46
0	-8	9	1.24	0.80
-1	8	-9	-0.11	0.64
-1	8	-9	0.30	0.41

-1	8	-9	0.31	0.25
-1	8	-9	0.35	0.29
1	-8	9	0.31	0.43
1	-8	9	1.02	0.38
1	-8	9	-0.70	0.75
1	-8	9	0.68	0.52
-2	8	-9	21.85	1.19
-2	8	-9	22.15	1.05
-2	8	-9	21.11	1.55
-2	8	-9	22.07	0.91
2	-8	9	24.61	1.43
2	-8	9	22.82	1.94
2	-8	9	24.71	1.26
2	-8	9	21.88	1.09
-3	8	-9	31.95	1.24
-3	8	-9	33.98	1.52
-3	8	-9	32.58	1.37
-3	8	-9	30.88	1.85
3	-8	9	33.36	1.71
3	-8	9	36.40	1.59
3	-8	9	30.57	2.30
-4	8	-9	7.73	0.67
-4	8	-9	5.63	0.58
-4	8	-9	8.44	1.33
-4	8	-9	8.17	0.87
4	-8	9	7.52	1.37
4	-8	9	6.34	1.07
4	-8	9	6.66	1.02
-5	8	-9	4.94	0.62
-5	8	-9	6.25	0.86
-5	8	-9	4.28	0.63
5	-8	9	5.27	0.82
5	-8	9	6.58	1.48
5	-8	9	5.20	0.87
-6	8	-9	16.33	0.94
-6	8	-9	15.59	0.99
-6	8	-9	17.48	1.22
6	-8	9	16.67	1.42
6	-8	9	16.02	2.29
6	-8	9	17.37	1.36
-7	8	-9	16.04	1.03
-7	8	-9	13.20	0.98
7	-8	9	15.82	1.39
7	-8	9	13.70	2.53

-8	8	-9	2.91	0.58
-8	8	-9	2.62	0.56
8	-8	9	2.90	0.85
8	-8	9	1.94	1.81
-9	8	-9	11.10	1.06
9	-8	9	12.23	1.50
9	-8	9	15.12	2.72
-10	8	-9	18.52	1.27
10	-8	9	13.54	2.93
10	-8	9	19.44	1.80
10	-8	9	17.15	1.67
-11	8	-9	8.28	1.15
11	-8	9	7.99	2.81
11	-8	9	12.61	1.94
11	-8	9	6.80	1.27
13	7	-9	0.80	0.72
13	7	-9	-0.83	1.25
13	7	-9	6.78	2.01
12	7	-9	2.90	0.69
12	7	-9	-0.58	1.05
12	7	-9	0.44	1.05
-12	-7	9	4.63	1.35
11	7	-9	1.65	0.97
11	7	-9	1.70	0.63
11	7	-9	4.01	0.95
-11	-7	9	3.73	1.21
10	7	-9	6.90	1.08
10	7	-9	9.01	2.19
10	7	-9	5.77	0.87
10	7	-9	4.64	0.75
10	7	-9	7.45	1.09
-10	-7	9	5.30	1.42
9	7	-9	1.79	0.59
9	7	-9	2.15	0.52
9	7	-9	1.66	1.24
9	7	-9	1.90	0.54
9	7	-9	2.55	0.80
-9	-7	9	1.15	0.86
8	7	-9	4.53	0.70
8	7	-9	5.50	1.11
8	7	-9	3.44	0.58
8	7	-9	4.02	0.66
8	7	-9	3.51	0.78
-8	-7	9	3.60	0.90

7	7	-9	1.27	0.59
7	7	-9	3.15	0.92
7	7	-9	1.42	0.38
7	7	-9	0.98	0.41
-7	-7	9	2.25	0.80
6	7	-9	2.58	1.02
6	7	-9	2.81	0.61
6	7	-9	2.03	0.55
6	7	-9	2.39	0.44
-6	-7	9	2.12	0.46
-6	-7	9	1.42	0.58
-6	-7	9	1.71	0.67
5	7	-9	1.66	0.73
5	7	-9	1.82	0.53
5	7	-9	0.62	0.50
5	7	-9	1.44	0.41
-5	-7	9	1.34	0.33
-5	-7	9	1.19	0.61
-5	-7	9	1.34	0.36
-5	-7	9	1.52	0.60
4	7	-9	1.55	0.39
4	7	-9	1.11	0.47
4	7	-9	1.58	0.68
4	7	-9	1.09	0.44
-4	-7	9	2.52	0.68
-4	-7	9	2.43	0.39
-4	-7	9	1.60	0.56
-4	-7	9	1.48	0.33
3	7	-9	14.27	0.94
3	7	-9	14.30	0.97
3	7	-9	16.03	0.84
-3	-7	9	14.04	0.76
-3	-7	9	14.75	0.75
-3	-7	9	15.27	1.25
-3	-7	9	13.36	1.13
2	7	-9	12.83	1.10
2	7	-9	12.89	0.78
2	7	-9	14.89	0.84
2	7	-9	12.78	0.90
-2	-7	9	12.58	1.28
-2	-7	9	14.13	0.77
-2	-7	9	12.27	0.72
-2	-7	9	13.59	1.07
1	7	-9	3.53	0.52

1	7	-9	4.95	0.56
1	7	-9	4.41	0.70
1	7	-9	4.63	0.66
-1	-7	9	3.00	0.58
-1	-7	9	5.12	0.92
-1	-7	9	3.95	0.57
-1	-7	9	5.00	0.54
0	7	-9	6.01	0.53
0	7	-9	6.74	0.60
0	7	-9	5.60	0.95
0	7	-9	7.35	0.71
0	-7	9	7.64	1.21
0	-7	9	5.85	0.59
0	-7	9	5.61	0.82
0	-7	9	6.87	0.72
-1	7	-9	12.57	0.84
-1	7	-9	11.42	0.73
-1	7	-9	11.74	1.12
-1	7	-9	12.46	0.67
1	-7	9	11.87	0.76
1	-7	9	12.27	1.01
1	-7	9	12.22	1.40
1	-7	9	13.49	0.96
-2	7	-9	20.01	0.89
-2	7	-9	21.46	1.40
-2	7	-9	20.63	1.09
-2	7	-9	19.78	0.96
2	-7	9	20.33	1.20
2	-7	9	19.10	1.00
2	-7	9	21.70	1.82
2	-7	9	19.23	1.23
-3	7	-9	15.24	0.80
-3	7	-9	15.87	0.98
-3	7	-9	16.22	0.86
3	-7	9	12.84	1.64
3	-7	9	17.07	1.17
3	-7	9	14.99	0.94
3	-7	9	11.81	1.09
-4	7	-9	20.39	1.01
-4	7	-9	19.66	0.96
-4	7	-9	20.47	1.17
-4	7	-9	20.92	1.51
4	-7	9	18.12	1.33
4	-7	9	17.72	1.97

4	-7	9	18.96	1.28
-5	7	-9	17.42	0.97
-5	7	-9	16.38	1.20
-5	7	-9	16.66	0.93
5	-7	9	14.83	2.00
5	-7	9	16.62	1.25
5	-7	9	18.09	1.37
-6	7	-9	7.85	0.76
-6	7	-9	6.89	0.77
-6	7	-9	7.18	1.04
6	-7	9	7.93	1.67
6	-7	9	7.95	1.12
6	-7	9	8.00	1.32
-7	7	-9	15.88	0.99
-7	7	-9	14.83	1.00
-7	7	-9	17.06	1.69
7	-7	9	12.93	1.34
7	-7	9	13.82	2.18
-8	7	-9	5.95	0.84
-8	7	-9	5.83	0.85
8	-7	9	6.36	1.85
8	-7	9	5.23	0.96
-9	7	-9	9.21	1.04
-9	7	-9	9.16	0.98
9	-7	9	9.24	1.43
9	-7	9	7.95	2.28
-10	7	-9	12.45	1.16
10	-7	9	12.81	1.66
10	-7	9	12.37	2.85
-11	7	-9	7.73	1.12
11	-7	9	7.75	2.71
11	-7	9	6.67	1.25
11	-7	9	9.41	1.54
14	6	-9	0.72	0.72
14	6	-9	0.08	1.33
14	6	-9	-1.68	1.76
13	6	-9	-0.63	1.65
13	6	-9	0.20	1.24
13	6	-9	1.33	0.64
12	6	-9	0.76	1.03
12	6	-9	0.22	0.56
12	6	-9	3.03	0.99
11	6	-9	1.55	0.80
11	6	-9	-0.05	0.48

11	6	-9	-0.08	0.85
-11	-6	9	0.39	0.95
10	6	-9	1.06	0.46
10	6	-9	0.95	1.17
10	6	-9	2.02	0.59
10	6	-9	1.27	0.80
10	6	-9	1.68	0.69
-10	-6	9	-0.10	0.81
9	6	-9	0.31	0.63
9	6	-9	-0.53	0.43
9	6	-9	-0.17	0.52
9	6	-9	-0.18	0.97
-9	-6	9	-0.16	0.72
8	6	-9	0.97	0.42
8	6	-9	0.93	0.80
8	6	-9	0.07	0.37
8	6	-9	0.15	0.60
8	6	-9	0.73	0.39
8	6	-9	1.45	0.56
-8	-6	9	0.97	0.68
7	6	-9	0.26	0.40
7	6	-9	0.66	0.51
7	6	-9	1.34	0.70
7	6	-9	0.86	0.38
7	6	-9	-0.30	0.49
-7	-6	9	0.51	0.62
-7	-6	9	0.84	0.43
6	6	-9	5.70	0.61
6	6	-9	5.38	0.72
6	6	-9	5.95	0.81
6	6	-9	5.56	0.98
-6	-6	9	5.47	0.66
-6	-6	9	4.95	0.55
-6	-6	9	5.47	0.82
5	6	-9	0.55	0.31
5	6	-9	1.01	0.40
5	6	-9	0.74	0.55
5	6	-9	1.15	0.50
-5	-6	9	1.56	0.58
-5	-6	9	-0.18	0.83
-5	-6	9	0.56	0.28
-5	-6	9	0.67	0.35
4	6	-9	10.05	0.87
4	6	-9	8.86	0.66

4	6	-9	8.72	0.82
4	6	-9	9.40	0.81
-4	-6	9	9.07	0.60
-4	-6	9	9.25	0.97
3	6	-9	39.27	1.47
3	6	-9	37.16	1.58
3	6	-9	36.42	1.57
3	6	-9	36.55	1.54
-3	-6	9	38.61	1.77
-3	-6	9	36.09	1.83
-3	-6	9	36.49	1.45
-3	-6	9	39.06	1.40
2	6	-9	15.76	0.99
2	6	-9	15.04	0.91
2	6	-9	15.22	0.90
2	6	-9	15.07	0.81
-2	-6	9	15.65	1.26
-2	-6	9	15.89	0.87
-2	-6	9	14.98	0.78
-2	-6	9	14.69	1.06
1	6	-9	25.65	1.10
1	6	-9	26.06	1.21
1	6	-9	25.81	1.11
1	6	-9	25.10	1.28
-1	-6	9	26.39	1.37
-1	-6	9	26.88	1.21
-1	-6	9	25.36	1.59
-1	-6	9	25.81	1.09
0	6	-9	46.60	1.78
0	6	-9	52.11	1.91
0	6	-9	48.55	1.76
0	6	-9	54.54	2.04
0	-6	9	50.55	2.34
0	-6	9	48.13	1.92
0	-6	9	46.67	2.02
0	-6	9	49.26	1.79
-1	6	-9	6.13	0.86
-1	6	-9	5.57	0.54
-1	6	-9	5.09	0.53
-1	6	-9	4.69	0.62
1	-6	9	5.66	1.02
1	-6	9	5.26	0.80
1	-6	9	4.72	0.73
1	-6	9	4.60	0.57

-2	6	-9	42.31	1.57
-2	6	-9	41.48	1.60
-2	6	-9	41.94	1.72
-2	6	-9	43.13	1.89
2	-6	9	41.66	2.30
2	-6	9	42.58	1.62
2	-6	9	43.98	1.86
2	-6	9	41.81	1.82
-3	6	-9	76.13	2.96
-3	6	-9	77.83	2.63
-3	6	-9	80.77	2.80
-3	6	-9	76.67	2.65
3	-6	9	74.63	3.46
3	-6	9	76.01	2.66
3	-6	9	79.18	2.92
3	-6	9	81.23	2.92
-4	6	-9	10.44	0.92
-4	6	-9	7.66	0.71
-4	6	-9	7.81	1.10
-4	6	-9	8.87	0.69
4	-6	9	9.13	0.95
4	-6	9	9.19	1.62
4	-6	9	7.18	1.10
-5	6	-9	2.82	0.45
-5	6	-9	3.97	0.66
-5	6	-9	3.90	0.69
5	-6	9	4.17	0.73
5	-6	9	3.65	0.80
5	-6	9	4.10	1.27
-6	6	-9	38.29	1.89
-6	6	-9	39.37	1.61
6	-6	9	40.48	2.36
6	-6	9	33.97	2.78
6	-6	9	37.51	1.90
-7	6	-9	22.41	1.18
-7	6	-9	22.95	1.23
7	-6	9	21.42	1.53
7	-6	9	23.28	2.67
-8	6	-9	3.50	0.65
-8	6	-9	4.15	0.61
8	-6	9	4.23	0.90
8	-6	9	3.67	1.76
-9	6	-9	16.86	1.25
-9	6	-9	14.56	1.10

9	-6	9	13.02	2.56
9	-6	9	14.72	1.53
-10	6	-9	12.55	1.18
-10	6	-9	15.17	1.29
10	-6	9	9.03	2.51
10	-6	9	9.74	1.56
-11	6	-9	6.49	0.87
11	-6	9	6.49	1.25
11	-6	9	3.59	2.61
11	-6	9	6.21	1.18
15	5	-9	-0.84	1.35
15	5	-9	-1.20	0.65
14	5	-9	0.06	1.26
14	5	-9	-0.66	0.60
14	5	-9	0.15	1.61
13	5	-9	-1.48	1.06
13	5	-9	-0.68	0.56
13	5	-9	1.72	1.16
12	5	-9	1.64	0.97
12	5	-9	2.99	0.61
12	5	-9	2.31	0.90
-12	-5	9	3.18	1.07
11	5	-9	2.04	1.03
11	5	-9	0.33	0.70
11	5	-9	1.56	0.52
11	5	-9	2.05	1.23
11	5	-9	2.43	0.92
-11	-5	9	0.83	0.88
10	5	-9	2.13	0.78
10	5	-9	1.05	0.96
10	5	-9	0.67	0.41
10	5	-9	1.80	0.61
10	5	-9	2.04	0.66
10	5	-9	1.76	0.51
-10	-5	9	2.09	0.82
9	5	-9	0.43	0.67
9	5	-9	0.17	0.55
9	5	-9	1.26	0.79
9	5	-9	1.84	0.54
9	5	-9	1.00	0.39
-9	-5	9	1.61	0.59
-9	-5	9	1.06	0.72
8	5	-9	2.81	0.69
8	5	-9	3.18	0.72

8	5	-9	2.98	0.58
8	5	-9	3.37	0.59
8	5	-9	3.83	0.50
-8	-5	9	4.48	0.79
-8	-5	9	3.08	0.59
7	5	-9	0.73	0.51
7	5	-9	0.80	0.53
7	5	-9	0.13	0.40
7	5	-9	0.30	0.33
7	5	-9	0.38	0.26
-7	-5	9	0.81	0.59
-7	-5	9	0.17	0.29
-7	-5	9	-0.10	0.42
6	5	-9	2.66	0.60
6	5	-9	1.30	0.34
6	5	-9	2.02	0.46
6	5	-9	1.89	0.45
-6	-5	9	1.52	0.55
-6	-5	9	1.80	0.42
-6	-5	9	1.98	0.33
5	5	-9	14.16	0.99
5	5	-9	14.46	0.91
5	5	-9	14.19	0.79
-5	-5	9	15.01	0.73
-5	-5	9	13.93	1.06
-5	-5	9	14.84	0.86
4	5	-9	17.45	0.85
4	5	-9	20.34	1.02
4	5	-9	17.44	1.07
4	5	-9	17.55	0.88
-4	-5	9	18.13	1.15
-4	-5	9	18.07	0.95
-4	-5	9	17.49	1.01
-4	-5	9	17.74	0.81
3	5	-9	9.21	0.84
3	5	-9	10.21	0.65
3	5	-9	10.18	0.72
3	5	-9	9.36	0.65
-3	-5	9	9.92	0.60
-3	-5	9	9.61	0.86
-3	-5	9	9.70	0.93
-3	-5	9	8.91	0.73
2	5	-9	21.07	1.04
2	5	-9	20.89	0.95

2	5	-9	20.32	1.08
2	5	-9	20.71	1.00
-2	-5	9	21.19	1.08
-2	-5	9	21.21	0.92
-2	-5	9	21.03	1.35
-2	-5	9	19.60	1.14
1	5	-9	16.66	0.92
1	5	-9	17.66	1.34
1	5	-9	16.94	0.91
1	5	-9	16.02	0.81
-1	-5	9	17.46	0.81
-1	-5	9	14.64	0.98
-1	-5	9	17.09	1.01
-1	-5	9	16.54	1.22
0	5	-9	51.82	1.96
0	5	-9	50.73	1.80
0	5	-9	54.46	1.94
0	5	-9	49.70	1.84
0	-5	9	49.77	2.02
0	-5	9	48.32	2.24
0	-5	9	49.97	1.80
0	-5	9	48.98	2.01
-1	5	-9	47.63	1.86
-1	5	-9	49.31	1.75
-1	5	-9	49.68	1.95
-1	5	-9	47.55	1.78
1	-5	9	46.32	1.96
1	-5	9	46.15	2.31
1	-5	9	50.61	1.77
1	-5	9	50.28	2.03
-2	5	-9	11.20	0.69
-2	5	-9	10.97	0.93
-2	5	-9	10.12	0.72
-2	5	-9	10.74	0.82
2	-5	9	10.78	0.73
2	-5	9	11.18	1.02
2	-5	9	9.70	0.85
2	-5	9	9.21	1.31
-3	5	-9	13.68	0.96
-3	5	-9	13.01	0.76
-3	5	-9	12.36	0.83
-3	5	-9	12.07	1.06
3	-5	9	13.99	0.99
3	-5	9	13.84	1.18

3	-5	9	12.67	0.83
3	-5	9	13.82	1.53
-4	5	-9	58.60	2.33
-4	5	-9	59.34	2.43
-4	5	-9	60.76	2.11
-4	5	-9	57.08	2.13
4	-5	9	58.62	2.36
4	-5	9	55.35	3.00
4	-5	9	60.45	2.45
-5	5	-9	16.26	0.99
-5	5	-9	19.26	1.35
-5	5	-9	17.42	0.94
5	-5	9	17.27	1.47
5	-5	9	14.69	2.00
5	-5	9	14.40	1.16
-6	5	-9	21.75	1.54
-6	5	-9	20.76	1.17
-6	5	-9	21.44	1.08
6	-5	9	24.29	2.38
6	-5	9	20.34	1.41
-7	5	-9	12.18	0.90
7	-5	9	10.01	1.23
7	-5	9	13.45	2.11
-8	5	-9	14.21	1.01
-8	5	-9	13.98	1.11
8	-5	9	9.27	1.95
8	-5	9	9.72	1.31
-9	5	-9	18.22	1.31
-9	5	-9	19.50	1.20
9	-5	9	22.74	3.32
9	-5	9	15.71	1.58
-10	5	-9	9.18	1.09
-10	5	-9	8.02	1.21
10	-5	9	6.71	1.30
10	-5	9	3.92	2.23
-11	5	-9	12.76	1.42
-11	5	-9	13.00	1.22
11	-5	9	7.89	2.58
15	4	-9	2.48	1.39
15	4	-9	1.68	0.69
14	4	-9	0.99	0.58
14	4	-9	-1.60	1.39
14	4	-9	0.18	1.16
13	4	-9	1.11	0.53

13	4	-9	-0.61	0.95
13	4	-9	1.39	1.06
-13	-4	9	0.80	0.95
12	4	-9	0.87	0.48
12	4	-9	0.58	0.88
12	4	-9	-0.25	0.67
12	4	-9	0.59	0.94
-12	-4	9	0.52	0.85
11	4	-9	2.07	0.46
11	4	-9	0.74	1.00
11	4	-9	1.32	0.77
11	4	-9	3.36	0.89
11	4	-9	1.95	0.62
-11	-4	9	2.44	0.84
10	4	-9	1.50	0.72
10	4	-9	2.18	0.85
10	4	-9	1.72	0.63
10	4	-9	0.81	0.54
-10	-4	9	1.51	0.74
-10	-4	9	1.58	0.62
9	4	-9	-0.05	0.39
9	4	-9	-0.69	0.56
9	4	-9	-0.10	0.42
9	4	-9	-0.12	0.31
9	4	-9	0.51	0.60
-9	-4	9	-0.61	0.50
-9	-4	9	-0.39	0.50
8	4	-9	2.11	0.50
8	4	-9	1.16	0.51
8	4	-9	2.38	0.40
8	4	-9	1.81	0.61
-8	-4	9	1.72	0.57
-8	-4	9	1.62	0.49
7	4	-9	12.61	1.02
7	4	-9	11.32	0.85
7	4	-9	11.71	0.72
-7	-4	9	11.32	0.68
-7	-4	9	10.78	0.86
-7	-4	9	11.05	1.01
6	4	-9	27.35	1.17
6	4	-9	28.91	1.45
6	4	-9	28.39	1.29
-6	-4	9	29.08	1.30
-6	-4	9	26.90	1.10

-6	-4	9	29.40	1.47
5	4	-9	6.26	0.55
5	4	-9	6.98	0.81
5	4	-9	6.57	0.62
-5	-4	9	7.07	0.69
-5	-4	9	6.63	0.49
-5	-4	9	7.46	0.79
4	4	-9	7.90	0.79
4	4	-9	8.57	0.59
4	4	-9	8.20	0.65
-4	-4	9	8.31	0.53
-4	-4	9	7.89	0.78
-4	-4	9	8.00	0.72
3	4	-9	11.67	0.66
3	4	-9	10.45	0.71
3	4	-9	10.52	0.56
3	4	-9	10.28	0.85
-3	-4	9	11.61	0.62
-3	-4	9	10.61	0.79
-3	-4	9	10.89	0.87
-3	-4	9	9.51	0.82
2	4	-9	11.69	0.68
2	4	-9	12.34	0.85
2	4	-9	11.72	0.74
2	4	-9	12.58	0.66
-2	-4	9	11.91	0.66
-2	-4	9	12.14	1.03
-2	-4	9	12.49	0.84
-2	-4	9	11.16	0.87
1	4	-9	32.36	1.40
1	4	-9	34.68	1.29
1	4	-9	32.98	1.38
-1	-4	9	32.55	1.46
-1	-4	9	31.32	1.25
-1	-4	9	33.71	1.61
-1	-4	9	34.95	1.53
0	4	-9	34.97	1.45
0	4	-9	33.34	1.33
0	4	-9	35.68	1.44
0	4	-9	35.24	1.47
0	-4	9	37.43	1.62
0	-4	9	35.47	1.34
0	-4	9	33.56	1.51
0	-4	9	36.17	1.77

-1	4	-9	3.86	0.66
-1	4	-9	3.96	0.62
-1	4	-9	5.26	0.63
-1	4	-9	4.93	0.47
1	-4	9	5.23	0.92
1	-4	9	5.15	0.54
1	-4	9	3.53	0.61
1	-4	9	5.88	0.77
-2	4	-9	42.21	1.73
-2	4	-9	39.95	1.74
-2	4	-9	40.12	1.62
-2	4	-9	41.51	1.54
2	-4	9	42.16	1.56
2	-4	9	40.44	1.74
2	-4	9	41.93	2.18
2	-4	9	42.08	1.87
-3	4	-9	20.94	1.26
-3	4	-9	20.47	1.09
-3	4	-9	21.83	1.01
-3	4	-9	24.84	1.30
3	-4	9	21.26	1.75
3	-4	9	22.22	1.04
3	-4	9	23.69	1.24
3	-4	9	24.97	1.45
-4	4	-9	16.43	0.98
-4	4	-9	14.62	1.20
-4	4	-9	15.36	0.85
-4	4	-9	16.29	1.26
4	-4	9	13.93	1.06
4	-4	9	16.74	0.91
4	-4	9	17.64	1.35
4	-4	9	17.46	1.76
-5	4	-9	7.23	1.20
-5	4	-9	9.97	0.71
-5	4	-9	9.16	0.83
5	-4	9	8.26	1.50
5	-4	9	7.30	0.91
5	-4	9	8.32	1.21
-6	4	-9	33.48	1.52
-6	4	-9	33.88	2.28
-6	4	-9	33.19	1.39
6	-4	9	30.53	1.66
-7	4	-9	14.15	1.06
-7	4	-9	13.81	0.94

7	-4	9	11.86	1.20
7	-4	9	14.10	2.40
-8	4	-9	3.71	0.61
-8	4	-9	3.50	0.70
8	-4	9	2.27	1.63
8	-4	9	4.37	0.83
-9	4	-9	6.61	0.94
-9	4	-9	5.02	0.82
9	-4	9	3.19	1.95
-10	4	-9	11.37	1.15
-10	4	-9	8.43	1.25
10	-4	9	9.11	1.48
10	-4	9	11.18	2.55
-11	4	-9	6.11	0.99
-11	4	-9	3.07	0.82
11	-4	9	6.92	2.60
11	-4	9	6.64	1.15
16	3	-9	1.93	1.44
16	3	-9	-0.88	0.70
15	3	-9	1.42	0.64
15	3	-9	-1.45	1.18
14	3	-9	-0.09	1.09
14	3	-9	-0.38	1.30
14	3	-9	0.39	0.55
14	3	-9	1.65	1.32
13	3	-9	-0.24	0.45
13	3	-9	0.82	1.00
13	3	-9	0.31	0.91
-13	-3	9	-0.25	0.87
12	3	-9	0.54	0.83
12	3	-9	0.73	0.58
12	3	-9	1.19	1.04
12	3	-9	-0.69	0.41
-12	-3	9	1.27	0.79
11	3	-9	0.63	0.96
11	3	-9	0.49	0.77
11	3	-9	0.91	0.70
11	3	-9	0.39	0.38
11	3	-9	-0.17	0.48
-11	-3	9	-0.27	0.64
10	3	-9	0.40	0.68
10	3	-9	0.54	0.46
10	3	-9	0.60	0.66
10	3	-9	1.18	0.64

10	3	-9	-0.10	0.30
-10	-3	9	0.86	0.64
-10	-3	9	-0.41	0.56
9	3	-9	9.36	0.81
9	3	-9	8.91	1.12
9	3	-9	7.84	0.67
-9	-3	9	8.28	1.04
-9	-3	9	9.13	0.95
8	3	-9	11.43	0.69
8	3	-9	10.13	0.76
8	3	-9	10.80	0.82
8	3	-9	9.58	1.05
-8	-3	9	11.22	1.00
-8	-3	9	10.71	0.89
7	3	-9	16.05	0.89
7	3	-9	14.13	1.08
7	3	-9	14.32	0.77
-7	-3	9	13.82	1.01
-7	-3	9	14.77	0.98
6	3	-9	12.11	0.71
6	3	-9	14.72	0.82
6	3	-9	14.22	1.05
-6	-3	9	12.99	0.67
-6	-3	9	13.79	0.92
-6	-3	9	12.63	0.92
5	3	-9	12.91	0.79
5	3	-9	12.59	0.99
5	3	-9	12.89	0.70
-5	-3	9	14.31	0.91
-5	-3	9	14.32	0.66
-5	-3	9	12.90	0.91
4	3	-9	32.27	1.47
4	3	-9	31.14	1.30
4	3	-9	31.54	1.22
-4	-3	9	33.09	1.42
-4	-3	9	30.95	1.17
-4	-3	9	28.97	1.38
3	3	-9	1.10	0.32
3	3	-9	0.81	0.22
3	3	-9	0.65	0.27
3	3	-9	1.09	0.45
-3	-3	9	0.47	0.25
-3	-3	9	1.32	0.44
-3	-3	9	0.47	0.40

-3	-3	9	0.29	0.31
2	3	-9	12.34	0.87
2	3	-9	12.71	0.68
2	3	-9	12.21	0.64
2	3	-9	13.13	0.77
-2	-3	9	12.86	0.95
-2	-3	9	10.94	0.78
-2	-3	9	14.01	0.98
-2	-3	9	12.86	0.67
1	3	-9	37.15	1.54
1	3	-9	35.04	1.33
1	3	-9	35.99	1.47
-1	-3	9	33.63	1.49
-1	-3	9	35.61	1.63
-1	-3	9	36.51	1.34
-1	-3	9	37.09	1.65
0	3	-9	23.43	1.17
0	3	-9	23.30	1.17
0	3	-9	25.42	1.13
0	3	-9	24.01	1.01
0	-3	9	24.24	1.02
0	-3	9	26.17	1.36
0	-3	9	24.59	1.17
0	-3	9	23.41	1.39
-1	3	-9	71.58	2.56
-1	3	-9	70.00	2.48
-1	3	-9	71.31	2.50
-1	3	-9	68.15	2.33
1	-3	9	67.59	2.83
1	-3	9	70.12	2.34
1	-3	9	65.83	2.51
1	-3	9	71.92	2.69
-2	3	-9	18.18	1.10
-2	3	-9	16.58	1.06
-2	3	-9	15.95	0.82
-2	3	-9	17.19	0.98
2	-3	9	16.69	1.21
2	-3	9	17.61	1.46
2	-3	9	15.88	0.99
2	-3	9	16.04	0.85
-3	3	-9	7.22	0.94
-3	3	-9	8.22	0.62
-3	3	-9	8.74	0.76
-3	3	-9	8.17	0.92

3	-3	9	8.51	0.81
3	-3	9	9.62	1.15
3	-3	9	8.00	0.68
3	-3	9	8.59	1.23
-4	3	-9	32.16	1.83
-4	3	-9	34.59	1.76
-4	3	-9	38.64	1.56
-4	3	-9	35.81	1.41
4	-3	9	35.67	1.46
4	-3	9	36.44	2.27
4	-3	9	35.59	1.88
4	-3	9	37.01	1.64
-5	3	-9	21.93	1.63
-5	3	-9	22.36	1.21
-5	3	-9	21.05	1.05
5	-3	9	22.48	1.30
5	-3	9	22.80	2.01
-6	3	-9	26.29	1.21
6	-3	9	25.32	2.43
6	-3	9	28.34	1.52
-7	3	-9	29.48	1.52
-7	3	-9	30.00	1.34
7	-3	9	29.38	1.62
-8	3	-9	11.25	1.15
-8	3	-9	10.68	0.95
8	-3	9	10.13	1.24
8	-3	9	11.40	2.00
-9	3	-9	9.78	1.04
-9	3	-9	9.24	1.17
9	-3	9	6.99	1.94
9	-3	9	10.40	1.33
-10	3	-9	9.68	1.09
-10	3	-9	8.59	1.28
10	-3	9	5.79	2.22
10	-3	9	9.21	1.51
-11	3	-9	2.27	0.94
-11	3	-9	4.66	0.88
11	-3	9	4.05	1.09
16	2	-9	-0.05	1.36
16	2	-9	0.74	0.63
15	2	-9	-0.27	1.20
15	2	-9	0.46	0.57
14	2	-9	3.11	0.62
14	2	-9	2.90	1.16

14	2	-9	1.78	1.23
14	2	-9	4.28	1.36
13	2	-9	1.00	0.56
13	2	-9	1.55	0.48
13	2	-9	1.55	0.94
13	2	-9	1.28	1.06
-13	-2	9	-0.06	0.77
12	2	-9	-0.99	0.83
12	2	-9	1.14	0.92
12	2	-9	0.29	0.48
12	2	-9	-0.24	0.38
12	2	-9	-0.75	0.83
-12	-2	9	0.57	0.72
11	2	-9	1.05	0.48
11	2	-9	1.98	0.74
11	2	-9	1.49	0.77
11	2	-9	0.34	0.45
11	2	-9	0.78	0.82
-11	-2	9	0.70	0.63
10	2	-9	20.37	0.97
10	2	-9	20.85	1.07
10	2	-9	21.41	1.49
-10	-2	9	21.12	1.33
-10	-2	9	17.87	1.28
9	2	-9	11.51	1.20
9	2	-9	14.61	0.85
9	2	-9	14.48	0.77
9	2	-9	14.30	0.97
-9	-2	9	14.29	1.07
-9	-2	9	13.69	1.07
8	2	-9	12.87	0.72
8	2	-9	10.95	1.10
8	2	-9	12.86	0.79
8	2	-9	13.36	0.80
-8	-2	9	12.77	0.97
-8	-2	9	12.31	0.94
7	2	-9	22.33	1.04
7	2	-9	21.74	1.05
7	2	-9	23.71	1.34
7	2	-9	22.22	0.97
-7	-2	9	22.27	1.17
-7	-2	9	22.48	1.21
6	2	-9	9.35	0.65
6	2	-9	8.48	0.89

6	2	-9	8.79	0.58
-6	-2	9	7.85	0.80
-6	-2	9	8.06	0.53
-6	-2	9	7.73	0.71
5	2	-9	22.36	0.96
5	2	-9	23.57	1.05
5	2	-9	23.70	1.29
-5	-2	9	23.27	1.20
-5	-2	9	22.15	1.11
-5	-2	9	22.81	0.92
4	2	-9	29.13	1.46
4	2	-9	32.45	1.30
4	2	-9	31.53	1.19
-4	-2	9	30.74	1.14
-4	-2	9	29.92	1.42
-4	-2	9	29.52	1.32
3	2	-9	26.42	1.31
3	2	-9	24.23	1.12
3	2	-9	23.77	1.00
-3	-2	9	26.00	0.98
-3	-2	9	25.90	1.31
-3	-2	9	24.15	1.11
-3	-2	9	24.32	1.04
-3	-2	9	25.08	1.16
2	2	-9	5.51	0.59
2	2	-9	5.41	0.72
2	2	-9	4.86	0.45
-2	-2	9	5.92	0.76
-2	-2	9	4.98	0.61
-2	-2	9	4.42	0.53
-2	-2	9	6.03	0.47
-2	-2	9	6.95	0.83
1	2	-9	60.38	2.12
1	2	-9	61.55	2.35
1	2	-9	62.41	2.20
1	2	-9	64.57	2.32
-1	-2	9	66.62	2.31
-1	-2	9	62.55	2.11
-1	-2	9	63.60	2.48
-1	-2	9	63.78	2.42
0	2	-9	3.06	0.47
0	2	-9	3.71	0.62
0	2	-9	3.55	0.43
0	2	-9	3.56	0.54

0	-2	9	3.65	0.45
0	-2	9	3.46	0.68
0	-2	9	3.16	0.61
0	-2	9	3.42	0.53
-1	2	-9	4.38	0.62
-1	2	-9	4.61	0.45
-1	2	-9	2.88	0.49
-1	2	-9	4.59	0.69
1	-2	9	4.60	0.76
1	-2	9	3.00	0.54
1	-2	9	5.29	0.75
1	-2	9	4.01	0.49
-2	2	-9	20.14	1.18
-2	2	-9	19.13	1.07
-2	2	-9	20.47	1.24
-2	2	-9	19.27	0.89
2	-2	9	19.66	1.07
2	-2	9	17.41	1.44
2	-2	9	18.55	0.91
2	-2	9	21.34	1.35
-3	2	-9	22.93	1.17
-3	2	-9	22.49	0.98
-3	2	-9	20.93	1.31
-3	2	-9	19.08	1.39
3	-2	9	22.10	1.66
3	-2	9	22.53	1.17
3	-2	9	21.61	1.01
3	-2	9	20.98	1.52
-4	2	-9	9.67	0.66
-4	2	-9	11.59	1.31
-4	2	-9	8.83	0.87
-4	2	-9	8.62	1.13
4	-2	9	8.55	0.76
4	-2	9	8.05	0.83
4	-2	9	9.23	1.43
-5	2	-9	22.00	1.03
-5	2	-9	20.82	1.22
5	-2	9	21.48	1.26
5	-2	9	21.82	1.31
5	-2	9	21.39	1.96
-6	2	-9	28.10	1.26
-6	2	-9	29.77	1.48
6	-2	9	25.22	2.30
6	-2	9	30.98	1.55

-7	2	-9	22.58	1.18
-7	2	-9	22.82	1.38
7	-2	9	25.72	1.47
7	-2	9	23.47	2.50
-8	2	-9	14.32	1.26
-8	2	-9	16.07	1.08
8	-2	9	15.01	2.59
8	-2	9	17.40	1.32
-9	2	-9	6.36	0.94
-9	2	-9	4.11	0.81
9	-2	9	4.44	0.86
9	-2	9	1.58	1.70
-10	2	-9	11.02	1.19
-10	2	-9	7.69	1.29
10	-2	9	5.00	2.09
10	-2	9	11.01	1.45
16	1	-9	4.63	1.38
15	1	-9	0.81	0.54
15	1	-9	1.27	1.24
14	1	-9	2.85	1.22
14	1	-9	-0.26	0.45
14	1	-9	1.42	1.02
-14	-1	9	1.20	0.81
13	1	-9	1.05	0.45
13	1	-9	2.38	0.91
13	1	-9	1.15	0.95
13	1	-9	2.39	1.06
13	1	-9	1.70	0.48
-13	-1	9	1.77	0.72
12	1	-9	9.27	1.42
12	1	-9	7.43	0.73
12	1	-9	8.05	1.39
12	1	-9	9.92	0.78
-12	-1	9	8.97	1.10
11	1	-9	15.49	1.11
11	1	-9	17.25	1.48
11	1	-9	14.59	0.87
11	1	-9	17.63	0.91
-11	-1	9	14.42	1.16
10	1	-9	2.44	0.41
10	1	-9	2.58	0.75
10	1	-9	3.00	0.54
-10	-1	9	2.59	0.58
-10	-1	9	2.79	0.66

9	1	-9	37.05	1.82
9	1	-9	35.36	1.36
9	1	-9	33.11	1.40
9	1	-9	35.68	1.40
-9	-1	9	34.82	1.65
-9	-1	9	37.56	1.62
8	1	-9	12.43	0.69
8	1	-9	12.83	1.10
8	1	-9	12.16	0.75
8	1	-9	13.22	0.75
-8	-1	9	11.76	0.97
-8	-1	9	11.60	0.86
7	1	-9	19.80	1.31
7	1	-9	22.12	1.00
7	1	-9	21.32	0.93
7	1	-9	23.62	1.01
-7	-1	9	20.16	1.08
-7	-1	9	23.55	1.22
6	1	-9	28.10	1.16
6	1	-9	27.34	1.43
6	1	-9	25.34	1.07
-6	-1	9	27.26	1.04
-6	-1	9	27.95	1.35
-6	-1	9	26.17	1.21
5	1	-9	8.04	0.86
5	1	-9	8.93	0.55
5	1	-9	9.40	0.65
-5	-1	9	10.72	0.87
-5	-1	9	7.96	0.65
-5	-1	9	8.57	0.51
4	1	-9	88.89	2.98
4	1	-9	85.47	2.83
4	1	-9	89.66	3.18
-4	-1	9	89.20	2.81
-4	-1	9	86.10	2.79
-4	-1	9	86.29	2.98
-4	-1	9	87.21	2.88
-4	-1	9	89.12	3.14
3	1	-9	219.47	6.98
3	1	-9	219.45	7.12
3	1	-9	225.27	6.80
-3	-1	9	225.67	6.77
-3	-1	9	221.14	6.96
-3	-1	9	223.88	6.91

-3	-1	9	216.32	6.91
-3	-1	9	231.13	7.16
2	1	-9	16.09	1.06
2	1	-9	16.73	0.78
2	1	-9	19.18	1.00
-2	-1	9	16.92	1.16
-2	-1	9	17.78	0.96
-2	-1	9	18.03	0.78
-2	-1	9	17.45	1.03
-2	-1	9	17.23	0.91
1	1	-9	2.97	0.50
1	1	-9	2.71	0.51
1	1	-9	3.28	0.41
-1	-1	9	3.26	0.41
-1	-1	9	2.31	0.61
-1	-1	9	2.66	0.56
-1	-1	9	3.97	0.60
-1	-1	9	2.51	0.39
0	1	-9	29.65	1.44
0	1	-9	32.32	1.50
0	1	-9	29.52	1.16
0	-1	9	30.53	1.54
0	-1	9	31.66	1.18
0	-1	9	28.94	1.60
0	-1	9	32.34	1.36
0	-1	9	32.36	1.57
-1	1	-9	17.43	0.82
-1	1	-9	19.09	1.20
-1	1	-9	17.48	1.06
1	-1	9	17.95	1.33
1	-1	9	20.96	1.32
1	-1	9	19.10	0.85
1	-1	9	20.24	1.32
1	-1	9	17.05	0.98
-2	1	-9	15.49	1.03
-2	1	-9	15.68	1.23
-2	1	-9	14.75	0.77
2	-1	9	16.62	1.40
2	-1	9	14.77	0.82
2	-1	9	18.07	0.99
2	-1	9	16.63	1.35
2	-1	9	16.26	1.28
-3	1	-9	19.49	1.58
-3	1	-9	22.53	1.21

-3	1	-9	22.08	0.96
3	-1	9	23.56	1.18
3	-1	9	21.40	1.02
3	-1	9	20.12	1.71
3	-1	9	23.12	1.65
3	-1	9	21.68	1.54
-4	1	-9	32.09	1.31
-4	1	-9	40.97	2.67
-4	1	-9	32.61	1.55
4	-1	9	34.51	2.15
4	-1	9	32.79	1.37
4	-1	9	33.78	1.57
-5	1	-9	28.84	1.23
5	-1	9	27.71	1.31
5	-1	9	26.85	2.38
5	-1	9	32.39	1.53
-6	1	-9	24.37	1.41
-6	1	-9	25.64	1.16
6	-1	9	25.85	1.44
6	-1	9	24.69	2.23
-7	1	-9	16.57	1.28
-7	1	-9	18.81	1.31
7	-1	9	18.58	1.30
7	-1	9	15.78	2.25
-8	1	-9	13.76	1.25
-8	1	-9	12.22	1.00
8	-1	9	12.53	1.26
8	-1	9	7.76	1.72
-9	1	-9	12.20	1.33
-9	1	-9	12.38	1.11
9	-1	9	12.83	1.45
9	-1	9	8.53	2.06
-10	1	-9	6.41	0.86
-10	1	-9	5.93	0.97
10	-1	9	6.00	2.05
10	-1	9	7.23	1.12
17	0	-9	0.61	1.46
17	0	-9	0.65	0.72
16	0	-9	2.23	1.31
16	0	-9	1.78	0.64
15	0	-9	5.17	1.27
15	0	-9	3.85	0.66
15	0	-9	3.30	1.53
14	0	-9	3.08	1.10

14	0	-9	3.98	0.61
14	0	-9	2.76	1.28
-14	0	9	4.38	0.80
13	0	-9	3.64	0.49
13	0	-9	3.55	0.95
13	0	-9	3.60	0.53
13	0	-9	4.81	1.12
13	0	-9	3.79	0.96
-13	0	9	3.61	0.72
12	0	-9	0.76	0.74
12	0	-9	-0.14	0.32
12	0	-9	-1.05	0.69
12	0	-9	0.33	0.84
12	0	-9	-0.25	0.34
-12	0	9	-0.39	0.53
11	0	-9	4.85	0.58
11	0	-9	3.98	0.88
11	0	-9	5.66	0.58
11	0	-9	5.10	0.65
-11	0	9	5.39	0.69
10	0	-9	47.66	1.70
10	0	-9	48.07	2.19
10	0	-9	46.07	1.70
-10	0	9	43.80	1.99
-10	0	9	45.59	1.87
9	0	-9	9.38	0.63
9	0	-9	10.05	0.68
9	0	-9	10.27	0.71
9	0	-9	8.76	1.08
-9	0	9	10.07	0.81
-9	0	9	10.41	0.99
8	0	-9	12.82	0.70
8	0	-9	14.09	1.15
8	0	-9	13.21	0.76
8	0	-9	13.89	0.78
-8	0	9	11.50	1.00
-8	0	9	13.96	0.85
7	0	-9	38.77	1.47
7	0	-9	38.70	1.42
7	0	-9	36.55	1.36
7	0	-9	36.80	1.79
-7	0	9	35.85	1.49
-7	0	9	35.22	1.67
6	0	-9	25.14	1.01

6	0	-9	25.10	1.40
6	0	-9	25.15	1.14
-6	0	9	24.63	0.97
-6	0	9	26.16	1.32
-6	0	9	24.09	1.11
5	0	-9	11.74	0.76
5	0	-9	11.05	0.60
5	0	-9	10.53	0.97
-5	0	9	11.90	0.58
-5	0	9	12.28	0.95
-5	0	9	11.21	0.70
4	0	-9	68.87	2.35
4	0	-9	70.77	2.73
4	0	-9	72.81	2.56
-4	0	9	68.83	2.54
-4	0	9	71.07	2.49
-4	0	9	75.10	2.35
-4	0	9	72.91	2.31
-4	0	9	73.62	2.74
3	0	-9	82.32	2.76
3	0	-9	84.81	3.13
3	0	-9	87.45	3.03
-3	0	9	88.65	3.19
-3	0	9	83.50	2.92
-3	0	9	86.51	2.74
-3	0	9	90.96	2.87
-3	0	9	85.21	3.04
2	0	-9	21.86	0.90
2	0	-9	19.81	1.22
2	0	-9	21.76	1.17
-2	0	9	22.05	1.35
-2	0	9	22.08	1.27
-2	0	9	22.40	0.89
-2	0	9	20.79	1.03
-2	0	9	23.61	1.08
1	0	-9	15.53	0.72
1	0	-9	15.48	1.07
1	0	-9	14.64	1.05
-1	0	9	16.38	0.88
-1	0	9	15.87	1.19
-1	0	9	15.64	0.72
-1	0	9	16.20	0.97
-1	0	9	13.86	1.15
0	0	-9	20.69	1.19

0	0	-9	20.51	1.27
0	0	-9	20.37	0.87
0	0	9	19.53	1.04
0	0	9	21.08	0.88
0	0	9	19.60	1.35
0	0	9	21.74	1.21
0	0	9	21.77	1.41
-1	0	-9	20.56	1.20
-1	0	-9	22.32	1.41
-1	0	-9	21.21	0.89
1	0	9	21.28	1.10
1	0	9	21.91	1.46
1	0	9	22.10	1.53
1	0	9	21.85	0.93
1	0	9	21.96	1.35
-2	0	-9	26.87	1.38
-2	0	-9	29.07	1.78
-2	0	-9	27.28	1.10
2	0	9	29.02	1.14
2	0	9	29.61	1.68
2	0	9	25.77	1.67
2	0	9	28.77	1.89
2	0	9	27.50	1.34
-3	0	-9	31.82	2.05
-3	0	-9	32.84	1.28
-3	0	-9	32.31	1.56
3	0	9	33.40	2.13
3	0	9	35.27	1.34
3	0	9	31.89	1.52
3	0	9	35.95	1.96
-4	0	-9	5.81	0.85
-4	0	-9	5.60	0.58
4	0	9	6.50	0.83
4	0	9	6.27	0.74
4	0	9	5.44	0.97
-5	0	-9	41.39	1.84
-5	0	-9	40.68	1.54
5	0	9	38.84	1.83
5	0	9	39.14	1.62
5	0	9	41.87	2.44
-6	0	-9	16.26	1.24
-6	0	-9	16.59	0.92
6	0	9	14.78	1.98
6	0	9	15.33	1.22

-7	0	-9	3.94	0.77
7	0	9	1.70	1.20
7	0	9	4.08	0.74
-8	0	-9	9.02	0.97
-8	0	-9	9.94	1.23
8	0	9	11.94	1.30
8	0	9	7.83	1.81
-9	0	-9	6.01	0.86
-9	0	-9	7.69	1.30
9	0	9	9.26	1.42
9	0	9	7.77	1.94
17	-1	-9	-0.34	1.30
17	-1	-9	-0.63	0.62
16	-1	-9	4.84	0.72
16	-1	-9	4.48	1.31
15	-1	-9	-0.99	1.38
15	-1	-9	2.06	1.12
15	-1	-9	2.36	0.58
14	-1	-9	1.57	1.03
14	-1	-9	0.48	1.18
14	-1	-9	0.81	0.50
-14	1	9	0.12	0.62
13	-1	-9	0.31	0.42
13	-1	-9	-0.72	0.77
13	-1	-9	1.91	1.06
13	-1	-9	0.08	0.39
13	-1	-9	1.29	0.75
-13	1	9	0.05	0.53
12	-1	-9	4.20	0.93
12	-1	-9	4.65	0.65
12	-1	-9	3.47	0.58
12	-1	-9	4.49	0.98
-12	1	9	4.17	0.60
11	-1	-9	13.44	0.77
11	-1	-9	11.49	1.29
11	-1	-9	12.60	0.81
11	-1	-9	11.24	1.71
-11	1	9	12.44	0.92
-11	1	9	12.55	0.76
10	-1	-9	0.72	0.37
10	-1	-9	1.41	0.39
10	-1	-9	1.04	0.60
10	-1	-9	1.40	0.62
10	-1	-9	1.33	0.32

-10	1	9	1.88	0.61
-10	1	9	0.23	0.39
-10	1	9	1.68	0.34
9	-1	-9	17.98	0.93
9	-1	-9	15.42	1.27
9	-1	-9	16.80	0.81
9	-1	-9	17.26	0.85
-9	1	9	16.28	1.18
-9	1	9	17.60	0.94
8	-1	-9	36.92	1.37
8	-1	-9	37.17	1.40
8	-1	-9	39.43	1.52
8	-1	-9	35.39	1.84
-8	1	9	38.72	1.76
-8	1	9	36.72	1.48
7	-1	-9	9.59	0.62
7	-1	-9	8.30	0.54
7	-1	-9	10.19	0.72
7	-1	-9	8.59	0.99
-7	1	9	9.29	0.91
-7	1	9	8.31	0.63
6	-1	-9	34.33	1.40
6	-1	-9	30.92	1.62
6	-1	-9	29.96	1.16
-6	1	9	31.34	1.14
-6	1	9	29.88	1.26
-6	1	9	30.76	1.30
-6	1	9	31.27	1.54
5	-1	-9	30.02	1.57
5	-1	-9	31.50	1.40
5	-1	-9	31.11	1.14
-5	1	9	29.94	1.10
-5	1	9	31.08	1.26
-5	1	9	29.23	1.11
-5	1	9	30.94	1.55
-5	1	9	30.24	1.42
4	-1	-9	5.40	0.71
4	-1	-9	3.96	0.41
4	-1	-9	3.37	0.65
-4	1	9	2.73	0.61
-4	1	9	2.61	0.35
-4	1	9	4.11	0.69
-4	1	9	3.87	0.47
-4	1	9	4.03	0.50

3	-1	-9	89.15	3.19
3	-1	-9	88.14	3.24
3	-1	-9	92.61	2.83
-3	1	9	86.63	2.99
-3	1	9	93.07	3.33
-3	1	9	90.35	2.92
-3	1	9	71.78	3.89
-3	1	9	90.10	2.78
2	-1	-9	21.89	1.32
2	-1	-9	23.89	0.93
2	-1	-9	22.91	1.32
-2	1	9	23.22	1.46
-2	1	9	24.32	1.12
-2	1	9	23.06	1.11
-2	1	9	25.92	1.46
-2	1	9	24.51	0.92
1	-1	-9	9.81	0.54
1	-1	-9	9.45	0.91
1	-1	-9	10.17	1.01
-1	1	9	9.90	0.73
-1	1	9	8.09	1.17
-1	1	9	10.94	1.09
-1	1	9	9.97	0.81
-1	1	9	10.96	0.57
0	-1	-9	38.61	1.97
0	-1	-9	39.19	1.76
0	-1	-9	38.14	1.38
0	1	9	40.17	1.65
0	1	9	38.18	1.72
0	1	9	38.46	1.96
0	1	9	40.40	2.09
0	1	9	40.30	1.40
-1	-1	-9	49.73	2.43
-1	-1	-9	53.02	2.10
-1	-1	-9	50.53	1.74
1	1	9	50.44	2.35
1	1	9	50.84	1.75
1	1	9	52.70	2.19
1	1	9	50.55	2.02
1	1	9	49.87	2.51
-2	-1	-9	16.71	1.12
-2	-1	-9	17.19	1.55
-2	-1	-9	16.17	0.76
2	1	9	17.84	1.31

2	1	9	14.23	1.75
2	1	9	16.60	1.06
2	1	9	16.37	0.83
-3	-1	-9	27.34	1.42
-3	-1	-9	27.47	1.10
3	1	9	27.31	1.74
3	1	9	27.01	1.16
3	1	9	27.17	1.44
-4	-1	-9	37.26	1.41
-4	-1	-9	38.62	1.75
4	1	9	39.01	1.79
4	1	9	36.90	1.48
4	1	9	33.44	2.23
-5	-1	-9	17.83	1.25
-5	-1	-9	15.85	0.88
5	1	9	16.31	1.30
5	1	9	15.00	1.04
5	1	9	19.42	1.87
-6	-1	-9	4.49	0.78
-6	-1	-9	4.52	0.56
6	1	9	2.99	1.18
6	1	9	3.87	0.75
-7	-1	-9	5.07	0.76
-7	-1	-9	6.94	1.15
7	1	9	6.48	1.60
7	1	9	5.19	0.92
-8	-1	-9	6.22	1.01
-8	-1	-9	7.62	0.97
8	1	9	3.55	1.67
8	1	9	5.91	0.98
-9	-1	-9	8.41	1.42
-9	-1	-9	8.73	1.18
9	1	9	6.43	1.07
9	1	9	5.69	1.79
17	-2	-9	3.87	1.37
17	-2	-9	1.98	0.69
16	-2	-9	-1.22	1.71
16	-2	-9	-2.41	1.06
16	-2	-9	1.32	0.60
15	-2	-9	2.94	1.08
15	-2	-9	3.01	1.48
15	-2	-9	1.11	0.52
14	-2	-9	3.94	0.57
14	-2	-9	4.73	1.12

14	-2	-9	5.20	1.37
-14	2	9	3.56	0.69
-14	2	9	5.20	0.82
13	-2	-9	9.28	1.38
13	-2	-9	12.68	1.51
13	-2	-9	9.55	1.15
13	-2	-9	10.15	0.79
-13	2	9	9.83	0.95
-13	2	9	11.42	1.01
12	-2	-9	3.68	1.02
12	-2	-9	4.07	1.15
12	-2	-9	3.42	0.54
12	-2	-9	3.45	0.47
-12	2	9	3.55	0.58
-12	2	9	2.28	0.55
11	-2	-9	7.18	1.07
11	-2	-9	7.20	0.75
11	-2	-9	6.08	0.59
11	-2	-9	8.34	1.20
-11	2	9	6.84	0.69
-11	2	9	5.60	0.67
10	-2	-9	17.76	0.89
10	-2	-9	16.51	1.38
10	-2	-9	17.52	0.83
10	-2	-9	17.67	1.02
-10	2	9	15.49	1.25
-10	2	9	15.66	0.91
-10	2	9	16.84	0.87
9	-2	-9	21.14	1.21
9	-2	-9	22.56	1.49
9	-2	-9	23.07	0.99
9	-2	-9	23.41	1.17
9	-2	-9	22.13	0.95
-9	2	9	19.95	1.37
-9	2	9	21.45	1.02
-9	2	9	22.33	1.00
8	-2	-9	19.59	1.10
8	-2	-9	19.39	0.85
8	-2	-9	20.10	0.90
8	-2	-9	19.22	1.38
8	-2	-9	21.65	1.08
-8	2	9	20.08	0.99
-8	2	9	18.34	0.93
-8	2	9	19.59	1.27

7	-2	-9	24.65	1.00
7	-2	-9	23.10	1.22
7	-2	-9	23.68	1.46
7	-2	-9	23.22	1.12
7	-2	-9	23.89	0.95
-7	2	9	23.85	1.20
-7	2	9	22.22	1.36
-7	2	9	24.11	1.06
6	-2	-9	25.41	1.00
6	-2	-9	24.15	1.27
6	-2	-9	22.62	1.41
6	-2	-9	23.84	1.18
6	-2	-9	23.77	0.94
-6	2	9	22.53	1.03
-6	2	9	22.36	1.34
-6	2	9	23.51	1.32
5	-2	-9	47.39	1.89
5	-2	-9	50.85	2.06
5	-2	-9	50.65	2.17
5	-2	-9	49.66	1.68
-5	2	9	49.07	2.13
-5	2	9	50.21	2.15
-5	2	9	47.48	1.82
-5	2	9	50.06	1.64
4	-2	-9	19.41	1.11
4	-2	-9	19.81	1.27
4	-2	-9	20.71	1.30
4	-2	-9	21.00	0.84
-4	2	9	22.47	1.37
-4	2	9	19.68	0.98
-4	2	9	19.67	0.78
3	-2	-9	25.71	1.46
3	-2	-9	25.76	0.97
3	-2	-9	25.07	1.42
-3	2	9	25.12	1.17
-3	2	9	26.35	0.94
-3	2	9	24.90	1.60
-3	2	9	24.18	1.48
-3	2	9	26.09	1.14
2	-2	-9	34.98	1.25
2	-2	-9	36.01	1.83
2	-2	-9	34.46	1.71
-2	2	9	33.96	1.81
-2	2	9	34.08	1.49

-2	2	9	37.80	1.24
-2	2	9	34.74	1.98
-2	2	9	35.66	1.49
1	-2	-9	29.08	1.72
1	-2	-9	29.81	1.09
1	-2	-9	28.53	1.51
-1	2	9	29.81	1.91
-1	2	9	30.80	1.10
-1	2	9	29.87	1.39
-1	2	9	32.62	1.72
-1	2	9	29.35	1.38
0	-2	-9	39.68	1.81
0	-2	-9	39.20	1.39
0	-2	-9	41.79	2.16
0	2	9	41.30	1.78
0	2	9	34.60	2.01
0	2	9	42.09	2.31
0	2	9	41.00	1.41
0	2	9	41.32	1.73
-1	-2	-9	24.14	1.38
-1	-2	-9	24.69	0.97
-1	-2	-9	23.11	1.79
1	2	9	25.69	1.02
1	2	9	25.04	1.32
1	2	9	28.28	2.07
1	2	9	24.24	1.40
1	2	9	25.25	1.76
-2	-2	-9	20.72	1.30
-2	-2	-9	23.22	0.95
2	2	9	23.24	1.01
2	2	9	22.61	1.47
2	2	9	26.07	1.35
2	2	9	27.15	2.55
-3	-2	-9	42.09	1.57
3	2	9	47.21	2.02
3	2	9	45.38	1.65
3	2	9	44.86	2.22
-4	-2	-9	21.07	1.34
-4	-2	-9	20.55	0.95
4	2	9	20.36	1.05
4	2	9	20.32	1.42
4	2	9	23.00	1.74
-5	-2	-9	10.22	0.73
-5	-2	-9	7.83	1.08

5	2	9	10.69	0.95
5	2	9	10.09	1.68
5	2	9	9.99	1.16
-6	-2	-9	5.30	0.85
-6	-2	-9	7.03	0.79
6	2	9	5.61	1.32
6	2	9	5.57	0.93
-7	-2	-9	14.40	1.00
-7	-2	-9	13.49	1.38
7	2	9	11.94	2.19
7	2	9	13.87	1.53
-8	-2	-9	10.89	1.12
-8	-2	-9	11.91	1.41
8	2	9	14.72	2.51
8	2	9	11.36	1.58
17	-3	-9	-0.70	1.18
17	-3	-9	1.11	0.64
16	-3	-9	6.16	1.85
16	-3	-9	3.41	1.29
16	-3	-9	3.87	0.67
15	-3	-9	5.87	1.64
15	-3	-9	6.96	1.39
15	-3	-9	7.78	0.87
14	-3	-9	0.03	0.94
14	-3	-9	1.61	0.48
14	-3	-9	3.36	1.28
-14	3	9	2.08	0.73
13	-3	-9	4.75	1.27
13	-3	-9	4.31	1.07
13	-3	-9	4.43	0.66
13	-3	-9	6.05	0.90
-13	3	9	3.96	0.60
-13	3	9	6.40	0.98
12	-3	-9	18.73	1.73
12	-3	-9	16.96	0.90
-12	3	9	16.64	1.12
-12	3	9	16.38	0.97
11	-3	-9	5.03	0.86
11	-3	-9	4.01	0.89
11	-3	-9	7.16	1.03
11	-3	-9	6.57	0.59
-11	3	9	5.15	0.63
10	-3	-9	4.53	0.51
10	-3	-9	6.26	0.61

10	-3	-9	4.00	0.69
10	-3	-9	5.27	0.91
10	-3	-9	5.10	0.85
-10	3	9	4.76	0.55
-10	3	9	5.12	0.79
9	-3	-9	26.54	1.67
9	-3	-9	29.55	1.12
9	-3	-9	24.35	1.41
9	-3	-9	26.22	1.60
9	-3	-9	29.92	1.16
-9	3	9	30.69	1.20
-9	3	9	29.14	1.60
-9	3	9	28.93	1.39
8	-3	-9	39.61	1.42
8	-3	-9	41.27	1.86
8	-3	-9	42.26	2.02
8	-3	-9	35.70	1.77
8	-3	-9	42.29	1.45
-8	3	9	38.53	1.86
-8	3	9	38.56	1.76
-8	3	9	39.27	1.51
7	-3	-9	17.80	1.37
7	-3	-9	21.28	0.88
7	-3	-9	19.42	1.25
7	-3	-9	19.69	0.82
7	-3	-9	20.06	1.29
-7	3	9	20.00	1.35
-7	3	9	19.37	0.93
-7	3	9	19.55	1.29
6	-3	-9	25.96	1.50
6	-3	-9	29.86	1.10
6	-3	-9	27.74	1.06
6	-3	-9	27.58	1.41
6	-3	-9	26.95	1.59
-6	3	9	29.73	1.65
-6	3	9	28.46	1.53
-6	3	9	27.99	1.20
5	-3	-9	11.32	0.58
5	-3	-9	11.76	1.12
5	-3	-9	11.25	0.99
5	-3	-9	10.94	1.10
-5	3	9	12.06	1.29
-5	3	9	11.42	0.75
-5	3	9	12.61	1.11

4	-3	-9	44.87	2.09
4	-3	-9	44.46	2.03
4	-3	-9	43.67	1.88
4	-3	-9	42.54	1.49
-4	3	9	42.78	1.72
-4	3	9	44.23	2.23
-4	3	9	43.71	2.04
-4	3	9	43.21	1.44
3	-3	-9	51.37	2.26
3	-3	-9	54.57	1.74
3	-3	-9	51.79	2.18
3	-3	-9	48.77	2.35
-3	3	9	54.42	2.38
-3	3	9	51.82	2.56
-3	3	9	53.73	1.70
-3	3	9	52.10	2.02
2	-3	-9	36.42	1.27
2	-3	-9	36.40	1.77
2	-3	-9	35.37	1.98
-2	3	9	35.58	1.57
-2	3	9	35.85	1.59
-2	3	9	39.36	1.95
-2	3	9	37.83	1.26
-2	3	9	40.12	2.26
1	-3	-9	33.15	2.03
1	-3	-9	34.88	1.22
1	-3	-9	35.81	1.71
-1	3	9	36.39	1.23
-1	3	9	35.31	1.61
-1	3	9	35.63	1.94
-1	3	9	33.24	2.22
-1	3	9	34.49	1.57
0	-3	-9	48.44	2.59
0	-3	-9	50.90	2.16
0	-3	-9	49.34	1.66
0	3	9	52.11	1.68
0	3	9	50.90	2.08
0	3	9	49.23	2.45
0	3	9	46.74	2.07
0	3	9	52.84	2.82
-1	-3	-9	37.18	1.79
-1	-3	-9	38.96	1.35
1	3	9	39.19	2.57
1	3	9	38.76	1.79

1	3	9	38.77	1.38
1	3	9	39.96	1.86
-2	-3	-9	14.44	0.71
-2	-3	-9	14.61	1.17
2	3	9	16.06	1.18
2	3	9	16.51	0.80
2	3	9	14.62	1.25
-3	-3	-9	15.13	0.75
-3	-3	-9	15.81	1.19
3	3	9	14.15	0.83
3	3	9	16.09	1.66
3	3	9	13.13	1.18
-4	-3	-9	20.49	0.95
-4	-3	-9	22.47	1.42
4	3	9	21.48	1.09
4	3	9	23.26	1.49
4	3	9	18.80	1.75
-5	-3	-9	16.58	0.93
-5	-3	-9	17.99	1.39
5	3	9	18.01	1.93
5	3	9	17.99	1.09
5	3	9	17.73	1.47
-6	-3	-9	11.74	0.91
-6	-3	-9	9.79	1.30
6	3	9	11.29	2.03
6	3	9	11.33	1.42
-7	-3	-9	10.82	1.00
-7	-3	-9	10.38	1.38
7	3	9	11.88	1.57
7	3	9	8.89	1.75
-8	-3	-9	7.47	1.09
-8	-3	-9	10.04	1.15
8	3	9	8.89	1.94
8	3	9	11.00	1.69
18	-4	-9	-2.39	2.58
18	-4	-9	4.94	0.97
17	-4	-9	5.65	1.62
17	-4	-9	5.51	0.75
16	-4	-9	2.28	0.59
16	-4	-9	5.32	1.81
16	-4	-9	1.53	1.16
15	-4	-9	12.59	0.98
15	-4	-9	14.28	2.48
15	-4	-9	15.09	1.91

14	-4	-9	22.96	1.19
14	-4	-9	24.08	2.39
14	-4	-9	22.25	2.00
-14	4	9	26.02	1.53
13	-4	-9	19.83	1.74
13	-4	-9	17.60	0.98
13	-4	-9	19.33	1.18
13	-4	-9	20.48	2.07
-13	4	9	17.70	1.37
-13	4	9	20.19	1.10
12	-4	-9	5.53	1.10
12	-4	-9	5.43	1.16
12	-4	-9	4.31	1.12
12	-4	-9	5.98	0.61
-12	4	9	6.35	0.86
-12	4	9	5.66	0.65
11	-4	-9	9.92	1.44
11	-4	-9	8.61	0.63
11	-4	-9	8.41	1.25
11	-4	-9	8.57	0.73
11	-4	-9	8.92	1.05
-11	4	9	9.51	1.09
-11	4	9	9.30	0.67
10	-4	-9	82.35	3.35
10	-4	-9	86.54	2.81
10	-4	-9	86.66	2.79
10	-4	-9	87.60	3.47
10	-4	-9	78.52	3.15
-10	4	9	89.07	3.18
-10	4	9	86.70	2.84
9	-4	-9	24.63	1.63
9	-4	-9	28.57	1.07
9	-4	-9	27.25	1.10
9	-4	-9	23.42	1.51
9	-4	-9	27.48	1.69
-9	4	9	25.00	1.54
-9	4	9	28.60	1.14
-9	4	9	27.57	1.60
8	-4	-9	13.49	0.70
8	-4	-9	12.07	1.24
8	-4	-9	12.78	1.21
8	-4	-9	12.78	0.63
8	-4	-9	11.38	1.21
-8	4	9	12.11	1.27

-8	4	9	12.30	1.19
-8	4	9	12.31	0.73
7	-4	-9	29.14	1.70
7	-4	-9	28.80	1.09
7	-4	-9	28.77	1.69
7	-4	-9	30.33	1.13
7	-4	-9	29.10	1.66
-7	4	9	28.93	1.23
-7	4	9	29.15	1.62
-7	4	9	31.21	1.79
6	-4	-9	36.66	1.28
6	-4	-9	38.32	1.31
6	-4	-9	35.36	1.90
6	-4	-9	34.15	1.87
6	-4	-9	35.70	1.79
-6	4	9	36.47	1.49
-6	4	9	37.31	2.04
-6	4	9	34.78	1.81
5	-4	-9	12.18	1.28
5	-4	-9	12.23	1.16
5	-4	-9	12.11	1.21
5	-4	-9	12.64	0.61
5	-4	-9	13.29	0.63
-5	4	9	11.96	1.19
-5	4	9	11.63	1.47
-5	4	9	11.72	0.82
4	-4	-9	40.07	1.97
4	-4	-9	42.33	1.98
4	-4	-9	41.88	1.41
4	-4	-9	40.40	2.13
-4	4	9	42.81	2.38
-4	4	9	42.72	1.72
-4	4	9	38.40	1.39
-4	4	9	40.70	2.02
3	-4	-9	12.63	1.14
3	-4	-9	13.46	1.21
3	-4	-9	14.34	1.42
3	-4	-9	11.94	0.58
-3	4	9	12.59	0.89
-3	4	9	12.31	1.31
-3	4	9	11.87	0.54
-3	4	9	14.24	1.71
2	-4	-9	40.03	2.28
2	-4	-9	38.43	1.34

2	-4	-9	40.74	1.91
2	-4	-9	42.21	2.04
-2	4	9	39.72	2.11
-2	4	9	40.34	1.32
-2	4	9	37.55	1.75
-2	4	9	42.47	2.57
1	-4	-9	60.61	1.97
1	-4	-9	67.16	3.40
1	-4	-9	58.47	2.52
-1	4	9	62.21	3.08
-1	4	9	63.57	1.97
-1	4	9	59.62	2.43
-1	4	9	64.29	3.26
-1	4	9	59.09	2.42
0	-4	-9	37.98	1.79
0	-4	-9	35.07	1.26
0	4	9	36.76	2.69
0	4	9	38.51	1.79
0	4	9	34.01	1.75
0	4	9	40.59	1.40
-1	-4	-9	24.08	1.40
-1	-4	-9	19.92	0.83
1	4	9	20.32	1.42
1	4	9	22.11	0.88
1	4	9	22.02	1.39
-2	-4	-9	36.13	1.78
-2	-4	-9	32.89	1.22
2	4	9	35.25	1.81
2	4	9	29.22	2.62
2	4	9	34.65	1.28
-3	-4	-9	18.29	0.83
-3	-4	-9	18.53	1.37
3	4	9	20.45	1.59
3	4	9	19.16	1.45
3	4	9	18.09	0.94
-4	-4	-9	17.91	1.45
-4	-4	-9	16.26	0.89
4	4	9	19.62	1.55
4	4	9	23.60	1.51
4	4	9	16.69	1.74
-5	-4	-9	23.03	1.56
-5	-4	-9	19.69	1.01
5	4	9	17.37	1.64
5	4	9	19.16	2.03

-6	-4	-9	11.22	1.42
-6	-4	-9	12.26	0.93
6	4	9	13.01	1.58
6	4	9	6.85	1.51
-7	-4	-9	12.49	1.55
7	4	9	12.48	2.01
7	4	9	10.46	1.70
18	-5	-9	-0.78	0.70
18	-5	-9	0.84	1.74
17	-5	-9	2.11	1.38
17	-5	-9	0.94	0.64
16	-5	-9	14.25	1.13
16	-5	-9	18.57	2.19
16	-5	-9	17.86	2.84
15	-5	-9	9.17	0.91
15	-5	-9	6.40	1.27
15	-5	-9	6.35	1.82
14	-5	-9	3.15	0.52
14	-5	-9	5.14	1.50
14	-5	-9	3.81	1.11
-14	5	9	2.68	0.91
13	-5	-9	21.99	2.27
13	-5	-9	22.31	1.08
13	-5	-9	22.19	1.90
13	-5	-9	20.01	1.29
-13	5	9	23.10	1.12
-13	5	9	24.04	1.63
12	-5	-9	27.62	2.26
12	-5	-9	30.74	1.24
12	-5	-9	28.46	1.92
-12	5	9	28.54	1.22
-12	5	9	32.64	1.83
11	-5	-9	23.22	1.74
11	-5	-9	23.48	1.07
11	-5	-9	22.25	1.89
11	-5	-9	22.20	1.47
11	-5	-9	22.83	1.00
-11	5	9	22.30	1.00
-11	5	9	22.46	1.66
10	-5	-9	38.08	2.18
10	-5	-9	44.72	2.35
10	-5	-9	43.78	1.58
10	-5	-9	42.87	1.53
10	-5	-9	43.59	2.07

-10	5	9	40.72	2.20
-10	5	9	42.76	1.58
9	-5	-9	35.92	1.94
9	-5	-9	34.85	2.02
9	-5	-9	32.67	1.96
9	-5	-9	36.81	1.35
9	-5	-9	36.99	1.32
-9	5	9	37.15	1.41
-9	5	9	36.30	2.06
8	-5	-9	29.51	1.84
8	-5	-9	31.26	1.16
8	-5	-9	31.61	1.82
8	-5	-9	29.82	1.12
8	-5	-9	28.39	1.76
-8	5	9	31.91	2.02
-8	5	9	27.44	1.65
-8	5	9	30.22	1.27
7	-5	-9	36.68	2.06
7	-5	-9	38.16	2.03
7	-5	-9	37.88	1.33
7	-5	-9	38.30	2.02
7	-5	-9	39.62	1.36
-7	5	9	40.12	2.25
-7	5	9	38.44	1.56
-7	5	9	34.97	1.91
6	-5	-9	29.57	1.72
6	-5	-9	26.74	1.72
6	-5	-9	27.94	1.02
6	-5	-9	28.11	1.03
6	-5	-9	26.87	1.80
-6	5	9	26.96	1.66
-6	5	9	27.16	1.28
-6	5	9	26.31	2.02
5	-5	-9	7.36	0.56
5	-5	-9	8.11	1.11
5	-5	-9	7.60	0.48
5	-5	-9	8.63	1.18
5	-5	-9	8.39	1.34
-5	5	9	7.22	1.20
-5	5	9	7.93	0.75
-5	5	9	9.23	1.65
4	-5	-9	27.43	0.98
4	-5	-9	25.89	0.95
4	-5	-9	25.12	1.56

4	-5	-9	26.13	1.70
4	-5	-9	24.50	1.86
-4	5	9	26.15	1.34
-4	5	9	26.04	2.19
-4	5	9	26.41	1.71
3	-5	-9	37.81	2.05
3	-5	-9	38.04	1.92
3	-5	-9	37.76	1.27
3	-5	-9	36.84	2.29
-3	5	9	34.12	2.55
-3	5	9	37.68	2.04
-3	5	9	40.72	1.76
2	-5	-9	34.24	1.78
2	-5	-9	32.09	1.10
2	-5	-9	32.84	1.95
-2	5	9	34.22	1.66
-2	5	9	32.01	2.58
-2	5	9	30.47	1.07
1	-5	-9	20.41	0.76
1	-5	-9	17.13	1.35
-1	5	9	20.01	2.38
-1	5	9	22.50	1.33
-1	5	9	19.23	0.73
0	-5	-9	44.74	2.08
0	5	9	44.98	2.10
0	5	9	44.76	2.09
0	5	9	44.91	1.47
-1	-5	-9	38.44	1.89
-1	-5	-9	38.85	1.29
1	5	9	38.67	1.96
1	5	9	35.21	1.97
-2	-5	-9	32.13	1.16
-2	-5	-9	30.89	1.76
2	5	9	34.01	1.22
2	5	9	30.10	1.90
2	5	9	34.74	1.92
-3	-5	-9	13.20	0.71
-3	-5	-9	13.52	1.31
3	5	9	12.23	1.41
3	5	9	12.17	1.53
-4	-5	-9	15.16	1.45
-4	-5	-9	15.26	0.84
4	5	9	14.83	1.58
4	5	9	16.02	1.76

-5	-5	-9	21.30	1.61
-5	-5	-9	17.61	1.02
5	5	9	18.92	2.10
5	5	9	21.82	1.85
-6	-5	-9	21.71	1.66
6	5	9	16.67	2.31
6	5	9	20.12	1.94
-7	-5	-9	5.84	1.49
7	5	9	11.64	1.76
18	-6	-9	0.68	0.74
18	-6	-9	3.71	2.15
17	-6	-9	5.46	2.22
17	-6	-9	7.74	1.07
17	-6	-9	5.71	1.47
16	-6	-9	5.53	0.75
16	-6	-9	7.97	1.54
16	-6	-9	9.46	2.04
15	-6	-9	3.13	1.06
15	-6	-9	2.20	1.63
15	-6	-9	3.03	0.58
14	-6	-9	12.32	1.88
14	-6	-9	9.12	0.82
14	-6	-9	11.96	1.79
-14	6	9	11.13	1.57
13	-6	-9	19.57	1.37
13	-6	-9	18.59	2.39
13	-6	-9	19.29	1.82
-13	6	9	20.99	1.83
-13	6	9	20.65	1.02
12	-6	-9	6.40	1.40
12	-6	-9	7.77	1.22
12	-6	-9	6.53	0.63
12	-6	-9	5.13	1.05
-12	6	9	5.39	1.16
-12	6	9	6.19	0.59
11	-6	-9	30.02	2.27
11	-6	-9	32.29	1.34
11	-6	-9	32.90	1.27
11	-6	-9	31.49	1.79
11	-6	-9	26.82	2.85
-11	6	9	35.33	2.16
-11	6	9	33.08	1.29
10	-6	-9	42.79	2.12
10	-6	-9	40.08	1.53

10	-6	-9	40.43	1.47
10	-6	-9	38.78	2.19
10	-6	-9	42.42	2.41
-10	6	9	43.34	2.42
-10	6	9	40.78	1.55
9	-6	-9	37.55	2.09
9	-6	-9	33.23	2.03
9	-6	-9	35.56	2.18
9	-6	-9	36.42	1.32
9	-6	-9	37.76	1.37
-9	6	9	37.58	1.47
-9	6	9	36.56	2.30
8	-6	-9	24.02	0.96
8	-6	-9	23.53	0.92
8	-6	-9	21.56	1.77
8	-6	-9	22.18	1.71
8	-6	-9	21.96	1.75
-8	6	9	23.77	1.15
-8	6	9	23.39	2.08
7	-6	-9	3.86	0.41
7	-6	-9	2.69	0.80
7	-6	-9	5.21	1.06
7	-6	-9	3.08	0.93
7	-6	-9	3.52	0.45
-7	6	9	1.35	1.10
-7	6	9	2.98	0.82
-7	6	9	3.96	0.54
6	-6	-9	6.85	1.18
6	-6	-9	9.76	1.38
6	-6	-9	11.18	1.48
6	-6	-9	8.97	0.51
6	-6	-9	9.37	0.51
-6	6	9	9.73	1.53
-6	6	9	8.54	0.80
-6	6	9	8.04	1.25
5	-6	-9	35.18	1.20
5	-6	-9	32.95	1.90
5	-6	-9	36.95	2.26
5	-6	-9	34.44	1.21
5	-6	-9	37.99	2.10
-5	6	9	29.79	1.95
-5	6	9	37.29	1.65
-5	6	9	36.93	2.56
4	-6	-9	22.04	1.51

4	-6	-9	19.09	1.85
4	-6	-9	21.34	0.78
4	-6	-9	20.22	1.69
-4	6	9	21.84	1.27
-4	6	9	20.18	2.26
3	-6	-9	18.50	1.43
3	-6	-9	18.80	1.68
-3	6	9	21.18	2.40
-3	6	9	18.93	1.27
2	-6	-9	13.97	1.29
2	-6	-9	17.67	1.64
-2	6	9	17.36	2.37
-2	6	9	16.04	1.25
1	-6	-9	24.40	1.89
1	-6	-9	27.07	1.58
-1	6	9	19.83	1.74
0	-6	-9	44.34	2.10
0	6	9	41.50	2.19
0	6	9	47.81	2.23
-1	-6	-9	7.52	1.14
1	6	9	8.20	1.27
1	6	9	8.80	1.30
-2	-6	-9	12.50	1.30
2	6	9	9.71	1.43
2	6	9	11.12	1.46
-3	-6	-9	18.94	0.84
-3	-6	-9	19.18	1.52
3	6	9	17.43	1.80
3	6	9	14.25	1.65
-4	-6	-9	14.56	1.49
4	6	9	12.96	1.84
4	6	9	11.56	1.67
-5	-6	-9	18.37	1.66
5	6	9	18.41	2.15
5	6	9	19.99	1.93
-6	-6	-9	13.01	1.63
6	6	9	9.83	1.44
6	6	9	7.23	1.73
18	-7	-9	0.97	0.73
18	-7	-9	-1.75	2.16
17	-7	-9	5.36	0.78
17	-7	-9	3.96	2.25
17	-7	-9	7.07	1.59
16	-7	-9	20.93	3.24

16	-7	-9	27.47	2.53
16	-7	-9	23.74	1.35
15	-7	-9	7.55	1.47
15	-7	-9	11.56	2.04
15	-7	-9	10.14	0.94
14	-7	-9	2.65	1.16
14	-7	-9	2.89	0.53
14	-7	-9	2.03	1.66
-14	7	9	2.18	1.19
13	-7	-9	12.62	1.37
13	-7	-9	15.13	1.80
13	-7	-9	15.80	2.35
13	-7	-9	15.52	0.91
-13	7	9	14.85	1.95
-13	7	9	15.20	0.80
12	-7	-9	39.85	1.50
12	-7	-9	36.73	2.34
12	-7	-9	37.14	2.06
12	-7	-9	40.67	2.79
-12	7	9	36.83	2.48
-12	7	9	40.28	1.47
11	-7	-9	41.97	2.35
11	-7	-9	38.65	1.54
11	-7	-9	41.00	1.48
11	-7	-9	39.20	2.14
11	-7	-9	39.19	2.66
-11	7	9	38.90	1.51
-11	7	9	40.40	2.59
10	-7	-9	17.37	1.67
10	-7	-9	12.77	1.89
10	-7	-9	11.48	1.50
10	-7	-9	13.77	0.81
10	-7	-9	13.08	0.71
-10	7	9	14.11	2.06
-10	7	9	14.74	0.84
9	-7	-9	34.19	1.25
9	-7	-9	28.98	2.23
9	-7	-9	32.87	2.02
9	-7	-9	30.34	1.18
9	-7	-9	29.53	2.10
-9	7	9	36.07	2.50
-9	7	9	32.55	1.41
8	-7	-9	32.34	1.97
8	-7	-9	34.17	2.21

8	-7	-9	35.51	1.26
8	-7	-9	32.83	2.27
8	-7	-9	33.57	1.22
-8	7	9	38.71	2.64
-8	7	9	33.37	1.51
7	-7	-9	33.61	1.18
7	-7	-9	33.13	2.27
7	-7	-9	31.64	1.97
7	-7	-9	31.05	2.22
7	-7	-9	33.46	1.18
-7	7	9	33.28	2.58
-7	7	9	33.96	1.55
6	-7	-9	54.25	1.75
6	-7	-9	53.01	2.73
6	-7	-9	51.66	2.55
6	-7	-9	53.69	2.92
6	-7	-9	54.66	1.73
-6	7	9	52.89	2.18
-6	7	9	53.15	3.20
5	-7	-9	27.25	2.30
5	-7	-9	30.61	2.12
5	-7	-9	30.17	1.85
-5	7	9	31.83	1.59
-5	7	9	32.61	2.73
4	-7	-9	39.81	2.14
4	-7	-9	41.31	2.42
-4	7	9	41.39	3.16
-4	7	9	43.60	1.99
3	-7	-9	77.11	3.12
3	-7	-9	79.22	3.46
-3	7	9	73.13	3.02
2	-7	-9	18.49	1.88
2	-7	-9	15.75	1.47
-2	7	9	16.63	1.72
1	-7	-9	20.86	1.50
-1	7	9	20.01	1.54
0	-7	-9	40.95	2.09
0	7	9	44.17	2.27
0	7	9	40.45	2.17
-1	-7	-9	12.53	1.35
1	7	9	10.82	1.52
1	7	9	15.82	1.59
-2	-7	-9	8.05	1.30
2	7	9	8.27	1.18

2	7	9	10.22	1.58
-3	-7	-9	11.30	1.47
3	7	9	13.73	1.85
3	7	9	10.47	1.72
-4	-7	-9	12.82	1.55
4	7	9	11.27	1.85
4	7	9	12.56	2.02
-5	-7	-9	13.05	1.65
5	7	9	8.06	1.57
5	7	9	12.70	1.98
17	-8	-9	7.72	1.07
17	-8	-9	2.36	1.50
17	-8	-9	7.22	2.38
16	-8	-9	10.07	2.22
16	-8	-9	4.61	1.54
16	-8	-9	5.52	0.72
15	-8	-9	7.31	0.90
15	-8	-9	7.43	1.50
15	-8	-9	7.35	2.09
14	-8	-9	19.93	1.09
14	-8	-9	17.53	2.09
14	-8	-9	21.44	2.83
-14	8	9	16.68	2.21
13	-8	-9	15.78	2.63
13	-8	-9	16.99	1.98
13	-8	-9	18.81	0.99
-13	8	9	19.36	2.38
-13	8	9	18.99	0.92
12	-8	-9	7.26	1.31
12	-8	-9	7.41	0.67
12	-8	-9	8.27	1.71
12	-8	-9	9.84	1.65
-12	8	9	6.26	1.52
-12	8	9	7.61	0.65
11	-8	-9	19.53	2.31
11	-8	-9	15.16	0.89
11	-8	-9	14.94	1.69
11	-8	-9	14.62	0.77
11	-8	-9	11.99	1.75
-11	8	9	12.92	2.31
-11	8	9	12.49	0.83
10	-8	-9	11.86	1.80
10	-8	-9	16.33	2.12
10	-8	-9	13.96	0.83

10	-8	-9	13.88	1.86
10	-8	-9	14.36	0.72
-10	8	9	13.96	0.91
-10	8	9	14.04	2.29
9	-8	-9	10.15	1.81
9	-8	-9	12.66	1.54
9	-8	-9	11.49	0.63
9	-8	-9	13.10	0.73
9	-8	-9	12.21	1.91
-9	8	9	12.20	0.91
-9	8	9	11.66	1.85
8	-8	-9	26.92	2.29
8	-8	-9	28.06	1.04
8	-8	-9	28.23	1.07
8	-8	-9	28.19	2.23
8	-8	-9	27.61	1.92
-8	8	9	28.44	1.43
-8	8	9	26.78	2.66
7	-8	-9	33.46	1.15
7	-8	-9	31.41	2.36
7	-8	-9	29.33	2.41
7	-8	-9	30.38	2.02
-7	8	9	33.88	1.63
-7	8	9	33.53	2.83
6	-8	-9	34.07	2.10
6	-8	-9	37.48	2.44
-6	8	9	31.68	3.06
-6	8	9	37.99	1.80
5	-8	-9	62.59	3.20
5	-8	-9	68.78	2.92
-5	8	9	66.69	3.87
-5	8	9	61.45	2.65
4	-8	-9	39.88	2.55
4	-8	-9	37.07	2.13
-4	8	9	40.98	2.02
3	-8	-9	20.07	1.91
3	-8	-9	22.45	2.06
-3	8	9	21.45	1.56
2	-8	-9	25.90	2.28
2	-8	-9	23.81	1.70
-2	8	9	24.79	1.71
1	-8	-9	39.19	2.65
1	-8	-9	36.26	2.02
-1	8	9	39.28	2.21

0	-8	-9	23.27	1.68
0	8	9	25.04	1.87
-1	-8	-9	11.47	1.45
1	8	9	12.55	1.72
1	8	9	12.65	1.65
-2	-8	-9	10.69	1.51
2	8	9	11.06	1.81
2	8	9	11.08	1.73
-3	-8	-9	16.16	1.68
3	8	9	16.24	1.97
3	8	9	13.23	2.03
-4	-8	-9	7.12	1.14
4	8	9	7.60	1.62
4	8	9	6.91	1.41
17	-9	-9	7.84	2.57
17	-9	-9	2.78	1.52
16	-9	-9	19.60	2.49
16	-9	-9	17.56	2.77
15	-9	-9	17.85	1.14
15	-9	-9	16.38	2.29
15	-9	-9	22.23	3.24
14	-9	-9	23.37	3.05
14	-9	-9	20.01	2.25
14	-9	-9	23.57	1.20
13	-9	-9	33.26	3.19
13	-9	-9	31.39	1.34
13	-9	-9	29.69	2.41
-13	9	9	32.61	1.31
12	-9	-9	16.80	1.05
12	-9	-9	16.50	0.92
12	-9	-9	17.82	1.98
12	-9	-9	18.17	2.63
-12	9	9	17.46	0.96
-12	9	9	17.22	2.99
11	-9	-9	18.97	1.03
11	-9	-9	20.54	2.12
11	-9	-9	16.21	2.47
11	-9	-9	19.38	0.90
11	-9	-9	20.48	1.93
-11	9	9	18.69	1.08
-11	9	9	15.25	2.63
10	-9	-9	29.35	2.16
10	-9	-9	26.37	2.62
10	-9	-9	30.94	1.18

10	-9	-9	33.68	1.28
10	-9	-9	29.27	2.42
-10	9	9	29.60	2.88
-10	9	9	30.17	1.45
9	-9	-9	28.09	1.09
9	-9	-9	29.21	1.16
9	-9	-9	28.57	2.42
9	-9	-9	31.97	2.57
9	-9	-9	28.53	2.09
-9	9	9	28.23	1.47
-9	9	9	30.05	2.84
8	-9	-9	26.59	1.00
8	-9	-9	20.21	2.36
8	-9	-9	26.13	0.97
8	-9	-9	26.84	2.44
-8	9	9	23.72	1.42
-8	9	9	23.41	2.93
7	-9	-9	73.02	3.70
-7	9	9	74.64	2.93
6	-9	-9	43.52	2.80
-6	9	9	46.52	2.17
5	-9	-9	32.44	2.16
5	-9	-9	31.17	2.46
4	-9	-9	43.13	2.32
-4	9	9	45.49	2.28
3	-9	-9	38.33	2.69
3	-9	-9	38.29	2.12
-3	9	9	37.52	2.16
2	-9	-9	27.02	2.48
2	-9	-9	24.83	1.78
-2	9	9	25.09	1.86
1	-9	-9	24.29	1.76
1	-9	-9	25.29	2.46
-1	9	9	23.84	1.92
0	-9	-9	11.45	1.51
0	-9	-9	15.92	2.41
0	9	9	13.29	1.78
-1	-9	-9	18.14	1.76
1	9	9	18.19	1.93
1	9	9	15.87	1.92
-2	-9	-9	13.50	1.63
2	9	9	14.72	1.92
2	9	9	12.53	2.01
-3	-9	-9	6.85	1.19

3	9	9	4.86	1.37
3	9	9	7.60	1.49
17	-10	-9	12.19	2.87
17	-10	-9	7.00	1.78
16	-10	-9	5.68	2.47
16	-10	-9	4.76	1.50
15	-10	-9	11.98	1.91
15	-10	-9	21.62	3.49
15	-10	-9	15.30	1.10
14	-10	-9	36.23	5.94
14	-10	-9	36.50	2.75
14	-10	-9	33.26	1.48
13	-10	-9	20.89	1.08
13	-10	-9	22.28	2.30
13	-10	-9	26.22	3.10
-13	10	9	21.29	1.09
12	-10	-9	2.30	0.56
12	-10	-9	1.01	1.09
12	-10	-9	2.47	1.63
12	-10	-9	1.68	0.42
-12	10	9	1.97	0.51
-12	10	9	3.67	1.04
11	-10	-9	13.81	2.12
11	-10	-9	15.48	1.93
11	-10	-9	12.75	0.91
11	-10	-9	12.89	0.77
-11	10	9	12.36	1.02
10	-10	-9	32.65	2.32
10	-10	-9	27.57	2.83
10	-10	-9	32.98	1.35
10	-10	-9	34.20	1.26
10	-10	-9	32.76	2.92
-10	10	9	36.15	1.67
9	-10	-9	30.19	1.12
9	-10	-9	31.70	2.26
9	-10	-9	29.42	1.17
9	-10	-9	29.57	2.77
-9	10	9	29.71	1.62
8	-10	-9	31.77	2.77
8	-10	-9	32.46	1.17
8	-10	-9	33.25	1.16
8	-10	-9	32.95	2.29
-8	10	9	31.67	1.76
7	-10	-9	35.94	2.85

7	-10	-9	39.83	2.37
-7	10	9	37.24	1.97
6	-10	-9	13.58	1.68
-6	10	9	18.74	1.49
5	-10	-9	57.55	2.78
5	-10	-9	56.37	3.33
-5	10	9	55.45	2.65
4	-10	-9	26.54	1.91
4	-10	-9	28.37	2.59
-4	10	9	25.96	1.90
3	-10	-9	6.59	1.19
3	-10	-9	11.84	1.82
-3	10	9	10.30	1.56
2	-10	-9	40.84	2.28
2	-10	-9	43.85	3.13
-2	10	9	41.12	2.47
1	-10	-9	27.49	2.00
1	-10	-9	28.55	2.82
-1	10	9	34.16	2.30
0	-10	-9	13.17	2.12
0	-10	-9	11.20	1.66
0	10	9	15.85	1.96
-1	-10	-9	9.69	1.65
1	10	9	11.48	1.95
1	10	9	12.19	1.97
-2	-10	-9	7.50	1.24
2	10	9	6.25	1.54
2	10	9	6.97	1.40
16	-11	-9	15.70	2.94
16	-11	-9	19.36	1.33
16	-11	-9	16.45	2.70
15	-11	-9	28.31	1.46
15	-11	-9	31.24	3.96
15	-11	-9	28.86	2.89
14	-11	-9	15.08	1.06
14	-11	-9	16.37	2.47
14	-11	-9	13.57	2.53
13	-11	-9	12.88	2.43
13	-11	-9	12.79	0.90
13	-11	-9	10.81	1.64
-13	11	9	11.96	0.99
12	-11	-9	18.57	2.30
12	-11	-9	18.65	3.00
12	-11	-9	16.65	0.91

-12	11	9	14.38	1.17
11	-11	-9	11.47	0.73
11	-11	-9	10.43	2.15
11	-11	-9	9.92	1.56
-11	11	9	11.38	1.11
-11	11	9	6.82	1.27
10	-11	-9	19.21	2.66
10	-11	-9	19.30	0.87
10	-11	-9	16.00	2.07
-10	11	9	18.77	1.39
9	-11	-9	22.96	2.79
9	-11	-9	26.03	2.29
9	-11	-9	25.51	0.99
-9	11	9	24.46	1.61
8	-11	-9	27.58	2.87
8	-11	-9	28.13	2.22
-8	11	9	26.68	2.01
7	-11	-9	45.71	2.62
7	-11	-9	41.14	3.19
-7	11	9	45.48	2.32
6	-11	-9	30.65	2.24
6	-11	-9	32.98	2.90
-6	11	9	29.11	2.00
5	-11	-9	7.67	1.33
5	-11	-9	7.27	1.74
-5	11	9	10.32	1.52
4	-11	-9	11.03	2.00
4	-11	-9	9.11	1.29
-4	11	9	11.90	1.64
3	-11	-9	29.04	2.07
3	-11	-9	34.74	2.91
-3	11	9	28.60	2.24
2	-11	-9	11.99	1.71
2	-11	-9	15.84	2.78
-2	11	9	18.66	1.99
1	-11	-9	18.29	1.81
1	-11	-9	18.39	2.86
-1	11	9	19.47	2.11
0	-11	-9	17.81	1.88
0	-11	-9	18.82	2.87
0	11	9	15.41	2.18
-1	-11	-9	6.47	1.27
1	11	9	7.93	2.11
1	11	9	5.17	1.48

16	-12	-9	11.34	1.23
16	-12	-9	14.32	3.03
16	-12	-9	8.63	1.91
15	-12	-9	13.01	2.10
15	-12	-9	14.82	3.00
15	-12	-9	13.01	1.18
14	-12	-9	33.81	3.03
14	-12	-9	34.79	1.53
14	-12	-9	31.63	4.10
13	-12	-9	28.85	3.76
13	-12	-9	23.27	1.22
13	-12	-9	25.19	2.75
-13	12	9	25.41	1.43
12	-12	-9	15.02	2.56
12	-12	-9	16.39	0.95
12	-12	-9	16.07	2.35
-12	12	9	18.21	1.35
11	-12	-9	13.27	2.39
11	-12	-9	13.09	0.81
11	-12	-9	11.80	1.81
-11	12	9	11.43	1.30
10	-12	-9	35.32	1.28
10	-12	-9	36.05	3.45
10	-12	-9	32.12	2.66
-10	12	9	31.75	1.99
9	-12	-9	17.10	3.08
9	-12	-9	19.06	2.25
-9	12	9	19.91	1.65
8	-12	-9	25.49	3.10
8	-12	-9	22.72	2.29
-8	12	9	23.75	1.81
7	-12	-9	39.21	2.64
7	-12	-9	42.25	3.40
-7	12	9	41.18	2.35
6	-12	-9	25.36	3.06
6	-12	-9	21.21	2.12
-6	12	9	25.60	2.05
5	-12	-9	19.74	2.85
5	-12	-9	20.85	2.05
-5	12	9	17.70	1.95
4	-12	-9	19.62	2.04
4	-12	-9	18.47	2.93
-4	12	9	16.13	2.08
3	-12	-9	15.08	2.73

3	-12	-9	12.92	1.83
-3	12	9	11.49	1.97
2	-12	-9	13.03	1.83
2	-12	-9	14.83	2.24
-2	12	9	12.95	2.00
1	-12	-9	16.76	2.01
1	-12	-9	21.57	3.11
-1	12	9	17.22	2.31
15	-13	-9	21.18	3.02
15	-13	-9	16.74	1.25
15	-13	-9	13.10	3.03
14	-13	-9	21.77	3.31
14	-13	-9	14.79	2.21
14	-13	-9	13.84	1.10
13	-13	-9	18.05	3.16
13	-13	-9	21.49	1.70
13	-13	-9	15.73	2.66
12	-13	-9	26.07	2.85
12	-13	-9	22.73	3.09
-12	13	9	24.78	1.70
11	-13	-9	31.42	3.83
11	-13	-9	36.89	2.91
-11	13	9	30.94	1.99
10	-13	-9	30.03	2.83
10	-13	-9	40.08	3.87
-10	13	9	33.56	2.11
9	-13	-9	11.71	1.77
9	-13	-9	12.36	2.40
-9	13	9	13.71	1.71
8	-13	-9	13.52	1.90
8	-13	-9	16.05	2.53
-8	13	9	16.78	1.83
7	-13	-9	21.71	2.40
7	-13	-9	27.93	3.29
-7	13	9	20.73	2.08
6	-13	-9	25.62	2.33
6	-13	-9	18.36	3.22
-6	13	9	26.77	2.26
5	-13	-9	10.93	2.22
5	-13	-9	15.75	2.09
-5	13	9	12.81	1.75
4	-13	-9	10.03	1.51
4	-13	-9	8.82	2.20
-4	13	9	6.98	1.50

3	-13	-9	9.44	1.51
3	-13	-9	10.15	2.30
-3	13	9	8.55	1.77
2	-13	-9	12.91	1.59
2	-13	-9	10.22	2.27
-2	13	9	9.65	2.02
14	-14	-9	18.24	2.44
14	-14	-9	21.42	3.35
13	-14	-9	28.51	4.27
13	-14	-9	28.34	3.22
12	-14	-9	26.20	3.06
12	-14	-9	27.13	4.04
-12	14	9	26.03	1.92
11	-14	-9	25.32	2.96
11	-14	-9	31.37	4.09
-11	14	9	27.65	2.10
10	-14	-9	32.46	4.15
10	-14	-9	37.32	3.10
-10	14	9	39.03	2.43
9	-14	-9	22.30	2.71
9	-14	-9	33.88	3.89
-9	14	9	23.32	2.19
8	-14	-9	26.90	2.76
8	-14	-9	26.55	3.68
-8	14	9	25.25	2.31
7	-14	-9	14.31	2.33
7	-14	-9	11.50	2.48
-7	14	9	12.90	2.13
6	-14	-9	18.12	2.30
6	-14	-9	17.51	2.69
-6	14	9	18.75	2.33
5	-14	-9	15.37	2.52
5	-14	-9	17.75	2.29
-5	14	9	15.48	2.33
4	-14	-9	5.94	2.24
-4	14	9	5.32	1.60
11	-15	-9	25.06	4.25
11	-15	-9	27.83	3.22
-11	15	9	24.11	2.32
10	-15	-9	27.30	3.06
10	-15	-9	32.08	4.25
-10	15	9	24.70	2.39
9	-15	-9	27.77	4.05
9	-15	-9	20.25	2.85

-9	15	9	15.47	2.29
8	-15	-9	20.25	2.69
8	-15	-9	21.59	3.89
-8	15	9	21.26	2.43
7	-15	-9	12.45	2.66
1	17	-10	0.32	0.76
-1	-17	10	1.49	1.62
-1	-17	10	1.33	2.66
0	17	-10	1.84	0.82
0	-17	10	2.42	1.55
0	-17	10	4.07	2.77
-1	17	-10	0.63	0.74
1	-17	10	-1.05	2.45
1	-17	10	2.61	1.55
-2	17	-10	1.67	0.78
2	-17	10	2.51	1.56
2	-17	10	2.75	2.59
-3	17	-10	6.80	0.92
3	-17	10	6.05	1.82
3	-17	10	3.39	2.66
-4	17	-10	6.24	0.92
4	-17	10	3.06	2.75
4	-17	10	3.85	1.66
-5	17	-10	1.98	0.80
5	-17	10	2.22	2.83
5	-17	10	2.55	1.67
-6	17	-10	3.69	0.88
6	-17	10	4.68	3.00
6	-17	10	4.89	1.74
4	16	-10	-0.09	0.90
-4	-16	10	0.59	2.34
3	16	-10	-0.71	0.85
-3	-16	10	1.38	1.60
-3	-16	10	-0.36	2.25
2	16	-10	0.67	0.80
-2	-16	10	-1.39	1.49
-2	-16	10	-1.46	2.23
1	16	-10	2.14	0.80
-1	-16	10	0.64	1.38
-1	-16	10	2.43	2.31
0	16	-10	1.70	0.73
0	-16	10	1.52	2.20
0	-16	10	1.92	1.36
-1	16	-10	8.97	1.17

1	-16	10	10.22	1.83
1	-16	10	7.39	2.49
-2	16	-10	6.08	0.87
2	-16	10	7.72	1.62
2	-16	10	8.63	2.51
-3	16	-10	2.44	0.74
3	-16	10	6.04	2.49
3	-16	10	1.76	1.40
-4	16	-10	4.27	0.80
4	-16	10	6.44	2.49
4	-16	10	1.59	1.44
-5	16	-10	7.64	1.15
5	-16	10	4.49	2.70
5	-16	10	3.59	1.58
-6	16	-10	6.71	0.90
6	-16	10	4.88	1.67
6	-16	10	6.75	2.91
-7	16	-10	1.58	0.75
7	-16	10	3.26	2.76
7	-16	10	4.09	1.60
-8	16	-10	2.44	0.82
8	-16	10	2.08	2.97
8	-16	10	2.11	1.60
5	15	-10	1.73	0.93
-5	-15	10	1.48	2.11
-5	-15	10	-0.29	1.63
4	15	-10	0.41	0.85
-4	-15	10	-0.46	1.42
-4	-15	10	2.57	2.06
3	15	-10	0.33	0.82
-3	-15	10	0.81	1.42
-3	-15	10	-0.18	1.91
2	15	-10	1.48	0.73
-2	-15	10	2.25	1.99
-2	-15	10	3.03	1.51
1	15	-10	10.49	1.20
-1	-15	10	8.20	1.55
-1	-15	10	8.08	2.15
0	15	-10	12.70	1.16
0	-15	10	9.83	2.32
0	-15	10	11.80	1.78
-1	15	-10	2.63	0.68
1	-15	10	3.18	1.29
1	-15	10	4.84	2.05

-2	15	-10	3.08	0.70
2	-15	10	4.81	1.42
2	-15	10	4.68	2.18
-3	15	-10	15.18	1.24
3	-15	10	16.99	2.27
3	-15	10	19.53	2.91
-4	15	-10	12.64	1.13
4	-15	10	8.66	1.58
4	-15	10	10.78	2.59
-5	15	-10	7.13	0.87
5	-15	10	2.54	2.24
5	-15	10	6.05	1.50
-6	15	-10	7.33	0.88
6	-15	10	7.77	1.56
6	-15	10	10.31	2.77
-7	15	-10	5.25	0.85
7	-15	10	5.84	1.53
7	-15	10	3.76	2.63
-8	15	-10	7.77	0.96
8	-15	10	7.32	1.65
8	-15	10	5.77	2.87
-9	15	-10	4.34	0.90
9	-15	10	5.40	2.94
9	-15	10	7.38	1.70
7	14	-10	-1.92	0.88
-7	-14	10	-0.73	1.64
6	14	-10	1.83	0.91
-6	-14	10	0.19	1.76
-6	-14	10	0.20	1.40
5	14	-10	1.08	0.81
-5	-14	10	3.71	1.43
-5	-14	10	0.50	1.77
4	14	-10	0.75	0.77
-4	-14	10	0.87	1.31
3	14	-10	0.52	0.66
-3	-14	10	0.77	1.26
-3	-14	10	0.87	1.77
2	14	-10	7.47	1.15
-2	-14	10	7.22	1.56
-2	-14	10	6.03	1.85
1	14	-10	8.41	1.06
-1	-14	10	1.47	1.75
-1	-14	10	5.18	1.40
0	14	-10	6.59	1.01

0	-14	10	4.40	1.25
-1	14	-10	5.45	0.75
1	-14	10	6.13	1.89
1	-14	10	5.90	1.29
-2	14	-10	8.26	0.98
2	-14	10	7.98	2.15
2	-14	10	9.99	1.60
-3	14	-10	11.88	1.08
3	-14	10	16.35	3.00
3	-14	10	15.39	2.05
-4	14	-10	6.65	0.99
4	-14	10	8.65	2.34
4	-14	10	7.69	1.47
-5	14	-10	9.83	1.08
5	-14	10	12.36	1.98
5	-14	10	9.64	2.51
-6	14	-10	15.35	1.24
6	-14	10	12.42	2.62
6	-14	10	16.61	2.20
-7	14	-10	10.91	1.19
7	-14	10	13.75	2.23
7	-14	10	10.86	2.96
-8	14	-10	7.11	0.91
8	-14	10	7.97	2.81
8	-14	10	6.51	1.58
-9	14	-10	6.17	0.91
9	-14	10	6.36	2.97
9	-14	10	10.21	1.65
8	13	-10	-0.22	0.89
-8	-13	10	2.23	1.60
7	13	-10	-0.44	0.81
-7	-13	10	-2.84	1.57
-7	-13	10	0.10	1.46
6	13	-10	-0.49	0.76
-6	-13	10	-2.45	1.24
-6	-13	10	1.97	1.51
5	13	-10	0.67	0.70
-5	-13	10	-0.28	1.40
-5	-13	10	1.14	1.27
4	13	-10	5.31	0.82
-4	-13	10	6.27	1.78
-4	-13	10	6.69	1.48
3	13	-10	1.90	0.65
-3	-13	10	1.90	1.06

-3	-13	10	3.34	1.52
2	13	-10	0.09	0.58
-2	-13	10	-0.57	1.00
-2	-13	10	0.39	1.43
1	13	-10	3.41	0.66
-1	-13	10	4.38	1.16
-1	-13	10	4.02	1.65
0	13	-10	16.73	1.14
0	-13	10	13.91	2.48
0	-13	10	15.32	1.96
-1	13	-10	6.85	0.89
1	-13	10	6.51	1.23
1	-13	10	7.39	1.82
-2	13	-10	0.89	0.54
2	-13	10	2.47	1.60
2	-13	10	1.92	1.02
-3	13	-10	22.47	1.28
3	-13	10	24.09	2.09
3	-13	10	22.36	2.98
-4	13	-10	4.39	0.66
4	-13	10	3.31	1.80
4	-13	10	4.33	1.22
-5	13	-10	3.30	0.68
5	-13	10	4.31	0.60
5	-13	10	3.99	2.00
5	-13	10	2.41	1.09
-6	13	-10	20.23	1.28
6	-13	10	17.05	2.03
6	-13	10	14.72	2.66
6	-13	10	19.85	1.24
-7	13	-10	14.58	1.21
7	-13	10	21.49	3.18
7	-13	10	15.17	2.05
7	-13	10	17.03	1.35
-8	13	-10	10.88	1.17
8	-13	10	11.44	1.42
8	-13	10	9.08	2.77
8	-13	10	10.35	1.53
-9	13	-10	15.85	1.32
9	-13	10	18.44	2.23
9	-13	10	11.32	2.97
9	-13	10	15.53	1.65
-10	13	-10	3.68	0.94
10	-13	10	7.06	1.73

10	-13	10	7.75	3.08
9	12	-10	0.46	0.89
-9	-12	10	-0.97	1.56
8	12	-10	-0.29	0.83
-8	-12	10	2.68	1.53
-8	-12	10	0.43	1.33
7	12	-10	1.48	0.79
-7	-12	10	2.61	1.43
-7	-12	10	-1.59	1.33
6	12	-10	1.83	0.70
-6	-12	10	3.23	1.40
-6	-12	10	0.57	1.17
5	12	-10	9.76	1.13
-5	-12	10	15.31	2.17
-5	-12	10	11.60	1.95
4	12	-10	9.54	1.10
4	12	-10	16.29	2.68
-4	-12	10	12.30	1.76
-4	-12	10	8.58	1.48
3	12	-10	5.79	0.76
3	12	-10	5.70	1.72
-3	-12	10	5.98	1.28
-3	-12	10	6.90	1.60
2	12	-10	0.87	0.52
2	12	-10	2.19	1.50
-2	-12	10	1.18	0.95
-2	-12	10	2.17	1.31
1	12	-10	4.62	0.65
1	12	-10	3.48	1.51
-1	-12	10	3.29	1.07
-1	-12	10	3.87	1.36
0	12	-10	3.54	1.42
0	12	-10	2.40	0.57
0	-12	10	5.37	1.17
0	-12	10	3.22	1.41
-1	12	-10	13.28	0.97
1	-12	10	13.49	1.65
1	-12	10	11.77	1.90
-2	12	-10	13.93	1.00
2	-12	10	14.63	1.68
2	-12	10	15.34	2.46
-3	12	-10	11.71	0.96
3	-12	10	13.23	2.04
3	-12	10	11.55	1.58

-4	12	-10	6.19	0.86
4	-12	10	5.43	0.77
4	-12	10	5.26	1.03
4	-12	10	5.44	0.66
4	-12	10	6.72	1.85
-5	12	-10	5.97	0.76
5	-12	10	5.92	1.96
5	-12	10	6.52	1.18
5	-12	10	5.43	0.65
-6	12	-10	10.42	1.03
6	-12	10	9.10	1.63
6	-12	10	9.11	1.05
6	-12	10	13.61	2.52
-7	12	-10	15.93	1.23
7	-12	10	18.15	1.89
7	-12	10	20.01	3.57
7	-12	10	18.22	1.35
-8	12	-10	12.34	1.14
8	-12	10	10.75	1.43
8	-12	10	9.19	2.57
8	-12	10	11.07	1.86
-9	12	-10	11.34	1.23
9	-12	10	15.69	2.06
9	-12	10	14.63	3.14
-10	12	-10	8.42	1.24
10	-12	10	12.94	2.12
10	-12	10	6.95	1.37
10	-12	10	5.76	2.98
10	11	-10	0.75	0.87
-10	-11	10	-0.03	1.54
9	11	-10	2.01	0.88
-9	-11	10	0.09	1.46
8	11	-10	0.11	0.70
-8	-11	10	0.21	1.15
-8	-11	10	2.26	1.33
7	11	-10	1.32	0.71
7	11	-10	2.24	1.29
-7	-11	10	1.37	1.18
-7	-11	10	-0.76	1.15
6	11	-10	2.95	0.69
6	11	-10	4.96	2.09
6	11	-10	5.29	0.77
-6	-11	10	4.18	1.23
-6	-11	10	1.51	1.09

5	11	-10	10.30	1.03
5	11	-10	11.91	1.87
-5	-11	10	14.56	1.83
-5	-11	10	8.51	1.32
4	11	-10	29.33	2.57
4	11	-10	30.27	1.50
-4	-11	10	28.31	2.28
-4	-11	10	29.32	2.14
3	11	-10	7.99	1.50
3	11	-10	6.60	1.16
-3	-11	10	5.90	1.32
-3	-11	10	4.00	1.01
2	11	-10	3.28	1.28
2	11	-10	1.27	0.65
-2	-11	10	2.18	1.17
-2	-11	10	1.44	0.76
1	11	-10	5.94	0.83
1	11	-10	7.68	1.49
-1	-11	10	6.45	1.16
-1	-11	10	4.71	1.28
0	11	-10	16.56	1.95
0	11	-10	13.08	1.17
0	-11	10	16.68	2.09
0	-11	10	16.45	1.62
-1	11	-10	13.49	2.01
1	-11	10	16.87	2.08
1	-11	10	16.84	2.24
-2	11	-10	9.67	1.50
-2	11	-10	10.40	0.87
2	-11	10	12.54	1.87
-3	11	-10	21.54	1.15
3	-11	10	18.63	0.91
3	-11	10	19.21	1.20
3	-11	10	22.91	2.54
-4	11	-10	17.41	1.10
4	-11	10	19.49	1.35
4	-11	10	16.32	0.94
4	-11	10	16.00	2.60
-5	11	-10	8.23	0.91
5	-11	10	9.80	1.99
5	-11	10	8.93	1.06
5	-11	10	6.30	0.82
-6	11	-10	15.95	1.14
6	-11	10	16.25	2.23

6	-11	10	15.07	1.13
6	-11	10	19.55	3.01
6	-11	10	17.33	1.67
-7	11	-10	27.67	1.49
7	-11	10	31.14	3.64
7	-11	10	28.65	1.58
7	-11	10	28.22	2.05
-8	11	-10	8.89	1.04
8	-11	10	8.41	1.32
8	-11	10	10.02	1.70
8	-11	10	10.78	2.58
-9	11	-10	18.03	1.35
9	-11	10	20.36	2.04
9	-11	10	16.29	3.13
9	-11	10	20.57	1.73
-10	11	-10	6.68	0.90
10	-11	10	12.05	1.86
10	-11	10	10.92	3.02
10	-11	10	10.23	1.51
11	10	-10	0.00	0.87
-11	-10	10	3.09	1.64
10	10	-10	-1.32	0.70
-10	-10	10	2.39	1.51
9	10	-10	2.06	0.84
9	10	-10	2.30	0.96
9	10	-10	1.45	0.70
-9	-10	10	2.19	1.33
8	10	-10	1.61	0.86
8	10	-10	0.16	0.66
-8	-10	10	0.87	1.14
7	10	-10	4.24	0.70
7	10	-10	2.98	0.82
7	10	-10	5.24	2.11
-7	-10	10	3.92	1.08
-7	-10	10	3.98	1.17
6	10	-10	9.14	1.64
6	10	-10	7.07	1.12
6	10	-10	8.25	1.00
6	10	-10	9.74	0.65
-6	-10	10	9.48	1.59
-6	-10	10	11.06	1.74
5	10	-10	7.87	1.46
5	10	-10	8.00	0.94
5	10	-10	7.66	1.10

-5	-10	10	6.09	1.14
-5	-10	10	4.83	1.04
4	10	-10	9.92	0.91
4	10	-10	10.76	1.85
-4	-10	10	10.64	1.47
-4	-10	10	9.48	1.59
3	10	-10	5.67	0.81
3	10	-10	8.42	1.34
-3	-10	10	6.26	1.30
-3	-10	10	6.94	1.30
2	10	-10	24.83	1.25
2	10	-10	26.95	2.07
-2	-10	10	24.82	2.02
-2	-10	10	22.46	1.93
1	10	-10	12.94	1.67
1	10	-10	13.47	0.94
-1	-10	10	13.58	1.39
-1	-10	10	14.00	1.82
0	10	-10	6.96	1.27
0	10	-10	5.84	0.73
0	-10	10	7.38	1.35
0	-10	10	6.78	1.17
-1	10	-10	14.11	1.70
-1	10	-10	14.09	0.94
1	-10	10	16.67	2.04
1	-10	10	14.44	0.82
1	-10	10	14.69	1.38
-2	10	-10	16.31	0.75
-2	10	-10	16.35	1.78
-2	10	-10	17.69	1.05
2	-10	10	18.70	1.12
2	-10	10	20.05	2.20
2	-10	10	19.32	1.51
-3	10	-10	13.22	0.69
-3	10	-10	13.38	0.91
-3	10	-10	11.92	2.16
3	-10	10	13.81	1.39
3	-10	10	12.21	1.16
3	-10	10	16.37	2.19
-4	10	-10	12.34	0.75
-4	10	-10	14.36	0.96
4	-10	10	16.61	1.50
4	-10	10	13.48	2.34
4	-10	10	14.19	1.34

-5	10	-10	13.82	1.02
-5	10	-10	14.44	0.86
5	-10	10	17.67	1.51
5	-10	10	15.27	1.49
-6	10	-10	25.72	1.36
6	-10	10	28.08	1.87
6	-10	10	27.19	3.07
6	-10	10	26.64	1.87
-7	10	-10	17.01	1.19
7	-10	10	18.89	2.02
7	-10	10	18.20	1.80
7	-10	10	21.27	1.40
7	-10	10	18.08	3.19
-8	10	-10	8.44	1.02
8	-10	10	7.96	1.29
8	-10	10	12.48	2.66
8	-10	10	7.74	1.72
-9	10	-10	11.24	1.14
9	-10	10	11.11	2.76
9	-10	10	10.11	1.55
9	-10	10	16.46	1.91
-10	10	-10	7.26	0.94
10	-10	10	8.80	1.38
10	-10	10	14.10	1.97
10	-10	10	10.19	3.04
-11	10	-10	8.23	1.33
11	-10	10	13.88	2.10
11	-10	10	7.00	3.43
12	9	-10	-0.51	0.79
12	9	-10	-0.19	1.43
-12	-9	10	0.28	1.59
11	9	-10	0.01	0.72
11	9	-10	1.07	1.10
-11	-9	10	-0.83	1.43
10	9	-10	1.08	0.94
10	9	-10	2.76	0.86
10	9	-10	2.66	0.73
-10	-9	10	1.27	1.34
9	9	-10	2.61	0.67
9	9	-10	3.66	0.79
9	9	-10	4.05	0.96
-9	-9	10	2.87	1.14
8	9	-10	0.94	0.80
8	9	-10	1.06	0.52

8	9	-10	2.16	0.61
8	9	-10	1.28	0.66
-8	-9	10	2.22	1.02
7	9	-10	4.96	1.38
7	9	-10	3.97	0.80
7	9	-10	3.74	0.51
7	9	-10	5.73	0.88
-7	-9	10	5.18	0.92
-7	-9	10	5.42	1.16
6	9	-10	11.23	1.77
6	9	-10	10.46	1.11
6	9	-10	11.08	0.68
6	9	-10	13.04	1.34
-6	-9	10	11.26	1.36
-6	-9	10	14.96	1.62
5	9	-10	8.34	0.96
5	9	-10	9.85	1.59
5	9	-10	9.18	0.87
5	9	-10	8.15	0.53
-5	-9	10	10.72	1.66
-5	-9	10	9.77	1.35
4	9	-10	25.19	1.89
4	9	-10	26.50	0.93
4	9	-10	26.97	1.29
-4	-9	10	25.18	1.78
-4	-9	10	25.41	1.81
3	9	-10	21.22	1.13
3	9	-10	24.48	1.76
-3	-9	10	17.56	1.55
-3	-9	10	23.51	1.77
2	9	-10	12.68	0.86
2	9	-10	10.93	1.40
-2	-9	10	11.25	1.51
-2	-9	10	9.62	1.20
1	9	-10	20.64	1.63
1	9	-10	19.77	1.07
-1	-9	10	20.64	1.51
-1	-9	10	21.58	1.88
-1	-9	10	19.42	0.88
0	9	-10	28.20	1.80
0	9	-10	27.23	1.26
0	-9	10	27.34	1.18
0	-9	10	29.69	1.71
0	-9	10	27.21	2.11

-1	9	-10	7.86	1.35
-1	9	-10	7.33	0.53
-1	9	-10	9.02	0.72
1	-9	10	9.13	0.86
1	-9	10	8.77	1.11
1	-9	10	6.82	1.34
-2	9	-10	21.60	1.66
-2	9	-10	18.64	1.03
-2	9	-10	18.40	0.85
2	-9	10	22.12	1.27
2	-9	10	20.17	1.49
2	-9	10	19.74	2.02
-3	9	-10	23.69	1.01
-3	9	-10	25.15	1.23
-3	9	-10	19.99	1.74
3	-9	10	24.68	2.33
3	-9	10	23.92	1.60
3	-9	10	27.02	1.53
-4	9	-10	19.44	0.97
-4	9	-10	20.75	1.11
4	-9	10	22.98	1.53
4	-9	10	21.54	2.33
4	-9	10	21.51	1.59
-5	9	-10	15.89	0.92
-5	9	-10	15.56	1.02
5	-9	10	16.44	1.47
5	-9	10	21.63	2.51
5	-9	10	16.57	1.56
-6	9	-10	10.90	0.86
-6	9	-10	10.37	0.94
6	-9	10	9.99	1.39
6	-9	10	10.81	1.56
6	-9	10	15.00	2.25
-7	9	-10	3.94	0.64
-7	9	-10	4.51	0.61
7	-9	10	4.84	1.00
7	-9	10	2.98	1.79
-8	9	-10	16.48	1.19
8	-9	10	16.04	2.04
8	-9	10	16.12	2.85
-9	9	-10	16.64	1.27
9	-9	10	21.57	3.92
9	-9	10	15.02	1.63
9	-9	10	17.33	1.93

-10	9	-10	12.07	1.23
10	-9	10	16.22	1.96
10	-9	10	8.57	2.83
10	-9	10	10.89	1.45
-11	9	-10	12.53	1.39
11	-9	10	13.92	2.07
11	-9	10	10.30	3.49
13	8	-10	0.68	0.81
13	8	-10	-0.44	1.38
13	8	-10	0.47	2.10
12	8	-10	2.79	1.21
12	8	-10	1.99	0.78
-12	-8	10	2.67	1.50
11	8	-10	3.51	0.75
11	8	-10	1.72	1.06
-11	-8	10	3.31	1.43
10	8	-10	1.15	0.62
10	8	-10	1.67	0.94
10	8	-10	2.09	0.73
-10	-8	10	-0.59	1.07
9	8	-10	-0.18	0.55
9	8	-10	0.42	0.52
9	8	-10	1.27	0.58
9	8	-10	2.48	0.84
-9	-8	10	-0.80	0.91
8	8	-10	0.66	0.49
8	8	-10	1.39	0.47
8	8	-10	1.09	0.75
8	8	-10	0.15	1.16
8	8	-10	1.30	0.57
-8	-8	10	1.15	0.88
7	8	-10	20.45	1.81
7	8	-10	20.54	0.96
7	8	-10	18.02	1.38
7	8	-10	17.99	1.35
-7	-8	10	18.39	1.72
-7	-8	10	21.34	1.32
6	8	-10	16.63	1.21
6	8	-10	18.54	0.81
6	8	-10	14.96	1.56
6	8	-10	17.60	1.06
-6	-8	10	17.86	1.56
-6	-8	10	16.12	1.29
5	8	-10	16.25	1.16

5	8	-10	16.40	1.02
5	8	-10	18.85	1.51
-5	-8	10	19.44	1.51
-5	-8	10	18.03	0.90
-5	-8	10	18.32	1.40
4	8	-10	7.63	1.17
4	8	-10	4.93	0.83
4	8	-10	5.51	0.70
-4	-8	10	6.10	0.99
-4	-8	10	6.32	0.59
-4	-8	10	5.03	0.90
3	8	-10	19.96	1.10
3	8	-10	21.31	1.11
3	8	-10	21.62	1.44
-3	-8	10	20.88	0.95
-3	-8	10	22.83	1.53
-3	-8	10	21.86	1.58
2	8	-10	64.18	2.30
2	8	-10	65.87	2.63
2	8	-10	66.33	2.20
-2	-8	10	61.42	2.85
-2	-8	10	64.26	2.18
-2	-8	10	60.88	2.70
1	8	-10	14.49	0.89
1	8	-10	15.22	0.80
1	8	-10	16.68	1.32
-1	-8	10	16.91	1.57
-1	-8	10	14.26	0.88
-1	-8	10	15.05	1.24
0	8	-10	6.01	1.08
0	8	-10	7.98	1.11
0	8	-10	7.03	0.67
0	8	-10	6.45	0.54
0	-8	10	7.57	1.21
0	-8	10	6.19	1.00
0	-8	10	8.20	0.81
-1	8	-10	31.23	1.35
-1	8	-10	31.22	1.21
-1	8	-10	33.28	1.79
1	-8	10	34.19	1.54
1	-8	10	28.99	2.19
1	-8	10	31.65	1.72
-2	8	-10	49.42	2.34
-2	8	-10	51.39	1.81

-2	8	-10	51.37	1.94
2	-8	10	52.67	2.86
2	-8	10	51.86	2.32
2	-8	10	54.58	2.18
-3	8	-10	25.54	1.20
-3	8	-10	25.69	1.09
-3	8	-10	26.63	1.72
3	-8	10	25.79	1.56
3	-8	10	22.48	1.56
3	-8	10	25.76	2.28
-4	8	-10	21.88	1.05
4	-8	10	22.48	1.54
4	-8	10	20.67	1.53
4	-8	10	20.63	2.27
-5	8	-10	7.52	0.74
-5	8	-10	7.92	0.79
5	-8	10	7.79	1.08
5	-8	10	8.40	1.20
5	-8	10	7.08	1.62
-6	8	-10	15.60	1.02
-6	8	-10	13.45	0.96
6	-8	10	13.94	2.22
6	-8	10	14.46	1.58
6	-8	10	12.63	1.40
-7	8	-10	6.00	0.87
-7	8	-10	6.11	0.83
7	-8	10	2.39	1.78
7	-8	10	5.03	0.97
-8	8	-10	10.07	1.00
-8	8	-10	7.72	1.00
8	-8	10	9.70	1.50
8	-8	10	6.48	2.20
-9	8	-10	10.84	1.18
-9	8	-10	10.93	1.09
9	-8	10	6.53	2.52
9	-8	10	10.73	1.71
-10	8	-10	12.64	1.25
10	-8	10	10.36	3.03
10	-8	10	13.92	1.90
10	-8	10	14.59	1.82
-11	8	-10	13.93	1.40
11	-8	10	8.26	3.49
11	-8	10	15.78	2.08
13	7	-10	2.89	0.77

13	7	-10	4.01	1.37
-13	-7	10	6.55	1.64
12	7	-10	1.35	1.11
12	7	-10	0.53	0.64
-12	-7	10	1.89	1.34
11	7	-10	0.84	0.95
11	7	-10	-0.09	0.78
11	7	-10	0.15	0.59
-11	-7	10	0.74	1.10
10	7	-10	1.30	0.64
10	7	-10	1.59	0.67
10	7	-10	0.69	0.83
10	7	-10	1.31	0.56
-10	-7	10	0.71	0.98
9	7	-10	6.41	0.90
9	7	-10	6.66	1.20
9	7	-10	6.41	0.84
9	7	-10	7.44	1.02
9	7	-10	6.60	0.84
-9	-7	10	4.82	1.10
8	7	-10	10.26	1.22
8	7	-10	13.48	0.81
8	7	-10	10.64	0.87
8	7	-10	9.90	1.65
8	7	-10	9.17	0.83
-8	-7	10	10.04	1.48
7	7	-10	14.06	1.37
7	7	-10	14.30	0.94
7	7	-10	13.11	1.18
-7	-7	10	14.59	1.41
-7	-7	10	14.54	0.97
6	7	-10	8.29	0.76
6	7	-10	7.07	0.99
6	7	-10	9.01	1.13
-6	-7	10	6.65	0.98
-6	-7	10	7.52	1.20
-6	-7	10	9.54	0.78
5	7	-10	2.28	0.61
5	7	-10	4.00	0.71
5	7	-10	3.36	0.52
-5	-7	10	3.68	0.75
-5	-7	10	2.37	0.74
-5	-7	10	2.79	0.47
4	7	-10	15.01	1.09

4	7	-10	18.09	0.97
4	7	-10	18.87	1.17
4	7	-10	18.50	1.12
-4	-7	10	17.30	0.91
-4	-7	10	17.93	0.96
-4	-7	10	18.23	1.35
-4	-7	10	16.61	1.31
3	7	-10	17.30	1.02
3	7	-10	19.50	0.98
3	7	-10	20.18	1.12
3	7	-10	15.18	1.69
-3	-7	10	18.06	0.91
-3	-7	10	19.41	0.97
-3	-7	10	17.83	1.29
-3	-7	10	17.43	1.38
2	7	-10	6.86	0.69
2	7	-10	6.43	0.76
2	7	-10	7.00	0.64
2	7	-10	6.77	0.90
-2	-7	10	6.15	0.90
-2	-7	10	6.23	0.62
-2	-7	10	6.83	0.72
-2	-7	10	8.12	1.23
1	7	-10	15.99	1.00
1	7	-10	17.80	1.16
1	7	-10	15.61	0.85
1	7	-10	15.90	0.87
-1	-7	10	14.40	1.44
-1	-7	10	15.39	1.15
-1	-7	10	14.64	0.85
0	7	-10	64.88	2.46
0	7	-10	66.73	2.59
0	7	-10	65.91	2.26
0	7	-10	64.34	2.32
0	-7	10	69.40	2.47
0	-7	10	67.43	3.02
0	-7	10	67.31	2.68
-1	7	-10	13.44	1.16
-1	7	-10	15.06	0.96
-1	7	-10	12.55	0.76
-1	7	-10	13.88	0.82
1	-7	10	13.04	1.11
1	-7	10	13.85	0.93
1	-7	10	11.88	1.07

1	-7	10	13.91	1.55
-2	7	-10	29.68	1.30
-2	7	-10	31.70	1.45
-2	7	-10	28.82	1.65
-2	7	-10	31.08	1.27
2	-7	10	30.56	2.16
2	-7	10	32.63	1.67
2	-7	10	29.82	1.62
-3	7	-10	43.84	1.72
-3	7	-10	44.18	1.67
-3	7	-10	41.68	2.07
-3	7	-10	45.60	1.93
3	-7	10	41.62	2.65
3	-7	10	43.27	2.04
3	-7	10	46.58	2.07
-4	7	-10	15.55	0.93
-4	7	-10	15.67	0.94
4	-7	10	14.42	1.43
4	-7	10	15.17	1.28
4	-7	10	17.73	2.05
-5	7	-10	43.30	1.72
-5	7	-10	42.22	1.69
5	-7	10	39.63	2.94
5	-7	10	42.06	2.19
5	-7	10	40.80	2.07
-6	7	-10	18.28	1.07
-6	7	-10	17.08	1.09
6	-7	10	13.95	2.52
6	-7	10	18.52	1.48
-7	7	-10	6.94	0.84
7	-7	10	5.99	1.01
7	-7	10	5.02	1.92
-8	7	-10	11.81	1.05
-8	7	-10	13.38	1.12
8	-7	10	11.16	1.76
8	-7	10	14.02	2.66
-9	7	-10	9.75	1.20
-9	7	-10	12.05	1.16
9	-7	10	12.65	1.70
9	-7	10	12.12	2.69
-10	7	-10	9.71	1.19
-10	7	-10	10.51	1.31
10	-7	10	10.02	1.77
10	-7	10	8.51	2.73

-11	7	-10	8.53	1.27
11	-7	10	4.43	1.27
11	-7	10	-1.53	3.34
14	6	-10	3.04	1.40
14	6	-10	2.06	0.75
14	6	-10	5.91	2.06
13	6	-10	-0.32	0.60
13	6	-10	0.05	1.20
-13	-6	10	-2.05	1.22
12	6	-10	7.00	1.00
12	6	-10	11.19	1.74
-12	-6	10	8.82	1.50
11	6	-10	6.88	1.14
11	6	-10	5.54	0.72
11	6	-10	6.67	1.11
-11	-6	10	6.04	1.13
10	6	-10	18.75	1.58
10	6	-10	18.83	1.10
10	6	-10	14.67	1.16
10	6	-10	16.62	1.12
-10	-6	10	17.03	1.76
9	6	-10	7.54	1.34
9	6	-10	10.03	0.88
9	6	-10	10.16	0.94
9	6	-10	10.31	0.86
9	6	-10	9.65	1.28
-9	-6	10	9.97	1.25
8	6	-10	17.13	1.02
8	6	-10	15.07	1.31
8	6	-10	16.59	0.85
-8	-6	10	17.12	1.48
-8	-6	10	17.39	1.10
7	6	-10	13.82	1.09
7	6	-10	14.61	0.90
7	6	-10	16.04	1.21
-7	-6	10	14.54	1.00
-7	-6	10	12.32	1.30
6	6	-10	23.46	1.14
6	6	-10	22.38	1.28
6	6	-10	21.68	1.34
6	6	-10	23.61	1.13
-6	-6	10	24.34	1.53
-6	-6	10	24.41	1.16
5	6	-10	20.10	0.99

5	6	-10	18.03	1.14
5	6	-10	19.54	0.99
5	6	-10	18.66	1.20
-5	-6	10	19.47	1.34
-5	-6	10	19.11	1.07
-5	-6	10	17.86	1.01
-5	-6	10	18.44	0.89
4	6	-10	2.11	0.56
4	6	-10	2.58	0.44
4	6	-10	1.84	0.46
4	6	-10	2.06	0.47
-4	-6	10	1.46	0.50
-4	-6	10	1.97	0.37
-4	-6	10	3.05	0.46
-4	-6	10	2.60	0.75
3	6	-10	5.91	0.78
3	6	-10	5.56	0.59
3	6	-10	6.82	0.71
3	6	-10	4.52	0.69
-3	-6	10	6.43	0.86
-3	-6	10	5.36	0.56
-3	-6	10	5.92	0.69
-3	-6	10	7.66	1.13
2	6	-10	21.41	1.12
2	6	-10	21.06	1.02
2	6	-10	21.42	1.13
-2	-6	10	22.42	1.00
-2	-6	10	21.63	1.31
-2	-6	10	23.78	1.15
-2	-6	10	19.35	1.43
1	6	-10	37.76	1.48
1	6	-10	36.84	1.52
1	6	-10	38.84	1.60
1	6	-10	37.72	1.61
-1	-6	10	37.42	1.46
-1	-6	10	36.84	1.75
-1	-6	10	35.85	1.95
-1	-6	10	39.98	1.65
0	6	-10	14.16	0.86
0	6	-10	16.22	1.05
0	6	-10	14.48	0.94
0	6	-10	14.64	0.82
0	-6	10	14.75	0.85
0	-6	10	12.49	1.37

0	-6	10	13.02	1.04
0	-6	10	15.41	1.13
-1	6	-10	51.41	2.07
-1	6	-10	53.63	1.94
-1	6	-10	51.62	2.16
-1	6	-10	53.00	1.94
1	-6	10	51.21	2.21
1	-6	10	57.59	2.26
1	-6	10	56.32	2.64
1	-6	10	53.11	1.95
-2	6	-10	30.97	1.33
-2	6	-10	30.54	1.48
-2	6	-10	31.00	1.60
-2	6	-10	33.38	1.34
2	-6	10	32.73	2.14
2	-6	10	33.82	1.65
2	-6	10	33.47	1.72
2	-6	10	30.06	1.38
-3	6	-10	14.64	0.88
-3	6	-10	15.65	1.22
-3	6	-10	14.04	0.91
-3	6	-10	14.60	1.09
3	-6	10	14.64	1.84
3	-6	10	15.51	1.15
3	-6	10	14.60	1.35
-4	6	-10	39.98	1.65
-4	6	-10	41.37	2.34
-4	6	-10	43.61	1.69
4	-6	10	44.57	2.14
4	-6	10	43.99	2.76
4	-6	10	38.91	1.96
-5	6	-10	21.24	1.17
5	-6	10	21.73	1.69
5	-6	10	21.03	1.47
5	-6	10	23.46	2.38
-6	6	-10	28.94	1.42
-6	6	-10	30.62	1.37
6	-6	10	25.48	1.71
6	-6	10	32.42	2.84
-7	6	-10	26.02	1.30
-7	6	-10	28.15	1.37
7	-6	10	19.57	2.87
7	-6	10	18.46	1.61
-8	6	-10	15.20	1.22

-8	6	-10	15.93	1.14
8	-6	10	11.21	2.23
-9	6	-10	13.11	1.28
-9	6	-10	13.08	1.17
9	-6	10	9.87	1.33
9	-6	10	13.16	2.82
-10	6	-10	16.69	1.32
-10	6	-10	14.00	1.43
10	-6	10	16.96	3.11
15	5	-10	-0.51	0.75
15	5	-10	0.65	1.41
14	5	-10	2.66	0.70
14	5	-10	0.84	1.29
13	5	-10	5.39	1.31
13	5	-10	6.70	0.98
-13	-5	10	4.59	1.36
12	5	-10	14.37	1.68
12	5	-10	13.64	1.06
12	5	-10	11.74	1.42
-12	-5	10	13.26	1.82
11	5	-10	8.34	1.15
11	5	-10	7.47	0.86
11	5	-10	7.14	1.15
-11	-5	10	6.03	1.16
10	5	-10	11.57	1.43
10	5	-10	12.31	0.92
10	5	-10	13.59	1.39
-10	-5	10	13.56	1.53
-10	-5	10	11.04	1.14
9	5	-10	18.77	1.43
9	5	-10	18.88	1.24
-9	-5	10	18.62	1.49
-9	-5	10	17.28	1.20
8	5	-10	5.83	0.89
8	5	-10	5.58	0.87
8	5	-10	5.14	0.64
-8	-5	10	6.28	0.76
-8	-5	10	5.37	0.85
7	5	-10	18.72	1.00
7	5	-10	18.84	1.16
7	5	-10	20.73	1.35
-7	-5	10	20.09	1.40
-7	-5	10	21.32	1.16
6	5	-10	7.45	0.80

6	5	-10	7.48	0.64
6	5	-10	6.62	0.97
-6	-5	10	6.58	0.95
-6	-5	10	8.20	0.81
-6	-5	10	6.71	0.57
5	5	-10	24.61	1.36
5	5	-10	24.63	1.11
5	5	-10	24.64	1.22
-5	-5	10	25.11	1.20
-5	-5	10	24.13	1.04
-5	-5	10	23.04	1.39
4	5	-10	31.07	1.50
4	5	-10	31.07	1.28
4	5	-10	30.86	1.38
4	5	-10	31.20	1.13
-4	-5	10	29.94	1.37
-4	-5	10	28.61	1.36
-4	-5	10	32.73	1.23
-4	-5	10	31.52	1.57
3	5	-10	9.05	0.65
3	5	-10	7.75	0.59
3	5	-10	7.78	0.71
3	5	-10	8.77	0.86
-3	-5	10	8.24	0.86
-3	-5	10	8.20	0.60
-3	-5	10	9.48	0.81
2	5	-10	3.62	0.60
2	5	-10	5.14	0.55
2	5	-10	4.70	0.62
2	5	-10	5.00	0.59
-2	-5	10	4.18	0.65
-2	-5	10	4.54	0.71
-2	-5	10	3.71	0.74
-2	-5	10	4.97	0.54
1	5	-10	52.36	1.95
1	5	-10	54.27	2.06
1	5	-10	55.04	2.01
1	5	-10	55.86	2.08
-1	-5	10	58.80	2.21
-1	-5	10	51.33	2.19
-1	-5	10	54.82	2.40
-1	-5	10	55.57	1.94
0	5	-10	92.77	3.25
0	5	-10	98.64	3.34

0	5	-10	93.62	3.29
0	5	-10	94.37	3.19
0	-5	10	101.89	3.48
0	-5	10	99.78	3.47
0	-5	10	95.89	3.70
0	-5	10	98.55	3.19
-1	5	-10	11.54	0.72
-1	5	-10	11.73	0.90
-1	5	-10	11.50	0.94
-1	5	-10	12.10	0.83
1	-5	10	10.62	1.35
1	-5	10	11.10	0.94
1	-5	10	13.28	1.17
1	-5	10	10.81	0.78
-2	5	-10	22.69	1.26
-2	5	-10	19.52	1.25
-2	5	-10	19.99	1.06
-2	5	-10	20.15	0.99
2	-5	10	22.94	1.45
2	-5	10	19.10	1.22
2	-5	10	20.19	1.03
2	-5	10	19.97	1.71
-3	5	-10	23.04	1.38
-3	5	-10	20.66	1.30
-3	5	-10	21.06	1.04
-3	5	-10	20.71	1.12
3	-5	10	21.97	1.31
3	-5	10	22.76	1.55
3	-5	10	20.56	1.93
-4	5	-10	32.79	1.45
-4	5	-10	30.82	1.34
4	-5	10	31.83	2.40
4	-5	10	31.17	1.94
4	-5	10	30.26	1.65
-5	5	-10	35.60	1.59
-5	5	-10	37.26	1.53
5	-5	10	35.15	1.84
5	-5	10	34.62	2.66
-6	5	-10	18.43	1.34
-6	5	-10	18.35	1.05
6	-5	10	17.15	2.41
6	-5	10	19.35	1.47
-7	5	-10	12.14	0.98
-7	5	-10	13.58	1.10

7	-5	10	10.94	1.35
7	-5	10	11.14	2.04
-8	5	-10	14.10	1.12
-8	5	-10	14.84	1.23
8	-5	10	15.40	2.56
8	-5	10	12.72	1.51
-9	5	-10	20.85	1.32
-9	5	-10	20.14	1.44
9	-5	10	19.73	1.82
9	-5	10	12.25	2.75
-10	5	-10	12.33	1.38
-10	5	-10	11.07	1.24
10	-5	10	10.19	1.79
10	-5	10	7.88	2.65
15	4	-10	3.97	0.77
15	4	-10	4.65	1.47
14	4	-10	0.03	1.24
14	4	-10	1.10	0.62
-14	-4	10	2.25	1.14
13	4	-10	5.58	1.25
13	4	-10	7.21	0.98
-13	-4	10	5.79	1.25
12	4	-10	17.89	1.37
12	4	-10	15.79	1.09
12	4	-10	14.18	1.43
12	4	-10	13.41	1.71
-12	-4	10	15.07	1.74
11	4	-10	22.67	1.19
11	4	-10	21.35	1.71
11	4	-10	24.04	1.44
-11	-4	10	21.94	1.76
10	4	-10	16.07	1.46
10	4	-10	18.38	1.19
10	4	-10	16.62	0.98
-10	-4	10	16.84	1.23
-10	-4	10	15.64	1.49
9	4	-10	10.07	0.77
9	4	-10	11.83	0.97
9	4	-10	11.43	1.24
-9	-4	10	11.59	1.23
-9	-4	10	11.29	1.08
8	4	-10	3.57	0.56
8	4	-10	3.54	0.75
8	4	-10	3.09	0.46

-8	-4	10	4.91	0.69
-8	-4	10	2.69	0.67
7	4	-10	7.51	0.66
7	4	-10	8.41	1.05
7	4	-10	8.81	0.79
-7	-4	10	8.16	0.62
-7	-4	10	8.00	0.87
-7	-4	10	6.67	0.95
6	4	-10	47.49	2.01
6	4	-10	46.32	1.81
6	4	-10	44.48	1.69
-6	-4	10	46.06	1.86
-6	-4	10	45.62	2.00
-6	-4	10	44.01	1.64
5	4	-10	20.42	1.03
5	4	-10	18.89	1.22
5	4	-10	19.30	0.93
-5	-4	10	18.12	1.17
-5	-4	10	17.89	0.85
-5	-4	10	18.97	1.12
4	4	-10	14.58	0.81
4	4	-10	15.59	1.08
4	4	-10	14.33	0.88
-4	-4	10	14.85	0.75
-4	-4	10	15.65	1.01
-4	-4	10	13.23	1.00
3	4	-10	45.85	1.66
3	4	-10	43.50	1.53
3	4	-10	45.22	1.87
3	4	-10	45.84	1.76
-3	-4	10	43.94	1.59
-3	-4	10	47.06	1.87
-3	-4	10	41.31	1.82
-3	-4	10	43.29	1.86
2	4	-10	45.55	1.66
2	4	-10	45.60	1.87
2	4	-10	44.64	1.62
2	4	-10	44.63	1.77
-2	-4	10	40.46	1.84
-2	-4	10	44.14	1.63
-2	-4	10	49.30	1.95
-2	-4	10	46.30	1.98
1	4	-10	49.49	1.88
1	4	-10	47.32	1.75

1	4	-10	48.40	1.90
1	4	-10	45.46	1.72
-1	-4	10	47.41	1.71
-1	-4	10	50.33	2.14
-1	-4	10	47.15	2.05
-1	-4	10	42.72	1.91
0	4	-10	12.20	0.88
0	4	-10	13.56	0.77
0	4	-10	14.37	0.93
0	4	-10	12.99	0.91
0	-4	10	13.87	1.15
0	-4	10	12.77	0.77
0	-4	10	13.04	0.94
0	-4	10	12.29	1.22
-1	4	-10	55.10	2.19
-1	4	-10	55.33	2.14
-1	4	-10	56.11	2.10
-1	4	-10	57.29	1.99
1	-4	10	51.98	2.20
1	-4	10	60.54	2.62
1	-4	10	54.81	2.36
1	-4	10	54.25	1.98
-2	4	-10	29.83	1.48
-2	4	-10	27.32	1.19
-2	4	-10	27.39	1.33
-2	4	-10	31.18	1.53
2	-4	10	26.78	1.91
2	-4	10	25.89	1.41
2	-4	10	28.76	1.24
2	-4	10	29.89	1.68
-3	4	-10	23.28	1.08
-3	4	-10	23.35	1.22
-3	4	-10	24.90	1.44
-3	4	-10	21.82	1.40
3	-4	10	23.38	1.59
3	-4	10	22.66	1.93
3	-4	10	24.45	1.32
3	-4	10	21.95	1.15
-4	4	-10	31.39	1.51
-4	4	-10	32.23	1.38
4	-4	10	37.52	2.33
4	-4	10	34.38	1.68
4	-4	10	32.81	2.44
-5	4	-10	17.70	1.00

-5	4	-10	19.46	1.17
5	-4	10	18.60	2.14
5	-4	10	20.48	1.34
-6	4	-10	22.12	1.28
-6	4	-10	22.38	1.13
6	-4	10	21.96	2.51
6	-4	10	18.94	1.44
-7	4	-10	19.72	1.32
-7	4	-10	20.07	1.16
7	-4	10	17.72	2.72
-8	4	-10	7.34	1.11
-8	4	-10	6.36	0.91
8	-4	10	4.72	1.84
8	-4	10	3.35	0.91
-9	4	-10	11.05	1.14
-9	4	-10	9.68	1.27
9	-4	10	11.86	1.54
9	-4	10	12.65	2.56
-10	4	-10	8.85	1.25
-10	4	-10	13.90	1.44
10	-4	10	14.18	2.77
10	-4	10	14.84	1.72
16	3	-10	0.78	1.42
16	3	-10	2.31	0.81
15	3	-10	-0.22	1.28
15	3	-10	0.47	0.59
14	3	-10	5.20	1.26
14	3	-10	1.39	0.62
-14	-3	10	1.33	1.09
13	3	-10	8.49	1.56
13	3	-10	8.11	0.91
13	3	-10	6.49	1.22
-13	-3	10	8.39	1.20
12	3	-10	10.42	0.89
12	3	-10	8.61	1.03
12	3	-10	10.62	1.43
-12	-3	10	11.11	1.46
11	3	-10	10.95	1.00
11	3	-10	12.53	0.88
11	3	-10	12.33	1.43
-11	-3	10	9.66	1.31
10	3	-10	15.85	1.33
10	3	-10	20.03	1.15
10	3	-10	21.87	1.56

-10	-3	10	19.32	1.31
-10	-3	10	20.35	1.44
9	3	-10	9.51	0.71
9	3	-10	9.07	0.83
9	3	-10	10.15	1.21
-9	-3	10	10.21	1.04
-9	-3	10	9.12	1.03
8	3	-10	14.54	0.90
8	3	-10	12.49	1.22
8	3	-10	13.25	0.80
-8	-3	10	13.68	1.08
-8	-3	10	10.63	1.04
7	3	-10	43.71	1.98
7	3	-10	44.13	1.70
7	3	-10	42.16	1.60
-7	-3	10	39.77	1.54
-7	-3	10	41.67	1.87
-7	-3	10	43.16	1.82
6	3	-10	16.07	0.88
6	3	-10	14.13	0.80
6	3	-10	13.41	1.11
-6	-3	10	13.82	0.73
-6	-3	10	15.04	1.05
-6	-3	10	15.45	1.03
5	3	-10	35.88	1.71
5	3	-10	36.68	1.40
5	3	-10	36.71	1.50
-5	-3	10	36.41	1.62
-5	-3	10	35.16	1.60
-5	-3	10	34.89	1.32
4	3	-10	42.00	1.57
4	3	-10	41.63	1.86
4	3	-10	41.93	1.67
-4	-3	10	43.67	1.82
-4	-3	10	41.46	1.50
-4	-3	10	41.46	1.76
3	3	-10	36.29	1.39
3	3	-10	36.78	1.68
3	3	-10	35.51	1.50
-3	-3	10	37.68	1.58
-3	-3	10	33.86	1.57
-3	-3	10	39.16	1.71
-3	-3	10	37.58	1.36
2	3	-10	9.57	0.73

2	3	-10	7.48	0.88
2	3	-10	9.20	0.63
2	3	-10	8.56	0.64
-2	-3	10	9.78	0.91
-2	-3	10	8.77	0.61
-2	-3	10	9.31	0.77
-2	-3	10	9.48	0.99
1	3	-10	78.01	2.84
1	3	-10	77.41	2.86
1	3	-10	79.08	2.70
1	3	-10	77.58	2.65
-1	-3	10	79.78	2.64
-1	-3	10	81.66	3.00
-1	-3	10	82.26	2.88
-1	-3	10	81.37	3.02
0	3	-10	63.48	2.34
0	3	-10	59.00	2.09
0	3	-10	60.09	2.22
0	3	-10	58.46	2.28
0	-3	10	60.04	2.57
0	-3	10	61.05	2.32
0	-3	10	58.18	2.09
0	-3	10	65.01	2.53
-1	3	-10	40.95	1.77
-1	3	-10	42.07	1.79
-1	3	-10	40.33	1.85
-1	3	-10	43.48	1.60
1	-3	10	41.32	1.80
1	-3	10	43.96	1.62
1	-3	10	46.29	2.21
1	-3	10	46.37	2.09
-2	3	-10	34.19	1.57
-2	3	-10	34.87	1.66
-2	3	-10	32.61	1.72
-2	3	-10	36.72	1.41
2	-3	10	35.56	1.43
2	-3	10	37.74	2.11
2	-3	10	36.13	1.91
2	-3	10	37.35	1.63
-3	3	-10	36.70	1.91
-3	3	-10	36.74	1.62
-3	3	-10	37.37	1.46
-3	3	-10	36.07	1.82
3	-3	10	37.21	1.99

3	-3	10	35.01	1.49
3	-3	10	34.57	2.24
3	-3	10	41.04	1.73
-4	3	-10	20.90	1.07
-4	3	-10	23.02	1.27
4	-3	10	24.34	1.37
4	-3	10	20.41	1.96
-5	3	-10	26.62	1.99
-5	3	-10	31.59	1.36
5	-3	10	35.51	1.69
-6	3	-10	26.86	1.51
-6	3	-10	27.51	1.30
6	-3	10	28.95	1.62
6	-3	10	28.77	2.64
-7	3	-10	17.27	1.27
-7	3	-10	14.59	1.06
7	-3	10	13.96	2.60
7	-3	10	16.46	1.34
-8	3	-10	14.54	1.31
-8	3	-10	13.03	1.08
8	-3	10	10.63	1.39
8	-3	10	11.09	2.17
-9	3	-10	14.37	1.22
-9	3	-10	15.51	1.42
9	-3	10	16.37	1.67
9	-3	10	13.91	2.57
-10	3	-10	8.72	1.25
-10	3	-10	5.87	0.99
10	-3	10	5.42	1.13
10	-3	10	5.54	2.32
16	2	-10	3.82	0.82
16	2	-10	3.26	1.52
15	2	-10	9.16	1.06
15	2	-10	8.74	1.52
14	2	-10	2.24	0.63
14	2	-10	3.40	1.16
-14	-2	10	1.77	0.97
13	2	-10	1.09	0.98
13	2	-10	0.10	0.56
13	2	-10	0.04	0.48
13	2	-10	-0.31	0.93
-13	-2	10	1.73	0.84
12	2	-10	8.54	0.81
12	2	-10	8.25	0.92

12	2	-10	8.75	1.21
-12	-2	10	9.32	1.32
11	2	-10	30.56	1.40
11	2	-10	31.41	1.94
11	2	-10	30.19	1.34
-11	-2	10	30.70	1.71
10	2	-10	12.35	0.80
10	2	-10	13.10	1.34
10	2	-10	12.51	0.89
-10	-2	10	10.07	1.12
-10	-2	10	12.91	1.14
9	2	-10	19.54	1.46
9	2	-10	18.28	0.93
9	2	-10	18.71	1.00
-9	-2	10	17.37	1.23
-9	-2	10	16.77	1.22
8	2	-10	26.21	1.59
8	2	-10	28.73	1.24
8	2	-10	27.30	1.23
8	2	-10	28.05	1.17
-8	-2	10	25.65	1.40
-8	-2	10	27.44	1.45
7	2	-10	23.45	1.06
7	2	-10	25.14	1.15
7	2	-10	24.63	1.48
-7	-2	10	24.13	1.29
-7	-2	10	24.67	1.02
-7	-2	10	25.01	1.33
6	2	-10	53.54	2.28
6	2	-10	53.67	2.00
6	2	-10	53.79	1.93
-6	-2	10	53.19	2.12
-6	-2	10	53.61	1.86
-6	-2	10	58.94	2.21
5	2	-10	16.30	0.91
5	2	-10	16.32	1.16
5	2	-10	16.48	0.82
-5	-2	10	16.40	0.97
-5	-2	10	15.87	0.76
-5	-2	10	17.71	1.13
4	2	-10	23.04	1.35
4	2	-10	23.41	1.11
4	2	-10	23.04	1.01
-4	-2	10	22.34	0.94

-4	-2	10	24.48	1.32
-4	-2	10	21.94	0.99
-4	-2	10	23.24	1.02
-4	-2	10	20.72	1.13
3	2	-10	72.09	2.73
3	2	-10	71.58	2.59
3	2	-10	71.04	2.42
-3	-2	10	72.27	2.50
-3	-2	10	71.51	2.61
-3	-2	10	74.79	2.78
-3	-2	10	72.64	2.38
2	2	-10	26.01	1.41
2	2	-10	27.61	1.13
2	2	-10	27.24	1.30
-2	-2	10	29.36	1.37
-2	-2	10	27.87	1.11
-2	-2	10	24.50	1.43
-2	-2	10	31.60	1.58
-2	-2	10	26.16	1.26
1	2	-10	22.62	1.22
1	2	-10	22.39	1.27
1	2	-10	22.31	1.00
-1	-2	10	24.41	1.18
-1	-2	10	21.25	1.41
-1	-2	10	22.80	1.28
-1	-2	10	23.38	0.99
-1	-2	10	24.73	1.34
0	2	-10	10.86	0.91
0	2	-10	10.20	0.95
0	2	-10	11.08	0.67
0	-2	10	13.89	1.15
0	-2	10	12.65	1.17
0	-2	10	10.22	0.81
0	-2	10	10.98	0.70
-1	2	-10	27.25	1.16
-1	2	-10	27.97	1.53
-1	2	-10	28.60	1.40
1	-2	10	31.17	1.77
1	-2	10	30.11	1.70
1	-2	10	26.74	1.17
1	-2	10	27.87	1.35
-2	2	-10	26.69	1.11
-2	2	-10	24.99	1.53
-2	2	-10	25.52	1.33

2	-2	10	25.57	1.31
2	-2	10	24.77	1.13
2	-2	10	22.34	1.66
2	-2	10	28.39	1.81
-3	2	-10	22.54	1.09
-3	2	-10	25.99	1.34
3	-2	10	25.12	1.89
3	-2	10	24.86	1.18
3	-2	10	25.88	1.33
-4	2	-10	14.29	0.83
-4	2	-10	14.54	1.08
4	-2	10	14.27	1.72
4	-2	10	14.50	1.09
4	-2	10	13.88	1.13
-5	2	-10	10.11	1.01
-5	2	-10	9.72	0.76
5	-2	10	6.68	1.30
5	-2	10	10.33	1.03
-6	2	-10	14.92	1.23
6	-2	10	14.73	1.21
6	-2	10	15.37	2.25
-7	2	-10	29.73	1.61
-7	2	-10	25.17	1.33
7	-2	10	29.49	1.65
7	-2	10	23.26	2.73
-8	2	-10	11.43	1.08
-8	2	-10	12.05	1.28
8	-2	10	14.92	2.76
8	-2	10	14.05	1.38
-9	2	-10	4.32	0.75
-9	2	-10	4.36	0.87
9	-2	10	3.15	0.93
9	-2	10	-1.29	1.92
16	1	-10	6.81	1.57
16	1	-10	6.99	1.09
15	1	-10	1.20	1.17
15	1	-10	1.31	0.62
14	1	-10	2.04	1.13
14	1	-10	2.89	1.27
14	1	-10	2.38	0.55
-14	-1	10	2.95	0.93
13	1	-10	5.52	0.79
13	1	-10	4.89	0.61
13	1	-10	5.06	1.18

13	1	-10	6.31	1.10
-13	-1	10	5.01	0.90
12	1	-10	12.40	0.91
12	1	-10	13.47	0.88
-12	-1	10	13.62	1.29
11	1	-10	27.96	1.82
11	1	-10	27.09	1.27
-11	-1	10	27.29	1.53
10	1	-10	30.98	1.80
10	1	-10	30.78	1.32
10	1	-10	30.08	1.27
-10	-1	10	28.24	1.58
-10	-1	10	29.12	1.52
9	1	-10	22.62	1.03
9	1	-10	22.15	1.55
9	1	-10	24.38	1.35
9	1	-10	23.03	1.08
-9	-1	10	21.82	1.26
-9	-1	10	22.16	1.39
8	1	-10	34.86	1.85
8	1	-10	35.95	1.46
8	1	-10	36.67	1.39
8	1	-10	37.22	1.47
-8	-1	10	35.78	1.60
-8	-1	10	33.20	1.69
7	1	-10	32.88	1.26
7	1	-10	30.17	1.65
7	1	-10	33.57	1.36
-7	-1	10	30.11	1.20
-7	-1	10	30.79	1.56
-7	-1	10	30.41	1.40
6	1	-10	25.90	1.51
6	1	-10	26.92	1.20
6	1	-10	26.42	1.09
-6	-1	10	25.64	1.03
-6	-1	10	27.30	1.41
-6	-1	10	24.75	1.22
5	1	-10	50.68	1.81
5	1	-10	51.03	2.20
5	1	-10	52.27	1.96
-5	-1	10	50.60	1.97
-5	-1	10	50.53	1.75
-5	-1	10	54.46	2.16
4	1	-10	275.22	9.06

4	1	-10	283.60	8.88
4	1	-10	291.40	8.70
-4	-1	10	287.58	8.87
-4	-1	10	290.96	8.70
-4	-1	10	285.35	9.05
-4	-1	10	287.58	8.79
-4	-1	10	283.63	8.64
3	1	-10	174.59	5.41
3	1	-10	172.05	5.76
3	1	-10	174.83	5.64
-3	-1	10	172.11	5.59
-3	-1	10	181.26	5.54
-3	-1	10	174.82	5.81
-3	-1	10	172.37	5.59
-3	-1	10	172.34	5.36
2	1	-10	29.44	1.55
2	1	-10	31.01	1.45
2	1	-10	30.39	1.20
-2	-1	10	31.36	1.18
-2	-1	10	30.68	1.52
-2	-1	10	31.29	1.66
-2	-1	10	31.40	1.43
-2	-1	10	29.60	1.35
1	1	-10	29.31	1.46
1	1	-10	28.17	1.44
1	1	-10	26.45	1.12
-1	-1	10	28.96	1.45
-1	-1	10	28.51	1.12
-1	-1	10	29.98	1.57
-1	-1	10	29.18	1.63
-1	-1	10	26.58	1.28
0	1	-10	52.64	1.82
0	1	-10	49.95	2.10
0	1	-10	49.89	2.20
0	-1	10	51.84	2.23
0	-1	10	50.85	2.29
0	-1	10	52.48	1.82
0	-1	10	55.65	2.38
0	-1	10	52.08	2.02
-1	1	-10	29.79	1.73
-1	1	-10	29.89	1.24
-1	1	-10	31.21	1.51
1	-1	10	32.16	1.27
1	-1	10	32.91	1.85

1	-1	10	33.43	1.78
1	-1	10	31.71	1.46
1	-1	10	34.15	1.86
-2	1	-10	36.56	1.74
-2	1	-10	39.82	1.47
2	-1	10	41.32	2.14
2	-1	10	39.20	2.20
2	-1	10	39.44	2.15
2	-1	10	39.45	1.70
2	-1	10	37.87	1.50
-3	1	-10	29.62	1.48
-3	1	-10	30.26	1.23
3	-1	10	30.49	1.99
3	-1	10	27.05	1.43
3	-1	10	29.66	1.27
-4	1	-10	19.01	1.50
-4	1	-10	19.92	0.97
4	-1	10	20.69	1.25
4	-1	10	17.94	1.07
4	-1	10	15.59	1.71
-5	1	-10	24.80	1.17
-5	1	-10	26.78	1.48
5	-1	10	24.35	1.67
5	-1	10	23.63	2.15
-6	1	-10	8.62	1.21
6	-1	10	4.80	1.31
6	-1	10	6.42	1.03
-7	1	-10	7.94	1.20
-7	1	-10	6.57	0.89
7	-1	10	7.58	1.19
7	-1	10	5.32	1.52
-8	1	-10	16.60	1.18
-8	1	-10	16.82	1.43
8	-1	10	14.44	1.49
8	-1	10	15.69	2.79
-9	1	-10	6.14	1.04
-9	1	-10	11.34	1.22
9	-1	10	9.22	2.36
9	-1	10	9.61	1.48
17	0	-10	0.80	1.49
17	0	-10	2.72	0.83
16	0	-10	4.46	0.74
16	0	-10	4.87	1.42
15	0	-10	9.90	1.57

15	0	-10	14.59	1.07
14	0	-10	10.22	0.91
14	0	-10	10.57	1.81
14	0	-10	7.73	1.37
-14	0	10	8.35	1.30
13	0	-10	2.71	1.09
13	0	-10	4.49	1.17
13	0	-10	5.01	0.73
13	0	-10	4.52	0.75
13	0	-10	4.92	0.86
-13	0	10	4.35	0.82
12	0	-10	4.43	0.65
12	0	-10	4.05	0.54
-12	0	10	3.96	0.71
11	0	-10	17.29	0.97
11	0	-10	16.16	1.48
11	0	-10	16.69	0.92
-11	0	10	16.91	1.16
10	0	-10	24.83	1.15
10	0	-10	24.72	1.65
10	0	-10	23.52	1.07
-10	0	10	22.02	1.28
-10	0	10	26.62	1.77
9	0	-10	9.14	0.73
9	0	-10	9.90	0.65
9	0	-10	8.41	1.13
9	0	-10	10.27	0.76
-9	0	10	8.61	1.06
-9	0	10	8.75	0.82
8	0	-10	5.72	0.62
8	0	-10	4.95	0.83
8	0	-10	5.65	0.53
8	0	-10	5.25	0.65
-8	0	10	4.98	0.65
-8	0	10	5.42	0.91
7	0	-10	8.51	0.68
7	0	-10	7.45	1.04
7	0	-10	7.76	0.58
7	0	-10	7.88	0.72
-7	0	10	7.42	0.68
-7	0	10	6.50	0.92
6	0	-10	23.74	1.17
6	0	-10	24.07	1.01
6	0	-10	22.60	1.44

-6	0	10	24.60	1.38
-6	0	10	23.56	0.96
-6	0	10	24.12	1.14
5	0	-10	31.72	1.23
5	0	-10	31.01	1.64
5	0	-10	31.81	1.44
-5	0	10	31.42	1.36
-5	0	10	31.17	1.61
-5	0	10	31.87	1.18
-5	0	10	34.20	1.19
-5	0	10	32.53	1.41
4	0	-10	158.99	4.92
4	0	-10	160.46	5.19
4	0	-10	155.13	5.35
-4	0	10	156.52	5.35
-4	0	10	153.94	5.08
-4	0	10	155.30	5.16
-4	0	10	157.47	4.87
-4	0	10	165.94	4.97
3	0	-10	66.56	2.64
3	0	-10	66.57	2.23
3	0	-10	68.44	2.55
-3	0	10	62.33	2.38
-3	0	10	67.55	2.19
-3	0	10	67.39	2.70
-3	0	10	67.35	2.35
2	0	-10	39.76	1.82
2	0	-10	37.70	1.41
2	0	-10	40.12	1.79
-2	0	10	37.35	1.85
-2	0	10	38.11	1.61
-2	0	10	38.56	1.59
-2	0	10	40.06	1.39
-2	0	10	37.28	1.91
1	0	-10	50.05	2.16
1	0	-10	52.61	2.24
1	0	-10	52.17	1.81
-1	0	10	55.02	2.38
-1	0	10	52.19	2.36
-1	0	10	51.72	2.01
-1	0	10	51.41	1.79
-1	0	10	53.20	2.11
0	0	-10	28.52	1.11
0	0	-10	27.39	1.65

0	0	-10	28.94	1.47
0	0	10	30.54	1.83
0	0	10	26.81	1.31
0	0	10	28.57	1.13
0	0	10	25.14	1.50
0	0	10	28.51	1.74
-1	0	-10	22.77	1.31
-1	0	-10	23.80	1.70
-1	0	-10	23.90	1.01
1	0	10	25.16	1.53
1	0	10	23.51	1.04
1	0	10	22.40	1.82
1	0	10	24.23	1.24
1	0	10	22.73	1.64
-2	0	-10	31.61	1.58
-2	0	-10	32.54	1.26
2	0	10	31.07	1.50
2	0	10	30.89	1.85
2	0	10	34.18	2.22
2	0	10	31.33	1.29
-3	0	-10	36.02	1.72
-3	0	-10	35.22	1.37
3	0	10	35.67	2.05
3	0	10	35.41	1.65
3	0	10	32.60	1.43
-4	0	-10	35.88	1.75
-4	0	-10	35.06	1.41
4	0	10	37.37	2.33
4	0	10	36.09	1.52
-5	0	-10	10.87	0.84
-5	0	-10	13.08	1.20
5	0	10	12.12	1.86
5	0	10	13.45	1.14
-6	0	-10	5.88	0.89
-6	0	-10	6.36	0.76
6	0	10	4.85	0.83
6	0	10	4.23	1.37
-7	0	-10	8.06	1.23
-7	0	-10	6.09	0.88
7	0	10	9.46	1.26
7	0	10	8.98	1.79
-8	0	-10	12.84	1.14
-8	0	-10	13.45	1.43
8	0	10	14.90	2.68

8	0	10	10.87	1.45
-9	0	-10	3.44	1.39
-9	0	-10	6.35	0.92
9	0	10	2.95	2.06
9	0	10	5.38	1.07
17	-1	-10	12.26	1.21
17	-1	-10	7.80	1.73
16	-1	-10	16.44	1.23
16	-1	-10	14.86	1.93
15	-1	-10	11.57	1.01
15	-1	-10	7.58	1.50
14	-1	-10	25.86	2.08
14	-1	-10	25.83	1.28
14	-1	-10	23.83	2.09
-14	1	10	23.78	1.50
13	-1	-10	23.65	1.84
13	-1	-10	24.62	1.22
13	-1	-10	22.69	1.89
13	-1	-10	25.09	1.19
13	-1	-10	19.97	1.38
-13	1	10	21.57	1.38
12	-1	-10	15.79	0.89
12	-1	-10	14.79	1.75
12	-1	-10	15.93	1.01
-12	1	10	14.70	1.12
11	-1	-10	29.73	1.88
11	-1	-10	30.41	1.28
11	-1	-10	33.07	1.40
-11	1	10	29.23	1.44
10	-1	-10	35.28	1.93
10	-1	-10	32.36	1.41
10	-1	-10	30.60	1.27
-10	1	10	29.40	1.42
9	-1	-10	24.04	1.14
9	-1	-10	25.74	1.07
9	-1	-10	23.19	1.62
9	-1	-10	27.41	1.25
-9	1	10	25.23	1.21
-9	1	10	26.34	1.53
8	-1	-10	39.32	2.02
8	-1	-10	40.74	1.50
8	-1	-10	41.90	1.55
8	-1	-10	41.45	1.68
-8	1	10	38.31	1.61

-8	1	10	42.07	1.92
7	-1	-10	56.88	1.96
7	-1	-10	53.05	2.42
7	-1	-10	56.83	1.92
7	-1	-10	57.04	2.14
-7	1	10	55.67	2.34
-7	1	10	52.70	2.04
6	-1	-10	40.01	1.47
6	-1	-10	42.33	1.75
6	-1	-10	39.53	1.96
-6	1	10	39.65	1.60
-6	1	10	40.52	1.43
-6	1	10	40.98	1.67
-6	1	10	40.03	1.38
-6	1	10	40.00	1.90
5	-1	-10	38.88	1.93
5	-1	-10	40.57	1.44
5	-1	-10	41.82	1.76
-5	1	10	39.56	1.89
-5	1	10	38.59	1.78
-5	1	10	40.05	1.39
-5	1	10	38.02	1.52
-5	1	10	38.48	1.57
4	-1	-10	48.00	2.19
4	-1	-10	49.47	1.72
4	-1	-10	48.01	2.07
-4	1	10	48.04	1.77
-4	1	10	51.57	2.25
-4	1	10	49.02	1.88
-4	1	10	48.75	1.66
-4	1	10	52.95	2.21
3	-1	-10	51.83	2.24
3	-1	-10	52.02	2.21
3	-1	-10	52.00	1.78
-3	1	10	51.81	1.91
-3	1	10	50.78	1.97
-3	1	10	51.97	1.75
-3	1	10	51.17	2.30
-3	1	10	53.26	2.35
2	-1	-10	46.40	1.64
2	-1	-10	47.39	2.08
2	-1	-10	43.85	2.10
-2	1	10	48.32	1.87
-2	1	10	49.89	2.27

-2	1	10	49.27	2.29
-2	1	10	48.28	1.62
-2	1	10	44.60	1.84
1	-1	-10	40.30	1.90
1	-1	-10	43.32	1.52
1	-1	-10	42.38	2.13
-1	1	10	43.92	1.77
-1	1	10	43.58	1.83
-1	1	10	44.39	1.52
-1	1	10	40.13	2.14
-1	1	10	44.19	2.30
0	-1	-10	59.92	2.35
0	-1	-10	53.28	1.89
0	-1	-10	55.77	2.59
0	1	10	57.08	2.73
0	1	10	56.31	2.19
0	1	10	55.89	1.91
0	1	10	55.58	2.29
0	1	10	54.77	2.55
-1	-1	-10	21.65	1.31
-1	-1	-10	19.85	0.91
1	1	10	22.77	1.21
1	1	10	20.38	1.41
1	1	10	19.10	1.94
1	1	10	21.50	0.95
-2	-1	-10	29.57	1.61
-2	-1	-10	32.65	1.24
2	1	10	31.15	1.56
2	1	10	34.33	1.31
2	1	10	31.80	1.85
-3	-1	-10	37.32	1.79
-3	-1	-10	37.66	1.40
3	1	10	34.79	1.48
3	1	10	38.10	2.14
-4	-1	-10	28.27	1.58
-4	-1	-10	27.18	1.16
4	1	10	26.69	2.02
4	1	10	24.11	1.28
4	1	10	27.57	1.56
-5	-1	-10	13.51	1.29
-5	-1	-10	14.88	0.91
5	1	10	13.58	1.27
5	1	10	14.59	1.88
-6	-1	-10	4.60	0.61

-6	-1	-10	4.07	0.84
6	1	10	5.17	1.42
6	1	10	4.58	0.87
-7	-1	-10	4.19	0.66
-7	-1	-10	4.16	0.86
7	1	10	4.60	1.62
7	1	10	4.44	0.92
-8	-1	-10	7.07	1.09
-8	-1	-10	6.42	0.84
8	1	10	5.87	1.02
8	1	10	3.54	1.71
17	-2	-10	2.16	1.40
17	-2	-10	2.97	0.76
16	-2	-10	7.65	1.02
16	-2	-10	8.29	1.56
15	-2	-10	5.91	1.30
15	-2	-10	8.82	0.97
15	-2	-10	9.94	1.65
14	-2	-10	10.75	1.78
14	-2	-10	14.36	0.98
14	-2	-10	14.13	1.86
-14	2	10	13.62	1.18
13	-2	-10	27.19	1.42
13	-2	-10	26.24	1.99
13	-2	-10	26.85	2.02
13	-2	-10	24.88	1.24
-13	2	10	28.64	1.42
-13	2	10	24.15	1.55
12	-2	-10	20.05	1.18
12	-2	-10	16.72	1.58
12	-2	-10	18.04	1.73
12	-2	-10	15.63	1.36
-12	2	10	15.08	0.93
-12	2	10	16.13	1.07
11	-2	-10	10.87	0.72
11	-2	-10	10.34	1.37
11	-2	-10	12.08	1.15
11	-2	-10	11.17	0.99
-11	2	10	11.07	0.71
-11	2	10	9.79	0.87
10	-2	-10	27.46	1.17
10	-2	-10	31.89	1.47
10	-2	-10	28.14	1.83
10	-2	-10	26.82	1.21

-10	2	10	28.79	1.29
-10	2	10	29.29	1.14
9	-2	-10	44.55	2.21
9	-2	-10	44.27	1.87
9	-2	-10	46.15	1.65
9	-2	-10	44.36	1.61
-9	2	10	43.97	1.71
-9	2	10	40.75	2.03
8	-2	-10	27.32	1.14
8	-2	-10	25.62	1.16
8	-2	-10	28.55	1.43
8	-2	-10	26.60	1.08
8	-2	-10	27.72	1.67
-8	2	10	25.48	1.22
-8	2	10	25.19	1.56
-8	2	10	27.84	1.21
7	-2	-10	57.69	1.96
7	-2	-10	61.47	2.01
7	-2	-10	55.77	2.30
7	-2	-10	58.21	2.55
7	-2	-10	55.18	2.02
-7	2	10	55.26	2.08
-7	2	10	59.14	2.23
-7	2	10	56.12	2.42
6	-2	-10	80.19	3.00
6	-2	-10	78.38	3.17
6	-2	-10	80.29	2.61
6	-2	-10	82.75	2.76
-6	2	10	79.99	2.77
-6	2	10	81.22	3.02
-6	2	10	77.22	3.07
5	-2	-10	28.32	1.09
5	-2	-10	28.95	1.54
5	-2	-10	26.98	1.64
-5	2	10	28.06	1.67
-5	2	10	26.80	1.24
-5	2	10	28.52	1.05
-5	2	10	26.77	1.62
4	-2	-10	34.82	1.81
4	-2	-10	35.98	1.31
4	-2	-10	37.89	1.83
-4	2	10	34.06	1.49
-4	2	10	37.34	1.95
-4	2	10	35.15	1.88

-4	2	10	34.06	1.42
-4	2	10	36.98	1.26
3	-2	-10	20.66	1.42
3	-2	-10	20.34	0.86
3	-2	-10	20.46	1.36
-3	2	10	20.66	1.04
-3	2	10	18.10	1.47
-3	2	10	19.70	1.06
-3	2	10	20.22	0.82
-3	2	10	20.52	1.61
2	-2	-10	29.13	1.80
2	-2	-10	28.97	1.61
2	-2	-10	29.36	1.13
-2	2	10	31.27	1.85
-2	2	10	30.65	1.40
-2	2	10	30.91	1.12
-2	2	10	32.63	2.03
-2	2	10	29.51	1.39
1	-2	-10	72.61	2.33
1	-2	-10	70.73	2.80
1	-2	-10	70.38	3.08
-1	2	10	68.98	3.00
-1	2	10	72.30	2.66
-1	2	10	77.07	3.31
-1	2	10	67.65	2.66
-1	2	10	72.27	2.33
0	-2	-10	47.48	2.16
0	-2	-10	49.87	1.73
0	2	10	49.82	2.15
0	2	10	53.19	1.75
0	2	10	54.19	2.13
0	2	10	49.19	2.79
-1	-2	-10	14.22	0.73
-1	-2	-10	16.09	1.18
1	2	10	18.56	2.05
1	2	10	14.46	1.07
1	2	10	13.98	1.23
1	2	10	14.02	0.79
-2	-2	-10	23.85	1.42
-2	-2	-10	21.39	0.96
2	2	10	23.09	1.04
2	2	10	21.86	1.57
-3	-2	-10	44.69	1.64
-3	-2	-10	44.17	2.08

3	2	10	48.39	2.13
3	2	10	46.96	1.73
3	2	10	45.42	2.36
-4	-2	-10	18.49	1.41
-4	-2	-10	17.59	0.93
4	2	10	19.01	1.42
4	2	10	17.20	1.08
4	2	10	18.69	1.84
-5	-2	-10	7.97	1.18
-5	-2	-10	7.51	0.77
5	2	10	4.85	1.32
5	2	10	5.13	0.88
5	2	10	8.43	1.30
-6	-2	-10	9.77	0.94
-6	-2	-10	10.04	1.35
6	2	10	12.46	1.42
6	2	10	13.44	2.15
-7	-2	-10	9.71	1.03
-7	-2	-10	5.08	0.96
7	2	10	3.57	1.51
7	2	10	6.95	1.14
-8	-2	-10	3.14	0.79
8	2	10	4.28	2.60
8	2	10	4.57	1.06
17	-3	-10	14.94	1.24
17	-3	-10	15.52	2.39
16	-3	-10	14.97	2.14
16	-3	-10	10.28	1.05
15	-3	-10	3.97	1.28
15	-3	-10	4.72	0.66
15	-3	-10	3.18	1.48
14	-3	-10	22.41	2.17
14	-3	-10	26.73	2.16
14	-3	-10	23.38	1.22
-14	3	10	22.57	1.28
-14	3	10	25.53	1.65
13	-3	-10	24.71	1.99
13	-3	-10	22.98	1.13
13	-3	-10	22.32	1.92
13	-3	-10	18.27	1.37
-13	3	10	23.37	1.22
-13	3	10	22.03	1.23
12	-3	-10	12.28	1.22
12	-3	-10	16.86	0.90

12	-3	-10	17.62	1.62
12	-3	-10	17.08	1.64
-12	3	10	17.85	1.01
11	-3	-10	40.98	2.03
11	-3	-10	49.52	1.74
11	-3	-10	46.61	2.44
11	-3	-10	47.79	2.26
-11	3	10	49.17	1.84
-11	3	10	48.33	1.83
10	-3	-10	27.64	1.82
10	-3	-10	29.42	1.15
10	-3	-10	27.76	1.21
10	-3	-10	27.04	1.65
10	-3	-10	24.32	1.51
-10	3	10	27.75	1.33
-10	3	10	26.87	1.21
9	-3	-10	11.62	1.19
9	-3	-10	10.22	0.65
9	-3	-10	12.62	0.75
9	-3	-10	8.50	1.08
9	-3	-10	9.82	1.27
-9	3	10	10.31	0.99
-9	3	10	9.02	0.72
8	-3	-10	72.29	2.99
8	-3	-10	81.17	2.62
8	-3	-10	80.10	2.89
8	-3	-10	81.88	2.59
8	-3	-10	78.03	3.22
-8	3	10	79.64	2.95
-8	3	10	76.59	3.08
-8	3	10	79.85	2.72
7	-3	-10	82.89	2.63
7	-3	-10	77.60	2.86
7	-3	-10	78.84	3.22
7	-3	-10	80.27	2.60
7	-3	-10	76.53	3.08
-7	3	10	78.39	3.14
-7	3	10	78.97	2.74
-7	3	10	82.71	3.11
6	-3	-10	20.66	1.47
6	-3	-10	21.88	0.92
6	-3	-10	23.38	0.99
6	-3	-10	21.99	1.29
6	-3	-10	21.63	1.48

-6	3	10	22.57	1.09
-6	3	10	22.91	1.63
-6	3	10	20.95	1.49
5	-3	-10	32.54	1.85
5	-3	-10	33.01	1.56
5	-3	-10	32.69	1.21
5	-3	-10	32.50	1.79
-5	3	10	32.35	1.16
-5	3	10	31.79	1.40
-5	3	10	32.28	1.96
-5	3	10	32.54	1.84
4	-3	-10	46.51	1.61
4	-3	-10	43.09	2.25
4	-3	-10	45.96	2.16
4	-3	-10	50.45	2.05
-4	3	10	48.10	2.48
-4	3	10	47.10	1.56
-4	3	10	43.89	1.85
-4	3	10	47.65	2.27
3	-3	-10	39.39	2.23
3	-3	-10	40.63	2.03
3	-3	-10	44.06	1.50
-3	3	10	41.51	2.19
-3	3	10	42.88	1.76
-3	3	10	43.85	2.47
-3	3	10	43.69	1.46
-3	3	10	46.08	1.82
2	-3	-10	46.74	2.48
2	-3	-10	47.35	1.65
2	-3	-10	47.70	2.20
-2	3	10	48.97	2.01
-2	3	10	54.60	2.78
-2	3	10	48.73	2.01
-2	3	10	47.87	2.40
-2	3	10	51.77	1.65
1	-3	-10	30.44	1.69
1	-3	-10	30.25	1.14
-1	3	10	31.36	2.35
-1	3	10	31.75	1.57
-1	3	10	32.55	1.16
-1	3	10	30.96	1.53
0	-3	-10	32.48	1.71
0	-3	-10	32.57	1.21
0	3	10	34.28	1.72

0	3	10	34.04	1.23
0	3	10	31.26	1.60
0	3	10	36.02	2.60
-1	-3	-10	13.37	1.79
-1	-3	-10	12.35	0.65
1	3	10	10.32	1.17
1	3	10	13.60	0.74
-2	-3	-10	14.00	0.73
-2	-3	-10	14.34	1.26
2	3	10	13.58	1.20
2	3	10	14.43	0.85
-3	-3	-10	9.37	0.68
-3	-3	-10	10.97	1.19
3	3	10	9.48	0.83
3	3	10	9.75	1.17
3	3	10	8.53	1.43
-4	-3	-10	17.59	1.43
-4	-3	-10	16.11	0.88
4	3	10	16.66	1.43
4	3	10	15.36	1.82
-5	-3	-10	16.52	1.00
-5	-3	-10	17.63	1.48
5	3	10	16.58	1.54
5	3	10	15.84	2.11
-6	-3	-10	2.43	0.60
-6	-3	-10	1.40	0.81
6	3	10	2.16	0.96
6	3	10	2.19	1.47
-7	-3	-10	0.79	0.86
7	3	10	3.24	1.03
7	3	10	2.15	1.50
18	-4	-10	7.78	1.37
17	-4	-10	9.23	1.84
17	-4	-10	11.38	1.13
16	-4	-10	9.82	1.06
16	-4	-10	8.45	1.51
15	-4	-10	17.52	2.06
15	-4	-10	14.47	1.04
15	-4	-10	17.24	2.42
14	-4	-10	24.62	2.20
14	-4	-10	27.95	1.29
14	-4	-10	31.31	2.37
-14	4	10	25.19	1.33
-14	4	10	25.50	1.54

13	-4	-10	29.66	2.13
13	-4	-10	30.23	2.24
13	-4	-10	28.10	1.28
13	-4	-10	26.42	1.64
-13	4	10	28.81	1.34
-13	4	10	29.47	1.55
12	-4	-10	30.82	1.27
12	-4	-10	24.32	1.67
12	-4	-10	32.08	2.04
12	-4	-10	29.73	2.04
-12	4	10	30.44	1.56
-12	4	10	29.24	1.30
11	-4	-10	25.43	2.28
11	-4	-10	28.98	1.91
11	-4	-10	30.80	1.24
11	-4	-10	26.57	1.71
-11	4	10	30.71	1.30
-11	4	10	33.16	1.62
10	-4	-10	24.01	1.70
10	-4	-10	29.93	1.25
10	-4	-10	30.23	1.19
10	-4	-10	28.47	1.87
10	-4	-10	30.28	1.78
-10	4	10	30.42	1.69
-10	4	10	30.87	1.27
9	-4	-10	73.61	2.39
9	-4	-10	69.18	2.83
9	-4	-10	72.34	3.09
9	-4	-10	62.36	2.88
9	-4	-10	71.77	2.36
-9	4	10	72.27	2.89
-9	4	10	72.72	2.48
8	-4	-10	40.56	2.00
8	-4	-10	39.50	2.19
8	-4	-10	44.18	1.56
8	-4	-10	38.09	2.09
8	-4	-10	43.27	1.52
-8	4	10	45.83	2.20
-8	4	10	43.65	1.66
7	-4	-10	35.98	1.79
7	-4	-10	36.60	1.34
7	-4	-10	34.02	2.02
7	-4	-10	39.58	2.02
7	-4	-10	39.89	1.39

-7	4	10	31.80	1.86
-7	4	10	37.87	2.12
-7	4	10	35.21	1.49
6	-4	-10	80.01	3.40
6	-4	-10	82.53	3.18
6	-4	-10	83.30	3.37
6	-4	-10	85.30	2.72
6	-4	-10	88.52	2.74
-6	4	10	84.32	2.95
-6	4	10	84.54	3.33
-6	4	10	86.48	3.55
5	-4	-10	35.97	1.93
5	-4	-10	38.29	1.36
5	-4	-10	35.61	2.10
5	-4	-10	36.05	1.31
5	-4	-10	35.27	1.84
-5	4	10	37.09	1.59
-5	4	10	34.30	1.98
-5	4	10	38.18	2.32
4	-4	-10	38.03	1.99
4	-4	-10	36.15	2.23
4	-4	-10	37.90	1.93
4	-4	-10	39.64	1.36
-4	4	10	39.36	1.68
-4	4	10	39.39	2.10
-4	4	10	39.19	2.49
-4	4	10	37.24	1.31
3	-4	-10	67.83	3.24
3	-4	-10	69.06	2.22
3	-4	-10	71.19	2.88
3	-4	-10	67.95	2.85
-3	4	10	68.81	2.19
-3	4	10	66.83	2.62
-3	4	10	72.70	3.42
2	-4	-10	59.02	2.52
2	-4	-10	55.68	1.88
-2	4	10	58.42	1.87
-2	4	10	56.26	3.21
-2	4	10	57.71	2.32
-2	4	10	59.97	2.35
1	-4	-10	29.89	1.08
1	-4	-10	28.01	1.67
-1	4	10	27.88	2.62
-1	4	10	32.31	1.09

-1	4	10	29.23	1.60
-1	4	10	29.76	1.55
0	-4	-10	26.35	0.99
0	4	10	25.37	1.56
0	4	10	27.56	1.02
-1	-4	-10	24.98	0.99
-1	-4	-10	27.62	1.60
1	4	10	26.03	1.04
1	4	10	22.92	1.72
1	4	10	25.58	1.54
-2	-4	-10	16.19	1.38
-2	-4	-10	14.63	0.76
2	4	10	12.68	1.41
2	4	10	16.75	0.88
2	4	10	17.87	1.37
-3	-4	-10	18.24	0.86
-3	-4	-10	19.32	1.49
3	4	10	12.90	1.64
3	4	10	17.23	1.48
-4	-4	-10	20.08	1.58
-4	-4	-10	19.45	0.98
4	4	10	15.78	1.99
4	4	10	20.40	1.65
-5	-4	-10	11.23	0.92
-5	-4	-10	14.24	1.49
5	4	10	12.87	1.56
5	4	10	15.78	2.08
-6	-4	-10	5.60	1.05
6	4	10	4.45	1.54
6	4	10	5.37	1.12
18	-5	-10	3.77	0.84
17	-5	-10	13.49	1.20
17	-5	-10	14.97	2.36
16	-5	-10	13.11	1.13
16	-5	-10	15.16	2.22
16	-5	-10	11.95	2.44
15	-5	-10	14.17	2.13
15	-5	-10	11.07	1.01
15	-5	-10	12.45	1.90
14	-5	-10	30.00	1.33
14	-5	-10	26.07	2.54
14	-5	-10	28.17	2.27
-14	5	10	28.94	1.35
-14	5	10	27.42	1.72

13	-5	-10	29.93	2.24
13	-5	-10	32.81	1.37
13	-5	-10	30.58	1.78
13	-5	-10	32.48	2.42
-13	5	10	32.65	1.40
-13	5	10	33.65	1.84
12	-5	-10	22.46	1.91
12	-5	-10	24.97	1.10
12	-5	-10	21.45	1.67
12	-5	-10	20.73	2.01
-12	5	10	24.73	1.13
-12	5	10	26.07	1.72
11	-5	-10	44.17	2.23
11	-5	-10	42.09	1.65
11	-5	-10	42.43	2.43
11	-5	-10	43.16	1.58
11	-5	-10	42.82	2.34
-11	5	10	42.93	2.20
-11	5	10	40.84	1.63
10	-5	-10	41.29	2.29
10	-5	-10	40.49	2.22
10	-5	-10	41.47	1.55
10	-5	-10	42.30	1.51
10	-5	-10	37.28	2.16
-10	5	10	42.43	2.25
-10	5	10	41.54	1.60
9	-5	-10	26.33	1.78
9	-5	-10	24.13	1.75
9	-5	-10	24.84	1.03
9	-5	-10	21.61	1.79
9	-5	-10	26.50	1.09
-9	5	10	24.90	1.14
-9	5	10	23.43	1.88
8	-5	-10	88.44	2.74
8	-5	-10	82.25	2.71
8	-5	-10	84.92	3.35
8	-5	-10	79.35	3.46
8	-5	-10	85.05	3.50
-8	5	10	84.71	2.90
-8	5	10	81.72	3.56
7	-5	-10	132.75	4.09
7	-5	-10	123.16	4.78
7	-5	-10	139.46	4.10
7	-5	-10	124.62	4.87

7	-5	-10	135.18	4.71
-7	5	10	131.23	4.33
-7	5	10	128.26	4.99
6	-5	-10	8.10	0.54
6	-5	-10	8.41	0.59
6	-5	-10	8.66	1.30
6	-5	-10	7.75	1.23
6	-5	-10	6.42	1.18
-6	5	10	10.04	1.84
-6	5	10	7.28	0.79
5	-5	-10	39.90	2.08
5	-5	-10	38.89	1.35
5	-5	-10	39.75	1.38
5	-5	-10	36.84	2.04
5	-5	-10	36.92	2.34
-5	5	10	38.35	2.58
-5	5	10	37.59	1.69
4	-5	-10	32.62	3.30
4	-5	-10	27.01	1.02
4	-5	-10	25.60	1.70
4	-5	-10	26.86	1.80
-4	5	10	28.14	1.42
-4	5	10	26.86	2.44
3	-5	-10	13.22	1.32
3	-5	-10	14.45	0.64
3	-5	-10	18.51	1.56
-3	5	10	15.44	2.26
-3	5	10	15.63	1.09
-3	5	10	15.85	0.64
2	-5	-10	18.83	1.43
2	-5	-10	17.67	0.71
-2	5	10	16.97	0.69
-2	5	10	17.67	1.24
-2	5	10	17.21	2.48
1	-5	-10	16.73	1.66
1	-5	-10	17.39	0.70
-1	5	10	17.35	0.71
-1	5	10	19.33	1.37
-1	5	10	16.05	1.34
0	-5	-10	14.32	1.30
0	-5	-10	12.43	0.62
0	5	10	15.43	1.28
0	5	10	12.37	1.28
-1	-5	-10	20.97	1.49

-1	-5	-10	17.97	0.78
1	5	10	17.73	1.48
1	5	10	18.71	0.83
1	5	10	18.65	1.56
-2	-5	-10	11.06	0.66
-2	-5	-10	11.74	1.31
2	5	10	11.49	1.55
2	5	10	11.12	1.35
-3	-5	-10	1.96	0.41
-3	-5	-10	2.54	0.84
3	5	10	3.28	1.02
3	5	10	0.29	0.79
-4	-5	-10	7.07	1.06
4	5	10	6.51	1.31
4	5	10	8.01	1.15
-5	-5	-10	12.04	1.50
5	5	10	8.87	1.33
5	5	10	10.51	1.66
-6	-5	-10	4.42	1.32
6	5	10	2.17	1.07
6	5	10	2.15	1.91
18	-6	-10	9.35	1.26
17	-6	-10	14.83	2.41
17	-6	-10	16.18	1.26
16	-6	-10	10.22	1.67
16	-6	-10	9.83	1.05
16	-6	-10	12.37	2.12
15	-6	-10	7.50	0.92
15	-6	-10	5.96	1.35
15	-6	-10	9.74	1.90
14	-6	-10	19.12	1.08
14	-6	-10	17.61	2.05
14	-6	-10	18.27	2.43
-14	6	10	18.55	1.74
-14	6	10	19.04	1.05
13	-6	-10	37.47	2.67
13	-6	-10	33.56	1.46
13	-6	-10	34.11	1.97
13	-6	-10	32.56	2.35
-13	6	10	37.16	2.18
-13	6	10	34.37	1.42
12	-6	-10	25.50	1.83
12	-6	-10	25.78	2.01
12	-6	-10	26.08	1.14

12	-6	-10	23.81	2.26
-12	6	10	24.81	1.99
-12	6	10	25.98	1.17
11	-6	-10	39.84	2.49
11	-6	-10	42.38	1.64
11	-6	-10	40.87	2.32
11	-6	-10	41.53	1.57
11	-6	-10	45.09	2.45
-11	6	10	43.10	1.64
-11	6	10	47.07	2.52
10	-6	-10	57.46	2.91
10	-6	-10	61.60	2.93
10	-6	-10	63.69	2.13
10	-6	-10	65.90	2.18
-10	6	10	63.45	2.26
-10	6	10	64.08	3.11
9	-6	-10	137.77	4.98
9	-6	-10	133.25	5.05
9	-6	-10	137.43	4.22
9	-6	-10	132.76	4.19
9	-6	-10	124.93	4.99
-9	6	10	131.74	4.37
-9	6	10	141.42	5.18
8	-6	-10	47.99	2.42
8	-6	-10	46.87	1.63
8	-6	-10	48.05	2.61
8	-6	-10	42.66	1.58
8	-6	-10	44.25	2.42
-8	6	10	46.40	1.84
-8	6	10	45.50	2.77
7	-6	-10	45.83	1.53
7	-6	-10	46.46	1.54
7	-6	-10	40.70	2.31
7	-6	-10	39.34	2.49
7	-6	-10	41.15	2.31
-7	6	10	45.23	1.85
-7	6	10	42.68	2.80
6	-6	-10	81.80	3.48
6	-6	-10	80.84	3.72
6	-6	-10	82.12	3.47
6	-6	-10	84.16	2.67
6	-6	-10	87.94	2.67
-6	6	10	84.14	3.05
-6	6	10	84.03	4.00

5	-6	-10	59.66	1.94
5	-6	-10	61.63	2.05
5	-6	-10	57.08	2.79
5	-6	-10	60.12	2.73
-5	6	10	56.66	3.34
-5	6	10	62.22	2.41
4	-6	-10	12.23	1.34
4	-6	-10	14.04	0.65
4	-6	-10	12.71	0.61
4	-6	-10	13.93	1.63
-4	6	10	13.44	1.10
-4	6	10	15.92	2.34
3	-6	-10	21.85	1.83
3	-6	-10	19.36	1.52
-3	6	10	19.60	1.33
-3	6	10	16.95	2.54
2	-6	-10	26.64	2.02
2	-6	-10	24.14	1.68
-2	6	10	27.18	1.60
1	-6	-10	36.00	2.05
-1	6	10	43.63	2.07
-1	6	10	37.67	2.04
0	-6	-10	18.88	1.51
0	6	10	20.04	1.53
0	6	10	19.00	1.60
-1	-6	-10	4.98	0.94
1	6	10	4.22	0.87
1	6	10	3.40	0.96
-2	-6	-10	6.72	1.04
2	6	10	6.36	1.14
2	6	10	6.01	1.00
-3	-6	-10	6.37	1.07
3	6	10	5.96	1.11
3	6	10	5.57	1.26
-4	-6	-10	4.39	1.01
4	6	10	3.65	1.31
4	6	10	4.19	1.10
-5	-6	-10	6.65	1.12
5	6	10	3.85	1.50
5	6	10	6.32	1.29
17	-7	-10	18.13	2.59
17	-7	-10	20.15	1.34
16	-7	-10	17.78	2.38
16	-7	-10	20.57	3.13

16	-7	-10	24.54	1.34
15	-7	-10	15.78	2.79
15	-7	-10	12.68	1.00
15	-7	-10	8.84	1.52
14	-7	-10	2.70	0.57
14	-7	-10	3.70	1.59
14	-7	-10	4.39	1.24
-14	7	10	3.98	0.51
-14	7	10	4.54	1.35
13	-7	-10	20.55	2.10
13	-7	-10	16.43	2.42
-13	7	10	17.74	0.97
-13	7	10	16.41	2.01
12	-7	-10	47.63	2.97
12	-7	-10	48.71	2.72
12	-7	-10	46.83	1.75
12	-7	-10	46.32	2.51
-12	7	10	48.24	1.78
-12	7	10	45.54	2.77
11	-7	-10	55.81	2.04
11	-7	-10	53.09	2.84
11	-7	-10	56.09	3.10
11	-7	-10	56.13	1.96
11	-7	-10	60.28	2.93
-11	7	10	56.10	2.07
-11	7	10	58.63	3.14
10	-7	-10	55.76	2.97
10	-7	-10	58.47	2.02
10	-7	-10	59.14	1.97
10	-7	-10	58.83	2.89
10	-7	-10	54.65	2.99
-10	7	10	55.75	3.18
-10	7	10	57.94	2.14
9	-7	-10	13.98	0.73
9	-7	-10	15.64	0.82
9	-7	-10	13.77	1.83
9	-7	-10	11.27	1.84
-9	7	10	11.84	2.20
-9	7	10	13.20	0.94
8	-7	-10	46.35	1.65
8	-7	-10	44.77	1.55
8	-7	-10	41.93	2.44
8	-7	-10	43.20	2.52
8	-7	-10	44.47	2.72

-8	7	10	44.14	1.87
-8	7	10	45.36	3.00
7	-7	-10	53.21	1.71
7	-7	-10	52.58	1.72
7	-7	-10	49.54	2.71
7	-7	-10	47.38	2.86
7	-7	-10	47.08	2.57
-7	7	10	52.75	2.12
-7	7	10	47.61	3.19
6	-7	-10	11.72	1.47
6	-7	-10	11.18	0.85
6	-7	-10	12.16	0.60
6	-7	-10	12.04	1.73
-6	7	10	13.74	2.43
-6	7	10	12.17	1.08
5	-7	-10	38.07	2.25
5	-7	-10	39.47	2.41
-5	7	10	33.31	3.08
-5	7	10	38.59	1.84
4	-7	-10	33.90	2.26
4	-7	-10	26.68	2.19
-4	7	10	28.67	1.92
3	-7	-10	19.39	1.62
3	-7	-10	21.48	2.01
-3	7	10	23.51	1.49
2	-7	-10	19.78	1.65
-2	7	10	20.86	1.54
1	-7	-10	23.34	1.70
-1	7	10	21.04	1.69
-1	7	10	28.28	1.78
0	-7	-10	6.41	1.05
0	7	10	6.55	1.10
0	7	10	5.51	1.00
-1	-7	-10	4.67	0.98
1	7	10	4.68	0.98
1	7	10	6.00	1.19
-2	-7	-10	4.46	0.96
2	7	10	4.37	1.19
2	7	10	3.36	1.00
-3	-7	-10	4.35	1.01
3	7	10	1.24	1.20
3	7	10	4.40	1.11
-4	-7	-10	3.15	1.06
4	7	10	2.56	1.27

4	7	10	1.47	1.09
17	-8	-10	20.58	2.69
17	-8	-10	19.90	1.38
16	-8	-10	33.24	3.50
16	-8	-10	31.15	1.55
16	-8	-10	24.49	2.76
15	-8	-10	16.58	1.12
15	-8	-10	17.16	2.36
15	-8	-10	16.09	2.40
14	-8	-10	33.89	3.13
14	-8	-10	31.82	1.46
14	-8	-10	33.31	2.64
-14	8	10	34.85	1.54
13	-8	-10	36.97	1.47
13	-8	-10	38.09	2.58
13	-8	-10	34.30	3.02
-13	8	10	35.31	1.45
-13	8	10	30.31	2.72
12	-8	-10	23.94	2.61
12	-8	-10	24.68	2.17
12	-8	-10	26.53	1.16
12	-8	-10	23.21	2.22
-12	8	10	24.42	1.18
-12	8	10	28.01	2.56
11	-8	-10	30.01	1.28
11	-8	-10	29.23	2.36
11	-8	-10	28.94	1.19
11	-8	-10	29.69	2.56
11	-8	-10	30.06	2.21
-11	8	10	28.11	2.71
-11	8	10	28.07	1.32
10	-8	-10	48.99	2.97
10	-8	-10	52.63	1.82
10	-8	-10	54.32	1.89
10	-8	-10	54.94	2.86
10	-8	-10	55.96	3.09
-10	8	10	47.65	3.28
-10	8	10	52.69	2.07
9	-8	-10	31.12	1.18
9	-8	-10	29.75	2.18
9	-8	-10	32.50	1.24
9	-8	-10	33.31	2.61
9	-8	-10	28.34	2.38
-9	8	10	29.92	2.84

-9	8	10	30.64	1.49
8	-8	-10	14.82	1.97
8	-8	-10	15.07	2.22
8	-8	-10	13.96	1.70
8	-8	-10	15.51	0.78
8	-8	-10	15.93	0.75
-8	8	10	14.24	1.10
-8	8	10	16.04	2.69
7	-8	-10	11.71	0.77
7	-8	-10	12.61	0.65
7	-8	-10	14.53	1.62
7	-8	-10	11.96	1.91
-7	8	10	11.92	1.08
-7	8	10	13.96	2.12
6	-8	-10	43.19	2.72
6	-8	-10	44.94	2.43
-6	8	10	52.05	5.28
-6	8	10	44.72	2.07
5	-8	-10	31.37	2.37
5	-8	-10	30.55	2.04
-5	8	10	32.94	2.01
4	-8	-10	19.86	2.12
4	-8	-10	19.18	1.70
-4	8	10	20.50	1.50
3	-8	-10	35.07	2.09
3	-8	-10	36.09	2.57
-3	8	10	39.70	2.09
2	-8	-10	17.32	2.17
2	-8	-10	18.99	1.67
-2	8	10	21.13	1.66
1	-8	-10	9.13	1.46
-1	8	10	8.87	1.48
0	-8	-10	5.44	1.09
0	8	10	4.82	1.12
0	8	10	7.64	1.26
-1	-8	-10	4.37	1.05
1	8	10	2.52	1.16
1	8	10	4.32	1.17
-2	-8	-10	6.53	1.11
2	8	10	7.73	1.37
2	8	10	5.23	1.11
-3	-8	-10	3.98	1.11
3	8	10	3.23	1.38
3	8	10	4.66	1.22

17	-9	-10	16.69	1.33
17	-9	-10	16.19	2.26
16	-9	-10	28.13	1.52
16	-9	-10	23.25	2.75
16	-9	-10	31.62	5.39
15	-9	-10	41.73	1.78
15	-9	-10	46.40	3.77
15	-9	-10	38.72	2.99
14	-9	-10	45.11	3.62
14	-9	-10	44.81	1.75
14	-9	-10	38.76	2.93
13	-9	-10	27.92	1.26
13	-9	-10	28.95	3.09
13	-9	-10	26.74	2.39
-13	9	10	27.37	1.28
12	-9	-10	22.48	1.07
12	-9	-10	22.19	1.24
12	-9	-10	21.65	2.20
12	-9	-10	20.65	2.75
-12	9	10	20.83	1.15
11	-9	-10	32.41	2.40
11	-9	-10	31.66	2.82
11	-9	-10	30.05	2.69
11	-9	-10	33.37	1.30
11	-9	-10	33.32	1.40
-11	9	10	27.15	3.02
-11	9	10	34.67	1.54
10	-9	-10	23.97	2.72
10	-9	-10	29.26	2.65
10	-9	-10	26.30	1.11
10	-9	-10	28.46	2.22
10	-9	-10	26.97	1.20
-10	9	10	32.37	3.62
-10	9	10	26.59	1.43
9	-9	-10	19.42	0.89
9	-9	-10	21.41	2.01
9	-9	-10	20.14	2.40
9	-9	-10	19.75	0.94
-9	9	10	18.53	1.25
8	-9	-10	43.92	2.62
8	-9	-10	43.12	1.52
8	-9	-10	44.32	1.52
8	-9	-10	43.50	2.96
-8	9	10	45.30	2.03

7	-9	-10	15.68	1.83
7	-9	-10	19.18	2.19
-7	9	10	15.76	1.35
6	-9	-10	16.69	1.80
6	-9	-10	15.94	2.17
-6	9	10	17.91	1.40
5	-9	-10	29.08	2.08
-5	9	10	30.35	1.83
4	-9	-10	19.88	1.76
4	-9	-10	16.79	2.23
-4	9	10	18.48	1.61
3	-9	-10	23.82	2.45
3	-9	-10	17.43	1.77
-3	9	10	19.40	1.73
2	-9	-10	21.36	2.49
2	-9	-10	18.95	1.77
-2	9	10	21.31	1.80
1	-9	-10	1.21	1.46
1	-9	-10	3.63	1.02
-1	9	10	3.29	1.01
0	-9	-10	2.45	1.04
0	9	10	3.71	1.14
0	9	10	0.15	1.03
-1	-9	-10	11.87	1.69
1	9	10	7.16	1.47
1	9	10	9.14	1.49
-2	-9	-10	5.23	1.17
2	9	10	6.78	1.44
2	9	10	5.01	1.23
17	-10	-10	12.22	2.25
17	-10	-10	17.34	1.36
16	-10	-10	19.56	1.33
16	-10	-10	18.64	2.84
16	-10	-10	17.72	2.71
15	-10	-10	24.97	2.71
15	-10	-10	24.12	3.55
15	-10	-10	27.75	1.42
14	-10	-10	40.02	2.94
14	-10	-10	37.06	1.60
13	-10	-10	35.26	1.46
13	-10	-10	29.27	2.67
13	-10	-10	31.85	3.36
-13	10	10	35.73	1.52
12	-10	-10	10.49	0.78

12	-10	-10	9.19	1.55
12	-10	-10	8.43	1.99
12	-10	-10	10.50	1.42
-12	10	10	9.08	0.98
11	-10	-10	11.33	1.84
11	-10	-10	14.52	0.82
11	-10	-10	13.16	1.77
11	-10	-10	14.84	1.25
11	-10	-10	14.99	2.56
-11	10	10	13.16	1.15
10	-10	-10	40.49	1.47
10	-10	-10	36.33	2.59
10	-10	-10	40.94	3.13
-10	10	10	40.45	1.88
9	-10	-10	39.39	3.04
9	-10	-10	41.06	1.46
9	-10	-10	43.02	2.72
-9	10	10	39.74	1.96
8	-10	-10	22.76	3.00
8	-10	-10	26.38	2.24
-8	10	10	27.34	1.86
7	-10	-10	10.12	1.49
7	-10	-10	8.08	1.62
-7	10	10	9.17	1.27
6	-10	-10	27.11	2.15
6	-10	-10	23.32	2.58
-6	10	10	25.71	1.79
5	-10	-10	12.55	2.26
5	-10	-10	11.53	1.71
-5	10	10	11.51	1.49
4	-10	-10	9.65	1.76
4	-10	-10	10.87	1.68
-4	10	10	10.74	1.53
3	-10	-10	14.56	1.78
3	-10	-10	14.08	2.54
-3	10	10	14.06	1.72
2	-10	-10	15.22	2.59
2	-10	-10	16.17	1.81
-2	10	10	18.87	1.88
1	-10	-10	6.30	1.24
1	-10	-10	4.90	1.69
-1	10	10	6.85	1.21
0	-10	-10	4.12	1.19
0	10	10	3.25	1.09

0	10	10	1.87	1.21
-1	-10	-10	1.84	1.07
1	10	10	3.56	1.37
1	10	10	2.29	1.38
16	-11	-10	28.00	1.55
16	-11	-10	23.13	4.16
16	-11	-10	23.28	2.98
15	-11	-10	23.93	3.87
15	-11	-10	27.48	2.88
15	-11	-10	28.14	1.46
14	-11	-10	22.49	3.64
14	-11	-10	21.62	1.25
14	-11	-10	25.19	2.77
13	-11	-10	19.45	1.08
13	-11	-10	20.18	2.60
13	-11	-10	18.62	2.67
-13	11	10	17.94	1.22
12	-11	-10	30.64	3.29
12	-11	-10	29.76	1.26
12	-11	-10	23.82	2.55
-12	11	10	28.06	1.57
11	-11	-10	21.69	3.05
11	-11	-10	22.75	1.06
11	-11	-10	22.42	2.46
-11	11	10	23.32	1.48
10	-11	-10	19.54	2.88
10	-11	-10	21.11	2.37
10	-11	-10	20.16	0.95
-10	11	10	19.88	1.52
9	-11	-10	20.79	2.31
9	-11	-10	20.20	2.84
-9	11	10	19.88	1.57
8	-11	-10	30.87	2.51
8	-11	-10	36.01	3.07
-8	11	10	34.50	1.99
7	-11	-10	14.03	2.58
7	-11	-10	14.64	2.02
-7	11	10	9.81	1.52
6	-11	-10	-0.41	1.06
6	-11	-10	1.15	1.52
-6	11	10	1.20	0.89
5	-11	-10	4.01	1.24
5	-11	-10	4.10	1.58
-5	11	10	5.60	1.11

4	-11	-10	11.84	1.58
4	-11	-10	11.72	1.98
-4	11	10	10.84	1.75
3	-11	-10	7.61	1.34
3	-11	-10	11.24	2.03
-3	11	10	6.60	1.25
2	-11	-10	6.27	1.82
2	-11	-10	8.52	1.33
-2	11	10	6.38	1.24
1	-11	-10	4.29	1.23
1	-11	-10	2.07	1.83
-1	11	10	4.10	1.20
0	-11	-10	1.60	1.15
0	11	10	1.06	1.43
15	-12	-10	18.29	3.17
15	-12	-10	19.18	1.31
15	-12	-10	22.77	2.95
14	-12	-10	29.07	1.44
14	-12	-10	30.28	3.09
14	-12	-10	29.01	4.05
13	-12	-10	29.95	2.92
13	-12	-10	29.56	3.93
13	-12	-10	31.23	1.38
-13	12	10	29.73	1.65
12	-12	-10	20.72	1.16
12	-12	-10	17.16	2.64
12	-12	-10	19.12	3.52
-12	12	10	18.22	1.46
11	-12	-10	22.70	2.64
11	-12	-10	25.62	3.49
-11	12	10	23.67	1.73
10	-12	-10	28.91	3.40
10	-12	-10	30.16	2.70
-10	12	10	26.10	1.82
9	-12	-10	9.62	1.70
9	-12	-10	11.32	2.15
-9	12	10	8.74	1.17
8	-12	-10	8.16	2.12
8	-12	-10	9.08	1.54
-8	12	10	7.46	1.16
7	-12	-10	16.50	2.38
7	-12	-10	14.24	2.19
-7	12	10	12.42	1.75
6	-12	-10	6.94	1.44

6	-12	-10	7.82	1.95
-6	12	10	4.19	1.10
5	-12	-10	3.43	1.29
5	-12	-10	3.68	1.81
-5	12	10	2.82	1.16
4	-12	-10	5.94	1.30
4	-12	-10	5.37	1.92
-4	12	10	3.80	1.22
3	-12	-10	0.37	1.80
3	-12	-10	1.57	1.21
-3	12	10	1.30	1.10
2	-12	-10	0.50	1.86
2	-12	-10	-0.67	1.14
-2	12	10	1.04	1.24
14	-13	-10	11.71	2.08
14	-13	-10	18.40	3.08
13	-13	-10	12.65	2.16
13	-13	-10	17.30	3.00
12	-13	-10	21.31	3.85
12	-13	-10	24.80	3.02
-12	13	10	23.00	1.73
11	-13	-10	21.10	3.79
11	-13	-10	24.74	2.88
-11	13	10	22.56	1.82
10	-13	-10	11.42	2.39
10	-13	-10	11.69	1.96
-10	13	10	9.92	1.65
9	-13	-10	4.95	1.66
9	-13	-10	6.69	2.18
-9	13	10	6.25	1.20
8	-13	-10	13.09	1.88
8	-13	-10	13.70	2.49
-8	13	10	12.40	1.86
7	-13	-10	18.63	2.32
7	-13	-10	16.54	2.55
-7	13	10	13.96	1.93
6	-13	-10	8.36	1.60
6	-13	-10	10.03	2.37
-6	13	10	12.01	1.62
5	-13	-10	-0.67	1.30
5	-13	-10	0.25	1.94
-5	13	10	1.73	1.20
4	-13	-10	-1.25	1.89
4	-13	-10	0.81	1.27

-4	13	10	-0.09	1.19
12	-14	-10	10.05	2.66
12	-14	-10	6.90	2.01
-12	14	10	8.57	1.23
11	-14	-10	9.20	2.62
11	-14	-10	0.93	1.72
-11	14	10	4.45	1.15
10	-14	-10	14.95	2.20
10	-14	-10	12.34	2.65
-10	14	10	11.25	1.92
9	-14	-10	13.78	2.70
9	-14	-10	13.29	2.08
-9	14	10	14.74	2.07
8	-14	-10	4.76	2.35
8	-14	-10	3.85	1.75
-8	14	10	5.86	1.42
7	-14	-10	10.63	1.90
7	-14	-10	8.69	2.46
-7	14	10	7.37	1.42
6	-14	-10	1.83	1.49
6	-14	-10	4.60	2.17
-6	14	10	2.87	1.36
0	17	-11	1.48	0.86
0	-17	11	3.66	1.86
0	-17	11	1.60	2.77
-1	17	-11	3.16	0.87
1	-17	11	9.63	2.96
1	-17	11	2.67	1.62
-2	17	-11	7.81	1.00
2	-17	11	13.89	3.17
2	-17	11	11.49	1.91
-3	17	-11	14.92	1.45
3	-17	11	26.10	4.51
3	-17	11	22.06	2.99
-4	17	-11	3.66	0.89
4	-17	11	2.37	1.74
4	-17	11	4.08	2.95
3	16	-11	1.65	0.91
-3	-16	11	1.20	1.76
-3	-16	11	3.58	2.44
2	16	-11	3.10	0.93
-2	-16	11	3.03	2.38
-2	-16	11	2.28	1.74
1	16	-11	10.12	1.33

-1	-16	11	7.89	1.82
-1	-16	11	9.10	2.64
0	16	-11	14.81	1.33
0	-16	11	13.78	2.07
0	-16	11	15.81	2.89
-1	16	-11	12.86	1.30
1	-16	11	12.58	1.93
1	-16	11	16.31	2.99
-2	16	-11	10.43	1.23
2	-16	11	11.57	2.81
2	-16	11	8.22	1.76
-3	16	-11	14.75	1.37
3	-16	11	12.99	3.11
3	-16	11	11.69	1.91
-4	16	-11	10.56	1.29
4	-16	11	14.26	1.98
4	-16	11	7.34	2.86
-5	16	-11	12.69	1.34
5	-16	11	15.96	2.71
5	-16	11	11.36	3.14
-6	16	-11	14.55	1.39
6	-16	11	11.57	1.84
6	-16	11	11.41	3.31
5	15	-11	8.11	1.12
-5	-15	11	7.97	1.92
-5	-15	11	5.54	2.16
4	15	-11	0.13	0.86
-4	-15	11	4.62	2.09
-4	-15	11	1.20	1.49
3	15	-11	3.06	0.93
-3	-15	11	4.59	2.18
-3	-15	11	3.15	1.45
2	15	-11	15.45	1.41
-2	-15	11	18.91	2.67
-2	-15	11	15.51	2.13
1	15	-11	14.85	1.36
-1	-15	11	11.68	2.51
-1	-15	11	12.16	1.83
0	15	-11	11.33	1.20
0	-15	11	9.28	2.33
0	-15	11	8.05	1.58
-1	15	-11	11.10	1.18
1	-15	11	8.02	2.37
1	-15	11	11.41	1.78

-2	15	-11	10.31	1.18
2	-15	11	13.56	2.30
2	-15	11	11.03	2.62
-3	15	-11	22.95	1.48
3	-15	11	23.86	2.55
3	-15	11	22.34	3.86
-4	15	-11	27.48	1.57
4	-15	11	26.11	4.05
4	-15	11	26.20	2.67
-5	15	-11	19.76	1.41
5	-15	11	17.29	2.53
5	-15	11	19.67	3.28
-6	15	-11	6.26	0.91
6	-15	11	6.79	1.61
6	-15	11	8.69	2.94
-7	15	-11	12.52	1.35
7	-15	11	19.89	2.58
7	-15	11	17.86	3.55
-8	15	-11	15.82	1.64
8	-15	11	21.32	3.85
7	14	-11	5.68	1.07
-7	-14	11	8.39	2.01
6	14	-11	3.94	0.96
-6	-14	11	4.26	1.69
5	14	-11	3.99	0.90
-5	-14	11	1.49	1.85
-5	-14	11	1.78	1.40
4	14	-11	8.33	1.02
-4	-14	11	7.86	1.76
-4	-14	11	10.32	2.01
3	14	-11	11.71	1.30
-3	-14	11	8.47	2.13
-3	-14	11	8.28	1.57
2	14	-11	7.41	0.94
-2	-14	11	5.89	1.45
-2	-14	11	10.22	2.11
1	14	-11	7.91	1.16
-1	-14	11	10.18	1.73
-1	-14	11	8.03	2.09
0	14	-11	8.79	1.09
0	-14	11	9.31	1.59
-1	14	-11	9.22	1.05
1	-14	11	9.43	1.57
1	-14	11	10.00	2.16

-2	14	-11	18.59	1.30
2	-14	11	25.58	2.33
2	-14	11	20.39	3.30
-3	14	-11	15.48	1.23
3	-14	11	19.65	2.17
3	-14	11	19.14	3.40
-4	14	-11	11.94	1.18
4	-14	11	13.61	2.12
4	-14	11	14.26	2.59
-5	14	-11	20.81	1.42
5	-14	11	22.56	3.93
5	-14	11	25.42	2.42
-6	14	-11	21.64	1.44
6	-14	11	20.43	3.31
6	-14	11	18.22	2.41
-7	14	-11	8.31	1.17
7	-14	11	13.04	3.04
7	-14	11	9.66	1.67
-8	14	-11	6.95	0.95
8	-14	11	7.76	1.69
8	-14	11	7.63	3.02
8	13	-11	4.02	1.05
-8	-13	11	6.46	1.88
7	13	-11	7.47	1.05
-7	-13	11	5.06	1.70
6	13	-11	3.62	0.89
-6	-13	11	4.10	1.72
-6	-13	11	3.48	1.44
5	13	-11	15.10	1.42
-5	-13	11	15.96	2.39
-5	-13	11	14.65	2.59
4	13	-11	10.56	1.25
-4	-13	11	13.01	1.93
-4	-13	11	8.07	1.52
3	13	-11	0.85	0.64
-3	-13	11	-0.55	1.15
-3	-13	11	-0.12	1.54
2	13	-11	9.68	1.15
-2	-13	11	11.48	1.74
-2	-13	11	11.98	2.05
1	13	-11	34.17	1.65
-1	-13	11	33.69	2.51
-1	-13	11	28.39	3.01
0	13	-11	17.37	1.28

0	-13	11	22.21	2.14
0	-13	11	19.23	2.68
-1	13	-11	15.78	1.17
1	-13	11	19.57	2.06
1	-13	11	15.45	2.71
-2	13	-11	16.55	1.18
2	-13	11	15.43	1.96
-3	13	-11	11.65	1.05
3	-13	11	9.56	2.04
3	-13	11	7.62	1.35
-4	13	-11	8.85	1.02
4	-13	11	9.46	2.34
4	-13	11	9.18	1.43
-5	13	-11	10.75	1.14
5	-13	11	10.72	1.90
5	-13	11	14.02	2.67
-6	13	-11	11.50	1.15
6	-13	11	10.49	2.63
6	-13	11	12.16	2.05
-7	13	-11	9.87	1.18
7	-13	11	12.64	2.85
7	-13	11	13.58	2.19
-8	13	-11	17.14	1.39
8	-13	11	15.51	3.35
8	-13	11	19.35	2.47
9	-13	11	10.44	3.22
9	-13	11	9.01	1.66
9	12	-11	2.02	0.96
-9	-12	11	0.58	1.69
8	12	-11	4.78	0.96
-8	-12	11	5.74	1.66
7	12	-11	14.41	1.43
-7	-12	11	11.11	1.86
-7	-12	11	15.82	2.20
6	12	-11	7.26	0.96
-6	-12	11	6.28	1.50
-6	-12	11	5.26	1.40
5	12	-11	9.71	1.20
-5	-12	11	11.81	2.09
-5	-12	11	10.43	1.67
4	12	-11	8.97	1.10
-4	-12	11	7.71	1.54
-4	-12	11	7.17	1.56
3	12	-11	12.01	1.83

3	12	-11	9.57	1.08
-3	-12	11	8.71	1.42
-3	-12	11	8.24	1.69
2	12	-11	20.97	2.49
2	12	-11	15.01	1.18
-2	-12	11	12.08	1.86
-2	-12	11	16.91	2.35
1	12	-11	27.07	2.58
1	12	-11	28.76	1.47
-1	-12	11	27.97	2.19
-1	-12	11	26.45	2.62
0	12	-11	43.09	2.92
0	12	-11	41.90	1.87
0	-12	11	46.23	3.21
0	-12	11	45.27	2.60
-1	12	-11	17.76	1.14
1	-12	11	17.06	1.88
1	-12	11	13.80	2.49
-2	12	-11	31.35	1.53
2	-12	11	32.43	2.24
2	-12	11	32.65	2.99
-3	12	-11	16.97	1.14
3	-12	11	15.32	0.92
3	-12	11	20.41	1.90
-4	12	-11	5.20	0.76
4	-12	11	6.25	2.01
4	-12	11	5.90	0.77
4	-12	11	5.84	1.19
-5	12	-11	6.95	0.98
5	-12	11	8.64	0.97
5	-12	11	8.42	1.35
5	-12	11	9.79	2.47
-6	12	-11	24.40	1.44
6	-12	11	23.63	2.18
6	-12	11	25.66	1.47
6	-12	11	21.07	3.06
-7	12	-11	20.68	1.37
7	-12	11	19.42	2.18
7	-12	11	17.47	2.91
7	-12	11	20.51	1.54
-8	12	-11	10.07	1.20
8	-12	11	10.43	1.63
8	-12	11	12.85	3.04
8	-12	11	13.46	1.60

9	-12	11	7.82	1.57
9	-12	11	5.58	3.07
9	-12	11	8.05	1.33
10	11	-11	2.96	0.94
-10	-11	11	1.12	1.65
9	11	-11	8.01	1.03
-9	-11	11	6.35	1.68
8	11	-11	7.67	0.98
-8	-11	11	7.05	1.63
7	11	-11	2.60	0.77
-7	-11	11	2.71	1.18
-7	-11	11	5.49	1.30
6	11	-11	15.56	1.26
-6	-11	11	15.11	1.96
-6	-11	11	18.69	2.16
5	11	-11	16.66	1.24
5	11	-11	20.71	2.56
-5	-11	11	16.37	2.00
-5	-11	11	16.03	1.96
4	11	-11	21.04	1.33
4	11	-11	19.64	2.36
-4	-11	11	22.09	2.17
-4	-11	11	22.62	2.04
3	11	-11	17.66	1.17
3	11	-11	13.29	2.14
-3	-11	11	19.59	1.82
-3	-11	11	15.56	2.08
2	11	-11	29.04	2.26
2	11	-11	25.22	1.37
-2	-11	11	21.99	1.96
-2	-11	11	29.77	2.31
1	11	-11	18.53	1.17
1	11	-11	20.58	2.07
-1	-11	11	21.43	1.81
-1	-11	11	17.60	2.19
0	11	-11	41.21	1.78
0	11	-11	42.01	2.73
0	-11	11	40.97	2.37
0	-11	11	41.83	2.87
-1	11	-11	27.25	2.25
-1	11	-11	31.65	1.44
1	-11	11	30.57	2.02
1	-11	11	29.75	2.63
-2	11	-11	18.04	1.16

2	-11	11	20.49	1.74
2	-11	11	17.12	2.50
2	-11	11	19.48	1.15
-3	11	-11	25.00	1.32
3	-11	11	25.10	1.91
3	-11	11	23.93	2.74
3	-11	11	25.75	1.50
-4	11	-11	25.05	1.36
4	-11	11	28.51	1.77
4	-11	11	25.36	1.94
4	-11	11	24.74	2.96
-5	11	-11	14.53	1.12
5	-11	11	15.28	1.73
5	-11	11	14.38	1.12
5	-11	11	13.73	2.37
-6	11	-11	36.09	1.74
6	-11	11	35.84	3.69
6	-11	11	35.48	1.78
6	-11	11	37.59	2.34
-7	11	-11	30.98	1.65
7	-11	11	40.01	2.43
7	-11	11	34.69	1.86
7	-11	11	28.70	3.88
-8	11	-11	16.36	1.28
8	-11	11	18.33	1.67
8	-11	11	19.59	3.21
8	-11	11	14.92	2.13
-9	11	-11	9.09	1.23
9	-11	11	13.98	1.80
9	-11	11	7.37	2.93
9	-11	11	9.97	1.61
10	-11	11	8.48	3.45
11	10	-11	3.55	0.91
-11	-10	11	6.63	1.85
10	10	-11	10.58	1.33
-10	-10	11	9.11	1.78
9	10	-11	0.63	0.70
9	10	-11	3.06	1.21
-9	-10	11	-1.01	1.24
8	10	-11	6.18	1.07
8	10	-11	6.97	1.14
8	10	-11	6.46	0.84
-8	-10	11	4.57	1.34
7	10	-11	26.18	1.51

7	10	-11	24.42	1.58
-7	-10	11	26.02	2.27
-7	-10	11	25.31	1.79
6	10	-11	24.35	2.26
6	10	-11	23.25	1.40
6	10	-11	25.10	1.47
-6	-10	11	24.62	1.84
-6	-10	11	25.61	2.17
5	10	-11	7.09	1.37
5	10	-11	5.70	0.50
5	10	-11	7.41	1.05
5	10	-11	6.87	0.93
-5	-10	11	8.48	1.21
4	10	-11	15.78	1.90
4	10	-11	17.07	1.14
-4	-10	11	16.44	1.76
-4	-10	11	17.98	1.75
3	10	-11	51.09	1.99
3	10	-11	51.58	2.59
-3	-10	11	47.49	2.57
-3	-10	11	46.15	2.63
2	10	-11	28.57	2.03
2	10	-11	26.75	1.44
-2	-10	11	28.43	1.89
-2	-10	11	28.30	2.17
1	10	-11	27.08	1.92
1	10	-11	24.46	1.47
-1	-10	11	27.15	1.85
-1	-10	11	26.56	2.24
0	10	-11	20.29	1.13
0	10	-11	19.96	1.78
0	-10	11	17.89	1.64
0	-10	11	18.77	2.12
-1	10	-11	6.85	0.54
-1	10	-11	7.41	0.79
-1	10	-11	8.82	1.51
1	-10	11	6.21	1.13
1	-10	11	6.72	0.91
1	-10	11	7.28	1.57
-2	10	-11	24.31	1.02
-2	10	-11	25.45	1.97
-2	10	-11	26.36	1.29
2	-10	11	23.48	1.85
2	-10	11	21.86	2.40

2	-10	11	27.89	1.48
-3	10	-11	23.45	1.23
-3	10	-11	21.15	0.97
3	-10	11	22.77	1.56
3	-10	11	23.68	2.58
3	-10	11	19.78	1.69
-4	10	-11	12.00	0.81
-4	10	-11	14.26	1.03
4	-10	11	12.06	1.52
4	-10	11	17.62	1.56
4	-10	11	11.81	2.01
-5	10	-11	23.01	1.13
-5	10	-11	24.14	1.30
5	-10	11	20.52	1.80
5	-10	11	25.25	2.95
5	-10	11	22.71	1.83
-6	10	-11	43.53	1.84
-6	10	-11	38.73	1.64
6	-10	11	39.78	2.38
6	-10	11	38.14	2.34
6	-10	11	40.45	3.68
-7	10	-11	14.40	1.18
7	-10	11	15.35	2.66
7	-10	11	19.16	1.86
-8	10	-11	12.11	1.18
8	-10	11	10.53	1.49
8	-10	11	11.84	1.56
8	-10	11	6.28	2.64
-9	10	-11	11.04	1.23
9	-10	11	9.73	1.55
9	-10	11	9.04	1.35
9	-10	11	9.32	3.09
-10	10	-11	10.30	1.44
10	-10	11	6.64	1.54
10	-10	11	10.50	1.50
10	-10	11	-0.24	3.03
12	9	-11	8.94	1.35
-12	-9	11	3.29	1.80
11	9	-11	2.97	0.81
11	9	-11	2.71	1.19
-11	-9	11	4.01	1.67
10	9	-11	3.16	1.07
10	9	-11	1.91	0.77
-10	-9	11	2.21	1.39

9	9	-11	12.70	1.18
9	9	-11	10.87	1.53
9	9	-11	13.46	1.18
-9	-9	11	11.25	1.61
8	9	-11	8.43	0.99
8	9	-11	9.51	1.07
8	9	-11	9.53	1.43
-8	-9	11	9.88	1.47
7	9	-11	21.16	1.29
7	9	-11	18.34	0.99
7	9	-11	20.56	1.48
7	9	-11	20.83	1.94
-7	-9	11	21.52	1.46
-7	-9	11	21.73	1.98
6	9	-11	22.70	1.85
6	9	-11	22.35	1.28
6	9	-11	21.33	0.95
6	9	-11	21.39	1.42
-6	-9	11	24.39	1.92
-6	-9	11	23.35	1.57
5	9	-11	25.54	1.39
5	9	-11	24.69	1.79
5	9	-11	24.71	1.31
-5	-9	11	27.08	1.71
-5	-9	11	23.63	1.87
4	9	-11	35.37	1.52
4	9	-11	34.18	1.94
4	9	-11	27.49	2.10
-4	-9	11	33.39	1.99
-4	-9	11	34.05	1.30
-4	-9	11	35.74	2.37
3	9	-11	32.72	1.51
3	9	-11	33.71	1.46
3	9	-11	34.04	1.93
-3	-9	11	33.95	2.13
-3	-9	11	33.87	1.29
2	9	-11	44.77	1.85
2	9	-11	48.24	1.83
2	9	-11	46.80	2.23
-2	-9	11	48.94	2.55
-2	-9	11	45.51	2.33
-2	-9	11	44.50	1.67
1	9	-11	50.13	2.39
1	9	-11	53.30	2.02

1	9	-11	51.85	1.78
-1	-9	11	53.77	2.50
-1	-9	11	51.23	2.78
-1	-9	11	51.10	1.90
0	9	-11	24.52	1.21
0	9	-11	23.99	1.68
0	9	-11	23.51	0.99
0	-9	11	24.81	2.11
0	-9	11	23.70	1.42
0	-9	11	25.44	1.64
-1	9	-11	45.66	1.63
-1	9	-11	51.10	2.35
1	-9	11	43.08	2.26
1	-9	11	42.90	2.79
1	-9	11	47.17	1.96
-2	9	-11	26.10	1.27
-2	9	-11	25.89	1.85
-2	9	-11	24.74	1.09
2	-9	11	24.87	2.38
2	-9	11	25.25	1.68
2	-9	11	29.10	1.61
-3	9	-11	15.54	0.87
-3	9	-11	15.60	1.01
3	-9	11	15.52	1.53
3	-9	11	16.07	1.51
3	-9	11	17.71	2.25
-4	9	-11	64.17	2.33
-4	9	-11	57.56	2.16
4	-9	11	63.90	2.71
4	-9	11	58.17	3.57
-5	9	-11	17.91	1.14
-5	9	-11	18.66	1.06
5	-9	11	14.22	2.65
5	-9	11	17.64	1.75
5	-9	11	16.88	1.76
-6	9	-11	8.09	0.99
-6	9	-11	8.59	0.92
6	-9	11	7.53	2.09
6	-9	11	8.59	1.23
-7	9	-11	18.64	1.25
-7	9	-11	12.78	1.33
7	-9	11	19.74	3.42
7	-9	11	18.22	1.83
-8	9	-11	13.25	1.18

-8	9	-11	13.31	1.18
8	-9	11	13.60	2.82
8	-9	11	12.27	1.57
-9	9	-11	8.10	1.16
9	-9	11	10.36	1.72
9	-9	11	7.11	2.92
9	-9	11	9.95	1.54
-10	9	-11	11.21	1.36
10	-9	11	11.12	3.26
10	-9	11	13.57	2.24
10	-9	11	9.03	1.48
13	8	-11	4.49	0.98
13	8	-11	3.16	1.47
-13	-8	11	6.13	1.80
12	8	-11	5.62	1.33
12	8	-11	4.38	0.87
-12	-8	11	6.98	1.75
11	8	-11	9.12	1.17
11	8	-11	11.95	1.80
-11	-8	11	7.54	1.57
10	8	-11	7.02	1.05
10	8	-11	5.93	1.11
-10	-8	11	6.38	1.44
9	8	-11	1.62	0.60
9	8	-11	1.09	0.58
9	8	-11	1.38	0.89
9	8	-11	0.23	0.55
-9	-8	11	0.24	0.99
8	8	-11	7.01	1.07
8	8	-11	4.19	0.76
8	8	-11	4.86	0.70
8	8	-11	6.41	0.79
-8	-8	11	7.11	1.22
7	8	-11	38.33	1.70
7	8	-11	37.83	1.99
7	8	-11	37.10	1.91
-7	-8	11	40.40	1.68
-7	-8	11	37.69	2.26
6	8	-11	10.32	1.15
6	8	-11	8.09	1.24
6	8	-11	9.46	0.91
-6	-8	11	10.38	0.85
-6	-8	11	9.55	1.75
-6	-8	11	9.36	1.08

5	8	-11	7.62	1.14
5	8	-11	7.74	0.98
5	8	-11	6.48	0.81
-5	-8	11	7.36	0.94
-5	-8	11	7.85	0.76
-5	-8	11	7.91	1.27
4	8	-11	23.36	1.51
4	8	-11	25.32	1.24
4	8	-11	23.97	1.32
-4	-8	11	26.36	1.70
-4	-8	11	27.78	1.18
-4	-8	11	26.16	1.69
3	8	-11	25.57	1.23
3	8	-11	25.11	1.23
3	8	-11	26.08	1.46
-3	-8	11	25.51	1.65
-3	-8	11	23.91	1.70
2	8	-11	26.01	1.24
2	8	-11	24.75	1.16
2	8	-11	24.99	1.48
-2	-8	11	25.88	1.60
-2	-8	11	26.82	1.86
1	8	-11	44.38	1.69
1	8	-11	45.59	1.80
1	8	-11	45.60	2.07
-1	-8	11	45.51	2.45
-1	-8	11	44.32	2.19
-1	-8	11	50.69	1.87
0	8	-11	20.92	0.99
0	8	-11	20.12	1.46
0	8	-11	21.84	1.10
0	-8	11	21.72	1.49
0	-8	11	24.07	1.97
0	-8	11	21.19	1.29
-1	8	-11	33.41	1.32
-1	8	-11	32.75	1.43
-1	8	-11	34.20	1.84
1	-8	11	34.64	1.74
1	-8	11	33.27	1.85
1	-8	11	30.68	2.30
-2	8	-11	34.79	1.72
-2	8	-11	37.83	1.99
-2	8	-11	37.93	1.48
2	-8	11	41.06	2.62

2	-8	11	38.18	2.01
2	-8	11	39.79	1.98
-3	8	-11	47.51	1.75
-3	8	-11	43.82	2.30
-3	8	-11	44.03	1.82
3	-8	11	44.27	2.24
3	-8	11	47.22	2.24
3	-8	11	46.94	2.96
-4	8	-11	20.26	1.15
-4	8	-11	19.97	1.09
4	-8	11	19.25	1.69
4	-8	11	22.62	2.42
4	-8	11	21.03	1.60
-5	8	-11	10.97	0.94
-5	8	-11	9.65	0.90
5	-8	11	10.81	2.01
5	-8	11	11.38	1.65
-6	8	-11	11.50	0.99
-6	8	-11	12.31	1.01
6	-8	11	13.25	1.57
6	-8	11	8.76	1.92
-7	8	-11	12.69	1.07
-7	8	-11	11.76	1.06
7	-8	11	12.18	2.49
7	-8	11	11.82	1.62
-8	8	-11	9.10	1.06
-8	8	-11	8.08	1.12
8	-8	11	7.48	2.64
8	-8	11	6.46	1.29
-9	8	-11	11.68	1.22
-9	8	-11	12.16	1.31
9	-8	11	11.87	2.97
9	-8	11	9.14	1.48
-10	8	-11	5.37	0.92
10	-8	11	8.50	1.45
10	-8	11	4.41	3.03
10	-8	11	12.69	2.17
13	7	-11	8.39	1.55
13	7	-11	8.07	1.22
-13	-7	11	9.54	1.82
12	7	-11	13.65	1.27
12	7	-11	15.54	2.03
-12	-7	11	14.62	2.38
11	7	-11	8.92	1.10

11	7	-11	10.34	1.66
-11	-7	11	10.89	1.54
10	7	-11	11.68	1.12
10	7	-11	13.09	1.11
10	7	-11	12.02	1.57
10	7	-11	9.39	1.06
-10	-7	11	14.86	1.88
9	7	-11	30.54	2.62
9	7	-11	32.04	1.53
9	7	-11	33.27	1.43
9	7	-11	33.14	1.92
-9	-7	11	28.64	2.36
8	7	-11	28.27	1.38
8	7	-11	28.50	1.77
-8	-7	11	27.00	1.43
-8	-7	11	29.00	1.98
7	7	-11	12.71	1.26
7	7	-11	12.62	1.01
-7	-7	11	11.06	0.99
-7	-7	11	12.06	1.41
6	7	-11	12.39	0.94
6	7	-11	14.15	1.21
-6	-7	11	13.95	1.01
-6	-7	11	13.22	1.35
-6	-7	11	13.04	0.98
5	7	-11	43.23	1.89
5	7	-11	45.30	1.76
5	7	-11	43.61	1.72
-5	-7	11	43.80	1.74
-5	-7	11	42.69	2.19
4	7	-11	64.58	2.40
4	7	-11	67.74	2.49
4	7	-11	66.76	2.40
-4	-7	11	65.75	2.83
-4	-7	11	69.52	2.70
-4	-7	11	65.90	2.38
3	7	-11	38.35	1.58
3	7	-11	36.47	1.63
-3	-7	11	38.67	1.99
-3	-7	11	40.09	1.61
-3	-7	11	35.75	1.91
2	7	-11	12.98	0.84
2	7	-11	10.97	0.98
2	7	-11	11.49	0.87

-2	-7	11	11.18	0.95
-2	-7	11	13.13	1.16
-2	-7	11	13.45	1.39
1	7	-11	28.13	1.29
1	7	-11	28.01	1.27
1	7	-11	28.64	1.48
-1	-7	11	30.73	1.51
-1	-7	11	29.78	1.65
-1	-7	11	30.49	1.92
0	7	-11	30.77	1.58
0	7	-11	30.08	1.33
0	7	-11	29.87	1.31
0	-7	11	31.10	2.06
0	-7	11	30.03	1.70
0	-7	11	30.39	1.61
-1	7	-11	60.38	2.42
-1	7	-11	56.97	2.12
-1	7	-11	58.92	2.08
1	-7	11	53.48	2.90
1	-7	11	58.76	2.44
1	-7	11	57.41	2.49
-2	7	-11	19.42	1.04
-2	7	-11	20.72	1.42
-2	7	-11	17.48	1.02
2	-7	11	21.47	2.01
2	-7	11	17.16	1.37
2	-7	11	20.82	1.57
-3	7	-11	34.98	1.48
-3	7	-11	32.49	1.85
3	-7	11	35.39	2.54
3	-7	11	33.60	1.86
3	-7	11	38.09	2.03
-4	7	-11	43.40	1.75
-4	7	-11	41.81	1.74
4	-7	11	40.57	2.15
4	-7	11	48.11	2.34
4	-7	11	39.89	2.94
-5	7	-11	23.42	1.24
-5	7	-11	21.78	1.22
5	-7	11	19.04	1.67
5	-7	11	22.87	2.63
-6	7	-11	7.30	0.86
-6	7	-11	7.39	0.92
6	-7	11	5.67	1.05

6	-7	11	9.21	2.13
-7	7	-11	7.62	1.03
-7	7	-11	10.49	1.00
7	-7	11	7.76	1.20
7	-7	11	8.42	2.25
-8	7	-11	19.59	1.40
-8	7	-11	20.51	1.33
8	-7	11	14.46	1.85
8	-7	11	24.47	3.80
-9	7	-11	11.34	1.22
-9	7	-11	11.94	1.33
9	-7	11	9.88	2.83
9	-7	11	7.77	1.44
-10	7	-11	4.48	0.96
-10	7	-11	3.80	0.85
10	-7	11	0.64	2.80
10	-7	11	4.43	1.37
14	6	-11	9.92	1.25
14	6	-11	6.38	1.62
-14	-6	11	7.63	1.74
13	6	-11	4.84	0.82
13	6	-11	4.01	1.39
-13	-6	11	5.70	1.54
12	6	-11	6.99	1.33
12	6	-11	5.56	0.82
-12	-6	11	6.93	1.41
11	6	-11	20.00	1.27
11	6	-11	21.30	1.84
10	6	-11	33.51	2.02
10	6	-11	34.55	1.59
10	6	-11	32.60	1.59
-10	-6	11	37.20	2.25
9	6	-11	8.86	1.34
9	6	-11	10.10	0.94
-9	-6	11	11.87	1.09
-9	-6	11	9.03	1.44
8	6	-11	23.08	1.19
8	6	-11	21.21	1.56
-8	-6	11	23.99	1.32
-8	-6	11	20.29	1.65
7	6	-11	71.72	2.53
7	6	-11	70.33	2.79
-7	-6	11	67.50	2.96
-7	-6	11	71.04	2.58

6	6	-11	53.25	2.06
6	6	-11	53.83	2.30
-6	-6	11	51.79	2.45
-6	-6	11	59.94	2.14
5	6	-11	57.81	2.15
5	6	-11	57.40	2.34
-5	-6	11	57.50	2.18
-5	-6	11	59.08	2.54
4	6	-11	59.87	2.21
4	6	-11	59.31	2.07
4	6	-11	60.75	2.37
4	6	-11	71.58	2.69
-4	-6	11	62.26	2.58
-4	-6	11	54.85	2.51
-4	-6	11	60.07	2.28
3	6	-11	43.96	1.70
3	6	-11	43.83	1.65
3	6	-11	39.96	1.82
3	6	-11	41.98	1.84
-3	-6	11	46.09	2.08
-3	-6	11	43.77	2.02
-3	-6	11	41.93	1.81
-3	-6	11	45.84	1.78
2	6	-11	25.35	1.13
2	6	-11	23.40	1.17
2	6	-11	24.46	1.25
2	6	-11	21.63	1.25
-2	-6	11	25.00	1.44
-2	-6	11	25.11	1.34
-2	-6	11	22.22	1.10
-2	-6	11	21.57	1.53
1	6	-11	29.63	1.38
1	6	-11	31.08	1.41
1	6	-11	29.53	1.67
1	6	-11	29.12	1.30
-1	-6	11	31.41	1.37
-1	-6	11	32.08	1.59
-1	-6	11	28.86	1.83
-1	-6	11	29.70	1.61
0	6	-11	23.69	1.28
0	6	-11	24.35	1.14
0	6	-11	23.55	1.19
0	-6	11	25.31	1.82
0	-6	11	21.13	1.38

0	-6	11	25.75	1.54
-1	6	-11	21.54	1.13
-1	6	-11	22.70	1.08
-1	6	-11	22.84	1.32
1	-6	11	24.10	1.56
1	-6	11	20.79	1.85
1	-6	11	18.52	1.34
-2	6	-11	49.33	2.23
-2	6	-11	52.94	1.98
-2	6	-11	51.98	2.01
2	-6	11	56.44	2.89
2	-6	11	54.28	2.34
2	-6	11	54.45	2.46
-3	6	-11	62.96	2.22
-3	6	-11	60.51	2.23
-3	6	-11	54.73	2.47
3	-6	11	57.32	2.54
3	-6	11	61.45	2.66
3	-6	11	60.41	3.24
-4	6	-11	13.42	0.91
-4	6	-11	12.33	0.98
4	-6	11	12.25	1.57
4	-6	11	12.44	1.29
4	-6	11	14.39	2.12
-5	6	-11	16.36	1.04
-5	6	-11	16.76	1.10
5	-6	11	14.12	2.37
5	-6	11	12.20	1.42
-6	6	-11	23.52	1.35
-6	6	-11	26.08	1.30
6	-6	11	21.08	1.68
6	-6	11	21.02	2.87
-7	6	-11	17.25	1.27
-7	6	-11	18.77	1.19
7	-6	11	17.12	2.78
7	-6	11	14.55	1.64
-8	6	-11	18.15	1.31
-8	6	-11	17.79	1.39
8	-6	11	18.54	1.87
8	-6	11	21.33	3.60
-9	6	-11	10.62	1.32
-9	6	-11	13.03	1.22
9	-6	11	8.99	1.39
9	-6	11	10.85	2.62

-10	6	-11	9.47	1.29
-10	6	-11	4.05	0.97
10	-6	11	4.36	1.29
10	-6	11	0.85	2.80
15	5	-11	4.48	0.90
15	5	-11	5.22	1.60
14	5	-11	5.23	0.79
14	5	-11	7.54	1.61
-14	-5	11	6.50	1.56
13	5	-11	16.86	1.25
13	5	-11	20.28	2.19
12	5	-11	16.69	1.77
12	5	-11	10.85	1.05
-12	-5	11	10.69	1.82
11	5	-11	6.82	0.91
11	5	-11	4.76	1.07
-11	-5	11	5.53	1.12
10	5	-11	16.77	1.08
10	5	-11	18.16	1.61
-10	-5	11	15.44	1.27
-10	-5	11	14.45	1.59
9	5	-11	39.81	2.14
9	5	-11	44.75	1.76
-9	-5	11	45.66	2.27
-9	-5	11	41.86	1.90
8	5	-11	56.75	2.40
8	5	-11	47.01	1.89
8	5	-11	43.95	2.24
-8	-5	11	48.56	2.02
-8	-5	11	46.58	2.29
7	5	-11	65.62	2.51
7	5	-11	66.24	2.71
7	5	-11	66.82	2.38
-7	-5	11	65.90	2.77
-7	-5	11	67.77	2.52
6	5	-11	77.21	2.69
6	5	-11	75.62	2.80
6	5	-11	77.59	3.00
-6	-5	11	82.97	2.84
-6	-5	11	74.00	3.03
5	5	-11	42.82	1.66
5	5	-11	40.55	1.75
5	5	-11	40.28	1.90
-5	-5	11	43.27	1.72

-5	-5	11	41.86	1.80
-5	-5	11	42.00	1.96
4	5	-11	56.22	2.05
4	5	-11	59.86	2.32
4	5	-11	54.76	2.16
-4	-5	11	55.93	2.15
-4	-5	11	57.50	2.21
-4	-5	11	55.32	2.35
-4	-5	11	54.67	1.98
3	5	-11	47.07	1.69
3	5	-11	48.81	1.93
3	5	-11	48.94	2.01
3	5	-11	49.53	1.81
-3	-5	11	46.18	2.07
-3	-5	11	48.41	2.01
-3	-5	11	49.41	2.13
-3	-5	11	45.18	1.74
2	5	-11	23.77	1.29
2	5	-11	26.35	1.10
2	5	-11	21.37	1.10
2	5	-11	22.87	1.20
-2	-5	11	22.10	1.46
-2	-5	11	23.11	1.08
-2	-5	11	21.93	1.29
-2	-5	11	21.74	1.37
1	5	-11	20.98	1.10
1	5	-11	20.55	1.04
1	5	-11	21.79	1.16
1	5	-11	21.50	1.21
-1	-5	11	23.92	1.54
-1	-5	11	21.36	1.42
-1	-5	11	19.91	1.25
-1	-5	11	21.63	1.04
0	5	-11	57.34	2.22
0	5	-11	58.80	2.23
0	5	-11	60.30	2.13
0	-5	11	60.64	2.72
0	-5	11	57.25	2.41
0	-5	11	57.22	2.12
0	-5	11	63.36	2.53
-1	5	-11	47.63	1.99
-1	5	-11	47.60	1.93
-1	5	-11	46.60	1.81
1	-5	11	47.04	2.54

1	-5	11	51.65	2.30
1	-5	11	48.95	2.12
-2	5	-11	21.54	1.19
-2	5	-11	20.76	1.36
-2	5	-11	21.27	1.07
2	-5	11	19.58	1.63
2	-5	11	22.18	1.39
2	-5	11	20.33	1.89
-3	5	-11	18.23	1.00
-3	5	-11	18.03	1.14
-3	5	-11	18.22	1.39
3	-5	11	18.05	1.33
3	-5	11	18.80	1.69
3	-5	11	21.51	2.06
-4	5	-11	11.42	0.99
4	-5	11	10.96	1.21
4	-5	11	10.59	1.68
-5	5	-11	14.58	1.26
-5	5	-11	18.51	1.06
5	-5	11	16.90	2.39
-6	5	-11	8.26	1.05
-6	5	-11	9.88	0.91
6	-5	11	10.43	1.34
6	-5	11	9.84	2.07
-7	5	-11	6.24	0.86
-7	5	-11	6.43	0.96
7	-5	11	6.22	1.04
7	-5	11	8.25	2.16
-8	5	-11	11.46	1.29
-8	5	-11	12.80	1.15
8	-5	11	7.60	1.24
8	-5	11	11.09	2.42
-9	5	-11	4.25	0.78
-9	5	-11	3.24	0.89
9	-5	11	1.72	2.34
9	-5	11	4.32	1.17
15	4	-11	7.44	1.71
15	4	-11	6.43	0.87
14	4	-11	9.62	1.65
14	4	-11	9.52	1.12
-14	-4	11	8.50	1.48
13	4	-11	12.39	1.93
13	4	-11	11.01	1.08
-13	-4	11	11.06	1.87

12	4	-11	10.54	1.59
12	4	-11	13.90	1.06
-12	-4	11	11.67	1.71
11	4	-11	38.36	2.23
11	4	-11	39.09	1.68
-11	-4	11	40.07	2.21
10	4	-11	41.94	1.86
10	4	-11	41.09	1.74
10	4	-11	42.58	2.26
-10	-4	11	43.45	2.02
-10	-4	11	43.74	2.21
9	4	-11	15.26	1.47
9	4	-11	13.70	1.06
-9	-4	11	13.10	1.18
-9	-4	11	13.80	1.31
8	4	-11	48.72	1.87
8	4	-11	50.52	2.01
8	4	-11	47.21	2.27
-8	-4	11	50.43	2.07
-8	-4	11	47.36	2.22
7	4	-11	71.90	2.51
7	4	-11	71.67	2.88
7	4	-11	71.31	2.62
-7	-4	11	72.93	2.69
-7	-4	11	69.22	2.82
6	4	-11	32.22	1.71
6	4	-11	31.72	1.44
6	4	-11	32.53	1.34
-6	-4	11	30.05	1.29
-6	-4	11	31.48	1.56
-6	-4	11	31.53	1.63
5	4	-11	39.18	1.87
5	4	-11	40.25	1.67
5	4	-11	39.86	1.56
-5	-4	11	38.20	1.83
-5	-4	11	41.37	1.78
-5	-4	11	38.58	1.48
4	4	-11	33.88	1.67
4	4	-11	34.26	1.37
4	4	-11	33.51	1.49
-4	-4	11	36.40	1.64
-4	-4	11	30.91	1.60
-4	-4	11	32.62	1.32
3	4	-11	22.19	1.18

3	4	-11	22.73	1.08
3	4	-11	21.89	0.97
3	4	-11	25.01	1.34
-3	-4	11	22.79	1.03
-3	-4	11	23.24	1.30
-3	-4	11	24.06	1.39
-3	-4	11	23.99	1.40
2	4	-11	54.83	2.09
2	4	-11	57.26	2.04
2	4	-11	58.02	2.26
2	4	-11	59.30	2.21
-2	-4	11	57.64	2.38
-2	-4	11	55.17	2.00
-2	-4	11	55.38	2.27
-2	-4	11	57.11	2.37
1	4	-11	74.21	2.53
1	4	-11	70.54	2.66
1	4	-11	71.50	2.47
1	4	-11	71.92	2.68
-1	-4	11	73.90	2.88
-1	-4	11	70.17	2.72
-1	-4	11	71.87	2.46
-1	-4	11	70.90	2.90
0	4	-11	25.62	1.30
0	4	-11	28.28	1.39
0	4	-11	25.72	1.38
0	4	-11	26.74	1.17
0	-4	11	27.73	1.42
0	-4	11	25.91	1.20
0	-4	11	24.94	1.71
0	-4	11	27.89	1.70
-1	4	-11	37.95	1.50
-1	4	-11	37.40	1.71
-1	4	-11	39.01	1.68
1	-4	11	37.71	1.77
1	-4	11	40.68	2.08
1	-4	11	38.85	2.19
1	-4	11	33.99	1.49
-2	4	-11	64.19	2.54
-2	4	-11	62.72	2.42
-2	4	-11	65.76	2.30
2	-4	11	64.61	3.11
2	-4	11	67.45	2.85
2	-4	11	61.67	2.56

-3	4	-11	17.55	0.99
-3	4	-11	17.80	1.67
-3	4	-11	19.38	1.19
3	-4	11	19.88	1.96
3	-4	11	20.41	1.30
-4	4	-11	11.81	0.85
-4	4	-11	12.56	1.06
4	-4	11	11.92	1.96
4	-4	11	12.06	1.16
-5	4	-11	28.29	1.33
-5	4	-11	29.45	1.52
5	-4	11	28.56	1.79
5	-4	11	26.62	2.59
-6	4	-11	17.08	1.31
-6	4	-11	16.12	1.09
6	-4	11	20.66	2.71
6	-4	11	17.60	1.43
-7	4	-11	13.71	1.09
-7	4	-11	12.84	1.27
7	-4	11	12.84	1.46
7	-4	11	20.62	2.96
-8	4	-11	7.28	1.06
-8	4	-11	8.06	1.25
8	-4	11	6.04	1.11
8	-4	11	5.80	2.16
-9	4	-11	5.11	0.94
-9	4	-11	5.87	0.85
9	-4	11	6.69	1.18
9	-4	11	3.98	2.24
16	3	-11	4.19	1.62
16	3	-11	8.37	1.39
15	3	-11	5.11	0.78
15	3	-11	5.14	1.54
14	3	-11	4.80	1.31
14	3	-11	3.01	0.66
-14	-3	11	1.12	1.16
13	3	-11	11.99	1.04
13	3	-11	10.59	1.81
-13	-3	11	11.32	1.69
12	3	-11	44.63	1.86
12	3	-11	44.99	1.96
12	3	-11	45.22	2.48
-12	-3	11	43.92	2.35
11	3	-11	38.10	2.21

11	3	-11	38.42	1.59
11	3	-11	34.43	1.69
-11	-3	11	36.88	2.02
10	3	-11	26.92	1.82
10	3	-11	26.65	1.36
10	3	-11	25.63	1.24
-10	-3	11	29.73	1.87
-10	-3	11	25.02	1.65
9	3	-11	46.93	2.29
9	3	-11	46.63	1.78
9	3	-11	45.82	1.85
-9	-3	11	43.72	2.03
-9	-3	11	45.21	2.11
8	3	-11	38.54	2.00
8	3	-11	39.82	1.66
8	3	-11	39.87	1.57
-8	-3	11	39.34	1.84
-8	-3	11	38.80	1.86
7	3	-11	34.29	1.83
7	3	-11	35.36	1.51
7	3	-11	34.23	1.41
-7	-3	11	33.76	1.50
-7	-3	11	33.20	1.69
-7	-3	11	35.75	1.69
6	3	-11	28.46	1.23
6	3	-11	29.38	1.67
6	3	-11	29.03	1.33
-6	-3	11	30.18	1.50
-6	-3	11	28.80	1.52
-6	-3	11	27.95	1.17
5	3	-11	18.19	0.92
5	3	-11	18.44	1.30
5	3	-11	17.01	1.00
-5	-3	11	16.16	1.09
-5	-3	11	19.24	1.24
-5	-3	11	18.02	0.86
4	3	-11	57.18	2.16
4	3	-11	57.50	2.02
4	3	-11	56.35	2.35
-4	-3	11	58.23	2.26
-4	-3	11	56.24	2.32
-4	-3	11	56.41	2.06
-4	-3	11	55.84	1.96
-4	-3	11	55.62	1.96

3	3	-11	31.23	1.27
3	3	-11	32.00	1.59
3	3	-11	28.13	1.38
-3	-3	11	29.82	1.64
-3	-3	11	30.25	1.54
-3	-3	11	30.04	1.21
-3	-3	11	30.40	1.32
-3	-3	11	30.75	1.47
2	3	-11	23.27	1.07
2	3	-11	24.91	1.37
2	3	-11	23.74	1.29
-2	-3	11	23.40	1.06
-2	-3	11	24.00	1.27
-2	-3	11	23.70	1.41
-2	-3	11	25.35	1.53
1	3	-11	17.48	1.02
1	3	-11	18.04	1.14
1	3	-11	19.16	1.18
1	3	-11	18.22	0.92
-1	-3	11	17.43	0.91
-1	-3	11	16.56	1.06
-1	-3	11	17.72	1.30
-1	-3	11	20.54	1.46
0	3	-11	31.90	1.32
0	3	-11	34.17	1.63
0	3	-11	34.48	1.58
0	3	-11	31.51	1.50
0	-3	11	34.49	1.88
0	-3	11	31.72	1.54
0	-3	11	31.78	1.34
0	-3	11	33.31	1.87
-1	3	-11	42.03	1.58
-1	3	-11	40.59	1.80
-1	3	-11	39.23	1.80
1	-3	11	40.67	2.17
1	-3	11	39.98	1.81
1	-3	11	40.90	2.22
1	-3	11	40.87	1.60
-2	3	-11	4.70	0.79
-2	3	-11	6.75	0.65
-2	3	-11	6.02	0.87
2	-3	11	5.70	0.83
2	-3	11	5.79	1.17
2	-3	11	6.05	0.75

2	-3	11	5.30	1.01
-3	3	-11	6.61	0.69
-3	3	-11	7.52	0.91
3	-3	11	5.63	0.89
3	-3	11	5.83	1.27
-4	3	-11	23.40	1.19
4	-3	11	28.73	2.11
4	-3	11	27.17	2.29
-5	3	-11	8.99	0.81
-5	3	-11	8.44	1.04
5	-3	11	9.53	1.14
5	-3	11	11.89	2.16
-6	3	-11	6.92	0.86
-6	3	-11	6.65	1.10
6	-3	11	7.00	0.94
6	-3	11	8.21	1.76
-7	3	-11	12.02	1.08
-7	3	-11	12.35	1.31
7	-3	11	13.36	1.43
7	-3	11	11.21	2.08
-8	3	-11	4.90	0.94
-8	3	-11	2.90	0.75
8	-3	11	4.27	0.98
8	-3	11	4.35	1.97
-9	3	-11	2.15	0.94
-9	3	-11	4.04	0.83
9	-3	11	4.11	1.07
9	-3	11	4.29	2.22
16	2	-11	6.71	1.66
16	2	-11	9.64	1.20
15	2	-11	11.16	1.16
15	2	-11	12.74	1.76
14	2	-11	12.44	1.08
14	2	-11	12.08	1.96
-14	-2	11	10.79	1.71
13	2	-11	7.78	1.29
13	2	-11	8.60	0.91
13	2	-11	9.86	1.40
-13	-2	11	7.75	1.15
12	2	-11	34.27	2.12
12	2	-11	31.48	1.42
12	2	-11	29.72	1.45
-12	-2	11	25.23	1.78
11	2	-11	41.37	1.70

11	2	-11	42.50	2.31
11	2	-11	41.07	1.77
-11	-2	11	46.21	2.13
10	2	-11	46.14	2.34
10	2	-11	46.55	1.86
-10	-2	11	45.91	2.11
9	2	-11	24.73	1.73
9	2	-11	27.43	1.22
9	2	-11	28.10	1.31
-9	-2	11	27.01	1.48
-9	-2	11	28.52	1.64
8	2	-11	57.81	2.61
8	2	-11	61.39	2.15
8	2	-11	60.12	2.23
-8	-2	11	59.87	2.40
-8	-2	11	60.84	2.47
7	2	-11	35.21	1.38
7	2	-11	34.85	1.84
7	2	-11	33.81	1.47
-7	-2	11	34.22	1.32
-7	-2	11	33.03	1.71
-7	-2	11	33.17	1.60
6	2	-11	45.95	2.13
6	2	-11	45.67	1.81
6	2	-11	44.71	1.69
-6	-2	11	44.46	1.89
-6	-2	11	46.66	2.02
-6	-2	11	43.81	1.62
5	2	-11	69.23	2.59
5	2	-11	73.39	2.47
5	2	-11	73.00	2.88
-5	-2	11	71.40	2.67
-5	-2	11	71.83	2.40
-5	-2	11	73.90	2.80
4	2	-11	89.05	3.10
4	2	-11	84.98	3.28
4	2	-11	87.54	2.91
-4	-2	11	88.78	3.13
-4	-2	11	86.16	2.95
-4	-2	11	88.29	2.86
-4	-2	11	86.17	2.96
-4	-2	11	90.40	3.28
3	2	-11	60.55	2.14
3	2	-11	63.57	2.51

3	2	-11	61.24	2.34
-3	-2	11	58.26	2.27
-3	-2	11	64.41	2.58
-3	-2	11	61.38	2.33
-3	-2	11	61.27	2.09
-3	-2	11	59.22	2.33
2	2	-11	50.76	2.11
2	2	-11	52.25	1.85
2	2	-11	50.12	2.16
-2	-2	11	50.63	2.04
-2	-2	11	52.93	2.14
-2	-2	11	53.19	2.33
-2	-2	11	51.42	2.10
-2	-2	11	50.29	1.81
1	2	-11	42.62	1.91
1	2	-11	42.38	1.61
1	2	-11	43.11	1.92
-1	-2	11	44.98	2.00
-1	-2	11	43.64	2.02
-1	-2	11	44.88	2.15
-1	-2	11	42.22	1.81
-1	-2	11	42.97	1.60
0	2	-11	17.38	0.87
0	2	-11	13.42	1.40
0	2	-11	16.28	1.16
0	-2	11	15.64	0.88
0	-2	11	17.91	1.09
0	-2	11	19.01	1.54
0	-2	11	19.03	1.45
0	-2	11	15.94	1.33
-1	2	-11	19.59	0.97
-1	2	-11	21.82	1.28
1	-2	11	20.16	1.20
1	-2	11	23.57	1.66
1	-2	11	19.64	1.01
1	-2	11	19.55	1.61
1	-2	11	20.14	1.65
-2	2	-11	70.75	2.44
-2	2	-11	72.44	2.71
2	-2	11	76.56	3.24
2	-2	11	71.12	2.70
2	-2	11	65.63	3.09
2	-2	11	67.03	2.44
-3	2	-11	21.83	1.07

-3	2	-11	21.79	1.32
3	-2	11	19.65	1.28
3	-2	11	21.84	1.92
-4	2	-11	8.85	0.77
-4	2	-11	11.14	1.08
4	-2	11	9.30	1.66
4	-2	11	9.34	1.03
-5	2	-11	11.93	1.19
5	-2	11	12.17	1.15
5	-2	11	8.02	1.55
-6	2	-11	8.86	0.91
-6	2	-11	9.39	1.22
6	-2	11	4.75	1.56
6	-2	11	8.08	1.16
-7	2	-11	11.25	1.31
-7	2	-11	8.84	1.04
7	-2	11	10.46	2.05
7	-2	11	9.63	1.34
-8	2	-11	3.81	0.89
-8	2	-11	3.77	0.76
8	-2	11	3.90	0.96
8	-2	11	4.75	1.96
-9	2	-11	0.81	0.81
-9	2	-11	0.11	0.95
9	-2	11	-0.19	0.89
9	-2	11	0.39	2.28
16	1	-11	12.97	1.91
16	1	-11	13.82	1.28
15	1	-11	13.60	2.19
15	1	-11	13.42	1.16
14	1	-11	24.49	2.26
14	1	-11	23.19	1.34
-14	-1	11	25.86	1.85
13	1	-11	29.05	1.46
13	1	-11	31.49	2.21
13	1	-11	28.91	1.43
-13	-1	11	33.90	1.90
12	1	-11	35.11	1.49
12	1	-11	30.76	2.14
12	1	-11	34.17	1.52
-12	-1	11	33.43	1.83
11	1	-11	24.41	1.17
11	1	-11	23.80	1.83
11	1	-11	25.20	1.25

-11	-1	11	23.73	1.49
10	1	-11	20.33	1.14
10	1	-11	22.60	1.74
10	1	-11	22.04	1.08
-10	-1	11	23.79	1.36
9	1	-11	54.37	2.55
9	1	-11	54.27	2.07
9	1	-11	56.60	2.00
-9	-1	11	53.21	2.34
-9	-1	11	56.22	2.25
8	1	-11	30.09	1.36
8	1	-11	29.04	1.32
8	1	-11	29.96	1.26
8	1	-11	32.25	1.83
-8	-1	11	29.04	1.44
-8	-1	11	27.89	1.62
7	1	-11	31.11	1.75
7	1	-11	28.72	1.22
7	1	-11	28.77	1.35
-7	-1	11	28.47	1.39
-7	-1	11	31.05	1.60
-7	-1	11	27.99	1.17
6	1	-11	34.22	1.83
6	1	-11	34.91	1.37
6	1	-11	35.59	1.53
-6	-1	11	34.35	1.74
-6	-1	11	33.49	1.52
-6	-1	11	33.54	1.29
5	1	-11	64.21	2.65
5	1	-11	65.34	2.40
5	1	-11	62.42	2.19
-5	-1	11	60.71	2.12
-5	-1	11	65.80	2.60
-5	-1	11	62.46	2.37
4	1	-11	179.07	5.95
4	1	-11	175.54	5.76
4	1	-11	176.87	5.52
-4	-1	11	174.71	5.95
-4	-1	11	183.67	5.65
-4	-1	11	178.60	5.73
-4	-1	11	174.28	5.68
-4	-1	11	173.95	5.47
3	1	-11	42.54	2.06
3	1	-11	47.86	1.98

3	1	-11	45.31	1.68
-3	-1	11	46.21	1.63
-3	-1	11	47.52	1.88
-3	-1	11	44.37	2.16
-3	-1	11	49.22	1.88
2	1	-11	18.36	0.89
2	1	-11	18.11	1.26
2	1	-11	16.59	1.22
-2	-1	11	17.41	1.04
-2	-1	11	20.62	1.49
-2	-1	11	17.62	0.86
-2	-1	11	19.66	1.36
-2	-1	11	18.06	1.15
1	1	-11	23.00	1.37
1	1	-11	22.33	1.00
1	1	-11	22.82	1.42
-1	-1	11	20.11	1.57
-1	-1	11	22.54	1.19
-1	-1	11	22.19	1.00
-1	-1	11	22.95	1.58
-1	-1	11	23.76	1.41
0	1	-11	40.19	1.83
0	1	-11	39.60	1.48
0	-1	11	37.51	2.09
0	-1	11	38.37	1.50
0	-1	11	39.13	1.71
0	-1	11	39.34	1.98
0	-1	11	40.88	2.14
-1	1	-11	34.71	1.33
-1	1	-11	32.36	1.65
1	-1	11	31.39	1.34
1	-1	11	32.72	1.90
1	-1	11	33.93	1.56
1	-1	11	33.46	2.11
-2	1	-11	29.67	1.56
-2	1	-11	26.65	1.22
2	-1	11	30.32	1.49
2	-1	11	27.87	1.28
2	-1	11	31.18	2.43
2	-1	11	33.15	2.00
-3	1	-11	22.12	1.05
-3	1	-11	24.41	1.42
3	-1	11	20.13	1.17
3	-1	11	23.73	1.87

3	-1	11	19.65	1.64
-4	1	-11	28.15	1.57
-4	1	-11	24.32	1.20
4	-1	11	26.89	1.52
4	-1	11	27.75	2.19
-5	1	-11	4.35	0.64
-5	1	-11	3.48	0.79
5	-1	11	3.42	0.71
5	-1	11	0.74	1.16
-6	1	-11	0.52	0.53
-6	1	-11	0.90	0.73
6	-1	11	0.42	0.65
6	-1	11	2.45	1.42
-7	1	-11	2.86	0.86
-7	1	-11	3.88	0.70
7	-1	11	6.12	0.93
7	-1	11	5.22	1.75
-8	1	-11	4.53	0.80
-8	1	-11	2.69	0.89
8	-1	11	1.72	1.87
8	-1	11	4.05	0.96
17	0	-11	8.48	1.85
17	0	-11	14.32	1.71
16	0	-11	7.67	1.56
16	0	-11	8.03	1.06
15	0	-11	23.92	1.37
15	0	-11	24.41	2.39
14	0	-11	48.95	2.88
14	0	-11	51.58	2.02
-14	0	11	48.03	2.35
13	0	-11	47.51	1.89
13	0	-11	50.80	2.71
13	0	-11	46.69	1.94
-13	0	11	48.23	2.22
12	0	-11	17.75	1.02
12	0	-11	18.88	1.13
12	0	-11	14.18	1.72
-12	0	11	17.07	1.35
11	0	-11	46.37	2.27
11	0	-11	50.11	1.93
11	0	-11	46.58	2.45
-11	0	11	47.60	2.06
10	0	-11	39.46	1.57
10	0	-11	39.72	2.23

10	0	-11	41.69	1.71
-10	0	11	41.12	1.81
9	0	-11	22.33	1.22
9	0	-11	23.39	1.08
9	0	-11	23.73	1.64
-9	0	11	23.30	1.50
-9	0	11	23.75	1.28
8	0	-11	51.36	1.82
8	0	-11	48.91	2.34
8	0	-11	49.80	1.88
8	0	-11	48.22	1.96
-8	0	11	48.57	2.23
-8	0	11	50.44	1.99
7	0	-11	18.19	1.45
7	0	-11	20.89	1.13
7	0	-11	20.42	0.95
-7	0	11	19.22	0.89
-7	0	11	19.91	1.09
-7	0	11	18.90	1.34
6	0	-11	45.22	1.65
6	0	-11	47.26	2.19
6	0	-11	45.89	1.87
-6	0	11	42.63	1.80
-6	0	11	44.33	2.08
-6	0	11	45.14	1.59
5	0	-11	73.20	3.01
5	0	-11	74.76	2.51
5	0	-11	78.44	2.80
-5	0	11	76.70	2.74
-5	0	11	74.02	2.68
-5	0	11	74.52	2.96
-5	0	11	71.69	2.44
-5	0	11	74.31	2.49
4	0	-11	29.09	1.67
4	0	-11	29.66	1.53
4	0	-11	30.69	1.22
-4	0	11	31.15	1.63
-4	0	11	30.93	1.76
-4	0	11	29.20	1.37
-4	0	11	30.18	1.16
-4	0	11	30.26	1.30
3	0	-11	30.17	1.59
3	0	-11	28.85	1.19
3	0	-11	27.23	1.61

-3	0	11	29.41	1.65
-3	0	11	28.71	1.34
-3	0	11	29.70	1.34
-3	0	11	29.19	1.75
-3	0	11	29.62	1.14
2	0	-11	45.34	2.12
2	0	-11	46.62	2.10
2	0	-11	47.40	1.69
-2	0	11	46.13	2.31
-2	0	11	48.43	2.28
-2	0	11	48.22	1.90
-2	0	11	45.91	1.65
-2	0	11	45.72	1.93
1	0	-11	46.68	2.13
1	0	-11	46.63	1.73
-1	0	11	51.64	2.45
-1	0	11	48.60	1.73
-1	0	11	47.82	2.38
-1	0	11	50.60	1.99
-1	0	11	50.50	2.11
0	0	-11	14.02	1.16
0	0	-11	15.67	0.81
0	0	11	14.62	1.00
0	0	11	16.35	1.28
0	0	11	18.95	1.73
0	0	11	16.08	0.86
-1	0	-11	13.72	1.15
-1	0	-11	13.10	0.75
1	0	11	14.29	1.89
1	0	11	12.23	0.97
1	0	11	12.28	1.35
1	0	11	12.76	0.84
-2	0	-11	36.58	1.41
2	0	11	34.08	2.04
2	0	11	36.20	1.45
-3	0	-11	17.19	1.27
-3	0	-11	15.25	0.88
3	0	11	16.03	1.18
3	0	11	16.29	1.03
-4	0	-11	5.58	0.84
-4	0	-11	5.94	0.71
4	0	11	4.95	0.82
4	0	11	5.16	1.23
-5	0	-11	6.98	1.15

-5	0	-11	4.41	0.59
5	0	11	4.55	1.38
5	0	11	3.13	0.72
-6	0	-11	0.16	0.54
-6	0	-11	-0.02	0.75
6	0	11	-0.73	1.35
6	0	11	0.63	0.69
-7	0	-11	2.73	0.68
-7	0	-11	0.91	0.79
7	0	11	2.25	1.52
7	0	11	1.55	0.82
-8	0	-11	3.01	0.79
-8	0	-11	0.98	1.00
8	0	11	1.42	0.90
8	0	11	3.06	1.83
17	-1	-11	11.80	1.90
17	-1	-11	10.83	1.26
16	-1	-11	22.64	1.37
16	-1	-11	20.57	2.48
15	-1	-11	16.07	2.16
15	-1	-11	14.92	1.12
14	-1	-11	45.26	2.68
14	-1	-11	40.97	1.76
-14	1	11	39.98	2.01
13	-1	-11	49.50	1.87
13	-1	-11	45.90	2.64
13	-1	-11	46.12	1.95
-13	1	11	44.35	2.07
12	-1	-11	58.42	2.17
12	-1	-11	52.25	2.71
12	-1	-11	54.82	2.02
-12	1	11	51.05	2.22
11	-1	-11	55.72	2.20
11	-1	-11	56.03	2.73
11	-1	-11	54.71	2.04
-11	1	11	58.02	2.25
10	-1	-11	18.52	1.10
10	-1	-11	16.23	1.55
10	-1	-11	15.38	0.88
-10	1	11	14.86	1.03
9	-1	-11	61.58	2.76
9	-1	-11	61.30	2.13
9	-1	-11	62.55	2.35
9	-1	-11	61.62	2.17

-9	1	11	56.99	2.28
8	-1	-11	72.74	2.65
8	-1	-11	72.51	2.45
8	-1	-11	69.94	2.99
8	-1	-11	71.24	2.41
-8	1	11	67.07	2.82
-8	1	11	68.71	2.55
7	-1	-11	43.19	2.17
7	-1	-11	41.61	1.83
7	-1	-11	42.26	1.56
-7	1	11	40.84	1.69
-7	1	11	41.08	2.05
6	-1	-11	22.65	1.56
6	-1	-11	21.80	1.00
6	-1	-11	25.64	1.37
-6	1	11	21.42	1.49
-6	1	11	21.49	0.94
-6	1	11	24.63	1.33
-6	1	11	21.94	1.14
-6	1	11	22.72	0.94
5	-1	-11	35.92	1.91
5	-1	-11	36.17	1.38
5	-1	-11	34.85	1.74
-5	1	11	36.03	1.31
-5	1	11	35.63	1.38
-5	1	11	38.08	1.86
-5	1	11	35.01	1.51
-5	1	11	34.28	1.90
4	-1	-11	18.21	1.36
4	-1	-11	18.23	0.86
4	-1	-11	19.10	1.30
-4	1	11	15.16	0.91
-4	1	11	17.48	0.79
-4	1	11	16.93	0.98
-4	1	11	18.11	1.50
-4	1	11	19.05	1.48
3	-1	-11	36.75	1.98
3	-1	-11	42.16	2.05
3	-1	-11	42.70	1.53
-3	1	11	41.61	1.74
-3	1	11	43.32	1.50
-3	1	11	44.17	2.22
-3	1	11	44.14	1.72
-3	1	11	41.66	2.23

2	-1	-11	14.82	1.61
2	-1	-11	11.83	1.18
2	-1	-11	14.55	0.75
-2	1	11	15.78	1.02
-2	1	11	14.12	0.74
-2	1	11	13.44	0.93
-2	1	11	13.57	1.44
-2	1	11	17.98	1.67
1	-1	-11	21.09	1.44
1	-1	-11	21.35	0.94
-1	1	11	22.55	0.96
-1	1	11	19.66	1.28
-1	1	11	22.87	1.20
-1	1	11	19.76	1.89
0	-1	-11	28.01	1.14
0	-1	-11	29.59	1.59
0	1	11	26.26	1.57
0	1	11	29.22	1.18
0	1	11	27.53	1.41
0	1	11	26.77	2.17
-1	-1	-11	10.40	1.29
-1	-1	-11	11.69	0.69
1	1	11	10.90	1.26
1	1	11	10.03	1.08
1	1	11	11.85	0.79
-2	-1	-11	3.01	0.43
-2	-1	-11	3.57	0.78
2	1	11	2.04	0.56
2	1	11	2.96	0.51
2	1	11	2.57	0.91
-3	-1	-11	7.59	1.11
-3	-1	-11	6.92	0.70
3	1	11	7.19	0.88
3	1	11	7.51	1.01
3	1	11	8.92	1.53
-4	-1	-11	7.29	1.17
-4	-1	-11	4.87	0.73
4	1	11	7.31	1.30
4	1	11	5.36	0.83
-5	-1	-11	2.11	0.80
-5	-1	-11	1.02	0.52
5	1	11	2.01	1.25
5	1	11	0.17	0.63
-6	-1	-11	1.98	0.87

-6	-1	-11	1.20	0.60
6	1	11	2.03	1.53
6	1	11	2.99	0.85
-7	-1	-11	1.49	0.86
-7	-1	-11	2.07	0.73
7	1	11	1.74	0.88
7	1	11	-0.65	1.53
17	-2	-11	5.84	1.73
17	-2	-11	11.44	1.20
16	-2	-11	13.17	2.17
16	-2	-11	5.47	0.78
15	-2	-11	20.31	1.23
15	-2	-11	17.92	2.21
14	-2	-11	34.63	1.55
14	-2	-11	36.50	2.50
14	-2	-11	31.27	2.19
-14	2	11	37.05	1.77
13	-2	-11	43.19	2.54
13	-2	-11	45.05	1.74
13	-2	-11	40.60	1.97
-13	2	11	40.55	1.89
12	-2	-11	17.66	1.01
12	-2	-11	18.89	1.84
12	-2	-11	19.96	1.36
-12	2	11	19.25	1.19
11	-2	-11	39.95	1.81
11	-2	-11	37.43	1.46
11	-2	-11	32.84	3.11
-11	2	11	34.12	1.61
10	-2	-11	40.63	1.54
10	-2	-11	36.38	2.16
10	-2	-11	41.10	1.87
-10	2	11	39.70	1.67
9	-2	-11	30.74	1.27
9	-2	-11	32.62	1.38
9	-2	-11	31.38	1.66
9	-2	-11	34.97	1.99
-9	2	11	30.78	1.41
8	-2	-11	35.35	1.42
8	-2	-11	33.90	1.74
8	-2	-11	35.31	2.01
8	-2	-11	35.23	1.37
-8	2	11	35.42	1.53
-8	2	11	34.30	1.49

7	-2	-11	57.72	2.44
7	-2	-11	57.98	2.03
7	-2	-11	57.98	2.64
7	-2	-11	59.60	2.09
-7	2	11	59.88	2.18
-7	2	11	58.38	2.36
-7	2	11	57.68	2.54
6	-2	-11	37.76	1.86
6	-2	-11	36.29	1.99
6	-2	-11	39.59	1.41
-6	2	11	36.45	1.97
-6	2	11	35.26	1.39
-6	2	11	34.70	1.33
-6	2	11	37.86	1.56
-6	2	11	35.93	1.93
5	-2	-11	37.62	1.98
5	-2	-11	35.87	1.36
5	-2	-11	35.49	1.90
-5	2	11	36.48	1.55
-5	2	11	35.70	1.44
-5	2	11	35.23	1.30
-5	2	11	38.07	2.06
-5	2	11	37.15	2.02
4	-2	-11	17.98	1.41
4	-2	-11	18.56	0.86
4	-2	-11	16.78	1.47
-4	2	11	20.63	1.55
-4	2	11	17.84	1.03
-4	2	11	19.45	1.73
-4	2	11	17.84	0.79
-4	2	11	17.07	0.98
3	-2	-11	13.13	1.31
3	-2	-11	14.62	1.51
3	-2	-11	14.87	0.77
-3	2	11	16.20	1.62
-3	2	11	16.77	0.99
-3	2	11	14.09	0.96
-3	2	11	15.20	0.72
-3	2	11	14.76	1.75
2	-2	-11	23.79	0.98
2	-2	-11	23.27	1.52
-2	2	11	23.94	0.97
-2	2	11	22.81	1.30
-2	2	11	23.17	2.05

-2	2	11	22.47	1.24
1	-2	-11	21.78	1.46
1	-2	-11	22.87	0.96
-1	2	11	24.26	0.99
-1	2	11	24.29	1.37
-1	2	11	22.07	1.26
-1	2	11	22.03	2.13
0	-2	-11	10.91	1.15
0	-2	-11	11.14	0.66
0	2	11	10.66	1.08
0	2	11	10.37	1.12
0	2	11	10.34	1.57
0	2	11	10.76	0.70
-1	-2	-11	8.43	1.11
-1	-2	-11	8.37	0.60
1	2	11	7.74	1.22
1	2	11	7.70	0.96
1	2	11	7.58	0.71
-2	-2	-11	9.29	0.66
-2	-2	-11	9.64	1.19
2	2	11	9.68	1.09
2	2	11	8.38	1.17
2	2	11	10.19	0.83
-3	-2	-11	1.98	0.46
-3	-2	-11	2.68	0.79
3	2	11	2.85	0.74
3	2	11	2.32	0.99
3	2	11	1.75	0.55
-4	-2	-11	1.20	0.76
-4	-2	-11	0.12	0.46
4	2	11	-0.26	1.07
4	2	11	0.72	0.69
-5	-2	-11	1.68	0.55
-5	-2	-11	2.04	0.84
5	2	11	2.43	0.84
5	2	11	-0.14	1.29
-6	-2	-11	-0.20	0.82
-6	-2	-11	-0.40	0.58
6	2	11	0.05	0.78
6	2	11	1.90	1.48
-7	-2	-11	-0.46	0.94
7	2	11	0.28	0.86
7	2	11	1.96	1.78
17	-3	-11	22.10	2.57

17	-3	-11	21.59	1.41
16	-3	-11	35.75	2.76
16	-3	-11	35.78	1.71
15	-3	-11	24.56	2.41
15	-3	-11	27.27	1.40
14	-3	-11	28.63	2.21
14	-3	-11	32.59	2.41
14	-3	-11	33.83	1.47
-14	3	11	30.32	1.58
13	-3	-11	33.27	1.41
13	-3	-11	31.22	2.01
13	-3	-11	28.93	1.82
13	-3	-11	35.14	2.36
-13	3	11	33.23	1.57
-13	3	11	31.73	1.43
12	-3	-11	38.05	2.01
12	-3	-11	37.78	2.37
12	-3	-11	32.03	1.89
12	-3	-11	34.44	1.71
-12	3	11	36.65	1.48
-12	3	11	38.26	1.62
11	-3	-11	37.45	1.48
11	-3	-11	36.69	1.96
11	-3	-11	37.70	2.28
-11	3	11	38.18	1.62
-11	3	11	37.90	1.55
10	-3	-11	23.73	1.62
10	-3	-11	24.84	1.81
10	-3	-11	22.20	1.05
10	-3	-11	24.27	1.12
-10	3	11	22.77	1.24
-10	3	11	22.58	1.16
9	-3	-11	42.77	1.61
9	-3	-11	41.29	2.08
9	-3	-11	44.01	1.93
9	-3	-11	41.74	2.27
9	-3	-11	42.36	1.56
-9	3	11	41.61	1.84
-9	3	11	40.21	1.67
8	-3	-11	64.94	2.45
8	-3	-11	66.41	2.96
8	-3	-11	68.58	2.25
8	-3	-11	59.64	2.73
8	-3	-11	67.48	2.28

-8	3	11	66.68	2.67
-8	3	11	65.62	2.38
7	-3	-11	30.00	1.50
7	-3	-11	30.77	1.92
7	-3	-11	31.82	1.25
7	-3	-11	32.60	1.88
7	-3	-11	32.27	1.31
-7	3	11	32.51	1.41
6	-3	-11	21.57	1.64
6	-3	-11	20.90	0.92
6	-3	-11	20.56	1.60
6	-3	-11	20.38	1.24
-6	3	11	17.69	1.67
-6	3	11	20.64	1.10
5	-3	-11	12.48	1.36
5	-3	-11	14.39	1.50
5	-3	-11	14.21	0.73
-5	3	11	12.95	0.89
-5	3	11	15.09	1.73
-5	3	11	12.87	0.66
4	-3	-11	30.41	1.16
4	-3	-11	27.55	2.00
4	-3	-11	29.47	1.77
-4	3	11	29.41	1.41
-4	3	11	29.34	1.38
-4	3	11	31.18	2.24
-4	3	11	28.69	1.09
3	-3	-11	38.72	1.98
3	-3	-11	37.21	1.36
-3	3	11	37.74	1.68
-3	3	11	36.62	1.32
-3	3	11	39.05	2.55
-3	3	11	37.25	1.69
2	-3	-11	11.83	1.24
2	-3	-11	12.94	0.68
-2	3	11	13.35	1.07
-2	3	11	14.71	2.15
-2	3	11	13.77	0.68
-2	3	11	13.44	1.01
1	-3	-11	13.02	0.67
1	-3	-11	11.45	1.37
-1	3	11	12.09	1.14
-1	3	11	14.18	1.23
-1	3	11	13.33	1.86

-1	3	11	13.30	0.71
0	-3	-11	14.57	1.31
0	-3	-11	13.13	0.71
0	3	11	14.82	1.24
0	3	11	15.04	1.18
0	3	11	14.85	0.78
-1	-3	-11	11.06	1.24
-1	-3	-11	9.65	0.64
1	3	11	10.13	0.75
1	3	11	4.59	1.46
1	3	11	8.93	1.08
-2	-3	-11	1.60	0.73
-2	-3	-11	2.04	0.41
2	3	11	3.17	0.77
2	3	11	2.31	0.91
2	3	11	2.04	0.52
-3	-3	-11	6.99	0.71
-3	-3	-11	7.29	1.02
3	3	11	4.72	0.90
3	3	11	5.47	1.19
-4	-3	-11	8.69	1.33
-4	-3	-11	5.95	0.78
4	3	11	7.20	1.05
4	3	11	4.15	1.32
-5	-3	-11	0.10	0.80
-5	-3	-11	-0.14	0.52
5	3	11	-0.34	0.79
5	3	11	0.29	1.30
-6	-3	-11	0.74	0.90
6	3	11	1.48	1.55
6	3	11	-0.36	0.91
17	-4	-11	20.98	1.38
17	-4	-11	24.33	2.68
16	-4	-11	15.07	1.20
16	-4	-11	18.01	2.42
15	-4	-11	33.52	1.58
15	-4	-11	35.90	2.69
14	-4	-11	44.02	1.81
14	-4	-11	47.08	2.84
14	-4	-11	44.36	2.60
-14	4	11	43.95	1.86
13	-4	-11	27.57	2.23
13	-4	-11	24.38	1.85
13	-4	-11	28.27	1.28

13	-4	-11	25.33	2.00
-13	4	11	30.11	1.47
-13	4	11	28.58	1.40
12	-4	-11	22.73	1.11
12	-4	-11	23.46	2.01
12	-4	-11	23.71	1.83
12	-4	-11	17.35	1.77
-12	4	11	24.49	1.38
-12	4	11	23.93	1.19
11	-4	-11	36.04	1.37
11	-4	-11	35.62	2.21
11	-4	-11	29.85	2.01
11	-4	-11	34.77	1.92
-11	4	11	34.14	1.47
-11	4	11	29.37	2.37
10	-4	-11	62.98	3.11
10	-4	-11	79.91	2.62
10	-4	-11	78.53	2.57
10	-4	-11	83.42	3.02
-10	4	11	78.12	2.69
-10	4	11	71.36	3.61
9	-4	-11	48.38	2.51
9	-4	-11	43.68	2.44
9	-4	-11	49.91	1.76
9	-4	-11	50.83	1.81
9	-4	-11	49.22	2.20
-9	4	11	50.44	1.90
-9	4	11	48.96	2.34
8	-4	-11	28.18	1.96
8	-4	-11	30.68	1.21
8	-4	-11	29.00	1.66
8	-4	-11	32.10	1.26
8	-4	-11	27.77	1.87
-8	4	11	29.42	1.99
-8	4	11	30.44	1.35
7	-4	-11	39.82	1.97
7	-4	-11	45.78	1.61
7	-4	-11	44.87	2.32
7	-4	-11	39.88	2.34
7	-4	-11	43.28	1.55
-7	4	11	42.55	1.75
-7	4	11	43.26	2.48
6	-4	-11	16.04	0.81
6	-4	-11	15.29	1.47

6	-4	-11	15.02	1.73
6	-4	-11	16.13	1.34
6	-4	-11	17.19	0.87
-6	4	11	15.45	0.98
-6	4	11	18.22	1.96
5	-4	-11	17.61	1.40
5	-4	-11	19.23	2.12
5	-4	-11	14.93	1.44
5	-4	-11	17.05	0.82
-5	4	11	19.21	2.07
-5	4	11	17.42	1.08
4	-4	-11	23.93	1.65
4	-4	-11	24.37	0.99
-4	4	11	24.58	1.33
-4	4	11	24.73	0.94
-4	4	11	24.61	2.37
3	-4	-11	29.86	1.78
3	-4	-11	29.29	1.11
-3	4	11	31.62	1.56
-3	4	11	28.86	2.62
-3	4	11	29.49	1.53
-3	4	11	30.42	1.09
2	-4	-11	22.72	0.90
-2	4	11	21.06	1.67
-2	4	11	21.33	1.38
-2	4	11	22.58	0.88
-2	4	11	21.84	2.59
1	-4	-11	10.59	1.22
1	-4	-11	9.28	0.56
-1	4	11	9.24	0.59
-1	4	11	9.26	1.01
-1	4	11	8.33	1.12
0	-4	-11	1.60	0.75
0	-4	-11	1.53	0.33
0	4	11	1.18	0.37
0	4	11	0.62	0.53
0	4	11	1.12	0.68
-1	-4	-11	3.21	0.42
-1	-4	-11	4.42	0.86
1	4	11	4.12	0.64
1	4	11	2.78	0.86
1	4	11	4.95	0.86
-2	-4	-11	1.34	0.41
-2	-4	-11	1.50	0.80

2	4	11	1.04	0.94
2	4	11	1.20	0.69
-3	-4	-11	1.50	0.44
-3	-4	-11	2.11	0.81
3	4	11	1.59	0.79
3	4	11	0.46	1.07
-4	-4	-11	2.04	0.57
-4	-4	-11	2.44	0.93
4	4	11	3.28	1.32
4	4	11	1.68	0.89
-5	-4	-11	0.23	0.90
5	4	11	-0.73	0.86
5	4	11	-0.71	1.28
17	-5	-11	16.42	2.68
17	-5	-11	21.52	1.40
16	-5	-11	22.07	1.36
16	-5	-11	24.34	2.57
15	-5	-11	10.60	1.00
15	-5	-11	13.19	1.93
15	-5	-11	11.36	1.73
14	-5	-11	25.22	1.26
14	-5	-11	25.31	2.34
14	-5	-11	22.13	2.30
-14	5	11	23.51	1.28
13	-5	-11	20.78	1.07
13	-5	-11	18.41	2.05
13	-5	-11	19.77	2.13
13	-5	-11	17.93	2.02
-13	5	11	20.03	1.54
-13	5	11	20.52	1.15
12	-5	-11	26.06	1.98
12	-5	-11	26.22	2.09
12	-5	-11	23.77	1.12
12	-5	-11	22.26	1.99
-12	5	11	23.94	1.20
-12	5	11	24.05	1.70
11	-5	-11	34.12	2.20
11	-5	-11	29.11	2.16
11	-5	-11	30.96	2.05
11	-5	-11	34.19	1.33
-11	5	11	32.38	1.42
-11	5	11	33.16	2.01
10	-5	-11	27.14	1.83
10	-5	-11	25.95	1.18

10	-5	-11	21.65	1.98
10	-5	-11	23.59	1.09
10	-5	-11	25.98	1.95
-10	5	11	25.88	1.21
-10	5	11	26.45	1.99
9	-5	-11	33.10	1.93
9	-5	-11	27.26	2.20
9	-5	-11	27.95	2.97
9	-5	-11	33.45	1.33
9	-5	-11	33.95	1.28
-9	5	11	34.18	2.24
-9	5	11	31.93	1.42
8	-5	-11	18.35	1.92
8	-5	-11	23.49	1.04
8	-5	-11	23.12	1.65
8	-5	-11	20.19	1.76
8	-5	-11	21.60	0.98
-8	5	11	21.58	1.16
-8	5	11	25.43	2.16
7	-5	-11	24.11	1.86
7	-5	-11	20.72	2.06
7	-5	-11	25.91	1.73
7	-5	-11	27.93	1.14
7	-5	-11	27.72	1.09
-7	5	11	31.44	2.38
-7	5	11	26.39	1.32
6	-5	-11	24.70	1.05
6	-5	-11	22.81	1.77
6	-5	-11	22.29	1.69
6	-5	-11	24.60	1.00
-6	5	11	24.32	1.30
-6	5	11	24.93	2.39
5	-5	-11	14.28	0.72
5	-5	-11	13.30	1.45
5	-5	-11	16.00	0.82
5	-5	-11	15.30	1.51
-5	5	11	13.95	1.07
-5	5	11	12.49	2.24
4	-5	-11	23.41	0.91
4	-5	-11	19.65	1.70
4	-5	-11	23.51	1.77
-4	5	11	22.41	1.33
-4	5	11	23.22	2.52
3	-5	-11	21.60	0.88

3	-5	-11	22.28	1.65
-3	5	11	22.37	2.66
-3	5	11	22.82	0.86
-3	5	11	22.53	1.42
2	-5	-11	4.25	0.90
2	-5	-11	4.98	0.45
-2	5	11	5.60	0.89
-2	5	11	4.17	0.69
-2	5	11	4.56	0.44
1	-5	-11	1.56	0.30
1	-5	-11	1.88	0.77
-1	5	11	1.77	0.67
-1	5	11	1.79	0.30
-1	5	11	0.78	0.70
0	-5	-11	2.99	0.82
0	-5	-11	2.45	0.35
0	5	11	4.12	0.89
0	5	11	2.19	0.70
0	5	11	4.79	0.71
-1	-5	-11	0.70	0.80
-1	-5	-11	0.70	0.33
1	5	11	2.41	0.91
1	5	11	0.62	0.73
-2	-5	-11	1.28	0.84
-2	-5	-11	0.49	0.38
2	5	11	0.93	0.78
2	5	11	1.51	0.96
-3	-5	-11	-0.33	0.86
3	5	11	1.02	0.78
3	5	11	0.86	1.12
-4	-5	-11	2.32	0.99
4	5	11	-0.87	0.90
4	5	11	1.13	1.24
-5	-5	-11	-3.27	1.53
5	5	11	0.55	1.00
5	5	11	0.01	1.55
17	-6	-11	28.26	2.87
17	-6	-11	26.55	1.55
16	-6	-11	5.20	1.55
16	-6	-11	8.25	1.07
15	-6	-11	16.46	1.13
15	-6	-11	13.06	2.17
15	-6	-11	16.44	2.59
14	-6	-11	25.26	2.44

14	-6	-11	32.62	1.43
14	-6	-11	30.55	2.64
-14	6	11	30.82	1.44
13	-6	-11	9.05	0.82
13	-6	-11	9.77	1.56
13	-6	-11	8.59	1.52
-13	6	11	8.35	1.26
-13	6	11	9.60	0.85
12	-6	-11	14.96	1.98
12	-6	-11	16.51	0.93
12	-6	-11	16.69	1.97
12	-6	-11	19.54	1.96
-12	6	11	15.45	1.00
-12	6	11	19.21	1.88
11	-6	-11	44.08	1.65
11	-6	-11	45.67	1.72
11	-6	-11	44.97	2.53
11	-6	-11	47.60	2.62
11	-6	-11	38.70	2.60
-11	6	11	45.13	2.61
-11	6	11	43.78	1.75
10	-6	-11	34.95	2.23
10	-6	-11	32.59	2.45
10	-6	-11	37.08	1.42
10	-6	-11	34.73	2.30
10	-6	-11	34.43	1.34
-10	6	11	33.97	2.47
-10	6	11	32.82	1.48
9	-6	-11	9.22	0.67
9	-6	-11	8.97	1.48
9	-6	-11	9.36	1.29
9	-6	-11	7.56	0.76
9	-6	-11	10.31	1.49
-9	6	11	12.40	2.15
-9	6	11	11.03	0.86
8	-6	-11	8.73	0.65
8	-6	-11	8.57	0.72
8	-6	-11	8.30	1.53
8	-6	-11	9.80	1.63
8	-6	-11	11.32	1.94
-8	6	11	8.93	0.84
-8	6	11	7.68	1.60
7	-6	-11	4.86	1.06
7	-6	-11	4.41	0.61

7	-6	-11	3.57	0.52
7	-6	-11	3.75	1.66
-7	6	11	3.57	0.59
-7	6	11	2.61	1.45
6	-6	-11	48.81	1.66
6	-6	-11	49.79	1.68
6	-6	-11	42.76	2.80
6	-6	-11	46.94	2.52
-6	6	11	50.73	3.27
-6	6	11	48.42	2.06
5	-6	-11	44.73	1.49
5	-6	-11	38.90	2.29
5	-6	-11	42.65	2.38
5	-6	-11	40.93	1.45
-5	6	11	40.48	1.91
-5	6	11	43.31	3.11
4	-6	-11	7.24	0.59
4	-6	-11	4.88	1.13
4	-6	-11	6.27	0.45
4	-6	-11	6.34	1.13
-4	6	11	4.97	0.81
-4	6	11	6.87	1.76
3	-6	-11	4.42	0.98
3	-6	-11	3.16	1.10
-3	6	11	3.58	0.74
2	-6	-11	4.33	0.99
-2	6	11	6.11	0.97
-2	6	11	5.02	0.84
1	-6	-11	1.24	0.77
-1	6	11	1.16	0.68
-1	6	11	1.10	0.77
0	-6	-11	1.38	0.87
0	6	11	-0.09	0.69
0	6	11	1.25	0.91
-1	-6	-11	-0.73	0.82
1	6	11	0.17	0.81
1	6	11	0.10	0.95
-2	-6	-11	-0.09	0.85
2	6	11	0.54	0.85
2	6	11	-0.10	1.08
-3	-6	-11	0.93	0.94
3	6	11	1.13	0.90
3	6	11	-0.11	1.17
-4	-6	-11	0.40	1.03

4	6	11	1.06	1.07
4	6	11	0.57	1.38
17	-7	-11	22.78	2.79
17	-7	-11	26.86	1.53
16	-7	-11	29.19	2.73
16	-7	-11	28.65	1.51
15	-7	-11	2.44	1.34
15	-7	-11	3.94	0.68
15	-7	-11	3.49	1.79
14	-7	-11	12.07	1.86
14	-7	-11	8.17	1.55
14	-7	-11	9.07	0.87
-14	7	11	8.43	0.91
13	-7	-11	14.09	2.06
13	-7	-11	16.25	1.06
13	-7	-11	16.11	2.34
-13	7	11	16.31	1.04
-13	7	11	16.85	2.13
12	-7	-11	28.46	2.44
12	-7	-11	30.79	2.51
12	-7	-11	28.19	2.36
12	-7	-11	30.64	1.28
-12	7	11	29.22	2.47
-12	7	11	30.50	1.37
11	-7	-11	12.61	2.14
11	-7	-11	16.69	2.13
11	-7	-11	18.07	0.91
11	-7	-11	16.06	1.03
11	-7	-11	18.31	2.00
-11	7	11	17.23	2.32
-11	7	11	17.23	1.04
10	-7	-11	27.43	2.18
10	-7	-11	24.83	2.24
10	-7	-11	25.44	1.12
10	-7	-11	22.67	2.52
10	-7	-11	28.26	1.22
-10	7	11	31.57	2.68
-10	7	11	27.95	1.33
9	-7	-11	4.38	1.18
9	-7	-11	4.71	1.35
9	-7	-11	4.55	1.13
9	-7	-11	3.84	0.59
9	-7	-11	5.23	0.68
-9	7	11	5.03	1.62

-9	7	11	4.35	0.77
8	-7	-11	5.28	1.23
8	-7	-11	5.77	0.65
8	-7	-11	5.65	1.20
8	-7	-11	3.83	0.54
-8	7	11	4.95	0.85
-8	7	11	3.25	1.61
7	-7	-11	7.31	0.62
7	-7	-11	6.82	0.57
7	-7	-11	7.63	1.21
7	-7	-11	8.16	1.34
-7	7	11	2.31	1.62
-7	7	11	4.83	0.72
6	-7	-11	13.73	0.70
6	-7	-11	13.37	1.69
6	-7	-11	16.17	0.76
6	-7	-11	12.38	1.79
-6	7	11	18.93	2.90
-6	7	11	12.23	1.14
5	-7	-11	7.83	1.26
5	-7	-11	6.58	1.33
-5	7	11	6.68	0.91
4	-7	-11	6.48	1.28
4	-7	-11	5.87	1.05
-4	7	11	6.82	1.09
3	-7	-11	4.05	0.97
-3	7	11	3.53	0.77
2	-7	-11	1.48	0.85
-2	7	11	0.36	0.70
-2	7	11	0.32	0.79
1	-7	-11	0.84	0.86
-1	7	11	1.79	0.85
-1	7	11	1.50	0.94
0	-7	-11	1.00	0.97
0	7	11	-0.54	0.81
0	7	11	-0.60	0.96
-1	-7	-11	1.00	0.90
1	7	11	-0.80	0.80
1	7	11	-0.35	1.05
-2	-7	-11	0.46	0.92
2	7	11	-0.56	1.20
2	7	11	0.18	0.96
-3	-7	-11	-1.31	0.94
3	7	11	-0.40	0.96

3	7	11	0.71	1.27
17	-8	-11	23.55	2.99
17	-8	-11	26.42	1.53
16	-8	-11	19.08	1.29
16	-8	-11	13.74	2.09
15	-8	-11	24.25	3.11
15	-8	-11	18.90	1.24
15	-8	-11	17.19	2.47
14	-8	-11	51.94	3.45
14	-8	-11	39.75	3.00
14	-8	-11	47.90	1.90
-14	8	11	52.69	2.00
13	-8	-11	18.42	2.56
13	-8	-11	18.88	1.43
13	-8	-11	17.05	2.20
-13	8	11	16.13	1.05
12	-8	-11	3.99	1.31
12	-8	-11	4.09	0.54
12	-8	-11	5.20	1.68
12	-8	-11	3.46	1.52
-12	8	11	4.15	0.74
-12	8	11	5.18	1.77
11	-8	-11	28.68	1.35
11	-8	-11	29.54	1.22
11	-8	-11	28.91	2.33
11	-8	-11	27.48	2.54
11	-8	-11	24.97	2.69
-11	8	11	30.69	2.83
-11	8	11	30.00	1.41
10	-8	-11	34.72	2.47
10	-8	-11	28.60	2.92
10	-8	-11	36.61	2.71
10	-8	-11	37.60	1.40
10	-8	-11	37.76	1.48
-10	8	11	36.40	3.14
-10	8	11	38.93	1.70
9	-8	-11	16.09	2.04
9	-8	-11	13.94	0.81
9	-8	-11	14.35	1.88
9	-8	-11	16.06	0.88
-9	8	11	12.97	2.29
-9	8	11	14.26	1.14
8	-8	-11	16.54	1.87
8	-8	-11	15.56	0.82

8	-8	-11	13.40	2.02
8	-8	-11	14.93	0.79
-8	8	11	14.96	2.24
-8	8	11	14.60	1.17
7	-8	-11	3.25	1.20
7	-8	-11	1.32	0.35
7	-8	-11	3.31	1.11
7	-8	-11	2.34	0.38
6	-8	-11	2.27	1.19
6	-8	-11	2.50	1.03
-6	8	11	3.95	0.77
5	-8	-11	8.74	1.53
5	-8	-11	10.06	1.39
-5	8	11	10.49	1.26
4	-8	-11	1.80	0.98
4	-8	-11	1.87	1.25
-4	8	11	1.74	0.70
3	-8	-11	0.46	1.20
3	-8	-11	0.18	0.92
-3	8	11	1.16	0.76
2	-8	-11	0.09	0.89
-2	8	11	0.53	0.75
1	-8	-11	3.40	1.08
-1	8	11	1.97	1.03
-1	8	11	1.66	0.91
0	-8	-11	-0.24	0.90
0	8	11	1.48	1.14
0	8	11	0.53	0.86
-1	-8	-11	2.15	1.00
1	8	11	0.71	0.93
1	8	11	1.54	1.22
-2	-8	-11	0.06	1.01
2	8	11	3.22	1.40
2	8	11	1.31	1.14
17	-9	-11	9.46	2.74
17	-9	-11	16.09	1.36
16	-9	-11	23.70	1.44
16	-9	-11	20.59	2.88
15	-9	-11	30.56	1.52
15	-9	-11	30.96	2.86
15	-9	-11	30.20	3.44
14	-9	-11	15.25	2.33
14	-9	-11	14.41	2.32
14	-9	-11	11.87	1.00

13	-9	-11	17.74	2.43
13	-9	-11	19.85	1.09
13	-9	-11	17.58	2.86
-13	9	11	18.16	1.14
12	-9	-11	5.61	1.41
12	-9	-11	12.08	1.99
12	-9	-11	7.83	0.81
-12	9	11	7.72	0.92
11	-9	-11	11.26	1.89
11	-9	-11	12.54	1.99
11	-9	-11	8.41	0.89
11	-9	-11	6.13	2.20
11	-9	-11	9.98	0.76
-11	9	11	8.52	1.01
10	-9	-11	16.28	2.49
10	-9	-11	15.36	2.14
10	-9	-11	16.83	0.96
10	-9	-11	14.50	0.88
-10	9	11	14.39	1.17
9	-9	-11	9.78	1.52
9	-9	-11	9.05	0.89
9	-9	-11	9.12	1.67
9	-9	-11	9.69	0.70
-9	9	11	9.49	1.07
8	-9	-11	8.47	0.63
8	-9	-11	6.96	1.53
8	-9	-11	8.44	1.45
-8	9	11	7.89	1.12
7	-9	-11	2.99	1.16
7	-9	-11	6.47	1.49
-7	9	11	7.93	1.16
6	-9	-11	0.19	1.02
6	-9	-11	0.97	1.20
5	-9	-11	-1.41	1.18
5	-9	-11	-1.05	0.95
-5	9	11	0.69	0.67
4	-9	-11	1.49	1.02
4	-9	-11	-0.46	1.34
-4	9	11	1.29	0.82
3	-9	-11	3.43	1.14
3	-9	-11	2.13	1.48
-3	9	11	3.29	1.03
2	-9	-11	0.81	1.44
2	-9	-11	0.56	1.01

-2	9	11	0.04	0.80
1	-9	-11	1.78	1.07
-1	9	11	2.55	1.21
-1	9	11	2.62	1.05
0	-9	-11	1.71	1.06
0	9	11	1.63	1.03
0	9	11	0.58	1.22
-1	-9	-11	-1.50	1.03
1	9	11	-0.74	1.11
1	9	11	1.66	1.27
16	-10	-11	8.08	0.93
16	-10	-11	6.35	1.79
15	-10	-11	21.69	3.53
15	-10	-11	23.52	2.84
15	-10	-11	20.62	1.33
14	-10	-11	15.97	2.54
14	-10	-11	12.45	1.03
13	-10	-11	9.39	1.77
13	-10	-11	12.65	0.97
13	-10	-11	15.26	2.36
-13	10	11	14.05	1.09
12	-10	-11	4.09	1.77
12	-10	-11	2.09	0.51
12	-10	-11	1.51	1.38
-12	10	11	3.32	0.70
11	-10	-11	3.47	1.74
11	-10	-11	3.27	0.50
11	-10	-11	3.33	1.35
-11	10	11	3.81	0.74
10	-10	-11	7.62	0.74
10	-10	-11	7.75	1.57
10	-10	-11	9.91	2.24
9	-10	-11	2.15	0.43
9	-10	-11	3.03	1.34
-9	10	11	1.40	0.76
8	-10	-11	-0.70	1.30
8	-10	-11	-0.71	1.12
-8	10	11	0.24	0.71
7	-10	-11	-0.18	1.10
7	-10	-11	0.54	1.42
-7	10	11	1.13	0.75
6	-10	-11	-0.42	1.39
6	-10	-11	-0.21	1.12
-6	10	11	0.44	0.78

5	-10	-11	-1.09	1.40
5	-10	-11	1.67	1.16
-5	10	11	0.06	0.82
4	-10	-11	0.24	1.16
4	-10	-11	-0.29	1.52
-4	10	11	0.40	0.87
3	-10	-11	-1.42	0.99
3	-10	-11	-0.37	1.49
-3	10	11	0.85	0.90
2	-10	-11	-0.53	1.58
2	-10	-11	-0.64	1.06
-2	10	11	-0.18	0.89
1	-10	-11	3.43	1.14
-1	10	11	2.03	1.26
-1	10	11	3.65	1.18
0	-10	-11	3.62	1.22
0	10	11	2.19	1.39
15	-11	-11	9.38	1.95
15	-11	-11	11.44	2.69
15	-11	-11	11.90	1.18
14	-11	-11	2.74	1.58
14	-11	-11	1.91	2.11
14	-11	-11	2.69	0.64
13	-11	-11	5.60	0.85
13	-11	-11	4.04	2.13
13	-11	-11	4.07	1.73
-13	11	11	3.96	0.76
12	-11	-11	7.49	0.85
12	-11	-11	6.73	1.75
12	-11	-11	5.73	2.17
-12	11	11	8.67	1.17
11	-11	-11	9.80	2.09
11	-11	-11	8.29	1.74
-11	11	11	7.05	1.19
10	-11	-11	0.64	1.40
10	-11	-11	1.70	1.78
-10	11	11	-0.23	0.70
9	-11	-11	3.09	1.71
9	-11	-11	1.56	1.40
-9	11	11	3.92	0.94
8	-11	-11	-0.09	1.59
8	-11	-11	1.38	1.33
-8	11	11	-0.22	0.80
7	-11	-11	1.01	1.65

7	-11	-11	0.54	1.25
-7	11	11	1.25	0.85
6	-11	-11	3.75	1.31
6	-11	-11	4.59	1.83
-6	11	11	4.42	1.14
5	-11	-11	0.59	1.62
5	-11	-11	2.63	1.30
-5	11	11	3.08	1.05
4	-11	-11	1.38	1.25
4	-11	-11	2.94	1.69
-4	11	11	3.15	1.06
3	-11	-11	0.71	1.76
3	-11	-11	0.99	1.13
-3	11	11	0.94	1.08
2	-11	-11	2.85	1.90
2	-11	-11	1.47	1.22
-2	11	11	2.23	1.17
15	-12	-11	11.17	2.80
14	-12	-11	2.87	2.36
14	-12	-11	1.99	1.73
13	-12	-11	-2.09	1.63
13	-12	-11	0.25	2.20
-13	12	11	1.29	0.88
12	-12	-11	1.46	2.20
12	-12	-11	5.36	1.75
-12	12	11	2.73	0.91
11	-12	-11	6.79	2.18
11	-12	-11	5.62	1.80
-11	12	11	6.84	1.06
10	-12	-11	3.02	1.58
10	-12	-11	3.39	2.07
-10	12	11	0.76	0.86
9	-12	-11	0.42	1.94
9	-12	-11	3.54	1.57
-9	12	11	2.95	0.98
8	-12	-11	0.83	1.39
8	-12	-11	1.90	1.82
-8	12	11	1.23	0.98
7	-12	-11	-0.66	1.31
7	-12	-11	0.66	1.88
-7	12	11	-0.82	0.94
6	-12	-11	0.08	1.79
6	-12	-11	-2.46	1.25
-6	12	11	0.26	1.07

5	-12	-11	-0.83	1.81
5	-12	-11	0.97	1.34
-5	12	11	0.32	1.06
4	-12	-11	7.24	2.16
4	-12	-11	5.19	1.42
-4	12	11	4.38	1.35
13	-13	-11	0.43	1.87
13	-13	-11	2.94	2.57
12	-13	-11	6.39	1.85
12	-13	-11	3.84	2.41
-12	13	11	1.88	0.97
11	-13	-11	3.03	1.70
11	-13	-11	0.14	2.24
-11	13	11	0.09	0.98
10	-13	-11	0.78	2.03
10	-13	-11	0.76	1.59
-10	13	11	0.12	1.00
9	-13	-11	-0.49	1.48
9	-13	-11	1.53	2.00
-9	13	11	-0.73	1.00
8	-13	-11	1.45	1.57
8	-13	-11	-0.01	2.03
-8	13	11	0.54	1.06
7	-13	-11	2.36	1.56
7	-13	-11	3.56	2.11
-7	13	11	0.54	1.13
6	-13	-11	-1.00	1.99
6	-13	-11	-1.32	1.35
-6	13	11	-1.23	1.08
2	16	-12	13.03	1.52
-2	-16	12	12.38	2.77
-2	-16	12	8.89	2.04
1	16	-12	10.06	1.41
-1	-16	12	4.48	2.56
-1	-16	12	8.72	1.83
0	16	-12	21.69	1.58
0	-16	12	19.77	2.88
0	-16	12	26.38	3.53
-1	16	-12	19.30	1.55
1	-16	12	23.33	3.37
1	-16	12	25.13	2.91
-2	16	-12	19.08	1.52
2	-16	12	20.60	3.31
2	-16	12	20.66	2.75

-3	16	-12	14.80	1.43
3	-16	12	9.66	1.88
3	-16	12	11.07	3.06
-4	16	-12	18.33	1.49
4	-16	12	18.85	3.44
4	-16	12	15.83	2.16
-5	16	-12	18.98	1.54
5	-16	12	21.17	2.86
5	-16	12	19.05	3.67
5	15	-12	7.26	1.14
4	15	-12	10.22	1.57
-4	-15	12	11.47	2.10
-4	-15	12	13.93	2.75
3	15	-12	10.69	1.50
-3	-15	12	10.66	2.43
-3	-15	12	10.50	1.96
2	15	-12	11.35	1.41
-2	-15	12	11.12	1.77
-2	-15	12	13.43	2.54
1	15	-12	20.17	1.54
-1	-15	12	21.53	3.64
-1	-15	12	21.69	2.65
0	15	-12	29.43	1.64
0	-15	12	22.53	3.75
0	-15	12	24.82	2.74
-1	15	-12	5.74	0.90
1	-15	12	5.21	1.51
1	-15	12	4.55	2.25
-2	15	-12	16.38	1.39
2	-15	12	17.44	2.47
2	-15	12	19.99	3.05
-3	15	-12	23.27	1.54
3	-15	12	25.30	2.71
3	-15	12	22.16	4.09
-4	15	-12	17.01	1.42
4	-15	12	27.46	4.26
4	-15	12	14.96	2.50
-5	15	-12	18.11	1.44
5	-15	12	18.54	3.35
5	-15	12	15.06	2.63
-6	15	-12	13.55	1.40
6	-15	12	18.37	2.75
6	-15	12	15.80	3.57
6	14	-12	12.26	1.58

-6	-14	12	13.36	2.02
5	14	-12	13.35	1.56
-5	-14	12	10.89	1.89
-5	-14	12	12.28	3.47
4	14	-12	11.37	1.42
-4	-14	12	14.21	2.44
-4	-14	12	12.33	2.17
3	14	-12	16.38	1.50
-3	-14	12	21.57	3.11
-3	-14	12	15.24	2.42
2	14	-12	21.53	1.51
-2	-14	12	18.18	3.17
-2	-14	12	21.90	2.48
1	14	-12	12.72	1.27
-1	-14	12	10.94	1.61
-1	-14	12	17.96	2.52
0	14	-12	14.16	1.23
0	-14	12	10.95	1.69
0	-14	12	14.18	2.86
-1	14	-12	20.73	1.40
1	-14	12	23.20	2.40
1	-14	12	20.53	3.32
-2	14	-12	16.77	1.34
2	-14	12	17.90	2.33
2	-14	12	17.02	2.73
-3	14	-12	8.60	1.16
3	-14	12	19.94	3.59
3	-14	12	7.63	1.61
-4	14	-12	14.23	1.31
4	-14	12	21.25	3.91
4	-14	12	17.68	2.41
-5	14	-12	23.40	1.52
5	-14	12	25.88	2.67
5	-14	12	29.72	4.32
-6	14	-12	22.35	1.53
6	-14	12	22.35	2.69
6	-14	12	24.68	4.38
-7	14	-12	9.11	1.29
7	-14	12	9.66	1.67
7	-14	12	8.87	3.36
8	13	-12	11.40	1.55
-8	-13	12	9.32	1.99
7	13	-12	14.85	1.60
-7	-13	12	9.48	1.84

6	13	-12	13.14	1.50
-6	-13	12	12.94	1.88
5	13	-12	25.43	1.66
-5	-13	12	25.72	2.71
-5	-13	12	26.21	2.71
4	13	-12	10.84	1.29
-4	-13	12	11.56	1.73
-4	-13	12	10.81	1.92
3	13	-12	10.31	1.22
-3	-13	12	8.34	1.89
-3	-13	12	8.66	1.53
2	13	-12	29.46	1.67
-2	-13	12	34.00	3.10
-2	-13	12	28.42	2.57
1	13	-12	37.52	1.80
-1	-13	12	33.62	3.26
-1	-13	12	35.84	2.66
0	13	-12	21.70	1.40
0	-13	12	18.65	2.90
0	-13	12	24.07	2.30
-1	13	-12	16.31	1.25
1	-13	12	21.45	2.20
1	-13	12	17.17	2.53
-2	13	-12	22.75	1.40
2	-13	12	22.82	2.27
2	-13	12	24.75	3.13
-3	13	-12	15.24	1.27
3	-13	12	24.11	3.47
3	-13	12	19.63	2.11
-4	13	-12	10.98	1.15
4	-13	12	10.37	1.57
4	-13	12	10.19	2.59
-5	13	-12	12.06	1.22
5	-13	12	20.33	3.76
5	-13	12	11.57	1.72
-6	13	-12	25.68	1.57
6	-13	12	26.46	2.56
6	-13	12	24.65	4.22
-7	13	-12	13.61	1.36
7	-13	12	14.64	2.40
7	-13	12	15.64	3.22
-8	13	-12	6.47	1.33
8	-13	12	7.85	1.66
8	-13	12	2.60	2.83

9	12	-12	13.64	1.40
-9	-12	12	17.05	2.93
8	12	-12	19.76	1.66
-8	-12	12	18.65	2.78
7	12	-12	21.06	1.57
-7	-12	12	18.02	2.61
6	12	-12	15.64	1.39
-6	-12	12	13.26	2.15
-6	-12	12	14.25	2.32
5	12	-12	17.91	1.41
-5	-12	12	16.31	2.25
-5	-12	12	21.67	2.31
4	12	-12	11.03	1.23
-4	-12	12	13.89	2.34
-4	-12	12	10.78	1.69
3	12	-12	22.44	1.43
-3	-12	12	22.54	2.19
-3	-12	12	22.14	2.52
2	12	-12	44.79	2.00
2	12	-12	40.89	2.90
-2	-12	12	47.77	3.22
-2	-12	12	47.50	2.83
1	12	-12	29.76	2.65
1	12	-12	31.78	1.60
-1	-12	12	29.66	2.81
-1	-12	12	30.29	2.38
0	12	-12	26.65	1.44
0	-12	12	23.28	2.12
0	-12	12	23.48	2.80
-1	12	-12	46.05	1.95
1	-12	12	42.03	2.62
1	-12	12	46.03	3.37
-2	12	-12	38.22	1.78
2	-12	12	39.61	2.51
2	-12	12	40.18	3.36
-3	12	-12	28.21	1.47
3	-12	12	28.64	2.26
3	-12	12	24.96	1.19
3	-12	12	21.84	3.15
-4	12	-12	11.75	1.13
4	-12	12	11.14	1.00
4	-12	12	10.71	1.90
4	-12	12	10.52	2.38
-5	12	-12	14.48	1.21

5	-12	12	18.66	2.82
5	-12	12	18.03	2.07
5	-12	12	14.77	1.24
-6	12	-12	39.82	1.88
6	-12	12	41.71	4.33
6	-12	12	37.21	2.73
6	-12	12	35.01	1.88
-7	12	-12	21.58	1.45
7	-12	12	22.54	2.47
7	-12	12	22.51	1.67
7	-12	12	23.68	3.54
-8	12	-12	6.48	0.96
8	-12	12	6.26	1.57
8	-12	12	6.35	1.31
8	-12	12	7.16	2.93
10	11	-12	28.50	1.87
-10	-11	12	29.18	3.19
9	11	-12	19.28	1.60
-9	-11	12	16.13	2.63
8	11	-12	24.51	1.63
-8	-11	12	22.27	2.67
7	11	-12	34.72	1.79
-7	-11	12	31.18	2.11
-7	-11	12	33.13	2.80
6	11	-12	5.22	0.83
-6	-11	12	5.48	1.33
-6	-11	12	8.37	1.34
5	11	-12	17.33	1.32
-5	-11	12	20.67	2.12
4	11	-12	60.36	2.35
-4	-11	12	53.69	3.03
-4	-11	12	57.60	3.10
3	11	-12	23.12	2.22
3	11	-12	21.82	1.38
-3	-11	12	24.96	2.13
-3	-11	12	25.98	2.39
2	11	-12	16.73	1.21
2	11	-12	17.98	2.03
-2	-11	12	17.02	2.17
-2	-11	12	18.79	1.94
1	11	-12	41.17	2.51
1	11	-12	43.79	1.85
-1	-11	12	39.96	2.89
-1	-11	12	40.48	2.48

0	11	-12	22.19	2.17
0	11	-12	25.68	1.36
0	-11	12	25.34	1.99
0	-11	12	20.23	2.44
-1	11	-12	23.19	2.61
-1	11	-12	31.01	1.47
1	-11	12	32.42	2.90
1	-11	12	30.79	2.15
1	-11	12	27.53	1.34
-2	11	-12	24.91	1.03
-2	11	-12	26.20	1.39
2	-11	12	25.12	2.05
2	-11	12	26.74	1.75
2	-11	12	25.22	2.81
-3	11	-12	17.95	1.21
-3	11	-12	19.40	0.93
3	-11	12	19.99	1.90
-4	11	-12	14.54	0.90
-4	11	-12	13.92	1.17
4	-11	12	16.42	1.90
4	-11	12	23.00	3.14
-5	11	-12	23.29	1.40
5	-11	12	21.15	2.10
5	-11	12	22.84	3.51
-6	11	-12	18.55	1.32
6	-11	12	17.13	2.10
6	-11	12	20.67	3.77
-7	11	-12	6.18	0.82
7	-11	12	8.33	1.31
7	-11	12	8.44	2.98
7	-11	12	9.50	1.54
-8	11	-12	6.67	0.96
8	-11	12	5.24	2.85
8	-11	12	13.18	2.33
8	-11	12	7.82	1.24
9	-11	12	2.98	3.20
11	10	-12	21.82	1.72
-11	-10	12	22.55	3.15
10	10	-12	16.04	1.48
-10	-10	12	18.37	2.20
9	10	-12	14.57	1.38
-9	-10	12	13.62	2.42
8	10	-12	21.51	2.02
8	10	-12	24.24	1.51

-8	-10	12	19.89	2.43
7	10	-12	27.57	1.73
7	10	-12	32.23	1.66
-7	-10	12	28.03	1.80
-7	-10	12	30.61	2.50
6	10	-12	20.00	1.30
6	10	-12	17.24	1.38
-6	-10	12	19.80	2.08
-6	-10	12	17.87	1.69
5	10	-12	32.98	1.67
5	10	-12	40.52	2.30
5	10	-12	33.72	1.57
-5	-10	12	35.64	2.17
-5	-10	12	31.98	2.29
4	10	-12	33.54	2.11
4	10	-12	31.22	1.56
-4	-10	12	32.13	2.26
-4	-10	12	29.84	1.20
-4	-10	12	29.72	2.22
3	10	-12	39.15	1.73
3	10	-12	40.14	2.21
-3	-10	12	36.74	2.40
-3	-10	12	35.73	2.27
2	10	-12	36.88	1.66
2	10	-12	34.26	2.12
-2	-10	12	33.67	1.36
-2	-10	12	38.58	2.52
-2	-10	12	38.10	2.25
1	10	-12	33.83	1.55
1	10	-12	33.66	2.07
-1	-10	12	33.11	2.07
-1	-10	12	31.76	2.45
-1	-10	12	31.13	1.35
0	10	-12	13.40	1.65
0	10	-12	15.46	1.06
0	10	-12	14.97	0.76
0	-10	12	16.80	1.25
0	-10	12	15.26	1.64
0	-10	12	16.27	2.25
-1	10	-12	24.55	1.29
-1	10	-12	23.34	1.02
-1	10	-12	25.39	1.98
1	-10	12	22.08	2.44
1	-10	12	26.63	1.84

1	-10	12	25.40	1.44
-2	10	-12	20.25	1.21
-2	10	-12	18.94	0.95
2	-10	12	20.50	1.54
2	-10	12	20.65	2.52
2	-10	12	23.12	1.80
-3	10	-12	13.29	0.86
-3	10	-12	15.11	1.06
3	-10	12	15.54	1.65
3	-10	12	12.77	1.57
3	-10	12	12.43	2.07
-4	10	-12	18.44	1.04
-4	10	-12	19.66	1.25
4	-10	12	20.61	1.86
4	-10	12	21.85	2.93
4	-10	12	16.89	1.84
-5	10	-12	25.45	1.43
-5	10	-12	25.63	1.26
5	-10	12	23.22	2.07
5	-10	12	26.22	3.39
-6	10	-12	11.07	1.04
-6	10	-12	12.47	1.14
6	-10	12	10.97	1.51
6	-10	12	13.06	2.51
-7	10	-12	4.21	0.73
-7	10	-12	3.40	0.76
7	-10	12	4.34	1.30
7	-10	12	4.06	2.56
-8	10	-12	3.48	0.75
8	-10	12	5.47	1.43
8	-10	12	5.55	2.77
-9	10	-12	2.24	0.84
9	-10	12	4.64	1.34
9	-10	12	4.36	1.50
9	-10	12	2.34	2.99
12	9	-12	12.28	1.47
-12	-9	12	11.94	2.15
11	9	-12	18.35	1.48
-11	-9	12	17.44	2.76
10	9	-12	14.49	1.30
10	9	-12	10.67	1.40
-10	-9	12	9.49	1.67
9	9	-12	13.76	1.69
9	9	-12	12.27	1.25

-9	-9	12	13.86	2.19
8	9	-12	22.28	1.76
8	9	-12	25.44	1.49
8	9	-12	24.68	1.77
-8	-9	12	21.21	2.21
7	9	-12	24.55	1.68
7	9	-12	25.20	1.45
-7	-9	12	26.10	2.15
-7	-9	12	26.66	1.49
6	9	-12	22.53	1.45
6	9	-12	22.47	1.31
-6	-9	12	20.28	1.51
-6	-9	12	20.77	1.14
-6	-9	12	21.22	1.92
5	9	-12	39.11	1.80
5	9	-12	41.02	1.75
5	9	-12	40.13	1.98
-5	-9	12	39.82	2.11
-5	-9	12	39.13	1.57
-5	-9	12	40.99	2.42
4	9	-12	46.15	2.16
4	9	-12	47.13	1.93
4	9	-12	47.71	1.98
-4	-9	12	47.29	1.88
-4	-9	12	51.72	2.43
-4	-9	12	49.25	2.55
3	9	-12	24.93	1.29
3	9	-12	21.70	1.57
3	9	-12	24.51	1.18
-3	-9	12	26.19	1.83
-3	-9	12	21.43	1.91
2	9	-12	8.37	0.75
2	9	-12	9.40	0.90
2	9	-12	7.71	1.24
-2	-9	12	8.50	1.36
-2	-9	12	10.57	1.34
1	9	-12	37.78	1.66
1	9	-12	37.53	1.48
1	9	-12	42.99	2.05
-1	-9	12	38.97	2.50
-1	-9	12	38.24	1.68
-1	-9	12	36.20	2.12
0	9	-12	33.91	1.37
0	9	-12	34.70	1.56

0	9	-12	33.97	1.92
0	-9	12	35.69	2.50
0	-9	12	37.22	1.73
0	-9	12	36.35	2.07
-1	9	-12	22.98	1.20
-1	9	-12	22.19	1.05
-1	9	-12	21.40	1.68
1	-9	12	20.81	2.27
1	-9	12	23.57	1.71
1	-9	12	24.02	1.57
-2	9	-12	16.86	1.06
-2	9	-12	15.25	1.65
-2	9	-12	16.49	0.95
2	-9	12	18.17	2.27
2	-9	12	16.07	1.56
2	-9	12	14.78	1.53
-3	9	-12	17.24	1.14
-3	9	-12	17.07	1.01
3	-9	12	18.21	2.51
3	-9	12	17.82	1.65
3	-9	12	17.59	1.71
-4	9	-12	15.73	1.02
-4	9	-12	17.30	1.15
4	-9	12	14.06	2.25
4	-9	12	18.85	1.75
4	-9	12	15.00	1.79
-5	9	-12	9.31	0.97
-5	9	-12	10.66	1.02
5	-9	12	10.60	1.66
5	-9	12	8.88	2.19
-6	9	-12	3.58	0.70
-6	9	-12	1.95	0.64
6	-9	12	4.29	1.18
6	-9	12	6.10	2.28
-7	9	-12	10.03	1.12
-7	9	-12	7.31	1.10
7	-9	12	10.85	1.94
7	-9	12	11.94	2.67
-8	9	-12	3.26	0.83
-8	9	-12	4.11	0.80
8	-9	12	6.61	1.45
8	-9	12	2.76	2.57
-9	9	-12	3.69	0.87
9	-9	12	6.79	1.57

9	-9	12	3.08	2.94
12	8	-12	26.26	2.38
12	8	-12	26.05	1.65
-12	-8	12	24.77	2.89
11	8	-12	17.20	2.04
11	8	-12	19.07	1.42
-11	-8	12	16.62	2.50
10	8	-12	12.04	1.79
10	8	-12	10.84	1.20
-10	-8	12	16.04	2.15
9	8	-12	20.89	1.81
9	8	-12	21.06	1.36
-9	-8	12	19.52	2.09
8	8	-12	23.95	1.72
8	8	-12	23.38	1.39
-8	-8	12	21.41	2.02
7	8	-12	18.77	1.52
7	8	-12	16.30	1.19
-7	-8	12	18.68	1.77
-7	-8	12	18.93	1.16
6	8	-12	46.12	1.86
6	8	-12	48.39	1.96
6	8	-12	48.67	2.15
-6	-8	12	46.22	1.87
-6	-8	12	45.12	2.49
-6	-8	12	48.22	2.14
5	8	-12	38.68	1.69
5	8	-12	35.30	1.66
5	8	-12	41.60	1.85
-5	-8	12	40.64	2.22
-5	-8	12	39.87	1.63
-5	-8	12	37.17	1.92
4	8	-12	14.21	1.02
4	8	-12	15.43	1.13
-4	-8	12	13.63	1.64
-4	-8	12	14.74	1.44
-4	-8	12	14.73	1.01
3	8	-12	21.31	1.20
3	8	-12	21.37	1.29
-3	-8	12	22.99	1.70
-3	-8	12	21.77	1.23
2	8	-12	20.75	1.13
2	8	-12	23.65	1.37
-2	-8	12	20.88	1.29

-2	-8	12	23.72	1.87
1	8	-12	24.86	1.49
1	8	-12	23.95	1.16
-1	-8	12	24.05	1.42
-1	-8	12	25.15	2.00
0	8	-12	40.90	1.71
0	8	-12	36.54	1.91
0	8	-12	36.96	1.55
0	-8	12	40.90	1.92
0	-8	12	38.01	2.46
-1	8	-12	51.05	2.30
-1	8	-12	48.52	1.87
-1	8	-12	50.13	1.96
1	-8	12	46.60	2.96
1	-8	12	53.19	2.35
1	-8	12	51.37	2.95
-2	8	-12	30.80	1.42
-2	8	-12	29.43	1.35
-2	8	-12	29.13	1.82
2	-8	12	32.67	1.93
2	-8	12	32.09	2.56
2	-8	12	27.95	1.81
-3	8	-12	22.04	1.21
-3	8	-12	22.44	1.18
3	-8	12	22.00	1.70
3	-8	12	18.57	1.85
3	-8	12	23.95	2.55
-4	8	-12	3.76	0.62
-4	8	-12	4.34	0.67
4	-8	12	3.01	0.89
4	-8	12	4.18	1.14
-5	8	-12	1.96	0.59
-5	8	-12	1.35	0.57
5	-8	12	3.17	0.92
5	-8	12	2.84	1.76
-6	8	-12	6.29	0.82
-6	8	-12	8.46	1.02
6	-8	12	5.91	1.20
6	-8	12	6.87	2.15
-7	8	-12	10.35	1.10
-7	8	-12	6.80	0.93
7	-8	12	7.51	1.36
7	-8	12	9.59	2.66
-8	8	-12	3.80	0.85

-8	8	-12	2.80	0.77
8	-8	12	3.74	2.61
8	-8	12	1.98	1.26
-9	8	-12	1.59	0.78
-9	8	-12	1.10	0.81
9	-8	12	-4.18	2.78
9	-8	12	0.58	1.29
13	7	-12	15.41	1.47
13	7	-12	21.66	2.36
-13	-7	12	25.39	2.97
12	7	-12	19.91	2.20
12	7	-12	21.87	1.48
-12	-7	12	20.61	2.56
11	7	-12	37.52	1.84
11	7	-12	38.29	2.42
-11	-7	12	37.28	2.76
10	7	-12	29.34	1.55
10	7	-12	25.04	2.03
-10	-7	12	30.07	2.35
9	7	-12	38.75	1.71
9	7	-12	35.72	2.13
-9	-7	12	35.49	2.31
-9	-7	12	33.87	1.73
8	7	-12	67.62	2.53
8	7	-12	67.37	2.84
-8	-7	12	64.89	2.52
-8	-7	12	66.92	3.11
7	7	-12	23.52	1.62
7	7	-12	22.12	1.25
-7	-7	12	21.04	1.29
6	7	-12	19.96	1.15
6	7	-12	17.09	1.39
-6	-7	12	18.64	1.62
-6	-7	12	22.20	1.23
5	7	-12	36.32	1.81
-5	-7	12	37.29	1.77
-5	-7	12	34.87	2.00
4	7	-12	84.80	3.06
4	7	-12	90.25	3.19
4	7	-12	87.42	2.94
-4	-7	12	90.99	3.13
-4	-7	12	88.45	3.32
-4	-7	12	88.81	3.51
3	7	-12	25.78	1.30

3	7	-12	28.60	1.39
3	7	-12	29.06	1.44
-3	-7	12	31.26	1.75
-3	-7	12	27.77	1.49
-3	-7	12	29.73	1.82
2	7	-12	15.11	1.08
2	7	-12	17.05	0.99
2	7	-12	16.72	1.11
-2	-7	12	16.04	1.23
-2	-7	12	16.22	1.57
-2	-7	12	15.51	1.32
1	7	-12	50.78	1.92
1	7	-12	48.71	2.01
1	7	-12	48.10	1.88
-1	-7	12	45.58	2.24
-1	-7	12	49.50	2.16
-1	-7	12	46.66	2.52
0	7	-12	53.09	2.25
0	7	-12	54.77	2.09
0	7	-12	54.19	2.09
0	-7	12	57.59	2.45
0	-7	12	56.92	2.85
0	-7	12	56.37	2.50
-1	7	-12	24.85	1.57
-1	7	-12	26.18	1.30
1	-7	12	26.97	2.21
1	-7	12	32.04	1.81
1	-7	12	26.89	1.71
-2	7	-12	26.75	1.33
-2	7	-12	26.63	1.31
-2	7	-12	29.18	1.74
2	-7	12	28.57	2.39
2	-7	12	28.44	1.91
-3	7	-12	17.57	1.09
-3	7	-12	17.94	1.12
3	-7	12	16.92	1.52
3	-7	12	18.07	2.30
3	-7	12	24.46	1.96
-4	7	-12	16.61	1.10
-4	7	-12	16.57	1.14
-5	7	-12	6.18	0.94
-5	7	-12	4.53	0.67
5	-7	12	6.07	1.07
5	-7	12	7.18	1.91

-6	7	-12	0.22	0.61
-6	7	-12	1.92	0.59
6	-7	12	0.41	0.91
6	-7	12	2.26	2.00
-7	7	-12	5.22	0.84
-7	7	-12	7.60	1.08
7	-7	12	4.30	1.14
7	-7	12	4.75	2.35
-8	7	-12	5.11	0.84
-8	7	-12	4.18	0.88
8	-7	12	4.80	2.62
8	-7	12	5.69	1.36
-9	7	-12	1.46	0.87
-9	7	-12	-0.96	0.73
9	-7	12	0.16	1.23
9	-7	12	4.33	2.86
14	6	-12	13.31	1.40
14	6	-12	17.29	2.39
-14	-6	12	13.26	2.00
13	6	-12	12.34	1.26
13	6	-12	13.52	2.20
-13	-6	12	10.95	1.82
12	6	-12	23.48	1.45
12	6	-12	20.94	2.14
-12	-6	12	25.23	2.36
11	6	-12	24.05	1.43
11	6	-12	24.18	2.04
-11	-6	12	26.18	2.26
10	6	-12	38.78	2.25
10	6	-12	35.59	1.71
-10	-6	12	36.72	2.38
9	6	-12	18.87	1.19
9	6	-12	19.16	1.68
-9	-6	12	19.23	1.34
-9	-6	12	17.23	2.09
8	6	-12	28.13	1.38
8	6	-12	27.62	1.76
-8	-6	12	28.61	1.50
-8	-6	12	25.82	1.85
7	6	-12	47.65	1.87
7	6	-12	43.31	2.19
-7	-6	12	45.93	1.96
-7	-6	12	45.98	2.31
6	6	-12	41.50	2.02

6	6	-12	42.07	1.77
-6	-6	12	40.87	2.12
-6	-6	12	43.41	1.85
5	6	-12	24.02	1.53
5	6	-12	24.59	1.23
-5	-6	12	25.97	1.61
-5	-6	12	26.11	1.41
4	6	-12	42.95	1.76
4	6	-12	44.35	1.99
4	6	-12	44.35	1.57
-4	-6	12	45.62	2.15
-4	-6	12	47.14	1.94
3	6	-12	44.97	1.83
3	6	-12	48.46	1.75
3	6	-12	48.25	2.03
-3	-6	12	48.33	2.22
-3	-6	12	45.17	2.02
-3	-6	12	49.77	2.20
2	6	-12	85.06	2.90
2	6	-12	83.02	3.00
2	6	-12	83.11	2.86
-2	-6	12	79.29	3.22
-2	-6	12	80.24	3.30
-2	-6	12	92.16	3.18
1	6	-12	12.77	0.86
1	6	-12	13.78	1.03
1	6	-12	13.51	0.98
-1	-6	12	11.32	1.45
-1	-6	12	13.04	1.33
-1	-6	12	12.64	1.16
0	6	-12	8.51	0.99
0	6	-12	8.31	0.77
0	6	-12	10.42	0.92
0	-6	12	7.80	1.03
0	-6	12	12.07	1.56
0	-6	12	8.95	1.09
-1	6	-12	54.19	2.33
-1	6	-12	57.20	2.21
-1	6	-12	59.51	2.14
1	-6	12	59.63	2.53
1	-6	12	55.60	2.93
1	-6	12	57.23	2.62
-2	6	-12	16.28	1.11
-2	6	-12	18.87	1.44

2	-6	12	14.51	1.65
2	-6	12	17.07	2.04
2	-6	12	21.99	1.77
-3	6	-12	3.41	0.65
-3	6	-12	3.45	0.58
3	-6	12	2.36	1.25
3	-6	12	2.50	1.21
3	-6	12	3.56	0.84
-4	6	-12	12.92	1.09
-4	6	-12	12.20	0.97
4	-6	12	9.83	1.34
-5	6	-12	1.87	0.55
-5	6	-12	3.16	0.69
5	-6	12	3.40	1.73
5	-6	12	1.44	0.79
-6	6	-12	3.94	0.78
-6	6	-12	2.93	0.64
6	-6	12	4.21	1.04
6	-6	12	2.80	1.96
-7	6	-12	3.23	0.75
-7	6	-12	2.59	0.83
7	-6	12	3.72	2.14
7	-6	12	1.82	1.02
-8	6	-12	-1.71	0.72
-8	6	-12	-0.17	0.67
8	-6	12	-1.45	2.18
8	-6	12	-0.80	1.04
-9	6	-12	2.15	0.91
-9	6	-12	-0.04	0.76
9	-6	12	-0.60	2.49
9	-6	12	0.61	1.19
14	5	-12	36.96	2.76
14	5	-12	32.54	1.75
-14	-5	12	32.37	2.88
13	5	-12	25.28	1.51
13	5	-12	24.59	2.37
-13	-5	12	25.98	2.45
12	5	-12	11.57	1.52
12	5	-12	9.80	1.11
-12	-5	12	13.18	2.37
11	5	-12	20.04	1.88
11	5	-12	20.10	1.29
-11	-5	12	25.44	1.94
10	5	-12	15.17	1.12

10	5	-12	17.82	1.74
-10	-5	12	15.13	1.63
9	5	-12	19.83	1.68
9	5	-12	21.56	1.20
-9	-5	12	20.45	1.64
-9	-5	12	19.67	1.43
8	5	-12	50.37	1.92
8	5	-12	45.95	2.32
-8	-5	12	45.43	2.32
-8	-5	12	49.55	2.10
7	5	-12	51.56	2.04
7	5	-12	51.83	2.39
-7	-5	12	51.86	2.18
-7	-5	12	53.81	2.41
6	5	-12	17.96	1.03
6	5	-12	19.54	1.45
-6	-5	12	18.02	1.37
-6	-5	12	18.45	1.26
5	5	-12	48.56	2.21
5	5	-12	48.55	1.87
-5	-5	12	50.05	2.09
-5	-5	12	46.51	2.21
4	5	-12	58.35	2.43
4	5	-12	57.72	1.95
4	5	-12	58.62	2.17
-4	-5	12	57.61	2.30
-4	-5	12	61.72	2.53
-4	-5	12	60.83	2.42
3	5	-12	8.98	0.74
3	5	-12	6.88	0.64
3	5	-12	7.56	1.04
-3	-5	12	8.09	1.11
-3	-5	12	7.11	1.09
-3	-5	12	7.25	0.94
2	5	-12	17.01	0.96
2	5	-12	17.41	1.19
2	5	-12	16.34	0.95
-2	-5	12	16.35	1.42
-2	-5	12	16.16	1.19
-2	-5	12	16.81	1.39
1	5	-12	21.52	1.21
1	5	-12	23.38	1.33
1	5	-12	22.89	1.12
-1	-5	12	23.13	1.66

-1	-5	12	20.53	1.37
-1	-5	12	24.54	1.64
0	5	-12	35.56	1.47
0	5	-12	34.33	1.64
0	5	-12	35.10	1.63
0	-5	12	35.72	2.01
0	-5	12	36.90	2.19
0	-5	12	33.66	1.75
-1	5	-12	10.67	0.79
-1	5	-12	11.06	1.14
-1	5	-12	9.79	0.99
1	-5	12	10.43	1.49
1	-5	12	9.25	1.60
1	-5	12	9.23	1.07
-2	5	-12	2.06	0.58
-2	5	-12	3.18	0.81
-2	5	-12	2.85	0.52
2	-5	12	2.69	0.72
2	-5	12	1.61	1.06
2	-5	12	2.37	1.00
-3	5	-12	-0.27	0.53
-3	5	-12	1.06	0.44
3	-5	12	-0.03	1.13
3	-5	12	1.59	0.69
-4	5	-12	4.10	1.04
-4	5	-12	4.53	0.61
4	-5	12	5.30	0.90
4	-5	12	5.91	1.88
-5	5	-12	4.11	0.64
-5	5	-12	3.57	0.72
5	-5	12	5.89	1.74
5	-5	12	3.16	0.87
-6	5	-12	0.53	0.66
-6	5	-12	-0.36	0.58
6	-5	12	2.55	1.81
6	-5	12	1.03	0.89
-7	5	-12	0.24	0.65
-7	5	-12	0.93	0.77
7	-5	12	0.84	0.93
7	-5	12	-1.98	1.97
-8	5	-12	0.16	0.83
-8	5	-12	1.75	0.74
8	-5	12	0.45	2.30
8	-5	12	0.61	1.04

15	4	-12	20.12	1.47
15	4	-12	21.93	2.56
14	4	-12	14.40	1.24
14	4	-12	13.95	2.23
-14	-4	12	12.27	2.04
13	4	-12	15.22	1.20
13	4	-12	20.42	2.13
-13	-4	12	17.12	2.10
12	4	-12	20.09	1.26
12	4	-12	18.84	1.97
-12	-4	12	18.95	1.90
11	4	-12	22.64	1.29
11	4	-12	21.50	1.87
-11	-4	12	21.82	1.79
10	4	-12	32.02	2.12
10	4	-12	32.40	1.50
-10	-4	12	32.27	1.96
9	4	-12	24.21	1.82
9	4	-12	28.99	1.34
-9	-4	12	28.03	1.74
-9	-4	12	26.15	1.60
8	4	-12	16.08	1.38
8	4	-12	16.54	1.53
-8	-4	12	17.23	1.32
-8	-4	12	17.86	1.36
7	4	-12	53.75	2.02
7	4	-12	53.40	2.46
-7	-4	12	53.50	2.26
-7	-4	12	53.09	2.36
6	4	-12	83.70	2.88
6	4	-12	85.07	3.28
-6	-4	12	83.33	3.12
-6	-4	12	81.48	3.19
5	4	-12	28.83	1.70
5	4	-12	26.91	1.25
-5	-4	12	26.08	1.50
-5	-4	12	29.26	1.58
4	4	-12	15.38	1.30
4	4	-12	17.72	0.94
-4	-4	12	22.12	1.41
-4	-4	12	16.91	0.91
-4	-4	12	13.32	1.10
-4	-4	12	15.92	1.16
-4	-4	12	15.82	1.29

3	4	-12	12.16	1.18
3	4	-12	13.57	0.83
-3	-4	12	11.65	0.81
-3	-4	12	12.78	1.27
-3	-4	12	12.87	1.05
-3	-4	12	12.27	1.18
2	4	-12	34.94	1.46
2	4	-12	36.60	1.48
2	4	-12	36.01	1.76
-2	-4	12	35.04	1.71
-2	-4	12	34.47	1.84
-2	-4	12	35.21	1.45
-2	-4	12	38.05	1.95
1	4	-12	17.03	0.92
1	4	-12	15.48	1.18
1	4	-12	14.20	1.04
-1	-4	12	14.87	1.50
-1	-4	12	14.68	1.40
-1	-4	12	14.41	1.12
-1	-4	12	17.53	1.14
0	4	-12	10.48	1.07
0	4	-12	11.55	1.04
0	4	-12	9.86	0.77
0	-4	12	10.49	1.02
0	-4	12	11.93	1.49
0	-4	12	10.77	1.47
-1	4	-12	22.79	1.47
-1	4	-12	23.30	1.37
-1	4	-12	22.99	1.12
1	-4	12	23.71	1.84
1	-4	12	23.13	1.42
1	-4	12	25.79	1.96
-2	4	-12	-0.11	0.71
-2	4	-12	0.20	0.55
-2	4	-12	-0.38	0.38
2	-4	12	0.66	0.94
2	-4	12	0.40	0.89
-3	4	-12	2.16	0.61
3	-4	12	0.97	0.57
3	-4	12	2.46	1.21
-4	4	-12	3.44	0.69
-4	4	-12	3.01	0.56
4	-4	12	3.19	0.77
4	-4	12	3.98	1.42

-5	4	-12	0.15	0.64
-5	4	-12	0.17	0.49
5	-4	12	3.74	1.58
5	-4	12	0.21	0.65
-6	4	-12	1.16	0.73
-6	4	-12	-0.45	0.55
6	-4	12	4.20	1.85
6	-4	12	-0.15	0.72
-7	4	-12	1.23	0.78
-7	4	-12	-0.57	0.58
7	-4	12	1.58	0.87
7	-4	12	-0.18	1.86
-8	4	-12	1.51	0.88
-8	4	-12	0.36	0.73
8	-4	12	0.68	0.97
8	-4	12	-2.99	1.91
15	3	-12	15.09	2.39
15	3	-12	12.39	1.26
14	3	-12	18.04	1.30
14	3	-12	17.29	2.25
-14	-3	12	14.89	2.07
13	3	-12	20.01	1.27
13	3	-12	19.03	2.14
-13	-3	12	19.33	2.04
12	3	-12	40.34	2.44
12	3	-12	39.70	1.73
-12	-3	12	37.28	2.24
11	3	-12	19.95	1.86
11	3	-12	23.96	1.62
11	3	-12	19.86	1.20
-11	-3	12	23.26	1.71
10	3	-12	20.23	1.28
10	3	-12	24.54	1.22
10	3	-12	22.58	1.86
-10	-3	12	23.94	1.60
9	3	-12	50.14	2.45
9	3	-12	48.51	2.07
9	3	-12	48.35	1.98
-9	-3	12	49.02	2.23
-9	-3	12	48.74	2.19
8	3	-12	34.26	1.54
8	3	-12	32.82	1.96
8	3	-12	33.06	1.42
-8	-3	12	32.94	1.73

-8	-3	12	30.23	1.71
7	3	-12	19.76	1.06
7	3	-12	21.72	1.58
7	3	-12	19.42	1.15
-7	-3	12	22.48	1.46
-7	-3	12	23.03	1.38
6	3	-12	18.55	1.00
6	3	-12	18.46	1.45
6	3	-12	17.19	1.08
-6	-3	12	19.25	1.22
-6	-3	12	18.56	1.36
5	3	-12	31.88	1.84
5	3	-12	35.42	1.42
5	3	-12	33.36	1.54
-5	-3	12	33.33	1.77
-5	-3	12	37.09	1.68
-5	-3	12	31.76	1.32
4	3	-12	23.60	1.11
4	3	-12	23.39	1.52
4	3	-12	22.30	1.31
-4	-3	12	23.04	1.33
-4	-3	12	21.76	1.04
-4	-3	12	24.63	1.16
-4	-3	12	25.11	1.29
-4	-3	12	23.99	1.54
3	3	-12	17.53	1.34
3	3	-12	18.09	0.95
-3	-3	12	16.94	1.14
-3	-3	12	19.97	0.94
-3	-3	12	15.37	1.45
-3	-3	12	16.81	1.16
-3	-3	12	18.81	1.25
2	3	-12	2.36	0.45
2	3	-12	2.16	0.68
-2	-3	12	2.44	0.87
-2	-3	12	2.19	0.44
-2	-3	12	1.35	0.45
-2	-3	12	2.11	0.68
-2	-3	12	2.58	0.70
1	3	-12	24.54	1.46
1	3	-12	23.35	1.09
-1	-3	12	23.90	1.35
-1	-3	12	22.11	1.58
-1	-3	12	23.57	1.10

-1	-3	12	23.66	1.56
-1	-3	12	24.69	1.77
0	3	-12	21.36	1.03
0	3	-12	21.19	1.34
0	-3	12	18.45	1.05
0	-3	12	20.32	1.65
0	-3	12	18.87	1.64
0	-3	12	20.97	1.25
0	-3	12	22.52	1.83
-1	3	-12	0.04	0.38
-1	3	-12	0.00	0.56
1	-3	12	1.06	0.52
1	-3	12	0.78	0.84
1	-3	12	-0.16	0.91
-2	3	-12	0.24	0.37
-2	3	-12	0.13	0.56
2	-3	12	0.82	1.01
2	-3	12	-1.16	1.21
-3	3	-12	0.68	0.63
-3	3	-12	1.62	0.49
3	-3	12	1.52	1.08
3	-3	12	0.36	0.53
-4	3	-12	0.39	0.45
-4	3	-12	0.40	0.62
4	-3	12	1.35	0.64
4	-3	12	1.16	1.20
-5	3	-12	0.29	0.50
-5	3	-12	0.43	0.66
5	-3	12	2.33	1.47
5	-3	12	-0.32	0.62
-6	3	-12	0.81	0.71
-6	3	-12	0.30	0.56
6	-3	12	-0.93	0.70
6	-3	12	0.15	1.64
-7	3	-12	-0.85	0.62
-7	3	-12	1.62	0.84
7	-3	12	-1.38	1.76
7	-3	12	-0.28	0.81
-8	3	-12	-1.06	0.84
-8	3	-12	-0.13	0.75
8	-3	12	-0.77	2.15
8	-3	12	0.38	0.89
16	2	-12	21.20	2.64
16	2	-12	17.47	1.48

15	2	-12	24.04	1.44
15	2	-12	20.05	2.47
14	2	-12	33.05	2.59
14	2	-12	35.50	1.68
-14	-2	12	32.63	2.31
13	2	-12	34.60	1.63
13	2	-12	35.48	2.47
-13	-2	12	35.34	2.14
12	2	-12	26.96	1.35
12	2	-12	26.69	2.08
12	2	-12	23.95	1.43
-12	-2	12	26.53	1.80
11	2	-12	44.17	2.48
11	2	-12	45.02	1.81
11	2	-12	42.64	1.88
-11	-2	12	45.76	2.19
10	2	-12	43.05	2.39
10	2	-12	46.04	1.78
10	2	-12	43.35	1.86
-10	-2	12	43.45	2.10
9	2	-12	51.48	2.09
9	2	-12	54.27	2.00
9	2	-12	52.05	2.54
-9	-2	12	54.42	2.30
-9	-2	12	50.48	2.36
8	2	-12	24.05	1.16
8	2	-12	26.08	1.74
8	2	-12	24.82	1.31
-8	-2	12	23.59	1.42
-8	-2	12	25.72	1.63
7	2	-12	53.40	2.50
7	2	-12	53.11	2.12
7	2	-12	56.04	2.00
-7	-2	12	53.34	2.23
-7	-2	12	52.85	2.34
6	2	-12	42.55	2.09
6	2	-12	40.76	1.60
6	2	-12	39.58	1.75
-6	-2	12	37.92	1.50
-6	-2	12	41.08	2.01
-6	-2	12	43.02	1.83
5	2	-12	11.28	1.26
5	2	-12	12.32	0.80
5	2	-12	12.07	0.98

-5	-2	12	9.81	0.91
-5	-2	12	11.38	0.74
-5	-2	12	13.09	1.29
4	2	-12	33.23	1.80
4	2	-12	33.38	1.37
4	2	-12	30.02	1.55
-4	-2	12	31.98	1.53
-4	-2	12	34.37	1.31
-4	-2	12	33.46	1.88
-4	-2	12	35.25	1.57
-4	-2	12	31.76	1.55
3	2	-12	37.67	1.91
3	2	-12	36.95	1.49
-3	-2	12	36.47	1.70
-3	-2	12	39.03	1.78
-3	-2	12	38.52	2.05
-3	-2	12	36.82	1.44
-3	-2	12	39.31	1.75
2	2	-12	24.44	1.12
2	2	-12	23.08	1.49
-2	-2	12	24.51	1.30
-2	-2	12	24.50	1.76
-2	-2	12	24.66	1.46
-2	-2	12	24.34	1.54
-2	-2	12	22.93	1.08
1	2	-12	8.01	0.68
1	2	-12	9.51	1.08
-1	-2	12	7.59	1.11
-1	-2	12	8.17	1.20
-1	-2	12	7.89	0.82
-1	-2	12	8.82	1.14
-1	-2	12	7.11	0.71
0	2	-12	4.52	0.59
0	2	-12	5.13	0.80
0	-2	12	4.01	0.57
0	-2	12	4.91	0.92
0	-2	12	6.42	1.10
0	-2	12	3.15	0.56
-1	2	-12	1.67	0.64
-1	2	-12	1.89	0.42
1	-2	12	1.47	0.87
1	-2	12	2.87	1.11
1	-2	12	1.79	0.54
1	-2	12	2.05	0.59

-2	2	-12	1.06	0.42
-2	2	-12	1.04	0.87
2	-2	12	1.74	0.56
2	-2	12	2.94	1.04
-3	2	-12	-0.08	0.60
-3	2	-12	-0.31	0.41
3	-2	12	-0.26	0.52
-4	2	-12	0.93	0.71
-4	2	-12	0.66	0.52
4	-2	12	0.24	0.57
4	-2	12	0.32	1.17
-5	2	-12	-0.86	0.49
-5	2	-12	0.57	0.75
5	-2	12	1.02	1.45
5	-2	12	0.24	0.63
-6	2	-12	0.24	0.62
-6	2	-12	-0.02	0.79
6	-2	12	1.94	0.80
6	-2	12	-0.69	1.57
-7	2	-12	-0.10	0.67
-7	2	-12	-0.43	0.79
7	-2	12	1.08	0.87
7	-2	12	0.15	1.86
-8	2	-12	0.28	0.79
-8	2	-12	0.32	0.95
8	-2	12	1.15	0.94
8	-2	12	5.71	2.29
16	1	-12	5.68	1.63
16	1	-12	5.02	0.85
15	1	-12	19.01	2.35
15	1	-12	16.59	1.27
14	1	-12	44.87	2.94
14	1	-12	49.24	2.01
-14	-1	12	46.84	2.49
13	1	-12	29.83	1.47
13	1	-12	26.44	2.27
13	1	-12	33.46	2.06
-13	-1	12	28.11	1.89
12	1	-12	18.62	1.23
12	1	-12	22.87	1.94
12	1	-12	20.60	1.17
-12	-1	12	19.32	1.52
11	1	-12	21.16	1.10
11	1	-12	17.97	2.11

11	1	-12	19.03	1.19
-11	-1	12	18.53	1.40
10	1	-12	6.91	1.14
10	1	-12	6.29	0.85
-10	-1	12	8.21	1.04
9	1	-12	25.97	1.21
9	1	-12	26.44	1.36
9	1	-12	27.32	1.86
-9	-1	12	26.31	1.46
8	1	-12	39.06	1.51
8	1	-12	37.18	2.07
8	1	-12	37.36	1.65
-8	-1	12	33.06	1.88
-8	-1	12	35.43	1.69
7	1	-12	5.64	0.95
7	1	-12	7.42	0.86
7	1	-12	5.59	0.65
-7	-1	12	6.68	0.82
-7	-1	12	5.71	0.59
-7	-1	12	6.48	0.97
6	1	-12	18.29	1.17
6	1	-12	18.75	1.55
6	1	-12	18.43	0.96
-6	-1	12	17.91	1.11
-6	-1	12	18.84	0.91
-6	-1	12	18.11	1.48
5	1	-12	49.42	2.06
5	1	-12	49.33	1.82
5	1	-12	50.45	2.33
-5	-1	12	47.52	1.73
-5	-1	12	47.67	1.81
-5	-1	12	48.40	2.00
-5	-1	12	49.34	1.98
-5	-1	12	49.03	2.31
4	1	-12	7.89	0.68
4	1	-12	6.80	0.95
4	1	-12	8.62	1.13
-4	-1	12	7.68	0.61
-4	-1	12	8.45	0.77
-4	-1	12	7.00	1.05
-4	-1	12	8.93	1.14
-4	-1	12	7.47	0.82
3	1	-12	2.11	0.41
3	1	-12	2.76	0.74

-3	-1	12	2.09	0.51
-3	-1	12	2.54	0.55
-3	-1	12	2.47	0.40
-3	-1	12	3.87	0.82
-3	-1	12	3.77	0.92
2	1	-12	1.71	0.61
2	1	-12	1.39	0.40
-2	-1	12	3.68	0.92
-2	-1	12	2.78	0.86
-2	-1	12	2.09	0.63
-2	-1	12	1.24	0.46
-2	-1	12	2.39	0.41
1	1	-12	1.17	0.34
1	1	-12	1.71	0.67
-1	-1	12	1.04	0.39
-1	-1	12	0.52	0.37
-1	-1	12	1.59	0.69
-1	-1	12	1.40	0.97
0	1	-12	0.25	0.56
0	1	-12	-0.59	0.33
0	-1	12	0.12	0.66
0	-1	12	0.66	0.41
0	-1	12	0.36	0.96
0	-1	12	0.18	0.40
-1	1	-12	1.82	0.41
-1	1	-12	1.67	0.62
1	-1	12	0.71	0.77
1	-1	12	0.84	0.51
1	-1	12	-1.35	1.00
1	-1	12	0.65	0.50
-2	1	-12	0.04	0.38
-2	1	-12	-0.61	0.58
2	-1	12	-0.39	0.43
2	-1	12	-0.34	0.82
-3	1	-12	0.30	0.64
-3	1	-12	0.00	0.39
3	-1	12	0.57	0.53
3	-1	12	0.15	1.00
-4	1	-12	-0.62	0.56
-4	1	-12	0.17	0.69
4	-1	12	0.12	1.09
4	-1	12	0.52	0.63
-5	1	-12	0.96	0.56
-5	1	-12	0.14	0.74

5	-1	12	0.91	1.37
5	-1	12	0.91	0.69
-6	1	-12	3.23	0.68
-6	1	-12	1.96	0.88
6	-1	12	2.32	0.81
6	-1	12	-0.15	1.53
-7	1	-12	0.00	0.71
-7	1	-12	0.08	0.84
7	-1	12	2.07	0.85
7	-1	12	-1.07	1.71
16	0	-12	18.16	2.49
16	0	-12	12.31	1.23
15	0	-12	21.90	2.45
15	0	-12	20.76	1.33
14	0	-12	40.10	2.73
14	0	-12	40.54	1.78
-14	0	12	38.86	2.16
13	0	-12	25.03	2.24
13	0	-12	28.58	1.38
13	0	-12	27.03	1.56
-13	0	12	26.68	1.74
12	0	-12	26.30	1.45
12	0	-12	26.67	2.13
12	0	-12	28.10	1.32
-12	0	12	26.46	1.61
11	0	-12	21.76	1.31
11	0	-12	23.03	1.15
-11	0	12	22.32	1.41
10	0	-12	17.97	1.70
10	0	-12	15.43	1.13
10	0	-12	17.33	0.97
-10	0	12	18.22	1.21
9	0	-12	20.37	1.22
9	0	-12	21.26	1.06
9	0	-12	22.10	1.75
-9	0	12	20.42	1.22
8	0	-12	8.74	1.33
8	0	-12	9.11	0.93
8	0	-12	8.92	0.72
8	0	-12	10.01	0.86
-8	0	12	9.00	0.86
7	0	-12	23.52	1.65
7	0	-12	24.90	1.10
7	0	-12	23.64	1.35

-7	0	12	21.52	1.56
-7	0	12	23.69	1.26
-7	0	12	24.16	1.06
6	0	-12	34.24	1.72
6	0	-12	35.86	1.44
6	0	-12	37.13	1.97
-6	0	12	38.03	1.97
-6	0	12	33.98	1.35
-6	0	12	37.19	1.62
5	0	-12	13.68	1.16
5	0	-12	15.56	0.86
5	0	-12	16.59	1.36
-5	0	12	16.66	1.46
-5	0	12	15.32	0.99
-5	0	12	15.66	0.95
-5	0	12	15.29	0.80
-5	0	12	16.81	1.32
4	0	-12	-0.02	0.30
4	0	-12	0.32	0.63
4	0	-12	-0.02	0.92
-4	0	12	0.18	0.77
-4	0	12	-0.22	0.32
-4	0	12	0.23	0.31
-4	0	12	0.53	0.48
-4	0	12	0.19	0.73
3	0	-12	5.44	0.88
3	0	-12	6.46	0.62
-3	0	12	6.96	0.85
-3	0	12	6.02	1.06
-3	0	12	6.70	1.07
-3	0	12	7.35	0.78
-3	0	12	7.97	0.61
2	0	-12	0.17	0.31
2	0	-12	0.40	0.62
-2	0	12	0.08	0.56
-2	0	12	-0.33	0.89
-2	0	12	0.02	0.35
-2	0	12	-0.23	0.36
1	0	-12	0.51	0.34
1	0	-12	0.08	0.62
-1	0	12	0.43	0.63
-1	0	12	-0.18	0.96
-1	0	12	0.00	0.37
-1	0	12	0.08	0.33

0	0	-12	0.17	0.33
0	0	-12	0.19	0.58
0	0	12	-0.14	0.39
0	0	12	1.07	1.09
0	0	12	0.20	0.66
-1	0	-12	0.05	0.34
-1	0	-12	0.12	0.59
1	0	12	0.53	0.48
1	0	12	0.76	0.81
1	0	12	1.23	0.49
-2	0	-12	1.21	0.43
-2	0	-12	1.18	0.64
2	0	12	0.31	1.06
2	0	12	1.14	0.52
2	0	12	1.39	0.56
-3	0	-12	0.81	0.73
-3	0	-12	0.74	0.44
3	0	12	1.10	1.06
3	0	12	0.96	0.63
-4	0	-12	3.49	0.57
-4	0	-12	4.34	0.91
4	0	12	2.49	1.26
4	0	12	3.86	0.78
-5	0	-12	5.63	0.97
-5	0	-12	4.53	0.69
5	0	12	6.27	1.71
5	0	12	7.28	1.22
-6	0	-12	1.58	0.69
-6	0	-12	2.02	0.80
6	0	12	2.17	1.53
6	0	12	-0.05	0.77
-7	0	-12	0.46	1.02
-7	0	-12	1.88	0.78
7	0	12	1.19	0.92
7	0	12	3.58	1.84
17	-1	-12	16.09	2.15
17	-1	-12	15.30	1.48
16	-1	-12	14.10	1.95
16	-1	-12	11.09	1.13
15	-1	-12	6.69	0.98
15	-1	-12	8.03	1.64
14	-1	-12	12.51	1.78
14	-1	-12	14.30	1.06
-14	1	12	12.20	1.44

13	-1	-12	12.67	1.23
13	-1	-12	11.95	1.90
13	-1	-12	13.66	1.00
-13	1	12	12.34	1.33
12	-1	-12	20.36	1.99
12	-1	-12	20.28	1.39
12	-1	-12	23.00	1.18
-12	1	12	23.55	1.41
11	-1	-12	21.24	1.21
11	-1	-12	21.51	1.85
11	-1	-12	19.17	1.34
-11	1	12	21.08	1.30
10	-1	-12	11.44	1.52
10	-1	-12	11.86	1.10
10	-1	-12	11.85	0.79
-10	1	12	10.67	0.96
9	-1	-12	14.30	1.16
9	-1	-12	12.35	0.85
9	-1	-12	13.21	1.51
-9	1	12	13.18	0.98
8	-1	-12	15.48	1.57
8	-1	-12	14.77	1.20
8	-1	-12	15.83	0.88
8	-1	-12	17.06	1.03
-8	1	12	15.73	1.03
7	-1	-12	5.46	0.61
7	-1	-12	6.81	1.05
7	-1	-12	4.19	0.92
-7	1	12	4.51	0.56
-7	1	12	5.09	0.69
6	-1	-12	0.83	0.64
6	-1	-12	-0.05	0.73
6	-1	-12	0.71	0.36
-6	1	12	0.58	0.32
-6	1	12	0.47	0.38
-6	1	12	0.84	1.23
-6	1	12	0.23	0.37
-6	1	12	0.76	0.75
5	-1	-12	14.71	1.31
5	-1	-12	18.18	1.48
5	-1	-12	15.75	0.84
-5	1	12	16.73	1.57
-5	1	12	16.13	0.78
-5	1	12	14.31	0.97

-5	1	12	16.04	0.90
4	-1	-12	8.64	0.68
4	-1	-12	7.18	1.01
-4	1	12	8.58	0.81
-4	1	12	8.48	0.61
-4	1	12	8.17	0.78
-4	1	12	13.92	1.62
3	-1	-12	0.13	0.58
3	-1	-12	0.27	0.35
-3	1	12	0.50	0.97
-3	1	12	-0.39	0.32
-3	1	12	-0.18	0.31
-3	1	12	-0.44	0.44
2	-1	-12	0.09	0.59
2	-1	-12	-0.19	0.31
-2	1	12	-0.05	0.36
-2	1	12	-0.25	0.34
-2	1	12	1.04	1.13
-2	1	12	0.61	0.55
1	-1	-12	1.74	0.69
1	-1	-12	2.47	0.41
-1	1	12	-1.82	1.09
-1	1	12	0.95	0.64
-1	1	12	2.33	0.42
0	-1	-12	0.46	0.59
0	-1	-12	-0.23	0.31
0	1	12	-0.10	1.23
0	1	12	0.87	0.68
0	1	12	-0.21	0.43
0	1	12	-0.18	0.41
-1	-1	-12	1.37	0.73
-1	-1	-12	0.96	0.40
1	1	12	0.39	0.78
1	1	12	1.47	0.49
1	1	12	1.00	0.50
-2	-1	-12	2.60	0.81
2	1	12	1.66	0.59
2	1	12	2.18	0.66
2	1	12	2.20	1.02
-3	-1	-12	4.72	0.95
-3	-1	-12	4.69	0.59
3	1	12	2.13	1.10
3	1	12	4.62	0.81
-4	-1	-12	2.22	0.57

-4	-1	-12	2.33	0.81
4	1	12	3.20	1.38
4	1	12	2.09	0.73
-5	-1	-12	2.30	0.86
-5	-1	-12	1.90	0.62
5	1	12	1.84	1.42
5	1	12	1.00	0.76
-6	-1	-12	1.21	0.96
-6	-1	-12	2.32	0.72
6	1	12	1.97	0.91
6	1	12	-1.37	1.45
17	-2	-12	6.91	1.70
17	-2	-12	6.56	0.92
16	-2	-12	15.27	2.45
16	-2	-12	16.71	1.27
15	-2	-12	21.34	2.34
15	-2	-12	19.17	1.27
14	-2	-12	12.85	1.02
14	-2	-12	15.26	2.07
-14	2	12	12.61	1.38
13	-2	-12	13.61	1.90
13	-2	-12	13.32	0.96
13	-2	-12	11.07	1.40
-13	2	12	12.37	1.24
12	-2	-12	15.68	1.45
12	-2	-12	16.04	1.89
12	-2	-12	16.46	0.99
-12	2	12	15.80	1.20
11	-2	-12	25.42	1.99
11	-2	-12	25.67	1.61
11	-2	-12	23.67	1.19
-11	2	12	24.28	1.34
10	-2	-12	38.68	1.53
10	-2	-12	40.40	1.98
-10	2	12	37.47	1.68
9	-2	-12	2.34	0.92
9	-2	-12	2.21	0.44
9	-2	-12	2.09	0.51
9	-2	-12	1.84	0.71
-9	2	12	2.17	0.48
8	-2	-12	1.68	0.82
8	-2	-12	0.82	0.65
8	-2	-12	1.60	0.48
8	-2	-12	0.86	0.38

-8	2	12	0.70	0.33
-8	2	12	1.13	0.42
-8	2	12	1.97	0.63
7	-2	-12	7.61	0.81
7	-2	-12	5.65	1.02
7	-2	-12	5.90	0.62
7	-2	-12	5.30	0.90
-7	2	12	5.69	0.63
-7	2	12	7.69	1.05
-7	2	12	7.37	0.75
6	-2	-12	13.21	0.80
6	-2	-12	10.49	1.32
6	-2	-12	13.02	1.43
-6	2	12	13.14	0.90
-6	2	12	12.47	0.80
-6	2	12	13.81	1.57
-6	2	12	11.86	0.69
5	-2	-12	0.29	0.32
5	-2	-12	1.29	0.71
-5	2	12	0.04	0.37
-5	2	12	0.64	0.32
-5	2	12	0.94	1.02
-5	2	12	0.49	0.39
4	-2	-12	1.00	0.36
4	-2	-12	1.17	0.73
-4	2	12	3.63	1.15
-4	2	12	1.24	0.34
-4	2	12	0.52	0.37
-4	2	12	0.41	0.42
3	-2	-12	0.45	0.31
3	-2	-12	-0.08	0.62
-3	2	12	0.06	0.39
-3	2	12	-0.12	1.08
-3	2	12	0.02	0.48
-3	2	12	-0.23	0.30
2	-2	-12	0.09	0.66
2	-2	-12	1.12	0.33
-2	2	12	0.92	0.34
-2	2	12	0.60	0.61
-2	2	12	1.26	1.22
-2	2	12	0.15	0.53
1	-2	-12	1.14	0.66
1	-2	-12	0.52	0.33
-1	2	12	0.59	0.61

-1	2	12	-0.21	1.30
-1	2	12	0.99	0.49
-1	2	12	0.31	0.37
0	-2	-12	0.20	0.69
0	-2	-12	0.67	0.33
0	2	12	0.16	0.70
0	2	12	1.48	0.43
0	2	12	0.98	0.49
-1	-2	-12	2.27	0.82
-1	-2	-12	3.92	0.58
1	2	12	3.29	0.56
1	2	12	2.28	1.00
1	2	12	2.54	0.64
-2	-2	-12	1.58	0.80
-2	-2	-12	2.51	0.48
2	2	12	2.36	0.74
2	2	12	2.58	0.59
2	2	12	3.73	1.08
-3	-2	-12	-0.95	0.70
-3	-2	-12	0.62	0.48
3	2	12	2.06	1.15
3	2	12	0.34	0.66
-4	-2	-12	3.45	0.60
-4	-2	-12	3.33	0.91
4	2	12	3.32	0.90
4	2	12	3.90	1.50
-5	-2	-12	6.08	1.23
-5	-2	-12	4.64	1.00
5	2	12	5.08	1.63
5	2	12	5.64	0.98
6	2	12	5.60	1.08
6	2	12	6.80	1.91
17	-3	-12	16.74	1.32
17	-3	-12	12.21	1.92
16	-3	-12	19.02	1.31
16	-3	-12	20.95	2.54
15	-3	-12	15.39	2.28
15	-3	-12	15.99	1.19
14	-3	-12	23.47	2.33
14	-3	-12	20.99	1.23
-14	3	12	21.86	1.45
13	-3	-12	8.96	1.44
13	-3	-12	8.91	1.40
13	-3	-12	10.51	0.88

-13	3	12	10.67	1.09
12	-3	-12	22.55	1.12
12	-3	-12	19.22	2.01
12	-3	-12	18.31	1.74
-12	3	12	22.91	1.28
11	-3	-12	35.09	1.43
11	-3	-12	32.90	2.28
11	-3	-12	34.79	2.01
-11	3	12	33.20	1.55
10	-3	-12	8.93	1.42
10	-3	-12	8.76	1.50
10	-3	-12	12.07	0.80
-10	3	12	11.71	1.03
-10	3	12	10.67	0.89
9	-3	-12	-0.08	0.60
9	-3	-12	-0.11	0.81
9	-3	-12	0.48	0.36
9	-3	-12	0.54	0.47
9	-3	-12	-0.41	0.85
-9	3	12	0.75	0.38
-9	3	12	0.28	0.69
8	-3	-12	26.24	1.32
8	-3	-12	23.42	1.82
8	-3	-12	25.28	1.20
8	-3	-12	25.57	1.12
8	-3	-12	19.97	1.78
-8	3	12	26.97	1.77
-8	3	12	25.37	1.27
7	-3	-12	5.27	0.98
7	-3	-12	4.66	0.72
7	-3	-12	4.60	1.02
7	-3	-12	5.42	0.62
-7	3	12	5.24	0.70
-7	3	12	5.88	1.14
6	-3	-12	2.46	0.43
6	-3	-12	2.63	0.89
-6	3	12	1.98	0.49
-6	3	12	5.27	1.19
-6	3	12	3.35	0.55
5	-3	-12	2.84	0.88
5	-3	-12	3.33	0.44
-5	3	12	3.86	0.67
-5	3	12	3.12	0.56
-5	3	12	4.28	0.51

-5	3	12	2.37	1.27
4	-3	-12	0.00	0.33
4	-3	-12	0.36	0.74
-4	3	12	0.01	0.41
-4	3	12	0.48	0.30
-4	3	12	-0.15	0.45
-4	3	12	-0.48	1.20
3	-3	-12	0.93	0.32
-3	3	12	1.10	0.32
-3	3	12	2.24	1.30
-3	3	12	0.44	0.54
-3	3	12	1.01	0.68
2	-3	-12	1.21	0.76
2	-3	-12	1.26	0.32
-2	3	12	1.36	1.43
-2	3	12	1.34	0.36
-2	3	12	1.29	0.48
-2	3	12	0.77	0.63
1	-3	-12	3.58	0.86
1	-3	-12	5.44	0.57
-1	3	12	6.69	1.08
-1	3	12	4.75	0.74
-1	3	12	6.38	0.64
0	-3	-12	3.09	0.44
0	-3	-12	3.57	0.86
0	3	12	3.67	0.73
0	3	12	3.43	0.97
0	3	12	5.37	0.69
-1	-3	-12	-0.39	0.38
-1	-3	-12	0.06	0.73
1	3	12	-0.51	0.49
1	3	12	1.16	0.65
1	3	12	2.38	0.91
-2	-3	-12	0.11	0.43
-2	-3	-12	-1.34	0.73
2	3	12	-0.41	0.91
2	3	12	0.77	0.67
-3	-3	-12	0.79	0.80
-3	-3	-12	0.32	0.48
3	3	12	0.62	1.11
3	3	12	0.65	0.75
-4	-3	-12	1.73	0.90
-4	-3	-12	1.39	0.59
4	3	12	1.26	1.36

4	3	12	1.23	0.84
-5	-3	-12	5.01	1.10
5	3	12	6.22	1.80
5	3	12	6.46	1.16
17	-4	-12	11.66	1.89
17	-4	-12	12.86	1.27
16	-4	-12	5.32	1.61
16	-4	-12	6.20	0.80
15	-4	-12	15.97	2.30
15	-4	-12	10.04	1.03
14	-4	-12	12.50	1.00
14	-4	-12	15.74	2.20
-14	4	12	12.37	1.16
13	-4	-12	3.35	1.00
13	-4	-12	3.84	0.58
13	-4	-12	5.16	1.34
-13	4	12	4.76	0.72
12	-4	-12	5.86	1.00
12	-4	-12	4.14	1.17
12	-4	-12	4.55	0.70
12	-4	-12	6.06	1.35
-12	4	12	5.41	0.81
-12	4	12	5.07	0.81
11	-4	-12	8.93	1.34
11	-4	-12	9.91	0.77
11	-4	-12	7.86	1.26
11	-4	-12	8.75	1.38
-11	4	12	8.37	1.46
-11	4	12	8.83	0.86
10	-4	-12	6.45	1.18
10	-4	-12	4.12	0.65
10	-4	-12	5.62	0.91
10	-4	-12	5.48	1.11
10	-4	-12	7.14	0.82
-10	4	12	7.13	0.75
-10	4	12	5.17	1.51
9	-4	-12	4.62	0.64
9	-4	-12	4.99	1.12
9	-4	-12	6.97	1.23
9	-4	-12	5.30	0.59
9	-4	-12	5.39	0.93
-9	4	12	5.13	0.73
-9	4	12	9.28	1.51
8	-4	-12	-0.21	0.65

8	-4	-12	-0.03	0.34
8	-4	-12	1.12	0.96
8	-4	-12	0.22	0.44
-8	4	12	0.47	0.38
-8	4	12	-1.32	1.05
7	-4	-12	1.46	1.17
7	-4	-12	0.26	0.45
7	-4	-12	-0.42	0.32
7	-4	-12	-1.29	0.65
-7	4	12	1.69	2.30
-7	4	12	0.00	0.40
6	-4	-12	5.62	0.59
6	-4	-12	5.08	0.73
6	-4	-12	4.28	0.85
-6	4	12	5.57	1.41
-6	4	12	4.44	0.73
5	-4	-12	3.86	0.55
-5	4	12	3.81	0.67
-5	4	12	3.81	1.33
-5	4	12	3.49	0.63
4	-4	-12	4.22	0.94
4	-4	-12	5.12	0.54
-4	4	12	5.89	0.52
-4	4	12	4.49	1.54
-4	4	12	5.07	0.92
3	-4	-12	1.73	0.79
3	-4	-12	2.64	0.37
-3	4	12	2.43	0.36
-3	4	12	2.03	1.60
-3	4	12	1.38	0.54
-3	4	12	1.97	0.66
2	-4	-12	11.58	1.43
2	-4	-12	13.29	0.71
-2	4	12	12.70	1.17
-2	4	12	13.65	0.71
-2	4	12	12.35	1.26
1	-4	-12	13.69	1.43
1	-4	-12	11.53	0.69
-1	4	12	10.23	1.27
-1	4	12	11.23	1.21
-1	4	12	10.60	0.71
0	-4	-12	2.40	0.43
0	-4	-12	4.35	0.95
0	4	12	2.96	0.88

0	4	12	3.81	0.86
-1	-4	-12	3.93	0.95
-1	-4	-12	6.17	0.68
1	4	12	7.45	1.04
1	4	12	6.40	1.15
-2	-4	-12	1.25	0.46
-2	-4	-12	0.70	0.84
2	4	12	4.06	0.95
2	4	12	2.61	1.15
-3	-4	-12	1.09	0.88
3	4	12	2.89	0.98
3	4	12	1.31	1.19
-4	-4	-12	6.70	1.10
4	4	12	5.36	1.04
4	4	12	7.63	1.59
17	-5	-12	11.38	2.00
17	-5	-12	10.83	1.19
16	-5	-12	8.25	1.72
16	-5	-12	8.19	1.09
15	-5	-12	9.58	1.75
15	-5	-12	8.08	0.99
14	-5	-12	2.81	1.39
14	-5	-12	2.43	0.56
-14	5	12	2.69	0.68
13	-5	-12	0.76	1.02
13	-5	-12	1.34	1.19
13	-5	-12	0.36	0.46
-13	5	12	1.14	0.58
12	-5	-12	6.36	1.34
12	-5	-12	4.54	0.58
12	-5	-12	5.65	1.11
12	-5	-12	11.26	1.97
-12	5	12	4.64	0.79
-12	5	12	6.39	1.05
11	-5	-12	7.36	0.72
11	-5	-12	7.67	1.39
11	-5	-12	7.15	1.40
11	-5	-12	10.38	1.49
-11	5	12	8.92	1.61
-11	5	12	8.41	0.83
10	-5	-12	-0.06	0.35
10	-5	-12	0.17	1.12
10	-5	-12	0.95	0.48
10	-5	-12	-0.32	0.84

10	-5	-12	-0.12	0.90
-10	5	12	0.24	0.40
-10	5	12	0.20	1.12
9	-5	-12	0.08	1.13
9	-5	-12	-0.67	0.44
9	-5	-12	-0.50	0.84
9	-5	-12	0.20	0.88
9	-5	-12	-0.01	0.35
-9	5	12	0.11	1.16
-9	5	12	0.12	0.39
8	-5	-12	-1.16	0.86
8	-5	-12	0.05	0.81
8	-5	-12	0.63	0.36
8	-5	-12	0.87	0.45
-8	5	12	0.81	0.42
-8	5	12	1.15	1.28
7	-5	-12	5.74	0.62
7	-5	-12	5.10	1.01
7	-5	-12	5.98	0.73
7	-5	-12	5.45	1.11
-7	5	12	5.97	1.54
-7	5	12	5.74	0.80
6	-5	-12	2.47	0.88
6	-5	-12	2.50	0.41
6	-5	-12	2.20	0.47
6	-5	-12	1.81	0.89
-6	5	12	1.77	1.49
-6	5	12	2.02	0.51
5	-5	-12	2.24	0.93
5	-5	-12	2.50	0.50
5	-5	-12	1.39	0.84
5	-5	-12	3.87	0.54
-5	5	12	1.25	1.46
4	-5	-12	3.87	0.49
4	-5	-12	1.17	0.82
-4	5	12	4.01	0.70
-4	5	12	5.10	0.81
-4	5	12	2.31	1.64
3	-5	-12	6.02	0.51
3	-5	-12	5.97	1.06
-3	5	12	5.44	0.87
-3	5	12	5.64	1.00
-3	5	12	4.98	0.52
2	-5	-12	6.29	1.06

2	-5	-12	7.90	0.57
-2	5	12	10.03	1.14
-2	5	12	7.32	1.20
-2	5	12	7.90	0.55
1	-5	-12	3.09	0.39
1	-5	-12	2.15	0.88
-1	5	12	2.39	0.88
-1	5	12	3.69	0.84
0	-5	-12	4.25	0.98
0	-5	-12	2.43	0.40
0	5	12	3.03	0.81
0	5	12	3.93	1.04
-1	-5	-12	5.87	1.08
1	5	12	4.75	1.18
1	5	12	4.59	0.95
-2	-5	-12	7.93	1.18
2	5	12	10.96	1.92
2	5	12	10.10	1.48
-3	-5	-12	12.79	1.75
3	5	12	17.43	2.28
3	5	12	10.78	1.61
4	5	12	7.70	1.77
17	-6	-12	6.14	0.84
17	-6	-12	3.81	1.69
16	-6	-12	0.39	0.64
16	-6	-12	-0.05	1.43
15	-6	-12	3.82	1.51
15	-6	-12	4.20	0.70
14	-6	-12	5.35	1.41
14	-6	-12	3.87	0.63
14	-6	-12	5.16	1.50
-14	6	12	5.04	0.72
13	-6	-12	0.67	1.17
13	-6	-12	0.81	1.17
13	-6	-12	1.65	0.48
-13	6	12	1.42	0.55
12	-6	-12	0.67	1.11
12	-6	-12	0.52	1.11
12	-6	-12	0.29	0.44
12	-6	-12	-2.24	1.31
-12	6	12	1.22	1.16
-12	6	12	-0.26	0.45
11	-6	-12	3.11	1.14
11	-6	-12	1.88	0.47

11	-6	-12	2.74	1.42
11	-6	-12	2.63	1.07
-11	6	12	1.27	1.26
-11	6	12	2.28	0.54
10	-6	-12	0.62	0.42
10	-6	-12	0.24	1.24
10	-6	-12	-0.13	0.50
10	-6	-12	-0.16	0.88
10	-6	-12	1.46	1.01
-10	6	12	2.96	1.38
-10	6	12	0.12	0.44
9	-6	-12	1.72	0.41
9	-6	-12	1.36	1.00
9	-6	-12	1.03	0.47
9	-6	-12	1.48	0.98
-9	6	12	1.51	0.48
-9	6	12	1.13	1.47
8	-6	-12	13.81	0.83
8	-6	-12	13.15	1.77
8	-6	-12	10.37	1.66
8	-6	-12	13.26	0.88
-8	6	12	14.11	1.04
-8	6	12	14.12	2.04
7	-6	-12	5.50	0.72
7	-6	-12	7.36	1.17
7	-6	-12	6.58	0.62
7	-6	-12	4.92	1.12
-7	6	12	6.16	0.88
-7	6	12	8.20	1.83
6	-6	-12	0.11	0.33
6	-6	-12	0.55	0.42
6	-6	-12	0.36	0.93
6	-6	-12	0.10	0.94
-6	6	12	0.05	0.51
-6	6	12	-0.72	1.54
5	-6	-12	8.78	0.70
5	-6	-12	7.53	1.24
5	-6	-12	6.61	0.57
5	-6	-12	9.25	1.63
-5	6	12	11.48	1.97
-5	6	12	7.22	1.02
4	-6	-12	1.96	0.95
4	-6	-12	3.72	0.47
4	-6	-12	4.55	0.64

-4	6	12	4.75	0.83
3	-6	-12	3.62	0.98
3	-6	-12	4.61	0.47
-3	6	12	4.36	0.87
-3	6	12	4.31	0.85
2	-6	-12	7.38	1.15
-2	6	12	4.33	0.84
-2	6	12	8.24	1.36
1	-6	-12	3.39	0.93
-1	6	12	3.76	1.03
-1	6	12	3.58	0.85
0	-6	-12	4.69	1.06
0	6	12	2.43	0.87
0	6	12	4.60	1.15
-1	-6	-12	7.76	1.20
1	6	12	13.06	1.95
1	6	12	9.64	1.46
-2	-6	-12	11.23	1.68
2	6	12	10.08	1.62
2	6	12	14.15	2.07
-3	-6	-12	6.78	1.62
3	6	12	6.07	1.61
3	6	12	5.51	1.21
17	-7	-12	5.78	2.03
17	-7	-12	4.33	0.82
16	-7	-12	2.36	0.68
16	-7	-12	3.33	1.60
15	-7	-12	1.69	1.45
15	-7	-12	1.32	0.64
14	-7	-12	1.70	1.52
14	-7	-12	2.21	1.28
14	-7	-12	0.63	0.59
-14	7	12	0.90	0.58
13	-7	-12	1.94	0.52
13	-7	-12	2.29	1.28
13	-7	-12	2.96	1.42
-13	7	12	3.97	0.64
12	-7	-12	3.15	1.35
12	-7	-12	5.92	1.38
12	-7	-12	1.92	0.49
12	-7	-12	2.44	1.70
-12	7	12	2.99	0.57
11	-7	-12	0.17	0.54
11	-7	-12	1.32	1.52

11	-7	-12	4.34	1.30
11	-7	-12	1.57	0.47
11	-7	-12	1.09	1.13
-11	7	12	1.99	0.55
-11	7	12	0.45	1.50
10	-7	-12	0.88	1.13
10	-7	-12	0.64	0.44
10	-7	-12	0.10	0.49
10	-7	-12	1.99	1.17
-10	7	12	-0.06	0.48
-10	7	12	0.69	1.52
9	-7	-12	4.99	1.27
9	-7	-12	3.80	0.51
9	-7	-12	4.29	0.57
9	-7	-12	4.25	1.23
-9	7	12	7.13	1.86
-9	7	12	2.96	0.62
8	-7	-12	2.43	0.44
8	-7	-12	1.73	0.47
8	-7	-12	0.48	1.04
8	-7	-12	1.63	1.18
-8	7	12	2.11	0.63
-8	7	12	2.88	1.69
7	-7	-12	0.86	0.98
7	-7	-12	2.45	1.17
7	-7	-12	1.42	0.45
7	-7	-12	1.88	0.40
-7	7	12	3.62	1.87
-7	7	12	1.63	0.61
6	-7	-12	6.35	1.31
6	-7	-12	3.54	1.05
6	-7	-12	3.99	0.54
6	-7	-12	4.36	0.47
-6	7	12	4.03	0.78
5	-7	-12	1.35	0.98
5	-7	-12	1.86	1.13
-5	7	12	0.76	0.60
4	-7	-12	2.93	1.06
-4	7	12	2.81	0.74
3	-7	-12	5.55	1.14
-3	7	12	5.11	0.91
-3	7	12	6.20	1.06
2	-7	-12	4.11	1.14
-2	7	12	3.00	1.02

-2	7	12	5.09	0.94
1	-7	-12	11.07	1.66
-1	7	12	13.12	1.50
-1	7	12	11.28	1.76
0	-7	-12	7.08	1.22
0	7	12	9.79	1.49
0	7	12	6.39	1.10
-1	-7	-12	4.16	1.16
1	7	12	2.68	1.08
1	7	12	2.27	1.31
-2	-7	-12	11.33	1.89
2	7	12	6.84	1.53
2	7	12	7.44	1.47
16	-8	-12	0.49	1.57
16	-8	-12	-0.30	0.65
15	-8	-12	1.30	0.62
15	-8	-12	0.29	1.41
14	-8	-12	7.68	1.77
14	-8	-12	6.32	0.93
14	-8	-12	4.46	1.53
-14	8	12	4.35	0.93
13	-8	-12	-1.89	1.43
13	-8	-12	-0.97	1.17
13	-8	-12	0.28	0.62
-13	8	12	0.00	0.53
12	-8	-12	4.92	1.58
12	-8	-12	1.84	1.24
12	-8	-12	3.81	0.58
-12	8	12	4.01	0.65
11	-8	-12	2.28	0.48
11	-8	-12	2.17	0.63
11	-8	-12	4.76	1.42
11	-8	-12	0.34	1.27
-11	8	12	3.11	1.89
-11	8	12	0.99	0.54
10	-8	-12	1.38	1.22
10	-8	-12	0.87	0.45
10	-8	-12	0.57	0.51
10	-8	-12	1.53	1.25
-10	8	12	-0.22	0.50
-10	8	12	1.36	1.97
9	-8	-12	1.10	1.24
9	-8	-12	2.05	1.25
9	-8	-12	1.29	0.51

9	-8	-12	1.03	0.46
-9	8	12	-2.53	1.85
8	-8	-12	4.76	1.34
8	-8	-12	2.63	0.44
8	-8	-12	2.47	1.39
-8	8	12	2.61	0.66
7	-8	-12	1.27	0.38
7	-8	-12	3.00	1.16
7	-8	-12	-0.04	1.19
-7	8	12	0.10	0.63
6	-8	-12	1.55	1.21
6	-8	-12	2.43	1.14
-6	8	12	0.27	0.64
5	-8	-12	6.86	1.34
5	-8	-12	5.64	1.45
-5	8	12	4.89	0.90
4	-8	-12	17.44	1.88
4	-8	-12	14.22	2.24
-4	8	12	14.23	1.50
3	-8	-12	2.69	1.15
-3	8	12	2.26	0.90
2	-8	-12	6.32	1.21
-2	8	12	5.37	1.02
-2	8	12	3.69	1.19
1	-8	-12	13.91	1.89
-1	8	12	14.59	1.74
-1	8	12	17.74	2.09
0	-8	-12	7.33	1.31
0	8	12	5.72	1.29
0	8	12	7.75	1.48
-1	-8	-12	13.98	1.95
1	8	12	11.74	1.74
16	-9	-12	2.50	0.79
16	-9	-12	-3.17	1.56
15	-9	-12	0.05	0.68
15	-9	-12	-0.65	1.46
14	-9	-12	6.25	1.73
14	-9	-12	4.08	0.65
14	-9	-12	3.05	1.91
13	-9	-12	0.28	1.44
13	-9	-12	1.24	1.74
13	-9	-12	-0.84	0.52
-13	9	12	0.02	0.60
12	-9	-12	0.18	0.49

12	-9	-12	-0.02	1.37
12	-9	-12	0.16	1.56
-12	9	12	-0.25	0.55
11	-9	-12	5.26	1.64
11	-9	-12	3.85	0.58
11	-9	-12	3.32	1.39
-11	9	12	3.80	0.74
10	-9	-12	12.09	1.81
10	-9	-12	8.85	1.67
10	-9	-12	10.33	0.84
-10	9	12	11.30	1.14
9	-9	-12	4.00	1.28
9	-9	-12	4.61	1.52
9	-9	-12	2.16	0.47
-9	9	12	1.82	0.71
8	-9	-12	5.69	1.35
8	-9	-12	5.78	1.58
-8	9	12	4.88	0.86
7	-9	-12	3.05	1.39
7	-9	-12	2.04	1.19
-7	9	12	1.84	0.77
6	-9	-12	12.20	2.02
6	-9	-12	10.62	1.69
-6	9	12	11.68	1.44
5	-9	-12	21.33	2.20
5	-9	-12	19.29	2.57
-5	9	12	18.83	1.67
4	-9	-12	13.32	2.01
4	-9	-12	13.45	2.50
-4	9	12	14.45	1.67
3	-9	-12	2.62	1.14
-3	9	12	2.11	0.92
2	-9	-12	5.66	1.25
-2	9	12	5.12	1.28
-2	9	12	5.51	1.16
1	-9	-12	16.28	2.01
-1	9	12	18.10	1.93
-1	9	12	23.06	2.30
15	-10	-12	0.22	0.68
15	-10	-12	3.01	1.77
14	-10	-12	-0.15	0.62
14	-10	-12	-1.09	1.45
13	-10	-12	-0.32	0.55
13	-10	-12	-1.15	2.11

13	-10	-12	2.33	1.55
-13	10	12	0.09	0.60
12	-10	-12	4.30	1.65
12	-10	-12	3.77	0.62
-12	10	12	4.17	0.80
11	-10	-12	5.96	0.83
11	-10	-12	3.40	1.58
-11	10	12	7.31	1.20
10	-10	-12	2.02	1.46
10	-10	-12	4.58	1.69
-10	10	12	5.34	0.94
9	-10	-12	16.02	2.39
9	-10	-12	13.51	2.03
-9	10	12	11.97	1.43
8	-10	-12	17.35	2.68
8	-10	-12	13.57	2.31
-8	10	12	16.29	1.55
7	-10	-12	9.73	1.76
7	-10	-12	10.28	1.99
-7	10	12	11.41	1.48
6	-10	-12	14.88	2.65
6	-10	-12	14.81	2.23
-6	10	12	17.11	1.74
5	-10	-12	9.15	1.93
5	-10	-12	13.31	2.08
-5	10	12	15.44	1.73
4	-10	-12	6.27	1.42
4	-10	-12	11.15	1.99
-4	10	12	5.95	1.18
3	-10	-12	5.61	1.96
3	-10	-12	4.55	1.30
-3	10	12	4.33	1.19
2	-10	-12	10.78	1.57
-2	10	12	11.22	1.66
-2	10	12	14.05	2.02
15	-11	-12	1.79	0.71
15	-11	-12	2.82	1.95
14	-11	-12	2.04	1.01
14	-11	-12	2.33	1.76
14	-11	-12	2.30	2.25
13	-11	-12	1.50	2.11
13	-11	-12	4.12	1.68
-13	11	12	3.36	1.43
12	-11	-12	1.93	1.62

12	-11	-12	-1.28	1.92
-12	11	12	0.20	0.74
11	-11	-12	6.59	2.06
11	-11	-12	10.45	1.95
-11	11	12	7.88	1.38
10	-11	-12	5.60	1.64
10	-11	-12	8.40	2.09
-10	11	12	10.42	1.44
9	-11	-12	10.16	1.85
9	-11	-12	13.03	2.24
-9	11	12	8.38	1.18
8	-11	-12	13.08	2.27
8	-11	-12	11.20	1.77
-8	11	12	11.49	1.59
7	-11	-12	11.85	1.82
7	-11	-12	12.57	2.16
-7	11	12	14.43	1.74
6	-11	-12	8.60	2.11
6	-11	-12	10.28	1.64
5	-11	-12	5.76	1.49
5	-11	-12	4.94	1.95
-5	11	12	7.39	1.27
4	-11	-12	11.12	2.17
4	-11	-12	7.04	1.47
-4	11	12	11.90	1.87
13	-12	-12	-0.03	1.83
13	-12	-12	4.62	2.29
12	-12	-12	4.41	1.86
12	-12	-12	5.16	2.24
-12	12	12	5.66	1.04
11	-12	-12	8.45	1.89
11	-12	-12	12.31	2.45
-11	12	12	9.25	1.59
10	-12	-12	1.43	2.08
10	-12	-12	1.85	1.64
-10	12	12	1.86	0.99
9	-12	-12	5.08	2.06
9	-12	-12	6.85	1.74
-9	12	12	4.55	1.13
8	-12	-12	12.93	2.42
8	-12	-12	9.38	1.89
-8	12	12	10.31	1.42
7	-12	-12	6.59	2.15
7	-12	-12	8.15	1.73

-7	12	12	10.32	1.81
6	-12	-12	2.11	1.46
6	-12	-12	2.88	2.04
-6	12	12	6.04	1.25
1	16	-13	17.65	1.59
-1	-16	13	24.39	4.34
0	16	-13	21.33	1.63
0	-16	13	24.90	3.44
0	-16	13	19.15	3.08
-1	16	-13	9.74	1.42
1	-16	13	13.87	3.12
1	-16	13	12.77	2.00
-2	16	-13	17.95	1.54
2	-16	13	19.42	3.44
2	-16	13	17.39	2.91
3	-16	13	16.58	3.53
4	15	-13	9.39	1.21
3	15	-13	10.55	1.19
-3	-15	13	13.39	2.18
2	15	-13	17.97	1.58
-2	-15	13	13.75	2.63
-2	-15	13	12.81	1.99
1	15	-13	17.08	1.51
-1	-15	13	14.63	2.12
-1	-15	13	17.50	2.86
0	15	-13	17.12	1.45
0	-15	13	12.45	1.90
0	-15	13	19.24	3.03
-1	15	-13	13.77	1.40
1	-15	13	16.67	2.54
1	-15	13	21.52	3.31
-2	15	-13	12.49	1.31
2	-15	13	13.05	3.03
2	-15	13	13.38	2.02
-3	15	-13	19.45	1.53
3	-15	13	21.90	2.76
3	-15	13	19.05	3.37
-4	15	-13	12.55	1.40
4	-15	13	13.47	2.00
4	-15	13	15.84	3.45
-5	15	-13	3.91	0.91
5	-15	13	4.78	1.72
5	-15	13	3.96	3.18
6	14	-13	19.22	1.73

-6	-14	13	19.89	3.30
5	14	-13	24.55	1.81
-5	-14	13	26.84	2.98
4	14	-13	14.13	1.53
-4	-14	13	16.12	2.64
3	14	-13	12.18	1.50
-3	-14	13	15.87	2.37
-3	-14	13	16.86	2.48
2	14	-13	13.55	1.41
-2	-14	13	18.36	3.24
-2	-14	13	10.17	1.88
1	14	-13	13.39	1.37
-1	-14	13	13.99	2.37
-1	-14	13	15.36	3.08
0	14	-13	10.64	1.20
0	-14	13	7.87	2.32
0	-14	13	9.93	1.64
-1	14	-13	25.60	1.60
1	-14	13	24.20	2.64
1	-14	13	31.74	3.77
-2	14	-13	12.05	1.29
2	-14	13	16.70	2.36
2	-14	13	15.26	2.84
-3	14	-13	3.52	0.78
3	-14	13	4.67	2.59
3	-14	13	2.92	1.48
-4	14	-13	3.75	0.82
4	-14	13	2.82	1.50
4	-14	13	5.00	2.70
-5	14	-13	12.34	1.33
5	-14	13	9.61	3.08
5	-14	13	15.63	2.57
-6	14	-13	4.38	1.06
6	-14	13	5.91	3.13
6	-14	13	7.59	1.75
7	13	-13	15.45	1.68
-7	-13	13	20.05	2.94
6	13	-13	25.60	1.78
-6	-13	13	19.04	2.74
5	13	-13	22.05	1.66
-5	-13	13	21.98	2.73
4	13	-13	12.74	1.42
-4	-13	13	12.15	2.01
-4	-13	13	11.81	1.81

3	13	-13	28.94	1.73
-3	-13	13	31.32	3.10
-3	-13	13	26.78	2.66
2	13	-13	32.72	1.78
-2	-13	13	33.38	2.66
-2	-13	13	28.80	3.23
1	13	-13	15.41	1.35
-1	-13	13	12.59	2.21
-1	-13	13	16.58	2.41
0	13	-13	11.82	1.19
0	-13	13	11.39	2.20
0	-13	13	12.73	2.17
-1	13	-13	13.66	1.24
1	-13	13	13.15	2.05
1	-13	13	15.01	2.89
-2	13	-13	3.50	0.71
2	-13	13	0.57	2.08
2	-13	13	3.79	1.27
-3	13	-13	6.82	0.93
3	-13	13	7.26	1.55
3	-13	13	11.98	2.63
-4	13	-13	1.99	0.74
4	-13	13	3.05	2.37
4	-13	13	4.36	1.39
-5	13	-13	1.90	0.75
5	-13	13	2.42	1.38
5	-13	13	2.40	2.70
-6	13	-13	3.90	0.84
6	-13	13	6.84	3.06
6	-13	13	2.91	1.54
7	-13	13	9.09	3.18
8	12	-13	30.69	1.91
-8	-12	13	28.98	3.05
7	12	-13	20.92	1.64
-7	-12	13	18.95	2.67
6	12	-13	10.30	1.34
-6	-12	13	6.96	1.67
5	12	-13	16.25	1.39
-5	-12	13	13.56	2.27
-5	-12	13	12.75	2.21
4	12	-13	35.11	1.80
-4	-12	13	31.29	2.63
-4	-12	13	32.00	2.76
3	12	-13	36.07	1.84

-3	-12	13	37.29	2.72
-3	-12	13	36.86	2.97
2	12	-13	33.13	1.73
-2	-12	13	33.21	2.58
-2	-12	13	32.89	2.96
1	12	-13	16.85	1.29
-1	-12	13	15.75	2.23
-1	-12	13	16.86	2.05
0	12	-13	8.85	1.09
0	-12	13	9.21	1.47
0	-12	13	12.54	2.16
-1	12	-13	16.72	1.24
1	-12	13	16.33	2.03
1	-12	13	13.97	2.30
-2	12	-13	7.59	1.10
2	-12	13	11.45	1.92
2	-12	13	9.62	2.26
3	-12	13	4.67	2.12
3	-12	13	5.25	1.28
-4	12	-13	3.97	0.79
4	-12	13	4.47	2.39
4	-12	13	5.65	1.30
-5	12	-13	5.35	0.87
5	-12	13	11.15	1.69
5	-12	13	4.96	2.65
-6	12	-13	4.26	0.81
6	-12	13	3.83	1.50
6	-12	13	4.22	2.57
-7	12	-13	-0.33	0.71
7	-12	13	-0.58	2.76
7	-12	13	3.98	1.50
10	11	-13	14.58	1.65
-10	-11	13	17.07	2.40
9	11	-13	25.61	1.79
-9	-11	13	23.68	2.92
8	11	-13	22.01	1.63
-8	-11	13	21.06	2.72
7	11	-13	8.61	0.99
-7	-11	13	7.93	1.56
6	11	-13	15.09	1.35
-6	-11	13	15.08	2.29
-6	-11	13	14.21	2.27
5	11	-13	25.85	1.57
-5	-11	13	23.28	2.17

-5	-11	13	29.20	2.47
4	11	-13	22.09	1.48
-4	-11	13	27.35	2.38
-4	-11	13	27.89	2.36
3	11	-13	5.96	0.84
-3	-11	13	5.57	1.34
-3	-11	13	6.81	1.63
2	11	-13	9.64	1.12
2	11	-13	10.04	1.87
-2	-11	13	12.31	1.81
-2	-11	13	11.83	2.18
1	11	-13	9.25	1.07
1	11	-13	8.89	1.38
-1	-11	13	7.85	1.77
-1	-11	13	10.57	1.77
0	11	-13	8.71	0.63
0	11	-13	10.14	1.06
0	11	-13	11.20	1.88
0	-11	13	9.52	1.91
0	-11	13	12.41	1.78
-1	11	-13	4.10	0.73
-1	11	-13	4.53	0.60
1	-11	13	3.58	1.06
1	-11	13	5.37	1.77
-2	11	-13	-0.73	0.57
-2	11	-13	0.57	0.41
2	-11	13	0.78	1.05
2	-11	13	-1.60	1.54
-3	11	-13	2.05	0.66
-3	11	-13	2.50	0.51
3	-11	13	5.54	1.99
3	-11	13	3.41	1.11
-4	11	-13	5.62	0.80
-4	11	-13	6.84	0.84
4	-11	13	7.75	1.42
4	-11	13	7.96	2.30
-5	11	-13	0.02	0.55
-5	11	-13	-0.90	0.61
5	-11	13	0.51	2.18
5	-11	13	1.00	1.20
-6	11	-13	0.93	0.67
6	-11	13	1.90	2.52
6	-11	13	3.02	1.34
-7	11	-13	-0.40	0.70

7	-11	13	-6.08	2.44
7	-11	13	-0.69	1.04
7	-11	13	3.64	1.47
11	10	-13	20.45	1.71
-11	-10	13	17.82	3.12
10	10	-13	16.84	1.54
-10	-10	13	13.91	2.02
9	10	-13	10.35	1.33
-9	-10	13	8.54	1.68
8	10	-13	37.36	1.91
-8	-10	13	35.47	2.87
7	10	-13	27.58	1.64
-7	-10	13	30.33	2.47
6	10	-13	24.01	1.48
6	10	-13	24.03	1.48
-6	-10	13	25.24	2.20
-6	-10	13	21.84	1.71
5	10	-13	23.23	1.41
5	10	-13	26.95	1.53
-5	-10	13	27.93	2.00
-5	-10	13	25.20	2.19
4	10	-13	13.23	1.15
4	10	-13	14.33	1.01
-4	-10	13	14.99	1.81
-4	-10	13	12.06	1.91
3	10	-13	16.51	1.22
3	10	-13	17.26	0.98
3	10	-13	18.25	1.62
-3	-10	13	18.40	1.86
-3	-10	13	19.29	1.03
-3	-10	13	20.01	2.04
2	10	-13	20.56	1.28
2	10	-13	16.44	0.94
2	10	-13	19.71	1.69
-2	-10	13	19.05	1.83
-2	-10	13	22.57	2.30
1	10	-13	7.65	0.72
1	10	-13	7.26	1.18
1	10	-13	8.92	0.98
-1	-10	13	11.92	2.13
-1	-10	13	7.02	1.00
-1	-10	13	10.15	1.57
0	10	-13	14.20	1.62
0	10	-13	8.67	0.72

0	10	-13	10.17	0.99
0	-10	13	8.19	0.93
0	-10	13	13.15	2.25
0	-10	13	9.91	1.55
-1	10	-13	2.87	0.66
-1	10	-13	3.91	1.36
-1	10	-13	4.90	0.68
1	-10	13	4.69	1.06
1	-10	13	4.42	0.93
1	-10	13	3.75	1.58
-2	10	-13	1.35	0.48
-2	10	-13	0.12	0.52
2	-10	13	3.02	1.26
2	-10	13	-0.21	1.57
2	-10	13	1.69	0.97
-3	10	-13	0.93	0.57
-3	10	-13	0.21	0.50
3	-10	13	0.59	0.98
3	-10	13	-0.28	1.64
-4	10	-13	2.66	0.61
-4	10	-13	3.02	0.70
4	-10	13	0.74	1.04
4	-10	13	5.41	1.97
-5	10	-13	0.03	0.58
-5	10	-13	0.73	0.63
5	-10	13	0.35	1.97
5	-10	13	0.52	1.12
-6	10	-13	0.30	0.67
-6	10	-13	0.85	0.69
6	-10	13	-0.27	1.22
6	-10	13	-1.38	2.31
-7	10	-13	1.84	0.75
-7	10	-13	-0.23	0.71
7	-10	13	1.28	1.40
7	-10	13	-0.66	2.62
-8	10	-13	-1.01	0.91
8	-10	13	-1.54	1.43
8	-10	13	-2.34	2.89
11	9	-13	7.78	1.05
-11	-9	13	10.19	1.90
10	9	-13	13.85	2.35
10	9	-13	10.84	1.36
-10	-9	13	16.61	2.48
9	9	-13	22.04	2.02

9	9	-13	28.06	1.59
-9	-9	13	21.90	2.44
8	9	-13	9.31	1.18
8	9	-13	10.29	1.32
-8	-9	13	9.50	1.54
7	9	-13	17.43	1.55
7	9	-13	16.16	1.28
-7	-9	13	16.34	2.00
6	9	-13	16.17	1.25
6	9	-13	16.30	1.44
-6	-9	13	19.35	1.20
-6	-9	13	14.89	1.86
5	9	-13	17.75	1.22
5	9	-13	19.46	1.33
-5	-9	13	18.35	1.83
-5	-9	13	19.79	1.58
-5	-9	13	17.42	1.12
4	9	-13	23.30	1.35
4	9	-13	22.38	1.41
4	9	-13	22.23	1.31
-4	-9	13	25.75	1.90
-4	-9	13	21.73	1.25
-4	-9	13	22.87	1.93
3	9	-13	5.24	0.87
3	9	-13	4.52	0.66
3	9	-13	4.11	0.65
-3	-9	13	5.31	1.16
-3	-9	13	5.09	1.06
-3	-9	13	4.42	0.67
2	9	-13	14.15	1.37
2	9	-13	12.48	0.93
2	9	-13	11.90	1.05
-2	-9	13	15.83	1.15
-2	-9	13	11.84	1.86
-2	-9	13	13.52	1.53
1	9	-13	7.67	1.29
1	9	-13	8.06	0.90
1	9	-13	6.59	0.80
-1	-9	13	10.28	1.62
-1	-9	13	8.81	0.93
-1	-9	13	8.62	1.42
0	9	-13	6.96	0.79
0	9	-13	6.80	0.88
0	9	-13	7.59	1.12

0	-9	13	6.78	1.00
0	-9	13	7.64	1.22
0	-9	13	6.54	1.49
-1	9	-13	1.44	0.99
-1	9	-13	0.90	0.49
-1	9	-13	0.30	0.47
1	-9	13	-0.48	0.90
1	-9	13	-0.61	1.31
1	-9	13	0.39	0.81
-2	9	-13	-0.40	0.47
-2	9	-13	0.01	0.50
2	-9	13	-1.10	0.76
2	-9	13	-2.02	1.31
2	-9	13	-0.75	0.99
-3	9	-13	0.58	0.54
-3	9	-13	0.51	0.58
3	-9	13	-1.05	1.16
3	-9	13	0.63	0.88
-4	9	-13	-0.52	0.54
-4	9	-13	-0.56	0.57
4	-9	13	0.24	0.95
4	-9	13	-4.29	1.58
-5	9	-13	0.04	0.64
-5	9	-13	-0.99	0.59
5	-9	13	0.32	1.03
5	-9	13	-2.45	1.91
-6	9	-13	-0.34	0.67
-6	9	-13	0.54	0.67
6	-9	13	-0.14	1.13
6	-9	13	-1.09	2.23
-7	9	-13	-0.29	0.76
-7	9	-13	-0.52	0.65
7	-9	13	2.39	2.57
7	-9	13	-0.59	1.27
-8	9	-13	2.74	0.84
-8	9	-13	1.81	0.86
8	-9	13	1.86	1.41
8	-9	13	3.37	3.01
12	8	-13	23.12	1.65
12	8	-13	21.35	2.39
-12	-8	13	24.43	3.01
11	8	-13	27.02	1.66
11	8	-13	31.19	2.42
-11	-8	13	26.63	2.79

10	8	-13	16.86	1.96
10	8	-13	12.42	1.29
-10	-8	13	19.56	2.36
9	8	-13	6.83	1.25
9	8	-13	4.67	0.82
-9	-8	13	4.61	1.24
8	8	-13	30.36	1.61
8	8	-13	29.28	2.00
-8	-8	13	31.18	2.32
-8	-8	13	27.97	1.57
7	8	-13	27.54	1.82
7	8	-13	27.54	1.51
-7	-8	13	27.45	1.51
-7	-8	13	27.12	2.13
6	8	-13	22.43	1.66
6	8	-13	24.30	1.39
-6	-8	13	24.89	1.37
-6	-8	13	24.72	1.92
5	8	-13	12.38	1.27
5	8	-13	12.92	1.03
5	8	-13	10.75	0.86
-5	-8	13	13.36	1.11
-5	-8	13	10.14	1.41
-5	-8	13	11.31	1.50
4	8	-13	11.80	1.16
4	8	-13	11.23	0.99
-4	-8	13	8.79	1.35
-4	-8	13	10.22	1.45
-4	-8	13	11.93	1.08
3	8	-13	5.62	0.95
3	8	-13	8.22	0.97
3	8	-13	5.89	0.82
-3	-8	13	7.98	1.07
-3	-8	13	7.09	1.17
-3	-8	13	5.11	0.92
2	8	-13	9.55	0.96
2	8	-13	10.15	1.11
2	8	-13	10.54	0.93
-2	-8	13	10.04	1.72
-2	-8	13	11.64	1.22
-2	-8	13	12.80	1.39
1	8	-13	4.81	0.82
1	8	-13	6.37	0.81
1	8	-13	5.17	0.85

-1	-8	13	6.46	0.96
-1	-8	13	5.96	1.36
-1	-8	13	7.67	1.24
0	8	-13	1.98	0.78
0	8	-13	2.02	0.56
0	8	-13	1.09	0.48
0	-8	13	1.11	0.79
0	-8	13	2.68	0.92
0	-8	13	3.39	1.21
-1	8	-13	0.02	0.83
-1	8	-13	0.92	0.53
-1	8	-13	0.87	0.47
1	-8	13	-0.97	1.13
1	-8	13	2.65	1.00
1	-8	13	0.17	0.71
-2	8	-13	1.47	0.52
-2	8	-13	2.28	0.61
2	-8	13	2.38	1.54
2	-8	13	1.89	1.07
2	-8	13	2.27	0.92
-3	8	-13	0.83	0.58
-3	8	-13	-0.71	0.50
3	-8	13	1.64	1.20
3	-8	13	2.04	1.42
3	-8	13	0.87	0.86
-4	8	-13	0.10	0.59
-4	8	-13	1.41	0.59
4	-8	13	-0.01	0.85
4	-8	13	3.19	1.87
-5	8	-13	-1.13	0.57
-5	8	-13	-0.18	0.63
5	-8	13	0.53	1.01
5	-8	13	1.33	2.01
-6	8	-13	0.10	0.73
-6	8	-13	0.58	0.61
6	-8	13	1.34	1.13
6	-8	13	-1.37	2.04
-7	8	-13	1.97	0.71
-7	8	-13	0.16	0.79
7	-8	13	-4.49	2.32
7	-8	13	0.00	1.20
-8	8	-13	2.69	0.83
-8	8	-13	1.48	0.88
8	-8	13	3.53	1.40

8	-8	13	0.18	2.82
13	7	-13	26.22	1.72
13	7	-13	30.50	2.65
-13	-7	13	22.98	3.03
12	7	-13	24.67	2.39
12	7	-13	24.00	1.60
-12	-7	13	30.08	2.84
11	7	-13	4.29	0.79
11	7	-13	7.33	1.44
-11	-7	13	5.93	1.48
10	7	-13	12.86	1.85
10	7	-13	11.62	1.20
-10	-7	13	11.38	1.57
9	7	-13	26.83	1.49
9	7	-13	27.01	1.96
-9	-7	13	26.33	2.17
-9	-7	13	26.15	1.64
8	7	-13	22.36	1.33
8	7	-13	19.81	1.74
-8	-7	13	19.44	1.90
-8	-7	13	21.56	1.40
7	7	-13	16.16	1.16
7	7	-13	15.74	1.56
-7	-7	13	17.44	1.66
-7	-7	13	16.75	1.28
6	7	-13	9.88	0.94
6	7	-13	8.47	1.35
-6	-7	13	8.14	1.38
-6	-7	13	7.85	1.10
5	7	-13	7.35	0.96
5	7	-13	6.24	0.84
-5	-7	13	7.14	1.21
-5	-7	13	8.69	1.08
4	7	-13	3.50	0.56
4	7	-13	3.41	0.63
4	7	-13	4.70	0.83
-4	-7	13	3.84	0.94
-4	-7	13	4.84	0.79
-4	-7	13	3.15	0.79
3	7	-13	-0.11	0.59
3	7	-13	0.96	0.47
3	7	-13	0.65	0.45
-3	-7	13	-0.71	0.67
-3	-7	13	0.01	0.61

-3	-7	13	0.07	0.88
2	7	-13	-0.31	0.60
2	7	-13	0.43	0.52
2	7	-13	-0.49	0.41
-2	-7	13	1.92	0.78
-2	-7	13	0.18	0.61
-2	-7	13	0.88	0.95
1	7	-13	2.52	0.52
1	7	-13	2.85	0.68
1	7	-13	2.59	0.63
-1	-7	13	3.60	0.91
-1	-7	13	3.20	1.06
-1	-7	13	2.81	0.81
0	7	-13	4.44	0.66
0	7	-13	3.08	0.56
0	7	-13	5.10	0.80
0	-7	13	3.91	1.25
0	-7	13	4.33	1.04
0	-7	13	3.75	0.78
-1	7	-13	-0.08	0.46
-1	7	-13	-0.76	0.49
-1	7	-13	-0.03	1.08
1	-7	13	-0.27	1.15
1	-7	13	0.54	0.67
1	-7	13	-1.37	0.96
-2	7	-13	-0.27	0.53
-2	7	-13	0.02	0.46
2	-7	13	0.06	1.16
2	-7	13	1.58	1.14
2	-7	13	0.11	0.69
-3	7	-13	2.37	0.66
-3	7	-13	2.60	0.58
3	-7	13	2.29	0.93
3	-7	13	0.66	1.40
-4	7	-13	1.33	0.56
-4	7	-13	1.46	0.71
4	-7	13	1.84	0.94
4	-7	13	2.14	1.74
-5	7	-13	3.86	0.75
-5	7	-13	3.57	0.70
5	-7	13	5.62	2.06
-6	7	-13	1.01	0.65
-6	7	-13	-0.45	0.70
6	-7	13	0.58	1.11

6	-7	13	4.31	2.25
-7	7	-13	0.20	0.81
-7	7	-13	0.61	0.68
7	-7	13	-2.31	2.35
7	-7	13	-0.38	1.13
-8	7	-13	3.68	0.89
-8	7	-13	2.47	0.94
8	-7	13	4.13	2.86
8	-7	13	2.79	1.34
14	6	-13	15.46	1.59
14	6	-13	17.29	2.67
-14	-6	13	19.73	2.98
13	6	-13	8.28	1.22
13	6	-13	9.23	1.63
-13	-6	13	10.56	1.77
12	6	-13	10.61	1.25
12	6	-13	10.55	1.60
-12	-6	13	13.20	2.32
11	6	-13	28.72	1.59
11	6	-13	26.64	2.21
-11	-6	13	29.78	2.35
10	6	-13	6.90	1.00
10	6	-13	5.44	1.21
-10	-6	13	5.03	1.15
9	6	-13	11.30	1.57
9	6	-13	10.53	1.05
-9	-6	13	9.07	1.61
-9	-6	13	12.91	1.33
8	6	-13	23.78	1.81
8	6	-13	23.17	1.33
-8	-6	13	23.52	1.81
-8	-6	13	23.06	1.47
7	6	-13	24.63	1.66
7	6	-13	21.95	1.25
-7	-6	13	21.18	1.43
-7	-6	13	20.84	1.77
6	6	-13	3.07	0.58
6	6	-13	3.34	0.91
-6	-6	13	2.18	0.74
5	6	-13	0.08	0.39
5	6	-13	-0.67	0.72
-5	-6	13	-0.72	0.66
4	6	-13	1.35	0.38
4	6	-13	2.33	0.76

-4	-6	13	1.47	1.30
-4	-6	13	2.36	0.77
3	6	-13	0.15	0.47
3	6	-13	0.41	0.66
3	6	-13	0.95	0.55
-3	-6	13	-0.48	0.80
-3	-6	13	0.32	0.73
2	6	-13	-0.05	0.58
2	6	-13	-0.83	0.45
2	6	-13	-0.62	0.48
-2	-6	13	-0.54	0.84
-2	-6	13	0.33	0.79
1	6	-13	0.66	0.57
1	6	-13	1.08	0.59
-1	-6	13	1.23	0.59
-1	-6	13	0.00	0.86
-1	-6	13	-0.30	0.93
0	6	-13	-0.41	0.63
0	6	-13	-0.70	0.55
0	6	-13	0.61	0.43
0	-6	13	0.84	1.02
0	-6	13	1.87	0.96
0	-6	13	0.45	0.56
-1	6	-13	1.15	0.74
-1	6	-13	-0.03	0.56
-1	6	-13	0.33	0.45
1	-6	13	-0.48	1.12
1	-6	13	0.42	0.68
1	-6	13	0.12	1.03
-2	6	-13	1.86	0.63
-2	6	-13	2.06	0.52
2	-6	13	1.72	0.76
2	-6	13	2.44	1.33
-3	6	-13	0.97	0.52
-3	6	-13	0.92	0.61
3	-6	13	1.93	1.45
3	-6	13	0.38	0.74
-4	6	-13	1.73	0.61
-4	6	-13	1.06	0.64
4	-6	13	0.30	1.55
4	-6	13	2.91	0.96
-5	6	-13	8.10	1.01
-5	6	-13	5.99	0.88
5	-6	13	7.93	1.26

5	-6	13	9.24	2.15
-6	6	-13	2.75	0.86
-6	6	-13	3.28	0.74
6	-6	13	2.89	1.07
6	-6	13	3.27	2.21
-7	6	-13	3.79	0.84
-7	6	-13	5.51	0.95
7	-6	13	6.78	2.49
7	-6	13	6.77	1.35
-8	6	-13	10.22	1.48
-8	6	-13	11.27	1.35
8	-6	13	8.43	1.45
8	-6	13	9.54	2.94
14	5	-13	14.18	1.96
14	5	-13	15.97	1.42
-14	-5	13	19.64	2.70
13	5	-13	7.49	0.93
13	5	-13	7.94	1.59
-13	-5	13	5.64	1.47
12	5	-13	3.02	0.71
12	5	-13	3.20	1.23
-12	-5	13	3.95	1.23
11	5	-13	10.39	1.45
11	5	-13	6.75	0.86
-11	-5	13	7.56	1.33
10	5	-13	10.08	1.38
10	5	-13	12.21	1.06
-10	-5	13	9.18	1.45
9	5	-13	31.01	2.07
9	5	-13	31.30	1.52
-9	-5	13	29.43	1.71
8	5	-13	9.24	1.47
8	5	-13	9.71	0.91
-8	-5	13	9.83	1.31
-8	-5	13	10.84	1.27
7	5	-13	2.79	0.57
7	5	-13	3.71	0.93
-7	-5	13	2.02	0.72
-7	-5	13	1.83	0.73
6	5	-13	1.83	0.87
6	5	-13	2.74	0.90
-6	-5	13	3.40	0.76
-6	-5	13	2.03	0.77
5	5	-13	-0.35	0.71

5	5	-13	0.71	0.41
-5	-5	13	1.05	0.61
-5	-5	13	0.51	0.69
4	5	-13	-0.31	0.67
4	5	-13	0.22	0.42
-4	-5	13	0.30	0.47
-4	-5	13	-0.18	0.55
-4	-5	13	0.38	0.75
3	5	-13	0.88	0.40
3	5	-13	0.73	0.64
-3	-5	13	0.28	0.70
-3	-5	13	-0.52	0.45
-3	-5	13	0.54	0.79
2	5	-13	1.44	0.45
2	5	-13	0.69	0.61
2	5	-13	0.47	0.49
-2	-5	13	1.00	0.61
-2	-5	13	1.17	0.87
-2	-5	13	1.63	0.84
1	5	-13	5.83	0.97
1	5	-13	3.39	0.67
1	5	-13	1.45	0.45
-1	-5	13	4.94	1.05
-1	-5	13	1.98	0.96
0	5	-13	4.64	0.79
0	5	-13	4.09	0.75
0	5	-13	2.59	0.50
0	-5	13	3.50	1.17
0	-5	13	4.15	0.82
0	-5	13	3.96	1.09
-1	5	-13	1.68	0.67
-1	5	-13	1.84	0.77
-1	5	-13	1.02	0.46
1	-5	13	1.38	0.63
1	-5	13	1.37	1.12
-2	5	-13	9.74	0.89
-2	5	-13	9.06	1.06
2	-5	13	8.49	1.19
2	-5	13	10.47	1.68
-3	5	-13	0.87	0.56
-3	5	-13	0.93	0.77
3	-5	13	2.76	2.28
3	-5	13	0.57	0.68
-4	5	-13	1.25	0.54

-4	5	-13	1.44	0.68
4	-5	13	0.78	0.78
4	-5	13	0.96	1.52
-5	5	-13	2.58	0.77
-5	5	-13	2.24	0.66
5	-5	13	1.48	0.89
5	-5	13	2.73	1.83
-6	5	-13	2.26	0.82
-6	5	-13	3.85	0.76
6	-5	13	2.87	2.27
6	-5	13	4.56	1.08
-7	5	-13	4.89	0.85
-7	5	-13	2.95	0.94
7	-5	13	3.41	2.28
7	-5	13	3.52	1.16
15	4	-13	17.37	1.54
15	4	-13	16.08	2.05
14	4	-13	11.37	1.82
-14	-4	13	11.12	1.72
13	4	-13	0.49	0.67
13	4	-13	0.71	1.26
-13	-4	13	1.68	1.29
12	4	-13	9.64	1.47
12	4	-13	7.39	1.06
11	4	-13	20.20	1.28
-11	-4	13	18.87	1.77
10	4	-13	8.44	1.33
10	4	-13	10.68	0.99
-10	-4	13	12.60	1.45
9	4	-13	1.19	0.95
9	4	-13	1.38	0.51
-9	-4	13	1.36	0.75
8	4	-13	0.60	0.47
8	4	-13	1.21	0.86
-8	-4	13	0.88	0.64
-8	-4	13	1.73	0.77
7	4	-13	3.80	0.95
-7	-4	13	3.39	0.82
-7	-4	13	3.80	0.78
6	4	-13	-0.68	0.72
6	4	-13	0.18	0.41
-6	-4	13	0.42	0.52
-6	-4	13	0.90	0.74
5	4	-13	0.99	0.76

5	4	-13	0.31	0.37
-5	-4	13	-1.09	0.72
-5	-4	13	0.95	0.52
4	4	-13	1.77	0.73
4	4	-13	1.80	0.46
-4	-4	13	1.70	0.54
-4	-4	13	1.55	0.55
-4	-4	13	1.34	0.80
3	4	-13	-0.01	0.36
3	4	-13	0.14	0.66
-3	-4	13	1.85	0.76
-3	-4	13	-0.52	0.80
-3	-4	13	0.11	0.46
-3	-4	13	0.50	0.64
2	4	-13	-0.02	0.37
2	4	-13	0.10	0.65
-2	-4	13	0.42	0.77
-2	-4	13	-0.08	0.74
-2	-4	13	0.67	0.52
-2	-4	13	-0.65	0.84
1	4	-13	1.25	0.66
1	4	-13	0.70	0.41
-1	-4	13	0.42	0.92
-1	-4	13	0.51	0.87
0	4	-13	16.22	0.99
0	4	-13	17.19	1.31
0	-4	13	20.42	2.14
0	-4	13	17.69	1.75
0	-4	13	18.52	1.32
-1	4	-13	5.62	0.84
-1	4	-13	5.78	0.85
1	-4	13	6.31	1.04
1	-4	13	7.47	1.32
-2	4	-13	2.88	0.72
-2	4	-13	3.78	0.57
2	-4	13	3.50	0.75
2	-4	13	5.25	1.36
-3	4	-13	6.86	0.86
-3	4	-13	6.94	1.11
3	-4	13	7.53	1.22
3	-4	13	7.56	1.76
-4	4	-13	4.99	0.68
-4	4	-13	5.00	0.87
4	-4	13	7.25	1.25

4	-4	13	5.24	1.69
-5	4	-13	9.41	1.06
-5	4	-13	11.35	1.36
5	-4	13	12.98	2.28
5	-4	13	11.44	1.48
-6	4	-13	6.08	0.85
-6	4	-13	5.02	0.96
6	-4	13	5.63	2.10
6	-4	13	5.15	1.07
-7	4	-13	2.10	0.94
-7	4	-13	4.77	0.88
7	-4	13	0.95	2.14
7	-4	13	3.63	1.10
15	3	-13	1.88	0.74
15	3	-13	0.11	1.48
14	3	-13	6.34	0.85
14	3	-13	7.70	1.71
-14	-3	13	9.00	1.47
13	3	-13	15.80	2.16
13	3	-13	17.87	1.25
-13	-3	13	19.57	2.04
12	3	-13	16.25	1.20
12	3	-13	19.49	2.06
-12	-3	13	18.18	1.80
11	3	-13	3.01	0.62
-11	-3	13	4.21	0.97
10	3	-13	5.40	1.14
10	3	-13	6.69	0.75
-10	-3	13	5.07	0.92
9	3	-13	10.41	0.91
9	3	-13	11.27	1.58
-9	-3	13	9.75	1.23
8	3	-13	1.31	0.54
8	3	-13	-0.06	0.82
-8	-3	13	1.16	0.78
-8	-3	13	1.62	0.65
7	3	-13	3.76	0.93
7	3	-13	3.31	0.52
-7	-3	13	2.66	0.63
-7	-3	13	2.36	0.82
6	3	-13	0.30	0.38
6	3	-13	0.22	0.83
-6	-3	13	0.35	0.69
-6	-3	13	0.04	0.47

5	3	-13	0.23	0.77
5	3	-13	0.47	0.39
-5	-3	13	0.46	0.49
-5	-3	13	0.77	0.74
4	3	-13	8.69	1.25
4	3	-13	8.18	0.78
-4	-3	13	8.71	1.12
-4	-3	13	6.38	0.88
-4	-3	13	7.55	0.99
-4	-3	13	7.26	1.06
3	3	-13	1.41	0.42
3	3	-13	0.09	0.68
-3	-3	13	1.28	0.64
-3	-3	13	0.76	0.50
-3	-3	13	1.00	0.89
-3	-3	13	0.20	0.70
2	3	-13	3.78	0.81
2	3	-13	4.14	0.56
-2	-3	13	3.32	0.69
-2	-3	13	1.70	0.87
-2	-3	13	4.99	0.92
-2	-3	13	2.48	0.99
1	3	-13	0.48	0.63
1	3	-13	0.97	0.42
-1	-3	13	1.84	0.94
-1	-3	13	-0.26	0.76
0	3	-13	2.04	0.68
0	3	-13	1.93	0.49
0	-3	13	1.88	0.98
0	-3	13	2.15	0.64
0	-3	13	1.58	0.94
-1	3	-13	9.33	0.81
-1	3	-13	11.43	1.18
1	-3	13	9.23	1.04
1	-3	13	12.35	1.72
1	-3	13	8.02	1.29
-2	3	-13	7.03	1.13
2	-3	13	7.52	1.08
2	-3	13	11.15	1.83
-3	3	-13	4.35	0.61
-3	3	-13	4.38	0.82
3	-3	13	4.06	0.76
3	-3	13	8.13	1.53
-4	3	-13	5.14	0.93

-4	3	-13	8.33	0.94
4	-3	13	4.02	1.57
4	-3	13	8.38	1.26
-5	3	-13	17.61	1.53
-5	3	-13	15.90	1.18
5	-3	13	20.05	1.57
5	-3	13	13.84	2.18
-6	3	-13	8.43	1.14
-6	3	-13	9.56	1.22
6	-3	13	10.28	1.50
6	-3	13	10.62	2.36
-7	3	-13	2.98	0.83
-7	3	-13	6.03	1.07
7	-3	13	4.95	1.08
7	-3	13	5.66	2.20
16	2	-13	1.19	1.58
15	2	-13	8.23	1.12
15	2	-13	7.71	1.62
14	2	-13	7.52	1.65
14	2	-13	11.51	1.14
-14	-2	13	12.39	1.95
13	2	-13	2.45	1.26
13	2	-13	3.37	0.70
-13	-2	13	0.32	1.03
12	2	-13	4.71	0.71
12	2	-13	6.47	1.32
-12	-2	13	4.99	1.04
11	2	-13	7.60	1.32
11	2	-13	7.01	0.91
-11	-2	13	8.28	1.30
10	2	-13	1.66	0.98
10	2	-13	2.47	0.56
-10	-2	13	1.12	0.67
9	2	-13	0.19	0.42
9	2	-13	-0.31	0.84
-9	-2	13	0.08	0.58
8	2	-13	3.08	0.52
8	2	-13	3.28	1.00
-8	-2	13	1.76	0.63
7	2	-13	0.70	0.82
7	2	-13	1.07	0.44
-7	-2	13	0.20	0.48
-7	-2	13	0.82	0.76
6	2	-13	-0.18	0.40

6	2	-13	-0.16	0.74
-6	-2	13	-0.30	0.73
-6	-2	13	0.02	0.47
5	2	-13	4.35	0.62
5	2	-13	3.74	0.92
-5	-2	13	3.85	0.94
-5	-2	13	2.58	0.55
4	2	-13	0.12	0.72
4	2	-13	-0.80	0.33
-4	-2	13	0.21	0.45
-4	-2	13	0.25	0.53
-4	-2	13	-0.05	0.47
-4	-2	13	-0.01	0.70
-4	-2	13	1.83	0.87
3	2	-13	2.72	0.81
3	2	-13	2.71	0.49
-3	-2	13	2.43	0.57
-3	-2	13	1.57	0.69
-3	-2	13	2.82	0.90
-3	-2	13	2.52	0.47
-3	-2	13	2.61	0.86
2	2	-13	1.39	0.68
2	2	-13	1.26	0.41
-2	-2	13	1.05	0.70
-2	-2	13	0.94	0.47
-2	-2	13	0.69	0.45
-2	-2	13	2.05	0.88
1	2	-13	3.26	0.48
1	2	-13	3.51	0.80
-1	-2	13	3.35	0.90
-1	-2	13	2.91	1.07
-1	-2	13	2.93	0.60
0	2	-13	10.02	1.21
0	2	-13	12.18	0.87
0	-2	13	13.50	1.77
0	-2	13	14.39	1.14
0	-2	13	14.01	1.54
-1	2	-13	6.58	1.09
-1	2	-13	4.83	0.72
1	-2	13	4.50	1.10
1	-2	13	5.55	0.93
-2	2	-13	6.91	0.79
-2	2	-13	8.35	1.21
2	-2	13	8.69	1.09

2	-2	13	7.62	1.38
-3	2	-13	14.63	1.41
-3	2	-13	15.96	1.05
3	-2	13	16.07	2.12
3	-2	13	15.87	1.33
-4	2	-13	13.02	1.43
-4	2	-13	14.96	1.10
4	-2	13	15.16	1.37
4	-2	13	14.25	2.42
-5	2	-13	19.73	1.64
-5	2	-13	22.17	1.33
5	-2	13	23.69	2.94
5	-2	13	20.63	1.66
-6	2	-13	10.08	1.22
-6	2	-13	10.10	1.51
6	-2	13	7.69	2.15
6	-2	13	14.09	1.57
-7	2	-13	4.58	1.14
7	-2	13	7.76	2.31
7	-2	13	10.02	1.65
16	1	-13	1.20	0.82
16	1	-13	0.16	1.57
15	1	-13	3.85	1.52
15	1	-13	2.22	0.73
14	1	-13	6.95	1.50
14	1	-13	8.46	1.04
-14	-1	13	8.78	1.27
13	1	-13	3.26	0.68
13	1	-13	5.75	1.44
-13	-1	13	3.23	1.02
12	1	-13	4.97	0.80
12	1	-13	2.94	1.17
12	1	-13	5.13	0.68
-12	-1	13	5.46	0.98
11	1	-13	1.85	1.03
11	1	-13	2.79	0.56
11	1	-13	1.30	0.65
-11	-1	13	0.66	0.67
10	1	-13	1.21	0.96
10	1	-13	-0.04	0.44
10	1	-13	-0.15	0.61
-10	-1	13	0.71	0.60
9	1	-13	1.43	0.80
9	1	-13	0.48	0.50

9	1	-13	1.53	0.85
-9	-1	13	1.14	0.55
8	1	-13	2.53	0.91
8	1	-13	2.58	0.48
-8	-1	13	3.25	0.64
7	1	-13	0.72	0.81
7	1	-13	-0.20	0.39
-7	-1	13	1.96	0.56
6	1	-13	-0.15	0.74
6	1	-13	-0.36	0.36
-6	-1	13	-1.31	0.90
-6	-1	13	0.09	0.44
5	1	-13	8.63	0.74
5	1	-13	8.88	1.27
-5	-1	13	8.00	0.93
-5	-1	13	8.80	0.90
-5	-1	13	6.65	0.66
-5	-1	13	8.69	1.19
-5	-1	13	10.29	1.64
4	1	-13	9.27	0.75
4	1	-13	9.67	1.32
-4	-1	13	8.19	0.71
-4	-1	13	9.46	0.96
-4	-1	13	8.42	0.86
-4	-1	13	7.77	1.36
3	1	-13	1.47	0.75
3	1	-13	0.26	0.40
-3	-1	13	1.19	0.62
-3	-1	13	2.01	0.43
-3	-1	13	0.45	0.39
-3	-1	13	-0.12	0.92
2	1	-13	11.35	1.26
2	1	-13	11.19	0.80
-2	-1	13	10.73	0.83
-2	-1	13	13.02	1.22
-2	-1	13	13.73	1.71
1	1	-13	14.01	1.30
1	1	-13	15.04	0.88
-1	-1	13	14.24	1.09
-1	-1	13	11.56	0.89
-1	-1	13	14.64	1.87
-1	-1	13	16.86	1.41
0	1	-13	1.60	0.74
0	1	-13	1.82	0.44

0	-1	13	1.36	0.55
0	-1	13	0.84	1.20
0	-1	13	2.34	0.88
-1	1	-13	7.58	0.77
-1	1	-13	6.59	0.96
1	-1	13	7.06	1.22
1	-1	13	7.49	0.99
-2	1	-13	13.75	0.96
-2	1	-13	15.53	1.40
2	-1	13	16.14	1.27
2	-1	13	14.26	1.88
-3	1	-13	10.08	1.16
-3	1	-13	11.80	1.39
3	-1	13	12.54	2.09
3	-1	13	12.33	1.26
-4	1	-13	16.02	1.14
-4	1	-13	18.43	1.64
4	-1	13	18.84	1.47
4	-1	13	16.11	2.40
-5	1	-13	19.46	1.71
-5	1	-13	20.32	1.33
5	-1	13	21.12	1.64
5	-1	13	17.29	2.73
-6	1	-13	11.18	1.26
-6	1	-13	6.80	1.18
6	-1	13	9.36	1.49
6	-1	13	8.64	2.04
16	0	-13	3.70	1.68
15	0	-13	1.93	1.45
14	0	-13	10.25	1.07
14	0	-13	10.09	1.67
-14	0	13	9.18	1.22
13	0	-13	1.03	1.22
13	0	-13	1.49	0.59
-13	0	13	0.72	0.81
12	0	-13	-0.28	0.46
12	0	-13	0.28	0.69
12	0	-13	-0.35	0.98
-12	0	13	0.63	0.67
11	0	-13	0.07	0.47
11	0	-13	-2.11	0.83
11	0	-13	0.55	0.64
-11	0	13	0.10	0.59
10	0	-13	0.61	0.47

10	0	-13	0.25	0.59
-10	0	13	0.77	0.54
9	0	-13	3.37	0.70
9	0	-13	4.08	1.04
9	0	-13	2.68	0.51
-9	0	13	4.14	0.69
8	0	-13	6.60	1.07
8	0	-13	5.55	0.69
-8	0	13	5.89	0.84
7	0	-13	1.93	0.90
7	0	-13	2.05	0.46
-7	0	13	1.98	0.53
6	0	-13	1.16	0.85
6	0	-13	1.54	0.42
-6	0	13	1.09	0.38
-6	0	13	1.03	0.73
-6	0	13	0.95	0.48
-6	0	13	1.34	0.48
5	0	-13	13.09	1.46
5	0	-13	14.60	0.88
-5	0	13	12.65	0.80
-5	0	13	12.95	0.95
-5	0	13	14.22	1.01
4	0	-13	16.36	1.50
4	0	-13	19.59	1.01
-4	0	13	18.23	0.93
-4	0	13	18.32	1.16
-4	0	13	20.31	1.18
-4	0	13	17.14	1.75
3	0	-13	4.78	0.66
3	0	-13	4.15	0.85
-3	0	13	3.21	0.59
-3	0	13	3.73	0.48
-3	0	13	3.72	1.14
-3	0	13	5.38	0.93
2	0	-13	9.81	0.76
2	0	-13	10.82	1.26
-2	0	13	7.95	0.76
-2	0	13	10.97	0.93
-2	0	13	8.96	1.15
-2	0	13	7.27	1.40
1	0	-13	12.11	0.81
1	0	-13	12.11	1.34
-1	0	13	12.56	1.05

-1	0	13	11.29	1.31
-1	0	13	10.95	1.53
-1	0	13	12.49	0.90
0	0	-13	15.82	1.42
0	0	-13	16.93	0.93
0	0	13	13.73	0.98
0	0	13	17.58	1.22
0	0	13	14.77	1.54
-1	0	-13	8.40	1.27
-1	0	-13	10.46	0.84
1	0	13	9.96	1.55
1	0	13	9.80	1.10
-2	0	-13	7.05	0.78
-2	0	-13	5.26	0.97
2	0	13	6.20	1.08
2	0	13	5.01	1.22
-3	0	-13	18.13	1.56
-3	0	-13	18.51	1.12
3	0	13	15.62	2.11
3	0	13	17.64	1.43
-4	0	-13	21.85	1.31
-4	0	-13	21.87	1.78
4	0	13	24.63	2.56
4	0	13	24.97	1.70
-5	0	-13	5.19	1.03
-5	0	-13	6.93	0.84
5	0	13	4.82	1.67
5	0	13	6.40	1.03
-6	0	-13	3.98	1.14
-6	0	-13	5.39	0.90
6	0	13	5.36	1.01
6	0	13	6.88	1.88
16	-1	-13	1.87	0.69
16	-1	-13	4.49	1.58
15	-1	-13	0.02	1.33
15	-1	-13	1.57	0.65
14	-1	-13	1.97	1.31
14	-1	-13	1.58	0.64
-14	1	13	3.11	0.92
13	-1	-13	0.84	0.52
13	-1	-13	1.79	1.25
-13	1	13	-0.02	0.75
12	-1	-13	-0.08	0.45
12	-1	-13	1.74	1.13

12	-1	-13	1.62	0.80
-12	1	13	0.13	0.64
11	-1	-13	-0.65	0.93
11	-1	-13	-0.05	0.44
11	-1	-13	0.61	0.73
-11	1	13	0.02	0.54
10	-1	-13	2.79	0.54
10	-1	-13	3.51	0.99
10	-1	-13	2.75	0.77
-10	1	13	2.02	0.58
9	-1	-13	3.67	0.82
9	-1	-13	3.02	1.15
9	-1	-13	2.74	0.51
-9	1	13	4.06	0.67
8	-1	-13	4.38	1.04
8	-1	-13	3.91	0.55
-8	1	13	4.77	0.77
7	-1	-13	11.68	1.49
7	-1	-13	10.38	0.81
-7	1	13	10.81	0.75
-7	1	13	10.85	0.75
-7	1	13	11.73	1.20
-7	1	13	10.87	0.94
6	-1	-13	2.16	0.89
6	-1	-13	2.71	0.48
-6	1	13	2.91	0.93
-6	1	13	2.39	0.51
-6	1	13	2.09	0.42
-6	1	13	2.70	0.47
5	-1	-13	2.14	0.85
5	-1	-13	2.90	0.49
-5	1	13	4.08	0.70
-5	1	13	3.20	0.61
-5	1	13	3.60	1.10
-5	1	13	4.43	0.56
4	-1	-13	9.19	0.76
4	-1	-13	10.67	1.34
-4	1	13	10.26	0.71
-4	1	13	11.02	0.94
-4	1	13	9.39	0.89
-4	1	13	13.84	1.89
3	-1	-13	9.97	1.57
3	-1	-13	11.29	0.77
-3	1	13	11.29	1.04

-3	1	13	10.91	0.75
-3	1	13	10.92	2.01
-3	1	13	11.35	1.07
2	-1	-13	12.37	0.79
2	-1	-13	11.23	1.34
-2	1	13	11.97	1.02
-2	1	13	12.77	0.82
-2	1	13	14.28	1.23
-2	1	13	13.41	2.11
1	-1	-13	12.07	1.37
1	-1	-13	12.67	0.79
-1	1	13	8.32	1.52
-1	1	13	11.66	1.05
-1	1	13	11.24	1.31
-1	1	13	11.27	0.84
0	-1	-13	9.89	1.38
0	-1	-13	11.91	0.81
0	1	13	10.82	1.10
0	1	13	10.06	0.92
0	1	13	12.70	1.46
-1	-1	-13	11.78	1.42
-1	-1	-13	11.90	0.88
1	1	13	11.61	1.00
1	1	13	14.12	1.24
1	1	13	12.83	1.70
-2	-1	-13	17.95	1.60
-2	-1	-13	18.20	1.07
2	1	13	18.46	1.99
2	1	13	19.02	1.43
-3	-1	-13	16.77	1.60
-3	-1	-13	18.19	1.11
3	1	13	12.58	2.12
3	1	13	18.37	1.51
-4	-1	-13	10.69	1.55
-4	-1	-13	8.01	1.03
4	1	13	9.14	1.81
4	1	13	11.11	1.39
-5	-1	-13	6.11	0.82
-5	-1	-13	5.34	1.07
5	1	13	6.57	1.08
5	1	13	6.64	1.65
17	-2	-13	0.46	0.76
17	-2	-13	0.06	1.69
16	-2	-13	0.71	1.52

16	-2	-13	-0.24	0.58
15	-2	-13	4.76	0.75
15	-2	-13	5.37	1.55
14	-2	-13	0.37	1.25
14	-2	-13	0.45	0.56
-14	2	13	0.55	0.79
13	-2	-13	2.19	0.57
13	-2	-13	0.74	1.16
-13	2	13	2.41	0.74
12	-2	-13	0.70	1.06
12	-2	-13	-0.74	0.85
12	-2	-13	1.71	0.51
-12	2	13	1.38	0.66
11	-2	-13	1.39	0.47
11	-2	-13	0.58	0.97
11	-2	-13	0.53	0.86
-11	2	13	0.97	0.55
10	-2	-13	6.85	1.03
10	-2	-13	7.31	0.78
10	-2	-13	5.11	1.11
-10	2	13	6.99	0.87
9	-2	-13	13.01	0.89
9	-2	-13	12.47	1.58
9	-2	-13	15.25	1.67
-9	2	13	14.05	1.04
8	-2	-13	5.09	0.64
8	-2	-13	3.77	0.54
-8	2	13	5.24	0.87
-8	2	13	5.79	0.79
7	-2	-13	5.32	0.68
7	-2	-13	4.09	1.13
-7	2	13	7.14	0.81
-7	2	13	5.41	0.62
-7	2	13	8.59	1.23
6	-2	-13	2.35	0.43
6	-2	-13	1.11	0.87
-6	2	13	1.54	0.38
-6	2	13	1.67	0.50
-6	2	13	2.07	1.14
-6	2	13	1.71	0.43
5	-2	-13	15.89	0.88
5	-2	-13	15.60	2.33
-5	2	13	15.51	0.98
-5	2	13	14.27	0.81

-5	2	13	14.11	1.04
-5	2	13	13.48	1.99
4	-2	-13	18.65	1.83
4	-2	-13	21.80	1.00
-4	2	13	14.10	1.42
-4	2	13	18.80	1.19
-4	2	13	24.01	2.35
-4	2	13	19.53	0.92
3	-2	-13	12.49	1.40
3	-2	-13	13.13	0.80
-3	2	13	13.14	0.77
-3	2	13	12.74	1.77
-3	2	13	10.32	1.09
-3	2	13	12.02	1.03
2	-2	-13	6.32	0.64
2	-2	-13	5.99	1.11
-2	2	13	6.66	0.68
-2	2	13	7.35	1.11
-2	2	13	9.36	1.78
-2	2	13	5.04	0.89
1	-2	-13	19.03	1.61
1	-2	-13	21.30	1.00
-1	2	13	20.56	1.55
-1	2	13	19.82	1.02
-1	2	13	17.22	1.30
0	-2	-13	16.85	0.93
0	-2	-13	15.78	1.57
0	2	13	17.69	1.33
0	2	13	15.97	1.02
-1	-2	-13	8.51	1.17
-1	-2	-13	8.96	0.79
1	2	13	8.24	1.15
1	2	13	9.58	1.38
-2	-2	-13	14.53	1.00
-2	-2	-13	14.97	1.59
2	2	13	15.77	2.00
2	2	13	15.21	1.43
-3	-2	-13	9.98	1.48
-3	-2	-13	9.93	0.95
3	2	13	8.88	1.35
3	2	13	8.44	1.66
-4	-2	-13	13.33	1.69
4	2	13	13.19	1.94
4	2	13	11.87	1.53

5	2	13	11.08	2.07
17	-3	-13	-0.11	1.73
17	-3	-13	0.72	0.74
16	-3	-13	1.84	1.43
16	-3	-13	-0.23	0.64
15	-3	-13	2.08	1.48
15	-3	-13	-0.38	0.61
14	-3	-13	1.18	0.55
14	-3	-13	0.45	1.31
-14	3	13	0.85	0.74
13	-3	-13	0.46	1.25
13	-3	-13	0.78	0.52
-13	3	13	0.75	0.68
12	-3	-13	1.14	1.03
12	-3	-13	1.53	0.49
12	-3	-13	0.34	1.07
-12	3	13	1.52	0.59
11	-3	-13	2.63	1.08
11	-3	-13	4.00	1.21
-11	3	13	5.59	0.87
10	-3	-13	3.38	0.56
10	-3	-13	3.50	1.11
10	-3	-13	3.73	1.06
-10	3	13	4.70	0.67
9	-3	-13	8.78	0.79
9	-3	-13	14.36	1.54
9	-3	-13	11.47	1.64
-9	3	13	9.66	0.90
-9	3	13	8.74	1.32
8	-3	-13	5.32	0.70
8	-3	-13	6.61	1.24
8	-3	-13	6.80	0.72
-8	3	13	4.59	0.81
-8	3	13	5.15	0.69
-8	3	13	5.75	1.23
7	-3	-13	4.93	0.64
7	-3	-13	4.54	0.66
7	-3	-13	5.98	1.12
-7	3	13	4.57	0.70
-7	3	13	5.46	1.30
-7	3	13	4.39	0.74
6	-3	-13	18.68	1.70
6	-3	-13	18.06	0.94
-6	3	13	16.42	1.11

-6	3	13	15.04	0.86
-6	3	13	17.00	1.04
-6	3	13	19.81	2.20
5	-3	-13	22.89	1.83
5	-3	-13	24.71	1.08
-5	3	13	22.28	1.01
-5	3	13	27.64	2.51
-5	3	13	20.82	1.63
-5	3	13	22.26	1.28
4	-3	-13	13.29	0.80
4	-3	-13	14.50	1.56
-4	3	13	13.57	1.08
-4	3	13	13.87	1.13
-4	3	13	14.21	0.77
-4	3	13	13.73	2.33
3	-3	-13	9.11	1.45
3	-3	-13	12.75	0.79
-3	3	13	13.05	0.76
-3	3	13	14.27	1.12
-3	3	13	12.27	1.15
-3	3	13	17.07	2.71
2	-3	-13	34.37	1.34
2	-3	-13	33.87	2.11
-2	3	13	31.80	1.80
-2	3	13	33.91	1.33
-2	3	13	35.77	1.79
1	-3	-13	31.45	1.27
1	-3	-13	32.61	2.00
-1	3	13	30.88	1.71
-1	3	13	30.49	1.29
-1	3	13	29.43	1.89
0	-3	-13	13.58	0.85
0	-3	-13	15.02	1.57
0	3	13	13.81	1.62
0	3	13	13.59	1.30
-1	-3	-13	11.21	1.58
-1	-3	-13	14.21	0.90
1	3	13	12.13	1.36
1	3	13	12.95	1.78
-2	-3	-13	15.70	1.70
-2	-3	-13	12.72	0.95
2	3	13	14.80	1.49
2	3	13	16.45	2.08
-3	-3	-13	7.78	1.19

3	3	13	10.88	1.70
3	3	13	9.74	1.47
-4	-3	-13	13.32	1.85
4	3	13	16.22	2.66
4	3	13	13.61	1.66
17	-4	-13	-0.06	0.76
17	-4	-13	4.34	1.85
16	-4	-13	0.60	1.49
16	-4	-13	3.54	0.73
15	-4	-13	-1.37	0.58
14	-4	-13	1.95	1.33
14	-4	-13	4.06	0.66
-14	4	13	4.20	0.81
13	-4	-13	-0.23	1.16
13	-4	-13	1.10	0.55
-13	4	13	2.26	0.66
12	-4	-13	5.79	1.39
12	-4	-13	3.87	1.31
12	-4	-13	5.26	0.77
-12	4	13	5.12	0.92
11	-4	-13	8.39	0.81
11	-4	-13	8.85	1.45
11	-4	-13	4.74	1.32
11	-4	-13	7.70	1.18
-11	4	13	8.18	0.93
-11	4	13	8.66	1.21
10	-4	-13	4.04	0.57
10	-4	-13	3.92	1.26
10	-4	-13	3.75	0.80
-10	4	13	3.92	0.60
-10	4	13	2.58	1.24
9	-4	-13	1.33	0.54
9	-4	-13	1.84	0.46
9	-4	-13	1.80	0.74
9	-4	-13	2.58	1.05
-9	4	13	2.55	0.57
-9	4	13	1.20	1.11
8	-4	-13	21.49	1.99
8	-4	-13	22.92	1.16
8	-4	-13	20.63	1.08
-8	4	13	21.37	1.24
-8	4	13	24.62	2.26
7	-4	-13	9.93	0.77
7	-4	-13	8.19	0.88

7	-4	-13	10.85	1.63
-7	4	13	13.72	2.17
-7	4	13	9.30	0.93
6	-4	-13	15.40	1.72
6	-4	-13	14.78	1.04
6	-4	-13	16.51	0.88
-6	4	13	13.98	2.38
-6	4	13	14.88	1.14
-6	4	13	17.02	1.29
5	-4	-13	13.99	0.84
5	-4	-13	15.07	1.67
-5	4	13	12.23	1.13
-5	4	13	14.17	2.51
-5	4	13	11.43	0.76
-5	4	13	11.98	1.06
4	-4	-13	20.08	1.77
4	-4	-13	20.30	0.94
-4	4	13	18.14	0.90
-4	4	13	18.15	1.30
-4	4	13	19.33	2.84
-4	4	13	19.04	1.34
3	-4	-13	28.67	1.19
3	-4	-13	33.64	2.07
-3	4	13	28.98	1.64
-3	4	13	30.56	1.72
-3	4	13	27.63	1.16
2	-4	-13	32.09	1.26
2	-4	-13	33.76	2.12
-2	4	13	30.98	1.84
-2	4	13	30.83	1.78
1	-4	-13	34.71	2.09
1	-4	-13	30.90	1.27
-1	4	13	30.82	1.95
-1	4	13	32.76	1.84
0	-4	-13	30.37	2.05
0	-4	-13	29.60	1.26
0	4	13	28.68	1.87
0	4	13	33.60	2.18
-1	-4	-13	20.01	1.04
-1	-4	-13	19.41	1.84
1	4	13	22.64	2.11
1	4	13	20.09	1.67
-2	-4	-13	7.67	1.18
2	4	13	7.72	1.15

2	4	13	6.74	1.46
-3	-4	-13	6.28	1.20
3	4	13	9.47	1.73
3	4	13	6.84	1.17
17	-5	-13	1.51	2.33
16	-5	-13	-0.21	0.66
16	-5	-13	2.04	1.58
15	-5	-13	4.06	1.53
14	-5	-13	6.16	1.57
14	-5	-13	6.36	0.94
-14	5	13	4.91	0.80
13	-5	-13	-0.92	1.27
13	-5	-13	0.42	0.51
-13	5	13	0.32	0.60
12	-5	-13	12.45	1.52
12	-5	-13	12.32	0.90
12	-5	-13	10.26	1.74
12	-5	-13	13.46	2.08
-12	5	13	11.80	1.07
11	-5	-13	14.63	1.52
11	-5	-13	13.83	2.03
11	-5	-13	10.40	2.01
11	-5	-13	14.43	0.95
-11	5	13	14.93	1.09
-11	5	13	14.62	1.96
10	-5	-13	8.57	0.83
10	-5	-13	8.77	1.15
10	-5	-13	6.44	1.30
-10	5	13	9.66	1.52
-10	5	13	8.46	0.92
9	-5	-13	5.51	0.64
9	-5	-13	8.10	1.41
9	-5	-13	5.83	0.71
9	-5	-13	5.99	1.02
-9	5	13	5.06	0.82
-9	5	13	5.02	1.46
8	-5	-13	9.71	0.89
8	-5	-13	10.17	0.80
8	-5	-13	8.24	1.36
8	-5	-13	7.81	1.40
-8	5	13	8.06	1.75
-8	5	13	11.05	0.97
7	-5	-13	10.47	1.50
7	-5	-13	8.92	0.75

7	-5	-13	10.02	1.50
7	-5	-13	12.43	0.91
-7	5	13	13.37	2.02
-7	5	13	8.94	0.98
6	-5	-13	15.18	0.90
6	-5	-13	18.29	1.87
6	-5	-13	18.08	1.02
-6	5	13	16.10	2.76
-6	5	13	15.58	1.19
5	-5	-13	22.00	0.99
5	-5	-13	21.60	1.94
5	-5	-13	22.89	1.14
-5	5	13	22.78	1.43
-5	5	13	19.68	1.44
-5	5	13	17.06	2.97
4	-5	-13	18.71	0.90
4	-5	-13	18.45	1.80
-4	5	13	18.81	1.48
-4	5	13	18.69	1.39
3	-5	-13	18.01	0.87
3	-5	-13	17.91	1.78
-3	5	13	20.55	1.47
-3	5	13	15.56	1.46
2	-5	-13	38.51	2.28
2	-5	-13	37.51	1.40
-2	5	13	37.06	2.14
-2	5	13	39.92	2.10
1	-5	-13	29.59	2.01
1	-5	-13	25.45	1.10
-1	5	13	23.59	1.96
-1	5	13	29.04	1.87
0	-5	-13	17.45	1.81
0	5	13	16.15	1.86
0	5	13	15.20	1.57
-1	-5	-13	19.48	1.87
1	5	13	22.83	2.24
1	5	13	22.86	1.84
-2	-5	-13	13.49	1.79
2	5	13	13.23	2.27
2	5	13	16.15	1.78
17	-6	-13	3.30	0.83
16	-6	-13	1.84	0.72
16	-6	-13	1.47	1.59
15	-6	-13	-0.50	0.59

15	-6	-13	-1.16	1.45
14	-6	-13	1.05	0.59
14	-6	-13	-0.26	1.34
-14	6	13	0.81	0.64
13	-6	-13	8.37	0.93
13	-6	-13	7.83	1.51
-13	6	13	8.10	1.05
12	-6	-13	9.42	1.44
12	-6	-13	9.54	1.75
12	-6	-13	5.28	2.39
12	-6	-13	8.94	1.67
-12	6	13	9.89	1.02
11	-6	-13	11.21	0.89
11	-6	-13	9.10	1.33
11	-6	-13	9.75	1.57
-11	6	13	9.26	0.99
10	-6	-13	18.73	1.16
10	-6	-13	18.53	2.13
10	-6	-13	18.89	1.86
10	-6	-13	20.54	1.07
-10	6	13	17.56	2.30
-10	6	13	18.70	1.22
9	-6	-13	8.82	1.30
9	-6	-13	9.40	0.93
9	-6	-13	11.45	1.64
9	-6	-13	10.28	0.84
-9	6	13	10.92	1.89
-9	6	13	9.42	1.02
8	-6	-13	12.28	0.86
8	-6	-13	11.88	0.91
8	-6	-13	9.88	1.54
8	-6	-13	11.43	1.68
-8	6	13	11.81	1.96
-8	6	13	10.91	1.05
7	-6	-13	14.07	1.88
7	-6	-13	15.73	1.84
7	-6	-13	15.14	0.97
7	-6	-13	15.32	0.90
-7	6	13	22.54	3.03
-7	6	13	14.57	1.19
6	-6	-13	11.97	0.80
6	-6	-13	11.31	1.79
6	-6	-13	13.74	0.91
6	-6	-13	12.00	1.81

-6	6	13	13.16	2.19
-6	6	13	14.31	1.23
5	-6	-13	25.35	1.05
5	-6	-13	25.11	2.14
-5	6	13	24.83	1.60
-5	6	13	26.20	1.64
4	-6	-13	21.86	1.99
4	-6	-13	22.05	0.93
-4	6	13	23.27	1.62
-4	6	13	21.46	1.95
3	-6	-13	14.01	1.85
-3	6	13	15.03	1.58
-3	6	13	13.82	1.44
2	-6	-13	18.26	1.83
-2	6	13	19.08	1.80
-2	6	13	16.36	1.53
1	-6	-13	11.03	1.78
-1	6	13	10.86	1.84
-1	6	13	12.95	1.53
0	-6	-13	9.33	1.28
0	6	13	7.95	1.38
0	6	13	13.27	1.59
-1	-6	-13	11.50	1.79
1	6	13	12.08	1.68
1	6	13	10.46	1.69
16	-7	-13	-1.36	1.57
16	-7	-13	0.23	0.71
15	-7	-13	2.38	1.53
15	-7	-13	1.83	0.69
14	-7	-13	4.51	1.52
14	-7	-13	1.64	0.61
-14	7	13	3.12	0.69
13	-7	-13	13.12	1.95
13	-7	-13	9.98	0.98
13	-7	-13	8.75	1.70
-13	7	13	10.52	1.13
12	-7	-13	7.88	1.49
12	-7	-13	8.73	0.93
12	-7	-13	8.97	1.71
-12	7	13	10.14	1.06
11	-7	-13	7.38	0.89
11	-7	-13	6.23	1.41
11	-7	-13	5.66	1.49
-11	7	13	6.72	0.81

10	-7	-13	26.27	1.35
10	-7	-13	26.02	2.25
10	-7	-13	27.03	1.27
10	-7	-13	29.05	2.53
-10	7	13	29.39	3.37
9	-7	-13	23.48	2.17
9	-7	-13	21.72	2.31
9	-7	-13	26.02	1.34
9	-7	-13	24.28	1.18
-9	7	13	22.80	3.17
-9	7	13	23.06	1.42
8	-7	-13	12.74	0.86
8	-7	-13	11.15	1.89
8	-7	-13	8.77	1.52
-8	7	13	14.11	1.20
-8	7	13	13.99	2.28
7	-7	-13	16.39	0.91
7	-7	-13	15.71	2.07
7	-7	-13	17.64	2.13
-7	7	13	17.63	1.40
6	-7	-13	22.20	0.96
6	-7	-13	21.85	2.16
6	-7	-13	20.55	2.17
-6	7	13	22.23	1.59
5	-7	-13	35.20	2.41
-5	7	13	32.57	1.92
4	-7	-13	18.76	2.04
-4	7	13	19.50	1.60
-4	7	13	17.21	1.74
3	-7	-13	4.84	1.19
-3	7	13	7.01	1.21
-3	7	13	5.08	0.96
2	-7	-13	24.60	2.11
-2	7	13	22.32	1.85
-2	7	13	20.26	2.07
1	-7	-13	17.75	1.91
-1	7	13	15.79	2.06
-1	7	13	18.96	1.74
0	-7	-13	2.97	1.17
0	7	13	1.66	0.98
0	7	13	0.52	1.31
16	-8	-13	5.89	1.96
16	-8	-13	3.49	0.82
15	-8	-13	4.86	1.72

15	-8	-13	4.17	0.78
14	-8	-13	8.57	1.08
14	-8	-13	10.52	1.90
13	-8	-13	18.74	1.17
13	-8	-13	18.63	2.57
13	-8	-13	18.52	2.47
-13	8	13	17.12	1.31
12	-8	-13	20.50	2.51
12	-8	-13	20.23	1.18
12	-8	-13	17.67	2.39
-12	8	13	19.89	1.33
11	-8	-13	9.63	1.72
11	-8	-13	9.06	1.63
11	-8	-13	9.13	0.93
10	-8	-13	18.43	2.44
10	-8	-13	14.84	2.23
10	-8	-13	13.26	0.95
-10	8	13	13.16	1.22
9	-8	-13	23.20	2.43
9	-8	-13	18.47	1.05
9	-8	-13	16.57	2.33
-9	8	13	18.14	1.37
8	-8	-13	23.49	2.39
8	-8	-13	24.19	1.11
8	-8	-13	23.99	2.39
-8	8	13	24.68	1.62
7	-8	-13	21.95	2.66
7	-8	-13	21.35	2.34
-7	8	13	22.43	1.65
6	-8	-13	22.20	2.33
6	-8	-13	23.27	2.44
-6	8	13	23.09	1.69
5	-8	-13	32.13	2.47
5	-8	-13	36.15	2.76
-5	8	13	30.99	2.01
4	-8	-13	17.46	2.08
-4	8	13	17.64	1.90
-4	8	13	16.86	1.62
3	-8	-13	13.49	1.95
-3	8	13	10.37	1.55
-3	8	13	12.66	1.97
2	-8	-13	11.45	1.53
-2	8	13	12.61	2.04
-2	8	13	12.84	1.70

1	-8	-13	13.99	2.00
-1	8	13	10.27	1.63
-1	8	13	12.77	1.78
15	-9	-13	20.98	2.95
15	-9	-13	21.51	1.44
14	-9	-13	18.81	1.30
14	-9	-13	15.99	2.73
13	-9	-13	7.11	1.90
13	-9	-13	5.86	1.62
-13	9	13	6.71	0.89
12	-9	-13	16.22	2.58
12	-9	-13	17.61	2.55
12	-9	-13	14.64	1.10
-12	9	13	14.23	1.30
11	-9	-13	18.11	2.62
11	-9	-13	22.91	1.20
11	-9	-13	21.39	2.65
-11	9	13	20.55	1.48
10	-9	-13	13.33	1.97
10	-9	-13	12.69	1.01
10	-9	-13	8.90	1.66
-10	9	13	13.62	1.34
9	-9	-13	19.31	3.92
9	-9	-13	23.04	2.48
-9	9	13	23.41	1.64
8	-9	-13	8.98	1.57
8	-9	-13	10.23	1.80
-8	9	13	9.98	1.42
7	-9	-13	15.37	2.23
7	-9	-13	14.28	2.45
-7	9	13	16.81	1.56
6	-9	-13	36.21	3.05
6	-9	-13	36.98	2.68
-6	9	13	35.95	2.18
5	-9	-13	20.69	2.74
5	-9	-13	18.06	2.29
-5	9	13	20.80	1.80
4	-9	-13	2.85	1.34
-4	9	13	4.59	1.07
3	-9	-13	6.78	1.43
-3	9	13	5.76	1.41
-3	9	13	6.63	1.23
2	-9	-13	13.49	2.13
-2	9	13	16.15	2.29

-2	9	13	12.91	1.99
15	-10	-13	16.90	3.80
14	-10	-13	3.41	0.71
14	-10	-13	3.47	1.67
13	-10	-13	6.32	1.21
13	-10	-13	7.65	1.84
13	-10	-13	5.40	2.34
-13	10	13	6.91	1.22
12	-10	-13	18.06	2.86
12	-10	-13	13.53	2.00
-12	10	13	18.76	1.48
11	-10	-13	17.78	2.89
11	-10	-13	20.72	2.80
-11	10	13	23.43	1.63
10	-10	-13	20.41	2.66
10	-10	-13	15.75	2.18
-10	10	13	14.99	1.56
9	-10	-13	25.39	2.75
9	-10	-13	29.18	3.08
-9	10	13	34.64	2.06
8	-10	-13	29.98	2.72
8	-10	-13	27.63	3.08
-8	10	13	29.02	2.00
7	-10	-13	29.28	2.71
7	-10	-13	31.00	3.13
-7	10	13	29.08	2.03
6	-10	-13	17.76	2.92
6	-10	-13	24.74	2.49
-6	10	13	26.65	1.97
5	-10	-13	5.40	1.83
5	-10	-13	9.11	1.62
-5	10	13	6.95	1.29
4	-10	-13	15.90	2.24
-4	10	13	16.67	1.91
13	-11	-13	2.21	2.46
13	-11	-13	0.84	1.81
-13	11	13	2.27	0.97
12	-11	-13	2.44	1.80
12	-11	-13	2.43	2.06
-12	11	13	1.91	0.91
11	-11	-13	3.17	1.74
11	-11	-13	8.07	2.17
-11	11	13	9.92	1.56
10	-11	-13	9.76	2.18

10	-11	-13	9.93	1.89
-10	11	13	10.78	1.64
9	-11	-13	25.84	2.78
9	-11	-13	27.15	3.26
-9	11	13	22.84	1.94
8	-11	-13	23.01	3.22
8	-11	-13	27.11	2.85
-8	11	13	27.41	2.05
7	-11	-13	13.23	2.24
7	-11	-13	11.14	1.86
-7	11	13	14.23	1.81
6	-11	-13	10.00	2.15
6	-11	-13	8.88	1.70
-6	11	13	7.55	1.28
10	-12	-13	7.05	2.27
2	15	-14	0.58	0.86
-2	-15	14	0.97	1.87
1	15	-14	3.08	0.90
-1	-15	14	1.65	2.48
-1	-15	14	3.58	1.69
0	15	-14	0.67	0.81
0	-15	14	1.07	2.53
0	-15	14	0.05	1.50
-1	15	-14	-1.68	0.75
1	-15	14	0.75	1.47
1	-15	14	-1.31	2.63
2	-15	14	4.97	1.71
2	-15	14	6.11	2.85
3	-15	14	3.77	3.07
5	14	-14	4.46	1.05
-5	-14	14	2.34	1.98
4	14	-14	3.11	0.98
-4	-14	14	1.59	1.58
3	14	-14	5.57	1.01
-3	-14	14	6.31	1.76
2	14	-14	1.22	0.83
-2	-14	14	4.50	2.14
-2	-14	14	0.22	1.35
1	14	-14	0.43	0.79
-1	-14	14	-0.55	4.02
-1	-14	14	0.64	1.35
0	14	-14	3.83	0.83
0	-14	14	0.68	1.30
0	-14	14	1.02	2.24

-1	14	-14	0.94	0.70
1	-14	14	3.38	2.50
1	-14	14	0.44	1.35
-2	14	-14	1.14	0.77
2	-14	14	0.71	2.54
2	-14	14	-0.03	1.42
-3	14	-14	0.45	0.78
3	-14	14	2.36	1.45
3	-14	14	-2.72	2.63
-4	14	-14	0.76	0.82
4	-14	14	3.54	2.88
4	-14	14	3.49	1.59
7	13	-14	4.29	1.05
-7	-13	14	-0.23	1.75
6	13	-14	3.77	0.98
-6	-13	14	4.20	1.65
5	13	-14	3.79	0.95
-5	-13	14	6.02	1.65
4	13	-14	3.56	0.89
-4	-13	14	2.08	1.38
3	13	-14	3.68	0.91
-3	-13	14	4.87	1.93
-3	-13	14	4.88	1.41
2	13	-14	3.43	0.83
-2	-13	14	2.25	1.43
-2	-13	14	2.83	1.92
1	13	-14	0.99	0.74
-1	-13	14	2.57	1.97
-1	-13	14	-0.38	1.20
0	13	-14	1.68	0.70
0	-13	14	1.77	1.18
0	-13	14	-0.87	2.07
-1	13	-14	-0.34	0.69
1	-13	14	2.49	2.12
1	-13	14	0.31	1.25
2	-13	14	2.37	2.28
2	-13	14	3.30	1.37
-3	13	-14	-0.35	0.68
3	-13	14	-1.05	1.23
3	-13	14	2.94	2.51
-4	13	-14	0.94	0.76
4	-13	14	4.75	2.76
4	-13	14	1.90	1.43
-5	13	-14	1.18	0.84

5	-13	14	8.32	2.89
5	-13	14	2.88	1.61
8	12	-14	5.46	1.03
-8	-12	14	6.17	1.88
7	12	-14	-0.22	0.80
-7	-12	14	-1.11	1.42
6	12	-14	1.94	0.85
-6	-12	14	-0.59	1.33
5	12	-14	1.11	0.77
-5	-12	14	-0.41	1.28
4	12	-14	4.71	0.91
-4	-12	14	8.21	2.72
-4	-12	14	7.11	1.51
3	12	-14	4.50	0.84
-3	-12	14	6.62	1.81
-3	-12	14	5.43	1.37
2	12	-14	0.88	0.68
-2	-12	14	1.68	1.20
-2	-12	14	1.54	1.67
1	12	-14	0.54	0.64
-1	-12	14	-0.71	1.75
-1	-12	14	1.35	1.12
0	12	-14	0.20	0.62
0	-12	14	0.66	1.83
0	-12	14	-0.44	1.09
-1	12	-14	0.91	0.67
1	-12	14	1.89	1.21
1	-12	14	2.12	2.33
-2	12	-14	3.56	0.73
2	-12	14	4.33	1.27
2	-12	14	5.51	2.33
-3	12	-14	2.01	0.75
3	-12	14	0.91	2.25
3	-12	14	1.23	1.23
-4	12	-14	2.31	0.73
4	-12	14	1.41	2.60
4	-12	14	2.23	1.43
-5	12	-14	3.23	0.83
5	-12	14	5.97	1.63
5	-12	14	4.02	2.82
6	-12	14	5.03	3.18
6	-12	14	5.70	1.70
9	11	-14	6.05	1.04
-9	-11	14	4.83	1.72

8	11	-14	4.95	0.93
-8	-11	14	5.98	1.65
7	11	-14	0.59	0.78
-7	-11	14	-0.12	1.36
6	11	-14	0.62	0.75
-6	-11	14	-1.06	1.14
5	11	-14	2.33	0.76
-5	-11	14	2.60	1.25
4	11	-14	3.37	0.79
-4	-11	14	0.66	1.11
-4	-11	14	2.55	1.37
3	11	-14	0.36	0.67
-3	-11	14	1.17	1.05
-3	-11	14	-1.22	1.41
2	11	-14	1.25	0.68
-2	-11	14	0.65	1.06
-2	-11	14	2.12	1.52
1	11	-14	0.56	0.64
-1	-11	14	-0.59	1.05
-1	-11	14	-0.37	1.52
0	11	-14	2.30	0.69
0	-11	14	1.24	1.13
0	-11	14	-0.36	1.62
-1	11	-14	1.62	0.66
1	-11	14	2.35	1.11
1	-11	14	2.03	1.88
-2	11	-14	4.24	0.74
2	-11	14	1.56	2.03
2	-11	14	2.28	1.19
-3	11	-14	0.37	0.67
3	-11	14	0.05	1.12
3	-11	14	-0.47	2.16
-4	11	-14	2.35	0.71
4	-11	14	2.95	2.33
4	-11	14	2.66	1.28
-5	11	-14	12.65	1.31
5	-11	14	14.84	3.14
5	-11	14	13.97	2.38
-6	11	-14	4.89	0.94
6	-11	14	7.84	1.67
6	-11	14	5.62	3.09
10	10	-14	2.16	0.90
-10	-10	14	2.19	1.68
9	10	-14	3.16	0.89

-9	-10	14	2.14	1.49
8	10	-14	1.89	0.79
-8	-10	14	-0.27	1.32
7	10	-14	3.71	0.79
-7	-10	14	6.06	1.47
6	10	-14	-0.23	0.68
-6	-10	14	-0.66	1.02
5	10	-14	-0.03	0.62
5	10	-14	0.14	0.85
-5	-10	14	0.36	1.05
-5	-10	14	1.67	1.03
4	10	-14	0.98	0.63
4	10	-14	-0.17	0.60
-4	-10	14	-0.13	0.96
-4	-10	14	1.90	1.25
3	10	-14	0.03	0.54
3	10	-14	0.72	0.61
-3	-10	14	-0.98	0.87
-3	-10	14	1.41	1.29
2	10	-14	0.51	0.48
2	10	-14	-0.36	0.58
-2	-10	14	0.62	1.42
-2	-10	14	0.13	0.91
1	10	-14	1.93	0.62
1	10	-14	1.53	0.50
-1	-10	14	2.49	1.46
-1	-10	14	1.91	1.03
0	10	-14	0.75	0.49
0	10	-14	1.54	0.56
0	10	-14	2.53	1.12
0	-10	14	1.40	1.01
0	-10	14	2.00	1.58
-1	10	-14	1.82	0.55
-1	10	-14	0.62	0.57
1	-10	14	2.61	1.67
1	-10	14	1.78	1.03
-2	10	-14	4.68	0.77
-2	10	-14	5.50	0.86
2	-10	14	5.15	1.25
2	-10	14	3.54	3.08
-3	10	-14	2.80	0.64
-3	10	-14	2.53	0.70
3	-10	14	2.82	2.14
3	-10	14	4.88	1.30

-4	10	-14	6.12	0.86
-4	10	-14	4.15	0.74
4	-10	14	5.63	1.34
4	-10	14	4.45	2.32
-5	10	-14	10.30	1.39
-5	10	-14	10.91	1.21
5	-10	14	6.76	2.74
5	-10	14	13.32	2.28
-6	10	-14	5.46	0.90
-6	10	-14	5.06	0.90
6	-10	14	6.42	2.94
6	-10	14	6.48	1.61
11	9	-14	0.44	0.84
-11	-9	14	0.03	1.66
10	9	-14	1.04	0.73
-10	-9	14	1.52	1.46
9	9	-14	1.97	1.63
9	9	-14	2.44	0.79
-9	-9	14	2.54	1.47
8	9	-14	0.15	0.70
8	9	-14	1.41	1.07
-8	-9	14	0.33	1.17
7	9	-14	0.70	0.68
7	9	-14	-1.39	0.92
-7	-9	14	0.20	1.03
6	9	-14	3.66	0.71
6	9	-14	2.66	0.87
-6	-9	14	3.32	1.11
5	9	-14	0.07	0.77
5	9	-14	1.71	0.66
-5	-9	14	2.87	1.02
-5	-9	14	0.96	0.91
4	9	-14	1.17	0.63
4	9	-14	-0.55	0.66
-4	-9	14	0.10	1.01
-4	-9	14	-0.28	0.89
-4	-9	14	0.94	0.71
3	9	-14	5.14	0.75
3	9	-14	4.94	0.77
-3	-9	14	5.27	1.33
-3	-9	14	5.07	0.84
-3	-9	14	4.80	1.12
2	9	-14	0.29	0.56
2	9	-14	-0.08	0.54

2	9	-14	0.27	0.77
-2	-9	14	1.45	0.93
-2	-9	14	1.49	1.25
-2	-9	14	0.41	0.76
1	9	-14	0.90	0.56
1	9	-14	0.24	0.55
1	9	-14	0.35	0.85
-1	-9	14	0.59	0.81
-1	-9	14	0.94	0.86
-1	-9	14	0.11	1.31
0	9	-14	4.55	1.02
0	9	-14	4.42	0.67
0	9	-14	4.71	0.69
0	-9	14	6.60	1.06
0	-9	14	8.41	1.73
0	-9	14	6.28	1.26
-1	9	-14	8.35	1.00
-1	9	-14	7.49	0.94
1	-9	14	9.00	1.86
1	-9	14	7.39	1.25
1	-9	14	7.49	1.31
-2	9	-14	8.12	0.99
-2	9	-14	7.82	0.99
2	-9	14	7.56	1.33
2	-9	14	10.61	2.15
-3	9	-14	6.27	1.05
-3	9	-14	9.26	1.11
3	-9	14	13.12	2.26
3	-9	14	6.78	1.39
-4	9	-14	6.63	0.88
-4	9	-14	8.62	1.11
4	-9	14	6.82	1.41
4	-9	14	7.88	2.36
-5	9	-14	3.67	0.79
-5	9	-14	3.38	0.78
5	-9	14	5.21	2.55
5	-9	14	1.49	1.32
-6	9	-14	8.97	1.30
-6	9	-14	11.85	1.30
6	-9	14	7.71	1.65
6	-9	14	8.64	2.90
-7	9	-14	12.18	1.44
-7	9	-14	9.90	1.47
7	-9	14	10.90	1.77

7	-9	14	14.69	3.35
12	8	-14	5.20	0.97
-12	-8	14	5.72	1.76
11	8	-14	3.50	1.36
11	8	-14	1.53	0.77
-11	-8	14	0.56	1.54
10	8	-14	3.96	1.28
10	8	-14	-0.10	0.68
-10	-8	14	-0.67	1.28
9	8	-14	0.87	1.16
9	8	-14	1.71	0.72
-9	-8	14	2.30	1.22
8	8	-14	0.15	0.64
8	8	-14	0.75	1.03
-8	-8	14	0.53	1.11
7	8	-14	-0.55	0.95
7	8	-14	-0.03	0.60
-7	-8	14	-0.86	1.07
-7	-8	14	1.20	0.99
6	8	-14	0.29	0.89
6	8	-14	0.10	0.56
-6	-8	14	0.51	0.71
-6	-8	14	0.49	0.86
5	8	-14	3.35	0.89
-5	-8	14	5.81	0.84
-5	-8	14	5.03	1.09
4	8	-14	15.05	1.14
4	8	-14	15.40	1.33
-4	-8	14	15.33	1.60
-4	-8	14	17.54	1.38
-4	-8	14	16.25	1.64
3	8	-14	-0.48	0.57
3	8	-14	0.39	0.68
3	8	-14	0.44	0.49
-3	-8	14	1.07	0.82
-3	-8	14	1.06	1.08
-3	-8	14	0.29	0.74
2	8	-14	0.50	0.60
2	8	-14	0.17	0.66
2	8	-14	-0.68	0.51
-2	-8	14	1.79	0.80
-2	-8	14	1.84	1.19
-2	-8	14	1.06	0.83
1	8	-14	3.85	0.71

1	8	-14	3.77	0.85
1	8	-14	2.49	0.58
-1	-8	14	2.75	1.34
-1	-8	14	3.10	1.01
-1	-8	14	4.00	0.99
0	8	-14	8.09	0.92
0	8	-14	7.82	1.01
0	8	-14	9.51	1.33
0	-8	14	10.26	1.74
0	-8	14	9.89	1.38
0	-8	14	9.34	1.23
-1	8	-14	19.47	1.26
-1	8	-14	21.23	1.29
-1	8	-14	20.44	2.00
1	-8	14	20.12	2.02
1	-8	14	21.35	1.86
1	-8	14	16.02	2.50
-2	8	-14	15.71	1.21
-2	8	-14	16.30	1.19
2	-8	14	16.87	1.79
2	-8	14	13.84	2.58
-3	8	-14	9.44	1.12
-3	8	-14	10.15	1.08
3	-8	14	12.48	2.20
3	-8	14	8.45	1.38
-4	8	-14	14.53	1.28
-4	8	-14	13.00	1.21
4	-8	14	15.60	2.50
4	-8	14	12.64	1.92
-5	8	-14	5.45	0.83
-5	8	-14	3.93	0.89
5	-8	14	2.53	2.23
5	-8	14	5.41	1.38
-6	8	-14	2.26	0.74
-6	8	-14	3.44	0.90
6	-8	14	3.36	1.36
6	-8	14	4.09	2.52
-7	8	-14	4.23	0.90
-7	8	-14	4.45	0.99
7	-8	14	6.02	1.45
7	-8	14	1.72	2.68
13	7	-14	5.23	1.72
13	7	-14	1.62	0.91
-13	-7	14	0.62	1.75

12	7	-14	3.03	1.39
12	7	-14	1.20	0.77
-12	-7	14	3.02	1.59
11	7	-14	0.29	0.73
-11	-7	14	1.82	1.39
10	7	-14	0.55	0.70
10	7	-14	-0.58	1.10
-10	-7	14	-0.87	1.09
9	7	-14	0.30	1.10
9	7	-14	0.80	0.69
-9	-7	14	1.29	1.09
-9	-7	14	2.23	1.11
8	7	-14	0.00	0.62
8	7	-14	1.35	1.01
-8	-7	14	0.43	0.79
-8	-7	14	-1.70	0.87
7	7	-14	0.44	0.55
7	7	-14	0.58	0.96
-7	-7	14	0.46	0.76
-7	-7	14	1.72	0.89
6	7	-14	1.80	0.58
6	7	-14	2.13	0.90
-6	-7	14	2.52	0.87
-6	-7	14	3.33	0.78
5	7	-14	0.19	0.81
5	7	-14	-0.19	0.47
-5	-7	14	1.14	0.80
-5	-7	14	-0.59	0.69
4	7	-14	2.25	0.47
4	7	-14	0.93	0.53
4	7	-14	2.35	0.82
-4	-7	14	2.21	0.82
-4	-7	14	0.67	0.73
-4	-7	14	0.94	0.95
3	7	-14	8.12	1.17
3	7	-14	9.83	0.98
3	7	-14	9.91	0.92
-3	-7	14	13.09	1.42
-3	-7	14	9.99	1.35
-3	-7	14	12.82	1.65
2	7	-14	9.41	0.95
2	7	-14	10.00	1.17
2	7	-14	10.47	1.06
-2	-7	14	8.01	1.39

-2	-7	14	11.86	1.51
-2	-7	14	10.64	1.77
1	7	-14	8.56	0.91
1	7	-14	7.84	1.15
1	7	-14	6.65	1.06
-1	-7	14	8.72	1.22
-1	-7	14	8.50	1.47
-1	-7	14	7.75	1.33
0	7	-14	19.36	1.35
0	7	-14	20.39	1.20
0	7	-14	19.01	1.49
0	-7	14	19.88	2.24
0	-7	14	19.60	1.69
0	-7	14	19.86	1.98
-1	7	-14	28.84	1.93
-1	7	-14	33.62	1.54
-1	7	-14	32.74	1.62
1	-7	14	31.78	2.03
1	-7	14	30.86	2.71
1	-7	14	26.90	2.29
-2	7	-14	11.21	1.18
-2	7	-14	12.69	1.09
2	-7	14	14.92	2.16
2	-7	14	10.77	1.56
-3	7	-14	11.59	1.09
3	-7	14	12.54	2.31
3	-7	14	10.22	1.68
-4	7	-14	12.35	1.21
-4	7	-14	13.68	1.31
4	-7	14	11.76	1.83
4	-7	14	15.98	2.62
-5	7	-14	13.02	1.40
-5	7	-14	9.86	1.23
5	-7	14	10.37	1.55
5	-7	14	15.86	2.74
-6	7	-14	8.13	1.21
-6	7	-14	6.20	1.00
6	-7	14	11.93	2.93
6	-7	14	7.28	1.43
-7	7	-14	7.41	1.11
-7	7	-14	5.35	0.90
7	-7	14	5.48	1.45
7	-7	14	8.60	3.00
13	6	-14	-1.17	1.44

13	6	-14	0.20	0.78
-13	-6	14	-0.97	1.54
12	6	-14	-1.13	1.30
12	6	-14	-0.83	0.69
-12	-6	14	-1.44	1.34
11	6	-14	3.07	1.46
11	6	-14	-0.03	0.64
-11	-6	14	-2.10	1.09
10	6	-14	-0.67	1.11
10	6	-14	-0.12	0.63
-10	-6	14	1.22	1.12
9	6	-14	0.79	0.59
9	6	-14	2.14	1.13
-9	-6	14	1.18	0.95
8	6	-14	0.71	0.99
8	6	-14	2.19	0.67
-8	-6	14	0.94	0.86
-8	-6	14	2.86	0.96
7	6	-14	0.23	0.95
7	6	-14	0.33	0.53
-7	-6	14	1.52	0.77
-7	-6	14	0.63	0.75
6	6	-14	2.29	0.55
6	6	-14	1.76	0.89
-6	-6	14	1.92	0.82
-6	-6	14	2.06	0.79
5	6	-14	2.95	0.59
5	6	-14	3.15	0.87
-5	-6	14	2.43	0.75
-5	-6	14	5.15	0.93
4	6	-14	7.99	0.89
4	6	-14	8.53	1.25
-4	-6	14	8.52	1.20
-4	-6	14	7.61	1.16
-4	-6	14	4.81	0.97
3	6	-14	8.32	0.87
3	6	-14	7.56	0.85
3	6	-14	9.30	1.22
-3	-6	14	6.95	1.04
-3	-6	14	9.54	1.43
-3	-6	14	8.39	1.15
2	6	-14	26.74	1.59
2	6	-14	27.75	1.35
2	6	-14	23.35	1.36

-2	-6	14	28.77	1.77
-2	-6	14	26.35	1.89
-2	-6	14	24.14	2.01
1	6	-14	31.54	1.68
1	6	-14	33.98	1.71
1	6	-14	35.16	1.56
-1	-6	14	35.28	2.40
-1	-6	14	32.82	1.95
-1	-6	14	30.19	2.19
0	6	-14	18.36	1.35
0	6	-14	18.69	1.14
0	6	-14	20.75	1.49
0	-6	14	18.45	1.60
0	-6	14	14.32	1.90
0	-6	14	18.86	2.13
-1	6	-14	12.58	1.81
-1	6	-14	13.59	1.24
-1	6	-14	14.43	1.07
1	-6	14	13.66	1.48
1	-6	14	15.44	2.28
-2	6	-14	11.32	1.04
-2	6	-14	14.17	1.23
2	-6	14	15.15	2.49
2	-6	14	13.88	1.55
-3	6	-14	11.29	1.25
-3	6	-14	14.21	1.15
3	-6	14	13.42	1.63
3	-6	14	14.43	2.25
-4	6	-14	15.60	1.42
-4	6	-14	16.45	1.26
4	-6	14	18.09	1.82
4	-6	14	16.55	3.03
-5	6	-14	14.67	1.48
-5	6	-14	15.67	1.30
5	-6	14	12.98	1.88
5	-6	14	15.26	2.66
-6	6	-14	8.91	1.44
-6	6	-14	8.01	1.21
6	-6	14	8.19	2.63
6	-6	14	7.50	1.36
14	5	-14	2.82	1.64
14	5	-14	0.45	0.85
-14	-5	14	0.28	1.63
13	5	-14	0.91	0.74

13	5	-14	1.12	1.45
-13	-5	14	0.32	1.45
12	5	-14	-0.06	0.67
12	5	-14	-0.65	1.23
-12	-5	14	-0.33	1.16
11	5	-14	0.68	1.17
11	5	-14	1.08	0.67
-11	-5	14	1.12	1.07
10	5	-14	-0.75	1.02
10	5	-14	0.09	0.56
-10	-5	14	-0.74	0.86
9	5	-14	-0.08	1.02
9	5	-14	-0.40	0.52
-9	-5	14	1.11	0.83
8	5	-14	0.61	0.55
8	5	-14	0.48	0.92
-8	-5	14	1.78	0.81
-8	-5	14	0.12	0.77
7	5	-14	7.88	0.94
7	5	-14	8.52	1.24
-7	-5	14	7.75	1.30
-7	-5	14	10.30	1.37
6	5	-14	9.72	1.44
6	5	-14	8.86	0.93
-6	-5	14	9.08	1.36
-6	-5	14	9.78	1.28
5	5	-14	1.98	0.87
5	5	-14	2.85	0.55
-5	-5	14	2.77	0.77
-5	-5	14	2.84	0.87
4	5	-14	16.18	1.01
4	5	-14	10.93	1.38
-4	-5	14	13.40	1.29
-4	-5	14	10.70	1.43
-4	-5	14	10.66	1.48
-4	-5	14	15.26	1.11
3	5	-14	31.59	1.44
3	5	-14	31.16	1.76
-3	-5	14	29.37	1.64
-3	-5	14	28.50	1.98
-3	-5	14	27.59	1.73
-3	-5	14	32.15	1.90
2	5	-14	28.51	1.39
2	5	-14	28.74	1.71

-2	-5	14	32.22	2.06
-2	-5	14	29.46	2.02
-2	-5	14	27.58	1.72
1	5	-14	20.08	1.13
1	5	-14	15.93	1.38
-1	-5	14	20.08	2.26
-1	-5	14	18.07	1.90
-1	-5	14	19.14	1.46
0	5	-14	9.26	1.29
0	5	-14	9.97	0.89
0	5	-14	10.20	1.21
0	-5	14	11.38	1.65
0	-5	14	12.02	1.33
-1	5	-14	10.91	0.98
-1	5	-14	10.40	1.22
1	-5	14	16.22	2.20
1	-5	14	12.20	1.40
-2	5	-14	24.85	1.58
-2	5	-14	28.96	1.45
2	-5	14	29.14	1.87
2	-5	14	27.27	2.64
-3	5	-14	16.27	1.17
-3	5	-14	15.11	1.42
3	-5	14	16.27	1.64
3	-5	14	17.23	2.68
-4	5	-14	10.53	1.12
-4	5	-14	12.86	1.39
4	-5	14	12.79	2.26
4	-5	14	8.73	1.54
-5	5	-14	15.22	1.30
-5	5	-14	16.19	1.59
5	-5	14	16.78	1.89
5	-5	14	16.29	2.81
-6	5	-14	5.42	1.07
-6	5	-14	6.27	0.95
6	-5	14	5.76	2.49
6	-5	14	7.81	1.41
14	4	-14	0.67	1.60
14	4	-14	2.49	0.84
-14	-4	14	3.08	1.50
13	4	-14	1.30	0.72
13	4	-14	0.31	1.45
-13	-4	14	2.43	1.28
12	4	-14	0.65	0.61

12	4	-14	0.00	1.20
-12	-4	14	0.16	1.03
11	4	-14	2.23	0.68
11	4	-14	2.97	1.16
-11	-4	14	3.35	1.09
10	4	-14	0.11	0.57
10	4	-14	0.17	0.94
-10	-4	14	-0.48	0.79
9	4	-14	10.53	0.99
9	4	-14	13.16	1.72
-9	-4	14	8.72	1.34
8	4	-14	11.32	1.61
8	4	-14	11.96	1.00
-8	-4	14	12.22	1.44
7	4	-14	1.41	0.92
7	4	-14	3.10	0.58
-7	-4	14	1.94	0.86
-7	-4	14	3.50	0.85
6	4	-14	7.90	0.84
6	4	-14	7.55	1.17
-6	-4	14	6.96	1.13
-6	-4	14	7.76	1.10
5	4	-14	28.12	1.83
5	4	-14	30.67	1.62
-5	-4	14	25.15	1.82
-5	-4	14	25.46	1.61
4	4	-14	12.56	1.46
4	4	-14	10.68	1.40
-4	-4	14	14.86	1.26
-4	-4	14	13.29	1.21
-4	-4	14	14.76	1.38
-4	-4	14	12.48	1.57
3	4	-14	18.94	1.33
3	4	-14	21.87	1.55
-3	-4	14	20.50	1.57
-3	-4	14	20.78	1.43
-3	-4	14	19.39	1.81
-3	-4	14	20.87	1.53
2	4	-14	29.64	1.79
-2	-4	14	31.10	2.01
-2	-4	14	31.28	1.90
-2	-4	14	30.88	1.73
1	4	-14	28.71	1.47
1	4	-14	27.50	1.66

-1	-4	14	30.23	1.71
-1	-4	14	30.13	2.00
-1	-4	14	23.88	1.99
0	4	-14	26.28	1.29
0	4	-14	24.22	1.59
0	-4	14	28.65	2.20
0	-4	14	22.66	1.61
0	-4	14	25.04	2.06
-1	4	-14	18.41	1.09
-1	4	-14	15.03	1.37
1	-4	14	13.66	1.39
1	-4	14	18.43	2.19
-2	4	-14	17.58	1.46
-2	4	-14	21.92	1.25
2	-4	14	21.11	1.63
-3	4	-14	18.65	1.54
-3	4	-14	20.53	1.28
3	-4	14	18.53	2.56
3	-4	14	21.19	1.71
-4	4	-14	22.82	1.41
-4	4	-14	22.09	1.69
4	-4	14	22.10	3.07
4	-4	14	23.66	1.81
-5	4	-14	17.56	1.33
-5	4	-14	20.57	1.68
5	-4	14	16.91	2.64
5	-4	14	18.83	1.83
-6	4	-14	3.83	0.90
-6	4	-14	1.26	0.90
6	-4	14	4.18	1.18
6	-4	14	-0.31	2.14
15	3	-14	-1.32	0.74
15	3	-14	-0.12	1.65
14	3	-14	2.18	1.55
14	3	-14	0.11	0.68
-14	-3	14	2.56	1.39
13	3	-14	-0.21	0.63
13	3	-14	0.00	1.41
-13	-3	14	-0.73	1.12
12	3	-14	1.42	1.22
12	3	-14	0.84	0.67
11	3	-14	6.49	1.27
11	3	-14	5.11	0.75
10	3	-14	11.26	2.17

10	3	-14	8.29	0.95
-10	-3	14	6.78	1.20
9	3	-14	1.87	1.01
9	3	-14	2.82	0.60
-9	-3	14	2.16	0.81
8	3	-14	6.58	1.17
8	3	-14	4.97	0.67
-8	-3	14	4.06	0.86
7	3	-14	26.65	1.32
7	3	-14	25.73	1.93
-7	-3	14	23.02	1.77
6	3	-14	5.32	1.08
5	3	-14	2.02	0.94
5	3	-14	2.33	0.52
-5	-3	14	3.08	1.12
-5	-3	14	1.85	0.79
4	3	-14	4.82	0.76
4	3	-14	5.06	1.01
-4	-3	14	6.47	0.92
-4	-3	14	5.40	0.98
-4	-3	14	6.51	0.89
3	3	-14	21.52	1.18
3	3	-14	21.83	1.65
-3	-3	14	21.07	1.42
-3	-3	14	23.76	1.72
-3	-3	14	25.28	1.63
2	3	-14	28.37	1.35
2	3	-14	29.19	1.79
-2	-3	14	29.75	1.65
-2	-3	14	26.95	1.87
-2	-3	14	28.15	1.99
1	3	-14	18.11	1.44
1	3	-14	16.29	1.01
-1	-3	14	15.17	1.87
-1	-3	14	16.22	1.32
-1	-3	14	19.05	1.74
0	3	-14	13.95	1.58
0	3	-14	11.71	1.36
0	-3	14	13.96	1.27
0	-3	14	10.45	1.75
0	-3	14	10.47	1.56
-1	3	-14	19.88	1.53
-1	3	-14	16.06	1.12
1	-3	14	19.44	1.43

1	-3	14	20.02	2.11
-2	3	-14	20.25	1.59
-2	3	-14	22.80	1.26
2	-3	14	22.29	1.58
2	-3	14	20.72	2.31
-3	3	-14	18.69	1.58
-3	3	-14	17.86	1.23
3	-3	14	16.69	2.54
3	-3	14	19.72	1.55
-4	3	-14	16.69	1.28
-4	3	-14	17.42	1.59
4	-3	14	19.56	2.97
4	-3	14	17.42	1.63
-5	3	-14	19.80	1.74
-5	3	-14	21.72	1.43
5	-3	14	20.53	2.74
5	-3	14	20.49	1.82
-6	3	-14	8.66	1.23
-6	3	-14	12.02	1.35
6	-3	14	7.57	2.28
6	-3	14	12.04	1.68
15	2	-14	2.35	1.69
15	2	-14	-0.42	0.69
14	2	-14	2.27	0.75
14	2	-14	1.33	1.52
-14	-2	14	2.28	1.21
13	2	-14	7.66	1.08
13	2	-14	8.53	1.66
-13	-2	14	8.57	1.35
12	2	-14	15.31	1.19
12	2	-14	12.15	1.69
-12	-2	14	15.12	1.67
11	2	-14	6.23	1.31
11	2	-14	10.02	1.02
-11	-2	14	9.89	1.38
10	2	-14	6.44	1.33
10	2	-14	6.50	0.90
-10	-2	14	5.88	0.96
9	2	-14	25.70	1.33
9	2	-14	23.84	2.01
-9	-2	14	25.34	1.61
8	2	-14	26.39	1.35
8	2	-14	27.89	2.04
-8	-2	14	27.75	1.62

7	2	-14	7.19	1.04
7	2	-14	6.83	1.21
-7	-2	14	6.90	0.99
6	2	-14	15.46	0.98
6	2	-14	14.04	1.59
-6	-2	14	14.76	1.33
5	2	-14	60.44	2.27
5	2	-14	61.56	2.81
-5	-2	14	62.14	2.48
4	2	-14	10.33	1.44
4	2	-14	12.23	0.93
-4	-2	14	13.56	1.50
-4	-2	14	11.99	1.25
-4	-2	14	12.60	1.11
3	2	-14	9.49	0.86
3	2	-14	10.39	1.37
-3	-2	14	9.14	1.26
-3	-2	14	8.18	1.02
-3	-2	14	11.12	1.64
2	2	-14	18.16	1.07
2	2	-14	19.20	1.58
-2	-2	14	21.58	1.66
-2	-2	14	20.87	1.36
-2	-2	14	19.74	1.92
1	2	-14	11.06	1.59
1	2	-14	12.18	0.88
-1	-2	14	11.83	1.59
-1	-2	14	10.70	1.11
-1	-2	14	12.32	1.59
0	2	-14	13.44	1.39
0	2	-14	14.67	0.99
0	-2	14	14.01	1.78
0	-2	14	18.48	2.65
0	-2	14	18.13	1.31
-1	2	-14	15.42	1.06
-1	2	-14	15.95	1.51
1	-2	14	15.14	1.31
1	-2	14	13.83	1.94
-2	2	-14	7.53	0.90
-2	2	-14	7.27	1.07
2	-2	14	7.58	1.52
2	-2	14	9.14	1.21
-3	2	-14	15.33	1.14
-3	2	-14	13.98	1.50

3	-2	14	11.14	1.79
3	-2	14	13.08	1.41
-4	2	-14	16.92	1.25
-4	2	-14	16.63	1.62
4	-2	14	16.97	2.80
4	-2	14	15.78	1.56
-5	2	-14	16.27	1.67
-5	2	-14	12.86	1.30
5	-2	14	12.19	2.29
5	-2	14	13.56	1.63
16	1	-14	-0.22	1.69
15	1	-14	0.56	0.69
15	1	-14	1.68	1.60
14	1	-14	0.28	1.39
14	1	-14	-0.46	0.60
-14	-1	14	-0.44	1.07
13	1	-14	4.65	0.79
13	1	-14	6.15	1.48
-13	-1	14	6.93	1.18
12	1	-14	9.99	1.05
12	1	-14	11.54	1.62
-12	-1	14	8.74	1.40
11	1	-14	8.80	1.54
11	1	-14	8.47	0.97
-11	-1	14	10.41	1.34
10	1	-14	13.15	1.80
10	1	-14	9.87	0.95
-10	-1	14	10.32	1.24
9	1	-14	5.07	0.69
-9	-1	14	5.88	1.01
8	1	-14	10.14	1.61
-8	-1	14	9.49	1.06
7	1	-14	19.43	1.84
7	1	-14	22.19	1.15
-7	-1	14	20.05	1.32
6	1	-14	37.81	1.55
6	1	-14	35.12	2.19
-6	-1	14	34.18	1.90
5	1	-14	71.23	2.50
5	1	-14	70.35	3.08
-5	-1	14	68.55	2.59
-5	-1	14	72.01	2.91
-5	-1	14	66.80	3.22
4	1	-14	33.85	2.07

4	1	-14	35.47	1.51
-4	-1	14	33.76	2.19
-4	-1	14	33.49	1.70
-4	-1	14	35.64	1.72
3	1	-14	16.22	1.01
3	1	-14	14.47	1.53
-3	-1	14	16.27	1.93
-3	-1	14	15.46	1.16
-3	-1	14	15.80	1.36
2	1	-14	27.81	1.82
2	1	-14	25.94	1.28
-2	-1	14	31.25	2.30
-2	-1	14	25.96	1.52
-2	-1	14	27.66	1.76
1	1	-14	13.16	1.42
1	1	-14	12.69	0.89
-1	-1	14	11.32	1.51
-1	-1	14	12.11	1.74
-1	-1	14	12.21	1.16
0	1	-14	17.82	1.04
0	1	-14	17.46	1.54
0	-1	14	17.34	1.35
0	-1	14	17.51	1.79
-1	1	-14	15.45	1.52
-1	1	-14	13.97	1.11
1	-1	14	14.51	1.30
1	-1	14	12.86	2.06
-2	1	-14	16.81	1.12
-2	1	-14	18.08	1.62
2	-1	14	17.68	1.44
2	-1	14	18.34	2.17
-3	1	-14	15.04	1.62
-3	1	-14	14.29	1.14
3	-1	14	16.08	1.48
3	-1	14	14.80	2.50
-4	1	-14	15.88	1.25
-4	1	-14	17.10	1.72
4	-1	14	17.31	1.60
4	-1	14	15.36	2.72
-5	1	-14	3.45	0.84
-5	1	-14	4.09	1.06
5	-1	14	3.03	1.83
5	-1	14	5.60	1.10
16	0	-14	3.19	1.72

16	0	-14	0.09	0.76
15	0	-14	1.82	0.69
15	0	-14	0.77	1.54
14	0	-14	2.21	1.40
14	0	-14	0.80	0.62
-14	0	14	1.22	1.06
13	0	-14	2.65	0.67
13	0	-14	1.52	1.34
-13	0	14	2.83	1.01
12	0	-14	20.59	1.30
12	0	-14	20.17	2.17
-12	0	14	22.99	1.64
11	0	-14	4.38	0.66
11	0	-14	5.41	1.30
-11	0	14	5.40	0.87
10	0	-14	4.25	1.18
10	0	-14	4.80	0.64
-10	0	14	4.65	0.77
9	0	-14	22.78	1.37
9	0	-14	21.67	1.97
-9	0	14	21.07	1.39
8	0	-14	25.93	1.25
-8	0	14	23.18	1.42
7	0	-14	5.91	0.76
7	0	-14	4.17	1.32
-7	0	14	4.26	0.72
6	0	-14	30.03	1.35
6	0	-14	28.24	2.02
-6	0	14	30.00	1.83
-6	0	14	28.69	1.42
5	0	-14	70.32	2.44
5	0	-14	58.05	3.30
-5	0	14	67.60	2.68
-5	0	14	72.90	3.07
-5	0	14	68.31	2.54
4	0	-14	35.19	2.91
4	0	-14	37.77	1.56
-4	0	14	37.72	2.47
-4	0	14	36.95	1.79
-4	0	14	39.62	1.70
-4	0	14	39.15	1.80
3	0	-14	14.53	0.93
3	0	-14	15.76	1.51
-3	0	14	14.02	1.30

-3	0	14	13.48	0.95
-3	0	14	12.56	1.13
-3	0	14	14.08	2.10
2	0	-14	28.55	1.87
2	0	-14	27.34	1.24
-2	0	14	25.06	2.47
-2	0	14	27.38	1.53
-2	0	14	26.04	1.74
1	0	-14	19.88	1.08
1	0	-14	20.57	1.64
-1	0	14	18.06	1.70
-1	0	14	18.81	1.35
-1	0	14	20.55	2.94
0	0	-14	18.24	1.59
0	0	-14	17.91	1.05
0	0	14	16.91	1.35
0	0	14	19.93	1.82
-1	0	-14	18.67	1.13
-1	0	-14	21.70	1.72
1	0	14	18.70	1.44
1	0	14	23.07	2.04
-2	0	-14	17.13	1.32
-2	0	-14	18.30	1.66
2	0	14	16.66	1.44
2	0	14	14.96	2.10
-3	0	-14	14.32	1.62
-3	0	-14	14.28	1.14
3	0	14	17.05	1.53
3	0	14	14.67	2.40
-4	0	-14	13.14	1.23
-4	0	-14	13.61	1.61
4	0	14	10.15	1.49
4	0	14	8.20	1.93
16	-1	-14	3.35	0.83
16	-1	-14	4.88	1.74
15	-1	-14	3.26	1.58
15	-1	-14	3.37	0.76
14	-1	-14	5.48	1.53
14	-1	-14	5.58	0.78
-14	1	14	7.28	1.18
13	-1	-14	2.97	1.36
13	-1	-14	3.92	0.69
-13	1	14	4.58	0.96
12	-1	-14	7.18	1.50

12	-1	-14	6.42	0.94
-12	1	14	8.14	1.29
11	-1	-14	16.12	2.05
11	-1	-14	11.56	0.99
-11	1	14	11.93	1.21
10	-1	-14	30.59	2.32
10	-1	-14	31.95	1.46
-10	1	14	29.97	1.64
9	-1	-14	17.04	1.04
9	-1	-14	15.33	1.84
-9	1	14	15.69	1.20
8	-1	-14	30.74	2.14
8	-1	-14	27.91	1.34
-8	1	14	28.72	1.54
7	-1	-14	32.97	1.45
7	-1	-14	32.66	2.18
-7	1	14	32.82	1.47
-7	1	14	32.63	1.88
-7	1	14	33.05	1.69
6	-1	-14	21.02	1.12
6	-1	-14	21.79	1.84
-6	1	14	20.54	1.18
-6	1	14	19.58	1.36
-6	1	14	23.47	1.96
-6	1	14	23.67	1.22
5	-1	-14	16.63	1.65
5	-1	-14	17.97	1.02
-5	1	14	18.64	1.23
-5	1	14	18.27	2.11
-5	1	14	15.84	0.92
-5	1	14	18.06	1.20
4	-1	-14	17.62	1.02
4	-1	-14	15.92	1.65
-4	1	14	17.93	1.30
-4	1	14	20.28	2.29
-4	1	14	19.64	1.25
-4	1	14	16.67	0.96
3	-1	-14	29.74	1.97
3	-1	-14	30.46	1.32
-3	1	14	26.79	1.28
-3	1	14	30.29	2.64
-3	1	14	30.04	1.69
-3	1	14	29.57	1.59
2	-1	-14	25.54	1.19

2	-1	-14	24.98	1.86
-2	1	14	26.71	2.74
-2	1	14	25.92	1.71
-2	1	14	24.94	1.49
-2	1	14	22.86	1.18
1	-1	-14	23.57	1.14
1	-1	-14	21.84	1.73
-1	1	14	21.47	1.45
-1	1	14	21.91	1.76
-1	1	14	19.78	1.18
0	-1	-14	14.05	0.97
0	-1	-14	13.26	1.52
0	1	14	13.20	1.24
0	1	14	13.92	1.67
-1	-1	-14	20.27	1.13
-1	-1	-14	17.56	1.75
1	1	14	21.02	1.53
1	1	14	21.56	2.08
-2	-1	-14	24.34	1.81
-2	-1	-14	24.06	1.29
2	1	14	20.76	2.43
2	1	14	24.49	1.69
-3	-1	-14	9.65	1.53
-3	-1	-14	9.78	1.09
3	1	14	7.36	1.12
3	1	14	9.24	1.81
-4	-1	-14	1.38	0.72
-4	-1	-14	2.73	1.04
4	1	14	1.87	1.64
4	1	14	5.24	1.03
16	-2	-14	1.62	0.76
16	-2	-14	2.10	1.74
15	-2	-14	5.95	0.86
15	-2	-14	4.43	1.62
14	-2	-14	7.56	1.05
14	-2	-14	9.07	1.68
-14	2	14	4.74	1.02
13	-2	-14	5.05	0.71
13	-2	-14	3.17	1.42
-13	2	14	3.73	0.89
12	-2	-14	14.39	1.09
12	-2	-14	16.09	2.10
-12	2	14	13.84	1.32
11	-2	-14	12.34	1.99

11	-2	-14	13.20	0.99
-11	2	14	11.18	1.15
10	-2	-14	19.29	2.02
-10	2	14	18.62	1.27
9	-2	-14	35.68	2.44
9	-2	-14	39.17	1.62
-9	2	14	39.01	1.81
8	-2	-14	33.04	1.45
8	-2	-14	28.79	2.25
-8	2	14	33.64	1.88
-8	2	14	31.24	1.35
-8	2	14	35.59	1.66
7	-2	-14	20.91	1.11
7	-2	-14	18.84	1.89
-7	2	14	19.73	1.09
-7	2	14	20.91	1.98
-7	2	14	21.82	1.71
6	-2	-14	20.75	1.86
6	-2	-14	19.71	1.07
-6	2	14	17.03	2.20
-6	2	14	17.86	1.12
-6	2	14	19.78	1.24
-6	2	14	19.41	0.99
5	-2	-14	33.48	1.40
5	-2	-14	32.67	2.17
-5	2	14	31.68	1.55
-5	2	14	31.05	1.32
-5	2	14	29.94	2.65
-5	2	14	32.80	1.63
4	-2	-14	24.27	1.84
4	-2	-14	22.74	1.12
-4	2	14	21.22	2.61
-4	2	14	22.44	1.05
-4	2	14	23.58	1.39
-4	2	14	21.77	1.40
3	-2	-14	17.75	1.71
3	-2	-14	17.58	0.97
-3	2	14	18.99	1.31
-3	2	14	17.78	1.41
-3	2	14	19.99	2.75
-3	2	14	18.42	0.98
2	-2	-14	34.41	2.11
2	-2	-14	33.02	1.40
-2	2	14	33.93	1.95

-2	2	14	34.55	1.79
-2	2	14	31.38	1.39
1	-2	-14	16.67	1.69
1	-2	-14	16.85	1.02
-1	2	14	20.28	1.45
-1	2	14	19.88	1.22
0	-2	-14	6.50	1.17
0	-2	-14	6.85	0.83
0	2	14	5.23	1.31
0	2	14	6.66	0.97
-1	-2	-14	13.17	1.00
-1	-2	-14	13.40	1.64
1	2	14	13.13	1.40
1	2	14	13.76	2.01
-2	-2	-14	11.06	1.61
-2	-2	-14	12.04	1.05
2	2	14	7.35	1.60
2	2	14	10.56	1.40
-3	-2	-14	6.83	1.18
3	2	14	4.90	1.63
3	2	14	6.33	1.11
16	-3	-14	7.04	0.91
16	-3	-14	6.24	1.84
15	-3	-14	8.70	1.76
15	-3	-14	11.16	1.18
14	-3	-14	6.54	1.02
14	-3	-14	9.18	1.66
-14	3	14	7.86	1.37
13	-3	-14	10.75	1.05
13	-3	-14	9.72	1.72
-13	3	14	10.90	1.31
12	-3	-14	12.35	1.81
12	-3	-14	14.59	1.07
-12	3	14	13.99	1.31
11	-3	-14	21.90	2.28
11	-3	-14	21.84	1.23
-11	3	14	22.99	1.39
10	-3	-14	24.60	1.26
10	-3	-14	27.55	2.26
-10	3	14	25.06	1.43
9	-3	-14	5.20	1.31
9	-3	-14	9.56	0.83
-9	3	14	9.08	0.96
-9	3	14	10.19	1.49

-9	3	14	6.95	0.74
8	-3	-14	19.65	1.33
8	-3	-14	16.34	1.03
8	-3	-14	12.86	1.66
-8	3	14	16.44	1.95
-8	3	14	16.11	0.99
7	-3	-14	15.89	1.93
7	-3	-14	17.56	1.01
7	-3	-14	16.83	1.19
-7	3	14	15.62	1.06
-7	3	14	17.32	2.25
-7	3	14	17.36	1.19
6	-3	-14	36.30	1.48
6	-3	-14	33.82	2.35
-6	3	14	35.27	1.41
-6	3	14	34.25	1.71
-6	3	14	39.81	2.83
-6	3	14	35.19	1.64
5	-3	-14	24.87	1.18
5	-3	-14	23.51	2.03
-5	3	14	22.83	1.09
-5	3	14	27.15	2.72
-5	3	14	22.95	1.38
-5	3	14	24.49	1.45
4	-3	-14	20.18	1.86
4	-3	-14	21.25	1.07
-4	3	14	19.59	2.89
-4	3	14	21.00	1.41
-4	3	14	20.56	1.01
-4	3	14	20.40	1.37
3	-3	-14	34.30	1.38
3	-3	-14	34.31	2.19
-3	3	14	33.67	1.35
-3	3	14	32.93	1.77
-3	3	14	31.09	1.84
2	-3	-14	22.44	1.11
2	-3	-14	22.15	1.88
-2	3	14	22.94	1.54
-2	3	14	24.76	1.72
1	-3	-14	11.28	0.88
1	-3	-14	11.69	1.66
-1	3	14	14.17	1.34
-1	3	14	9.13	1.62
0	-3	-14	10.81	1.61

0	-3	-14	11.86	0.90
0	3	14	10.48	1.31
0	3	14	8.18	1.41
-1	-3	-14	10.31	0.92
-1	-3	-14	11.61	1.69
1	3	14	8.87	1.51
1	3	14	9.02	1.36
-2	-3	-14	7.25	1.24
2	3	14	7.94	1.68
2	3	14	10.16	1.46
3	3	14	14.41	1.74
3	3	14	15.38	2.74
16	-4	-14	17.40	2.75
16	-4	-14	14.68	1.32
15	-4	-14	12.83	1.23
15	-4	-14	11.64	1.94
14	-4	-14	7.80	1.05
14	-4	-14	6.38	1.61
-14	4	14	5.55	0.94
13	-4	-14	22.71	1.30
13	-4	-14	23.68	2.49
-13	4	14	21.34	1.51
12	-4	-14	38.20	2.83
12	-4	-14	36.51	1.63
-12	4	14	36.96	1.82
11	-4	-14	10.58	1.14
11	-4	-14	12.78	2.07
-11	4	14	11.99	1.11
10	-4	-14	13.84	2.06
10	-4	-14	15.32	1.01
-10	4	14	13.08	1.11
9	-4	-14	17.42	1.07
9	-4	-14	17.22	2.13
-9	4	14	20.97	2.09
-9	4	14	17.38	1.09
8	-4	-14	22.69	1.28
8	-4	-14	21.39	1.15
8	-4	-14	25.16	2.21
-8	4	14	22.56	2.51
-8	4	14	20.35	1.25
-8	4	14	22.47	1.34
7	-4	-14	12.78	1.85
7	-4	-14	12.28	0.93
7	-4	-14	14.32	1.06

-7	4	14	11.08	1.10
-7	4	14	11.90	1.10
-7	4	14	14.83	1.97
6	-4	-14	14.06	0.93
6	-4	-14	14.99	1.84
6	-4	-14	13.66	1.08
-6	4	14	14.07	1.97
-6	4	14	13.07	1.15
-6	4	14	13.20	1.16
5	-4	-14	18.70	2.02
5	-4	-14	21.82	1.05
-5	4	14	26.27	3.10
-5	4	14	19.48	1.01
-5	4	14	18.88	1.41
-5	4	14	21.57	1.40
4	-4	-14	25.64	2.05
4	-4	-14	24.58	1.12
-4	4	14	23.61	1.54
-4	4	14	23.32	1.62
3	-4	-14	19.68	1.89
3	-4	-14	19.05	1.00
-3	4	14	20.24	1.45
-3	4	14	17.57	1.54
2	-4	-14	10.37	1.71
2	-4	-14	12.51	0.84
-2	4	14	11.14	1.33
-2	4	14	12.97	1.56
1	-4	-14	16.29	0.94
1	-4	-14	17.18	1.81
-1	4	14	12.84	1.80
-1	4	14	16.08	1.46
0	-4	-14	19.51	1.90
0	4	14	17.54	1.62
0	4	14	16.92	2.06
-1	-4	-14	14.28	1.85
1	4	14	11.07	1.69
1	4	14	16.30	1.64
-2	-4	-14	7.65	1.74
2	4	14	6.03	1.17
2	4	14	7.04	1.67
16	-5	-14	4.84	0.85
16	-5	-14	8.63	1.90
15	-5	-14	25.09	1.46
15	-5	-14	20.65	2.73

14	-5	-14	23.86	1.40
14	-5	-14	25.30	2.68
-14	5	14	24.06	1.59
13	-5	-14	20.79	2.49
13	-5	-14	19.72	1.26
-13	5	14	19.79	1.43
12	-5	-14	13.64	1.93
12	-5	-14	12.99	1.06
-12	5	14	14.35	1.22
11	-5	-14	32.74	1.86
11	-5	-14	35.29	2.66
11	-5	-14	35.96	1.54
-11	5	14	34.58	1.72
10	-5	-14	23.68	1.69
10	-5	-14	24.30	1.25
10	-5	-14	26.95	2.41
9	-5	-14	23.48	1.23
9	-5	-14	23.32	2.28
9	-5	-14	23.06	1.28
9	-5	-14	24.13	1.72
-9	5	14	23.38	1.40
8	-5	-14	20.12	1.09
8	-5	-14	19.08	2.13
8	-5	-14	18.38	1.17
-8	5	14	25.88	2.82
-8	5	14	16.84	1.26
7	-5	-14	16.51	1.00
7	-5	-14	17.54	1.11
7	-5	-14	19.41	2.06
-7	5	14	17.01	1.32
-7	5	14	16.81	2.31
-7	5	14	15.33	1.26
6	-5	-14	13.24	1.06
6	-5	-14	13.14	1.90
6	-5	-14	12.92	0.92
-6	5	14	12.44	2.32
-6	5	14	11.50	1.30
-6	5	14	12.41	1.19
5	-5	-14	13.18	1.87
5	-5	-14	11.94	0.85
-5	5	14	13.72	1.28
-5	5	14	11.92	1.37
4	-5	-14	9.19	0.77
4	-5	-14	9.65	1.43

-4	5	14	9.12	1.40
-4	5	14	11.45	1.24
3	-5	-14	9.80	1.48
3	-5	-14	11.94	0.80
-3	5	14	16.46	1.60
-3	5	14	13.55	1.40
2	-5	-14	16.69	1.84
-2	5	14	14.66	1.75
-2	5	14	16.40	1.55
1	-5	-14	12.73	1.81
-1	5	14	15.26	1.59
-1	5	14	13.29	1.91
0	-5	-14	12.73	1.81
0	5	14	14.01	1.64
0	5	14	13.77	2.08
-1	-5	-14	7.26	1.42
1	5	14	8.63	1.28
1	5	14	8.95	1.61
16	-6	-14	4.96	0.89
16	-6	-14	3.82	1.98
15	-6	-14	10.18	2.01
15	-6	-14	12.88	1.23
14	-6	-14	13.39	1.16
14	-6	-14	10.08	2.07
-14	6	14	12.20	1.31
13	-6	-14	25.75	2.86
13	-6	-14	31.64	1.56
-13	6	14	32.42	1.70
12	-6	-14	20.29	2.45
12	-6	-14	22.82	1.27
-12	6	14	20.76	1.41
11	-6	-14	19.08	1.17
11	-6	-14	17.88	1.85
11	-6	-14	21.52	2.42
-11	6	14	18.39	2.10
10	-6	-14	19.22	2.43
10	-6	-14	18.76	1.17
10	-6	-14	22.66	1.91
-10	6	14	19.88	1.36
9	-6	-14	35.96	2.30
9	-6	-14	40.76	1.64
9	-6	-14	39.33	2.81
-9	6	14	38.81	1.88
8	-6	-14	21.94	1.97

8	-6	-14	21.37	2.24
8	-6	-14	21.44	1.16
-8	6	14	20.42	1.45
-8	6	14	26.94	3.71
7	-6	-14	20.09	1.08
7	-6	-14	20.11	2.15
-7	6	14	19.56	1.43
-7	6	14	23.40	3.20
6	-6	-14	19.57	2.19
6	-6	-14	20.77	1.01
-6	6	14	17.84	1.46
-6	6	14	16.72	1.54
5	-6	-14	10.61	1.56
5	-6	-14	12.13	0.78
-5	6	14	13.44	1.34
-5	6	14	10.23	1.80
4	-6	-14	9.00	1.43
-4	6	14	11.64	1.42
-4	6	14	9.32	1.56
3	-6	-14	16.62	1.95
-3	6	14	14.95	1.56
-3	6	14	11.59	1.79
2	-6	-14	18.85	1.99
-2	6	14	18.37	1.73
-2	6	14	21.54	2.14
1	-6	-14	6.72	1.27
-1	6	14	5.50	1.40
-1	6	14	7.58	1.19
0	-6	-14	4.65	1.35
0	6	14	2.88	1.38
0	6	14	4.77	1.19
16	-7	-14	-0.70	0.78
15	-7	-14	6.28	1.84
15	-7	-14	6.38	0.92
14	-7	-14	27.28	2.81
14	-7	-14	23.60	1.45
-14	7	14	26.14	1.80
13	-7	-14	13.23	1.15
13	-7	-14	8.92	1.78
-13	7	14	11.47	1.26
12	-7	-14	7.26	1.72
-12	7	14	9.21	1.43
11	-7	-14	15.24	2.10
11	-7	-14	19.03	1.19

11	-7	-14	19.03	2.48
-11	7	14	20.77	1.38
10	-7	-14	20.08	1.19
10	-7	-14	17.34	2.09
10	-7	-14	16.81	2.38
-10	7	14	19.05	1.38
9	-7	-14	21.07	1.15
9	-7	-14	21.12	2.42
9	-7	-14	18.77	2.12
-9	7	14	20.39	1.45
8	-7	-14	24.93	1.19
8	-7	-14	21.83	2.40
8	-7	-14	19.76	2.24
-8	7	14	22.94	1.57
7	-7	-14	24.28	2.33
7	-7	-14	24.87	1.12
7	-7	-14	24.36	2.35
-7	7	14	22.15	1.63
6	-7	-14	21.68	2.29
-6	7	14	21.34	1.65
-6	7	14	20.02	1.76
5	-7	-14	15.71	2.11
-5	7	14	19.26	1.64
-5	7	14	17.15	1.82
4	-7	-14	15.12	2.10
-4	7	14	17.34	1.67
-4	7	14	15.10	1.92
3	-7	-14	17.38	1.99
-3	7	14	12.20	1.60
-3	7	14	11.27	1.90
2	-7	-14	15.47	2.04
-2	7	14	18.36	1.83
-2	7	14	14.21	2.17
1	-7	-14	7.99	1.64
-1	7	14	9.76	1.64
-1	7	14	10.99	1.88
15	-8	-14	11.29	1.32
15	-8	-14	10.66	2.23
14	-8	-14	12.20	2.03
14	-8	-14	8.98	1.23
13	-8	-14	7.63	1.14
13	-8	-14	9.32	1.87
-13	8	14	9.50	1.52
12	-8	-14	27.52	1.41

12	-8	-14	21.90	2.82
-12	8	14	25.84	1.61
11	-8	-14	14.12	2.24
11	-8	-14	11.46	1.06
11	-8	-14	11.48	1.97
-11	8	14	9.39	1.26
10	-8	-14	12.94	2.31
10	-8	-14	13.54	1.05
10	-8	-14	12.31	1.91
-10	8	14	11.20	1.32
9	-8	-14	23.42	2.52
9	-8	-14	23.42	2.57
-9	8	14	25.74	1.68
8	-8	-14	13.53	2.28
-8	8	14	16.63	1.52
7	-8	-14	17.67	2.43
7	-8	-14	15.25	2.45
-7	8	14	20.51	1.68
6	-8	-14	17.67	2.30
-6	8	14	15.77	1.64
5	-8	-14	10.15	1.66
-5	8	14	11.36	1.89
-5	8	14	11.53	1.62
4	-8	-14	9.46	1.59
-4	8	14	14.83	1.70
-4	8	14	15.39	2.11
3	-8	-14	6.63	1.42
-3	8	14	8.87	1.55
-3	8	14	11.98	1.65
-2	8	14	1.14	1.38
14	-9	-14	6.74	1.99
14	-9	-14	11.39	1.29
13	-9	-14	6.25	1.83
13	-9	-14	6.07	0.84
-13	9	14	4.55	0.90
12	-9	-14	16.34	2.77
-12	9	14	15.37	1.47
11	-9	-14	24.63	2.77
11	-9	-14	23.70	2.78
-11	9	14	24.28	1.69
10	-9	-14	24.00	2.81
-10	9	14	29.82	1.86
9	-9	-14	20.48	2.63
9	-9	-14	22.31	2.66

-9	9	14	22.88	1.73
8	-9	-14	18.88	2.72
8	-9	-14	15.87	2.46
-8	9	14	18.84	1.70
7	-9	-14	24.15	2.86
7	-9	-14	19.96	2.55
-7	9	14	27.44	1.98
6	-9	-14	16.68	2.81
6	-9	-14	14.46	2.31
-6	9	14	15.59	1.75
5	-9	-14	14.57	2.32
-5	9	14	8.31	1.25
-5	9	14	7.11	1.47
4	-9	-14	10.96	1.71
-4	9	14	14.13	2.34
-4	9	14	17.66	1.90
13	-10	-14	4.59	2.08
-13	10	14	6.40	1.00
-12	10	14	17.76	1.67
11	-10	-14	13.42	2.21
11	-10	-14	16.55	2.81
-11	10	14	16.00	1.63
10	-10	-14	21.27	2.88
10	-10	-14	16.54	2.95
-10	10	14	22.62	1.82
9	-10	-14	20.88	2.77
9	-10	-14	18.76	2.91
-9	10	14	18.35	1.82
8	-10	-14	16.41	2.97
8	-10	-14	13.32	2.11
-8	10	14	16.08	1.81
7	-10	-14	6.54	1.69
7	-10	-14	9.62	2.10
-7	10	14	11.58	1.69
6	-10	-14	5.75	1.60
6	-10	-14	6.11	1.95
-6	10	14	3.91	1.09
3	14	-15	2.00	0.90
-3	-14	15	4.68	2.29
2	14	-15	2.50	0.91
-2	-14	15	4.03	1.65
1	14	-15	3.62	0.91
-1	-14	15	4.08	1.53
0	14	-15	2.51	0.82

0	-14	15	4.52	1.76
0	-14	15	2.39	2.39
-1	14	-15	6.00	0.98
1	-14	15	10.74	1.96
1	-14	15	12.23	2.94
-2	14	-15	12.58	1.68
2	-14	15	10.21	1.84
2	-14	15	11.73	3.18
6	13	-15	1.79	0.93
-6	-13	15	3.05	2.24
5	13	-15	1.69	0.91
-5	-13	15	0.43	1.44
4	13	-15	3.11	0.95
-4	-13	15	-0.27	1.47
3	13	-15	3.91	0.91
-3	-13	15	0.89	1.38
2	13	-15	4.25	0.90
-2	-13	15	7.98	1.67
1	13	-15	8.21	1.21
-1	-13	15	7.07	1.65
-1	-13	15	5.68	2.20
0	13	-15	8.03	1.21
0	-13	15	6.92	1.58
0	-13	15	6.10	2.78
-1	13	-15	13.26	1.40
1	-13	15	17.18	2.88
1	-13	15	14.08	2.45
-2	13	-15	3.12	0.85
2	-13	15	3.77	1.48
2	-13	15	3.70	2.70
-3	13	-15	1.21	0.83
3	-13	15	0.65	1.56
3	-13	15	2.64	2.88
7	12	-15	2.94	0.98
-7	-12	15	4.26	1.62
6	12	-15	12.34	1.49
-6	-12	15	13.29	1.99
5	12	-15	5.22	0.92
-5	-12	15	6.95	1.55
4	12	-15	0.46	0.79
-4	-12	15	1.65	1.35
3	12	-15	2.52	0.79
-3	-12	15	4.56	1.38
2	12	-15	12.74	1.34

-2	-12	15	13.18	2.22
-2	-12	15	16.42	2.86
1	12	-15	19.18	1.46
-1	-12	15	18.48	3.23
-1	-12	15	18.07	2.34
0	12	-15	15.94	1.37
0	-12	15	18.02	3.30
0	-12	15	18.12	2.32
-1	12	-15	11.58	1.29
1	-12	15	13.85	2.29
1	-12	15	11.05	2.62
-2	12	-15	14.16	1.34
2	-12	15	11.64	1.85
2	-12	15	15.48	2.96
-3	12	-15	8.76	1.30
3	-12	15	9.64	1.80
3	-12	15	11.58	2.97
-4	12	-15	5.58	1.03
4	-12	15	10.35	1.84
4	-12	15	3.34	2.93
8	11	-15	2.99	0.92
-8	-11	15	5.42	1.59
7	11	-15	5.57	0.96
-7	-11	15	8.05	1.68
6	11	-15	5.37	0.97
-6	-11	15	7.80	1.70
5	11	-15	11.21	1.31
-5	-11	15	5.65	1.57
4	11	-15	15.02	1.38
-4	-11	15	9.10	1.51
3	11	-15	4.05	0.81
-3	-11	15	1.43	1.78
-3	-11	15	0.89	1.17
2	11	-15	8.92	1.19
-2	-11	15	9.33	1.58
-2	-11	15	7.47	1.94
1	11	-15	9.43	1.18
-1	-11	15	6.14	1.98
-1	-11	15	6.88	1.32
0	11	-15	8.46	1.15
0	-11	15	5.45	1.42
0	-11	15	8.99	2.18
-1	11	-15	19.73	1.42
1	-11	15	19.67	2.26

1	-11	15	15.74	4.80
-2	11	-15	13.81	1.25
2	-11	15	12.59	2.68
2	-11	15	11.28	1.75
-3	11	-15	3.05	0.78
3	-11	15	9.34	2.59
3	-11	15	5.91	1.46
-4	11	-15	8.60	1.05
4	-11	15	10.55	3.02
4	-11	15	11.39	1.77
-5	11	-15	25.95	2.69
5	-11	15	27.08	2.87
5	-11	15	26.03	4.84
10	10	-15	1.73	1.03
-10	-10	15	5.51	1.82
9	10	-15	4.41	0.97
-9	-10	15	2.48	1.52
8	10	-15	11.04	1.35
-8	-10	15	9.27	1.72
7	10	-15	10.33	1.33
-7	-10	15	10.42	1.69
6	10	-15	14.69	1.40
-6	-10	15	17.37	2.28
5	10	-15	22.59	1.50
-5	-10	15	21.88	2.22
4	10	-15	22.27	1.48
-4	-10	15	20.23	2.21
3	10	-15	13.73	1.34
-3	-10	15	14.73	1.98
-3	-10	15	16.99	2.28
2	10	-15	16.41	1.32
2	10	-15	17.70	1.28
-2	-10	15	17.24	2.07
-2	-10	15	16.03	2.56
1	10	-15	11.03	1.04
1	10	-15	14.23	1.23
-1	-10	15	12.53	1.92
-1	-10	15	13.50	2.08
0	10	-15	15.81	1.13
0	10	-15	15.96	1.24
0	-10	15	11.78	2.04
0	-10	15	20.08	2.55
-1	10	-15	9.97	1.08
-1	10	-15	10.82	1.16

1	-10	15	14.65	2.39
1	-10	15	12.89	2.02
-2	10	-15	15.68	1.32
-2	10	-15	15.57	1.18
2	-10	15	16.55	2.57
2	-10	15	15.79	2.15
-3	10	-15	15.78	1.34
-3	10	-15	13.33	1.22
3	-10	15	15.69	2.79
3	-10	15	17.14	2.33
-4	10	-15	5.21	0.90
-4	10	-15	9.58	1.39
4	-10	15	6.11	1.55
4	-10	15	11.67	2.93
-5	10	-15	5.52	0.96
-5	10	-15	7.34	1.01
5	-10	15	6.24	2.84
5	-10	15	8.35	1.74
11	9	-15	2.66	1.08
-11	-9	15	2.70	1.78
10	9	-15	0.07	0.81
-10	-9	15	-0.41	1.42
9	9	-15	4.24	0.87
-9	-9	15	5.70	1.55
8	9	-15	22.69	1.58
-8	-9	15	16.84	2.44
7	9	-15	10.09	1.33
7	9	-15	8.94	1.20
-7	-9	15	9.96	1.60
6	9	-15	8.39	1.14
6	9	-15	6.15	0.92
-6	-9	15	7.25	1.48
5	9	-15	12.53	1.38
5	9	-15	13.07	1.25
-5	-9	15	9.24	1.45
4	9	-15	19.24	1.42
4	9	-15	16.50	1.34
-4	-9	15	20.05	2.03
-4	-9	15	18.90	1.96
3	9	-15	30.12	1.69
3	9	-15	34.59	1.67
-3	-9	15	34.96	2.56
-3	-9	15	33.78	2.33
2	9	-15	21.60	1.34

2	9	-15	19.72	1.36
-2	-9	15	22.62	2.49
-2	-9	15	21.89	2.02
1	9	-15	10.56	1.12
1	9	-15	7.33	1.05
-1	-9	15	7.57	1.26
-1	-9	15	10.49	1.94
0	9	-15	19.35	1.29
0	9	-15	18.69	1.31
0	-9	15	19.23	2.81
0	-9	15	21.21	2.03
-1	9	-15	24.12	1.42
-1	9	-15	25.96	1.49
1	-9	15	25.36	2.24
1	-9	15	22.31	3.03
-2	9	-15	13.20	1.24
-2	9	-15	14.19	1.23
2	-9	15	15.41	2.47
2	-9	15	11.70	2.06
-3	9	-15	8.05	1.17
-3	9	-15	5.08	1.00
3	-9	15	7.49	1.49
3	-9	15	5.34	2.36
-4	9	-15	5.78	0.83
-4	9	-15	6.24	0.92
4	-9	15	4.80	1.46
4	-9	15	7.63	2.55
-5	9	-15	9.19	1.38
-5	9	-15	10.97	1.34
5	-9	15	9.84	3.00
5	-9	15	9.54	1.70
11	8	-15	-0.70	0.77
-11	-8	15	1.14	1.56
10	8	-15	3.30	0.82
10	8	-15	2.87	1.35
-10	-8	15	1.78	1.43
9	8	-15	23.14	2.13
9	8	-15	21.34	1.54
-9	-8	15	20.97	2.43
8	8	-15	4.07	0.84
8	8	-15	3.98	1.26
-8	-8	15	2.84	1.28
7	8	-15	4.40	0.81
7	8	-15	4.28	1.14

-7	-8	15	2.90	1.08
6	8	-15	6.61	0.87
6	8	-15	8.97	1.48
-6	-8	15	5.49	1.19
5	8	-15	18.89	1.57
5	8	-15	16.87	1.28
-5	-8	15	18.07	1.86
4	8	-15	18.03	1.33
4	8	-15	20.50	1.53
-4	-8	15	21.25	1.83
-4	-8	15	17.58	1.56
-4	-8	15	22.96	1.99
3	8	-15	17.29	1.41
3	8	-15	17.54	1.29
-3	-8	15	17.73	2.04
-3	-8	15	18.22	1.78
-3	-8	15	18.12	1.62
2	8	-15	7.94	1.15
2	8	-15	7.67	1.01
-2	-8	15	8.34	1.64
-2	-8	15	7.22	1.17
-2	-8	15	9.24	1.32
1	8	-15	19.47	1.31
1	8	-15	18.54	1.36
-1	-8	15	20.90	2.47
-1	-8	15	17.62	1.91
-1	-8	15	17.08	1.83
0	8	-15	15.69	1.21
0	8	-15	18.28	1.32
0	-8	15	20.44	2.63
0	-8	15	17.23	1.86
0	-8	15	18.99	1.96
-1	8	-15	7.87	1.08
-1	8	-15	8.47	1.17
1	-8	15	10.22	2.05
1	-8	15	8.32	1.36
-2	8	-15	14.62	1.33
-2	8	-15	17.88	1.30
2	-8	15	22.58	3.07
2	-8	15	12.92	1.93
-3	8	-15	12.53	1.36
-3	8	-15	12.87	1.25
3	-8	15	12.79	2.49
3	-8	15	8.93	1.48

-4	8	-15	9.34	1.36
-4	8	-15	8.29	1.20
4	-8	15	8.83	1.59
4	-8	15	5.62	2.57
-5	8	-15	8.96	1.31
-5	8	-15	6.91	1.03
5	-8	15	11.10	2.88
5	-8	15	7.15	1.55
12	7	-15	3.89	0.89
12	7	-15	5.13	1.63
-12	-7	15	3.91	1.80
11	7	-15	13.68	1.41
11	7	-15	15.94	2.26
-11	-7	15	15.70	2.53
10	7	-15	3.19	1.37
10	7	-15	3.14	0.82
-10	-7	15	5.84	1.46
9	7	-15	7.88	1.46
9	7	-15	11.46	1.23
-9	-7	15	8.82	1.52
8	7	-15	12.13	1.28
8	7	-15	15.77	1.90
-8	-7	15	15.47	1.97
7	7	-15	9.56	1.12
7	7	-15	9.48	1.31
-7	-7	15	8.04	1.30
-7	-7	15	10.09	1.47
6	7	-15	19.86	1.74
6	7	-15	18.84	1.31
-6	-7	15	18.26	1.80
-6	-7	15	15.97	1.53
5	7	-15	24.36	1.43
5	7	-15	22.30	1.75
-5	-7	15	22.28	1.88
-5	-7	15	23.79	1.70
4	7	-15	6.87	1.00
4	7	-15	7.78	1.30
-4	-7	15	8.11	1.13
-4	-7	15	9.01	1.53
-4	-7	15	7.04	1.10
3	7	-15	19.17	1.28
3	7	-15	18.62	1.51
-3	-7	15	18.21	1.73
-3	-7	15	22.06	1.98

-3	-7	15	20.13	1.80
2	7	-15	24.07	1.56
2	7	-15	25.04	1.38
-2	-7	15	22.31	1.88
-2	-7	15	22.90	2.03
-2	-7	15	24.78	2.28
1	7	-15	9.25	1.23
1	7	-15	9.63	1.04
1	7	-15	11.13	1.61
-1	-7	15	13.19	2.17
-1	-7	15	9.06	1.30
-1	-7	15	11.25	1.57
0	7	-15	11.86	1.24
0	7	-15	11.65	1.08
0	7	-15	12.38	1.62
0	-7	15	10.33	1.57
0	-7	15	14.72	2.04
-1	7	-15	26.16	1.62
-1	7	-15	30.81	1.56
1	-7	15	30.10	2.11
1	-7	15	29.25	2.94
-2	7	-15	19.44	1.38
-2	7	-15	23.68	1.54
2	-7	15	21.83	2.00
2	-7	15	17.07	2.95
-3	7	-15	20.61	1.52
-3	7	-15	17.44	1.40
3	-7	15	18.65	2.06
3	-7	15	18.54	3.20
-4	7	-15	22.35	1.67
-4	7	-15	19.65	1.47
4	-7	15	18.37	2.14
4	-7	15	17.08	2.83
-5	7	-15	16.11	1.39
-5	7	-15	15.95	1.59
5	-7	15	12.92	2.94
5	-7	15	15.33	2.21
13	6	-15	9.52	1.90
13	6	-15	13.18	1.49
-13	-6	15	6.96	1.88
12	6	-15	5.66	1.59
12	6	-15	4.75	0.89
-12	-6	15	6.68	1.59
11	6	-15	3.49	0.77

11	6	-15	5.36	1.51
-11	-6	15	5.05	1.47
10	6	-15	13.29	1.27
10	6	-15	12.83	2.08
-10	-6	15	11.30	1.98
9	6	-15	18.36	1.37
9	6	-15	18.43	2.01
-9	-6	15	18.22	1.95
8	6	-15	11.05	1.17
8	6	-15	11.20	1.86
-8	-6	15	9.85	1.66
7	6	-15	21.87	1.90
7	6	-15	17.81	1.31
-7	-6	15	19.50	1.64
-7	-6	15	17.15	1.73
6	6	-15	14.40	1.17
6	6	-15	14.21	1.65
-6	-6	15	14.36	1.60
-6	-6	15	15.65	1.63
5	6	-15	15.72	1.19
5	6	-15	17.05	1.65
-5	-6	15	19.89	1.75
-5	-6	15	14.49	1.60
4	6	-15	15.66	1.17
4	6	-15	15.89	1.53
-4	-6	15	18.15	1.79
-4	-6	15	18.41	1.66
-4	-6	15	16.08	1.25
-4	-6	15	15.21	1.60
3	6	-15	16.77	1.46
3	6	-15	14.58	1.12
-3	-6	15	11.45	1.76
-3	-6	15	13.07	1.43
-3	-6	15	12.56	1.47
-3	-6	15	16.07	1.76
2	6	-15	13.06	1.05
2	6	-15	10.32	1.33
-2	-6	15	11.95	1.89
-2	-6	15	11.98	1.46
1	6	-15	25.93	1.62
1	6	-15	21.93	1.33
1	6	-15	21.64	1.60
-1	-6	15	26.14	1.88
-1	-6	15	24.22	2.31

0	6	-15	13.72	1.11
0	6	-15	12.49	1.33
0	6	-15	12.77	1.58
0	-6	15	14.97	1.99
0	-6	15	13.67	1.59
-1	6	-15	13.96	1.14
-1	6	-15	12.54	1.35
1	-6	15	11.22	1.57
1	-6	15	14.23	2.50
-2	6	-15	23.53	1.44
-2	6	-15	24.27	1.63
2	-6	15	23.46	1.96
2	-6	15	24.27	2.99
-3	6	-15	16.97	1.34
-3	6	-15	15.35	1.50
3	-6	15	20.30	1.94
3	-6	15	16.91	3.15
-4	6	-15	16.89	1.61
-4	6	-15	18.07	1.40
4	-6	15	17.19	1.98
4	-6	15	22.55	3.50
-5	6	-15	5.93	0.92
-5	6	-15	6.98	1.09
5	-6	15	4.65	2.54
5	-6	15	6.93	1.41
13	5	-15	2.42	1.66
13	5	-15	4.59	0.91
-13	-5	15	4.55	1.62
12	5	-15	9.89	1.24
12	5	-15	9.91	1.69
-12	-5	15	7.54	1.57
11	5	-15	17.75	1.38
11	5	-15	19.46	2.22
-11	-5	15	18.85	2.19
10	5	-15	4.98	0.84
-10	-5	15	5.90	1.24
9	5	-15	13.77	1.86
9	5	-15	13.03	1.19
-9	-5	15	11.35	1.69
8	5	-15	31.52	1.67
8	5	-15	35.02	2.29
-8	-5	15	31.57	2.13
7	5	-15	23.01	2.01
7	5	-15	23.70	1.37

-7	-5	15	24.55	1.84
6	5	-15	17.26	1.74
6	5	-15	16.50	1.18
-6	-5	15	15.09	1.57
5	5	-15	21.23	1.78
5	5	-15	21.09	1.29
-5	-5	15	21.74	1.65
-5	-5	15	22.58	2.08
4	5	-15	11.76	1.47
4	5	-15	10.78	1.02
-4	-5	15	9.92	1.22
-4	-5	15	10.25	1.42
-4	-5	15	7.91	1.31
3	5	-15	32.62	1.56
3	5	-15	31.84	1.91
-3	-5	15	32.63	1.91
-3	-5	15	29.09	1.90
-3	-5	15	33.97	2.09
2	5	-15	49.32	2.26
2	5	-15	47.97	1.97
-2	-5	15	46.25	2.63
-2	-5	15	48.98	2.40
-2	-5	15	44.78	2.41
1	5	-15	8.63	1.26
1	5	-15	9.18	0.96
-1	-5	15	7.01	1.33
-1	-5	15	8.56	1.33
-1	-5	15	10.37	1.91
0	5	-15	16.75	1.91
0	5	-15	20.29	1.25
0	-5	15	22.25	2.43
0	-5	15	19.71	2.18
0	-5	15	21.59	1.71
-1	5	-15	32.37	1.61
-1	5	-15	30.89	1.86
1	-5	15	36.13	2.89
1	-5	15	33.57	2.07
-2	5	-15	18.73	1.58
-2	5	-15	17.77	1.29
2	-5	15	18.05	2.66
2	-5	15	18.05	1.77
-3	5	-15	14.19	1.24
-3	5	-15	12.73	1.54
3	-5	15	15.08	1.72

3	-5	15	13.13	2.40
-4	5	-15	16.44	1.65
-4	5	-15	16.21	1.35
4	-5	15	20.02	1.94
4	-5	15	14.91	2.67
-5	5	-15	6.76	1.00
-5	5	-15	7.16	1.15
5	-5	15	10.35	1.81
5	-5	15	8.55	2.69
14	4	-15	10.29	1.39
14	4	-15	10.42	1.87
-14	-4	15	10.66	1.89
13	4	-15	20.07	1.46
13	4	-15	18.27	2.58
-13	-4	15	18.28	2.43
12	4	-15	5.96	0.88
12	4	-15	8.72	1.62
-12	-4	15	5.83	1.41
11	4	-15	4.81	1.36
11	4	-15	3.37	0.74
-11	-4	15	1.25	1.05
10	4	-15	12.82	1.89
10	4	-15	12.26	1.18
-10	-4	15	10.08	1.66
9	4	-15	29.53	1.53
9	4	-15	22.37	2.08
-9	-4	15	28.69	1.98
8	4	-15	23.02	2.00
8	4	-15	19.49	1.31
-8	-4	15	21.41	1.71
7	4	-15	9.40	1.00
7	4	-15	8.36	1.33
-7	-4	15	6.48	1.00
6	4	-15	19.81	1.76
6	4	-15	15.58	1.14
-6	-4	15	17.21	1.49
5	4	-15	12.06	1.04
5	4	-15	14.54	1.62
-5	-4	15	14.70	1.40
4	4	-15	16.06	1.09
4	4	-15	15.56	1.56
-4	-4	15	15.73	1.41
-4	-4	15	16.13	1.50
3	4	-15	22.45	1.69

3	4	-15	21.03	1.26
-3	-4	15	23.34	1.84
-3	-4	15	19.70	1.79
-3	-4	15	22.82	1.62
2	4	-15	15.24	1.51
2	4	-15	14.22	1.06
-2	-4	15	17.14	1.83
-2	-4	15	13.83	1.36
-2	-4	15	15.15	1.87
1	4	-15	17.11	1.83
1	4	-15	19.31	1.17
-1	-4	15	18.18	2.08
-1	-4	15	21.15	2.12
-1	-4	15	20.33	1.60
0	4	-15	25.56	1.72
0	4	-15	28.00	1.41
0	-4	15	24.98	1.80
0	-4	15	26.02	2.46
-1	4	-15	12.47	1.45
-1	4	-15	10.36	1.07
1	-4	15	11.09	1.85
1	-4	15	11.31	1.43
-2	4	-15	10.91	1.10
-2	4	-15	6.60	1.04
2	-4	15	7.10	1.12
2	-4	15	10.40	1.94
-3	4	-15	16.73	1.61
-3	4	-15	15.76	1.29
3	-4	15	15.12	1.69
3	-4	15	11.48	2.22
-4	4	-15	16.11	1.70
-4	4	-15	16.95	1.37
4	-4	15	16.80	2.75
4	-4	15	19.82	1.88
-5	4	-15	4.87	1.08
-5	4	-15	8.97	1.31
5	-4	15	6.57	2.45
5	-4	15	9.10	1.38
14	3	-15	2.97	1.64
14	3	-15	2.56	0.83
-14	-3	15	2.61	1.42
13	3	-15	8.85	1.72
13	3	-15	8.70	1.23
-13	-3	15	8.19	1.47

12	3	-15	21.98	2.44
12	3	-15	24.54	1.52
-12	-3	15	27.35	2.14
11	3	-15	14.23	2.12
11	3	-15	15.09	1.25
-11	-3	15	14.46	1.76
10	3	-15	17.77	1.28
10	3	-15	20.69	2.04
-10	-3	15	18.55	1.73
9	3	-15	19.84	1.29
9	3	-15	22.37	2.51
-9	-3	15	20.43	1.66
8	3	-15	25.66	1.38
8	3	-15	24.32	2.08
-8	-3	15	23.71	1.71
7	3	-15	25.00	1.32
7	3	-15	23.78	1.97
-7	-3	15	20.68	1.58
6	3	-15	22.68	1.28
6	3	-15	21.80	1.86
-6	-3	15	23.36	1.61
5	3	-15	14.90	1.06
5	3	-15	13.86	1.63
-5	-3	15	14.59	1.34
4	3	-15	14.99	1.03
4	3	-15	12.68	1.55
-4	-3	15	14.55	1.58
-4	-3	15	12.10	1.26
-4	-3	15	12.65	1.44
3	3	-15	22.10	1.26
3	3	-15	23.82	1.76
-3	-3	15	22.31	1.57
-3	-3	15	26.40	1.97
-3	-3	15	23.85	1.83
2	3	-15	12.11	1.45
2	3	-15	10.23	0.97
-2	-3	15	12.45	1.70
-2	-3	15	10.81	1.49
-2	-3	15	13.98	1.30
1	3	-15	9.54	0.94
1	3	-15	9.79	1.39
-1	-3	15	10.07	1.28
-1	-3	15	12.46	1.87
-1	-3	15	10.17	1.62

0	3	-15	15.84	1.12
0	3	-15	17.11	1.55
0	-3	15	17.12	1.49
0	-3	15	17.40	2.13
-1	3	-15	12.96	1.55
-1	3	-15	16.24	1.18
1	-3	15	15.92	1.51
1	-3	15	18.34	2.70
-2	3	-15	9.57	1.04
-2	3	-15	8.55	1.44
2	-3	15	9.96	1.39
2	-3	15	8.86	1.82
-3	3	-15	5.62	1.07
-3	3	-15	9.09	1.16
3	-3	15	6.59	1.92
3	-3	15	9.40	1.43
-4	3	-15	11.25	1.68
-4	3	-15	10.42	1.27
4	-3	15	10.64	1.61
4	-3	15	10.64	2.28
15	2	-15	2.43	1.64
15	2	-15	0.89	0.87
14	2	-15	6.17	0.90
14	2	-15	8.61	1.78
-14	-2	15	6.78	1.47
13	2	-15	21.00	2.56
13	2	-15	24.26	1.52
-13	-2	15	23.01	2.14
12	2	-15	10.15	1.70
12	2	-15	13.73	1.23
-12	-2	15	10.09	1.69
11	2	-15	13.07	2.07
11	2	-15	14.96	1.20
-11	-2	15	11.61	1.60
10	2	-15	23.83	1.41
10	2	-15	25.25	2.23
-10	-2	15	26.42	1.80
9	2	-15	24.96	1.38
9	2	-15	26.47	2.16
-9	-2	15	23.24	1.68
8	2	-15	21.23	2.34
8	2	-15	22.36	1.26
-8	-2	15	19.55	1.50
7	2	-15	14.33	2.03

7	2	-15	12.70	1.01
-7	-2	15	12.39	1.25
6	2	-15	48.09	1.92
6	2	-15	47.08	2.58
-6	-2	15	43.13	2.17
5	2	-15	60.71	3.36
5	2	-15	70.41	2.63
-5	-2	15	68.74	2.81
-5	-2	15	65.29	2.79
-5	-2	15	66.88	2.65
-4	-2	15	21.25	1.46
-4	-2	15	17.73	1.54
-4	-2	15	22.36	1.91
3	2	-15	8.47	1.18
-3	-2	15	6.53	1.12
-3	-2	15	7.84	1.16
-3	-2	15	7.25	1.38
2	2	-15	6.97	0.90
2	2	-15	6.58	1.06
-2	-2	15	6.46	0.89
-2	-2	15	6.94	1.46
-2	-2	15	7.79	1.24
1	2	-15	17.85	1.61
1	2	-15	14.80	1.06
-1	-2	15	15.50	1.35
-1	-2	15	22.00	2.43
-1	-2	15	14.09	1.90
0	2	-15	29.71	1.89
0	2	-15	28.17	1.41
0	-2	15	27.63	1.73
0	-2	15	25.98	2.31
-1	2	-15	15.42	1.16
-1	2	-15	15.01	1.61
1	-2	15	14.26	2.23
1	-2	15	14.53	1.45
-2	2	-15	10.41	1.11
-2	2	-15	9.25	1.25
2	-2	15	11.65	1.98
2	-2	15	10.63	1.42
-3	2	-15	6.97	1.15
-3	2	-15	7.81	1.14
3	-2	15	8.70	1.43
3	-2	15	8.65	2.03
-4	2	-15	10.72	1.64

-4	2	-15	6.95	0.94
4	-2	15	7.87	1.19
4	-2	15	5.95	2.13
15	1	-15	7.51	1.96
15	1	-15	10.42	1.34
14	1	-15	5.73	1.70
14	1	-15	4.94	0.85
-14	-1	15	5.70	1.57
13	1	-15	6.49	1.60
13	1	-15	8.20	1.17
-13	-1	15	7.09	1.47
12	1	-15	24.83	1.48
12	1	-15	28.36	2.52
-12	-1	15	25.30	1.95
11	1	-15	17.95	1.28
11	1	-15	18.92	2.29
-11	-1	15	23.05	1.72
10	1	-15	15.38	1.14
10	1	-15	17.82	2.08
-10	-1	15	14.88	1.46
9	1	-15	9.41	0.99
9	1	-15	8.36	1.34
-9	-1	15	8.59	1.19
8	1	-15	7.03	1.42
8	1	-15	9.91	0.97
-8	-1	15	8.96	1.15
7	1	-15	24.88	1.26
7	1	-15	23.11	2.01
-7	-1	15	20.58	1.47
6	1	-15	64.06	3.03
6	1	-15	69.49	3.19
-6	-1	15	72.04	2.85
5	1	-15	39.21	1.68
5	1	-15	40.40	2.36
-5	-1	15	40.34	1.89
-5	-1	15	38.29	1.96
-5	-1	15	40.10	2.31
4	1	-15	25.00	1.88
4	1	-15	22.43	1.25
-4	-1	15	24.78	2.21
-4	-1	15	22.98	1.63
-4	-1	15	22.99	1.50
3	1	-15	16.91	1.67
3	1	-15	14.93	1.04

-3	-1	15	16.69	1.30
-3	-1	15	15.77	1.52
-3	-1	15	14.23	2.20
2	1	-15	18.21	1.14
2	1	-15	20.64	1.76
-2	-1	15	24.02	1.50
-2	-1	15	18.15	2.43
-2	-1	15	20.65	1.83
1	1	-15	13.78	1.58
-1	-1	15	12.27	1.82
-1	-1	15	15.56	1.35
0	1	-15	17.27	1.17
0	1	-15	20.17	1.73
0	-1	15	21.05	1.53
-1	1	-15	24.60	1.89
-1	1	-15	26.87	1.38
1	-1	15	23.92	1.72
1	-1	15	26.84	2.49
-2	1	-15	16.09	1.22
-2	1	-15	14.14	1.61
2	-1	15	16.44	1.51
2	-1	15	13.88	2.51
-3	1	-15	10.27	1.64
-3	1	-15	13.06	1.25
3	-1	15	14.79	1.54
3	-1	15	10.14	2.04
-4	1	-15	3.29	0.86
-4	1	-15	2.90	1.08
4	-1	15	2.85	1.00
4	-1	15	2.47	1.85
15	0	-15	8.07	1.97
15	0	-15	9.63	1.32
14	0	-15	17.64	2.57
14	0	-15	14.19	1.32
-14	0	15	14.93	1.89
13	0	-15	23.58	1.47
13	0	-15	25.36	2.59
-13	0	15	20.88	1.89
12	0	-15	16.01	1.25
12	0	-15	16.39	2.31
11	0	-15	16.94	2.14
11	0	-15	12.06	1.11
10	0	-15	21.26	1.29
10	0	-15	23.55	2.24

-10	0	15	22.51	1.58
9	0	-15	16.80	1.13
9	0	-15	15.05	1.99
-9	0	15	14.19	1.29
8	0	-15	11.85	1.85
8	0	-15	10.51	0.98
-8	0	15	11.43	1.17
7	0	-15	14.31	1.05
7	0	-15	12.04	1.76
-7	0	15	13.96	1.24
6	0	-15	31.81	1.47
6	0	-15	33.85	2.21
-6	0	15	27.49	3.11
-6	0	15	30.41	1.52
-6	0	15	28.67	1.66
5	0	-15	35.42	1.56
5	0	-15	34.31	2.27
-5	0	15	33.61	1.80
-5	0	15	40.92	2.53
-5	0	15	33.49	1.76
4	0	-15	17.80	1.80
4	0	-15	17.56	1.09
-4	0	15	17.60	1.33
-4	0	15	20.39	2.32
-4	0	15	18.96	1.46
3	0	-15	24.80	1.91
3	0	-15	24.35	1.24
-3	0	15	23.37	1.70
-3	0	15	24.63	1.53
-3	0	15	19.77	2.54
2	0	-15	14.56	1.68
2	0	-15	16.11	1.03
-2	0	15	13.96	1.62
-2	0	15	15.91	1.31
-2	0	15	15.03	2.63
1	0	-15	15.35	1.70
1	0	-15	15.52	1.07
-1	0	15	16.54	1.80
-1	0	15	16.00	1.39
0	0	-15	13.71	1.67
0	0	15	14.46	1.35
0	0	15	13.97	1.93
-1	0	-15	20.55	1.84
-1	0	-15	18.71	1.23

1	0	15	16.88	1.49
1	0	15	16.52	2.26
-2	0	-15	13.95	1.19
-2	0	-15	9.97	1.60
2	0	15	12.64	1.42
2	0	15	10.25	1.80
-3	0	-15	9.72	1.23
-3	0	-15	13.67	1.76
3	0	15	7.37	1.89
3	0	15	10.57	1.51
16	-1	-15	10.41	2.08
16	-1	-15	5.54	0.95
15	-1	-15	25.31	1.55
15	-1	-15	18.92	2.83
14	-1	-15	6.74	0.91
14	-1	-15	9.87	1.85
-14	1	15	8.65	1.29
13	-1	-15	9.83	1.71
13	-1	-15	12.87	1.20
-13	1	15	10.79	1.58
12	-1	-15	21.72	2.41
12	-1	-15	19.52	1.36
-12	1	15	19.94	1.67
11	-1	-15	16.05	1.16
11	-1	-15	16.74	2.22
10	-1	-15	17.27	2.11
10	-1	-15	13.31	1.09
-10	1	15	15.05	1.31
9	-1	-15	20.90	1.22
9	-1	-15	20.67	2.21
-9	1	15	21.46	1.44
8	-1	-15	14.69	1.26
8	-1	-15	14.68	2.00
-8	1	15	15.91	1.27
7	-1	-15	25.77	2.20
7	-1	-15	27.84	1.35
-7	1	15	28.48	1.98
-7	1	15	27.43	1.59
-7	1	15	26.65	1.34
6	-1	-15	14.23	0.99
6	-1	-15	11.34	1.78
-6	1	15	13.13	1.13
-6	1	15	13.55	1.16
-6	1	15	13.97	2.04

5	-1	-15	13.32	1.01
5	-1	-15	15.25	1.83
-5	1	15	14.01	1.23
-5	1	15	17.07	1.28
-5	1	15	15.14	2.32
4	-1	-15	14.85	1.02
4	-1	-15	17.91	1.76
-4	1	15	14.71	1.36
-4	1	15	14.69	1.25
-4	1	15	14.60	2.53
3	-1	-15	20.87	1.83
3	-1	-15	18.53	1.09
-3	1	15	14.89	2.25
-3	1	15	20.17	1.62
-3	1	15	20.35	1.43
2	-1	-15	26.20	1.97
2	-1	-15	25.98	1.28
-2	1	15	25.25	1.88
-2	1	15	25.13	1.60
1	-1	-15	9.81	1.61
1	-1	-15	10.79	0.95
-1	1	15	12.80	2.00
-1	1	15	9.93	1.27
0	-1	-15	10.28	1.62
0	-1	-15	9.51	0.99
0	1	15	11.07	1.97
0	1	15	10.23	1.28
-1	-1	-15	17.18	1.77
-1	-1	-15	18.07	1.39
1	1	15	18.11	1.56
1	1	15	16.23	2.35
-2	-1	-15	7.52	1.08
-2	-1	-15	6.90	1.25
2	1	15	5.84	1.72
2	1	15	6.00	1.00
-3	-1	-15	2.85	1.51
3	1	15	4.16	1.08
3	1	15	1.75	1.71
16	-2	-15	5.81	0.91
16	-2	-15	5.33	1.94
15	-2	-15	8.34	1.93
15	-2	-15	12.99	1.30
14	-2	-15	14.54	1.26
14	-2	-15	10.17	2.01

-14	2	15	11.33	1.65
13	-2	-15	18.38	2.54
13	-2	-15	17.75	1.29
-13	2	15	16.67	1.65
12	-2	-15	7.41	1.62
-12	2	15	4.52	0.93
11	-2	-15	13.92	1.14
11	-2	-15	11.39	1.78
10	-2	-15	23.79	1.31
10	-2	-15	21.13	2.26
-10	2	15	24.21	1.50
9	-2	-15	21.93	2.25
-9	2	15	22.11	1.44
8	-2	-15	20.21	1.18
8	-2	-15	18.50	2.10
-8	2	15	20.80	1.36
-8	2	15	18.89	1.11
7	-2	-15	13.55	1.03
7	-2	-15	14.96	1.91
-7	2	15	15.67	1.25
-7	2	15	13.78	1.07
-7	2	15	18.42	2.18
6	-2	-15	14.75	1.86
6	-2	-15	14.08	1.03
-6	2	15	11.70	1.17
-6	2	15	13.38	2.36
-6	2	15	14.93	1.18
5	-2	-15	20.04	1.16
5	-2	-15	18.44	1.97
-5	2	15	20.86	1.40
-5	2	15	18.65	2.69
-5	2	15	20.24	1.42
4	-2	-15	17.18	1.09
4	-2	-15	18.94	1.90
-4	2	15	16.50	1.06
-4	2	15	19.14	2.91
-4	2	15	18.40	1.36
-4	2	15	20.51	1.51
3	-2	-15	15.09	1.03
3	-2	-15	17.45	1.86
-3	2	15	15.89	1.63
-3	2	15	15.96	1.35
-3	2	15	16.62	1.17
2	-2	-15	17.60	1.09

2	-2	-15	17.52	1.88
-2	2	15	17.40	1.82
-2	2	15	16.42	1.42
1	-2	-15	13.33	1.74
1	-2	-15	12.57	1.01
-1	2	15	14.33	1.40
-1	2	15	14.47	1.89
0	-2	-15	12.64	1.01
0	-2	-15	12.52	1.73
0	2	15	10.96	1.42
0	2	15	11.05	1.74
-1	-2	-15	10.65	1.67
-1	-2	-15	10.18	1.05
1	2	15	8.30	1.67
1	2	15	9.17	1.40
-2	-2	-15	4.27	1.23
2	2	15	3.46	1.00
2	2	15	2.05	1.67
16	-3	-15	16.29	1.45
16	-3	-15	10.11	2.13
15	-3	-15	26.26	1.58
15	-3	-15	24.82	2.89
14	-3	-15	9.83	1.76
14	-3	-15	8.80	1.20
-14	3	15	10.61	1.56
13	-3	-15	11.70	1.20
13	-3	-15	13.08	1.92
-13	3	15	11.27	1.46
12	-3	-15	22.19	1.36
12	-3	-15	19.82	2.49
-12	3	15	22.01	1.82
11	-3	-15	20.38	2.31
11	-3	-15	20.55	1.24
-11	3	15	18.18	1.43
10	-3	-15	19.08	2.33
10	-3	-15	21.56	1.24
-10	3	15	19.60	1.40
9	-3	-15	31.77	2.54
9	-3	-15	29.06	1.42
-9	3	15	27.63	1.61
-9	3	15	27.83	1.26
8	-3	-15	16.15	1.07
8	-3	-15	16.03	2.09
-8	3	15	14.86	1.06

-8	3	15	16.25	1.27
7	-3	-15	11.62	1.91
7	-3	-15	9.38	0.96
-7	3	15	8.99	1.03
-7	3	15	8.38	1.07
6	-3	-15	16.36	1.06
6	-3	-15	12.11	1.90
-6	3	15	13.85	1.25
-6	3	15	19.04	2.71
-6	3	15	13.72	0.98
-6	3	15	15.88	1.25
5	-3	-15	25.20	2.12
5	-3	-15	23.37	1.20
-5	3	15	24.17	3.02
-5	3	15	23.19	1.17
-5	3	15	21.12	1.53
-5	3	15	24.27	1.53
4	-3	-15	26.57	2.06
4	-3	-15	21.05	1.13
-4	3	15	19.78	1.47
-4	3	15	19.74	1.64
3	-3	-15	15.29	1.03
3	-3	-15	13.55	1.89
-3	3	15	15.63	1.64
-3	3	15	17.38	1.44
2	-3	-15	15.68	1.86
2	-3	-15	16.45	1.07
-2	3	15	20.63	1.87
-2	3	15	19.53	1.51
1	-3	-15	10.58	0.96
1	-3	-15	10.29	1.44
-1	3	15	11.74	1.39
-1	3	15	12.17	1.93
0	-3	-15	6.00	1.20
0	3	15	4.25	0.93
0	3	15	5.17	1.45
-1	-3	-15	4.07	1.18
1	3	15	4.00	0.93
1	3	15	2.20	1.63
16	-4	-15	13.13	2.74
16	-4	-15	16.29	1.43
15	-4	-15	19.18	1.43
15	-4	-15	14.47	2.16
14	-4	-15	23.29	1.49

14	-4	-15	21.43	2.75
-14	4	15	20.50	1.69
13	-4	-15	21.34	2.65
13	-4	-15	21.62	1.42
-13	4	15	21.11	1.60
12	-4	-15	16.11	2.36
12	-4	-15	14.14	1.21
-12	4	15	16.79	1.72
11	-4	-15	29.90	1.48
11	-4	-15	29.63	2.70
-11	4	15	27.67	1.66
10	-4	-15	34.18	2.79
10	-4	-15	35.44	1.83
-10	4	15	34.98	1.83
-10	4	15	39.57	1.82
9	-4	-15	38.90	1.67
9	-4	-15	38.67	2.79
-9	4	15	39.10	1.90
-9	4	15	34.78	1.64
8	-4	-15	24.51	1.34
8	-4	-15	24.17	2.35
-8	4	15	27.56	1.57
-8	4	15	24.81	1.44
7	-4	-15	8.68	1.45
7	-4	-15	8.25	0.92
7	-4	-15	8.49	1.04
-7	4	15	8.23	1.10
-7	4	15	6.23	0.91
6	-4	-15	9.68	0.94
6	-4	-15	6.92	1.39
-6	4	15	11.03	1.20
-6	4	15	11.19	1.31
5	-4	-15	23.37	1.21
5	-4	-15	25.07	2.24
-5	4	15	24.00	1.60
-5	4	15	22.96	1.65
4	-4	-15	24.81	1.20
4	-4	-15	22.62	2.14
-4	4	15	27.23	1.70
-4	4	15	23.96	1.76
3	-4	-15	13.27	0.94
3	-4	-15	14.43	1.92
-3	4	15	13.07	1.40
-3	4	15	10.41	1.62

2	-4	-15	8.99	1.41
-2	4	15	10.74	1.86
-2	4	15	9.03	1.37
1	-4	-15	12.79	1.82
-1	4	15	8.97	1.39
-1	4	15	7.35	1.47
0	-4	-15	5.74	1.31
0	4	15	4.73	1.00
0	4	15	7.50	1.62
15	-5	-15	27.83	1.64
15	-5	-15	24.63	3.00
14	-5	-15	9.92	1.85
14	-5	-15	8.24	1.20
-14	5	15	11.12	1.43
13	-5	-15	14.04	1.23
13	-5	-15	11.16	1.87
12	-5	-15	30.69	1.57
12	-5	-15	28.43	2.80
-12	5	15	30.17	1.77
11	-5	-15	28.57	1.45
11	-5	-15	30.21	2.71
-11	5	15	25.14	1.61
10	-5	-15	25.78	1.38
10	-5	-15	25.77	2.64
-10	5	15	23.68	1.55
-10	5	15	22.98	1.39
9	-5	-15	34.66	2.77
9	-5	-15	34.31	1.59
-9	5	15	32.58	1.73
-9	5	15	33.39	1.80
8	-5	-15	14.46	1.11
8	-5	-15	11.35	1.80
-8	5	15	16.14	1.36
-8	5	15	13.60	1.29
7	-5	-15	20.37	2.28
7	-5	-15	17.65	1.12
-7	5	15	18.83	1.45
-7	5	15	20.20	1.55
6	-5	-15	18.31	1.06
6	-5	-15	17.04	2.20
-6	5	15	16.61	1.57
-6	5	15	18.27	1.47
5	-5	-15	9.57	0.84
5	-5	-15	8.40	1.48

-5	5	15	9.17	1.52
-5	5	15	11.00	1.35
4	-5	-15	10.58	1.57
-4	5	15	9.63	1.33
-4	5	15	12.36	1.66
3	-5	-15	10.26	1.50
-3	5	15	10.36	1.75
-3	5	15	14.69	1.50
2	-5	-15	12.40	1.89
-2	5	15	13.93	1.98
-2	5	15	12.57	1.54
1	-5	-15	4.31	1.30
-1	5	15	4.32	1.46
-1	5	15	4.80	1.08
15	-6	-15	9.57	1.30
15	-6	-15	4.02	2.02
14	-6	-15	14.96	1.33
14	-6	-15	11.09	1.94
-14	6	15	11.67	1.49
13	-6	-15	30.86	1.62
13	-6	-15	29.80	2.93
-13	6	15	29.53	1.83
12	-6	-15	27.90	2.87
12	-6	-15	23.89	1.44
-12	6	15	26.32	1.63
11	-6	-15	18.30	1.44
11	-6	-15	16.29	2.68
-11	6	15	18.96	1.47
10	-6	-15	10.20	1.82
10	-6	-15	12.45	1.11
-10	6	15	11.26	1.28
9	-6	-15	6.16	0.95
9	-6	-15	7.86	1.63
-9	6	15	4.74	0.89
8	-6	-15	17.33	2.37
8	-6	-15	21.53	1.21
-8	6	15	23.58	1.59
-8	6	15	18.41	1.63
7	-6	-15	21.00	1.15
7	-6	-15	19.65	2.38
-7	6	15	21.95	1.75
-7	6	15	19.90	1.56
6	-6	-15	8.56	1.55
-6	6	15	8.98	1.44

-6	6	15	10.22	1.37
5	-6	-15	9.21	1.58
-5	6	15	7.08	1.03
-5	6	15	10.22	1.66
4	-6	-15	11.83	2.00
-4	6	15	10.64	1.47
-4	6	15	10.22	1.84
3	-6	-15	9.01	1.46
-3	6	15	9.46	1.51
-3	6	15	8.89	1.46
2	-6	-15	4.46	1.29
-2	6	15	4.22	1.12
-2	6	15	6.58	1.43
15	-7	-15	15.42	1.52
14	-7	-15	17.03	1.46
14	-7	-15	12.58	2.22
13	-7	-15	16.35	1.36
13	-7	-15	16.27	2.75
-13	7	15	14.62	1.49
12	-7	-15	25.83	1.52
12	-7	-15	25.63	2.81
-12	7	15	27.43	1.74
11	-7	-15	8.92	1.81
11	-7	-15	11.39	1.17
-11	7	15	11.13	1.33
10	-7	-15	10.86	1.79
10	-7	-15	10.48	1.11
-10	7	15	10.55	1.34
9	-7	-15	12.82	1.09
9	-7	-15	11.08	2.06
9	-7	-15	7.28	1.70
-9	7	15	9.61	1.35
8	-7	-15	10.86	1.88
-8	7	15	8.61	1.15
7	-7	-15	16.30	2.39
-7	7	15	17.10	1.83
-7	7	15	17.23	1.60
6	-7	-15	17.07	2.34
-6	7	15	23.94	2.01
-6	7	15	19.58	1.71
5	-7	-15	15.33	2.15
-5	7	15	12.80	1.55
-5	7	15	12.97	1.97
4	-7	-15	7.35	1.52

-4	7	15	9.31	1.60
-4	7	15	8.00	1.49
3	-7	-15	4.07	1.47
-3	7	15	4.26	1.09
-3	7	15	5.28	1.43
14	-8	-15	4.93	0.94
13	-8	-15	7.00	2.01
13	-8	-15	7.29	0.90
-13	8	15	5.84	1.04
12	-8	-15	21.67	2.96
-12	8	15	16.81	1.58
11	-8	-15	11.99	2.13
-11	8	15	12.10	1.47
10	-8	-15	3.53	1.64
-10	8	15	8.03	1.39
9	-8	-15	11.11	3.22
9	-8	-15	11.47	1.82
-9	8	15	9.35	1.42
8	-8	-15	13.75	2.43
8	-8	-15	11.54	1.85
-8	8	15	15.93	1.59
7	-8	-15	11.34	1.87
-7	8	15	11.88	1.94
-7	8	15	12.71	1.55
6	-8	-15	9.64	1.71
-6	8	15	8.95	1.55
-6	8	15	10.96	1.62
5	-8	-15	16.58	2.38
-5	8	15	8.91	1.63
-5	8	15	13.45	1.74
12	-9	-15	13.11	2.39
-12	9	15	12.89	1.58
11	-9	-15	17.66	2.89
-11	9	15	18.18	1.73
10	-9	-15	23.76	2.88
-10	9	15	21.82	1.79
9	-9	-15	18.17	2.76
9	-9	-15	15.53	2.18
-9	9	15	18.28	1.78
8	-9	-15	8.27	1.88
8	-9	-15	9.84	1.95
-8	9	15	10.04	1.59
7	-9	-15	14.16	2.17
-7	9	15	17.20	1.88

4	13	-16	4.90	1.05
3	13	-16	22.63	1.76
-3	-13	16	22.22	3.19
2	13	-16	6.57	1.06
-2	-13	16	7.04	1.80
1	13	-16	9.36	1.37
-1	-13	16	6.24	1.67
0	13	-16	20.48	1.66
0	-13	16	20.46	2.88
1	-13	16	11.37	2.00
1	-13	16	18.24	3.77
6	12	-16	11.75	1.60
-6	-12	16	8.63	1.91
5	12	-16	10.12	1.19
-5	-12	16	7.90	1.74
4	12	-16	5.99	1.03
-4	-12	16	6.62	1.67
3	12	-16	13.38	1.45
-3	-12	16	11.07	1.83
2	12	-16	23.34	1.67
-2	-12	16	24.93	2.69
1	12	-16	16.23	1.46
-1	-12	16	20.12	2.57
0	12	-16	10.79	1.36
0	-12	16	11.11	1.73
-1	12	-16	15.82	1.50
1	-12	16	15.28	2.61
1	-12	16	23.66	4.07
-2	12	-16	9.59	1.38
2	-12	16	13.18	2.03
2	-12	16	5.52	2.80
8	11	-16	8.52	1.41
-8	-11	16	9.34	1.99
7	11	-16	6.06	1.04
-7	-11	16	7.42	1.75
6	11	-16	15.33	1.48
-6	-11	16	12.71	1.91
5	11	-16	8.08	1.09
-5	-11	16	10.78	1.71
4	11	-16	19.99	1.60
-4	-11	16	21.59	2.44
3	11	-16	22.80	1.64
-3	-11	16	23.03	2.47
2	11	-16	12.84	1.37

-2	-11	16	11.61	1.79
1	11	-16	8.76	1.24
-1	-11	16	13.12	1.87
-1	-11	16	13.54	2.41
0	11	-16	17.40	1.37
0	-11	16	26.55	3.58
0	-11	16	16.46	2.28
-1	11	-16	14.94	1.42
1	-11	16	17.35	2.41
1	-11	16	15.98	2.84
-2	11	-16	8.73	1.09
2	-11	16	4.12	2.66
2	-11	16	10.02	1.76
-3	11	-16	9.99	1.56
3	-11	16	16.18	3.24
3	-11	16	13.05	1.97
9	10	-16	14.81	1.70
-9	-10	16	17.16	2.81
8	10	-16	13.91	1.51
-8	-10	16	9.94	1.74
7	10	-16	5.24	0.96
-7	-10	16	7.26	1.56
6	10	-16	12.42	1.39
-6	-10	16	18.07	2.34
5	10	-16	32.52	1.85
-5	-10	16	30.98	2.69
4	10	-16	13.97	1.40
-4	-10	16	14.81	1.92
3	10	-16	19.75	1.51
-3	-10	16	19.39	2.27
2	10	-16	18.56	1.43
-2	-10	16	21.65	3.60
-2	-10	16	18.62	2.19
1	10	-16	7.68	0.99
-1	-10	16	12.18	2.10
-1	-10	16	10.27	1.70
0	10	-16	9.41	1.19
0	-10	16	11.62	2.39
0	-10	16	8.25	1.43
-1	10	-16	11.05	1.26
1	-10	16	9.05	1.54
1	-10	16	13.20	2.67
-2	10	-16	10.66	1.34
2	-10	16	14.98	2.86

2	-10	16	14.93	2.33
-3	10	-16	20.05	1.57
3	-10	16	19.71	2.65
3	-10	16	21.69	3.43
10	9	-16	4.19	1.04
-10	-9	16	2.67	1.62
9	9	-16	7.37	1.06
-9	-9	16	12.16	1.92
8	9	-16	9.22	1.31
-8	-9	16	8.06	1.75
7	9	-16	20.33	1.62
-7	-9	16	21.59	2.39
6	9	-16	10.66	1.34
-6	-9	16	11.70	2.07
5	9	-16	8.76	1.29
5	9	-16	9.18	1.25
-5	-9	16	11.19	1.96
4	9	-16	15.85	1.46
4	9	-16	14.00	1.32
-4	-9	16	17.29	1.99
3	9	-16	15.41	1.39
3	9	-16	18.35	1.37
-3	-9	16	15.97	2.01
2	9	-16	7.16	0.94
2	9	-16	7.56	1.15
-2	-9	16	6.92	1.29
-2	-9	16	11.52	1.87
1	9	-16	20.18	1.38
1	9	-16	16.04	1.36
-1	-9	16	17.76	2.07
-1	-9	16	18.53	2.83
0	9	-16	26.35	1.56
0	9	-16	23.65	1.53
0	-9	16	25.48	2.37
0	-9	16	26.95	3.20
-1	9	-16	13.88	1.32
-1	9	-16	13.27	1.29
1	-9	16	13.93	2.14
-2	9	-16	12.74	1.31
-2	9	-16	12.09	1.57
2	-9	16	14.18	2.29
2	-9	16	13.73	2.70
-3	9	-16	5.39	0.93
-3	9	-16	5.76	0.97

3	-9	16	8.46	2.69
3	-9	16	5.32	1.50
-4	9	-16	4.82	0.96
-4	9	-16	4.80	1.01
4	-9	16	3.54	2.75
4	-9	16	3.79	1.48
11	8	-16	5.60	1.14
-11	-8	16	5.20	1.83
10	8	-16	17.61	1.62
-10	-8	16	17.31	2.63
9	8	-16	13.77	2.33
9	8	-16	17.14	1.50
-9	-8	16	17.52	2.48
8	8	-16	6.38	0.99
8	8	-16	6.19	1.37
-8	-8	16	8.14	1.63
7	8	-16	10.21	1.26
7	8	-16	9.93	1.36
-7	-8	16	8.47	1.46
6	8	-16	21.93	1.48
6	8	-16	19.62	1.89
-6	-8	16	19.90	2.14
5	8	-16	22.11	1.83
5	8	-16	25.62	1.57
-5	-8	16	29.27	2.25
4	8	-16	6.43	1.09
4	8	-16	6.41	0.88
-4	-8	16	5.07	1.16
3	8	-16	5.33	0.86
3	8	-16	6.36	0.96
-3	-8	16	6.02	1.51
-3	-8	16	5.07	1.17
2	8	-16	29.88	1.66
2	8	-16	29.47	1.77
-2	-8	16	29.50	2.33
-2	-8	16	31.87	2.78
1	8	-16	27.92	1.53
1	8	-16	20.90	1.63
-1	-8	16	30.29	2.82
-1	-8	16	24.30	2.18
0	8	-16	20.34	1.54
0	8	-16	20.76	1.43
0	-8	16	26.09	3.09
0	-8	16	22.58	2.15

-1	8	-16	20.95	1.41
-1	8	-16	13.93	2.03
1	-8	16	14.69	2.06
-2	8	-16	11.95	1.42
-2	8	-16	11.91	1.28
2	-8	16	10.72	2.49
2	-8	16	8.78	1.47
-3	8	-16	6.17	0.99
-3	8	-16	4.01	0.89
3	-8	16	9.51	2.68
3	-8	16	2.03	1.26
-4	8	-16	0.84	0.93
-4	8	-16	0.39	0.84
4	-8	16	3.28	1.40
4	-8	16	-0.44	2.55
-12	-7	16	11.79	2.07
11	7	-16	15.42	1.54
11	7	-16	9.50	1.71
-11	-7	16	12.88	1.91
10	7	-16	24.99	2.84
10	7	-16	25.89	1.70
-10	-7	16	26.40	2.66
9	7	-16	8.65	1.57
9	7	-16	10.27	1.31
-9	-7	16	9.18	1.57
8	7	-16	17.77	2.11
8	7	-16	19.92	1.46
-8	-7	16	19.93	2.24
7	7	-16	22.87	1.50
7	7	-16	22.67	2.02
-7	-7	16	23.48	2.12
6	7	-16	24.24	2.01
6	7	-16	26.13	1.56
-6	-7	16	23.15	2.10
5	7	-16	18.81	1.39
5	7	-16	17.95	1.74
-5	-7	16	19.86	1.90
4	7	-16	18.30	1.32
4	7	-16	16.78	1.61
-4	-7	16	18.26	1.88
-4	-7	16	16.76	1.79
3	7	-16	23.87	1.41
3	7	-16	18.19	1.66
-3	-7	16	20.90	1.99

-3	-7	16	20.63	2.26
2	7	-16	12.71	1.48
2	7	-16	14.23	1.22
-2	-7	16	18.89	1.88
-2	-7	16	17.58	2.36
1	7	-16	23.98	1.61
1	7	-16	23.14	1.43
-1	-7	16	18.33	1.96
-1	-7	16	25.28	2.72
0	7	-16	19.34	1.39
0	-7	16	20.39	2.95
0	-7	16	20.07	1.99
-1	7	-16	13.25	1.45
-1	7	-16	12.37	1.23
1	-7	16	10.45	1.79
-2	7	-16	8.80	1.51
-2	7	-16	14.38	1.29
2	-7	16	8.74	1.42
2	-7	16	11.51	2.48
-3	7	-16	6.63	0.96
-3	7	-16	7.58	1.12
3	-7	16	10.18	2.52
3	-7	16	9.45	1.58
-4	7	-16	2.14	1.04
-4	7	-16	3.35	0.85
4	-7	16	9.77	2.96
4	-7	16	2.79	1.33
12	6	-16	19.09	1.65
12	6	-16	19.31	2.64
-12	-6	16	21.59	2.79
11	6	-16	8.28	1.04
11	6	-16	9.27	1.72
-11	-6	16	13.59	1.88
10	6	-16	15.25	2.24
10	6	-16	16.92	1.46
-10	-6	16	17.15	2.21
9	6	-16	41.35	2.02
9	6	-16	39.29	2.73
-9	-6	16	41.74	2.64
8	6	-16	17.73	2.10
8	6	-16	19.91	1.45
-8	-6	16	22.17	2.07
7	6	-16	9.31	1.19
7	6	-16	11.16	1.46

-7	-6	16	10.45	1.68
6	6	-16	24.19	2.02
6	6	-16	25.63	1.53
-6	-6	16	27.96	2.06
5	6	-16	26.07	1.92
5	6	-16	26.55	1.51
-5	-6	16	23.63	1.96
4	6	-16	10.88	1.53
4	6	-16	12.87	1.17
-4	-6	16	10.21	1.59
3	6	-16	14.73	1.62
3	6	-16	18.48	1.29
-3	-6	16	18.59	2.11
-3	-6	16	18.07	1.73
2	6	-16	16.43	1.21
2	6	-16	13.47	1.51
-2	-6	16	13.21	2.23
-2	-6	16	14.34	1.64
1	6	-16	12.87	1.16
-1	-6	16	16.64	1.74
-1	-6	16	14.40	2.49
0	6	-16	18.30	1.58
0	6	-16	17.64	1.30
0	-6	16	17.89	2.76
0	-6	16	20.73	1.86
-1	6	-16	6.64	1.08
-1	6	-16	7.32	1.14
1	-6	16	5.37	1.07
1	-6	16	7.78	2.35
-2	6	-16	7.36	1.15
-2	6	-16	5.33	0.89
2	-6	16	7.52	1.30
2	-6	16	6.07	2.24
-3	6	-16	5.03	1.09
-3	6	-16	8.60	1.25
3	-6	16	9.69	2.59
3	-6	16	7.43	1.35
-4	6	-16	9.44	1.57
-4	6	-16	9.33	1.37
4	-6	16	16.94	3.05
4	-6	16	8.58	1.48
13	5	-16	12.25	2.05
13	5	-16	11.01	1.57
-13	-5	16	14.48	2.06

12	5	-16	16.16	2.66
12	5	-16	18.37	1.50
-12	-5	16	15.86	2.45
11	5	-16	23.49	2.56
11	5	-16	19.77	1.53
-11	-5	16	20.17	2.36
10	5	-16	9.74	1.63
10	5	-16	13.07	1.32
-10	-5	16	13.11	2.03
9	5	-16	16.76	2.17
9	5	-16	16.92	1.37
-9	-5	16	14.65	1.84
8	5	-16	34.66	1.77
8	5	-16	33.60	2.46
-8	-5	16	34.01	2.33
7	5	-16	21.99	1.41
7	5	-16	20.45	2.02
-7	-5	16	22.34	1.90
6	5	-16	20.30	1.91
6	5	-16	21.18	1.39
-6	-5	16	18.26	1.80
5	5	-16	6.87	0.87
5	5	-16	9.11	1.23
-5	-5	16	11.01	1.50
4	5	-16	20.32	1.32
4	5	-16	20.02	1.74
-4	-5	16	18.85	1.85
-4	-5	16	18.66	1.68
3	5	-16	17.37	1.27
3	5	-16	18.15	1.73
-3	-5	16	14.58	1.96
-3	-5	16	19.03	1.69
2	5	-16	10.50	1.09
2	5	-16	8.42	1.41
-2	-5	16	8.25	1.43
-2	-5	16	11.11	1.72
1	5	-16	21.62	1.32
1	5	-16	19.01	1.66
-1	-5	16	18.63	1.75
-1	-5	16	18.18	2.45
0	5	-16	13.14	1.58
0	5	-16	14.01	1.23
0	-5	16	21.77	2.67
0	-5	16	10.58	1.55

-1	5	-16	4.28	0.78
-1	5	-16	2.90	0.97
1	-5	16	6.09	1.87
1	-5	16	4.31	1.07
-2	5	-16	8.65	1.20
-2	5	-16	5.99	1.12
2	-5	16	5.80	1.20
2	-5	16	5.82	2.06
-3	5	-16	5.78	0.94
-3	5	-16	4.81	1.08
3	-5	16	8.62	2.41
3	-5	16	7.60	1.24
-4	5	-16	3.01	0.84
-4	5	-16	3.21	1.11
4	-5	16	6.19	2.57
4	-5	16	3.22	1.18
13	4	-16	19.07	2.21
13	4	-16	18.17	1.54
-13	-4	16	18.57	2.65
12	4	-16	23.63	2.65
12	4	-16	21.89	1.54
-12	-4	16	17.26	2.31
11	4	-16	3.92	1.48
11	4	-16	4.71	0.86
-11	-4	16	5.31	1.32
10	4	-16	10.96	1.25
10	4	-16	11.01	1.64
-10	-4	16	11.67	1.76
9	4	-16	21.82	1.43
9	4	-16	22.85	2.26
-9	-4	16	15.90	1.83
8	4	-16	11.43	1.91
8	4	-16	10.16	1.16
-8	-4	16	9.68	1.58
7	4	-16	12.31	1.15
-7	-4	16	9.20	1.47
6	4	-16	23.73	1.42
6	4	-16	21.21	2.26
-6	-4	16	22.14	1.76
5	4	-16	14.81	2.33
5	4	-16	17.42	1.24
-5	-4	16	19.65	1.64
4	4	-16	8.68	1.05
4	4	-16	16.03	2.53

-4	-4	16	8.63	1.33
-4	-4	16	8.37	1.56
3	4	-16	15.43	1.66
3	4	-16	13.27	1.16
-3	-4	16	18.47	1.95
-3	-4	16	15.30	1.51
2	4	-16	14.04	1.14
2	4	-16	14.10	1.58
-2	-4	16	13.98	2.15
-2	-4	16	11.91	1.45
1	4	-16	22.86	1.81
1	4	-16	24.80	1.41
-1	-4	16	24.27	1.85
-1	-4	16	31.01	2.58
0	4	-16	11.84	1.16
0	4	-16	8.42	1.21
0	-4	16	14.01	1.55
0	-4	16	13.07	1.97
-1	4	-16	4.11	0.79
-1	4	-16	4.08	1.05
1	-4	16	5.45	1.07
1	-4	16	6.51	1.88
-2	4	-16	4.27	0.84
-2	4	-16	4.87	1.05
2	-4	16	3.72	1.03
2	-4	16	2.05	1.91
-3	4	-16	6.62	1.23
-3	4	-16	6.23	0.97
3	-4	16	5.39	1.15
3	-4	16	8.61	2.34
14	3	-16	11.62	1.50
14	3	-16	9.16	1.85
-14	-3	16	12.35	1.88
13	3	-16	22.20	1.59
13	3	-16	23.05	2.80
-13	-3	16	20.61	2.44
12	3	-16	11.29	1.30
12	3	-16	15.28	2.00
-12	-3	16	10.45	1.55
11	3	-16	28.16	2.57
11	3	-16	26.88	1.61
-11	-3	16	27.41	2.18
10	3	-16	20.11	2.27
10	3	-16	15.03	1.36

-10	-3	16	17.69	1.83
9	3	-16	5.44	1.37
9	3	-16	3.07	0.70
-9	-3	16	5.11	1.06
8	3	-16	16.11	2.01
8	3	-16	18.53	1.30
-8	-3	16	17.79	1.66
7	3	-16	24.64	2.06
7	3	-16	25.14	1.42
-7	-3	16	22.46	1.74
6	3	-16	18.36	1.88
6	3	-16	18.00	1.26
-6	-3	16	16.25	1.54
5	3	-16	31.14	1.56
5	3	-16	29.15	2.13
-5	-3	16	31.33	1.91
4	3	-16	19.36	1.83
4	3	-16	17.65	1.26
-4	-3	16	18.14	1.53
-4	-3	16	19.81	1.78
3	3	-16	13.36	1.65
3	3	-16	13.04	1.12
-3	-3	16	11.87	1.38
-3	-3	16	14.52	1.89
2	3	-16	17.26	1.19
2	3	-16	19.47	1.74
-2	-3	16	16.85	1.54
-2	-3	16	13.79	2.04
1	3	-16	14.95	1.19
1	3	-16	14.11	1.64
-1	-3	16	14.38	1.52
-1	-3	16	15.73	2.25
0	3	-16	4.72	1.05
0	3	-16	4.77	0.78
0	-3	16	7.72	1.96
0	-3	16	4.93	0.98
-1	3	-16	6.15	1.12
-1	3	-16	6.08	0.90
1	-3	16	8.47	1.43
1	-3	16	7.65	1.84
-2	3	-16	10.55	1.25
-2	3	-16	7.81	1.18
2	-3	16	8.60	2.12
2	-3	16	11.03	1.57

-3	3	-16	6.67	0.92
-3	3	-16	3.93	1.15
3	-3	16	7.31	1.25
3	-3	16	4.81	2.21
14	2	-16	13.66	2.13
14	2	-16	12.81	1.42
-14	-2	16	9.89	1.75
13	2	-16	19.13	1.50
13	2	-16	22.27	2.69
-13	-2	16	17.74	2.26
12	2	-16	14.84	1.34
12	2	-16	16.07	2.46
-12	-2	16	13.58	1.89
11	2	-16	16.48	1.37
11	2	-16	16.12	2.33
-11	-2	16	16.24	1.85
10	2	-16	18.48	1.38
10	2	-16	22.16	2.39
-10	-2	16	19.01	1.83
9	2	-16	19.21	1.36
9	2	-16	17.48	2.17
-9	-2	16	20.37	1.70
8	2	-16	18.82	2.07
8	2	-16	18.66	1.30
-8	-2	16	21.10	1.63
7	2	-16	18.52	1.98
7	2	-16	17.35	1.25
-7	-2	16	15.61	1.46
6	2	-16	48.44	2.02
6	2	-16	46.94	2.66
-6	-2	16	47.34	2.34
5	2	-16	45.00	2.52
5	2	-16	43.85	1.88
-5	-2	16	42.10	2.21
-5	-2	16	43.28	2.21
4	2	-16	6.33	1.23
4	2	-16	9.41	0.99
-4	-2	16	9.15	1.55
-4	-2	16	6.58	0.99
3	2	-16	9.32	1.01
3	2	-16	10.31	1.67
-3	-2	16	11.37	1.77
-3	-2	16	8.63	1.28
2	2	-16	12.78	1.68

2	2	-16	11.98	1.03
-2	-2	16	11.90	1.39
-2	-2	16	13.94	1.95
1	2	-16	7.33	1.03
1	2	-16	6.28	1.16
-1	-2	16	8.14	1.52
-1	-2	16	4.85	0.91
0	2	-16	14.17	1.23
0	2	-16	15.07	1.70
0	-2	16	16.49	1.53
0	-2	16	17.56	2.42
-1	2	-16	8.17	1.27
-1	2	-16	11.19	1.17
1	-2	16	9.12	1.43
1	-2	16	10.31	1.92
-2	2	-16	3.02	0.81
-2	2	-16	1.04	1.00
2	-2	16	4.17	1.84
2	-2	16	3.34	0.94
-3	2	-16	13.66	1.38
-3	2	-16	7.12	1.31
3	-2	16	7.98	1.24
3	-2	16	10.06	2.40
14	1	-16	8.03	1.89
14	1	-16	7.80	1.01
-14	-1	16	5.61	1.46
13	1	-16	17.41	1.39
13	1	-16	20.10	2.63
-13	-1	16	14.68	2.02
12	1	-16	9.31	1.83
12	1	-16	11.87	1.27
-12	-1	16	11.69	1.75
11	1	-16	19.37	1.35
11	1	-16	17.25	2.36
-11	-1	16	19.54	1.80
10	1	-16	14.66	2.20
10	1	-16	17.30	1.34
-10	-1	16	17.23	1.63
9	1	-16	9.24	1.61
9	1	-16	8.18	1.05
-9	-1	16	10.40	1.38
8	1	-16	16.50	2.19
8	1	-16	19.10	1.29
-8	-1	16	20.98	1.58

7	1	-16	48.72	2.84
7	1	-16	52.16	2.07
-7	-1	16	47.50	2.34
6	1	-16	43.29	1.83
6	1	-16	39.99	2.56
-6	-1	16	40.63	2.13
-6	-1	16	41.76	1.99
5	1	-16	8.83	1.02
5	1	-16	10.92	1.81
-5	-1	16	11.03	1.40
-5	-1	16	10.86	1.28
4	1	-16	11.89	1.72
4	1	-16	9.46	1.01
-4	-1	16	9.51	1.56
-4	-1	16	10.26	1.27
3	1	-16	13.91	1.76
3	1	-16	12.34	1.09
-3	-1	16	13.95	1.36
-3	-1	16	13.91	1.79
2	1	-16	9.63	1.02
2	1	-16	11.27	1.67
-2	-1	16	10.42	1.81
-2	-1	16	10.32	1.29
1	1	-16	9.43	1.04
1	1	-16	6.24	1.17
-1	-1	16	10.46	1.28
-1	-1	16	8.89	1.51
0	1	-16	10.99	1.13
0	1	-16	7.73	1.27
0	-1	16	8.09	1.65
0	-1	16	9.53	1.39
-1	1	-16	9.79	1.19
-1	1	-16	12.05	1.72
1	-1	16	10.26	2.00
1	-1	16	10.46	1.40
-2	1	-16	7.20	1.28
-2	1	-16	10.69	1.29
2	-1	16	8.69	1.54
2	-1	16	11.21	2.08
15	0	-16	17.22	1.60
15	0	-16	22.24	3.01
14	0	-16	13.41	2.05
14	0	-16	11.05	1.35
-14	0	16	16.39	2.06

13	0	-16	18.18	1.43
13	0	-16	21.36	2.65
-13	0	16	16.46	1.94
12	0	-16	10.38	1.77
12	0	-16	12.20	1.23
-12	0	16	10.84	1.65
11	0	-16	10.65	1.15
11	0	-16	7.05	1.52
-11	0	16	7.99	1.42
10	0	-16	18.88	2.34
10	0	-16	17.93	1.28
-10	0	16	15.48	1.53
9	0	-16	16.67	2.23
9	0	-16	18.45	1.31
-9	0	16	22.72	1.63
8	0	-16	9.62	1.50
8	0	-16	14.11	1.13
-8	0	16	14.10	1.36
7	0	-16	16.06	1.20
7	0	-16	14.72	1.97
-7	0	16	17.08	1.44
6	0	-16	12.06	1.60
6	0	-16	13.72	1.07
-6	0	16	10.55	1.26
-6	0	16	10.56	1.27
5	0	-16	9.53	1.41
5	0	-16	9.36	1.04
-5	0	16	8.94	1.36
-5	0	16	9.74	1.22
4	0	-16	7.13	0.98
4	0	-16	8.37	1.38
-4	0	16	6.98	0.96
-4	0	16	8.14	1.50
3	0	-16	6.75	0.93
3	0	-16	7.41	1.27
-3	0	16	9.49	1.64
-3	0	16	8.49	1.25
2	0	-16	7.73	0.98
2	0	-16	8.26	1.30
-2	0	16	6.45	0.94
-2	0	16	11.00	1.77
1	0	-16	4.55	1.17
1	0	-16	5.58	0.77
-1	0	16	3.57	0.83

-1	0	16	4.97	1.40
0	0	-16	6.89	1.06
0	0	-16	4.04	1.09
0	0	16	6.59	1.56
0	0	16	7.58	1.30
-1	0	-16	5.65	0.85
-1	0	-16	5.58	1.22
1	0	16	9.28	1.84
1	0	16	3.68	0.92
-2	0	-16	2.82	0.83
-2	0	-16	0.06	1.10
2	0	16	3.34	1.04
2	0	16	2.66	1.80
15	-1	-16	18.30	2.98
15	-1	-16	19.39	1.57
14	-1	-16	10.16	1.97
14	-1	-16	16.10	1.44
-14	1	16	15.06	1.94
13	-1	-16	12.92	1.30
13	-1	-16	17.59	2.67
-13	1	16	14.34	1.79
12	-1	-16	13.07	1.23
12	-1	-16	10.88	1.86
-12	1	16	12.99	1.57
11	-1	-16	22.54	1.44
11	-1	-16	20.53	2.44
-11	1	16	22.61	1.69
10	-1	-16	19.47	2.39
10	-1	-16	19.72	1.35
-10	1	16	21.14	1.57
9	-1	-16	18.65	2.40
9	-1	-16	23.08	1.36
-9	1	16	21.88	1.59
8	-1	-16	10.49	1.02
8	-1	-16	9.43	1.60
-8	1	16	10.05	1.25
7	-1	-16	9.64	1.54
7	-1	-16	14.27	1.09
-7	1	16	10.98	1.30
-7	1	16	10.59	1.16
6	-1	-16	13.63	2.04
-6	1	16	13.60	1.33
-6	1	16	13.34	1.31
5	-1	-16	8.79	1.41

5	-1	-16	10.07	1.02
-5	1	16	10.39	1.38
-5	1	16	6.90	1.15
4	-1	-16	9.94	1.00
4	-1	-16	10.90	1.77
-4	1	16	9.90	1.27
-4	1	16	9.52	1.53
3	-1	-16	11.07	1.03
3	-1	-16	12.91	1.87
-3	1	16	9.18	1.38
-3	1	16	10.72	1.29
2	-1	-16	11.37	1.80
-2	1	16	15.96	1.46
1	-1	-16	5.47	0.78
1	-1	-16	6.08	1.24
-1	1	16	8.68	1.33
-1	1	16	6.15	1.47
0	-1	-16	0.82	1.00
0	-1	-16	0.85	0.67
0	1	16	0.52	0.75
0	1	16	0.29	1.40
-1	-1	-16	3.19	0.76
-1	-1	-16	1.58	1.13
1	1	16	4.30	1.66
1	1	16	3.07	0.92
15	-2	-16	13.30	2.14
15	-2	-16	12.30	1.39
14	-2	-16	10.17	1.92
14	-2	-16	9.16	1.32
-14	2	16	14.22	1.81
13	-2	-16	18.51	2.66
13	-2	-16	14.04	1.34
-13	2	16	15.69	1.76
12	-2	-16	24.36	1.53
12	-2	-16	23.72	2.65
-12	2	16	24.74	1.78
11	-2	-16	8.60	1.11
11	-2	-16	10.33	1.77
-11	2	16	8.32	1.33
10	-2	-16	18.05	1.31
10	-2	-16	17.70	2.46
-10	2	16	17.80	1.51
9	-2	-16	30.41	1.60
9	-2	-16	35.36	2.73

-9	2	16	32.64	1.85
8	-2	-16	8.62	1.56
8	-2	-16	8.10	1.00
-8	2	16	10.13	1.21
7	-2	-16	7.03	1.40
7	-2	-16	6.92	0.82
-7	2	16	6.30	0.97
-7	2	16	6.46	0.92
6	-2	-16	12.28	1.68
6	-2	-16	12.44	1.07
-6	2	16	11.98	1.26
-6	2	16	9.86	1.35
5	-2	-16	7.04	1.41
5	-2	-16	9.01	1.00
-5	2	16	8.23	1.11
-5	2	16	7.07	1.19
4	-2	-16	13.68	1.03
4	-2	-16	10.13	1.48
-4	2	16	11.10	1.64
-4	2	16	14.44	1.36
3	-2	-16	7.46	1.30
3	-2	-16	6.83	0.97
-3	2	16	8.03	1.35
-3	2	16	7.35	1.22
2	-2	-16	5.04	0.73
2	-2	-16	5.26	1.21
-2	2	16	6.52	1.38
-2	2	16	6.43	1.06
1	-2	-16	4.00	1.16
1	-2	-16	2.51	0.69
-1	2	16	4.47	1.43
-1	2	16	2.01	0.80
0	-2	-16	3.29	1.15
0	2	16	3.12	0.87
0	2	16	3.67	1.67
15	-3	-16	12.33	2.19
15	-3	-16	12.74	1.41
14	-3	-16	17.46	2.87
14	-3	-16	15.05	1.45
-14	3	16	17.75	1.85
13	-3	-16	14.79	2.20
13	-3	-16	15.34	1.34
-13	3	16	16.36	1.69
12	-3	-16	23.71	1.50

12	-3	-16	21.84	2.65
-12	3	16	24.59	1.76
11	-3	-16	26.09	1.50
11	-3	-16	25.38	2.61
-11	3	16	26.56	1.76
10	-3	-16	24.23	2.63
10	-3	-16	24.90	1.43
-10	3	16	21.84	1.64
9	-3	-16	14.85	2.33
9	-3	-16	16.87	1.23
-9	3	16	17.41	1.44
8	-3	-16	21.56	1.40
8	-3	-16	23.33	2.44
-8	3	16	22.17	1.57
-8	3	16	23.10	1.52
7	-3	-16	13.52	1.13
7	-3	-16	13.83	1.86
-7	3	16	10.83	1.30
-7	3	16	13.53	1.36
6	-3	-16	10.81	1.63
6	-3	-16	13.40	1.11
-6	3	16	13.70	1.34
-6	3	16	11.80	1.38
5	-3	-16	17.29	2.08
5	-3	-16	15.85	1.14
-5	3	16	13.33	1.60
-5	3	16	15.97	1.42
4	-3	-16	8.91	1.42
4	-3	-16	11.91	1.04
-4	3	16	10.36	1.32
-4	3	16	10.65	1.67
3	-3	-16	5.17	0.72
3	-3	-16	5.07	1.26
-3	3	16	4.92	0.88
-3	3	16	7.59	1.30
2	-3	-16	5.40	1.26
2	-3	-16	8.22	1.25
-2	3	16	4.22	1.39
-2	3	16	6.05	1.06
1	-3	-16	3.25	1.24
-1	3	16	1.36	1.50
-1	3	16	1.11	0.86
0	3	16	4.58	1.81
15	-4	-16	8.45	2.18

15	-4	-16	7.09	1.02
14	-4	-16	6.05	1.84
14	-4	-16	5.10	0.92
-14	4	16	6.81	1.14
13	-4	-16	23.75	2.68
13	-4	-16	22.61	1.48
-13	4	16	17.80	1.72
12	-4	-16	23.39	2.70
12	-4	-16	21.97	1.48
-12	4	16	23.53	1.73
11	-4	-16	6.20	1.72
11	-4	-16	6.87	0.87
-11	4	16	6.29	1.00
10	-4	-16	18.03	2.49
10	-4	-16	19.60	1.34
-10	4	16	16.33	1.47
9	-4	-16	16.60	2.39
9	-4	-16	14.77	1.47
-9	4	16	20.65	1.54
8	-4	-16	8.72	0.88
8	-4	-16	5.41	1.50
-8	4	16	8.73	1.24
-8	4	16	8.33	1.20
7	-4	-16	15.00	1.17
7	-4	-16	13.86	2.19
-7	4	16	15.61	1.43
-7	4	16	13.48	1.39
6	-4	-16	8.48	1.51
6	-4	-16	8.65	1.01
-6	4	16	9.34	1.44
-6	4	16	8.12	1.29
5	-4	-16	6.75	1.49
5	-4	-16	10.81	0.96
-5	4	16	8.92	1.34
-5	4	16	7.08	1.18
4	-4	-16	4.08	1.31
-4	4	16	4.33	1.26
-4	4	16	6.54	1.02
3	-4	-16	4.75	1.27
-3	4	16	3.50	0.90
-3	4	16	4.96	1.35
2	-4	-16	3.01	1.29
-2	4	16	3.26	0.86
-2	4	16	2.56	1.49

-1	4	16	3.20	1.53
14	-5	-16	8.30	2.01
14	-5	-16	9.66	1.40
-14	5	16	8.99	1.56
13	-5	-16	15.26	2.31
13	-5	-16	17.81	1.44
-13	5	16	13.82	1.63
12	-5	-16	12.83	2.00
12	-5	-16	11.37	1.30
-12	5	16	11.00	1.50
11	-5	-16	17.34	2.69
11	-5	-16	19.92	1.40
-11	5	16	17.65	1.56
10	-5	-16	6.95	1.01
10	-5	-16	8.80	1.81
-10	5	16	7.62	1.33
9	-5	-16	2.01	0.69
9	-5	-16	3.10	1.43
-9	5	16	2.41	0.82
8	-5	-16	13.84	2.31
8	-5	-16	13.34	1.17
-8	5	16	12.81	1.41
-8	5	16	10.94	1.43
7	-5	-16	14.19	1.11
7	-5	-16	11.36	1.84
-7	5	16	13.72	1.45
-7	5	16	13.46	1.58
6	-5	-16	3.08	1.38
-6	5	16	1.88	0.80
-6	5	16	2.03	1.02
5	-5	-16	4.04	1.44
-5	5	16	5.77	1.21
-5	5	16	3.58	0.87
4	-5	-16	-0.10	1.29
-4	5	16	3.16	1.24
-4	5	16	1.37	0.87
3	-5	-16	3.77	1.36
-3	5	16	0.68	0.82
-3	5	16	0.33	1.31
-2	5	16	3.04	1.54
14	-6	-16	9.21	2.97
14	-6	-16	7.08	1.03
13	-6	-16	25.05	1.66
13	-6	-16	26.52	3.05

-13	6	16	22.13	1.85
12	-6	-16	26.43	1.62
12	-6	-16	22.88	3.01
-12	6	16	27.01	1.85
11	-6	-16	7.67	1.18
11	-6	-16	6.74	1.79
-11	6	16	5.19	0.99
10	-6	-16	3.83	1.60
10	-6	-16	5.57	0.82
-10	6	16	6.16	1.01
9	-6	-16	7.64	0.88
9	-6	-16	7.14	1.64
-9	6	16	6.29	0.98
8	-6	-16	11.34	1.75
-8	6	16	10.65	1.59
-8	6	16	9.97	1.41
7	-6	-16	9.47	1.67
-7	6	16	12.74	2.06
-7	6	16	14.87	1.54
6	-6	-16	3.03	1.41
-6	6	16	3.09	0.88
-6	6	16	3.40	1.19
5	-6	-16	6.89	1.56
-5	6	16	6.26	1.12
-5	6	16	5.21	1.34
4	-6	-16	1.77	1.45
-4	6	16	2.57	1.00
-4	6	16	4.14	1.42
13	-7	-16	13.17	1.42
-13	7	16	9.49	1.63
12	-7	-16	19.35	1.45
12	-7	-16	13.58	2.26
-12	7	16	20.53	1.75
11	-7	-16	7.81	1.85
-11	7	16	11.27	1.53
10	-7	-16	2.49	1.65
-10	7	16	5.81	1.04
9	-7	-16	4.84	1.69
-9	7	16	3.91	0.94
8	-7	-16	3.57	1.54
-8	7	16	3.19	1.00
-8	7	16	3.48	1.20
7	-7	-16	4.47	1.63
-7	7	16	7.28	1.37

-7	7	16	4.20	1.00
6	-7	-16	-0.46	1.41
-6	7	16	2.59	0.95
-6	7	16	5.43	1.34
-5	7	16	-0.40	0.93
-5	7	16	-0.90	1.35
-11	8	16	5.27	1.08
10	-8	-16	6.53	2.39
-10	8	16	4.99	1.05
9	-8	-16	11.20	3.05
-8	8	16	4.37	1.39
-8	8	16	3.01	1.00
3	12	-17	9.80	1.51
-2	-12	17	12.39	2.54
6	11	-17	16.79	1.81
-6	-11	17	12.01	2.12
5	11	-17	11.33	1.57
-5	-11	17	10.89	1.92
4	11	-17	3.98	0.99
-4	-11	17	8.78	1.78
3	11	-17	9.75	1.40
-3	-11	17	7.46	1.58
2	11	-17	4.75	0.90
-2	-11	17	7.06	1.51
1	11	-17	7.24	0.98
-1	-11	17	5.41	1.59
0	11	-17	10.08	1.43
0	-11	17	10.46	1.87
1	-11	17	5.72	1.78
-8	-10	17	4.35	1.64
7	10	-17	17.72	1.70
-7	-10	17	17.62	2.69
6	10	-17	17.41	1.63
-6	-10	17	17.40	2.51
5	10	-17	7.93	1.08
-5	-10	17	10.54	1.73
4	10	-17	9.43	1.37
-4	-10	17	9.25	1.60
3	10	-17	9.14	1.30
-3	-10	17	8.50	1.63
2	10	-17	9.38	1.30
-2	-10	17	8.26	1.52
1	10	-17	11.78	1.34
-1	-10	17	12.24	1.86

0	10	-17	3.60	0.89
0	-10	17	3.17	1.47
-1	10	-17	1.59	0.88
1	-10	17	1.05	2.49
1	-10	17	3.50	1.41
9	9	-17	17.08	2.34
-9	-9	17	9.88	1.89
8	9	-17	13.75	1.58
-8	-9	17	18.28	2.56
7	9	-17	11.20	1.47
-7	-9	17	13.11	2.26
6	9	-17	17.11	1.48
-6	-9	17	21.27	2.36
5	9	-17	13.60	1.36
-5	-9	17	10.48	1.58
4	9	-17	8.81	1.29
-4	-9	17	7.32	1.34
3	9	-17	12.85	1.35
-3	-9	17	13.61	2.06
2	9	-17	18.84	1.48
-2	-9	17	21.73	2.24
1	9	-17	5.75	0.86
-1	-9	17	6.35	1.41
0	9	-17	6.15	1.44
0	9	-17	10.77	1.26
0	-9	17	8.86	2.36
0	-9	17	6.49	1.41
-1	9	-17	12.43	1.38
1	-9	17	13.68	2.38
1	-9	17	14.12	2.70
-2	9	-17	4.97	0.94
2	-9	17	1.99	2.69
2	-9	17	4.06	1.51
10	8	-17	21.42	2.53
-10	-8	17	19.53	2.78
9	8	-17	9.30	1.44
-9	-8	17	8.04	1.68
8	8	-17	12.39	1.42
-8	-8	17	9.20	1.61
7	8	-17	20.12	2.16
7	8	-17	14.35	1.48
-7	-8	17	15.10	1.93
6	8	-17	26.36	2.02
6	8	-17	20.98	1.59

-6	-8	17	20.93	2.25
5	8	-17	10.02	1.63
5	8	-17	13.02	1.35
-5	-8	17	14.29	2.04
4	8	-17	9.68	1.54
4	8	-17	11.83	1.33
-4	-8	17	7.92	1.40
3	8	-17	11.17	1.29
3	8	-17	11.23	1.50
-3	-8	17	15.79	2.01
2	8	-17	13.55	1.50
2	8	-17	16.88	1.40
-2	-8	17	13.99	2.11
1	8	-17	20.67	1.90
1	8	-17	19.16	1.45
-1	-8	17	19.30	2.20
-1	-8	17	24.95	3.08
0	8	-17	8.51	1.09
0	8	-17	10.63	1.23
0	-8	17	12.75	2.39
0	-8	17	8.25	1.36
-1	8	-17	14.81	1.38
-1	8	-17	14.39	1.62
1	-8	17	15.77	2.22
1	-8	17	17.27	2.75
-2	8	-17	11.78	1.40
-2	8	-17	9.36	1.20
2	-8	17	10.88	1.66
2	-8	17	6.24	2.71
-11	-7	17	9.44	1.97
10	7	-17	10.84	1.85
-10	-7	17	11.99	1.86
9	7	-17	17.03	1.50
9	7	-17	17.63	2.37
-9	-7	17	17.47	2.39
8	7	-17	17.36	1.48
8	7	-17	13.31	2.13
-8	-7	17	17.35	2.28
7	7	-17	14.20	1.44
7	7	-17	12.55	1.99
-7	-7	17	14.29	2.04
6	7	-17	23.53	2.06
6	7	-17	18.94	1.49
-6	-7	17	18.96	2.07

5	7	-17	11.07	1.26
5	7	-17	10.80	1.73
-5	-7	17	13.89	1.88
4	7	-17	8.56	1.28
4	7	-17	6.41	0.91
-4	-7	17	7.11	1.27
3	7	-17	10.01	1.57
3	7	-17	10.29	1.26
-3	-7	17	11.20	1.62
2	7	-17	15.25	1.32
2	7	-17	10.51	1.60
-2	-7	17	18.83	3.47
-2	-7	17	10.92	1.54
1	7	-17	10.39	1.24
1	7	-17	9.14	1.56
-1	-7	17	14.69	1.92
-1	-7	17	13.41	2.11
0	7	-17	10.69	1.30
0	7	-17	10.02	1.54
0	-7	17	12.01	2.36
0	-7	17	14.28	1.91
-1	7	-17	13.86	1.64
-1	7	-17	9.47	1.31
1	-7	17	14.16	2.60
1	-7	17	12.66	1.94
-2	7	-17	8.58	1.32
-2	7	-17	9.56	1.60
2	-7	17	8.03	2.68
2	-7	17	9.83	1.65
11	6	-17	5.96	1.05
11	6	-17	7.19	1.82
-11	-6	17	5.61	1.67
10	6	-17	19.21	1.60
10	6	-17	16.56	2.55
-10	-6	17	21.31	2.44
9	6	-17	30.93	1.85
9	6	-17	29.14	2.60
-9	-6	17	31.07	2.50
8	6	-17	19.13	1.47
8	6	-17	20.06	2.21
-8	-6	17	19.31	2.17
7	6	-17	15.15	1.40
-7	-6	17	15.56	2.00
6	6	-17	14.76	2.26

6	6	-17	15.43	1.35
-6	-6	17	15.85	1.88
5	6	-17	8.99	1.23
5	6	-17	11.01	1.64
-5	-6	17	12.21	1.82
4	6	-17	17.32	1.40
-4	-6	17	13.53	1.78
3	6	-17	9.39	1.19
3	6	-17	5.92	1.15
-3	-6	17	7.33	1.25
2	6	-17	8.63	1.20
2	6	-17	10.67	1.20
-2	-6	17	6.85	1.25
-2	-6	17	9.48	1.79
1	6	-17	23.43	1.51
1	6	-17	24.42	1.88
-1	-6	17	21.77	2.03
-1	-6	17	28.89	3.03
0	6	-17	7.10	0.96
0	6	-17	6.67	1.17
0	-6	17	3.81	1.12
0	-6	17	6.11	2.08
-1	6	-17	3.80	1.07
-1	6	-17	1.71	0.76
1	-6	17	0.37	2.08
1	-6	17	0.86	1.01
-2	6	-17	2.76	1.08
-2	6	-17	5.23	0.94
2	-6	17	1.08	2.48
2	-6	17	3.16	1.24
12	5	-17	9.73	1.52
12	5	-17	19.58	2.73
-12	-5	17	9.30	1.77
11	5	-17	9.60	1.45
11	5	-17	15.91	2.64
-11	-5	17	17.39	2.36
10	5	-17	12.68	1.44
10	5	-17	16.57	2.60
-10	-5	17	14.77	2.11
9	5	-17	18.05	2.39
9	5	-17	19.27	1.54
-9	-5	17	19.17	2.14
8	5	-17	19.85	2.28
8	5	-17	23.05	1.49

-8	-5	17	18.63	2.06
7	5	-17	15.03	2.05
7	5	-17	17.46	1.44
-7	-5	17	15.88	1.91
6	5	-17	7.91	1.45
6	5	-17	10.13	1.24
-6	-5	17	12.59	1.77
5	5	-17	7.52	1.29
5	5	-17	9.41	1.18
-5	-5	17	8.83	1.58
4	5	-17	4.62	1.17
4	5	-17	5.99	0.85
-4	-5	17	3.60	0.95
3	5	-17	16.41	1.82
3	5	-17	21.24	1.41
-3	-5	17	21.09	2.40
-3	-5	17	21.07	1.85
2	5	-17	16.53	1.30
2	5	-17	16.82	1.81
-2	-5	17	17.26	1.80
-2	-5	17	20.57	2.53
1	5	-17	3.81	1.14
1	5	-17	5.70	0.88
-1	-5	17	3.37	1.02
-1	-5	17	5.74	1.77
0	5	-17	3.50	1.09
0	5	-17	4.64	0.84
0	-5	17	3.99	1.05
-1	5	-17	3.39	1.12
-1	5	-17	3.15	0.83
1	-5	17	2.65	2.06
1	-5	17	2.55	1.02
-2	5	-17	1.34	0.80
-2	5	-17	1.61	1.06
2	-5	17	2.19	1.15
2	-5	17	4.80	2.31
13	4	-17	19.08	2.87
-13	-4	17	14.03	2.06
12	4	-17	8.13	1.91
12	4	-17	13.31	1.49
-12	-4	17	10.66	1.70
11	4	-17	18.53	2.64
11	4	-17	15.98	1.48
-11	-4	17	17.82	2.25

10	4	-17	17.86	1.51
10	4	-17	21.84	2.57
-10	-4	17	14.69	2.06
9	4	-17	20.75	1.52
9	4	-17	19.76	2.50
-9	-4	17	21.52	2.04
8	4	-17	15.51	2.23
8	4	-17	17.46	1.38
-8	-4	17	14.54	1.85
7	4	-17	8.12	1.21
7	4	-17	7.51	1.44
-7	-4	17	6.31	1.13
6	4	-17	10.97	1.19
6	4	-17	10.12	1.46
-6	-4	17	7.04	1.17
5	4	-17	8.48	1.33
5	4	-17	7.72	0.94
-5	-4	17	9.78	1.55
4	4	-17	10.59	1.75
4	4	-17	7.63	0.93
-4	-4	17	9.98	1.51
3	4	-17	10.84	1.17
3	4	-17	11.64	1.77
-3	-4	17	12.34	1.54
-3	-4	17	15.70	2.24
2	4	-17	12.45	1.71
2	4	-17	10.23	1.17
-2	-4	17	11.88	1.85
-2	-4	17	10.37	1.57
1	4	-17	4.08	1.17
1	4	-17	4.71	0.81
-1	-4	17	4.13	1.00
-1	-4	17	8.28	1.76
0	4	-17	2.76	1.11
0	4	-17	0.53	0.72
0	-4	17	1.90	1.78
0	-4	17	2.08	0.91
-1	4	-17	-0.08	1.03
-1	4	-17	0.43	0.76
1	-4	17	3.30	2.06
1	-4	17	1.62	0.94
-2	4	-17	2.93	1.14
-2	4	-17	5.00	0.96
2	-4	17	2.72	2.35

2	-4	17	3.17	1.09
13	3	-17	17.41	1.56
13	3	-17	13.44	2.07
-13	-3	17	14.20	1.90
12	3	-17	23.97	2.88
12	3	-17	27.07	1.72
-12	-3	17	24.84	2.44
11	3	-17	28.07	2.78
11	3	-17	23.50	1.63
-11	-3	17	23.45	2.27
10	3	-17	18.94	1.50
10	3	-17	23.28	2.56
-10	-3	17	19.63	2.06
9	3	-17	13.61	1.36
9	3	-17	14.66	2.31
-9	-3	17	17.84	1.89
8	3	-17	8.98	1.18
8	3	-17	10.81	1.71
-8	-3	17	11.04	1.59
7	3	-17	8.19	1.51
7	3	-17	11.32	1.25
-7	-3	17	9.57	1.51
6	3	-17	12.00	1.62
6	3	-17	12.94	1.26
-6	-3	17	12.73	1.57
5	3	-17	10.03	1.17
5	3	-17	13.13	1.92
-5	-3	17	11.57	1.53
4	3	-17	6.76	1.25
4	3	-17	5.18	0.80
-4	-3	17	7.10	1.13
-4	-3	17	5.59	1.36
3	3	-17	14.86	1.90
3	3	-17	17.07	1.31
-3	-3	17	14.56	1.57
-3	-3	17	21.16	2.19
2	3	-17	10.05	1.14
2	3	-17	9.59	1.40
-2	-3	17	9.38	1.50
-2	-3	17	9.35	1.76
1	3	-17	-1.24	1.00
1	3	-17	0.98	0.71
-1	-3	17	-0.08	0.80
-1	-3	17	1.25	1.58

0	3	-17	3.90	1.12
0	3	-17	0.29	0.74
0	-3	17	2.65	1.77
0	-3	17	0.78	0.86
-1	3	-17	1.92	0.80
-1	3	-17	-0.05	1.08
1	-3	17	3.05	2.10
1	-3	17	2.35	1.01
13	2	-17	18.81	1.60
13	2	-17	19.34	2.88
-13	-2	17	18.60	2.42
12	2	-17	10.19	1.34
12	2	-17	10.55	1.82
-12	-2	17	8.91	1.49
11	2	-17	11.52	1.87
11	2	-17	14.17	1.42
-11	-2	17	13.12	1.93
10	2	-17	17.54	2.49
10	2	-17	20.99	1.51
-10	-2	17	18.01	1.92
9	2	-17	14.35	2.34
9	2	-17	13.23	1.30
-9	-2	17	14.82	1.67
8	2	-17	13.54	2.13
8	2	-17	14.66	1.26
-8	-2	17	10.82	1.54
7	2	-17	8.46	1.18
7	2	-17	12.62	2.05
-7	-2	17	11.14	1.49
6	2	-17	13.19	1.22
6	2	-17	12.31	1.70
-6	-2	17	14.91	1.53
5	2	-17	13.16	1.24
5	2	-17	13.58	1.94
-5	-2	17	14.64	1.55
4	2	-17	10.77	1.85
4	2	-17	15.40	1.23
-4	-2	17	16.02	1.92
-4	-2	17	11.17	1.43
3	2	-17	2.09	1.15
3	2	-17	2.39	0.72
-3	-2	17	2.15	0.86
-3	-2	17	2.87	1.32
2	2	-17	3.43	1.14

2	2	-17	3.32	0.76
-2	-2	17	4.45	1.55
-2	-2	17	2.60	0.92
1	2	-17	5.43	1.20
1	2	-17	3.70	0.80
-1	-2	17	5.78	1.10
-1	-2	17	3.62	1.91
0	2	-17	2.31	0.79
0	2	-17	1.98	1.15
0	-2	17	1.22	1.79
0	-2	17	1.90	0.91
-1	2	-17	-1.50	1.08
-1	2	-17	0.81	0.84
1	-2	17	-0.95	0.87
1	-2	17	0.15	1.92
14	1	-17	6.85	1.93
14	1	-17	6.20	1.05
-14	-1	17	8.19	1.62
13	1	-17	7.68	1.91
13	1	-17	12.03	1.41
-13	-1	17	13.02	2.12
12	1	-17	8.79	1.29
12	1	-17	9.10	1.81
-12	-1	17	8.69	1.38
11	1	-17	8.37	1.78
11	1	-17	9.90	1.33
-11	-1	17	8.12	1.30
10	1	-17	8.41	1.73
10	1	-17	8.96	1.24
-10	-1	17	11.44	1.62
9	1	-17	2.49	1.49
9	1	-17	4.18	0.84
-9	-1	17	5.19	1.00
8	1	-17	11.02	1.27
8	1	-17	14.86	2.23
-8	-1	17	16.17	1.57
7	1	-17	21.98	1.44
7	1	-17	21.68	2.29
-7	-1	17	22.58	1.72
6	1	-17	4.92	0.80
6	1	-17	6.01	1.45
-6	-1	17	3.71	0.92
5	1	-17	2.70	0.74
5	1	-17	2.51	1.21

-5	-1	17	3.00	0.83
4	1	-17	7.17	1.36
4	1	-17	8.01	1.12
-4	-1	17	7.86	1.33
-4	-1	17	6.07	0.97
3	1	-17	2.72	1.14
3	1	-17	1.68	0.69
-3	-1	17	0.68	0.74
-3	-1	17	3.68	1.28
2	1	-17	-0.26	1.08
2	1	-17	0.39	0.66
-2	-1	17	0.65	0.75
-2	-1	17	0.07	1.37
1	1	-17	-1.32	1.09
1	1	-17	-1.19	0.69
-1	-1	17	0.47	0.73
-1	-1	17	0.63	1.51
0	1	-17	1.39	0.80
0	1	-17	0.64	1.12
0	-1	17	2.79	1.78
0	-1	17	-0.75	0.77
-1	1	-17	-2.97	1.08
-1	1	-17	0.05	0.83
1	-1	17	0.00	1.11
1	-1	17	0.77	1.94
14	0	-17	22.87	1.68
14	0	-17	22.27	3.10
-14	0	17	23.59	2.38
13	0	-17	17.63	1.49
13	0	-17	13.95	2.13
-13	0	17	16.85	2.17
12	0	-17	2.65	0.82
12	0	-17	4.72	1.73
-12	0	17	4.52	1.20
11	0	-17	10.51	1.30
11	0	-17	13.52	2.02
-11	0	17	10.45	1.67
10	0	-17	11.95	1.83
10	0	-17	13.54	1.32
-10	0	17	10.27	1.57
9	0	-17	6.03	1.50
9	0	-17	3.51	0.81
-9	0	17	4.35	0.95
8	0	-17	16.52	1.31

8	0	-17	12.47	1.78
-8	0	17	17.95	1.58
7	0	-17	11.43	1.21
7	0	-17	12.48	2.04
-7	0	17	10.58	1.43
6	0	-17	3.09	1.29
6	0	-17	1.95	0.71
-6	0	17	2.58	0.89
5	0	-17	11.58	1.96
5	0	-17	11.75	1.17
-5	0	17	9.17	1.31
-5	0	17	11.07	1.37
4	0	-17	3.27	0.73
4	0	-17	0.08	1.10
-4	0	17	2.52	0.86
-4	0	17	2.62	1.16
3	0	-17	1.58	1.14
3	0	-17	0.66	0.67
-3	0	17	-1.46	1.13
-3	0	17	0.52	0.73
2	0	-17	0.76	1.07
2	0	-17	0.57	0.70
-2	0	17	1.65	1.38
-2	0	17	0.88	0.69
1	0	-17	0.22	0.72
1	0	-17	1.34	1.15
-1	0	17	2.82	1.54
-1	0	17	-0.09	0.83
0	0	-17	0.31	0.77
0	0	-17	0.24	1.13
0	0	17	-0.61	1.67
0	0	17	-0.24	0.84
14	-1	-17	10.29	1.37
14	-1	-17	10.04	2.08
-14	1	17	9.77	1.51
13	-1	-17	16.43	1.51
13	-1	-17	23.43	2.91
-13	1	17	20.20	2.11
12	-1	-17	21.23	2.80
12	-1	-17	21.41	1.51
-12	1	17	16.86	1.91
11	-1	-17	6.74	0.94
11	-1	-17	5.83	1.67
-11	1	17	7.96	1.19

10	-1	-17	5.70	1.69
10	-1	-17	7.63	0.95
-10	1	17	7.24	1.08
9	-1	-17	10.30	1.21
9	-1	-17	5.05	1.55
-9	1	17	9.75	1.42
8	-1	-17	6.68	0.87
8	-1	-17	3.93	1.45
-8	1	17	5.35	1.00
7	-1	-17	11.61	1.22
7	-1	-17	10.81	1.64
-7	1	17	11.29	1.45
6	-1	-17	7.73	1.12
6	-1	-17	5.40	1.44
-6	1	17	7.31	1.10
5	-1	-17	1.84	0.69
5	-1	-17	-0.80	1.13
-5	1	17	0.87	0.74
-5	1	17	1.15	0.98
4	-1	-17	1.76	1.16
4	-1	-17	1.65	0.64
-4	1	17	0.94	0.74
-4	1	17	1.28	1.11
3	-1	-17	4.21	0.77
3	-1	-17	5.81	1.32
-3	1	17	3.35	1.31
-3	1	17	3.10	0.94
2	-1	-17	-0.44	0.66
2	-1	-17	-1.97	1.03
-2	1	17	0.71	1.40
-2	1	17	0.22	0.83
1	-1	-17	-0.14	0.72
1	-1	-17	-0.20	1.15
-1	1	17	-0.10	1.60
-1	1	17	0.17	0.81
14	-2	-17	17.55	1.51
14	-2	-17	11.09	2.23
-14	2	17	14.03	2.06
13	-2	-17	22.79	2.87
13	-2	-17	21.50	1.61
-13	2	17	19.21	2.01
12	-2	-17	8.02	1.05
12	-2	-17	5.00	1.74
-12	2	17	6.74	1.19

11	-2	-17	3.00	0.82
11	-2	-17	4.86	1.72
-11	2	17	3.17	0.96
10	-2	-17	5.42	0.88
10	-2	-17	7.45	1.70
-10	2	17	3.53	0.94
9	-2	-17	5.67	1.61
9	-2	-17	4.81	0.83
-9	2	17	8.05	1.37
8	-2	-17	12.68	1.88
8	-2	-17	15.77	1.30
-8	2	17	12.01	1.48
7	-2	-17	3.45	1.40
7	-2	-17	0.88	0.71
-7	2	17	4.21	0.91
6	-2	-17	-0.34	0.65
6	-2	-17	-0.48	1.16
-6	2	17	-0.27	0.70
5	-2	-17	2.94	1.28
5	-2	-17	2.22	0.71
-5	2	17	1.83	0.83
-5	2	17	1.38	1.06
4	-2	-17	0.55	1.19
4	-2	-17	0.09	0.69
-4	2	17	2.62	1.25
-4	2	17	0.30	0.77
3	-2	-17	1.92	1.19
3	-2	-17	0.40	0.67
-3	2	17	-0.35	1.28
-3	2	17	-0.40	0.75
2	-2	-17	0.37	0.82
2	-2	-17	-1.53	1.08
-2	2	17	2.51	1.50
-2	2	17	1.04	0.76
-1	2	17	1.68	1.69
14	-3	-17	5.55	1.91
14	-3	-17	5.85	0.98
-14	3	17	11.05	1.90
13	-3	-17	10.33	2.05
13	-3	-17	8.35	1.06
-13	3	17	10.70	1.76
12	-3	-17	10.92	1.40
12	-3	-17	9.02	1.99
-12	3	17	10.14	1.66

11	-3	-17	2.58	0.85
11	-3	-17	3.71	1.67
-11	3	17	2.75	0.98
10	-3	-17	3.36	1.55
10	-3	-17	2.49	0.81
-10	3	17	2.02	0.87
9	-3	-17	3.24	1.54
9	-3	-17	2.20	0.76
-9	3	17	1.87	0.83
8	-3	-17	2.43	1.39
8	-3	-17	3.10	0.74
-8	3	17	0.14	0.74
7	-3	-17	2.27	1.47
7	-3	-17	1.01	0.73
-7	3	17	1.60	0.77
6	-3	-17	0.81	1.30
6	-3	-17	1.11	0.87
-6	3	17	-0.22	0.71
-6	3	17	0.83	1.64
5	-3	-17	2.59	1.37
5	-3	-17	0.99	0.66
-5	3	17	-2.61	1.07
-5	3	17	-0.25	0.72
4	-3	-17	0.66	1.25
4	-3	-17	0.07	0.69
-4	3	17	0.05	0.74
-4	3	17	0.25	1.26
3	-3	-17	-0.02	1.19
-3	3	17	1.21	1.44
-3	3	17	-1.28	0.77
-2	3	17	-1.56	1.57
13	-4	-17	2.69	0.94
13	-4	-17	2.05	1.80
-13	4	17	5.71	1.15
12	-4	-17	5.60	0.97
12	-4	-17	4.74	1.78
-12	4	17	2.71	1.05
11	-4	-17	4.37	1.65
11	-4	-17	8.35	1.03
-11	4	17	7.61	1.15
10	-4	-17	-1.84	1.41
10	-4	-17	0.60	0.78
-10	4	17	0.16	0.83
9	-4	-17	3.15	1.47

9	-4	-17	0.42	0.76
-9	4	17	-0.43	0.75
8	-4	-17	-1.80	1.30
-8	4	17	-0.65	0.73
7	-4	-17	-0.62	1.26
7	-4	-17	0.45	0.73
-7	4	17	-1.07	0.71
6	-4	-17	0.83	0.94
6	-4	-17	2.08	1.35
-6	4	17	0.38	0.76
-6	4	17	-1.22	1.44
5	-4	-17	0.08	1.26
-5	4	17	-1.89	1.11
-5	4	17	0.94	0.87
4	-4	-17	2.16	1.33
-4	4	17	1.65	1.33
-4	4	17	2.07	0.91
13	-5	-17	7.50	2.73
13	-5	-17	4.35	1.01
-13	5	17	2.76	1.08
12	-5	-17	0.47	0.92
12	-5	-17	0.70	1.70
-12	5	17	1.10	0.95
11	-5	-17	1.52	0.83
11	-5	-17	1.69	1.66
-11	5	17	0.82	0.90
10	-5	-17	1.16	0.80
10	-5	-17	1.95	1.57
-10	5	17	1.93	0.89
9	-5	-17	-1.24	1.47
-9	5	17	0.85	0.84
8	-5	-17	0.55	1.44
-8	5	17	0.85	0.81
7	-5	-17	-0.94	1.40
-7	5	17	-0.27	0.73
6	-5	-17	0.86	1.39
-6	5	17	-1.59	0.75
-6	5	17	-3.04	1.40
5	-5	-17	0.26	1.56
-5	5	17	-0.35	1.31
-5	5	17	-0.51	0.87
-12	6	17	1.86	0.99
11	-6	-17	2.74	2.00
10	-6	-17	-1.94	1.68

-10	6	17	-0.25	0.89
9	-6	-17	3.40	1.74
-9	6	17	0.61	0.87
8	-6	-17	-2.80	1.60
-8	6	17	2.43	0.89
-7	6	17	0.73	0.88
-5	-10	18	8.48	2.32
4	10	-18	6.93	1.27
-4	-10	18	2.50	1.64
3	10	-18	5.76	1.07
-3	-10	18	8.86	2.37
2	10	-18	4.88	1.30
7	9	-18	7.66	1.31
-7	-9	18	12.32	1.95
6	9	-18	5.29	0.99
-6	-9	18	4.07	1.46
5	9	-18	1.55	0.90
-5	-9	18	4.52	1.38
4	9	-18	7.61	1.05
-4	-9	18	7.02	1.45
3	9	-18	10.10	1.37
-3	-9	18	6.50	1.49
2	9	-18	3.06	0.86
-2	-9	18	2.28	1.40
1	9	-18	0.68	0.79
-1	-9	18	2.35	1.36
8	8	-18	5.12	1.02
-8	-8	18	4.34	1.59
7	8	-18	5.08	0.95
-7	-8	18	2.97	1.36
6	8	-18	4.24	0.97
-6	-8	18	5.62	1.38
5	8	-18	3.13	0.93
-5	-8	18	0.55	1.17
4	8	-18	0.41	1.39
4	8	-18	1.84	0.84
-4	-8	18	1.65	1.25
3	8	-18	7.02	1.31
3	8	-18	6.43	0.97
-3	-8	18	10.25	1.65
2	8	-18	5.13	0.93
2	8	-18	0.81	1.03
-2	-8	18	3.37	1.33
1	8	-18	1.38	0.81

1	8	-18	1.95	1.00
-1	-8	18	-1.29	1.20
0	8	-18	0.29	0.83
0	8	-18	0.82	1.07
0	-8	18	0.78	1.35
9	7	-18	5.87	1.05
-9	-7	18	6.04	1.54
8	7	-18	9.57	1.63
8	7	-18	5.52	1.03
-8	-7	18	5.94	1.53
7	7	-18	4.46	1.44
7	7	-18	9.19	1.35
-7	-7	18	4.98	1.28
6	7	-18	2.77	0.82
6	7	-18	1.31	1.22
-6	-7	18	0.69	1.18
5	7	-18	4.61	1.20
5	7	-18	2.22	0.83
-5	-7	18	4.17	1.22
4	7	-18	4.33	0.85
4	7	-18	2.31	1.15
-4	-7	18	3.86	1.22
3	7	-18	0.29	0.75
3	7	-18	1.95	1.09
-3	-7	18	-0.87	1.02
2	7	-18	1.11	1.05
2	7	-18	0.35	0.71
-2	-7	18	0.54	1.18
1	7	-18	-0.42	0.99
1	7	-18	0.68	0.78
-1	-7	18	1.81	1.24
0	7	-18	-0.44	1.03
0	7	-18	-0.08	0.80
0	-7	18	1.20	1.22
10	6	-18	13.86	1.96
10	6	-18	13.95	1.56
-10	-6	18	10.33	1.77
9	6	-18	8.77	1.79
9	6	-18	11.95	1.47
-9	-6	18	7.63	1.49
8	6	-18	0.91	0.78
8	6	-18	2.25	1.41
-8	-6	18	2.96	1.32
7	6	-18	2.57	1.41

7	6	-18	0.46	0.78
-7	-6	18	0.67	1.12
6	6	-18	3.36	0.86
6	6	-18	1.52	1.24
-6	-6	18	6.90	1.38
5	6	-18	2.54	0.83
5	6	-18	1.18	1.23
-5	-6	18	1.64	1.11
4	6	-18	-0.43	1.12
4	6	-18	-1.56	0.71
-4	-6	18	2.47	1.15
3	6	-18	1.63	1.11
3	6	-18	2.40	0.81
-3	-6	18	1.24	1.04
2	6	-18	0.01	1.04
2	6	-18	1.98	0.80
-2	-6	18	2.17	1.07
1	6	-18	0.99	1.09
1	6	-18	1.07	0.77
-1	-6	18	-1.09	1.00
0	6	-18	-0.30	1.03
0	6	-18	0.52	0.79
0	-6	18	-1.06	1.05
11	5	-18	13.13	2.09
11	5	-18	13.03	1.66
-11	-5	18	13.70	1.96
10	5	-18	4.39	1.74
10	5	-18	4.05	0.98
-10	-5	18	5.43	1.46
9	5	-18	2.17	1.50
9	5	-18	1.79	0.80
-9	-5	18	1.03	1.21
8	5	-18	5.52	1.67
8	5	-18	5.93	0.98
-8	-5	18	6.95	1.36
7	5	-18	3.95	1.50
7	5	-18	2.31	0.82
-7	-5	18	4.52	1.25
6	5	-18	0.47	1.30
6	5	-18	-0.58	0.75
-6	-5	18	0.53	1.00
5	5	-18	1.12	0.78
5	5	-18	-1.25	1.22
-5	-5	18	1.49	0.96

4	5	-18	1.42	1.19
4	5	-18	4.06	0.84
-4	-5	18	1.89	0.94
3	5	-18	1.51	1.16
3	5	-18	-0.50	0.76
2	5	-18	1.90	1.16
2	5	-18	0.61	0.72
-2	-5	18	-0.03	0.95
1	5	-18	0.60	0.78
1	5	-18	0.12	1.06
-1	-5	18	-0.32	0.93
0	5	-18	2.10	1.17
0	5	-18	2.21	0.88
0	-5	18	1.93	2.46
0	-5	18	3.11	1.12
11	4	-18	6.59	1.83
11	4	-18	4.72	1.00
-11	-4	18	4.92	1.49
10	4	-18	4.57	0.93
10	4	-18	6.28	1.77
-10	-4	18	3.24	1.37
9	4	-18	12.46	1.40
9	4	-18	5.95	1.72
-9	-4	18	11.04	1.96
8	4	-18	0.39	0.78
8	4	-18	2.65	1.48
-8	-4	18	1.25	1.07
7	4	-18	2.38	0.81
7	4	-18	3.21	1.39
-7	-4	18	1.56	1.02
6	4	-18	2.99	1.38
6	4	-18	2.68	0.83
-6	-4	18	2.05	1.14
5	4	-18	1.70	0.81
5	4	-18	0.43	1.26
-5	-4	18	1.54	0.94
4	4	-18	-0.93	1.21
4	4	-18	-0.26	0.75
-4	-4	18	1.78	0.94
3	4	-18	1.08	0.75
3	4	-18	0.08	1.14
2	4	-18	1.31	1.21
2	4	-18	0.34	0.72
-2	-4	18	0.61	0.95

1	4	-18	1.18	1.10
1	4	-18	2.28	0.85
-1	-4	18	1.07	0.99
0	4	-18	0.30	0.83
0	4	-18	1.88	1.18
0	-4	18	5.82	2.25
0	-4	18	-0.66	1.13
12	3	-18	1.95	0.92
12	3	-18	3.75	1.70
-12	-3	18	2.00	1.39
11	3	-18	7.29	1.83
11	3	-18	7.09	1.06
-11	-3	18	5.52	1.39
10	3	-18	3.15	0.89
10	3	-18	2.42	1.62
-10	-3	18	1.82	1.18
9	3	-18	1.02	1.48
9	3	-18	1.82	0.78
-9	-3	18	0.93	1.12
8	3	-18	1.77	0.82
8	3	-18	1.93	1.47
-8	-3	18	2.57	1.01
7	3	-18	1.15	1.35
7	3	-18	2.48	0.78
-7	-3	18	-0.31	0.89
6	3	-18	2.39	0.81
6	3	-18	4.24	1.34
-6	-3	18	0.92	0.89
5	3	-18	0.68	1.20
5	3	-18	2.02	0.79
-5	-3	18	2.15	0.97
4	3	-18	-0.29	1.21
4	3	-18	-0.66	0.73
-4	-3	18	1.58	0.95
3	3	-18	2.22	0.81
3	3	-18	2.63	1.20
-3	-3	18	1.52	0.87
2	3	-18	1.99	0.83
2	3	-18	0.18	1.15
-2	-3	18	0.58	0.89
1	3	-18	1.36	1.21
1	3	-18	3.00	0.85
-1	-3	18	1.31	1.95
-1	-3	18	0.02	0.92

0	-3	18	2.15	2.15
12	2	-18	1.79	0.95
12	2	-18	0.76	1.69
-12	-2	18	-0.61	1.23
11	2	-18	-0.88	0.79
11	2	-18	1.01	1.67
-11	-2	18	1.88	1.18
10	2	-18	4.61	1.72
10	2	-18	2.44	0.87
-10	-2	18	0.18	1.05
9	2	-18	2.48	0.80
9	2	-18	2.43	1.57
-9	-2	18	2.56	1.05
8	2	-18	1.02	0.78
8	2	-18	1.23	1.42
-8	-2	18	-0.19	0.97
7	2	-18	0.14	1.33
7	2	-18	0.53	0.75
-7	-2	18	0.76	0.84
6	2	-18	-0.37	0.73
6	2	-18	2.03	1.31
-6	-2	18	0.75	0.81
5	2	-18	0.16	1.22
5	2	-18	1.69	0.80
-5	-2	18	0.76	0.86
4	2	-18	1.86	0.82
4	2	-18	0.41	1.19
-4	-2	18	1.41	0.84
3	2	-18	1.22	1.19
3	2	-18	1.58	0.76
-3	-2	18	2.42	0.95
2	2	-18	-1.18	1.13
2	2	-18	-0.31	0.78
-2	-2	18	0.99	0.90
1	2	-18	-0.81	0.81
1	2	-18	-0.81	1.14
-1	-2	18	3.16	1.84
-1	-2	18	-0.69	0.93
12	1	-18	1.12	0.89
12	1	-18	3.08	1.77
-12	-1	18	0.48	1.18
11	1	-18	0.82	0.87
11	1	-18	2.61	1.73
-11	-1	18	2.08	1.16

10	1	-18	2.27	1.56
10	1	-18	0.26	0.82
-10	-1	18	0.84	0.96
9	1	-18	0.85	1.38
9	1	-18	-0.30	0.73
-9	-1	18	0.70	0.94
8	1	-18	2.53	0.80
8	1	-18	3.07	1.50
-8	-1	18	4.30	1.02
7	1	-18	0.18	1.37
7	1	-18	1.76	0.81
-7	-1	18	2.34	0.95
6	1	-18	9.01	1.56
6	1	-18	8.74	1.29
-6	-1	18	8.53	1.18
5	1	-18	-0.08	1.21
5	1	-18	-0.03	0.73
-5	-1	18	0.03	0.85
4	1	-18	6.50	1.51
4	1	-18	5.72	0.92
-4	-1	18	3.75	0.96
3	1	-18	1.00	1.20
3	1	-18	2.81	0.84
-3	-1	18	2.50	0.90
2	1	-18	1.83	1.25
2	1	-18	-0.29	0.80
-2	-1	18	2.05	1.86
-2	-1	18	0.23	0.85
1	1	-18	1.52	1.19
1	1	-18	4.03	0.95
-1	-1	18	6.71	1.99
13	0	-18	1.29	0.93
13	0	-18	4.95	1.92
12	0	-18	-0.35	0.87
12	0	-18	-0.75	1.67
-12	0	18	1.66	1.24
11	0	-18	-0.05	0.80
11	0	-18	-0.29	1.67
-11	0	18	1.39	1.08
10	0	-18	-0.46	1.59
10	0	-18	-0.07	0.77
-10	0	18	-1.22	0.88
9	0	-18	1.00	0.81
9	0	-18	1.51	1.51

-9	0	18	0.91	0.89
8	0	-18	0.81	1.37
8	0	-18	-0.04	0.74
-8	0	18	1.65	0.91
7	0	-18	-2.05	1.33
7	0	-18	-1.10	0.72
-7	0	18	-0.39	0.84
6	0	-18	4.23	1.48
6	0	-18	5.11	0.90
-6	0	18	3.38	0.91
5	0	-18	1.29	1.33
5	0	-18	0.90	0.81
-5	0	18	1.50	0.81
4	0	-18	3.50	0.84
4	0	-18	-1.22	1.27
-4	0	18	0.99	0.83
3	0	-18	2.24	1.30
3	0	-18	1.47	0.85
-3	0	18	2.06	0.88
2	0	-18	4.76	0.93
2	0	-18	4.92	1.35
-2	0	18	3.53	1.82
-2	0	18	2.78	1.48
13	-1	-18	-1.72	1.70
12	-1	-18	-2.07	0.85
12	-1	-18	0.78	1.70
-12	1	18	1.40	1.17
11	-1	-18	2.35	1.67
11	-1	-18	2.66	0.89
-11	1	18	-0.03	1.02
10	-1	-18	2.94	0.90
10	-1	-18	5.29	1.77
-10	1	18	3.51	1.03
9	-1	-18	5.11	0.95
9	-1	-18	5.52	1.64
-9	1	18	4.73	1.06
8	-1	-18	1.17	0.82
8	-1	-18	0.73	1.46
-8	1	18	-0.01	0.86
7	-1	-18	0.73	1.33
7	-1	-18	-0.66	0.77
-7	1	18	1.03	0.86
6	-1	-18	5.15	0.87
6	-1	-18	4.69	1.50

-6	1	18	2.79	0.87
5	-1	-18	0.59	1.27
5	-1	-18	3.45	0.85
-5	1	18	4.28	0.89
4	-1	-18	4.40	0.89
4	-1	-18	4.27	1.41
-4	1	18	1.52	0.85
3	-1	-18	7.02	0.95
3	-1	-18	5.98	1.43
-3	1	18	6.31	1.17
12	-2	-18	-1.66	1.72
12	-2	-18	0.94	0.89
-12	2	18	-0.03	1.04
11	-2	-18	4.43	0.95
11	-2	-18	1.26	1.64
-11	2	18	2.64	1.03
10	-2	-18	-1.09	0.79
10	-2	-18	0.79	1.60
-10	2	18	0.90	0.92
9	-2	-18	1.60	1.49
9	-2	-18	2.48	0.89
-9	2	18	1.52	0.93
8	-2	-18	5.74	1.56
-8	2	18	8.54	1.51
7	-2	-18	4.27	1.59
7	-2	-18	5.65	0.98
-7	2	18	7.28	1.14
6	-2	-18	5.30	0.89
6	-2	-18	6.44	1.58
-6	2	18	2.68	0.90
5	-2	-18	8.55	1.24
5	-2	-18	5.75	1.49
-5	2	18	5.05	1.11
4	-2	-18	6.15	1.53
4	-2	-18	10.39	1.37
-4	2	18	6.88	1.21
12	-3	-18	-1.62	1.78
12	-3	-18	0.03	0.92
-12	3	18	1.21	1.06
11	-3	-18	-2.09	0.83
11	-3	-18	1.17	1.67
-11	3	18	1.43	1.02
10	-3	-18	0.97	0.90
10	-3	-18	-0.31	1.63

-10	3	18	1.24	1.01
9	-3	-18	1.67	1.60
9	-3	-18	1.84	0.86
-9	3	18	3.50	1.04
8	-3	-18	0.55	1.57
8	-3	-18	3.06	0.89
-8	3	18	2.80	0.92
7	-3	-18	10.22	1.33
7	-3	-18	8.63	1.71
-7	3	18	10.46	1.55
6	-3	-18	6.63	1.55
6	-3	-18	8.11	1.29
-6	3	18	4.84	1.04
5	-3	-18	5.42	1.65
-5	3	18	6.80	1.17
11	-4	-18	-0.21	0.90
11	-4	-18	-3.42	1.83
-11	4	18	1.36	1.02
10	-4	-18	2.62	1.79
10	-4	-18	2.46	0.91
-10	4	18	3.25	1.08
9	-4	-18	5.09	0.97
9	-4	-18	2.99	1.71
-9	4	18	4.77	1.04
8	-4	-18	7.13	1.89
-8	4	18	5.93	1.14
7	-4	-18	9.30	1.93
-7	4	18	11.35	1.64
7	7	-19	-1.13	0.94
-7	-7	19	0.75	1.40
6	7	-19	-0.02	0.88
6	7	-19	4.58	2.08
-6	-7	19	-0.42	1.26
5	7	-19	2.71	0.97
5	7	-19	6.06	1.44
-5	-7	19	5.21	1.55
4	7	-19	3.77	1.29
4	7	-19	4.65	1.03
-4	-7	19	4.39	1.81
3	7	-19	1.45	0.89
3	7	-19	4.00	1.27
8	6	-19	0.50	0.88
8	6	-19	-0.20	1.60
-8	-6	19	-0.06	1.21

7	6	-19	0.62	1.42
7	6	-19	2.52	0.92
-7	-6	19	1.36	1.29
6	6	-19	4.34	1.46
6	6	-19	5.54	1.01
-6	-6	19	3.34	1.30
5	6	-19	7.62	1.53
5	6	-19	6.59	1.05
-5	-6	19	4.87	1.36
4	6	-19	9.18	1.52
4	6	-19	11.65	1.43
-4	-6	19	6.64	1.45
3	6	-19	7.42	1.01
3	6	-19	10.16	1.48
-3	-6	19	8.92	2.00
9	5	-19	0.13	0.90
9	5	-19	-0.28	1.62
-9	-5	19	1.65	1.41
8	5	-19	1.67	1.62
8	5	-19	1.89	0.89
-8	-5	19	2.84	1.30
7	5	-19	1.64	1.50
7	5	-19	2.54	0.92
-7	-5	19	4.19	1.32
6	5	-19	4.69	0.94
6	5	-19	5.91	1.52
-6	-5	19	5.63	1.43
5	5	-19	7.31	1.51
5	5	-19	7.16	1.05
-5	-5	19	7.56	1.30
4	5	-19	16.11	1.47
4	5	-19	15.44	2.12
-4	-5	19	11.47	2.02
3	5	-19	3.21	1.35
3	5	-19	3.18	0.92
-3	-5	19	5.68	1.36
10	4	-19	4.99	0.99
10	4	-19	2.12	1.73
-10	-4	19	2.89	1.37
9	4	-19	3.85	1.72
9	4	-19	2.83	0.95
-9	-4	19	2.47	1.26
8	4	-19	3.66	1.64
8	4	-19	4.02	0.96

-8	-4	19	2.17	1.20
7	4	-19	4.86	1.58
7	4	-19	3.50	0.92
-7	-4	19	4.71	1.35
6	4	-19	0.80	0.88
6	4	-19	3.53	1.45
-6	-4	19	1.56	1.20
5	4	-19	2.41	0.87
5	4	-19	3.55	1.48
-5	-4	19	3.96	1.11
4	4	-19	16.98	1.57
4	4	-19	18.55	2.24
-4	-4	19	16.90	2.00
3	4	-19	9.27	1.44
3	4	-19	7.47	1.03
-3	-4	19	4.98	1.36
10	3	-19	1.69	1.67
10	3	-19	5.83	0.98
-10	-3	19	3.50	1.30
9	3	-19	6.43	1.09
9	3	-19	6.51	1.74
-9	-3	19	5.31	1.32
8	3	-19	2.76	1.62
8	3	-19	4.16	0.98
-8	-3	19	3.71	1.16
7	3	-19	3.48	0.94
7	3	-19	2.18	1.51
-7	-3	19	3.97	1.14
6	3	-19	2.41	0.92
6	3	-19	2.76	1.48
-6	-3	19	2.76	1.18
5	3	-19	8.03	1.57
5	3	-19	6.78	1.03
-5	-3	19	7.24	1.26
4	3	-19	6.16	1.04
4	3	-19	7.05	1.52
-4	-3	19	7.39	1.31
3	3	-19	0.63	0.86
3	3	-19	3.28	1.33
10	2	-19	6.32	1.04
10	2	-19	5.88	1.83
-10	-2	19	6.07	1.38
9	2	-19	5.04	1.06
9	2	-19	3.39	1.70

-9	-2	19	7.47	1.33
8	2	-19	9.08	1.13
8	2	-19	9.34	1.89
-8	-2	19	10.47	1.46
7	2	-19	26.69	2.72
7	2	-19	24.34	1.75
-7	-2	19	23.10	2.12
6	2	-19	21.33	1.67
6	2	-19	20.24	2.52
-6	-2	19	20.23	2.00
5	2	-19	5.56	1.54
5	2	-19	6.02	1.00
-5	-2	19	4.85	1.15
4	2	-19	7.70	1.05
4	2	-19	8.33	1.58
-4	-2	19	7.78	1.32
10	1	-19	9.66	1.49
10	1	-19	10.38	1.88
-10	-1	19	7.11	1.38
9	1	-19	4.74	1.07
9	1	-19	4.91	1.73
-9	-1	19	5.78	1.24
8	1	-19	10.34	1.92
8	1	-19	14.16	1.58
-8	-1	19	14.20	1.85
7	1	-19	28.32	2.80
7	1	-19	26.50	1.78
-7	-1	19	22.31	2.09
6	1	-19	21.40	1.69
6	1	-19	18.29	2.51
-6	-1	19	20.31	1.94
5	1	-19	14.28	2.37
5	1	-19	11.41	1.54
-5	-1	19	14.14	1.84
4	1	-19	15.13	1.52
10	0	-19	18.24	2.79
10	0	-19	13.87	1.63
-10	0	19	13.03	1.97
9	0	-19	13.02	1.57
9	0	-19	13.04	2.07
-9	0	19	10.62	1.83
8	0	-19	10.77	1.53
8	0	-19	10.23	1.87
-8	0	19	12.42	1.77

```
   7   0 -19   27.00    1.84
   7   0 -19   22.01    2.65
  -7   0  19   21.61    2.04
   6   0 -19   20.58    1.68
   6   0 -19   17.01    2.52
  -6   0  19   19.78    1.93
   5   0 -19   10.46    1.71
   5   0 -19   11.67    1.51
  -5   0  19   10.84    1.80
  10  -1 -19   16.22    1.68
  10  -1 -19   11.26    2.03
 -10   1  19   16.16    1.95
   9  -1 -19   20.61    2.84
   9  -1 -19   18.95    1.69
  -9   1  19   16.57    1.92
   8  -1 -19   11.43    1.59
   8  -1 -19    8.61    2.00
  -8   1  19   14.52    1.82
   7  -1 -19   12.62    1.98
   7  -1 -19   15.51    1.63
  -7   1  19   15.81    1.86
   6  -1 -19    9.07    1.73
   0   0   0    0.00    0.00
;
_shelx_hkl_checksum   79788
```
