# Supplementary material for: Structure-property relationships of photofunctional diiridium(II) complexes with tetracationic charge and an unsupported Ir–Ir bond
Source: Commun Chem. 2022 Nov 23;5:159. doi: 10.1038/s42004-022-00775-4 (PMC9814866; doi:10.1038/s42004-022-00775-4)
Supplement: Supplementary file 9 — Supplementary Data 6 [file 42004_2022_775_MOESM9_ESM.pdf]

```
data_Ir(III)(t-Bu-terpyr)(PPh3)(CO)I[OTf]2

_audit_creation_method            'SHELXL-2014/7'
_shelx_SHELXL_version_number      '2014/7'
_chemical_name_systematic         ?
_chemical_name_common             ?
_chemical_melting_point           ?
_chemical_formula_moiety          ?
_chemical_formula_sum
 'C101 H95 F12 I2 Ir2 N9 O15 P2 S4'
_chemical_formula_weight          2731.23

loop_
 _atom_type_symbol
 _atom_type_description
 _atom_type_scat_dispersion_real
 _atom_type_scat_dispersion_imag
 _atom_type_scat_source
 'C'  'C'   0.0181   0.0091
 'International Tables Vol C Tables 4.2.6.8 and 6.1.1.4'
 'H'  'H'   0.0000   0.0000
 'International Tables Vol C Tables 4.2.6.8 and 6.1.1.4'
 'N'  'N'   0.0311   0.0180
 'International Tables Vol C Tables 4.2.6.8 and 6.1.1.4'
 'O'  'O'   0.0492   0.0322
 'International Tables Vol C Tables 4.2.6.8 and 6.1.1.4'
 'F'  'F'   0.0727   0.0534
 'International Tables Vol C Tables 4.2.6.8 and 6.1.1.4'
 'P'  'P'   0.2955   0.4335
 'International Tables Vol C Tables 4.2.6.8 and 6.1.1.4'
 'S'  'S'   0.3331   0.5567
 'International Tables Vol C Tables 4.2.6.8 and 6.1.1.4'
 'I'  'I'  -0.3257   6.8362
 'International Tables Vol C Tables 4.2.6.8 and 6.1.1.4'
 'Ir'  'Ir'  -4.7710   6.5667
 'International Tables Vol C Tables 4.2.6.8 and 6.1.1.4'

_space_group_crystal_system       triclinic
_space_group_IT_number            2
_space_group_name_H-M_alt         'P -1'
_space_group_name_Hall            '-P 1'

_shelx_space_group_comment
```

```
;
The symmetry employed for this shelxl refinement is uniquely defined
by the following loop, which should always be used as a source of
symmetry information in preference to the above space-group names.
They are only intended as comments.
;

loop_
 _space_group_symop_operation_xyz
 'x, y, z'
 '-x, -y, -z'

_cell_length_a                    10.8622(3)
_cell_length_b                    21.4480(6)
_cell_length_c                    23.7518(7)
_cell_angle_alpha                 74.084(2)
_cell_angle_beta                  82.940(2)
_cell_angle_gamma                 84.797(2)
_cell_volume                      5271.6(3)
_cell_formula_units_Z             2
_cell_measurement_temperature     100(2)
_cell_measurement_reflns_used     9887
_cell_measurement_theta_min       3.26
_cell_measurement_theta_max       65.28

_exptl_crystal_description        block
_exptl_crystal_colour             colorless
_exptl_crystal_density_meas       ?
_exptl_crystal_density_method     ?
_exptl_crystal_density_diffrn     1.721
_exptl_crystal_F_000              2692
_exptl_transmission_factor_min    ?
_exptl_transmission_factor_max    ?
_exptl_crystal_size_max           0.210
_exptl_crystal_size_mid           0.190
_exptl_crystal_size_min           0.170
_exptl_absorpt_coefficient_mu     11.195
_shelx_estimated_absorpt_T_min    0.202
_shelx_estimated_absorpt_T_max    0.252
_exptl_absorpt_correction_type    'multi-scan'
_exptl_absorpt_correction_T_min   0.4060
_exptl_absorpt_correction_T_max   0.7526
_exptl_absorpt_process_details
'SADABS-2014/5  (Bruker,2014/5)  was  used  for  absorption  correction.
```

```
wR2(int) was 0.1198 before and 0.0661 after correction. The Ratio of
minimum to maximum transmission is 0.5395. The \l/2 correction factor
is Not present.'
_exptl_absorpt_special_details    ?
_diffrn_ambient_temperature       100(2)
_diffrn_radiation_wavelength      1.54178
_diffrn_radiation_type            CuK\a
_diffrn_source                    ?
_diffrn_measurement_device_type   'Bruker APEX-II CCD'
_diffrn_measurement_method        '\f and \w scans'
_diffrn_detector_area_resol_mean  ?
_diffrn_reflns_number             81908
_diffrn_reflns_av_unetI/netI      0.0458
_diffrn_reflns_av_R_equivalents   0.0606
_diffrn_reflns_limit_h_min        -12
_diffrn_reflns_limit_h_max        12
_diffrn_reflns_limit_k_min        -25
_diffrn_reflns_limit_k_max        25
_diffrn_reflns_limit_l_min        -27
_diffrn_reflns_limit_l_max        27
_diffrn_reflns_theta_min          2.146
_diffrn_reflns_theta_max          65.364
_diffrn_reflns_theta_full         65.364
_diffrn_measured_fraction_theta_max   0.988
_diffrn_measured_fraction_theta_full  0.988
_diffrn_reflns_Laue_measured_fraction_max    0.988
_diffrn_reflns_Laue_measured_fraction_full   0.988
_diffrn_reflns_point_group_measured_fraction_max   0.988
_diffrn_reflns_point_group_measured_fraction_full  0.988
_reflns_number_total              17879
_reflns_number_gt                 15240
_reflns_threshold_expression      'I > 2\s(I)'
_reflns_Friedel_coverage          0.000
_reflns_Friedel_fraction_max      .
_reflns_Friedel_fraction_full     .

_reflns_special_details
;
 Reflections were merged by SHELXL according to the crystal
 class for the calculation of statistics and refinement.

 _reflns_Friedel_fraction is defined as the number of unique
 Friedel pairs measured divided by the number that would be
 possible theoretically, ignoring centric projections and
```

```
 systematic absences.
;

_computing_data_collection        'Bruker APEX2'
_computing_cell_refinement        'Bruker SAINT'
_computing_data_reduction         'Bruker SAINT'
_computing_structure_solution     'SHELXS-97 (Sheldrick 2008)'
_computing_structure_refinement 'SHELXL-2014/7 (Sheldrick, 2014)'
_computing_molecular_graphics     'Bruker SHELXTL'
_computing_publication_material   'Bruker SHELXTL'
_refine_special_details           ?
_refine_ls_structure_factor_coef  Fsqd
_refine_ls_matrix_type            full
_refine_ls_weighting_scheme       calc
_refine_ls_weighting_details
'w=1/[\s^2^(Fo^2^)+(0.0545P)^2^+66.6561P] where P=(Fo^2^+2Fc^2^)/3'
_atom_sites_solution_primary      ?
_atom_sites_solution_secondary    ?
_atom_sites_solution_hydrogens    geom
_refine_ls_hydrogen_treatment     constr
_refine_ls_extinction_method      none
_refine_ls_extinction_coef        .
_refine_ls_number_reflns          17879
_refine_ls_number_parameters      1455
_refine_ls_number_restraints      329
_refine_ls_R_factor_all           0.0673
_refine_ls_R_factor_gt            0.0551
_refine_ls_wR_factor_ref          0.1374
_refine_ls_wR_factor_gt           0.1309
_refine_ls_goodness_of_fit_ref    1.051
_refine_ls_restrained_S_all       1.076
_refine_ls_shift/su_max           0.001
_refine_ls_shift/su_mean          0.000

loop_
 _atom_site_label
 _atom_site_type_symbol
 _atom_site_fract_x
 _atom_site_fract_y
 _atom_site_fract_z
 _atom_site_U_iso_or_equiv
 _atom_site_adp_type
 _atom_site_occupancy
 _atom_site_site_symmetry_order
```

```
 _atom_site_calc_flag
 _atom_site_refinement_flags_posn
 _atom_site_refinement_flags_adp
 _atom_site_refinement_flags_occupancy
 _atom_site_disorder_assembly
 _atom_site_disorder_group
Ir1 Ir -0.54728(3) 0.86892(2) 0.25207(2) 0.01552(9) Uani 1 1 d . . . . .
I1 I -0.73653(5) 0.84437(2) 0.33836(2) 0.02378(13) Uani 1 1 d . . . . .
P1 P -0.37661(18) 0.88873(9) 0.17927(9) 0.0196(4) Uani 1 1 d . . . . .
O1 O -0.6066(6) 1.0130(3) 0.2395(3) 0.0342(15) Uani 1 1 d . . . . .
N2 N -0.5181(6) 0.7729(3) 0.2670(3) 0.0176(13) Uani 1 1 d . . . . .
N1 N -0.6638(6) 0.8511(3) 0.1961(3) 0.0190(14) Uani 1 1 d . . . . .
N3 N -0.4296(6) 0.8489(3) 0.3175(3) 0.0173(13) Uani 1 1 d . . . . .
C1 C -0.7453(7) 0.8916(4) 0.1658(3) 0.0221(17) Uani 1 1 d . . . . .
H1 H -0.7505 0.9358 0.1668 0.027 Uiso 1 1 calc R U . . .
C2 C -0.8239(8) 0.8731(4) 0.1323(4) 0.0269(19) Uani 1 1 d . . . . .
H2 H -0.8824 0.9041 0.1119 0.032 Uiso 1 1 calc R U . . .
C3 C -0.8174(8) 0.8091(4) 0.1285(4) 0.0254(18) Uani 1 1 d . . . . .
C4 C -0.7318(7) 0.7662(4) 0.1611(3) 0.0216(17) Uani 1 1 d . . . . .
H4 H -0.7235 0.7221 0.1596 0.026 Uiso 1 1 calc R U . . .
C5 C -0.6588(7) 0.7865(4) 0.1954(3) 0.0190(16) Uani 1 1 d . . . . .
C6 C -0.5785(7) 0.7424(4) 0.2366(3) 0.0182(16) Uani 1 1 d . . . . .
C7 C -0.5645(7) 0.6755(4) 0.2484(4) 0.0228(17) Uani 1 1 d . . . . .
H7 H -0.6056 0.6537 0.2270 0.027 Uiso 1 1 calc R U . . .
C8 C -0.4904(8) 0.6398(4) 0.2916(4) 0.0287(19) Uani 1 1 d . . . . .
C9 C -0.4315(8) 0.6740(4) 0.3219(4) 0.0246(18) Uani 1 1 d . . . . .
H9 H -0.3809 0.6510 0.3517 0.029 Uiso 1 1 calc R U . . .
C10 C -0.4461(7) 0.7406(4) 0.3092(3) 0.0178(16) Uani 1 1 d . . . . .
C11 C -0.3938(7) 0.7845(4) 0.3373(3) 0.0182(16) Uani 1 1 d . . . . .
C12 C -0.3137(7) 0.7633(4) 0.3808(3) 0.0202(17) Uani 1 1 d . . . . .
H12 H -0.2891 0.7185 0.3937 0.024 Uiso 1 1 calc R U . . .
C13 C -0.2688(7) 0.8081(4) 0.4058(4) 0.0223(17) Uani 1 1 d . . . . .
C14 C -0.3098(7) 0.8727(4) 0.3849(4) 0.0242(18) Uani 1 1 d . . . . .
H14 H -0.2827 0.9044 0.4011 0.029 Uiso 1 1 calc R U . . .
C15 C -0.3881(8) 0.8919(4) 0.3417(4) 0.0245(18) Uani 1 1 d . . . . .
H15 H -0.4138 0.9366 0.3285 0.029 Uiso 1 1 calc R U . . .
C16 C -0.9000(9) 0.7866(4) 0.0913(4) 0.031(2) Uani 1 1 d . . . . .
C17 C -1.0103(9) 0.8355(5) 0.0745(4) 0.041(2) Uani 1 1 d . . . . .
H17A H -1.0583 0.8422 0.1103 0.061 Uiso 1 1 calc R U . . .
H17B H -1.0635 0.8185 0.0523 0.061 Uiso 1 1 calc R U . . .
H17C H -0.9794 0.8769 0.0501 0.061 Uiso 1 1 calc R U . . .
C18 C -0.9524(9) 0.7212(5) 0.1268(4) 0.040(2) Uani 1 1 d . . . . .
H18A H -0.8843 0.6876 0.1336 0.059 Uiso 1 1 calc R U . . .
H18B H -1.0123 0.7089 0.1047 0.059 Uiso 1 1 calc R U . . .
```

H18C H -0.9939 0.7255 0.1648 0.059 Uiso 1 1 calc R U . . .
C19 C -0.8221(9) 0.7798(6) 0.0371(4) 0.044(3) Uani 1 1 d . . . . .
H19A H -0.7953 0.8225 0.0139 0.066 Uiso 1 1 calc R U . . .
H19B H -0.8706 0.7618 0.0138 0.066 Uiso 1 1 calc R U . . .
H19C H -0.7490 0.7506 0.0480 0.066 Uiso 1 1 calc R U . . .
C20 C -0.4806(9) 0.5659(4) 0.3072(5) 0.044(3) Uani 1 1 d . . . . .
C21 C -0.4829(18) 0.5421(7) 0.2496(8) 0.095(5) Uani 1 1 d . U . . .
H21A H -0.5646 0.5538 0.2348 0.143 Uiso 1 1 calc R U . . .
H21B H -0.4671 0.4948 0.2590 0.143 Uiso 1 1 calc R U . . .
H21C H -0.4186 0.5629 0.2194 0.143 Uiso 1 1 calc R U . . .
C22 C -0.3511(13) 0.5381(5) 0.3202(7) 0.073(4) Uani 1 1 d . U . . .
H22A H -0.2905 0.5606 0.2889 0.110 Uiso 1 1 calc R U . . .
H22B H -0.3447 0.4917 0.3223 0.110 Uiso 1 1 calc R U . . .
H22C H -0.3341 0.5441 0.3579 0.110 Uiso 1 1 calc R U . . .
C24 C -0.1819(8) 0.7892(4) 0.4541(4) 0.0263(18) Uani 1 1 d . . . . .
C25 C -0.2473(10) 0.8124(5) 0.5072(4) 0.040(2) Uani 1 1 d . . . . .
H25A H -0.2651 0.8595 0.4950 0.059 Uiso 1 1 calc R U . . .
H25B H -0.1929 0.8016 0.5389 0.059 Uiso 1 1 calc R U . . .
H25C H -0.3252 0.7907 0.5212 0.059 Uiso 1 1 calc R U . . .
C26 C -0.1541(9) 0.7151(4) 0.4746(4) 0.031(2) Uani 1 1 d . . . . .
H26A H -0.2322 0.6936 0.4880 0.047 Uiso 1 1 calc R U . . .
H26B H -0.1014 0.7050 0.5070 0.047 Uiso 1 1 calc R U . . .
H26C H -0.1108 0.6998 0.4417 0.047 Uiso 1 1 calc R U . . .
C27 C -0.0611(8) 0.8219(5) 0.4320(4) 0.035(2) Uani 1 1 d . . . . .
H27A H -0.0220 0.8079 0.3977 0.052 Uiso 1 1 calc R U . . .
H27B H -0.0052 0.8098 0.4632 0.052 Uiso 1 1 calc R U . . .
H27C H -0.0776 0.8691 0.4209 0.052 Uiso 1 1 calc R U . . .
C28 C -0.5843(7) 0.9596(4) 0.2435(4) 0.0240(18) Uani 1 1 d . . . . .
C29 C -0.3882(9) 0.9603(4) 0.1167(4) 0.030(2) Uani 1 1 d . . . . .
C30 C -0.4963(9) 0.9968(4) 0.1039(4) 0.032(2) Uani 1 1 d . . . . .
H30 H -0.5712 0.9861 0.1286 0.038 Uiso 1 1 calc R U . . .
C31 C -0.4957(10) 1.0499(5) 0.0541(4) 0.039(2) Uani 1 1 d . . . . .
H31 H -0.5703 1.0756 0.0450 0.046 Uiso 1 1 calc R U . . .
C32 C -0.3876(10) 1.0650(5) 0.0183(4) 0.039(2) Uani 1 1 d . . . . .
H32 H -0.3870 1.1014 -0.0152 0.047 Uiso 1 1 calc R U . . .
C33 C -0.2824(11) 1.0280(6) 0.0307(6) 0.063(4) Uani 1 1 d . . . . .
H33 H -0.2081 1.0381 0.0053 0.076 Uiso 1 1 calc R U . . .
C34 C -0.2806(11) 0.9753(5) 0.0802(5) 0.058(3) Uani 1 1 d . . . . .
H34 H -0.2056 0.9499 0.0887 0.069 Uiso 1 1 calc R U . . .
C35 C -0.2421(8) 0.9023(4) 0.2115(4) 0.0257(18) Uani 1 1 d . . . . .
C36 C -0.2406(9) 0.9641(5) 0.2202(5) 0.041(2) Uani 1 1 d . . . . .
H36 H -0.3050 0.9960 0.2073 0.050 Uiso 1 1 calc R U . . .
C37 C -0.1459(11) 0.9790(5) 0.2473(6) 0.053(3) Uani 1 1 d . . . . .
H37 H -0.1458 1.0210 0.2533 0.063 Uiso 1 1 calc R U . . .

C38 C -0.0499(9) 0.9321(5) 0.2660(5) 0.044(3) Uani 1 1 d . . . . .
H38 H 0.0158 0.9425 0.2842 0.053 Uiso 1 1 calc R U . . .
C39 C -0.0515(8) 0.8706(5) 0.2578(4) 0.035(2) Uani 1 1 d . . . . .
H39 H 0.0127 0.8386 0.2707 0.042 Uiso 1 1 calc R U . . .
C40 C -0.1481(8) 0.8559(4) 0.2304(4) 0.029(2) Uani 1 1 d . . . . .
H40 H -0.1491 0.8138 0.2248 0.035 Uiso 1 1 calc R U . . .
C41 C -0.3514(7) 0.8210(4) 0.1461(4) 0.0240(18) Uani 1 1 d . . . . .
C42 C -0.4017(10) 0.8280(5) 0.0939(4) 0.040(2) Uani 1 1 d . . . . .
H42 H -0.4316 0.8698 0.0726 0.048 Uiso 1 1 calc R U . . .
C43 C -0.4091(13) 0.7747(6) 0.0721(5) 0.059(3) Uani 1 1 d . . . . .
H43 H -0.4442 0.7801 0.0362 0.070 Uiso 1 1 calc R U . . .
C44 C -0.3655(10) 0.7142(5) 0.1026(5) 0.046(3) Uani 1 1 d . . . . .
H44 H -0.3735 0.6774 0.0887 0.055 Uiso 1 1 calc R U . . .
C45 C -0.3104(9) 0.7071(5) 0.1530(5) 0.039(2) Uani 1 1 d . . . . .
H45 H -0.2762 0.6657 0.1729 0.047 Uiso 1 1 calc R U . . .
C46 C -0.3043(7) 0.7604(4) 0.1754(4) 0.0261(19) Uani 1 1 d . . . . .
H46 H -0.2677 0.7549 0.2109 0.031 Uiso 1 1 calc R U . . .
Ir2 Ir 0.16867(3) 0.36429(2) 0.26890(2) 0.02045(10) Uani 1 1 d . . . . .
I2 I 0.32345(5) 0.34924(3) 0.35363(2) 0.02610(13) Uani 1 1 d . . . . .
P2 P 0.0321(2) 0.37546(10) 0.19555(9) 0.0219(4) Uani 1 1 d . . . . .
O2 O 0.2314(7) 0.5062(3) 0.2320(4) 0.060(2) Uani 1 1 d . . . . .
N5 N 0.1369(6) 0.2704(3) 0.3007(3) 0.0171(13) Uani 1 1 d . . . . .
N4 N 0.3107(6) 0.3230(3) 0.2226(3) 0.0247(15) Uani 1 1 d . . . . .
N6 N 0.0223(6) 0.3684(3) 0.3324(3) 0.0211(14) Uani 1 1 d . . . . .
C47 C 0.4035(8) 0.3523(5) 0.1844(4) 0.029(2) Uani 1 1 d . . . . .
H47 H 0.4079 0.3980 0.1765 0.035 Uiso 1 1 calc R U . . .
C48 C 0.4919(8) 0.3179(5) 0.1568(4) 0.035(2) Uani 1 1 d . . . . .
H48 H 0.5562 0.3406 0.1305 0.042 Uiso 1 1 calc R U . . .
C49 C 0.4914(8) 0.2520(5) 0.1654(4) 0.030(2) Uani 1 1 d . . . . .
C50 C 0.3967(8) 0.2220(5) 0.2058(4) 0.0267(19) Uani 1 1 d . . . . .
H50 H 0.3921 0.1763 0.2142 0.032 Uiso 1 1 calc R U . . .
C51 C 0.3091(8) 0.2568(4) 0.2341(3) 0.0232(18) Uani 1 1 d . . . . .
C52 C 0.2098(7) 0.2272(4) 0.2793(3) 0.0181(16) Uani 1 1 d . . . . .
C53 C 0.1921(7) 0.1615(4) 0.3019(4) 0.0199(16) Uani 1 1 d . . . . .
H53 H 0.2425 0.1305 0.2860 0.024 Uiso 1 1 calc R U . . .
C54 C 0.0992(7) 0.1409(4) 0.3483(3) 0.0169(16) Uani 1 1 d . . . . .
C55 C 0.0269(7) 0.1884(4) 0.3697(3) 0.0180(16) Uani 1 1 d . . . . .
H55 H -0.0359 0.1760 0.4015 0.022 Uiso 1 1 calc R U . . .
C56 C 0.0463(7) 0.2525(4) 0.3451(3) 0.0162(15) Uani 1 1 d . . . . .
C57 C -0.0200(7) 0.3088(4) 0.3624(3) 0.0205(17) Uani 1 1 d . . . . .
C58 C -0.1177(7) 0.3026(4) 0.4068(4) 0.0216(17) Uani 1 1 d . . . . .
H58 H -0.1458 0.2608 0.4267 0.026 Uiso 1 1 calc R U . . .
C59 C -0.1755(8) 0.3577(4) 0.4228(4) 0.0275(19) Uani 1 1 d . . . . .
C60 C -0.1283(9) 0.4161(4) 0.3934(4) 0.030(2) Uani 1 1 d . . . . .

H60 H -0.1628 0.4542 0.4039 0.036 Uiso 1 1 calc R U . . .
C61 C -0.0310(8) 0.4214(4) 0.3485(4) 0.0268(19) Uani 1 1 d . . . . .
H61 H -0.0015 0.4630 0.3288 0.032 Uiso 1 1 calc R U . . .
C62 C 0.5827(8) 0.2137(5) 0.1317(4) 0.035(2) Uani 1 1 d . . . . .
C63 C 0.5933(11) 0.1421(6) 0.1616(5) 0.060(4) Uani 1 1 d . . . . .
H63A H 0.6266 0.1352 0.1995 0.090 Uiso 1 1 calc R U . . .
H63B H 0.6492 0.1199 0.1366 0.090 Uiso 1 1 calc R U . . .
H63C H 0.5110 0.1245 0.1681 0.090 Uiso 1 1 calc R U . . .
C64 C 0.5346(10) 0.2230(5) 0.0713(4) 0.043(2) Uani 1 1 d . . . . .
H64A H 0.4511 0.2070 0.0771 0.065 Uiso 1 1 calc R U . . .
H64B H 0.5906 0.1987 0.0482 0.065 Uiso 1 1 calc R U . . .
H64C H 0.5315 0.2693 0.0504 0.065 Uiso 1 1 calc R U . . .
C65 C 0.7123(10) 0.2416(8) 0.1215(6) 0.076(5) Uani 1 1 d . . . . .
H65A H 0.7056 0.2879 0.1008 0.114 Uiso 1 1 calc R U . . .
H65B H 0.7692 0.2185 0.0977 0.114 Uiso 1 1 calc R U . . .
H65C H 0.7442 0.2362 0.1595 0.114 Uiso 1 1 calc R U . . .
C66 C 0.0790(7) 0.0692(4) 0.3769(4) 0.0212(17) Uani 1 1 d . . . . .
C67 C 0.1072(9) 0.0534(4) 0.4407(4) 0.031(2) Uani 1 1 d . . . . .
H67A H 0.1908 0.0663 0.4421 0.047 Uiso 1 1 calc R U . . .
H67B H 0.1026 0.0067 0.4587 0.047 Uiso 1 1 calc R U . . .
H67C H 0.0462 0.0772 0.4623 0.047 Uiso 1 1 calc R U . . .
C68 C 0.1611(8) 0.0245(4) 0.3455(4) 0.028(2) Uani 1 1 d . . . . .
H68A H 0.1427 0.0345 0.3045 0.043 Uiso 1 1 calc R U . . .
H68B H 0.1446 -0.0208 0.3654 0.043 Uiso 1 1 calc R U . . .
H68C H 0.2487 0.0313 0.3466 0.043 Uiso 1 1 calc R U . . .
C69 C -0.0589(8) 0.0574(4) 0.3750(5) 0.036(2) Uani 1 1 d . . . . .
H69A H -0.1124 0.0837 0.3971 0.054 Uiso 1 1 calc R U . . .
H69B H -0.0730 0.0113 0.3928 0.054 Uiso 1 1 calc R U . . .
H69C H -0.0783 0.0697 0.3341 0.054 Uiso 1 1 calc R U . . .
C70 C -0.2828(9) 0.3496(5) 0.4711(4) 0.034(2) Uani 1 1 d . . . . .
C71 C -0.2387(11) 0.3048(6) 0.5270(5) 0.055(3) Uani 1 1 d . . . . .
H71A H -0.2141 0.2618 0.5209 0.083 Uiso 1 1 calc R U . . .
H71B H -0.3060 0.3009 0.5590 0.083 Uiso 1 1 calc R U . . .
H71C H -0.1673 0.3227 0.5373 0.083 Uiso 1 1 calc R U . . .
C72 C -0.3858(12) 0.3204(9) 0.4514(7) 0.087(5) Uani 1 1 d . . . . .
H72A H -0.4097 0.3487 0.4139 0.130 Uiso 1 1 calc R U . . .
H72B H -0.4577 0.3161 0.4812 0.130 Uiso 1 1 calc R U . . .
H72C H -0.3568 0.2775 0.4464 0.130 Uiso 1 1 calc R U . . .
C73 C -0.3249(13) 0.4151(6) 0.4839(6) 0.072(4) Uani 1 1 d . . . . .
H73A H -0.2524 0.4361 0.4892 0.107 Uiso 1 1 calc R U . . .
H73B H -0.3827 0.4079 0.5199 0.107 Uiso 1 1 calc R U . . .
H73C H -0.3666 0.4430 0.4509 0.107 Uiso 1 1 calc R U . . .
C74 C 0.2086(9) 0.4535(5) 0.2438(4) 0.037(2) Uani 1 1 d . . . . .
C75 C -0.0259(9) 0.4572(4) 0.1618(4) 0.034(2) Uani 1 1 d . . . . .

S2 S -0.0734(3) 0.62600(11) 0.31697(10) 0.0392(6) Uani 1 1 d . . . . .
F4 F 0.1367(6) 0.6788(5) 0.2748(3) 0.083(3) Uani 1 1 d . . . . .
F5 F 0.1305(9) 0.6252(5) 0.3642(4) 0.108(3) Uani 1 1 d . . . . .
F6 F 0.0397(9) 0.7160(5) 0.3420(5) 0.111(4) Uani 1 1 d . . . . .
O6 O -0.1313(7) 0.6760(4) 0.2722(4) 0.070(3) Uani 1 1 d . U . . .
O7 O -0.1421(10) 0.6166(5) 0.3733(4) 0.088(3) Uani 1 1 d . U . . .
O8 O -0.0245(10) 0.5712(4) 0.2973(4) 0.071(3) Uani 1 1 d . U . . .
C94 C 0.0681(11) 0.6628(6) 0.3240(5) 0.049(3) Uani 1 1 d . . . . .
S1 S 0.5856(2) 0.06150(11) 0.35565(12) 0.0382(6) Uani 1 1 d . . . . .
F2 F 0.5799(6) 0.1552(3) 0.4072(3) 0.0471(15) Uani 1 1 d . . . . .
F3 F 0.4228(6) 0.0960(3) 0.4321(3) 0.065(2) Uani 1 1 d . . . . .
F1 F 0.4428(6) 0.1676(3) 0.3460(3) 0.0606(18) Uani 1 1 d . . . . .
O4 O 0.6731(7) 0.0958(4) 0.3111(3) 0.0491(18) Uani 1 1 d . . . . .
O5 O 0.6399(6) 0.0174(3) 0.4083(4) 0.052(2) Uani 1 1 d . . . . .
O3 O 0.4882(7) 0.0340(4) 0.3364(4) 0.052(2) Uani 1 1 d . . . . .
C93 C 0.5023(9) 0.1237(4) 0.3864(5) 0.038(2) Uani 1 1 d . . . . .
O1W O -0.4101(7) 0.5118(3) 0.1308(3) 0.0415(16) Uani 1 1 d G . . . .
H0AA H -0.4521 0.4903 0.1627 0.062 Uiso 1 1 d G U . . .
H0AB H -0.3726 0.5422 0.1385 0.062 Uiso 1 1 d G U . . .
N7 N -0.1895(8) 0.1451(4) 0.4900(4) 0.0393(19) Uani 1 1 d . . . . .
C95 C -0.2709(8) 0.1140(4) 0.5108(4) 0.031(2) Uani 1 1 d . . . . .
C96 C -0.3746(9) 0.0742(5) 0.5360(5) 0.041(2) Uani 1 1 d . . . . .
H96A H -0.3779 0.0419 0.5141 0.061 Uiso 1 1 calc R U . . .
H96B H -0.4520 0.1017 0.5338 0.061 Uiso 1 1 calc R U . . .
H96C H -0.3644 0.0521 0.5773 0.061 Uiso 1 1 calc R U . . .
N8 N 0.360(2) 0.4118(11) 0.5250(10) 0.160(8) Uani 1 1 d . U . . .
C97 C 0.2791(18) 0.4347(9) 0.4959(8) 0.092(5) Uani 1 1 d . U . . .
C98 C 0.1546(16) 0.4575(6) 0.4712(6) 0.083(5) Uani 1 1 d . . . . .
H98A H 0.1692 0.4823 0.4299 0.124 Uiso 1 1 calc R U . . .
H98B H 0.1071 0.4851 0.4939 0.124 Uiso 1 1 calc R U . . .
H98C H 0.1077 0.4196 0.4738 0.124 Uiso 1 1 calc R U . . .
C80 C -0.045(2) 0.4741(10) 0.1001(10) 0.025(5) Uani 0.509(14) 1 d . U P
A 1
C78 C -0.098(2) 0.5861(10) 0.1013(10) 0.031(5) Uani 0.509(14) 1 d . U P
A 1
C79 C -0.084(2) 0.5386(10) 0.0709(10) 0.038(5) Uani 0.509(14) 1 d . U P
A 1
C87 C 0.088(2) 0.3448(13) 0.1365(11) 0.022(6) Uani 0.509(14) 1 d . U P
A 1
C92 C 0.039(2) 0.2924(9) 0.1226(8) 0.029(4) Uani 0.509(14) 1 d . U P A
1
C91 C 0.093(2) 0.2730(11) 0.0736(10) 0.048(6) Uani 0.509(14) 1 d . U P
A 1
C90 C 0.213(3) 0.3044(16) 0.0418(13) 0.055(8) Uani 0.509(14) 1 d . U P

A 1
C89 C 0.246(3) 0.3529(15) 0.0522(13) 0.051(7) Uani 0.509(14) 1 d . U P
A 1
C88 C 0.194(2) 0.3757(12) 0.1009(9) 0.026(5) Uani 0.509(14) 1 d . U P A
1
F9 F 0.0229(14) 0.1616(8) 0.0706(6) 0.079(5) Uani 0.509(14) 1 d . U P A
1
F8 F -0.0430(17) 0.0702(10) 0.1151(9) 0.098(6) Uani 0.509(14) 1 d . U P
A 1
C23 C -0.544(3) 0.5398(12) 0.3768(16) 0.080(9) Uani 0.509(14) 1 d . U P
A 1
C23A C -0.609(3) 0.5444(10) 0.3374(12) 0.058(7) Uani 0.491(14) 1 d . U
P A 2
C78A C -0.151(3) 0.5733(14) 0.1130(13) 0.050(7) Uani 0.491(14) 1 d . U
P A 2
C79A C -0.144(3) 0.5233(13) 0.0838(12) 0.049(6) Uani 0.491(14) 1 d . U
P A 2
C80A C -0.089(3) 0.4612(15) 0.1117(13) 0.048(8) Uani 0.491(14) 1 d . U
P A 2
C87A C 0.128(2) 0.3541(11) 0.1313(12) 0.015(5) Uani 0.491(14) 1 d . U P
A 2
C91A C 0.180(3) 0.2878(13) 0.0622(14) 0.050(7) Uani 0.491(14) 1 d . U P
A 2
C90A C 0.276(3) 0.3373(14) 0.0340(11) 0.042(6) Uani 0.491(14) 1 d . U P
A 2
C89A C 0.2790(19) 0.3919(11) 0.0501(9) 0.036(5) Uani 0.491(14) 1 d . U
P A 2
C92A C 0.119(2) 0.2983(9) 0.1136(9) 0.034(5) Uani 0.491(14) 1 d . U P A
2
C88A C 0.208(2) 0.4041(12) 0.0996(10) 0.030(5) Uani 0.491(14) 1 d . U P
A 2
F9A F 0.031(4) 0.0369(18) 0.0883(16) 0.170(12) Uani 0.491(14) 1 d . U P
A 2
F8A F 0.139(4) 0.121(2) 0.0456(17) 0.194(15) Uani 0.491(14) 1 d . U P A
2
C76 C -0.0391(8) 0.5069(4) 0.1898(4) 0.031(2) Uani 1 1 d . . . . .
C77 C -0.0866(11) 0.5688(5) 0.1617(5) 0.049(3) Uani 1 1 d . . . . .
S3 S 0.1510(4) 0.09646(16) 0.15716(17) 0.0689(9) Uani 1 1 d D U . . .
O10 O 0.1660(11) 0.0292(5) 0.1858(6) 0.099(3) Uani 1 1 d . U . . .
O9 O 0.2492(14) 0.1320(7) 0.1351(7) 0.144(5) Uani 1 1 d . U . . .
C99 C 0.048(3) 0.1031(18) 0.0998(12) 0.171(11) Uani 1 1 d D U . . .
F7 F 0.1252(12) 0.0690(5) 0.0832(6) 0.117(4) Uani 1 1 d . U . . .
S4 S 0.4083(4) 0.57539(17) 0.09772(18) 0.0775(11) Uani 1 1 d . U . . .
N9 N 0.3813(7) 0.5665(3) 0.0622(3) 0.0261(16) Uani 1 1 d . U . . .

```
O13 O 0.3585(10) 0.6229(4) 0.1292(4) 0.079(3) Uani 1 1 d . U . . .
C101 C 0.1690(14) 0.6112(6) -0.0020(7) 0.070(3) Uani 1 1 d . U . . .
O12 O 0.3965(16) 0.5093(6) 0.1299(7) 0.139(5) Uani 1 1 d . U . . .
F10 F 0.3330(14) 0.5401(7) 0.0102(6) 0.159(4) Uani 1 1 d . U . . .
O14 O 0.5183(14) 0.5938(8) 0.0594(7) 0.137(5) Uani 1 1 d . U . . .
F11 F 0.2918(15) 0.6394(7) 0.0054(7) 0.165(4) Uani 1 1 d . U . . .
C100 C 0.294(2) 0.5839(11) 0.0430(10) 0.105(5) Uani 1 1 d . U . . .
F12 F 0.1851(16) 0.5675(8) 0.0683(7) 0.176(5) Uani 1 1 d . U . . .
C81 C -0.1043(9) 0.3318(5) 0.2277(4) 0.042(2) Uani 1 1 d . U . . .
C82 C -0.2116(11) 0.3694(7) 0.2403(6) 0.060(3) Uani 1 1 d . U . . .
H82 H -0.2102 0.4153 0.2275 0.073 Uiso 1 1 calc R U . . .
C86 C -0.1057(12) 0.2657(6) 0.2438(6) 0.061(3) Uani 1 1 d . U . . .
H86 H -0.0350 0.2391 0.2352 0.074 Uiso 1 1 calc R U . . .
O11 O 0.0603(16) 0.1272(7) 0.1892(7) 0.142(5) Uani 1 1 d . U . . .
C85 C -0.2207(14) 0.2376(8) 0.2748(6) 0.077(4) Uani 1 1 d . U . . .
H85 H -0.2276 0.1919 0.2872 0.092 Uiso 1 1 calc R U . . .
C83 C -0.3186(14) 0.3420(8) 0.2705(7) 0.080(4) Uani 1 1 d . U . . .
H83 H -0.3894 0.3682 0.2798 0.096 Uiso 1 1 calc R U . . .
C84 C -0.3188(14) 0.2795(8) 0.2856(7) 0.076(4) Uani 1 1 d . U . . .
H84 H -0.3932 0.2608 0.3058 0.091 Uiso 1 1 calc R U . . .

loop_
 _atom_site_aniso_label
 _atom_site_aniso_U_11
 _atom_site_aniso_U_22
 _atom_site_aniso_U_33
 _atom_site_aniso_U_23
 _atom_site_aniso_U_13
 _atom_site_aniso_U_12
Ir1  0.01689(17)  0.01293(16)  0.01787(17)  -0.00671(12)  -0.00158(13)
0.00103(12)
I1 0.0220(3) 0.0230(3) 0.0265(3) -0.0097(2) 0.0029(2) 0.0000(2)
P1 0.0210(10) 0.0165(9) 0.0220(10) -0.0073(8) 0.0001(8) -0.0010(8)
O1 0.049(4) 0.016(3) 0.037(4) -0.008(3) -0.005(3) 0.007(3)
N2 0.016(3) 0.021(3) 0.018(3) -0.010(3) -0.001(3) 0.000(3)
N1 0.021(3) 0.023(3) 0.015(3) -0.009(3) 0.000(3) -0.003(3)
N3 0.016(3) 0.014(3) 0.022(3) -0.008(3) 0.000(3) 0.002(2)
C1 0.025(4) 0.020(4) 0.019(4) -0.002(3) -0.002(3) -0.002(3)
C2 0.020(4) 0.036(5) 0.020(4) 0.002(4) -0.004(3) -0.009(4)
C3 0.026(4) 0.033(5) 0.018(4) -0.006(4) -0.003(3) -0.007(4)
C4 0.028(4) 0.022(4) 0.018(4) -0.009(3) 0.001(3) -0.007(3)
C5 0.016(4) 0.015(4) 0.024(4) -0.007(3) 0.004(3) 0.000(3)
C6 0.015(4) 0.021(4) 0.021(4) -0.011(3) 0.004(3) -0.003(3)
C7 0.026(4) 0.016(4) 0.030(5) -0.013(3) -0.001(4) -0.001(3)
```

C8 0.026(5) 0.022(4) 0.039(5) -0.009(4) -0.002(4) -0.004(4)
C9 0.024(4) 0.014(4) 0.034(5) -0.003(3) -0.004(4) -0.002(3)
C10 0.013(4) 0.019(4) 0.021(4) -0.006(3) 0.001(3) -0.002(3)
C11 0.017(4) 0.015(4) 0.022(4) -0.007(3) 0.002(3) 0.000(3)
C12 0.023(4) 0.016(4) 0.021(4) -0.006(3) -0.001(3) 0.000(3)
C13 0.021(4) 0.024(4) 0.020(4) -0.005(3) 0.001(3) -0.001(3)
C14 0.024(4) 0.025(4) 0.029(5) -0.013(4) -0.005(4) -0.007(3)
C15 0.028(4) 0.021(4) 0.026(5) -0.009(4) -0.004(4) -0.001(3)
C16 0.037(5) 0.036(5) 0.023(5) -0.009(4) -0.007(4) -0.013(4)
C17 0.035(5) 0.055(6) 0.035(6) -0.012(5) -0.013(4) -0.004(5)
C18 0.041(6) 0.044(6) 0.035(5) -0.011(5) 0.000(4) -0.016(5)
C19 0.033(5) 0.068(7) 0.038(6) -0.024(5) 0.004(4) -0.022(5)
C20 0.042(6) 0.014(4) 0.078(8) -0.013(5) -0.017(5) -0.003(4)
C21 0.133(12) 0.046(7) 0.118(12) -0.029(8) -0.048(10) 0.012(8)
C22 0.092(9) 0.026(5) 0.111(11) -0.019(6) -0.055(8) 0.005(6)
C24 0.028(5) 0.029(4) 0.022(4) -0.008(4) -0.008(4) 0.005(4)
C25 0.052(6) 0.042(6) 0.030(5) -0.019(4) -0.012(5) 0.010(5)
C26 0.037(5) 0.031(5) 0.027(5) -0.009(4) -0.007(4) 0.003(4)
C27 0.033(5) 0.036(5) 0.036(5) -0.007(4) -0.014(4) -0.002(4)
C28 0.021(4) 0.032(5) 0.019(4) -0.006(4) -0.004(3) -0.004(4)
C29 0.044(5) 0.020(4) 0.025(5) -0.003(4) 0.003(4) -0.005(4)
C30 0.041(5) 0.028(5) 0.029(5) -0.007(4) -0.010(4) -0.015(4)
C31 0.048(6) 0.030(5) 0.037(6) 0.003(4) -0.021(5) -0.011(4)
C32 0.055(7) 0.028(5) 0.030(5) 0.001(4) -0.001(5) -0.011(5)
C33 0.051(7) 0.046(7) 0.067(8) 0.016(6) 0.016(6) -0.001(6)
C34 0.051(7) 0.038(6) 0.059(7) 0.012(5) 0.023(6) 0.010(5)
C35 0.023(4) 0.024(4) 0.033(5) -0.013(4) 0.006(4) -0.011(3)
C36 0.035(5) 0.030(5) 0.064(7) -0.017(5) -0.009(5) -0.005(4)
C37 0.052(7) 0.044(6) 0.076(8) -0.033(6) -0.011(6) -0.021(5)
C38 0.028(5) 0.059(7) 0.055(7) -0.026(6) -0.002(5) -0.017(5)
C39 0.024(5) 0.053(6) 0.034(5) -0.019(5) -0.010(4) 0.002(4)
C40 0.023(4) 0.034(5) 0.035(5) -0.018(4) -0.001(4) -0.004(4)
C41 0.022(4) 0.026(4) 0.025(4) -0.010(4) 0.006(3) -0.007(3)
C42 0.056(6) 0.037(5) 0.029(5) -0.020(4) 0.002(5) 0.007(5)
C43 0.087(9) 0.057(7) 0.042(7) -0.032(6) -0.010(6) 0.004(7)
C44 0.052(6) 0.047(6) 0.049(7) -0.034(5) 0.008(5) -0.006(5)
C45 0.041(6) 0.027(5) 0.052(6) -0.019(5) 0.013(5) -0.006(4)
C46 0.017(4) 0.026(4) 0.036(5) -0.013(4) 0.007(4) -0.003(3)
Ir2 0.02622(19) 0.01739(17) 0.01823(18) -0.00323(13) -0.00679(14) -0.00250(13)
I2 0.0304(3) 0.0257(3) 0.0244(3) -0.0066(2) -0.0102(2) -0.0034(2)
P2 0.0293(11) 0.0187(10) 0.0178(10) -0.0036(8) -0.0059(9) -0.0007(8)
O2 0.061(5) 0.018(3) 0.096(7) 0.007(4) -0.033(5) -0.018(3)
N5 0.015(3) 0.019(3) 0.021(3) -0.009(3) -0.006(3) 0.000(3)

N4 0.028(4) 0.030(4) 0.019(4) -0.009(3) -0.006(3) -0.003(3)
N6 0.028(4) 0.017(3) 0.020(3) -0.006(3) -0.009(3) 0.003(3)
C47 0.023(4) 0.037(5) 0.024(5) -0.002(4) -0.002(4) -0.007(4)
C48 0.029(5) 0.051(6) 0.017(4) 0.003(4) 0.003(4) -0.011(4)
C49 0.025(5) 0.049(6) 0.018(4) -0.013(4) 0.000(4) -0.003(4)
C50 0.023(4) 0.042(5) 0.021(4) -0.016(4) -0.004(3) -0.006(4)
C51 0.024(4) 0.032(5) 0.015(4) -0.007(3) -0.003(3) -0.006(4)
C52 0.018(4) 0.029(4) 0.010(4) -0.011(3) -0.002(3) 0.001(3)
C53 0.018(4) 0.020(4) 0.025(4) -0.013(3) 0.000(3) 0.003(3)
C54 0.018(4) 0.020(4) 0.015(4) -0.007(3) -0.006(3) 0.001(3)
C55 0.018(4) 0.024(4) 0.014(4) -0.008(3) -0.002(3) -0.001(3)
C56 0.016(4) 0.021(4) 0.013(4) -0.006(3) -0.005(3) 0.003(3)
C57 0.023(4) 0.025(4) 0.017(4) -0.009(3) -0.010(3) 0.003(3)
C58 0.024(4) 0.022(4) 0.022(4) -0.010(3) -0.007(3) 0.006(3)
C59 0.028(5) 0.032(5) 0.025(4) -0.013(4) -0.015(4) 0.019(4)
C60 0.047(6) 0.025(4) 0.019(4) -0.011(4) -0.013(4) 0.020(4)
C61 0.045(5) 0.016(4) 0.021(4) -0.007(3) -0.017(4) 0.010(4)
C62 0.016(4) 0.064(7) 0.025(5) -0.018(5) 0.008(4) -0.002(4)
C63 0.055(7) 0.073(8) 0.036(6) -0.008(6) 0.014(5) 0.037(6)
C64 0.047(6) 0.056(7) 0.031(5) -0.022(5) -0.004(5) -0.003(5)
C65 0.027(6) 0.145(14) 0.081(10) -0.074(10) 0.007(6) -0.009(7)
C66 0.015(4) 0.015(4) 0.036(5) -0.011(3) 0.000(3) 0.001(3)
C67 0.036(5) 0.022(4) 0.033(5) -0.005(4) 0.001(4) -0.003(4)
C68 0.027(5) 0.022(4) 0.041(5) -0.021(4) 0.006(4) 0.003(3)
C69 0.017(4) 0.030(5) 0.064(7) -0.020(5) -0.002(4) 0.001(4)
C70 0.035(5) 0.040(5) 0.031(5) -0.019(4) -0.007(4) 0.018(4)
C71 0.057(7) 0.057(7) 0.040(6) -0.003(5) 0.015(5) -0.001(6)
C72 0.045(7) 0.149(15) 0.089(11) -0.082(11) 0.030(7) -0.020(8)
C73 0.082(10) 0.058(8) 0.068(9) -0.025(7) 0.007(7) 0.034(7)
C74 0.034(5) 0.045(6) 0.034(5) -0.005(5) -0.018(4) -0.001(4)
C75 0.038(5) 0.032(5) 0.034(5) -0.012(4) -0.014(4) 0.015(4)
S2 0.0590(16) 0.0324(12) 0.0309(12) -0.0178(10) 0.0115(11) -0.0201(11)
F4 0.044(4) 0.151(8) 0.045(4) -0.011(4) 0.015(3) -0.032(4)
F5 0.115(8) 0.138(9) 0.071(6) -0.007(6) -0.045(6) -0.023(6)
F6 0.099(7) 0.104(7) 0.164(10) -0.095(7) 0.035(6) -0.062(6)
O6 0.046(5) 0.052(5) 0.098(7) 0.004(5) -0.017(5) 0.006(4)
O7 0.105(7) 0.127(8) 0.045(5) -0.038(5) 0.028(5) -0.073(6)
O8 0.121(7) 0.034(4) 0.068(6) -0.032(4) -0.018(5) 0.005(4)
C94 0.055(7) 0.060(7) 0.031(6) -0.013(5) 0.009(5) -0.011(6)
S1 0.0332(12) 0.0285(11) 0.0584(16) -0.0243(11) 0.0082(11) -0.0076(9)
F2 0.058(4) 0.042(3) 0.049(4) -0.018(3) -0.016(3) -0.013(3)
F3 0.059(4) 0.058(4) 0.086(5) -0.041(4) 0.033(4) -0.027(3)
F1 0.055(4) 0.045(4) 0.089(5) -0.022(4) -0.036(4) 0.014(3)
O4 0.041(4) 0.058(5) 0.048(4) -0.015(4) 0.005(3) -0.004(3)

O5 0.038(4) 0.024(3) 0.090(6) -0.007(4) -0.014(4) 0.007(3)
O3 0.044(4) 0.057(5) 0.069(5) -0.042(4) -0.014(4) 0.015(4)
C93 0.040(6) 0.027(5) 0.053(6) -0.018(5) -0.013(5) 0.000(4)
O1W 0.044(4) 0.038(4) 0.042(4) -0.006(3) -0.004(3) -0.015(3)
N7 0.036(5) 0.046(5) 0.038(5) -0.017(4) 0.008(4) -0.014(4)
C95 0.029(5) 0.034(5) 0.028(5) -0.010(4) 0.002(4) 0.002(4)
C96 0.032(5) 0.037(5) 0.047(6) 0.001(5) -0.003(5) -0.013(4)
N8 0.152(11) 0.164(11) 0.159(11) -0.036(8) -0.013(9) -0.006(9)
C97 0.095(9) 0.092(8) 0.089(8) -0.027(7) -0.018(7) 0.019(7)
C98 0.136(15) 0.045(7) 0.064(9) -0.007(7) -0.013(9) -0.007(8)
C80 0.024(8) 0.023(8) 0.028(8) -0.003(6) -0.012(7) -0.006(7)
C78 0.035(9) 0.027(8) 0.031(8) -0.005(6) -0.009(7) 0.002(7)
C79 0.038(9) 0.035(8) 0.039(9) -0.004(7) -0.017(7) 0.003(7)
C87 0.018(9) 0.025(9) 0.017(8) 0.003(6) 0.000(7) 0.000(7)
C92 0.040(8) 0.029(7) 0.022(7) -0.012(6) -0.011(7) 0.006(7)
C91 0.057(10) 0.047(9) 0.040(9) -0.016(7) -0.011(7) 0.011(7)
C90 0.048(11) 0.053(11) 0.052(11) -0.007(8) 0.007(8) 0.015(8)
C89 0.049(10) 0.055(10) 0.043(10) -0.005(8) -0.010(8) 0.011(8)
C88 0.023(8) 0.031(9) 0.019(8) 0.001(7) -0.006(6) 0.004(7)
F9 0.068(8) 0.093(9) 0.054(7) 0.024(6) -0.032(6) 0.009(6)
F8 0.072(8) 0.118(10) 0.115(10) -0.025(8) -0.043(7) -0.033(7)
C23 0.059(13) 0.042(12) 0.112(17) 0.007(12) 0.026(13) 0.002(10)
C23A 0.085(15) 0.021(9) 0.066(14) -0.009(9) 0.013(12) -0.022(10)
C78A 0.054(11) 0.051(10) 0.048(10) -0.019(8) -0.013(8) 0.013(8)
C79A 0.059(10) 0.045(9) 0.044(10) -0.015(7) -0.017(8) 0.016(8)
C80A 0.045(11) 0.050(11) 0.050(11) -0.011(8) -0.018(8) 0.012(8)
C87A 0.019(9) 0.010(8) 0.020(9) -0.009(6) -0.005(8) 0.000(7)
C91A 0.054(11) 0.036(10) 0.055(11) -0.010(8) 0.006(8) 0.005(8)
C90A 0.048(10) 0.047(10) 0.030(9) -0.010(8) -0.010(8) 0.018(8)
C89A 0.036(8) 0.045(9) 0.028(8) -0.006(7) -0.016(7) 0.001(7)
C92A 0.050(9) 0.030(8) 0.025(8) -0.018(6) -0.002(7) 0.001(7)
C88A 0.033(9) 0.025(8) 0.034(9) -0.011(7) 0.000(7) -0.008(7)
F9A 0.164(15) 0.178(15) 0.178(15) -0.053(10) -0.043(10) -0.011(10)
F8A 0.210(17) 0.200(17) 0.172(17) -0.051(10) -0.025(10) -0.003(10)
C76 0.032(5) 0.027(5) 0.034(5) -0.011(4) -0.007(4) 0.010(4)
C77 0.065(7) 0.037(6) 0.046(6) -0.014(5) -0.021(6) 0.018(5)
S3 0.077(2) 0.0487(17) 0.080(2) -0.0092(16) -0.0262(19) 0.0036(16)
O10 0.122(7) 0.050(5) 0.129(7) -0.008(5) -0.063(6) -0.007(5)
O9 0.133(8) 0.114(8) 0.179(10) -0.005(7) -0.041(7) -0.055(7)
C99 0.163(14) 0.171(14) 0.176(14) -0.039(10) -0.017(10) -0.019(10)
F7 0.133(7) 0.067(5) 0.137(7) -0.004(5) -0.025(6) 0.011(6)
S4 0.119(3) 0.055(2) 0.058(2) -0.0229(17) 0.020(2) -0.020(2)
N9 0.043(4) 0.023(3) 0.020(3) -0.009(3) -0.014(3) -0.012(3)
O13 0.116(8) 0.049(5) 0.081(6) -0.035(5) 0.012(6) -0.023(5)

```
C101 0.085(5) 0.057(5) 0.082(5) -0.036(4) -0.022(4) -0.011(4)
O12 0.200(10) 0.083(7) 0.130(8) -0.038(6) 0.015(7) -0.007(7)
F10 0.191(8) 0.159(7) 0.154(8) -0.087(7) -0.032(6) 0.006(6)
O14 0.135(8) 0.147(9) 0.137(8) -0.066(7) 0.035(7) -0.036(7)
F11 0.189(8) 0.140(6) 0.174(9) -0.033(6) -0.057(7) -0.035(6)
C100 0.119(8) 0.103(8) 0.111(9) -0.052(6) -0.023(6) -0.007(7)
F12 0.171(6) 0.188(6) 0.168(6) -0.047(4) -0.010(4) -0.026(5)
C81 0.039(4) 0.052(4) 0.033(4) -0.001(3) -0.015(3) -0.014(3)
C82 0.050(5) 0.074(5) 0.059(5) -0.012(4) -0.016(4) -0.015(4)
C86 0.058(5) 0.067(5) 0.059(5) -0.003(4) -0.025(4) -0.021(4)
O11 0.179(9) 0.112(8) 0.134(8) -0.059(7) 0.008(7) 0.041(7)
C85 0.082(6) 0.077(6) 0.069(5) -0.005(4) -0.025(4) -0.018(4)
C83 0.073(6) 0.090(6) 0.076(6) -0.017(4) -0.011(4) -0.011(4)
C84 0.067(5) 0.092(6) 0.072(6) -0.016(4) -0.022(4) -0.016(4)

_geom_special_details
;
 All esds (except the esd in the dihedral angle between two l.s. planes)
 are estimated using the full covariance matrix.  The cell esds are
taken
 into account individually in the estimation of esds in distances,
angles
 and torsion angles; correlations between esds in cell parameters are
only
 used when they are defined by crystal symmetry.  An approximate
(isotropic)
 treatment of cell esds is used for estimating esds involving l.s.
planes.
;

loop_
 _geom_bond_atom_site_label_1
 _geom_bond_atom_site_label_2
 _geom_bond_distance
 _geom_bond_site_symmetry_2
 _geom_bond_publ_flag
Ir1 C28 1.910(9) . ?
Ir1 N2 1.994(6) . ?
Ir1 N3 2.063(6) . ?
Ir1 N1 2.072(6) . ?
Ir1 P1 2.358(2) . ?
Ir1 I1 2.6968(6) . ?
P1 C35 1.812(9) . ?
P1 C41 1.818(8) . ?
```

P1 C29 1.827(9) . ?
O1 C28 1.129(10) . ?
N2 C10 1.344(10) . ?
N2 C6 1.354(10) . ?
N1 C1 1.317(10) . ?
N1 C5 1.386(10) . ?
N3 C15 1.348(10) . ?
N3 C11 1.369(9) . ?
C1 C2 1.386(12) . ?
C1 H1 0.9500 . ?
C2 C3 1.395(12) . ?
C2 H2 0.9500 . ?
C3 C4 1.394(12) . ?
C3 C16 1.525(12) . ?
C4 C5 1.378(11) . ?
C4 H4 0.9500 . ?
C5 C6 1.472(11) . ?
C6 C7 1.382(11) . ?
C7 C8 1.394(12) . ?
C7 H7 0.9500 . ?
C8 C9 1.398(12) . ?
C8 C20 1.524(11) . ?
C9 C10 1.375(11) . ?
C9 H9 0.9500 . ?
C10 C11 1.480(11) . ?
C11 C12 1.387(11) . ?
C12 C13 1.409(11) . ?
C12 H12 0.9500 . ?
C13 C14 1.389(11) . ?
C13 C24 1.520(11) . ?
C14 C15 1.365(12) . ?
C14 H14 0.9500 . ?
C15 H15 0.9500 . ?
C16 C19 1.485(13) . ?
C16 C17 1.534(13) . ?
C16 C18 1.543(12) . ?
C17 H17A 0.9800 . ?
C17 H17B 0.9800 . ?
C17 H17C 0.9800 . ?
C18 H18A 0.9800 . ?
C18 H18B 0.9800 . ?
C18 H18C 0.9800 . ?
C19 H19A 0.9800 . ?
C19 H19B 0.9800 . ?

C19 H19C 0.9800 . ?
C20 C22 1.510(16) . ?
C20 C23A 1.53(3) . ?
C20 C21 1.591(19) . ?
C20 C23 1.67(3) . ?
C21 H21A 0.9800 . ?
C21 H21B 0.9800 . ?
C21 H21C 0.9800 . ?
C22 H22A 0.9800 . ?
C22 H22B 0.9800 . ?
C22 H22C 0.9800 . ?
C24 C27 1.511(12) . ?
C24 C26 1.542(12) . ?
C24 C25 1.548(12) . ?
C25 H25A 0.9800 . ?
C25 H25B 0.9800 . ?
C25 H25C 0.9800 . ?
C26 H26A 0.9800 . ?
C26 H26B 0.9800 . ?
C26 H26C 0.9800 . ?
C27 H27A 0.9800 . ?
C27 H27B 0.9800 . ?
C27 H27C 0.9800 . ?
C29 C30 1.369(13) . ?
C29 C34 1.374(14) . ?
C30 C31 1.400(12) . ?
C30 H30 0.9500 . ?
C31 C32 1.370(14) . ?
C31 H31 0.9500 . ?
C32 C33 1.347(15) . ?
C32 H32 0.9500 . ?
C33 C34 1.390(15) . ?
C33 H33 0.9500 . ?
C34 H34 0.9500 . ?
C35 C40 1.385(12) . ?
C35 C36 1.396(12) . ?
C36 C37 1.383(14) . ?
C36 H36 0.9500 . ?
C37 C38 1.405(16) . ?
C37 H37 0.9500 . ?
C38 C39 1.386(14) . ?
C38 H38 0.9500 . ?
C39 C40 1.402(12) . ?
C39 H39 0.9500 . ?

C40 H40 0.9500 . ?
C41 C46 1.382(12) . ?
C41 C42 1.382(13) . ?
C42 C43 1.390(14) . ?
C42 H42 0.9500 . ?
C43 C44 1.375(16) . ?
C43 H43 0.9500 . ?
C44 C45 1.370(15) . ?
C44 H44 0.9500 . ?
C45 C46 1.395(12) . ?
C45 H45 0.9500 . ?
C46 H46 0.9500 . ?
Ir2 C74 1.914(10) . ?
Ir2 N5 1.993(6) . ?
Ir2 N4 2.061(7) . ?
Ir2 N6 2.067(7) . ?
Ir2 P2 2.374(2) . ?
Ir2 I2 2.7126(6) . ?
P2 C87 1.73(3) . ?
P2 C81 1.799(10) . ?
P2 C75 1.808(9) . ?
P2 C87A 1.88(3) . ?
O2 C74 1.131(12) . ?
N5 C52 1.332(10) . ?
N5 C56 1.348(10) . ?
N4 C47 1.346(11) . ?
N4 C51 1.371(11) . ?
N6 C61 1.356(10) . ?
N6 C57 1.371(10) . ?
C47 C48 1.372(13) . ?
C47 H47 0.9500 . ?
C48 C49 1.371(14) . ?
C48 H48 0.9500 . ?
C49 C50 1.390(12) . ?
C49 C62 1.523(12) . ?
C50 C51 1.379(12) . ?
C50 H50 0.9500 . ?
C51 C52 1.478(11) . ?
C52 C53 1.386(11) . ?
C53 C54 1.403(11) . ?
C53 H53 0.9500 . ?
C54 C55 1.397(11) . ?
C54 C66 1.526(10) . ?
C55 C56 1.363(11) . ?

C55 H55 0.9500 . ?
C56 C57 1.478(11) . ?
C57 C58 1.390(11) . ?
C58 C59 1.411(11) . ?
C58 H58 0.9500 . ?
C59 C60 1.367(13) . ?
C59 C70 1.517(13) . ?
C60 C61 1.395(13) . ?
C60 H60 0.9500 . ?
C61 H61 0.9500 . ?
C62 C63 1.505(15) . ?
C62 C64 1.541(13) . ?
C62 C65 1.545(14) . ?
C63 H63A 0.9800 . ?
C63 H63B 0.9800 . ?
C63 H63C 0.9800 . ?
C64 H64A 0.9800 . ?
C64 H64B 0.9800 . ?
C64 H64C 0.9800 . ?
C65 H65A 0.9800 . ?
C65 H65B 0.9800 . ?
C65 H65C 0.9800 . ?
C66 C67 1.523(12) . ?
C66 C68 1.529(10) . ?
C66 C69 1.549(11) . ?
C67 H67A 0.9800 . ?
C67 H67B 0.9800 . ?
C67 H67C 0.9800 . ?
C68 H68A 0.9800 . ?
C68 H68B 0.9800 . ?
C68 H68C 0.9800 . ?
C69 H69A 0.9800 . ?
C69 H69B 0.9800 . ?
C69 H69C 0.9800 . ?
C70 C72 1.509(16) . ?
C70 C71 1.513(14) . ?
C70 C73 1.536(14) . ?
C71 H71A 0.9800 . ?
C71 H71B 0.9800 . ?
C71 H71C 0.9800 . ?
C72 H72A 0.9800 . ?
C72 H72B 0.9800 . ?
C72 H72C 0.9800 . ?
C73 H73A 0.9800 . ?

C73 H73B 0.9800 . ?
C73 H73C 0.9800 . ?
C75 C76 1.392(13) . ?
C75 C80A 1.42(3) . ?
C75 C80 1.45(2) . ?
S2 O8 1.417(8) . ?
S2 O7 1.421(8) . ?
S2 O6 1.447(8) . ?
S2 C94 1.836(12) . ?
F4 C94 1.285(12) . ?
F5 C94 1.289(14) . ?
F6 C94 1.322(14) . ?
S1 O4 1.421(8) . ?
S1 O3 1.433(8) . ?
S1 O5 1.496(8) . ?
S1 C93 1.813(10) . ?
F2 C93 1.341(11) . ?
F3 C93 1.336(12) . ?
F1 C93 1.332(11) . ?
O1W H0AA 0.8695 . ?
O1W H0AB 0.8701 . ?
N7 C95 1.136(12) . ?
C95 C96 1.444(13) . ?
C96 H96A 0.9800 . ?
C96 H96B 0.9800 . ?
C96 H96C 0.9800 . ?
N8 C97 1.18(2) . ?
C97 C98 1.53(2) . ?
C98 H98A 0.9800 . ?
C98 H98B 0.9800 . ?
C98 H98C 0.9800 . ?
C80 C79 1.42(3) . ?
C78 C79 1.39(3) . ?
C78 C77 1.40(2) . ?
C87 C92 1.42(3) . ?
C87 C88 1.44(3) . ?
C92 C91 1.39(3) . ?
C91 C90 1.54(4) . ?
C90 C89 1.23(5) . ?
C89 C88 1.41(4) . ?
F9 C99 1.28(3) . ?
F8 C99 1.23(3) . ?
C78A C77 1.40(3) . ?
C78A C79A 1.42(4) . ?

C79A C80A 1.43(4) . ?
C87A C92A 1.39(3) . ?
C87A C88A 1.43(3) . ?
C91A C92A 1.38(4) . ?
C91A C90A 1.52(4) . ?
C90A C89A 1.33(4) . ?
C89A C88A 1.40(3) . ?
F9A F7 1.26(3) . ?
F9A C99 1.55(4) . ?
F8A F7 1.23(4) . ?
F8A C99 1.50(5) . ?
C76 C77 1.396(13) . ?
S3 O9 1.336(13) . ?
S3 O11 1.399(13) . ?
S3 O10 1.421(10) . ?
S3 C99 1.835(10) . ?
S3 F7 2.056(14) . ?
C99 F7 1.16(4) . ?
S4 N9 0.996(7) . ?
S4 O12 1.423(14) . ?
S4 O14 1.426(14) . ?
S4 O13 1.451(9) . ?
S4 C100 1.87(2) . ?
N9 C100 1.09(2) . ?
N9 O14 1.635(16) . ?
N9 F10 1.650(15) . ?
N9 O12 1.751(16) . ?
C101 F11 1.561(18) . ?
C101 F12 1.70(2) . ?
C101 C100 1.79(3) . ?
F10 C100 1.38(2) . ?
F11 C100 1.28(2) . ?
C100 F12 1.29(2) . ?
C81 C86 1.363(16) . ?
C81 C82 1.405(17) . ?
C82 C83 1.378(19) . ?
C82 H82 0.9500 . ?
C86 C85 1.461(19) . ?
C86 H86 0.9500 . ?
C85 C84 1.38(2) . ?
C85 H85 0.9500 . ?
C83 C84 1.29(2) . ?
C83 H83 0.9500 . ?
C84 H84 0.9500 . ?

```
loop_
 _geom_angle_atom_site_label_1
 _geom_angle_atom_site_label_2
 _geom_angle_atom_site_label_3
 _geom_angle
 _geom_angle_site_symmetry_1
 _geom_angle_site_symmetry_3
 _geom_angle_publ_flag
C28 Ir1 N2 175.3(3) . . ?
C28 Ir1 N3 99.9(3) . . ?
N2 Ir1 N3 79.0(2) . . ?
C28 Ir1 N1 101.1(3) . . ?
N2 Ir1 N1 79.6(3) . . ?
N3 Ir1 N1 158.3(2) . . ?
C28 Ir1 P1 92.1(2) . . ?
N2 Ir1 P1 92.49(18) . . ?
N3 Ir1 P1 90.66(18) . . ?
N1 Ir1 P1 93.90(18) . . ?
C28 Ir1 I1 88.7(2) . . ?
N2 Ir1 I1 86.68(18) . . ?
N3 Ir1 I1 87.26(17) . . ?
N1 Ir1 I1 87.88(17) . . ?
P1 Ir1 I1 177.87(5) . . ?
C35 P1 C41 113.2(4) . . ?
C35 P1 C29 103.1(4) . . ?
C41 P1 C29 104.2(4) . . ?
C35 P1 Ir1 109.5(3) . . ?
C41 P1 Ir1 108.7(3) . . ?
C29 P1 Ir1 118.1(3) . . ?
C10 N2 C6 122.6(7) . . ?
C10 N2 Ir1 119.1(5) . . ?
C6 N2 Ir1 118.2(5) . . ?
C1 N1 C5 118.0(7) . . ?
C1 N1 Ir1 128.4(5) . . ?
C5 N1 Ir1 113.4(5) . . ?
C15 N3 C11 119.1(7) . . ?
C15 N3 Ir1 126.8(5) . . ?
C11 N3 Ir1 114.0(5) . . ?
N1 C1 C2 123.2(8) . . ?
N1 C1 H1 118.4 . . ?
C2 C1 H1 118.4 . . ?
C1 C2 C3 120.2(8) . . ?
C1 C2 H2 119.9 . . ?
```

C3 C2 H2 119.9 . . ?
C4 C3 C2 116.5(7) . . ?
C4 C3 C16 121.3(8) . . ?
C2 C3 C16 122.2(8) . . ?
C5 C4 C3 121.1(7) . . ?
C5 C4 H4 119.5 . . ?
C3 C4 H4 119.5 . . ?
C4 C5 N1 120.9(7) . . ?
C4 C5 C6 123.9(7) . . ?
N1 C5 C6 115.1(7) . . ?
N2 C6 C7 119.1(7) . . ?
N2 C6 C5 113.6(7) . . ?
C7 C6 C5 127.2(7) . . ?
C6 C7 C8 120.5(8) . . ?
C6 C7 H7 119.7 . . ?
C8 C7 H7 119.7 . . ?
C7 C8 C9 117.7(7) . . ?
C7 C8 C20 120.7(8) . . ?
C9 C8 C20 121.5(8) . . ?
C10 C9 C8 120.8(8) . . ?
C10 C9 H9 119.6 . . ?
C8 C9 H9 119.6 . . ?
N2 C10 C9 119.3(7) . . ?
N2 C10 C11 112.4(6) . . ?
C9 C10 C11 128.3(7) . . ?
N3 C11 C12 121.0(7) . . ?
N3 C11 C10 115.4(7) . . ?
C12 C11 C10 123.6(7) . . ?
C11 C12 C13 120.1(7) . . ?
C11 C12 H12 119.9 . . ?
C13 C12 H12 119.9 . . ?
C14 C13 C12 116.5(7) . . ?
C14 C13 C24 119.7(7) . . ?
C12 C13 C24 123.8(7) . . ?
C15 C14 C13 121.7(7) . . ?
C15 C14 H14 119.1 . . ?
C13 C14 H14 119.1 . . ?
N3 C15 C14 121.4(7) . . ?
N3 C15 H15 119.3 . . ?
C14 C15 H15 119.3 . . ?
C19 C16 C3 107.5(7) . . ?
C19 C16 C17 109.5(8) . . ?
C3 C16 C17 111.6(8) . . ?
C19 C16 C18 111.1(8) . . ?

C3 C16 C18 109.3(7) . . ?
C17 C16 C18 107.9(8) . . ?
C16 C17 H17A 109.5 . . ?
C16 C17 H17B 109.5 . . ?
H17A C17 H17B 109.5 . . ?
C16 C17 H17C 109.5 . . ?
H17A C17 H17C 109.5 . . ?
H17B C17 H17C 109.5 . . ?
C16 C18 H18A 109.5 . . ?
C16 C18 H18B 109.5 . . ?
H18A C18 H18B 109.5 . . ?
C16 C18 H18C 109.5 . . ?
H18A C18 H18C 109.5 . . ?
H18B C18 H18C 109.5 . . ?
C16 C19 H19A 109.5 . . ?
C16 C19 H19B 109.5 . . ?
H19A C19 H19B 109.5 . . ?
C16 C19 H19C 109.5 . . ?
H19A C19 H19C 109.5 . . ?
H19B C19 H19C 109.5 . . ?
C22 C20 C8 112.3(8) . . ?
C22 C20 C23A 131.4(14) . . ?
C8 C20 C23A 105.7(11) . . ?
C22 C20 C21 96.6(10) . . ?
C8 C20 C21 109.8(9) . . ?
C23A C20 C21 98.0(14) . . ?
C22 C20 C23 94.8(14) . . ?
C8 C20 C23 106.8(12) . . ?
C21 C20 C23 133.7(15) . . ?
C20 C21 H21A 109.5 . . ?
C20 C21 H21B 109.5 . . ?
H21A C21 H21B 109.5 . . ?
C20 C21 H21C 109.5 . . ?
H21A C21 H21C 109.5 . . ?
H21B C21 H21C 109.5 . . ?
C20 C22 H22A 109.5 . . ?
C20 C22 H22B 109.5 . . ?
H22A C22 H22B 109.5 . . ?
C20 C22 H22C 109.5 . . ?
H22A C22 H22C 109.5 . . ?
H22B C22 H22C 109.5 . . ?
C27 C24 C13 109.6(7) . . ?
C27 C24 C26 109.0(7) . . ?
C13 C24 C26 112.2(7) . . ?

C27 C24 C25 110.3(8) . . ?
C13 C24 C25 107.4(7) . . ?
C26 C24 C25 108.4(7) . . ?
C24 C25 H25A 109.5 . . ?
C24 C25 H25B 109.5 . . ?
H25A C25 H25B 109.5 . . ?
C24 C25 H25C 109.5 . . ?
H25A C25 H25C 109.5 . . ?
H25B C25 H25C 109.5 . . ?
C24 C26 H26A 109.5 . . ?
C24 C26 H26B 109.5 . . ?
H26A C26 H26B 109.5 . . ?
C24 C26 H26C 109.5 . . ?
H26A C26 H26C 109.5 . . ?
H26B C26 H26C 109.5 . . ?
C24 C27 H27A 109.5 . . ?
C24 C27 H27B 109.5 . . ?
H27A C27 H27B 109.5 . . ?
C24 C27 H27C 109.5 . . ?
H27A C27 H27C 109.5 . . ?
H27B C27 H27C 109.5 . . ?
O1 C28 Ir1 178.8(8) . . ?
C30 C29 C34 119.9(9) . . ?
C30 C29 P1 124.0(7) . . ?
C34 C29 P1 116.0(7) . . ?
C29 C30 C31 119.5(9) . . ?
C29 C30 H30 120.2 . . ?
C31 C30 H30 120.2 . . ?
C32 C31 C30 120.1(10) . . ?
C32 C31 H31 120.0 . . ?
C30 C31 H31 120.0 . . ?
C33 C32 C31 119.8(9) . . ?
C33 C32 H32 120.1 . . ?
C31 C32 H32 120.1 . . ?
C32 C33 C34 121.0(11) . . ?
C32 C33 H33 119.5 . . ?
C34 C33 H33 119.5 . . ?
C29 C34 C33 119.5(11) . . ?
C29 C34 H34 120.2 . . ?
C33 C34 H34 120.2 . . ?
C40 C35 C36 119.6(8) . . ?
C40 C35 P1 125.1(6) . . ?
C36 C35 P1 115.2(7) . . ?
C37 C36 C35 120.4(10) . . ?

C37 C36 H36 119.8 . . ?
C35 C36 H36 119.8 . . ?
C36 C37 C38 120.0(9) . . ?
C36 C37 H37 120.0 . . ?
C38 C37 H37 120.0 . . ?
C39 C38 C37 119.7(9) . . ?
C39 C38 H38 120.1 . . ?
C37 C38 H38 120.1 . . ?
C38 C39 C40 119.8(9) . . ?
C38 C39 H39 120.1 . . ?
C40 C39 H39 120.1 . . ?
C35 C40 C39 120.4(8) . . ?
C35 C40 H40 119.8 . . ?
C39 C40 H40 119.8 . . ?
C46 C41 C42 118.8(8) . . ?
C46 C41 P1 122.8(7) . . ?
C42 C41 P1 117.5(7) . . ?
C41 C42 C43 120.9(10) . . ?
C41 C42 H42 119.6 . . ?
C43 C42 H42 119.6 . . ?
C44 C43 C42 119.8(11) . . ?
C44 C43 H43 120.1 . . ?
C42 C43 H43 120.1 . . ?
C45 C44 C43 119.9(9) . . ?
C45 C44 H44 120.1 . . ?
C43 C44 H44 120.1 . . ?
C44 C45 C46 120.4(10) . . ?
C44 C45 H45 119.8 . . ?
C46 C45 H45 119.8 . . ?
C41 C46 C45 120.2(9) . . ?
C41 C46 H46 119.9 . . ?
C45 C46 H46 119.9 . . ?
C74 Ir2 N5 174.9(3) . . ?
C74 Ir2 N4 100.8(4) . . ?
N5 Ir2 N4 79.1(3) . . ?
C74 Ir2 N6 100.5(4) . . ?
N5 Ir2 N6 79.2(3) . . ?
N4 Ir2 N6 157.8(3) . . ?
C74 Ir2 P2 94.2(3) . . ?
N5 Ir2 P2 90.92(18) . . ?
N4 Ir2 P2 92.46(19) . . ?
N6 Ir2 P2 92.03(18) . . ?
C74 Ir2 I2 86.9(3) . . ?
N5 Ir2 I2 88.03(17) . . ?

N4 Ir2 I2 87.47(18) . . ?
N6 Ir2 I2 87.65(18) . . ?
P2 Ir2 I2 178.94(5) . . ?
C87 P2 C81 104.5(9) . . ?
C87 P2 C75 104.0(9) . . ?
C81 P2 C75 105.1(5) . . ?
C81 P2 C87A 118.9(9) . . ?
C75 P2 C87A 101.2(8) . . ?
C87 P2 Ir2 116.6(8) . . ?
C81 P2 Ir2 109.3(3) . . ?
C75 P2 Ir2 116.2(3) . . ?
C87A P2 Ir2 106.4(7) . . ?
C52 N5 C56 122.1(7) . . ?
C52 N5 Ir2 118.8(5) . . ?
C56 N5 Ir2 119.0(5) . . ?
C47 N4 C51 117.6(7) . . ?
C47 N4 Ir2 128.4(6) . . ?
C51 N4 Ir2 114.0(5) . . ?
C61 N6 C57 118.3(7) . . ?
C61 N6 Ir2 128.0(6) . . ?
C57 N6 Ir2 113.6(5) . . ?
N4 C47 C48 121.6(8) . . ?
N4 C47 H47 119.2 . . ?
C48 C47 H47 119.2 . . ?
C49 C48 C47 122.7(8) . . ?
C49 C48 H48 118.6 . . ?
C47 C48 H48 118.6 . . ?
C48 C49 C50 115.2(8) . . ?
C48 C49 C62 123.5(8) . . ?
C50 C49 C62 121.2(9) . . ?
C51 C50 C49 121.7(9) . . ?
C51 C50 H50 119.1 . . ?
C49 C50 H50 119.1 . . ?
N4 C51 C50 121.1(8) . . ?
N4 C51 C52 114.8(7) . . ?
C50 C51 C52 124.1(8) . . ?
N5 C52 C53 120.0(7) . . ?
N5 C52 C51 113.3(7) . . ?
C53 C52 C51 126.6(7) . . ?
C52 C53 C54 119.6(7) . . ?
C52 C53 H53 120.2 . . ?
C54 C53 H53 120.2 . . ?
C55 C54 C53 118.0(7) . . ?
C55 C54 C66 120.0(7) . . ?

C53 C54 C66 122.0(7) . . ?
C56 C55 C54 120.2(7) . . ?
C56 C55 H55 119.9 . . ?
C54 C55 H55 119.9 . . ?
N5 C56 C55 120.2(7) . . ?
N5 C56 C57 112.3(7) . . ?
C55 C56 C57 127.5(7) . . ?
N6 C57 C58 121.2(7) . . ?
N6 C57 C56 115.8(7) . . ?
C58 C57 C56 122.9(7) . . ?
C57 C58 C59 120.7(8) . . ?
C57 C58 H58 119.6 . . ?
C59 C58 H58 119.6 . . ?
C60 C59 C58 116.5(8) . . ?
C60 C59 C70 124.0(8) . . ?
C58 C59 C70 119.6(8) . . ?
C59 C60 C61 121.9(8) . . ?
C59 C60 H60 119.0 . . ?
C61 C60 H60 119.0 . . ?
N6 C61 C60 121.3(8) . . ?
N6 C61 H61 119.4 . . ?
C60 C61 H61 119.4 . . ?
C63 C62 C49 113.5(8) . . ?
C63 C62 C64 108.4(9) . . ?
C49 C62 C64 106.8(8) . . ?
C63 C62 C65 109.6(10) . . ?
C49 C62 C65 109.9(9) . . ?
C64 C62 C65 108.4(9) . . ?
C62 C63 H63A 109.5 . . ?
C62 C63 H63B 109.5 . . ?
H63A C63 H63B 109.5 . . ?
C62 C63 H63C 109.5 . . ?
H63A C63 H63C 109.5 . . ?
H63B C63 H63C 109.5 . . ?
C62 C64 H64A 109.5 . . ?
C62 C64 H64B 109.5 . . ?
H64A C64 H64B 109.5 . . ?
C62 C64 H64C 109.5 . . ?
H64A C64 H64C 109.5 . . ?
H64B C64 H64C 109.5 . . ?
C62 C65 H65A 109.5 . . ?
C62 C65 H65B 109.5 . . ?
H65A C65 H65B 109.5 . . ?
C62 C65 H65C 109.5 . . ?

H65A C65 H65C 109.5 . . ?
H65B C65 H65C 109.5 . . ?
C67 C66 C54 108.2(6) . . ?
C67 C66 C68 109.5(7) . . ?
C54 C66 C68 112.6(7) . . ?
C67 C66 C69 109.4(7) . . ?
C54 C66 C69 108.5(6) . . ?
C68 C66 C69 108.6(7) . . ?
C66 C67 H67A 109.5 . . ?
C66 C67 H67B 109.5 . . ?
H67A C67 H67B 109.5 . . ?
C66 C67 H67C 109.5 . . ?
H67A C67 H67C 109.5 . . ?
H67B C67 H67C 109.5 . . ?
C66 C68 H68A 109.5 . . ?
C66 C68 H68B 109.5 . . ?
H68A C68 H68B 109.5 . . ?
C66 C68 H68C 109.5 . . ?
H68A C68 H68C 109.5 . . ?
H68B C68 H68C 109.5 . . ?
C66 C69 H69A 109.5 . . ?
C66 C69 H69B 109.5 . . ?
H69A C69 H69B 109.5 . . ?
C66 C69 H69C 109.5 . . ?
H69A C69 H69C 109.5 . . ?
H69B C69 H69C 109.5 . . ?
C72 C70 C71 110.5(11) . . ?
C72 C70 C59 107.7(8) . . ?
C71 C70 C59 108.7(8) . . ?
C72 C70 C73 112.1(10) . . ?
C71 C70 C73 107.3(9) . . ?
C59 C70 C73 110.5(9) . . ?
C70 C71 H71A 109.5 . . ?
C70 C71 H71B 109.5 . . ?
H71A C71 H71B 109.5 . . ?
C70 C71 H71C 109.5 . . ?
H71A C71 H71C 109.5 . . ?
H71B C71 H71C 109.5 . . ?
C70 C72 H72A 109.5 . . ?
C70 C72 H72B 109.5 . . ?
H72A C72 H72B 109.5 . . ?
C70 C72 H72C 109.5 . . ?
H72A C72 H72C 109.5 . . ?
H72B C72 H72C 109.5 . . ?

C70 C73 H73A 109.5 . . ?
C70 C73 H73B 109.5 . . ?
H73A C73 H73B 109.5 . . ?
C70 C73 H73C 109.5 . . ?
H73A C73 H73C 109.5 . . ?
H73B C73 H73C 109.5 . . ?
O2 C74 Ir2 176.4(9) . . ?
C76 C75 C80A 121.6(14) . . ?
C76 C75 C80 117.8(11) . . ?
C76 C75 P2 123.5(7) . . ?
C80A C75 P2 112.6(13) . . ?
C80 C75 P2 118.2(10) . . ?
O8 S2 O7 119.4(6) . . ?
O8 S2 O6 113.2(6) . . ?
O7 S2 O6 112.4(7) . . ?
O8 S2 C94 102.2(6) . . ?
O7 S2 C94 104.3(5) . . ?
O6 S2 C94 102.8(5) . . ?
F4 C94 F5 110.6(11) . . ?
F4 C94 F6 107.6(10) . . ?
F5 C94 F6 104.4(11) . . ?
F4 C94 S2 111.9(8) . . ?
F5 C94 S2 111.4(9) . . ?
F6 C94 S2 110.6(8) . . ?
O4 S1 O3 116.7(5) . . ?
O4 S1 O5 115.2(4) . . ?
O3 S1 O5 113.6(5) . . ?
O4 S1 C93 104.2(5) . . ?
O3 S1 C93 102.7(4) . . ?
O5 S1 C93 101.7(5) . . ?
F1 C93 F3 110.7(9) . . ?
F1 C93 F2 107.6(7) . . ?
F3 C93 F2 106.5(8) . . ?
F1 C93 S1 111.1(7) . . ?
F3 C93 S1 109.4(6) . . ?
F2 C93 S1 111.3(7) . . ?
H0AA O1W H0AB 109.5 . . ?
N7 C95 C96 178.7(11) . . ?
C95 C96 H96A 109.5 . . ?
C95 C96 H96B 109.5 . . ?
H96A C96 H96B 109.5 . . ?
C95 C96 H96C 109.5 . . ?
H96A C96 H96C 109.5 . . ?
H96B C96 H96C 109.5 . . ?

N8 C97 C98 167(2) . . ?
C97 C98 H98A 109.5 . . ?
C97 C98 H98B 109.5 . . ?
H98A C98 H98B 109.5 . . ?
C97 C98 H98C 109.5 . . ?
H98A C98 H98C 109.5 . . ?
H98B C98 H98C 109.5 . . ?
C79 C80 C75 120.3(18) . . ?
C79 C78 C77 119.9(17) . . ?
C78 C79 C80 119.4(19) . . ?
C92 C87 C88 120(2) . . ?
C92 C87 P2 125.2(18) . . ?
C88 C87 P2 115(2) . . ?
C91 C92 C87 119(2) . . ?
C92 C91 C90 117(2) . . ?
C89 C90 C91 121(3) . . ?
C90 C89 C88 123(3) . . ?
C89 C88 C87 118(3) . . ?
C77 C78A C79A 123(2) . . ?
C78A C79A C80A 118(2) . . ?
C75 C80A C79A 117(2) . . ?
C92A C87A C88A 122(2) . . ?
C92A C87A P2 125(2) . . ?
C88A C87A P2 112.3(16) . . ?
C92A C91A C90A 112(2) . . ?
C89A C90A C91A 122(2) . . ?
C90A C89A C88A 124(2) . . ?
C91A C92A C87A 124(2) . . ?
C89A C88A C87A 115(2) . . ?
F7 F9A C99 47.7(19) . . ?
F7 F8A C99 49(2) . . ?
C75 C76 C77 120.8(9) . . ?
C76 C77 C78 120.6(12) . . ?
C76 C77 C78A 116.1(14) . . ?
O9 S3 O11 112.8(11) . . ?
O9 S3 O10 121.1(9) . . ?
O11 S3 O10 110.7(9) . . ?
O9 S3 C99 110.2(13) . . ?
O11 S3 C99 91.7(14) . . ?
O10 S3 C99 106.0(12) . . ?
O9 S3 F7 98.4(9) . . ?
O11 S3 F7 125.4(8) . . ?
O10 S3 F7 86.8(6) . . ?
C99 S3 F7 34.2(12) . . ?

F7 C99 F8 104(3) . . ?
F7 C99 F9 123(3) . . ?
F8 C99 F9 114(3) . . ?
F7 C99 F8A 53(2) . . ?
F7 C99 F9A 53(2) . . ?
F8A C99 F9A 91(3) . . ?
F7 C99 S3 83.4(14) . . ?
F8 C99 S3 115(2) . . ?
F9 C99 S3 114(2) . . ?
F8A C99 S3 100(2) . . ?
F9A C99 S3 113(2) . . ?
C99 F7 F8A 78(2) . . ?
C99 F7 F9A 79(2) . . ?
F8A F7 F9A 122(3) . . ?
C99 F7 S3 62.4(8) . . ?
F8A F7 S3 100.0(19) . . ?
F9A F7 S3 115(2) . . ?
N9 S4 O12 91.0(8) . . ?
N9 S4 O14 82.9(8) . . ?
O12 S4 O14 119.7(10) . . ?
N9 S4 O13 129.9(8) . . ?
O12 S4 O13 115.4(7) . . ?
O14 S4 O13 113.8(7) . . ?
N9 S4 C100 27.9(8) . . ?
O12 S4 C100 100.6(10) . . ?
O14 S4 C100 100.6(10) . . ?
O13 S4 C100 102.6(8) . . ?
S4 N9 C100 126.7(14) . . ?
S4 N9 O14 59.9(7) . . ?
C100 N9 O14 137.6(15) . . ?
S4 N9 F10 170.9(9) . . ?
C100 N9 F10 56.4(12) . . ?
O14 N9 F10 124.9(9) . . ?
S4 N9 O12 54.4(6) . . ?
C100 N9 O12 125.6(16) . . ?
O14 N9 O12 93.3(9) . . ?
F10 N9 O12 116.5(8) . . ?
F11 C101 F12 82.1(10) . . ?
F11 C101 C100 44.2(9) . . ?
F12 C101 C100 43.4(9) . . ?
S4 O12 N9 34.7(4) . . ?
C100 F10 N9 41.0(10) . . ?
S4 O14 N9 37.2(5) . . ?
C100 F11 C101 77.4(14) . . ?

```
N9 C100 F11 117(2) . . ?
N9 C100 F12 125(2) . . ?
F11 C100 F12 113(2) . . ?
N9 C100 F10 82.6(16) . . ?
F11 C100 F10 104.7(19) . . ?
F12 C100 F10 105.8(18) . . ?
N9 C100 C101 168(2) . . ?
F11 C100 C101 58.3(12) . . ?
F12 C100 C101 64.5(13) . . ?
F10 C100 C101 88.3(13) . . ?
N9 C100 S4 25.3(7) . . ?
F11 C100 S4 113.4(15) . . ?
F12 C100 S4 111.9(17) . . ?
F10 C100 S4 107.5(15) . . ?
C101 C100 S4 164.0(13) . . ?
C100 F12 C101 72.1(14) . . ?
C86 C81 C82 119.7(11) . . ?
C86 C81 P2 123.9(9) . . ?
C82 C81 P2 116.2(9) . . ?
C83 C82 C81 122.5(13) . . ?
C83 C82 H82 118.8 . . ?
C81 C82 H82 118.8 . . ?
C81 C86 C85 117.1(13) . . ?
C81 C86 H86 121.5 . . ?
C85 C86 H86 121.5 . . ?
C84 C85 C86 117.8(14) . . ?
C84 C85 H85 121.1 . . ?
C86 C85 H85 121.1 . . ?
C84 C83 C82 117.3(16) . . ?
C84 C83 H83 121.4 . . ?
C82 C83 H83 121.4 . . ?
C83 C84 C85 125.7(16) . . ?
C83 C84 H84 117.2 . . ?
C85 C84 H84 117.2 . . ?

loop_
 _geom_torsion_atom_site_label_1
 _geom_torsion_atom_site_label_2
 _geom_torsion_atom_site_label_3
 _geom_torsion_atom_site_label_4
 _geom_torsion
 _geom_torsion_site_symmetry_1
 _geom_torsion_site_symmetry_2
 _geom_torsion_site_symmetry_3
```

_geom_torsion_site_symmetry_4
_geom_torsion_publ_flag
C5 N1 C1 C2 -1.4(12) . . . . ?
Ir1 N1 C1 C2 -176.0(6) . . . . ?
N1 C1 C2 C3 -1.4(13) . . . . ?
C1 C2 C3 C4 1.8(12) . . . . ?
C1 C2 C3 C16 -178.7(8) . . . . ?
C2 C3 C4 C5 0.6(12) . . . . ?
C16 C3 C4 C5 -178.9(8) . . . . ?
C3 C4 C5 N1 -3.4(12) . . . . ?
C3 C4 C5 C6 171.8(7) . . . . ?
C1 N1 C5 C4 3.8(11) . . . . ?
Ir1 N1 C5 C4 179.2(6) . . . . ?
C1 N1 C5 C6 -171.8(7) . . . . ?
Ir1 N1 C5 C6 3.5(8) . . . . ?
C10 N2 C6 C7 -1.0(11) . . . . ?
Ir1 N2 C6 C7 -176.8(6) . . . . ?
C10 N2 C6 C5 176.0(7) . . . . ?
Ir1 N2 C6 C5 0.3(8) . . . . ?
C4 C5 C6 N2 -178.1(7) . . . . ?
N1 C5 C6 N2 -2.6(9) . . . . ?
C4 C5 C6 C7 -1.3(13) . . . . ?
N1 C5 C6 C7 174.2(7) . . . . ?
N2 C6 C7 C8 0.7(12) . . . . ?
C5 C6 C7 C8 -175.9(8) . . . . ?
C6 C7 C8 C9 0.0(12) . . . . ?
C6 C7 C8 C20 176.4(8) . . . . ?
C7 C8 C9 C10 -0.4(13) . . . . ?
C20 C8 C9 C10 -176.8(8) . . . . ?
C6 N2 C10 C9 0.6(11) . . . . ?
Ir1 N2 C10 C9 176.3(6) . . . . ?
C6 N2 C10 C11 -178.1(7) . . . . ?
Ir1 N2 C10 C11 -2.4(8) . . . . ?
C8 C9 C10 N2 0.1(12) . . . . ?
C8 C9 C10 C11 178.6(8) . . . . ?
C15 N3 C11 C12 -1.4(11) . . . . ?
Ir1 N3 C11 C12 179.3(6) . . . . ?
C15 N3 C11 C10 178.6(7) . . . . ?
Ir1 N3 C11 C10 -0.6(8) . . . . ?
N2 C10 C11 N3 1.9(9) . . . . ?
C9 C10 C11 N3 -176.6(8) . . . . ?
N2 C10 C11 C12 -178.0(7) . . . . ?
C9 C10 C11 C12 3.4(13) . . . . ?
N3 C11 C12 C13 0.7(12) . . . . ?

C10 C11 C12 C13 -179.4(7) . . . . ?
C11 C12 C13 C14 0.5(11) . . . . ?
C11 C12 C13 C24 179.4(7) . . . . ?
C12 C13 C14 C15 -1.0(12) . . . . ?
C24 C13 C14 C15 -179.9(8) . . . . ?
C11 N3 C15 C14 0.9(12) . . . . ?
Ir1 N3 C15 C14 -179.9(6) . . . . ?
C13 C14 C15 N3 0.3(13) . . . . ?
C4 C3 C16 C19 -76.5(10) . . . . ?
C2 C3 C16 C19 104.0(10) . . . . ?
C4 C3 C16 C17 163.4(8) . . . . ?
C2 C3 C16 C17 -16.1(12) . . . . ?
C4 C3 C16 C18 44.1(11) . . . . ?
C2 C3 C16 C18 -135.4(9) . . . . ?
C7 C8 C20 C22 142.5(11) . . . . ?
C9 C8 C20 C22 -41.1(14) . . . . ?
C7 C8 C20 C23A -68.5(16) . . . . ?
C9 C8 C20 C23A 107.9(15) . . . . ?
C7 C8 C20 C21 36.3(14) . . . . ?
C9 C8 C20 C21 -147.3(11) . . . . ?
C7 C8 C20 C23 -114.8(15) . . . . ?
C9 C8 C20 C23 61.5(16) . . . . ?
C14 C13 C24 C27 -62.9(10) . . . . ?
C12 C13 C24 C27 118.3(9) . . . . ?
C14 C13 C24 C26 175.9(8) . . . . ?
C12 C13 C24 C26 -2.9(11) . . . . ?
C14 C13 C24 C25 56.9(10) . . . . ?
C12 C13 C24 C25 -121.9(9) . . . . ?
C35 P1 C29 C30 -132.8(8) . . . . ?
C41 P1 C29 C30 108.8(8) . . . . ?
Ir1 P1 C29 C30 -11.9(9) . . . . ?
C35 P1 C29 C34 49.7(9) . . . . ?
C41 P1 C29 C34 -68.7(9) . . . . ?
Ir1 P1 C29 C34 170.6(8) . . . . ?
C34 C29 C30 C31 -1.1(14) . . . . ?
P1 C29 C30 C31 -178.5(7) . . . . ?
C29 C30 C31 C32 0.4(14) . . . . ?
C30 C31 C32 C33 0.9(16) . . . . ?
C31 C32 C33 C34 -1(2) . . . . ?
C30 C29 C34 C33 0.7(18) . . . . ?
P1 C29 C34 C33 178.3(10) . . . . ?
C32 C33 C34 C29 1(2) . . . . ?
C41 P1 C35 C40 -23.6(9) . . . . ?
C29 P1 C35 C40 -135.5(8) . . . . ?

Ir1 P1 C35 C40 97.9(8) . . . . ?
C41 P1 C35 C36 160.1(7) . . . . ?
C29 P1 C35 C36 48.2(8) . . . . ?
Ir1 P1 C35 C36 -78.3(7) . . . . ?
C40 C35 C36 C37 0.1(15) . . . . ?
P1 C35 C36 C37 176.6(9) . . . . ?
C35 C36 C37 C38 0.5(17) . . . . ?
C36 C37 C38 C39 -0.9(17) . . . . ?
C37 C38 C39 C40 0.6(16) . . . . ?
C36 C35 C40 C39 -0.3(14) . . . . ?
P1 C35 C40 C39 -176.4(7) . . . . ?
C38 C39 C40 C35 -0.1(14) . . . . ?
C35 P1 C41 C46 50.5(8) . . . . ?
C29 P1 C41 C46 161.7(7) . . . . ?
Ir1 P1 C41 C46 -71.5(7) . . . . ?
C35 P1 C41 C42 -141.0(7) . . . . ?
C29 P1 C41 C42 -29.8(8) . . . . ?
Ir1 P1 C41 C42 97.0(7) . . . . ?
C46 C41 C42 C43 2.3(15) . . . . ?
P1 C41 C42 C43 -166.6(9) . . . . ?
C41 C42 C43 C44 -0.3(18) . . . . ?
C42 C43 C44 C45 -2.6(18) . . . . ?
C43 C44 C45 C46 3.5(16) . . . . ?
C42 C41 C46 C45 -1.5(12) . . . . ?
P1 C41 C46 C45 166.9(7) . . . . ?
C44 C45 C46 C41 -1.4(13) . . . . ?
C51 N4 C47 C48 1.6(12) . . . . ?
Ir2 N4 C47 C48 -180.0(6) . . . . ?
N4 C47 C48 C49 0.7(14) . . . . ?
C47 C48 C49 C50 -1.9(13) . . . . ?
C47 C48 C49 C62 175.1(8) . . . . ?
C48 C49 C50 C51 0.9(12) . . . . ?
C62 C49 C50 C51 -176.1(8) . . . . ?
C47 N4 C51 C50 -2.5(11) . . . . ?
Ir2 N4 C51 C50 178.8(6) . . . . ?
C47 N4 C51 C52 176.1(7) . . . . ?
Ir2 N4 C51 C52 -2.6(8) . . . . ?
C49 C50 C51 N4 1.2(12) . . . . ?
C49 C50 C51 C52 -177.2(7) . . . . ?
C56 N5 C52 C53 1.3(11) . . . . ?
Ir2 N5 C52 C53 178.1(6) . . . . ?
C56 N5 C52 C51 -175.5(6) . . . . ?
Ir2 N5 C52 C51 1.3(8) . . . . ?
N4 C51 C52 N5 0.9(10) . . . . ?

C50 C51 C52 N5 179.5(7) . . . . ?
N4 C51 C52 C53 -175.6(7) . . . . ?
C50 C51 C52 C53 2.9(13) . . . . ?
N5 C52 C53 C54 -1.6(11) . . . . ?
C51 C52 C53 C54 174.7(7) . . . . ?
C52 C53 C54 C55 0.5(11) . . . . ?
C52 C53 C54 C66 -177.2(7) . . . . ?
C53 C54 C55 C56 0.9(11) . . . . ?
C66 C54 C55 C56 178.7(7) . . . . ?
C52 N5 C56 C55 0.1(11) . . . . ?
Ir2 N5 C56 C55 -176.6(5) . . . . ?
C52 N5 C56 C57 178.5(6) . . . . ?
Ir2 N5 C56 C57 1.7(8) . . . . ?
C54 C55 C56 N5 -1.3(11) . . . . ?
C54 C55 C56 C57 -179.3(7) . . . . ?
C61 N6 C57 C58 2.1(11) . . . . ?
Ir2 N6 C57 C58 179.7(6) . . . . ?
C61 N6 C57 C56 -176.7(7) . . . . ?
Ir2 N6 C57 C56 0.9(8) . . . . ?
N5 C56 C57 N6 -1.7(9) . . . . ?
C55 C56 C57 N6 176.5(7) . . . . ?
N5 C56 C57 C58 179.5(7) . . . . ?
C55 C56 C57 C58 -2.3(12) . . . . ?
N6 C57 C58 C59 -0.6(12) . . . . ?
C56 C57 C58 C59 178.1(7) . . . . ?
C57 C58 C59 C60 -1.5(12) . . . . ?
C57 C58 C59 C70 179.4(7) . . . . ?
C58 C59 C60 C61 2.2(12) . . . . ?
C70 C59 C60 C61 -178.7(8) . . . . ?
C57 N6 C61 C60 -1.4(11) . . . . ?
Ir2 N6 C61 C60 -178.7(6) . . . . ?
C59 C60 C61 N6 -0.8(13) . . . . ?
C48 C49 C62 C63 160.5(10) . . . . ?
C50 C49 C62 C63 -22.6(13) . . . . ?
C48 C49 C62 C64 -80.1(11) . . . . ?
C50 C49 C62 C64 96.8(10) . . . . ?
C48 C49 C62 C65 37.4(13) . . . . ?
C50 C49 C62 C65 -145.8(10) . . . . ?
C55 C54 C66 C67 -62.1(9) . . . . ?
C53 C54 C66 C67 115.6(8) . . . . ?
C55 C54 C66 C68 176.8(7) . . . . ?
C53 C54 C66 C68 -5.5(11) . . . . ?
C55 C54 C66 C69 56.5(10) . . . . ?
C53 C54 C66 C69 -125.8(8) . . . . ?

C60 C59 C70 C72 119.9(11) . . . . ?
C58 C59 C70 C72 -61.1(12) . . . . ?
C60 C59 C70 C71 -120.4(10) . . . . ?
C58 C59 C70 C71 58.7(11) . . . . ?
C60 C59 C70 C73 -2.9(13) . . . . ?
C58 C59 C70 C73 176.1(9) . . . . ?
C87 P2 C75 C76 -156.7(12) . . . . ?
C81 P2 C75 C76 93.8(9) . . . . ?
C87A P2 C75 C76 -141.9(11) . . . . ?
Ir2 P2 C75 C76 -27.2(10) . . . . ?
C81 P2 C75 C80A -69.2(16) . . . . ?
C87A P2 C75 C80A 55.1(17) . . . . ?
Ir2 P2 C75 C80A 169.8(15) . . . . ?
C87 P2 C75 C80 15.3(15) . . . . ?
C81 P2 C75 C80 -94.3(12) . . . . ?
Ir2 P2 C75 C80 144.8(11) . . . . ?
O8 S2 C94 F4 59.7(10) . . . . ?
O7 S2 C94 F4 -175.3(10) . . . . ?
O6 S2 C94 F4 -57.8(10) . . . . ?
O8 S2 C94 F5 -64.6(10) . . . . ?
O7 S2 C94 F5 60.4(11) . . . . ?
O6 S2 C94 F5 177.9(9) . . . . ?
O8 S2 C94 F6 179.7(9) . . . . ?
O7 S2 C94 F6 -55.3(11) . . . . ?
O6 S2 C94 F6 62.2(10) . . . . ?
O4 S1 C93 F1 61.9(8) . . . . ?
O3 S1 C93 F1 -60.3(8) . . . . ?
O5 S1 C93 F1 -178.0(7) . . . . ?
O4 S1 C93 F3 -175.5(7) . . . . ?
O3 S1 C93 F3 62.3(8) . . . . ?
O5 S1 C93 F3 -55.4(8) . . . . ?
O4 S1 C93 F2 -58.0(8) . . . . ?
O3 S1 C93 F2 179.8(7) . . . . ?
O5 S1 C93 F2 62.1(8) . . . . ?
C76 C75 C80 C79 -4(2) . . . . ?
P2 C75 C80 C79 -176.9(16) . . . . ?
C77 C78 C79 C80 -7(3) . . . . ?
C75 C80 C79 C78 4(3) . . . . ?
C81 P2 C87 C92 -5(2) . . . . ?
C75 P2 C87 C92 -115.4(19) . . . . ?
Ir2 P2 C87 C92 115.4(18) . . . . ?
C81 P2 C87 C88 176.2(15) . . . . ?
C75 P2 C87 C88 66.2(18) . . . . ?
Ir2 P2 C87 C88 -63.1(18) . . . . ?

C88 C87 C92 C91 -2(3) . . . . ?
P2 C87 C92 C91 179.7(17) . . . . ?
C87 C92 C91 C90 7(3) . . . . ?
C92 C91 C90 C89 -14(4) . . . . ?
C91 C90 C89 C88 13(4) . . . . ?
C90 C89 C88 C87 -7(4) . . . . ?
C92 C87 C88 C89 1(3) . . . . ?
P2 C87 C88 C89 179.3(17) . . . . ?
C77 C78A C79A C80A 13(5) . . . . ?
C76 C75 C80A C79A 14(3) . . . . ?
P2 C75 C80A C79A 177(2) . . . . ?
C78A C79A C80A C75 -11(4) . . . . ?
C81 P2 C87A C92A -15(2) . . . . ?
C75 P2 C87A C92A -129(2) . . . . ?
Ir2 P2 C87A C92A 109(2) . . . . ?
C81 P2 C87A C88A 162.1(15) . . . . ?
C75 P2 C87A C88A 47.8(18) . . . . ?
Ir2 P2 C87A C88A -74.1(17) . . . . ?
C92A C91A C90A C89A -14(4) . . . . ?
C91A C90A C89A C88A 10(4) . . . . ?
C90A C91A C92A C87A 11(4) . . . . ?
C88A C87A C92A C91A -4(4) . . . . ?
P2 C87A C92A C91A 172(2) . . . . ?
C90A C89A C88A C87A -2(3) . . . . ?
C92A C87A C88A C89A -1(3) . . . . ?
P2 C87A C88A C89A -177.9(15) . . . . ?
C80A C75 C76 C77 -17(2) . . . . ?
C80 C75 C76 C77 9.2(17) . . . . ?
P2 C75 C76 C77 -178.9(8) . . . . ?
C75 C76 C77 C78 -13(2) . . . . ?
C75 C76 C77 C78A 17(2) . . . . ?
C79 C78 C77 C76 12(3) . . . . ?
C79A C78A C77 C76 -15(4) . . . . ?
F7 F8A C99 F9A -40(2) . . . . ?
F7 F8A C99 S3 73(2) . . . . ?
F7 F9A C99 F8A 40(2) . . . . ?
F7 F9A C99 S3 -62(2) . . . . ?
O9 S3 C99 F7 75(2) . . . . ?
O11 S3 C99 F7 -170.3(19) . . . . ?
O10 S3 C99 F7 -58(2) . . . . ?
O9 S3 C99 F8 177(3) . . . . ?
O11 S3 C99 F8 -67(3) . . . . ?
O10 S3 C99 F8 45(3) . . . . ?
F7 S3 C99 F8 103(4) . . . . ?

O9 S3 C99 F9 -49(3) . . . . ?
O11 S3 C99 F9 66(3) . . . . ?
O10 S3 C99 F9 178(2) . . . . ?
F7 S3 C99 F9 -123(4) . . . . ?
O9 S3 C99 F8A 24(3) . . . . ?
O11 S3 C99 F8A 139(3) . . . . ?
O10 S3 C99 F8A -109(2) . . . . ?
F7 S3 C99 F8A -51(2) . . . . ?
O9 S3 C99 F9A 120(3) . . . . ?
O11 S3 C99 F9A -125(3) . . . . ?
O10 S3 C99 F9A -13(3) . . . . ?
F7 S3 C99 F9A 45(2) . . . . ?
F9A C99 F7 F8A 126(3) . . . . ?
S3 C99 F7 F8A -108(2) . . . . ?
F8A C99 F7 F9A -126(3) . . . . ?
S3 C99 F7 F9A 125(2) . . . . ?
F8 C99 F7 S3 -114(2) . . . . ?
F9 C99 F7 S3 114(2) . . . . ?
F8A C99 F7 S3 108(2) . . . . ?
F9A C99 F7 S3 -125(2) . . . . ?
C99 F8A F7 F9A 69(3) . . . . ?
C99 F8A F7 S3 -58.7(10) . . . . ?
C99 F9A F7 F8A -68(3) . . . . ?
C99 F9A F7 S3 52.7(15) . . . . ?
O12 S4 N9 C100 111.0(19) . . . . ?
O14 S4 N9 C100 -129.2(19) . . . . ?
O13 S4 N9 C100 -14(2) . . . . ?
O12 S4 N9 O14 -119.8(10) . . . . ?
O13 S4 N9 O14 115.2(10) . . . . ?
C100 S4 N9 O14 129.2(19) . . . . ?
O14 S4 N9 O12 119.8(10) . . . . ?
O13 S4 N9 O12 -125.0(10) . . . . ?
C100 S4 N9 O12 -111.0(19) . . . . ?
O14 S4 O12 N9 -82.3(10) . . . . ?
O13 S4 O12 N9 135.9(10) . . . . ?
C100 S4 O12 N9 26.4(9) . . . . ?
C100 N9 O12 S4 -113.1(17) . . . . ?
O14 N9 O12 S4 48.8(7) . . . . ?
F10 N9 O12 S4 -179.2(11) . . . . ?
O14 N9 F10 C100 -128.1(19) . . . . ?
O12 N9 F10 C100 116.7(18) . . . . ?
O12 S4 O14 N9 87.1(10) . . . . ?
O13 S4 O14 N9 -130.7(9) . . . . ?
C100 S4 O14 N9 -21.7(8) . . . . ?

C100 N9 O14 S4 113(2) . . . . ?
F10 N9 O14 S4 -170.8(11) . . . . ?
O12 N9 O14 S4 -44.9(7) . . . . ?
F12 C101 F11 C100 24.6(13) . . . . ?
S4 N9 C100 F11 88(2) . . . . ?
O14 N9 C100 F11 4(3) . . . . ?
F10 N9 C100 F11 -103(2) . . . . ?
O12 N9 C100 F11 156.6(15) . . . . ?
S4 N9 C100 F12 -65(3) . . . . ?
O14 N9 C100 F12 -149.1(18) . . . . ?
F10 N9 C100 F12 104(3) . . . . ?
O12 N9 C100 F12 4(3) . . . . ?
S4 N9 C100 F10 -169.5(11) . . . . ?
O14 N9 C100 F10 106.9(18) . . . . ?
O12 N9 C100 F10 -100.6(15) . . . . ?
S4 N9 C100 C101 152(9) . . . . ?
O14 N9 C100 C101 68(10) . . . . ?
F10 N9 C100 C101 -39(9) . . . . ?
O12 N9 C100 C101 -140(9) . . . . ?
O14 N9 C100 S4 -83.7(19) . . . . ?
F10 N9 C100 S4 169.5(11) . . . . ?
O12 N9 C100 S4 68.9(15) . . . . ?
C101 F11 C100 N9 168(2) . . . . ?
C101 F11 C100 F12 -36.0(18) . . . . ?
C101 F11 C100 F10 78.6(16) . . . . ?
C101 F11 C100 S4 -164.5(15) . . . . ?
N9 F10 C100 F11 116(2) . . . . ?
N9 F10 C100 F12 -124(3) . . . . ?
N9 F10 C100 C101 173(2) . . . . ?
N9 F10 C100 S4 -4.7(5) . . . . ?
F11 C101 C100 N9 -70(10) . . . . ?
F12 C101 C100 N9 147(10) . . . . ?
F12 C101 C100 F11 -143.1(19) . . . . ?
F11 C101 C100 F12 143.1(19) . . . . ?
F11 C101 C100 F10 -108.5(19) . . . . ?
F12 C101 C100 F10 108.4(18) . . . . ?
F11 C101 C100 S4 63(6) . . . . ?
F12 C101 C100 S4 -81(6) . . . . ?
O12 S4 C100 N9 -71.7(19) . . . . ?
O14 S4 C100 N9 51.5(19) . . . . ?
O13 S4 C100 N9 169.1(16) . . . . ?
N9 S4 C100 F11 -104(3) . . . . ?
O12 S4 C100 F11 -176.0(17) . . . . ?
O14 S4 C100 F11 -52.8(19) . . . . ?

```
O13 S4 C100 F11 64.8(18) . . . . ?
N9 S4 C100 F12 127(3) . . . . ?
O12 S4 C100 F12 54.9(18) . . . . ?
O14 S4 C100 F12 178.1(16) . . . . ?
O13 S4 C100 F12 -64.3(17) . . . . ?
N9 S4 C100 F10 11.0(12) . . . . ?
O12 S4 C100 F10 -60.7(17) . . . . ?
O14 S4 C100 F10 62.4(17) . . . . ?
O13 S4 C100 F10 -180.0(14) . . . . ?
N9 S4 C100 C101 -160(7) . . . . ?
O12 S4 C100 C101 129(6) . . . . ?
O14 S4 C100 C101 -108(6) . . . . ?
O13 S4 C100 C101 9(6) . . . . ?
N9 C100 F12 C101 -172(2) . . . . ?
F11 C100 F12 C101 33.6(16) . . . . ?
F10 C100 F12 C101 -80.3(16) . . . . ?
S4 C100 F12 C101 162.9(14) . . . . ?
F11 C101 F12 C100 -25.0(13) . . . . ?
C87 P2 C81 C86 52.8(13) . . . . ?
C75 P2 C81 C86 161.9(9) . . . . ?
C87A P2 C81 C86 49.7(13) . . . . ?
Ir2 P2 C81 C86 -72.7(10) . . . . ?
C87 P2 C81 C82 -132.1(11) . . . . ?
C75 P2 C81 C82 -22.9(9) . . . . ?
C87A P2 C81 C82 -135.2(11) . . . . ?
Ir2 P2 C81 C82 102.4(8) . . . . ?
C86 C81 C82 C83 2.3(18) . . . . ?
P2 C81 C82 C83 -173.0(11) . . . . ?
C82 C81 C86 C85 -1.2(16) . . . . ?
P2 C81 C86 C85 173.8(9) . . . . ?
C81 C86 C85 C84 0.0(18) . . . . ?
C81 C82 C83 C84 -2(2) . . . . ?
C82 C83 C84 C85 1(2) . . . . ?
C86 C85 C84 C83 0(2) . . . . ?

_refine_diff_density_max    2.161
_refine_diff_density_min   -1.457
_refine_diff_density_rms    0.166

_shelx_res_file
;

    z.res created by SHELXL-2014/7
```

```
TITL z_a.res in P-1
REM Old TITL z in P-1
REM SHELXT solution in P-1
REM R1 0.121, Rweak 0.011, Alpha 0.113, Orientation as input
REM Formula found by SHELXT: C105 N11 O15 F4 P S4 I2 Ir2
CELL 1.54178 10.8622 21.448 23.7518 74.084 82.94 84.797
ZERR 2 0.0003 0.0006 0.0007 0.002 0.002 0.002
LATT 1
SFAC C H N O F P S I Ir
UNIT 202 190 18 30 24 4 8 4 4
DFIX 1.82 0.01 C99 S3
ISOR 0.01 0.02 C21 C22 C23 C23A
ISOR 0.01 0.02 S3 C99 F7 F8A F9A
ISOR 0.01 0.02 C78 C78A C79 C79A C80 C80A
ISOR 0.005 0.01 C81 C82 C83 C84 C85 C86 N8 F9 N9 O9 O10 O11 F8 C101 F12
ISOR 0.01 0.02 C87 C87A C88 C88A C89 C89A C90 C90A C91 C91A C92 C92A
ISOR 0.01 0.02 S4 O12 O13 O14 C100 F10 F11 F12 O6 O7 O8 C97
delu 0.02 0.01 C101 F12 C100 F11 F12 F10

L.S. 10
PLAN  1
TEMP -173.15
BOND $H
htab
size 0.21 0.19 0.17
conf
list 4
fmap 2
ACTA
REM <olex2.extras>
REM <HklSrc "%.\\z.hkl">
REM </olex2.extras>

WGHT    0.054500   66.656097
FVAR       0.15340   0.50861
IR1    9    -0.547283     0.868921     0.252069     11.00000     0.01689
0.01293 =
         0.01787   -0.00671   -0.00158    0.00103
I1     8    -0.736535     0.844367     0.338365     11.00000     0.02198
0.02304 =
         0.02650   -0.00966    0.00294   -0.00001
P1     6    -0.376613     0.888733     0.179268     11.00000     0.02102
0.01653 =
```

```
         0.02200   -0.00729    0.00013   -0.00098
O1     4    -0.606602     1.013005     0.239465     11.00000     0.04876
0.01591 =
         0.03726   -0.00847   -0.00461    0.00690
N2     3    -0.518147     0.772917     0.267007     11.00000     0.01645
0.02095 =
         0.01771   -0.00982   -0.00056   -0.00038
N1     3    -0.663825     0.851083     0.196144     11.00000     0.02112
0.02307 =
         0.01492   -0.00890   -0.00007   -0.00256
N3     3    -0.429613     0.848936     0.317492     11.00000     0.01650
0.01387 =
         0.02221   -0.00765   -0.00049    0.00242
C1     1    -0.745322     0.891635     0.165764     11.00000     0.02486
0.02032 =
         0.01901   -0.00157   -0.00207   -0.00207
AFIX  43
H1    2   -0.750478    0.935752    0.166843    11.00000   -1.20000
AFIX   0
C2     1    -0.823930     0.873119     0.132298     11.00000     0.02040
0.03575 =
         0.02048    0.00216   -0.00383   -0.00862
AFIX  43
H2    2   -0.882387    0.904092    0.111862    11.00000   -1.20000
AFIX   0
C3     1    -0.817432     0.809099     0.128543     11.00000     0.02570
0.03301 =
         0.01823   -0.00577   -0.00284   -0.00742
C4     1    -0.731761     0.766154     0.161096     11.00000     0.02769
0.02221 =
         0.01758   -0.00936    0.00058   -0.00720
AFIX  43
H4    2   -0.723524    0.722145    0.159601    11.00000   -1.20000
AFIX   0
C5     1    -0.658801     0.786507     0.195421     11.00000     0.01646
0.01549 =
         0.02408   -0.00671    0.00410    0.00009
C6     1    -0.578486     0.742393     0.236599     11.00000     0.01486
0.02098 =
         0.02068   -0.01081    0.00425   -0.00301
C7     1    -0.564459     0.675528     0.248432     11.00000     0.02569
0.01598 =
         0.03035   -0.01300   -0.00078   -0.00101
AFIX  43
```

```
H7    2   -0.605626    0.653695    0.227017    11.00000   -1.20000
AFIX   0
C8     1    -0.490354     0.639843     0.291561     11.00000     0.02621
0.02232 =
         0.03857   -0.00904   -0.00249   -0.00427
C9     1    -0.431549     0.674030     0.321923     11.00000     0.02395
0.01411 =
         0.03381   -0.00254   -0.00385   -0.00244
AFIX  43
H9    2   -0.380911    0.650979    0.351726    11.00000   -1.20000
AFIX   0
C10    1    -0.446136     0.740646     0.309159     11.00000     0.01330
0.01928 =
         0.02076   -0.00644    0.00143   -0.00219
C11    1    -0.393814     0.784508     0.337286     11.00000     0.01742
0.01501 =
         0.02227   -0.00704    0.00215   -0.00012
C12    1    -0.313711     0.763303     0.380797     11.00000     0.02318
0.01608 =
         0.02139   -0.00572   -0.00149    0.00014
AFIX  43
H12   2   -0.289054    0.718465    0.393748    11.00000   -1.20000
AFIX   0
C13    1    -0.268811     0.808136     0.405841     11.00000     0.02134
0.02406 =
         0.02009   -0.00544    0.00131   -0.00051
C14    1    -0.309835     0.872741     0.384929     11.00000     0.02416
0.02497 =
         0.02857   -0.01326   -0.00477   -0.00660
AFIX  43
H14   2   -0.282727    0.904390    0.401116    11.00000   -1.20000
AFIX   0
C15    1    -0.388095     0.891943     0.341723     11.00000     0.02804
0.02113 =
         0.02634   -0.00941   -0.00401   -0.00083
AFIX  43
H15   2   -0.413837    0.936602    0.328454    11.00000   -1.20000
AFIX   0
C16    1    -0.899991     0.786611     0.091322     11.00000     0.03730
0.03606 =
         0.02320   -0.00889   -0.00738   -0.01322
C17    1    -1.010255     0.835456     0.074506     11.00000     0.03482
0.05480 =
         0.03520   -0.01153   -0.01260   -0.00390
```

```
AFIX 137
H17A  2   -1.058348    0.842184    0.110286    11.00000   -1.50000
H17B  2   -1.063459    0.818529    0.052347    11.00000   -1.50000
H17C  2   -0.979417    0.876867    0.050139    11.00000   -1.50000
AFIX   0
C18    1   -0.952405     0.721194     0.126848     11.00000     0.04142
0.04382 =
         0.03524   -0.01085    0.00023   -0.01577
AFIX 137
H18A  2   -0.884286    0.687642    0.133613    11.00000   -1.50000
H18B  2   -1.012338    0.708899    0.104733    11.00000   -1.50000
H18C  2   -0.993870    0.725505    0.164753    11.00000   -1.50000
AFIX   0
C19    1   -0.822119     0.779805     0.037149     11.00000     0.03329
0.06838 =
         0.03789   -0.02367    0.00446   -0.02211
AFIX 137
H19A  2   -0.795341    0.822519    0.013885    11.00000   -1.50000
H19B  2   -0.870635    0.761769    0.013829    11.00000   -1.50000
H19C  2   -0.748992    0.750636    0.047986    11.00000   -1.50000
AFIX   0
C20    1   -0.480558     0.565863     0.307242     11.00000     0.04195
0.01428 =
         0.07841   -0.01258   -0.01678   -0.00344
C21    1   -0.482917     0.542068     0.249559     11.00000     0.13336
0.04554 =
         0.11750   -0.02946   -0.04787    0.01186
AFIX 137
H21A  2   -0.564550    0.553833    0.234786    11.00000   -1.50000
H21B  2   -0.467101    0.494810    0.259000    11.00000   -1.50000
H21C  2   -0.418553    0.562910    0.219371    11.00000   -1.50000
AFIX   0
C22    1   -0.351106     0.538136     0.320220     11.00000     0.09221
0.02627 =
         0.11141   -0.01856   -0.05485    0.00533
AFIX 137
H22A  2   -0.290531    0.560616    0.288856    11.00000   -1.50000
H22B  2   -0.344718    0.491702    0.322304    11.00000   -1.50000
H22C  2   -0.334057    0.544097    0.357922    11.00000   -1.50000
AFIX   0
C24    1   -0.181927     0.789231     0.454087     11.00000     0.02827
0.02937 =
         0.02213   -0.00817   -0.00805    0.00474
C25    1   -0.247273     0.812354     0.507152     11.00000     0.05235
```

```
0.04204 =
         0.02970   -0.01940   -0.01152    0.00977
AFIX 137
H25A  2   -0.265145    0.859468    0.494977    11.00000   -1.50000
H25B  2   -0.192862    0.801562    0.538896    11.00000   -1.50000
H25C  2   -0.325173    0.790666    0.521169    11.00000   -1.50000
AFIX   0
C26    1    -0.154062     0.715133     0.474565     11.00000     0.03664
0.03110 =
         0.02650   -0.00902   -0.00717    0.00307
AFIX 137
H26A  2   -0.232212    0.693553    0.488023    11.00000   -1.50000
H26B  2   -0.101438    0.704966    0.506976    11.00000   -1.50000
H26C  2   -0.110831    0.699798    0.441709    11.00000   -1.50000
AFIX   0
C27    1    -0.061058     0.821864     0.432025     11.00000     0.03324
0.03605 =
         0.03598   -0.00716   -0.01365   -0.00230
AFIX 137
H27A  2   -0.021983    0.807898    0.397699    11.00000   -1.50000
H27B  2   -0.005226    0.809752    0.463215    11.00000   -1.50000
H27C  2   -0.077597    0.869101    0.420855    11.00000   -1.50000
AFIX   0
C28    1    -0.584336     0.959616     0.243499     11.00000     0.02103
0.03176 =
         0.01939   -0.00561   -0.00355   -0.00361
C29    1    -0.388165     0.960297     0.116718     11.00000     0.04352
0.01984 =
         0.02504   -0.00328    0.00297   -0.00464
C30    1    -0.496293     0.996787     0.103885     11.00000     0.04064
0.02823 =
         0.02910   -0.00652   -0.00966   -0.01466
AFIX  43
H30   2   -0.571212    0.986109    0.128628    11.00000   -1.20000
AFIX   0
C31    1    -0.495664     1.049920     0.054063     11.00000     0.04756
0.03011 =
         0.03693    0.00304   -0.02107   -0.01110
AFIX  43
H31   2   -0.570264    1.075577    0.045041    11.00000   -1.20000
AFIX   0
C32    1    -0.387626     1.064978     0.018292     11.00000     0.05545
0.02756 =
         0.03016    0.00125   -0.00128   -0.01079
```

```
AFIX  43
H32   2   -0.386956    1.101422   -0.015203    11.00000   -1.20000
AFIX   0
C33    1    -0.282369     1.027999     0.030718     11.00000     0.05136
0.04603 =
         0.06708    0.01590    0.01616   -0.00148
AFIX  43
H33   2   -0.208139    1.038081    0.005263    11.00000   -1.20000
AFIX   0
C34    1    -0.280628     0.975319     0.080195     11.00000     0.05119
0.03837 =
         0.05863    0.01223    0.02258    0.00970
AFIX  43
H34   2   -0.205580    0.949872    0.088698    11.00000   -1.20000
AFIX   0
C35    1    -0.242098     0.902332     0.211538     11.00000     0.02271
0.02379 =
         0.03301   -0.01284    0.00629   -0.01055
C36    1    -0.240573     0.964067     0.220174     11.00000     0.03541
0.02999 =
         0.06371   -0.01747   -0.00927   -0.00520
AFIX  43
H36   2   -0.304971    0.996000    0.207323    11.00000   -1.20000
AFIX   0
C37    1    -0.145915     0.979012     0.247316     11.00000     0.05250
0.04408 =
         0.07553   -0.03251   -0.01081   -0.02073
AFIX  43
H37   2   -0.145758    1.021003    0.253333    11.00000   -1.20000
AFIX   0
C38    1    -0.049941     0.932105     0.266003     11.00000     0.02781
0.05869 =
         0.05498   -0.02645   -0.00191   -0.01682
AFIX  43
H38   2    0.015772    0.942476    0.284177    11.00000   -1.20000
AFIX   0
C39    1    -0.051523     0.870636     0.257817     11.00000     0.02417
0.05271 =
         0.03445   -0.01926   -0.00980    0.00151
AFIX  43
H39   2    0.012672    0.838602    0.270732    11.00000   -1.20000
AFIX   0
C40    1    -0.148101     0.855880     0.230430     11.00000     0.02296
0.03441 =
```

```
         0.03545   -0.01783   -0.00114   -0.00408
AFIX  43
H40   2   -0.149109    0.813766    0.224788    11.00000   -1.20000
AFIX   0
C41    1    -0.351443     0.821046     0.146096     11.00000     0.02173
0.02621 =
         0.02468   -0.00962    0.00559   -0.00668
C42    1    -0.401709     0.828021     0.093861     11.00000     0.05605
0.03722 =
         0.02872   -0.01973    0.00198    0.00732
AFIX  43
H42   2   -0.431591    0.869779    0.072568    11.00000   -1.20000
AFIX   0
C43    1    -0.409118     0.774711     0.072065     11.00000     0.08719
0.05712 =
         0.04165   -0.03180   -0.00964    0.00390
AFIX  43
H43   2   -0.444243    0.780071    0.036204    11.00000   -1.20000
AFIX   0
C44    1    -0.365467     0.714226     0.102558     11.00000     0.05173
0.04704 =
         0.04891   -0.03437    0.00779   -0.00556
AFIX  43
H44   2   -0.373481    0.677406    0.088667    11.00000   -1.20000
AFIX   0
C45    1    -0.310404     0.707136     0.152998     11.00000     0.04058
0.02693 =
         0.05231   -0.01894    0.01270   -0.00638
AFIX  43
H45   2   -0.276173    0.665740    0.172851    11.00000   -1.20000
AFIX   0
C46    1    -0.304320     0.760385     0.175378     11.00000     0.01661
0.02645 =
         0.03578   -0.01282    0.00702   -0.00345
AFIX  43
H46   2   -0.267659    0.754916    0.210862    11.00000   -1.20000
AFIX   0
IR2    9     0.168674     0.364286     0.268902     11.00000     0.02622
0.01739 =
         0.01823   -0.00323   -0.00679   -0.00250
I2     8     0.323453     0.349239     0.353633     11.00000     0.03041
0.02567 =
         0.02445   -0.00663   -0.01017   -0.00336
P2     6     0.032080     0.375461     0.195555     11.00000     0.02932
```

```
0.01870 =
         0.01778   -0.00359   -0.00588   -0.00071
O2     4     0.231386     0.506209     0.231962     11.00000     0.06142
0.01768 =
         0.09586    0.00733   -0.03299   -0.01763
N5     3     0.136859     0.270416     0.300732     11.00000     0.01473
0.01855 =
         0.02098   -0.00888   -0.00555   -0.00026
N4     3     0.310716     0.322953     0.222559     11.00000     0.02777
0.02997 =
         0.01875   -0.00867   -0.00574   -0.00329
N6     3     0.022311     0.368418     0.332449     11.00000     0.02827
0.01728 =
         0.01951   -0.00611   -0.00949    0.00309
C47    1     0.403503     0.352306     0.184427     11.00000     0.02342
0.03741 =
         0.02370   -0.00206   -0.00157   -0.00676
AFIX  43
H47    2    0.407883    0.397985    0.176467    11.00000   -1.20000
AFIX   0
C48    1     0.491933     0.317875     0.156766     11.00000     0.02937
0.05115 =
         0.01664    0.00301    0.00284   -0.01118
AFIX  43
H48    2    0.556222    0.340619    0.130504    11.00000   -1.20000
AFIX   0
C49    1     0.491396     0.252024     0.165441     11.00000     0.02501
0.04859 =
         0.01828   -0.01308   -0.00034   -0.00345
C50    1     0.396669     0.221984     0.205826     11.00000     0.02276
0.04186 =
         0.02137   -0.01610   -0.00400   -0.00605
AFIX  43
H50    2    0.392147    0.176277    0.214169    11.00000   -1.20000
AFIX   0
C51    1     0.309121     0.256766     0.234089     11.00000     0.02390
0.03231 =
         0.01525   -0.00740   -0.00344   -0.00551
C52    1     0.209790     0.227162     0.279298     11.00000     0.01816
0.02909 =
         0.01004   -0.01085   -0.00167    0.00113
C53    1     0.192072     0.161460     0.301861     11.00000     0.01815
0.01960 =
         0.02505   -0.01332   -0.00008    0.00257
```

```
AFIX  43
H53   2    0.242473    0.130520    0.286001    11.00000   -1.20000
AFIX   0
C54   1     0.099171     0.140922     0.348301     11.00000     0.01752
0.01977 =
         0.01548   -0.00665   -0.00623    0.00109
C55   1     0.026929     0.188401     0.369716     11.00000     0.01801
0.02367 =
         0.01383   -0.00776   -0.00158   -0.00092
AFIX  43
H55   2   -0.035860    0.175965    0.401510    11.00000   -1.20000
AFIX   0
C56   1     0.046298     0.252506     0.345115     11.00000     0.01598
0.02105 =
         0.01282   -0.00599   -0.00529    0.00280
C57   1    -0.019964     0.308780     0.362398     11.00000     0.02262
0.02527 =
         0.01697   -0.00893   -0.01018    0.00313
C58   1    -0.117663     0.302603     0.406830     11.00000     0.02392
0.02194 =
         0.02155   -0.01045   -0.00703    0.00573
AFIX  43
H58   2   -0.145822    0.260784    0.426656    11.00000   -1.20000
AFIX   0
C59   1    -0.175520     0.357748     0.422832     11.00000     0.02802
0.03241 =
         0.02468   -0.01347   -0.01472    0.01887
C60   1    -0.128312     0.416141     0.393435     11.00000     0.04713
0.02523 =
         0.01875   -0.01127   -0.01277    0.01973
AFIX  43
H60   2   -0.162773    0.454239    0.403892    11.00000   -1.20000
AFIX   0
C61   1    -0.030974     0.421442     0.348510     11.00000     0.04541
0.01644 =
         0.02088   -0.00701   -0.01660    0.00985
AFIX  43
H61   2   -0.001479    0.463004    0.328801    11.00000   -1.20000
AFIX   0
C62   1     0.582685     0.213700     0.131701     11.00000     0.01617
0.06425 =
         0.02474   -0.01808    0.00808   -0.00231
C63   1     0.593293     0.142092     0.161557     11.00000     0.05521
0.07262 =
```

```
         0.03553   -0.00770    0.01411    0.03662
AFIX 137
H63A  2    0.626601    0.135194    0.199456    11.00000   -1.50000
H63B  2    0.649206    0.119931    0.136581    11.00000   -1.50000
H63C  2    0.510979    0.124528    0.168072    11.00000   -1.50000
AFIX   0
C64    1     0.534578     0.223021     0.071345     11.00000     0.04717
0.05643 =
         0.03143   -0.02154   -0.00437   -0.00337
AFIX 137
H64A  2    0.451062    0.206979    0.077083    11.00000   -1.50000
H64B  2    0.590606    0.198742    0.048222    11.00000   -1.50000
H64C  2    0.531480    0.269269    0.050357    11.00000   -1.50000
AFIX   0
C65    1     0.712289     0.241610     0.121478     11.00000     0.02677
0.14470 =
         0.08073   -0.07444    0.00653   -0.00912
AFIX 137
H65A  2    0.705619    0.287898    0.100800    11.00000   -1.50000
H65B  2    0.769207    0.218478    0.097672    11.00000   -1.50000
H65C  2    0.744229    0.236151    0.159479    11.00000   -1.50000
AFIX   0
C66    1     0.078984     0.069166     0.376863     11.00000     0.01519
0.01474 =
         0.03551   -0.01119   -0.00003    0.00063
C67    1     0.107171     0.053433     0.440656     11.00000     0.03614
0.02166 =
         0.03312   -0.00491    0.00104   -0.00306
AFIX 137
H67A  2    0.190818    0.066277    0.442058    11.00000   -1.50000
H67B  2    0.102593    0.006667    0.458719    11.00000   -1.50000
H67C  2    0.046245    0.077229    0.462251    11.00000   -1.50000
AFIX   0
C68    1     0.161124     0.024545     0.345534     11.00000     0.02676
0.02158 =
         0.04121   -0.02073    0.00553    0.00262
AFIX 137
H68A  2    0.142697    0.034524    0.304526    11.00000   -1.50000
H68B  2    0.144587   -0.020813    0.365395    11.00000   -1.50000
H68C  2    0.248729    0.031328    0.346603    11.00000   -1.50000
AFIX   0
C69    1    -0.058860     0.057374     0.375037     11.00000     0.01707
0.02980 =
         0.06352   -0.01951   -0.00242    0.00071
```

```
AFIX 137
H69A  2   -0.112432    0.083696    0.397125    11.00000   -1.50000
H69B  2   -0.073042    0.011314    0.392751    11.00000   -1.50000
H69C  2   -0.078334    0.069650    0.334081    11.00000   -1.50000
AFIX   0
C70    1    -0.282780     0.349583     0.471088     11.00000     0.03494
0.04024 =
         0.03109   -0.01880   -0.00720    0.01756
C71    1    -0.238665     0.304797     0.526982     11.00000     0.05665
0.05665 =
         0.04031   -0.00332    0.01547   -0.00059
AFIX 137
H71A  2   -0.214106    0.261846    0.520855    11.00000   -1.50000
H71B  2   -0.306049    0.300879    0.559000    11.00000   -1.50000
H71C  2   -0.167262    0.322653    0.537302    11.00000   -1.50000
AFIX   0
C72    1    -0.385778     0.320383     0.451417     11.00000     0.04488
0.14930 =
         0.08860   -0.08167    0.03036   -0.02030
AFIX 137
H72A  2   -0.409733    0.348661    0.413932    11.00000   -1.50000
H72B  2   -0.457696    0.316079    0.481192    11.00000   -1.50000
H72C  2   -0.356778    0.277451    0.446354    11.00000   -1.50000
AFIX   0
C73    1    -0.324950     0.415055     0.483906     11.00000     0.08213
0.05837 =
         0.06808   -0.02504    0.00710    0.03402
AFIX 137
H73A  2   -0.252418    0.436116    0.489187    11.00000   -1.50000
H73B  2   -0.382710    0.407859    0.519863    11.00000   -1.50000
H73C  2   -0.366633    0.442974    0.450850    11.00000   -1.50000
AFIX   0
C74    1     0.208574     0.453479     0.243788     11.00000     0.03372
0.04480 =
         0.03390   -0.00516   -0.01797   -0.00080
C75    1    -0.025914     0.457213     0.161777     11.00000     0.03798
0.03212 =
         0.03444   -0.01231   -0.01418    0.01543
S2     7    -0.073402     0.626001     0.316971     11.00000     0.05903
0.03238 =
         0.03087   -0.01781    0.01149   -0.02006
F4     5     0.136695     0.678777     0.274764     11.00000     0.04382
0.15135 =
         0.04465   -0.01137    0.01477   -0.03174
```

```
F5     5     0.130539     0.625183     0.364178     11.00000     0.11455
0.13827 =
         0.07137   -0.00709   -0.04454   -0.02260
F6     5     0.039679     0.715960     0.341990     11.00000     0.09882
0.10422 =
         0.16433   -0.09481    0.03466   -0.06164
O6     4    -0.131341     0.675971     0.272164     11.00000     0.04552
0.05192 =
         0.09781    0.00393   -0.01743    0.00629
O7     4    -0.142077     0.616630     0.373284     11.00000     0.10474
0.12740 =
         0.04516   -0.03849    0.02806   -0.07343
O8     4    -0.024528     0.571203     0.297339     11.00000     0.12134
0.03447 =
         0.06800   -0.03191   -0.01759    0.00545
C94    1     0.068089     0.662838     0.323973     11.00000     0.05527
0.05959 =
         0.03128   -0.01335    0.00939   -0.01062
S1     7     0.585568     0.061495     0.355650     11.00000     0.03324
0.02846 =
         0.05836   -0.02432    0.00822   -0.00757
F2     5     0.579943     0.155188     0.407226     11.00000     0.05828
0.04159 =
         0.04942   -0.01782   -0.01550   -0.01320
F3     5     0.422762     0.096050     0.432124     11.00000     0.05883
0.05782 =
         0.08564   -0.04114    0.03277   -0.02652
F1     5     0.442779     0.167648     0.345992     11.00000     0.05505
0.04511 =
         0.08866   -0.02247   -0.03612    0.01369
O4     4     0.673119     0.095816     0.311110     11.00000     0.04084
0.05773 =
         0.04755   -0.01547    0.00498   -0.00434
O5     4     0.639865     0.017419     0.408326     11.00000     0.03838
0.02399 =
         0.08978   -0.00749   -0.01375    0.00695
O3     4     0.488185     0.033999     0.336432     11.00000     0.04403
0.05745 =
         0.06941   -0.04203   -0.01405    0.01529
C93    1     0.502282     0.123738     0.386419     11.00000     0.03976
0.02734 =
         0.05294   -0.01816   -0.01293   -0.00010
AFIX   6
O1W    4    -0.410135     0.511783     0.130835     11.00000     0.04383
```

```
0.03770 =
         0.04234   -0.00554   -0.00418   -0.01506
H0AA  2   -0.452130    0.490309    0.162735    11.00000   -1.50000
H0AB  2   -0.372641    0.542230    0.138498    11.00000   -1.50000
AFIX   0
N7     3    -0.189528     0.145134     0.490006     11.00000     0.03595
0.04606 =
         0.03833   -0.01679    0.00766   -0.01405
C95    1    -0.270894     0.114006     0.510838     11.00000     0.02926
0.03396 =
         0.02774   -0.00967    0.00219    0.00153
C96    1    -0.374641     0.074205     0.536030     11.00000     0.03201
0.03749 =
         0.04749    0.00062   -0.00293   -0.01310
AFIX 137
H96A  2   -0.377883    0.041854    0.514123    11.00000   -1.50000
H96B  2   -0.452017    0.101677    0.533767    11.00000   -1.50000
H96C  2   -0.364430    0.052072    0.577298    11.00000   -1.50000
AFIX   0
N8     3     0.360462     0.411804     0.525025     11.00000     0.15200
0.16359 =
         0.15894   -0.03639   -0.01264   -0.00601
C97    1     0.279076     0.434712     0.495905     11.00000     0.09505
0.09168 =
         0.08932   -0.02665   -0.01788    0.01929
C98    1     0.154648     0.457457     0.471186     11.00000     0.13577
0.04482 =
         0.06353   -0.00712   -0.01261   -0.00687
AFIX 137
H98A  2    0.169173    0.482264    0.429906    11.00000   -1.50000
H98B  2    0.107129    0.485070    0.493927    11.00000   -1.50000
H98C  2    0.107718    0.419648    0.473847    11.00000   -1.50000
AFIX   0
PART   1
C80    1    -0.044799     0.474068     0.100089     21.00000     0.02439
0.02290 =
         0.02822   -0.00251   -0.01243   -0.00618
C78    1    -0.098131     0.586140     0.101313     21.00000     0.03533
0.02731 =
         0.03058   -0.00542   -0.00934    0.00164
C79    1    -0.084361     0.538599     0.070881     21.00000     0.03819
0.03527 =
         0.03937   -0.00420   -0.01747    0.00314
C87    1     0.087854     0.344817     0.136529     21.00000     0.01782
```

```
0.02550 =
         0.01726    0.00298    0.00005   -0.00010
C92    1     0.039346     0.292432     0.122551     21.00000     0.04031
0.02857 =
         0.02184   -0.01213   -0.01143    0.00650
C91    1     0.092934     0.273019     0.073594     21.00000     0.05723
0.04684 =
         0.04020   -0.01604   -0.01089    0.01066
C90    1     0.212827     0.304414     0.041828     21.00000     0.04823
0.05282 =
         0.05180   -0.00690    0.00682    0.01483
C89    1     0.246040     0.352885     0.052237     21.00000     0.04934
0.05454 =
         0.04272   -0.00455   -0.01002    0.01077
C88    1     0.194316     0.375749     0.100875     21.00000     0.02339
0.03100 =
         0.01864    0.00129   -0.00562    0.00367
F9     5     0.022926     0.161600     0.070561     21.00000     0.06786
0.09307 =
         0.05358    0.02407   -0.03201    0.00875
F8     5    -0.043035     0.070154     0.115059     21.00000     0.07201
0.11802 =
         0.11498   -0.02521   -0.04288   -0.03329
C23    1    -0.543559     0.539761     0.376778     21.00000     0.05909
0.04198 =
         0.11161    0.00668    0.02584    0.00203
PART   2
C23A   1    -0.608704     0.544413     0.337430    -21.00000     0.08537
0.02084 =
         0.06596   -0.00904    0.01264   -0.02169
C78A   1    -0.150678     0.573267     0.113007    -21.00000     0.05431
0.05083 =
         0.04787   -0.01852   -0.01309    0.01330
C79A   1    -0.143523     0.523291     0.083828    -21.00000     0.05949
0.04526 =
         0.04423   -0.01471   -0.01744    0.01579
C80A   1    -0.089177     0.461154     0.111747    -21.00000     0.04467
0.04970 =
         0.04978   -0.01060   -0.01799    0.01163
C87A   1     0.128009     0.354129     0.131334    -21.00000     0.01873
0.01012 =
         0.02001   -0.00911   -0.00455    0.00035
C91A   1     0.180296     0.287824     0.062233    -21.00000     0.05443
0.03617 =
```

```
         0.05465   -0.01009    0.00581    0.00484
C90A  1     0.276452     0.337275     0.033958    -21.00000     0.04830
0.04680 =
         0.02970   -0.00988   -0.01032    0.01834
C89A  1     0.279050     0.391895     0.050108    -21.00000     0.03599
0.04537 =
         0.02781   -0.00596   -0.01598    0.00102
C92A  1     0.119287     0.298328     0.113569    -21.00000     0.05044
0.03013 =
         0.02550   -0.01771   -0.00228    0.00130
C88A  1     0.208472     0.404145     0.099593    -21.00000     0.03319
0.02524 =
         0.03356   -0.01141   -0.00002   -0.00841
F9A   5     0.031016     0.036940     0.088290    -21.00000     0.16409
0.17840 =
         0.17842   -0.05340   -0.04337   -0.01126
F8A   5     0.138592     0.121162     0.045555    -21.00000     0.21028
0.19957 =
         0.17217   -0.05050   -0.02504   -0.00325
PART   0
C76   1    -0.039107     0.506890     0.189801     11.00000     0.03195
0.02709 =
         0.03411   -0.01127   -0.00721    0.01019
C77   1    -0.086560     0.568817     0.161738     11.00000     0.06517
0.03710 =
         0.04553   -0.01367   -0.02111    0.01777
S3    7     0.150964     0.096464     0.157160     11.00000     0.07675
0.04870 =
         0.07999   -0.00922   -0.02625    0.00364
O10   4     0.165968     0.029181     0.185793     11.00000     0.12233
0.04997 =
         0.12941   -0.00760   -0.06283   -0.00672
O9    4     0.249222     0.131963     0.135119     11.00000     0.13251
0.11416 =
         0.17901   -0.00468   -0.04073   -0.05450
C99   1     0.048086     0.103067     0.099762     11.00000     0.16328
0.17080 =
         0.17617   -0.03874   -0.01716   -0.01851
F7    5     0.125167     0.068963     0.083233     11.00000     0.13314
0.06748 =
         0.13659   -0.00405   -0.02480    0.01084
S4    7     0.408322     0.575393     0.097717     11.00000     0.11893
0.05539 =
         0.05822   -0.02295    0.02005   -0.01989
```

```
N9     3     0.381293     0.566546     0.062212     11.00000     0.04290
0.02255 =
         0.01953   -0.00906   -0.01355   -0.01232
O13    4     0.358525     0.622893     0.129158     11.00000     0.11557
0.04948 =
         0.08056   -0.03479    0.01150   -0.02285
C101   1     0.169026     0.611222    -0.002017     11.00000     0.08481
0.05684 =
         0.08222   -0.03579   -0.02222   -0.01138
O12    4     0.396464     0.509292     0.129927     11.00000     0.20000
0.08320 =
         0.13001   -0.03772    0.01495   -0.00682
F10    5     0.333028     0.540103     0.010179     11.00000     0.19070
0.15855 =
         0.15444   -0.08672   -0.03238    0.00610
O14    4     0.518310     0.593804     0.059364     11.00000     0.13482
0.14735 =
         0.13739   -0.06550    0.03489   -0.03558
F11    5     0.291805     0.639393     0.005365     11.00000     0.18940
0.14045 =
         0.17403   -0.03311   -0.05733   -0.03514
C100   1     0.294097     0.583861     0.043045     11.00000     0.11867
0.10315 =
         0.11057   -0.05160   -0.02272   -0.00662
F12    5     0.185127     0.567461     0.068301     11.00000     0.17147
0.18803 =
         0.16800   -0.04704   -0.01019   -0.02552
C81    1    -0.104312     0.331755     0.227711     11.00000     0.03945
0.05177 =
         0.03255   -0.00122   -0.01512   -0.01385
C82    1    -0.211570     0.369361     0.240311     11.00000     0.04997
0.07390 =
         0.05870   -0.01152   -0.01633   -0.01515
AFIX  43
H82    2   -0.210219    0.415348    0.227547    11.00000   -1.20000
AFIX   0
C86    1    -0.105695     0.265711     0.243792     11.00000     0.05815
0.06692 =
         0.05870   -0.00252   -0.02536   -0.02133
AFIX  43
H86    2   -0.034984    0.239133    0.235192    11.00000   -1.20000
AFIX   0
O11    4     0.060259     0.127205     0.189154     11.00000     0.17948
0.11187 =
```

```
         0.13397   -0.05922    0.00760    0.04079
C85    1    -0.220749     0.237601     0.274842     11.00000     0.08165
0.07700 =
         0.06912   -0.00466   -0.02511   -0.01817
AFIX  43
H85   2   -0.227593    0.191901    0.287220    11.00000   -1.20000
AFIX   0
C83    1    -0.318626     0.342041     0.270484     11.00000     0.07330
0.08959 =
         0.07555   -0.01727   -0.01051   -0.01089
AFIX  43
H83   2   -0.389406    0.368179    0.279810    11.00000   -1.20000
AFIX   0
C84    1    -0.318817     0.279503     0.285556     11.00000     0.06690
0.09159 =
         0.07158   -0.01612   -0.02243   -0.01562
AFIX  43
H84   2   -0.393154    0.260755    0.305818    11.00000   -1.20000

AFIX   0
HKLF 4

REM  z_a.res in P-1
REM R1 =  0.0551 for   15240 Fo > 4sig(Fo)  and  0.0673 for all   17879
data
REM   1455 parameters refined using    329 restraints

END

WGHT      0.0424     68.9479

REM Instructions for potential hydrogen bonds
EQIV $1 x-1, y+1, z
HTAB C1 O10_$1
EQIV $2 x-1, y, z
HTAB C4 O13_$2
HTAB C14 O5_$1
HTAB C15 S1_$1
HTAB C15 O3_$1
HTAB C46 O6
HTAB C47 O12
HTAB C55 N7
HTAB C58 N7
HTAB C61 O8
```

```
REM  Highest  difference  peak   2.161,   deepest  hole  -1.457,   1-sigma
level  0.166
Q1    1   0.5311  0.0749  0.3283  11.00000  0.05    2.16
;
_shelx_res_checksum   15863

_shelx_hkl_file
;
  -1   0   0  317.17   14.50
  -1   0   0  330.47   14.60
   1   0   0  325.37   14.60
   1   0   0  325.77   14.50
   1   0   0  309.77   14.50
  -2   0   0   13.60    1.90
  -2   0   0   12.60    1.50
   2   0   0   14.00    1.70
   2   0   0   12.90    1.30
   2   0   0   13.90    1.90
  -3   0   0  118.29    6.80
  -3   0   0  126.09    6.30
   3   0   0  122.49    6.30
   3   0   0  122.59    6.20
   3   0   0  122.09    6.90
  -4   0   0   75.79    5.80
  -4   0   0   77.99    5.00
   4   0   0   87.09    6.00
   4   0   0   76.79    5.00
   4   0   0   80.79    4.80
  -5   0   0   20.70    3.10
  -5   0   0   17.60    3.80
   5   0   0   25.70    3.60
   5   0   0   19.00    4.50
   5   0   0   23.30    3.80
  -6   0   0   43.20    6.90
  -6   0   0   58.59    6.70
  -6   0   0   64.69    7.30
   6   0   0   76.89   11.40
   6   0   0   64.49    6.80
  -7   0   0  411.66   25.50
  -7   0   0  437.26   23.70
  -7   0   0  453.85   22.60
  -7   0   0  417.16   26.20
  -7   0   0  474.65   26.90
```

-7	0	0	423.56	24.10
7	0	0	427.96	22.50
-8	0	0	194.28	24.70
-8	0	0	225.58	16.30
-8	0	0	232.88	14.70
-8	0	0	251.97	20.50
-8	0	0	233.18	20.00
-8	0	0	235.58	17.00
8	0	0	208.98	16.20
-9	0	0	77.89	10.60
-9	0	0	82.19	11.80
-9	0	0	73.29	12.50
-9	0	0	72.49	12.40
-9	0	0	63.59	13.10
9	0	0	84.49	11.80
9	0	0	97.69	13.70
-10	0	0	192.18	18.10
-10	0	0	214.78	16.70
-10	0	0	218.18	17.50
-10	0	0	167.58	21.70
10	0	0	170.68	19.20
-11	0	0	51.19	12.80
-11	0	0	56.79	12.00
-11	0	0	72.49	10.10
11	0	0	83.29	12.70
-12	0	0	32.30	8.10
12	0	0	33.00	11.40
12	-1	0	265.17	32.50
12	-1	0	207.88	20.50
-12	1	0	245.38	17.40
11	-1	0	46.30	11.70
-11	1	0	87.19	12.70
-11	1	0	79.39	10.50
-11	1	0	75.79	13.70
10	-1	0	20.00	10.00
-10	1	0	32.90	9.50
-10	1	0	29.30	10.00
-10	1	0	19.60	8.00
-10	1	0	4.10	13.80
9	-1	0	38.10	9.90
-9	1	0	43.50	9.10
-9	1	0	35.50	8.00
-9	1	0	49.90	12.80
-9	1	0	38.80	7.60

8	-1	0	218.48	16.60
-8	1	0	237.98	21.30
-8	1	0	241.38	14.90
-8	1	0	217.98	20.10
-8	1	0	233.98	16.70
-8	1	0	170.78	25.90
7	-1	0	389.16	27.20
-7	1	0	391.46	22.20
-7	1	0	360.86	25.30
-7	1	0	382.66	24.60
-7	1	0	398.16	22.60
-7	1	0	398.36	26.10
-7	1	0	407.26	20.80
6	-1	0	126.99	13.30
6	-1	0	117.69	9.70
6	-1	0	135.09	8.40
-6	1	0	112.39	12.10
-6	1	0	138.29	9.70
5	-1	0	1080.59	48.30
5	-1	0	1041.00	48.30
5	-1	0	1064.19	49.60
-5	1	0	1086.69	48.60
-5	1	0	1050.19	49.40
-5	1	0	1017.40	48.70
4	-1	0	721.33	31.90
4	-1	0	709.13	32.70
-4	1	0	617.94	32.00
-4	1	0	709.03	32.60
-4	1	0	711.93	32.10
3	-1	0	6.30	1.80
3	-1	0	6.80	1.50
-3	1	0	10.40	1.80
-3	1	0	9.40	2.00
-3	1	0	7.30	2.00
2	-1	0	1423.96	62.19
2	-1	0	1371.16	61.99
-2	1	0	1401.76	62.19
-2	1	0	1406.86	62.09
-2	1	0	1315.47	61.89
1	-1	0	240.08	10.80
1	-1	0	235.38	10.90
1	-1	0	241.28	11.00
-1	1	0	219.38	10.80
-1	1	0	233.78	10.90

-1	1	0	248.78	11.00
0	1	0	9.00	0.80
1	1	0	2276.77	101.19
-2	-1	0	119.89	5.70
2	1	0	119.49	6.40
2	1	0	121.29	6.00
2	1	0	129.29	5.70
-3	-1	0	121.59	5.80
3	1	0	109.19	6.00
3	1	0	123.69	5.70
3	1	0	115.59	6.70
-4	-1	0	8.00	1.50
4	1	0	8.80	1.50
4	1	0	9.50	2.70
4	1	0	8.10	1.80
-5	-1	0	1104.39	49.90
-5	-1	0	1102.39	50.89
5	1	0	1120.79	50.49
5	1	0	1087.59	49.80
5	1	0	1125.49	51.79
-6	-1	0	1749.03	74.19
-6	-1	0	1479.95	76.69
-6	-1	0	1576.24	74.69
6	1	0	1685.33	73.69
6	1	0	1607.74	75.89
-7	-1	0	76.89	10.10
-7	-1	0	88.19	14.80
-7	-1	0	113.89	12.50
-7	-1	0	96.09	11.40
-7	-1	0	84.69	8.80
-7	-1	0	106.69	10.80
-8	-1	0	4.20	9.50
-8	-1	0	9.40	9.00
-8	-1	0	4.20	5.70
-8	-1	0	13.90	7.10
-8	-1	0	6.40	9.60
-8	-1	0	3.40	5.40
8	1	0	1.90	6.20
-9	-1	0	162.58	14.70
-9	-1	0	163.18	15.50
-9	-1	0	172.48	18.10
-9	-1	0	158.48	19.90
-9	-1	0	155.98	13.70
9	1	0	133.69	19.10

9	1	0	150.88	18.30
-10	-1	0	109.79	18.80
-10	-1	0	145.89	15.90
-10	-1	0	121.89	14.30
-10	-1	0	113.99	14.70
10	1	0	127.19	17.50
-11	-1	0	61.19	22.90
-11	-1	0	68.99	12.80
-11	-1	0	86.99	13.30
-11	-1	0	73.59	11.80
11	1	0	96.79	17.20
-12	-1	0	14.10	7.50
12	1	0	22.90	11.80
12	-2	0	37.30	11.60
12	-2	0	60.09	27.80
-12	2	0	46.10	9.30
11	-2	0	139.39	17.70
-11	2	0	97.19	15.10
-11	2	0	140.69	19.40
-11	2	0	128.59	19.00
10	-2	0	93.09	16.20
-10	2	0	104.49	14.50
-10	2	0	77.49	10.90
-10	2	0	84.09	11.70
9	-2	0	30.90	10.00
-9	2	0	36.50	9.10
-9	2	0	22.10	7.20
-9	2	0	30.10	8.30
8	-2	0	22.30	10.60
8	-2	0	39.10	8.70
-8	2	0	35.70	10.90
-8	2	0	51.29	8.30
-8	2	0	35.50	6.70
7	-2	0	1283.67	62.39
7	-2	0	1274.07	62.79
-7	2	0	1296.47	63.79
-7	2	0	1373.86	61.49
-7	2	0	1272.57	63.79
-7	2	0	1383.76	62.29
-7	2	0	1360.46	62.49
6	-2	0	367.26	17.20
6	-2	0	320.17	21.20
6	-2	0	301.87	18.20
-6	2	0	346.97	17.80

-6	2	0	309.67	20.20
-6	2	0	336.07	19.50
-6	2	0	326.17	18.80
5	-2	0	-0.40	4.10
5	-2	0	-0.80	2.40
5	-2	0	-4.30	3.30
-5	2	0	-1.60	3.70
-5	2	0	-1.00	3.40
-5	2	0	4.10	3.60
4	-2	0	40.00	3.70
4	-2	0	38.40	4.30
4	-2	0	39.00	7.00
-4	2	0	41.60	4.40
-4	2	0	30.80	5.20
-4	2	0	32.50	5.90
3	-2	0	18.10	3.20
3	-2	0	21.10	2.70
3	-2	0	17.90	2.80
-3	2	0	25.40	3.00
-3	2	0	21.90	2.90
-3	2	0	21.70	3.30
2	-2	0	818.62	36.90
2	-2	0	819.92	37.30
2	-2	0	815.62	37.10
-2	2	0	796.42	36.80
-2	2	0	817.62	37.30
-2	2	0	839.52	37.20
1	-2	0	1236.98	57.19
1	-2	0	1314.07	57.39
1	-2	0	1283.57	57.49
-1	2	0	1210.28	57.19
-1	2	0	1300.07	57.59
-1	2	0	1340.47	57.39
0	-2	0	293.57	12.20
0	-2	0	231.08	12.60
0	2	0	242.98	12.70
0	2	0	271.97	12.00
0	2	0	290.67	12.20
-1	-2	0	986.40	44.90
1	2	0	942.61	45.00
1	2	0	1134.39	45.40
1	2	0	972.70	44.90
2	2	0	68.89	3.70
2	2	0	67.39	4.20

3	2	0	92.99	5.10
3	2	0	93.39	5.80
4	2	0	485.15	22.30
4	2	0	474.35	23.50
-5	-2	0	326.27	16.50
5	2	0	319.37	17.50
5	2	0	323.27	15.90
-6	-2	0	715.83	36.70
-6	-2	0	771.72	36.00
-6	-2	0	755.82	38.60
6	2	0	793.52	35.60
6	2	0	755.02	38.20
-7	-2	0	493.95	24.10
-7	-2	0	476.05	25.00
-7	-2	0	491.05	27.20
-7	-2	0	448.56	25.20
-7	-2	0	447.76	27.00
-7	-2	0	483.85	26.80
7	2	0	464.25	23.80
-8	-2	0	15.30	9.10
-8	-2	0	61.69	9.90
-8	-2	0	42.20	9.50
-8	-2	0	41.00	7.20
-8	-2	0	39.90	6.90
-8	-2	0	33.50	9.30
8	2	0	24.60	6.90
-9	-2	0	13.00	6.70
-9	-2	0	-0.80	8.30
-9	-2	0	3.60	8.00
-9	-2	0	0.40	6.00
-9	-2	0	6.90	9.90
9	2	0	3.90	10.50
9	2	0	9.60	9.00
-10	-2	0	37.10	7.50
-10	-2	0	37.00	14.30
-10	-2	0	28.30	9.40
-10	-2	0	40.00	10.90
-10	-2	0	45.70	10.00
10	2	0	30.00	9.70
-11	-2	0	166.78	18.60
-11	-2	0	165.18	19.10
-11	-2	0	211.68	21.50
-11	-2	0	184.98	15.60
11	2	0	176.38	19.50

-12	-2	0	28.40	7.70
12	2	0	26.10	11.60
12	-3	0	104.89	16.10
-12	3	0	79.49	13.90
11	-3	0	257.57	20.40
-11	3	0	235.98	21.60
-11	3	0	195.78	22.70
-11	3	0	253.77	18.90
10	-3	0	33.70	10.20
-10	3	0	33.70	9.70
-10	3	0	39.60	9.80
-10	3	0	35.60	10.40
9	-3	0	52.29	10.70
9	-3	0	59.99	12.30
-9	3	0	63.09	9.90
-9	3	0	99.29	13.90
-9	3	0	70.49	12.40
8	-3	0	-4.80	9.40
-8	3	0	18.20	8.20
-8	3	0	30.60	11.70
-8	3	0	15.40	7.80
-8	3	0	3.50	6.20
-8	3	0	14.20	15.40
7	-3	0	13.50	8.50
7	-3	0	4.30	6.30
-7	3	0	9.50	11.30
-7	3	0	9.60	11.10
-7	3	0	8.90	7.40
-7	3	0	7.90	6.60
-7	3	0	7.40	4.70
6	-3	0	1685.03	77.79
6	-3	0	1729.93	77.09
-6	3	0	1635.64	78.29
-6	3	0	1688.13	78.39
-6	3	0	1637.54	78.09
-6	3	0	1760.42	77.49
5	-3	0	519.25	23.50
5	-3	0	460.25	24.10
5	-3	0	503.65	25.50
-5	3	0	491.25	25.30
-5	3	0	482.85	24.50
-5	3	0	479.65	24.10
4	-3	0	487.25	22.80
4	-3	0	483.05	23.30

4	-3	0	507.85	24.00
-4	3	0	448.36	22.80
-4	3	0	487.15	23.90
-4	3	0	500.95	23.50
3	-3	0	19.00	2.60
3	-3	0	17.40	2.70
-3	3	0	16.10	2.30
-3	3	0	14.20	2.60
-3	3	0	11.40	2.40
2	-3	0	666.93	30.90
2	-3	0	658.43	30.70
2	-3	0	675.33	30.40
-2	3	0	681.93	31.00
-2	3	0	675.63	30.80
-2	3	0	656.63	30.30
1	-3	0	1510.05	69.19
1	-3	0	1411.36	68.69
1	-3	0	1598.54	68.99
-1	3	0	1572.44	68.89
-1	3	0	1522.45	68.59
-1	3	0	1602.54	69.29
0	-3	0	26.80	2.10
0	-3	0	29.20	3.60
0	3	0	28.20	1.80
0	3	0	28.70	3.50
0	3	0	29.70	2.00
-1	-3	0	443.66	20.40
-1	-3	0	458.25	21.00
1	3	0	421.76	20.30
1	3	0	441.16	20.40
1	3	0	495.45	21.00
2	3	0	3739.53	154.78
2	3	0	3189.98	154.08
3	3	0	9.00	1.60
3	3	0	9.50	2.00
4	3	0	1727.23	78.69
4	3	0	1726.83	77.59
-5	-3	0	27.70	4.30
5	3	0	31.30	4.00
5	3	0	36.80	5.60
-6	-3	0	572.14	30.00
-6	-3	0	626.44	29.10
-6	-3	0	591.64	31.70
6	3	0	560.54	31.60

6	3	0	641.64	28.70
-7	-3	0	47.20	8.00
-7	-3	0	47.30	9.50
-7	-3	0	57.19	7.90
-7	-3	0	56.49	9.90
-7	-3	0	43.50	6.70
-7	-3	0	47.20	8.30
7	3	0	54.79	7.50
-8	-3	0	143.59	12.10
-8	-3	0	164.28	17.00
-8	-3	0	164.28	19.40
-8	-3	0	169.28	13.50
-9	-3	0	47.20	8.40
-9	-3	0	29.40	9.10
-9	-3	0	45.90	8.00
-9	-3	0	27.10	10.10
-9	-3	0	45.00	9.10
9	3	0	44.30	10.70
9	3	0	49.00	10.90
-10	-3	0	194.88	16.60
-10	-3	0	198.68	15.90
-10	-3	0	166.78	22.10
-10	-3	0	200.58	17.60
-10	-3	0	165.08	18.10
10	3	0	199.38	19.60
-11	-3	0	141.59	19.40
-11	-3	0	130.29	18.40
-11	-3	0	139.29	14.00
-11	-3	0	146.79	17.60
11	3	0	123.49	19.20
-12	-3	0	157.58	13.30
12	3	0	128.09	19.80
12	-4	0	12.00	10.30
-12	4	0	14.50	9.30
11	-4	0	337.77	23.70
-11	4	0	336.67	25.70
-11	4	0	384.26	24.00
10	-4	0	129.49	22.40
10	-4	0	166.18	17.30
-10	4	0	166.58	18.20
-10	4	0	168.78	17.40
-10	4	0	166.58	17.10
9	-4	0	21.60	10.30
9	-4	0	5.50	9.20

-9	4	0	25.80	8.90
-9	4	0	20.60	9.20
-9	4	0	13.20	8.50
8	-4	0	258.27	20.40
-8	4	0	291.57	17.80
-8	4	0	301.77	19.50
-8	4	0	285.97	20.00
-8	4	0	237.58	23.90
7	-4	0	98.69	11.40
7	-4	0	138.89	13.60
-7	4	0	104.19	10.20
-7	4	0	135.79	21.40
-7	4	0	92.39	13.00
-7	4	0	128.49	13.20
-7	4	0	107.19	14.90
6	-4	0	377.96	22.90
6	-4	0	367.26	22.90
6	-4	0	389.86	20.20
-6	4	0	381.06	22.00
-6	4	0	433.66	20.60
-6	4	0	386.36	23.70
-6	4	0	356.56	22.20
5	-4	0	56.69	9.90
5	-4	0	54.49	6.30
5	-4	0	70.99	8.40
-5	4	0	80.69	10.00
-5	4	0	76.99	8.90
-5	4	0	70.59	7.80
4	-4	0	1.40	2.40
4	-4	0	3.70	3.30
4	-4	0	1.50	3.70
-4	4	0	5.20	3.00
-4	4	0	5.50	3.80
-4	4	0	6.50	4.10
-4	4	0	4.00	3.00
3	-4	0	24.10	4.40
3	-4	0	14.10	2.60
3	-4	0	17.40	2.90
-3	4	0	13.10	2.70
-3	4	0	18.10	2.90
-3	4	0	15.00	2.40
2	-4	0	59.69	5.00
2	-4	0	58.99	4.30
2	-4	0	60.09	4.40

-2	4	0	56.39	4.40
-2	4	0	60.09	5.00
-2	4	0	60.99	3.70
1	-4	0	716.73	34.00
1	-4	0	670.73	33.30
1	-4	0	771.72	33.50
-1	4	0	755.02	33.50
-1	4	0	711.03	33.00
-1	4	0	770.02	34.30
0	-4	0	5104.79	245.08
0	-4	0	5596.64	244.38
0	4	0	5769.22	245.38
-1	-4	0	684.83	30.10
-1	-4	0	660.53	30.90
1	4	0	705.23	30.90
1	4	0	628.04	30.00
1	4	0	655.23	30.10
-2	-4	0	71.69	3.50
2	4	0	68.79	4.00
2	4	0	70.99	3.50
2	4	0	78.69	5.10
3	4	0	729.23	33.90
3	4	0	763.52	34.80
-4	-4	0	49.70	6.90
4	4	0	32.60	5.30
4	4	0	33.40	4.70
4	4	0	31.20	3.50
-5	-4	0	46.30	5.40
5	4	0	36.60	5.70
5	4	0	38.80	4.50
-6	-4	0	-11.20	7.40
-6	-4	0	7.90	7.40
-6	-4	0	2.70	4.30
-6	-4	0	-4.00	7.90
-6	-4	0	5.50	7.80
-6	-4	0	5.10	5.30
6	4	0	1.20	8.10
6	4	0	5.50	3.30
-7	-4	0	347.87	20.70
-7	-4	0	360.36	20.10
-7	-4	0	352.96	21.50
-7	-4	0	361.46	22.70
-7	-4	0	358.96	19.30
-7	-4	0	362.06	21.60

7	4	0	383.36	19.30
-8	-4	0	184.18	16.10
-8	-4	0	204.78	13.70
-8	-4	0	198.18	17.40
-8	-4	0	190.88	14.50
-8	-4	0	194.88	16.70
-8	-4	0	194.58	16.20
8	4	0	193.08	14.60
8	4	0	213.48	18.70
-9	-4	0	26.30	9.80
-9	-4	0	-3.70	5.80
-9	-4	0	4.50	6.70
-9	-4	0	4.40	8.00
-9	-4	0	0.00	7.00
9	4	0	4.70	10.50
9	4	0	3.70	9.70
-10	-4	0	445.46	25.50
-10	-4	0	425.26	26.00
-10	-4	0	409.36	25.00
-10	-4	0	400.16	28.90
-10	-4	0	469.55	24.90
10	4	0	430.36	27.70
-11	-4	0	97.29	17.50
-11	-4	0	69.39	12.30
-11	-4	0	96.69	12.40
-11	-4	0	72.39	13.90
-11	-4	0	105.79	17.30
11	4	0	75.49	13.30
12	4	0	55.99	13.70
12	-5	0	199.08	18.80
-12	5	0	235.08	18.70
11	-5	0	2.00	9.80
-11	5	0	-11.10	13.20
-11	5	0	5.80	9.90
10	-5	0	-6.00	9.60
-10	5	0	2.30	10.00
-10	5	0	-0.50	9.40
9	-5	0	5.00	9.90
9	-5	0	-13.60	9.10
-9	5	0	3.90	8.50
-9	5	0	5.40	9.20
-9	5	0	-0.10	10.10
8	-5	0	67.39	10.30
-8	5	0	69.89	11.00

-8	5	0	69.39	10.50
-8	5	0	50.39	9.40
7	-5	0	237.88	18.10
7	-5	0	232.88	27.30
-7	5	0	255.07	15.80
-7	5	0	224.78	17.90
-7	5	0	253.27	17.50
6	-5	0	64.99	10.20
6	-5	0	83.29	13.30
-6	5	0	80.79	9.90
-6	5	0	82.09	9.30
-6	5	0	87.69	12.60
-6	5	0	60.99	14.50
5	-5	0	1319.47	62.19
5	-5	0	1273.77	63.19
5	-5	0	1348.97	61.09
-5	5	0	1335.17	63.29
-5	5	0	1320.07	62.59
-5	5	0	1403.06	61.79
4	-5	0	116.59	9.60
4	-5	0	104.89	8.60
4	-5	0	107.59	7.50
-4	5	0	113.69	8.50
-4	5	0	115.99	9.70
-4	5	0	89.69	8.50
3	-5	0	148.09	9.90
3	-5	0	162.78	9.30
3	-5	0	150.28	8.90
-3	5	0	163.28	8.30
-3	5	0	157.78	10.00
-3	5	0	159.68	9.40
2	-5	0	395.86	20.30
2	-5	0	405.46	20.90
2	-5	0	407.46	20.10
-2	5	0	437.66	20.40
-2	5	0	441.76	21.30
-2	5	0	454.85	20.00
1	-5	0	16.20	2.80
1	-5	0	14.10	3.00
1	-5	0	14.30	3.30
-1	5	0	13.50	3.70
-1	5	0	15.00	2.10
-1	5	0	15.20	2.70
0	-5	0	10.50	3.10

0	-5	0	5.60	1.50
0	5	0	12.30	3.60
0	5	0	8.30	1.20
0	5	0	6.40	1.50
-1	-5	0	405.16	18.10
-1	-5	0	366.46	19.30
1	5	0	384.36	18.10
1	5	0	405.96	19.30
-2	-5	0	158.78	7.60
2	5	0	155.48	7.80
2	5	0	160.48	7.60
2	5	0	164.58	9.10
3	5	0	16.70	3.00
3	5	0	21.60	2.50
-4	-5	0	152.18	9.50
4	5	0	147.39	8.30
4	5	0	164.08	10.20
4	5	0	156.08	10.40
-5	-5	0	515.95	25.10
5	5	0	530.05	24.60
5	5	0	521.35	26.90
5	5	0	502.35	27.00
-6	-5	0	853.91	41.90
-6	-5	0	872.61	43.30
-6	-5	0	930.01	41.30
-6	-5	0	817.32	42.20
-6	-5	0	853.21	42.10
-6	-5	0	879.71	42.60
6	5	0	922.41	41.40
-7	-5	0	149.29	13.60
-7	-5	0	142.89	12.70
-7	-5	0	129.49	10.30
-7	-5	0	106.99	11.30
-7	-5	0	127.09	14.70
-7	-5	0	131.29	12.60
7	5	0	129.29	10.10
-8	-5	0	192.38	14.60
-8	-5	0	147.49	14.10
-8	-5	0	162.98	15.70
-8	-5	0	165.28	13.40
-8	-5	0	174.68	12.50
8	5	0	189.48	18.30
8	5	0	168.88	14.00
-9	-5	0	551.44	31.90

-9	-5	0	555.24	30.30
-9	-5	0	563.14	30.00
-9	-5	0	551.84	29.80
-9	-5	0	597.44	29.60
9	5	0	566.54	32.30
-10	-5	0	148.49	20.20
-10	-5	0	92.79	15.20
-10	-5	0	104.39	14.30
-10	-5	0	118.39	15.00
-10	-5	0	113.39	13.30
10	5	0	105.99	22.90
-11	-5	0	4.70	13.50
-11	-5	0	4.70	11.30
-11	-5	0	18.10	13.00
-11	-5	0	-0.60	10.30
-11	-5	0	-7.00	7.20
11	5	0	-0.50	12.90
-12	-5	0	15.70	7.00
12	5	0	31.80	20.70
12	-6	0	152.88	16.90
-12	6	0	110.69	16.50
11	-6	0	3.90	9.00
-11	6	0	9.20	9.80
-11	6	0	-6.00	14.40
10	-6	0	133.79	21.10
-10	6	0	116.19	23.50
-10	6	0	140.19	17.90
-10	6	0	143.59	19.60
9	-6	0	29.80	10.10
9	-6	0	61.69	10.50
-9	6	0	44.40	10.40
-9	6	0	55.99	10.30
-9	6	0	41.40	9.90
8	-6	0	-0.20	8.00
-8	6	0	5.40	8.50
-8	6	0	3.40	7.90
-8	6	0	2.80	6.80
7	-6	0	1119.09	51.19
-7	6	0	954.40	54.99
-7	6	0	980.30	49.70
-7	6	0	1026.60	49.30
6	-6	0	38.90	8.40
6	-6	0	58.09	8.80
-6	6	0	54.19	9.40

-6	6	0	39.90	6.60
-6	6	0	38.40	8.30
5	-6	0	113.69	10.30
5	-6	0	106.99	12.50
5	-6	0	71.69	10.70
5	-6	0	100.79	13.30
-5	6	0	93.59	13.90
-5	6	0	80.79	14.10
-5	6	0	108.59	9.70
-5	6	0	116.69	12.70
4	-6	0	41.10	7.10
4	-6	0	38.60	5.20
4	-6	0	27.60	5.40
-4	6	0	41.40	5.20
-4	6	0	42.90	5.70
-4	6	0	35.80	5.60
-4	6	0	43.00	7.40
3	-6	0	43.00	7.10
3	-6	0	39.50	5.60
3	-6	0	40.20	6.10
-3	6	0	38.60	3.80
-3	6	0	38.50	5.80
-3	6	0	31.20	5.20
-3	6	0	41.90	7.40
2	-6	0	213.98	13.40
2	-6	0	229.38	13.40
2	-6	0	227.08	12.70
-2	6	0	254.57	14.10
-2	6	0	240.08	11.40
1	-6	0	722.43	32.50
1	-6	0	616.64	32.40
1	-6	0	669.13	33.50
-1	6	0	763.42	34.20
-1	6	0	724.33	32.40
-1	6	0	691.13	31.70
0	-6	0	977.30	43.50
0	-6	0	940.31	44.70
0	6	0	1011.00	45.00
0	6	0	955.80	43.40
0	6	0	879.91	42.80
-1	-6	0	1296.77	54.49
-1	-6	0	1065.19	55.59
1	6	0	1197.38	54.09
1	6	0	1263.17	54.39

1	6	0	1196.18	55.59
-2	-6	0	552.04	24.90
-2	-6	0	521.85	26.50
2	6	0	576.74	26.40
2	6	0	537.05	24.90
2	6	0	533.95	24.80
3	6	0	92.29	7.50
3	6	0	90.39	5.40
-4	-6	0	463.15	22.80
4	6	0	476.55	22.20
4	6	0	465.65	24.20
-5	-6	0	275.07	14.50
5	6	0	275.37	17.50
5	6	0	260.37	13.90
5	6	0	261.77	17.60
-6	-6	0	292.97	19.80
-6	-6	0	270.77	18.30
-6	-6	0	309.37	16.20
-6	-6	0	275.67	17.70
-6	-6	0	310.07	17.60
-6	-6	0	309.17	18.10
6	6	0	307.47	16.00
-7	-6	0	256.07	16.20
-7	-6	0	257.27	16.70
-7	-6	0	286.67	15.20
-7	-6	0	250.47	16.40
-7	-6	0	253.97	17.50
-7	-6	0	251.37	18.70
7	6	0	233.38	22.20
7	6	0	258.47	15.30
-8	-6	0	53.49	9.20
-8	-6	0	42.80	7.30
-8	-6	0	31.90	8.50
-8	-6	0	53.99	8.00
-8	-6	0	37.70	7.60
8	6	0	35.70	7.70
8	6	0	28.40	10.80
-9	-6	0	36.20	9.90
-9	-6	0	23.30	7.30
-9	-6	0	17.90	7.70
-9	-6	0	18.30	8.10
-9	-6	0	35.40	8.20
9	6	0	22.80	11.50
-10	-6	0	6.30	11.80

-10	-6	0	7.90	6.90
-10	-6	0	9.70	8.20
-10	-6	0	20.30	9.50
-10	-6	0	11.40	9.30
10	6	0	-4.60	20.70
10	6	0	-9.40	12.50
-11	-6	0	156.68	13.80
-11	-6	0	151.18	21.10
-11	-6	0	171.98	21.50
-11	-6	0	129.69	16.90
-11	-6	0	139.69	19.00
11	6	0	130.89	22.40
-12	-6	0	-4.80	13.90
-12	-6	0	-1.70	7.00
12	6	0	-5.10	11.60
11	-7	0	117.99	21.70
11	-7	0	149.39	16.10
-11	7	0	129.69	18.60
10	-7	0	18.30	11.80
10	-7	0	27.00	10.20
-10	7	0	21.10	11.90
-10	7	0	34.70	12.10
-10	7	0	35.70	12.30
9	-7	0	83.09	15.50
9	-7	0	80.79	11.50
-9	7	0	73.09	12.10
-9	7	0	78.89	12.10
-9	7	0	74.19	12.30
8	-7	0	7.20	7.80
8	-7	0	12.70	8.90
-8	7	0	5.40	8.70
-8	7	0	9.50	7.90
-8	7	0	10.00	10.00
7	-7	0	212.08	16.30
-7	7	0	198.48	21.50
-7	7	0	215.38	15.90
-7	7	0	202.78	18.40
6	-7	0	1129.69	53.59
-6	7	0	1109.39	53.49
-6	7	0	1152.48	54.99
-6	7	0	1092.59	54.29
5	-7	0	17.90	6.40
5	-7	0	21.30	7.80
-5	7	0	28.90	5.90

-5	7	0	20.90	9.20
4	-7	0	204.78	14.20
4	-7	0	208.68	14.60
4	-7	0	206.68	16.10
-4	7	0	218.58	11.70
-4	7	0	201.88	13.60
-4	7	0	217.78	14.20
-4	7	0	226.28	16.40
3	-7	0	4.80	4.90
3	-7	0	-5.80	4.00
3	-7	0	2.20	5.10
-3	7	0	7.80	5.60
-3	7	0	2.10	3.10
-3	7	0	0.30	4.40
-3	7	0	6.90	2.40
2	-7	0	92.59	9.60
2	-7	0	90.59	9.30
-2	7	0	97.49	7.10
-2	7	0	99.09	5.70
-2	7	0	94.89	9.90
-2	7	0	99.29	10.30
1	-7	0	434.16	22.50
-1	7	0	440.26	20.30
-1	7	0	435.66	21.80
0	-7	0	12.30	4.20
0	-7	0	4.20	2.00
0	7	0	5.90	1.90
0	7	0	14.10	4.40
0	7	0	7.60	1.50
-1	-7	0	317.67	14.80
-1	-7	0	250.97	16.80
1	7	0	315.37	14.70
1	7	0	321.87	17.00
1	7	0	301.97	14.20
-2	-7	0	703.43	36.20
-2	-7	0	777.32	34.30
2	7	0	753.12	34.20
2	7	0	745.33	34.20
2	7	0	773.82	36.20
-3	-7	0	3.70	3.40
3	7	0	4.80	1.80
3	7	0	6.40	3.80
-4	-7	0	798.12	37.80
4	7	0	818.22	37.30

-5	-7	0	103.89	12.50
-5	-7	0	114.29	16.50
-5	-7	0	127.49	11.50
-5	-7	0	116.69	8.80
-5	-7	0	118.49	10.10
5	7	0	85.09	14.30
5	7	0	125.99	8.10
5	7	0	128.09	14.80
-6	-7	0	121.59	14.70
-6	-7	0	122.99	12.30
-6	-7	0	135.39	13.20
-6	-7	0	151.68	12.10
-6	-7	0	145.79	11.40
-6	-7	0	155.38	10.30
6	7	0	149.88	10.00
-7	-7	0	0.50	4.80
-7	-7	0	16.50	7.50
-7	-7	0	2.70	6.30
-7	-7	0	6.30	4.70
-7	-7	0	9.10	5.80
7	7	0	6.60	5.00
-8	-7	0	514.05	28.00
-8	-7	0	529.75	27.30
-8	-7	0	532.55	28.40
-8	-7	0	532.35	29.50
-8	-7	0	520.75	28.20
8	7	0	525.55	32.60
8	7	0	556.84	28.60
-9	-7	0	126.09	17.40
-9	-7	0	100.09	13.50
-9	-7	0	109.49	12.90
-9	-7	0	117.89	16.80
-9	-7	0	130.79	13.80
9	7	0	91.99	15.80
9	7	0	123.09	22.10
-10	-7	0	228.28	16.80
-10	-7	0	189.18	21.90
-10	-7	0	218.48	18.20
-10	-7	0	222.58	18.30
10	7	0	170.18	25.50
10	7	0	204.98	38.70
-11	-7	0	124.59	15.30
-11	-7	0	90.19	14.10
-11	-7	0	123.89	12.90

-11	-7	0	99.49	21.10
-11	-7	0	116.09	19.20
-12	-7	0	164.78	14.30
-12	-7	0	173.28	20.80
12	7	0	183.18	18.80
11	-8	0	296.67	23.70
11	-8	0	309.07	20.40
-11	8	0	227.18	21.90
10	-8	0	90.29	15.70
10	-8	0	102.89	17.50
-10	8	0	73.69	14.60
-10	8	0	93.49	14.20
-10	8	0	82.19	14.00
9	-8	0	10.90	8.80
9	-8	0	6.30	9.40
-9	8	0	-1.20	10.70
-9	8	0	15.90	10.90
-9	8	0	10.90	10.70
8	-8	0	381.86	23.70
8	-8	0	369.16	24.10
-8	8	0	390.46	24.50
-8	8	0	366.16	24.70
-8	8	0	386.26	26.70
7	-8	0	14.60	7.70
-7	8	0	33.20	10.10
-7	8	0	14.90	7.80
-7	8	0	25.00	9.20
6	-8	0	324.37	20.20
-6	8	0	329.87	23.10
-6	8	0	342.57	20.70
-6	8	0	334.87	23.10
5	-8	0	32.30	11.70
5	-8	0	26.60	7.40
-5	8	0	22.70	6.70
-5	8	0	24.90	9.20
4	-8	0	11.30	8.40
4	-8	0	28.40	9.90
4	-8	0	9.60	6.70
-4	8	0	23.20	9.40
-4	8	0	19.00	3.90
-4	8	0	32.40	6.70
-4	8	0	16.10	6.50
3	-8	0	195.68	13.60
3	-8	0	174.98	15.70

-3	8	0	208.38	14.10
-3	8	0	227.58	13.00
-3	8	0	205.68	10.90
-3	8	0	224.28	17.40
2	-8	0	130.59	12.70
-2	8	0	154.38	8.00
-2	8	0	127.09	12.20
-2	8	0	149.69	13.70
-2	8	0	155.18	9.70
1	-8	0	1466.15	70.49
1	-8	0	1465.45	69.09
-1	8	0	1645.74	71.59
-1	8	0	1485.25	68.09
-1	8	0	1509.25	69.09
0	-8	0	1753.72	81.59
0	-8	0	1745.83	80.49
0	8	0	1707.83	80.09
0	8	0	1678.33	79.29
0	8	0	1960.10	82.29
-1	-8	0	335.57	18.00
-1	-8	0	324.87	16.00
-1	-8	0	282.67	18.30
1	8	0	317.87	15.30
1	8	0	320.17	15.90
1	8	0	363.36	20.40
-2	-8	0	318.07	14.60
-2	-8	0	257.47	17.60
-2	-8	0	298.57	16.10
2	8	0	301.47	14.40
2	8	0	288.47	17.50
2	8	0	303.47	14.30
-3	-8	0	627.74	29.60
-3	-8	0	664.03	31.10
3	8	0	676.13	30.20
3	8	0	640.44	29.60
-4	-8	0	27.80	4.90
4	8	0	24.40	3.50
4	8	0	23.90	7.00
-5	-8	0	-1.90	5.50
-5	-8	0	-1.00	6.70
-5	-8	0	1.50	6.60
-5	-8	0	-5.00	5.70
-5	-8	0	3.10	4.50
5	8	0	5.20	3.50

-6	-8	0	81.59	10.90
-6	-8	0	107.19	11.10
-6	-8	0	101.59	8.90
-6	-8	0	88.49	14.90
-6	-8	0	102.99	12.90
-6	-8	0	89.99	10.20
6	8	0	94.69	8.70
-7	-8	0	194.08	14.50
-7	-8	0	188.08	13.30
-7	-8	0	198.98	14.60
-7	-8	0	203.38	17.70
-7	-8	0	189.88	17.30
7	8	0	202.98	14.00
-8	-8	0	52.69	8.10
-8	-8	0	57.19	8.70
-8	-8	0	68.09	11.00
-8	-8	0	60.69	9.60
-8	-8	0	60.19	10.20
8	8	0	50.59	9.10
-9	-8	0	9.00	8.20
-9	-8	0	9.20	9.50
-9	-8	0	16.10	7.20
-9	-8	0	-5.30	6.70
-9	-8	0	14.80	8.40
9	8	0	6.30	12.60
-10	-8	0	192.78	19.50
-10	-8	0	207.68	26.30
-10	-8	0	186.08	29.20
-10	-8	0	167.98	17.60
-10	-8	0	129.29	22.10
10	8	0	150.68	26.90
-11	-8	0	30.10	11.90
-11	-8	0	33.10	12.50
-11	-8	0	15.40	9.00
-11	-8	0	46.80	15.10
-12	-8	0	4.60	7.40
-12	-8	0	-2.20	11.90
12	8	0	0.80	10.40
11	-9	0	17.10	10.60
11	-9	0	39.00	20.40
-11	9	0	2.50	10.90
10	-9	0	9.60	9.70
10	-9	0	27.80	10.60
-10	9	0	25.40	13.10

-10	9	0	15.00	16.80
-10	9	0	30.70	11.50
9	-9	0	-5.70	8.60
9	-9	0	6.90	10.10
-9	9	0	-0.80	13.20
-9	9	0	1.80	11.20
-9	9	0	4.80	11.80
8	-9	0	29.70	10.20
8	-9	0	36.60	8.80
-8	9	0	18.50	12.80
-8	9	0	39.80	10.90
-8	9	0	48.60	12.80
7	-9	0	55.99	9.00
7	-9	0	43.60	9.90
-7	9	0	35.40	10.00
-7	9	0	31.60	9.20
-7	9	0	61.09	12.90
6	-9	0	596.74	33.70
6	-9	0	547.15	33.80
-6	9	0	536.55	32.20
-6	9	0	537.25	30.30
5	-9	0	1276.27	65.29
5	-9	0	1390.96	64.29
-5	9	0	1397.36	66.19
-5	9	0	1414.96	65.39
4	-9	0	-3.90	9.10
4	-9	0	-4.10	6.10
-4	9	0	-0.30	10.20
-4	9	0	-3.00	10.10
-4	9	0	2.00	3.90
3	-9	0	8.50	6.40
3	-9	0	-1.40	8.50
-3	9	0	7.80	2.80
-3	9	0	10.50	6.40
-3	9	0	11.50	5.70
-3	9	0	17.30	10.40
2	-9	0	519.95	26.40
2	-9	0	488.15	28.80
-2	9	0	563.14	30.30
-2	9	0	475.65	27.30
-2	9	0	519.95	26.10
-2	9	0	561.34	24.60
1	-9	0	181.68	14.00
-1	9	0	154.98	8.80

-1	9	0	183.38	15.10
-1	9	0	164.48	10.40
0	-9	0	55.09	6.00
0	-9	0	33.80	6.50
0	9	0	46.30	3.80
0	9	0	42.40	5.40
-1	-9	0	1597.44	79.19
-1	-9	0	1725.03	77.29
1	9	0	1727.23	76.89
1	9	0	1724.63	76.29
-2	-9	0	399.46	18.60
-2	-9	0	307.87	22.40
-2	-9	0	363.36	19.80
2	9	0	392.66	18.00
2	9	0	401.46	18.40
-3	-9	0	9.70	4.50
-3	-9	0	4.80	2.90
3	9	0	-0.80	2.30
3	9	0	1.60	2.10
3	9	0	3.50	7.00
-4	-9	0	191.88	13.80
-4	-9	0	216.68	17.50
-4	-9	0	210.48	15.70
-4	-9	0	223.18	12.80
4	9	0	182.08	23.00
4	9	0	212.48	11.50
-5	-9	0	648.64	30.70
-5	-9	0	592.14	31.20
-5	-9	0	609.34	31.60
-5	-9	0	633.64	32.10
-5	-9	0	627.04	33.20
-5	-9	0	622.74	31.10
5	9	0	655.93	30.40
-6	-9	0	16.50	6.80
-6	-9	0	7.30	7.30
-6	-9	0	24.20	5.50
-6	-9	0	12.90	4.90
-6	-9	0	20.00	9.30
6	9	0	15.40	4.90
-7	-9	0	186.18	15.00
-7	-9	0	196.98	14.90
-7	-9	0	192.18	16.80
-7	-9	0	205.48	13.70
7	9	0	185.38	14.70

-8	-9	0	331.77	20.00
-8	-9	0	320.27	20.10
-8	-9	0	321.67	20.20
-8	-9	0	307.07	19.30
-8	-9	0	311.67	21.60
8	9	0	296.57	21.70
-9	-9	0	370.46	23.90
-9	-9	0	431.86	26.00
-9	-9	0	413.56	23.60
-9	-9	0	397.96	23.40
-9	-9	0	388.36	23.80
9	9	0	359.76	30.10
-10	-9	0	14.70	11.00
-10	-9	0	1.90	7.80
-10	-9	0	0.10	10.70
-10	-9	0	-7.60	7.80
-10	-9	0	6.60	10.70
-11	-9	0	-5.00	6.80
-11	-9	0	1.50	8.20
-11	-9	0	24.10	12.60
-11	-9	0	-8.10	22.60
11	9	0	-12.40	10.50
-12	-9	0	11.10	12.10
-12	-9	0	5.80	10.90
12	9	0	24.30	13.70
11	-10	0	0.00	12.50
11	-10	0	2.60	11.80
-11	10	0	-6.40	10.30
10	-10	0	75.59	11.40
10	-10	0	63.49	11.60
-10	10	0	76.09	14.30
-10	10	0	95.19	17.60
-10	10	0	77.39	13.20
9	-10	0	12.90	10.50
9	-10	0	47.20	11.40
-9	10	0	42.90	13.50
-9	10	0	36.20	17.20
-9	10	0	34.90	13.30
8	-10	0	190.68	16.60
8	-10	0	188.48	17.80
-8	10	0	160.48	21.00
-8	10	0	147.49	20.30
-8	10	0	172.88	22.50
7	-10	0	936.51	46.70

7	-10	0	860.11	47.10
-7	10	0	996.40	51.09
-7	10	0	961.80	49.40
-7	10	0	992.30	49.00
6	-10	0	64.59	12.60
-6	10	0	62.19	11.90
-6	10	0	49.00	12.90
-6	10	0	54.49	10.70
5	-10	0	23.30	9.00
5	-10	0	0.10	6.10
-5	10	0	3.20	10.20
-5	10	0	9.00	7.70
4	-10	0	186.48	16.40
4	-10	0	184.48	15.00
-4	10	0	205.88	19.90
-4	10	0	187.48	12.20
-4	10	0	186.98	16.10
3	-10	0	206.18	17.40
3	-10	0	230.98	17.00
-3	10	0	249.97	16.90
-3	10	0	260.47	12.90
-3	10	0	210.28	21.20
2	-10	0	7.20	9.00
2	-10	0	-4.20	6.20
-2	10	0	6.90	2.60
-2	10	0	1.80	9.40
-2	10	0	14.70	7.00
-2	10	0	7.90	5.00
1	-10	0	-0.60	4.90
1	-10	0	-0.30	8.00
-1	10	0	12.40	7.50
-1	10	0	4.30	2.20
-1	10	0	3.70	3.90
0	-10	0	2143.39	98.99
0	10	0	2157.58	95.89
0	10	0	2100.99	96.99
-1	-10	0	452.95	27.90
-1	-10	0	519.95	24.90
1	10	0	519.05	23.60
1	10	0	502.95	24.50
-2	-10	0	406.86	20.60
-2	-10	0	419.06	21.40
2	10	0	416.46	19.60
2	10	0	398.16	20.00

2	10	0	389.16	25.00
-3	-10	0	429.16	22.10
-3	-10	0	502.55	23.70
3	10	0	442.26	21.70
3	10	0	492.75	22.10
3	10	0	401.86	28.50
-4	-10	0	673.93	34.10
-4	-10	0	711.03	35.20
-4	-10	0	661.73	35.00
-4	-10	0	670.63	35.00
-4	-10	0	782.52	34.70
-4	-10	0	672.13	34.60
-5	-10	0	192.78	12.90
-5	-10	0	208.28	13.50
-5	-10	0	172.38	16.30
-5	-10	0	190.08	14.70
-5	-10	0	172.18	14.60
-5	-10	0	190.38	15.30
5	10	0	193.78	12.00
-6	-10	0	224.78	14.70
-6	-10	0	209.68	18.00
-6	-10	0	232.68	18.70
-6	-10	0	228.88	18.90
-6	-10	0	203.78	15.90
-6	-10	0	238.28	15.90
6	10	0	239.08	14.80
-7	-10	0	172.88	16.00
-7	-10	0	165.88	14.60
-7	-10	0	161.28	13.20
-7	-10	0	145.09	13.50
-7	-10	0	190.38	14.70
7	10	0	175.28	15.00
-8	-10	0	144.49	15.70
-8	-10	0	161.48	15.70
-8	-10	0	161.28	15.20
-8	-10	0	167.18	16.70
-8	-10	0	151.78	14.60
8	10	0	148.19	18.10
-9	-10	0	11.70	8.90
-9	-10	0	24.90	7.40
-9	-10	0	11.20	7.50
-9	-10	0	32.60	9.80
-9	-10	0	16.40	9.20
9	10	0	4.30	16.30

-10	-10	0	10.50	11.20
-10	-10	0	6.90	8.00
-10	-10	0	-4.20	11.80
-10	-10	0	7.70	7.70
-10	-10	0	-7.80	10.10
-11	-10	0	101.19	12.50
-11	-10	0	70.39	14.50
-11	-10	0	92.59	12.60
11	10	0	61.89	11.80
10	-11	0	34.00	9.60
10	-11	0	13.90	11.80
-10	11	0	30.00	14.00
-10	11	0	22.20	11.80
9	-11	0	47.60	10.90
-9	11	0	48.00	13.50
-9	11	0	81.79	14.80
-9	11	0	54.99	14.90
8	-11	0	179.58	19.00
8	-11	0	206.68	16.30
-8	11	0	197.68	21.70
-8	11	0	160.18	21.90
-8	11	0	194.28	22.80
7	-11	0	402.16	23.10
7	-11	0	358.36	24.90
-7	11	0	373.86	27.60
-7	11	0	358.26	28.60
-7	11	0	393.46	28.50
6	-11	0	895.01	48.20
6	-11	0	951.30	46.60
6	-11	0	931.61	48.50
-6	11	0	976.20	50.79
-6	11	0	974.90	50.49
-6	11	0	989.10	49.60
5	-11	0	155.98	17.10
5	-11	0	160.78	15.00
-5	11	0	148.49	23.10
-5	11	0	145.09	16.80
4	-11	0	264.07	17.90
4	-11	0	286.67	19.20
-4	11	0	246.58	23.60
-4	11	0	282.37	20.40
-4	11	0	280.37	16.40
3	-11	0	13.00	7.00
3	-11	0	11.00	7.30

-3	11	0	5.80	10.30
-3	11	0	7.10	6.30
-3	11	0	15.80	4.10
2	-11	0	216.98	16.70
2	-11	0	224.18	19.40
-2	11	0	249.88	12.80
-2	11	0	248.78	17.80
1	-11	0	1236.28	60.69
1	-11	0	1433.66	65.49
1	-11	0	1204.88	60.89
-1	11	0	1241.08	58.69
-1	11	0	1451.55	67.79
-1	11	0	1212.28	60.49
0	-11	0	2.00	5.90
0	-11	0	13.50	8.30
0	11	0	-7.80	8.90
0	11	0	2.70	4.50
0	11	0	1.50	2.20
-1	-11	0	353.86	24.90
1	11	0	350.56	19.50
1	11	0	398.86	25.60
1	11	0	392.46	18.30
-2	-11	0	436.36	23.50
-2	-11	0	449.06	22.60
-2	-11	0	375.06	24.10
-2	-11	0	437.96	22.70
2	11	0	436.16	21.80
2	11	0	449.46	20.90
-3	-11	0	105.49	10.50
-3	-11	0	90.19	11.30
-3	-11	0	83.29	14.00
-3	-11	0	78.69	12.60
-3	-11	0	89.19	13.20
-3	-11	0	88.99	11.40
3	11	0	94.49	7.50
-4	-11	0	107.49	12.30
-4	-11	0	95.49	10.10
-4	-11	0	92.99	13.00
-4	-11	0	121.99	11.20
-4	-11	0	89.79	12.80
-4	-11	0	100.59	13.00
4	11	0	106.39	8.00
-5	-11	0	-1.80	6.80
-5	-11	0	3.20	7.60

-5	-11	0	10.80	5.90
-5	-11	0	2.50	7.40
-5	-11	0	5.60	6.60
-5	-11	0	17.00	6.10
5	11	0	6.50	4.40
-6	-11	0	170.38	15.40
-6	-11	0	182.98	15.50
-6	-11	0	199.78	17.40
-6	-11	0	170.68	16.30
-6	-11	0	197.38	14.10
-6	-11	0	200.98	14.30
6	11	0	185.58	14.10
-7	-11	0	246.68	16.80
-7	-11	0	268.37	18.20
-7	-11	0	259.97	18.90
-7	-11	0	225.08	16.90
-7	-11	0	225.98	21.80
-7	-11	0	262.67	18.20
7	11	0	242.08	18.50
-8	-11	0	405.56	24.30
-8	-11	0	338.87	23.20
-8	-11	0	385.96	23.00
-8	-11	0	423.66	24.60
-8	-11	0	401.66	24.30
-9	-11	0	365.56	21.60
-9	-11	0	350.86	22.70
-9	-11	0	332.57	24.40
-9	-11	0	383.16	23.90
-9	-11	0	356.96	23.60
9	11	0	316.97	33.50
-10	-11	0	114.69	18.00
-10	-11	0	125.29	12.90
-10	-11	0	139.69	14.90
10	11	0	104.29	14.90
-11	-11	0	35.50	8.70
-11	-11	0	23.60	18.40
-11	-11	0	19.10	7.80
11	11	0	1.80	11.00
10	-12	0	16.50	8.80
-10	12	0	24.50	16.00
-10	12	0	8.50	10.40
9	-12	0	25.80	7.90
9	-12	0	23.60	12.50
-9	12	0	24.90	14.70

-9	12	0	6.90	11.00
-9	12	0	14.00	13.40
8	-12	0	193.48	16.30
-8	12	0	200.58	23.20
-8	12	0	178.48	26.20
-8	12	0	193.38	23.00
7	-12	0	145.79	14.30
7	-12	0	139.39	25.60
7	-12	0	95.59	14.80
-7	12	0	112.39	16.80
-7	12	0	122.59	20.80
-7	12	0	121.49	21.30
6	-12	0	307.57	23.80
6	-12	0	308.27	19.90
-6	12	0	308.67	25.70
-6	12	0	279.07	26.40
-6	12	0	304.77	24.80
5	-12	0	78.79	12.10
5	-12	0	83.29	15.10
-5	12	0	54.39	11.70
-5	12	0	76.79	13.50
4	-12	0	54.09	8.40
4	-12	0	76.99	13.20
-4	12	0	43.40	9.80
-4	12	0	33.30	11.80
-4	12	0	37.50	8.00
3	-12	0	581.54	31.00
3	-12	0	532.15	34.60
3	-12	0	581.14	31.00
-3	12	0	627.64	32.40
-3	12	0	608.24	34.00
-3	12	0	613.64	29.10
2	-12	0	285.47	21.00
2	-12	0	276.67	18.50
2	-12	0	257.77	20.80
2	-12	0	263.67	20.20
-2	12	0	290.97	15.20
-2	12	0	319.17	19.00
1	-12	0	899.41	45.90
1	-12	0	893.61	45.90
1	-12	0	1000.30	47.00
-1	12	0	1000.90	44.00
0	-12	0	383.76	24.10
0	-12	0	418.36	23.90

0	-12	0	425.06	24.50
0	-12	0	424.26	23.20
0	-12	0	439.36	23.80
0	12	0	374.66	22.90
0	12	0	439.06	20.40
-1	-12	0	1194.18	56.09
-1	-12	0	1196.08	56.79
-1	-12	0	1205.98	56.39
-1	-12	0	1149.98	55.59
-1	-12	0	1151.08	55.49
-1	-12	0	1206.48	56.69
1	12	0	1117.19	55.59
1	12	0	1240.08	54.29
-2	-12	0	37.00	7.00
-2	-12	0	49.90	7.30
-2	-12	0	48.50	6.40
-2	-12	0	65.79	9.40
-2	-12	0	52.29	8.30
2	12	0	46.80	7.50
-3	-12	0	410.56	23.40
-3	-12	0	381.06	24.40
-3	-12	0	399.56	23.40
-3	-12	0	445.16	22.20
-3	-12	0	391.56	22.20
-3	-12	0	409.56	22.70
3	12	0	414.26	20.50
-4	-12	0	187.08	15.50
-4	-12	0	187.78	13.80
-4	-12	0	194.98	14.70
-4	-12	0	186.18	15.80
-4	-12	0	190.98	14.00
-4	-12	0	182.78	16.00
4	12	0	184.98	11.70
-5	-12	0	67.29	10.70
-5	-12	0	64.89	10.20
-5	-12	0	82.19	10.40
-5	-12	0	89.79	12.10
5	12	0	87.69	9.50
-6	-12	0	27.20	8.10
-6	-12	0	17.70	9.00
-6	-12	0	9.70	6.50
-6	-12	0	19.30	7.50
-6	-12	0	18.10	8.30
-6	-12	0	20.50	7.60

6	12	0	12.90	6.80
-7	-12	0	115.99	15.60
-7	-12	0	129.09	18.00
-7	-12	0	153.48	15.80
-7	-12	0	142.69	13.50
-7	-12	0	145.69	15.30
-7	-12	0	137.79	14.20
7	12	0	109.69	15.80
-8	-12	0	57.89	9.90
-8	-12	0	32.80	8.50
-8	-12	0	32.60	7.80
-8	-12	0	43.30	10.30
-8	-12	0	44.60	10.30
8	12	0	41.40	13.50
-9	-12	0	33.00	10.80
-9	-12	0	20.50	11.90
-9	-12	0	27.40	12.20
-9	-12	0	20.00	10.50
-9	-12	0	19.90	14.30
9	12	0	29.00	20.50
-10	-12	0	155.88	18.30
-10	-12	0	162.98	13.70
-10	-12	0	123.39	18.90
-10	-12	0	159.88	15.20
10	12	0	158.38	20.30
-11	-12	0	40.10	15.00
-11	-12	0	-10.80	6.70
-11	-12	0	1.70	7.90
11	12	0	7.10	12.40
10	-13	0	13.90	8.10
-10	13	0	22.00	9.90
9	-13	0	-2.20	6.90
-9	13	0	3.50	13.10
-9	13	0	11.50	15.30
-9	13	0	26.70	10.60
8	-13	0	12.30	8.40
-8	13	0	7.70	13.80
-8	13	0	14.00	12.60
-8	13	0	-5.90	12.90
7	-13	0	24.90	14.20
7	-13	0	7.40	6.70
-7	13	0	25.40	13.50
-7	13	0	20.10	13.40
-7	13	0	24.70	12.50

6	-13	0	443.16	27.50
6	-13	0	428.56	24.50
-6	13	0	338.07	30.00
-6	13	0	487.35	29.80
-6	13	0	365.96	29.80
5	-13	0	462.85	26.80
5	-13	0	458.85	25.80
-5	13	0	431.36	30.20
-5	13	0	443.06	29.50
4	-13	0	-1.40	6.60
4	-13	0	-3.80	7.40
-4	13	0	21.30	9.40
-4	13	0	0.40	11.00
3	-13	0	117.29	13.20
3	-13	0	93.39	14.00
3	-13	0	133.89	19.60
-3	13	0	113.59	16.50
-3	13	0	110.39	10.50
-3	13	0	115.99	14.10
2	-13	0	760.42	41.60
2	-13	0	720.03	38.40
2	-13	0	762.22	39.20
2	-13	0	716.73	37.40
2	-13	0	718.53	37.90
-2	13	0	753.42	35.60
-2	13	0	801.42	38.20
1	-13	0	8.80	6.50
1	-13	0	-3.10	8.30
1	-13	0	15.00	7.80
1	-13	0	-4.30	11.00
1	-13	0	3.50	10.50
-1	13	0	5.70	6.10
-1	13	0	-9.60	16.90
-1	13	0	5.20	3.20
0	-13	0	49.40	8.30
0	-13	0	28.80	7.50
0	-13	0	50.49	9.00
0	-13	0	22.20	8.60
0	-13	0	35.40	8.70
0	13	0	46.90	5.50
0	13	0	32.80	25.00
-1	-13	0	242.98	16.70
-1	-13	0	230.98	17.80
-1	-13	0	238.48	15.90

-1	-13	0	188.08	23.20
-1	-13	0	269.17	18.70
-1	-13	0	213.08	16.40
1	13	0	248.88	13.00
-2	-13	0	540.95	28.40
-2	-13	0	540.05	29.30
-2	-13	0	524.95	27.70
-2	-13	0	500.65	31.00
-2	-13	0	566.94	27.70
-2	-13	0	541.55	28.00
2	13	0	550.74	26.20
-3	-13	0	374.66	22.20
-3	-13	0	380.96	23.00
-3	-13	0	394.56	21.70
-3	-13	0	363.56	21.30
-3	-13	0	384.06	21.10
-3	-13	0	362.46	32.70
3	13	0	389.36	19.60
-4	-13	0	307.97	21.40
-4	-13	0	346.87	19.60
-4	-13	0	330.57	19.30
-4	-13	0	330.57	20.60
-4	-13	0	325.87	20.70
-4	-13	0	342.47	20.00
4	13	0	336.07	18.20
-5	-13	0	331.47	19.70
-5	-13	0	308.87	19.10
-5	-13	0	318.07	18.70
-5	-13	0	279.47	20.70
-5	-13	0	295.77	20.50
-5	-13	0	265.57	19.80
5	13	0	336.57	18.50
5	13	0	316.67	29.50
-6	-13	0	15.30	8.00
-6	-13	0	16.50	8.50
-6	-13	0	7.90	6.50
-6	-13	0	11.10	8.60
-6	-13	0	-1.70	7.00
-6	-13	0	1.40	7.80
6	13	0	6.90	7.10
-7	-13	0	6.40	8.80
-7	-13	0	15.00	7.00
-7	-13	0	20.40	9.00
-7	-13	0	23.30	9.40

-7	-13	0	16.00	10.00
-7	-13	0	25.00	14.80
7	13	0	5.00	10.10
-8	-13	0	316.67	19.50
-8	-13	0	331.27	21.70
-8	-13	0	304.47	21.00
-8	-13	0	304.47	21.10
-8	-13	0	313.17	21.40
8	13	0	268.87	26.10
8	13	0	276.77	27.30
-9	-13	0	128.89	16.40
-9	-13	0	145.49	12.60
-9	-13	0	120.49	17.00
-9	-13	0	123.09	16.00
9	13	0	128.39	21.60
-10	-13	0	36.90	7.10
-10	-13	0	40.00	10.90
-10	-13	0	29.60	8.80
-10	-13	0	38.10	13.00
10	13	0	23.90	13.20
-11	-13	0	8.70	8.70
-11	-13	0	-3.90	8.30
9	-14	0	3.90	7.30
-9	14	0	-11.20	16.60
-9	14	0	23.40	13.10
-9	14	0	6.10	9.70
8	-14	0	77.39	13.00
-8	14	0	72.89	13.20
-8	14	0	80.39	14.50
-8	14	0	69.89	15.70
7	-14	0	526.65	28.10
-7	14	0	506.85	32.90
-7	14	0	490.95	33.90
-7	14	0	513.25	35.10
6	-14	0	31.30	8.10
-6	14	0	40.80	13.50
-6	14	0	28.60	13.40
-6	14	0	25.70	11.90
5	-14	0	29.30	7.60
5	-14	0	38.80	9.50
-5	14	0	30.00	12.20
-5	14	0	22.10	11.40
4	-14	0	793.32	42.10
4	-14	0	817.12	42.60

-4	14	0	915.61	45.00
-4	14	0	868.51	45.50
3	-14	0	172.18	20.30
3	-14	0	129.69	14.60
3	-14	0	106.99	19.40
-3	14	0	150.08	12.80
-3	14	0	177.28	15.80
-3	14	0	162.68	17.60
2	-14	0	30.80	10.10
2	-14	0	25.80	9.50
2	-14	0	26.30	9.40
2	-14	0	19.20	9.20
2	-14	0	18.30	13.30
-2	14	0	9.90	6.90
-2	14	0	26.10	5.40
1	-14	0	31.80	14.80
1	-14	0	10.20	7.50
1	-14	0	23.20	7.50
1	-14	0	21.40	10.70
1	-14	0	8.40	8.40
-1	14	0	13.10	4.10
-1	14	0	8.90	6.70
-1	14	0	5.70	17.90
0	-14	0	969.90	48.10
0	-14	0	1057.09	48.80
0	-14	0	1008.80	48.40
0	-14	0	980.40	47.90
0	14	0	955.50	50.19
0	14	0	989.10	46.30
-1	-14	0	14.50	7.20
-1	-14	0	20.40	8.20
-1	-14	0	4.10	6.70
-1	-14	0	7.50	7.90
-1	-14	0	1.00	5.80
1	14	0	10.80	3.70
-2	-14	0	119.99	13.40
-2	-14	0	136.49	12.50
-2	-14	0	123.09	18.30
-2	-14	0	140.89	12.50
-2	-14	0	120.59	14.50
2	14	0	138.59	9.60
-3	-14	0	664.33	41.40
-3	-14	0	801.02	39.10
-3	-14	0	775.22	38.00

-3	-14	0	814.22	38.30
-3	-14	0	772.12	42.90
3	14	0	803.12	37.20
-4	-14	0	476.35	26.80
-4	-14	0	462.85	25.60
-4	-14	0	445.16	26.90
-4	-14	0	489.25	26.10
-4	-14	0	480.15	25.50
4	14	0	495.45	24.80
-5	-14	0	10.50	6.30
-5	-14	0	18.50	7.50
-5	-14	0	15.20	8.30
-5	-14	0	17.50	7.30
-5	-14	0	15.30	8.00
5	14	0	15.90	6.80
-6	-14	0	96.09	12.90
-6	-14	0	78.29	11.00
-6	-14	0	92.39	14.00
-6	-14	0	91.99	11.20
-6	-14	0	104.99	12.00
6	14	0	139.19	25.50
6	14	0	88.19	14.50
-7	-14	0	149.99	13.20
-7	-14	0	135.99	17.10
-7	-14	0	120.79	16.10
-7	-14	0	132.79	14.90
-7	-14	0	154.08	15.70
7	14	0	147.09	24.60
7	14	0	108.99	19.10
-8	-14	0	220.58	25.20
-8	-14	0	180.28	14.20
-8	-14	0	168.08	16.90
-8	-14	0	149.79	20.60
-9	-14	0	2.40	8.70
-9	-14	0	-10.70	10.10
-9	-14	0	0.40	9.50
-9	-14	0	0.60	5.50
9	14	0	-5.50	13.80
-10	-14	0	-23.80	13.10
-10	-14	0	13.40	6.20
-10	-14	0	7.50	8.10
-10	-14	0	-8.60	10.10
10	14	0	-0.10	13.40
9	-15	0	124.09	12.50

-9	15	0	122.99	19.90
-9	15	0	91.49	14.60
8	-15	0	89.19	12.10
-8	15	0	66.39	12.30
-8	15	0	111.39	19.60
7	-15	0	90.49	13.80
7	-15	0	84.39	15.70
-7	15	0	102.89	16.30
-7	15	0	96.29	15.60
-7	15	0	80.09	13.60
6	-15	0	179.88	15.80
6	-15	0	205.78	20.10
-6	15	0	180.78	22.10
-6	15	0	194.28	23.30
-6	15	0	205.58	21.50
5	-15	0	361.76	24.00
5	-15	0	364.96	22.50
-5	15	0	342.17	26.20
-5	15	0	372.26	27.40
4	-15	0	38.00	8.30
4	-15	0	44.10	8.90
-4	15	0	30.40	10.00
-4	15	0	37.60	11.30
3	-15	0	73.09	12.80
3	-15	0	45.30	8.60
3	-15	0	36.10	9.10
-3	15	0	69.59	11.60
-3	15	0	53.29	9.10
-3	15	0	58.79	10.30
2	-15	0	472.35	30.30
2	-15	0	440.26	26.30
2	-15	0	457.15	25.60
2	-15	0	459.45	26.90
-2	15	0	427.16	23.70
-2	15	0	434.76	25.40
1	-15	0	319.37	23.20
1	-15	0	331.37	21.20
-1	15	0	336.67	18.30
-1	15	0	332.37	20.10
0	-15	0	146.59	14.70
0	-15	0	150.38	15.40
0	15	0	109.39	19.50
0	15	0	151.88	10.80
-1	-15	0	362.86	22.40

-1	-15	0	349.87	23.40
-1	-15	0	320.57	20.50
-1	-15	0	360.96	21.70
-1	-15	0	334.57	21.50
1	15	0	361.96	18.90
-2	-15	0	513.55	27.80
-2	-15	0	517.65	28.10
-2	-15	0	495.75	30.20
2	15	0	546.75	26.30
-3	-15	0	25.40	7.60
-3	-15	0	15.60	8.10
-3	-15	0	33.90	13.00
-3	-15	0	16.20	6.70
-3	-15	0	22.40	8.10
3	15	0	25.20	5.90
-4	-15	0	157.48	15.40
-4	-15	0	146.89	12.70
-4	-15	0	131.39	13.70
-4	-15	0	113.79	14.20
-4	-15	0	113.79	12.60
4	15	0	137.79	11.90
-5	-15	0	-0.70	7.80
-5	-15	0	-3.40	7.60
-5	-15	0	3.30	7.10
-5	-15	0	6.00	5.90
-5	-15	0	21.80	7.60
5	15	0	6.00	7.30
-6	-15	0	47.60	8.70
-6	-15	0	32.20	8.90
-6	-15	0	19.30	6.20
-6	-15	0	27.10	8.90
-6	-15	0	9.30	8.40
6	15	0	29.90	14.10
6	15	0	72.89	29.10
-7	-15	0	103.79	14.80
-7	-15	0	88.89	11.90
-7	-15	0	100.89	16.10
-7	-15	0	81.79	13.70
-7	-15	0	97.19	11.50
7	15	0	71.69	14.40
7	15	0	124.89	19.00
-8	-15	0	24.40	9.50
-8	-15	0	21.10	6.40
-8	-15	0	19.90	8.90

-8	-15	0	45.90	14.80
8	15	0	29.80	16.50
8	15	0	46.80	21.50
-9	-15	0	626.64	31.20
-9	-15	0	634.74	32.70
-9	-15	0	598.54	32.50
9	15	0	619.34	37.50
-10	-15	0	84.49	10.20
-10	-15	0	82.79	14.50
-10	-15	0	88.79	12.60
10	15	0	64.89	16.60
9	-16	0	2.60	7.90
-9	16	0	8.50	9.00
-9	16	0	0.10	14.90
8	-16	0	67.99	11.60
-8	16	0	75.49	15.80
-8	16	0	109.59	18.40
7	-16	0	157.88	14.50
7	-16	0	163.78	22.70
-7	16	0	168.18	22.50
-7	16	0	165.88	21.00
-7	16	0	154.48	17.60
6	-16	0	17.90	12.80
6	-16	0	13.70	15.70
-6	16	0	9.00	12.40
5	-16	0	127.49	14.80
5	-16	0	108.59	16.60
-5	16	0	144.49	18.40
-5	16	0	128.39	19.20
4	-16	0	127.59	16.50
4	-16	0	134.79	14.90
-4	16	0	137.89	18.60
-4	16	0	149.99	19.80
3	-16	0	444.06	30.40
3	-16	0	431.06	26.70
3	-16	0	485.45	27.00
-3	16	0	470.25	27.60
-3	16	0	473.25	28.30
2	-16	0	652.93	37.60
2	-16	0	651.73	35.20
2	-16	0	630.34	35.50
2	-16	0	685.23	35.60
2	-16	0	652.93	34.70
-2	16	0	661.13	34.50

-2	16	0	663.33	33.70
1	-16	0	221.38	17.60
1	-16	0	182.48	19.60
1	-16	0	219.28	17.10
1	-16	0	194.18	17.40
-1	16	0	214.28	14.80
-1	16	0	201.38	15.90
0	-16	0	51.29	8.90
0	-16	0	35.20	8.10
0	-16	0	51.19	8.70
0	-16	0	39.30	9.30
0	16	0	53.09	16.80
0	16	0	56.79	9.40
-1	-16	0	238.28	19.00
-1	-16	0	274.37	17.60
-1	-16	0	239.68	17.60
-1	-16	0	240.48	17.90
1	16	0	267.97	15.80
1	16	0	226.28	21.10
-2	-16	0	452.65	24.60
-2	-16	0	431.26	25.00
-2	-16	0	449.76	27.50
-2	-16	0	445.56	24.90
-2	-16	0	410.66	24.00
2	16	0	443.76	23.40
-3	-16	0	41.50	8.20
-3	-16	0	30.20	8.10
-3	-16	0	42.40	7.80
-3	-16	0	24.40	12.70
-3	-16	0	36.70	8.10
3	16	0	37.10	6.70
-4	-16	0	114.99	11.80
-4	-16	0	111.59	13.70
-4	-16	0	117.89	13.60
-4	-16	0	114.59	14.00
4	16	0	111.19	12.70
-5	-16	0	7.20	6.30
-5	-16	0	3.70	8.40
-5	-16	0	28.50	8.50
-5	-16	0	9.30	7.90
-5	-16	0	-5.50	7.10
5	16	0	1.60	8.90
-6	-16	0	11.80	7.90
-6	-16	0	1.10	7.70

-6	-16	0	2.90	5.90
-6	-16	0	-9.40	7.50
-6	-16	0	13.90	8.40
6	16	0	-4.40	10.50
-7	-16	0	169.88	19.70
-7	-16	0	162.18	15.50
-7	-16	0	164.78	18.20
7	16	0	153.28	24.90
-8	-16	0	105.69	19.10
-8	-16	0	97.79	14.00
-8	-16	0	103.59	14.30
-8	-16	0	95.09	11.10
-8	-16	0	84.39	13.70
8	16	0	78.59	19.40
-9	-16	0	5.40	7.90
-9	-16	0	11.80	5.90
-9	-16	0	-0.90	9.10
9	16	0	20.90	18.40
-10	-16	0	74.39	11.20
-10	-16	0	56.49	8.80
-10	-16	0	69.79	10.80
8	-17	0	93.19	10.50
-8	17	0	58.39	16.20
-8	17	0	93.09	14.20
7	-17	0	54.19	14.10
7	-17	0	39.30	7.90
-7	17	0	35.50	10.20
-7	17	0	42.70	12.10
-7	17	0	69.69	15.90
6	-17	0	208.38	17.10
6	-17	0	231.08	21.10
-6	17	0	228.68	22.30
-6	17	0	235.48	20.20
-6	17	0	226.98	22.20
5	-17	0	78.49	11.90
5	-17	0	62.69	10.70
-5	17	0	61.79	12.80
-5	17	0	75.29	12.50
-5	17	0	58.49	12.70
4	-17	0	15.30	8.50
4	-17	0	24.70	9.20
-4	17	0	21.90	10.30
-4	17	0	13.10	10.30
3	-17	0	93.09	14.60

3	-17	0	113.09	15.90
3	-17	0	95.69	14.00
-3	17	0	92.29	15.60
-3	17	0	92.19	15.70
2	-17	0	461.05	32.70
2	-17	0	487.05	27.70
2	-17	0	541.45	28.70
2	-17	0	422.56	29.20
-2	17	0	466.65	27.70
-2	17	0	455.15	28.70
-2	17	0	497.95	27.40
1	-17	0	0.00	8.50
1	-17	0	-8.30	8.10
1	-17	0	-0.70	10.70
1	-17	0	-3.40	7.70
-1	17	0	-4.10	7.30
-1	17	0	1.20	7.00
0	-17	0	26.20	8.30
0	-17	0	52.09	9.80
0	-17	0	24.20	8.60
0	-17	0	30.30	8.60
0	17	0	50.89	8.30
0	17	0	47.40	13.60
0	17	0	30.00	7.50
-1	-17	0	152.88	14.10
-1	-17	0	156.58	15.20
-1	-17	0	167.48	14.70
-1	-17	0	137.99	16.20
1	17	0	128.19	19.20
1	17	0	149.09	13.60
-2	-17	0	493.95	29.00
-2	-17	0	485.55	26.10
-2	-17	0	489.05	26.60
-2	-17	0	468.15	26.20
-2	-17	0	481.85	26.80
2	17	0	413.76	28.70
2	17	0	509.05	26.20
-3	-17	0	314.37	19.50
-3	-17	0	319.67	19.90
-3	-17	0	287.77	24.80
-3	-17	0	322.57	20.30
-3	-17	0	290.67	19.10
3	17	0	305.37	19.40
-4	-17	0	259.07	17.40

-4	-17	0	212.38	17.60
-4	-17	0	235.38	17.10
-4	-17	0	239.88	15.90
4	17	0	195.58	24.60
-5	-17	0	0.30	7.30
-5	-17	0	17.80	8.90
-5	-17	0	15.90	6.30
5	17	0	12.30	9.60
-6	-17	0	16.60	8.40
-6	-17	0	26.50	8.50
-6	-17	0	18.60	8.60
-6	-17	0	18.90	6.40
6	17	0	21.10	12.90
-7	-17	0	13.70	8.70
-7	-17	0	21.00	8.80
-7	-17	0	11.60	6.10
-7	-17	0	17.00	8.50
-8	-17	0	397.96	28.20
-8	-17	0	475.45	25.80
-8	-17	0	482.35	25.60
-8	-17	0	463.75	23.90
-9	-17	0	17.30	5.40
-9	-17	0	17.30	6.90
-9	-17	0	9.30	8.90
7	-18	0	230.48	15.50
-7	18	0	225.08	18.00
-7	18	0	210.28	19.80
-7	18	0	204.18	21.70
6	-18	0	67.49	12.00
6	-18	0	56.89	11.40
-6	18	0	36.90	13.30
-6	18	0	66.49	10.70
-6	18	0	45.50	12.30
5	-18	0	43.80	8.90
5	-18	0	38.70	9.80
-5	18	0	33.50	11.20
-5	18	0	43.30	11.60
-5	18	0	26.00	10.10
4	-18	0	374.76	31.10
4	-18	0	379.76	23.60
-4	18	0	367.06	24.00
-4	18	0	334.47	24.20
3	-18	0	0.50	12.20
3	-18	0	14.60	9.90

3	-18	0	13.10	8.70
-3	18	0	-1.40	8.30
-3	18	0	-6.50	8.70
2	-18	0	87.99	14.60
2	-18	0	94.29	16.70
-2	18	0	77.09	12.30
-2	18	0	64.19	11.00
1	-18	0	21.10	8.60
1	-18	0	16.70	8.60
1	-18	0	42.70	11.70
1	-18	0	8.60	8.60
-1	18	0	13.10	8.40
-1	18	0	38.50	11.30
0	-18	0	179.68	16.00
0	-18	0	203.08	16.90
0	-18	0	156.08	17.60
0	-18	0	176.38	17.80
0	18	0	185.28	23.90
0	18	0	206.08	17.90
0	18	0	230.28	25.20
-1	-18	0	87.59	15.20
-1	-18	0	89.49	12.60
-1	-18	0	75.19	9.90
-1	-18	0	92.89	12.80
1	18	0	67.79	12.80
1	18	0	64.69	10.30
-2	-18	0	82.89	12.80
-2	-18	0	104.99	13.10
-2	-18	0	75.59	12.50
-2	-18	0	65.99	11.30
2	18	0	104.09	12.90
2	18	0	118.29	17.40
-3	-18	0	882.21	43.00
-3	-18	0	863.51	42.50
-3	-18	0	875.31	42.60
-3	-18	0	824.92	45.60
-3	-18	0	861.41	42.30
3	18	0	863.41	43.20
-4	-18	0	306.87	19.10
-4	-18	0	332.87	20.30
-4	-18	0	295.87	19.40
-4	-18	0	323.47	20.20
4	18	0	312.07	22.70
-5	-18	0	69.49	9.70

-5	-18	0	56.09	9.70
-5	-18	0	54.69	8.10
-5	-18	0	61.89	9.70
5	18	0	52.69	13.20
-6	-18	0	214.88	16.20
-6	-18	0	218.98	17.40
-6	-18	0	215.68	17.00
-6	-18	0	216.68	15.30
6	18	0	218.28	37.50
-7	-18	0	10.90	5.60
-7	-18	0	16.00	7.90
-7	-18	0	6.90	8.30
-7	-18	0	2.60	7.40
-8	-18	0	136.49	13.90
-8	-18	0	143.79	11.10
-8	-18	0	116.69	13.70
-9	-18	0	1.00	5.30
-9	-18	0	18.10	8.70
-9	-18	0	6.60	7.70
7	-19	0	15.30	8.30
-7	19	0	8.10	11.10
-7	19	0	29.00	9.20
6	-19	0	6.80	7.80
6	-19	0	3.80	9.90
-6	19	0	12.10	12.20
-6	19	0	-3.30	10.20
-6	19	0	21.70	9.40
5	-19	0	219.08	18.40
5	-19	0	206.58	16.80
-5	19	0	205.28	20.00
-5	19	0	224.68	19.40
-5	19	0	199.68	17.60
4	-19	0	-5.40	9.30
4	-19	0	2.20	8.00
-4	19	0	5.40	9.70
-4	19	0	-8.30	8.70
3	-19	0	3.50	11.70
3	-19	0	4.50	9.40
-3	19	0	9.60	8.70
-3	19	0	8.80	10.60
2	-19	0	87.89	12.20
2	-19	0	103.49	14.60
-2	19	0	94.59	13.30
-2	19	0	85.19	11.40

1	-19	0	52.49	12.30
1	-19	0	67.19	10.10
1	-19	0	54.49	9.70
1	-19	0	52.69	10.00
-1	19	0	37.60	9.00
0	-19	0	14.30	8.00
0	-19	0	33.10	9.00
0	-19	0	14.40	8.80
0	-19	0	31.90	10.20
0	19	0	33.70	12.90
0	19	0	33.10	8.80
-1	-19	0	446.76	25.70
-1	-19	0	415.76	25.30
-1	-19	0	439.76	26.90
-1	-19	0	454.85	25.30
1	19	0	465.85	28.70
1	19	0	433.36	30.20
1	19	0	423.86	25.50
-2	-19	0	85.39	13.50
-2	-19	0	117.29	17.30
-2	-19	0	136.09	13.60
-2	-19	0	110.09	13.10
2	19	0	129.99	17.60
2	19	0	134.99	18.30
-3	-19	0	39.70	13.50
-3	-19	0	9.40	7.20
-3	-19	0	13.00	6.80
-3	-19	0	12.00	7.50
3	19	0	3.10	10.00
3	19	0	5.60	11.20
-4	-19	0	104.49	12.80
-4	-19	0	97.39	12.30
-4	-19	0	87.49	13.50
4	19	0	79.49	16.40
4	19	0	99.69	14.90
-5	-19	0	8.20	7.90
-5	-19	0	-3.90	6.10
-5	-19	0	-5.60	7.90
-5	-19	0	-4.70	7.20
5	19	0	52.89	24.50
-6	-19	0	15.60	8.10
-6	-19	0	24.30	7.70
-6	-19	0	5.00	5.90
-6	-19	0	20.10	7.40

-7	-19	0	77.09	9.70
-7	-19	0	73.69	13.20
-7	-19	0	81.39	12.50
-7	-19	0	80.89	9.70
-8	-19	0	50.89	7.80
-8	-19	0	34.70	10.10
6	-20	0	6.20	12.70
-6	20	0	13.20	10.90
-6	20	0	5.20	13.00
-6	20	0	8.50	11.90
5	-20	0	70.89	13.80
5	-20	0	86.79	14.40
-5	20	0	127.79	14.20
-5	20	0	116.49	15.20
-5	20	0	100.79	13.50
4	-20	0	239.68	20.20
4	-20	0	285.57	18.30
-4	20	0	244.18	19.40
-4	20	0	248.38	18.70
-4	20	0	200.28	18.90
3	-20	0	395.36	23.30
3	-20	0	364.36	28.80
-3	20	0	380.66	23.90
-3	20	0	372.36	23.30
2	-20	0	153.88	18.50
2	-20	0	163.58	16.50
-2	20	0	173.88	16.90
-2	20	0	134.49	15.30
1	-20	0	34.30	14.10
1	-20	0	25.20	16.20
-1	20	0	14.90	9.10
-1	20	0	18.40	8.30
0	-20	0	19.30	8.50
0	-20	0	23.20	10.60
0	-20	0	24.10	9.10
0	20	0	15.60	7.70
-1	-20	0	20.20	7.60
-1	-20	0	15.20	8.80
-1	-20	0	25.60	11.10
1	20	0	11.30	7.80
-2	-20	0	486.35	35.10
-2	-20	0	480.85	25.50
2	20	0	446.66	34.10
2	20	0	355.96	30.00

2	20	0	427.96	25.00
-3	-20	0	-1.80	8.40
-3	-20	0	-2.90	14.80
-3	-20	0	0.40	7.80
-3	-20	0	8.80	9.90
3	20	0	9.50	10.80
3	20	0	28.70	15.60
-4	-20	0	320.47	19.90
-4	-20	0	308.27	19.20
-4	-20	0	301.17	18.60
4	20	0	288.37	21.60
-5	-20	0	-0.80	6.20
-5	-20	0	3.30	7.10
-5	-20	0	0.60	6.90
5	20	0	3.90	9.30
-6	-20	0	5.10	7.50
-6	-20	0	6.30	5.70
-6	-20	0	-4.60	6.90
6	20	0	10.30	8.30
-7	-20	0	199.88	17.80
-7	-20	0	202.58	13.20
-7	-20	0	190.38	16.90
7	20	0	213.98	16.20
5	-21	0	-6.60	8.20
5	-21	0	-21.20	11.20
-5	21	0	-7.00	9.10
-5	21	0	20.70	11.10
-5	21	0	4.00	11.80
4	-21	0	1.50	7.70
-4	21	0	2.40	8.30
-4	21	0	16.80	10.80
-4	21	0	-7.60	8.80
3	-21	0	229.98	16.30
-3	21	0	182.28	17.20
-3	21	0	221.98	16.70
2	-21	0	82.29	12.60
2	-21	0	90.39	12.60
-2	21	0	85.09	13.50
-2	21	0	86.29	13.10
1	-21	0	33.10	9.50
1	-21	0	22.00	8.60
-1	21	0	30.10	8.60
-1	21	0	27.40	9.10
0	-21	0	57.59	12.40

0	-21	0	73.69	10.10
0	21	0	62.09	9.40
-1	-21	0	53.79	10.40
-1	-21	0	76.09	11.30
-1	-21	0	87.19	12.50
-1	-21	0	87.49	13.50
1	21	0	79.79	12.30
-2	-21	0	409.46	25.70
-2	-21	0	387.26	23.60
-2	-21	0	446.16	23.70
-2	-21	0	423.96	24.10
2	21	0	399.86	28.10
2	21	0	429.46	24.10
-3	-21	0	69.19	10.80
-3	-21	0	70.49	11.70
3	21	0	50.99	9.10
3	21	0	68.09	12.20
-4	-21	0	15.40	6.90
-4	-21	0	21.20	6.60
-4	-21	0	6.30	7.80
4	21	0	38.40	10.50
4	21	0	41.20	9.60
-5	-21	0	56.59	10.40
-5	-21	0	64.19	10.20
-5	-21	0	65.99	9.00
5	21	0	63.49	10.30
-6	-21	0	4.50	6.80
-6	-21	0	0.90	10.80
6	21	0	4.20	8.70
-7	-21	0	97.59	14.10
7	21	0	83.59	13.40
4	-22	0	103.19	12.30
-4	22	0	92.29	15.40
-4	22	0	134.89	15.50
-4	22	0	102.39	13.30
3	-22	0	80.59	11.90
-3	22	0	105.19	14.00
-3	22	0	89.39	14.80
-3	22	0	78.79	12.40
2	-22	0	131.29	13.30
2	-22	0	96.49	17.50
-2	22	0	115.99	13.30
-2	22	0	124.49	18.40
1	-22	0	6.00	7.70

1	-22	0	-14.70	8.80
-1	22	0	16.30	7.40
-1	22	0	1.70	8.20
0	-22	0	41.00	9.10
0	-22	0	48.90	9.00
0	22	0	69.29	11.50
0	22	0	55.79	8.90
-1	-22	0	12.00	7.20
-1	-22	0	8.60	8.20
-1	-22	0	19.50	10.50
-1	-22	0	6.40	8.80
1	22	0	8.10	7.70
-2	-22	0	4.20	8.60
-2	-22	0	2.80	8.00
-2	-22	0	-5.10	10.80
-2	-22	0	5.60	6.70
2	22	0	-1.50	7.60
2	22	0	-9.30	16.90
-3	-22	0	654.03	32.00
-3	-22	0	606.44	32.00
-3	-22	0	646.84	31.90
3	22	0	641.24	32.70
3	22	0	609.64	34.50
-4	-22	0	32.40	6.80
-4	-22	0	46.80	8.70
-4	-22	0	59.09	8.30
4	22	0	41.90	8.80
4	22	0	66.39	11.30
-5	-22	0	4.90	7.60
-5	-22	0	10.60	6.20
-5	-22	0	6.60	6.40
5	22	0	14.60	8.90
5	22	0	-9.00	9.80
-6	-22	0	165.08	13.30
-6	-22	0	158.08	12.00
6	22	0	177.88	14.90
3	-23	0	6.70	6.80
-3	23	0	21.50	11.10
-3	23	0	8.20	9.40
-3	23	0	6.20	10.10
2	-23	0	205.68	20.30
2	-23	0	235.98	15.60
-2	23	0	228.28	17.20
-2	23	0	224.38	16.10

1	-23	0	19.10	7.50
1	-23	0	36.30	9.70
-1	23	0	23.00	8.50
-1	23	0	32.90	7.40
0	-23	0	12.30	7.90
0	-23	0	13.10	8.30
0	23	0	9.60	6.70
0	23	0	9.30	8.20
-1	-23	0	398.66	24.40
-1	-23	0	430.26	22.90
1	23	0	442.06	23.30
1	23	0	406.96	23.40
-2	-23	0	69.99	10.50
2	23	0	67.99	10.00
-3	-23	0	9.70	6.10
-3	-23	0	1.50	9.40
3	23	0	-2.70	8.20
-4	-23	0	41.00	7.10
-4	-23	0	39.60	7.90
-4	-23	0	47.70	10.00
4	23	0	44.80	11.80
4	23	0	31.80	9.40
1	-24	0	10.50	7.70
1	-24	0	6.40	10.20
-1	24	0	21.50	10.20
-1	24	0	8.70	8.70
0	-24	0	11.20	8.40
0	-24	0	5.10	8.60
0	24	0	-2.00	8.50
0	24	0	-10.00	6.70
-1	-24	0	-6.60	10.40
-1	-24	0	-15.30	8.30
1	24	0	1.50	6.20
1	24	0	-3.00	8.40
-2	-24	0	167.18	13.10
2	24	0	146.99	14.20
2	24	0	184.78	13.20
1	24	-1	6.20	8.30
-1	-24	1	14.00	11.10
4	23	-1	31.10	9.60
4	23	-1	25.80	10.90
-4	-23	1	13.90	8.40
-4	-23	1	15.20	6.20
3	23	-1	30.10	8.80

3	23	-1	21.50	12.40
-3	-23	1	-2.70	9.60
-3	-23	1	10.50	6.10
2	23	-1	407.26	21.20
2	23	-1	355.86	21.60
-2	-23	1	357.26	20.30
-2	-23	1	367.56	26.90
1	23	-1	23.60	7.10
1	23	-1	19.70	8.40
-1	-23	1	15.90	7.20
-1	-23	1	19.80	9.20
0	23	-1	107.99	11.90
0	23	-1	104.59	13.00
0	-23	1	106.09	12.60
0	-23	1	76.99	12.70
-1	23	-1	14.20	8.90
-1	23	-1	1.70	6.80
1	-23	1	-2.60	6.90
1	-23	1	4.30	15.00
-2	23	-1	45.00	9.80
-2	23	-1	36.40	8.20
2	-23	1	34.60	7.20
5	22	-1	18.60	13.90
5	22	-1	10.00	9.50
-5	-22	1	2.40	5.50
4	22	-1	199.78	18.60
4	22	-1	224.58	16.30
-4	-22	1	222.68	14.50
-4	-22	1	210.08	14.90
-4	-22	1	201.58	19.40
3	22	-1	44.10	10.30
3	22	-1	23.30	10.50
-3	-22	1	32.20	6.70
-3	-22	1	35.20	9.80
2	22	-1	154.58	19.00
2	22	-1	157.78	14.00
-2	-22	1	177.78	18.20
-2	-22	1	118.09	15.50
-2	-22	1	172.68	16.30
-2	-22	1	169.28	13.00
1	22	-1	0.50	7.40
-1	-22	1	6.20	8.90
-1	-22	1	13.80	7.30
0	22	-1	23.40	7.50

0	22	-1	12.20	8.00
0	-22	1	12.60	8.10
0	-22	1	16.80	8.20
-1	22	-1	57.89	10.20
-1	22	-1	55.09	9.00
1	-22	1	56.79	10.20
1	-22	1	54.89	9.10
-2	22	-1	3.00	7.60
-2	22	-1	3.80	9.00
2	-22	1	3.50	7.20
2	-22	1	28.00	12.50
-3	22	-1	260.37	16.90
-3	22	-1	212.98	17.40
-3	22	-1	205.18	20.50
3	-22	1	229.68	16.00
-4	22	-1	451.35	27.90
-4	22	-1	491.65	25.30
-4	22	-1	420.16	26.40
4	-22	1	481.65	24.90
6	21	-1	-9.20	10.20
6	21	-1	2.80	8.40
-6	-21	1	9.40	6.00
-6	-21	1	-8.90	8.90
-6	-21	1	6.80	6.60
5	21	-1	26.50	9.50
5	21	-1	25.30	9.40
-5	-21	1	16.80	6.70
-5	-21	1	17.00	6.50
-5	-21	1	30.20	8.80
4	21	-1	18.50	10.00
-4	-21	1	0.70	6.30
-4	-21	1	-0.40	8.30
-4	-21	1	-7.30	5.90
3	21	-1	61.09	9.40
3	21	-1	50.99	11.50
-3	-21	1	64.19	9.00
-3	-21	1	55.19	9.50
2	21	-1	69.69	12.50
2	21	-1	55.99	9.50
-2	-21	1	47.00	11.10
-2	-21	1	73.99	11.00
-2	-21	1	58.49	9.70
-2	-21	1	63.89	10.20
1	21	-1	712.53	39.00

1	21	-1	749.82	36.70
-1	-21	1	673.53	36.30
-1	-21	1	707.53	37.10
-1	-21	1	706.73	36.90
-1	-21	1	726.63	36.20
0	21	-1	50.69	9.20
0	21	-1	39.60	8.70
0	-21	1	41.10	9.30
0	-21	1	27.10	8.90
-1	21	-1	45.90	9.20
-1	21	-1	49.80	8.80
1	-21	1	47.20	10.10
1	-21	1	64.99	12.10
-2	21	-1	324.77	20.80
-2	21	-1	323.07	21.10
2	-21	1	325.67	20.70
2	-21	1	328.37	23.30
-3	21	-1	4.50	7.50
-3	21	-1	16.10	8.90
3	-21	1	22.70	7.50
-4	21	-1	26.60	10.30
-4	21	-1	36.60	8.80
-4	21	-1	26.90	10.10
4	-21	1	36.60	8.40
-5	21	-1	31.30	11.50
-5	21	-1	14.50	11.20
-5	21	-1	37.90	9.40
5	-21	1	25.90	8.70
5	-21	1	4.50	16.40
7	20	-1	2.90	7.80
-7	-20	1	9.30	11.50
-7	-20	1	6.90	5.40
-7	-20	1	-17.00	8.40
6	20	-1	150.48	14.70
-6	-20	1	141.39	12.60
-6	-20	1	112.39	13.80
-6	-20	1	136.89	11.60
5	20	-1	-10.10	8.80
-5	-20	1	1.50	8.20
-5	-20	1	2.20	6.50
-5	-20	1	2.30	6.40
4	20	-1	74.39	11.10
-4	-20	1	67.69	11.40
-4	-20	1	74.99	10.00

-4	-20	1	82.79	11.90
3	20	-1	732.53	37.00
3	20	-1	693.73	35.70
-3	-20	1	654.13	34.60
-3	-20	1	656.13	35.00
-3	-20	1	674.63	47.10
-3	-20	1	702.73	35.10
2	20	-1	139.69	13.90
2	20	-1	179.18	19.80
-2	-20	1	155.98	16.00
-2	-20	1	162.28	22.20
-2	-20	1	159.78	15.80
-2	-20	1	137.89	18.00
1	20	-1	6.40	7.90
1	20	-1	-2.90	13.20
-1	-20	1	22.20	10.20
-1	-20	1	9.80	10.80
0	20	-1	92.39	12.40
0	-20	1	96.09	13.00
0	-20	1	88.69	13.90
-1	20	-1	3.80	12.40
-1	20	-1	4.80	7.20
1	-20	1	5.80	10.00
1	-20	1	-4.30	9.60
-2	20	-1	47.20	9.60
-2	20	-1	29.10	8.70
2	-20	1	33.90	11.50
-3	20	-1	64.89	10.50
-3	20	-1	79.39	13.10
3	-20	1	69.39	24.80
3	-20	1	83.79	12.80
-4	20	-1	198.68	18.70
-4	20	-1	251.47	18.30
-4	20	-1	235.48	18.80
4	-20	1	219.88	20.60
4	-20	1	246.58	17.30
-5	20	-1	224.88	17.70
-5	20	-1	185.38	17.70
-5	20	-1	220.08	16.90
5	-20	1	194.38	16.70
5	-20	1	182.58	17.30
-6	20	-1	53.99	12.70
-6	20	-1	30.30	10.90
-6	20	-1	7.90	10.60

6	-20	1	27.60	13.20
6	-20	1	30.40	9.00
8	19	-1	299.57	18.60
-8	-19	1	283.67	19.30
-8	-19	1	319.47	16.80
7	19	-1	107.19	12.90
-7	-19	1	78.79	12.80
-7	-19	1	71.19	12.70
-7	-19	1	80.69	9.60
-6	-19	1	-3.80	7.70
-6	-19	1	-6.30	5.80
-6	-19	1	15.10	7.30
-6	-19	1	6.50	7.70
5	19	-1	129.79	16.00
-5	-19	1	115.09	12.10
-5	-19	1	124.59	14.10
-5	-19	1	130.49	13.10
4	19	-1	13.60	15.10
4	19	-1	16.80	15.30
-4	-19	1	-8.70	8.20
-4	-19	1	9.40	11.70
3	19	-1	94.39	15.10
3	19	-1	125.19	16.30
-3	-19	1	94.19	12.50
-3	-19	1	121.59	14.40
-3	-19	1	98.09	12.90
2	19	-1	430.26	31.40
2	19	-1	493.45	27.60
2	19	-1	520.65	29.60
-2	-19	1	488.05	26.80
-2	-19	1	511.15	27.40
-2	-19	1	509.45	29.20
-2	-19	1	496.45	27.30
1	19	-1	5.30	7.20
1	19	-1	2.50	13.00
1	19	-1	-5.20	11.30
-1	-19	1	5.20	7.70
-1	-19	1	-3.20	8.80
-1	-19	1	18.80	8.10
-1	-19	1	13.40	9.10
0	19	-1	143.09	16.10
0	19	-1	148.49	14.50
0	-19	1	126.59	16.50
0	-19	1	158.08	18.40

0	-19	1	127.99	15.20
0	-19	1	129.09	15.30
-1	19	-1	111.69	13.80
1	-19	1	136.09	15.60
1	-19	1	136.19	16.30
-2	19	-1	134.79	15.20
-2	19	-1	143.59	15.00
2	-19	1	167.48	16.40
2	-19	1	150.98	20.60
-3	19	-1	421.86	24.80
3	-19	1	424.56	30.60
3	-19	1	385.06	27.30
-4	19	-1	11.20	9.20
-4	19	-1	0.30	9.10
4	-19	1	-2.90	9.00
4	-19	1	-5.90	8.40
-5	19	-1	50.29	9.90
-5	19	-1	54.09	10.70
-5	19	-1	43.70	11.70
5	-19	1	38.30	9.20
5	-19	1	54.29	10.00
-6	19	-1	93.39	14.70
-6	19	-1	81.29	12.90
-6	19	-1	63.79	11.00
6	-19	1	75.49	12.20
6	-19	1	83.29	11.50
-7	19	-1	32.80	21.40
-7	19	-1	8.00	10.40
-7	19	-1	-8.50	9.60
7	-19	1	-1.80	15.40
7	-19	1	18.90	8.70
-8	-18	1	25.00	5.80
-8	-18	1	13.00	8.00
-8	-18	1	31.00	13.20
-8	-18	1	18.50	8.10
-7	-18	1	212.18	14.30
-7	-18	1	213.78	16.20
-7	-18	1	222.58	16.80
-7	-18	1	209.18	17.20
-6	-18	1	16.90	8.00
-6	-18	1	11.10	6.00
-6	-18	1	1.60	8.20
-6	-18	1	9.40	7.70
5	18	-1	20.50	14.00

-5	-18	1	6.10	11.00
-5	-18	1	15.40	11.30
-5	-18	1	13.70	8.80
4	18	-1	376.06	27.30
-4	-18	1	348.67	21.20
-4	-18	1	380.36	22.30
-4	-18	1	388.66	22.20
-4	-18	1	361.96	22.50
3	18	-1	11.60	9.60
3	18	-1	6.10	10.10
-3	-18	1	27.00	7.40
-3	-18	1	4.20	12.10
-3	-18	1	16.60	7.10
-3	-18	1	-3.90	7.20
2	18	-1	142.39	17.70
2	18	-1	151.88	18.00
-2	-18	1	141.29	13.90
-2	-18	1	141.69	14.50
-2	-18	1	120.19	17.80
-2	-18	1	133.89	14.30
1	18	-1	20.60	10.90
1	18	-1	15.20	12.20
1	18	-1	7.20	7.90
-1	-18	1	5.80	7.90
-1	-18	1	13.20	7.90
-1	-18	1	17.60	9.30
-1	-18	1	9.70	7.20
0	18	-1	26.00	8.50
0	18	-1	-5.80	12.50
0	18	-1	41.60	12.40
0	-18	1	28.30	8.00
0	-18	1	21.40	8.70
0	-18	1	11.20	8.10
0	-18	1	18.60	10.20
-1	18	-1	73.29	12.70
1	-18	1	39.60	9.40
1	-18	1	61.89	10.10
1	-18	1	62.99	10.30
1	-18	1	68.49	15.80
-2	18	-1	97.99	14.10
-2	18	-1	80.09	13.20
2	-18	1	90.89	12.30
2	-18	1	88.29	14.70
-3	18	-1	839.82	43.50

-3	18	-1	843.52	43.10
3	-18	1	837.12	42.80
3	-18	1	779.72	45.00
3	-18	1	913.31	43.00
-4	18	-1	447.06	27.90
-4	18	-1	479.75	31.70
4	-18	1	489.05	30.40
4	-18	1	476.95	30.70
-5	18	-1	48.30	11.70
-5	18	-1	54.89	11.50
-5	18	-1	44.40	10.70
5	-18	1	53.39	10.20
5	-18	1	45.00	8.90
-6	18	-1	13.40	9.50
-6	18	-1	8.30	10.30
-6	18	-1	-3.80	11.80
6	-18	1	6.40	10.20
6	-18	1	24.20	7.60
-7	18	-1	36.50	10.70
-7	18	-1	45.20	13.00
-7	18	-1	34.40	9.70
7	-18	1	5.40	16.50
-9	-17	1	411.06	24.70
-9	-17	1	478.55	22.90
-9	-17	1	438.16	24.60
-8	-17	1	182.38	12.80
-8	-17	1	160.48	15.50
-8	-17	1	195.38	15.60
-8	-17	1	170.28	19.10
-7	-17	1	22.40	8.50
-7	-17	1	43.30	9.50
-7	-17	1	42.30	9.10
-7	-17	1	36.60	7.10
6	17	-1	22.90	14.50
-6	-17	1	1.90	9.20
-6	-17	1	4.30	12.60
5	17	-1	90.69	13.60
-5	-17	1	88.49	13.30
-5	-17	1	85.09	10.60
-5	-17	1	82.09	12.60
-5	-17	1	74.79	11.30
4	17	-1	9.80	9.40
-4	-17	1	3.20	6.30
-4	-17	1	6.60	7.20

-4	-17	1	11.60	7.80
-4	-17	1	4.10	7.30
3	17	-1	14.90	8.10
-3	-17	1	6.70	7.20
-3	-17	1	16.20	7.70
-3	-17	1	12.20	7.00
-3	-17	1	-2.90	12.20
-3	-17	1	-3.20	6.60
2	17	-1	174.28	18.00
2	17	-1	158.48	15.50
-2	-17	1	153.28	14.60
-2	-17	1	156.18	14.90
-2	-17	1	189.08	18.20
-2	-17	1	153.28	14.60
1	17	-1	364.96	23.70
1	17	-1	406.56	26.20
-1	-17	1	416.26	24.10
-1	-17	1	436.96	25.40
-1	-17	1	420.56	24.00
-1	-17	1	392.36	23.70
0	17	-1	17.50	8.10
0	17	-1	11.40	11.90
0	17	-1	8.30	7.50
0	-17	1	-9.00	9.30
0	-17	1	-4.70	8.20
0	-17	1	8.40	7.50
0	-17	1	3.90	8.40
-1	17	-1	131.59	16.30
-1	17	-1	111.29	13.50
-1	17	-1	143.39	17.20
1	-17	1	143.09	16.10
1	-17	1	131.69	14.80
1	-17	1	156.58	16.60
1	-17	1	149.39	16.00
-2	17	-1	215.68	18.20
2	-17	1	261.37	19.40
2	-17	1	258.37	22.40
2	-17	1	232.18	20.30
-3	17	-1	-6.40	7.90
-3	17	-1	-3.80	8.30
3	-17	1	5.20	12.30
3	-17	1	4.30	9.50
3	-17	1	13.20	8.50
-4	17	-1	3.90	9.30

-4	17	-1	-1.00	8.90
4	-17	1	12.10	9.20
4	-17	1	14.60	8.60
-5	17	-1	72.59	12.30
-5	17	-1	76.49	16.00
-6	17	-1	14.20	9.80
-6	17	-1	13.60	11.50
-6	17	-1	6.40	11.90
6	-17	1	8.30	7.70
-7	17	-1	1.80	11.10
-7	17	-1	42.70	17.20
-7	17	-1	7.90	9.70
7	-17	1	5.20	7.40
7	-17	1	31.70	13.10
-8	17	-1	130.69	20.00
-8	17	-1	139.99	17.70
-8	17	-1	144.39	22.60
8	-17	1	162.28	12.90
9	16	-1	8.30	31.00
-9	-16	1	15.90	8.20
-9	-16	1	4.50	5.50
-9	-16	1	12.60	9.80
8	16	-1	119.39	20.10
-8	-16	1	168.68	19.10
-8	-16	1	130.99	11.70
-8	-16	1	126.59	15.40
-8	-16	1	115.29	15.20
7	16	-1	-1.10	18.40
-7	-16	1	0.80	9.60
-7	-16	1	-0.30	9.20
-7	-16	1	8.60	7.60
-7	-16	1	-5.20	8.50
6	16	-1	152.78	20.90
-6	-16	1	96.79	14.50
-6	-16	1	118.39	12.00
-6	-16	1	96.59	15.10
-6	-16	1	110.59	14.30
5	16	-1	3.20	9.90
-5	-16	1	8.70	7.90
-5	-16	1	-5.00	7.90
-5	-16	1	3.60	7.30
-5	-16	1	4.40	6.10
4	16	-1	306.37	20.40
-4	-16	1	300.37	19.20

-4	-16	1	316.07	20.90
-4	-16	1	315.27	20.50
-4	-16	1	320.77	20.00
3	16	-1	973.50	46.70
-3	-16	1	971.00	50.09
-3	-16	1	999.00	47.30
-3	-16	1	920.71	47.10
-3	-16	1	944.61	46.50
-3	-16	1	984.50	47.10
2	16	-1	7.90	6.20
-2	-16	1	6.30	6.90
-2	-16	1	-16.10	9.20
-2	-16	1	6.80	7.40
-2	-16	1	9.00	7.10
-2	-16	1	3.80	6.40
1	16	-1	122.49	12.00
1	16	-1	127.09	16.90
-1	-16	1	138.39	14.80
-1	-16	1	115.79	13.80
-1	-16	1	111.39	15.70
-1	-16	1	141.09	13.40
0	16	-1	154.28	13.10
0	16	-1	144.89	13.20
0	16	-1	178.48	19.40
0	-16	1	145.79	15.50
0	-16	1	152.28	15.10
0	-16	1	116.99	16.00
0	-16	1	129.49	14.20
-1	16	-1	-10.60	13.30
-1	16	-1	-3.50	6.80
-1	16	-1	-0.30	6.70
1	-16	1	1.50	8.00
1	-16	1	19.50	11.00
1	-16	1	-1.30	7.60
1	-16	1	-1.10	8.50
-2	16	-1	100.59	15.60
-2	16	-1	123.99	13.20
2	-16	1	119.29	19.20
2	-16	1	139.19	15.20
2	-16	1	95.79	16.40
-3	16	-1	24.00	9.20
-3	16	-1	17.80	8.20
3	-16	1	20.00	7.80
3	-16	1	18.70	9.20

-4	16	-1	417.06	26.80
-4	16	-1	410.16	26.70
4	-16	1	377.76	24.00
4	-16	1	408.86	25.20
-5	16	-1	46.00	11.50
-5	16	-1	39.60	11.70
-5	16	-1	62.39	11.60
5	-16	1	48.50	8.70
5	-16	1	51.79	9.90
-6	16	-1	62.69	19.00
-6	16	-1	47.30	12.30
-6	16	-1	41.50	12.90
6	-16	1	36.60	12.10
6	-16	1	48.20	11.20
-7	16	-1	284.67	23.10
-7	16	-1	283.67	21.20
-7	16	-1	254.37	24.00
7	-16	1	246.98	24.60
7	-16	1	289.47	18.10
-8	16	-1	139.69	16.10
-8	16	-1	149.88	18.90
-8	16	-1	113.19	17.00
8	-16	1	164.08	13.20
10	15	-1	38.90	15.30
-10	-15	1	45.80	16.10
-10	-15	1	50.39	9.40
-10	-15	1	64.69	9.10
9	15	-1	38.30	15.10
-9	-15	1	19.90	8.40
-9	-15	1	15.70	5.90
-9	-15	1	14.20	9.50
8	15	-1	779.12	43.50
-8	-15	1	753.92	39.20
-8	-15	1	719.13	38.30
-8	-15	1	782.42	37.40
-8	-15	1	759.12	40.20
-8	-15	1	751.32	38.90
7	15	-1	19.20	18.70
7	15	-1	35.70	16.10
-7	-15	1	42.80	9.70
-7	-15	1	45.50	9.80
-7	-15	1	37.50	7.50
-7	-15	1	45.50	10.00
-7	-15	1	35.50	9.60

6	15	-1	-11.80	9.70
6	15	-1	-1.70	18.00
-6	-15	1	3.70	6.10
-6	-15	1	5.70	8.00
-6	-15	1	0.00	7.50
-6	-15	1	15.80	8.60
-6	-15	1	2.40	8.00
5	15	-1	53.59	9.60
-5	-15	1	56.29	9.90
-5	-15	1	58.79	10.30
-5	-15	1	51.59	8.40
-5	-15	1	48.80	8.30
-5	-15	1	82.99	13.40
4	15	-1	25.60	7.30
-4	-15	1	34.50	8.50
-4	-15	1	30.10	9.30
-4	-15	1	21.50	7.10
-4	-15	1	27.80	6.90
3	15	-1	94.29	10.30
-3	-15	1	72.99	12.70
-3	-15	1	74.09	11.60
-3	-15	1	81.19	11.60
-3	-15	1	91.09	13.60
2	15	-1	599.24	29.80
-2	-15	1	568.04	30.50
-2	-15	1	585.14	30.70
-2	-15	1	622.54	31.60
-2	-15	1	588.84	30.80
1	15	-1	215.38	13.40
1	15	-1	177.68	19.40
-1	-15	1	202.48	15.10
-1	-15	1	169.98	16.10
-1	-15	1	200.88	18.60
0	15	-1	57.79	8.80
0	15	-1	56.49	11.50
0	-15	1	43.10	8.60
0	-15	1	52.79	9.40
0	-15	1	61.99	9.70
0	-15	1	38.50	11.10
-1	15	-1	66.99	13.90
-1	15	-1	42.30	7.70
-1	15	-1	57.29	7.40
1	-15	1	64.09	13.30
1	-15	1	55.69	9.10

1	-15	1	66.69	9.30
1	-15	1	52.19	11.80
-2	15	-1	418.96	23.80
-2	15	-1	476.55	25.10
2	-15	1	410.96	26.70
2	-15	1	408.86	34.40
2	-15	1	436.66	27.00
2	-15	1	437.06	25.00
-3	15	-1	439.36	26.10
-3	15	-1	402.56	24.80
3	-15	1	388.96	24.70
3	-15	1	414.06	24.60
3	-15	1	424.16	28.30
-4	15	-1	232.48	20.50
-4	15	-1	218.98	19.00
4	-15	1	189.78	17.70
4	-15	1	221.68	18.10
-5	15	-1	149.39	20.10
-5	15	-1	127.49	19.30
5	-15	1	124.09	14.70
5	-15	1	91.29	16.20
-6	15	-1	213.98	24.40
-6	15	-1	261.27	22.10
-6	15	-1	252.67	23.80
6	-15	1	242.98	21.70
6	-15	1	261.47	17.90
-7	15	-1	35.50	10.90
-7	15	-1	20.10	13.80
7	-15	1	19.00	7.70
7	-15	1	19.30	14.00
-8	15	-1	123.19	17.10
-8	15	-1	107.19	15.40
8	-15	1	151.08	13.20
-9	15	-1	28.70	17.70
-9	15	-1	24.10	9.80
9	-15	1	18.80	7.00
10	14	-1	165.58	21.30
-10	-14	1	190.68	13.20
-10	-14	1	198.48	20.90
-10	-14	1	165.88	16.00
9	14	-1	27.10	14.00
-9	-14	1	37.70	11.40
-9	-14	1	-2.00	10.10
-9	-14	1	17.40	9.40

-9	-14	1	26.10	6.40
8	14	-1	131.99	24.30
8	14	-1	108.99	20.50
-8	-14	1	161.08	18.20
-8	-14	1	110.29	17.80
-8	-14	1	104.99	14.30
-8	-14	1	121.69	15.60
7	14	-1	219.98	25.10
7	14	-1	148.49	24.30
-7	-14	1	229.58	18.40
-7	-14	1	187.18	17.80
-7	-14	1	174.28	17.60
-7	-14	1	178.58	17.20
-7	-14	1	207.78	14.60
6	14	-1	-9.30	8.80
6	14	-1	7.30	15.50
-6	-14	1	-3.50	6.00
-6	-14	1	-7.90	8.50
-6	-14	1	16.30	8.40
-6	-14	1	-5.60	7.60
-6	-14	1	6.10	8.20
5	14	-1	237.68	31.60
5	14	-1	202.38	15.50
-5	-14	1	183.08	16.90
-5	-14	1	204.28	15.40
-5	-14	1	220.78	15.90
-5	-14	1	159.88	17.40
-5	-14	1	203.58	14.80
4	14	-1	220.48	14.90
-4	-14	1	222.28	16.60
-4	-14	1	212.88	16.20
-4	-14	1	232.98	18.10
-4	-14	1	246.68	15.60
-4	-14	1	200.48	15.70
3	14	-1	237.68	15.30
-3	-14	1	244.98	18.80
-3	-14	1	299.47	19.50
-3	-14	1	281.87	17.50
-3	-14	1	211.48	24.40
2	14	-1	506.15	25.40
-2	-14	1	503.35	27.60
-2	-14	1	521.55	29.80
-2	-14	1	520.35	27.40
-2	-14	1	493.85	26.70

-2	-14	1	489.85	26.60
1	14	-1	105.89	8.90
-1	-14	1	127.69	15.30
-1	-14	1	94.09	13.70
-1	-14	1	113.09	11.80
-1	-14	1	103.49	12.40
-1	-14	1	104.19	13.20
0	14	-1	24.00	10.70
0	14	-1	14.50	4.50
0	-14	1	17.10	7.10
0	-14	1	11.30	8.60
0	-14	1	23.60	7.90
0	-14	1	19.20	8.50
-1	14	-1	294.57	27.50
-1	14	-1	353.36	18.70
-1	14	-1	354.26	20.80
1	-14	1	364.66	22.90
1	-14	1	366.36	21.80
1	-14	1	339.37	25.30
1	-14	1	358.26	21.50
-2	14	-1	437.26	24.20
-2	14	-1	417.96	22.00
2	-14	1	435.76	25.50
2	-14	1	427.26	26.10
2	-14	1	378.16	26.20
2	-14	1	393.06	25.40
-3	14	-1	583.84	32.20
-3	14	-1	668.13	35.50
-3	14	-1	681.23	34.50
3	-14	1	606.44	33.20
3	-14	1	631.84	33.40
3	-14	1	656.73	36.20
-4	14	-1	27.90	11.70
-4	14	-1	34.80	10.20
4	-14	1	37.30	8.30
4	-14	1	32.20	8.70
-5	14	-1	527.35	33.70
-5	14	-1	545.75	33.10
5	-14	1	518.15	29.80
5	-14	1	510.55	28.70
-6	14	-1	40.10	13.80
-6	14	-1	32.90	13.80
-6	14	-1	15.20	11.70
6	-14	1	36.90	8.80

6	-14	1	43.70	11.30
-7	14	-1	20.60	13.20
-7	14	-1	5.80	13.40
-7	14	-1	17.60	15.70
7	-14	1	35.90	23.40
7	-14	1	15.00	7.60
-8	14	-1	62.39	11.90
-8	14	-1	42.10	13.90
-8	14	-1	2.30	14.80
8	-14	1	44.20	8.00
-9	14	-1	11.10	15.70
-9	14	-1	23.90	12.60
-9	14	-1	-2.00	9.70
9	-14	1	6.60	7.00
10	13	-1	78.89	14.00
-10	-13	1	100.89	25.60
-10	-13	1	64.59	10.40
-10	-13	1	65.39	12.70
-10	-13	1	64.59	10.30
9	13	-1	549.74	34.10
-9	-13	1	534.85	30.60
-9	-13	1	561.14	28.30
-9	-13	1	498.55	29.80
-9	-13	1	535.75	30.50
8	13	-1	18.30	16.80
8	13	-1	46.50	14.50
-8	-13	1	13.10	8.70
-8	-13	1	8.80	9.50
-8	-13	1	2.70	6.20
-8	-13	1	8.60	9.60
7	13	-1	345.67	28.10
7	13	-1	367.16	32.80
-7	-13	1	364.56	22.70
-7	-13	1	349.17	22.80
-7	-13	1	361.86	21.20
-7	-13	1	359.36	23.30
-7	-13	1	327.47	23.20
6	13	-1	64.59	10.00
6	13	-1	66.79	15.10
-6	-13	1	79.59	12.60
-6	-13	1	66.59	9.70
-6	-13	1	72.49	10.80
-6	-13	1	62.59	10.30
-6	-13	1	82.79	11.20

5	13	-1	55.89	8.10
-5	-13	1	48.60	9.00
-5	-13	1	36.30	7.50
-5	-13	1	64.79	10.10
-5	-13	1	42.80	7.80
-5	-13	1	52.59	7.90
4	13	-1	104.79	9.70
-4	-13	1	92.29	11.00
-4	-13	1	100.59	15.20
-4	-13	1	87.59	11.00
3	13	-1	9.60	4.20
-3	-13	1	-0.60	8.10
-3	-13	1	9.00	6.10
-3	-13	1	11.90	7.50
-3	-13	1	2.00	5.80
-3	-13	1	8.90	12.60
-3	-13	1	2.70	5.60
2	13	-1	605.74	28.70
-2	-13	1	594.84	30.20
-2	-13	1	581.94	30.70
-2	-13	1	592.74	33.10
-2	-13	1	584.54	31.30
-2	-13	1	581.94	30.10
1	13	-1	604.64	28.50
-1	-13	1	605.04	31.00
-1	-13	1	595.14	31.70
-1	-13	1	571.44	30.10
-1	-13	1	561.54	30.30
-1	-13	1	596.74	30.50
0	13	-1	11.20	3.50
0	13	-1	14.80	10.60
0	-13	1	3.20	8.30
0	-13	1	16.80	6.70
0	-13	1	11.30	8.60
0	-13	1	11.60	7.40
-1	13	-1	138.69	12.80
-1	13	-1	149.19	9.90
-1	13	-1	150.88	22.80
1	-13	1	143.89	19.60
1	-13	1	143.89	14.20
1	-13	1	156.38	16.10
1	-13	1	130.39	19.30
1	-13	1	126.39	15.10
-2	13	-1	302.87	18.50

-2	13	-1	285.77	15.90
2	-13	1	256.37	20.40
2	-13	1	293.37	21.30
2	-13	1	261.17	20.40
2	-13	1	277.47	18.70
-3	13	-1	231.38	15.50
-3	13	-1	230.58	17.90
-3	13	-1	242.18	20.10
3	-13	1	237.08	22.50
3	-13	1	227.18	17.40
3	-13	1	227.18	17.30
-4	13	-1	67.09	12.80
4	-13	1	38.10	8.70
4	-13	1	31.60	8.00
-5	13	-1	624.04	35.60
-5	13	-1	596.64	36.30
5	-13	1	597.74	32.10
5	-13	1	619.04	33.30
-6	13	-1	2.90	13.10
-6	13	-1	17.40	11.30
6	-13	1	7.20	7.00
6	-13	1	-2.80	10.10
-7	13	-1	91.59	15.80
-7	13	-1	78.69	15.80
-7	13	-1	127.19	21.30
7	-13	1	99.39	13.30
7	-13	1	132.29	23.30
-8	13	-1	139.09	17.80
-8	13	-1	123.59	17.90
-8	13	-1	113.49	15.40
8	-13	1	145.69	14.90
-9	13	-1	129.39	20.60
-9	13	-1	91.59	17.70
-9	13	-1	76.79	12.50
9	-13	1	79.09	12.20
-10	13	-1	14.70	14.30
-10	13	-1	27.90	13.40
10	-13	1	16.30	8.20
11	12	-1	17.80	11.90
-11	-12	1	9.40	6.60
10	12	-1	29.10	11.80
-10	-12	1	-1.10	5.90
-10	-12	1	14.90	8.50
-10	-12	1	36.30	14.80

-10	-12	1	10.00	12.00
9	12	-1	19.10	26.20
9	12	-1	26.30	13.40
-9	-12	1	24.60	9.70
-9	-12	1	30.90	11.30
-9	-12	1	41.70	12.00
-9	-12	1	34.20	7.80
8	12	-1	7.00	12.50
8	12	-1	-11.50	14.10
-8	-12	1	16.00	9.90
-8	-12	1	-0.80	8.30
-8	-12	1	-0.30	9.40
-8	-12	1	27.70	7.50
-8	-12	1	18.60	8.60
7	12	-1	28.40	10.40
-7	-12	1	46.60	10.10
-7	-12	1	44.00	9.70
-7	-12	1	24.80	9.90
-7	-12	1	31.10	8.60
-7	-12	1	23.50	8.60
-7	-12	1	25.70	7.10
6	12	-1	404.56	23.40
-6	-12	1	402.96	24.30
-6	-12	1	410.06	22.70
-6	-12	1	395.66	24.10
-6	-12	1	410.86	23.00
-6	-12	1	351.86	24.00
-6	-12	1	399.16	23.80
5	12	-1	15.30	5.80
-5	-12	1	32.20	13.30
-5	-12	1	20.70	7.40
-5	-12	1	26.60	6.80
-5	-12	1	17.30	6.50
4	12	-1	364.96	19.00
-4	-12	1	336.87	21.20
-4	-12	1	375.16	20.80
-4	-12	1	309.77	22.10
-4	-12	1	366.66	20.30
-4	-12	1	348.77	21.40
-4	-12	1	352.36	20.20
3	12	-1	1070.99	47.90
-3	-12	1	1079.79	49.00
-3	-12	1	929.01	49.99
-3	-12	1	1016.10	48.70

-3	-12	1	992.70	49.50
-3	-12	1	1066.69	49.20
2	12	-1	507.65	23.90
-2	-12	1	529.55	25.20
-2	-12	1	482.75	29.10
-2	-12	1	451.05	25.40
-2	-12	1	479.95	25.70
-2	-12	1	475.75	26.10
-2	-12	1	462.75	27.10
1	12	-1	38.80	5.60
1	12	-1	31.90	7.10
-1	-12	1	37.50	7.90
-1	-12	1	41.30	9.30
-1	-12	1	34.90	7.90
-1	-12	1	26.60	8.80
-1	-12	1	23.10	8.40
-1	-12	1	28.40	7.00
0	12	-1	472.25	31.10
0	12	-1	508.55	29.20
0	12	-1	545.45	25.30
0	-12	1	570.44	35.40
0	-12	1	538.85	28.50
0	-12	1	539.95	28.10
0	-12	1	475.15	27.40
0	-12	1	542.15	29.30
-1	12	-1	51.89	5.70
-1	12	-1	54.29	7.90
1	-12	1	60.69	9.50
1	-12	1	50.49	8.90
1	-12	1	31.00	8.00
-2	12	-1	41.40	7.40
-2	12	-1	21.60	4.10
2	-12	1	38.10	7.80
2	-12	1	29.40	9.80
-3	12	-1	12.60	4.80
-3	12	-1	17.40	7.30
-3	12	-1	10.00	10.20
3	-12	1	26.40	7.90
3	-12	1	9.90	7.10
3	-12	1	15.60	12.70
-4	12	-1	681.63	37.40
-4	12	-1	662.13	35.80
4	-12	1	655.93	33.30
4	-12	1	590.04	33.60

-5	12	-1	1.00	11.00
-5	12	-1	1.90	9.80
5	-12	1	11.00	6.20
5	-12	1	-0.20	8.20
-6	12	-1	52.09	14.30
-6	12	-1	83.59	14.10
-6	12	-1	96.29	14.50
6	-12	1	79.09	12.80
6	-12	1	131.79	18.80
-7	12	-1	691.03	41.30
-7	12	-1	716.03	40.30
-7	12	-1	718.93	41.20
7	-12	1	609.84	37.80
7	-12	1	697.43	40.30
7	-12	1	711.93	35.80
-8	12	-1	137.29	24.10
-8	12	-1	146.09	23.30
-8	12	-1	157.68	22.90
8	-12	1	161.38	14.90
8	-12	1	146.79	22.90
-9	12	-1	27.80	13.70
-9	12	-1	7.00	15.20
-9	12	-1	7.20	11.10
9	-12	1	6.30	12.30
9	-12	1	3.60	7.50
-10	12	-1	126.69	20.30
-10	12	-1	126.29	16.40
-10	12	-1	133.39	23.00
10	-12	1	126.39	14.40
10	-12	1	124.59	18.00
11	11	-1	9.50	10.60
-11	-11	1	2.40	8.00
-11	-11	1	7.80	6.70
10	11	-1	4.80	11.30
-10	-11	1	18.10	8.60
-10	-11	1	25.00	12.30
-10	-11	1	35.60	7.70
-10	-11	1	17.40	18.20
9	11	-1	121.99	22.30
-9	-11	1	167.28	18.00
-9	-11	1	144.19	17.70
-9	-11	1	149.88	14.40
-9	-11	1	155.48	18.10
8	11	-1	1391.06	68.49

-8	-11	1	1304.37	66.39
-8	-11	1	1475.55	66.09
-8	-11	1	1319.17	66.39
-8	-11	1	1411.06	66.39
-8	-11	1	1454.85	66.89
7	11	-1	22.90	8.90
-7	-11	1	21.10	8.20
-7	-11	1	26.30	10.50
-7	-11	1	30.50	8.20
-7	-11	1	17.00	6.60
-7	-11	1	35.30	9.30
-7	-11	1	22.80	7.90
6	11	-1	28.60	7.00
-6	-11	1	27.80	6.80
-6	-11	1	41.30	8.20
-6	-11	1	26.30	7.60
-6	-11	1	25.40	9.10
-6	-11	1	25.90	7.20
5	11	-1	254.07	15.30
-5	-11	1	271.77	17.70
-5	-11	1	230.08	17.40
-5	-11	1	252.27	18.40
-5	-11	1	257.67	16.30
-5	-11	1	259.17	16.10
-5	-11	1	242.98	18.00
4	11	-1	76.49	7.50
-4	-11	1	74.39	11.30
-4	-11	1	66.19	8.20
-4	-11	1	63.49	11.60
-4	-11	1	75.69	10.40
-4	-11	1	67.79	9.50
-4	-11	1	78.69	10.40
-3	-11	1	103.59	9.90
-3	-11	1	71.79	12.80
-3	-11	1	87.79	11.00
-3	-11	1	109.39	11.80
-3	-11	1	81.89	12.50
-3	-11	1	107.19	11.90
2	11	-1	551.24	28.30
2	11	-1	595.74	27.50
-2	-11	1	569.24	30.40
-2	-11	1	587.34	29.60
-2	-11	1	610.44	28.90
-2	-11	1	559.84	29.50

-2	-11	1	566.24	28.90
1	11	-1	573.34	25.90
1	11	-1	535.75	27.20
-1	-11	1	552.94	28.50
-1	-11	1	510.05	27.90
0	11	-1	226.18	11.80
0	11	-1	242.18	26.50
0	11	-1	214.28	14.00
0	-11	1	212.08	15.10
0	-11	1	219.48	16.00
-1	11	-1	1.30	2.50
-1	11	-1	-3.20	5.30
1	-11	1	5.40	7.20
1	-11	1	9.90	7.70
-2	11	-1	872.21	40.20
-2	11	-1	821.42	37.50
2	-11	1	843.22	42.80
2	-11	1	692.63	41.00
2	-11	1	782.32	39.60
-3	11	-1	41.40	6.80
-3	11	-1	41.00	7.80
-3	11	-1	39.40	10.70
3	-11	1	37.30	7.50
3	-11	1	53.79	8.50
-4	11	-1	170.78	14.60
-4	11	-1	182.58	17.40
-4	11	-1	193.38	20.40
4	-11	1	192.18	16.60
4	-11	1	194.98	15.00
-5	11	-1	509.45	39.30
-5	11	-1	427.06	27.50
5	-11	1	420.56	24.40
5	-11	1	422.46	26.10
-6	11	-1	269.37	26.10
-6	11	-1	323.37	25.10
-6	11	-1	335.27	24.70
6	-11	1	353.26	24.60
6	-11	1	321.17	24.50
6	-11	1	322.77	20.80
-7	11	-1	-3.80	12.10
-7	11	-1	33.20	23.60
-7	11	-1	15.40	12.80
7	-11	1	11.20	7.30
7	-11	1	21.40	11.00

-8	11	-1	125.89	20.70
-8	11	-1	111.69	16.80
-8	11	-1	143.69	21.50
8	-11	1	114.29	14.30
8	-11	1	133.89	18.00
-9	11	-1	9.60	13.90
-9	11	-1	24.70	13.20
-9	11	-1	14.60	12.10
9	-11	1	-6.00	10.00
9	-11	1	16.20	8.40
-10	11	-1	9.60	13.50
-10	11	-1	-8.80	16.80
-10	11	-1	12.20	10.70
10	-11	1	-1.80	11.50
10	-11	1	5.40	9.10
11	10	-1	17.50	10.80
-11	-10	1	10.00	7.30
-11	-10	1	18.70	8.30
10	10	-1	210.38	21.30
-10	-10	1	168.98	21.30
-10	-10	1	195.38	21.70
-10	-10	1	224.08	16.40
-10	-10	1	217.88	17.50
9	10	-1	11.30	18.10
-9	-10	1	41.30	8.40
-9	-10	1	39.30	8.40
-9	-10	1	58.79	11.30
-9	-10	1	52.29	10.40
-9	-10	1	39.80	11.20
8	10	-1	88.39	13.10
-8	-10	1	67.89	10.30
-8	-10	1	63.69	11.30
-8	-10	1	80.49	13.30
7	10	-1	474.55	26.90
-7	-10	1	427.86	26.60
-7	-10	1	458.45	26.20
-7	-10	1	458.25	25.70
-7	-10	1	491.15	25.70
-7	-10	1	498.65	29.10
-7	-10	1	505.65	27.80
6	10	-1	-1.50	5.50
-6	-10	1	4.20	7.90
-6	-10	1	7.50	8.50
-6	-10	1	-14.50	7.20

-6	-10	1	7.50	5.90
-6	-10	1	1.80	5.70
-6	-10	1	-11.50	7.80
5	10	-1	80.69	8.20
-5	-10	1	71.89	12.20
-5	-10	1	87.19	11.90
-5	-10	1	86.79	9.70
-5	-10	1	64.99	8.90
-5	-10	1	78.49	13.50
-5	-10	1	77.09	9.60
4	10	-1	10.80	3.80
-4	-10	1	16.00	5.80
-4	-10	1	9.30	8.20
-4	-10	1	14.50	7.60
-4	-10	1	7.40	7.20
-4	-10	1	8.00	8.10
-4	-10	1	22.80	5.70
3	10	-1	696.13	31.90
-3	-10	1	722.33	35.40
-3	-10	1	647.84	33.50
-3	-10	1	623.24	31.90
-3	-10	1	712.33	34.10
-3	-10	1	649.43	34.20
-3	-10	1	691.23	33.20
2	10	-1	379.16	24.80
2	10	-1	399.56	18.40
2	10	-1	362.66	19.00
-2	-10	1	362.26	21.80
-2	-10	1	330.27	25.00
-2	-10	1	404.36	20.20
1	10	-1	615.14	32.30
1	10	-1	575.44	28.50
1	10	-1	574.64	27.70
-1	-10	1	636.74	29.30
-1	-10	1	551.14	32.40
-1	-10	1	595.04	28.90
0	10	-1	276.97	13.50
0	10	-1	259.87	14.90
0	-10	1	266.17	18.20
-1	10	-1	907.71	43.50
-1	10	-1	783.32	39.80
-1	10	-1	832.72	38.50
1	-10	1	843.32	42.50
1	-10	1	810.52	40.20

-2	10	-1	350.16	25.90
-2	10	-1	355.16	20.30
-2	10	-1	381.36	17.70
-2	10	-1	375.06	20.80
2	-10	1	320.17	24.00
2	-10	1	345.97	20.80
-3	10	-1	275.27	14.30
-3	10	-1	240.18	21.20
3	-10	1	283.07	18.50
3	-10	1	253.47	18.10
-4	10	-1	48.00	12.60
-4	10	-1	45.00	6.60
-4	10	-1	57.19	9.70
4	-10	1	45.70	9.20
-5	10	-1	180.28	19.40
-5	10	-1	174.18	16.80
5	-10	1	152.18	18.30
5	-10	1	182.78	15.00
-6	10	-1	138.99	20.00
-6	10	-1	125.09	17.10
-6	10	-1	136.69	19.20
6	-10	1	123.19	13.70
-7	10	-1	8.90	11.50
-7	10	-1	-11.40	11.10
-7	10	-1	20.90	10.60
7	-10	1	9.90	7.60
7	-10	1	20.10	10.10
-8	10	-1	58.69	12.90
-8	10	-1	43.30	13.50
-8	10	-1	60.89	13.60
8	-10	1	74.29	13.40
8	-10	1	59.19	11.50
-9	10	-1	-13.60	12.90
-9	10	-1	4.00	13.00
-9	10	-1	-2.90	12.50
9	-10	1	-8.40	10.40
9	-10	1	-5.20	9.20
-10	10	-1	28.90	15.80
-10	10	-1	4.30	12.60
-10	10	-1	7.50	10.90
10	-10	1	9.80	9.50
10	-10	1	-10.70	10.00
-11	10	-1	144.89	17.20
11	-10	1	177.08	18.00

11	-10	1	137.49	18.80
11	9	-1	19.80	10.80
-11	-9	1	29.50	8.60
-11	-9	1	23.30	8.30
-10	-9	1	227.08	17.60
-10	-9	1	188.58	21.10
-10	-9	1	185.58	21.60
-10	-9	1	241.58	16.90
-10	-9	1	234.28	19.90
9	9	-1	598.24	43.70
-9	-9	1	509.35	31.50
-9	-9	1	578.44	32.20
-9	-9	1	577.34	30.90
-9	-9	1	596.24	31.20
-9	-9	1	622.74	32.40
8	9	-1	130.69	17.10
-8	-9	1	146.29	17.00
-8	-9	1	128.09	13.80
-8	-9	1	142.79	14.20
-8	-9	1	123.89	13.30
-8	-9	1	144.99	15.40
7	9	-1	221.18	16.40
-7	-9	1	237.08	16.40
-7	-9	1	235.68	16.30
-7	-9	1	211.28	15.00
-7	-9	1	215.98	15.70
-7	-9	1	239.78	18.10
6	9	-1	28.30	5.70
-6	-9	1	31.10	8.60
-6	-9	1	11.70	9.10
-6	-9	1	22.30	5.60
-6	-9	1	33.50	7.90
-6	-9	1	30.60	7.40
-6	-9	1	21.00	6.10
5	9	-1	9.80	4.10
-5	-9	1	-8.00	8.00
-5	-9	1	0.40	6.80
-5	-9	1	0.80	4.80
-5	-9	1	14.20	7.80
-5	-9	1	3.60	7.00
-5	-9	1	3.00	4.90
4	9	-1	151.78	9.20
-4	-9	1	179.58	21.80
-4	-9	1	111.99	13.50

-4	-9	1	159.78	15.10
-4	-9	1	148.49	10.40
-4	-9	1	121.69	14.20
-4	-9	1	130.09	11.50
3	9	-1	53.09	9.80
3	9	-1	46.30	4.80
3	9	-1	39.40	4.80
-3	-9	1	48.20	5.50
2	9	-1	788.52	37.50
2	9	-1	825.62	37.40
-2	-9	1	796.12	38.70
-2	-9	1	836.42	37.80
1	9	-1	610.34	33.70
1	9	-1	742.33	32.20
-1	-9	1	677.83	35.90
-1	-9	1	729.13	33.60
-1	-9	1	720.73	33.20
0	9	-1	127.89	7.20
0	9	-1	139.09	8.70
0	-9	1	137.39	12.50
0	-9	1	116.09	9.00
-1	9	-1	1039.10	47.70
-1	9	-1	1066.39	49.00
1	-9	1	1038.10	50.79
-2	9	-1	75.79	11.30
-2	9	-1	64.49	8.00
-2	9	-1	65.29	5.40
-2	9	-1	61.69	9.80
2	-9	1	66.29	8.90
2	-9	1	74.89	11.00
-3	9	-1	502.85	26.40
-3	9	-1	503.85	26.10
-3	9	-1	528.65	23.70
-3	9	-1	493.45	31.10
3	-9	1	426.16	29.00
3	-9	1	474.35	26.50
-4	9	-1	216.68	15.70
-4	9	-1	209.48	12.80
-4	9	-1	176.28	19.10
4	-9	1	193.88	18.10
4	-9	1	197.98	15.20
-5	9	-1	249.68	20.40
-5	9	-1	245.68	18.00
5	-9	1	254.57	17.10

5	-9	1	248.68	21.10
-6	9	-1	456.55	26.20
6	-9	1	427.56	24.50
6	-9	1	384.36	29.10
-7	9	-1	382.56	24.70
-7	9	-1	349.47	25.90
-7	9	-1	347.37	25.70
7	-9	1	381.06	22.60
-8	9	-1	225.28	21.10
-8	9	-1	208.08	21.70
-8	9	-1	211.98	22.70
8	-9	1	204.28	18.80
8	-9	1	198.58	17.80
-9	9	-1	76.79	13.60
-9	9	-1	77.79	13.50
-9	9	-1	74.69	14.80
9	-9	1	72.39	12.10
9	-9	1	85.29	14.40
-10	9	-1	-0.10	10.90
-10	9	-1	4.50	14.50
-10	9	-1	7.70	12.50
10	-9	1	26.10	10.40
10	-9	1	19.10	10.00
-11	9	-1	-2.70	10.20
-11	9	-1	0.70	15.30
11	-9	1	3.90	14.30
12	8	-1	11.80	10.80
-12	-8	1	24.40	10.40
-12	-8	1	18.50	10.90
11	8	-1	46.70	11.10
-11	-8	1	60.89	9.80
-11	-8	1	49.00	8.60
-10	-8	1	18.80	12.10
-10	-8	1	25.70	11.50
-10	-8	1	21.00	8.90
-10	-8	1	9.50	8.60
-10	-8	1	2.30	11.30
9	8	-1	39.80	15.40
-9	-8	1	25.50	7.30
-9	-8	1	31.90	10.00
-9	-8	1	34.20	10.40
-9	-8	1	28.00	9.60
8	8	-1	184.18	17.00
-8	-8	1	196.58	15.00

-8	-8	1	197.68	15.00
-8	-8	1	203.28	14.80
-8	-8	1	196.28	18.40
-8	-8	1	163.18	15.80
7	8	-1	91.49	11.50
-7	-8	1	101.89	11.80
-7	-8	1	118.99	14.30
-7	-8	1	105.39	10.50
-7	-8	1	88.39	11.30
6	8	-1	446.16	22.90
-6	-8	1	438.26	24.00
-6	-8	1	468.95	24.10
-6	-8	1	418.76	27.10
-6	-8	1	454.35	22.90
-6	-8	1	430.26	24.80
-5	-8	1	102.09	11.60
-5	-8	1	95.09	11.60
-5	-8	1	78.49	12.60
-5	-8	1	85.59	9.60
-5	-8	1	78.59	12.50
-5	-8	1	128.99	18.40
4	8	-1	866.11	39.70
4	8	-1	825.52	42.70
-4	-8	1	869.51	40.20
3	8	-1	282.37	13.50
3	8	-1	260.47	12.80
-3	-8	1	247.88	12.90
-3	-8	1	281.97	15.00
2	8	-1	734.13	39.40
2	8	-1	747.03	34.20
2	8	-1	749.42	34.20
-2	-8	1	696.63	35.40
-2	-8	1	786.02	34.40
1	8	-1	37.90	4.30
1	8	-1	53.89	9.00
1	8	-1	38.80	3.50
-1	-8	1	43.10	4.70
-1	-8	1	50.99	9.00
-1	-8	1	49.20	5.90
0	8	-1	735.23	35.30
0	8	-1	713.13	34.10
0	-8	1	812.62	37.00
0	-8	1	724.73	35.10
-1	8	-1	20.90	3.10

-1	8	-1	23.10	3.80
1	-8	1	27.00	4.30
1	-8	1	7.90	5.70
-2	8	-1	47.40	8.50
-2	8	-1	42.30	4.20
-2	8	-1	39.60	6.00
-2	8	-1	33.70	7.50
2	-8	1	32.00	7.10
2	-8	1	49.40	7.30
-3	8	-1	411.56	22.20
-3	8	-1	439.26	22.80
-3	8	-1	445.16	25.90
-3	8	-1	429.76	20.30
3	-8	1	406.46	22.40
3	-8	1	418.06	22.70
3	-8	1	392.86	24.30
-4	8	-1	757.32	40.00
-4	8	-1	794.72	36.30
-4	8	-1	826.52	38.40
4	-8	1	724.63	38.00
4	-8	1	756.82	38.60
-5	8	-1	11.20	8.90
-5	8	-1	1.00	6.10
5	-8	1	13.00	6.10
5	-8	1	15.20	10.90
-6	8	-1	-10.90	9.50
-6	8	-1	7.60	7.30
-6	8	-1	3.80	8.90
6	-8	1	3.40	6.70
-7	8	-1	690.53	37.20
-7	8	-1	689.53	36.60
-7	8	-1	724.03	38.10
7	-8	1	672.53	35.80
-8	8	-1	53.09	10.60
-8	8	-1	40.10	10.20
-8	8	-1	42.20	11.00
8	-8	1	52.79	9.00
-9	8	-1	48.40	11.90
-9	8	-1	35.90	11.40
9	-8	1	19.80	9.20
9	-8	1	31.00	10.10
-10	8	-1	144.19	20.10
-10	8	-1	160.88	20.70
-10	8	-1	140.49	19.10

10	-8	1	138.19	16.90
10	-8	1	145.39	19.20
-11	8	-1	-9.10	16.50
-11	8	-1	-10.20	11.00
11	-8	1	14.60	13.20
11	-8	1	22.30	9.50
12	7	-1	21.50	11.30
-12	-7	1	7.40	7.40
-12	-7	1	10.20	11.70
-11	-7	1	0.80	7.40
-11	-7	1	3.20	8.80
-11	-7	1	12.00	22.30
10	7	-1	20.40	26.20
-10	-7	1	11.00	11.00
-10	-7	1	0.40	9.80
-10	-7	1	24.50	12.50
-10	-7	1	15.10	14.10
9	7	-1	328.87	25.60
-9	-7	1	301.77	20.40
-9	-7	1	278.57	19.00
-9	-7	1	307.67	19.90
-9	-7	1	294.07	20.40
-9	-7	1	258.67	22.40
8	7	-1	516.55	30.00
-8	-7	1	559.34	29.60
-8	-7	1	591.24	30.90
-8	-7	1	590.24	29.60
-8	-7	1	542.55	29.50
-8	-7	1	557.54	28.80
7	7	-1	214.98	14.90
-7	-7	1	216.08	15.70
-7	-7	1	215.98	14.00
-7	-7	1	250.47	18.70
6	7	-1	34.90	5.30
-6	-7	1	33.20	5.30
-6	-7	1	39.10	6.90
-6	-7	1	32.70	6.70
-6	-7	1	29.10	5.40
-6	-7	1	63.09	16.30
-6	-7	1	41.10	7.80
5	7	-1	932.21	45.90
5	7	-1	839.42	45.60
5	7	-1	963.60	42.40
-5	-7	1	898.21	43.30

-5	-7	1	958.20	42.50
-5	-7	1	884.21	43.00
-5	-7	1	859.51	43.80
4	7	-1	126.39	12.50
4	7	-1	146.29	8.30
-4	-7	1	134.69	9.30
3	7	-1	159.88	8.60
3	7	-1	170.18	11.80
-3	-7	1	152.98	10.20
2	7	-1	3.80	1.60
2	7	-1	2.00	1.70
2	7	-1	1.30	3.70
-2	-7	1	3.00	4.00
-2	-7	1	2.70	1.90
1	7	-1	875.91	38.50
1	7	-1	776.32	41.60
1	7	-1	894.71	38.90
-1	-7	1	709.23	41.70
-1	-7	1	962.20	39.10
0	7	-1	9.90	1.60
0	7	-1	15.50	2.70
0	-7	1	8.20	2.40
-1	7	-1	15.40	2.10
-1	7	-1	14.00	2.90
-1	7	-1	18.90	5.50
1	-7	1	14.70	4.50
1	-7	1	13.20	3.20
1	-7	1	13.30	4.80
-2	7	-1	1121.79	50.29
-2	7	-1	1115.89	48.30
-2	7	-1	945.21	48.80
-2	7	-1	1057.39	47.20
2	-7	1	981.10	49.30
2	-7	1	1009.90	48.00
-3	7	-1	32.60	6.10
-3	7	-1	33.80	4.30
-3	7	-1	35.60	7.60
-3	7	-1	32.00	5.20
3	-7	1	32.30	6.20
3	-7	1	25.90	6.60
3	-7	1	36.20	5.70
-4	7	-1	-6.10	7.50
-4	7	-1	1.60	5.40
-4	7	-1	7.50	3.90

-4	7	-1	-3.30	4.70
4	-7	1	6.00	7.10
4	-7	1	14.10	5.50
4	-7	1	4.60	5.70
-5	7	-1	1213.98	59.29
-5	7	-1	1177.38	59.09
-5	7	-1	1409.86	61.69
5	-7	1	1154.08	57.99
5	-7	1	1218.88	57.79
-6	7	-1	454.65	25.70
-6	7	-1	447.06	23.60
-6	7	-1	371.86	25.00
6	-7	1	399.96	23.80
-7	7	-1	10.80	8.30
-7	7	-1	10.20	6.70
-7	7	-1	-1.40	7.80
7	-7	1	-11.90	6.90
-8	7	-1	-5.20	7.90
-8	7	-1	0.40	8.50
-8	7	-1	-3.20	9.20
8	-7	1	2.60	7.70
-9	7	-1	32.10	10.90
-9	7	-1	26.90	11.60
-9	7	-1	40.40	10.50
9	-7	1	27.50	10.00
9	-7	1	27.20	9.70
-10	7	-1	8.90	11.70
-10	7	-1	7.70	10.80
-10	7	-1	28.80	11.40
10	-7	1	6.90	9.90
-11	7	-1	-7.60	18.10
-11	7	-1	-6.30	10.40
-11	7	-1	17.20	13.40
11	-7	1	25.30	9.90
11	-7	1	7.50	13.80
12	6	-1	13.50	11.50
-12	-6	1	7.40	12.90
-12	-6	1	-8.30	6.90
11	6	-1	-17.10	14.90
-11	-6	1	-0.40	9.00
-11	-6	1	15.70	7.20
-11	-6	1	-1.50	16.60
-10	-6	1	284.07	28.80
-10	-6	1	278.27	26.80

-10	-6	1	299.67	19.50
-10	-6	1	308.07	21.40
-10	-6	1	255.57	20.00
9	6	-1	15.00	11.70
-9	-6	1	16.50	7.20
-9	-6	1	19.80	7.10
-9	-6	1	23.80	10.20
-9	-6	1	21.20	8.70
8	6	-1	278.77	22.90
8	6	-1	263.17	17.80
-8	-6	1	236.88	15.80
-8	-6	1	241.88	19.60
-8	-6	1	235.58	17.40
-8	-6	1	248.08	16.70
7	6	-1	440.06	30.50
7	6	-1	539.85	26.50
-7	-6	1	514.05	27.10
-7	-6	1	521.15	28.30
-7	-6	1	506.75	27.20
-7	-6	1	529.55	26.80
-7	-6	1	532.55	26.10
6	6	-1	1.70	3.80
-6	-6	1	-1.50	6.90
-6	-6	1	-6.40	7.00
-6	-6	1	4.40	5.20
-6	-6	1	13.50	4.50
-6	-6	1	8.20	5.50
5	6	-1	375.46	22.20
5	6	-1	386.66	18.80
5	6	-1	346.87	22.10
-5	-6	1	392.36	19.30
4	6	-1	58.79	5.00
4	6	-1	58.39	8.10
-4	-6	1	55.19	6.60
3	6	-1	883.21	40.90
3	6	-1	847.02	39.40
2	6	-1	164.48	8.30
2	6	-1	164.18	10.20
2	6	-1	168.68	8.20
-2	-6	1	163.08	8.20
-2	-6	1	160.18	10.40
1	6	-1	765.82	38.50
1	6	-1	790.92	36.80
1	6	-1	838.02	37.10

-1	-6	1	854.21	37.20
0	6	-1	1576.84	68.29
0	6	-1	1404.66	66.19
0	6	-1	1458.45	66.69
0	-6	1	1472.55	68.09
0	-6	1	1476.35	66.79
-1	6	-1	3466.15	158.58
-1	6	-1	3843.72	160.78
-1	6	-1	3587.84	159.28
1	-6	1	3632.04	159.28
1	-6	1	3407.66	160.18
1	-6	1	3403.16	158.98
-2	6	-1	365.66	19.30
-2	6	-1	357.56	16.80
-2	6	-1	366.06	17.80
2	-6	1	357.46	17.80
2	-6	1	345.17	18.80
2	-6	1	346.17	17.70
-3	6	-1	3.20	2.20
-3	6	-1	5.80	4.50
-3	6	-1	-1.80	2.90
-3	6	-1	-0.10	4.60
3	-6	1	4.60	3.40
3	-6	1	1.90	3.60
3	-6	1	-5.20	3.60
-4	6	-1	28.30	5.30
-4	6	-1	15.50	5.60
-4	6	-1	25.70	6.10
-4	6	-1	32.30	5.70
4	-6	1	20.30	5.20
4	-6	1	23.20	6.10
4	-6	1	22.70	4.40
-5	6	-1	146.89	15.10
-5	6	-1	138.49	15.20
-5	6	-1	137.19	14.10
-5	6	-1	151.88	11.40
5	-6	1	156.28	14.20
5	-6	1	153.58	11.20
5	-6	1	146.09	14.20
-6	6	-1	364.06	20.00
-6	6	-1	335.07	21.60
-6	6	-1	368.06	22.40
6	-6	1	344.47	20.60
6	-6	1	332.77	21.00

-7	6	-1	15.40	6.10
-7	6	-1	7.10	7.40
-8	6	-1	186.48	14.90
-8	6	-1	173.08	16.20
-8	6	-1	161.48	18.00
8	-6	1	168.88	16.90
-9	6	-1	6.40	9.50
-9	6	-1	-1.30	9.00
-9	6	-1	-3.30	9.10
9	-6	1	-1.10	8.90
9	-6	1	-15.70	9.40
-10	6	-1	47.40	18.60
-10	6	-1	36.20	12.10
-10	6	-1	37.60	11.60
10	-6	1	25.40	12.60
10	-6	1	24.00	16.70
-11	6	-1	195.18	21.00
-11	6	-1	213.28	20.20
-11	6	-1	180.98	23.00
11	-6	1	199.18	18.40
-12	6	-1	20.50	11.40
12	-6	1	49.99	11.00
12	5	-1	14.60	12.80
12	5	-1	-7.90	18.30
-12	-5	1	24.60	7.40
-12	-5	1	46.30	15.90
11	5	-1	100.09	15.10
-11	-5	1	105.59	16.30
-11	-5	1	131.79	18.00
-11	-5	1	148.89	23.70
-11	-5	1	126.09	12.90
10	5	-1	206.88	22.60
-10	-5	1	210.28	16.20
-10	-5	1	210.68	23.50
-10	-5	1	205.28	18.40
-10	-5	1	221.78	17.90
-10	-5	1	218.38	19.10
9	5	-1	173.18	20.60
-9	-5	1	196.08	16.70
-9	-5	1	208.78	20.30
-9	-5	1	177.48	15.50
-9	-5	1	205.18	15.20
-9	-5	1	186.38	16.10
8	5	-1	576.74	32.10

8	5	-1	555.24	29.90
-8	-5	1	585.14	30.00
-8	-5	1	574.34	28.80
-8	-5	1	569.94	29.90
-8	-5	1	555.94	31.00
-8	-5	1	556.94	29.50
7	5	-1	28.20	5.50
7	5	-1	44.10	10.60
-7	-5	1	28.20	6.70
-7	-5	1	26.80	5.40
-7	-5	1	22.30	6.10
-7	-5	1	20.00	7.70
6	5	-1	245.48	14.10
-6	-5	1	212.18	16.60
-6	-5	1	212.08	14.80
-6	-5	1	249.48	16.30
-6	-5	1	244.58	13.50
-6	-5	1	214.68	17.40
-6	-5	1	239.38	15.60
5	5	-1	184.08	10.10
5	5	-1	161.78	13.30
5	5	-1	182.38	13.30
-5	-5	1	177.68	10.90
4	5	-1	205.78	12.10
4	5	-1	189.38	10.20
4	5	-1	198.48	12.00
-4	-5	1	187.18	11.20
3	5	-1	456.65	21.70
3	5	-1	420.96	20.40
2	5	-1	484.15	23.30
2	5	-1	533.25	24.50
2	5	-1	495.95	23.10
-2	-5	1	521.55	23.20
1	5	-1	85.89	4.70
1	5	-1	79.99	4.50
1	5	-1	86.39	6.60
-1	-5	1	77.69	6.70
-1	-5	1	89.09	4.90
0	5	-1	16.20	2.00
0	5	-1	16.60	2.60
0	5	-1	31.00	5.30
0	-5	1	17.80	3.50
0	-5	1	16.20	2.80
0	-5	1	16.50	2.70

-1	5	-1	1258.17	56.09
-1	5	-1	1333.37	57.29
-1	5	-1	1230.18	55.59
1	-5	1	1235.08	56.09
1	-5	1	1169.28	56.89
1	-5	1	1196.08	55.99
-2	5	-1	225.68	11.70
-2	5	-1	231.38	12.80
-2	5	-1	224.98	11.00
2	-5	1	221.88	11.60
2	-5	1	224.58	11.80
2	-5	1	224.38	12.50
-3	5	-1	19.10	2.70
-3	5	-1	20.00	3.50
-3	5	-1	9.70	3.50
3	-5	1	11.90	3.10
3	-5	1	17.80	3.00
3	-5	1	14.50	3.70
-4	5	-1	231.68	12.50
-4	5	-1	243.98	14.90
-4	5	-1	248.98	14.00
-4	5	-1	225.48	13.70
4	-5	1	246.68	13.80
4	-5	1	246.08	12.70
4	-5	1	237.38	14.80
-5	5	-1	28.40	6.50
-5	5	-1	15.60	7.10
5	-5	1	32.80	6.90
5	-5	1	37.40	7.70
5	-5	1	31.10	4.60
-6	5	-1	1685.83	77.69
-6	5	-1	1697.53	76.79
-6	5	-1	1608.24	77.09
6	-5	1	1618.94	77.19
6	-5	1	1696.53	76.19
-7	5	-1	261.07	18.40
-7	5	-1	250.07	17.80
-7	5	-1	200.48	34.50
-7	5	-1	244.18	16.20
7	-5	1	251.37	18.10
-8	5	-1	135.89	24.00
-8	5	-1	87.59	12.00
-8	5	-1	93.59	13.90
-8	5	-1	92.79	12.40

-9	5	-1	19.40	10.00
-9	5	-1	16.30	8.70
-9	5	-1	25.20	9.30
9	-5	1	11.50	9.80
-10	5	-1	-11.70	9.40
-10	5	-1	-15.70	9.10
10	-5	1	7.30	10.00
-11	5	-1	41.10	15.80
-11	5	-1	24.10	10.40
-11	5	-1	22.90	12.20
11	-5	1	29.80	10.50
-12	5	-1	200.08	19.00
12	-5	1	245.78	19.20
12	4	-1	6.70	13.60
12	4	-1	12.20	15.30
-12	-4	1	20.00	17.20
-12	-4	1	-1.50	7.00
11	4	-1	64.29	13.60
-11	-4	1	80.69	12.10
-11	-4	1	74.99	11.30
-11	-4	1	84.09	15.80
10	4	-1	52.29	11.50
-10	-4	1	36.20	9.40
-10	-4	1	39.70	10.50
-10	-4	1	43.90	10.70
-10	-4	1	23.00	14.30
-10	-4	1	41.50	7.80
9	4	-1	63.49	13.00
9	4	-1	105.29	16.70
-9	-4	1	52.99	8.80
-9	-4	1	64.39	8.90
-9	-4	1	68.49	12.00
-9	-4	1	68.39	9.70
-9	-4	1	72.89	11.10
8	4	-1	143.69	16.80
8	4	-1	150.18	13.90
-8	-4	1	133.89	13.70
-8	-4	1	137.49	14.70
-8	-4	1	149.29	11.90
-8	-4	1	132.59	12.90
-8	-4	1	153.18	16.30
7	4	-1	286.17	21.00
7	4	-1	297.27	16.40
-7	-4	1	274.57	17.10

-7	-4	1	264.47	19.00
-7	-4	1	276.67	19.50
-7	-4	1	295.37	18.30
-7	-4	1	277.87	18.60
-7	-4	1	287.07	16.20
6	4	-1	452.25	20.90
6	4	-1	422.76	28.90
6	4	-1	359.66	24.40
-6	-4	1	379.06	21.80
-6	-4	1	411.66	22.60
-6	-4	1	432.16	23.90
-6	-4	1	408.56	24.30
-6	-4	1	430.06	21.00
5	4	-1	576.84	29.50
5	4	-1	599.24	27.70
-5	-4	1	583.94	28.10
4	4	-1	688.73	31.90
4	4	-1	670.13	32.00
4	4	-1	644.24	30.80
3	4	-1	7.30	1.70
3	4	-1	3.60	2.20
2	4	-1	212.58	10.30
2	4	-1	190.78	9.40
2	4	-1	186.48	9.00
-2	-4	1	188.78	9.00
1	4	-1	5211.38	223.98
1	4	-1	4947.80	223.08
1	4	-1	4903.11	222.98
-1	-4	1	4813.92	223.98
-1	-4	1	5195.98	223.18
0	4	-1	4892.71	204.18
0	-4	1	4878.41	203.28
0	-4	1	4020.20	202.98
0	-4	1	4453.15	204.08
-1	4	-1	1223.78	51.69
-1	4	-1	1087.89	50.39
-1	4	-1	1135.39	50.89
1	-4	1	1151.58	50.89
1	-4	1	1099.59	51.39
1	-4	1	1042.40	50.69
-2	4	-1	188.18	9.60
-2	4	-1	200.78	10.30
-2	4	-1	201.28	10.80
2	-4	1	190.98	9.90

2	-4	1	207.88	10.80
2	-4	1	198.28	10.30
-3	4	-1	181.28	9.60
-3	4	-1	193.28	10.40
-3	4	-1	196.38	11.00
3	-4	1	189.38	9.70
3	-4	1	187.78	10.30
3	-4	1	192.38	10.90
-4	4	-1	288.17	16.00
-4	4	-1	316.87	16.00
-4	4	-1	290.87	15.10
-4	4	-1	302.87	16.60
4	-4	1	297.97	14.90
4	-4	1	316.57	15.90
4	-4	1	298.97	16.50
-5	4	-1	269.27	17.40
-5	4	-1	287.17	15.60
-5	4	-1	274.37	16.30
5	-4	1	288.07	14.40
5	-4	1	276.37	17.40
5	-4	1	256.37	15.90
-6	4	-1	63.99	9.50
-6	4	-1	81.89	11.50
-6	4	-1	87.09	13.60
-6	4	-1	75.39	12.00
6	-4	1	53.29	9.20
6	-4	1	84.89	8.10
6	-4	1	76.29	12.60
-7	4	-1	956.70	46.90
-7	4	-1	976.50	46.20
-7	4	-1	957.10	49.70
7	-4	1	968.60	47.50
-8	4	-1	3.20	11.80
-8	4	-1	6.20	6.50
-8	4	-1	11.50	8.40
-8	4	-1	15.70	8.40
8	-4	1	-7.20	8.50
8	-4	1	-19.40	12.80
-9	4	-1	9.40	8.40
-9	4	-1	-0.90	8.00
-9	4	-1	1.80	8.20
9	-4	1	12.80	10.10
-10	4	-1	72.49	11.10
-10	4	-1	86.79	14.30

-10	4	-1	91.49	15.90
10	-4	1	83.79	16.20
10	-4	1	73.49	12.00
-11	4	-1	23.20	12.00
-11	4	-1	8.50	8.80
-11	4	-1	31.40	13.60
11	-4	1	36.50	10.20
-12	4	-1	297.97	21.60
12	-4	1	326.77	22.20
12	3	-1	14.20	13.60
12	3	-1	16.10	13.40
-12	-3	1	9.40	6.90
11	3	-1	6.50	11.60
-11	-3	1	-21.40	14.50
-11	-3	1	-12.70	10.20
-11	-3	1	4.40	7.10
10	3	-1	126.39	17.90
-10	-3	1	102.99	15.20
-10	-3	1	88.49	15.10
-10	-3	1	72.09	11.40
-10	-3	1	95.79	12.40
-10	-3	1	80.39	15.90
9	3	-1	530.05	32.80
9	3	-1	577.94	32.00
-9	-3	1	545.05	33.00
-9	-3	1	609.24	29.80
-9	-3	1	540.65	30.60
-9	-3	1	561.04	30.10
-9	-3	1	582.74	30.70
8	3	-1	183.28	17.80
-8	-3	1	191.38	14.20
-8	-3	1	174.88	15.90
-8	-3	1	169.28	13.10
7	3	-1	399.86	21.00
-7	-3	1	407.36	21.10
-7	-3	1	389.66	23.90
-7	-3	1	421.16	22.00
-7	-3	1	390.56	22.90
-7	-3	1	396.36	24.10
-7	-3	1	398.86	22.80
6	3	-1	510.85	28.50
6	3	-1	507.45	28.50
6	3	-1	522.95	25.00
-6	-3	1	479.35	28.10

-6	-3	1	551.84	25.40
-6	-3	1	516.25	27.30
-6	-3	1	503.55	26.40
5	3	-1	1302.17	59.09
5	3	-1	1295.57	60.49
-5	-3	1	1301.07	59.49
4	3	-1	525.25	28.80
4	3	-1	513.95	24.80
4	3	-1	562.74	26.10
3	3	-1	62.19	4.80
3	3	-1	58.59	3.90
3	3	-1	59.19	4.70
2	3	-1	360.46	17.40
2	3	-1	396.16	18.00
1	3	-1	489.25	22.10
1	3	-1	457.55	21.50
1	3	-1	459.35	21.40
-1	-3	1	486.55	21.50
-1	-3	1	480.75	22.20
0	3	-1	87.89	5.90
0	3	-1	96.69	4.70
0	3	-1	100.89	4.90
0	-3	1	92.79	6.30
0	-3	1	100.59	4.90
0	-3	1	77.79	5.20
-1	3	-1	248.68	12.20
-1	3	-1	267.37	12.60
-1	3	-1	279.27	13.10
1	-3	1	257.27	12.50
1	-3	1	262.27	13.00
1	-3	1	268.07	12.60
-2	3	-1	444.36	20.10
-2	3	-1	414.86	20.30
-2	3	-1	413.26	19.60
2	-3	1	438.96	20.10
2	-3	1	423.96	20.30
2	-3	1	426.96	19.70
-3	3	-1	389.16	18.60
-3	3	-1	398.86	18.30
-3	3	-1	356.46	17.60
3	-3	1	377.16	18.50
3	-3	1	364.66	17.70
3	-3	1	375.36	18.20
-4	3	-1	5.10	3.10

-4	3	-1	11.20	3.70
-4	3	-1	6.30	2.90
-4	3	-1	2.10	3.10
4	-3	1	9.20	3.60
4	-3	1	7.70	2.20
4	-3	1	1.00	2.70
-5	3	-1	1776.72	80.89
-5	3	-1	1837.72	81.29
-5	3	-1	1751.92	81.99
5	-3	1	1782.62	80.39
5	-3	1	1794.42	80.99
5	-3	1	1715.03	81.89
-6	3	-1	223.28	13.10
-6	3	-1	193.58	15.50
-6	3	-1	184.58	15.50
-6	3	-1	215.08	15.60
6	-3	1	220.58	15.10
6	-3	1	200.48	12.20
6	-3	1	242.08	19.40
-7	3	-1	102.09	19.00
-7	3	-1	97.99	12.50
-7	3	-1	78.49	12.00
-7	3	-1	97.19	9.80
7	-3	1	90.79	15.60
7	-3	1	96.99	12.70
-8	3	-1	126.39	13.90
-8	3	-1	106.49	11.60
-8	3	-1	150.78	26.50
-8	3	-1	122.09	14.10
8	-3	1	106.19	15.10
8	-3	1	161.28	24.70
-9	3	-1	-4.20	7.30
-9	3	-1	5.70	7.70
-9	3	-1	0.60	7.60
9	-3	1	19.40	10.70
-10	3	-1	28.30	9.00
-10	3	-1	8.50	8.60
-10	3	-1	4.40	9.10
10	-3	1	-3.30	9.80
-11	3	-1	0.20	11.20
-11	3	-1	11.30	9.40
-11	3	-1	12.60	12.00
11	-3	1	10.30	9.80
-12	3	-1	304.17	20.70

12	-3	1	299.47	21.90
12	2	-1	-7.30	11.70
12	2	-1	5.40	15.90
-12	-2	1	-7.40	6.70
11	2	-1	86.79	13.40
-11	-2	1	65.59	12.30
-11	-2	1	54.69	9.00
-11	-2	1	64.19	17.00
10	2	-1	276.57	21.60
-10	-2	1	257.27	17.80
-10	-2	1	254.47	20.40
-10	-2	1	250.37	20.60
-10	-2	1	244.28	19.60
9	2	-1	67.89	11.00
9	2	-1	42.10	13.00
-9	-2	1	60.09	10.20
-9	-2	1	39.10	8.40
-9	-2	1	76.89	11.40
8	2	-1	57.89	8.80
8	2	-1	83.69	14.80
-8	-2	1	69.59	12.20
-8	-2	1	71.69	9.90
-8	-2	1	64.19	10.60
-8	-2	1	64.19	12.80
-8	-2	1	68.39	10.90
-7	-2	1	371.06	21.10
-7	-2	1	388.76	23.60
-7	-2	1	373.36	22.30
-7	-2	1	375.66	20.10
-7	-2	1	356.76	23.20
-7	-2	1	394.36	22.00
6	2	-1	224.88	17.20
6	2	-1	237.48	13.30
-6	-2	1	296.27	23.70
-6	-2	1	234.08	18.30
-6	-2	1	254.77	14.10
-6	-2	1	240.88	15.20
5	2	-1	16.90	2.90
5	2	-1	13.60	4.30
-5	-2	1	18.10	4.00
4	2	-1	377.16	18.80
4	2	-1	380.26	18.00
3	2	-1	41.90	3.20
3	2	-1	43.50	3.80

2	2	-1	895.51	38.70
2	2	-1	806.52	38.30
1	2	-1	77.79	3.80
1	2	-1	70.49	3.90
1	2	-1	88.99	4.40
-1	-2	1	81.49	4.30
-1	-2	1	75.69	3.80
0	2	-1	1614.14	71.39
0	2	-1	1685.43	70.89
0	2	-1	1612.84	70.79
0	-2	1	1703.03	70.89
0	-2	1	1471.15	71.39
0	-2	1	1440.16	70.99
-1	2	-1	1857.61	80.09
-1	2	-1	1705.93	79.59
-1	2	-1	1879.41	79.89
1	-2	1	1757.52	79.99
1	-2	1	1793.12	79.89
1	-2	1	1720.13	79.69
-2	2	-1	110.39	5.90
-2	2	-1	102.69	5.50
-2	2	-1	109.59	6.10
2	-2	1	106.59	6.10
2	-2	1	104.89	5.50
2	-2	1	107.99	5.90
-3	2	-1	20.10	3.30
-3	2	-1	20.60	3.00
-3	2	-1	18.30	3.10
3	-2	1	19.60	3.00
3	-2	1	18.70	2.40
3	-2	1	18.50	2.70
-4	2	-1	55.59	5.00
-4	2	-1	53.19	5.80
4	-2	1	49.30	4.80
4	-2	1	55.29	4.10
4	-2	1	59.29	5.70
-5	2	-1	15.20	4.80
-5	2	-1	11.30	3.80
-5	2	-1	15.50	4.70
5	-2	1	17.50	4.90
5	-2	1	7.50	4.70
5	-2	1	17.90	2.80
-6	2	-1	276.47	16.30
-6	2	-1	257.67	14.70

-6	2	-1	235.38	17.50
-6	2	-1	227.18	16.70
-6	2	-1	281.57	19.90
6	-2	1	266.87	15.90
6	-2	1	244.08	19.20
6	-2	1	277.67	14.00
-7	2	-1	34.30	14.40
-7	2	-1	35.70	11.70
-7	2	-1	28.40	7.10
-7	2	-1	36.00	7.70
-7	2	-1	23.10	5.30
7	-2	1	31.60	5.80
7	-2	1	21.80	9.90
-8	2	-1	286.17	18.80
-8	2	-1	324.57	26.50
-8	2	-1	299.67	19.20
-8	2	-1	285.67	17.40
8	-2	1	256.27	21.70
8	-2	1	262.77	18.60
-9	2	-1	12.00	7.00
-9	2	-1	20.80	7.80
-9	2	-1	-0.50	10.50
-9	2	-1	14.00	8.20
9	-2	1	-7.60	12.90
-10	2	-1	134.79	16.00
-10	2	-1	174.78	16.50
-10	2	-1	169.88	15.40
10	-2	1	134.69	19.00
-11	2	-1	398.06	26.40
-11	2	-1	426.16	26.20
-11	2	-1	434.96	25.90
11	-2	1	427.56	26.80
-12	2	-1	14.20	8.60
12	-2	1	18.30	10.10
12	1	-1	121.59	18.40
12	1	-1	169.78	24.60
-12	-1	1	141.49	13.40
11	1	-1	160.08	18.80
-11	-1	1	168.88	14.90
-11	-1	1	140.59	21.00
-11	-1	1	143.59	23.80
10	1	-1	140.39	17.80
-10	-1	1	120.09	13.70
-10	-1	1	117.89	18.30

-10	-1	1	148.29	17.10
-10	-1	1	101.29	15.80
9	1	-1	-1.50	12.70
9	1	-1	20.10	9.30
-9	-1	1	11.60	7.50
-9	-1	1	15.10	6.70
-9	-1	1	34.60	9.70
-9	-1	1	7.50	12.10
-9	-1	1	12.10	8.90
8	1	-1	487.45	28.10
8	1	-1	479.85	26.10
-8	-1	1	483.75	25.40
-8	-1	1	456.95	28.40
-8	-1	1	501.55	26.60
-8	-1	1	487.35	26.40
7	1	-1	14.70	5.00
-7	-1	1	25.60	8.10
-7	-1	1	-2.70	8.70
-7	-1	1	5.70	7.00
-7	-1	1	26.70	10.50
-7	-1	1	18.60	5.00
-7	-1	1	16.20	5.80
6	1	-1	416.26	19.80
6	1	-1	379.66	23.00
-6	-1	1	406.36	20.20
-6	-1	1	374.36	24.00
-6	-1	1	387.26	21.40
5	1	-1	574.74	27.10
5	1	-1	582.74	28.40
-5	-1	1	576.44	27.70
-5	-1	1	579.74	26.50
4	1	-1	324.07	16.10
4	1	-1	338.67	16.90
-4	-1	1	351.56	15.80
3	1	-1	107.39	5.70
3	1	-1	106.19	6.10
-3	-1	1	110.79	5.30
-3	-1	1	93.39	5.90
2	1	-1	254.77	11.70
2	1	-1	258.47	12.00
-2	-1	1	236.18	11.70
-2	-1	1	252.87	11.40
1	1	-1	1406.56	58.09
1	1	-1	1268.57	57.89

-1	-1	1	1338.67	57.79
-1	-1	1	1181.88	57.89
0	1	-1	38.70	2.00
0	1	-1	40.50	2.00
0	1	-1	37.80	2.40
0	-1	1	43.90	2.10
0	-1	1	34.50	2.40
-1	1	-1	2623.34	115.49
-1	1	-1	2622.64	115.59
1	-1	1	2580.04	115.49
1	-1	1	2493.25	115.29
1	-1	1	2645.34	115.59
-2	1	-1	478.75	21.50
-2	1	-1	471.85	21.70
2	-1	1	463.25	21.70
2	-1	1	462.15	21.50
-3	1	-1	259.27	12.20
-3	1	-1	250.37	12.60
3	-1	1	246.88	12.50
3	-1	1	243.28	12.10
-4	1	-1	180.28	9.20
-4	1	-1	164.78	9.90
4	-1	1	177.68	9.10
4	-1	1	161.18	10.20
-5	1	-1	269.57	14.10
-5	1	-1	258.57	14.40
-5	1	-1	267.27	15.30
5	-1	1	254.37	13.90
5	-1	1	296.17	13.70
5	-1	1	281.17	15.60
-6	1	-1	3538.25	146.39
-6	1	-1	3224.88	146.89
-6	1	-1	2999.00	146.89
6	-1	1	3075.99	147.69
6	-1	1	3388.96	145.89
-7	1	-1	114.49	9.70
-7	1	-1	96.39	12.10
-7	1	-1	115.49	13.00
-7	1	-1	118.39	12.50
-7	1	-1	107.99	16.50
-7	1	-1	84.09	12.30
7	-1	1	98.59	9.60
7	-1	1	100.19	12.90
-8	1	-1	33.50	7.90

-8	1	-1	33.50	10.80
-8	1	-1	24.90	6.00
-8	1	-1	20.40	12.10
8	-1	1	39.20	7.70
-9	1	-1	40.20	7.40
-9	1	-1	34.20	19.50
-9	1	-1	32.60	8.40
-9	1	-1	43.10	11.00
-9	1	-1	19.00	7.60
-9	1	-1	32.30	13.80
9	-1	1	28.90	12.10
-10	1	-1	140.39	15.50
-10	1	-1	136.99	15.80
-10	1	-1	117.99	14.40
10	-1	1	106.79	14.00
-11	1	-1	129.69	17.40
-11	1	-1	155.48	17.50
-11	1	-1	148.59	15.00
11	-1	1	110.29	17.40
-12	1	-1	49.50	9.00
12	-1	1	37.80	11.10
12	0	-1	27.20	19.00
12	0	-1	-1.30	10.50
-12	0	1	5.00	7.20
11	0	-1	107.19	17.30
-11	0	1	108.79	14.00
-11	0	1	94.99	16.10
10	0	-1	26.00	9.90
-10	0	1	19.00	10.60
-10	0	1	18.50	9.60
-10	0	1	21.00	8.30
-10	0	1	34.60	11.30
9	0	-1	26.40	10.00
-9	0	1	26.20	7.90
-9	0	1	26.80	10.30
-9	0	1	25.70	7.30
-9	0	1	28.80	9.40
8	0	-1	55.09	8.70
-8	0	1	49.20	8.10
-8	0	1	46.80	7.10
-8	0	1	36.00	12.70
-8	0	1	46.40	12.40
-8	0	1	53.49	9.30
-7	0	1	490.95	23.50

-7	0	1	457.95	25.10
-7	0	1	382.66	28.80
-7	0	1	446.96	26.00
-7	0	1	483.85	24.70
-7	0	1	454.55	27.10
6	0	-1	397.46	23.10
-6	0	1	382.86	21.80
-6	0	1	426.06	20.50
-6	0	1	391.46	19.80
5	0	-1	250.67	14.00
5	0	-1	225.88	12.10
5	0	-1	241.08	11.80
-5	0	1	238.58	12.10
-5	0	1	223.48	13.00
4	0	-1	992.10	41.30
4	0	-1	929.11	42.20
4	0	-1	880.21	41.50
-4	0	1	958.20	41.50
-4	0	1	800.72	41.50
3	0	-1	148.39	8.00
3	0	-1	167.18	7.70
3	0	-1	160.38	8.30
-3	0	1	146.79	8.00
-3	0	1	161.08	8.30
-3	0	1	163.08	7.90
2	0	-1	564.94	26.10
2	0	-1	586.54	26.00
2	0	-1	583.94	26.30
-2	0	1	550.54	26.10
-2	0	1	579.74	26.30
-2	0	1	594.54	26.10
1	0	-1	1904.71	88.69
1	0	-1	2045.80	88.69
1	0	-1	2070.09	88.59
-1	0	1	1947.41	88.69
-1	0	-1	2441.46	108.09
1	0	1	2401.16	107.79
1	0	1	2436.36	107.99
-2	0	-1	10.20	1.60
-2	0	-1	8.20	1.40
2	0	1	11.70	1.50
2	0	1	8.30	1.50
2	0	1	9.50	1.60
-3	0	-1	12.10	1.90

-3	0	-1	7.70	1.80
3	0	1	10.10	1.50
3	0	1	7.20	1.80
3	0	1	9.50	1.50
-4	0	-1	1094.89	49.40
4	0	1	1096.59	49.30
4	0	1	1082.79	49.30
-5	0	-1	19.70	3.00
-5	0	-1	23.70	4.00
-5	0	-1	21.40	4.50
5	0	1	21.30	4.40
5	0	1	18.40	3.00
5	0	1	18.20	2.80
-6	0	-1	403.46	20.90
-6	0	-1	458.85	21.70
-6	0	-1	432.76	23.00
6	0	1	406.66	24.40
6	0	1	430.86	20.90
-7	0	-1	1004.20	50.49
-7	0	-1	1048.10	48.60
-7	0	-1	1062.09	49.50
-7	0	-1	1005.80	50.99
-7	0	-1	1024.80	49.40
-7	0	-1	1001.90	50.59
7	0	1	1069.79	48.30
-8	0	-1	-5.20	9.70
-8	0	-1	17.30	6.60
-8	0	-1	13.40	5.20
-8	0	-1	14.30	10.90
8	0	1	7.60	6.20
-9	0	-1	91.49	17.20
-9	0	-1	89.49	12.70
-9	0	-1	85.69	13.80
-9	0	-1	69.89	11.50
-9	0	-1	84.19	13.30
-9	0	-1	98.09	13.20
9	0	1	109.09	17.70
-10	0	-1	140.19	15.10
-10	0	-1	134.59	15.60
-10	0	-1	141.49	14.70
10	0	1	144.19	19.00
-11	0	-1	112.89	16.10
-11	0	-1	98.39	16.50
-11	0	-1	142.89	14.10

11	0	1	96.89	13.30
-12	0	-1	140.59	14.20
12	0	1	132.29	17.80
12	-1	-1	-7.10	10.60
12	-1	-1	48.50	29.90
-12	1	1	2.30	7.40
11	-1	-1	146.49	18.40
-11	1	1	145.69	15.10
10	-1	-1	342.47	23.20
-10	1	1	271.37	25.30
-10	1	1	307.17	23.20
-10	1	1	373.76	21.90
-10	1	1	320.07	22.30
9	-1	-1	409.16	27.20
-9	1	1	454.05	25.50
-9	1	1	451.75	27.60
-9	1	1	428.56	25.80
-9	1	1	406.56	24.40
8	-1	-1	13.60	8.70
-8	1	1	4.00	5.40
-8	1	1	25.50	9.70
-8	1	1	15.70	6.80
-8	1	1	14.80	9.70
7	-1	-1	94.79	10.60
-7	1	1	84.99	15.90
-7	1	1	94.19	14.10
-7	1	1	122.19	15.30
-7	1	1	99.69	9.60
-7	1	1	86.39	11.30
6	-1	-1	842.72	43.80
6	-1	-1	912.01	41.70
6	-1	-1	902.01	41.60
-6	1	1	888.71	43.00
-6	1	1	948.41	42.10
-6	1	1	864.01	41.90
5	-1	-1	496.75	25.10
5	-1	-1	522.85	23.90
5	-1	-1	502.65	23.80
-5	1	1	490.65	24.10
-5	1	1	494.05	24.40
-5	1	1	501.35	24.90
4	-1	-1	51.59	5.30
4	-1	-1	63.19	4.40
-4	1	1	56.39	5.30

-4	1	1	58.59	5.20
-4	1	1	57.99	4.80
3	-1	-1	79.49	5.10
3	-1	-1	85.99	4.60
3	-1	-1	68.49	4.90
-3	1	1	84.59	5.10
-3	1	1	78.49	4.80
-3	1	1	80.29	4.80
2	-1	-1	402.56	19.10
2	-1	-1	423.96	19.30
2	-1	-1	437.46	19.10
-2	1	1	392.46	19.10
-2	1	1	430.66	19.20
-2	1	1	419.16	19.30
1	-1	-1	1061.89	47.90
1	-1	-1	1121.29	47.90
1	-1	-1	1053.29	47.80
-1	1	1	1081.79	47.90
-1	1	1	1077.99	47.80
-1	1	1	1028.30	47.90
0	-1	-1	3.10	0.50
0	-1	-1	2.20	0.40
0	1	1	2.80	0.40
-1	-1	-1	57.49	2.60
1	1	1	51.39	2.90
1	1	1	55.49	2.60
-2	-1	-1	68.49	4.00
-2	-1	-1	71.49	3.40
2	1	1	68.69	3.50
2	1	1	64.89	4.00
2	1	1	66.39	3.50
-3	-1	-1	11.30	1.60
-3	-1	-1	12.30	2.20
3	1	1	10.80	2.00
3	1	1	12.10	1.60
3	1	1	11.50	1.60
-4	-1	-1	278.37	12.80
4	1	1	266.97	15.10
4	1	1	275.27	13.10
4	1	1	267.07	12.80
-5	-1	-1	2119.89	94.49
-5	-1	-1	2031.60	95.29
5	1	1	2095.69	94.89
5	1	1	2139.99	96.29

5	1	1	2167.58	94.49
-6	-1	-1	25.10	13.30
-6	-1	-1	15.80	5.70
-6	-1	-1	10.80	3.00
-6	-1	-1	10.70	3.80
6	1	1	19.30	7.60
6	1	1	17.20	3.20
-7	-1	-1	377.06	24.40
-7	-1	-1	380.06	24.90
-7	-1	-1	376.86	21.80
-7	-1	-1	414.86	20.70
-7	-1	-1	414.46	24.40
-7	-1	-1	403.86	21.90
-8	-1	-1	241.48	17.10
-8	-1	-1	216.78	15.80
-8	-1	-1	179.28	23.90
-8	-1	-1	206.48	20.10
-8	-1	-1	178.68	18.50
-8	-1	-1	201.08	13.70
8	1	1	205.88	14.20
-9	-1	-1	45.00	8.10
-9	-1	-1	46.70	7.80
-9	-1	-1	34.80	8.60
-9	-1	-1	12.00	10.40
-9	-1	-1	25.70	10.30
-9	-1	-1	35.40	11.10
9	1	1	15.10	9.90
-10	-1	-1	50.29	13.00
-10	-1	-1	26.50	12.50
-10	-1	-1	45.40	9.60
-10	-1	-1	39.60	9.20
-10	-1	-1	29.80	8.00
10	1	1	46.80	11.70
-11	-1	-1	69.59	10.90
-11	-1	-1	48.60	8.50
-11	-1	-1	31.10	10.40
11	1	1	40.50	11.30
-12	-1	-1	290.17	18.20
-12	-1	-1	245.78	26.30
12	1	1	252.67	21.20
12	-2	-1	-9.40	33.10
12	-2	-1	6.30	11.10
-12	2	1	-0.10	8.00
11	-2	-1	110.29	16.80

-11	2	1	94.99	14.30
10	-2	-1	294.57	22.10
-10	2	1	306.97	22.50
-10	2	1	308.97	26.80
-10	2	1	328.77	21.20
-10	2	1	284.67	21.20
9	-2	-1	2.30	9.20
-9	2	1	14.80	9.10
-9	2	1	6.50	7.30
-9	2	1	22.40	11.90
-9	2	1	17.10	7.60
8	-2	-1	88.99	11.70
8	-2	-1	96.19	15.20
-8	2	1	70.29	9.50
-8	2	1	110.19	16.00
-8	2	1	81.99	10.90
7	-2	-1	166.98	12.90
7	-2	-1	127.29	17.40
-7	2	1	159.48	11.70
-7	2	1	145.09	18.70
-7	2	1	168.28	13.70
-7	2	1	150.38	15.40
-7	2	1	154.68	18.40
6	-2	-1	154.18	10.00
6	-2	-1	130.69	11.40
6	-2	-1	136.89	15.00
-6	2	1	178.48	10.90
-6	2	1	147.79	14.00
-6	2	1	143.89	12.70
-6	2	1	163.08	12.50
5	-2	-1	40.70	6.00
5	-2	-1	41.20	5.00
5	-2	-1	43.30	5.70
-5	2	1	51.49	6.50
-5	2	1	45.10	6.70
-5	2	1	45.00	7.10
4	-2	-1	175.38	10.20
4	-2	-1	181.38	9.60
4	-2	-1	172.18	9.30
-4	2	1	181.88	9.90
-4	2	1	176.58	9.70
-4	2	1	181.78	10.30
3	-2	-1	104.29	5.70
3	-2	-1	97.89	5.70

3	-2	-1	103.59	6.20
-3	2	1	106.29	6.10
-3	2	1	98.39	5.70
-3	2	1	95.99	5.90
2	-2	-1	2524.65	116.59
2	-2	-1	2685.83	116.89
2	-2	-1	2680.13	116.79
-2	2	1	2517.15	116.59
-2	2	1	2630.74	116.89
-2	2	1	2652.43	116.89
1	-2	-1	24.20	1.80
1	-2	-1	22.90	2.00
-1	2	1	25.00	1.90
-1	2	1	21.40	2.00
-1	2	1	28.90	1.70
0	-2	-1	4243.68	189.38
0	2	1	4264.77	189.48
1	2	1	283.87	12.80
1	2	1	265.87	12.40
2	2	1	305.77	14.50
2	2	1	332.37	15.10
-3	-2	-1	42.30	2.30
3	2	1	42.50	2.30
3	2	1	44.30	3.90
3	2	1	44.80	2.90
-4	-2	-1	566.04	26.30
4	2	1	611.34	26.30
4	2	1	579.54	26.90
-5	-2	-1	266.37	14.30
5	2	1	275.97	13.50
5	2	1	270.97	15.40
-6	-2	-1	244.38	15.60
-6	-2	-1	240.28	18.70
-6	-2	-1	262.07	14.00
6	2	1	234.68	17.60
6	2	1	270.97	13.50
-7	-2	-1	462.65	23.20
-7	-2	-1	434.26	26.40
-7	-2	-1	449.95	24.30
-7	-2	-1	447.56	26.00
-7	-2	-1	454.05	26.40
-7	-2	-1	454.05	24.40
7	2	1	473.75	22.90
-8	-2	-1	136.69	11.60

-8	-2	-1	114.89	16.20
-8	-2	-1	140.29	16.00
-8	-2	-1	167.08	17.10
-8	-2	-1	151.68	15.60
-8	-2	-1	142.09	12.90
8	2	1	143.69	12.20
-9	-2	-1	7.70	9.30
-9	-2	-1	-6.70	6.10
-9	-2	-1	-2.30	7.30
-9	-2	-1	3.00	6.20
-9	-2	-1	8.50	9.60
-9	-2	-1	-10.50	9.20
9	2	1	5.90	9.30
-10	-2	-1	279.97	19.40
-10	-2	-1	267.67	19.90
-10	-2	-1	288.47	22.70
-10	-2	-1	286.67	23.80
-10	-2	-1	266.07	19.30
10	2	1	338.87	44.90
10	2	1	295.77	22.50
-11	-2	-1	241.78	17.30
-11	-2	-1	210.08	18.60
-11	-2	-1	208.78	18.80
11	2	1	223.48	20.30
-12	-2	-1	-0.30	6.90
-12	-2	-1	-34.90	18.20
12	2	1	14.10	11.70
12	-3	-1	259.87	21.00
-12	3	1	307.37	19.50
11	-3	-1	87.49	16.00
-11	3	1	65.59	10.60
10	-3	-1	10.50	9.60
-10	3	1	21.40	11.40
-10	3	1	16.00	10.50
-10	3	1	22.60	9.20
9	-3	-1	31.70	12.50
9	-3	-1	32.60	9.90
-9	3	1	16.70	9.40
-9	3	1	27.40	8.00
-9	3	1	26.60	8.00
-9	3	1	17.70	12.90
-8	3	1	614.44	33.30
-8	3	1	652.13	34.10
-8	3	1	635.44	32.30

7	-3	-1	132.79	15.40
7	-3	-1	111.99	12.50
-7	3	1	128.19	13.20
-7	3	1	160.58	19.00
-7	3	1	144.39	11.10
-7	3	1	114.19	14.10
-7	3	1	124.69	15.30
6	-3	-1	156.98	16.80
6	-3	-1	166.68	13.40
6	-3	-1	172.18	11.20
-6	3	1	181.28	13.80
-6	3	1	164.48	14.30
-6	3	1	160.48	11.50
-6	3	1	177.98	14.90
5	-3	-1	1582.84	73.69
5	-3	-1	1606.14	75.09
5	-3	-1	1650.23	74.19
-5	3	1	1654.73	74.59
-5	3	1	1643.84	74.89
-5	3	1	1618.44	74.09
4	-3	-1	975.20	45.50
4	-3	-1	1045.40	45.90
4	-3	-1	999.70	46.50
-4	3	1	1032.60	46.50
-4	3	1	954.30	45.50
-4	3	1	987.20	46.10
3	-3	-1	7.50	1.90
3	-3	-1	10.70	2.50
3	-3	-1	6.30	1.90
-3	3	1	10.60	2.30
-3	3	1	7.10	1.80
-3	3	1	9.90	2.40
2	-3	-1	638.54	29.40
2	-3	-1	612.94	29.10
2	-3	-1	639.74	29.60
-2	3	1	655.93	29.10
-2	3	1	626.74	29.50
-2	3	1	667.23	29.50
1	-3	-1	283.77	14.20
1	-3	-1	320.67	14.30
1	-3	-1	297.17	14.60
-1	3	1	307.17	14.60
-1	3	1	304.97	13.90
-1	3	1	314.37	14.30

0	-3	-1	35.30	2.30
0	-3	-1	32.80	3.20
0	3	1	36.90	1.90
0	3	1	36.50	3.50
0	3	1	35.60	2.20
-1	-3	-1	1584.34	73.99
-1	-3	-1	1611.54	74.49
1	3	1	1800.22	74.59
1	3	1	1650.43	73.99
2	3	1	276.87	13.60
2	3	1	325.47	14.50
3	3	1	222.68	11.20
3	3	1	207.98	10.20
4	3	1	542.35	26.40
4	3	1	550.54	25.20
-5	-3	-1	640.14	30.00
5	3	1	628.04	29.40
5	3	1	627.64	31.10
-6	-3	-1	1698.63	79.99
-6	-3	-1	1625.54	78.79
-6	-3	-1	1795.02	78.29
6	3	1	1640.64	80.19
6	3	1	1816.72	77.99
-7	-3	-1	127.29	12.00
-7	-3	-1	101.09	15.30
-7	-3	-1	119.69	11.20
-7	-3	-1	113.29	14.10
-7	-3	-1	108.09	9.60
-7	-3	-1	107.39	14.40
7	3	1	126.89	9.20
-8	-3	-1	53.59	8.20
-8	-3	-1	66.49	9.50
-8	-3	-1	38.90	9.70
-8	-3	-1	49.40	9.60
-8	-3	-1	52.99	9.00
-9	-3	-1	104.09	16.20
-9	-3	-1	109.29	15.70
-9	-3	-1	93.09	13.10
-9	-3	-1	85.99	12.20
-9	-3	-1	91.89	14.60
-9	-3	-1	97.79	11.70
9	3	1	97.29	14.90
9	3	1	122.59	17.50
-10	-3	-1	83.69	14.40

-10	-3	-1	73.69	12.80
-10	-3	-1	77.89	12.60
-10	-3	-1	68.49	11.80
-10	-3	-1	104.39	18.80
10	3	1	97.49	17.40
10	3	1	106.19	18.80
-11	-3	-1	114.49	18.30
-11	-3	-1	100.99	15.10
-11	-3	-1	122.39	13.20
-11	-3	-1	102.19	16.00
-11	-3	-1	110.09	15.90
11	3	1	109.19	18.80
-12	-3	-1	25.00	7.50
-12	-3	-1	46.00	18.60
12	3	1	11.20	12.60
12	-4	-1	128.19	15.60
-12	4	1	130.39	15.10
11	-4	-1	90.69	12.40
-11	4	1	93.99	14.90
10	-4	-1	86.19	16.10
-10	4	1	66.49	11.40
-10	4	1	76.89	17.90
-10	4	1	59.99	12.00
9	-4	-1	15.30	9.80
9	-4	-1	1.30	11.00
-9	4	1	52.39	14.10
-9	4	1	26.30	9.00
-9	4	1	36.50	9.10
-9	4	1	13.30	9.30
8	-4	-1	32.80	8.80
-8	4	1	-3.20	15.00
-8	4	1	21.70	7.20
-8	4	1	4.50	7.30
-8	4	1	15.60	8.70
7	-4	-1	648.94	33.70
7	-4	-1	634.14	33.20
-7	4	1	580.34	36.90
-7	4	1	663.83	36.50
-7	4	1	659.23	33.70
-7	4	1	695.63	32.60
-7	4	1	663.93	33.70
6	-4	-1	362.06	18.80
6	-4	-1	320.87	21.20
6	-4	-1	314.37	21.00

-6	4	1	361.26	20.40
-6	4	1	327.27	21.80
-6	4	1	336.67	21.30
-6	4	1	376.66	18.80
5	-4	-1	4.80	3.50
5	-4	-1	4.40	4.70
5	-4	-1	5.10	5.60
-5	4	1	8.70	5.00
-5	4	1	8.80	5.40
-5	4	1	10.60	4.90
-5	4	1	10.70	4.50
4	-4	-1	360.06	17.90
4	-4	-1	340.97	17.30
4	-4	-1	342.77	18.40
-4	4	1	364.86	18.20
-4	4	1	358.56	19.30
-4	4	1	362.06	18.50
3	-4	-1	73.09	5.40
3	-4	-1	65.49	5.80
3	-4	-1	65.69	5.00
-3	4	1	69.99	5.60
-3	4	1	77.39	5.50
2	-4	-1	6.10	2.30
2	-4	-1	7.40	1.80
2	-4	-1	8.50	2.20
-2	4	1	10.80	1.60
-2	4	1	11.90	2.50
1	-4	-1	634.44	31.70
1	-4	-1	679.53	31.20
-1	4	1	701.03	31.20
-1	4	1	685.13	30.80
-1	4	1	709.83	32.00
0	-4	-1	533.75	23.60
0	-4	-1	481.85	24.40
0	4	1	509.45	24.60
0	4	1	535.75	23.60
0	4	1	516.85	23.20
-1	-4	-1	615.94	27.20
-1	-4	-1	591.04	27.90
1	4	1	571.04	26.90
1	4	1	633.34	27.90
1	4	1	600.94	27.20
2	4	1	4.50	0.90
2	4	1	2.60	0.70

2	4	1	4.40	1.90
3	4	1	31.40	3.90
3	4	1	31.10	4.00
3	4	1	26.00	2.40
-4	-4	-1	29.00	4.60
4	4	1	35.50	4.70
4	4	1	28.80	3.10
4	4	1	28.90	5.00
-5	-4	-1	94.29	7.80
5	4	1	80.19	8.90
5	4	1	92.89	6.10
-6	-4	-1	1315.17	64.69
-6	-4	-1	1378.46	64.29
-6	-4	-1	1473.55	63.79
-6	-4	-1	1366.66	65.49
-6	-4	-1	1332.87	64.19
6	4	1	1456.35	63.49
-7	-4	-1	817.82	41.10
-7	-4	-1	804.12	39.50
-7	-4	-1	806.32	40.40
-7	-4	-1	828.72	40.40
-7	-4	-1	786.62	39.50
-7	-4	-1	843.12	38.90
7	4	1	809.02	38.60
-8	-4	-1	9.90	7.90
-8	-4	-1	-2.00	9.30
-8	-4	-1	9.40	6.00
-8	-4	-1	4.50	8.40
-8	-4	-1	7.80	5.60
8	4	1	7.90	5.80
-9	-4	-1	51.69	8.50
-9	-4	-1	31.80	7.40
-9	-4	-1	63.99	8.40
-9	-4	-1	44.70	9.60
-9	-4	-1	48.60	8.70
9	4	1	60.89	11.00
9	4	1	45.60	10.10
-10	-4	-1	-2.30	8.80
-10	-4	-1	6.50	6.60
-10	-4	-1	6.50	8.20
-10	-4	-1	-20.50	11.20
-10	-4	-1	14.60	8.00
10	4	1	7.00	9.30
10	4	1	-13.40	16.80

-11	-4	-1	211.98	15.50
-11	-4	-1	209.78	25.80
-11	-4	-1	150.78	21.80
-11	-4	-1	156.68	17.70
-11	-4	-1	173.08	17.60
11	4	1	206.48	20.70
-12	-4	-1	61.59	24.10
-12	-4	-1	33.80	7.60
12	4	1	27.20	12.60
12	-5	-1	32.10	10.40
-12	5	1	46.90	10.00
11	-5	-1	66.09	11.60
-11	5	1	76.39	12.10
10	-5	-1	322.07	30.30
10	-5	-1	284.17	22.40
-10	5	1	287.77	26.70
-10	5	1	335.07	22.80
-10	5	1	291.27	23.00
9	-5	-1	97.19	16.10
9	-5	-1	119.99	18.50
-9	5	1	129.29	15.80
-9	5	1	126.39	16.70
-9	5	1	131.49	19.60
8	-5	-1	29.70	9.40
8	-5	-1	8.30	8.20
-8	5	1	17.10	13.50
-8	5	1	22.30	7.30
-8	5	1	12.60	8.20
-8	5	1	36.80	9.10
7	-5	-1	25.40	8.00
-7	5	1	26.70	6.40
-7	5	1	23.20	11.70
-7	5	1	44.60	8.80
-7	5	1	36.40	8.00
6	-5	-1	1321.67	63.99
6	-5	-1	1378.06	62.29
6	-5	-1	1366.86	62.99
-6	5	1	1245.18	64.49
-6	5	1	1315.97	63.09
-6	5	1	1397.26	63.49
5	-5	-1	262.57	17.80
5	-5	-1	255.47	16.00
5	-5	-1	261.47	14.70
-5	5	1	247.38	16.40

-5	5	1	257.87	14.70
-5	5	1	267.77	15.20
-5	5	1	277.57	17.20
4	-5	-1	105.19	7.80
4	-5	-1	113.59	9.70
4	-5	-1	127.29	8.80
-4	5	1	114.29	8.80
-4	5	1	126.19	9.40
-4	5	1	102.99	9.10
-4	5	1	109.59	7.30
3	-5	-1	82.69	6.30
3	-5	-1	70.49	6.20
3	-5	-1	80.79	7.10
-3	5	1	81.59	6.80
-3	5	1	87.59	6.50
-3	5	1	81.19	5.00
2	-5	-1	358.36	18.60
2	-5	-1	387.86	19.40
2	-5	-1	397.26	18.80
-2	5	1	415.46	19.00
-2	5	1	395.96	19.50
-2	5	1	390.56	18.10
1	-5	-1	12.00	2.20
1	-5	-1	10.10	3.00
-1	5	1	10.70	3.50
-1	5	1	10.70	2.00
0	-5	-1	18.80	3.50
0	-5	-1	8.70	1.60
0	5	1	13.40	1.60
0	5	1	16.50	2.30
0	5	1	12.60	3.40
-1	-5	-1	0.70	1.10
-1	-5	-1	2.30	2.30
1	5	1	2.30	1.20
1	5	1	3.10	2.30
1	5	1	2.80	0.80
-2	-5	-1	38.60	2.40
2	5	1	37.20	2.40
2	5	1	36.50	2.40
2	5	1	37.30	4.10
3	5	1	12.80	2.10
3	5	1	12.60	2.60
-4	-5	-1	521.45	25.80
4	5	1	555.44	26.90

4	5	1	547.85	25.20
4	5	1	548.55	26.70
-5	-5	-1	1634.54	73.29
5	5	1	1685.53	74.89
5	5	1	1537.35	74.79
5	5	1	1586.04	72.89
-6	-5	-1	142.99	9.70
-6	-5	-1	142.89	14.60
-6	-5	-1	150.68	13.00
-6	-5	-1	142.09	13.50
-6	-5	-1	130.39	12.40
-6	-5	-1	112.19	11.30
6	5	1	141.59	9.40
-7	-5	-1	84.89	10.90
-7	-5	-1	77.19	10.30
-7	-5	-1	86.99	8.40
-7	-5	-1	92.99	13.50
-7	-5	-1	64.69	8.50
-7	-5	-1	93.89	11.90
7	5	1	84.69	8.10
-8	-5	-1	216.48	16.40
-8	-5	-1	224.08	15.40
-8	-5	-1	205.88	18.10
-8	-5	-1	218.28	14.00
-8	-5	-1	224.38	15.00
-8	-5	-1	190.78	15.80
8	5	1	197.28	14.70
-9	-5	-1	8.60	8.60
-9	-5	-1	18.30	7.60
-9	-5	-1	17.10	6.90
-9	-5	-1	8.00	6.90
-9	-5	-1	24.80	8.40
9	5	1	30.60	10.60
-10	-5	-1	91.69	13.00
-10	-5	-1	68.19	10.40
-10	-5	-1	76.29	13.60
-10	-5	-1	73.89	12.20
-10	-5	-1	75.69	11.80
10	5	1	118.39	18.00
-11	-5	-1	94.79	15.10
-11	-5	-1	54.89	11.90
-11	-5	-1	85.59	12.70
-11	-5	-1	95.69	15.60
-11	-5	-1	89.59	12.40

11	5	1	78.99	13.70
-12	-5	-1	193.08	14.90
-12	-5	-1	168.78	27.60
12	5	1	230.08	29.30
12	-6	-1	22.50	12.80
-12	6	1	-2.60	9.90
11	-6	-1	236.18	19.50
-11	6	1	231.08	20.10
10	-6	-1	208.28	24.00
10	-6	-1	197.28	22.10
-10	6	1	227.48	35.20
-10	6	1	219.78	22.70
-10	6	1	218.08	20.60
9	-6	-1	1.80	9.30
9	-6	-1	4.60	9.10
-9	6	1	1.80	8.60
-9	6	1	23.30	10.10
-9	6	1	12.90	9.80
8	-6	-1	236.98	18.30
8	-6	-1	228.38	19.10
-8	6	1	222.28	19.20
-8	6	1	210.98	19.10
-8	6	1	229.68	16.90
7	-6	-1	139.69	13.60
-7	6	1	121.49	12.20
-7	6	1	121.19	17.50
-7	6	1	142.39	14.80
-7	6	1	126.29	21.20
6	-6	-1	144.29	14.30
-6	6	1	187.08	12.60
-6	6	1	159.98	15.80
-6	6	1	163.08	15.60
5	-6	-1	12.40	6.20
5	-6	-1	6.70	7.20
5	-6	-1	15.70	8.60
5	-6	-1	8.00	6.60
-5	6	1	18.30	6.70
-5	6	1	23.50	7.80
-5	6	1	14.90	7.70
-5	6	1	14.40	5.30
-5	6	1	1.80	4.90
4	-6	-1	31.40	6.40
4	-6	-1	31.50	5.60
-4	6	1	27.10	6.20

-4	6	1	32.90	4.00
-4	6	1	31.50	5.10
-4	6	1	20.70	4.90
3	-6	-1	205.98	11.70
3	-6	-1	197.48	11.70
3	-6	-1	194.78	12.40
-3	6	1	196.98	10.20
-3	6	1	207.48	11.60
-3	6	1	195.28	12.50
2	-6	-1	8.60	4.00
2	-6	-1	9.10	3.60
-2	6	1	10.30	2.80
-2	6	1	10.10	3.80
-2	6	1	14.40	2.00
1	-6	-1	1280.57	57.49
1	-6	-1	1184.48	58.29
-1	6	1	1230.68	56.69
-1	6	1	1365.96	57.59
-1	6	1	1257.57	58.89
0	-6	-1	6274.27	284.37
0	-6	-1	6221.38	285.37
0	6	1	6222.68	284.37
0	6	1	6819.02	285.77
-1	-6	-1	45.10	3.40
-1	-6	-1	42.20	5.50
1	6	1	45.20	3.30
1	6	1	45.60	5.50
1	6	1	44.80	2.70
-2	-6	-1	490.25	22.70
2	6	1	526.95	24.20
2	6	1	475.65	22.60
2	6	1	500.05	22.70
3	6	1	378.26	18.60
3	6	1	428.76	20.40
-4	-6	-1	28.30	4.10
4	6	1	24.00	3.30
4	6	1	26.00	5.00
-5	-6	-1	158.68	9.90
5	6	1	144.19	12.90
5	6	1	160.88	9.10
5	6	1	146.59	12.90
-6	-6	-1	21.40	4.40
-6	-6	-1	26.00	6.60
-6	-6	-1	15.10	6.10

-6	-6	-1	13.40	6.40
-6	-6	-1	19.60	7.30
-6	-6	-1	18.60	5.40
6	6	1	19.60	4.10
-7	-6	-1	461.45	23.60
-7	-6	-1	440.56	26.40
-7	-6	-1	470.15	25.40
-7	-6	-1	472.15	24.70
-7	-6	-1	464.65	24.50
-7	-6	-1	460.05	24.50
7	6	1	462.65	23.60
-8	-6	-1	199.78	14.70
-8	-6	-1	203.78	13.60
-8	-6	-1	211.38	15.10
-8	-6	-1	165.48	17.50
-8	-6	-1	196.38	16.20
-8	-6	-1	188.88	15.40
8	6	1	199.38	14.60
8	6	1	200.28	20.80
-9	-6	-1	-4.80	7.10
-9	-6	-1	14.10	9.00
-9	-6	-1	9.30	6.80
-9	-6	-1	-6.10	6.10
-9	-6	-1	11.00	7.50
9	6	1	11.50	10.30
-10	-6	-1	328.97	20.50
-10	-6	-1	309.87	20.00
-10	-6	-1	306.27	21.50
-10	-6	-1	301.87	23.50
-10	-6	-1	322.67	20.10
10	6	1	279.57	24.90
10	6	1	278.37	31.90
-11	-6	-1	76.89	12.30
-11	-6	-1	96.79	16.70
-11	-6	-1	94.99	12.00
-11	-6	-1	87.59	14.30
-11	-6	-1	71.49	12.10
11	6	1	71.09	15.50
-12	-6	-1	22.20	17.10
-12	-6	-1	12.10	7.50
11	-7	-1	29.80	10.10
11	-7	-1	8.20	15.50
-11	7	1	19.70	10.90
10	-7	-1	2.70	9.60

10	-7	-1	-16.20	11.40
-10	7	1	-2.70	12.70
-10	7	1	15.20	11.00
-10	7	1	1.10	12.60
9	-7	-1	5.00	9.10
9	-7	-1	0.30	9.20
-9	7	1	4.40	10.20
-9	7	1	-13.70	9.60
-9	7	1	-6.30	10.40
8	-7	-1	49.20	10.30
8	-7	-1	31.70	8.70
-8	7	1	26.90	9.70
-8	7	1	38.20	9.70
-8	7	1	38.50	8.80
7	-7	-1	225.88	17.10
-7	7	1	227.18	16.40
-7	7	1	223.58	18.60
6	-7	-1	6.70	6.70
-6	7	1	1.40	5.80
-6	7	1	4.50	7.70
-6	7	1	25.50	8.50
5	-7	-1	1779.52	80.19
-5	7	1	1786.02	80.09
-5	7	1	1700.83	79.09
-5	7	1	1676.73	80.99
4	-7	-1	225.88	15.10
4	-7	-1	224.88	16.80
4	-7	-1	213.88	15.30
-4	7	1	244.78	14.80
-4	7	1	230.28	16.50
-4	7	1	221.78	14.20
-4	7	1	244.28	12.40
3	-7	-1	63.59	9.50
3	-7	-1	56.89	7.30
3	-7	-1	78.69	8.00
-3	7	1	67.09	8.80
-3	7	1	75.29	5.20
-3	7	1	72.49	7.40
-3	7	1	89.59	10.00
2	-7	-1	1269.87	62.19
-2	7	1	1381.76	62.99
-2	7	1	1319.47	60.19
-2	7	1	1373.06	61.29
1	-7	-1	118.59	9.40

1	-7	-1	108.59	7.10
-1	7	1	109.99	5.90
-1	7	1	111.99	10.50
-1	7	1	106.89	6.90
0	-7	-1	2.50	3.60
0	-7	-1	4.10	2.20
0	7	1	4.50	1.20
0	7	1	7.70	3.90
0	7	1	4.00	1.80
-1	-7	-1	568.44	25.30
-1	-7	-1	458.55	26.60
1	7	1	535.35	26.90
1	7	1	573.34	25.20
1	7	1	565.24	24.70
-2	-7	-1	75.89	4.80
-2	-7	-1	82.29	7.70
2	7	1	77.89	4.50
2	7	1	82.59	7.40
2	7	1	82.09	4.80
3	7	1	13.00	2.10
3	7	1	17.80	4.60
-4	-7	-1	15.90	4.20
4	7	1	8.90	5.40
4	7	1	10.30	2.40
4	7	1	5.30	5.10
-5	-7	-1	1477.75	70.09
-5	-7	-1	1548.65	69.09
-5	-7	-1	1573.54	70.19
5	7	1	1548.65	68.69
5	7	1	1400.06	71.49
-6	-7	-1	419.36	20.80
-6	-7	-1	417.86	22.80
-6	-7	-1	409.96	21.80
-6	-7	-1	378.06	22.00
-6	-7	-1	393.56	24.00
-6	-7	-1	394.26	21.90
6	7	1	424.66	20.70
-7	-7	-1	295.67	18.30
-7	-7	-1	316.17	17.20
-7	-7	-1	297.17	18.40
-7	-7	-1	323.97	18.40
-7	-7	-1	295.17	19.10
-7	-7	-1	302.47	20.70
7	7	1	308.07	17.30

-8	-7	-1	19.70	6.80
-8	-7	-1	16.30	7.50
-8	-7	-1	27.80	7.00
-8	-7	-1	14.40	6.90
-8	-7	-1	18.40	6.20
8	7	1	16.30	6.90
8	7	1	14.30	11.60
-9	-7	-1	153.28	17.70
-9	-7	-1	177.38	14.70
-9	-7	-1	166.38	15.60
-9	-7	-1	187.18	15.00
9	7	1	172.88	22.00
9	7	1	183.18	19.50
-10	-7	-1	20.70	9.10
-10	-7	-1	18.70	8.90
-10	-7	-1	31.40	11.00
-10	-7	-1	41.40	8.20
-10	-7	-1	21.60	9.10
10	7	1	58.59	20.50
10	7	1	16.40	19.20
-11	-7	-1	24.40	12.10
-11	-7	-1	30.60	10.30
-11	-7	-1	24.10	10.60
-11	-7	-1	33.00	12.10
-11	-7	-1	38.60	8.00
-12	-7	-1	14.30	7.10
-12	-7	-1	-32.80	18.60
-12	-7	-1	32.30	23.30
12	7	1	12.40	11.10
11	-8	-1	0.10	14.60
11	-8	-1	6.10	10.20
-11	8	1	-15.10	10.70
10	-8	-1	83.99	11.90
10	-8	-1	96.39	13.10
-10	8	1	71.19	14.10
-10	8	1	76.59	14.80
-10	8	1	75.59	17.80
9	-8	-1	8.40	9.30
9	-8	-1	27.90	9.90
-9	8	1	31.40	11.50
-9	8	1	37.00	11.70
-9	8	1	35.20	11.70
8	-8	-1	35.60	9.70
8	-8	-1	25.10	7.90

-8	8	1	10.50	10.40
-8	8	1	32.50	10.20
-8	8	1	20.90	9.30
7	-8	-1	1082.59	51.99
7	-8	-1	1054.69	52.49
-7	8	1	1059.89	52.29
-7	8	1	1059.89	53.09
-7	8	1	1133.09	54.59
6	-8	-1	126.69	13.60
-6	8	1	121.89	16.40
-6	8	1	151.88	13.40
-6	8	1	171.58	16.60
5	-8	-1	92.49	12.90
5	-8	-1	76.59	9.00
-5	8	1	59.99	10.20
-5	8	1	86.19	11.70
-5	8	1	71.39	9.50
4	-8	-1	11.60	9.20
4	-8	-1	18.70	7.10
4	-8	-1	21.70	7.60
-4	8	1	6.70	5.40
-4	8	1	24.20	8.80
-4	8	1	15.00	3.60
-4	8	1	13.50	6.30
3	-8	-1	206.88	15.30
3	-8	-1	184.38	13.20
-3	8	1	191.58	10.40
-3	8	1	214.68	16.40
-3	8	1	190.28	13.70
-3	8	1	196.68	12.60
2	-8	-1	277.37	18.30
-2	8	1	302.27	14.50
-2	8	1	312.27	16.00
-2	8	1	314.17	19.50
-2	8	1	283.87	18.00
1	-8	-1	1954.90	90.49
1	-8	-1	1892.41	89.09
-1	8	1	2175.68	91.59
-1	8	1	1953.40	89.19
-1	8	1	1867.01	88.19
0	-8	-1	1198.38	56.59
0	-8	-1	1186.28	55.09
0	8	1	1158.88	54.29
0	8	1	1269.57	57.09

0	8	1	1218.68	55.09
-1	-8	-1	1686.83	71.99
-1	-8	-1	1380.16	73.39
1	8	1	1667.33	71.89
1	8	1	1629.64	71.29
1	8	1	1575.44	73.69
-2	-8	-1	78.69	9.20
-2	-8	-1	76.79	5.40
2	8	1	78.89	4.70
2	8	1	78.09	5.20
-3	-8	-1	7.90	3.60
3	8	1	-5.20	5.70
3	8	1	2.70	1.60
3	8	1	1.50	1.70
-4	-8	-1	703.93	32.60
4	8	1	677.53	31.90
4	8	1	673.43	34.80
-5	-8	-1	111.79	15.40
-5	-8	-1	89.89	10.70
-5	-8	-1	108.59	8.30
-5	-8	-1	97.79	10.40
-5	-8	-1	104.19	11.60
-5	-8	-1	103.49	10.70
5	8	1	101.49	7.80
-6	-8	-1	116.99	12.70
-6	-8	-1	137.89	12.30
-6	-8	-1	142.89	10.10
-6	-8	-1	137.39	11.80
-6	-8	-1	139.19	15.10
-6	-8	-1	143.19	11.90
6	8	1	152.98	10.10
-7	-8	-1	184.58	12.70
-7	-8	-1	186.98	21.10
-7	-8	-1	177.98	14.40
7	8	1	192.88	13.10
-8	-8	-1	106.99	14.60
-8	-8	-1	93.19	11.60
-8	-8	-1	91.79	10.40
-8	-8	-1	102.99	12.10
-8	-8	-1	107.49	11.70
8	8	1	81.09	12.60
-9	-8	-1	46.80	8.70
-9	-8	-1	61.89	9.10
-9	-8	-1	49.40	9.20

-9	-8	-1	37.10	7.30
-9	-8	-1	45.10	9.80
9	8	1	49.20	13.40
-10	-8	-1	43.80	16.80
-10	-8	-1	41.30	8.50
-10	-8	-1	35.10	9.70
-11	-8	-1	118.39	13.00
-11	-8	-1	99.39	16.10
-11	-8	-1	136.49	17.60
-11	-8	-1	152.58	17.60
-12	-8	-1	8.70	7.20
-12	-8	-1	20.70	13.70
12	8	1	-6.80	10.80
11	-9	-1	85.49	23.10
11	-9	-1	68.89	11.70
-11	9	1	57.09	12.70
10	-9	-1	105.59	15.70
10	-9	-1	85.19	12.30
-10	9	1	91.89	14.10
-10	9	1	129.89	16.20
9	-9	-1	48.30	10.20
9	-9	-1	36.50	11.00
-9	9	1	45.30	18.70
-9	9	1	48.10	12.80
-9	9	1	36.60	12.80
8	-9	-1	0.10	7.30
8	-9	-1	1.10	8.80
-8	9	1	2.50	16.10
-8	9	1	7.10	10.40
-8	9	1	-2.60	11.00
7	-9	-1	107.69	15.20
7	-9	-1	109.99	13.00
-7	9	1	77.69	11.70
-7	9	1	114.69	19.30
-7	9	1	81.49	14.00
6	-9	-1	1153.08	61.89
6	-9	-1	1290.97	63.29
-6	9	1	1264.27	59.99
-6	9	1	1271.07	60.99
5	-9	-1	-14.10	9.50
5	-9	-1	4.80	6.00
-5	9	1	-2.00	11.20
-5	9	1	14.70	9.90
-5	9	1	8.60	6.50

4	-9	-1	21.90	8.90
4	-9	-1	21.50	6.60
-4	9	1	39.20	12.20
-4	9	1	33.10	7.40
-4	9	1	25.30	4.10
3	-9	-1	10.70	6.70
3	-9	-1	2.40	8.70
-3	9	1	15.40	6.30
-3	9	1	8.70	5.10
-3	9	1	9.10	2.80
-3	9	1	13.60	9.80
2	-9	-1	106.19	13.30
-2	9	1	129.99	12.20
-2	9	1	109.49	14.80
-2	9	1	107.19	9.10
-2	9	1	114.39	6.70
1	-9	-1	905.21	43.60
-1	9	1	959.20	44.90
-1	9	1	849.81	40.60
-1	9	1	860.71	41.80
0	-9	-1	0.80	5.30
0	-9	-1	6.60	3.50
0	9	1	12.20	5.50
0	9	1	10.90	2.00
0	9	1	7.00	2.70
-1	-9	-1	228.88	16.10
-1	-9	-1	239.38	12.80
1	9	1	238.08	11.60
1	9	1	243.88	12.50
-2	-9	-1	800.22	45.40
-2	-9	-1	975.30	43.00
2	9	1	977.70	42.40
2	9	1	968.00	42.80
-3	-9	-1	257.87	13.60
3	9	1	234.88	11.90
3	9	1	224.38	11.60
3	9	1	224.68	16.80
-4	-9	-1	766.02	42.30
-4	-9	-1	934.31	46.20
-4	-9	-1	878.01	40.50
4	9	1	892.31	39.80
4	9	1	829.52	43.60
-5	-9	-1	394.26	19.80
-5	-9	-1	403.56	26.00

-5	-9	-1	375.96	21.30
-5	-9	-1	356.96	20.60
-5	-9	-1	355.46	20.50
-5	-9	-1	372.76	21.60
5	9	1	399.26	19.40
-6	-9	-1	968.60	47.30
-6	-9	-1	985.90	46.30
-6	-9	-1	955.40	48.60
-6	-9	-1	1012.40	47.10
-6	-9	-1	967.60	46.60
6	9	1	984.20	46.20
-7	-9	-1	85.19	11.30
-7	-9	-1	78.69	12.80
-7	-9	-1	88.29	9.30
-7	-9	-1	90.79	10.80
-7	-9	-1	74.19	10.80
7	9	1	74.19	10.60
-8	-9	-1	116.99	13.00
-8	-9	-1	130.39	12.00
-8	-9	-1	132.69	15.60
-8	-9	-1	112.39	13.50
-8	-9	-1	125.69	12.90
8	9	1	109.09	14.80
-9	-9	-1	233.68	19.10
-9	-9	-1	175.08	16.10
-9	-9	-1	188.38	16.30
-9	-9	-1	227.58	16.90
-9	-9	-1	217.08	16.90
9	9	1	206.08	23.00
-10	-9	-1	140.19	17.10
-10	-9	-1	145.99	15.90
-10	-9	-1	147.79	16.10
-10	-9	-1	109.89	18.30
-10	-9	-1	140.39	17.50
-11	-9	-1	125.09	13.20
-11	-9	-1	118.79	15.00
-11	-9	-1	111.39	18.00
-11	-9	-1	153.48	17.60
-11	-9	-1	115.59	15.60
-12	-9	-1	112.49	19.10
-12	-9	-1	80.09	11.90
12	9	1	74.89	12.60
11	-10	-1	164.98	21.40
11	-10	-1	178.28	20.10

-11	10	1	108.69	16.50
10	-10	-1	90.39	15.80
10	-10	-1	55.69	11.20
-10	10	1	54.59	13.00
-10	10	1	57.49	16.00
9	-10	-1	-12.20	11.00
9	-10	-1	-12.90	10.80
-9	10	1	-0.90	15.20
-9	10	1	14.90	13.30
-9	10	1	8.20	13.80
8	-10	-1	350.36	22.50
8	-10	-1	363.06	23.50
-8	10	1	358.56	25.90
-8	10	1	328.87	24.90
-8	10	1	363.46	28.60
7	-10	-1	40.30	7.70
7	-10	-1	68.49	10.90
-7	10	1	54.59	12.50
-7	10	1	55.09	10.80
-7	10	1	58.99	11.50
6	-10	-1	351.76	21.60
6	-10	-1	344.17	26.30
-6	10	1	370.36	26.20
-6	10	1	359.76	23.70
-6	10	1	412.06	27.20
5	-10	-1	36.20	9.40
5	-10	-1	37.40	7.80
-5	10	1	32.20	9.00
-5	10	1	47.50	11.60
4	-10	-1	-1.90	5.80
4	-10	-1	0.70	8.60
-4	10	1	-2.90	3.80
-4	10	1	4.60	7.50
-4	10	1	17.30	11.40
3	-10	-1	214.18	16.50
3	-10	-1	217.28	16.40
-3	10	1	213.08	11.60
-3	10	1	199.98	15.70
-3	10	1	217.18	21.50
2	-10	-1	180.98	14.40
2	-10	-1	201.98	17.90
-2	10	1	196.08	10.40
-2	10	1	197.78	20.00
-2	10	1	215.58	15.10

-2	10	1	182.18	13.00
1	-10	-1	1571.94	74.89
1	-10	-1	1685.23	76.69
-1	10	1	1558.64	74.69
-1	10	1	1749.72	77.79
-1	10	1	1571.24	73.29
0	-10	-1	2406.66	113.19
0	10	1	2444.66	110.39
0	10	1	2410.16	111.49
0	10	1	2590.34	113.89
-1	-10	-1	253.17	18.10
-1	-10	-1	253.57	14.40
1	10	1	248.68	13.70
1	10	1	263.87	12.60
-2	-10	-1	36.10	5.20
-2	-10	-1	49.10	6.30
-2	-10	-1	44.60	9.30
2	10	1	34.90	3.60
2	10	1	32.00	5.10
-3	-10	-1	763.12	35.30
3	10	1	772.42	34.00
3	10	1	690.93	38.80
3	10	1	698.73	33.80
-4	-10	-1	48.90	8.10
-4	-10	-1	50.49	7.90
-4	-10	-1	46.80	7.80
-4	-10	-1	49.99	8.10
-4	-10	-1	46.00	7.00
4	10	1	45.90	6.10
-5	-10	-1	8.50	4.80
-5	-10	-1	4.30	6.20
-5	-10	-1	4.40	6.80
-5	-10	-1	7.60	6.30
-5	-10	-1	7.90	7.80
-5	-10	-1	11.50	6.00
5	10	1	9.60	3.90
-6	-10	-1	6.50	9.50
-6	-10	-1	7.90	5.80
-6	-10	-1	-2.30	6.40
-6	-10	-1	7.80	5.90
-6	-10	-1	7.30	6.90
-6	-10	-1	9.30	4.80
6	10	1	-1.70	4.70
-7	-10	-1	523.95	27.50

-7	-10	-1	514.75	29.00
-7	-10	-1	545.65	28.70
-7	-10	-1	539.85	28.10
7	10	1	532.55	28.40
-8	-10	-1	203.48	16.20
-8	-10	-1	212.18	18.10
-8	-10	-1	200.58	15.50
-8	-10	-1	216.18	16.00
-8	-10	-1	201.88	16.20
8	10	1	218.08	18.80
-9	-10	-1	7.90	9.10
-9	-10	-1	20.00	8.60
-9	-10	-1	-0.30	7.10
-9	-10	-1	16.60	8.50
-9	-10	-1	8.80	7.00
9	10	1	-5.00	14.00
-10	-10	-1	205.08	18.80
-10	-10	-1	208.78	17.10
-10	-10	-1	237.18	17.80
-10	-10	-1	236.78	20.00
-10	-10	-1	219.68	19.50
-11	-10	-1	19.60	7.50
-11	-10	-1	0.50	13.90
-11	-10	-1	10.00	11.90
-11	-10	-1	23.20	8.30
11	10	1	4.00	10.90
10	-11	-1	45.10	11.70
10	-11	-1	49.20	10.00
-10	11	1	58.69	12.70
9	-11	-1	0.10	8.10
9	-11	-1	-4.30	10.10
-9	11	1	13.10	13.20
-9	11	1	-1.80	14.30
-9	11	1	-5.10	11.90
8	-11	-1	49.60	11.90
8	-11	-1	90.79	13.80
-8	11	1	52.89	13.00
-8	11	1	61.99	14.70
-8	11	1	78.89	13.40
7	-11	-1	24.30	7.80
7	-11	-1	29.80	10.60
-7	11	1	23.10	11.40
-7	11	1	23.60	11.90
-7	11	1	7.40	12.60

6	-11	-1	257.87	22.20
6	-11	-1	296.27	18.50
6	-11	-1	274.27	23.10
-6	11	1	252.47	21.50
-6	11	1	264.67	24.00
-6	11	1	254.37	23.20
5	-11	-1	960.40	56.59
5	-11	-1	1030.90	51.29
-5	11	1	1100.49	53.89
-5	11	1	1096.79	52.79
4	-11	-1	7.20	6.30
4	-11	-1	28.10	16.60
4	-11	-1	33.90	8.90
-4	11	1	12.40	11.40
-4	11	1	15.20	8.00
-4	11	1	18.80	5.10
3	-11	-1	6.00	6.50
3	-11	-1	18.70	7.50
-3	11	1	8.40	7.20
-3	11	1	10.80	3.60
-3	11	1	21.40	11.00
2	-11	-1	345.07	22.00
-2	11	1	426.66	22.70
-2	11	1	384.16	18.90
1	-11	-1	4.00	6.70
1	-11	-1	7.00	8.80
-1	11	1	10.80	5.00
-1	11	1	5.30	9.10
-1	11	1	8.60	2.60
0	-11	-1	0.10	5.40
0	-11	-1	1.80	7.70
0	11	1	2.20	2.10
0	11	1	5.70	4.30
0	11	1	-2.90	8.60
-1	-11	-1	207.98	12.50
-1	-11	-1	180.38	17.70
1	11	1	173.68	18.60
1	11	1	186.68	9.90
1	11	1	178.88	11.70
-2	-11	-1	64.39	11.20
-2	-11	-1	60.69	13.00
-2	-11	-1	73.99	8.40
-2	-11	-1	62.99	9.10
2	11	1	57.39	11.80

2	11	1	69.09	5.10
2	11	1	64.49	7.10
-3	-11	-1	12.80	7.70
-3	-11	-1	-1.40	8.30
-3	-11	-1	13.00	6.00
-3	-11	-1	8.30	6.80
-3	-11	-1	-0.10	8.10
3	11	1	10.50	3.20
-4	-11	-1	305.17	17.20
-4	-11	-1	291.27	19.00
-4	-11	-1	294.47	17.50
-4	-11	-1	293.47	18.70
-4	-11	-1	301.17	19.10
-4	-11	-1	287.27	19.20
4	11	1	304.07	15.70
-5	-11	-1	860.11	42.80
-5	-11	-1	864.01	42.40
-5	-11	-1	859.01	41.60
-5	-11	-1	839.72	42.00
-5	-11	-1	894.21	42.60
-5	-11	-1	861.91	41.70
5	11	1	863.51	41.10
-6	-11	-1	207.88	16.70
-6	-11	-1	201.38	14.70
-6	-11	-1	197.08	15.80
-6	-11	-1	210.48	16.60
-6	-11	-1	212.08	18.70
-6	-11	-1	201.88	14.00
6	11	1	215.68	14.40
-7	-11	-1	327.27	19.50
-7	-11	-1	318.97	20.80
-7	-11	-1	335.47	20.20
-7	-11	-1	287.57	19.90
-7	-11	-1	291.47	18.80
7	11	1	321.27	20.50
-8	-11	-1	555.44	29.00
-8	-11	-1	539.05	28.40
-8	-11	-1	510.75	29.10
-8	-11	-1	494.95	28.40
-8	-11	-1	576.44	31.20
8	11	1	422.16	30.90
-9	-11	-1	265.97	19.30
-9	-11	-1	267.07	19.20
-9	-11	-1	327.87	21.10

-9	-11	-1	321.87	21.00
-9	-11	-1	261.77	19.90
9	11	1	253.17	29.20
-10	-11	-1	16.10	7.50
-10	-11	-1	26.00	11.40
-10	-11	-1	7.20	8.20
-10	-11	-1	36.20	10.70
-10	-11	-1	15.20	9.70
-11	-11	-1	-2.60	14.40
-11	-11	-1	-0.20	7.50
-11	-11	-1	26.80	11.60
-11	-11	-1	-11.60	7.00
11	11	1	-12.60	10.20
10	-12	-1	143.29	15.00
10	-12	-1	123.39	21.20
-10	12	1	99.89	17.00
9	-12	-1	19.30	8.10
9	-12	-1	31.70	12.10
-9	12	1	-1.80	11.10
-9	12	1	24.40	16.50
-9	12	1	29.70	13.90
8	-12	-1	99.19	13.90
-8	12	1	84.59	15.50
-8	12	1	80.19	14.90
-8	12	1	49.60	20.30
7	-12	-1	890.21	43.40
7	-12	-1	769.12	49.00
7	-12	-1	875.71	45.10
-7	12	1	884.91	47.30
-7	12	1	855.71	48.30
-7	12	1	927.91	47.90
6	-12	-1	5.20	7.00
6	-12	-1	6.00	13.80
6	-12	-1	-3.80	10.40
-6	12	1	-7.70	10.90
-6	12	1	-3.50	12.60
-6	12	1	-5.20	12.50
5	-12	-1	26.90	9.60
5	-12	-1	2.30	7.10
-5	12	1	6.50	10.00
-5	12	1	15.10	12.20
4	-12	-1	324.87	19.50
4	-12	-1	307.57	39.70
4	-12	-1	325.57	20.60

-4	12	1	302.37	25.60
-4	12	1	305.47	22.50
-4	12	1	322.77	20.60
3	-12	-1	118.59	19.80
3	-12	-1	115.19	13.50
3	-12	-1	109.19	16.90
3	-12	-1	101.49	13.00
-3	12	1	111.19	18.60
-3	12	1	124.29	9.60
-3	12	1	109.39	14.50
2	-12	-1	151.88	13.90
2	-12	-1	131.99	21.80
2	-12	-1	135.49	16.00
2	-12	-1	137.19	16.80
2	-12	-1	143.09	16.50
-2	12	1	181.68	14.40
-2	12	1	164.48	9.90
1	-12	-1	80.09	11.20
1	-12	-1	78.29	12.30
-1	12	1	77.79	6.30
0	-12	-1	1357.06	59.79
0	-12	-1	1312.47	59.39
0	-12	-1	1177.28	58.69
0	-12	-1	1187.98	59.59
0	-12	-1	1245.88	59.29
0	12	1	1286.27	57.19
0	12	1	1207.18	58.99
-1	-12	-1	317.27	20.20
-1	-12	-1	346.87	21.00
-1	-12	-1	311.57	25.10
-1	-12	-1	311.67	18.80
-1	-12	-1	330.17	19.70
-1	-12	-1	302.77	19.50
1	12	1	316.17	18.40
1	12	1	337.17	16.20
-2	-12	-1	467.15	26.50
-2	-12	-1	448.36	25.40
-2	-12	-1	451.05	24.90
-2	-12	-1	486.95	24.70
-2	-12	-1	496.55	24.40
2	12	1	451.25	24.10
-3	-12	-1	142.79	14.90
-3	-12	-1	141.09	12.90
-3	-12	-1	134.19	13.60

-3	-12	-1	148.09	11.30
-3	-12	-1	116.89	14.20
-3	-12	-1	130.69	11.80
3	12	1	145.79	8.80
-4	-12	-1	443.66	25.90
-4	-12	-1	475.05	24.80
-4	-12	-1	478.75	24.90
-4	-12	-1	457.25	26.00
-4	-12	-1	421.66	26.20
-4	-12	-1	523.95	25.70
4	12	1	481.65	23.40
-5	-12	-1	171.98	15.00
-5	-12	-1	142.99	14.80
-5	-12	-1	168.98	13.80
-5	-12	-1	165.98	14.50
-5	-12	-1	174.38	12.90
-5	-12	-1	172.88	15.10
5	12	1	155.88	11.70
-6	-12	-1	410.86	24.30
-6	-12	-1	430.46	23.50
-6	-12	-1	390.76	25.10
-6	-12	-1	407.36	24.90
-6	-12	-1	427.16	23.50
-6	-12	-1	426.36	24.60
6	12	1	434.26	23.50
-7	-12	-1	115.29	13.00
-7	-12	-1	113.09	15.50
-7	-12	-1	113.59	12.40
-7	-12	-1	102.29	18.60
-7	-12	-1	120.49	14.00
-7	-12	-1	115.99	14.60
7	12	1	106.29	15.00
-8	-12	-1	166.18	15.60
-8	-12	-1	198.18	17.10
-8	-12	-1	184.08	16.50
-8	-12	-1	143.39	14.10
-8	-12	-1	182.18	16.60
8	12	1	127.79	20.30
-9	-12	-1	0.20	9.40
-9	-12	-1	3.80	9.90
-9	-12	-1	10.40	10.20
-9	-12	-1	-7.50	9.90
9	12	1	-1.00	17.90
-10	-12	-1	149.19	14.80

-10	-12	-1	144.49	13.60
-10	-12	-1	140.89	17.00
-10	-12	-1	127.79	17.20
10	12	1	164.18	20.10
-11	-12	-1	81.89	11.90
-11	-12	-1	51.19	16.50
-11	-12	-1	73.89	12.40
-11	-12	-1	52.09	18.70
11	12	1	47.20	12.90
10	-13	-1	18.10	8.70
-10	13	1	-2.90	9.60
9	-13	-1	10.20	7.70
9	-13	-1	11.40	15.20
-9	13	1	4.20	13.50
-9	13	1	20.20	17.10
-9	13	1	-17.80	10.20
8	-13	-1	12.20	13.60
8	-13	-1	12.00	8.70
-8	13	1	15.40	13.60
-8	13	1	13.30	13.90
-8	13	1	20.70	14.30
7	-13	-1	118.89	16.00
7	-13	-1	111.39	13.80
-7	13	1	101.79	16.30
-7	13	1	109.19	16.30
-7	13	1	100.99	16.20
6	-13	-1	454.05	25.80
6	-13	-1	452.25	28.80
-6	13	1	454.25	32.50
-6	13	1	498.55	31.60
-6	13	1	444.46	31.30
5	-13	-1	50.49	8.50
5	-13	-1	62.59	10.10
-5	13	1	36.10	11.80
-5	13	1	35.70	13.10
4	-13	-1	14.30	6.70
4	-13	-1	18.30	7.60
-4	13	1	17.00	9.80
-4	13	1	21.10	12.00
-4	13	1	24.90	7.10
3	-13	-1	53.49	8.30
3	-13	-1	76.99	16.80
3	-13	-1	57.89	8.80
-3	13	1	68.49	10.30

-3	13	1	60.59	11.60
-3	13	1	84.79	8.80
2	-13	-1	260.47	23.40
2	-13	-1	234.48	16.90
2	-13	-1	245.78	20.10
2	-13	-1	262.17	19.20
2	-13	-1	227.28	19.00
-2	13	1	249.97	13.80
-2	13	1	238.78	17.30
1	-13	-1	761.42	38.20
1	-13	-1	703.53	40.40
1	-13	-1	759.22	38.40
1	-13	-1	792.02	39.70
1	-13	-1	787.92	39.10
-1	13	1	792.82	36.10
0	-13	-1	8.70	6.80
0	-13	-1	9.40	7.90
0	-13	-1	11.50	7.20
0	-13	-1	6.50	7.80
0	-13	-1	3.60	7.60
0	13	1	0.60	2.70
-1	-13	-1	197.18	18.90
-1	-13	-1	238.08	16.20
-1	-13	-1	234.58	16.90
-1	-13	-1	217.18	15.80
-1	-13	-1	220.38	15.40
-1	-13	-1	216.68	18.30
1	13	1	197.68	16.40
1	13	1	219.88	11.90
-2	-13	-1	435.46	30.00
-2	-13	-1	522.75	28.00
-2	-13	-1	549.15	26.50
-2	-13	-1	497.65	26.90
-2	-13	-1	520.55	27.40
-2	-13	-1	488.75	26.60
2	13	1	551.44	24.90
-3	-13	-1	134.39	11.30
-3	-13	-1	106.49	13.20
-3	-13	-1	134.59	12.70
-3	-13	-1	127.39	14.10
-3	-13	-1	130.39	11.90
3	13	1	136.19	8.80
-4	-13	-1	195.08	13.60
-4	-13	-1	188.98	13.50

-4	-13	-1	188.98	14.70
-4	-13	-1	176.78	15.60
-4	-13	-1	170.48	15.30
-4	-13	-1	167.08	16.00
4	13	1	179.98	11.60
-5	-13	-1	48.30	8.20
-5	-13	-1	60.29	9.90
-5	-13	-1	32.70	8.10
-5	-13	-1	43.00	7.10
-5	-13	-1	41.10	9.50
-5	-13	-1	58.49	9.60
5	13	1	36.80	6.40
-6	-13	-1	199.58	17.80
-6	-13	-1	207.78	17.70
-6	-13	-1	225.58	17.70
-6	-13	-1	201.38	15.90
-6	-13	-1	228.48	15.50
-6	-13	-1	208.28	17.30
6	13	1	227.78	16.50
-7	-13	-1	177.88	17.40
-7	-13	-1	181.98	19.40
-7	-13	-1	200.88	17.70
-7	-13	-1	202.48	17.30
-7	-13	-1	212.68	17.10
-7	-13	-1	181.58	14.80
7	13	1	177.08	18.20
-8	-13	-1	35.70	9.90
-8	-13	-1	40.50	9.90
-8	-13	-1	46.10	9.50
-8	-13	-1	25.30	7.40
8	13	1	48.00	13.60
-9	-13	-1	270.97	19.70
-9	-13	-1	287.57	20.50
-9	-13	-1	211.28	19.40
-9	-13	-1	266.27	17.10
-9	-13	-1	229.68	20.10
9	13	1	234.58	24.60
-10	-13	-1	185.88	18.30
-10	-13	-1	186.58	17.80
-10	-13	-1	176.18	16.20
-10	-13	-1	224.88	14.70
10	13	1	224.48	22.20
-11	-13	-1	93.69	13.70
-11	-13	-1	103.19	13.30

-11	-13	-1	114.59	17.20
11	13	1	138.49	20.10
9	-14	-1	15.20	7.60
-9	14	1	-6.50	9.60
-9	14	1	38.80	14.60
8	-14	-1	111.39	13.90
-8	14	1	118.79	19.80
-8	14	1	114.19	16.80
-8	14	1	126.39	20.90
7	-14	-1	13.40	7.90
-7	14	1	5.40	13.50
-7	14	1	24.60	13.90
-7	14	1	8.80	12.00
6	-14	-1	246.88	18.00
6	-14	-1	204.18	21.80
-6	14	1	316.07	25.00
-6	14	1	263.87	25.40
-6	14	1	245.88	25.70
5	-14	-1	265.77	18.30
5	-14	-1	253.27	20.40
-5	14	1	240.48	22.90
-5	14	1	267.17	24.50
4	-14	-1	-1.80	7.30
4	-14	-1	19.70	7.90
-4	14	1	13.70	11.20
-4	14	1	0.70	9.10
3	-14	-1	180.18	25.40
3	-14	-1	105.49	19.60
3	-14	-1	144.59	14.40
-3	14	1	138.49	12.00
-3	14	1	140.59	15.20
-3	14	1	133.99	17.70
2	-14	-1	187.48	21.10
2	-14	-1	141.09	16.00
2	-14	-1	160.18	14.10
2	-14	-1	128.19	17.50
2	-14	-1	111.69	15.80
-2	14	1	135.19	10.50
-2	14	1	150.58	14.30
1	-14	-1	945.11	45.40
1	-14	-1	832.12	46.00
1	-14	-1	908.01	44.60
1	-14	-1	863.51	44.30
1	-14	-1	954.90	45.20

-1	14	1	932.91	42.60
-1	14	1	914.11	44.50
0	-14	-1	375.86	22.50
0	-14	-1	371.86	21.90
0	-14	-1	409.96	22.60
0	-14	-1	378.36	22.40
0	14	1	305.67	28.00
0	14	1	396.46	19.20
-1	-14	-1	646.64	33.30
-1	-14	-1	662.93	33.30
-1	-14	-1	664.53	33.70
-1	-14	-1	672.43	34.00
-1	-14	-1	662.43	34.50
1	14	1	659.83	31.50
-2	-14	-1	11.40	7.50
-2	-14	-1	13.60	10.60
-2	-14	-1	7.70	7.40
-2	-14	-1	3.20	6.00
-2	-14	-1	12.40	6.20
2	14	1	17.30	4.00
-3	-14	-1	520.25	26.80
-3	-14	-1	480.15	26.90
-3	-14	-1	503.55	26.20
-3	-14	-1	468.75	36.10
-3	-14	-1	509.35	26.20
-3	-14	-1	497.65	27.60
3	14	1	500.55	24.80
-4	-14	-1	-1.90	7.90
-4	-14	-1	14.70	7.90
-4	-14	-1	-1.80	6.10
-4	-14	-1	6.80	6.60
-4	-14	-1	6.80	5.50
4	14	1	10.00	4.70
-5	-14	-1	96.39	13.50
-5	-14	-1	97.29	11.70
-5	-14	-1	81.79	13.20
-5	-14	-1	88.69	11.30
-5	-14	-1	109.49	14.10
-5	-14	-1	88.69	13.50
5	14	1	113.69	11.10
-6	-14	-1	30.70	6.70
-6	-14	-1	39.00	9.00
-6	-14	-1	31.30	9.10
-6	-14	-1	29.40	8.20

-6	-14	-1	19.00	8.80
-6	-14	-1	24.40	8.40
6	14	1	35.60	16.60
6	14	1	36.20	8.40
-7	-14	-1	215.58	17.90
-7	-14	-1	201.48	17.10
-7	-14	-1	169.48	19.70
-7	-14	-1	188.88	18.10
-7	-14	-1	225.78	15.60
7	14	1	207.88	19.70
7	14	1	233.68	26.00
-8	-14	-1	235.48	17.80
-8	-14	-1	191.18	15.00
-8	-14	-1	195.58	17.90
-8	-14	-1	196.38	18.10
-8	-14	-1	196.68	17.20
8	14	1	149.09	25.90
8	14	1	197.88	28.00
-9	-14	-1	81.39	14.10
-9	-14	-1	80.89	14.20
-9	-14	-1	87.09	14.20
-9	-14	-1	76.59	10.90
9	14	1	73.79	15.30
-10	-14	-1	116.89	13.60
-10	-14	-1	107.59	16.70
-10	-14	-1	121.19	15.20
-10	-14	-1	121.89	11.90
10	14	1	125.49	20.60
-9	15	1	8.80	20.80
-9	15	1	14.30	9.10
8	-15	-1	31.20	7.70
-8	15	1	47.60	25.20
-8	15	1	14.60	10.50
-8	15	1	18.60	13.30
7	-15	-1	5.30	8.00
7	-15	-1	6.30	14.40
-7	15	1	16.70	12.00
-7	15	1	12.40	13.00
-7	15	1	2.40	13.80
6	-15	-1	354.16	21.70
6	-15	-1	345.97	24.70
-6	15	1	340.17	27.70
-6	15	1	359.16	28.70
-6	15	1	362.56	26.70

5	-15	-1	233.88	20.00
5	-15	-1	233.18	18.40
-5	15	1	290.07	23.60
-5	15	1	283.57	29.30
4	-15	-1	324.47	22.20
4	-15	-1	318.97	21.10
-4	15	1	326.67	25.30
-4	15	1	364.16	23.90
3	-15	-1	78.89	10.40
3	-15	-1	65.19	16.90
3	-15	-1	57.59	12.30
3	-15	-1	51.49	9.20
-3	15	1	39.80	9.10
-3	15	1	60.79	8.60
-3	15	1	62.89	11.00
2	-15	-1	165.38	17.80
2	-15	-1	149.69	15.30
2	-15	-1	147.69	20.00
2	-15	-1	155.48	17.50
2	-15	-1	207.88	20.70
-2	15	1	149.09	14.50
-2	15	1	176.08	12.10
1	-15	-1	22.60	9.60
1	-15	-1	7.90	8.10
1	-15	-1	2.50	10.10
1	-15	-1	12.10	9.40
-1	15	1	11.50	7.30
0	-15	-1	51.09	8.90
0	-15	-1	73.89	9.90
0	-15	-1	69.09	9.70
0	-15	-1	72.89	11.70
0	15	1	68.49	7.60
-1	-15	-1	363.06	20.50
-1	-15	-1	334.77	20.90
-1	-15	-1	340.17	22.60
-1	-15	-1	348.87	25.30
1	15	1	362.16	18.50
-2	-15	-1	133.19	18.00
-2	-15	-1	136.59	16.50
-2	-15	-1	130.09	13.80
-2	-15	-1	124.19	14.90
-2	-15	-1	134.09	17.70
2	15	1	116.49	9.60
-3	-15	-1	113.69	11.30

-3	-15	-1	121.69	15.50
-3	-15	-1	127.69	13.20
-3	-15	-1	128.19	12.90
-3	-15	-1	107.79	13.70
-3	-15	-1	98.49	11.70
3	15	1	120.09	9.80
-4	-15	-1	296.27	18.50
-4	-15	-1	294.27	18.30
-4	-15	-1	311.97	19.60
-4	-15	-1	280.07	19.10
-4	-15	-1	323.97	20.70
4	15	1	293.67	17.40
-5	-15	-1	671.13	33.30
-5	-15	-1	658.53	33.90
-5	-15	-1	665.23	34.20
-5	-15	-1	644.94	34.50
-5	-15	-1	622.34	34.80
5	15	1	668.03	33.50
-6	-15	-1	17.10	8.50
-6	-15	-1	6.00	8.00
-6	-15	-1	24.00	9.10
-6	-15	-1	10.10	5.90
-6	-15	-1	28.90	10.30
6	15	1	12.10	9.50
-7	-15	-1	17.50	8.90
-7	-15	-1	19.30	9.90
-7	-15	-1	29.40	8.70
-7	-15	-1	23.50	8.70
-7	-15	-1	10.20	6.10
7	15	1	56.59	17.30
7	15	1	-0.30	11.60
-8	-15	-1	506.35	29.20
-8	-15	-1	526.45	30.50
-8	-15	-1	574.44	29.00
-8	-15	-1	536.75	30.50
8	15	1	533.95	37.60
-9	-15	-1	123.69	15.20
-9	-15	-1	164.48	15.50
-9	-15	-1	113.29	14.90
-9	-15	-1	152.18	12.10
9	15	1	101.19	18.10
-10	-15	-1	-11.70	11.40
-10	-15	-1	16.30	6.20
-10	-15	-1	-1.10	7.30

-10	-15	-1	8.40	9.80
10	15	1	30.30	23.70
9	-16	-1	15.40	7.50
-9	16	1	14.60	9.40
8	-16	-1	230.38	15.50
-8	16	1	246.08	19.10
-8	16	1	184.98	24.00
-8	16	1	211.08	21.90
7	-16	-1	463.15	25.80
7	-16	-1	504.35	31.10
-7	16	1	476.95	29.00
-7	16	1	469.05	30.00
-7	16	1	409.26	30.80
6	-16	-1	60.99	9.70
-6	16	1	74.39	14.20
-6	16	1	32.20	20.00
-6	16	1	57.29	13.10
5	-16	-1	21.20	9.50
5	-16	-1	0.30	7.60
-5	16	1	28.50	12.40
-5	16	1	14.70	11.40
4	-16	-1	259.37	18.90
4	-16	-1	247.38	19.40
-4	16	1	253.37	22.40
3	-16	-1	15.70	15.60
3	-16	-1	1.70	11.40
3	-16	-1	15.30	8.00
3	-16	-1	-3.30	8.30
-3	16	1	10.10	9.00
-3	16	1	13.80	8.70
-3	16	1	2.00	9.40
2	-16	-1	42.20	8.30
2	-16	-1	100.59	15.80
2	-16	-1	65.09	10.80
2	-16	-1	58.69	11.00
2	-16	-1	56.69	13.10
-2	16	1	77.99	12.00
-2	16	1	72.49	11.10
1	-16	-1	140.09	16.90
1	-16	-1	112.09	14.80
1	-16	-1	126.09	14.60
1	-16	-1	141.39	15.60
-1	16	1	123.49	11.20
-1	16	1	108.39	13.00

0	-16	-1	506.15	27.50
0	-16	-1	466.45	26.90
0	-16	-1	512.85	27.50
0	-16	-1	484.65	27.70
0	16	1	494.05	25.10
0	16	1	470.55	31.70
-1	-16	-1	37.50	8.40
-1	-16	-1	32.90	7.60
-1	-16	-1	29.70	7.60
-1	-16	-1	23.60	9.10
-1	-16	-1	26.50	7.00
1	16	1	37.80	6.20
-2	-16	-1	58.19	10.70
-2	-16	-1	24.40	8.00
-2	-16	-1	13.30	7.20
-2	-16	-1	39.10	8.30
-2	-16	-1	31.70	7.50
2	16	1	30.60	5.80
-3	-16	-1	412.66	24.10
-3	-16	-1	418.86	28.60
-3	-16	-1	415.16	24.10
-3	-16	-1	417.86	23.80
-3	-16	-1	421.26	23.30
3	16	1	417.96	22.40
-4	-16	-1	362.16	21.80
-4	-16	-1	375.26	21.90
-4	-16	-1	364.46	20.70
-4	-16	-1	355.46	21.00
-4	-16	-1	343.27	22.50
4	16	1	351.56	20.70
-5	-16	-1	-0.90	8.00
-5	-16	-1	-4.40	7.40
-5	-16	-1	-4.30	7.40
-5	-16	-1	3.20	11.10
5	16	1	7.70	7.60
-6	-16	-1	303.17	19.60
-6	-16	-1	293.57	19.80
-6	-16	-1	273.07	20.50
-6	-16	-1	304.87	18.30
-6	-16	-1	303.77	20.80
6	16	1	287.87	22.10
-7	-16	-1	28.60	9.30
-7	-16	-1	18.40	8.60
-7	-16	-1	37.60	9.20

-7	-16	-1	17.50	7.00
7	16	1	41.00	14.00
-8	-16	-1	113.39	13.90
-8	-16	-1	83.79	11.00
-8	-16	-1	78.09	13.50
-8	-16	-1	81.19	13.30
8	16	1	99.29	20.00
-9	-16	-1	-8.20	9.00
-9	-16	-1	7.10	9.90
-9	-16	-1	8.20	5.80
-9	-16	-1	4.50	7.80
9	16	1	-10.70	18.00
-10	-16	-1	44.50	10.50
-10	-16	-1	49.90	9.70
-10	-16	-1	48.80	6.90
8	-17	-1	134.39	12.60
-8	17	1	139.09	15.10
-8	17	1	111.19	20.00
7	-17	-1	118.89	12.80
7	-17	-1	136.89	21.20
-7	17	1	102.59	15.70
-7	17	1	126.69	21.30
-7	17	1	87.09	14.20
6	-17	-1	117.29	18.80
6	-17	-1	154.88	14.60
-6	17	1	152.78	21.10
-6	17	1	158.88	21.00
-6	17	1	161.28	18.70
5	-17	-1	154.68	17.40
5	-17	-1	119.89	14.40
-5	17	1	146.69	20.00
-5	17	1	150.78	19.60
4	-17	-1	8.20	8.20
4	-17	-1	1.80	8.10
-4	17	1	-14.30	9.80
-4	17	1	0.10	10.00
3	-17	-1	2.70	8.80
3	-17	-1	4.90	7.60
-3	17	1	-0.40	9.70
-3	17	1	11.60	11.00
2	-17	-1	382.86	27.40
2	-17	-1	413.16	25.20
2	-17	-1	385.86	26.00
2	-17	-1	403.26	24.60

2	-17	-1	374.46	23.90
-2	17	1	390.86	23.80
-2	17	1	377.66	23.70
1	-17	-1	331.47	21.80
1	-17	-1	327.87	22.90
1	-17	-1	330.77	22.00
1	-17	-1	326.27	21.20
-1	17	1	348.37	21.30
-1	17	1	311.17	19.80
0	-17	-1	16.00	8.50
0	-17	-1	34.60	8.50
0	-17	-1	39.20	8.90
0	-17	-1	36.10	8.70
0	17	1	36.10	8.20
0	17	1	24.10	6.40
0	17	1	40.10	21.00
-1	-17	-1	187.18	15.90
-1	-17	-1	182.88	15.50
-1	-17	-1	182.68	17.80
-1	-17	-1	178.18	15.60
1	17	1	217.28	21.70
1	17	1	196.88	14.00
-2	-17	-1	216.08	15.80
-2	-17	-1	188.48	19.10
-2	-17	-1	194.78	16.10
-2	-17	-1	201.08	16.30
-2	-17	-1	223.38	16.20
2	17	1	209.98	14.60
-3	-17	-1	78.69	13.70
-3	-17	-1	67.89	12.00
-3	-17	-1	74.49	12.30
-3	-17	-1	65.69	8.60
-3	-17	-1	90.19	13.10
3	17	1	81.59	11.30
-4	-17	-1	72.09	10.50
-4	-17	-1	78.99	11.20
-4	-17	-1	88.29	12.70
-4	-17	-1	71.59	12.00
4	17	1	79.09	12.00
-5	-17	-1	17.30	7.80
-5	-17	-1	4.50	6.00
-5	-17	-1	-9.10	7.10
-5	-17	-1	10.30	8.00
5	17	1	9.20	8.80

-6	-17	-1	34.60	9.00
-6	-17	-1	47.50	9.20
-6	-17	-1	33.70	7.20
-6	-17	-1	31.30	8.80
6	17	1	29.40	11.60
-7	-17	-1	88.89	11.60
-7	-17	-1	104.49	14.70
-7	-17	-1	111.79	11.60
-7	-17	-1	97.39	11.40
7	17	1	68.39	16.50
-8	-17	-1	17.20	8.30
-8	-17	-1	11.40	7.60
-8	-17	-1	24.50	6.00
-9	-17	-1	361.46	21.40
-9	-17	-1	371.56	21.30
-9	-17	-1	372.36	19.90
8	-18	-1	71.69	15.80
-8	18	1	79.29	18.10
7	-18	-1	147.89	22.30
7	-18	-1	192.38	13.90
-7	18	1	180.38	19.10
-7	18	1	131.89	15.70
-7	18	1	122.69	21.60
6	-18	-1	226.28	16.30
6	-18	-1	201.88	19.70
-6	18	1	248.68	21.60
-6	18	1	232.38	18.60
-6	18	1	209.08	22.40
5	-18	-1	38.90	9.90
5	-18	-1	24.70	8.50
-5	18	1	49.20	11.90
-5	18	1	45.80	10.70
-5	18	1	64.99	12.80
4	-18	-1	129.99	15.80
4	-18	-1	115.09	15.70
-4	18	1	98.09	16.70
-4	18	1	97.29	16.80
3	-18	-1	295.37	20.00
3	-18	-1	257.07	23.90
3	-18	-1	269.17	21.30
-3	18	1	271.57	21.20
-3	18	1	280.47	21.10
2	-18	-1	163.18	18.20
2	-18	-1	187.48	16.90

2	-18	-1	129.39	20.90
2	-18	-1	170.68	21.70
-2	18	1	154.68	16.90
-2	18	1	175.18	16.40
1	-18	-1	210.78	18.30
1	-18	-1	224.48	18.00
1	-18	-1	241.48	20.50
1	-18	-1	242.28	18.70
-1	18	1	225.28	19.10
-1	18	1	231.58	27.60
0	-18	-1	13.00	7.80
0	-18	-1	15.10	8.80
0	-18	-1	-15.50	7.60
0	-18	-1	-13.60	7.20
0	18	1	9.60	7.60
0	18	1	-1.40	7.90
-1	-18	-1	176.08	18.00
-1	-18	-1	197.58	16.20
-1	-18	-1	169.28	15.50
-1	-18	-1	178.68	15.60
1	18	1	183.28	15.60
1	18	1	202.28	22.60
-2	-18	-1	178.48	15.50
-2	-18	-1	167.38	15.10
-2	-18	-1	164.08	17.80
-2	-18	-1	164.08	14.50
2	18	1	180.38	15.60
2	18	1	158.88	19.90
-3	-18	-1	132.09	13.10
-3	-18	-1	116.09	21.20
-3	-18	-1	107.69	13.60
-3	-18	-1	142.19	13.20
-3	-18	-1	130.39	13.80
3	18	1	134.29	18.90
-4	-18	-1	85.09	12.80
-4	-18	-1	80.79	12.70
-4	-18	-1	113.39	13.50
-4	-18	-1	91.79	11.90
4	18	1	108.29	14.50
-5	-18	-1	4.50	7.30
-5	-18	-1	-5.60	7.60
-5	-18	-1	10.90	6.30
-5	-18	-1	0.10	7.40
5	18	1	16.10	10.40

-6	-18	-1	60.69	10.40
-6	-18	-1	50.59	10.00
-6	-18	-1	60.99	10.80
6	18	1	59.09	14.30
-7	-18	-1	266.47	18.30
-7	-18	-1	241.38	18.80
-7	-18	-1	263.17	16.00
-7	-18	-1	255.77	17.80
-8	-18	-1	20.50	7.70
-8	-18	-1	8.90	5.50
-8	-18	-1	11.30	8.30
-9	-18	-1	-2.20	8.00
-9	-18	-1	3.70	5.70
-9	-18	-1	11.50	8.30
7	-19	-1	30.60	7.70
7	-19	-1	46.50	15.80
-7	19	1	3.50	11.90
-7	19	1	47.70	12.60
6	-19	-1	399.66	21.60
6	-19	-1	398.26	24.20
-6	19	1	360.66	23.00
-6	19	1	353.26	25.50
-6	19	1	363.16	24.70
5	-19	-1	168.38	16.10
5	-19	-1	170.68	17.40
-5	19	1	191.28	17.60
-5	19	1	185.88	19.90
-5	19	1	183.28	19.10
4	-19	-1	-4.10	8.40
4	-19	-1	13.20	9.70
-4	19	1	5.40	9.90
-4	19	1	-2.60	9.60
3	-19	-1	185.58	16.50
-3	19	1	161.78	17.20
-3	19	1	175.18	16.70
2	-19	-1	121.19	14.50
2	-19	-1	148.79	22.50
2	-19	-1	126.99	17.00
-2	19	1	119.99	15.30
-2	19	1	97.89	14.00
1	-19	-1	43.40	9.30
1	-19	-1	68.79	10.20
1	-19	-1	46.10	9.20
1	-19	-1	52.39	11.30

-1	19	1	43.10	9.80
-1	19	1	46.40	12.60
0	-19	-1	54.89	10.20
0	-19	-1	77.19	13.30
0	-19	-1	52.79	9.30
0	-19	-1	98.49	15.00
0	19	1	76.09	13.00
0	19	1	76.79	12.00
-1	-19	-1	42.40	8.90
-1	-19	-1	59.99	9.30
-1	-19	-1	51.79	8.90
-1	-19	-1	25.40	9.20
1	19	1	45.10	10.50
1	19	1	45.60	13.70
1	19	1	48.60	9.00
-2	-19	-1	72.79	11.60
-2	-19	-1	44.80	8.00
-2	-19	-1	58.49	9.00
-2	-19	-1	72.59	11.50
2	19	1	70.39	12.60
2	19	1	60.79	11.10
-3	-19	-1	279.27	18.30
-3	-19	-1	259.27	18.20
-3	-19	-1	243.88	24.80
-3	-19	-1	267.77	17.70
3	19	1	263.47	20.80
3	19	1	271.97	21.30
-4	-19	-1	188.38	15.90
-4	-19	-1	192.28	16.00
-4	-19	-1	213.08	15.80
-4	-19	-1	179.18	14.90
4	19	1	204.38	20.30
-5	-19	-1	112.69	13.80
-5	-19	-1	103.89	12.60
-5	-19	-1	97.39	13.70
-5	-19	-1	136.09	14.80
5	19	1	108.89	15.20
-6	-19	-1	50.89	9.00
-6	-19	-1	33.50	8.30
-6	-19	-1	69.09	11.10
-6	-19	-1	48.50	7.50
-7	-19	-1	16.30	5.80
-7	-19	-1	-1.70	7.50
-7	-19	-1	3.40	8.90

-7	-19	-1	32.80	7.50
-8	-19	-1	396.06	20.20
-8	-19	-1	406.86	21.70
-8	-19	-1	356.06	21.20
6	-20	-1	19.80	10.10
6	-20	-1	17.30	8.10
-6	20	1	20.50	14.00
-6	20	1	17.20	9.00
-6	20	1	22.40	11.30
5	-20	-1	86.09	13.50
5	-20	-1	57.29	10.10
-5	20	1	55.69	12.60
-5	20	1	48.30	11.40
-5	20	1	38.70	9.30
4	-20	-1	287.47	21.20
4	-20	-1	312.37	20.30
-4	20	1	292.67	21.90
-4	20	1	282.17	21.20
3	-20	-1	31.60	17.50
3	-20	-1	7.50	8.10
3	-20	-1	22.90	13.30
-3	20	1	17.00	9.20
-3	20	1	8.60	9.30
2	-20	-1	12.50	10.80
-2	20	1	-1.50	8.00
1	-20	-1	107.29	21.70
1	-20	-1	92.89	14.20
1	-20	-1	91.79	16.00
-1	20	1	93.09	12.80
-1	20	1	87.89	14.10
0	-20	-1	540.75	29.50
0	-20	-1	553.34	29.80
0	-20	-1	555.44	30.40
0	-20	-1	541.85	30.90
0	20	1	518.35	35.00
0	20	1	524.65	29.50
-1	-20	-1	17.10	8.90
-1	-20	-1	9.00	7.20
-1	-20	-1	18.90	8.10
-1	-20	-1	21.90	9.50
1	20	1	0.00	7.60
1	20	1	-3.40	12.60
-2	-20	-1	1.00	7.50
-2	-20	-1	13.10	7.40

-2	-20	-1	11.20	7.80
-2	-20	-1	26.90	12.50
2	20	1	14.10	8.10
2	20	1	18.00	20.60
-3	-20	-1	305.97	26.20
-3	-20	-1	319.97	25.30
3	20	1	331.37	25.00
3	20	1	315.27	26.90
-4	-20	-1	207.68	16.00
-4	-20	-1	194.68	15.80
-4	-20	-1	189.58	14.70
4	20	1	238.48	25.10
4	20	1	176.38	18.70
-5	-20	-1	14.80	6.50
-5	-20	-1	21.60	7.00
-5	-20	-1	11.80	7.40
-5	-20	-1	13.80	7.50
5	20	1	10.00	9.80
-6	-20	-1	51.49	8.30
-6	-20	-1	66.39	9.70
-6	-20	-1	45.60	8.20
-7	-20	-1	6.30	7.10
-7	-20	-1	13.40	5.70
-7	-20	-1	1.70	7.30
-7	-20	-1	18.10	9.10
7	20	1	6.00	8.40
-8	-20	-1	140.39	13.30
8	20	1	96.59	16.40
5	-21	-1	190.58	17.40
5	-21	-1	186.68	15.70
-5	21	1	172.78	17.00
-5	21	1	148.79	15.60
-5	21	1	178.68	18.50
4	-21	-1	-19.40	14.90
4	-21	-1	-1.30	7.70
-4	21	1	13.20	10.60
-4	21	1	-5.40	8.20
-4	21	1	-4.30	9.30
3	-21	-1	-12.10	7.40
3	-21	-1	5.40	14.20
-3	21	1	5.20	8.50
-3	21	1	8.00	9.80
2	-21	-1	314.97	21.40
2	-21	-1	336.27	23.10

-2	21	1	324.77	21.80
-2	21	1	332.97	21.70
1	-21	-1	54.49	10.70
1	-21	-1	55.29	15.10
1	-21	-1	75.79	10.20
-1	21	1	77.49	12.20
-1	21	1	80.09	12.80
0	-21	-1	17.20	9.00
0	-21	-1	2.30	8.20
0	-21	-1	8.20	8.00
0	-21	-1	0.90	10.50
0	21	1	1.70	7.70
-1	-21	-1	345.47	20.90
-1	-21	-1	315.47	20.20
-1	-21	-1	320.57	21.40
-1	-21	-1	320.27	22.30
1	21	1	312.07	20.50
-2	-21	-1	186.88	15.40
-2	-21	-1	178.78	15.60
-2	-21	-1	234.48	19.10
-2	-21	-1	203.78	16.50
2	21	1	177.88	23.20
2	21	1	199.58	15.70
-3	-21	-1	31.70	7.90
-3	-21	-1	36.60	8.00
-3	-21	-1	28.50	7.70
3	21	1	29.80	8.30
3	21	1	22.70	11.50
-4	-21	-1	-5.30	6.10
-4	-21	-1	-5.10	7.30
-4	-21	-1	13.00	6.50
4	21	1	4.20	9.80
-5	-21	-1	13.30	7.00
-5	-21	-1	2.30	6.70
-5	-21	-1	10.30	6.00
5	21	1	7.70	10.00
-6	-21	-1	19.60	7.80
-6	-21	-1	34.20	7.60
-6	-21	-1	28.80	6.10
6	21	1	33.10	9.80
-7	-21	-1	76.59	11.20
-7	-21	-1	81.79	14.40
-7	-21	-1	67.99	8.70
7	21	1	63.69	14.30

4	-22	-1	159.18	12.70
-4	22	1	138.09	15.00
-4	22	1	116.49	18.50
-4	22	1	126.79	16.70
3	-22	-1	222.18	16.60
-3	22	1	265.97	17.90
-3	22	1	234.58	18.60
2	-22	-1	112.59	13.40
2	-22	-1	148.99	16.80
-2	22	1	102.79	14.30
-2	22	1	142.59	14.00
1	-22	-1	72.59	10.00
1	-22	-1	71.49	10.60
-1	22	1	79.29	12.80
-1	22	1	78.59	13.10
0	-22	-1	8.30	8.40
0	-22	-1	-6.90	11.30
0	-22	-1	-4.50	7.90
0	22	1	1.20	7.90
0	22	1	-5.30	7.30
-1	-22	-1	47.10	8.90
-1	-22	-1	50.99	10.20
-1	-22	-1	55.09	9.90
-1	-22	-1	47.90	8.20
1	22	1	67.59	11.40
-2	-22	-1	199.38	15.20
-2	-22	-1	211.58	16.00
-2	-22	-1	197.68	19.50
-2	-22	-1	195.48	14.80
2	22	1	188.08	15.40
-3	-22	-1	4.40	7.30
-3	-22	-1	2.50	6.30
-3	-22	-1	10.30	7.40
3	22	1	-3.80	12.40
3	22	1	2.10	7.60
-4	-22	-1	284.77	17.50
-4	-22	-1	273.07	17.50
-4	-22	-1	277.87	17.20
4	22	1	303.27	21.30
-5	-22	-1	34.50	7.90
-5	-22	-1	20.50	7.20
-5	-22	-1	16.40	6.20
5	22	1	34.30	9.90
5	22	1	25.40	10.60

-6	-22	-1	20.60	9.90
-6	-22	-1	18.30	5.80
-6	-22	-1	9.00	7.30
6	22	1	26.30	10.30
6	22	1	-16.30	10.00
3	-23	-1	163.08	12.80
-3	23	1	122.29	14.60
-3	23	1	140.29	14.20
-3	23	1	169.58	16.00
2	-23	-1	53.79	8.70
2	-23	-1	41.80	13.20
-2	23	1	85.79	11.70
-2	23	1	65.79	11.00
1	-23	-1	28.30	8.00
1	-23	-1	11.60	9.20
-1	23	1	17.20	7.80
-1	23	1	20.20	8.70
0	-23	-1	24.30	8.30
0	-23	-1	39.20	8.90
0	23	1	37.90	9.20
0	23	1	31.70	8.10
-1	-23	-1	1.40	6.80
-1	-23	-1	3.70	8.90
-1	-23	-1	7.60	10.10
1	23	1	4.30	8.20
1	23	1	10.90	7.10
-2	-23	-1	421.66	23.50
-2	-23	-1	398.16	22.90
-2	-23	-1	431.66	22.60
-2	-23	-1	384.36	28.30
2	23	1	412.96	23.20
-3	-23	-1	259.87	16.80
-3	-23	-1	252.47	16.10
-3	-23	-1	271.87	17.20
3	23	1	242.38	17.40
-4	-23	-1	58.79	10.50
-4	-23	-1	79.69	10.40
-4	-23	-1	83.29	10.20
4	23	1	103.79	18.80
4	23	1	91.49	12.80
-5	-23	-1	-0.90	8.50
-5	-23	-1	3.60	7.30
-5	-23	-1	19.00	6.30
5	23	1	18.30	9.90

5	23	1	19.70	11.70
1	-24	-1	34.50	10.00
1	-24	-1	33.60	10.80
-1	24	1	10.30	9.40
-1	24	1	35.30	8.30
0	-24	-1	55.79	11.30
0	24	1	54.69	8.40
0	24	1	67.19	10.00
-1	-24	-1	-3.70	6.60
-1	-24	-1	-6.30	10.90
1	24	1	4.50	6.70
1	24	1	-3.90	8.40
-2	-24	-1	39.20	10.50
-2	-24	-1	32.50	7.10
2	24	1	38.20	7.50
2	24	1	44.40	9.60
-3	-24	-1	326.17	20.10
-3	-24	-1	360.96	18.70
3	24	1	322.07	20.30
3	23	-2	39.30	9.70
3	23	-2	57.19	12.00
3	23	-2	34.80	8.00
-3	-23	2	50.89	7.20
2	23	-2	10.90	7.10
2	23	-2	18.70	8.40
-2	-23	2	0.60	6.20
1	23	-2	207.08	15.80
1	23	-2	195.58	14.70
-1	-23	2	188.58	14.50
-1	-23	2	244.28	23.90
0	23	-2	4.30	7.10
0	23	-2	26.00	9.00
0	-23	2	8.40	8.00
0	-23	2	0.70	8.00
-1	23	-2	16.60	7.30
-1	23	-2	21.10	9.10
1	-23	2	6.50	7.20
1	-23	2	8.60	10.20
-2	23	-2	259.37	16.50
-2	23	-2	228.58	17.00
2	-23	2	273.07	16.90
5	22	-2	-7.10	10.20
5	22	-2	2.60	8.80
-5	-22	2	19.70	7.30

-5	-22	2	-0.70	6.40
4	22	-2	21.40	9.20
4	22	-2	14.40	9.70
-4	-22	2	21.80	6.30
3	22	-2	277.67	18.30
3	22	-2	322.37	20.40
-3	-22	2	299.77	17.80
-3	-22	2	300.07	21.90
2	22	-2	65.69	12.20
2	22	-2	79.69	12.40
-2	-22	2	75.89	12.60
-2	-22	2	75.29	10.60
1	22	-2	32.50	12.30
-1	-22	2	42.10	8.10
-1	-22	2	41.10	9.30
0	22	-2	59.29	10.90
0	22	-2	42.30	8.30
0	-22	2	66.59	12.20
0	-22	2	58.59	9.30
-1	22	-2	5.90	9.40
-1	22	-2	-4.70	7.10
1	-22	2	-0.80	7.50
1	-22	2	2.40	9.30
-2	22	-2	-3.80	8.40
-2	22	-2	-3.60	6.60
2	-22	2	-3.70	7.00
-3	22	-2	203.58	16.80
-3	22	-2	211.38	15.90
3	-22	2	240.88	15.60
-4	22	-2	24.10	11.60
-4	22	-2	38.10	10.50
6	21	-2	39.10	9.00
6	21	-2	40.90	11.80
-6	-21	2	42.50	8.90
-6	-21	2	28.80	10.80
5	21	-2	181.08	15.70
5	21	-2	175.68	15.40
-5	-21	2	149.69	13.00
-5	-21	2	179.98	13.10
4	21	-2	2.20	14.20
4	21	-2	-4.60	9.20
-4	-21	2	-3.10	6.00
-4	-21	2	0.40	9.70
-4	-21	2	-10.30	6.50

3	21	-2	6.50	8.30
3	21	-2	4.90	9.70
-3	-21	2	-1.00	6.00
-3	-21	2	9.40	9.50
-3	-21	2	21.70	8.50
2	21	-2	436.06	27.00
2	21	-2	471.85	25.70
-2	-21	2	488.75	27.30
-2	-21	2	494.85	34.60
-2	-21	2	429.16	27.30
-2	-21	2	474.85	25.10
1	21	-2	15.40	12.10
1	21	-2	19.90	7.90
-1	-21	2	8.80	8.70
-1	-21	2	3.70	6.80
0	21	-2	4.90	7.50
0	21	-2	8.60	7.30
0	-21	2	4.40	7.90
0	-21	2	15.80	8.20
-1	21	-2	4.10	7.70
-1	21	-2	12.10	7.50
1	-21	2	-7.40	9.40
1	-21	2	-3.00	7.90
-2	21	-2	60.29	10.00
-2	21	-2	56.89	9.20
2	-21	2	84.79	12.00
2	-21	2	57.29	12.60
-3	21	-2	424.26	23.70
-3	21	-2	385.36	29.00
-3	21	-2	369.16	26.00
3	-21	2	456.45	23.60
-4	21	-2	9.40	12.50
-4	21	-2	26.30	10.00
-4	21	-2	17.00	8.50
-5	21	-2	30.40	9.80
-5	21	-2	26.60	10.50
5	-21	2	33.30	11.80
7	20	-2	119.09	12.00
-7	-20	2	128.39	13.30
-7	-20	2	122.49	12.50
6	20	-2	42.30	8.70
6	20	-2	15.90	11.10
-6	-20	2	34.90	12.70
-6	-20	2	18.50	6.60

-6	-20	2	21.80	6.50
5	20	-2	16.90	8.50
5	20	-2	19.10	10.20
-5	-20	2	13.20	6.70
-5	-20	2	14.10	6.80
-5	-20	2	30.60	9.80
4	20	-2	236.08	18.00
4	20	-2	270.87	19.20
-4	-20	2	267.07	17.30
-4	-20	2	262.57	19.10
-4	-20	2	265.27	17.40
3	20	-2	18.90	10.10
3	20	-2	20.60	8.80
-3	-20	2	10.60	8.90
-3	-20	2	11.70	6.70
-3	-20	2	9.10	7.50
2	20	-2	174.48	15.70
2	20	-2	201.48	19.60
-2	-20	2	187.48	17.10
-2	-20	2	177.98	17.20
-2	-20	2	221.78	19.10
-2	-20	2	204.78	15.70
1	20	-2	16.10	7.90
1	20	-2	12.30	11.50
-1	-20	2	10.20	11.00
-1	-20	2	6.10	8.00
-1	-20	2	13.80	9.70
-1	-20	2	9.10	9.60
0	20	-2	45.10	9.10
0	20	-2	56.59	14.50
0	-20	2	55.39	9.90
0	-20	2	41.30	10.10
-1	20	-2	80.39	13.10
-1	20	-2	49.60	9.40
1	-20	2	48.40	10.90
1	-20	2	63.49	10.30
-2	20	-2	7.20	8.00
-2	20	-2	5.10	7.90
2	-20	2	5.30	8.00
2	-20	2	2.70	10.90
-3	20	-2	238.18	17.70
-3	20	-2	249.97	18.10
3	-20	2	243.78	17.50
-4	20	-2	668.53	37.60

-4	20	-2	739.73	37.30
-4	20	-2	763.52	37.20
4	-20	2	672.03	38.70
4	-20	2	771.42	36.80
-5	20	-2	16.00	8.60
-5	20	-2	-1.60	10.20
5	-20	2	-15.00	8.20
5	-20	2	0.00	12.70
-6	20	-2	12.70	15.20
-6	20	-2	11.00	11.70
6	-20	2	-1.10	9.80
6	-20	2	5.50	9.20
7	19	-2	0.60	7.60
-7	-19	2	2.90	7.40
-7	-19	2	-1.50	5.20
-7	-19	2	-9.00	10.50
6	19	-2	32.40	8.50
-6	-19	2	17.70	7.20
-6	-19	2	27.00	9.00
-6	-19	2	36.60	6.70
5	19	-2	85.39	13.70
-5	-19	2	85.49	13.60
-5	-19	2	96.29	11.80
-5	-19	2	76.59	11.40
4	19	-2	227.38	18.50
-4	-19	2	206.68	15.50
-4	-19	2	186.68	15.70
-4	-19	2	156.78	16.90
3	19	-2	214.28	26.20
-3	-19	2	234.28	25.40
-3	-19	2	218.08	24.10
-3	-19	2	213.28	16.20
2	19	-2	-2.90	7.40
2	19	-2	11.20	10.20
-2	-19	2	-3.00	8.40
-2	-19	2	8.00	7.00
-2	-19	2	7.40	10.50
-2	-19	2	15.40	8.50
1	19	-2	249.08	22.00
1	19	-2	281.87	18.60
-1	-19	2	273.77	18.70
-1	-19	2	233.18	20.10
-1	-19	2	242.18	18.90
-1	-19	2	265.77	19.90

0	19	-2	-1.80	7.00
0	19	-2	8.80	11.70
0	-19	2	0.80	7.80
0	-19	2	10.40	9.30
0	-19	2	5.50	9.20
-1	19	-2	39.20	8.80
-1	19	-2	30.70	8.90
1	-19	2	28.00	8.70
1	-19	2	25.40	9.50
-2	19	-2	463.65	27.30
-2	19	-2	452.95	28.10
2	-19	2	532.45	28.90
2	-19	2	549.35	38.40
-3	19	-2	77.79	13.60
-3	19	-2	72.49	11.50
3	-19	2	87.19	25.60
3	-19	2	91.89	14.70
-4	19	-2	9.10	8.80
-4	19	-2	4.00	10.00
-4	19	-2	11.80	8.90
4	-19	2	11.50	9.80
4	-19	2	1.40	8.20
-5	19	-2	86.09	13.70
-5	19	-2	81.99	12.10
-5	19	-2	95.49	15.30
5	-19	2	85.99	14.40
5	-19	2	74.29	10.20
-6	19	-2	-0.20	11.10
-6	19	-2	6.80	12.70
-6	19	-2	27.80	9.70
6	-19	2	-4.50	9.90
6	-19	2	10.90	7.90
8	18	-2	33.60	7.90
-8	-18	2	47.40	10.10
-8	-18	2	26.90	6.00
-8	-18	2	40.00	11.30
-7	-18	2	-6.90	8.40
-7	-18	2	8.30	7.50
-7	-18	2	0.10	5.20
-7	-18	2	4.30	8.60
-6	-18	2	147.89	14.00
-6	-18	2	155.28	14.40
-6	-18	2	165.38	12.90
-6	-18	2	126.89	15.50

-5	-18	2	-2.00	8.60
-5	-18	2	-8.20	7.90
-5	-18	2	-1.40	8.70
-5	-18	2	19.00	7.50
4	18	-2	2.30	14.40
4	18	-2	16.80	9.10
-4	-18	2	18.70	7.20
-4	-18	2	39.10	8.70
-4	-18	2	14.40	7.20
3	18	-2	850.61	43.40
3	18	-2	882.01	45.60
-3	-18	2	848.92	42.50
-3	-18	2	838.92	41.90
-3	-18	2	864.21	45.50
-3	-18	2	855.41	42.30
2	18	-2	84.99	11.50
2	18	-2	72.99	13.00
-2	-18	2	62.59	9.70
-2	-18	2	66.79	10.00
-2	-18	2	71.39	12.50
-2	-18	2	94.79	16.90
1	18	-2	14.60	10.60
1	18	-2	-4.80	11.70
1	18	-2	-13.70	6.50
-1	-18	2	-7.20	8.60
-1	-18	2	2.70	8.80
-1	-18	2	-4.70	8.00
-1	-18	2	0.70	7.20
0	18	-2	16.80	11.40
0	18	-2	3.80	6.90
0	-18	2	6.70	10.60
0	-18	2	1.90	7.80
0	-18	2	4.40	8.30
0	-18	2	11.00	8.70
-1	18	-2	28.40	13.00
-1	18	-2	18.30	7.40
1	-18	2	8.60	8.70
1	-18	2	10.80	8.70
-2	18	-2	198.28	16.70
-2	18	-2	200.88	17.50
2	-18	2	246.68	20.20
2	-18	2	191.78	17.80
-3	18	-2	88.99	13.80
-3	18	-2	97.29	14.60

3	-18	2	83.39	16.90
3	-18	2	72.59	12.50
-4	18	-2	290.77	21.10
-4	18	-2	317.87	30.20
4	-18	2	297.97	21.00
4	-18	2	325.97	28.00
-5	18	-2	249.48	20.00
-5	18	-2	216.28	19.90
-5	18	-2	249.58	19.40
5	-18	2	268.17	19.10
5	-18	2	221.18	17.60
-6	18	-2	9.40	9.90
-6	18	-2	-1.00	9.50
-6	18	-2	3.40	11.40
6	-18	2	9.60	7.80
6	-18	2	4.90	10.00
-7	18	-2	119.19	17.90
-7	18	-2	109.79	15.30
-7	18	-2	137.99	16.00
7	-18	2	107.59	12.00
7	-18	2	98.89	15.20
-9	-17	2	48.90	7.50
-9	-17	2	30.00	10.20
-8	-17	2	385.76	20.30
-8	-17	2	346.57	22.40
-8	-17	2	378.46	22.90
-8	-17	2	411.76	24.20
-7	-17	2	180.38	13.20
-7	-17	2	171.28	16.70
-7	-17	2	180.38	15.70
-7	-17	2	196.98	16.20
6	17	-2	-6.10	22.70
-6	-17	2	46.40	8.20
-6	-17	2	50.89	9.70
-6	-17	2	55.39	9.70
-6	-17	2	41.50	9.80
5	17	-2	175.98	23.90
-5	-17	2	189.38	14.20
-5	-17	2	195.28	15.70
-5	-17	2	156.48	16.30
-5	-17	2	176.28	15.50
4	17	-2	1.40	11.10
-4	-17	2	4.80	6.40
-4	-17	2	-5.50	7.90

-4	-17	2	16.60	7.60
-4	-17	2	9.20	6.90
3	17	-2	19.10	9.40
3	17	-2	34.20	11.30
-3	-17	2	32.20	8.60
-3	-17	2	14.40	7.30
-3	-17	2	28.20	12.80
-3	-17	2	7.90	8.00
-3	-17	2	21.30	7.50
2	17	-2	531.55	29.30
2	17	-2	518.55	30.20
-2	-17	2	517.35	28.50
-2	-17	2	553.24	28.20
-2	-17	2	464.95	27.70
-2	-17	2	527.35	30.00
1	17	-2	39.30	7.90
1	17	-2	37.30	10.30
1	17	-2	36.30	9.90
-1	-17	2	37.50	8.90
-1	-17	2	53.89	9.10
-1	-17	2	37.40	8.10
-1	-17	2	45.50	9.50
0	17	-2	50.19	10.60
0	17	-2	25.30	7.90
0	17	-2	62.19	11.90
0	-17	2	21.80	7.90
0	-17	2	36.80	10.50
0	-17	2	33.50	9.40
0	-17	2	40.20	9.40
-1	17	-2	14.10	8.20
-1	17	-2	44.20	12.50
1	-17	2	13.40	9.60
1	-17	2	17.70	8.40
1	-17	2	22.10	8.40
-2	17	-2	287.07	20.40
-2	17	-2	285.57	20.10
2	-17	2	300.47	20.70
2	-17	2	276.47	22.00
2	-17	2	246.88	28.30
-3	17	-2	295.77	21.10
-3	17	-2	274.37	20.30
3	-17	2	287.17	21.30
3	-17	2	288.37	20.40
3	-17	2	280.27	25.40

-4	17	-2	5.70	9.30
-4	17	-2	12.20	8.80
4	-17	2	7.20	8.70
4	-17	2	23.60	8.90
-5	17	-2	1.60	9.60
-5	17	-2	1.50	10.40
5	-17	2	15.40	9.00
5	-17	2	-1.70	9.50
-6	17	-2	117.09	17.00
-6	17	-2	127.69	17.40
-6	17	-2	118.79	18.20
6	-17	2	101.39	13.70
6	-17	2	137.09	17.60
-7	17	-2	37.70	12.50
-7	17	-2	53.99	10.60
-7	17	-2	16.80	10.90
7	-17	2	52.99	13.70
7	-17	2	41.50	8.30
-8	17	-2	263.17	20.20
-8	17	-2	218.48	21.70
-8	17	-2	250.37	32.80
8	-17	2	300.07	16.90
9	16	-2	12.00	17.30
-9	-16	2	4.90	11.10
-9	-16	2	8.30	5.30
8	16	-2	144.49	26.70
-8	-16	2	86.19	14.00
-8	-16	2	96.49	14.10
-8	-16	2	81.29	10.10
-8	-16	2	91.49	12.40
7	16	-2	188.88	31.90
-7	-16	2	135.19	15.60
-7	-16	2	150.28	12.70
-7	-16	2	136.79	15.50
-7	-16	2	112.89	16.20
6	16	-2	9.40	14.20
-6	-16	2	-1.90	8.60
-6	-16	2	-8.40	6.20
-6	-16	2	8.80	8.90
-6	-16	2	6.60	7.70
5	16	-2	80.09	12.80
-5	-16	2	62.29	9.00
-5	-16	2	54.79	10.10
-5	-16	2	59.69	8.60

-5	-16	2	65.69	9.80
4	16	-2	203.88	18.80
-4	-16	2	208.58	17.80
-4	-16	2	220.28	15.90
-4	-16	2	219.98	16.30
-4	-16	2	233.48	17.00
3	16	-2	38.40	8.60
-3	-16	2	19.70	6.50
-3	-16	2	30.50	7.20
-3	-16	2	32.70	7.70
-3	-16	2	52.69	12.80
-3	-16	2	30.00	8.50
2	16	-2	642.34	32.10
2	16	-2	594.94	33.30
-2	-16	2	640.44	32.10
-2	-16	2	593.84	32.30
-2	-16	2	596.64	34.00
-2	-16	2	630.74	32.10
1	16	-2	19.70	9.60
1	16	-2	1.30	7.10
-1	-16	2	-2.00	7.40
-1	-16	2	8.50	6.80
-1	-16	2	-11.00	8.10
-1	-16	2	3.40	8.00
0	16	-2	15.70	10.50
0	16	-2	-4.30	7.10
0	16	-2	10.90	6.90
0	-16	2	0.20	7.90
0	-16	2	-14.90	9.30
0	-16	2	12.00	7.50
0	-16	2	5.40	8.60
-1	16	-2	8.70	8.60
-1	16	-2	4.20	6.30
-1	16	-2	13.50	12.50
1	-16	2	12.00	7.80
1	-16	2	5.50	8.00
1	-16	2	3.00	9.10
-2	16	-2	36.70	8.80
-2	16	-2	39.90	8.20
-2	16	-2	46.10	18.50
2	-16	2	34.10	11.50
2	-16	2	19.50	10.20
2	-16	2	24.20	7.90
-3	16	-2	544.95	29.30

-3	16	-2	464.75	30.00
3	-16	2	448.46	28.30
3	-16	2	516.25	28.50
3	-16	2	490.75	31.60
-4	16	-2	503.85	30.50
-4	16	-2	529.05	30.20
4	-16	2	494.45	29.10
4	-16	2	555.14	30.10
-5	16	-2	75.69	12.20
-5	16	-2	63.39	12.50
-5	16	-2	46.40	11.40
5	-16	2	36.50	10.00
5	-16	2	55.09	9.50
-6	16	-2	0.80	10.70
-6	16	-2	-6.50	11.00
-6	16	-2	-5.50	12.40
6	-16	2	-3.50	7.70
6	-16	2	-5.40	10.30
-7	16	-2	93.39	16.30
-7	16	-2	92.89	15.30
-7	16	-2	111.59	18.20
7	-16	2	118.99	15.80
7	-16	2	109.59	12.80
-8	16	-2	61.19	12.00
-8	16	-2	66.49	11.20
-8	16	-2	60.09	13.10
8	-16	2	64.99	11.30
10	15	-2	50.49	15.20
-10	-15	2	1.20	5.60
-10	-15	2	-8.50	9.10
9	15	-2	653.63	38.80
-9	-15	2	717.43	35.70
-9	-15	2	697.93	33.60
-9	-15	2	661.33	35.20
8	15	-2	308.37	27.80
-8	-15	2	308.77	18.30
-8	-15	2	302.17	21.10
-8	-15	2	309.87	22.90
-8	-15	2	313.57	21.50
7	15	-2	25.20	26.80
7	15	-2	28.80	17.40
-7	-15	2	-10.50	5.60
-7	-15	2	1.70	16.50
6	15	-2	-21.80	16.50

6	15	-2	17.20	12.10
-6	-15	2	-1.60	8.80
-6	-15	2	7.20	6.00
-6	-15	2	7.80	7.40
-6	-15	2	12.90	9.20
5	15	-2	116.09	15.90
-5	-15	2	94.59	11.60
-5	-15	2	98.79	12.80
-5	-15	2	98.99	14.20
-5	-15	2	95.49	12.10
4	15	-2	107.69	13.70
-4	-15	2	136.69	15.40
-4	-15	2	117.99	14.20
-4	-15	2	113.09	13.00
-4	-15	2	127.49	12.40
3	15	-2	116.49	11.90
-3	-15	2	101.49	11.80
-3	-15	2	93.19	14.50
-3	-15	2	101.69	12.70
-3	-15	2	124.59	12.40
-3	-15	2	81.09	14.30
2	15	-2	540.35	27.90
-2	-15	2	519.75	28.30
-2	-15	2	541.15	30.70
-2	-15	2	544.55	28.80
-2	-15	2	526.35	28.60
-2	-15	2	567.74	29.50
1	15	-2	825.82	42.90
1	15	-2	867.71	41.10
-1	-15	2	839.62	41.70
-1	-15	2	853.01	42.60
0	15	-2	1.10	5.70
0	15	-2	2.00	5.40
0	-15	2	19.10	9.30
0	-15	2	1.20	7.40
0	-15	2	11.90	7.60
-1	15	-2	179.78	14.50
-1	15	-2	200.68	14.90
1	-15	2	186.18	16.60
1	-15	2	183.38	16.90
1	-15	2	164.08	29.00
1	-15	2	222.88	18.10
-2	15	-2	238.28	17.60
-2	15	-2	235.68	25.80

-2	15	-2	255.57	20.10
2	-15	2	251.47	18.60
2	-15	2	255.27	21.20
2	-15	2	242.98	20.60
-3	15	-2	82.39	11.90
-3	15	-2	70.69	10.60
3	-15	2	48.90	9.40
3	-15	2	53.29	13.40
-4	15	-2	154.38	17.80
-4	15	-2	181.78	19.30
4	-15	2	198.48	16.80
4	-15	2	168.48	17.60
-5	15	-2	186.48	20.40
-5	15	-2	163.58	19.20
-5	15	-2	151.58	19.90
5	-15	2	128.99	15.90
5	-15	2	185.28	17.40
-6	15	-2	16.00	12.50
-6	15	-2	24.20	10.90
-6	15	-2	31.00	12.50
6	-15	2	34.00	10.90
6	-15	2	20.50	8.00
-7	15	-2	21.70	13.00
-7	15	-2	13.00	10.40
-7	15	-2	15.40	12.90
7	-15	2	13.90	13.20
7	-15	2	16.10	7.70
-8	15	-2	118.39	16.50
-8	15	-2	75.79	23.20
-8	15	-2	96.49	14.70
8	-15	2	109.49	12.50
-9	15	-2	286.97	22.50
-9	15	-2	261.27	24.10
-9	15	-2	288.17	27.20
9	-15	2	339.17	18.50
10	14	-2	1.90	14.20
-10	-14	2	3.70	5.40
-10	-14	2	-3.10	11.70
9	14	-2	5.80	11.90
-9	-14	2	9.10	9.00
-9	-14	2	3.40	5.30
-9	-14	2	-7.10	10.80
8	14	-2	37.40	14.80
-8	-14	2	47.40	10.80

-8	-14	2	66.89	11.30
-8	-14	2	84.89	11.20
-8	-14	2	66.99	12.00
-8	-14	2	66.99	11.20
7	14	-2	-10.80	14.90
7	14	-2	25.10	14.90
-7	-14	2	35.50	9.70
-7	-14	2	15.60	6.30
-7	-14	2	28.40	9.40
-7	-14	2	16.70	8.60
-7	-14	2	12.00	9.00
6	14	-2	78.09	12.50
6	14	-2	90.19	17.20
-6	-14	2	65.59	11.40
-6	-14	2	74.79	12.10
-6	-14	2	81.79	12.40
-6	-14	2	68.59	11.30
-6	-14	2	77.49	11.10
5	14	-2	-3.00	7.60
5	14	-2	18.10	16.70
-5	-14	2	-2.60	6.70
-5	-14	2	-6.10	8.20
-5	-14	2	-0.20	6.80
-5	-14	2	0.20	6.10
-5	-14	2	12.50	7.90
4	14	-2	704.23	34.00
-4	-14	2	652.03	33.60
-4	-14	2	624.84	35.20
-4	-14	2	678.93	34.40
-4	-14	2	653.73	34.20
-4	-14	2	687.03	33.90
3	14	-2	1532.95	70.39
-3	-14	2	1484.55	72.79
-3	-14	2	1561.74	71.19
-3	-14	2	1492.65	70.59
-3	-14	2	1511.15	70.99
2	14	-2	3.80	4.80
-2	-14	2	12.80	9.30
-2	-14	2	8.80	6.60
-2	-14	2	-5.20	7.80
1	14	-2	12.30	8.90
1	14	-2	13.10	4.70
-1	-14	2	19.00	8.10
-1	-14	2	19.40	7.80

-1	-14	2	17.00	8.30
-1	-14	2	15.40	6.70
-1	-14	2	8.30	7.60
0	14	-2	2.50	4.30
0	14	-2	1.40	9.50
0	-14	2	-12.00	8.80
0	-14	2	-4.50	7.00
0	-14	2	0.70	6.70
0	-14	2	-11.10	8.30
-1	14	-2	19.60	11.60
-1	14	-2	8.70	5.40
-1	14	-2	8.30	6.10
1	-14	2	-3.00	8.90
1	-14	2	18.70	14.80
1	-14	2	15.50	7.80
-2	14	-2	76.19	11.20
-2	14	-2	90.79	11.60
-2	14	-2	87.39	14.70
2	-14	2	68.49	10.70
2	-14	2	94.79	16.10
2	-14	2	82.99	11.40
2	-14	2	84.79	13.00
-3	14	-2	79.79	12.50
-3	14	-2	54.59	10.20
3	-14	2	54.09	9.20
3	-14	2	51.69	9.30
-4	14	-2	615.24	34.70
-4	14	-2	654.43	35.80
4	-14	2	612.64	33.10
4	-14	2	600.84	32.50
-5	14	-2	185.98	20.40
-5	14	-2	217.38	21.60
5	-14	2	179.18	18.30
5	-14	2	164.38	16.00
-6	14	-2	51.19	13.70
-6	14	-2	54.09	14.00
-6	14	-2	41.70	11.90
6	-14	2	57.99	9.20
6	-14	2	43.10	11.20
-7	14	-2	121.69	23.30
-7	14	-2	123.99	20.40
7	-14	2	134.69	23.90
7	-14	2	135.29	17.00
-8	14	-2	124.89	16.90

-8	14	-2	126.89	17.90
-8	14	-2	101.69	18.20
8	-14	2	107.39	13.00
-9	14	-2	7.80	9.80
-9	14	-2	0.20	12.00
-9	14	-2	-11.40	14.70
9	-14	2	13.20	7.20
10	13	-2	118.69	17.90
-10	-13	2	158.68	11.90
-10	-13	2	139.19	16.00
9	13	-2	46.60	13.80
-9	-13	2	33.40	6.70
-9	-13	2	48.80	11.40
-9	-13	2	13.90	9.40
-9	-13	2	44.20	12.70
8	13	-2	548.15	41.90
8	13	-2	642.64	45.10
-8	-13	2	608.64	36.30
-8	-13	2	559.74	37.80
-8	-13	2	547.35	32.00
-8	-13	2	596.24	30.60
7	13	-2	118.49	16.00
7	13	-2	155.88	22.90
-7	-13	2	120.19	15.70
-7	-13	2	162.68	13.80
-7	-13	2	117.89	16.50
-7	-13	2	124.99	16.00
-7	-13	2	134.89	15.80
6	13	-2	13.70	13.70
6	13	-2	6.50	8.90
-6	-13	2	18.10	8.90
-6	-13	2	8.60	8.10
-6	-13	2	7.00	8.90
-6	-13	2	-9.20	6.00
-6	-13	2	18.80	7.70
5	13	-2	15.10	7.00
5	13	-2	32.10	15.10
-5	-13	2	9.60	6.90
-5	-13	2	14.20	8.10
-5	-13	2	3.40	8.20
-5	-13	2	2.50	6.30
-5	-13	2	8.20	6.50
4	13	-2	56.89	9.40
-4	-13	2	43.80	7.40

-4	-13	2	37.20	7.20
-4	-13	2	34.40	7.40
-4	-13	2	28.00	9.50
-4	-13	2	46.10	8.90
3	13	-2	96.49	9.30
-3	-13	2	69.79	8.80
-3	-13	2	108.69	15.40
-3	-13	2	104.79	11.50
-3	-13	2	101.19	11.80
-3	-13	2	91.19	10.30
-3	-13	2	91.49	12.30
2	13	-2	683.83	32.30
-2	-13	2	666.63	33.80
-2	-13	2	669.73	35.70
-2	-13	2	673.43	33.50
-2	-13	2	649.34	34.70
-2	-13	2	640.54	33.20
1	13	-2	49.20	6.90
-1	-13	2	79.09	10.50
-1	-13	2	51.39	8.50
-1	-13	2	44.20	7.00
-1	-13	2	55.49	9.00
-1	-13	2	70.49	9.20
0	13	-2	26.70	10.00
0	13	-2	27.10	4.50
0	-13	2	27.20	7.30
0	-13	2	25.30	9.60
0	-13	2	12.40	9.40
0	-13	2	26.50	7.40
-1	13	-2	48.60	12.40
-1	13	-2	45.30	6.10
-1	13	-2	37.10	6.50
1	-13	2	25.60	7.40
1	-13	2	65.19	16.20
1	-13	2	30.70	8.50
1	-13	2	23.00	14.90
1	-13	2	42.10	9.80
-2	13	-2	663.23	35.50
-2	13	-2	680.23	40.40
-2	13	-2	723.93	35.70
2	-13	2	700.63	37.00
2	-13	2	678.23	35.30
2	-13	2	678.83	36.60
2	-13	2	728.63	36.40

-3	13	-2	641.54	33.70
3	-13	2	657.23	33.10
3	-13	2	579.94	36.40
-4	13	-2	119.89	14.90
-4	13	-2	85.29	14.70
4	-13	2	108.49	14.00
4	-13	2	105.99	13.90
-5	13	-2	14.80	11.40
-5	13	-2	28.30	10.50
5	-13	2	-0.60	7.50
5	-13	2	4.00	8.60
-6	13	-2	297.07	27.70
-6	13	-2	322.97	25.30
-6	13	-2	333.37	28.20
6	-13	2	326.87	24.80
6	-13	2	315.27	20.80
-7	13	-2	78.49	15.10
-7	13	-2	134.19	23.00
-7	13	-2	106.59	14.40
7	-13	2	81.79	14.90
7	-13	2	81.79	10.30
-8	13	-2	109.89	18.60
-8	13	-2	86.69	16.30
-8	13	-2	100.39	15.80
8	-13	2	106.79	13.50
-9	13	-2	15.90	13.00
-9	13	-2	12.20	10.70
-9	13	-2	-19.40	14.00
9	-13	2	14.20	7.70
-10	13	-2	155.48	20.40
-10	13	-2	152.38	16.20
10	-13	2	173.28	14.00
11	12	-2	33.40	16.80
-11	-12	2	39.80	9.10
-11	-12	2	19.10	9.60
10	12	-2	119.29	19.00
-10	-12	2	87.19	11.50
-10	-12	2	113.19	22.00
-10	-12	2	100.79	11.20
9	12	-2	50.49	13.10
-9	-12	2	33.10	7.40
-9	-12	2	32.00	12.30
-9	-12	2	53.09	10.50
-9	-12	2	42.30	12.90

8	12	-2	55.49	13.30
8	12	-2	39.80	17.30
-8	-12	2	47.90	7.70
-8	-12	2	34.60	10.80
-8	-12	2	62.09	13.40
-8	-12	2	37.40	10.20
-8	-12	2	46.70	9.90
7	12	-2	132.69	19.60
7	12	-2	114.39	22.10
-7	-12	2	155.88	13.60
-7	-12	2	179.98	17.10
-7	-12	2	145.59	16.60
-7	-12	2	143.59	16.90
6	12	-2	58.59	14.10
6	12	-2	61.29	9.50
-6	-12	2	67.59	9.40
-6	-12	2	74.39	10.70
-6	-12	2	66.29	10.40
-6	-12	2	64.59	9.30
-6	-12	2	52.69	8.00
5	12	-2	31.90	13.90
5	12	-2	25.20	6.60
-5	-12	2	15.50	10.20
-5	-12	2	9.20	7.10
-5	-12	2	9.60	6.50
-5	-12	2	21.50	6.70
-5	-12	2	34.60	8.70
4	12	-2	653.13	40.30
4	12	-2	648.14	31.50
-4	-12	2	618.14	33.10
-4	-12	2	694.13	32.70
-4	-12	2	631.94	32.10
-4	-12	2	641.64	32.20
-4	-12	2	560.84	33.80
3	12	-2	154.68	10.60
-3	-12	2	152.68	13.30
-3	-12	2	180.78	17.90
-3	-12	2	141.59	14.40
-3	-12	2	139.89	16.50
-3	-12	2	161.78	13.30
-3	-12	2	150.58	12.20
2	12	-2	824.42	36.90
-2	-12	2	721.83	38.00
-2	-12	2	796.52	38.20

-2	-12	2	746.13	40.30
-2	-12	2	771.82	39.80
-2	-12	2	790.12	38.50
1	12	-2	117.09	13.20
1	12	-2	138.79	8.90
-1	-12	2	111.29	15.40
-1	-12	2	111.89	12.70
-1	-12	2	126.69	12.20
-1	-12	2	123.29	16.00
-1	-12	2	93.29	15.60
-1	-12	2	110.09	13.90
0	12	-2	8.20	3.60
0	12	-2	2.20	9.70
0	-12	2	33.00	16.10
0	-12	2	7.50	6.60
0	-12	2	15.80	7.60
0	-12	2	6.70	9.30
-1	12	-2	115.59	8.70
-1	12	-2	121.59	11.70
-1	12	-2	131.39	20.10
1	-12	2	137.19	16.40
1	-12	2	129.29	13.60
1	-12	2	122.69	14.40
-2	12	-2	869.61	39.70
-2	12	-2	722.63	44.40
-2	12	-2	802.12	37.30
2	-12	2	834.92	39.30
2	-12	2	734.03	40.00
2	-12	2	770.32	39.60
2	-12	2	754.72	40.50
-3	12	-2	884.31	43.00
-3	12	-2	982.60	45.80
-3	12	-2	946.21	46.40
3	-12	2	880.11	44.40
3	-12	2	910.51	44.40
3	-12	2	825.22	51.29
-4	12	-2	264.27	20.40
-5	12	-2	305.17	25.30
-5	12	-2	324.77	23.90
5	-12	2	342.47	20.90
5	-12	2	337.47	22.90
-6	12	-2	27.80	11.20
-6	12	-2	26.10	13.60
-6	12	-2	39.30	12.30

6	-12	2	21.50	7.20
-7	12	-2	45.60	14.00
-7	12	-2	46.10	12.60
-7	12	-2	51.79	14.40
7	-12	2	57.19	13.60
7	-12	2	36.70	8.30
7	-12	2	71.49	14.90
-8	12	-2	103.49	17.50
8	-12	2	85.59	14.50
-9	12	-2	15.10	13.30
-9	12	-2	-18.20	10.20
-9	12	-2	18.10	14.60
9	-12	2	9.50	7.20
9	-12	2	-2.00	12.90
-10	12	-2	30.80	14.20
-10	12	-2	17.50	9.70
-10	12	-2	44.60	17.00
10	-12	2	16.80	16.30
10	-12	2	14.20	8.40
11	11	-2	30.50	11.50
-11	-11	2	3.80	10.20
-11	-11	2	8.60	6.60
10	11	-2	77.09	13.50
-10	-11	2	101.99	11.80
-10	-11	2	93.09	15.10
-10	-11	2	60.99	10.40
-9	-11	2	653.23	35.90
-9	-11	2	685.53	37.10
-9	-11	2	582.04	35.00
8	11	-2	44.20	15.60
-8	-11	2	73.69	11.60
-8	-11	2	70.59	12.10
-8	-11	2	102.59	15.50
-8	-11	2	45.20	10.90
-8	-11	2	58.69	9.70
7	11	-2	166.88	18.10
-7	-11	2	131.89	15.30
-7	-11	2	185.48	14.10
-7	-11	2	134.99	17.50
-7	-11	2	175.78	16.60
-7	-11	2	183.68	17.50
-7	-11	2	182.98	16.40
6	11	-2	43.00	7.90
-6	-11	2	38.70	6.90

-6	-11	2	56.59	10.70
-6	-11	2	36.00	8.30
-6	-11	2	49.80	8.90
-6	-11	2	44.80	8.60
5	11	-2	1.20	5.20
-5	-11	2	1.00	7.70
-5	-11	2	-0.50	7.40
-5	-11	2	5.70	6.80
-5	-11	2	0.80	6.40
-5	-11	2	0.00	8.40
-5	-11	2	-3.10	6.00
4	11	-2	83.89	8.60
4	11	-2	100.89	16.10
-4	-11	2	92.39	14.00
-4	-11	2	85.49	12.20
-4	-11	2	95.29	12.30
-4	-11	2	90.49	15.10
-4	-11	2	90.29	10.50
-4	-11	2	81.29	10.50
3	11	-2	3.90	3.60
-3	-11	2	1.10	6.20
-3	-11	2	-0.50	7.80
-3	-11	2	10.70	8.50
-3	-11	2	10.40	5.40
-3	-11	2	1.10	6.10
2	11	-2	1072.09	48.70
2	11	-2	995.50	49.30
-2	-11	2	1029.40	50.09
-2	-11	2	1071.09	49.90
-2	-11	2	1068.39	50.49
-2	-11	2	1076.99	50.09
1	11	-2	531.05	27.80
1	11	-2	588.44	26.50
-1	-11	2	526.75	28.40
-1	-11	2	560.44	29.50
-1	-11	2	553.04	28.50
0	11	-2	6.30	2.80
0	11	-2	11.90	5.30
0	-11	2	13.80	7.00
0	-11	2	13.20	8.00
0	-11	2	0.90	6.40
0	-11	2	9.80	7.90
-1	11	-2	186.68	10.30
1	-11	2	154.68	15.20

-2	11	-2	124.79	8.40
-2	11	-2	120.89	12.10
2	-11	2	131.89	13.40
2	-11	2	114.29	17.00
2	-11	2	105.79	15.40
-3	11	-2	176.88	11.70
-3	11	-2	187.18	14.80
-3	11	-2	148.79	17.60
3	-11	2	169.38	15.60
3	-11	2	176.18	15.20
-4	11	-2	15.60	8.10
-4	11	-2	-7.60	9.90
4	-11	2	8.00	7.40
4	-11	2	4.30	6.10
-5	11	-2	145.19	16.90
-5	11	-2	132.99	19.00
5	-11	2	138.69	14.40
-6	11	-2	201.08	21.70
-6	11	-2	194.68	20.90
-6	11	-2	199.98	23.40
6	-11	2	218.28	16.60
6	-11	2	215.98	21.30
-7	11	-2	267.77	32.80
-7	11	-2	296.67	26.30
-7	11	-2	237.28	24.40
7	-11	2	277.37	19.50
7	-11	2	244.68	22.60
-8	11	-2	340.67	28.40
-8	11	-2	374.66	28.90
-8	11	-2	402.56	28.40
8	-11	2	369.96	23.20
8	-11	2	371.26	25.40
-9	11	-2	190.08	23.50
-9	11	-2	187.18	23.60
-9	11	-2	172.48	21.70
9	-11	2	193.28	19.00
9	-11	2	209.48	17.40
-10	11	-2	3.00	10.00
-10	11	-2	24.40	13.70
-10	11	-2	33.40	17.30
10	-11	2	8.00	12.10
10	-11	2	8.90	8.90
-11	-10	2	12.50	6.60
-11	-10	2	15.30	8.50

10	10	-2	9.10	10.80
-10	-10	2	4.80	14.10
-10	-10	2	5.00	6.80
-10	-10	2	13.20	8.40
-9	-10	2	16.00	18.00
-9	-10	2	4.40	11.60
-9	-10	2	-4.80	6.60
-9	-10	2	4.20	8.00
8	10	-2	126.49	24.30
-8	-10	2	143.99	16.40
-8	-10	2	140.59	14.30
-8	-10	2	137.89	13.30
-8	-10	2	148.89	18.40
-8	-10	2	127.49	18.40
7	10	-2	46.30	9.50
-7	-10	2	62.69	10.60
-7	-10	2	67.19	9.60
-7	-10	2	44.70	8.00
-7	-10	2	57.89	10.00
-7	-10	2	53.59	10.50
-7	-10	2	37.50	8.40
6	10	-2	113.89	11.70
-6	-10	2	97.39	12.00
-6	-10	2	116.69	11.40
-6	-10	2	79.99	14.40
-6	-10	2	124.19	13.80
5	10	-2	33.60	5.80
-5	-10	2	27.20	7.30
-5	-10	2	30.90	6.10
-5	-10	2	40.30	7.10
-5	-10	2	46.50	9.50
-5	-10	2	33.70	7.60
-5	-10	2	35.20	8.00
4	10	-2	1086.69	46.80
-4	-10	2	1050.69	48.20
-4	-10	2	900.31	48.20
-4	-10	2	1010.20	47.40
-4	-10	2	960.60	47.80
-4	-10	2	986.40	47.20
-4	-10	2	966.30	48.40
3	10	-2	1523.55	66.89
-3	-10	2	1419.76	67.69
-3	-10	2	1519.85	67.69
-3	-10	2	1512.75	68.19

-3	-10	2	1385.66	67.99
-3	-10	2	1407.36	68.39
2	10	-2	0.80	4.00
2	10	-2	9.00	2.90
2	10	-2	-1.40	9.60
-2	-10	2	12.10	5.10
-2	-10	2	10.40	10.90
-2	-10	2	15.90	5.20
1	10	-2	26.20	4.90
1	10	-2	26.20	4.10
1	10	-2	33.40	8.30
-1	-10	2	33.80	5.70
-1	-10	2	19.40	8.30
0	10	-2	764.82	38.30
0	10	-2	796.72	37.20
0	10	-2	852.71	41.90
-1	10	-2	562.84	32.10
-1	10	-2	581.54	26.90
-1	10	-2	567.14	28.60
1	-10	2	570.74	31.90
1	-10	2	555.14	29.00
-2	10	-2	6.20	3.00
-2	10	-2	-1.60	5.00
2	-10	2	-6.40	7.30
-3	10	-2	55.29	6.80
-3	10	-2	64.89	8.90
-3	10	-2	62.09	11.30
3	-10	2	64.09	9.00
3	-10	2	48.20	9.10
-4	10	-2	972.40	49.10
-4	10	-2	990.30	47.80
4	-10	2	918.41	46.80
4	-10	2	933.21	46.10
-5	10	-2	175.48	17.60
-5	10	-2	212.18	19.80
5	-10	2	211.08	18.70
5	-10	2	176.98	14.90
-6	10	-2	9.70	9.80
-6	10	-2	7.10	9.60
-6	10	-2	20.40	11.40
6	-10	2	13.70	7.30
6	-10	2	8.60	18.60
-7	10	-2	720.23	40.30
-7	10	-2	708.33	39.30

-7	10	-2	663.93	39.50
7	-10	2	714.43	36.50
-8	10	-2	300.17	25.00
-8	10	-2	240.18	24.30
-8	10	-2	248.38	24.70
8	-10	2	253.87	20.90
8	-10	2	279.17	19.50
-9	10	-2	-3.50	12.80
-9	10	-2	11.10	13.40
-9	10	-2	-2.30	12.30
9	-10	2	6.90	8.90
9	-10	2	-6.20	9.90
-10	10	-2	97.69	16.80
-10	10	-2	53.59	11.90
-10	10	-2	69.39	15.10
10	-10	2	75.69	11.00
10	-10	2	74.99	11.70
-11	10	-2	-4.10	14.40
11	-10	2	28.10	11.30
11	-10	2	9.00	12.50
11	9	-2	20.10	10.70
-11	-9	2	1.50	8.00
-11	-9	2	-2.30	7.60
10	9	-2	103.69	18.80
-10	-9	2	104.49	16.50
-10	-9	2	108.79	14.10
-10	-9	2	124.39	13.60
9	9	-2	83.89	20.50
-9	-9	2	93.09	13.70
-9	-9	2	90.39	12.50
-9	-9	2	58.09	12.80
-9	-9	2	95.69	13.00
-9	-9	2	93.29	16.90
8	9	-2	656.53	36.90
-8	-9	2	670.33	35.50
-8	-9	2	699.43	34.40
-8	-9	2	639.94	34.70
-8	-9	2	649.54	34.50
-8	-9	2	675.63	35.80
7	9	-2	10.20	7.50
-7	-9	2	31.40	11.20
-7	-9	2	13.60	6.80
-7	-9	2	30.30	9.20
-7	-9	2	17.80	9.10

-7	-9	2	9.30	5.70
-7	-9	2	11.60	6.90
6	9	-2	55.29	7.30
-6	-9	2	47.70	7.40
-6	-9	2	51.89	8.40
-6	-9	2	51.99	9.30
-6	-9	2	54.19	9.20
-6	-9	2	51.19	9.70
-6	-9	2	55.99	9.70
5	9	-2	332.07	17.40
-5	-9	2	327.67	21.10
-5	-9	2	305.37	19.80
-5	-9	2	339.17	18.10
-5	-9	2	288.47	20.10
-5	-9	2	303.07	20.00
4	9	-2	119.79	8.80
-4	-9	2	120.79	10.00
-4	-9	2	101.49	10.60
-4	-9	2	91.09	12.90
-4	-9	2	100.89	13.60
-4	-9	2	123.79	13.80
-4	-9	2	125.39	14.60
3	9	-2	195.58	10.50
3	9	-2	173.78	18.40
3	9	-2	200.78	10.80
-3	-9	2	183.18	10.70
-3	-9	2	184.38	12.70
2	9	-2	605.94	32.80
2	9	-2	610.74	28.60
2	9	-2	596.64	28.90
-2	-9	2	616.14	30.00
-2	-9	2	622.54	29.10
1	9	-2	1308.77	60.99
1	9	-2	1353.26	60.49
-1	-9	2	1376.26	61.29
-1	-9	2	1382.16	61.59
-1	-9	2	1240.88	63.69
0	9	-2	95.19	6.10
0	9	-2	102.89	7.70
0	-9	2	92.79	11.90
0	-9	2	89.39	8.10
-1	9	-2	52.29	4.70
-1	9	-2	68.09	7.00
1	-9	2	47.70	8.40

-2	9	-2	964.50	42.40
-2	9	-2	937.31	44.10
-2	9	-2	918.01	47.20
-2	9	-2	923.41	44.50
2	-9	2	851.01	46.30
2	-9	2	925.21	44.30
-3	9	-2	416.96	26.90
-3	9	-2	466.65	24.50
-3	9	-2	452.25	21.80
-4	9	-2	171.78	14.70
-4	9	-2	175.68	18.20
4	-9	2	208.78	17.20
4	-9	2	175.58	14.50
-5	9	-2	505.45	27.90
-5	9	-2	520.95	29.30
5	-9	2	483.15	26.40
5	-9	2	444.26	28.80
-6	9	-2	765.92	39.10
-6	9	-2	732.03	40.40
6	-9	2	772.32	37.60
6	-9	2	670.33	40.20
-7	9	-2	28.60	9.90
-7	9	-2	30.70	10.80
-7	9	-2	22.50	11.20
7	-9	2	14.30	7.10
-8	9	-2	98.09	14.80
-8	9	-2	112.69	14.70
-8	9	-2	89.19	13.50
8	-9	2	115.49	14.80
-9	9	-2	36.20	12.70
-9	9	-2	54.49	20.30
-9	9	-2	48.40	12.90
9	-9	2	63.09	14.60
9	-9	2	54.19	10.20
-10	9	-2	139.09	16.80
-10	9	-2	112.09	19.10
-10	9	-2	124.99	20.90
10	-9	2	162.08	17.00
10	-9	2	106.39	16.20
-11	9	-2	35.60	10.70
-11	9	-2	40.90	16.00
-11	9	-2	-2.30	22.60
11	-9	2	39.90	10.60
11	-9	2	23.60	13.10

11	8	-2	19.40	11.20
-11	-8	2	37.80	9.30
-11	-8	2	23.80	8.30
-10	-8	2	147.59	15.50
-10	-8	2	159.08	22.30
-10	-8	2	142.29	21.80
-10	-8	2	139.49	14.70
-9	-8	2	-4.60	10.30
-9	-8	2	11.70	7.20
-9	-8	2	9.30	6.60
-9	-8	2	9.20	10.30
-9	-8	2	16.70	9.70
8	8	-2	22.20	10.20
-8	-8	2	1.80	9.00
-8	-8	2	12.20	6.70
-8	-8	2	10.70	7.00
-8	-8	2	14.40	9.00
-8	-8	2	9.70	7.90
7	8	-2	117.99	12.70
-7	-8	2	108.49	11.40
-7	-8	2	107.29	13.80
-7	-8	2	108.29	15.10
-7	-8	2	119.09	12.30
6	8	-2	7.00	4.80
-6	-8	2	4.50	4.90
-6	-8	2	-0.50	7.40
-6	-8	2	6.00	6.50
-6	-8	2	-8.10	7.70
-6	-8	2	-3.10	5.40
-6	-8	2	14.40	9.80
5	8	-2	188.08	13.90
-5	-8	2	196.68	13.10
-5	-8	2	182.48	15.80
-5	-8	2	194.18	17.00
-5	-8	2	191.18	15.40
-5	-8	2	212.38	15.10
4	8	-2	427.46	26.20
4	8	-2	474.45	21.20
-4	-8	2	368.36	23.80
-4	-8	2	398.16	22.20
-4	-8	2	446.86	21.90
3	8	-2	780.12	36.90
3	8	-2	834.42	37.50
-3	-8	2	799.72	36.90

-3	-8	2	819.12	38.40
2	8	-2	776.52	35.90
2	8	-2	761.52	35.80
-2	-8	2	803.12	36.00
-2	-8	2	767.82	37.10
1	8	-2	112.59	6.60
1	8	-2	115.49	7.30
1	8	-2	114.59	11.00
-1	-8	2	110.89	7.60
-1	-8	2	112.69	9.50
-1	-8	2	110.29	11.60
0	8	-2	1989.50	86.99
0	8	-2	1861.41	84.99
0	8	-2	1794.52	84.19
0	-8	2	1952.40	86.89
0	-8	2	1778.62	84.99
-1	8	-2	711.23	32.80
-1	8	-2	717.53	31.80
1	-8	2	607.74	32.50
1	-8	2	677.53	34.80
1	-8	2	724.43	33.00
-2	8	-2	1143.69	49.20
-2	8	-2	1129.49	50.59
2	-8	2	987.50	51.99
2	-8	2	1048.90	50.29
-3	8	-2	893.21	41.00
-3	8	-2	930.21	43.10
-3	8	-2	904.01	45.90
-3	8	-2	897.41	42.80
3	-8	2	920.81	42.40
3	-8	2	774.62	44.20
3	-8	2	899.41	42.90
-4	8	-2	41.10	7.20
-4	8	-2	37.80	10.60
4	-8	2	38.30	7.60
4	-8	2	39.00	7.80
-5	8	-2	267.07	17.90
-5	8	-2	267.57	20.40
-5	8	-2	271.27	20.30
5	-8	2	253.87	23.20
5	-8	2	269.97	17.70
-6	8	-2	88.49	13.20
-6	8	-2	83.09	11.50
-6	8	-2	94.89	11.90

6	-8	2	77.69	11.80
-7	8	-2	4.90	9.40
-7	8	-2	-9.90	8.00
-7	8	-2	6.70	9.40
7	-8	2	4.60	7.80
-8	8	-2	288.87	21.80
-8	8	-2	238.58	21.80
-8	8	-2	274.87	23.20
8	-8	2	262.27	19.80
-9	8	-2	-3.30	10.90
-9	8	-2	-9.70	10.50
-9	8	-2	5.20	10.90
9	-8	2	-5.40	9.90
9	-8	2	-0.30	9.00
-10	8	-2	6.40	13.20
-10	8	-2	32.30	11.80
-10	8	-2	12.40	11.60
10	-8	2	1.40	9.60
10	-8	2	21.40	10.50
-11	8	-2	71.19	12.30
-11	8	-2	94.49	14.70
-11	8	-2	45.20	17.90
11	-8	2	93.69	14.70
11	-8	2	73.89	14.30
12	7	-2	37.30	11.90
-12	-7	2	58.99	11.50
-12	-7	2	35.60	12.00
11	7	-2	32.40	11.60
-11	-7	2	-7.70	7.10
-11	-7	2	12.40	8.50
-10	-7	2	244.08	23.90
-10	-7	2	232.18	21.10
-10	-7	2	275.97	23.10
-10	-7	2	210.98	23.10
-10	-7	2	207.18	23.40
-9	-7	2	556.04	30.40
-9	-7	2	573.34	32.30
-9	-7	2	559.44	31.60
-9	-7	2	589.84	30.90
-9	-7	2	605.64	33.00
8	7	-2	119.79	15.70
-8	-7	2	103.79	12.60
-8	-7	2	106.89	12.60
-8	-7	2	136.59	14.90

-8	-7	2	113.89	12.90
-8	-7	2	111.29	16.60
7	7	-2	217.18	15.80
-7	-7	2	217.58	14.90
-7	-7	2	241.58	16.90
-7	-7	2	234.58	15.40
-7	-7	2	265.47	19.50
6	7	-2	33.30	5.40
-6	-7	2	10.40	10.40
-6	-7	2	28.90	6.50
-6	-7	2	29.20	8.40
-6	-7	2	25.70	7.80
-6	-7	2	38.40	5.90
-6	-7	2	39.40	6.90
5	7	-2	0.30	9.00
5	7	-2	17.20	13.40
5	7	-2	9.50	3.80
-5	-7	2	9.30	7.20
-5	-7	2	9.20	4.30
-5	-7	2	13.10	6.80
-5	-7	2	9.50	4.60
4	7	-2	105.29	7.00
4	7	-2	88.29	12.10
-4	-7	2	106.69	8.70
3	7	-2	55.89	4.70
-3	-7	2	59.09	4.10
-3	-7	2	50.49	7.40
2	7	-2	2276.47	96.29
2	7	-2	2240.48	96.19
2	7	-2	2058.99	98.09
-2	-7	2	2073.39	97.19
-2	-7	2	1848.02	98.19
-2	-7	2	2355.56	96.39
1	7	-2	327.67	17.30
1	7	-2	294.17	14.70
1	7	-2	304.67	14.50
-1	-7	2	311.07	15.00
-1	-7	2	259.47	17.20
0	7	-2	1.70	2.10
0	7	-2	4.50	1.60
0	7	-2	3.70	4.50
0	-7	2	0.90	4.10
0	-7	2	6.10	3.70
0	-7	2	-2.40	2.30

-1	7	-2	767.12	35.00
-1	7	-2	812.42	37.80
-1	7	-2	788.22	35.90
1	-7	2	747.73	35.80
1	-7	2	729.73	37.40
1	-7	2	752.12	35.90
-2	7	-2	15.00	2.50
-2	7	-2	12.20	3.30
2	-7	2	11.30	4.70
2	-7	2	15.50	4.60
-3	7	-2	208.48	13.60
-3	7	-2	212.68	11.20
-3	7	-2	212.88	13.00
-3	7	-2	226.28	15.90
3	-7	2	194.08	14.70
3	-7	2	195.98	12.50
3	-7	2	209.28	13.30
-4	7	-2	8.00	6.00
-4	7	-2	5.70	8.50
-4	7	-2	12.90	5.30
-4	7	-2	1.20	5.30
4	-7	2	9.40	5.10
4	-7	2	-5.20	5.70
4	-7	2	9.10	8.00
-5	7	-2	206.28	17.90
-5	7	-2	237.58	15.30
-5	7	-2	225.88	18.80
5	-7	2	212.28	15.50
5	-7	2	218.68	16.20
-6	7	-2	294.07	20.80
-6	7	-2	283.77	18.60
-6	7	-2	294.97	20.70
6	-7	2	312.67	20.60
6	-7	2	266.97	18.90
-7	7	-2	587.44	30.80
-7	7	-2	554.74	32.10
-7	7	-2	547.25	31.40
7	-7	2	570.94	30.90
-8	7	-2	372.66	26.50
-8	7	-2	391.26	24.70
-8	7	-2	381.06	23.90
8	-7	2	391.46	24.10
-9	7	-2	163.88	18.80
-9	7	-2	157.88	18.90

-9	7	-2	151.38	18.20
9	-7	2	136.09	17.70
9	-7	2	184.38	17.70
-10	7	-2	37.70	11.30
-10	7	-2	47.20	12.70
-10	7	-2	58.49	13.10
10	-7	2	57.59	12.20
10	-7	2	41.30	10.70
-11	7	-2	25.00	10.80
-11	7	-2	33.70	13.40
-11	7	-2	-4.30	15.60
11	-7	2	-0.50	13.40
11	-7	2	27.80	10.40
12	6	-2	-4.80	11.50
-12	-6	2	5.40	7.40
-12	-6	2	5.80	13.50
11	6	-2	60.09	20.60
11	6	-2	39.40	12.50
-11	-6	2	39.70	8.30
-11	-6	2	43.30	9.70
10	6	-2	33.40	16.50
-10	-6	2	24.70	14.50
-10	-6	2	4.00	12.00
-10	-6	2	-3.20	13.00
-10	-6	2	10.30	20.80
-10	-6	2	1.50	7.60
9	6	-2	24.10	12.60
-9	-6	2	19.80	10.10
-9	-6	2	12.70	7.10
-9	-6	2	23.70	11.60
-9	-6	2	33.50	10.90
-9	-6	2	24.70	7.30
8	6	-2	28.80	11.20
8	6	-2	47.30	9.10
-8	-6	2	33.80	9.50
-8	-6	2	25.10	6.80
-8	-6	2	41.10	9.00
-8	-6	2	43.10	7.70
-8	-6	2	30.70	7.50
7	6	-2	121.99	13.80
7	6	-2	92.69	10.80
-7	-6	2	91.69	11.00
-7	-6	2	93.79	14.80
-7	-6	2	97.69	14.30

-7	-6	2	82.79	12.70
-7	-6	2	95.69	10.00
6	6	-2	189.38	12.10
-6	-6	2	173.58	16.40
-6	-6	2	209.18	12.50
-6	-6	2	175.08	15.00
-6	-6	2	190.28	13.50
-6	-6	2	202.28	14.20
5	6	-2	52.59	5.90
5	6	-2	36.60	8.30
5	6	-2	48.90	8.90
-5	-6	2	56.19	7.70
-5	-6	2	58.09	9.40
-5	-6	2	47.10	7.00
4	6	-2	661.93	30.40
4	6	-2	654.63	32.50
-4	-6	2	632.24	30.90
3	6	-2	893.31	40.80
3	6	-2	823.52	39.20
-3	-6	2	857.11	40.30
2	6	-2	862.81	39.40
2	6	-2	857.21	41.10
2	6	-2	891.91	39.60
-2	-6	2	788.32	41.20
-2	-6	2	941.01	39.60
1	6	-2	78.09	4.70
1	6	-2	69.89	7.40
1	6	-2	74.99	4.80
-1	-6	2	79.29	5.20
0	6	-2	1710.93	76.89
0	6	-2	1821.82	78.39
0	6	-2	1586.84	76.39
0	-6	2	1779.52	78.39
0	-6	2	1773.12	76.99
0	-6	2	1553.14	76.59
-1	6	-2	212.58	12.80
-1	6	-2	220.28	11.00
-1	6	-2	200.88	10.20
1	-6	2	190.98	12.60
1	-6	2	194.38	11.40
1	-6	2	213.88	11.10
-2	6	-2	5.30	1.90
-2	6	-2	3.70	2.50
-2	6	-2	-0.10	4.00

2	-6	2	2.90	2.80
2	-6	2	4.90	4.00
2	-6	2	7.80	3.20
-3	6	-2	272.67	15.30
-3	6	-2	295.27	17.20
3	-6	2	280.17	14.70
3	-6	2	287.17	15.60
-4	6	-2	1079.99	52.49
-4	6	-2	1108.39	50.89
-4	6	-2	1065.49	49.70
-4	6	-2	1087.09	51.09
4	-6	2	1111.79	49.99
4	-6	2	1084.49	51.19
4	-6	2	1046.30	51.99
-5	6	-2	7.40	7.90
-5	6	-2	16.70	8.50
-5	6	-2	5.40	4.80
-5	6	-2	4.60	7.00
5	-6	2	12.10	6.70
5	-6	2	2.50	4.70
5	-6	2	7.30	7.50
-6	6	-2	12.30	8.10
-6	6	-2	20.00	7.90
-6	6	-2	19.90	6.00
6	-6	2	25.90	7.00
6	-6	2	12.70	6.90
-7	6	-2	890.31	43.70
7	-6	2	872.41	46.10
-8	6	-2	100.59	16.30
-8	6	-2	110.79	12.90
-8	6	-2	102.79	14.40
8	-6	2	74.59	10.30
-9	6	-2	7.30	9.40
-9	6	-2	-4.80	9.40
-9	6	-2	2.00	8.70
9	-6	2	-1.50	9.30
-10	6	-2	249.28	21.90
-10	6	-2	274.17	26.40
-10	6	-2	283.57	23.10
10	-6	2	329.47	28.30
10	-6	2	272.57	24.70
-11	6	-2	-7.10	13.70
-11	6	-2	16.50	12.20
-11	6	-2	5.00	10.30

11	-6	2	9.40	9.60
11	-6	2	20.90	15.20
-12	6	-2	126.69	19.50
12	-6	2	185.68	20.40
12	5	-2	-3.10	12.50
-12	-5	2	-9.60	13.00
-12	-5	2	19.50	7.80
11	5	-2	17.00	13.60
-11	-5	2	20.50	7.50
10	5	-2	20.70	14.50
-10	-5	2	11.80	7.50
-10	-5	2	3.80	8.10
-10	-5	2	8.70	12.40
-10	-5	2	20.60	18.20
9	5	-2	703.23	38.80
9	5	-2	629.64	41.00
-9	-5	2	707.83	37.00
-9	-5	2	717.53	39.30
-9	-5	2	720.73	36.20
-9	-5	2	784.32	37.30
-9	-5	2	711.63	36.60
8	5	-2	846.12	41.20
8	5	-2	771.72	42.40
-8	-5	2	812.22	40.70
-8	-5	2	846.82	40.00
-8	-5	2	817.12	41.90
-8	-5	2	818.72	40.40
-8	-5	2	818.92	40.80
7	5	-2	112.09	18.20
7	5	-2	100.09	10.30
-7	-5	2	104.09	13.10
-7	-5	2	111.19	10.30
-7	-5	2	88.39	14.90
-7	-5	2	104.99	12.00
-7	-5	2	84.69	10.40
6	5	-2	272.87	24.10
6	5	-2	276.97	17.00
-6	-5	2	325.77	16.10
-6	-5	2	271.27	17.00
-6	-5	2	293.07	18.80
-6	-5	2	278.17	19.60
-6	-5	2	276.37	17.40
5	5	-2	358.26	17.60
5	5	-2	327.37	20.30

5	5	-2	344.07	20.30
-5	-5	2	360.76	18.10
4	5	-2	11.10	4.20
4	5	-2	12.60	2.70
4	5	-2	10.70	4.10
-4	-5	2	15.40	4.30
3	5	-2	397.56	18.50
3	5	-2	382.16	20.50
2	5	-2	535.35	25.80
2	5	-2	528.25	24.50
2	5	-2	532.55	24.70
-2	-5	2	568.84	24.60
-2	-5	2	512.95	25.90
1	5	-2	1164.58	51.09
1	5	-2	1152.38	51.19
1	5	-2	1122.59	52.39
-1	-5	2	1213.28	51.29
-1	-5	2	1022.50	51.19
0	5	-2	22.90	2.40
0	5	-2	21.40	2.80
0	5	-2	23.10	3.90
0	-5	2	25.20	3.30
0	-5	2	24.20	4.40
0	-5	2	23.10	3.00
-1	5	-2	271.17	13.00
-1	5	-2	298.27	15.10
-1	5	-2	272.97	13.60
1	-5	2	261.07	14.70
1	-5	2	275.27	13.70
1	-5	2	273.37	13.80
-2	5	-2	2142.99	90.89
-2	5	-2	2009.50	89.99
-2	5	-2	1956.60	89.19
2	-5	2	1910.41	89.49
2	-5	2	2014.20	89.99
2	-5	2	1914.11	90.39
-3	5	-2	7.10	4.10
-3	5	-2	7.60	2.60
-3	5	-2	2.10	2.60
-3	5	-2	5.10	3.50
3	-5	2	7.70	3.40
3	-5	2	8.00	2.70
3	-5	2	2.70	3.00
-4	5	-2	67.69	9.20

-4	5	-2	50.19	6.70
-4	5	-2	75.19	7.40
-4	5	-2	61.89	7.20
4	-5	2	58.19	5.70
4	-5	2	66.29	7.60
4	-5	2	61.39	8.80
-5	5	-2	1011.80	50.59
-5	5	-2	1079.09	48.70
-5	5	-2	1059.39	49.50
5	-5	2	1072.79	47.70
5	-5	2	978.80	49.10
5	-5	2	972.30	50.29
-6	5	-2	541.85	28.60
-6	5	-2	568.44	27.60
-6	5	-2	516.05	28.90
6	-5	2	512.55	26.90
6	-5	2	539.85	28.90
-7	5	-2	35.80	8.40
-7	5	-2	50.69	9.60
-7	5	-2	32.30	7.10
-7	5	-2	32.20	13.90
7	-5	2	43.20	8.60
7	-5	2	24.60	8.00
-8	5	-2	36.90	13.70
-8	5	-2	5.60	6.90
-8	5	-2	3.20	8.00
-8	5	-2	14.80	8.60
8	-5	2	-9.50	8.60
-9	5	-2	126.69	15.30
-9	5	-2	100.29	14.90
-9	5	-2	116.19	16.90
9	-5	2	97.39	17.00
-10	5	-2	15.60	10.00
-10	5	-2	26.50	10.80
10	-5	2	15.90	11.20
-11	5	-2	48.20	17.10
-11	5	-2	44.00	11.20
-11	5	-2	27.30	11.80
11	-5	2	37.30	10.60
-12	5	-2	70.79	18.40
-12	5	-2	70.79	11.50
12	4	-2	2.30	18.10
12	4	-2	17.10	13.30
-12	-4	2	5.00	6.70

-12	-4	2	-8.20	14.60
11	4	-2	37.30	13.00
-11	-4	2	51.39	8.20
-11	-4	2	57.49	10.20
10	4	-2	377.76	26.70
-10	-4	2	364.06	28.90
-10	-4	2	360.56	26.30
-10	-4	2	347.07	26.20
-10	-4	2	408.46	24.10
-10	-4	2	414.46	23.10
9	4	-2	71.59	11.70
9	4	-2	62.89	14.60
-9	-4	2	48.50	12.80
-9	-4	2	75.79	9.90
-9	-4	2	46.60	8.70
-9	-4	2	70.59	11.20
-9	-4	2	79.19	11.50
8	4	-2	191.08	18.20
-8	-4	2	158.88	18.70
-8	-4	2	147.29	13.60
-8	-4	2	179.38	13.30
-8	-4	2	156.88	14.80
-8	-4	2	142.69	15.10
7	4	-2	84.19	11.20
7	4	-2	60.49	9.30
-7	-4	2	75.09	10.10
-7	-4	2	64.09	10.00
-7	-4	2	77.59	9.00
-7	-4	2	65.39	9.70
-7	-4	2	69.89	9.20
6	4	-2	33.30	9.80
6	4	-2	16.70	8.90
6	4	-2	30.10	4.80
6	4	-2	27.10	9.90
-6	-4	2	25.60	5.70
-6	-4	2	27.90	5.10
-6	-4	2	27.10	7.90
-6	-4	2	21.20	5.90
5	4	-2	7.00	5.30
5	4	-2	19.20	5.40
5	4	-2	17.40	3.30
-5	-4	2	13.40	4.30
4	4	-2	27.50	4.30
4	4	-2	33.20	4.00

4	4	-2	34.00	5.70
3	4	-2	3877.61	166.48
3	4	-2	3549.54	165.68
2	4	-2	75.09	4.50
2	4	-2	74.99	4.10
2	4	-2	87.09	5.70
-2	-4	2	77.89	4.20
1	4	-2	1151.28	49.99
1	4	-2	1093.39	50.99
1	4	-2	1166.98	50.09
-1	-4	2	1059.79	51.09
-1	-4	2	1197.98	50.09
-1	-4	2	1000.10	50.09
0	4	-2	1143.39	50.59
0	4	-2	1098.79	49.50
0	4	-2	1122.89	49.20
0	-4	2	1153.28	49.60
0	-4	2	994.20	49.50
0	-4	2	1060.09	50.59
-1	4	-2	69.29	4.10
-1	4	-2	70.69	4.50
-1	4	-2	66.79	5.70
1	-4	2	68.59	4.60
1	-4	2	63.89	5.00
1	-4	2	68.99	5.60
-2	4	-2	1351.16	60.99
-2	4	-2	1299.97	60.29
-2	4	-2	1378.66	61.49
2	-4	2	1354.06	61.29
2	-4	2	1353.06	60.99
2	-4	2	1319.37	60.49
-3	4	-2	343.37	16.50
-3	4	-2	318.07	15.50
-3	4	-2	319.27	16.80
3	-4	2	310.97	15.50
3	-4	2	314.87	16.70
3	-4	2	333.87	16.60
-4	4	-2	301.67	16.30
-4	4	-2	280.07	15.50
-4	4	-2	331.07	16.40
-4	4	-2	308.77	17.20
4	-4	2	301.77	16.30
4	-4	2	314.67	17.10
4	-4	2	320.77	15.30

-5	4	-2	146.99	10.60
-5	4	-2	143.09	11.40
-5	4	-2	146.59	13.10
5	-4	2	163.18	11.60
5	-4	2	146.89	9.00
5	-4	2	130.89	12.70
-6	4	-2	381.56	22.50
-6	4	-2	369.96	21.90
-6	4	-2	405.16	20.80
-6	4	-2	376.46	23.50
6	-4	2	361.16	23.50
6	-4	2	418.06	20.10
6	-4	2	386.06	23.40
-7	4	-2	27.30	19.80
-7	4	-2	31.80	6.40
-7	4	-2	19.30	7.70
-7	4	-2	38.30	12.50
-7	4	-2	23.20	7.60
7	-4	2	30.40	7.10
7	-4	2	22.40	8.50
-8	4	-2	162.38	13.40
-8	4	-2	136.19	15.70
-8	4	-2	167.88	16.00
-8	4	-2	168.68	15.40
8	-4	2	124.39	17.00
8	-4	2	143.99	18.00
-9	4	-2	22.60	8.80
-9	4	-2	3.30	12.60
-9	4	-2	6.30	9.50
-9	4	-2	19.30	8.50
9	-4	2	19.40	10.20
-10	4	-2	53.69	10.10
-10	4	-2	91.89	15.60
-10	4	-2	38.90	9.80
10	-4	2	48.20	11.80
10	-4	2	55.19	15.60
-11	4	-2	406.96	24.90
-11	4	-2	347.17	25.00
-11	4	-2	362.86	25.50
11	-4	2	411.86	24.80
-12	4	-2	56.79	10.40
-12	4	-2	61.59	15.10
12	-4	2	88.29	14.70
12	3	-2	86.69	14.50

12	3	-2	97.49	16.30
-12	-3	2	82.99	17.90
-12	-3	2	90.19	10.90
11	3	-2	72.39	12.70
-11	-3	2	74.49	12.40
-11	-3	2	82.39	11.60
10	3	-2	475.15	28.70
-10	-3	2	445.86	27.40
-10	-3	2	449.36	26.80
-10	-3	2	504.65	25.80
-10	-3	2	428.76	28.00
-10	-3	2	384.16	39.20
9	3	-2	309.07	23.50
9	3	-2	339.87	28.10
-9	-3	2	327.07	26.30
-9	-3	2	324.17	20.70
-9	-3	2	324.27	20.00
-9	-3	2	340.77	21.20
-9	-3	2	304.37	22.10
8	3	-2	650.33	34.60
-8	-3	2	661.93	32.90
-8	-3	2	655.23	32.40
-8	-3	2	619.14	33.70
7	3	-2	295.97	20.30
7	3	-2	291.67	16.50
-7	-3	2	265.17	20.00
-7	-3	2	293.77	16.40
-7	-3	2	272.97	19.00
-7	-3	2	272.17	16.90
-7	-3	2	268.97	18.00
6	3	-2	17.90	9.20
6	3	-2	1.40	8.10
6	3	-2	3.40	3.40
6	3	-2	11.70	7.80
-6	-3	2	-0.50	5.80
-6	-3	2	10.60	7.30
-6	-3	2	10.50	6.00
-6	-3	2	12.30	4.90
5	3	-2	23.20	5.20
5	3	-2	17.50	3.20
-5	-3	2	19.10	4.90
4	3	-2	722.33	34.50
4	3	-2	782.22	37.80
3	3	-2	217.38	11.10

3	3	-2	208.88	11.00
3	3	-2	208.48	10.50
2	3	-2	596.94	27.80
-2	-3	2	558.74	27.50
-2	-3	2	657.33	27.20
1	3	-2	81.29	4.20
1	3	-2	79.49	4.40
1	3	-2	80.89	5.50
-1	-3	2	85.39	4.30
-1	-3	2	72.69	4.90
-1	-3	2	80.09	5.30
0	3	-2	331.67	15.40
0	3	-2	317.37	14.40
0	3	-2	325.77	14.20
0	-3	2	267.97	15.40
0	-3	2	278.77	14.70
0	-3	2	323.87	14.40
-1	3	-2	894.91	40.40
-1	3	-2	945.41	41.00
-1	3	-2	855.01	40.10
1	-3	2	914.21	40.50
1	-3	2	863.21	40.30
1	-3	2	874.21	40.80
-2	3	-2	157.98	8.10
-2	3	-2	173.38	9.10
-2	3	-2	159.88	8.60
2	-3	2	154.78	8.60
2	-3	2	168.08	9.00
2	-3	2	166.68	8.20
-3	3	-2	15.20	2.80
-3	3	-2	19.70	2.90
-3	3	-2	18.70	2.60
3	-3	2	17.10	2.90
3	-3	2	15.80	2.80
3	-3	2	17.10	2.60
-4	3	-2	903.81	44.20
-4	3	-2	947.01	44.80
-4	3	-2	988.50	44.50
4	-3	2	953.60	43.50
4	-3	2	948.21	44.80
4	-3	2	976.20	44.30
-5	3	-2	158.18	10.60
-5	3	-2	144.29	12.20
-5	3	-2	163.38	10.20

5	-3	2	153.48	10.60
5	-3	2	131.49	8.70
5	-3	2	150.38	12.00
-6	3	-2	1478.95	67.59
-6	3	-2	1419.16	67.09
-6	3	-2	1418.96	67.59
6	-3	2	1537.25	66.09
6	-3	2	1409.16	67.99
6	-3	2	1388.06	67.29
-7	3	-2	230.78	16.30
-7	3	-2	243.18	17.10
-7	3	-2	232.68	14.60
-7	3	-2	218.48	23.70
-7	3	-2	209.08	21.40
7	-3	2	243.58	19.10
7	-3	2	221.08	14.70
-8	3	-2	108.59	14.10
-8	3	-2	136.49	14.70
-8	3	-2	119.19	13.80
-8	3	-2	142.09	15.30
-8	3	-2	141.19	12.00
8	-3	2	135.39	14.70
8	-3	2	115.59	17.20
-9	3	-2	34.80	12.50
-9	3	-2	27.70	7.90
-9	3	-2	10.00	8.20
-9	3	-2	36.20	9.30
9	-3	2	27.70	11.10
-10	3	-2	1.10	9.00
-10	3	-2	10.40	8.90
-10	3	-2	-4.60	8.20
10	-3	2	30.50	17.10
-11	3	-2	122.49	16.30
-11	3	-2	114.79	15.20
-11	3	-2	71.19	12.60
11	-3	2	108.69	16.40
-12	3	-2	340.37	22.80
-12	3	-2	331.17	26.20
12	-3	2	406.36	24.20
12	2	-2	4.70	15.90
12	2	-2	-14.50	11.70
-12	-2	2	0.10	6.80
-12	-2	2	-20.60	20.00
11	2	-2	39.30	11.90

-11	-2	2	44.20	11.60
-11	-2	2	36.40	8.10
10	2	-2	42.80	10.90
-10	-2	2	18.40	9.80
-10	-2	2	17.40	7.10
-10	-2	2	-5.30	11.40
-10	-2	2	18.10	11.60
9	2	-2	41.50	10.20
-9	-2	2	23.00	6.80
-9	-2	2	41.70	10.60
-9	-2	2	9.10	13.80
-9	-2	2	27.30	9.10
-9	-2	2	40.50	8.70
8	2	-2	-3.90	8.20
8	2	-2	7.60	8.20
-8	-2	2	12.50	7.20
-8	-2	2	2.00	9.40
-8	-2	2	11.10	6.30
-8	-2	2	26.60	10.50
-8	-2	2	23.10	6.80
7	2	-2	138.19	15.90
-7	-2	2	153.98	13.30
-7	-2	2	105.39	14.40
-7	-2	2	147.49	12.20
-7	-2	2	111.39	12.60
-7	-2	2	135.49	11.00
6	2	-2	683.83	36.20
6	2	-2	738.83	33.70
-6	-2	2	686.53	35.20
-6	-2	2	739.53	34.00
-6	-2	2	706.43	36.50
-6	-2	2	697.03	34.70
5	2	-2	777.82	35.70
5	2	-2	767.22	36.90
-5	-2	2	761.22	36.20
4	2	-2	573.44	26.70
4	2	-2	576.64	27.40
3	2	-2	21.70	2.70
3	2	-2	24.80	3.20
3	2	-2	25.70	3.40
-3	-2	2	19.80	2.90
-3	-2	2	22.90	1.70
-3	-2	2	24.10	1.90
2	2	-2	1496.75	67.49

2	2	-2	1658.03	67.79
-2	-2	2	1464.65	67.19
-2	-2	2	1408.56	67.49
1	2	-2	1582.04	68.59
1	2	-2	1603.04	68.99
1	2	-2	1560.64	68.69
-1	-2	2	1353.86	68.69
-1	-2	2	1535.05	68.59
-1	-2	2	1587.74	68.99
0	2	-2	1242.68	52.49
0	2	-2	1199.78	51.99
0	2	-2	1201.28	51.89
0	-2	2	1071.69	52.19
0	-2	2	1044.10	52.49
0	-2	2	1201.98	51.99
-1	2	-2	256.67	12.00
-1	2	-2	237.88	11.70
-1	2	-2	241.78	11.40
1	-2	2	239.18	11.70
1	-2	2	252.17	11.60
1	-2	2	259.87	12.00
-2	2	-2	426.46	19.70
-2	2	-2	394.96	19.00
-2	2	-2	426.66	19.50
2	-2	2	417.96	19.10
2	-2	2	410.96	19.70
2	-2	2	417.76	19.40
-3	2	-2	1299.07	57.79
-3	2	-2	1266.07	58.19
3	-2	2	1222.58	57.79
3	-2	2	1282.47	57.39
3	-2	2	1304.87	58.19
-4	2	-2	538.25	25.50
-4	2	-2	547.95	24.70
-4	2	-2	495.55	25.00
4	-2	2	524.45	24.70
4	-2	2	496.75	24.00
4	-2	2	503.35	25.20
-5	2	-2	89.29	8.80
-5	2	-2	70.39	6.90
-5	2	-2	72.29	7.10
5	-2	2	56.29	6.90
5	-2	2	77.09	8.50
5	-2	2	78.59	5.60

-6	2	-2	51.59	7.50
-6	2	-2	64.19	8.40
-6	2	-2	56.79	7.20
-6	2	-2	65.69	11.30
6	-2	2	73.19	6.40
6	-2	2	50.79	7.70
-7	2	-2	1413.36	66.19
-7	2	-2	1467.95	66.59
-7	2	-2	1358.66	67.69
-7	2	-2	1413.36	65.69
-7	2	-2	1443.66	67.79
7	-2	2	1320.37	66.99
7	-2	2	1471.95	65.29
-8	2	-2	10.80	7.00
-8	2	-2	10.10	5.50
-8	2	-2	0.30	10.80
-8	2	-2	10.60	6.90
8	-2	2	12.70	9.90
8	-2	2	9.20	7.10
-9	2	-2	-9.00	7.00
-9	2	-2	-7.90	10.90
-9	2	-2	17.70	6.90
-9	2	-2	-1.30	7.20
-9	2	-2	7.60	13.50
9	-2	2	-23.30	11.60
9	-2	2	-5.80	12.50
-10	2	-2	169.28	17.70
-10	2	-2	188.48	16.10
-10	2	-2	201.48	17.00
10	-2	2	164.08	19.80
-11	2	-2	32.40	10.00
-11	2	-2	16.80	10.40
-11	2	-2	29.40	9.80
11	-2	2	32.00	10.60
-12	2	-2	118.19	14.80
-12	2	-2	86.89	18.00
12	-2	2	99.09	16.60
12	1	-2	32.90	17.70
12	1	-2	10.10	11.60
-12	-1	2	12.30	7.30
11	1	-2	7.30	11.20
-11	-1	2	11.40	11.70
-11	-1	2	19.60	7.90
10	1	-2	58.29	11.80

-10	-1	2	50.99	12.20
-10	-1	2	32.40	7.80
-10	-1	2	49.99	11.00
-10	-1	2	36.90	12.20
9	1	-2	818.62	44.10
-9	-1	2	859.31	43.80
-9	-1	2	902.21	42.90
-9	-1	2	845.02	43.30
-9	-1	2	871.91	42.40
8	1	-2	357.06	23.20
8	1	-2	360.16	22.40
-8	-1	2	396.26	25.20
-8	-1	2	357.26	20.70
-8	-1	2	378.86	21.50
-8	-1	2	355.36	22.80
-8	-1	2	353.86	22.20
7	1	-2	302.47	20.10
-7	-1	2	289.37	17.60
-7	-1	2	307.77	16.90
-7	-1	2	287.87	18.60
-7	-1	2	280.57	22.20
-7	-1	2	270.77	19.40
6	1	-2	92.99	13.10
6	1	-2	104.19	7.80
-6	-1	2	80.59	12.50
-6	-1	2	106.19	11.10
-6	-1	2	90.29	15.00
-6	-1	2	105.49	8.90
5	1	-2	1554.54	70.39
5	1	-2	1551.24	71.39
-5	-1	2	1567.04	69.79
-5	-1	2	1539.45	70.69
4	1	-2	375.66	17.80
4	1	-2	397.06	18.50
-4	-1	2	332.87	18.00
-4	-1	2	385.56	17.40
3	1	-2	152.18	7.70
3	1	-2	150.98	8.00
-3	-1	2	142.19	7.80
-3	-1	2	152.78	7.30
2	1	-2	719.03	32.80
2	1	-2	754.82	33.00
-2	-1	2	715.13	32.60
-2	-1	2	715.13	32.80

1	1	-2	2451.75	112.99
1	1	-2	2757.52	113.09
-1	-1	2	2346.77	112.99
-1	-1	2	2590.84	112.89
0	1	-2	114.89	5.40
0	1	-2	109.89	5.60
0	1	-2	113.29	5.40
0	-1	2	104.29	5.70
0	-1	2	116.99	5.40
-1	1	-2	449.36	20.90
-1	1	-2	460.95	20.80
1	-1	2	457.05	20.90
1	-1	2	462.15	20.80
1	-1	2	453.05	20.60
-2	1	-2	501.25	23.20
-2	1	-2	498.35	22.90
2	-1	2	481.45	22.90
2	-1	2	503.35	23.20
2	-1	2	511.35	22.60
-3	1	-2	250.77	11.60
-3	1	-2	232.48	12.00
3	-1	2	230.58	12.00
3	-1	2	227.18	11.60
-4	1	-2	196.58	9.80
-4	1	-2	177.68	10.70
4	-1	2	182.88	9.90
4	-1	2	180.78	9.30
4	-1	2	180.98	10.60
-5	1	-2	1545.35	70.79
-5	1	-2	1520.85	69.99
-5	1	-2	1545.45	69.69
5	-1	2	1540.65	69.39
5	-1	2	1570.04	70.89
5	-1	2	1477.75	69.59
-6	1	-2	265.87	18.10
-6	1	-2	287.67	16.20
-6	1	-2	354.46	26.90
-6	1	-2	303.47	15.70
-6	1	-2	294.87	18.40
6	-1	2	262.07	14.60
6	-1	2	298.07	16.10
6	-1	2	235.08	19.60
-7	1	-2	261.57	15.20
-7	1	-2	234.68	20.20

-7	1	-2	263.97	17.10
-7	1	-2	248.78	21.00
-7	1	-2	256.67	17.10
7	-1	2	239.78	21.90
7	-1	2	264.17	14.80
-8	1	-2	7.30	5.00
-8	1	-2	12.10	7.70
-8	1	-2	2.20	9.70
-8	1	-2	13.40	6.50
8	-1	2	21.00	7.00
8	-1	2	27.50	11.20
-9	1	-2	-10.30	11.60
-9	1	-2	21.80	7.30
-9	1	-2	-10.80	9.70
-9	1	-2	29.80	8.30
-9	1	-2	9.80	6.40
9	-1	2	9.70	10.60
-10	1	-2	-0.90	7.70
-10	1	-2	6.80	8.30
-10	1	-2	10.40	8.00
-11	1	-2	17.30	10.30
-11	1	-2	10.70	8.30
-11	1	-2	21.30	10.00
11	-1	2	28.10	10.30
-12	1	-2	208.68	19.90
-12	1	-2	149.19	23.70
-12	1	-2	239.98	17.00
12	-1	2	198.08	19.50
12	0	-2	22.30	11.50
12	0	-2	-5.40	19.00
-12	0	2	-1.70	6.80
11	0	-2	16.30	11.20
-11	0	2	12.60	13.10
-11	0	2	12.20	8.00
10	0	-2	339.27	23.20
-10	0	2	311.37	29.30
-10	0	2	372.06	21.50
-10	0	2	302.47	24.00
-10	0	2	302.87	26.10
9	0	-2	34.60	9.10
-9	0	2	40.20	8.40
-9	0	2	36.60	10.60
-9	0	2	37.90	9.80
-9	0	2	20.00	8.70

8	0	-2	246.18	19.30
8	0	-2	257.57	19.10
-8	0	2	268.77	16.60
-8	0	2	233.38	18.70
-8	0	2	246.48	19.60
-8	0	2	256.17	17.40
7	0	-2	220.18	14.50
-7	0	2	226.08	14.00
-7	0	2	230.28	18.60
-7	0	2	231.68	15.60
-7	0	2	207.18	17.80
-7	0	2	198.88	17.20
6	0	-2	11.60	7.20
-6	0	2	6.00	8.60
-6	0	2	0.30	9.50
-6	0	2	3.60	6.10
-6	0	2	1.00	4.20
5	0	-2	34.10	5.00
5	0	-2	37.40	4.60
-5	0	2	33.70	5.20
-5	0	2	33.30	4.70
4	0	-2	238.38	12.20
4	0	-2	235.58	11.60
-4	0	2	215.88	12.00
-4	0	2	234.78	11.60
3	0	-2	1692.03	72.69
3	0	-2	1668.53	73.19
-3	0	2	1594.54	72.89
-3	0	2	1545.05	72.89
2	0	-2	64.89	3.70
2	0	-2	62.39	3.70
-2	0	2	61.09	3.40
-2	0	2	59.59	3.70
1	0	-2	42.60	2.20
1	0	-2	41.20	2.40
-1	0	2	38.60	2.40
-1	0	2	42.30	2.30
-1	0	-2	231.58	10.70
-1	0	-2	232.78	10.80
1	0	2	236.08	10.80
1	0	2	230.28	10.70
1	0	2	229.88	10.40
-2	0	-2	985.20	44.30
-2	0	-2	970.40	44.60

2	0	2	996.70	44.60
2	0	2	971.60	44.30
2	0	2	1005.20	44.10
-3	0	-2	67.69	5.00
-3	0	-2	73.09	4.30
3	0	2	72.59	4.40
3	0	2	78.59	4.10
3	0	2	73.79	5.00
-4	0	-2	123.89	8.00
-4	0	-2	122.09	6.90
4	0	2	114.09	7.00
4	0	2	136.19	6.80
4	0	2	134.69	8.10
-5	0	-2	0.10	3.00
-5	0	-2	2.10	2.60
-5	0	-2	-3.10	3.80
5	0	2	-1.00	3.70
5	0	2	2.80	2.70
-6	0	-2	371.56	18.60
-6	0	-2	366.06	20.60
-6	0	-2	349.67	18.20
6	0	2	364.86	18.30
6	0	2	333.07	21.30
6	0	2	360.16	17.80
-7	0	-2	141.49	12.60
-7	0	-2	157.38	16.30
-7	0	-2	121.69	9.90
-7	0	-2	110.59	16.30
-7	0	-2	127.59	15.50
-7	0	-2	125.79	12.50
7	0	2	134.29	9.50
-8	0	-2	231.38	18.80
-8	0	-2	281.57	32.20
-8	0	-2	195.18	16.00
-8	0	-2	227.08	14.60
8	0	2	205.88	14.80
-9	0	-2	5.80	7.10
-9	0	-2	7.80	10.00
-9	0	-2	1.10	5.90
-9	0	-2	7.10	7.40
-9	0	-2	-8.20	10.60
9	0	2	-8.90	9.50
-10	0	-2	210.88	16.80
-10	0	-2	206.18	17.10

-10	0	-2	218.68	22.70
-10	0	-2	214.38	17.20
10	0	2	212.88	20.80
-11	0	-2	123.99	14.40
-11	0	-2	112.19	14.90
-11	0	-2	98.59	15.40
11	0	2	120.29	17.40
-12	0	-2	19.40	11.80
-12	0	-2	0.80	7.90
12	0	2	33.40	10.70
12	-1	-2	115.59	29.60
12	-1	-2	104.99	16.80
-12	1	2	104.89	12.80
11	-1	-2	126.99	18.40
-11	1	2	137.09	17.60
-11	1	2	173.78	15.50
10	-1	-2	365.46	25.10
-10	1	2	384.96	23.10
-10	1	2	440.06	28.90
-10	1	2	296.77	26.20
-10	1	2	385.96	32.70
9	-1	-2	13.70	9.10
-9	1	2	11.70	10.20
-9	1	2	21.10	10.10
-9	1	2	-9.70	8.10
-9	1	2	14.20	7.40
8	-1	-2	614.94	32.70
8	-1	-2	561.34	32.40
-8	1	2	582.34	32.50
-8	1	2	598.64	31.30
-8	1	2	621.54	32.30
-8	1	2	596.24	30.40
7	-1	-2	10.80	6.40
-7	1	2	14.50	9.30
-7	1	2	4.00	6.00
-7	1	2	6.20	5.10
-7	1	2	-4.10	8.30
-7	1	2	-0.70	8.30
6	-1	-2	264.97	13.90
6	-1	-2	249.58	17.70
-6	1	2	231.98	14.80
-6	1	2	264.17	16.20
-6	1	2	246.98	14.60
5	-1	-2	626.64	30.20

5	-1	-2	658.23	31.30
5	-1	-2	650.33	30.00
-5	1	2	632.14	30.70
-5	1	2	654.13	30.50
4	-1	-2	393.46	19.00
4	-1	-2	398.66	18.30
-4	1	2	403.46	18.90
-4	1	2	361.06	18.50
-4	1	2	370.46	18.50
3	-1	-2	343.77	16.70
3	-1	-2	360.46	16.40
-3	1	2	351.56	16.70
-3	1	2	337.77	16.50
-3	1	2	362.46	16.60
2	-1	-2	305.97	14.10
2	-1	-2	304.07	14.20
-2	1	2	308.87	14.10
-2	1	2	290.67	14.10
-2	1	2	308.57	14.20
1	-1	-2	24.90	1.50
1	-1	-2	28.60	1.70
1	-1	-2	23.10	1.80
-1	1	2	24.30	1.50
-1	1	2	22.30	1.70
-1	1	2	23.60	1.70
0	1	2	11.30	0.80
-1	-1	-2	1338.47	59.49
-1	-1	-2	1351.86	59.59
1	1	2	1307.77	59.59
1	1	2	1332.17	59.49
-2	-1	-2	18.80	2.30
-2	-1	-2	21.50	1.60
2	1	2	19.60	2.40
2	1	2	20.10	1.70
2	1	2	22.20	1.50
-3	-1	-2	22.10	2.90
-3	-1	-2	16.60	1.90
3	1	2	14.30	2.00
3	1	2	15.60	1.90
3	1	2	9.90	2.00
-4	-1	-2	764.82	35.30
-4	-1	-2	788.62	36.40
4	1	2	790.62	35.40
4	1	2	787.22	36.40

4	1	2	756.32	35.30
-5	-1	-2	31.80	3.60
-5	-1	-2	31.50	4.30
5	1	2	35.20	3.80
5	1	2	41.80	6.50
5	1	2	24.90	3.80
-6	-1	-2	2324.17	106.89
-6	-1	-2	2486.85	107.99
-6	-1	-2	2296.37	108.39
6	1	2	2463.25	107.39
6	1	2	2448.66	107.09
6	1	2	2281.07	109.59
-7	-1	-2	176.38	16.80
-7	-1	-2	157.38	19.80
-7	-1	-2	206.08	12.10
-7	-1	-2	174.58	13.90
-7	-1	-2	183.48	17.00
-7	-1	-2	171.88	13.80
7	1	2	186.88	11.20
-8	-1	-2	23.80	11.50
-8	-1	-2	34.80	7.70
-8	-1	-2	37.50	11.00
-8	-1	-2	16.00	8.60
-8	-1	-2	25.60	6.00
8	1	2	50.39	8.70
-9	-1	-2	9.40	11.10
-9	-1	-2	1.50	7.10
-9	-1	-2	-9.10	5.80
-9	-1	-2	-13.60	9.30
-9	-1	-2	4.90	9.70
-9	-1	-2	6.40	6.60
9	1	2	7.30	9.00
-10	-1	-2	12.80	11.70
-10	-1	-2	2.50	7.20
-10	-1	-2	-10.10	7.60
-10	-1	-2	6.00	7.90
10	1	2	7.90	10.20
10	1	2	49.30	17.50
-11	-1	-2	29.30	9.80
-11	-1	-2	39.20	8.40
-11	-1	-2	38.40	10.20
11	1	2	32.80	11.00
-12	-1	-2	90.79	11.70
-12	-1	-2	66.59	12.80

12	-2	-2	10.00	10.50
-12	2	2	-9.10	7.30
11	-2	-2	108.39	17.10
-11	2	2	96.59	14.10
10	-2	-2	13.30	9.90
-10	2	2	-0.40	7.80
-10	2	2	-0.70	12.30
-10	2	2	-3.50	14.60
9	-2	-2	46.50	10.30
-9	2	2	68.99	11.50
-9	2	2	77.89	12.60
-9	2	2	45.70	11.40
-9	2	2	50.59	9.50
8	-2	-2	17.90	8.70
8	-2	-2	8.60	16.30
-8	2	2	10.30	8.70
-8	2	2	14.60	7.40
-8	2	2	14.00	6.70
-8	2	2	18.90	10.40
7	-2	-2	406.36	25.10
7	-2	-2	398.96	22.60
-7	2	2	397.16	25.90
-7	2	2	422.36	25.60
-7	2	2	414.46	23.10
-7	2	2	414.46	22.00
-7	2	2	426.06	24.80
6	-2	-2	449.16	21.40
6	-2	-2	395.16	21.90
6	-2	-2	401.16	24.60
-6	2	2	412.36	22.90
-6	2	2	428.76	23.00
-6	2	2	441.26	21.80
5	-2	-2	49.70	7.50
5	-2	-2	63.69	6.10
5	-2	-2	53.99	5.60
-5	2	2	46.10	6.00
-5	2	2	62.39	7.20
-5	2	2	61.09	7.10
-5	2	2	58.39	7.30
4	-2	-2	683.13	31.50
4	-2	-2	669.53	32.10
4	-2	-2	718.63	31.70
-4	2	2	659.43	31.50
-4	2	2	695.53	32.10

-4	2	2	670.83	31.90
3	-2	-2	56.59	4.40
3	-2	-2	58.49	4.10
3	-2	-2	59.69	4.00
-3	2	2	59.39	4.30
-3	2	2	60.69	4.30
-3	2	2	57.49	4.00
2	-2	-2	74.19	4.50
2	-2	-2	76.19	4.40
2	-2	-2	75.99	4.30
-2	2	2	74.99	4.30
-2	2	2	75.39	4.20
-2	2	2	73.99	4.40
1	-2	-2	1302.57	55.79
1	-2	-2	1202.38	55.79
-1	2	2	1239.98	55.79
-1	2	2	1263.17	55.59
-1	2	2	1217.38	55.69
0	-2	-2	48.60	2.80
0	-2	-2	52.89	2.90
0	2	2	59.29	3.10
0	2	2	53.29	2.80
-2	-2	-2	2032.30	89.39
2	2	2	1938.71	89.39
2	2	2	2049.59	89.99
-3	-2	-2	320.57	14.30
3	2	2	314.97	15.40
3	2	2	303.97	14.40
3	2	2	314.37	14.40
-4	-2	-2	17.10	2.00
4	2	2	20.10	2.10
-5	-2	-2	108.89	5.60
-5	-2	-2	105.39	7.60
5	2	2	98.09	8.70
5	2	2	109.79	5.70
5	2	2	104.49	6.40
-6	-2	-2	195.58	11.60
-6	-2	-2	178.88	13.40
-6	-2	-2	195.48	16.40
6	2	2	153.88	14.80
6	2	2	215.28	10.80
-7	-2	-2	1045.80	52.09
-7	-2	-2	1070.39	51.29
-7	-2	-2	1109.09	49.99

-7	-2	-2	1102.09	50.79
-7	-2	-2	1039.20	50.29
-7	-2	-2	1025.00	52.29
-8	-2	-2	14.90	8.70
-8	-2	-2	9.60	6.10
-8	-2	-2	24.20	10.00
-8	-2	-2	22.00	5.80
-8	-2	-2	29.30	9.50
8	2	2	22.90	5.90
-9	-2	-2	8.40	9.60
-9	-2	-2	1.70	9.50
-9	-2	-2	-0.20	5.90
-9	-2	-2	0.00	7.00
-9	-2	-2	15.40	6.60
-9	-2	-2	1.70	10.00
9	2	2	24.20	8.70
-10	-2	-2	90.09	14.20
-10	-2	-2	111.79	13.50
-10	-2	-2	97.89	13.50
-10	-2	-2	104.29	13.20
-10	-2	-2	102.79	14.00
10	2	2	111.79	17.10
10	2	2	82.49	17.70
-11	-2	-2	162.08	14.90
-11	-2	-2	123.99	15.70
-11	-2	-2	109.69	16.00
-11	-2	-2	207.38	26.00
11	2	2	176.98	18.50
-12	-2	-2	91.69	12.30
-12	-2	-2	92.59	12.90
-12	-2	-2	72.29	15.40
12	2	2	113.29	17.00
12	-3	-2	12.40	10.00
-12	3	2	4.80	7.90
11	-3	-2	65.29	23.20
11	-3	-2	101.99	16.20
-11	3	2	94.09	14.30
10	-3	-2	15.70	9.80
-10	3	2	15.60	8.50
-10	3	2	10.00	14.80
9	-3	-2	328.07	29.10
9	-3	-2	445.96	25.70
-9	3	2	406.96	24.10
-9	3	2	432.16	27.70

-9	3	2	401.46	25.90
-9	3	2	409.16	24.90
8	-3	-2	25.80	12.40
8	-3	-2	24.40	8.80
-8	3	2	21.00	7.40
-8	3	2	20.40	7.30
-8	3	2	21.60	10.10
7	-3	-2	293.77	20.80
7	-3	-2	293.37	19.40
-7	3	2	325.57	18.20
-7	3	2	334.37	21.70
-7	3	2	304.97	19.30
-7	3	2	311.67	22.50
6	-3	-2	958.80	43.80
6	-3	-2	855.41	46.50
6	-3	-2	968.10	44.60
-6	3	2	879.21	45.60
-6	3	2	936.51	45.10
-6	3	2	922.81	45.50
-6	3	2	1001.60	44.10
5	-3	-2	1132.59	51.89
5	-3	-2	1122.79	50.59
5	-3	-2	1136.99	50.99
-5	3	2	1020.10	50.59
-5	3	2	1096.59	51.49
-5	3	2	1082.59	50.99
-5	3	2	1122.09	51.59
4	-3	-2	17.30	3.50
4	-3	-2	16.90	3.20
4	-3	-2	14.60	2.80
-4	3	2	17.10	3.50
-4	3	2	12.90	3.30
3	-3	-2	243.18	11.70
3	-3	-2	224.88	11.50
3	-3	-2	227.98	11.90
-3	3	2	233.28	11.90
-3	3	2	233.58	11.80
-3	3	2	229.58	11.90
2	-3	-2	20.20	3.00
2	-3	-2	26.50	2.70
2	-3	-2	26.80	3.10
-2	3	2	24.40	2.70
-2	3	2	24.10	2.30
-2	3	2	24.60	2.70

1	-3	-2	5530.85	248.68
-1	3	2	5635.64	248.68
0	-3	-2	40.60	2.90
0	-3	-2	38.70	2.40
0	3	2	37.70	2.40
0	3	2	36.00	3.30
1	3	2	1378.96	65.39
1	3	2	1555.34	65.89
2	3	2	605.24	27.80
2	3	2	631.44	27.80
2	3	2	623.94	28.60
3	3	2	42.30	4.00
3	3	2	39.80	2.60
4	3	2	734.53	33.10
4	3	2	720.63	34.40
-5	-3	-2	1763.62	81.69
5	3	2	1854.31	81.19
5	3	2	1788.12	82.79
-6	-3	-2	16.20	4.20
-6	-3	-2	24.30	8.30
-6	-3	-2	20.30	5.80
-6	-3	-2	27.30	8.20
6	3	2	13.80	6.80
-7	-3	-2	236.08	18.00
-7	-3	-2	259.27	15.50
-7	-3	-2	215.88	15.40
-7	-3	-2	228.58	17.40
-7	-3	-2	239.68	14.00
-7	-3	-2	257.87	19.30
7	3	2	234.48	13.50
-8	-3	-2	55.89	9.90
-8	-3	-2	22.40	12.00
-8	-3	-2	44.30	9.80
-8	-3	-2	50.59	7.90
-8	-3	-2	44.00	6.60
-9	-3	-2	1.00	7.10
-9	-3	-2	5.70	6.50
-9	-3	-2	12.30	9.40
-9	-3	-2	9.30	8.80
-9	-3	-2	11.10	6.80
-9	-3	-2	17.00	9.70
9	3	2	10.10	8.30
-10	-3	-2	9.00	8.10
-10	-3	-2	8.70	10.50

-10	-3	-2	20.30	7.00
-10	-3	-2	9.10	11.00
-10	-3	-2	8.70	8.10
10	3	2	9.00	10.20
10	3	2	41.60	15.90
-11	-3	-2	7.40	7.10
-11	-3	-2	35.10	14.50
-11	-3	-2	6.90	9.20
-11	-3	-2	9.50	9.50
11	3	2	11.70	10.80
-12	-3	-2	199.88	23.10
-12	-3	-2	249.28	20.30
-12	-3	-2	280.17	17.30
12	3	2	218.58	22.40
12	-4	-2	22.90	10.80
-12	4	2	12.80	9.00
11	-4	-2	144.19	17.30
-11	4	2	174.58	16.40
10	-4	-2	190.18	19.10
-10	4	2	180.58	17.80
9	-4	-2	27.80	11.30
9	-4	-2	38.80	9.80
-9	4	2	36.30	8.90
-9	4	2	26.40	10.70
-9	4	2	42.60	12.90
-9	4	2	33.80	10.20
8	-4	-2	351.36	23.20
8	-4	-2	363.96	23.40
-8	4	2	327.97	22.90
-8	4	2	359.86	22.60
-8	4	2	331.87	21.00
-8	4	2	363.76	26.20
7	-4	-2	96.89	14.00
-7	4	2	87.29	11.90
-7	4	2	91.09	10.70
-7	4	2	82.29	11.90
-7	4	2	109.09	12.30
6	-4	-2	11.80	10.20
6	-4	-2	16.50	6.10
6	-4	-2	15.60	8.20
-6	4	2	12.40	9.00
-6	4	2	25.70	5.30
-6	4	2	13.40	8.80
-6	4	2	27.10	7.00

5	-4	-2	71.89	10.40
5	-4	-2	91.59	8.80
5	-4	-2	85.69	7.70
-5	4	2	84.49	8.30
-5	4	2	85.49	10.00
-5	4	2	88.29	8.70
-5	4	2	86.19	9.90
4	-4	-2	94.29	8.10
4	-4	-2	94.59	7.20
4	-4	-2	102.99	7.40
-4	4	2	100.79	6.70
-4	4	2	112.89	7.90
-4	4	2	94.09	7.70
3	-4	-2	72.99	5.80
3	-4	-2	71.39	5.40
3	-4	-2	69.49	5.40
-3	4	2	78.59	4.80
-3	4	2	76.49	5.70
-3	4	2	74.99	5.60
2	-4	-2	125.79	7.60
2	-4	-2	154.48	7.70
2	-4	-2	140.39	8.00
-2	4	2	134.99	7.80
-2	4	2	158.98	7.80
-2	4	2	127.79	7.30
1	-4	-2	272.17	14.70
1	-4	-2	298.87	14.10
-1	4	2	304.97	14.10
-1	4	2	308.87	13.60
-1	4	2	285.97	14.80
0	-4	-2	3200.78	138.79
0	-4	-2	2968.70	139.19
0	4	2	3174.88	138.69
0	4	2	3096.89	139.49
-1	-4	-2	88.99	4.70
-1	-4	-2	91.19	5.50
1	4	2	103.89	5.60
1	4	2	89.09	4.70
2	4	2	23.70	3.20
2	4	2	23.70	1.70
3	4	2	3.10	1.10
3	4	2	5.50	2.40
-4	-4	-2	10.30	3.40
4	4	2	3.90	1.60

4	4	2	0.90	2.60
-5	-4	-2	21.80	4.00
5	4	2	15.50	5.10
5	4	2	12.20	2.50
-6	-4	-2	101.19	8.30
-6	-4	-2	102.19	10.20
-6	-4	-2	96.59	13.50
-6	-4	-2	99.69	13.00
-6	-4	-2	87.49	10.70
6	4	2	110.49	7.50
-7	-4	-2	246.48	14.30
-7	-4	-2	241.78	17.40
-7	-4	-2	231.28	15.30
-7	-4	-2	245.38	15.60
-7	-4	-2	237.68	18.10
-7	-4	-2	239.88	18.10
7	4	2	238.58	13.70
-8	-4	-2	41.60	6.50
-8	-4	-2	44.40	9.20
-8	-4	-2	60.79	8.30
-8	-4	-2	77.89	14.20
-8	-4	-2	45.40	8.20
-8	-4	-2	58.89	7.80
8	4	2	57.19	9.10
-9	-4	-2	8.00	7.20
-9	-4	-2	7.10	6.40
-9	-4	-2	5.50	8.30
-9	-4	-2	-2.30	8.50
-9	-4	-2	11.00	6.20
-9	-4	-2	12.70	9.50
9	4	2	13.60	8.50
-10	-4	-2	472.55	26.10
-10	-4	-2	465.75	26.70
-10	-4	-2	493.45	26.30
-10	-4	-2	440.06	28.60
-10	-4	-2	439.26	28.20
10	4	2	555.14	41.70
10	4	2	471.35	29.40
-11	-4	-2	161.68	17.60
-11	-4	-2	207.28	17.70
-11	-4	-2	240.88	16.80
-11	-4	-2	225.58	22.80
11	4	2	192.58	20.60
-12	-4	-2	28.50	11.70

-12	-4	-2	29.90	15.00
-12	-4	-2	11.90	7.10
12	4	2	49.80	13.40
12	-5	-2	82.89	12.50
-12	5	2	111.99	14.80
11	-5	-2	24.50	10.30
-11	5	2	31.10	9.70
10	-5	-2	124.49	16.90
10	-5	-2	107.69	19.30
-10	5	2	111.19	18.90
9	-5	-2	68.39	11.70
9	-5	-2	81.19	14.90
-9	5	2	78.89	11.30
-9	5	2	66.99	15.20
-9	5	2	72.69	11.60
-9	5	2	85.19	13.60
8	-5	-2	414.56	24.40
8	-5	-2	405.36	25.20
-8	5	2	384.36	23.10
-8	5	2	399.66	25.10
-8	5	2	387.96	24.70
-8	5	2	385.66	28.40
7	-5	-2	94.89	13.80
-7	5	2	114.59	15.30
-7	5	2	121.59	11.80
-7	5	2	139.39	13.80
-7	5	2	121.99	27.90
6	-5	-2	110.69	16.80
6	-5	-2	134.79	13.50
-6	5	2	122.69	13.50
-6	5	2	121.59	10.60
-6	5	2	97.39	15.30
5	-5	-2	1096.49	53.19
5	-5	-2	1169.88	53.89
5	-5	-2	1107.19	54.89
-5	5	2	1186.28	54.69
-5	5	2	1149.98	52.99
-5	5	2	1204.08	53.49
-5	5	2	1135.79	54.59
4	-5	-2	333.77	17.50
4	-5	-2	358.26	18.60
4	-5	-2	356.76	18.00
-4	5	2	351.66	18.30
-4	5	2	354.56	16.90

-4	5	2	346.57	18.30
-4	5	2	305.47	18.00
3	-5	-2	9.50	3.50
3	-5	-2	8.90	3.30
3	-5	-2	10.30	3.00
-3	5	2	9.10	3.00
-3	5	2	12.40	3.20
-3	5	2	11.50	2.10
2	-5	-2	826.82	37.80
2	-5	-2	743.53	36.90
2	-5	-2	791.72	37.20
-2	5	2	818.22	36.60
-2	5	2	837.42	37.40
-2	5	2	829.22	37.70
1	-5	-2	14.10	3.30
-1	5	2	8.00	1.90
-1	5	2	10.40	3.40
-1	5	2	11.10	1.60
0	-5	-2	490.25	25.50
0	-5	-2	564.74	24.80
0	5	2	582.04	24.90
0	5	2	519.95	26.00
0	5	2	531.15	24.20
-1	-5	-2	173.48	9.70
-1	-5	-2	179.88	8.80
1	5	2	181.08	8.80
1	5	2	185.48	9.70
2	5	2	248.28	11.30
2	5	2	249.28	11.50
2	5	2	260.47	12.70
2	5	2	234.38	11.20
3	5	2	86.79	6.10
3	5	2	75.89	4.30
-4	-5	-2	112.99	8.30
4	5	2	132.79	9.10
4	5	2	121.79	6.70
4	5	2	136.99	9.10
-5	-5	-2	320.57	17.00
5	5	2	314.57	18.80
5	5	2	350.76	19.20
5	5	2	329.07	16.10
-6	-5	-2	3059.89	138.29
-6	-5	-2	3073.69	137.69
-6	-5	-2	2961.80	137.49

-6	-5	-2	2965.90	137.89
-6	-5	-2	2949.00	137.19
-6	-5	-2	3074.49	137.19
6	5	2	3169.78	136.69
-7	-5	-2	14.30	7.40
-7	-5	-2	11.40	6.90
-7	-5	-2	13.60	4.60
-7	-5	-2	15.10	5.60
-7	-5	-2	19.50	7.30
-7	-5	-2	10.50	6.30
7	5	2	11.90	4.10
-8	-5	-2	46.80	7.40
-8	-5	-2	35.10	7.10
-8	-5	-2	31.00	6.00
-8	-5	-2	36.30	8.20
-8	-5	-2	22.50	8.60
8	5	2	28.40	6.20
-9	-5	-2	165.78	18.50
-9	-5	-2	165.58	13.50
-9	-5	-2	167.18	14.90
-9	-5	-2	144.89	15.90
-9	-5	-2	178.68	14.50
-9	-5	-2	167.28	16.50
9	5	2	161.48	19.10
-10	-5	-2	28.80	7.70
-10	-5	-2	29.30	8.20
-10	-5	-2	41.60	10.50
-10	-5	-2	22.90	9.90
-10	-5	-2	22.50	7.90
10	5	2	42.10	11.10
10	5	2	56.59	15.50
-11	-5	-2	42.10	8.30
-11	-5	-2	68.69	13.70
-11	-5	-2	65.89	10.70
11	5	2	55.49	12.40
-12	-5	-2	27.50	7.60
-12	-5	-2	26.10	12.00
-12	-5	-2	45.70	15.50
12	5	2	35.80	13.90
12	-6	-2	9.80	17.50
-12	6	2	6.60	10.80
11	-6	-2	35.20	10.30
-11	6	2	58.79	11.10
10	-6	-2	69.59	13.60

10	-6	-2	58.89	12.60
-10	6	2	69.69	14.50
9	-6	-2	35.20	9.70
9	-6	-2	28.40	10.40
-9	6	2	8.10	15.70
-9	6	2	21.90	11.30
-9	6	2	20.60	10.90
-9	6	2	13.60	8.90
8	-6	-2	43.40	10.00
8	-6	-2	50.09	8.70
-8	6	2	92.69	15.40
-8	6	2	62.49	10.30
-8	6	2	48.00	9.20
7	-6	-2	667.73	43.40
7	-6	-2	683.33	38.10
-7	6	2	710.93	35.40
-7	6	2	720.53	36.60
-7	6	2	743.93	37.30
6	-6	-2	403.06	22.50
-6	6	2	409.16	23.70
-6	6	2	363.26	23.70
-6	6	2	394.86	21.40
5	-6	-2	92.49	12.80
5	-6	-2	102.99	14.80
5	-6	-2	80.59	11.30
-5	6	2	98.59	12.20
-5	6	2	112.89	12.90
-5	6	2	84.59	8.70
-5	6	2	104.09	10.00
-5	6	2	98.79	13.00
4	-6	-2	759.12	38.40
4	-6	-2	827.62	37.80
4	-6	-2	748.63	37.20
-4	6	2	792.52	38.20
-4	6	2	826.52	38.00
-4	6	2	756.02	37.30
-4	6	2	796.92	36.40
3	-6	-2	108.19	7.90
3	-6	-2	103.59	9.20
3	-6	-2	114.19	9.00
-3	6	2	103.39	8.60
-3	6	2	109.49	8.10
-3	6	2	109.79	6.50
2	-6	-2	12.60	3.80

-2	6	2	14.50	2.30
-2	6	2	9.30	2.90
-2	6	2	15.50	4.10
1	-6	-2	2147.09	99.89
1	-6	-2	2286.37	99.19
-1	6	2	2202.48	100.49
-1	6	2	2244.98	99.19
-1	6	2	2137.59	98.39
0	-6	-2	136.79	7.50
0	-6	-2	144.09	9.00
0	6	2	152.38	9.80
0	6	2	138.39	7.50
0	6	2	132.19	6.70
-1	-6	-2	3227.38	139.89
-1	-6	-2	3063.19	140.79
1	6	2	2983.80	139.59
1	6	2	3091.39	139.89
1	6	2	3299.27	140.99
-2	-6	-2	6.20	1.40
2	6	2	9.10	1.10
2	6	2	6.10	1.40
2	6	2	5.50	2.50
3	6	2	679.83	32.10
3	6	2	738.63	33.70
-4	-6	-2	4.40	3.10
4	6	2	5.20	1.90
4	6	2	10.60	3.90
-5	-6	-2	14.70	4.00
5	6	2	1.30	5.40
5	6	2	2.30	5.90
5	6	2	7.90	2.60
-6	-6	-2	393.26	23.30
-6	-6	-2	398.06	21.40
-6	-6	-2	387.96	22.10
-6	-6	-2	365.66	21.10
-6	-6	-2	392.56	21.70
-6	-6	-2	405.06	20.20
6	6	2	412.76	19.90
-7	-6	-2	538.55	27.40
-7	-6	-2	505.85	26.90
-7	-6	-2	507.15	25.60
-7	-6	-2	499.15	28.00
-7	-6	-2	486.95	26.30
-7	-6	-2	500.45	26.40

7	6	2	507.55	25.40
-8	-6	-2	42.00	8.10
-8	-6	-2	24.60	9.10
-8	-6	-2	34.50	7.30
-8	-6	-2	36.40	7.60
-8	-6	-2	30.00	8.10
-8	-6	-2	33.20	6.60
8	6	2	28.20	6.60
-9	-6	-2	54.69	8.90
-9	-6	-2	73.19	11.80
-9	-6	-2	42.40	7.40
-9	-6	-2	66.99	9.80
-9	-6	-2	45.90	7.60
9	6	2	68.29	12.00
-10	-6	-2	17.80	8.50
-10	-6	-2	40.20	8.40
-10	-6	-2	22.80	9.90
-10	-6	-2	27.90	7.80
-10	-6	-2	24.20	10.50
10	6	2	27.20	15.80
10	6	2	44.10	11.70
-11	-6	-2	269.07	19.00
-11	-6	-2	252.07	17.60
-11	-6	-2	261.17	23.30
-11	-6	-2	254.47	22.90
-11	-6	-2	224.28	19.30
11	6	2	230.58	23.60
-12	-6	-2	36.80	7.80
-12	-6	-2	34.10	14.20
-12	-6	-2	15.90	16.30
11	-7	-2	125.99	16.70
-11	7	2	136.79	17.70
10	-7	-2	206.98	18.70
10	-7	-2	221.38	21.10
-10	7	2	214.98	21.50
9	-7	-2	199.28	19.20
9	-7	-2	214.38	18.50
-9	7	2	166.48	19.20
-9	7	2	187.88	20.10
-9	7	2	198.48	20.30
8	-7	-2	5.40	7.50
8	-7	-2	14.10	9.10
-8	7	2	10.20	10.40
-8	7	2	5.30	8.20

-8	7	2	44.40	15.30
-8	7	2	10.70	8.90
7	-7	-2	23.60	7.80
7	-7	-2	32.70	9.10
-7	7	2	26.60	7.40
-7	7	2	24.40	9.50
-7	7	2	26.40	9.00
6	-7	-2	1372.96	62.39
-6	7	2	1356.06	63.59
-6	7	2	1311.07	61.99
-6	7	2	1255.87	62.89
5	-7	-2	66.69	11.60
5	-7	-2	58.69	11.80
-5	7	2	63.19	10.20
-5	7	2	87.89	9.70
4	-7	-2	79.39	10.10
4	-7	-2	66.09	12.20
-4	7	2	76.19	9.60
-4	7	2	78.79	9.00
-4	7	2	87.49	6.40
-4	7	2	83.89	11.10
3	-7	-2	-2.30	4.90
3	-7	-2	4.60	3.90
-3	7	2	6.10	5.10
-3	7	2	2.50	4.90
-3	7	2	0.80	3.50
-3	7	2	8.00	2.30
2	-7	-2	27.50	5.60
2	-7	-2	25.60	6.60
-2	7	2	9.60	8.70
-2	7	2	29.60	4.10
-2	7	2	21.70	2.90
1	-7	-2	840.32	40.60
1	-7	-2	857.41	39.40
-1	7	2	849.72	41.30
-1	7	2	831.02	38.50
-1	7	2	879.31	39.50
0	-7	-2	105.09	8.10
0	-7	-2	101.09	6.30
0	7	2	103.99	6.20
0	7	2	97.19	5.20
0	7	2	100.59	9.10
-1	-7	-2	341.97	16.50
-1	-7	-2	309.67	17.80

1	7	2	349.07	15.80
1	7	2	360.36	16.50
1	7	2	353.96	18.20
-2	-7	-2	693.03	32.20
2	7	2	704.93	32.20
2	7	2	695.23	31.80
2	7	2	729.13	33.80
3	7	2	7.10	1.60
3	7	2	7.30	1.30
-4	-7	-2	977.30	45.10
4	7	2	976.40	44.40
4	7	2	973.90	46.90
-5	-7	-2	895.81	41.20
-5	-7	-2	883.61	44.40
-5	-7	-2	934.51	44.80
5	7	2	847.92	43.60
5	7	2	892.71	40.70
5	7	2	830.02	43.90
-6	-7	-2	254.87	14.40
-6	-7	-2	260.07	15.90
-6	-7	-2	262.87	18.20
-6	-7	-2	252.97	16.00
-6	-7	-2	262.87	16.70
-6	-7	-2	250.67	16.30
6	7	2	254.57	14.10
-7	-7	-2	343.37	19.40
-7	-7	-2	361.46	18.70
-7	-7	-2	340.27	19.80
-7	-7	-2	347.77	20.70
-7	-7	-2	327.47	21.90
7	7	2	342.07	18.70
-8	-7	-2	208.28	15.40
-8	-7	-2	240.18	18.80
-8	-7	-2	218.08	16.40
-8	-7	-2	211.78	14.00
-8	-7	-2	220.98	15.20
-8	-7	-2	191.58	15.70
8	7	2	213.38	15.30
8	7	2	179.68	22.40
-9	-7	-2	8.30	7.20
-9	-7	-2	6.60	9.20
-9	-7	-2	10.60	8.60
-9	-7	-2	6.50	6.40
-9	-7	-2	15.00	7.50

9	7	2	9.40	9.40
9	7	2	0.60	11.40
-10	-7	-2	16.70	10.30
-10	-7	-2	20.80	9.40
-10	-7	-2	45.20	9.00
-10	-7	-2	29.20	7.30
-10	-7	-2	36.80	8.70
10	7	2	36.90	14.20
10	7	2	30.80	17.60
-11	-7	-2	108.99	13.00
-11	-7	-2	134.19	19.20
-11	-7	-2	106.59	15.70
-11	-7	-2	98.49	15.00
-11	-7	-2	106.29	13.80
11	7	2	104.59	22.10
-12	-7	-2	265.27	16.90
-12	-7	-2	198.68	29.10
11	-8	-2	210.68	18.40
11	-8	-2	163.28	30.40
-11	8	2	185.08	19.60
10	-8	-2	61.89	11.10
10	-8	-2	49.30	11.20
-10	8	2	41.20	12.30
9	-8	-2	22.80	9.70
9	-8	-2	20.40	9.50
-9	8	2	45.50	12.70
-9	8	2	17.60	11.60
-9	8	2	3.80	10.10
8	-8	-2	137.29	15.80
8	-8	-2	145.59	15.30
-8	8	2	110.69	15.50
-8	8	2	117.09	18.10
-8	8	2	61.39	24.90
-8	8	2	99.99	13.20
7	-8	-2	33.60	7.00
7	-8	-2	27.20	9.30
-7	8	2	36.10	9.30
-7	8	2	41.60	8.70
-7	8	2	41.60	10.00
6	-8	-2	38.10	9.30
6	-8	-2	41.30	7.00
-6	8	2	42.20	9.80
-6	8	2	32.40	7.40
-6	8	2	25.30	8.40

5	-8	-2	9.60	11.70
5	-8	-2	7.40	6.70
5	-8	-2	2.40	12.60
-5	8	2	21.70	10.40
-5	8	2	19.00	5.40
4	-8	-2	90.59	11.90
4	-8	-2	98.29	15.50
4	-8	-2	88.69	11.60
-4	8	2	66.19	10.60
-4	8	2	78.49	6.50
-4	8	2	70.59	9.90
-4	8	2	74.89	10.50
3	-8	-2	18.50	7.10
3	-8	-2	11.70	4.90
-3	8	2	9.40	2.40
-3	8	2	12.10	6.80
-3	8	2	9.80	4.30
-3	8	2	11.00	5.70
2	-8	-2	234.78	15.80
-2	8	2	200.88	16.10
-2	8	2	244.18	13.40
-2	8	2	265.07	17.20
-2	8	2	247.08	11.90
1	-8	-2	325.47	18.60
1	-8	-2	319.37	17.00
-1	8	2	367.86	20.40
-1	8	2	322.77	15.80
-1	8	2	345.87	17.00
0	-8	-2	3926.31	178.78
0	8	2	3700.13	177.88
0	8	2	4393.16	180.68
0	8	2	3962.10	178.78
-1	-8	-2	13.30	2.80
-1	-8	-2	14.90	4.60
1	8	2	19.00	2.70
1	8	2	22.20	5.40
1	8	2	16.10	2.00
-2	-8	-2	470.85	22.50
2	8	2	476.95	22.00
2	8	2	492.95	22.50
3	8	2	113.59	6.20
3	8	2	109.59	6.00
-4	-8	-2	215.38	12.00
4	8	2	200.08	10.90

4	8	2	222.78	15.30
-5	-8	-2	6.30	6.50
-5	-8	-2	15.90	4.40
-5	-8	-2	9.80	5.00
-5	-8	-2	0.50	9.50
-5	-8	-2	8.60	6.00
-5	-8	-2	27.70	7.10
-6	-8	-2	55.69	7.50
-6	-8	-2	65.19	10.70
-6	-8	-2	73.99	13.30
-6	-8	-2	47.10	6.90
-6	-8	-2	53.89	7.40
-6	-8	-2	52.59	9.30
6	8	2	60.59	6.90
-7	-8	-2	200.88	23.00
-7	-8	-2	175.58	13.60
-7	-8	-2	172.88	19.50
-7	-8	-2	182.88	13.60
-7	-8	-2	177.08	12.20
7	8	2	180.28	12.50
-8	-8	-2	205.18	16.00
-8	-8	-2	166.08	14.60
-8	-8	-2	183.48	13.00
-8	-8	-2	161.58	14.00
-8	-8	-2	178.88	14.30
8	8	2	168.08	14.70
-9	-8	-2	15.30	7.80
-9	-8	-2	19.10	7.40
-9	-8	-2	26.80	9.20
-9	-8	-2	9.70	7.60
-9	-8	-2	32.20	7.50
-10	-8	-2	496.85	31.80
-10	-8	-2	484.55	28.50
-10	-8	-2	515.75	27.90
-10	-8	-2	473.35	27.40
-10	-8	-2	507.15	29.70
-11	-8	-2	97.59	15.70
-11	-8	-2	68.49	12.30
-11	-8	-2	84.69	12.60
-11	-8	-2	88.29	15.70
-11	-8	-2	110.69	12.90
-12	-8	-2	40.70	8.30
-12	-8	-2	34.30	12.90
11	-9	-2	17.10	16.20

11	-9	-2	-5.70	10.40
-11	9	2	-9.70	10.30
10	-9	-2	-1.10	10.60
10	-9	-2	2.60	9.50
-10	9	2	12.10	10.90
9	-9	-2	15.60	9.50
9	-9	-2	47.30	10.50
-9	9	2	25.90	13.20
-9	9	2	25.50	12.00
8	-9	-2	159.88	17.20
8	-9	-2	170.68	16.20
-8	9	2	165.88	20.90
-8	9	2	139.49	19.10
-8	9	2	181.58	25.70
7	-9	-2	175.68	17.50
7	-9	-2	231.58	16.70
-7	9	2	191.98	19.30
-7	9	2	207.38	21.60
-7	9	2	221.08	17.50
6	-9	-2	17.40	13.80
6	-9	-2	20.50	9.30
-6	9	2	37.40	8.10
-6	9	2	13.50	10.20
5	-9	-2	737.23	40.90
5	-9	-2	817.92	39.30
5	-9	-2	771.12	41.00
-5	9	2	841.82	40.00
-5	9	2	833.82	38.30
-5	9	2	792.02	41.80
4	-9	-2	51.49	9.90
4	-9	-2	76.99	11.70
4	-9	-2	62.69	18.90
-4	9	2	57.59	8.20
-4	9	2	92.39	13.90
-4	9	2	60.09	16.30
-4	9	2	69.39	6.60
3	-9	-2	2.80	8.90
3	-9	-2	7.30	6.00
-3	9	2	7.80	9.40
-3	9	2	-4.20	4.70
-3	9	2	12.10	6.70
-3	9	2	2.80	2.40
2	-9	-2	1601.44	73.39
-2	9	2	1562.64	71.69

-2	9	2	1619.44	70.29
-2	9	2	1572.84	74.59
-2	9	2	1417.66	72.49
1	-9	-2	0.80	5.80
-1	9	2	8.80	6.80
-1	9	2	3.00	1.60
-1	9	2	-0.70	3.10
0	-9	-2	111.59	8.20
0	-9	-2	115.99	10.70
0	9	2	135.99	12.20
0	9	2	115.99	6.30
0	9	2	110.49	7.60
-1	-9	-2	449.16	21.90
-1	-9	-2	418.36	24.10
1	9	2	465.25	20.90
1	9	2	439.86	24.50
1	9	2	471.25	21.80
-2	-9	-2	22.00	6.40
-2	-9	-2	16.60	3.40
2	9	2	20.10	2.60
2	9	2	23.90	3.90
-3	-9	-2	19.90	5.20
3	9	2	22.70	3.10
3	9	2	24.80	3.60
-4	-9	-2	9.20	3.90
4	9	2	4.40	2.50
4	9	2	1.20	7.20
-5	-9	-2	832.32	43.40
-5	-9	-2	835.32	41.10
-5	-9	-2	930.01	40.60
-5	-9	-2	839.82	41.30
-5	-9	-2	818.82	40.80
-5	-9	-2	835.12	41.30
5	9	2	892.11	40.10
-6	-9	-2	780.92	38.60
-6	-9	-2	813.82	37.90
-6	-9	-2	778.42	38.80
-6	-9	-2	787.32	40.10
-6	-9	-2	786.22	38.20
6	9	2	788.42	37.70
-7	-9	-2	265.67	17.10
-7	-9	-2	260.47	15.80
-7	-9	-2	261.97	17.80
-7	-9	-2	251.37	16.50

-7	-9	-2	282.67	17.00
7	9	2	250.07	16.20
-8	-9	-2	22.90	6.60
-8	-9	-2	24.80	8.00
-8	-9	-2	35.70	7.80
-8	-9	-2	40.50	7.60
-8	-9	-2	26.90	7.20
8	9	2	21.00	8.10
-9	-9	-2	310.27	20.40
-9	-9	-2	355.56	22.20
-9	-9	-2	296.27	20.00
-9	-9	-2	352.06	21.20
-9	-9	-2	337.77	20.70
9	9	2	287.27	24.30
-10	-9	-2	16.50	9.90
-10	-9	-2	34.80	17.90
-10	-9	-2	12.70	8.80
-10	-9	-2	8.30	11.10
-11	-9	-2	92.79	12.60
-11	-9	-2	78.99	12.80
-11	-9	-2	63.09	17.60
-11	-9	-2	117.49	15.20
-11	-9	-2	80.69	11.60
-12	-9	-2	-7.10	13.10
-12	-9	-2	11.00	7.50
12	9	2	23.20	10.80
11	-10	-2	22.80	14.20
11	-10	-2	1.80	13.90
-11	10	2	-13.10	10.50
10	-10	-2	208.38	18.30
10	-10	-2	172.18	18.20
-10	10	2	128.39	20.00
9	-10	-2	3.90	10.90
9	-10	-2	15.60	11.30
-9	10	2	2.00	13.90
-9	10	2	38.10	14.50
-9	10	2	19.40	18.20
8	-10	-2	17.70	8.10
8	-10	-2	21.90	9.90
-8	10	2	27.60	11.90
-8	10	2	13.50	11.00
-8	10	2	8.40	12.20
7	-10	-2	835.12	40.80
7	-10	-2	779.72	41.60

-7	10	2	887.11	46.10
-7	10	2	792.02	42.10
-7	10	2	788.42	42.80
6	-10	-2	240.08	22.00
-6	10	2	284.07	23.10
-6	10	2	235.18	19.10
5	-10	-2	-12.10	14.10
5	-10	-2	30.60	11.50
5	-10	-2	2.40	5.70
5	-10	-2	-3.50	9.30
-5	10	2	1.60	7.60
-5	10	2	-5.60	6.10
-5	10	2	-0.80	10.80
4	-10	-2	21.00	15.00
4	-10	-2	0.70	6.30
4	-10	-2	8.60	7.60
-4	10	2	11.90	7.10
-4	10	2	13.90	4.40
-4	10	2	32.50	11.80
3	-10	-2	59.79	8.70
3	-10	-2	79.79	13.40
3	-10	-2	60.89	11.50
3	-10	-2	91.29	9.90
-3	10	2	95.29	12.10
-3	10	2	90.89	6.30
-3	10	2	52.09	13.50
2	-10	-2	-4.40	7.90
2	-10	-2	11.70	6.30
-2	10	2	15.90	10.50
-2	10	2	5.40	4.40
-2	10	2	10.70	6.50
-2	10	2	9.40	2.50
1	-10	-2	1021.10	48.60
-1	10	2	931.11	46.60
-1	10	2	1079.09	50.19
-1	10	2	952.20	45.20
0	-10	-2	1390.86	67.59
0	10	2	1375.26	64.59
0	10	2	1433.16	65.89
0	10	2	1542.55	68.69
-1	-10	-2	905.21	42.50
-1	-10	-2	804.92	44.50
1	10	2	926.01	45.30
1	10	2	948.41	42.30

1	10	2	943.51	41.30
-2	-10	-2	70.09	13.00
-2	-10	-2	92.19	7.70
2	10	2	93.09	5.50
2	10	2	88.99	6.80
-3	-10	-2	206.98	12.20
3	10	2	195.78	10.20
3	10	2	182.98	10.40
3	10	2	188.08	17.20
-4	-10	-2	115.99	11.30
-4	-10	-2	101.39	12.90
-4	-10	-2	105.09	12.50
-4	-10	-2	101.79	11.00
-4	-10	-2	137.59	13.20
4	10	2	131.39	7.90
-5	-10	-2	74.99	8.90
-5	-10	-2	60.49	8.20
-5	-10	-2	59.99	7.70
-5	-10	-2	56.29	9.40
-5	-10	-2	58.79	7.80
-5	-10	-2	56.49	9.40
5	10	2	56.49	6.60
-6	-10	-2	372.56	21.40
-6	-10	-2	380.86	19.80
-6	-10	-2	329.47	20.50
-6	-10	-2	363.66	21.00
-6	-10	-2	380.36	28.90
-6	-10	-2	366.66	20.40
6	10	2	364.86	19.80
-7	-10	-2	451.05	25.50
-7	-10	-2	418.06	24.10
-7	-10	-2	443.26	25.00
-7	-10	-2	456.65	24.80
-7	-10	-2	476.35	23.90
7	10	2	447.86	24.60
-8	-10	-2	4.90	6.20
-8	-10	-2	6.20	7.00
-8	-10	-2	7.70	7.00
-8	-10	-2	7.20	7.20
-8	-10	-2	14.50	7.90
8	10	2	-3.30	8.30
-9	-10	-2	116.39	13.40
-9	-10	-2	123.09	15.30
-9	-10	-2	85.89	13.90

-9	-10	-2	128.69	14.10
-9	-10	-2	115.79	13.70
9	10	2	88.89	15.00
-10	-10	-2	3.60	8.10
-10	-10	-2	-1.10	9.50
-10	-10	-2	5.00	9.90
-10	-10	-2	20.00	8.10
-10	-10	-2	2.70	9.30
-11	-10	-2	244.48	16.80
-11	-10	-2	231.38	19.20
-11	-10	-2	217.58	20.10
-11	-10	-2	260.97	18.20
-11	-10	-2	210.18	18.90
-12	-10	-2	22.80	13.60
-12	-10	-2	49.60	15.90
-12	-10	-2	50.69	14.40
12	10	2	14.70	15.80
10	-11	-2	136.89	16.20
10	-11	-2	98.09	17.20
-10	11	2	103.29	18.40
9	-11	-2	46.40	9.70
9	-11	-2	46.00	11.40
-9	11	2	68.49	14.40
-9	11	2	44.00	15.20
-9	11	2	51.29	19.00
8	-11	-2	18.20	7.90
8	-11	-2	36.60	10.80
-8	11	2	36.10	12.70
-8	11	2	25.40	12.80
-8	11	2	17.10	13.10
7	-11	-2	70.99	12.20
7	-11	-2	53.19	11.80
-7	11	2	75.89	13.70
-7	11	2	73.69	13.30
-7	11	2	77.69	15.00
6	-11	-2	834.02	43.80
6	-11	-2	882.21	41.90
6	-11	-2	816.02	43.50
-6	11	2	851.21	46.60
-6	11	2	852.11	45.20
-6	11	2	816.82	43.70
5	-11	-2	13.40	9.40
5	-11	-2	-0.50	12.30
5	-11	-2	-7.70	15.40

-5	11	2	21.20	9.10
-5	11	2	11.50	11.40
4	-11	-2	142.39	25.80
4	-11	-2	199.98	14.90
4	-11	-2	188.18	16.40
-4	11	2	205.98	12.80
-4	11	2	215.58	17.30
-4	11	2	203.88	21.30
3	-11	-2	10.20	14.90
3	-11	-2	-11.60	7.10
3	-11	-2	-4.10	9.50
3	-11	-2	-2.80	7.10
-3	11	2	2.60	3.10
-3	11	2	7.70	8.40
-3	11	2	-15.70	11.60
2	-11	-2	94.99	13.30
2	-11	-2	130.79	13.10
2	-11	-2	123.99	20.00
-2	11	2	109.39	12.80
-2	11	2	95.29	15.80
-2	11	2	104.79	7.10
1	-11	-2	1065.29	55.29
1	-11	-2	1192.78	57.49
-1	11	2	1114.39	55.09
-1	11	2	1201.58	53.39
-1	11	2	1298.67	59.49
0	-11	-2	11.70	8.30
0	-11	-2	3.90	5.70
0	11	2	17.40	4.60
0	11	2	0.00	8.60
0	11	2	9.00	2.30
-1	-11	-2	239.68	18.90
-1	-11	-2	259.87	15.10
1	11	2	255.97	12.50
1	11	2	243.58	20.20
1	11	2	248.08	14.30
-2	-11	-2	140.79	18.70
-2	-11	-2	208.58	12.70
-2	-11	-2	175.88	12.50
2	11	2	149.39	19.10
2	11	2	200.38	9.80
2	11	2	193.68	11.40
-3	-11	-2	18.50	5.80
-3	-11	-2	41.90	9.00

-3	-11	-2	8.50	6.50
-3	-11	-2	21.40	7.60
-3	-11	-2	17.20	5.30
-3	-11	-2	21.30	7.90
3	11	2	19.60	3.30
-4	-11	-2	1000.40	45.20
-4	-11	-2	977.40	46.10
-4	-11	-2	933.41	45.30
-4	-11	-2	915.31	45.90
-4	-11	-2	933.01	45.50
-4	-11	-2	934.51	46.20
4	11	2	970.60	44.20
-5	-11	-2	273.47	17.40
-5	-11	-2	299.27	18.20
-5	-11	-2	287.37	17.40
-5	-11	-2	274.97	18.10
-5	-11	-2	295.87	19.80
-5	-11	-2	271.27	16.30
5	11	2	270.47	15.50
-6	-11	-2	390.46	22.90
-6	-11	-2	329.07	24.20
-6	-11	-2	337.37	23.60
-6	-11	-2	323.47	25.00
-6	-11	-2	333.37	19.30
6	11	2	336.47	19.20
-7	-11	-2	18.20	7.00
-7	-11	-2	9.10	7.80
-7	-11	-2	3.30	5.90
-7	-11	-2	12.90	6.50
-7	-11	-2	12.50	7.00
7	11	2	-0.90	7.00
-8	-11	-2	402.96	23.50
-8	-11	-2	422.56	24.10
-8	-11	-2	373.66	29.30
-8	-11	-2	419.66	24.30
-8	-11	-2	392.56	23.30
8	11	2	372.06	25.70
-9	-11	-2	58.19	9.80
-9	-11	-2	88.29	13.20
-9	-11	-2	95.09	15.00
-9	-11	-2	87.69	13.20
-9	-11	-2	64.49	9.70
9	11	2	54.29	15.10
-10	-11	-2	35.70	10.70

-10	-11	-2	89.19	13.10
-10	-11	-2	64.49	9.40
-10	-11	-2	61.59	10.70
-10	-11	-2	48.60	10.90
-11	-11	-2	54.49	13.70
-11	-11	-2	84.49	12.40
-11	-11	-2	96.79	12.50
-11	-11	-2	83.09	12.90
11	11	2	70.89	13.10
10	-12	-2	14.00	16.80
10	-12	-2	15.80	9.40
-10	12	2	22.70	11.10
9	-12	-2	-2.50	11.40
9	-12	-2	0.60	7.50
-9	12	2	-10.60	14.30
-9	12	2	-2.70	11.40
8	-12	-2	124.59	14.20
-8	12	2	118.99	20.60
-8	12	2	85.99	16.00
7	-12	-2	-4.00	21.10
7	-12	-2	11.90	11.60
-7	12	2	7.80	12.70
-7	12	2	40.60	13.60
-7	12	2	19.70	12.10
6	-12	-2	334.97	20.40
6	-12	-2	342.07	24.10
6	-12	-2	298.47	25.10
-6	12	2	337.27	27.00
-6	12	2	306.57	27.90
-6	12	2	314.17	24.50
5	-12	-2	3.60	6.90
5	-12	-2	8.40	8.40
-5	12	2	-3.40	11.30
-5	12	2	-1.10	9.40
4	-12	-2	30.70	7.40
4	-12	-2	11.80	8.20
4	-12	-2	16.00	15.80
-4	12	2	2.30	8.40
-4	12	2	3.60	11.60
-4	12	2	14.60	5.70
3	-12	-2	-6.50	11.60
3	-12	-2	31.70	13.70
3	-12	-2	3.00	6.40
3	-12	-2	-11.20	6.50

-3	12	2	-16.50	10.80
-3	12	2	0.50	7.00
-3	12	2	5.40	6.60
2	-12	-2	13.10	8.60
2	-12	-2	12.20	9.40
2	-12	-2	-1.50	7.20
2	-12	-2	0.90	11.80
2	-12	-2	4.10	8.70
-2	12	2	2.30	3.10
-2	12	2	1.60	7.10
1	-12	-2	1695.13	83.69
1	-12	-2	1695.23	81.09
1	-12	-2	1697.03	80.99
1	-12	-2	1861.31	82.19
-1	12	2	1821.52	79.49
0	-12	-2	1488.45	72.29
0	-12	-2	1593.24	72.29
0	-12	-2	1489.15	71.89
0	-12	-2	1511.15	71.59
0	-12	-2	1615.44	72.59
0	12	2	1634.44	70.09
0	12	2	1481.25	71.69
-1	-12	-2	143.59	13.80
-1	-12	-2	184.38	15.70
-1	-12	-2	166.28	15.20
-1	-12	-2	162.18	14.20
-1	-12	-2	175.58	12.00
-1	-12	-2	166.48	13.60
1	12	2	162.88	9.10
1	12	2	147.09	12.10
-2	-12	-2	-5.90	7.80
-2	-12	-2	2.00	6.10
-2	-12	-2	16.00	6.20
-2	-12	-2	5.80	5.60
-2	-12	-2	11.10	8.00
-2	-12	-2	5.20	9.00
2	12	2	9.10	5.10
2	12	2	8.90	2.80
-3	-12	-2	80.19	14.60
-3	-12	-2	75.79	12.80
-3	-12	-2	94.09	9.80
-3	-12	-2	98.79	10.40
-3	-12	-2	91.49	11.60
-3	-12	-2	73.39	12.40

3	12	2	99.19	6.70
-4	-12	-2	-1.60	5.30
-4	-12	-2	0.40	7.00
-4	-12	-2	-5.10	6.70
-4	-12	-2	1.40	5.20
-4	-12	-2	2.50	6.50
-4	-12	-2	6.80	7.30
4	12	2	4.50	3.20
-5	-12	-2	39.80	6.50
-5	-12	-2	49.60	8.60
-5	-12	-2	44.40	7.20
-5	-12	-2	53.19	8.90
-5	-12	-2	30.60	7.30
-5	-12	-2	39.90	7.80
5	12	2	51.59	7.50
-6	-12	-2	54.79	8.80
-6	-12	-2	34.60	7.40
-6	-12	-2	40.00	8.00
-6	-12	-2	45.70	7.60
-6	-12	-2	45.70	8.90
6	12	2	39.20	7.00
-7	-12	-2	150.28	14.90
-7	-12	-2	160.58	21.80
-7	-12	-2	143.89	13.40
-7	-12	-2	152.38	13.60
-7	-12	-2	127.59	15.60
-7	-12	-2	147.79	14.70
7	12	2	137.99	15.30
-8	-12	-2	65.89	8.90
-8	-12	-2	52.49	9.70
-8	-12	-2	47.50	9.10
-8	-12	-2	64.39	10.30
-8	-12	-2	49.80	9.00
8	12	2	53.29	11.90
-9	-12	-2	32.00	9.30
-9	-12	-2	22.90	8.80
-9	-12	-2	13.00	7.80
-9	-12	-2	17.60	9.10
-9	-12	-2	26.50	9.30
9	12	2	6.90	16.70
-10	-12	-2	400.36	24.10
-10	-12	-2	403.96	25.40
-10	-12	-2	359.56	25.40
-10	-12	-2	441.16	23.50

-10	-12	-2	469.15	25.30
-11	-12	-2	9.00	7.70
-11	-12	-2	5.10	7.50
-11	-12	-2	1.50	10.80
-11	-12	-2	-10.40	12.60
11	12	2	4.60	11.70
10	-13	-2	8.70	8.40
-10	13	2	12.30	10.10
9	-13	-2	4.80	7.80
9	-13	-2	1.10	14.40
-9	13	2	-14.50	21.60
-9	13	2	9.20	10.90
8	-13	-2	25.80	8.50
8	-13	-2	41.10	13.40
-8	13	2	45.80	14.80
-8	13	2	37.50	14.60
-8	13	2	29.70	14.00
7	-13	-2	25.80	14.30
7	-13	-2	56.69	15.00
7	-13	-2	38.80	8.40
-7	13	2	51.29	14.10
-7	13	2	29.10	13.40
-7	13	2	26.50	12.70
6	-13	-2	313.47	18.90
6	-13	-2	290.37	22.30
-6	13	2	253.27	26.10
-6	13	2	231.98	25.20
-6	13	2	206.18	23.50
5	-13	-2	505.85	26.60
5	-13	-2	470.15	28.10
-5	13	2	479.15	30.30
-5	13	2	444.66	31.30
4	-13	-2	18.30	23.50
4	-13	-2	39.40	9.10
4	-13	-2	33.90	16.20
4	-13	-2	35.70	7.30
-4	13	2	48.90	10.60
-4	13	2	34.20	7.10
3	-13	-2	-1.20	6.60
3	-13	-2	13.20	14.30
3	-13	-2	8.00	11.40
3	-13	-2	10.80	7.50
-3	13	2	14.40	8.70
-3	13	2	10.30	4.50

-3	13	2	3.10	11.00
2	-13	-2	673.63	36.30
2	-13	-2	701.93	38.80
2	-13	-2	706.73	37.40
2	-13	-2	693.13	36.50
2	-13	-2	677.43	35.60
-2	13	2	727.73	33.60
-2	13	2	759.22	36.70
1	-13	-2	5.60	8.20
1	-13	-2	18.60	9.60
1	-13	-2	10.90	10.80
1	-13	-2	8.40	7.20
1	-13	-2	10.40	6.20
-1	13	2	2.50	2.90
0	-13	-2	3.10	7.70
0	-13	-2	16.20	7.20
0	-13	-2	-6.00	7.70
0	-13	-2	10.80	6.40
0	-13	-2	5.60	7.90
0	13	2	5.20	2.80
-1	-13	-2	152.88	16.10
-1	-13	-2	159.48	12.80
-1	-13	-2	118.99	12.90
-1	-13	-2	119.49	13.80
-1	-13	-2	140.39	16.90
-1	-13	-2	148.39	14.20
1	13	2	136.59	14.10
1	13	2	158.48	8.70
-2	-13	-2	-0.90	7.80
-2	-13	-2	-2.20	5.10
-2	-13	-2	-0.10	6.20
-2	-13	-2	5.30	7.20
-2	-13	-2	13.20	10.70
2	13	2	0.10	2.40
-3	-13	-2	16.00	5.80
-3	-13	-2	3.10	7.60
-3	-13	-2	9.40	6.60
-3	-13	-2	3.40	7.50
-3	-13	-2	8.80	5.80
-3	-13	-2	25.70	8.40
3	13	2	10.60	3.20
-4	-13	-2	156.88	14.50
-4	-13	-2	141.99	12.50
-4	-13	-2	167.48	14.20

-4	-13	-2	138.09	14.70
-4	-13	-2	144.69	14.60
-4	-13	-2	154.18	12.70
4	13	2	171.78	10.40
-5	-13	-2	1084.29	54.69
-5	-13	-2	1095.89	52.09
-5	-13	-2	1031.40	52.79
-5	-13	-2	1142.09	51.89
-5	-13	-2	1075.69	52.59
-5	-13	-2	1105.39	52.49
5	13	2	1075.39	51.19
-6	-13	-2	253.07	19.60
-6	-13	-2	295.67	17.60
-6	-13	-2	216.58	18.70
-6	-13	-2	279.97	19.40
-6	-13	-2	230.28	18.50
-6	-13	-2	274.97	17.20
6	13	2	243.88	17.30
-7	-13	-2	108.39	15.20
-7	-13	-2	156.48	15.60
-7	-13	-2	120.79	14.50
-7	-13	-2	112.79	13.00
-7	-13	-2	153.78	13.70
7	13	2	130.79	16.10
-8	-13	-2	86.29	13.70
-8	-13	-2	117.49	14.40
-8	-13	-2	89.99	11.40
-8	-13	-2	73.09	10.70
-8	-13	-2	72.39	11.90
8	13	2	72.89	14.20
-9	-13	-2	224.48	19.30
-9	-13	-2	214.78	18.20
-9	-13	-2	264.27	20.40
-9	-13	-2	239.98	19.80
-9	-13	-2	271.57	17.70
-10	-13	-2	31.50	7.60
-10	-13	-2	33.20	8.50
-10	-13	-2	12.50	11.70
-10	-13	-2	8.30	9.40
10	13	2	34.70	13.40
-11	-13	-2	-2.30	11.30
-11	-13	-2	1.30	7.60
-11	-13	-2	12.70	10.40
-11	-13	-2	19.20	16.60

11	13	2	4.10	12.70
9	-14	-2	15.60	7.70
-9	14	2	-1.00	10.30
8	-14	-2	68.59	12.50
-8	14	2	84.39	16.80
-8	14	2	55.19	14.60
-8	14	2	59.39	14.10
7	-14	-2	550.44	49.50
7	-14	-2	555.44	29.10
-7	14	2	492.45	33.90
-7	14	2	520.05	34.20
-7	14	2	535.25	34.70
6	-14	-2	72.89	12.30
6	-14	-2	45.50	10.80
-6	14	2	41.90	14.40
-6	14	2	77.29	14.80
-6	14	2	52.19	14.60
5	-14	-2	8.90	8.80
5	-14	-2	8.20	6.80
-5	14	2	8.40	12.00
-5	14	2	10.60	11.10
4	-14	-2	67.99	9.70
4	-14	-2	69.39	19.10
4	-14	-2	87.89	12.40
-4	14	2	86.09	13.10
-4	14	2	65.99	11.70
-4	14	2	85.59	13.80
3	-14	-2	5.70	10.80
3	-14	-2	-6.80	7.20
3	-14	-2	32.10	14.10
-3	14	2	8.70	5.20
-3	14	2	-3.90	8.30
2	-14	-2	19.40	8.70
2	-14	-2	18.90	11.20
2	-14	-2	5.50	8.90
2	-14	-2	3.60	6.80
2	-14	-2	14.60	8.90
-2	14	2	7.90	4.30
-2	14	2	7.80	7.10
1	-14	-2	356.36	21.30
1	-14	-2	282.57	23.80
1	-14	-2	314.07	20.30
1	-14	-2	335.77	22.10
1	-14	-2	335.97	20.60

-1	14	2	332.87	17.10
-1	14	2	342.07	20.20
0	-14	-2	1002.90	47.80
0	-14	-2	1032.10	48.10
0	-14	-2	964.30	47.40
0	-14	-2	950.10	47.30
0	-14	-2	960.60	47.70
0	14	2	970.90	45.40
-1	-14	-2	260.17	16.90
-1	-14	-2	253.07	17.60
-1	-14	-2	246.68	17.50
-1	-14	-2	239.98	16.50
-1	-14	-2	272.57	19.70
1	14	2	260.67	13.70
-2	-14	-2	116.59	11.80
-2	-14	-2	97.59	13.80
-2	-14	-2	116.79	12.00
-2	-14	-2	118.99	11.00
-2	-14	-2	121.49	13.20
-2	-14	-2	117.39	17.90
2	14	2	116.39	8.00
-3	-14	-2	71.39	15.20
-3	-14	-2	81.49	12.00
-3	-14	-2	81.99	10.00
-3	-14	-2	95.99	13.20
-3	-14	-2	89.19	12.30
-3	-14	-2	87.49	10.40
3	14	2	83.89	7.30
-4	-14	-2	330.67	20.70
-4	-14	-2	331.87	19.40
-4	-14	-2	328.97	24.20
-4	-14	-2	327.57	19.70
4	14	2	361.46	18.30
-5	-14	-2	36.70	8.80
-5	-14	-2	59.89	10.20
-5	-14	-2	41.80	9.00
-5	-14	-2	40.20	6.90
-5	-14	-2	36.50	8.20
-5	-14	-2	40.80	9.40
5	14	2	35.30	6.70
-6	-14	-2	211.98	17.60
-6	-14	-2	197.18	17.90
-6	-14	-2	218.78	17.90
-6	-14	-2	235.18	15.50

-6	-14	-2	198.58	17.60
-6	-14	-2	206.08	16.20
6	14	2	213.48	16.40
-7	-14	-2	199.38	16.20
-7	-14	-2	204.98	16.70
-7	-14	-2	173.48	16.80
-7	-14	-2	205.98	14.80
-7	-14	-2	170.78	17.20
-7	-14	-2	173.28	20.50
7	14	2	145.09	18.20
-8	-14	-2	10.60	8.10
-8	-14	-2	23.20	8.80
-8	-14	-2	15.80	8.70
-8	-14	-2	23.20	9.00
-8	-14	-2	14.90	6.90
8	14	2	-7.70	13.50
-9	-14	-2	20.20	8.80
-9	-14	-2	21.20	6.90
-9	-14	-2	24.00	9.90
-9	-14	-2	19.90	9.00
-9	-14	-2	38.90	9.80
9	14	2	19.70	14.00
-10	-14	-2	44.40	7.50
-10	-14	-2	26.00	11.10
-10	-14	-2	30.10	8.10
-10	-14	-2	51.09	10.50
10	14	2	75.39	15.10
-11	-14	-2	86.39	11.80
-11	-14	-2	78.99	13.80
11	14	2	66.99	15.30
9	-15	-2	10.10	7.40
-9	15	2	26.30	9.50
8	-15	-2	-11.00	6.60
-8	15	2	-0.10	16.30
-8	15	2	-0.30	10.60
-8	15	2	-3.40	13.40
7	-15	-2	172.48	24.30
7	-15	-2	191.88	16.50
-7	15	2	161.18	23.60
-7	15	2	182.48	23.00
-7	15	2	138.69	22.90
6	-15	-2	541.55	28.80
6	-15	-2	529.45	31.30
-6	15	2	491.35	32.90

-6	15	2	518.55	34.40
-6	15	2	536.25	33.50
5	-15	-2	25.60	8.10
5	-15	-2	42.70	10.20
-5	15	2	10.90	12.40
4	-15	-2	45.30	17.40
4	-15	-2	63.99	10.10
4	-15	-2	44.30	8.30
4	-15	-2	50.29	17.30
-4	15	2	69.29	12.90
-4	15	2	57.19	11.80
3	-15	-2	98.79	15.60
3	-15	-2	96.09	13.30
3	-15	-2	91.49	12.80
3	-15	-2	61.79	9.50
-3	15	2	92.99	12.70
-3	15	2	81.29	11.20
-3	15	2	72.79	11.30
2	-15	-2	404.66	24.60
2	-15	-2	444.46	27.80
2	-15	-2	453.25	26.60
2	-15	-2	376.36	25.70
-2	15	2	447.86	25.30
-2	15	2	440.06	22.30
1	-15	-2	523.55	29.00
1	-15	-2	565.04	30.90
1	-15	-2	527.05	31.70
1	-15	-2	523.35	29.70
-1	15	2	545.85	29.10
-1	15	2	561.94	26.80
0	-15	-2	118.59	13.60
0	-15	-2	106.69	13.30
0	-15	-2	108.79	14.00
0	-15	-2	111.79	13.60
0	15	2	124.39	8.70
-1	-15	-2	191.28	17.10
-1	-15	-2	187.28	14.30
-1	-15	-2	186.88	14.80
-1	-15	-2	178.98	14.60
-1	-15	-2	186.78	15.40
1	15	2	185.38	11.30
-2	-15	-2	245.58	17.60
-2	-15	-2	232.88	16.70
-2	-15	-2	238.48	20.10

2	15	2	230.78	13.60
-3	-15	-2	128.69	13.20
-3	-15	-2	160.48	12.30
-3	-15	-2	152.98	21.40
-3	-15	-2	138.69	12.60
-3	-15	-2	128.89	13.70
-3	-15	-2	130.29	14.60
3	15	2	146.69	10.20
-4	-15	-2	187.88	15.70
-4	-15	-2	214.08	17.20
-4	-15	-2	193.68	14.90
-4	-15	-2	211.28	16.30
-4	-15	-2	198.68	14.60
4	15	2	214.08	13.40
-5	-15	-2	18.40	7.20
-5	-15	-2	17.10	7.50
-5	-15	-2	32.10	7.00
-5	-15	-2	24.10	8.30
-5	-15	-2	21.40	7.90
5	15	2	24.30	6.80
-6	-15	-2	493.85	28.00
-6	-15	-2	462.05	28.20
-6	-15	-2	500.55	27.90
-6	-15	-2	496.05	26.30
-6	-15	-2	482.85	27.80
6	15	2	487.45	27.70
-7	-15	-2	17.00	8.60
-7	-15	-2	4.00	8.10
-7	-15	-2	4.90	8.20
-7	-15	-2	19.70	8.20
-7	-15	-2	1.10	5.60
-7	-15	-2	-18.00	9.70
7	15	2	3.80	10.70
7	15	2	20.90	17.10
-8	-15	-2	79.39	14.00
-8	-15	-2	100.99	13.50
-8	-15	-2	128.49	21.50
8	15	2	58.29	17.30
8	15	2	76.39	18.30
-9	-15	-2	154.68	15.50
-9	-15	-2	120.59	14.80
-9	-15	-2	120.39	15.50
-10	-15	-2	108.19	10.80
-10	-15	-2	71.29	11.30

-10	-15	-2	104.29	13.80
10	15	2	88.29	17.00
9	-16	-2	-11.90	6.90
-9	16	2	-14.60	10.70
8	-16	-2	216.98	15.00
-8	16	2	212.68	23.60
-8	16	2	165.28	17.80
7	-16	-2	14.00	7.70
7	-16	-2	15.60	14.10
-7	16	2	20.90	14.70
-7	16	2	14.60	11.30
-7	16	2	20.80	13.10
6	-16	-2	205.28	31.00
6	-16	-2	174.68	16.20
-6	16	2	210.18	27.60
-6	16	2	192.08	21.90
-6	16	2	214.08	20.90
5	-16	-2	2.60	7.30
5	-16	-2	-2.80	8.70
-5	16	2	11.30	12.00
-5	16	2	-9.50	12.20
4	-16	-2	-7.10	7.70
4	-16	-2	0.90	8.10
4	-16	-2	-15.40	18.90
4	-16	-2	14.40	16.60
-4	16	2	9.10	11.70
-4	16	2	7.00	10.70
3	-16	-2	58.09	14.60
3	-16	-2	37.00	8.80
3	-16	-2	56.59	9.50
3	-16	-2	46.70	11.90
-3	16	2	28.40	8.00
-3	16	2	36.30	11.00
-3	16	2	48.00	10.00
2	-16	-2	306.97	22.20
2	-16	-2	274.97	23.80
2	-16	-2	311.77	22.10
2	-16	-2	299.47	20.50
2	-16	-2	346.37	22.00
-2	16	2	331.17	20.90
-2	16	2	307.37	18.30
1	-16	-2	1150.88	53.99
1	-16	-2	1081.59	53.39
1	-16	-2	1077.59	53.59

1	-16	-2	1071.79	53.09
-1	16	2	1077.29	51.59
-1	16	2	1145.99	53.59
0	-16	-2	907.11	45.90
0	-16	-2	956.80	46.40
0	-16	-2	944.81	46.60
0	-16	-2	936.91	46.10
0	16	2	961.20	44.60
-1	-16	-2	340.87	22.20
-1	-16	-2	350.66	20.80
-1	-16	-2	350.06	21.40
-1	-16	-2	346.57	20.50
-1	-16	-2	322.87	20.70
1	16	2	320.37	25.70
1	16	2	342.27	18.40
-2	-16	-2	14.10	7.00
-2	-16	-2	22.10	10.20
-2	-16	-2	25.40	8.00
-2	-16	-2	19.30	7.70
-2	-16	-2	31.10	7.10
2	16	2	21.70	5.20
-3	-16	-2	80.09	13.00
-3	-16	-2	46.90	13.30
-3	-16	-2	76.79	12.60
-3	-16	-2	93.19	11.40
3	16	2	70.99	8.80
-4	-16	-2	49.30	9.00
-4	-16	-2	34.50	7.90
-4	-16	-2	50.79	7.80
-4	-16	-2	36.90	8.30
-4	-16	-2	51.19	8.60
4	16	2	44.00	7.10
-5	-16	-2	214.08	15.80
-5	-16	-2	199.18	16.70
-5	-16	-2	209.48	16.90
-5	-16	-2	181.98	16.90
-5	-16	-2	186.78	14.80
5	16	2	207.48	15.80
-6	-16	-2	20.80	8.40
-6	-16	-2	26.70	8.40
-6	-16	-2	17.70	8.00
-6	-16	-2	18.20	8.10
-6	-16	-2	17.50	6.50
6	16	2	33.10	9.40

-7	-16	-2	390.96	27.00
-7	-16	-2	364.36	22.90
-7	-16	-2	392.26	21.80
-7	-16	-2	393.76	23.80
-7	-16	-2	391.16	24.10
7	16	2	351.86	26.80
-8	-16	-2	136.79	12.30
-8	-16	-2	141.59	15.50
-8	-16	-2	140.69	14.80
-8	-16	-2	135.79	15.30
8	16	2	108.19	21.60
-9	-16	-2	19.70	8.70
-9	-16	-2	13.50	7.40
-9	-16	-2	21.70	13.50
-9	-16	-2	12.70	6.20
9	16	2	55.19	18.70
-10	-16	-2	143.89	13.70
-10	-16	-2	160.08	20.70
-10	-16	-2	159.78	11.90
10	16	2	182.18	28.30
8	-17	-2	9.30	7.00
-8	17	2	-10.70	22.70
-8	17	2	-2.10	8.60
7	-17	-2	10.40	7.00
7	-17	-2	-6.00	14.20
-7	17	2	12.00	9.70
-7	17	2	-9.30	15.30
-7	17	2	16.40	12.60
6	-17	-2	360.26	25.10
6	-17	-2	385.06	22.00
-6	17	2	347.57	27.20
-6	17	2	341.87	27.00
-6	17	2	352.26	25.50
5	-17	-2	454.95	27.90
5	-17	-2	470.35	26.00
-5	17	2	459.75	30.20
-5	17	2	448.16	30.10
4	-17	-2	48.60	19.60
4	-17	-2	20.80	8.50
4	-17	-2	29.10	9.10
-4	17	2	42.80	11.90
-4	17	2	19.60	11.10
3	-17	-2	-4.50	11.30
3	-17	-2	-1.50	7.90

3	-17	-2	16.10	8.60
3	-17	-2	3.30	14.00
-3	17	2	-7.60	10.20
-3	17	2	-11.20	11.10
-3	17	2	4.70	9.80
2	-17	-2	128.09	19.80
2	-17	-2	160.88	17.70
2	-17	-2	180.08	19.60
2	-17	-2	166.28	16.50
2	-17	-2	133.59	16.40
-2	17	2	172.98	16.00
-2	17	2	168.58	14.60
1	-17	-2	22.70	8.30
1	-17	-2	26.30	8.60
1	-17	-2	20.00	9.80
1	-17	-2	13.40	8.30
-1	17	2	29.10	8.00
-1	17	2	28.00	6.60
0	-17	-2	101.19	13.60
0	-17	-2	100.79	14.20
0	-17	-2	102.09	13.60
0	17	2	108.19	10.70
-1	-17	-2	296.17	21.20
-1	-17	-2	301.07	19.50
-1	-17	-2	283.17	19.30
-1	-17	-2	298.07	19.90
1	17	2	306.97	17.60
1	17	2	301.77	25.40
-2	-17	-2	37.20	9.90
-2	-17	-2	59.79	9.60
-2	-17	-2	54.69	8.60
-2	-17	-2	47.80	8.00
-2	-17	-2	61.89	8.70
2	17	2	56.89	9.30
-3	-17	-2	9.10	6.80
-3	-17	-2	17.20	7.50
-3	-17	-2	2.60	12.20
-3	-17	-2	8.30	7.50
-3	-17	-2	4.90	6.40
3	17	2	15.90	6.10
-4	-17	-2	255.87	18.30
-4	-17	-2	273.97	17.30
-4	-17	-2	268.27	18.50
-4	-17	-2	259.67	18.10

4	17	2	248.88	17.20
-5	-17	-2	534.75	28.30
-5	-17	-2	537.85	29.60
-5	-17	-2	553.64	29.30
-5	-17	-2	560.14	29.70
-5	-17	-2	519.85	30.00
5	17	2	559.14	29.80
-6	-17	-2	20.00	7.90
-6	-17	-2	16.10	6.20
-6	-17	-2	2.90	8.10
-6	-17	-2	8.70	8.10
-6	-17	-2	3.10	8.40
6	17	2	5.50	10.00
-7	-17	-2	289.77	17.60
-7	-17	-2	229.28	19.30
-7	-17	-2	232.48	20.20
-7	-17	-2	237.08	17.20
-7	-17	-2	274.27	18.70
7	17	2	233.48	25.90
-8	-17	-2	244.48	18.20
-8	-17	-2	259.27	16.20
-8	-17	-2	253.17	17.40
-8	-17	-2	262.87	17.90
-9	-17	-2	95.49	12.30
-9	-17	-2	104.19	12.40
-9	-17	-2	106.59	10.30
8	-18	-2	167.78	18.50
-8	18	2	123.19	15.80
7	-18	-2	450.95	23.90
7	-18	-2	475.25	29.90
-7	18	2	452.35	25.80
-7	18	2	343.47	30.40
-7	18	2	440.96	28.30
6	-18	-2	17.00	7.80
6	-18	-2	29.60	10.90
-6	18	2	8.90	12.40
-6	18	2	22.70	13.70
-6	18	2	18.60	9.20
5	-18	-2	18.20	10.10
5	-18	-2	-4.20	8.40
-5	18	2	-3.50	11.60
-5	18	2	8.60	10.20
-5	18	2	16.00	12.60
4	-18	-2	50.49	10.20

4	-18	-2	73.39	11.20
-4	18	2	45.50	11.90
-4	18	2	56.09	11.60
3	-18	-2	31.40	14.70
3	-18	-2	20.30	10.00
3	-18	-2	-9.90	11.40
3	-18	-2	18.50	8.10
-3	18	2	12.60	9.60
-3	18	2	4.60	9.30
2	-18	-2	8.50	7.80
2	-18	-2	-3.90	9.20
2	-18	-2	8.50	11.40
2	-18	-2	-7.70	12.00
-2	18	2	12.10	16.80
-2	18	2	12.50	9.10
-2	18	2	-8.80	8.60
1	-18	-2	16.10	9.90
1	-18	-2	8.70	8.10
1	-18	-2	5.30	8.30
1	-18	-2	4.70	8.40
-1	18	2	-1.80	7.10
-1	18	2	7.80	9.80
0	-18	-2	683.13	35.90
0	-18	-2	673.43	35.30
0	-18	-2	669.83	35.50
0	-18	-2	682.73	35.50
0	18	2	719.83	36.10
0	18	2	668.83	34.60
-1	-18	-2	21.90	8.00
-1	-18	-2	32.80	8.10
-1	-18	-2	28.60	9.40
-1	-18	-2	29.70	7.90
1	18	2	32.20	13.60
1	18	2	31.80	7.10
-2	-18	-2	83.29	13.20
-2	-18	-2	100.39	17.30
-2	-18	-2	105.69	13.30
-2	-18	-2	86.99	12.60
-2	-18	-2	125.79	13.60
2	18	2	118.29	14.30
2	18	2	99.29	12.00
-3	-18	-2	613.54	35.80
-3	-18	-2	556.44	30.60
-3	-18	-2	573.34	30.20

-3	-18	-2	572.44	31.20
-3	-18	-2	582.74	31.20
3	18	2	625.54	36.10
-4	-18	-2	250.97	18.10
-4	-18	-2	251.87	17.20
-4	-18	-2	279.17	18.50
-4	-18	-2	270.07	18.20
4	18	2	273.47	18.20
-5	-18	-2	131.69	14.40
-5	-18	-2	109.79	13.80
-5	-18	-2	120.09	12.10
-5	-18	-2	132.59	13.70
5	18	2	141.09	16.50
-6	-18	-2	297.57	21.30
6	18	2	232.98	23.60
-7	-18	-2	60.79	10.30
-7	-18	-2	68.69	11.80
-7	-18	-2	36.10	10.30
-7	-18	-2	68.19	11.70
-8	-18	-2	108.79	13.10
-8	-18	-2	114.89	10.90
-8	-18	-2	127.89	13.00
-9	-18	-2	1.70	7.20
-9	-18	-2	11.20	5.50
-9	-18	-2	7.80	7.70
7	-19	-2	100.19	11.50
7	-19	-2	93.89	16.50
-7	19	2	88.29	14.20
-7	19	2	94.49	13.20
6	-19	-2	201.78	18.20
6	-19	-2	200.78	15.20
-6	19	2	158.98	17.20
-6	19	2	165.18	19.60
-6	19	2	179.58	21.60
5	-19	-2	66.49	9.80
5	-19	-2	84.99	15.00
-5	19	2	75.99	14.20
-5	19	2	75.29	11.10
-5	19	2	64.99	12.60
4	-19	-2	18.30	9.30
4	-19	-2	2.80	9.50
-4	19	2	29.70	11.50
-4	19	2	16.40	11.00
3	-19	-2	8.20	8.30

3	-19	-2	10.90	18.10
3	-19	-2	5.10	15.30
-3	19	2	2.60	9.50
-3	19	2	-9.70	9.60
2	-19	-2	247.78	20.60
2	-19	-2	247.38	22.20
2	-19	-2	253.87	18.80
-2	19	2	223.28	18.00
-2	19	2	230.88	18.80
1	-19	-2	334.57	21.30
1	-19	-2	317.17	20.90
1	-19	-2	276.77	20.60
1	-19	-2	301.97	21.80
-1	19	2	286.17	23.00
-1	19	2	284.47	20.20
0	-19	-2	13.00	8.40
0	-19	-2	19.60	8.50
0	-19	-2	1.90	7.40
0	-19	-2	0.80	7.80
0	19	2	15.10	8.60
0	19	2	21.90	8.00
-1	-19	-2	458.25	25.50
-1	-19	-2	473.65	27.30
-1	-19	-2	429.66	25.50
-1	-19	-2	436.96	25.50
1	19	2	434.26	25.80
1	19	2	460.75	25.90
-2	-19	-2	285.97	18.70
-2	-19	-2	295.07	21.90
-2	-19	-2	260.27	18.20
-2	-19	-2	258.97	18.60
2	19	2	290.37	19.80
2	19	2	235.18	23.60
-3	-19	-2	104.89	12.30
-3	-19	-2	90.69	14.90
-3	-19	-2	80.59	12.50
-3	-19	-2	95.89	12.90
-3	-19	-2	97.79	12.30
3	19	2	87.79	10.90
3	19	2	66.29	12.70
-4	-19	-2	44.00	8.50
-4	-19	-2	49.99	8.50
-4	-19	-2	26.40	8.10
4	19	2	56.39	10.50

-5	-19	-2	36.10	11.60
-5	-19	-2	16.70	12.00
5	19	2	54.29	12.50
-6	-19	-2	108.09	13.40
-6	-19	-2	118.29	12.00
-6	-19	-2	130.49	14.00
-6	-19	-2	139.59	14.40
-7	-19	-2	95.59	15.80
-7	-19	-2	109.99	10.30
-7	-19	-2	92.89	11.80
-7	-19	-2	100.79	12.40
-8	-19	-2	65.99	10.50
-8	-19	-2	39.60	7.50
-8	-19	-2	62.29	8.70
6	-20	-2	61.89	11.60
6	-20	-2	85.89	12.10
-6	20	2	61.59	15.70
-6	20	2	86.49	18.40
-6	20	2	104.09	13.20
5	-20	-2	0.90	8.90
5	-20	-2	16.00	8.70
-5	20	2	13.50	8.50
-5	20	2	23.60	10.70
-5	20	2	-11.30	12.00
4	-20	-2	-0.80	7.80
4	-20	-2	10.10	9.70
-4	20	2	21.50	9.90
-4	20	2	9.00	11.30
3	-20	-2	283.97	22.50
3	-20	-2	291.77	20.50
3	-20	-2	272.17	24.20
-3	20	2	287.47	20.60
-3	20	2	302.17	21.10
2	-20	-2	333.27	28.10
2	-20	-2	336.57	31.70
2	-20	-2	348.57	29.70
-2	20	2	334.27	34.80
-2	20	2	353.76	22.60
1	-20	-2	340.57	21.70
1	-20	-2	302.87	20.80
1	-20	-2	302.57	21.70
-1	20	2	348.07	31.00
-1	20	2	303.97	20.70
0	-20	-2	85.69	13.70

0	-20	-2	92.19	14.20
0	-20	-2	105.09	15.40
0	-20	-2	71.29	10.80
0	20	2	74.79	13.10
0	20	2	98.99	13.50
-1	-20	-2	457.75	27.60
-1	-20	-2	473.45	25.90
-1	-20	-2	421.56	25.70
-1	-20	-2	475.85	25.70
1	20	2	453.25	26.20
1	20	2	431.96	28.50
-2	-20	-2	85.59	12.80
-2	-20	-2	82.99	12.80
-2	-20	-2	90.79	12.60
-2	-20	-2	79.99	12.30
2	20	2	74.09	12.50
2	20	2	91.19	14.70
-3	-20	-2	103.49	13.50
-3	-20	-2	102.29	12.80
-3	-20	-2	89.29	12.40
-3	-20	-2	91.09	17.60
3	20	2	82.59	12.60
-4	-20	-2	55.89	10.00
-4	-20	-2	65.49	9.80
-4	-20	-2	75.99	13.20
-4	-20	-2	42.00	8.30
4	20	2	53.29	13.00
4	20	2	44.80	11.50
-5	-20	-2	20.70	7.90
-5	-20	-2	19.10	7.90
-5	-20	-2	10.60	7.30
-5	-20	-2	13.80	6.60
-6	-20	-2	-2.50	6.80
-6	-20	-2	-2.10	5.60
-6	-20	-2	2.90	7.40
-6	-20	-2	-5.40	6.90
-7	-20	-2	259.87	15.70
-7	-20	-2	230.08	15.00
-7	-20	-2	240.68	19.00
-7	-20	-2	250.37	16.40
-8	-20	-2	61.79	8.00
-8	-20	-2	79.79	11.20
-8	-20	-2	45.60	10.40
5	-21	-2	110.39	13.70

-5	21	2	87.39	12.70
-5	21	2	102.99	13.20
4	-21	-2	0.90	7.80
4	-21	-2	24.50	11.90
-4	21	2	-6.40	8.20
-4	21	2	-13.10	9.50
-4	21	2	10.70	10.90
3	-21	-2	79.89	12.90
3	-21	-2	100.79	15.50
-3	21	2	84.69	14.80
-3	21	2	108.69	14.70
2	-21	-2	201.68	18.00
2	-21	-2	171.98	16.50
-2	21	2	198.08	17.20
-2	21	2	177.48	17.30
1	-21	-2	17.50	9.30
1	-21	-2	8.30	11.40
1	-21	-2	34.70	9.50
-1	21	2	52.59	9.70
-1	21	2	39.70	9.10
0	-21	-2	48.70	10.60
0	-21	-2	50.99	9.90
0	-21	-2	71.09	11.10
0	-21	-2	52.69	9.80
0	21	2	56.69	9.80
-1	-21	-2	231.18	18.10
-1	-21	-2	241.38	18.00
-1	-21	-2	226.58	19.90
-1	-21	-2	264.47	17.90
1	21	2	178.38	26.60
1	21	2	232.38	17.40
-2	-21	-2	174.78	15.40
-2	-21	-2	185.38	18.80
-2	-21	-2	158.78	15.40
-2	-21	-2	170.78	15.40
2	21	2	182.48	25.50
2	21	2	154.98	15.20
-3	-21	-2	168.98	14.20
-3	-21	-2	160.08	13.90
-3	-21	-2	176.58	14.50
3	21	2	147.19	21.40
-4	-21	-2	247.78	16.70
-4	-21	-2	252.97	16.70
-4	-21	-2	221.98	15.70

4	21	2	208.98	20.60
-5	-21	-2	225.48	14.80
-5	-21	-2	224.98	16.30
-5	-21	-2	214.08	15.40
5	21	2	242.88	19.30
-6	-21	-2	-2.90	5.30
-6	-21	-2	2.50	6.70
6	21	2	13.50	10.10
-7	-21	-2	13.40	5.50
-7	-21	-2	13.90	9.30
7	21	2	12.30	9.10
5	-22	-2	16.90	8.70
-5	22	2	-16.70	10.50
4	-22	-2	145.19	13.30
-4	22	2	113.19	17.10
-4	22	2	123.99	13.50
-4	22	2	117.69	15.10
3	-22	-2	5.90	7.80
-3	22	2	11.40	9.00
-3	22	2	11.40	10.20
2	-22	-2	5.00	10.70
2	-22	-2	10.90	7.40
-2	22	2	8.80	9.30
-2	22	2	1.10	8.20
1	-22	-2	40.60	8.70
1	-22	-2	32.10	9.50
-1	22	2	42.90	9.40
-1	22	2	27.10	8.60
0	-22	-2	394.26	23.90
0	-22	-2	375.86	23.00
0	-22	-2	370.76	24.30
0	22	2	421.66	23.50
0	22	2	408.76	23.30
-1	-22	-2	-3.20	7.30
-1	-22	-2	6.90	9.30
-1	-22	-2	-2.30	8.90
-1	-22	-2	-2.30	6.60
1	22	2	9.70	7.80
-2	-22	-2	-2.00	6.50
-2	-22	-2	1.30	10.60
-2	-22	-2	4.50	7.60
-2	-22	-2	-0.60	7.60
2	22	2	4.80	7.70
-3	-22	-2	600.34	31.00

-3	-22	-2	640.94	31.50
-3	-22	-2	618.74	31.10
3	22	2	606.44	36.50
3	22	2	583.74	31.70
-4	-22	-2	183.38	14.40
-4	-22	-2	207.48	14.40
-4	-22	-2	175.08	13.70
4	22	2	187.28	20.30
4	22	2	190.08	15.40
-5	-22	-2	-3.70	6.60
-5	-22	-2	16.20	6.20
-5	-22	-2	15.00	7.10
5	22	2	0.20	10.90
-6	-22	-2	74.79	11.20
-6	-22	-2	111.49	9.80
-6	-22	-2	109.39	18.60
3	-23	-2	-3.90	6.80
-3	23	2	-12.90	8.50
-3	23	2	-3.60	11.00
-3	23	2	-6.00	11.20
2	-23	-2	180.88	13.90
2	-23	-2	201.98	21.50
-2	23	2	183.28	15.50
-2	23	2	162.58	16.10
1	-23	-2	85.09	11.50
1	-23	-2	110.39	12.90
-1	23	2	98.39	13.20
-1	23	2	120.69	12.80
0	-23	-2	3.10	11.40
0	-23	-2	20.10	8.70
0	-23	-2	21.60	8.40
0	23	2	34.50	11.70
0	23	2	29.50	7.90
-1	-23	-2	348.87	22.90
-1	-23	-2	394.76	22.00
-1	-23	-2	389.66	23.40
1	23	2	414.96	24.70
-2	-23	-2	138.99	13.70
-2	-23	-2	185.08	18.90
-2	-23	-2	177.88	13.20
-2	-23	-2	156.68	14.30
2	23	2	124.39	18.40
-3	-23	-2	5.70	7.40
-3	-23	-2	12.00	6.50

-3	-23	-2	10.30	7.10
3	23	2	8.20	8.00
-4	-23	-2	25.30	6.90
-4	-23	-2	14.70	7.20
-4	-23	-2	17.20	6.60
4	23	2	29.60	9.30
-5	-23	-2	6.20	7.00
-5	-23	-2	1.90	6.40
-5	-23	-2	13.30	7.70
5	23	2	-0.40	12.90
5	23	2	25.90	9.50
2	-24	-2	59.69	15.70
2	-24	-2	75.29	10.60
-2	24	2	52.19	10.30
-2	24	2	69.49	11.50
1	-24	-2	53.59	10.80
1	-24	-2	59.29	10.40
-1	24	2	72.69	11.80
-1	24	2	77.19	10.90
0	-24	-2	7.70	8.50
0	-24	-2	-10.60	7.50
0	24	2	-1.00	8.80
0	24	2	12.20	7.20
-1	-24	-2	38.90	7.80
-1	-24	-2	43.80	15.80
-1	-24	-2	40.40	11.00
1	24	2	48.10	7.90
1	24	2	59.19	9.50
-2	-24	-2	177.78	12.40
-2	-24	-2	165.18	14.60
2	24	2	144.39	14.10
-3	-24	-2	3.60	7.70
-3	-24	-2	2.50	6.20
-3	-24	-2	3.90	7.40
3	24	2	8.50	8.40
2	23	-3	80.39	10.70
2	23	-3	106.59	12.20
-2	-23	3	112.39	11.00
1	23	-3	26.50	7.10
1	23	-3	4.00	8.70
-1	-23	3	33.00	10.10
-1	-23	3	25.40	8.40
0	23	-3	20.50	7.00
0	23	-3	11.80	8.20

0	-23	3	-0.60	8.50
0	-23	3	-4.00	10.90
-1	23	-3	12.80	8.50
-1	23	-3	22.10	7.00
1	-23	3	19.40	11.50
1	-23	3	29.60	11.10
4	22	-3	21.30	10.20
4	22	-3	49.30	13.60
-4	-22	3	46.00	9.80
-4	-22	3	54.39	8.90
3	22	-3	68.19	10.40
3	22	-3	72.49	12.70
-3	-22	3	80.39	9.80
2	22	-3	18.80	7.80
2	22	-3	32.80	8.60
2	22	-3	29.00	10.50
-2	-22	3	26.80	6.90
1	22	-3	0.90	8.00
1	22	-3	38.30	21.20
1	22	-3	6.10	7.00
-1	-22	3	16.60	7.50
-1	-22	3	13.50	8.80
0	22	-3	135.59	13.00
0	22	-3	102.59	13.40
0	-22	3	149.09	13.50
0	-22	3	138.19	14.30
-1	22	-3	110.69	12.80
-1	22	-3	138.19	12.80
1	-22	3	116.49	12.70
1	-22	3	127.59	15.50
-2	22	-3	36.70	7.60
-2	22	-3	43.00	9.20
-3	22	-3	32.50	8.10
-3	22	-3	18.40	9.60
3	-22	3	32.00	7.50
5	21	-3	38.80	8.90
5	21	-3	32.70	10.90
-5	-21	3	21.20	8.80
-5	-21	3	19.60	5.90
4	21	-3	332.17	20.60
4	21	-3	303.77	19.80
-4	-21	3	315.17	18.10
-4	-21	3	271.57	18.30
3	21	-3	15.00	10.30

3	21	-3	7.90	9.50
-3	-21	3	0.80	8.80
-3	-21	3	2.90	6.10
2	21	-3	-10.50	9.50
2	21	-3	11.20	8.10
-2	-21	3	1.70	6.00
-2	-21	3	-13.80	10.20
1	21	-3	523.45	28.00
1	21	-3	479.15	29.70
1	21	-3	512.35	27.90
-1	-21	3	544.55	27.60
-1	-21	3	525.05	28.40
0	21	-3	19.80	7.70
0	21	-3	25.80	8.00
0	21	-3	34.50	19.40
0	-21	3	21.60	8.30
0	-21	3	22.10	8.60
-1	21	-3	-1.60	7.10
-1	21	-3	11.90	8.10
1	-21	3	-3.90	7.50
1	-21	3	1.40	9.40
-2	21	-3	211.18	16.60
-2	21	-3	209.38	16.60
2	-21	3	258.07	16.60
2	-21	3	246.98	21.90
-3	21	-3	21.80	8.00
-3	21	-3	17.60	9.30
3	-21	3	31.60	7.80
-4	21	-3	22.10	9.70
-4	21	-3	6.70	7.70
4	-21	3	17.40	7.50
6	20	-3	178.88	14.00
6	20	-3	163.18	17.00
-6	-20	3	142.29	12.30
-6	-20	3	182.38	13.00
5	20	-3	1.50	8.30
5	20	-3	-20.20	9.60
-5	-20	3	-0.90	6.70
-5	-20	3	4.10	6.30
4	20	-3	6.10	9.00
4	20	-3	6.00	8.40
-4	-20	3	1.50	6.20
-4	-20	3	6.90	7.00
3	20	-3	528.75	28.60

3	20	-3	543.45	29.70
-3	-20	3	514.95	27.80
-3	-20	3	527.45	35.90
-3	-20	3	515.85	28.00
2	20	-3	220.98	19.30
2	20	-3	209.18	17.10
-2	-20	3	244.18	19.20
-2	-20	3	206.38	16.30
-2	-20	3	266.07	19.60
-2	-20	3	245.48	19.30
1	20	-3	6.50	10.80
1	20	-3	0.00	7.50
-1	-20	3	16.50	11.00
-1	-20	3	9.80	9.00
-1	-20	3	19.10	7.60
0	20	-3	74.49	11.80
0	20	-3	86.09	13.50
0	-20	3	44.00	9.30
0	-20	3	55.49	9.80
-1	20	-3	66.49	9.70
-1	20	-3	77.29	12.10
1	-20	3	101.89	16.00
1	-20	3	67.29	10.30
-2	20	-3	14.70	7.90
-2	20	-3	-5.90	7.80
2	-20	3	12.50	8.00
2	-20	3	4.40	11.60
-3	20	-3	323.67	20.20
-3	20	-3	320.87	20.60
-3	20	-3	274.37	23.80
3	-20	3	336.67	20.10
-4	20	-3	55.09	20.30
-4	20	-3	34.70	13.60
-4	20	-3	21.50	8.70
4	-20	3	27.00	8.10
-5	20	-3	208.68	17.80
-5	20	-3	220.68	16.50
5	-20	3	242.58	17.80
5	-20	3	211.38	19.10
7	19	-3	34.30	14.20
7	19	-3	61.29	10.70
-7	-19	3	47.50	8.20
-7	-19	3	61.19	8.90
6	19	-3	106.09	13.20

-6	-19	3	87.79	11.80
-6	-19	3	103.39	11.00
5	19	-3	234.58	17.70
-5	-19	3	208.38	15.10
-5	-19	3	241.38	18.60
-5	-19	3	203.28	15.10
4	19	-3	17.00	9.00
-4	-19	3	24.70	7.40
-4	-19	3	20.40	10.10
-4	-19	3	14.10	7.60
3	19	-3	7.90	8.30
3	19	-3	-3.70	9.70
-3	-19	3	1.90	10.20
-3	-19	3	21.20	13.20
-3	-19	3	-2.10	7.80
-3	-19	3	8.00	9.90
2	19	-3	457.05	34.60
2	19	-3	474.15	26.50
-2	-19	3	475.95	29.40
-2	-19	3	503.15	34.40
-2	-19	3	470.25	26.50
1	19	-3	27.60	8.50
1	19	-3	5.50	10.80
-1	-19	3	7.60	9.60
-1	-19	3	27.10	7.90
0	19	-3	62.89	15.00
0	19	-3	39.40	8.40
0	-19	3	50.29	9.30
0	-19	3	31.80	9.40
-1	19	-3	67.39	19.40
-1	19	-3	69.29	10.10
-1	19	-3	52.49	9.00
1	-19	3	22.10	14.10
1	-19	3	64.49	10.80
-2	19	-3	134.89	24.40
-2	19	-3	152.28	15.50
2	-19	3	179.88	20.40
2	-19	3	199.18	24.00
-3	19	-3	332.27	21.70
-3	19	-3	344.67	22.00
3	-19	3	360.16	22.20
-4	19	-3	44.60	9.90
-4	19	-3	32.30	9.10
-4	19	-3	46.30	12.00

4	-19	3	47.30	10.90
4	-19	3	32.10	8.60
-5	19	-3	56.89	9.60
-5	19	-3	65.29	12.70
-5	19	-3	68.39	11.80
5	-19	3	53.49	9.90
5	-19	3	58.59	9.90
-6	19	-3	8.20	8.50
-6	19	-3	-15.10	10.20
6	-19	3	13.70	8.30
6	-19	3	-12.20	9.60
8	18	-3	27.20	7.50
-8	-18	3	24.70	5.50
-8	-18	3	56.39	11.20
7	18	-3	170.98	14.80
-7	-18	3	171.08	12.30
-7	-18	3	151.38	19.10
-7	-18	3	160.18	14.20
6	18	-3	24.50	8.20
-6	-18	3	28.70	10.10
-6	-18	3	22.60	6.40
5	18	-3	30.80	9.00
-5	-18	3	21.10	9.40
-5	-18	3	9.50	7.00
-5	-18	3	24.20	7.00
4	18	-3	296.47	20.70
-4	-18	3	260.57	23.30
-4	-18	3	289.27	18.80
-4	-18	3	254.87	23.20
3	18	-3	93.29	15.50
-3	-18	3	62.59	9.40
-3	-18	3	92.89	12.10
-3	-18	3	85.19	14.00
-3	-18	3	88.69	14.60
2	18	-3	135.49	14.70
2	18	-3	153.78	17.20
-2	-18	3	160.58	18.10
-2	-18	3	130.19	15.50
-2	-18	3	136.59	15.00
-2	-18	3	141.39	14.30
1	18	-3	54.49	9.70
1	18	-3	75.29	11.50
-1	-18	3	70.59	10.60
-1	-18	3	70.29	12.10

-1	-18	3	62.59	10.30
-1	-18	3	43.70	10.30
0	18	-3	132.99	14.30
0	18	-3	126.29	18.60
0	-18	3	143.69	15.30
0	-18	3	108.49	15.60
0	-18	3	146.89	17.10
-1	18	-3	72.99	12.00
-1	18	-3	69.49	13.20
-1	18	-3	72.49	10.60
1	-18	3	87.39	14.80
1	-18	3	85.49	15.20
-2	18	-3	22.40	8.20
-2	18	-3	31.70	8.10
2	-18	3	58.09	11.10
2	-18	3	30.30	9.20
-3	18	-3	448.26	26.60
-3	18	-3	466.35	27.40
3	-18	3	468.25	27.10
-4	18	-3	461.15	27.40
-4	18	-3	467.15	26.90
-4	18	-3	418.36	28.30
4	-18	3	494.05	27.30
4	-18	3	419.96	26.50
-5	18	-3	9.10	10.80
-5	18	-3	7.70	9.20
-5	18	-3	5.20	8.80
5	-18	3	1.80	8.00
5	-18	3	-11.40	8.40
-6	18	-3	2.60	11.40
-6	18	-3	13.90	9.30
6	-18	3	25.50	10.50
6	-18	3	11.20	7.70
-7	18	-3	25.30	11.90
-7	18	-3	17.70	13.50
-7	18	-3	16.40	9.40
7	-18	3	29.30	14.10
7	-18	3	8.90	7.40
-8	-17	3	153.98	19.30
-8	-17	3	156.48	11.60
-7	-17	3	14.30	8.00
-7	-17	3	4.90	9.80
-7	-17	3	17.80	8.90
-7	-17	3	5.50	5.70

-6	-17	3	7.10	6.30
-6	-17	3	-4.20	8.70
-6	-17	3	6.80	7.20
-6	-17	3	7.70	9.20
-5	-17	3	42.20	7.90
-5	-17	3	39.20	7.50
-5	-17	3	35.70	9.00
4	17	-3	178.58	23.30
-4	-17	3	201.18	16.90
-4	-17	3	172.68	15.20
-4	-17	3	169.58	14.60
-4	-17	3	190.18	15.30
3	17	-3	9.80	8.60
3	17	-3	18.00	12.40
-3	-17	3	4.40	6.60
-3	-17	3	2.20	12.20
-3	-17	3	22.10	7.40
-3	-17	3	7.10	8.30
2	17	-3	32.40	9.60
2	17	-3	39.20	11.60
-2	-17	3	8.10	9.40
-2	-17	3	16.10	9.10
-2	-17	3	20.70	8.20
-2	-17	3	33.70	7.60
1	17	-3	404.46	24.80
1	17	-3	386.76	25.90
-1	-17	3	374.36	23.70
-1	-17	3	339.87	23.70
-1	-17	3	373.96	22.90
-1	-17	3	395.46	24.20
0	17	-3	5.10	10.50
0	-17	3	-1.10	7.60
0	-17	3	-1.80	8.10
0	-17	3	-2.10	9.40
-1	17	-3	-10.90	11.00
-1	17	-3	1.30	8.40
1	-17	3	2.40	8.40
1	-17	3	6.60	8.70
-2	17	-3	709.63	37.40
-2	17	-3	717.03	41.40
-2	17	-3	703.83	37.90
2	-17	3	772.82	38.10
2	-17	3	738.83	38.70
-3	17	-3	10.80	8.70

-3	17	-3	10.40	8.40
3	-17	3	10.60	14.30
3	-17	3	9.30	10.20
3	-17	3	-2.50	7.90
-4	17	-3	32.20	9.30
-4	17	-3	35.40	9.70
4	-17	3	31.50	9.60
4	-17	3	35.30	9.20
-5	17	-3	9.10	10.60
-5	17	-3	24.40	11.20
-5	17	-3	21.80	9.70
5	-17	3	12.40	9.70
5	-17	3	20.20	8.40
-6	17	-3	-2.00	9.60
-6	17	-3	23.60	10.90
-6	17	-3	13.50	10.20
6	-17	3	-0.60	9.70
6	-17	3	-1.70	7.30
-7	17	-3	14.20	9.80
-7	17	-3	5.50	11.20
-7	17	-3	-13.30	11.20
7	-17	3	12.80	13.20
7	-17	3	3.20	7.00
-8	17	-3	-2.00	13.00
-8	17	-3	19.90	12.90
-8	17	-3	35.10	12.20
8	-17	3	21.10	8.00
9	16	-3	28.00	23.60
-9	-16	3	45.80	6.50
-9	-16	3	35.10	12.00
8	16	-3	89.99	17.70
-8	-16	3	77.59	11.50
-8	-16	3	95.79	15.00
-8	-16	3	82.89	10.10
-8	-16	3	101.69	11.60
7	16	-3	-30.20	27.10
-7	-16	3	4.20	6.20
-7	-16	3	18.30	10.00
-7	-16	3	9.00	7.60
-7	-16	3	9.40	8.80
-6	-16	3	122.29	12.50
-6	-16	3	145.39	22.00
-6	-16	3	132.19	15.90
5	16	-3	100.79	15.80

-5	-16	3	89.39	11.40
-5	-16	3	71.59	12.30
-5	-16	3	77.09	11.10
-5	-16	3	86.89	13.30
4	16	-3	2.20	15.90
-4	-16	3	22.00	7.30
-4	-16	3	16.70	8.70
-4	-16	3	9.20	6.70
-4	-16	3	16.40	7.50
3	16	-3	982.60	49.10
-3	-16	3	942.51	47.60
-3	-16	3	990.70	48.50
-3	-16	3	1012.10	48.30
-3	-16	3	1017.30	50.59
-3	-16	3	1003.20	48.30
2	16	-3	262.07	19.20
2	16	-3	269.47	20.10
-2	-16	3	271.67	19.30
-2	-16	3	274.57	18.10
-2	-16	3	266.87	21.10
-2	-16	3	266.17	18.70
1	16	-3	7.10	9.30
1	16	-3	23.70	8.50
-1	-16	3	2.20	8.20
-1	-16	3	2.50	7.00
-1	-16	3	21.30	9.20
-1	-16	3	-4.50	8.60
0	16	-3	31.30	9.80
0	16	-3	49.60	11.30
0	16	-3	45.10	8.20
0	-16	3	40.10	8.20
0	-16	3	52.49	11.30
0	-16	3	57.39	10.00
0	-16	3	41.80	9.80
-1	16	-3	31.20	12.30
-1	16	-3	49.50	8.20
1	-16	3	62.79	11.20
1	-16	3	57.69	9.90
1	-16	3	48.30	8.90
-2	16	-3	80.19	12.00
-2	16	-3	84.59	11.20
-2	16	-3	82.99	14.50
2	-16	3	88.89	13.10
2	-16	3	73.89	11.80

2	-16	3	62.19	9.50
-3	16	-3	112.19	15.60
-3	16	-3	124.29	15.50
3	-16	3	146.29	15.30
3	-16	3	89.09	11.70
3	-16	3	117.09	15.80
-4	16	-3	384.16	24.90
-4	16	-3	380.66	24.90
4	-16	3	397.56	24.20
4	-16	3	394.06	25.00
-5	16	-3	388.76	25.50
-5	16	-3	381.86	26.40
-5	16	-3	382.56	26.40
5	-16	3	399.56	25.40
5	-16	3	367.36	23.80
-6	16	-3	1.00	10.70
-6	16	-3	5.50	10.90
-6	16	-3	-2.40	10.60
6	-16	3	3.90	10.60
6	-16	3	-8.40	7.20
-7	16	-3	93.69	12.90
-7	16	-3	107.19	13.90
-7	16	-3	79.89	14.30
7	-16	3	91.69	14.60
7	-16	3	108.49	12.80
-8	16	-3	95.49	16.50
-8	16	-3	98.59	14.60
8	-16	3	134.19	12.50
9	15	-3	121.39	21.60
-9	-15	3	46.70	10.90
-9	-15	3	75.19	9.30
8	15	-3	227.48	25.70
-8	-15	3	282.97	21.30
-8	-15	3	295.27	20.50
-8	-15	3	280.67	16.30
-8	-15	3	234.88	20.00
7	15	-3	70.19	17.20
-7	-15	3	77.59	11.90
-7	-15	3	98.29	12.60
-7	-15	3	63.59	10.50
6	15	-3	67.49	17.20
6	15	-3	37.80	15.00
-6	-15	3	27.80	7.10
-6	-15	3	44.80	8.70

-6	-15	3	46.70	9.90
-6	-15	3	33.50	10.20
5	15	-3	96.19	19.50
-5	-15	3	118.69	14.80
-5	-15	3	80.59	12.10
-5	-15	3	109.69	16.00
-5	-15	3	97.49	13.20
4	15	-3	16.50	8.70
-4	-15	3	14.50	7.80
-4	-15	3	4.20	6.70
-4	-15	3	6.50	6.70
-4	-15	3	15.00	8.80
3	15	-3	12.10	7.40
-3	-15	3	8.00	7.40
-3	-15	3	12.20	12.20
-3	-15	3	-6.70	8.20
-3	-15	3	3.40	6.40
-3	-15	3	4.60	6.20
2	15	-3	732.93	39.50
2	15	-3	792.02	38.30
-2	-15	3	773.42	38.50
-2	-15	3	784.32	38.80
-2	-15	3	809.52	40.10
-2	-15	3	758.02	38.30
-2	-15	3	757.92	39.10
1	15	-3	5.60	6.50
-1	-15	3	-2.00	8.20
-1	-15	3	2.80	6.20
-1	-15	3	14.70	8.10
-1	-15	3	2.60	8.90
0	15	-3	47.50	8.10
0	15	-3	54.29	10.70
0	-15	3	26.40	7.40
0	-15	3	22.10	7.60
0	-15	3	28.30	9.40
-1	15	-3	12.50	6.90
-1	15	-3	8.70	11.00
-1	15	-3	3.90	7.90
1	-15	3	29.80	8.20
1	-15	3	30.70	10.20
1	-15	3	14.10	7.20
-2	15	-3	707.73	39.50
-2	15	-3	726.43	36.90
-2	15	-3	724.13	37.50

2	-15	3	694.83	38.10
2	-15	3	727.93	37.30
2	-15	3	722.73	38.30
-3	15	-3	660.43	34.10
-3	15	-3	675.13	34.60
-3	15	-3	555.54	51.09
3	-15	3	602.54	33.60
3	-15	3	669.83	34.00
3	-15	3	599.54	37.40
-4	15	-3	-0.40	9.30
4	-15	3	29.90	8.90
4	-15	3	10.80	7.90
-5	15	-3	19.10	10.60
-5	15	-3	32.30	11.00
-5	15	-3	20.60	10.60
5	-15	3	17.80	9.40
5	-15	3	21.20	8.10
-6	15	-3	75.49	20.90
-6	15	-3	52.99	13.40
-6	15	-3	57.39	12.30
6	-15	3	45.90	8.90
-7	15	-3	14.80	12.00
-7	15	-3	15.30	10.80
-7	15	-3	7.10	12.40
7	-15	3	44.30	13.80
7	-15	3	23.00	7.60
-8	15	-3	239.28	24.90
-8	15	-3	237.88	21.80
-8	15	-3	210.88	20.20
8	-15	3	275.37	17.00
-9	15	-3	32.00	14.40
-9	15	-3	12.70	11.10
-9	15	-3	61.89	15.60
9	-15	3	27.10	6.80
10	14	-3	243.88	22.70
-10	-14	3	208.88	13.80
-10	-14	3	204.78	18.50
9	14	-3	7.10	13.40
-9	-14	3	51.59	13.00
-9	-14	3	30.70	10.20
8	14	-3	117.99	17.70
-8	-14	3	126.69	17.10
-8	-14	3	109.79	11.40
-8	-14	3	90.89	16.40

7	14	-3	155.58	24.80
7	14	-3	81.39	22.90
-7	-14	3	142.39	17.50
-7	-14	3	159.88	16.30
-7	-14	3	147.29	13.20
-7	-14	3	144.89	17.00
6	14	-3	22.40	15.00
6	14	-3	5.90	12.10
-6	-14	3	10.80	6.50
-6	-14	3	13.70	9.70
-6	-14	3	19.90	7.60
-6	-14	3	21.60	9.50
-6	-14	3	7.60	9.10
5	14	-3	36.60	9.50
5	14	-3	-10.20	15.90
-5	-14	3	1.80	9.20
-5	-14	3	33.80	7.90
-5	-14	3	16.90	6.80
-5	-14	3	13.00	8.90
4	14	-3	561.94	29.70
-4	-14	3	567.84	31.00
-4	-14	3	510.05	29.10
-4	-14	3	571.44	30.00
-4	-14	3	571.14	29.60
3	14	-3	8.80	6.10
-3	-14	3	-14.80	13.30
-3	-14	3	1.50	9.00
-3	-14	3	5.80	6.50
-3	-14	3	-0.10	7.20
-3	-14	3	4.20	6.40
2	14	-3	488.65	25.10
-2	-14	3	455.95	27.80
-2	-14	3	491.25	27.10
-2	-14	3	479.45	25.90
-2	-14	3	464.85	25.70
-2	-14	3	489.45	28.00
1	14	-3	36.80	8.90
1	14	-3	36.80	6.50
-1	-14	3	44.80	10.90
-1	-14	3	34.70	9.30
-1	-14	3	46.80	8.90
-1	-14	3	35.10	8.50
0	14	-3	59.59	10.40
0	14	-3	69.19	9.50

0	14	-3	48.00	7.40
0	-14	3	66.59	11.50
0	-14	3	79.89	11.30
0	-14	3	58.29	10.30
-1	14	-3	25.90	6.80
-1	14	-3	13.60	11.10
-1	14	-3	8.40	6.30
1	-14	3	31.10	9.90
1	-14	3	8.00	8.50
1	-14	3	0.10	7.30
-2	14	-3	1.00	6.10
-2	14	-3	1.50	11.80
2	-14	3	7.90	9.60
2	-14	3	-5.60	6.40
-3	14	-3	575.74	40.70
-3	14	-3	666.93	34.10
-3	14	-3	686.13	33.30
3	-14	3	536.65	37.20
3	-14	3	570.24	32.40
3	-14	3	607.64	32.70
-4	14	-3	847.52	43.10
-4	14	-3	831.92	43.60
4	-14	3	804.32	41.50
4	-14	3	794.22	40.90
-5	14	-3	69.79	12.50
-5	14	-3	62.79	11.90
-5	14	-3	87.19	18.20
5	-14	3	62.09	10.30
5	-14	3	48.20	9.10
-6	14	-3	3.30	11.00
-6	14	-3	-2.10	11.80
-6	14	-3	14.70	11.80
6	-14	3	5.00	7.20
6	-14	3	1.10	10.20
-7	14	-3	35.10	19.20
-7	14	-3	30.00	11.90
-7	14	-3	19.30	12.90
7	-14	3	18.60	9.50
-8	14	-3	75.69	12.10
-8	14	-3	63.39	15.50
-8	14	-3	88.89	14.90
8	-14	3	83.49	12.00
-9	14	-3	16.00	12.20
-9	14	-3	20.90	14.50

-9	14	-3	28.60	10.00
9	-14	3	27.60	7.60
10	13	-3	66.09	13.10
-10	-13	3	54.09	8.90
-10	-13	3	27.10	14.90
9	13	-3	371.36	26.90
-9	-13	3	372.86	20.50
-9	-13	3	361.86	25.40
-9	-13	3	337.97	23.80
8	13	-3	102.89	18.10
-8	-13	3	98.59	17.10
-8	-13	3	88.79	13.40
-8	-13	3	84.49	12.30
-8	-13	3	93.69	12.80
7	13	-3	17.00	14.10
7	13	-3	14.60	15.50
-7	-13	3	32.90	10.00
-7	-13	3	32.10	10.00
-7	-13	3	47.50	10.80
-7	-13	3	53.29	9.20
-7	-13	3	29.80	7.30
6	13	-3	120.79	17.30
6	13	-3	100.39	16.20
-6	-13	3	93.69	15.30
-6	-13	3	78.19	11.60
-6	-13	3	114.29	13.10
-6	-13	3	76.19	13.50
-6	-13	3	91.59	12.20
5	13	-3	205.48	16.30
5	13	-3	174.88	24.90
-5	-13	3	199.58	15.60
-5	-13	3	191.58	18.70
-5	-13	3	191.98	16.30
-5	-13	3	205.28	15.20
-5	-13	3	194.58	15.20
4	13	-3	134.69	18.10
4	13	-3	93.89	11.80
-4	-13	3	111.89	12.50
-4	-13	3	106.19	19.20
-4	-13	3	119.99	12.40
-4	-13	3	113.39	14.00
-4	-13	3	99.99	11.80
3	13	-3	220.88	14.20
-3	-13	3	222.68	16.00

-3	-13	3	203.88	18.00
-3	-13	3	189.68	15.00
2	13	-3	326.67	17.60
-2	-13	3	284.77	19.60
-2	-13	3	284.17	20.80
-2	-13	3	335.67	19.40
-2	-13	3	320.17	19.10
-2	-13	3	320.77	22.30
1	13	-3	843.32	39.40
-1	-13	3	831.82	40.90
-1	-13	3	841.42	40.60
-1	-13	3	768.02	40.40
-1	-13	3	827.42	41.50
-1	-13	3	838.02	41.40
0	13	-3	17.20	4.80
0	13	-3	12.90	9.00
0	-13	3	23.50	10.10
0	-13	3	16.40	6.80
0	-13	3	11.90	9.70
0	-13	3	17.20	8.10
-1	13	-3	170.98	13.50
-1	13	-3	165.18	19.20
-1	13	-3	162.48	13.50
1	-13	3	179.58	16.00
1	-13	3	153.08	18.60
1	-13	3	167.48	15.10
-2	13	-3	867.51	45.60
-2	13	-3	912.01	48.60
-2	13	-3	1002.10	45.80
2	-13	3	906.91	46.50
2	-13	3	911.71	46.40
2	-13	3	980.70	46.20
2	-13	3	891.51	46.10
-3	13	-3	48.60	8.70
-3	13	-3	55.19	15.40
-3	13	-3	65.89	10.90
3	-13	3	28.20	16.60
3	-13	3	42.70	8.80
-4	13	-3	6.30	8.60
-4	13	-3	17.60	10.00
4	-13	3	13.90	7.40
4	-13	3	0.20	7.20
-5	13	-3	352.16	26.40
-5	13	-3	368.76	26.60

-5	13	-3	406.86	26.00
5	-13	3	365.46	24.00
5	-13	3	373.86	22.70
-6	13	-3	21.60	12.90
-6	13	-3	11.50	12.80
-6	13	-3	-7.80	10.50
6	-13	3	2.60	7.20
6	-13	3	-6.50	10.00
-7	13	-3	-18.20	10.90
-7	13	-3	-3.70	13.00
-7	13	-3	-6.30	12.50
7	-13	3	-5.00	7.10
7	-13	3	16.80	15.30
-8	13	-3	83.79	16.30
-8	13	-3	84.39	15.80
-8	13	-3	99.09	13.50
8	-13	3	97.19	13.30
-9	13	-3	428.96	26.60
-9	13	-3	453.15	29.70
-9	13	-3	410.46	30.00
9	-13	3	483.25	24.90
-10	13	-3	29.70	12.30
-10	13	-3	44.80	10.70
10	12	-3	7.10	11.80
-10	-12	3	4.60	6.00
-10	-12	3	13.40	9.90
9	12	-3	78.59	14.30
-9	-12	3	90.29	22.40
-9	-12	3	96.59	14.40
-9	-12	3	84.59	11.40
-9	-12	3	72.19	11.40
8	12	-3	36.60	13.40
-8	-12	3	14.40	9.60
-8	-12	3	25.90	7.20
-8	-12	3	9.20	12.40
-8	-12	3	13.60	11.00
-8	-12	3	4.70	11.40
7	12	-3	29.40	13.70
-7	-12	3	54.79	10.30
-7	-12	3	44.60	9.40
-7	-12	3	35.80	11.20
-7	-12	3	45.40	7.70
-7	-12	3	59.19	11.90
6	12	-3	124.09	22.00

6	12	-3	108.49	15.20
-6	-12	3	105.49	16.00
-6	-12	3	97.19	13.20
-6	-12	3	83.49	10.70
-6	-12	3	102.59	15.70
-6	-12	3	111.59	12.10
5	12	-3	24.60	13.90
5	12	-3	16.20	6.80
-5	-12	3	32.10	9.60
-5	-12	3	16.40	6.90
-5	-12	3	14.70	6.60
-5	-12	3	21.60	9.20
-5	-12	3	22.60	7.20
4	12	-3	119.89	10.90
4	12	-3	122.69	17.70
-4	-12	3	92.09	12.00
-4	-12	3	109.59	14.10
-4	-12	3	118.39	11.50
-4	-12	3	89.49	14.20
-4	-12	3	125.89	16.10
3	12	-3	1871.91	82.39
-3	-12	3	1814.52	82.89
-3	-12	3	1787.72	83.09
-3	-12	3	1666.63	84.39
-3	-12	3	1829.52	82.79
-3	-12	3	1869.71	83.09
-3	-12	3	1715.93	83.69
2	12	-3	142.69	10.20
-2	-12	3	160.78	17.30
-2	-12	3	118.69	13.90
-2	-12	3	154.38	12.70
-2	-12	3	112.39	13.40
-2	-12	3	149.69	17.80
1	12	-3	-1.30	8.70
1	12	-3	5.00	4.20
-1	-12	3	22.60	12.20
-1	-12	3	16.70	7.60
-1	-12	3	7.60	6.60
-1	-12	3	6.30	7.40
-1	-12	3	-13.50	7.90
-1	-12	3	2.10	11.50
0	12	-3	173.68	16.10
0	12	-3	174.88	10.70
0	-12	3	129.79	20.20

0	-12	3	161.38	18.50
0	-12	3	142.79	13.70
0	-12	3	162.28	15.20
-1	12	-3	5.60	10.30
-1	12	-3	5.90	3.80
-1	12	-3	1.10	5.20
1	-12	3	6.00	7.50
1	-12	3	-8.10	9.70
1	-12	3	22.90	10.50
1	-12	3	-4.20	7.30
-2	12	-3	158.48	13.30
-2	12	-3	125.49	22.40
-2	12	-3	172.98	11.70
2	-12	3	172.68	18.80
2	-12	3	181.58	14.90
2	-12	3	116.39	16.40
2	-12	3	156.78	18.90
-3	12	-3	61.99	11.40
-3	12	-3	78.49	10.60
-3	12	-3	73.79	16.40
3	-12	3	70.39	9.60
3	-12	3	68.59	12.60
-4	12	-3	968.80	48.30
-4	12	-3	1015.60	49.50
4	-12	3	896.11	46.80
4	-12	3	917.81	46.50
-5	12	-3	49.40	10.80
-5	12	-3	75.79	12.90
-5	12	-3	39.80	11.80
5	-12	3	84.99	14.50
5	-12	3	71.29	9.60
-6	12	-3	23.60	13.10
-6	12	-3	5.90	10.80
-6	12	-3	7.40	11.40
6	-12	3	-1.00	6.70
6	-12	3	14.10	10.20
-7	12	-3	166.08	22.90
-7	12	-3	124.39	17.40
-7	12	-3	135.39	19.80
7	-12	3	129.89	24.10
7	-12	3	108.39	14.20
7	-12	3	108.59	18.70
-8	12	-3	138.29	21.70
-8	12	-3	129.89	21.30

-8	12	-3	135.59	22.10
8	-12	3	167.68	25.40
8	-12	3	101.49	15.00
-9	12	-3	-25.30	13.70
-9	12	-3	13.00	10.50
-9	12	-3	-4.00	12.90
9	-12	3	8.70	7.60
9	-12	3	7.70	13.20
-10	12	-3	49.30	13.80
-10	12	-3	24.70	10.40
-10	12	-3	77.09	16.70
10	-12	3	36.00	8.40
-11	-11	3	92.59	15.40
-11	-11	3	81.39	9.90
-10	-11	3	146.99	13.00
-10	-11	3	146.59	16.50
9	11	-3	0.10	12.90
-9	-11	3	23.80	14.20
-9	-11	3	-0.90	9.50
-9	-11	3	7.20	7.40
-9	-11	3	22.20	14.90
8	11	-3	645.94	41.90
8	11	-3	671.13	38.30
-8	-11	3	675.53	36.60
-8	-11	3	695.43	34.10
-8	-11	3	730.83	35.50
-8	-11	3	576.34	35.50
-8	-11	3	624.44	36.00
7	11	-3	153.98	20.00
7	11	-3	155.68	21.90
-7	-11	3	170.88	17.10
-7	-11	3	158.18	18.70
-7	-11	3	210.08	17.00
-7	-11	3	203.58	17.00
-7	-11	3	186.88	14.90
6	11	-3	37.50	8.40
6	11	-3	23.30	12.30
-6	-11	3	21.60	10.00
-6	-11	3	39.90	9.30
-6	-11	3	33.20	7.00
-6	-11	3	34.10	8.60
-6	-11	3	19.40	7.80
5	11	-3	82.99	11.10
5	11	-3	93.49	14.60

-5	-11	3	90.09	11.60
-5	-11	3	106.89	12.00
-5	-11	3	118.89	15.70
-5	-11	3	92.89	14.30
4	11	-3	-0.60	4.60
4	11	-3	18.70	14.00
-4	-11	3	2.90	8.80
-4	-11	3	2.50	6.00
-4	-11	3	5.80	6.30
-4	-11	3	-8.00	8.60
-4	-11	3	7.70	6.30
3	11	-3	439.96	21.60
3	11	-3	343.47	35.00
-3	-11	3	443.06	23.30
-3	-11	3	385.66	26.00
-3	-11	3	451.85	22.60
-3	-11	3	418.26	23.00
-3	-11	3	412.06	23.90
2	11	-3	1111.19	50.39
-2	-11	3	1061.09	51.49
-2	-11	3	1078.89	53.09
-2	-11	3	1162.78	59.99
-2	-11	3	1075.09	51.79
-2	-11	3	1033.30	51.49
1	11	-3	86.29	7.00
1	11	-3	81.29	10.40
-1	-11	3	98.79	12.70
-1	-11	3	78.99	12.00
-1	-11	3	80.99	14.70
-1	-11	3	79.79	11.30
0	11	-3	34.60	9.70
0	11	-3	34.30	7.20
0	11	-3	40.10	5.50
0	-11	3	20.40	7.60
0	-11	3	46.10	7.50
0	-11	3	46.90	8.70
-1	11	-3	20.40	11.20
-1	11	-3	9.80	3.60
-1	11	-3	-0.60	5.20
1	-11	3	-0.70	8.20
1	-11	3	10.00	6.60
-2	11	-3	629.74	39.10
-2	11	-3	672.83	30.90
-2	11	-3	670.23	32.90

2	-11	3	573.04	35.00
2	-11	3	633.94	34.90
2	-11	3	641.44	32.90
2	-11	3	658.13	33.40
-3	11	-3	499.25	35.00
-3	11	-3	496.45	25.60
-3	11	-3	472.55	28.20
-3	11	-3	462.25	26.70
3	-11	3	446.46	26.10
3	-11	3	482.55	26.40
-4	11	-3	-6.00	8.10
-4	11	-3	11.30	10.40
-4	11	-3	8.40	18.10
4	-11	3	6.10	6.70
4	-11	3	6.30	7.20
-5	11	-3	81.39	12.60
-5	11	-3	96.09	15.70
5	-11	3	83.89	15.60
5	-11	3	116.39	13.00
-6	11	-3	275.27	24.70
-6	11	-3	222.58	22.10
-6	11	-3	254.17	22.70
6	-11	3	300.97	23.30
6	-11	3	278.07	19.10
-7	11	-3	329.57	28.40
-7	11	-3	346.07	25.30
-7	11	-3	307.97	28.30
7	-11	3	354.86	22.20
7	-11	3	363.06	43.80
-8	11	-3	269.47	25.00
-8	11	-3	284.37	24.50
-8	11	-3	198.38	23.60
8	-11	3	260.37	22.00
8	-11	3	232.48	18.80
-9	11	-3	36.10	14.10
-9	11	-3	41.20	15.80
-9	11	-3	28.60	11.30
9	-11	3	38.30	11.60
9	-11	3	26.20	8.40
-10	11	-3	179.58	22.60
-10	11	-3	174.18	24.60
-10	11	-3	247.68	19.10
10	-11	3	212.18	21.00
10	-11	3	240.98	17.40

11	10	-3	53.29	11.90
-11	-10	3	45.20	7.70
-11	-10	3	22.10	10.50
10	10	-3	305.77	23.10
-10	-10	3	266.87	17.90
-10	-10	3	278.07	20.30
9	10	-3	4.40	14.00
-9	-10	3	3.20	7.50
-9	-10	3	-12.90	14.40
-9	-10	3	2.80	13.10
8	10	-3	8.20	14.80
8	10	-3	-16.50	11.40
-8	-10	3	19.70	8.10
-8	-10	3	15.30	6.40
-8	-10	3	20.70	11.90
-8	-10	3	8.30	10.90
7	10	-3	318.97	22.90
-7	-10	3	349.96	21.40
-7	-10	3	318.37	22.60
-7	-10	3	287.97	23.30
-7	-10	3	318.47	21.50
-7	-10	3	373.26	20.50
-7	-10	3	344.47	22.50
6	10	-3	174.38	14.30
-6	-10	3	166.98	13.10
-6	-10	3	151.18	14.30
-6	-10	3	163.98	15.80
-6	-10	3	173.58	15.20
-6	-10	3	168.58	15.90
-6	-10	3	136.09	19.10
5	10	-3	8.40	5.40
-5	-10	3	22.50	7.40
-5	-10	3	9.70	6.60
-5	-10	3	20.90	8.40
-5	-10	3	8.80	5.90
-5	-10	3	-0.50	9.80
-5	-10	3	5.10	7.30
4	10	-3	441.36	21.80
4	10	-3	368.06	30.40
-4	-10	3	417.26	23.50
-4	-10	3	421.56	23.60
-4	-10	3	428.56	22.50
-4	-10	3	366.06	24.40
-4	-10	3	433.06	25.80

-4	-10	3	446.06	22.60
3	10	-3	226.88	12.90
-3	-10	3	222.38	15.70
-3	-10	3	224.58	15.20
-3	-10	3	203.68	15.20
-3	-10	3	232.38	17.70
2	10	-3	1099.79	53.59
2	10	-3	922.21	47.60
2	10	-3	1019.00	47.30
-2	-10	3	1017.30	48.90
-2	-10	3	998.90	48.00
-2	-10	3	1063.69	48.30
-2	-10	3	1066.49	54.89
1	10	-3	50.49	7.40
1	10	-3	35.50	4.80
1	10	-3	34.70	9.70
-1	-10	3	27.40	9.60
0	10	-3	51.89	7.80
0	10	-3	42.90	5.00
0	10	-3	49.00	10.40
0	-10	3	36.70	9.00
0	-10	3	38.80	6.50
-1	10	-3	29.10	6.40
-1	10	-3	17.60	5.50
-1	10	-3	26.60	10.70
-1	10	-3	19.00	3.60
1	-10	3	0.20	11.10
1	-10	3	20.70	7.10
1	-10	3	16.50	7.10
-2	10	-3	54.49	6.10
-2	10	-3	56.09	9.70
2	-10	3	39.30	7.30
-3	10	-3	1274.07	59.99
-3	10	-3	1264.77	60.99
-3	10	-3	1248.18	57.89
3	-10	3	1236.28	59.39
3	-10	3	1248.18	59.29
-4	10	-3	301.87	22.60
-4	10	-3	313.27	20.30
-4	10	-3	258.37	30.10
4	-10	3	325.87	20.70
4	-10	3	298.07	19.00
-5	10	-3	161.58	16.30
-5	10	-3	166.38	18.40

-5	10	-3	155.48	21.90
5	-10	3	134.29	17.00
5	-10	3	149.39	19.50
-6	10	-3	69.09	12.20
-6	10	-3	62.49	11.50
-6	10	-3	73.29	13.20
6	-10	3	71.89	12.90
6	-10	3	48.00	8.50
-7	10	-3	70.69	14.40
-7	10	-3	85.19	13.80
-7	10	-3	83.29	13.10
7	-10	3	90.89	10.90
-8	10	-3	87.69	15.40
-8	10	-3	96.09	15.00
-8	10	-3	78.99	13.80
8	-10	3	64.89	11.70
8	-10	3	93.29	14.10
-9	10	-3	28.90	12.00
-9	10	-3	51.49	14.60
-9	10	-3	36.20	14.00
9	-10	3	47.40	11.20
9	-10	3	38.30	9.00
-10	10	-3	-12.40	15.40
-10	10	-3	5.10	13.80
-10	10	-3	26.40	10.80
10	-10	3	10.30	10.90
10	-10	3	2.10	9.40
-11	10	-3	139.99	16.20
-11	10	-3	115.09	20.30
11	-10	3	102.49	16.70
11	-10	3	112.59	19.20
11	9	-3	13.40	10.30
-11	-9	3	0.20	8.80
-11	-9	3	-6.50	7.00
10	9	-3	41.00	12.50
-10	-9	3	28.50	8.60
-10	-9	3	20.20	7.30
9	9	-3	704.63	38.70
-9	-9	3	696.63	35.10
-9	-9	3	576.54	37.30
-9	-9	3	686.73	34.50
8	9	-3	24.80	13.00
-8	-9	3	22.10	11.70
-8	-9	3	9.10	7.80

-8	-9	3	8.50	6.70
-8	-9	3	-2.80	17.40
-8	-9	3	1.50	9.30
7	9	-3	533.95	30.10
-7	-9	3	478.35	29.30
-7	-9	3	573.14	29.60
-7	-9	3	585.34	30.70
-7	-9	3	589.04	29.00
-7	-9	3	546.45	31.60
-7	-9	3	518.85	31.10
6	9	-3	59.49	8.10
-6	-9	3	74.79	12.90
-6	-9	3	75.89	11.30
-6	-9	3	80.69	13.90
-6	-9	3	76.69	13.90
-6	-9	3	66.19	10.90
-6	-9	3	79.79	10.80
5	9	-3	39.70	6.00
-5	-9	3	37.60	8.10
-5	-9	3	38.20	7.10
-5	-9	3	40.80	8.50
-5	-9	3	47.50	10.30
-5	-9	3	27.00	5.70
-5	-9	3	45.30	8.70
4	9	-3	21.20	4.90
-4	-9	3	22.70	10.20
-4	-9	3	28.20	7.70
-4	-9	3	23.70	6.20
-4	-9	3	27.20	6.60
-4	-9	3	29.20	9.20
-4	-9	3	30.00	7.40
3	9	-3	29.60	5.20
3	9	-3	5.10	9.80
3	9	-3	40.50	6.50
-3	-9	3	27.50	5.90
-3	-9	3	40.70	7.70
-3	-9	3	19.10	6.30
-3	-9	3	29.10	4.80
2	9	-3	533.65	26.80
2	9	-3	575.54	26.60
2	9	-3	565.14	31.10
-2	-9	3	582.54	27.70
-2	-9	3	537.55	28.20
1	9	-3	1564.24	72.49

1	9	-3	1700.53	75.09
1	9	-3	1597.94	71.89
-1	-9	3	1452.65	75.09
-1	-9	3	1614.94	72.69
0	9	-3	67.49	5.40
0	9	-3	69.59	7.00
0	9	-3	61.49	8.90
0	-9	3	66.09	9.20
-1	9	-3	521.05	29.70
-1	9	-3	540.75	26.90
-1	9	-3	550.24	27.60
-1	9	-3	534.65	25.40
1	-9	3	530.05	29.60
1	-9	3	546.35	27.30
-2	9	-3	393.56	25.80
-2	9	-3	407.16	21.90
-2	9	-3	405.16	22.10
-2	9	-3	429.56	19.90
2	-9	3	383.96	25.90
2	-9	3	393.76	22.50
-3	9	-3	771.32	36.40
-3	9	-3	702.03	36.90
-3	9	-3	674.63	33.00
3	-9	3	641.74	35.20
-4	9	-3	9.70	9.40
-4	9	-3	15.80	6.70
-5	9	-3	190.28	18.70
-5	9	-3	216.88	16.70
-5	9	-3	211.38	19.70
5	-9	3	200.08	15.60
5	-9	3	176.18	19.40
-6	9	-3	47.10	10.00
-6	9	-3	34.10	11.40
-6	9	-3	38.60	11.00
6	-9	3	38.60	12.90
6	-9	3	35.20	8.40
-7	9	-3	49.50	10.90
-7	9	-3	64.69	12.30
-7	9	-3	35.30	10.60
-8	9	-3	338.27	26.00
-8	9	-3	355.86	26.90
-8	9	-3	398.96	26.90
8	-9	3	341.57	23.00
-9	9	-3	424.66	30.40

9	-9	3	489.55	30.40
-10	9	-3	22.00	11.10
-10	9	-3	15.20	14.80
-10	9	-3	12.90	13.10
10	-9	3	23.70	10.00
10	-9	3	6.20	9.90
-11	9	-3	27.40	22.30
-11	9	-3	-17.60	16.60
-11	9	-3	14.20	10.30
11	-9	3	26.50	10.90
11	-9	3	14.00	16.00
11	8	-3	37.90	11.90
-11	-8	3	35.50	8.20
-11	-8	3	39.80	9.00
10	8	-3	11.20	11.60
-10	-8	3	2.30	8.30
-10	-8	3	9.90	7.20
-9	-8	3	5.60	12.90
-9	-8	3	14.80	7.20
-9	-8	3	10.30	12.60
-9	-8	3	14.70	10.10
-9	-8	3	6.60	7.30
8	8	-3	47.20	12.50
-8	-8	3	62.79	10.10
-8	-8	3	48.00	10.00
-8	-8	3	54.29	11.70
-8	-8	3	54.29	8.40
-8	-8	3	35.10	7.20
7	8	-3	287.37	18.70
-7	-8	3	236.18	20.20
-7	-8	3	261.07	17.90
-7	-8	3	271.97	19.90
-7	-8	3	277.67	17.40
-7	-8	3	307.07	18.30
6	8	-3	229.58	14.60
-6	-8	3	232.48	14.90
-6	-8	3	192.58	15.80
-6	-8	3	214.78	18.80
-6	-8	3	227.68	17.30
-6	-8	3	186.68	17.90
5	8	-3	4.90	4.40
-5	-8	3	5.80	5.30
-5	-8	3	5.80	8.10
-5	-8	3	-3.20	4.80

-5	-8	3	-3.30	7.10
-5	-8	3	17.30	8.30
-5	-8	3	-8.10	8.60
4	8	-3	1301.27	55.49
4	8	-3	1055.99	58.89
-4	-8	3	1160.28	55.89
-4	-8	3	1162.18	56.69
-4	-8	3	1270.17	56.89
-4	-8	3	1244.18	55.99
3	8	-3	2405.96	101.29
3	8	-3	2220.98	100.69
3	8	-3	2061.19	103.69
-3	-8	3	2240.08	100.69
-3	-8	3	2282.47	101.99
2	8	-3	364.76	17.90
2	8	-3	351.96	17.70
-2	-8	3	368.76	19.40
-2	-8	3	373.16	18.20
1	8	-3	220.48	11.00
-1	-8	3	193.78	14.40
-1	-8	3	213.38	15.70
0	8	-3	47.40	4.20
0	8	-3	47.80	5.40
0	-8	3	34.30	6.10
0	-8	3	39.90	5.80
0	-8	3	54.29	9.80
-1	8	-3	30.30	3.90
-1	8	-3	37.10	4.90
1	-8	3	23.70	6.20
1	-8	3	29.20	5.60
-2	8	-3	16.90	4.10
-2	8	-3	-10.90	8.20
-2	8	-3	10.90	2.80
2	-8	3	12.20	5.10
2	-8	3	7.60	6.40
2	-8	3	12.30	9.80
-3	8	-3	38.10	6.20
-3	8	-3	42.10	9.90
-3	8	-3	51.49	8.60
-3	8	-3	51.99	8.70
3	-8	3	30.80	8.10
3	-8	3	38.20	6.80
-4	8	-3	2095.49	99.09
-4	8	-3	2253.47	99.19

4	-8	3	2137.49	99.39
4	-8	3	2066.99	97.59
-5	8	-3	7.50	16.10
-5	8	-3	16.20	9.00
-5	8	-3	11.40	9.80
-5	8	-3	14.70	6.70
5	-8	3	11.40	10.80
5	-8	3	7.20	8.70
5	-8	3	16.20	7.10
-6	8	-3	10.00	9.70
-6	8	-3	16.70	9.70
-6	8	-3	17.60	7.70
6	-8	3	12.60	17.90
-7	8	-3	459.95	28.50
-7	8	-3	440.46	27.40
-7	8	-3	457.55	28.50
7	-8	3	463.25	26.50
-8	8	-3	216.78	20.20
-8	8	-3	177.78	20.10
8	-8	3	172.98	17.00
-9	8	-3	5.10	10.70
-9	8	-3	15.80	11.20
-9	8	-3	40.20	11.60
9	-8	3	9.40	10.00
9	-8	3	1.40	8.80
-10	8	-3	94.09	15.70
-10	8	-3	115.49	17.70
-10	8	-3	129.39	20.00
10	-8	3	106.39	15.50
10	-8	3	147.69	18.30
-11	8	-3	54.69	11.80
-11	8	-3	73.29	17.60
-11	8	-3	58.19	14.40
11	-8	3	93.79	15.70
11	-8	3	71.09	13.90
-12	-7	3	-8.10	9.30
11	7	-3	53.59	12.30
-11	-7	3	43.50	8.90
-11	-7	3	53.39	9.20
10	7	-3	142.69	21.50
-10	-7	3	187.68	16.10
-10	-7	3	160.18	15.70
-9	-7	3	129.69	17.70
-9	-7	3	106.89	18.70

-9	-7	3	100.09	13.20
-9	-7	3	89.89	14.00
-9	-7	3	98.89	13.00
8	7	-3	1432.16	70.89
-8	-7	3	1501.25	69.69
-8	-7	3	1526.45	69.89
-8	-7	3	1420.86	69.89
-8	-7	3	1512.75	70.59
-8	-7	3	1504.05	69.59
7	7	-3	13.40	6.80
-7	-7	3	17.70	6.70
-7	-7	3	0.70	9.00
-7	-7	3	0.00	6.70
-7	-7	3	36.80	14.00
-7	-7	3	3.40	8.50
-7	-7	3	4.00	6.00
6	7	-3	27.70	5.70
-6	-7	3	20.90	6.00
-6	-7	3	29.40	7.30
-6	-7	3	16.40	5.70
-6	-7	3	11.80	8.60
-6	-7	3	21.50	9.30
-6	-7	3	8.50	9.70
5	7	-3	26.80	13.50
5	7	-3	20.90	4.70
-5	-7	3	13.70	5.00
-5	-7	3	25.50	8.30
-5	-7	3	22.70	8.90
-5	-7	3	17.40	5.30
-5	-7	3	13.90	8.50
-5	-7	3	29.20	7.30
4	7	-3	-1.60	2.70
4	7	-3	8.30	7.10
-4	-7	3	2.50	4.70
3	7	-3	397.46	18.50
-3	-7	3	356.76	19.80
-3	-7	3	372.66	17.80
2	7	-3	377.36	18.00
2	7	-3	379.96	18.00
2	7	-3	364.26	21.00
-2	-7	3	391.06	18.20
-2	-7	3	352.46	19.70
1	7	-3	911.51	40.50
1	7	-3	844.32	38.40

1	7	-3	859.91	38.10
-1	-7	3	753.02	38.30
-1	-7	3	886.11	38.60
-1	-7	3	730.43	40.50
0	7	-3	14.60	2.40
0	7	-3	15.30	4.90
0	-7	3	7.20	4.60
0	-7	3	19.40	4.20
-1	7	-3	17.10	5.10
-1	7	-3	23.10	3.30
-1	7	-3	23.10	3.50
1	-7	3	25.60	4.20
1	-7	3	11.70	5.20
1	-7	3	24.30	4.60
-2	7	-3	805.12	36.00
-2	7	-3	790.42	37.20
2	-7	3	794.22	36.90
2	-7	3	738.23	38.60
-3	7	-3	390.76	20.20
-3	7	-3	402.26	19.20
3	-7	3	375.76	20.60
3	-7	3	332.57	21.80
3	-7	3	382.46	19.60
-4	7	-3	188.18	14.20
-4	7	-3	197.58	13.50
-4	7	-3	190.68	17.40
-4	7	-3	267.17	27.30
4	-7	3	185.68	15.30
4	-7	3	204.28	13.10
4	-7	3	209.08	24.00
-5	7	-3	99.49	11.30
-5	7	-3	126.49	16.30
-5	7	-3	104.89	14.60
5	-7	3	116.89	11.90
-6	7	-3	487.85	27.00
-6	7	-3	503.25	29.00
-6	7	-3	482.55	28.30
6	-7	3	466.55	27.70
6	-7	3	499.75	26.90
-7	7	-3	234.68	17.70
-7	7	-3	205.08	18.60
-7	7	-3	229.68	19.80
7	-7	3	236.28	18.10
-8	7	-3	70.29	10.80

-8	7	-3	74.69	12.20
-8	7	-3	67.89	11.00
8	-7	3	67.79	10.20
-9	7	-3	26.40	10.90
-9	7	-3	9.00	9.90
-9	7	-3	23.80	10.50
9	-7	3	17.40	9.10
-10	7	-3	123.99	19.30
-10	7	-3	114.89	19.20
-10	7	-3	111.39	18.70
10	-7	3	100.79	17.20
-11	7	-3	41.80	11.30
-11	7	-3	44.50	13.60
-11	7	-3	22.70	14.90
11	-7	3	55.39	10.30
11	-7	3	36.00	14.20
12	6	-3	44.70	12.00
-12	-6	3	37.00	9.40
-12	-6	3	34.60	11.40
11	6	-3	7.40	11.10
-11	-6	3	6.20	7.80
-11	-6	3	3.50	8.30
10	6	-3	226.18	26.70
-10	-6	3	283.17	20.00
9	6	-3	9.80	12.50
-9	-6	3	10.50	6.70
-9	-6	3	8.70	11.60
-9	-6	3	16.50	11.60
-9	-6	3	18.00	11.10
8	6	-3	55.19	10.40
8	6	-3	32.20	12.40
-8	-6	3	65.69	11.00
-8	-6	3	50.49	8.70
-8	-6	3	48.90	10.40
-8	-6	3	69.89	11.50
7	6	-3	131.69	12.50
7	6	-3	157.58	20.50
-7	-6	3	130.59	14.70
-7	-6	3	144.89	17.90
-7	-6	3	130.79	15.90
-7	-6	3	116.79	11.60
-7	-6	3	137.29	12.00
6	6	-3	58.39	8.40
-6	-6	3	63.09	8.70

-6	-6	3	54.89	9.20
-6	-6	3	60.79	9.80
-6	-6	3	74.39	10.00
-6	-6	3	57.39	11.00
-6	-6	3	64.99	10.90
5	6	-3	314.17	15.90
5	6	-3	275.77	20.80
5	6	-3	273.97	20.00
-5	-6	3	275.37	24.10
-5	-6	3	290.57	17.40
-5	-6	3	328.57	16.50
-5	-6	3	301.27	20.20
4	6	-3	217.48	11.30
4	6	-3	186.28	14.00
-4	-6	3	204.88	12.50
3	6	-3	913.11	42.90
3	6	-3	910.61	41.30
-3	-6	3	844.72	41.90
-3	-6	3	925.21	40.70
2	6	-3	729.03	33.80
2	6	-3	721.03	33.70
-2	-6	3	755.32	33.80
1	6	-3	667.83	31.30
1	6	-3	673.83	31.10
1	6	-3	728.03	33.10
-1	-6	3	717.03	31.40
-1	-6	3	602.64	31.40
0	6	-3	189.78	11.80
0	6	-3	183.78	9.70
0	6	-3	177.38	9.20
0	-6	3	191.78	12.20
0	-6	3	185.68	9.90
0	-6	3	167.88	10.20
-1	6	-3	811.72	35.80
-1	6	-3	751.92	32.90
1	-6	3	635.44	34.90
1	-6	3	651.43	35.00
1	-6	3	734.13	33.50
-2	6	-3	1138.89	50.79
-2	6	-3	1066.09	52.79
-2	6	-3	1173.68	51.69
2	-6	3	1132.89	51.19
2	-6	3	1074.69	52.49
-3	6	-3	1596.74	73.09

-3	6	-3	1643.14	74.29
3	-6	3	1612.94	73.49
3	-6	3	1673.93	74.39
3	-6	3	1567.04	74.99
-4	6	-3	14.90	4.70
-4	6	-3	17.80	5.00
-4	6	-3	19.20	6.70
4	-6	3	14.30	5.90
4	-6	3	29.20	7.20
4	-6	3	24.90	4.40
-5	6	-3	602.64	33.40
-5	6	-3	613.44	34.80
-5	6	-3	640.64	34.20
-5	6	-3	625.64	32.70
5	-6	3	617.84	30.30
-6	6	-3	61.09	8.50
-6	6	-3	59.19	14.10
-6	6	-3	78.89	10.60
-6	6	-3	78.49	10.90
6	-6	3	44.70	8.50
6	-6	3	50.99	7.80
-7	6	-3	18.90	7.40
-7	6	-3	10.00	8.50
-7	6	-3	20.90	9.70
7	-6	3	1.00	9.90
7	-6	3	7.70	7.70
-8	6	-3	259.17	21.00
-8	6	-3	271.67	19.60
-8	6	-3	282.97	18.80
8	-6	3	213.28	19.80
-9	6	-3	-6.00	9.30
-9	6	-3	-0.60	8.70
-9	6	-3	-3.40	9.30
9	-6	3	5.40	9.20
-10	6	-3	13.50	11.60
-10	6	-3	28.70	11.60
-10	6	-3	-3.70	11.40
10	-6	3	16.80	11.70
-11	6	-3	207.78	20.20
-11	6	-3	207.48	21.20
-11	6	-3	189.68	19.20
11	-6	3	192.68	24.00
11	-6	3	194.38	17.30
-12	6	-3	104.39	16.50

-12	6	-3	107.89	17.30
12	-6	3	110.89	19.40
12	5	-3	40.90	13.50
-12	-5	3	59.69	9.20
-12	-5	3	57.09	12.60
11	5	-3	-6.60	11.60
11	5	-3	30.90	15.20
-11	-5	3	6.60	7.60
-11	-5	3	7.20	8.60
10	5	-3	220.68	28.60
-10	-5	3	165.18	26.60
-10	-5	3	162.88	16.90
-10	-5	3	144.59	18.40
9	5	-3	1072.09	57.49
-9	-5	3	1161.28	61.79
-9	-5	3	1192.98	55.39
-9	-5	3	1131.39	56.09
-9	-5	3	1173.98	57.39
-9	-5	3	1199.98	55.99
8	5	-3	47.10	11.00
8	5	-3	33.70	9.90
-8	-5	3	43.80	10.90
-8	-5	3	43.80	7.60
-8	-5	3	56.19	10.30
-8	-5	3	48.60	8.60
-8	-5	3	58.19	8.60
7	5	-3	302.87	18.10
7	5	-3	272.67	22.90
-7	-5	3	307.27	22.00
-7	-5	3	338.17	18.30
-7	-5	3	291.57	19.30
-7	-5	3	281.37	20.00
-7	-5	3	307.17	17.70
6	5	-3	3.90	5.40
6	5	-3	21.90	11.30
-6	-5	3	15.80	8.00
-6	-5	3	7.70	6.00
-6	-5	3	6.90	5.20
-6	-5	3	7.20	5.90
-6	-5	3	-12.30	8.70
5	5	-3	2.60	3.10
5	5	-3	7.10	6.60
5	5	-3	-2.20	6.50
-5	-5	3	0.50	5.90

-5	-5	3	2.80	4.50
4	5	-3	81.19	8.30
4	5	-3	97.49	8.70
4	5	-3	76.09	5.90
-4	-5	3	61.39	8.00
3	5	-3	11.20	2.40
-3	-5	3	12.50	1.80
2	5	-3	845.82	39.60
2	5	-3	842.62	38.10
2	5	-3	870.21	38.30
-2	-5	3	809.92	39.50
-2	-5	3	916.71	38.20
-2	-5	3	755.62	38.30
1	5	-3	1732.23	79.69
1	5	-3	1803.72	79.89
1	5	-3	1888.61	81.19
-1	-5	3	1575.74	79.79
-1	-5	3	1887.81	79.99
0	5	-3	250.47	12.40
0	5	-3	252.37	12.00
0	5	-3	264.27	13.90
0	-5	3	254.17	12.50
0	-5	3	248.88	14.30
0	-5	3	227.88	12.60
-1	5	-3	1105.19	48.50
-1	5	-3	1050.49	46.80
-1	5	-3	1037.20	47.40
1	-5	3	1047.90	47.40
1	-5	3	998.30	47.30
1	-5	3	979.10	48.20
-2	5	-3	2.20	1.90
-2	5	-3	1.30	3.00
-2	5	-3	1.20	2.30
2	-5	3	-0.40	2.40
2	-5	3	2.90	2.40
2	-5	3	0.40	3.10
-3	5	-3	780.12	35.20
-3	5	-3	716.43	34.10
3	-5	3	743.23	35.80
3	-5	3	737.93	34.40
3	-5	3	734.93	35.20
-4	5	-3	67.49	7.50
-4	5	-3	72.89	7.70
-4	5	-3	65.59	9.70

4	-5	3	66.19	6.20
4	-5	3	90.59	8.70
4	-5	3	49.80	9.10
-5	5	-3	257.67	14.80
-5	5	-3	239.78	16.40
-5	5	-3	233.68	17.00
5	-5	3	232.38	13.50
5	-5	3	248.28	16.70
-6	5	-3	426.76	31.50
-6	5	-3	400.16	29.40
-6	5	-3	396.26	24.20
-6	5	-3	428.46	23.90
-6	5	-3	413.16	22.60
6	-5	3	390.96	24.10
6	-5	3	440.96	22.00
-7	5	-3	268.37	26.40
-7	5	-3	263.77	19.30
-7	5	-3	304.47	18.50
-7	5	-3	296.67	20.10
-7	5	-3	308.47	27.60
7	-5	3	300.07	19.40
7	-5	3	311.07	20.20
-8	5	-3	409.16	24.50
-8	5	-3	406.96	30.40
-8	5	-3	438.86	25.70
-8	5	-3	388.46	23.50
8	-5	3	424.26	31.20
8	-5	3	388.26	25.50
-9	5	-3	138.29	18.00
-9	5	-3	164.28	17.10
-9	5	-3	184.48	18.60
-9	5	-3	139.99	16.60
9	-5	3	190.48	18.50
-10	5	-3	70.19	12.10
-10	5	-3	65.19	15.80
10	-5	3	42.50	16.20
10	-5	3	56.79	13.00
-11	5	-3	9.40	11.00
-11	5	-3	15.10	9.80
-11	5	-3	24.40	16.10
11	-5	3	5.90	10.00
11	-5	3	25.90	17.30
-12	5	-3	230.08	19.30
-12	5	-3	180.58	22.30

12	-5	3	239.58	18.80
12	4	-3	-1.30	16.10
12	4	-3	30.00	13.20
-12	-4	3	8.40	13.80
-12	-4	3	20.90	8.00
11	4	-3	45.30	13.20
11	4	-3	20.30	12.90
-11	-4	3	29.60	7.80
-11	-4	3	29.70	9.20
10	4	-3	-7.40	13.60
-10	-4	3	14.50	7.00
-10	-4	3	2.20	8.10
9	4	-3	22.10	10.50
-9	-4	3	21.80	12.90
-9	-4	3	-0.10	10.90
-9	-4	3	31.80	7.90
-9	-4	3	6.50	7.30
-9	-4	3	26.80	10.20
8	4	-3	73.29	11.20
-8	-4	3	80.19	12.20
-8	-4	3	55.09	11.50
-8	-4	3	58.89	10.90
-8	-4	3	49.90	7.50
-8	-4	3	63.09	8.80
7	4	-3	320.57	22.40
7	4	-3	315.67	18.30
-7	-4	3	292.27	21.90
-7	-4	3	333.77	18.60
-7	-4	3	314.17	17.90
-7	-4	3	282.37	20.70
-7	-4	3	302.07	18.60
6	4	-3	289.77	21.80
6	4	-3	291.47	22.00
6	4	-3	307.07	16.50
6	4	-3	323.97	24.90
-6	-4	3	316.97	16.50
-6	-4	3	287.67	17.50
-6	-4	3	293.17	17.80
-6	-4	3	255.87	20.70
5	4	-3	309.17	18.40
5	4	-3	308.17	15.80
-5	-4	3	306.07	16.50
4	4	-3	1966.20	90.19
4	4	-3	1954.20	91.09

4	4	-3	2088.49	91.19
3	4	-3	818.92	35.20
3	4	-3	778.72	34.40
-3	-4	3	717.53	33.70
-3	-4	3	658.63	34.40
-3	-4	3	769.52	33.70
2	4	-3	871.61	39.90
2	4	-3	891.31	40.90
2	4	-3	874.81	40.10
-2	-4	3	897.11	39.90
1	4	-3	30.00	2.80
1	4	-3	33.90	2.70
1	4	-3	39.30	5.20
-1	-4	3	30.20	4.90
-1	-4	3	26.30	4.30
-1	-4	3	33.60	2.80
0	4	-3	43.10	5.20
0	4	-3	43.40	3.20
0	4	-3	45.10	3.40
0	-4	3	42.20	3.50
0	-4	3	36.00	4.70
0	-4	3	45.80	6.00
-1	4	-3	253.87	12.10
-1	4	-3	240.28	11.50
-1	4	-3	248.18	12.90
1	-4	3	241.28	12.00
1	-4	3	228.38	12.00
1	-4	3	230.98	12.80
-2	4	-3	19.90	3.30
-2	4	-3	21.80	3.10
-2	4	-3	23.20	3.70
2	-4	3	22.30	3.20
2	-4	3	16.50	3.00
2	-4	3	22.50	3.70
-3	4	-3	1166.88	51.99
-3	4	-3	1065.79	50.39
-3	4	-3	1135.99	51.29
3	-4	3	1105.19	50.59
3	-4	3	1096.89	51.29
3	-4	3	1116.29	51.69
-4	4	-3	703.63	34.00
-4	4	-3	662.63	31.50
4	-4	3	632.04	31.50
4	-4	3	633.04	30.30

4	-4	3	635.44	32.10
-5	4	-3	53.99	9.40
-5	4	-3	72.49	12.60
-5	4	-3	66.19	8.20
5	-4	3	68.29	11.80
5	-4	3	83.29	6.30
5	-4	3	62.19	9.70
-6	4	-3	31.80	9.50
-6	4	-3	30.00	14.70
-6	4	-3	25.70	8.80
-6	4	-3	11.90	4.80
-6	4	-3	22.40	16.20
-6	4	-3	25.20	8.10
-6	4	-3	17.20	6.40
6	-4	3	16.60	4.50
6	-4	3	12.40	9.20
-7	4	-3	1012.60	54.39
-7	4	-3	1053.49	54.39
-7	4	-3	1076.79	52.19
-7	4	-3	1113.79	51.39
-7	4	-3	1051.29	51.59
7	-4	3	1104.19	50.89
7	-4	3	1091.29	52.39
-8	4	-3	50.29	8.70
-8	4	-3	83.69	11.00
-8	4	-3	19.80	15.30
-8	4	-3	60.99	10.00
-8	4	-3	42.10	12.70
8	-4	3	48.00	10.80
8	-4	3	56.59	10.20
-9	4	-3	2.50	13.10
-9	4	-3	-4.80	7.90
-9	4	-3	9.10	8.80
9	-4	3	2.60	9.90
-10	4	-3	87.59	12.50
-10	4	-3	116.89	16.30
-10	4	-3	154.38	17.10
10	-4	3	125.49	18.10
-11	4	-3	12.90	11.40
-11	4	-3	3.60	10.60
-11	4	-3	18.70	9.60
11	-4	3	17.10	9.30
-12	4	-3	93.09	22.80
-12	4	-3	106.29	19.10

-12	4	-3	130.89	14.60
12	-4	3	105.99	15.70
12	3	-3	9.70	22.30
12	3	-3	1.40	14.70
-12	-3	3	0.50	14.00
-12	-3	3	10.70	7.60
11	3	-3	1.70	11.70
11	3	-3	-13.40	12.80
-11	-3	3	12.40	9.20
-11	-3	3	7.90	6.90
10	3	-3	30.40	12.00
-10	-3	3	52.89	8.90
-10	-3	3	28.50	9.00
9	3	-3	364.96	24.00
-9	-3	3	332.27	20.40
-9	-3	3	342.47	21.80
-9	-3	3	302.87	29.10
-9	-3	3	334.77	25.80
-9	-3	3	319.17	21.60
8	3	-3	1038.80	51.09
8	3	-3	1022.30	51.39
-8	-3	3	1076.99	50.59
-8	-3	3	1022.50	49.50
-8	-3	3	1047.70	52.39
-8	-3	3	1037.30	49.90
7	3	-3	110.99	15.20
7	3	-3	107.49	11.30
-7	-3	3	112.59	17.80
-7	-3	3	96.19	12.00
-7	-3	3	123.79	16.10
-7	-3	3	111.09	10.80
-7	-3	3	111.09	11.30
6	3	-3	1.50	8.20
6	3	-3	3.80	3.80
6	3	-3	5.50	8.60
6	3	-3	-1.90	8.50
-6	-3	3	19.60	9.40
-6	-3	3	9.70	5.70
-6	-3	3	8.60	5.00
-6	-3	3	6.00	5.90
5	3	-3	410.16	22.00
5	3	-3	419.76	20.40
-5	-3	3	419.56	21.10
4	3	-3	93.39	6.10

4	3	-3	91.39	7.60
3	3	-3	304.67	14.20
3	3	-3	284.67	13.60
3	3	-3	284.07	14.10
-3	-3	3	250.17	13.80
-3	-3	3	275.17	12.90
-3	-3	3	275.87	12.90
2	3	-3	59.59	4.50
-2	-3	3	51.89	4.30
-2	-3	3	53.89	3.20
1	3	-3	115.69	5.50
1	3	-3	99.69	6.80
1	3	-3	101.09	5.50
-1	-3	3	109.69	5.50
-1	-3	3	101.29	6.60
-1	-3	3	93.09	6.20
0	3	-3	1914.11	84.29
0	3	-3	2035.20	85.19
0	3	-3	1966.40	84.59
0	-3	3	1693.83	84.59
0	-3	3	1693.73	85.29
0	-3	3	2014.30	84.59
-1	3	-3	7.60	1.50
-1	3	-3	4.80	2.10
-1	3	-3	5.60	1.60
1	-3	3	6.00	1.90
1	-3	3	2.40	1.50
1	-3	3	11.80	2.50
-2	3	-3	1212.88	53.79
-2	3	-3	1129.09	52.79
-2	3	-3	1191.88	53.39
2	-3	3	1165.68	52.89
2	-3	3	1182.18	53.39
2	-3	3	1169.88	53.69
-3	3	-3	72.19	5.30
3	-3	3	66.89	4.40
3	-3	3	69.89	5.60
-4	3	-3	93.19	11.40
-4	3	-3	91.09	7.00
4	-3	3	87.29	7.40
4	-3	3	93.39	8.20
4	-3	3	90.59	5.80
-5	3	-3	1276.17	58.19
-5	3	-3	1222.58	59.19

-5	3	-3	1254.27	57.79
5	-3	3	1187.58	58.09
5	-3	3	1287.47	57.09
5	-3	3	1270.97	59.19
-6	3	-3	472.75	26.00
-6	3	-3	461.75	25.20
-6	3	-3	459.05	26.40
-6	3	-3	459.15	28.00
-6	3	-3	515.65	24.30
6	-3	3	462.05	23.10
6	-3	3	457.45	26.10
-7	3	-3	4.00	7.10
-7	3	-3	20.10	8.00
-7	3	-3	15.80	10.90
-7	3	-3	2.60	11.20
-7	3	-3	6.60	5.30
7	-3	3	7.60	5.00
7	-3	3	10.50	9.00
-8	3	-3	7.80	6.10
-8	3	-3	-4.80	7.60
-8	3	-3	9.00	8.40
-8	3	-3	-8.50	10.70
8	-3	3	2.00	9.50
8	-3	3	8.50	8.00
-9	3	-3	95.49	13.60
-9	3	-3	110.39	12.80
-9	3	-3	93.69	12.90
-9	3	-3	114.39	20.30
-9	3	-3	112.19	15.20
9	-3	3	120.79	18.20
-10	3	-3	163.58	18.00
-10	3	-3	161.78	15.40
-10	3	-3	152.18	16.50
10	-3	3	127.99	22.80
-11	3	-3	94.19	15.20
-11	3	-3	57.49	11.90
-11	3	-3	53.89	11.90
11	-3	3	75.99	12.00
-12	3	-3	31.10	15.90
-12	3	-3	13.10	15.80
-12	3	-3	29.20	9.70
12	-3	3	35.30	10.20
12	2	-3	18.50	13.00
12	2	-3	24.90	15.90

-12	-2	3	-11.40	15.80
-12	-2	3	12.20	7.20
11	2	-3	-8.10	10.80
-11	-2	3	-1.80	6.70
-11	-2	3	-2.10	10.20
10	2	-3	253.97	22.00
-10	-2	3	271.57	18.30
-10	-2	3	244.78	20.10
9	2	-3	5.30	9.20
-9	-2	3	12.20	6.50
-9	-2	3	14.80	8.30
8	2	-3	116.99	16.00
8	2	-3	93.69	15.70
-8	-2	3	91.99	22.70
-8	-2	3	79.79	13.10
-8	-2	3	105.49	15.30
-8	-2	3	88.29	11.50
-8	-2	3	101.39	12.50
7	2	-3	119.79	15.30
-7	-2	3	156.88	16.50
-7	-2	3	120.89	11.90
-7	-2	3	170.28	19.70
-7	-2	3	121.89	12.80
-7	-2	3	118.49	11.20
6	2	-3	16.10	8.10
6	2	-3	7.60	7.70
-6	-2	3	27.60	7.20
-6	-2	3	17.90	5.60
-6	-2	3	17.70	6.20
5	2	-3	34.40	4.80
5	2	-3	28.20	5.30
5	2	-3	45.80	6.10
-5	-2	3	44.70	5.70
4	2	-3	26.50	4.80
4	2	-3	31.80	4.80
4	2	-3	34.90	3.90
-4	-2	3	26.30	2.90
-4	-2	3	37.20	6.20
-4	-2	3	30.10	2.40
3	2	-3	1676.03	70.19
3	2	-3	1528.95	69.79
3	2	-3	1592.74	70.19
-3	-2	3	1377.06	69.79
-3	-2	3	1579.34	69.39

-3	-2	3	1558.94	69.39
2	2	-3	12.90	1.80
2	2	-3	15.10	2.10
-2	-2	3	15.90	1.70
-2	-2	3	10.70	1.90
1	2	-3	101.99	5.60
1	2	-3	102.69	6.20
1	2	-3	109.89	5.50
-1	-2	3	112.69	5.40
-1	-2	3	107.49	5.90
0	2	-3	1969.80	83.89
0	2	-3	2043.90	84.19
0	2	-3	1908.21	83.59
0	-2	3	1602.44	83.89
0	-2	3	1722.23	84.29
0	-2	3	1998.50	83.89
-1	2	-3	3168.58	147.19
-1	2	-3	3583.84	147.89
-1	2	-3	3300.47	147.59
1	-2	3	3247.48	147.79
1	-2	3	3255.77	147.39
1	-2	3	3292.87	147.59
-2	2	-3	1643.84	73.29
-2	2	-3	1581.74	73.59
2	-2	3	1653.43	73.39
2	-2	3	1637.44	73.59
-3	2	-3	560.84	24.70
-3	2	-3	497.65	24.90
3	-2	3	520.45	24.00
3	-2	3	516.15	25.00
3	-2	3	520.55	24.60
-4	2	-3	23.00	4.30
-4	2	-3	30.10	4.30
-4	2	-3	35.30	4.80
4	-2	3	20.20	3.50
4	-2	3	30.80	3.10
4	-2	3	24.70	3.80
-5	2	-3	187.18	11.30
-5	2	-3	182.48	12.80
-5	2	-3	177.18	10.80
5	-2	3	178.28	9.60
5	-2	3	156.58	12.30
5	-2	3	176.58	11.40
-6	2	-3	327.77	22.50

-6	2	-3	311.67	19.60
-6	2	-3	331.27	19.20
-6	2	-3	304.27	17.90
-6	2	-3	320.47	19.00
6	-2	3	300.77	18.90
6	-2	3	333.77	16.40
-7	2	-3	16.00	5.00
-7	2	-3	21.80	10.80
-7	2	-3	11.10	10.30
-7	2	-3	27.10	6.60
-7	2	-3	10.10	7.70
7	-2	3	21.70	10.00
7	-2	3	22.20	5.10
-8	2	-3	331.37	18.60
-8	2	-3	277.17	23.50
-8	2	-3	312.17	19.60
-8	2	-3	337.67	23.80
-8	2	-3	331.07	20.70
8	-2	3	250.77	23.30
8	-2	3	308.17	19.10
-9	2	-3	21.70	11.00
-9	2	-3	52.79	12.10
-9	2	-3	29.10	8.30
-9	2	-3	36.10	7.80
-9	2	-3	25.90	8.70
9	-2	3	31.50	12.50
9	-2	3	22.00	11.90
-10	2	-3	22.40	8.20
-10	2	-3	30.40	8.90
-10	2	-3	26.50	9.30
-11	2	-3	341.27	22.20
-11	2	-3	305.47	22.10
-11	2	-3	340.57	22.40
11	-2	3	307.87	24.10
-12	2	-3	7.90	8.70
-12	2	-3	10.20	14.30
-12	2	-3	31.90	12.00
12	-2	3	5.90	10.60
12	1	-3	61.99	18.00
12	1	-3	42.80	12.80
-12	-1	3	34.20	7.80
-12	-1	3	43.00	18.30
11	1	-3	-27.20	14.40
11	1	-3	-4.80	10.10

-11	-1	3	2.80	7.00
-11	-1	3	10.70	10.90
10	1	-3	237.78	20.60
-10	-1	3	204.68	16.90
-10	-1	3	201.98	18.80
9	1	-3	529.95	30.20
-9	-1	3	479.15	28.40
-9	-1	3	551.94	28.10
-9	-1	3	488.85	29.20
8	1	-3	372.96	23.40
8	1	-3	378.46	24.40
-8	-1	3	366.66	21.30
-8	-1	3	375.06	21.80
-8	-1	3	371.36	22.50
-8	-1	3	382.86	23.90
7	1	-3	97.19	14.80
7	1	-3	83.59	13.30
-7	-1	3	91.69	11.40
-7	-1	3	68.39	11.40
-7	-1	3	99.79	10.40
-7	-1	3	96.49	11.20
-7	-1	3	137.59	21.60
6	1	-3	338.67	21.40
6	1	-3	339.27	21.90
-6	-1	3	345.37	20.50
-6	-1	3	372.06	18.80
-6	-1	3	332.87	19.70
5	1	-3	328.47	16.90
5	1	-3	350.26	18.30
-5	-1	3	346.77	16.10
-5	-1	3	339.97	18.10
-5	-1	3	342.47	16.50
4	1	-3	714.43	32.00
4	1	-3	746.03	32.60
-4	-1	3	677.73	31.70
-4	-1	3	633.04	32.10
3	1	-3	494.25	22.80
3	1	-3	482.45	22.60
-3	-1	3	490.45	22.30
-3	-1	3	482.05	22.70
2	1	-3	8069.99	345.67
-2	-1	3	7476.35	345.67
1	1	-3	1056.29	49.40
1	1	-3	1098.49	49.50

-1	-1	3	1116.09	49.40
-1	-1	3	1135.69	49.40
0	1	-3	3.50	0.90
0	-1	3	0.70	1.30
0	-1	3	2.20	0.70
-1	1	-3	1318.47	58.59
-1	1	-3	1339.07	58.69
1	-1	3	1291.77	58.69
1	-1	3	1327.77	58.69
1	-1	3	1254.97	58.29
-2	1	-3	24.40	3.00
-2	1	-3	29.60	2.70
2	-1	3	26.40	3.00
2	-1	3	26.00	2.10
2	-1	3	29.00	2.80
-3	1	-3	399.06	18.60
-3	1	-3	384.16	19.10
3	-1	3	389.66	18.20
3	-1	3	396.26	19.10
3	-1	3	395.06	18.70
-4	1	-3	516.45	24.50
-4	1	-3	468.05	23.90
-4	1	-3	505.55	23.60
4	-1	3	517.15	24.40
4	-1	3	500.25	23.10
4	-1	3	480.85	23.70
-5	1	-3	22.90	5.10
-5	1	-3	22.10	3.80
-5	1	-3	27.10	4.80
5	-1	3	22.50	4.80
5	-1	3	18.10	4.70
5	-1	3	23.00	3.40
-6	1	-3	1286.77	63.69
-6	1	-3	1312.87	60.89
-6	1	-3	1347.27	60.79
-6	1	-3	1366.06	61.89
-6	1	-3	1244.88	61.29
6	-1	3	1251.77	60.89
6	-1	3	1397.06	60.19
-7	1	-3	542.75	31.40
-7	1	-3	557.44	28.90
-7	1	-3	577.94	29.30
-7	1	-3	542.05	31.60
-7	1	-3	594.34	28.10

7	-1	3	490.65	32.10
7	-1	3	564.14	27.30
-8	1	-3	274.87	18.20
-8	1	-3	262.17	17.20
-8	1	-3	257.27	23.50
-8	1	-3	220.68	20.20
-8	1	-3	263.87	15.90
8	-1	3	248.58	16.00
8	-1	3	205.88	22.50
-9	1	-3	21.00	11.20
-9	1	-3	29.40	10.90
-9	1	-3	26.70	7.40
-9	1	-3	31.40	6.80
-9	1	-3	36.80	8.50
9	-1	3	29.00	10.50
-10	1	-3	7.10	8.30
-10	1	-3	39.70	11.80
-10	1	-3	17.10	8.20
-10	1	-3	18.20	8.00
-10	1	-3	13.10	13.30
-11	1	-3	16.30	9.90
-11	1	-3	13.50	9.80
-11	1	-3	22.40	8.60
11	-1	3	30.00	11.90
-12	1	-3	147.79	15.10
-12	1	-3	110.99	19.40
-12	1	-3	133.29	16.90
12	-1	3	148.59	18.10
12	0	-3	54.79	29.30
-12	0	3	23.10	7.60
11	0	-3	11.90	16.20
11	0	-3	-1.40	9.90
-11	0	3	26.00	7.50
-11	0	3	11.30	12.20
10	0	-3	23.80	10.20
-10	0	3	14.20	10.30
-10	0	3	14.40	7.30
9	0	-3	16.00	9.10
-9	0	3	10.60	6.80
-9	0	3	9.00	8.70
-9	0	3	5.00	9.80
8	0	-3	174.28	16.60
-8	0	3	148.49	14.00
-8	0	3	148.69	17.00

-8	0	3	154.28	15.20
-8	0	3	167.58	14.20
7	0	-3	126.49	12.70
7	0	-3	153.28	15.50
-7	0	3	140.99	11.90
-7	0	3	124.79	16.50
-7	0	3	156.38	13.10
-7	0	3	130.89	14.20
6	0	-3	263.47	14.10
6	0	-3	243.98	18.70
6	0	-3	262.67	17.80
-6	0	3	235.38	16.10
-6	0	3	233.18	17.40
-6	0	3	249.68	14.70
5	0	-3	317.57	17.20
-5	0	3	318.77	16.10
-5	0	3	318.97	16.90
4	0	-3	2097.09	90.09
4	0	-3	2010.70	89.59
-4	0	3	1937.71	89.69
-4	0	3	1933.01	89.59
3	0	-3	520.65	23.60
3	0	-3	520.85	23.70
-3	0	3	487.25	23.40
-3	0	3	511.35	23.60
2	0	-3	351.26	15.90
2	0	-3	346.17	15.90
-2	0	3	328.97	15.90
-2	0	3	345.17	15.90
1	0	-3	499.05	21.00
1	0	-3	446.96	21.10
-1	0	3	493.95	21.20
-1	0	3	424.96	21.20
0	0	-3	1030.70	43.70
0	0	-3	983.40	43.40
0	0	3	928.01	43.40
0	0	3	895.31	43.50
0	0	3	1023.50	43.60
-1	0	-3	268.67	12.40
-1	0	-3	264.27	12.40
1	0	3	258.97	12.40
1	0	3	267.37	12.00
1	0	3	271.77	12.40
-2	0	-3	351.46	16.30

-2	0	-3	343.37	16.00
2	0	3	350.86	15.60
2	0	3	332.77	16.00
2	0	3	342.67	16.30
-3	0	-3	555.44	25.30
-3	0	-3	540.75	25.80
3	0	3	541.15	25.40
3	0	3	558.64	25.90
3	0	3	551.14	25.00
-4	0	-3	952.60	43.00
-4	0	-3	931.21	43.80
4	0	3	954.80	42.70
4	0	3	914.11	43.10
4	0	3	960.50	43.90
-5	0	-3	77.49	6.10
-5	0	-3	82.99	8.20
-5	0	-3	81.89	6.90
5	0	3	73.69	6.50
5	0	3	80.99	8.30
-6	0	-3	42.00	7.30
-6	0	-3	63.39	10.60
-6	0	-3	42.10	7.20
-6	0	-3	52.89	10.30
-6	0	-3	46.70	6.50
6	0	3	47.40	6.10
6	0	3	53.99	5.00
-7	0	-3	933.01	44.50
-7	0	-3	925.61	45.70
-7	0	-3	936.41	44.90
-7	0	-3	923.41	46.60
-7	0	-3	965.80	44.00
7	0	3	824.02	47.90
7	0	3	945.61	43.30
-8	0	-3	1.50	12.50
-8	0	-3	14.20	9.10
-8	0	-3	9.30	7.00
-8	0	-3	-2.80	6.30
-8	0	-3	8.60	4.90
8	0	3	3.60	5.60
-9	0	-3	17.60	7.30
-9	0	-3	20.80	6.40
-9	0	-3	10.10	10.00
-9	0	-3	14.00	10.10
-9	0	-3	26.30	8.30

9	0	3	-5.60	9.10
-10	0	-3	189.08	16.00
-10	0	-3	186.38	21.40
-10	0	-3	185.78	16.20
-10	0	-3	185.28	16.70
-10	0	-3	169.78	21.50
10	0	3	183.98	29.00
-11	0	-3	21.40	9.80
-11	0	-3	8.00	9.40
-11	0	-3	16.80	8.20
11	0	3	20.00	10.90
-12	0	-3	108.79	16.20
-12	0	-3	132.99	14.20
-12	0	-3	93.99	14.60
12	0	3	152.98	17.50
12	-1	-3	-32.90	23.90
12	-1	-3	1.40	10.80
-12	1	3	16.90	7.60
11	-1	-3	2.60	17.30
11	-1	-3	22.00	10.60
-11	1	3	26.50	7.60
-11	1	3	39.00	14.30
10	-1	-3	125.39	17.80
-10	1	3	139.09	15.00
-10	1	3	114.29	14.20
9	-1	-3	739.33	39.60
-9	1	3	782.02	41.40
-9	1	3	760.22	38.50
-9	1	3	711.83	39.50
8	-1	-3	205.68	17.90
-8	1	3	181.58	15.70
-8	1	3	188.58	17.00
-8	1	3	230.88	15.90
-8	1	3	211.48	18.80
7	-1	-3	145.19	15.80
7	-1	-3	124.19	13.30
-7	1	3	141.19	13.00
-7	1	3	156.58	11.90
-7	1	3	154.78	15.40
-7	1	3	149.49	17.60
6	-1	-3	26.00	8.00
6	-1	-3	36.10	5.40
-6	1	3	42.50	8.60
-6	1	3	56.09	8.00

-6	1	3	49.40	8.30
5	-1	-3	328.07	16.10
5	-1	-3	337.17	17.30
-5	1	3	299.97	17.00
-5	1	3	318.77	17.10
-5	1	3	302.27	16.50
4	-1	-3	10.20	2.80
-4	1	3	9.80	2.60
-4	1	3	3.80	2.70
3	-1	-3	3.70	1.60
3	-1	-3	1.40	1.50
-3	1	3	2.60	1.70
-3	1	3	2.60	1.50
-3	1	3	2.10	1.50
2	-1	-3	124.39	6.30
2	-1	-3	124.59	6.20
-2	1	3	122.29	6.10
-2	1	3	118.59	6.30
-2	1	3	129.49	6.40
1	-1	-3	793.82	36.10
-1	1	3	790.42	36.30
-1	1	3	822.22	36.50
-1	1	3	829.72	36.10
0	1	3	296.37	13.20
0	1	3	275.07	12.80
-1	-1	-3	144.19	7.00
-1	-1	-3	145.29	7.00
1	1	3	147.39	7.00
1	1	3	145.49	7.00
-2	-1	-3	4350.76	194.78
-2	-1	-3	4393.06	194.48
2	1	3	4330.97	194.48
2	1	3	4404.36	194.88
-3	-1	-3	33.70	3.60
-3	-1	-3	38.00	2.70
3	1	3	36.30	3.60
3	1	3	33.70	2.50
3	1	3	37.80	3.00
-4	-1	-3	7.20	2.80
-4	-1	-3	7.80	1.80
4	1	3	9.20	2.90
4	1	3	6.50	2.10
4	1	3	7.00	1.60
-5	-1	-3	1448.36	67.09

-5	-1	-3	1484.65	67.99
-5	-1	-3	1460.15	66.39
5	1	3	1442.56	66.49
5	1	3	1495.25	66.49
5	1	3	1497.05	68.09
-6	-1	-3	189.98	10.80
-6	-1	-3	140.49	18.50
-6	-1	-3	165.28	13.30
-6	-1	-3	166.38	16.50
-6	-1	-3	164.08	9.90
-6	-1	-3	150.08	13.10
6	1	3	184.08	18.80
6	1	3	171.48	10.20
-7	-1	-3	168.88	16.50
-7	-1	-3	142.99	13.10
-7	-1	-3	169.88	16.10
-7	-1	-3	156.18	11.10
-7	-1	-3	168.48	13.10
7	1	3	159.98	10.10
-8	-1	-3	-10.30	8.40
-8	-1	-3	-1.90	4.50
-8	-1	-3	5.20	8.40
-8	-1	-3	6.90	8.60
-8	-1	-3	4.00	6.10
8	1	3	18.40	5.20
-9	-1	-3	74.19	10.40
-9	-1	-3	94.39	11.90
-9	-1	-3	70.29	11.80
-9	-1	-3	88.09	10.60
-9	-1	-3	73.99	12.10
9	1	3	58.39	10.10
-10	-1	-3	43.80	8.80
-10	-1	-3	36.90	11.90
-10	-1	-3	35.10	11.50
-10	-1	-3	51.99	9.10
-10	-1	-3	34.80	8.70
10	1	3	42.60	16.10
-11	-1	-3	63.29	9.90
-11	-1	-3	45.80	10.30
-11	-1	-3	31.40	14.80
-11	-1	-3	39.20	10.10
11	1	3	27.20	11.30
-12	-1	-3	78.09	13.80
-12	-1	-3	87.89	15.60

-12	-1	-3	96.89	13.10
12	1	3	117.49	17.70
12	-2	-3	11.30	10.60
-12	2	3	10.40	7.80
11	-2	-3	48.20	19.90
11	-2	-3	15.40	10.10
-11	2	3	14.70	15.80
-11	2	3	17.90	7.90
10	-2	-3	342.87	24.80
-10	2	3	406.06	23.40
-10	2	3	364.76	27.40
9	-2	-3	19.60	9.30
-9	2	3	14.70	7.30
-9	2	3	6.50	9.60
-9	2	3	14.80	13.10
8	-2	-3	128.39	16.00
-8	2	3	151.18	17.20
-8	2	3	116.89	13.40
-8	2	3	104.99	15.30
-8	2	3	153.08	14.10
7	-2	-3	85.69	13.40
7	-2	-3	92.19	13.00
7	-2	-3	79.59	11.20
-7	2	3	98.09	10.80
-7	2	3	112.09	12.30
-7	2	3	89.49	14.00
-7	2	3	80.59	11.70
6	-2	-3	9.80	4.90
6	-2	-3	-0.50	7.90
-6	2	3	-4.40	7.70
-6	2	3	5.10	5.20
-6	2	3	4.30	8.00
-6	2	3	1.90	5.80
5	-2	-3	185.98	13.90
5	-2	-3	173.78	10.50
5	-2	-3	180.28	12.30
-5	2	3	171.38	11.60
-5	2	3	196.98	11.90
-5	2	3	187.48	11.70
-5	2	3	180.78	15.10
4	-2	-3	198.88	9.80
4	-2	-3	175.58	9.60
4	-2	-3	182.68	10.30
-4	2	3	181.38	10.10

-4	2	3	162.38	10.20
-4	2	3	183.78	10.30
3	-2	-3	2435.06	109.19
3	-2	-3	2502.55	109.09
3	-2	-3	2390.86	108.89
-3	2	3	2391.36	109.19
-3	2	3	2456.05	109.09
2	-2	-3	198.98	9.50
2	-2	-3	194.38	9.50
2	-2	-3	197.58	9.50
-2	2	3	190.78	9.40
-2	2	3	195.18	9.40
-2	2	3	191.08	9.60
1	-2	-3	521.65	23.70
1	-2	-3	499.95	23.50
-1	2	3	540.65	23.40
-1	2	3	530.55	23.70
-1	2	3	499.45	23.40
1	2	3	537.25	24.30
1	2	3	529.05	24.10
-2	-2	-3	551.84	24.80
2	2	3	548.25	24.90
2	2	3	542.85	25.30
-3	-2	-3	160.78	7.60
3	2	3	151.38	7.70
3	2	3	160.28	7.50
3	2	3	155.08	8.60
-4	-2	-3	267.47	12.70
4	2	3	274.17	14.30
4	2	3	273.27	12.90
4	2	3	267.87	13.00
-5	-2	-3	271.77	13.50
-5	-2	-3	283.67	14.80
5	2	3	282.97	13.60
5	2	3	290.27	13.80
5	2	3	281.67	15.90
-6	-2	-3	12.60	5.50
-6	-2	-3	24.40	14.80
-6	-2	-3	12.10	7.80
-6	-2	-3	14.60	3.60
-6	-2	-3	15.00	5.90
6	2	3	21.20	9.50
-7	-2	-3	332.77	25.20
-7	-2	-3	394.16	20.70

-7	-2	-3	356.86	22.20
-7	-2	-3	330.67	22.60
-7	-2	-3	349.96	19.00
-7	-2	-3	365.26	20.10
-8	-2	-3	219.18	18.20
-8	-2	-3	223.08	21.00
-8	-2	-3	194.98	18.60
-8	-2	-3	230.28	15.80
-8	-2	-3	212.88	14.00
8	2	3	208.78	13.90
-9	-2	-3	11.50	7.20
-9	-2	-3	-1.90	9.00
-9	-2	-3	-1.90	5.70
-9	-2	-3	15.90	9.30
-9	-2	-3	0.30	6.70
9	2	3	0.70	7.90
-10	-2	-3	255.17	17.90
-10	-2	-3	231.18	17.70
-10	-2	-3	251.07	22.20
-10	-2	-3	210.38	20.60
-10	-2	-3	232.38	17.40
10	2	3	263.37	26.80
-11	-2	-3	379.26	23.70
-11	-2	-3	371.26	23.70
-11	-2	-3	325.77	28.30
-11	-2	-3	424.26	24.10
11	2	3	431.76	26.60
-12	-2	-3	-6.30	10.60
-12	-2	-3	3.10	7.50
-12	-2	-3	3.80	12.30
12	2	3	1.60	11.30
12	-3	-3	25.60	11.10
-12	3	3	50.89	8.80
11	-3	-3	23.40	10.90
11	-3	-3	44.30	23.90
-11	3	3	28.90	8.50
-11	3	3	18.40	17.70
10	-3	-3	173.98	18.40
-10	3	3	198.98	31.10
-10	3	3	164.78	16.90
9	-3	-3	60.39	10.80
-9	3	3	84.09	18.20
-9	3	3	86.59	13.10
-9	3	3	43.10	11.20

8	-3	-3	962.10	47.00
8	-3	-3	908.61	47.30
-8	3	3	905.21	47.40
-8	3	3	921.91	45.30
-8	3	3	972.80	46.40
7	-3	-3	54.89	9.50
7	-3	-3	51.49	9.60
-7	3	3	44.90	7.60
-7	3	3	59.69	9.70
-7	3	3	39.20	10.80
-7	3	3	47.90	7.10
6	-3	-3	218.28	17.20
6	-3	-3	209.08	18.60
6	-3	-3	226.08	14.60
6	-3	-3	207.48	18.40
-6	3	3	230.98	15.60
-6	3	3	263.07	14.40
-6	3	3	200.78	17.40
5	-3	-3	660.13	31.80
5	-3	-3	665.33	33.10
5	-3	-3	702.23	32.20
-5	3	3	679.23	32.20
-5	3	3	651.13	31.90
-5	3	3	673.43	32.90
-5	3	3	677.43	32.60
4	-3	-3	323.57	16.10
4	-3	-3	337.27	16.70
-4	3	3	322.57	16.60
-4	3	3	309.97	15.80
-4	3	3	321.87	16.50
3	-3	-3	8.00	2.30
3	-3	-3	5.00	2.30
3	-3	-3	4.30	1.90
-3	3	3	8.10	2.10
-3	3	3	8.30	1.80
-3	3	3	7.80	2.30
2	-3	-3	922.61	42.20
2	-3	-3	860.91	41.90
2	-3	-3	991.30	42.30
-2	3	3	945.61	42.30
-2	3	3	929.81	42.10
1	-3	-3	46.60	3.80
1	-3	-3	39.40	3.00
-1	3	3	49.50	3.20

-1	3	3	43.60	2.70
-1	3	3	38.90	3.00
0	-3	-3	3.50	1.10
0	-3	-3	4.10	1.10
0	3	3	1.80	1.40
0	3	3	2.10	0.80
1	3	3	602.24	28.10
1	3	3	644.54	28.50
2	3	3	8.40	1.20
2	3	3	8.00	1.70
3	3	3	31.50	2.40
3	3	3	35.00	3.70
3	3	3	29.40	2.40
3	3	3	31.30	2.20
-4	-3	-3	1388.06	61.49
4	3	3	1342.57	60.49
4	3	3	1351.76	61.89
4	3	3	1304.97	60.29
-5	-3	-3	143.49	9.10
5	3	3	139.09	10.60
5	3	3	150.08	7.90
-6	-3	-3	2112.99	99.79
-6	-3	-3	2098.29	98.69
-6	-3	-3	2054.59	98.39
-6	-3	-3	2262.77	99.69
-6	-3	-3	2217.98	98.19
6	3	3	2235.68	97.59
-7	-3	-3	247.08	15.80
-7	-3	-3	240.78	17.70
-7	-3	-3	240.58	16.10
-7	-3	-3	261.27	18.20
-7	-3	-3	266.27	14.60
-7	-3	-3	222.38	19.60
7	3	3	250.67	13.80
-8	-3	-3	288.37	20.40
-8	-3	-3	299.37	20.80
-8	-3	-3	293.37	17.00
-8	-3	-3	266.47	20.70
-8	-3	-3	294.97	18.40
-9	-3	-3	21.10	7.50
-9	-3	-3	-0.70	10.40
-9	-3	-3	25.90	9.50
-9	-3	-3	29.30	7.30
-9	-3	-3	22.50	8.80

-9	-3	-3	25.70	7.20
9	3	3	19.10	7.60
-10	-3	-3	16.20	9.40
-10	-3	-3	16.30	7.50
-10	-3	-3	6.70	7.90
-10	-3	-3	1.80	7.40
-10	-3	-3	-9.80	10.00
10	3	3	-6.80	13.90
10	3	3	2.50	10.40
-11	-3	-3	117.69	13.70
-11	-3	-3	111.59	20.60
-11	-3	-3	112.69	14.50
-11	-3	-3	129.29	14.90
-12	-3	-3	163.38	14.50
-12	-3	-3	136.39	16.80
-12	-3	-3	140.59	18.60
12	3	3	187.18	19.10
12	-4	-3	8.70	10.70
-12	4	3	3.70	8.30
11	-4	-3	60.49	11.20
-11	4	3	62.99	10.10
10	-4	-3	224.18	20.00
-10	4	3	199.08	18.50
-10	4	3	188.38	32.10
9	-4	-3	95.79	13.70
9	-4	-3	109.89	16.20
-9	4	3	113.39	14.70
-9	4	3	108.49	14.80
8	-4	-3	14.50	9.20
8	-4	-3	16.40	8.90
-8	4	3	-5.70	10.30
-8	4	3	15.90	8.10
-8	4	3	6.70	7.70
-8	4	3	11.40	10.00
7	-4	-3	303.87	20.40
7	-4	-3	280.47	19.60
-7	4	3	286.07	18.80
-7	4	3	284.37	17.80
-7	4	3	330.37	22.30
-7	4	3	282.07	21.20
6	-4	-3	130.19	14.10
6	-4	-3	165.28	15.30
6	-4	-3	140.19	18.90
6	-4	-3	123.89	14.50

-6	4	3	151.58	16.80
-6	4	3	177.78	13.20
-6	4	3	156.68	16.50
-6	4	3	149.09	11.60
5	-4	-3	307.47	17.00
5	-4	-3	298.27	18.40
5	-4	-3	296.97	16.60
-5	4	3	310.37	18.10
-5	4	3	326.47	17.70
-5	4	3	303.67	16.40
-5	4	3	314.07	17.00
4	-4	-3	482.65	24.00
4	-4	-3	507.65	24.40
-4	4	3	512.55	23.80
-4	4	3	515.35	24.90
3	-4	-3	91.09	5.70
3	-4	-3	64.29	5.90
3	-4	-3	71.89	5.90
-3	4	3	75.59	4.90
-3	4	3	78.09	5.60
-3	4	3	82.19	5.80
2	-4	-3	955.20	44.30
2	-4	-3	944.61	44.10
-2	4	3	986.00	44.10
-2	4	3	1022.00	44.20
-2	4	3	930.91	43.60
1	-4	-3	64.09	5.70
1	-4	-3	83.09	4.80
-1	4	3	84.09	4.10
-1	4	3	72.79	5.40
-1	4	3	84.69	4.80
0	-4	-3	3264.67	142.49
0	-4	-3	3292.27	142.79
0	4	3	3040.70	142.99
0	4	3	3181.98	142.49
1	4	3	546.45	23.70
1	4	3	472.55	23.10
2	4	3	68.79	3.70
2	4	3	67.49	3.80
2	4	3	73.99	4.80
3	4	3	15.00	1.50
3	4	3	15.00	2.60
3	4	3	14.70	1.50
3	4	3	12.30	1.70

3	4	3	13.30	1.80
-4	-4	-3	502.55	24.20
4	4	3	495.05	22.90
4	4	3	497.75	24.50
-5	-4	-3	42.40	5.80
5	4	3	44.20	7.50
5	4	3	44.10	4.10
-6	-4	-3	573.84	30.30
-6	-4	-3	602.44	31.60
-6	-4	-3	627.74	32.30
-6	-4	-3	629.04	29.50
-6	-4	-3	586.24	30.20
6	4	3	630.94	28.90
-7	-4	-3	692.33	33.70
-7	-4	-3	686.23	33.90
-7	-4	-3	672.83	34.30
-7	-4	-3	683.13	35.40
-7	-4	-3	625.54	34.60
-7	-4	-3	725.43	33.20
7	4	3	681.73	32.60
-8	-4	-3	4.60	4.90
-8	-4	-3	3.00	6.00
-8	-4	-3	1.90	5.90
-8	-4	-3	3.80	8.10
-8	-4	-3	6.10	7.80
-8	-4	-3	3.50	8.00
8	4	3	5.00	5.00
-9	-4	-3	63.59	10.30
-9	-4	-3	52.39	9.80
-9	-4	-3	78.29	12.10
-9	-4	-3	75.19	12.00
-9	-4	-3	61.49	11.10
-9	-4	-3	66.99	11.30
9	4	3	59.39	9.70
-10	-4	-3	143.09	16.40
-10	-4	-3	135.79	14.20
-10	-4	-3	122.39	13.70
-10	-4	-3	97.49	14.60
-10	-4	-3	127.29	13.40
10	4	3	159.28	19.10
10	4	3	128.49	16.60
-11	-4	-3	151.78	16.20
-11	-4	-3	174.78	16.10
-11	-4	-3	167.98	21.60

-11	-4	-3	200.38	15.30
11	4	3	184.88	19.70
-12	-4	-3	288.57	18.60
-12	-4	-3	283.07	20.80
-12	-4	-3	248.08	33.30
12	4	3	330.97	23.90
12	-5	-3	8.10	10.20
-12	5	3	7.60	8.70
11	-5	-3	49.40	10.70
-11	5	3	45.40	9.90
10	-5	-3	85.59	16.10
-10	5	3	74.69	16.40
9	-5	-3	550.64	32.30
9	-5	-3	550.54	31.60
-9	5	3	585.24	34.60
-9	5	3	570.24	31.80
8	-5	-3	14.40	9.20
8	-5	-3	25.20	8.40
-8	5	3	26.60	11.50
-8	5	3	19.10	7.60
-8	5	3	20.80	8.30
-8	5	3	22.40	10.80
7	-5	-3	145.29	21.00
7	-5	-3	155.48	14.80
-7	5	3	157.78	18.30
-7	5	3	146.79	17.20
-7	5	3	137.79	13.00
6	-5	-3	361.76	21.60
6	-5	-3	343.77	20.60
6	-5	-3	402.46	31.40
-6	5	3	302.87	22.90
-6	5	3	352.36	21.00
-6	5	3	351.96	19.20
-6	5	3	308.17	26.00
5	-5	-3	514.75	28.60
5	-5	-3	538.35	27.40
5	-5	-3	520.05	27.50
-5	5	3	539.85	28.80
-5	5	3	538.15	26.90
-5	5	3	556.94	28.40
-5	5	3	555.24	28.00
-5	5	3	542.85	26.20
4	-5	-3	70.69	7.10
4	-5	-3	71.09	8.20

4	-5	-3	55.99	8.10
-4	5	3	64.99	7.20
-4	5	3	75.19	7.60
-4	5	3	60.49	5.40
3	-5	-3	130.89	9.30
3	-5	-3	156.98	8.80
3	-5	-3	137.89	9.10
-3	5	3	137.19	8.70
-3	5	3	143.59	7.70
-3	5	3	152.18	8.90
2	-5	-3	22.00	3.60
2	-5	-3	33.60	3.70
-2	5	3	28.80	2.60
-2	5	3	22.10	3.30
-2	5	3	32.80	3.60
1	-5	-3	2175.08	103.79
1	-5	-3	2390.76	103.39
-1	5	3	2316.57	102.79
-1	5	3	2261.67	104.09
-1	5	3	2375.46	103.39
0	-5	-3	741.73	32.20
0	-5	-3	682.33	32.60
0	5	3	728.63	31.50
0	5	3	703.83	32.20
0	5	3	662.13	33.10
-1	-5	-3	17.80	3.20
1	5	3	14.00	1.90
1	5	3	22.30	3.30
2	5	3	153.68	7.70
2	5	3	167.28	8.90
3	5	3	66.49	5.60
3	5	3	64.59	3.60
3	5	3	62.69	3.70
3	5	3	68.39	3.50
3	5	3	63.29	3.40
3	5	3	64.79	6.20
4	5	3	112.49	6.00
4	5	3	112.19	8.40
-5	-5	-3	1757.12	79.29
5	5	3	1692.83	78.59
5	5	3	1844.72	80.99
5	5	3	1686.43	80.69
-6	-5	-3	13.40	6.00
-6	-5	-3	13.50	5.90

-6	-5	-3	12.30	5.90
-6	-5	-3	5.60	8.20
6	5	3	8.60	3.30
-7	-5	-3	384.36	22.80
-7	-5	-3	396.56	20.80
-7	-5	-3	384.06	21.60
-7	-5	-3	411.06	23.70
-7	-5	-3	393.36	22.70
-7	-5	-3	399.96	21.80
7	5	3	391.86	20.10
-8	-5	-3	9.60	5.20
-8	-5	-3	2.80	6.10
-8	-5	-3	15.00	6.40
-8	-5	-3	9.20	7.30
-8	-5	-3	5.40	9.70
-8	-5	-3	9.80	8.40
8	5	3	9.10	5.20
-9	-5	-3	15.70	7.20
-9	-5	-3	0.20	9.50
-9	-5	-3	4.50	6.00
-9	-5	-3	13.70	8.20
-9	-5	-3	1.60	6.90
-9	-5	-3	5.10	8.70
-10	-5	-3	10.80	9.10
-10	-5	-3	15.80	7.20
-10	-5	-3	13.40	7.40
-10	-5	-3	-0.60	10.80
-10	-5	-3	13.90	7.60
10	5	3	15.00	10.70
10	5	3	3.70	13.40
-11	-5	-3	6.10	8.70
-11	-5	-3	23.30	7.40
-11	-5	-3	26.50	11.90
-11	-5	-3	-0.60	9.00
11	5	3	-12.20	11.10
-12	-5	-3	276.67	17.80
-12	-5	-3	209.88	21.70
-12	-5	-3	299.07	20.10
-12	-5	-3	283.17	25.60
12	5	3	239.58	23.90
11	-6	-3	238.38	19.20
-11	6	3	203.68	19.10
10	-6	-3	214.78	20.80
10	-6	-3	250.57	23.50

-10	6	3	272.27	21.50
9	-6	-3	16.90	10.10
9	-6	-3	11.20	9.20
-9	6	3	26.10	14.20
-9	6	3	2.30	8.80
8	-6	-3	171.48	16.30
8	-6	-3	131.79	16.10
-8	6	3	123.99	21.80
-8	6	3	121.89	18.30
-8	6	3	149.49	16.50
-8	6	3	131.59	15.30
7	-6	-3	397.66	24.90
-7	6	3	409.86	23.20
-7	6	3	413.36	24.60
-7	6	3	422.06	25.60
6	-6	-3	7.30	7.90
6	-6	-3	10.10	6.60
-6	6	3	-7.00	9.60
-6	6	3	4.30	6.90
5	-6	-3	45.00	9.60
5	-6	-3	49.40	8.30
5	-6	-3	68.99	11.30
-5	6	3	49.80	8.10
-5	6	3	37.60	8.30
-5	6	3	47.20	8.10
-5	6	3	48.00	7.20
-5	6	3	52.49	9.00
4	-6	-3	606.64	32.80
4	-6	-3	705.23	32.20
-4	6	3	635.24	31.70
-4	6	3	686.43	32.50
-4	6	3	641.84	32.30
-4	6	3	672.93	30.80
3	-6	-3	1263.17	56.39
3	-6	-3	1110.79	56.79
3	-6	-3	1239.18	57.49
-3	6	3	1272.67	56.59
-3	6	3	1222.18	55.39
-3	6	3	1234.88	56.69
2	-6	-3	168.88	10.80
2	-6	-3	161.88	10.20
-2	6	3	178.38	8.70
-2	6	3	180.68	10.80
1	-6	-3	76.19	6.70

1	-6	-3	74.69	5.20
-1	6	3	56.99	7.50
-1	6	3	69.99	4.00
-1	6	3	74.89	5.00
0	-6	-3	3304.57	144.89
0	-6	-3	3064.29	145.39
0	6	3	3308.47	144.89
0	6	3	3097.39	144.09
0	6	3	3423.86	146.09
-1	-6	-3	123.89	7.70
-1	-6	-3	117.19	6.50
1	6	3	122.29	7.80
1	6	3	123.49	6.50
2	6	3	125.49	6.50
2	6	3	132.19	8.10
3	6	3	87.39	4.70
3	6	3	91.69	5.20
3	6	3	97.09	7.20
3	6	3	91.59	4.90
3	6	3	97.59	4.80
4	6	3	396.46	18.90
4	6	3	416.56	21.30
-5	-6	-3	128.09	8.80
5	6	3	124.99	7.50
5	6	3	142.39	12.30
5	6	3	115.19	11.70
-6	-6	-3	274.47	14.50
-6	-6	-3	255.07	15.70
-6	-6	-3	226.58	16.70
-6	-6	-3	247.28	15.80
-6	-6	-3	263.37	17.10
-6	-6	-3	270.87	18.20
6	6	3	246.38	13.60
-7	-6	-3	274.27	16.70
-7	-6	-3	266.87	17.50
-7	-6	-3	267.47	15.20
-7	-6	-3	263.37	17.60
-7	-6	-3	255.67	16.40
-7	-6	-3	258.37	18.70
7	6	3	255.37	14.80
-8	-6	-3	54.79	8.60
-8	-6	-3	29.70	7.80
-8	-6	-3	68.49	8.60
-8	-6	-3	52.79	8.70

-8	-6	-3	55.49	7.70
-8	-6	-3	53.39	10.00
8	6	3	42.40	6.90
-9	-6	-3	0.50	6.40
-9	-6	-3	8.60	7.00
-9	-6	-3	-1.30	7.70
-9	-6	-3	-4.20	8.90
-9	-6	-3	10.50	6.30
-9	-6	-3	14.70	8.10
-10	-6	-3	372.56	25.10
-10	-6	-3	404.56	22.50
-10	-6	-3	382.36	22.00
-10	-6	-3	399.36	24.30
-10	-6	-3	357.96	21.70
10	6	3	315.77	25.70
10	6	3	351.36	28.90
-11	-6	-3	390.46	23.80
-11	-6	-3	378.66	23.30
-11	-6	-3	402.56	23.30
-11	-6	-3	399.96	28.40
-11	-6	-3	394.66	26.50
11	6	3	400.36	27.80
-12	-6	-3	17.10	14.40
-12	-6	-3	35.60	10.90
-12	-6	-3	23.40	7.40
-12	-6	-3	35.40	13.60
12	6	3	44.00	24.50
11	-7	-3	118.49	15.90
-11	7	3	142.49	17.20
10	-7	-3	100.19	16.20
10	-7	-3	113.29	18.90
-10	7	3	95.49	16.90
9	-7	-3	30.90	10.10
9	-7	-3	19.00	9.30
-9	7	3	27.00	10.10
-9	7	3	56.29	16.20
8	-7	-3	598.94	30.90
8	-7	-3	577.94	31.20
-8	7	3	498.75	36.50
-8	7	3	600.94	33.10
-8	7	3	550.44	31.10
-8	7	3	562.04	33.00
7	-7	-3	140.49	13.80
7	-7	-3	145.19	16.30

-7	7	3	143.79	15.50
-7	7	3	169.48	16.90
-7	7	3	139.19	14.20
6	-7	-3	38.00	8.90
6	-7	-3	51.59	7.80
-6	7	3	47.80	8.50
-6	7	3	38.30	10.10
-6	7	3	64.89	10.40
5	-7	-3	531.95	27.10
5	-7	-3	514.55	29.10
5	-7	-3	473.05	31.00
-5	7	3	461.65	29.10
-5	7	3	549.45	26.60
-5	7	3	550.54	25.50
4	-7	-3	169.78	15.20
4	-7	-3	156.78	12.20
-4	7	3	165.28	13.30
-4	7	3	153.58	12.60
-4	7	3	168.88	9.50
-4	7	3	157.68	11.80
3	-7	-3	97.19	10.40
3	-7	-3	107.79	8.60
-3	7	3	103.39	9.70
-3	7	3	101.09	8.50
-3	7	3	105.89	6.30
-3	7	3	98.79	10.00
2	-7	-3	864.51	43.80
-2	7	3	937.31	41.90
-2	7	3	945.81	43.00
-2	7	3	939.11	44.20
1	-7	-3	215.88	13.60
1	-7	-3	254.27	14.40
-1	7	3	250.57	13.20
-1	7	3	263.57	11.90
-1	7	3	273.37	16.00
0	-7	-3	229.08	12.00
0	-7	-3	222.78	13.20
0	7	3	245.78	12.00
0	7	3	244.18	14.30
0	7	3	241.18	11.10
-1	-7	-3	121.19	8.20
-1	-7	-3	118.69	6.60
1	7	3	108.09	8.40
1	7	3	117.99	5.50

1	7	3	99.99	6.00
1	7	3	116.39	6.40
2	7	3	72.69	4.60
2	7	3	74.39	3.80
2	7	3	85.39	7.30
2	7	3	72.09	4.30
3	7	3	60.79	6.70
3	7	3	56.89	3.90
3	7	3	52.29	3.40
3	7	3	56.49	3.60
4	7	3	1576.04	74.19
4	7	3	1728.03	76.69
-5	-7	-3	676.63	31.20
5	7	3	643.84	33.90
5	7	3	598.14	33.80
5	7	3	690.33	30.60
-6	-7	-3	2156.58	93.49
-6	-7	-3	1913.01	93.89
-6	-7	-3	2048.20	93.69
-6	-7	-3	2048.79	94.89
-6	-7	-3	2055.09	94.19
-6	-7	-3	2017.90	93.89
6	7	3	2137.89	93.19
-7	-7	-3	16.10	6.80
-7	-7	-3	7.70	5.80
-7	-7	-3	14.90	5.00
-7	-7	-3	1.60	6.50
-7	-7	-3	16.90	6.40
7	7	3	7.20	4.30
-8	-7	-3	37.70	6.60
-8	-7	-3	33.80	7.60
-8	-7	-3	42.40	7.60
-8	-7	-3	39.00	8.50
-8	-7	-3	39.00	9.30
-8	-7	-3	31.80	7.40
8	7	3	29.40	6.70
-9	-7	-3	30.20	7.30
-9	-7	-3	26.70	8.40
-9	-7	-3	30.00	8.50
-9	-7	-3	28.00	10.00
-9	-7	-3	30.10	7.30
-9	-7	-3	23.50	7.60
9	7	3	29.10	9.30
9	7	3	34.00	12.10

-10	-7	-3	16.00	6.90
-10	-7	-3	25.80	10.30
-10	-7	-3	29.40	9.70
-10	-7	-3	11.10	7.50
-10	-7	-3	18.00	7.30
10	7	3	8.30	15.30
10	7	3	27.30	12.50
-11	-7	-3	186.08	16.30
-11	-7	-3	132.89	20.40
-11	-7	-3	153.08	16.60
-11	-7	-3	198.38	19.60
-11	-7	-3	168.08	15.00
11	7	3	165.58	23.70
-12	-7	-3	38.90	7.90
-12	-7	-3	43.20	13.20
-12	-7	-3	74.19	14.50
11	-8	-3	32.40	10.60
11	-8	-3	26.50	20.80
-11	8	3	30.00	10.90
10	-8	-3	92.29	16.00
10	-8	-3	76.89	12.30
-10	8	3	79.09	13.40
9	-8	-3	61.89	11.20
9	-8	-3	63.79	11.10
-9	8	3	63.99	12.60
-9	8	3	38.60	24.10
8	-8	-3	16.10	8.90
8	-8	-3	3.40	8.00
-8	8	3	-3.70	9.80
-8	8	3	5.00	11.70
-8	8	3	28.80	15.70
-8	8	3	-5.50	10.00
7	-8	-3	471.85	27.20
7	-8	-3	502.35	26.40
-7	8	3	452.65	28.20
-7	8	3	474.75	26.70
6	-8	-3	445.76	24.30
6	-8	-3	437.66	26.30
-6	8	3	431.66	26.80
-6	8	3	456.05	24.60
-6	8	3	445.56	26.70
5	-8	-3	522.55	30.40
5	-8	-3	476.15	29.40
5	-8	-3	507.95	27.10

-5	8	3	508.65	32.20
-5	8	3	526.15	25.70
-5	8	3	552.94	27.10
4	-8	-3	61.09	11.10
4	-8	-3	48.20	8.10
4	-8	-3	59.89	8.10
-4	8	3	57.19	5.70
-4	8	3	53.19	8.20
-4	8	3	67.49	7.70
-4	8	3	70.19	9.70
3	-8	-3	144.29	10.80
3	-8	-3	104.19	13.30
-3	8	3	131.89	10.40
-3	8	3	134.19	7.70
-3	8	3	124.19	11.60
-3	8	3	147.49	13.20
2	-8	-3	16.40	5.90
-2	8	3	13.40	6.70
-2	8	3	8.90	3.60
-2	8	3	13.20	2.20
-2	8	3	12.80	7.20
1	-8	-3	1771.72	78.89
1	-8	-3	1696.73	77.69
-1	8	3	1619.64	76.59
-1	8	3	1790.72	79.99
-1	8	3	1668.43	77.69
0	-8	-3	36.90	4.80
0	8	3	45.20	3.00
0	8	3	47.60	4.60
-1	-8	-3	1923.41	83.19
-1	-8	-3	1748.23	84.29
1	8	3	1834.42	82.29
1	8	3	1942.11	83.19
1	8	3	1763.92	84.59
-2	-8	-3	246.08	12.70
-2	-8	-3	257.27	13.90
2	8	3	257.97	12.60
2	8	3	252.07	11.80
2	8	3	257.17	15.00
3	8	3	4.50	1.80
3	8	3	3.10	1.50
-4	-8	-3	185.48	10.80
4	8	3	183.58	9.40
4	8	3	162.58	13.70

4	8	3	188.08	13.70
-5	-8	-3	76.89	7.10
-5	-8	-3	79.99	10.00
-5	-8	-3	77.59	10.20
-5	-8	-3	84.39	9.90
-5	-8	-3	73.69	11.30
5	8	3	67.69	6.30
-6	-8	-3	164.18	12.50
-6	-8	-3	158.58	12.90
-6	-8	-3	183.78	15.80
-6	-8	-3	164.28	13.50
-6	-8	-3	164.18	10.90
6	8	3	164.68	10.60
-7	-8	-3	201.38	17.60
-7	-8	-3	200.08	14.60
-7	-8	-3	215.18	20.40
-7	-8	-3	192.18	14.50
-7	-8	-3	200.68	13.00
-7	-8	-3	186.68	16.60
7	8	3	188.48	12.80
-8	-8	-3	18.70	5.60
-8	-8	-3	32.10	7.40
-8	-8	-3	27.20	8.00
-8	-8	-3	17.30	6.50
-8	-8	-3	15.80	9.10
-8	-8	-3	17.30	7.10
8	8	3	17.40	6.80
-9	-8	-3	84.19	12.20
-9	-8	-3	72.19	12.00
-9	-8	-3	82.79	14.30
-9	-8	-3	76.09	13.40
-9	-8	-3	81.39	12.00
9	8	3	71.99	15.30
-10	-8	-3	67.39	11.30
-10	-8	-3	82.39	14.90
-10	-8	-3	87.69	12.80
-10	-8	-3	104.09	13.10
-10	-8	-3	77.49	12.80
-11	-8	-3	170.68	19.30
-11	-8	-3	183.48	18.50
-11	-8	-3	186.58	16.30
-11	-8	-3	175.08	14.80
-11	-8	-3	155.68	16.20
-12	-8	-3	131.99	18.40

-12	-8	-3	155.18	25.20
-12	-8	-3	89.99	12.40
11	-9	-3	212.78	24.40
11	-9	-3	229.68	19.00
-11	9	3	204.68	19.60
10	-9	-3	179.08	18.30
10	-9	-3	238.88	19.80
-10	9	3	187.08	20.40
9	-9	-3	166.58	19.10
9	-9	-3	173.88	17.40
-9	9	3	143.39	20.40
8	-9	-3	11.00	7.80
8	-9	-3	9.00	9.20
-8	9	3	22.00	18.00
-8	9	3	12.30	11.40
-8	9	3	-5.70	16.40
-8	9	3	18.00	11.50
7	-9	-3	27.60	7.40
-7	9	3	26.40	9.20
-7	9	3	28.50	10.70
-7	9	3	44.90	11.80
6	-9	-3	867.71	42.60
6	-9	-3	823.52	43.80
6	-9	-3	813.32	47.40
-6	9	3	877.91	42.20
-6	9	3	852.31	43.50
-6	9	3	839.92	47.50
5	-9	-3	143.99	22.70
5	-9	-3	165.88	18.70
5	-9	-3	199.48	14.30
5	-9	-3	194.38	18.90
-5	9	3	170.78	14.80
-5	9	3	194.58	20.00
-5	9	3	207.38	12.90
4	-9	-3	119.19	21.40
4	-9	-3	127.79	12.70
4	-9	-3	122.09	15.30
-4	9	3	156.18	18.70
-4	9	3	153.08	9.00
-4	9	3	142.09	12.80
3	-9	-3	42.40	7.00
3	-9	-3	60.99	10.50
-3	9	3	62.89	10.40
-3	9	3	58.89	5.00

-3	9	3	54.99	9.20
-3	9	3	63.99	10.90
2	-9	-3	32.60	7.50
-2	9	3	28.60	3.50
-2	9	3	22.50	7.60
-2	9	3	39.10	6.70
-2	9	3	27.50	8.70
1	-9	-3	2137.69	96.79
-1	9	3	2286.07	98.29
-1	9	3	1991.80	94.39
-1	9	3	2018.10	95.59
0	-9	-3	430.56	21.90
0	-9	-3	451.55	23.60
0	-9	-3	445.76	23.10
0	9	3	425.36	20.50
0	9	3	441.46	21.80
0	9	3	474.25	24.80
-1	-9	-3	141.69	11.50
-1	-9	-3	153.08	11.20
-1	-9	-3	144.79	9.10
1	9	3	149.99	7.40
1	9	3	141.19	12.50
1	9	3	149.59	8.70
-2	-9	-3	443.66	21.30
2	9	3	438.56	20.30
2	9	3	432.36	21.00
2	9	3	448.76	24.00
3	9	3	5.30	1.80
3	9	3	8.00	2.40
-4	-9	-3	649.24	30.10
4	9	3	611.54	32.90
4	9	3	612.04	29.00
-5	-9	-3	1154.18	54.49
-5	-9	-3	1148.69	54.39
-5	-9	-3	1151.68	54.59
-5	-9	-3	1116.59	55.89
-5	-9	-3	1284.27	53.89
-5	-9	-3	1170.68	54.19
5	9	3	1087.09	53.59
-6	-9	-3	456.85	24.50
-6	-9	-3	462.75	23.50
-6	-9	-3	473.35	24.70
-6	-9	-3	462.05	24.20
-6	-9	-3	467.75	24.90

-6	-9	-3	456.45	26.30
6	9	3	453.55	23.30
-7	-9	-3	394.46	24.70
-7	-9	-3	320.57	19.80
-7	-9	-3	317.57	19.10
-7	-9	-3	340.57	18.50
-7	-9	-3	328.17	20.50
7	9	3	320.97	18.90
-8	-9	-3	49.20	8.40
-8	-9	-3	78.09	11.70
-8	-9	-3	75.69	11.30
-8	-9	-3	49.20	7.60
-8	-9	-3	62.19	9.40
8	9	3	52.99	9.10
-9	-9	-3	7.70	8.00
-9	-9	-3	19.60	7.90
-9	-9	-3	8.40	8.40
-9	-9	-3	10.80	6.80
-9	-9	-3	12.00	7.00
9	9	3	7.50	10.20
-10	-9	-3	32.40	19.20
-10	-9	-3	17.30	8.30
-10	-9	-3	19.20	9.60
-10	-9	-3	8.60	12.30
-11	-9	-3	32.90	9.70
-11	-9	-3	45.90	8.20
-11	-9	-3	38.80	11.00
-11	-9	-3	20.50	10.20
-11	-9	-3	40.20	11.20
-12	-9	-3	145.39	12.80
-12	-9	-3	105.89	17.30
11	-10	-3	203.38	22.70
11	-10	-3	168.48	23.20
-11	10	3	150.08	18.20
10	-10	-3	318.17	21.60
10	-10	-3	277.57	21.00
-10	10	3	251.87	22.90
9	-10	-3	-7.20	9.50
9	-10	-3	12.70	11.00
-9	10	3	-1.90	15.50
8	-10	-3	194.88	18.20
8	-10	-3	208.98	17.40
-8	10	3	187.58	21.20
-8	10	3	221.38	31.70

-8	10	3	235.28	23.40
-8	10	3	182.58	21.40
7	-10	-3	51.09	10.60
-7	10	3	63.99	12.70
-7	10	3	86.19	13.80
-7	10	3	90.79	15.40
6	-10	-3	161.48	20.80
6	-10	-3	157.18	13.80
6	-10	-3	154.18	27.80
-6	10	3	147.89	16.00
-6	10	3	181.58	19.70
5	-10	-3	61.39	10.90
5	-10	-3	60.99	12.10
5	-10	-3	95.09	11.60
-5	10	3	73.49	9.70
-5	10	3	86.99	10.80
4	-10	-3	483.05	25.10
4	-10	-3	441.96	26.10
-4	10	3	445.56	29.80
-4	10	3	486.65	23.10
3	-10	-3	209.78	21.80
3	-10	-3	209.68	15.80
3	-10	-3	200.78	16.10
-3	10	3	202.58	22.80
-3	10	3	209.58	11.40
-3	10	3	224.58	16.30
2	-10	-3	15.50	6.40
2	-10	-3	12.80	8.60
-2	10	3	10.20	4.80
-2	10	3	19.80	7.30
-2	10	3	10.30	2.40
-2	10	3	-2.70	10.30
1	-10	-3	713.03	36.70
1	-10	-3	797.02	38.40
-1	10	3	831.02	40.50
-1	10	3	748.53	35.00
-1	10	3	731.83	36.50
0	-10	-3	5024.40	227.58
0	10	3	5120.09	226.18
0	10	3	5409.16	228.38
0	10	3	4642.34	224.88
-1	-10	-3	114.59	8.90
-1	-10	-3	111.09	12.20
1	10	3	119.49	8.00

1	10	3	117.89	13.40
1	10	3	116.59	6.30
-2	-10	-3	161.48	11.60
-2	-10	-3	161.68	10.40
2	10	3	168.48	8.50
2	10	3	171.98	9.80
2	10	3	159.08	15.30
-3	-10	-3	128.49	9.30
3	10	3	110.39	7.50
3	10	3	126.59	6.90
3	10	3	120.09	14.70
-4	-10	-3	158.18	12.40
-4	-10	-3	152.58	12.50
-4	-10	-3	165.58	13.80
-4	-10	-3	154.08	12.20
-4	-10	-3	148.39	13.40
4	10	3	159.78	9.10
-5	-10	-3	6.00	5.80
-5	-10	-3	14.80	6.20
-5	-10	-3	10.30	9.80
-5	-10	-3	-17.10	10.10
-5	-10	-3	15.10	4.80
-5	-10	-3	16.00	6.90
-5	-10	-3	4.10	6.20
5	10	3	14.00	3.60
-6	-10	-3	54.09	7.80
-6	-10	-3	60.19	8.00
-6	-10	-3	42.80	6.60
-6	-10	-3	50.29	7.50
-6	-10	-3	58.19	7.90
6	10	3	42.40	7.50
-7	-10	-3	380.06	21.50
-7	-10	-3	335.07	19.70
-7	-10	-3	367.36	21.20
-7	-10	-3	356.26	21.20
7	10	3	343.27	20.40
-8	-10	-3	-3.80	6.80
-8	-10	-3	8.40	6.90
-8	-10	-3	-2.70	7.20
-8	-10	-3	3.50	6.20
-8	-10	-3	-1.60	7.30
8	10	3	-6.60	8.00
-9	-10	-3	-9.20	6.90
-9	-10	-3	0.30	7.00

-9	-10	-3	-8.10	7.70
-9	-10	-3	-1.90	8.30
-9	-10	-3	-1.60	7.10
9	10	3	-2.90	11.30
-10	-10	-3	415.56	25.20
-10	-10	-3	424.16	26.50
-10	-10	-3	418.96	24.70
-10	-10	-3	449.66	26.10
-10	-10	-3	440.46	25.70
-11	-10	-3	66.49	11.10
-11	-10	-3	40.90	10.10
-11	-10	-3	37.10	10.10
-11	-10	-3	54.39	13.20
-11	-10	-3	39.50	8.10
-12	-10	-3	79.69	12.10
-12	-10	-3	44.40	12.40
12	10	3	49.80	12.90
10	-11	-3	16.70	9.80
10	-11	-3	-2.70	10.90
-10	11	3	12.50	10.90
9	-11	-3	32.40	9.20
9	-11	-3	38.80	11.10
-9	11	3	29.20	12.80
8	-11	-3	309.47	20.20
8	-11	-3	271.97	21.40
-8	11	3	272.07	24.20
-8	11	3	284.97	24.60
-8	11	3	276.67	25.60
7	-11	-3	105.29	16.70
7	-11	-3	97.39	13.00
-7	11	3	98.49	15.40
-7	11	3	111.99	17.40
-7	11	3	80.79	14.20
6	-11	-3	61.69	11.70
6	-11	-3	62.69	11.40
6	-11	-3	34.10	11.80
-6	11	3	54.19	11.10
-6	11	3	52.59	12.50
-6	11	3	41.00	11.80
5	-11	-3	852.81	47.50
5	-11	-3	923.31	46.10
-5	11	3	931.61	48.10
-5	11	3	927.71	44.60
-5	11	3	990.30	46.70

4	-11	-3	188.98	16.00
4	-11	-3	150.28	18.10
4	-11	-3	195.58	14.60
4	-11	-3	198.78	33.20
-4	11	3	185.48	21.70
-4	11	3	184.18	16.40
-4	11	3	206.58	16.70
3	-11	-3	106.49	19.60
3	-11	-3	140.99	13.20
3	-11	-3	126.39	13.70
3	-11	-3	145.69	20.90
3	-11	-3	147.79	14.00
-3	11	3	107.49	15.40
-3	11	3	139.29	8.70
-3	11	3	127.19	15.60
2	-11	-3	928.11	46.30
2	-11	-3	1041.10	49.70
2	-11	-3	835.62	47.90
-2	11	3	970.70	52.19
-2	11	3	992.00	47.30
-2	11	3	989.60	44.20
1	-11	-3	9.50	7.40
1	-11	-3	-5.00	5.80
-1	11	3	5.90	4.90
-1	11	3	14.60	10.30
-1	11	3	6.20	2.20
0	-11	-3	197.28	16.40
0	-11	-3	171.08	13.10
0	11	3	176.78	12.10
0	11	3	194.08	9.80
0	11	3	208.48	18.30
-1	-11	-3	71.39	13.30
-1	-11	-3	82.49	9.20
1	11	3	78.59	5.10
1	11	3	80.19	7.70
1	11	3	77.89	11.30
-2	-11	-3	246.08	14.70
-2	-11	-3	223.78	14.50
2	11	3	256.67	13.50
2	11	3	248.88	12.00
2	11	3	210.78	20.40
-3	-11	-3	-5.10	7.30
-3	-11	-3	10.00	4.60
-3	-11	-3	-3.50	7.00

-3	-11	-3	15.30	6.20
-3	-11	-3	2.00	6.60
3	11	3	5.80	2.30
3	11	3	8.40	4.40
-4	-11	-3	47.00	7.90
-4	-11	-3	56.09	7.80
-4	-11	-3	42.40	6.60
-4	-11	-3	52.29	7.60
-4	-11	-3	47.00	8.20
4	11	3	42.10	5.10
-5	-11	-3	1513.05	69.79
-5	-11	-3	1496.95	69.19
-5	-11	-3	1430.26	69.89
-5	-11	-3	1558.34	68.99
-5	-11	-3	1465.35	68.99
-5	-11	-3	1506.35	69.49
5	11	3	1453.75	68.29
-6	-11	-3	950.90	44.30
-6	-11	-3	906.41	46.30
6	11	3	911.01	44.10
-7	-11	-3	138.09	13.70
-7	-11	-3	126.89	13.70
-7	-11	-3	131.29	12.90
-7	-11	-3	153.58	11.80
-7	-11	-3	154.08	14.50
7	11	3	133.39	13.70
-8	-11	-3	15.00	7.50
-8	-11	-3	14.80	7.70
-8	-11	-3	16.90	8.40
-8	-11	-3	2.00	6.90
-8	-11	-3	14.90	7.60
8	11	3	14.40	9.50
-9	-11	-3	79.59	12.80
-9	-11	-3	83.09	12.10
-9	-11	-3	53.99	9.40
-9	-11	-3	49.99	9.10
9	11	3	51.39	13.90
-10	-11	-3	33.70	9.70
-10	-11	-3	17.30	8.00
-10	-11	-3	12.40	8.30
-10	-11	-3	-0.10	8.80
-10	-11	-3	5.80	8.80
-11	-11	-3	63.59	12.30
-11	-11	-3	49.70	9.90

-11	-11	-3	48.40	10.00
-11	-11	-3	59.39	11.40
-11	-11	-3	76.09	12.10
10	-12	-3	191.58	16.10
10	-12	-3	192.38	26.30
-10	12	3	149.09	18.60
9	-12	-3	-2.60	11.50
9	-12	-3	21.70	8.30
-9	12	3	3.80	11.40
8	-12	-3	8.00	7.50
-8	12	3	-0.80	13.00
-8	12	3	8.70	14.00
7	-12	-3	863.91	39.40
7	-12	-3	748.23	40.80
-7	12	3	755.12	45.30
-7	12	3	771.72	42.80
-7	12	3	720.13	42.30
6	-12	-3	319.77	26.00
6	-12	-3	313.47	25.50
6	-12	-3	385.06	22.00
-6	12	3	408.26	29.00
-6	12	3	353.76	27.80
-6	12	3	387.16	25.20
5	-12	-3	152.68	14.00
5	-12	-3	185.58	17.70
-5	12	3	185.68	15.60
-5	12	3	142.19	17.60
4	-12	-3	104.59	17.10
4	-12	-3	59.99	15.80
4	-12	-3	84.89	13.50
4	-12	-3	90.29	12.20
-4	12	3	95.09	14.90
-4	12	3	83.89	14.50
-4	12	3	104.49	9.40
3	-12	-3	18.10	7.70
3	-12	-3	13.00	13.50
3	-12	-3	-1.40	9.90
3	-12	-3	11.20	7.30
3	-12	-3	11.00	11.00
-3	12	3	10.60	4.00
-3	12	3	24.50	8.10
-3	12	3	6.70	11.80
2	-12	-3	38.70	9.10
2	-12	-3	29.40	11.20

2	-12	-3	29.60	9.20
2	-12	-3	29.30	8.00
2	-12	-3	25.30	9.60
-2	12	3	21.10	4.40
-2	12	3	29.30	8.40
1	-12	-3	671.03	35.30
1	-12	-3	731.33	35.50
1	-12	-3	627.54	34.40
1	-12	-3	648.64	34.30
-1	12	3	714.23	31.80
0	-12	-3	653.23	34.40
0	-12	-3	715.43	34.90
0	-12	-3	656.33	34.50
0	-12	-3	665.33	34.20
0	-12	-3	666.63	34.90
0	-12	-3	711.73	34.80
0	12	3	692.73	31.40
0	12	3	638.84	33.50
-1	-12	-3	693.63	31.70
-1	-12	-3	616.54	32.90
-1	-12	-3	611.94	32.10
-1	-12	-3	625.74	32.40
-1	-12	-3	649.24	33.40
-1	-12	-3	600.34	32.10
1	12	3	622.54	31.60
1	12	3	678.73	29.70
-2	-12	-3	15.50	6.40
-2	-12	-3	18.30	8.30
-2	-12	-3	8.50	8.10
-2	-12	-3	16.40	6.40
2	12	3	8.70	2.60
2	12	3	12.10	5.20
-3	-12	-3	130.59	13.90
-3	-12	-3	138.79	13.20
-3	-12	-3	120.59	11.40
-3	-12	-3	104.39	13.30
-3	-12	-3	138.19	11.00
-3	-12	-3	125.79	16.20
3	12	3	135.29	7.90
-4	-12	-3	309.47	17.60
-4	-12	-3	306.97	19.40
-4	-12	-3	261.97	19.20
-4	-12	-3	331.37	17.70
-4	-12	-3	303.77	19.40

-4	-12	-3	288.27	18.60
4	12	3	299.57	15.60
-5	-12	-3	17.00	8.10
-5	-12	-3	11.00	7.00
-5	-12	-3	-0.50	6.10
-5	-12	-3	-2.20	5.10
-5	-12	-3	11.70	7.60
-5	-12	-3	-2.20	5.80
5	12	3	1.70	4.00
-6	-12	-3	290.77	18.90
-6	-12	-3	253.27	17.00
-6	-12	-3	288.47	18.30
-6	-12	-3	260.37	16.50
-6	-12	-3	271.17	17.70
-6	-12	-3	255.67	20.60
6	12	3	245.78	16.50
-7	-12	-3	151.88	15.20
-7	-12	-3	180.68	14.40
-7	-12	-3	186.78	16.20
-7	-12	-3	161.88	13.40
-7	-12	-3	173.38	14.80
7	12	3	140.19	15.40
-8	-12	-3	97.39	13.50
-8	-12	-3	68.89	9.90
-8	-12	-3	98.99	12.40
-8	-12	-3	90.99	12.60
-8	-12	-3	98.89	14.00
8	12	3	65.79	12.40
-9	-12	-3	25.00	7.70
-9	-12	-3	14.70	8.30
-9	-12	-3	17.00	8.60
-9	-12	-3	28.00	9.20
-9	-12	-3	32.80	8.60
9	12	3	34.30	15.10
-10	-12	-3	29.30	8.70
-10	-12	-3	37.00	10.50
-10	-12	-3	49.20	10.20
-10	-12	-3	30.50	7.80
-10	-12	-3	40.90	8.80
-11	-12	-3	225.18	17.00
-11	-12	-3	222.58	16.10
-11	-12	-3	153.78	18.20
-11	-12	-3	206.28	17.90
11	12	3	209.38	20.50

10	-13	-3	57.19	10.10
9	-13	-3	73.59	16.30
9	-13	-3	66.59	12.30
-9	13	3	60.79	13.00
8	-13	-3	34.60	9.50
8	-13	-3	6.10	13.20
-8	13	3	31.90	15.10
-8	13	3	7.80	15.50
-8	13	3	31.70	14.80
7	-13	-3	-1.30	7.10
7	-13	-3	18.10	13.10
7	-13	-3	51.49	15.30
-7	13	3	11.80	13.00
-7	13	3	-19.50	12.40
-7	13	3	-19.30	12.50
6	-13	-3	1079.09	53.29
6	-13	-3	1110.39	51.59
-6	13	3	1042.10	54.49
-6	13	3	1067.39	56.69
-6	13	3	1004.60	54.99
5	-13	-3	7.10	8.80
5	-13	-3	-4.90	15.80
5	-13	-3	25.60	7.60
-5	13	3	12.10	12.40
-5	13	3	15.40	10.40
4	-13	-3	65.49	15.60
4	-13	-3	36.00	8.10
4	-13	-3	43.70	7.30
4	-13	-3	32.10	15.70
-4	13	3	58.09	10.70
-4	13	3	39.20	13.30
3	-13	-3	-0.40	7.00
3	-13	-3	6.10	13.10
3	-13	-3	-3.50	7.10
3	-13	-3	14.10	10.80
-3	13	3	7.70	11.50
-3	13	3	9.20	4.20
-3	13	3	8.50	8.70
2	-13	-3	180.48	17.20
2	-13	-3	207.48	15.50
2	-13	-3	213.78	18.60
2	-13	-3	196.88	20.40
2	-13	-3	209.48	17.30
-2	13	3	201.98	16.00

-2	13	3	191.78	11.40
1	-13	-3	865.71	44.00
1	-13	-3	851.31	43.10
1	-13	-3	828.22	44.50
1	-13	-3	950.90	44.50
1	-13	-3	864.41	43.10
-1	13	3	940.31	41.10
0	-13	-3	104.09	13.50
0	-13	-3	117.09	13.90
0	-13	-3	100.79	14.30
0	-13	-3	109.29	12.70
0	-13	-3	107.49	13.60
0	13	3	127.89	7.50
-1	-13	-3	74.99	12.70
-1	-13	-3	73.29	11.60
-1	-13	-3	80.79	14.90
-1	-13	-3	82.89	11.10
-1	-13	-3	100.19	12.50
-1	-13	-3	95.29	11.40
1	13	3	71.89	9.30
1	13	3	92.59	6.10
-2	-13	-3	183.68	13.70
-2	-13	-3	206.18	15.50
-2	-13	-3	192.18	13.30
-2	-13	-3	185.28	14.40
-2	-13	-3	199.48	15.80
-2	-13	-3	176.08	20.10
2	13	3	202.68	10.60
-3	-13	-3	12.00	5.50
-3	-13	-3	12.30	7.90
-3	-13	-3	26.20	9.30
-3	-13	-3	7.30	7.60
-3	-13	-3	8.30	6.50
-3	-13	-3	20.70	6.30
3	13	3	14.60	3.10
-4	-13	-3	587.44	28.70
-4	-13	-3	538.85	27.40
-4	-13	-3	507.35	28.70
-4	-13	-3	524.75	28.90
-4	-13	-3	544.35	27.90
-4	-13	-3	483.95	29.10
4	13	3	551.54	26.10
-5	-13	-3	348.57	21.30
-5	-13	-3	357.46	20.40

-5	-13	-3	350.06	21.90
-5	-13	-3	386.26	20.60
-5	-13	-3	349.87	24.20
-5	-13	-3	330.77	22.00
5	13	3	341.67	19.10
-6	-13	-3	692.83	35.60
-6	-13	-3	719.73	34.90
-6	-13	-3	687.63	36.10
-6	-13	-3	686.33	36.10
-6	-13	-3	716.63	35.10
-6	-13	-3	704.33	36.70
6	13	3	649.43	34.90
-7	-13	-3	50.79	9.10
-7	-13	-3	49.50	9.40
-7	-13	-3	72.29	11.20
-7	-13	-3	54.19	8.50
-7	-13	-3	23.60	14.70
-7	-13	-3	57.79	9.60
7	13	3	41.70	9.60
-8	-13	-3	26.10	8.20
-8	-13	-3	20.50	8.40
-8	-13	-3	41.60	7.90
-8	-13	-3	25.40	8.20
-8	-13	-3	27.10	8.90
8	13	3	27.80	12.20
-9	-13	-3	12.40	9.90
-9	-13	-3	13.60	8.80
-9	-13	-3	40.30	14.70
-9	-13	-3	26.70	9.00
-9	-13	-3	35.40	11.40
9	13	3	7.50	17.50
-10	-13	-3	40.60	10.30
-10	-13	-3	23.10	8.80
-10	-13	-3	29.80	7.90
-10	-13	-3	39.00	8.20
-10	-13	-3	26.70	10.00
-11	-13	-3	96.69	15.10
-11	-13	-3	90.89	11.90
-11	-13	-3	81.79	13.60
-11	-13	-3	131.09	22.40
11	13	3	116.69	19.60
9	-14	-3	22.60	7.90
-9	14	3	21.60	10.80
8	-14	-3	123.09	14.20

-8	14	3	148.79	23.40
-8	14	3	168.08	21.90
7	-14	-3	26.00	7.60
-7	14	3	4.60	14.60
-7	14	3	14.10	14.00
-7	14	3	25.40	13.10
6	-14	-3	248.58	21.80
6	-14	-3	241.88	17.70
-6	14	3	265.07	25.20
-6	14	3	226.08	24.70
-6	14	3	181.98	24.90
5	-14	-3	8.90	9.00
5	-14	-3	5.90	7.40
5	-14	-3	-0.10	17.30
-5	14	3	-0.90	12.20
-5	14	3	0.80	11.10
4	-14	-3	217.98	26.60
4	-14	-3	194.78	16.80
4	-14	-3	213.68	28.60
4	-14	-3	175.78	15.60
-4	14	3	215.08	21.80
-4	14	3	154.58	18.80
-4	14	3	185.78	15.10
3	-14	-3	6.50	10.90
3	-14	-3	1.90	6.80
3	-14	-3	7.30	10.70
3	-14	-3	20.90	13.20
-3	14	3	13.50	5.10
-3	14	3	-4.90	12.20
-3	14	3	22.50	9.40
2	-14	-3	3.40	8.80
2	-14	-3	6.50	6.40
2	-14	-3	11.00	11.10
2	-14	-3	23.90	9.60
2	-14	-3	22.60	8.90
-2	14	3	9.30	7.40
-2	14	3	9.80	4.00
1	-14	-3	701.63	40.30
1	-14	-3	863.21	40.20
1	-14	-3	775.02	39.60
1	-14	-3	758.82	39.00
1	-14	-3	799.72	39.10
-1	14	3	796.22	36.80
0	-14	-3	1719.73	77.49

0	-14	-3	1619.54	77.19
0	-14	-3	1653.43	76.89
0	-14	-3	1684.33	77.29
0	-14	-3	1596.64	76.89
0	14	3	1677.53	75.39
-1	-14	-3	66.59	11.40
-1	-14	-3	116.99	13.20
-1	-14	-3	96.09	14.90
-1	-14	-3	98.89	11.60
-1	-14	-3	89.99	12.80
1	14	3	90.19	6.70
-2	-14	-3	8.50	5.60
-2	-14	-3	19.90	6.60
-2	-14	-3	4.10	7.30
-2	-14	-3	-4.30	10.10
-2	-14	-3	-0.20	6.00
-2	-14	-3	5.40	7.30
2	14	3	9.20	3.00
-3	-14	-3	264.97	17.70
-3	-14	-3	205.08	24.50
-3	-14	-3	257.57	16.50
-3	-14	-3	249.18	19.60
-3	-14	-3	259.47	18.30
-3	-14	-3	273.07	16.50
-3	-14	-3	272.67	17.20
3	14	3	272.97	14.20
-4	-14	-3	50.59	8.20
-4	-14	-3	56.09	12.00
-4	-14	-3	23.10	11.70
-4	-14	-3	23.20	8.10
-4	-14	-3	35.10	7.50
-4	-14	-3	29.00	9.50
-5	-14	-3	26.40	8.90
-5	-14	-3	17.70	6.30
-5	-14	-3	4.50	7.70
-5	-14	-3	26.10	7.70
-5	-14	-3	8.50	8.60
-5	-14	-3	10.30	7.00
5	14	3	23.80	5.70
-6	-14	-3	7.70	6.90
-6	-14	-3	4.60	5.80
-6	-14	-3	19.20	8.10
-6	-14	-3	12.20	8.90
-6	-14	-3	13.00	8.00

-6	-14	-3	12.70	8.10
6	14	3	0.40	7.30
-7	-14	-3	244.08	16.70
-7	-14	-3	254.67	18.60
-7	-14	-3	234.88	18.50
-7	-14	-3	225.28	18.10
-7	-14	-3	184.08	22.00
-7	-14	-3	251.27	17.80
-8	-14	-3	29.20	8.60
-8	-14	-3	29.30	9.10
-8	-14	-3	41.20	8.90
-8	-14	-3	27.40	6.80
8	14	3	14.20	13.20
-9	-14	-3	76.99	12.20
-9	-14	-3	61.89	10.70
-9	-14	-3	68.19	10.70
-9	-14	-3	79.99	14.20
-9	-14	-3	70.19	10.30
-10	-14	-3	190.68	14.80
-10	-14	-3	175.78	16.60
10	14	3	177.88	22.50
-11	-14	-3	29.80	9.70
-11	-14	-3	9.70	7.70
-11	-14	-3	1.80	8.00
11	14	3	7.00	14.40
9	-15	-3	54.39	8.70
-9	15	3	49.70	10.70
8	-15	-3	25.50	7.70
-8	15	3	35.20	10.80
-8	15	3	28.40	17.80
7	-15	-3	105.69	17.50
7	-15	-3	145.09	15.80
-7	15	3	97.59	16.70
-7	15	3	119.09	17.10
-7	15	3	92.09	16.00
6	-15	-3	77.09	12.90
-6	15	3	39.80	14.20
-6	15	3	46.80	14.40
-6	15	3	61.59	13.60
5	-15	-3	697.43	35.40
5	-15	-3	663.73	34.30
5	-15	-3	560.14	42.10
-5	15	3	683.73	38.40
4	-15	-3	15.30	15.50

4	-15	-3	-1.20	7.10
4	-15	-3	5.40	8.60
4	-15	-3	-17.40	15.20
-4	15	3	1.40	8.50
-4	15	3	-1.10	11.40
-4	15	3	-12.90	9.80
3	-15	-3	46.40	11.70
3	-15	-3	72.19	14.40
3	-15	-3	43.20	8.70
3	-15	-3	51.89	9.30
-3	15	3	25.00	11.10
-3	15	3	58.19	7.50
-3	15	3	54.79	10.50
2	-15	-3	533.55	29.60
2	-15	-3	502.85	28.80
2	-15	-3	575.34	31.30
2	-15	-3	539.35	32.00
-2	15	3	524.35	29.40
-2	15	3	537.95	26.70
1	-15	-3	18.50	11.10
1	-15	-3	-5.90	8.30
1	-15	-3	9.60	9.20
1	-15	-3	6.80	7.50
-1	15	3	5.80	6.70
-1	15	3	11.40	3.90
0	-15	-3	-8.70	7.10
0	-15	-3	5.50	7.80
0	-15	-3	-8.10	6.80
0	-15	-3	-0.40	6.20
0	15	3	0.80	3.10
-1	-15	-3	204.48	15.70
-1	-15	-3	197.98	17.30
-1	-15	-3	208.68	15.80
-1	-15	-3	217.98	15.30
-1	-15	-3	198.98	16.10
1	15	3	202.68	11.70
-2	-15	-3	13.70	6.70
-2	-15	-3	16.60	7.60
-2	-15	-3	17.20	6.40
-2	-15	-3	20.80	7.30
-2	-15	-3	7.70	9.20
2	15	3	12.10	3.60
-3	-15	-3	4.40	7.70
-3	-15	-3	0.00	5.70

-3	-15	-3	1.10	6.20
-3	-15	-3	-14.00	12.30
-3	-15	-3	3.90	7.30
-3	-15	-3	-1.50	7.00
3	15	3	4.10	3.70
-4	-15	-3	143.69	12.50
-4	-15	-3	121.69	14.90
-4	-15	-3	127.79	12.20
-4	-15	-3	147.29	14.50
-4	-15	-3	143.19	14.30
-4	-15	-3	123.29	13.70
4	15	3	137.39	10.50
-5	-15	-3	1457.65	68.49
-5	-15	-3	1491.05	67.59
-5	-15	-3	1498.95	68.39
-5	-15	-3	1377.26	68.19
-5	-15	-3	1429.16	68.09
-5	-15	-3	1366.56	68.59
5	15	3	1475.05	67.39
-6	-15	-3	530.25	27.30
-6	-15	-3	511.75	29.30
-6	-15	-3	550.74	29.20
-6	-15	-3	483.25	29.20
-6	-15	-3	524.05	28.80
-6	-15	-3	491.15	28.70
6	15	3	498.55	28.40
-7	-15	-3	67.59	10.20
-7	-15	-3	49.80	9.50
-7	-15	-3	68.89	10.00
-7	-15	-3	57.99	12.30
-7	-15	-3	51.09	7.90
7	15	3	38.60	11.60
-8	-15	-3	20.10	9.70
-8	-15	-3	27.10	13.40
-8	-15	-3	15.30	9.50
-8	-15	-3	26.30	8.60
8	15	3	7.60	14.60
8	15	3	11.80	18.70
-9	-15	-3	79.19	14.00
-9	-15	-3	133.49	12.10
-9	-15	-3	109.19	14.10
-9	-15	-3	152.68	14.20
9	15	3	107.99	17.70
-10	-15	-3	19.30	7.50

-10	-15	-3	14.90	7.00
-10	-15	-3	-4.00	9.90
-10	-15	-3	16.50	8.90
10	15	3	-15.00	15.70
9	-16	-3	9.50	7.40
-9	16	3	-17.60	8.90
8	-16	-3	54.19	8.10
-8	16	3	27.60	10.60
7	-16	-3	536.15	34.50
7	-16	-3	536.55	28.60
-7	16	3	493.85	33.10
-7	16	3	546.75	33.10
-7	16	3	535.35	34.60
6	-16	-3	124.09	19.20
6	-16	-3	114.09	13.90
-6	16	3	128.19	16.90
-6	16	3	134.29	20.50
5	-16	-3	5.90	8.60
5	-16	-3	13.80	7.90
-5	16	3	26.90	12.60
-5	16	3	-1.30	11.80
4	-16	-3	54.19	9.10
4	-16	-3	36.00	8.80
4	-16	-3	49.40	16.20
4	-16	-3	48.50	16.00
-4	16	3	51.49	12.60
-4	16	3	43.10	12.30
3	-16	-3	32.20	11.90
3	-16	-3	57.19	13.80
3	-16	-3	52.19	9.10
3	-16	-3	45.10	8.70
-3	16	3	56.79	8.50
-3	16	3	37.60	9.90
-3	16	3	55.79	11.60
2	-16	-3	129.99	14.70
2	-16	-3	159.88	17.10
2	-16	-3	132.59	16.20
2	-16	-3	101.59	15.90
2	-16	-3	171.48	18.60
-2	16	3	130.59	14.50
-2	16	3	124.19	11.40
1	-16	-3	21.40	8.90
1	-16	-3	7.60	6.80
1	-16	-3	15.90	13.20

1	-16	-3	17.10	8.60
1	-16	-3	9.00	7.40
-1	16	3	14.20	5.00
-1	16	3	12.30	7.30
0	-16	-3	732.33	38.20
0	-16	-3	768.92	38.30
0	-16	-3	767.12	38.60
0	-16	-3	769.62	38.40
0	16	3	742.43	36.10
-1	-16	-3	560.04	30.40
-1	-16	-3	575.44	31.50
-1	-16	-3	580.04	30.60
-1	-16	-3	577.84	30.20
-1	-16	-3	591.94	30.70
1	16	3	587.34	28.40
-2	-16	-3	75.99	12.50
-2	-16	-3	71.19	11.00
-2	-16	-3	87.69	10.30
-2	-16	-3	73.49	11.90
2	16	3	63.49	7.80
-3	-16	-3	12.40	6.50
-3	-16	-3	-4.40	6.50
-3	-16	-3	0.50	6.40
-3	-16	-3	3.60	7.10
-3	-16	-3	6.70	7.70
3	16	3	0.10	4.30
-4	-16	-3	237.88	16.30
-4	-16	-3	237.68	17.60
-4	-16	-3	262.67	17.40
-4	-16	-3	242.78	16.10
-4	-16	-3	224.38	17.60
4	16	3	230.38	14.90
-5	-16	-3	9.60	7.10
-5	-16	-3	3.50	7.90
-5	-16	-3	16.50	7.80
-5	-16	-3	8.30	8.10
-5	-16	-3	14.90	6.30
5	16	3	16.70	6.60
-6	-16	-3	351.06	23.50
-6	-16	-3	362.26	21.20
-6	-16	-3	355.36	22.50
-6	-16	-3	369.46	21.90
-6	-16	-3	370.66	23.00
6	16	3	368.56	23.20

-7	-16	-3	122.69	13.70
-7	-16	-3	108.89	14.10
-7	-16	-3	100.29	14.70
-7	-16	-3	140.09	18.50
-7	-16	-3	111.19	12.00
7	16	3	105.79	14.40
-8	-16	-3	31.10	7.20
-8	-16	-3	33.80	9.00
-8	-16	-3	21.80	8.10
-8	-16	-3	31.60	9.00
8	16	3	21.30	18.50
8	16	3	31.40	22.10
-9	-16	-3	14.20	8.10
-9	-16	-3	-2.50	6.00
-9	-16	-3	6.00	8.60
-9	-16	-3	13.20	7.60
9	16	3	-6.90	18.80
-10	-16	-3	83.49	11.50
-10	-16	-3	97.29	10.70
10	16	3	102.79	23.60
8	-17	-3	4.80	7.30
-8	17	3	20.90	9.10
7	-17	-3	59.49	8.60
7	-17	-3	63.89	15.40
-7	17	3	40.70	16.80
-7	17	3	41.30	10.80
6	-17	-3	361.76	21.20
6	-17	-3	346.17	24.80
-6	17	3	350.96	27.20
-6	17	3	291.67	26.80
-6	17	3	348.27	25.40
5	-17	-3	50.59	10.20
5	-17	-3	90.49	13.20
-5	17	3	54.19	13.40
-5	17	3	59.89	13.40
4	-17	-3	61.29	10.10
4	-17	-3	106.39	17.50
4	-17	-3	104.49	17.20
4	-17	-3	94.99	14.20
-4	17	3	82.69	13.40
-4	17	3	86.69	13.40
3	-17	-3	18.80	13.30
3	-17	-3	17.90	7.90
3	-17	-3	14.80	8.70

3	-17	-3	0.80	11.30
-3	17	3	13.70	10.10
-3	17	3	5.80	8.70
-3	17	3	24.60	11.30
2	-17	-3	121.39	14.80
2	-17	-3	161.18	18.80
2	-17	-3	115.19	18.90
2	-17	-3	144.39	16.00
2	-17	-3	143.69	16.30
-2	17	3	132.19	13.00
-2	17	3	126.29	14.80
1	-17	-3	203.08	17.60
1	-17	-3	184.98	17.10
1	-17	-3	191.68	18.10
-1	17	3	215.58	16.30
-1	17	3	183.48	13.60
0	-17	-3	17.50	7.20
0	-17	-3	0.50	7.90
0	-17	-3	0.50	7.70
0	-17	-3	-0.50	7.70
0	17	3	12.60	5.40
-1	-17	-3	10.90	7.00
-1	-17	-3	2.40	8.10
-1	-17	-3	11.10	7.50
-1	-17	-3	14.00	8.10
-1	-17	-3	12.50	7.50
1	17	3	2.00	4.60
-2	-17	-3	272.27	18.50
-2	-17	-3	286.57	19.00
-2	-17	-3	260.77	18.30
-2	-17	-3	275.57	17.90
-2	-17	-3	282.67	21.50
2	17	3	280.77	16.30
-3	-17	-3	145.59	22.20
-3	-17	-3	131.09	13.70
-3	-17	-3	111.99	13.30
-3	-17	-3	110.39	12.40
-3	-17	-3	106.19	13.70
3	17	3	111.19	10.70
-4	-17	-3	327.67	19.50
-4	-17	-3	328.67	20.90
-4	-17	-3	297.17	20.70
-4	-17	-3	312.57	19.80
-4	-17	-3	322.37	20.50

4	17	3	332.67	19.10
-5	-17	-3	63.69	9.30
-5	-17	-3	39.50	8.90
-5	-17	-3	47.50	7.60
-5	-17	-3	73.99	11.90
-5	-17	-3	44.30	8.60
5	17	3	75.39	12.50
-6	-17	-3	363.36	22.40
-6	-17	-3	362.46	23.10
-6	-17	-3	378.06	23.40
-6	-17	-3	361.46	20.90
-6	-17	-3	338.97	21.60
6	17	3	389.06	24.00
-7	-17	-3	-0.30	12.90
-7	-17	-3	17.30	10.90
-7	-17	-3	12.10	7.90
-7	-17	-3	13.40	10.80
7	17	3	16.20	13.30
-8	-17	-3	125.89	12.90
-8	-17	-3	84.99	10.70
-8	-17	-3	128.59	13.70
-8	-17	-3	114.89	12.70
-9	-17	-3	112.79	13.10
-9	-17	-3	85.09	12.30
-9	-17	-3	86.89	11.90
-9	-17	-3	88.69	10.40
8	-18	-3	103.79	11.70
7	-18	-3	41.90	15.70
7	-18	-3	43.10	8.00
-7	18	3	65.59	10.40
-7	18	3	58.49	17.80
6	-18	-3	418.36	26.10
6	-18	-3	409.36	23.00
-6	18	3	361.26	27.50
-6	18	3	379.26	27.40
-6	18	3	394.86	25.10
5	-18	-3	7.00	10.30
5	-18	-3	12.70	8.90
-5	18	3	23.90	12.20
-5	18	3	21.00	12.80
4	-18	-3	-12.00	8.50
4	-18	-3	4.40	16.90
4	-18	-3	-8.60	19.50
4	-18	-3	-12.20	8.00

-4	18	3	-0.20	10.80
-4	18	3	8.60	10.80
3	-18	-3	2.50	11.50
3	-18	-3	9.00	13.10
3	-18	-3	-0.70	9.30
3	-18	-3	-6.10	8.00
-3	18	3	2.30	9.70
-3	18	3	-4.00	9.40
2	-18	-3	17.50	9.50
2	-18	-3	14.50	11.00
2	-18	-3	8.40	11.40
2	-18	-3	13.90	7.90
-2	18	3	8.10	9.70
-2	18	3	23.60	9.20
-2	18	3	22.10	9.60
1	-18	-3	471.45	28.00
1	-18	-3	495.95	27.80
1	-18	-3	476.35	27.50
1	-18	-3	498.05	27.40
-1	18	3	476.75	27.90
-1	18	3	470.95	26.10
0	-18	-3	391.86	23.80
0	-18	-3	392.46	23.70
0	-18	-3	377.36	24.00
0	-18	-3	418.66	25.10
0	18	3	402.06	24.30
0	18	3	405.06	22.20
-1	-18	-3	284.57	18.90
-1	-18	-3	257.67	18.90
-1	-18	-3	277.67	18.80
-1	-18	-3	285.47	20.80
1	18	3	268.07	17.20
-2	-18	-3	44.10	8.10
-2	-18	-3	61.39	8.80
-2	-18	-3	56.29	11.50
-2	-18	-3	38.20	8.10
-2	-18	-3	72.19	9.80
2	18	3	57.19	7.60
-3	-18	-3	143.49	14.30
-3	-18	-3	139.19	14.50
-3	-18	-3	124.49	12.60
-3	-18	-3	126.49	14.30
-3	-18	-3	118.29	21.90
3	18	3	147.19	12.90

-4	-18	-3	18.70	7.50
-4	-18	-3	-2.20	7.50
-4	-18	-3	11.80	6.30
-4	-18	-3	21.00	8.00
4	18	3	13.50	7.40
-5	-18	-3	8.20	7.70
-5	-18	-3	-8.00	7.70
-5	-18	-3	13.40	7.50
-5	-18	-3	7.60	6.40
5	18	3	-10.40	8.40
-6	-18	-3	0.00	8.10
-6	-18	-3	1.20	8.70
-6	-18	-3	0.90	6.40
6	18	3	17.00	11.00
-7	-18	-3	235.08	15.80
-7	-18	-3	227.58	16.90
-7	-18	-3	217.18	17.20
-7	-18	-3	221.68	16.40
-7	-18	-3	254.37	20.20
-8	-18	-3	20.80	6.00
-8	-18	-3	12.50	7.10
-8	-18	-3	13.10	7.40
-9	-18	-3	64.49	10.90
-9	-18	-3	53.49	8.90
-9	-18	-3	57.39	9.20
7	-19	-3	20.60	16.00
7	-19	-3	3.30	6.80
-7	19	3	7.90	7.70
6	-19	-3	463.85	24.50
6	-19	-3	477.25	27.30
-6	19	3	432.46	28.50
-6	19	3	419.26	26.00
-6	19	3	395.16	29.60
5	-19	-3	494.95	28.70
5	-19	-3	515.75	27.30
-5	19	3	502.15	29.10
-5	19	3	516.15	30.30
-5	19	3	465.15	30.40
4	-19	-3	5.60	9.30
4	-19	-3	-9.60	25.20
4	-19	-3	-7.80	9.20
-4	19	3	2.10	11.30
-4	19	3	-7.00	11.00
3	-19	-3	12.70	12.30

3	-19	-3	34.50	11.20
3	-19	-3	21.50	8.60
3	-19	-3	8.70	15.60
-3	19	3	18.40	10.20
-3	19	3	28.50	10.50
2	-19	-3	233.48	19.50
2	-19	-3	219.58	18.00
2	-19	-3	218.88	20.90
2	-19	-3	176.08	25.60
-2	19	3	237.08	22.40
-2	19	3	185.78	18.10
-2	19	3	204.68	18.70
1	-19	-3	37.20	9.20
1	-19	-3	33.40	9.70
1	-19	-3	26.00	8.90
1	-19	-3	33.00	8.60
-1	19	3	36.70	10.00
-1	19	3	34.20	9.10
0	-19	-3	69.99	10.80
0	-19	-3	57.59	9.60
0	-19	-3	43.50	8.90
0	-19	-3	63.29	10.30
0	19	3	64.99	10.10
0	19	3	63.59	9.10
-1	-19	-3	88.39	13.40
-1	-19	-3	104.89	12.80
-1	-19	-3	87.59	16.10
-1	-19	-3	79.49	12.70
1	19	3	97.39	12.90
-2	-19	-3	35.60	8.10
-2	-19	-3	45.20	11.10
-2	-19	-3	25.80	8.00
-2	-19	-3	31.40	7.50
2	19	3	44.70	13.80
2	19	3	35.90	7.80
-3	-19	-3	13.90	6.80
-3	-19	-3	19.70	13.70
-3	-19	-3	14.40	7.60
-3	-19	-3	13.50	7.10
-3	-19	-3	10.70	7.20
3	19	3	9.70	7.80
-4	-19	-3	86.39	12.40
-4	-19	-3	73.59	10.90
-4	-19	-3	75.19	12.80

-4	-19	-3	87.79	12.80
4	19	3	98.89	13.90
-5	-19	-3	398.16	22.60
-5	-19	-3	445.96	26.80
-5	-19	-3	368.56	23.10
-5	-19	-3	387.46	28.50
5	19	3	390.96	25.90
-6	-19	-3	11.90	7.40
-6	-19	-3	17.60	6.00
-6	-19	-3	14.40	8.50
-6	-19	-3	15.10	7.40
-7	-19	-3	203.98	14.10
-7	-19	-3	212.98	18.80
-7	-19	-3	220.98	15.20
-7	-19	-3	200.28	15.50
-8	-19	-3	286.57	15.70
-8	-19	-3	240.88	16.80
-8	-19	-3	240.28	16.50
6	-20	-3	15.80	7.90
6	-20	-3	23.70	10.90
-6	20	3	30.10	16.90
-6	20	3	19.90	8.70
-6	20	3	-1.90	29.80
5	-20	-3	-9.10	8.30
5	-20	-3	-1.20	8.60
-5	20	3	5.20	8.30
-5	20	3	-8.00	13.00
-5	20	3	15.40	11.00
4	-20	-3	-5.70	9.30
4	-20	-3	-0.60	8.20
-4	20	3	2.20	10.60
-4	20	3	13.60	11.60
3	-20	-3	4.00	12.60
3	-20	-3	-4.50	11.40
3	-20	-3	29.00	15.70
3	-20	-3	21.00	9.00
-3	20	3	-1.80	10.10
-3	20	3	24.10	10.30
2	-20	-3	32.40	9.10
2	-20	-3	41.90	16.50
-2	20	3	41.80	10.10
-2	20	3	30.10	13.90
1	-20	-3	7.00	8.80
1	-20	-3	6.70	7.90

1	-20	-3	-14.40	10.00
1	-20	-3	7.10	8.70
-1	20	3	-15.00	12.40
-1	20	3	-9.60	7.90
0	-20	-3	379.56	23.80
0	-20	-3	358.26	23.10
0	-20	-3	355.66	22.70
0	-20	-3	372.46	23.00
0	20	3	380.06	25.00
0	20	3	350.86	22.50
-1	-20	-3	129.89	16.80
-1	-20	-3	149.39	14.80
-1	-20	-3	117.89	14.00
-1	-20	-3	140.89	14.50
1	20	3	131.89	14.00
1	20	3	149.29	16.90
-2	-20	-3	86.09	12.60
-2	-20	-3	81.59	12.80
-2	-20	-3	88.89	12.30
-2	-20	-3	70.09	9.50
2	20	3	95.39	16.20
-3	-20	-3	124.49	13.50
-3	-20	-3	95.39	13.30
-3	-20	-3	103.29	12.50
3	20	3	148.09	16.50
3	20	3	111.79	23.90
-4	-20	-3	191.48	21.50
-4	-20	-3	198.18	22.30
-4	-20	-3	233.88	20.80
4	20	3	231.78	20.20
-5	-20	-3	-1.80	7.20
-5	-20	-3	6.50	6.20
-5	-20	-3	5.20	7.20
-5	-20	-3	-5.40	7.50
-6	-20	-3	184.48	13.80
-6	-20	-3	164.68	14.10
-6	-20	-3	190.38	13.20
-6	-20	-3	171.58	15.80
-7	-20	-3	91.69	9.70
-7	-20	-3	79.99	11.80
-7	-20	-3	117.29	11.30
-7	-20	-3	104.19	11.40
-8	-20	-3	20.70	5.60
-8	-20	-3	22.90	7.20

-8	-20	-3	29.10	7.20
6	-21	-3	86.59	12.60
6	-21	-3	50.09	11.60
-6	21	3	28.40	20.10
-6	21	3	69.79	12.10
5	-21	-3	112.19	13.60
5	-21	-3	88.99	15.00
-5	21	3	119.29	16.30
-5	21	3	73.39	12.10
-5	21	3	69.49	15.70
4	-21	-3	59.99	12.20
-4	21	3	42.00	11.30
-4	21	3	46.30	12.80
3	-21	-3	-11.90	7.50
3	-21	-3	4.60	12.80
-3	21	3	3.80	9.90
-3	21	3	5.60	10.40
2	-21	-3	261.07	22.50
2	-21	-3	216.98	20.00
2	-21	-3	234.18	17.80
-2	21	3	208.28	18.90
-2	21	3	216.78	19.10
1	-21	-3	160.58	16.50
1	-21	-3	144.29	18.10
1	-21	-3	115.19	16.00
-1	21	3	146.29	16.00
-1	21	3	157.28	17.10
0	-21	-3	55.99	10.30
0	-21	-3	61.39	11.00
0	-21	-3	77.49	13.40
0	-21	-3	67.49	11.00
0	21	3	79.39	12.40
0	21	3	63.49	14.70
-1	-21	-3	149.79	16.00
-1	-21	-3	142.79	23.20
-1	-21	-3	172.98	16.20
-1	-21	-3	139.89	18.20
1	21	3	171.48	15.30
1	21	3	159.98	22.30
-2	-21	-3	273.87	21.90
-2	-21	-3	288.27	18.90
-2	-21	-3	280.87	19.30
2	21	3	232.08	23.60
2	21	3	282.07	18.80

-3	-21	-3	1.00	6.90
-3	-21	-3	0.70	7.20
-3	-21	-3	11.50	7.30
3	21	3	1.20	15.70
3	21	3	14.20	13.40
-4	-21	-3	158.08	14.10
-4	-21	-3	163.28	14.60
-4	-21	-3	160.18	13.00
4	21	3	162.28	19.70
-5	-21	-3	5.60	7.10
-5	-21	-3	27.30	6.30
-5	-21	-3	13.40	6.80
5	21	3	2.10	10.60
-6	-21	-3	172.28	15.70
-6	-21	-3	194.68	14.20
-6	-21	-3	223.58	13.50
-6	-21	-3	175.38	13.80
6	21	3	217.08	18.10
-7	-21	-3	137.39	16.20
-7	-21	-3	112.89	11.30
-7	-21	-3	98.49	9.70
-7	-21	-3	85.09	10.70
7	21	3	92.59	14.60
5	-22	-3	2.90	15.40
5	-22	-3	-30.50	15.00
-5	22	3	-8.10	8.00
-5	22	3	12.00	11.90
4	-22	-3	67.89	11.70
-4	22	3	50.99	13.80
-4	22	3	66.19	11.80
-4	22	3	41.80	8.90
3	-22	-3	69.49	11.70
3	-22	-3	88.09	16.10
-3	22	3	86.09	12.40
-3	22	3	85.69	11.30
2	-22	-3	97.69	13.40
2	-22	-3	88.99	15.60
-2	22	3	109.49	15.30
-2	22	3	93.49	14.10
1	-22	-3	494.55	27.50
1	-22	-3	533.15	28.20
1	-22	-3	494.25	29.10
-1	22	3	491.55	27.90
-1	22	3	478.55	27.70

0	-22	-3	42.60	8.90
0	-22	-3	23.30	7.70
0	-22	-3	24.10	8.10
0	-22	-3	19.20	9.70
0	22	3	38.00	9.20
-1	-22	-3	164.08	14.30
-1	-22	-3	182.58	17.10
-1	-22	-3	155.68	15.60
-1	-22	-3	149.88	14.40
1	22	3	169.08	15.30
-2	-22	-3	22.70	7.70
-2	-22	-3	11.90	11.60
-2	-22	-3	20.70	7.20
-2	-22	-3	14.70	7.00
2	22	3	13.20	7.90
-3	-22	-3	15.50	6.70
-3	-22	-3	18.70	7.30
-3	-22	-3	18.40	7.30
3	22	3	21.20	8.20
-4	-22	-3	72.59	10.80
-4	-22	-3	82.19	11.20
-4	-22	-3	77.09	10.40
4	22	3	128.79	19.70
-5	-22	-3	2.60	5.70
-5	-22	-3	-1.60	7.00
-5	-22	-3	7.30	6.50
5	22	3	18.10	11.00
-6	-22	-3	6.10	5.30
-6	-22	-3	1.00	6.20
-6	-22	-3	6.60	7.70
3	-23	-3	111.29	11.30
-3	23	3	107.49	16.50
-3	23	3	85.59	13.90
2	-23	-3	55.79	11.60
2	-23	-3	50.89	8.90
-2	23	3	36.00	10.90
-2	23	3	62.39	10.00
1	-23	-3	52.69	10.10
1	-23	-3	86.19	17.70
1	-23	-3	70.59	12.30
-1	23	3	63.39	10.60
0	-23	-3	8.20	9.90
0	-23	-3	4.80	8.80
0	-23	-3	-10.00	7.60

0	23	3	14.10	9.30
0	23	3	13.10	7.80
-1	-23	-3	45.50	8.30
-1	-23	-3	49.80	9.50
-1	-23	-3	40.50	7.70
-1	-23	-3	50.29	9.90
1	23	3	31.80	8.60
-2	-23	-3	115.49	13.20
-2	-23	-3	92.79	13.80
-2	-23	-3	92.29	11.30
-2	-23	-3	113.89	11.90
2	23	3	93.89	13.20
-3	-23	-3	167.88	13.80
-3	-23	-3	171.48	13.50
-3	-23	-3	170.18	13.50
3	23	3	196.88	16.30
-4	-23	-3	90.79	10.40
-4	-23	-3	113.59	11.40
-4	-23	-3	86.49	10.90
4	23	3	77.09	12.50
-5	-23	-3	148.39	11.90
-5	-23	-3	157.68	13.90
-5	-23	-3	184.98	13.20
5	23	3	146.19	15.00
5	23	3	112.19	20.80
2	-24	-3	24.10	12.60
2	-24	-3	12.20	7.00
-2	24	3	30.60	11.10
-2	24	3	26.50	10.60
1	-24	-3	27.00	13.00
1	-24	-3	30.40	8.10
-1	24	3	32.20	8.50
-1	24	3	26.00	10.30
0	-24	-3	323.87	20.20
0	-24	-3	329.77	20.00
0	-24	-3	319.87	26.10
0	24	3	339.77	21.10
0	24	3	360.66	20.10
-1	-24	-3	5.60	7.00
-1	-24	-3	16.00	11.40
1	24	3	22.80	8.80
1	24	3	12.80	7.10
-2	-24	-3	16.50	9.20
-2	-24	-3	9.10	7.10

-2	-24	-3	15.20	6.50
2	24	3	39.50	9.20
-3	-24	-3	197.38	19.40
-3	-24	-3	221.38	19.70
-3	-24	-3	216.98	13.80
3	24	3	195.48	15.90
-4	-24	-3	97.79	11.20
-4	-24	-3	97.89	10.80
-4	-24	-3	88.49	10.50
4	24	3	80.19	12.20
1	23	-4	78.69	12.10
1	23	-4	99.49	13.80
-1	-23	4	100.29	14.80
0	23	-4	58.59	9.90
0	-23	4	44.30	8.90
4	22	-4	-9.60	7.80
4	22	-4	20.10	8.70
-4	-22	4	0.20	5.90
3	22	-4	194.78	14.90
3	22	-4	174.18	21.50
3	22	-4	199.18	16.20
-3	-22	4	198.78	13.10
2	22	-4	123.89	12.70
2	22	-4	169.78	25.20
2	22	-4	125.69	13.90
-2	-22	4	86.09	14.80
-2	-22	4	138.29	11.70
1	22	-4	250.87	17.50
1	22	-4	210.28	25.60
1	22	-4	260.27	16.70
-1	-22	4	267.67	16.70
-1	-22	4	239.28	17.50
0	22	-4	173.78	14.20
0	22	-4	175.48	13.40
0	-22	4	169.58	14.50
0	-22	4	174.08	15.40
-1	22	-4	16.60	7.40
-1	22	-4	15.40	8.80
1	-22	4	15.30	13.10
1	-22	4	21.10	7.30
-2	22	-4	109.99	13.00
-2	22	-4	121.99	11.60
2	-22	4	114.19	12.10
-3	22	-4	83.39	12.40

-3	22	-4	78.19	11.00
3	-22	4	86.89	13.60
5	21	-4	284.57	18.50
5	21	-4	265.47	24.80
-5	-21	4	292.47	18.00
-5	-21	4	304.07	16.80
4	21	-4	23.60	10.40
4	21	-4	10.80	8.50
-4	-21	4	19.70	10.90
-4	-21	4	16.90	6.20
3	21	-4	13.70	8.90
3	21	-4	34.60	9.70
-3	-21	4	17.30	6.40
2	21	-4	142.79	15.10
2	21	-4	108.49	15.70
-2	-21	4	129.59	13.70
-2	-21	4	141.09	12.20
1	21	-4	42.10	10.80
1	21	-4	39.00	8.20
1	21	-4	40.10	8.50
-1	-21	4	36.30	7.90
-1	-21	4	48.40	9.70
0	21	-4	4.60	12.60
0	21	-4	19.20	7.50
0	21	-4	8.90	8.00
0	-21	4	28.30	8.70
0	-21	4	14.10	8.10
-1	21	-4	19.10	8.50
-1	21	-4	14.70	7.40
1	-21	4	8.90	10.10
1	-21	4	15.00	7.80
-2	21	-4	-4.90	9.30
-2	21	-4	-1.40	7.10
2	-21	4	1.60	6.90
-3	21	-4	289.77	18.80
-3	21	-4	301.57	19.40
3	-21	4	295.97	18.40
-4	21	-4	53.39	10.60
-4	21	-4	56.99	8.80
4	-21	4	72.79	11.70
6	20	-4	87.89	11.60
-6	-20	4	69.99	9.80
5	20	-4	169.88	16.60
5	20	-4	169.68	14.10

-5	-20	4	133.59	12.30
-5	-20	4	168.18	13.20
4	20	-4	115.59	17.00
4	20	-4	141.99	14.50
-4	-20	4	132.69	11.80
-4	-20	4	113.99	13.20
3	20	-4	110.39	13.10
3	20	-4	107.19	14.80
-3	-20	4	110.39	11.60
-3	-20	4	77.09	10.90
2	20	-4	6.80	9.40
2	20	-4	9.80	7.80
-2	-20	4	6.60	6.50
-2	-20	4	5.10	9.80
1	20	-4	-3.90	9.20
1	20	-4	-7.50	8.00
-1	-20	4	3.40	7.60
-1	-20	4	-0.50	8.60
0	20	-4	174.08	19.80
0	20	-4	171.88	14.50
0	20	-4	159.78	14.90
0	-20	4	160.88	15.40
0	-20	4	163.88	16.40
-1	20	-4	323.67	27.20
-1	20	-4	263.77	18.50
-1	20	-4	243.78	18.30
1	-20	4	295.17	20.50
1	-20	4	274.37	19.00
-2	20	-4	16.90	8.20
-2	20	-4	19.60	7.60
2	-20	4	27.40	12.70
2	-20	4	21.70	8.10
-3	20	-4	29.10	7.90
-3	20	-4	20.40	8.50
3	-20	4	28.20	7.70
-4	20	-4	562.74	29.70
-4	20	-4	537.35	29.90
4	-20	4	583.54	29.50
-5	20	-4	37.30	10.70
-5	20	-4	48.90	9.20
5	-20	4	57.09	9.60
5	-20	4	71.59	17.20
7	19	-4	11.60	7.60
-7	-19	4	37.50	8.50

-7	-19	4	21.00	8.70
6	19	-4	120.59	16.90
6	19	-4	107.89	12.70
-6	-19	4	108.39	11.50
-6	-19	4	100.79	11.10
5	19	-4	2.20	11.60
5	19	-4	12.70	8.10
-5	-19	4	13.80	6.70
-5	-19	4	6.30	7.00
4	19	-4	521.95	28.40
4	19	-4	500.35	28.00
-4	-19	4	497.15	27.00
-4	-19	4	520.05	27.30
3	19	-4	73.19	10.50
3	19	-4	70.99	10.40
-3	-19	4	79.19	14.60
-3	-19	4	60.49	9.70
-3	-19	4	56.39	8.90
2	19	-4	13.90	10.80
2	19	-4	12.80	8.20
-2	-19	4	14.90	8.60
-2	-19	4	15.70	10.30
-2	-19	4	4.80	10.00
1	19	-4	185.58	22.40
1	19	-4	203.68	16.30
-1	-19	4	230.68	26.60
0	19	-4	33.20	12.80
0	19	-4	59.49	17.10
0	19	-4	45.30	9.10
0	-19	4	43.00	11.90
-1	19	-4	32.10	14.90
-1	19	-4	36.20	13.80
-1	19	-4	32.20	8.50
1	-19	4	49.30	12.60
1	-19	4	19.30	10.40
-2	19	-4	492.35	27.30
-2	19	-4	471.15	27.80
2	-19	4	474.75	27.90
2	-19	4	486.55	29.70
-3	19	-4	48.40	9.50
-3	19	-4	75.79	13.00
3	-19	4	88.59	13.60
-4	19	-4	19.20	8.20
-4	19	-4	1.00	8.60

4	-19	4	20.30	11.10
4	-19	4	20.20	8.10
-5	19	-4	-6.50	8.00
5	-19	4	-0.60	9.50
5	-19	4	-0.50	8.60
-6	19	-4	76.19	12.80
-6	19	-4	93.59	14.90
6	-19	4	79.59	14.30
6	-19	4	74.69	16.30
8	18	-4	17.00	8.10
-8	-18	4	4.70	7.60
-8	-18	4	33.10	10.40
7	18	-4	-23.60	19.00
7	18	-4	-3.70	7.10
-7	-18	4	0.80	6.90
-7	-18	4	15.20	5.80
6	18	-4	281.67	18.70
-6	-18	4	229.88	16.90
-6	-18	4	272.97	16.40
5	18	-4	2.20	8.40
-5	-18	4	6.10	7.00
-5	-18	4	-2.30	6.70
4	18	-4	75.49	12.60
-4	-18	4	60.99	9.40
-4	-18	4	64.99	9.90
3	18	-4	240.78	17.80
3	18	-4	234.48	19.60
-3	-18	4	250.27	17.10
-3	-18	4	246.28	21.70
2	18	-4	191.78	18.40
2	18	-4	222.28	16.30
-2	-18	4	211.58	16.20
-2	-18	4	196.28	19.60
-2	-18	4	198.68	17.70
-2	-18	4	199.58	23.30
1	18	-4	162.38	15.60
1	18	-4	198.88	17.90
-1	-18	4	224.68	23.50
-1	-18	4	216.68	18.30
-1	-18	4	184.78	15.90
0	18	-4	12.20	7.40
0	18	-4	23.70	10.60
0	-18	4	22.90	8.00
0	-18	4	-3.90	8.20

-1	18	-4	31.90	8.70
-1	18	-4	24.20	7.50
-1	18	-4	46.30	11.90
1	-18	4	21.80	9.70
1	-18	4	24.50	8.70
-2	18	-4	46.30	9.10
-2	18	-4	52.49	20.60
-2	18	-4	55.99	9.40
2	-18	4	65.79	14.20
2	-18	4	28.10	8.80
-3	18	-4	194.08	23.20
-3	18	-4	242.58	17.40
3	-18	4	216.98	21.00
-4	18	-4	0.00	8.10
-4	18	-4	-0.80	8.00
4	-18	4	3.60	8.50
4	-18	4	10.50	9.20
-5	18	-4	248.68	18.70
-5	18	-4	255.97	23.70
-5	18	-4	277.37	19.40
5	-18	4	239.28	19.30
5	-18	4	253.37	18.40
-6	18	-4	30.70	9.40
-6	18	-4	34.70	10.80
6	-18	4	18.40	10.00
6	-18	4	27.70	8.10
-7	18	-4	37.60	9.60
-7	18	-4	52.69	11.80
7	-18	4	71.39	11.90
7	-18	4	48.20	14.70
-8	-17	4	57.99	10.60
-8	-17	4	34.70	6.00
7	17	-4	211.28	16.20
-7	-17	4	193.48	15.70
-7	-17	4	194.88	13.60
6	17	-4	171.68	16.10
-6	-17	4	132.09	18.70
-6	-17	4	187.58	14.30
-6	-17	4	164.48	13.20
-5	-17	4	560.34	32.70
-5	-17	4	643.44	33.60
4	17	-4	35.80	9.20
-4	-17	4	30.30	9.80
-4	-17	4	21.10	7.40

-4	-17	4	14.20	6.80
3	17	-4	55.19	10.20
-3	-17	4	50.59	8.60
-3	-17	4	33.80	10.00
-3	-17	4	49.60	12.50
-3	-17	4	30.40	7.20
2	17	-4	291.97	19.20
2	17	-4	280.57	20.30
-2	-17	4	271.67	19.40
-2	-17	4	272.17	21.70
-2	-17	4	279.17	21.00
-2	-17	4	299.97	18.90
1	17	-4	32.40	8.70
1	17	-4	14.00	9.60
-1	-17	4	44.60	10.20
-1	-17	4	11.20	8.80
-1	-17	4	24.90	7.80
-1	-17	4	10.30	9.90
0	17	-4	-0.50	8.00
0	17	-4	0.10	9.80
0	-17	4	4.30	8.00
0	-17	4	6.50	10.30
0	-17	4	-8.60	8.10
-1	17	-4	40.40	11.20
-1	17	-4	45.50	8.50
1	-17	4	36.50	8.60
1	-17	4	42.90	9.40
-2	17	-4	23.00	8.60
-2	17	-4	23.30	8.20
-2	17	-4	33.10	13.10
2	-17	4	33.00	8.80
2	-17	4	24.20	10.60
-3	17	-4	308.57	20.70
-3	17	-4	334.37	21.30
3	-17	4	302.27	23.50
3	-17	4	331.47	21.70
-4	17	-4	5.40	15.30
-4	17	-4	4.80	9.30
-4	17	-4	2.20	8.10
4	-17	4	13.00	10.80
-5	17	-4	90.39	14.40
-5	17	-4	76.99	13.40
-5	17	-4	87.19	11.70
5	-17	4	79.79	14.10

5	-17	4	87.09	15.40
-6	17	-4	24.90	12.60
-6	17	-4	55.59	11.50
-6	17	-4	43.90	10.10
6	-17	4	37.50	10.80
6	-17	4	42.40	8.70
-7	17	-4	-18.50	10.90
-7	17	-4	9.00	9.00
7	-17	4	46.80	20.40
7	-17	4	-5.50	7.20
9	16	-4	42.00	8.60
-9	-16	4	51.49	8.20
8	16	-4	-3.50	16.70
-8	-16	4	-8.30	9.60
-8	-16	4	0.70	5.60
7	16	-4	288.07	36.40
-7	-16	4	235.78	15.60
-7	-16	4	260.57	20.50
-7	-16	4	240.18	17.10
-7	-16	4	200.38	18.20
-6	-16	4	42.40	8.50
-6	-16	4	27.30	9.70
-6	-16	4	49.30	12.00
-6	-16	4	40.50	9.10
-5	-16	4	125.79	13.60
-5	-16	4	97.69	13.50
-5	-16	4	120.19	12.80
-5	-16	4	102.89	16.60
4	16	-4	372.76	30.80
-4	-16	4	371.16	22.50
-4	-16	4	341.47	21.50
-4	-16	4	376.16	22.10
-4	-16	4	373.76	23.70
3	16	-4	54.59	12.50
3	16	-4	46.20	9.40
-3	-16	4	46.30	9.80
-3	-16	4	22.00	12.30
-3	-16	4	43.60	8.00
-3	-16	4	47.80	8.10
2	16	-4	120.59	15.90
2	16	-4	141.99	18.60
-2	-16	4	118.09	14.70
-2	-16	4	140.99	17.30
-2	-16	4	108.39	16.50

-2	-16	4	134.89	13.30
1	16	-4	82.79	12.40
1	16	-4	92.29	11.50
-1	-16	4	99.29	15.00
-1	-16	4	124.39	15.20
-1	-16	4	77.69	11.50
-1	-16	4	57.09	8.70
0	16	-4	94.59	12.70
0	16	-4	110.49	16.30
0	-16	4	115.39	15.00
0	-16	4	83.39	12.50
0	-16	4	107.19	14.10
-1	16	-4	33.20	10.50
-1	16	-4	22.20	7.40
1	-16	4	10.50	8.40
1	-16	4	25.20	8.80
-2	16	-4	48.80	9.20
-2	16	-4	48.20	13.40
-2	16	-4	64.69	14.80
2	-16	4	83.59	13.70
2	-16	4	62.79	11.40
2	-16	4	45.50	13.00
-3	16	-4	247.68	19.50
-3	16	-4	260.77	28.00
-3	16	-4	224.88	18.30
3	-16	4	237.78	18.60
3	-16	4	215.78	20.10
-4	16	-4	598.24	33.20
-4	16	-4	580.84	33.10
4	-16	4	580.04	32.20
4	-16	4	617.34	32.70
-5	16	-4	7.90	9.50
-5	16	-4	-3.20	10.40
-5	16	-4	15.60	18.40
5	-16	4	0.80	11.00
5	-16	4	0.80	10.30
-6	16	-4	-12.90	10.00
-6	16	-4	-3.70	10.00
-6	16	-4	1.70	11.20
6	-16	4	-0.50	10.20
6	-16	4	-10.50	7.50
-7	16	-4	2.20	12.10
-7	16	-4	0.00	9.50
-7	16	-4	-6.90	11.00

7	-16	4	2.90	7.40
7	-16	4	20.60	12.90
-8	16	-4	11.00	11.90
-8	16	-4	25.50	10.10
-8	16	-4	14.60	15.50
8	-16	4	21.20	7.00
-9	-15	4	136.49	11.10
8	15	-4	262.17	25.70
-8	-15	4	240.78	15.10
-8	-15	4	190.18	19.80
-8	-15	4	244.38	20.40
-8	-15	4	249.58	19.60
7	15	-4	54.39	16.20
-7	-15	4	25.60	6.90
-7	-15	4	19.70	8.20
-7	-15	4	11.00	10.50
-7	-15	4	42.00	11.10
6	15	-4	0.30	15.80
-6	-15	4	-1.10	6.20
-6	-15	4	4.60	7.30
-6	-15	4	11.10	10.20
-6	-15	4	22.70	9.40
5	15	-4	173.18	23.10
-5	-15	4	173.58	14.80
-5	-15	4	163.58	14.40
-5	-15	4	154.58	15.60
-5	-15	4	196.38	18.50
4	15	-4	236.38	21.40
-4	-15	4	241.98	17.20
-4	-15	4	239.68	17.20
-4	-15	4	240.98	16.50
-4	-15	4	225.98	19.90
3	15	-4	52.19	10.00
-3	-15	4	29.90	9.70
-3	-15	4	30.60	12.00
-3	-15	4	35.90	7.80
-3	-15	4	30.70	8.70
-3	-15	4	30.60	7.80
2	15	-4	157.08	15.50
2	15	-4	143.39	15.70
-2	-15	4	142.29	13.90
-2	-15	4	161.98	17.00
-2	-15	4	139.99	14.50
-2	-15	4	178.48	16.40

1	15	-4	353.06	23.30
1	15	-4	391.96	22.80
-1	-15	4	372.06	22.00
-1	-15	4	368.36	24.40
-1	-15	4	371.56	23.20
-1	-15	4	398.76	23.30
0	15	-4	39.30	8.20
0	15	-4	38.40	9.80
0	15	-4	43.70	9.00
0	-15	4	23.70	8.00
0	-15	4	31.90	7.90
0	-15	4	18.50	10.40
-1	15	-4	9.20	10.20
-1	15	-4	11.90	6.40
1	-15	4	7.80	7.20
1	-15	4	37.20	11.20
1	-15	4	10.00	8.40
-2	15	-4	262.77	24.90
-2	15	-4	340.77	20.60
-2	15	-4	324.37	21.50
2	-15	4	359.36	23.90
2	-15	4	313.37	24.30
2	-15	4	349.37	21.80
-3	15	-4	30.80	9.40
-3	15	-4	2.90	7.90
-3	15	-4	22.40	14.50
3	-15	4	5.70	8.50
3	-15	4	23.30	8.20
-4	15	-4	177.68	17.80
-4	15	-4	187.48	18.10
4	-15	4	132.39	16.40
4	-15	4	197.88	17.00
-5	15	-4	-11.70	9.00
-5	15	-4	13.30	11.30
-5	15	-4	-1.80	9.90
5	-15	4	2.40	7.80
5	-15	4	12.40	9.00
-6	15	-4	-0.30	16.70
-6	15	-4	7.10	12.20
-6	15	-4	-5.80	9.70
6	-15	4	-11.00	13.80
-7	15	-4	12.90	11.10
-7	15	-4	7.30	11.80
-7	15	-4	-1.70	11.00

7	-15	4	18.30	13.20
7	-15	4	-4.60	6.90
-8	15	-4	7.00	11.50
-8	15	-4	-5.10	10.40
8	-15	4	12.60	7.00
-9	15	-4	386.76	24.20
-9	15	-4	340.97	25.90
9	-15	4	422.96	21.90
10	14	-4	2.70	12.60
10	14	-4	13.40	8.90
-10	-14	4	5.20	6.80
9	14	-4	72.19	14.10
-9	-14	4	86.69	9.70
-9	-14	4	80.49	12.40
8	14	-4	-4.10	13.30
-8	-14	4	9.80	9.60
-8	-14	4	12.70	6.30
-8	-14	4	17.70	10.70
-8	-14	4	11.30	11.80
-7	-14	4	8.20	6.30
6	14	-4	438.76	31.60
6	14	-4	397.16	31.60
-6	-14	4	378.66	25.10
-6	-14	4	445.86	24.30
-6	-14	4	402.36	23.30
-6	-14	4	406.36	26.80
5	14	-4	3.30	15.60
5	14	-4	-3.40	10.60
-5	-14	4	4.10	6.50
-5	-14	4	-0.90	8.50
-5	-14	4	-0.60	10.00
-5	-14	4	2.70	6.70
4	14	-4	3.60	8.70
-4	-14	4	14.00	8.40
-4	-14	4	15.90	9.40
-4	-14	4	18.00	6.40
-4	-14	4	11.20	6.80
3	14	-4	1108.09	50.49
-3	-14	4	1057.39	50.29
-3	-14	4	1007.50	49.80
-3	-14	4	1004.00	52.19
-3	-14	4	1009.60	50.89
-3	-14	4	1058.29	50.39
2	14	-4	296.67	18.30

-2	-14	4	282.87	18.90
-2	-14	4	305.17	18.80
-2	-14	4	291.37	21.30
-2	-14	4	275.17	20.80
-2	-14	4	312.77	22.80
1	14	-4	12.50	6.30
1	14	-4	6.70	8.30
-1	-14	4	5.70	8.60
-1	-14	4	8.40	7.60
-1	-14	4	4.40	6.50
-1	-14	4	21.20	10.00
0	14	-4	22.10	12.50
0	14	-4	17.40	6.80
0	14	-4	6.80	6.80
0	-14	4	19.00	7.80
0	-14	4	23.70	10.10
0	-14	4	14.20	7.10
-1	14	-4	66.79	10.90
-1	14	-4	83.09	15.00
1	-14	4	67.19	9.40
1	-14	4	65.09	10.10
1	-14	4	88.39	12.20
-2	14	-4	57.69	9.90
-2	14	-4	62.69	10.80
-2	14	-4	56.29	12.00
2	-14	4	52.29	10.50
2	-14	4	75.39	12.90
2	-14	4	61.69	13.60
-3	14	-4	264.07	26.70
-3	14	-4	297.27	19.60
-3	14	-4	285.27	20.90
3	-14	4	283.67	19.90
3	-14	4	276.47	20.70
-4	14	-4	110.29	16.40
-4	14	-4	87.59	22.00
-4	14	-4	95.59	15.00
4	-14	4	95.39	14.70
4	-14	4	92.09	14.90
-5	14	-4	439.46	29.00
-5	14	-4	429.76	29.10
-5	14	-4	470.55	29.20
5	-14	4	431.86	27.20
5	-14	4	430.66	25.90
-6	14	-4	59.29	13.60

-6	14	-4	49.40	12.50
-6	14	-4	53.59	12.30
6	-14	4	56.59	11.30
6	-14	4	70.39	12.80
-7	14	-4	45.40	13.00
-7	14	-4	39.40	13.70
-7	14	-4	52.99	12.20
7	-14	4	66.39	14.70
7	-14	4	23.80	8.90
-8	14	-4	216.88	21.30
-8	14	-4	192.78	21.90
-8	14	-4	197.68	20.20
8	-14	4	218.48	15.60
-9	14	-4	1.90	10.90
-9	14	-4	16.50	11.50
-9	14	-4	3.00	13.70
9	-14	4	12.00	7.20
10	13	-4	24.40	11.70
-10	-13	4	10.40	11.50
-10	-13	4	4.00	5.40
9	13	-4	23.10	12.30
-9	-13	4	0.70	5.40
-9	-13	4	-0.90	10.20
8	13	-4	219.58	24.30
-8	-13	4	256.37	22.40
-8	-13	4	220.08	21.10
-8	-13	4	227.08	20.40
-8	-13	4	259.97	16.60
7	13	-4	342.47	27.90
-7	-13	4	352.46	24.30
-7	-13	4	340.47	22.60
-7	-13	4	350.96	24.00
-7	-13	4	367.76	21.20
6	13	-4	83.29	15.20
6	13	-4	85.39	14.60
-6	-13	4	58.19	11.50
-6	-13	4	90.39	17.70
-6	-13	4	78.89	12.90
-6	-13	4	68.69	11.20
-6	-13	4	80.09	11.30
5	13	-4	41.20	14.30
5	13	-4	43.10	9.80
-5	-13	4	62.19	9.10
-5	-13	4	54.49	8.20

-5	-13	4	49.30	9.80
-5	-13	4	53.49	10.80
4	13	-4	147.39	14.10
4	13	-4	178.18	24.20
-4	-13	4	145.39	13.70
-4	-13	4	145.29	17.80
-4	-13	4	164.08	15.40
-4	-13	4	146.49	13.40
3	13	-4	4.20	6.00
-3	-13	4	10.00	6.10
-3	-13	4	0.80	9.50
-3	-13	4	-10.30	10.80
-3	-13	4	-0.90	8.10
2	13	-4	783.22	38.30
-2	-13	4	802.12	42.80
-2	-13	4	844.12	40.90
-2	-13	4	793.52	38.90
-2	-13	4	766.02	40.30
-2	-13	4	740.33	38.90
1	13	-4	39.50	6.20
1	13	-4	38.60	8.50
-1	-13	4	40.40	9.00
-1	-13	4	50.09	10.90
-1	-13	4	38.60	6.90
-1	-13	4	32.80	8.70
0	13	-4	83.89	14.10
0	13	-4	72.99	10.50
0	13	-4	57.79	9.70
0	-13	4	56.89	11.40
0	-13	4	46.50	7.90
0	-13	4	50.59	8.50
-1	13	-4	13.60	5.60
-1	13	-4	11.70	10.10
1	-13	4	15.00	7.80
1	-13	4	16.90	7.80
1	-13	4	2.00	10.80
-2	13	-4	342.67	20.10
-2	13	-4	342.67	24.50
2	-13	4	317.57	23.10
2	-13	4	316.47	21.90
2	-13	4	274.27	22.50
-3	13	-4	767.82	40.00
-3	13	-4	731.93	43.90
-3	13	-4	793.92	39.30

3	-13	4	713.63	38.40
3	-13	4	784.02	38.80
-4	13	-4	40.50	16.30
-4	13	-4	50.99	10.30
-4	13	-4	44.70	11.50
4	-13	4	34.70	8.80
4	-13	4	31.20	7.60
-5	13	-4	104.89	18.30
-5	13	-4	85.99	13.00
-5	13	-4	79.69	12.80
5	-13	4	92.59	14.00
5	-13	4	85.09	14.90
-6	13	-4	20.70	11.60
-6	13	-4	23.50	11.20
-6	13	-4	10.90	12.40
6	-13	4	12.30	7.20
6	-13	4	23.20	11.10
-7	13	-4	20.40	13.50
-7	13	-4	11.20	13.40
-7	13	-4	38.30	13.00
7	-13	4	25.10	7.90
-8	13	-4	328.87	24.80
-8	13	-4	336.77	27.50
-8	13	-4	327.07	26.60
8	-13	4	343.97	21.20
-9	13	-4	41.30	12.20
-9	13	-4	58.59	14.30
-9	13	-4	46.80	11.40
9	-13	4	75.19	11.90
10	12	-4	186.48	18.70
-10	-12	4	139.59	19.50
-10	-12	4	170.18	12.90
9	12	-4	25.50	12.60
-9	-12	4	34.30	10.60
-9	-12	4	19.00	6.40
8	12	-4	134.09	22.90
-8	-12	4	104.09	12.30
-8	-12	4	103.79	15.00
-8	-12	4	98.49	14.00
-8	-12	4	87.69	18.10
7	12	-4	250.87	25.00
7	12	-4	304.37	27.80
-7	-12	4	252.97	21.20
-7	-12	4	296.17	18.30

-7	-12	4	259.37	20.70
-7	-12	4	287.57	19.80
-7	-12	4	265.77	22.20
6	12	-4	0.60	10.30
6	12	-4	-9.30	12.00
-6	-12	4	11.80	8.70
-6	-12	4	3.90	9.50
-6	-12	4	-7.20	6.30
-6	-12	4	1.50	9.80
-6	-12	4	-7.90	6.70
5	12	-4	22.40	13.50
5	12	-4	26.60	7.90
-5	-12	4	25.30	6.80
-5	-12	4	11.70	6.50
-5	-12	4	16.50	7.60
-5	-12	4	23.70	9.20
-5	-12	4	32.80	10.20
4	12	-4	828.22	48.40
4	12	-4	882.21	43.50
-4	-12	4	944.31	43.70
-4	-12	4	839.02	45.10
-4	-12	4	906.01	44.60
-4	-12	4	875.11	50.19
-4	-12	4	991.10	44.40
3	12	-4	79.39	9.60
3	12	-4	40.60	22.70
-3	-12	4	69.39	9.30
-3	-12	4	89.89	13.20
-3	-12	4	77.99	11.20
-3	-12	4	52.49	11.90
-3	-12	4	113.19	13.90
2	12	-4	708.53	33.60
-2	-12	4	704.13	34.60
-2	-12	4	644.74	36.50
-2	-12	4	689.33	34.70
-2	-12	4	681.83	35.00
-2	-12	4	708.33	36.80
1	12	-4	218.18	13.90
-1	-12	4	196.08	17.40
-1	-12	4	237.78	19.60
-1	-12	4	232.68	17.70
-1	-12	4	208.98	16.20
-1	-12	4	212.78	15.40
0	12	-4	164.08	11.20

0	12	-4	168.98	15.60
0	-12	4	124.39	19.40
0	-12	4	160.68	14.90
0	-12	4	172.58	14.10
-1	12	-4	44.60	11.10
-1	12	-4	51.69	6.60
-1	12	-4	55.29	8.00
1	-12	4	20.70	11.30
1	-12	4	62.59	9.30
1	-12	4	42.50	11.50
1	-12	4	40.60	7.40
-2	12	-4	0.60	7.00
-2	12	-4	6.50	5.60
-2	12	-4	10.90	11.60
2	-12	4	-7.70	7.30
2	-12	4	5.50	10.20
2	-12	4	6.90	8.40
-3	12	-4	628.24	37.20
-3	12	-4	623.94	31.80
-3	12	-4	574.14	33.80
3	-12	4	546.05	33.50
3	-12	4	570.64	31.00
-4	12	-4	1366.16	64.99
-4	12	-4	1259.37	67.19
-4	12	-4	1361.46	64.39
4	-12	4	1278.37	62.89
4	-12	4	1323.37	62.49
-5	12	-4	137.39	20.20
-5	12	-4	159.68	17.70
-5	12	-4	169.38	19.60
5	-12	4	153.18	15.10
5	-12	4	129.59	17.10
-6	12	-4	17.30	11.10
-6	12	-4	17.80	17.20
-6	12	-4	6.00	12.00
6	-12	4	1.30	7.10
6	-12	4	-10.70	10.60
-7	12	-4	47.90	13.20
-7	12	-4	35.00	12.00
-7	12	-4	47.40	13.70
7	-12	4	63.09	15.30
-8	12	-4	90.49	16.00
-8	12	-4	80.49	16.40
-8	12	-4	84.99	13.10

8	-12	4	75.49	11.00
8	-12	4	70.59	15.00
-9	12	-4	9.10	14.60
-9	12	-4	25.10	11.00
-9	12	-4	19.90	13.90
9	-12	4	27.30	14.90
9	-12	4	38.80	7.80
-10	12	-4	23.30	11.40
-10	12	-4	12.00	15.70
-10	12	-4	0.30	10.70
10	-12	4	19.60	8.30
11	11	-4	-1.90	13.20
-11	-11	4	-2.80	8.90
10	11	-4	-11.10	11.00
-10	-11	4	3.70	6.30
-10	-11	4	-2.50	10.50
9	11	-4	307.27	24.70
-9	-11	4	256.67	21.30
-9	-11	4	298.37	18.40
8	11	-4	428.56	29.30
-8	-11	4	380.86	27.40
-8	-11	4	415.96	23.20
-8	-11	4	417.26	26.20
-8	-11	4	405.96	27.50
-8	-11	4	379.16	27.00
7	11	-4	11.80	13.20
7	11	-4	16.70	12.50
-7	-11	4	40.60	10.40
-7	-11	4	30.10	7.10
-7	-11	4	41.90	19.40
-7	-11	4	17.00	11.30
-7	-11	4	17.50	8.50
6	11	-4	31.60	12.40
6	11	-4	44.00	9.80
-6	-11	4	51.99	8.90
-6	-11	4	43.00	9.60
-6	-11	4	69.79	11.60
-6	-11	4	70.19	11.00
-6	-11	4	35.60	7.00
5	11	-4	253.67	17.10
-5	-11	4	217.68	23.40
-5	-11	4	264.47	17.60
-5	-11	4	265.67	17.10
-5	-11	4	241.78	16.40

4	11	-4	180.28	13.20
4	11	-4	181.08	24.80
-4	-11	4	171.88	14.80
-4	-11	4	197.88	16.80
-4	-11	4	180.28	19.20
-4	-11	4	199.78	14.00
-4	-11	4	205.48	14.20
3	11	-4	188.78	12.50
3	11	-4	168.58	26.70
-3	-11	4	190.58	13.10
-3	-11	4	190.18	21.80
-3	-11	4	169.38	14.80
-3	-11	4	175.88	15.90
-3	-11	4	147.99	18.90
-3	-11	4	183.98	14.30
2	11	-4	592.94	27.70
-2	-11	4	558.74	31.60
-2	-11	4	529.35	29.50
-2	-11	4	562.04	29.20
-2	-11	4	578.44	32.30
-2	-11	4	547.25	29.70
1	11	-4	339.07	21.50
1	11	-4	416.06	19.40
-1	-11	4	380.66	21.90
-1	-11	4	379.46	21.50
-1	-11	4	398.66	22.80
-1	-11	4	318.57	22.40
0	11	-4	1.90	3.50
0	11	-4	12.40	8.80
0	-11	4	11.50	6.30
0	-11	4	-8.80	6.50
-1	11	-4	17.70	5.40
-1	11	-4	20.00	4.40
-1	11	-4	31.70	10.30
1	-11	4	9.50	8.00
1	-11	4	18.60	7.90
-2	11	-4	484.75	25.60
-2	11	-4	548.25	27.40
-2	11	-4	497.05	32.20
2	-11	4	551.34	27.70
2	-11	4	502.45	29.80
2	-11	4	469.15	30.30
-3	11	-4	45.80	8.60
-3	11	-4	59.29	15.60

-3	11	-4	49.60	10.50
3	-11	4	39.50	8.70
3	-11	4	36.80	8.40
-4	11	-4	218.58	17.20
-4	11	-4	185.58	28.00
-4	11	-4	206.38	24.10
4	-11	4	179.88	15.90
4	-11	4	219.88	16.80
-5	11	-4	0.70	9.70
-5	11	-4	-9.20	16.90
-5	11	-4	18.00	8.90
-5	11	-4	11.60	10.70
5	-11	4	11.90	8.90
5	-11	4	18.70	7.20
-6	11	-4	7.20	10.40
-6	11	-4	2.90	11.80
-6	11	-4	-1.80	10.80
6	-11	4	-8.20	7.00
6	-11	4	18.10	10.80
-7	11	-4	16.60	12.20
-7	11	-4	10.70	11.10
-7	11	-4	-4.40	12.40
7	-11	4	-12.30	17.70
7	-11	4	1.60	7.10
-8	11	-4	35.70	13.80
-8	11	-4	19.70	13.70
-8	11	-4	35.60	14.00
8	-11	4	22.70	8.00
-9	11	-4	588.24	37.20
-9	11	-4	662.83	34.90
-9	11	-4	537.25	37.20
9	-11	4	585.64	33.80
9	-11	4	687.13	32.60
-10	11	-4	131.59	22.70
-10	11	-4	120.19	17.00
-10	11	-4	134.19	20.10
10	-11	4	182.58	15.10
10	-11	4	143.89	21.50
11	10	-4	34.30	11.20
-11	-10	4	8.40	10.70
-11	-10	4	30.30	6.70
10	10	-4	25.70	11.50
-10	-10	4	28.50	7.20
-10	-10	4	14.80	10.00

9	10	-4	42.00	14.20
-9	-10	4	37.00	9.00
-9	-10	4	47.90	10.40
8	10	-4	14.40	11.30
-8	-10	4	20.90	8.80
-8	-10	4	-6.90	12.40
-8	-10	4	27.50	11.50
-8	-10	4	12.20	12.80
7	10	-4	40.90	13.20
7	10	-4	51.49	12.30
-7	-10	4	60.89	10.30
-7	-10	4	44.20	12.40
-7	-10	4	50.39	9.70
-7	-10	4	49.70	10.40
-7	-10	4	50.79	7.90
6	10	-4	409.56	29.20
6	10	-4	427.86	25.40
-6	-10	4	463.25	24.10
-6	-10	4	413.76	26.90
-6	-10	4	451.75	25.80
-6	-10	4	444.16	25.60
-6	-10	4	434.66	25.20
5	10	-4	55.39	13.10
5	10	-4	68.49	10.20
-5	-10	4	63.19	8.60
-5	-10	4	60.19	8.40
-5	-10	4	78.69	11.40
-5	-10	4	42.70	6.90
-5	-10	4	66.39	10.30
4	10	-4	37.20	13.00
4	10	-4	34.40	6.10
-4	-10	4	47.20	10.50
-4	-10	4	38.50	10.70
-4	-10	4	29.60	6.80
-4	-10	4	31.10	6.80
-4	-10	4	39.50	7.20
3	10	-4	1941.31	97.49
3	10	-4	2198.18	93.79
-3	-10	4	2113.29	94.19
-3	-10	4	2047.00	94.09
-3	-10	4	1966.70	97.19
-3	-10	4	2024.80	94.59
-3	-10	4	2096.39	94.49
2	10	-4	101.19	10.80

2	10	-4	126.19	8.40
-2	-10	4	115.09	11.80
-2	-10	4	112.19	14.50
-2	-10	4	110.29	11.80
1	10	-4	3.10	5.10
1	10	-4	8.30	12.80
1	10	-4	6.40	3.10
-1	-10	4	0.80	8.50
-1	-10	4	10.80	6.50
-1	-10	4	11.30	16.10
-1	-10	4	3.30	7.00
0	10	-4	149.99	11.90
0	10	-4	158.78	9.40
0	-10	4	139.19	14.80
0	-10	4	146.29	13.20
-1	10	-4	-2.90	4.60
-1	10	-4	3.20	3.30
-1	10	-4	1.50	6.30
1	-10	4	-2.90	6.30
-2	10	-4	129.49	11.50
-2	10	-4	117.79	9.30
-2	10	-4	123.79	22.40
2	-10	4	101.99	13.10
-3	10	-4	141.69	16.00
-3	10	-4	163.68	25.20
-3	10	-4	150.88	13.20
3	-10	4	146.09	14.50
-4	10	-4	1247.78	59.19
-4	10	-4	1144.59	62.09
-4	10	-4	1253.67	58.29
4	-10	4	1167.58	56.79
4	-10	4	1161.68	57.19
-5	10	-4	424.36	25.70
-5	10	-4	400.86	27.10
-5	10	-4	413.46	28.50
-5	10	-4	387.26	32.70
5	-10	4	398.96	25.10
5	-10	4	404.26	23.70
-6	10	-4	-18.20	10.10
-6	10	-4	-1.30	10.90
-6	10	-4	9.20	9.60
6	-10	4	8.30	7.70
6	-10	4	9.60	10.70
-7	10	-4	403.66	28.70

-7	10	-4	328.97	25.30
-7	10	-4	342.77	27.50
7	-10	4	366.76	23.20
-8	10	-4	127.59	20.60
-8	10	-4	130.69	15.30
-8	10	-4	137.79	19.90
8	-10	4	124.69	15.60
-9	10	-4	14.60	12.70
-9	10	-4	4.70	13.10
-9	10	-4	15.90	11.50
9	-10	4	22.20	10.90
9	-10	4	16.00	8.40
-10	10	-4	59.39	13.60
-10	10	-4	63.39	16.10
-10	10	-4	48.90	11.30
10	-10	4	85.39	14.80
10	-10	4	43.30	11.70
-11	10	-4	24.60	11.10
-11	10	-4	40.80	13.50
11	-10	4	22.50	11.10
11	-10	4	45.20	14.20
11	9	-4	93.09	16.30
-11	-9	4	66.09	10.50
-11	-9	4	87.69	13.20
10	9	-4	104.89	17.70
-10	-9	4	95.89	14.90
-10	-9	4	104.49	12.30
9	9	-4	31.30	12.20
-9	-9	4	34.80	7.90
-9	-9	4	34.80	13.50
8	9	-4	629.64	36.80
-8	-9	4	637.54	36.90
-8	-9	4	679.83	33.20
-8	-9	4	676.53	33.70
-8	-9	4	551.54	36.50
7	9	-4	322.47	22.20
-7	-9	4	321.17	22.00
-7	-9	4	256.97	20.80
-7	-9	4	319.47	20.40
-7	-9	4	259.87	23.90
-7	-9	4	294.27	21.30
-7	-9	4	316.97	18.90
6	9	-4	25.80	7.40
-6	-9	4	37.50	7.00

-6	-9	4	28.40	8.10
-6	-9	4	41.70	11.40
-6	-9	4	29.30	8.70
5	9	-4	258.77	15.30
-5	-9	4	244.68	16.00
-5	-9	4	223.38	15.10
-5	-9	4	222.78	18.00
-5	-9	4	232.98	17.10
-5	-9	4	188.08	20.10
-5	-9	4	264.37	17.30
4	9	-4	28.20	13.00
4	9	-4	29.10	5.50
-4	-9	4	30.50	10.90
-4	-9	4	28.40	5.80
-4	-9	4	39.70	17.10
-4	-9	4	36.90	7.00
3	9	-4	121.09	8.90
-3	-9	4	125.59	10.90
-3	-9	4	126.39	12.00
-3	-9	4	118.99	12.10
2	9	-4	573.14	31.70
2	9	-4	554.24	26.50
2	9	-4	537.45	27.00
-2	-9	4	479.55	32.50
-2	-9	4	600.24	27.70
-2	-9	4	569.34	27.40
1	9	-4	122.99	8.00
1	9	-4	111.59	14.70
1	9	-4	130.69	9.30
-1	-9	4	118.09	14.70
-1	-9	4	132.89	11.40
0	9	-4	124.69	8.10
0	9	-4	128.99	9.90
0	9	-4	131.59	14.50
0	-9	4	141.29	14.40
-1	9	-4	16.40	7.40
-1	9	-4	1.60	4.30
-1	9	-4	12.90	3.20
1	-9	4	12.20	5.80
1	-9	4	9.60	7.30
-2	9	-4	1120.59	52.69
-2	9	-4	1034.40	52.79
-2	9	-4	1122.99	51.09
-2	9	-4	1150.78	60.09

2	-9	4	961.40	52.19
2	-9	4	1128.89	53.39
2	-9	4	1221.68	55.29
2	-9	4	1157.48	61.69
-3	9	-4	794.02	37.40
-3	9	-4	728.83	38.70
3	-9	4	733.73	37.50
3	-9	4	719.33	37.70
-4	9	-4	58.39	8.40
-4	9	-4	52.99	17.00
-4	9	-4	51.79	10.30
4	-9	4	50.99	10.10
-5	9	-4	123.99	15.90
-5	9	-4	125.99	18.10
-5	9	-4	126.49	13.90
-5	9	-4	116.09	17.30
5	-9	4	129.29	13.20
5	-9	4	96.89	16.20
-6	9	-4	49.70	20.90
-6	9	-4	43.80	9.50
-6	9	-4	43.50	11.70
-6	9	-4	34.80	11.00
6	-9	4	40.40	13.10
6	-9	4	40.70	7.90
-7	9	-4	8.30	9.90
-7	9	-4	30.40	11.50
-7	9	-4	12.50	10.00
7	-9	4	19.80	8.00
-8	9	-4	324.17	24.30
-8	9	-4	295.67	24.90
-8	9	-4	312.47	25.70
8	-9	4	294.87	21.20
-9	9	-4	34.60	13.20
-9	9	-4	80.59	29.20
-9	9	-4	19.00	13.90
9	-9	4	53.29	18.10
9	-9	4	29.80	11.90
-10	9	-4	288.67	24.80
-10	9	-4	289.87	21.80
-10	9	-4	222.28	25.00
10	-9	4	325.77	20.90
10	-9	4	283.07	20.90
-11	9	-4	1.30	16.30
-11	9	-4	43.40	22.00

11	-9	4	13.70	14.40
11	-9	4	36.30	14.10
11	8	-4	44.80	11.50
-11	-8	4	72.69	11.70
-11	-8	4	48.70	10.50
10	8	-4	130.49	19.70
-10	-8	4	155.58	14.10
-10	-8	4	142.69	15.70
9	8	-4	21.30	11.70
-9	-8	4	4.60	6.40
-9	-8	4	10.00	8.00
8	8	-4	128.49	17.30
-8	-8	4	140.59	13.30
-8	-8	4	136.59	14.70
-8	-8	4	159.88	18.00
-8	-8	4	112.09	15.70
-8	-8	4	123.49	18.60
7	8	-4	257.27	20.00
-7	-8	4	282.87	18.40
-7	-8	4	276.87	20.30
-7	-8	4	261.97	22.80
-7	-8	4	255.67	19.50
-7	-8	4	267.27	21.90
6	8	-4	7.10	6.10
-6	-8	4	8.80	11.00
-6	-8	4	5.30	7.40
-6	-8	4	5.10	7.80
-6	-8	4	6.00	7.40
-6	-8	4	7.60	8.40
5	8	-4	24.80	5.50
-5	-8	4	16.60	8.10
-5	-8	4	29.50	11.20
-5	-8	4	26.00	7.20
-5	-8	4	22.00	7.50
-5	-8	4	16.90	4.60
-5	-8	4	21.30	8.10
4	8	-4	767.42	47.50
4	8	-4	982.10	42.60
-4	-8	4	888.11	43.90
-4	-8	4	941.31	43.90
-4	-8	4	926.11	43.20
3	8	-4	652.83	39.00
3	8	-4	786.02	35.00
-3	-8	4	751.72	34.60

-3	-8	4	771.02	35.90
2	8	-4	1676.83	76.59
2	8	-4	1624.44	76.79
-2	-8	4	1739.93	77.39
-2	-8	4	1724.93	76.89
1	8	-4	3.40	2.40
1	8	-4	1.30	3.00
-1	-8	4	4.70	3.70
-1	-8	4	-3.10	5.90
0	8	-4	224.58	12.80
0	8	-4	221.78	11.70
0	-8	4	228.48	13.40
0	-8	4	247.98	16.40
0	-8	4	213.28	13.70
-1	8	-4	3.40	5.20
-1	8	-4	7.40	2.70
-1	8	-4	-3.90	5.60
-1	8	-4	8.80	3.90
1	-8	4	13.10	7.10
1	-8	4	-4.20	4.50
1	-8	4	-0.90	4.80
-2	8	-4	298.87	20.20
-2	8	-4	272.67	14.90
-2	8	-4	282.87	16.60
-2	8	-4	315.47	17.20
2	-8	4	312.07	17.10
2	-8	4	285.97	20.00
2	-8	4	269.47	17.20
-3	8	-4	1436.86	64.39
-3	8	-4	1263.97	62.79
-3	8	-4	1347.17	65.59
-3	8	-4	1316.27	66.09
3	-8	4	1446.46	72.79
3	-8	4	1429.06	63.59
-4	8	-4	432.16	22.70
-4	8	-4	372.96	24.10
4	-8	4	399.56	22.40
4	-8	4	382.76	24.50
-5	8	-4	303.27	18.60
-5	8	-4	284.27	20.40
-5	8	-4	263.77	26.70
-5	8	-4	291.57	22.10
5	-8	4	271.37	21.80
5	-8	4	255.87	19.80

-6	8	-4	-11.10	6.90
-6	8	-4	-14.00	14.40
-6	8	-4	-10.20	14.70
-6	8	-4	-2.60	9.30
6	-8	4	3.20	6.80
6	-8	4	-11.40	12.70
-7	8	-4	44.40	10.60
-7	8	-4	25.60	8.80
-7	8	-4	26.80	10.10
7	-8	4	24.70	8.10
-8	8	-4	228.58	22.20
-8	8	-4	212.18	21.80
-8	8	-4	205.28	21.50
8	-8	4	209.98	18.00
-9	8	-4	15.80	12.00
-9	8	-4	10.80	11.50
-9	8	-4	3.10	11.20
9	-8	4	20.60	9.10
-10	8	-4	4.90	10.30
-10	8	-4	2.80	11.90
-10	8	-4	-12.90	12.90
10	-8	4	7.90	10.10
10	-8	4	13.30	10.20
-11	8	-4	57.99	11.20
-11	8	-4	77.29	14.60
-11	8	-4	68.09	16.90
11	-8	4	58.19	10.60
11	-8	4	63.99	13.40
11	7	-4	28.20	11.30
-11	-7	4	22.60	9.00
-11	-7	4	28.80	7.90
10	7	-4	18.10	11.80
-10	-7	4	19.10	7.70
-10	-7	4	19.70	8.80
-9	-7	4	586.14	30.90
-9	-7	4	574.64	30.90
8	7	-4	153.58	21.30
-8	-7	4	116.99	17.80
-8	-7	4	103.79	16.30
-8	-7	4	129.09	13.70
-8	-7	4	85.49	13.80
7	7	-4	188.28	16.80
-7	-7	4	207.88	16.30
-7	-7	4	246.98	15.90

-7	-7	4	237.48	19.00
-7	-7	4	228.08	31.60
6	7	-4	16.30	6.00
-6	-7	4	42.20	11.00
-6	-7	4	20.40	9.50
-6	-7	4	21.60	5.80
-6	-7	4	13.30	7.90
-6	-7	4	26.40	7.40
-6	-7	4	27.10	7.10
5	7	-4	131.49	10.40
-5	-7	4	111.19	14.60
-5	-7	4	107.99	13.90
-5	-7	4	138.99	11.20
-5	-7	4	144.99	14.10
-5	-7	4	134.09	11.40
-5	-7	4	98.99	17.70
4	7	-4	45.20	10.10
4	7	-4	60.99	6.20
-4	-7	4	76.69	12.80
-4	-7	4	59.09	9.20
-4	-7	4	53.09	6.90
3	7	-4	146.29	8.80
-3	-7	4	147.69	8.50
-3	-7	4	132.29	11.30
2	7	-4	523.65	25.00
2	7	-4	517.05	24.90
2	7	-4	516.95	27.80
-2	-7	4	558.54	25.20
-2	-7	4	517.75	26.30
1	7	-4	434.46	20.60
1	7	-4	442.86	21.00
1	7	-4	469.45	23.50
-1	-7	4	445.36	21.20
-1	-7	4	413.26	23.80
-1	-7	4	395.26	21.50
0	7	-4	46.50	5.20
0	7	-4	50.29	4.40
0	7	-4	63.19	9.00
0	-7	4	49.50	5.80
0	-7	4	41.90	6.70
0	-7	4	60.09	9.50
-1	7	-4	733.43	34.60
-1	7	-4	693.93	33.60
-1	7	-4	686.23	31.70

-1	7	-4	693.83	32.60
1	-7	4	724.93	32.90
1	-7	4	577.24	33.50
1	-7	4	682.83	34.60
-2	7	-4	1478.85	64.89
-2	7	-4	1454.05	65.99
2	-7	4	1343.47	67.09
2	-7	4	1434.46	65.39
-3	7	-4	39.80	6.10
-3	7	-4	54.99	9.40
-3	7	-4	37.80	6.60
-3	7	-4	53.59	6.90
3	-7	4	55.19	8.70
3	-7	4	58.79	7.70
3	-7	4	45.90	7.20
-4	7	-4	54.29	8.80
-4	7	-4	62.09	10.20
4	-7	4	36.70	5.80
-5	7	-4	87.59	13.10
-5	7	-4	82.89	13.80
-5	7	-4	108.99	15.50
-5	7	-4	108.69	11.00
-5	7	-4	78.09	11.60
5	-7	4	82.99	11.20
5	-7	4	75.99	12.10
-6	7	-4	65.49	11.50
-6	7	-4	68.19	14.80
-6	7	-4	68.29	9.80
-6	7	-4	86.09	14.30
6	-7	4	51.29	12.80
6	-7	4	44.90	8.50
-7	7	-4	202.68	16.00
-7	7	-4	163.68	18.40
-7	7	-4	184.88	17.20
7	-7	4	142.19	16.20
-8	7	-4	488.55	30.00
-8	7	-4	473.65	28.40
-8	7	-4	492.75	29.10
8	-7	4	440.66	27.90
-9	7	-4	473.85	29.80
-9	7	-4	494.45	31.40
-9	7	-4	506.85	30.60
9	-7	4	511.75	29.30
-10	7	-4	12.70	10.70

-10	7	-4	18.80	12.60
-10	7	-4	13.10	12.20
10	-7	4	14.60	10.50
10	-7	4	11.50	10.90
-11	7	-4	58.29	13.50
-11	7	-4	49.20	14.80
11	-7	4	14.20	14.10
11	-7	4	51.59	10.80
11	6	-4	24.00	11.80
-11	-6	4	5.00	8.50
-11	-6	4	27.40	8.20
10	6	-4	3.50	12.40
10	6	-4	-13.20	16.30
-10	-6	4	30.00	8.30
-10	-6	4	12.20	8.00
9	6	-4	40.30	13.60
-9	-6	4	6.40	11.80
-9	-6	4	8.50	7.30
-9	-6	4	18.00	7.60
8	6	-4	-6.80	11.70
8	6	-4	24.50	13.00
-8	-6	4	16.40	16.60
-8	-6	4	13.80	6.50
-8	-6	4	8.60	6.70
-8	-6	4	-7.40	9.00
-8	-6	4	-2.40	9.80
7	6	-4	8.70	12.70
7	6	-4	3.10	7.00
-7	-6	4	14.30	14.90
-7	-6	4	2.30	5.40
-7	-6	4	5.10	6.80
-7	-6	4	13.30	10.60
-7	-6	4	16.80	11.40
6	6	-4	169.28	12.20
-6	-6	4	157.88	13.50
-6	-6	4	146.09	12.40
-6	-6	4	152.08	17.20
-6	-6	4	131.89	17.00
-6	-6	4	149.79	12.40
-6	-6	4	136.79	16.50
5	6	-4	121.19	9.10
5	6	-4	114.69	17.20
5	6	-4	99.99	15.20
-5	-6	4	138.19	15.10

-5	-6	4	118.29	11.00
-5	-6	4	129.89	14.90
-5	-6	4	123.89	10.70
-5	-6	4	103.79	13.10
4	6	-4	783.62	39.00
4	6	-4	794.62	36.90
-4	-6	4	785.52	37.50
3	6	-4	2145.79	92.89
3	6	-4	1997.10	94.39
-3	-6	4	1937.11	93.39
-3	-6	4	2151.18	92.39
2	6	-4	34.30	6.10
2	6	-4	36.30	4.00
2	6	-4	35.20	4.00
-2	-6	4	41.90	6.40
-2	-6	4	29.10	4.00
-2	-6	4	28.00	5.40
1	6	-4	197.98	12.80
1	6	-4	179.48	9.40
1	6	-4	177.38	9.20
-1	-6	4	163.58	9.50
-1	-6	4	158.98	10.40
0	6	-4	298.57	14.70
0	6	-4	292.57	14.10
0	-6	4	270.97	14.80
0	-6	4	301.87	14.80
-1	6	-4	15.40	4.20
-1	6	-4	13.80	3.20
-1	6	-4	16.10	3.20
1	-6	4	12.70	3.60
1	-6	4	14.20	3.30
1	-6	4	11.90	4.50
-2	6	-4	28.90	5.40
-2	6	-4	41.20	5.50
-2	6	-4	32.80	5.50
-2	6	-4	32.40	3.70
2	-6	4	21.70	3.90
2	-6	4	30.10	5.90
-3	6	-4	17.40	4.10
-3	6	-4	28.00	4.80
3	-6	4	24.10	4.00
3	-6	4	22.30	5.10
3	-6	4	15.60	5.10
-4	6	-4	1229.08	60.49

-4	6	-4	1221.98	58.79
-4	6	-4	1333.77	58.59
4	-6	4	1184.78	59.89
4	-6	4	1316.77	57.59
4	-6	4	1204.58	58.99
-5	6	-4	50.09	10.90
-5	6	-4	40.70	8.30
-5	6	-4	48.90	10.20
-5	6	-4	30.10	6.10
5	-6	4	28.80	5.40
-6	6	-4	7.70	8.50
-6	6	-4	-6.20	5.90
-6	6	-4	8.00	12.20
-6	6	-4	7.50	8.00
6	-6	4	-11.60	6.90
-7	6	-4	343.67	28.20
-7	6	-4	355.26	22.70
-7	6	-4	355.36	23.10
-7	6	-4	335.77	21.30
7	-6	4	328.07	22.50
7	-6	4	349.37	23.40
-8	6	-4	126.69	15.00
-8	6	-4	140.09	16.70
-8	6	-4	119.19	17.90
8	-6	4	132.69	16.30
-9	6	-4	14.10	9.40
-9	6	-4	12.40	9.70
-9	6	-4	23.20	10.20
9	-6	4	22.00	9.40
-10	6	-4	108.89	18.10
-10	6	-4	86.69	13.60
-10	6	-4	84.99	13.60
10	-6	4	119.89	19.80
10	-6	4	97.59	18.10
-11	6	-4	28.90	14.10
-11	6	-4	35.50	10.70
-11	6	-4	61.99	13.40
11	-6	4	20.80	10.10
11	-6	4	13.10	15.30
12	-6	4	38.00	19.10
12	5	-4	59.19	14.00
-12	-5	4	50.79	9.20
-12	-5	4	73.49	11.90
11	5	-4	84.39	17.90

11	5	-4	154.98	19.50
-11	-5	4	117.29	13.90
-11	-5	4	103.49	14.20
10	5	-4	128.39	20.30
10	5	-4	136.29	23.20
-10	-5	4	133.59	18.60
-10	-5	4	126.19	15.40
9	5	-4	221.08	23.40
-9	-5	4	243.28	17.20
-9	-5	4	239.68	17.00
-9	-5	4	225.88	24.10
8	5	-4	651.23	38.30
-8	-5	4	732.73	35.60
-8	-5	4	695.03	35.90
-8	-5	4	738.13	36.00
-8	-5	4	703.63	38.30
7	5	-4	64.89	8.80
7	5	-4	73.49	12.10
-7	-5	4	60.99	10.70
-7	-5	4	71.79	10.50
-7	-5	4	69.29	8.80
-7	-5	4	50.49	11.70
-7	-5	4	55.19	10.90
6	5	-4	-2.10	10.60
6	5	-4	7.20	6.00
-6	-5	4	6.20	5.80
-6	-5	4	4.20	9.70
-6	-5	4	-11.40	9.70
-6	-5	4	11.80	6.10
-6	-5	4	7.80	5.60
5	5	-4	299.27	19.30
5	5	-4	303.47	20.30
5	5	-4	302.77	15.80
-5	-5	4	300.87	17.30
-5	-5	4	246.38	23.70
-5	-5	4	315.07	16.70
4	5	-4	9.40	4.80
4	5	-4	3.80	5.00
4	5	-4	9.30	2.80
-4	-5	4	6.00	5.00
3	5	-4	516.25	26.80
3	5	-4	540.55	24.30
-3	-5	4	445.66	24.60
-3	-5	4	553.94	23.80

2	5	-4	409.86	18.80
2	5	-4	384.16	20.50
2	5	-4	433.16	18.90
-2	-5	4	377.16	20.20
-2	-5	4	343.47	19.10
-2	-5	4	425.06	18.70
1	5	-4	425.66	20.40
1	5	-4	438.76	20.60
1	5	-4	452.35	22.10
-1	-5	4	402.76	20.90
-1	-5	4	459.25	20.60
0	5	-4	9.30	3.70
0	5	-4	7.40	1.80
0	5	-4	3.10	1.80
0	-5	4	4.00	2.00
0	-5	4	5.60	3.70
0	-5	4	3.10	2.70
-1	5	-4	7.80	3.40
-1	5	-4	1.60	1.80
-1	5	-4	6.30	2.20
1	-5	4	0.00	2.30
1	-5	4	7.00	3.30
1	-5	4	2.50	2.70
-2	5	-4	1026.10	46.00
-2	5	-4	1025.20	46.90
-2	5	-4	984.90	45.10
2	-5	4	942.41	46.50
2	-5	4	986.20	45.50
2	-5	4	988.90	45.90
-3	5	-4	716.53	32.50
-3	5	-4	631.64	31.30
3	-5	4	659.73	33.00
3	-5	4	673.53	31.50
3	-5	4	695.13	32.60
-4	5	-4	432.66	20.80
-4	5	-4	331.07	19.90
-4	5	-4	353.36	21.90
4	-5	4	426.86	19.40
4	-5	4	404.96	21.10
4	-5	4	378.96	21.70
-5	5	-4	439.66	23.90
-5	5	-4	404.86	27.60
-5	5	-4	420.46	22.80
-5	5	-4	433.56	24.10

5	-5	4	391.66	24.10
5	-5	4	450.75	21.50
-6	5	-4	320.47	23.40
-6	5	-4	355.36	19.60
-6	5	-4	326.77	26.10
-6	5	-4	336.17	20.80
-6	5	-4	333.57	21.90
-6	5	-4	318.77	27.30
6	-5	4	362.16	19.20
-7	5	-4	94.29	13.80
-7	5	-4	90.49	15.10
-7	5	-4	95.19	11.20
-7	5	-4	81.39	11.00
7	-5	4	113.89	20.20
7	-5	4	77.39	13.00
-8	5	-4	119.19	15.40
-8	5	-4	87.09	14.50
-8	5	-4	131.69	16.40
-8	5	-4	117.39	15.70
-8	5	-4	109.69	13.20
8	-5	4	90.79	14.20
8	-5	4	92.19	15.60
-9	5	-4	246.18	25.20
-9	5	-4	161.48	16.90
-9	5	-4	200.28	17.60
-9	5	-4	180.78	18.60
9	-5	4	158.78	18.80
-10	5	-4	337.47	23.20
10	-5	4	335.07	26.90
-11	5	-4	47.60	12.30
-11	5	-4	28.30	13.10
-11	5	-4	32.80	10.60
11	-5	4	32.60	10.40
11	-5	4	25.00	17.00
-12	5	-4	1.20	12.80
-12	5	-4	20.50	14.80
12	-5	4	12.00	10.10
12	4	-4	18.60	14.00
12	4	-4	30.50	19.60
-12	-4	4	31.40	12.30
-12	-4	4	47.50	11.90
11	4	-4	68.49	14.20
11	4	-4	123.29	21.60
-11	-4	4	80.29	10.50

-11	-4	4	64.89	9.40
10	4	-4	266.47	27.20
9	4	-4	21.20	11.30
-9	-4	4	7.90	7.20
-9	-4	4	14.50	6.90
-9	-4	4	8.30	13.70
8	4	-4	171.58	18.60
-8	-4	4	168.38	14.70
-8	-4	4	194.18	15.60
-8	-4	4	182.98	21.40
-8	-4	4	161.88	13.90
7	4	-4	69.29	10.70
7	4	-4	95.19	12.00
-7	-4	4	85.09	10.80
-7	-4	4	86.99	14.00
-7	-4	4	91.69	11.30
-7	-4	4	128.49	18.70
6	4	-4	37.10	5.90
6	4	-4	54.59	11.40
6	4	-4	69.49	11.00
6	4	-4	37.40	12.20
-6	-4	4	63.79	11.00
-6	-4	4	41.70	11.20
-6	-4	4	50.29	7.60
-6	-4	4	41.20	6.60
-6	-4	4	49.80	7.70
5	4	-4	31.50	7.00
5	4	-4	36.00	5.60
-5	-4	4	30.60	5.90
-5	-4	4	28.10	6.00
4	4	-4	218.48	13.10
4	4	-4	207.48	11.50
4	4	-4	220.98	12.70
3	4	-4	1382.16	63.79
3	4	-4	1400.36	63.09
-3	-4	4	1400.06	62.59
2	4	-4	982.60	46.70
2	4	-4	1024.10	45.70
2	4	-4	1007.20	45.80
-2	-4	4	1018.70	45.60
1	4	-4	166.18	8.50
1	4	-4	170.28	8.60
1	4	-4	181.38	10.20
-1	-4	4	145.49	9.30

-1	-4	4	176.38	8.60
0	4	-4	93.19	5.70
0	4	-4	117.99	7.40
0	4	-4	97.39	5.50
0	-4	4	91.09	7.90
0	-4	4	84.79	6.50
0	-4	4	103.19	5.80
-1	4	-4	58.89	4.00
-1	4	-4	49.99	5.30
-1	4	-4	58.39	4.30
1	-4	4	49.99	4.30
1	-4	4	52.89	5.40
1	-4	4	52.59	4.50
-2	4	-4	697.93	32.40
-2	4	-4	708.63	33.10
-2	4	-4	719.33	33.70
2	-4	4	698.93	33.20
2	-4	4	717.13	33.60
2	-4	4	714.73	32.70
-3	4	-4	691.73	31.20
-3	4	-4	600.54	33.20
3	-4	4	643.24	31.60
3	-4	4	663.23	30.30
3	-4	4	671.13	31.30
-4	4	-4	212.18	12.10
4	-4	4	200.68	12.60
4	-4	4	188.08	13.10
4	-4	4	188.58	10.60
-5	4	-4	185.98	13.70
-5	4	-4	176.48	11.90
-5	4	-4	183.78	18.50
5	-4	4	170.28	14.10
5	-4	4	171.28	10.30
5	-4	4	176.88	15.40
-6	4	-4	108.69	17.30
-6	4	-4	103.19	12.60
-6	4	-4	139.79	10.70
-6	4	-4	134.19	13.70
-6	4	-4	130.99	16.60
-6	4	-4	146.59	15.00
6	-4	4	122.19	10.10
-7	4	-4	24.90	10.90
-7	4	-4	6.80	6.60
-7	4	-4	6.50	8.20

-7	4	-4	6.70	5.50
-7	4	-4	-20.70	11.40
7	-4	4	11.40	8.60
7	-4	4	-5.70	5.90
-8	4	-4	508.75	31.20
-8	4	-4	497.25	27.40
-8	4	-4	454.55	30.50
-8	4	-4	451.75	25.50
-8	4	-4	413.66	26.20
8	-4	4	464.45	27.10
8	-4	4	420.56	27.30
-9	4	-4	-19.60	13.60
-9	4	-4	5.60	8.80
-9	4	-4	-5.10	8.60
-9	4	-4	-18.90	12.70
-9	4	-4	7.40	10.40
9	-4	4	-8.40	9.60
-10	4	-4	17.90	9.60
-10	4	-4	15.00	9.20
-10	4	-4	22.30	10.50
-11	4	-4	268.57	22.20
-11	4	-4	263.27	21.20
-11	4	-4	308.47	21.80
11	-4	4	283.77	21.50
-12	4	-4	186.58	25.50
-12	4	-4	169.78	17.10
-12	4	-4	128.59	22.40
12	-4	4	147.49	17.10
12	3	-4	51.89	15.80
12	3	-4	47.90	15.20
-12	-3	4	48.80	9.70
-12	-3	4	46.90	13.60
11	3	-4	3.90	13.30
11	3	-4	5.30	12.60
-11	-3	4	20.50	7.90
-11	-3	4	-2.60	8.90
10	3	-4	133.39	20.20
-10	-3	4	118.89	14.10
-10	-3	4	97.29	14.30
9	3	-4	501.85	31.80
-9	-3	4	535.35	28.80
-9	-3	4	571.84	29.70
8	3	-4	30.40	12.50
8	3	-4	26.40	9.00

-8	-3	4	31.00	6.70
-8	-3	4	29.00	7.50
-8	-3	4	22.70	9.10
-8	-3	4	8.90	12.70
7	3	-4	406.56	25.20
7	3	-4	428.66	23.10
-7	-3	4	400.66	22.20
-7	-3	4	395.56	22.90
-7	-3	4	396.06	22.30
-7	-3	4	402.26	27.40
6	3	-4	105.89	11.80
6	3	-4	77.99	11.30
6	3	-4	84.29	8.80
-6	-3	4	91.09	10.80
-6	-3	4	91.19	9.90
-6	-3	4	88.99	10.90
-6	-3	4	87.39	13.30
5	3	-4	-8.80	5.00
5	3	-4	-0.80	3.00
-5	-3	4	9.10	5.10
4	3	-4	233.68	12.50
4	3	-4	240.28	11.70
4	3	-4	226.18	13.80
-4	-3	4	196.88	10.60
-4	-3	4	199.78	13.60
-4	-3	4	227.98	10.50
3	3	-4	24.60	3.40
3	3	-4	29.10	3.50
3	3	-4	25.20	3.90
-3	-3	4	23.60	2.60
-3	-3	4	22.50	2.50
-3	-3	4	21.60	3.30
2	3	-4	1088.79	47.50
2	3	-4	946.91	47.10
-2	-3	4	1072.69	46.70
1	3	-4	139.99	6.80
1	3	-4	144.79	8.20
1	3	-4	129.89	6.80
-1	-3	4	112.79	7.70
-1	-3	4	106.29	7.50
-1	-3	4	136.09	6.70
0	3	-4	14.50	2.70
0	3	-4	13.00	1.90
0	3	-4	8.20	1.70

0	-3	4	5.60	2.30
0	-3	4	9.10	1.70
0	-3	4	13.20	3.30
-1	3	-4	39.60	4.20
-1	3	-4	41.00	3.50
-1	3	-4	37.60	3.30
1	-3	4	39.70	4.30
1	-3	4	38.90	3.50
-2	3	-4	309.57	14.70
-2	3	-4	313.27	15.20
2	-3	4	277.67	14.90
2	-3	4	305.37	14.80
2	-3	4	282.17	14.20
-3	3	-4	137.89	9.10
3	-3	4	142.69	9.00
3	-3	4	143.79	7.50
-4	3	-4	69.79	6.40
4	-3	4	59.99	7.40
4	-3	4	62.69	7.20
4	-3	4	59.39	4.80
-5	3	-4	820.92	37.50
-5	3	-4	810.92	36.80
-5	3	-4	740.63	38.60
5	-3	4	709.33	38.10
5	-3	4	767.12	37.40
5	-3	4	762.92	35.70
-6	3	-4	146.69	15.00
-6	3	-4	149.09	10.80
-6	3	-4	147.69	15.70
-6	3	-4	155.98	17.70
-6	3	-4	136.39	18.40
-6	3	-4	140.89	15.10
-6	3	-4	137.99	12.90
6	-3	4	160.28	10.20
6	-3	4	130.19	15.20
-7	3	-4	71.79	9.40
-7	3	-4	69.09	11.80
-7	3	-4	66.39	9.50
-7	3	-4	76.19	12.00
-7	3	-4	71.29	9.20
7	-3	4	76.19	9.20
7	-3	4	84.29	11.40
-8	3	-4	638.74	32.20
-8	3	-4	592.94	35.60

-8	3	-4	618.34	32.80
-8	3	-4	614.54	35.30
-8	3	-4	632.24	33.60
8	-3	4	620.04	34.50
8	-3	4	635.44	32.50
-9	3	-4	324.07	22.00
-9	3	-4	353.96	21.60
-9	3	-4	302.57	25.80
-9	3	-4	306.57	20.60
-9	3	-4	269.67	26.60
9	-3	4	296.77	27.10
9	-3	4	306.57	23.60
-10	3	-4	25.20	8.80
-10	3	-4	12.40	9.30
-10	3	-4	9.50	8.30
-10	3	-4	23.10	14.10
10	-3	4	16.50	13.90
-11	3	-4	5.90	10.80
-11	3	-4	11.30	9.80
-11	3	-4	22.40	9.20
11	-3	4	15.00	10.30
-12	3	-4	202.88	19.90
-12	3	-4	214.38	22.10
-12	3	-4	246.88	18.40
12	-3	4	249.78	19.70
12	2	-4	1.70	17.40
-12	-2	4	10.80	7.60
-12	-2	4	25.90	14.70
11	2	-4	5.30	11.50
11	2	-4	-9.90	14.00
-11	-2	4	15.80	7.70
-11	-2	4	3.70	9.60
10	2	-4	34.60	11.20
-10	-2	4	26.70	7.90
-10	-2	4	19.80	9.00
9	2	-4	103.29	17.00
-9	-2	4	65.69	9.10
8	2	-4	5.60	8.40
-8	-2	4	9.80	13.30
-8	-2	4	12.90	7.10
-8	-2	4	11.10	6.40
7	2	-4	29.50	8.40
-7	-2	4	12.70	12.30
-7	-2	4	27.40	6.80

-7	-2	4	43.90	7.50
-7	-2	4	29.60	6.50
6	2	-4	21.60	9.00
6	2	-4	33.90	8.80
6	2	-4	37.80	8.50
6	2	-4	40.70	5.50
-6	-2	4	43.00	6.80
-6	-2	4	40.10	6.60
-6	-2	4	47.10	7.70
5	2	-4	23.00	5.20
5	2	-4	27.10	5.50
5	2	-4	24.90	3.80
-5	-2	4	24.80	5.70
4	2	-4	1470.55	64.29
4	2	-4	1448.76	63.79
4	2	-4	1502.75	64.29
-4	-2	4	1340.27	63.29
-4	-2	4	1257.77	63.89
-4	-2	4	1426.46	63.09
3	2	-4	2566.04	108.59
3	2	-4	2495.25	108.49
3	2	-4	2432.66	108.29
-3	-2	4	2424.16	107.89
-3	-2	4	2340.37	107.99
-3	-2	4	2243.68	108.29
2	2	-4	53.89	4.00
2	2	-4	50.89	3.80
-2	-2	4	46.60	3.20
-2	-2	4	44.10	4.00
1	2	-4	102.29	5.80
1	2	-4	116.19	5.90
1	2	-4	102.59	6.70
-1	-2	4	107.99	5.70
-1	-2	4	115.79	6.30
0	2	-4	37.90	3.60
0	2	-4	38.40	3.00
0	2	-4	40.70	3.00
0	-2	4	38.00	4.50
0	-2	4	38.90	3.00
0	-2	4	37.10	4.10
-1	2	-4	135.09	7.00
-1	2	-4	136.39	7.30
1	-2	4	127.79	7.30
1	-2	4	123.39	6.70

1	-2	4	133.69	7.00
-2	2	-4	559.34	26.30
-2	2	-4	567.54	26.10
2	-2	4	551.94	26.30
2	-2	4	549.45	25.50
2	-2	4	568.74	26.10
-3	2	-4	91.89	6.70
-3	2	-4	94.89	6.10
3	-2	4	85.69	6.20
3	-2	4	99.69	5.30
3	-2	4	95.89	6.70
-4	2	-4	2085.99	91.39
4	-2	4	2010.10	91.49
4	-2	4	1951.70	90.59
4	-2	4	2023.20	91.99
-5	2	-4	54.99	6.60
-5	2	-4	62.09	7.50
-5	2	-4	55.19	9.40
5	-2	4	61.29	8.20
5	-2	4	64.49	9.00
5	-2	4	48.00	4.70
-6	2	-4	-14.70	10.20
-6	2	-4	2.60	5.90
-6	2	-4	-3.10	9.00
-6	2	-4	-9.10	9.50
-6	2	-4	3.80	4.20
-6	2	-4	7.30	6.90
-6	2	-4	18.20	8.30
6	-2	4	5.60	3.20
6	-2	4	16.20	7.40
-7	2	-4	872.11	45.20
-7	2	-4	934.01	43.80
-7	2	-4	885.31	42.60
-7	2	-4	842.42	45.20
-7	2	-4	888.21	43.10
7	-2	4	915.61	41.90
7	-2	4	833.72	44.60
-8	2	-4	50.09	7.80
-8	2	-4	36.00	8.10
-8	2	-4	40.60	10.60
-8	2	-4	67.49	11.40
-8	2	-4	78.09	16.60
8	-2	4	32.90	18.40
8	-2	4	29.90	7.40

-9	2	-4	19.70	11.00
-9	2	-4	27.30	8.70
-9	2	-4	11.30	11.40
-9	2	-4	14.70	7.60
-9	2	-4	15.50	7.00
9	-2	4	21.00	11.10
9	-2	4	39.40	11.90
-10	2	-4	174.28	18.80
-10	2	-4	246.78	23.80
-10	2	-4	217.08	17.50
-10	2	-4	178.18	16.30
-10	2	-4	196.48	18.70
-11	2	-4	40.10	10.10
-11	2	-4	22.10	10.00
-11	2	-4	38.20	10.70
11	-2	4	36.00	12.40
-12	2	-4	103.89	15.70
-12	2	-4	133.29	15.00
-12	2	-4	125.79	16.90
12	-2	4	93.09	17.00
12	1	-4	40.70	13.80
-12	-1	4	48.40	8.70
11	1	-4	29.00	15.50
11	1	-4	20.60	11.40
-11	-1	4	5.10	10.20
-11	-1	4	7.60	7.80
10	1	-4	11.80	11.20
-10	-1	4	-1.30	7.10
-10	-1	4	-2.40	8.80
9	1	-4	703.83	36.60
-9	-1	4	692.93	35.00
-9	-1	4	640.84	35.80
8	1	-4	1280.47	61.79
-8	-1	4	1315.27	60.79
-8	-1	4	1255.27	61.19
7	1	-4	-3.40	7.80
7	1	-4	4.30	8.10
-7	-1	4	-0.60	6.40
-7	-1	4	-3.60	5.80
-7	-1	4	-5.80	6.40
6	1	-4	14.80	8.50
6	1	-4	5.10	7.20
6	1	-4	1.40	7.90
-6	-1	4	3.80	6.30

-6	-1	4	2.20	5.90
-6	-1	4	4.20	6.30
5	1	-4	274.87	15.70
5	1	-4	284.07	15.90
5	1	-4	275.57	14.50
-5	-1	4	279.37	13.50
-5	-1	4	265.77	14.10
-5	-1	4	296.67	16.00
4	1	-4	20.00	3.20
4	1	-4	15.60	2.80
4	1	-4	21.70	3.50
-4	-1	4	11.40	3.80
-4	-1	4	28.00	3.60
-4	-1	4	26.60	2.80
3	1	-4	650.23	29.50
3	1	-4	627.34	29.50
-3	-1	4	632.24	29.20
-3	-1	4	647.24	29.10
2	1	-4	881.21	40.70
2	1	-4	890.91	40.60
2	1	-4	952.30	40.80
-2	-1	4	881.11	40.70
-2	-1	4	896.41	40.60
1	1	-4	284.47	14.60
1	1	-4	295.97	14.30
-1	-1	4	323.67	14.30
-1	-1	4	326.27	14.40
0	1	-4	267.37	12.10
0	1	-4	276.77	12.00
0	-1	4	267.67	12.10
0	-1	4	234.48	12.20
0	-1	4	219.58	12.20
-1	1	-4	11.80	1.70
-1	1	-4	10.70	1.60
1	-1	4	11.00	1.70
1	-1	4	13.10	2.00
1	-1	4	9.50	1.60
-2	1	-4	867.11	37.90
-2	1	-4	830.52	38.10
2	-1	4	826.32	38.10
2	-1	4	792.82	37.60
2	-1	4	841.82	38.00
-3	1	-4	8.30	1.90
-3	1	-4	9.70	2.30

3	-1	4	5.40	2.00
3	-1	4	7.00	1.30
-4	1	-4	327.77	15.60
4	-1	4	293.57	15.60
4	-1	4	304.67	16.30
4	-1	4	307.17	14.70
-5	1	-4	809.52	39.20
-5	1	-4	875.21	39.30
-5	1	-4	875.11	40.60
5	-1	4	790.42	39.40
5	-1	4	796.92	40.30
5	-1	4	870.31	38.50
-6	1	-4	667.83	33.70
-6	1	-4	622.74	35.80
-6	1	-4	631.84	32.70
-6	1	-4	713.13	32.60
-6	1	-4	647.64	36.10
6	-1	4	686.43	31.10
6	-1	4	633.34	32.70
-7	1	-4	61.79	11.00
-7	1	-4	63.49	8.50
-7	1	-4	83.89	11.20
-7	1	-4	51.09	8.10
7	-1	4	58.19	7.20
7	-1	4	63.99	11.90
-8	1	-4	76.69	11.80
-8	1	-4	88.59	13.20
-8	1	-4	98.69	10.30
-8	1	-4	100.59	17.10
8	-1	4	92.39	12.60
8	-1	4	72.39	10.50
-9	1	-4	252.17	21.70
-9	1	-4	264.27	19.40
-9	1	-4	245.18	17.80
-9	1	-4	234.08	16.90
-9	1	-4	222.88	21.90
9	-1	4	262.97	20.00
9	-1	4	225.28	23.50
-10	1	-4	119.99	15.20
-10	1	-4	169.98	21.70
-10	1	-4	114.79	13.70
-10	1	-4	125.39	14.70
-10	1	-4	102.39	15.60
-11	1	-4	34.30	9.60

11	-1	4	30.00	11.50
-12	1	-4	97.99	15.20
-12	1	-4	91.69	14.10
-12	1	-4	64.69	14.00
12	-1	4	59.39	11.40
12	0	-4	10.80	12.50
12	0	-4	28.80	20.50
-12	0	4	27.20	18.20
-12	0	4	11.70	7.80
11	0	-4	81.79	17.90
11	0	-4	120.69	17.50
-11	0	4	75.39	12.20
-11	0	4	79.99	13.20
10	0	-4	327.17	24.90
-10	0	4	340.67	21.70
-10	0	4	348.57	23.70
9	0	-4	84.89	15.80
-9	0	4	62.49	10.90
-9	0	4	54.29	8.80
-8	0	4	42.80	7.60
-8	0	4	35.90	7.90
7	0	-4	91.59	12.40
7	0	-4	81.99	13.80
-7	0	4	77.49	11.10
-7	0	4	69.69	9.10
-7	0	4	64.09	11.10
6	0	-4	54.19	8.20
6	0	-4	64.89	9.30
6	0	-4	76.29	9.70
-6	0	4	71.59	9.00
-6	0	4	59.19	9.90
-6	0	4	66.39	8.20
5	0	-4	34.30	5.20
-5	0	4	30.50	4.60
4	0	-4	7.80	2.70
4	0	-4	10.70	2.70
-4	0	4	11.10	2.80
-4	0	4	7.30	3.00
3	0	-4	4239.88	189.38
3	0	-4	4373.76	189.48
-3	0	4	4142.69	189.48
-3	0	4	4236.58	189.48
2	0	-4	155.98	7.10
2	0	-4	151.98	7.40

2	0	-4	139.69	7.00
-2	0	4	132.79	7.40
-2	0	4	135.59	7.20
1	0	-4	554.74	27.70
1	0	-4	679.73	28.60
-1	0	4	690.03	28.10
-1	0	4	542.95	28.00
0	0	-4	41.00	2.80
0	0	-4	38.80	2.30
0	0	4	43.50	2.70
0	0	4	37.70	2.90
0	0	4	38.10	2.50
-1	0	-4	1625.04	73.59
-1	0	-4	1671.83	73.69
1	0	4	1632.04	73.59
1	0	4	1599.44	73.19
1	0	4	1694.03	73.69
-2	0	-4	966.70	43.70
-2	0	-4	964.90	43.90
2	0	4	966.40	43.90
2	0	4	976.70	43.70
2	0	4	952.10	43.20
-3	0	-4	1147.09	52.09
-3	0	-4	1160.28	52.59
3	0	4	1124.09	51.59
3	0	4	1135.69	52.19
3	0	4	1189.18	52.69
-4	0	-4	21.00	3.50
4	0	4	25.00	4.20
4	0	4	25.40	4.70
-5	0	-4	513.85	25.00
-5	0	-4	521.55	26.20
-5	0	-4	542.65	24.70
5	0	4	494.05	24.90
5	0	4	496.65	26.20
-6	0	-4	189.88	11.60
-6	0	-4	170.38	18.30
-6	0	-4	188.68	19.80
-6	0	-4	198.48	14.00
-6	0	-4	184.38	15.00
-6	0	-4	184.08	12.20
6	0	4	177.28	12.60
6	0	4	195.48	10.40
-7	0	-4	20.80	4.80

-7	0	-4	17.00	8.90
-7	0	-4	28.70	7.40
-7	0	-4	37.70	9.00
-7	0	-4	20.80	8.60
-7	0	-4	37.20	6.50
7	0	4	35.10	5.90
7	0	4	19.40	12.20
-8	0	-4	312.47	23.30
-8	0	-4	386.46	27.50
-8	0	-4	345.87	19.60
-8	0	-4	343.57	21.20
-8	0	-4	352.56	20.80
8	0	4	344.17	19.20
8	0	4	290.97	33.40
-9	0	-4	-0.40	5.70
-9	0	-4	1.50	7.60
-9	0	-4	2.30	6.30
-9	0	-4	10.30	9.40
9	0	4	16.70	8.70
-10	0	-4	20.90	11.50
-10	0	-4	19.60	8.20
-10	0	-4	41.90	11.80
-10	0	-4	33.50	8.50
-10	0	-4	21.50	8.50
10	0	4	8.00	16.80
-11	0	-4	340.97	28.70
-11	0	-4	286.87	21.20
-11	0	-4	322.17	21.10
-11	0	-4	310.57	21.40
11	0	4	304.17	24.30
-12	0	-4	117.89	14.00
-12	0	-4	127.09	17.30
-12	0	-4	101.09	15.40
12	0	4	126.99	17.30
12	-1	-4	29.30	12.50
12	-1	-4	20.10	25.10
-12	1	4	42.00	9.20
11	-1	-4	-8.00	17.40
11	-1	-4	13.60	10.00
-11	1	4	10.00	7.20
-11	1	4	14.20	12.00
10	-1	-4	221.08	20.30
-10	1	4	224.38	17.80
-10	1	4	226.28	20.90

9	-1	-4	341.57	23.90
-9	1	4	378.76	24.40
-9	1	4	379.36	22.90
8	-1	-4	337.87	21.50
-8	1	4	295.57	20.60
-8	1	4	305.77	20.00
7	-1	-4	146.39	20.10
7	-1	-4	149.79	15.70
-7	1	4	159.88	15.20
-7	1	4	164.58	13.60
-7	1	4	164.38	13.70
6	-1	-4	84.49	9.10
6	-1	-4	77.89	13.60
6	-1	-4	97.29	10.60
-6	1	4	90.69	10.10
-6	1	4	65.49	10.90
-6	1	4	102.19	12.10
5	-1	-4	7.00	4.30
-5	1	4	-1.20	4.30
-5	1	4	5.40	6.40
4	-1	-4	18.60	3.10
-4	1	4	16.50	3.20
-4	1	4	16.90	3.20
3	-1	-4	247.08	12.20
-3	1	4	247.98	12.40
-3	1	4	259.47	12.30
2	-1	-4	6717.93	298.67
-1	1	4	1174.58	56.69
-1	1	4	1358.66	56.99
0	1	4	3.50	1.00
0	1	4	2.20	0.70
0	1	4	3.30	0.60
-1	-1	-4	98.09	5.00
-1	-1	-4	91.29	5.00
1	1	4	98.59	5.00
1	1	4	96.79	5.10
-2	-1	-4	68.89	4.40
-2	-1	-4	70.19	4.00
2	1	4	72.99	4.10
2	1	4	61.09	4.30
-3	-1	-4	837.62	38.70
-3	-1	-4	836.42	38.10
3	1	4	817.42	38.20
3	1	4	846.22	37.60

3	1	4	853.21	38.70
-4	-1	-4	13.20	3.10
-4	-1	-4	10.20	2.10
-4	-1	-4	6.70	4.10
4	1	4	7.40	2.70
4	1	4	11.50	2.60
4	1	4	14.60	2.30
-5	-1	-4	76.89	8.20
-5	-1	-4	82.69	5.90
-5	-1	-4	65.99	7.40
5	1	4	78.59	5.20
5	1	4	75.39	6.60
5	1	4	84.39	8.50
-6	-1	-4	1434.26	68.89
-6	-1	-4	1425.26	66.09
-6	-1	-4	1479.35	67.39
-6	-1	-4	1382.26	69.99
-6	-1	-4	1560.24	66.99
-6	-1	-4	1431.76	67.59
6	1	4	1461.75	66.39
-7	-1	-4	495.15	26.40
-7	-1	-4	492.35	25.90
-7	-1	-4	503.05	29.70
-7	-1	-4	497.35	27.60
-7	-1	-4	479.55	25.10
-7	-1	-4	493.45	28.90
7	1	4	498.45	24.30
-8	-1	-4	242.18	15.90
-8	-1	-4	189.88	25.80
-8	-1	-4	221.18	14.40
-8	-1	-4	215.18	19.30
-8	-1	-4	238.68	17.00
8	1	4	222.68	14.40
-9	-1	-4	28.40	7.30
-9	-1	-4	17.90	6.20
-9	-1	-4	29.80	8.30
-9	-1	-4	4.30	8.90
-9	-1	-4	30.60	10.30
9	1	4	51.79	9.10
-10	-1	-4	111.49	13.60
-10	-1	-4	76.89	13.30
-10	-1	-4	106.89	18.50
-10	-1	-4	95.89	13.60
-10	-1	-4	74.39	12.70

10	1	4	75.09	16.10
-11	-1	-4	44.90	14.00
-11	-1	-4	18.90	8.20
-11	-1	-4	21.90	9.10
-11	-1	-4	11.80	8.90
11	1	4	9.40	11.70
-12	-1	-4	222.48	17.70
-12	-1	-4	226.08	16.70
-12	-1	-4	179.88	19.30
12	1	4	227.98	20.50
12	-2	-4	4.20	11.10
-12	2	4	-6.20	7.30
11	-2	-4	-22.70	20.10
11	-2	-4	12.10	10.00
-11	2	4	19.70	7.90
-11	2	4	22.50	14.20
-10	2	4	80.99	13.60
-10	2	4	83.39	15.20
9	-2	-4	57.39	10.00
-9	2	4	76.49	13.00
-9	2	4	37.80	10.10
8	-2	-4	9.70	8.10
-8	2	4	11.40	6.50
-8	2	4	-0.50	7.70
7	-2	-4	86.39	12.10
7	-2	-4	93.79	10.50
-7	2	4	58.79	8.20
-7	2	4	77.49	11.40
-7	2	4	65.19	9.00
6	-2	-4	132.69	11.50
6	-2	-4	122.19	15.20
6	-2	-4	138.99	15.20
-6	2	4	135.69	11.50
-6	2	4	122.59	13.60
-6	2	4	124.99	12.20
5	-2	-4	34.20	5.70
5	-2	-4	30.90	4.70
-5	2	4	62.29	10.20
-5	2	4	36.10	5.90
-5	2	4	33.90	5.20
4	-2	-4	1981.70	95.29
4	-2	-4	2172.68	94.99
-4	2	4	2068.39	94.59
-4	2	4	2218.08	94.89

-4	2	4	2080.19	95.09
3	-2	-4	38.10	3.40
3	-2	-4	25.60	3.40
3	-2	-4	34.50	3.90
-3	2	4	31.80	3.10
-3	2	4	28.90	3.40
-3	2	4	31.10	3.10
2	-2	-4	2836.42	120.59
2	-2	-4	2688.73	120.59
-2	2	4	2764.62	120.89
-2	2	4	2360.16	120.79
-2	2	4	2862.41	120.49
1	-2	-4	214.68	11.60
-1	2	4	265.47	11.40
-1	2	4	281.97	12.00
-1	2	4	243.48	11.70
0	2	4	170.08	8.00
1	2	4	405.76	19.20
1	2	4	430.46	19.30
-2	-2	-4	7.90	1.40
2	2	4	8.40	1.40
2	2	4	7.00	1.70
-3	-2	-4	1852.91	78.49
-3	-2	-4	1703.93	77.69
3	2	4	1729.53	77.39
3	2	4	1745.13	77.79
3	2	4	1820.22	78.49
3	2	4	1558.64	77.29
-4	-2	-4	424.96	20.00
4	2	4	433.76	19.90
4	2	4	429.06	20.30
4	2	4	436.36	21.40
-5	-2	-4	0.20	4.10
-5	-2	-4	1.10	2.20
5	2	4	3.60	2.70
5	2	4	2.80	1.80
5	2	4	4.60	3.80
-6	-2	-4	108.19	8.10
-6	-2	-4	144.39	15.40
-6	-2	-4	119.59	12.10
-6	-2	-4	124.19	8.90
-6	-2	-4	141.39	18.20
-6	-2	-4	118.59	11.40
-6	-2	-4	119.59	14.40

6	2	4	137.29	7.90
-7	-2	-4	1090.69	52.59
-7	-2	-4	1120.79	51.29
-7	-2	-4	1095.69	51.99
-7	-2	-4	1073.79	51.59
-7	-2	-4	1078.89	53.49
-7	-2	-4	1086.69	54.39
-8	-2	-4	6.90	6.10
-8	-2	-4	2.70	8.50
-8	-2	-4	7.60	4.70
-8	-2	-4	2.80	6.20
8	2	4	3.70	4.80
-9	-2	-4	20.60	7.80
-9	-2	-4	22.90	8.90
-9	-2	-4	27.10	7.30
-9	-2	-4	22.70	6.70
-9	-2	-4	29.10	9.70
9	2	4	28.40	8.30
-10	-2	-4	319.47	25.00
-10	-2	-4	332.77	20.60
-10	-2	-4	306.17	21.00
-10	-2	-4	316.57	20.30
-10	-2	-4	309.57	23.20
10	2	4	322.87	27.90
-11	-2	-4	0.30	8.70
-11	-2	-4	20.80	9.10
-11	-2	-4	21.80	12.90
11	2	4	31.90	11.70
-12	-2	-4	128.89	14.00
-12	-2	-4	159.08	15.50
-12	-2	-4	154.58	16.90
12	2	4	141.99	18.40
12	-3	-4	25.30	11.40
-12	3	4	-6.50	8.10
11	-3	-4	85.69	26.80
11	-3	-4	10.00	9.90
-11	3	4	28.70	8.20
-11	3	4	-14.60	15.30
10	-3	-4	36.10	10.40
10	-3	-4	41.60	24.30
-10	3	4	17.40	8.60
9	-3	-4	591.54	33.90
-9	3	4	602.74	34.30
-9	3	4	621.84	32.60

8	-3	-4	249.48	20.00
8	-3	-4	243.28	22.20
-8	3	4	291.17	20.90
-8	3	4	270.87	18.70
7	-3	-4	308.37	22.70
7	-3	-4	351.56	21.90
-7	3	4	351.96	22.40
-7	3	4	352.66	20.50
-7	3	4	368.76	21.00
6	-3	-4	27.60	12.70
6	-3	-4	13.50	7.60
6	-3	-4	16.20	9.70
6	-3	-4	31.20	8.90
-6	3	4	16.20	6.30
-6	3	4	14.00	6.10
5	-3	-4	382.46	19.50
5	-3	-4	380.56	19.40
5	-3	-4	378.16	20.40
-5	3	4	361.46	19.90
-5	3	4	390.26	19.80
-5	3	4	372.76	20.50
-5	3	4	359.06	19.10
4	-3	-4	36.10	5.40
4	-3	-4	37.40	5.70
4	-3	-4	33.90	5.50
-4	3	4	40.20	5.00
-4	3	4	42.90	4.50
3	-3	-4	56.29	4.50
3	-3	-4	46.10	5.10
3	-3	-4	45.40	4.50
-3	3	4	51.59	3.90
-3	3	4	58.09	4.50
-3	3	4	52.79	4.10
2	-3	-4	2.30	1.70
2	-3	-4	3.30	1.60
-2	3	4	5.10	1.30
-2	3	4	3.50	1.50
-2	3	4	2.70	1.20
1	-3	-4	527.95	26.70
1	-3	-4	631.04	26.90
-1	3	4	638.14	26.90
-1	3	4	560.64	26.50
-1	3	4	570.64	26.40
0	3	4	425.16	19.90

0	3	4	438.56	19.90
1	3	4	622.24	28.40
2	3	4	1593.74	73.09
2	3	4	1671.83	73.59
3	3	4	71.19	4.30
3	3	4	69.69	5.20
4	3	4	4.20	2.50
4	3	4	5.60	1.50
4	3	4	7.20	2.00
-5	-3	-4	1655.43	83.29
5	3	4	1884.11	82.09
5	3	4	1924.11	82.39
5	3	4	1862.31	84.29
-6	-3	-4	469.45	25.00
-6	-3	-4	511.65	24.20
-6	-3	-4	471.25	25.40
-6	-3	-4	481.55	27.40
-6	-3	-4	497.95	35.40
-6	-3	-4	471.55	26.30
6	3	4	504.75	23.40
-7	-3	-4	3.10	9.30
-7	-3	-4	12.30	7.80
-7	-3	-4	7.00	4.00
-7	-3	-4	11.00	5.40
-7	-3	-4	6.90	7.80
-7	-3	-4	16.50	5.90
7	3	4	6.00	3.30
-8	-3	-4	4.00	8.00
-8	-3	-4	4.40	4.80
-8	-3	-4	8.50	5.90
-8	-3	-4	5.20	6.20
-8	-3	-4	7.60	9.50
-9	-3	-4	270.67	17.60
-9	-3	-4	274.27	21.90
-9	-3	-4	284.87	18.40
-9	-3	-4	294.47	18.80
-9	-3	-4	258.07	19.80
9	3	4	290.37	18.50
-10	-3	-4	12.30	7.60
-10	-3	-4	20.90	10.60
-10	-3	-4	11.20	7.70
-10	-3	-4	14.60	9.40
-10	-3	-4	15.60	7.60
10	3	4	-3.70	12.70

-11	-3	-4	36.70	8.20
-11	-3	-4	54.59	9.60
-11	-3	-4	54.69	9.50
-11	-3	-4	24.10	11.50
11	3	4	37.10	11.90
-12	-3	-4	215.08	15.30
-12	-3	-4	154.98	17.60
-12	-3	-4	197.18	16.90
-12	-3	-4	130.79	21.40
12	3	4	169.48	19.60
12	-4	-4	5.30	10.90
-12	4	4	-1.50	8.20
11	-4	-4	96.89	15.10
-11	4	4	74.09	18.40
-11	4	4	74.59	13.30
10	-4	-4	253.17	41.00
-10	4	4	328.77	27.80
-10	4	4	293.07	25.60
9	-4	-4	22.70	9.40
9	-4	-4	14.60	12.10
-9	4	4	28.50	8.10
-9	4	4	35.00	11.30
8	-4	-4	50.29	10.10
-8	4	4	38.20	8.10
-8	4	4	68.09	10.80
7	-4	-4	32.80	8.20
7	-4	-4	27.10	9.30
-7	4	4	37.80	9.00
-7	4	4	31.80	7.80
-7	4	4	14.30	7.50
6	-4	-4	2.90	12.70
6	-4	-4	3.20	7.30
6	-4	-4	8.50	7.30
-6	4	4	5.80	6.60
-6	4	4	9.10	14.20
-6	4	4	6.40	5.80
5	-4	-4	25.90	7.30
5	-4	-4	44.10	10.00
5	-4	-4	33.20	6.00
-5	4	4	30.30	5.80
-5	4	4	26.10	6.90
-5	4	4	27.60	6.10
-5	4	4	35.00	4.90
4	-4	-4	82.69	7.70

4	-4	-4	91.09	7.20
4	-4	-4	79.09	8.10
-4	4	4	86.79	7.80
-4	4	4	101.99	7.30
-4	4	4	90.89	6.20
3	-4	-4	1871.41	83.29
3	-4	-4	1831.52	83.39
3	-4	-4	1690.73	82.89
-3	4	4	1877.61	83.09
-3	4	4	1791.72	82.79
-3	4	4	2004.80	83.59
2	-4	-4	136.19	8.40
2	-4	-4	157.18	8.20
-2	4	4	144.49	7.90
-2	4	4	162.28	8.20
-2	4	4	155.98	7.60
1	-4	-4	370.46	18.20
1	-4	-4	377.76	18.00
-1	4	4	384.36	18.20
-1	4	4	393.66	18.00
0	-4	-4	1936.61	83.49
0	-4	-4	1871.41	83.49
0	4	4	1862.01	83.49
0	4	4	1780.22	83.79
1	4	4	4486.05	187.38
1	4	4	3911.21	186.98
2	4	4	1006.50	47.30
2	4	4	1028.70	46.70
2	4	4	1015.00	46.80
2	4	4	1102.39	47.40
3	4	4	5.20	1.50
3	4	4	4.80	2.30
3	4	4	6.30	1.30
4	4	4	207.38	11.80
4	4	4	198.18	9.70
4	4	4	201.18	9.80
-5	-4	-4	325.37	17.10
5	4	4	348.97	16.10
5	4	4	329.37	18.60
-6	-4	-4	297.57	15.80
-6	-4	-4	286.77	18.70
-6	-4	-4	292.67	19.60
-6	-4	-4	276.77	17.20
-6	-4	-4	273.57	16.90

6	4	4	301.67	14.90
-7	-4	-4	403.16	24.40
-7	-4	-4	420.96	23.90
-7	-4	-4	420.56	22.30
-7	-4	-4	419.56	22.80
-7	-4	-4	418.56	21.60
-7	-4	-4	402.76	24.70
7	4	4	415.96	20.90
-8	-4	-4	170.28	13.50
-8	-4	-4	130.39	14.70
-8	-4	-4	144.99	11.60
-8	-4	-4	173.48	17.80
-8	-4	-4	152.28	13.30
-8	-4	-4	121.99	16.00
8	4	4	144.89	11.60
-9	-4	-4	24.10	8.40
-9	-4	-4	25.10	7.10
-9	-4	-4	36.70	8.00
-9	-4	-4	45.10	10.20
-9	-4	-4	28.20	7.60
9	4	4	31.40	8.00
-10	-4	-4	74.59	10.60
-10	-4	-4	68.79	12.00
-10	-4	-4	54.89	9.20
-10	-4	-4	80.09	12.60
-10	-4	-4	66.79	11.70
10	4	4	68.69	14.20
-11	-4	-4	244.98	18.80
-11	-4	-4	294.77	19.20
-11	-4	-4	259.87	18.50
-11	-4	-4	257.07	22.70
11	4	4	277.87	22.50
-12	-4	-4	37.80	9.80
-12	-4	-4	26.40	16.50
-12	-4	-4	35.80	8.00
-12	-4	-4	4.60	11.00
12	-5	-4	62.89	18.00
11	-5	-4	12.50	9.70
-11	5	4	28.10	8.90
10	-5	-4	385.16	26.50
-10	5	4	277.37	30.50
-10	5	4	363.76	26.40
9	-5	-4	133.89	16.90
-9	5	4	109.09	14.70

-9	5	4	118.79	20.50
8	-5	-4	478.05	28.70
8	-5	-4	491.35	28.80
-8	5	4	506.25	30.70
-8	5	4	528.35	28.70
7	-5	-4	44.50	8.60
7	-5	-4	28.70	10.50
-7	5	4	35.60	8.20
-7	5	4	36.70	11.50
6	-5	-4	168.98	15.60
6	-5	-4	151.98	13.40
-6	5	4	137.89	12.30
-6	5	4	142.19	13.70
5	-5	-4	468.95	27.50
5	-5	-4	493.85	26.70
5	-5	-4	486.85	25.10
-5	5	4	480.15	25.30
-5	5	4	458.85	26.10
-5	5	4	479.45	26.10
-5	5	4	487.85	24.60
-5	5	4	490.85	23.80
4	-5	-4	539.65	26.10
4	-5	-4	521.35	26.60
-4	5	4	538.15	25.20
-4	5	4	523.25	26.10
-4	5	4	538.25	26.60
3	-5	-4	126.49	8.80
3	-5	-4	124.29	8.20
-3	5	4	147.49	8.40
-3	5	4	129.79	7.10
-3	5	4	121.09	8.00
2	-5	-4	1786.12	78.99
2	-5	-4	1618.94	79.19
-2	5	4	1765.72	78.39
-2	5	4	1730.73	78.89
-2	5	4	1840.22	79.09
1	-5	-4	720.93	33.20
1	-5	-4	701.63	33.60
-1	5	4	726.33	33.30
-1	5	4	717.13	33.90
-1	5	4	745.13	32.60
0	-5	-4	140.59	7.40
0	-5	-4	150.08	7.80
0	5	4	141.89	7.30

0	5	4	127.49	8.60
1	5	4	9.00	1.50
1	5	4	10.30	2.30
2	5	4	298.57	14.50
2	5	4	279.37	13.50
3	5	4	52.19	3.10
3	5	4	47.60	3.50
4	5	4	449.06	22.20
4	5	4	424.56	20.00
-5	-5	-4	11.70	3.40
5	5	4	27.80	5.70
5	5	4	17.20	2.60
-6	-5	-4	2183.98	100.99
-6	-5	-4	2204.08	102.09
-6	-5	-4	2149.19	100.79
-6	-5	-4	2198.58	101.29
-6	-5	-4	2341.17	101.19
6	5	4	2220.28	100.99
-7	-5	-4	551.74	28.40
-7	-5	-4	573.04	28.30
-7	-5	-4	546.85	30.10
-7	-5	-4	498.05	29.50
-7	-5	-4	585.24	27.70
-7	-5	-4	549.45	28.90
7	5	4	557.44	26.90
-8	-5	-4	91.49	14.30
-8	-5	-4	90.09	11.50
-8	-5	-4	98.89	15.00
-8	-5	-4	89.59	11.80
-8	-5	-4	97.59	13.60
-8	-5	-4	91.99	9.80
8	5	4	93.29	9.40
-9	-5	-4	13.70	9.80
-9	-5	-4	2.90	9.00
-9	-5	-4	23.60	8.80
-9	-5	-4	22.40	7.00
-9	-5	-4	12.40	6.10
-9	-5	-4	13.30	7.10
-10	-5	-4	31.80	8.00
-10	-5	-4	13.90	16.80
-10	-5	-4	32.70	7.70
-10	-5	-4	13.70	7.20
-10	-5	-4	10.60	10.20
-10	-5	-4	25.30	9.30

10	5	4	25.40	11.10
10	5	4	43.20	13.40
-11	-5	-4	28.70	8.90
-11	-5	-4	45.80	9.00
-11	-5	-4	25.30	11.70
-11	-5	-4	30.80	8.90
11	5	4	32.90	11.60
-12	-5	-4	90.29	13.20
-12	-5	-4	89.29	16.90
-12	-5	-4	90.49	12.60
-12	-5	-4	102.19	14.10
11	-6	-4	80.19	11.90
-11	6	4	83.29	14.40
10	-6	-4	116.09	16.70
10	-6	-4	52.89	14.70
-10	6	4	83.19	12.10
-10	6	4	87.69	17.20
9	-6	-4	4.50	10.10
9	-6	-4	14.90	9.30
-9	6	4	14.00	13.30
-9	6	4	21.40	8.60
8	-6	-4	-8.00	8.20
8	-6	-4	21.70	8.90
-8	6	4	18.90	11.50
-8	6	4	10.60	8.80
7	-6	-4	202.98	16.90
-7	6	4	168.28	15.30
-7	6	4	166.58	15.60
6	-6	-4	417.06	23.80
6	-6	-4	424.36	25.20
-6	6	4	444.06	25.00
-6	6	4	453.65	23.80
5	-6	-4	286.27	20.60
5	-6	-4	271.17	21.00
5	-6	-4	290.77	17.80
5	-6	-4	276.07	18.20
-5	6	4	276.97	17.30
-5	6	4	281.07	19.70
-5	6	4	327.57	18.70
-5	6	4	299.47	15.60
-5	6	4	268.27	18.70
4	-6	-4	1713.43	77.29
4	-6	-4	1681.03	77.89
-4	6	4	1713.13	77.79

-4	6	4	1728.53	76.29
-4	6	4	1565.14	76.79
-4	6	4	1707.63	77.29
3	-6	-4	13.30	4.60
3	-6	-4	10.70	3.40
-3	6	4	4.10	3.30
-3	6	4	13.20	3.50
-3	6	4	9.10	2.20
2	-6	-4	93.69	6.90
2	-6	-4	93.79	8.30
-2	6	4	115.19	7.80
-2	6	4	103.39	6.80
-2	6	4	105.99	5.70
1	-6	-4	2648.03	118.49
1	-6	-4	2696.23	118.09
-1	6	4	2630.84	117.29
-1	6	4	2423.36	118.89
-1	6	4	2748.72	118.09
0	-6	-4	323.27	15.30
0	-6	-4	332.27	15.90
0	6	4	317.87	16.80
0	6	4	341.67	15.30
0	6	4	270.67	14.80
-1	-6	-4	37.10	3.30
-1	-6	-4	30.50	4.20
1	6	4	43.50	4.70
1	6	4	40.70	3.10
2	6	4	348.27	16.50
2	6	4	359.56	17.70
3	6	4	780.22	38.50
3	6	4	864.91	38.40
3	6	4	907.01	40.00
3	6	4	853.81	40.00
4	6	4	288.07	16.20
4	6	4	287.17	13.60
-5	-6	-4	41.10	6.10
5	6	4	33.60	7.10
5	6	4	32.00	6.50
5	6	4	34.50	4.00
-6	-6	-4	69.99	7.50
-6	-6	-4	65.09	9.90
-6	-6	-4	83.19	13.20
-6	-6	-4	64.39	8.50
-6	-6	-4	65.99	9.60

-6	-6	-4	72.09	10.80
6	6	4	70.49	6.10
-7	-6	-4	335.27	21.40
-7	-6	-4	354.06	20.40
-7	-6	-4	352.96	21.10
-7	-6	-4	364.36	19.30
-7	-6	-4	371.56	22.60
-7	-6	-4	377.46	20.20
7	6	4	348.87	18.60
-8	-6	-4	14.40	6.90
-8	-6	-4	8.90	5.90
-8	-6	-4	21.70	8.20
-8	-6	-4	9.00	5.60
-8	-6	-4	-7.40	6.60
8	6	4	-0.70	5.10
-9	-6	-4	58.39	8.80
-9	-6	-4	46.80	8.00
-9	-6	-4	32.30	9.60
-9	-6	-4	34.30	6.90
-9	-6	-4	36.70	8.50
-9	-6	-4	58.09	10.90
-10	-6	-4	166.88	14.70
-10	-6	-4	176.18	21.40
-10	-6	-4	176.98	15.70
-10	-6	-4	197.28	17.60
-10	-6	-4	164.88	14.10
10	6	4	152.88	21.50
10	6	4	200.88	21.90
-11	-6	-4	144.79	14.00
-11	-6	-4	129.59	14.80
-11	-6	-4	113.19	13.80
11	6	4	109.29	19.70
-12	-6	-4	230.58	16.10
-12	-6	-4	231.28	17.30
-12	-6	-4	204.98	22.50
-12	-6	-4	192.88	19.40
12	6	4	202.88	28.10
11	-7	-4	14.00	10.20
-11	7	4	23.00	9.80
10	-7	-4	107.09	16.30
10	-7	-4	76.39	13.20
-10	7	4	56.49	11.10
-10	7	4	33.90	18.60
9	-7	-4	633.14	33.60

9	-7	-4	624.34	33.80
-9	7	4	565.24	39.10
-9	7	4	606.24	34.60
8	-7	-4	98.29	14.80
8	-7	-4	100.59	15.20
-8	7	4	62.09	13.90
-8	7	4	60.19	11.20
7	-7	-4	99.39	12.80
7	-7	-4	115.09	14.70
-7	7	4	81.19	14.10
-7	7	4	97.99	13.50
6	-7	-4	202.48	17.50
6	-7	-4	205.28	15.10
-6	7	4	202.88	15.20
-6	7	4	239.18	17.40
5	-7	-4	274.87	17.20
5	-7	-4	242.38	19.80
5	-7	-4	239.88	23.80
-5	7	4	301.07	15.20
-5	7	4	280.57	17.10
-5	7	4	270.47	20.00
4	-7	-4	135.29	12.10
4	-7	-4	151.68	14.50
-4	7	4	147.79	12.10
-4	7	4	140.09	11.60
-4	7	4	135.19	12.20
-4	7	4	144.09	8.60
3	-7	-4	621.64	32.70
3	-7	-4	698.03	31.90
-3	7	4	672.43	30.40
-3	7	4	621.34	31.90
-3	7	4	660.03	31.90
2	-7	-4	63.19	8.30
-2	7	4	62.79	4.20
-2	7	4	69.39	8.10
-2	7	4	60.49	5.70
1	-7	-4	246.58	13.00
1	-7	-4	257.67	13.90
-1	7	4	249.88	11.60
-1	7	4	248.48	13.20
-1	7	4	220.68	15.20
0	-7	-4	141.99	8.00
0	-7	-4	137.09	9.00
0	7	4	142.89	10.30

0	7	4	138.79	7.80
0	7	4	139.29	6.70
-1	-7	-4	1297.67	59.99
-1	-7	-4	1310.67	60.79
1	7	4	1354.26	61.09
1	7	4	1339.07	59.99
2	7	4	148.49	10.00
2	7	4	151.88	7.80
-3	-7	-4	5.20	3.00
3	7	4	8.70	1.50
3	7	4	6.20	1.10
3	7	4	6.60	2.20
3	7	4	7.60	1.70
3	7	4	4.70	3.60
4	7	4	15.60	2.70
4	7	4	13.20	4.40
-5	-7	-4	1749.92	76.39
-5	-7	-4	1571.24	72.99
-5	-7	-4	1640.84	75.89
5	7	4	1562.14	72.29
5	7	4	1461.85	75.19
5	7	4	1623.54	75.99
-6	-7	-4	23.60	6.40
-6	-7	-4	47.80	9.50
-6	-7	-4	24.70	6.70
-6	-7	-4	31.80	7.40
-6	-7	-4	40.70	6.40
-6	-7	-4	34.50	6.30
6	7	4	25.50	4.10
-7	-7	-4	563.64	29.30
-7	-7	-4	556.24	31.20
-7	-7	-4	554.24	29.60
-7	-7	-4	581.64	29.00
-7	-7	-4	573.74	29.70
-7	-7	-4	591.84	28.50
7	7	4	568.64	28.00
-8	-7	-4	17.50	6.60
-8	-7	-4	31.30	8.10
-8	-7	-4	21.90	7.50
-8	-7	-4	19.90	9.00
-8	-7	-4	22.80	6.60
-8	-7	-4	20.40	5.70
8	7	4	21.60	6.10
-9	-7	-4	86.69	14.40

-9	-7	-4	85.79	15.10
-9	-7	-4	97.19	12.50
-9	-7	-4	97.89	13.00
-9	-7	-4	93.99	12.40
-9	-7	-4	110.19	16.20
9	7	4	94.89	14.10
-10	-7	-4	1.00	6.90
-10	-7	-4	18.40	7.70
-10	-7	-4	16.50	11.20
-10	-7	-4	8.80	7.10
-10	-7	-4	0.00	8.70
10	7	4	-24.10	13.40
10	7	4	11.30	12.20
-11	-7	-4	9.10	7.80
-11	-7	-4	26.10	10.50
-11	-7	-4	12.40	7.90
-11	-7	-4	11.20	8.80
11	7	4	20.40	14.70
-12	-7	-4	161.38	16.00
-12	-7	-4	167.08	13.70
-12	-7	-4	112.29	18.90
-12	-7	-4	105.09	16.50
11	-8	-4	187.38	17.90
-11	8	4	159.48	17.80
10	-8	-4	149.19	19.10
10	-8	-4	184.48	17.30
-10	8	4	149.29	18.50
9	-8	-4	9.10	9.80
9	-8	-4	3.60	9.50
-9	8	4	-21.20	17.40
-9	8	4	-2.40	9.50
8	-8	-4	35.60	8.60
8	-8	-4	43.00	9.60
-8	8	4	36.90	10.20
-8	8	4	0.60	13.60
7	-8	-4	120.19	13.70
7	-8	-4	127.69	16.00
-7	8	4	140.99	16.30
-7	8	4	109.39	14.80
6	-8	-4	21.10	8.20
6	-8	-4	6.00	6.40
-6	8	4	14.20	7.20
-6	8	4	-5.40	8.30
-6	8	4	9.10	10.60

5	-8	-4	9.00	11.10
5	-8	-4	15.40	9.40
5	-8	-4	16.30	6.40
5	-8	-4	-2.50	11.10
-5	8	4	11.40	6.60
-5	8	4	11.40	12.80
-5	8	4	17.10	4.70
-5	8	4	31.40	15.80
4	-8	-4	642.04	31.50
4	-8	-4	538.35	28.20
4	-8	-4	533.55	31.60
-4	8	4	522.85	29.50
-4	8	4	567.84	26.30
-4	8	4	517.25	27.80
-4	8	4	530.65	30.40
3	-8	-4	440.56	25.40
-3	8	4	469.05	21.40
-3	8	4	428.86	23.70
-3	8	4	473.35	24.70
-3	8	4	431.66	23.40
2	-8	-4	177.68	13.40
-2	8	4	188.58	9.40
-2	8	4	181.38	14.30
-2	8	4	182.98	11.20
1	-8	-4	36.00	6.50
1	-8	-4	34.30	5.30
-1	8	4	35.10	7.90
-1	8	4	40.80	4.80
-1	8	4	36.90	3.00
0	-8	-4	2111.99	93.29
0	-8	-4	1788.62	93.19
0	8	4	2018.10	91.29
0	8	4	2260.87	94.19
-1	-8	-4	458.95	23.00
-1	-8	-4	455.25	21.70
1	8	4	396.76	20.80
1	8	4	468.65	20.50
1	8	4	490.65	21.70
1	8	4	449.06	23.40
-2	-8	-4	191.68	11.60
2	8	4	198.48	12.60
2	8	4	193.38	9.40
2	8	4	202.68	10.20
2	8	4	197.08	9.10

3	8	4	12.00	1.60
3	8	4	10.90	2.70
3	8	4	10.80	2.20
3	8	4	13.30	5.00
-4	-8	-4	18.10	4.10
4	8	4	24.40	5.80
4	8	4	21.60	3.10
4	8	4	14.60	5.90
-5	-8	-4	481.05	24.80
-5	-8	-4	470.25	24.50
-5	-8	-4	457.45	24.90
-5	-8	-4	508.65	23.80
-5	-8	-4	445.56	25.50
5	8	4	486.05	22.80
-6	-8	-4	10.80	6.10
-6	-8	-4	18.70	6.30
-6	-8	-4	16.40	4.60
-6	-8	-4	17.60	6.20
-6	-8	-4	25.30	8.70
-6	-8	-4	16.30	6.20
6	8	4	14.90	4.00
-7	-8	-4	242.08	17.80
-7	-8	-4	239.48	19.20
-7	-8	-4	220.18	13.60
-7	-8	-4	189.68	17.60
-7	-8	-4	213.98	15.40
-7	-8	-4	201.78	15.30
7	8	4	201.48	13.30
-8	-8	-4	275.57	16.60
-8	-8	-4	253.77	20.70
-8	-8	-4	281.67	18.90
-8	-8	-4	307.07	18.90
-8	-8	-4	290.17	18.30
-8	-8	-4	268.17	17.60
8	8	4	254.77	17.30
-9	-8	-4	9.20	6.90
-9	-8	-4	1.20	6.90
-9	-8	-4	4.60	8.50
-9	-8	-4	-8.40	7.50
-9	-8	-4	3.10	9.50
-9	-8	-4	5.90	6.60
9	8	4	22.80	14.40
9	8	4	-26.00	13.10
-10	-8	-4	403.16	23.20

-10	-8	-4	396.56	29.40
-10	-8	-4	421.26	24.00
-10	-8	-4	420.46	25.20
-10	-8	-4	345.57	22.50
10	8	4	373.86	30.30
-11	-8	-4	311.17	19.90
-11	-8	-4	300.67	20.50
-11	-8	-4	351.66	22.20
-11	-8	-4	274.27	24.70
-11	-8	-4	326.37	20.60
-12	-8	-4	24.80	11.80
-12	-8	-4	5.20	12.00
-12	-8	-4	25.20	7.60
-12	-8	-4	40.50	10.50
11	-9	-4	76.79	12.70
11	-9	-4	67.09	19.20
-11	9	4	75.09	11.80
10	-9	-4	209.48	18.80
10	-9	-4	166.18	17.80
-10	9	4	161.18	19.80
9	-9	-4	29.50	11.20
9	-9	-4	28.70	13.70
-9	9	4	5.40	19.70
-9	9	4	14.50	11.10
8	-9	-4	222.68	19.10
8	-9	-4	232.18	17.80
-8	9	4	207.28	22.50
-8	9	4	220.18	27.40
7	-9	-4	7.10	7.10
7	-9	-4	13.60	9.10
-7	9	4	7.90	9.30
-7	9	4	-0.20	9.30
-7	9	4	-2.00	11.10
6	-9	-4	22.20	16.90
6	-9	-4	31.50	6.40
6	-9	-4	28.50	9.30
-6	9	4	39.70	8.80
-6	9	4	46.00	11.90
-6	9	4	25.90	9.20
5	-9	-4	554.04	28.50
5	-9	-4	573.44	31.00
5	-9	-4	530.75	31.20
-5	9	4	555.54	27.30
-5	9	4	545.75	28.80

-5	9	4	536.15	32.80
4	-9	-4	330.47	22.70
4	-9	-4	307.37	25.40
4	-9	-4	312.27	27.20
4	-9	-4	378.36	20.30
-4	9	4	334.47	24.20
-4	9	4	381.76	17.90
-4	9	4	363.56	20.40
3	-9	-4	11.20	5.90
3	-9	-4	1.00	9.40
-3	9	4	15.00	8.40
-3	9	4	5.90	6.50
-3	9	4	-1.10	5.70
2	-9	-4	1086.89	49.10
-2	9	4	1025.00	45.90
-2	9	4	1006.50	47.50
-2	9	4	911.11	48.50
-2	9	4	1003.80	49.99
1	-9	-4	175.18	12.50
-1	9	4	171.08	15.40
-1	9	4	149.39	10.00
-1	9	4	155.48	8.20
0	-9	-4	166.98	13.20
0	-9	-4	163.78	10.40
0	-9	-4	176.58	12.20
0	9	4	169.98	8.50
0	9	4	183.08	14.20
0	9	4	177.68	10.10
-1	-9	-4	50.49	7.10
-1	-9	-4	49.20	6.80
1	9	4	43.10	3.00
1	9	4	43.90	7.10
2	9	4	86.69	5.90
2	9	4	80.09	4.40
2	9	4	76.29	10.00
3	9	4	54.79	4.80
3	9	4	68.79	9.90
3	9	4	58.09	3.80
-4	-9	-4	256.97	13.60
4	9	4	254.37	18.00
4	9	4	221.68	17.00
4	9	4	239.08	12.20
-5	-9	-4	855.21	37.40
-5	-9	-4	732.73	38.10

-5	-9	-4	761.22	40.40
-5	-9	-4	767.42	38.20
-5	-9	-4	820.02	38.30
-5	-9	-4	766.22	37.70
-6	-9	-4	2287.57	102.79
-6	-9	-4	2214.38	103.09
-6	-9	-4	2273.87	103.19
-6	-9	-4	2191.38	102.99
-6	-9	-4	2237.98	103.89
-6	-9	-4	2358.26	102.59
6	9	4	2217.98	102.19
-7	-9	-4	69.09	10.60
-7	-9	-4	57.09	7.90
-7	-9	-4	44.50	14.00
-7	-9	-4	67.29	11.80
-7	-9	-4	50.49	8.30
7	9	4	58.99	8.60
-8	-9	-4	21.70	6.90
-8	-9	-4	34.70	9.90
-8	-9	-4	35.30	7.10
-8	-9	-4	36.60	8.10
-8	-9	-4	27.70	7.60
-8	-9	-4	33.50	6.50
8	9	4	37.70	7.90
-9	-9	-4	13.70	6.50
-9	-9	-4	12.70	8.30
-9	-9	-4	10.50	8.60
-9	-9	-4	24.70	8.20
-9	-9	-4	10.50	7.00
9	9	4	12.30	9.90
-10	-9	-4	17.30	11.50
-10	-9	-4	2.10	11.50
-10	-9	-4	5.70	8.10
-10	-9	-4	12.70	7.80
-10	-9	-4	-3.60	11.30
-11	-9	-4	145.09	17.20
-11	-9	-4	119.39	13.50
-11	-9	-4	109.89	14.90
-11	-9	-4	98.69	14.90
-11	-9	-4	164.68	19.00
-12	-9	-4	7.00	12.70
-12	-9	-4	45.10	17.80
-12	-9	-4	37.50	8.40
-12	-9	-4	10.60	13.50

11	-10	-4	7.00	12.60
-11	10	4	2.20	9.90
10	-10	-4	193.08	18.80
10	-10	-4	168.38	17.90
-10	10	4	142.19	19.20
9	-10	-4	5.80	10.60
9	-10	-4	27.20	9.90
-9	10	4	14.80	12.50
8	-10	-4	18.50	9.80
8	-10	-4	18.50	7.90
-8	10	4	-4.00	16.00
-8	10	4	20.20	11.60
7	-10	-4	344.57	20.40
7	-10	-4	288.57	21.70
-7	10	4	332.77	26.00
-7	10	4	272.57	24.30
-7	10	4	299.07	22.20
6	-10	-4	254.47	22.80
6	-10	-4	255.87	20.50
6	-10	-4	273.87	17.80
-6	10	4	304.67	23.20
-6	10	4	234.98	19.60
-6	10	4	262.77	22.50
5	-10	-4	209.98	19.00
5	-10	-4	218.28	15.40
5	-10	-4	242.98	21.10
-5	10	4	207.88	21.90
-5	10	4	210.48	16.70
4	-10	-4	137.99	21.70
4	-10	-4	130.49	15.90
4	-10	-4	131.39	12.90
4	-10	-4	127.29	15.70
-4	10	4	179.48	14.10
-4	10	4	164.18	25.50
-4	10	4	161.38	9.90
3	-10	-4	263.17	21.40
3	-10	-4	300.37	19.00
3	-10	-4	290.07	19.60
3	-10	-4	308.97	24.20
-3	10	4	333.47	15.50
-3	10	4	294.17	24.40
-3	10	4	331.27	19.90
2	-10	-4	91.39	11.40
2	-10	-4	69.89	10.60

-2	10	4	105.79	13.70
-2	10	4	75.29	13.10
-2	10	4	87.29	5.80
-2	10	4	85.59	9.40
1	-10	-4	1653.93	74.49
1	-10	-4	1503.55	73.19
-1	10	4	1560.64	71.59
-1	10	4	1505.35	73.09
-1	10	4	1737.03	76.39
0	-10	-4	27.50	6.90
0	10	4	19.70	4.20
0	10	4	22.70	2.80
0	10	4	26.50	8.30
-1	-10	-4	605.44	33.20
-1	-10	-4	649.54	31.40
1	10	4	655.03	34.40
1	10	4	685.03	31.20
1	10	4	669.73	29.80
-2	-10	-4	81.39	8.80
-2	-10	-4	93.19	8.90
2	10	4	99.19	5.30
2	10	4	97.09	12.70
2	10	4	99.19	7.10
-3	-10	-4	130.09	9.30
3	10	4	114.69	7.70
3	10	4	111.69	6.40
3	10	4	118.29	14.00
-4	-10	-4	532.65	26.90
-4	-10	-4	512.25	27.30
-4	-10	-4	528.25	27.20
-4	-10	-4	529.25	26.50
-4	-10	-4	491.45	27.40
4	10	4	537.75	24.60
-5	-10	-4	15.90	6.70
-5	-10	-4	33.90	10.60
-5	-10	-4	34.80	6.70
-5	-10	-4	31.50	6.80
-5	-10	-4	32.70	5.60
-5	-10	-4	34.30	7.50
-5	-10	-4	31.10	10.70
5	10	4	24.70	4.20
-6	-10	-4	422.96	22.40
-6	-10	-4	426.36	21.80
-6	-10	-4	413.96	25.00

-6	-10	-4	402.46	23.10
-6	-10	-4	431.26	23.20
-6	-10	-4	402.36	22.90
6	10	4	405.96	21.50
-7	-10	-4	561.14	29.40
-7	-10	-4	557.74	28.10
-7	-10	-4	579.24	29.50
-7	-10	-4	574.94	31.20
-7	-10	-4	540.45	28.50
-7	-10	-4	538.35	29.20
7	10	4	532.25	28.60
-8	-10	-4	15.90	7.20
-8	-10	-4	22.20	7.00
-8	-10	-4	33.40	7.70
-8	-10	-4	26.20	8.20
-8	-10	-4	19.30	7.80
8	10	4	18.50	8.00
-9	-10	-4	72.99	9.60
-9	-10	-4	54.69	9.30
-9	-10	-4	63.89	10.00
-9	-10	-4	36.20	7.90
-9	-10	-4	54.79	8.30
9	10	4	54.29	12.10
-10	-10	-4	42.70	9.60
-10	-10	-4	36.40	9.40
-10	-10	-4	53.59	10.20
-10	-10	-4	35.10	8.90
-10	-10	-4	63.39	11.50
-11	-10	-4	329.87	22.90
-11	-10	-4	314.67	20.50
-11	-10	-4	331.57	21.70
-11	-10	-4	320.67	20.00
-11	-10	-4	280.37	25.70
-12	-10	-4	77.19	12.10
-12	-10	-4	84.09	13.70
-12	-10	-4	90.09	14.80
10	-11	-4	271.67	19.60
10	-11	-4	254.97	21.40
-10	11	4	192.48	20.70
9	-11	-4	302.07	22.50
9	-11	-4	353.36	21.90
-9	11	4	318.77	26.10
8	-11	-4	30.50	10.60
8	-11	-4	43.30	8.90

-8	11	4	19.40	13.00
-8	11	4	7.10	17.40
7	-11	-4	70.99	12.50
7	-11	-4	44.30	10.20
-7	11	4	50.09	13.80
-7	11	4	38.70	11.90
-7	11	4	27.20	12.80
6	-11	-4	256.97	22.60
6	-11	-4	277.37	21.80
-6	11	4	286.17	20.70
-6	11	4	278.97	24.80
-6	11	4	243.98	23.40
5	-11	-4	168.18	18.90
5	-11	-4	206.58	17.90
5	-11	-4	168.88	21.50
5	-11	-4	179.28	17.70
-5	11	4	209.88	14.40
-5	11	4	212.98	22.00
-5	11	4	210.08	17.20
4	-11	-4	115.09	16.10
4	-11	-4	137.19	16.80
4	-11	-4	126.09	15.10
4	-11	-4	125.49	12.50
-4	11	4	137.79	20.50
-4	11	4	111.29	9.40
-4	11	4	133.79	14.50
3	-11	-4	6.50	11.90
3	-11	-4	8.90	7.00
3	-11	-4	1.40	9.60
3	-11	-4	1.10	7.30
3	-11	-4	-18.10	11.50
-3	11	4	14.70	8.00
-3	11	4	-0.10	2.90
-3	11	4	5.00	13.20
2	-11	-4	19.60	7.00
2	-11	-4	28.50	10.80
2	-11	-4	17.50	10.20
-2	11	4	24.40	3.90
-2	11	4	19.00	13.30
-2	11	4	20.90	8.40
1	-11	-4	740.33	37.80
1	-11	-4	771.82	39.60
-1	11	4	708.13	37.30
-1	11	4	780.72	35.30

-1	11	4	840.02	42.30
0	-11	-4	231.48	17.90
0	-11	-4	224.78	15.20
0	11	4	229.98	14.20
0	11	4	247.48	11.90
0	11	4	271.67	20.10
-1	-11	-4	356.86	18.70
-1	-11	-4	322.17	21.70
1	11	4	331.57	23.30
1	11	4	339.67	17.80
1	11	4	329.37	16.00
-2	-11	-4	300.77	16.40
-2	-11	-4	269.27	16.10
2	11	4	293.87	13.50
2	11	4	229.58	21.80
2	11	4	277.77	15.10
-3	-11	-4	13.30	5.90
-3	-11	-4	9.80	7.00
-3	-11	-4	7.90	7.90
-3	-11	-4	19.10	4.80
3	11	4	17.20	2.50
3	11	4	13.00	4.30
-4	-11	-4	590.84	31.70
-4	-11	-4	606.84	30.40
-4	-11	-4	594.34	31.10
-4	-11	-4	604.74	31.50
-4	-11	-4	593.54	30.50
-4	-11	-4	658.83	30.10
4	11	4	621.84	28.70
-5	-11	-4	953.80	45.90
-5	-11	-4	942.41	48.50
-5	-11	-4	923.81	45.70
-5	-11	-4	975.90	46.30
-5	-11	-4	974.20	47.30
-5	-11	-4	1031.80	45.70
-5	-11	-4	955.20	46.60
5	11	4	936.41	44.80
-6	-11	-4	46.70	10.40
-6	-11	-4	52.19	8.90
-6	-11	-4	57.99	8.40
6	11	4	43.10	6.60
-7	-11	-4	225.58	17.10
-7	-11	-4	243.18	15.00
-7	-11	-4	225.48	15.70

-7	-11	-4	239.98	17.00
-7	-11	-4	229.28	16.60
7	11	4	220.38	16.30
-8	-11	-4	-2.90	7.30
-8	-11	-4	-4.60	7.40
-8	-11	-4	2.30	6.50
-8	-11	-4	-7.00	6.90
-8	-11	-4	8.50	6.90
8	11	4	4.40	8.90
-9	-11	-4	55.89	8.90
-9	-11	-4	39.10	7.90
-9	-11	-4	31.10	8.40
-9	-11	-4	48.70	9.00
-9	-11	-4	47.80	9.30
9	11	4	38.30	12.70
-10	-11	-4	44.30	9.70
-10	-11	-4	26.00	8.50
-10	-11	-4	19.90	8.70
-10	-11	-4	25.70	8.80
-10	-11	-4	23.50	8.30
-11	-11	-4	34.90	8.40
-11	-11	-4	34.60	10.40
-11	-11	-4	41.10	9.90
-11	-11	-4	32.20	10.60
-11	-11	-4	39.40	9.60
10	-12	-4	148.39	26.70
10	-12	-4	106.69	15.10
-10	12	4	64.69	12.40
9	-12	-4	11.00	8.30
9	-12	-4	37.00	11.70
-9	12	4	8.90	11.30
8	-12	-4	39.80	8.70
-8	12	4	34.80	13.60
7	-12	-4	86.39	13.00
7	-12	-4	89.29	13.00
-7	12	4	63.49	14.10
-7	12	4	82.99	15.70
-7	12	4	69.09	14.30
6	-12	-4	11.70	11.10
6	-12	-4	12.60	6.90
6	-12	-4	1.90	11.90
-6	12	4	13.80	10.70
-6	12	4	15.30	12.50
-6	12	4	5.60	12.60

5	-12	-4	125.69	22.40
5	-12	-4	93.89	12.20
5	-12	-4	91.79	15.20
-5	12	4	99.89	12.60
-5	12	4	79.39	12.60
4	-12	-4	205.88	25.10
4	-12	-4	267.57	16.40
4	-12	-4	241.78	18.40
4	-12	-4	214.58	25.70
-4	12	4	228.68	14.10
-4	12	4	216.48	23.20
-4	12	4	249.97	18.90
3	-12	-4	394.66	27.40
3	-12	-4	437.06	24.70
3	-12	-4	363.76	27.00
3	-12	-4	388.86	28.70
3	-12	-4	476.05	24.50
-3	12	4	485.95	22.00
-3	12	4	463.05	30.40
-3	12	4	487.25	26.30
2	-12	-4	173.58	16.30
2	-12	-4	206.08	15.20
2	-12	-4	181.78	19.30
2	-12	-4	223.68	17.30
2	-12	-4	181.68	18.10
-2	12	4	213.28	11.90
-2	12	4	200.68	19.90
1	-12	-4	162.88	16.90
1	-12	-4	159.88	15.30
1	-12	-4	147.39	14.60
1	-12	-4	211.98	16.40
-1	12	4	177.28	9.60
0	-12	-4	1583.94	73.69
0	-12	-4	1537.15	73.59
0	-12	-4	1738.93	74.49
0	-12	-4	1489.95	74.09
0	-12	-4	1529.75	73.79
0	-12	-4	1549.54	76.49
0	12	4	1511.85	73.69
0	12	4	1668.23	71.89
0	12	4	1699.13	78.09
-1	-12	-4	54.09	8.60
-1	-12	-4	63.79	9.50
-1	-12	-4	71.39	9.40

-1	-12	-4	71.59	10.00
-1	-12	-4	82.79	10.90
-1	-12	-4	61.69	11.30
1	12	4	67.59	9.00
1	12	4	78.19	5.10
-2	-12	-4	18.80	8.30
-2	-12	-4	9.50	6.30
-2	-12	-4	4.60	6.00
-2	-12	-4	3.90	5.40
-2	-12	-4	9.30	7.70
2	12	4	8.80	5.30
2	12	4	12.30	2.60
-3	-12	-4	116.59	15.40
-3	-12	-4	116.59	13.30
-3	-12	-4	128.39	13.30
-3	-12	-4	137.29	11.70
-3	-12	-4	121.29	13.70
-3	-12	-4	129.49	11.00
3	12	4	134.99	7.70
-4	-12	-4	15.40	7.60
-4	-12	-4	23.20	5.40
-4	-12	-4	30.90	7.50
-4	-12	-4	25.80	6.50
-4	-12	-4	25.20	6.40
-4	-12	-4	30.40	7.90
4	12	4	28.60	4.80
-5	-12	-4	1.20	7.50
-5	-12	-4	13.80	7.10
-5	-12	-4	18.00	8.60
-5	-12	-4	14.00	6.00
-5	-12	-4	12.20	5.90
-5	-12	-4	8.60	5.10
5	12	4	7.10	4.00
-6	-12	-4	47.90	7.60
-6	-12	-4	53.29	7.40
-6	-12	-4	47.70	8.10
-6	-12	-4	39.90	7.70
-6	-12	-4	37.10	11.00
-6	-12	-4	51.39	8.60
6	12	4	40.20	7.00
-7	-12	-4	245.88	16.00
-7	-12	-4	262.07	18.10
-7	-12	-4	230.38	17.40
-7	-12	-4	221.18	16.80

-7	-12	-4	228.68	16.60
7	12	4	244.18	17.90
-8	-12	-4	140.09	14.00
-8	-12	-4	155.58	14.70
-8	-12	-4	161.58	15.30
-8	-12	-4	152.08	15.60
-8	-12	-4	151.98	14.30
8	12	4	114.99	19.50
-9	-12	-4	-0.20	7.80
-9	-12	-4	-16.50	7.90
-9	-12	-4	5.60	8.00
-9	-12	-4	0.70	7.30
-9	-12	-4	-3.20	7.30
9	12	4	-28.80	13.40
-10	-12	-4	580.14	29.20
-10	-12	-4	542.25	29.90
-10	-12	-4	553.04	29.30
-10	-12	-4	509.75	29.50
-10	-12	-4	510.75	29.20
-11	-12	-4	96.69	15.10
-11	-12	-4	91.99	15.70
-11	-12	-4	72.19	12.10
-11	-12	-4	95.79	14.60
10	-13	-4	-35.50	25.80
10	-13	-4	-0.40	9.40
-10	13	4	11.20	9.90
9	-13	-4	45.00	15.90
9	-13	-4	80.89	13.20
-9	13	4	63.19	12.60
8	-13	-4	261.87	23.30
8	-13	-4	283.77	19.20
-8	13	4	234.28	27.40
7	-13	-4	98.59	16.90
7	-13	-4	92.19	13.20
7	-13	-4	77.99	14.20
-7	13	4	45.00	13.90
-7	13	4	49.40	14.20
-7	13	4	69.99	15.10
6	-13	-4	2.40	10.50
-6	13	4	5.10	12.80
-6	13	4	15.20	13.20
-6	13	4	-5.60	11.60
5	-13	-4	685.73	36.20
5	-13	-4	685.23	34.60

-5	13	4	684.03	37.60
-5	13	4	673.13	38.80
-5	13	4	672.33	35.50
4	-13	-4	183.68	25.40
4	-13	-4	204.58	20.10
4	-13	-4	219.48	15.60
4	-13	-4	169.78	25.10
-4	13	4	191.58	18.20
-4	13	4	161.08	21.90
-4	13	4	220.18	13.70
3	-13	-4	81.69	12.30
3	-13	-4	76.39	13.80
3	-13	-4	78.39	12.60
3	-13	-4	71.99	12.30
-3	13	4	85.99	7.80
-3	13	4	80.29	10.90
-3	13	4	60.09	13.20
2	-13	-4	603.94	32.30
2	-13	-4	634.74	33.30
2	-13	-4	589.44	34.00
2	-13	-4	628.54	34.00
2	-13	-4	592.24	31.90
-2	13	4	646.74	29.70
-2	13	4	649.74	33.00
1	-13	-4	77.69	11.40
1	-13	-4	86.09	12.90
1	-13	-4	79.09	12.00
1	-13	-4	98.99	14.00
1	-13	-4	88.49	13.70
-1	13	4	69.39	6.00
0	-13	-4	6.50	7.40
0	-13	-4	10.80	7.00
0	-13	-4	14.70	7.30
0	-13	-4	2.90	8.70
0	-13	-4	14.20	7.30
0	13	4	14.50	2.90
-1	-13	-4	46.10	9.90
-1	-13	-4	44.20	7.30
-1	-13	-4	59.99	10.30
-1	-13	-4	50.49	8.00
-1	-13	-4	50.49	7.50
-1	-13	-4	49.90	8.20
-1	-13	-4	39.60	9.20
1	13	4	31.40	7.90

1	13	4	37.60	4.30
-2	-13	-4	65.59	9.20
-2	-13	-4	61.59	9.70
-2	-13	-4	67.89	8.60
-2	-13	-4	76.29	12.00
-2	-13	-4	66.29	8.70
-2	-13	-4	82.99	11.40
-2	-13	-4	75.99	10.00
2	13	4	62.89	5.20
-3	-13	-4	30.30	7.10
-3	-13	-4	45.20	6.80
-3	-13	-4	36.20	8.20
-3	-13	-4	57.29	9.60
-3	-13	-4	61.59	10.10
-3	-13	-4	44.50	8.50
3	13	4	45.40	4.80
-4	-13	-4	185.38	15.90
-4	-13	-4	253.17	17.40
-4	-13	-4	181.98	16.60
-4	-13	-4	198.38	14.40
-4	-13	-4	235.38	14.60
-4	-13	-4	230.48	16.40
4	13	4	212.28	12.00
-5	-13	-4	497.55	27.00
-5	-13	-4	497.35	26.00
-5	-13	-4	502.45	28.50
-5	-13	-4	477.55	26.70
-5	-13	-4	500.15	27.40
5	13	4	478.15	24.80
-6	-13	-4	1668.53	75.59
-6	-13	-4	1520.35	76.19
-6	-13	-4	1611.14	75.69
-6	-13	-4	1724.23	76.59
-6	-13	-4	1634.64	75.89
-6	-13	-4	1693.93	75.49
6	13	4	1491.05	75.29
-7	-13	-4	30.40	7.70
-7	-13	-4	39.70	7.80
-7	-13	-4	31.10	8.40
-7	-13	-4	37.60	8.40
-7	-13	-4	39.40	8.50
7	13	4	30.90	9.20
-8	-13	-4	47.40	9.10
-8	-13	-4	43.10	8.60

-8	-13	-4	58.29	9.20
-8	-13	-4	77.69	13.00
8	13	4	68.59	12.50
-9	-13	-4	65.69	11.40
-9	-13	-4	45.10	8.70
9	13	4	27.30	16.10
-10	-13	-4	5.70	7.50
-10	-13	-4	19.80	7.80
-10	-13	-4	30.40	10.10
-10	-13	-4	9.30	7.40
-10	-13	-4	10.60	9.20
-11	-13	-4	89.19	14.00
-11	-13	-4	92.99	12.60
-11	-13	-4	149.49	24.40
-11	-13	-4	93.09	15.70
11	13	4	110.29	20.00
9	-14	-4	28.20	8.10
-9	14	4	1.80	10.20
8	-14	-4	15.00	7.70
-8	14	4	20.40	12.90
7	-14	-4	275.27	19.20
-7	14	4	268.67	26.20
-7	14	4	269.57	27.00
6	-14	-4	172.68	20.40
6	-14	-4	214.38	16.40
-6	14	4	209.78	24.30
-6	14	4	217.88	25.00
-6	14	4	215.78	24.00
5	-14	-4	118.49	15.60
5	-14	-4	93.79	13.40
-5	14	4	116.59	20.40
-5	14	4	129.59	18.90
4	-14	-4	43.70	7.90
4	-14	-4	49.10	15.40
4	-14	-4	60.29	10.00
4	-14	-4	57.89	15.30
-4	14	4	51.89	11.00
-4	14	4	52.29	13.40
3	-14	-4	112.09	13.40
3	-14	-4	114.69	13.60
3	-14	-4	91.79	15.40
-3	14	4	115.39	9.80
-3	14	4	120.79	19.60
-3	14	4	127.19	15.80

2	-14	-4	-4.50	8.70
2	-14	-4	9.20	8.70
2	-14	-4	1.40	7.10
2	-14	-4	-0.70	9.90
2	-14	-4	13.20	8.30
-2	14	4	12.40	7.60
-2	14	4	8.10	3.80
1	-14	-4	945.71	49.00
1	-14	-4	960.60	47.80
1	-14	-4	1045.70	48.90
1	-14	-4	1021.70	48.50
1	-14	-4	986.40	48.00
-1	14	4	966.70	45.70
0	-14	-4	344.57	21.10
0	-14	-4	378.26	22.50
0	-14	-4	403.76	22.10
0	-14	-4	351.76	21.50
0	-14	-4	355.56	21.70
0	14	4	350.06	18.00
-1	-14	-4	1168.18	57.09
-1	-14	-4	1236.28	56.99
-1	-14	-4	1196.88	56.49
-1	-14	-4	1185.98	56.19
-1	-14	-4	1176.88	56.79
1	14	4	1191.68	54.59
-2	-14	-4	30.20	6.60
-2	-14	-4	32.80	10.70
-2	-14	-4	15.80	8.00
-2	-14	-4	33.30	6.90
-2	-14	-4	29.90	8.10
-2	-14	-4	21.50	7.10
2	14	4	29.60	4.40
-3	-14	-4	69.69	12.30
-3	-14	-4	72.59	10.30
-3	-14	-4	85.49	11.40
-3	-14	-4	85.09	10.50
-3	-14	-4	93.39	15.50
-3	-14	-4	85.39	11.40
-3	-14	-4	84.09	13.00
3	14	4	82.09	6.60
-4	-14	-4	239.38	16.70
-4	-14	-4	196.38	18.00
-4	-14	-4	243.58	15.80
4	14	4	225.18	13.00

-5	-14	-4	23.90	8.00
-5	-14	-4	15.10	8.00
-5	-14	-4	25.60	6.60
-5	-14	-4	25.00	8.50
-5	-14	-4	45.50	8.90
-5	-14	-4	30.20	7.50
5	14	4	18.10	5.40
-6	-14	-4	443.46	26.40
-6	-14	-4	443.26	26.20
-6	-14	-4	475.05	25.60
-6	-14	-4	465.25	27.30
-6	-14	-4	448.76	26.30
-6	-14	-4	470.15	24.90
6	14	4	432.66	25.30
-7	-14	-4	145.19	14.70
-7	-14	-4	162.48	13.60
-7	-14	-4	149.69	16.40
-7	-14	-4	159.78	15.10
-7	-14	-4	175.68	24.90
-7	-14	-4	131.29	15.40
-8	-14	-4	1.50	7.40
-8	-14	-4	-0.30	7.90
-8	-14	-4	5.30	6.70
-8	-14	-4	8.70	7.50
-8	-14	-4	-4.70	7.90
8	14	4	8.00	12.80
-9	-14	-4	110.29	13.70
-9	-14	-4	79.69	12.60
-9	-14	-4	67.79	10.50
9	14	4	64.69	20.90
-10	-14	-4	13.20	7.70
-10	-14	-4	20.10	7.40
-10	-14	-4	29.20	8.70
-10	-14	-4	10.30	12.70
-10	-14	-4	29.70	8.60
10	14	4	21.60	14.40
-11	-14	-4	229.68	16.50
-11	-14	-4	228.28	19.70
-11	-14	-4	241.38	17.30
11	14	4	250.37	23.90
9	-15	-4	128.69	12.10
-9	15	4	105.29	15.70
8	-15	-4	90.09	12.30
-8	15	4	106.69	18.50

7	-15	-4	14.60	8.10
7	-15	-4	1.30	15.80
-7	15	4	18.00	17.10
-7	15	4	27.20	15.60
-7	15	4	30.00	14.70
6	-15	-4	485.95	26.60
6	-15	-4	472.05	29.60
-6	15	4	459.95	32.30
-6	15	4	503.75	32.10
-6	15	4	446.16	31.50
5	-15	-4	54.29	18.10
5	-15	-4	86.39	14.40
-5	15	4	86.09	14.50
-5	15	4	79.39	17.20
4	-15	-4	27.40	9.30
4	-15	-4	29.00	8.30
4	-15	-4	28.70	14.90
4	-15	-4	26.70	14.90
-4	15	4	29.70	8.50
-4	15	4	43.80	12.30
-4	15	4	21.80	10.70
3	-15	-4	102.59	15.00
3	-15	-4	79.69	12.90
3	-15	-4	92.09	13.10
3	-15	-4	77.09	13.40
-3	15	4	78.59	11.50
-3	15	4	85.59	13.10
-3	15	4	98.29	10.40
2	-15	-4	183.88	17.70
2	-15	-4	191.18	17.10
-2	15	4	175.28	11.90
-2	15	4	183.78	15.90
1	-15	-4	993.50	52.99
1	-15	-4	1084.39	53.19
1	-15	-4	1048.30	51.19
1	-15	-4	1093.99	52.79
1	-15	-4	1039.20	51.39
-1	15	4	1113.29	51.89
-1	15	4	1062.89	49.30
0	-15	-4	77.49	13.00
0	-15	-4	94.79	13.50
0	-15	-4	106.59	12.50
0	-15	-4	116.09	13.10
0	15	4	98.89	7.50

-1	-15	-4	293.47	19.90
-1	-15	-4	298.27	18.50
-1	-15	-4	306.57	20.60
-1	-15	-4	285.97	19.30
-1	-15	-4	297.47	18.90
1	15	4	314.27	15.60
-2	-15	-4	63.39	11.70
-2	-15	-4	96.19	12.40
-2	-15	-4	84.79	12.40
-2	-15	-4	83.19	10.30
-2	-15	-4	83.09	11.80
-2	-15	-4	91.29	11.20
2	15	4	90.39	7.00
-3	-15	-4	12.30	8.80
-3	-15	-4	0.10	6.90
-3	-15	-4	9.80	13.70
-3	-15	-4	14.80	6.30
-3	-15	-4	-3.30	5.80
-3	-15	-4	-6.60	7.20
3	15	4	11.70	3.80
-4	-15	-4	186.08	15.80
-4	-15	-4	198.78	14.90
-4	-15	-4	197.18	16.70
-4	-15	-4	190.78	17.50
-4	-15	-4	183.78	16.30
-4	-15	-4	216.98	14.70
4	15	4	204.98	12.50
-5	-15	-4	234.48	18.70
-5	-15	-4	228.28	16.40
-5	-15	-4	283.77	18.60
-5	-15	-4	236.78	18.20
-5	-15	-4	246.58	17.20
-5	-15	-4	272.87	19.10
5	15	4	253.17	15.80
-6	-15	-4	464.35	26.40
-6	-15	-4	431.26	25.50
-6	-15	-4	439.16	27.00
-6	-15	-4	477.55	25.10
-6	-15	-4	471.15	26.80
-6	-15	-4	432.46	26.60
6	15	4	455.35	25.80
-7	-15	-4	275.47	23.30
-7	-15	-4	231.48	16.40
-7	-15	-4	204.68	18.10

-7	-15	-4	243.68	18.60
-7	-15	-4	203.78	17.40
-7	-15	-4	242.38	18.60
7	15	4	215.28	20.40
-8	-15	-4	39.40	9.60
-8	-15	-4	28.00	8.20
-8	-15	-4	21.20	8.30
-8	-15	-4	28.00	9.30
-8	-15	-4	48.00	10.10
8	15	4	21.40	14.30
-9	-15	-4	12.30	6.80
-9	-15	-4	-11.10	7.20
-9	-15	-4	-7.00	7.80
-9	-15	-4	16.00	8.90
-9	-15	-4	6.40	8.10
9	15	4	-13.90	15.70
-10	-15	-4	30.90	8.80
-10	-15	-4	21.70	7.40
-10	-15	-4	18.10	9.80
-10	-15	-4	35.00	7.50
10	15	4	70.79	16.50
9	-16	-4	-0.80	7.50
-9	16	4	-2.40	8.70
8	-16	-4	126.49	13.20
-8	16	4	127.39	17.20
7	-16	-4	40.50	15.50
7	-16	-4	17.50	7.60
-7	16	4	23.90	11.90
-7	16	4	5.70	14.90
6	-16	-4	94.89	13.70
-6	16	4	133.09	19.50
-6	16	4	83.59	16.00
5	-16	-4	169.48	27.30
5	-16	-4	146.59	17.60
5	-16	-4	147.59	15.50
-5	16	4	160.48	21.70
-5	16	4	152.98	21.40
4	-16	-4	202.48	26.30
4	-16	-4	191.98	18.30
4	-16	-4	252.37	25.80
4	-16	-4	204.08	17.40
-4	16	4	225.88	21.00
-4	16	4	210.38	21.60
-4	16	4	182.28	19.20

3	-16	-4	-6.00	10.90
3	-16	-4	-11.00	12.10
3	-16	-4	11.00	8.40
3	-16	-4	13.50	7.80
-3	16	4	14.30	7.00
-3	16	4	1.30	9.10
-3	16	4	18.00	10.80
2	-16	-4	230.38	17.70
2	-16	-4	230.08	19.60
2	-16	-4	250.67	19.30
2	-16	-4	230.78	19.90
2	-16	-4	245.48	19.40
-2	16	4	230.38	14.80
-2	16	4	222.08	18.30
1	-16	-4	500.25	27.40
1	-16	-4	514.15	28.00
1	-16	-4	423.56	30.10
1	-16	-4	472.35	27.20
1	-16	-4	501.05	27.20
-1	16	4	467.15	27.00
-1	16	4	489.65	24.40
0	-16	-4	1273.47	62.39
0	-16	-4	1330.17	62.89
0	-16	-4	1325.67	62.39
0	-16	-4	1359.46	62.89
0	16	4	1308.07	60.79
-1	-16	-4	8.90	9.70
-1	-16	-4	-3.10	7.70
-1	-16	-4	-8.50	6.20
-1	-16	-4	9.50	6.90
-1	-16	-4	-1.00	6.60
1	16	4	5.40	3.60
-2	-16	-4	7.00	6.40
-2	-16	-4	7.40	6.70
-2	-16	-4	24.10	7.90
-2	-16	-4	18.90	7.80
2	16	4	17.60	4.10
-3	-16	-4	27.10	7.70
-3	-16	-4	17.00	6.40
-3	-16	-4	31.00	8.10
-3	-16	-4	16.20	6.50
-3	-16	-4	26.50	7.90
3	16	4	22.10	4.70
-4	-16	-4	32.90	6.80

-4	-16	-4	23.20	7.80
-4	-16	-4	16.10	7.00
-4	-16	-4	21.40	8.00
-4	-16	-4	42.70	8.60
4	16	4	37.90	6.00
-5	-16	-4	140.39	13.60
-5	-16	-4	174.28	15.70
-5	-16	-4	146.79	15.40
-5	-16	-4	135.19	16.20
-5	-16	-4	136.89	12.60
-5	-16	-4	136.99	15.20
5	16	4	165.08	12.90
-6	-16	-4	30.20	9.20
-6	-16	-4	3.50	7.30
-6	-16	-4	7.50	6.40
-6	-16	-4	11.80	8.30
-6	-16	-4	9.90	8.00
-6	-16	-4	10.20	7.70
6	16	4	2.30	8.30
-7	-16	-4	229.98	17.70
-7	-16	-4	201.78	15.60
-7	-16	-4	204.08	17.60
-7	-16	-4	202.68	22.60
-7	-16	-4	222.18	17.50
-7	-16	-4	202.88	16.50
7	16	4	200.98	21.10
-8	-16	-4	107.19	14.40
-8	-16	-4	104.79	14.20
-8	-16	-4	89.89	13.30
-8	-16	-4	104.09	14.40
-8	-16	-4	106.19	12.30
8	16	4	62.89	17.20
8	16	4	110.69	35.20
-9	-16	-4	25.50	7.60
-9	-16	-4	38.80	9.50
-9	-16	-4	51.09	8.90
9	16	4	-1.30	19.40
-10	-16	-4	431.56	21.70
-10	-16	-4	331.47	22.80
-10	-16	-4	367.86	21.90
-10	-16	-4	414.46	22.30
10	16	4	393.76	40.90
8	-17	-4	111.89	12.30
-8	17	4	108.59	14.20

7	-17	-4	17.60	15.10
7	-17	-4	19.10	7.50
-7	17	4	18.30	10.20
6	-17	-4	194.58	16.00
6	-17	-4	185.88	20.30
-6	17	4	136.89	22.50
-6	17	4	163.98	23.40
-6	17	4	146.79	19.40
5	-17	-4	833.32	42.00
5	-17	-4	833.42	41.00
-5	17	4	786.02	44.10
-5	17	4	796.02	44.20
4	-17	-4	53.09	9.50
4	-17	-4	95.29	16.10
4	-17	-4	55.19	10.50
4	-17	-4	87.69	16.10
-4	17	4	58.99	12.70
-4	17	4	64.19	12.70
3	-17	-4	19.70	12.70
3	-17	-4	48.50	12.10
3	-17	-4	25.80	8.00
3	-17	-4	32.60	9.20
-3	17	4	40.00	11.20
-3	17	4	34.10	9.20
-3	17	4	43.30	11.40
2	-17	-4	190.18	16.90
2	-17	-4	189.58	18.10
2	-17	-4	173.58	19.10
2	-17	-4	181.48	19.10
2	-17	-4	218.28	18.60
-2	17	4	180.38	14.10
-2	17	4	184.08	17.30
1	-17	-4	13.20	8.20
1	-17	-4	12.70	7.40
1	-17	-4	18.40	8.90
-1	17	4	30.10	8.40
-1	17	4	19.60	5.50
0	-17	-4	90.49	13.50
0	-17	-4	95.49	14.00
0	-17	-4	81.59	13.50
0	-17	-4	98.69	13.60
0	17	4	96.29	9.50
-1	-17	-4	173.38	15.00
-1	-17	-4	150.18	14.20

-1	-17	-4	140.79	16.30
-1	-17	-4	160.48	14.50
-1	-17	-4	141.99	14.40
1	17	4	150.18	11.00
-2	-17	-4	5.80	7.00
-2	-17	-4	4.40	7.80
-2	-17	-4	-11.70	7.00
-2	-17	-4	-6.20	10.10
-2	-17	-4	1.50	6.60
2	17	4	3.50	4.50
-3	-17	-4	29.90	8.70
-3	-17	-4	41.70	7.50
-3	-17	-4	45.30	13.80
-3	-17	-4	40.00	8.70
-3	-17	-4	32.60	7.40
3	17	4	57.59	8.40
-4	-17	-4	-0.60	6.30
-4	-17	-4	0.90	7.50
-4	-17	-4	-5.20	7.50
-4	-17	-4	0.50	7.40
-4	-17	-4	15.30	7.00
4	17	4	4.20	5.80
-5	-17	-4	995.90	49.80
-5	-17	-4	984.20	50.09
-5	-17	-4	1071.09	50.29
-5	-17	-4	1024.20	49.10
-5	-17	-4	1022.80	49.99
5	17	4	1019.90	50.29
-6	-17	-4	182.98	15.30
-6	-17	-4	196.38	16.30
-6	-17	-4	193.58	16.20
-6	-17	-4	167.78	13.80
-6	-17	-4	184.88	17.80
6	17	4	169.08	17.60
-7	-17	-4	326.97	25.10
-7	-17	-4	359.76	21.10
-7	-17	-4	354.76	28.70
-7	-17	-4	355.06	26.00
7	17	4	339.87	26.20
-8	-17	-4	20.50	7.70
-8	-17	-4	30.20	6.90
-8	-17	-4	27.70	7.60
-8	-17	-4	37.30	8.40
-9	-17	-4	81.19	11.70

-9	-17	-4	80.19	10.40
-9	-17	-4	57.39	8.90
-9	-17	-4	86.89	12.40
-10	-17	-4	13.50	7.50
-10	-17	-4	18.30	9.00
-10	-17	-4	28.40	8.40
8	-18	-4	64.39	11.00
-8	18	4	54.79	10.30
7	-18	-4	492.05	31.80
7	-18	-4	478.55	25.30
-7	18	4	447.56	27.30
6	-18	-4	286.97	22.50
6	-18	-4	283.47	18.80
-6	18	4	291.17	33.70
-6	18	4	285.27	21.10
-6	18	4	266.37	25.10
5	-18	-4	18.80	9.10
5	-18	-4	-1.30	10.30
-5	18	4	20.70	13.70
-5	18	4	19.60	12.70
4	-18	-4	22.70	14.90
4	-18	-4	3.20	8.60
4	-18	-4	13.60	15.50
4	-18	-4	-2.60	8.80
-4	18	4	5.00	11.20
-4	18	4	-8.50	11.30
3	-18	-4	9.40	9.20
3	-18	-4	-1.00	12.60
3	-18	-4	2.80	11.60
3	-18	-4	16.10	8.00
-3	18	4	15.20	10.50
-3	18	4	8.50	10.40
-3	18	4	11.30	10.80
2	-18	-4	85.09	14.30
2	-18	-4	60.59	11.30
2	-18	-4	97.29	16.10
2	-18	-4	79.59	12.80
2	-18	-4	87.99	11.70
-2	18	4	94.79	15.70
-2	18	4	74.09	11.80
-2	18	4	55.59	9.00
1	-18	-4	72.29	10.40
1	-18	-4	73.09	10.30
1	-18	-4	50.39	9.30

1	-18	-4	89.49	11.10
-1	18	4	80.49	10.80
-1	18	4	104.09	13.50
0	-18	-4	818.72	40.40
0	-18	-4	788.72	39.60
0	-18	-4	741.03	39.00
0	-18	-4	755.42	39.50
0	18	4	770.32	39.70
0	18	4	774.12	37.80
-1	-18	-4	753.92	38.50
-1	-18	-4	778.22	38.80
-1	-18	-4	779.42	40.00
-1	-18	-4	737.63	38.50
1	18	4	769.62	37.20
-2	-18	-4	14.50	8.20
-2	-18	-4	18.20	11.00
-2	-18	-4	29.30	8.10
-2	-18	-4	24.50	7.60
-2	-18	-4	16.00	7.10
2	18	4	26.80	6.40
-3	-18	-4	30.00	8.00
-3	-18	-4	30.60	8.60
-3	-18	-4	47.40	14.00
-3	-18	-4	26.40	8.10
-3	-18	-4	25.80	7.50
3	18	4	31.40	6.80
-4	-18	-4	32.20	8.00
-4	-18	-4	22.20	6.60
-4	-18	-4	29.30	8.00
-4	-18	-4	19.40	8.10
4	18	4	28.30	7.40
-5	-18	-4	7.30	7.90
-5	-18	-4	-2.80	7.20
-5	-18	-4	6.70	8.00
-5	-18	-4	2.20	7.40
-5	-18	-4	1.20	6.40
5	18	4	-12.80	7.90
-6	-18	-4	119.39	14.50
-6	-18	-4	105.79	12.70
-6	-18	-4	129.59	14.40
-6	-18	-4	148.79	18.30
-6	-18	-4	111.99	12.90
6	18	4	135.59	17.70
-7	-18	-4	31.20	8.20

-7	-18	-4	37.40	8.40
-7	-18	-4	34.50	10.90
-7	-18	-4	40.20	7.20
-7	-18	-4	25.80	8.20
-8	-18	-4	32.80	6.50
-8	-18	-4	24.10	7.80
-8	-18	-4	39.70	7.80
-8	-18	-4	29.80	8.40
-9	-18	-4	19.30	7.00
-9	-18	-4	40.60	6.60
-9	-18	-4	29.00	7.20
7	-19	-4	95.39	17.60
7	-19	-4	107.39	12.10
-7	19	4	124.19	13.10
6	-19	-4	403.46	22.60
6	-19	-4	406.26	26.00
-6	19	4	379.56	24.20
-6	19	4	397.26	27.80
5	-19	-4	6.40	8.70
5	-19	-4	-0.50	8.20
-5	19	4	-0.90	9.10
-5	19	4	15.80	11.80
-5	19	4	-0.40	13.80
4	-19	-4	65.29	17.20
4	-19	-4	71.99	18.70
4	-19	-4	50.89	11.10
4	-19	-4	60.59	10.80
-4	19	4	56.19	12.80
-4	19	4	65.09	13.10
3	-19	-4	2.80	7.80
3	-19	-4	30.70	13.20
3	-19	-4	22.80	11.60
3	-19	-4	-13.40	10.50
-3	19	4	-4.90	9.80
-3	19	4	-17.00	10.20
2	-19	-4	318.77	23.20
2	-19	-4	318.37	21.30
2	-19	-4	326.17	22.90
2	-19	-4	301.77	25.40
-2	19	4	318.17	22.80
-2	19	4	322.87	23.60
-2	19	4	307.17	22.30
1	-19	-4	422.16	25.50
1	-19	-4	419.06	25.30

1	-19	-4	417.66	24.90
1	-19	-4	366.36	25.20
-1	19	4	455.05	25.60
-1	19	4	420.06	24.30
0	-19	-4	0.30	8.60
0	-19	-4	1.50	8.30
0	-19	-4	12.50	7.60
0	-19	-4	3.10	7.60
0	19	4	4.10	9.20
0	19	4	-7.90	7.10
-1	-19	-4	107.39	13.00
-1	-19	-4	102.19	13.80
-1	-19	-4	112.99	13.60
-1	-19	-4	105.09	16.40
1	19	4	113.69	13.20
-2	-19	-4	79.49	13.00
-2	-19	-4	76.99	11.70
-2	-19	-4	88.99	12.80
-2	-19	-4	62.29	11.80
2	19	4	85.89	11.60
-3	-19	-4	155.88	23.10
-3	-19	-4	147.59	14.20
-3	-19	-4	163.48	15.00
-3	-19	-4	151.18	14.50
-3	-19	-4	152.88	13.70
3	19	4	157.28	14.40
-4	-19	-4	172.88	15.00
-4	-19	-4	157.78	14.80
-4	-19	-4	164.98	14.90
-4	-19	-4	145.39	13.10
4	19	4	139.59	15.00
-5	-19	-4	7.30	7.40
-5	-19	-4	8.20	8.40
-5	-19	-4	12.30	7.70
-5	-19	-4	12.60	6.50
5	19	4	-2.80	9.50
-6	-19	-4	453.75	27.40
-6	-19	-4	502.85	26.30
-6	-19	-4	479.95	26.50
-6	-19	-4	471.15	25.50
6	19	4	455.25	30.10
-7	-19	-4	12.10	5.90
-7	-19	-4	8.40	7.10
-7	-19	-4	-0.20	10.50

-7	-19	-4	7.30	6.70
-8	-19	-4	148.39	12.50
-8	-19	-4	133.79	11.00
-8	-19	-4	131.09	12.40
-9	-19	-4	62.99	10.20
-9	-19	-4	63.29	12.60
-9	-19	-4	48.20	9.50
6	-20	-4	165.28	14.30
6	-20	-4	163.58	18.50
-6	20	4	183.48	15.50
-6	20	4	159.48	22.70
5	-20	-4	32.80	9.40
5	-20	-4	39.40	9.20
-5	20	4	31.60	9.00
-5	20	4	42.80	15.10
-5	20	4	15.20	12.40
4	-20	-4	-5.60	9.50
-4	20	4	0.00	10.60
-4	20	4	13.10	12.40
3	-20	-4	44.00	12.20
3	-20	-4	79.99	11.40
3	-20	-4	41.40	14.50
3	-20	-4	69.99	13.40
-3	20	4	73.89	12.10
-3	20	4	65.09	12.40
2	-20	-4	26.00	16.20
2	-20	-4	22.70	8.70
-2	20	4	30.10	10.00
-2	20	4	10.20	10.00
-2	20	4	12.90	14.70
1	-20	-4	689.73	35.70
1	-20	-4	690.93	36.20
1	-20	-4	660.93	36.00
1	-20	-4	709.23	35.50
-1	20	4	686.43	37.30
-1	20	4	625.44	35.90
0	-20	-4	298.97	20.00
0	-20	-4	272.47	19.50
0	-20	-4	300.97	20.00
0	-20	-4	291.67	21.20
0	20	4	288.77	19.80
0	20	4	243.68	20.50
-1	-20	-4	214.28	19.30
-1	-20	-4	187.58	16.30

-1	-20	-4	179.68	16.40
-1	-20	-4	232.48	16.60
1	20	4	217.88	17.40
1	20	4	200.78	17.00
-2	-20	-4	8.50	7.10
-2	-20	-4	13.40	7.50
-2	-20	-4	2.20	10.70
-2	-20	-4	-3.30	6.70
2	20	4	3.40	8.20
-3	-20	-4	30.30	22.10
-3	-20	-4	26.90	7.90
-3	-20	-4	27.80	7.30
-3	-20	-4	33.70	8.10
-3	-20	-4	32.50	8.40
3	20	4	23.80	14.20
3	20	4	23.10	9.20
-4	-20	-4	82.79	15.50
-4	-20	-4	92.49	15.10
-4	-20	-4	82.49	12.00
4	20	4	85.99	11.50
-5	-20	-4	97.29	12.10
-5	-20	-4	105.19	12.60
-5	-20	-4	85.89	13.70
-5	-20	-4	93.69	11.40
5	20	4	82.69	13.50
-6	-20	-4	18.60	8.90
-6	-20	-4	4.20	5.50
-6	-20	-4	14.40	6.50
-6	-20	-4	3.40	6.80
-7	-20	-4	236.88	14.50
-7	-20	-4	227.08	18.90
-7	-20	-4	211.38	15.10
-7	-20	-4	219.28	15.30
-8	-20	-4	24.20	5.90
-8	-20	-4	32.40	7.00
-8	-20	-4	29.00	7.60
6	-21	-4	252.17	19.00
6	-21	-4	208.38	16.40
-6	21	4	193.48	19.00
5	-21	-4	289.27	19.20
5	-21	-4	286.27	19.50
-5	21	4	281.97	30.10
-5	21	4	281.47	18.70
-5	21	4	232.18	25.60

4	-21	-4	6.70	7.80
4	-21	-4	5.50	11.10
-4	21	4	-5.20	10.80
-4	21	4	-1.60	12.70
3	-21	-4	33.30	14.80
3	-21	-4	12.10	7.90
3	-21	-4	7.00	18.50
3	-21	-4	16.30	13.10
-3	21	4	16.50	11.70
-3	21	4	23.40	10.40
2	-21	-4	323.07	23.30
2	-21	-4	369.56	22.50
2	-21	-4	332.17	24.60
-2	21	4	328.57	22.70
-2	21	4	374.06	23.60
1	-21	-4	96.49	14.70
1	-21	-4	57.69	11.30
1	-21	-4	89.79	15.10
-1	21	4	90.49	15.20
-1	21	4	81.99	13.80
-1	21	4	45.80	15.90
0	-21	-4	7.00	15.60
0	-21	-4	28.80	11.10
0	-21	-4	32.20	12.30
0	21	4	9.00	8.70
0	21	4	-0.30	11.90
-1	-21	-4	62.49	15.50
-1	-21	-4	57.79	17.30
-1	-21	-4	19.70	12.50
1	21	4	49.40	11.80
1	21	4	46.20	9.50
-2	-21	-4	197.88	17.70
-2	-21	-4	150.18	21.20
-2	-21	-4	183.38	20.30
-2	-21	-4	196.68	16.60
2	21	4	149.09	14.80
2	21	4	150.78	19.50
-3	-21	-4	27.90	20.80
-3	-21	-4	42.80	8.20
-3	-21	-4	61.29	9.80
3	21	4	65.69	20.40
3	21	4	35.50	11.90
-4	-21	-4	183.48	15.40
-4	-21	-4	173.48	14.10

-4	-21	-4	171.38	13.90
4	21	4	160.58	21.70
-5	-21	-4	604.44	31.40
-5	-21	-4	628.54	31.20
-5	-21	-4	614.54	32.30
-5	-21	-4	646.64	31.70
5	21	4	617.34	34.60
-6	-21	-4	48.60	9.20
-6	-21	-4	59.29	10.00
-6	-21	-4	59.19	9.10
-6	-21	-4	59.89	10.20
-7	-21	-4	103.29	9.60
-7	-21	-4	107.29	17.30
-7	-21	-4	121.39	10.90
-7	-21	-4	116.49	11.10
5	-22	-4	8.20	12.50
-5	22	4	3.10	10.20
-5	22	4	-0.20	14.20
4	-22	-4	45.00	8.50
-4	22	4	49.10	14.70
-4	22	4	63.69	11.40
-4	22	4	42.90	8.70
3	-22	-4	-9.60	13.50
3	-22	-4	-8.50	7.40
-3	22	4	-11.10	9.60
-3	22	4	-15.40	11.30
2	-22	-4	16.00	10.40
2	-22	-4	2.30	7.90
2	-22	-4	34.00	13.10
-2	22	4	12.50	9.30
-2	22	4	22.40	10.50
1	-22	-4	9.10	10.30
1	-22	-4	6.80	8.60
1	-22	-4	24.90	8.40
-1	22	4	11.90	8.90
-1	22	4	22.50	10.00
0	-22	-4	816.22	41.70
0	-22	-4	869.41	42.50
0	-22	-4	862.51	41.70
0	-22	-4	821.02	41.80
0	22	4	807.32	42.00
-1	-22	-4	101.39	13.20
-1	-22	-4	137.49	13.30
-1	-22	-4	113.09	14.40

-1	-22	-4	107.69	16.30
1	22	4	129.19	14.50
-2	-22	-4	3.50	7.20
-2	-22	-4	-16.50	11.80
-2	-22	-4	12.10	6.90
-2	-22	-4	6.20	7.00
2	22	4	5.60	8.40
-3	-22	-4	179.28	14.40
-3	-22	-4	169.18	13.80
-3	-22	-4	194.48	13.90
3	22	4	169.38	15.10
-4	-22	-4	74.69	11.00
-4	-22	-4	89.29	12.10
-4	-22	-4	114.99	10.80
4	22	4	102.49	15.60
-5	-22	-4	11.60	5.80
-5	-22	-4	1.30	6.40
-5	-22	-4	19.70	7.20
5	22	4	6.70	11.90
-6	-22	-4	46.00	7.20
-6	-22	-4	45.30	6.60
-6	-22	-4	46.50	9.10
6	22	4	25.60	17.60
4	-23	-4	37.90	12.40
-4	23	4	20.80	11.90
-4	23	4	42.90	8.30
3	-23	-4	8.50	6.90
-3	23	4	-10.70	10.00
-3	23	4	23.90	12.90
2	-23	-4	171.68	17.80
2	-23	-4	183.28	14.40
-2	23	4	177.78	16.00
-2	23	4	162.08	17.00
1	-23	-4	194.98	17.80
1	-23	-4	155.28	15.00
1	-23	-4	154.78	15.40
-1	23	4	147.09	16.20
-1	23	4	187.58	15.50
0	-23	-4	12.50	7.70
0	-23	-4	-2.10	8.40
0	-23	-4	3.30	8.70
0	23	4	-3.00	9.50
0	23	4	2.20	7.80
-1	-23	-4	152.78	16.70

-1	-23	-4	137.49	14.30
-1	-23	-4	161.98	13.70
-1	-23	-4	173.68	13.50
1	23	4	135.49	14.10
-2	-23	-4	83.79	13.80
-2	-23	-4	112.69	11.50
-2	-23	-4	83.09	11.00
-2	-23	-4	88.39	12.50
2	23	4	81.09	12.50
-3	-23	-4	62.49	8.50
-3	-23	-4	85.99	10.80
-3	-23	-4	50.39	8.10
-4	-23	-4	61.29	8.50
-4	-23	-4	57.49	9.70
-4	-23	-4	51.39	8.20
4	23	4	57.29	10.40
-5	-23	-4	14.50	5.80
-5	-23	-4	19.90	7.50
-5	-23	-4	27.80	6.80
5	23	4	7.20	9.20
2	-24	-4	14.40	7.50
2	-24	-4	18.20	12.10
-2	24	4	33.60	11.10
-2	24	4	26.20	11.70
1	-24	-4	307.97	19.80
1	-24	-4	299.87	18.80
-1	24	4	303.07	20.20
-1	24	4	320.37	19.70
0	-24	-4	123.79	13.50
0	-24	-4	113.59	17.60
0	-24	-4	113.69	12.80
0	24	4	109.79	12.20
0	24	4	108.09	14.00
-1	-24	-4	93.89	12.90
-1	-24	-4	96.69	11.60
-1	-24	-4	84.89	12.00
1	24	4	103.59	13.20
1	24	4	106.69	12.00
-2	-24	-4	52.69	7.90
-2	-24	-4	44.20	7.70
-2	-24	-4	57.69	16.90
-2	-24	-4	40.50	8.40
2	24	4	58.89	9.80
-3	-24	-4	19.90	6.80

-3	-24	-4	19.60	7.30
-3	-24	-4	17.90	6.70
3	24	4	11.80	8.60
-4	-24	-4	111.49	14.20
-4	-24	-4	82.29	13.10
-4	-24	-4	103.69	11.10
4	24	4	98.29	13.60
-1	-25	-4	25.30	12.50
1	25	4	48.20	9.90
1	25	4	41.10	10.00
3	22	-5	32.40	8.10
3	22	-5	13.20	8.60
-3	-22	5	21.20	6.80
2	22	-5	25.30	9.70
2	22	-5	-3.90	9.80
2	22	-5	-1.80	7.70
-2	-22	5	13.30	6.70
1	22	-5	23.50	9.90
1	22	-5	0.80	7.30
1	22	-5	38.20	11.30
-1	-22	5	12.20	7.70
-1	-22	5	5.30	11.60
0	22	-5	81.19	11.30
0	-22	5	55.49	14.00
0	-22	5	60.19	11.30
-1	22	-5	354.36	19.80
-1	22	-5	315.57	20.60
1	-22	5	346.57	19.10
1	-22	5	313.67	22.50
-2	22	-5	7.90	9.00
-2	22	-5	3.50	7.00
2	-22	5	-12.70	6.80
4	21	-5	290.47	21.60
4	21	-5	345.17	19.90
4	21	-5	279.57	28.80
-4	-21	5	340.47	18.10
3	21	-5	-11.20	9.80
3	21	-5	2.30	8.20
3	21	-5	4.00	8.30
-3	-21	5	4.30	5.90
2	21	-5	24.70	9.50
2	21	-5	-1.70	7.60
2	21	-5	11.60	8.90
-2	-21	5	3.20	6.40

1	21	-5	212.28	17.70
1	21	-5	190.18	14.90
1	21	-5	172.78	15.40
-1	-21	5	172.18	14.60
-1	-21	5	201.48	16.40
0	21	-5	39.30	8.50
0	21	-5	42.10	10.20
0	21	-5	62.59	12.40
0	-21	5	32.90	8.50
0	-21	5	31.20	9.00
-1	21	-5	0.10	8.20
-1	21	-5	-3.90	7.00
1	-21	5	8.10	10.40
1	-21	5	-12.50	7.10
-2	21	-5	263.67	18.10
-2	21	-5	240.58	17.30
2	-21	5	276.97	17.10
-3	21	-5	75.09	12.70
-3	21	-5	52.49	8.80
3	-21	5	68.79	10.00
-4	21	-5	36.20	10.00
-4	21	-5	24.30	8.20
4	-21	5	27.80	12.30
6	20	-5	118.29	14.00
-6	-20	5	106.99	11.90
-6	-20	5	135.29	15.70
5	20	-5	14.00	7.80
5	20	-5	8.10	12.70
-5	-20	5	14.60	6.10
4	20	-5	31.70	10.90
4	20	-5	39.80	8.80
-4	-20	5	33.20	9.20
-4	-20	5	40.10	7.10
3	20	-5	115.19	14.80
3	20	-5	109.79	17.10
-3	-20	5	134.89	11.90
2	20	-5	70.49	10.00
2	20	-5	85.19	14.90
-2	-20	5	74.79	11.70
-2	-20	5	69.29	11.10
1	20	-5	118.39	13.30
1	20	-5	130.09	15.30
1	20	-5	98.09	13.50
-1	-20	5	112.59	12.90

-1	-20	5	107.39	14.30
0	20	-5	357.76	22.10
0	20	-5	348.07	22.20
0	20	-5	413.66	25.30
0	-20	5	367.26	22.40
0	-20	5	369.96	22.80
-1	20	-5	1.60	8.10
-1	20	-5	24.00	13.60
-1	20	-5	1.90	7.40
1	-20	5	5.90	9.40
1	-20	5	2.90	7.60
-2	20	-5	163.48	14.90
-2	20	-5	173.88	15.30
2	-20	5	152.98	22.40
2	-20	5	165.38	15.10
-3	20	-5	144.79	13.70
-3	20	-5	136.09	18.00
3	-20	5	140.59	13.70
-4	20	-5	3.50	7.30
-4	20	-5	5.90	9.10
4	-20	5	8.20	7.40
-5	20	-5	86.29	14.40
-5	20	-5	105.89	12.50
5	-20	5	89.59	15.50
6	19	-5	63.09	11.50
-6	-19	5	53.59	9.80
-6	-19	5	83.79	10.30
5	19	-5	316.47	23.20
5	19	-5	360.36	21.30
-5	-19	5	353.56	20.40
-5	-19	5	369.46	20.20
4	19	-5	29.10	8.20
4	19	-5	13.90	9.70
-4	-19	5	2.40	7.70
-4	-19	5	13.30	6.50
3	19	-5	95.59	13.20
3	19	-5	93.29	14.30
-3	-19	5	71.79	9.40
-3	-19	5	83.39	14.30
2	19	-5	125.19	15.70
2	19	-5	142.89	14.50
-2	-19	5	156.78	13.50
-2	-19	5	129.99	17.30
1	19	-5	-1.60	9.50

1	19	-5	-0.80	7.80
-1	-19	5	3.50	7.70
-1	-19	5	5.50	9.10
0	19	-5	37.00	9.20
0	19	-5	31.30	8.00
0	19	-5	56.09	11.10
0	-19	5	31.70	9.30
0	-19	5	46.00	9.40
-1	19	-5	41.70	13.10
-1	19	-5	39.70	8.70
1	-19	5	45.00	9.20
1	-19	5	40.10	10.60
-2	19	-5	5.80	8.00
-2	19	-5	-2.80	15.00
-2	19	-5	11.70	8.00
2	-19	5	-8.10	12.60
2	-19	5	1.60	7.90
-3	19	-5	169.48	16.10
-3	19	-5	185.18	15.90
3	-19	5	204.08	16.00
-4	19	-5	70.09	9.90
-4	19	-5	79.09	12.50
4	-19	5	64.39	15.30
4	-19	5	65.09	12.30
-5	19	-5	168.08	14.30
-5	19	-5	120.59	14.90
5	-19	5	175.58	15.10
5	-19	5	136.49	19.70
-6	19	-5	69.79	11.80
-6	19	-5	68.49	12.00
6	-19	5	71.89	12.70
7	18	-5	293.37	17.30
-7	-18	5	262.97	16.10
-7	-18	5	266.47	17.10
6	18	-5	154.88	14.10
6	18	-5	143.89	19.60
-6	-18	5	136.79	12.60
-6	-18	5	136.99	12.80
5	18	-5	92.19	12.50
5	18	-5	92.59	13.20
-5	-18	5	94.39	11.10
-5	-18	5	84.79	11.70
4	18	-5	20.20	8.40
4	18	-5	43.00	9.60

-4	-18	5	11.10	6.60
-4	-18	5	31.30	8.30
3	18	-5	83.99	19.40
3	18	-5	88.89	13.20
-3	-18	5	64.19	10.00
-3	-18	5	59.19	13.60
2	18	-5	41.70	8.80
2	18	-5	53.39	10.40
-2	-18	5	40.10	8.20
1	18	-5	33.50	10.00
1	18	-5	25.40	8.10
-1	-18	5	8.70	7.60
-1	-18	5	23.20	9.20
0	18	-5	210.18	19.50
0	18	-5	251.47	17.70
0	-18	5	226.78	19.40
0	-18	5	268.67	18.30
-1	18	-5	271.87	18.50
-1	18	-5	276.07	19.30
-1	18	-5	205.58	22.80
1	-18	5	291.57	21.00
1	-18	5	273.27	19.40
-2	18	-5	-2.80	7.50
-2	18	-5	16.30	9.00
-2	18	-5	12.10	15.50
2	-18	5	10.90	9.20
2	-18	5	12.50	13.00
-3	18	-5	-2.50	8.80
-3	18	-5	6.20	7.90
3	-18	5	15.90	8.60
-4	18	-5	416.36	24.80
-4	18	-5	424.46	24.70
4	-18	5	382.86	24.90
4	-18	5	413.36	24.50
-5	18	-5	126.69	14.70
-5	18	-5	117.19	13.90
5	-18	5	130.79	14.30
5	-18	5	115.89	15.20
-6	18	-5	7.30	8.20
-6	18	-5	8.30	9.70
6	-18	5	11.30	10.00
6	-18	5	4.70	7.90
-7	18	-5	38.70	11.40
-7	18	-5	45.70	12.20

7	-18	5	36.00	19.00
7	-18	5	43.60	8.30
8	17	-5	2.70	7.70
-8	-17	5	25.30	10.10
-8	-17	5	13.30	5.60
7	17	-5	32.60	8.20
-7	-17	5	27.30	7.70
-7	-17	5	40.80	6.90
6	17	-5	73.09	12.90
-6	-17	5	81.49	10.50
-6	-17	5	90.49	11.50
5	17	-5	18.60	8.70
-5	-17	5	-0.90	6.80
-5	-17	5	3.50	6.70
4	17	-5	495.55	28.30
-4	-17	5	491.35	27.10
3	17	-5	4.10	8.50
3	17	-5	10.70	8.30
-3	-17	5	21.10	8.20
-3	-17	5	10.30	6.90
-3	-17	5	38.30	12.60
2	17	-5	3.40	8.20
2	17	-5	2.20	8.10
-2	-17	5	5.90	9.60
-2	-17	5	-3.80	6.80
-2	-17	5	4.40	8.80
1	17	-5	368.06	22.30
1	17	-5	366.56	24.30
-1	-17	5	380.46	22.50
-1	-17	5	358.16	23.90
0	17	-5	71.09	11.00
0	17	-5	54.09	9.10
0	-17	5	64.29	9.60
0	-17	5	47.50	9.60
-1	17	-5	-1.40	6.90
-1	17	-5	-10.00	9.50
-1	17	-5	-1.60	7.80
1	-17	5	-1.90	8.80
1	-17	5	12.20	8.00
-2	17	-5	460.55	27.50
-2	17	-5	477.55	28.00
-2	17	-5	494.65	31.20
2	-17	5	477.75	29.90
2	-17	5	542.65	28.20

-3	17	-5	29.20	17.60
-3	17	-5	0.80	8.00
-3	17	-5	16.30	8.20
3	-17	5	10.00	13.40
3	-17	5	19.70	8.40
-4	17	-5	5.80	15.10
-4	17	-5	16.90	8.90
4	-17	5	18.80	11.10
4	-17	5	18.90	9.10
-5	17	-5	10.20	9.20
-5	17	-5	16.30	9.10
5	-17	5	15.70	8.40
5	-17	5	5.20	9.20
-6	17	-5	104.39	15.20
6	-17	5	136.99	13.90
6	-17	5	118.09	16.20
-7	17	-5	8.30	10.90
-7	17	-5	4.60	8.60
7	-17	5	9.60	13.90
7	-17	5	3.70	7.60
8	16	-5	11.50	7.60
-8	-16	5	11.40	9.90
-8	-16	5	9.80	5.40
7	16	-5	-0.20	23.20
7	16	-5	4.30	7.90
-7	-16	5	6.40	7.30
-7	-16	5	-3.10	5.90
-6	-16	5	327.37	19.80
-6	-16	5	349.27	21.00
5	16	-5	28.20	8.80
-5	-16	5	3.10	6.90
-5	-16	5	7.50	7.20
4	16	-5	40.20	8.90
-4	-16	5	28.30	7.40
-4	-16	5	20.80	7.10
3	16	-5	553.04	30.40
-3	-16	5	553.64	28.90
-3	-16	5	522.95	28.60
-3	-16	5	524.75	33.10
2	16	-5	263.57	25.20
2	16	-5	292.27	21.20
-2	-16	5	325.27	20.80
-2	-16	5	316.07	22.40
-2	-16	5	325.27	19.50

1	16	-5	475.45	27.30
1	16	-5	475.45	28.10
-1	-16	5	499.65	27.00
-1	-16	5	491.05	28.10
0	16	-5	113.69	16.70
0	16	-5	106.29	13.40
0	-16	5	113.69	14.40
0	-16	5	131.39	14.70
-1	16	-5	208.68	19.20
-1	16	-5	201.38	22.90
1	-16	5	223.68	18.90
1	-16	5	253.47	18.50
-2	16	-5	227.18	22.30
-2	16	-5	183.28	17.00
-2	16	-5	191.18	17.00
2	-16	5	215.08	17.60
2	-16	5	177.78	20.60
-3	16	-5	275.47	19.50
-3	16	-5	257.17	27.30
-3	16	-5	269.37	19.90
3	-16	5	298.87	21.80
3	-16	5	292.87	20.50
-4	16	-5	56.19	10.00
-4	16	-5	53.39	14.00
-4	16	-5	50.89	10.40
4	-16	5	43.80	9.30
4	-16	5	46.40	9.60
-5	16	-5	162.88	16.80
-5	16	-5	153.48	24.00
5	-16	5	222.28	17.70
5	-16	5	179.48	21.30
-6	16	-5	61.59	15.60
-6	16	-5	55.89	11.30
-6	16	-5	47.50	10.90
6	-16	5	56.09	9.30
6	-16	5	38.40	11.30
-7	16	-5	80.89	11.30
-7	16	-5	70.89	15.00
7	-16	5	87.59	14.60
7	-16	5	74.59	12.00
-8	16	-5	90.09	14.50
-8	16	-5	83.49	13.20
8	-16	5	107.69	11.50
9	15	-5	-16.20	13.30

9	15	-5	-1.20	7.60
-9	-15	5	10.10	5.40
8	15	-5	15.70	13.70
-8	-15	5	33.40	9.70
-8	-15	5	31.00	6.50
7	15	-5	68.89	15.90
-7	-15	5	62.19	12.00
-7	-15	5	88.99	12.90
-7	-15	5	71.99	11.00
6	15	-5	283.57	45.30
-6	-15	5	343.07	21.10
-6	-15	5	306.67	31.40
-6	-15	5	372.76	28.30
-6	-15	5	368.46	23.10
5	15	-5	420.66	43.60
-5	-15	5	397.56	28.70
-5	-15	5	462.55	25.60
-5	-15	5	448.56	25.00
-5	-15	5	454.45	24.40
-4	-15	5	7.60	8.00
-4	-15	5	26.70	8.00
-4	-15	5	8.40	14.80
-4	-15	5	5.40	7.20
3	15	-5	23.70	9.30
-3	-15	5	29.70	12.10
-3	-15	5	23.00	7.60
-3	-15	5	24.40	7.80
2	15	-5	205.88	17.10
-2	-15	5	198.78	18.10
-2	-15	5	187.28	15.20
-2	-15	5	190.88	16.80
1	15	-5	3.30	6.70
1	15	-5	-1.10	8.20
-1	-15	5	3.80	7.60
-1	-15	5	24.50	9.60
-1	-15	5	2.10	6.80
0	15	-5	73.49	10.70
0	15	-5	90.69	11.90
0	-15	5	92.79	13.30
0	-15	5	64.79	9.10
-1	15	-5	-0.60	9.80
-1	15	-5	5.60	6.40
1	-15	5	-1.20	8.00
1	-15	5	-2.30	8.30

-2	15	-5	249.18	17.00
-2	15	-5	222.98	18.10
-2	15	-5	229.18	21.40
2	-15	5	200.88	22.80
2	-15	5	225.78	17.80
2	-15	5	243.18	21.50
-3	15	-5	240.18	23.30
-3	15	-5	288.67	26.50
-3	15	-5	267.97	20.60
3	-15	5	295.47	19.90
3	-15	5	280.97	21.20
-4	15	-5	186.28	18.90
-4	15	-5	172.18	24.50
-4	15	-5	237.28	28.30
-4	15	-5	235.48	18.80
4	-15	5	234.08	18.80
4	-15	5	234.88	18.40
-5	15	-5	58.29	11.10
-5	15	-5	92.49	14.30
-5	15	-5	76.79	12.70
5	-15	5	70.69	11.10
5	-15	5	67.19	10.40
-6	15	-5	40.60	11.00
-6	15	-5	45.00	13.20
-6	15	-5	46.10	12.80
6	-15	5	27.90	11.50
6	-15	5	29.30	9.00
-7	15	-5	0.30	10.40
-7	15	-5	23.20	13.20
-7	15	-5	21.00	11.20
7	-15	5	37.30	13.30
7	-15	5	2.00	7.50
-8	15	-5	73.89	13.20
-8	15	-5	61.29	11.30
8	-15	5	49.60	8.30
9	14	-5	31.70	12.90
-9	-14	5	29.90	5.80
8	14	-5	74.89	14.90
-8	-14	5	60.39	13.90
-8	-14	5	65.49	10.00
-8	-14	5	61.79	10.30
7	14	-5	300.97	26.60
-7	-14	5	297.57	20.10
-7	-14	5	292.27	24.10

-7	-14	5	285.17	19.70
-7	-14	5	257.47	22.90
6	14	-5	46.00	15.00
-6	-14	5	66.69	11.30
-6	-14	5	42.40	10.70
-6	-14	5	19.80	11.40
-6	-14	5	45.10	8.80
5	14	-5	55.19	14.80
-5	-14	5	35.60	9.70
-5	-14	5	34.10	7.60
-5	-14	5	30.40	11.00
-5	-14	5	31.60	7.20
4	14	-5	235.88	31.00
4	14	-5	251.07	21.40
-4	-14	5	251.27	17.50
-4	-14	5	202.08	20.50
-4	-14	5	226.28	17.10
-4	-14	5	246.98	17.00
3	14	-5	157.18	17.30
-3	-14	5	190.48	15.50
-3	-14	5	149.69	20.00
-3	-14	5	169.38	14.40
-3	-14	5	195.28	15.30
-3	-14	5	202.58	25.30
2	14	-5	81.19	13.20
2	14	-5	108.29	14.40
-2	-14	5	77.69	11.70
-2	-14	5	69.29	9.50
-2	-14	5	66.89	10.40
1	14	-5	72.39	9.80
1	14	-5	47.10	8.90
-1	-14	5	68.59	11.60
-1	-14	5	75.39	13.20
-1	-14	5	58.89	9.30
0	14	-5	233.68	16.70
0	14	-5	239.28	18.40
0	-14	5	241.88	17.40
0	-14	5	244.58	18.40
-1	14	-5	330.97	19.30
-1	14	-5	269.07	21.80
1	-14	5	303.97	20.70
1	-14	5	298.27	20.20
-2	14	-5	216.28	20.40
-2	14	-5	230.48	17.50

-2	14	-5	230.48	16.00
2	-14	5	205.38	20.00
2	-14	5	205.48	21.20
2	-14	5	227.08	17.50
-3	14	-5	74.49	14.00
-3	14	-5	74.19	12.30
-3	14	-5	93.29	14.50
3	-14	5	76.69	13.20
3	-14	5	61.79	9.70
-4	14	-5	666.13	43.00
-4	14	-5	727.93	38.60
-4	14	-5	697.93	38.20
4	-14	5	735.83	37.40
4	-14	5	719.83	37.20
-5	14	-5	30.20	9.90
-5	14	-5	-0.20	10.20
-5	14	-5	26.10	12.30
5	-14	5	22.30	9.10
5	-14	5	10.80	7.80
-6	14	-5	8.60	10.80
-6	14	-5	21.30	10.50
-6	14	-5	34.20	13.50
6	-14	5	8.20	7.60
6	-14	5	14.10	10.70
-7	14	-5	28.10	12.20
-7	14	-5	46.80	12.50
-7	14	-5	29.00	12.90
7	-14	5	52.59	14.10
7	-14	5	46.30	8.30
-8	14	-5	124.59	18.00
-8	14	-5	86.09	13.00
-8	14	-5	114.79	14.40
8	-14	5	114.39	12.40
-9	14	-5	78.69	12.40
-9	14	-5	65.99	14.20
9	-14	5	90.39	11.60
10	13	-5	95.59	18.20
10	13	-5	61.89	10.60
-10	-13	5	83.09	9.00
9	13	-5	62.59	13.50
-9	-13	5	51.79	8.90
-9	-13	5	51.79	13.30
8	13	-5	145.19	21.50
-8	-13	5	139.59	12.80

-8	-13	5	138.59	16.50
-8	-13	5	123.39	20.00
7	13	-5	126.69	22.20
-7	-13	5	159.58	22.40
-7	-13	5	107.89	15.90
-7	-13	5	136.89	15.40
-7	-13	5	127.19	13.40
6	13	-5	78.19	15.30
-6	-13	5	68.29	12.30
-6	-13	5	50.99	8.50
-6	-13	5	104.19	16.60
5	13	-5	54.99	14.40
5	13	-5	82.09	12.70
-5	-13	5	71.59	11.50
-5	-13	5	69.19	11.00
-5	-13	5	54.29	12.00
-5	-13	5	59.69	8.90
4	13	-5	189.48	25.40
4	13	-5	219.78	17.80
-4	-13	5	213.08	15.80
-4	-13	5	200.48	16.90
-4	-13	5	208.48	15.80
-4	-13	5	162.48	20.10
3	13	-5	186.78	15.10
-3	-13	5	165.68	15.10
-3	-13	5	170.88	14.90
-3	-13	5	181.48	14.40
-3	-13	5	153.58	19.40
-3	-13	5	133.59	18.00
2	13	-5	149.09	13.80
-2	-13	5	134.09	15.10
-2	-13	5	164.78	13.80
-2	-13	5	166.08	16.70
1	13	-5	913.61	43.90
-1	-13	5	867.81	44.60
-1	-13	5	952.40	45.40
0	13	-5	1.90	5.80
0	13	-5	16.40	7.10
0	13	-5	4.20	8.00
0	-13	5	3.20	9.40
0	-13	5	18.90	7.60
-1	13	-5	148.59	17.60
-1	13	-5	180.98	13.70
1	-13	5	156.78	18.60

1	-13	5	167.58	16.00
-2	13	-5	606.84	31.40
-2	13	-5	599.54	32.20
2	-13	5	570.14	32.30
2	-13	5	586.74	31.70
2	-13	5	603.84	32.90
-3	13	-5	68.49	10.10
-3	13	-5	64.89	13.60
-3	13	-5	65.09	10.80
3	-13	5	47.40	8.60
-4	13	-5	44.30	14.90
-4	13	-5	41.30	9.60
-4	13	-5	37.00	10.30
4	-13	5	42.50	8.40
4	-13	5	30.90	8.40
-5	13	-5	1.80	11.90
-5	13	-5	13.80	11.80
-5	13	-5	9.20	10.50
-5	13	-5	-9.80	16.90
5	-13	5	13.60	9.30
5	-13	5	3.10	7.50
-6	13	-5	146.39	20.00
-6	13	-5	186.38	21.60
-6	13	-5	170.48	17.10
6	-13	5	139.89	19.30
6	-13	5	160.88	15.90
-7	13	-5	-9.40	14.00
-7	13	-5	-5.30	13.50
-7	13	-5	5.10	11.30
7	-13	5	-14.40	15.00
7	-13	5	-8.30	11.00
-8	13	-5	8.40	12.50
-8	13	-5	39.60	12.10
-8	13	-5	5.00	11.20
8	-13	5	10.90	7.10
-9	13	-5	399.16	29.60
-9	13	-5	370.16	27.40
-9	13	-5	400.06	26.40
9	-13	5	396.96	22.40
10	12	-5	1.00	11.70
-10	-12	5	6.70	5.20
9	12	-5	61.99	13.00
-9	-12	5	35.70	7.10
-9	-12	5	40.00	12.10

8	12	-5	-2.50	14.20
-8	-12	5	34.40	10.10
-8	-12	5	39.30	12.30
-8	-12	5	20.20	10.00
7	12	-5	36.20	13.50
-7	-12	5	46.60	12.20
-7	-12	5	34.30	7.50
-7	-12	5	44.00	8.70
-7	-12	5	27.30	11.40
6	12	-5	126.69	21.30
6	12	-5	137.49	22.10
-6	-12	5	122.99	18.50
-6	-12	5	104.99	17.90
-6	-12	5	127.09	13.50
-6	-12	5	130.79	13.00
5	12	-5	19.60	13.40
5	12	-5	24.60	9.50
-5	-12	5	15.10	9.70
-5	-12	5	11.00	7.10
-5	-12	5	10.70	6.70
-5	-12	5	-9.90	10.40
4	12	-5	6.30	7.10
-4	-12	5	12.70	6.40
-4	-12	5	8.90	9.00
-4	-12	5	19.20	10.70
-4	-12	5	12.70	6.70
3	12	-5	1035.80	59.49
3	12	-5	1222.38	55.09
-3	-12	5	1209.58	55.39
-3	-12	5	1177.78	55.19
-3	-12	5	1260.07	57.49
-3	-12	5	1042.70	57.39
2	12	-5	475.05	24.90
-2	-12	5	489.25	26.10
-2	-12	5	497.55	28.00
-2	-12	5	470.75	25.70
-2	-12	5	436.66	26.30
1	12	-5	98.99	10.20
1	12	-5	99.39	13.30
-1	-12	5	100.19	14.00
-1	-12	5	119.19	11.90
-1	-12	5	103.09	15.30
0	12	-5	26.00	6.30
0	12	-5	37.20	8.50

0	12	-5	33.90	6.40
0	-12	5	34.60	7.50
0	-12	5	27.90	8.40
-1	12	-5	263.47	20.50
-1	12	-5	247.68	16.80
-1	12	-5	272.07	17.30
1	-12	5	271.37	23.90
1	-12	5	260.67	18.40
1	-12	5	287.57	18.60
-2	12	-5	9.60	5.90
-2	12	-5	-10.40	10.50
2	-12	5	0.60	7.90
2	-12	5	12.90	10.00
2	-12	5	5.90	7.30
-3	12	-5	214.78	16.30
-3	12	-5	245.18	23.50
-3	12	-5	184.68	18.00
3	-12	5	225.38	18.00
3	-12	5	186.78	16.70
-4	12	-5	403.76	25.50
-4	12	-5	369.76	24.00
-4	12	-5	366.06	31.20
4	-12	5	370.76	23.50
4	-12	5	372.66	22.80
-5	12	-5	325.77	33.50
-5	12	-5	370.36	25.10
-5	12	-5	384.36	26.50
-5	12	-5	390.16	26.70
5	-12	5	365.36	24.20
5	-12	5	357.36	22.70
-6	12	-5	-0.60	11.80
-6	12	-5	31.70	12.70
-6	12	-5	28.90	13.00
6	-12	5	17.70	8.60
6	-12	5	21.20	10.80
-7	12	-5	44.70	13.00
-7	12	-5	33.40	13.70
-7	12	-5	34.10	12.20
7	-12	5	51.79	15.60
7	-12	5	22.10	7.60
-8	12	-5	132.09	21.10
-8	12	-5	147.89	19.90
-8	12	-5	104.99	16.70
8	-12	5	157.88	15.00

-9	12	-5	11.60	11.20
-9	12	-5	-0.80	13.90
-9	12	-5	24.50	12.30
9	-12	5	16.20	7.40
-10	12	-5	-17.50	14.70
-10	12	-5	23.70	13.10
-10	12	-5	31.50	12.90
10	-12	5	24.00	8.10
10	11	-5	30.30	10.70
-10	-11	5	24.00	6.30
-10	-11	5	23.60	15.50
9	11	-5	4.20	11.50
-9	-11	5	20.30	10.90
-9	-11	5	5.80	6.70
8	11	-5	59.29	13.60
-8	-11	5	72.79	11.40
-8	-11	5	75.39	17.00
7	11	-5	236.68	23.10
-7	-11	5	230.18	20.60
-7	-11	5	229.38	16.30
-7	-11	5	177.68	21.70
-7	-11	5	227.28	17.90
-7	-11	5	219.58	21.50
6	11	-5	80.89	14.30
6	11	-5	80.89	12.90
-6	-11	5	61.49	10.40
-6	-11	5	86.39	12.70
-6	-11	5	80.39	12.50
-6	-11	5	84.59	13.10
-6	-11	5	76.59	11.80
5	11	-5	155.28	14.90
5	11	-5	178.18	22.70
-5	-11	5	146.19	19.70
-5	-11	5	133.79	14.10
-5	-11	5	133.69	14.00
-5	-11	5	155.68	16.90
-5	-11	5	141.99	12.80
4	11	-5	58.29	14.30
4	11	-5	68.89	10.60
-4	-11	5	61.19	8.20
-4	-11	5	69.99	10.70
-4	-11	5	54.49	11.90
3	11	-5	34.90	14.10
3	11	-5	7.50	5.60

-3	-11	5	35.00	13.30
-3	-11	5	17.40	11.20
-3	-11	5	12.10	8.40
-3	-11	5	12.80	7.20
-3	-11	5	7.70	5.90
2	11	-5	699.93	33.00
-2	-11	5	669.03	33.80
-2	-11	5	642.64	34.70
-2	-11	5	685.13	34.20
-2	-11	5	659.63	35.60
1	11	-5	29.30	12.00
1	11	-5	32.90	5.40
1	11	-5	37.40	8.90
-1	-11	5	37.70	7.10
-1	-11	5	30.30	9.90
-1	-11	5	39.50	8.80
0	11	-5	68.89	7.90
0	11	-5	68.99	9.50
0	-11	5	53.39	8.00
0	-11	5	62.09	9.10
-1	11	-5	186.68	13.00
-1	11	-5	188.68	17.60
-1	11	-5	209.98	13.90
1	-11	5	171.68	16.50
1	-11	5	192.68	15.30
-2	11	-5	180.78	20.00
-2	11	-5	167.18	13.60
2	-11	5	172.78	14.90
2	-11	5	179.18	19.20
-3	11	-5	1481.55	72.89
-3	11	-5	1511.25	70.49
-3	11	-5	1483.15	71.19
3	-11	5	1467.85	69.89
-4	11	-5	185.28	16.40
-4	11	-5	175.88	18.40
-4	11	-5	158.08	26.40
4	-11	5	188.38	21.10
-5	11	-5	535.15	31.10
-5	11	-5	529.15	32.50
-5	11	-5	517.55	37.60
-5	11	-5	549.74	32.50
5	-11	5	537.55	29.20
5	-11	5	533.65	30.30
-6	11	-5	166.08	28.10

-6	11	-5	105.29	14.30
-6	11	-5	114.19	15.50
-6	11	-5	118.89	16.40
6	-11	5	148.19	19.00
6	-11	5	112.19	14.60
-7	11	-5	-6.80	12.10
-7	11	-5	12.80	12.30
-7	11	-5	0.10	11.30
7	-11	5	-0.20	7.60
7	-11	5	30.80	15.40
-8	11	-5	158.08	26.60
-8	11	-5	127.49	17.70
-8	11	-5	131.29	17.30
8	-11	5	114.09	15.60
8	-11	5	129.39	19.10
-9	11	-5	73.69	12.20
-9	11	-5	55.69	13.70
-9	11	-5	52.59	14.50
9	-11	5	95.79	13.60
9	-11	5	56.29	13.20
-10	11	-5	263.67	25.80
-10	11	-5	330.77	31.80
-10	11	-5	344.37	24.40
10	-11	5	350.26	20.30
10	-11	5	263.67	26.20
11	10	-5	14.20	12.30
-11	-10	5	23.80	7.00
10	10	-5	208.18	19.40
-10	-10	5	202.38	19.60
-10	-10	5	223.68	15.30
9	10	-5	22.20	11.70
-9	-10	5	6.90	10.70
-9	-10	5	13.50	6.70
8	10	-5	51.79	13.00
-8	-10	5	62.59	11.80
-8	-10	5	35.80	7.60
-8	-10	5	45.40	10.60
7	10	-5	259.47	24.90
7	10	-5	297.47	36.50
-7	-10	5	304.17	18.70
-7	-10	5	320.37	22.00
-7	-10	5	310.07	22.50
-7	-10	5	279.87	20.80
-7	-10	5	281.27	25.00

6	10	-5	17.40	9.60
6	10	-5	28.00	11.70
-6	-10	5	33.40	12.00
-6	-10	5	15.30	9.10
-6	-10	5	40.80	8.70
-6	-10	5	29.60	6.80
-6	-10	5	4.20	9.40
5	10	-5	218.68	15.30
-5	-10	5	206.88	20.00
-5	-10	5	172.78	18.80
-5	-10	5	191.58	14.60
-5	-10	5	215.28	17.10
-5	-10	5	185.38	15.50
4	10	-5	1033.60	54.89
4	10	-5	1098.09	50.79
-4	-10	5	1148.99	51.59
-4	-10	5	1108.79	50.99
-4	-10	5	1086.79	50.89
-4	-10	5	899.71	52.89
-4	-10	5	1125.49	52.19
3	10	-5	11.70	5.20
3	10	-5	1.80	13.30
-3	-10	5	4.00	9.30
-3	-10	5	7.30	6.50
-3	-10	5	14.20	5.90
-3	-10	5	20.40	12.30
2	10	-5	581.24	27.70
-2	-10	5	560.44	29.90
-2	-10	5	553.34	30.30
-2	-10	5	556.94	29.00
-2	-10	5	575.34	31.80
1	10	-5	105.89	8.20
1	10	-5	93.19	11.10
-1	-10	5	135.59	14.60
-1	-10	5	111.39	12.80
-1	-10	5	107.79	15.60
-1	-10	5	116.09	11.90
0	10	-5	409.66	20.40
0	10	-5	396.16	24.10
0	10	-5	377.36	22.60
0	-10	5	397.76	23.70
0	-10	5	404.56	23.20
0	-10	5	438.66	23.60
-1	10	-5	173.28	13.50

-1	10	-5	171.38	11.50
-1	10	-5	170.48	17.60
1	-10	5	193.38	14.90
1	-10	5	180.78	16.20
-2	10	-5	127.39	11.40
-2	10	-5	136.89	19.50
2	-10	5	96.29	13.00
-3	10	-5	1069.99	55.79
-3	10	-5	1068.99	51.79
-3	10	-5	1063.19	52.69
3	-10	5	1063.49	51.49
-4	10	-5	1087.09	52.39
-4	10	-5	1042.50	52.99
-4	10	-5	969.70	56.39
4	-10	5	1138.79	51.79
-5	10	-5	41.50	8.90
-5	10	-5	48.80	10.20
-5	10	-5	66.59	12.30
-5	10	-5	69.49	15.90
5	-10	5	44.10	8.00
-6	10	-5	17.80	11.60
-6	10	-5	45.80	16.50
-6	10	-5	35.90	10.50
-6	10	-5	33.30	11.90
6	-10	5	45.00	11.40
6	-10	5	51.49	8.90
-7	10	-5	30.20	10.90
-7	10	-5	17.80	13.60
-7	10	-5	27.40	12.70
7	-10	5	40.20	9.20
-8	10	-5	210.38	22.60
-8	10	-5	264.87	24.40
-8	10	-5	193.08	22.70
8	-10	5	233.78	18.00
-9	10	-5	93.09	15.50
-9	10	-5	82.89	15.40
-9	10	-5	107.79	17.30
9	-10	5	136.89	17.30
9	-10	5	115.09	14.90
-10	10	-5	11.00	10.90
-10	10	-5	-15.60	12.20
-10	10	-5	-8.40	14.70
10	-10	5	-17.00	8.80
10	-10	5	7.20	11.10

11	9	-5	13.90	10.40
-11	-9	5	5.80	6.50
-11	-9	5	2.10	11.60
10	9	-5	54.89	12.50
-10	-9	5	42.60	11.10
-10	-9	5	74.39	11.10
9	9	-5	279.07	30.40
-9	-9	5	278.47	21.80
-9	-9	5	288.77	25.20
8	9	-5	115.29	20.70
-8	-9	5	166.88	13.80
-8	-9	5	159.38	20.30
-8	-9	5	137.89	16.50
7	9	-5	-9.50	12.00
7	9	-5	-7.60	10.80
-7	-9	5	5.20	13.10
-7	-9	5	-10.60	8.20
-7	-9	5	5.20	5.90
-7	-9	5	-9.20	9.80
-7	-9	5	14.30	8.80
6	9	-5	7.70	10.70
6	9	-5	-2.70	7.80
-6	-9	5	7.60	9.00
-6	-9	5	7.50	6.10
-6	-9	5	24.10	8.70
-6	-9	5	9.70	7.40
5	9	-5	451.15	23.00
5	9	-5	422.86	28.00
-5	-9	5	411.76	23.50
-5	-9	5	389.96	25.50
-5	-9	5	429.66	23.80
-5	-9	5	419.56	22.70
-5	-9	5	384.66	26.50
4	9	-5	549.35	26.60
4	9	-5	511.45	31.60
-4	-9	5	469.05	29.80
-4	-9	5	464.65	30.80
-4	-9	5	507.95	26.50
-4	-9	5	552.94	27.30
-4	-9	5	515.45	26.80
3	9	-5	2.90	13.60
3	9	-5	11.30	4.20
-3	-9	5	27.00	9.90
-3	-9	5	13.80	5.10

-3	-9	5	16.50	6.80
-3	-9	5	13.90	5.50
2	9	-5	865.61	42.20
2	9	-5	947.61	41.70
2	9	-5	875.71	46.30
-2	-9	5	913.81	42.50
-2	-9	5	822.02	47.30
-2	-9	5	903.61	42.80
1	9	-5	953.50	44.40
1	9	-5	909.51	45.30
1	9	-5	1023.50	48.60
-1	-9	5	958.80	48.80
0	9	-5	58.69	6.10
0	9	-5	57.39	9.50
0	9	-5	53.49	8.20
0	-9	5	46.10	9.20
0	-9	5	67.49	8.20
-1	9	-5	138.59	11.20
-1	9	-5	127.89	9.00
-1	9	-5	125.09	11.40
-1	9	-5	124.69	15.90
1	-9	5	123.79	12.90
1	-9	5	150.18	13.40
1	-9	5	137.89	17.00
-2	9	-5	535.85	28.00
-2	9	-5	509.25	26.50
-2	9	-5	591.44	38.40
2	-9	5	537.95	28.00
2	-9	5	552.14	28.80
-3	9	-5	70.79	10.40
-3	9	-5	100.09	15.40
-3	9	-5	74.19	10.60
3	-9	5	56.69	8.50
3	-9	5	81.99	14.00
-4	9	-5	25.80	14.80
-4	9	-5	58.29	9.10
-4	9	-5	56.09	10.90
4	-9	5	63.99	9.80
4	-9	5	38.40	16.20
-5	9	-5	15.70	11.30
-5	9	-5	6.10	14.10
-5	9	-5	-1.60	9.80
-5	9	-5	-1.10	7.60
5	-9	5	10.90	8.70

-6	9	-5	24.10	20.30
-6	9	-5	34.10	11.30
-6	9	-5	47.90	10.30
-6	9	-5	37.80	11.90
6	-9	5	18.40	12.40
6	-9	5	16.70	7.10
-7	9	-5	-42.30	25.60
-7	9	-5	-1.70	11.50
-7	9	-5	5.40	9.90
-7	9	-5	-1.30	10.00
7	-9	5	-0.20	7.50
-8	9	-5	50.59	12.50
-8	9	-5	65.09	13.10
-8	9	-5	34.60	12.80
8	-9	5	47.70	9.00
-9	9	-5	613.74	39.60
-9	9	-5	716.63	41.40
-9	9	-5	698.43	39.00
9	-9	5	778.42	37.90
-10	9	-5	160.08	17.40
-10	9	-5	137.19	20.70
-10	9	-5	129.29	21.90
10	-9	5	158.98	16.60
-11	9	-5	50.99	12.00
-11	9	-5	44.40	16.70
11	-9	5	45.30	12.00
11	-9	5	53.59	13.60
11	8	-5	61.09	11.90
-11	-8	5	52.79	8.40
-11	-8	5	51.59	20.30
10	8	-5	9.90	10.60
-10	-8	5	23.10	7.20
-10	-8	5	27.60	9.70
9	8	-5	51.89	13.00
-9	-8	5	54.59	13.80
8	8	-5	25.80	11.90
-8	-8	5	2.30	8.40
-8	-8	5	23.40	13.10
-8	-8	5	11.80	6.80
7	8	-5	97.99	13.10
7	8	-5	117.69	14.90
-7	-8	5	112.79	14.50
-7	-8	5	113.39	13.10
-7	-8	5	113.09	11.80

-7	-8	5	104.39	12.80
6	8	-5	139.39	14.50
-6	-8	5	173.98	13.10
-6	-8	5	156.68	21.40
-6	-8	5	169.28	15.40
-6	-8	5	168.08	19.00
-6	-8	5	168.68	16.20
5	8	-5	44.10	11.70
5	8	-5	43.30	6.70
-5	-8	5	48.70	9.90
-5	-8	5	57.09	8.60
-5	-8	5	37.70	12.40
-5	-8	5	50.89	8.40
-5	-8	5	50.09	8.70
4	8	-5	139.29	15.00
4	8	-5	132.39	10.00
-4	-8	5	116.09	11.80
-4	-8	5	134.19	12.30
-4	-8	5	117.59	11.10
-4	-8	5	132.59	20.70
3	8	-5	2210.78	107.89
3	8	-5	2450.85	104.99
-3	-8	5	2225.28	104.59
-3	-8	5	2351.96	105.89
-3	-8	5	2351.06	105.39
2	8	-5	419.76	23.80
2	8	-5	401.76	19.00
2	8	-5	374.56	19.40
-2	-8	5	380.86	19.70
-2	-8	5	319.07	23.90
1	8	-5	24.00	4.20
1	8	-5	16.30	3.10
-1	-8	5	19.20	4.70
-1	-8	5	26.40	7.60
0	8	-5	36.30	4.90
0	8	-5	27.20	4.60
0	-8	5	37.70	7.90
0	-8	5	36.40	5.80
-1	8	-5	97.69	8.90
-1	8	-5	108.39	7.40
-1	8	-5	106.79	10.00
-1	8	-5	96.69	12.60
1	-8	5	121.29	13.70
1	-8	5	115.49	10.50

1	-8	5	82.19	9.40
-2	8	-5	2.40	7.20
-2	8	-5	13.40	5.40
-2	8	-5	1.50	4.80
-2	8	-5	10.70	4.10
2	-8	5	14.50	5.60
2	-8	5	3.20	6.50
2	-8	5	-6.80	8.60
-3	8	-5	171.08	16.90
-3	8	-5	182.98	13.30
3	-8	5	183.18	16.20
3	-8	5	181.48	13.50
-4	8	-5	693.83	39.00
-4	8	-5	671.03	34.90
-4	8	-5	676.63	33.80
4	-8	5	670.83	33.50
4	-8	5	604.24	34.60
-5	8	-5	438.66	29.90
-5	8	-5	416.86	31.60
-5	8	-5	465.85	25.60
-5	8	-5	429.26	28.70
-5	8	-5	463.15	27.00
5	-8	5	460.45	26.10
5	-8	5	448.66	26.80
-6	8	-5	89.19	13.30
-6	8	-5	64.49	12.60
-6	8	-5	71.59	14.90
-6	8	-5	70.89	12.10
6	-8	5	79.59	10.20
6	-8	5	76.69	13.80
-7	8	-5	260.57	21.50
-7	8	-5	244.58	21.50
-7	8	-5	245.28	20.00
-7	8	-5	239.28	28.00
7	-8	5	232.78	19.10
-8	8	-5	172.48	20.10
-8	8	-5	186.88	20.90
-8	8	-5	167.78	20.50
8	-8	5	172.78	17.90
-9	8	-5	5.90	11.40
-9	8	-5	7.60	11.10
-9	8	-5	16.20	13.80
9	-8	5	13.80	9.10
-10	8	-5	44.50	12.10

-10	8	-5	45.30	13.80
-10	8	-5	35.10	11.80
10	-8	5	46.50	10.90
-11	8	-5	59.99	11.40
-11	8	-5	56.19	16.30
-11	8	-5	45.90	13.20
11	-8	5	67.59	11.30
11	-8	5	60.29	14.30
11	7	-5	232.18	20.40
-11	-7	5	267.47	17.30
-11	-7	5	256.47	19.60
10	7	-5	81.19	13.70
-10	-7	5	90.59	12.20
-10	-7	5	90.99	14.60
9	7	-5	21.70	11.30
-9	-7	5	7.20	6.70
-9	-7	5	-0.20	8.10
-8	-7	5	482.85	26.10
-8	-7	5	463.85	26.60
7	7	-5	182.18	17.70
-7	-7	5	177.48	13.80
-7	-7	5	174.48	18.60
-7	-7	5	120.39	17.60
-7	-7	5	165.08	17.60
-7	-7	5	183.98	15.20
6	7	-5	89.69	11.70
-6	-7	5	92.89	10.30
-6	-7	5	97.29	13.80
-6	-7	5	86.69	11.10
-6	-7	5	74.19	10.30
-6	-7	5	89.09	16.80
5	7	-5	18.00	5.60
-5	-7	5	8.30	7.80
-5	-7	5	9.20	7.40
-5	-7	5	0.90	4.80
-5	-7	5	9.00	8.40
-5	-7	5	2.10	6.80
-5	-7	5	20.00	13.40
4	7	-5	40.10	12.00
4	7	-5	72.69	7.20
-4	-7	5	66.29	11.80
-4	-7	5	92.79	10.20
-4	-7	5	71.79	8.30
-4	-7	5	67.29	10.40

3	7	-5	43.50	5.60
-3	-7	5	41.60	6.70
-3	-7	5	45.30	6.10
2	7	-5	1184.78	52.59
2	7	-5	1130.89	52.69
2	7	-5	1275.97	56.19
-2	-7	5	1010.80	55.19
-2	-7	5	1154.68	52.69
-2	-7	5	1130.69	53.29
1	7	-5	130.89	12.20
1	7	-5	131.69	8.70
1	7	-5	144.69	8.20
-1	-7	5	157.78	13.10
-1	-7	5	140.99	9.20
-1	-7	5	134.09	10.30
0	7	-5	40.80	6.70
0	7	-5	36.50	5.20
0	7	-5	32.00	4.40
0	-7	5	28.40	4.30
0	-7	5	46.10	7.50
-1	7	-5	8.30	2.90
-1	7	-5	9.30	3.50
1	-7	5	2.80	5.90
1	-7	5	11.20	4.30
-2	7	-5	1963.30	85.19
-2	7	-5	1840.42	83.39
-2	7	-5	1792.22	82.09
-2	7	-5	1762.42	83.29
2	-7	5	1739.93	84.69
2	-7	5	1845.22	82.69
2	-7	5	1779.62	83.49
-3	7	-5	1067.19	47.50
-3	7	-5	992.40	49.80
-3	7	-5	957.50	47.40
3	-7	5	972.40	47.90
3	-7	5	1021.70	46.50
3	-7	5	937.91	49.00
-4	7	-5	126.49	11.50
-4	7	-5	121.19	14.10
4	-7	5	123.49	11.00
4	-7	5	122.59	19.20
-5	7	-5	105.89	18.70
-5	7	-5	91.69	11.40
-5	7	-5	131.29	15.30

-5	7	-5	91.49	14.00
-5	7	-5	103.29	13.40
5	-7	5	100.59	11.90
5	-7	5	99.49	13.50
-6	7	-5	54.49	8.80
-6	7	-5	37.50	10.20
-6	7	-5	72.19	13.90
-6	7	-5	47.70	10.30
6	-7	5	37.80	9.30
-7	7	-5	176.88	17.70
-7	7	-5	171.88	25.00
-7	7	-5	180.38	15.90
-7	7	-5	151.08	18.70
7	-7	5	133.19	16.80
-8	7	-5	319.07	24.60
-8	7	-5	361.86	33.00
-8	7	-5	323.37	25.00
-8	7	-5	386.16	35.60
8	-7	5	341.47	23.20
-9	7	-5	13.30	10.90
-9	7	-5	16.10	10.10
-9	7	-5	19.40	10.80
9	-7	5	16.70	9.60
-10	7	-5	321.07	23.20
-10	7	-5	323.17	25.20
-10	7	-5	344.57	25.40
10	-7	5	340.07	24.10
10	-7	5	304.07	22.90
-11	7	-5	29.40	14.70
-11	7	-5	10.10	12.50
-11	7	-5	5.00	10.00
11	-7	5	0.50	9.20
11	-7	5	-10.20	13.40
11	6	-5	59.59	12.30
-11	-6	5	37.70	8.20
-11	-6	5	36.40	9.50
10	6	-5	380.76	26.50
10	6	-5	388.56	35.50
-10	-6	5	343.17	21.50
-10	-6	5	340.57	22.30
9	6	-5	33.30	15.10
9	6	-5	58.99	12.80
-9	-6	5	47.70	8.30
-9	-6	5	30.50	8.20

8	6	-5	69.89	14.70
-8	-6	5	62.39	8.90
-8	-6	5	49.60	7.90
7	6	-5	117.09	16.00
7	6	-5	176.68	20.10
-7	-6	5	138.99	15.40
-7	-6	5	142.29	13.80
-7	-6	5	148.19	12.90
-7	-6	5	153.58	18.00
6	6	-5	216.28	15.70
-6	-6	5	238.28	16.70
-6	-6	5	209.78	19.20
-6	-6	5	219.18	15.00
-6	-6	5	214.48	16.30
-6	-6	5	235.58	18.30
5	6	-5	17.80	10.20
5	6	-5	-14.80	12.00
5	6	-5	7.80	4.40
-5	-6	5	18.10	8.60
-5	-6	5	20.70	8.10
-5	-6	5	1.40	5.40
-5	-6	5	11.30	6.50
-5	-6	5	15.80	9.00
4	6	-5	821.72	37.60
4	6	-5	778.32	39.80
-4	-6	5	836.42	39.20
-4	-6	5	763.42	38.50
3	6	-5	430.86	20.00
3	6	-5	376.26	22.90
-3	-6	5	388.56	21.60
-3	-6	5	421.86	19.60
2	6	-5	1169.68	52.29
2	6	-5	1221.78	54.09
2	6	-5	1186.98	52.09
-2	-6	5	1052.59	53.99
-2	-6	5	1224.68	52.29
-2	-6	5	995.80	52.29
1	6	-5	94.19	6.10
1	6	-5	96.69	6.50
1	6	-5	98.49	9.30
-1	-6	5	95.99	6.70
-1	-6	5	99.19	10.00
-1	-6	5	78.39	7.80
0	6	-5	271.57	15.90

0	6	-5	281.97	14.10
0	6	-5	272.57	13.60
0	-6	5	274.57	14.30
0	-6	5	243.48	14.10
0	-6	5	297.17	16.70
-1	6	-5	4.40	4.20
-1	6	-5	9.90	2.90
-1	6	-5	9.10	2.60
-1	6	-5	2.10	4.50
1	-6	5	10.50	3.40
1	-6	5	9.10	4.90
1	-6	5	3.50	3.40
-2	6	-5	-1.10	3.00
-2	6	-5	2.60	3.30
2	-6	5	-1.90	5.00
2	-6	5	1.00	3.30
-3	6	-5	557.64	28.70
-3	6	-5	507.75	26.70
-3	6	-5	566.74	26.90
3	-6	5	473.35	27.80
3	-6	5	534.05	27.20
3	-6	5	552.44	25.90
-4	6	-5	497.65	24.20
-4	6	-5	473.95	25.80
-4	6	-5	429.16	24.90
4	-6	5	466.75	23.10
4	-6	5	441.26	25.50
-5	6	-5	121.59	11.30
-5	6	-5	174.78	18.70
-5	6	-5	135.19	14.50
-5	6	-5	110.39	13.80
-5	6	-5	105.49	19.10
5	-6	5	118.39	10.60
-6	6	-5	4.20	11.10
-6	6	-5	-9.50	9.20
-6	6	-5	-5.40	6.00
-6	6	-5	0.00	7.80
-6	6	-5	1.70	8.60
6	-6	5	-6.90	6.70
-7	6	-5	16.20	8.20
-7	6	-5	16.40	7.10
-7	6	-5	15.40	9.60
7	-6	5	20.00	9.60
7	-6	5	15.10	8.40

-8	6	-5	142.09	19.10
-8	6	-5	153.88	17.40
-8	6	-5	162.88	15.90
-8	6	-5	154.48	25.80
8	-6	5	133.49	17.60
-9	6	-5	55.59	11.80
-9	6	-5	37.30	11.00
-9	6	-5	56.39	11.90
9	-6	5	50.49	10.40
-10	6	-5	16.20	11.10
-10	6	-5	-7.80	10.10
-10	6	-5	13.70	11.60
10	-6	5	-2.20	11.60
-11	6	-5	186.38	18.30
-11	6	-5	204.08	20.80
-11	6	-5	160.98	21.00
11	-6	5	199.48	18.40
11	-6	5	222.08	24.30
11	5	-5	4.80	11.70
11	5	-5	24.00	18.40
-11	-5	5	30.80	8.30
-11	-5	5	38.80	9.10
10	5	-5	40.80	15.60
10	5	-5	32.50	13.30
-10	-5	5	30.40	8.50
-10	-5	5	32.30	8.60
9	5	-5	845.32	48.30
-9	-5	5	983.00	46.30
-9	-5	5	963.50	45.80
8	5	-5	29.80	12.20
-8	-5	5	38.00	7.80
-8	-5	5	12.20	11.50
-8	-5	5	34.30	7.50
7	5	-5	65.29	12.20
7	5	-5	51.29	9.60
-7	-5	5	36.50	6.80
-7	-5	5	58.59	14.90
-7	-5	5	46.20	10.80
-7	-5	5	54.69	11.80
6	5	-5	135.99	20.90
6	5	-5	95.29	13.10
-6	-5	5	104.09	12.70
-6	-5	5	91.99	11.80
-6	-5	5	113.69	10.80

-6	-5	5	83.29	10.80
-6	-5	5	84.29	11.10
5	5	-5	598.34	34.30
5	5	-5	629.54	30.60
5	5	-5	618.84	33.40
-5	-5	5	675.03	31.50
-5	-5	5	662.53	33.00
-5	-5	5	630.54	31.50
-5	-5	5	622.04	33.90
4	5	-5	194.58	11.00
4	5	-5	194.18	14.80
4	5	-5	185.08	13.40
-4	-5	5	200.18	13.60
3	5	-5	92.19	10.10
3	5	-5	104.59	6.70
-3	-5	5	103.49	6.10
-3	-5	5	79.49	8.80
2	5	-5	1325.77	57.49
2	5	-5	1300.87	58.99
2	5	-5	1277.57	57.49
-2	-5	5	1361.56	57.49
-2	-5	5	1210.88	58.69
-2	-5	5	1124.49	57.59
1	5	-5	459.45	21.40
1	5	-5	477.05	21.10
1	5	-5	487.35	22.80
-1	-5	5	468.75	21.40
-1	-5	5	413.06	21.60
-1	-5	5	372.76	22.80
0	5	-5	57.19	6.70
0	5	-5	60.39	4.50
0	5	-5	62.59	4.80
0	-5	5	58.99	7.80
0	-5	5	46.10	5.50
0	-5	5	58.99	4.90
-1	5	-5	110.09	6.70
-1	5	-5	113.79	7.10
1	-5	5	106.19	7.30
1	-5	5	113.19	7.50
-2	5	-5	84.89	6.70
-2	5	-5	97.59	7.00
-2	5	-5	78.19	8.10
-2	5	-5	91.49	8.30
2	-5	5	92.09	6.70

2	-5	5	91.09	8.40
-3	5	-5	682.33	33.00
3	-5	5	645.34	33.10
3	-5	5	695.03	33.80
3	-5	5	708.43	32.10
-4	5	-5	527.25	25.60
-4	5	-5	538.95	26.40
4	-5	5	446.96	26.90
4	-5	5	504.35	24.50
4	-5	5	520.45	26.70
-5	5	-5	55.89	13.70
-5	5	-5	45.10	7.60
-5	5	-5	55.09	8.50
-5	5	-5	64.89	11.30
5	-5	5	45.20	6.90
-6	5	-5	26.00	11.30
-6	5	-5	2.50	12.20
-6	5	-5	5.40	5.40
-6	5	-5	-2.20	9.10
-6	5	-5	14.10	9.50
-6	5	-5	25.90	7.50
6	-5	5	-2.10	5.40
-7	5	-5	74.69	11.10
-7	5	-5	81.89	13.30
-7	5	-5	115.09	14.20
-7	5	-5	112.39	21.00
-7	5	-5	70.99	11.80
7	-5	5	74.19	10.50
7	-5	5	75.09	12.70
-8	5	-5	304.37	20.30
-8	5	-5	315.57	22.80
-8	5	-5	305.77	21.50
-8	5	-5	341.87	27.10
-8	5	-5	325.57	26.80
8	-5	5	333.47	25.40
8	-5	5	296.97	22.70
-9	5	-5	760.22	38.80
-9	5	-5	672.03	41.90
-9	5	-5	638.74	41.80
-9	5	-5	767.52	39.20
-9	5	-5	782.02	39.80
9	-5	5	739.43	39.20
-10	5	-5	15.60	10.20
-10	5	-5	21.20	11.70

-10	5	-5	31.00	12.50
10	-5	5	29.00	13.50
-11	5	-5	32.90	14.20
-11	5	-5	33.90	11.90
-11	5	-5	21.20	10.00
11	-5	5	26.90	16.70
11	-5	5	24.10	10.40
-12	5	-5	95.69	16.50
-12	5	-5	114.99	15.70
12	-5	5	82.29	15.50
12	4	-5	5.40	17.80
-12	-4	5	0.50	8.20
-12	-4	5	-19.20	10.30
11	4	-5	123.49	22.30
11	4	-5	112.69	21.20
-11	-4	5	131.19	14.30
-11	-4	5	132.49	14.80
10	4	-5	31.70	14.80
10	4	-5	70.09	14.10
-10	-4	5	57.59	9.80
-10	-4	5	51.19	10.10
9	4	-5	100.79	13.60
-9	-4	5	92.89	13.20
-9	-4	5	116.09	13.10
8	4	-5	-7.40	10.20
-8	-4	5	14.20	6.60
-8	-4	5	-13.50	12.40
-8	-4	5	3.70	6.40
7	4	-5	368.46	23.80
7	4	-5	386.86	25.30
-7	-4	5	402.46	22.30
-7	-4	5	375.36	26.60
-7	-4	5	411.06	22.30
6	4	-5	142.09	18.10
6	4	-5	160.98	12.10
-6	-4	5	156.38	12.70
-6	-4	5	142.59	19.10
-6	-4	5	140.69	13.30
-6	-4	5	166.18	17.10
-6	-4	5	155.28	12.60
5	4	-5	17.20	4.30
5	4	-5	4.00	7.80
5	4	-5	13.50	7.00
-5	-4	5	18.60	6.30

-5	-4	5	28.10	6.30
4	4	-5	787.82	36.50
4	4	-5	805.52	37.70
4	4	-5	815.32	37.40
-4	-4	5	697.73	35.60
-4	-4	5	810.02	35.50
3	4	-5	1206.98	56.49
3	4	-5	1223.58	55.59
-3	-4	5	1235.48	55.19
2	4	-5	2.60	1.80
2	4	-5	2.40	2.00
2	4	-5	6.10	3.70
-2	-4	5	0.90	2.80
-2	-4	5	2.30	1.70
1	4	-5	322.27	15.70
1	4	-5	306.57	14.40
1	4	-5	300.37	14.20
-1	-4	5	237.08	15.50
-1	-4	5	301.07	14.30
0	4	-5	4.80	2.00
0	4	-5	8.00	3.00
0	4	-5	7.50	2.10
0	-4	5	7.80	2.80
0	-4	5	8.90	3.80
0	-4	5	8.20	2.00
-1	4	-5	10.80	3.00
-1	4	-5	10.90	2.20
-1	4	-5	11.00	2.40
1	-4	5	12.40	3.40
1	-4	5	11.70	2.60
1	-4	5	12.90	2.70
-2	4	-5	37.80	4.50
-2	4	-5	39.50	5.40
-2	4	-5	37.00	5.60
2	-4	5	37.00	4.10
2	-4	5	37.00	4.80
2	-4	5	35.50	5.50
-3	4	-5	19.20	4.30
-3	4	-5	14.50	3.90
-3	4	-5	13.80	3.30
3	-4	5	18.90	4.60
3	-4	5	12.90	3.80
3	-4	5	19.30	2.90
-4	4	-5	961.60	45.20

4	-4	5	926.31	45.30
4	-4	5	991.40	43.90
4	-4	5	932.91	45.80
-5	4	-5	140.89	14.00
-5	4	-5	136.59	10.90
-5	4	-5	173.28	23.40
-5	4	-5	148.89	12.90
5	-4	5	136.39	14.10
5	-4	5	140.69	9.20
-6	4	-5	-1.40	8.30
-6	4	-5	0.80	9.20
-6	4	-5	10.60	10.10
-6	4	-5	0.90	6.70
-6	4	-5	-1.70	4.50
-6	4	-5	-9.50	10.90
6	-4	5	-0.30	4.70
-7	4	-5	485.65	28.70
-7	4	-5	499.85	30.90
-7	4	-5	487.85	30.00
-7	4	-5	487.45	31.30
-7	4	-5	506.65	26.60
-7	4	-5	489.65	30.40
7	-4	5	477.65	27.90
7	-4	5	508.35	26.00
-8	4	-5	147.99	15.30
-8	4	-5	174.28	22.20
-8	4	-5	174.88	14.30
-8	4	-5	161.58	17.60
-8	4	-5	171.68	22.10
8	-4	5	154.48	17.60
8	-4	5	195.38	18.80
-9	4	-5	-8.90	13.00
-9	4	-5	29.50	10.20
-9	4	-5	0.40	12.10
-9	4	-5	2.60	8.20
-9	4	-5	4.90	8.40
9	-4	5	0.00	9.80
-10	4	-5	101.29	15.50
-10	4	-5	70.29	20.90
-10	4	-5	97.39	14.60
-10	4	-5	78.79	13.40
-11	4	-5	67.89	11.30
-11	4	-5	64.49	12.60
-11	4	-5	77.79	12.40

11	-4	5	53.19	12.20
11	-4	5	77.79	29.70
-12	4	-5	32.60	14.70
-12	4	-5	12.80	10.70
-12	4	-5	-19.50	19.20
12	-4	5	5.00	9.50
12	3	-5	76.09	20.70
12	3	-5	49.80	16.60
-12	-3	5	61.29	9.60
11	3	-5	57.39	13.70
11	3	-5	31.30	13.90
-11	-3	5	30.10	8.40
-11	-3	5	45.00	9.70
10	3	-5	98.99	15.20
-10	-3	5	95.19	13.20
9	3	-5	96.59	18.00
-9	-3	5	77.99	11.90
-9	-3	5	91.19	13.60
8	3	-5	851.01	43.90
-8	-3	5	870.91	42.00
-8	-3	5	816.22	46.00
-8	-3	5	872.91	42.60
-7	-3	5	113.09	14.30
-7	-3	5	98.69	11.50
6	3	-5	6.40	8.50
6	3	-5	4.90	5.30
-6	-3	5	-6.10	5.50
-6	-3	5	4.90	6.10
-6	-3	5	-5.10	9.00
-6	-3	5	2.60	11.50
-6	-3	5	11.50	9.90
5	3	-5	25.10	7.10
5	3	-5	22.60	4.30
5	3	-5	14.60	6.20
-5	-3	5	24.30	6.50
-5	-3	5	25.60	6.50
4	3	-5	72.89	6.90
4	3	-5	67.19	7.50
4	3	-5	72.89	6.00
-4	-3	5	75.49	4.70
-4	-3	5	57.59	8.60
-4	-3	5	63.69	5.30
3	3	-5	630.54	28.00
3	3	-5	610.94	28.50

3	3	-5	623.54	28.20
-3	-3	5	616.94	27.50
-3	-3	5	520.15	28.10
-3	-3	5	592.14	27.70
2	3	-5	228.98	11.10
2	3	-5	209.78	12.00
-2	-3	5	220.88	10.80
1	3	-5	296.47	13.90
1	3	-5	280.27	14.60
1	3	-5	297.47	13.70
-1	-3	5	251.67	14.50
-1	-3	5	301.97	13.80
0	3	-5	164.78	8.50
0	3	-5	157.38	8.30
0	3	-5	185.58	9.20
0	-3	5	147.49	8.90
0	-3	5	151.68	9.90
0	-3	5	157.88	8.40
-1	3	-5	3.40	1.80
-1	3	-5	1.80	2.50
-1	3	-5	0.90	1.90
1	-3	5	1.20	1.70
1	-3	5	-0.30	1.50
1	-3	5	2.00	2.10
-2	3	-5	3163.58	142.29
-2	3	-5	3356.26	142.79
2	-3	5	3058.59	142.49
2	-3	5	3006.00	141.69
2	-3	5	3279.07	142.39
-3	3	-5	346.97	18.30
-3	3	-5	397.16	18.30
-3	3	-5	370.56	18.80
3	-3	5	352.06	17.20
3	-3	5	350.46	18.50
3	-3	5	353.36	18.30
-4	3	-5	81.49	7.50
4	-3	5	77.29	8.20
4	-3	5	86.19	8.60
4	-3	5	71.59	5.40
-5	3	-5	132.99	9.80
-5	3	-5	135.29	14.10
-5	3	-5	129.99	11.60
5	-3	5	137.39	13.00
5	-3	5	138.89	8.30

5	-3	5	125.09	12.20
-6	3	-5	140.49	15.70
-6	3	-5	98.09	12.00
-6	3	-5	96.29	14.70
-6	3	-5	105.89	16.70
-6	3	-5	113.79	16.60
-6	3	-5	124.99	10.00
-6	3	-5	138.79	19.40
6	-3	5	121.09	16.80
-7	3	-5	67.09	11.70
-7	3	-5	71.59	10.20
-7	3	-5	65.29	9.30
-7	3	-5	60.69	10.40
-7	3	-5	62.29	12.20
-7	3	-5	80.09	11.60
7	-3	5	71.19	9.00
-8	3	-5	182.08	14.50
-8	3	-5	194.48	17.20
-8	3	-5	179.18	16.10
-8	3	-5	194.68	20.90
-8	3	-5	170.48	21.60
8	-3	5	162.08	15.60
8	-3	5	209.18	19.80
-9	3	-5	30.80	13.10
-9	3	-5	36.40	9.90
-9	3	-5	36.50	8.60
-9	3	-5	61.49	13.00
-9	3	-5	45.40	12.40
9	-3	5	63.19	12.10
9	-3	5	47.40	14.30
-10	3	-5	482.45	28.30
-10	3	-5	424.96	44.00
-10	3	-5	438.56	33.30
-10	3	-5	481.75	27.80
-10	3	-5	488.85	27.30
10	-3	5	442.26	34.00
-11	3	-5	96.59	13.20
-11	3	-5	87.99	15.10
-11	3	-5	83.69	11.80
11	-3	5	68.39	12.30
-12	3	-5	26.60	14.00
-12	3	-5	27.30	11.20
-12	3	-5	14.50	9.80
12	-3	5	14.30	11.00

12	2	-5	31.40	19.90
12	2	-5	20.10	13.80
-12	-2	5	14.00	10.60
-12	-2	5	32.90	13.50
11	2	-5	48.70	13.30
11	2	-5	27.00	14.20
-11	-2	5	19.80	9.70
-11	-2	5	24.20	8.10
9	2	-5	9.70	10.20
-9	-2	5	15.40	7.00
-9	-2	5	8.00	7.60
8	2	-5	340.17	22.70
-8	-2	5	324.07	20.10
-8	-2	5	333.47	20.90
7	2	-5	157.28	16.40
7	2	-5	190.68	26.80
-7	-2	5	168.18	14.00
-7	-2	5	176.58	13.40
6	2	-5	3.30	5.10
6	2	-5	2.90	7.60
-6	-2	5	5.00	6.30
-6	-2	5	9.10	6.30
5	2	-5	14.10	5.70
5	2	-5	26.80	5.70
5	2	-5	23.30	4.20
-5	-2	5	23.80	3.80
-5	-2	5	33.50	6.70
-5	-2	5	23.20	3.90
4	2	-5	569.64	25.60
4	2	-5	550.84	25.10
4	2	-5	553.44	25.40
-4	-2	5	484.65	24.60
-4	-2	5	456.85	25.40
-4	-2	5	550.84	24.40
3	2	-5	1855.61	78.79
3	2	-5	1840.32	78.69
3	2	-5	1761.72	78.59
-3	-2	5	1472.75	79.09
-3	-2	5	1830.82	78.29
-3	-2	5	1705.33	78.39
2	2	-5	2530.85	117.39
2	2	-5	2663.33	117.19
-2	-2	5	2664.13	117.09
1	2	-5	67.79	4.80

1	2	-5	82.09	5.50
1	2	-5	78.79	4.70
-1	-2	5	77.39	4.60
-1	-2	5	74.39	5.00
0	2	-5	359.36	16.40
0	2	-5	345.17	16.30
0	2	-5	354.66	16.20
0	-2	5	341.47	16.20
0	-2	5	309.97	16.90
-1	2	-5	149.88	7.80
-1	2	-5	152.68	8.10
1	-2	5	142.29	7.60
1	-2	5	146.79	7.80
1	-2	5	142.89	8.10
-2	2	-5	17.50	3.00
-2	2	-5	19.50	3.00
2	-2	5	17.40	2.30
2	-2	5	20.00	3.50
2	-2	5	12.00	2.40
-3	2	-5	504.45	23.70
-3	2	-5	501.05	24.20
3	-2	5	473.75	23.90
3	-2	5	497.05	24.10
3	-2	5	498.65	22.90
-4	2	-5	115.19	7.60
4	-2	5	98.09	8.30
4	-2	5	105.09	8.10
4	-2	5	92.19	6.00
-5	2	-5	357.16	20.80
-5	2	-5	346.77	19.20
-5	2	-5	356.86	18.50
5	-2	5	352.16	19.70
5	-2	5	363.06	17.40
5	-2	5	357.06	20.30
-6	2	-5	321.27	17.10
-6	2	-5	307.67	18.10
-6	2	-5	298.77	23.00
-6	2	-5	283.57	19.10
-6	2	-5	276.47	20.20
-6	2	-5	327.57	21.40
-6	2	-5	284.27	21.70
6	-2	5	284.37	20.20
6	-2	5	311.97	15.90
-7	2	-5	17.70	8.70

-7	2	-5	11.20	9.30
-7	2	-5	6.80	8.20
-7	2	-5	14.40	6.50
-7	2	-5	2.60	4.50
-7	2	-5	-2.80	9.70
7	-2	5	9.20	4.40
-8	2	-5	377.86	24.30
-8	2	-5	404.66	22.70
-8	2	-5	408.56	27.50
-8	2	-5	437.56	26.90
-8	2	-5	406.66	23.50
8	-2	5	387.36	26.90
8	-2	5	406.46	22.70
-9	2	-5	-5.20	10.60
-9	2	-5	11.10	7.50
-9	2	-5	17.70	11.30
-9	2	-5	-3.40	8.30
-9	2	-5	11.90	7.10
9	-2	5	6.60	11.00
9	-2	5	18.20	11.70
-10	2	-5	31.50	9.30
-10	2	-5	38.50	8.90
-10	2	-5	28.00	13.40
-10	2	-5	41.70	14.20
-10	2	-5	18.90	9.10
10	-2	5	25.40	15.60
-11	2	-5	157.28	16.80
-11	2	-5	180.98	17.10
-11	2	-5	169.78	17.40
11	-2	5	178.38	20.00
-12	2	-5	52.59	13.80
-12	2	-5	38.60	11.90
-12	2	-5	24.60	9.90
12	-2	5	40.00	11.60
12	1	-5	56.39	19.10
12	1	-5	61.79	18.10
-12	-1	5	86.69	14.00
-12	-1	5	76.99	15.50
11	1	-5	26.30	15.60
11	1	-5	52.89	12.40
-11	-1	5	55.19	11.20
-11	-1	5	80.09	12.70
10	1	-5	184.48	32.80
-10	-1	5	123.79	16.80

9	1	-5	681.03	38.00
-9	-1	5	696.03	36.60
-9	-1	5	722.13	36.00
8	1	-5	87.39	14.90
-8	-1	5	72.69	9.70
-8	-1	5	81.39	11.80
7	1	-5	45.50	17.20
7	1	-5	43.60	8.90
-7	-1	5	44.40	7.70
-7	-1	5	45.30	7.80
6	1	-5	38.10	8.50
-6	-1	5	42.10	7.20
-6	-1	5	54.99	7.90
5	1	-5	89.39	8.70
5	1	-5	69.19	7.10
5	1	-5	84.19	8.90
-5	-1	5	82.59	5.90
-5	-1	5	73.79	7.50
4	1	-5	1224.68	53.89
4	1	-5	1240.18	54.09
4	1	-5	1225.38	54.09
-4	-1	5	1127.59	53.69
-4	-1	5	1059.19	54.89
-4	-1	5	1213.18	53.49
3	1	-5	1373.26	61.29
3	1	-5	1438.46	61.49
3	1	-5	1316.87	61.39
-3	-1	5	1372.26	61.19
-3	-1	5	1295.27	61.29
2	1	-5	4105.89	178.98
2	1	-5	3984.10	179.08
2	1	-5	3994.40	179.08
-2	-1	5	3987.30	179.18
1	1	-5	32.40	3.20
1	1	-5	26.70	3.30
-1	-1	5	35.30	2.80
-1	-1	5	37.20	2.70
0	1	-5	325.07	14.50
0	1	-5	322.37	14.70
0	-1	5	331.07	14.70
0	-1	5	289.07	14.80
0	-1	5	267.67	14.70
-1	1	-5	872.51	38.30
-1	1	-5	879.11	38.20

1	-1	5	742.13	37.80
1	-1	5	868.71	38.30
1	-1	5	830.52	38.20
-2	1	-5	377.46	17.60
-2	1	-5	372.56	17.80
2	-1	5	366.36	16.90
2	-1	5	368.86	17.60
2	-1	5	355.36	17.70
-3	1	-5	2085.69	93.39
-3	1	-5	2053.59	92.89
3	-1	5	2043.90	92.99
3	-1	5	2096.09	93.29
3	-1	5	2035.50	92.29
-4	1	-5	18.50	4.20
4	-1	5	27.10	2.80
4	-1	5	17.20	4.50
4	-1	5	27.60	4.50
-5	1	-5	229.98	13.00
-5	1	-5	199.88	14.60
-5	1	-5	232.48	12.90
5	-1	5	226.38	13.70
5	-1	5	210.78	14.40
5	-1	5	234.38	11.50
-6	1	-5	37.50	9.20
-6	1	-5	34.80	8.40
-6	1	-5	35.40	9.20
-6	1	-5	32.90	6.80
-6	1	-5	41.70	7.20
-6	1	-5	22.00	4.70
-6	1	-5	10.20	8.60
6	-1	5	23.10	7.50
6	-1	5	29.70	4.20
-7	1	-5	121.89	16.70
-7	1	-5	126.89	12.30
-7	1	-5	118.89	17.50
-7	1	-5	111.29	13.60
-7	1	-5	130.79	17.00
-7	1	-5	111.79	10.20
7	-1	5	121.29	9.10
-8	1	-5	580.14	32.10
-8	1	-5	560.04	30.10
-8	1	-5	585.14	30.80
-8	1	-5	567.64	29.40
-8	1	-5	560.94	33.30

8	-1	5	563.44	29.10
-9	1	-5	411.36	28.40
-9	1	-5	421.16	24.00
-9	1	-5	407.66	27.10
-9	1	-5	430.66	24.60
-9	1	-5	437.76	25.40
9	-1	5	388.26	25.60
9	-1	5	403.36	28.50
-10	1	-5	34.50	12.70
-10	1	-5	53.39	13.50
-10	1	-5	34.60	8.60
-10	1	-5	40.40	9.20
-10	1	-5	44.90	9.20
10	-1	5	41.80	18.90
-11	1	-5	29.50	9.40
-11	1	-5	17.40	9.50
-11	1	-5	18.10	9.00
-11	1	-5	15.30	16.00
11	-1	5	15.90	11.80
-12	1	-5	103.69	15.50
-12	1	-5	101.29	15.10
-12	1	-5	102.39	17.50
12	-1	5	117.59	17.30
12	0	-5	66.19	21.80
12	0	-5	25.70	16.70
-12	0	5	-12.80	17.10
11	0	-5	22.00	11.60
11	0	-5	31.20	16.60
-11	0	5	33.50	11.50
-11	0	5	15.10	7.60
10	0	-5	8.90	11.60
-10	0	5	13.70	13.90
-10	0	5	3.10	8.40
9	0	-5	1.70	9.40
-9	0	5	9.40	8.30
-9	0	5	-1.50	7.30
8	0	-5	16.20	8.90
-8	0	5	7.70	6.90
-8	0	5	1.30	6.70
7	0	-5	91.99	13.90
-7	0	5	94.59	12.80
-7	0	5	102.29	12.20
6	0	-5	25.90	8.50
6	0	-5	33.60	6.30

-6	0	5	34.00	7.30
-6	0	5	41.10	7.40
5	0	-5	1.90	4.80
5	0	-5	1.80	4.40
-5	0	5	1.40	4.60
-5	0	5	3.00	3.20
-5	0	5	2.00	7.70
4	0	-5	2400.56	107.49
4	0	-5	2338.97	107.39
4	0	-5	2559.94	107.59
-4	0	5	2286.67	107.49
-4	0	5	2511.05	107.29
-4	0	5	2251.07	107.49
3	0	-5	3255.17	138.09
3	0	-5	3110.19	138.09
3	0	-5	3048.99	138.09
-3	0	5	3232.98	138.19
-3	0	5	2861.11	138.19
-3	0	5	3036.70	138.29
2	0	-5	163.38	8.70
2	0	-5	176.48	8.30
2	0	-5	174.48	8.10
-2	0	5	170.88	8.70
-2	0	5	157.78	8.70
-2	0	5	179.78	8.70
1	0	-5	62.39	3.20
-1	0	5	58.09	3.90
0	0	-5	1607.04	72.09
0	0	-5	1677.93	72.59
0	0	5	1551.14	72.39
0	0	5	1530.65	72.19
0	0	5	1707.13	72.49
-1	0	-5	28.00	2.60
-1	0	-5	24.70	2.70
1	0	5	25.50	2.60
1	0	5	30.20	1.90
-2	0	-5	180.78	9.30
-2	0	-5	184.98	9.50
2	0	5	177.88	9.40
2	0	5	188.28	9.40
-3	0	-5	129.69	7.20
-3	0	-5	120.09	7.80
3	0	5	132.09	7.70
3	0	5	129.59	7.40

-4	0	-5	1930.11	86.09
4	0	5	1836.92	86.29
4	0	5	1956.20	86.99
-5	0	-5	102.49	7.40
5	0	5	75.29	8.00
5	0	5	82.59	5.40
5	0	5	83.39	9.30
-6	0	-5	9.20	5.60
-6	0	-5	-1.00	5.30
-6	0	-5	14.90	7.10
-6	0	-5	-2.90	8.10
-6	0	-5	0.00	9.10
-6	0	-5	18.80	8.90
6	0	5	5.00	6.40
6	0	5	6.70	2.90
-7	0	-5	289.67	21.60
-7	0	-5	298.47	17.10
-7	0	-5	314.67	21.20
-7	0	-5	306.87	19.20
-7	0	-5	309.07	22.60
-7	0	-5	307.37	18.50
7	0	5	290.27	16.00
-8	0	-5	3.80	8.80
-8	0	-5	12.60	7.30
-8	0	-5	11.50	9.30
-8	0	-5	11.40	5.20
-8	0	-5	15.00	6.40
8	0	5	-0.70	12.10
8	0	5	2.80	5.10
-9	0	-5	112.09	11.90
-9	0	-5	96.79	17.80
-9	0	-5	116.09	17.60
-9	0	-5	101.39	14.90
-9	0	-5	121.29	13.30
9	0	5	115.09	14.70
-10	0	-5	103.89	13.60
-10	0	-5	81.09	13.00
-10	0	-5	108.09	15.00
-10	0	-5	89.39	13.00
-10	0	-5	75.29	13.70
10	0	5	113.39	18.10
-11	0	-5	76.09	11.10
-11	0	-5	57.59	10.40
-11	0	-5	86.19	15.00

-11	0	-5	42.80	14.60
11	0	5	59.29	12.50
-12	0	-5	23.40	9.80
-12	0	-5	21.80	8.90
-12	0	-5	8.90	11.30
12	0	5	39.40	11.50
12	-1	-5	44.40	12.80
12	-1	-5	68.99	31.50
-12	1	5	83.99	20.40
-12	1	5	60.49	11.10
11	-1	-5	46.00	18.60
11	-1	-5	17.50	10.90
-11	1	5	30.30	8.10
-11	1	5	45.50	12.50
10	-1	-5	44.60	11.80
-10	1	5	46.50	10.20
9	-1	-5	231.38	19.50
-9	1	5	179.38	17.60
-9	1	5	194.38	16.60
8	-1	-5	587.04	32.80
-8	1	5	591.84	32.40
-8	1	5	640.44	32.00
7	-1	-5	20.40	8.40
-7	1	5	-2.20	7.00
-7	1	5	3.90	6.00
6	-1	-5	13.00	6.80
6	-1	-5	27.40	8.30
-6	1	5	48.80	7.60
-6	1	5	9.90	6.40
5	-1	-5	179.08	12.50
5	-1	-5	204.38	11.60
-5	1	5	152.58	12.10
-5	1	5	191.78	12.00
4	-1	-5	21.40	5.50
4	-1	-5	27.40	3.60
-4	1	5	31.90	4.60
-4	1	5	27.90	4.60
3	-1	-5	222.98	11.00
-3	1	5	223.88	11.40
-3	1	5	229.48	11.30
2	-1	-5	267.27	11.70
2	-1	-5	244.98	12.20
-2	1	5	235.98	12.10
-2	1	5	260.47	12.30

-1	1	5	1662.13	67.49
-1	1	5	1340.07	67.29
0	-1	-5	535.45	23.70
0	1	5	498.95	23.90
0	1	5	518.35	23.80
0	1	5	560.84	24.30
-1	-1	-5	65.19	4.00
-1	-1	-5	64.99	3.80
1	1	5	63.29	4.00
1	1	5	62.39	3.70
-2	-1	-5	9999.00	444.66
-3	-1	-5	389.86	17.60
-3	-1	-5	378.06	18.20
3	1	5	359.76	18.10
3	1	5	358.46	16.90
3	1	5	372.06	17.80
-4	-1	-5	971.50	44.50
-4	-1	-5	935.71	45.50
4	1	5	946.61	44.70
4	1	5	1020.30	45.50
4	1	5	993.90	44.00
-5	-1	-5	316.67	16.10
5	1	5	301.47	16.70
5	1	5	335.67	15.50
5	1	5	325.37	18.20
-6	-1	-5	250.97	16.90
-6	-1	-5	254.37	14.30
-6	-1	-5	253.77	19.80
-6	-1	-5	261.37	15.70
-6	-1	-5	271.37	18.60
-6	-1	-5	201.88	20.80
-6	-1	-5	234.98	15.60
6	1	5	243.58	16.90
-7	-1	-5	4.80	9.40
-7	-1	-5	12.60	4.20
-7	-1	-5	12.90	6.90
-7	-1	-5	16.90	9.30
-7	-1	-5	11.80	5.70
-7	-1	-5	25.50	8.00
7	1	5	7.60	3.20
-8	-1	-5	18.00	7.30
-8	-1	-5	44.50	9.70
-8	-1	-5	35.50	7.30
-8	-1	-5	40.30	10.20

-8	-1	-5	31.10	6.10
8	1	5	24.90	5.50
-9	-1	-5	325.87	19.20
-9	-1	-5	330.57	20.80
-9	-1	-5	277.77	21.80
-9	-1	-5	315.87	20.00
-9	-1	-5	300.87	23.50
9	1	5	306.17	20.20
-10	-1	-5	213.48	22.40
-10	-1	-5	226.88	17.20
-10	-1	-5	222.38	17.30
-10	-1	-5	253.77	21.70
-10	-1	-5	244.08	18.00
10	1	5	201.08	24.50
-11	-1	-5	196.08	17.10
-11	-1	-5	178.08	22.60
-11	-1	-5	214.08	17.80
-11	-1	-5	204.78	17.40
11	1	5	208.98	21.50
-12	-1	-5	20.50	9.30
-12	-1	-5	9.40	7.90
-12	-1	-5	10.10	10.80
12	1	5	19.90	11.80
12	-2	-5	34.40	12.10
-12	2	5	14.30	12.60
11	-2	-5	80.79	21.50
11	-2	-5	92.49	16.50
-11	2	5	50.09	13.90
-11	2	5	78.39	12.80
10	-2	-5	389.86	39.80
10	-2	-5	473.45	31.70
-10	2	5	475.25	29.30
9	-2	-5	40.90	10.20
-9	2	5	28.20	9.80
8	-2	-5	154.48	16.90
-8	2	5	155.58	14.80
-8	2	5	149.99	15.70
7	-2	-5	18.10	7.80
7	-2	-5	27.80	16.60
-7	2	5	36.10	8.60
-7	2	5	27.40	6.90
6	-2	-5	5.40	7.40
6	-2	-5	3.60	18.30
-6	2	5	5.90	6.40

-6	2	5	8.60	6.00
5	-2	-5	196.28	12.50
5	-2	-5	195.68	13.10
-5	2	5	189.98	12.20
-5	2	5	211.98	13.60
4	-2	-5	67.69	6.10
4	-2	-5	72.09	6.60
-4	2	5	69.29	6.30
-4	2	5	70.89	5.60
3	-2	-5	2125.09	93.09
3	-2	-5	1998.80	93.09
-3	2	5	2102.69	93.49
2	-2	-5	2274.07	98.09
-2	2	5	2197.68	98.59
-2	2	5	2119.59	98.29
-1	2	5	1875.51	80.09
-1	2	5	1689.63	79.69
0	2	5	1445.26	61.59
0	2	5	1379.26	62.09
0	2	5	1315.27	61.49
1	2	5	322.87	15.10
1	2	5	325.77	15.30
-2	-2	-5	116.59	6.70
-2	-2	-5	125.89	6.40
2	2	5	124.69	6.50
2	2	5	119.89	6.70
-3	-2	-5	3127.19	140.79
-3	-2	-5	3197.98	141.39
3	2	5	3241.38	140.89
3	2	5	2996.80	140.09
3	2	5	3196.08	141.49
-4	-2	-5	77.09	7.10
-4	-2	-5	98.69	5.70
4	2	5	84.29	6.10
4	2	5	97.49	5.10
4	2	5	95.49	7.00
-5	-2	-5	369.46	17.70
5	2	5	379.86	17.30
5	2	5	360.86	19.80
5	2	5	344.07	18.10
-6	-2	-5	1.40	7.00
-6	-2	-5	-3.10	4.00
-6	-2	-5	8.10	8.20
-6	-2	-5	0.90	10.10

-6	-2	-5	10.60	7.60
-6	-2	-5	2.60	3.50
-6	-2	-5	6.00	5.10
6	2	5	2.70	2.60
-7	-2	-5	86.69	11.90
-7	-2	-5	99.19	8.90
-7	-2	-5	99.19	14.30
-7	-2	-5	107.99	13.50
-7	-2	-5	109.19	17.60
-7	-2	-5	84.69	10.70
-8	-2	-5	522.65	29.80
-8	-2	-5	539.65	29.00
-8	-2	-5	563.24	28.10
-8	-2	-5	545.85	31.00
8	2	5	546.15	27.30
-9	-2	-5	5.00	6.50
-9	-2	-5	-1.00	7.30
-9	-2	-5	-3.00	8.30
-9	-2	-5	24.10	10.30
-9	-2	-5	10.50	5.90
9	2	5	4.60	7.00
-10	-2	-5	24.50	9.90
-10	-2	-5	43.90	8.40
-10	-2	-5	31.50	8.00
-10	-2	-5	39.90	11.70
-10	-2	-5	20.40	7.70
10	2	5	55.39	13.60
-11	-2	-5	310.27	20.60
-11	-2	-5	322.67	20.60
-11	-2	-5	303.87	20.10
-11	-2	-5	290.67	24.90
11	2	5	338.67	24.80
-12	-2	-5	100.39	13.80
-12	-2	-5	96.69	15.70
12	2	5	91.99	17.30
12	-3	-5	-4.80	11.00
-12	3	5	-0.30	13.70
11	-3	-5	19.20	10.30
-11	3	5	8.60	14.30
-11	3	5	32.50	8.30
-10	3	5	88.89	15.10
9	-3	-5	424.86	25.90
-9	3	5	405.36	24.50
-9	3	5	401.66	26.30

8	-3	-5	205.28	17.90
8	-3	-5	166.38	20.20
-8	3	5	191.78	18.40
-8	3	5	187.18	16.10
7	-3	-5	157.58	18.20
-7	3	5	221.18	16.20
-7	3	5	204.28	15.40
6	-3	-5	18.60	8.00
6	-3	-5	-3.10	8.60
-6	3	5	1.20	6.30
5	-3	-5	38.00	6.50
-5	3	5	44.30	7.40
-5	3	5	37.70	5.20
4	-3	-5	107.59	8.80
4	-3	-5	113.69	7.80
-4	3	5	102.79	7.00
-4	3	5	92.19	7.80
-4	3	5	101.19	6.80
3	-3	-5	14.20	3.60
3	-3	-5	23.70	3.70
-3	3	5	16.50	2.90
-3	3	5	20.50	3.60
-3	3	5	18.20	2.40
2	-3	-5	945.21	45.90
-2	3	5	1044.40	46.40
-2	3	5	1011.00	45.90
-2	3	5	1066.09	45.70
1	-3	-5	434.06	19.20
-1	3	5	404.66	19.10
-1	3	5	429.26	19.80
0	3	5	131.19	6.90
0	3	5	142.79	7.10
1	3	5	248.28	12.10
1	3	5	251.87	11.90
2	3	5	60.99	3.90
2	3	5	57.79	4.20
3	3	5	1398.96	62.89
3	3	5	1362.56	62.19
4	3	5	641.64	30.80
4	3	5	690.53	30.20
5	3	5	52.19	4.00
5	3	5	45.40	7.40
5	3	5	46.60	5.40
-6	-3	-5	585.74	28.50

-6	-3	-5	564.84	29.80
-6	-3	-5	590.74	31.00
-6	-3	-5	514.65	32.90
-6	-3	-5	498.65	29.10
-6	-3	-5	544.15	29.00
6	3	5	642.34	26.90
-7	-3	-5	593.14	28.10
-7	-3	-5	554.94	28.50
-7	-3	-5	561.24	29.10
-7	-3	-5	554.64	32.60
-7	-3	-5	568.44	29.70
-7	-3	-5	550.64	30.90
7	3	5	549.15	27.00
-8	-3	-5	723.33	35.80
-8	-3	-5	719.43	38.30
-8	-3	-5	714.13	36.30
-8	-3	-5	755.32	36.90
-8	-3	-5	724.93	37.30
8	3	5	727.13	35.30
-9	-3	-5	252.37	16.00
-9	-3	-5	253.17	17.60
-9	-3	-5	238.08	16.80
-9	-3	-5	235.28	20.60
-9	-3	-5	204.48	18.70
9	3	5	238.38	16.90
-10	-3	-5	54.19	11.60
-10	-3	-5	55.79	9.50
-10	-3	-5	45.00	8.50
-10	-3	-5	59.79	10.60
-10	-3	-5	50.89	8.70
10	3	5	68.99	13.30
-11	-3	-5	52.79	8.90
-11	-3	-5	49.10	9.20
-11	-3	-5	49.60	9.30
-11	-3	-5	49.90	11.90
11	3	5	54.39	13.20
-12	-3	-5	265.17	17.90
-12	-3	-5	225.18	18.90
-12	-3	-5	274.37	25.70
-12	-3	-5	260.47	18.30
12	3	5	245.78	21.40
12	-4	-5	14.20	17.70
-12	4	5	6.60	10.10
11	-4	-5	25.70	10.00

-11	4	5	30.70	8.10
-11	4	5	24.00	16.40
10	-4	-5	109.89	30.40
10	-4	-5	104.49	19.50
-10	4	5	67.99	15.10
-10	4	5	67.29	11.50
9	-4	-5	137.99	21.20
9	-4	-5	152.28	16.60
-9	4	5	165.28	15.90
-9	4	5	112.29	18.50
8	-4	-5	31.60	9.40
8	-4	-5	23.50	10.20
-8	4	5	17.40	7.30
-8	4	5	37.20	9.80
7	-4	-5	110.79	15.50
7	-4	-5	134.59	16.70
-7	4	5	95.19	14.30
6	-4	-5	79.29	12.90
6	-4	-5	66.39	9.10
-6	4	5	102.99	11.60
5	-4	-5	152.68	14.80
5	-4	-5	112.59	12.50
-5	4	5	128.29	9.40
-5	4	5	135.39	10.90
-5	4	5	109.89	12.70
4	-4	-5	2152.88	92.09
4	-4	-5	1946.81	91.99
-4	4	5	2098.69	91.79
-4	4	5	1986.80	92.29
-4	4	5	1961.90	91.39
3	-4	-5	811.92	37.60
3	-4	-5	802.22	37.60
-3	4	5	819.12	37.90
-3	4	5	792.72	37.00
-3	4	5	826.02	37.20
2	-4	-5	1778.12	87.89
2	-4	-5	1992.80	88.09
-2	4	5	2088.59	88.19
-2	4	5	1970.70	87.59
-2	4	5	1950.30	87.59
1	-4	-5	45.30	3.80
1	-4	-5	45.50	3.60
-1	4	5	51.59	2.90
-1	4	5	48.90	3.70

-1	4	5	38.40	3.50
0	4	5	212.08	10.20
0	4	5	199.68	10.60
1	4	5	12.00	2.10
1	4	5	18.40	2.60
2	4	5	137.79	7.10
2	4	5	128.79	7.40
3	4	5	1172.68	52.09
3	4	5	1093.39	51.19
4	4	5	1288.17	58.49
4	4	5	1149.19	58.09
4	4	5	1386.06	58.09
4	4	5	1362.26	59.99
-5	-4	-5	2.50	3.40
5	4	5	6.80	5.00
5	4	5	5.90	2.00
5	4	5	9.00	2.80
-6	-4	-5	70.89	12.30
-6	-4	-5	68.89	11.50
-6	-4	-5	69.59	10.60
-6	-4	-5	77.39	13.70
-6	-4	-5	59.59	9.10
-6	-4	-5	62.49	9.20
6	4	5	65.09	5.80
-7	-4	-5	348.57	21.40
-7	-4	-5	339.27	26.00
-7	-4	-5	341.87	22.60
-7	-4	-5	353.56	19.00
-7	-4	-5	371.06	20.20
-7	-4	-5	352.46	20.40
7	4	5	364.16	18.20
-8	-4	-5	-0.70	6.70
-8	-4	-5	5.90	5.00
-8	-4	-5	8.00	8.30
-8	-4	-5	-1.50	6.00
-8	-4	-5	10.10	6.70
-9	-4	-5	15.60	8.10
-9	-4	-5	22.00	9.10
-9	-4	-5	10.00	6.70
-9	-4	-5	1.30	5.70
-9	-4	-5	23.00	8.00
9	4	5	9.60	6.90
-10	-4	-5	421.86	24.60
-10	-4	-5	440.56	25.40

-10	-4	-5	441.66	29.00
-10	-4	-5	414.56	26.40
-10	-4	-5	460.95	25.20
10	4	5	438.96	28.40
-11	-4	-5	0.60	7.50
-11	-4	-5	4.30	10.80
-11	-4	-5	16.20	7.90
-11	-4	-5	3.00	7.90
11	4	5	12.10	12.30
-12	-4	-5	258.57	17.80
-12	-4	-5	255.37	17.90
-12	-4	-5	245.48	19.00
-12	-4	-5	284.77	24.60
12	4	5	213.18	22.30
11	-5	-5	5.50	9.80
-11	5	5	13.00	8.20
-11	5	5	18.20	18.00
10	-5	-5	61.49	11.80
-10	5	5	41.40	14.80
-10	5	5	29.10	9.60
9	-5	-5	989.30	48.50
9	-5	-5	971.40	48.60
-9	5	5	881.81	49.50
-9	5	5	963.80	47.80
8	-5	-5	260.37	20.90
8	-5	-5	274.47	20.50
-8	5	5	282.97	19.60
-8	5	5	272.77	22.60
7	-5	-5	55.89	8.80
7	-5	-5	48.80	8.90
-7	5	5	54.19	9.40
6	-5	-5	48.80	8.50
6	-5	-5	53.09	8.20
-6	5	5	51.09	7.90
5	-5	-5	98.09	11.30
5	-5	-5	115.59	12.30
5	-5	-5	134.59	14.10
5	-5	-5	126.89	14.30
-5	5	5	85.09	13.40
-5	5	5	127.69	12.80
-5	5	5	126.69	11.40
-5	5	5	105.69	8.70
4	-5	-5	14.20	4.10
4	-5	-5	10.00	5.40

-4	5	5	7.30	2.60
-4	5	5	3.40	4.40
-4	5	5	17.50	4.00
3	-5	-5	54.89	5.50
3	-5	-5	43.50	6.60
-3	5	5	50.49	4.10
-3	5	5	51.49	5.60
-3	5	5	53.89	5.00
2	-5	-5	4.80	2.30
2	-5	-5	7.80	3.30
-2	5	5	9.40	2.30
-2	5	5	8.80	1.60
-2	5	5	7.60	2.20
1	-5	-5	686.13	32.50
1	-5	-5	703.83	32.60
-1	5	5	727.83	32.60
-1	5	5	689.23	32.80
-1	5	5	717.13	31.80
0	-5	-5	26.80	3.10
0	5	5	23.60	2.70
0	5	5	26.60	4.60
1	5	5	1581.44	69.29
1	5	5	1482.65	68.89
2	5	5	3553.34	157.58
2	5	5	3578.74	157.18
2	5	5	3425.66	156.98
3	5	5	60.99	3.80
3	5	5	61.39	5.90
3	5	5	53.19	4.20
3	5	5	57.49	5.40
4	5	5	18.90	2.60
4	5	5	19.30	3.20
4	5	5	18.70	4.20
4	5	5	16.80	2.50
4	5	5	16.80	2.20
-5	-5	-5	1302.87	58.29
5	5	5	1199.98	59.59
5	5	5	1297.77	57.29
-6	-5	-5	150.58	24.80
-6	-5	-5	190.68	16.00
-6	-5	-5	184.78	12.80
-6	-5	-5	159.38	14.60
-6	-5	-5	201.58	13.60
-6	-5	-5	194.78	11.50

-7	-5	-5	-7.80	8.10
-7	-5	-5	14.70	6.10
-7	-5	-5	12.90	6.80
-7	-5	-5	9.60	6.00
-7	-5	-5	8.90	4.80
-7	-5	-5	5.50	7.60
7	5	5	14.70	3.80
-8	-5	-5	18.40	6.50
-8	-5	-5	12.30	5.30
-8	-5	-5	14.30	6.40
-8	-5	-5	5.00	6.90
-8	-5	-5	29.50	8.40
-8	-5	-5	-7.20	9.10
8	5	5	19.40	5.10
-9	-5	-5	286.57	18.90
-9	-5	-5	265.67	18.00
-9	-5	-5	291.17	21.70
-9	-5	-5	262.07	19.50
-9	-5	-5	256.67	17.20
9	5	5	272.07	18.10
-10	-5	-5	15.00	7.20
-10	-5	-5	-0.80	10.20
-10	-5	-5	12.30	7.90
-10	-5	-5	9.30	7.60
-10	-5	-5	22.90	8.70
10	5	5	10.50	12.00
-11	-5	-5	44.90	9.10
-11	-5	-5	48.60	8.80
-11	-5	-5	19.40	10.50
-11	-5	-5	34.60	8.20
11	5	5	36.80	12.20
-12	-5	-5	228.78	16.50
-12	-5	-5	198.88	21.10
-12	-5	-5	190.48	15.10
-12	-5	-5	166.78	16.90
12	5	5	216.48	22.00
11	-6	-5	153.78	17.00
-11	6	5	127.59	15.30
10	-6	-5	333.67	22.40
10	-6	-5	289.37	27.40
-10	6	5	310.67	29.10
-10	6	5	321.27	22.20
9	-6	-5	15.20	9.40
9	-6	-5	33.10	10.90

-9	6	5	30.10	9.40
-9	6	5	18.10	13.10
8	-6	-5	31.60	9.10
8	-6	-5	10.50	8.50
-8	6	5	7.30	8.20
-8	6	5	13.60	10.80
7	-6	-5	74.89	14.20
7	-6	-5	78.09	13.80
6	-6	-5	11.60	6.80
6	-6	-5	5.70	7.70
-6	6	5	13.60	7.80
5	-6	-5	13.30	6.40
5	-6	-5	6.70	7.00
5	-6	-5	31.20	8.70
5	-6	-5	9.20	9.90
-5	6	5	13.00	7.50
-5	6	5	4.80	6.60
-5	6	5	17.10	4.50
-5	6	5	1.00	8.60
-5	6	5	10.90	7.20
4	-6	-5	139.59	10.30
4	-6	-5	116.29	12.40
-4	6	5	116.89	7.80
-4	6	5	129.59	9.90
-4	6	5	138.29	11.20
3	-6	-5	2957.10	127.59
3	-6	-5	2682.13	127.79
-3	6	5	2864.11	127.79
-3	6	5	2843.82	126.69
-3	6	5	2794.82	127.19
2	-6	-5	11.70	4.00
2	-6	-5	6.40	2.50
-2	6	5	13.20	2.30
-2	6	5	8.90	2.50
-2	6	5	17.20	3.30
1	-6	-5	1768.52	79.29
-1	6	5	1773.62	78.29
-1	6	5	1789.62	79.19
-1	6	5	1674.73	79.89
0	-6	-5	483.95	22.50
0	-6	-5	467.85	22.30
0	6	5	444.16	23.30
0	6	5	492.15	22.30
1	6	5	1628.04	73.89

1	6	5	1626.84	73.29
2	6	5	187.18	10.40
2	6	5	183.78	9.30
-3	-6	-5	1476.45	64.79
3	6	5	1310.37	63.79
3	6	5	1450.05	63.59
3	6	5	1363.16	64.89
3	6	5	1475.85	64.99
4	6	5	115.79	6.80
4	6	5	112.29	6.60
4	6	5	119.99	6.20
4	6	5	149.49	11.40
4	6	5	114.39	9.30
4	6	5	127.29	6.30
-5	-6	-5	124.69	9.00
5	6	5	122.89	12.00
5	6	5	133.79	7.30
5	6	5	131.79	13.40
-6	-6	-5	63.59	10.30
-6	-6	-5	70.79	9.70
-6	-6	-5	66.99	8.10
-6	-6	-5	72.59	13.50
-6	-6	-5	82.69	7.70
-6	-6	-5	52.59	14.50
6	6	5	78.79	6.10
-7	-6	-5	150.38	15.30
-7	-6	-5	170.98	13.00
-7	-6	-5	141.69	12.40
-7	-6	-5	152.38	13.50
-7	-6	-5	155.48	15.30
-7	-6	-5	146.19	10.90
7	6	5	148.69	10.00
-8	-6	-5	100.99	12.10
-8	-6	-5	126.39	15.90
-8	-6	-5	125.39	13.30
-8	-6	-5	111.99	10.50
-8	-6	-5	94.99	12.00
8	6	5	112.59	10.20
-9	-6	-5	20.90	11.20
-9	-6	-5	-2.70	6.30
-9	-6	-5	10.00	7.00
-9	-6	-5	10.30	8.30
-9	-6	-5	9.40	9.00
-9	-6	-5	7.50	6.80

-10	-6	-5	47.30	9.70
-10	-6	-5	67.39	11.40
-10	-6	-5	29.60	8.00
-10	-6	-5	46.90	7.80
10	6	5	55.49	12.70
-11	-6	-5	417.56	24.00
-11	-6	-5	412.36	24.70
-11	-6	-5	441.56	26.40
-11	-6	-5	403.36	23.70
11	6	5	397.26	27.30
-12	-6	-5	32.80	13.30
-12	-6	-5	8.90	10.80
-12	-6	-5	21.80	9.10
-12	-6	-5	5.90	7.60
12	6	5	7.60	18.70
11	-7	-5	23.70	10.70
-11	7	5	0.90	8.80
10	-7	-5	229.88	19.50
10	-7	-5	239.58	23.20
-10	7	5	228.38	19.80
-10	7	5	206.18	29.50
9	-7	-5	36.60	10.30
9	-7	-5	26.80	10.00
-9	7	5	34.10	15.10
-9	7	5	34.40	9.40
8	-7	-5	550.74	29.90
8	-7	-5	542.05	30.20
-8	7	5	524.75	30.60
-8	7	5	494.55	33.10
7	-7	-5	151.88	16.10
-7	7	5	125.29	15.30
6	-7	-5	31.40	8.30
6	-7	-5	19.10	6.70
-6	7	5	28.70	7.60
5	-7	-5	132.59	12.50
5	-7	-5	142.79	21.20
5	-7	-5	131.59	15.90
-5	7	5	142.39	10.10
-5	7	5	149.59	19.20
-5	7	5	102.29	13.10
-5	7	5	157.68	14.30
4	-7	-5	291.17	17.80
4	-7	-5	291.27	22.60
-4	7	5	302.97	17.80

-4	7	5	312.07	18.60
-4	7	5	324.57	15.40
-4	7	5	273.77	17.70
3	-7	-5	9.70	4.80
-3	7	5	16.00	8.30
-3	7	5	12.60	4.00
-3	7	5	9.90	2.40
2	-7	-5	241.28	15.00
-2	7	5	264.77	12.70
-2	7	5	267.97	14.90
-2	7	5	270.67	14.10
1	-7	-5	26.50	4.20
1	-7	-5	31.30	5.50
-1	7	5	29.60	2.50
-1	7	5	31.00	4.00
-1	7	5	33.60	6.00
0	-7	-5	17.90	3.30
0	-7	-5	22.10	3.70
0	7	5	23.20	3.30
0	7	5	11.20	4.40
0	7	5	17.80	2.20
0	7	5	10.30	3.10
1	7	5	1085.39	48.40
1	7	5	1038.00	49.30
2	7	5	74.29	7.00
2	7	5	70.79	4.70
-3	-7	-5	257.17	14.60
3	7	5	305.97	15.80
3	7	5	266.37	14.00
3	7	5	285.47	13.60
3	7	5	301.97	15.70
4	7	5	825.42	38.80
4	7	5	775.02	35.80
4	7	5	831.82	38.70
4	7	5	797.62	35.80
4	7	5	739.23	36.00
-5	-7	-5	27.60	8.40
-5	-7	-5	21.80	5.80
-5	-7	-5	21.70	6.50
-5	-7	-5	19.70	3.80
5	7	5	26.30	4.00
5	7	5	30.70	10.00
-6	-7	-5	936.41	41.70
-6	-7	-5	867.51	43.70

-6	-7	-5	892.81	42.10
-6	-7	-5	786.52	42.40
-6	-7	-5	871.41	42.30
-6	-7	-5	872.41	42.40
6	7	5	926.01	40.90
-7	-7	-5	406.76	22.10
-7	-7	-5	400.16	20.80
-7	-7	-5	388.96	21.50
-7	-7	-5	399.66	22.30
-7	-7	-5	353.76	22.90
-7	-7	-5	409.16	23.90
7	7	5	381.96	20.20
-8	-7	-5	285.87	19.70
-8	-7	-5	251.47	17.10
-8	-7	-5	261.57	16.10
-8	-7	-5	277.67	17.60
-8	-7	-5	267.97	19.70
-8	-7	-5	242.98	18.00
8	7	5	258.17	16.10
-9	-7	-5	14.20	8.90
-9	-7	-5	29.00	10.40
-9	-7	-5	16.50	7.10
-9	-7	-5	22.20	9.60
-9	-7	-5	30.20	7.70
-9	-7	-5	22.70	7.90
9	7	5	16.60	8.20
-10	-7	-5	9.50	7.60
-10	-7	-5	45.00	12.90
-10	-7	-5	14.50	10.20
-10	-7	-5	10.00	7.20
-10	-7	-5	10.60	8.50
-10	-7	-5	13.90	7.40
10	7	5	18.30	12.90
10	7	5	25.30	13.00
-11	-7	-5	85.29	12.90
-11	-7	-5	53.29	9.00
-11	-7	-5	55.69	9.50
11	7	5	36.70	13.90
-12	-7	-5	111.99	12.80
-12	-7	-5	106.89	18.50
-12	-7	-5	97.09	14.80
-12	-7	-5	67.69	10.50
11	-8	-5	27.00	11.00
10	-8	-5	154.08	17.00

10	-8	-5	132.99	20.30
-10	8	5	115.89	16.80
9	-8	-5	34.40	10.40
9	-8	-5	16.40	10.20
-9	8	5	44.60	23.10
-9	8	5	46.40	11.20
8	-8	-5	-3.50	7.80
8	-8	-5	-10.20	8.80
-8	8	5	-12.60	12.30
-8	8	5	-2.10	9.10
7	-8	-5	209.08	17.20
7	-8	-5	238.98	18.20
-7	8	5	205.08	18.60
6	-8	-5	88.29	14.60
6	-8	-5	91.09	11.90
-6	8	5	101.29	15.60
-6	8	5	116.09	13.80
5	-8	-5	553.24	29.50
5	-8	-5	488.75	30.90
5	-8	-5	512.75	27.20
5	-8	-5	502.75	30.70
-5	8	5	532.05	25.90
-5	8	5	532.55	27.70
4	-8	-5	1130.79	53.79
4	-8	-5	1089.59	55.89
4	-8	-5	1134.69	56.89
-4	8	5	1221.08	52.59
-4	8	5	1110.69	53.49
-4	8	5	1177.28	54.79
3	-8	-5	68.09	12.60
3	-8	-5	80.69	8.80
-3	8	5	65.89	8.80
-3	8	5	78.29	10.20
-3	8	5	73.19	10.80
-3	8	5	76.19	5.40
2	-8	-5	753.22	37.90
-2	8	5	745.13	35.80
-2	8	5	778.32	34.40
-2	8	5	720.23	38.50
1	-8	-5	17.00	4.30
1	-8	-5	17.30	3.30
-1	8	5	18.30	2.40
-1	8	5	20.70	6.40
-1	8	5	29.30	4.10

0	-8	-5	17.50	3.80
0	-8	-5	8.20	2.70
0	8	5	22.00	5.50
0	8	5	9.60	2.60
0	8	5	12.00	1.80
-1	-8	-5	74.39	8.20
-1	-8	-5	73.29	5.90
1	8	5	77.89	5.50
1	8	5	77.89	9.40
-2	-8	-5	51.49	7.20
2	8	5	69.59	4.80
2	8	5	71.89	7.80
3	8	5	132.69	8.20
3	8	5	138.99	7.60
3	8	5	136.49	10.70
-4	-8	-5	479.55	23.00
4	8	5	461.85	21.90
4	8	5	457.75	21.60
4	8	5	482.05	25.40
4	8	5	457.35	21.60
4	8	5	483.95	25.40
-5	-8	-5	160.68	12.50
-5	-8	-5	170.28	13.10
-5	-8	-5	162.88	13.10
-5	-8	-5	176.98	13.40
5	8	5	183.28	9.60
-6	-8	-5	118.09	10.90
-6	-8	-5	129.89	15.50
-6	-8	-5	97.99	12.60
-6	-8	-5	131.09	9.50
-6	-8	-5	113.29	11.90
-6	-8	-5	134.39	11.60
6	8	5	120.19	8.50
-7	-8	-5	321.57	23.80
-7	-8	-5	288.77	16.40
-7	-8	-5	258.47	18.70
-7	-8	-5	289.77	20.50
-7	-8	-5	297.27	21.40
7	8	5	277.57	16.10
-8	-8	-5	3.80	8.10
-8	-8	-5	11.10	5.60
-8	-8	-5	3.90	6.50
-8	-8	-5	3.10	8.40
-8	-8	-5	5.90	6.80

-8	-8	-5	10.40	6.40
8	8	5	-2.30	5.50
-9	-8	-5	23.70	8.10
-9	-8	-5	21.80	7.50
-9	-8	-5	24.60	9.60
-9	-8	-5	15.10	9.90
-9	-8	-5	13.40	7.10
-9	-8	-5	11.10	7.50
9	8	5	18.70	13.00
-10	-8	-5	282.37	20.00
-10	-8	-5	290.87	18.20
-10	-8	-5	266.47	19.60
-10	-8	-5	279.47	18.80
10	8	5	259.97	25.00
-11	-8	-5	80.49	12.40
-11	-8	-5	62.29	10.40
-11	-8	-5	116.29	15.90
-11	-8	-5	95.89	12.90
-12	-8	-5	162.18	17.70
-12	-8	-5	177.18	15.90
-12	-8	-5	125.49	16.70
-12	-8	-5	195.58	14.80
11	-9	-5	33.70	11.70
-11	9	5	21.80	10.20
10	-9	-5	26.20	12.00
-10	9	5	30.40	10.70
9	-9	-5	798.32	48.70
-9	9	5	687.43	48.00
-9	9	5	884.01	47.10
8	-9	-5	41.00	9.80
8	-9	-5	32.30	8.70
-8	9	5	59.59	14.60
7	-9	-5	88.39	13.20
7	-9	-5	127.59	15.70
-7	9	5	95.09	16.90
6	-9	-5	76.09	15.70
6	-9	-5	75.09	12.20
-6	9	5	92.69	12.30
-6	9	5	73.39	10.30
5	-9	-5	209.98	14.50
5	-9	-5	157.38	20.50
5	-9	-5	198.08	18.60
5	-9	-5	184.98	19.20
-5	9	5	210.08	13.10

4	-9	-5	13.80	5.70
4	-9	-5	18.40	8.50
4	-9	-5	35.40	14.20
4	-9	-5	27.40	10.90
-4	9	5	25.00	4.10
-4	9	5	6.90	10.00
-4	9	5	22.10	7.00
3	-9	-5	107.09	15.50
-3	9	5	83.29	5.90
-3	9	5	65.59	9.80
-3	9	5	82.49	10.80
-3	9	5	68.59	11.10
2	-9	-5	64.19	8.70
-2	9	5	59.19	7.50
-2	9	5	37.00	8.80
-2	9	5	51.09	9.20
-2	9	5	52.89	4.30
1	-9	-5	1115.19	52.99
-1	9	5	1101.19	50.79
-1	9	5	1113.39	52.19
-1	9	5	1172.68	54.79
0	-9	-5	53.29	6.30
0	-9	-5	66.59	8.20
0	-9	-5	64.09	10.60
0	9	5	66.59	11.00
0	9	5	67.99	3.90
0	9	5	61.69	5.80
-1	-9	-5	509.95	26.80
-1	-9	-5	546.75	25.70
-1	-9	-5	540.75	27.00
1	9	5	526.75	28.00
1	9	5	570.84	24.10
1	9	5	560.04	25.50
1	9	5	458.45	25.50
2	9	5	787.52	35.60
2	9	5	801.72	35.50
2	9	5	795.12	36.70
2	9	5	779.32	38.80
-3	-9	-5	277.87	15.00
3	9	5	282.47	17.30
3	9	5	276.07	12.90
3	9	5	276.17	13.90
-4	-9	-5	0.30	3.80
4	9	5	2.70	7.20

4	9	5	7.20	6.80
4	9	5	3.00	1.90
-5	-9	-5	1378.86	59.39
-5	-9	-5	1289.77	60.09
-5	-9	-5	1302.27	61.89
-5	-9	-5	1200.18	59.89
-5	-9	-5	1258.27	60.09
-5	-9	-5	1273.27	59.59
-6	-9	-5	21.60	4.80
-6	-9	-5	16.80	6.30
-6	-9	-5	8.40	6.90
-6	-9	-5	16.00	6.60
-6	-9	-5	16.70	6.10
-6	-9	-5	28.70	9.10
6	9	5	13.60	4.00
-7	-9	-5	23.70	5.20
-7	-9	-5	26.20	9.30
-7	-9	-5	31.80	6.80
-7	-9	-5	29.40	6.30
-7	-9	-5	28.40	7.00
-7	-9	-5	49.60	12.50
7	9	5	33.50	5.80
-8	-9	-5	24.80	7.20
-8	-9	-5	17.00	8.90
-8	-9	-5	36.60	7.80
-8	-9	-5	22.20	7.60
-8	-9	-5	22.80	8.00
-8	-9	-5	17.50	6.10
8	9	5	23.20	7.30
-9	-9	-5	47.60	9.10
-9	-9	-5	53.29	7.80
-9	-9	-5	41.10	7.90
-9	-9	-5	74.29	12.90
-9	-9	-5	66.09	11.90
-9	-9	-5	57.29	9.80
9	9	5	62.89	10.80
-10	-9	-5	3.60	7.10
-10	-9	-5	12.70	12.80
-10	-9	-5	14.20	10.90
-10	-9	-5	15.90	8.70
-10	-9	-5	7.50	11.90
-11	-9	-5	56.49	9.60
-11	-9	-5	57.69	9.40
-11	-9	-5	59.29	10.60

-11	-9	-5	49.20	9.80
-12	-9	-5	150.98	16.50
-12	-9	-5	114.99	16.40
-12	-9	-5	150.38	17.30
-12	-9	-5	178.68	13.60
11	-10	-5	158.68	22.20
11	-10	-5	204.78	19.10
-11	10	5	162.68	19.90
10	-10	-5	264.37	21.30
10	-10	-5	290.47	21.50
-10	10	5	281.97	22.20
9	-10	-5	31.10	9.80
9	-10	-5	19.70	10.10
-9	10	5	3.20	17.90
-9	10	5	15.90	12.00
8	-10	-5	44.90	8.70
8	-10	-5	30.70	10.40
-8	10	5	36.00	12.30
-8	10	5	9.30	15.60
7	-10	-5	42.00	10.00
7	-10	-5	33.70	8.00
-7	10	5	61.09	12.70
6	-10	-5	31.60	7.70
6	-10	-5	26.50	9.40
-6	10	5	10.90	10.00
-6	10	5	22.10	9.70
5	-10	-5	4.10	5.40
5	-10	-5	-4.50	10.20
5	-10	-5	-3.60	9.30
5	-10	-5	-10.60	11.70
-5	10	5	-7.70	14.40
-5	10	5	7.40	5.50
-5	10	5	14.10	8.20
4	-10	-5	421.96	24.30
4	-10	-5	432.36	22.70
4	-10	-5	374.66	27.50
4	-10	-5	352.16	28.00
4	-10	-5	441.16	29.40
-4	10	5	416.16	23.20
-4	10	5	453.15	21.80
-4	10	5	354.16	28.20
3	-10	-5	1597.54	75.19
3	-10	-5	1409.76	73.59
3	-10	-5	1559.84	72.19

3	-10	-5	1521.15	72.59
-3	10	5	1509.75	72.79
-3	10	5	1703.43	70.99
-3	10	5	1619.74	75.29
2	-10	-5	65.39	9.80
2	-10	-5	90.69	13.80
-2	10	5	70.29	5.10
-2	10	5	52.09	9.80
-2	10	5	58.39	11.60
-2	10	5	77.99	9.70
1	-10	-5	447.56	24.90
1	-10	-5	408.56	23.10
-1	10	5	446.76	21.10
-1	10	5	529.95	28.10
-1	10	5	431.56	23.00
0	-10	-5	2423.06	111.99
0	10	5	2405.66	109.39
0	10	5	2321.27	110.79
0	10	5	2646.04	113.29
-1	-10	-5	638.44	31.20
-1	-10	-5	607.54	32.70
1	10	5	666.13	29.40
1	10	5	644.44	34.10
1	10	5	673.03	31.00
-2	-10	-5	89.39	8.90
2	10	5	97.59	5.30
2	10	5	104.89	7.30
2	10	5	97.69	13.00
-3	-10	-5	613.64	28.80
3	10	5	569.64	31.80
3	10	5	572.74	27.90
3	10	5	608.54	27.00
-4	-10	-5	67.79	8.00
-4	-10	-5	57.49	7.60
-4	-10	-5	94.29	12.60
-4	-10	-5	68.19	9.00
-4	-10	-5	62.89	10.20
4	10	5	64.89	4.90
-5	-10	-5	94.49	10.20
-5	-10	-5	74.39	11.30
-5	-10	-5	87.19	11.00
-5	-10	-5	97.09	8.50
-5	-10	-5	106.39	17.40
-5	-10	-5	108.59	11.90

5	10	5	96.99	7.40
-6	-10	-5	158.88	13.90
-6	-10	-5	152.28	13.50
-6	-10	-5	142.79	13.60
-6	-10	-5	177.68	16.70
-6	-10	-5	166.98	11.20
-6	-10	-5	145.59	12.20
6	10	5	167.88	11.00
-7	-10	-5	400.06	22.10
-7	-10	-5	357.36	21.90
-7	-10	-5	357.06	20.70
-7	-10	-5	379.76	20.10
-7	-10	-5	370.96	21.80
-7	-10	-5	364.56	23.60
7	10	5	338.37	20.40
-8	-10	-5	41.10	8.10
-8	-10	-5	28.10	6.90
-8	-10	-5	31.40	8.50
-8	-10	-5	23.20	7.60
-8	-10	-5	35.80	6.30
-8	-10	-5	32.30	10.00
8	10	5	25.90	7.90
-9	-10	-5	1.80	6.60
-9	-10	-5	-0.60	8.30
-9	-10	-5	7.00	6.50
-9	-10	-5	5.90	7.30
-9	-10	-5	-1.50	8.30
9	10	5	4.60	10.50
-10	-10	-5	280.77	20.80
-10	-10	-5	217.68	17.40
-10	-10	-5	272.77	20.40
-10	-10	-5	255.37	20.50
-10	-10	-5	268.77	18.50
-11	-10	-5	338.97	21.30
-11	-10	-5	321.97	20.90
-11	-10	-5	334.47	20.70
-11	-10	-5	317.27	24.90
-11	-10	-5	350.96	21.70
-12	-10	-5	31.10	11.60
-12	-10	-5	21.00	12.40
-12	-10	-5	26.60	8.30
-12	-10	-5	27.20	12.90
10	-11	-5	194.18	18.10
10	-11	-5	176.18	19.40

-10	11	5	130.29	18.10
9	-11	-5	16.60	10.80
9	-11	-5	15.20	9.30
-9	11	5	-0.60	11.30
8	-11	-5	445.96	25.50
8	-11	-5	398.26	26.60
-8	11	5	430.96	30.40
7	-11	-5	9.20	7.60
7	-11	-5	-4.40	9.50
-7	11	5	6.80	11.60
6	-11	-5	-12.60	11.60
6	-11	-5	0.40	6.60
6	-11	-5	-5.30	10.00
-6	11	5	-7.50	10.70
-6	11	5	5.30	9.90
-6	11	5	-29.10	19.40
5	-11	-5	384.76	27.00
5	-11	-5	443.36	25.50
5	-11	-5	362.66	29.40
-5	11	5	421.86	22.10
-5	11	5	388.46	24.50
-5	11	5	410.76	29.00
4	-11	-5	431.66	36.40
4	-11	-5	391.36	23.50
4	-11	-5	383.96	22.00
4	-11	-5	425.36	28.90
4	-11	-5	362.56	27.80
-4	11	5	379.36	28.30
-4	11	5	399.76	20.20
-4	11	5	391.36	23.50
3	-11	-5	122.59	19.00
3	-11	-5	174.48	13.60
3	-11	-5	158.98	14.90
3	-11	-5	167.58	17.50
3	-11	-5	199.88	35.30
3	-11	-5	211.78	18.20
-3	11	5	169.58	15.60
-3	11	5	125.39	26.10
-3	11	5	151.48	23.20
2	-11	-5	377.76	22.80
2	-11	-5	336.77	24.70
2	-11	-5	431.66	25.10
2	-11	-5	348.97	24.70
2	-11	-5	408.16	26.10

2	-11	-5	360.76	22.30
-2	11	5	439.66	19.30
-2	11	5	398.66	29.30
-2	11	5	421.56	24.00
1	-11	-5	44.10	7.70
1	-11	-5	39.60	9.90
-1	11	5	87.29	13.20
-1	11	5	54.89	4.50
-1	11	5	56.99	9.30
0	-11	-5	82.59	10.10
0	-11	-5	104.89	13.50
0	11	5	90.99	8.90
0	11	5	100.39	16.30
0	11	5	98.39	5.80
-1	-11	-5	5.70	5.70
-1	-11	-5	35.90	8.60
-1	-11	-5	4.40	6.90
1	11	5	10.30	4.30
1	11	5	13.20	2.00
1	11	5	10.60	8.90
-2	-11	-5	486.35	24.90
2	11	5	482.55	24.10
2	11	5	506.15	22.40
2	11	5	457.15	29.40
-3	-11	-5	-3.90	7.60
-3	-11	-5	6.70	5.10
-3	-11	-5	4.90	5.70
-3	-11	-5	-1.90	7.30
3	11	5	1.90	1.80
3	11	5	7.80	4.20
-4	-11	-5	986.70	47.40
-4	-11	-5	929.51	46.10
-4	-11	-5	951.00	46.60
-4	-11	-5	1081.79	46.00
-4	-11	-5	937.01	47.00
-4	-11	-5	949.40	46.10
-5	-11	-5	239.18	16.20
-5	-11	-5	228.48	20.30
-5	-11	-5	196.08	19.00
-5	-11	-5	223.48	15.80
-5	-11	-5	230.78	14.80
-5	-11	-5	233.88	13.90
-5	-11	-5	231.18	15.00
5	11	5	217.98	12.70

-6	-11	-5	1172.58	57.39
-6	-11	-5	1194.28	55.99
-6	-11	-5	1240.38	55.69
6	11	5	1127.29	55.29
-7	-11	-5	199.28	13.90
-7	-11	-5	192.68	15.50
-7	-11	-5	204.98	16.20
-7	-11	-5	215.88	18.70
-7	-11	-5	204.08	15.90
7	11	5	185.78	14.90
-8	-11	-5	7.50	7.50
-8	-11	-5	16.60	7.10
-8	-11	-5	18.40	8.00
-8	-11	-5	3.40	6.20
-8	-11	-5	6.80	6.50
8	11	5	6.30	8.60
-9	-11	-5	34.90	8.70
-9	-11	-5	26.10	8.90
-9	-11	-5	17.50	7.10
-9	-11	-5	13.90	7.50
-9	-11	-5	27.20	8.50
9	11	5	29.60	12.20
-10	-11	-5	17.40	8.90
-10	-11	-5	1.10	8.10
-10	-11	-5	19.00	8.20
-10	-11	-5	15.80	8.60
10	11	5	31.60	25.30
-11	-11	-5	146.99	14.50
-11	-11	-5	114.59	13.70
-11	-11	-5	166.88	14.80
-11	-11	-5	114.49	18.90
-11	-11	-5	124.99	15.00
10	-12	-5	160.18	24.20
10	-12	-5	185.48	17.40
-10	12	5	149.19	17.10
9	-12	-5	41.90	12.40
9	-12	-5	39.90	9.30
-9	12	5	43.50	12.00
8	-12	-5	7.90	8.90
8	-12	-5	4.50	16.40
-8	12	5	9.70	18.00
-8	12	5	-2.10	13.00
7	-12	-5	184.08	20.60
7	-12	-5	258.47	17.50

-7	12	5	205.38	24.20
6	-12	-5	357.56	24.40
6	-12	-5	318.57	20.00
6	-12	-5	281.37	24.20
-6	12	5	297.77	23.60
-6	12	5	271.17	26.00
-6	12	5	345.27	27.20
5	-12	-5	398.76	23.80
5	-12	-5	390.96	22.10
5	-12	-5	306.87	29.50
5	-12	-5	348.97	27.00
-5	12	5	349.67	24.40
-5	12	5	383.86	21.40
-5	12	5	376.56	28.40
4	-12	-5	241.68	24.90
4	-12	-5	293.07	25.10
4	-12	-5	265.27	19.20
-4	12	5	294.57	24.90
-4	12	5	308.07	20.30
-4	12	5	270.07	15.70
3	-12	-5	143.69	14.60
3	-12	-5	151.38	13.60
3	-12	-5	174.98	18.60
3	-12	-5	145.99	19.00
-3	12	5	120.29	16.40
-3	12	5	140.09	9.10
-3	12	5	118.19	15.60
2	-12	-5	522.05	30.70
2	-12	-5	545.75	29.10
2	-12	-5	509.55	28.80
2	-12	-5	545.05	31.30
2	-12	-5	524.65	29.70
-2	12	5	585.64	26.30
-2	12	5	615.94	30.50
1	-12	-5	399.36	23.00
1	-12	-5	381.56	23.30
1	-12	-5	448.56	25.10
1	-12	-5	392.16	27.10
1	-12	-5	415.36	23.90
-1	12	5	413.36	19.70
-1	12	5	392.96	29.80
0	-12	-5	5.00	6.90
0	-12	-5	36.10	9.80
0	-12	-5	14.80	7.60

0	-12	-5	35.50	8.30
0	-12	-5	22.40	7.40
0	-12	-5	4.70	7.80
0	12	5	23.70	6.30
0	12	5	23.50	3.60
0	12	5	46.60	12.70
-1	-12	-5	651.03	35.10
-1	-12	-5	673.73	34.20
-1	-12	-5	631.54	34.70
-1	-12	-5	782.62	42.20
-1	-12	-5	675.33	39.60
-1	-12	-5	617.34	34.20
-1	-12	-5	704.53	34.10
1	12	5	820.32	42.80
1	12	5	691.13	34.10
1	12	5	714.63	31.80
-2	-12	-5	158.08	15.90
-2	-12	-5	164.28	12.00
-2	-12	-5	148.29	14.70
-2	-12	-5	136.59	13.70
-2	-12	-5	155.28	12.50
2	12	5	161.48	8.40
2	12	5	158.68	11.80
-3	-12	-5	208.78	14.30
-3	-12	-5	236.08	14.60
-3	-12	-5	238.58	16.40
-3	-12	-5	222.08	16.20
-3	-12	-5	218.68	17.00
-3	-12	-5	194.88	18.30
-4	-12	-5	175.68	15.90
-4	-12	-5	188.78	12.40
-4	-12	-5	164.08	14.80
-4	-12	-5	166.48	13.80
-4	-12	-5	182.98	13.00
-4	-12	-5	160.98	15.10
4	12	5	178.48	9.90
-5	-12	-5	72.99	8.90
-5	-12	-5	91.19	12.10
-5	-12	-5	51.59	8.30
-5	-12	-5	77.49	13.50
-5	-12	-5	81.89	10.20
-5	-12	-5	59.09	10.30
-5	-12	-5	99.39	15.10
5	12	5	65.09	7.40

-6	-12	-5	245.18	21.90
-6	-12	-5	245.98	16.90
-6	-12	-5	262.77	16.50
-6	-12	-5	219.98	21.30
-6	-12	-5	240.18	15.20
-6	-12	-5	237.98	16.00
-6	-12	-5	232.08	17.00
6	12	5	205.68	15.00
-7	-12	-5	181.28	15.90
-7	-12	-5	174.08	14.40
-7	-12	-5	182.98	15.80
-7	-12	-5	196.98	13.30
-7	-12	-5	152.88	14.20
7	12	5	173.48	15.60
-8	-12	-5	2.00	7.10
-8	-12	-5	-7.70	6.80
-8	-12	-5	1.30	7.40
-8	-12	-5	12.50	7.40
-8	-12	-5	-1.40	7.20
8	12	5	9.70	10.50
-9	-12	-5	50.79	9.40
-9	-12	-5	43.50	8.90
-9	-12	-5	62.49	9.30
-9	-12	-5	57.89	8.90
-9	-12	-5	59.99	9.10
9	12	5	48.70	13.80
-10	-12	-5	119.79	15.50
-10	-12	-5	117.49	14.40
-10	-12	-5	137.79	15.10
-10	-12	-5	114.09	13.70
-10	-12	-5	130.19	13.80
-11	-12	-5	185.48	15.20
-11	-12	-5	217.38	20.70
-11	-12	-5	212.48	15.80
-11	-12	-5	197.88	16.30
-11	-12	-5	148.89	17.00
10	-13	-5	100.99	20.30
10	-13	-5	91.49	15.80
-10	13	5	65.09	11.50
9	-13	-5	339.77	28.20
9	-13	-5	387.46	22.00
-9	13	5	343.27	25.30
8	-13	-5	27.60	13.90
8	-13	-5	24.70	8.20

-8	13	5	14.90	13.20
-8	13	5	5.40	18.60
7	-13	-5	191.48	15.80
7	-13	-5	157.48	19.50
7	-13	-5	143.09	21.60
-7	13	5	163.28	24.00
6	-13	-5	438.86	30.60
6	-13	-5	436.66	29.00
6	-13	-5	501.35	26.10
-6	13	5	409.86	31.10
-6	13	5	439.36	29.10
-6	13	5	460.15	32.50
5	-13	-5	136.39	19.10
5	-13	-5	202.48	15.10
5	-13	-5	196.38	17.40
-5	13	5	175.58	16.10
-5	13	5	143.59	19.10
-5	13	5	178.98	22.80
4	-13	-5	24.10	7.80
4	-13	-5	25.40	8.40
4	-13	-5	29.00	13.80
-4	13	5	54.89	10.40
-4	13	5	34.20	6.20
-4	13	5	18.10	12.90
3	-13	-5	175.58	18.70
3	-13	-5	160.18	20.60
3	-13	-5	151.38	14.70
3	-13	-5	143.89	13.80
-3	13	5	162.28	16.60
-3	13	5	149.88	10.20
-3	13	5	145.59	21.50
2	-13	-5	45.80	10.30
2	-13	-5	46.20	9.80
2	-13	-5	44.60	8.30
2	-13	-5	47.70	8.40
2	-13	-5	33.30	9.90
-2	13	5	37.10	5.10
-2	13	5	31.90	8.60
1	-13	-5	1144.79	53.79
1	-13	-5	1237.38	54.49
1	-13	-5	1068.29	53.19
1	-13	-5	1032.30	54.19
1	-13	-5	1068.39	53.19
-1	13	5	1152.18	51.19

0	-13	-5	38.50	7.90
0	-13	-5	41.20	8.50
0	-13	-5	42.70	10.60
0	-13	-5	39.40	8.00
0	-13	-5	44.70	7.30
0	13	5	39.90	4.30
-1	-13	-5	254.57	16.40
-1	-13	-5	266.67	17.30
-1	-13	-5	223.18	16.10
-1	-13	-5	216.88	18.80
-1	-13	-5	226.38	18.60
-1	-13	-5	255.47	18.00
-1	-13	-5	256.57	15.50
1	13	5	247.68	12.40
1	13	5	233.18	17.20
-2	-13	-5	290.67	23.20
-2	-13	-5	312.17	19.10
-2	-13	-5	306.77	19.50
-2	-13	-5	298.67	18.60
-2	-13	-5	265.77	28.20
-2	-13	-5	356.16	18.60
-2	-13	-5	334.97	20.30
2	13	5	313.27	15.40
-3	-13	-5	100.79	13.20
-3	-13	-5	115.49	10.90
-3	-13	-5	126.09	12.90
-3	-13	-5	126.69	17.50
-3	-13	-5	113.29	11.20
-3	-13	-5	108.79	13.70
3	13	5	123.69	7.30
-4	-13	-5	356.36	23.10
-4	-13	-5	415.16	21.90
-4	-13	-5	381.06	21.60
-4	-13	-5	404.46	22.90
-4	-13	-5	424.86	22.90
-4	-13	-5	362.16	23.20
4	13	5	393.86	19.40
-5	-13	-5	728.03	39.30
-5	-13	-5	713.13	38.20
-5	-13	-5	779.62	38.30
-5	-13	-5	770.42	37.00
-5	-13	-5	767.72	38.30
5	13	5	725.63	35.80
-6	-13	-5	321.77	19.70

-6	-13	-5	282.37	20.00
-6	-13	-5	314.37	18.50
-6	-13	-5	341.77	20.20
-6	-13	-5	289.17	23.30
-6	-13	-5	308.77	18.60
6	13	5	245.38	18.10
-7	-13	-5	268.47	17.20
-7	-13	-5	253.87	17.00
-7	-13	-5	240.08	17.70
-7	-13	-5	282.87	18.80
-7	-13	-5	261.27	18.70
7	13	5	212.78	18.90
-8	-13	-5	16.70	8.30
-8	-13	-5	1.40	7.10
-8	-13	-5	12.20	8.00
-8	-13	-5	6.20	7.80
-8	-13	-5	1.60	7.20
8	13	5	-11.70	10.80
-9	-13	-5	35.70	8.20
-9	-13	-5	14.90	14.20
-9	-13	-5	38.00	13.10
9	13	5	22.10	14.70
-10	-13	-5	-0.10	8.20
-10	-13	-5	1.00	8.10
-10	-13	-5	9.60	9.50
-10	-13	-5	2.90	8.50
-10	-13	-5	-13.70	6.90
-11	-13	-5	22.90	8.20
-11	-13	-5	6.10	11.30
-11	-13	-5	25.10	9.20
-11	-13	-5	32.40	10.60
-11	-13	-5	16.70	7.30
9	-14	-5	68.79	12.40
-9	14	5	63.29	11.90
8	-14	-5	100.19	13.30
-8	14	5	74.59	15.00
7	-14	-5	74.19	10.00
-7	14	5	83.69	15.50
6	-14	-5	18.70	11.40
6	-14	-5	19.50	8.20
-6	14	5	33.10	12.60
-6	14	5	22.40	14.60
-6	14	5	7.50	14.20
5	-14	-5	46.90	10.00

5	-14	-5	74.29	12.30
5	-14	-5	49.80	16.70
-5	14	5	64.19	12.80
-5	14	5	47.10	14.60
4	-14	-5	594.74	36.70
4	-14	-5	586.24	31.20
4	-14	-5	603.24	37.60
4	-14	-5	616.84	32.30
-4	14	5	603.94	33.80
-4	14	5	609.14	30.30
-4	14	5	569.74	35.50
3	-14	-5	312.17	20.60
3	-14	-5	318.27	23.80
3	-14	-5	296.47	27.90
-3	14	5	325.77	17.40
-3	14	5	319.57	22.10
-3	14	5	336.97	25.40
2	-14	-5	140.79	17.50
2	-14	-5	189.18	17.90
2	-14	-5	165.28	14.60
2	-14	-5	191.38	16.70
2	-14	-5	152.48	16.50
-2	14	5	164.58	15.90
-2	14	5	156.88	10.30
1	-14	-5	84.19	10.80
1	-14	-5	75.99	10.00
1	-14	-5	95.29	12.40
1	-14	-5	81.39	11.70
1	-14	-5	70.29	10.40
-1	14	5	58.39	6.00
0	-14	-5	2557.94	110.79
0	-14	-5	2394.56	110.49
0	-14	-5	2434.66	110.19
0	-14	-5	2332.27	110.09
0	-14	-5	2347.17	110.29
0	14	5	2397.86	108.59
-1	-14	-5	227.58	16.50
-1	-14	-5	227.18	18.00
-1	-14	-5	226.98	15.80
-1	-14	-5	201.68	17.20
-1	-14	-5	212.58	15.70
1	14	5	230.78	11.80
-2	-14	-5	16.10	6.30
-2	-14	-5	8.70	6.50

-2	-14	-5	5.80	10.70
-2	-14	-5	12.80	6.40
-2	-14	-5	1.20	7.70
-2	-14	-5	1.30	7.30
2	14	5	13.50	2.90
-3	-14	-5	10.60	13.90
-3	-14	-5	47.90	8.80
-3	-14	-5	50.89	8.20
-3	-14	-5	35.10	6.50
-3	-14	-5	25.50	10.00
-3	-14	-5	46.90	8.90
-3	-14	-5	42.80	7.30
3	14	5	44.90	5.10
-4	-14	-5	212.68	18.40
-4	-14	-5	227.88	15.40
-4	-14	-5	237.98	17.00
4	14	5	220.58	12.60
-5	-14	-5	62.89	9.80
-5	-14	-5	67.19	10.50
-5	-14	-5	67.09	8.30
-5	-14	-5	59.79	9.60
-5	-14	-5	53.69	9.50
5	14	5	77.19	8.60
-6	-14	-5	20.40	8.40
-6	-14	-5	11.10	7.80
-6	-14	-5	11.80	7.30
-6	-14	-5	6.30	6.40
-6	-14	-5	24.50	10.20
-6	-14	-5	14.30	7.90
6	14	5	15.90	7.30
-7	-14	-5	102.49	14.80
-7	-14	-5	99.99	13.90
-7	-14	-5	97.59	12.20
-7	-14	-5	85.19	14.10
-7	-14	-5	121.49	14.90
7	14	5	80.69	17.10
-8	-14	-5	65.69	10.20
-8	-14	-5	58.99	10.10
-8	-14	-5	73.99	9.60
-8	-14	-5	62.79	9.60
-9	-14	-5	19.60	8.30
-9	-14	-5	16.30	7.30
-9	-14	-5	23.60	8.80
-9	-14	-5	10.80	8.50

-9	-14	-5	13.70	8.00
9	14	5	-10.40	16.40
-10	-14	-5	258.37	17.30
-10	-14	-5	257.47	17.10
-10	-14	-5	232.08	17.30
-10	-14	-5	198.88	18.60
-10	-14	-5	230.38	17.40
-11	-14	-5	96.49	12.10
-11	-14	-5	102.19	15.40
-11	-14	-5	96.29	14.70
-11	-14	-5	98.69	14.30
-9	15	5	42.70	10.20
8	-15	-5	117.49	13.30
-8	15	5	103.19	18.40
7	-15	-5	85.79	13.60
7	-15	-5	62.09	18.20
-7	15	5	66.59	15.80
6	-15	-5	25.70	8.00
6	-15	-5	-7.00	10.90
-6	15	5	9.80	11.70
-6	15	5	10.60	14.50
-6	15	5	11.40	14.10
5	-15	-5	645.34	33.80
5	-15	-5	641.44	32.50
5	-15	-5	627.14	39.80
-5	15	5	528.85	43.70
-5	15	5	561.54	36.80
4	-15	-5	274.37	20.70
4	-15	-5	320.47	27.10
4	-15	-5	307.37	19.70
4	-15	-5	281.47	27.50
-4	15	5	324.97	19.80
-4	15	5	298.47	22.90
-4	15	5	271.87	25.20
3	-15	-5	81.19	13.30
3	-15	-5	70.99	12.20
3	-15	-5	57.09	12.40
3	-15	-5	47.80	8.40
-3	15	5	46.90	10.90
-3	15	5	61.89	13.00
-3	15	5	57.19	9.30
2	-15	-5	765.62	39.10
2	-15	-5	724.73	37.70
2	-15	-5	769.72	45.60

2	-15	-5	695.03	38.90
2	-15	-5	700.73	37.40
-2	15	5	751.02	38.40
-2	15	5	756.22	35.30
1	-15	-5	6.80	8.20
1	-15	-5	9.00	12.90
1	-15	-5	15.00	10.60
1	-15	-5	7.20	7.80
1	-15	-5	8.10	11.00
-1	15	5	1.20	7.10
-1	15	5	8.40	3.60
0	-15	-5	46.40	8.80
0	-15	-5	51.89	8.30
0	-15	-5	60.49	8.80
0	-15	-5	46.40	9.50
0	-15	-5	53.59	8.40
0	15	5	50.49	5.70
-1	-15	-5	19.50	7.10
-1	-15	-5	12.90	8.00
-1	-15	-5	16.80	7.10
-1	-15	-5	26.80	7.30
-1	-15	-5	7.80	8.10
1	15	5	14.30	3.40
-2	-15	-5	115.59	17.80
-2	-15	-5	108.39	12.80
-2	-15	-5	76.49	11.80
-2	-15	-5	115.19	11.70
-2	-15	-5	99.09	10.90
-2	-15	-5	110.69	13.20
2	15	5	103.19	7.30
-3	-15	-5	5.40	6.30
-3	-15	-5	7.40	7.20
-3	-15	-5	4.40	8.30
-3	-15	-5	23.60	13.90
-3	-15	-5	0.20	6.20
-3	-15	-5	5.00	10.00
3	15	5	7.30	3.50
-4	-15	-5	94.69	13.50
-4	-15	-5	79.49	10.60
-4	-15	-5	90.89	13.60
-4	-15	-5	107.39	11.50
-4	-15	-5	97.19	12.80
-4	-15	-5	93.89	11.20
4	15	5	87.89	8.20

-5	-15	-5	1118.99	52.09
-5	-15	-5	1094.89	52.69
-5	-15	-5	1115.19	53.09
-5	-15	-5	1064.69	52.99
-5	-15	-5	1029.90	53.19
-5	-15	-5	1092.89	53.09
5	15	5	1120.19	51.49
-6	-15	-5	1256.87	59.89
-6	-15	-5	1160.98	60.19
-6	-15	-5	1199.88	59.99
-6	-15	-5	1242.18	59.79
-6	-15	-5	1315.27	59.19
-6	-15	-5	1329.87	60.39
6	15	5	1204.88	59.49
-7	-15	-5	7.20	6.90
-7	-15	-5	-7.40	16.30
-7	-15	-5	20.70	8.30
-7	-15	-5	1.80	8.40
-7	-15	-5	16.70	8.40
-7	-15	-5	2.40	7.50
7	15	5	-0.20	9.90
-8	-15	-5	0.80	7.20
-8	-15	-5	6.40	8.10
-8	-15	-5	0.80	8.20
-8	-15	-5	13.80	8.70
-8	-15	-5	-0.60	7.70
8	15	5	9.40	13.80
-9	-15	-5	28.20	9.10
-9	-15	-5	26.00	8.20
-9	-15	-5	37.40	7.70
-9	-15	-5	33.60	9.20
-9	-15	-5	15.90	8.00
-10	-15	-5	6.90	7.70
-10	-15	-5	7.10	7.40
-10	-15	-5	7.00	8.90
-10	-15	-5	-4.80	7.00
10	15	5	1.20	15.60
9	-16	-5	-4.70	8.00
-9	16	5	14.30	9.40
8	-16	-5	-6.30	7.00
-8	16	5	14.30	10.00
7	-16	-5	269.07	18.00
7	-16	-5	261.47	26.80
-7	16	5	224.08	22.50

6	-16	-5	234.88	17.70
-6	16	5	253.27	31.00
-6	16	5	237.78	25.20
-6	16	5	217.88	22.70
5	-16	-5	106.79	16.10
5	-16	-5	87.59	17.60
5	-16	-5	64.99	9.50
-5	16	5	66.29	15.40
-5	16	5	87.49	14.70
4	-16	-5	94.09	15.90
4	-16	-5	105.69	15.10
4	-16	-5	107.59	16.40
4	-16	-5	83.09	15.30
-4	16	5	115.39	16.70
-4	16	5	109.99	18.80
-4	16	5	120.49	19.90
3	-16	-5	110.39	19.10
3	-16	-5	85.49	13.20
3	-16	-5	85.09	15.10
3	-16	-5	101.69	14.30
-3	16	5	91.19	16.20
-3	16	5	113.89	19.10
-3	16	5	108.19	11.90
2	-16	-5	55.59	10.70
2	-16	-5	67.59	10.10
2	-16	-5	53.39	10.00
2	-16	-5	76.49	13.80
2	-16	-5	82.59	11.70
-2	16	5	70.99	8.90
-2	16	5	82.59	13.80
1	-16	-5	871.31	43.90
1	-16	-5	864.11	45.80
1	-16	-5	865.21	44.10
1	-16	-5	889.71	44.40
1	-16	-5	953.00	44.90
-1	16	5	883.11	44.30
-1	16	5	876.71	41.80
0	-16	-5	244.48	17.20
0	-16	-5	239.18	17.20
0	-16	-5	217.48	17.90
0	-16	-5	241.68	17.30
0	16	5	217.08	13.10
-1	-16	-5	790.42	40.10
-1	-16	-5	847.72	40.40

-1	-16	-5	812.42	45.10
-1	-16	-5	792.52	39.90
-1	-16	-5	794.32	39.70
1	16	5	778.22	37.70
-2	-16	-5	9.10	6.90
-2	-16	-5	-4.10	6.00
-2	-16	-5	6.30	7.60
-2	-16	-5	7.20	7.30
2	16	5	5.70	3.50
-3	-16	-5	128.09	13.70
-3	-16	-5	123.59	12.40
-3	-16	-5	136.09	12.50
-3	-16	-5	119.59	14.20
-3	-16	-5	125.99	13.40
3	16	5	134.29	9.60
-4	-16	-5	232.78	17.80
-4	-16	-5	242.38	17.10
-4	-16	-5	224.78	16.00
-4	-16	-5	230.58	16.20
-4	-16	-5	210.38	17.60
4	16	5	228.08	14.00
-5	-16	-5	2.50	6.20
-5	-16	-5	20.70	8.60
-5	-16	-5	7.30	7.90
-5	-16	-5	-5.30	7.80
-5	-16	-5	-4.70	8.50
-5	-16	-5	14.50	7.40
5	16	5	10.20	6.10
-6	-16	-5	255.77	16.60
-6	-16	-5	286.07	20.50
-6	-16	-5	230.88	17.90
-6	-16	-5	200.18	18.90
-6	-16	-5	231.58	18.60
-6	-16	-5	230.48	18.40
6	16	5	226.28	18.00
-7	-16	-5	96.49	14.50
-7	-16	-5	120.19	15.20
-7	-16	-5	117.09	14.00
-7	-16	-5	98.09	11.70
-7	-16	-5	80.39	13.70
-7	-16	-5	101.99	12.40
7	16	5	73.39	13.30
-8	-16	-5	31.00	8.40
-8	-16	-5	23.90	7.50

-8	-16	-5	24.20	9.00
-8	-16	-5	17.20	8.90
-8	-16	-5	18.90	8.80
8	16	5	26.10	15.40
-9	-16	-5	39.10	9.20
-9	-16	-5	53.29	8.80
-9	-16	-5	30.10	7.40
9	16	5	78.89	19.90
-10	-16	-5	31.00	9.40
-10	-16	-5	25.00	6.70
-10	-16	-5	25.10	7.70
-10	-16	-5	17.60	6.70
10	16	5	-55.99	30.70
8	-17	-5	54.69	8.50
-8	17	5	35.50	10.20
7	-17	-5	-4.30	6.90
7	-17	-5	44.90	15.90
-7	17	5	1.00	10.00
6	-17	-5	346.57	25.30
6	-17	-5	334.07	20.60
-6	17	5	253.67	28.70
-6	17	5	333.97	24.60
5	-17	-5	9.10	7.80
5	-17	-5	2.10	9.40
5	-17	-5	4.20	15.90
-5	17	5	14.90	13.60
-5	17	5	1.10	13.40
4	-17	-5	84.69	15.10
4	-17	-5	62.39	10.90
4	-17	-5	85.69	15.60
4	-17	-5	68.79	10.50
-4	17	5	64.19	12.90
-4	17	5	60.59	13.20
-4	17	5	44.40	13.00
3	-17	-5	34.70	11.70
3	-17	-5	28.20	11.90
3	-17	-5	26.10	8.00
3	-17	-5	35.70	9.30
-3	17	5	58.19	11.70
-3	17	5	53.79	12.00
-3	17	5	36.80	8.80
2	-17	-5	168.08	16.90
2	-17	-5	211.18	18.40
2	-17	-5	168.98	17.50

2	-17	-5	187.88	18.80
2	-17	-5	219.98	19.90
-2	17	5	189.78	14.20
-2	17	5	197.78	17.60
1	-17	-5	311.07	21.80
1	-17	-5	306.77	22.00
1	-17	-5	312.27	26.70
1	-17	-5	340.07	21.80
1	-17	-5	312.17	21.30
-1	17	5	343.77	18.40
-1	17	5	372.36	22.20
0	-17	-5	93.59	15.30
0	-17	-5	82.39	13.70
0	-17	-5	113.79	13.60
0	-17	-5	102.89	14.00
0	17	5	122.69	9.40
-1	-17	-5	258.07	18.30
-1	-17	-5	252.57	20.30
-1	-17	-5	279.97	18.70
-1	-17	-5	234.38	17.40
-1	-17	-5	275.57	18.40
1	17	5	259.07	14.80
-2	-17	-5	168.08	13.90
-2	-17	-5	149.69	18.70
-2	-17	-5	170.28	15.00
-2	-17	-5	160.38	15.60
-2	-17	-5	178.28	15.00
2	17	5	164.48	11.20
-3	-17	-5	45.10	8.20
-3	-17	-5	60.59	9.20
-3	-17	-5	38.80	7.30
-3	-17	-5	50.99	9.30
3	17	5	35.90	5.90
-4	-17	-5	432.76	26.10
-4	-17	-5	447.36	24.70
-4	-17	-5	441.46	25.80
-4	-17	-5	460.65	25.80
-4	-17	-5	458.65	25.40
4	17	5	458.95	23.80
-5	-17	-5	137.59	12.90
-5	-17	-5	128.29	14.60
-5	-17	-5	164.68	15.10
-5	-17	-5	149.59	15.70
-5	-17	-5	143.09	15.40

-6	-17	-5	538.25	30.50
-6	-17	-5	521.35	29.60
-6	-17	-5	538.75	28.00
-6	-17	-5	493.75	28.50
-6	-17	-5	558.14	29.20
6	17	5	513.55	29.70
-7	-17	-5	78.49	12.30
-7	-17	-5	64.49	11.20
-7	-17	-5	62.89	13.40
-7	-17	-5	62.39	10.80
-7	-17	-5	92.99	14.40
7	17	5	78.89	13.70
-8	-17	-5	0.50	8.20
-8	-17	-5	5.20	7.80
-8	-17	-5	4.10	6.20
-8	-17	-5	11.80	7.90
-9	-17	-5	11.00	6.90
-9	-17	-5	9.70	7.70
-9	-17	-5	10.30	8.80
-9	-17	-5	20.70	6.80
-10	-17	-5	12.40	6.40
-10	-17	-5	30.80	9.40
-10	-17	-5	-0.90	7.30
-10	-17	-5	-5.10	9.40
8	-18	-5	86.09	11.40
-8	18	5	57.89	9.90
7	-18	-5	18.00	7.30
7	-18	-5	-11.10	15.50
-7	18	5	-1.90	8.60
6	-18	-5	28.90	7.80
6	-18	-5	34.90	12.10
-6	18	5	30.60	9.80
-6	18	5	8.10	16.20
5	-18	-5	102.19	15.10
-5	18	5	91.89	15.70
-5	18	5	96.49	15.70
4	-18	-5	134.59	22.50
4	-18	-5	171.38	26.20
4	-18	-5	135.89	16.40
4	-18	-5	117.79	15.50
-4	18	5	142.29	19.30
-4	18	5	126.49	20.40
3	-18	-5	21.50	9.60
3	-18	-5	-5.20	11.50

3	-18	-5	15.60	11.10
3	-18	-5	5.50	8.20
-3	18	5	20.40	11.50
-3	18	5	20.30	10.50
-3	18	5	17.70	10.70
2	-18	-5	9.20	10.10
2	-18	-5	7.70	8.40
2	-18	-5	12.40	8.30
2	-18	-5	-5.90	9.20
2	-18	-5	-3.50	11.20
-2	18	5	8.20	9.80
-2	18	5	4.60	7.60
1	-18	-5	256.37	19.20
1	-18	-5	244.48	19.70
1	-18	-5	284.87	19.30
1	-18	-5	238.28	19.50
-1	18	5	234.18	18.70
-1	18	5	238.08	16.20
0	-18	-5	985.50	47.50
0	-18	-5	894.11	46.40
0	-18	-5	929.91	46.20
0	-18	-5	965.10	46.50
0	18	5	952.20	47.20
0	18	5	919.31	44.70
-1	-18	-5	20.30	7.50
-1	-18	-5	24.10	7.80
-1	-18	-5	15.20	7.30
-1	-18	-5	22.30	9.10
1	18	5	9.40	5.00
-2	-18	-5	11.70	7.40
-2	-18	-5	-3.70	10.40
-2	-18	-5	9.60	6.90
-2	-18	-5	-9.80	7.80
-2	-18	-5	-1.80	7.10
2	18	5	10.00	5.30
-3	-18	-5	73.39	9.90
-3	-18	-5	44.40	7.70
-3	-18	-5	41.60	8.00
-3	-18	-5	38.70	14.30
-3	-18	-5	53.29	8.60
3	18	5	62.59	9.60
-4	-18	-5	96.89	12.90
-4	-18	-5	89.89	10.70
-4	-18	-5	69.89	11.00

-4	-18	-5	64.69	9.60
4	18	5	85.19	11.40
-5	-18	-5	22.00	7.80
-5	-18	-5	21.30	6.40
-5	-18	-5	29.30	8.30
-5	-18	-5	7.80	8.30
-5	-18	-5	11.10	7.50
5	18	5	30.90	8.50
-6	-18	-5	18.80	7.80
-6	-18	-5	16.10	6.90
-6	-18	-5	14.70	12.60
-6	-18	-5	15.60	7.80
-6	-18	-5	29.80	8.60
6	18	5	19.40	10.40
-7	-18	-5	105.49	12.60
-7	-18	-5	123.99	11.90
-7	-18	-5	120.29	18.80
-7	-18	-5	115.99	13.40
-7	-18	-5	128.89	13.60
7	18	5	88.89	21.40
-8	-18	-5	29.00	8.40
-8	-18	-5	39.20	7.60
-8	-18	-5	59.79	9.70
-8	-18	-5	54.49	8.40
-9	-18	-5	61.79	10.30
-9	-18	-5	45.70	7.10
-9	-18	-5	67.89	10.70
-9	-18	-5	37.20	8.90
7	-19	-5	22.40	7.60
7	-19	-5	2.80	16.50
-7	19	5	37.20	8.40
6	-19	-5	93.39	12.80
6	-19	-5	120.89	17.60
-6	19	5	112.59	15.10
5	-19	-5	311.97	21.20
5	-19	-5	285.07	19.00
-5	19	5	276.37	24.60
-5	19	5	289.67	21.90
-5	19	5	241.08	25.80
4	-19	-5	36.00	11.80
4	-19	-5	79.19	17.50
4	-19	-5	40.30	15.30
4	-19	-5	44.20	10.60
-4	19	5	56.89	13.50

-4	19	5	36.30	12.50
3	-19	-5	-5.10	11.30
3	-19	-5	10.60	10.00
3	-19	-5	-4.20	8.00
3	-19	-5	16.30	12.30
-3	19	5	13.20	10.40
-3	19	5	-4.50	10.50
-3	19	5	2.10	14.00
2	-19	-5	171.68	18.50
2	-19	-5	183.78	22.10
2	-19	-5	195.38	17.10
2	-19	-5	172.68	18.60
-2	19	5	185.38	17.70
-2	19	5	183.18	18.80
-2	19	5	195.98	18.50
1	-19	-5	8.60	8.90
1	-19	-5	-3.70	8.00
1	-19	-5	11.30	8.30
1	-19	-5	20.30	8.40
-1	19	5	-4.60	8.90
-1	19	5	0.90	6.90
0	-19	-5	102.19	14.30
0	-19	-5	121.19	16.60
0	-19	-5	88.59	14.20
0	-19	-5	114.99	13.70
0	19	5	157.58	17.00
0	19	5	134.49	16.10
-1	-19	-5	33.80	10.00
-1	-19	-5	20.70	7.70
-1	-19	-5	34.70	8.00
-1	-19	-5	38.80	8.40
-2	-19	-5	41.60	8.30
-2	-19	-5	50.59	8.90
-2	-19	-5	31.30	8.40
-2	-19	-5	40.80	8.20
-2	-19	-5	34.80	11.40
2	19	5	36.20	7.30
-3	-19	-5	93.79	15.60
-3	-19	-5	82.19	12.50
-3	-19	-5	72.79	12.50
-3	-19	-5	88.19	11.90
-3	-19	-5	77.29	13.00
3	19	5	91.99	11.70
-4	-19	-5	-0.20	7.60

-4	-19	-5	7.70	7.80
-4	-19	-5	9.80	7.00
-4	-19	-5	11.40	6.60
4	19	5	2.90	7.70
-5	-19	-5	931.51	45.30
-5	-19	-5	891.51	43.60
-5	-19	-5	905.51	44.30
-5	-19	-5	884.71	44.30
5	19	5	873.01	44.80
-6	-19	-5	376.56	21.70
-6	-19	-5	371.16	22.20
-6	-19	-5	378.86	24.10
-6	-19	-5	386.26	22.70
6	19	5	349.96	26.30
-7	-19	-5	23.70	7.50
-7	-19	-5	12.90	6.30
-7	-19	-5	31.30	11.70
-7	-19	-5	48.80	8.20
-8	-19	-5	81.59	11.10
-8	-19	-5	98.99	10.10
-8	-19	-5	64.19	10.90
-9	-19	-5	72.89	11.10
-9	-19	-5	60.29	10.30
-9	-19	-5	52.39	7.80
6	-20	-5	93.09	12.50
6	-20	-5	73.79	12.50
-6	20	5	82.29	13.30
5	-20	-5	1.80	9.20
5	-20	-5	-0.70	8.60
-5	20	5	0.30	7.90
-5	20	5	-17.20	13.50
4	-20	-5	-2.50	14.80
4	-20	-5	7.50	9.60
4	-20	-5	-15.10	16.70
4	-20	-5	9.80	8.70
-4	20	5	13.90	11.70
-4	20	5	9.90	13.30
3	-20	-5	1.10	9.00
3	-20	-5	40.20	11.70
3	-20	-5	35.20	12.50
3	-20	-5	4.50	12.90
-3	20	5	25.60	11.10
-3	20	5	14.50	11.40
2	-20	-5	55.09	17.60

2	-20	-5	38.30	12.40
2	-20	-5	49.70	9.90
-2	20	5	46.50	12.60
-2	20	5	29.80	11.90
-2	20	5	44.60	11.00
1	-20	-5	226.88	17.80
1	-20	-5	191.08	17.10
1	-20	-5	188.98	17.30
1	-20	-5	180.88	17.20
-1	20	5	198.38	18.10
-1	20	5	206.28	19.10
0	-20	-5	180.88	16.50
0	-20	-5	196.18	15.60
0	-20	-5	170.48	16.50
0	-20	-5	194.18	18.10
0	20	5	166.68	16.90
0	20	5	169.08	16.30
-1	-20	-5	565.34	30.30
-1	-20	-5	514.15	30.00
-1	-20	-5	590.84	32.00
-1	-20	-5	582.94	30.10
1	20	5	548.25	31.10
1	20	5	577.04	29.90
-2	-20	-5	2.40	11.00
-2	-20	-5	23.50	7.50
-2	-20	-5	18.50	7.60
-2	-20	-5	25.80	7.70
2	20	5	16.40	8.00
-3	-20	-5	196.88	14.90
-3	-20	-5	144.89	26.10
-3	-20	-5	174.68	14.90
-3	-20	-5	165.78	14.10
-3	-20	-5	170.38	15.90
3	20	5	223.38	29.70
3	20	5	190.88	16.40
-4	-20	-5	89.09	13.10
-4	-20	-5	114.39	12.10
-4	-20	-5	101.69	13.20
-4	-20	-5	85.79	15.50
4	20	5	96.79	15.10
-5	-20	-5	-9.90	7.50
-5	-20	-5	-0.80	6.80
-5	-20	-5	6.80	8.10
-5	-20	-5	1.20	6.40

5	20	5	12.70	11.40
-6	-20	-5	217.38	15.40
-6	-20	-5	191.88	15.10
-6	-20	-5	228.48	17.50
-6	-20	-5	190.78	14.20
-7	-20	-5	183.58	13.80
-7	-20	-5	165.68	13.20
-7	-20	-5	235.68	20.60
-7	-20	-5	188.28	14.20
-8	-20	-5	-8.70	6.40
-8	-20	-5	7.60	5.70
-8	-20	-5	1.30	5.90
6	-21	-5	127.89	13.40
6	-21	-5	109.09	17.60
-6	21	5	98.99	15.80
5	-21	-5	4.30	9.10
5	-21	-5	19.80	9.40
-5	21	5	20.90	16.50
-5	21	5	10.30	7.70
4	-21	-5	39.10	10.80
4	-21	-5	36.30	8.50
-4	21	5	26.00	15.00
-4	21	5	56.29	12.10
3	-21	-5	-0.30	12.30
3	-21	-5	11.40	8.20
3	-21	-5	26.80	12.60
3	-21	-5	-5.60	13.60
-3	21	5	19.40	10.60
-3	21	5	23.90	12.30
2	-21	-5	281.87	19.40
2	-21	-5	260.77	21.60
2	-21	-5	260.67	21.20
-2	21	5	257.27	21.00
-2	21	5	282.67	21.30
1	-21	-5	458.95	27.80
1	-21	-5	460.65	27.80
1	-21	-5	470.25	28.30
-1	21	5	468.55	28.10
-1	21	5	452.85	30.60
-1	21	5	468.45	27.60
0	-21	-5	40.80	12.80
0	21	5	47.80	12.20
0	21	5	59.29	10.60
-1	-21	-5	156.48	17.60

-1	-21	-5	151.48	18.30
1	21	5	151.38	15.10
1	21	5	146.59	17.60
-2	-21	-5	83.69	14.50
-2	-21	-5	104.29	18.90
2	21	5	89.09	15.90
-3	-21	-5	0.60	8.10
-3	-21	-5	14.90	18.80
-3	-21	-5	2.30	7.30
-3	-21	-5	-3.40	7.20
3	21	5	-2.40	10.20
-4	-21	-5	328.07	19.30
-4	-21	-5	321.07	19.70
-4	-21	-5	309.67	20.40
-4	-21	-5	334.87	19.90
4	21	5	312.57	27.20
-5	-21	-5	17.20	7.80
-5	-21	-5	25.60	6.30
-6	-21	-5	415.66	22.00
-6	-21	-5	418.46	22.40
-6	-21	-5	411.56	24.00
-6	-21	-5	401.46	22.30
-7	-21	-5	2.80	6.10
-7	-21	-5	4.10	5.50
-7	-21	-5	-0.60	13.00
-7	-21	-5	2.50	5.30
5	-22	-5	69.69	12.80
-5	22	5	57.79	9.20
4	-22	-5	41.20	15.60
4	-22	-5	28.50	8.50
-4	22	5	55.39	20.60
-4	22	5	78.79	21.20
-4	22	5	30.70	7.90
3	-22	-5	-16.90	15.70
3	-22	-5	2.60	8.10
3	-22	-5	28.90	12.90
-3	22	5	15.50	13.10
-3	22	5	38.70	10.40
2	-22	-5	-6.80	7.50
2	-22	-5	11.90	11.50
2	-22	-5	0.10	9.80
-2	22	5	-0.40	10.70
-2	22	5	3.80	9.50
1	-22	-5	568.54	31.40

1	-22	-5	598.34	31.60
1	-22	-5	586.84	31.30
-1	22	5	581.54	31.70
-1	22	5	562.54	31.90
0	-22	-5	452.55	26.10
0	-22	-5	426.06	24.40
0	-22	-5	434.36	25.30
0	-22	-5	426.06	24.90
0	22	5	401.76	25.70
-1	-22	-5	37.70	8.90
-1	-22	-5	29.60	8.40
-1	-22	-5	30.30	10.10
-1	-22	-5	39.80	8.30
1	22	5	21.00	9.50
-2	-22	-5	-8.70	7.10
-2	-22	-5	-5.20	11.20
-2	-22	-5	-5.70	7.00
-2	-22	-5	-8.40	6.80
2	22	5	-2.40	8.40
-3	-22	-5	51.09	8.00
-3	-22	-5	72.29	10.90
-3	-22	-5	69.29	11.40
-4	-22	-5	137.49	11.90
-4	-22	-5	122.89	11.90
-4	-22	-5	138.79	13.30
-5	-22	-5	2.80	5.60
-5	-22	-5	15.50	7.50
-5	-22	-5	-0.60	6.10
5	22	5	22.30	14.10
-6	-22	-5	-0.70	5.70
-6	-22	-5	1.10	8.60
-6	-22	-5	-3.90	5.30
-7	-22	-5	102.19	10.00
-7	-22	-5	74.19	11.40
7	22	5	78.19	12.40
4	-23	-5	-5.00	10.40
-4	23	5	9.00	8.70
-4	23	5	-10.20	16.90
3	-23	-5	23.80	7.60
3	-23	-5	25.50	15.40
-3	23	5	40.30	15.60
-3	23	5	45.00	14.00
2	-23	-5	159.68	16.50
2	-23	-5	131.59	19.20

2	-23	-5	154.28	13.80
-2	23	5	151.48	15.50
-2	23	5	136.19	17.70
1	-23	-5	21.60	8.40
1	-23	-5	20.30	9.00
1	-23	-5	18.20	10.50
-1	23	5	25.70	9.20
-1	23	5	18.70	10.10
0	-23	-5	3.20	8.80
0	-23	-5	11.30	8.90
0	-23	-5	-3.80	7.40
0	23	5	13.70	8.50
0	23	5	10.60	9.50
-1	-23	-5	46.70	9.10
-1	-23	-5	54.09	8.40
-1	-23	-5	41.60	7.80
1	23	5	54.69	10.00
-2	-23	-5	3.00	7.10
-2	-23	-5	5.50	12.40
-2	-23	-5	2.60	6.40
-2	-23	-5	4.00	6.30
2	23	5	4.80	8.60
-3	-23	-5	-2.20	6.00
-3	-23	-5	2.00	6.80
-3	-23	-5	5.30	6.50
3	23	5	16.70	10.60
-4	-23	-5	25.30	6.20
-4	-23	-5	28.60	7.60
-4	-23	-5	28.30	6.80
4	23	5	17.90	9.20
-5	-23	-5	534.45	26.60
-5	-23	-5	524.95	27.50
-5	-23	-5	529.95	26.80
-6	-23	-5	50.49	8.80
-6	-23	-5	48.20	9.30
-6	-23	-5	41.50	10.40
2	-24	-5	12.50	7.60
2	-24	-5	44.20	11.90
-2	24	5	12.20	15.40
-2	24	5	27.30	9.70
1	-24	-5	19.60	7.80
1	-24	-5	20.50	11.30
1	-24	-5	34.60	9.20
-1	24	5	26.00	11.10

-1	24	5	16.10	8.90
0	-24	-5	345.47	22.60
0	-24	-5	400.66	22.80
0	-24	-5	425.36	22.10
0	24	5	353.06	23.10
0	24	5	387.76	22.60
-1	-24	-5	108.19	13.00
-1	-24	-5	117.09	12.10
-1	-24	-5	121.19	15.50
1	24	5	131.99	13.20
1	24	5	125.39	13.80
-2	-24	-5	7.00	6.70
-2	-24	-5	28.80	14.90
-2	-24	-5	20.20	7.50
-2	-24	-5	4.70	6.40
2	24	5	18.40	9.10
-3	-24	-5	106.99	10.70
-3	-24	-5	128.89	11.30
-3	-24	-5	97.89	10.70
3	24	5	72.99	12.30
-4	-24	-5	138.49	11.40
-4	-24	-5	143.59	12.50
-4	-24	-5	132.19	11.40
4	24	5	146.29	14.30
0	-25	-5	24.80	9.30
0	-25	-5	0.80	11.00
0	-25	-5	27.80	8.00
0	25	5	11.20	8.40
0	25	5	-9.20	15.00
-1	-25	-5	58.59	9.90
-1	-25	-5	31.50	12.00
-1	-25	-5	46.30	9.50
1	25	5	31.80	9.80
1	25	5	35.10	10.30
-2	-25	-5	4.80	7.50
-2	-25	-5	6.20	7.70
2	25	5	14.50	9.60
2	25	5	17.40	11.70
2	22	-6	3.90	8.70
2	22	-6	15.80	9.90
-2	-22	6	3.00	6.90
1	22	-6	97.39	16.40
1	22	-6	89.79	14.70
1	22	-6	95.69	14.50

-1	-22	6	58.99	9.10
-1	-22	6	62.59	10.60
0	22	-6	394.86	24.70
0	22	-6	388.76	22.60
0	-22	6	369.76	21.80
0	-22	6	368.96	21.50
-1	22	-6	101.99	15.10
-1	22	-6	67.39	10.90
1	-22	6	89.49	15.10
4	21	-6	110.79	16.00
4	21	-6	88.09	14.70
-4	-21	6	89.39	10.10
3	21	-6	10.90	12.90
3	21	-6	13.70	8.00
3	21	-6	-2.10	11.30
-3	-21	6	28.70	6.60
2	21	-6	82.69	13.10
2	21	-6	39.90	10.70
2	21	-6	54.39	8.90
-2	-21	6	27.60	13.10
-2	-21	6	61.79	10.20
1	21	-6	70.69	11.90
1	21	-6	62.79	11.10
1	21	-6	52.19	10.40
-1	-21	6	62.09	10.00
0	21	-6	28.90	7.70
0	21	-6	45.10	11.80
0	21	-6	20.30	9.10
0	-21	6	15.40	8.10
-1	21	-6	36.40	9.40
-1	21	-6	14.70	7.40
1	-21	6	5.80	7.50
1	-21	6	30.70	16.40
-2	21	-6	-1.00	9.10
-2	21	-6	6.90	7.30
2	-21	6	16.20	6.80
-3	21	-6	111.39	14.50
-3	21	-6	150.68	13.00
3	-21	6	145.59	11.80
5	20	-6	30.30	12.30
5	20	-6	9.30	7.40
-5	-20	6	7.90	12.30
-5	-20	6	11.20	5.80
4	20	-6	14.10	7.60

4	20	-6	24.90	12.40
-4	-20	6	27.60	6.70
3	20	-6	-9.70	10.50
3	20	-6	1.40	7.90
3	20	-6	8.20	8.50
-3	-20	6	2.00	6.50
2	20	-6	5.90	8.10
2	20	-6	-6.70	15.70
2	20	-6	-3.60	8.20
-2	-20	6	1.90	6.50
-2	-20	6	5.00	10.60
1	20	-6	25.80	9.30
1	20	-6	22.30	8.20
1	20	-6	19.60	8.90
-1	-20	6	23.90	8.90
-1	-20	6	27.30	7.70
0	20	-6	95.39	17.00
0	20	-6	106.39	12.90
0	20	-6	79.79	13.30
0	-20	6	100.89	13.80
0	-20	6	76.59	10.50
-1	20	-6	663.53	36.60
-1	20	-6	638.84	33.90
-1	20	-6	684.23	33.90
1	-20	6	656.23	34.00
1	-20	6	658.23	34.90
-2	20	-6	8.20	10.00
-2	20	-6	27.90	7.90
2	-20	6	23.20	7.90
-3	20	-6	43.30	9.80
-3	20	-6	61.59	9.10
3	-20	6	34.50	8.00
-4	20	-6	148.69	14.90
-4	20	-6	132.79	13.30
4	-20	6	132.99	12.90
6	19	-6	292.37	17.60
-6	-19	6	272.97	16.30
-6	-19	6	249.38	16.50
5	19	-6	40.20	14.30
5	19	-6	47.80	8.40
-5	-19	6	20.40	8.40
-5	-19	6	33.00	6.60
4	19	-6	341.17	23.10
4	19	-6	373.96	21.60

-4	-19	6	334.37	21.90
-4	-19	6	372.86	20.60
3	19	-6	12.80	9.40
3	19	-6	11.30	8.30
-3	-19	6	2.00	6.30
2	19	-6	11.80	8.90
2	19	-6	9.40	8.40
-2	-19	6	9.60	9.90
-2	-19	6	7.90	6.90
1	19	-6	241.88	17.60
1	19	-6	230.68	17.00
1	19	-6	207.98	18.30
-1	-19	6	246.78	17.20
-1	-19	6	232.38	17.80
0	19	-6	174.68	15.00
0	19	-6	164.88	15.50
0	19	-6	161.18	18.20
0	-19	6	171.68	16.10
0	-19	6	179.48	16.20
-1	19	-6	-1.30	8.20
-1	19	-6	8.80	8.10
-1	19	-6	-18.50	10.80
1	-19	6	-3.70	9.70
1	-19	6	-1.50	7.90
-2	19	-6	266.17	24.20
-2	19	-6	219.48	16.90
-2	19	-6	238.08	17.20
2	-19	6	196.08	22.70
2	-19	6	236.78	17.40
-3	19	-6	7.30	8.60
-3	19	-6	24.70	8.10
3	-19	6	15.90	7.90
-4	19	-6	11.30	12.60
-4	19	-6	15.20	8.10
4	-19	6	12.00	7.90
-5	19	-6	8.80	9.40
-5	19	-6	16.50	7.60
5	-19	6	44.30	11.80
5	-19	6	36.40	8.50
7	18	-6	11.80	6.90
-7	-18	6	15.20	9.20
-7	-18	6	4.90	7.70
6	18	-6	195.28	15.50
-6	-18	6	204.28	14.20

-6	-18	6	203.68	14.40
5	18	-6	-1.10	12.20
5	18	-6	8.60	7.80
-5	-18	6	0.90	6.10
-5	-18	6	10.30	7.40
4	18	-6	71.29	9.70
4	18	-6	74.99	11.40
-4	-18	6	63.39	11.10
-4	-18	6	72.69	12.80
3	18	-6	21.40	9.20
3	18	-6	37.30	9.50
-3	-18	6	8.10	12.60
-3	-18	6	21.50	9.60
-3	-18	6	8.40	6.80
2	18	-6	17.20	8.50
2	18	-6	5.90	14.10
-2	-18	6	11.50	10.10
-2	-18	6	21.60	10.80
1	18	-6	139.99	13.80
1	18	-6	133.99	22.20
-1	-18	6	132.39	18.80
0	18	-6	424.16	24.40
0	18	-6	457.35	27.20
0	-18	6	417.86	31.60
-1	18	-6	20.70	15.50
-1	18	-6	10.60	7.90
-1	18	-6	30.40	14.40
1	-18	6	-3.40	10.50
-2	18	-6	344.37	28.10
-2	18	-6	361.76	22.60
-2	18	-6	367.66	22.00
2	-18	6	361.26	23.00
2	-18	6	320.57	26.40
-3	18	-6	132.89	14.90
-3	18	-6	152.48	14.90
-3	18	-6	194.18	25.10
3	-18	6	163.58	16.30
-4	18	-6	12.30	8.40
-4	18	-6	16.80	8.30
4	-18	6	-6.40	10.70
4	-18	6	-9.10	8.10
-5	18	-6	66.19	9.90
-5	18	-6	83.59	13.20
5	-18	6	75.59	13.50

5	-18	6	82.19	14.40
-6	18	-6	25.20	10.20
-6	18	-6	9.90	7.70
6	-18	6	8.60	8.50
6	-18	6	10.70	10.00
7	17	-6	17.40	7.70
-7	-17	6	-3.20	5.50
-7	-17	6	-1.20	6.90
6	17	-6	13.10	7.70
-6	-17	6	2.70	6.30
-6	-17	6	-1.50	6.30
5	17	-6	598.54	31.70
5	17	-6	554.94	32.30
-5	-17	6	589.04	30.50
-5	-17	6	598.04	29.90
4	17	-6	-3.80	9.40
4	17	-6	8.20	10.30
-4	-17	6	14.20	7.60
-4	-17	6	16.90	8.10
3	17	-6	58.49	10.00
3	17	-6	62.29	9.60
-3	-17	6	66.89	9.00
2	17	-6	94.09	14.40
2	17	-6	77.59	13.10
-2	-17	6	97.19	12.60
-2	-17	6	72.39	11.50
1	17	-6	85.39	12.50
1	17	-6	98.29	15.70
-1	-17	6	121.69	16.00
-1	-17	6	86.59	13.30
0	17	-6	8.80	9.00
0	17	-6	6.60	7.30
0	-17	6	6.40	8.30
0	-17	6	9.60	8.00
-1	17	-6	19.10	7.00
-1	17	-6	15.00	8.00
-1	17	-6	10.80	10.00
1	-17	6	16.90	8.30
-2	17	-6	23.20	12.10
-2	17	-6	13.50	8.10
-2	17	-6	2.10	7.90
2	-17	6	-11.90	11.80
2	-17	6	12.00	8.30
-3	17	-6	445.66	26.20

-3	17	-6	455.75	25.50
-3	17	-6	437.06	35.30
3	-17	6	465.65	26.80
3	-17	6	378.26	28.20
-4	17	-6	24.50	8.80
-4	17	-6	41.60	9.60
4	-17	6	32.90	9.40
4	-17	6	44.90	10.30
-5	17	-6	453.05	28.00
-5	17	-6	515.05	28.20
5	-17	6	482.85	27.40
5	-17	6	556.74	28.70
-6	17	-6	123.69	18.20
-6	17	-6	129.19	14.60
6	-17	6	144.29	16.40
6	-17	6	160.78	13.90
-7	17	-6	13.10	10.70
-7	17	-6	40.80	9.00
7	-17	6	27.20	14.20
7	-17	6	23.40	7.70
8	16	-6	8.50	7.60
-8	-16	6	-11.00	9.10
-8	-16	6	0.40	5.40
7	16	-6	295.87	19.20
-7	-16	6	274.97	17.40
-7	-16	6	317.97	18.70
6	16	-6	90.59	14.30
-6	-16	6	68.79	11.00
-6	-16	6	84.29	11.70
-5	-16	6	127.09	14.30
-5	-16	6	106.59	19.40
4	16	-6	55.19	9.60
-4	-16	6	55.49	8.50
-4	-16	6	67.89	11.00
3	16	-6	17.60	8.80
3	16	-6	14.60	9.10
-3	-16	6	2.00	8.00
-3	-16	6	12.60	11.90
-3	-16	6	7.40	6.60
2	16	-6	0.60	8.20
2	16	-6	20.20	8.40
-2	-16	6	5.10	9.00
-2	-16	6	-2.60	6.50
-2	-16	6	19.90	9.50

1	16	-6	16.30	8.80
1	16	-6	13.30	8.80
-1	-16	6	13.70	7.20
-1	-16	6	-1.60	7.90
0	16	-6	302.97	21.40
0	16	-6	303.07	20.30
0	-16	6	281.07	19.80
0	-16	6	285.97	20.50
-1	16	-6	661.43	37.00
-1	16	-6	719.73	36.40
-1	16	-6	687.53	36.10
1	-16	6	702.03	36.80
1	-16	6	716.23	36.60
-2	16	-6	23.10	8.40
-2	16	-6	14.10	7.80
-2	16	-6	30.20	11.60
2	-16	6	6.90	7.80
2	-16	6	28.80	11.60
-3	16	-6	18.00	8.10
-3	16	-6	21.40	12.90
3	-16	6	24.60	10.30
3	-16	6	8.20	7.90
-4	16	-6	353.06	23.40
-4	16	-6	393.86	24.20
4	-16	6	343.57	26.10
4	-16	6	385.46	23.80
-5	16	-6	30.20	10.40
5	-16	6	46.70	9.00
5	-16	6	45.00	10.00
-6	16	-6	48.50	10.50
6	-16	6	31.80	11.00
6	-16	6	31.90	8.40
-7	16	-6	22.10	9.40
-7	16	-6	33.40	11.10
7	-16	6	30.60	13.90
7	-16	6	16.80	7.30
-8	16	-6	21.00	9.20
-8	16	-6	17.20	11.10
8	-16	6	8.40	7.40
9	15	-6	-4.90	9.00
-9	-15	6	-5.90	8.30
8	15	-6	22.30	8.20
8	15	-6	20.00	13.50
-8	-15	6	37.50	9.20

-8	-15	6	33.10	6.60
7	15	-6	-6.70	7.60
7	15	-6	-2.20	13.80
-7	-15	6	-0.40	5.70
-7	-15	6	7.50	7.00
6	15	-6	76.09	16.10
-6	-15	6	98.79	13.30
-6	-15	6	107.39	12.60
5	15	-6	96.69	17.60
-5	-15	6	126.19	13.30
-5	-15	6	140.99	13.90
4	15	-6	508.15	28.50
-4	-15	6	504.75	27.30
-4	-15	6	477.35	26.80
3	15	-6	14.30	8.70
-3	-15	6	11.00	11.30
-3	-15	6	11.20	7.00
-3	-15	6	6.60	7.70
2	15	-6	7.00	8.20
2	15	-6	-4.20	7.60
-2	-15	6	17.50	9.00
-2	-15	6	7.50	6.70
-2	-15	6	14.70	9.10
1	15	-6	134.09	16.50
1	15	-6	155.48	14.80
-1	-15	6	185.48	15.90
-1	-15	6	162.08	14.60
0	15	-6	93.49	15.40
0	15	-6	77.99	12.30
0	-15	6	92.69	13.30
0	-15	6	63.39	9.20
-1	15	-6	15.10	8.90
-1	15	-6	15.90	8.40
-1	15	-6	15.80	7.70
1	-15	6	15.10	8.80
1	-15	6	23.60	7.80
-2	15	-6	314.27	23.40
-2	15	-6	306.17	20.70
-2	15	-6	314.27	21.00
2	-15	6	324.87	21.30
2	-15	6	328.87	24.30
2	-15	6	301.17	26.40
-3	15	-6	36.70	9.20
-3	15	-6	44.80	13.00

-3	15	-6	31.60	9.20
3	-15	6	46.10	10.30
3	-15	6	29.90	8.70
-4	15	-6	70.99	11.20
-4	15	-6	42.10	15.10
-4	15	-6	62.69	11.00
4	-15	6	57.99	9.90
4	-15	6	53.89	10.00
-5	15	-6	28.60	10.30
-5	15	-6	38.20	9.80
5	-15	6	23.90	9.10
5	-15	6	24.70	9.80
-6	15	-6	100.89	16.40
-6	15	-6	132.09	17.90
6	-15	6	102.49	13.60
6	-15	6	82.49	13.00
-7	15	-6	32.20	11.20
-7	15	-6	17.20	9.90
7	-15	6	63.59	13.50
7	-15	6	12.60	7.70
-8	15	-6	36.00	11.40
-8	15	-6	37.00	10.00
8	-15	6	31.90	7.40
9	14	-6	94.79	15.20
9	14	-6	93.09	13.20
-9	-14	6	85.69	8.90
8	14	-6	-2.50	12.60
-8	-14	6	9.20	8.90
-8	-14	6	-0.20	5.60
7	14	-6	11.40	13.60
-7	-14	6	-7.30	6.00
-7	-14	6	-13.40	7.10
6	14	-6	507.25	33.80
-6	-14	6	530.85	27.30
-6	-14	6	524.85	28.00
-6	-14	6	447.86	30.70
5	14	-6	17.80	13.60
-5	-14	6	-1.60	9.20
-5	-14	6	0.10	6.60
-5	-14	6	2.80	6.70
4	14	-6	21.10	14.30
-4	-14	6	19.00	6.60
-4	-14	6	20.60	7.50
3	14	-6	264.97	18.90

-3	-14	6	218.68	21.50
-3	-14	6	235.28	17.80
-3	-14	6	260.47	17.50
2	14	-6	204.48	17.30
-2	-14	6	200.98	15.90
-2	-14	6	226.38	18.60
-2	-14	6	200.28	17.30
1	14	-6	382.36	22.80
1	14	-6	388.56	23.90
-1	-14	6	403.06	24.00
-1	-14	6	391.56	22.80
0	14	-6	394.56	22.40
0	14	-6	343.87	23.60
0	-14	6	409.46	23.40
0	-14	6	378.06	22.70
-1	14	-6	172.98	14.50
-1	14	-6	189.58	18.10
1	-14	6	201.88	16.80
1	-14	6	173.48	15.90
-2	14	-6	237.98	16.80
-2	14	-6	201.28	17.80
-2	14	-6	233.28	20.10
2	-14	6	223.48	21.00
2	-14	6	214.68	17.10
2	-14	6	192.28	21.10
-3	14	-6	719.03	37.70
-3	14	-6	724.63	40.20
-3	14	-6	723.73	37.30
3	-14	6	725.53	37.40
3	-14	6	685.93	37.20
-4	14	-6	68.39	11.80
-4	14	-6	46.50	15.20
-4	14	-6	77.09	11.50
4	-14	6	58.69	10.10
4	-14	6	96.19	15.40
-5	14	-6	322.37	37.30
-5	14	-6	241.58	30.30
-5	14	-6	292.77	22.20
-5	14	-6	303.17	21.70
5	-14	6	282.87	21.10
5	-14	6	266.07	20.00
-6	14	-6	-5.20	18.20
-6	14	-6	2.60	10.70
-6	14	-6	-4.80	9.50

6	-14	6	-2.10	8.60
-7	14	-6	73.29	13.60
7	-14	6	45.90	8.40
7	-14	6	66.39	14.40
-8	14	-6	156.38	17.50
8	-14	6	181.28	14.50
-9	14	-6	12.60	9.80
-9	14	-6	-9.70	12.10
9	-14	6	16.70	7.00
9	13	-6	7.20	11.80
9	13	-6	4.40	8.60
-9	-13	6	0.90	5.70
8	13	-6	66.09	13.40
-8	-13	6	56.29	9.80
-8	-13	6	65.49	10.50
7	13	-6	94.19	19.10
-7	-13	6	92.69	18.20
6	13	-6	185.08	23.40
-6	-13	6	177.28	18.50
-6	-13	6	202.28	15.70
-6	-13	6	198.08	16.30
5	13	-6	670.73	42.10
-5	-13	6	766.42	37.80
-5	-13	6	746.03	38.40
-5	-13	6	792.12	38.00
4	13	-6	0.30	13.20
-4	-13	6	8.70	6.10
-4	-13	6	9.80	7.40
-4	-13	6	24.40	8.90
3	13	-6	23.00	9.70
3	13	-6	21.50	16.10
-3	-13	6	33.80	11.80
-3	-13	6	9.90	7.70
-3	-13	6	27.50	6.50
-3	-13	6	16.10	8.40
2	13	-6	302.37	19.60
2	13	-6	289.27	20.60
-2	-13	6	270.67	20.80
-2	-13	6	294.87	18.80
1	13	-6	11.20	6.40
1	13	-6	9.30	7.40
-1	-13	6	10.10	6.90
-1	-13	6	-1.70	7.50
0	13	-6	55.59	9.80

0	13	-6	69.69	8.90
0	-13	6	65.89	9.10
0	-13	6	66.89	9.60
-1	13	-6	75.99	11.30
-1	13	-6	92.59	15.10
1	-13	6	72.79	13.30
-2	13	-6	1.90	6.80
-2	13	-6	-13.40	9.00
-2	13	-6	6.10	8.30
2	-13	6	9.00	15.60
2	-13	6	18.40	7.50
2	-13	6	-5.80	15.80
-3	13	-6	435.36	24.70
-3	13	-6	417.16	25.70
-3	13	-6	491.15	28.90
3	-13	6	422.46	24.80
3	-13	6	348.07	24.90
-4	13	-6	11.80	13.90
-4	13	-6	12.10	8.40
-4	13	-6	11.40	9.20
4	-13	6	10.00	7.70
-5	13	-6	279.87	31.50
-5	13	-6	307.57	27.50
-5	13	-6	297.37	23.40
-5	13	-6	312.27	23.00
5	-13	6	308.67	21.90
5	-13	6	322.77	21.30
-6	13	-6	35.40	10.90
-6	13	-6	33.20	14.20
-6	13	-6	41.60	11.90
6	-13	6	35.80	8.70
6	-13	6	32.40	10.80
-7	13	-6	209.18	23.30
-7	13	-6	160.18	23.50
-7	13	-6	205.28	19.60
7	-13	6	133.89	24.90
7	-13	6	150.98	16.60
-8	13	-6	57.79	12.40
-8	13	-6	55.89	13.90
-8	13	-6	51.19	13.00
8	-13	6	82.99	12.50
-9	13	-6	160.18	19.30
-9	13	-6	166.58	17.90
9	-13	6	202.18	14.70

10	12	-6	182.28	18.50
10	12	-6	215.58	18.20
-10	-12	6	222.08	13.30
9	12	-6	48.80	12.60
-9	-12	6	42.10	6.80
8	12	-6	59.29	13.30
-8	-12	6	51.09	8.00
-8	-12	6	57.69	14.30
-8	-12	6	63.99	10.50
7	12	-6	283.87	24.90
-7	-12	6	268.67	19.30
-7	-12	6	265.67	25.00
-7	-12	6	274.97	17.90
6	12	-6	13.20	12.70
-6	-12	6	9.50	7.80
-6	-12	6	9.90	12.30
-6	-12	6	2.40	11.60
-6	-12	6	16.40	7.00
5	12	-6	117.69	21.60
-5	-12	6	100.69	16.70
-5	-12	6	119.69	13.40
-5	-12	6	122.29	13.10
4	12	-6	28.90	13.10
4	12	-6	22.40	9.50
-4	-12	6	30.40	7.10
-4	-12	6	22.90	7.60
-4	-12	6	30.70	9.30
3	12	-6	13.60	13.30
3	12	-6	6.10	7.90
-3	-12	6	7.80	5.90
-3	-12	6	6.90	10.70
2	12	-6	5.50	7.30
-2	-12	6	16.90	8.50
-2	-12	6	13.20	7.70
1	12	-6	110.79	13.80
1	12	-6	132.79	14.00
-1	-12	6	138.39	12.80
-1	-12	6	156.28	14.20
0	12	-6	294.67	20.30
0	12	-6	330.07	18.90
0	-12	6	290.77	19.40
0	-12	6	310.07	19.80
-1	12	-6	251.57	16.70
-1	12	-6	253.07	20.00

1	-12	6	255.27	18.70
1	-12	6	258.57	18.00
-2	12	-6	87.29	11.40
-2	12	-6	100.19	12.70
2	-12	6	115.89	17.30
2	-12	6	77.49	11.80
-3	12	-6	42.60	8.90
-3	12	-6	52.99	10.90
-3	12	-6	46.70	18.70
3	-12	6	45.20	9.10
3	-12	6	47.40	8.90
-4	12	-6	970.20	48.40
-4	12	-6	991.70	49.00
-4	12	-6	969.60	52.29
-4	12	-6	970.10	52.19
4	-12	6	926.41	47.50
4	-12	6	920.81	47.10
-5	12	-6	149.88	19.30
-5	12	-6	139.59	19.50
-5	12	-6	174.48	18.00
-5	12	-6	160.98	22.90
5	-12	6	151.98	16.20
5	-12	6	165.28	17.60
-6	12	-6	115.99	19.20
-6	12	-6	97.99	17.10
-6	12	-6	110.59	15.00
-6	12	-6	144.89	19.90
6	-12	6	93.09	15.20
6	-12	6	131.89	18.70
-7	12	-6	113.69	20.50
-7	12	-6	68.89	14.20
-7	12	-6	78.69	13.50
7	-12	6	54.99	22.60
7	-12	6	69.39	10.20
-8	12	-6	86.59	14.30
-8	12	-6	97.59	15.40
-8	12	-6	84.19	15.20
8	-12	6	127.89	14.10
-9	12	-6	108.99	19.30
-9	12	-6	99.39	14.40
9	-12	6	122.99	13.10
-10	12	-6	24.50	14.40
-10	12	-6	42.80	17.30
10	-12	6	25.10	9.10

10	11	-6	51.79	20.70
10	11	-6	58.79	12.10
-10	-11	6	45.40	7.00
9	11	-6	45.40	12.30
-9	-11	6	33.10	7.00
-9	-11	6	37.50	13.60
8	11	-6	139.19	25.60
-8	-11	6	103.29	13.10
-8	-11	6	108.79	15.60
7	11	-6	50.39	13.00
-7	-11	6	44.70	7.70
-7	-11	6	46.70	12.30
-7	-11	6	35.30	8.90
6	11	-6	46.30	13.20
-6	-11	6	68.79	11.50
-6	-11	6	33.20	8.00
-6	-11	6	39.90	12.40
-6	-11	6	34.90	12.50
5	11	-6	73.59	13.40
5	11	-6	78.19	11.80
-5	-11	6	77.79	12.10
-5	-11	6	51.09	8.20
-5	-11	6	71.29	14.40
-5	-11	6	65.29	11.00
4	11	-6	512.05	29.20
-4	-11	6	556.44	29.00
-4	-11	6	553.64	30.10
-4	-11	6	560.14	28.90
3	11	-6	22.60	13.00
3	11	-6	15.70	7.10
-3	-11	6	15.60	5.80
-3	-11	6	15.60	9.00
-3	-11	6	20.00	7.50
-3	-11	6	31.80	10.30
2	11	-6	37.50	14.10
2	11	-6	6.40	6.50
-2	-11	6	23.10	9.20
-2	-11	6	1.20	7.90
-2	-11	6	0.50	9.10
-2	-11	6	3.00	5.70
1	11	-6	401.86	22.90
1	11	-6	371.46	21.00
-1	-11	6	388.66	23.30
-1	-11	6	397.36	22.20

0	11	-6	3.30	5.70
0	11	-6	17.80	8.40
0	11	-6	6.60	5.70
0	-11	6	5.10	7.00
0	-11	6	13.40	7.00
-1	11	-6	-7.10	8.40
-1	11	-6	1.60	5.60
1	-11	6	5.90	7.60
1	-11	6	1.20	7.10
-2	11	-6	717.03	35.50
-2	11	-6	706.43	36.30
-2	11	-6	694.13	37.90
2	-11	6	730.23	35.80
2	-11	6	649.34	37.20
-3	11	-6	9.40	6.30
-3	11	-6	3.10	11.80
-3	11	-6	5.20	8.30
3	-11	6	-0.30	6.60
-4	11	-6	38.90	10.50
-4	11	-6	26.80	8.80
4	-11	6	12.90	7.70
-5	11	-6	38.10	16.10
-5	11	-6	54.69	13.80
-5	11	-6	32.50	8.90
-5	11	-6	28.60	10.20
5	-11	6	43.80	9.40
5	-11	6	31.80	8.30
-6	11	-6	68.79	13.30
-6	11	-6	61.29	14.60
-6	11	-6	54.39	17.30
-6	11	-6	55.09	13.80
6	-11	6	42.50	8.50
-7	11	-6	21.40	12.40
-7	11	-6	11.20	11.60
-7	11	-6	21.20	13.00
7	-11	6	21.70	14.80
7	-11	6	12.20	8.40
-8	11	-6	55.39	21.70
-8	11	-6	24.90	12.60
-8	11	-6	53.19	13.20
8	-11	6	62.49	11.20
8	-11	6	27.20	12.90
-9	11	-6	383.26	28.60
-9	11	-6	398.76	28.60

-9	11	-6	389.46	27.40
9	-11	6	462.65	24.30
9	-11	6	399.96	27.40
-10	11	-6	356.36	24.90
-10	11	-6	345.97	26.10
-10	11	-6	321.17	25.50
10	-11	6	417.76	21.90
10	-11	6	340.67	26.80
10	10	-6	12.50	10.60
-10	-10	6	6.90	6.00
9	10	-6	56.29	12.70
-9	-10	6	63.49	10.90
-9	-10	6	57.99	13.20
8	10	-6	26.60	12.50
-8	-10	6	11.70	12.60
-8	-10	6	-6.90	10.40
-8	-10	6	8.80	6.40
7	10	-6	-4.60	11.60
-7	-10	6	-2.30	7.80
-7	-10	6	5.60	6.00
-7	-10	6	18.60	11.30
6	10	-6	739.63	41.10
-6	-10	6	743.73	39.60
-6	-10	6	779.72	37.20
-6	-10	6	747.93	37.90
-6	-10	6	716.23	39.70
-6	-10	6	684.93	37.60
5	10	-6	21.50	11.80
5	10	-6	18.70	8.50
-5	-10	6	17.80	6.00
-5	-10	6	18.10	7.80
-5	-10	6	8.00	11.90
-5	-10	6	10.30	6.40
-5	-10	6	6.00	11.50
4	10	-6	6.20	6.50
4	10	-6	15.50	12.50
-4	-10	6	11.00	10.40
-4	-10	6	3.30	6.40
-4	-10	6	15.70	6.60
3	10	-6	1531.55	79.99
3	10	-6	1736.43	77.39
-3	-10	6	1739.33	77.89
-3	-10	6	1655.33	79.19
-3	-10	6	1677.83	77.59

-3	-10	6	1717.93	78.29
2	10	-6	467.75	32.60
2	10	-6	493.45	25.50
-2	-10	6	496.25	27.50
-2	-10	6	501.65	29.20
-2	-10	6	515.55	26.50
-2	-10	6	536.75	28.40
1	10	-6	97.39	8.90
1	10	-6	92.59	12.80
1	10	-6	94.29	13.50
-1	-10	6	78.09	14.30
-1	-10	6	97.99	11.60
0	10	-6	14.00	4.80
0	10	-6	13.90	5.80
0	10	-6	20.60	8.30
0	-10	6	18.20	6.90
0	-10	6	34.80	7.80
-1	10	-6	788.12	44.50
-1	10	-6	977.90	43.90
-1	10	-6	910.61	45.20
1	-10	6	920.31	44.20
1	-10	6	915.31	44.50
-2	10	-6	110.19	11.10
-2	10	-6	113.09	14.10
-2	10	-6	100.89	16.80
2	-10	6	80.49	12.80
2	-10	6	111.99	13.20
-3	10	-6	439.26	25.10
-3	10	-6	439.76	26.60
-3	10	-6	450.45	30.60
3	-10	6	460.45	25.40
-4	10	-6	393.56	28.80
-4	10	-6	414.36	24.20
-4	10	-6	420.46	31.50
4	-10	6	397.56	24.30
-5	10	-6	348.97	33.00
-5	10	-6	350.56	23.60
-5	10	-6	424.36	34.90
-5	10	-6	354.46	25.40
5	-10	6	356.56	22.30
5	-10	6	358.46	23.20
-6	10	-6	4.10	11.00
-6	10	-6	-29.70	14.80
-6	10	-6	-2.00	11.20

-6	10	-6	-5.50	9.00
6	-10	6	7.60	10.60
6	-10	6	-2.50	7.00
-7	10	-6	161.38	30.40
-7	10	-6	266.97	22.40
-7	10	-6	235.08	23.10
-7	10	-6	220.58	23.10
7	-10	6	201.98	18.70
-8	10	-6	152.48	18.30
-8	10	-6	156.08	21.90
-8	10	-6	96.69	15.40
8	-10	6	142.79	15.50
-9	10	-6	-12.00	11.80
-9	10	-6	9.30	12.00
-9	10	-6	5.00	12.80
9	-10	6	12.70	8.00
9	-10	6	-2.70	10.80
-10	10	-6	85.19	16.80
-10	10	-6	101.29	14.90
-10	10	-6	75.69	13.30
10	-10	6	118.59	17.30
10	-10	6	88.59	13.80
11	9	-6	137.99	16.40
-11	-9	6	166.08	12.80
10	9	-6	15.90	10.80
-10	-9	6	15.50	12.50
-10	-9	6	1.30	6.30
9	9	-6	8.40	11.50
-9	-9	6	1.30	6.50
-9	-9	6	20.50	11.40
8	9	-6	223.58	22.20
-8	-9	6	200.18	15.70
-8	-9	6	191.28	20.70
-8	-9	6	248.78	20.20
7	9	-6	275.77	24.00
-7	-9	6	267.87	20.30
-7	-9	6	287.87	21.30
-7	-9	6	260.97	17.90
6	9	-6	162.48	21.40
6	9	-6	206.18	18.90
-6	-9	6	170.18	15.20
-6	-9	6	174.28	17.10
-6	-9	6	160.78	19.00
-6	-9	6	163.08	13.80

5	9	-6	846.32	41.90
-5	-9	6	844.22	41.90
-5	-9	6	818.72	44.40
-5	-9	6	893.71	46.40
-5	-9	6	862.11	44.00
4	9	-6	80.19	13.80
4	9	-6	75.39	9.70
-4	-9	6	66.09	10.30
-4	-9	6	77.69	11.10
-4	-9	6	76.89	12.20
-4	-9	6	83.89	10.70
3	9	-6	17.30	11.80
3	9	-6	1.80	4.70
-3	-9	6	-2.60	6.70
-3	-9	6	-2.10	11.60
-3	-9	6	9.10	5.80
2	9	-6	343.57	18.30
2	9	-6	315.67	20.00
2	9	-6	360.96	27.50
-2	-9	6	351.26	26.20
-2	-9	6	372.96	24.30
-2	-9	6	333.27	28.10
-2	-9	6	351.46	19.60
1	9	-6	15.10	9.60
1	9	-6	18.10	6.00
1	9	-6	13.50	4.10
-1	-9	6	12.50	10.00
-1	-9	6	7.70	8.10
-1	-9	6	17.30	6.30
-1	-9	6	10.40	6.90
0	9	-6	253.07	14.00
0	9	-6	277.87	20.50
0	9	-6	269.87	15.90
0	9	-6	226.78	15.60
0	9	-6	243.48	18.70
0	-9	6	219.48	17.20
0	-9	6	246.18	19.30
0	-9	6	256.37	17.30
0	-9	6	266.37	20.80
-1	9	-6	200.28	17.80
-1	9	-6	178.28	12.30
-1	9	-6	192.98	13.30
1	-9	6	199.98	14.90
1	-9	6	173.88	14.70

-2	9	-6	249.48	20.80
-2	9	-6	204.38	14.50
-2	9	-6	219.18	17.50
2	-9	6	223.08	16.00
2	-9	6	193.88	16.10
-3	9	-6	1745.53	78.89
-3	9	-6	1603.54	81.49
-3	9	-6	1718.83	79.59
3	-9	6	1653.43	78.29
3	-9	6	1717.13	78.69
-4	9	-6	26.20	7.10
-4	9	-6	19.90	8.70
-4	9	-6	49.10	13.90
-4	9	-6	35.90	14.00
4	-9	6	34.40	10.40
4	-9	6	31.00	8.10
-5	9	-6	252.97	20.80
-5	9	-6	227.98	25.00
-5	9	-6	264.87	24.70
-5	9	-6	246.38	27.30
-5	9	-6	274.87	18.90
5	-9	6	275.17	20.20
-6	9	-6	5.90	8.90
-6	9	-6	32.90	14.60
-6	9	-6	24.90	11.30
-6	9	-6	19.70	10.70
6	-9	6	7.80	11.50
-7	9	-6	8.70	10.90
-7	9	-6	11.10	16.40
-7	9	-6	-0.30	11.30
-7	9	-6	0.00	11.00
7	-9	6	8.40	8.50
-8	9	-6	307.67	25.30
-8	9	-6	331.87	25.30
-8	9	-6	252.67	23.50
8	-9	6	309.07	21.20
-9	9	-6	85.09	15.10
-9	9	-6	91.89	15.40
-9	9	-6	74.39	13.40
9	-9	6	118.59	15.90
-10	9	-6	172.38	26.50
-10	9	-6	166.38	21.10
-10	9	-6	220.38	19.10
10	-9	6	169.08	18.60

10	-9	6	225.98	18.10
-11	9	-6	39.10	13.40
-11	9	-6	31.60	15.90
-11	9	-6	16.80	14.90
11	-9	6	26.70	11.90
11	-9	6	30.00	11.40
11	8	-6	19.90	9.80
-11	-8	6	24.30	7.20
-11	-8	6	5.00	15.70
10	8	-6	154.28	20.00
-10	-8	6	189.08	14.60
-10	-8	6	219.08	19.60
-9	-8	6	29.40	12.10
-9	-8	6	56.29	11.20
8	8	-6	77.59	13.60
-8	-8	6	98.19	13.40
-8	-8	6	80.19	11.50
-8	-8	6	92.39	11.60
7	8	-6	387.16	27.40
-7	-8	6	393.86	22.40
-7	-8	6	383.56	24.80
-7	-8	6	380.66	24.60
6	8	-6	22.70	10.60
6	8	-6	16.20	9.20
-6	-8	6	27.50	15.80
-6	-8	6	26.30	8.20
-6	-8	6	42.20	10.00
-6	-8	6	15.80	5.90
5	8	-6	231.38	21.00
5	8	-6	186.68	15.10
-5	-8	6	205.68	14.10
-5	-8	6	216.28	15.50
-5	-8	6	230.68	19.90
-5	-8	6	178.58	15.90
4	8	-6	301.67	23.70
4	8	-6	327.47	17.80
-4	-8	6	307.07	18.70
-4	-8	6	338.57	19.30
-4	-8	6	276.47	24.20
-4	-8	6	323.37	18.40
3	8	-6	20.20	12.90
3	8	-6	20.20	11.90
3	8	-6	24.30	4.70
-3	-8	6	19.00	5.80

-3	-8	6	25.50	6.30
-3	-8	6	29.40	6.80
2	8	-6	144.49	17.00
2	8	-6	143.89	11.10
2	8	-6	158.98	9.90
-2	-8	6	132.69	12.70
-2	-8	6	155.28	17.80
-2	-8	6	150.78	11.70
1	8	-6	573.74	29.50
1	8	-6	645.84	32.60
1	8	-6	602.84	28.60
-1	-8	6	575.34	32.60
0	8	-6	63.89	6.40
0	8	-6	52.19	7.60
0	8	-6	66.99	12.30
0	-8	6	62.59	9.10
0	-8	6	60.39	9.30
0	-8	6	69.19	12.60
-1	8	-6	314.27	17.20
-1	8	-6	297.07	17.20
-1	8	-6	321.37	20.30
-1	8	-6	270.17	15.80
1	-8	6	301.07	18.00
1	-8	6	278.97	16.50
1	-8	6	304.57	20.90
-2	8	-6	131.29	15.90
-2	8	-6	82.39	9.70
2	-8	6	115.29	11.00
2	-8	6	101.89	12.50
-3	8	-6	501.15	28.20
-3	8	-6	462.75	32.20
-3	8	-6	514.05	26.20
3	-8	6	492.05	27.30
3	-8	6	484.15	25.70
-4	8	-6	1300.87	58.49
-4	8	-6	1229.88	58.89
-4	8	-6	1402.56	62.99
-4	8	-6	1161.28	61.59
-4	8	-6	1102.79	61.29
4	-8	6	1199.78	57.69
4	-8	6	1157.68	58.29
-5	8	-6	86.29	15.20
-5	8	-6	75.39	13.20
-5	8	-6	57.09	9.60

-5	8	-6	56.09	12.90
-5	8	-6	71.09	11.80
5	-8	6	77.89	12.10
5	-8	6	57.59	11.20
-6	8	-6	67.09	11.10
-6	8	-6	54.49	9.80
6	-8	6	61.39	17.50
6	-8	6	58.89	12.70
-7	8	-6	72.89	16.90
-7	8	-6	71.19	13.10
-7	8	-6	100.29	13.70
-7	8	-6	86.89	14.70
7	-8	6	85.69	14.90
-8	8	-6	102.99	14.50
-8	8	-6	155.38	19.20
-8	8	-6	135.99	18.60
-8	8	-6	152.58	19.80
8	-8	6	97.09	15.70
-9	8	-6	90.89	14.00
-9	8	-6	99.99	15.10
-10	8	-6	5.60	10.80
-10	8	-6	2.60	11.40
-10	8	-6	8.20	16.20
10	-8	6	-9.90	9.90
10	-8	6	4.50	10.20
-11	8	-6	90.69	13.50
-11	8	-6	62.99	17.00
-11	8	-6	74.99	14.60
11	-8	6	109.69	15.40
11	-8	6	93.39	21.20
11	7	-6	9.00	11.20
-11	-7	6	17.60	6.80
-11	-7	6	20.90	11.60
10	7	-6	97.99	18.10
-10	-7	6	112.59	12.20
-10	-7	6	83.49	11.80
9	7	-6	105.89	19.70
-9	-7	6	73.89	11.90
8	7	-6	120.39	20.20
-8	-7	6	120.89	15.10
-8	-7	6	133.49	15.40
-8	-7	6	127.99	13.10
7	7	-6	12.50	10.90
-7	-7	6	-12.70	8.00

-7	-7	6	0.20	5.60
-7	-7	6	3.80	9.30
6	7	-6	42.20	11.40
6	7	-6	56.69	9.30
-6	-7	6	36.30	6.70
-6	-7	6	48.60	15.50
-6	-7	6	52.39	9.40
-6	-7	6	34.80	9.30
5	7	-6	193.18	20.50
5	7	-6	230.28	15.00
-5	-7	6	198.08	19.70
-5	-7	6	217.38	16.20
-5	-7	6	218.48	19.20
-5	-7	6	217.38	14.50
4	7	-6	226.58	24.50
4	7	-6	281.07	21.80
4	7	-6	259.47	14.60
-4	-7	6	237.38	16.10
-4	-7	6	248.08	16.70
-4	-7	6	279.07	16.70
3	7	-6	59.39	6.70
3	7	-6	68.19	10.80
-3	-7	6	71.39	8.00
-3	-7	6	55.99	7.60
-3	-7	6	60.79	10.80
2	7	-6	712.03	32.20
2	7	-6	689.63	32.70
-2	-7	6	620.84	35.20
-2	-7	6	700.33	32.60
1	7	-6	797.62	36.60
1	7	-6	781.72	37.20
-1	-7	6	766.22	37.20
-1	-7	6	802.62	39.80
0	7	-6	133.79	8.40
0	7	-6	140.49	9.20
0	-7	6	125.49	9.50
0	-7	6	137.59	12.90
-1	7	-6	244.88	14.00
-1	7	-6	241.48	13.10
1	-7	6	228.38	17.10
1	-7	6	235.68	13.80
1	-7	6	247.88	14.60
-2	7	-6	1468.15	67.89
-2	7	-6	1453.55	68.09

-2	7	-6	1491.45	69.79
2	-7	6	1408.36	69.69
2	-7	6	1466.75	68.29
2	-7	6	1509.85	67.39
-3	7	-6	540.25	27.30
-3	7	-6	516.05	29.40
-3	7	-6	547.45	38.70
3	-7	6	533.25	26.60
-4	7	-6	136.49	20.80
-4	7	-6	122.59	14.00
-4	7	-6	138.79	11.80
4	-7	6	124.49	16.50
4	-7	6	133.59	11.40
-5	7	-6	8.40	6.00
-5	7	-6	13.80	12.20
-5	7	-6	6.80	12.00
-5	7	-6	0.50	9.90
-5	7	-6	1.20	7.80
5	-7	6	-6.80	9.90
5	-7	6	1.50	7.20
-6	7	-6	111.99	15.10
-6	7	-6	107.49	13.20
-6	7	-6	128.09	15.60
-6	7	-6	123.29	18.20
-6	7	-6	114.89	17.90
6	-7	6	89.79	17.90
6	-7	6	105.19	16.10
-7	7	-6	41.90	14.80
-7	7	-6	59.49	9.40
-7	7	-6	41.10	9.90
7	-7	6	38.50	8.60
7	-7	6	20.00	14.70
-8	7	-6	14.00	11.00
-8	7	-6	-15.40	14.90
-8	7	-6	21.30	11.10
-8	7	-6	25.20	10.50
8	-7	6	-2.70	9.00
-9	7	-6	662.43	38.60
-9	7	-6	702.53	38.80
-9	7	-6	725.23	39.70
9	-7	6	688.83	37.20
-10	7	-6	180.88	19.50
-10	7	-6	203.88	21.30
-10	7	-6	173.18	20.30

10	-7	6	228.68	20.80
-11	7	-6	-14.00	13.90
-11	7	-6	12.30	12.40
-11	7	-6	0.60	10.60
11	-7	6	8.70	13.80
11	-7	6	-3.40	9.50
11	6	-6	55.69	12.80
-11	-6	6	72.39	11.60
-11	-6	6	65.39	11.40
10	6	-6	13.40	11.60
-10	-6	6	22.10	7.30
-10	-6	6	16.60	9.10
9	6	-6	-4.30	11.70
9	6	-6	38.00	16.40
-9	-6	6	26.70	9.00
-9	-6	6	21.90	7.40
8	6	-6	-7.90	14.10
8	6	-6	0.70	10.60
-8	-6	6	10.10	6.20
-8	-6	6	8.40	7.80
7	6	-6	-2.30	13.30
-7	-6	6	20.40	8.20
-7	-6	6	-7.20	9.20
-7	-6	6	3.80	5.50
6	6	-6	674.93	36.00
-6	-6	6	706.03	36.10
-6	-6	6	752.52	39.40
-6	-6	6	720.13	36.70
5	6	-6	136.89	11.10
-5	-6	6	103.39	14.00
-5	-6	6	136.19	14.10
-5	-6	6	172.78	16.00
-5	-6	6	128.59	12.00
4	6	-6	205.08	16.60
4	6	-6	217.88	12.30
4	6	-6	190.18	21.20
-4	-6	6	206.38	15.00
-4	-6	6	192.18	15.20
-4	-6	6	189.78	14.10
3	6	-6	2231.68	101.49
3	6	-6	2220.48	99.49
-3	-6	6	2127.99	99.99
-3	-6	6	2226.88	99.19
2	6	-6	376.06	20.10

2	6	-6	346.37	17.10
2	6	-6	322.77	17.30
-2	-6	6	327.27	19.70
-2	-6	6	342.37	17.30
1	6	-6	19.40	3.40
1	6	-6	24.00	3.90
1	6	-6	23.70	5.80
-1	-6	6	21.20	5.90
-1	-6	6	24.40	4.00
-1	-6	6	16.90	4.20
0	6	-6	16.90	3.30
0	6	-6	19.40	5.10
0	6	-6	24.70	4.00
0	-6	6	14.80	3.50
0	-6	6	18.50	3.90
0	-6	6	19.80	5.70
-1	6	-6	1020.10	45.70
-1	6	-6	989.60	44.50
-1	6	-6	920.71	44.70
-1	6	-6	924.11	43.50
1	-6	6	914.31	44.00
1	-6	6	923.01	45.90
-3	6	-6	154.48	14.10
-3	6	-6	167.98	11.40
3	-6	6	154.68	13.80
3	-6	6	168.78	10.60
3	-6	6	160.68	13.10
-4	6	-6	1347.67	62.09
-4	6	-6	1214.08	71.79
-4	6	-6	1306.27	62.19
4	-6	6	1374.16	60.79
-5	6	-6	267.77	23.20
-5	6	-6	289.97	22.70
-5	6	-6	242.78	24.10
-5	6	-6	300.57	17.70
-5	6	-6	318.47	40.50
-5	6	-6	304.57	19.50
5	-6	6	289.47	23.80
5	-6	6	284.87	17.30
-6	6	-6	0.80	9.60
-6	6	-6	12.50	9.60
-6	6	-6	-5.20	13.60
-6	6	-6	3.50	6.00
-6	6	-6	-3.10	11.40

-6	6	-6	9.90	7.60
-7	6	-6	233.78	24.80
-7	6	-6	264.27	18.00
-7	6	-6	280.17	22.10
-7	6	-6	240.08	19.30
7	-6	6	211.48	19.10
7	-6	6	244.08	21.00
-8	6	-6	60.19	14.90
-8	6	-6	61.59	11.20
-8	6	-6	81.29	13.70
-8	6	-6	59.29	17.40
-8	6	-6	73.09	12.00
8	-6	6	51.79	10.20
-9	6	-6	0.00	10.30
-9	6	-6	11.40	15.40
-9	6	-6	18.20	10.40
-9	6	-6	24.40	9.40
9	-6	6	13.70	9.60
-10	6	-6	36.20	11.70
-10	6	-6	53.79	12.40
-10	6	-6	32.00	10.80
10	-6	6	41.00	12.40
-11	6	-6	0.80	13.00
-11	6	-6	29.60	12.10
-11	6	-6	28.10	10.40
11	-6	6	31.30	14.80
11	5	-6	190.48	20.70
-11	-5	6	230.68	16.30
-11	-5	6	189.58	17.80
10	5	-6	36.00	13.30
10	5	-6	40.00	16.00
-10	-5	6	42.50	8.70
-10	-5	6	43.50	9.60
9	5	-6	11.80	11.40
9	5	-6	15.70	13.60
-9	-5	6	6.50	6.40
-9	-5	6	3.80	7.70
8	5	-6	326.87	26.40
8	5	-6	312.77	25.80
-8	-5	6	346.97	21.10
-8	-5	6	365.26	20.70
7	5	-6	154.58	21.70
-7	-5	6	151.08	13.70
-7	-5	6	184.08	14.60

6	5	-6	109.79	13.40
6	5	-6	114.59	14.30
-6	-5	6	123.49	17.50
-6	-5	6	125.99	15.10
-6	-5	6	96.99	13.20
-6	-5	6	92.49	11.00
5	5	-6	314.97	17.10
-5	-5	6	292.97	20.50
-5	-5	6	291.07	21.50
-5	-5	6	292.77	19.40
-5	-5	6	318.87	17.90
4	5	-6	72.29	13.30
4	5	-6	82.39	10.60
4	5	-6	70.09	7.20
-4	-5	6	79.79	6.40
-4	-5	6	66.99	11.20
-4	-5	6	86.19	12.10
-4	-5	6	83.89	7.50
3	5	-6	210.88	11.30
3	5	-6	193.18	14.50
-3	-5	6	174.28	12.70
-3	-5	6	219.68	10.90
2	5	-6	391.96	19.10
2	5	-6	414.46	21.20
2	5	-6	390.06	19.30
-2	-5	6	429.66	20.90
-2	-5	6	384.56	19.10
-2	-5	6	360.26	20.50
1	5	-6	51.49	7.20
1	5	-6	42.50	4.90
1	5	-6	53.79	5.20
-1	-5	6	45.30	4.90
-1	-5	6	36.00	5.80
-1	-5	6	50.59	6.30
0	5	-6	6.10	2.60
0	5	-6	4.40	3.80
0	5	-6	-1.00	2.40
0	-5	6	2.30	2.40
0	-5	6	1.80	2.90
0	-5	6	-1.80	4.10
-1	5	-6	46.50	6.80
-1	5	-6	50.19	5.40
-1	5	-6	43.80	5.00
1	-5	6	39.70	5.60

1	-5	6	50.39	5.70
1	-5	6	49.40	7.90
-2	5	-6	1163.48	55.09
-2	5	-6	1199.58	55.09
-2	5	-6	1291.17	56.09
2	-5	6	1152.08	55.89
2	-5	6	1149.39	54.49
-3	5	-6	1790.22	99.59
-3	5	-6	2290.27	100.39
3	-5	6	2321.27	100.49
3	-5	6	2399.96	99.49
-4	5	-6	-1.10	5.20
-4	5	-6	3.70	3.80
-4	5	-6	9.30	7.00
4	-5	6	-4.70	6.60
4	-5	6	-0.70	3.20
4	-5	6	-6.90	6.50
-5	5	-6	232.58	19.60
-5	5	-6	230.28	14.50
-5	5	-6	218.08	16.20
-5	5	-6	212.88	20.50
5	-5	6	217.78	13.40
-6	5	-6	-4.80	8.00
-6	5	-6	-3.70	10.10
-6	5	-6	11.60	9.70
-6	5	-6	2.70	12.50
-6	5	-6	5.10	6.90
-6	5	-6	0.00	5.10
6	-5	6	2.90	5.40
-7	5	-6	15.20	7.70
-7	5	-6	3.60	6.60
-7	5	-6	1.50	8.80
-7	5	-6	16.40	12.20
7	-5	6	18.00	8.00
-8	5	-6	207.98	26.10
-8	5	-6	240.28	20.50
-8	5	-6	228.28	17.30
-8	5	-6	219.08	18.70
-8	5	-6	261.07	24.60
8	-5	6	208.28	19.30
8	-5	6	206.18	23.70
-9	5	-6	29.20	15.00
-9	5	-6	30.30	9.60
-9	5	-6	2.00	9.70

-9	5	-6	14.60	14.00
-9	5	-6	22.90	9.30
9	-5	6	14.90	10.30
-10	5	-6	492.75	30.70
-10	5	-6	484.25	30.30
-10	5	-6	490.85	29.70
10	-5	6	534.15	32.10
-11	5	-6	9.40	16.50
-11	5	-6	22.30	10.30
-11	5	-6	8.60	10.80
11	-5	6	11.40	10.60
11	-5	6	41.20	15.70
-12	5	-6	-17.40	11.80
-12	5	-6	-12.40	11.70
12	-5	6	3.50	17.00
11	4	-6	17.50	12.90
11	4	-6	13.50	16.30
-11	-4	6	26.50	8.50
-11	-4	6	20.80	8.90
10	4	-6	246.38	24.90
10	4	-6	236.38	24.30
-10	-4	6	248.08	19.10
-10	-4	6	275.27	18.30
9	4	-6	16.90	12.10
9	4	-6	17.20	12.40
-9	-4	6	17.80	6.70
-9	-4	6	19.00	7.60
8	4	-6	102.29	14.00
-8	-4	6	100.69	13.00
-8	-4	6	104.79	12.70
-7	-4	6	114.49	20.50
-7	-4	6	177.28	18.40
-7	-4	6	147.09	13.40
-7	-4	6	181.78	19.20
6	4	-6	261.67	22.00
6	4	-6	252.37	16.70
-6	-4	6	261.57	16.90
-6	-4	6	248.98	19.20
-6	-4	6	217.38	21.80
-6	-4	6	254.67	16.20
5	4	-6	21.60	5.00
5	4	-6	24.40	8.20
-5	-4	6	10.00	7.40
-5	-4	6	24.10	11.00

-5	-4	6	22.00	6.10
4	4	-6	1231.78	61.09
4	4	-6	1361.06	59.79
4	4	-6	1389.66	60.59
-4	-4	6	1206.78	59.29
-4	-4	6	1340.67	59.09
3	4	-6	152.98	8.90
3	4	-6	122.19	11.20
-3	-4	6	160.48	8.40
2	4	-6	675.33	31.50
2	4	-6	682.83	32.40
-2	-4	6	659.13	31.10
1	4	-6	67.29	5.00
1	4	-6	67.89	6.50
1	4	-6	59.99	5.10
-1	-4	6	68.09	4.90
-1	-4	6	61.09	6.90
-1	-4	6	57.49	6.20
0	4	-6	89.29	6.00
0	4	-6	96.79	7.10
0	4	-6	77.69	6.10
0	-4	6	88.99	6.70
0	-4	6	93.39	6.10
0	-4	6	102.59	8.40
-1	4	-6	87.59	6.40
-1	4	-6	101.79	7.40
-1	4	-6	104.79	6.50
1	-4	6	77.39	7.70
1	-4	6	96.39	6.50
1	-4	6	97.89	6.70
-2	4	-6	5.80	3.70
2	-4	6	6.40	3.50
2	-4	6	6.20	2.50
-3	4	-6	1692.43	73.09
3	-4	6	1536.85	73.39
3	-4	6	1568.94	73.09
3	-4	6	1566.64	71.99
-4	4	-6	1343.67	59.19
-4	4	-6	1297.77	59.59
4	-4	6	1282.87	59.79
4	-4	6	1167.68	59.99
4	-4	6	1279.87	58.19
-5	4	-6	178.88	19.00
-5	4	-6	138.29	13.70

-5	4	-6	163.28	15.10
-5	4	-6	158.08	17.30
-5	4	-6	145.79	14.20
5	-4	6	176.48	10.30
5	-4	6	201.68	19.90
-6	4	-6	32.80	12.30
-6	4	-6	36.70	6.70
-6	4	-6	24.40	10.20
-6	4	-6	23.10	8.80
-6	4	-6	19.50	9.80
-6	4	-6	21.20	7.90
6	-4	6	31.80	5.50
-7	4	-6	42.40	11.40
-7	4	-6	27.00	10.90
-7	4	-6	26.20	11.50
-7	4	-6	42.00	10.50
7	-4	6	27.80	6.70
-8	4	-6	474.15	26.50
-8	4	-6	447.86	32.30
-8	4	-6	481.65	27.30
-8	4	-6	493.25	28.30
-8	4	-6	493.05	31.40
8	-4	6	458.95	28.10
8	-4	6	452.35	29.10
-9	4	-6	63.29	11.70
-9	4	-6	67.49	11.60
-9	4	-6	66.69	14.30
-9	4	-6	75.59	13.10
-9	4	-6	75.09	14.70
9	-4	6	60.59	12.00
9	-4	6	33.50	25.10
-10	4	-6	-21.60	17.00
-10	4	-6	13.20	9.00
-10	4	-6	29.90	16.30
-10	4	-6	29.90	16.80
10	-4	6	14.90	14.10
-11	4	-6	63.39	10.80
-11	4	-6	56.19	11.90
-11	4	-6	62.19	12.60
11	-4	6	35.50	11.80
11	-4	6	64.89	18.70
-12	4	-6	35.40	15.30
-12	4	-6	-1.00	19.30
-12	4	-6	19.40	11.20

12	-4	6	-0.90	10.50
11	3	-6	44.20	13.80
11	3	-6	8.30	13.80
-11	-3	6	47.00	9.20
-11	-3	6	33.10	8.80
10	3	-6	40.90	14.70
10	3	-6	46.90	12.90
-10	-3	6	16.90	8.30
-10	-3	6	38.50	8.70
9	3	-6	175.38	20.80
9	3	-6	202.68	22.50
-9	-3	6	226.28	16.90
-9	-3	6	206.58	16.30
8	3	-6	163.78	19.00
-8	-3	6	146.89	14.20
-8	-3	6	152.28	13.40
-7	-3	6	1.30	13.20
-7	-3	6	40.60	7.80
-7	-3	6	38.90	8.90
-7	-3	6	28.80	12.30
6	3	-6	32.20	7.10
6	3	-6	24.30	9.60
-6	-3	6	24.20	12.20
-6	-3	6	25.00	9.10
-6	-3	6	21.10	6.90
-6	-3	6	24.60	6.40
5	3	-6	208.08	13.00
5	3	-6	238.08	15.20
5	3	-6	212.88	16.30
-5	-3	6	203.28	14.50
4	3	-6	474.05	23.10
4	3	-6	489.45	24.20
4	3	-6	504.85	23.70
-4	-3	6	473.75	22.40
-4	-3	6	441.96	22.80
3	3	-6	22.70	3.40
3	3	-6	26.50	4.40
3	3	-6	24.20	4.50
-3	-3	6	30.20	5.20
-3	-3	6	22.20	3.20
-3	-3	6	21.80	3.60
2	3	-6	1709.03	74.89
2	3	-6	1684.63	74.19
-2	-3	6	1798.92	74.29

-2	-3	6	1392.06	74.49
1	3	-6	244.48	12.50
1	3	-6	233.78	11.80
1	3	-6	239.68	11.90
-1	-3	6	227.38	12.60
-1	-3	6	228.18	11.80
0	3	-6	9.20	2.50
0	3	-6	14.10	2.80
0	3	-6	9.30	2.20
0	-3	6	5.50	1.90
0	-3	6	8.90	3.50
0	-3	6	8.40	2.60
-1	3	-6	891.51	39.90
-1	3	-6	883.81	39.60
1	-3	6	821.12	39.30
1	-3	6	860.21	39.60
1	-3	6	831.32	39.90
-2	3	-6	2059.69	91.09
-2	3	-6	2011.60	90.59
2	-3	6	1911.51	89.99
2	-3	6	1965.00	90.99
2	-3	6	2068.69	90.69
-3	3	-6	1188.18	53.49
-3	3	-6	1178.08	54.09
-3	3	-6	1131.09	53.39
3	-3	6	1179.18	53.69
3	-3	6	1137.19	52.59
3	-3	6	1136.89	53.79
-4	3	-6	7.30	4.10
-4	3	-6	13.30	5.60
-4	3	-6	13.50	4.40
4	-3	6	9.80	4.70
4	-3	6	7.90	2.50
-5	3	-6	52.39	8.80
-5	3	-6	44.90	6.10
-5	3	-6	48.20	7.40
-5	3	-6	60.99	11.40
5	-3	6	26.30	7.30
5	-3	6	38.70	5.00
-6	3	-6	8.80	10.40
-6	3	-6	15.00	9.90
-6	3	-6	18.00	5.50
-6	3	-6	5.00	9.10
-6	3	-6	-1.00	6.70

-6	3	-6	14.00	8.60
6	-3	6	10.90	4.00
-7	3	-6	19.30	10.50
-7	3	-6	27.40	5.70
-7	3	-6	29.80	10.30
-7	3	-6	46.60	10.70
-7	3	-6	23.70	7.20
7	-3	6	39.20	6.30
-8	3	-6	356.56	23.20
-8	3	-6	311.97	20.40
-8	3	-6	345.07	26.70
-8	3	-6	340.67	21.60
-8	3	-6	358.76	25.10
8	-3	6	336.17	24.50
8	-3	6	355.06	21.60
-9	3	-6	823.92	41.10
-9	3	-6	833.02	41.70
-9	3	-6	796.12	41.10
-9	3	-6	802.22	43.50
-9	3	-6	770.82	44.20
9	-3	6	771.92	45.00
9	-3	6	761.32	42.60
-10	3	-6	29.70	8.80
-10	3	-6	19.80	10.20
-10	3	-6	21.30	9.50
-11	3	-6	118.89	16.10
-11	3	-6	106.19	16.40
-11	3	-6	125.59	16.60
11	-3	6	143.39	18.20
-12	3	-6	37.40	11.10
-12	3	-6	30.10	11.50
-12	3	-6	25.30	14.10
12	-3	6	11.50	10.00
12	2	-6	8.10	15.40
-12	-2	6	-1.10	12.00
11	2	-6	59.09	14.20
11	2	-6	57.49	14.20
-11	-2	6	49.70	10.20
-11	-2	6	76.89	13.10
10	2	-6	60.89	13.70
10	2	-6	56.59	16.00
-10	-2	6	43.40	9.20
-10	-2	6	55.29	10.40
9	2	-6	103.69	18.10

9	2	-6	85.59	15.20
-9	-2	6	97.19	13.50
-9	-2	6	75.29	9.70
8	2	-6	5.10	9.70
-8	-2	6	7.00	6.60
-8	-2	6	0.70	6.60
-7	-2	6	188.98	14.80
-7	-2	6	192.38	14.40
6	2	-6	200.38	18.00
-6	-2	6	182.88	15.10
-6	-2	6	213.38	14.30
5	2	-6	195.28	12.20
5	2	-6	191.28	14.00
5	2	-6	196.38	13.20
-5	-2	6	184.48	13.70
4	2	-6	445.16	21.00
4	2	-6	415.86	20.80
4	2	-6	442.16	21.40
-4	-2	6	446.06	20.20
-4	-2	6	382.76	21.40
-4	-2	6	420.86	20.60
3	2	-6	2737.73	118.29
3	2	-6	2866.11	118.09
3	2	-6	2489.65	119.29
-3	-2	6	2668.63	118.09
-3	-2	6	2795.12	117.89
-3	-2	6	2231.28	118.09
2	2	-6	447.56	20.80
2	2	-6	423.46	21.10
-2	-2	6	450.65	20.70
1	2	-6	118.99	7.00
1	2	-6	123.89	6.90
1	2	-6	128.19	7.10
-1	-2	6	113.59	6.70
-1	-2	6	121.79	7.10
0	2	-6	152.18	8.00
0	2	-6	155.58	8.00
0	-2	6	144.79	7.90
0	-2	6	137.59	8.90
0	-2	6	145.99	8.40
-1	2	-6	61.29	4.50
-1	2	-6	56.29	4.40
1	-2	6	60.99	4.50
1	-2	6	54.49	4.70

1	-2	6	53.29	4.20
-2	2	-6	155.58	9.00
-2	2	-6	167.88	8.70
2	-2	6	159.18	8.90
2	-2	6	143.89	7.90
2	-2	6	151.18	8.90
-3	2	-6	214.58	12.40
-3	2	-6	183.28	11.50
-3	2	-6	203.68	11.00
3	-2	6	201.98	11.50
3	-2	6	194.88	9.90
3	-2	6	195.18	11.30
-4	2	-6	3122.59	138.19
4	-2	6	2980.00	138.29
4	-2	6	3055.79	138.69
4	-2	6	3097.99	137.19
-5	2	-6	84.29	11.70
-5	2	-6	86.79	7.70
-5	2	-6	67.39	9.10
5	-2	6	73.29	11.10
5	-2	6	75.49	5.80
5	-2	6	78.29	10.70
-6	2	-6	6.40	9.60
-6	2	-6	0.30	8.40
-6	2	-6	10.40	9.00
-6	2	-6	25.50	9.00
-6	2	-6	6.80	6.00
-6	2	-6	-1.10	8.90
-6	2	-6	12.30	4.70
6	-2	6	6.60	3.20
6	-2	6	10.90	10.10
-7	2	-6	654.93	38.60
-7	2	-6	737.83	37.70
-7	2	-6	701.83	39.00
-7	2	-6	743.93	37.00
7	-2	6	719.63	34.50
-8	2	-6	158.08	18.50
-8	2	-6	164.08	14.50
-8	2	-6	155.68	20.20
-8	2	-6	138.69	15.70
-8	2	-6	130.59	12.40
8	-2	6	125.99	12.90
8	-2	6	123.19	19.10
-9	2	-6	58.19	11.90

-9	2	-6	54.09	9.40
-9	2	-6	46.90	12.60
-9	2	-6	48.50	11.70
-9	2	-6	69.69	10.50
9	-2	6	47.20	12.60
9	-2	6	56.09	11.90
-10	2	-6	52.49	9.30
-10	2	-6	62.09	14.50
-10	2	-6	59.59	10.90
-10	2	-6	38.40	13.20
-10	2	-6	61.39	10.20
10	-2	6	34.30	16.40
-11	2	-6	55.99	19.80
-11	2	-6	46.20	10.20
-11	2	-6	46.00	10.90
-11	2	-6	42.90	10.30
-12	2	-6	10.90	12.70
-12	2	-6	18.00	10.90
-12	2	-6	6.20	9.90
12	-2	6	21.80	10.60
12	1	-6	111.29	20.90
12	1	-6	98.19	15.60
-12	-1	6	95.39	15.70
-12	-1	6	118.69	13.90
11	1	-6	96.79	14.70
11	1	-6	151.88	23.30
-11	-1	6	116.79	16.30
-11	-1	6	118.19	13.60
10	1	-6	80.79	14.30
10	1	-6	60.89	17.70
-10	-1	6	90.69	13.70
-10	-1	6	65.39	11.90
9	1	-6	58.59	11.20
-9	-1	6	46.80	9.00
-9	-1	6	43.70	8.40
8	1	-6	788.22	40.90
-8	-1	6	773.22	39.60
-8	-1	6	789.62	39.10
-7	-1	6	95.29	16.70
-7	-1	6	153.68	12.80
6	1	-6	134.19	12.90
6	1	-6	95.49	14.60
-6	-1	6	95.69	12.90
-6	-1	6	120.09	11.90

5	1	-6	82.09	15.80
5	1	-6	70.29	9.40
-5	-1	6	75.49	10.40
-5	-1	6	64.09	8.50
-5	-1	6	66.69	6.40
4	1	-6	22.20	3.60
4	1	-6	34.70	4.40
4	1	-6	24.50	3.90
-4	-1	6	24.00	3.90
-4	-1	6	13.30	3.90
-4	-1	6	34.30	4.10
3	1	-6	1175.18	52.09
3	1	-6	1104.19	52.09
3	1	-6	1200.68	51.89
-3	-1	6	1028.00	52.19
-3	-1	6	1220.68	52.09
-3	-1	6	1151.88	52.19
2	1	-6	848.82	40.30
2	1	-6	826.52	40.30
2	1	-6	939.71	40.00
-2	-1	6	931.21	40.50
1	1	-6	23.20	2.60
-1	-1	6	19.70	3.10
-1	-1	6	24.00	2.70
-1	-1	6	21.80	2.40
0	1	-6	8.20	1.60
0	-1	6	4.50	1.50
0	-1	6	4.70	2.20
0	-1	6	4.50	2.00
-1	1	-6	58.89	4.10
-1	1	-6	61.19	4.30
1	-1	6	54.49	4.20
1	-1	6	61.29	3.70
1	-1	6	55.89	4.20
-2	1	-6	2972.40	130.89
-2	1	-6	2906.91	130.79
2	-1	6	2858.71	130.09
2	-1	6	2997.10	130.89
2	-1	6	2848.22	130.79
-3	1	-6	670.03	29.60
-3	1	-6	643.04	29.50
3	-1	6	621.74	28.20
3	-1	6	619.44	29.40
3	-1	6	524.65	30.20

-4	1	-6	11.70	3.30
4	-1	6	4.30	3.10
4	-1	6	7.00	3.70
4	-1	6	6.90	1.70
-5	1	-6	368.46	18.30
-5	1	-6	365.06	18.70
-5	1	-6	312.77	20.10
5	-1	6	319.87	19.20
5	-1	6	339.57	19.90
5	-1	6	366.36	16.90
-6	1	-6	245.88	18.30
-6	1	-6	258.47	14.50
-6	1	-6	275.37	20.80
-6	1	-6	244.88	15.90
-6	1	-6	216.48	20.40
-6	1	-6	223.28	17.10
6	-1	6	229.38	18.60
6	-1	6	240.38	14.20
-7	1	-6	19.00	9.50
-7	1	-6	29.40	6.80
-7	1	-6	26.20	5.30
-7	1	-6	24.50	10.20
-7	1	-6	43.30	9.50
7	-1	6	30.90	4.90
-8	1	-6	259.27	16.30
-8	1	-6	242.68	17.40
-8	1	-6	243.28	21.90
-8	1	-6	252.07	18.80
-8	1	-6	261.37	20.50
8	-1	6	242.68	15.70
8	-1	6	206.78	22.50
-9	1	-6	114.69	12.90
-9	1	-6	116.89	20.00
-9	1	-6	132.59	16.10
-9	1	-6	132.19	18.10
-9	1	-6	138.19	14.40
9	-1	6	139.49	16.40
9	-1	6	93.49	14.60
-10	1	-6	441.96	29.10
-10	1	-6	430.76	25.10
-10	1	-6	404.26	24.30
-10	1	-6	398.06	29.40
-10	1	-6	427.36	25.60
10	-1	6	348.37	35.00

-11	1	-6	34.90	9.90
-11	1	-6	31.70	9.70
-11	1	-6	27.90	9.30
-11	1	-6	17.10	14.70
-12	1	-6	1.40	9.10
-12	1	-6	0.00	11.70
-12	1	-6	-16.70	9.20
12	-1	6	8.60	11.30
12	0	-6	46.60	13.30
12	0	-6	0.40	22.50
-12	0	6	43.40	16.30
-12	0	6	58.79	9.10
11	0	-6	31.50	12.40
11	0	-6	51.89	16.70
-11	0	6	52.09	8.60
-11	0	6	51.89	11.30
10	0	-6	153.78	28.10
10	0	-6	173.98	22.50
-10	0	6	178.18	18.80
-10	0	6	169.58	19.60
9	0	-6	101.99	17.20
-9	0	6	94.19	14.60
-9	0	6	108.39	13.70
8	0	-6	83.69	11.70
-8	0	6	79.99	12.30
-8	0	6	70.39	12.20
7	0	-6	11.60	9.90
-7	0	6	15.30	6.70
6	0	-6	33.00	8.30
-6	0	6	39.40	7.40
-6	0	6	39.30	7.90
5	0	-6	52.99	8.20
5	0	-6	49.90	8.40
5	0	-6	42.10	8.90
-5	0	6	73.09	9.00
-5	0	6	70.69	9.90
-5	0	6	54.39	9.30
-5	0	6	56.49	6.60
4	0	-6	1524.05	67.69
4	0	-6	1543.65	67.79
-4	0	6	1426.76	67.99
-4	0	6	1459.15	67.99
-4	0	6	1521.35	67.69
3	0	-6	2500.35	109.09

3	0	-6	2522.95	109.19
3	0	-6	2387.96	109.29
-3	0	6	2260.57	109.49
-3	0	6	2416.16	109.59
-3	0	6	2549.34	109.49
2	0	-6	2635.94	119.79
2	0	-6	2442.36	119.59
2	0	-6	2812.92	119.39
-2	0	6	2530.65	120.09
-2	0	6	2866.41	120.09
-2	0	6	2800.62	120.19
-1	0	6	71.09	4.60
0	0	-6	11.40	1.70
0	0	-6	11.60	1.80
0	0	6	14.70	2.40
0	0	6	14.20	1.90
0	0	6	13.80	2.30
-1	0	-6	3.60	1.50
-1	0	-6	3.00	1.60
1	0	6	6.90	1.70
1	0	6	2.70	1.40
-2	0	-6	11.30	2.10
-2	0	-6	16.20	2.90
2	0	6	20.60	3.10
2	0	6	20.00	3.00
2	0	6	15.60	1.60
-3	0	-6	2506.15	110.89
-3	0	-6	2408.26	111.69
3	0	6	2559.34	111.79
3	0	6	2465.65	111.09
3	0	6	2403.46	110.09
-4	0	-6	280.27	14.50
4	0	6	265.57	13.10
4	0	6	272.57	15.10
4	0	6	276.67	15.00
-5	0	-6	385.06	21.60
-5	0	-6	414.56	20.20
-5	0	-6	398.86	20.00
5	0	6	400.76	18.80
5	0	6	369.26	20.60
5	0	6	390.76	21.60
-6	0	-6	25.00	7.10
-6	0	-6	40.30	8.30
-6	0	-6	39.00	8.60

-6	0	-6	26.80	10.10
-6	0	-6	23.90	8.90
-6	0	-6	31.60	5.30
-6	0	-6	48.80	7.40
6	0	6	39.20	8.20
-7	0	-6	115.29	13.50
-7	0	-6	76.39	13.20
-7	0	-6	98.59	9.10
-7	0	-6	87.09	11.20
-7	0	-6	66.79	11.90
-7	0	-6	93.89	11.10
7	0	6	88.89	7.50
-8	0	-6	443.96	24.80
-8	0	-6	454.85	28.40
-8	0	-6	468.45	26.00
-8	0	-6	441.56	24.00
-8	0	-6	472.35	27.20
8	0	6	443.36	23.30
8	0	6	365.96	29.70
-9	0	-6	25.20	10.40
-9	0	-6	34.60	9.90
-9	0	-6	14.50	6.80
-9	0	-6	19.00	8.60
-9	0	-6	11.80	5.90
9	0	6	26.10	8.70
-10	0	-6	14.70	7.70
-10	0	-6	13.90	11.40
-10	0	-6	14.00	8.50
-10	0	-6	20.60	8.30
-10	0	-6	19.00	11.70
10	0	6	11.20	15.60
-11	0	-6	131.59	17.30
-11	0	-6	136.29	14.80
-11	0	-6	135.89	15.80
-11	0	-6	116.79	14.90
-11	0	-6	145.19	21.40
-12	0	-6	60.79	10.80
-12	0	-6	75.89	10.70
-12	0	-6	71.19	12.70
12	0	6	74.59	12.70
12	-1	-6	70.39	16.50
-12	1	6	63.69	18.90
-12	1	6	63.89	9.70
11	-1	-6	66.19	12.40

11	-1	-6	68.19	19.00
-11	1	6	50.69	9.10
-11	1	6	64.29	12.70
10	-1	-6	85.59	18.80
10	-1	-6	68.99	15.10
-10	1	6	45.30	11.00
-10	1	6	49.40	12.20
9	-1	-6	449.95	33.20
9	-1	-6	430.76	27.60
-9	1	6	436.96	26.20
-9	1	6	439.26	25.40
8	-1	-6	19.00	8.90
-8	1	6	8.30	7.10
-8	1	6	4.80	6.80
-7	1	6	330.87	20.20
6	-1	-6	18.70	7.50
-6	1	6	52.19	7.60
-6	1	6	35.50	7.80
5	-1	-6	174.98	12.30
5	-1	-6	180.88	12.30
5	-1	-6	159.18	15.80
5	-1	-6	211.18	12.30
-5	1	6	170.98	12.80
-5	1	6	181.18	12.30
4	-1	-6	589.24	26.80
4	-1	-6	592.24	26.80
4	-1	-6	578.24	27.20
-4	1	6	544.15	27.00
-4	1	6	546.95	27.30
3	-1	-6	18.80	2.80
3	-1	-6	15.40	2.70
3	-1	-6	25.90	3.80
-2	1	6	3156.98	152.38
-2	1	6	3441.06	152.68
-2	1	6	3641.64	152.68
-1	1	6	56.59	3.80
-1	1	6	42.10	4.00
0	-1	-6	182.28	8.20
0	1	6	171.18	8.30
0	1	6	185.78	9.00
0	1	6	165.08	8.70
-1	-1	-6	712.53	33.00
-1	-1	-6	713.73	33.20
1	1	6	716.73	33.00

1	1	6	758.82	33.30
-2	-1	-6	2930.01	133.69
-2	-1	-6	3001.80	133.59
2	1	6	2885.11	133.59
2	1	6	3124.79	133.69
-3	-1	-6	5154.98	231.88
-3	-1	-6	5277.67	231.48
3	1	6	5332.07	231.98
3	1	6	5075.59	231.58
3	1	6	5104.39	230.68
-4	-1	-6	89.79	6.30
-4	-1	-6	92.79	7.50
-4	-1	-6	87.89	7.00
4	1	6	82.29	4.90
4	1	6	81.59	6.80
4	1	6	93.79	7.60
-5	-1	-6	284.17	14.60
-5	-1	-6	262.27	16.20
-5	-1	-6	276.87	14.30
5	1	6	265.47	16.10
5	1	6	249.88	14.90
-6	-1	-6	203.58	12.40
-6	-1	-6	193.68	14.30
-6	-1	-6	217.68	16.50
-6	-1	-6	203.28	13.30
-6	-1	-6	204.28	18.20
-6	-1	-6	214.08	16.30
-6	-1	-6	175.68	19.70
6	1	6	211.38	12.20
6	1	6	186.98	14.80
-7	-1	-6	76.69	12.30
-7	-1	-6	75.99	13.00
-7	-1	-6	75.59	10.30
-7	-1	-6	73.69	8.40
-7	-1	-6	74.79	10.90
-7	-1	-6	78.99	13.80
7	1	6	75.49	6.50
-8	-1	-6	751.82	38.20
-8	-1	-6	759.82	37.80
-8	-1	-6	693.33	39.60
-8	-1	-6	756.52	36.60
-8	-1	-6	745.43	37.10
8	1	6	719.83	35.80
-9	-1	-6	497.65	31.90

-9	-1	-6	546.95	29.50
-9	-1	-6	535.45	28.10
-9	-1	-6	499.55	30.30
-9	-1	-6	519.05	28.60
9	1	6	546.35	28.60
-10	-1	-6	7.10	7.20
-10	-1	-6	5.10	8.20
-10	-1	-6	20.00	7.60
-10	-1	-6	1.30	10.10
-10	-1	-6	23.10	11.30
10	1	6	28.90	13.30
-11	-1	-6	41.90	14.00
-11	-1	-6	22.50	9.10
-11	-1	-6	44.80	12.50
-11	-1	-6	23.40	9.10
-11	-1	-6	39.20	9.90
-12	-1	-6	117.59	15.30
-12	-1	-6	105.29	14.00
-12	-1	-6	86.89	13.80
12	1	6	109.99	14.20
12	-2	-6	-27.70	15.40
-12	2	6	41.30	20.70
-12	2	6	-5.80	7.90
11	-2	-6	-7.90	21.80
11	-2	-6	9.10	10.80
-11	2	6	10.10	8.00
-11	2	6	7.50	12.10
10	-2	-6	67.49	18.10
10	-2	-6	76.49	14.20
-10	2	6	61.69	12.30
-10	2	6	59.59	10.40
9	-2	-6	239.58	21.20
9	-2	-6	280.27	30.30
-9	2	6	263.07	18.40
-9	2	6	239.18	20.20
8	-2	-6	105.19	15.20
-8	2	6	77.59	12.80
7	-2	-6	342.47	27.60
-7	2	6	321.27	20.20
6	-2	-6	21.60	10.60
6	-2	-6	38.30	8.10
-6	2	6	27.60	6.20
-6	2	6	24.40	7.10
5	-2	-6	150.08	11.70

5	-2	-6	161.68	15.40
-5	2	6	163.68	10.70
-5	2	6	129.39	12.40
4	-2	-6	1602.74	72.19
4	-2	-6	1516.75	72.29
-4	2	6	1594.94	72.29
-4	2	6	1662.03	72.79
3	-2	-6	1604.14	76.39
3	-2	-6	1798.12	76.29
-3	2	6	1701.93	76.89
-3	2	6	1689.63	76.59
-2	2	6	440.06	21.70
-2	2	6	498.75	22.20
-1	2	6	435.66	22.70
-1	2	6	555.14	23.20
0	2	6	3.10	0.80
0	2	6	4.30	1.00
0	2	6	2.20	1.40
0	2	6	3.70	1.10
1	2	6	503.45	22.80
1	2	6	475.75	22.50
-2	-2	-6	587.74	27.50
-2	-2	-6	601.84	27.60
2	2	6	603.64	27.60
2	2	6	588.34	27.60
-3	-2	-6	24.80	3.40
-3	-2	-6	32.90	3.30
3	2	6	20.20	3.70
3	2	6	30.50	3.70
-4	-2	-6	2138.79	103.39
-4	-2	-6	2382.86	104.09
-4	-2	-6	2292.77	103.09
4	2	6	2244.88	103.29
4	2	6	2379.36	104.19
4	2	6	2326.27	102.39
-5	-2	-6	18.00	3.70
-5	-2	-6	27.60	3.90
5	2	6	20.60	4.50
5	2	6	34.00	5.90
5	2	6	24.70	3.20
-6	-2	-6	81.39	12.90
-6	-2	-6	110.39	8.40
-6	-2	-6	90.79	14.80
-6	-2	-6	86.39	11.70

-6	-2	-6	82.79	10.30
-6	-2	-6	88.59	13.20
6	2	6	101.39	6.90
-7	-2	-6	502.85	25.10
-7	-2	-6	475.85	25.70
-7	-2	-6	510.85	27.30
-7	-2	-6	483.95	28.10
-7	-2	-6	505.85	26.60
-7	-2	-6	452.15	29.90
7	2	6	486.35	23.90
-8	-2	-6	76.99	11.80
-8	-2	-6	77.89	10.90
-8	-2	-6	82.79	9.50
-8	-2	-6	79.39	13.90
-8	-2	-6	60.09	8.90
8	2	6	48.90	8.10
-9	-2	-6	50.99	9.40
-9	-2	-6	46.50	8.90
-9	-2	-6	34.80	7.80
-9	-2	-6	44.50	10.70
-9	-2	-6	37.90	7.00
9	2	6	20.30	7.60
-10	-2	-6	258.27	18.70
-10	-2	-6	235.18	17.30
-10	-2	-6	201.08	16.50
-10	-2	-6	216.88	22.00
-10	-2	-6	205.78	20.50
10	2	6	243.88	23.30
-11	-2	-6	64.29	10.30
-11	-2	-6	63.29	9.90
-11	-2	-6	59.69	12.30
-11	-2	-6	65.49	13.90
-11	-2	-6	68.89	12.10
-12	-2	-6	23.40	8.60
-12	-2	-6	8.40	16.70
-12	-2	-6	22.40	10.50
-12	-2	-6	35.60	9.40
12	2	6	36.70	12.20
11	-3	-6	31.20	10.80
-11	3	6	37.40	13.90
-11	3	6	20.20	8.50
10	-3	-6	44.70	19.50
10	-3	-6	14.20	11.80
-10	3	6	13.60	8.60

-10	3	6	23.20	11.70
9	-3	-6	307.27	31.20
9	-3	-6	315.97	22.60
-9	3	6	297.57	22.30
-9	3	6	257.77	19.80
8	-3	-6	797.82	40.30
8	-3	-6	696.43	44.70
-8	3	6	779.92	39.30
-8	3	6	802.62	40.30
7	-3	-6	33.80	10.10
7	-3	-6	24.70	8.80
6	-3	-6	10.60	7.60
6	-3	-6	15.30	9.40
-6	3	6	9.00	6.60
5	-3	-6	6.20	7.20
-5	3	6	10.20	4.30
-5	3	6	9.20	7.10
4	-3	-6	263.57	14.20
-4	3	6	267.77	13.70
-4	3	6	268.77	14.80
3	-3	-6	17.30	2.90
-3	3	6	20.70	3.10
2	-3	-6	51.59	3.80
-2	3	6	54.99	3.00
-2	3	6	55.29	3.80
-2	3	6	50.19	4.30
-1	3	6	272.67	13.00
-1	3	6	256.97	12.50
0	3	6	98.99	5.40
0	3	6	105.99	5.10
0	3	6	105.09	5.90
1	3	6	11.60	1.70
1	3	6	12.30	2.10
2	3	6	4018.70	181.88
2	3	6	4122.59	181.78
3	3	6	243.78	12.50
3	3	6	256.37	13.10
-4	-3	-6	25.50	3.90
4	3	6	31.60	4.30
4	3	6	17.50	3.50
4	3	6	26.40	2.40
4	3	6	24.30	3.50
-5	-3	-6	213.58	12.00
5	3	6	218.58	10.50

5	3	6	194.68	11.90
-6	-3	-6	234.58	16.70
-6	-3	-6	240.28	21.50
-6	-3	-6	244.88	17.00
-6	-3	-6	235.68	13.70
-6	-3	-6	245.88	18.90
-6	-3	-6	232.18	15.20
6	3	6	249.38	12.80
-7	-3	-6	61.39	9.60
-7	-3	-6	80.89	12.60
-7	-3	-6	75.69	11.30
-7	-3	-6	79.29	9.70
-7	-3	-6	89.79	8.30
-7	-3	-6	85.29	12.30
-8	-3	-6	10.20	5.20
-8	-3	-6	3.10	8.40
-8	-3	-6	13.30	8.10
-8	-3	-6	7.00	6.70
-8	-3	-6	11.90	6.10
8	3	6	6.80	4.40
-9	-3	-6	303.07	19.90
-9	-3	-6	271.27	21.00
-9	-3	-6	314.77	19.10
-9	-3	-6	304.07	18.50
-9	-3	-6	299.07	22.70
9	3	6	306.47	19.00
-10	-3	-6	176.18	15.10
-10	-3	-6	142.49	18.10
-10	-3	-6	155.58	14.30
-10	-3	-6	171.28	16.00
-10	-3	-6	159.78	19.40
10	3	6	137.89	20.30
-11	-3	-6	75.79	13.70
-11	-3	-6	43.60	9.10
-11	-3	-6	43.60	9.10
-11	-3	-6	37.90	8.90
-11	-3	-6	34.40	11.30
11	3	6	66.39	13.10
-12	-3	-6	27.90	8.90
-12	-3	-6	19.30	10.10
-12	-3	-6	37.20	8.70
-12	-3	-6	44.10	14.50
12	3	6	38.40	12.50
11	-4	-6	47.70	10.90

-11	4	6	37.10	9.10
-11	4	6	55.79	15.90
10	-4	-6	319.57	22.70
-10	4	6	253.67	25.00
-10	4	6	289.27	21.10
9	-4	-6	18.00	9.60
9	-4	-6	42.90	21.10
-9	4	6	37.40	8.80
-9	4	6	39.70	10.80
8	-4	-6	35.50	10.80
8	-4	-6	39.10	9.40
-8	4	6	21.40	9.70
-8	4	6	31.30	8.10
7	-4	-6	105.89	13.70
7	-4	-6	68.19	10.50
-7	4	6	98.79	12.90
-7	4	6	82.49	14.30
6	-4	-6	117.39	14.50
6	-4	-6	104.09	14.80
-6	4	6	125.59	12.90
5	-4	-6	27.20	7.90
5	-4	-6	24.60	7.80
-5	4	6	19.90	7.90
-5	4	6	29.90	4.80
-5	4	6	34.40	7.90
4	-4	-6	280.87	15.20
-4	4	6	272.27	13.80
-4	4	6	281.97	14.10
-4	4	6	244.08	15.30
3	-4	-6	2853.01	134.09
-3	4	6	3132.59	133.69
-3	4	6	2970.60	133.89
-3	4	6	3008.20	134.59
2	-4	-6	1501.55	71.39
-2	4	6	1569.94	71.29
-2	4	6	1652.43	71.99
-2	4	6	1625.74	71.09
1	-4	-6	175.58	8.40
-1	4	6	171.68	8.50
-1	4	6	182.08	9.20
-1	4	6	168.78	8.50
0	4	6	361.36	18.00
0	4	6	394.36	17.90
1	4	6	274.67	13.70

1	4	6	309.37	13.70
1	4	6	274.97	13.60
1	4	6	267.27	13.50
2	4	6	779.82	35.40
2	4	6	761.72	35.50
3	4	6	752.12	34.60
4	4	6	504.45	23.40
4	4	6	507.75	22.30
4	4	6	443.06	26.90
4	4	6	508.85	24.60
-5	-4	-6	1097.49	48.20
5	4	6	957.60	47.50
5	4	6	1061.89	47.00
-6	-4	-6	122.99	10.90
-6	-4	-6	107.89	12.60
-6	-4	-6	119.49	15.20
-6	-4	-6	131.89	9.30
-6	-4	-6	122.29	12.40
-6	-4	-6	124.49	14.40
-7	-4	-6	138.89	13.00
-7	-4	-6	124.89	19.60
-7	-4	-6	152.18	12.40
-7	-4	-6	155.88	15.60
-7	-4	-6	148.79	10.80
-7	-4	-6	142.49	14.30
7	4	6	152.98	9.50
-8	-4	-6	582.74	29.80
-8	-4	-6	587.54	30.80
-8	-4	-6	591.54	30.20
-8	-4	-6	584.34	31.40
-8	-4	-6	600.74	32.80
-9	-4	-6	19.90	6.80
-9	-4	-6	4.00	8.30
-9	-4	-6	4.70	7.70
-9	-4	-6	2.70	8.80
-9	-4	-6	9.50	6.00
9	4	6	2.40	6.80
-10	-4	-6	25.20	10.50
-10	-4	-6	16.20	7.10
-10	-4	-6	28.90	8.70
-10	-4	-6	25.30	7.70
-10	-4	-6	22.80	8.90
10	4	6	13.70	11.60
-11	-4	-6	287.67	20.00

-11	-4	-6	299.07	19.60
-11	-4	-6	303.37	20.30
-11	-4	-6	301.37	22.90
11	4	6	305.77	24.30
-12	-4	-6	109.99	14.40
-12	-4	-6	102.89	15.00
-12	-4	-6	96.69	13.70
-12	-4	-6	100.39	13.70
12	4	6	134.89	19.30
11	-5	-6	35.20	11.10
-11	5	6	5.60	17.00
-11	5	6	32.50	8.60
10	-5	-6	278.57	20.80
-10	5	6	247.98	19.50
-10	5	6	211.18	25.00
9	-5	-6	734.23	40.60
9	-5	-6	807.02	40.70
-9	5	6	765.62	39.50
-9	5	6	733.83	41.70
8	-5	-6	169.28	17.00
8	-5	-6	147.39	17.50
-8	5	6	145.19	15.50
-8	5	6	168.38	18.10
7	-5	-6	225.68	17.90
-7	5	6	211.98	16.60
6	-5	-6	11.90	7.40
6	-5	-6	1.90	7.20
-6	5	6	3.80	6.90
5	-5	-6	467.95	25.90
5	-5	-6	484.35	26.90
5	-5	-6	478.75	28.70
-5	5	6	465.65	27.10
-5	5	6	485.15	26.10
-5	5	6	495.65	23.90
4	-5	-6	245.88	15.70
4	-5	-6	251.27	15.00
-4	5	6	258.47	15.40
-4	5	6	263.37	14.00
-4	5	6	262.27	13.30
3	-5	-6	19.90	4.40
3	-5	-6	11.80	3.80
-3	5	6	9.90	2.60
-3	5	6	12.20	2.40
-3	5	6	8.30	3.20

2	-5	-6	1135.79	52.19
2	-5	-6	1146.89	51.99
-2	5	6	1170.18	52.29
-2	5	6	1157.58	51.39
-2	5	6	1084.99	51.49
1	-5	-6	5.40	1.90
-1	5	6	4.90	1.20
-1	5	6	5.40	2.30
-1	5	6	6.40	1.80
0	5	6	36.50	5.00
0	5	6	34.90	3.30
1	5	6	216.88	10.80
1	5	6	195.88	10.30
2	5	6	267.07	13.20
2	5	6	256.47	13.40
2	5	6	287.37	13.70
2	5	6	258.57	12.90
3	5	6	1425.16	63.99
3	5	6	1324.27	63.09
3	5	6	1438.36	62.99
4	5	6	124.49	6.70
4	5	6	121.89	9.00
4	5	6	108.79	7.30
-5	-5	-6	267.57	13.80
5	5	6	254.07	12.10
5	5	6	244.18	12.10
5	5	6	241.98	12.90
5	5	6	227.18	15.40
-6	-5	-6	358.76	27.00
-6	-5	-6	385.56	22.10
-6	-5	-6	395.76	21.30
-6	-5	-6	414.26	22.00
-6	-5	-6	413.76	20.50
-6	-5	-6	395.66	23.70
-7	-5	-6	220.28	22.80
-7	-5	-6	235.18	15.70
-7	-5	-6	225.28	18.10
-7	-5	-6	235.58	16.40
-7	-5	-6	227.78	14.70
-7	-5	-6	216.38	13.80
7	5	6	228.28	12.70
-8	-5	-6	951.90	45.00
-8	-5	-6	900.81	47.30
-8	-5	-6	957.70	46.10

-8	-5	-6	941.01	45.80
-8	-5	-6	935.51	45.30
8	5	6	934.11	44.40
-9	-5	-6	284.07	18.50
-9	-5	-6	276.57	21.80
-9	-5	-6	271.17	19.40
-9	-5	-6	289.57	20.60
-9	-5	-6	295.37	17.80
9	5	6	288.27	18.20
-10	-5	-6	104.19	12.40
-10	-5	-6	104.99	13.70
-10	-5	-6	115.49	14.90
-10	-5	-6	95.69	17.80
-10	-5	-6	123.89	13.50
10	5	6	125.89	19.30
-11	-5	-6	12.50	8.00
-11	-5	-6	11.90	7.80
-11	-5	-6	2.80	7.70
-11	-5	-6	1.80	9.70
11	5	6	22.20	12.30
-12	-5	-6	155.18	14.60
-12	-5	-6	161.08	19.60
-12	-5	-6	118.79	15.10
-12	-5	-6	149.79	14.60
12	5	6	153.88	20.30
11	-6	-6	27.10	11.20
-11	6	6	28.60	8.80
-10	6	6	95.59	15.80
-10	6	6	100.99	15.20
9	-6	-6	15.00	9.60
9	-6	-6	26.00	11.10
-9	6	6	25.20	8.40
-9	6	6	14.40	12.30
8	-6	-6	-10.30	8.90
8	-6	-6	-11.50	8.20
-8	6	6	-6.00	8.00
7	-6	-6	76.89	10.90
7	-6	-6	98.59	13.00
-7	6	6	60.69	9.20
6	-6	-6	49.50	7.90
6	-6	-6	60.89	9.60
-6	6	6	81.69	12.00
-6	6	6	54.89	15.30
5	-6	-6	-0.40	6.70

5	-6	-6	22.10	8.00
-5	6	6	7.60	7.10
-5	6	6	12.10	4.40
4	-6	-6	1969.60	87.99
4	-6	-6	1779.62	92.19
-4	6	6	2021.60	87.59
-4	6	6	1965.10	86.69
-4	6	6	1846.42	88.19
3	-6	-6	804.12	40.30
3	-6	-6	832.72	40.00
-3	6	6	835.02	40.20
-3	6	6	889.11	38.90
-3	6	6	877.21	39.40
2	-6	-6	1568.24	73.69
2	-6	-6	1678.33	73.69
-2	6	6	1630.14	72.79
-2	6	6	1652.63	73.79
-2	6	6	1573.74	73.29
1	-6	-6	13.80	2.70
1	-6	-6	15.70	2.80
-1	6	6	20.40	3.90
-1	6	6	15.70	2.10
-1	6	6	13.00	2.60
0	-6	-6	11.50	2.50
0	6	6	7.20	3.70
0	6	6	9.40	1.90
1	6	6	47.10	3.70
1	6	6	43.30	4.90
2	6	6	2.70	2.00
2	6	6	2.00	1.60
2	6	6	1.00	2.40
-3	-6	-6	24.70	4.20
3	6	6	28.90	5.10
3	6	6	24.10	3.10
3	6	6	26.30	4.10
3	6	6	21.50	3.90
4	6	6	1171.98	56.29
4	6	6	1182.68	54.29
4	6	6	1356.86	56.79
4	6	6	1107.19	54.59
-5	-6	-6	60.49	7.10
5	6	6	68.59	12.50
5	6	6	66.59	5.40
5	6	6	64.99	4.70

5	6	6	64.59	4.30
-6	-6	-6	201.08	22.60
-6	-6	-6	189.18	14.20
-6	-6	-6	179.48	15.00
-6	-6	-6	213.48	17.10
-6	-6	-6	197.58	12.40
-6	-6	-6	208.28	13.80
6	6	6	217.28	11.10
-7	-6	-6	426.96	29.30
-7	-6	-6	414.76	22.50
-7	-6	-6	406.26	23.10
-7	-6	-6	420.06	24.60
-7	-6	-6	406.06	21.70
-7	-6	-6	405.56	24.70
-7	-6	-6	404.96	22.80
7	6	6	417.06	20.70
-8	-6	-6	-0.50	7.80
-8	-6	-6	13.80	5.20
-8	-6	-6	5.20	12.10
-8	-6	-6	-6.00	6.80
-8	-6	-6	-0.10	6.30
-8	-6	-6	4.70	6.20
8	6	6	-1.90	4.70
-9	-6	-6	14.50	6.70
-9	-6	-6	22.40	8.90
-9	-6	-6	12.90	7.40
-9	-6	-6	14.80	7.00
-9	-6	-6	25.10	8.10
-10	-6	-6	265.47	19.70
-10	-6	-6	252.17	17.20
-10	-6	-6	231.98	21.70
-10	-6	-6	301.07	18.90
-10	-6	-6	250.37	17.70
10	6	6	251.77	21.70
-11	-6	-6	7.80	7.90
-11	-6	-6	6.10	7.70
-11	-6	-6	9.10	9.30
-11	-6	-6	17.70	8.30
11	6	6	19.00	12.20
-12	-6	-6	127.59	15.60
-12	-6	-6	164.78	14.90
-12	-6	-6	177.38	18.90
-12	-6	-6	165.78	14.80
12	6	6	159.78	20.60

11	-7	-6	14.10	10.80
-11	7	6	3.10	9.30
10	-7	-6	8.20	14.50
10	-7	-6	23.70	10.00
-10	7	6	13.90	16.00
-10	7	6	-11.10	8.30
9	-7	-6	589.14	33.10
9	-7	-6	630.64	33.50
-9	7	6	540.35	32.80
-9	7	6	573.94	37.80
8	-7	-6	245.78	22.90
8	-7	-6	240.88	19.80
-8	7	6	254.67	19.60
-8	7	6	272.57	23.80
7	-7	-6	-1.00	7.60
7	-7	-6	9.60	8.40
-7	7	6	7.70	7.60
6	-7	-6	13.40	7.80
6	-7	-6	-1.20	6.20
-6	7	6	4.40	7.00
-6	7	6	-3.30	14.50
5	-7	-6	316.07	29.40
5	-7	-6	301.87	18.70
5	-7	-6	311.57	20.50
-5	7	6	294.47	20.00
-5	7	6	308.87	16.70
4	-7	-6	7.00	9.20
4	-7	-6	-11.50	9.00
4	-7	-6	-6.30	5.90
4	-7	-6	9.70	6.60
-4	7	6	3.00	5.50
-4	7	6	2.80	2.80
-4	7	6	2.40	6.70
3	-7	-6	357.16	18.70
3	-7	-6	341.17	19.90
-3	7	6	367.46	16.90
-3	7	6	334.87	18.70
-3	7	6	338.87	18.10
2	-7	-6	51.79	7.10
-2	7	6	48.50	3.80
-2	7	6	51.29	5.90
-2	7	6	53.79	6.60
1	-7	-6	1307.37	60.89
1	-7	-6	1348.47	60.89

-1	7	6	1284.17	61.79
-1	7	6	1376.86	59.79
-1	7	6	1342.57	60.89
0	-7	-6	606.94	27.80
0	-7	-6	520.85	28.00
0	7	6	591.94	29.10
0	7	6	559.84	26.80
0	7	6	609.64	26.30
0	7	6	623.54	29.50
1	7	6	1209.48	54.39
1	7	6	1178.48	55.19
2	7	6	1144.69	51.79
2	7	6	1133.69	52.99
-3	-7	-6	54.89	6.10
3	7	6	61.29	7.20
3	7	6	61.39	6.70
3	7	6	62.19	4.50
3	7	6	63.29	5.50
-4	-7	-6	1217.28	54.29
4	7	6	1170.28	55.69
4	7	6	1180.18	53.29
4	7	6	1100.99	53.59
4	7	6	1230.88	55.79
-5	-7	-6	769.52	37.40
-5	-7	-6	705.33	35.70
-5	-7	-6	773.32	35.10
-5	-7	-6	694.73	36.30
5	7	6	747.13	33.90
-6	-7	-6	71.99	15.00
-6	-7	-6	59.19	9.50
-6	-7	-6	48.70	9.30
-6	-7	-6	59.59	7.70
-6	-7	-6	91.49	14.00
-6	-7	-6	74.99	10.40
-6	-7	-6	81.99	7.60
6	7	6	58.89	6.00
-7	-7	-6	56.49	9.30
-7	-7	-6	63.39	9.90
-7	-7	-6	54.89	9.80
-7	-7	-6	68.49	11.20
-7	-7	-6	68.79	8.70
-7	-7	-6	60.79	8.00
7	7	6	63.89	7.20
-8	-7	-6	24.30	7.30

-8	-7	-6	49.70	8.30
-8	-7	-6	28.30	5.90
-8	-7	-6	42.20	7.50
-8	-7	-6	36.50	10.30
-8	-7	-6	47.70	10.10
8	7	6	56.39	8.60
-9	-7	-6	180.88	18.40
-9	-7	-6	180.08	16.40
-9	-7	-6	152.08	14.80
-9	-7	-6	205.88	20.40
-9	-7	-6	178.48	15.40
9	7	6	164.08	15.20
-10	-7	-6	39.00	7.60
-10	-7	-6	36.20	8.00
-10	-7	-6	19.50	8.90
-10	-7	-6	36.40	10.70
-10	-7	-6	43.30	9.00
10	7	6	16.50	12.10
-11	-7	-6	193.28	14.80
-11	-7	-6	173.38	15.50
-11	-7	-6	159.28	17.60
-11	-7	-6	170.28	14.70
11	7	6	186.78	22.30
-12	-7	-6	40.60	11.00
-12	-7	-6	82.29	12.30
-12	-7	-6	65.79	9.70
-12	-7	-6	61.29	12.30
11	-8	-6	118.79	16.40
-11	8	6	83.89	11.50
10	-8	-6	313.27	21.70
10	-8	-6	288.27	24.60
-10	8	6	218.18	30.40
-10	8	6	288.37	21.10
9	-8	-6	42.50	10.90
-9	8	6	15.40	9.90
8	-8	-6	82.69	10.90
8	-8	-6	74.09	11.80
-8	8	6	59.59	13.70
-8	8	6	79.29	11.90
7	-8	-6	47.00	9.60
7	-8	-6	40.60	8.00
-7	8	6	38.30	8.90
6	-8	-6	32.20	9.40
5	-8	-6	54.79	12.30

5	-8	-6	37.60	7.70
5	-8	-6	43.90	9.90
5	-8	-6	39.60	11.00
-5	8	6	37.80	6.40
4	-8	-6	141.59	19.50
4	-8	-6	129.19	17.60
4	-8	-6	177.68	13.40
4	-8	-6	193.98	21.40
-4	8	6	165.38	14.60
-4	8	6	172.38	10.00
3	-8	-6	2406.36	106.39
-3	8	6	2220.08	105.39
-3	8	6	2342.17	104.19
-3	8	6	2303.37	105.89
2	-8	-6	78.99	9.00
-2	8	6	61.29	7.00
-2	8	6	76.19	9.10
-2	8	6	62.19	4.50
1	-8	-6	29.20	5.10
1	-8	-6	27.90	6.80
-1	8	6	29.50	2.80
-1	8	6	28.60	6.60
-1	8	6	33.40	4.30
0	-8	-6	546.05	26.70
0	-8	-6	512.25	26.20
0	8	6	543.85	28.10
0	8	6	589.44	24.80
0	8	6	570.14	26.20
0	8	6	523.35	26.20
-2	-8	-6	194.18	12.50
2	8	6	228.78	11.30
2	8	6	225.78	13.50
3	8	6	230.08	12.90
3	8	6	238.68	12.20
3	8	6	247.78	14.90
3	8	6	252.17	14.60
-4	-8	-6	151.58	9.50
4	8	6	147.69	9.30
4	8	6	138.69	7.20
4	8	6	153.18	12.70
4	8	6	149.19	8.10
4	8	6	143.69	12.50
-5	-8	-6	724.13	37.10
-5	-8	-6	763.02	37.00

-5	-8	-6	771.52	37.30
-5	-8	-6	774.72	40.40
5	8	6	782.72	35.10
-6	-8	-6	23.60	8.00
-6	-8	-6	14.30	6.20
-6	-8	-6	6.60	6.00
-6	-8	-6	8.90	4.40
-6	-8	-6	3.00	5.90
-6	-8	-6	13.60	8.20
6	8	6	4.30	3.40
-7	-8	-6	124.89	20.90
-7	-8	-6	99.89	14.30
-7	-8	-6	116.49	9.90
7	8	6	105.59	9.10
-8	-8	-6	315.67	22.10
-8	-8	-6	312.57	19.10
-8	-8	-6	300.37	21.10
-8	-8	-6	310.77	19.70
-8	-8	-6	310.07	19.70
-8	-8	-6	302.17	18.00
8	8	6	315.07	18.30
-9	-8	-6	82.79	13.20
-9	-8	-6	58.69	8.50
-9	-8	-6	54.49	11.10
-9	-8	-6	37.00	11.20
-9	-8	-6	69.09	11.50
-9	-8	-6	37.30	7.70
-10	-8	-6	52.89	8.90
-10	-8	-6	40.60	7.70
-10	-8	-6	54.19	9.50
-10	-8	-6	89.19	14.70
-10	-8	-6	67.39	12.10
10	8	6	46.10	13.30
-11	-8	-6	264.47	18.40
-11	-8	-6	233.98	18.00
-11	-8	-6	282.07	18.20
-11	-8	-6	280.27	19.60
-12	-8	-6	34.90	10.70
-12	-8	-6	26.60	9.10
-12	-8	-6	-2.80	10.80
-12	-8	-6	-2.60	7.00
11	-9	-6	9.80	13.00
-11	9	6	2.10	9.40
10	-9	-6	299.67	23.10

10	-9	-6	279.67	21.10
-10	9	6	256.67	20.70
-10	9	6	218.38	30.70
9	-9	-6	180.28	19.30
9	-9	-6	138.19	19.20
-9	9	6	178.48	21.90
8	-9	-6	260.77	20.30
8	-9	-6	233.88	18.40
-8	9	6	206.38	20.30
-8	9	6	231.58	25.70
7	-9	-6	240.88	19.90
7	-9	-6	286.87	19.30
-7	9	6	281.27	21.30
6	-9	-6	36.10	7.60
6	-9	-6	32.60	9.50
6	-9	-6	14.60	13.70
-6	9	6	55.39	10.30
5	-9	-6	356.66	19.90
5	-9	-6	363.06	22.90
5	-9	-6	307.37	23.50
5	-9	-6	312.37	24.60
-5	9	6	332.67	18.50
4	-9	-6	249.28	20.30
4	-9	-6	248.98	18.90
4	-9	-6	257.07	16.40
4	-9	-6	243.58	24.60
4	-9	-6	280.47	21.30
4	-9	-6	205.98	21.70
-4	9	6	229.88	17.20
-4	9	6	279.07	14.00
-4	9	6	234.48	28.50
3	-9	-6	359.86	22.30
3	-9	-6	319.07	19.30
-3	9	6	314.17	20.50
-3	9	6	329.27	16.30
-3	9	6	323.37	19.80
2	-9	-6	1773.02	74.59
2	-9	-6	1588.14	73.79
-2	9	6	1670.83	72.19
-2	9	6	1546.95	73.79
-2	9	6	1612.54	74.99
-2	9	6	1405.96	74.09
1	-9	-6	567.74	28.20
-1	9	6	560.24	25.70

-1	9	6	598.64	30.30
-1	9	6	512.95	27.30
0	-9	-6	10.30	4.60
0	-9	-6	7.60	4.30
0	9	6	8.10	3.40
0	9	6	8.40	6.50
0	9	6	7.30	1.50
-1	-9	-6	771.32	39.70
1	9	6	933.11	38.90
1	9	6	720.13	37.30
1	9	6	799.82	40.80
1	9	6	920.31	37.20
2	9	6	52.09	7.20
-3	-9	-6	733.03	34.70
3	9	6	697.43	34.60
3	9	6	737.53	36.60
3	9	6	768.92	34.20
-4	-9	-6	295.37	15.30
4	9	6	270.47	13.20
4	9	6	308.87	19.50
4	9	6	302.17	18.90
4	9	6	248.78	15.00
4	9	6	272.47	13.90
-5	-9	-6	6.50	9.90
-5	-9	-6	4.30	5.90
-5	-9	-6	2.70	5.80
-5	-9	-6	6.00	3.90
-5	-9	-6	-1.00	6.40
-5	-9	-6	3.30	6.90
5	9	6	5.20	3.30
-6	-9	-6	624.14	31.60
-6	-9	-6	616.14	30.90
-6	-9	-6	633.34	31.70
-6	-9	-6	589.54	31.80
-6	-9	-6	642.14	30.50
-6	-9	-6	633.84	34.00
6	9	6	610.94	29.80
-7	-9	-6	240.28	18.00
-7	-9	-6	259.47	17.20
-7	-9	-6	261.77	15.30
-7	-9	-6	270.27	20.20
-7	-9	-6	266.37	17.40
-7	-9	-6	239.18	16.00
7	9	6	244.98	15.00

-8	-9	-6	391.26	24.80
-8	-9	-6	378.36	23.70
-8	-9	-6	395.06	21.50
-8	-9	-6	395.16	23.10
-8	-9	-6	389.16	22.30
-8	-9	-6	375.56	23.20
8	9	6	393.26	22.10
-9	-9	-6	18.10	6.20
-9	-9	-6	15.40	9.80
-9	-9	-6	20.60	7.30
-9	-9	-6	19.10	8.30
-9	-9	-6	25.50	8.00
-9	-9	-6	10.60	9.80
9	9	6	29.90	9.10
-10	-9	-6	95.29	15.20
-10	-9	-6	65.59	12.10
-10	-9	-6	81.89	10.90
-10	-9	-6	80.89	13.40
-10	-9	-6	83.19	14.70
-11	-9	-6	24.80	8.30
-11	-9	-6	38.00	8.10
-11	-9	-6	25.20	8.80
-11	-9	-6	20.60	9.30
-12	-9	-6	91.19	14.70
-12	-9	-6	94.39	14.60
-12	-9	-6	85.49	13.20
-12	-9	-6	72.39	12.40
10	-10	-6	206.08	19.40
10	-10	-6	167.08	18.50
-10	10	6	145.99	18.30
9	-10	-6	35.40	10.80
-9	10	6	23.30	17.10
-9	10	6	45.40	11.30
8	-10	-6	32.00	10.40
8	-10	-6	9.50	8.40
-8	10	6	18.20	10.70
-8	10	6	21.40	14.20
7	-10	-6	13.60	9.60
-7	10	6	19.80	10.40
6	-10	-6	72.49	23.10
6	-10	-6	67.89	10.90
6	-10	-6	96.29	12.90
-6	10	6	95.49	16.80
5	-10	-6	174.98	20.80

5	-10	-6	172.08	19.10
5	-10	-6	177.18	18.90
5	-10	-6	192.28	14.60
5	-10	-6	212.98	25.70
-5	10	6	196.48	12.90
-5	10	6	196.88	18.40
4	-10	-6	1617.44	75.19
4	-10	-6	1556.24	76.39
4	-10	-6	1596.74	76.69
4	-10	-6	1690.03	74.69
4	-10	-6	1475.25	76.69
-4	10	6	1564.24	74.79
-4	10	6	1774.52	73.79
3	-10	-6	273.27	19.90
3	-10	-6	218.38	16.10
3	-10	-6	257.47	19.30
3	-10	-6	219.78	17.10
3	-10	-6	210.68	21.60
-3	10	6	233.18	17.20
-3	10	6	242.28	12.60
2	-10	-6	522.85	27.20
-2	10	6	503.15	22.60
-2	10	6	431.36	26.50
-2	10	6	429.46	25.10
-2	10	6	486.25	28.90
1	-10	-6	114.99	12.10
1	-10	-6	92.19	9.30
-1	10	6	92.49	5.60
-1	10	6	93.99	15.30
-1	10	6	89.49	8.60
0	-10	-6	153.58	12.90
0	-10	-6	180.68	20.50
0	10	6	177.88	16.10
0	10	6	165.58	8.30
0	10	6	160.38	10.60
1	10	6	345.67	17.60
1	10	6	342.47	15.60
1	10	6	316.57	21.40
-2	-10	-6	157.28	11.40
2	10	6	159.18	15.00
2	10	6	171.28	10.10
2	10	6	169.78	8.00
-3	-10	-6	312.37	16.00
3	10	6	287.17	15.10

3	10	6	255.77	15.80
3	10	6	295.37	13.50
3	10	6	298.67	19.80
-4	-10	-6	931.91	45.50
-4	-10	-6	932.11	44.60
-4	-10	-6	964.80	44.90
-4	-10	-6	906.01	45.30
4	10	6	968.60	42.70
-5	-10	-6	235.68	15.50
-5	-10	-6	227.88	13.20
-5	-10	-6	230.98	14.50
-5	-10	-6	219.38	20.10
-5	-10	-6	211.38	15.50
-5	-10	-6	212.08	15.50
5	10	6	205.58	12.10
-6	-10	-6	82.99	8.50
-6	-10	-6	61.19	10.50
-6	-10	-6	65.89	11.50
-6	-10	-6	80.19	10.20
-6	-10	-6	64.09	10.70
-6	-10	-6	79.99	11.30
6	10	6	82.29	8.00
-7	-10	-6	298.37	18.80
-7	-10	-6	301.57	18.10
-7	-10	-6	316.77	18.90
-7	-10	-6	279.47	19.00
-7	-10	-6	295.67	17.20
-7	-10	-6	288.17	20.90
7	10	6	276.47	17.30
-8	-10	-6	75.29	9.90
-8	-10	-6	90.39	9.90
-8	-10	-6	88.09	13.80
-8	-10	-6	82.99	15.70
-8	-10	-6	86.69	13.10
-8	-10	-6	77.49	11.10
8	10	6	85.69	12.40
-9	-10	-6	-0.90	9.70
-9	-10	-6	-4.30	7.60
-9	-10	-6	8.30	7.00
-9	-10	-6	5.40	9.00
-9	-10	-6	8.70	6.60
-9	-10	-6	6.30	7.50
9	10	6	5.80	10.00
-10	-10	-6	262.17	21.40

-10	-10	-6	237.58	18.80
-10	-10	-6	216.18	15.80
-10	-10	-6	205.58	19.20
-10	-10	-6	206.08	17.20
-11	-10	-6	28.30	9.00
-11	-10	-6	28.80	8.20
-11	-10	-6	42.70	8.40
-11	-10	-6	38.20	9.10
-12	-10	-6	113.29	12.50
-12	-10	-6	132.49	21.40
-12	-10	-6	102.49	15.30
10	-11	-6	121.99	19.60
10	-11	-6	128.89	17.20
9	-11	-6	863.91	42.10
9	-11	-6	875.01	41.20
-9	11	6	769.52	43.50
-9	11	6	697.53	47.60
8	-11	-6	145.09	15.90
8	-11	-6	110.09	17.70
-8	11	6	133.19	20.10
-8	11	6	113.39	19.00
7	-11	-6	20.90	9.90
7	-11	-6	16.00	7.50
-7	11	6	14.80	11.30
6	-11	-6	79.29	12.60
6	-11	-6	69.19	11.30
6	-11	-6	51.39	12.50
-6	11	6	45.30	11.50
5	-11	-6	125.09	19.90
5	-11	-6	114.69	12.90
5	-11	-6	136.49	18.80
-5	11	6	146.69	17.00
-5	11	6	124.09	11.40
4	-11	-6	129.89	15.20
4	-11	-6	138.49	14.80
4	-11	-6	110.09	15.20
4	-11	-6	128.89	19.50
4	-11	-6	149.49	12.90
-4	11	6	119.29	14.70
-4	11	6	138.39	9.70
3	-11	-6	125.99	17.60
3	-11	-6	114.79	16.70
3	-11	-6	120.89	12.40
3	-11	-6	162.08	17.20

3	-11	-6	99.89	13.40
-3	11	6	125.09	8.30
-3	11	6	120.89	14.30
-3	11	6	92.09	17.00
2	-11	-6	154.48	18.80
2	-11	-6	200.88	15.10
2	-11	-6	202.18	17.30
2	-11	-6	178.98	18.10
2	-11	-6	195.08	17.40
2	-11	-6	200.18	15.70
-2	11	6	190.78	10.30
-2	11	6	135.09	22.00
1	-11	-6	480.85	26.90
1	-11	-6	426.16	26.10
1	-11	-6	540.75	34.50
1	-11	-6	528.05	28.50
1	-11	-6	559.24	30.70
-1	11	6	488.55	23.30
-1	11	6	444.36	25.90
-1	11	6	563.14	31.70
0	-11	-6	304.27	20.30
0	-11	-6	290.77	18.30
0	11	6	364.36	23.60
0	11	6	320.37	15.20
0	11	6	310.97	17.70
-1	-11	-6	384.96	23.30
-1	-11	-6	431.26	23.30
1	11	6	484.95	28.10
1	11	6	471.65	20.60
1	11	6	449.86	22.80
-2	-11	-6	1130.19	52.39
2	11	6	1172.18	50.29
2	11	6	1031.70	55.59
2	11	6	1120.59	51.89
-3	-11	-6	100.29	9.30
-3	-11	-6	81.29	11.00
-3	-11	-6	118.79	12.40
-3	-11	-6	88.59	13.40
3	11	6	100.19	8.70
3	11	6	102.49	5.70
-4	-11	-6	159.68	12.40
-4	-11	-6	159.48	14.00
-4	-11	-6	129.39	12.30
-4	-11	-6	144.49	15.80

-4	-11	-6	169.78	14.20
-4	-11	-6	172.88	11.00
4	11	6	145.29	9.30
-5	-11	-6	1157.08	56.69
-5	-11	-6	1163.68	55.09
-5	-11	-6	1205.98	55.19
-5	-11	-6	1126.99	57.39
-5	-11	-6	1270.57	54.99
-5	-11	-6	1150.08	55.89
-5	-11	-6	1180.68	55.39
5	11	6	1130.09	53.89
-6	-11	-6	24.20	6.60
-6	-11	-6	11.20	5.10
-6	-11	-6	9.40	9.00
-6	-11	-6	21.30	9.80
6	11	6	5.00	5.10
-7	-11	-6	101.99	14.90
-7	-11	-6	122.39	11.50
-7	-11	-6	91.59	9.90
-7	-11	-6	94.49	12.40
-7	-11	-6	86.49	12.70
-7	-11	-6	98.69	12.80
7	11	6	61.79	11.20
-8	-11	-6	2.00	7.10
-8	-11	-6	21.30	7.20
-8	-11	-6	5.80	9.60
-8	-11	-6	8.10	7.80
-8	-11	-6	16.90	7.40
-8	-11	-6	22.40	8.20
8	11	6	9.70	8.40
-9	-11	-6	137.49	14.90
-9	-11	-6	150.38	14.10
-9	-11	-6	134.89	15.90
-9	-11	-6	158.38	15.70
-9	-11	-6	128.49	12.90
9	11	6	134.29	18.60
-10	-11	-6	53.89	8.70
-10	-11	-6	79.69	13.00
-10	-11	-6	49.10	9.30
-10	-11	-6	65.79	10.90
-10	-11	-6	41.20	8.50
-11	-11	-6	75.09	12.10
-11	-11	-6	79.99	13.40
-11	-11	-6	60.89	12.30

-11	-11	-6	55.39	9.40
10	-12	-6	162.08	16.70
10	-12	-6	137.19	21.70
-10	12	6	143.59	17.00
9	-12	-6	-19.80	12.40
9	-12	-6	13.10	8.80
-9	12	6	4.20	10.60
8	-12	-6	31.80	12.90
-8	12	6	-8.30	17.20
-8	12	6	3.20	18.30
7	-12	-6	41.80	11.50
7	-12	-6	30.40	8.30
-7	12	6	49.99	13.60
6	-12	-6	-13.50	11.20
6	-12	-6	-3.80	7.30
6	-12	-6	21.40	11.30
-6	12	6	-0.40	11.30
5	-12	-6	30.20	7.60
5	-12	-6	47.00	22.20
5	-12	-6	23.70	10.10
5	-12	-6	40.10	13.60
-5	12	6	22.30	7.00
-5	12	6	45.00	10.90
4	-12	-6	166.18	20.40
4	-12	-6	185.88	16.40
4	-12	-6	199.88	14.80
4	-12	-6	194.28	22.40
-4	12	6	198.58	12.00
-4	12	6	191.78	17.40
-4	12	6	128.59	23.40
3	-12	-6	1573.64	70.49
3	-12	-6	1516.55	70.89
3	-12	-6	1528.05	71.89
3	-12	-6	1507.55	72.59
-3	12	6	1632.14	69.29
-3	12	6	1371.86	73.39
-3	12	6	1452.35	71.29
2	-12	-6	114.49	15.70
2	-12	-6	104.09	15.50
2	-12	-6	102.79	12.70
2	-12	-6	131.89	13.50
2	-12	-6	110.29	14.70
-2	12	6	94.19	11.30
-2	12	6	87.19	6.90

1	-12	-6	126.29	16.10
1	-12	-6	130.09	19.60
1	-12	-6	145.29	14.00
1	-12	-6	175.58	14.80
1	-12	-6	134.29	13.90
1	-12	-6	156.88	19.60
-1	12	6	172.68	23.40
0	-12	-6	545.25	33.40
0	-12	-6	536.95	30.70
0	-12	-6	683.73	31.70
0	-12	-6	587.64	30.30
0	-12	-6	577.84	31.20
0	-12	-6	583.64	30.20
0	12	6	638.74	27.50
0	12	6	602.14	36.10
0	12	6	534.95	30.10
-1	-12	-6	1073.99	47.40
-1	-12	-6	968.90	47.50
-1	-12	-6	982.10	47.30
-1	-12	-6	1042.20	48.40
-1	-12	-6	1059.29	51.39
-1	-12	-6	914.41	48.00
1	12	6	959.40	52.19
1	12	6	939.71	47.40
1	12	6	1009.70	45.20
-2	-12	-6	87.09	15.30
-2	-12	-6	111.69	10.70
-2	-12	-6	126.39	12.70
-2	-12	-6	104.19	11.20
-2	-12	-6	103.49	13.20
2	12	6	110.19	6.20
2	12	6	93.89	10.60
-3	-12	-6	256.07	15.50
-3	-12	-6	227.28	17.40
-3	-12	-6	268.47	18.00
-3	-12	-6	259.17	19.20
-3	-12	-6	243.48	15.40
-3	-12	-6	231.78	17.50
-4	-12	-6	102.29	12.70
-4	-12	-6	72.79	13.10
-4	-12	-6	107.49	9.90
-4	-12	-6	72.09	10.10
-4	-12	-6	112.99	10.90
-4	-12	-6	95.59	11.60

4	12	6	95.89	6.50
-5	-12	-6	187.28	14.30
-5	-12	-6	209.98	13.20
-5	-12	-6	174.58	14.00
-5	-12	-6	185.08	15.10
-5	-12	-6	209.48	18.50
-5	-12	-6	204.38	14.90
-5	-12	-6	195.48	20.70
5	12	6	170.38	11.50
-6	-12	-6	1.20	5.60
-6	-12	-6	4.50	6.50
-6	-12	-6	11.10	6.50
-6	-12	-6	15.90	6.70
-6	-12	-6	21.90	13.90
-6	-12	-6	-1.40	6.40
6	12	6	1.70	5.50
-7	-12	-6	298.67	22.70
-7	-12	-6	345.77	20.20
-7	-12	-6	325.37	20.80
-7	-12	-6	342.47	20.30
-7	-12	-6	315.17	19.70
-7	-12	-6	332.87	19.20
7	12	6	285.37	20.00
-8	-12	-6	93.09	10.50
-8	-12	-6	93.99	11.40
-8	-12	-6	82.19	9.90
-8	-12	-6	86.49	12.30
-8	-12	-6	93.39	13.10
8	12	6	71.69	12.30
-9	-12	-6	11.70	7.00
-9	-12	-6	16.10	8.00
-9	-12	-6	7.80	8.70
-9	-12	-6	11.40	8.10
-9	-12	-6	-3.00	7.50
9	12	6	-0.10	12.00
-10	-12	-6	221.88	17.30
-10	-12	-6	235.98	18.50
-10	-12	-6	217.58	16.60
-10	-12	-6	210.58	17.70
-10	-12	-6	189.08	16.20
-11	-12	-6	284.67	18.70
-11	-12	-6	291.97	18.40
-11	-12	-6	280.87	19.50
-11	-12	-6	277.27	18.70

10	-13	-6	131.59	19.90
10	-13	-6	85.69	14.80
-10	13	6	83.29	11.70
9	-13	-6	-17.00	14.80
9	-13	-6	32.60	8.70
-9	13	6	11.00	10.30
8	-13	-6	308.57	19.80
8	-13	-6	297.07	24.90
-8	13	6	232.18	29.40
-8	13	6	305.57	26.40
7	-13	-6	25.90	14.00
7	-13	-6	22.00	17.20
-7	13	6	33.60	13.00
6	-13	-6	9.90	7.30
6	-13	-6	4.30	11.70
6	-13	-6	10.90	13.50
-6	13	6	1.50	12.00
5	-13	-6	253.97	20.10
5	-13	-6	313.67	32.20
5	-13	-6	278.77	18.40
5	-13	-6	206.68	26.90
-5	13	6	269.77	22.10
-5	13	6	266.37	18.40
-5	13	6	195.38	35.00
4	-13	-6	132.09	20.50
4	-13	-6	101.29	15.80
4	-13	-6	99.19	13.20
4	-13	-6	86.59	14.50
-4	13	6	110.59	9.80
-4	13	6	89.09	15.60
-4	13	6	104.19	16.10
3	-13	-6	12.10	7.70
3	-13	-6	31.20	10.90
3	-13	-6	14.30	10.70
3	-13	-6	18.60	6.90
-3	13	6	21.20	13.20
-3	13	6	2.10	8.60
-3	13	6	12.00	4.00
2	-13	-6	699.63	36.30
2	-13	-6	678.13	35.30
2	-13	-6	726.03	36.10
2	-13	-6	712.13	36.60
2	-13	-6	727.63	37.30
-2	13	6	649.74	36.40

-2	13	6	690.63	32.90
1	-13	-6	10.50	9.80
1	-13	-6	40.90	8.50
1	-13	-6	22.40	8.60
1	-13	-6	33.90	8.40
1	-13	-6	25.40	7.20
-1	13	6	26.30	4.20
0	-13	-6	39.40	7.60
0	-13	-6	43.80	8.20
0	-13	-6	38.60	8.10
0	-13	-6	37.10	11.20
0	13	6	38.40	4.20
-1	-13	-6	65.49	8.00
-1	-13	-6	60.69	10.90
-1	-13	-6	65.09	10.70
-1	-13	-6	68.89	12.20
-1	-13	-6	66.49	10.40
-1	-13	-6	54.89	8.20
1	13	6	58.09	4.80
-2	-13	-6	345.77	22.30
-2	-13	-6	380.66	21.10
-2	-13	-6	321.07	24.60
-2	-13	-6	375.46	21.90
-2	-13	-6	351.06	20.20
-2	-13	-6	394.46	20.80
2	13	6	379.86	17.60
-3	-13	-6	97.39	13.00
-3	-13	-6	91.29	12.80
-3	-13	-6	97.09	10.60
-3	-13	-6	79.59	17.50
-3	-13	-6	127.09	16.10
-3	-13	-6	83.89	10.90
-3	-13	-6	101.19	13.30
3	13	6	92.69	6.20
-4	-13	-6	531.75	26.50
-4	-13	-6	522.25	27.90
-4	-13	-6	543.55	26.60
-4	-13	-6	486.05	27.80
-4	-13	-6	499.35	27.30
-4	-13	-6	462.45	27.90
4	13	6	510.75	24.30
-5	-13	-6	160.78	23.10
-5	-13	-6	141.49	14.90
-5	-13	-6	154.78	16.80

-5	-13	-6	158.68	12.10
-5	-13	-6	151.48	14.50
5	13	6	137.09	10.50
-6	-13	-6	1326.37	63.19
-6	-13	-6	1406.26	63.09
-6	-13	-6	1361.26	62.89
-6	-13	-6	1448.56	66.09
-6	-13	-6	1365.16	63.19
-6	-13	-6	1306.87	63.89
6	13	6	1152.38	62.49
-7	-13	-6	79.99	13.20
-7	-13	-6	73.49	10.40
-7	-13	-6	53.19	8.10
-7	-13	-6	77.79	11.80
-7	-13	-6	74.09	10.60
7	13	6	42.00	9.40
-8	-13	-6	170.18	15.90
-8	-13	-6	160.38	15.10
-8	-13	-6	151.88	14.10
-8	-13	-6	150.48	15.40
-8	-13	-6	177.18	15.60
-9	-13	-6	16.10	12.50
-9	-13	-6	3.30	7.20
-9	-13	-6	7.80	10.60
9	13	6	-7.00	13.50
-10	-13	-6	33.20	8.10
-10	-13	-6	28.60	9.00
-10	-13	-6	29.30	8.90
-10	-13	-6	12.50	8.30
-10	-13	-6	36.40	8.50
-11	-13	-6	85.79	12.30
-11	-13	-6	106.69	17.90
-11	-13	-6	80.99	13.00
-11	-13	-6	84.79	12.10
-11	-13	-6	66.49	10.30
9	-14	-6	71.09	12.40
-9	14	6	49.00	10.90
8	-14	-6	33.40	8.40
8	-14	-6	32.40	19.60
-8	14	6	29.10	12.10
-8	14	6	80.89	18.00
7	-14	-6	57.79	9.50
7	-14	-6	80.49	30.20
-7	14	6	52.19	14.70

6	-14	-6	80.09	10.10
6	-14	-6	64.09	12.60
-6	14	6	64.09	14.80
5	-14	-6	208.08	16.50
5	-14	-6	235.38	33.30
5	-14	-6	217.28	18.40
5	-14	-6	172.68	25.80
-5	14	6	198.88	24.70
-5	14	6	211.68	21.10
-5	14	6	191.28	18.80
4	-14	-6	451.95	31.80
4	-14	-6	457.85	31.40
4	-14	-6	481.25	26.20
4	-14	-6	460.95	26.70
-4	14	6	432.86	31.30
-4	14	6	478.05	24.70
-4	14	6	469.35	28.70
3	-14	-6	169.38	17.10
3	-14	-6	184.08	22.40
3	-14	-6	161.58	15.60
3	-14	-6	192.88	31.50
-3	14	6	165.68	17.20
-3	14	6	138.89	21.60
-3	14	6	162.58	11.10
2	-14	-6	123.19	13.90
2	-14	-6	145.19	15.90
2	-14	-6	164.58	17.30
2	-14	-6	122.29	15.80
2	-14	-6	156.08	16.80
-2	14	6	152.28	9.40
-2	14	6	140.99	15.50
1	-14	-6	192.18	16.30
1	-14	-6	213.48	16.80
1	-14	-6	243.58	17.50
1	-14	-6	223.68	20.00
1	-14	-6	220.58	17.00
-1	14	6	196.68	11.50
0	-14	-6	18.80	7.80
0	-14	-6	14.10	7.20
0	-14	-6	11.40	7.30
0	-14	-6	20.00	9.40
0	-14	-6	13.90	18.50
0	-14	-6	18.30	8.20
0	14	6	11.10	2.70

-1	-14	-6	366.86	21.00
-1	-14	-6	372.56	21.30
-1	-14	-6	374.86	22.80
-1	-14	-6	349.77	23.20
-1	-14	-6	365.66	21.90
1	14	6	376.16	18.00
-2	-14	-6	245.48	22.10
-2	-14	-6	264.77	18.20
-2	-14	-6	284.37	17.20
-2	-14	-6	265.77	17.50
-2	-14	-6	276.77	17.20
-2	-14	-6	284.17	28.10
-2	-14	-6	275.07	19.10
2	14	6	282.57	13.90
-3	-14	-6	349.77	19.60
-3	-14	-6	335.07	27.80
-3	-14	-6	347.97	21.50
-3	-14	-6	295.27	24.60
-3	-14	-6	347.27	21.30
-3	-14	-6	346.37	20.00
-3	-14	-6	350.86	20.80
3	14	6	343.77	16.90
-4	-14	-6	471.55	27.40
-4	-14	-6	493.25	26.90
-4	-14	-6	478.05	26.00
4	14	6	486.75	23.60
-5	-14	-6	7.30	6.30
-5	-14	-6	9.20	8.00
-5	-14	-6	8.60	7.40
-5	-14	-6	3.90	8.60
-5	-14	-6	2.00	7.90
-5	-14	-6	12.50	6.40
5	14	6	1.70	4.90
-6	-14	-6	154.38	16.70
-6	-14	-6	186.88	16.60
-6	-14	-6	195.68	15.00
-6	-14	-6	183.08	16.00
-6	-14	-6	211.78	20.90
-6	-14	-6	200.08	14.90
6	14	6	153.58	14.50
-7	-14	-6	131.29	14.20
-7	-14	-6	142.29	14.10
-7	-14	-6	137.19	14.60
-7	-14	-6	159.48	15.70

-7	-14	-6	154.08	15.50
-8	-14	-6	10.30	7.50
-8	-14	-6	-8.70	7.50
-8	-14	-6	-0.20	8.00
-8	-14	-6	-2.90	6.90
-8	-14	-6	10.80	8.20
8	14	6	9.30	11.70
-9	-14	-6	64.29	10.10
-9	-14	-6	81.39	13.90
-9	-14	-6	98.59	13.80
-9	-14	-6	50.79	9.30
-9	-14	-6	73.19	9.60
9	14	6	49.50	16.20
-10	-14	-6	2.70	7.50
-10	-14	-6	12.50	8.40
-10	-14	-6	3.70	8.60
-10	-14	-6	7.20	7.40
-10	-14	-6	20.20	7.70
-11	-14	-6	173.98	16.30
-11	-14	-6	220.98	15.30
-11	-14	-6	184.98	14.70
-11	-14	-6	201.28	14.30
-11	-14	-6	160.08	18.70
9	-15	-6	305.67	19.10
-9	15	6	300.07	20.50
8	-15	-6	30.80	7.90
-8	15	6	11.90	10.90
7	-15	-6	37.00	17.60
7	-15	-6	39.60	8.60
-7	15	6	42.90	13.40
6	-15	-6	120.39	14.50
6	-15	-6	108.39	19.10
-6	15	6	147.99	21.70
5	-15	-6	22.30	9.30
5	-15	-6	8.90	7.70
5	-15	-6	14.50	21.50
5	-15	-6	-4.40	14.90
-5	15	6	-5.80	14.40
4	-15	-6	110.29	15.70
4	-15	-6	86.49	14.30
4	-15	-6	98.09	13.70
4	-15	-6	82.79	15.40
-4	15	6	84.69	15.10
-4	15	6	87.59	13.10

-4	15	6	82.69	12.60
3	-15	-6	44.00	11.70
3	-15	-6	27.80	8.60
3	-15	-6	26.40	8.10
3	-15	-6	25.40	11.10
-3	15	6	35.70	10.50
-3	15	6	46.60	8.20
-3	15	6	29.20	12.30
2	-15	-6	46.40	10.10
2	-15	-6	27.40	9.60
2	-15	-6	22.10	7.40
2	-15	-6	44.90	9.50
-2	15	6	28.90	5.10
-2	15	6	25.80	8.90
1	-15	-6	565.84	28.90
1	-15	-6	475.25	27.60
1	-15	-6	486.65	32.60
-1	15	6	473.55	27.70
-1	15	6	483.55	24.30
0	-15	-6	106.69	13.60
0	-15	-6	102.29	12.80
0	-15	-6	102.39	13.80
0	-15	-6	106.99	12.00
0	-15	-6	111.99	12.70
0	15	6	115.99	7.60
-1	-15	-6	292.97	21.10
-1	-15	-6	296.87	20.40
-1	-15	-6	315.27	18.80
-1	-15	-6	349.37	19.80
-1	-15	-6	297.47	19.10
1	15	6	303.97	15.70
-2	-15	-6	220.08	16.40
-2	-15	-6	210.98	16.60
-2	-15	-6	255.97	15.40
-2	-15	-6	198.48	20.20
-2	-15	-6	216.08	16.10
-2	-15	-6	228.58	15.50
2	15	6	212.28	11.90
-3	-15	-6	34.50	8.50
-3	-15	-6	39.30	11.00
-3	-15	-6	43.00	7.60
-3	-15	-6	22.30	12.50
-3	-15	-6	48.30	14.50
-3	-15	-6	28.30	6.80

3	15	6	34.40	5.30
-4	-15	-6	102.59	13.40
-4	-15	-6	86.69	13.50
-4	-15	-6	89.89	16.20
-4	-15	-6	76.49	10.50
-4	-15	-6	79.29	11.00
-4	-15	-6	80.69	11.20
4	15	6	72.09	7.70
-5	-15	-6	1153.78	58.09
-5	-15	-6	1214.18	57.19
-5	-15	-6	1200.28	58.09
-5	-15	-6	1182.28	57.79
-5	-15	-6	1322.07	58.49
-5	-15	-6	1148.39	58.29
5	15	6	1233.78	56.59
-6	-15	-6	104.49	14.50
-6	-15	-6	85.19	12.30
-6	-15	-6	98.59	12.00
-6	-15	-6	91.09	13.80
-6	-15	-6	87.39	12.40
6	15	6	81.79	13.10
-7	-15	-6	145.89	14.70
-7	-15	-6	159.98	16.60
-7	-15	-6	164.08	16.30
-7	-15	-6	174.38	16.20
-7	-15	-6	170.08	13.90
7	15	6	117.09	17.70
-8	-15	-6	14.50	8.50
-8	-15	-6	9.40	8.80
-8	-15	-6	2.00	7.20
-8	-15	-6	8.40	8.10
-8	-15	-6	1.40	8.30
8	15	6	-2.10	13.00
-9	-15	-6	13.40	8.00
-9	-15	-6	15.50	8.50
-9	-15	-6	7.60	7.90
-9	-15	-6	25.50	7.60
-9	-15	-6	28.30	8.50
-10	-15	-6	6.90	7.50
-10	-15	-6	4.70	7.80
-10	-15	-6	11.30	8.60
-10	-15	-6	-5.60	7.30
-10	-15	-6	-7.30	7.70
10	15	6	-8.30	16.00

8	-16	-6	49.70	8.50
-8	16	6	36.90	10.90
7	-16	-6	25.80	7.80
7	-16	-6	29.20	16.10
-7	16	6	29.00	11.80
6	-16	-6	55.09	9.70
-6	16	6	75.59	14.20
5	-16	-6	202.88	19.10
5	-16	-6	169.08	26.60
5	-16	-6	187.38	16.20
-5	16	6	208.98	24.60
-5	16	6	175.48	24.20
4	-16	-6	215.68	23.80
4	-16	-6	203.08	18.50
4	-16	-6	228.88	25.30
4	-16	-6	226.58	18.10
-4	16	6	229.88	23.20
-4	16	6	242.08	18.80
-4	16	6	206.38	21.60
3	-16	-6	339.77	22.60
3	-16	-6	371.96	25.70
3	-16	-6	376.76	23.30
3	-16	-6	368.36	26.40
-3	16	6	385.06	20.80
-3	16	6	337.37	26.90
-3	16	6	337.67	24.50
2	-16	-6	783.32	39.80
2	-16	-6	752.92	40.00
2	-16	-6	760.22	40.00
2	-16	-6	790.82	40.60
2	-16	-6	776.12	39.40
-2	16	6	751.92	37.00
-2	16	6	765.52	40.00
1	-16	-6	7.10	7.30
1	-16	-6	5.80	7.90
1	-16	-6	-0.70	8.00
1	-16	-6	8.40	20.10
1	-16	-6	13.60	8.30
-1	16	6	12.10	4.10
-1	16	6	24.20	9.10
0	-16	-6	899.31	44.80
0	-16	-6	914.21	45.40
0	-16	-6	931.91	45.20
0	-16	-6	908.11	45.00

0	16	6	915.71	42.80
-1	-16	-6	242.48	16.60
-1	-16	-6	218.38	17.70
-1	-16	-6	205.08	16.80
-1	-16	-6	219.28	19.30
-1	-16	-6	214.98	15.70
1	16	6	213.58	12.30
-2	-16	-6	0.50	7.10
-2	-16	-6	5.10	6.50
-2	-16	-6	-6.60	7.70
-2	-16	-6	-5.10	6.90
2	16	6	4.80	3.30
-3	-16	-6	57.59	8.50
-3	-16	-6	45.70	7.30
-3	-16	-6	49.20	8.40
-3	-16	-6	43.20	7.80
-3	-16	-6	59.49	9.60
3	16	6	46.60	6.70
-4	-16	-6	79.29	13.80
-4	-16	-6	105.39	14.10
-4	-16	-6	110.79	11.80
-4	-16	-6	121.59	16.30
-4	-16	-6	75.09	13.60
-4	-16	-6	110.19	12.00
4	16	6	102.19	9.20
-5	-16	-6	232.88	17.80
-5	-16	-6	222.18	18.80
-5	-16	-6	236.58	16.60
-5	-16	-6	219.38	15.70
-5	-16	-6	221.28	17.80
-5	-16	-6	212.38	19.10
5	16	6	238.08	15.10
-6	-16	-6	10.80	8.60
-6	-16	-6	7.10	8.40
-6	-16	-6	16.20	6.40
-6	-16	-6	-5.80	7.30
-6	-16	-6	8.80	8.10
-6	-16	-6	12.60	10.30
6	16	6	13.00	8.50
-7	-16	-6	164.48	23.10
-7	-16	-6	159.28	15.80
-7	-16	-6	140.89	15.20
-7	-16	-6	139.99	14.30
-7	-16	-6	130.89	16.10

7	16	6	138.09	18.90
-8	-16	-6	96.79	12.50
-8	-16	-6	81.69	14.40
-8	-16	-6	87.39	11.80
-8	-16	-6	68.29	12.30
-8	-16	-6	96.89	12.00
-9	-16	-6	20.60	8.90
-9	-16	-6	2.60	7.50
-9	-16	-6	4.80	7.10
-9	-16	-6	5.90	7.70
-9	-16	-6	8.20	7.10
9	16	6	-23.40	21.20
-10	-16	-6	242.28	16.00
-10	-16	-6	243.18	16.40
-10	-16	-6	254.17	16.00
-10	-16	-6	222.78	16.60
8	-17	-6	248.68	16.60
-8	17	6	254.77	18.60
7	-17	-6	8.50	7.30
7	-17	-6	2.80	15.60
-7	17	6	5.00	9.80
6	-17	-6	-0.40	7.80
6	-17	-6	31.60	11.90
-6	17	6	9.90	10.70
5	-17	-6	464.25	34.60
5	-17	-6	507.35	29.90
5	-17	-6	537.45	28.50
-5	17	6	491.35	32.90
-5	17	6	497.05	41.70
4	-17	-6	15.00	8.50
4	-17	-6	-22.60	14.50
4	-17	-6	10.50	13.30
4	-17	-6	-0.60	9.00
-4	17	6	35.60	13.30
-4	17	6	30.20	13.20
-4	17	6	10.50	11.60
3	-17	-6	36.50	8.60
3	-17	-6	55.29	9.80
3	-17	-6	40.80	11.80
3	-17	-6	30.70	11.60
-3	17	6	31.20	8.50
-3	17	6	56.69	12.50
-3	17	6	66.19	12.50
2	-17	-6	625.44	34.00

2	-17	-6	591.74	32.70
2	-17	-6	574.84	33.60
2	-17	-6	637.24	34.10
2	-17	-6	602.44	33.50
-2	17	6	618.44	30.70
-2	17	6	608.84	33.70
1	-17	-6	56.99	14.10
1	-17	-6	56.19	9.60
1	-17	-6	60.19	10.20
1	-17	-6	54.79	9.30
1	-17	-6	44.70	9.50
-1	17	6	51.99	10.00
-1	17	6	38.50	6.10
0	-17	-6	81.49	10.40
0	-17	-6	89.89	10.70
0	-17	-6	78.89	13.30
0	-17	-6	85.89	14.70
0	17	6	75.59	8.30
-1	-17	-6	46.40	9.80
-1	-17	-6	39.20	8.80
-1	-17	-6	27.10	7.60
-1	-17	-6	37.60	8.00
-1	-17	-6	35.00	8.00
1	17	6	33.90	5.10
-2	-17	-6	19.30	7.20
-2	-17	-6	20.80	7.10
-2	-17	-6	10.20	7.50
-2	-17	-6	21.90	8.50
-2	-17	-6	9.10	10.20
2	17	6	38.30	6.70
-3	-17	-6	48.60	9.60
-3	-17	-6	62.49	9.60
-3	-17	-6	53.69	8.40
-3	-17	-6	33.50	14.70
3	17	6	49.00	7.90
-4	-17	-6	262.97	19.50
-4	-17	-6	307.37	19.70
-4	-17	-6	270.57	18.60
-4	-17	-6	276.77	17.80
-4	-17	-6	304.47	20.00
4	17	6	270.57	16.60
-5	-17	-6	558.14	29.40
-5	-17	-6	555.64	30.80
-5	-17	-6	570.74	30.90

-5	-17	-6	587.44	31.50
-5	-17	-6	565.14	31.10
-6	-17	-6	383.86	25.10
-6	-17	-6	411.86	26.50
-6	-17	-6	407.76	23.30
-6	-17	-6	447.36	24.90
-6	-17	-6	449.46	25.10
-6	-17	-6	406.06	24.30
6	17	6	365.76	24.90
-7	-17	-6	3.40	13.60
-7	-17	-6	9.50	7.90
-7	-17	-6	-2.30	10.50
-7	-17	-6	11.70	8.50
-7	-17	-6	10.90	8.40
-7	-17	-6	2.30	7.90
7	17	6	12.80	11.80
-8	-17	-6	45.80	8.90
-8	-17	-6	62.09	10.00
-8	-17	-6	66.59	8.60
-8	-17	-6	48.40	8.90
-9	-17	-6	17.30	7.40
-9	-17	-6	29.30	7.20
-9	-17	-6	6.50	11.00
-9	-17	-6	23.60	7.70
-10	-17	-6	18.60	7.60
-10	-17	-6	6.60	9.30
-10	-17	-6	-9.00	7.40
-10	-17	-6	1.70	6.50
8	-18	-6	31.00	9.40
-8	18	6	19.50	10.30
7	-18	-6	303.67	30.00
7	-18	-6	339.57	19.70
-7	18	6	315.97	21.70
6	-18	-6	313.87	19.70
6	-18	-6	301.57	24.50
-6	18	6	326.27	22.50
5	-18	-6	30.10	10.80
5	-18	-6	18.30	9.20
-5	18	6	5.60	15.60
-5	18	6	18.70	19.60
4	-18	-6	94.69	15.30
4	-18	-6	25.30	15.10
4	-18	-6	44.30	10.00
4	-18	-6	79.29	14.80

-4	18	6	74.89	14.20
-4	18	6	61.09	13.40
3	-18	-6	396.26	27.00
3	-18	-6	405.26	24.50
3	-18	-6	382.76	24.80
3	-18	-6	436.86	27.20
-3	18	6	367.86	27.10
-3	18	6	384.36	24.70
-3	18	6	402.76	26.60
2	-18	-6	15.60	10.10
2	-18	-6	29.30	11.60
2	-18	-6	15.00	8.50
2	-18	-6	0.00	9.20
2	-18	-6	30.10	8.80
-2	18	6	19.20	10.40
-2	18	6	29.10	18.30
-2	18	6	13.60	8.50
1	-18	-6	231.98	19.00
1	-18	-6	255.47	18.60
1	-18	-6	208.68	18.40
1	-18	-6	255.97	32.10
1	-18	-6	225.78	18.70
-1	18	6	205.68	18.70
-1	18	6	229.38	15.10
0	-18	-6	122.79	13.50
0	-18	-6	110.09	14.60
0	-18	-6	143.99	16.60
0	-18	-6	108.69	14.30
0	18	6	80.29	14.50
0	18	6	111.49	10.20
-1	-18	-6	328.27	22.80
-1	-18	-6	315.17	20.40
-1	-18	-6	301.67	19.80
-1	-18	-6	317.17	20.40
1	18	6	312.97	17.50
-2	-18	-6	84.59	12.60
-2	-18	-6	76.09	10.00
-2	-18	-6	79.59	13.50
-2	-18	-6	76.29	11.90
-2	-18	-6	82.39	10.60
2	18	6	86.59	9.20
-3	-18	-6	-18.70	13.70
-3	-18	-6	-1.90	6.60
-3	-18	-6	0.60	6.80

-3	-18	-6	14.40	8.60
-3	-18	-6	1.80	7.70
3	18	6	5.10	5.50
-4	-18	-6	121.49	14.20
-4	-18	-6	81.19	14.10
-4	-18	-6	105.79	12.10
-4	-18	-6	94.79	13.10
4	18	6	103.79	12.10
-5	-18	-6	85.79	13.40
-5	-18	-6	62.09	9.50
-5	-18	-6	70.69	10.70
-5	-18	-6	77.89	12.70
-5	-18	-6	75.29	10.40
5	18	6	85.09	12.70
-6	-18	-6	49.50	8.20
-6	-18	-6	75.79	11.50
-6	-18	-6	86.69	12.80
-6	-18	-6	64.09	9.20
6	18	6	92.89	16.00
-7	-18	-6	40.30	13.00
-7	-18	-6	26.90	6.90
-7	-18	-6	30.10	8.90
-7	-18	-6	31.00	7.80
-7	-18	-6	27.90	8.30
7	18	6	-1.70	13.30
-8	-18	-6	19.40	7.80
-8	-18	-6	5.60	6.70
-8	-18	-6	24.40	8.70
-9	-18	-6	90.09	11.30
-9	-18	-6	94.29	10.90
-9	-18	-6	77.99	11.00
-9	-18	-6	79.89	10.80
7	-19	-6	38.60	17.80
7	-19	-6	3.70	7.30
-7	19	6	0.10	7.80
6	-19	-6	129.59	13.60
6	-19	-6	128.89	18.20
-6	19	6	130.69	15.80
5	-19	-6	34.10	10.10
5	-19	-6	27.70	8.60
-5	19	6	35.00	16.00
-5	19	6	46.90	10.40
4	-19	-6	158.38	17.10
4	-19	-6	170.18	17.90

4	-19	-6	162.28	22.30
4	-19	-6	133.69	18.30
-4	19	6	146.49	20.90
-4	19	6	127.69	20.90
3	-19	-6	19.60	11.40
3	-19	-6	-0.20	11.70
3	-19	-6	42.90	11.00
3	-19	-6	5.00	8.40
-3	19	6	25.40	11.80
-3	19	6	-0.10	12.70
-3	19	6	2.10	11.50
2	-19	-6	33.50	10.40
2	-19	-6	10.70	8.20
2	-19	-6	27.80	10.30
2	-19	-6	44.20	13.20
-2	19	6	13.40	9.40
-2	19	6	16.30	10.50
-2	19	6	24.30	10.60
1	-19	-6	411.86	25.00
1	-19	-6	364.76	23.80
1	-19	-6	387.86	23.70
1	-19	-6	383.96	24.20
-1	19	6	375.36	22.40
-1	19	6	417.36	25.10
0	-19	-6	-0.30	7.90
0	-19	-6	3.30	7.40
0	-19	-6	3.00	7.80
0	-19	-6	4.10	9.00
0	19	6	10.10	7.10
0	19	6	11.10	10.20
-1	-19	-6	148.39	14.70
-1	-19	-6	132.49	14.80
-1	-19	-6	151.78	15.00
-1	-19	-6	156.48	18.30
1	19	6	174.48	17.00
-2	-19	-6	59.69	9.30
-2	-19	-6	48.90	11.80
-2	-19	-6	42.70	8.30
-2	-19	-6	55.89	9.50
-2	-19	-6	50.79	9.00
2	19	6	65.79	10.10
-3	-19	-6	51.59	8.70
-3	-19	-6	43.00	15.40
-3	-19	-6	64.19	8.70

-3	-19	-6	56.19	9.60
-3	-19	-6	57.69	8.70
3	19	6	64.69	10.80
-4	-19	-6	204.18	15.90
-4	-19	-6	203.48	15.40
-4	-19	-6	206.08	17.70
-4	-19	-6	221.78	16.80
4	19	6	200.88	16.10
-5	-19	-6	375.86	21.80
-5	-19	-6	368.66	23.60
-5	-19	-6	325.87	20.70
-5	-19	-6	364.86	21.60
5	19	6	331.77	22.60
-6	-19	-6	161.08	14.00
-6	-19	-6	165.18	14.00
-6	-19	-6	160.88	17.60
-6	-19	-6	191.98	15.80
-6	-19	-6	174.48	15.10
6	19	6	158.78	20.00
-7	-19	-6	23.10	7.30
-7	-19	-6	44.20	13.30
-7	-19	-6	23.00	7.70
-7	-19	-6	29.80	7.60
-7	-19	-6	24.50	6.60
-8	-19	-6	35.90	7.20
-8	-19	-6	25.90	6.60
-9	-19	-6	52.49	8.40
-9	-19	-6	47.80	8.00
-9	-19	-6	54.29	9.40
6	-20	-6	109.29	12.80
6	-20	-6	98.79	17.70
-6	20	6	111.49	13.70
5	-20	-6	115.69	16.00
5	-20	-6	110.19	14.30
-5	20	6	121.29	15.20
4	-20	-6	95.49	15.20
4	-20	-6	77.79	14.60
4	-20	-6	108.99	15.80
4	-20	-6	102.69	17.70
-4	20	6	71.59	14.20
-4	20	6	84.79	16.20
3	-20	-6	102.39	18.80
3	-20	-6	109.79	19.90
3	-20	-6	123.69	18.70

3	-20	-6	140.69	16.10
-3	20	6	122.19	18.80
-3	20	6	115.99	18.40
2	-20	-6	200.78	19.60
2	-20	-6	162.78	26.80
2	-20	-6	202.58	24.60
2	-20	-6	193.88	17.70
-2	20	6	222.68	23.40
-2	20	6	207.98	19.20
-2	20	6	191.08	20.10
1	-20	-6	245.58	18.60
1	-20	-6	258.67	19.50
1	-20	-6	269.67	19.90
1	-20	-6	242.08	19.50
-1	20	6	249.08	19.10
-1	20	6	216.58	19.80
0	-20	-6	746.43	37.20
0	-20	-6	708.53	38.40
0	-20	-6	696.93	37.30
0	-20	-6	723.63	37.30
0	20	6	758.02	36.60
0	20	6	706.03	38.10
-1	-20	-6	7.80	7.40
-1	-20	-6	3.30	7.40
-1	-20	-6	15.30	9.90
-1	-20	-6	5.30	7.20
1	20	6	7.20	7.40
1	20	6	9.20	9.10
-2	-20	-6	-2.50	7.40
-2	-20	-6	4.60	11.10
-2	-20	-6	8.70	7.00
-2	-20	-6	14.00	7.20
2	20	6	14.70	7.60
-3	-20	-6	1.80	6.40
-3	-20	-6	-2.00	7.60
-3	-20	-6	9.30	7.70
-3	-20	-6	11.90	15.80
-3	-20	-6	14.70	7.30
3	20	6	9.90	7.80
-4	-20	-6	130.69	15.40
-4	-20	-6	143.89	14.40
-4	-20	-6	118.89	12.50
-4	-20	-6	136.29	13.20
4	20	6	133.49	15.40

-5	-20	-6	110.39	13.60
-5	-20	-6	95.89	12.40
-5	-20	-6	76.49	11.40
-5	-20	-6	96.69	14.20
5	20	6	73.89	12.80
-6	-20	-6	20.20	6.90
-6	-20	-6	20.70	9.40
-6	-20	-6	28.10	7.30
-7	-20	-6	196.38	13.80
-7	-20	-6	202.48	14.60
-7	-20	-6	195.18	21.90
-7	-20	-6	217.18	14.60
-8	-20	-6	11.40	6.00
-8	-20	-6	6.60	6.50
-8	-20	-6	14.20	5.90
6	-21	-6	119.59	17.60
6	-21	-6	160.38	14.20
-6	21	6	137.69	15.50
5	-21	-6	487.25	27.20
5	-21	-6	467.65	26.50
-5	21	6	474.45	26.40
4	-21	-6	18.20	11.00
4	-21	-6	36.40	9.30
4	-21	-6	65.59	20.30
-4	21	6	44.10	13.80
-4	21	6	38.20	27.60
3	-21	-6	24.90	13.30
3	-21	-6	35.40	12.30
3	-21	-6	17.40	8.10
3	-21	-6	25.20	12.00
-3	21	6	48.60	13.60
-3	21	6	10.40	11.80
2	-21	-6	260.67	18.40
2	-21	-6	227.28	20.40
2	-21	-6	219.58	20.00
-2	21	6	241.28	20.20
-2	21	6	194.28	20.70
1	-21	-6	88.69	12.20
1	-21	-6	103.39	15.60
1	-21	-6	110.19	18.00
-1	21	6	82.99	13.80
-1	21	6	88.69	12.70
-1	21	6	99.19	16.60
0	-21	-6	-15.50	11.80

0	-21	-6	-1.10	9.50
0	21	6	13.30	9.50
0	21	6	-19.70	10.00
-1	-21	-6	63.69	9.80
-1	-21	-6	73.69	13.10
-1	-21	-6	101.19	18.20
1	21	6	95.89	14.60
1	21	6	104.49	15.20
-2	-21	-6	56.39	12.40
-2	-21	-6	75.09	14.60
-2	-21	-6	52.29	12.00
2	21	6	74.29	10.70
-3	-21	-6	1.60	7.60
-3	-21	-6	-2.90	7.80
-3	-21	-6	-3.90	17.10
-3	-21	-6	2.90	7.20
3	21	6	10.10	9.80
-4	-21	-6	8.30	6.60
-4	-21	-6	-0.50	7.60
-4	-21	-6	1.50	7.30
-4	-21	-6	-1.90	6.20
4	21	6	22.70	13.90
4	21	6	22.70	24.40
-5	-21	-6	536.85	27.90
-5	-21	-6	505.05	27.10
-5	-21	-6	483.75	26.90
-5	-21	-6	516.85	26.50
-6	-21	-6	314.47	17.80
-6	-21	-6	306.97	18.00
-6	-21	-6	320.87	18.30
-6	-21	-6	284.07	20.60
-7	-21	-6	57.49	9.50
-7	-21	-6	44.70	6.80
-7	-21	-6	72.89	10.00
-7	-21	-6	80.09	15.90
5	-22	-6	15.00	11.50
-5	22	6	29.10	7.50
4	-22	-6	11.70	14.70
4	-22	-6	17.10	8.50
-4	22	6	29.00	16.90
-4	22	6	21.10	7.80
3	-22	-6	73.59	13.20
3	-22	-6	90.89	13.70
-3	22	6	97.09	12.90

-3	22	6	117.19	18.40
2	-22	-6	19.50	8.20
2	-22	-6	5.60	10.20
2	-22	-6	16.60	10.60
-2	22	6	9.40	10.10
-2	22	6	21.60	11.50
1	-22	-6	143.89	15.40
1	-22	-6	119.69	14.60
1	-22	-6	128.39	15.70
-1	22	6	98.19	16.40
-1	22	6	138.39	16.90
0	-22	-6	392.46	23.70
0	-22	-6	409.46	24.30
0	-22	-6	430.16	25.20
0	-22	-6	394.66	24.00
0	22	6	407.26	25.10
-1	-22	-6	338.67	23.50
-1	-22	-6	338.17	21.50
-1	-22	-6	337.67	21.10
-1	-22	-6	345.67	20.80
1	22	6	354.06	22.80
-2	-22	-6	10.50	12.10
-2	-22	-6	9.90	7.60
-2	-22	-6	-1.50	6.90
-2	-22	-6	3.00	7.60
2	22	6	-2.80	8.90
-3	-22	-6	79.79	12.40
-3	-22	-6	103.39	11.40
-3	-22	-6	98.19	11.10
-4	-22	-6	176.78	13.40
-4	-22	-6	170.28	13.80
-4	-22	-6	184.88	15.20
-5	-22	-6	-0.10	8.30
-5	-22	-6	4.70	6.00
-5	-22	-6	10.80	6.10
5	22	6	6.00	19.00
-6	-22	-6	50.49	10.50
-6	-22	-6	57.39	8.50
-6	-22	-6	59.99	9.90
-6	-22	-6	54.49	9.30
6	22	6	62.79	18.60
-7	-22	-6	46.00	7.00
-7	-22	-6	69.59	9.10
-7	-22	-6	56.29	9.10

7	22	6	63.09	12.50
4	-23	-6	80.59	12.60
-4	23	6	72.69	12.10
3	-23	-6	45.40	15.00
3	-23	-6	23.90	7.60
-3	23	6	35.20	12.80
2	-23	-6	268.67	19.10
2	-23	-6	235.28	16.80
2	-23	-6	193.18	19.70
-2	23	6	218.98	20.20
-2	23	6	216.58	18.70
1	-23	-6	337.27	21.40
1	-23	-6	329.07	21.20
1	-23	-6	332.87	22.00
-1	23	6	372.46	22.50
-1	23	6	333.67	22.80
0	-23	-6	38.20	9.00
0	-23	-6	21.20	9.30
0	-23	-6	12.90	7.60
0	23	6	24.70	11.20
-1	-23	-6	108.29	12.90
-1	-23	-6	121.09	12.00
-1	-23	-6	129.69	13.30
-1	-23	-6	123.79	16.70
1	23	6	114.49	14.30
-2	-23	-6	17.90	7.10
-2	-23	-6	39.60	13.10
-2	-23	-6	41.10	7.20
-2	-23	-6	38.10	7.90
2	23	6	31.00	9.70
-3	-23	-6	-7.10	6.00
-3	-23	-6	0.20	6.60
-3	-23	-6	-1.00	6.30
3	23	6	-9.20	9.60
-4	-23	-6	164.58	13.00
-4	-23	-6	195.18	14.70
-4	-23	-6	190.78	13.10
4	23	6	185.18	15.80
-5	-23	-6	70.69	9.40
-5	-23	-6	64.19	9.00
-6	-23	-6	398.16	21.90
-6	-23	-6	342.77	19.40
-6	-23	-6	360.66	22.80
2	-24	-6	0.10	10.90

2	-24	-6	29.90	7.90
-2	24	6	17.80	10.70
1	-24	-6	480.75	25.30
1	-24	-6	492.35	26.20
-1	24	6	462.05	27.10
-1	24	6	444.36	26.10
0	-24	-6	332.37	20.00
0	-24	-6	281.47	18.60
0	-24	-6	304.87	19.50
0	24	6	261.87	20.50
0	24	6	329.37	20.00
-1	-24	-6	25.10	8.30
-1	-24	-6	26.20	7.60
-1	-24	-6	24.30	6.70
-1	-24	-6	54.79	10.70
1	24	6	26.20	10.00
1	24	6	33.90	9.00
-2	-24	-6	-3.60	6.30
-2	-24	-6	-8.40	6.60
-2	-24	-6	-3.30	6.80
-2	-24	-6	-28.70	16.70
2	24	6	-5.90	8.80
-3	-24	-6	6.90	7.00
-3	-24	-6	13.20	6.30
-3	-24	-6	13.10	6.60
3	24	6	-16.30	9.00
-4	-24	-6	77.49	9.80
-4	-24	-6	67.39	10.90
-4	-24	-6	66.19	8.90
4	24	6	73.39	11.30
0	-25	-6	11.30	9.90
0	-25	-6	-1.30	11.40
0	-25	-6	-0.10	9.00
0	25	6	0.60	8.80
-1	-25	-6	-14.00	6.70
-1	-25	-6	1.80	11.70
-1	-25	-6	7.20	7.90
1	25	6	-14.40	8.90
1	25	6	-15.40	10.30
-2	-25	-6	16.90	6.70
-2	-25	-6	3.00	7.00
2	25	6	3.70	9.30
2	25	6	10.50	9.90
3	21	-7	30.90	10.60

3	21	-7	24.00	10.00
2	21	-7	26.20	12.70
2	21	-7	-8.20	9.10
2	21	-7	9.00	12.80
-2	-21	7	-2.10	6.70
1	21	-7	168.18	17.60
1	21	-7	155.08	16.40
1	21	-7	173.48	14.80
-1	-21	7	171.98	13.90
-1	-21	7	169.68	22.10
0	21	-7	116.79	12.90
0	21	-7	122.49	20.30
0	21	-7	96.89	14.30
0	-21	7	77.89	12.00
-1	21	-7	5.30	7.60
-1	21	-7	7.80	9.90
-1	21	-7	12.40	19.10
1	-21	7	-2.40	7.00
-2	21	-7	155.28	13.00
-2	21	-7	124.29	13.90
2	-21	7	182.68	12.90
4	20	-7	14.70	11.70
4	20	-7	4.30	7.10
-4	-20	7	-2.60	5.40
3	20	-7	91.09	14.70
3	20	-7	101.29	17.00
3	20	-7	95.19	12.60
-3	-20	7	96.19	10.60
2	20	-7	11.90	10.50
2	20	-7	-1.50	8.00
2	20	-7	9.60	8.20
-2	-20	7	6.90	6.70
-2	-20	7	3.50	10.90
1	20	-7	32.80	8.20
1	20	-7	39.80	9.70
1	20	-7	43.50	9.90
-1	-20	7	43.40	8.50
-1	-20	7	31.50	9.10
0	20	-7	557.24	31.50
0	20	-7	577.04	31.70
0	20	-7	607.34	30.80
0	-20	7	633.04	30.90
0	-20	7	582.44	30.90
-1	20	-7	119.59	18.40

-1	20	-7	138.49	14.20
-1	20	-7	136.19	13.70
1	-20	7	111.69	13.20
1	-20	7	132.59	17.00
-2	20	-7	249.28	17.00
-2	20	-7	227.38	17.50
2	-20	7	238.08	16.50
-3	20	-7	-0.70	7.10
-3	20	-7	1.90	9.00
3	-20	7	-1.10	6.70
-4	20	-7	7.30	7.20
-4	20	-7	-6.60	9.40
4	-20	7	-0.40	7.30
6	19	-7	9.90	8.80
-6	-19	7	26.30	8.40
-6	-19	7	8.10	7.20
5	19	-7	285.87	17.50
-5	-19	7	277.37	16.40
-5	-19	7	239.38	19.30
4	19	-7	109.99	18.90
4	19	-7	133.59	13.50
-4	-19	7	126.49	11.70
3	19	-7	45.00	8.50
3	19	-7	35.30	11.50
3	19	-7	56.69	10.00
-3	-19	7	59.69	10.50
2	19	-7	52.39	9.50
2	19	-7	43.00	9.00
2	19	-7	34.70	9.50
-2	-19	7	41.30	7.70
-2	-19	7	51.09	10.70
1	19	-7	20.60	8.90
1	19	-7	3.90	8.30
1	19	-7	21.10	9.00
-1	-19	7	23.10	7.80
-1	-19	7	11.60	8.70
0	19	-7	45.40	10.40
0	19	-7	5.30	8.40
0	19	-7	29.20	8.20
0	-19	7	23.00	9.10
0	-19	7	11.70	8.20
-1	19	-7	18.10	8.80
-1	19	-7	30.50	8.20
-1	19	-7	11.90	11.00

1	-19	7	18.80	8.00
1	-19	7	31.50	10.60
-2	19	-7	-2.20	7.60
-2	19	-7	18.20	13.10
-2	19	-7	17.20	9.00
2	-19	7	20.40	7.60
-3	19	-7	88.69	14.80
-3	19	-7	122.89	13.30
3	-19	7	96.29	12.80
-4	19	-7	-8.90	7.30
-4	19	-7	8.40	9.00
4	-19	7	-0.90	7.30
-5	19	-7	161.38	16.00
-5	19	-7	182.28	14.50
5	-19	7	179.08	15.00
6	18	-7	241.78	15.90
-6	-18	7	221.58	14.90
-6	-18	7	226.88	18.00
5	18	-7	50.69	8.20
-5	-18	7	34.50	6.80
-5	-18	7	37.90	8.20
4	18	-7	13.80	7.60
-4	-18	7	2.90	6.00
-4	-18	7	14.60	9.00
3	18	-7	9.00	8.50
-3	-18	7	-24.20	13.10
-3	-18	7	-8.90	6.40
2	18	-7	7.80	8.30
2	18	-7	4.30	8.90
-2	-18	7	-5.70	9.40
-2	-18	7	9.10	7.00
1	18	-7	112.99	14.90
1	18	-7	100.09	14.10
1	18	-7	128.09	16.70
-1	-18	7	141.19	15.20
-1	-18	7	146.19	15.60
0	18	-7	24.80	9.50
0	18	-7	24.00	8.50
0	-18	7	29.80	9.10
0	-18	7	35.50	9.70
-1	18	-7	673.63	35.40
-1	18	-7	679.93	36.90
-1	18	-7	660.93	35.00
1	-18	7	642.34	36.10

1	-18	7	688.13	35.60
-2	18	-7	20.20	8.60
-2	18	-7	30.50	9.00
-2	18	-7	7.00	11.50
2	-18	7	35.00	13.80
2	-18	7	14.50	8.00
-3	18	-7	4.80	14.30
-3	18	-7	4.00	8.40
-3	18	-7	16.60	7.80
3	-18	7	27.20	8.10
-4	18	-7	238.08	18.10
-4	18	-7	261.67	18.00
4	-18	7	253.07	20.80
4	-18	7	254.87	18.00
-5	18	-7	29.30	9.80
5	-18	7	22.50	10.30
5	-18	7	35.90	9.40
-6	18	-7	37.60	8.40
-6	18	-7	39.00	10.50
6	-18	7	36.60	12.80
6	-18	7	46.90	9.50
7	17	-7	7.50	7.30
-7	-17	7	-5.60	6.90
-7	-17	7	1.60	6.10
6	17	-7	171.08	14.90
-6	-17	7	180.58	13.60
-6	-17	7	189.78	13.60
5	17	-7	80.49	17.80
5	17	-7	87.79	13.20
-5	-17	7	93.09	12.50
-5	-17	7	88.99	11.00
4	17	-7	424.56	23.90
4	17	-7	374.96	24.70
-4	-17	7	388.06	23.10
-4	-17	7	384.76	22.30
3	17	-7	30.30	9.30
3	17	-7	37.70	16.80
-3	-17	7	33.90	10.20
-3	-17	7	18.80	9.90
-3	-17	7	36.70	12.20
2	17	-7	-11.60	8.20
2	17	-7	-3.20	8.40
-2	-17	7	10.40	7.40
1	17	-7	242.48	18.50

1	17	-7	219.28	17.50
-1	-17	7	271.27	20.00
-1	-17	7	234.98	17.70
0	17	-7	78.69	14.10
0	17	-7	100.19	15.10
0	17	-7	98.89	12.60
0	-17	7	95.39	14.40
0	-17	7	67.29	10.20
-1	17	-7	12.20	9.80
-1	17	-7	4.70	7.60
-1	17	-7	2.20	8.10
1	-17	7	-4.70	7.80
1	-17	7	13.40	10.00
-2	17	-7	36.60	9.30
-2	17	-7	44.00	8.90
2	-17	7	58.69	15.80
2	-17	7	60.69	9.90
-3	17	-7	0.50	8.70
-3	17	-7	-7.50	13.30
-3	17	-7	5.50	8.50
3	-17	7	5.00	9.10
3	-17	7	-5.00	13.30
-4	17	-7	26.40	9.40
-4	17	-7	27.80	9.30
-4	17	-7	17.50	16.50
4	-17	7	25.50	9.30
4	-17	7	39.30	10.30
-5	17	-7	59.19	9.80
-5	17	-7	86.09	13.00
5	-17	7	83.59	14.10
5	-17	7	58.09	10.00
-6	17	-7	64.69	10.90
-6	17	-7	97.49	12.90
6	-17	7	58.49	11.00
6	-17	7	75.59	11.40
-7	17	-7	70.69	11.90
-7	17	-7	106.09	11.60
7	-17	7	92.89	17.80
7	-17	7	114.09	14.10
8	16	-7	5.30	7.90
-8	-16	7	10.80	9.00
7	16	-7	6.90	7.60
-7	-16	7	5.90	6.70
-7	-16	7	2.40	5.80

6	16	-7	365.46	22.10
-6	-16	7	341.37	20.70
-6	-16	7	388.06	21.20
5	16	-7	208.78	19.60
5	16	-7	220.88	16.90
-5	-16	7	169.88	14.60
-5	-16	7	169.08	15.10
4	16	-7	8.80	8.20
4	16	-7	-0.40	9.50
-4	-16	7	6.50	6.80
3	16	-7	154.38	15.80
3	16	-7	161.18	15.90
-3	-16	7	184.38	21.30
-3	-16	7	158.78	14.20
-3	-16	7	176.68	16.80
2	16	-7	-8.60	7.70
2	16	-7	-0.10	8.00
-2	-16	7	6.20	6.80
-2	-16	7	5.10	8.70
1	16	-7	131.49	14.80
1	16	-7	105.39	13.70
-1	-16	7	104.49	13.30
-1	-16	7	86.19	11.00
0	16	-7	503.85	29.30
0	16	-7	549.45	30.50
0	-16	7	534.95	29.20
0	-16	7	559.54	30.00
-1	16	-7	35.70	8.50
-1	16	-7	42.40	9.80
-1	16	-7	38.30	9.10
1	-16	7	25.30	7.90
1	-16	7	30.30	9.40
-2	16	-7	374.76	23.40
-2	16	-7	385.96	25.70
-2	16	-7	351.46	23.00
2	-16	7	355.26	26.40
2	-16	7	396.66	23.50
-3	16	-7	74.19	14.20
-3	16	-7	85.79	13.00
-3	16	-7	71.69	10.80
3	-16	7	70.59	12.40
3	-16	7	85.29	14.40
-4	16	-7	32.90	15.30
-4	16	-7	1.90	8.20

4	-16	7	-5.40	9.60
-5	16	-7	30.20	9.40
-5	16	-7	26.10	9.20
5	-16	7	20.20	8.40
5	-16	7	23.90	10.10
-6	16	-7	23.30	10.00
-6	16	-7	18.70	9.00
6	-16	7	23.20	10.90
6	-16	7	5.20	7.80
-7	16	-7	53.59	11.40
-7	16	-7	52.29	9.50
7	-16	7	40.80	8.80
7	-16	7	71.29	17.40
8	15	-7	69.99	12.00
-8	-15	7	46.20	6.90
-8	-15	7	45.20	10.10
7	15	-7	23.50	13.80
7	15	-7	7.60	8.20
-7	-15	7	9.60	6.40
-7	-15	7	37.90	7.30
6	15	-7	-25.00	13.50
6	15	-7	6.20	8.10
-6	-15	7	0.90	6.90
-6	-15	7	7.30	7.00
5	15	-7	470.95	37.10
5	15	-7	550.94	30.40
-5	-15	7	578.54	29.50
-5	-15	7	564.34	33.50
4	15	-7	247.58	18.60
-4	-15	7	210.08	16.80
-4	-15	7	208.88	16.20
3	15	-7	140.59	15.30
3	15	-7	137.49	15.50
-3	-15	7	112.49	14.60
-3	-15	7	129.39	19.10
-3	-15	7	121.79	13.80
2	15	-7	87.19	14.50
2	15	-7	63.69	15.30
-2	-15	7	58.09	10.50
-2	-15	7	35.00	17.40
-2	-15	7	68.49	12.00
1	15	-7	4.90	8.40
1	15	-7	7.60	8.20
-1	-15	7	-0.80	6.90

-1	-15	7	14.10	8.40
0	15	-7	179.08	17.20
0	15	-7	154.58	15.30
0	-15	7	179.18	16.10
0	-15	7	180.48	16.00
-1	15	-7	-6.00	10.00
-1	15	-7	13.80	7.80
-1	15	-7	-3.60	8.40
1	-15	7	-3.60	8.40
1	-15	7	7.70	7.50
-2	15	-7	-9.20	9.80
-2	15	-7	10.30	8.20
-2	15	-7	10.30	7.80
2	-15	7	-5.10	11.00
2	-15	7	-7.60	12.80
2	-15	7	2.30	7.40
-3	15	-7	170.18	21.20
-3	15	-7	158.08	15.50
-3	15	-7	118.99	16.00
3	-15	7	185.48	19.30
3	-15	7	132.99	15.80
-4	15	-7	47.00	14.40
-4	15	-7	53.39	10.00
-4	15	-7	56.09	10.60
4	-15	7	59.09	10.70
4	-15	7	48.90	9.90
-5	15	-7	336.17	35.70
-5	15	-7	378.46	34.80
-5	15	-7	387.56	25.00
5	-15	7	424.36	28.60
5	-15	7	478.55	38.10
-6	15	-7	210.78	19.00
-6	15	-7	205.88	18.10
6	-15	7	201.48	16.90
6	-15	7	207.78	19.40
-7	15	-7	12.70	10.10
-7	15	-7	22.80	9.50
7	-15	7	23.40	13.40
7	-15	7	12.10	7.50
-8	15	-7	13.90	10.80
-8	15	-7	-1.60	8.60
8	-15	7	8.40	6.70
9	14	-7	39.30	12.30
9	14	-7	32.50	12.10

-9	-14	7	32.70	5.80
8	14	-7	20.50	8.70
8	14	-7	19.10	12.30
-8	-14	7	20.80	8.30
-8	-14	7	8.20	6.00
7	14	-7	307.27	25.00
7	14	-7	290.87	21.00
-7	-14	7	317.97	19.50
-7	-14	7	328.37	19.00
6	14	-7	224.68	24.80
5	14	-7	73.09	14.10
5	14	-7	71.39	11.10
-5	-14	7	76.29	11.90
-5	-14	7	69.69	9.10
4	14	-7	136.79	22.10
4	14	-7	105.59	14.80
-4	-14	7	94.39	12.10
-4	-14	7	67.19	9.70
3	14	-7	-1.60	8.10
-3	-14	7	-2.50	8.00
-3	-14	7	0.30	6.40
-3	-14	7	7.90	10.80
2	14	-7	42.00	9.10
2	14	-7	39.00	8.60
-2	-14	7	49.00	9.50
-2	-14	7	32.50	7.40
1	14	-7	-8.80	6.70
1	14	-7	-9.40	7.30
-1	-14	7	-11.90	6.90
-1	-14	7	16.60	8.40
0	14	-7	524.35	28.20
0	14	-7	529.45	29.30
0	-14	7	529.65	28.70
0	-14	7	479.45	28.10
-1	14	-7	842.52	41.50
-1	14	-7	815.22	41.80
-1	14	-7	831.02	42.10
1	-14	7	845.62	41.80
1	-14	7	788.42	41.50
-2	14	-7	197.48	16.90
-2	14	-7	181.18	15.40
-2	14	-7	169.78	17.60
2	-14	7	177.08	20.30
2	-14	7	157.98	16.10

2	-14	7	199.78	20.80
-3	14	-7	18.60	10.90
-3	14	-7	10.10	11.40
-3	14	-7	37.00	9.60
3	-14	7	20.60	9.10
3	-14	7	9.40	7.60
-4	14	-7	221.88	25.60
-4	14	-7	213.38	19.10
-4	14	-7	233.68	18.70
4	-14	7	256.87	19.30
4	-14	7	216.88	18.80
-5	14	-7	18.60	9.10
-5	14	-7	38.60	15.70
-5	14	-7	16.70	9.60
5	-14	7	36.90	9.60
5	-14	7	29.00	9.90
-6	14	-7	7.30	10.10
-6	14	-7	-10.90	12.70
6	-14	7	5.60	8.40
6	-14	7	4.80	11.30
-7	14	-7	93.89	15.60
-7	14	-7	103.19	15.80
7	-14	7	95.69	15.20
7	-14	7	78.79	12.50
-8	14	-7	3.50	10.90
-8	14	-7	22.20	9.70
8	-14	7	18.30	7.40
-9	14	-7	32.90	12.40
9	-14	7	52.19	9.40
9	13	-7	0.10	11.30
9	13	-7	17.20	9.30
-9	-13	7	3.90	5.40
8	13	-7	33.10	12.70
8	13	-7	27.60	9.30
-8	-13	7	21.10	9.20
-8	-13	7	28.30	6.60
7	13	-7	-8.00	12.50
-7	-13	7	14.70	7.80
-7	-13	7	19.20	7.30
6	13	-7	230.48	23.70
-6	-13	7	263.87	17.90
-6	-13	7	269.37	18.00
5	13	-7	7.20	12.30
-5	-13	7	3.00	7.20

-5	-13	7	19.20	6.90
4	13	-7	505.95	32.20
-4	-13	7	527.45	28.00
-4	-13	7	497.05	27.80
3	13	-7	6.00	7.60
3	13	-7	27.90	13.50
-3	-13	7	-5.00	7.60
-3	-13	7	24.70	10.70
-3	-13	7	9.10	6.40
2	13	-7	164.68	15.90
-2	-13	7	159.78	17.20
-2	-13	7	181.38	14.80
-2	-13	7	190.68	17.50
1	13	-7	655.03	32.20
1	13	-7	623.74	33.10
-1	-13	7	595.04	32.80
-1	-13	7	609.64	32.10
0	13	-7	22.80	6.70
0	13	-7	15.10	8.60
0	-13	7	16.70	7.50
0	-13	7	1.40	7.30
-1	13	-7	19.30	7.00
-1	13	-7	16.80	8.80
1	-13	7	19.10	8.30
1	-13	7	22.90	8.00
-2	13	-7	324.37	19.70
-2	13	-7	318.07	21.10
-2	13	-7	296.47	21.40
2	-13	7	318.57	20.40
2	-13	7	274.07	21.80
2	-13	7	241.58	22.50
-3	13	-7	7.70	11.00
-3	13	-7	5.30	7.60
-3	13	-7	18.80	9.20
3	-13	7	18.50	8.10
3	-13	7	13.60	8.40
-4	13	-7	6.30	8.70
-4	13	-7	1.20	8.30
-4	13	-7	17.30	13.00
4	-13	7	9.50	7.80
4	-13	7	3.50	7.70
-5	13	-7	-2.50	8.70
-5	13	-7	-3.60	15.00
-5	13	-7	-0.50	9.60

5	-13	7	8.60	9.10
5	-13	7	6.40	8.30
-6	13	-7	220.48	19.90
-6	13	-7	213.98	21.40
-6	13	-7	224.28	34.30
6	-13	7	245.18	18.90
6	-13	7	262.27	21.50
-7	13	-7	81.99	13.70
-7	13	-7	76.79	13.00
7	-13	7	124.69	14.10
7	-13	7	109.89	16.00
-8	13	-7	100.79	16.40
8	-13	7	113.49	13.00
-9	13	-7	104.99	15.70
9	-13	7	111.99	12.20
9	12	-7	17.70	8.70
9	12	-7	7.60	11.40
-9	-12	7	12.40	5.60
8	12	-7	-5.80	11.50
-8	-12	7	-9.20	8.80
-8	-12	7	5.00	6.30
7	12	-7	21.40	13.50
-7	-12	7	16.60	7.20
-7	-12	7	1.10	12.10
6	12	-7	430.36	29.40
-6	-12	7	433.26	24.30
-6	-12	7	415.36	25.00
5	12	-7	55.99	13.00
-5	-12	7	43.10	7.60
-5	-12	7	51.49	11.40
-5	-12	7	73.99	12.40
4	12	-7	13.90	12.10
-4	-12	7	44.00	9.70
-4	-12	7	27.40	7.70
-4	-12	7	17.50	7.40
3	12	-7	77.49	13.60
-3	-12	7	60.99	9.70
-3	-12	7	65.39	11.70
-3	-12	7	80.09	14.90
2	12	-7	152.18	15.00
2	12	-7	158.78	22.90
-2	-12	7	132.29	16.50
-2	-12	7	145.09	13.50
1	12	-7	441.66	26.10

1	12	-7	459.55	25.10
-1	-12	7	469.45	28.30
0	12	-7	345.27	20.50
0	-12	7	346.47	21.10
-1	12	-7	219.48	15.50
1	-12	7	202.68	16.20
1	-12	7	201.88	17.10
-2	12	-7	192.78	17.40
-2	12	-7	188.18	15.10
-2	12	-7	200.28	18.50
2	-12	7	180.98	16.00
2	-12	7	213.08	19.70
-3	12	-7	178.68	20.70
-3	12	-7	186.08	15.20
-3	12	-7	171.18	16.90
3	-12	7	191.18	16.60
-4	12	-7	39.30	13.90
-4	12	-7	61.09	11.10
-4	12	-7	40.10	9.40
4	-12	7	24.20	8.70
4	-12	7	15.90	8.00
-5	12	-7	213.38	20.50
-5	12	-7	236.98	23.60
-5	12	-7	233.48	27.90
5	-12	7	222.68	18.40
5	-12	7	231.48	18.90
-6	12	-7	-19.20	16.30
-6	12	-7	-14.00	10.10
-6	12	-7	-15.70	10.40
6	-12	7	8.10	9.70
6	-12	7	5.60	7.80
-7	12	-7	56.39	14.20
-7	12	-7	74.29	12.80
7	-12	7	67.19	11.90
7	-12	7	107.19	17.70
-8	12	-7	169.08	20.10
-8	12	-7	189.98	20.00
8	-12	7	200.98	16.30
-9	12	-7	-6.60	10.10
-9	12	-7	-10.00	12.00
9	-12	7	17.30	7.60
10	11	-7	76.89	14.30
10	11	-7	62.49	12.50
-10	-11	7	57.29	9.40

9	11	-7	8.60	11.30
-9	-11	7	1.20	5.90
8	11	-7	23.60	12.10
-8	-11	7	28.00	9.60
-8	-11	7	33.20	7.40
7	11	-7	11.40	12.00
-7	-11	7	21.10	8.50
-7	-11	7	18.50	6.90
6	11	-7	41.10	12.80
-6	-11	7	46.70	7.90
-6	-11	7	45.20	8.40
5	11	-7	532.05	33.00
-5	-11	7	513.55	28.90
-5	-11	7	525.25	31.00
-5	-11	7	556.84	28.60
4	11	-7	89.49	13.80
-4	-11	7	79.89	12.20
-4	-11	7	77.69	11.30
-4	-11	7	63.69	13.50
3	11	-7	15.70	11.90
3	11	-7	11.60	10.20
-3	-11	7	26.20	11.00
-3	-11	7	22.50	8.70
-3	-11	7	-6.00	7.60
-3	-11	7	2.90	5.80
2	11	-7	390.56	23.60
2	11	-7	319.77	27.80
2	11	-7	399.56	23.50
-2	-11	7	397.96	24.60
-2	-11	7	419.36	24.70
-2	-11	7	411.36	22.50
1	11	-7	0.70	7.10
1	11	-7	6.10	6.80
-1	-11	7	4.80	7.10
-1	-11	7	6.40	6.10
0	11	-7	369.46	20.30
0	11	-7	329.07	21.60
0	-11	7	349.07	21.30
0	-11	7	335.97	21.30
-1	11	-7	79.19	10.50
-1	11	-7	61.19	10.40
1	-11	7	65.89	9.70
1	-11	7	49.80	8.10
-2	11	-7	47.90	9.10

-2	11	-7	52.39	7.80
-2	11	-7	80.19	11.30
2	-11	7	74.09	11.60
2	-11	7	54.29	9.00
-3	11	-7	634.74	33.80
-3	11	-7	654.23	42.50
-3	11	-7	624.64	34.90
3	-11	7	659.83	33.80
-4	11	-7	337.67	28.70
-4	11	-7	342.17	23.20
-4	11	-7	332.57	21.90
4	-11	7	344.47	22.20
-5	11	-7	678.63	37.10
-5	11	-7	655.23	37.90
-5	11	-7	727.23	42.90
5	-11	7	665.03	35.70
5	-11	7	706.83	36.40
-6	11	-7	73.79	15.70
-6	11	-7	44.80	16.60
-6	11	-7	60.39	13.00
-6	11	-7	68.29	13.00
-7	11	-7	122.09	21.20
-7	11	-7	196.18	22.00
-7	11	-7	162.18	18.10
-7	11	-7	179.28	19.80
7	-11	7	176.78	16.30
7	-11	7	146.89	25.90
-8	11	-7	-12.50	11.60
-8	11	-7	-7.20	13.20
-8	11	-7	1.40	11.40
8	-11	7	-1.30	7.50
-9	11	-7	220.08	21.80
-9	11	-7	262.27	22.90
9	-11	7	294.57	18.60
9	-11	7	294.37	23.70
-10	11	-7	59.49	12.60
-10	11	-7	46.50	15.40
10	-11	7	72.39	12.80
10	10	-7	181.08	22.40
10	10	-7	192.68	18.90
-10	-10	7	170.28	13.30
9	10	-7	11.90	11.20
-9	-10	7	2.10	6.00
8	10	-7	49.20	21.60

-8	-10	7	109.89	14.80
-8	-10	7	103.99	17.20
7	10	-7	397.76	28.60
-7	-10	7	426.36	23.30
-7	-10	7	405.36	24.90
6	10	-7	183.98	21.40
-6	-10	7	159.98	15.60
-6	-10	7	190.08	14.90
5	10	-7	321.57	25.30
-5	-10	7	307.67	19.60
-5	-10	7	303.87	18.90
4	10	-7	339.07	22.60
-4	-10	7	380.36	21.40
-4	-10	7	367.16	24.90
3	10	-7	13.00	7.60
3	10	-7	20.70	12.20
-3	-10	7	3.30	9.30
-3	-10	7	25.40	11.10
-3	-10	7	3.40	5.10
-3	-10	7	2.00	7.00
2	10	-7	52.79	12.70
2	10	-7	33.40	6.90
-2	-10	7	59.69	10.50
-2	-10	7	28.50	6.60
-2	-10	7	40.90	9.70
1	10	-7	45.90	7.40
1	10	-7	46.80	8.80
1	10	-7	50.39	13.70
-1	-10	7	39.10	7.10
-1	-10	7	48.40	9.10
0	10	-7	1064.99	49.10
0	10	-7	987.30	49.80
0	-10	7	1030.30	49.40
0	-10	7	1020.00	49.50
-1	10	-7	467.35	26.50
-1	10	-7	439.06	24.00
1	-10	7	418.46	25.60
1	-10	7	452.85	25.10
-2	10	-7	62.59	8.50
-2	10	-7	80.29	10.80
-2	10	-7	74.99	11.60
2	-10	7	53.29	11.70
2	-10	7	44.10	7.90
-3	10	-7	51.29	12.10

-3	10	-7	28.80	7.10
-3	10	-7	36.80	9.10
3	-10	7	24.30	7.00
-4	10	-7	684.33	37.10
-4	10	-7	735.03	43.10
-4	10	-7	676.73	36.50
4	-10	7	702.03	39.90
-5	10	-7	43.70	11.70
-5	10	-7	20.60	15.20
-5	10	-7	47.70	10.10
5	-10	7	34.00	8.90
-6	10	-7	48.80	14.30
-6	10	-7	91.69	17.30
-6	10	-7	72.49	12.10
-6	10	-7	57.99	12.40
6	-10	7	72.09	10.60
6	-10	7	69.89	12.30
-7	10	-7	-4.50	11.10
-7	10	-7	12.60	10.60
-7	10	-7	-13.30	13.20
-7	10	-7	5.90	16.30
7	-10	7	6.50	8.30
7	-10	7	49.00	19.10
-8	10	-7	182.98	21.70
-8	10	-7	218.68	24.90
-8	10	-7	247.58	24.20
8	-10	7	250.57	19.50
-9	10	-7	64.79	12.80
-9	10	-7	125.49	19.40
-9	10	-7	111.69	19.00
9	-10	7	70.39	12.80
-10	10	-7	4.80	10.90
-10	10	-7	13.40	14.10
10	-10	7	16.70	8.80
10	-10	7	-11.60	16.00
10	9	-7	38.50	10.80
10	9	-7	28.30	26.50
9	9	-7	2.20	11.50
-9	-9	7	4.80	6.40
-9	-9	7	17.20	13.80
8	9	-7	9.70	11.80
-8	-9	7	20.90	6.80
-8	-9	7	14.00	10.50
-8	-9	7	-8.90	13.10

7	9	-7	141.99	21.00
-7	-9	7	157.18	13.40
-7	-9	7	139.19	15.20
-7	-9	7	132.29	16.00
6	9	-7	51.69	12.40
-6	-9	7	39.10	7.10
-6	-9	7	57.49	9.00
5	9	-7	125.39	20.50
5	9	-7	153.18	24.70
-5	-9	7	95.99	11.40
-5	-9	7	93.29	12.30
4	9	-7	1239.48	62.99
4	9	-7	1320.97	64.19
-4	-9	7	1375.76	61.89
-4	-9	7	1350.96	61.39
-4	-9	7	1241.38	65.39
3	9	-7	24.70	6.50
3	9	-7	26.50	12.00
-3	-9	7	36.50	10.80
-3	-9	7	17.70	11.30
-3	-9	7	18.60	6.30
-3	-9	7	34.90	7.50
2	9	-7	317.37	17.90
2	9	-7	296.97	26.20
-2	-9	7	303.57	21.20
-2	-9	7	343.97	22.40
-2	-9	7	307.57	18.60
-2	-9	7	332.07	22.20
1	9	-7	171.78	15.20
1	9	-7	183.98	12.70
1	9	-7	192.88	15.40
-1	-9	7	145.49	15.80
-1	-9	7	191.68	13.70
0	9	-7	72.79	9.80
0	9	-7	78.49	10.40
0	9	-7	106.09	10.40
0	-9	7	77.29	12.50
0	-9	7	78.49	12.70
-1	9	-7	6.80	7.50
1	-9	7	0.60	6.20
1	-9	7	0.90	7.00
1	-9	7	4.90	8.40
-2	9	-7	842.32	43.10
-2	9	-7	888.31	44.60

-2	9	-7	891.51	42.20
2	-9	7	874.81	42.70
2	-9	7	826.62	41.90
-3	9	-7	94.39	11.30
-3	9	-7	115.79	19.50
-3	9	-7	68.09	10.10
3	-9	7	88.39	14.80
3	-9	7	111.79	13.70
-4	9	-7	30.40	9.10
-4	9	-7	26.70	13.40
-4	9	-7	16.20	7.30
4	-9	7	41.60	11.10
4	-9	7	15.60	7.60
-5	9	-7	44.70	9.80
-5	9	-7	41.10	13.40
-5	9	-7	39.00	8.40
5	-9	7	33.30	15.30
-6	9	-7	174.98	20.00
-6	9	-7	162.98	22.50
-6	9	-7	207.38	18.00
-6	9	-7	170.28	27.50
6	-9	7	210.88	20.80
6	-9	7	198.98	17.00
-7	9	-7	7.70	15.90
-7	9	-7	3.30	13.10
-7	9	-7	-7.40	10.80
-7	9	-7	10.00	11.60
7	-9	7	0.40	7.80
-8	9	-7	76.09	13.60
-8	9	-7	54.59	12.70
-8	9	-7	104.99	18.50
-8	9	-7	93.69	14.50
8	-9	7	87.99	15.60
-9	9	-7	275.47	24.20
-9	9	-7	243.58	22.90
-9	9	-7	279.47	24.80
9	-9	7	317.07	20.70
-10	9	-7	339.77	24.90
-10	9	-7	314.97	28.70
-10	9	-7	283.47	24.60
10	-9	7	369.26	22.30
10	-9	7	340.27	22.60
11	8	-7	120.49	15.80
-11	-8	7	90.49	11.10

10	8	-7	14.20	11.30
-10	-8	7	31.10	18.80
-10	-8	7	10.80	6.40
9	8	-7	80.29	14.10
-9	-8	7	67.59	11.70
-9	-8	7	87.89	13.50
8	8	-7	24.20	11.50
-8	-8	7	16.40	10.60
-8	-8	7	0.70	6.00
-8	-8	7	10.70	10.40
7	8	-7	53.99	12.90
-7	-8	7	35.10	7.20
-7	-8	7	57.89	9.80
-7	-8	7	46.30	11.30
6	8	-7	325.77	25.50
-6	-8	7	360.26	24.20
-6	-8	7	328.47	19.60
-6	-8	7	304.17	20.70
5	8	-7	67.19	10.20
-5	-8	7	104.39	16.60
4	8	-7	108.09	18.70
4	8	-7	100.59	11.00
-4	-8	7	81.59	11.50
-4	-8	7	102.49	11.20
3	8	-7	604.14	28.90
3	8	-7	560.84	34.20
3	8	-7	537.65	35.50
-3	-8	7	577.34	30.00
-3	-8	7	614.54	29.90
2	8	-7	547.15	26.50
2	8	-7	572.74	32.10
2	8	-7	536.25	27.70
2	8	-7	537.05	33.50
-2	-8	7	464.05	31.80
-2	-8	7	548.65	27.70
1	8	-7	625.84	34.20
1	8	-7	609.04	31.20
1	8	-7	621.54	30.20
1	8	-7	629.34	32.60
-1	-8	7	630.94	34.60
-1	-8	7	613.44	31.40
-1	-8	7	638.54	31.60
0	8	-7	257.57	15.20
0	8	-7	276.47	16.60

0	8	-7	257.67	18.90
0	8	-7	245.08	16.10
0	8	-7	286.27	19.80
0	-8	7	264.47	16.10
0	-8	7	272.07	17.20
0	-8	7	288.87	20.50
-1	8	-7	307.77	21.70
-1	8	-7	304.17	17.50
-1	8	-7	310.77	20.80
-1	8	-7	286.77	18.40
1	-8	7	300.67	17.00
1	-8	7	293.67	22.50
1	-8	7	310.07	23.60
-2	8	-7	256.57	15.50
-2	8	-7	226.58	20.80
-2	8	-7	275.57	19.20
2	-8	7	228.48	16.70
2	-8	7	234.58	15.60
-3	8	-7	522.25	31.80
-3	8	-7	509.85	26.70
-3	8	-7	489.75	28.30
3	-8	7	504.35	26.50
3	-8	7	512.95	27.80
-4	8	-7	209.98	15.20
-4	8	-7	224.38	17.10
-4	8	-7	191.68	23.60
-4	8	-7	169.38	24.90
4	-8	7	194.58	17.70
4	-8	7	212.28	16.40
-5	8	-7	594.14	36.20
-5	8	-7	639.44	45.30
-5	8	-7	672.03	33.80
-5	8	-7	613.64	40.80
-5	8	-7	640.74	38.90
5	-8	7	666.53	34.60
5	-8	7	604.74	34.50
-6	8	-7	5.50	9.10
-6	8	-7	1.20	10.60
-6	8	-7	19.10	14.60
-6	8	-7	-4.00	7.50
-6	8	-7	11.30	13.90
6	-8	7	13.10	13.20
6	-8	7	4.10	11.30
-7	8	-7	148.99	18.70

-7	8	-7	115.99	16.20
-7	8	-7	136.79	15.90
-7	8	-7	131.09	16.00
7	-8	7	110.99	15.50
-8	8	-7	321.37	26.40
-8	8	-7	318.37	30.90
-8	8	-7	281.57	24.10
-8	8	-7	360.56	25.30
8	-8	7	294.87	22.20
-9	8	-7	37.30	13.90
-9	8	-7	21.60	11.70
-9	8	-7	22.60	12.20
9	-8	7	34.30	10.30
-10	8	-7	2.50	10.90
-10	8	-7	16.80	13.00
-10	8	-7	16.60	11.50
10	-8	7	23.80	10.60
10	-8	7	23.80	10.40
-11	8	-7	-4.70	15.20
-11	8	-7	-4.70	16.30
-11	8	-7	1.30	12.00
11	-8	7	21.90	13.90
11	7	-7	268.07	20.20
-11	-7	7	232.68	16.20
10	7	-7	25.20	11.40
-10	-7	7	35.10	12.20
-10	-7	7	25.60	7.00
9	7	-7	21.20	20.50
-9	-7	7	3.30	11.80
-9	-7	7	10.80	10.20
8	7	-7	64.59	13.60
-8	-7	7	83.29	11.70
-8	-7	7	76.59	11.80
-8	-7	7	115.69	19.40
7	7	-7	85.89	13.50
-7	-7	7	102.89	16.70
-7	-7	7	106.59	12.10
-7	-7	7	101.89	11.80
-6	-7	7	71.29	11.50
-6	-7	7	35.10	8.40
-6	-7	7	48.80	7.20
5	7	-7	730.63	37.80
5	7	-7	723.13	40.10
-5	-7	7	749.03	37.10

-5	-7	7	801.42	37.70
4	7	-7	34.40	6.30
4	7	-7	35.80	10.80
-4	-7	7	26.00	6.80
-4	-7	7	39.60	7.40
-4	-7	7	57.09	8.60
3	7	-7	-12.60	10.80
3	7	-7	-12.00	10.30
3	7	-7	-0.80	4.50
-3	-7	7	6.80	7.00
-3	-7	7	-5.80	6.10
2	7	-7	656.53	32.20
2	7	-7	656.13	31.40
-2	-7	7	677.53	32.90
-2	-7	7	633.64	35.00
-2	-7	7	670.93	32.20
1	7	-7	91.69	7.90
1	7	-7	82.49	8.50
-1	-7	7	91.79	12.60
-1	-7	7	71.49	9.40
0	7	-7	99.49	8.80
0	-7	7	99.99	9.70
0	-7	7	110.89	12.50
0	-7	7	82.69	8.50
-1	7	-7	405.16	21.60
-1	7	-7	387.66	21.30
-1	7	-7	437.66	23.90
1	-7	7	407.46	24.50
1	-7	7	380.56	21.80
1	-7	7	410.76	20.80
-2	7	-7	548.35	26.50
-2	7	-7	471.05	26.60
-2	7	-7	538.45	29.50
2	-7	7	478.35	29.70
2	-7	7	525.65	27.70
2	-7	7	527.25	25.90
-3	7	-7	812.02	40.80
-3	7	-7	782.82	42.90
-3	7	-7	804.82	39.80
3	-7	7	847.82	38.90
3	-7	7	794.42	40.10
-4	7	-7	108.69	11.00
-4	7	-7	120.99	20.70
-4	7	-7	130.59	19.60

-4	7	-7	110.29	13.70
4	-7	7	106.29	15.20
4	-7	7	106.19	11.30
-5	7	-7	342.37	31.60
-5	7	-7	388.76	27.80
-5	7	-7	381.56	23.70
-5	7	-7	378.26	26.40
-5	7	-7	398.86	22.60
5	-7	7	378.46	22.60
5	-7	7	377.56	25.00
-6	7	-7	7.00	8.40
-6	7	-7	12.80	11.50
-6	7	-7	-6.30	7.00
6	-7	7	15.50	12.00
6	-7	7	9.00	9.30
-7	7	-7	41.40	11.60
-7	7	-7	66.69	14.90
-7	7	-7	68.79	13.50
7	-7	7	27.30	13.90
7	-7	7	36.80	8.90
-8	7	-7	130.39	16.90
-8	7	-7	92.59	14.10
-8	7	-7	112.49	17.40
8	-7	7	87.69	16.30
-9	7	-7	168.18	17.70
-9	7	-7	132.99	19.00
-9	7	-7	99.69	23.00
9	-7	7	130.69	18.20
-10	7	-7	236.38	21.50
-10	7	-7	199.38	20.70
-10	7	-7	175.88	20.30
10	-7	7	248.58	21.20
-11	7	-7	38.60	13.70
-11	7	-7	36.80	15.60
-11	7	-7	58.39	13.10
11	-7	7	57.49	16.60
11	-7	7	47.50	10.60
-11	-6	7	72.99	11.30
-11	-6	7	50.59	15.90
10	6	-7	196.78	21.70
-10	-6	7	195.48	19.10
-10	-6	7	246.98	16.00
9	6	-7	83.19	28.20
9	6	-7	25.80	12.60

-9	-6	7	38.30	8.00
8	6	-7	225.18	27.30
8	6	-7	219.28	22.50
-8	-6	7	214.48	15.60
-8	-6	7	206.28	23.10
-8	-6	7	183.88	18.50
7	6	-7	295.47	23.90
7	6	-7	216.78	26.70
-7	-6	7	282.67	19.70
-7	-6	7	255.87	20.50
-7	-6	7	283.07	17.50
-6	-6	7	29.80	6.80
-6	-6	7	29.10	9.90
-6	-6	7	28.00	9.70
5	6	-7	256.67	17.50
5	6	-7	278.67	22.00
-5	-6	7	272.67	18.00
-5	-6	7	277.87	16.80
4	6	-7	143.09	17.90
4	6	-7	171.08	24.80
4	6	-7	182.38	19.00
4	6	-7	188.08	12.20
-4	-6	7	193.18	13.60
-4	-6	7	165.78	13.50
3	6	-7	16.40	11.50
3	6	-7	29.30	14.00
3	6	-7	5.00	4.10
-3	-6	7	7.90	6.30
-3	-6	7	9.80	7.20
2	6	-7	610.24	29.80
2	6	-7	554.44	27.70
2	6	-7	567.64	27.30
-2	-6	7	543.15	27.90
-2	-6	7	562.64	29.60
-2	-6	7	576.24	27.50
1	6	-7	187.48	10.80
1	6	-7	194.78	13.40
1	6	-7	166.78	11.10
-1	-6	7	183.18	11.10
-1	-6	7	193.68	14.00
-1	-6	7	164.48	11.40
0	6	-7	594.04	31.20
0	6	-7	618.04	30.40
0	6	-7	598.34	29.90

0	6	-7	668.43	31.80
0	-6	7	605.64	30.00
0	-6	7	711.73	32.50
-1	6	-7	8.90	3.50
1	-6	7	12.90	6.20
1	-6	7	10.60	4.50
1	-6	7	9.20	4.10
-2	6	-7	607.94	31.10
-2	6	-7	576.74	29.40
-2	6	-7	602.44	29.30
2	-6	7	571.54	31.20
2	-6	7	601.14	28.60
2	-6	7	586.04	29.80
-3	6	-7	428.86	25.00
-3	6	-7	467.85	27.00
-3	6	-7	484.05	24.20
3	-6	7	425.76	27.00
3	-6	7	501.25	28.20
3	-6	7	501.85	23.60
-4	6	-7	905.01	47.20
-4	6	-7	992.30	52.29
-4	6	-7	945.91	44.30
-4	6	-7	871.91	44.90
4	-6	7	951.20	43.50
4	-6	7	851.81	45.40
-5	6	-7	35.60	12.10
-5	6	-7	50.19	7.90
-5	6	-7	33.40	14.10
-5	6	-7	32.70	10.60
-5	6	-7	14.50	13.20
-5	6	-7	57.39	9.40
5	-6	7	39.80	7.00
5	-6	7	40.90	10.60
-6	6	-7	128.29	19.30
-6	6	-7	133.09	19.70
-6	6	-7	77.39	15.90
-6	6	-7	106.99	12.40
-6	6	-7	101.39	17.50
6	-6	7	98.29	13.10
-7	6	-7	-8.30	10.90
-7	6	-7	0.30	9.00
-7	6	-7	39.90	12.90
-7	6	-7	21.70	13.90
-7	6	-7	-3.90	7.00

-7	6	-7	-1.40	8.10
7	-6	7	7.10	11.00
-8	6	-7	343.07	23.70
-8	6	-7	323.07	24.90
-8	6	-7	291.17	31.60
-8	6	-7	313.47	28.50
8	-6	7	318.67	23.30
-9	6	-7	217.88	20.60
-9	6	-7	204.08	18.90
-9	6	-7	210.08	27.30
-9	6	-7	218.38	20.30
9	-6	7	228.08	20.20
-10	6	-7	32.00	11.70
-10	6	-7	42.70	12.10
-10	6	-7	24.90	11.10
10	-6	7	37.90	12.00
-11	6	-7	122.09	19.70
-11	6	-7	124.49	17.90
-11	6	-7	89.29	15.80
11	-6	7	116.59	21.30
11	-6	7	127.49	16.20
11	5	-7	7.00	11.80
-11	-5	7	18.50	7.60
-11	-5	7	47.00	14.60
10	5	-7	84.39	18.60
10	5	-7	131.69	19.10
-10	-5	7	99.19	12.40
-10	-5	7	99.09	15.80
9	5	-7	51.29	13.20
9	5	-7	36.40	16.20
-9	-5	7	53.99	8.70
-9	-5	7	46.70	10.10
8	5	-7	61.39	14.60
8	5	-7	70.79	13.80
-8	-5	7	71.69	11.60
-8	-5	7	46.10	18.20
-8	-5	7	108.79	14.90
7	5	-7	47.80	12.50
7	5	-7	79.49	13.30
-7	-5	7	64.39	9.20
-7	-5	7	53.09	10.60
-7	-5	7	36.80	7.40
6	5	-7	17.20	11.60
-6	-5	7	13.30	8.20

-6	-5	7	-3.40	8.40
5	5	-7	277.87	21.60
5	5	-7	186.28	24.90
5	5	-7	218.88	15.70
-5	-5	7	215.58	15.00
-5	-5	7	254.87	16.90
4	5	-7	206.98	12.50
4	5	-7	196.98	19.00
4	5	-7	172.78	14.80
4	5	-7	194.48	19.30
-4	-5	7	193.38	14.30
-4	-5	7	203.18	14.00
3	5	-7	319.47	19.90
3	5	-7	333.07	16.80
-3	-5	7	337.57	16.60
-3	-5	7	293.97	17.80
2	5	-7	275.87	14.10
2	5	-7	268.27	17.00
-2	-5	7	240.98	15.00
-2	-5	7	273.57	14.00
1	5	-7	714.63	32.80
1	5	-7	651.63	31.30
1	5	-7	677.33	31.80
-1	-5	7	626.74	31.70
-1	-5	7	676.63	33.20
-1	-5	7	670.83	31.70
0	5	-7	7.90	3.70
0	5	-7	8.90	3.20
0	5	-7	12.20	4.40
0	-5	7	9.80	3.40
0	-5	7	13.90	3.40
0	-5	7	13.90	5.10
-1	5	-7	45.00	5.70
-1	5	-7	47.50	6.40
-1	5	-7	41.40	5.90
1	-5	7	47.50	7.10
1	-5	7	40.10	5.80
-2	5	-7	929.81	43.60
2	-5	7	923.71	44.60
2	-5	7	914.31	42.80
-3	5	-7	6.30	6.00
-3	5	-7	13.40	4.90
-3	5	-7	8.90	4.40
3	-5	7	8.60	3.40

3	-5	7	11.90	5.40
3	-5	7	9.80	5.70
-4	5	-7	144.29	17.30
-4	5	-7	159.08	11.00
-4	5	-7	125.59	14.20
4	-5	7	139.89	9.80
-5	5	-7	61.89	9.40
-5	5	-7	46.10	7.20
-5	5	-7	38.10	10.20
-5	5	-7	42.10	10.40
-5	5	-7	95.19	21.20
5	-5	7	38.10	9.30
5	-5	7	10.40	12.60
-6	5	-7	182.98	20.20
-6	5	-7	170.48	13.30
-6	5	-7	165.48	15.20
-6	5	-7	170.08	17.90
-6	5	-7	143.59	23.30
6	-5	7	155.58	12.60
-7	5	-7	-1.70	11.40
-7	5	-7	24.30	11.60
-7	5	-7	-5.70	9.40
-7	5	-7	8.30	6.90
-7	5	-7	10.20	8.20
7	-5	7	7.90	8.20
-8	5	-7	14.70	8.00
-8	5	-7	-3.50	10.00
-8	5	-7	-3.70	13.90
-8	5	-7	16.70	9.30
-8	5	-7	28.60	13.10
8	-5	7	28.40	13.50
8	-5	7	10.40	9.20
-9	5	-7	906.21	46.10
-9	5	-7	900.41	48.60
-9	5	-7	809.02	45.30
-9	5	-7	901.81	48.90
-9	5	-7	886.31	45.70
9	-5	7	897.21	46.20
-10	5	-7	346.87	24.20
-10	5	-7	298.77	23.80
-10	5	-7	360.46	43.80
-10	5	-7	326.07	23.60
-10	5	-7	306.57	32.70
10	-5	7	379.36	26.60

-11	5	-7	34.80	12.20
-11	5	-7	35.20	11.10
-11	5	-7	4.90	14.30
11	-5	7	50.49	15.80
11	4	-7	85.79	15.00
-11	-4	7	110.89	12.00
-11	-4	7	83.89	12.10
10	4	-7	34.20	13.00
10	4	-7	57.19	15.30
-10	-4	7	65.19	10.30
-10	-4	7	87.49	12.70
9	4	-7	89.29	14.70
9	4	-7	60.19	14.30
-9	-4	7	100.19	12.50
-9	-4	7	105.09	15.10
8	4	-7	15.40	12.30
8	4	-7	26.40	11.70
-8	-4	7	15.60	7.70
-8	-4	7	9.70	6.70
7	4	-7	204.18	21.30
7	4	-7	237.58	23.10
-7	-4	7	232.08	15.80
-7	-4	7	234.58	20.20
-7	-4	7	212.38	16.80
6	4	-7	176.08	20.80
-6	-4	7	240.38	15.50
-6	-4	7	249.97	17.80
-6	-4	7	233.18	17.40
5	4	-7	288.37	33.30
5	4	-7	391.96	25.50
5	4	-7	366.96	20.30
-5	-4	7	373.36	21.60
-5	-4	7	391.56	20.50
4	4	-7	27.50	5.90
4	4	-7	23.00	8.20
4	4	-7	43.30	12.00
4	4	-7	23.50	4.80
-4	-4	7	12.50	5.30
-4	-4	7	13.30	5.80
-4	-4	7	15.60	3.90
3	4	-7	1236.28	53.39
3	4	-7	1194.88	54.59
-3	-4	7	1046.00	53.79
-3	-4	7	1171.88	53.09

2	4	-7	464.75	22.10
2	4	-7	484.25	23.40
-2	-4	7	408.56	22.70
-2	-4	7	478.35	22.10
1	4	-7	83.89	6.60
1	4	-7	81.99	7.20
1	4	-7	69.79	6.80
-1	-4	7	79.89	7.60
-1	-4	7	69.89	6.90
-1	-4	7	73.49	5.90
0	4	-7	23.90	4.20
0	4	-7	30.40	5.30
0	4	-7	22.40	4.00
0	-4	7	20.80	4.70
0	-4	7	24.90	3.90
0	-4	7	14.60	2.90
-1	4	-7	1002.90	45.90
1	-4	7	951.50	45.50
1	-4	7	1015.20	46.00
-2	4	-7	22.60	4.10
-2	4	-7	18.80	4.20
-2	4	-7	17.00	4.70
2	-4	7	21.40	3.30
2	-4	7	20.00	4.40
-3	4	-7	591.74	28.50
3	-4	7	569.24	27.40
3	-4	7	615.44	29.00
3	-4	7	536.65	29.10
-4	4	-7	570.54	30.90
-4	4	-7	587.64	29.40
-4	4	-7	601.84	28.80
4	-4	7	577.84	30.70
4	-4	7	587.94	27.70
4	-4	7	549.65	30.10
-5	4	-7	111.09	14.50
-5	4	-7	117.89	17.10
-5	4	-7	119.29	10.40
-5	4	-7	101.59	12.60
-5	4	-7	101.69	15.30
5	-4	7	114.69	8.90
-6	4	-7	20.30	6.60
-6	4	-7	6.00	9.90
-6	4	-7	14.20	9.30
-6	4	-7	9.20	11.90

-6	4	-7	12.80	5.00
-6	4	-7	15.50	11.40
6	-4	7	13.10	4.80
-7	4	-7	352.16	20.30
-7	4	-7	338.87	21.10
-7	4	-7	309.57	24.00
-7	4	-7	328.17	24.30
-7	4	-7	298.87	25.80
7	-4	7	327.47	19.60
-8	4	-7	491.45	26.50
-8	4	-7	480.35	30.90
-8	4	-7	413.76	31.50
-8	4	-7	447.16	27.10
-8	4	-7	481.05	28.50
8	-4	7	475.55	28.90
8	-4	7	457.25	28.10
-9	4	-7	43.50	10.60
-9	4	-7	32.70	9.40
-9	4	-7	50.39	13.70
-9	4	-7	48.10	10.60
-9	4	-7	33.20	14.20
9	-4	7	40.40	11.60
9	-4	7	57.69	18.50
-10	4	-7	101.29	18.60
-10	4	-7	132.69	16.50
-10	4	-7	115.49	18.60
-10	4	-7	139.79	18.90
-10	4	-7	153.48	25.60
10	-4	7	143.49	22.60
-11	4	-7	8.80	10.10
-11	4	-7	9.30	11.10
-11	4	-7	7.70	10.30
11	-4	7	1.80	10.40
11	-4	7	15.60	17.70
-12	4	-7	-20.60	15.40
12	-4	7	-8.80	9.80
11	3	-7	268.47	25.00
11	3	-7	247.28	25.50
-11	-3	7	291.77	19.80
-11	-3	7	298.47	19.20
10	3	-7	10.40	13.50
10	3	-7	9.70	12.80
-10	-3	7	24.10	8.90
-10	-3	7	16.50	7.40

9	3	-7	72.09	12.90
9	3	-7	36.10	13.20
-9	-3	7	33.70	8.50
-9	-3	7	41.90	8.00
8	3	-7	428.26	29.10
8	3	-7	464.65	31.90
-8	-3	7	489.55	26.30
-8	-3	7	498.65	26.40
7	3	-7	511.35	31.70
-7	-3	7	574.44	28.90
-7	-3	7	519.55	35.00
-7	-3	7	562.74	29.20
6	3	-7	57.09	11.30
-6	-3	7	70.89	9.10
-6	-3	7	45.70	6.70
-6	-3	7	49.10	9.60
5	3	-7	130.59	11.50
5	3	-7	128.39	18.90
-5	-3	7	135.99	11.70
4	3	-7	103.49	8.60
4	3	-7	104.39	8.90
4	3	-7	84.99	11.60
-4	-3	7	87.79	10.60
-4	-3	7	107.09	8.70
-4	-3	7	115.19	7.50
3	3	-7	15.80	4.90
3	3	-7	17.50	3.40
3	3	-7	21.10	4.40
-3	-3	7	19.70	3.70
-3	-3	7	13.30	4.40
-3	-3	7	21.60	3.20
2	3	-7	112.99	7.50
2	3	-7	107.09	8.10
-2	-3	7	95.69	8.10
-2	-3	7	112.39	6.90
1	3	-7	246.08	12.50
1	3	-7	244.68	12.70
1	3	-7	233.98	12.40
-1	-3	7	214.48	12.80
-1	-3	7	250.27	13.10
-1	-3	7	243.98	12.30
0	3	-7	360.36	17.30
0	-3	7	360.56	18.30
0	-3	7	317.27	17.10

-1	3	-7	335.07	16.50
-1	3	-7	342.07	16.30
1	-3	7	307.17	15.90
1	-3	7	303.17	16.70
1	-3	7	326.17	16.30
-2	3	-7	446.16	22.40
-2	3	-7	474.45	22.90
2	-3	7	468.15	21.80
2	-3	7	447.46	22.80
-3	3	-7	1005.50	46.30
3	-3	7	1004.80	45.40
3	-3	7	1029.90	46.70
3	-3	7	926.21	46.70
-4	3	-7	3.60	4.50
-4	3	-7	2.00	4.30
-4	3	-7	8.30	5.90
4	-3	7	3.60	5.30
4	-3	7	3.70	2.30
4	-3	7	12.30	5.50
-5	3	-7	80.39	11.80
-5	3	-7	111.29	14.50
-5	3	-7	116.79	13.60
-5	3	-7	71.99	11.70
5	-3	7	59.99	9.60
5	-3	7	88.49	7.00
-6	3	-7	4.70	4.80
-6	3	-7	-6.20	9.40
-6	3	-7	21.00	9.20
-6	3	-7	3.80	6.30
-6	3	-7	-5.90	10.60
-6	3	-7	9.00	10.50
6	-3	7	-2.00	3.70
-7	3	-7	20.30	10.50
-7	3	-7	16.50	7.10
-7	3	-7	41.10	10.40
-7	3	-7	17.80	6.00
-7	3	-7	10.40	14.20
-7	3	-7	33.50	10.10
7	-3	7	20.00	5.80
-8	3	-7	658.53	34.50
-8	3	-7	628.54	35.20
-8	3	-7	645.64	33.80
-8	3	-7	641.24	37.90
-8	3	-7	668.73	37.40

8	-3	7	644.04	36.20
8	-3	7	677.23	34.20
-9	3	-7	78.19	13.00
-9	3	-7	58.49	13.20
-9	3	-7	105.09	16.50
-9	3	-7	59.09	10.30
-9	3	-7	56.29	12.50
9	-3	7	61.39	12.30
9	-3	7	58.99	21.30
-10	3	-7	395.86	24.80
-10	3	-7	423.66	34.20
-10	3	-7	326.47	34.10
-10	3	-7	454.95	39.00
-10	3	-7	448.76	26.90
10	-3	7	408.06	31.60
-11	3	-7	-0.30	9.80
-11	3	-7	17.10	9.60
-11	3	-7	11.30	10.70
-12	3	-7	34.10	14.50
-12	3	-7	12.60	11.40
12	-3	7	6.50	11.00
11	2	-7	97.49	15.10
11	2	-7	87.79	16.40
-11	-2	7	85.59	13.70
-11	-2	7	89.29	14.20
10	2	-7	187.88	25.00
10	2	-7	193.38	22.10
-10	-2	7	221.58	17.20
-10	-2	7	224.28	16.70
9	2	-7	86.09	15.30
9	2	-7	56.39	12.40
-9	-2	7	79.69	13.20
-9	-2	7	56.99	9.10
8	2	-7	294.87	26.80
8	2	-7	303.27	23.10
-8	-2	7	278.67	19.60
-8	-2	7	332.27	19.80
7	2	-7	169.18	17.60
-7	-2	7	118.69	12.60
-7	-2	7	132.59	19.20
6	2	-7	140.89	16.80
-6	-2	7	141.39	14.10
-6	-2	7	155.58	12.50
5	2	-7	112.09	12.40

5	2	-7	106.59	10.90
-5	-2	7	103.99	11.20
4	2	-7	1058.09	45.00
4	2	-7	1009.90	45.10
4	2	-7	929.21	45.50
-4	-2	7	1024.30	44.70
-4	-2	7	827.12	45.40
-4	-2	7	982.70	45.20
3	2	-7	23.90	4.00
3	2	-7	18.10	2.90
3	2	-7	18.30	4.20
-3	-2	7	21.70	3.00
-3	-2	7	16.70	3.30
-3	-2	7	13.20	3.80
2	2	-7	1803.02	83.79
2	2	-7	1890.81	83.99
-2	-2	7	1895.71	83.99
1	2	-7	213.68	11.80
1	2	-7	193.88	10.70
1	2	-7	214.78	10.50
-1	-2	7	204.58	10.70
-1	-2	7	199.38	10.60
0	2	-7	362.16	16.60
0	2	-7	343.57	16.80
0	-2	7	344.77	16.80
0	-2	7	323.37	16.90
0	-2	7	343.27	17.50
-1	2	-7	134.39	9.10
-1	2	-7	144.89	7.80
1	-2	7	131.29	8.10
1	-2	7	119.19	7.40
-2	2	-7	187.08	10.10
-2	2	-7	184.28	10.30
2	-2	7	173.28	9.20
2	-2	7	179.68	10.30
2	-2	7	174.08	10.30
-3	2	-7	1957.10	87.69
-3	2	-7	1879.91	87.09
-3	2	-7	1929.21	87.29
3	-2	7	1911.91	87.49
3	-2	7	1936.21	87.39
3	-2	7	1910.51	86.29
-4	2	-7	672.93	31.60
-4	2	-7	685.03	31.40

-4	2	-7	638.94	32.50
4	-2	7	625.34	30.10
4	-2	7	670.13	32.00
4	-2	7	606.14	32.10
-5	2	-7	66.89	9.30
-5	2	-7	47.90	12.10
-5	2	-7	70.29	11.00
-5	2	-7	63.89	10.20
-5	2	-7	59.09	7.30
-5	2	-7	73.39	11.50
5	-2	7	70.19	5.50
5	-2	7	62.99	8.70
-6	2	-7	78.39	11.90
-6	2	-7	113.59	11.80
-6	2	-7	116.69	17.00
-6	2	-7	76.29	11.40
-6	2	-7	97.49	9.50
-6	2	-7	75.89	11.30
6	-2	7	95.29	7.60
-7	2	-7	1.00	9.60
-7	2	-7	3.50	8.60
-7	2	-7	-4.50	9.50
-7	2	-7	6.10	4.70
-7	2	-7	6.00	6.50
-7	2	-7	5.80	8.80
7	-2	7	5.00	4.50
-8	2	-7	707.23	39.00
-8	2	-7	699.83	36.50
-8	2	-7	689.23	35.80
-8	2	-7	680.03	37.50
-8	2	-7	672.33	38.30
-8	2	-7	687.83	35.00
8	-2	7	717.13	34.80
8	-2	7	612.94	37.40
-9	2	-7	121.39	20.50
-9	2	-7	116.19	17.00
-9	2	-7	153.48	19.70
-9	2	-7	128.09	14.80
-9	2	-7	135.69	14.60
9	-2	7	146.59	19.70
9	-2	7	96.99	14.60
-10	2	-7	7.30	14.50
-10	2	-7	2.60	7.80
-10	2	-7	14.40	14.80

-10	2	-7	14.20	8.70
10	-2	7	10.00	23.20
-11	2	-7	80.89	16.60
-11	2	-7	78.49	18.10
-11	2	-7	101.79	15.40
-11	2	-7	104.49	15.40
-11	2	-7	97.29	15.00
-12	2	-7	-8.20	12.50
-12	2	-7	1.10	10.70
-12	2	-7	4.30	12.20
12	-2	7	-4.50	10.90
11	1	-7	42.60	16.70
11	1	-7	56.69	13.20
-11	-1	7	47.70	10.10
10	1	-7	24.50	12.20
10	1	-7	36.70	14.40
-10	-1	7	11.70	7.90
-10	-1	7	30.70	8.80
9	1	-7	302.27	28.00
9	1	-7	307.57	23.50
-9	-1	7	287.27	19.80
-9	-1	7	314.07	20.30
8	1	-7	22.90	13.20
8	1	-7	34.40	9.90
-8	-1	7	25.80	7.50
-8	-1	7	28.70	7.50
7	1	-7	15.30	8.90
-7	-1	7	15.20	6.80
6	1	-7	117.59	15.00
-6	-1	7	83.29	13.70
-6	-1	7	88.29	11.10
5	1	-7	241.28	19.20
-5	-1	7	231.38	15.10
4	1	-7	381.86	17.80
4	1	-7	359.46	18.30
-4	-1	7	369.26	17.80
-4	-1	7	329.57	18.50
-4	-1	7	357.26	18.40
3	1	-7	17.70	3.60
3	1	-7	23.70	4.20
3	1	-7	25.00	3.20
-3	-1	7	21.30	3.80
-3	-1	7	27.90	4.30
-3	-1	7	27.70	4.00

2	1	-7	739.43	31.90
2	1	-7	677.73	32.10
2	1	-7	734.33	31.60
-2	-1	7	598.54	32.50
-2	-1	7	732.53	32.30
1	1	-7	84.49	4.50
-1	-1	7	72.99	5.20
-1	-1	7	78.19	4.50
0	1	-7	172.68	8.30
0	1	-7	170.48	8.90
0	-1	7	157.08	9.30
0	-1	7	167.18	8.90
0	-1	7	155.38	9.00
-1	1	-7	1642.84	73.29
-1	1	-7	1680.83	73.09
1	-1	7	1576.34	73.09
1	-1	7	1643.64	73.29
1	-1	7	1555.34	72.69
-2	1	-7	1199.28	54.19
2	-1	7	1160.68	53.29
2	-1	7	1195.28	54.19
-3	1	-7	491.05	24.10
-3	1	-7	542.25	25.90
-3	1	-7	497.15	24.60
3	-1	7	479.35	24.40
3	-1	7	509.05	23.10
3	-1	7	486.15	24.30
-4	1	-7	391.96	19.50
4	-1	7	370.76	19.80
4	-1	7	372.06	20.10
4	-1	7	372.56	17.90
-5	1	-7	15.20	4.20
-5	1	-7	16.60	7.00
-5	1	-7	19.80	5.80
5	-1	7	18.50	6.50
5	-1	7	27.20	6.30
-6	1	-7	44.90	6.20
-6	1	-7	46.20	9.40
-6	1	-7	39.50	9.40
-6	1	-7	54.39	7.80
-6	1	-7	20.50	9.30
-6	1	-7	55.09	11.20
-6	1	-7	35.50	9.50
6	-1	7	35.60	5.10

6	-1	7	39.80	11.20
-7	1	-7	30.10	6.80
-7	1	-7	43.30	9.40
-7	1	-7	25.90	9.30
-7	1	-7	31.00	5.40
-7	1	-7	60.09	11.30
7	-1	7	37.10	6.60
-8	1	-7	170.38	19.90
-8	1	-7	227.28	18.70
-8	1	-7	216.08	21.60
-8	1	-7	191.18	15.80
-8	1	-7	203.68	18.00
-8	1	-7	185.18	14.40
8	-1	7	190.48	14.10
-9	1	-7	895.81	45.00
-9	1	-7	893.31	46.00
-9	1	-7	909.51	44.60
-9	1	-7	883.61	47.00
9	-1	7	879.41	44.80
9	-1	7	839.42	46.90
-10	1	-7	104.19	13.10
-10	1	-7	92.19	12.40
-10	1	-7	118.99	21.30
-10	1	-7	95.19	14.30
-10	1	-7	130.69	20.30
10	-1	7	177.58	30.20
-11	1	-7	26.80	9.60
-11	1	-7	42.00	14.60
-11	1	-7	20.80	14.20
-11	1	-7	33.20	9.80
-11	1	-7	28.10	9.50
-12	1	-7	75.89	11.60
-12	1	-7	78.29	12.80
-12	1	-7	85.89	14.90
12	-1	7	63.29	13.60
11	0	-7	47.30	12.70
11	0	-7	18.40	17.00
-11	0	7	36.70	9.10
-11	0	7	49.99	10.80
10	0	-7	37.10	15.70
10	0	-7	5.10	11.90
-10	0	7	3.80	7.90
-10	0	7	18.90	8.80
9	0	-7	7.70	10.10

9	0	-7	-2.20	13.80
-9	0	7	2.10	7.30
-9	0	7	18.90	7.50
8	0	-7	1.50	13.30
8	0	-7	2.20	9.40
-8	0	7	10.10	6.90
-8	0	7	10.80	6.80
7	0	-7	338.87	23.60
-7	0	7	374.96	28.60
-7	0	7	375.06	21.60
6	0	-7	318.77	21.30
-6	0	7	303.27	20.00
-6	0	7	326.67	18.90
5	0	-7	58.29	8.90
-5	0	7	46.60	7.20
-5	0	7	83.39	9.60
-5	0	7	55.09	8.40
-5	0	7	63.39	7.60
4	0	-7	329.67	15.60
4	0	-7	316.77	15.80
-4	0	7	310.57	18.70
-4	0	7	310.97	16.40
-4	0	7	295.77	17.70
3	0	-7	3342.77	161.38
3	0	-7	3718.83	161.18
-3	0	7	3722.43	161.98
-3	0	7	3689.03	162.18
-2	0	7	305.17	14.30
-2	0	7	296.57	14.40
-2	0	7	264.07	14.50
-1	0	7	118.79	6.80
0	0	-7	492.65	22.20
0	0	7	514.85	23.00
0	0	7	474.85	22.60
0	0	7	463.45	22.80
-1	0	-7	8.90	1.90
-1	0	-7	10.80	1.90
1	0	7	12.40	2.10
1	0	7	14.60	2.60
1	0	7	13.20	2.10
-2	0	-7	185.48	9.60
-2	0	-7	167.88	9.50
2	0	7	184.48	9.70
2	0	7	173.78	8.50

2	0	7	175.68	9.50
-3	0	-7	74.89	5.60
-3	0	-7	67.09	6.20
3	0	7	65.19	4.00
3	0	7	64.19	5.80
3	0	7	63.99	5.90
-4	0	-7	2519.75	111.79
4	0	7	2433.16	111.99
4	0	7	2464.85	112.49
4	0	7	2487.45	110.79
-5	0	-7	247.48	14.40
-5	0	-7	229.08	16.40
-5	0	-7	270.77	13.80
5	0	7	226.38	15.90
5	0	7	233.88	15.30
5	0	7	239.78	12.20
-6	0	-7	57.19	9.20
-6	0	-7	46.60	8.10
-6	0	-7	39.60	10.20
-6	0	-7	45.70	7.10
-6	0	-7	40.50	9.90
-6	0	-7	40.30	7.30
-6	0	-7	49.10	9.60
6	0	7	28.00	9.80
6	0	7	38.80	4.70
-7	0	-7	454.55	26.30
-7	0	-7	409.56	28.30
-7	0	-7	445.66	25.90
-7	0	-7	429.76	27.50
-7	0	-7	459.65	23.60
-7	0	-7	463.55	24.50
7	0	7	435.26	22.20
-8	0	-7	165.38	14.30
-8	0	-7	156.68	18.50
-8	0	-7	162.98	16.40
-8	0	-7	170.68	19.90
-8	0	-7	179.38	17.20
-8	0	-7	179.78	13.30
8	0	7	152.88	12.30
-9	0	-7	208.88	19.10
-9	0	-7	205.98	16.10
-9	0	-7	171.28	20.50
-9	0	-7	196.88	17.70
-9	0	-7	193.58	15.40

9	0	7	197.08	25.00
9	0	7	205.88	17.10
-10	0	-7	88.19	14.40
-10	0	-7	74.89	11.20
-10	0	-7	90.99	12.70
-10	0	-7	69.69	10.40
-10	0	-7	80.89	12.40
10	0	7	85.29	16.90
-11	0	-7	30.70	9.40
-11	0	-7	30.80	9.50
-11	0	-7	39.70	13.90
-11	0	-7	42.00	9.60
-11	0	-7	13.30	14.20
-12	0	-7	28.50	11.20
-12	0	-7	4.40	9.30
-12	0	-7	1.80	8.90
12	0	7	-12.10	12.00
11	-1	-7	173.88	28.20
11	-1	-7	147.29	18.80
-11	1	7	155.28	18.10
-11	1	7	131.69	14.50
10	-1	-7	111.99	18.00
10	-1	-7	103.19	19.00
-10	1	7	108.89	15.10
-10	1	7	106.29	15.90
9	-1	-7	-12.90	14.70
9	-1	-7	3.30	9.60
-9	1	7	0.00	7.10
-9	1	7	3.40	7.90
8	-1	-7	254.87	21.60
8	-1	-7	310.87	28.00
-8	1	7	287.37	19.40
-8	1	7	286.57	19.50
7	-1	-7	195.58	18.20
-7	1	7	215.68	16.10
6	-1	-7	83.49	13.70
-6	1	7	93.79	11.50
-6	1	7	95.89	10.40
5	-1	-7	46.70	9.20
-5	1	7	41.60	7.90
-5	1	7	48.10	6.80
-5	1	7	40.60	6.10
4	-1	-7	113.19	7.20
4	-1	-7	97.99	7.20

-4	1	7	115.69	8.70
-4	1	7	111.39	8.00
-4	1	7	103.39	7.70
3	-1	-7	7.80	2.00
-3	1	7	11.80	2.70
-3	1	7	11.30	2.90
-3	1	7	13.90	2.80
-2	1	7	176.08	9.50
-2	1	7	183.78	9.50
-2	1	7	176.48	9.60
0	-1	-7	21.30	1.70
0	1	7	18.60	2.80
0	1	7	17.80	3.00
0	1	7	18.50	2.20
-1	-1	-7	55.59	3.70
-1	-1	-7	55.99	4.20
1	1	7	56.49	3.30
1	1	7	58.79	4.30
1	1	7	55.09	3.70
-2	-1	-7	4755.12	212.68
-2	-1	-7	4905.41	212.68
2	1	7	4700.83	211.68
2	1	7	4810.22	212.78
2	1	7	4644.64	212.58
-3	-1	-7	1551.94	67.29
-3	-1	-7	1393.26	68.49
3	1	7	1496.25	67.39
-4	-1	-7	32.30	4.90
4	1	7	37.20	5.90
4	1	7	34.20	6.00
-5	-1	-7	66.79	7.60
-5	-1	-7	61.49	10.00
-5	-1	-7	53.99	6.50
5	1	7	61.59	9.30
5	1	7	66.09	9.00
-6	-1	-7	37.90	7.50
-6	-1	-7	50.09	11.10
-6	-1	-7	55.89	10.60
-6	-1	-7	55.09	9.60
-6	-1	-7	39.00	7.00
-6	-1	-7	60.19	8.30
-6	-1	-7	57.49	8.30
6	1	7	38.90	4.60
6	1	7	52.89	12.80

-7	-1	-7	-1.40	7.40
-7	-1	-7	20.40	9.60
-7	-1	-7	2.50	4.10
-7	-1	-7	-1.10	7.50
-7	-1	-7	6.90	9.30
-7	-1	-7	0.90	5.20
7	1	7	7.50	3.40
-8	-1	-7	237.98	16.60
-8	-1	-7	220.18	22.40
-8	-1	-7	243.28	20.60
-8	-1	-7	221.48	17.50
-8	-1	-7	225.88	18.80
8	1	7	226.08	14.30
-9	-1	-7	56.59	10.40
-9	-1	-7	52.49	9.80
-9	-1	-7	51.29	11.90
-9	-1	-7	61.09	10.50
-9	-1	-7	66.09	9.50
9	1	7	82.79	12.70
-10	-1	-7	440.76	28.70
-10	-1	-7	460.55	25.80
-10	-1	-7	486.95	26.90
-10	-1	-7	404.86	29.00
-10	-1	-7	453.25	25.30
10	1	7	459.75	31.40
-11	-1	-7	44.70	14.20
-11	-1	-7	34.10	9.50
-11	-1	-7	57.89	12.90
-11	-1	-7	38.30	9.30
-11	-1	-7	20.30	9.00
-12	-1	-7	-7.50	8.40
-12	-1	-7	-8.30	9.30
-12	-1	-7	-5.80	8.90
-12	-1	-7	-14.40	28.40
12	1	7	4.40	12.20
11	-2	-7	71.79	23.20
-11	2	7	58.39	9.40
-11	2	7	76.39	12.80
10	-2	-7	152.88	21.60
10	-2	-7	152.88	19.40
-10	2	7	150.78	17.80
-10	2	7	183.18	16.40
9	-2	-7	25.40	17.00
9	-2	-7	29.30	9.90

-9	2	7	35.20	9.10
-9	2	7	48.00	8.80
8	-2	-7	268.97	28.70
8	-2	-7	277.57	20.40
-8	2	7	276.87	19.10
-8	2	7	249.68	19.20
7	-2	-7	8.60	7.70
-7	2	7	7.80	6.80
6	-2	-7	291.77	23.90
6	-2	-7	359.26	21.50
-6	2	7	342.87	20.00
-6	2	7	351.16	20.70
5	-2	-7	15.20	8.10
-5	2	7	0.50	4.60
-5	2	7	11.10	7.40
4	-2	-7	368.76	17.30
4	-2	-7	360.86	17.70
-4	2	7	344.47	17.90
-4	2	7	357.46	18.80
-3	2	7	2102.79	97.19
-3	2	7	2220.78	97.69
-2	2	7	962.60	42.90
-2	2	7	900.91	42.40
-1	2	7	18.30	2.90
-1	2	7	15.30	2.40
-1	2	7	18.60	3.00
0	2	7	1079.69	44.80
0	2	7	944.81	45.10
0	2	7	1015.50	45.70
0	2	7	961.70	44.80
1	2	7	20.20	2.30
1	2	7	23.30	3.00
-2	-2	-7	46.30	3.90
-2	-2	-7	44.90	4.00
2	2	7	46.50	3.90
2	2	7	48.50	4.20
-3	-2	-7	4293.37	186.18
3	2	7	4036.70	186.28
-4	-2	-7	211.58	10.80
4	2	7	191.98	9.50
4	2	7	186.78	11.70
4	2	7	191.08	11.30
-5	-2	-7	15.30	4.10
-5	-2	-7	18.60	4.10

5	2	7	13.40	2.20
5	2	7	12.50	5.30
5	2	7	11.20	4.90
-6	-2	-7	13.40	7.40
-6	-2	-7	15.90	3.90
-6	-2	-7	16.70	8.00
-6	-2	-7	2.60	7.90
-6	-2	-7	10.40	5.00
-6	-2	-7	31.30	10.70
-7	-2	-7	79.99	10.00
-7	-2	-7	82.89	11.80
-7	-2	-7	91.29	17.10
-7	-2	-7	78.99	10.60
-7	-2	-7	83.99	10.00
7	2	7	80.19	6.60
-8	-2	-7	703.83	35.50
-8	-2	-7	644.84	38.60
-8	-2	-7	701.03	34.40
-8	-2	-7	682.43	37.70
-8	-2	-7	705.73	35.90
-8	-2	-7	679.03	34.80
8	2	7	694.03	33.50
-9	-2	-7	8.30	10.00
-9	-2	-7	13.30	7.90
-9	-2	-7	5.10	7.10
-9	-2	-7	10.40	9.00
-9	-2	-7	11.60	6.90
9	2	7	4.00	6.90
-10	-2	-7	18.00	10.60
-10	-2	-7	10.00	6.80
-10	-2	-7	20.70	8.50
-10	-2	-7	18.00	9.90
-10	-2	-7	9.20	7.40
10	2	7	21.00	12.40
-11	-2	-7	50.69	14.00
-11	-2	-7	56.79	10.00
-11	-2	-7	54.79	9.80
-11	-2	-7	71.39	10.40
-11	-2	-7	54.69	12.00
-12	-2	-7	28.80	9.10
-12	-2	-7	28.90	10.80
-12	-2	-7	49.00	15.00
-12	-2	-7	26.30	9.40
12	2	7	39.00	12.40

11	-3	-7	141.29	18.20
-11	3	7	135.49	20.50
-11	3	7	124.79	14.20
10	-3	-7	12.40	10.10
10	-3	-7	-4.00	20.00
-10	3	7	9.20	8.00
-10	3	7	11.70	10.40
9	-3	-7	319.67	33.70
9	-3	-7	395.36	24.60
-9	3	7	375.56	22.50
-9	3	7	335.97	24.10
8	-3	-7	123.69	18.50
8	-3	-7	105.99	16.80
-8	3	7	114.39	15.60
-8	3	7	111.09	14.30
7	-3	-7	25.90	10.20
7	-3	-7	34.00	9.20
-7	3	7	22.80	6.80
-7	3	7	24.10	7.70
6	-3	-7	-4.10	8.90
6	-3	-7	22.20	7.40
-6	3	7	15.40	7.40
5	-3	-7	214.98	17.00
-5	3	7	241.58	17.30
-5	3	7	200.98	16.80
-5	3	7	219.98	13.20
4	-3	-7	454.45	22.10
-4	3	7	445.86	23.00
3	-3	-7	10.30	2.70
-3	3	7	7.30	2.90
-3	3	7	7.50	2.20
-2	3	7	668.03	32.60
-2	3	7	689.23	31.20
-1	3	7	329.47	15.40
-1	3	7	297.87	14.80
0	3	7	19.70	2.40
0	3	7	21.10	2.90
0	3	7	18.40	1.70
0	3	7	23.30	3.00
1	3	7	976.00	44.80
1	3	7	987.10	44.40
2	3	7	117.39	6.80
2	3	7	118.79	6.90
3	3	7	1411.36	64.09

4	3	7	103.09	8.20
4	3	7	106.19	7.20
4	3	7	114.99	5.60
4	3	7	111.79	7.90
-5	-3	-7	2.10	3.00
5	3	7	7.00	5.00
5	3	7	7.30	1.90
5	3	7	10.60	4.70
-6	-3	-7	-3.50	3.60
-6	-3	-7	-2.00	6.30
-6	-3	-7	3.80	8.00
-6	-3	-7	7.80	7.20
-6	-3	-7	9.20	5.80
-6	-3	-7	-1.90	9.50
6	3	7	1.90	2.60
-7	-3	-7	173.58	19.50
-7	-3	-7	152.78	15.00
-7	-3	-7	171.38	12.70
-7	-3	-7	149.69	14.40
-7	-3	-7	159.18	16.10
-8	-3	-7	589.24	30.40
-8	-3	-7	602.04	30.90
-8	-3	-7	554.64	35.50
-8	-3	-7	587.04	33.70
-8	-3	-7	623.74	31.80
-8	-3	-7	614.74	32.20
8	3	7	604.74	29.50
-9	-3	-7	737.63	41.10
-9	-3	-7	796.22	39.20
-9	-3	-7	764.22	39.40
-9	-3	-7	755.52	38.20
-9	-3	-7	754.32	38.00
9	3	7	738.83	37.80
-10	-3	-7	24.30	9.10
-10	-3	-7	22.30	7.70
-10	-3	-7	24.10	10.80
-10	-3	-7	9.40	6.70
-10	-3	-7	12.30	8.40
10	3	7	26.60	11.70
-11	-3	-7	4.90	10.70
-11	-3	-7	6.70	7.70
-11	-3	-7	5.80	12.50
-11	-3	-7	6.40	8.50
-11	-3	-7	2.30	7.90

-12	-3	-7	73.29	11.20
-12	-3	-7	92.49	15.00
-12	-3	-7	77.59	13.50
12	3	7	62.59	13.60
11	-4	-7	5.80	10.60
-11	4	7	15.30	8.10
-11	4	7	21.80	13.90
10	-4	-7	49.40	11.00
-10	4	7	29.90	9.30
-10	4	7	35.50	12.10
9	-4	-7	68.39	20.70
9	-4	-7	81.79	12.10
-9	4	7	92.59	13.30
-9	4	7	64.19	11.50
8	-4	-7	7.60	18.80
8	-4	-7	17.00	10.90
8	-4	-7	2.20	8.80
-8	4	7	17.80	9.00
-8	4	7	0.00	7.30
7	-4	-7	181.28	30.40
7	-4	-7	191.98	16.80
-7	4	7	189.98	16.20
-7	4	7	199.28	16.40
6	-4	-7	110.99	14.30
6	-4	-7	99.49	11.10
-6	4	7	123.69	13.00
5	-4	-7	62.79	9.50
5	-4	-7	67.09	12.70
-5	4	7	78.19	9.80
-5	4	7	69.19	10.20
-5	4	7	58.49	7.80
4	-4	-7	1170.08	57.19
-4	4	7	1263.57	58.09
-4	4	7	1297.97	56.89
3	-4	-7	2951.90	143.49
-3	4	7	3419.96	144.59
-3	4	7	3260.37	143.69
-2	4	7	98.19	5.80
-2	4	7	97.69	6.70
-2	4	7	108.69	5.10
-1	4	7	46.30	3.30
-1	4	7	44.20	4.00
0	4	7	26.80	3.60
0	4	7	24.70	3.00

0	4	7	26.40	2.20
1	4	7	27.20	2.70
1	4	7	25.40	3.30
1	4	7	28.80	3.20
1	4	7	23.50	3.10
2	4	7	64.79	5.00
2	4	7	69.59	4.90
3	4	7	191.08	10.60
3	4	7	181.88	10.10
4	4	7	2607.04	122.89
4	4	7	2862.41	123.99
-5	-4	-7	138.79	9.00
5	4	7	122.49	9.80
5	4	7	134.59	7.10
5	4	7	131.89	11.20
-6	-4	-7	19.70	4.70
-6	-4	-7	15.70	7.60
-6	-4	-7	17.50	6.20
-6	-4	-7	23.10	7.60
-6	-4	-7	26.90	6.30
6	4	7	18.60	3.50
-7	-4	-7	360.06	19.00
-7	-4	-7	341.97	20.80
-7	-4	-7	326.57	22.50
-7	-4	-7	351.36	19.70
-7	-4	-7	323.67	25.70
-7	-4	-7	353.96	20.70
7	4	7	346.17	17.60
-8	-4	-7	4.40	7.20
-8	-4	-7	-0.10	4.90
-8	-4	-7	10.60	5.80
-8	-4	-7	13.30	7.00
-8	-4	-7	8.10	10.40
-8	-4	-7	14.80	8.90
-9	-4	-7	158.58	15.90
-9	-4	-7	167.78	14.60
-9	-4	-7	158.08	16.60
-9	-4	-7	167.48	17.80
-9	-4	-7	150.88	13.40
9	4	7	157.68	13.70
-10	-4	-7	196.98	16.30
-10	-4	-7	218.48	20.10
-10	-4	-7	213.48	18.80
-10	-4	-7	177.08	16.90

-10	-4	-7	188.68	15.10
10	4	7	184.58	20.20
-11	-4	-7	20.10	12.10
-11	-4	-7	8.00	8.30
-11	-4	-7	23.40	10.40
-11	-4	-7	14.30	7.70
-11	-4	-7	14.40	8.20
-12	-4	-7	59.79	10.10
-12	-4	-7	61.09	13.40
-12	-4	-7	57.09	9.40
-12	-4	-7	52.29	10.30
12	4	7	63.29	13.70
11	-5	-7	50.19	11.70
-11	5	7	82.59	13.40
-11	5	7	67.59	16.80
10	-5	-7	178.28	19.30
-10	5	7	161.88	21.30
-10	5	7	141.59	16.50
9	-5	-7	261.07	25.30
9	-5	-7	242.28	20.10
-9	5	7	236.88	18.80
-9	5	7	202.28	21.40
8	-5	-7	605.84	34.40
8	-5	-7	622.64	34.10
-8	5	7	639.74	33.50
-8	5	7	610.44	34.40
7	-5	-7	46.40	9.70
7	-5	-7	54.89	10.00
-7	5	7	63.29	9.00
6	-5	-7	28.10	8.10
6	-5	-7	22.40	7.80
-6	5	7	47.40	12.50
-6	5	7	26.50	7.20
5	-5	-7	72.09	13.30
5	-5	-7	63.99	9.40
-5	5	7	107.69	12.60
-5	5	7	96.89	15.20
-5	5	7	81.89	8.10
4	-5	-7	29.80	6.80
-4	5	7	31.70	4.50
-4	5	7	26.70	4.40
-4	5	7	24.50	6.20
3	-5	-7	452.95	24.00
-3	5	7	506.25	24.60

-3	5	7	511.45	23.20
-3	5	7	517.55	23.50
2	-5	-7	711.43	33.00
-2	5	7	716.73	33.90
-2	5	7	721.83	32.80
-2	5	7	735.63	33.00
-1	5	7	34.70	3.50
-1	5	7	34.20	3.80
-1	5	7	39.60	2.90
0	5	7	104.79	6.20
0	5	7	94.99	7.10
1	5	7	36.60	3.30
1	5	7	34.10	4.00
1	5	7	34.50	3.40
2	5	7	2779.12	124.89
2	5	7	2714.73	125.19
2	5	7	2915.51	125.29
2	5	7	2741.83	125.09
3	5	7	359.66	16.50
3	5	7	333.57	17.20
3	5	7	319.67	16.70
-4	-5	-7	1.30	4.60
4	5	7	4.70	3.50
4	5	7	6.00	3.30
-5	-5	-7	83.99	7.30
5	5	7	66.69	8.00
5	5	7	86.39	5.00
5	5	7	74.19	6.10
5	5	7	62.09	10.00
-6	-5	-7	83.89	13.00
-6	-5	-7	103.89	8.50
-6	-5	-7	108.89	14.60
-6	-5	-7	90.49	12.20
-6	-5	-7	84.09	10.40
-6	-5	-7	85.59	11.60
6	5	7	104.49	6.70
-7	-5	-7	219.98	14.60
-7	-5	-7	239.58	16.00
-7	-5	-7	219.78	18.10
-7	-5	-7	229.38	22.10
-7	-5	-7	210.38	16.10
-7	-5	-7	207.88	13.60
7	5	7	217.18	12.40
-8	-5	-7	5.80	5.40

-8	-5	-7	15.90	6.40
-8	-5	-7	3.60	7.30
-8	-5	-7	4.30	6.90
-8	-5	-7	16.00	8.70
-9	-5	-7	302.17	19.70
-9	-5	-7	285.57	18.40
-9	-5	-7	287.57	22.10
-9	-5	-7	278.57	17.70
-9	-5	-7	271.97	20.20
9	5	7	259.07	17.90
-10	-5	-7	469.75	28.50
-10	-5	-7	451.75	25.40
-10	-5	-7	423.36	25.80
-10	-5	-7	458.05	24.80
-10	-5	-7	459.15	27.20
10	5	7	436.16	28.20
-11	-5	-7	10.70	7.70
-11	-5	-7	-3.60	8.00
-11	-5	-7	22.20	11.80
-11	-5	-7	10.20	8.10
-11	-5	-7	2.50	9.80
11	5	7	5.30	11.80
-12	-5	-7	60.89	12.70
-12	-5	-7	95.49	13.30
-12	-5	-7	69.69	12.00
-12	-5	-7	72.39	12.80
12	5	7	92.89	15.10
11	-6	-7	61.59	12.10
-11	6	7	81.09	13.40
-11	6	7	36.00	28.00
10	-6	-7	174.38	18.50
-10	6	7	179.68	17.00
-10	6	7	181.48	22.80
9	-6	-7	20.20	9.90
9	-6	-7	38.10	12.60
-9	6	7	1.10	11.60
-9	6	7	17.20	8.40
8	-6	-7	316.87	22.40
8	-6	-7	334.57	23.20
-8	6	7	322.87	24.50
-8	6	7	312.47	21.60
7	-6	-7	6.20	8.20
7	-6	-7	-12.70	7.70
-7	6	7	6.30	7.80

6	-6	-7	51.99	9.40
6	-6	-7	67.39	12.20
-6	6	7	66.59	11.60
-6	6	7	39.80	9.20
5	-6	-7	215.78	26.50
5	-6	-7	137.99	13.80
5	-6	-7	139.99	15.50
-5	6	7	149.79	10.70
-5	6	7	150.48	14.00
4	-6	-7	422.96	24.20
4	-6	-7	364.26	23.10
4	-6	-7	420.46	26.10
-4	6	7	421.16	20.80
-4	6	7	390.66	22.60
-4	6	7	420.76	20.00
3	-6	-7	1485.45	68.39
-3	6	7	1490.95	67.69
-3	6	7	1555.94	67.69
-3	6	7	1449.85	68.89
2	-6	-7	1094.79	50.39
-2	6	7	1068.99	50.19
-2	6	7	1135.49	50.09
-2	6	7	1123.69	51.19
1	-6	-7	6.10	2.20
-1	6	7	8.50	1.40
-1	6	7	3.10	2.10
-1	6	7	7.00	3.00
0	6	7	77.79	6.40
0	6	7	73.79	7.40
1	6	7	55.79	4.50
1	6	7	56.39	5.40
2	6	7	274.17	14.20
2	6	7	284.57	14.60
2	6	7	270.87	14.30
2	6	7	284.67	13.80
-3	-6	-7	2107.79	97.59
3	6	7	2207.08	97.29
3	6	7	2141.99	98.19
3	6	7	2076.09	97.49
3	6	7	2284.97	98.39
4	6	7	92.69	7.60
4	6	7	124.49	10.60
4	6	7	92.69	9.00
4	6	7	102.49	6.40

-5	-6	-7	535.85	28.50
-5	-6	-7	572.14	27.00
-5	-6	-7	516.95	28.50
5	6	7	518.65	26.50
5	6	7	572.44	25.50
5	6	7	607.64	31.10
5	6	7	548.05	25.80
-6	-6	-7	43.60	7.60
-6	-6	-7	31.20	8.10
-6	-6	-7	49.10	11.90
-6	-6	-7	48.80	6.20
-6	-6	-7	45.30	7.20
-6	-6	-7	36.20	7.40
6	6	7	42.90	5.00
-7	-6	-7	76.29	9.10
-7	-6	-7	107.49	11.20
-7	-6	-7	91.99	12.20
-7	-6	-7	109.99	14.30
-7	-6	-7	77.39	12.00
-7	-6	-7	91.69	14.30
7	6	7	93.09	7.50
-8	-6	-7	505.85	29.50
-8	-6	-7	505.35	28.10
-8	-6	-7	538.85	26.90
-8	-6	-7	500.65	27.90
-8	-6	-7	507.25	27.30
8	6	7	529.35	26.10
-9	-6	-7	6.80	7.70
-9	-6	-7	1.20	9.40
-9	-6	-7	-7.20	7.10
-9	-6	-7	8.90	7.90
-9	-6	-7	9.90	6.90
-10	-6	-7	7.80	7.50
-10	-6	-7	3.50	8.00
-10	-6	-7	6.80	9.90
-10	-6	-7	9.70	8.30
-10	-6	-7	6.00	6.80
10	6	7	-2.40	10.80
-11	-6	-7	240.78	18.70
-11	-6	-7	249.88	17.10
-11	-6	-7	230.48	18.00
-11	-6	-7	197.68	16.50
11	6	7	187.18	22.70
-12	-6	-7	40.60	8.90

-12	-6	-7	62.09	10.80
-12	-6	-7	42.50	11.20
-12	-6	-7	27.60	9.70
12	6	7	47.00	16.80
11	-7	-7	105.59	17.60
-11	7	7	119.99	21.60
-11	7	7	110.99	14.30
10	-7	-7	148.69	25.70
10	-7	-7	204.38	18.30
-10	7	7	142.39	18.20
-10	7	7	172.68	16.60
9	-7	-7	493.65	29.10
-9	7	7	419.36	33.40
-9	7	7	475.45	28.20
8	-7	-7	3.60	8.80
8	-7	-7	9.50	9.30
-8	7	7	15.40	9.10
-8	7	7	22.90	11.10
7	-7	-7	200.28	18.60
7	-7	-7	205.58	18.00
-7	7	7	249.48	18.30
6	-7	-7	14.60	7.60
6	-7	-7	4.30	10.90
-6	7	7	-13.00	9.80
-6	7	7	-4.40	7.80
5	-7	-7	346.07	29.60
5	-7	-7	329.97	19.70
5	-7	-7	309.77	25.60
5	-7	-7	316.37	21.30
-5	7	7	327.07	17.90
-5	7	7	320.97	20.30
4	-7	-7	340.77	20.00
4	-7	-7	309.37	23.20
4	-7	-7	331.27	21.40
4	-7	-7	322.67	22.00
-4	7	7	356.56	17.00
3	-7	-7	49.80	7.10
-3	7	7	43.20	6.20
-3	7	7	49.80	4.30
-3	7	7	49.80	6.70
2	-7	-7	2247.28	97.49
2	-7	-7	2035.10	97.89
-2	7	7	2296.37	96.79
-2	7	7	2051.79	97.29

-2	7	7	2149.08	97.99
1	-7	-7	11.00	3.00
-1	7	7	3.80	2.50
-1	7	7	7.70	4.20
-1	7	7	8.00	1.60
0	7	7	113.59	9.90
0	7	7	129.29	7.50
0	7	7	113.79	5.70
1	7	7	464.05	23.10
2	7	7	1.80	2.00
2	7	7	4.10	3.20
2	7	7	4.60	2.60
-3	-7	-7	2467.75	115.59
3	7	7	2430.46	114.99
3	7	7	2608.24	115.99
3	7	7	2650.03	115.99
3	7	7	2621.04	114.69
-4	-7	-7	126.29	8.30
4	7	7	102.69	8.50
4	7	7	97.19	9.90
4	7	7	110.09	6.90
4	7	7	124.09	11.10
-5	-7	-7	37.20	7.60
-5	-7	-7	31.30	7.20
-5	-7	-7	31.30	7.80
-5	-7	-7	38.60	12.60
-5	-7	-7	49.30	6.40
5	7	7	30.50	4.00
-6	-7	-7	243.78	14.80
-6	-7	-7	224.18	15.40
-6	-7	-7	238.88	13.50
-6	-7	-7	217.48	17.90
-6	-7	-7	208.18	18.20
-6	-7	-7	210.78	15.70
-6	-7	-7	187.58	23.40
6	7	7	225.78	12.00
-7	-7	-7	259.07	17.40
-7	-7	-7	250.37	20.20
-7	-7	-7	270.67	15.40
-7	-7	-7	247.98	18.20
-7	-7	-7	254.27	26.70
-7	-7	-7	260.97	17.80
-7	-7	-7	264.87	19.70
7	7	7	258.57	14.40

-8	-7	-7	416.56	23.30
-8	-7	-7	403.36	25.60
-8	-7	-7	402.76	23.40
-8	-7	-7	396.76	21.90
-8	-7	-7	392.26	26.20
-8	-7	-7	391.66	22.70
8	7	7	392.76	21.40
-9	-7	-7	615.94	32.40
-9	-7	-7	616.94	32.60
-9	-7	-7	617.64	33.90
-9	-7	-7	584.74	34.50
-9	-7	-7	608.34	31.80
-10	-7	-7	47.40	10.70
-10	-7	-7	78.99	14.20
-10	-7	-7	67.69	10.90
-10	-7	-7	60.29	9.70
-10	-7	-7	50.99	8.50
10	7	7	64.19	12.40
-11	-7	-7	53.09	9.80
-11	-7	-7	51.39	8.60
-11	-7	-7	50.89	8.50
-11	-7	-7	54.39	9.20
11	7	7	33.50	14.30
-12	-7	-7	92.39	12.30
-12	-7	-7	88.99	13.00
-12	-7	-7	106.99	16.00
-12	-7	-7	57.39	10.70
11	-8	-7	27.50	10.70
-11	8	7	26.90	9.30
10	-8	-7	90.19	15.40
-10	8	7	67.69	10.80
-10	8	7	72.29	17.00
9	-8	-7	54.79	12.70
-9	8	7	34.30	17.10
8	-8	-7	47.90	9.90
-8	8	7	36.80	9.70
-8	8	7	41.10	12.40
7	-8	-7	21.80	8.10
7	-8	-7	21.60	9.30
-7	8	7	27.60	8.70
6	-8	-7	9.10	8.30
6	-8	-7	18.60	6.90
-6	8	7	31.60	8.30
-6	8	7	24.40	8.90

5	-8	-7	2.40	8.90
5	-8	-7	0.10	6.90
5	-8	-7	9.60	14.30
5	-8	-7	-1.50	10.80
-5	8	7	10.30	7.40
-5	8	7	7.80	5.20
4	-8	-7	2183.88	103.19
4	-8	-7	2215.78	103.09
4	-8	-7	2228.38	102.89
4	-8	-7	2188.78	101.99
4	-8	-7	2210.98	105.49
-4	8	7	2390.66	101.19
3	-8	-7	490.05	27.00
-3	8	7	495.55	27.10
-3	8	7	568.74	24.70
2	-8	-7	639.04	30.30
-2	8	7	603.54	30.30
-2	8	7	589.94	30.30
-2	8	7	642.34	28.70
1	-8	-7	27.30	4.50
-1	8	7	29.10	2.70
-1	8	7	15.60	6.10
-1	8	7	34.00	5.30
0	-8	-7	13.40	3.80
0	8	7	11.00	5.60
0	8	7	13.10	1.90
0	8	7	8.20	4.10
0	8	7	8.90	2.80
1	8	7	18.80	3.30
-2	-8	-7	80.69	8.40
2	8	7	79.09	8.50
3	8	7	525.15	24.40
3	8	7	540.15	26.40
3	8	7	521.85	26.20
3	8	7	466.95	24.80
-4	-8	-7	1324.17	58.49
4	8	7	1284.47	60.39
4	8	7	1165.18	58.39
4	8	7	1276.67	57.79
4	8	7	1316.07	60.59
-5	-8	-7	5.80	10.00
-5	-8	-7	14.40	6.10
-5	-8	-7	12.70	5.80
-5	-8	-7	7.30	6.40

-5	-8	-7	17.60	3.90
5	8	7	11.50	2.80
-6	-8	-7	154.28	13.60
-6	-8	-7	174.78	16.90
-6	-8	-7	177.08	11.70
-6	-8	-7	223.18	27.00
-6	-8	-7	166.48	13.80
-6	-8	-7	175.88	15.70
-6	-8	-7	188.28	13.00
6	8	7	179.58	10.40
-7	-8	-7	437.26	22.50
-7	-8	-7	408.16	25.10
-7	-8	-7	426.06	24.10
-7	-8	-7	416.16	23.90
7	8	7	431.16	21.60
-8	-8	-7	-1.20	6.10
-8	-8	-7	5.20	8.60
-8	-8	-7	2.10	10.00
-8	-8	-7	0.50	6.40
-8	-8	-7	2.90	6.80
-8	-8	-7	10.00	5.30
8	8	7	8.70	5.60
-9	-8	-7	1.50	7.60
-9	-8	-7	11.00	6.70
-9	-8	-7	26.10	11.70
-9	-8	-7	9.10	6.90
-9	-8	-7	12.10	7.70
-9	-8	-7	4.70	8.70
9	8	7	7.00	8.10
-10	-8	-7	258.17	17.40
-10	-8	-7	261.67	18.30
-10	-8	-7	282.37	20.10
-10	-8	-7	288.17	22.80
-10	-8	-7	298.37	20.20
10	8	7	247.78	22.80
-11	-8	-7	26.60	8.10
-11	-8	-7	13.20	8.90
-11	-8	-7	30.30	7.90
-11	-8	-7	13.20	8.00
11	8	7	49.40	19.50
-12	-8	-7	174.98	16.90
-12	-8	-7	163.08	14.10
-12	-8	-7	145.39	14.30
-12	-8	-7	123.79	15.00

11	-9	-7	20.60	13.10
-11	9	7	17.40	13.00
10	-9	-7	4.70	10.40
10	-9	-7	27.90	13.30
-10	9	7	-29.60	17.10
-10	9	7	11.20	9.30
9	-9	-7	490.25	28.70
9	-9	-7	491.85	28.80
-9	9	7	451.95	28.50
-9	9	7	356.66	34.20
8	-9	-7	244.78	19.40
8	-9	-7	249.38	20.20
-8	9	7	220.58	25.20
-8	9	7	240.88	20.20
7	-9	-7	5.00	9.00
7	-9	-7	-4.50	7.30
-7	9	7	13.70	9.70
6	-9	-7	3.10	8.70
6	-9	-7	-6.10	13.40
6	-9	-7	6.20	6.90
-6	9	7	11.00	8.90
-6	9	7	3.30	9.00
5	-9	-7	79.79	11.50
5	-9	-7	57.19	11.20
5	-9	-7	67.49	11.60
5	-9	-7	63.59	10.50
5	-9	-7	39.70	15.00
-5	9	7	63.49	8.80
4	-9	-7	33.00	9.70
4	-9	-7	25.30	9.20
4	-9	-7	31.90	6.80
4	-9	-7	25.70	10.30
4	-9	-7	22.70	11.50
3	-9	-7	257.67	17.50
3	-9	-7	259.47	20.00
3	-9	-7	284.67	19.70
3	-9	-7	270.57	18.30
-3	9	7	285.97	13.90
-3	9	7	252.87	17.60
2	-9	-7	59.79	9.10
2	-9	-7	83.99	10.80
-2	9	7	81.09	5.30
-2	9	7	86.29	11.40
-2	9	7	86.99	9.10

1	-9	-7	177.58	12.00
1	-9	-7	183.88	12.40
-1	9	7	191.68	9.30
-1	9	7	178.08	15.00
-1	9	7	180.58	11.60
0	-9	-7	11.90	4.60
0	9	7	14.20	7.10
0	9	7	12.40	3.50
0	9	7	12.30	2.10
-1	-9	-7	868.11	44.80
1	9	7	1051.69	44.10
1	9	7	909.81	45.80
2	9	7	2895.01	131.79
2	9	7	2896.71	130.29
-3	-9	-7	46.20	6.80
3	9	7	51.59	8.30
3	9	7	50.69	7.60
3	9	7	44.50	7.10
3	9	7	48.40	5.70
-4	-9	-7	322.27	19.50
-4	-9	-7	348.07	17.30
-4	-9	-7	312.47	22.50
4	9	7	324.97	22.10
4	9	7	380.66	24.10
4	9	7	271.67	17.80
4	9	7	337.37	16.40
-5	-9	-7	542.15	25.50
-5	-9	-7	488.55	29.80
-5	-9	-7	499.15	27.10
-5	-9	-7	522.05	27.10
-5	-9	-7	497.85	26.90
-5	-9	-7	525.85	26.40
5	9	7	511.15	24.30
-6	-9	-7	133.19	10.00
-6	-9	-7	110.29	12.30
-6	-9	-7	124.09	11.40
-6	-9	-7	123.89	14.00
-6	-9	-7	146.19	16.20
-6	-9	-7	119.79	12.40
6	9	7	128.69	8.90
-7	-9	-7	47.90	8.00
-7	-9	-7	31.90	8.90
-7	-9	-7	43.80	9.30
-7	-9	-7	28.10	6.80

-7	-9	-7	41.70	8.10
-7	-9	-7	47.20	7.70
7	9	7	43.50	7.70
-8	-9	-7	24.00	8.70
-8	-9	-7	28.00	7.00
-8	-9	-7	26.40	9.20
-8	-9	-7	29.10	7.80
8	9	7	29.90	6.90
-9	-9	-7	181.68	16.00
-9	-9	-7	172.28	13.90
-9	-9	-7	161.08	19.70
-9	-9	-7	169.08	14.90
-9	-9	-7	186.38	16.10
-9	-9	-7	188.08	17.90
9	9	7	179.28	16.30
-10	-9	-7	85.29	15.40
-10	-9	-7	99.69	11.10
-10	-9	-7	137.89	26.20
-10	-9	-7	90.49	15.80
-10	-9	-7	125.89	20.60
10	9	7	93.19	14.70
-11	-9	-7	96.29	12.30
-11	-9	-7	92.49	12.50
-11	-9	-7	109.09	14.80
-11	-9	-7	109.99	13.70
-12	-9	-7	133.99	15.10
-12	-9	-7	120.89	13.30
-12	-9	-7	142.99	14.70
-12	-9	-7	96.19	13.30
10	-10	-7	264.47	21.20
10	-10	-7	213.18	20.70
-10	10	7	228.88	36.60
-10	10	7	236.58	19.30
9	-10	-7	1.00	9.90
9	-10	-7	10.20	11.10
-9	10	7	-10.70	15.30
-9	10	7	-2.80	10.20
8	-10	-7	31.50	10.90
8	-10	-7	16.60	8.80
-8	10	7	33.70	14.50
-8	10	7	2.10	10.20
7	-10	-7	1.00	7.60
7	-10	-7	14.40	9.50
-7	10	7	5.00	9.20

6	-10	-7	29.90	7.60
6	-10	-7	46.50	10.10
6	-10	-7	27.30	13.30
-6	10	7	26.00	9.20
5	-10	-7	14.10	10.90
5	-10	-7	15.60	10.00
5	-10	-7	1.40	14.80
5	-10	-7	23.40	10.60
-5	10	7	14.90	5.70
4	-10	-7	76.99	11.60
4	-10	-7	72.89	11.50
4	-10	-7	94.69	14.40
4	-10	-7	84.89	12.40
4	-10	-7	53.39	12.30
-4	10	7	77.69	7.40
3	-10	-7	1903.41	89.69
3	-10	-7	1880.91	86.69
3	-10	-7	1866.71	86.29
3	-10	-7	1909.71	87.39
3	-10	-7	1885.41	87.39
-3	10	7	1854.21	87.99
-3	10	7	1854.11	86.59
2	-10	-7	153.28	15.20
2	-10	-7	128.59	13.80
2	-10	-7	118.59	15.30
2	-10	-7	154.48	15.40
2	-10	-7	148.79	18.50
-2	10	7	143.39	16.70
-2	10	7	153.18	8.10
-2	10	7	131.29	15.10
1	-10	-7	372.26	22.20
1	-10	-7	418.96	23.30
-1	10	7	400.56	21.90
-1	10	7	436.76	19.60
-1	10	7	455.35	25.70
0	-10	-7	200.08	13.60
0	-10	-7	201.18	15.80
0	-10	-7	208.18	14.60
0	10	7	209.18	12.80
0	10	7	215.78	10.10
0	10	7	209.98	17.70
1	10	7	174.48	8.20
1	10	7	165.98	10.90
-2	-10	-7	445.06	23.30

2	10	7	441.86	26.00
2	10	7	475.45	22.70
-3	-10	-7	909.51	40.90
3	10	7	910.81	40.60
3	10	7	839.32	43.50
3	10	7	802.72	41.10
-4	-10	-7	26.20	7.70
-4	-10	-7	7.90	6.10
-4	-10	-7	10.10	6.70
-4	-10	-7	3.70	6.30
-4	-10	-7	14.50	7.00
4	10	7	13.50	2.20
-5	-10	-7	129.99	13.00
-5	-10	-7	136.89	9.90
-5	-10	-7	142.09	11.80
-5	-10	-7	131.39	12.80
-5	-10	-7	133.29	18.90
-5	-10	-7	134.99	12.80
5	10	7	126.39	8.60
-6	-10	-7	2.00	6.50
-6	-10	-7	4.40	5.90
-6	-10	-7	11.30	7.30
-6	-10	-7	6.50	5.70
-6	-10	-7	5.90	9.50
-6	-10	-7	-1.70	4.20
6	10	7	4.50	4.40
-7	-10	-7	249.58	17.00
-7	-10	-7	214.48	16.50
-7	-10	-7	221.78	14.80
-7	-10	-7	245.38	16.10
-7	-10	-7	257.37	19.50
-7	-10	-7	233.68	17.70
7	10	7	222.28	14.80
-8	-10	-7	275.27	20.20
-8	-10	-7	274.47	18.00
-8	-10	-7	302.87	19.60
-8	-10	-7	289.37	21.00
-8	-10	-7	277.37	19.40
-8	-10	-7	280.47	17.20
8	10	7	286.57	18.40
-9	-10	-7	20.40	10.20
-9	-10	-7	29.10	7.50
-9	-10	-7	12.60	6.70
-9	-10	-7	10.60	7.60

-9	-10	-7	13.80	9.90
-9	-10	-7	11.70	7.80
9	10	7	-2.80	9.30
-10	-10	-7	39.70	12.90
-10	-10	-7	32.50	8.60
-10	-10	-7	30.80	9.70
-10	-10	-7	29.30	7.70
-10	-10	-7	35.10	9.70
-11	-10	-7	332.67	19.60
-11	-10	-7	335.17	20.80
-11	-10	-7	313.87	20.50
-11	-10	-7	299.97	19.70
-12	-10	-7	15.80	10.80
-12	-10	-7	17.40	11.50
-12	-10	-7	6.70	7.80
-12	-10	-7	32.00	11.10
10	-11	-7	210.98	20.40
10	-11	-7	218.58	20.10
-10	11	7	179.18	18.20
9	-11	-7	135.79	18.60
9	-11	-7	128.19	16.90
-9	11	7	110.29	18.80
-9	11	7	132.69	17.20
8	-11	-7	253.97	18.80
8	-11	-7	247.88	21.20
-8	11	7	272.97	40.00
-8	11	7	196.28	21.80
7	-11	-7	69.99	11.60
7	-11	-7	68.99	10.00
-7	11	7	62.79	12.40
6	-11	-7	7.50	7.40
6	-11	-7	-16.50	12.10
6	-11	-7	-6.10	10.20
-6	11	7	1.20	9.40
5	-11	-7	396.46	27.20
5	-11	-7	381.36	32.30
5	-11	-7	417.16	28.50
5	-11	-7	438.46	24.00
-5	11	7	433.96	22.40
4	-11	-7	378.96	25.90
4	-11	-7	366.16	26.50
4	-11	-7	393.96	22.50
4	-11	-7	379.76	28.10
4	-11	-7	385.46	23.90

-4	11	7	428.96	20.50
3	-11	-7	42.00	9.70
3	-11	-7	18.20	7.50
3	-11	-7	24.00	10.20
3	-11	-7	34.70	8.00
-3	11	7	38.40	9.10
-3	11	7	38.20	5.00
2	-11	-7	739.43	36.50
2	-11	-7	732.73	38.00
2	-11	-7	715.73	36.20
2	-11	-7	744.93	37.00
2	-11	-7	705.43	36.90
2	-11	-7	811.42	39.30
-2	11	7	644.94	37.00
-2	11	7	700.13	40.80
1	-11	-7	16.10	7.80
1	-11	-7	31.50	9.00
1	-11	-7	27.20	7.90
1	-11	-7	20.20	9.00
1	-11	-7	11.50	7.00
-1	11	7	35.70	11.50
-1	11	7	21.60	3.40
0	-11	-7	5.00	6.30
0	-11	-7	26.10	8.10
0	11	7	14.90	4.90
0	11	7	26.80	10.70
0	11	7	20.60	3.00
-1	-11	-7	446.06	23.10
1	11	7	388.46	21.90
1	11	7	404.26	27.00
1	11	7	446.06	19.60
-2	-11	-7	477.75	25.70
-2	-11	-7	511.05	25.50
-2	-11	-7	450.55	31.00
2	11	7	531.55	22.70
2	11	7	499.95	25.10
2	11	7	492.15	30.50
-3	-11	-7	346.57	23.90
-3	-11	-7	437.96	21.20
-3	-11	-7	407.36	22.00
-3	-11	-7	409.06	22.80
-3	-11	-7	416.86	25.50
3	11	7	346.87	22.00
3	11	7	409.56	20.90

3	11	7	427.46	18.50
-4	-11	-7	280.57	15.40
-4	-11	-7	265.07	20.20
-4	-11	-7	268.57	16.80
-4	-11	-7	241.08	18.30
-4	-11	-7	275.47	16.50
-4	-11	-7	255.97	18.20
4	11	7	275.67	13.20
-5	-11	-7	4.90	6.10
-5	-11	-7	0.80	7.00
-5	-11	-7	14.10	6.50
-5	-11	-7	7.70	11.00
-5	-11	-7	8.10	4.70
-5	-11	-7	4.10	11.50
-5	-11	-7	2.30	5.80
5	11	7	7.00	3.60
-6	-11	-7	670.43	31.40
-6	-11	-7	665.73	32.30
-6	-11	-7	611.54	33.50
-6	-11	-7	647.34	34.60
-6	-11	-7	595.64	36.70
6	11	7	581.54	30.80
-7	-11	-7	385.96	22.40
-7	-11	-7	379.86	20.30
-7	-11	-7	345.67	23.20
-7	-11	-7	348.87	20.80
-7	-11	-7	368.86	21.60
-7	-11	-7	374.66	22.00
7	11	7	331.87	20.60
-8	-11	-7	229.58	17.40
-8	-11	-7	237.38	17.90
-8	-11	-7	219.98	19.60
-8	-11	-7	226.68	20.30
-8	-11	-7	218.38	17.70
-8	-11	-7	240.28	16.30
8	11	7	214.88	17.20
-9	-11	-7	170.48	15.40
-9	-11	-7	169.98	18.70
-9	-11	-7	145.49	13.20
-9	-11	-7	176.48	16.00
-9	-11	-7	178.58	18.60
-9	-11	-7	188.58	14.50
9	11	7	149.99	18.30
-10	-11	-7	131.39	14.00

-10	-11	-7	134.19	15.40
-10	-11	-7	139.79	15.50
-10	-11	-7	105.99	12.90
-10	-11	-7	107.69	13.60
-11	-11	-7	21.80	8.50
-11	-11	-7	7.40	7.70
-11	-11	-7	19.50	10.00
-11	-11	-7	17.90	8.10
10	-12	-7	150.28	20.50
-10	12	7	105.39	14.80
9	-12	-7	64.29	10.70
9	-12	-7	71.39	13.70
-9	12	7	84.39	12.60
-9	12	7	65.39	18.40
8	-12	-7	-10.30	12.20
8	-12	-7	4.50	9.20
-8	12	7	-20.80	13.30
-8	12	7	-6.70	14.90
7	-12	-7	5.70	7.40
7	-12	-7	-8.40	11.00
-7	12	7	3.90	11.40
6	-12	-7	104.09	18.80
6	-12	-7	67.19	12.90
6	-12	-7	54.99	8.70
-6	12	7	75.79	14.10
5	-12	-7	98.89	15.60
5	-12	-7	95.39	13.40
5	-12	-7	103.39	15.00
5	-12	-7	74.69	17.10
5	-12	-7	101.39	14.90
-5	12	7	89.39	11.40
4	-12	-7	1240.18	60.09
4	-12	-7	1143.49	58.99
4	-12	-7	1223.98	68.79
4	-12	-7	1176.18	57.29
-4	12	7	1139.69	57.49
-4	12	7	1246.58	55.59
3	-12	-7	35.90	10.40
3	-12	-7	28.20	7.90
3	-12	-7	42.20	10.60
3	-12	-7	30.40	8.60
-3	12	7	36.80	5.80
2	-12	-7	1408.66	69.09
2	-12	-7	1362.66	65.49

2	-12	-7	1413.36	64.99
2	-12	-7	1436.66	66.79
2	-12	-7	1371.26	65.09
2	-12	-7	1333.37	65.79
-2	12	7	1376.26	65.79
-2	12	7	1443.36	63.19
-2	12	7	1319.27	71.89
1	-12	-7	27.00	8.40
1	-12	-7	45.20	10.40
1	-12	-7	10.90	11.20
1	-12	-7	28.40	7.90
1	-12	-7	32.00	8.00
1	-12	-7	22.80	8.90
-1	12	7	26.30	3.90
-1	12	7	24.60	14.40
0	-12	-7	89.69	13.60
0	-12	-7	70.39	8.80
0	-12	-7	85.39	10.10
0	-12	-7	74.79	11.90
0	-12	-7	77.89	12.40
0	12	7	78.89	5.50
0	12	7	81.59	14.70
-1	-12	-7	99.49	10.40
-1	-12	-7	104.69	11.50
-1	-12	-7	108.89	12.20
-1	-12	-7	105.29	13.30
-1	-12	-7	103.99	15.30
1	12	7	75.49	14.40
1	12	7	109.89	6.20
1	12	7	111.89	11.20
-2	-12	-7	55.29	7.40
-2	-12	-7	49.99	8.40
-2	-12	-7	39.10	6.80
-2	-12	-7	59.29	11.10
-2	-12	-7	46.50	8.30
2	12	7	45.40	6.80
2	12	7	50.69	4.00
-3	-12	-7	1002.60	44.20
-3	-12	-7	916.51	45.00
-3	-12	-7	858.41	45.20
-3	-12	-7	900.51	44.80
-3	-12	-7	958.60	44.10
-3	-12	-7	837.72	45.00
3	12	7	964.30	41.90

-4	-12	-7	552.94	30.10
-4	-12	-7	575.04	29.50
-4	-12	-7	547.65	30.70
-4	-12	-7	606.34	28.90
-4	-12	-7	573.24	29.00
-4	-12	-7	565.34	30.90
4	12	7	581.84	26.70
-5	-12	-7	31.00	6.60
-5	-12	-7	26.40	12.60
-5	-12	-7	53.59	11.40
-5	-12	-7	38.00	7.10
-5	-12	-7	33.30	7.40
-5	-12	-7	41.00	6.00
-5	-12	-7	43.50	8.70
5	12	7	35.10	6.30
-6	-12	-7	187.98	14.60
-6	-12	-7	176.58	13.10
-6	-12	-7	207.08	14.40
-6	-12	-7	198.38	15.70
-6	-12	-7	192.28	15.30
6	12	7	153.68	13.10
-7	-12	-7	206.98	18.50
-7	-12	-7	208.88	16.10
-7	-12	-7	190.68	15.20
-7	-12	-7	187.88	14.10
-7	-12	-7	184.98	16.20
-7	-12	-7	210.58	16.00
7	12	7	170.28	15.20
-8	-12	-7	-0.70	9.70
-8	-12	-7	10.00	7.10
-8	-12	-7	15.30	8.00
-8	-12	-7	7.50	8.00
-8	-12	-7	4.10	7.60
8	12	7	3.40	9.00
-9	-12	-7	0.90	7.50
-9	-12	-7	-2.60	6.70
-9	-12	-7	15.30	9.40
-9	-12	-7	8.10	7.90
-9	-12	-7	9.50	8.00
9	12	7	5.40	11.40
-10	-12	-7	338.97	22.50
-10	-12	-7	340.77	21.30
-10	-12	-7	408.36	24.20
-10	-12	-7	354.76	22.10

-10	-12	-7	363.46	21.70
-11	-12	-7	35.40	8.60
-11	-12	-7	51.69	10.20
-11	-12	-7	46.60	8.40
-11	-12	-7	77.19	11.50
10	-13	-7	11.10	18.90
10	-13	-7	26.60	10.90
-10	13	7	-3.70	16.40
9	-13	-7	528.15	33.50
9	-13	-7	581.74	29.70
-9	13	7	497.05	31.20
8	-13	-7	111.29	21.10
8	-13	-7	106.29	14.00
7	-13	-7	59.29	19.20
7	-13	-7	29.20	8.10
7	-13	-7	19.90	14.00
-7	13	7	28.00	12.30
6	-13	-7	28.10	7.80
6	-13	-7	41.80	12.40
6	-13	-7	23.90	14.60
-6	13	7	24.60	11.40
5	-13	-7	16.60	19.00
5	-13	-7	30.80	7.80
5	-13	-7	38.80	14.20
5	-13	-7	38.30	10.00
5	-13	-7	13.60	16.80
-5	13	7	43.50	8.60
4	-13	-7	85.69	14.20
4	-13	-7	79.69	12.80
4	-13	-7	75.39	14.00
-4	13	7	98.29	9.20
-4	13	7	92.09	12.40
3	-13	-7	235.18	19.30
3	-13	-7	186.48	16.10
3	-13	-7	192.18	19.60
3	-13	-7	183.38	15.80
-3	13	7	197.18	11.60
-3	13	7	177.78	17.80
-3	13	7	164.18	19.90
2	-13	-7	7.90	6.80
2	-13	-7	11.30	8.70
2	-13	-7	3.20	8.50
2	-13	-7	17.00	8.00
2	-13	-7	-0.20	9.10

-2	13	7	5.30	3.10
-2	13	7	16.90	8.80
1	-13	-7	585.84	30.60
1	-13	-7	580.24	31.80
1	-13	-7	560.74	30.30
1	-13	-7	551.94	30.40
1	-13	-7	603.24	31.70
-1	13	7	589.24	27.40
0	-13	-7	53.39	12.20
0	-13	-7	33.00	8.80
0	-13	-7	29.90	8.20
0	-13	-7	21.20	7.40
0	-13	-7	38.50	7.70
0	-13	-7	4.30	13.70
0	13	7	40.40	4.20
-1	-13	-7	658.23	31.90
-1	-13	-7	603.34	33.40
-1	-13	-7	614.34	32.40
-1	-13	-7	642.54	32.90
-1	-13	-7	667.73	32.00
-1	-13	-7	571.44	33.30
1	13	7	673.13	29.40
-2	-13	-7	888.21	42.70
-2	-13	-7	896.81	43.30
-2	-13	-7	873.41	43.80
-2	-13	-7	931.11	43.20
-2	-13	-7	864.51	45.10
-2	-13	-7	827.42	44.10
2	13	7	931.71	40.60
-3	-13	-7	315.67	18.90
-3	-13	-7	320.47	17.80
-3	-13	-7	247.78	21.10
-3	-13	-7	285.57	19.30
-3	-13	-7	282.87	17.70
-3	-13	-7	274.27	19.60
3	13	7	295.67	14.30
-4	-13	-7	4.70	8.20
-4	-13	-7	6.90	6.00
-4	-13	-7	6.00	7.20
-4	-13	-7	7.80	7.80
-4	-13	-7	9.90	6.00
-4	-13	-7	9.00	8.20
4	13	7	3.60	2.90
-5	-13	-7	523.45	29.70

-5	-13	-7	573.04	28.30
-5	-13	-7	565.24	31.10
-5	-13	-7	573.24	34.90
-5	-13	-7	534.15	28.80
5	13	7	519.35	26.80
-6	-13	-7	33.80	12.20
-6	-13	-7	13.60	7.80
-6	-13	-7	11.60	7.40
-6	-13	-7	8.50	6.30
-6	-13	-7	3.30	6.50
-6	-13	-7	23.20	7.80
6	13	7	7.70	6.50
-7	-13	-7	17.00	8.00
-7	-13	-7	12.50	6.80
-7	-13	-7	19.50	7.30
-7	-13	-7	8.20	7.90
-7	-13	-7	-4.50	7.00
7	13	7	3.90	8.00
-8	-13	-7	54.29	8.80
-8	-13	-7	47.70	9.00
-8	-13	-7	38.00	8.10
-8	-13	-7	52.79	9.00
-8	-13	-7	59.89	9.40
-9	-13	-7	187.58	17.90
-9	-13	-7	160.58	14.20
-10	-13	-7	30.50	8.20
-10	-13	-7	18.80	8.20
-10	-13	-7	30.20	8.70
-10	-13	-7	23.50	8.40
-10	-13	-7	20.70	10.90
-11	-13	-7	27.70	8.50
-11	-13	-7	20.80	7.90
-11	-13	-7	12.00	7.90
-11	-13	-7	11.50	10.10
9	-14	-7	-3.20	8.20
-9	14	7	7.10	9.80
8	-14	-7	69.59	17.20
8	-14	-7	91.29	12.90
-8	14	7	60.39	12.50
7	-14	-7	34.50	10.60
7	-14	-7	15.90	17.40
7	-14	-7	5.30	19.00
-7	14	7	28.10	13.40
6	-14	-7	-6.10	11.50

6	-14	-7	-7.10	7.50
-6	14	7	4.30	11.20
5	-14	-7	4.90	9.50
5	-14	-7	8.80	7.80
5	-14	-7	-28.70	13.40
5	-14	-7	-4.70	17.10
-5	14	7	14.50	9.80
4	-14	-7	467.35	25.80
4	-14	-7	383.06	29.70
4	-14	-7	471.85	25.50
4	-14	-7	455.45	30.30
-4	14	7	438.46	23.30
-4	14	7	376.96	27.10
3	-14	-7	1548.84	69.99
3	-14	-7	1444.76	68.19
3	-14	-7	1435.86	70.29
3	-14	-7	1477.95	67.89
-3	14	7	1330.87	70.49
-3	14	7	1488.85	66.39
-3	14	7	1346.17	68.69
2	-14	-7	32.10	9.10
2	-14	-7	12.70	8.30
2	-14	-7	21.20	7.90
2	-14	-7	17.60	8.90
2	-14	-7	29.70	10.10
-2	14	7	14.90	3.70
-2	14	7	16.70	8.90
1	-14	-7	2.60	7.70
1	-14	-7	-7.30	6.90
1	-14	-7	3.80	10.50
1	-14	-7	4.70	8.20
1	-14	-7	1.70	8.20
-1	14	7	10.10	3.00
0	-14	-7	566.64	36.20
0	-14	-7	559.44	31.00
0	-14	-7	586.14	31.70
0	-14	-7	620.94	33.20
0	-14	-7	637.34	31.40
0	-14	-7	647.04	31.80
0	14	7	591.04	28.30
-1	-14	-7	155.58	15.30
-1	-14	-7	188.28	17.30
-1	-14	-7	194.68	14.30
-1	-14	-7	204.98	17.50

-1	-14	-7	184.38	14.80
1	14	7	170.08	9.70
-2	-14	-7	87.19	10.70
-2	-14	-7	81.69	12.00
-2	-14	-7	82.69	12.40
-2	-14	-7	93.29	14.50
-2	-14	-7	81.09	11.10
-2	-14	-7	96.99	13.00
2	14	7	81.49	5.90
-3	-14	-7	118.79	19.30
-3	-14	-7	60.79	10.50
-3	-14	-7	85.09	11.30
-3	-14	-7	81.29	12.50
-3	-14	-7	86.79	10.60
-3	-14	-7	51.19	14.90
-3	-14	-7	87.49	12.60
3	14	7	72.29	5.80
-4	-14	-7	67.49	12.10
-4	-14	-7	90.39	11.20
-4	-14	-7	92.89	13.90
-4	-14	-7	65.39	10.40
4	14	7	90.59	7.00
-5	-14	-7	178.78	16.30
-5	-14	-7	195.28	14.50
-5	-14	-7	197.68	14.40
-5	-14	-7	206.98	18.20
-5	-14	-7	160.38	15.90
-5	-14	-7	200.28	16.20
5	14	7	186.38	12.40
-6	-14	-7	65.49	13.30
-6	-14	-7	59.09	10.50
-6	-14	-7	64.09	8.90
-6	-14	-7	40.70	8.70
-6	-14	-7	56.19	8.20
-6	-14	-7	73.59	12.80
6	14	7	53.09	8.70
-7	-14	-7	116.59	13.80
-7	-14	-7	137.49	14.80
-7	-14	-7	137.39	14.00
-7	-14	-7	120.09	15.70
-7	-14	-7	123.59	14.00
-8	-14	-7	86.89	13.50
-8	-14	-7	73.29	10.70
-8	-14	-7	80.49	12.00

-8	-14	-7	88.09	13.70
-8	-14	-7	92.59	13.30
-9	-14	-7	16.00	8.30
-9	-14	-7	9.90	8.50
-9	-14	-7	22.60	8.90
-9	-14	-7	9.10	7.90
-9	-14	-7	20.00	8.40
9	14	7	15.50	14.70
-10	-14	-7	130.19	13.50
-10	-14	-7	117.19	13.90
-10	-14	-7	144.79	13.60
-10	-14	-7	119.79	14.40
-10	-14	-7	137.09	15.90
-11	-14	-7	265.07	16.70
-11	-14	-7	248.48	19.00
-11	-14	-7	257.57	17.70
-11	-14	-7	254.77	17.40
11	14	7	321.77	24.40
9	-15	-7	15.30	8.20
-9	15	7	3.20	9.30
8	-15	-7	295.67	18.90
-8	15	7	298.07	30.40
-8	15	7	278.87	22.70
7	-15	-7	-11.10	7.30
7	-15	-7	5.10	17.50
-7	15	7	-7.30	12.30
6	-15	-7	14.20	7.60
6	-15	-7	3.50	12.00
-6	15	7	6.80	11.60
5	-15	-7	169.28	17.50
5	-15	-7	147.29	15.30
5	-15	-7	141.89	20.60
5	-15	-7	151.48	18.40
-5	15	7	134.79	23.50
4	-15	-7	390.76	25.30
4	-15	-7	375.56	29.80
4	-15	-7	450.15	24.80
4	-15	-7	445.06	29.90
-4	15	7	426.26	23.50
-4	15	7	419.26	28.00
-4	15	7	386.76	30.70
3	-15	-7	44.10	8.60
3	-15	-7	40.20	8.80
3	-15	-7	51.19	11.20

3	-15	-7	62.29	11.90
-3	15	7	37.70	11.10
-3	15	7	45.50	13.90
-3	15	7	37.50	6.00
2	-15	-7	780.52	40.20
2	-15	-7	866.51	41.00
2	-15	-7	752.32	39.20
2	-15	-7	779.42	40.20
2	-15	-7	751.32	40.90
-2	15	7	749.03	36.80
-2	15	7	759.12	40.10
1	-15	-7	24.10	12.30
1	-15	-7	29.00	11.20
1	-15	-7	29.80	11.30
-1	15	7	13.40	8.60
-1	15	7	23.50	4.10
0	-15	-7	20.00	8.40
0	-15	-7	15.40	18.20
0	-15	-7	18.70	9.80
0	-15	-7	12.00	7.40
0	-15	-7	-3.30	6.80
0	-15	-7	7.00	7.10
0	15	7	7.30	3.00
-1	-15	-7	50.39	8.60
-1	-15	-7	43.40	9.90
-1	-15	-7	55.99	8.30
-1	-15	-7	65.59	10.60
-1	-15	-7	59.69	10.10
-2	-15	-7	603.04	30.10
-2	-15	-7	577.94	30.50
-2	-15	-7	567.54	33.10
-2	-15	-7	584.24	31.40
-2	-15	-7	577.24	31.50
-2	-15	-7	553.74	30.90
2	15	7	569.34	27.30
-3	-15	-7	293.97	17.50
-3	-15	-7	289.17	25.30
-3	-15	-7	230.88	23.80
-3	-15	-7	279.77	22.90
-3	-15	-7	255.57	26.50
-3	-15	-7	284.37	19.20
-3	-15	-7	286.87	18.20
3	15	7	290.17	14.60
-4	-15	-7	137.99	12.90

-4	-15	-7	150.48	15.10
-4	-15	-7	145.29	15.20
-4	-15	-7	115.39	12.40
-4	-15	-7	148.59	18.10
-4	-15	-7	159.18	15.50
4	15	7	133.49	9.60
-5	-15	-7	162.78	13.70
-5	-15	-7	147.89	15.40
-5	-15	-7	158.18	13.70
-5	-15	-7	132.09	15.20
-5	-15	-7	159.28	17.60
5	15	7	143.99	11.70
-6	-15	-7	593.44	31.50
-6	-15	-7	536.85	31.80
-6	-15	-7	607.64	33.60
-6	-15	-7	615.84	30.50
-6	-15	-7	582.54	31.10
-6	-15	-7	615.04	31.50
6	15	7	530.65	30.70
-7	-15	-7	127.39	13.80
-7	-15	-7	124.89	15.00
-7	-15	-7	120.49	13.60
-7	-15	-7	122.09	15.40
-7	-15	-7	122.59	15.50
-8	-15	-7	32.70	9.00
-8	-15	-7	29.60	8.20
-8	-15	-7	39.40	9.80
-8	-15	-7	36.40	9.10
-8	-15	-7	45.00	10.00
-9	-15	-7	3.70	8.10
-9	-15	-7	17.90	8.00
-9	-15	-7	15.90	8.10
-9	-15	-7	2.50	7.90
-9	-15	-7	2.10	7.80
-10	-15	-7	6.40	7.00
-10	-15	-7	14.60	7.40
-10	-15	-7	9.10	8.80
-10	-15	-7	12.20	7.00
-10	-15	-7	-3.20	8.10
8	-16	-7	3.30	7.60
-8	16	7	-0.70	9.50
7	-16	-7	135.79	19.90
7	-16	-7	148.69	14.30
-7	16	7	113.39	18.30

6	-16	-7	85.89	17.20
-6	16	7	88.19	17.30
5	-16	-7	177.18	31.80
5	-16	-7	183.28	16.90
5	-16	-7	199.58	19.40
5	-16	-7	190.18	25.10
4	-16	-7	425.16	24.00
4	-16	-7	398.46	24.60
4	-16	-7	416.16	29.10
4	-16	-7	372.36	29.20
-4	16	7	335.67	29.20
-4	16	7	364.46	27.70
-4	16	7	391.06	24.20
3	-16	-7	9.00	7.70
3	-16	-7	9.30	10.70
3	-16	-7	28.90	11.30
3	-16	-7	10.10	8.60
-3	16	7	25.60	13.20
-3	16	7	6.70	10.80
-3	16	7	23.60	6.60
2	-16	-7	379.06	24.80
2	-16	-7	403.46	25.20
2	-16	-7	365.96	25.10
2	-16	-7	387.76	23.60
2	-16	-7	380.56	25.50
-2	16	7	396.46	20.60
-2	16	7	409.26	25.10
1	-16	-7	674.03	36.80
1	-16	-7	759.42	37.70
1	-16	-7	712.63	37.00
1	-16	-7	711.53	36.70
-1	16	7	700.43	34.00
-1	16	7	713.43	37.30
0	-16	-7	10.60	7.40
0	-16	-7	-1.80	7.20
0	-16	-7	14.40	8.50
0	-16	-7	-3.50	7.10
0	16	7	-1.60	3.30
-1	-16	-7	318.07	19.90
-1	-16	-7	315.07	20.00
-1	-16	-7	309.07	21.70
-1	-16	-7	319.77	19.50
-1	-16	-7	320.57	21.60
1	16	7	321.87	16.50

-2	-16	-7	46.10	8.00
-2	-16	-7	59.59	9.20
-2	-16	-7	82.39	13.60
-2	-16	-7	69.29	16.00
-2	-16	-7	53.49	7.80
2	16	7	58.69	6.20
-3	-16	-7	634.84	33.30
-3	-16	-7	627.14	33.30
-3	-16	-7	645.84	32.90
-3	-16	-7	636.14	32.40
-3	-16	-7	637.54	33.70
3	16	7	636.14	30.40
-4	-16	-7	423.76	23.60
-4	-16	-7	421.16	24.80
-4	-16	-7	457.35	24.50
-4	-16	-7	418.16	25.30
-4	-16	-7	425.86	25.30
-4	-16	-7	394.26	26.50
4	16	7	429.86	21.90
-5	-16	-7	12.40	7.70
-5	-16	-7	11.00	9.30
-5	-16	-7	11.00	7.10
-5	-16	-7	27.80	8.30
-5	-16	-7	-3.40	8.90
-5	-16	-7	9.80	8.60
5	16	7	14.30	6.20
-6	-16	-7	57.69	8.50
-6	-16	-7	73.59	10.90
-6	-16	-7	74.79	10.60
-6	-16	-7	79.29	12.20
-6	-16	-7	70.79	10.30
-6	-16	-7	93.29	15.80
6	16	7	72.39	10.20
-7	-16	-7	22.20	7.80
-7	-16	-7	9.10	6.90
-7	-16	-7	16.00	7.60
-7	-16	-7	17.20	8.50
-7	-16	-7	27.70	9.10
7	16	7	5.60	10.60
-8	-16	-7	80.69	13.00
-8	-16	-7	79.39	11.40
-8	-16	-7	58.99	10.80
-8	-16	-7	81.99	11.70
-8	-16	-7	85.29	12.30

-9	-16	-7	23.50	8.10
-9	-16	-7	28.40	7.50
-9	-16	-7	28.30	8.00
-9	-16	-7	22.70	8.50
-9	-16	-7	13.40	7.60
-10	-16	-7	125.09	12.80
-10	-16	-7	104.59	13.80
-10	-16	-7	107.29	11.60
-10	-16	-7	82.99	15.00
8	-17	-7	14.10	7.40
-8	17	7	25.60	9.00
7	-17	-7	-2.70	7.10
7	-17	-7	8.80	16.80
-7	17	7	-6.30	9.20
6	-17	-7	154.88	21.00
6	-17	-7	161.68	15.00
-6	17	7	154.18	19.00
5	-17	-7	5.00	10.20
5	-17	-7	18.30	15.20
5	-17	-7	16.90	8.70
4	-17	-7	51.09	13.60
4	-17	-7	44.50	9.60
4	-17	-7	54.59	14.70
4	-17	-7	58.79	10.50
-4	17	7	47.60	14.20
-4	17	7	51.99	12.00
-4	17	7	43.40	14.90
3	-17	-7	4.60	10.70
3	-17	-7	-8.50	10.80
3	-17	-7	0.10	8.00
3	-17	-7	5.10	9.30
-3	17	7	3.40	11.50
-3	17	7	9.30	7.60
-3	17	7	-13.70	12.80
2	-17	-7	221.98	19.30
2	-17	-7	173.08	18.70
2	-17	-7	197.18	17.40
2	-17	-7	199.28	17.70
2	-17	-7	194.78	18.70
-2	17	7	152.98	13.10
-2	17	7	151.58	18.10
1	-17	-7	522.65	28.80
1	-17	-7	501.85	29.30
1	-17	-7	526.25	29.10

1	-17	-7	551.74	33.30
1	-17	-7	508.55	29.10
-1	17	7	506.35	25.80
-1	17	7	483.65	29.10
0	-17	-7	129.39	14.60
0	-17	-7	132.19	14.20
0	-17	-7	141.09	15.60
0	-17	-7	125.89	14.20
0	17	7	126.89	9.60
-1	-17	-7	620.04	33.30
-1	-17	-7	602.94	33.20
-1	-17	-7	651.53	32.80
-1	-17	-7	643.94	34.40
-1	-17	-7	619.74	32.90
1	17	7	646.14	30.50
-2	-17	-7	141.39	19.30
-2	-17	-7	173.68	15.10
-2	-17	-7	155.98	14.20
-2	-17	-7	169.28	15.60
-2	-17	-7	168.28	14.90
2	17	7	170.78	10.80
-3	-17	-7	217.88	17.30
-3	-17	-7	220.88	15.80
-3	-17	-7	211.78	16.70
-3	-17	-7	202.38	15.10
-3	-17	-7	148.59	25.40
-3	-17	-7	168.48	16.90
3	17	7	206.48	12.60
-4	-17	-7	11.10	7.70
-4	-17	-7	22.70	6.70
-4	-17	-7	9.10	8.70
-4	-17	-7	13.60	7.70
4	17	7	16.20	5.60
-5	-17	-7	83.49	13.60
-5	-17	-7	98.69	15.60
-5	-17	-7	96.09	14.30
-5	-17	-7	107.99	16.20
-5	-17	-7	91.49	13.80
-5	-17	-7	74.69	11.10
5	17	7	82.59	13.00
-6	-17	-7	33.80	8.60
-6	-17	-7	30.90	7.20
-6	-17	-7	52.39	9.20
-6	-17	-7	47.00	10.00

-6	-17	-7	23.10	10.70
6	17	7	38.30	10.10
-7	-17	-7	187.48	16.20
-7	-17	-7	193.88	23.90
-7	-17	-7	168.68	16.30
-7	-17	-7	179.78	16.80
-7	-17	-7	162.68	14.60
7	17	7	144.59	20.10
-8	-17	-7	55.19	9.10
-8	-17	-7	43.80	9.30
-8	-17	-7	72.19	12.20
-8	-17	-7	48.00	7.90
-8	-17	-7	44.40	9.20
-9	-17	-7	34.00	8.30
-9	-17	-7	35.90	11.60
-9	-17	-7	18.00	7.30
-9	-17	-7	23.30	7.30
-10	-17	-7	-3.60	8.20
-10	-17	-7	16.80	8.90
-10	-17	-7	15.70	6.90
-10	-17	-7	13.00	7.20
7	-18	-7	40.50	24.60
7	-18	-7	13.40	7.60
-7	18	7	20.90	9.10
6	-18	-7	-4.80	7.60
6	-18	-7	-0.60	11.40
-6	18	7	1.40	9.10
5	-18	-7	42.70	9.40
5	-18	-7	55.29	11.80
4	-18	-7	336.67	26.30
4	-18	-7	349.47	22.80
4	-18	-7	363.36	28.20
4	-18	-7	322.67	21.90
-4	18	7	277.37	27.50
-4	18	7	323.67	26.80
-4	18	7	255.97	40.10
3	-18	-7	346.57	23.10
3	-18	-7	338.57	24.90
3	-18	-7	343.07	24.90
3	-18	-7	326.17	21.90
-3	18	7	315.57	22.40
-3	18	7	333.47	24.90
-3	18	7	288.37	25.30
2	-18	-7	51.49	10.30

2	-18	-7	25.10	8.80
2	-18	-7	10.10	11.30
2	-18	-7	28.10	9.60
2	-18	-7	22.70	9.90
-2	18	7	13.50	16.50
-2	18	7	13.30	11.40
-2	18	7	20.70	11.10
1	-18	-7	1.70	8.00
1	-18	-7	-20.70	14.20
1	-18	-7	-4.80	8.00
1	-18	-7	19.80	8.80
1	-18	-7	6.40	8.40
-1	18	7	6.70	9.90
-1	18	7	7.40	5.90
0	-18	-7	565.64	32.80
0	-18	-7	609.84	32.00
0	-18	-7	612.34	31.60
0	-18	-7	592.74	31.90
0	18	7	581.24	32.60
0	18	7	583.54	29.30
-1	-18	-7	320.87	21.10
-1	-18	-7	347.27	23.40
-1	-18	-7	355.76	21.10
-1	-18	-7	326.57	21.10
1	18	7	333.57	18.30
-2	-18	-7	12.10	10.90
-2	-18	-7	-2.10	6.70
-2	-18	-7	7.70	8.30
-2	-18	-7	6.80	7.20
-2	-18	-7	5.20	7.40
2	18	7	17.00	5.00
-3	-18	-7	77.59	16.00
-3	-18	-7	97.59	12.80
-3	-18	-7	88.49	13.90
-3	-18	-7	79.49	10.50
-3	-18	-7	88.29	12.20
3	18	7	98.59	9.90
-4	-18	-7	52.59	9.90
-4	-18	-7	69.59	11.30
-4	-18	-7	64.79	9.80
-4	-18	-7	77.99	12.80
-4	-18	-7	48.10	9.50
4	18	7	76.79	10.50
-5	-18	-7	100.19	14.20

-5	-18	-7	100.49	13.20
-5	-18	-7	102.89	13.20
-5	-18	-7	88.79	15.40
-5	-18	-7	100.59	11.70
5	18	7	76.79	13.00
-6	-18	-7	14.10	8.40
-6	-18	-7	23.20	8.00
-6	-18	-7	27.90	15.40
-6	-18	-7	15.70	7.00
-6	-18	-7	12.60	8.00
6	18	7	9.00	10.20
-7	-18	-7	222.68	17.20
-7	-18	-7	217.28	16.50
-7	-18	-7	188.78	26.00
-7	-18	-7	216.98	15.60
-7	-18	-7	202.68	15.90
7	18	7	168.48	22.60
-8	-18	-7	47.40	8.90
-8	-18	-7	44.90	7.80
-8	-18	-7	45.00	7.70
-8	-18	-7	48.60	8.40
-9	-18	-7	14.10	6.90
-9	-18	-7	12.60	7.10
-9	-18	-7	21.40	6.80
-9	-18	-7	18.80	8.70
7	-19	-7	-36.30	20.50
7	-19	-7	-1.60	7.40
-7	19	7	5.90	8.20
6	-19	-7	0.50	11.70
6	-19	-7	8.10	7.90
-6	19	7	10.90	8.30
5	-19	-7	327.07	22.40
5	-19	-7	316.57	21.30
-5	19	7	331.47	23.20
4	-19	-7	55.89	13.70
4	-19	-7	49.90	10.70
4	-19	-7	44.80	10.50
4	-19	-7	26.80	15.50
-4	19	7	37.50	22.80
-4	19	7	39.50	15.10
3	-19	-7	3.60	11.40
3	-19	-7	13.90	8.50
3	-19	-7	16.50	11.20
3	-19	-7	8.90	11.40

-3	19	7	13.00	11.70
-3	19	7	16.50	12.00
-3	19	7	22.10	13.00
2	-19	-7	129.59	17.50
2	-19	-7	114.19	17.20
2	-19	-7	170.68	16.60
2	-19	-7	161.18	21.00
-2	19	7	112.49	17.70
-2	19	7	131.19	19.20
-2	19	7	148.29	15.30
1	-19	-7	23.10	9.10
1	-19	-7	9.50	8.60
1	-19	-7	13.10	8.60
1	-19	-7	4.10	8.80
-1	19	7	15.90	7.10
-1	19	7	13.00	9.80
0	-19	-7	5.20	7.70
0	-19	-7	-1.20	7.80
0	-19	-7	9.10	8.00
0	-19	-7	-4.80	9.10
0	19	7	3.40	6.00
0	19	7	9.20	10.80
-1	-19	-7	6.20	7.70
-1	-19	-7	6.10	7.80
-1	-19	-7	15.70	9.80
-1	-19	-7	10.20	7.70
1	19	7	15.60	8.20
-2	-19	-7	132.49	13.10
-2	-19	-7	122.09	14.70
-2	-19	-7	123.79	19.90
-2	-19	-7	124.49	13.50
-2	-19	-7	147.69	15.00
2	19	7	131.29	11.50
-3	-19	-7	15.30	7.30
-3	-19	-7	6.20	7.60
-3	-19	-7	43.20	15.30
-3	-19	-7	25.10	8.50
-3	-19	-7	12.00	6.90
3	19	7	19.00	6.60
-4	-19	-7	198.68	16.50
-4	-19	-7	191.68	14.80
-4	-19	-7	190.28	16.90
-4	-19	-7	198.48	15.50
4	19	7	182.58	15.40

-5	-19	-7	479.75	27.60
-5	-19	-7	468.85	25.90
-5	-19	-7	440.56	25.00
-5	-19	-7	440.16	25.90
5	19	7	480.45	26.50
-6	-19	-7	574.04	30.70
-6	-19	-7	594.74	30.70
-6	-19	-7	595.04	29.90
-6	-19	-7	569.84	32.10
-6	-19	-7	537.55	30.00
6	19	7	547.75	32.60
-7	-19	-7	30.10	6.80
-7	-19	-7	36.20	7.40
-7	-19	-7	24.30	7.30
-7	-19	-7	11.80	7.30
-7	-19	-7	42.90	15.40
-8	-19	-7	65.39	10.90
-8	-19	-7	70.19	10.50
-8	-19	-7	65.09	11.20
-8	-19	-7	93.39	12.80
-9	-19	-7	27.90	6.40
-9	-19	-7	36.40	11.80
6	-20	-7	156.98	14.20
6	-20	-7	146.89	18.20
-6	20	7	154.98	15.00
5	-20	-7	185.98	18.00
5	-20	-7	200.78	16.60
-5	20	7	228.78	17.20
4	-20	-7	252.07	25.30
4	-20	-7	217.08	18.40
4	-20	-7	232.28	22.70
4	-20	-7	266.07	19.30
-4	20	7	212.28	24.30
3	-20	-7	157.18	16.70
3	-20	-7	157.28	19.00
3	-20	-7	154.38	19.00
-3	20	7	119.09	19.80
-3	20	7	119.09	21.10
2	-20	-7	43.70	11.90
2	-20	-7	48.90	19.50
2	-20	-7	56.89	14.30
2	-20	-7	79.49	14.60
-2	20	7	47.50	12.00
-2	20	7	35.20	11.40

1	-20	-7	129.79	16.50
1	-20	-7	142.69	15.20
1	-20	-7	130.69	16.30
1	-20	-7	131.09	16.10
-1	20	7	151.78	15.30
-1	20	7	129.19	17.10
0	-20	-7	217.68	17.00
0	-20	-7	241.38	18.00
0	-20	-7	211.08	17.90
0	-20	-7	273.97	20.30
0	20	7	215.18	19.30
0	20	7	212.18	16.20
-1	-20	-7	607.64	32.80
-1	-20	-7	573.34	30.00
-1	-20	-7	560.74	30.30
-1	-20	-7	552.64	29.80
1	20	7	542.65	29.10
1	20	7	520.55	30.80
-2	-20	-7	17.30	7.30
-2	-20	-7	5.20	7.10
-2	-20	-7	19.40	7.40
-2	-20	-7	-0.30	12.00
2	20	7	18.20	7.20
-3	-20	-7	183.48	17.00
-3	-20	-7	263.57	26.60
-3	-20	-7	235.18	16.80
-3	-20	-7	203.88	15.80
-3	-20	-7	203.48	15.60
3	20	7	196.88	16.30
-4	-20	-7	50.39	10.20
-4	-20	-7	70.89	11.50
-4	-20	-7	69.69	12.20
-4	-20	-7	70.29	11.50
4	20	7	93.29	10.80
-5	-20	-7	7.30	6.70
-5	-20	-7	13.90	8.60
-5	-20	-7	7.20	8.40
-5	-20	-7	8.60	7.30
5	20	7	12.60	10.30
-6	-20	-7	94.79	11.50
-6	-20	-7	95.29	10.70
-6	-20	-7	70.39	11.70
-6	-20	-7	92.89	16.10
-7	-20	-7	13.30	7.00

-7	-20	-7	32.40	18.30
-7	-20	-7	12.70	6.00
-7	-20	-7	14.30	6.40
-8	-20	-7	7.30	5.80
-8	-20	-7	8.90	6.70
-8	-20	-7	4.70	5.80
6	-21	-7	148.39	14.10
6	-21	-7	122.49	18.20
-6	21	7	151.88	15.50
5	-21	-7	13.10	9.40
5	-21	-7	-5.20	9.00
-5	21	7	3.60	7.50
4	-21	-7	79.69	17.50
4	-21	-7	95.59	15.20
3	-21	-7	18.70	8.80
3	-21	-7	39.00	11.40
3	-21	-7	37.20	12.30
3	-21	-7	31.20	13.20
-3	21	7	44.20	17.40
-3	21	7	31.00	12.60
2	-21	-7	132.09	15.20
2	-21	-7	145.19	16.90
2	-21	-7	137.39	17.40
-2	21	7	122.99	18.50
-2	21	7	104.99	14.40
1	-21	-7	347.37	23.90
1	-21	-7	333.97	23.30
1	-21	-7	347.27	23.10
-1	21	7	319.17	24.30
-1	21	7	348.97	23.60
-1	21	7	367.56	24.40
0	-21	-7	182.58	25.10
0	21	7	233.58	19.00
0	21	7	242.08	19.30
-1	-21	-7	320.67	21.50
-1	-21	-7	355.56	22.00
-1	-21	-7	314.57	22.60
1	21	7	318.27	22.10
1	21	7	293.27	20.80
-2	-21	-7	102.69	17.80
-2	-21	-7	92.29	15.50
-2	-21	-7	86.29	15.00
-2	-21	-7	78.69	15.10
2	21	7	92.49	14.10

-3	-21	-7	87.79	12.90
-3	-21	-7	65.49	18.00
-3	-21	-7	80.79	14.00
-3	-21	-7	83.09	13.40
3	21	7	111.69	15.50
-4	-21	-7	159.38	13.10
-4	-21	-7	131.19	12.70
-4	-21	-7	155.88	14.70
-4	-21	-7	126.99	13.50
4	21	7	144.29	22.40
-5	-21	-7	264.57	17.50
-5	-21	-7	271.07	17.10
-5	-21	-7	306.77	17.90
-5	-21	-7	294.67	19.80
-6	-21	-7	143.39	12.60
-6	-21	-7	170.08	13.30
-6	-21	-7	163.08	12.30
-6	-21	-7	165.58	17.30
-7	-21	-7	184.08	12.70
-7	-21	-7	164.38	13.20
-7	-21	-7	165.48	12.80
-8	-21	-7	-7.20	8.40
5	-22	-7	97.69	15.60
5	-22	-7	54.59	12.90
-5	22	7	76.49	11.40
4	-22	-7	118.59	14.30
4	-22	-7	134.39	19.80
-4	22	7	151.48	13.90
3	-22	-7	41.40	8.70
3	-22	-7	34.20	12.40
3	-22	-7	26.30	12.60
-3	22	7	42.60	13.30
2	-22	-7	72.79	11.70
2	-22	-7	59.69	11.10
2	-22	-7	84.29	13.30
-2	22	7	41.20	25.30
-2	22	7	83.59	12.60
1	-22	-7	97.49	14.60
1	-22	-7	117.59	14.40
1	-22	-7	90.39	11.60
-1	22	7	125.69	16.30
-1	22	7	117.99	16.10
0	-22	-7	637.34	33.30
0	-22	-7	655.73	34.50

0	-22	-7	631.14	33.30
0	-22	-7	623.74	33.60
0	22	7	656.63	34.70
-1	-22	-7	11.30	8.00
-1	-22	-7	9.90	8.10
-1	-22	-7	5.80	10.40
-1	-22	-7	18.90	7.50
1	22	7	18.90	10.30
-2	-22	-7	12.20	7.70
-2	-22	-7	-19.40	11.90
-2	-22	-7	-13.00	6.80
-2	-22	-7	-2.70	7.30
2	22	7	-18.90	9.20
-3	-22	-7	39.20	7.40
-3	-22	-7	49.00	7.60
-3	-22	-7	33.30	7.80
-4	-22	-7	161.08	12.90
-4	-22	-7	157.58	14.50
-4	-22	-7	150.18	12.70
-5	-22	-7	60.39	9.40
-5	-22	-7	33.30	9.10
-5	-22	-7	46.50	8.20
-5	-22	-7	33.40	6.50
-6	-22	-7	2.50	5.70
-6	-22	-7	18.30	10.20
-6	-22	-7	12.20	6.90
-6	-22	-7	20.20	6.10
6	22	7	9.10	16.00
-7	-22	-7	154.58	12.20
-7	-22	-7	154.38	11.90
-7	-22	-7	175.78	11.90
7	22	7	203.18	19.90
4	-23	-7	-0.30	9.60
-4	23	7	11.40	7.30
3	-23	-7	-0.80	12.80
3	-23	-7	-4.50	7.30
3	-23	-7	-2.00	15.10
2	-23	-7	198.18	18.30
2	-23	-7	210.98	16.10
2	-23	-7	194.18	18.20
-2	23	7	247.48	19.50
-2	23	7	180.38	22.20
1	-23	-7	31.30	9.70
1	-23	-7	19.60	9.10

1	-23	-7	32.20	9.00
-1	23	7	6.90	10.50
-1	23	7	53.59	12.80
0	-23	-7	13.70	8.30
0	-23	-7	-0.20	7.40
0	-23	-7	9.40	9.20
0	23	7	14.60	9.50
-1	-23	-7	8.10	7.30
-1	-23	-7	10.10	6.70
-1	-23	-7	6.20	10.30
-1	-23	-7	0.50	7.30
1	23	7	18.20	9.80
-2	-23	-7	69.89	10.90
-2	-23	-7	52.99	8.60
-2	-23	-7	42.00	13.50
-2	-23	-7	76.59	10.30
2	23	7	82.69	14.20
-3	-23	-7	21.50	7.60
-3	-23	-7	30.80	6.90
-3	-23	-7	29.40	6.90
3	23	7	22.50	13.50
-4	-23	-7	37.10	8.40
-4	-23	-7	31.00	6.70
-4	-23	-7	53.29	9.30
-5	-23	-7	590.44	30.80
-5	-23	-7	608.04	29.50
-5	-23	-7	586.04	29.50
-6	-23	-7	322.07	17.40
-6	-23	-7	310.47	21.60
-6	-23	-7	317.27	17.50
2	-24	-7	-9.60	11.30
2	-24	-7	3.00	7.80
2	-24	-7	17.20	11.70
-2	24	7	-9.10	12.30
1	-24	-7	80.49	11.70
1	-24	-7	93.89	13.00
1	-24	-7	90.19	12.80
-1	24	7	87.49	18.00
-1	24	7	49.70	13.90
0	-24	-7	238.88	17.40
0	-24	-7	232.08	16.50
0	-24	-7	275.67	18.40
0	24	7	251.27	20.20
0	24	7	211.38	18.50

-1	-24	-7	223.78	15.90
-1	-24	-7	230.98	15.60
-1	-24	-7	217.28	14.80
-1	-24	-7	244.68	18.90
1	24	7	213.98	16.90
1	24	7	203.58	17.70
-2	-24	-7	-21.90	18.00
-2	-24	-7	1.70	5.90
-2	-24	-7	11.70	7.10
-2	-24	-7	8.30	7.40
2	24	7	-5.20	9.60
-3	-24	-7	96.79	11.90
-3	-24	-7	100.49	10.30
-3	-24	-7	99.79	10.50
3	24	7	140.49	14.70
-4	-24	-7	82.79	9.70
-4	-24	-7	114.99	10.30
-4	-24	-7	70.79	11.70
4	24	7	96.49	14.30
5	24	7	8.40	10.30
0	-25	-7	-4.30	9.10
0	-25	-7	-3.60	8.90
0	25	7	27.10	10.50
-1	-25	-7	38.90	8.10
-1	-25	-7	41.80	7.80
1	25	7	49.40	14.00
1	25	7	45.10	9.30
-2	-25	-7	6.20	6.20
-2	-25	-7	-2.20	7.80
2	25	7	-5.50	8.50
2	25	7	-14.00	10.30
2	21	-8	6.90	10.80
2	21	-8	17.90	8.30
-2	-21	8	14.10	6.90
1	21	-8	15.90	9.20
1	21	-8	24.00	9.00
-1	-21	8	23.30	9.70
-1	-21	8	17.90	8.20
0	21	-8	49.40	11.00
0	21	-8	49.00	10.00
0	21	-8	54.79	13.40
0	-21	8	37.40	9.90
0	-21	8	43.30	10.10
-1	21	-8	12.20	8.50

-1	21	-8	25.10	11.60
-1	21	-8	20.40	15.20
1	-21	8	2.60	10.80
4	20	-8	-9.80	8.80
-4	-20	8	1.90	5.80
3	20	-8	35.10	8.00
3	20	-8	16.20	11.60
-3	-20	8	37.10	6.80
2	20	-8	113.79	18.60
2	20	-8	76.69	10.80
2	20	-8	97.59	12.90
-2	-20	8	129.19	18.40
-2	-20	8	91.69	11.40
1	20	-8	4.60	9.00
1	20	-8	14.20	8.40
1	20	-8	11.50	11.20
-1	-20	8	13.10	8.60
-1	-20	8	-3.30	7.80
0	20	-8	51.79	10.30
0	20	-8	31.50	8.80
0	20	-8	35.80	10.90
0	-20	8	41.10	9.80
0	-20	8	39.70	8.40
-1	20	-8	419.16	22.80
-1	20	-8	394.86	24.70
-1	20	-8	400.06	23.60
1	-20	8	335.47	26.80
1	-20	8	400.76	22.30
-2	20	-8	77.09	12.40
-2	20	-8	55.29	10.90
-2	20	-8	81.89	15.40
2	-20	8	82.39	11.20
-3	20	-8	19.10	9.80
-3	20	-8	-3.20	7.40
3	-20	8	-9.90	6.70
5	19	-8	135.49	12.90
-5	-19	8	139.59	11.10
4	19	-8	36.40	7.90
4	19	-8	64.69	16.30
-4	-19	8	54.19	9.60
3	19	-8	65.79	11.40
3	19	-8	60.89	14.10
3	19	-8	55.79	8.60
-3	-19	8	62.19	10.20

2	19	-8	9.90	9.10
2	19	-8	25.50	11.40
2	19	-8	0.30	7.50
-2	-19	8	16.40	10.30
-2	-19	8	6.10	6.60
1	19	-8	277.67	18.70
1	19	-8	248.28	18.70
1	19	-8	290.57	25.90
-1	-19	8	250.57	19.20
-1	-19	8	278.77	18.30
0	19	-8	255.47	18.70
0	19	-8	263.67	20.10
0	19	-8	282.97	18.60
0	-19	8	251.37	18.30
0	-19	8	288.27	19.30
-1	19	-8	-1.70	10.20
-1	19	-8	-11.10	7.60
1	-19	8	7.10	7.60
1	-19	8	-19.60	10.40
-2	19	-8	167.58	15.00
-2	19	-8	172.48	19.30
2	-19	8	185.58	15.00
-3	19	-8	3.70	7.80
-3	19	-8	18.30	9.20
3	-19	8	-1.10	7.20
-4	19	-8	6.10	7.70
-4	19	-8	-17.60	9.20
4	-19	8	-5.40	7.00
-5	19	-8	126.99	14.40
-5	19	-8	106.49	14.90
5	-19	8	131.29	12.80
6	18	-8	113.09	12.00
-6	-18	8	89.29	9.90
-6	-18	8	113.19	13.30
5	18	-8	25.90	7.80
-5	-18	8	35.20	6.50
-5	-18	8	38.70	12.30
4	18	-8	-4.60	11.50
4	18	-8	18.50	7.80
-4	-18	8	11.30	6.20
3	18	-8	95.49	17.80
3	18	-8	68.89	9.40
3	18	-8	69.99	11.00
-3	-18	8	60.29	10.60

2	18	-8	16.60	9.50
2	18	-8	2.50	9.10
2	18	-8	-4.50	7.60
-2	-18	8	-11.60	9.10
-2	-18	8	-4.20	6.70
1	18	-8	14.40	8.60
1	18	-8	6.70	8.80
1	18	-8	1.90	8.50
-1	-18	8	25.00	8.30
-1	-18	8	22.30	9.00
0	18	-8	798.82	41.60
0	18	-8	810.32	41.10
0	18	-8	824.22	41.20
0	-18	8	815.22	41.30
0	-18	8	843.12	41.40
-1	18	-8	117.59	17.30
-1	18	-8	137.19	15.10
-1	18	-8	122.59	14.90
1	-18	8	161.08	17.30
1	-18	8	129.89	14.70
-2	18	-8	268.07	22.50
-2	18	-8	280.07	19.00
-2	18	-8	276.97	19.40
2	-18	8	256.07	18.60
-3	18	-8	25.50	8.80
-3	18	-8	9.80	8.80
-3	18	-8	44.60	13.80
3	-18	8	19.60	7.90
-4	18	-8	17.90	11.80
-4	18	-8	20.80	8.10
4	-18	8	17.20	7.40
-5	18	-8	12.10	9.50
-5	18	-8	37.10	8.60
5	-18	8	25.00	8.70
5	-18	8	12.10	12.40
-6	18	-8	2.80	9.90
-6	18	-8	22.10	9.90
6	-18	8	3.60	10.00
6	-18	8	9.10	9.90
7	17	-8	-0.50	7.40
-7	-17	8	2.70	7.70
-7	-17	8	5.60	6.70
6	17	-8	33.70	8.00
-6	-17	8	7.10	7.10

-6	-17	8	19.30	6.40
5	17	-8	376.86	21.60
-5	-17	8	347.17	21.40
-5	-17	8	372.36	20.60
4	17	-8	159.68	14.70
4	17	-8	165.28	21.00
-4	-17	8	151.78	13.00
-4	-17	8	135.49	16.40
3	17	-8	-0.30	7.80
3	17	-8	0.60	10.60
-3	-17	8	5.50	6.50
-3	-17	8	17.10	11.90
2	17	-8	67.89	13.00
2	17	-8	33.40	9.60
-2	-17	8	34.80	10.40
-2	-17	8	48.20	10.00
1	17	-8	5.20	8.00
0	17	-8	119.59	21.40
0	17	-8	89.59	13.50
-1	17	-8	8.40	18.30
-1	17	-8	5.40	8.10
-1	17	-8	6.00	16.10
1	-17	8	11.10	11.00
-2	17	-8	-5.70	10.80
-2	17	-8	25.60	8.80
-2	17	-8	12.40	9.20
2	-17	8	-6.70	13.70
2	-17	8	5.80	9.00
-3	17	-8	118.19	19.90
-3	17	-8	109.19	14.50
-3	17	-8	124.59	14.90
3	-17	8	79.09	14.80
-4	17	-8	-12.80	14.70
-4	17	-8	-7.00	8.10
-4	17	-8	-6.70	8.00
4	-17	8	1.60	8.00
4	-17	8	1.90	10.80
-5	17	-8	348.17	23.10
-5	17	-8	372.16	22.00
5	-17	8	339.47	21.70
5	-17	8	362.16	22.30
-6	17	-8	391.16	22.80
-6	17	-8	382.06	23.90
6	-17	8	391.36	24.00

6	-17	8	419.96	22.40
7	16	-8	231.98	16.10
-7	-16	8	211.28	14.50
-7	-16	8	249.38	15.40
6	16	-8	258.87	17.70
-6	-16	8	238.58	15.90
-6	-16	8	243.48	16.10
5	16	-8	3.80	7.80
-5	-16	8	3.70	7.40
-5	-16	8	9.20	6.50
4	16	-8	17.40	8.80
4	16	-8	-2.10	11.60
-4	-16	8	12.70	8.80
-4	-16	8	0.70	6.90
3	16	-8	-0.70	8.00
3	16	-8	-4.80	8.90
-3	-16	8	-1.20	6.40
-3	-16	8	-4.10	19.30
2	16	-8	17.40	9.10
2	16	-8	7.90	8.20
-2	-16	8	-6.30	8.90
-2	-16	8	-3.40	6.60
1	16	-8	7.10	8.40
1	16	-8	6.10	8.10
-1	-16	8	9.40	7.70
-1	-16	8	-8.10	8.10
0	16	-8	79.79	12.10
0	16	-8	110.09	14.10
0	16	-8	71.29	10.50
0	-16	8	88.79	13.70
0	-16	8	71.99	9.90
-1	16	-8	848.72	44.20
-1	16	-8	841.02	43.80
-1	16	-8	886.11	44.20
1	-16	8	912.41	44.70
1	-16	8	896.01	44.10
-2	16	-8	53.49	9.40
-2	16	-8	44.50	9.40
-2	16	-8	40.50	10.80
2	-16	8	49.50	13.60
2	-16	8	37.40	8.70
-3	16	-8	19.60	8.40
-3	16	-8	28.30	10.20
3	-16	8	21.00	9.70

3	-16	8	6.50	12.70
-4	16	-8	262.57	20.00
-4	16	-8	248.48	26.40
-4	16	-8	310.97	21.30
4	-16	8	307.67	21.00
4	-16	8	260.17	20.80
-5	16	-8	7.60	8.30
-5	16	-8	4.90	8.80
5	-16	8	-1.80	8.50
5	-16	8	10.80	9.20
-6	16	-8	60.99	10.00
-6	16	-8	65.99	12.30
6	-16	8	48.20	8.80
6	-16	8	52.99	11.30
-7	16	-8	74.89	11.70
-7	16	-8	81.89	12.20
7	-16	8	70.39	11.70
8	15	-8	5.50	8.00
8	15	-8	2.40	13.10
-8	-15	8	-4.50	8.30
-8	-15	8	2.90	5.50
7	15	-8	26.00	13.20
7	15	-8	14.40	7.90
-7	-15	8	24.90	7.00
-7	-15	8	16.90	6.70
6	15	-8	185.58	23.20
6	15	-8	222.28	17.40
-6	-15	8	242.78	16.40
-6	-15	8	261.57	16.50
5	15	-8	87.69	16.90
5	15	-8	120.39	16.00
-5	-15	8	115.99	13.30
-5	-15	8	90.19	13.40
4	15	-8	128.99	16.90
4	15	-8	124.09	15.60
-4	-15	8	117.69	15.00
-4	-15	8	138.89	13.90
3	15	-8	28.30	10.20
3	15	-8	51.39	9.70
-3	-15	8	30.80	7.30
-3	-15	8	22.20	11.00
-3	-15	8	29.90	9.20
2	15	-8	20.50	8.60
2	15	-8	-4.50	7.50

-2	-15	8	-0.80	6.70
1	15	-8	416.76	25.40
1	15	-8	410.36	25.00
-1	-15	8	411.16	24.10
-1	-15	8	414.56	25.50
0	15	-8	396.06	24.20
0	15	-8	391.36	24.40
0	15	-8	390.76	24.20
0	-15	8	418.96	23.90
0	-15	8	385.76	24.20
-1	15	-8	21.20	9.10
-1	15	-8	39.90	8.90
-1	15	-8	16.90	8.40
1	-15	8	21.90	8.50
1	-15	8	22.50	9.40
-2	15	-8	206.48	17.20
-2	15	-8	183.78	16.40
-2	15	-8	208.18	20.10
2	-15	8	239.78	17.50
2	-15	8	198.78	22.00
-3	15	-8	-11.10	7.70
-3	15	-8	0.00	8.30
-3	15	-8	-4.90	10.80
3	-15	8	1.80	7.70
3	-15	8	-0.40	10.40
-4	15	-8	18.10	15.50
-4	15	-8	16.80	8.90
-4	15	-8	22.70	9.30
4	-15	8	14.50	11.70
4	-15	8	36.30	9.90
-5	15	-8	65.99	10.90
-5	15	-8	98.29	16.20
-5	15	-8	48.30	16.60
5	-15	8	118.29	16.80
5	-15	8	85.09	10.50
-6	15	-8	152.58	16.30
-6	15	-8	158.38	16.40
6	-15	8	129.89	18.30
6	-15	8	130.79	14.50
-7	15	-8	184.78	18.30
-7	15	-8	252.97	17.60
7	-15	8	222.48	15.70
7	-15	8	183.58	23.00
-8	15	-8	14.80	8.30

8	-15	8	8.90	7.10
8	14	-8	-0.10	8.40
8	14	-8	12.30	12.00
-8	-14	8	10.20	8.10
-8	-14	8	3.70	5.70
7	14	-8	52.39	9.60
7	14	-8	83.09	13.30
-7	-14	8	71.79	10.90
-7	-14	8	73.79	11.70
6	14	-8	223.28	23.00
6	14	-8	264.47	19.50
-6	-14	8	252.67	17.60
-6	-14	8	262.57	18.00
5	14	-8	99.29	14.80
5	14	-8	112.09	21.00
-5	-14	8	93.29	12.30
-5	-14	8	84.79	13.40
4	14	-8	15.20	8.50
4	14	-8	12.30	13.00
4	14	-8	36.00	10.80
-4	-14	8	14.80	6.60
-4	-14	8	7.70	7.70
3	14	-8	91.29	14.40
3	14	-8	116.59	18.70
-3	-14	8	94.89	11.10
-3	-14	8	103.59	13.10
-3	-14	8	84.99	12.50
2	14	-8	7.90	7.80
2	14	-8	3.10	8.60
-2	-14	8	4.90	6.50
1	14	-8	127.19	14.00
1	14	-8	129.29	14.80
-1	-14	8	140.49	15.30
-1	-14	8	129.49	14.10
0	14	-8	937.31	48.10
0	14	-8	1014.50	47.70
0	-14	8	1009.60	47.90
0	-14	8	900.91	47.30
-1	14	-8	34.40	8.70
-1	14	-8	37.40	9.30
-1	14	-8	13.00	7.50
1	-14	8	11.80	8.80
1	-14	8	12.80	8.20
-2	14	-8	515.05	30.00

-2	14	-8	538.35	31.00
-2	14	-8	532.85	31.10
2	-14	8	511.45	31.90
2	-14	8	524.05	29.40
2	-14	8	546.75	31.90
-3	14	-8	-4.80	10.60
-3	14	-8	-7.80	8.10
-3	14	-8	8.70	8.60
3	-14	8	-5.30	7.20
3	-14	8	12.20	9.30
-4	14	-8	57.89	10.50
-4	14	-8	51.19	12.90
-4	14	-8	44.90	10.00
4	-14	8	43.30	9.20
4	-14	8	33.60	8.80
-5	14	-8	90.99	23.10
-5	14	-8	43.80	9.90
5	-14	8	59.29	11.00
5	-14	8	47.40	9.90
-6	14	-8	35.30	10.60
-6	14	-8	28.70	18.10
-6	14	-8	35.20	10.50
6	-14	8	48.30	11.80
6	-14	8	31.50	8.50
-7	14	-8	30.50	10.00
-7	14	-8	33.70	10.70
7	-14	8	15.20	13.80
7	-14	8	31.20	8.00
-8	14	-8	200.18	18.40
-8	14	-8	214.88	17.40
8	-14	8	197.38	15.10
9	13	-8	20.80	18.70
9	13	-8	30.20	10.10
-9	-13	8	15.10	5.50
8	13	-8	77.99	11.00
8	13	-8	84.89	13.40
-8	-13	8	77.59	13.60
-8	-13	8	89.79	10.30
7	13	-8	-1.60	8.70
7	13	-8	11.90	11.80
-7	-13	8	0.10	6.80
-7	-13	8	3.90	7.30
6	13	-8	13.40	8.80
6	13	-8	-12.50	17.40

-6	-13	8	0.70	8.00
-6	-13	8	2.70	7.40
5	13	-8	610.94	36.10
5	13	-8	636.54	34.30
-5	-13	8	656.73	33.00
-5	-13	8	622.44	32.90
4	13	-8	105.09	14.40
-4	-13	8	91.79	13.10
-4	-13	8	79.99	13.90
3	13	-8	22.70	12.70
3	13	-8	0.50	7.60
3	13	-8	-14.10	8.40
-3	-13	8	20.70	10.30
-3	-13	8	5.60	6.80
-3	-13	8	-1.60	8.30
2	13	-8	108.09	14.00
2	13	-8	105.59	13.30
-2	-13	8	103.59	15.30
-2	-13	8	101.29	12.60
1	13	-8	9.80	7.30
1	13	-8	12.20	8.00
-1	-13	8	12.20	8.10
-1	-13	8	18.50	6.90
0	13	-8	211.88	16.30
0	13	-8	234.98	18.00
0	-13	8	197.48	16.80
0	-13	8	202.78	16.20
-1	13	-8	15.00	8.10
-1	13	-8	17.30	8.90
-1	13	-8	19.90	7.20
1	-13	8	18.90	8.70
1	-13	8	28.00	8.10
-2	13	-8	-4.50	8.80
-2	13	-8	4.00	7.40
-2	13	-8	-3.70	8.30
2	-13	8	-7.20	10.50
2	-13	8	5.70	9.30
2	-13	8	-2.70	6.70
-3	13	-8	89.09	14.20
-3	13	-8	130.19	19.20
-3	13	-8	139.79	15.90
3	-13	8	116.09	15.80
3	-13	8	131.99	15.30
-4	13	-8	8.40	12.20

-4	13	-8	2.10	9.20
-4	13	-8	0.20	9.30
4	-13	8	11.60	8.20
4	-13	8	17.30	8.10
-5	13	-8	386.46	30.90
-5	13	-8	395.96	25.60
-5	13	-8	378.96	25.00
5	-13	8	343.87	23.80
5	-13	8	384.66	24.70
-6	13	-8	248.48	36.60
-6	13	-8	244.08	20.90
-6	13	-8	283.57	23.10
6	-13	8	276.07	21.20
-7	13	-8	129.39	18.00
-7	13	-8	157.18	17.40
7	-13	8	131.39	14.90
7	-13	8	132.39	23.70
-8	13	-8	42.80	10.30
-8	13	-8	58.89	13.20
8	-13	8	47.10	8.60
-9	13	-8	124.39	15.80
-9	13	-8	124.99	14.10
9	-13	8	119.79	12.40
9	12	-8	51.09	11.30
9	12	-8	39.30	10.80
8	12	-8	-7.40	10.80
8	12	-8	10.50	9.00
-8	-12	8	0.40	6.10
-8	-12	8	-4.50	8.40
7	12	-8	325.87	26.50
-7	-12	8	328.97	21.20
-7	-12	8	314.57	20.50
6	12	-8	70.29	12.90
-6	-12	8	59.19	9.30
-6	-12	8	98.39	12.80
5	12	-8	58.39	12.70
-5	-12	8	55.99	8.80
-5	-12	8	67.79	11.60
4	12	-8	25.80	11.40
-4	-12	8	-0.30	6.30
-4	-12	8	-4.20	7.30
3	12	-8	11.00	11.70
3	12	-8	7.30	7.80
-3	-12	8	-4.60	6.30

-3	-12	8	-11.10	8.20
-3	-12	8	19.50	10.30
2	12	-8	10.80	7.60
2	12	-8	15.10	8.00
-2	-12	8	26.80	7.40
-2	-12	8	23.20	8.90
1	12	-8	19.00	7.80
1	12	-8	31.60	7.70
-1	-12	8	34.30	7.30
-1	-12	8	24.90	8.00
0	12	-8	229.28	16.50
0	12	-8	265.17	18.50
0	-12	8	254.37	17.50
0	-12	8	214.08	17.50
-1	12	-8	1430.76	68.49
-1	12	-8	1489.85	67.79
-1	12	-8	1466.05	68.19
1	-12	8	1313.47	68.69
1	-12	8	1469.55	67.99
-2	12	-8	2.70	8.90
-2	12	-8	0.70	6.80
-2	12	-8	5.40	8.70
2	-12	8	7.10	12.90
2	-12	8	-2.40	6.80
-3	12	-8	5.20	8.20
-3	12	-8	4.60	10.50
3	-12	8	-0.80	7.50
-4	12	-8	772.02	39.20
-4	12	-8	789.82	43.00
-4	12	-8	751.32	39.50
4	-12	8	689.63	38.00
4	-12	8	730.93	38.60
-5	12	-8	20.40	14.60
-5	12	-8	6.40	10.00
-5	12	-8	-0.50	9.80
5	-12	8	3.40	7.80
5	-12	8	9.30	8.30
-6	12	-8	-0.60	10.70
-6	12	-8	-11.90	9.20
-6	12	-8	-0.30	14.90
6	-12	8	-4.10	7.80
6	-12	8	13.10	10.50
-7	12	-8	116.59	16.30
-7	12	-8	86.59	20.20

-7	12	-8	84.09	15.30
7	-12	8	93.09	12.40
7	-12	8	86.79	17.00
-8	12	-8	51.49	11.50
-8	12	-8	70.39	12.50
8	-12	8	43.90	8.30
-9	12	-8	70.49	11.40
-9	12	-8	49.70	12.80
9	-12	8	80.19	12.00
10	11	-8	-2.50	16.60
-10	-11	8	1.30	5.60
9	11	-8	17.20	10.60
9	11	-8	10.00	10.60
-9	-11	8	-1.60	5.80
8	11	-8	-12.30	11.40
-8	-11	8	-1.40	6.40
-8	-11	8	0.50	8.70
7	11	-8	31.60	12.60
-7	-11	8	21.30	7.40
-7	-11	8	11.00	8.60
6	11	-8	314.97	25.30
-6	-11	8	305.57	20.50
-6	-11	8	318.67	19.50
5	11	-8	43.80	12.50
-5	-11	8	35.20	7.50
-5	-11	8	37.40	8.00
4	11	-8	328.57	25.10
-4	-11	8	328.87	20.40
-4	-11	8	318.07	20.30
3	11	-8	6.50	7.90
3	11	-8	15.20	11.50
-3	-11	8	10.10	6.60
2	11	-8	65.79	12.30
2	11	-8	30.20	8.20
-2	-11	8	35.50	10.50
-2	-11	8	33.10	6.90
-2	-11	8	36.40	8.90
1	11	-8	871.91	41.40
1	11	-8	809.72	45.00
-1	-11	8	842.32	42.30
-1	-11	8	841.82	41.70
0	11	-8	20.00	6.60
0	11	-8	34.00	8.20
0	-11	8	19.00	7.00

0	-11	8	25.20	7.30
-1	11	-8	6.10	8.30
-1	11	-8	5.20	6.20
1	-11	8	11.10	8.60
-2	11	-8	708.83	42.00
-2	11	-8	621.04	32.60
2	-11	8	648.74	33.20
2	-11	8	552.24	34.40
-3	11	-8	32.40	10.90
-3	11	-8	24.60	8.10
-3	11	-8	22.40	9.10
3	-11	8	25.50	7.60
-4	11	-8	65.19	14.80
-4	11	-8	92.89	15.70
-4	11	-8	115.59	14.10
4	-11	8	79.59	14.70
-5	11	-8	1.30	9.50
-5	11	-8	10.20	14.40
-5	11	-8	23.30	8.90
5	-11	8	7.30	8.30
5	-11	8	15.30	7.60
-6	11	-8	564.14	37.30
-6	11	-8	515.45	31.30
-6	11	-8	529.65	32.60
6	-11	8	474.65	31.00
6	-11	8	546.85	29.70
-7	11	-8	111.89	16.80
-7	11	-8	72.49	13.80
-7	11	-8	132.39	27.90
7	-11	8	87.99	14.70
-8	11	-8	79.89	13.30
-8	11	-8	63.89	13.00
8	-11	8	94.59	14.20
-9	11	-8	19.30	14.80
-9	11	-8	48.80	11.00
9	-11	8	74.29	12.20
9	-11	8	15.60	18.80
-10	11	-8	318.87	21.40
-10	11	-8	269.87	23.10
10	-11	8	280.77	18.70
10	10	-8	26.40	10.10
-10	-10	8	11.80	5.90
9	10	-8	55.09	11.40
9	10	-8	94.99	17.00

-9	-10	8	78.29	10.60
8	10	-8	26.80	12.50
-8	-10	8	26.30	7.60
-8	-10	8	39.80	9.80
7	10	-8	20.80	12.00
-7	-10	8	11.40	6.60
6	10	-8	771.62	44.40
-6	-10	8	845.52	41.10
-6	-10	8	860.61	42.00
5	10	-8	140.29	19.60
-5	-10	8	111.09	12.70
-5	-10	8	91.39	12.80
4	10	-8	5.50	11.00
-4	-10	8	6.90	6.80
-4	-10	8	-6.20	5.40
3	10	-8	92.79	13.50
-3	-10	8	120.89	13.50
-3	-10	8	91.49	12.60
-3	-10	8	100.19	18.70
2	10	-8	82.49	12.50
2	10	-8	105.69	13.00
-2	-10	8	76.79	11.00
-2	-10	8	88.19	15.00
-2	-10	8	92.09	11.70
1	10	-8	193.08	23.10
1	10	-8	219.18	15.60
1	10	-8	203.48	16.40
-1	-10	8	206.98	15.60
-1	-10	8	206.78	16.50
0	10	-8	405.46	22.60
0	10	-8	378.66	30.30
0	10	-8	409.96	24.20
0	-10	8	417.76	23.60
0	-10	8	415.26	23.40
-1	10	-8	227.58	15.60
-1	10	-8	226.98	18.50
1	-10	8	212.08	18.30
1	-10	8	237.98	16.60
-2	10	-8	677.63	36.10
-2	10	-8	672.93	34.20
-2	10	-8	666.73	35.50
2	-10	8	697.43	34.70
2	-10	8	633.84	36.30
-3	10	-8	143.19	18.80

-3	10	-8	119.39	16.20
-3	10	-8	114.49	12.80
-4	10	-8	253.97	19.70
-4	10	-8	266.27	18.50
-4	10	-8	241.58	26.00
4	-10	8	235.48	18.90
-5	10	-8	150.68	15.70
-5	10	-8	133.99	18.00
-5	10	-8	141.69	25.30
5	-10	8	122.79	17.00
-6	10	-8	24.80	15.50
-6	10	-8	4.80	10.40
-6	10	-8	18.30	14.80
6	-10	8	6.70	7.70
6	-10	8	11.30	10.00
-7	10	-8	279.27	30.10
-7	10	-8	275.87	22.30
-7	10	-8	269.97	23.10
7	-10	8	271.97	20.10
7	-10	8	261.87	29.20
-8	10	-8	235.48	32.20
-8	10	-8	230.18	21.70
-8	10	-8	215.48	28.10
8	-10	8	275.07	21.80
-9	10	-8	13.70	12.10
-9	10	-8	-0.50	10.30
9	-10	8	3.40	7.60
-10	10	-8	53.69	15.00
-10	10	-8	94.69	16.60
10	-10	8	88.69	13.10
10	9	-8	110.79	13.70
-10	-9	8	108.39	10.50
9	9	-8	43.60	11.40
-9	-9	8	41.60	7.20
8	9	-8	93.29	21.20
-8	-9	8	111.39	14.40
-8	-9	8	89.79	13.70
7	9	-8	22.60	12.00
-7	-9	8	18.70	6.60
-7	-9	8	39.30	9.10
6	9	-8	65.39	13.20
-6	-9	8	49.50	8.50
-6	-9	8	62.49	10.90
5	9	-8	756.82	42.10

-5	-9	8	789.02	38.40
-5	-9	8	770.22	38.90
-4	-9	8	55.19	7.70
-4	-9	8	66.89	8.70
3	9	-8	79.99	13.10
-3	-9	8	67.89	11.70
-3	-9	8	68.89	10.40
-3	-9	8	71.29	9.10
2	9	-8	135.39	14.10
2	9	-8	124.79	18.70
-2	-9	8	128.89	12.00
-2	-9	8	132.29	16.70
1	9	-8	24.60	7.20
1	9	-8	16.60	11.50
-1	-9	8	32.10	7.00
0	9	-8	237.78	24.70
0	9	-8	255.07	17.60
0	9	-8	222.58	17.10
0	-9	8	256.87	17.60
-1	9	-8	64.39	10.50
-1	9	-8	75.09	10.10
-1	9	-8	55.09	18.00
1	-9	8	74.49	12.00
1	-9	8	80.79	10.40
1	-9	8	74.29	11.40
-2	9	-8	8.90	5.60
-2	9	-8	2.30	8.40
-2	9	-8	13.90	9.40
2	-9	8	0.70	7.50
2	-9	8	-2.50	10.70
2	-9	8	5.60	6.80
-3	9	-8	447.66	25.30
-3	9	-8	475.05	26.90
-3	9	-8	490.35	29.60
3	-9	8	463.75	27.00
3	-9	8	442.36	26.30
-4	9	-8	361.16	28.40
-4	9	-8	370.66	22.10
4	-9	8	350.76	25.90
4	-9	8	358.96	22.60
-5	9	-8	1155.48	55.89
-5	9	-8	1084.89	58.79
-5	9	-8	1145.99	55.29
5	-9	8	1105.99	55.09

-6	9	-8	165.98	26.00
-6	9	-8	177.98	17.00
-6	9	-8	142.89	18.60
6	-9	8	156.08	20.10
-7	9	-8	229.18	19.60
-7	9	-8	153.78	28.20
-7	9	-8	181.78	20.70
7	-9	8	190.38	17.90
-8	9	-8	32.80	11.00
-8	9	-8	27.60	11.10
8	-9	8	31.90	9.40
-9	9	-8	239.48	32.60
-9	9	-8	221.78	22.20
-9	9	-8	238.78	21.80
9	-9	8	261.17	18.90
-10	9	-8	25.40	11.40
-10	9	-8	19.30	13.40
10	-9	8	7.40	11.00
10	-9	8	33.20	9.90
10	8	-8	151.38	18.10
-10	-8	8	224.78	14.30
9	8	-8	19.40	11.60
-9	-8	8	18.90	6.70
8	8	-8	24.40	11.80
-8	-8	8	23.20	10.60
-8	-8	8	17.20	7.00
7	8	-8	745.43	42.10
-7	-8	8	761.32	38.10
-7	-8	8	765.72	39.20
6	8	-8	21.30	11.20
-6	-8	8	20.60	6.20
-6	-8	8	14.90	7.70
5	8	-8	375.86	26.00
-5	-8	8	305.87	20.40
-5	-8	8	332.77	19.80
4	8	-8	38.20	11.70
-4	-8	8	42.80	7.50
-4	-8	8	29.60	7.00
3	8	-8	20.40	10.90
-3	-8	8	4.10	6.10
-3	-8	8	20.50	7.30
2	8	-8	36.50	11.50
2	8	-8	28.50	12.10
-2	-8	8	23.20	13.00

-2	-8	8	33.90	9.70
-2	-8	8	39.90	6.60
1	8	-8	97.89	16.70
1	8	-8	101.59	13.10
1	8	-8	126.19	15.60
1	8	-8	107.89	13.50
-1	-8	8	102.39	13.40
-1	-8	8	99.79	17.90
-1	-8	8	79.49	11.20
0	8	-8	962.00	47.00
0	8	-8	921.41	48.70
0	8	-8	992.60	46.00
0	-8	8	1013.00	45.60
0	-8	8	929.81	48.90
0	-8	8	945.51	46.30
-1	8	-8	1538.45	73.69
-1	8	-8	1610.54	72.59
1	-8	8	1544.75	71.79
-2	8	-8	819.42	42.80
-2	8	-8	836.32	42.30
-2	8	-8	864.61	40.70
2	-8	8	832.02	40.40
2	-8	8	826.02	41.20
-3	8	-8	34.40	11.50
-3	8	-8	21.80	5.90
-3	8	-8	46.30	8.80
3	-8	8	25.10	7.30
-4	8	-8	493.65	26.50
-4	8	-8	515.85	33.30
-4	8	-8	500.75	32.30
-4	8	-8	458.25	27.30
4	-8	8	433.26	35.70
4	-8	8	470.15	27.40
-5	8	-8	31.90	9.20
-5	8	-8	16.70	11.00
-5	8	-8	32.00	8.20
5	-8	8	27.50	10.20
5	-8	8	21.70	10.30
-6	8	-8	98.49	16.90
-6	8	-8	98.89	13.80
-6	8	-8	107.09	18.60
-6	8	-8	110.39	16.80
6	-8	8	134.99	23.20
6	-8	8	76.29	12.70

-7	8	-8	103.49	15.80
-7	8	-8	82.09	13.50
-7	8	-8	79.79	17.10
-7	8	-8	87.89	20.60
7	-8	8	84.89	15.60
-8	8	-8	71.89	16.60
-8	8	-8	37.60	14.00
-8	8	-8	44.50	11.40
-8	8	-8	67.49	13.20
8	-8	8	46.00	9.90
-9	8	-8	93.79	13.60
-9	8	-8	30.30	18.50
-9	8	-8	65.39	12.90
-9	8	-8	71.49	12.70
9	-8	8	100.19	15.30
-10	8	-8	13.20	13.40
-10	8	-8	39.40	18.20
-10	8	-8	4.40	11.00
10	-8	8	9.30	10.50
10	-8	8	28.10	10.30
-11	8	-8	128.69	19.90
-11	8	-8	185.98	20.90
11	-8	8	129.29	16.70
11	-8	8	196.18	19.60
11	7	-8	146.09	20.60
-11	-7	8	122.89	14.50
10	7	-8	17.70	10.20
-10	-7	8	27.30	7.00
9	7	-8	-3.90	11.30
-9	-7	8	0.00	6.50
-9	-7	8	5.90	16.00
8	7	-8	44.70	12.50
-8	-7	8	52.79	12.00
-8	-7	8	47.10	12.30
-8	-7	8	49.50	8.10
7	7	-8	6.80	10.90
-7	-7	8	20.90	8.70
-7	-7	8	24.60	6.80
6	7	-8	190.98	20.30
-6	-7	8	169.98	15.40
-6	-7	8	182.78	14.20
-5	-7	8	265.97	22.70
4	7	-8	1320.07	64.99
-4	-7	8	1340.97	62.49

-4	-7	8	1322.77	62.19
3	7	-8	19.90	6.50
3	7	-8	10.20	11.00
-3	-7	8	17.40	5.50
-3	-7	8	3.50	7.00
2	7	-8	387.06	23.40
2	7	-8	350.16	18.70
2	7	-8	325.97	26.30
2	7	-8	337.67	19.80
-2	-7	8	340.77	23.20
-2	-7	8	298.87	19.40
-2	-7	8	343.67	19.50
1	7	-8	1105.19	50.49
1	7	-8	1012.90	49.80
1	7	-8	977.90	48.60
-1	-7	8	965.10	47.90
-1	-7	8	1018.70	48.70
-1	-7	8	1023.70	48.70
-1	-7	8	1073.89	50.89
0	7	-8	-0.70	7.50
0	7	-8	20.60	6.40
0	7	-8	18.10	7.60
0	7	-8	16.90	5.70
0	-7	8	7.50	5.50
0	-7	8	12.40	8.10
0	-7	8	20.50	4.90
-1	7	-8	49.80	9.20
-1	7	-8	42.70	6.60
-1	7	-8	42.20	9.50
1	-7	8	36.60	5.50
1	-7	8	34.60	7.40
1	-7	8	56.99	10.40
-2	7	-8	504.85	36.40
-2	7	-8	489.05	30.70
-2	7	-8	406.96	27.60
-2	7	-8	485.15	29.00
-2	7	-8	468.95	24.40
2	-7	8	468.85	24.10
-3	7	-8	104.99	17.40
-3	7	-8	77.49	10.10
-3	7	-8	92.99	14.40
3	-7	8	93.29	10.30
3	-7	8	78.39	12.70
-4	7	-8	3.40	7.30

-4	7	-8	19.20	16.40
-4	7	-8	19.70	12.20
-4	7	-8	18.00	6.10
-4	7	-8	32.80	12.00
4	-7	8	11.70	7.20
4	-7	8	1.00	7.80
-5	7	-8	71.59	13.40
-5	7	-8	116.19	16.70
-5	7	-8	76.89	11.30
-5	7	-8	103.89	13.50
-5	7	-8	97.79	13.40
5	-7	8	62.09	11.20
5	-7	8	111.49	13.60
-6	7	-8	537.95	30.50
-6	7	-8	533.05	31.80
-6	7	-8	511.25	34.70
-6	7	-8	536.25	29.40
-6	7	-8	521.45	39.20
6	-7	8	511.65	30.70
-7	7	-8	45.50	13.90
-7	7	-8	30.00	9.40
-7	7	-8	25.90	10.50
-7	7	-8	37.30	14.90
7	-7	8	25.60	14.90
-8	7	-8	113.59	16.00
-8	7	-8	106.59	18.10
-8	7	-8	120.19	15.70
-8	7	-8	125.09	15.40
8	-7	8	95.19	16.80
-9	7	-8	270.77	24.00
-9	7	-8	274.87	22.30
-9	7	-8	261.27	32.40
9	-7	8	255.87	34.40
-10	7	-8	337.07	25.20
-10	7	-8	356.56	26.00
-10	7	-8	311.57	25.70
10	-7	8	416.36	25.40
-11	7	-8	17.50	11.00
-11	7	-8	25.50	14.50
11	-7	8	18.00	10.00
11	-7	8	24.30	19.50
11	6	-8	178.38	18.20
-11	-6	8	174.18	13.70
10	6	-8	-4.00	11.20

-10	-6	8	1.20	6.20
9	6	-8	103.49	18.00
-9	-6	8	113.49	14.10
-9	-6	8	91.39	14.40
8	6	-8	-8.90	10.90
8	6	-8	0.00	17.50
-8	-6	8	-21.30	10.20
-8	-6	8	0.80	5.90
-8	-6	8	-0.30	11.30
7	6	-8	81.09	13.00
7	6	-8	64.69	17.30
-7	-6	8	99.29	11.10
-7	-6	8	92.89	15.00
-7	-6	8	102.49	17.60
6	6	-8	996.60	52.79
-6	-6	8	1073.29	50.69
-6	-6	8	1049.99	49.99
5	6	-8	2.00	10.10
-5	-6	8	15.50	6.60
-5	-6	8	8.00	5.60
4	6	-8	46.50	11.20
4	6	-8	28.80	10.10
-4	-6	8	40.10	7.30
-4	-6	8	40.10	7.00
3	6	-8	301.17	23.30
3	6	-8	267.87	16.20
-3	-6	8	269.87	17.00
-3	-6	8	268.07	18.00
-3	-6	8	282.47	17.10
2	6	-8	246.88	17.00
2	6	-8	227.78	13.60
-2	-6	8	207.18	14.80
-2	-6	8	214.98	14.30
1	6	-8	468.85	24.90
1	6	-8	490.45	25.10
1	6	-8	534.95	26.60
-1	-6	8	509.25	27.10
-1	-6	8	481.15	24.70
0	6	-8	141.39	10.50
0	-6	8	143.59	13.10
0	-6	8	139.59	9.80
0	-6	8	142.89	10.80
-1	6	-8	550.34	28.70
1	-6	8	595.44	31.10

1	-6	8	553.44	29.00
1	-6	8	597.74	28.00
-2	6	-8	446.66	21.80
-2	6	-8	398.46	23.70
-2	6	-8	392.96	22.00
2	-6	8	375.66	22.60
2	-6	8	427.96	21.00
2	-6	8	394.76	25.00
-3	6	-8	388.36	25.90
-3	6	-8	414.16	25.90
-3	6	-8	405.16	24.30
-3	6	-8	432.96	22.00
3	-6	8	400.86	21.30
-4	6	-8	579.04	33.80
-4	6	-8	643.44	40.60
-4	6	-8	571.24	34.00
-4	6	-8	591.94	31.10
-4	6	-8	590.54	30.00
4	-6	8	635.34	29.90
4	-6	8	519.05	31.50
-5	6	-8	264.87	23.00
-5	6	-8	253.27	18.20
-5	6	-8	249.48	16.70
-5	6	-8	251.37	22.00
-5	6	-8	249.88	26.00
5	-6	8	236.88	16.10
5	-6	8	214.18	21.50
-6	6	-8	65.19	10.10
-6	6	-8	22.40	15.00
-6	6	-8	42.70	14.10
-6	6	-8	53.79	8.70
6	-6	8	51.99	8.70
6	-6	8	38.30	21.50
-7	6	-8	206.88	21.40
-7	6	-8	219.48	25.40
-7	6	-8	225.28	25.70
-7	6	-8	270.07	24.60
-7	6	-8	227.98	17.60
-7	6	-8	215.28	19.10
7	-6	8	229.08	22.10
-8	6	-8	231.88	24.30
-8	6	-8	280.07	22.20
-8	6	-8	337.67	31.50
-8	6	-8	310.47	28.10

-8	6	-8	297.97	22.00
8	-6	8	313.57	22.60
8	-6	8	265.47	47.30
-9	6	-8	52.49	22.20
-9	6	-8	42.10	10.30
-9	6	-8	21.00	10.10
-9	6	-8	32.30	18.20
9	-6	8	45.40	11.10
-10	6	-8	42.90	11.60
-10	6	-8	18.00	11.60
-10	6	-8	41.50	12.30
-10	6	-8	70.09	25.40
10	-6	8	34.20	11.70
-11	6	-8	-16.00	12.90
-11	6	-8	-23.70	14.00
-11	6	-8	26.30	11.40
11	-6	8	4.30	10.00
11	-6	8	3.40	14.60
11	5	-8	181.98	20.00
-11	-5	8	182.08	14.30
10	5	-8	50.39	12.80
10	5	-8	49.30	18.90
-10	-5	8	73.59	12.70
-10	-5	8	55.89	8.10
9	5	-8	19.00	17.40
9	5	-8	-2.80	17.60
-9	-5	8	12.20	7.40
-9	-5	8	26.10	11.70
8	5	-8	182.38	24.50
8	5	-8	143.59	20.90
-8	-5	8	157.78	13.70
-8	-5	8	123.89	20.40
-8	-5	8	199.28	17.60
7	5	-8	34.20	13.60
7	5	-8	67.49	13.30
-7	-5	8	63.09	8.30
-7	-5	8	95.79	16.10
-7	-5	8	65.49	10.50
6	5	-8	195.78	23.50
6	5	-8	273.57	22.50
-6	-5	8	235.88	15.90
5	5	-8	511.35	30.50
5	5	-8	466.85	33.00
-5	-5	8	537.15	27.40

-5	-5	8	531.85	26.90
4	5	-8	36.30	7.40
4	5	-8	50.49	11.10
-4	-5	8	44.00	6.60
-4	-5	8	60.09	10.10
3	5	-8	3.50	5.10
3	5	-8	12.90	11.30
3	5	-8	-0.60	7.50
-3	-5	8	12.60	6.40
-3	-5	8	-8.90	4.40
2	5	-8	67.29	10.50
2	5	-8	65.19	11.00
-2	-5	8	65.29	8.50
-2	-5	8	67.09	7.20
1	5	-8	12.00	5.00
1	5	-8	24.00	5.50
1	5	-8	24.60	4.50
-1	-5	8	23.60	5.40
-1	-5	8	12.50	5.70
-1	-5	8	21.90	4.60
0	5	-8	337.27	18.70
0	5	-8	370.46	19.50
0	-5	8	392.76	20.60
0	-5	8	343.77	18.20
-1	5	-8	230.18	14.50
-1	5	-8	236.78	13.60
-1	5	-8	257.87	14.80
1	-5	8	227.38	16.20
1	-5	8	203.58	13.60
1	-5	8	234.98	13.00
-2	5	-8	483.15	24.40
2	-5	8	478.55	23.50
2	-5	8	482.35	24.80
2	-5	8	458.45	25.90
-3	5	-8	981.30	48.50
-3	5	-8	961.00	47.10
-3	5	-8	1067.19	46.70
3	-5	8	871.21	48.00
3	-5	8	951.60	47.90
3	-5	8	1064.39	46.00
-4	5	-8	674.63	34.50
-4	5	-8	675.23	34.10
-4	5	-8	680.63	36.20
-4	5	-8	636.14	37.30

-4	5	-8	660.53	35.90
4	-5	8	660.73	36.70
4	-5	8	703.43	32.50
-5	5	-8	487.15	29.40
-5	5	-8	403.86	32.80
-5	5	-8	490.85	26.80
-5	5	-8	485.45	29.60
-5	5	-8	498.15	25.80
5	-5	8	502.15	24.70
5	-5	8	468.05	29.80
-6	5	-8	66.79	13.30
-6	5	-8	43.00	13.40
-6	5	-8	40.60	8.10
-6	5	-8	69.69	14.50
-6	5	-8	63.99	12.30
-6	5	-8	68.59	11.70
6	-5	8	45.90	7.40
-7	5	-8	119.69	13.30
-7	5	-8	96.79	14.70
-7	5	-8	136.69	21.30
-7	5	-8	156.98	21.80
-7	5	-8	130.59	18.20
-7	5	-8	139.69	21.50
7	-5	8	123.99	15.90
-8	5	-8	57.49	12.70
-8	5	-8	39.00	11.80
-8	5	-8	59.79	13.70
-8	5	-8	57.69	14.30
-8	5	-8	40.00	9.60
8	-5	8	69.89	14.30
8	-5	8	38.50	10.90
-9	5	-8	348.37	32.70
-9	5	-8	435.36	26.70
-9	5	-8	396.26	25.60
-9	5	-8	377.96	32.00
-9	5	-8	426.76	27.30
9	-5	8	438.26	27.90
-10	5	-8	141.19	18.40
-10	5	-8	135.99	17.30
-10	5	-8	138.49	18.20
-10	5	-8	127.49	20.60
-10	5	-8	138.09	21.40
10	-5	8	134.89	21.20
-11	5	-8	41.60	12.80

-11	5	-8	38.20	12.70
-11	5	-8	41.70	11.40
11	-5	8	38.40	16.30
11	-5	8	38.40	11.10
11	4	-8	22.60	12.30
11	4	-8	4.00	24.80
-11	-4	8	20.10	7.30
10	4	-8	164.08	21.40
10	4	-8	163.98	25.30
-10	-4	8	224.68	18.80
-10	-4	8	223.28	15.40
9	4	-8	28.30	12.80
9	4	-8	40.10	19.90
-9	-4	8	25.90	7.60
8	4	-8	84.19	13.90
8	4	-8	115.49	15.10
-8	-4	8	82.49	14.50
-8	-4	8	50.89	13.30
-8	-4	8	70.99	11.90
7	4	-8	841.02	44.00
7	4	-8	762.72	42.40
-7	-4	8	796.72	41.00
-7	-4	8	805.92	39.60
-7	-4	8	776.72	40.10
6	4	-8	128.59	18.80
6	4	-8	88.59	13.80
-6	-4	8	82.79	11.20
-6	-4	8	77.39	13.20
-6	-4	8	75.69	10.90
5	4	-8	527.95	31.20
5	4	-8	495.75	31.50
-5	-4	8	529.45	26.80
-5	-4	8	511.05	27.50
4	4	-8	342.47	22.50
4	4	-8	317.37	23.50
-4	-4	8	300.07	18.40
3	4	-8	46.80	7.70
3	4	-8	51.29	7.80
-3	-4	8	51.39	9.00
-3	-4	8	49.60	6.80
2	4	-8	174.38	11.30
2	4	-8	149.39	10.50
-2	-4	8	158.38	9.70
-2	-4	8	130.79	10.60

1	4	-8	145.29	9.40
1	4	-8	145.89	10.10
1	4	-8	158.88	10.20
-1	-4	8	153.08	9.30
-1	-4	8	138.69	10.50
-1	-4	8	144.69	10.00
0	4	-8	728.53	34.30
0	4	-8	679.63	34.00
0	-4	8	794.02	35.30
0	-4	8	675.93	33.90
0	-4	8	712.73	33.70
-1	4	-8	285.97	14.80
-1	4	-8	277.87	15.20
1	-4	8	257.47	14.30
1	-4	8	262.97	16.00
-2	4	-8	831.12	37.70
-2	4	-8	757.32	37.00
2	-4	8	740.73	37.60
2	-4	8	772.72	36.20
-3	4	-8	422.06	21.40
-3	4	-8	394.56	21.80
-3	4	-8	423.26	23.10
3	-4	8	425.36	22.50
3	-4	8	369.86	22.50
3	-4	8	446.56	20.50
-4	4	-8	300.87	22.30
-4	4	-8	324.77	22.10
-4	4	-8	364.96	18.40
-4	4	-8	321.07	20.50
4	-4	8	325.37	17.00
-5	4	-8	7.90	7.10
-5	4	-8	24.40	10.50
-5	4	-8	18.40	12.00
-5	4	-8	15.80	8.40
-5	4	-8	6.30	5.00
5	-4	8	9.80	4.20
-6	4	-8	67.49	13.40
-6	4	-8	28.40	12.40
-6	4	-8	52.09	10.50
-6	4	-8	33.60	6.70
-6	4	-8	54.69	10.50
-6	4	-8	30.60	7.60
6	-4	8	43.80	6.40
-7	4	-8	12.20	7.90

-7	4	-8	10.90	10.20
-7	4	-8	18.00	6.90
-7	4	-8	23.40	12.30
-7	4	-8	17.90	10.80
7	-4	8	1.10	6.80
-8	4	-8	280.67	25.90
-8	4	-8	264.37	20.60
-8	4	-8	342.47	22.70
-8	4	-8	274.27	19.60
-8	4	-8	271.87	25.40
8	-4	8	307.57	23.00
8	-4	8	276.17	22.50
-9	4	-8	172.28	16.80
-9	4	-8	168.38	23.90
-9	4	-8	151.98	19.70
-9	4	-8	179.98	24.50
-9	4	-8	223.58	17.90
9	-4	8	156.58	21.70
9	-4	8	192.18	21.40
-10	4	-8	6.70	9.40
-10	4	-8	1.10	15.60
-10	4	-8	51.19	16.00
-10	4	-8	21.60	10.90
-10	4	-8	0.70	10.00
10	-4	8	13.70	13.10
-11	4	-8	109.49	21.00
-11	4	-8	88.09	15.90
-11	4	-8	115.49	17.60
11	-4	8	111.79	18.90
11	3	-8	0.30	23.10
11	3	-8	55.89	14.20
-11	-3	8	38.60	8.20
-11	-3	8	56.29	14.10
10	3	-8	70.99	14.20
10	3	-8	50.29	15.10
-10	-3	8	85.89	11.80
-10	-3	8	101.39	15.80
9	3	-8	21.40	13.00
9	3	-8	39.30	13.00
-9	-3	8	31.40	8.20
-9	-3	8	29.20	9.30
8	3	-8	134.39	18.90
8	3	-8	95.29	15.30
-8	-3	8	88.49	12.10

-8	-3	8	78.79	14.10
7	3	-8	19.50	11.40
7	3	-8	24.10	10.40
-7	-3	8	-1.20	9.20
-7	-3	8	6.30	5.90
-7	-3	8	9.00	7.80
6	3	-8	36.50	11.80
6	3	-8	33.10	10.80
-6	-3	8	16.70	7.80
-6	-3	8	9.20	8.20
-6	-3	8	18.00	8.00
5	3	-8	615.64	35.50
5	3	-8	653.53	35.90
-5	-3	8	653.73	32.10
4	3	-8	886.51	45.80
-4	-3	8	798.02	42.90
-4	-3	8	992.10	43.10
3	3	-8	66.59	8.40
3	3	-8	86.29	9.20
3	3	-8	82.19	6.60
-3	-3	8	78.59	6.90
-3	-3	8	58.99	8.50
-3	-3	8	84.29	7.70
2	3	-8	761.42	32.90
2	3	-8	685.73	32.70
-2	-3	8	591.84	33.00
-2	-3	8	737.63	32.70
1	3	-8	561.44	27.20
1	3	-8	603.74	27.10
-1	-3	8	566.64	27.30
-1	-3	8	540.05	27.00
0	3	-8	142.79	8.80
0	3	-8	139.69	8.70
0	-3	8	154.98	10.30
0	-3	8	134.89	8.70
0	-3	8	142.69	8.90
-1	3	-8	9.30	3.20
-1	3	-8	10.90	3.30
1	-3	8	4.30	3.60
1	-3	8	5.40	2.60
1	-3	8	1.00	2.50
-2	3	-8	992.70	44.40
-2	3	-8	936.21	44.30
-2	3	-8	955.30	44.60

2	-3	8	923.61	44.70
2	-3	8	932.31	43.60
-3	3	-8	37.30	6.70
3	-3	8	34.30	5.80
3	-3	8	30.90	4.40
3	-3	8	38.40	6.00
-4	3	-8	25.50	7.30
-4	3	-8	26.00	5.70
-4	3	-8	30.00	5.40
4	-3	8	17.80	3.20
4	-3	8	17.60	6.50
4	-3	8	10.10	6.40
-5	3	-8	297.07	23.00
-5	3	-8	339.57	23.70
-5	3	-8	361.46	18.50
-5	3	-8	306.27	26.10
-5	3	-8	334.17	21.60
-5	3	-8	316.47	20.30
-6	3	-8	388.06	22.30
-6	3	-8	357.76	26.20
-6	3	-8	398.06	27.80
-6	3	-8	387.26	24.90
-6	3	-8	403.66	27.20
-6	3	-8	388.26	21.30
6	-3	8	352.36	19.70
-7	3	-8	162.48	13.10
-7	3	-8	156.08	19.40
-7	3	-8	164.28	14.70
-7	3	-8	144.29	18.90
-7	3	-8	119.69	19.20
7	-3	8	185.68	13.10
-8	3	-8	16.60	6.60
-8	3	-8	19.40	10.30
-8	3	-8	8.00	11.30
-8	3	-8	9.60	7.30
-8	3	-8	15.20	10.20
-8	3	-8	17.80	11.10
8	-3	8	16.50	8.70
-9	3	-8	705.03	39.40
-9	3	-8	749.33	39.00
-9	3	-8	742.33	42.50
-9	3	-8	757.72	41.90
-9	3	-8	732.53	38.70
9	-3	8	761.32	42.70

9	-3	8	751.52	41.30
-10	3	-8	165.18	19.50
-10	3	-8	184.58	27.10
-10	3	-8	212.58	29.00
-10	3	-8	128.99	18.30
-10	3	-8	167.58	16.10
10	-3	8	215.78	23.90
-11	3	-8	-8.30	10.30
-11	3	-8	27.10	17.70
-11	3	-8	-2.80	8.90
-11	3	-8	18.30	10.70
-11	3	-8	-8.10	16.00
-12	3	-8	-2.30	11.70
-12	3	-8	10.80	13.80
-12	3	-8	30.00	12.90
12	-3	8	4.10	11.10
11	2	-8	64.59	14.80
-11	-2	8	63.99	14.50
10	2	-8	29.20	14.20
10	2	-8	39.30	13.20
-10	-2	8	12.10	7.40
-10	-2	8	18.90	9.70
9	2	-8	-8.20	12.40
9	2	-8	1.30	11.80
-9	-2	8	4.70	7.10
-9	-2	8	5.70	8.10
8	2	-8	20.70	12.60
8	2	-8	33.20	11.40
-8	-2	8	33.90	7.50
-8	-2	8	19.30	7.80
7	2	-8	259.87	25.00
7	2	-8	275.77	22.40
-7	-2	8	298.47	19.50
-7	-2	8	322.57	22.40
-7	-2	8	288.37	18.40
6	2	-8	718.23	38.80
6	2	-8	778.72	41.20
-6	-2	8	765.22	37.10
-6	-2	8	740.03	37.60
5	2	-8	21.70	11.50
5	2	-8	17.30	10.10
-5	-2	8	20.30	6.30
4	2	-8	57.39	6.70
4	2	-8	86.09	13.30

4	2	-8	36.70	7.10
-4	-2	8	52.69	8.70
-4	-2	8	65.49	7.00
-4	-2	8	58.19	9.60
3	2	-8	986.40	51.19
3	2	-8	1119.29	48.80
3	2	-8	1076.99	49.40
-3	-2	8	1134.69	49.50
-3	-2	8	1051.99	49.70
2	2	-8	585.84	26.60
-2	-2	8	551.84	26.90
1	2	-8	27.50	3.70
-1	-2	8	24.60	4.20
-1	-2	8	25.80	4.00
-1	-2	8	34.30	4.00
0	2	-8	97.39	6.10
0	2	-8	97.29	6.60
0	-2	8	97.29	7.80
0	-2	8	95.49	6.60
0	-2	8	89.39	6.80
-1	2	-8	1148.39	52.89
-1	2	-8	1201.48	52.69
1	-2	8	1195.48	52.99
1	-2	8	1146.09	52.39
1	-2	8	1061.09	52.79
-2	2	-8	11.80	3.10
-2	2	-8	10.10	3.40
2	-2	8	8.80	3.90
2	-2	8	8.90	2.30
-3	2	-8	325.97	17.10
3	-2	8	324.07	17.50
3	-2	8	317.87	15.60
3	-2	8	318.57	17.40
-4	2	-8	769.42	37.80
-4	2	-8	773.62	38.80
-4	2	-8	815.82	37.30
4	-2	8	805.62	36.20
4	-2	8	763.42	38.20
4	-2	8	766.82	38.40
-5	2	-8	118.49	14.10
-5	2	-8	97.59	12.90
-5	2	-8	122.09	17.10
-5	2	-8	128.69	12.50
-5	2	-8	117.39	13.60

-5	2	-8	127.29	19.60
5	-2	8	97.19	14.10
5	-2	8	125.39	8.00
-6	2	-8	-6.20	10.90
-6	2	-8	7.60	6.70
-6	2	-8	5.60	9.80
-6	2	-8	13.30	8.10
-6	2	-8	-2.20	10.70
-6	2	-8	14.80	5.40
6	-2	8	6.70	3.50
-7	2	-8	345.57	25.70
-7	2	-8	351.06	23.60
-7	2	-8	292.27	25.80
-7	2	-8	310.07	18.90
-7	2	-8	298.17	20.00
7	-2	8	324.27	17.90
-8	2	-8	167.18	15.30
-8	2	-8	159.68	13.60
-8	2	-8	138.09	19.70
-8	2	-8	134.19	20.50
-8	2	-8	161.18	17.90
-8	2	-8	151.48	19.20
8	-2	8	152.48	14.30
-9	2	-8	134.09	19.80
-9	2	-8	104.79	13.50
-9	2	-8	122.99	16.90
-9	2	-8	102.29	14.90
-9	2	-8	91.89	14.00
9	-2	8	77.19	14.10
9	-2	8	91.29	14.60
-10	2	-8	22.40	9.50
-10	2	-8	20.70	8.20
10	-2	8	31.20	21.90
10	-2	8	24.00	17.00
-11	2	-8	3.00	14.70
-11	2	-8	-4.80	10.20
-11	2	-8	35.50	16.20
-11	2	-8	5.00	10.10
-11	2	-8	12.00	9.30
-12	2	-8	8.20	11.60
-12	2	-8	4.20	10.50
12	-2	8	-6.30	11.50
11	1	-8	241.28	26.40
-11	-1	8	255.77	18.50

-11	-1	8	233.58	18.40
10	1	-8	33.50	12.60
10	1	-8	28.10	14.50
-10	-1	8	18.90	8.30
-10	-1	8	1.40	8.20
9	1	-8	91.79	14.00
9	1	-8	85.39	15.60
-9	-1	8	108.49	13.40
-9	-1	8	116.59	14.40
8	1	-8	62.89	14.80
8	1	-8	77.59	12.20
-8	-1	8	83.69	12.50
-8	-1	8	104.99	13.70
7	1	-8	137.59	16.30
7	1	-8	96.89	17.40
-7	-1	8	135.19	13.90
-7	-1	8	106.99	12.50
6	1	-8	370.06	27.20
6	1	-8	370.76	24.60
-6	-1	8	412.76	22.20
-6	-1	8	403.76	23.00
5	1	-8	1181.78	59.29
5	1	-8	1278.07	61.29
-5	-1	8	1258.37	58.09
4	1	-8	60.89	8.50
4	1	-8	44.60	5.50
-4	-1	8	33.70	5.60
-4	-1	8	39.20	6.40
-4	-1	8	49.00	8.10
3	1	-8	23.70	3.20
3	1	-8	31.60	4.90
-3	-1	8	25.60	4.30
-3	-1	8	26.50	4.20
-3	-1	8	30.20	3.90
2	1	-8	3.70	1.30
2	1	-8	6.10	1.80
-2	-1	8	2.20	3.10
1	1	-8	118.69	5.90
-1	-1	8	88.99	7.70
-1	-1	8	120.29	6.00
0	1	-8	85.99	7.40
0	1	-8	105.99	5.70
0	-1	8	98.49	7.10
0	-1	8	92.69	6.80

0	-1	8	99.19	6.60
-1	1	-8	20.00	3.20
-1	1	-8	13.80	2.50
1	-1	8	11.80	2.70
1	-1	8	9.40	2.20
1	-1	8	13.20	2.60
-2	1	-8	702.93	33.30
-2	1	-8	702.13	33.30
2	-1	8	730.03	32.40
2	-1	8	720.03	33.30
2	-1	8	685.03	33.40
-3	1	-8	2702.43	120.59
-3	1	-8	2738.03	120.79
-3	1	-8	2641.14	121.09
3	-1	8	2547.84	119.69
3	-1	8	2716.03	120.99
3	-1	8	2713.53	121.09
-4	1	-8	624.14	29.50
-4	1	-8	632.64	30.00
-4	1	-8	604.54	30.80
4	-1	8	636.54	30.50
4	-1	8	573.34	30.30
4	-1	8	595.74	28.30
-5	1	-8	115.69	13.70
-5	1	-8	105.99	8.90
-5	1	-8	123.39	12.10
-5	1	-8	103.29	11.70
-5	1	-8	100.69	16.80
-5	1	-8	102.69	13.50
-5	1	-8	92.19	12.00
5	-1	8	104.09	6.90
5	-1	8	108.49	12.90
-6	1	-8	136.39	15.20
-6	1	-8	125.69	12.10
-6	1	-8	93.59	9.60
-6	1	-8	107.79	17.70
-6	1	-8	131.59	18.20
-6	1	-8	100.09	12.30
6	-1	8	107.49	7.70
-7	1	-8	6.70	8.30
-7	1	-8	7.50	9.60
-7	1	-8	4.10	9.50
-7	1	-8	9.50	10.00
-7	1	-8	3.00	5.10

7	-1	8	9.30	4.30
-8	1	-8	204.38	15.80
-8	1	-8	232.78	22.40
-8	1	-8	195.68	19.10
-8	1	-8	216.68	20.00
-8	1	-8	243.58	22.40
-8	1	-8	247.98	17.40
8	-1	8	251.57	15.70
-9	1	-8	63.39	10.60
-9	1	-8	87.39	13.00
-9	1	-8	73.29	11.80
-9	1	-8	89.39	14.10
-9	1	-8	54.39	9.20
9	-1	8	62.79	13.90
9	-1	8	48.90	13.90
-10	1	-8	476.35	27.70
-10	1	-8	533.65	30.20
-10	1	-8	484.05	28.30
-10	1	-8	559.44	32.50
-10	1	-8	464.85	33.30
10	-1	8	437.46	38.60
-11	1	-8	-24.70	14.60
-11	1	-8	16.30	9.20
-11	1	-8	0.20	14.10
-11	1	-8	-3.40	9.00
-11	1	-8	-4.10	9.80
-12	1	-8	1.70	9.50
-12	1	-8	1.90	17.60
-12	1	-8	28.80	11.90
12	-1	8	-6.90	12.00
11	0	-8	23.00	16.80
11	0	-8	40.60	13.40
-11	0	8	21.50	9.40
-11	0	8	20.40	9.40
10	0	-8	190.08	22.10
10	0	-8	226.08	27.10
-10	0	8	203.88	17.30
-10	0	8	212.08	17.60
9	0	-8	34.50	11.40
9	0	-8	32.10	14.50
-9	0	8	28.50	8.20
-9	0	8	50.59	9.10
8	0	-8	122.49	16.70
8	0	-8	125.69	14.40

-8	0	8	118.89	13.60
-8	0	8	105.39	14.20
7	0	-8	363.16	24.10
7	0	-8	359.96	28.10
-7	0	8	378.46	21.60
-7	0	8	344.47	21.60
6	0	-8	142.99	15.00
6	0	-8	144.29	21.90
-6	0	8	134.89	12.30
-6	0	8	124.79	15.20
5	0	-8	170.88	21.30
5	0	-8	178.68	16.20
-5	0	8	142.99	14.00
-5	0	8	161.38	12.70
4	0	-8	1210.38	60.79
4	0	-8	1426.76	60.59
-4	0	8	1356.66	62.09
-4	0	8	1427.86	61.59
-4	0	8	1294.17	61.79
-3	0	8	254.57	13.70
-3	0	8	272.37	13.50
-3	0	8	233.58	13.60
-2	0	8	496.25	24.00
-1	0	8	631.14	29.60
0	0	-8	1229.18	54.79
0	0	8	1301.87	55.89
0	0	8	1167.08	55.29
0	0	8	1188.68	55.49
-1	0	-8	7.80	2.00
1	0	8	9.70	1.90
1	0	8	7.60	3.20
1	0	8	12.00	2.30
-2	0	-8	1093.29	51.69
-2	0	-8	1169.28	51.69
2	0	8	1130.19	51.59
2	0	8	1103.29	51.79
2	0	8	1138.79	50.69
-3	0	-8	576.84	29.30
-3	0	-8	616.34	29.60
-3	0	-8	617.44	29.20
3	0	8	622.24	28.10
3	0	8	630.94	29.50
3	0	8	619.94	29.60
-4	0	-8	1065.89	48.20

4	0	8	992.20	48.70
4	0	8	1024.40	48.50
4	0	8	1044.20	46.80
-5	0	-8	174.98	17.90
-5	0	-8	155.38	20.60
-5	0	-8	135.89	18.90
-5	0	-8	165.88	15.70
-5	0	-8	163.28	12.70
-5	0	-8	191.78	11.30
-5	0	-8	184.88	15.40
5	0	8	186.88	9.50
5	0	8	177.18	13.90
5	0	8	185.98	15.40
-6	0	-8	16.20	8.20
-6	0	-8	16.70	5.10
-6	0	-8	37.80	10.60
-6	0	-8	25.00	7.10
-6	0	-8	30.50	10.90
-6	0	-8	26.90	9.20
-6	0	-8	7.80	9.70
6	0	8	19.90	13.50
6	0	8	17.30	3.30
-7	0	-8	51.39	8.30
-7	0	-8	47.30	11.20
-7	0	-8	34.30	8.40
-7	0	-8	29.10	9.30
-7	0	-8	36.70	6.70
-7	0	-8	54.59	10.30
7	0	8	26.40	4.50
-8	0	-8	636.44	37.50
-8	0	-8	658.83	33.80
-8	0	-8	675.93	35.40
-8	0	-8	671.03	34.40
-8	0	-8	677.23	38.90
-8	0	-8	675.63	35.80
8	0	8	654.03	33.00
-9	0	-8	89.79	11.90
-9	0	-8	106.49	17.30
-9	0	-8	123.79	16.70
-9	0	-8	80.49	13.00
-9	0	-8	67.89	13.20
9	0	8	106.59	14.50
9	0	8	102.99	15.50
-10	0	-8	25.60	9.60

-10	0	-8	7.70	8.20
-10	0	-8	6.10	12.70
-10	0	-8	11.40	7.90
-10	0	-8	11.50	10.90
10	0	8	0.30	15.50
-11	0	-8	61.29	10.80
-11	0	-8	66.79	10.30
-11	0	-8	80.59	15.50
-11	0	-8	70.69	10.90
-11	0	-8	62.99	14.30
-12	0	-8	21.40	17.90
-12	0	-8	-4.10	9.90
-12	0	-8	-6.50	8.80
-12	0	-8	1.30	11.40
12	0	8	1.20	11.70
11	-1	-8	33.60	12.10
11	-1	-8	12.60	19.60
-11	1	8	29.40	10.50
-11	1	8	34.60	9.40
10	-1	-8	94.89	17.50
10	-1	-8	81.79	14.20
-10	1	8	83.29	10.60
-10	1	8	94.29	15.20
9	-1	-8	79.39	16.70
9	-1	-8	91.19	16.80
-9	1	8	95.99	14.30
-9	1	8	79.89	13.70
8	-1	-8	143.29	17.90
8	-1	-8	135.79	18.00
-8	1	8	151.28	14.90
-8	1	8	170.98	15.30
7	-1	-8	24.80	9.00
7	-1	-8	13.50	13.30
-7	1	8	19.70	7.70
-7	1	8	21.50	7.20
6	-1	-8	2.10	8.00
6	-1	-8	21.50	13.60
-6	1	8	16.30	6.40
-6	1	8	13.50	7.80
5	-1	-8	1013.70	48.70
5	-1	-8	975.60	51.89
-5	1	8	1035.40	47.70
-5	1	8	964.50	47.20
-4	1	8	851.61	41.10

-4	1	8	862.11	41.70
-4	1	8	920.41	41.30
-3	1	8	21.40	3.80
-3	1	8	18.90	3.80
-2	1	8	1405.66	68.49
-2	1	8	1594.64	68.79
-2	1	8	1537.35	68.99
-1	1	8	394.56	17.00
-1	1	8	290.67	16.70
0	1	8	270.77	13.40
0	1	8	258.87	13.20
0	1	8	270.77	12.70
-1	-1	-8	13.90	2.60
1	1	8	19.30	3.40
1	1	8	15.30	2.70
1	1	8	14.00	2.10
-2	-1	-8	1754.92	75.19
-2	-1	-8	1627.34	77.19
2	1	8	1587.64	74.29
2	1	8	1659.73	75.19
2	1	8	1668.53	75.99
2	1	8	1671.73	74.19
-3	-1	-8	2846.72	127.29
-3	-1	-8	2848.31	127.09
3	1	8	2726.93	127.19
3	1	8	2865.51	127.39
3	1	8	2838.02	125.99
-4	-1	-8	22.00	4.10
4	1	8	19.20	2.60
4	1	8	22.50	4.80
4	1	8	17.80	4.60
-5	-1	-8	99.89	10.00
-5	-1	-8	105.89	12.10
-5	-1	-8	104.09	16.80
-5	-1	-8	101.69	8.40
-5	-1	-8	102.09	14.00
5	1	8	110.09	6.30
5	1	8	112.49	11.90
5	1	8	106.69	11.30
-6	-1	-8	137.99	17.20
-6	-1	-8	153.48	13.20
-6	-1	-8	171.48	11.20
-6	-1	-8	162.58	18.00
-6	-1	-8	164.08	19.60

-6	-1	-8	141.49	14.20
-6	-1	-8	163.28	17.20
6	1	8	157.48	9.10
-7	-1	-8	26.80	5.10
-7	-1	-8	43.70	8.50
-7	-1	-8	26.20	5.90
-7	-1	-8	47.50	10.60
-7	-1	-8	35.70	9.30
7	1	8	42.30	5.80
-8	-1	-8	134.29	16.10
-8	-1	-8	107.69	15.50
-8	-1	-8	131.99	11.80
-8	-1	-8	145.49	18.20
-8	-1	-8	129.99	13.30
-8	-1	-8	140.99	20.30
8	1	8	122.49	10.60
-9	-1	-8	665.83	34.50
-9	-1	-8	637.04	36.90
-9	-1	-8	655.03	37.60
-9	-1	-8	670.83	35.20
-9	-1	-8	649.34	35.70
9	1	8	669.93	34.20
-10	-1	-8	132.59	16.30
-10	-1	-8	151.78	15.10
-10	-1	-8	113.39	19.40
-10	-1	-8	139.79	20.80
-10	-1	-8	158.18	14.30
10	1	8	158.38	22.50
-11	-1	-8	8.30	8.30
-11	-1	-8	-3.40	11.80
-11	-1	-8	8.50	9.00
-11	-1	-8	5.50	9.00
-11	-1	-8	2.90	12.70
-12	-1	-8	22.30	23.50
-12	-1	-8	15.50	10.90
-12	-1	-8	17.40	9.10
-12	-1	-8	18.20	9.70
12	1	8	14.90	12.30
11	-2	-8	0.10	11.40
-11	2	8	32.90	10.90
-11	2	8	5.90	8.30
10	-2	-8	101.19	19.80
10	-2	-8	113.69	19.10
-10	2	8	94.49	14.40

-10	2	8	126.59	16.30
9	-2	-8	50.89	11.30
9	-2	-8	46.30	16.80
-9	2	8	59.39	9.30
-9	2	8	52.39	9.90
8	-2	-8	16.20	9.90
8	-2	-8	-6.80	14.40
-8	2	8	7.60	7.60
-8	2	8	-8.40	7.30
7	-2	-8	366.06	23.60
7	-2	-8	344.17	29.10
-7	2	8	361.86	22.00
-7	2	8	341.57	21.50
6	-2	-8	44.60	9.10
6	-2	-8	58.79	12.00
6	-2	-8	62.99	15.60
-6	2	8	45.10	8.50
-6	2	8	72.49	10.60
5	-2	-8	635.74	34.50
5	-2	-8	713.33	38.10
5	-2	-8	656.43	39.90
-5	2	8	671.03	33.20
-5	2	8	717.43	37.40
-5	2	8	636.44	35.00
-5	2	8	621.24	32.20
-4	2	8	812.72	37.10
-4	2	8	762.42	37.90
-3	2	8	2387.26	104.09
-3	2	8	2294.17	104.19
-3	2	8	2227.98	103.69
-2	2	8	81.99	5.80
-2	2	8	73.09	5.80
-2	2	8	68.59	5.40
-1	2	8	47.00	4.70
-1	2	8	64.39	5.40
-1	2	8	65.89	6.40
0	2	8	83.59	5.30
0	2	8	91.89	4.40
0	2	8	85.19	5.60
0	2	8	79.19	4.40
1	2	8	259.37	12.70
1	2	8	269.07	13.40
2	2	8	491.85	22.90
2	2	8	474.15	23.10

-3	-2	-8	79.89	6.10
-3	-2	-8	80.29	6.60
3	2	8	80.39	4.70
3	2	8	81.59	4.20
3	2	8	85.29	6.50
3	2	8	84.49	6.50
-4	-2	-8	4289.87	191.58
4	2	8	4450.55	192.48
4	2	8	4270.27	190.48
4	2	8	4095.39	191.78
-5	-2	-8	156.18	10.00
-5	-2	-8	146.19	10.60
5	2	8	160.58	12.20
5	2	8	130.29	12.50
-6	-2	-8	51.19	10.00
-6	-2	-8	62.89	8.30
-6	-2	-8	36.80	6.50
-6	-2	-8	37.70	8.50
-6	-2	-8	58.89	7.30
-6	-2	-8	38.90	14.60
-6	-2	-8	57.49	11.40
6	2	8	52.89	4.80
-7	-2	-8	356.66	24.40
-7	-2	-8	376.16	20.20
-7	-2	-8	372.56	23.00
-7	-2	-8	369.16	21.00
-7	-2	-8	376.76	22.70
-7	-2	-8	355.86	26.00
7	2	8	370.26	18.70
-8	-2	-8	80.49	11.60
-8	-2	-8	94.89	14.20
-8	-2	-8	100.49	16.30
-8	-2	-8	102.29	14.10
-8	-2	-8	93.99	10.10
8	2	8	67.39	8.50
-9	-2	-8	114.29	18.60
-9	-2	-8	103.69	15.20
-9	-2	-8	118.69	13.40
-9	-2	-8	112.59	15.80
-9	-2	-8	110.89	12.60
9	2	8	80.79	12.60
-10	-2	-8	179.18	18.80
-10	-2	-8	196.18	17.60
-10	-2	-8	180.58	15.70

-10	-2	-8	201.78	21.60
-10	-2	-8	162.88	14.40
10	2	8	153.78	21.20
-11	-2	-8	22.40	8.70
-11	-2	-8	29.10	8.70
-11	-2	-8	31.20	9.10
-11	-2	-8	26.80	13.00
-11	-2	-8	26.30	11.80
-12	-2	-8	-7.70	9.30
-12	-2	-8	15.10	8.70
-12	-2	-8	13.00	10.10
12	2	8	8.30	13.10
11	-3	-8	214.78	20.10
-11	3	8	254.57	21.90
-11	3	8	248.98	17.70
10	-3	-8	52.29	21.50
10	-3	-8	41.10	12.30
-10	3	8	19.80	10.20
-10	3	8	24.40	8.40
9	-3	-8	-0.60	9.90
9	-3	-8	-1.10	17.80
-9	3	8	-2.60	8.20
-9	3	8	1.60	7.90
8	-3	-8	450.45	27.70
8	-3	-8	382.16	34.50
-8	3	8	465.85	26.30
-8	3	8	475.45	27.00
7	-3	-8	333.87	29.90
7	-3	-8	326.67	22.90
-7	3	8	371.26	21.50
-7	3	8	351.26	21.80
6	-3	-8	106.19	16.30
6	-3	-8	109.09	14.10
6	-3	-8	149.88	25.90
-6	3	8	80.89	13.30
5	-3	-8	68.19	16.40
5	-3	-8	38.80	9.50
5	-3	-8	65.79	13.30
5	-3	-8	57.29	10.30
-5	3	8	47.70	10.40
-5	3	8	77.79	13.40
-5	3	8	59.79	7.90
-4	3	8	324.17	16.30
-4	3	8	308.97	18.00

-3	3	8	17.30	3.70
-3	3	8	25.00	3.90
-2	3	8	183.08	9.90
-2	3	8	201.88	10.80
-1	3	8	668.83	33.30
-1	3	8	793.42	34.10
-1	3	8	726.73	34.00
0	3	8	6.00	1.00
0	3	8	4.60	1.50
0	3	8	4.00	1.80
0	3	8	2.80	1.80
1	3	8	25.90	2.70
1	3	8	27.80	3.60
2	3	8	931.31	42.20
2	3	8	898.31	42.00
3	3	8	2141.79	91.29
3	3	8	1877.21	92.19
4	3	8	6.80	3.80
4	3	8	5.70	3.60
4	3	8	7.70	1.50
-5	-3	-8	27.80	4.40
5	3	8	34.00	6.40
5	3	8	34.10	7.10
5	3	8	32.00	3.60
-6	-3	-8	32.90	8.60
-6	-3	-8	27.90	7.40
-6	-3	-8	39.90	9.00
-6	-3	-8	22.50	9.80
-6	-3	-8	27.10	5.00
-6	-3	-8	30.80	6.70
6	3	8	24.90	4.00
-7	-3	-8	77.79	14.40
-7	-3	-8	84.49	13.00
-7	-3	-8	74.39	12.30
-7	-3	-8	65.59	8.10
-7	-3	-8	89.09	10.10
-7	-3	-8	60.49	8.80
7	3	8	63.49	6.30
-8	-3	-8	341.57	26.40
-8	-3	-8	359.26	20.60
-8	-3	-8	346.57	22.10
-8	-3	-8	347.07	23.80
-8	-3	-8	363.06	22.40
-8	-3	-8	334.77	19.80

8	3	8	349.87	18.70
-9	-3	-8	18.20	8.30
-9	-3	-8	18.40	8.50
-9	-3	-8	2.40	9.30
-9	-3	-8	11.90	6.80
-9	-3	-8	24.30	7.10
9	3	8	7.70	6.90
-10	-3	-8	578.54	33.70
-10	-3	-8	568.14	30.00
-10	-3	-8	557.24	31.80
-10	-3	-8	541.75	31.20
-10	-3	-8	569.34	29.60
10	3	8	513.65	32.30
-11	-3	-8	-9.60	7.90
-11	-3	-8	-6.30	8.30
-11	-3	-8	19.10	12.80
-11	-3	-8	3.70	10.40
-11	-3	-8	13.10	8.40
-12	-3	-8	0.70	8.90
-12	-3	-8	5.60	8.00
-12	-3	-8	10.30	12.80
-12	-3	-8	16.60	9.10
12	3	8	5.80	12.90
-11	4	8	100.19	15.60
-11	4	8	94.09	13.90
10	-4	-8	115.99	18.30
-10	4	8	179.48	18.90
-10	4	8	159.08	16.00
9	-4	-8	42.40	10.80
9	-4	-8	24.00	21.70
-9	4	8	28.50	10.20
-9	4	8	53.69	9.50
8	-4	-8	55.29	10.90
8	-4	-8	59.29	13.00
8	-4	-8	66.69	19.00
-8	4	8	55.49	9.20
-8	4	8	36.70	9.00
7	-4	-8	-26.00	17.30
7	-4	-8	12.80	9.70
7	-4	-8	0.60	8.90
-7	4	8	6.10	6.60
-7	4	8	12.80	7.40
6	-4	-8	468.55	26.40
6	-4	-8	493.75	27.00

6	-4	-8	434.76	36.90
6	-4	-8	456.15	35.40
-6	4	8	443.06	31.00
-6	4	8	461.65	25.70
5	-4	-8	42.10	13.20
5	-4	-8	28.40	8.00
5	-4	-8	43.00	9.10
-5	4	8	49.40	7.90
-5	4	8	39.10	11.00
-5	4	8	45.30	8.30
4	-4	-8	484.65	28.40
4	-4	-8	394.46	21.80
-4	4	8	438.46	23.80
-4	4	8	478.05	21.90
-3	4	8	1087.19	50.09
-3	4	8	1062.99	49.10
-2	4	8	473.05	22.30
-2	4	8	437.76	21.30
-1	4	8	18.40	2.90
-1	4	8	17.50	3.10
0	4	8	100.79	5.00
0	4	8	91.09	6.10
1	4	8	58.49	5.00
1	4	8	61.89	4.20
1	4	8	65.99	4.60
2	4	8	289.47	15.10
2	4	8	310.77	15.00
3	4	8	1903.81	87.89
3	4	8	1981.60	88.29
-5	-4	-8	33.60	6.00
-5	-4	-8	60.69	11.50
-5	-4	-8	36.90	10.30
5	4	8	47.70	7.50
5	4	8	41.10	3.70
5	4	8	32.10	6.20
-6	-4	-8	269.87	17.50
-6	-4	-8	270.27	14.90
-6	-4	-8	255.87	22.70
-6	-4	-8	231.08	18.10
-6	-4	-8	258.37	19.60
-6	-4	-8	242.78	15.80
6	4	8	272.07	13.30
-7	-4	-8	70.09	10.00
-7	-4	-8	52.49	8.10

-7	-4	-8	47.80	11.00
-7	-4	-8	63.89	8.40
-7	-4	-8	54.89	9.70
-7	-4	-8	63.49	9.00
7	4	8	63.69	6.30
-8	-4	-8	720.53	41.50
-8	-4	-8	753.42	37.10
-8	-4	-8	775.12	36.80
-8	-4	-8	730.63	37.90
-8	-4	-8	738.53	38.30
-8	-4	-8	728.23	39.80
8	4	8	748.23	35.80
-9	-4	-8	37.80	7.90
-9	-4	-8	20.00	8.00
-9	-4	-8	30.20	8.00
-9	-4	-8	16.90	8.00
-9	-4	-8	22.40	9.30
9	4	8	16.70	7.20
-10	-4	-8	91.49	14.10
-10	-4	-8	111.99	16.10
-10	-4	-8	108.79	12.90
-10	-4	-8	85.99	11.60
-10	-4	-8	107.79	17.80
10	4	8	84.49	12.70
-11	-4	-8	242.48	23.80
-11	-4	-8	248.68	18.50
-11	-4	-8	275.77	20.40
-11	-4	-8	234.68	17.40
-11	-4	-8	234.88	17.30
-12	-4	-8	28.60	9.80
-12	-4	-8	44.30	9.00
-12	-4	-8	19.60	12.20
-12	-4	-8	44.90	10.00
12	4	8	24.60	13.70
11	-5	-8	92.09	15.50
-11	5	8	75.79	16.50
-11	5	8	66.19	10.30
10	-5	-8	16.70	10.90
-10	5	8	9.20	8.20
9	-5	-8	331.57	38.50
9	-5	-8	373.26	25.90
-9	5	8	372.36	26.20
-9	5	8	394.06	24.00
8	-5	-8	33.20	10.70

8	-5	-8	3.50	9.00
-8	5	8	11.00	7.80
-8	5	8	-2.80	8.40
7	-5	-8	246.48	19.70
7	-5	-8	262.67	20.00
-7	5	8	241.68	18.40
-7	5	8	274.97	19.20
6	-5	-8	33.00	16.80
6	-5	-8	-0.60	7.50
-6	5	8	23.30	9.20
-6	5	8	11.30	7.10
5	-5	-8	596.34	31.50
5	-5	-8	572.54	30.20
-5	5	8	580.84	30.60
-5	5	8	574.34	28.50
4	-5	-8	167.08	16.80
-4	5	8	185.08	14.00
-4	5	8	194.08	10.60
3	-5	-8	112.89	7.00
-3	5	8	116.29	9.20
-3	5	8	130.09	7.20
-2	5	8	963.80	44.00
-2	5	8	973.70	45.10
-1	5	8	65.39	5.30
-1	5	8	57.09	3.80
-1	5	8	64.99	4.10
0	5	8	28.40	5.20
0	5	8	31.50	3.70
0	5	8	29.70	2.80
1	5	8	891.81	40.70
1	5	8	891.61	41.00
2	5	8	394.66	19.90
2	5	8	414.96	20.00
2	5	8	403.16	19.80
2	5	8	419.66	19.70
-3	-5	-8	731.63	35.80
3	5	8	748.73	35.90
3	5	8	819.22	36.40
-4	-5	-8	6.20	3.40
4	5	8	12.50	4.00
4	5	8	11.90	4.20
-5	-5	-8	-0.10	6.40
-5	-5	-8	7.10	6.80
-5	-5	-8	2.00	3.60

-5	-5	-8	-0.40	7.90
5	5	8	2.20	1.90
5	5	8	2.40	5.60
-6	-5	-8	3.80	6.50
-6	-5	-8	11.00	8.20
-6	-5	-8	6.40	7.30
-6	-5	-8	3.50	5.30
-6	-5	-8	15.50	11.20
-6	-5	-8	6.20	3.80
6	5	8	8.70	2.70
-7	-5	-8	-0.40	7.50
-7	-5	-8	4.90	4.50
-7	-5	-8	12.70	10.20
-7	-5	-8	5.10	6.80
-7	-5	-8	-0.20	5.20
-7	-5	-8	2.20	6.50
7	5	8	4.10	3.40
-8	-5	-8	444.26	25.60
-8	-5	-8	475.35	25.20
-8	-5	-8	465.85	28.10
-8	-5	-8	448.26	24.30
-8	-5	-8	435.66	30.20
-8	-5	-8	453.15	26.40
-9	-5	-8	686.73	40.10
-9	-5	-8	683.13	35.80
-9	-5	-8	699.03	38.30
-9	-5	-8	708.43	36.80
-9	-5	-8	708.43	36.60
9	5	8	711.33	35.20
-10	-5	-8	16.50	7.20
-10	-5	-8	14.80	8.40
-10	-5	-8	20.00	8.70
-10	-5	-8	21.40	10.10
-10	-5	-8	23.40	7.80
-11	-5	-8	-4.00	7.30
-11	-5	-8	9.70	11.10
-11	-5	-8	16.50	9.50
-11	-5	-8	3.10	7.60
-11	-5	-8	-1.00	8.20
-12	-5	-8	96.79	17.00
-12	-5	-8	63.69	9.80
-12	-5	-8	102.29	13.10
-12	-5	-8	82.99	14.20
12	5	8	91.59	14.90

11	-6	-8	17.00	10.80
-11	6	8	18.00	8.90
-11	6	8	10.40	19.80
10	-6	-8	63.89	12.20
-10	6	8	49.30	9.70
-10	6	8	67.29	13.70
9	-6	-8	75.29	13.00
-9	6	8	93.59	14.40
-9	6	8	109.39	15.50
8	-6	-8	2.50	9.50
8	-6	-8	20.50	9.70
-8	6	8	18.40	8.30
-8	6	8	1.00	9.50
7	-6	-8	421.86	26.20
7	-6	-8	466.45	27.60
-7	6	8	475.95	26.50
6	-6	-8	29.20	24.20
6	-6	-8	8.40	7.60
-6	6	8	23.10	8.80
-6	6	8	4.00	7.00
5	-6	-8	122.69	14.80
5	-6	-8	124.29	17.20
5	-6	-8	151.78	23.50
5	-6	-8	105.19	13.00
-5	6	8	131.59	10.50
-5	6	8	125.69	14.30
4	-6	-8	669.73	36.40
4	-6	-8	697.83	34.80
4	-6	-8	726.63	36.10
-4	6	8	666.13	35.60
-4	6	8	726.73	32.90
3	-6	-8	942.91	44.50
-3	6	8	1017.90	44.20
-3	6	8	988.70	44.50
-3	6	8	924.91	45.90
2	-6	-8	296.57	14.30
-2	6	8	304.67	14.30
-2	6	8	300.27	15.90
-2	6	8	280.47	14.10
-1	6	8	820.12	39.70
-1	6	8	899.01	40.60
-1	6	8	889.01	39.30
0	6	8	553.84	26.40
0	6	8	527.35	25.70

1	6	8	198.78	10.90
1	6	8	208.68	11.50
1	6	8	213.88	10.80
2	6	8	76.39	5.90
2	6	8	70.79	6.50
-3	-6	-8	265.17	14.50
3	6	8	260.87	14.50
3	6	8	279.37	15.00
3	6	8	269.77	13.50
3	6	8	240.78	14.00
-4	-6	-8	2061.09	92.39
4	6	8	2236.58	94.19
4	6	8	1858.41	92.49
4	6	8	1927.71	93.49
4	6	8	2122.99	92.09
-5	-6	-8	-2.30	8.90
-5	-6	-8	19.60	6.70
-5	-6	-8	8.80	4.20
-5	-6	-8	7.70	6.30
5	6	8	10.50	3.80
5	6	8	6.00	2.10
5	6	8	0.80	5.70
-6	-6	-8	118.39	15.60
-6	-6	-8	113.79	13.60
-6	-6	-8	116.79	21.20
-6	-6	-8	120.89	9.80
-6	-6	-8	127.09	11.80
-6	-6	-8	135.99	12.90
6	6	8	132.79	7.80
-7	-6	-8	375.66	23.60
-7	-6	-8	390.06	27.00
-7	-6	-8	357.36	21.80
-7	-6	-8	368.16	20.70
-7	-6	-8	338.67	21.50
-7	-6	-8	378.56	20.00
7	6	8	362.16	18.60
-8	-6	-8	85.99	13.40
-8	-6	-8	93.89	15.60
-8	-6	-8	120.69	11.00
-8	-6	-8	115.99	20.80
-8	-6	-8	113.49	12.40
-8	-6	-8	110.39	13.30
8	6	8	96.39	9.30
-9	-6	-8	67.39	11.80

-9	-6	-8	54.89	9.40
-9	-6	-8	71.79	10.50
-9	-6	-8	89.49	16.20
-9	-6	-8	77.89	11.70
9	6	8	66.19	11.00
-10	-6	-8	63.19	9.90
-10	-6	-8	85.59	14.10
-10	-6	-8	60.49	11.30
-10	-6	-8	65.99	11.40
-10	-6	-8	79.19	12.60
10	6	8	63.79	12.00
-11	-6	-8	38.00	9.80
-11	-6	-8	37.40	8.10
-11	-6	-8	34.20	8.20
-11	-6	-8	23.20	8.70
-11	-6	-8	45.20	11.80
11	6	8	47.00	13.80
-12	-6	-8	17.40	10.10
-12	-6	-8	23.40	8.00
-12	-6	-8	28.20	8.90
-12	-6	-8	22.10	9.10
12	6	8	-0.60	21.80
11	-7	-8	94.59	16.50
-11	7	8	64.99	19.40
-11	7	8	77.49	11.40
10	-7	-8	6.00	10.80
-10	7	8	27.50	13.70
-10	7	8	17.10	9.00
9	-7	-8	259.17	32.90
9	-7	-8	257.97	25.00
8	-7	-8	526.05	30.70
8	-7	-8	560.84	31.60
-8	7	8	531.05	30.90
-8	7	8	561.64	32.70
7	-7	-8	90.39	14.70
7	-7	-8	81.39	15.20
-7	7	8	81.29	14.40
6	-7	-8	4.10	11.40
-6	7	8	-1.20	8.40
5	-7	-8	19.30	12.50
5	-7	-8	0.90	8.30
5	-7	-8	6.90	6.60
5	-7	-8	22.20	13.20
-5	7	8	2.90	6.90

-5	7	8	4.40	5.00
4	-7	-8	96.59	15.50
4	-7	-8	93.39	12.10
4	-7	-8	93.89	12.50
4	-7	-8	89.19	13.00
4	-7	-8	83.59	13.60
-4	7	8	96.69	7.40
3	-7	-8	6.20	4.90
-3	7	8	5.50	5.20
-3	7	8	5.90	3.80
-3	7	8	8.80	2.50
2	-7	-8	1.10	3.40
-2	7	8	2.50	3.40
-2	7	8	2.90	1.60
-2	7	8	4.90	2.90
1	-7	-8	376.16	18.60
-1	7	8	398.26	17.90
-1	7	8	391.06	19.40
-1	7	8	368.66	19.30
0	7	8	24.30	3.90
0	7	8	33.40	5.70
0	7	8	32.10	4.50
1	7	8	-1.80	3.60
1	7	8	5.00	2.40
2	7	8	2732.33	121.69
2	7	8	2680.53	121.89
2	7	8	2588.44	121.49
2	7	8	2782.32	121.29
3	7	8	805.42	36.20
3	7	8	798.72	36.00
3	7	8	628.04	36.50
-4	-7	-8	415.06	20.20
4	7	8	423.16	22.80
4	7	8	361.66	20.60
4	7	8	400.56	19.70
4	7	8	408.66	22.00
-5	-7	-8	68.99	9.10
-5	-7	-8	68.09	10.60
-5	-7	-8	52.29	7.70
-5	-7	-8	73.59	10.30
5	7	8	61.09	4.70
-6	-7	-8	19.80	6.90
-6	-7	-8	19.60	4.70
-6	-7	-8	26.80	7.40

-6	-7	-8	38.20	12.70
-6	-7	-8	2.80	12.40
-6	-7	-8	7.90	5.90
-6	-7	-8	26.00	7.90
6	7	8	20.10	3.40
-7	-7	-8	28.20	12.80
-7	-7	-8	13.40	7.60
-7	-7	-8	14.20	4.80
-7	-7	-8	11.80	8.70
7	7	8	20.10	4.40
-8	-7	-8	172.98	17.70
-8	-7	-8	176.68	14.80
-8	-7	-8	168.38	12.60
-8	-7	-8	146.39	15.20
-8	-7	-8	143.19	14.00
8	7	8	165.88	11.90
-9	-7	-8	71.89	13.20
-9	-7	-8	92.49	16.30
-9	-7	-8	53.79	9.20
-9	-7	-8	76.29	11.30
-9	-7	-8	77.69	12.20
-10	-7	-8	324.57	22.20
-10	-7	-8	331.37	23.90
-10	-7	-8	321.87	21.40
-10	-7	-8	331.67	19.80
-10	-7	-8	343.97	20.40
10	7	8	300.17	23.50
-11	-7	-8	29.70	10.80
-11	-7	-8	19.40	8.90
-11	-7	-8	44.70	8.60
-11	-7	-8	42.50	8.90
11	7	8	29.90	14.40
-12	-7	-8	30.10	10.70
-12	-7	-8	26.90	8.00
-12	-7	-8	34.50	9.30
12	7	8	42.90	22.20
11	-8	-8	68.79	14.50
-11	8	8	63.19	11.40
10	-8	-8	162.08	18.00
10	-8	-8	219.08	23.80
-10	8	8	154.18	24.60
-10	8	8	165.48	16.40
9	-8	-8	89.39	13.70
9	-8	-8	72.09	12.60

-9	8	8	88.29	12.40
-9	8	8	69.29	15.70
8	-8	-8	64.29	11.30
8	-8	-8	85.79	15.00
-8	8	8	75.69	11.20
-8	8	8	79.79	13.70
7	-8	-8	31.50	9.30
7	-8	-8	18.00	8.30
-7	8	8	27.70	9.10
6	-8	-8	229.38	18.50
6	-8	-8	233.88	17.30
-6	8	8	244.58	18.50
-6	8	8	191.38	17.80
5	-8	-8	59.19	8.50
5	-8	-8	83.29	14.10
5	-8	-8	70.99	14.30
5	-8	-8	64.89	12.60
-5	8	8	48.70	8.80
-5	8	8	59.29	9.60
4	-8	-8	196.08	17.80
4	-8	-8	198.48	19.80
4	-8	-8	186.68	16.80
4	-8	-8	164.88	18.90
4	-8	-8	192.68	14.80
3	-8	-8	2052.79	87.59
3	-8	-8	1832.92	87.59
3	-8	-8	1847.72	88.39
-3	8	8	1984.30	86.49
-3	8	8	1850.41	86.99
2	-8	-8	454.45	23.00
-2	8	8	450.85	23.60
-2	8	8	485.65	21.60
-2	8	8	438.66	22.80
1	-8	-8	5.10	3.60
-1	8	8	4.20	3.30
-1	8	8	5.20	1.70
-1	8	8	4.70	5.30
0	8	8	24.10	3.90
0	8	8	11.50	3.90
0	8	8	22.20	2.30
0	8	8	17.00	6.30
1	8	8	176.38	9.80
1	8	8	144.79	13.10
-2	-8	-8	43.80	6.00

2	8	8	42.50	6.00
2	8	8	35.00	4.90
2	8	8	52.59	7.70
2	8	8	38.30	5.90
3	8	8	1676.03	74.49
3	8	8	1536.95	74.89
3	8	8	1689.13	75.99
3	8	8	1662.33	75.99
-4	-8	-8	3.10	4.00
4	8	8	7.40	3.60
4	8	8	11.60	6.50
4	8	8	5.00	6.90
4	8	8	7.20	5.40
-5	-8	-8	224.08	16.80
-5	-8	-8	235.38	20.30
-5	-8	-8	219.78	16.20
-5	-8	-8	248.58	13.60
-5	-8	-8	212.48	15.00
5	8	8	235.98	11.60
-6	-8	-8	188.58	14.20
-6	-8	-8	190.98	15.00
-6	-8	-8	157.78	17.00
-6	-8	-8	193.78	13.50
-6	-8	-8	194.78	12.30
-6	-8	-8	196.38	23.60
-6	-8	-8	184.38	17.20
6	8	8	191.08	10.70
-7	-8	-8	287.87	17.00
-7	-8	-8	281.97	18.70
-7	-8	-8	260.97	20.60
-7	-8	-8	269.17	16.10
-7	-8	-8	261.57	17.90
-7	-8	-8	226.18	28.50
-7	-8	-8	261.17	21.30
7	8	8	276.97	15.00
-8	-8	-8	512.45	26.70
-8	-8	-8	527.55	27.50
-8	-8	-8	518.55	28.00
-8	-8	-8	521.05	28.10
-8	-8	-8	514.85	29.90
-8	-8	-8	425.86	37.00
8	8	8	518.75	26.20
-9	-8	-8	9.50	6.90
-9	-8	-8	3.60	7.80

-9	-8	-8	23.20	8.00
-9	-8	-8	1.50	6.30
-9	-8	-8	16.10	9.20
-10	-8	-8	28.90	7.50
-10	-8	-8	21.80	9.60
-10	-8	-8	33.30	10.90
-10	-8	-8	37.20	9.00
-10	-8	-8	36.50	8.30
10	8	8	25.30	11.60
-11	-8	-8	183.98	16.60
-11	-8	-8	237.08	17.60
-11	-8	-8	205.48	15.30
-11	-8	-8	178.78	15.00
11	8	8	184.18	25.20
-12	-8	-8	41.10	10.60
-12	-8	-8	36.90	8.10
-12	-8	-8	28.40	9.70
-12	-8	-8	16.80	8.60
10	-9	-8	56.49	14.90
10	-9	-8	70.89	12.60
9	-9	-8	334.67	23.20
9	-9	-8	285.57	23.40
-9	9	8	301.47	22.10
-9	9	8	218.68	28.00
8	-9	-8	68.99	11.70
8	-9	-8	47.90	9.60
-8	9	8	32.60	13.60
-8	9	8	56.49	10.70
7	-9	-8	193.38	18.90
7	-9	-8	193.98	17.10
-7	9	8	179.48	18.40
6	-9	-8	46.80	10.00
6	-9	-8	41.00	8.40
-6	9	8	35.80	9.00
-6	9	8	36.40	9.40
5	-9	-8	568.44	35.50
5	-9	-8	529.85	31.50
5	-9	-8	577.14	29.90
5	-9	-8	587.04	32.60
-5	9	8	569.94	28.30
-5	9	8	564.24	31.00
4	-9	-8	113.59	12.70
4	-9	-8	135.79	18.10
4	-9	-8	99.49	17.10

4	-9	-8	106.29	15.60
4	-9	-8	82.49	14.50
-4	9	8	125.29	8.60
3	-9	-8	213.38	19.10
3	-9	-8	225.98	17.70
3	-9	-8	204.18	16.70
3	-9	-8	235.58	20.60
3	-9	-8	218.38	15.90
-3	9	8	220.38	11.80
-2	9	8	816.12	41.90
-2	9	8	914.41	39.80
1	-9	-8	26.40	5.90
-1	9	8	31.00	5.20
-1	9	8	27.60	3.00
-1	9	8	27.10	7.70
0	9	8	2.10	7.00
0	9	8	8.00	3.90
0	9	8	10.80	1.70
-1	-9	-8	370.66	21.90
1	9	8	437.16	20.00
1	9	8	360.76	22.40
2	9	8	12.50	6.10
2	9	8	17.40	3.70
-3	-9	-8	1207.28	55.89
3	9	8	1252.97	55.79
3	9	8	1209.58	57.49
3	9	8	1160.78	56.39
3	9	8	1242.98	57.89
-4	-9	-8	98.59	13.50
-4	-9	-8	123.19	8.90
-4	-9	-8	105.59	12.00
4	9	8	110.19	9.50
4	9	8	102.49	11.90
-5	-9	-8	253.27	13.70
-5	-9	-8	231.18	17.10
-5	-9	-8	224.88	16.80
-5	-9	-8	218.38	15.60
-5	-9	-8	236.18	14.80
-5	-9	-8	218.08	20.60
5	9	8	224.98	12.00
-6	-9	-8	6.50	5.00
-6	-9	-8	20.60	6.50
-6	-9	-8	14.50	6.90
-6	-9	-8	-0.20	7.90

-6	-9	-8	14.60	8.80
-6	-9	-8	12.10	6.60
6	9	8	11.20	3.90
-7	-9	-8	62.59	8.80
-7	-9	-8	70.49	10.20
-7	-9	-8	77.29	11.00
-7	-9	-8	65.79	8.10
-7	-9	-8	66.59	11.90
-7	-9	-8	70.99	8.80
7	9	8	63.29	8.10
-8	-9	-8	532.85	31.00
-8	-9	-8	547.25	29.40
-8	-9	-8	562.34	28.80
-8	-9	-8	546.45	28.30
-8	-9	-8	557.24	30.10
-8	-9	-8	537.05	31.10
8	9	8	548.55	28.10
-9	-9	-8	369.16	22.50
-9	-9	-8	352.76	22.60
-9	-9	-8	347.67	21.00
-9	-9	-8	373.06	24.60
-9	-9	-8	366.76	21.80
9	9	8	355.46	22.20
-10	-9	-8	58.29	13.50
-10	-9	-8	60.29	10.00
-10	-9	-8	43.50	14.70
-11	-9	-8	-5.70	6.90
-11	-9	-8	0.50	7.60
-11	-9	-8	3.90	7.90
-11	-9	-8	-9.80	7.80
-12	-9	-8	63.99	10.10
-12	-9	-8	60.39	11.40
-12	-9	-8	78.39	12.00
-12	-9	-8	88.09	14.10
10	-10	-8	62.69	12.70
10	-10	-8	38.60	14.10
-10	10	8	52.89	10.30
-10	10	8	18.40	23.30
9	-10	-8	91.09	16.00
9	-10	-8	106.69	18.10
-9	10	8	57.69	11.10
-9	10	8	72.19	16.70
8	-10	-8	125.49	18.00
8	-10	-8	105.89	16.00

-8	10	8	99.09	16.80
-8	10	8	151.38	24.00
7	-10	-8	89.79	14.10
7	-10	-8	74.09	11.90
-7	10	8	104.59	16.00
6	-10	-8	127.59	14.10
6	-10	-8	92.89	17.00
6	-10	-8	142.99	22.00
-6	10	8	100.19	18.10
-6	10	8	120.69	16.30
5	-10	-8	11.00	10.20
5	-10	-8	5.80	9.70
5	-10	-8	2.80	13.40
5	-10	-8	-9.20	13.20
5	-10	-8	-8.00	6.70
-5	10	8	2.90	5.70
4	-10	-8	1645.14	78.39
4	-10	-8	1699.23	77.79
4	-10	-8	1671.53	78.59
4	-10	-8	1597.74	78.69
4	-10	-8	1637.74	76.99
-4	10	8	1717.33	75.99
3	-10	-8	390.06	34.30
3	-10	-8	480.75	26.20
3	-10	-8	435.76	26.80
3	-10	-8	430.96	25.50
3	-10	-8	475.85	24.90
-3	10	8	492.05	22.30
2	-10	-8	136.99	17.50
2	-10	-8	174.58	14.70
2	-10	-8	152.58	14.00
2	-10	-8	162.58	14.80
2	-10	-8	157.58	15.20
2	-10	-8	139.09	15.40
-2	10	8	136.89	15.90
-2	10	8	161.58	8.80
1	-10	-8	385.16	21.50
-1	10	8	364.36	20.90
-1	10	8	369.66	24.30
-1	10	8	389.06	17.90
0	10	8	4.90	4.00
0	10	8	8.30	8.80
1	10	8	176.98	11.10
1	10	8	132.69	16.00

-2	-10	-8	0.40	5.70
2	10	8	4.40	4.30
2	10	8	6.40	7.70
-3	-10	-8	14.20	5.10
3	10	8	20.90	7.60
3	10	8	18.70	4.80
3	10	8	8.60	8.40
3	10	8	16.20	6.20
-4	-10	-8	1538.25	67.09
-4	-10	-8	1401.56	68.19
-4	-10	-8	1462.15	67.89
-4	-10	-8	1464.15	67.39
-4	-10	-8	1412.46	67.39
-5	-10	-8	6.70	6.90
-5	-10	-8	2.10	4.70
-5	-10	-8	-0.60	6.40
-5	-10	-8	15.30	7.30
-5	-10	-8	-6.30	10.00
-5	-10	-8	-10.10	6.70
5	10	8	-0.60	3.30
-6	-10	-8	32.30	7.90
-6	-10	-8	38.20	7.60
-6	-10	-8	40.60	6.90
-6	-10	-8	31.40	5.70
-6	-10	-8	22.50	7.10
-6	-10	-8	28.80	10.10
6	10	8	24.30	5.00
-7	-10	-8	298.37	18.00
-7	-10	-8	283.47	17.00
-7	-10	-8	300.47	19.40
-7	-10	-8	275.37	18.70
-7	-10	-8	261.27	19.50
-7	-10	-8	287.77	21.20
7	10	8	284.37	16.60
-8	-10	-8	114.39	15.80
-8	-10	-8	78.49	13.10
-8	-10	-8	86.19	15.00
-8	-10	-8	97.89	14.20
-8	-10	-8	73.59	10.20
-8	-10	-8	93.59	12.00
8	10	8	87.09	11.50
-9	-10	-8	100.19	12.40
-9	-10	-8	86.79	13.80
-9	-10	-8	74.29	10.40

-9	-10	-8	118.09	16.60
-9	-10	-8	92.09	11.10
-9	-10	-8	79.49	13.10
9	10	8	70.59	11.00
-10	-10	-8	197.18	17.20
-10	-10	-8	157.08	14.10
-10	-10	-8	193.98	16.00
-10	-10	-8	185.58	20.10
-10	-10	-8	150.38	16.60
-11	-10	-8	9.60	8.00
-11	-10	-8	23.00	7.70
-11	-10	-8	3.90	8.00
-12	-10	-8	99.89	15.60
-12	-10	-8	81.79	12.10
-12	-10	-8	116.99	16.60
-12	-10	-8	124.69	18.30
10	-11	-8	23.40	15.10
-10	11	8	-18.30	20.90
-10	11	8	5.50	9.10
9	-11	-8	416.66	26.50
9	-11	-8	418.86	25.00
-9	11	8	395.56	25.90
-9	11	8	311.77	31.70
-8	11	8	516.55	34.50
-8	11	8	405.06	48.20
7	-11	-8	13.40	8.30
7	-11	-8	33.90	11.10
-7	11	8	54.59	11.70
6	-11	-8	8.60	10.30
6	-11	-8	16.30	12.70
6	-11	-8	-8.10	6.80
-6	11	8	1.90	9.50
5	-11	-8	14.70	10.10
5	-11	-8	23.10	11.60
5	-11	-8	25.80	12.40
5	-11	-8	18.80	7.10
-5	11	8	32.90	6.80
4	-11	-8	134.69	14.90
4	-11	-8	114.29	15.90
4	-11	-8	95.29	16.40
4	-11	-8	106.69	12.90
4	-11	-8	142.59	18.20
-4	11	8	114.49	9.30
3	-11	-8	79.79	11.10

3	-11	-8	55.39	17.50
3	-11	-8	84.09	11.40
3	-11	-8	94.99	12.50
3	-11	-8	77.89	10.00
-3	11	8	72.59	6.60
2	-11	-8	17.80	10.90
2	-11	-8	39.50	7.70
2	-11	-8	50.19	8.90
2	-11	-8	19.40	7.50
2	-11	-8	41.30	10.20
2	-11	-8	35.60	9.00
-2	11	8	46.30	9.90
-2	11	8	28.80	13.50
-2	11	8	28.40	4.20
1	-11	-8	18.80	9.10
1	-11	-8	31.20	8.20
1	-11	-8	17.50	7.10
1	-11	-8	29.60	10.30
1	-11	-8	38.10	8.50
-1	11	8	44.30	12.00
-1	11	8	38.40	4.00
0	-11	-8	82.69	12.60
0	-11	-8	96.19	13.10
0	-11	-8	88.79	10.10
0	11	8	104.79	5.70
0	11	8	85.89	10.60
0	11	8	123.19	18.90
-1	-11	-8	130.59	13.40
-1	-11	-8	130.69	14.10
-1	-11	-8	193.78	14.10
1	11	8	156.88	12.00
1	11	8	171.38	8.10
1	11	8	202.78	19.50
-2	-11	-8	1967.40	86.69
-2	-11	-8	1860.51	85.79
-2	-11	-8	1955.50	85.89
-2	-11	-8	1774.82	86.69
2	11	8	1716.83	88.69
2	11	8	1955.30	85.89
-3	-11	-8	-8.90	8.40
-3	-11	-8	5.40	8.30
-3	-11	-8	3.10	5.20
-3	-11	-8	-12.90	6.20
3	11	8	1.00	5.20

3	11	8	5.60	7.10
-4	-11	-8	305.17	18.20
-4	-11	-8	285.37	19.70
-4	-11	-8	283.77	22.10
-4	-11	-8	290.07	18.50
-4	-11	-8	321.67	17.10
-4	-11	-8	308.37	19.90
4	11	8	327.27	14.50
-5	-11	-8	321.87	19.10
-5	-11	-8	327.77	24.80
-5	-11	-8	321.87	18.40
-5	-11	-8	302.67	19.20
-5	-11	-8	325.37	17.50
-5	-11	-8	317.17	20.10
5	11	8	277.77	15.80
-6	-11	-8	9.30	5.00
-6	-11	-8	5.90	7.10
-6	-11	-8	10.40	6.00
-6	-11	-8	8.50	9.20
-6	-11	-8	17.00	6.70
-6	-11	-8	12.40	7.10
6	11	8	9.00	5.20
-7	-11	-8	52.99	7.40
-7	-11	-8	70.89	9.70
-7	-11	-8	55.89	10.30
-7	-11	-8	38.80	7.90
-7	-11	-8	55.99	9.40
-7	-11	-8	54.79	8.20
7	11	8	40.90	7.40
-8	-11	-8	0.90	10.70
-8	-11	-8	13.80	7.30
-8	-11	-8	27.40	9.10
-8	-11	-8	2.30	8.90
-8	-11	-8	-9.10	8.70
-8	-11	-8	-4.40	7.10
8	11	8	9.30	7.70
-9	-11	-8	278.57	21.80
-9	-11	-8	292.57	18.10
-9	-11	-8	278.57	19.70
-9	-11	-8	285.87	18.80
-9	-11	-8	274.77	21.90
-9	-11	-8	229.68	16.80
9	11	8	237.48	20.40
-10	-11	-8	84.59	11.90

-10	-11	-8	97.79	13.40
-10	-11	-8	94.09	13.10
-10	-11	-8	74.79	11.20
-11	-11	-8	63.29	9.20
-11	-11	-8	62.29	9.80
-11	-11	-8	50.99	9.30
-11	-11	-8	74.69	11.00
10	-12	-8	224.18	21.30
10	-12	-8	190.28	18.20
-10	12	8	158.88	16.10
9	-12	-8	2.80	9.40
9	-12	-8	-0.60	12.50
-9	12	8	5.40	10.00
-9	12	8	-0.30	15.90
8	-12	-8	66.39	13.50
8	-12	-8	66.79	10.90
-8	12	8	48.40	12.50
7	-12	-8	-17.40	11.80
7	-12	-8	1.70	8.10
-7	12	8	11.50	10.90
6	-12	-8	23.60	12.50
6	-12	-8	46.20	13.00
6	-12	-8	47.80	8.90
-6	12	8	59.99	12.10
5	-12	-8	142.19	14.80
5	-12	-8	142.89	21.60
5	-12	-8	127.09	16.70
5	-12	-8	145.49	25.10
5	-12	-8	121.49	23.30
-5	12	8	161.28	13.10
4	-12	-8	21.00	7.10
4	-12	-8	-11.60	15.10
4	-12	-8	6.90	11.10
4	-12	-8	11.70	11.10
-4	12	8	11.20	4.80
3	-12	-8	1805.62	84.69
3	-12	-8	1826.02	85.49
3	-12	-8	1805.12	85.79
3	-12	-8	1855.21	84.49
-3	12	8	1836.02	82.99
2	-12	-8	163.98	15.80
2	-12	-8	138.09	18.10
2	-12	-8	160.48	16.50
2	-12	-8	151.78	14.40

-2	12	8	181.98	9.60
-2	12	8	151.48	16.70
1	-12	-8	6.90	8.80
1	-12	-8	20.10	11.40
1	-12	-8	6.80	7.90
1	-12	-8	-3.40	6.90
1	-12	-8	8.20	8.10
1	-12	-8	-1.60	10.00
-1	12	8	-8.50	14.80
0	-12	-8	292.47	19.20
0	-12	-8	262.37	17.60
0	-12	-8	277.27	17.90
0	-12	-8	224.08	18.00
0	12	8	285.17	26.80
-1	-12	-8	168.18	16.50
-1	-12	-8	145.49	15.30
-1	-12	-8	143.79	13.80
-1	-12	-8	142.29	13.40
-1	-12	-8	148.99	13.20
1	12	8	161.28	23.20
1	12	8	167.58	8.20
1	12	8	136.89	13.20
-2	-12	-8	145.99	17.90
-2	-12	-8	146.59	13.90
-2	-12	-8	149.09	14.30
-2	-12	-8	128.99	18.30
-2	-12	-8	146.59	12.20
-2	-12	-8	125.39	12.60
2	12	8	143.29	7.30
2	12	8	113.49	12.30
-3	-12	-8	599.64	32.10
-3	-12	-8	598.44	32.30
-3	-12	-8	643.64	30.50
-3	-12	-8	535.25	32.20
-3	-12	-8	590.14	31.70
-3	-12	-8	619.24	30.60
3	12	8	658.43	27.80
-4	-12	-8	93.09	13.40
-4	-12	-8	75.29	9.20
-4	-12	-8	67.79	8.90
-4	-12	-8	50.69	10.20
-4	-12	-8	57.19	9.10
-4	-12	-8	57.09	7.40
4	12	8	62.29	5.00

-5	-12	-8	250.27	17.70
-5	-12	-8	222.68	21.50
-5	-12	-8	245.18	16.20
-5	-12	-8	237.28	16.80
-5	-12	-8	248.98	16.30
-5	-12	-8	267.97	15.40
-5	-12	-8	267.77	23.20
5	12	8	204.88	13.10
-6	-12	-8	25.10	7.90
-6	-12	-8	33.20	7.40
-6	-12	-8	38.40	7.70
-6	-12	-8	33.10	7.60
-6	-12	-8	41.00	7.00
6	12	8	30.10	6.50
-7	-12	-8	578.34	30.60
-7	-12	-8	583.04	31.40
-7	-12	-8	589.24	30.30
-7	-12	-8	569.94	32.00
-7	-12	-8	611.94	30.60
-7	-12	-8	587.64	29.90
7	12	8	511.75	29.80
-8	-12	-8	282.07	19.10
-8	-12	-8	275.97	18.90
-8	-12	-8	306.77	20.70
-8	-12	-8	285.67	22.00
-8	-12	-8	308.97	20.50
-8	-12	-8	273.87	18.20
8	12	8	260.37	19.80
-9	-12	-8	37.30	7.70
-9	-12	-8	31.50	8.50
-9	-12	-8	26.20	17.30
-9	-12	-8	23.50	8.60
-9	-12	-8	23.70	8.10
9	12	8	31.00	11.30
-10	-12	-8	16.80	8.00
-10	-12	-8	17.40	12.30
-10	-12	-8	36.20	8.40
-10	-12	-8	29.50	8.80
-10	-12	-8	11.70	7.90
-11	-12	-8	260.17	18.10
-11	-12	-8	272.77	18.40
-11	-12	-8	284.67	18.50
-11	-12	-8	278.27	17.80
9	-13	-8	156.68	22.80

9	-13	-8	189.08	16.30
-9	13	8	127.49	17.40
-9	13	8	192.68	26.70
8	-13	-8	167.08	15.90
8	-13	-8	136.29	16.70
-8	13	8	153.28	24.90
-8	13	8	159.48	20.10
7	-13	-8	68.59	9.90
7	-13	-8	49.00	15.80
-7	13	8	67.79	13.50
6	-13	-8	-6.80	12.00
6	-13	-8	7.70	8.10
6	-13	-8	-14.40	14.40
-6	13	8	-1.70	10.30
5	-13	-8	357.16	28.60
5	-13	-8	348.47	30.80
5	-13	-8	342.27	31.00
5	-13	-8	368.86	24.20
5	-13	-8	367.36	22.00
-5	13	8	337.67	21.50
4	-13	-8	212.58	21.40
4	-13	-8	200.98	18.10
4	-13	-8	177.88	21.30
4	-13	-8	207.08	16.90
-4	13	8	229.18	13.80
3	-13	-8	61.89	11.10
3	-13	-8	52.79	17.00
3	-13	-8	56.89	11.80
-3	13	8	57.79	7.00
-3	13	8	47.40	11.50
2	-13	-8	415.66	23.50
2	-13	-8	392.56	25.10
2	-13	-8	400.86	24.40
2	-13	-8	404.56	24.10
2	-13	-8	379.86	23.00
-2	13	8	352.16	24.50
-2	13	8	398.86	19.60
1	-13	-8	24.90	10.90
1	-13	-8	30.00	7.70
1	-13	-8	31.00	9.10
1	-13	-8	16.80	7.80
1	-13	-8	44.80	8.40
-1	13	8	39.80	4.50
0	-13	-8	20.60	13.50

0	-13	-8	10.10	12.60
0	-13	-8	22.70	7.50
0	-13	-8	32.80	7.90
0	-13	-8	28.40	8.00
0	13	8	37.60	4.00
-1	-13	-8	332.97	21.40
-1	-13	-8	336.17	21.90
-1	-13	-8	353.86	20.10
-1	-13	-8	309.77	22.80
-1	-13	-8	346.57	20.70
-1	-13	-8	363.86	20.10
1	13	8	356.86	16.60
-2	-13	-8	253.27	21.70
-2	-13	-8	199.88	20.10
-2	-13	-8	234.18	17.10
-2	-13	-8	245.18	17.70
-2	-13	-8	250.37	16.00
-2	-13	-8	268.67	16.50
2	13	8	254.27	12.20
-3	-13	-8	1099.99	60.09
-3	-13	-8	1313.07	59.89
-3	-13	-8	1233.18	60.09
-3	-13	-8	1327.67	59.09
-3	-13	-8	1290.97	59.49
-3	-13	-8	1229.18	62.39
-3	-13	-8	1261.07	60.19
3	13	8	1316.57	56.89
-4	-13	-8	162.98	16.70
-4	-13	-8	138.19	12.90
-4	-13	-8	168.38	14.60
-4	-13	-8	106.39	15.30
-4	-13	-8	152.28	12.40
-4	-13	-8	137.39	15.00
4	13	8	149.19	8.50
-5	-13	-8	8.20	6.50
-5	-13	-8	12.00	6.10
-5	-13	-8	9.20	10.80
-5	-13	-8	-2.70	6.80
-5	-13	-8	7.10	8.30
5	13	8	8.30	4.40
-6	-13	-8	320.97	20.40
-6	-13	-8	351.16	20.00
-6	-13	-8	347.67	20.90
-6	-13	-8	355.46	24.90

-6	-13	-8	339.57	20.50
-6	-13	-8	326.97	21.70
-7	-13	-8	242.08	16.90
-7	-13	-8	227.98	18.60
-7	-13	-8	239.18	16.20
-7	-13	-8	256.37	17.50
-7	-13	-8	231.08	17.30
-7	-13	-8	232.28	21.00
-8	-13	-8	302.27	20.10
-8	-13	-8	324.77	20.80
-8	-13	-8	331.67	21.00
-8	-13	-8	338.87	21.10
-8	-13	-8	327.07	22.30
-9	-13	-8	99.29	12.10
-9	-13	-8	155.78	21.40
9	13	8	91.29	13.70
-10	-13	-8	60.99	12.10
-10	-13	-8	82.29	13.00
-10	-13	-8	63.59	9.80
-10	-13	-8	60.59	9.70
-10	-13	-8	55.69	9.20
-11	-13	-8	3.40	9.70
-11	-13	-8	11.10	7.70
-11	-13	-8	1.80	7.70
-11	-13	-8	9.90	7.80
9	-14	-8	75.19	13.70
-9	14	8	96.99	14.40
8	-14	-8	39.70	17.20
8	-14	-8	15.60	8.20
-8	14	8	-9.80	10.30
-8	14	8	-1.40	14.70
7	-14	-8	183.18	30.20
7	-14	-8	107.89	18.00
7	-14	-8	110.99	20.40
-7	14	8	126.09	22.90
6	-14	-8	20.10	19.50
6	-14	-8	28.10	8.60
6	-14	-8	-1.50	11.90
-6	14	8	9.20	11.00
5	-14	-8	0.60	9.60
5	-14	-8	-1.10	13.00
5	-14	-8	4.50	7.30
5	-14	-8	-13.30	16.60
-5	14	8	13.80	9.40

4	-14	-8	1309.27	63.99
4	-14	-8	1241.68	65.49
4	-14	-8	1385.46	63.59
4	-14	-8	1415.36	66.49
-4	14	8	1313.17	62.19
3	-14	-8	9.60	10.00
3	-14	-8	16.50	10.40
3	-14	-8	25.50	8.80
3	-14	-8	28.70	8.20
-3	14	8	29.50	11.00
-3	14	8	22.10	4.90
2	-14	-8	648.04	36.10
2	-14	-8	750.52	41.60
2	-14	-8	640.24	34.70
-2	14	8	669.13	31.70
-2	14	8	624.74	35.50
1	-14	-8	15.10	8.20
1	-14	-8	9.70	7.20
1	-14	-8	13.30	7.60
1	-14	-8	21.10	11.20
1	-14	-8	13.20	8.80
-1	14	8	16.10	9.00
-1	14	8	11.40	3.20
0	-14	-8	25.20	7.80
0	-14	-8	56.69	8.50
0	-14	-8	50.89	8.30
0	-14	-8	35.10	14.60
0	-14	-8	52.79	8.80
0	14	8	42.90	4.80
-1	-14	-8	145.09	13.00
-1	-14	-8	148.59	15.80
-1	-14	-8	136.99	14.10
-1	-14	-8	146.79	13.80
-1	-14	-8	148.19	17.40
1	14	8	151.18	8.40
-2	-14	-8	102.29	15.00
-2	-14	-8	78.59	9.70
-2	-14	-8	75.79	12.40
-2	-14	-8	88.99	11.50
-2	-14	-8	108.39	13.20
-2	-14	-8	69.89	10.90
2	14	8	79.39	5.70
-3	-14	-8	237.88	17.30
-3	-14	-8	254.77	16.30

-3	-14	-8	259.87	16.50
-3	-14	-8	265.57	25.30
-3	-14	-8	231.58	22.30
-3	-14	-8	234.58	17.50
-3	-14	-8	229.48	18.50
3	14	8	245.88	12.50
-4	-14	-8	1135.29	53.09
-4	-14	-8	1184.58	53.79
-4	-14	-8	1027.10	53.39
-4	-14	-8	1107.79	53.49
-4	-14	-8	1065.29	53.79
4	14	8	1127.09	50.79
-5	-14	-8	11.50	7.80
-5	-14	-8	10.00	6.70
-5	-14	-8	5.00	6.10
-5	-14	-8	2.70	8.20
-5	-14	-8	16.20	9.90
-5	-14	-8	10.40	7.80
5	14	8	11.80	4.90
-6	-14	-8	137.59	15.80
-6	-14	-8	158.98	21.60
-6	-14	-8	158.28	13.60
-6	-14	-8	154.38	13.60
-6	-14	-8	138.19	14.90
-6	-14	-8	139.19	15.30
-7	-14	-8	279.97	18.80
-7	-14	-8	256.67	19.30
-7	-14	-8	275.57	18.60
-7	-14	-8	284.87	18.70
-7	-14	-8	260.57	20.40
-8	-14	-8	86.99	13.50
-8	-14	-8	82.09	13.80
-8	-14	-8	98.89	12.40
-8	-14	-8	86.59	14.30
-8	-14	-8	86.09	14.30
-9	-14	-8	-7.60	8.10
-9	-14	-8	5.70	8.10
-9	-14	-8	3.10	8.70
-9	-14	-8	13.40	8.10
-9	-14	-8	8.20	7.70
9	14	8	-5.30	13.30
-10	-14	-8	161.68	14.30
-10	-14	-8	164.48	14.00
-10	-14	-8	148.29	17.80

-10	-14	-8	124.19	14.80
-10	-14	-8	127.39	14.00
-11	-14	-8	65.09	11.10
-11	-14	-8	39.10	8.00
-11	-14	-8	50.69	8.80
-11	-14	-8	27.90	10.30
9	-15	-8	282.47	18.40
-9	15	8	257.57	18.60
8	-15	-8	203.38	15.90
-8	15	8	221.08	19.40
-8	15	8	160.68	25.00
7	-15	-8	69.79	19.00
7	-15	-8	41.90	8.50
-7	15	8	55.69	12.80
6	-15	-8	35.50	13.00
6	-15	-8	24.10	8.10
-6	15	8	51.69	12.50
5	-15	-8	-15.70	17.50
5	-15	-8	24.30	8.40
5	-15	-8	-3.30	13.40
5	-15	-8	22.90	10.00
-5	15	8	34.50	13.10
4	-15	-8	262.57	18.50
4	-15	-8	200.98	23.80
4	-15	-8	231.78	19.10
4	-15	-8	255.57	23.60
-4	15	8	241.28	16.50
3	-15	-8	155.08	16.00
3	-15	-8	128.59	15.70
3	-15	-8	104.19	18.00
3	-15	-8	176.08	19.10
-3	15	8	138.79	18.30
-3	15	8	136.19	10.90
2	-15	-8	74.89	10.80
2	-15	-8	93.49	15.30
2	-15	-8	55.39	10.40
2	-15	-8	47.10	10.10
2	-15	-8	61.59	10.10
-2	15	8	31.30	10.70
-2	15	8	57.59	7.00
1	-15	-8	161.28	15.40
1	-15	-8	157.88	16.90
1	-15	-8	147.79	15.30
1	-15	-8	164.38	19.90

1	-15	-8	180.78	15.60
-1	15	8	147.39	16.50
-1	15	8	156.78	9.80
0	-15	-8	52.69	10.90
0	-15	-8	35.30	10.80
0	-15	-8	28.50	15.30
0	-15	-8	38.60	11.40
0	-15	-8	44.20	11.30
0	15	8	43.10	5.10
-1	-15	-8	617.44	34.30
-1	-15	-8	605.14	33.90
-1	-15	-8	653.13	34.40
-1	-15	-8	755.62	35.80
-1	-15	-8	632.44	34.00
1	15	8	615.14	30.20
-2	-15	-8	1603.34	72.59
-2	-15	-8	1513.45	73.89
-2	-15	-8	1531.95	72.99
2	15	8	1561.54	70.39
-3	-15	-8	-7.30	12.30
-3	-15	-8	3.30	10.20
-3	-15	-8	-0.20	6.00
-3	-15	-8	4.30	7.30
-3	-15	-8	6.00	13.80
-3	-15	-8	-5.60	7.70
-3	-15	-8	1.50	7.20
3	15	8	6.80	3.20
-4	-15	-8	4.30	8.40
-4	-15	-8	15.60	6.40
-4	-15	-8	13.50	7.60
-4	-15	-8	5.20	10.40
-4	-15	-8	11.60	7.20
-4	-15	-8	-0.80	8.10
4	15	8	9.80	4.00
-5	-15	-8	472.55	25.40
-5	-15	-8	490.65	27.60
-5	-15	-8	472.85	26.00
-5	-15	-8	462.45	27.20
-5	-15	-8	467.25	26.80
-5	-15	-8	433.86	26.70
5	15	8	465.55	24.00
-6	-15	-8	47.20	9.30
-6	-15	-8	58.49	9.80
-6	-15	-8	42.00	12.60

-6	-15	-8	37.00	8.70
-6	-15	-8	42.00	9.20
-6	-15	-8	22.60	7.20
6	15	8	29.20	8.50
-7	-15	-8	11.10	8.10
-7	-15	-8	5.00	7.80
-7	-15	-8	-4.00	8.10
-7	-15	-8	5.50	7.90
-7	-15	-8	11.50	7.30
7	15	8	-1.50	9.60
-8	-15	-8	35.20	9.50
-8	-15	-8	46.10	9.30
-8	-15	-8	57.29	14.30
-8	-15	-8	44.20	8.60
-8	-15	-8	46.40	10.00
-9	-15	-8	174.38	15.40
-9	-15	-8	133.39	14.80
-9	-15	-8	159.28	14.90
-9	-15	-8	129.19	14.50
-9	-15	-8	164.58	15.40
-10	-15	-8	18.70	7.60
-10	-15	-8	-2.80	8.40
-10	-15	-8	4.40	7.00
-10	-15	-8	7.10	7.70
-10	-15	-8	11.10	10.00
8	-16	-8	13.50	7.70
-8	16	8	16.50	9.70
7	-16	-8	-4.10	7.30
7	-16	-8	22.00	18.20
-7	16	8	-2.30	9.80
6	-16	-8	2.80	13.30
6	-16	-8	4.50	12.40
-6	16	8	28.20	16.70
5	-16	-8	123.99	16.80
5	-16	-8	110.69	15.10
5	-16	-8	86.49	20.00
5	-16	-8	90.79	17.20
4	-16	-8	150.48	21.40
4	-16	-8	138.29	15.60
4	-16	-8	151.18	17.20
4	-16	-8	110.59	15.80
-4	16	8	124.69	16.90
3	-16	-8	1325.27	63.09
3	-16	-8	1385.36	63.69

3	-16	-8	1335.67	61.99
3	-16	-8	1305.57	61.99
-3	16	8	1305.07	60.19
-3	16	8	1139.79	64.49
-3	16	8	1205.88	62.69
2	-16	-8	66.89	10.90
2	-16	-8	71.79	11.20
2	-16	-8	64.19	11.10
2	-16	-8	83.09	14.20
2	-16	-8	62.89	10.90
-2	16	8	61.49	8.10
-2	16	8	43.20	11.40
1	-16	-8	-11.00	8.20
1	-16	-8	10.10	7.60
1	-16	-8	10.00	7.80
1	-16	-8	5.80	12.30
1	-16	-8	17.60	8.60
-1	16	8	-1.80	9.50
-1	16	8	8.10	4.10
0	-16	-8	507.45	27.70
0	-16	-8	477.55	28.60
0	-16	-8	488.45	27.80
0	-16	-8	538.15	28.40
0	-16	-8	531.05	37.80
0	16	8	498.95	24.60
-1	-16	-8	87.39	15.20
-1	-16	-8	72.09	12.40
-1	-16	-8	94.89	13.20
-1	-16	-8	104.59	12.80
-1	-16	-8	107.39	15.30
1	16	8	83.09	7.00
-2	-16	-8	25.10	8.40
-2	-16	-8	10.50	10.70
-2	-16	-8	8.90	7.30
-2	-16	-8	32.00	9.30
-2	-16	-8	13.00	6.60
2	16	8	13.70	3.70
-3	-16	-8	410.66	23.20
-3	-16	-8	404.56	23.90
-3	-16	-8	427.36	31.00
-3	-16	-8	386.66	22.60
-3	-16	-8	424.86	24.20
-3	-16	-8	383.86	24.20
3	16	8	393.66	20.20

-4	-16	-8	264.87	18.00
-4	-16	-8	246.38	16.90
-4	-16	-8	275.47	18.60
-4	-16	-8	256.27	19.40
-4	-16	-8	244.98	19.10
4	16	8	255.97	14.80
-5	-16	-8	137.09	13.40
-5	-16	-8	137.49	17.60
-5	-16	-8	145.99	14.70
-5	-16	-8	164.88	16.90
-5	-16	-8	146.89	15.20
-5	-16	-8	151.48	16.20
5	16	8	138.19	12.40
-6	-16	-8	12.40	8.80
-6	-16	-8	3.70	11.80
-6	-16	-8	21.20	9.60
-6	-16	-8	12.60	7.20
-6	-16	-8	5.30	8.10
-6	-16	-8	1.70	7.40
6	16	8	16.50	8.60
-7	-16	-8	436.36	23.90
-7	-16	-8	410.36	24.80
-7	-16	-8	394.46	25.10
-7	-16	-8	377.66	24.20
-7	-16	-8	431.26	25.20
-8	-16	-8	97.39	14.00
-8	-16	-8	102.99	15.50
-8	-16	-8	88.59	13.00
-8	-16	-8	84.89	14.40
-9	-16	-8	-0.80	7.90
-9	-16	-8	-9.90	7.70
-9	-16	-8	14.00	7.20
-9	-16	-8	-4.60	7.20
-9	-16	-8	5.30	8.10
-10	-16	-8	97.39	11.70
-10	-16	-8	71.19	12.60
-10	-16	-8	81.29	15.60
-10	-16	-8	95.59	11.60
-10	-16	-8	71.79	11.60
8	-17	-8	246.08	17.10
-8	17	8	254.27	18.90
7	-17	-8	73.19	18.60
7	-17	-8	46.60	8.20
-7	17	8	52.49	10.90

6	-17	-8	10.60	8.20
6	-17	-8	22.20	12.50
-6	17	8	10.00	10.50
5	-17	-8	222.18	32.90
5	-17	-8	230.88	22.30
5	-17	-8	247.98	19.00
5	-17	-8	267.27	34.20
4	-17	-8	69.59	10.60
4	-17	-8	34.80	13.80
4	-17	-8	78.59	13.90
-4	17	8	107.19	17.80
3	-17	-8	37.60	11.50
3	-17	-8	8.40	9.30
3	-17	-8	4.10	10.50
3	-17	-8	8.60	9.00
-3	17	8	16.70	13.80
-3	17	8	24.90	9.10
-3	17	8	-12.90	17.30
2	-17	-8	333.07	22.30
2	-17	-8	316.27	22.80
2	-17	-8	272.87	21.50
2	-17	-8	306.87	21.90
2	-17	-8	320.37	21.50
-2	17	8	314.17	17.70
-2	17	8	277.07	22.50
-2	17	8	277.37	24.50
1	-17	-8	12.20	8.60
1	-17	-8	5.10	13.10
1	-17	-8	8.00	8.10
1	-17	-8	20.00	8.30
1	-17	-8	14.50	8.30
-1	17	8	-8.70	9.10
-1	17	8	5.70	4.70
0	-17	-8	254.27	18.40
0	-17	-8	250.27	18.20
0	-17	-8	246.28	18.60
0	-17	-8	249.08	19.60
0	17	8	239.78	14.10
-1	-17	-8	29.90	9.00
-1	-17	-8	21.40	9.70
-1	-17	-8	12.00	7.60
-1	-17	-8	17.40	7.30
-1	-17	-8	16.50	7.40
1	17	8	19.70	4.40

-2	-17	-8	88.69	13.40
-2	-17	-8	105.79	13.50
-2	-17	-8	129.59	15.00
-2	-17	-8	105.29	12.20
-2	-17	-8	79.69	12.60
2	17	8	108.19	8.40
-3	-17	-8	516.45	27.70
-3	-17	-8	590.14	34.90
-3	-17	-8	504.65	28.20
-3	-17	-8	493.75	28.40
-3	-17	-8	477.25	27.00
-3	-17	-8	479.45	27.80
3	17	8	515.75	25.20
-4	-17	-8	323.17	20.10
-4	-17	-8	339.67	21.90
-4	-17	-8	331.27	20.90
-4	-17	-8	323.17	21.80
-4	-17	-8	329.17	21.60
4	17	8	333.27	18.40
-5	-17	-8	29.10	9.10
-5	-17	-8	28.80	7.60
-5	-17	-8	33.80	8.80
-5	-17	-8	45.70	9.00
-5	-17	-8	33.70	10.30
-5	-17	-8	40.00	9.60
5	17	8	24.30	8.10
-6	-17	-8	421.36	24.50
-6	-17	-8	390.76	23.90
-6	-17	-8	419.56	27.00
-6	-17	-8	363.26	24.60
-6	-17	-8	396.96	22.90
-6	-17	-8	405.86	24.30
6	17	8	378.66	24.50
-7	-17	-8	298.37	20.80
-7	-17	-8	283.97	25.30
-7	-17	-8	292.17	23.00
-7	-17	-8	258.47	18.80
-8	-17	-8	341.87	19.90
-8	-17	-8	312.27	20.70
-8	-17	-8	287.77	20.10
-8	-17	-8	327.07	20.40
-8	-17	-8	308.27	20.50
-9	-17	-8	25.50	7.80
-9	-17	-8	35.00	8.30

-9	-17	-8	26.00	7.80
-9	-17	-8	27.70	7.60
-10	-17	-8	24.10	7.40
-10	-17	-8	-6.30	9.30
-10	-17	-8	14.40	6.50
-10	-17	-8	31.70	9.40
7	-18	-8	9.30	22.60
7	-18	-8	17.80	7.90
-7	18	8	16.90	9.10
6	-18	-8	39.40	8.80
6	-18	-8	27.50	12.30
-6	18	8	26.70	10.20
5	-18	-8	68.39	10.30
5	-18	-8	57.29	11.60
5	-18	-8	95.79	22.20
4	-18	-8	320.17	29.00
4	-18	-8	380.16	23.40
4	-18	-8	320.47	23.50
4	-18	-8	379.16	26.90
-4	18	8	282.07	27.30
3	-18	-8	133.99	17.20
3	-18	-8	121.09	15.20
3	-18	-8	91.99	13.60
3	-18	-8	101.19	18.50
-3	18	8	83.29	15.70
-3	18	8	109.29	15.50
-3	18	8	66.99	14.90
2	-18	-8	315.97	21.70
2	-18	-8	286.57	24.10
2	-18	-8	355.76	23.10
2	-18	-8	311.07	22.60
-2	18	8	300.37	26.90
-2	18	8	322.87	21.30
-2	18	8	293.87	25.40
1	-18	-8	101.19	14.40
1	-18	-8	88.49	15.10
1	-18	-8	86.89	11.20
1	-18	-8	72.69	15.70
1	-18	-8	101.59	13.90
-1	18	8	76.49	10.10
-1	18	8	73.69	12.20
0	-18	-8	17.70	7.80
0	-18	-8	24.10	8.40
0	-18	-8	23.50	9.30

0	-18	-8	10.70	7.60
0	18	8	20.90	9.50
0	18	8	12.60	5.10
-1	-18	-8	7.50	7.20
-1	-18	-8	19.20	7.40
-1	-18	-8	23.50	8.70
-1	-18	-8	26.50	10.40
1	18	8	26.20	5.50
-2	-18	-8	108.99	15.10
-2	-18	-8	124.89	13.10
-2	-18	-8	97.09	18.10
-2	-18	-8	135.89	14.40
-2	-18	-8	141.19	14.20
2	18	8	116.49	9.80
-3	-18	-8	31.30	7.90
-3	-18	-8	29.10	7.50
-3	-18	-8	17.40	7.80
-3	-18	-8	35.90	9.20
-3	-18	-8	61.69	15.70
3	18	8	32.20	6.00
-4	-18	-8	301.57	19.70
-4	-18	-8	319.37	19.40
-4	-18	-8	273.97	19.00
-4	-18	-8	287.97	20.70
-4	-18	-8	273.97	18.40
4	18	8	265.07	17.20
-5	-18	-8	43.90	7.80
-5	-18	-8	53.89	9.50
-5	-18	-8	45.30	9.40
-5	-18	-8	34.40	9.70
-5	-18	-8	31.40	8.20
5	18	8	48.90	8.80
-6	-18	-8	151.18	15.30
-6	-18	-8	152.98	14.30
-6	-18	-8	169.48	14.50
-6	-18	-8	184.28	15.60
6	18	8	121.09	18.00
-7	-18	-8	95.79	12.80
-7	-18	-8	84.29	13.60
-7	-18	-8	93.19	12.30
-7	-18	-8	81.29	12.80
7	18	8	53.39	14.40
-8	-18	-8	24.80	7.90
-8	-18	-8	31.60	7.50

-8	-18	-8	25.20	7.10
-8	-18	-8	20.80	8.20
-9	-18	-8	-1.10	8.20
-9	-18	-8	28.10	6.70
-9	-18	-8	13.00	7.20
-9	-18	-8	14.10	7.00
7	-19	-8	67.49	11.30
-7	19	8	55.29	9.20
6	-19	-8	26.90	8.40
6	-19	-8	6.40	12.20
-6	19	8	24.60	9.20
5	-19	-8	20.80	10.40
5	-19	-8	13.40	8.80
-5	19	8	21.00	8.90
4	-19	-8	106.19	15.10
4	-19	-8	128.39	17.30
4	-19	-8	119.19	17.00
4	-19	-8	136.89	16.80
3	-19	-8	37.90	12.10
3	-19	-8	42.40	9.80
3	-19	-8	43.60	12.10
3	-19	-8	50.99	12.90
-3	19	8	64.09	14.80
-3	19	8	60.39	12.20
2	-19	-8	50.29	10.40
2	-19	-8	23.90	13.00
2	-19	-8	32.20	10.10
2	-19	-8	37.70	9.00
-2	19	8	34.70	12.20
-2	19	8	30.10	12.40
-2	19	8	31.90	9.10
1	-19	-8	190.58	17.50
1	-19	-8	182.38	18.50
1	-19	-8	182.08	17.10
1	-19	-8	172.08	17.80
1	-19	-8	181.98	23.20
-1	19	8	188.78	19.50
-1	19	8	236.18	15.00
0	-19	-8	137.79	17.80
0	-19	-8	141.09	15.00
0	-19	-8	126.69	15.10
0	-19	-8	144.79	15.40
0	19	8	166.98	18.20
0	19	8	145.99	12.10

-1	-19	-8	331.57	21.60
-1	-19	-8	344.07	22.50
-1	-19	-8	340.47	25.10
-1	-19	-8	384.36	22.20
1	19	8	359.16	19.50
-2	-19	-8	211.08	16.20
-2	-19	-8	199.78	16.50
-2	-19	-8	192.48	17.20
-2	-19	-8	210.98	22.40
-2	-19	-8	224.88	17.60
2	19	8	228.28	14.30
-3	-19	-8	147.39	13.50
-3	-19	-8	117.89	14.50
-3	-19	-8	126.99	18.10
-3	-19	-8	115.29	14.60
-3	-19	-8	119.09	13.10
3	19	8	125.29	11.80
-4	-19	-8	11.90	6.80
-4	-19	-8	12.90	7.10
-4	-19	-8	12.50	8.40
-4	-19	-8	12.00	8.20
4	19	8	26.20	7.60
-5	-19	-8	585.94	31.50
-5	-19	-8	605.14	32.80
-5	-19	-8	565.14	31.00
-5	-19	-8	580.54	30.50
-5	-19	-8	616.34	31.70
5	19	8	592.44	31.70
-6	-19	-8	18.60	8.60
-6	-19	-8	24.70	11.10
-6	-19	-8	8.50	7.50
-6	-19	-8	6.60	7.00
-6	-19	-8	2.80	7.40
6	19	8	7.10	11.30
-7	-19	-8	15.50	6.70
-7	-19	-8	15.50	7.10
-7	-19	-8	13.60	7.40
-7	-19	-8	2.60	7.40
-8	-19	-8	16.60	8.70
-8	-19	-8	32.70	7.30
-8	-19	-8	22.70	7.00
-9	-19	-8	101.69	10.70
-9	-19	-8	90.29	11.80
-9	-19	-8	120.49	10.80

-9	-19	-8	156.18	18.20
6	-20	-8	17.20	13.10
6	-20	-8	21.10	8.30
-6	20	8	23.70	8.30
5	-20	-8	12.10	9.10
5	-20	-8	23.50	9.90
-5	20	8	24.10	8.30
4	-20	-8	346.57	25.80
4	-20	-8	357.36	28.30
4	-20	-8	354.76	22.70
4	-20	-8	361.56	22.70
3	-20	-8	463.45	28.30
3	-20	-8	448.96	27.70
3	-20	-8	462.25	29.60
3	-20	-8	447.36	27.40
-3	20	8	432.26	31.10
2	-20	-8	154.18	15.80
2	-20	-8	182.58	24.60
2	-20	-8	109.99	17.80
-2	20	8	115.69	20.00
-2	20	8	110.39	19.30
-2	20	8	108.79	13.80
1	-20	-8	15.20	8.20
1	-20	-8	3.60	8.90
1	-20	-8	29.10	9.60
1	-20	-8	6.50	8.70
-1	20	8	8.90	11.30
-1	20	8	3.10	8.10
0	-20	-8	347.97	21.90
0	-20	-8	364.56	21.80
0	-20	-8	340.57	23.50
0	-20	-8	340.37	22.00
0	20	8	324.17	23.20
0	20	8	348.57	20.40
-1	-20	-8	187.58	15.90
-1	-20	-8	173.48	16.30
-1	-20	-8	204.38	16.10
-1	-20	-8	213.68	20.40
1	20	8	175.48	17.90
1	20	8	189.98	14.50
-2	-20	-8	3.00	7.80
-2	-20	-8	13.40	12.40
-2	-20	-8	2.60	6.80
-2	-20	-8	25.20	7.80

2	20	8	16.90	7.10
-3	-20	-8	75.89	11.80
-3	-20	-8	56.69	16.90
-3	-20	-8	67.49	9.00
-3	-20	-8	51.89	9.50
3	20	8	96.39	12.40
-4	-20	-8	174.68	16.10
-4	-20	-8	177.08	18.60
-4	-20	-8	173.88	15.10
-4	-20	-8	159.68	14.10
4	20	8	147.49	16.30
-5	-20	-8	17.20	7.30
-5	-20	-8	14.30	8.30
-5	-20	-8	8.00	7.40
-5	-20	-8	10.70	9.20
5	20	8	14.90	10.70
-6	-20	-8	0.70	7.10
-6	-20	-8	-13.30	10.70
-6	-20	-8	7.50	6.30
-6	-20	-8	10.30	6.10
-7	-20	-8	278.17	16.80
-7	-20	-8	250.67	17.10
-7	-20	-8	272.97	17.10
-8	-20	-8	12.70	5.70
-8	-20	-8	1.90	6.80
-8	-20	-8	15.80	6.20
5	-21	-8	294.57	20.40
5	-21	-8	272.57	19.20
-5	21	8	302.27	18.80
4	-21	-8	33.20	15.40
4	-21	-8	39.10	8.90
4	-21	-8	17.50	10.70
4	-21	-8	35.20	11.90
3	-21	-8	39.00	11.00
3	-21	-8	40.10	12.70
3	-21	-8	33.90	8.80
3	-21	-8	41.10	12.50
-3	21	8	50.79	20.60
2	-21	-8	165.38	18.10
2	-21	-8	143.59	17.60
2	-21	-8	174.28	16.80
-2	21	8	143.09	21.20
-2	21	8	140.49	19.10
1	-21	-8	-4.10	9.40

1	-21	-8	4.60	9.80
1	-21	-8	0.10	9.40
-1	21	8	32.40	11.50
-1	21	8	27.20	11.30
-1	21	8	14.90	10.00
0	21	8	7.50	8.90
0	21	8	15.20	10.60
-1	-21	-8	21.30	12.90
-1	-21	-8	20.90	17.80
-1	-21	-8	24.00	10.20
-1	-21	-8	20.90	8.90
1	21	8	33.20	10.70
1	21	8	18.10	8.20
-2	-21	-8	129.39	16.20
-2	-21	-8	89.59	14.10
-2	-21	-8	130.19	17.40
2	21	8	135.59	14.70
-3	-21	-8	57.49	10.10
-3	-21	-8	94.29	17.90
-3	-21	-8	90.89	12.90
-3	-21	-8	84.39	12.40
-4	-21	-8	217.58	15.60
-4	-21	-8	195.68	15.50
-4	-21	-8	229.68	16.80
-4	-21	-8	229.58	18.10
4	21	8	257.27	26.30
-5	-21	-8	63.99	10.20
-5	-21	-8	78.19	13.80
-5	-21	-8	51.39	7.80
-5	-21	-8	88.59	11.00
-6	-21	-8	365.86	25.50
-6	-21	-8	406.46	22.70
-6	-21	-8	401.66	22.00
-6	-21	-8	433.36	22.00
-7	-21	-8	35.00	6.60
-7	-21	-8	30.40	6.60
-7	-21	-8	36.20	7.40
-8	-21	-8	54.39	10.90
-8	-21	-8	42.30	7.20
5	-22	-8	84.89	14.10
5	-22	-8	86.99	15.90
-5	22	8	105.89	11.20
4	-22	-8	85.19	13.40
4	-22	-8	103.39	22.50

4	-22	-8	91.59	29.60
-4	22	8	123.79	12.40
3	-22	-8	129.49	14.40
3	-22	-8	150.78	16.90
3	-22	-8	140.59	18.60
2	-22	-8	44.80	9.50
2	-22	-8	46.50	10.50
2	-22	-8	41.00	10.80
-2	22	8	62.59	15.40
-2	22	8	45.10	13.00
1	-22	-8	130.79	15.50
1	-22	-8	114.19	15.20
1	-22	-8	130.69	14.50
-1	22	8	132.69	18.60
-1	22	8	154.68	18.50
0	-22	-8	48.30	9.40
0	-22	-8	52.09	9.70
0	-22	-8	75.59	12.30
0	22	8	43.30	11.70
-1	-22	-8	302.57	18.80
-1	-22	-8	287.47	19.00
-1	-22	-8	291.97	19.50
-1	-22	-8	294.97	22.40
1	22	8	259.67	21.10
-2	-22	-8	23.00	7.80
-2	-22	-8	17.50	8.40
-2	-22	-8	15.80	12.50
-2	-22	-8	24.30	7.70
2	22	8	23.80	10.60
-3	-22	-8	84.39	12.50
-3	-22	-8	71.59	10.70
-3	-22	-8	77.09	11.00
-4	-22	-8	281.77	17.20
-4	-22	-8	266.37	18.90
-4	-22	-8	280.47	17.10
-5	-22	-8	32.10	6.60
-5	-22	-8	20.50	7.30
-5	-22	-8	23.50	9.60
-5	-22	-8	32.40	6.70
-6	-22	-8	75.99	10.60
-6	-22	-8	84.89	15.30
-6	-22	-8	78.59	10.00
-6	-22	-8	85.89	9.80
-7	-22	-8	27.60	6.00

-7	-22	-8	30.70	6.20
-7	-22	-8	30.30	6.90
7	22	8	-7.60	17.90
4	-23	-8	51.99	12.20
-4	23	8	56.29	8.70
3	-23	-8	-2.40	12.60
3	-23	-8	-3.70	16.00
3	-23	-8	13.50	8.00
2	-23	-8	87.69	14.70
2	-23	-8	71.29	11.60
2	-23	-8	86.09	13.00
-2	23	8	43.30	20.00
1	-23	-8	211.98	17.20
1	-23	-8	204.18	17.20
1	-23	-8	217.58	17.10
-1	23	8	207.88	19.90
-1	23	8	234.28	19.40
0	-23	-8	77.49	12.20
0	-23	-8	72.49	12.90
0	-23	-8	92.69	14.60
0	23	8	96.09	15.80
0	23	8	85.49	13.60
-1	-23	-8	299.87	19.50
-1	-23	-8	305.17	18.80
-1	-23	-8	308.67	19.20
-1	-23	-8	333.37	22.80
1	23	8	319.07	24.20
-2	-23	-8	22.00	12.90
-2	-23	-8	34.20	7.80
-2	-23	-8	25.60	7.30
-2	-23	-8	31.50	7.20
2	23	8	18.90	11.90
-3	-23	-8	160.48	12.80
-3	-23	-8	148.19	14.20
-3	-23	-8	155.78	12.50
3	23	8	140.49	21.10
-4	-23	-8	81.59	12.20
-4	-23	-8	67.19	9.70
-4	-23	-8	62.89	9.80
-5	-23	-8	172.58	15.60
-5	-23	-8	184.78	12.90
-5	-23	-8	195.08	12.60
-6	-23	-8	257.97	19.50
-6	-23	-8	222.48	13.80

-6	-23	-8	218.48	13.70
2	-24	-8	54.89	9.10
2	-24	-8	54.59	11.40
1	-24	-8	122.99	14.20
1	-24	-8	130.89	14.50
1	-24	-8	147.89	14.20
-1	24	8	106.29	17.60
0	-24	-8	245.18	16.90
0	-24	-8	238.58	17.80
0	-24	-8	242.58	16.50
0	24	8	207.58	20.00
0	24	8	222.68	18.70
-1	-24	-8	23.40	6.70
-1	-24	-8	25.40	7.60
-1	-24	-8	-2.00	10.50
-1	-24	-8	19.50	7.70
1	24	8	18.50	11.10
1	24	8	38.30	9.60
-2	-24	-8	-0.50	7.00
-2	-24	-8	9.00	6.80
-2	-24	-8	43.50	25.80
2	24	8	-13.80	9.90
-3	-24	-8	0.70	6.90
-3	-24	-8	5.10	6.20
-3	-24	-8	6.00	5.80
3	24	8	-7.30	10.20
-4	-24	-8	132.29	13.20
-4	-24	-8	134.39	11.60
-4	-24	-8	149.59	11.00
4	24	8	142.69	15.60
-5	-24	-8	83.89	13.70
-5	-24	-8	112.39	12.70
5	24	8	97.79	15.00
0	-25	-8	13.00	11.50
0	-25	-8	24.00	9.30
0	-25	-8	15.40	8.50
0	25	8	12.50	11.00
-1	-25	-8	15.50	7.00
-1	-25	-8	20.00	11.30
-1	-25	-8	29.70	12.10
1	25	8	5.00	9.70
-2	-25	-8	92.19	10.10
-2	-25	-8	96.29	11.10
2	25	8	97.49	13.00

3	20	-9	177.68	15.70
-3	-20	9	134.99	13.60
2	20	-9	32.90	12.40
-2	-20	9	45.30	14.90
1	20	-9	23.50	11.90
1	20	-9	11.50	8.30
1	20	-9	-17.10	13.30
-1	-20	9	22.70	8.10
0	20	-9	298.97	19.50
0	20	-9	262.47	18.70
0	20	-9	219.98	20.40
0	-20	9	238.88	19.70
0	-20	9	290.17	17.30
-1	20	-9	259.37	22.10
-1	20	-9	257.17	17.60
-1	20	-9	258.57	19.60
1	-20	9	259.17	16.70
-2	20	-9	21.90	10.90
-2	20	-9	8.40	13.30
-2	20	-9	10.60	13.60
2	-20	9	6.80	6.80
4	19	-9	153.98	13.20
-4	-19	9	130.99	11.30
3	19	-9	0.10	7.00
3	19	-9	-27.00	14.20
-3	-19	9	6.50	6.00
2	19	-9	94.39	15.80
2	19	-9	79.89	15.40
2	19	-9	114.99	12.40
-2	-19	9	127.49	16.10
-2	-19	9	72.99	11.20
1	19	-9	88.29	13.40
1	19	-9	112.89	16.40
1	19	-9	109.89	14.70
-1	-19	9	107.19	12.60
-1	-19	9	85.49	13.60
0	19	-9	46.60	11.40
0	19	-9	44.00	9.50
0	19	-9	31.40	9.30
0	-19	9	16.90	8.20
0	-19	9	32.00	9.30
-1	19	-9	7.60	8.20
-1	19	-9	-1.90	9.70
1	-19	9	12.60	7.50

1	-19	9	21.10	12.00
-2	19	-9	-0.30	11.00
-2	19	-9	0.40	8.30
-2	19	-9	-18.40	9.90
2	-19	9	0.60	7.00
-3	19	-9	146.49	13.40
-3	19	-9	133.79	22.90
-3	19	-9	121.69	15.10
3	-19	9	106.59	12.50
-4	19	-9	3.30	10.20
-4	19	-9	-8.00	7.70
4	-19	9	13.10	7.40
5	18	-9	-0.40	6.70
-5	-18	9	-5.00	5.30
4	18	-9	33.90	7.90
-4	-18	9	27.20	6.60
3	18	-9	11.40	7.40
3	18	-9	16.20	11.70
-3	-18	9	15.30	6.00
2	18	-9	199.78	18.30
2	18	-9	226.68	16.70
2	18	-9	219.78	24.30
-2	-18	9	208.18	15.30
1	18	-9	69.39	11.70
1	18	-9	72.29	13.10
1	18	-9	48.10	10.40
-1	-18	9	68.49	9.90
-1	-18	9	53.09	8.90
0	18	-9	20.60	9.60
0	18	-9	36.20	9.50
0	18	-9	34.00	9.00
0	-18	9	44.80	9.10
0	-18	9	40.70	9.20
-1	18	-9	379.86	24.90
-1	18	-9	393.36	23.60
-1	18	-9	394.96	23.30
1	-18	9	408.86	23.20
1	-18	9	376.76	24.80
-2	18	-9	114.49	13.80
-2	18	-9	93.79	13.00
-2	18	-9	98.99	14.10
2	-18	9	93.29	12.90
-3	18	-9	23.70	13.80
-3	18	-9	6.90	8.00

3	-18	9	6.90	7.40
-4	18	-9	107.99	13.10
-4	18	-9	107.39	14.10
4	-18	9	98.09	12.60
-5	18	-9	3.50	7.80
-5	18	-9	-3.60	10.00
5	-18	9	2.30	8.20
5	-18	9	-10.00	15.30
6	17	-9	22.10	7.50
-6	-17	9	34.00	6.40
-6	-17	9	29.90	8.80
5	17	-9	125.19	13.00
-5	-17	9	125.79	11.40
4	17	-9	81.99	10.00
-4	-17	9	94.19	10.70
3	17	-9	4.90	7.90
3	17	-9	22.80	12.40
3	17	-9	-17.40	11.00
-3	-17	9	14.30	6.50
-3	-17	9	11.90	12.30
2	17	-9	3.00	7.40
2	17	-9	-12.80	9.20
2	17	-9	0.00	9.70
-2	-17	9	-5.80	6.80
-2	-17	9	-7.90	8.90
1	17	-9	312.57	21.70
1	17	-9	351.36	22.30
1	17	-9	341.97	21.80
-1	-17	9	362.96	21.50
-1	-17	9	315.47	21.60
0	17	-9	184.98	16.50
0	17	-9	149.29	16.70
0	17	-9	204.78	17.40
0	-17	9	160.18	17.40
0	-17	9	188.18	16.70
-1	17	-9	0.90	8.20
-1	17	-9	-9.00	8.30
-1	17	-9	-13.40	8.90
1	-17	9	-4.30	7.70
1	-17	9	9.70	10.40
-2	17	-9	48.40	9.90
-2	17	-9	46.10	9.90
-2	17	-9	50.99	11.50
2	-17	9	49.00	9.00

-3	17	-9	-4.40	8.60
-3	17	-9	9.20	8.00
-3	17	-9	4.60	11.30
3	-17	9	-3.00	7.60
-4	17	-9	-11.10	7.80
-4	17	-9	10.50	13.40
-4	17	-9	-2.30	8.70
4	-17	9	12.50	8.00
4	-17	9	-2.00	11.30
-5	17	-9	192.68	15.70
-5	17	-9	203.38	16.60
5	-17	9	187.48	17.40
5	-17	9	195.98	16.40
-6	17	-9	7.50	7.90
-6	17	-9	-6.80	9.60
6	-17	9	-3.60	10.00
6	-17	9	11.10	9.00
7	16	-9	64.49	11.70
-7	-16	9	69.09	10.70
-7	-16	9	87.39	12.10
6	16	-9	140.39	13.10
-6	-16	9	112.99	12.70
-6	-16	9	146.09	12.10
5	16	-9	142.39	14.00
-5	-16	9	140.99	14.20
-5	-16	9	142.49	12.40
4	16	-9	32.80	9.00
-4	-16	9	31.60	7.30
-4	-16	9	34.70	10.60
3	16	-9	93.99	14.50
3	16	-9	87.99	13.20
-3	-16	9	91.49	13.30
-3	-16	9	80.89	13.10
2	16	-9	-8.80	9.30
2	16	-9	6.20	8.50
-2	-16	9	5.10	7.80
1	16	-9	-16.40	7.90
1	16	-9	5.10	8.10
1	16	-9	-9.80	9.20
-1	-16	9	-11.00	8.40
-1	-16	9	-8.10	7.20
0	16	-9	572.84	31.10
0	16	-9	534.35	30.50
0	16	-9	555.94	31.40

0	-16	9	587.74	31.30
0	-16	9	576.04	31.00
-1	16	-9	114.59	15.40
-1	16	-9	123.59	15.80
-1	16	-9	110.29	13.70
1	-16	9	109.59	14.90
1	-16	9	107.19	14.40
-2	16	-9	42.20	9.50
-2	16	-9	38.80	18.20
-2	16	-9	48.60	17.70
2	-16	9	41.50	10.70
2	-16	9	66.39	16.60
-3	16	-9	-2.90	8.40
-3	16	-9	-16.50	10.70
-3	16	-9	-2.80	9.10
3	-16	9	1.80	8.70
3	-16	9	-21.10	11.80
-4	16	-9	8.30	12.80
-4	16	-9	-7.20	8.60
-4	16	-9	8.30	8.80
4	-16	9	-3.20	8.50
4	-16	9	9.30	9.70
-5	16	-9	86.59	14.40
5	-16	9	91.29	15.40
5	-16	9	65.19	10.20
-6	16	-9	23.40	10.20
-6	16	-9	12.70	8.40
6	-16	9	-1.50	9.80
6	-16	9	4.70	8.30
-7	16	-9	-6.30	9.90
-7	16	-9	14.40	7.50
7	-16	9	25.80	25.10
7	-16	9	18.40	8.30
7	15	-9	41.40	8.50
7	15	-9	28.90	21.80
-7	-15	9	31.50	6.90
-7	-15	9	18.20	6.60
6	15	-9	73.79	10.50
6	15	-9	115.49	20.50
-6	-15	9	67.89	11.90
-6	-15	9	61.09	10.60
5	15	-9	362.66	23.00
5	15	-9	404.36	29.50
-5	-15	9	395.06	22.00

-5	-15	9	349.77	21.90
-4	-15	9	183.48	17.90
3	15	-9	14.10	8.30
3	15	-9	-7.80	10.10
-3	-15	9	1.60	6.70
-3	-15	9	5.10	11.50
2	15	-9	88.59	11.50
2	15	-9	82.19	13.80
-2	-15	9	92.19	13.00
-2	-15	9	84.79	16.00
1	15	-9	-2.10	8.00
1	15	-9	-2.10	8.60
1	15	-9	1.30	7.80
-1	-15	9	6.00	7.00
-1	-15	9	10.20	8.50
0	15	-9	199.48	16.50
0	15	-9	196.58	17.70
0	15	-9	189.78	17.20
0	-15	9	205.28	16.60
0	-15	9	191.28	17.40
-1	15	-9	-1.30	8.00
-1	15	-9	13.00	9.10
-1	15	-9	-0.50	8.40
1	-15	9	7.30	7.30
1	-15	9	12.30	9.30
-2	15	-9	-2.40	7.90
-2	15	-9	1.40	8.40
-2	15	-9	2.40	9.10
2	-15	9	-21.10	11.90
2	-15	9	13.10	7.30
-3	15	-9	42.50	9.80
-3	15	-9	59.49	11.90
-3	15	-9	47.50	9.60
3	-15	9	41.20	8.80
3	-15	9	68.69	13.80
-4	15	-9	40.10	15.00
-4	15	-9	23.80	9.40
-4	15	-9	47.50	14.10
4	-15	9	23.70	13.20
4	-15	9	17.00	10.50
-5	15	-9	361.96	23.80
-5	15	-9	332.57	28.80
-5	15	-9	392.46	23.80
5	-15	9	368.46	23.20

5	-15	9	399.16	24.20
-6	15	-9	522.45	29.70
-6	15	-9	549.45	30.10
6	-15	9	564.74	30.60
6	-15	9	526.95	28.60
-7	15	-9	14.00	8.10
-7	15	-9	-9.80	9.50
7	-15	9	-0.30	18.50
7	-15	9	12.30	7.60
8	14	-9	-20.60	15.40
8	14	-9	-2.50	8.10
-8	-14	9	2.00	6.20
-8	-14	9	9.80	9.70
7	14	-9	202.18	19.60
7	14	-9	224.88	17.20
-7	-14	9	224.78	15.20
-7	-14	9	232.98	15.90
6	14	-9	318.57	21.10
6	14	-9	343.97	24.80
-6	-14	9	292.57	19.80
-6	-14	9	308.17	19.40
5	14	-9	21.40	13.40
-5	-14	9	34.70	9.00
4	14	-9	11.50	13.40
4	14	-9	20.90	8.50
-4	-14	9	2.40	8.40
-4	-14	9	6.30	6.30
3	14	-9	138.59	16.30
3	14	-9	194.78	26.00
3	14	-9	183.98	17.90
-3	-14	9	167.58	19.40
-3	-14	9	155.58	17.60
-3	-14	9	163.28	14.20
2	14	-9	135.49	16.20
2	14	-9	114.09	15.40
-2	-14	9	112.29	13.30
-2	-14	9	137.09	16.10
1	14	-9	59.39	9.80
-1	-14	9	38.30	8.60
-1	-14	9	39.90	8.00
0	14	-9	16.10	9.60
0	14	-9	8.90	8.40
0	14	-9	14.90	8.60
0	-14	9	-0.50	7.60

0	-14	9	2.80	7.40
-1	14	-9	639.64	34.30
-1	14	-9	636.44	34.10
1	-14	9	661.13	34.30
1	-14	9	613.04	34.10
-2	14	-9	36.70	9.00
-2	14	-9	29.40	10.10
-2	14	-9	23.80	8.80
2	-14	9	22.10	7.80
2	-14	9	41.40	11.70
2	-14	9	42.70	12.30
-3	14	-9	9.80	10.60
-3	14	-9	22.20	9.20
-3	14	-9	4.10	8.70
3	-14	9	-2.40	9.70
3	-14	9	-1.90	7.20
-4	14	-9	99.09	15.00
-4	14	-9	63.49	13.60
-4	14	-9	65.09	11.10
4	-14	9	101.89	16.40
4	-14	9	72.59	13.80
-5	14	-9	7.70	14.70
-5	14	-9	4.80	8.90
5	-14	9	-15.40	10.70
5	-14	9	3.00	11.50
-6	14	-9	13.10	9.10
-6	14	-9	14.90	16.60
-6	14	-9	21.80	9.80
6	-14	9	7.90	7.80
6	-14	9	27.90	10.60
-7	14	-9	108.59	14.90
-7	14	-9	86.49	13.90
7	-14	9	96.39	12.90
7	-14	9	72.09	15.30
-8	14	-9	12.30	10.60
-8	14	-9	4.10	8.10
8	-14	9	21.80	6.70
8	13	-9	-1.90	11.00
8	13	-9	-11.10	8.40
-8	-13	9	0.70	5.80
7	13	-9	102.19	15.00
7	13	-9	106.69	17.90
-7	-13	9	100.39	12.80
-7	-13	9	120.79	12.30

6	13	-9	78.59	11.60
6	13	-9	111.59	19.20
-6	-13	9	108.39	13.20
-6	-13	9	123.49	14.00
5	13	-9	7.50	8.30
5	13	-9	18.90	12.00
-5	-13	9	10.80	7.70
-5	-13	9	20.30	7.20
4	13	-9	395.66	27.60
4	13	-9	394.36	24.60
-4	-13	9	366.66	23.80
-4	-13	9	395.96	23.10
3	13	-9	-0.10	12.20
3	13	-9	13.10	10.50
3	13	-9	-3.80	8.00
-3	-13	9	10.90	10.00
-3	-13	9	-3.80	8.90
-3	-13	9	1.50	6.90
2	13	-9	57.99	10.10
2	13	-9	52.29	9.30
2	13	-9	47.30	15.10
-2	-13	9	46.00	8.50
-2	-13	9	46.00	9.30
1	13	-9	409.36	23.70
1	13	-9	383.76	24.40
-1	-13	9	405.86	24.30
-1	-13	9	396.96	23.10
0	13	-9	80.49	10.70
0	13	-9	78.39	12.60
0	13	-9	101.39	14.00
0	-13	9	73.09	9.90
0	-13	9	80.29	13.50
-1	13	-9	10.30	8.90
-1	13	-9	16.80	8.50
-1	13	-9	3.20	7.00
1	-13	9	11.30	8.80
1	-13	9	13.90	7.00
-2	13	-9	80.19	11.40
-2	13	-9	90.69	12.80
-2	13	-9	83.99	11.50
2	-13	9	63.69	9.60
2	-13	9	80.19	11.60
2	-13	9	74.29	12.50
-3	13	-9	28.80	10.90

-3	13	-9	21.90	9.00
3	-13	9	27.90	8.60
3	-13	9	28.50	8.80
-4	13	-9	49.60	12.70
-4	13	-9	63.69	10.80
-4	13	-9	53.09	10.50
4	-13	9	39.80	8.90
4	-13	9	41.30	9.20
-5	13	-9	112.59	15.80
-5	13	-9	146.99	23.60
-5	13	-9	153.18	17.30
5	-13	9	128.79	16.70
5	-13	9	105.79	16.40
-6	13	-9	96.89	15.10
-6	13	-9	125.29	18.60
-6	13	-9	94.89	15.30
6	-13	9	110.99	18.60
6	-13	9	74.99	11.20
-7	13	-9	536.75	31.50
-7	13	-9	539.25	31.40
7	-13	9	566.74	29.70
7	-13	9	590.74	35.10
-8	13	-9	30.80	9.80
-8	13	-9	43.20	11.40
8	-13	9	37.80	7.80
-9	13	-9	-5.00	11.80
-9	13	-9	-17.30	9.40
9	-13	9	4.00	8.90
9	12	-9	33.10	11.50
9	12	-9	20.80	10.60
-9	-12	9	29.40	5.80
8	12	-9	-12.30	10.80
8	12	-9	-0.70	9.70
-8	-12	9	-6.80	8.30
-8	-12	9	0.70	6.00
7	12	-9	63.89	10.70
7	12	-9	75.69	13.10
-7	-12	9	80.79	12.80
-7	-12	9	65.79	9.10
6	12	-9	194.88	21.70
6	12	-9	217.28	17.80
-6	-12	9	215.98	16.10
-6	-12	9	188.28	16.50
5	12	-9	221.18	21.40

5	12	-9	231.68	18.30
-5	-12	9	245.38	17.50
-5	-12	9	217.58	16.50
4	12	-9	50.09	9.60
4	12	-9	42.60	11.60
-4	-12	9	53.29	9.60
3	12	-9	50.79	9.00
3	12	-9	84.99	14.70
3	12	-9	59.19	12.50
-3	-12	9	53.19	10.40
-3	-12	9	53.29	8.90
-3	-12	9	87.89	14.50
2	12	-9	3.40	11.80
2	12	-9	-1.20	7.90
2	12	-9	-8.10	7.60
-2	-12	9	-6.10	6.30
-2	-12	9	-8.00	7.80
1	12	-9	122.09	18.00
1	12	-9	122.99	15.00
1	12	-9	125.89	14.00
-1	-12	9	141.09	15.00
-1	-12	9	151.38	13.80
0	12	-9	1177.98	54.29
0	12	-9	1120.69	54.79
0	-12	9	1071.79	53.99
0	-12	9	1133.39	54.69
-1	12	-9	58.29	10.10
-1	12	-9	53.69	8.70
-1	12	-9	46.70	9.20
1	-12	9	38.10	9.40
1	-12	9	41.20	8.00
-2	12	-9	354.26	23.80
-2	12	-9	360.46	22.80
-2	12	-9	352.96	21.50
2	-12	9	330.47	21.70
2	-12	9	329.57	24.80
-3	12	-9	43.10	11.00
-3	12	-9	52.39	9.90
-3	12	-9	58.49	11.00
3	-12	9	53.89	9.20
-4	12	-9	18.10	11.80
-4	12	-9	14.00	10.80
-4	12	-9	1.80	8.70
4	-12	9	-0.80	8.00

4	-12	9	13.90	8.60
-5	12	-9	58.09	10.80
-5	12	-9	74.39	11.30
-5	12	-9	50.09	14.30
5	-12	9	45.00	9.50
5	-12	9	31.20	8.60
-6	12	-9	24.60	10.60
-6	12	-9	-0.30	9.40
-6	12	-9	-4.00	14.30
6	-12	9	14.00	10.20
6	-12	9	-1.60	7.80
-7	12	-9	66.19	12.30
-7	12	-9	61.09	18.40
-7	12	-9	60.89	11.80
7	-12	9	52.79	9.30
7	-12	9	62.19	15.40
-8	12	-9	256.07	20.10
-8	12	-9	228.98	20.00
8	-12	9	263.57	17.60
-9	12	-9	-14.20	11.20
-9	12	-9	9.70	9.00
9	-12	9	4.80	7.10
9	11	-9	80.59	12.00
9	11	-9	45.70	15.80
-9	-11	9	84.89	10.20
8	11	-9	104.59	15.80
8	11	-9	109.89	18.10
-8	-11	9	111.09	14.80
-8	-11	9	109.49	11.80
7	11	-9	13.40	12.70
7	11	-9	19.70	10.70
-7	-11	9	5.70	7.60
-7	-11	9	-0.80	8.20
6	11	-9	25.30	11.50
-6	-11	9	15.00	7.00
-6	-11	9	19.00	7.90
5	11	-9	1009.30	54.99
-5	-11	9	1132.79	52.79
-5	-11	9	1137.99	53.29
4	11	-9	265.67	22.50
4	11	-9	265.07	19.80
-4	-11	9	287.57	18.40
-4	-11	9	268.17	19.10
3	11	-9	24.30	7.90

3	11	-9	14.80	10.40
-3	-11	9	13.20	6.40
-3	-11	9	27.40	10.30
-3	-11	9	23.70	8.80
2	11	-9	94.19	13.60
2	11	-9	103.69	14.70
2	11	-9	109.99	13.40
-2	-11	9	99.99	12.10
-2	-11	9	71.29	10.60
1	11	-9	22.40	7.80
1	11	-9	27.40	12.20
1	11	-9	16.00	7.10
-1	-11	9	8.10	7.10
-1	-11	9	11.80	6.60
0	11	-9	455.25	24.80
0	11	-9	399.06	39.00
0	11	-9	441.26	25.60
0	-11	9	445.16	30.00
0	-11	9	440.66	25.50
-1	11	-9	12.30	8.90
-1	11	-9	21.20	7.00
1	-11	9	11.80	9.40
1	-11	9	12.60	7.20
-2	11	-9	18.50	9.20
-2	11	-9	13.10	8.60
-2	11	-9	5.90	6.80
2	-11	9	9.50	10.40
2	-11	9	3.20	7.60
-3	11	-9	101.69	18.20
-3	11	-9	101.89	13.50
-3	11	-9	114.89	15.40
3	-11	9	117.79	14.60
-4	11	-9	6.30	9.00
-4	11	-9	10.20	8.30
-4	11	-9	14.10	12.50
4	-11	9	-6.90	7.20
-5	11	-9	573.14	36.40
-5	11	-9	539.45	31.90
-5	11	-9	508.45	38.50
5	-11	9	542.95	31.00
-6	11	-9	457.45	33.70
-6	11	-9	446.36	28.80
-6	11	-9	431.06	28.20
6	-11	9	456.45	26.60

6	-11	9	437.96	28.40
-7	11	-9	184.38	18.50
-7	11	-9	203.48	29.60
-7	11	-9	222.28	26.40
7	-11	9	149.99	27.10
7	-11	9	180.98	26.80
-8	11	-9	13.70	10.60
-8	11	-9	25.80	10.40
8	-11	9	26.10	8.20
-9	11	-9	120.49	17.70
-9	11	-9	133.99	16.60
9	-11	9	157.68	14.20
10	10	-9	245.78	25.30
-10	-10	9	178.68	15.50
9	10	-9	61.49	13.10
9	10	-9	38.40	10.90
-9	-10	9	71.49	10.10
8	10	-9	-1.70	9.80
8	10	-9	-17.70	11.70
-8	-10	9	-1.70	8.60
-8	-10	9	1.20	6.50
7	10	-9	402.26	28.50
-7	-10	9	423.96	23.80
-7	-10	9	421.86	25.20
6	10	-9	445.96	29.80
-6	-10	9	477.05	25.40
-6	-10	9	427.16	25.80
5	10	-9	147.79	20.60
-5	-10	9	176.68	14.80
-5	-10	9	146.89	14.30
4	10	-9	21.90	11.10
-4	-10	9	21.90	7.50
-4	-10	9	22.20	7.00
3	10	-9	38.30	10.90
3	10	-9	13.50	7.80
-3	-10	9	26.20	12.10
-3	-10	9	33.80	6.20
-3	-10	9	46.90	13.30
2	10	-9	0.90	10.00
2	10	-9	6.30	9.50
2	10	-9	14.00	8.20
-2	-10	9	-1.20	7.40
-2	-10	9	11.50	8.60
1	10	-9	106.89	14.30

1	10	-9	135.69	19.60
1	10	-9	152.68	13.70
-1	-10	9	154.18	14.60
-1	-10	9	149.19	13.30
0	10	-9	226.38	15.70
0	10	-9	234.78	22.90
0	10	-9	209.88	16.80
0	-10	9	210.48	16.80
0	-10	9	222.18	17.00
-1	10	-9	1401.76	64.79
-1	10	-9	1370.66	65.59
-1	10	-9	1366.76	65.59
1	-10	9	1360.36	64.99
1	-10	9	1370.66	65.09
-2	10	-9	-4.10	8.80
-2	10	-9	2.80	6.30
2	-10	9	6.00	10.60
-3	10	-9	12.10	9.70
-3	10	-9	9.70	8.50
-3	10	-9	-8.20	6.60
3	-10	9	-6.00	7.50
-4	10	-9	303.77	21.80
-4	10	-9	315.57	27.00
-4	10	-9	311.07	20.50
4	-10	9	295.77	20.80
-5	10	-9	18.10	13.60
-5	10	-9	21.70	9.30
-5	10	-9	-1.20	7.90
5	-10	9	8.00	8.60
-6	10	-9	-8.30	9.60
-6	10	-9	16.80	14.80
-6	10	-9	7.90	10.00
6	-10	9	15.70	7.90
6	-10	9	-13.80	10.00
-7	10	-9	63.59	12.30
-7	10	-9	99.49	13.90
-7	10	-9	87.79	18.20
7	-10	9	102.89	12.10
7	-10	9	83.19	16.10
-8	10	-9	71.69	12.10
-8	10	-9	72.29	18.40
-8	10	-9	34.60	11.10
8	-10	9	42.80	9.70
-9	10	-9	31.20	11.10

-9	10	-9	46.30	11.30
9	-10	9	51.79	8.70
9	-10	9	45.90	13.10
-10	10	-9	37.90	10.70
-10	10	-9	22.90	13.60
10	-10	9	20.60	8.70
10	-10	9	-1.90	14.40
10	9	-9	35.80	10.90
-10	-9	9	22.30	6.50
9	9	-9	-11.00	10.20
9	9	-9	-2.40	12.90
-9	-9	9	10.10	6.00
8	9	-9	21.60	11.60
-8	-9	9	20.60	7.20
-8	-9	9	18.50	9.80
7	9	-9	-11.70	10.40
-7	-9	9	8.10	8.00
-7	-9	9	14.50	6.50
6	9	-9	184.78	21.50
-6	-9	9	211.58	15.30
-6	-9	9	196.58	16.60
5	9	-9	148.69	18.80
-5	-9	9	132.49	13.10
-5	-9	9	119.49	13.30
4	9	-9	693.73	40.70
-4	-9	9	776.42	37.50
-4	-9	9	773.92	37.70
3	9	-9	-10.40	9.90
-3	-9	9	-2.20	9.80
-3	-9	9	6.40	7.80
2	9	-9	144.09	14.80
2	9	-9	109.69	18.40
2	9	-9	119.89	14.30
-2	-9	9	131.29	17.60
-2	-9	9	131.19	12.20
1	9	-9	833.32	45.70
1	9	-9	922.31	44.00
1	9	-9	881.21	43.10
-1	-9	9	899.21	43.10
0	9	-9	225.58	15.00
0	9	-9	199.78	15.90
0	9	-9	177.48	22.30
0	-9	9	200.38	16.00
-1	9	-9	23.50	10.30

-1	9	-9	40.90	7.00
-1	9	-9	10.40	12.80
-1	9	-9	32.40	9.10
1	-9	9	40.50	8.00
1	-9	9	29.40	9.00
-2	9	-9	451.95	26.40
-2	9	-9	436.36	24.10
-2	9	-9	476.65	26.90
-2	9	-9	412.16	34.10
2	-9	9	442.76	24.80
2	-9	9	396.46	24.70
2	-9	9	430.96	28.10
-3	9	-9	80.29	12.10
-3	9	-9	47.60	9.20
-3	9	-9	60.39	8.30
3	-9	9	44.90	21.20
3	-9	9	54.09	8.80
-4	9	-9	46.90	10.30
-4	9	-9	31.80	8.40
-4	9	-9	43.80	13.20
4	-9	9	48.00	13.50
4	-9	9	29.80	8.30
-5	9	-9	29.70	9.80
-5	9	-9	19.10	8.30
-5	9	-9	9.80	13.80
-5	9	-9	22.00	12.60
5	-9	9	10.20	9.00
-6	9	-9	555.24	39.60
-6	9	-9	660.63	35.40
-6	9	-9	620.64	34.20
6	-9	9	561.34	34.10
-7	9	-9	318.47	24.70
-7	9	-9	292.47	23.40
-7	9	-9	293.17	31.90
7	-9	9	322.37	21.70
-8	9	-9	100.59	16.90
-8	9	-9	132.59	20.80
8	-9	9	99.19	16.00
-9	9	-9	21.50	10.70
-9	9	-9	6.20	10.80
9	-9	9	20.70	8.40
-10	9	-9	229.08	22.10
-10	9	-9	219.78	20.00
10	-9	9	261.87	20.60

10	-9	9	269.87	18.70
10	8	-9	23.20	10.50
-10	-8	9	-1.20	5.90
9	8	-9	42.20	14.20
9	8	-9	30.20	10.40
-9	-8	9	46.10	7.20
-8	-8	9	7.50	10.90
-8	-8	9	31.10	9.40
7	8	-9	2.20	11.30
-7	-8	9	5.90	6.30
-7	-8	9	11.50	8.00
6	8	-9	722.43	40.30
-6	-8	9	725.73	36.00
-6	-8	9	686.93	36.60
5	8	-9	258.17	23.30
-5	-8	9	223.38	16.90
-5	-8	9	253.77	16.50
-4	-8	9	19.90	6.70
-4	-8	9	19.50	6.60
3	8	-9	170.18	20.00
-3	-8	9	185.08	13.60
-3	-8	9	167.68	15.20
2	8	-9	40.40	8.40
2	8	-9	23.50	10.10
-2	-8	9	38.20	7.20
-2	-8	9	51.99	10.20
1	8	-9	366.86	33.10
1	8	-9	364.46	21.10
1	8	-9	335.07	24.50
1	8	-9	361.76	22.00
-1	-8	9	304.67	30.10
-1	-8	9	372.36	20.60
0	8	-9	870.71	40.30
0	8	-9	826.32	41.30
0	8	-9	791.82	43.40
0	-8	9	802.12	40.40
0	-8	9	841.32	39.90
-1	8	-9	191.68	18.50
-1	8	-9	192.18	30.20
-1	8	-9	167.68	16.40
-1	8	-9	156.28	16.10
-1	8	-9	227.58	25.40
1	-8	9	173.68	13.20
1	-8	9	193.48	16.90

-2	8	-9	990.90	50.69
-2	8	-9	1034.40	50.49
-2	8	-9	1078.49	49.20
-2	8	-9	1052.69	52.09
-2	8	-9	955.40	53.49
2	-8	9	1026.00	48.80
-3	8	-9	124.59	14.60
-3	8	-9	121.39	17.40
-3	8	-9	110.19	17.70
-3	8	-9	128.29	11.90
3	-8	9	97.79	13.80
3	-8	9	113.99	14.70
-4	8	-9	194.88	28.50
-4	8	-9	180.78	22.80
-4	8	-9	150.78	21.10
-4	8	-9	154.88	15.80
-4	8	-9	174.98	14.30
4	-8	9	153.08	15.80
4	-8	9	156.78	17.00
-5	8	-9	220.48	18.70
-5	8	-9	211.98	17.20
-5	8	-9	229.18	24.80
5	-8	9	215.08	19.40
5	-8	9	248.18	21.20
-6	8	-9	7.80	8.30
-6	8	-9	20.20	11.00
-6	8	-9	-6.30	13.50
-6	8	-9	6.50	9.90
6	-8	9	2.80	10.80
6	-8	9	2.10	15.30
-7	8	-9	214.38	19.50
-7	8	-9	164.78	27.30
-7	8	-9	199.58	20.00
7	-8	9	206.38	18.60
-8	8	-9	221.48	20.40
-8	8	-9	180.58	27.90
-8	8	-9	194.78	18.40
8	-8	9	152.58	18.40
-9	8	-9	-18.00	10.00
-9	8	-9	-3.20	10.10
-9	8	-9	-1.70	17.10
9	-8	9	7.60	9.30
-10	8	-9	69.59	11.80
-10	8	-9	68.09	14.20

10	-8	9	77.99	11.70
10	7	-9	96.89	16.40
-10	-7	9	65.49	10.50
9	7	-9	6.50	10.90
-9	-7	9	5.10	6.50
8	7	-9	33.40	12.50
-8	-7	9	21.30	11.50
-8	-7	9	25.20	7.10
7	7	-9	16.10	11.00
-7	-7	9	8.30	6.50
-7	-7	9	7.50	8.20
6	7	-9	38.10	11.70
-6	-7	9	18.00	7.70
-6	-7	9	17.00	6.90
5	7	-9	1351.76	67.69
-5	-7	9	1373.26	64.99
-5	-7	9	1436.06	65.59
4	7	-9	12.10	10.30
-4	-7	9	13.20	6.70
-4	-7	9	14.30	5.80
3	7	-9	38.40	11.10
-3	-7	9	59.99	9.70
-3	-7	9	49.80	8.40
2	7	-9	230.88	20.20
2	7	-9	201.08	17.10
-2	-7	9	223.88	14.70
-2	-7	9	204.38	16.40
1	7	-9	82.39	13.50
1	7	-9	78.49	11.40
1	7	-9	87.49	12.50
1	7	-9	114.39	12.70
1	7	-9	103.89	12.60
-1	-7	9	66.99	11.50
-1	-7	9	85.29	11.30
-1	-7	9	71.69	12.30
-1	-7	9	81.29	10.40
0	7	-9	458.65	23.00
0	7	-9	451.05	30.20
0	7	-9	415.56	24.50
0	7	-9	415.26	25.60
0	-7	9	425.06	21.60
0	-7	9	395.76	26.30
0	-7	9	366.46	23.10
-1	7	-9	87.29	11.90

-1	7	-9	87.39	13.70
-1	7	-9	79.39	10.40
1	-7	9	73.19	9.00
1	-7	9	77.59	12.30
-2	7	-9	128.69	18.60
-2	7	-9	88.19	13.20
-2	7	-9	140.39	16.50
-2	7	-9	126.69	18.40
-2	7	-9	97.29	15.70
2	-7	9	103.99	10.70
-3	7	-9	376.86	31.80
-3	7	-9	400.16	25.70
-3	7	-9	357.16	23.10
-3	7	-9	371.76	25.30
-3	7	-9	381.06	30.60
-3	7	-9	390.66	21.50
3	-7	9	392.06	21.70
-4	7	-9	289.37	26.50
-4	7	-9	352.86	21.90
-4	7	-9	334.57	20.30
-4	7	-9	392.86	35.10
-4	7	-9	386.66	26.50
4	-7	9	340.77	22.30
4	-7	9	332.07	21.00
-5	7	-9	1464.55	72.79
-5	7	-9	1441.66	71.09
-5	7	-9	1497.65	71.09
-5	7	-9	1462.25	69.39
5	-7	9	1460.85	69.69
5	-7	9	1439.26	68.99
-6	7	-9	121.09	18.70
-6	7	-9	160.78	25.80
-6	7	-9	150.78	17.40
-6	7	-9	163.58	24.20
-6	7	-9	157.08	14.90
6	-7	9	167.88	16.60
6	-7	9	149.79	19.90
-7	7	-9	577.84	30.80
-7	7	-9	493.45	37.40
-7	7	-9	484.85	36.00
-7	7	-9	490.75	30.10
-7	7	-9	516.85	31.80
7	-7	9	457.95	35.40
-8	7	-9	2.80	15.70

-8	7	-9	6.70	9.30
-8	7	-9	19.60	10.10
8	-7	9	-2.60	8.80
-9	7	-9	151.88	28.60
-9	7	-9	195.18	19.30
-9	7	-9	199.88	21.40
9	-7	9	250.77	20.30
-10	7	-9	75.79	13.90
-10	7	-9	61.89	12.40
10	-7	9	68.49	11.90
-11	7	-9	8.20	10.80
-11	7	-9	-3.30	14.10
11	-7	9	11.10	13.10
10	6	-9	125.39	17.50
-10	-6	9	103.89	11.30
9	6	-9	6.90	11.20
-9	-6	9	-2.60	6.20
8	6	-9	4.20	11.60
-8	-6	9	2.70	10.30
-8	-6	9	9.20	6.30
7	6	-9	334.27	43.50
7	6	-9	420.26	28.50
-7	-6	9	379.56	24.10
-7	-6	9	395.16	22.40
6	6	-9	223.18	20.80
-6	-6	9	162.18	14.90
-6	-6	9	163.78	14.00
5	6	-9	531.35	31.80
4	6	-9	30.40	10.90
-4	-6	9	34.60	7.30
-4	-6	9	42.60	7.50
3	6	-9	38.20	11.10
-3	-6	9	23.90	5.70
-3	-6	9	28.40	7.80
2	6	-9	15.70	8.50
2	6	-9	10.00	10.90
-2	-6	9	6.40	6.30
-2	-6	9	20.20	6.40
-2	-6	9	13.30	6.00
1	6	-9	8.10	8.60
1	6	-9	3.90	5.40
-1	-6	9	-4.80	5.70
-1	-6	9	-3.80	4.40
-1	-6	9	5.40	5.70

-1	-6	9	4.50	7.20
0	6	-9	1450.95	69.89
0	6	-9	1393.46	70.69
0	-6	9	1533.65	68.89
0	-6	9	1694.03	71.49
0	-6	9	1455.65	69.89
-1	6	-9	1831.92	77.09
-1	6	-9	1640.24	77.49
-1	6	-9	1769.92	78.59
-1	6	-9	1606.64	78.09
1	-6	9	1545.05	77.79
1	-6	9	1586.54	78.79
1	-6	9	1703.33	76.29
-2	6	-9	79.19	14.00
-2	6	-9	90.99	9.60
-2	6	-9	104.19	17.80
-2	6	-9	84.29	14.70
2	-6	9	64.19	12.10
2	-6	9	97.79	9.30
-3	6	-9	51.59	9.00
-3	6	-9	80.89	11.80
-3	6	-9	62.19	9.40
-3	6	-9	51.69	15.30
-3	6	-9	55.49	9.70
3	-6	9	64.59	8.90
-4	6	-9	886.61	48.80
-4	6	-9	882.61	41.90
-4	6	-9	846.02	42.70
-4	6	-9	827.12	45.70
-4	6	-9	863.41	45.00
4	-6	9	828.62	42.80
4	-6	9	867.21	41.50
-5	6	-9	133.49	12.90
-5	6	-9	118.99	16.70
-5	6	-9	151.98	19.00
-5	6	-9	187.08	20.50
-5	6	-9	138.29	14.70
5	-6	9	141.69	13.40
5	-6	9	145.09	17.90
-6	6	-9	-9.80	8.30
-6	6	-9	-3.00	10.00
-6	6	-9	-7.20	11.40
-6	6	-9	1.20	6.40
6	-6	9	3.90	8.20

6	-6	9	-6.50	12.80
-7	6	-9	16.90	8.40
-7	6	-9	51.69	10.50
-7	6	-9	39.40	14.00
-7	6	-9	27.70	10.80
-7	6	-9	39.80	13.80
7	-6	9	30.30	12.00
-8	6	-9	258.57	19.20
-8	6	-9	245.68	27.50
-8	6	-9	267.47	21.00
-8	6	-9	263.87	29.50
-8	6	-9	210.18	24.00
8	-6	9	229.78	20.90
8	-6	9	199.08	31.70
-9	6	-9	98.19	17.50
-9	6	-9	102.79	21.70
-9	6	-9	134.89	28.40
-9	6	-9	136.99	21.80
-9	6	-9	87.89	15.10
9	-6	9	94.99	22.50
-10	6	-9	74.09	12.80
-10	6	-9	70.59	16.20
-10	6	-9	110.49	17.00
10	-6	9	121.89	19.40
-11	6	-9	190.98	21.30
-11	6	-9	190.68	19.20
11	-6	9	201.88	18.10
11	-6	9	195.88	29.80
11	5	-9	131.09	20.80
-11	-5	9	94.79	11.80
10	5	-9	16.90	11.00
-10	-5	9	8.90	6.10
9	5	-9	66.79	17.80
9	5	-9	51.29	14.20
-9	-5	9	64.89	9.50
8	5	-9	-17.70	11.20
8	5	-9	-1.90	14.70
-8	-5	9	-0.40	5.90
-8	-5	9	-14.60	10.40
7	5	-9	20.50	11.70
7	5	-9	33.80	14.40
-7	-5	9	22.50	8.60
-7	-5	9	22.50	7.20
6	5	-9	182.28	27.10

6	5	-9	239.88	22.00
-6	-5	9	219.98	16.50
-6	-5	9	217.28	15.30
5	5	-9	26.20	14.60
5	5	-9	32.80	10.70
-5	-5	9	20.00	6.40
-5	-5	9	14.70	5.60
4	5	-9	681.33	36.90
-4	-5	9	650.73	33.10
-4	-5	9	646.24	32.90
3	5	-9	11.50	10.00
-3	-5	9	3.70	7.00
-3	-5	9	3.70	6.90
2	5	-9	260.77	16.70
2	5	-9	253.37	22.40
-2	-5	9	251.07	15.60
1	5	-9	1099.09	49.70
1	5	-9	993.40	49.30
-1	-5	9	1029.50	49.99
-1	-5	9	1083.59	48.80
-1	-5	9	1030.10	49.20
0	5	-9	8.50	4.40
0	5	-9	18.10	7.00
0	5	-9	3.50	5.10
0	-5	9	1.20	5.90
0	-5	9	7.80	4.80
0	-5	9	5.70	3.40
-1	5	-9	98.09	9.60
1	-5	9	94.89	10.00
1	-5	9	83.39	12.50
1	-5	9	98.59	8.20
-2	5	-9	644.64	32.30
-2	5	-9	669.13	30.80
-2	5	-9	591.84	31.30
2	-5	9	632.04	30.10
2	-5	9	603.74	31.80
2	-5	9	585.64	32.90
-3	5	-9	309.07	20.70
-3	5	-9	243.68	22.40
-3	5	-9	299.67	16.60
-3	5	-9	318.67	21.60
-3	5	-9	309.27	27.30
3	-5	9	284.87	15.90
3	-5	9	242.18	21.60

-4	5	-9	10.50	9.00
-4	5	-9	7.60	8.30
-4	5	-9	35.40	14.40
-4	5	-9	6.30	5.20
-4	5	-9	14.70	7.70
-4	5	-9	4.90	10.70
-5	5	-9	62.89	11.40
-5	5	-9	70.29	10.10
-5	5	-9	62.59	14.80
-5	5	-9	64.79	11.30
-5	5	-9	68.09	9.90
5	-5	9	82.79	9.70
5	-5	9	47.50	11.30
-6	5	-9	425.86	25.00
-6	5	-9	386.66	31.60
-6	5	-9	411.26	23.90
-6	5	-9	417.76	30.80
6	-5	9	425.26	23.60
-7	5	-9	55.89	13.80
-7	5	-9	80.09	14.20
-7	5	-9	64.99	12.00
-7	5	-9	57.69	10.30
-7	5	-9	72.69	12.20
-7	5	-9	94.39	15.80
7	-5	9	53.69	10.60
-8	5	-9	68.89	14.00
-8	5	-9	73.09	15.10
-8	5	-9	92.49	14.90
-8	5	-9	55.59	14.60
-8	5	-9	71.49	10.80
8	-5	9	64.99	15.90
8	-5	9	57.69	10.80
-9	5	-9	267.17	25.20
-9	5	-9	284.97	21.20
-9	5	-9	245.98	27.60
-9	5	-9	281.97	31.00
-9	5	-9	250.67	20.00
9	-5	9	265.17	23.20
-10	5	-9	418.56	32.10
-10	5	-9	322.17	33.50
-10	5	-9	328.77	25.40
-10	5	-9	305.37	23.80
-10	5	-9	334.37	24.10
10	-5	9	346.67	26.00

-11	5	-9	13.00	12.60
-11	5	-9	21.30	11.10
11	-5	9	30.00	10.60
11	-5	9	10.90	16.00
11	4	-9	123.69	19.10
-11	-4	9	125.79	12.60
10	4	-9	32.00	15.90
10	4	-9	2.60	12.40
-10	-4	9	-0.40	6.40
9	4	-9	166.08	22.70
9	4	-9	121.69	17.80
-9	-4	9	149.19	20.50
8	4	-9	24.70	13.10
8	4	-9	16.10	11.80
-8	-4	9	-3.60	10.20
-8	-4	9	11.60	11.10
-8	-4	9	-0.20	6.30
7	4	-9	31.80	12.20
7	4	-9	34.10	11.50
-7	-4	9	13.30	8.80
-7	-4	9	27.30	6.90
6	4	-9	1360.46	67.99
-6	-4	9	1396.36	65.99
-6	-4	9	1424.46	65.49
5	4	-9	10.00	9.30
5	4	-9	26.10	11.20
-5	-4	9	8.60	5.10
-5	-4	9	6.40	5.90
4	4	-9	96.59	17.10
4	4	-9	41.40	17.70
-4	-4	9	82.39	10.10
3	4	-9	183.28	13.00
3	4	-9	165.08	20.50
3	4	-9	194.28	18.40
-3	-4	9	179.08	14.00
-3	-4	9	202.18	12.80
2	4	-9	129.29	10.40
-2	-4	9	109.29	10.00
1	4	-9	995.90	45.60
1	4	-9	964.80	45.80
-1	-4	9	1000.40	45.50
-1	-4	9	935.61	46.00
0	4	-9	252.27	16.60
0	4	-9	243.98	14.80

0	-4	9	286.07	21.60
0	-4	9	281.87	14.20
-1	4	-9	572.04	28.30
-1	4	-9	609.14	28.60
-1	4	-9	540.95	28.20
1	-4	9	559.84	29.40
1	-4	9	560.04	28.30
1	-4	9	587.04	27.60
-2	4	-9	180.28	12.00
2	-4	9	182.08	10.70
2	-4	9	173.08	12.50
2	-4	9	168.18	13.50
-3	4	-9	350.96	20.10
-3	4	-9	367.26	19.10
-3	4	-9	354.56	21.30
-3	4	-9	362.76	26.20
3	-4	9	364.06	18.20
3	-4	9	323.37	21.10
3	-4	9	344.07	21.30
-4	4	-9	124.29	15.50
-4	4	-9	148.59	14.20
-4	4	-9	107.19	13.50
-4	4	-9	127.69	16.20
4	-4	9	137.09	9.70
-5	4	-9	312.37	27.40
-5	4	-9	351.36	20.50
-5	4	-9	333.37	18.50
-5	4	-9	315.97	22.10
-5	4	-9	303.87	23.20
5	-4	9	318.17	17.30
5	-4	9	253.97	24.30
-6	4	-9	97.09	12.60
-6	4	-9	101.89	11.30
-6	4	-9	109.69	14.00
-6	4	-9	75.99	13.90
-6	4	-9	105.99	13.80
6	-4	9	81.79	10.30
-7	4	-9	211.78	25.70
-7	4	-9	168.08	19.50
-7	4	-9	156.88	16.20
-7	4	-9	129.99	20.90
-7	4	-9	147.79	13.90
-7	4	-9	124.59	22.00
7	-4	9	154.78	15.10

-8	4	-9	242.28	19.40
-8	4	-9	249.38	19.50
-8	4	-9	259.17	25.40
-8	4	-9	253.07	23.10
-8	4	-9	245.88	23.30
-8	4	-9	254.07	25.30
8	-4	9	292.97	23.60
-9	4	-9	43.80	14.00
-9	4	-9	31.30	8.60
-9	4	-9	77.19	12.50
-9	4	-9	47.30	9.90
-9	4	-9	75.09	15.60
9	-4	9	53.39	12.10
9	-4	9	50.99	20.10
-10	4	-9	5.50	9.70
-10	4	-9	24.90	10.00
-10	4	-9	29.60	16.10
-10	4	-9	30.00	12.00
-10	4	-9	41.80	15.40
10	-4	9	-0.30	12.70
-11	4	-9	29.90	10.50
-11	4	-9	17.90	11.40
-11	4	-9	36.40	13.90
11	-4	9	38.20	17.60
11	3	-9	254.87	24.00
11	3	-9	209.48	26.30
-11	-3	9	248.48	16.70
10	3	-9	30.80	13.90
10	3	-9	50.19	15.00
-10	-3	9	92.99	13.20
-10	-3	9	63.59	11.00
9	3	-9	2.80	16.40
-9	-3	9	18.00	12.30
-9	-3	9	16.40	7.10
8	3	-9	29.90	12.40
8	3	-9	11.80	11.50
-8	-3	9	28.60	7.40
-8	-3	9	14.90	9.30
-8	-3	9	57.79	12.50
7	3	-9	72.29	12.60
7	3	-9	65.29	12.90
-7	-3	9	65.19	10.40
-7	-3	9	78.39	11.00
-7	-3	9	59.19	9.90

6	3	-9	255.17	22.30
6	3	-9	229.18	28.00
-6	-3	9	229.38	15.80
-6	-3	9	231.08	21.10
5	3	-9	974.00	47.40
5	3	-9	809.12	46.10
-5	-3	9	946.31	43.80
4	3	-9	4.50	10.90
4	3	-9	10.40	9.80
-4	-3	9	13.50	7.70
-4	-3	9	4.90	5.80
3	3	-9	78.59	12.50
3	3	-9	85.59	7.20
3	3	-9	87.09	8.90
-3	-3	9	87.49	8.20
-3	-3	9	69.69	9.70
-3	-3	9	79.09	9.30
2	3	-9	160.78	9.70
-2	-3	9	130.29	10.80
-2	-3	9	159.38	9.80
1	3	-9	260.87	13.80
-1	-3	9	258.47	14.50
-1	-3	9	275.77	15.20
-1	-3	9	260.57	14.20
0	3	-9	42.80	5.60
0	3	-9	32.70	5.90
0	-3	9	44.80	5.70
0	-3	9	57.29	7.70
-1	3	-9	158.98	9.90
1	-3	9	120.89	16.10
1	-3	9	144.79	9.00
-2	3	-9	12.40	4.50
-2	3	-9	10.80	4.60
-2	3	-9	5.70	4.10
2	-3	9	4.90	2.90
2	-3	9	14.00	4.80
-3	3	-9	662.63	30.80
-3	3	-9	606.54	31.20
-3	3	-9	642.14	32.00
3	-3	9	638.94	29.80
3	-3	9	616.14	31.60
3	-3	9	609.84	31.80
-4	3	-9	214.48	17.50
-4	3	-9	237.48	13.60

-4	3	-9	173.28	32.40
-4	3	-9	212.08	21.20
-4	3	-9	184.58	18.90
-4	3	-9	222.88	16.60
4	-3	9	208.98	23.20
4	-3	9	219.08	12.00
-5	3	-9	1112.99	53.89
-5	3	-9	1170.38	52.39
-5	3	-9	1039.60	56.09
-5	3	-9	1055.19	55.29
-5	3	-9	1135.19	55.19
-5	3	-9	1118.39	53.09
5	-3	9	1096.29	51.19
-6	3	-9	20.30	7.40
-6	3	-9	17.80	10.00
-6	3	-9	20.20	11.70
-6	3	-9	6.40	8.90
-6	3	-9	4.70	5.70
6	-3	9	15.10	4.70
-7	3	-9	311.37	24.30
-7	3	-9	348.57	20.50
-7	3	-9	351.26	25.60
-7	3	-9	341.17	26.40
-7	3	-9	355.36	25.60
-7	3	-9	332.27	21.40
7	-3	9	331.07	19.70
-8	3	-9	70.29	12.80
-8	3	-9	91.99	11.80
-8	3	-9	95.79	14.20
-8	3	-9	80.39	14.00
-8	3	-9	66.29	12.60
-8	3	-9	78.99	12.20
8	-3	9	80.99	10.90
-9	3	-9	184.88	16.90
-9	3	-9	183.58	22.90
-9	3	-9	189.98	17.60
-9	3	-9	152.18	24.30
-9	3	-9	174.08	19.90
9	-3	9	214.18	22.70
9	-3	9	155.68	20.50
-10	3	-9	363.86	30.20
-10	3	-9	334.77	23.50
-10	3	-9	291.57	21.70
-10	3	-9	334.47	24.30

-10	3	-9	359.46	29.70
10	-3	9	296.77	28.50
-11	3	-9	36.00	11.70
-11	3	-9	55.59	17.10
-11	3	-9	23.30	12.90
-11	3	-9	20.80	9.60
-11	3	-9	8.60	16.30
11	-3	9	51.39	20.30
-12	3	-9	34.10	14.50
-12	3	-9	18.90	17.00
12	-3	9	48.70	13.10
11	2	-9	73.79	15.50
-11	-2	9	56.39	8.80
10	2	-9	132.59	22.00
10	2	-9	165.38	23.10
-10	-2	9	201.28	19.50
-10	-2	9	202.68	14.60
9	2	-9	-7.30	13.10
9	2	-9	21.30	13.60
-9	-2	9	14.80	6.80
8	2	-9	114.99	14.50
8	2	-9	133.09	21.90
-8	-2	9	96.99	12.80
-8	-2	9	157.98	27.40
-8	-2	9	103.19	15.90
7	2	-9	307.27	25.10
7	2	-9	273.97	22.60
-7	-2	9	249.18	20.00
-7	-2	9	313.37	21.10
-7	-2	9	304.67	18.70
6	2	-9	24.70	9.30
6	2	-9	1.20	14.40
-6	-2	9	19.60	6.20
5	2	-9	158.98	17.30
5	2	-9	120.39	18.80
-5	-2	9	109.89	11.40
4	2	-9	380.96	27.30
4	2	-9	463.85	27.90
-4	-2	9	417.16	23.70
-4	-2	9	456.45	23.30
-4	-2	9	437.46	22.30
3	2	-9	187.58	10.10
3	2	-9	203.48	10.90
-3	-2	9	181.78	12.40

-3	-2	9	212.48	11.90
2	2	-9	175.58	8.90
-2	-2	9	146.79	9.90
1	2	-9	22.00	3.00
-1	-2	9	20.50	3.70
-1	-2	9	19.70	4.10
-1	-2	9	19.10	3.10
0	2	-9	978.70	47.00
0	2	-9	1038.90	46.30
0	-2	9	951.20	46.60
0	-2	9	1085.99	47.30
0	-2	9	1002.70	46.90
-1	2	-9	224.48	11.80
-1	2	-9	231.08	12.20
1	-2	9	192.68	11.20
1	-2	9	198.18	12.30
-2	2	-9	41.10	6.10
-2	2	-9	38.30	6.20
2	-2	9	37.20	4.40
2	-2	9	43.90	6.40
-3	2	-9	162.78	11.30
3	-2	9	153.98	11.70
3	-2	9	161.28	9.00
3	-2	9	168.08	11.70
-4	2	-9	1066.89	51.29
-4	2	-9	953.10	51.29
-4	2	-9	1101.19	49.40
-4	2	-9	1080.39	50.39
-4	2	-9	1100.59	57.09
4	-2	9	1102.99	48.40
4	-2	9	1001.30	50.69
4	-2	9	1022.20	50.79
-5	2	-9	106.79	12.50
-5	2	-9	125.99	9.90
-5	2	-9	103.89	13.00
-5	2	-9	116.89	18.90
-5	2	-9	105.29	13.00
-5	2	-9	95.99	14.00
5	-2	9	100.99	7.70
-6	2	-9	51.89	11.60
-6	2	-9	37.80	6.40
-6	2	-9	31.30	9.10
-6	2	-9	48.30	11.30
-6	2	-9	37.60	7.40

6	-2	9	49.30	6.60
-7	2	-9	37.00	7.80
-7	2	-9	34.40	11.40
-7	2	-9	48.50	12.30
-7	2	-9	51.99	12.10
-7	2	-9	30.30	6.50
-7	2	-9	27.80	9.20
7	-2	9	35.90	6.30
-8	2	-9	597.14	33.00
-8	2	-9	631.74	34.70
-8	2	-9	589.84	32.50
-8	2	-9	628.34	35.40
-8	2	-9	605.24	37.10
-8	2	-9	680.03	37.40
8	-2	9	619.44	32.60
-9	2	-9	106.39	18.00
-9	2	-9	156.68	15.00
-9	2	-9	155.28	15.90
-9	2	-9	171.48	23.90
-9	2	-9	159.08	21.70
9	-2	9	134.29	22.60
9	-2	9	143.09	21.40
-10	2	-9	27.40	14.90
-10	2	-9	59.49	17.20
-10	2	-9	41.40	11.50
-10	2	-9	59.09	15.90
-10	2	-9	44.80	9.70
10	-2	9	37.10	16.10
10	-2	9	43.60	23.90
-11	2	-9	226.88	18.10
-11	2	-9	217.08	18.80
-11	2	-9	244.28	26.10
-11	2	-9	183.18	20.00
-11	2	-9	246.08	26.70
-12	2	-9	-12.20	12.70
-12	2	-9	-1.30	9.90
-12	2	-9	41.90	14.90
12	-2	9	-3.90	11.80
11	1	-9	9.50	13.20
11	1	-9	14.10	15.70
-11	-1	9	58.69	13.00
-11	-1	9	31.90	8.30
10	1	-9	39.00	14.70
10	1	-9	53.99	13.40

-10	-1	9	29.30	10.10
-10	-1	9	24.10	7.10
9	1	-9	10.70	14.50
9	1	-9	7.10	11.70
-9	-1	9	-13.30	10.70
-9	-1	9	0.90	6.80
8	1	-9	84.59	15.30
8	1	-9	95.89	17.80
-8	-1	9	96.49	12.30
-8	-1	9	94.89	15.10
7	1	-9	2.80	10.00
7	1	-9	-4.40	11.80
-7	-1	9	3.40	9.10
-7	-1	9	6.50	8.10
-7	-1	9	6.80	6.00
6	1	-9	12.20	12.30
6	1	-9	20.90	9.30
-6	-1	9	15.40	8.90
5	1	-9	152.78	17.30
5	1	-9	159.48	18.60
-5	-1	9	148.79	12.60
4	1	-9	456.55	29.70
4	1	-9	517.35	28.80
-4	-1	9	495.45	24.50
-4	-1	9	470.15	25.60
-4	-1	9	437.26	25.00
-4	-1	9	526.95	25.40
-3	-1	9	27.80	4.50
-3	-1	9	15.10	4.50
-3	-1	9	20.30	4.70
1	1	-9	407.96	18.20
-1	-1	9	398.46	19.60
-1	-1	9	380.56	19.90
0	1	-9	131.19	8.50
0	1	-9	134.69	7.30
0	-1	9	144.79	8.90
0	-1	9	132.09	8.40
0	-1	9	133.89	8.40
-1	1	-9	2.80	2.60
-1	1	-9	0.90	2.20
1	-1	9	4.60	2.80
1	-1	9	3.70	2.30
-2	1	-9	291.97	14.90
2	-1	9	305.57	16.50

2	-1	9	276.27	13.60
2	-1	9	233.08	16.10
-3	1	-9	47.00	6.60
3	-1	9	34.70	3.70
3	-1	9	40.90	6.70
3	-1	9	31.10	5.00
-4	1	-9	114.19	10.50
-4	1	-9	103.59	8.70
-4	1	-9	107.49	11.40
4	-1	9	100.79	10.80
4	-1	9	110.19	6.70
4	-1	9	106.69	11.10
-5	1	-9	292.37	22.30
-5	1	-9	244.58	21.30
-5	1	-9	265.27	22.20
-5	1	-9	275.67	17.40
-5	1	-9	248.88	17.30
-5	1	-9	247.88	17.90
-5	1	-9	265.67	18.30
5	-1	9	276.67	20.00
-6	1	-9	239.88	21.40
-6	1	-9	220.98	20.70
-6	1	-9	206.98	18.00
-6	1	-9	240.98	15.90
-6	1	-9	233.68	14.30
-6	1	-9	167.28	22.00
6	-1	9	214.08	12.40
-7	1	-9	29.40	6.60
-7	1	-9	45.10	17.60
-7	1	-9	39.70	8.30
-7	1	-9	32.90	11.30
-7	1	-9	39.70	10.00
-7	1	-9	38.70	11.50
7	-1	9	33.90	5.90
-8	1	-9	38.10	11.70
-8	1	-9	50.99	8.50
-8	1	-9	73.69	10.60
-8	1	-9	55.89	11.20
-8	1	-9	57.89	12.50
-8	1	-9	60.19	10.90
8	-1	9	52.09	8.20
-9	1	-9	619.74	33.40
-9	1	-9	602.54	37.00
-9	1	-9	630.94	34.80

9	-1	9	485.15	36.10
9	-1	9	596.24	33.30
-10	1	-9	404.36	25.20
-10	1	-9	377.06	23.60
-10	1	-9	467.85	29.70
-10	1	-9	349.47	42.00
10	-1	9	314.17	34.40
10	-1	9	426.16	35.10
-11	1	-9	10.30	9.70
-11	1	-9	0.80	14.00
-11	1	-9	-6.60	14.60
-11	1	-9	-5.90	8.70
-12	1	-9	18.80	12.10
-12	1	-9	38.30	10.00
12	-1	9	21.40	12.90
11	0	-9	85.39	14.80
-11	0	9	67.79	12.40
-11	0	9	80.99	13.80
10	0	-9	48.80	12.80
10	0	-9	52.39	16.20
-10	0	9	47.80	8.60
-10	0	9	29.80	9.80
9	0	-9	80.09	17.00
9	0	-9	81.39	13.80
-9	0	9	99.39	11.70
-9	0	9	79.49	12.70
8	0	-9	37.30	14.10
8	0	-9	31.60	11.00
-8	0	9	29.60	7.40
-8	0	9	24.80	8.70
7	0	-9	277.07	25.60
7	0	-9	321.07	22.60
-7	0	9	307.67	20.30
-7	0	9	332.77	19.60
6	0	-9	1627.84	76.69
6	0	-9	1592.14	78.29
-6	0	9	1641.14	75.99
-6	0	9	1647.64	76.99
5	0	-9	77.59	10.70
5	0	-9	53.19	12.10
-5	0	9	61.69	10.00
-5	0	9	52.99	9.10
4	0	-9	60.09	10.40
4	0	-9	69.39	12.90

-4	0	9	58.59	8.20
-4	0	9	61.99	8.10
-4	0	9	69.09	9.80
-3	0	9	1146.99	50.39
-3	0	9	1107.99	50.69
-3	0	9	1008.80	50.39
-2	0	9	160.98	9.50
-2	0	9	155.18	9.10
-2	0	9	127.79	9.60
-1	0	9	504.85	22.50
-1	0	9	419.66	22.40
0	0	-9	2.80	1.40
0	0	9	2.50	2.60
0	0	9	3.60	2.50
0	0	9	1.40	2.20
-1	0	-9	2027.30	89.29
-1	0	-9	2009.20	88.59
1	0	9	1924.71	88.39
1	0	9	1916.61	88.69
-2	0	-9	213.28	11.70
-2	0	-9	208.58	11.90
2	0	9	218.38	12.20
2	0	9	215.38	11.70
2	0	9	216.08	10.60
-3	0	-9	434.86	21.60
-3	0	-9	432.76	21.90
-3	0	-9	434.66	21.70
3	0	9	434.66	20.20
3	0	9	433.56	22.00
3	0	9	421.26	21.70
-4	0	-9	385.66	21.40
-4	0	-9	382.26	20.50
-4	0	-9	391.26	20.00
4	0	9	383.66	21.20
4	0	9	382.66	21.10
4	0	9	405.16	18.50
-5	0	-9	155.08	18.60
-5	0	-9	194.58	11.80
-5	0	-9	175.68	14.30
-5	0	-9	184.88	18.20
-5	0	-9	197.58	15.40
-5	0	-9	174.58	14.20
-5	0	-9	169.88	20.80
5	0	9	148.89	15.30

5	0	9	197.98	10.10
-6	0	-9	9.20	10.40
-6	0	-9	16.00	9.00
-6	0	-9	3.80	7.10
-6	0	-9	7.50	4.20
-6	0	-9	6.80	10.90
-6	0	-9	15.60	10.50
-6	0	-9	11.40	5.90
6	0	9	1.20	2.70
-7	0	-9	503.65	37.00
-7	0	-9	562.64	31.30
-7	0	-9	548.95	28.30
-7	0	-9	562.94	40.00
7	0	9	544.55	26.90
-8	0	-9	180.98	20.90
-8	0	-9	198.78	18.70
-8	0	-9	185.08	22.00
-8	0	-9	201.18	18.80
8	0	9	188.28	13.70
-9	0	-9	15.90	9.60
-9	0	-9	44.10	10.30
-9	0	-9	17.30	12.00
-9	0	-9	29.40	8.70
-9	0	-9	32.30	8.80
9	0	9	17.30	14.10
-10	0	-9	38.70	13.00
-10	0	-9	50.19	9.50
-10	0	-9	61.39	14.70
-10	0	-9	50.69	15.50
-10	0	-9	70.39	10.70
10	0	9	47.10	16.80
-11	0	-9	38.40	9.60
-11	0	-9	35.80	9.30
-11	0	-9	52.49	15.10
-11	0	-9	42.60	14.30
-12	0	-9	10.80	9.00
-12	0	-9	31.50	17.30
-12	0	-9	12.30	12.70
-12	0	-9	8.70	11.20
12	0	9	-3.70	12.60
11	-1	-9	224.58	21.00
11	-1	-9	161.38	31.70
-11	1	9	216.78	25.50
-11	1	9	269.67	18.00

10	-1	-9	26.00	11.80
10	-1	-9	27.60	16.10
-10	1	9	10.40	9.00
-10	1	9	17.70	7.90
9	-1	-9	-0.40	10.90
9	-1	-9	25.90	15.70
-9	1	9	6.40	7.50
-9	1	9	6.50	8.70
8	-1	-9	52.89	11.20
8	-1	-9	16.50	14.10
-8	1	9	62.69	9.10
-8	1	9	41.30	7.70
7	-1	-9	159.68	17.90
7	-1	-9	144.59	22.80
-7	1	9	150.18	15.80
-7	1	9	157.58	14.70
6	-1	-9	245.78	18.10
6	-1	-9	185.58	23.70
-6	1	9	233.78	15.60
-6	1	9	173.98	17.90
5	-1	-9	409.36	30.50
5	-1	-9	452.85	26.50
-5	1	9	461.85	24.10
-5	1	9	427.26	24.40
-5	1	9	502.55	28.90
4	-1	-9	19.60	9.30
-4	1	9	8.60	6.10
-4	1	9	3.10	3.90
-4	1	9	-1.70	4.90
-3	1	9	166.58	10.50
-3	1	9	177.28	11.40
-3	1	9	184.08	10.90
-2	1	9	534.65	25.80
-2	1	9	562.24	25.70
-2	1	9	497.65	25.50
-1	1	9	688.73	35.10
-1	1	9	822.82	35.40
0	1	9	97.99	6.70
0	1	9	99.49	6.60
0	1	9	102.99	6.10
-1	-1	-9	285.27	13.80
1	1	9	287.17	14.60
1	1	9	273.97	13.50
1	1	9	286.37	13.90

-2	-1	-9	1527.15	69.29
-2	-1	-9	1555.24	69.59
2	1	9	1510.05	69.69
2	1	9	1494.35	69.29
2	1	9	1543.35	68.29
-3	-1	-9	2560.04	114.09
-3	-1	-9	2350.56	113.49
-3	-1	-9	2570.24	113.89
3	1	9	2561.24	112.59
3	1	9	2512.65	114.19
3	1	9	2578.84	114.09
-4	-1	-9	216.58	14.50
-4	-1	-9	233.48	13.20
-4	-1	-9	237.08	13.70
4	1	9	232.58	14.40
4	1	9	238.98	11.40
4	1	9	228.38	14.40
-5	-1	-9	227.88	16.20
-5	-1	-9	201.78	20.80
-5	-1	-9	229.18	19.80
-5	-1	-9	245.28	13.80
-5	-1	-9	243.48	17.50
-5	-1	-9	220.38	15.20
5	1	9	240.48	16.90
5	1	9	254.87	12.20
-6	-1	-9	4.40	10.70
-6	-1	-9	13.40	6.60
-6	-1	-9	9.30	6.10
-6	-1	-9	7.10	8.60
-6	-1	-9	22.00	12.20
-6	-1	-9	24.70	10.40
-6	-1	-9	8.10	4.30
6	1	9	15.60	3.10
-7	-1	-9	198.78	23.10
-7	-1	-9	216.78	16.20
-7	-1	-9	221.48	14.40
-7	-1	-9	260.67	33.00
-7	-1	-9	213.68	19.20
-7	-1	-9	213.68	18.20
7	1	9	213.88	12.50
-8	-1	-9	381.36	26.70
-8	-1	-9	401.66	25.40
-8	-1	-9	408.86	23.80
-8	-1	-9	408.36	24.90

-8	-1	-9	414.46	29.90
8	1	9	414.46	21.80
-9	-1	-9	97.99	13.40
-9	-1	-9	119.99	16.40
-9	-1	-9	122.59	16.60
-9	-1	-9	98.79	15.00
-9	-1	-9	108.79	13.30
9	1	9	78.69	14.60
9	1	9	76.19	16.50
-10	-1	-9	352.66	22.60
-10	-1	-9	374.06	23.20
-10	-1	-9	380.46	25.80
-10	-1	-9	407.56	28.70
-10	-1	-9	382.46	25.10
10	1	9	348.77	29.10
-11	-1	-9	11.60	8.60
-11	-1	-9	-3.90	7.90
-11	-1	-9	12.70	9.50
-11	-1	-9	17.00	13.60
-12	-1	-9	39.60	11.10
-12	-1	-9	15.80	19.70
-12	-1	-9	38.30	15.50
-12	-1	-9	49.40	9.80
12	1	9	36.60	13.60
11	-2	-9	35.80	11.70
-11	2	9	42.10	10.50
10	-2	-9	135.99	18.40
10	-2	-9	172.18	28.90
-10	2	9	129.69	15.00
-10	2	9	134.39	15.90
9	-2	-9	13.80	11.50
9	-2	-9	18.20	17.30
-9	2	9	14.60	7.80
-9	2	9	35.80	9.40
8	-2	-9	177.98	25.70
8	-2	-9	192.38	19.20
-8	2	9	160.58	15.70
-8	2	9	138.29	16.40
7	-2	-9	100.89	16.60
7	-2	-9	99.39	16.00
-7	2	9	93.29	14.70
-7	2	9	102.59	13.10
6	-2	-9	353.76	26.40
6	-2	-9	285.07	27.30

6	-2	-9	287.47	21.30
-6	2	9	289.77	20.70
-6	2	9	327.27	19.50
5	-2	-9	340.07	25.50
5	-2	-9	361.86	25.60
5	-2	-9	333.47	28.60
5	-2	-9	350.26	22.20
-5	2	9	330.47	19.60
-5	2	9	365.26	22.90
4	-2	-9	245.48	18.90
-4	2	9	231.18	16.10
-4	2	9	262.67	15.30
-4	2	9	248.68	13.80
-3	2	9	100.89	8.50
-3	2	9	95.89	7.10
-2	2	9	316.77	16.40
-2	2	9	347.37	17.00
-2	2	9	327.67	17.00
-1	2	9	758.62	33.20
-1	2	9	720.03	33.20
-1	2	9	632.44	32.60
0	2	9	2111.99	96.79
0	2	9	2198.08	97.29
1	2	9	472.95	23.70
1	2	9	465.65	22.50
1	2	9	517.65	22.50
1	2	9	518.15	23.00
-2	-2	-9	342.87	17.00
2	2	9	340.47	17.00
2	2	9	341.57	15.80
2	2	9	354.46	15.70
2	2	9	349.96	17.50
-3	-2	-9	849.41	40.50
-3	-2	-9	849.61	40.10
-3	-2	-9	871.81	40.40
3	2	9	857.51	39.00
3	2	9	883.41	40.50
3	2	9	854.71	40.50
-4	-2	-9	1109.19	50.79
4	2	9	1085.09	51.59
4	2	9	1089.59	51.29
-5	-2	-9	118.79	10.50
-5	-2	-9	107.99	15.30
-5	-2	-9	106.29	8.70

-5	-2	-9	80.09	12.20
-5	-2	-9	96.69	11.80
-5	-2	-9	114.39	13.10
5	2	9	105.39	12.40
5	2	9	115.99	6.50
-6	-2	-9	99.29	11.90
-6	-2	-9	130.19	15.60
-6	-2	-9	108.09	9.00
-6	-2	-9	111.89	12.30
-6	-2	-9	71.39	10.60
-6	-2	-9	63.29	14.50
-6	-2	-9	94.49	14.00
6	2	9	93.79	6.50
-7	-2	-9	3.50	7.20
-7	-2	-9	7.50	8.90
-7	-2	-9	18.30	9.80
-7	-2	-9	3.00	5.60
-7	-2	-9	3.10	9.30
-7	-2	-9	11.00	4.70
7	2	9	9.20	3.70
-8	-2	-9	316.07	22.40
-8	-2	-9	345.77	20.70
-8	-2	-9	332.67	24.40
-8	-2	-9	296.47	27.00
-8	-2	-9	368.86	22.40
-8	-2	-9	335.37	19.50
8	2	9	336.27	18.50
-9	-2	-9	121.39	19.80
-9	-2	-9	102.39	15.90
-9	-2	-9	142.19	14.50
-9	-2	-9	138.09	16.70
-9	-2	-9	166.88	18.40
9	2	9	112.49	14.60
-10	-2	-9	20.60	8.30
-10	-2	-9	-4.80	9.40
-10	-2	-9	11.00	7.50
-10	-2	-9	-3.40	9.50
-10	-2	-9	22.30	11.30
10	2	9	9.00	12.30
-11	-2	-9	85.69	14.60
-11	-2	-9	57.79	9.60
-11	-2	-9	41.30	9.70
-11	-2	-9	46.40	12.40
-11	-2	-9	54.89	10.40

-12	-2	-9	20.20	14.10
-12	-2	-9	11.90	9.90
-12	-2	-9	-17.40	10.00
-12	-2	-9	-1.90	8.20
12	2	9	5.70	13.20
11	-3	-9	33.50	12.10
-11	3	9	27.40	13.40
-11	3	9	46.10	11.30
10	-3	-9	68.79	22.50
10	-3	-9	64.39	12.30
-10	3	9	88.09	14.80
-10	3	9	79.49	10.70
9	-3	-9	59.09	12.30
9	-3	-9	65.49	22.20
-9	3	9	58.19	9.80
-9	3	9	50.29	9.50
8	-3	-9	131.49	18.60
8	-3	-9	127.59	17.20
-8	3	9	121.99	15.40
-8	3	9	145.49	15.80
7	-3	-9	33.60	9.90
7	-3	-9	39.80	12.70
7	-3	-9	38.00	15.50
-7	3	9	22.70	7.60
-7	3	9	24.90	8.40
6	-3	-9	11.90	10.90
6	-3	-9	7.50	10.60
6	-3	-9	2.40	15.00
-6	3	9	3.30	8.00
5	-3	-9	537.75	28.80
5	-3	-9	445.06	34.50
5	-3	-9	537.75	31.70
5	-3	-9	531.65	28.50
-5	3	9	502.05	25.80
-5	3	9	478.05	27.90
4	-3	-9	619.44	33.50
-4	3	9	625.64	32.40
-4	3	9	668.33	32.00
-3	3	9	116.29	7.50
-3	3	9	110.79	9.30
-2	3	9	884.31	44.10
-2	3	9	1026.40	45.10
-2	3	9	990.80	45.10
-1	3	9	278.07	14.80

-1	3	9	313.47	15.60
-1	3	9	319.87	15.60
0	3	9	47.00	4.80
0	3	9	46.40	4.20
1	3	9	205.88	11.00
1	3	9	195.28	10.00
2	3	9	1409.66	64.79
2	3	9	1444.06	65.19
-3	-3	-9	910.81	44.30
3	3	9	990.30	44.10
3	3	9	930.01	44.10
4	3	9	204.98	13.20
4	3	9	218.48	10.10
4	3	9	203.88	13.20
-5	-3	-9	230.58	14.90
-5	-3	-9	204.58	15.90
-5	-3	-9	193.08	17.90
-5	-3	-9	227.68	12.90
-5	-3	-9	172.38	21.10
5	3	9	222.18	15.80
5	3	9	248.18	11.10
-6	-3	-9	110.69	15.80
-6	-3	-9	102.59	19.50
-6	-3	-9	128.89	9.60
-6	-3	-9	152.58	26.00
-6	-3	-9	108.99	11.40
-6	-3	-9	103.99	15.50
-6	-3	-9	132.29	13.60
-7	-3	-9	13.40	8.50
-7	-3	-9	7.30	8.50
-7	-3	-9	5.20	9.80
-7	-3	-9	19.70	7.00
-7	-3	-9	3.10	5.80
-7	-3	-9	17.60	5.10
7	3	9	9.80	3.50
-8	-3	-9	4.50	5.80
-8	-3	-9	7.80	6.80
-8	-3	-9	7.90	9.80
-8	-3	-9	11.20	7.90
-8	-3	-9	4.80	11.00
-8	-3	-9	4.40	8.20
8	3	9	2.30	4.60
-9	-3	-9	1144.69	54.59
-9	-3	-9	1017.00	54.89

-9	-3	-9	1139.09	53.49
-9	-3	-9	1144.29	53.99
-9	-3	-9	1087.89	56.09
9	3	9	1077.99	52.89
-10	-3	-9	127.69	16.70
-10	-3	-9	120.99	19.30
-10	-3	-9	169.58	15.10
-10	-3	-9	139.29	13.50
-10	-3	-9	156.98	17.90
10	3	9	132.09	19.50
-11	-3	-9	0.50	7.70
-11	-3	-9	16.40	10.50
-11	-3	-9	18.00	7.90
-11	-3	-9	20.70	12.20
-12	-3	-9	53.89	11.20
-12	-3	-9	62.29	10.70
-12	-3	-9	81.99	10.10
-12	-3	-9	59.59	13.80
12	3	9	62.19	14.80
11	-4	-9	19.60	11.40
-11	4	9	11.60	12.10
-11	4	9	-3.40	13.60
10	-4	-9	53.79	11.90
-10	4	9	18.50	10.20
-10	4	9	43.60	9.40
9	-4	-9	26.70	11.60
-9	4	9	14.00	9.60
-9	4	9	0.90	8.50
8	-4	-9	52.29	19.30
8	-4	-9	48.20	11.30
8	-4	-9	69.99	13.80
-8	4	9	32.90	9.40
-8	4	9	41.40	9.10
7	-4	-9	260.07	22.80
7	-4	-9	220.08	31.50
7	-4	-9	276.67	21.20
-7	4	9	280.87	19.50
-7	4	9	279.07	18.80
6	-4	-9	524.15	30.10
6	-4	-9	536.15	37.90
6	-4	-9	546.75	35.90
-6	4	9	576.74	29.80
5	-4	-9	460.95	30.10
5	-4	-9	397.46	35.90

5	-4	-9	482.65	26.20
5	-4	-9	485.45	26.30
-5	4	9	457.95	23.90
-5	4	9	465.85	26.20
4	-4	-9	121.99	17.70
4	-4	-9	115.49	17.60
4	-4	-9	121.59	15.60
-4	4	9	113.39	8.20
-4	4	9	91.89	12.00
-3	4	9	1711.93	74.19
-3	4	9	1556.64	75.09
-2	4	9	60.09	6.20
-2	4	9	57.29	4.50
-1	4	9	6.30	2.10
-1	4	9	7.00	2.80
-1	4	9	8.20	2.50
0	4	9	49.70	4.60
0	4	9	47.10	3.00
0	4	9	54.49	7.90
0	4	9	49.30	4.80
0	4	9	48.70	4.10
1	4	9	931.41	42.70
1	4	9	973.70	42.90
1	4	9	913.31	43.50
2	4	9	211.48	11.20
2	4	9	211.38	11.80
-3	-4	-9	13.20	3.90
3	4	9	25.30	3.90
3	4	9	16.40	3.80
4	4	9	2124.39	95.59
4	4	9	2053.59	95.09
-5	-4	-9	88.59	12.70
-5	-4	-9	62.19	7.70
-5	-4	-9	92.09	14.70
-5	-4	-9	69.39	7.20
-5	-4	-9	86.19	12.10
5	4	9	73.99	4.90
5	4	9	79.19	11.50
-6	-4	-9	56.29	9.70
-6	-4	-9	77.79	10.10
-6	-4	-9	72.69	12.00
-6	-4	-9	67.99	12.30
-6	-4	-9	73.89	10.60
-6	-4	-9	56.89	7.70

6	4	9	75.19	5.60
-7	-4	-9	505.55	28.30
-7	-4	-9	515.25	28.60
-7	-4	-9	514.35	27.30
-7	-4	-9	534.55	32.10
-7	-4	-9	537.05	30.00
-7	-4	-9	528.55	26.80
-8	-4	-9	13.30	11.10
-8	-4	-9	22.50	8.30
-8	-4	-9	21.80	5.90
-8	-4	-9	25.20	9.30
-8	-4	-9	23.10	7.70
-8	-4	-9	16.00	6.50
8	4	9	11.70	4.70
-9	-4	-9	163.98	19.10
-9	-4	-9	157.58	16.30
-9	-4	-9	170.28	14.70
-9	-4	-9	127.59	16.20
-9	-4	-9	168.18	16.90
9	4	9	126.29	13.10
-10	-4	-9	76.49	11.00
-10	-4	-9	54.89	9.20
-10	-4	-9	74.69	12.70
-10	-4	-9	56.79	9.00
-10	-4	-9	55.79	10.20
10	4	9	75.09	12.10
-11	-4	-9	8.00	11.30
-11	-4	-9	15.20	7.90
-11	-4	-9	41.60	9.50
-11	-4	-9	30.90	10.10
-11	-4	-9	22.00	7.60
-12	-4	-9	9.00	8.00
-12	-4	-9	3.60	9.80
-12	-4	-9	-9.30	11.30
-12	-4	-9	-3.60	8.20
12	4	9	10.90	13.50
11	-5	-9	174.38	18.60
-11	5	9	172.08	25.00
-11	5	9	138.89	23.10
10	-5	-9	43.70	11.50
-10	5	9	45.80	9.40
-10	5	9	34.80	11.50
9	-5	-9	20.70	11.30
-9	5	9	-3.20	10.80

8	-5	-9	381.46	29.30
8	-5	-9	474.45	27.30
-8	5	9	444.36	26.50
-8	5	9	411.06	25.80
7	-5	-9	249.97	20.40
7	-5	-9	245.28	20.50
7	-5	-9	268.97	50.49
-7	5	9	255.77	19.10
-7	5	9	231.08	18.60
6	-5	-9	214.98	27.50
6	-5	-9	248.38	19.40
6	-5	-9	237.88	18.30
6	-5	-9	319.97	44.70
-6	5	9	224.88	18.00
-6	5	9	230.08	19.20
5	-5	-9	268.77	23.60
5	-5	-9	277.27	19.50
5	-5	-9	256.87	18.60
-5	5	9	277.77	16.40
-5	5	9	264.07	18.60
4	-5	-9	316.87	21.20
4	-5	-9	266.37	18.40
4	-5	-9	278.67	20.30
-4	5	9	228.18	18.00
-4	5	9	276.87	14.50
-3	5	9	219.98	11.80
-3	5	9	233.58	14.20
-2	5	9	97.29	5.90
-2	5	9	89.69	7.70
-1	5	9	678.13	30.70
-1	5	9	604.44	29.30
0	5	9	15.60	3.50
0	5	9	10.60	4.10
0	5	9	11.70	1.80
0	5	9	12.50	2.30
0	5	9	14.20	3.00
0	5	9	12.80	2.10
1	5	9	26.50	3.90
1	5	9	32.60	4.40
1	5	9	26.40	4.10
1	5	9	25.70	3.40
2	5	9	1866.71	84.69
2	5	9	1903.31	84.79
2	5	9	1913.91	84.79

2	5	9	1801.02	84.89
-3	-5	-9	1040.30	50.19
3	5	9	1146.19	50.79
3	5	9	1085.99	50.59
-4	-5	-9	19.00	4.00
4	5	9	14.80	5.10
4	5	9	32.90	5.70
-5	-5	-9	0.00	3.90
-5	-5	-9	3.80	12.50
-5	-5	-9	0.10	6.50
-5	-5	-9	3.40	6.60
-5	-5	-9	11.30	7.70
5	5	9	2.70	2.00
5	5	9	2.90	6.90
-6	-5	-9	101.29	14.40
-6	-5	-9	100.59	10.80
-6	-5	-9	100.49	14.90
-6	-5	-9	80.19	14.20
-6	-5	-9	91.39	8.70
-6	-5	-9	105.59	12.10
6	5	9	98.89	6.50
-7	-5	-9	-1.90	8.10
-7	-5	-9	11.90	6.20
-7	-5	-9	0.00	11.40
-7	-5	-9	5.30	10.50
-7	-5	-9	11.50	8.00
-7	-5	-9	7.10	4.70
7	5	9	2.60	3.20
-8	-5	-9	269.37	18.10
-8	-5	-9	277.97	17.00
-8	-5	-9	262.27	19.50
-8	-5	-9	276.57	24.80
-8	-5	-9	268.87	19.10
-8	-5	-9	269.27	20.80
-9	-5	-9	84.49	12.60
-9	-5	-9	90.59	12.20
-9	-5	-9	87.59	12.80
-9	-5	-9	54.59	9.30
-9	-5	-9	83.39	14.30
9	5	9	66.49	12.20
-10	-5	-9	674.33	35.30
-10	-5	-9	668.73	35.00
-10	-5	-9	639.54	36.60
-10	-5	-9	621.54	33.40

-10	-5	-9	668.83	34.00
10	5	9	633.54	35.50
-11	-5	-9	13.50	10.90
-11	-5	-9	29.30	7.90
-11	-5	-9	30.90	9.10
-11	-5	-9	4.40	7.80
-11	-5	-9	18.70	9.20
-12	-5	-9	51.99	11.90
-12	-5	-9	48.50	9.10
-12	-5	-9	48.30	9.70
-12	-5	-9	58.79	10.70
12	5	9	25.80	14.30
11	-6	-9	25.70	10.80
-11	6	9	30.30	15.30
-11	6	9	12.30	9.20
10	-6	-9	128.99	17.50
-10	6	9	110.99	15.50
-10	6	9	153.48	19.80
9	-6	-9	47.40	15.90
-9	6	9	42.80	11.90
8	-6	-9	269.07	22.50
8	-6	-9	227.18	21.10
-8	6	9	243.98	20.70
-8	6	9	251.57	19.30
7	-6	-9	218.98	18.40
7	-6	-9	203.88	18.60
-7	6	9	208.78	18.10
6	-6	-9	79.99	14.30
6	-6	-9	100.59	16.60
-6	6	9	55.89	10.20
5	-6	-9	70.29	14.80
5	-6	-9	30.30	7.80
5	-6	-9	30.90	12.80
5	-6	-9	36.80	8.90
-5	6	9	52.49	8.40
-5	6	9	52.69	9.00
4	-6	-9	412.36	32.70
4	-6	-9	455.05	26.90
4	-6	-9	434.66	26.30
4	-6	-9	455.65	25.30
4	-6	-9	424.66	24.40
-4	6	9	431.86	25.70
-4	6	9	479.85	22.10
-3	6	9	548.75	28.30

-3	6	9	558.44	26.20
-2	6	9	1073.59	50.39
-2	6	9	1077.79	48.90
-1	6	9	530.05	25.70
-1	6	9	555.34	24.30
-1	6	9	493.25	24.10
0	6	9	210.28	10.30
0	6	9	253.57	12.80
0	6	9	224.98	10.80
0	6	9	206.78	11.80
0	6	9	193.88	10.30
1	6	9	11.80	3.40
1	6	9	16.90	2.80
1	6	9	13.20	3.10
1	6	9	14.70	3.20
2	6	9	7.70	3.40
2	6	9	12.10	3.00
2	6	9	9.50	3.60
2	6	9	6.00	2.80
-3	-6	-9	138.29	10.10
3	6	9	130.99	9.60
3	6	9	140.09	9.70
3	6	9	153.88	9.00
3	6	9	145.09	10.70
-4	-6	-9	138.79	9.40
4	6	9	128.99	8.90
4	6	9	124.49	10.60
4	6	9	103.49	10.20
4	6	9	144.19	12.60
-5	-6	-9	312.57	18.70
-5	-6	-9	312.27	19.00
-5	-6	-9	320.07	21.30
-5	-6	-9	292.57	19.00
5	6	9	324.07	14.80
-6	-6	-9	7.00	6.20
-6	-6	-9	9.90	6.30
-6	-6	-9	-4.80	11.30
-6	-6	-9	-2.20	7.90
-6	-6	-9	12.80	8.10
-6	-6	-9	3.70	4.70
6	6	9	2.40	2.60
-7	-6	-9	31.60	7.90
-7	-6	-9	29.10	9.30
-7	-6	-9	31.10	5.80

-7	-6	-9	34.80	10.30
7	6	9	32.90	4.60
-8	-6	-9	825.92	40.20
-8	-6	-9	829.42	41.30
-8	-6	-9	853.11	40.70
-8	-6	-9	808.32	44.90
-8	-6	-9	776.82	41.00
-8	-6	-9	815.72	42.80
-9	-6	-9	52.79	9.60
-9	-6	-9	51.29	8.50
-9	-6	-9	43.50	10.10
-9	-6	-9	47.70	8.30
-9	-6	-9	54.49	9.20
9	6	9	37.80	12.00
-10	-6	-9	28.30	10.00
-10	-6	-9	-4.20	8.10
-10	-6	-9	3.30	8.80
-10	-6	-9	15.60	7.90
-10	-6	-9	10.20	7.10
10	6	9	23.50	11.00
-11	-6	-9	20.60	8.00
-11	-6	-9	25.10	9.10
-11	-6	-9	41.70	11.50
-11	-6	-9	31.30	9.20
-11	-6	-9	39.20	7.90
-12	-6	-9	-3.30	7.50
-12	-6	-9	7.80	9.00
-12	-6	-9	33.10	9.80
-12	-6	-9	10.40	10.50
12	6	9	19.10	20.00
11	-7	-9	105.19	16.50
-11	7	9	81.69	13.90
10	-7	-9	27.70	10.90
-10	7	9	14.00	8.90
-10	7	9	28.80	13.20
9	-7	-9	257.77	23.10
9	-7	-9	255.37	24.50
-9	7	9	245.58	24.10
-9	7	9	252.57	20.70
8	-7	-9	57.29	10.60
8	-7	-9	44.30	11.20
-8	7	9	31.50	10.00
-8	7	9	26.10	9.20
7	-7	-9	33.10	9.20

7	-7	-9	26.20	10.00
-7	7	9	23.80	8.80
6	-7	-9	47.90	16.10
6	-7	-9	34.70	8.70
6	-7	-9	32.70	9.30
-6	7	9	32.90	8.00
-6	7	9	43.50	9.30
5	-7	-9	324.17	25.90
5	-7	-9	285.17	20.80
5	-7	-9	309.57	18.80
5	-7	-9	316.67	25.10
-5	7	9	282.97	16.90
-5	7	9	270.07	19.70
4	-7	-9	450.05	25.10
4	-7	-9	398.56	23.50
4	-7	-9	453.35	27.10
4	-7	-9	406.86	26.40
4	-7	-9	427.26	26.20
3	-7	-9	32.10	8.20
3	-7	-9	36.20	8.10
-3	7	9	32.80	4.40
-3	7	9	58.09	10.10
-2	7	9	1252.57	57.59
-2	7	9	1223.38	59.39
-2	7	9	1348.77	57.89
-1	7	9	78.59	7.10
-1	7	9	87.59	5.00
-1	7	9	73.79	6.60
0	7	9	25.50	2.60
0	7	9	19.50	5.40
0	7	9	16.50	2.70
1	7	9	515.75	24.80
1	7	9	497.75	25.40
1	7	9	517.55	24.50
2	7	9	44.10	5.60
2	7	9	46.10	5.80
2	7	9	41.20	5.20
2	7	9	46.30	6.60
3	7	9	2710.33	120.79
3	7	9	2656.33	120.89
3	7	9	2758.82	119.99
3	7	9	2520.95	120.29
-4	-7	-9	533.05	26.00
4	7	9	516.95	27.60

4	7	9	565.84	25.90
4	7	9	547.85	28.60
4	7	9	493.05	27.00
-5	-7	-9	89.39	12.10
-5	-7	-9	93.59	8.50
-5	-7	-9	81.99	11.10
-5	-7	-9	109.49	14.80
-5	-7	-9	120.29	16.30
5	7	9	93.69	5.70
-6	-7	-9	108.89	11.00
-6	-7	-9	88.89	15.00
-6	-7	-9	97.89	12.00
-6	-7	-9	93.39	9.10
-6	-7	-9	86.79	13.00
6	7	9	108.09	6.90
-7	-7	-9	9.40	6.60
-7	-7	-9	23.70	9.40
-7	-7	-9	10.20	5.50
-7	-7	-9	3.10	6.80
-7	-7	-9	5.80	11.00
7	7	9	7.50	3.80
-8	-7	-9	136.19	13.30
-8	-7	-9	153.78	14.50
-8	-7	-9	134.99	16.90
-8	-7	-9	154.58	15.30
-8	-7	-9	125.69	22.90
-8	-7	-9	150.08	11.90
8	7	9	155.28	11.20
-9	-7	-9	659.33	34.50
-9	-7	-9	692.33	35.60
-9	-7	-9	649.74	34.20
-9	-7	-9	676.43	37.00
-9	-7	-9	675.63	35.20
9	7	9	663.23	34.00
-10	-7	-9	34.30	10.20
-10	-7	-9	18.70	8.00
-10	-7	-9	42.00	9.80
-10	-7	-9	39.70	10.40
10	7	9	37.80	11.00
-11	-7	-9	24.60	11.10
-11	-7	-9	18.10	9.00
-11	-7	-9	18.80	8.80
-11	-7	-9	12.90	7.50
-11	-7	-9	2.10	7.40

11	7	9	-16.80	13.50
-12	-7	-9	56.09	10.00
-12	-7	-9	49.30	9.20
-12	-7	-9	54.49	10.90
-12	-7	-9	46.90	10.20
12	7	9	27.50	18.20
10	-8	-9	102.49	20.80
-10	8	9	68.69	10.60
-10	8	9	95.19	15.80
9	-8	-9	24.40	11.30
9	-8	-9	34.40	13.00
-9	8	9	29.60	9.80
-9	8	9	29.70	13.00
8	-8	-9	59.29	11.80
8	-8	-9	51.79	10.70
-8	8	9	53.99	11.90
-8	8	9	88.79	15.40
7	-8	-9	259.37	20.90
7	-8	-9	228.98	19.10
-7	8	9	257.17	19.30
6	-8	-9	71.69	10.90
6	-8	-9	61.99	9.60
-6	8	9	75.29	10.80
-6	8	9	101.69	13.40
5	-8	-9	447.46	28.60
5	-8	-9	415.96	23.60
5	-8	-9	404.16	29.40
5	-8	-9	411.56	25.70
-5	8	9	429.66	24.70
-5	8	9	413.56	22.30
4	-8	-9	617.14	33.20
4	-8	-9	612.24	31.20
4	-8	-9	629.94	34.10
4	-8	-9	584.74	33.50
4	-8	-9	588.04	32.10
-4	8	9	618.64	29.30
3	-8	-9	2075.19	95.29
3	-8	-9	1930.71	92.39
3	-8	-9	1978.30	92.39
3	-8	-9	1946.11	92.69
3	-8	-9	1983.60	91.89
-3	8	9	2123.09	90.69
2	-8	-9	41.30	6.90
-2	8	9	51.19	4.30

-2	8	9	43.50	6.60
-1	8	9	643.14	27.60
-1	8	9	560.34	29.10
-1	8	9	592.44	29.40
0	8	9	121.19	6.10
0	8	9	104.19	11.80
0	8	9	114.79	8.50
1	8	9	103.09	10.50
1	8	9	104.69	7.80
-2	-8	-9	32.00	6.20
2	8	9	41.90	7.00
2	8	9	43.80	5.60
2	8	9	42.00	6.50
2	8	9	31.20	6.00
3	8	9	298.77	17.80
3	8	9	273.87	16.80
3	8	9	284.47	16.50
3	8	9	302.77	15.40
-4	-8	-9	1073.19	48.10
4	8	9	1061.19	50.69
4	8	9	932.51	49.10
4	8	9	997.80	50.09
4	8	9	1103.29	48.00
-5	-8	-9	34.10	5.20
-5	-8	-9	45.10	7.10
-5	-8	-9	45.90	8.80
-5	-8	-9	35.30	7.60
-5	-8	-9	34.00	10.10
-6	-8	-9	54.29	8.10
-6	-8	-9	50.59	10.20
-6	-8	-9	61.59	9.00
-6	-8	-9	53.49	10.60
-6	-8	-9	43.50	14.00
-6	-8	-9	58.99	8.70
-6	-8	-9	50.49	7.30
6	8	9	63.99	5.90
-7	-8	-9	423.06	31.80
-7	-8	-9	462.05	26.80
-7	-8	-9	433.06	24.10
-7	-8	-9	435.06	24.90
-7	-8	-9	457.85	25.50
-7	-8	-9	441.36	23.60
7	8	9	447.86	22.20
-8	-8	-9	12.90	6.80

-8	-8	-9	32.50	8.60
-8	-8	-9	33.90	7.60
-8	-8	-9	30.20	6.60
-8	-8	-9	36.00	9.70
8	8	9	24.20	6.00
-9	-8	-9	140.09	14.00
-9	-8	-9	151.08	17.00
-9	-8	-9	161.88	16.60
-9	-8	-9	170.08	15.90
-9	-8	-9	148.39	13.00
-10	-8	-9	22.90	14.20
-10	-8	-9	13.70	7.10
-10	-8	-9	29.90	15.50
-10	-8	-9	1.40	12.90
10	8	9	9.40	11.10
-11	-8	-9	23.30	9.10
-11	-8	-9	10.00	7.50
-11	-8	-9	2.40	8.00
-11	-8	-9	3.70	10.30
11	8	9	26.40	16.60
-12	-8	-9	-1.70	8.60
-12	-8	-9	-4.10	9.20
-12	-8	-9	15.80	7.80
-12	-8	-9	-8.00	9.20
10	-9	-9	30.10	11.80
10	-9	-9	38.50	18.80
-10	9	9	46.40	15.90
-10	9	9	34.00	9.80
9	-9	-9	124.79	19.20
9	-9	-9	151.68	19.00
-9	9	9	101.29	15.70
-9	9	9	88.19	15.80
8	-9	-9	384.66	25.20
8	-9	-9	384.76	26.60
-8	9	9	377.06	25.00
-8	9	9	410.66	28.80
7	-9	-9	135.19	15.80
7	-9	-9	111.69	17.70
-7	9	9	146.49	16.20
6	-9	-9	63.59	15.50
6	-9	-9	40.90	8.50
6	-9	-9	16.60	17.50
6	-9	-9	45.80	10.40
-6	9	9	30.10	10.10

-6	9	9	45.50	9.80
5	-9	-9	135.89	21.80
5	-9	-9	151.88	19.20
5	-9	-9	203.18	21.20
5	-9	-9	199.08	18.50
-5	9	9	168.28	12.60
-5	9	9	169.48	16.30
4	-9	-9	28.80	9.70
4	-9	-9	46.60	11.30
4	-9	-9	24.10	8.50
4	-9	-9	39.10	8.10
4	-9	-9	48.50	10.80
-4	9	9	26.80	4.60
3	-9	-9	61.99	10.20
3	-9	-9	80.89	14.10
3	-9	-9	60.69	8.30
3	-9	-9	52.09	9.30
3	-9	-9	58.79	9.60
-3	9	9	58.89	5.50
2	-9	-9	124.19	14.20
2	-9	-9	113.89	13.60
2	-9	-9	121.59	13.90
2	-9	-9	102.69	10.90
2	-9	-9	115.99	13.50
-2	9	9	131.29	7.30
-2	9	9	119.09	10.90
-1	9	9	258.87	17.60
-1	9	9	313.77	13.90
-1	9	9	277.37	16.50
0	9	9	77.29	4.50
0	9	9	69.39	7.70
-1	-9	-9	91.79	13.00
1	9	9	97.59	12.50
1	9	9	98.89	8.10
2	9	9	2745.63	123.89
2	9	9	2746.13	123.19
2	9	9	2712.43	123.69
2	9	9	2706.03	124.49
-3	-9	-9	543.35	26.40
3	9	9	523.95	28.90
3	9	9	506.25	27.40
3	9	9	551.44	28.30
3	9	9	563.74	26.40
-4	-9	-9	40.90	6.80

-4	-9	-9	27.80	9.00
-4	-9	-9	30.00	7.80
-4	-9	-9	49.30	8.70
4	9	9	38.50	6.90
4	9	9	12.70	8.60
-5	-9	-9	5.00	7.70
-5	-9	-9	29.00	10.30
-5	-9	-9	4.50	8.20
-5	-9	-9	7.10	4.00
-5	-9	-9	-4.10	6.50
5	9	9	2.80	3.10
-6	-9	-9	-2.50	7.20
-6	-9	-9	-13.30	16.80
-6	-9	-9	-5.60	8.90
-6	-9	-9	-7.30	6.00
-6	-9	-9	1.60	8.80
-6	-9	-9	-2.90	5.50
-6	-9	-9	-0.40	4.30
6	9	9	1.50	3.60
-7	-9	-9	-2.10	15.50
-7	-9	-9	1.10	10.40
-7	-9	-9	15.80	5.50
-7	-9	-9	9.90	6.40
-7	-9	-9	16.60	7.60
-7	-9	-9	12.50	6.40
-7	-9	-9	20.00	8.90
7	9	9	6.20	4.90
-8	-9	-9	147.09	17.00
-8	-9	-9	133.19	14.40
-8	-9	-9	149.69	15.60
-8	-9	-9	142.49	13.40
-8	-9	-9	131.79	12.00
8	9	9	139.69	11.90
-9	-9	-9	52.39	8.40
-9	-9	-9	58.29	8.90
-9	-9	-9	54.09	10.10
9	9	9	80.79	13.10
-10	-9	-9	310.17	24.40
-10	-9	-9	306.87	21.90
-10	-9	-9	303.67	20.90
-10	-9	-9	321.97	21.30
-10	-9	-9	269.17	18.40
10	9	9	279.07	23.30
-11	-9	-9	0.40	8.40

-11	-9	-9	15.80	7.60
-11	-9	-9	21.00	8.00
-11	-9	-9	17.60	8.30
-12	-9	-9	12.10	10.00
-12	-9	-9	13.70	7.70
-12	-9	-9	-2.70	9.20
-12	-9	-9	10.00	10.70
10	-10	-9	152.18	25.20
10	-10	-9	113.69	18.10
-10	10	9	140.49	15.20
9	-10	-9	64.89	12.60
9	-10	-9	39.00	12.00
-9	10	9	59.29	14.90
-9	10	9	72.49	11.10
8	-10	-9	104.09	12.70
8	-10	-9	100.79	18.50
-8	10	9	75.49	12.10
-8	10	9	84.19	16.00
7	-10	-9	36.20	9.30
7	-10	-9	52.69	12.00
-7	10	9	25.20	9.80
6	-10	-9	234.38	20.50
6	-10	-9	169.58	16.40
6	-10	-9	248.98	25.20
6	-10	-9	267.87	32.10
-6	10	9	181.98	19.00
-6	10	9	161.98	18.10
5	-10	-9	54.69	8.50
5	-10	-9	51.29	13.00
5	-10	-9	80.69	16.70
-5	10	9	56.99	10.20
4	-10	-9	106.09	14.90
4	-10	-9	139.19	15.90
4	-10	-9	158.78	17.90
4	-10	-9	131.99	13.50
4	-10	-9	131.79	15.60
-4	10	9	140.49	9.80
3	-10	-9	1471.25	68.19
3	-10	-9	1449.15	68.09
3	-10	-9	1319.67	70.49
3	-10	-9	1429.86	67.39
3	-10	-9	1475.05	67.79
-3	10	9	1498.85	65.79
2	-10	-9	1118.99	53.69

2	-10	-9	1132.79	53.49
2	-10	-9	1071.59	54.59
2	-10	-9	1170.38	53.79
2	-10	-9	1121.49	53.59
1	-10	-9	7.10	7.90
1	-10	-9	0.30	7.70
1	-10	-9	3.20	7.70
-1	10	9	3.20	1.90
-1	10	9	-2.70	8.90
0	10	9	49.99	8.40
0	10	9	52.89	3.80
0	10	9	48.90	10.50
1	10	9	174.78	12.00
1	10	9	164.28	17.50
-2	-10	-9	20.60	6.60
2	10	9	29.90	8.90
2	10	9	11.70	4.90
-3	-10	-9	650.83	37.50
-3	-10	-9	753.42	34.00
3	10	9	632.54	36.90
3	10	9	665.53	35.10
3	10	9	728.53	36.50
3	10	9	779.52	34.30
-4	-10	-9	26.40	7.00
-4	-10	-9	23.10	7.30
-4	-10	-9	19.50	4.80
-4	-10	-9	15.70	8.80
-4	-10	-9	30.20	8.30
-5	-10	-9	366.76	22.30
-5	-10	-9	366.56	19.60
-5	-10	-9	356.56	21.30
-5	-10	-9	366.86	21.60
-5	-10	-9	369.76	20.80
-5	-10	-9	367.96	25.60
-6	-10	-9	5.60	9.20
-6	-10	-9	7.30	8.40
-6	-10	-9	4.20	7.40
-6	-10	-9	2.90	5.20
-6	-10	-9	4.00	8.50
6	10	9	-7.30	4.10
-7	-10	-9	104.99	11.60
-7	-10	-9	79.19	13.20
-7	-10	-9	106.89	16.00
-7	-10	-9	84.19	15.80

-7	-10	-9	75.59	9.70
7	10	9	100.99	9.50
-8	-10	-9	909.51	47.80
-8	-10	-9	951.30	46.50
-8	-10	-9	949.01	47.00
-8	-10	-9	971.70	46.70
-8	-10	-9	973.20	47.70
-8	-10	-9	962.60	48.40
8	10	9	985.70	46.10
-9	-10	-9	60.89	10.30
-9	-10	-9	37.90	7.40
-9	-10	-9	50.49	8.90
-9	-10	-9	69.49	11.80
9	10	9	45.10	9.70
-10	-10	-9	16.00	7.30
-10	-10	-9	39.80	10.70
-10	-10	-9	22.00	9.20
-10	-10	-9	14.20	8.40
-10	-10	-9	25.60	8.00
10	10	9	26.00	12.40
-11	-10	-9	78.99	13.70
-11	-10	-9	75.09	12.90
-11	-10	-9	98.39	13.00
-11	-10	-9	102.49	11.70
-12	-10	-9	13.30	9.10
-12	-10	-9	19.40	10.00
-12	-10	-9	24.50	11.20
-12	-10	-9	35.60	12.40
10	-11	-9	59.29	19.10
10	-11	-9	23.90	14.10
-10	11	9	9.20	18.50
-10	11	9	33.90	9.60
9	-11	-9	142.99	19.00
9	-11	-9	137.09	17.20
-9	11	9	109.39	17.50
-9	11	9	144.39	16.60
8	-11	-9	23.20	12.90
8	-11	-9	-0.40	10.00
-8	11	9	16.40	14.30
-8	11	9	13.00	11.80
7	-11	-9	164.88	15.80
7	-11	-9	135.79	19.70
-7	11	9	158.68	18.40
6	-11	-9	204.88	35.90

6	-11	-9	124.89	14.80
6	-11	-9	136.39	20.10
6	-11	-9	154.18	23.90
-6	11	9	131.39	17.90
5	-11	-9	319.27	22.50
5	-11	-9	301.27	19.60
5	-11	-9	264.47	24.30
5	-11	-9	285.77	23.90
5	-11	-9	269.77	26.10
-5	11	9	262.67	17.50
4	-11	-9	362.46	25.10
4	-11	-9	287.67	32.50
4	-11	-9	372.46	23.40
4	-11	-9	351.76	29.50
-4	11	9	419.86	19.80
3	-11	-9	-10.60	15.60
3	-11	-9	10.90	6.70
3	-11	-9	16.80	8.20
3	-11	-9	12.60	8.60
3	-11	-9	5.30	9.20
-3	11	9	10.80	3.50
2	-11	-9	636.24	36.70
2	-11	-9	675.33	35.10
2	-11	-9	692.73	35.40
2	-11	-9	699.43	35.40
2	-11	-9	693.63	35.10
-2	11	9	727.13	32.20
1	-11	-9	-12.40	9.50
1	-11	-9	3.40	7.50
1	-11	-9	1.20	7.70
1	-11	-9	11.40	9.50
1	-11	-9	15.90	9.10
1	-11	-9	5.10	7.00
-1	11	9	11.30	11.70
0	-11	-9	352.26	23.40
0	-11	-9	317.37	21.10
0	-11	-9	383.16	21.70
0	-11	-9	333.97	20.80
0	11	9	375.56	17.00
0	11	9	384.66	26.50
-1	-11	-9	1270.87	61.99
-1	-11	-9	1271.27	62.79
-1	-11	-9	1341.07	61.99
-1	-11	-9	1264.77	61.79

-1	-11	-9	1453.95	65.59
1	11	9	1269.77	61.99
1	11	9	1273.37	64.99
1	11	9	1440.16	59.39
-2	-11	-9	109.09	12.80
-2	-11	-9	108.29	14.90
-2	-11	-9	95.29	14.40
-2	-11	-9	116.19	12.00
2	11	9	111.39	11.10
-3	-11	-9	877.31	43.30
-3	-11	-9	924.41	43.90
-3	-11	-9	844.22	43.70
-3	-11	-9	900.51	42.70
-3	-11	-9	834.12	44.20
3	11	9	833.82	43.80
3	11	9	971.40	42.80
-4	-11	-9	73.89	12.20
-4	-11	-9	88.39	12.00
-4	-11	-9	77.39	14.10
-4	-11	-9	104.69	14.50
-4	-11	-9	78.59	11.90
-4	-11	-9	99.69	9.40
-5	-11	-9	18.80	6.30
-5	-11	-9	21.90	8.20
-5	-11	-9	44.70	11.80
-5	-11	-9	16.40	7.40
-5	-11	-9	10.20	7.00
-5	-11	-9	16.90	5.10
5	11	9	14.50	3.90
-6	-11	-9	81.89	9.50
-6	-11	-9	85.59	12.80
-6	-11	-9	78.99	11.20
-6	-11	-9	114.59	14.00
-6	-11	-9	89.29	12.80
-6	-11	-9	67.19	12.20
6	11	9	80.29	9.00
-7	-11	-9	29.40	9.60
-7	-11	-9	21.20	5.90
-7	-11	-9	32.60	7.40
-7	-11	-9	16.10	6.70
-7	-11	-9	29.50	8.90
-7	-11	-9	10.00	7.80
7	11	9	26.40	6.60
-8	-11	-9	611.44	32.40

-8	-11	-9	637.94	31.90
-8	-11	-9	569.84	33.40
-8	-11	-9	618.04	32.00
-8	-11	-9	573.44	33.10
-8	-11	-9	632.64	33.50
-9	-11	-9	457.55	27.50
-9	-11	-9	482.95	26.60
-9	-11	-9	457.55	25.90
-9	-11	-9	435.36	25.10
-9	-11	-9	479.05	29.90
-9	-11	-9	518.95	29.00
9	11	9	409.16	26.80
-10	-11	-9	60.09	10.40
-10	-11	-9	91.19	12.10
-10	-11	-9	71.69	10.30
-10	-11	-9	81.69	11.20
-11	-11	-9	26.90	8.40
-11	-11	-9	23.00	8.90
-11	-11	-9	15.80	8.40
-11	-11	-9	30.20	8.00
10	-12	-9	105.49	19.70
-10	12	9	70.59	11.20
9	-12	-9	35.80	11.10
9	-12	-9	55.89	12.90
-9	12	9	24.40	15.40
-9	12	9	54.89	10.50
8	-12	-9	18.90	9.20
8	-12	-9	18.60	12.70
-8	12	9	38.10	11.30
-8	12	9	0.40	13.70
7	-12	-9	127.79	15.60
7	-12	-9	120.69	20.80
-7	12	9	121.19	18.50
6	-12	-9	-27.50	28.70
6	-12	-9	8.70	7.60
6	-12	-9	4.60	12.00
6	-12	-9	4.60	13.30
-6	12	9	7.70	9.60
5	-12	-9	112.59	17.00
5	-12	-9	140.79	20.80
5	-12	-9	150.28	14.70
5	-12	-9	115.99	22.20
5	-12	-9	160.58	18.00
-5	12	9	122.99	12.90

4	-12	-9	881.11	48.80
4	-12	-9	950.20	46.20
4	-12	-9	908.11	44.80
4	-12	-9	811.02	52.19
4	-12	-9	903.11	43.90
-4	12	9	878.31	42.30
3	-12	-9	392.96	26.40
3	-12	-9	457.15	26.80
3	-12	-9	418.06	25.30
3	-12	-9	450.85	24.80
-3	12	9	467.15	22.00
2	-12	-9	302.37	20.40
2	-12	-9	335.37	21.10
2	-12	-9	317.17	22.70
2	-12	-9	327.87	20.70
2	-12	-9	331.77	21.30
-2	12	9	323.57	16.30
1	-12	-9	24.00	10.20
1	-12	-9	11.60	7.90
1	-12	-9	7.90	7.20
1	-12	-9	11.80	7.60
1	-12	-9	21.10	8.90
-1	12	9	8.10	2.40
0	-12	-9	67.09	11.50
0	-12	-9	75.29	12.30
0	-12	-9	88.79	14.10
0	-12	-9	61.19	9.80
0	12	9	63.19	5.00
0	12	9	63.99	16.50
-1	-12	-9	43.20	8.20
-1	-12	-9	29.80	9.20
-1	-12	-9	27.80	7.80
-1	-12	-9	46.90	9.20
-1	-12	-9	56.39	8.40
1	12	9	49.30	4.10
1	12	9	45.10	15.60
-2	-12	-9	71.89	12.80
-2	-12	-9	85.59	13.00
-2	-12	-9	112.59	12.90
-2	-12	-9	83.89	13.80
-2	-12	-9	79.09	11.70
-2	-12	-9	81.59	11.80
2	12	9	81.29	12.70
2	12	9	88.09	5.10

-3	-12	-9	188.98	13.70
-3	-12	-9	176.18	18.80
-3	-12	-9	192.88	14.50
-3	-12	-9	136.59	24.30
-3	-12	-9	200.18	16.20
-3	-12	-9	164.48	17.20
-3	-12	-9	202.38	16.90
3	12	9	201.38	9.30
-4	-12	-9	1008.50	50.49
-4	-12	-9	1110.09	51.39
-4	-12	-9	1064.99	49.60
-4	-12	-9	949.90	50.89
-4	-12	-9	1080.89	50.19
-4	-12	-9	1057.49	49.80
4	12	9	1058.19	47.30
-5	-12	-9	11.40	7.00
-5	-12	-9	0.20	5.40
-5	-12	-9	-11.70	7.60
-5	-12	-9	28.00	12.10
-5	-12	-9	8.20	6.80
-5	-12	-9	15.50	12.90
-5	-12	-9	5.50	8.10
-6	-12	-9	158.28	12.20
-6	-12	-9	138.19	13.30
-6	-12	-9	158.88	13.90
-6	-12	-9	148.49	14.50
-6	-12	-9	103.19	18.60
-6	-12	-9	127.69	14.60
-7	-12	-9	351.86	21.40
-7	-12	-9	338.07	22.30
-7	-12	-9	360.76	21.80
-7	-12	-9	368.06	20.30
-7	-12	-9	353.66	20.80
-7	-12	-9	341.17	23.30
7	12	9	320.47	20.20
-8	-12	-9	-0.20	6.40
-8	-12	-9	9.70	9.70
-8	-12	-9	4.10	7.00
-8	-12	-9	16.60	7.50
-8	-12	-9	11.50	8.50
-8	-12	-9	1.30	8.80
8	12	9	1.00	8.10
-9	-12	-9	52.89	8.80
-9	-12	-9	57.09	9.60

-9	-12	-9	86.39	19.70
-9	-12	-9	39.40	9.70
-9	-12	-9	43.30	7.90
9	12	9	45.50	11.00
-10	-12	-9	138.49	14.00
-10	-12	-9	168.88	14.40
-10	-12	-9	153.48	15.20
-10	-12	-9	154.28	15.00
-11	-12	-9	2.10	7.90
-11	-12	-9	12.40	7.50
-11	-12	-9	-10.90	7.70
-11	-12	-9	10.10	8.90
9	-13	-9	308.07	20.40
9	-13	-9	291.07	25.20
-9	13	9	209.88	19.50
8	-13	-9	320.37	21.30
8	-13	-9	323.07	26.30
-8	13	9	342.47	28.00
-8	13	9	323.37	23.80
7	-13	-9	59.19	9.80
-7	13	9	61.89	12.40
6	-13	-9	4.20	7.80
6	-13	-9	-4.20	14.00
6	-13	-9	7.50	12.70
-6	13	9	13.30	10.70
5	-13	-9	-2.80	14.50
5	-13	-9	15.80	12.00
5	-13	-9	24.10	15.90
5	-13	-9	33.70	10.40
5	-13	-9	17.30	7.70
-5	13	9	23.00	8.30
4	-13	-9	224.08	21.10
4	-13	-9	217.18	22.00
4	-13	-9	229.08	17.20
4	-13	-9	203.88	18.50
-4	13	9	209.88	14.00
3	-13	-9	16.40	8.70
3	-13	-9	4.40	8.00
3	-13	-9	4.70	9.60
-3	13	9	4.40	3.90
2	-13	-9	61.79	10.00
2	-13	-9	101.39	12.70
2	-13	-9	83.99	10.60
2	-13	-9	84.89	11.20

2	-13	-9	70.89	10.30
-2	13	9	62.49	6.30
1	-13	-9	155.48	14.60
1	-13	-9	145.69	16.10
1	-13	-9	159.18	15.20
1	-13	-9	155.88	15.10
1	-13	-9	164.88	18.50
-1	13	9	161.18	9.20
0	-13	-9	26.60	14.00
0	-13	-9	13.20	7.60
0	-13	-9	19.10	7.30
0	-13	-9	3.20	8.00
0	-13	-9	8.30	8.10
0	-13	-9	8.00	12.70
0	13	9	12.60	2.70
-1	-13	-9	52.89	8.10
-1	-13	-9	46.00	8.80
-1	-13	-9	49.30	20.30
-1	-13	-9	53.99	12.50
-1	-13	-9	63.59	8.70
-1	-13	-9	33.10	8.80
1	13	9	55.39	4.50
-2	-13	-9	1361.36	64.19
-2	-13	-9	1352.56	63.79
-2	-13	-9	1347.77	63.59
-2	-13	-9	1288.77	64.89
-2	-13	-9	1329.27	64.99
-2	-13	-9	1408.46	63.39
2	13	9	1393.96	60.99
-3	-13	-9	40.20	8.80
-3	-13	-9	48.30	8.00
-3	-13	-9	53.19	9.50
-3	-13	-9	45.90	10.00
-3	-13	-9	67.89	11.40
-3	-13	-9	54.69	7.90
3	13	9	45.60	4.30
-4	-13	-9	45.10	9.30
-4	-13	-9	42.80	7.30
-4	-13	-9	45.70	9.60
-4	-13	-9	41.80	8.50
-4	-13	-9	59.19	9.50
-4	-13	-9	51.49	8.90
4	13	9	50.89	4.90
-5	-13	-9	39.30	8.30

-5	-13	-9	47.70	7.70
-5	-13	-9	52.69	7.60
-5	-13	-9	66.89	12.50
-5	-13	-9	32.70	10.40
-5	-13	-9	35.40	9.10
5	13	9	40.20	7.10
-6	-13	-9	16.50	12.00
-6	-13	-9	9.80	7.70
-6	-13	-9	23.60	7.80
-6	-13	-9	19.10	6.80
-6	-13	-9	29.00	8.30
-6	-13	-9	17.90	7.60
6	13	9	6.60	6.50
-7	-13	-9	21.90	7.90
-7	-13	-9	47.00	11.00
-7	-13	-9	34.20	9.10
-7	-13	-9	25.60	8.20
-7	-13	-9	37.30	8.30
-7	-13	-9	30.70	7.80
7	13	9	17.70	8.00
-8	-13	-9	-3.50	8.60
-8	-13	-9	8.20	7.40
-8	-13	-9	20.00	8.00
-8	-13	-9	5.90	10.50
-8	-13	-9	17.30	8.50
-8	-13	-9	2.60	7.00
8	13	9	2.00	9.20
-9	-13	-9	155.58	14.30
-9	-13	-9	188.78	18.60
-9	-13	-9	206.58	21.60
-9	-13	-9	180.88	18.40
-9	-13	-9	164.98	18.50
9	13	9	137.49	19.20
-10	-13	-9	172.18	14.80
-10	-13	-9	188.58	16.70
-10	-13	-9	162.38	14.90
-10	-13	-9	180.68	15.20
-11	-13	-9	50.89	10.70
-11	-13	-9	47.30	8.50
-11	-13	-9	74.09	11.70
-11	-13	-9	31.00	8.70
9	-14	-9	32.10	10.70
-9	14	9	14.90	17.60
-9	14	9	16.30	8.80

8	-14	-9	6.60	16.90
8	-14	-9	44.80	9.00
-8	14	9	37.00	11.10
-8	14	9	42.40	14.60
7	-14	-9	3.20	20.20
7	-14	-9	43.50	16.90
7	-14	-9	16.00	9.50
-7	14	9	28.10	12.20
6	-14	-9	-4.50	17.50
6	-14	-9	24.60	13.10
6	-14	-9	4.40	7.80
-6	14	9	39.40	11.40
5	-14	-9	31.40	13.10
5	-14	-9	25.40	8.20
5	-14	-9	20.30	15.80
5	-14	-9	49.90	11.30
-5	14	9	28.60	10.20
4	-14	-9	3.90	8.90
4	-14	-9	-3.10	11.20
4	-14	-9	7.20	12.00
4	-14	-9	5.30	8.30
-4	14	9	7.70	6.00
3	-14	-9	1643.84	77.49
3	-14	-9	1668.53	77.99
3	-14	-9	1575.94	76.49
3	-14	-9	1656.53	76.39
-3	14	9	1593.84	74.59
2	-14	-9	191.58	19.50
2	-14	-9	207.68	16.90
2	-14	-9	201.48	17.80
2	-14	-9	226.48	22.50
-2	14	9	189.88	19.60
-2	14	9	217.48	12.20
1	-14	-9	58.19	9.70
1	-14	-9	42.20	8.70
1	-14	-9	36.30	11.00
1	-14	-9	46.30	8.70
-1	14	9	45.00	5.50
-1	14	9	51.89	10.80
0	-14	-9	94.89	12.80
0	-14	-9	79.49	13.60
0	-14	-9	93.49	15.30
0	-14	-9	75.79	12.80
0	-14	-9	89.39	13.80

0	-14	-9	85.99	10.50
0	14	9	88.09	6.40
-1	-14	-9	59.59	10.50
-1	-14	-9	69.09	9.80
-1	-14	-9	67.29	11.50
-1	-14	-9	73.29	9.30
-1	-14	-9	74.39	12.10
1	14	9	73.49	5.50
-2	-14	-9	85.09	12.90
-2	-14	-9	93.69	12.00
-2	-14	-9	65.69	11.40
-2	-14	-9	137.09	18.30
-2	-14	-9	106.49	16.00
-2	-14	-9	120.39	13.00
2	14	9	90.49	6.10
-3	-14	-9	348.17	24.10
-3	-14	-9	394.16	22.80
-3	-14	-9	371.16	22.00
-3	-14	-9	397.86	22.70
-3	-14	-9	306.57	30.00
-3	-14	-9	391.56	21.50
-3	-14	-9	357.36	29.00
3	14	9	386.96	18.00
-4	-14	-9	36.10	10.40
-4	-14	-9	21.90	7.30
-4	-14	-9	31.30	9.60
-4	-14	-9	25.10	9.00
-4	-14	-9	42.60	8.90
-4	-14	-9	19.60	6.70
4	14	9	29.70	5.20
-5	-14	-9	270.17	17.10
-5	-14	-9	240.38	18.10
-5	-14	-9	267.87	21.40
-5	-14	-9	238.98	18.30
-5	-14	-9	254.87	16.80
-5	-14	-9	253.87	19.00
5	14	9	224.18	14.40
-6	-14	-9	25.80	7.60
-6	-14	-9	22.00	8.50
-6	-14	-9	26.30	7.90
-6	-14	-9	23.70	7.40
-6	-14	-9	19.90	9.20
6	14	9	11.50	7.30
-7	-14	-9	167.48	16.00

-7	-14	-9	188.08	15.70
-7	-14	-9	193.28	15.80
-7	-14	-9	160.58	17.70
-7	-14	-9	187.88	16.60
-8	-14	-9	646.94	33.30
-8	-14	-9	625.64	33.00
-8	-14	-9	598.14	34.50
-8	-14	-9	658.83	34.00
-8	-14	-9	670.03	34.00
-9	-14	-9	8.30	7.60
-9	-14	-9	11.50	8.50
-9	-14	-9	7.20	8.00
-9	-14	-9	20.70	9.50
-9	-14	-9	19.90	7.60
9	14	9	0.50	16.90
-10	-14	-9	24.50	8.60
-10	-14	-9	27.90	12.60
-10	-14	-9	28.50	8.50
-10	-14	-9	15.80	7.50
-10	-14	-9	24.60	8.20
-11	-14	-9	190.48	15.00
-11	-14	-9	181.48	16.40
-11	-14	-9	194.98	14.70
-11	-14	-9	176.38	14.80
-9	15	9	76.69	17.50
8	-15	-9	162.78	14.80
-8	15	9	177.88	22.50
-8	15	9	123.99	17.40
7	-15	-9	72.89	10.00
7	-15	-9	82.79	20.10
-7	15	9	112.19	18.60
6	-15	-9	9.50	8.00
6	-15	-9	28.80	19.20
-6	15	9	12.60	11.10
5	-15	-9	149.09	19.20
5	-15	-9	169.88	15.90
5	-15	-9	183.58	23.70
5	-15	-9	166.58	20.00
-5	15	9	162.58	20.50
4	-15	-9	223.28	20.00
4	-15	-9	227.38	22.10
4	-15	-9	241.98	18.70
4	-15	-9	226.18	23.40
-4	15	9	212.98	15.80

3	-15	-9	-12.90	10.10
3	-15	-9	11.30	9.20
3	-15	-9	-0.80	10.10
3	-15	-9	16.10	9.40
-3	15	9	4.20	5.10
2	-15	-9	654.73	34.00
2	-15	-9	624.24	33.40
2	-15	-9	626.04	34.00
2	-15	-9	606.34	33.40
2	-15	-9	591.94	32.80
-2	15	9	592.34	29.80
1	-15	-9	2.80	7.70
1	-15	-9	-6.20	7.60
1	-15	-9	-26.40	13.50
1	-15	-9	-4.30	7.90
1	-15	-9	-7.40	8.50
-1	15	9	2.30	3.20
-1	15	9	-6.80	10.00
0	-15	-9	1.90	14.60
0	-15	-9	6.60	10.30
0	-15	-9	4.00	7.50
0	-15	-9	0.80	7.60
0	15	9	3.00	3.00
-1	-15	-9	346.07	23.30
-1	-15	-9	318.77	23.00
1	15	9	364.46	17.20
-2	-15	-9	272.97	17.20
-2	-15	-9	225.88	19.30
-2	-15	-9	243.08	17.10
-2	-15	-9	271.97	20.40
-2	-15	-9	288.87	21.70
-2	-15	-9	244.28	21.40
2	15	9	252.57	13.20
-3	-15	-9	854.91	42.00
-3	-15	-9	748.93	46.40
-3	-15	-9	888.51	41.70
-3	-15	-9	869.41	42.70
-3	-15	-9	869.81	42.40
-3	-15	-9	815.32	42.60
3	15	9	871.31	39.10
-4	-15	-9	7.50	10.60
-4	-15	-9	9.80	9.10
-4	-15	-9	19.70	8.00
-4	-15	-9	10.50	7.50

-4	-15	-9	10.00	6.40
-4	-15	-9	1.90	8.20
4	15	9	4.10	3.90
-5	-15	-9	96.99	12.10
-5	-15	-9	95.89	14.50
-5	-15	-9	116.49	15.00
-5	-15	-9	87.29	11.40
-5	-15	-9	87.09	12.50
5	15	9	80.89	9.70
-6	-15	-9	181.18	22.90
-6	-15	-9	180.08	19.40
-6	-15	-9	179.08	16.40
-6	-15	-9	196.68	15.00
-6	-15	-9	140.99	16.90
-6	-15	-9	192.38	17.30
6	15	9	142.29	14.70
-7	-15	-9	86.49	11.20
-7	-15	-9	121.39	13.00
-7	-15	-9	92.29	12.10
-7	-15	-9	88.69	13.20
-7	-15	-9	103.99	14.30
-8	-15	-9	294.17	19.70
-8	-15	-9	229.58	20.20
-8	-15	-9	258.17	23.10
-8	-15	-9	230.88	17.40
-9	-15	-9	197.38	15.50
-9	-15	-9	183.48	15.20
-9	-15	-9	192.18	17.10
-9	-15	-9	183.58	15.80
-9	-15	-9	176.18	16.20
-10	-15	-9	22.70	9.00
-10	-15	-9	-1.80	18.20
-10	-15	-9	19.00	7.40
-10	-15	-9	26.00	7.90
-10	-15	-9	17.40	7.60
8	-16	-9	73.89	12.50
-8	16	9	68.79	17.20
-8	16	9	85.89	11.70
7	-16	-9	183.88	15.60
7	-16	-9	172.88	29.30
-7	16	9	194.28	18.20
6	-16	-9	14.60	12.70
6	-16	-9	7.50	8.80
-6	16	9	18.80	11.70

5	-16	-9	16.10	10.90
5	-16	-9	10.30	8.30
5	-16	-9	5.40	13.20
5	-16	-9	38.70	17.30
4	-16	-9	975.20	48.60
4	-16	-9	1007.20	51.59
4	-16	-9	951.90	49.20
4	-16	-9	951.80	51.29
-4	16	9	1027.70	49.20
3	-16	-9	21.90	10.90
3	-16	-9	7.70	10.80
3	-16	-9	22.60	9.20
-3	16	9	24.20	6.60
2	-16	-9	550.34	29.90
2	-16	-9	552.34	30.20
2	-16	-9	503.95	29.90
2	-16	-9	521.05	30.00
2	-16	-9	493.45	28.70
-2	16	9	495.85	25.50
-2	16	9	448.76	30.00
1	-16	-9	15.70	8.90
1	-16	-9	15.00	11.90
1	-16	-9	22.80	9.00
1	-16	-9	2.90	8.70
1	-16	-9	21.10	8.60
-1	16	9	13.80	4.10
0	-16	-9	4.80	8.40
0	-16	-9	0.20	7.20
0	-16	-9	23.50	8.30
0	-16	-9	9.60	7.50
0	16	9	7.80	3.50
-1	-16	-9	115.99	16.90
-1	-16	-9	133.89	14.20
-1	-16	-9	135.69	13.70
-1	-16	-9	130.69	13.80
-1	-16	-9	99.99	16.50
1	16	9	135.19	8.60
-2	-16	-9	33.40	7.80
-2	-16	-9	35.70	10.90
-2	-16	-9	29.80	7.80
-2	-16	-9	34.40	9.40
-2	-16	-9	31.80	7.90
2	16	9	33.40	5.50
-3	-16	-9	210.08	15.70

-3	-16	-9	203.58	17.10
-3	-16	-9	173.08	19.50
-3	-16	-9	206.28	15.80
-3	-16	-9	213.18	17.10
-3	-16	-9	206.78	16.70
3	16	9	221.98	11.90
-4	-16	-9	482.35	27.00
-4	-16	-9	400.16	29.20
-4	-16	-9	448.06	27.00
-4	-16	-9	472.75	26.10
-4	-16	-9	489.05	25.50
-4	-16	-9	461.35	26.50
4	16	9	460.75	23.10
-5	-16	-9	24.60	7.20
-5	-16	-9	14.50	8.50
-5	-16	-9	25.70	10.40
-5	-16	-9	24.30	9.50
-5	-16	-9	25.60	8.70
-5	-16	-9	31.60	10.20
5	16	9	25.90	6.80
-6	-16	-9	109.69	15.10
-6	-16	-9	136.19	14.00
-6	-16	-9	131.39	13.80
-6	-16	-9	134.09	21.80
-6	-16	-9	165.58	17.10
-6	-16	-9	111.19	15.10
6	16	9	107.79	14.80
-7	-16	-9	165.38	17.30
-7	-16	-9	155.58	15.50
-7	-16	-9	122.19	15.10
-7	-16	-9	155.58	14.50
-7	-16	-9	140.99	14.70
-8	-16	-9	69.29	10.70
-8	-16	-9	63.59	9.50
-8	-16	-9	60.09	9.90
-8	-16	-9	78.19	14.20
8	16	9	40.80	13.30
-9	-16	-9	23.10	9.10
-9	-16	-9	19.20	7.50
-9	-16	-9	20.10	8.20
-9	-16	-9	10.10	8.40
-9	-16	-9	21.80	8.00
-10	-16	-9	117.99	12.40
-10	-16	-9	121.89	12.50

-10	-16	-9	142.69	17.30
-10	-16	-9	101.79	12.50
-10	-16	-9	109.69	14.20
8	-17	-9	130.49	13.60
-8	17	9	121.69	14.50
7	-17	-9	29.40	19.00
7	-17	-9	-5.50	7.70
-7	17	9	4.10	9.00
6	-17	-9	-15.70	12.30
6	-17	-9	-3.00	8.20
-6	17	9	5.60	10.20
5	-17	-9	36.10	19.60
5	-17	-9	7.90	11.80
4	-17	-9	73.19	14.50
4	-17	-9	56.09	10.50
4	-17	-9	40.80	10.10
4	-17	-9	51.69	13.00
-4	17	9	53.59	11.70
3	-17	-9	-13.80	9.20
3	-17	-9	0.90	8.50
3	-17	-9	-3.30	10.30
3	-17	-9	-0.50	11.10
-3	17	9	7.30	8.20
2	-17	-9	140.49	16.10
2	-17	-9	140.69	16.80
2	-17	-9	160.28	17.80
2	-17	-9	155.18	18.90
2	-17	-9	152.38	17.10
-2	17	9	129.29	18.40
-2	17	9	130.89	22.50
-2	17	9	124.29	11.60
1	-17	-9	126.99	15.00
1	-17	-9	130.69	15.90
1	-17	-9	101.59	15.20
1	-17	-9	87.79	15.20
1	-17	-9	100.59	15.10
-1	17	9	115.59	17.10
-1	17	9	105.49	9.40
0	-17	-9	42.80	22.10
0	-17	-9	44.00	8.40
0	-17	-9	54.09	9.20
0	-17	-9	51.49	10.70
0	-17	-9	39.50	8.90
0	17	9	40.80	5.60

-1	-17	-9	250.47	18.30
-1	-17	-9	236.68	18.80
-1	-17	-9	234.78	20.50
-1	-17	-9	237.98	17.70
-1	-17	-9	255.67	18.00
1	17	9	240.08	13.70
-2	-17	-9	377.46	22.10
-2	-17	-9	407.86	23.80
-2	-17	-9	403.46	26.80
-2	-17	-9	362.56	23.30
-2	-17	-9	362.16	23.10
2	17	9	372.06	19.30
-3	-17	-9	30.60	8.40
-3	-17	-9	38.00	8.90
-3	-17	-9	54.19	9.70
-3	-17	-9	30.90	7.60
-3	-17	-9	39.90	8.90
-3	-17	-9	44.20	16.10
3	17	9	52.09	7.30
-4	-17	-9	280.37	23.90
-4	-17	-9	295.77	19.30
-4	-17	-9	284.07	19.50
-4	-17	-9	272.07	18.00
-4	-17	-9	277.97	20.50
-4	-17	-9	265.17	20.20
4	17	9	283.27	16.50
-5	-17	-9	23.20	9.40
-5	-17	-9	28.60	8.30
-5	-17	-9	25.30	7.40
-5	-17	-9	20.50	10.70
-5	-17	-9	49.50	10.60
-5	-17	-9	30.50	9.30
5	17	9	31.80	7.80
-6	-17	-9	47.20	9.20
-6	-17	-9	49.99	10.00
-6	-17	-9	55.49	12.70
-6	-17	-9	32.30	10.20
-6	-17	-9	44.10	9.00
-6	-17	-9	33.30	7.80
6	17	9	38.60	9.90
-7	-17	-9	-0.70	14.20
-7	-17	-9	-10.20	12.00
-7	-17	-9	10.90	8.20
-7	-17	-9	-5.10	15.20

7	17	9	-10.10	11.00
-8	-17	-9	12.70	8.00
-8	-17	-9	20.40	8.10
-8	-17	-9	11.10	7.40
-8	-17	-9	15.90	8.30
-8	-17	-9	-3.40	7.20
-9	-17	-9	202.08	18.30
-9	-17	-9	211.58	14.90
-9	-17	-9	171.58	14.90
-9	-17	-9	189.98	15.90
-9	-17	-9	192.78	15.60
-10	-17	-9	137.49	16.70
-10	-17	-9	73.39	11.30
-10	-17	-9	114.29	11.50
7	-18	-9	42.80	19.30
7	-18	-9	-3.70	7.70
-7	18	9	11.80	9.00
6	-18	-9	18.30	12.80
6	-18	-9	25.20	8.50
-6	18	9	33.80	9.40
5	-18	-9	-1.50	10.70
5	-18	-9	3.20	8.70
5	-18	-9	29.50	19.90
-5	18	9	5.20	9.50
4	-18	-9	112.79	17.10
4	-18	-9	140.29	20.90
4	-18	-9	113.59	17.10
-4	18	9	160.18	22.10
3	-18	-9	1219.38	59.09
3	-18	-9	1177.98	58.09
3	-18	-9	1191.08	59.19
3	-18	-9	1154.58	57.89
-3	18	9	1191.68	56.89
2	-18	-9	23.00	9.80
2	-18	-9	25.10	9.50
2	-18	-9	-0.10	11.70
2	-18	-9	16.80	9.80
-2	18	9	7.80	13.70
-2	18	9	12.40	7.10
-2	18	9	21.30	14.50
1	-18	-9	12.10	14.60
1	-18	-9	0.60	8.50
1	-18	-9	11.60	9.40
1	-18	-9	16.10	8.80

1	-18	-9	3.40	8.30
-1	18	9	17.90	11.30
-1	18	9	6.90	6.00
0	-18	-9	172.98	18.30
0	-18	-9	192.78	16.70
0	-18	-9	199.68	16.30
0	-18	-9	202.58	17.00
0	18	9	194.08	19.00
0	18	9	190.48	12.70
-1	-18	-9	0.30	9.80
-1	-18	-9	8.30	7.90
-1	-18	-9	14.20	7.30
-1	-18	-9	4.30	7.30
1	18	9	0.40	4.70
-2	-18	-9	55.69	10.00
-2	-18	-9	58.19	9.00
-2	-18	-9	50.19	9.50
-2	-18	-9	50.89	8.60
-2	-18	-9	55.69	12.40
2	18	9	48.40	6.20
-3	-18	-9	129.59	15.40
-3	-18	-9	149.79	15.00
-3	-18	-9	148.79	26.00
-3	-18	-9	133.89	13.20
-3	-18	-9	125.99	13.90
3	18	9	128.29	11.00
-4	-18	-9	86.09	14.90
-4	-18	-9	48.30	8.30
-4	-18	-9	78.69	12.30
-4	-18	-9	68.69	9.60
4	18	9	59.19	10.30
-5	-18	-9	56.09	8.20
-5	-18	-9	47.10	9.20
-5	-18	-9	41.30	10.50
-5	-18	-9	76.29	12.70
-5	-18	-9	30.80	9.50
5	18	9	44.30	9.10
-6	-18	-9	9.50	8.30
-6	-18	-9	11.90	8.10
-6	-18	-9	-10.30	8.30
-6	-18	-9	13.90	7.30
-6	-18	-9	25.00	19.50
6	18	9	-2.00	10.20
-7	-18	-9	176.88	17.00

-7	-18	-9	166.08	15.20
-7	-18	-9	193.98	14.70
-7	-18	-9	176.78	15.60
-7	-18	-9	161.18	14.90
-8	-18	-9	116.39	12.70
-8	-18	-9	102.29	13.30
-8	-18	-9	104.09	12.90
-8	-18	-9	102.59	12.40
-9	-18	-9	106.19	11.40
-9	-18	-9	102.39	12.60
-9	-18	-9	81.39	10.70
-9	-18	-9	107.39	11.70
7	-19	-9	40.30	8.50
-7	19	9	28.10	8.50
6	-19	-9	9.10	11.90
6	-19	-9	5.30	8.00
-6	19	9	-2.70	8.50
5	-19	-9	327.47	41.50
5	-19	-9	310.77	20.70
5	-19	-9	317.67	22.70
-5	19	9	314.77	21.90
4	-19	-9	82.19	15.40
4	-19	-9	94.39	15.20
4	-19	-9	61.59	15.10
3	-19	-9	6.60	16.30
3	-19	-9	5.60	9.00
3	-19	-9	-0.80	10.70
3	-19	-9	11.70	13.30
-3	19	9	10.50	11.10
2	-19	-9	118.89	20.60
2	-19	-9	98.09	15.80
2	-19	-9	120.79	17.30
2	-19	-9	88.89	16.10
-2	19	9	135.89	16.80
-2	19	9	106.49	14.60
-2	19	9	154.28	20.70
1	-19	-9	-13.70	15.50
1	-19	-9	17.40	8.50
1	-19	-9	-3.10	8.10
1	-19	-9	15.70	9.00
1	-19	-9	8.00	8.80
-1	19	9	21.70	11.90
-1	19	9	2.50	6.80
0	-19	-9	66.19	10.00

0	-19	-9	94.19	13.80
0	-19	-9	81.99	10.60
0	19	9	89.29	10.30
0	19	9	82.69	12.60
-1	-19	-9	7.30	10.70
-1	-19	-9	13.10	7.70
-1	-19	-9	20.10	7.60
-1	-19	-9	11.40	7.90
1	19	9	10.40	5.50
-2	-19	-9	363.86	22.20
-2	-19	-9	356.36	23.10
-2	-19	-9	373.96	22.60
-2	-19	-9	370.66	23.30
-2	-19	-9	393.36	27.40
2	19	9	365.96	20.00
-3	-19	-9	502.55	29.90
-3	-19	-9	533.35	30.50
-3	-19	-9	553.34	29.30
-3	-19	-9	555.14	37.10
-3	-19	-9	574.24	29.90
3	19	9	576.54	28.10
-4	-19	-9	185.48	17.80
-4	-19	-9	150.28	15.40
-4	-19	-9	185.38	14.50
-4	-19	-9	198.08	15.70
4	19	9	190.88	15.00
-5	-19	-9	23.10	11.70
-5	-19	-9	14.20	8.00
-5	-19	-9	11.80	8.40
-5	-19	-9	11.50	7.50
-5	-19	-9	1.40	6.80
5	19	9	14.70	9.00
-6	-19	-9	84.09	13.20
-6	-19	-9	105.39	12.60
-6	-19	-9	82.59	13.20
-6	-19	-9	78.19	11.90
-6	-19	-9	108.89	12.00
6	19	9	73.99	13.10
-7	-19	-9	92.09	12.10
-7	-19	-9	106.69	11.70
-7	-19	-9	84.29	11.30
-7	-19	-9	98.89	12.40
-8	-19	-9	199.98	14.60
-8	-19	-9	166.68	15.00

-8	-19	-9	209.38	14.30
-8	-19	-9	195.08	14.30
-9	-19	-9	89.59	10.20
-9	-19	-9	70.79	9.20
-9	-19	-9	50.89	9.50
-9	-19	-9	79.29	10.40
6	-20	-9	14.70	8.30
-6	20	9	-11.40	7.90
5	-20	-9	211.38	18.50
5	-20	-9	187.88	16.60
-5	20	9	188.38	16.60
4	-20	-9	615.84	34.30
4	-20	-9	597.24	36.40
4	-20	-9	600.94	32.70
4	-20	-9	598.64	32.70
3	-20	-9	-11.20	11.60
3	-20	-9	0.80	11.60
3	-20	-9	18.40	9.00
3	-20	-9	-14.50	9.90
2	-20	-9	265.27	28.90
2	-20	-9	230.88	25.50
-2	20	9	234.78	26.00
-2	20	9	267.27	20.70
-2	20	9	215.08	24.10
1	-20	-9	0.90	7.80
1	-20	-9	7.50	8.80
1	-20	-9	-0.50	9.40
1	-20	-9	0.80	8.50
-1	20	9	5.70	12.80
-1	20	9	12.80	8.00
-1	20	9	11.10	11.60
0	-20	-9	4.60	8.40
0	-20	-9	19.50	8.30
0	-20	-9	23.70	10.40
0	-20	-9	16.90	8.40
0	20	9	26.20	7.60
0	20	9	52.49	11.90
-1	-20	-9	101.39	13.10
-1	-20	-9	115.09	14.50
-1	-20	-9	118.79	19.00
-1	-20	-9	123.29	13.00
1	20	9	94.89	16.40
1	20	9	108.79	11.80
-2	-20	-9	167.18	15.80

-2	-20	-9	194.68	15.60
-2	-20	-9	180.88	22.90
-2	-20	-9	187.18	14.90
2	20	9	177.88	14.30
-3	-20	-9	186.48	15.10
-3	-20	-9	191.18	15.20
-3	-20	-9	203.68	16.70
-3	-20	-9	220.28	16.90
-3	-20	-9	179.98	29.20
3	20	9	186.28	15.40
-4	-20	-9	200.38	16.70
-4	-20	-9	215.88	28.40
-4	-20	-9	206.88	16.50
-4	-20	-9	275.07	26.40
4	20	9	211.88	17.40
-5	-20	-9	6.30	9.70
-5	-20	-9	13.60	8.40
-5	-20	-9	7.70	7.30
-5	-20	-9	11.40	6.90
5	20	9	7.50	10.50
-6	-20	-9	8.50	11.40
-6	-20	-9	39.60	7.20
-6	-20	-9	30.20	6.90
-6	-20	-9	36.80	8.00
-7	-20	-9	10.60	6.10
-7	-20	-9	17.80	7.40
-7	-20	-9	16.90	6.60
-8	-20	-9	51.89	8.00
-8	-20	-9	71.39	9.70
-8	-20	-9	76.39	10.90
5	-21	-9	-0.90	9.40
5	-21	-9	-4.20	9.80
-5	21	9	20.40	7.70
4	-21	-9	57.89	10.00
4	-21	-9	52.09	11.40
4	-21	-9	49.99	15.10
3	-21	-9	18.40	12.50
3	-21	-9	11.40	11.60
3	-21	-9	-14.70	8.10
3	-21	-9	-4.30	10.10
2	-21	-9	3.40	10.20
2	-21	-9	15.90	9.40
2	-21	-9	-7.50	9.60
2	-21	-9	-2.00	16.50

-2	21	9	18.00	14.20
1	-21	-9	194.18	17.50
1	-21	-9	198.28	18.10
1	-21	-9	200.68	18.80
-1	21	9	187.68	18.80
-1	21	9	160.28	20.20
-1	21	9	173.08	21.10
0	-21	-9	150.48	22.00
0	-21	-9	127.59	17.70
0	-21	-9	115.09	15.40
0	21	9	129.89	14.60
0	21	9	112.09	17.70
-1	-21	-9	465.55	32.50
-1	-21	-9	555.34	39.10
1	21	9	477.35	28.80
1	21	9	453.65	26.40
-2	-21	-9	249.48	24.10
2	21	9	268.57	18.40
-3	-21	-9	141.89	15.90
-3	-21	-9	116.29	13.30
-3	-21	-9	103.99	18.60
-3	-21	-9	134.19	14.00
3	21	9	114.19	15.40
-4	-21	-9	7.70	8.00
-4	-21	-9	1.70	8.70
-4	-21	-9	7.80	6.40
-4	-21	-9	8.70	6.70
-5	-21	-9	198.08	14.40
-5	-21	-9	209.38	15.30
-5	-21	-9	195.38	14.40
-5	-21	-9	219.08	17.90
-6	-21	-9	0.70	5.90
-6	-21	-9	22.90	7.50
-6	-21	-9	9.70	6.40
-6	-21	-9	12.80	11.60
-7	-21	-9	67.69	9.20
-7	-21	-9	75.79	10.00
-7	-21	-9	65.19	10.50
4	-22	-9	194.88	23.30
4	-22	-9	241.08	17.30
4	-22	-9	247.78	25.90
-4	22	9	219.58	16.40
3	-22	-9	598.84	35.80
3	-22	-9	594.44	31.60

3	-22	-9	586.94	32.50
3	-22	-9	608.64	33.30
2	-22	-9	15.30	8.50
2	-22	-9	23.00	10.20
2	-22	-9	1.30	9.00
1	-22	-9	21.10	8.80
1	-22	-9	22.80	8.80
1	-22	-9	5.60	8.70
-1	22	9	35.90	17.00
-1	22	9	17.20	12.60
0	-22	-9	336.07	21.20
0	-22	-9	345.77	23.70
0	-22	-9	362.46	21.10
0	-22	-9	342.27	21.60
0	22	9	319.27	23.90
-1	-22	-9	16.30	8.00
-1	-22	-9	30.70	8.00
-1	-22	-9	17.90	10.80
-1	-22	-9	17.40	8.30
1	22	9	18.10	11.30
-2	-22	-9	37.00	8.10
-2	-22	-9	36.00	7.80
-2	-22	-9	13.50	12.90
-2	-22	-9	21.00	8.50
2	22	9	45.60	11.50
-3	-22	-9	93.69	11.50
-3	-22	-9	97.79	13.30
-3	-22	-9	101.59	11.30
-4	-22	-9	191.78	14.00
-4	-22	-9	197.18	14.20
-4	-22	-9	203.68	16.70
-5	-22	-9	114.59	15.40
-5	-22	-9	142.39	11.60
-5	-22	-9	113.89	11.20
-5	-22	-9	126.99	12.50
-6	-22	-9	25.30	6.10
-6	-22	-9	24.40	6.50
-6	-22	-9	24.70	7.40
-6	-22	-9	56.49	14.70
-7	-22	-9	220.78	14.10
-7	-22	-9	220.48	14.20
-7	-22	-9	208.38	13.60
3	-23	-9	27.00	16.40
3	-23	-9	42.80	19.40

3	-23	-9	23.20	8.10
2	-23	-9	144.89	14.80
2	-23	-9	158.68	15.80
2	-23	-9	161.78	16.90
1	-23	-9	-2.10	8.50
1	-23	-9	2.00	8.50
1	-23	-9	15.30	9.20
-1	23	9	6.00	12.90
0	-23	-9	-8.30	7.50
0	-23	-9	6.80	7.30
0	-23	-9	0.90	9.60
0	23	9	14.30	11.00
0	23	9	-1.30	13.00
-1	-23	-9	-3.60	10.50
-1	-23	-9	11.20	7.40
-1	-23	-9	-8.70	6.80
-1	-23	-9	-3.30	6.20
1	23	9	25.30	14.10
-2	-23	-9	281.97	17.20
-2	-23	-9	283.07	23.70
-2	-23	-9	296.07	17.60
-2	-23	-9	280.77	18.40
2	23	9	226.78	23.70
-3	-23	-9	62.29	9.60
-3	-23	-9	83.09	10.70
-3	-23	-9	96.59	10.50
3	23	9	92.69	15.20
-4	-23	-9	70.49	12.80
-4	-23	-9	81.59	10.30
-4	-23	-9	66.29	10.10
-5	-23	-9	122.59	10.80
-5	-23	-9	152.08	14.90
-5	-23	-9	136.99	11.00
-6	-23	-9	304.17	16.60
-6	-23	-9	254.77	22.80
-6	-23	-9	289.27	16.10
2	-24	-9	63.59	11.00
2	-24	-9	55.79	14.30
2	-24	-9	40.50	10.20
1	-24	-9	51.79	9.70
1	-24	-9	41.70	8.90
1	-24	-9	55.79	9.50
0	-24	-9	26.20	9.40
0	-24	-9	26.30	7.90

0	24	9	66.99	17.40
0	24	9	27.00	19.60
-1	-24	-9	186.68	14.20
-1	-24	-9	176.88	13.70
-1	-24	-9	193.88	14.20
-1	-24	-9	187.18	18.50
1	24	9	183.08	19.30
1	24	9	214.18	17.60
-2	-24	-9	26.20	7.50
-2	-24	-9	36.10	7.50
-2	-24	-9	30.60	6.60
2	24	9	24.50	11.70
-3	-24	-9	225.98	13.70
-3	-24	-9	210.18	13.90
-3	-24	-9	184.88	15.00
3	24	9	171.58	17.80
-4	-24	-9	140.49	12.40
-4	-24	-9	128.19	10.80
-4	-24	-9	119.39	11.30
4	24	9	142.79	16.20
-5	-24	-9	2.90	9.40
5	24	9	8.90	12.20
0	-25	-9	64.89	9.20
0	-25	-9	38.00	12.20
0	-25	-9	48.50	9.90
0	25	9	19.80	20.60
-1	-25	-9	191.28	20.90
-1	-25	-9	220.18	14.90
-1	-25	-9	216.78	20.20
1	25	9	212.68	19.10
-2	-25	-9	60.09	9.30
-2	-25	-9	81.09	11.70
-2	-25	-9	63.89	10.50
2	25	9	97.19	14.50
1	20	-10	2.20	12.90
1	20	-10	-3.90	9.20
-1	-20	10	18.10	9.50
-1	-20	10	-0.40	8.10
0	20	-10	98.69	13.30
0	20	-10	76.29	12.40
0	-20	10	97.59	15.00
0	-20	10	99.69	13.10
-1	20	-10	194.68	15.70
-1	20	-10	183.78	17.30

1	-20	10	155.78	17.60
3	19	-10	17.40	7.40
-3	-19	10	19.90	6.20
2	19	-10	16.60	20.40
2	19	-10	4.40	7.40
-2	-19	10	-1.60	11.40
-2	-19	10	12.50	6.70
1	19	-10	229.38	23.60
1	19	-10	317.87	19.40
1	19	-10	343.77	27.70
-1	-19	10	270.97	19.30
-1	-19	10	312.77	18.60
0	19	-10	175.48	20.30
0	19	-10	198.48	16.20
0	19	-10	177.28	16.50
0	-19	10	204.38	15.00
0	-19	10	156.68	15.60
-1	19	-10	-1.70	9.30
-1	19	-10	15.10	12.50
-1	19	-10	-1.00	9.10
1	-19	10	7.50	7.00
-2	19	-10	231.58	22.40
-2	19	-10	173.08	15.20
-2	19	-10	153.68	17.50
2	-19	10	185.78	13.60
-3	19	-10	-0.60	10.10
-3	19	-10	-15.30	14.50
-3	19	-10	10.90	10.90
3	-19	10	-3.20	6.90
5	18	-10	22.20	7.60
-5	-18	10	21.50	5.90
4	18	-10	73.19	10.90
-4	-18	10	73.29	9.40
3	18	-10	198.38	15.60
-3	-18	10	227.48	14.60
2	18	-10	37.60	8.00
2	18	-10	20.50	11.60
-2	-18	10	35.50	7.30
-2	-18	10	20.80	10.10
1	18	-10	119.09	18.70
1	18	-10	108.69	13.40
1	18	-10	96.39	15.80
-1	-18	10	102.49	14.20
-1	-18	10	105.29	13.00

0	18	-10	189.18	19.90
0	18	-10	207.38	17.50
0	18	-10	232.88	17.90
0	-18	10	209.18	17.00
0	-18	10	256.57	17.00
-1	18	-10	266.07	19.80
-1	18	-10	238.38	21.40
-1	18	-10	270.67	18.60
1	-18	10	251.37	17.80
1	-18	10	268.47	21.30
-2	18	-10	6.90	10.20
-2	18	-10	18.20	8.70
-2	18	-10	2.80	10.30
2	-18	10	-0.70	7.50
-3	18	-10	-8.20	11.80
-3	18	-10	11.30	8.30
-3	18	-10	-15.30	9.70
3	-18	10	2.40	7.00
-4	18	-10	22.30	10.80
-4	18	-10	-13.50	21.60
-4	18	-10	22.80	8.40
4	-18	10	13.80	7.30
-5	18	-10	81.19	11.90
6	17	-10	31.60	10.00
-6	-17	10	23.60	12.70
5	17	-10	103.19	12.10
-5	-17	10	96.39	9.80
4	17	-10	132.79	13.10
-4	-17	10	122.79	11.50
3	17	-10	-1.50	7.10
-3	-17	10	-6.20	5.90
2	17	-10	24.30	7.70
2	17	-10	30.10	16.60
2	17	-10	34.30	11.00
-2	-17	10	40.20	7.50
-2	-17	10	17.10	9.50
1	17	-10	54.49	9.50
1	17	-10	57.89	10.30
1	17	-10	49.00	12.00
-1	-17	10	34.20	9.10
-1	-17	10	33.80	8.30
0	17	-10	88.99	14.50
0	17	-10	115.69	15.40
0	17	-10	89.89	15.40

0	-17	10	98.89	13.90
0	-17	10	87.99	14.20
-1	17	-10	-0.60	8.70
-1	17	-10	-6.20	9.30
-1	17	-10	4.30	8.80
1	-17	10	8.90	10.60
1	-17	10	0.50	7.50
-2	17	-10	21.50	9.10
-2	17	-10	31.90	10.30
-2	17	-10	20.30	9.80
2	-17	10	25.30	7.90
-3	17	-10	63.69	10.00
-3	17	-10	68.39	11.20
-3	17	-10	81.49	12.60
3	-17	10	49.40	9.10
-4	17	-10	3.20	8.40
-4	17	-10	28.60	13.20
-4	17	-10	3.30	9.90
4	-17	10	0.10	7.60
-5	17	-10	0.50	8.00
-5	17	-10	-8.10	9.40
5	-17	10	12.80	8.30
5	-17	10	40.90	12.00
-6	17	-10	339.37	22.30
-6	17	-10	440.26	25.70
6	16	-10	214.78	15.40
-6	-16	10	217.18	16.20
-6	-16	10	196.38	13.90
5	16	-10	45.80	8.60
-5	-16	10	51.29	7.50
4	16	-10	29.10	7.80
-4	-16	10	55.19	9.90
3	16	-10	-9.60	18.30
3	16	-10	-5.80	7.70
-3	-16	10	16.70	12.00
-3	-16	10	4.60	6.60
2	16	-10	272.57	21.50
2	16	-10	261.27	19.50
2	16	-10	274.87	21.80
-2	-16	10	280.67	18.20
-2	-16	10	276.77	20.70
1	16	-10	10.90	9.50
1	16	-10	-1.40	11.00
1	16	-10	1.30	9.60

-1	-16	10	-6.10	9.00
-1	-16	10	1.60	8.10
0	16	-10	21.10	10.20
0	16	-10	44.50	15.00
0	16	-10	47.40	10.20
0	-16	10	38.30	12.30
0	-16	10	38.90	10.10
-1	16	-10	413.96	25.20
-1	16	-10	405.66	26.50
-1	16	-10	375.96	24.20
1	-16	10	384.86	26.00
1	-16	10	387.76	26.60
-2	16	-10	200.08	17.20
-2	16	-10	167.98	16.60
-2	16	-10	182.18	19.80
2	-16	10	211.18	17.20
-3	16	-10	49.20	11.20
-3	16	-10	34.00	9.80
-3	16	-10	26.10	9.70
3	-16	10	35.20	9.00
-4	16	-10	72.49	13.20
-4	16	-10	59.39	10.80
-4	16	-10	67.69	10.40
4	-16	10	47.80	10.90
4	-16	10	57.79	9.60
-5	16	-10	-11.20	8.10
-5	16	-10	13.30	14.40
5	-16	10	3.20	8.80
5	-16	10	1.90	8.80
-6	16	-10	-3.60	9.80
-6	16	-10	2.40	8.40
6	-16	10	4.90	8.40
6	-16	10	5.60	10.30
7	15	-10	76.39	15.90
7	15	-10	120.89	12.90
-7	-15	10	107.69	11.50
6	15	-10	65.39	9.60
6	15	-10	38.00	14.90
-6	-15	10	47.80	8.20
5	15	-10	115.09	13.80
5	15	-10	122.19	17.40
-5	-15	10	142.49	12.60
-5	-15	10	122.99	14.50
4	15	-10	52.89	9.10

-4	-15	10	61.79	11.10
3	15	-10	42.90	14.50
-3	-15	10	29.80	11.40
2	15	-10	21.60	8.50
2	15	-10	-6.40	9.50
2	15	-10	7.20	15.30
-2	-15	10	8.30	7.20
-2	-15	10	-0.30	9.30
1	15	-10	517.15	29.60
1	15	-10	538.05	29.70
1	15	-10	565.54	31.10
-1	-15	10	531.25	30.40
-1	-15	10	547.25	29.30
0	15	-10	191.28	17.30
0	15	-10	199.98	16.80
0	15	-10	203.68	18.00
0	-15	10	218.58	17.20
0	-15	10	191.48	17.70
-1	15	-10	-2.60	8.10
-1	15	-10	-0.80	8.60
-1	15	-10	1.60	8.60
1	-15	10	2.60	7.60
-2	15	-10	81.29	11.10
-2	15	-10	79.29	11.80
-2	15	-10	85.59	11.70
-3	15	-10	23.20	17.70
-3	15	-10	33.60	9.40
-3	15	-10	5.50	15.40
3	-15	10	29.70	18.10
3	-15	10	32.70	12.70
-4	15	-10	4.10	9.00
-4	15	-10	24.40	9.40
4	-15	10	31.40	10.10
4	-15	10	20.90	8.70
-5	15	-10	231.88	18.30
-5	15	-10	199.18	23.40
-5	15	-10	249.08	18.30
5	-15	10	203.08	18.10
5	-15	10	277.27	19.60
-6	15	-10	16.70	18.80
-6	15	-10	32.60	9.30
-6	15	-10	19.80	12.80
6	-15	10	27.20	8.00
6	-15	10	9.80	10.00

-7	15	-10	274.87	18.10
-7	15	-10	267.47	19.70
7	-15	10	291.57	17.60
7	-15	10	258.97	25.00
8	14	-10	1.20	8.90
8	14	-10	8.50	11.50
-8	-14	10	11.40	8.20
-8	-14	10	-11.20	5.70
7	14	-10	-9.50	11.70
7	14	-10	25.10	8.50
-7	-14	10	16.70	6.80
-7	-14	10	18.10	6.70
6	14	-10	96.89	14.10
6	14	-10	126.89	13.80
-6	-14	10	109.69	12.00
-6	-14	10	95.89	12.90
5	14	-10	152.68	15.30
5	14	-10	163.98	20.80
-5	-14	10	120.89	14.90
-5	-14	10	164.98	13.60
4	14	-10	121.89	15.50
4	14	-10	157.48	20.90
-4	-14	10	117.19	13.60
-4	-14	10	104.29	18.00
3	14	-10	162.48	18.20
3	14	-10	171.98	16.60
-3	-14	10	167.38	20.00
-3	-14	10	191.08	15.10
2	14	-10	38.20	10.60
2	14	-10	42.40	9.40
-2	-14	10	50.59	9.60
-2	-14	10	43.00	7.80
1	14	-10	97.29	13.60
1	14	-10	99.89	12.20
1	14	-10	71.99	10.40
-1	-14	10	90.19	14.80
-1	-14	10	72.69	12.90
0	14	-10	310.17	21.00
0	14	-10	331.97	22.50
0	14	-10	340.07	21.90
0	-14	10	283.97	20.30
0	-14	10	354.96	21.60
-1	14	-10	53.69	9.90
-1	14	-10	36.60	9.20

-1	14	-10	56.59	9.80
1	-14	10	71.69	10.10
1	-14	10	52.69	9.90
-2	14	-10	149.29	16.60
-2	14	-10	171.18	17.30
-2	14	-10	171.48	17.50
2	-14	10	198.18	23.10
2	-14	10	179.48	22.50
2	-14	10	177.68	16.20
-3	14	-10	-9.00	8.60
-3	14	-10	8.10	8.60
-3	14	-10	0.70	9.80
3	-14	10	-2.20	10.30
3	-14	10	12.50	8.00
-4	14	-10	65.59	10.80
-4	14	-10	87.59	19.20
-4	14	-10	75.39	12.00
4	-14	10	59.59	10.50
-5	14	-10	166.38	17.40
-5	14	-10	143.79	16.40
-5	14	-10	183.98	22.90
5	-14	10	181.28	17.50
5	-14	10	158.28	17.70
-6	14	-10	16.40	15.10
-6	14	-10	23.60	9.70
-6	14	-10	22.20	9.40
6	-14	10	29.20	10.60
6	-14	10	17.30	8.40
-7	14	-10	24.90	8.70
-7	14	-10	17.20	10.20
7	-14	10	11.30	18.80
7	-14	10	31.30	8.00
-8	14	-10	129.99	12.90
-8	14	-10	122.39	14.50
8	-14	10	155.88	13.10
8	13	-10	201.08	18.50
8	13	-10	212.68	16.90
-8	-13	10	218.38	14.10
-8	-13	10	171.78	22.60
7	13	-10	23.10	9.30
7	13	-10	13.60	10.60
-7	-13	10	2.90	7.00
-7	-13	10	23.60	6.90
6	13	-10	42.70	12.30

6	13	-10	45.50	9.70
-6	-13	10	46.90	8.50
-6	-13	10	52.99	8.70
5	13	-10	256.67	22.80
5	13	-10	215.78	32.20
-5	-13	10	230.08	21.00
-5	-13	10	264.17	20.40
4	13	-10	336.77	21.60
4	13	-10	291.97	24.20
-4	-13	10	335.37	20.40
-4	-13	10	300.07	21.80
3	13	-10	-0.80	7.80
3	13	-10	-10.30	12.10
3	13	-10	-3.80	18.00
-3	-13	10	7.10	9.90
-3	-13	10	8.60	7.00
2	13	-10	146.39	16.10
2	13	-10	148.79	22.10
-2	-13	10	149.29	14.40
-2	-13	10	169.78	16.70
1	13	-10	37.20	8.90
1	13	-10	37.80	9.30
-1	-13	10	49.30	9.20
-1	-13	10	44.40	8.60
0	13	-10	223.38	19.50
0	13	-10	232.18	17.70
0	13	-10	241.68	18.30
0	-13	10	242.08	17.80
0	-13	10	206.98	17.60
-1	13	-10	15.40	9.20
-1	13	-10	9.60	8.40
-1	13	-10	8.00	8.40
1	-13	10	5.10	7.90
1	-13	10	-8.90	8.40
-2	13	-10	77.09	11.50
-2	13	-10	58.99	11.40
2	-13	10	76.59	13.50
2	-13	10	59.99	11.60
2	-13	10	59.49	12.40
-3	13	-10	67.69	11.00
-3	13	-10	82.09	11.10
-3	13	-10	83.99	12.50
3	-13	10	58.79	9.40
3	-13	10	99.69	15.90

-4	13	-10	11.70	9.40
-4	13	-10	17.90	8.80
-4	13	-10	13.80	11.40
4	-13	10	9.30	8.10
4	-13	10	17.90	8.60
-5	13	-10	163.68	17.60
-5	13	-10	189.98	33.60
-5	13	-10	189.98	18.70
5	-13	10	191.58	21.00
5	-13	10	174.38	19.80
-6	13	-10	644.64	39.60
-6	13	-10	652.33	35.60
-6	13	-10	650.23	35.80
6	-13	10	666.13	34.80
6	-13	10	679.23	36.20
-7	13	-10	29.20	9.40
-7	13	-10	37.20	10.90
-7	13	-10	8.80	16.90
-8	13	-10	166.48	16.00
-8	13	-10	183.28	17.60
8	-13	10	184.38	14.40
9	12	-10	29.00	10.30
8	12	-10	12.90	10.70
8	12	-10	-9.70	9.00
-8	-12	10	3.20	8.20
-8	-12	10	4.40	6.20
7	12	-10	176.08	17.10
7	12	-10	195.78	19.60
-7	-12	10	192.98	15.20
-7	-12	10	195.38	15.60
6	12	-10	270.37	21.10
6	12	-10	264.87	22.60
-6	-12	10	265.97	19.30
-6	-12	10	273.17	19.00
5	12	-10	35.60	11.50
5	12	-10	52.29	10.00
-5	-12	10	62.59	9.00
-5	-12	10	43.10	8.80
4	12	-10	-0.50	10.80
4	12	-10	-2.00	8.00
-4	-12	10	-1.60	6.20
-4	-12	10	10.40	8.80
3	12	-10	134.69	15.10
3	12	-10	104.79	18.10

-3	-12	10	141.29	17.40
-3	-12	10	92.09	12.90
-3	-12	10	103.59	17.40
2	12	-10	83.79	11.10
2	12	-10	78.49	10.50
2	12	-10	84.09	13.90
-2	-12	10	87.69	11.10
-2	-12	10	70.99	12.80
1	12	-10	100.19	14.30
1	12	-10	112.09	21.00
1	12	-10	97.59	14.20
-1	-12	10	115.59	13.70
-1	-12	10	110.49	15.10
0	12	-10	7.30	9.30
0	12	-10	7.80	7.50
0	12	-10	-7.60	7.40
0	-12	10	-11.70	7.00
0	-12	10	6.10	7.20
-1	12	-10	1403.66	67.29
-1	12	-10	1372.86	66.99
-1	12	-10	1428.46	66.59
1	-12	10	1443.16	66.99
1	-12	10	1363.06	66.49
-2	12	-10	192.28	18.60
-2	12	-10	189.48	16.10
-2	12	-10	173.38	16.90
2	-12	10	203.68	16.30
2	-12	10	173.18	20.80
-3	12	-10	16.00	10.80
-3	12	-10	28.00	9.00
-3	12	-10	26.20	9.30
3	-12	10	18.20	8.00
-4	12	-10	123.19	15.00
-4	12	-10	109.99	16.60
-4	12	-10	98.29	15.50
4	-12	10	95.99	15.30
-5	12	-10	19.90	9.50
-5	12	-10	9.60	12.80
-5	12	-10	32.80	10.40
5	-12	10	9.30	8.70
5	-12	10	9.60	8.40
-6	12	-10	-15.30	16.50
-6	12	-10	-2.30	9.50
-6	12	-10	7.40	16.10

6	-12	10	-7.40	12.60
-7	12	-10	74.49	11.80
-7	12	-10	87.79	17.30
-7	12	-10	76.29	12.30
7	-12	10	82.59	10.80
7	-12	10	84.29	15.80
-8	12	-10	27.50	9.90
8	-12	10	18.10	7.70
-9	12	-10	40.00	9.20
-9	12	-10	27.50	11.60
9	-12	10	16.90	7.40
9	11	-10	49.90	10.80
9	11	-10	33.70	14.40
-9	-11	10	55.89	9.30
8	11	-10	11.60	10.50
8	11	-10	9.00	10.00
-8	-11	10	4.60	6.50
-8	-11	10	9.20	8.60
7	11	-10	149.59	18.90
7	11	-10	131.79	16.30
-7	-11	10	123.89	13.70
-7	-11	10	120.59	14.50
6	11	-10	179.78	18.40
6	11	-10	200.98	21.00
-6	-11	10	205.88	16.10
-6	-11	10	188.68	16.40
5	11	-10	21.70	8.60
5	11	-10	15.00	10.80
-5	-11	10	19.90	7.50
-5	-11	10	2.00	7.60
4	11	-10	202.38	18.00
4	11	-10	197.58	20.20
-4	-11	10	177.38	15.80
-4	-11	10	217.88	17.60
3	11	-10	87.79	14.00
-3	-11	10	80.19	11.70
-3	-11	10	96.29	12.10
2	11	-10	-2.10	7.50
2	11	-10	8.20	10.40
2	11	-10	12.70	9.30
-2	-11	10	-5.40	8.10
-2	-11	10	3.20	6.30
1	11	-10	900.81	44.80
1	11	-10	921.81	45.40

1	11	-10	892.61	47.00
-1	-11	10	910.81	45.20
-1	-11	10	915.81	44.80
0	11	-10	182.38	15.40
0	11	-10	177.58	18.30
0	11	-10	212.78	16.70
0	-11	10	198.68	16.50
0	-11	10	161.58	15.90
-1	11	-10	13.10	7.00
-1	11	-10	4.00	8.70
-1	11	-10	0.60	7.60
1	-11	10	-5.40	8.20
1	-11	10	10.50	7.40
-2	11	-10	243.68	18.00
-2	11	-10	233.88	19.30
-2	11	-10	269.57	20.50
2	-11	10	262.57	21.90
2	-11	10	257.57	18.90
-3	11	-10	10.40	7.60
-3	11	-10	0.10	9.70
-3	11	-10	4.10	8.30
3	-11	10	-3.30	7.50
-4	11	-10	198.48	17.60
-4	11	-10	240.48	22.20
-4	11	-10	191.98	18.70
4	-11	10	164.08	17.90
-5	11	-10	183.98	19.20
-5	11	-10	183.48	24.80
-5	11	-10	183.18	18.40
5	-11	10	217.98	19.00
-6	11	-10	224.18	19.50
-6	11	-10	300.87	27.30
-6	11	-10	263.07	21.20
6	-11	10	242.38	19.60
6	-11	10	218.28	21.50
-7	11	-10	572.94	38.80
-7	11	-10	575.14	33.40
-7	11	-10	564.24	34.10
7	-11	10	659.33	33.00
7	-11	10	565.84	37.70
-8	11	-10	-1.40	10.30
-8	11	-10	19.50	16.80
-8	11	-10	-5.40	9.50
8	-11	10	-2.20	7.40

-9	11	-10	12.60	11.00
-9	11	-10	11.60	9.10
9	-11	10	8.30	7.40
9	10	-10	68.09	11.40
9	10	-10	50.39	18.80
-9	-10	10	47.20	7.40
8	10	-10	13.20	10.30
8	10	-10	16.30	10.60
-8	-10	10	-6.00	7.90
-8	-10	10	4.60	6.40
7	10	-10	6.00	12.70
7	10	-10	8.40	10.20
-7	-10	10	-6.90	8.10
-7	-10	10	9.50	7.60
6	10	-10	226.28	21.30
6	10	-10	223.58	19.70
-6	-10	10	232.88	16.50
-6	-10	10	199.18	17.00
5	10	-10	616.54	34.40
5	10	-10	574.44	32.40
-5	-10	10	571.04	30.60
-5	-10	10	563.94	30.90
4	10	-10	16.10	8.60
4	10	-10	7.70	10.30
-4	-10	10	14.40	7.60
-4	-10	10	9.40	6.60
3	10	-10	2.60	8.00
3	10	-10	-7.00	9.60
-3	-10	10	4.10	9.50
-3	-10	10	10.00	5.90
-3	-10	10	5.90	9.00
2	10	-10	11.00	7.50
2	10	-10	-1.80	9.60
2	10	-10	-4.10	8.80
-2	-10	10	3.60	7.80
-2	-10	10	3.50	6.40
1	10	-10	12.70	7.80
1	10	-10	12.50	7.90
1	10	-10	-0.50	10.00
-1	-10	10	3.80	7.20
-1	-10	10	7.10	6.50
0	10	-10	1720.43	81.09
0	10	-10	1638.04	81.59
0	10	-10	1768.02	80.19

0	10	-10	1793.42	81.19
0	-10	10	1750.82	80.99
0	-10	10	1683.83	83.09
-1	10	-10	20.30	13.40
-1	10	-10	24.20	8.60
-1	10	-10	13.50	10.10
-1	10	-10	15.50	6.90
1	-10	10	10.10	8.10
1	-10	10	1.60	6.30
-2	10	-10	433.76	23.50
-2	10	-10	422.96	25.60
-2	10	-10	395.96	25.60
2	-10	10	410.36	24.20
2	-10	10	362.56	26.60
-3	10	-10	7.10	7.80
-3	10	-10	4.20	8.60
-3	10	-10	-4.80	10.10
3	-10	10	-2.70	7.40
-4	10	-10	7.10	11.70
-4	10	-10	15.40	8.10
-4	10	-10	17.60	9.50
4	-10	10	4.10	8.40
-5	10	-10	104.89	17.10
-5	10	-10	100.99	15.90
-5	10	-10	153.78	22.90
5	-10	10	111.89	16.20
-6	10	-10	84.29	16.40
-6	10	-10	98.79	16.90
-6	10	-10	85.89	12.80
6	-10	10	102.49	14.60
6	-10	10	65.89	11.90
-7	10	-10	116.29	17.90
-7	10	-10	152.88	34.70
-7	10	-10	76.39	12.70
7	-10	10	110.59	15.80
7	-10	10	103.69	20.10
-8	10	-10	121.49	17.60
-8	10	-10	112.89	14.50
-8	10	-10	103.39	17.90
8	-10	10	129.89	15.50
-9	10	-10	1.50	9.10
-9	10	-10	5.60	11.60
9	-10	10	-3.10	7.70
-10	10	-10	53.19	13.40

-10	10	-10	55.69	11.20
10	9	-10	51.99	12.90
9	9	-10	103.79	15.30
-9	-9	10	65.09	8.30
8	9	-10	210.88	20.00
8	9	-10	198.88	20.60
-8	-9	10	203.28	17.90
-8	-9	10	198.28	15.20
7	9	-10	-8.20	10.30
7	9	-10	-0.40	10.70
-7	-9	10	5.40	8.20
-7	-9	10	21.60	7.20
6	9	-10	6.70	10.90
-6	-9	10	20.90	6.90
-6	-9	10	5.80	7.00
5	9	-10	928.01	48.90
-5	-9	10	925.51	45.60
-5	-9	10	958.80	46.30
4	9	-10	389.76	26.00
4	9	-10	389.86	23.80
-4	-9	10	362.16	22.40
-4	-9	10	388.66	22.40
3	9	-10	66.39	11.60
3	9	-10	74.29	13.60
-3	-9	10	68.19	11.80
-3	-9	10	52.49	9.30
-3	-9	10	53.59	8.20
2	9	-10	105.59	17.20
2	9	-10	103.79	15.20
1	9	-10	18.80	10.00
1	9	-10	6.20	7.90
1	9	-10	5.70	7.50
-1	-9	10	0.70	5.80
0	9	-10	613.94	30.80
0	9	-10	499.75	32.80
0	9	-10	576.74	29.60
0	-9	10	563.84	30.00
-1	9	-10	17.00	10.50
-1	9	-10	3.10	6.30
-1	9	-10	7.60	8.10
-1	9	-10	-3.80	11.80
1	-9	10	19.90	8.30
-2	9	-10	14.20	9.50
-2	9	-10	-1.90	13.30

-2	9	-10	18.20	7.00
-2	9	-10	13.70	8.90
2	-9	10	8.60	9.80
2	-9	10	14.10	8.60
2	-9	10	29.00	11.40
-3	9	-10	173.88	26.80
-3	9	-10	173.78	19.40
-3	9	-10	176.38	14.60
3	-9	10	169.68	18.80
3	-9	10	159.08	15.80
-4	9	-10	2.50	7.00
-4	9	-10	8.70	8.20
-4	9	-10	-2.60	11.10
4	-9	10	-2.90	7.20
-5	9	-10	278.87	27.10
-5	9	-10	214.18	25.00
-5	9	-10	265.37	20.90
-5	9	-10	261.87	19.10
5	-9	10	219.68	19.50
-6	9	-10	766.32	40.00
-6	9	-10	706.03	44.30
-6	9	-10	739.03	39.90
-6	9	-10	676.13	41.40
6	-9	10	729.33	39.50
-7	9	-10	61.09	12.10
-7	9	-10	59.99	11.60
-7	9	-10	75.29	17.10
7	-9	10	40.20	9.40
-8	9	-10	28.20	11.20
-8	9	-10	40.40	12.70
8	-9	10	25.80	9.40
-9	9	-10	128.79	16.60
-9	9	-10	100.69	19.20
9	-9	10	101.69	14.20
-10	9	-10	8.30	13.30
-10	9	-10	17.60	9.60
10	-9	10	23.90	11.10
10	-9	10	41.90	12.60
10	8	-10	161.38	16.30
-10	-8	10	162.28	12.00
9	8	-10	22.50	10.40
-9	-8	10	11.00	5.90
8	8	-10	11.20	10.50
8	8	-10	19.40	11.70

-8	-8	10	9.40	9.50
-8	-8	10	3.50	6.70
7	8	-10	438.86	29.90
-7	-8	10	466.35	25.60
-7	-8	10	478.25	27.00
6	8	-10	369.36	26.40
-6	-8	10	357.06	22.70
-6	-8	10	386.16	22.00
5	8	-10	29.50	11.50
-5	-8	10	42.10	8.00
-5	-8	10	35.40	7.80
4	8	-10	12.90	10.20
-4	-8	10	11.10	7.10
-4	-8	10	8.00	6.40
3	8	-10	51.09	11.40
3	8	-10	68.19	10.10
-3	-8	10	53.39	8.90
-3	-8	10	64.09	11.30
2	8	-10	114.19	13.80
2	8	-10	102.79	17.00
-2	-8	10	110.29	11.50
1	8	-10	140.69	17.80
1	8	-10	107.19	13.70
1	8	-10	125.39	14.10
-1	-8	10	130.09	12.30
0	8	-10	192.98	15.70
0	8	-10	228.28	20.60
0	8	-10	192.58	18.50
0	-8	10	190.78	14.90
0	-8	10	181.68	15.30
-1	8	-10	1248.08	58.99
-1	8	-10	1268.57	60.29
-1	8	-10	1325.57	63.19
-1	8	-10	1241.38	60.69
-1	8	-10	1215.28	61.39
1	-8	10	1228.78	58.49
1	-8	10	1242.98	59.69
-2	8	-10	65.09	10.30
-2	8	-10	54.59	11.40
-2	8	-10	86.79	14.40
-2	8	-10	55.99	10.00
-2	8	-10	61.59	10.50
2	-8	10	51.19	8.80
-3	8	-10	8.10	5.80

-3	8	-10	10.30	13.90
-3	8	-10	-5.20	9.20
-3	8	-10	-1.10	8.10
3	-8	10	5.10	8.30
3	-8	10	-1.00	6.00
-4	8	-10	141.09	13.80
-4	8	-10	184.38	21.30
-4	8	-10	134.39	15.60
-4	8	-10	189.18	31.30
-4	8	-10	144.99	22.80
4	-8	10	145.59	15.90
4	-8	10	140.19	18.00
-5	8	-10	24.00	12.80
-5	8	-10	13.00	11.70
-5	8	-10	7.00	7.50
-5	8	-10	3.10	15.10
-5	8	-10	27.80	9.20
5	-8	10	9.30	8.90
5	-8	10	16.20	12.00
-6	8	-10	8.50	9.90
-6	8	-10	3.80	8.80
-6	8	-10	6.50	13.50
-6	8	-10	9.10	11.10
6	-8	10	-6.20	10.80
-7	8	-10	66.29	11.70
-7	8	-10	108.59	17.70
-7	8	-10	63.69	17.10
-8	8	-10	33.00	11.60
-8	8	-10	23.20	9.80
8	-8	10	41.40	10.30
-9	8	-10	48.70	17.10
-9	8	-10	52.89	10.60
-9	8	-10	42.20	11.20
-10	8	-10	5.80	10.00
-10	8	-10	19.90	12.50
10	-8	10	42.10	10.10
10	7	-10	13.50	10.20
-10	-7	10	27.50	6.50
9	7	-10	18.70	10.60
-9	-7	10	-5.70	6.00
8	7	-10	38.80	13.20
-8	-7	10	13.80	10.50
7	7	-10	72.09	13.10
-7	-7	10	82.59	11.00

-7	-7	10	52.39	9.70
6	7	-10	253.87	22.80
-6	-7	10	278.47	19.20
-6	-7	10	303.07	18.10
5	7	-10	-2.30	10.50
-5	-7	10	-0.40	6.30
-5	-7	10	1.40	6.60
4	7	-10	530.85	32.60
-4	-7	10	560.54	28.90
-4	-7	10	558.04	29.00
3	7	-10	-0.90	8.50
3	7	-10	-3.50	9.80
-3	-7	10	-0.30	7.60
-3	-7	10	2.30	5.30
2	7	-10	30.90	9.70
2	7	-10	29.90	9.00
-2	-7	10	25.50	6.50
1	7	-10	823.12	44.90
1	7	-10	885.91	43.00
1	7	-10	901.11	43.90
-1	-7	10	887.21	42.40
-1	-7	10	909.61	42.90
0	7	-10	20.80	7.50
0	7	-10	35.10	10.60
0	7	-10	21.30	7.40
0	7	-10	16.10	10.70
0	-7	10	29.20	5.90
0	-7	10	21.90	6.60
0	-7	10	28.00	11.40
-1	7	-10	87.99	10.90
-1	7	-10	87.89	12.90
-1	7	-10	75.79	13.40
-1	7	-10	100.39	13.70
-1	7	-10	128.99	19.90
1	-7	10	84.89	9.90
1	-7	10	74.59	9.90
-2	7	-10	329.27	24.30
-2	7	-10	303.97	26.50
-2	7	-10	324.57	21.50
-2	7	-10	332.77	18.90
-2	7	-10	309.07	22.40
2	-7	10	318.97	19.30
-3	7	-10	180.38	24.90
-3	7	-10	140.79	17.90

-3	7	-10	144.99	17.70
-3	7	-10	135.29	23.10
-3	7	-10	153.98	12.80
-3	7	-10	168.88	16.10
3	-7	10	135.49	14.10
-4	7	-10	168.88	16.30
-4	7	-10	264.17	27.80
-4	7	-10	210.38	15.10
-4	7	-10	198.78	20.80
-4	7	-10	167.08	21.20
4	-7	10	184.08	16.30
4	-7	10	197.38	17.10
-5	7	-10	28.50	12.10
-5	7	-10	28.00	14.60
-5	7	-10	31.50	8.90
-5	7	-10	0.20	14.30
-5	7	-10	17.70	7.10
5	-7	10	11.90	9.70
5	-7	10	22.20	9.30
-6	7	-10	549.25	37.10
-6	7	-10	577.94	32.40
-6	7	-10	556.14	36.80
-6	7	-10	554.14	33.40
-6	7	-10	578.54	31.10
6	-7	10	497.55	33.20
6	-7	10	582.94	34.10
-7	7	-10	332.47	24.30
-7	7	-10	340.67	30.30
-7	7	-10	332.67	24.20
-7	7	-10	359.26	32.00
-7	7	-10	351.66	26.30
7	-7	10	337.07	35.60
-8	7	-10	27.30	11.30
-8	7	-10	16.40	9.60
-8	7	-10	-3.50	15.40
8	-7	10	8.90	8.90
-9	7	-10	38.60	10.90
-9	7	-10	24.90	16.20
-9	7	-10	16.30	10.70
9	-7	10	12.70	10.00
-10	7	-10	254.27	35.50
-10	7	-10	186.28	19.10
10	-7	10	229.38	19.80
-11	7	-10	64.99	14.40

-11	7	-10	61.79	13.30
11	-7	10	38.70	11.00
11	-7	10	72.59	14.10
10	6	-10	-9.30	9.30
-10	-6	10	4.20	6.10
9	6	-10	142.29	18.10
-9	-6	10	115.09	12.10
8	6	-10	17.80	11.60
-8	-6	10	1.40	6.30
7	6	-10	16.90	11.60
-7	-6	10	-7.90	7.80
-7	-6	10	7.60	6.50
6	6	-10	453.35	29.60
-6	-6	10	473.35	25.20
-6	-6	10	441.46	25.70
5	6	-10	176.18	19.00
-5	-6	10	131.99	13.30
-5	-6	10	161.98	13.50
4	6	-10	8.20	10.00
-4	-6	10	10.60	7.00
-4	-6	10	12.20	6.00
3	6	-10	-5.80	10.60
3	6	-10	-8.20	8.70
-3	-6	10	0.80	5.00
-3	-6	10	-6.50	7.50
2	6	-10	28.80	9.40
2	6	-10	31.20	8.90
-2	-6	10	19.20	6.10
-2	-6	10	9.60	6.60
-2	-6	10	15.50	8.30
1	6	-10	111.49	13.20
1	6	-10	117.99	15.00
1	6	-10	128.79	17.20
-1	-6	10	121.69	11.60
-1	-6	10	105.29	14.40
-1	-6	10	105.89	16.30
-1	-6	10	129.49	10.50
0	6	-10	1109.49	54.89
0	6	-10	1158.18	54.09
0	6	-10	1147.69	56.59
0	6	-10	1110.79	55.39
0	-6	10	1229.58	53.29
0	-6	10	1173.48	56.09
0	-6	10	1086.09	54.09

-1	6	-10	143.19	15.90
-1	6	-10	155.48	15.30
-1	6	-10	107.09	20.50
-1	6	-10	159.48	12.50
-1	6	-10	132.69	20.50
1	-6	10	158.18	10.90
1	-6	10	137.39	18.20
-2	6	-10	694.63	37.70
-2	6	-10	763.62	41.00
-2	6	-10	756.52	35.40
-2	6	-10	700.63	37.40
-2	6	-10	648.04	38.70
2	-6	10	745.33	35.10
-3	6	-10	53.19	10.80
-3	6	-10	87.69	14.60
-3	6	-10	81.59	14.90
-3	6	-10	64.19	7.90
-3	6	-10	60.79	15.20
-4	6	-10	121.49	19.90
-4	6	-10	110.09	12.00
-4	6	-10	170.08	25.60
-4	6	-10	95.09	17.50
-4	6	-10	106.99	16.00
-4	6	-10	124.19	14.40
4	-6	10	113.99	15.00
4	-6	10	137.39	12.50
-5	6	-10	153.48	25.30
-5	6	-10	148.49	19.30
-5	6	-10	161.28	13.90
-5	6	-10	139.49	25.70
5	-6	10	176.78	18.30
5	-6	10	144.49	14.50
-6	6	-10	28.40	10.50
-6	6	-10	54.99	14.20
-6	6	-10	47.50	14.60
-6	6	-10	55.09	11.20
-6	6	-10	46.80	9.30
6	-6	10	41.20	12.70
6	-6	10	47.50	10.20
-7	6	-10	137.59	15.90
-7	6	-10	165.88	19.70
-7	6	-10	132.59	24.20
-7	6	-10	153.98	19.60
-7	6	-10	149.19	17.30

7	-6	10	136.69	23.20
-8	6	-10	275.17	29.20
-8	6	-10	322.47	21.80
-8	6	-10	257.67	21.90
-8	6	-10	298.77	29.90
8	-6	10	317.07	22.10
-9	6	-10	65.59	17.50
-9	6	-10	88.29	21.20
-9	6	-10	94.39	14.10
-9	6	-10	95.59	12.50
9	-6	10	73.29	13.00
-10	6	-10	157.88	17.50
-10	6	-10	132.99	17.80
10	-6	10	205.88	20.30
-11	6	-10	3.90	10.10
-11	6	-10	14.50	13.50
11	-6	10	1.80	9.60
11	-6	10	11.60	14.30
10	5	-10	85.39	13.90
-10	-5	10	100.39	11.10
9	5	-10	27.00	17.60
9	5	-10	30.60	12.00
-9	-5	10	18.90	7.20
8	5	-10	181.28	22.50
8	5	-10	159.48	26.00
-8	-5	10	236.38	15.50
-8	-5	10	171.68	20.10
7	5	-10	6.40	11.40
7	5	-10	-5.40	14.30
-7	-5	10	5.30	7.70
-7	-5	10	14.60	6.80
6	5	-10	145.89	20.50
6	5	-10	147.39	24.50
-6	-5	10	153.78	13.20
-6	-5	10	149.29	14.20
5	5	-10	599.94	38.80
5	5	-10	662.83	37.20
-5	-5	10	667.03	33.50
-5	-5	10	686.33	33.80
4	5	-10	21.40	10.20
4	5	-10	47.00	13.80
-4	-5	10	24.40	6.50
-4	-5	10	23.00	6.30
3	5	-10	32.40	14.20

3	5	-10	42.80	11.10
-3	-5	10	25.10	8.70
-3	-5	10	25.70	7.60
-3	-5	10	27.40	6.50
2	5	-10	104.69	12.50
2	5	-10	98.59	12.00
-2	-5	10	78.29	11.40
-2	-5	10	72.79	10.30
1	5	-10	127.79	16.10
1	5	-10	149.99	12.60
1	5	-10	143.69	17.10
1	5	-10	110.49	12.00
-1	-5	10	151.68	12.40
-1	-5	10	125.79	13.00
-1	-5	10	146.49	10.60
-1	-5	10	126.99	12.10
0	5	-10	1032.60	48.40
0	5	-10	1004.60	47.90
0	5	-10	896.61	48.10
0	5	-10	962.30	48.90
0	-5	10	930.81	48.20
0	-5	10	1111.69	49.40
0	-5	10	1119.79	47.10
-1	5	-10	41.50	6.70
-1	5	-10	56.19	9.90
1	-5	10	36.20	9.20
1	-5	10	45.30	5.80
-2	5	-10	27.00	10.40
-2	5	-10	32.50	6.30
-2	5	-10	29.50	7.90
-2	5	-10	16.90	8.30
-2	5	-10	1.90	14.10
2	-5	10	15.70	4.80
2	-5	10	13.90	9.60
-3	5	-10	51.49	9.30
-3	5	-10	78.49	10.70
-3	5	-10	79.99	11.20
-3	5	-10	62.89	10.20
-3	5	-10	73.29	13.40
-3	5	-10	93.49	14.90
3	-5	10	52.79	8.60
-4	5	-10	169.68	20.60
-4	5	-10	185.08	16.30
-4	5	-10	178.48	17.90

-4	5	-10	170.18	13.50
-4	5	-10	203.38	18.50
4	-5	10	208.98	13.20
-5	5	-10	731.63	39.80
-5	5	-10	733.43	39.60
-5	5	-10	705.53	36.40
-5	5	-10	737.93	37.50
-5	5	-10	777.82	43.70
5	-5	10	726.83	36.00
5	-5	10	659.53	38.30
-6	5	-10	262.07	24.10
-6	5	-10	276.57	20.10
-6	5	-10	271.07	22.80
-6	5	-10	267.67	18.30
-6	5	-10	281.27	27.50
6	-5	10	248.68	27.50
6	-5	10	278.37	18.80
-7	5	-10	303.97	23.70
-7	5	-10	297.17	19.40
-7	5	-10	277.77	26.90
-7	5	-10	269.47	20.60
-7	5	-10	265.47	26.80
7	-5	10	271.87	22.70
-8	5	-10	2.00	7.80
-8	5	-10	6.70	10.80
-8	5	-10	8.50	14.00
-8	5	-10	7.60	8.60
-8	5	-10	-4.10	13.80
8	-5	10	23.70	15.50
-9	5	-10	254.07	36.60
-9	5	-10	235.48	20.10
-9	5	-10	220.38	37.70
-9	5	-10	201.38	18.30
9	-5	10	280.07	35.00
-10	5	-10	11.60	9.80
-10	5	-10	-4.20	9.90
-10	5	-10	16.50	15.90
-10	5	-10	-4.60	18.90
10	-5	10	10.80	12.50
-11	5	-10	34.10	11.00
11	-5	10	41.60	23.60
10	4	-10	267.57	28.10
10	4	-10	265.57	22.90
-10	-4	10	243.38	16.60

9	4	-10	22.40	15.10
9	4	-10	45.50	13.10
-9	-4	10	40.20	7.90
8	4	-10	12.10	14.00
8	4	-10	7.50	11.30
-8	-4	10	10.70	6.60
-8	-4	10	13.40	10.30
7	4	-10	723.33	43.40
7	4	-10	779.72	43.60
-7	-4	10	800.22	40.30
-7	-4	10	828.22	39.20
6	4	-10	87.09	13.90
-6	-4	10	55.39	8.70
-6	-4	10	66.69	10.50
5	4	-10	293.87	26.50
-5	-4	10	344.17	19.40
-5	-4	10	334.67	20.10
4	4	-10	-17.70	12.30
4	4	-10	17.80	11.00
-4	-4	10	10.20	5.80
3	4	-10	116.99	28.50
3	4	-10	107.19	13.30
3	4	-10	146.39	26.30
-3	-4	10	104.29	11.40
-3	-4	10	68.39	12.20
-3	-4	10	103.69	12.40
2	4	-10	17.10	5.90
2	4	-10	29.30	10.50
-2	-4	10	17.70	5.80
-2	-4	10	11.10	5.40
1	4	-10	67.79	8.40
-1	-4	10	69.09	8.60
-1	-4	10	44.50	5.70
-1	-4	10	58.99	8.40
0	4	-10	1084.39	50.09
0	4	-10	983.00	50.19
0	-4	10	1106.09	49.50
0	-4	10	1188.68	51.09
0	-4	10	978.30	50.19
-1	4	-10	1110.89	51.49
-1	4	-10	1056.39	51.99
-1	4	-10	1153.68	52.09
1	-4	10	1125.79	51.09
1	-4	10	1096.49	53.09

1	-4	10	1068.49	52.09
-2	4	-10	516.15	29.70
-2	4	-10	591.04	27.40
-2	4	-10	566.64	29.90
-2	4	-10	559.24	28.60
2	-4	10	469.15	29.00
2	-4	10	546.35	26.60
-3	4	-10	-4.80	7.30
-3	4	-10	3.10	8.00
-3	4	-10	6.80	14.00
-3	4	-10	4.00	13.30
-3	4	-10	4.80	9.20
3	-4	10	2.50	3.70
3	-4	10	-0.20	7.90
-4	4	-10	153.68	16.40
-4	4	-10	174.58	12.10
-4	4	-10	117.79	15.60
-4	4	-10	145.19	16.30
-4	4	-10	141.29	19.60
-4	4	-10	192.08	23.50
4	-4	10	148.09	10.80
-5	4	-10	4.30	7.60
-5	4	-10	5.50	5.20
-5	4	-10	6.60	11.30
-5	4	-10	-5.70	7.60
-5	4	-10	8.70	8.80
-5	4	-10	7.60	9.50
5	-4	10	7.30	4.80
5	-4	10	6.60	10.70
-6	4	-10	39.30	8.10
-6	4	-10	37.20	10.60
-6	4	-10	46.90	8.70
-6	4	-10	55.19	11.80
-6	4	-10	65.29	18.70
6	-4	10	51.19	7.40
-7	4	-10	115.89	15.50
-7	4	-10	109.69	14.60
-7	4	-10	105.39	18.10
-7	4	-10	110.59	15.70
-7	4	-10	113.19	12.90
7	-4	10	102.79	14.80
-8	4	-10	59.39	12.10
-8	4	-10	86.49	13.90
-8	4	-10	71.49	11.30

-8	4	-10	78.49	14.90
-8	4	-10	82.29	14.70
8	-4	10	83.39	15.80
-9	4	-10	92.19	15.70
-9	4	-10	68.49	19.30
-9	4	-10	69.39	10.80
-9	4	-10	99.29	16.20
9	-4	10	93.39	22.10
9	-4	10	73.69	13.50
-10	4	-10	119.99	15.90
-10	4	-10	189.68	25.20
-10	4	-10	122.99	18.20
-10	4	-10	122.99	25.10
-10	4	-10	159.18	17.00
10	-4	10	104.29	15.90
-11	4	-10	323.37	22.00
-11	4	-10	312.97	22.80
-11	4	-10	326.47	47.70
11	-4	10	260.27	34.70
11	3	-10	185.98	22.20
11	3	-10	155.38	28.40
-11	-3	10	200.88	13.90
10	3	-10	34.60	15.00
10	3	-10	6.50	12.70
-10	-3	10	6.00	6.50
9	3	-10	-12.00	13.70
9	3	-10	17.40	13.50
-9	-3	10	5.30	6.60
8	3	-10	52.99	13.40
8	3	-10	38.60	13.40
-8	-3	10	33.60	7.10
-8	-3	10	51.59	11.70
7	3	-10	24.50	11.60
7	3	-10	17.30	12.30
-7	-3	10	14.20	6.00
-7	-3	10	6.80	7.90
6	3	-10	272.07	24.10
6	3	-10	347.57	24.60
-6	-3	10	322.57	20.30
-6	-3	10	328.27	19.20
5	3	-10	31.10	11.00
5	3	-10	35.40	10.60
4	3	-10	775.12	39.30
4	3	-10	656.63	38.60

-4	-3	10	611.54	36.70
-4	-3	10	777.92	35.00
3	3	-10	8.20	10.40
-3	-3	10	7.40	5.70
-3	-3	10	11.60	9.00
-3	-3	10	2.80	6.30
-3	-3	10	5.40	5.70
2	3	-10	248.48	12.90
2	3	-10	216.38	25.40
-2	-3	10	220.28	14.50
-2	-3	10	242.98	14.30
1	3	-10	977.40	46.00
-1	-3	10	1059.59	46.80
-1	-3	10	961.60	46.30
0	3	-10	61.19	6.80
0	3	-10	68.09	7.80
0	-3	10	61.89	6.70
0	-3	10	64.09	8.50
0	-3	10	62.49	7.60
-1	3	-10	4.20	4.40
-1	3	-10	9.90	4.20
1	-3	10	8.50	3.40
1	-3	10	0.80	5.70
1	-3	10	3.30	4.40
-2	3	-10	729.33	36.10
2	-3	10	764.92	35.00
2	-3	10	753.32	36.50
2	-3	10	713.53	36.70
-3	3	-10	76.19	11.40
-3	3	-10	47.10	8.10
-3	3	-10	76.79	13.40
-3	3	-10	48.30	7.90
3	-3	10	43.90	8.10
3	-3	10	49.90	5.90
-4	3	-10	36.70	9.70
-4	3	-10	44.50	10.90
-4	3	-10	27.20	8.70
-4	3	-10	38.00	9.20
-4	3	-10	45.90	13.20
4	-3	10	30.80	7.30
-5	3	-10	151.78	12.10
-5	3	-10	142.79	14.90
-5	3	-10	137.79	21.50
-5	3	-10	161.88	18.20

-5	3	-10	143.79	18.30
-5	3	-10	185.18	16.90
5	-3	10	166.08	10.70
-6	3	-10	540.55	35.60
-6	3	-10	564.14	30.00
-6	3	-10	589.74	36.00
-6	3	-10	591.94	32.30
-6	3	-10	641.84	36.80
6	-3	10	541.55	28.80
-7	3	-10	38.70	7.70
-7	3	-10	21.50	12.10
-7	3	-10	30.60	11.60
-7	3	-10	29.40	8.60
-7	3	-10	50.89	11.90
7	-3	10	33.70	7.50
-8	3	-10	126.69	20.20
-8	3	-10	135.29	15.60
-8	3	-10	150.78	15.90
-8	3	-10	107.89	15.60
-8	3	-10	111.49	15.60
-8	3	-10	144.59	21.30
8	-3	10	135.09	18.30
-9	3	-10	166.78	23.10
-9	3	-10	171.58	25.90
-9	3	-10	183.98	15.50
-9	3	-10	141.19	21.80
-9	3	-10	163.58	16.30
9	-3	10	167.58	28.50
9	-3	10	124.09	21.60
-10	3	-10	288.47	24.60
-10	3	-10	295.07	21.30
-10	3	-10	328.27	28.90
-10	3	-10	320.57	28.70
-10	3	-10	328.27	22.20
10	-3	10	286.37	28.10
-11	3	-10	72.59	13.50
-11	3	-10	75.19	11.30
-11	3	-10	85.99	17.50
11	-3	10	59.19	20.90
11	2	-10	76.59	16.00
11	2	-10	68.59	20.00
-11	-2	10	90.39	11.90
10	2	-10	14.60	13.90
10	2	-10	15.10	13.40

-10	-2	10	14.30	6.90
9	2	-10	21.60	14.70
9	2	-10	6.30	15.20
-9	-2	10	32.30	9.40
-9	-2	10	48.90	17.40
8	2	-10	103.79	14.40
8	2	-10	60.09	14.30
-8	-2	10	73.29	15.40
-8	-2	10	78.19	13.00
-8	-2	10	94.19	12.10
7	2	-10	71.69	12.40
7	2	-10	95.19	14.70
-7	-2	10	125.79	15.10
-7	-2	10	106.99	12.10
6	2	-10	1102.59	55.79
6	2	-10	1053.79	54.09
-6	-2	10	1129.49	52.49
5	2	-10	47.30	10.60
5	2	-10	72.19	12.50
-5	-2	10	61.29	9.90
4	2	-10	36.70	11.10
4	2	-10	32.30	12.00
-4	-2	10	34.10	6.50
-4	-2	10	19.80	7.50
3	2	-10	163.38	20.80
-3	-2	10	139.39	11.70
-3	-2	10	105.89	11.10
1	2	-10	906.71	43.80
-1	-2	10	1043.60	45.20
-1	-2	10	941.81	43.90
0	2	-10	394.96	22.10
0	2	-10	437.46	21.20
0	-2	10	493.75	22.50
0	-2	10	437.26	21.70
-1	2	-10	440.26	21.20
-1	2	-10	415.86	21.60
1	-2	10	431.76	20.90
1	-2	10	411.96	22.00
-2	2	-10	434.06	21.50
-2	2	-10	403.16	21.40
-2	2	-10	428.96	21.40
2	-2	10	356.56	21.40
2	-2	10	402.36	21.60
2	-2	10	436.26	20.20

-3	2	-10	25.60	6.10
-3	2	-10	21.10	6.20
-3	2	-10	31.20	5.60
3	-2	10	17.50	6.10
3	-2	10	21.60	6.10
3	-2	10	26.50	4.60
-4	2	-10	132.39	17.80
-4	2	-10	142.19	18.40
-4	2	-10	114.29	10.20
-4	2	-10	145.19	16.70
-4	2	-10	148.19	20.50
4	-2	10	137.29	8.70
-5	2	-10	105.89	14.00
-5	2	-10	103.69	9.90
-5	2	-10	98.49	14.40
-5	2	-10	93.29	16.10
-5	2	-10	99.09	13.00
-5	2	-10	128.99	18.30
5	-2	10	103.49	7.80
-6	2	-10	6.40	9.70
-6	2	-10	9.10	7.00
-6	2	-10	9.20	11.90
-6	2	-10	15.10	8.60
-6	2	-10	13.30	5.60
6	-2	10	11.80	4.30
-7	2	-10	130.89	20.20
-7	2	-10	131.69	21.60
-7	2	-10	154.28	12.90
-7	2	-10	150.88	14.50
-7	2	-10	160.78	18.50
7	-2	10	173.08	12.80
-8	2	-10	272.37	18.70
-8	2	-10	239.18	25.20
-8	2	-10	270.37	23.60
-8	2	-10	273.07	19.50
-8	2	-10	280.47	24.50
-8	2	-10	320.67	24.20
8	-2	10	281.07	19.80
-9	2	-10	6.50	11.30
-9	2	-10	20.00	11.60
-9	2	-10	17.90	8.50
-9	2	-10	20.90	8.40
-9	2	-10	30.70	13.20
9	-2	10	42.10	13.60

9	-2	10	6.00	12.50
-10	2	-10	71.99	11.30
-10	2	-10	86.09	13.90
-10	2	-10	65.99	13.10
-10	2	-10	100.39	17.30
-10	2	-10	91.39	15.20
10	-2	10	50.59	16.70
-11	2	-10	22.80	15.00
-11	2	-10	18.50	9.00
-11	2	-10	28.00	13.00
-11	2	-10	2.50	10.30
-11	2	-10	38.10	16.30
-12	2	-10	37.30	15.10
-12	2	-10	37.60	13.20
12	-2	10	47.10	14.90
11	1	-10	126.29	18.60
11	1	-10	117.99	22.20
-11	-1	10	169.48	13.50
10	1	-10	91.99	16.20
10	1	-10	87.99	15.60
-10	-1	10	94.29	11.90
-10	-1	10	147.39	21.70
9	1	-10	10.90	14.70
9	1	-10	18.30	18.20
-9	-1	10	2.90	11.20
8	1	-10	80.49	15.60
8	1	-10	123.39	19.40
-8	-1	10	119.19	16.50
-8	-1	10	79.19	14.20
-8	-1	10	86.59	12.30
7	1	-10	2.30	10.10
7	1	-10	15.80	12.20
-7	-1	10	-20.60	10.50
-7	-1	10	8.30	8.40
-7	-1	10	13.40	6.30
6	1	-10	342.67	26.30
6	1	-10	331.07	23.50
-6	-1	10	318.37	19.70
5	1	-10	1436.16	70.79
5	1	-10	1432.76	69.59
-5	-1	10	1510.75	68.09
4	1	-10	15.70	11.00
4	1	-10	31.40	9.50
-4	-1	10	38.90	6.50

-4	-1	10	40.40	7.20
-4	-1	10	34.30	10.70
-4	-1	10	33.30	7.10
3	1	-10	116.39	20.00
-3	-1	10	121.69	10.40
-3	-1	10	123.29	10.90
-2	-1	10	4.90	4.30
-1	-1	10	92.89	8.00
0	1	-10	813.12	38.60
0	-1	10	879.91	39.40
0	-1	10	843.52	39.40
-1	1	-10	116.29	8.40
-1	1	-10	117.59	7.50
1	-1	10	111.69	7.20
1	-1	10	104.29	8.40
-2	1	-10	463.35	23.70
-2	1	-10	483.85	23.50
2	-1	10	417.56	23.70
2	-1	10	511.65	22.60
2	-1	10	479.45	23.70
-3	1	-10	745.43	36.60
3	-1	10	753.12	36.90
3	-1	10	787.62	35.00
3	-1	10	736.73	36.80
-4	1	-10	188.68	11.90
-4	1	-10	154.58	15.10
-4	1	-10	150.48	20.90
-4	1	-10	193.38	21.20
-4	1	-10	175.88	19.10
-4	1	-10	169.98	14.10
-4	1	-10	178.68	15.30
4	-1	10	173.18	15.00
4	-1	10	200.68	10.20
4	-1	10	200.08	15.00
-5	1	-10	850.01	41.70
-5	1	-10	802.62	43.70
-5	1	-10	842.72	41.00
-5	1	-10	791.22	44.10
-5	1	-10	892.61	40.20
-5	1	-10	788.52	43.90
5	-1	10	850.81	38.90
-6	1	-10	-0.40	8.50
-6	1	-10	19.50	7.10
-6	1	-10	11.70	11.40

-6	1	-10	8.10	5.20
-6	1	-10	4.20	9.40
-6	1	-10	-2.00	10.30
6	-1	10	10.50	3.80
-7	1	-10	442.96	25.10
-7	1	-10	386.26	29.70
-7	1	-10	470.05	24.50
-7	1	-10	432.06	27.10
-7	1	-10	456.65	30.30
-7	1	-10	439.26	28.60
7	-1	10	456.65	23.20
-8	1	-10	17.40	7.60
-8	1	-10	11.80	9.70
-8	1	-10	-6.10	11.30
-8	1	-10	6.50	6.40
-8	1	-10	0.10	11.20
-8	1	-10	3.50	11.00
8	-1	10	18.90	7.10
-9	1	-10	465.65	29.00
-9	1	-10	410.06	31.20
-9	1	-10	431.76	27.70
-9	1	-10	423.66	25.20
-9	1	-10	440.86	25.00
9	-1	10	410.96	28.30
9	-1	10	344.27	31.00
-10	1	-10	223.58	19.50
-10	1	-10	202.38	27.60
-10	1	-10	182.08	20.30
-10	1	-10	250.97	24.70
-10	1	-10	212.98	16.90
10	-1	10	238.78	33.10
10	-1	10	160.48	21.30
-11	1	-10	14.90	15.10
-11	1	-10	17.90	15.50
-11	1	-10	28.10	12.40
-11	1	-10	7.20	8.90
-11	1	-10	29.10	10.50
-12	1	-10	16.80	9.60
-12	1	-10	8.10	11.60
-12	1	-10	27.10	19.70
12	-1	10	3.60	12.80
11	0	-10	21.90	17.60
11	0	-10	-17.50	15.50
-11	0	10	36.40	8.80

10	0	-10	172.38	24.70
10	0	-10	153.88	20.60
-10	0	10	129.39	13.60
-10	0	10	128.69	19.00
9	0	-10	8.70	24.60
-9	0	10	-5.00	10.50
-9	0	10	7.20	10.90
8	0	-10	109.39	16.90
8	0	-10	113.09	17.90
-8	0	10	104.79	12.80
-8	0	10	109.19	12.70
7	0	-10	490.95	29.40
7	0	-10	460.85	32.50
-7	0	10	511.15	26.90
-7	0	10	486.15	28.10
6	0	-10	11.70	12.80
6	0	-10	31.70	9.80
-6	0	10	18.60	6.50
5	0	-10	237.38	22.00
5	0	-10	234.28	19.70
-5	0	10	257.57	16.00
-5	0	10	201.68	18.80
4	0	-10	108.59	14.50
4	0	-10	92.39	15.20
-4	0	10	90.79	10.70
-4	0	10	89.89	10.80
-4	0	10	75.09	13.50
-4	0	10	84.19	10.10
-3	0	10	5.90	4.30
-3	0	10	14.80	5.30
-3	0	10	9.60	4.20
-2	0	10	277.17	17.10
-2	0	10	365.86	17.60
-1	0	10	162.68	10.30
0	0	-10	1990.50	91.99
0	0	10	2083.59	92.89
0	0	10	2095.69	92.59
-1	0	-10	844.22	38.40
-1	0	-10	847.72	39.20
1	0	10	821.52	38.30
1	0	10	820.52	38.70
-2	0	-10	284.87	17.40
-2	0	-10	313.17	16.60
2	0	10	320.57	16.50

2	0	10	321.27	15.10
2	0	10	309.47	16.70
-3	0	-10	557.04	27.90
3	0	10	561.74	28.00
3	0	10	585.54	26.20
3	0	10	551.64	28.20
-4	0	-10	648.24	33.00
-4	0	-10	684.43	39.00
-4	0	-10	622.84	34.30
-4	0	-10	645.44	33.60
-4	0	-10	713.13	32.10
4	0	10	619.44	33.20
4	0	10	693.53	30.70
4	0	10	661.83	33.60
-5	0	-10	102.99	12.40
-5	0	-10	100.79	9.10
-5	0	-10	81.79	12.40
-5	0	-10	120.89	16.70
-5	0	-10	94.19	13.20
-5	0	-10	102.79	17.30
5	0	10	104.19	7.00
-6	0	-10	9.70	7.30
-6	0	-10	9.20	9.60
-6	0	-10	0.50	10.00
-6	0	-10	12.50	4.80
-6	0	-10	14.10	6.70
-6	0	-10	-2.50	10.30
6	0	10	7.40	3.20
-7	0	-10	2.70	10.40
-7	0	-10	20.60	7.30
-7	0	-10	12.50	10.60
-7	0	-10	2.30	5.70
-7	0	-10	7.30	11.20
-7	0	-10	16.30	8.70
7	0	10	6.90	4.30
-8	0	-10	231.88	15.70
-8	0	-10	227.28	20.40
-8	0	-10	190.98	23.30
-8	0	-10	202.68	20.10
-8	0	-10	229.48	16.80
-8	0	-10	214.48	23.00
8	0	10	212.98	15.10
-9	0	-10	52.89	11.60
-9	0	-10	52.49	9.20

-9	0	-10	74.79	12.50
-9	0	-10	48.40	8.30
-9	0	-10	70.79	14.70
-9	0	-10	66.09	14.80
9	0	10	91.99	15.60
-10	0	-10	52.79	14.60
-10	0	-10	33.20	8.50
-10	0	-10	34.70	12.00
10	0	10	73.69	17.30
10	0	10	86.39	20.00
-11	0	-10	189.58	24.30
-11	0	-10	230.98	17.70
-11	0	-10	220.68	17.70
-11	0	-10	239.48	21.00
-11	0	-10	255.77	24.80
-12	0	-10	-10.70	11.20
-12	0	-10	-1.60	9.30
-12	0	-10	-24.80	15.00
-12	0	-10	0.50	17.10
12	0	10	-9.90	12.90
11	-1	-10	63.19	20.80
11	-1	-10	31.00	12.30
-11	1	10	15.70	8.60
10	-1	-10	56.09	17.30
-10	1	10	44.80	8.60
9	-1	-10	52.19	21.00
-9	1	10	19.80	11.10
-9	1	10	10.90	8.00
8	-1	-10	28.70	14.20
8	-1	-10	8.00	10.60
-8	1	10	35.80	9.50
7	-1	-10	94.89	15.40
7	-1	-10	98.29	17.80
-7	1	10	97.69	13.00
-7	1	10	132.59	15.60
6	-1	-10	140.19	17.10
-6	1	10	115.09	17.60
-6	1	10	128.49	12.50
5	-1	-10	743.83	38.50
5	-1	-10	694.83	40.90
5	-1	-10	803.82	41.80
-5	1	10	674.03	37.00
-5	1	10	770.22	36.50
4	-1	-10	666.53	33.60

4	-1	-10	616.34	37.20
4	-1	-10	614.74	33.10
-4	1	10	641.04	30.80
-4	1	10	576.14	32.10
-4	1	10	604.04	31.10
-3	1	10	32.80	6.10
-3	1	10	44.30	6.50
-3	1	10	53.89	6.30
-2	1	10	613.04	31.10
-2	1	10	688.73	31.60
-1	1	10	589.14	28.10
0	1	10	14.30	4.20
0	1	10	16.00	3.30
-1	-1	-10	55.39	4.50
1	1	10	52.39	4.30
1	1	10	50.09	5.00
-2	-1	-10	799.52	37.60
-2	-1	-10	791.22	38.00
2	1	10	823.12	36.60
2	1	10	788.22	38.10
2	1	10	828.12	37.70
-3	-1	-10	-0.10	3.50
-3	-1	-10	5.20	4.10
-3	-1	-10	2.60	4.40
3	1	10	5.20	4.30
3	1	10	9.10	1.90
3	1	10	6.00	3.70
-4	-1	-10	679.73	31.00
-4	-1	-10	612.14	32.20
-4	-1	-10	635.54	31.60
4	1	10	629.84	32.30
4	1	10	615.04	32.00
4	1	10	660.73	29.50
-5	-1	-10	278.77	20.90
-5	-1	-10	274.57	18.10
-5	-1	-10	291.97	17.90
-5	-1	-10	236.78	24.60
-5	-1	-10	289.37	18.50
-5	-1	-10	262.17	22.60
5	1	10	252.07	19.70
-6	-1	-10	646.54	35.70
-6	-1	-10	606.44	37.50
-6	-1	-10	655.73	37.00
-6	-1	-10	635.74	33.30

-6	-1	-10	656.53	32.40
-6	-1	-10	534.15	44.10
-6	-1	-10	660.63	31.60
6	1	10	652.53	30.20
-7	-1	-10	22.20	10.40
-7	-1	-10	1.10	10.70
-7	-1	-10	18.40	10.80
-7	-1	-10	21.70	5.90
-7	-1	-10	4.30	7.80
-7	-1	-10	15.20	6.80
7	1	10	14.30	4.20
-8	-1	-10	240.68	15.60
-8	-1	-10	225.58	22.30
-8	-1	-10	213.18	16.90
-8	-1	-10	198.98	19.30
-8	-1	-10	222.78	20.00
-8	-1	-10	207.18	24.30
8	1	10	240.78	14.90
-9	-1	-10	304.17	23.80
-9	-1	-10	352.56	24.30
-9	-1	-10	340.87	21.00
-9	-1	-10	357.36	27.80
-9	-1	-10	341.67	21.30
-9	-1	-10	376.36	30.00
9	1	10	280.37	30.70
9	1	10	368.96	22.20
-10	-1	-10	265.27	18.10
-10	-1	-10	266.07	35.40
-10	-1	-10	175.68	29.70
-10	-1	-10	263.47	19.20
-10	-1	-10	265.37	29.80
10	1	10	268.17	27.00
-11	-1	-10	16.30	13.70
-11	-1	-10	6.10	12.60
-11	-1	-10	-1.00	10.70
-11	-1	-10	-9.00	7.70
-11	-1	-10	10.20	9.00
-12	-1	-10	63.59	16.80
-12	-1	-10	84.59	14.40
-12	-1	-10	62.29	10.20
-12	-1	-10	59.89	11.30
12	1	10	70.19	14.70
11	-2	-10	57.89	12.70
-11	2	10	44.40	9.80

-11	2	10	36.10	18.70
10	-2	-10	-12.30	18.90
10	-2	-10	20.20	11.40
-10	2	10	18.80	8.20
-10	2	10	18.00	9.80
9	-2	-10	0.40	17.40
9	-2	-10	26.80	18.90
-9	2	10	20.50	11.70
-9	2	10	18.50	11.00
8	-2	-10	23.60	15.90
8	-2	-10	22.20	10.80
-8	2	10	23.90	8.90
-8	2	10	24.30	7.30
7	-2	-10	104.29	16.20
7	-2	-10	95.69	17.40
-7	2	10	103.69	13.00
-7	2	10	114.39	12.10
6	-2	-10	748.83	43.70
6	-2	-10	837.02	41.50
6	-2	-10	771.22	45.60
-6	2	10	804.32	41.00
-6	2	10	849.61	40.00
5	-2	-10	18.10	13.60
5	-2	-10	-3.10	11.10
5	-2	-10	15.80	9.80
5	-2	-10	10.70	8.70
-5	2	10	3.60	7.00
4	-2	-10	-6.00	8.80
4	-2	-10	7.30	8.40
4	-2	-10	20.40	14.60
-4	2	10	3.80	7.50
-4	2	10	5.90	4.20
-4	2	10	15.20	6.80
-3	2	10	53.39	8.00
-3	2	10	44.20	5.90
-3	2	10	37.00	6.30
-2	2	10	392.16	19.30
-2	2	10	347.97	18.50
-1	2	10	816.82	39.00
-1	2	10	867.11	39.70
0	2	10	0.90	2.50
1	2	10	123.99	6.70
1	2	10	124.79	7.20
2	2	10	178.88	10.20

2	2	10	176.78	8.70
2	2	10	183.08	11.00
2	2	10	187.48	10.30
-3	-2	-10	39.40	5.60
3	2	10	36.40	5.00
3	2	10	37.50	5.20
3	2	10	29.40	2.80
-4	-2	-10	1059.69	48.60
4	2	10	1014.80	49.70
4	2	10	985.60	49.60
4	2	10	1116.29	47.30
-5	-2	-10	497.85	26.50
-5	-2	-10	481.85	31.70
-5	-2	-10	508.25	27.30
-5	-2	-10	495.15	34.40
-5	-2	-10	518.55	29.20
-5	-2	-10	530.95	26.90
5	2	10	520.85	23.80
5	2	10	456.05	28.00
-6	-2	-10	105.79	19.30
-6	-2	-10	127.79	22.50
-6	-2	-10	106.59	9.70
-6	-2	-10	127.29	13.40
-6	-2	-10	108.19	16.20
-6	-2	-10	95.69	11.40
-6	-2	-10	109.69	18.00
6	2	10	113.79	7.40
-7	-2	-10	168.28	23.40
-7	-2	-10	201.88	16.10
-7	-2	-10	186.08	18.90
-7	-2	-10	183.68	13.10
-7	-2	-10	200.08	19.80
-7	-2	-10	168.68	16.60
7	2	10	192.28	11.40
-8	-2	-10	291.97	23.40
-8	-2	-10	308.97	18.00
-8	-2	-10	268.57	21.70
-8	-2	-10	296.87	19.00
-8	-2	-10	269.77	26.40
-8	-2	-10	309.67	20.90
8	2	10	282.87	16.90
-9	-2	-10	24.40	9.20
-9	-2	-10	25.20	11.60
-9	-2	-10	10.20	7.30

-9	-2	-10	13.70	9.60
-9	-2	-10	1.90	12.10
-9	-2	-10	18.80	7.40
9	2	10	18.20	8.40
-10	-2	-10	17.50	7.50
-10	-2	-10	-3.40	12.30
-10	-2	-10	10.90	10.30
-10	-2	-10	13.80	8.30
10	2	10	0.00	13.10
-11	-2	-10	29.70	10.60
-11	-2	-10	45.90	11.90
-11	-2	-10	30.00	9.10
-11	-2	-10	25.80	13.10
-11	-2	-10	40.70	9.00
-12	-2	-10	16.40	8.40
-12	-2	-10	1.40	10.10
-12	-2	-10	-10.30	16.20
-12	-2	-10	31.30	12.50
12	2	10	43.20	14.20
11	-3	-10	262.17	21.20
-11	3	10	275.97	20.30
-11	3	10	216.28	20.30
10	-3	-10	-3.90	25.90
10	-3	-10	17.00	11.40
-10	3	10	18.50	9.80
-10	3	10	2.40	8.20
9	-3	-10	17.80	19.50
9	-3	-10	37.10	20.00
-9	3	10	7.10	9.90
8	-3	-10	118.49	18.20
8	-3	-10	159.88	29.10
-8	3	10	137.79	14.40
-8	3	10	109.59	16.00
7	-3	-10	114.09	17.80
7	-3	-10	76.49	14.90
-7	3	10	94.49	15.40
-7	3	10	79.49	13.00
6	-3	-10	270.17	20.50
6	-3	-10	291.07	22.70
6	-3	-10	216.98	27.60
-6	3	10	232.28	19.90
-6	3	10	256.37	17.40
5	-3	-10	448.76	26.40
5	-3	-10	407.56	33.80

5	-3	-10	448.96	28.40
5	-3	-10	457.55	27.10
-5	3	10	422.26	23.80
-5	3	10	409.46	26.80
4	-3	-10	243.38	19.20
4	-3	-10	165.58	31.30
4	-3	-10	194.68	19.80
4	-3	-10	275.07	19.80
-4	3	10	246.48	13.60
-4	3	10	253.77	17.90
-4	3	10	216.48	16.50
-3	3	10	2.70	3.00
-3	3	10	8.30	5.00
-3	3	10	1.90	5.00
-2	3	10	179.48	11.20
-2	3	10	172.28	9.90
-1	3	10	506.75	24.80
-1	3	10	542.65	25.70
0	3	10	62.49	5.20
1	3	10	882.71	40.50
1	3	10	842.02	38.90
1	3	10	874.41	38.90
1	3	10	860.71	39.50
2	3	10	702.53	33.70
2	3	10	740.13	34.30
-3	-3	-10	1068.59	50.59
3	3	10	1103.69	51.19
3	3	10	1116.29	50.99
-4	-3	-10	19.60	4.30
4	3	10	14.00	5.20
4	3	10	18.30	5.90
-5	-3	-10	709.33	41.80
-5	-3	-10	782.32	37.50
-5	-3	-10	779.22	38.00
-5	-3	-10	745.13	39.30
-5	-3	-10	816.42	36.80
5	3	10	737.53	38.80
-6	-3	-10	35.20	5.60
-6	-3	-10	38.80	8.90
-6	-3	-10	55.69	10.60
-6	-3	-10	51.79	15.60
-6	-3	-10	43.60	7.10
-6	-3	-10	53.89	11.90
6	3	10	52.89	4.90

-7	-3	-10	163.18	17.10
-7	-3	-10	126.89	17.70
-7	-3	-10	140.29	16.70
-7	-3	-10	153.18	11.70
-7	-3	-10	159.08	19.40
7	3	10	152.58	9.70
-8	-3	-10	152.08	16.40
-8	-3	-10	162.08	17.30
-8	-3	-10	175.28	18.40
-8	-3	-10	168.78	13.10
-8	-3	-10	162.88	22.70
-8	-3	-10	161.38	14.20
8	3	10	156.48	11.60
-9	-3	-10	106.59	15.30
-9	-3	-10	64.59	12.00
-9	-3	-10	54.79	14.40
-9	-3	-10	87.69	12.70
-9	-3	-10	64.99	10.70
9	3	10	74.49	10.30
-10	-3	-10	445.96	30.90
-10	-3	-10	430.56	24.90
-10	-3	-10	454.55	25.70
-10	-3	-10	434.76	30.40
-10	-3	-10	460.05	27.50
10	3	10	433.16	28.90
-11	-3	-10	7.70	7.80
-11	-3	-10	5.30	10.70
-11	-3	-10	22.60	12.40
-11	-3	-10	26.70	10.20
-11	-3	-10	24.90	8.00
-12	-3	-10	-0.50	14.50
-12	-3	-10	29.40	10.10
-12	-3	-10	16.10	12.70
-12	-3	-10	28.20	8.70
12	3	10	5.50	13.70
11	-4	-10	52.49	11.90
-11	4	10	42.90	11.30
-11	4	10	55.69	19.70
-10	4	10	70.29	11.00
-10	4	10	68.29	10.70
9	-4	-10	15.50	23.20
-9	4	10	14.20	9.50
-9	4	10	9.90	16.40
8	-4	-10	77.19	16.60

8	-4	-10	115.39	20.60
8	-4	-10	84.19	13.00
-8	4	10	113.59	15.20
-8	4	10	81.19	13.90
7	-4	-10	331.67	24.00
7	-4	-10	316.27	25.30
7	-4	-10	291.27	34.60
-7	4	10	351.36	21.20
-7	4	10	334.57	22.20
6	-4	-10	192.68	21.20
6	-4	-10	201.98	19.30
6	-4	-10	171.78	23.30
6	-4	-10	197.78	18.70
-6	4	10	153.08	17.10
5	-4	-10	261.17	18.70
5	-4	-10	200.58	18.10
5	-4	-10	210.78	29.50
5	-4	-10	179.88	20.80
-5	4	10	207.38	16.00
-5	4	10	216.48	18.70
4	-4	-10	340.07	22.40
4	-4	-10	361.26	21.80
4	-4	-10	312.77	22.90
-4	4	10	339.87	17.90
-3	4	10	145.19	11.10
-3	4	10	129.89	8.50
-3	4	10	132.79	11.30
-2	4	10	496.15	24.70
-2	4	10	480.25	23.10
-1	4	10	290.47	14.90
-1	4	10	260.87	13.60
0	4	10	606.04	27.40
0	4	10	575.84	28.40
1	4	10	305.37	15.00
1	4	10	307.27	16.00
1	4	10	300.77	14.80
2	4	10	159.28	9.30
2	4	10	162.28	10.00
-3	-4	-10	815.22	40.00
3	4	10	913.41	40.70
3	4	10	848.72	40.70
-4	-4	-10	285.17	15.00
4	4	10	266.17	16.50
4	4	10	261.87	16.30

4	4	10	282.27	12.70
-5	-4	-10	35.40	8.40
-5	-4	-10	43.10	7.50
-5	-4	-10	44.50	5.80
-5	-4	-10	39.40	11.60
-5	-4	-10	40.10	7.90
5	4	10	43.60	3.90
5	4	10	22.10	8.70
-6	-4	-10	-4.20	7.20
-6	-4	-10	-13.40	15.90
-6	-4	-10	2.60	4.20
-6	-4	-10	-3.50	10.90
-6	-4	-10	0.70	5.50
-6	-4	-10	9.30	9.50
-6	-4	-10	4.00	6.60
-7	-4	-10	14.30	9.60
-7	-4	-10	22.20	9.20
-7	-4	-10	4.30	8.40
-7	-4	-10	16.60	5.10
-7	-4	-10	3.80	6.90
7	4	10	8.40	3.50
-8	-4	-10	515.85	33.50
-8	-4	-10	516.35	29.00
-8	-4	-10	478.25	30.60
-8	-4	-10	503.15	26.90
-8	-4	-10	508.55	27.50
-8	-4	-10	511.55	28.60
8	4	10	515.65	25.70
-9	-4	-10	389.36	23.20
-9	-4	-10	388.26	26.90
-9	-4	-10	407.16	22.90
-9	-4	-10	375.66	24.60
-9	-4	-10	400.16	24.90
9	4	10	375.56	23.40
-10	-4	-10	155.98	19.90
-10	-4	-10	156.28	22.70
-10	-4	-10	133.89	17.20
-10	-4	-10	142.49	15.10
-10	-4	-10	152.08	14.00
10	4	10	141.19	18.80
-11	-4	-10	177.28	14.60
-11	-4	-10	168.38	17.50
-11	-4	-10	170.48	14.60
-11	-4	-10	141.49	17.30

-11	-4	-10	163.18	21.20
-12	-4	-10	24.20	7.90
-12	-4	-10	-1.60	8.80
-12	-4	-10	-14.10	18.70
-12	-4	-10	8.30	12.00
12	4	10	35.50	14.60
11	-5	-10	39.10	11.30
-11	5	10	26.70	10.70
-11	5	10	15.70	12.20
10	-5	-10	92.39	16.90
-10	5	10	81.09	12.10
-10	5	10	92.69	15.30
9	-5	-10	53.59	14.70
-9	5	10	58.69	11.10
-9	5	10	66.69	10.60
8	-5	-10	10.00	10.50
8	-5	-10	20.60	13.20
-8	5	10	11.60	8.30
-8	5	10	25.90	8.70
7	-5	-10	29.40	25.00
7	-5	-10	23.80	11.00
7	-5	-10	11.80	9.90
-7	5	10	22.60	7.80
-7	5	10	15.00	9.00
6	-5	-10	50.09	20.80
6	-5	-10	121.49	19.60
6	-5	-10	80.39	15.40
6	-5	-10	71.89	10.80
-6	5	10	69.99	11.30
5	-5	-10	414.06	24.10
5	-5	-10	285.07	46.60
5	-5	-10	384.36	24.80
5	-5	-10	443.56	26.70
5	-5	-10	388.56	31.60
-5	5	10	411.46	24.50
-5	5	10	418.66	21.90
4	-5	-10	213.78	19.00
4	-5	-10	208.78	23.60
4	-5	-10	208.08	19.80
4	-5	-10	211.08	16.80
-4	5	10	234.28	13.00
3	-5	-10	67.39	9.20
3	-5	-10	46.60	10.10
-3	5	10	63.69	5.70

-3	5	10	70.79	10.10
-2	5	10	753.32	35.10
-2	5	10	766.12	36.70
-1	5	10	318.97	15.40
-1	5	10	316.57	16.80
0	5	10	81.39	6.40
0	5	10	92.59	7.00
0	5	10	93.49	5.90
0	5	10	100.09	5.10
1	5	10	167.28	10.20
1	5	10	159.48	9.40
1	5	10	152.58	8.80
1	5	10	171.88	9.90
2	5	10	1314.67	59.19
2	5	10	1235.78	59.39
2	5	10	1302.07	58.99
2	5	10	1304.37	59.39
-3	-5	-10	59.09	7.70
3	5	10	65.19	7.60
3	5	10	63.59	6.90
-4	-5	-10	78.29	7.70
4	5	10	74.29	9.10
4	5	10	76.09	9.80
-5	-5	-10	586.54	36.60
-5	-5	-10	622.24	31.50
-5	-5	-10	622.84	31.10
-5	-5	-10	605.64	32.90
5	5	10	577.34	32.80
5	5	10	666.93	28.20
-6	-5	-10	168.08	11.80
-6	-5	-10	176.48	14.80
-6	-5	-10	171.08	21.40
-6	-5	-10	150.68	13.50
-6	-5	-10	179.48	17.30
6	5	10	178.18	9.60
-7	-5	-10	4.10	6.60
-7	-5	-10	15.10	8.60
-7	-5	-10	15.10	5.90
-7	-5	-10	7.90	9.00
-7	-5	-10	5.70	4.80
-8	-5	-10	10.70	6.70
-8	-5	-10	6.50	8.30
-8	-5	-10	10.40	7.80
-8	-5	-10	13.20	5.60

-8	-5	-10	6.30	9.60
-8	-5	-10	15.30	12.00
8	5	10	6.00	4.80
-9	-5	-10	902.41	45.70
-9	-5	-10	925.71	47.50
-9	-5	-10	912.61	44.90
-9	-5	-10	900.11	45.80
-9	-5	-10	950.10	45.10
9	5	10	879.71	44.40
-10	-5	-10	98.99	13.80
-10	-5	-10	84.89	15.40
-10	-5	-10	95.29	12.20
-10	-5	-10	100.19	18.80
-10	-5	-10	99.99	18.20
10	5	10	109.09	17.60
-11	-5	-10	-4.20	8.90
-11	-5	-10	1.30	11.10
-11	-5	-10	6.40	7.40
-11	-5	-10	-1.70	9.60
-11	-5	-10	-6.40	7.50
-12	-5	-10	31.00	9.40
-12	-5	-10	23.30	8.50
-12	-5	-10	6.60	11.30
-12	-5	-10	55.99	15.10
12	5	10	10.00	16.80
11	-6	-10	11.30	14.00
-11	6	10	18.60	9.90
-11	6	10	36.40	14.10
10	-6	-10	17.30	11.30
-10	6	10	14.40	9.10
-10	6	10	-2.60	10.80
9	-6	-10	101.89	17.40
9	-6	-10	84.59	14.00
-9	6	10	107.29	17.00
-9	6	10	83.79	11.30
8	-6	-10	5.50	10.80
8	-6	-10	15.50	11.60
-8	6	10	0.10	8.80
-8	6	10	20.10	8.50
7	-6	-10	783.02	40.90
7	-6	-10	778.72	40.10
-7	6	10	724.53	39.00
6	-6	-10	319.07	22.50
6	-6	-10	305.57	20.70

-6	6	10	269.97	21.50
-6	6	10	335.87	21.20
5	-6	-10	333.57	25.00
5	-6	-10	359.56	20.90
5	-6	-10	320.87	22.50
5	-6	-10	351.06	28.40
-5	6	10	323.57	18.60
-5	6	10	298.17	21.10
4	-6	-10	36.20	8.20
4	-6	-10	38.80	10.40
4	-6	-10	28.00	9.90
4	-6	-10	49.50	12.00
-4	6	10	16.70	9.90
-4	6	10	20.40	4.20
3	-6	-10	904.41	48.00
3	-6	-10	921.51	46.00
3	-6	-10	937.71	46.60
3	-6	-10	976.00	46.30
-3	6	10	1016.50	44.10
-2	6	10	148.79	8.40
-2	6	10	146.19	10.90
-1	6	10	3.50	2.00
-1	6	10	9.20	3.90
0	6	10	23.80	3.40
0	6	10	28.60	3.20
0	6	10	23.10	3.70
0	6	10	35.00	5.00
0	6	10	21.20	4.30
0	6	10	21.30	2.90
1	6	10	705.23	31.90
1	6	10	694.33	32.80
1	6	10	680.23	32.40
1	6	10	655.73	32.40
2	6	10	17.80	4.90
2	6	10	23.00	4.50
2	6	10	16.60	3.50
3	6	10	23.10	4.50
3	6	10	18.70	5.40
3	6	10	11.50	4.00
3	6	10	20.80	4.90
-4	-6	-10	802.42	36.90
4	6	10	879.31	39.80
4	6	10	678.13	37.90
4	6	10	818.52	37.30

4	6	10	723.73	38.20
-5	-6	-10	109.49	15.50
-5	-6	-10	101.99	12.00
-5	-6	-10	100.69	8.90
-5	-6	-10	67.49	12.30
-5	-6	-10	107.39	16.90
-5	-6	-10	96.39	12.10
5	6	10	101.79	5.80
-6	-6	-10	88.09	11.10
-6	-6	-10	70.79	10.60
-6	-6	-10	90.69	10.70
-6	-6	-10	76.39	8.60
-6	-6	-10	107.09	14.40
-6	-6	-10	68.49	11.60
6	6	10	78.99	6.00
-7	-6	-10	513.35	29.20
-7	-6	-10	518.65	30.60
-7	-6	-10	542.75	28.80
-7	-6	-10	550.24	27.50
-7	-6	-10	536.85	28.10
7	6	10	531.55	25.80
-8	-6	-10	164.98	16.00
-8	-6	-10	132.09	22.50
-8	-6	-10	158.78	17.40
-8	-6	-10	145.49	16.40
-8	-6	-10	152.98	13.90
-8	-6	-10	183.08	12.90
-9	-6	-10	114.89	16.30
-9	-6	-10	170.38	15.80
-9	-6	-10	152.78	14.40
-9	-6	-10	125.19	17.80
-9	-6	-10	129.19	13.20
9	6	10	137.89	13.00
-10	-6	-10	23.40	8.20
-10	-6	-10	6.40	8.80
-10	-6	-10	3.90	6.70
-10	-6	-10	5.20	10.40
10	6	10	14.60	10.80
-11	-6	-10	17.40	11.00
-11	-6	-10	47.70	10.10
-11	-6	-10	30.10	8.40
-11	-6	-10	42.60	9.80
-11	-6	-10	41.00	8.20
-12	-6	-10	-3.20	10.20

-12	-6	-10	24.30	9.00
-12	-6	-10	18.80	12.70
12	6	10	18.20	16.50
10	-7	-10	11.40	11.30
-10	7	10	11.20	9.20
-10	7	10	3.80	11.80
9	-7	-10	36.30	14.20
9	-7	-10	40.70	11.60
-9	7	10	14.20	10.40
-9	7	10	8.10	9.70
8	-7	-10	321.07	25.00
8	-7	-10	332.67	25.10
-8	7	10	324.37	22.50
-8	7	10	336.27	24.00
7	-7	-10	67.29	11.80
7	-7	-10	111.39	16.10
-7	7	10	97.99	15.40
6	-7	-10	187.78	26.40
6	-7	-10	241.68	20.20
6	-7	-10	250.67	18.00
-6	7	10	182.58	18.40
-6	7	10	226.98	18.20
5	-7	-10	42.60	8.30
5	-7	-10	35.00	10.10
5	-7	-10	58.29	12.30
5	-7	-10	50.29	14.00
-5	7	10	55.99	9.40
-5	7	10	60.79	8.80
4	-7	-10	196.38	19.90
4	-7	-10	204.38	19.00
4	-7	-10	200.58	19.30
4	-7	-10	203.48	16.90
4	-7	-10	215.38	16.00
-4	7	10	188.58	17.60
-4	7	10	192.88	11.80
3	-7	-10	6.40	9.40
3	-7	-10	4.60	6.90
3	-7	-10	-0.80	8.00
3	-7	-10	3.40	7.50
3	-7	-10	9.20	11.20
-3	7	10	5.40	2.80
-2	7	10	9.60	2.40
-2	7	10	6.40	5.10
-1	7	10	207.68	10.00

-1	7	10	199.28	9.80
-1	7	10	177.98	10.10
-1	7	10	203.88	12.40
0	7	10	43.20	3.60
0	7	10	43.20	6.00
0	7	10	58.09	6.30
0	7	10	46.60	4.60
1	7	10	892.51	41.90
1	7	10	875.21	40.90
1	7	10	877.91	41.80
1	7	10	890.21	41.50
2	7	10	1343.97	62.29
2	7	10	1336.87	61.99
2	7	10	1333.07	62.39
2	7	10	1374.66	61.99
3	7	10	1117.69	52.19
3	7	10	1068.29	51.69
3	7	10	1108.59	51.19
3	7	10	1108.69	51.79
-4	-7	-10	137.69	10.00
4	7	10	143.99	13.00
4	7	10	132.79	13.20
4	7	10	148.29	15.00
4	7	10	133.69	10.40
-5	-7	-10	214.88	18.60
-5	-7	-10	241.28	13.40
-5	-7	-10	214.08	18.60
-5	-7	-10	193.98	24.10
-5	-7	-10	210.78	15.80
-5	-7	-10	207.48	15.00
-6	-7	-10	49.90	8.40
-6	-7	-10	53.19	7.70
-6	-7	-10	57.79	9.60
-6	-7	-10	41.60	6.40
-6	-7	-10	34.40	12.80
-6	-7	-10	46.30	9.70
6	7	10	54.89	5.30
-7	-7	-10	5.20	6.50
-7	-7	-10	-12.20	7.10
-7	-7	-10	4.70	5.50
-7	-7	-10	6.70	9.00
-7	-7	-10	-8.70	8.00
7	7	10	7.90	3.90
-8	-7	-10	591.54	37.60

-8	-7	-10	623.14	32.60
-8	-7	-10	568.14	31.60
-8	-7	-10	639.84	33.20
-8	-7	-10	619.74	31.30
-8	-7	-10	639.04	34.50
8	7	10	603.74	30.20
-9	-7	-10	26.90	7.00
-9	-7	-10	13.80	7.60
-9	-7	-10	21.50	9.40
-9	-7	-10	25.70	7.60
-9	-7	-10	40.30	9.40
9	7	10	16.90	7.30
-10	-7	-10	474.15	37.00
-10	-7	-10	514.95	37.10
-10	-7	-10	437.16	33.30
-10	-7	-10	472.95	30.00
-10	-7	-10	460.85	25.40
10	7	10	449.06	28.30
-11	-7	-10	26.00	7.80
-11	-7	-10	24.40	11.10
-11	-7	-10	25.30	9.70
-11	-7	-10	8.90	8.60
-11	-7	-10	6.40	7.90
-12	-7	-10	50.29	8.50
-12	-7	-10	58.59	10.70
-12	-7	-10	72.29	14.70
-12	-7	-10	43.30	9.80
12	7	10	43.50	18.20
10	-8	-10	131.49	17.80
-10	8	10	152.78	21.60
-10	8	10	132.59	15.30
9	-8	-10	25.40	12.00
9	-8	-10	1.40	12.60
-9	8	10	14.50	11.60
-9	8	10	8.10	9.00
8	-8	-10	156.68	19.20
8	-8	-10	104.19	14.20
-8	8	10	118.39	17.00
7	-8	-10	130.09	15.90
7	-8	-10	105.89	18.50
-7	8	10	107.69	15.60
6	-8	-10	246.58	18.90
6	-8	-10	301.27	29.00
6	-8	-10	267.27	37.30

6	-8	-10	272.17	22.10
-6	8	10	273.97	20.10
-6	8	10	269.67	19.50
5	-8	-10	39.70	12.20
5	-8	-10	21.30	12.40
-5	8	10	22.50	6.10
-5	8	10	22.90	8.10
4	-8	-10	303.77	22.40
4	-8	-10	310.37	22.30
4	-8	-10	343.47	20.90
4	-8	-10	310.57	23.80
4	-8	-10	311.27	19.60
-4	8	10	315.37	16.70
3	-8	-10	238.88	18.10
3	-8	-10	243.98	18.50
3	-8	-10	252.57	22.20
3	-8	-10	199.68	16.40
3	-8	-10	230.18	16.40
-3	8	10	226.88	12.40
2	-8	-10	488.45	26.90
2	-8	-10	459.85	26.50
2	-8	-10	486.15	26.70
2	-8	-10	474.05	27.40
-2	8	10	546.05	23.50
-1	8	10	583.54	25.90
-1	8	10	531.95	26.80
0	8	10	316.57	14.70
0	8	10	317.57	18.60
0	8	10	306.97	17.00
1	8	10	22.30	4.50
1	8	10	28.00	4.40
1	8	10	25.40	6.60
1	8	10	31.30	5.60
-2	-8	-10	365.36	20.10
2	8	10	380.56	19.40
2	8	10	365.96	20.20
2	8	10	368.96	20.80
2	8	10	383.46	19.60
-3	-8	-10	757.22	36.30
3	8	10	819.72	36.70
3	8	10	720.53	37.30
3	8	10	774.72	38.00
3	8	10	750.62	37.30
-4	-8	-10	97.09	8.70

-4	-8	-10	83.69	9.90
-4	-8	-10	93.69	13.50
4	8	10	101.09	9.60
4	8	10	129.39	19.80
4	8	10	107.29	13.30
4	8	10	93.19	15.80
-5	-8	-10	96.59	8.70
-5	-8	-10	100.59	11.40
-5	-8	-10	73.09	15.40
-5	-8	-10	76.69	12.30
-5	-8	-10	86.79	10.80
-5	-8	-10	91.99	16.90
-6	-8	-10	103.89	14.30
-6	-8	-10	105.09	16.10
-6	-8	-10	89.19	15.00
-6	-8	-10	84.79	9.40
-6	-8	-10	100.39	12.40
-6	-8	-10	82.09	11.20
6	8	10	98.69	7.00
-7	-8	-10	30.30	8.70
-7	-8	-10	36.80	9.30
-7	-8	-10	47.00	7.70
-7	-8	-10	32.10	7.90
-7	-8	-10	32.40	6.50
7	8	10	40.00	6.60
-8	-8	-10	462.95	24.80
-8	-8	-10	437.76	32.40
-8	-8	-10	455.65	26.60
-8	-8	-10	457.95	25.40
-8	-8	-10	469.35	26.10
-8	-8	-10	460.65	28.30
8	8	10	474.65	24.00
-9	-8	-10	169.38	13.90
-9	-8	-10	173.88	16.00
-9	-8	-10	153.38	17.60
-9	-8	-10	148.79	14.70
-9	-8	-10	150.78	17.40
-10	-8	-10	12.80	16.80
-10	-8	-10	7.40	6.80
-10	-8	-10	9.00	9.60
-10	-8	-10	18.30	11.60
-10	-8	-10	22.90	10.40
10	8	10	-14.80	11.00
-11	-8	-10	97.79	17.10

-11	-8	-10	91.29	11.60
-11	-8	-10	100.99	12.20
-11	-8	-10	88.09	11.70
-11	-8	-10	97.19	13.40
-12	-8	-10	-14.00	8.90
-12	-8	-10	7.30	7.40
-12	-8	-10	9.90	9.00
-12	-8	-10	-14.90	12.10
10	-9	-10	46.30	19.70
10	-9	-10	11.70	10.40
-10	9	10	34.90	14.50
-10	9	10	22.10	9.30
9	-9	-10	159.18	18.70
9	-9	-10	160.88	20.70
-9	9	10	144.79	17.00
-9	9	10	123.59	16.70
8	-9	-10	-2.20	10.70
8	-9	-10	-8.20	10.80
-8	9	10	-10.50	9.20
-8	9	10	33.70	11.60
7	-9	-10	26.50	11.50
7	-9	-10	26.20	9.20
-7	9	10	21.60	9.40
6	-9	-10	119.19	15.20
6	-9	-10	105.29	18.30
6	-9	-10	119.89	17.30
6	-9	-10	126.59	18.60
-6	9	10	117.09	15.10
-6	9	10	104.59	16.80
5	-9	-10	454.15	27.80
5	-9	-10	450.75	40.70
5	-9	-10	433.06	24.80
5	-9	-10	449.26	29.20
-5	9	10	422.16	22.90
-5	9	10	354.46	27.60
4	-9	-10	199.68	15.90
4	-9	-10	212.58	17.00
4	-9	-10	200.98	18.60
4	-9	-10	207.68	18.90
4	-9	-10	175.38	20.60
-4	9	10	218.18	12.40
3	-9	-10	138.29	13.30
3	-9	-10	110.79	15.20
3	-9	-10	113.39	13.70

3	-9	-10	123.69	21.50
3	-9	-10	105.99	14.60
-3	9	10	114.49	7.60
2	-9	-10	522.55	28.50
2	-9	-10	516.05	28.80
2	-9	-10	521.75	28.40
2	-9	-10	534.35	28.60
-2	9	10	564.04	27.10
1	-9	-10	5.00	7.70
1	-9	-10	17.10	12.50
1	-9	-10	5.10	7.20
1	-9	-10	21.20	8.10
-1	9	10	10.90	6.10
-1	9	10	5.00	2.00
0	9	10	185.08	8.80
0	9	10	162.98	15.60
0	9	10	172.88	12.10
-1	-9	-10	186.48	17.10
1	9	10	232.68	13.30
1	9	10	224.98	12.80
1	9	10	185.88	16.70
2	9	10	186.98	13.00
2	9	10	179.98	12.30
2	9	10	194.88	13.50
2	9	10	209.08	15.80
-3	-9	-10	774.42	36.20
3	9	10	769.42	38.70
3	9	10	806.62	36.70
3	9	10	723.43	37.90
3	9	10	718.73	37.50
-4	-9	-10	194.18	17.70
-4	-9	-10	202.38	15.30
-4	-9	-10	202.38	18.20
-5	-9	-10	134.09	17.70
-5	-9	-10	104.59	14.90
-5	-9	-10	106.39	13.60
-5	-9	-10	88.99	16.00
-5	-9	-10	114.39	9.60
-5	-9	-10	124.99	11.60
-5	-9	-10	104.99	17.50
-6	-9	-10	29.90	8.50
-6	-9	-10	31.00	6.90
-6	-9	-10	31.70	10.00
-6	-9	-10	25.90	5.80

-6	-9	-10	40.40	10.30
-6	-9	-10	35.20	7.50
6	9	10	33.80	4.50
-7	-9	-10	30.00	16.20
-7	-9	-10	1.00	5.10
-7	-9	-10	5.70	8.50
-7	-9	-10	6.10	6.10
-7	-9	-10	20.00	7.20
-7	-9	-10	-4.00	7.80
7	9	10	6.20	4.60
-8	-9	-10	150.18	17.70
-8	-9	-10	142.59	12.50
-8	-9	-10	146.79	13.40
-8	-9	-10	151.78	16.50
-8	-9	-10	148.89	15.30
8	9	10	139.39	12.00
-9	-9	-10	541.25	28.60
-9	-9	-10	531.45	28.10
-9	-9	-10	464.25	27.30
-9	-9	-10	520.75	30.10
-9	-9	-10	524.35	30.50
-10	-9	-10	30.20	7.60
-10	-9	-10	6.00	10.70
-10	-9	-10	15.60	8.80
-10	-9	-10	17.10	8.30
-10	-9	-10	19.80	9.40
10	9	10	11.10	11.50
-11	-9	-10	-13.40	7.40
-11	-9	-10	12.10	7.10
-11	-9	-10	3.60	7.40
-11	-9	-10	12.20	9.00
-12	-9	-10	64.19	10.70
-12	-9	-10	42.90	10.40
-12	-9	-10	65.89	11.20
-12	-9	-10	67.19	11.40
10	-10	-10	43.50	17.00
10	-10	-10	48.20	12.40
-10	10	10	63.29	20.00
-10	10	10	52.29	10.80
9	-10	-10	90.29	13.50
9	-10	-10	97.29	17.50
-9	10	10	91.59	15.20
-9	10	10	92.09	15.00
8	-10	-10	29.60	13.60

8	-10	-10	27.10	14.50
-8	10	10	49.50	15.20
-8	10	10	29.00	12.70
7	-10	-10	645.84	34.90
7	-10	-10	658.63	36.90
-7	10	10	649.74	35.80
6	-10	-10	105.59	18.80
6	-10	-10	70.89	16.10
6	-10	-10	127.19	14.60
6	-10	-10	86.69	18.50
-6	10	10	115.69	16.60
-6	10	10	100.09	17.30
5	-10	-10	179.68	20.90
5	-10	-10	202.98	23.60
5	-10	-10	208.78	21.10
5	-10	-10	191.28	16.40
-5	10	10	189.48	17.80
-5	10	10	175.48	13.80
4	-10	-10	317.67	22.30
4	-10	-10	335.87	22.90
4	-10	-10	332.07	20.80
4	-10	-10	373.46	26.30
4	-10	-10	355.66	23.40
-4	10	10	329.37	18.20
3	-10	-10	580.74	30.40
3	-10	-10	578.94	30.90
3	-10	-10	516.65	36.20
3	-10	-10	565.44	31.30
3	-10	-10	595.84	31.50
-3	10	10	610.64	27.70
2	-10	-10	4.10	9.60
2	-10	-10	23.50	8.50
2	-10	-10	18.70	8.50
2	-10	-10	6.10	6.80
2	-10	-10	16.30	7.70
-2	10	10	14.60	2.80
1	-10	-10	8.70	9.40
1	-10	-10	7.30	8.00
1	-10	-10	11.60	7.60
1	-10	-10	-3.70	7.10
-1	10	10	6.70	2.30
0	-10	-10	6.20	7.80
0	-10	-10	8.30	7.80
0	-10	-10	25.20	7.80

0	10	10	38.20	10.60
0	10	10	12.60	2.20
-1	-10	-10	207.18	15.30
1	10	10	207.18	13.90
1	10	10	184.18	19.30
-2	-10	-10	4.50	7.10
2	10	10	10.50	6.40
2	10	10	11.60	5.60
2	10	10	15.90	9.40
2	10	10	16.90	6.30
-3	-10	-10	104.99	9.90
-3	-10	-10	104.29	17.50
-3	-10	-10	111.89	13.50
3	10	10	106.59	13.70
3	10	10	74.49	10.70
3	10	10	80.49	12.10
3	10	10	123.19	10.80
-4	-10	-10	1417.86	65.69
-4	-10	-10	1521.05	64.59
-4	-10	-10	1408.76	65.19
-4	-10	-10	1298.37	66.39
-4	-10	-10	1369.66	65.19
-4	-10	-10	1364.66	67.49
-5	-10	-10	29.20	5.40
-5	-10	-10	17.00	8.70
-5	-10	-10	39.30	7.10
-5	-10	-10	30.90	7.90
-5	-10	-10	25.20	11.10
-5	-10	-10	13.40	8.30
-6	-10	-10	57.59	8.90
-6	-10	-10	49.70	7.00
-6	-10	-10	46.50	10.00
-6	-10	-10	37.20	11.10
-6	-10	-10	37.50	10.70
6	10	10	45.40	6.90
-7	-10	-10	730.63	37.60
-7	-10	-10	713.43	38.80
-7	-10	-10	752.42	39.40
-7	-10	-10	755.82	37.30
-7	-10	-10	761.22	36.80
-7	-10	-10	707.13	38.40
7	10	10	795.12	35.90
-8	-10	-10	19.60	9.40
-8	-10	-10	27.10	7.90

-8	-10	-10	24.00	9.10
-8	-10	-10	30.40	8.00
8	10	10	22.70	6.80
-9	-10	-10	203.88	16.70
-9	-10	-10	190.28	14.60
-9	-10	-10	209.68	15.90
-9	-10	-10	210.18	18.90
-9	-10	-10	200.68	18.70
9	10	10	192.88	16.80
-10	-10	-10	29.10	11.00
-10	-10	-10	54.99	9.10
-10	-10	-10	29.20	7.70
-10	-10	-10	31.50	7.60
-10	-10	-10	37.40	10.00
-11	-10	-10	6.20	8.00
-11	-10	-10	-4.40	10.60
-11	-10	-10	0.00	8.60
-11	-10	-10	9.50	7.20
10	-11	-10	92.39	16.60
10	-11	-10	65.89	14.40
-10	11	10	64.69	17.50
-10	11	10	54.79	10.40
9	-11	-10	70.19	13.40
9	-11	-10	83.39	13.00
-9	11	10	56.59	10.60
8	-11	-10	332.67	24.90
8	-11	-10	338.47	22.60
-8	11	10	284.07	25.60
-8	11	10	313.77	23.70
7	-11	-10	27.10	9.00
7	-11	-10	24.60	11.90
-7	11	10	14.90	9.80
6	-11	-10	71.29	12.80
6	-11	-10	58.89	15.00
6	-11	-10	23.80	19.30
6	-11	-10	63.39	10.10
-6	11	10	45.50	10.90
-6	11	10	24.50	20.60
5	-11	-10	84.89	14.50
5	-11	-10	68.89	13.20
5	-11	-10	61.09	11.90
5	-11	-10	81.39	13.70
-5	11	10	69.49	11.40
-5	11	10	92.89	15.70

4	-11	-10	294.77	31.00
4	-11	-10	235.58	25.30
4	-11	-10	244.48	20.20
4	-11	-10	231.18	17.90
-4	11	10	252.37	14.80
3	-11	-10	19.00	15.70
3	-11	-10	8.80	7.40
3	-11	-10	4.90	8.00
3	-11	-10	12.10	8.30
3	-11	-10	4.80	8.40
-3	11	10	10.70	3.50
2	-11	-10	34.80	8.90
2	-11	-10	31.80	9.80
2	-11	-10	44.40	9.30
2	-11	-10	55.89	18.80
2	-11	-10	28.60	8.40
2	-11	-10	41.30	8.80
-2	11	10	40.90	4.60
1	-11	-10	808.42	41.50
1	-11	-10	809.62	40.00
1	-11	-10	767.22	39.60
1	-11	-10	791.02	40.70
1	-11	-10	767.02	39.70
-1	11	10	864.31	37.00
0	-11	-10	2.70	7.60
0	-11	-10	-4.40	11.20
0	-11	-10	10.60	7.10
0	-11	-10	-5.20	6.90
0	-11	-10	-2.50	9.80
0	11	10	5.30	12.30
0	11	10	3.00	2.10
-1	-11	-10	47.40	9.30
-1	-11	-10	44.10	8.40
-1	-11	-10	57.19	9.20
-1	-11	-10	42.60	10.50
-1	-11	-10	54.99	8.60
1	11	10	30.00	13.10
-2	-11	-10	1912.31	87.39
-2	-11	-10	2061.79	87.89
-2	-11	-10	2023.80	87.49
-2	-11	-10	1931.61	88.39
-2	-11	-10	1951.30	88.99
2	11	10	1757.52	88.19
2	11	10	1992.30	87.99

2	11	10	1796.92	88.59
2	11	10	1691.13	90.69
-3	-11	-10	450.15	25.50
-3	-11	-10	416.36	26.30
-3	-11	-10	470.35	24.30
-3	-11	-10	424.76	25.40
3	11	10	414.36	25.40
-4	-11	-10	11.60	9.50
-4	-11	-10	-3.60	7.50
-4	-11	-10	9.80	9.10
-4	-11	-10	12.30	7.60
-4	-11	-10	9.30	5.30
-4	-11	-10	1.50	8.50
-5	-11	-10	84.79	9.40
-5	-11	-10	78.99	12.30
-5	-11	-10	95.99	11.30
-5	-11	-10	81.69	13.40
-5	-11	-10	90.89	13.60
-5	-11	-10	89.79	11.30
-6	-11	-10	24.10	5.80
-6	-11	-10	27.90	6.70
-6	-11	-10	25.90	7.90
-6	-11	-10	51.69	18.50
-6	-11	-10	20.30	8.60
6	11	10	21.90	5.40
-7	-11	-10	16.30	8.80
-7	-11	-10	17.50	7.90
-7	-11	-10	12.50	7.10
-7	-11	-10	44.20	10.50
-7	-11	-10	8.10	5.90
-7	-11	-10	13.00	9.20
7	11	10	21.20	6.20
-8	-11	-10	157.48	15.10
-8	-11	-10	182.18	14.50
-8	-11	-10	155.28	16.70
-8	-11	-10	160.48	17.50
-8	-11	-10	155.28	13.50
-8	-11	-10	167.88	19.30
8	11	10	154.68	14.10
-9	-11	-10	74.19	11.80
-9	-11	-10	63.19	10.90
-9	-11	-10	53.49	9.30
-9	-11	-10	50.99	8.40
-9	-11	-10	91.79	16.60

9	11	10	49.10	10.20
-10	-11	-10	385.16	23.10
-10	-11	-10	400.66	25.50
-10	-11	-10	351.06	21.50
-10	-11	-10	350.76	23.60
-10	-11	-10	370.06	21.90
-11	-11	-10	-6.40	7.10
-11	-11	-10	-1.40	7.70
-11	-11	-10	14.80	8.70
-11	-11	-10	6.60	8.30
9	-12	-10	50.39	11.40
9	-12	-10	30.80	13.60
-9	12	10	39.80	10.20
-9	12	10	26.10	14.10
8	-12	-10	171.28	20.00
8	-12	-10	165.18	17.20
-8	12	10	134.99	17.80
-8	12	10	142.19	17.10
7	-12	-10	7.70	13.00
7	-12	-10	-5.30	8.70
-7	12	10	10.70	10.20
6	-12	-10	381.46	29.20
6	-12	-10	333.07	27.10
6	-12	-10	331.17	22.00
6	-12	-10	418.86	37.30
-6	12	10	304.67	24.00
5	-12	-10	11.70	11.40
5	-12	-10	51.39	14.30
5	-12	-10	30.00	14.00
5	-12	-10	39.80	11.30
5	-12	-10	20.20	7.90
-5	12	10	22.90	10.00
-5	12	10	16.60	7.80
4	-12	-10	32.80	11.40
4	-12	-10	25.70	9.10
4	-12	-10	37.50	10.70
4	-12	-10	43.40	9.30
-4	12	10	43.60	6.20
3	-12	-10	1035.00	48.90
3	-12	-10	914.71	49.90
3	-12	-10	925.61	54.19
-3	12	10	1014.10	45.60
2	-12	-10	873.11	43.60
2	-12	-10	874.71	43.90

2	-12	-10	843.82	44.40
2	-12	-10	884.41	43.70
2	-12	-10	925.91	44.30
-2	12	10	899.31	41.00
1	-12	-10	49.00	8.50
1	-12	-10	60.89	9.40
1	-12	-10	74.19	10.90
1	-12	-10	57.69	9.10
1	-12	-10	51.89	10.60
-1	12	10	60.29	5.00
0	-12	-10	204.48	21.30
0	-12	-10	198.18	15.40
0	-12	-10	175.38	17.30
0	-12	-10	179.68	15.00
0	-12	-10	156.98	15.70
0	12	10	196.98	9.80
-1	-12	-10	3.50	8.70
-1	-12	-10	9.00	7.60
-1	-12	-10	14.70	8.40
-1	-12	-10	16.60	9.00
-1	-12	-10	2.00	7.10
-1	-12	-10	-9.10	15.70
1	12	10	18.30	17.30
1	12	10	6.60	3.90
-2	-12	-10	54.29	14.50
-2	-12	-10	53.49	9.50
-2	-12	-10	53.09	8.10
-2	-12	-10	35.50	11.60
-2	-12	-10	51.29	8.00
-2	-12	-10	74.29	13.40
-3	-12	-10	457.35	27.50
-3	-12	-10	497.25	25.60
-3	-12	-10	482.05	32.30
-3	-12	-10	462.25	28.10
-3	-12	-10	450.05	29.90
-3	-12	-10	520.25	26.10
-3	-12	-10	507.45	27.60
-4	-12	-10	12.00	5.80
-4	-12	-10	20.40	7.30
-4	-12	-10	8.10	9.00
-4	-12	-10	23.20	10.60
-4	-12	-10	16.70	8.70
-4	-12	-10	13.60	7.80
-5	-12	-10	43.10	13.50

-5	-12	-10	53.59	7.50
-5	-12	-10	46.00	8.30
-5	-12	-10	59.99	8.70
5	12	10	41.90	6.40
-6	-12	-10	72.79	12.70
-6	-12	-10	53.89	9.40
-6	-12	-10	84.99	9.70
-6	-12	-10	77.39	12.70
-6	-12	-10	68.69	9.60
-6	-12	-10	73.19	9.30
6	12	10	56.39	9.70
-7	-12	-10	282.57	18.90
-7	-12	-10	279.87	19.70
-7	-12	-10	258.67	18.20
-7	-12	-10	246.18	19.50
-7	-12	-10	260.17	21.10
-7	-12	-10	267.47	17.30
7	12	10	256.67	16.70
-8	-12	-10	478.05	26.60
-8	-12	-10	457.05	28.40
-8	-12	-10	478.65	26.30
-8	-12	-10	499.35	28.30
-8	-12	-10	494.85	28.50
-8	-12	-10	460.05	25.90
8	12	10	459.05	26.20
-9	-12	-10	14.50	8.90
-9	-12	-10	12.00	7.10
-9	-12	-10	30.20	12.10
9	12	10	6.40	9.80
-10	-12	-10	-0.90	7.30
-10	-12	-10	7.00	8.50
-10	-12	-10	14.60	7.90
-10	-12	-10	10.10	7.40
-11	-12	-10	25.70	9.10
-11	-12	-10	28.40	8.00
-11	-12	-10	30.00	8.90
-11	-12	-10	45.00	8.60
9	-13	-10	162.88	16.40
9	-13	-10	226.08	35.00
-9	13	10	141.29	25.20
-9	13	10	126.39	15.70
8	-13	-10	9.40	8.80
8	-13	-10	12.40	14.00
-8	13	10	25.50	13.10

-8	13	10	-2.90	10.00
7	-13	-10	146.49	23.50
7	-13	-10	210.68	32.40
-7	13	10	163.98	23.00
6	-13	-10	43.60	14.90
6	-13	-10	59.69	9.60
6	-13	-10	50.09	14.10
-6	13	10	48.30	11.40
5	-13	-10	329.77	21.10
5	-13	-10	345.67	29.40
5	-13	-10	314.47	26.20
5	-13	-10	308.77	23.90
5	-13	-10	294.77	28.40
-5	13	10	302.07	20.40
4	-13	-10	104.09	16.20
4	-13	-10	90.29	19.50
4	-13	-10	109.99	18.90
4	-13	-10	131.59	19.30
4	-13	-10	144.39	15.70
-4	13	10	142.19	11.50
3	-13	-10	294.17	21.30
3	-13	-10	284.87	20.20
3	-13	-10	294.77	22.10
3	-13	-10	248.38	19.70
-3	13	10	283.57	15.50
2	-13	-10	423.56	26.00
2	-13	-10	446.96	25.80
2	-13	-10	416.06	25.20
2	-13	-10	453.15	27.00
2	-13	-10	444.56	25.80
-2	13	10	451.75	21.80
1	-13	-10	19.50	8.40
1	-13	-10	12.70	7.60
1	-13	-10	7.50	7.80
1	-13	-10	21.80	9.30
1	-13	-10	13.00	10.50
-1	13	10	13.80	3.10
0	-13	-10	88.89	13.50
0	-13	-10	80.19	12.10
0	-13	-10	89.09	14.20
0	-13	-10	71.19	10.10
0	-13	-10	100.59	12.70
0	13	10	84.79	5.90
-1	-13	-10	339.77	20.90

-1	-13	-10	331.87	25.80
-1	-13	-10	312.47	31.90
-1	-13	-10	361.36	21.80
-1	-13	-10	369.06	22.60
-1	-13	-10	362.26	21.50
-1	-13	-10	358.96	21.30
1	13	10	382.46	17.10
-2	-13	-10	23.40	8.40
-2	-13	-10	11.40	11.20
-2	-13	-10	28.60	7.80
-2	-13	-10	19.80	7.20
-2	-13	-10	39.20	11.20
-2	-13	-10	29.80	8.20
2	13	10	22.70	3.70
-3	-13	-10	1379.66	68.79
-3	-13	-10	1400.16	70.89
-3	-13	-10	1487.45	67.69
-3	-13	-10	1501.15	68.39
-3	-13	-10	1471.65	68.59
-3	-13	-10	1439.56	67.99
3	13	10	1476.55	65.09
-4	-13	-10	91.69	12.10
-4	-13	-10	95.49	12.60
-4	-13	-10	108.79	13.60
-4	-13	-10	104.69	16.30
-4	-13	-10	105.09	11.10
-4	-13	-10	79.29	14.10
4	13	10	92.19	6.20
-5	-13	-10	62.79	11.40
-5	-13	-10	47.20	8.50
-5	-13	-10	33.50	9.50
-5	-13	-10	35.40	7.30
-5	-13	-10	40.30	8.50
-5	-13	-10	38.70	7.70
5	13	10	43.20	7.10
-6	-13	-10	21.10	7.70
-6	-13	-10	15.60	7.70
-6	-13	-10	15.30	6.50
-6	-13	-10	24.00	7.70
-6	-13	-10	12.90	11.90
-6	-13	-10	23.50	9.50
6	13	10	17.40	6.70
-7	-13	-10	16.40	7.80
-7	-13	-10	6.60	8.80

-7	-13	-10	6.40	7.90
-7	-13	-10	9.80	10.70
-7	-13	-10	1.00	7.40
7	13	10	-1.00	7.40
-8	-13	-10	195.98	20.10
-8	-13	-10	272.47	19.40
-8	-13	-10	205.18	16.10
-8	-13	-10	249.97	17.50
-8	-13	-10	218.08	17.00
-8	-13	-10	217.18	18.50
8	13	10	206.98	17.80
-9	-13	-10	572.04	29.90
-9	-13	-10	555.64	30.20
-9	-13	-10	541.95	32.00
-9	-13	-10	564.64	31.70
-9	-13	-10	526.85	29.20
-10	-13	-10	2.60	7.80
-10	-13	-10	8.50	8.20
-10	-13	-10	4.20	7.60
-10	-13	-10	8.10	7.40
-11	-13	-10	10.20	9.50
-11	-13	-10	18.80	7.60
-11	-13	-10	3.10	7.50
-11	-13	-10	16.20	7.60
9	-14	-10	20.20	10.00
-9	14	10	19.90	9.60
-9	14	10	53.89	16.30
8	-14	-10	58.29	18.10
8	-14	-10	75.29	13.70
-8	14	10	79.69	14.50
-8	14	10	55.79	11.20
7	-14	-10	304.37	36.20
7	-14	-10	276.87	28.20
7	-14	-10	331.57	21.40
-7	14	10	283.37	23.70
6	-14	-10	39.60	9.10
6	-14	-10	48.50	17.80
6	-14	-10	39.90	13.90
-6	14	10	63.59	11.80
5	-14	-10	76.39	23.20
5	-14	-10	70.29	12.20
5	-14	-10	53.69	15.60
5	-14	-10	54.39	13.40
4	-14	-10	961.40	48.70

4	-14	-10	979.50	46.80
4	-14	-10	930.01	49.00
4	-14	-10	929.31	47.00
-4	14	10	879.51	44.80
3	-14	-10	799.12	41.40
3	-14	-10	733.03	39.80
3	-14	-10	772.82	40.80
3	-14	-10	793.32	39.90
-3	14	10	787.72	37.40
2	-14	-10	-6.20	8.90
2	-14	-10	-2.10	8.50
2	-14	-10	6.30	8.80
2	-14	-10	9.60	8.70
2	-14	-10	-12.70	7.70
-2	14	10	8.90	3.70
1	-14	-10	11.20	10.80
1	-14	-10	21.60	9.40
-1	14	10	23.20	3.90
-1	14	10	29.60	12.20
0	-14	-10	79.99	9.80
0	-14	-10	78.39	12.70
0	-14	-10	76.79	14.40
0	-14	-10	53.09	23.30
0	-14	-10	54.09	9.60
0	-14	-10	62.49	8.80
0	14	10	65.09	5.70
-1	-14	-10	312.37	23.80
-1	-14	-10	311.07	38.90
-1	-14	-10	322.27	22.10
-1	-14	-10	335.87	19.80
-1	-14	-10	311.77	20.00
-1	-14	-10	338.37	20.40
1	14	10	325.67	15.90
-2	-14	-10	7.80	7.50
-2	-14	-10	13.20	10.70
-2	-14	-10	9.20	6.80
-2	-14	-10	23.70	10.90
-2	-14	-10	-9.00	7.30
2	14	10	2.60	2.60
-3	-14	-10	214.88	16.90
-3	-14	-10	237.98	25.90
-3	-14	-10	228.88	18.20
-3	-14	-10	261.77	19.30
-3	-14	-10	250.47	20.00

3	14	10	236.08	12.00
-4	-14	-10	1143.29	53.89
-4	-14	-10	1005.10	53.29
-4	-14	-10	1138.79	53.09
-4	-14	-10	1106.89	53.59
-4	-14	-10	1111.19	52.39
4	14	10	1108.09	50.19
-5	-14	-10	46.60	9.20
-5	-14	-10	66.49	10.50
-5	-14	-10	39.80	9.10
-5	-14	-10	70.39	11.80
-5	-14	-10	58.19	9.70
-5	-14	-10	68.69	11.20
5	14	10	59.89	8.10
-6	-14	-10	113.69	12.40
-6	-14	-10	131.09	14.10
-6	-14	-10	120.69	14.40
-6	-14	-10	102.79	16.10
-6	-14	-10	103.59	13.60
-7	-14	-10	189.38	15.70
-7	-14	-10	171.28	15.80
-7	-14	-10	177.28	14.30
-7	-14	-10	166.88	15.00
-7	-14	-10	134.19	16.60
-7	-14	-10	175.18	21.20
7	14	10	120.09	15.00
-8	-14	-10	112.59	15.60
-8	-14	-10	85.59	12.60
-8	-14	-10	107.19	14.10
-8	-14	-10	94.29	16.20
-8	-14	-10	122.99	13.70
8	14	10	98.09	16.00
-9	-14	-10	143.99	14.80
-9	-14	-10	113.49	15.90
-9	-14	-10	141.09	14.00
-9	-14	-10	126.59	13.20
-9	-14	-10	113.79	17.50
-10	-14	-10	42.50	8.60
-10	-14	-10	33.40	9.00
-10	-14	-10	70.29	11.40
-10	-14	-10	39.20	11.10
-11	-14	-10	-1.00	7.30
-11	-14	-10	1.70	9.50
-11	-14	-10	-15.00	9.70

-11	-14	-10	7.00	7.80
8	-15	-10	304.87	19.40
-8	15	10	267.77	24.30
-8	15	10	259.97	20.60
7	-15	-10	14.10	8.10
7	-15	-10	7.70	19.90
-7	15	10	9.40	9.90
6	-15	-10	91.39	15.10
6	-15	-10	104.29	24.70
5	-15	-10	46.10	16.10
5	-15	-10	35.20	11.20
5	-15	-10	24.70	13.00
5	-15	-10	36.50	9.20
-5	15	10	29.80	12.20
4	-15	-10	60.19	12.80
4	-15	-10	68.99	11.40
4	-15	-10	108.39	16.80
4	-15	-10	63.59	13.00
-4	15	10	82.39	11.90
3	-15	-10	90.09	12.80
3	-15	-10	91.29	12.90
3	-15	-10	100.49	15.00
3	-15	-10	102.29	16.10
-3	15	10	81.29	9.60
2	-15	-10	102.19	15.70
2	-15	-10	63.19	11.00
2	-15	-10	84.49	11.20
2	-15	-10	93.69	16.00
2	-15	-10	56.09	9.90
-2	15	10	78.89	7.90
1	-15	-10	192.98	17.30
1	-15	-10	196.28	20.90
1	-15	-10	169.88	17.40
1	-15	-10	191.88	16.90
1	-15	-10	211.18	16.90
-1	15	10	186.18	11.40
0	-15	-10	-5.60	8.10
0	-15	-10	7.40	7.60
0	-15	-10	2.30	7.40
0	-15	-10	-6.70	7.90
0	15	10	2.10	3.20
-1	-15	-10	129.39	19.10
-1	-15	-10	125.29	13.90
-1	-15	-10	133.89	13.60

-1	-15	-10	164.18	15.20
1	15	10	136.09	8.50
-2	-15	-10	2847.42	142.39
-2	-15	-10	2867.91	136.79
-2	-15	-10	3083.69	136.79
-2	-15	-10	2996.80	136.19
-2	-15	-10	3081.09	136.69
-2	-15	-10	3116.69	136.49
2	15	10	2984.80	134.29
-3	-15	-10	157.78	15.50
-3	-15	-10	157.08	25.90
-3	-15	-10	144.29	13.70
-3	-15	-10	101.19	15.50
-3	-15	-10	160.58	14.10
-3	-15	-10	134.09	14.50
3	15	10	151.28	8.80
-4	-15	-10	37.10	8.90
-4	-15	-10	47.90	11.80
-4	-15	-10	37.20	9.00
-4	-15	-10	28.30	7.40
-4	-15	-10	39.00	10.00
-4	-15	-10	35.10	9.90
4	15	10	38.80	6.40
-5	-15	-10	-3.20	7.70
-5	-15	-10	5.00	8.00
-5	-15	-10	20.70	10.20
-5	-15	-10	9.40	9.30
-5	-15	-10	19.00	9.00
-5	-15	-10	5.10	7.10
5	15	10	-2.60	5.50
-6	-15	-10	9.90	8.40
-6	-15	-10	12.80	7.40
-6	-15	-10	4.20	8.30
-6	-15	-10	2.90	13.80
-6	-15	-10	6.70	8.60
6	15	10	3.10	7.80
-7	-15	-10	34.60	9.40
-7	-15	-10	15.80	8.40
-7	-15	-10	27.40	8.10
-7	-15	-10	3.40	8.50
-7	-15	-10	13.80	8.80
7	15	10	12.30	9.10
-8	-15	-10	-13.10	9.30
-8	-15	-10	9.20	7.70

-8	-15	-10	2.80	13.50
8	15	10	24.80	10.70
-9	-15	-10	223.88	19.40
-9	-15	-10	252.47	18.00
-9	-15	-10	261.07	17.70
-9	-15	-10	234.88	17.50
-9	-15	-10	239.08	17.40
-10	-15	-10	202.98	15.60
-10	-15	-10	191.88	15.70
-10	-15	-10	193.78	16.20
-10	-15	-10	218.58	16.30
8	-16	-10	51.49	9.10
-8	16	10	73.19	15.10
-8	16	10	40.10	10.00
7	-16	-10	20.50	8.50
-7	16	10	15.10	9.90
6	-16	-10	86.29	15.70
6	-16	-10	103.19	15.10
-6	16	10	129.79	17.80
5	-16	-10	161.38	17.20
5	-16	-10	156.58	19.20
5	-16	-10	161.18	27.50
-5	16	10	179.28	28.10
4	-16	-10	1.60	12.30
4	-16	-10	2.20	9.10
4	-16	-10	17.80	9.10
4	-16	-10	1.70	11.20
-4	16	10	13.40	8.70
3	-16	-10	799.42	41.60
3	-16	-10	814.22	42.90
3	-16	-10	815.12	41.60
3	-16	-10	809.62	42.90
-3	16	10	793.72	39.20
2	-16	-10	389.06	25.40
2	-16	-10	412.26	26.50
2	-16	-10	425.96	26.40
2	-16	-10	449.76	26.60
2	-16	-10	394.46	26.30
-2	16	10	415.76	21.60
1	-16	-10	46.90	9.80
1	-16	-10	44.60	9.60
1	-16	-10	26.30	12.10
1	-16	-10	32.30	9.70
1	-16	-10	38.80	9.30

-1	16	10	32.90	11.40
-1	16	10	37.70	6.50
0	-16	-10	52.29	9.50
0	-16	-10	52.49	10.20
0	-16	-10	51.59	9.20
0	-16	-10	54.09	9.70
0	-16	-10	48.40	16.60
0	16	10	51.29	6.20
-1	-16	-10	20.00	9.30
-1	-16	-10	23.80	7.80
-1	-16	-10	31.00	7.80
-1	-16	-10	31.40	7.70
-1	-16	-10	26.20	11.40
1	16	10	33.10	5.50
-2	-16	-10	21.70	11.00
-2	-16	-10	26.90	8.00
-2	-16	-10	29.70	10.10
-2	-16	-10	24.30	8.00
-2	-16	-10	31.80	7.70
2	16	10	34.00	5.40
-3	-16	-10	278.47	18.30
-3	-16	-10	245.18	17.00
-3	-16	-10	234.38	19.10
-3	-16	-10	260.37	19.20
-3	-16	-10	261.27	26.80
-3	-16	-10	274.27	17.80
3	16	10	243.38	13.70
-4	-16	-10	194.18	22.90
-4	-16	-10	189.28	17.60
-4	-16	-10	189.28	18.20
-4	-16	-10	203.78	16.10
-4	-16	-10	195.38	15.70
-4	-16	-10	181.58	16.40
4	16	10	188.18	12.10
-5	-16	-10	127.99	14.70
-5	-16	-10	161.28	17.00
-5	-16	-10	117.79	16.00
-5	-16	-10	109.59	18.10
-5	-16	-10	100.79	18.00
-5	-16	-10	109.39	12.80
5	16	10	115.09	11.80
-6	-16	-10	14.80	7.50
-6	-16	-10	7.40	7.90
-6	-16	-10	14.20	8.90

-6	-16	-10	19.40	8.50
-6	-16	-10	0.60	13.20
-6	-16	-10	4.10	9.30
6	16	10	26.50	8.70
-7	-16	-10	171.98	17.60
-7	-16	-10	173.98	15.70
-7	-16	-10	126.69	15.10
-7	-16	-10	161.88	16.60
-7	-16	-10	155.28	15.90
-8	-16	-10	340.07	21.60
-8	-16	-10	346.77	21.90
-8	-16	-10	380.86	22.00
-8	-16	-10	339.27	22.00
-8	-16	-10	329.77	22.00
-9	-16	-10	2.60	9.30
-9	-16	-10	-8.20	8.00
-9	-16	-10	16.10	8.20
-9	-16	-10	2.00	7.60
-9	-16	-10	17.60	7.40
-10	-16	-10	16.80	9.30
-10	-16	-10	25.10	7.60
-10	-16	-10	19.00	7.80
-10	-16	-10	15.00	7.40
8	-17	-10	44.80	9.70
-8	17	10	50.69	13.50
7	-17	-10	101.59	13.30
7	-17	-10	170.08	29.30
-7	17	10	116.29	15.70
6	-17	-10	40.70	9.00
6	-17	-10	49.20	13.40
-6	17	10	25.80	10.10
5	-17	-10	232.88	30.70
5	-17	-10	275.37	24.90
5	-17	-10	275.47	27.10
-5	17	10	257.67	26.20
4	-17	-10	85.09	14.50
4	-17	-10	92.49	14.30
4	-17	-10	91.29	16.20
4	-17	-10	88.39	11.70
-4	17	10	74.59	12.10
3	-17	-10	33.00	10.30
3	-17	-10	20.40	9.10
3	-17	-10	18.40	11.20
3	-17	-10	22.80	10.80

-3	17	10	6.40	7.30
2	-17	-10	180.68	20.30
2	-17	-10	215.18	19.10
2	-17	-10	205.38	18.40
2	-17	-10	177.08	18.70
-2	17	10	174.08	13.20
1	-17	-10	7.30	8.50
1	-17	-10	-12.10	8.10
1	-17	-10	-3.10	8.40
1	-17	-10	12.00	8.20
1	-17	-10	4.40	12.80
-1	17	10	3.10	4.50
-1	17	10	-3.70	10.90
0	-17	-10	8.00	17.00
0	-17	-10	-5.60	7.60
0	-17	-10	7.20	7.70
0	-17	-10	2.10	8.00
0	-17	-10	-10.00	8.70
0	17	10	5.20	4.40
-1	-17	-10	171.78	15.70
-1	-17	-10	196.28	16.60
-1	-17	-10	164.28	15.60
-1	-17	-10	185.18	18.40
-1	-17	-10	142.49	17.80
1	17	10	196.08	11.20
-2	-17	-10	175.38	20.10
-2	-17	-10	125.59	14.80
-2	-17	-10	156.48	14.40
-2	-17	-10	157.78	15.20
-2	-17	-10	186.38	16.80
2	17	10	119.09	9.80
-3	-17	-10	1553.04	71.49
-3	-17	-10	1444.56	70.59
-3	-17	-10	1505.05	74.49
-3	-17	-10	1545.65	71.29
-3	-17	-10	1452.15	71.29
-3	-17	-10	1532.75	71.59
3	17	10	1550.04	69.09
-4	-17	-10	3.90	8.70
-4	-17	-10	6.50	9.00
-4	-17	-10	6.50	7.40
-4	-17	-10	6.40	8.70
-4	-17	-10	17.10	9.60
4	17	10	13.90	5.70

-5	-17	-10	16.80	9.90
-5	-17	-10	-1.10	10.90
-5	-17	-10	-1.10	8.20
-5	-17	-10	5.60	6.80
-5	-17	-10	10.50	8.00
-5	-17	-10	-4.20	8.50
5	17	10	14.10	7.40
-6	-17	-10	104.89	17.50
-6	-17	-10	139.69	22.00
-6	-17	-10	126.69	13.20
-6	-17	-10	136.49	15.10
-6	-17	-10	112.89	15.00
-6	-17	-10	124.29	13.70
6	17	10	98.39	15.80
-7	-17	-10	82.99	14.00
-7	-17	-10	75.09	12.00
-7	-17	-10	78.49	14.00
-7	-17	-10	73.89	12.60
-7	-17	-10	74.19	13.90
-8	-17	-10	330.07	21.90
-8	-17	-10	410.96	21.80
-8	-17	-10	356.66	22.00
-8	-17	-10	312.07	21.50
-8	-17	-10	352.66	21.70
-9	-17	-10	157.78	14.20
-9	-17	-10	147.69	15.50
-9	-17	-10	146.59	15.90
-9	-17	-10	152.18	13.90
-9	-17	-10	183.68	14.50
-10	-17	-10	46.40	10.10
-10	-17	-10	21.00	8.00
-10	-17	-10	25.10	8.00
7	-18	-10	40.30	8.50
-7	18	10	43.40	9.50
6	-18	-10	47.10	13.40
6	-18	-10	25.80	8.60
-6	18	10	31.50	9.90
5	-18	-10	-7.20	17.90
5	-18	-10	2.70	8.90
5	-18	-10	20.20	11.00
-5	18	10	-6.90	9.60
4	-18	-10	716.23	42.40
4	-18	-10	526.85	40.30
4	-18	-10	740.33	50.19

-4	18	10	568.14	36.30
3	-18	-10	150.58	18.50
3	-18	-10	159.88	17.40
3	-18	-10	142.19	18.00
3	-18	-10	147.59	19.70
-3	18	10	147.19	15.90
2	-18	-10	225.18	19.50
2	-18	-10	262.87	21.80
2	-18	-10	180.48	19.20
2	-18	-10	188.08	19.00
-2	18	10	224.28	15.40
1	-18	-10	10.40	8.60
1	-18	-10	19.90	9.20
1	-18	-10	6.20	9.10
1	-18	-10	7.00	8.60
0	-18	-10	-12.80	8.90
0	-18	-10	8.70	8.00
0	-18	-10	4.10	7.70
0	-18	-10	10.60	8.40
0	18	10	2.90	5.10
0	18	10	16.90	11.20
-1	-18	-10	33.40	8.40
-1	-18	-10	35.90	8.30
-1	-18	-10	47.70	11.60
-1	-18	-10	32.10	8.40
1	18	10	49.00	7.60
-2	-18	-10	12.40	7.30
-2	-18	-10	0.60	9.10
-2	-18	-10	4.80	6.90
-2	-18	-10	22.70	12.40
-2	-18	-10	0.60	7.70
2	18	10	6.20	4.90
-3	-18	-10	235.68	16.80
-3	-18	-10	255.57	18.50
-3	-18	-10	185.38	27.10
-3	-18	-10	228.98	17.00
-3	-18	-10	204.28	17.60
-4	-18	-10	564.24	30.10
-4	-18	-10	569.34	30.80
-4	-18	-10	547.55	29.20
-4	-18	-10	553.74	31.30
-4	-18	-10	534.35	30.00
4	18	10	565.34	28.30
-5	-18	-10	-3.40	7.10

-5	-18	-10	9.80	10.10
-5	-18	-10	2.00	8.80
-5	-18	-10	-1.20	8.00
-5	-18	-10	3.90	6.90
5	18	10	11.80	8.20
-6	-18	-10	233.28	18.10
-6	-18	-10	242.88	19.10
-6	-18	-10	223.88	17.20
-6	-18	-10	217.98	25.00
6	18	10	183.78	19.40
-7	-18	-10	127.29	14.50
-7	-18	-10	116.89	14.10
-7	-18	-10	133.29	14.80
-7	-18	-10	118.29	15.60
-7	-18	-10	126.09	13.70
-8	-18	-10	131.19	15.00
-8	-18	-10	104.69	13.30
-8	-18	-10	155.98	14.00
-8	-18	-10	157.48	13.80
-8	-18	-10	151.48	13.70
-9	-18	-10	35.30	7.20
-9	-18	-10	58.19	11.90
-9	-18	-10	23.50	7.60
-9	-18	-10	44.20	9.50
7	-19	-10	4.00	16.10
6	-19	-10	5.90	12.60
6	-19	-10	18.70	8.70
-6	19	10	15.10	9.10
5	-19	-10	26.50	9.30
5	-19	-10	11.10	19.60
5	-19	-10	26.50	11.00
-5	19	10	-6.90	8.20
4	-19	-10	75.79	11.60
4	-19	-10	82.79	15.50
4	-19	-10	73.49	15.10
4	-19	-10	72.89	13.60
3	-19	-10	-1.20	17.80
3	-19	-10	0.50	10.90
-3	19	10	15.40	11.30
2	-19	-10	124.19	16.70
2	-19	-10	122.09	16.70
2	-19	-10	122.79	16.40
-2	19	10	102.59	14.50
1	-19	-10	32.50	9.30

1	-19	-10	38.10	9.80
1	-19	-10	18.80	9.40
1	-19	-10	17.30	15.60
1	-19	-10	43.30	9.20
-1	19	10	45.70	14.30
-1	19	10	43.10	8.00
0	-19	-10	6.50	9.50
0	-19	-10	9.50	7.80
0	-19	-10	17.40	8.40
0	-19	-10	12.20	8.00
0	19	10	6.40	6.10
0	19	10	6.80	11.70
-1	-19	-10	303.57	21.10
-1	-19	-10	341.57	24.50
-1	-19	-10	320.87	20.80
-1	-19	-10	309.97	20.20
1	19	10	306.97	17.90
-2	-19	-10	992.90	49.30
-2	-19	-10	990.20	51.99
-2	-19	-10	1025.50	49.20
-2	-19	-10	1016.60	49.40
-2	-19	-10	1009.60	49.80
2	19	10	1012.80	47.50
-3	-19	-10	4.60	6.80
-3	-19	-10	10.10	7.40
-3	-19	-10	11.20	8.90
-3	-19	-10	-1.20	7.90
-3	-19	-10	5.20	15.30
3	19	10	19.60	6.50
-4	-19	-10	67.59	10.60
-4	-19	-10	45.10	8.10
-4	-19	-10	43.00	8.50
4	19	10	38.00	8.40
-5	-19	-10	160.58	14.50
-5	-19	-10	149.39	14.70
-5	-19	-10	189.38	16.80
-5	-19	-10	139.59	14.60
5	19	10	157.38	16.40
-6	-19	-10	-1.00	7.00
-6	-19	-10	-1.50	12.90
-6	-19	-10	-6.00	6.70
-6	-19	-10	-2.70	7.60
-6	-19	-10	1.60	7.80
6	19	10	-1.60	11.40

-7	-19	-10	32.70	8.70
-7	-19	-10	24.20	7.40
-7	-19	-10	20.60	7.40
-7	-19	-10	46.70	8.50
-8	-19	-10	48.40	9.70
-8	-19	-10	80.49	11.00
-8	-19	-10	59.89	10.40
-9	-19	-10	247.08	15.10
-9	-19	-10	215.88	14.80
-9	-19	-10	195.18	16.10
-9	-19	-10	233.68	16.50
6	-20	-10	60.29	12.90
6	-20	-10	26.10	8.60
-6	20	10	40.70	9.00
5	-20	-10	9.30	8.80
5	-20	-10	12.00	10.30
-5	20	10	21.70	8.50
4	-20	-10	112.79	17.60
4	-20	-10	126.99	16.00
4	-20	-10	111.89	16.60
4	-20	-10	131.99	15.60
3	-20	-10	676.43	38.00
3	-20	-10	672.13	36.70
3	-20	-10	699.53	36.60
3	-20	-10	660.03	36.40
2	-20	-10	76.99	14.90
2	-20	-10	107.19	16.10
2	-20	-10	96.89	15.50
-2	20	10	106.59	17.10
1	-20	-10	7.10	8.70
1	-20	-10	2.30	7.70
1	-20	-10	9.90	8.90
1	-20	-10	16.70	10.10
-1	20	10	8.60	13.40
-1	20	10	17.60	8.30
0	-20	-10	12.60	8.20
0	-20	-10	12.20	8.30
0	-20	-10	34.60	10.80
0	-20	-10	36.10	8.70
0	20	10	21.10	7.50
0	20	10	24.90	12.00
-1	-20	-10	27.40	8.10
-1	-20	-10	13.30	11.30
-1	-20	-10	31.50	8.70

-1	-20	-10	30.30	8.20
1	20	10	8.40	11.50
-2	-20	-10	-15.10	12.40
-2	-20	-10	8.80	7.20
-2	-20	-10	-0.70	7.20
-2	-20	-10	4.40	8.00
2	20	10	4.40	6.60
-3	-20	-10	218.98	18.90
-3	-20	-10	226.78	16.40
-3	-20	-10	269.27	18.50
-3	-20	-10	236.08	17.10
3	20	10	212.98	16.60
-4	-20	-10	54.89	12.90
4	20	10	54.79	9.70
-5	-20	-10	-14.30	9.60
-5	-20	-10	-5.20	6.50
-5	-20	-10	10.30	6.70
-5	-20	-10	-12.90	7.80
5	20	10	4.40	10.80
-6	-20	-10	-1.00	6.20
-6	-20	-10	6.40	6.80
-6	-20	-10	-3.10	12.50
-7	-20	-10	352.76	19.80
-7	-20	-10	318.07	19.40
-7	-20	-10	331.47	19.70
-8	-20	-10	132.19	12.60
-8	-20	-10	137.29	12.20
-8	-20	-10	138.89	11.80
5	-21	-10	189.58	17.20
5	-21	-10	169.68	16.30
-5	21	10	177.48	15.20
4	-21	-10	77.79	11.60
4	-21	-10	92.49	14.10
4	-21	-10	95.69	15.30
3	-21	-10	26.30	12.70
3	-21	-10	20.70	9.10
3	-21	-10	27.90	10.00
3	-21	-10	32.40	11.70
2	-21	-10	128.09	23.20
2	-21	-10	142.19	18.10
2	-21	-10	152.78	16.90
2	-21	-10	148.79	16.50
1	-21	-10	47.40	9.90
1	-21	-10	39.90	9.50

1	-21	-10	49.30	10.60
-1	21	10	24.90	10.30
-1	21	10	61.89	14.80
0	-21	-10	14.10	9.10
0	-21	-10	13.70	9.90
0	-21	-10	19.30	9.10
0	-21	-10	17.20	11.60
0	21	10	23.40	8.70
0	21	10	30.10	12.70
-1	-21	-10	133.89	20.50
-1	-21	-10	114.69	18.90
-1	-21	-10	114.69	18.80
1	21	10	93.69	13.80
1	21	10	107.69	14.20
-2	-21	-10	239.38	23.40
-2	-21	-10	239.28	19.40
-2	-21	-10	227.58	18.80
-2	-21	-10	241.28	19.20
2	21	10	190.68	17.00
-3	-21	-10	424.56	32.60
-3	-21	-10	407.06	23.20
-3	-21	-10	395.96	24.50
-3	-21	-10	394.66	23.10
3	21	10	400.36	24.10
-4	-21	-10	161.08	17.30
-4	-21	-10	155.68	14.20
-4	-21	-10	171.88	13.90
-4	-21	-10	170.28	15.20
-5	-21	-10	23.40	8.10
-5	-21	-10	13.80	6.70
-5	-21	-10	24.00	9.90
-5	-21	-10	24.30	6.80
-6	-21	-10	120.39	12.80
-6	-21	-10	117.09	19.40
-6	-21	-10	146.39	12.10
-6	-21	-10	119.79	11.70
-7	-21	-10	120.89	11.90
-7	-21	-10	99.29	11.10
-7	-21	-10	125.79	11.60
4	-22	-10	332.87	25.60
4	-22	-10	388.56	22.30
4	-22	-10	386.66	29.00
-4	22	10	408.86	22.40
3	-22	-10	29.60	12.00

3	-22	-10	49.99	15.40
3	-22	-10	34.10	10.10
3	-22	-10	36.70	8.80
2	-22	-10	219.38	18.40
2	-22	-10	221.88	17.60
2	-22	-10	188.38	17.00
1	-22	-10	-11.80	8.30
1	-22	-10	4.20	8.30
1	-22	-10	2.60	9.20
-1	22	10	8.70	21.50
0	-22	-10	17.80	7.70
0	-22	-10	15.10	7.40
0	-22	-10	19.90	10.20
0	-22	-10	-3.90	7.80
0	22	10	19.20	12.60
0	22	10	23.10	13.70
-1	-22	-10	187.28	15.70
-1	-22	-10	152.28	14.80
-1	-22	-10	191.18	14.70
-1	-22	-10	152.38	18.40
1	22	10	155.98	18.90
-2	-22	-10	18.80	8.20
-2	-22	-10	15.50	6.90
-2	-22	-10	35.80	12.70
-2	-22	-10	4.40	7.20
2	22	10	20.00	11.60
-3	-22	-10	167.28	13.60
-3	-22	-10	181.18	16.30
-3	-22	-10	180.88	13.90
-4	-22	-10	302.97	18.00
-4	-22	-10	296.37	20.20
-4	-22	-10	293.17	18.00
-5	-22	-10	17.70	8.20
-5	-22	-10	19.50	6.40
-5	-22	-10	37.20	10.10
-5	-22	-10	33.70	6.80
-6	-22	-10	96.89	10.10
-6	-22	-10	106.99	10.60
-6	-22	-10	43.90	22.10
-7	-22	-10	45.60	13.10
-7	-22	-10	10.20	8.00
3	-23	-10	7.80	15.90
3	-23	-10	12.90	8.00
2	-23	-10	29.80	10.90

2	-23	-10	41.20	9.40
2	-23	-10	22.40	9.60
1	-23	-10	45.40	9.50
1	-23	-10	56.19	9.90
1	-23	-10	55.99	10.10
0	-23	-10	10.60	9.70
0	-23	-10	5.30	7.20
0	-23	-10	21.20	8.10
0	23	10	20.70	13.10
-1	-23	-10	304.57	18.00
-1	-23	-10	298.47	18.60
-1	-23	-10	303.27	17.70
-1	-23	-10	257.97	21.30
1	23	10	242.18	22.30
-2	-23	-10	240.58	17.20
-2	-23	-10	256.37	16.10
-2	-23	-10	248.18	16.50
-2	-23	-10	251.57	23.10
-3	-23	-10	119.09	11.80
-3	-23	-10	99.69	13.40
-3	-23	-10	116.29	11.30
3	23	10	124.89	16.30
-4	-23	-10	22.10	6.20
-4	-23	-10	36.50	8.90
-4	-23	-10	11.20	6.30
-5	-23	-10	127.19	15.40
-5	-23	-10	126.89	11.50
-5	-23	-10	134.39	10.80
-6	-23	-10	15.20	13.60
-6	-23	-10	15.80	6.30
2	-24	-10	-9.20	8.90
2	-24	-10	18.90	10.10
2	-24	-10	14.00	8.00
1	-24	-10	6.50	8.60
1	-24	-10	10.80	7.90
1	-24	-10	7.50	9.40
0	-24	-10	81.49	14.40
0	-24	-10	77.99	10.10
0	-24	-10	77.09	12.00
0	24	10	78.29	21.40
-1	-24	-10	26.30	7.50
-1	-24	-10	36.30	11.10
-1	-24	-10	14.50	6.50
-1	-24	-10	35.20	8.00

1	24	10	4.60	15.60
1	24	10	31.90	17.60
-2	-24	-10	-3.10	7.30
-2	-24	-10	-6.00	6.40
-2	-24	-10	-10.30	16.60
-2	-24	-10	-8.40	5.90
2	24	10	-2.50	12.30
-3	-24	-10	104.89	12.40
-3	-24	-10	97.99	10.80
-3	-24	-10	103.39	10.30
3	24	10	106.79	14.80
-4	-24	-10	80.79	12.50
-4	-24	-10	71.19	9.50
-4	-24	-10	84.59	9.80
4	24	10	74.59	13.60
0	-25	-10	5.40	9.90
-1	-25	-10	16.60	11.80
-1	-25	-10	0.30	7.20
-2	-25	-10	223.68	19.60
-2	-25	-10	264.77	16.80
-2	-25	-10	262.97	15.20
2	25	10	277.97	20.30
2	19	-11	75.79	11.70
-2	-19	11	86.89	17.20
-2	-19	11	65.59	12.40
1	19	-11	94.19	12.20
-1	-19	11	100.69	13.80
0	19	-11	72.99	13.00
0	19	-11	80.39	13.10
0	-19	11	70.39	11.70
0	-19	11	75.49	11.40
-1	19	-11	37.20	10.50
-1	19	-11	20.20	15.20
1	-19	11	16.20	7.30
-2	19	-11	28.90	9.70
-2	19	-11	22.60	10.90
2	-19	11	15.60	7.00
4	18	-11	159.08	13.00
-4	-18	11	133.49	11.30
3	18	-11	11.60	6.90
-3	-18	11	-7.00	5.80
2	18	-11	243.68	17.00
-2	-18	11	229.28	25.40
-2	-18	11	259.67	16.20

1	18	-11	38.00	8.50
1	18	-11	57.79	16.30
-1	-18	11	18.00	8.10
-1	-18	11	21.70	8.60
0	18	-11	28.30	15.00
0	18	-11	30.90	11.30
0	18	-11	50.99	9.00
0	-18	11	35.50	9.60
0	-18	11	41.50	8.40
-1	18	-11	105.99	14.50
-1	18	-11	92.69	15.20
-1	18	-11	94.79	14.80
1	-18	11	84.29	12.20
-2	18	-11	157.88	15.10
-2	18	-11	148.79	16.10
-2	18	-11	138.59	18.40
2	-18	11	116.99	12.90
-3	18	-11	53.69	9.60
3	-18	11	73.99	11.20
-4	18	-11	133.29	16.60
-4	18	-11	106.59	16.00
4	-18	11	113.49	12.50
5	17	-11	89.89	11.60
-5	-17	11	82.89	9.00
4	17	-11	-0.50	6.80
-4	-17	11	0.50	5.50
3	17	-11	-0.60	7.00
-3	-17	11	-4.00	5.80
2	17	-11	2.70	7.30
-2	-17	11	-0.30	6.40
-2	-17	11	-1.40	9.20
1	17	-11	201.58	23.10
1	17	-11	250.77	20.70
1	17	-11	247.28	17.40
-1	-17	11	235.18	16.80
-1	-17	11	242.98	18.10
0	17	-11	199.08	32.60
0	17	-11	228.18	17.50
0	17	-11	226.48	19.20
0	-17	11	224.98	17.60
0	-17	11	242.58	17.70
-1	17	-11	50.79	10.50
-1	17	-11	56.49	15.10
-1	17	-11	68.69	10.40

1	-17	11	64.79	12.80
1	-17	11	73.09	12.30
-2	17	-11	260.97	19.60
-2	17	-11	279.17	20.40
-2	17	-11	280.77	25.80
2	-17	11	305.67	18.70
-3	17	-11	7.40	10.50
-3	17	-11	-7.70	10.50
-4	17	-11	33.40	11.00
-4	17	-11	-1.20	13.10
-4	17	-11	41.70	8.80
4	-17	11	31.20	8.00
-5	17	-11	177.48	16.20
-5	17	-11	117.69	13.80
5	-17	11	98.69	18.80
5	-17	11	172.28	17.90
6	16	-11	8.70	7.40
-6	-16	11	7.50	5.50
5	16	-11	33.00	7.60
-5	-16	11	9.00	5.90
4	16	-11	67.49	9.20
-4	-16	11	50.09	7.40
3	16	-11	469.35	25.80
-3	-16	11	457.05	24.80
-3	-16	11	460.75	28.90
2	16	-11	70.09	18.70
2	16	-11	72.29	12.90
-2	-16	11	76.19	14.00
-2	-16	11	81.59	12.20
1	16	-11	194.98	17.30
1	16	-11	227.68	19.80
1	16	-11	221.78	19.90
-1	-16	11	168.28	17.30
-1	-16	11	181.38	15.50
0	16	-11	96.29	16.40
0	16	-11	134.89	17.30
0	16	-11	132.19	15.20
0	-16	11	120.09	14.50
0	-16	11	170.58	15.90
-1	16	-11	273.07	20.80
-1	16	-11	253.17	20.20
-1	16	-11	265.47	20.80
1	-16	11	308.77	22.00
1	-16	11	302.67	19.80

-2	16	-11	-8.20	8.80
-2	16	-11	-1.60	9.50
-2	16	-11	6.60	9.30
2	-16	11	16.20	8.00
-3	16	-11	-3.70	10.00
-3	16	-11	4.30	10.00
-3	16	-11	15.30	9.00
3	-16	11	-6.10	7.10
-4	16	-11	10.80	11.20
-4	16	-11	23.60	9.00
-4	16	-11	12.40	11.10
4	-16	11	17.70	11.20
4	-16	11	3.90	7.70
-5	16	-11	249.78	18.50
-5	16	-11	230.28	17.50
-5	16	-11	242.08	27.00
5	-16	11	235.88	17.70
5	-16	11	211.28	17.70
-6	16	-11	12.30	10.50
-6	16	-11	5.40	7.90
6	-16	11	12.20	10.20
6	-16	11	-5.20	8.90
7	15	-11	189.88	14.70
-7	-15	11	144.69	14.60
-7	-15	11	178.98	12.70
6	15	-11	54.49	8.60
6	15	-11	91.49	17.50
-6	-15	11	45.60	7.10
-6	-15	11	43.20	9.70
5	15	-11	146.09	14.30
5	15	-11	204.18	23.90
-5	-15	11	158.48	12.90
4	15	-11	130.69	13.90
-4	-15	11	129.79	12.80
3	15	-11	1.30	7.50
-3	-15	11	-17.10	10.60
-3	-15	11	-2.50	6.40
2	15	-11	67.99	14.10
2	15	-11	56.79	10.70
-2	-15	11	59.99	9.80
-2	-15	11	56.09	10.80
1	15	-11	15.00	12.10
1	15	-11	34.10	9.60
1	15	-11	44.10	11.60

0	15	-11	100.69	15.50
0	15	-11	121.89	15.40
0	-15	11	96.19	17.20
-1	15	-11	-18.60	18.90
-1	15	-11	5.60	9.20
-1	15	-11	8.50	9.80
1	-15	11	4.00	11.90
1	-15	11	7.90	9.40
-2	15	-11	51.59	11.40
-2	15	-11	61.69	12.60
-2	15	-11	43.10	10.50
2	-15	11	50.19	14.20
-3	15	-11	109.39	17.80
-3	15	-11	118.19	15.90
-3	15	-11	134.89	16.40
3	-15	11	150.58	15.40
3	-15	11	166.08	20.80
-4	15	-11	3.00	8.90
-4	15	-11	16.00	9.90
-4	15	-11	17.40	11.10
4	-15	11	28.80	8.30
4	-15	11	19.90	10.50
-5	15	-11	-11.50	9.40
-5	15	-11	3.40	12.10
-5	15	-11	-8.90	8.30
5	-15	11	-13.90	8.10
5	-15	11	4.70	8.80
-6	15	-11	467.65	27.30
-6	15	-11	484.05	26.70
-6	15	-11	439.66	39.20
6	-15	11	500.25	27.70
6	-15	11	506.95	26.00
-7	15	-11	108.69	15.30
-7	15	-11	125.39	15.80
7	-15	11	116.59	15.00
7	14	-11	47.40	21.80
7	14	-11	27.60	8.90
-7	-14	11	37.50	9.00
-7	-14	11	34.50	7.00
6	14	-11	161.98	14.50
6	14	-11	122.09	19.80
-6	-14	11	147.59	12.80
5	14	-11	0.40	13.10
5	14	-11	11.90	7.80

-5	-14	11	20.50	6.50
-5	-14	11	25.00	9.60
4	14	-11	60.09	14.70
4	14	-11	50.29	9.90
-4	-14	11	66.29	11.60
3	14	-11	81.09	14.10
3	14	-11	80.39	17.70
-3	-14	11	90.39	18.40
-3	-14	11	63.99	11.30
2	14	-11	290.97	20.70
2	14	-11	336.47	25.10
-2	-14	11	286.77	21.50
-2	-14	11	329.17	20.30
1	14	-11	14.90	11.10
1	14	-11	19.00	10.10
1	14	-11	18.60	8.10
-1	-14	11	22.10	8.30
-1	-14	11	10.20	7.40
0	14	-11	95.19	14.90
0	14	-11	89.19	11.70
0	14	-11	95.89	14.10
0	-14	11	101.79	14.20
0	-14	11	91.89	14.10
-1	14	-11	311.07	21.70
-1	14	-11	276.37	20.50
-1	14	-11	297.27	21.30
1	-14	11	293.57	20.20
1	-14	11	299.27	21.40
-2	14	-11	270.57	19.90
-2	14	-11	229.38	19.50
-2	14	-11	218.18	18.20
2	-14	11	242.18	18.40
-3	14	-11	69.59	11.80
-3	14	-11	59.09	11.90
-3	14	-11	48.10	10.50
3	-14	11	50.09	9.80
-4	14	-11	123.59	16.30
-4	14	-11	135.99	17.50
-4	14	-11	125.29	20.90
4	-14	11	150.38	16.30
4	-14	11	155.48	18.20
-5	14	-11	17.90	9.40
-5	14	-11	21.50	12.50
-5	14	-11	16.50	9.90

5	-14	11	0.40	8.90
5	-14	11	1.60	8.90
-6	14	-11	6.80	8.70
-6	14	-11	12.10	9.80
-6	14	-11	-26.00	13.10
6	-14	11	4.30	10.10
6	-14	11	-1.20	7.50
-7	14	-11	57.29	9.10
-7	14	-11	62.69	11.20
7	-14	11	33.80	15.40
7	-14	11	38.30	8.00
8	13	-11	25.40	11.00
8	13	-11	52.49	10.60
-8	-13	11	38.00	7.20
7	13	-11	214.88	16.80
7	13	-11	226.38	19.80
-7	-13	11	234.48	15.50
-7	-13	11	238.78	15.80
6	13	-11	15.90	8.50
6	13	-11	27.50	11.30
-6	-13	11	22.60	6.70
-6	-13	11	19.70	7.90
5	13	-11	148.39	15.30
5	13	-11	124.09	19.40
-5	-13	11	114.49	16.30
-5	-13	11	144.89	13.80
4	13	-11	62.09	10.30
-4	-13	11	72.99	17.70
-4	-13	11	84.49	12.00
3	13	-11	12.90	8.00
3	13	-11	15.30	12.30
-3	-13	11	1.40	6.40
-3	-13	11	2.90	9.80
2	13	-11	19.40	13.20
2	13	-11	38.90	8.70
2	13	-11	41.80	15.40
-2	-13	11	28.40	8.70
-2	-13	11	24.30	7.40
1	13	-11	429.26	29.50
1	13	-11	467.75	28.00
1	13	-11	491.85	27.40
-1	-13	11	484.85	26.90
-1	-13	11	460.05	26.30
0	13	-11	69.89	11.40

0	13	-11	86.29	12.10
0	13	-11	94.29	13.90
0	-13	11	118.19	14.20
0	-13	11	84.39	13.70
-1	13	-11	35.60	9.10
-1	13	-11	16.20	9.50
-1	13	-11	27.10	9.50
1	-13	11	21.50	9.30
1	-13	11	26.10	8.30
-2	13	-11	235.18	19.10
-2	13	-11	212.28	18.50
-2	13	-11	237.38	19.30
2	-13	11	174.88	22.90
2	-13	11	253.17	18.10
-3	13	-11	23.00	9.60
-3	13	-11	29.40	10.20
-3	13	-11	17.30	9.20
3	-13	11	31.00	8.90
3	-13	11	33.70	9.80
-4	13	-11	73.59	11.00
-4	13	-11	88.29	15.50
-4	13	-11	94.99	13.40
4	-13	11	65.19	10.60
4	-13	11	53.89	9.70
-5	13	-11	263.17	21.30
-5	13	-11	223.88	19.70
-5	13	-11	256.17	24.80
5	-13	11	243.98	20.20
5	-13	11	239.78	20.00
-6	13	-11	37.70	14.20
-6	13	-11	11.20	9.90
-6	13	-11	4.70	9.20
6	-13	11	8.30	8.00
6	-13	11	8.20	9.90
-7	13	-11	362.76	22.80
-7	13	-11	346.67	27.70
-7	13	-11	366.06	29.20
7	-13	11	393.16	27.70
7	-13	11	373.46	21.90
-8	13	-11	36.60	11.10
-8	13	-11	23.30	7.90
8	-13	11	27.80	7.60
8	12	-11	13.50	10.00
8	12	-11	15.70	14.50

-8	-12	11	24.70	6.80
-8	-12	11	23.70	11.40
7	12	-11	91.29	14.20
7	12	-11	93.39	12.90
-7	-12	11	99.09	12.00
-7	-12	11	73.69	11.80
6	12	-11	105.49	18.30
6	12	-11	127.49	15.30
-6	-12	11	121.49	14.30
-6	-12	11	121.39	13.50
5	12	-11	54.19	10.40
-5	-12	11	40.70	7.90
4	12	-11	139.89	18.40
4	12	-11	146.29	15.90
-4	-12	11	120.49	13.20
-4	-12	11	153.18	16.70
3	12	-11	301.27	23.30
3	12	-11	283.27	20.50
-3	-12	11	277.87	19.10
-3	-12	11	290.67	21.80
2	12	-11	33.50	8.30
2	12	-11	39.60	12.20
-2	-12	11	46.40	9.50
-2	-12	11	25.10	7.80
1	12	-11	158.58	16.30
1	12	-11	154.38	16.30
1	12	-11	156.98	20.90
1	12	-11	157.18	16.60
-1	-12	11	157.98	16.50
-1	-12	11	161.08	14.90
0	12	-11	656.93	34.40
0	12	-11	675.93	35.20
0	12	-11	643.24	44.10
0	12	-11	608.24	35.30
0	-12	11	671.83	35.00
0	-12	11	665.93	34.20
-1	12	-11	277.57	20.30
-1	12	-11	260.67	21.20
-1	12	-11	303.17	20.20
1	-12	11	304.57	20.50
1	-12	11	288.37	20.60
-2	12	-11	32.70	9.30
-2	12	-11	42.50	9.80
-2	12	-11	7.50	9.30

2	-12	11	25.60	8.80
2	-12	11	24.10	13.00
-3	12	-11	61.09	11.10
-3	12	-11	35.10	10.80
-3	12	-11	35.30	10.20
3	-12	11	31.90	8.30
-4	12	-11	54.49	10.70
-4	12	-11	23.70	10.00
-4	12	-11	45.70	11.70
4	-12	11	50.79	9.70
-5	12	-11	276.17	21.60
-5	12	-11	258.27	20.50
-5	12	-11	247.08	25.60
5	-12	11	257.27	19.60
5	-12	11	244.58	20.40
-6	12	-11	14.20	9.90
-6	12	-11	16.70	10.20
-6	12	-11	2.10	14.00
6	-12	11	-12.80	10.40
6	-12	11	16.50	8.50
-7	12	-11	34.20	10.60
-7	12	-11	29.90	9.70
-7	12	-11	18.40	14.60
7	-12	11	12.40	15.20
7	-12	11	21.80	8.50
-8	12	-11	328.67	21.80
-8	12	-11	276.17	20.60
8	-12	11	335.87	19.60
9	11	-11	3.80	10.30
8	11	-11	335.37	22.60
8	11	-11	334.37	23.00
-8	-11	11	334.47	21.40
-8	-11	11	357.26	19.80
7	11	-11	66.09	10.60
7	11	-11	67.59	11.60
-7	-11	11	53.49	8.50
-7	-11	11	48.80	8.60
6	11	-11	25.70	12.30
6	11	-11	20.40	10.90
-6	-11	11	13.90	8.60
-6	-11	11	11.60	8.50
5	11	-11	374.86	25.70
5	11	-11	386.06	24.30
-5	-11	11	393.76	22.80

-5	-11	11	341.17	23.10
4	11	-11	33.90	8.90
4	11	-11	57.19	11.40
-4	-11	11	23.60	7.00
3	11	-11	4.10	7.70
3	11	-11	17.50	9.90
-3	-11	11	-6.30	6.40
-3	-11	11	-9.70	9.40
2	11	-11	8.60	7.50
2	11	-11	-1.40	10.30
-2	-11	11	0.20	8.30
-2	-11	11	-3.80	6.40
1	11	-11	-10.90	11.40
1	11	-11	-9.20	10.40
1	11	-11	-3.70	8.30
1	11	-11	7.20	7.70
-1	-11	11	-4.90	6.60
-1	-11	11	-1.60	7.70
0	11	-11	209.48	22.20
0	11	-11	171.38	16.10
0	11	-11	200.38	17.10
0	11	-11	179.28	19.70
0	-11	11	172.48	16.00
0	-11	11	184.88	16.30
-1	11	-11	15.00	8.20
-1	11	-11	13.90	7.50
-1	11	-11	39.90	14.20
-1	11	-11	28.20	9.80
1	-11	11	7.80	8.40
1	-11	11	7.20	7.00
-2	11	-11	69.19	10.90
-2	11	-11	57.69	9.50
-2	11	-11	66.09	11.30
2	-11	11	40.60	12.40
2	-11	11	57.09	9.80
-3	11	-11	143.09	15.90
-3	11	-11	151.08	17.00
-3	11	-11	157.68	19.40
3	-11	11	153.08	16.10
-4	11	-11	77.49	12.00
-4	11	-11	82.89	13.00
4	-11	11	63.49	11.00
-5	11	-11	112.19	16.10
-5	11	-11	149.19	17.30

-5	11	-11	153.78	22.80
5	-11	11	141.49	16.60
-6	11	-11	873.41	46.50
-6	11	-11	1005.60	57.39
-6	11	-11	863.21	47.00
6	-11	11	911.21	45.80
-7	11	-11	10.00	9.90
-7	11	-11	12.90	9.70
-7	11	-11	-11.50	13.30
7	-11	11	19.50	8.60
7	-11	11	17.90	15.50
-8	11	-11	1.30	9.70
-8	11	-11	-3.40	14.90
-8	11	-11	8.90	10.70
8	-11	11	-4.10	7.50
-9	11	-11	-9.50	11.50
-9	11	-11	9.20	8.80
9	-11	11	28.80	7.80
9	10	-11	114.79	15.30
-9	-10	11	115.19	10.80
8	10	-11	27.80	10.00
-8	-10	11	26.40	8.60
-8	-10	11	29.20	7.10
7	10	-11	196.88	19.50
7	10	-11	149.99	18.10
-7	-10	11	174.78	15.50
-7	-10	11	182.98	14.70
6	10	-11	265.87	21.10
6	10	-11	254.37	22.10
-6	-10	11	292.47	18.80
-6	-10	11	270.27	19.10
5	10	-11	55.39	10.30
5	10	-11	39.70	11.10
-5	-10	11	48.20	8.70
-5	-10	11	68.19	11.70
4	10	-11	24.10	10.20
4	10	-11	22.80	8.70
-4	-10	11	18.30	8.20
3	10	-11	181.98	16.90
3	10	-11	164.48	19.20
-3	-10	11	206.18	19.10
-3	-10	11	188.48	15.20
-3	-10	11	199.38	18.90
2	10	-11	203.88	20.00

2	10	-11	207.98	17.40
-2	-10	11	238.48	18.50
-2	-10	11	225.88	16.10
1	10	-11	49.80	9.10
1	10	-11	61.79	9.60
1	10	-11	55.29	11.50
-1	-10	11	60.89	8.60
-1	-10	11	52.69	8.60
0	10	-11	-3.40	7.10
0	10	-11	4.40	11.80
0	10	-11	0.00	7.20
0	-10	11	-4.90	6.80
0	-10	11	-2.60	6.90
-1	10	-11	982.20	48.50
-1	10	-11	1007.90	49.90
-1	10	-11	1014.50	49.70
1	-10	11	981.20	49.00
1	-10	11	1006.10	49.10
-2	10	-11	313.47	20.00
-2	10	-11	325.27	21.70
-2	10	-11	282.87	29.90
-2	10	-11	308.37	21.70
2	-10	11	232.38	25.10
2	-10	11	308.97	20.20
-3	10	-11	88.69	12.10
-3	10	-11	77.99	12.00
-3	10	-11	81.69	11.10
3	-10	11	80.39	14.10
-4	10	-11	97.29	14.70
-4	10	-11	117.89	14.90
-4	10	-11	99.89	12.60
4	-10	11	100.39	15.20
-5	10	-11	6.80	12.40
-5	10	-11	21.10	10.10
-5	10	-11	6.60	9.70
5	-10	11	14.40	8.80
-6	10	-11	4.70	10.30
-6	10	-11	20.30	14.00
-6	10	-11	2.00	8.60
6	-10	11	18.90	10.40
-7	10	-11	70.19	13.30
-7	10	-11	118.79	18.60
-7	10	-11	83.99	12.80
7	-10	11	120.49	18.00

7	-10	11	100.39	16.90
-8	10	-11	45.30	15.40
-8	10	-11	26.90	10.60
-8	10	-11	13.20	9.70
8	-10	11	15.80	8.40
-9	10	-11	12.90	9.10
-9	10	-11	20.30	11.20
9	-10	11	-19.10	16.90
9	-10	11	5.10	7.80
9	9	-11	91.29	15.00
-9	-9	11	81.59	10.90
8	9	-11	11.60	10.10
8	9	-11	-17.00	12.10
-8	-9	11	-8.50	8.30
-8	-9	11	2.50	6.20
7	9	-11	218.88	23.40
7	9	-11	178.98	19.30
-7	-9	11	182.38	17.20
-7	-9	11	164.68	15.40
6	9	-11	102.79	17.20
6	9	-11	134.39	18.60
-6	-9	11	111.39	13.20
-6	-9	11	125.09	13.70
5	9	-11	72.79	10.90
5	9	-11	19.30	10.60
-5	-9	11	48.10	8.10
-5	-9	11	39.50	7.50
4	9	-11	361.86	24.90
4	9	-11	334.67	23.30
-4	-9	11	363.86	21.60
-4	-9	11	360.06	22.10
3	9	-11	35.20	10.30
3	9	-11	29.00	8.80
-3	-9	11	34.60	6.80
-3	-9	11	53.29	10.90
-3	-9	11	33.60	9.50
2	9	-11	30.00	8.10
2	9	-11	37.00	9.80
1	9	-11	915.61	43.50
1	9	-11	817.72	43.00
1	9	-11	855.61	45.10
-1	-9	11	870.81	42.80
0	9	-11	216.18	21.50
0	9	-11	161.88	19.10

0	9	-11	175.48	16.30
0	9	-11	191.28	15.20
0	-9	11	177.48	16.10
-1	9	-11	17.00	7.50
-1	9	-11	5.70	7.00
-1	9	-11	3.30	11.30
-1	9	-11	3.80	10.50
1	-9	11	15.30	8.10
-2	9	-11	42.60	8.10
-2	9	-11	52.69	12.50
-2	9	-11	43.20	9.60
-2	9	-11	54.29	18.00
2	-9	11	71.09	16.40
2	-9	11	65.89	9.50
-3	9	-11	6.50	9.20
-3	9	-11	5.50	7.10
-3	9	-11	-0.60	9.90
3	-9	11	3.00	7.30
-4	9	-11	23.00	11.30
-4	9	-11	31.70	9.00
-4	9	-11	33.40	10.20
-4	9	-11	42.70	20.00
4	-9	11	14.00	8.50
-5	9	-11	267.77	27.20
-5	9	-11	300.17	26.90
-5	9	-11	300.47	22.00
-5	9	-11	296.77	21.00
5	-9	11	290.57	21.60
-6	9	-11	131.89	16.60
-6	9	-11	144.69	24.80
-6	9	-11	158.68	18.80
-6	9	-11	126.99	24.20
6	-9	11	145.39	19.20
-7	9	-11	642.74	36.60
-7	9	-11	660.63	37.00
-7	9	-11	664.23	41.50
7	-9	11	681.93	35.70
-8	9	-11	9.30	10.10
-8	9	-11	-5.00	9.80
-8	9	-11	11.90	16.30
8	-9	11	-0.90	8.70
-9	9	-11	16.40	10.00
-9	9	-11	21.90	12.10
-9	9	-11	11.70	16.00

9	-9	11	9.00	8.30
-10	9	-11	37.00	12.60
-10	9	-11	81.49	12.70
10	-9	11	58.99	10.70
9	8	-11	63.09	11.40
-9	-8	11	68.39	8.30
8	8	-11	13.90	13.30
8	8	-11	-7.10	9.70
-8	-8	11	5.80	8.60
-8	-8	11	14.10	6.30
7	8	-11	70.59	12.60
-7	-8	11	34.00	9.30
-7	-8	11	46.90	7.90
6	8	-11	653.53	38.40
6	8	-11	692.93	37.70
-6	-8	11	732.93	36.20
-6	-8	11	720.93	35.60
5	8	-11	558.64	30.90
5	8	-11	508.25	31.80
-5	-8	11	532.75	29.20
-5	-8	11	546.05	28.80
4	8	-11	199.78	20.30
4	8	-11	212.38	18.80
-4	-8	11	231.18	16.80
-4	-8	11	212.78	15.80
3	8	-11	213.28	19.10
3	8	-11	197.98	17.60
-3	-8	11	203.88	15.10
-3	-8	11	205.68	17.60
2	8	-11	148.19	16.90
1	8	-11	10.60	9.10
1	8	-11	-7.40	7.40
1	8	-11	-1.70	9.80
-1	-8	11	5.60	5.60
0	8	-11	884.51	47.80
0	8	-11	768.02	39.90
0	8	-11	763.12	44.50
0	8	-11	741.03	38.90
0	8	-11	816.12	41.30
0	-8	11	796.12	39.20
0	-8	11	748.63	38.70
-1	8	-11	16.20	10.60
-1	8	-11	31.80	11.10
-1	8	-11	9.60	6.60

-1	8	-11	8.30	8.10
-1	8	-11	10.50	12.10
1	-8	11	5.00	7.90
1	-8	11	3.10	9.20
-2	8	-11	614.64	35.80
-2	8	-11	636.44	36.70
-2	8	-11	630.54	34.30
-2	8	-11	591.64	34.10
-2	8	-11	648.74	32.30
2	-8	11	617.24	32.20
-3	8	-11	6.90	13.20
-3	8	-11	7.50	9.00
-3	8	-11	2.90	6.40
-3	8	-11	-13.60	9.10
3	-8	11	15.60	9.20
3	-8	11	8.60	7.80
-4	8	-11	24.90	18.00
-4	8	-11	4.90	8.50
-4	8	-11	2.00	6.90
-4	8	-11	-2.00	10.30
4	-8	11	2.00	10.50
4	-8	11	8.70	7.40
-5	8	-11	257.47	25.30
-5	8	-11	289.47	21.30
-5	8	-11	254.17	19.00
-5	8	-11	216.48	24.90
-5	8	-11	250.17	29.30
5	-8	11	248.68	20.20
5	-8	11	245.98	25.50
-6	8	-11	45.90	13.20
-6	8	-11	-1.60	11.50
-6	8	-11	-2.30	17.70
-6	8	-11	-2.70	13.70
-6	8	-11	14.40	10.10
6	-8	11	23.70	11.50
-7	8	-11	78.19	12.80
-7	8	-11	88.79	17.70
-7	8	-11	75.89	11.60
-8	8	-11	206.78	21.50
-8	8	-11	207.18	19.00
-8	8	-11	186.28	28.60
8	-8	11	226.48	19.90
-9	8	-11	4.20	9.60
-9	8	-11	11.20	16.30

-9	8	-11	17.90	11.00
9	-8	11	6.50	8.70
-10	8	-11	113.69	18.40
-10	8	-11	154.68	16.50
10	-8	11	183.08	16.80
10	7	-11	32.00	10.10
-10	-7	11	30.90	6.40
9	7	-11	36.10	10.90
-9	-7	11	58.59	9.70
8	7	-11	281.67	22.80
-8	-7	11	227.58	20.20
-8	-7	11	259.67	17.20
7	7	-11	19.30	11.40
7	7	-11	2.50	10.40
-7	-7	11	14.50	6.60
-7	-7	11	9.10	8.20
6	7	-11	29.90	10.60
6	7	-11	21.70	10.70
-6	-7	11	25.30	6.70
-6	-7	11	17.30	7.60
5	7	-11	676.23	37.70
5	7	-11	730.03	39.60
-5	-7	11	706.43	36.20
-5	-7	11	717.93	35.80
4	7	-11	175.68	17.50
4	7	-11	158.78	18.40
-4	-7	11	151.88	13.40
-4	-7	11	148.79	14.60
3	7	-11	21.60	9.00
-3	-7	11	14.60	8.40
-3	-7	11	17.50	8.90
2	7	-11	105.29	16.10
2	7	-11	84.09	14.10
-2	-7	11	121.49	11.70
1	7	-11	36.10	9.60
1	7	-11	24.00	7.40
-1	-7	11	27.80	7.10
-1	-7	11	35.10	6.80
0	7	-11	661.53	39.90
0	7	-11	624.84	33.60
0	7	-11	635.34	32.50
0	7	-11	638.94	35.20
0	-7	11	612.94	32.50
0	-7	11	660.63	31.90

-1	7	-11	55.49	9.60
-1	7	-11	57.49	12.30
-1	7	-11	42.30	12.50
-1	7	-11	50.99	7.80
1	-7	11	37.70	8.30
1	-7	11	35.70	6.90
-2	7	-11	36.50	12.30
-2	7	-11	52.99	10.70
-2	7	-11	28.70	7.00
-2	7	-11	28.00	10.60
-2	7	-11	16.20	17.80
-2	7	-11	43.80	9.90
2	-7	11	27.60	7.40
-3	7	-11	218.38	15.70
-3	7	-11	204.48	20.00
-3	7	-11	226.88	24.90
-3	7	-11	194.18	18.10
-3	7	-11	234.38	19.20
-3	7	-11	196.38	24.90
3	-7	11	226.48	16.80
-4	7	-11	6.40	13.70
-4	7	-11	-8.60	10.50
-4	7	-11	-7.50	13.60
-4	7	-11	6.70	6.30
-4	7	-11	2.90	10.90
4	-7	11	-9.60	12.20
4	-7	11	17.80	8.60
-5	7	-11	305.77	30.70
-5	7	-11	357.56	27.40
-5	7	-11	326.67	25.80
-5	7	-11	335.77	22.80
-5	7	-11	310.27	20.90
5	-7	11	309.87	22.90
5	-7	11	322.87	23.20
-6	7	-11	1010.70	56.19
-6	7	-11	987.50	55.99
-6	7	-11	1068.09	51.79
-6	7	-11	1024.80	53.29
-6	7	-11	1112.99	52.59
6	-7	11	1029.10	53.59
6	-7	11	1005.80	52.59
-7	7	-11	45.60	11.20
-7	7	-11	68.29	15.90
-7	7	-11	45.60	12.70

-7	7	-11	69.59	16.40
-7	7	-11	65.19	11.00
-8	7	-11	22.10	10.00
-8	7	-11	12.60	9.10
-8	7	-11	21.90	19.60
8	-7	11	21.10	9.60
-9	7	-11	26.60	16.90
-9	7	-11	33.50	10.70
-9	7	-11	34.30	10.20
9	-7	11	42.20	10.20
-10	7	-11	29.70	25.60
-10	7	-11	-11.10	11.40
-10	7	-11	6.30	9.80
10	-7	11	3.00	9.60
10	6	-11	105.49	16.00
-10	-6	11	86.19	10.50
9	6	-11	29.60	11.20
-9	-6	11	24.20	6.60
8	6	-11	22.70	11.90
-8	-6	11	18.70	7.60
-8	-6	11	11.80	9.30
7	6	-11	318.97	26.70
7	6	-11	290.37	23.90
-7	-6	11	311.17	19.10
-7	-6	11	283.97	20.60
6	6	-11	308.27	25.30
6	6	-11	339.67	24.80
-6	-6	11	331.47	21.00
-6	-6	11	327.17	19.70
5	6	-11	0.90	9.50
5	6	-11	3.50	10.30
-5	-6	11	-3.50	5.70
-5	-6	11	4.70	6.40
4	6	-11	72.09	13.30
4	6	-11	49.10	11.80
-4	-6	11	34.60	10.30
-4	-6	11	34.90	6.50
3	6	-11	263.07	22.10
3	6	-11	262.27	20.70
-3	-6	11	271.77	19.20
-3	-6	11	283.07	17.10
2	6	-11	72.79	11.20
2	6	-11	64.19	10.20
-2	-6	11	71.09	9.20

-2	-6	11	57.59	10.10
1	6	-11	46.90	9.00
1	6	-11	51.89	9.70
-1	-6	11	40.50	6.60
-1	-6	11	46.20	7.00
0	6	-11	419.66	25.70
0	6	-11	389.36	22.70
0	6	-11	414.46	23.80
0	6	-11	380.56	29.10
0	-6	11	360.36	22.20
0	-6	11	411.26	21.10
-1	6	-11	1617.94	77.29
-1	6	-11	1687.53	74.69
-1	6	-11	1643.04	85.39
-1	6	-11	1687.93	77.09
-1	6	-11	1638.14	75.79
-1	6	-11	1455.95	76.49
1	-6	11	1496.55	77.99
1	-6	11	1764.92	74.29
1	-6	11	1512.45	74.89
-2	6	-11	83.49	12.10
-2	6	-11	91.69	14.40
-2	6	-11	88.99	13.70
-2	6	-11	79.19	11.50
-2	6	-11	87.49	14.50
-2	6	-11	82.89	10.90
2	-6	11	69.39	11.80
-3	6	-11	40.20	13.00
-3	6	-11	39.30	7.30
-3	6	-11	38.80	9.50
-3	6	-11	37.20	13.40
-3	6	-11	47.50	10.90
-3	6	-11	35.90	10.30
3	-6	11	35.20	6.80
-4	6	-11	252.27	26.30
-4	6	-11	224.38	22.20
-4	6	-11	174.48	19.20
-4	6	-11	249.68	15.80
-4	6	-11	196.08	20.00
-4	6	-11	231.08	18.00
4	-6	11	215.58	17.80
4	-6	11	196.08	15.90
-5	6	-11	1.40	9.80
-5	6	-11	-3.80	8.90

-5	6	-11	10.40	10.70
-5	6	-11	-4.70	20.30
-5	6	-11	-3.10	6.60
5	-6	11	-3.10	9.90
5	-6	11	-1.30	7.80
-6	6	-11	40.10	8.80
-6	6	-11	27.50	10.70
-6	6	-11	44.50	15.00
-6	6	-11	49.99	13.70
-6	6	-11	28.20	9.60
6	-6	11	1.10	11.70
6	-6	11	31.20	10.70
-7	6	-11	312.57	28.70
-7	6	-11	276.97	22.00
-7	6	-11	313.07	24.90
-7	6	-11	322.87	22.30
-7	6	-11	252.77	29.30
7	-6	11	316.97	28.00
-8	6	-11	35.20	9.60
-8	6	-11	91.79	17.40
-8	6	-11	43.40	16.40
-8	6	-11	55.69	10.70
8	-6	11	51.29	10.70
-9	6	-11	20.90	16.10
-9	6	-11	32.20	10.60
-9	6	-11	39.10	19.00
-9	6	-11	26.10	10.50
9	-6	11	32.20	10.90
-10	6	-11	16.20	13.30
-10	6	-11	40.60	16.80
-10	6	-11	24.80	9.50
10	-6	11	26.30	11.10
-11	6	-11	256.17	22.30
-11	6	-11	275.47	20.70
11	-6	11	309.07	21.80
11	-6	11	275.47	23.70
10	5	-11	93.79	17.00
-10	-5	11	88.79	10.20
9	5	-11	16.20	11.40
-9	-5	11	3.70	6.00
8	5	-11	29.50	13.10
8	5	-11	16.20	18.10
-8	-5	11	26.10	7.90
-8	-5	11	19.80	11.90

7	5	-11	199.28	24.90
7	5	-11	130.09	20.10
-7	-5	11	152.88	15.30
-7	-5	11	153.08	13.70
6	5	-11	332.57	25.40
6	5	-11	301.37	26.10
6	5	-11	305.57	27.70
-6	-5	11	317.37	19.70
-6	-5	11	337.67	20.80
5	5	-11	17.10	11.10
5	5	-11	-11.00	10.20
-5	-5	11	6.00	6.10
-5	-5	11	0.00	6.10
4	5	-11	763.52	46.00
4	5	-11	885.71	43.50
-4	-5	11	893.31	41.00
-4	-5	11	797.82	44.80
3	5	-11	36.60	13.50
3	5	-11	36.40	10.60
3	5	-11	30.40	10.70
-3	-5	11	10.40	9.70
2	5	-11	262.17	27.80
2	5	-11	289.27	21.50
2	5	-11	301.37	21.10
-2	-5	11	320.37	18.30
1	5	-11	1088.69	54.99
1	5	-11	1133.29	55.89
-1	-5	11	1232.98	53.49
-1	-5	11	1113.99	53.89
0	5	-11	59.59	9.00
0	5	-11	64.19	10.70
0	-5	11	52.59	9.60
0	-5	11	50.19	7.90
0	-5	11	63.39	7.90
-1	5	-11	23.70	6.70
-1	5	-11	9.20	7.30
-1	5	-11	15.30	8.70
-1	5	-11	22.00	10.50
-1	5	-11	14.80	10.30
1	-5	11	14.20	4.70
1	-5	11	4.30	9.90
-2	5	-11	141.29	16.90
-2	5	-11	116.09	15.00
-2	5	-11	145.19	18.50

-2	5	-11	109.39	20.40
-2	5	-11	137.19	20.60
-2	5	-11	125.49	11.50
2	-5	11	101.39	15.70
2	-5	11	143.09	11.30
-3	5	-11	69.19	10.80
-3	5	-11	75.19	10.90
-3	5	-11	73.19	11.70
-3	5	-11	61.89	10.20
-3	5	-11	93.89	14.60
-3	5	-11	73.69	13.50
3	-5	11	68.09	9.30
-4	5	-11	27.40	8.90
-4	5	-11	11.90	8.10
-4	5	-11	22.80	11.40
-4	5	-11	31.50	6.70
-4	5	-11	19.50	13.20
-4	5	-11	44.40	9.20
4	-5	11	16.50	6.10
-5	5	-11	43.80	13.30
-5	5	-11	40.10	11.50
-5	5	-11	62.99	10.90
-5	5	-11	44.90	13.10
5	-5	11	35.50	7.80
5	-5	11	49.90	11.00
-6	5	-11	483.65	31.00
-6	5	-11	499.95	28.60
-6	5	-11	477.25	27.20
-6	5	-11	477.65	34.80
-6	5	-11	476.85	32.00
6	-5	11	450.35	31.60
6	-5	11	494.35	27.70
-7	5	-11	477.15	28.60
-7	5	-11	482.25	27.70
-7	5	-11	490.35	33.70
-7	5	-11	444.26	33.90
-7	5	-11	490.65	30.80
7	-5	11	457.45	30.10
-8	5	-11	112.69	17.90
-8	5	-11	117.19	16.10
-8	5	-11	107.89	20.20
-8	5	-11	100.79	15.10
-8	5	-11	127.29	16.70
8	-5	11	61.09	18.90

-9	5	-11	-4.70	9.10
-9	5	-11	18.20	16.80
-9	5	-11	39.70	16.50
-9	5	-11	16.90	11.10
9	-5	11	13.60	12.10
-10	5	-11	109.79	16.60
-10	5	-11	146.79	16.50
-10	5	-11	201.18	26.70
-10	5	-11	101.19	22.70
10	-5	11	151.58	20.40
-11	5	-11	18.00	12.60
-11	5	-11	9.40	9.80
11	-5	11	42.20	15.90
10	4	-11	19.70	11.70
-10	-4	11	10.80	6.50
9	4	-11	81.99	13.30
9	4	-11	94.99	17.30
-9	-4	11	95.09	11.50
8	4	-11	-11.20	15.40
8	4	-11	14.30	11.70
-8	-4	11	3.80	6.00
-8	-4	11	-3.40	11.90
7	4	-11	3.50	12.90
7	4	-11	34.70	12.30
-7	-4	11	21.00	6.70
-7	-4	11	24.30	8.20
6	4	-11	609.84	39.00
6	4	-11	708.03	48.50
6	4	-11	670.63	38.70
-6	-4	11	720.63	34.70
-6	-4	11	702.53	35.30
5	4	-11	69.79	20.70
5	4	-11	88.19	13.10
-5	-4	11	84.99	10.60
-5	-4	11	59.89	11.10
4	4	-11	24.90	11.60
4	4	-11	44.10	11.60
4	4	-11	36.60	12.50
-4	-4	11	36.10	6.70
3	4	-11	82.69	14.60
3	4	-11	50.49	14.30
3	4	-11	81.79	11.80
3	4	-11	100.09	13.00
-3	-4	11	56.99	9.20

-3	-4	11	85.69	12.20
2	4	-11	45.50	11.20
2	4	-11	46.40	10.70
2	4	-11	62.59	17.10
2	4	-11	50.69	10.80
-2	-4	11	52.19	7.50
-2	-4	11	60.19	8.60
1	4	-11	349.27	23.30
1	4	-11	295.87	21.80
1	4	-11	331.17	24.10
-1	-4	11	295.77	19.40
-1	-4	11	373.26	18.30
0	4	-11	1333.97	62.29
0	4	-11	1346.87	64.79
0	4	-11	1266.97	63.59
0	4	-11	1315.67	64.79
0	4	-11	1275.87	63.49
0	-4	11	1290.87	64.39
0	-4	11	1489.55	61.89
0	-4	11	1397.86	63.19
-1	4	-11	2.30	8.00
-1	4	-11	11.00	10.70
-1	4	-11	10.70	8.20
1	-4	11	12.50	4.30
1	-4	11	1.40	8.40
-2	4	-11	403.66	27.70
-2	4	-11	500.65	23.70
-2	4	-11	473.15	25.80
-2	4	-11	393.16	39.00
-2	4	-11	418.96	25.80
-2	4	-11	461.95	30.50
2	-4	11	465.05	22.90
2	-4	11	406.46	27.80
-3	4	-11	59.09	14.10
-3	4	-11	43.10	10.30
-3	4	-11	51.59	10.20
-3	4	-11	67.19	12.80
-3	4	-11	81.99	13.70
-3	4	-11	59.19	9.10
3	-4	11	50.59	7.60
-4	4	-11	682.93	33.60
-4	4	-11	647.34	35.20
-4	4	-11	699.33	38.50
-4	4	-11	650.13	40.40

-4	4	-11	693.63	36.00
-4	4	-11	670.73	36.40
4	-4	11	664.13	32.90
-5	4	-11	347.47	24.80
-5	4	-11	352.06	20.70
-5	4	-11	376.36	29.90
-5	4	-11	360.66	25.50
-5	4	-11	386.46	24.50
-5	4	-11	339.47	22.80
5	-4	11	333.67	25.00
5	-4	11	369.66	20.20
-6	4	-11	25.20	12.50
-6	4	-11	15.30	10.50
-6	4	-11	39.50	9.00
-6	4	-11	34.60	7.30
-6	4	-11	43.10	12.00
6	-4	11	25.20	7.60
-7	4	-11	138.09	14.00
-7	4	-11	147.39	17.10
-7	4	-11	119.79	19.50
-7	4	-11	130.59	17.80
-7	4	-11	179.48	16.20
7	-4	11	121.59	17.30
-8	4	-11	40.20	13.80
-8	4	-11	44.30	14.10
-8	4	-11	27.80	10.80
-8	4	-11	46.40	9.90
-8	4	-11	54.19	10.50
8	-4	11	21.10	14.20
-9	4	-11	20.90	19.70
-9	4	-11	14.80	8.60
-9	4	-11	29.70	22.30
-9	4	-11	1.50	9.20
9	-4	11	4.20	21.50
-10	4	-11	48.90	10.80
-10	4	-11	52.19	16.00
-10	4	-11	49.50	10.90
-10	4	-11	46.60	16.30
10	-4	11	39.10	14.00
-11	4	-11	-5.10	18.80
-11	4	-11	9.60	9.50
-11	4	-11	11.30	12.30
11	-4	11	32.30	18.90
10	3	-11	61.49	16.40

10	3	-11	62.99	13.60
-10	-3	11	79.09	10.90
9	3	-11	-18.60	12.10
9	3	-11	28.00	13.90
-9	-3	11	16.50	7.00
8	3	-11	101.29	15.40
8	3	-11	97.49	15.40
-8	-3	11	70.39	11.90
-8	-3	11	88.69	11.60
7	3	-11	8.10	12.50
7	3	-11	6.10	12.20
-7	-3	11	17.20	8.20
-7	-3	11	18.50	6.30
6	3	-11	12.50	11.10
6	3	-11	15.50	12.10
-6	-3	11	26.50	6.90
-6	-3	11	21.30	7.60
5	3	-11	969.10	52.49
5	3	-11	1110.59	53.69
4	3	-11	45.60	11.90
4	3	-11	47.30	11.80
4	3	-11	30.60	12.70
-4	-3	11	44.70	11.30
-4	-3	11	46.70	6.80
3	3	-11	147.39	21.10
3	3	-11	89.69	12.40
3	3	-11	122.39	22.10
-3	-3	11	91.09	12.70
-3	-3	11	86.49	13.70
-3	-3	11	92.89	12.20
-3	-3	11	97.89	12.20
2	3	-11	301.37	23.40
2	3	-11	383.76	25.40
-2	-3	11	304.87	19.00
-2	-3	11	327.67	18.70
1	3	-11	141.79	16.90
1	3	-11	99.69	8.50
-1	-3	11	117.19	10.90
-1	-3	11	111.29	9.00
0	3	-11	621.14	30.00
0	3	-11	564.64	30.90
0	-3	11	590.64	31.00
0	-3	11	641.04	30.90
0	-3	11	667.33	29.90

-1	3	-11	26.80	5.80
-1	3	-11	25.20	5.70
-1	3	-11	18.70	6.50
1	-3	11	22.80	7.70
1	-3	11	37.00	6.20
1	-3	11	19.30	5.70
-2	3	-11	0.30	9.40
-2	3	-11	9.60	7.60
-2	3	-11	0.20	6.00
-2	3	-11	9.90	6.00
-2	3	-11	-4.60	8.50
-2	3	-11	5.10	12.00
2	-3	11	4.20	7.30
2	-3	11	6.90	3.40
-3	3	-11	165.08	22.60
-3	3	-11	157.28	15.40
-3	3	-11	170.58	18.40
-3	3	-11	164.28	16.60
-3	3	-11	122.19	19.60
-3	3	-11	171.78	12.10
3	-3	11	150.18	17.20
3	-3	11	172.48	11.20
-4	3	-11	106.49	18.40
-4	3	-11	91.19	15.20
-4	3	-11	88.89	14.50
-4	3	-11	76.29	11.00
-4	3	-11	106.99	10.10
-4	3	-11	127.59	16.80
4	-3	11	114.59	8.70
-5	3	-11	1150.98	59.99
-5	3	-11	1212.08	62.19
-5	3	-11	1195.58	60.49
-5	3	-11	1252.67	57.49
-5	3	-11	1197.08	59.19
-5	3	-11	1266.87	58.29
5	-3	11	1220.28	56.69
-6	3	-11	562.24	32.90
-6	3	-11	583.24	39.70
-6	3	-11	527.45	33.20
-6	3	-11	565.04	29.50
-6	3	-11	557.04	30.50
6	-3	11	542.95	28.40
-7	3	-11	355.86	27.00
-7	3	-11	365.46	28.80

-7	3	-11	339.97	22.60
-7	3	-11	343.77	26.80
-7	3	-11	346.87	21.50
7	-3	11	365.96	21.90
-8	3	-11	52.39	9.80
-8	3	-11	40.50	9.00
-8	3	-11	40.90	12.10
-8	3	-11	32.60	12.70
-8	3	-11	39.80	14.00
-9	3	-11	127.89	15.40
-9	3	-11	116.89	17.50
-9	3	-11	141.69	23.50
-9	3	-11	127.29	14.60
9	-3	11	89.79	20.10
9	-3	11	124.79	20.60
-10	3	-11	113.69	17.60
-10	3	-11	84.29	14.50
-10	3	-11	95.79	15.40
-10	3	-11	137.69	24.00
10	-3	11	61.09	16.20
-11	3	-11	15.70	11.10
-11	3	-11	47.20	17.00
-11	3	-11	16.00	22.10
-11	3	-11	30.60	10.10
11	-3	11	-8.00	31.30
10	2	-11	152.08	21.00
10	2	-11	143.79	19.20
-10	-2	11	143.69	13.20
9	2	-11	-23.80	12.90
9	2	-11	16.70	13.20
-9	-2	11	-2.50	6.40
8	2	-11	24.10	12.30
8	2	-11	17.90	12.70
-8	-2	11	11.80	10.70
7	2	-11	476.45	30.80
7	2	-11	469.65	32.50
-7	-2	11	480.15	26.20
-7	-2	11	492.55	27.60
6	2	-11	306.27	24.10
6	2	-11	353.56	26.00
-6	-2	11	305.37	19.20
-5	-2	11	61.49	10.60
4	2	-11	35.40	10.90
4	2	-11	47.30	12.20

4	2	-11	41.00	17.40
-4	-2	11	25.70	9.40
-4	-2	11	44.10	7.30
3	2	-11	113.09	21.30
3	2	-11	152.88	19.00
3	2	-11	134.29	19.40
-3	-2	11	106.29	11.90
-3	-2	11	109.29	13.50
-3	-2	11	109.39	13.10
-3	-2	11	118.69	12.10
2	2	-11	40.30	11.80
-2	-2	11	27.60	5.60
-2	-2	11	26.80	6.00
1	2	-11	12.70	2.90
-1	-2	11	9.00	4.40
-1	-2	11	12.40	4.20
0	2	-11	949.01	44.80
0	-2	11	968.90	45.60
0	-2	11	941.31	46.10
0	-2	11	1037.80	45.40
-1	2	-11	569.84	29.90
1	-2	11	583.84	30.10
1	-2	11	649.54	29.00
-2	2	-11	7.40	5.40
-2	2	-11	8.00	6.60
-2	2	-11	9.20	6.80
2	-2	11	3.20	5.20
2	-2	11	-1.60	5.40
2	-2	11	3.20	3.00
-3	2	-11	2.90	4.90
-3	2	-11	0.70	7.10
-3	2	-11	-9.60	12.10
-3	2	-11	2.70	6.80
-3	2	-11	13.90	10.60
-3	2	-11	-10.60	8.80
3	-2	11	10.80	3.60
3	-2	11	1.50	6.90
-4	2	-11	354.96	28.70
-4	2	-11	416.86	24.30
-4	2	-11	406.56	26.10
-4	2	-11	335.67	25.00
-4	2	-11	381.76	22.80
-4	2	-11	419.66	26.70
4	-2	11	398.76	19.70

-5	2	-11	47.10	7.00
-5	2	-11	55.19	11.60
-5	2	-11	24.70	8.20
-5	2	-11	22.80	12.70
-5	2	-11	43.90	9.30
5	-2	11	41.00	6.30
-6	2	-11	8.20	16.70
-6	2	-11	11.10	6.00
6	-2	11	-0.80	4.50
-7	2	-11	116.79	14.10
-7	2	-11	118.99	12.50
-7	2	-11	125.89	19.80
-7	2	-11	125.29	17.80
-7	2	-11	120.69	22.80
7	-2	11	101.89	12.20
-8	2	-11	121.49	15.00
-8	2	-11	102.79	15.60
-8	2	-11	145.09	19.80
-8	2	-11	148.29	20.60
8	-2	11	125.29	16.70
-9	2	-11	18.50	11.90
-9	2	-11	6.80	7.50
-9	2	-11	8.40	7.80
9	-2	11	-11.40	12.40
9	-2	11	4.20	15.00
-10	2	-11	149.19	23.10
-10	2	-11	114.39	14.30
-10	2	-11	124.39	15.20
-10	2	-11	105.49	17.40
10	-2	11	88.09	17.60
-11	2	-11	384.06	25.20
-11	2	-11	450.05	31.30
-11	2	-11	428.56	25.00
-11	2	-11	395.36	32.10
10	1	-11	1.40	13.70
10	1	-11	4.70	12.70
-10	-1	11	7.00	6.20
9	1	-11	6.50	13.30
9	1	-11	-13.80	12.50
-9	-1	11	-3.30	6.30
8	1	-11	11.40	13.40
8	1	-11	27.50	12.20
-8	-1	11	10.50	6.20
-8	-1	11	12.10	11.60

7	1	-11	41.40	13.70
7	1	-11	38.00	10.70
-7	-1	11	48.40	9.40
-7	-1	11	45.40	7.50
6	1	-11	472.05	28.70
6	1	-11	434.56	31.20
-6	-1	11	484.85	25.50
5	1	-11	78.29	13.20
5	1	-11	89.59	15.90
-5	-1	11	91.79	10.70
4	1	-11	851.61	42.40
4	1	-11	770.52	44.40
4	1	-11	758.72	40.60
-4	-1	11	827.02	37.80
-4	-1	11	645.94	38.60
3	1	-11	0.60	10.70
3	1	-11	10.70	10.80
-3	-1	11	12.50	5.80
-3	-1	11	16.00	6.20
-3	-1	11	-10.20	6.40
-3	-1	11	-1.10	6.40
2	1	-11	221.38	27.50
-2	-1	11	187.48	13.60
-2	-1	11	226.28	13.50
-1	-1	11	1111.49	53.69
-1	-1	11	1191.38	53.49
0	1	-11	4.80	2.70
0	-1	11	2.20	3.60
0	-1	11	7.40	3.80
0	-1	11	6.40	3.10
-1	1	-11	97.39	8.90
-1	1	-11	97.29	7.70
1	-1	11	92.89	9.00
1	-1	11	100.09	7.20
1	-1	11	105.59	9.10
-2	1	-11	399.56	21.40
-2	1	-11	415.56	21.10
-2	1	-11	398.76	21.00
2	-1	11	410.86	19.90
2	-1	11	399.36	21.40
2	-1	11	399.46	21.70
-3	1	-11	89.09	11.60
-3	1	-11	106.59	9.80
-3	1	-11	106.69	11.40

-3	1	-11	109.49	13.80
-3	1	-11	146.59	27.10
-3	1	-11	98.19	10.60
3	-1	11	100.49	7.00
3	-1	11	102.99	11.40
3	-1	11	84.69	11.50
-4	1	-11	11.60	10.20
-4	1	-11	16.00	8.60
-4	1	-11	2.00	7.20
-4	1	-11	8.00	9.90
-4	1	-11	17.70	10.20
-4	1	-11	16.10	4.90
4	-1	11	17.40	4.20
-5	1	-11	0.40	8.00
-5	1	-11	8.00	10.20
-5	1	-11	7.80	7.20
-5	1	-11	22.50	10.70
-5	1	-11	9.30	4.80
-5	1	-11	8.30	10.70
5	-1	11	6.80	3.30
-6	1	-11	1517.75	70.39
-6	1	-11	1574.84	71.99
-6	1	-11	1456.15	73.49
-6	1	-11	1449.95	74.09
-6	1	-11	1546.55	70.89
-6	1	-11	1477.75	74.19
6	-1	11	1518.95	69.29
-7	1	-11	177.08	21.90
-7	1	-11	181.98	13.80
-7	1	-11	142.49	22.90
-7	1	-11	188.78	15.10
-7	1	-11	193.28	18.00
7	-1	11	150.48	12.60
-8	1	-11	192.38	15.90
-8	1	-11	169.58	22.50
-8	1	-11	144.69	22.20
-8	1	-11	230.98	31.90
-8	1	-11	170.08	20.50
-8	1	-11	173.58	15.00
8	-1	11	162.48	15.60
-9	1	-11	114.69	16.30
-9	1	-11	96.29	14.30
-9	1	-11	79.89	14.30
-9	1	-11	79.39	12.90

-9	1	-11	75.59	12.90
-9	1	-11	87.39	14.70
9	-1	11	75.89	13.60
9	-1	11	91.99	15.70
-10	1	-11	108.59	13.60
-10	1	-11	93.39	14.10
-10	1	-11	130.09	21.00
-10	1	-11	87.29	17.00
-10	1	-11	106.59	14.80
10	-1	11	106.19	18.90
-11	1	-11	40.60	16.00
-11	1	-11	29.60	8.60
-11	1	-11	22.80	15.60
-11	1	-11	15.90	12.10
11	0	-11	9.20	13.00
11	0	-11	13.20	19.20
-11	0	11	38.20	11.10
10	0	-11	-8.50	12.30
10	0	-11	4.40	14.70
-10	0	11	11.10	6.80
9	0	-11	118.79	15.70
9	0	-11	95.39	16.30
-9	0	11	117.39	13.30
8	0	-11	11.00	11.40
8	0	-11	28.40	14.60
-8	0	11	2.20	11.80
-8	0	11	4.80	6.40
7	0	-11	38.10	13.70
7	0	-11	29.90	10.80
-7	0	11	38.80	8.00
6	0	-11	1525.55	75.29
6	0	-11	1481.55	73.39
-6	0	11	1614.04	72.29
5	0	-11	89.49	14.00
5	0	-11	94.49	16.00
-5	0	11	102.69	10.90
-5	0	11	89.19	13.70
4	0	-11	5.20	8.30
4	0	-11	14.00	13.80
4	0	-11	29.50	11.80
4	0	-11	35.60	15.20
-4	0	11	5.10	6.30
-4	0	11	0.40	6.30
3	0	-11	7.50	9.10

3	0	-11	8.10	11.40
3	0	-11	25.30	8.70
-3	0	11	14.00	6.70
-3	0	11	10.30	5.80
-3	0	11	21.60	5.70
-3	0	11	7.20	6.80
-2	0	11	117.19	10.70
-2	0	11	119.49	10.10
-2	0	11	125.69	10.00
-1	0	11	350.86	18.60
-1	0	11	351.06	18.20
0	0	-11	601.64	27.70
0	0	11	621.24	28.90
0	0	11	610.04	28.10
0	0	11	593.04	29.50
-1	0	-11	1331.47	58.19
1	0	11	1195.28	58.49
1	0	11	1303.17	58.29
-2	0	-11	237.98	13.90
-2	0	-11	250.17	14.70
2	0	11	234.28	14.80
2	0	11	262.47	12.70
2	0	11	252.57	14.40
-3	0	-11	58.79	7.20
3	0	11	46.60	8.60
3	0	11	32.60	6.40
3	0	11	43.70	4.50
-4	0	-11	492.85	27.50
-4	0	-11	475.95	31.10
-4	0	-11	505.45	28.60
-4	0	-11	548.55	31.20
-4	0	-11	491.15	31.40
-4	0	-11	542.65	26.00
4	0	11	487.35	28.30
4	0	11	577.14	24.80
-5	0	-11	435.26	25.70
-5	0	-11	463.05	28.80
-5	0	-11	439.36	29.40
-5	0	-11	447.56	29.80
-5	0	-11	445.56	25.00
-5	0	-11	472.05	23.50
5	0	11	449.95	22.00
-6	0	-11	12.20	6.70
-6	0	-11	2.40	7.60

-6	0	-11	4.40	10.80
-6	0	-11	11.10	10.40
-6	0	-11	-3.60	10.20
-6	0	-11	9.70	5.00
6	0	11	4.30	3.50
-7	0	-11	251.97	17.40
-7	0	-11	269.27	19.70
-7	0	-11	275.37	25.10
-7	0	-11	241.18	24.30
-7	0	-11	250.17	16.50
7	0	11	239.08	15.00
-8	0	-11	137.59	18.80
-8	0	-11	172.48	23.00
-8	0	-11	176.48	22.40
-8	0	-11	182.88	14.70
-8	0	-11	170.88	21.30
-8	0	-11	177.58	15.80
8	0	11	175.98	14.40
-9	0	-11	6.20	7.90
-9	0	-11	-8.50	11.60
-9	0	-11	31.50	13.20
-9	0	-11	18.70	10.60
-9	0	-11	9.40	12.40
-9	0	-11	6.20	7.90
9	0	11	-11.40	10.00
9	0	11	2.90	14.10
-10	0	-11	64.89	14.10
-10	0	-11	55.99	15.80
-10	0	-11	58.89	12.40
-10	0	-11	42.40	10.50
10	0	11	28.00	18.10
10	0	11	66.49	19.50
-11	0	-11	84.39	14.80
-11	0	-11	83.19	13.40
-11	0	-11	83.99	16.70
-11	0	-11	85.39	13.20
-11	0	-11	72.69	15.50
-12	0	-11	79.49	17.50
-12	0	-11	48.40	9.70
-12	0	-11	42.50	11.80
12	0	11	41.10	12.90
11	-1	-11	112.19	19.40
-11	1	11	117.89	18.90
10	-1	-11	67.89	17.40

10	-1	-11	74.19	13.50
-10	1	11	94.19	12.00
9	-1	-11	59.99	16.10
9	-1	-11	63.39	13.60
-9	1	11	51.59	12.10
-9	1	11	32.30	8.30
8	-1	-11	64.39	16.00
8	-1	-11	50.79	12.20
-8	1	11	83.29	12.30
-8	1	11	76.89	11.70
-8	1	11	112.69	20.20
7	-1	-11	18.40	13.70
7	-1	-11	27.70	11.10
-7	1	11	21.10	7.30
6	-1	-11	362.36	28.30
-6	1	11	346.57	21.00
5	-1	-11	1014.60	52.69
5	-1	-11	1039.50	52.89
5	-1	-11	1037.70	50.59
-5	1	11	1050.59	48.90
-5	1	11	939.91	53.29
4	-1	-11	29.20	12.30
4	-1	-11	10.50	7.60
4	-1	-11	26.80	13.10
4	-1	-11	18.70	8.50
4	-1	-11	29.50	14.30
-4	1	11	5.70	7.70
-4	1	11	26.10	10.10
-4	1	11	18.50	6.50
3	-1	-11	27.30	9.00
3	-1	-11	5.00	12.90
3	-1	-11	8.30	8.60
-3	1	11	20.40	6.50
-3	1	11	20.40	4.90
-3	1	11	5.70	5.50
-2	1	11	513.55	25.20
-2	1	11	480.35	24.90
-2	1	11	501.45	25.60
-1	1	11	273.97	15.50
-1	1	11	291.67	15.20
1	1	11	15.20	2.90
1	1	11	12.10	3.30
-2	-1	-11	42.70	6.20
2	1	11	31.90	6.00

2	1	11	36.30	3.90
2	1	11	34.80	5.40
-3	-1	-11	117.69	10.10
3	1	11	110.49	9.60
3	1	11	130.99	6.80
3	1	11	107.09	10.80
-4	-1	-11	155.58	14.40
-4	-1	-11	167.48	13.70
-4	-1	-11	140.19	18.10
-4	-1	-11	169.98	20.50
-4	-1	-11	149.88	14.80
-4	-1	-11	173.18	11.30
-4	-1	-11	158.28	20.30
4	1	11	167.58	14.40
4	1	11	166.18	8.80
4	1	11	143.19	13.40
-5	-1	-11	992.40	44.80
-5	-1	-11	940.01	45.70
-5	-1	-11	919.41	49.60
-5	-1	-11	858.31	49.20
-5	-1	-11	941.31	47.90
-5	-1	-11	927.01	46.00
5	1	11	989.00	43.40
-6	-1	-11	185.28	16.20
-6	-1	-11	176.68	20.00
-6	-1	-11	185.68	21.50
-6	-1	-11	158.58	13.90
-6	-1	-11	175.28	12.50
-6	-1	-11	171.58	21.40
6	1	11	173.88	10.40
-7	-1	-11	654.43	39.10
-7	-1	-11	658.13	32.90
-7	-1	-11	671.13	33.40
-7	-1	-11	636.54	37.70
-7	-1	-11	616.94	38.70
-7	-1	-11	646.14	35.00
7	1	11	652.43	31.60
-8	-1	-11	43.40	11.70
-8	-1	-11	15.40	11.20
-8	-1	-11	33.80	6.70
-8	-1	-11	33.30	7.60
-8	-1	-11	49.90	13.20
-8	-1	-11	35.20	9.60
8	1	11	44.50	7.10

-9	-1	-11	291.87	22.30
-9	-1	-11	266.07	18.90
-9	-1	-11	237.58	23.30
-9	-1	-11	308.87	27.90
-9	-1	-11	308.77	26.50
-9	-1	-11	274.57	19.70
9	1	11	289.37	20.60
-10	-1	-11	155.18	19.50
-10	-1	-11	188.38	17.30
-10	-1	-11	146.19	14.40
-10	-1	-11	190.18	23.10
-10	-1	-11	130.59	25.00
10	1	11	120.19	17.70
-11	-1	-11	32.00	9.70
-11	-1	-11	53.79	15.20
-11	-1	-11	44.40	13.30
-11	-1	-11	31.80	8.60
-12	-1	-11	38.10	9.00
-12	-1	-11	33.10	15.50
-12	-1	-11	32.10	11.70
12	1	11	29.30	13.40
11	-2	-11	38.40	14.50
-11	2	11	40.50	13.00
10	-2	-11	99.29	17.10
10	-2	-11	101.39	19.70
-10	2	11	81.69	18.40
-10	2	11	76.59	12.70
9	-2	-11	1.60	11.80
-9	2	11	9.50	11.10
-9	2	11	-1.70	7.40
8	-2	-11	3.30	15.20
8	-2	-11	-13.80	10.40
-8	2	11	1.40	6.80
-8	2	11	20.60	10.10
7	-2	-11	279.77	27.80
7	-2	-11	200.48	21.10
-7	2	11	224.38	17.20
-7	2	11	248.38	19.60
6	-2	-11	72.59	14.90
6	-2	-11	83.79	14.80
6	-2	-11	75.19	11.60
-6	2	11	70.19	8.90
5	-2	-11	688.03	36.90
5	-2	-11	663.13	35.30

5	-2	-11	683.33	39.00
-5	2	11	611.94	34.20
-5	2	11	644.54	33.10
4	-2	-11	-17.10	13.90
4	-2	-11	22.00	13.70
4	-2	-11	27.50	10.40
4	-2	-11	12.30	9.50
4	-2	-11	13.10	9.10
-4	2	11	5.30	5.40
3	-2	-11	-2.00	8.00
3	-2	-11	-1.30	9.20
-3	2	11	-0.30	6.50
-3	2	11	1.90	3.70
-3	2	11	7.20	6.00
-2	2	11	25.50	5.30
-2	2	11	29.90	5.50
-2	2	11	21.90	4.20
-1	2	11	40.20	5.60
-1	2	11	36.90	6.00
-1	2	11	37.60	6.60
1	2	11	1066.09	49.40
1	2	11	1100.49	49.20
2	2	11	293.37	15.70
2	2	11	308.37	14.30
2	2	11	299.67	16.60
-3	-2	-11	7.10	5.10
3	2	11	11.40	4.40
3	2	11	10.10	1.90
3	2	11	7.30	4.60
-4	-2	-11	934.81	52.39
-4	-2	-11	1098.09	47.20
-4	-2	-11	988.70	50.09
-4	-2	-11	978.10	48.50
4	2	11	943.51	48.60
4	2	11	1106.89	45.80
4	2	11	975.70	48.50
-5	-2	-11	300.67	20.40
-5	-2	-11	328.87	22.60
-5	-2	-11	319.17	25.70
-5	-2	-11	317.97	17.60
-5	-2	-11	308.77	19.40
-5	-2	-11	344.97	27.80
5	2	11	338.07	15.90
-6	-2	-11	21.10	5.50

-6	-2	-11	25.70	12.20
-6	-2	-11	26.30	10.40
-6	-2	-11	33.40	7.00
-6	-2	-11	16.80	6.70
-6	-2	-11	19.40	12.30
6	2	11	23.80	3.80
-7	-2	-11	108.69	14.00
-7	-2	-11	144.19	11.90
-7	-2	-11	96.79	15.40
-7	-2	-11	134.29	15.10
-7	-2	-11	134.29	13.10
-7	-2	-11	144.19	19.00
7	2	11	137.89	9.50
-8	-2	-11	64.89	9.90
-8	-2	-11	84.69	13.30
-8	-2	-11	64.79	11.90
-8	-2	-11	76.59	12.40
-8	-2	-11	95.69	15.80
8	2	11	76.69	9.70
-9	-2	-11	81.09	14.30
-9	-2	-11	68.39	10.20
-9	-2	-11	66.59	11.20
-9	-2	-11	71.09	15.80
-9	-2	-11	72.09	11.70
-9	-2	-11	43.00	9.90
9	2	11	77.09	13.50
9	2	11	74.09	18.40
-10	-2	-11	141.09	19.10
-10	-2	-11	109.59	24.90
-10	-2	-11	138.09	13.80
-10	-2	-11	163.88	24.60
10	2	11	156.88	23.00
-11	-2	-11	198.78	21.30
-11	-2	-11	221.88	24.00
-11	-2	-11	176.88	19.60
-11	-2	-11	170.28	15.70
-11	-2	-11	195.78	15.70
-12	-2	-11	12.40	17.10
-12	-2	-11	4.20	22.00
-12	-2	-11	22.20	8.30
-12	-2	-11	7.60	10.40
12	2	11	27.60	13.10
11	-3	-11	76.09	23.30
-11	3	11	117.19	16.30

10	-3	-11	14.40	11.30
-10	3	11	-4.70	7.80
-10	3	11	37.00	11.40
9	-3	-11	10.40	11.80
-9	3	11	-11.50	10.70
-9	3	11	6.20	8.20
8	-3	-11	-2.80	10.60
8	-3	-11	29.20	17.70
-8	3	11	21.30	9.80
-8	3	11	4.60	6.90
7	-3	-11	151.08	17.50
7	-3	-11	187.18	25.50
7	-3	-11	102.79	18.00
-7	3	11	128.39	13.60
-7	3	11	112.49	17.60
6	-3	-11	24.90	15.30
6	-3	-11	7.10	8.70
-6	3	11	14.60	10.00
-6	3	11	16.00	7.00
5	-3	-11	446.66	29.20
5	-3	-11	542.85	30.10
5	-3	-11	500.25	28.50
5	-3	-11	466.05	34.90
-5	3	11	472.95	26.60
4	-3	-11	465.95	34.40
4	-3	-11	448.46	27.60
4	-3	-11	470.35	34.80
4	-3	-11	499.95	27.40
4	-3	-11	446.26	27.30
-4	3	11	468.35	25.30
3	-3	-11	135.79	18.20
3	-3	-11	88.49	16.30
3	-3	-11	107.49	14.70
-3	3	11	138.29	12.50
-3	3	11	125.19	12.50
-3	3	11	112.69	8.50
-2	3	11	546.25	27.20
-2	3	11	548.75	27.10
-2	3	11	517.95	25.80
-1	3	11	568.94	25.80
-1	3	11	515.85	26.70
-1	3	11	532.65	26.70
0	3	11	443.46	21.30
1	3	11	40.20	5.40

1	3	11	27.60	3.40
1	3	11	27.80	4.10
2	3	11	1523.95	67.69
2	3	11	1514.95	69.79
2	3	11	1533.55	68.09
2	3	11	1484.65	68.99
-3	-3	-11	10.30	4.70
3	3	11	11.30	1.70
3	3	11	23.20	4.80
3	3	11	12.20	5.10
-4	-3	-11	8.00	8.60
-4	-3	-11	12.10	13.30
-4	-3	-11	28.40	8.20
-4	-3	-11	16.70	4.40
4	3	11	20.40	6.30
4	3	11	18.40	6.70
4	3	11	17.20	2.90
-5	-3	-11	49.80	13.90
-5	-3	-11	28.70	5.10
-5	-3	-11	38.20	8.10
-5	-3	-11	37.10	9.60
-5	-3	-11	27.00	7.30
-5	-3	-11	25.30	10.50
5	3	11	36.80	11.10
5	3	11	33.80	4.20
-6	-3	-11	1345.77	60.89
-6	-3	-11	1315.87	61.89
-6	-3	-11	1196.18	65.29
-6	-3	-11	1299.07	63.49
-6	-3	-11	1362.16	61.29
-6	-3	-11	1191.28	66.39
6	3	11	1391.96	59.59
-7	-3	-11	46.90	10.40
-7	-3	-11	51.49	13.00
-7	-3	-11	68.39	10.10
-7	-3	-11	55.89	11.80
-7	-3	-11	69.59	9.20
-7	-3	-11	52.19	8.80
7	3	11	56.79	6.70
-8	-3	-11	19.30	7.00
-8	-3	-11	37.80	10.50
-8	-3	-11	24.00	13.20
-8	-3	-11	23.10	6.50
-8	-3	-11	26.40	10.50

-8	-3	-11	33.50	9.20
8	3	11	14.60	5.40
-9	-3	-11	514.35	27.30
-9	-3	-11	470.45	28.90
-9	-3	-11	509.25	32.10
-9	-3	-11	463.05	29.50
-9	-3	-11	521.25	35.00
-9	-3	-11	507.45	27.60
9	3	11	517.65	27.30
-10	-3	-11	178.88	24.80
-10	-3	-11	177.98	19.70
-10	-3	-11	192.98	15.00
10	3	11	165.88	21.60
-11	-3	-11	20.90	8.80
-11	-3	-11	22.70	12.60
-11	-3	-11	41.90	11.50
-11	-3	-11	22.60	11.70
-12	-3	-11	99.19	12.20
-12	-3	-11	97.99	16.40
-12	-3	-11	70.19	20.20
12	3	11	109.19	20.00
10	-4	-11	44.00	11.70
-10	4	11	36.30	11.00
-10	4	11	18.80	9.30
9	-4	-11	71.39	31.60
9	-4	-11	66.29	12.60
-9	4	11	49.00	11.10
-9	4	11	44.70	9.30
8	-4	-11	18.70	10.60
8	-4	-11	30.70	19.40
-8	4	11	10.10	7.90
-8	4	11	7.70	9.30
7	-4	-11	94.09	18.10
7	-4	-11	107.79	14.60
7	-4	-11	120.09	19.70
-7	4	11	102.49	13.60
-7	4	11	117.99	16.90
6	-4	-11	1245.48	60.29
6	-4	-11	1257.47	60.89
6	-4	-11	1213.08	66.09
5	-4	-11	41.90	9.40
5	-4	-11	47.50	11.00
5	-4	-11	72.19	17.10
5	-4	-11	22.10	19.10

5	-4	-11	59.59	10.80
-5	4	11	46.40	7.60
-5	4	11	59.09	12.00
4	-4	-11	88.79	11.40
4	-4	-11	79.99	15.10
4	-4	-11	96.19	14.50
4	-4	-11	112.19	18.10
4	-4	-11	79.09	13.70
-4	4	11	85.49	8.40
3	-4	-11	19.90	8.70
3	-4	-11	11.40	8.60
3	-4	-11	10.00	10.00
-3	4	11	17.70	3.60
-3	4	11	14.00	6.90
-3	4	11	6.90	6.50
-2	4	11	127.19	8.10
-2	4	11	131.99	10.80
-2	4	11	133.69	10.50
-1	4	11	400.06	19.50
-1	4	11	417.36	21.10
-1	4	11	395.46	20.80
0	4	11	24.30	4.10
0	4	11	28.60	4.40
1	4	11	1049.59	47.60
1	4	11	990.10	46.20
1	4	11	1049.99	46.40
1	4	11	996.40	45.60
1	4	11	987.90	45.50
2	4	11	8.70	4.50
2	4	11	14.60	3.50
-3	-4	-11	56.59	8.30
3	4	11	79.49	8.90
3	4	11	77.99	7.90
-4	-4	-11	655.93	30.70
-4	-4	-11	622.84	33.50
-4	-4	-11	595.94	39.00
-4	-4	-11	572.14	33.10
4	4	11	592.14	32.40
4	4	11	677.33	32.60
4	4	11	708.53	28.80
-5	-4	-11	144.79	16.40
-5	-4	-11	160.08	19.20
-5	-4	-11	170.38	10.90
-5	-4	-11	158.08	21.10

-5	-4	-11	146.99	13.90
-5	-4	-11	156.88	13.80
5	4	11	151.58	19.30
-6	-4	-11	98.39	18.30
-6	-4	-11	60.39	9.60
-6	-4	-11	68.89	11.90
-6	-4	-11	72.69	8.70
-6	-4	-11	83.09	12.80
-6	-4	-11	61.99	10.70
-6	-4	-11	78.59	14.50
-7	-4	-11	479.25	29.30
-7	-4	-11	465.15	32.70
-7	-4	-11	467.65	28.80
-7	-4	-11	481.55	26.20
-7	-4	-11	503.45	27.40
-7	-4	-11	485.65	25.60
7	4	11	506.35	23.90
-8	-4	-11	455.75	24.50
-8	-4	-11	470.45	23.90
-8	-4	-11	402.16	30.80
-8	-4	-11	403.46	27.70
-8	-4	-11	392.06	26.30
-8	-4	-11	424.26	25.50
8	4	11	426.36	22.40
-9	-4	-11	26.90	7.50
-9	-4	-11	18.00	8.70
-9	-4	-11	14.10	13.50
-9	-4	-11	18.00	9.90
-9	-4	-11	16.30	11.10
-9	-4	-11	24.70	7.90
9	4	11	29.10	7.80
-10	-4	-11	33.30	11.50
-10	-4	-11	43.20	8.10
-10	-4	-11	40.00	15.90
-10	-4	-11	3.10	18.00
10	4	11	29.60	11.80
-11	-4	-11	77.99	12.40
-11	-4	-11	93.59	12.60
-11	-4	-11	111.69	15.90
-11	-4	-11	98.09	12.90
-12	-4	-11	35.40	12.50
-12	-4	-11	4.50	8.60
-12	-4	-11	23.60	8.10
-12	-4	-11	12.20	13.80

12	4	11	46.10	15.30
10	-5	-11	15.10	10.90
-10	5	11	25.10	10.50
-10	5	11	28.00	9.90
9	-5	-11	37.10	11.90
-9	5	11	14.20	8.30
-9	5	11	18.20	9.90
8	-5	-11	169.48	19.40
8	-5	-11	195.98	23.60
-8	5	11	163.18	18.10
-8	5	11	179.48	16.40
7	-5	-11	80.49	12.70
7	-5	-11	105.39	13.50
7	-5	-11	122.29	24.20
-7	5	11	106.19	16.10
-7	5	11	113.59	13.60
6	-5	-11	164.78	19.20
6	-5	-11	190.18	19.50
6	-5	-11	187.88	23.20
-6	5	11	176.88	18.40
-6	5	11	190.08	23.10
5	-5	-11	392.96	24.70
5	-5	-11	351.56	25.80
5	-5	-11	367.46	24.10
5	-5	-11	399.56	36.00
5	-5	-11	388.16	30.50
-5	5	11	388.66	21.50
-5	5	11	419.46	25.00
4	-5	-11	273.67	25.00
4	-5	-11	328.87	21.20
4	-5	-11	282.47	20.00
4	-5	-11	318.57	21.50
4	-5	-11	239.88	34.50
-4	5	11	297.77	16.20
3	-5	-11	85.09	13.60
3	-5	-11	76.29	9.50
3	-5	-11	98.19	17.80
3	-5	-11	63.69	10.80
-3	5	11	90.19	7.20
-2	5	11	3.40	5.30
-2	5	11	2.20	4.50
-2	5	11	5.30	2.50
-1	5	11	424.76	22.40
-1	5	11	434.96	20.80

-1	5	11	452.25	22.70
0	5	11	485.35	22.50
0	5	11	471.45	23.90
1	5	11	148.49	10.20
1	5	11	146.79	9.10
1	5	11	143.59	8.30
1	5	11	149.88	9.90
2	5	11	147.19	9.90
2	5	11	132.89	10.20
2	5	11	135.69	8.90
2	5	11	146.69	10.00
3	5	11	1041.60	48.40
3	5	11	1014.20	48.70
-4	-5	-11	441.96	21.00
-4	-5	-11	406.26	24.40
-4	-5	-11	373.56	24.50
-4	-5	-11	385.46	33.70
4	5	11	406.36	23.00
4	5	11	413.26	22.70
-5	-5	-11	380.46	21.50
-5	-5	-11	343.97	28.10
-5	-5	-11	343.37	34.80
-5	-5	-11	385.46	19.60
-5	-5	-11	345.37	22.10
-5	-5	-11	371.66	23.80
5	5	11	403.26	17.70
-6	-5	-11	-8.80	10.20
-6	-5	-11	-6.00	7.50
-6	-5	-11	4.50	19.60
-6	-5	-11	2.40	6.80
-6	-5	-11	4.90	6.20
-6	-5	-11	7.90	4.80
-6	-5	-11	1.80	11.20
6	5	11	3.50	2.60
-7	-5	-11	203.38	23.60
-7	-5	-11	230.28	19.00
-7	-5	-11	189.98	16.80
-7	-5	-11	202.08	15.20
-7	-5	-11	227.78	14.50
-7	-5	-11	223.08	19.60
-8	-5	-11	118.89	17.20
-8	-5	-11	127.99	21.70
-8	-5	-11	152.98	15.40
-8	-5	-11	139.69	17.80

-8	-5	-11	136.79	12.80
-8	-5	-11	167.88	14.30
8	5	11	160.48	11.10
-9	-5	-11	45.60	9.20
-9	-5	-11	43.30	10.10
-9	-5	-11	41.90	8.40
-9	-5	-11	36.90	7.60
-9	-5	-11	41.10	13.90
-9	-5	-11	43.60	10.80
9	5	11	32.70	7.90
-10	-5	-11	210.88	27.40
-10	-5	-11	279.77	17.40
-10	-5	-11	266.37	21.90
10	5	11	235.38	21.50
-11	-5	-11	83.89	11.60
-11	-5	-11	69.79	13.10
-11	-5	-11	74.99	11.80
-11	-5	-11	44.40	9.30
-11	-5	-11	49.20	10.20
-12	-5	-11	75.09	11.70
-12	-5	-11	61.39	13.60
-12	-5	-11	33.20	11.60
-12	-5	-11	59.69	10.30
12	5	11	45.20	24.40
10	-6	-11	57.59	11.80
-10	6	11	27.40	10.30
-10	6	11	40.00	11.10
9	-6	-11	14.70	11.20
-9	6	11	19.20	9.50
-9	6	11	3.30	9.00
8	-6	-11	58.69	13.40
8	-6	-11	56.39	12.00
-8	6	11	92.29	11.60
-8	6	11	67.69	10.90
7	-6	-11	678.63	37.00
7	-6	-11	598.64	35.90
-7	6	11	645.84	35.20
-7	6	11	680.33	34.80
6	-6	-11	394.86	26.30
6	-6	-11	403.36	30.10
6	-6	-11	407.76	25.30
-6	6	11	371.86	25.80
-6	6	11	398.56	24.70
5	-6	-11	849.91	41.30

5	-6	-11	754.52	45.20
5	-6	-11	836.92	43.60
-5	6	11	829.22	41.90
-5	6	11	853.01	40.10
4	-6	-11	106.19	15.50
4	-6	-11	83.89	13.10
4	-6	-11	90.89	16.20
4	-6	-11	78.79	13.60
-4	6	11	84.59	8.00
-4	6	11	100.09	12.10
3	-6	-11	145.49	15.20
3	-6	-11	183.18	18.00
3	-6	-11	141.09	14.70
3	-6	-11	153.48	14.60
3	-6	-11	188.28	19.10
2	-6	-11	182.68	15.80
2	-6	-11	201.18	16.10
-2	6	11	194.58	14.10
-2	6	11	224.68	11.10
-1	6	11	66.79	8.10
-1	6	11	71.09	5.30
-1	6	11	74.29	8.10
0	6	11	787.72	37.30
0	6	11	794.92	35.50
0	6	11	799.02	37.60
0	6	11	792.22	35.30
0	6	11	724.73	35.70
1	6	11	509.65	26.30
1	6	11	552.04	25.30
1	6	11	545.45	26.10
1	6	11	529.55	26.80
2	6	11	240.08	14.00
2	6	11	238.18	13.40
2	6	11	244.48	14.60
3	6	11	113.49	9.90
3	6	11	122.69	10.90
3	6	11	116.99	9.90
3	6	11	131.79	11.60
-4	-6	-11	327.57	22.40
-4	-6	-11	279.87	20.10
-4	-6	-11	327.17	17.30
-4	-6	-11	373.06	42.20
4	6	11	354.06	18.60
4	6	11	298.17	19.50

4	6	11	333.67	21.80
4	6	11	292.87	19.60
-5	-6	-11	90.89	12.70
-5	-6	-11	82.79	10.70
-5	-6	-11	80.79	15.20
-5	-6	-11	102.69	10.50
-5	-6	-11	102.69	9.00
-5	-6	-11	87.69	24.30
-5	-6	-11	113.89	12.40
5	6	11	103.29	5.90
-6	-6	-11	222.38	16.30
-6	-6	-11	223.28	19.70
-6	-6	-11	206.38	23.50
-6	-6	-11	219.68	18.50
-6	-6	-11	206.48	15.30
-6	-6	-11	220.28	14.10
6	6	11	227.88	11.50
-7	-6	-11	163.98	14.00
-7	-6	-11	185.08	12.90
-7	-6	-11	169.88	15.30
-7	-6	-11	139.99	22.60
-7	-6	-11	183.18	17.60
-8	-6	-11	465.15	27.20
-8	-6	-11	465.15	25.70
-8	-6	-11	500.55	28.10
-8	-6	-11	447.56	28.80
-8	-6	-11	454.65	32.70
-8	-6	-11	512.35	26.50
8	6	11	479.95	24.30
-9	-6	-11	145.09	14.60
-9	-6	-11	143.59	17.20
-9	-6	-11	143.59	13.20
-9	-6	-11	108.99	17.20
-9	-6	-11	137.09	15.70
9	6	11	127.09	13.40
-10	-6	-11	168.48	21.60
-10	-6	-11	188.28	19.30
-10	-6	-11	171.48	14.30
-10	-6	-11	198.98	22.70
-10	-6	-11	177.08	29.10
10	6	11	137.69	19.10
-11	-6	-11	100.79	12.60
-11	-6	-11	137.39	17.60
-11	-6	-11	127.39	12.70

-11	-6	-11	109.49	14.40
-11	-6	-11	104.39	18.40
-12	-6	-11	31.50	10.20
-12	-6	-11	41.80	9.50
-12	-6	-11	-5.70	11.80
-12	-6	-11	20.60	8.00
12	6	11	3.70	15.50
10	-7	-11	60.59	12.30
-10	7	11	58.69	12.50
-10	7	11	87.79	14.80
9	-7	-11	20.70	11.50
9	-7	-11	11.30	16.00
-9	7	11	19.20	9.70
-9	7	11	21.20	9.90
8	-7	-11	31.90	11.30
8	-7	-11	9.30	12.30
-8	7	11	16.00	9.00
-8	7	11	-3.60	9.30
7	-7	-11	51.59	12.00
7	-7	-11	39.90	10.80
-7	7	11	48.50	10.70
6	-7	-11	29.30	14.20
6	-7	-11	27.80	10.80
6	-7	-11	49.30	9.60
-6	7	11	22.70	10.50
-6	7	11	40.20	9.60
5	-7	-11	288.77	22.40
5	-7	-11	252.67	22.30
5	-7	-11	247.48	24.90
-5	7	11	211.08	18.60
-5	7	11	264.97	16.00
4	-7	-11	512.05	30.40
4	-7	-11	514.25	31.30
4	-7	-11	535.65	27.80
4	-7	-11	506.05	29.70
-4	7	11	464.45	28.90
-4	7	11	547.75	25.60
3	-7	-11	11.80	7.70
3	-7	-11	10.40	9.10
3	-7	-11	6.50	7.10
3	-7	-11	26.40	11.10
3	-7	-11	4.60	7.60
2	-7	-11	1450.35	70.49
2	-7	-11	1500.35	70.39

2	-7	-11	1451.45	69.99
2	-7	-11	1457.75	69.89
2	-7	-11	1558.54	73.69
-2	7	11	1585.14	68.09
-1	7	11	1135.39	48.70
-1	7	11	1003.30	50.59
0	7	11	731.83	35.50
0	7	11	739.13	33.50
0	7	11	708.93	34.80
0	7	11	750.42	35.90
0	7	11	749.13	33.80
1	7	11	19.50	4.10
1	7	11	34.70	6.00
1	7	11	22.30	5.30
1	7	11	31.60	5.10
-2	-7	-11	293.47	17.20
2	7	11	277.97	15.50
2	7	11	274.97	16.30
2	7	11	267.47	16.40
2	7	11	271.57	15.20
-3	-7	-11	110.29	10.60
3	7	11	126.39	11.80
3	7	11	141.29	11.20
3	7	11	135.59	10.50
3	7	11	149.49	12.90
-4	-7	-11	83.29	8.70
-4	-7	-11	63.69	11.40
-4	-7	-11	101.39	10.90
4	7	11	90.59	15.40
4	7	11	78.29	13.80
4	7	11	95.59	10.40
4	7	11	68.29	12.10
-5	-7	-11	331.37	19.40
-5	-7	-11	317.77	19.40
-5	-7	-11	275.37	27.90
-5	-7	-11	310.37	21.50
-5	-7	-11	333.77	17.70
-6	-7	-11	308.77	21.40
-6	-7	-11	259.47	26.40
-6	-7	-11	320.77	17.60
-6	-7	-11	308.07	18.30
-6	-7	-11	311.97	21.90
-6	-7	-11	308.27	19.60
6	7	11	315.67	15.30

-7	-7	-11	106.89	16.60
-7	-7	-11	109.99	11.80
-7	-7	-11	80.09	15.00
-7	-7	-11	97.29	10.30
-7	-7	-11	102.99	15.30
-7	-7	-11	114.29	14.00
7	7	11	110.99	8.30
-8	-7	-11	43.30	13.50
-8	-7	-11	39.30	8.50
-8	-7	-11	38.20	9.50
-8	-7	-11	38.90	9.00
-8	-7	-11	39.10	7.40
-8	-7	-11	43.40	10.60
-9	-7	-11	509.15	28.50
-9	-7	-11	526.05	31.20
-9	-7	-11	555.54	31.00
-9	-7	-11	560.34	30.00
-9	-7	-11	549.15	28.90
9	7	11	519.45	28.10
-10	-7	-11	82.69	12.20
-10	-7	-11	64.39	12.30
-10	-7	-11	93.79	14.10
-10	-7	-11	78.49	11.40
-10	-7	-11	101.49	13.30
10	7	11	66.79	12.40
-11	-7	-11	12.90	7.50
-11	-7	-11	-12.80	9.80
-11	-7	-11	11.50	10.60
-11	-7	-11	8.40	7.70
-11	-7	-11	0.90	8.00
-12	-7	-11	29.50	10.10
-12	-7	-11	33.60	9.40
-12	-7	-11	31.90	8.10
-12	-7	-11	26.70	11.70
12	7	11	45.60	18.40
10	-8	-11	32.00	11.40
-10	8	11	34.70	10.70
-10	8	11	57.29	12.30
9	-8	-11	20.40	11.40
9	-8	-11	14.20	14.40
-9	8	11	6.60	10.00
-9	8	11	15.40	9.50
8	-8	-11	15.10	11.70
8	-8	-11	12.90	16.70

-8	8	11	-1.60	13.20
-8	8	11	27.00	10.00
7	-8	-11	771.42	40.20
7	-8	-11	764.22	41.40
-7	8	11	749.23	39.90
6	-8	-11	472.55	39.00
6	-8	-11	584.74	29.90
6	-8	-11	490.55	35.80
6	-8	-11	524.65	31.10
-6	8	11	539.75	29.80
-6	8	11	497.25	30.10
5	-8	-11	23.10	13.30
5	-8	-11	38.70	10.70
5	-8	-11	36.90	8.60
-5	8	11	30.40	6.80
4	-8	-11	20.90	10.30
4	-8	-11	30.00	7.60
4	-8	-11	44.50	10.40
4	-8	-11	18.70	9.20
4	-8	-11	40.20	8.60
-4	8	11	37.50	6.60
-4	8	11	43.00	9.50
3	-8	-11	419.26	29.20
3	-8	-11	454.35	26.50
3	-8	-11	462.85	25.50
3	-8	-11	433.66	26.20
3	-8	-11	450.85	25.20
-3	8	11	477.45	22.20
2	-8	-11	115.89	13.50
2	-8	-11	99.19	15.60
2	-8	-11	118.79	14.70
2	-8	-11	140.19	14.30
2	-8	-11	120.59	13.80
1	-8	-11	378.26	23.70
1	-8	-11	399.46	23.00
1	-8	-11	360.86	23.40
-1	8	11	437.86	19.00
0	8	11	100.29	5.80
0	8	11	105.29	9.90
0	8	11	92.79	11.10
0	8	11	105.69	7.40
1	8	11	688.93	31.40
1	8	11	676.13	32.90
1	8	11	612.14	33.10

1	8	11	644.84	32.10
-2	-8	-11	34.40	7.20
2	8	11	17.60	6.90
2	8	11	22.70	5.10
2	8	11	23.90	6.10
2	8	11	29.10	5.30
-3	-8	-11	43.60	6.20
3	8	11	29.90	6.40
3	8	11	35.70	6.30
3	8	11	35.20	7.30
3	8	11	28.70	7.70
-4	-8	-11	600.94	32.30
-4	-8	-11	546.55	29.80
-4	-8	-11	585.24	28.20
-4	-8	-11	573.04	30.50
4	8	11	581.44	29.50
4	8	11	559.64	31.80
-5	-8	-11	11.00	9.20
-5	-8	-11	26.40	5.00
-5	-8	-11	32.50	7.20
-5	-8	-11	34.10	10.60
-5	-8	-11	24.70	7.70
-5	-8	-11	12.40	13.50
-6	-8	-11	-5.70	6.80
-6	-8	-11	-6.80	5.90
-6	-8	-11	2.60	4.80
-6	-8	-11	-8.90	9.10
-6	-8	-11	14.90	12.90
-6	-8	-11	-4.60	8.70
6	8	11	4.40	3.20
-7	-8	-11	456.95	27.30
-7	-8	-11	443.96	26.50
-7	-8	-11	474.75	25.30
-7	-8	-11	437.86	28.10
-7	-8	-11	473.25	24.70
-7	-8	-11	459.95	31.20
7	8	11	494.55	23.10
-8	-8	-11	25.00	8.20
-8	-8	-11	19.80	13.30
-8	-8	-11	32.70	9.40
-8	-8	-11	36.40	7.70
-8	-8	-11	35.40	9.40
-8	-8	-11	28.10	7.70
8	8	11	29.70	5.90

-9	-8	-11	151.58	13.60
-9	-8	-11	169.28	18.20
-9	-8	-11	154.68	18.40
-9	-8	-11	164.28	14.70
-9	-8	-11	161.28	15.80
9	8	11	120.09	13.90
-10	-8	-11	2.80	8.70
-10	-8	-11	5.40	9.60
-10	-8	-11	-0.40	7.60
-10	-8	-11	3.00	10.60
-10	-8	-11	9.90	7.00
10	8	11	-12.80	11.00
-11	-8	-11	31.20	8.80
-11	-8	-11	41.70	10.90
-11	-8	-11	24.50	7.70
-12	-8	-11	8.30	7.30
-12	-8	-11	12.10	11.70
-12	-8	-11	-13.30	11.40
-12	-8	-11	-11.80	9.00
10	-9	-11	13.10	11.50
-10	9	11	12.20	12.80
-10	9	11	-2.60	11.60
9	-9	-11	-18.90	11.00
9	-9	-11	6.90	13.30
-9	9	11	-18.60	9.10
-9	9	11	-6.90	10.90
8	-9	-11	259.57	35.10
8	-9	-11	277.97	24.60
-8	9	11	253.77	23.10
7	-9	-11	234.68	20.10
7	-9	-11	240.38	21.20
-7	9	11	188.48	19.40
6	-9	-11	455.75	27.70
6	-9	-11	410.96	33.10
6	-9	-11	381.36	32.30
6	-9	-11	427.76	25.40
-6	9	11	395.96	25.40
-6	9	11	396.76	26.60
5	-9	-11	234.98	23.30
5	-9	-11	273.67	23.80
5	-9	-11	231.48	21.70
5	-9	-11	215.08	17.40
-5	9	11	234.18	18.90
-5	9	11	208.88	15.50

4	-9	-11	209.58	17.00
4	-9	-11	249.08	21.30
4	-9	-11	195.88	16.10
4	-9	-11	186.48	19.20
4	-9	-11	201.18	18.50
-4	9	11	186.38	12.40
3	-9	-11	-1.60	7.30
3	-9	-11	0.70	12.30
3	-9	-11	7.00	6.90
3	-9	-11	0.70	8.50
3	-9	-11	-2.30	8.30
-3	9	11	4.40	2.90
2	-9	-11	1.10	14.30
2	-9	-11	2.20	8.00
2	-9	-11	26.20	8.60
2	-9	-11	12.80	7.50
2	-9	-11	3.20	8.20
-2	9	11	16.80	3.00
1	-9	-11	622.44	33.70
1	-9	-11	652.53	34.20
1	-9	-11	642.54	33.90
1	-9	-11	644.54	33.40
-1	9	11	732.33	30.70
0	-9	-11	0.70	8.10
0	-9	-11	3.60	7.00
0	9	11	1.30	2.00
0	9	11	6.00	8.00
0	9	11	1.70	4.20
1	9	11	427.96	23.90
1	9	11	497.35	23.50
1	9	11	419.76	26.70
1	9	11	475.75	24.70
2	9	11	411.46	21.70
2	9	11	404.36	21.70
2	9	11	383.46	24.10
2	9	11	393.46	22.90
-3	-9	-11	774.42	35.10
3	9	11	670.63	36.80
3	9	11	726.23	38.00
3	9	11	765.02	36.10
3	9	11	704.13	37.60
-4	-9	-11	39.90	11.10
-4	-9	-11	21.70	10.80
-4	-9	-11	29.00	8.40

-4	-9	-11	39.80	5.80
-4	-9	-11	53.59	10.90
-4	-9	-11	41.90	9.40
-5	-9	-11	16.90	6.80
-5	-9	-11	35.50	10.30
-5	-9	-11	39.20	8.60
-5	-9	-11	33.80	10.60
-5	-9	-11	41.30	15.10
-5	-9	-11	25.70	5.40
-5	-9	-11	26.80	10.70
-6	-9	-11	47.30	10.70
-6	-9	-11	45.60	9.70
-6	-9	-11	32.90	5.90
-6	-9	-11	16.00	14.40
6	9	11	42.60	5.90
-7	-9	-11	219.88	15.60
-7	-9	-11	184.98	24.80
-7	-9	-11	190.78	16.50
-7	-9	-11	184.88	18.30
-7	-9	-11	233.48	19.70
-7	-9	-11	210.68	14.40
7	9	11	219.28	12.80
-8	-9	-11	416.76	26.90
-8	-9	-11	430.06	28.00
-8	-9	-11	422.46	25.00
-8	-9	-11	422.56	24.10
-8	-9	-11	486.45	32.60
-8	-9	-11	427.26	27.20
8	9	11	433.66	22.90
-9	-9	-11	-0.90	8.90
-9	-9	-11	13.10	7.50
-9	-9	-11	8.20	7.10
-9	-9	-11	6.90	6.30
-9	-9	-11	7.50	9.60
-10	-9	-11	478.35	28.40
-10	-9	-11	442.56	27.40
-10	-9	-11	447.16	25.10
-10	-9	-11	467.85	25.80
-10	-9	-11	501.15	26.60
10	9	11	448.36	29.90
-11	-9	-11	13.30	9.90
-11	-9	-11	28.50	9.70
-11	-9	-11	28.60	7.60
-11	-9	-11	32.80	8.70

-11	-9	-11	28.40	10.90
10	-10	-11	50.49	16.40
10	-10	-11	-6.30	24.50
-10	10	11	12.50	9.60
-10	10	11	40.30	14.20
9	-10	-11	29.10	12.40
9	-10	-11	18.40	11.80
-9	10	11	25.20	9.80
-9	10	11	20.30	12.00
8	-10	-11	133.89	20.00
8	-10	-11	123.69	18.90
-8	10	11	106.29	16.40
-8	10	11	137.79	20.10
7	-10	-11	235.28	22.00
7	-10	-11	206.48	19.20
-7	10	11	157.28	18.90
6	-10	-11	210.38	21.20
6	-10	-11	231.08	27.70
6	-10	-11	173.68	27.90
6	-10	-11	219.78	17.70
-6	10	11	179.98	18.40
5	-10	-11	174.08	16.40
5	-10	-11	120.79	21.40
5	-10	-11	201.08	21.60
5	-10	-11	187.28	20.40
-5	10	11	149.99	16.60
-5	10	11	169.08	13.80
4	-10	-11	453.85	25.50
4	-10	-11	414.86	29.50
4	-10	-11	457.85	32.60
4	-10	-11	481.55	33.50
4	-10	-11	442.76	26.80
-4	10	11	443.86	22.90
3	-10	-11	55.09	9.20
3	-10	-11	51.69	10.10
3	-10	-11	64.19	10.30
3	-10	-11	73.79	9.80
3	-10	-11	91.89	15.50
-3	10	11	64.29	6.30
2	-10	-11	238.78	18.40
2	-10	-11	277.97	27.70
2	-10	-11	232.88	18.00
2	-10	-11	266.87	18.70
2	-10	-11	252.17	18.40

-2	10	11	286.57	13.60
1	-10	-11	959.20	47.20
1	-10	-11	964.20	47.70
1	-10	-11	944.11	47.30
1	-10	-11	952.10	46.90
1	-10	-11	976.30	48.80
-1	10	11	1038.00	44.50
0	-10	-11	842.32	43.60
0	-10	-11	823.42	42.00
0	-10	-11	840.42	42.00
0	-10	-11	833.42	42.40
0	10	11	936.11	39.00
-1	-10	-11	73.99	10.00
-1	-10	-11	63.89	10.30
-1	-10	-11	63.49	9.20
1	10	11	52.69	9.90
1	10	11	48.50	12.10
-2	-10	-11	374.46	21.90
-2	-10	-11	344.87	24.40
-2	-10	-11	355.86	20.80
-2	-10	-11	340.77	21.60
2	10	11	358.76	25.40
2	10	11	345.37	20.90
2	10	11	323.37	21.20
2	10	11	325.17	20.20
-3	-10	-11	380.36	21.10
-3	-10	-11	385.96	26.40
-3	-10	-11	390.06	24.40
-3	-10	-11	407.06	24.60
-3	-10	-11	402.86	23.50
3	10	11	404.56	22.70
3	10	11	379.06	25.20
-4	-10	-11	44.50	9.20
-4	-10	-11	37.00	11.40
-4	-10	-11	40.80	9.50
-4	-10	-11	58.39	12.30
-4	-10	-11	45.40	6.30
-4	-10	-11	53.49	9.00
-5	-10	-11	144.09	13.50
-5	-10	-11	102.29	13.60
-5	-10	-11	143.29	11.10
-5	-10	-11	170.28	18.80
-5	-10	-11	139.79	16.30
-5	-10	-11	133.39	15.80

-6	-10	-11	6.60	10.00
-6	-10	-11	10.90	17.20
-6	-10	-11	17.50	7.10
-6	-10	-11	12.70	7.20
-6	-10	-11	18.50	6.20
-6	-10	-11	7.40	9.30
6	10	11	17.10	4.40
-7	-10	-11	22.50	7.40
-7	-10	-11	31.10	9.30
-7	-10	-11	15.80	7.10
-7	-10	-11	19.40	9.60
-7	-10	-11	19.30	6.50
-7	-10	-11	65.19	17.60
7	10	11	24.90	5.50
-8	-10	-11	663.73	34.20
-8	-10	-11	662.33	34.90
-8	-10	-11	672.33	34.30
-8	-10	-11	683.23	36.20
-8	-10	-11	672.03	36.90
-9	-10	-11	37.20	10.20
-9	-10	-11	47.10	7.90
-9	-10	-11	32.10	10.20
-9	-10	-11	37.10	8.20
-9	-10	-11	55.29	9.30
-10	-10	-11	59.59	12.00
-10	-10	-11	77.69	12.60
-10	-10	-11	47.60	10.40
-10	-10	-11	49.10	9.60
-10	-10	-11	38.30	8.00
-11	-10	-11	32.00	8.70
-11	-10	-11	34.40	8.80
-11	-10	-11	32.10	9.80
-11	-10	-11	45.10	8.30
9	-11	-11	33.30	12.30
9	-11	-11	66.19	13.00
-9	11	11	47.90	12.80
-9	11	11	41.00	10.30
8	-11	-11	-3.80	11.80
8	-11	-11	9.20	10.60
-8	11	11	26.70	12.00
-8	11	11	11.10	10.20
7	-11	-11	71.79	14.30
7	-11	-11	93.99	16.50
-7	11	11	88.79	12.30

6	-11	-11	13.90	8.40
6	-11	-11	25.30	12.10
6	-11	-11	-2.20	16.70
6	-11	-11	-7.20	14.60
-6	11	11	11.60	9.60
-6	11	11	20.40	22.70
5	-11	-11	307.07	25.50
5	-11	-11	333.37	24.60
5	-11	-11	307.37	20.80
5	-11	-11	321.87	27.00
-5	11	11	273.87	21.60
-5	11	11	312.27	19.20
4	-11	-11	192.88	25.80
4	-11	-11	153.78	19.50
4	-11	-11	186.68	16.60
4	-11	-11	163.38	18.10
4	-11	-11	181.58	18.30
-4	11	11	202.88	12.70
3	-11	-11	-9.80	7.90
3	-11	-11	-14.20	7.10
3	-11	-11	-7.70	8.90
3	-11	-11	-11.80	18.00
3	-11	-11	10.10	8.10
-3	11	11	3.60	3.40
2	-11	-11	932.71	52.29
2	-11	-11	977.80	46.90
2	-11	-11	879.71	47.00
2	-11	-11	949.40	46.70
2	-11	-11	933.41	46.70
2	-11	-11	991.00	46.80
-2	11	11	980.00	43.90
1	-11	-11	65.49	11.40
1	-11	-11	65.59	9.20
1	-11	-11	40.30	8.10
1	-11	-11	58.99	10.10
1	-11	-11	37.20	7.80
-1	11	11	52.99	4.80
0	-11	-11	72.99	9.40
0	-11	-11	100.49	13.50
0	-11	-11	80.29	11.40
0	-11	-11	105.39	18.50
0	-11	-11	89.99	12.40
0	11	11	83.59	6.70
-1	-11	-11	143.69	16.90

-1	-11	-11	152.78	15.70
-1	-11	-11	115.79	17.70
-1	-11	-11	120.49	16.20
-1	-11	-11	95.89	14.00
1	11	11	120.29	15.10
1	11	11	130.29	6.80
-2	-11	-11	514.25	28.40
-2	-11	-11	552.04	31.40
-2	-11	-11	557.94	28.60
-2	-11	-11	574.64	28.90
-2	-11	-11	524.85	29.80
2	11	11	517.15	36.80
2	11	11	500.75	30.20
-3	-11	-11	497.35	26.90
-3	-11	-11	490.85	32.20
-3	-11	-11	515.25	25.70
-3	-11	-11	458.05	28.30
-3	-11	-11	452.95	28.10
-3	-11	-11	511.55	27.50
-4	-11	-11	218.58	20.80
-4	-11	-11	297.77	18.50
-4	-11	-11	249.58	20.20
-4	-11	-11	260.07	18.30
-4	-11	-11	254.27	15.90
-4	-11	-11	243.58	21.20
-5	-11	-11	104.89	13.70
-5	-11	-11	107.29	14.10
-5	-11	-11	97.09	14.60
-5	-11	-11	87.19	10.10
-5	-11	-11	103.59	12.40
-5	-11	-11	97.39	15.60
-6	-11	-11	124.39	12.90
-6	-11	-11	107.29	15.40
-6	-11	-11	117.39	14.10
-6	-11	-11	117.19	11.30
-6	-11	-11	129.99	18.30
6	11	11	121.29	9.70
-7	-11	-11	49.30	8.40
-7	-11	-11	58.39	8.80
-7	-11	-11	65.39	9.80
-7	-11	-11	65.09	10.50
-7	-11	-11	90.79	16.40
-7	-11	-11	69.89	11.50
7	11	11	74.39	9.30

-8	-11	-11	92.09	13.40
-8	-11	-11	51.79	10.80
-8	-11	-11	68.69	10.20
-8	-11	-11	56.89	9.30
-8	-11	-11	60.19	11.50
8	11	11	50.29	8.30
-9	-11	-11	760.52	40.10
-9	-11	-11	734.23	39.70
-9	-11	-11	717.33	37.00
-9	-11	-11	747.23	37.40
-9	-11	-11	766.82	38.30
9	11	11	718.83	37.80
-10	-11	-11	16.90	9.10
-10	-11	-11	22.00	7.80
-10	-11	-11	17.90	10.60
-10	-11	-11	18.00	7.60
-10	-11	-11	21.90	8.00
-11	-11	-11	-2.60	7.80
-11	-11	-11	5.60	7.20
-11	-11	-11	-10.10	9.00
-11	-11	-11	9.00	8.30
9	-12	-11	5.20	12.40
9	-12	-11	7.10	11.10
-9	12	11	-0.20	13.20
-9	12	11	5.10	9.00
8	-12	-11	43.70	10.40
8	-12	-11	57.39	13.20
-8	12	11	44.40	10.90
-8	12	11	12.20	12.00
7	-12	-11	437.06	28.70
6	-12	-11	106.59	14.50
6	-12	-11	75.89	16.00
6	-12	-11	70.69	19.40
6	-12	-11	115.89	20.40
-6	12	11	82.19	12.20
5	-12	-11	185.38	24.40
5	-12	-11	204.28	23.90
5	-12	-11	230.48	21.70
5	-12	-11	206.08	17.60
-5	12	11	222.78	19.30
-5	12	11	199.88	16.50
4	-12	-11	132.49	17.10
4	-12	-11	112.99	17.80
4	-12	-11	135.19	19.30

4	-12	-11	145.19	15.40
4	-12	-11	148.29	25.10
-4	12	11	136.29	11.20
3	-12	-11	529.05	29.80
3	-12	-11	513.15	31.60
3	-12	-11	529.95	30.50
3	-12	-11	544.95	37.30
3	-12	-11	531.55	29.60
-3	12	11	547.35	26.30
2	-12	-11	32.30	8.70
2	-12	-11	16.90	8.40
2	-12	-11	25.10	8.80
2	-12	-11	46.70	9.30
2	-12	-11	24.50	9.40
-2	12	11	29.60	4.90
1	-12	-11	-3.70	7.70
1	-12	-11	10.20	7.50
1	-12	-11	-6.00	7.40
1	-12	-11	-14.50	9.00
-1	12	11	1.00	2.40
0	-12	-11	124.89	19.90
0	-12	-11	131.29	14.10
0	-12	-11	158.98	15.20
0	-12	-11	127.99	16.60
0	-12	-11	145.49	14.30
0	12	11	153.88	8.10
-1	-12	-11	278.97	18.30
-1	-12	-11	273.37	18.90
-1	-12	-11	258.67	20.60
-1	-12	-11	262.47	19.70
-1	-12	-11	314.77	20.50
1	12	11	312.07	13.90
-2	-12	-11	48.00	8.30
-2	-12	-11	25.20	7.30
-2	-12	-11	30.00	21.90
-2	-12	-11	28.80	11.50
-2	-12	-11	40.20	13.70
-2	-12	-11	25.70	9.20
-3	-12	-11	119.39	15.70
-3	-12	-11	82.69	10.80
-3	-12	-11	111.49	13.50
-3	-12	-11	118.79	13.00
-3	-12	-11	78.99	11.70
-4	-12	-11	1130.49	56.99

-4	-12	-11	1162.48	56.19
-4	-12	-11	1213.48	56.89
-4	-12	-11	1137.29	56.39
-4	-12	-11	1182.78	55.69
-4	-12	-11	1228.88	55.59
-5	-12	-11	3.20	9.30
-5	-12	-11	-8.00	12.40
-5	-12	-11	-1.00	6.10
-5	-12	-11	11.10	9.40
-5	-12	-11	-1.00	7.50
-6	-12	-11	-11.20	7.90
-6	-12	-11	-2.00	8.80
-6	-12	-11	-0.80	7.30
-6	-12	-11	-2.80	6.30
-6	-12	-11	-3.70	7.30
-6	-12	-11	17.80	10.90
6	12	11	0.00	5.80
-7	-12	-11	590.24	30.60
-7	-12	-11	588.04	30.10
-7	-12	-11	549.25	32.00
-7	-12	-11	583.64	32.90
-7	-12	-11	583.34	30.60
-7	-12	-11	581.34	31.70
7	12	11	556.84	29.00
-8	-12	-11	3.60	8.90
-8	-12	-11	-4.00	7.30
-8	-12	-11	15.20	7.30
-8	-12	-11	21.00	10.20
-8	-12	-11	5.50	11.30
-8	-12	-11	18.50	7.80
8	12	11	-1.60	7.80
-9	-12	-11	71.69	11.30
-9	-12	-11	88.39	15.00
-9	-12	-11	62.89	13.20
-9	-12	-11	76.09	13.10
-9	-12	-11	139.89	24.30
9	12	11	71.09	11.00
-10	-12	-11	11.90	7.90
-10	-12	-11	3.90	7.30
-10	-12	-11	1.80	8.90
-10	-12	-11	8.20	7.60
-11	-12	-11	-10.10	8.80
-11	-12	-11	19.80	8.90
-11	-12	-11	-0.20	7.50

-11	-12	-11	-0.80	7.40
9	-13	-11	12.50	11.10
9	-13	-11	51.89	15.40
-9	13	11	0.90	21.50
-9	13	11	-3.80	8.50
8	-13	-11	252.47	18.50
8	-13	-11	217.18	23.30
-8	13	11	192.98	19.50
-8	13	11	217.98	22.00
7	-13	-11	75.19	11.60
7	-13	-11	68.89	15.20
-7	13	11	77.99	12.60
6	-13	-11	74.49	16.10
6	-13	-11	95.99	14.90
6	-13	-11	116.59	21.70
-6	13	11	73.19	11.80
5	-13	-11	33.20	15.90
5	-13	-11	51.49	14.60
5	-13	-11	27.70	11.70
5	-13	-11	44.70	9.10
-5	13	11	54.49	11.20
-5	13	11	40.60	9.90
4	-13	-11	271.47	21.00
4	-13	-11	255.37	21.40
4	-13	-11	270.87	19.60
4	-13	-11	235.58	29.40
4	-13	-11	256.17	23.20
-4	13	11	253.87	16.30
3	-13	-11	21.60	8.90
3	-13	-11	4.90	9.10
3	-13	-11	20.10	9.10
3	-13	-11	1.30	9.10
-3	13	11	3.90	4.40
2	-13	-11	13.10	9.00
2	-13	-11	18.10	8.50
2	-13	-11	23.10	9.60
2	-13	-11	9.10	8.20
-2	13	11	11.60	3.60
1	-13	-11	312.57	20.50
1	-13	-11	319.97	20.60
1	-13	-11	313.87	20.20
1	-13	-11	315.27	22.70
1	-13	-11	329.47	22.00
-1	13	11	312.27	15.90

0	-13	-11	-3.90	8.60
0	-13	-11	16.30	8.00
0	-13	-11	10.70	7.70
0	-13	-11	26.40	12.50
0	-13	-11	31.30	8.60
0	13	11	14.70	2.90
-1	-13	-11	11.40	16.10
-1	-13	-11	4.60	8.60
-1	-13	-11	10.60	8.40
-1	-13	-11	14.00	7.00
-1	-13	-11	15.20	7.20
1	13	11	6.10	2.40
-2	-13	-11	1558.74	78.79
-2	-13	-11	1703.83	77.59
-2	-13	-11	1670.83	77.89
-2	-13	-11	1641.64	78.19
-2	-13	-11	1674.33	77.79
-2	-13	-11	1699.53	79.09
2	13	11	1761.42	75.09
-3	-13	-11	928.11	46.30
-3	-13	-11	892.81	47.00
-3	-13	-11	977.70	49.70
-3	-13	-11	935.51	45.30
-3	-13	-11	922.91	45.60
-3	-13	-11	965.00	46.10
-4	-13	-11	-1.40	8.60
-4	-13	-11	-12.10	8.70
-4	-13	-11	6.50	9.80
-4	-13	-11	0.90	6.90
-4	-13	-11	0.30	8.90
-5	-13	-11	66.09	8.80
-5	-13	-11	71.79	10.80
-5	-13	-11	51.09	9.10
-5	-13	-11	63.39	10.00
-5	-13	-11	53.79	8.40
5	13	11	47.90	7.50
-6	-13	-11	43.80	8.50
-6	-13	-11	23.70	12.70
-6	-13	-11	26.70	7.30
-6	-13	-11	49.40	9.00
-6	-13	-11	29.30	8.30
-6	-13	-11	42.40	10.20
6	13	11	12.80	6.70
-7	-13	-11	7.90	7.70

-7	-13	-11	3.10	7.80
-7	-13	-11	-1.30	7.40
-7	-13	-11	4.10	8.80
-7	-13	-11	7.10	9.50
-7	-13	-11	-0.30	10.40
-8	-13	-11	275.27	19.00
-8	-13	-11	259.57	21.20
-8	-13	-11	233.68	21.60
-8	-13	-11	259.17	21.00
-8	-13	-11	310.37	18.50
-8	-13	-11	225.58	17.20
8	13	11	235.68	18.40
-9	-13	-11	68.99	12.40
-9	-13	-11	70.29	11.70
-9	-13	-11	82.09	12.70
-9	-13	-11	87.99	13.40
-10	-13	-11	310.87	20.30
-10	-13	-11	336.37	22.50
-10	-13	-11	353.16	20.80
-10	-13	-11	350.36	20.20
-11	-13	-11	0.10	8.30
-11	-13	-11	6.30	7.80
-11	-13	-11	4.60	7.30
-11	-13	-11	-8.10	9.30
9	-14	-11	2.30	13.20
-9	14	11	40.50	14.90
-9	14	11	9.60	9.30
8	-14	-11	62.29	18.00
8	-14	-11	57.89	10.40
-8	14	11	45.10	10.90
-8	14	11	49.70	12.40
7	-14	-11	-9.90	15.90
7	-14	-11	10.80	9.20
7	-14	-11	1.20	25.80
-7	14	11	5.80	10.50
6	-14	-11	261.57	19.30
6	-14	-11	303.77	31.60
-6	14	11	217.48	21.10
5	-14	-11	-1.30	7.90
5	-14	-11	-5.20	10.90
5	-14	-11	16.40	14.70
5	-14	-11	28.00	18.00
-5	14	11	-3.50	9.80
-5	14	11	14.20	10.40

4	-14	-11	278.67	24.10
4	-14	-11	258.77	22.30
4	-14	-11	280.27	19.90
4	-14	-11	262.87	20.80
-4	14	11	264.07	17.10
3	-14	-11	377.36	24.30
3	-14	-11	372.06	23.90
3	-14	-11	353.36	23.80
3	-14	-11	367.56	24.60
-3	14	11	343.27	19.50
2	-14	-11	230.48	20.50
2	-14	-11	242.18	20.00
2	-14	-11	270.87	20.60
2	-14	-11	252.37	19.80
2	-14	-11	265.37	19.20
-2	14	11	256.67	14.20
1	-14	-11	232.38	18.30
1	-14	-11	224.78	17.60
1	-14	-11	244.48	18.40
1	-14	-11	212.78	32.00
-1	14	11	223.28	12.60
0	-14	-11	351.66	28.20
0	-14	-11	351.46	27.30
0	-14	-11	372.56	23.30
0	14	11	390.26	18.40
-1	-14	-11	30.70	8.20
-1	-14	-11	43.60	8.60
-1	-14	-11	61.79	13.50
-1	-14	-11	33.00	8.70
-1	-14	-11	51.49	9.80
-1	-14	-11	45.50	16.50
1	14	11	38.00	4.80
-2	-14	-11	55.49	9.40
-2	-14	-11	51.49	12.60
-2	-14	-11	77.89	12.30
-2	-14	-11	64.79	11.10
-2	-14	-11	62.89	12.50
-2	-14	-11	41.90	9.10
2	14	11	44.00	4.90
-3	-14	-11	400.66	29.90
-3	-14	-11	388.66	23.00
-3	-14	-11	399.26	25.50
-3	-14	-11	403.56	31.10
-3	-14	-11	372.36	23.20

3	14	11	388.36	18.50
-4	-14	-11	51.29	10.30
-4	-14	-11	38.50	8.50
-4	-14	-11	50.39	8.80
-4	-14	-11	45.90	9.30
-4	-14	-11	45.00	18.60
-4	-14	-11	45.50	7.80
4	14	11	40.90	5.40
-5	-14	-11	17.40	9.10
-5	-14	-11	44.00	12.10
-5	-14	-11	34.00	7.60
-5	-14	-11	39.10	8.50
-5	-14	-11	24.00	9.10
-5	-14	-11	20.40	8.60
5	14	11	21.70	5.60
-6	-14	-11	82.39	12.30
-6	-14	-11	94.09	12.00
-6	-14	-11	96.99	13.90
-6	-14	-11	93.99	13.60
-6	-14	-11	79.29	10.30
-7	-14	-11	66.49	11.00
-7	-14	-11	82.99	12.90
-7	-14	-11	114.59	14.50
-7	-14	-11	92.09	12.00
-7	-14	-11	104.09	15.00
7	14	11	69.39	9.70
-8	-14	-11	771.72	38.50
-8	-14	-11	783.22	38.80
-8	-14	-11	692.33	39.70
-8	-14	-11	791.12	38.70
-9	-14	-11	65.39	16.40
-9	-14	-11	94.49	12.70
-9	-14	-11	80.39	12.20
-9	-14	-11	89.19	13.40
-10	-14	-11	59.39	9.40
-10	-14	-11	53.19	9.10
-10	-14	-11	39.70	11.20
-10	-14	-11	69.59	11.80
-11	-14	-11	36.70	10.60
-11	-14	-11	54.79	10.20
-11	-14	-11	40.20	11.30
8	-15	-11	18.30	9.00
-8	15	11	21.70	9.80
7	-15	-11	99.59	14.20

7	-15	-11	98.79	23.40
-7	15	11	102.29	17.50
6	-15	-11	9.00	14.20
6	-15	-11	18.10	10.40
6	-15	-11	14.60	24.70
-6	15	11	7.70	11.50
5	-15	-11	200.28	18.40
5	-15	-11	238.98	27.50
5	-15	-11	242.08	22.20
-5	15	11	173.18	21.90
-5	15	11	222.38	20.30
4	-15	-11	31.80	10.50
4	-15	-11	56.39	12.90
4	-15	-11	37.40	10.20
4	-15	-11	46.40	12.10
3	-15	-11	31.20	9.30
3	-15	-11	44.70	11.30
3	-15	-11	59.69	11.20
3	-15	-11	44.00	10.00
-3	15	11	42.90	6.80
2	-15	-11	521.65	30.40
2	-15	-11	523.35	30.90
2	-15	-11	540.05	30.70
2	-15	-11	545.35	31.10
2	-15	-11	544.35	30.30
-2	15	11	527.95	26.50
1	-15	-11	23.20	8.60
1	-15	-11	36.10	8.60
1	-15	-11	14.90	11.10
1	-15	-11	26.40	8.50
1	-15	-11	32.70	9.70
-1	15	11	44.30	6.30
0	-15	-11	6.40	8.00
0	-15	-11	5.10	8.30
0	-15	-11	10.90	8.80
0	-15	-11	13.30	8.10
0	15	11	14.40	3.70
-1	-15	-11	648.84	33.30
-1	-15	-11	647.24	42.00
-1	-15	-11	631.94	33.10
-1	-15	-11	653.33	33.30
-1	-15	-11	624.24	34.90
-1	-15	-11	629.04	35.80
1	15	11	618.44	29.90

-2	-15	-11	64.09	9.80
-2	-15	-11	47.10	12.60
-2	-15	-11	43.60	8.20
-2	-15	-11	44.40	19.90
-2	-15	-11	55.99	9.40
-2	-15	-11	66.99	9.70
2	15	11	53.79	5.50
-3	-15	-11	828.22	40.70
-3	-15	-11	751.82	41.90
-3	-15	-11	847.22	41.30
-3	-15	-11	831.42	41.30
-3	-15	-11	808.52	46.60
-3	-15	-11	840.32	41.30
3	15	11	852.31	38.00
-4	-15	-11	181.48	17.00
-4	-15	-11	162.68	14.60
-4	-15	-11	169.28	15.60
-4	-15	-11	162.98	20.70
-4	-15	-11	182.38	16.60
-4	-15	-11	184.98	17.80
4	15	11	173.78	10.50
-5	-15	-11	28.70	8.90
-5	-15	-11	27.20	9.50
-5	-15	-11	31.90	10.60
-5	-15	-11	34.90	10.00
-5	-15	-11	24.20	8.70
-5	-15	-11	22.60	7.40
5	15	11	19.50	6.10
-6	-15	-11	61.19	10.30
-6	-15	-11	70.69	10.80
-6	-15	-11	72.89	10.60
-6	-15	-11	78.09	12.80
-6	-15	-11	50.89	9.80
-7	-15	-11	13.80	9.00
-7	-15	-11	34.10	8.90
-7	-15	-11	29.00	9.40
-7	-15	-11	44.20	9.20
-7	-15	-11	39.90	9.70
-8	-15	-11	193.58	18.10
-8	-15	-11	187.98	16.40
-8	-15	-11	211.28	18.30
-8	-15	-11	196.78	19.30
-8	-15	-11	222.08	19.80
-9	-15	-11	349.77	22.80

-9	-15	-11	375.76	21.80
-9	-15	-11	355.16	21.90
-9	-15	-11	323.47	21.70
-9	-15	-11	411.86	25.00
-10	-15	-11	12.50	7.70
-10	-15	-11	9.80	8.10
-10	-15	-11	4.80	7.80
-10	-15	-11	-18.50	9.10
8	-16	-11	140.39	14.30
-8	16	11	90.19	14.60
-8	16	11	129.09	18.60
7	-16	-11	256.87	33.50
7	-16	-11	263.97	18.30
-7	16	11	252.27	20.20
6	-16	-11	23.10	13.50
6	-16	-11	13.60	8.80
-6	16	11	-2.90	10.60
5	-16	-11	163.28	17.20
5	-16	-11	179.98	21.80
-5	16	11	188.48	24.70
-5	16	11	185.78	20.00
4	-16	-11	346.87	26.40
4	-16	-11	326.77	23.30
4	-16	-11	358.26	25.60
4	-16	-11	349.07	22.90
-4	16	11	272.67	21.10
3	-16	-11	196.28	20.80
3	-16	-11	274.27	21.50
3	-16	-11	237.18	20.00
3	-16	-11	246.98	20.00
-3	16	11	221.88	15.70
2	-16	-11	13.10	9.30
2	-16	-11	7.30	10.60
2	-16	-11	-1.10	9.20
2	-16	-11	6.90	8.40
-2	16	11	2.60	5.00
1	-16	-11	18.70	11.90
1	-16	-11	19.80	9.30
1	-16	-11	16.90	8.50
1	-16	-11	19.20	8.80
1	-16	-11	13.10	8.40
-1	16	11	23.10	4.70
0	-16	-11	64.99	15.60
0	-16	-11	63.59	9.70

0	-16	-11	97.09	15.10
0	-16	-11	71.99	10.10
0	-16	-11	56.19	9.40
0	16	11	63.69	7.00
-1	-16	-11	283.87	18.90
-1	-16	-11	283.37	19.70
-1	-16	-11	271.27	21.80
-1	-16	-11	253.27	22.60
-1	-16	-11	276.97	18.90
1	16	11	277.17	14.60
-2	-16	-11	7.20	7.20
-2	-16	-11	5.60	11.40
-2	-16	-11	5.60	7.50
-2	-16	-11	-3.40	7.40
-2	-16	-11	1.70	10.20
2	16	11	11.90	3.60
-3	-16	-11	128.39	16.90
-3	-16	-11	133.09	13.30
-3	-16	-11	122.19	16.50
-3	-16	-11	114.69	14.40
-3	-16	-11	117.09	15.00
-3	-16	-11	158.38	15.20
3	16	11	135.19	9.00
-4	-16	-11	574.84	42.00
-4	-16	-11	615.84	33.10
-4	-16	-11	646.84	33.30
-4	-16	-11	582.54	33.60
-4	-16	-11	663.53	33.80
-4	-16	-11	637.04	32.20
4	16	11	605.44	29.70
-5	-16	-11	-1.00	7.80
-5	-16	-11	4.70	7.20
-5	-16	-11	3.00	9.10
-5	-16	-11	16.80	11.00
-5	-16	-11	12.90	10.30
5	16	11	-6.80	6.50
-6	-16	-11	97.69	14.50
-6	-16	-11	126.59	15.70
-6	-16	-11	116.09	13.90
-6	-16	-11	114.39	17.00
-6	-16	-11	113.89	14.70
6	16	11	86.19	14.40
-7	-16	-11	384.36	24.30
-7	-16	-11	378.86	24.40

-7	-16	-11	383.76	24.50
-7	-16	-11	393.96	24.10
-7	-16	-11	362.96	23.40
-8	-16	-11	19.90	8.80
-8	-16	-11	33.30	9.30
-8	-16	-11	36.30	8.80
-8	-16	-11	20.80	8.80
-8	-16	-11	6.90	8.60
-9	-16	-11	177.38	18.40
-9	-16	-11	123.79	14.20
-9	-16	-11	148.09	14.50
-9	-16	-11	111.79	14.60
-9	-16	-11	135.39	14.00
-10	-16	-11	12.90	7.60
-10	-16	-11	18.00	7.70
-10	-16	-11	6.40	9.60
-10	-16	-11	8.60	7.50
7	-17	-11	-11.30	8.10
-7	17	11	18.70	9.60
6	-17	-11	-1.20	8.20
6	-17	-11	-1.60	13.40
-6	17	11	17.00	10.10
5	-17	-11	22.80	18.00
5	-17	-11	24.30	9.50
5	-17	-11	-7.40	10.90
-5	17	11	17.60	10.60
4	-17	-11	140.89	16.90
4	-17	-11	156.58	18.80
4	-17	-11	138.29	20.20
4	-17	-11	164.48	22.70
-4	17	11	163.98	19.00
3	-17	-11	12.30	10.40
3	-17	-11	21.20	10.10
3	-17	-11	8.50	9.40
3	-17	-11	16.70	10.70
-3	17	11	18.60	7.60
2	-17	-11	4.30	10.00
2	-17	-11	10.50	11.20
2	-17	-11	10.90	9.40
2	-17	-11	12.50	9.30
-2	17	11	-8.20	9.20
1	-17	-11	61.29	10.10
1	-17	-11	67.19	13.90
1	-17	-11	61.09	10.50

1	-17	-11	80.79	11.90
1	-17	-11	68.29	11.00
-1	17	11	63.39	8.30
0	-17	-11	214.18	28.90
0	-17	-11	250.37	19.40
0	-17	-11	289.27	20.10
0	-17	-11	258.57	21.30
0	-17	-11	300.77	19.30
0	17	11	262.77	15.00
0	17	11	270.17	22.00
-1	-17	-11	2.10	7.40
-1	-17	-11	8.50	8.20
-1	-17	-11	9.30	7.80
-1	-17	-11	2.20	10.10
1	17	11	6.70	4.30
-2	-17	-11	1134.19	54.19
-2	-17	-11	1084.99	54.59
-2	-17	-11	1148.49	56.49
-2	-17	-11	1088.39	53.69
-2	-17	-11	1146.79	54.49
2	17	11	1118.39	51.59
-3	-17	-11	222.38	19.20
-3	-17	-11	252.07	18.20
-3	-17	-11	248.28	18.10
-3	-17	-11	222.58	26.20
-3	-17	-11	224.78	16.70
-3	-17	-11	208.48	18.30
3	17	11	228.18	13.40
-4	-17	-11	102.19	13.30
-4	-17	-11	91.19	13.60
-4	-17	-11	117.69	15.50
-4	-17	-11	96.59	15.50
4	17	11	104.09	10.30
-5	-17	-11	52.29	9.80
-5	-17	-11	35.10	8.00
-5	-17	-11	70.89	11.70
-5	-17	-11	39.90	9.80
-5	-17	-11	45.40	9.20
-5	-17	-11	37.10	12.20
5	17	11	51.69	8.20
-6	-17	-11	15.40	8.20
-6	-17	-11	22.60	14.80
-6	-17	-11	21.70	11.30
-6	-17	-11	14.40	9.10

-6	-17	-11	13.20	8.60
-6	-17	-11	19.40	8.00
6	17	11	23.10	9.80
-7	-17	-11	0.10	10.10
-7	-17	-11	9.10	8.90
-7	-17	-11	22.00	9.90
-7	-17	-11	-4.70	8.60
-7	-17	-11	1.20	8.40
7	17	11	-0.30	10.50
-8	-17	-11	52.99	10.20
-8	-17	-11	46.90	9.40
-8	-17	-11	37.30	9.10
-8	-17	-11	47.30	8.70
-8	-17	-11	73.99	12.50
-9	-17	-11	225.58	16.10
-9	-17	-11	212.78	15.80
-9	-17	-11	211.18	16.30
-9	-17	-11	236.88	19.40
-9	-17	-11	217.68	16.20
-10	-17	-11	304.97	19.50
-10	-17	-11	247.48	18.40
7	-18	-11	32.30	8.50
-7	18	11	22.80	9.30
-7	18	11	19.90	13.00
6	-18	-11	199.88	21.70
6	-18	-11	194.98	16.20
-6	18	11	198.58	17.80
5	-18	-11	3.00	10.80
5	-18	-11	-6.40	17.80
5	-18	-11	10.90	8.90
-5	18	11	11.60	9.10
4	-18	-11	11.70	11.90
4	-18	-11	21.90	14.70
4	-18	-11	14.30	11.00
4	-18	-11	-2.00	13.60
-4	18	11	-9.90	13.20
3	-18	-11	913.41	44.20
3	-18	-11	799.42	43.20
3	-18	-11	824.82	44.00
3	-18	-11	835.02	43.40
-3	18	11	821.32	41.90
2	-18	-11	383.36	25.20
2	-18	-11	362.26	26.70
2	-18	-11	368.06	24.70

2	-18	-11	415.66	25.30
-2	18	11	395.76	22.10
1	-18	-11	40.60	9.50
1	-18	-11	39.20	9.20
1	-18	-11	50.49	10.50
1	-18	-11	52.99	9.40
1	-18	-11	44.60	14.30
-1	18	11	35.80	6.70
0	-18	-11	117.99	15.10
0	-18	-11	133.79	17.40
0	-18	-11	113.99	19.90
0	-18	-11	108.19	15.20
0	-18	-11	131.59	15.40
0	18	11	148.49	17.70
-1	-18	-11	27.30	8.40
-1	-18	-11	40.80	11.50
-1	-18	-11	35.30	9.00
-1	-18	-11	41.10	8.60
1	18	11	28.10	5.80
-2	-18	-11	39.70	12.70
-2	-18	-11	40.00	8.80
-2	-18	-11	41.80	9.40
-2	-18	-11	30.50	8.40
-2	-18	-11	40.40	10.30
2	18	11	27.30	7.20
-3	-18	-11	125.39	14.20
-3	-18	-11	116.79	12.90
-3	-18	-11	111.79	15.60
-3	-18	-11	137.09	25.00
-3	-18	-11	132.59	15.70
3	18	11	86.89	9.90
-4	-18	-11	-0.30	8.10
-4	-18	-11	-8.00	6.90
-4	-18	-11	8.80	9.10
-4	-18	-11	-13.50	7.20
4	18	11	-3.30	6.50
-5	-18	-11	18.60	7.60
-5	-18	-11	25.90	9.00
-5	-18	-11	20.20	9.60
-5	-18	-11	23.50	10.80
-5	-18	-11	16.10	7.90
5	18	11	12.80	8.30
-6	-18	-11	8.90	11.90
-6	-18	-11	6.40	8.30

-6	-18	-11	5.00	13.80
6	18	11	-5.30	10.20
-7	-18	-11	182.48	15.70
-7	-18	-11	188.58	16.80
-7	-18	-11	188.28	17.60
-7	-18	-11	228.58	16.00
-7	-18	-11	163.38	15.70
-8	-18	-11	305.77	20.50
-8	-18	-11	347.67	20.20
-8	-18	-11	387.26	20.80
-8	-18	-11	267.57	20.00
-8	-18	-11	341.27	20.30
-9	-18	-11	28.00	10.20
-9	-18	-11	20.20	7.20
-9	-18	-11	29.60	9.40
6	-19	-11	7.00	8.60
6	-19	-11	11.60	13.00
-6	19	11	9.10	9.20
5	-19	-11	209.98	16.70
5	-19	-11	186.08	22.50
5	-19	-11	177.98	19.60
-5	19	11	174.88	17.30
4	-19	-11	61.29	11.30
4	-19	-11	59.49	11.00
4	-19	-11	85.09	12.90
4	-19	-11	83.09	14.90
3	-19	-11	11.60	13.60
3	-19	-11	14.00	12.80
3	-19	-11	16.00	12.50
3	-19	-11	3.80	12.40
-3	19	11	28.00	11.40
2	-19	-11	209.18	19.10
2	-19	-11	205.58	18.90
2	-19	-11	211.58	18.50
2	-19	-11	240.48	22.80
-2	19	11	165.68	16.50
1	-19	-11	-3.60	7.90
1	-19	-11	1.50	8.80
1	-19	-11	2.30	8.10
1	-19	-11	-27.40	15.00
1	-19	-11	2.50	9.50
-1	19	11	0.20	6.90
0	-19	-11	12.90	8.70
0	-19	-11	7.30	8.10

0	-19	-11	19.60	10.50
0	-19	-11	4.80	8.10
0	19	11	11.80	6.30
0	19	11	-3.60	13.30
-1	-19	-11	177.38	16.20
-1	-19	-11	190.68	16.30
-1	-19	-11	187.58	16.70
-1	-19	-11	210.28	21.20
1	19	11	163.68	12.80
-2	-19	-11	70.69	12.20
-2	-19	-11	79.99	13.00
-2	-19	-11	82.59	11.40
-2	-19	-11	113.49	20.40
-2	-19	-11	85.29	14.00
2	19	11	74.69	9.90
-3	-19	-11	569.34	36.10
-3	-19	-11	558.24	29.20
-3	-19	-11	530.55	28.40
-3	-19	-11	517.25	29.70
-3	-19	-11	473.05	29.50
3	19	11	548.85	27.30
-4	-19	-11	3.20	7.20
-4	-19	-11	2.10	7.00
-4	-19	-11	1.20	9.10
-4	-19	-11	17.80	9.00
4	19	11	4.60	7.70
-5	-19	-11	0.20	12.80
-5	-19	-11	-2.30	12.50
-5	-19	-11	-0.70	8.20
-5	-19	-11	-12.30	12.20
5	19	11	10.40	9.10
-6	-19	-11	-1.10	8.10
-6	-19	-11	5.60	7.30
-6	-19	-11	2.60	7.00
-6	-19	-11	23.90	13.90
-6	-19	-11	4.70	8.20
-7	-19	-11	15.30	7.80
-7	-19	-11	7.20	8.20
-7	-19	-11	3.50	6.80
-7	-19	-11	14.00	7.80
-8	-19	-11	245.28	16.00
-8	-19	-11	224.18	17.10
-8	-19	-11	258.67	16.70
-8	-19	-11	240.88	17.40

-9	-19	-11	192.28	16.30
-9	-19	-11	181.88	18.60
-9	-19	-11	230.58	16.80
-9	-19	-11	259.07	16.10
6	-20	-11	-0.60	9.10
6	-20	-11	30.90	13.00
-6	20	11	20.20	9.30
5	-20	-11	4.60	9.20
5	-20	-11	44.40	11.20
-5	20	11	26.20	8.60
4	-20	-11	389.86	24.60
4	-20	-11	373.66	24.30
4	-20	-11	400.66	25.20
4	-20	-11	387.06	28.10
3	-20	-11	113.29	16.00
3	-20	-11	86.29	13.40
3	-20	-11	97.79	15.90
3	-20	-11	118.49	18.20
2	-20	-11	152.38	19.60
2	-20	-11	166.18	17.80
2	-20	-11	119.99	22.50
2	-20	-11	159.08	18.90
-2	20	11	182.68	18.40
1	-20	-11	21.80	18.70
1	-20	-11	29.70	11.10
1	-20	-11	34.30	10.30
1	-20	-11	22.60	9.90
-1	20	11	31.50	8.70
0	-20	-11	3.00	10.50
0	-20	-11	14.10	8.10
0	-20	-11	5.70	8.70
0	-20	-11	14.00	8.10
0	20	11	12.30	13.50
0	20	11	-3.40	7.10
-1	-20	-11	73.89	13.30
-1	-20	-11	64.19	10.00
-1	-20	-11	72.09	12.50
-1	-20	-11	88.79	12.80
1	20	11	72.49	13.60
1	20	11	69.59	11.10
-2	-20	-11	19.30	8.60
-2	-20	-11	15.50	7.40
-2	-20	-11	16.30	13.30
-2	-20	-11	-1.60	7.40

2	20	11	20.40	7.10
-3	-20	-11	13.60	24.20
-3	-20	-11	14.00	7.40
-3	-20	-11	22.20	7.80
-3	-20	-11	38.90	13.30
-3	-20	-11	15.20	9.00
3	20	11	17.20	7.90
-4	-20	-11	448.46	29.00
-4	-20	-11	507.45	31.00
-4	-20	-11	546.85	31.10
-4	-20	-11	458.15	27.60
4	20	11	473.25	27.10
-5	-20	-11	4.00	6.60
-5	-20	-11	1.40	6.80
-5	-20	-11	6.60	10.10
-5	-20	-11	0.40	8.40
-6	-20	-11	118.69	12.40
-6	-20	-11	130.19	13.30
-6	-20	-11	128.09	12.70
-6	-20	-11	118.79	16.00
-7	-20	-11	169.18	14.00
-7	-20	-11	182.18	13.60
-7	-20	-11	181.48	14.20
-8	-20	-11	130.99	11.80
-8	-20	-11	146.89	17.40
-8	-20	-11	120.99	12.00
5	-21	-11	1.70	10.40
5	-21	-11	8.60	11.00
-5	21	11	11.30	7.60
4	-21	-11	114.19	16.20
4	-21	-11	120.09	15.00
4	-21	-11	134.79	16.80
-4	21	11	124.89	14.00
3	-21	-11	15.70	8.60
3	-21	-11	15.40	10.90
3	-21	-11	3.60	9.40
3	-21	-11	17.30	12.10
2	-21	-11	72.19	15.90
2	-21	-11	62.19	10.30
2	-21	-11	63.39	10.70
2	-21	-11	51.29	10.70
1	-21	-11	223.98	19.70
1	-21	-11	233.28	18.60
1	-21	-11	249.58	18.70

0	-21	-11	14.40	8.10
0	-21	-11	-2.30	9.00
0	-21	-11	1.80	10.60
0	-21	-11	7.60	8.50
0	21	11	8.90	21.90
0	21	11	9.60	8.80
-1	-21	-11	151.28	20.60
-1	-21	-11	171.28	17.20
-1	-21	-11	199.18	15.90
-1	-21	-11	175.88	15.90
1	21	11	174.58	15.20
1	21	11	169.88	20.20
-2	-21	-11	970.40	49.00
-2	-21	-11	1014.70	48.60
-2	-21	-11	1021.40	51.19
-2	-21	-11	996.60	48.50
2	21	11	948.41	47.70
-3	-21	-11	16.50	8.60
-3	-21	-11	2.40	6.70
-3	-21	-11	-5.90	6.80
-3	-21	-11	-5.00	16.60
3	21	11	1.80	9.30
-4	-21	-11	112.09	12.60
-4	-21	-11	100.89	15.40
-4	-21	-11	114.69	12.40
-4	-21	-11	92.19	13.00
-5	-21	-11	64.59	11.30
-5	-21	-11	61.99	10.50
-5	-21	-11	71.19	11.00
-5	-21	-11	84.49	12.00
-6	-21	-11	-3.20	6.00
-6	-21	-11	-0.50	6.30
-6	-21	-11	2.80	7.60
-7	-21	-11	6.40	6.00
-7	-21	-11	5.80	6.90
-7	-21	-11	0.00	7.20
4	-22	-11	78.89	16.30
4	-22	-11	46.10	9.50
4	-22	-11	62.59	12.50
-4	22	11	56.09	8.70
3	-22	-11	687.83	51.09
3	-22	-11	782.72	40.70
3	-22	-11	783.52	39.20
3	-22	-11	829.32	39.60

2	-22	-11	121.69	14.40
2	-22	-11	105.19	14.00
2	-22	-11	122.89	16.60
1	-22	-11	9.80	8.40
1	-22	-11	13.00	9.00
1	-22	-11	20.20	9.50
0	-22	-11	53.59	8.90
0	-22	-11	47.10	9.10
0	-22	-11	37.30	7.90
0	-22	-11	20.50	10.00
0	22	11	34.40	18.50
-1	-22	-11	10.00	8.00
-1	-22	-11	3.00	7.00
-1	-22	-11	12.60	7.10
-1	-22	-11	1.50	10.60
1	22	11	-7.00	13.50
-2	-22	-11	35.90	8.60
-2	-22	-11	24.10	12.60
-2	-22	-11	29.10	7.30
2	22	11	13.30	12.30
-3	-22	-11	60.49	10.40
-3	-22	-11	63.59	8.70
-3	-22	-11	56.59	9.40
-4	-22	-11	105.49	11.60
-4	-22	-11	104.29	11.10
-4	-22	-11	115.49	14.50
-5	-22	-11	45.00	7.50
-5	-22	-11	48.90	10.70
-5	-22	-11	55.69	7.50
-6	-22	-11	8.00	6.10
-6	-22	-11	14.10	7.30
-6	-22	-11	4.60	6.30
-6	-22	-11	0.10	13.80
-7	-22	-11	208.68	17.70
-7	-22	-11	247.78	16.40
-7	-22	-11	250.27	15.90
3	-23	-11	41.40	9.00
3	-23	-11	39.10	11.90
2	-23	-11	129.69	14.30
2	-23	-11	116.59	15.40
2	-23	-11	124.39	14.20
1	-23	-11	3.20	8.10
1	-23	-11	-5.40	8.10
1	-23	-11	-7.70	8.50

0	-23	-11	10.50	7.70
0	-23	-11	15.70	10.00
0	-23	-11	9.30	7.90
-1	-23	-11	39.30	7.20
-1	-23	-11	34.70	8.10
-1	-23	-11	49.10	7.90
-1	-23	-11	47.70	11.70
-2	-23	-11	314.07	18.10
-2	-23	-11	276.57	24.90
-2	-23	-11	318.27	17.90
-2	-23	-11	282.97	19.00
2	23	11	233.58	22.30
-3	-23	-11	371.46	22.60
-3	-23	-11	389.76	21.30
-3	-23	-11	376.56	21.10
3	23	11	381.06	25.10
-4	-23	-11	0.40	6.10
-4	-23	-11	5.30	6.00
-4	-23	-11	9.20	8.70
4	23	11	-8.10	12.40
-5	-23	-11	2.70	12.60
-5	-23	-11	0.70	5.40
-5	-23	-11	4.70	6.20
-6	-23	-11	120.69	11.10
2	-24	-11	135.89	13.90
2	-24	-11	139.79	14.10
2	-24	-11	121.99	15.20
1	-24	-11	17.00	8.60
1	-24	-11	13.10	7.90
1	-24	-11	8.00	8.50
0	-24	-11	31.70	9.90
0	-24	-11	24.40	7.50
0	-24	-11	20.50	8.10
-1	-24	-11	120.59	13.00
-1	-24	-11	143.79	12.00
-1	-24	-11	142.19	17.60
1	24	11	150.58	22.60
-2	-24	-11	30.60	7.00
-2	-24	-11	37.20	16.00
-2	-24	-11	20.40	6.50
-2	-24	-11	22.80	7.50
2	24	11	26.40	24.20
-3	-24	-11	155.28	14.10
-3	-24	-11	162.98	12.40

-3	-24	-11	169.18	12.30
3	24	11	122.69	19.90
-4	-24	-11	174.08	12.00
-4	-24	-11	166.88	16.30
-4	-24	-11	159.68	12.10
4	24	11	147.59	24.70
-1	-25	-11	304.37	23.20
-1	-25	-11	276.77	20.20
3	18	-12	270.17	17.30
-3	-18	12	282.97	18.00
2	18	-12	265.37	17.20
-2	-18	12	270.17	16.60
-2	-18	12	232.18	20.30
1	18	-12	153.28	12.80
-1	-18	12	127.89	13.60
-1	-18	12	141.69	13.80
0	18	-12	10.70	7.90
0	18	-12	9.90	20.20
0	-18	12	21.10	7.60
0	-18	12	15.60	11.40
-1	18	-12	93.59	14.00
-1	18	-12	107.29	18.00
1	-18	12	96.79	11.80
-2	18	-12	56.59	10.80
-2	18	-12	-11.50	23.40
-2	18	-12	32.50	11.00
2	-18	12	33.60	7.20
-3	18	-12	64.09	10.70
-3	18	-12	95.49	15.70
4	17	-12	123.39	12.50
-4	-17	12	119.79	10.80
3	17	-12	9.00	7.20
-3	-17	12	9.80	6.30
2	17	-12	75.59	9.50
-2	-17	12	99.59	15.80
-2	-17	12	82.89	10.90
1	17	-12	48.80	8.80
-1	-17	12	39.70	8.40
-1	-17	12	39.40	8.80
0	17	-12	83.79	13.30
0	17	-12	97.79	12.80
0	-17	12	78.19	12.40
0	-17	12	65.09	10.30
-1	17	-12	20.80	10.80

-1	17	-12	20.40	14.40
-1	17	-12	17.90	8.70
1	-17	12	37.40	8.10
-2	17	-12	30.80	10.10
-2	17	-12	5.10	13.50
-2	17	-12	31.30	9.30
2	-17	12	31.10	7.80
-3	17	-12	201.78	18.90
-3	17	-12	188.08	19.30
-3	17	-12	212.18	16.70
3	-17	12	200.88	15.30
-4	17	-12	11.30	11.70
-4	17	-12	18.60	11.60
-4	17	-12	-23.00	11.80
4	-17	12	10.60	7.30
5	16	-12	6.10	7.20
-5	-16	12	1.30	5.30
4	16	-12	78.59	11.80
-4	-16	12	79.89	9.90
3	16	-12	56.79	8.50
-3	-16	12	58.19	9.80
2	16	-12	565.44	29.60
-2	-16	12	537.85	30.10
-2	-16	12	550.74	28.90
1	16	-12	166.78	15.00
-1	-16	12	140.69	15.50
-1	-16	12	171.08	14.50
0	16	-12	64.99	19.80
0	16	-12	94.69	13.30
0	16	-12	96.79	13.50
0	-16	12	86.19	13.40
0	-16	12	93.89	14.80
-1	16	-12	55.29	13.10
-1	16	-12	56.19	11.40
-1	16	-12	58.29	9.60
1	-16	12	79.79	13.00
1	-16	12	61.29	8.90
-2	16	-12	22.70	9.20
-2	16	-12	55.69	15.90
-2	16	-12	24.80	9.80
2	-16	12	28.10	8.00
-3	16	-12	87.89	15.50
-3	16	-12	81.09	14.50
-3	16	-12	94.19	13.70

3	-16	12	91.09	13.30
-4	16	-12	162.88	17.50
-4	16	-12	141.49	14.60
-4	16	-12	96.89	13.70
4	-16	12	127.29	13.50
-5	16	-12	62.09	14.20
-5	16	-12	6.40	8.60
-5	16	-12	25.10	11.40
5	-16	12	20.70	10.80
5	-16	12	23.80	9.00
6	15	-12	42.80	8.40
-6	-15	12	22.80	6.20
5	15	-12	11.00	7.40
-5	-15	12	20.40	6.20
4	15	-12	39.10	8.50
-4	-15	12	24.50	6.60
3	15	-12	86.79	12.60
-3	-15	12	80.99	11.10
2	15	-12	-11.70	7.20
-2	-15	12	10.40	8.80
-2	-15	12	6.40	7.10
1	15	-12	240.48	23.50
1	15	-12	248.88	18.50
-1	-15	12	229.38	18.90
-1	-15	12	241.88	17.10
0	15	-12	39.50	9.80
0	15	-12	40.90	11.70
0	15	-12	52.29	12.60
0	-15	12	44.10	9.30
0	-15	12	45.50	9.10
-1	15	-12	37.10	9.30
-1	15	-12	60.49	11.50
-1	15	-12	35.80	11.60
1	-15	12	22.90	11.10
1	-15	12	20.30	8.00
-2	15	-12	181.88	18.70
-2	15	-12	200.78	18.40
-2	15	-12	201.68	18.80
2	-15	12	219.58	16.80
-3	15	-12	-15.30	10.00
-3	15	-12	11.60	9.30
-3	15	-12	-10.60	8.60
3	-15	12	12.60	7.60
3	-15	12	19.20	15.70

-4	15	-12	74.79	12.10
-4	15	-12	54.59	10.20
-4	15	-12	67.39	12.10
4	-15	12	55.29	11.50
4	-15	12	68.29	12.90
-5	15	-12	58.99	10.20
-5	15	-12	55.59	11.60
-5	15	-12	64.89	12.80
5	-15	12	69.59	10.10
5	-15	12	88.99	14.00
-6	15	-12	46.60	11.30
-6	15	-12	41.10	9.10
-6	15	-12	46.70	14.60
6	-15	12	50.99	14.60
6	-15	12	45.90	9.30
7	14	-12	10.60	12.20
7	14	-12	0.40	8.70
-7	-14	12	18.70	6.80
-7	-14	12	-3.30	8.90
6	14	-12	16.10	8.20
6	14	-12	12.20	12.40
-6	-14	12	25.30	6.40
-6	-14	12	19.10	13.60
5	14	-12	53.59	13.40
5	14	-12	41.00	8.80
-5	-14	12	37.70	6.90
4	14	-12	87.29	13.10
4	14	-12	105.89	19.80
-4	-14	12	58.19	10.80
3	14	-12	556.04	30.90
3	14	-12	537.85	35.60
-3	-14	12	570.44	30.10
-3	-14	12	583.94	32.30
2	14	-12	62.59	13.30
-2	-14	12	62.79	11.50
1	14	-12	374.86	22.80
1	14	-12	273.37	36.30
-1	-14	12	334.97	22.00
-1	-14	12	344.07	23.60
0	14	-12	73.59	17.40
0	14	-12	27.40	11.20
0	14	-12	52.49	9.80
0	-14	12	31.00	8.70
0	-14	12	43.00	9.60

-1	14	-12	97.59	16.50
-1	14	-12	118.49	15.80
-1	14	-12	145.49	25.50
1	-14	12	131.89	15.10
-2	14	-12	27.00	12.50
-2	14	-12	5.10	9.50
2	-14	12	1.30	9.60
-3	14	-12	-13.20	9.90
-3	14	-12	-6.10	9.70
-3	14	-12	-2.00	10.10
3	-14	12	-8.60	8.60
-4	14	-12	25.20	9.50
-4	14	-12	16.20	10.70
-4	14	-12	29.20	10.50
4	-14	12	23.40	8.90
4	-14	12	30.10	9.90
-5	14	-12	169.18	17.70
-5	14	-12	141.09	18.50
-5	14	-12	181.48	17.30
5	-14	12	199.58	17.50
5	-14	12	194.58	17.10
-6	14	-12	-1.40	12.50
-6	14	-12	7.40	10.40
-6	14	-12	2.20	8.30
6	-14	12	18.20	10.10
6	-14	12	15.90	8.00
-7	14	-12	12.40	10.80
-7	14	-12	8.90	8.50
7	13	-12	161.68	17.50
7	13	-12	160.18	15.10
-7	-13	12	92.69	16.80
-7	-13	12	130.59	12.20
6	13	-12	0.80	8.40
6	13	-12	28.50	11.30
-6	-13	12	-5.50	8.20
-6	-13	12	-6.60	5.80
5	13	-12	66.59	9.80
5	13	-12	49.99	12.20
-5	-13	12	65.39	10.80
-5	-13	12	72.19	11.00
4	13	-12	385.36	26.80
4	13	-12	384.16	24.20
-4	-13	12	379.96	23.00
3	13	-12	2.50	8.40

-3	-13	12	-6.50	6.30
2	13	-12	74.49	14.60
2	13	-12	74.99	13.30
-2	-13	12	95.99	15.20
-2	-13	12	113.19	13.20
1	13	-12	91.89	18.70
1	13	-12	110.19	14.80
1	13	-12	95.49	16.70
1	13	-12	75.59	15.90
-1	-13	12	89.99	13.30
-1	-13	12	89.19	14.10
0	13	-12	124.79	16.70
0	13	-12	145.89	19.40
0	13	-12	157.78	15.60
0	-13	12	150.98	15.00
0	-13	12	105.49	14.80
-1	13	-12	15.60	8.80
-1	13	-12	7.50	9.80
-1	13	-12	2.20	10.00
1	-13	12	6.70	9.20
1	-13	12	15.80	8.10
-2	13	-12	53.79	10.90
-2	13	-12	40.80	10.20
-2	13	-12	67.09	11.00
2	-13	12	72.39	13.20
2	-13	12	74.99	13.60
-3	13	-12	212.58	19.10
-3	13	-12	167.98	17.70
-3	13	-12	196.98	18.40
3	-13	12	196.58	20.60
3	-13	12	188.28	17.00
-4	13	-12	60.49	14.70
-4	13	-12	48.90	10.70
-4	13	-12	57.69	12.40
4	-13	12	39.80	13.20
4	-13	12	48.40	10.70
-5	13	-12	8.20	11.90
-5	13	-12	16.80	9.90
-5	13	-12	6.80	9.60
5	-13	12	9.30	9.10
5	-13	12	3.00	8.60
-6	13	-12	488.25	27.70
-6	13	-12	458.05	27.70
-6	13	-12	470.35	30.70

6	-13	12	508.25	28.80
6	-13	12	474.15	27.20
-7	13	-12	152.68	16.30
-7	13	-12	156.98	15.50
-7	13	-12	134.79	20.20
7	-13	12	155.68	14.00
7	-13	12	159.18	26.40
-8	13	-12	9.00	10.70
-8	13	-12	-10.40	14.80
8	12	-12	16.60	12.70
8	12	-12	11.80	11.90
-8	-12	12	12.80	6.70
-8	-12	12	7.50	7.90
7	12	-12	32.10	9.80
7	12	-12	46.60	11.30
-7	-12	12	34.70	7.80
-7	-12	12	47.80	7.70
6	12	-12	229.08	19.70
6	12	-12	210.58	17.30
-6	-12	12	227.38	15.50
-6	-12	12	196.38	16.50
5	12	-12	64.49	10.20
5	12	-12	62.09	12.20
-5	-12	12	86.99	11.80
-5	-12	12	69.49	10.90
4	12	-12	117.89	18.70
4	12	-12	104.79	15.10
-4	-12	12	74.19	12.50
3	12	-12	-0.50	10.80
3	12	-12	7.50	8.10
-3	-12	12	-2.70	8.90
-3	-12	12	-8.50	6.40
2	12	-12	539.65	34.00
2	12	-12	591.74	32.20
-2	-12	12	608.44	32.20
-2	-12	12	628.04	31.90
1	12	-12	31.70	12.40
1	12	-12	17.50	8.20
-1	-12	12	25.40	8.40
-1	-12	12	14.80	7.30
0	12	-12	90.79	14.80
0	12	-12	105.69	18.20
0	12	-12	100.19	14.40
0	-12	12	105.19	14.20

0	-12	12	73.39	10.20
-1	12	-12	515.65	29.50
-1	12	-12	496.35	34.30
-1	12	-12	519.75	30.10
1	-12	12	490.35	29.00
1	-12	12	510.45	29.00
-2	12	-12	71.69	10.90
-2	12	-12	91.09	15.90
-2	12	-12	74.39	11.40
2	-12	12	71.59	13.40
-3	12	-12	39.40	10.20
-3	12	-12	40.60	10.60
-3	12	-12	52.99	10.80
3	-12	12	23.60	8.30
-4	12	-12	55.99	11.20
-4	12	-12	76.99	12.80
-4	12	-12	68.19	11.00
4	-12	12	52.79	9.90
-5	12	-12	2.00	9.40
-5	12	-12	-9.60	14.10
-5	12	-12	12.60	13.70
5	-12	12	15.80	11.10
5	-12	12	3.30	9.80
-6	12	-12	53.49	13.90
-6	12	-12	53.49	11.00
6	-12	12	53.89	11.40
6	-12	12	55.89	10.10
-7	12	-12	90.19	15.20
-7	12	-12	87.29	14.60
-7	12	-12	99.79	15.40
7	-12	12	85.09	13.40
-8	12	-12	22.60	10.70
-8	12	-12	13.50	8.10
8	-12	12	0.00	7.10
8	11	-12	39.20	13.40
-8	-11	12	37.30	9.00
-8	-11	12	47.80	7.60
7	11	-12	216.08	18.40
7	11	-12	172.58	17.20
-7	-11	12	181.58	14.90
-7	-11	12	194.88	14.60
6	11	-12	9.80	9.00
6	11	-12	19.40	11.00
-6	-11	12	20.80	7.50

-6	-11	12	26.90	8.10
5	11	-12	38.80	10.10
5	11	-12	35.60	14.10
-5	-11	12	22.00	7.60
4	11	-12	19.70	10.20
4	11	-12	6.80	8.20
-4	-11	12	1.30	6.70
-4	-11	12	11.40	10.10
3	11	-12	22.80	8.70
3	11	-12	27.00	10.90
-3	-11	12	15.00	7.10
-3	-11	12	31.10	9.80
2	11	-12	47.40	11.30
2	11	-12	34.20	9.10
-2	-11	12	29.50	8.00
-2	-11	12	26.90	8.80
1	11	-12	585.54	34.20
1	11	-12	586.84	32.50
-1	-11	12	601.14	32.00
-1	-11	12	632.34	32.60
0	11	-12	126.39	15.80
0	11	-12	99.09	14.10
0	11	-12	155.98	15.90
0	11	-12	124.09	21.00
0	-11	12	126.69	14.70
0	-11	12	129.49	14.00
-1	11	-12	208.98	17.90
-1	11	-12	210.98	17.90
-1	11	-12	245.18	19.90
-1	11	-12	168.88	23.40
1	-11	12	212.38	17.50
1	-11	12	200.78	17.80
-2	11	-12	390.76	24.40
-2	11	-12	399.26	23.80
-2	11	-12	301.17	23.80
2	-11	12	364.06	23.00
-3	11	-12	15.80	9.50
-3	11	-12	19.80	10.50
-3	11	-12	18.50	14.30
3	-11	12	8.10	7.70
-4	11	-12	23.00	10.40
-4	11	-12	15.10	9.50
-4	11	-12	28.80	10.70
4	-11	12	18.60	8.80

-5	11	-12	123.89	21.80
-5	11	-12	111.19	18.30
-5	11	-12	112.99	16.30
5	-11	12	145.19	17.40
-6	11	-12	11.20	10.80
-6	11	-12	44.20	14.00
-6	11	-12	3.50	10.00
6	-11	12	15.30	10.90
6	-11	12	6.60	8.90
-7	11	-12	400.16	25.50
-7	11	-12	441.46	30.30
-7	11	-12	412.56	25.40
7	-11	12	409.76	31.60
7	-11	12	398.96	24.20
-8	11	-12	9.00	14.20
-8	11	-12	18.80	8.90
-8	11	-12	12.10	10.30
8	-11	12	-2.50	7.20
-9	11	-12	79.19	17.00
-9	11	-12	53.79	12.10
9	-11	12	96.19	12.70
8	10	-12	25.60	9.80
-8	-10	12	19.30	6.40
-8	-10	12	19.70	8.50
7	10	-12	25.90	10.20
7	10	-12	36.30	9.90
-7	-10	12	20.90	7.30
-7	-10	12	32.90	7.70
6	10	-12	47.60	11.10
6	10	-12	44.70	12.70
-6	-10	12	60.89	10.10
-6	-10	12	52.89	9.40
5	10	-12	78.19	12.50
5	10	-12	66.79	11.30
-5	-10	12	75.39	9.90
-5	-10	12	66.39	11.60
4	10	-12	102.79	15.40
4	10	-12	102.59	13.00
-4	-10	12	69.89	11.90
-4	-10	12	111.49	15.80
3	10	-12	462.55	26.60
3	10	-12	416.76	27.60
-3	-10	12	463.75	25.30
-3	-10	12	439.86	27.50

2	10	-12	63.69	11.00
2	10	-12	81.99	13.80
-2	-10	12	48.90	8.10
-2	-10	12	52.19	9.40
1	10	-12	99.49	15.20
1	10	-12	135.19	17.90
-1	-10	12	117.49	14.90
-1	-10	12	121.99	13.30
0	10	-12	769.92	41.70
0	10	-12	848.52	41.20
0	10	-12	755.42	40.50
0	10	-12	770.02	42.50
0	-10	12	803.92	40.60
0	-10	12	829.42	40.30
-1	10	-12	255.57	19.30
-1	10	-12	242.78	18.30
-1	10	-12	219.28	22.90
-1	10	-12	239.78	21.00
1	-10	12	250.17	19.30
1	-10	12	258.37	18.30
-2	10	-12	7.40	9.80
-2	10	-12	49.10	13.80
-2	10	-12	22.20	9.40
-2	10	-12	20.50	8.10
2	-10	12	10.90	7.70
-3	10	-12	5.60	8.20
-3	10	-12	-0.30	9.00
-3	10	-12	2.40	9.90
-3	10	-12	17.00	16.60
3	-10	12	13.30	7.20
-4	10	-12	42.80	16.40
-4	10	-12	58.49	10.70
-4	10	-12	63.69	12.40
4	-10	12	66.09	10.20
-5	10	-12	302.87	22.60
-5	10	-12	323.57	23.90
5	-10	12	318.77	22.60
-6	10	-12	10.20	13.40
-6	10	-12	1.30	10.30
-6	10	-12	8.60	9.90
6	-10	12	2.80	12.70
-7	10	-12	55.49	15.50
-7	10	-12	56.39	11.60
-7	10	-12	63.39	11.70

7	-10	12	77.19	15.50
7	-10	12	56.09	9.70
-8	10	-12	223.08	24.30
-8	10	-12	207.58	17.90
-8	10	-12	182.08	17.80
8	-10	12	203.78	16.30
-9	10	-12	-4.40	10.60
-9	10	-12	6.00	8.80
9	-10	12	-6.60	7.30
9	9	-12	-2.10	9.40
-9	-9	12	-3.10	6.30
8	9	-12	324.87	21.90
-8	-9	12	335.57	19.50
-8	-9	12	321.27	21.00
7	9	-12	48.90	11.00
7	9	-12	49.20	11.10
-7	-9	12	45.90	8.20
-7	-9	12	53.29	9.20
6	9	-12	5.70	10.40
6	9	-12	-6.00	10.10
-6	-9	12	4.50	7.20
-6	-9	12	8.40	7.10
5	9	-12	195.88	18.70
5	9	-12	186.78	19.00
-5	-9	12	217.78	15.70
-5	-9	12	185.28	16.30
4	9	-12	457.35	29.10
4	9	-12	475.75	28.30
-4	-9	12	469.05	27.20
-4	-9	12	485.05	26.50
-4	-9	12	509.65	33.20
3	9	-12	7.10	8.00
3	9	-12	23.70	10.10
-3	-9	12	16.30	9.60
-3	-9	12	6.00	6.90
2	9	-12	160.88	18.00
2	9	-12	152.28	16.00
-2	-9	12	180.98	14.30
1	9	-12	75.09	13.60
1	9	-12	76.79	11.40
-1	-9	12	74.09	11.90
0	9	-12	495.35	27.70
0	9	-12	474.05	29.50
0	9	-12	521.15	28.20

0	-9	12	471.35	27.20
-1	9	-12	23.40	9.10
-1	9	-12	23.70	8.10
-1	9	-12	12.10	11.10
1	-9	12	17.40	8.70
-2	9	-12	309.97	25.10
-2	9	-12	339.57	23.20
-2	9	-12	326.47	23.20
-2	9	-12	324.87	21.00
2	-9	12	330.27	21.00
-3	9	-12	208.38	20.50
-3	9	-12	178.88	18.60
-3	9	-12	237.28	25.50
-3	9	-12	221.48	17.30
3	-9	12	216.08	17.80
-4	9	-12	126.79	18.90
-4	9	-12	107.39	16.50
-4	9	-12	122.99	14.90
-4	9	-12	120.39	19.00
4	-9	12	99.29	15.70
-5	9	-12	79.49	15.60
-5	9	-12	121.19	18.20
-5	9	-12	117.19	21.40
-5	9	-12	110.19	17.30
5	-9	12	125.79	16.80
-6	9	-12	730.43	39.90
-6	9	-12	732.73	40.60
-6	9	-12	730.63	44.00
-6	9	-12	788.82	44.40
6	-9	12	775.02	40.60
-7	9	-12	21.20	10.20
-7	9	-12	22.70	17.50
-7	9	-12	26.70	16.20
7	-9	12	14.80	15.90
7	-9	12	44.30	15.80
-8	9	-12	28.80	9.70
-8	9	-12	27.10	15.70
-8	9	-12	14.30	10.00
8	-9	12	16.30	8.30
-9	9	-12	9.30	15.30
-9	9	-12	1.30	10.60
-9	9	-12	9.10	9.30
9	-9	12	3.30	8.00
9	8	-12	45.70	10.30

-9	-8	12	46.90	7.40
8	8	-12	47.60	10.30
-8	-8	12	72.89	11.30
-8	-8	12	41.70	9.60
7	8	-12	76.89	13.50
7	8	-12	118.69	18.30
-7	-8	12	77.99	13.20
-7	-8	12	100.29	14.10
6	8	-12	57.69	11.70
6	8	-12	62.69	11.60
-6	-8	12	94.29	12.60
5	8	-12	16.10	10.00
5	8	-12	9.10	9.40
-5	-8	12	2.10	6.90
-5	-8	12	1.40	7.00
4	8	-12	85.09	12.20
4	8	-12	65.89	11.20
-4	-8	12	64.69	9.30
-4	-8	12	65.79	11.40
3	8	-12	26.00	9.60
3	8	-12	19.70	9.20
-3	-8	12	34.40	9.90
-3	-8	12	28.00	6.40
2	8	-12	465.95	27.40
2	8	-12	480.15	28.50
-2	-8	12	500.85	26.40
1	8	-12	217.78	17.70
-1	-8	12	220.78	15.90
0	8	-12	0.30	8.20
0	8	-12	-6.90	15.00
0	8	-12	9.90	9.40
0	8	-12	4.10	7.40
0	-8	12	-6.00	6.50
-1	8	-12	767.12	40.90
-1	8	-12	803.12	42.90
-1	8	-12	786.32	39.80
-1	8	-12	790.42	41.00
-1	8	-12	758.02	38.40
1	-8	12	713.23	38.40
1	-8	12	758.32	39.10
-2	8	-12	186.58	19.10
-2	8	-12	219.28	24.80
-2	8	-12	206.18	15.70
-2	8	-12	169.98	17.80

2	-8	12	178.98	16.30
-3	8	-12	210.28	18.70
-3	8	-12	162.68	23.90
-3	8	-12	214.68	19.70
-3	8	-12	230.08	16.40
-3	8	-12	192.08	31.30
3	-8	12	209.28	17.20
3	-8	12	226.38	18.70
-4	8	-12	258.57	22.90
-4	8	-12	290.37	30.30
-4	8	-12	229.88	25.00
-4	8	-12	272.27	20.30
-4	8	-12	256.67	19.00
4	-8	12	268.07	19.30
4	-8	12	232.58	23.70
-5	8	-12	3.80	12.70
-5	8	-12	36.60	15.80
-5	8	-12	24.70	10.70
-5	8	-12	16.10	9.20
-5	8	-12	17.80	12.50
5	-8	12	13.00	14.70
5	-8	12	9.80	8.80
-6	8	-12	9.00	10.10
-6	8	-12	9.70	9.20
-6	8	-12	1.50	14.10
-6	8	-12	-2.50	11.70
-6	8	-12	24.70	15.30
6	-8	12	19.30	10.50
-7	8	-12	41.70	11.50
-7	8	-12	48.00	10.70
-7	8	-12	57.79	16.10
-8	8	-12	48.90	10.90
-8	8	-12	61.99	11.20
-8	8	-12	66.39	17.30
8	-8	12	77.19	10.90
-9	8	-12	-1.70	11.00
-9	8	-12	-22.40	14.40
-9	8	-12	7.80	9.30
9	-8	12	7.00	8.80
-10	8	-12	15.10	9.50
-10	8	-12	20.90	12.40
10	-8	12	17.00	9.10
9	7	-12	100.09	15.90
-9	-7	12	70.49	10.50

8	7	-12	16.50	10.20
-8	-7	12	6.80	9.00
-8	-7	12	16.90	6.50
7	7	-12	146.59	23.90
7	7	-12	181.28	19.70
-7	-7	12	188.68	16.70
-7	-7	12	151.88	22.40
6	7	-12	22.50	10.50
-6	-7	12	30.90	7.50
-6	-7	12	37.60	7.60
5	7	-12	27.10	10.70
5	7	-12	32.50	10.40
-5	-7	12	36.90	8.20
-5	-7	12	36.70	7.70
4	7	-12	117.59	18.20
4	7	-12	121.69	13.40
-4	-7	12	123.69	14.20
-4	-7	12	138.09	13.00
3	7	-12	-14.20	8.90
3	7	-12	2.10	8.10
-3	-7	12	-7.80	9.00
-3	-7	12	7.90	6.10
-2	-7	12	10.70	7.20
1	7	-12	1141.49	55.79
1	7	-12	1145.09	55.09
-1	-7	12	1151.08	54.69
-1	-7	12	1120.49	54.19
0	7	-12	374.06	30.10
0	7	-12	359.26	22.40
0	7	-12	342.37	23.90
0	-7	12	345.47	21.00
0	-7	12	368.36	22.20
-1	7	-12	95.69	13.20
-1	7	-12	108.09	14.50
-1	7	-12	93.29	15.70
-1	7	-12	90.49	13.60
-1	7	-12	116.49	16.60
1	-7	12	98.29	11.70
1	-7	12	94.19	14.20
-2	7	-12	145.89	17.90
-2	7	-12	139.39	13.60
-2	7	-12	151.38	19.90
-2	7	-12	121.99	18.20
-2	7	-12	143.89	18.10

-2	7	-12	136.59	18.80
2	-7	12	152.18	13.60
-3	7	-12	26.30	13.80
-3	7	-12	48.20	12.80
-3	7	-12	29.50	9.50
-3	7	-12	35.90	10.00
-3	7	-12	41.60	8.30
-3	7	-12	22.80	13.50
3	-7	12	50.79	9.10
-4	7	-12	18.80	10.70
-4	7	-12	8.70	8.80
-4	7	-12	26.30	11.60
-4	7	-12	26.60	13.80
-4	7	-12	0.90	7.00
4	-7	12	5.30	9.00
4	-7	12	4.50	7.90
-5	7	-12	166.28	28.30
-5	7	-12	210.68	16.90
-5	7	-12	226.38	22.30
-5	7	-12	227.98	19.60
-5	7	-12	222.58	23.30
5	-7	12	218.98	22.00
5	-7	12	183.88	18.80
-6	7	-12	179.28	19.40
-6	7	-12	172.48	27.90
-6	7	-12	203.18	24.60
-6	7	-12	187.68	21.90
6	-7	12	181.78	21.50
6	-7	12	200.18	27.40
-7	7	-12	672.53	38.20
-7	7	-12	644.74	35.40
-7	7	-12	666.43	36.20
-7	7	-12	621.54	40.20
-7	7	-12	627.84	41.80
-8	7	-12	-1.70	10.60
-8	7	-12	-0.40	32.70
-8	7	-12	6.90	17.30
-8	7	-12	-10.20	9.10
8	-7	12	15.00	10.60
-9	7	-12	23.80	11.00
-9	7	-12	38.10	15.80
9	-7	12	20.10	9.30
-10	7	-12	42.90	12.50
-10	7	-12	58.59	18.20

-10	7	-12	58.49	10.90
10	-7	12	40.50	10.60
10	6	-12	14.10	12.60
-10	-6	12	37.90	8.30
9	6	-12	124.39	16.60
-9	-6	12	95.29	11.10
8	6	-12	7.20	10.40
-8	-6	12	8.60	8.90
-8	-6	12	8.40	6.80
7	6	-12	21.90	10.80
-7	-6	12	31.90	8.80
-7	-6	12	21.70	7.20
6	6	-12	172.58	20.80
6	6	-12	168.38	22.50
-6	-6	12	167.88	14.90
-6	-6	12	159.58	14.20
5	6	-12	295.67	23.80
5	6	-12	263.37	23.30
-5	-6	12	293.77	18.80
-5	-6	12	299.57	18.90
4	6	-12	133.79	18.10
4	6	-12	135.89	18.70
-4	-6	12	119.99	14.30
-4	-6	12	133.69	12.70
3	6	-12	146.09	17.20
3	6	-12	124.09	16.40
-3	-6	12	156.18	12.60
-3	-6	12	123.99	15.90
2	6	-12	84.19	11.30
2	6	-12	92.79	11.20
-2	-6	12	62.49	10.20
-2	-6	12	65.89	10.30
1	6	-12	61.09	9.80
1	6	-12	48.80	8.50
-1	-6	12	55.39	7.70
-1	-6	12	71.89	9.00
0	6	-12	1459.45	69.19
0	6	-12	1464.75	70.69
0	6	-12	1445.56	68.59
0	6	-12	1414.36	71.39
0	-6	12	1457.35	67.69
-1	6	-12	75.49	13.90
-1	6	-12	52.59	8.60
-1	6	-12	63.89	11.70

-1	6	-12	43.70	8.50
-1	6	-12	34.40	9.90
1	-6	12	29.20	8.10
1	-6	12	54.09	7.20
-2	6	-12	233.58	21.20
-2	6	-12	217.08	15.50
-2	6	-12	195.18	20.30
-2	6	-12	222.98	20.20
-2	6	-12	246.18	24.00
-2	6	-12	181.58	17.90
2	-6	12	231.18	15.40
-3	6	-12	3.10	8.40
-3	6	-12	-14.90	9.20
-3	6	-12	10.90	12.80
-3	6	-12	1.40	8.50
-3	6	-12	7.60	6.80
3	-6	12	1.60	6.50
-4	6	-12	122.29	13.20
-4	6	-12	142.49	18.00
-4	6	-12	137.29	20.00
-4	6	-12	95.89	17.00
4	-6	12	132.39	14.40
-5	6	-12	730.73	41.80
-5	6	-12	838.02	45.80
-5	6	-12	765.92	39.00
-5	6	-12	786.32	43.20
-5	6	-12	769.52	40.00
5	-6	12	724.43	40.00
5	-6	12	739.43	39.50
-6	6	-12	9.20	8.20
-6	6	-12	28.40	12.80
-6	6	-12	13.80	11.20
-6	6	-12	15.60	14.90
-6	6	-12	8.90	9.10
6	-6	12	18.20	12.00
6	-6	12	18.30	11.70
-7	6	-12	101.39	15.00
-7	6	-12	89.79	13.80
-7	6	-12	134.79	17.10
-7	6	-12	124.29	17.30
-7	6	-12	125.79	17.90
7	-6	12	101.59	19.20
-8	6	-12	219.68	19.40
-8	6	-12	215.78	18.20

-8	6	-12	183.78	28.80
-9	6	-12	14.00	17.80
-9	6	-12	3.90	9.50
-9	6	-12	15.80	15.70
-9	6	-12	10.30	9.30
9	-6	12	3.00	9.60
-10	6	-12	158.68	24.30
-10	6	-12	95.69	16.40
-10	6	-12	113.09	15.80
10	-6	12	105.99	17.70
10	5	-12	18.70	10.60
-10	-5	12	1.80	6.00
9	5	-12	53.39	11.90
-9	-5	12	62.69	10.20
8	5	-12	361.06	25.90
8	5	-12	296.07	30.90
-8	-5	12	299.37	22.60
-8	-5	12	357.36	20.70
7	5	-12	30.70	11.30
-7	-5	12	14.20	7.60
-7	-5	12	15.60	6.70
6	5	-12	9.80	13.80
6	5	-12	2.50	14.10
6	5	-12	11.40	10.80
-6	-5	12	16.30	6.50
-6	-5	12	14.20	6.90
5	5	-12	306.87	28.40
5	5	-12	340.87	26.80
5	5	-12	409.56	26.60
-5	-5	12	396.66	22.00
-5	-5	12	407.76	22.10
4	5	-12	112.59	17.30
4	5	-12	102.49	17.50
4	5	-12	119.69	22.10
-4	-5	12	115.09	11.70
3	5	-12	152.48	17.40
3	5	-12	130.89	17.30
3	5	-12	129.69	15.90
-3	-5	12	131.39	12.00
-3	-5	12	132.69	15.10
2	5	-12	117.59	15.30
2	5	-12	139.19	15.80
2	5	-12	132.09	16.60
-2	-5	12	116.69	11.60

-2	-5	12	127.19	13.50
1	5	-12	5.10	7.80
1	5	-12	-0.50	13.20
1	5	-12	31.40	12.40
1	5	-12	-0.90	8.60
-1	-5	12	7.20	6.00
-1	-5	12	7.20	5.90
0	5	-12	395.96	25.00
0	5	-12	392.56	23.30
0	5	-12	336.97	27.10
0	5	-12	355.86	28.40
0	-5	12	362.76	21.70
0	-5	12	422.96	20.80
-1	5	-12	-0.70	9.60
-1	5	-12	7.60	9.80
-1	5	-12	15.70	8.20
-1	5	-12	-9.10	6.90
-1	5	-12	-2.70	11.00
1	-5	12	6.10	5.40
1	-5	12	-1.50	7.30
-2	5	-12	160.18	20.10
-2	5	-12	162.18	19.00
-2	5	-12	131.99	20.10
-2	5	-12	133.69	18.90
-2	5	-12	131.79	15.20
-2	5	-12	147.09	14.30
2	-5	12	150.78	12.00
-3	5	-12	-2.70	8.10
-3	5	-12	23.10	11.50
-3	5	-12	23.00	8.20
-3	5	-12	33.20	12.80
-3	5	-12	16.80	6.40
-3	5	-12	11.30	9.80
3	-5	12	15.80	6.10
-4	5	-12	79.19	12.50
-4	5	-12	65.99	12.50
-4	5	-12	74.89	14.10
-4	5	-12	47.50	10.00
-4	5	-12	52.49	7.90
-4	5	-12	47.30	11.90
4	-5	12	56.69	8.40
-5	5	-12	512.05	27.50
-5	5	-12	510.75	31.40
-5	5	-12	526.05	29.40

-5	5	-12	470.45	31.00
-5	5	-12	530.15	35.50
5	-5	12	524.35	27.80
5	-5	12	428.46	29.40
-6	5	-12	1614.64	76.89
-6	5	-12	1541.35	75.89
-6	5	-12	1564.64	74.09
-6	5	-12	1603.14	77.69
-6	5	-12	1599.54	73.79
6	-5	12	1427.86	74.79
6	-5	12	1515.25	73.69
-7	5	-12	34.40	14.20
-7	5	-12	19.70	10.40
-7	5	-12	51.69	10.70
-7	5	-12	34.20	10.60
-7	5	-12	49.90	15.50
7	-5	12	48.00	15.70
-8	5	-12	46.90	15.50
-8	5	-12	49.20	13.80
-8	5	-12	50.99	10.40
-8	5	-12	32.30	14.60
-8	5	-12	40.90	10.60
8	-5	12	22.10	19.70
-9	5	-12	5.50	9.10
-9	5	-12	-13.90	16.20
-9	5	-12	-20.30	15.40
-9	5	-12	9.10	9.20
9	-5	12	1.00	10.90
-10	5	-12	-7.30	30.40
-10	5	-12	20.30	15.20
-10	5	-12	16.80	9.40
-10	5	-12	26.00	13.20
10	-5	12	20.50	12.40
-11	5	-12	25.00	9.60
-11	5	-12	1.30	12.80
11	-5	12	30.80	15.90
10	4	-12	168.88	18.50
-10	-4	12	166.88	12.90
9	4	-12	21.40	11.50
-9	-4	12	12.40	6.40
8	4	-12	61.19	15.80
8	4	-12	97.49	18.30
-8	-4	12	44.90	10.70
7	4	-12	217.08	24.30

7	4	-12	244.68	22.80
-7	-4	12	218.68	17.60
-7	-4	12	247.58	16.20
6	4	-12	219.18	20.60
6	4	-12	176.18	22.90
-6	-4	12	208.28	14.80
-6	-4	12	148.99	15.30
5	4	-12	-2.10	10.30
5	4	-12	1.90	11.80
5	4	-12	-2.30	12.90
-5	-4	12	0.70	5.80
-5	-4	12	-7.80	6.30
4	4	-12	10.80	12.30
4	4	-12	12.00	10.40
4	4	-12	5.50	11.90
-4	-4	12	22.40	6.40
3	4	-12	109.89	20.20
3	4	-12	104.49	17.00
3	4	-12	101.79	18.10
3	4	-12	150.68	21.70
-3	-4	12	95.09	16.10
-3	-4	12	94.79	13.20
2	4	-12	154.18	22.00
2	4	-12	179.68	16.80
2	4	-12	149.59	18.00
2	4	-12	163.08	20.40
-2	-4	12	174.18	13.80
1	4	-12	479.75	29.60
1	4	-12	472.85	27.90
1	4	-12	485.65	31.70
1	4	-12	449.95	30.80
1	4	-12	517.35	28.70
-1	-4	12	536.95	25.50
0	4	-12	113.69	15.90
0	4	-12	84.49	12.70
0	4	-12	95.39	14.70
0	4	-12	76.29	10.90
0	4	-12	114.09	21.30
0	-4	12	109.59	9.50
-1	4	-12	1632.24	75.79
-1	4	-12	1397.26	76.39
-1	4	-12	1562.44	75.69
-1	4	-12	1657.73	76.29
-1	4	-12	1621.14	74.69

-1	4	-12	1511.65	76.39
1	-4	12	1785.62	73.89
1	-4	12	1568.24	74.29
-2	4	-12	82.19	9.00
-2	4	-12	76.19	9.80
-2	4	-12	52.99	11.00
-2	4	-12	65.39	12.50
-2	4	-12	86.69	14.20
-2	4	-12	73.99	11.20
2	-4	12	41.80	14.50
2	-4	12	69.39	8.50
-3	4	-12	77.69	13.20
-3	4	-12	76.19	10.90
-3	4	-12	94.99	12.00
-3	4	-12	77.59	12.80
-3	4	-12	70.39	10.50
-3	4	-12	102.69	15.70
3	-4	12	68.69	8.80
-4	4	-12	118.39	16.80
-4	4	-12	85.79	10.90
-4	4	-12	126.39	17.20
-4	4	-12	118.29	19.50
-4	4	-12	92.39	15.50
-4	4	-12	83.79	15.60
4	-4	12	105.09	10.40
-5	4	-12	46.60	10.50
-5	4	-12	42.60	11.20
-5	4	-12	36.70	7.30
5	-4	12	22.20	6.70
-6	4	-12	22.00	7.90
-6	4	-12	26.10	9.50
-6	4	-12	14.70	12.50
-6	4	-12	19.80	11.50
-6	4	-12	10.40	10.10
6	-4	12	9.50	8.00
6	-4	12	9.70	13.20
-7	4	-12	121.09	19.70
-7	4	-12	150.68	21.60
-7	4	-12	139.99	16.40
-7	4	-12	116.99	14.60
-7	4	-12	141.39	24.40
7	-4	12	128.89	18.40
-8	4	-12	100.69	14.90
-8	4	-12	131.79	17.70

-8	4	-12	109.39	16.60
-8	4	-12	127.09	16.30
-8	4	-12	131.09	20.60
8	-4	12	113.79	17.50
-9	4	-12	7.20	8.50
-9	4	-12	13.70	15.70
-9	4	-12	0.90	15.30
-9	4	-12	5.90	9.50
9	-4	12	-11.10	35.60
9	-4	12	27.20	12.90
-10	4	-12	36.80	9.90
-10	4	-12	74.29	16.80
-10	4	-12	22.90	16.40
-10	4	-12	40.20	10.70
10	-4	12	52.09	14.50
-11	4	-12	360.46	30.00
-11	4	-12	368.16	22.20
-11	4	-12	342.07	23.30
11	-4	12	352.06	29.30
10	3	-12	66.39	12.80
10	3	-12	70.69	20.20
-10	-3	12	66.59	10.10
9	3	-12	48.10	12.20
9	3	-12	16.70	14.50
-9	-3	12	37.30	6.70
8	3	-12	50.49	16.00
8	3	-12	79.09	25.10
-8	-3	12	60.69	12.20
-8	-3	12	56.39	9.20
7	3	-12	111.39	19.90
7	3	-12	80.89	14.40
-7	-3	12	84.99	11.10
-7	-3	12	60.09	9.50
6	3	-12	143.09	21.50
6	3	-12	194.08	21.00
-6	-3	12	182.98	15.60
-6	-3	12	214.18	14.90
5	3	-12	28.00	11.20
5	3	-12	7.80	11.20
-5	-3	12	18.70	6.60
4	3	-12	458.55	33.30
4	3	-12	439.06	29.70
4	3	-12	418.16	28.00
4	3	-12	423.76	30.40

-4	-3	12	414.56	23.30
3	3	-12	3.70	11.40
3	3	-12	13.90	11.90
3	3	-12	3.10	11.30
3	3	-12	4.60	9.30
-3	-3	12	8.80	8.30
-3	-3	12	0.90	6.60
2	3	-12	-8.30	10.90
2	3	-12	10.80	8.80
2	3	-12	11.50	10.20
2	3	-12	7.40	11.10
-2	-3	12	0.40	5.80
1	3	-12	1088.19	53.69
1	3	-12	1090.89	54.39
1	3	-12	1045.80	53.29
1	3	-12	996.40	54.39
-1	-3	12	1245.18	51.49
0	3	-12	77.09	11.10
0	3	-12	105.09	15.20
0	3	-12	112.39	15.70
0	3	-12	105.29	16.60
0	3	-12	86.99	15.30
0	-3	12	97.29	8.60
-1	3	-12	16.90	7.50
-1	3	-12	11.50	9.30
-1	3	-12	8.70	6.40
-1	3	-12	17.50	9.70
-1	3	-12	-1.60	8.40
-1	3	-12	-2.30	10.70
1	-3	12	5.20	4.00
1	-3	12	9.50	8.60
-2	3	-12	81.29	12.60
-2	3	-12	70.89	12.90
-2	3	-12	79.69	13.70
-2	3	-12	84.49	12.20
-2	3	-12	78.49	11.60
-2	3	-12	91.59	11.20
-2	3	-12	94.19	12.80
2	-3	12	67.99	12.20
-3	3	-12	-3.70	6.70
-3	3	-12	12.10	11.90
-3	3	-12	6.80	8.90
-3	3	-12	3.30	10.20
-3	3	-12	4.80	5.70

-3	3	-12	2.10	10.70
3	-3	12	8.10	4.10
-4	3	-12	132.49	19.70
-4	3	-12	145.89	15.30
-4	3	-12	147.09	17.60
-4	3	-12	159.78	18.30
-4	3	-12	146.99	12.10
4	-3	12	136.59	10.80
-5	3	-12	121.29	11.90
-5	3	-12	98.99	15.20
-5	3	-12	129.19	17.50
-5	3	-12	139.59	18.30
-5	3	-12	133.19	16.80
-5	3	-12	92.59	13.60
5	-3	12	112.09	10.60
-6	3	-12	524.85	35.70
-6	3	-12	531.95	29.40
-6	3	-12	515.55	32.50
-6	3	-12	505.15	28.10
-6	3	-12	524.05	31.70
6	-3	12	518.95	27.80
-7	3	-12	755.62	43.70
-7	3	-12	796.32	46.20
-7	3	-12	800.12	40.30
-7	3	-12	802.12	41.00
-7	3	-12	806.82	44.30
7	-3	12	782.82	40.50
-8	3	-12	90.29	15.00
-8	3	-12	63.89	13.70
-8	3	-12	84.09	14.30
-8	3	-12	87.59	12.80
-8	3	-12	68.79	15.90
8	-3	12	125.79	21.40
-9	3	-12	56.89	13.30
-9	3	-12	74.89	11.00
9	-3	12	65.09	20.50
-10	3	-12	184.58	26.50
-10	3	-12	183.18	16.50
-10	3	-12	153.98	16.30
-10	3	-12	164.38	24.40
10	-3	12	164.88	25.40
-11	3	-12	-10.70	10.80
-11	3	-12	13.30	9.20
-11	3	-12	21.00	16.40

-11	3	-12	21.20	23.20
11	-3	12	8.70	19.10
10	2	-12	-5.80	14.30
10	2	-12	-2.30	12.80
-10	-2	12	-2.40	6.30
9	2	-12	146.99	21.30
9	2	-12	144.19	22.40
-9	-2	12	145.19	12.80
8	2	-12	13.40	12.60
8	2	-12	-3.60	14.00
-8	-2	12	10.40	6.90
7	2	-12	3.40	12.00
7	2	-12	-3.40	11.60
-7	-2	12	6.00	7.90
-7	-2	12	15.00	6.40
6	2	-12	585.64	35.10
6	2	-12	526.55	34.10
-6	-2	12	605.74	30.20
5	2	-12	378.66	27.10
5	2	-12	356.06	26.70
-5	-2	12	423.56	22.10
4	2	-12	126.79	17.10
4	2	-12	101.39	17.80
4	2	-12	116.79	13.60
-4	-2	12	111.59	11.50
-4	-2	12	76.19	13.50
3	2	-12	65.79	13.90
3	2	-12	30.70	10.60
3	2	-12	55.99	13.30
3	2	-12	55.19	11.60
-3	-2	12	45.00	7.70
2	2	-12	7.40	8.90
2	2	-12	22.10	10.00
2	2	-12	6.10	10.60
-2	-2	12	6.30	5.40
1	2	-12	55.59	10.30
1	2	-12	77.19	13.20
1	2	-12	53.59	10.50
-1	-2	12	58.49	8.10
-1	-2	12	71.69	9.90
0	2	-12	1110.59	55.89
0	2	-12	1076.39	52.29
0	2	-12	1117.69	55.59
0	-2	12	1260.37	52.99

-1	2	-12	8.30	9.60
-1	2	-12	0.70	8.50
-1	2	-12	2.10	8.60
-1	2	-12	2.40	5.20
1	-2	12	6.70	3.70
1	-2	12	3.00	7.10
-2	2	-12	169.18	17.20
-2	2	-12	132.19	16.50
-2	2	-12	136.39	18.40
-2	2	-12	176.28	14.90
-2	2	-12	162.18	20.70
2	-2	12	155.18	10.40
2	-2	12	142.39	15.40
-3	2	-12	32.50	10.40
-3	2	-12	31.00	6.20
-3	2	-12	4.90	8.80
-3	2	-12	11.20	10.60
-3	2	-12	22.40	9.40
-3	2	-12	10.40	11.80
-3	2	-12	16.80	6.60
3	-2	12	14.20	9.80
3	-2	12	19.60	4.20
-4	2	-12	47.70	10.10
-4	2	-12	54.49	9.40
-4	2	-12	60.39	11.10
-4	2	-12	74.89	12.70
-4	2	-12	56.29	9.10
-4	2	-12	36.00	12.70
4	-2	12	47.60	6.80
-5	2	-12	414.76	23.60
-5	2	-12	353.66	24.20
-5	2	-12	371.96	28.70
-5	2	-12	376.56	26.80
-5	2	-12	357.76	25.40
-5	2	-12	395.66	21.40
5	-2	12	383.66	20.10
-6	2	-12	16.70	7.50
-6	2	-12	7.40	10.40
-6	2	-12	22.60	9.70
-6	2	-12	-5.20	9.10
-6	2	-12	12.30	6.10
-6	2	-12	19.60	12.00
6	-2	12	24.20	6.00
-7	2	-12	1.10	9.80

-7	2	-12	13.50	13.00
-7	2	-12	25.30	11.50
-7	2	-12	5.90	7.40
-7	2	-12	6.90	6.60
7	-2	12	18.10	7.60
-8	2	-12	368.36	27.70
-8	2	-12	365.96	22.50
-8	2	-12	320.07	22.50
-8	2	-12	392.46	27.70
-8	2	-12	343.77	29.40
8	-2	12	347.97	25.20
-9	2	-12	18.90	13.50
-9	2	-12	0.80	7.90
-9	2	-12	-7.20	11.80
-9	2	-12	29.20	15.10
-9	2	-12	11.90	8.80
9	-2	12	5.10	16.00
9	-2	12	12.20	13.30
-10	2	-12	146.89	15.60
-10	2	-12	166.88	23.80
-10	2	-12	145.79	16.10
-10	2	-12	166.88	26.70
10	-2	12	149.69	27.20
-11	2	-12	19.30	15.40
-11	2	-12	4.70	9.00
-11	2	-12	6.70	15.00
-11	2	-12	29.90	10.80
10	1	-12	54.19	14.50
10	1	-12	53.69	14.10
-10	-1	12	84.49	10.90
9	1	-12	112.49	20.20
9	1	-12	85.69	14.90
-9	-1	12	97.19	11.90
8	1	-12	312.47	27.40
8	1	-12	303.47	25.80
-8	-1	12	278.77	26.00
-8	-1	12	349.27	20.10
7	1	-12	30.70	13.00
7	1	-12	35.00	12.10
-7	-1	12	29.10	9.10
-7	-1	12	37.60	7.40
6	1	-12	137.79	19.30
6	1	-12	149.88	21.80
-6	-1	12	168.98	13.70

5	1	-12	854.61	46.00
5	1	-12	883.01	47.60
-5	-1	12	936.11	43.50
4	1	-12	8.90	13.90
4	1	-12	29.40	11.00
4	1	-12	0.50	10.70
-4	-1	12	12.70	6.60
3	1	-12	106.79	14.80
3	1	-12	108.39	17.70
3	1	-12	97.19	12.90
3	1	-12	141.99	16.40
-3	-1	12	82.39	12.80
-3	-1	12	104.29	12.50
2	1	-12	30.00	10.80
2	1	-12	47.90	9.80
2	1	-12	30.50	11.50
-2	-1	12	36.80	7.10
-2	-1	12	29.00	6.40
1	1	-12	163.28	17.50
1	1	-12	145.89	18.20
-1	-1	12	147.59	12.20
-1	-1	12	178.38	11.20
0	1	-12	1002.70	48.50
0	-1	12	1010.90	50.49
0	-1	12	1068.59	48.90
0	-1	12	1187.88	49.70
-1	1	-12	54.49	13.80
-1	1	-12	42.40	7.20
1	-1	12	39.00	6.90
1	-1	12	33.90	6.80
1	-1	12	45.30	5.80
-2	1	-12	11.20	8.40
-2	1	-12	13.40	10.60
-2	1	-12	30.50	11.10
-2	1	-12	13.40	6.30
-2	1	-12	16.90	6.10
-2	1	-12	21.70	10.80
2	-1	12	19.10	3.90
2	-1	12	22.30	6.60
-3	1	-12	9.10	11.70
-3	1	-12	6.40	5.50
-3	1	-12	26.00	11.90
-3	1	-12	28.10	7.70
-3	1	-12	-0.20	9.80

-3	1	-12	-0.70	8.90
-3	1	-12	11.80	7.80
3	-1	12	-2.70	7.20
-4	1	-12	50.89	12.30
-4	1	-12	48.00	10.60
-4	1	-12	49.50	10.20
-4	1	-12	41.50	7.60
-4	1	-12	53.89	12.00
4	-1	12	45.10	5.90
-5	1	-12	666.23	36.80
-5	1	-12	633.04	32.10
-5	1	-12	602.94	34.00
-5	1	-12	665.73	38.10
-5	1	-12	636.54	37.10
-5	1	-12	644.64	33.50
5	-1	12	645.44	30.90
-6	1	-12	581.34	37.10
-6	1	-12	585.64	32.80
-6	1	-12	563.94	35.00
-6	1	-12	624.64	30.50
-6	1	-12	606.04	35.90
6	-1	12	545.25	28.90
-7	1	-12	373.96	23.00
-7	1	-12	394.56	29.20
-7	1	-12	361.16	30.40
-7	1	-12	395.56	26.10
-7	1	-12	368.56	22.10
7	-1	12	387.16	21.60
-8	1	-12	6.90	11.40
-8	1	-12	0.00	10.50
-8	1	-12	8.50	7.90
-8	1	-12	9.50	8.80
-8	1	-12	-3.00	13.20
8	-1	12	-14.20	9.40
-9	1	-12	77.99	16.10
-9	1	-12	70.19	14.90
-9	1	-12	90.49	12.90
-9	1	-12	65.49	10.00
-10	1	-12	96.59	13.70
-10	1	-12	120.29	21.60
-10	1	-12	88.29	16.50
-10	1	-12	72.09	11.10
10	-1	12	68.79	18.00
-11	1	-12	42.20	10.10

-11	1	-12	30.80	15.60
-11	1	-12	8.50	14.90
10	0	-12	151.18	20.50
10	0	-12	138.69	20.30
-10	0	12	106.59	11.70
9	0	-12	-19.20	13.40
9	0	-12	9.90	12.30
-9	0	12	-4.90	6.40
8	0	-12	30.20	12.20
8	0	-12	22.90	13.90
-8	0	12	-0.90	11.10
7	0	-12	614.64	40.40
7	0	-12	713.23	38.60
-7	0	12	711.93	35.20
6	0	-12	420.96	28.00
6	0	-12	460.25	31.10
-6	0	12	444.86	24.70
5	0	-12	97.29	14.80
-5	0	12	86.89	12.00
4	0	-12	79.49	14.30
4	0	-12	93.79	14.70
4	0	-12	82.19	13.30
4	0	-12	69.89	11.90
-4	0	12	94.89	10.90
-4	0	12	58.49	11.00
3	0	-12	12.20	11.70
3	0	-12	21.10	10.90
3	0	-12	51.49	11.00
3	0	-12	47.40	12.30
-3	0	12	38.00	7.50
-3	0	12	27.80	7.20
2	0	-12	178.68	19.80
2	0	-12	213.88	19.70
2	0	-12	149.79	19.60
2	0	-12	199.18	16.90
-2	0	12	226.38	13.80
-2	0	12	203.78	14.20
-1	0	12	98.89	10.00
-1	0	12	114.39	9.00
0	0	12	1366.86	59.79
0	0	12	1234.98	60.59
-1	0	-12	2398.06	106.49
1	0	12	2369.86	107.99
1	0	12	2452.55	106.79

1	0	12	2236.88	106.99
-2	0	-12	-0.10	6.60
-2	0	-12	4.20	5.00
2	0	12	2.60	4.90
2	0	12	3.30	2.80
2	0	12	2.90	5.50
-3	0	-12	205.38	13.30
-3	0	-12	183.08	15.20
-3	0	-12	191.18	14.90
-3	0	-12	206.38	22.00
-3	0	-12	160.48	20.30
-3	0	-12	174.28	18.40
3	0	12	183.78	15.00
3	0	12	200.88	10.90
-4	0	-12	192.38	20.30
-4	0	-12	173.28	19.30
-4	0	-12	143.79	25.50
-4	0	-12	195.18	21.10
-4	0	-12	177.28	17.20
-4	0	-12	195.78	12.60
-4	0	-12	179.68	15.00
4	0	12	180.48	10.50
-5	0	-12	102.99	19.00
-5	0	-12	63.59	12.50
-5	0	-12	83.49	12.60
-5	0	-12	76.89	9.80
-5	0	-12	73.39	12.50
-5	0	-12	98.49	9.50
5	0	12	80.19	7.00
-6	0	-12	13.50	13.00
-6	0	-12	3.40	11.60
-6	0	-12	6.70	12.50
-6	0	-12	9.50	6.80
-6	0	-12	5.70	5.50
6	0	12	4.10	3.90
-7	0	-12	87.39	15.80
-7	0	-12	75.29	14.40
-7	0	-12	88.99	12.50
-7	0	-12	102.19	11.20
-7	0	-12	83.89	14.30
7	0	12	96.19	10.00
-8	0	-12	21.70	13.30
-8	0	-12	-11.40	8.80
-8	0	-12	1.80	9.40

-8	0	-12	4.20	9.30
-8	0	-12	-6.10	11.70
8	0	12	-0.50	7.50
-9	0	-12	37.30	8.90
-9	0	-12	21.00	13.30
-9	0	-12	29.00	11.20
-9	0	-12	16.30	7.50
-9	0	-12	18.30	13.70
9	0	12	32.70	11.60
-10	0	-12	160.88	16.10
-10	0	-12	145.09	19.60
-10	0	-12	185.08	21.90
-10	0	-12	134.99	15.10
10	0	12	142.69	21.70
-11	0	-12	571.64	33.50
-11	0	-12	539.65	35.00
-11	0	-12	417.26	32.30
-11	0	-12	525.65	28.30
10	-1	-12	0.10	12.20
10	-1	-12	29.20	16.20
-10	1	12	14.30	6.80
9	-1	-12	-13.80	12.40
9	-1	-12	-0.10	14.70
-9	1	12	-7.30	6.60
8	-1	-12	60.19	13.30
8	-1	-12	77.39	16.20
-8	1	12	81.99	10.00
-8	1	12	65.39	12.80
7	-1	-12	25.90	13.80
7	-1	-12	5.20	10.70
-7	1	12	-9.40	6.00
6	-1	-12	69.89	11.50
6	-1	-12	83.99	14.80
-6	1	12	66.39	11.00
5	-1	-12	260.77	26.70
5	-1	-12	229.88	27.30
5	-1	-12	197.48	25.20
-5	1	12	181.38	20.00
4	-1	-12	421.66	28.60
4	-1	-12	414.46	28.90
4	-1	-12	391.66	25.80
4	-1	-12	373.36	27.70
-4	1	12	349.27	23.00
-4	1	12	414.76	22.00

3	-1	-12	83.79	12.90
3	-1	-12	120.99	20.30
3	-1	-12	76.49	13.90
3	-1	-12	97.19	17.00
3	-1	-12	86.99	12.40
-3	1	12	89.09	10.60
2	-1	-12	157.48	18.50
2	-1	-12	159.38	15.90
2	-1	-12	156.08	19.70
2	-1	-12	203.78	19.10
-2	1	12	179.38	13.60
-2	1	12	167.48	11.60
-2	1	12	141.69	12.50
-1	1	12	1746.93	84.79
-1	1	12	1950.90	84.29
0	1	12	14.90	4.40
0	1	12	17.70	3.80
1	1	12	8.50	4.00
1	1	12	15.60	3.20
1	1	12	3.90	4.00
-2	-1	-12	148.49	10.60
2	1	12	170.68	9.00
2	1	12	144.49	10.90
2	1	12	143.89	11.80
-3	-1	-12	-1.20	5.30
-3	-1	-12	-2.40	9.20
-3	-1	-12	5.00	10.00
-3	-1	-12	-5.70	8.60
-3	-1	-12	13.80	11.80
-3	-1	-12	5.80	6.10
3	1	12	2.60	2.30
3	1	12	-2.60	5.60
-4	-1	-12	13.50	5.10
-4	-1	-12	10.90	8.80
-4	-1	-12	21.90	7.40
-4	-1	-12	13.10	12.00
-4	-1	-12	12.40	15.00
-4	-1	-12	15.80	10.30
-4	-1	-12	10.70	9.10
4	1	12	9.50	3.10
4	1	12	16.60	8.10
-5	-1	-12	38.40	6.40
-5	-1	-12	20.50	7.70
-5	-1	-12	24.00	9.10

-5	-1	-12	27.10	10.70
-5	-1	-12	19.50	11.50
-5	-1	-12	31.80	8.80
5	1	12	33.60	4.90
-6	-1	-12	1029.40	53.79
-6	-1	-12	936.11	52.59
-6	-1	-12	993.50	48.20
-6	-1	-12	965.70	61.29
-6	-1	-12	1024.00	48.80
-6	-1	-12	1088.79	59.29
6	1	12	1031.70	47.10
-7	-1	-12	283.07	18.20
-7	-1	-12	273.87	26.80
-7	-1	-12	260.27	24.30
-7	-1	-12	287.57	17.30
-7	-1	-12	268.67	20.60
7	1	12	242.78	15.50
-8	-1	-12	368.96	30.70
-8	-1	-12	381.06	25.60
-8	-1	-12	397.86	23.50
-8	-1	-12	386.86	22.90
-8	-1	-12	393.76	29.30
8	1	12	371.86	21.90
-9	-1	-12	68.99	13.80
-9	-1	-12	39.30	8.70
-9	-1	-12	51.99	13.90
-9	-1	-12	59.19	11.20
-9	-1	-12	57.99	9.80
-9	-1	-12	53.59	15.80
9	1	12	57.69	10.90
-10	-1	-12	161.08	15.50
-10	-1	-12	147.09	14.60
-10	-1	-12	130.19	20.70
-10	-1	-12	149.99	23.40
-10	-1	-12	189.68	24.60
10	1	12	94.89	19.60
-11	-1	-12	32.40	8.60
-11	-1	-12	27.40	13.70
-11	-1	-12	10.50	10.10
-11	-1	-12	31.30	15.40
-12	-1	-12	90.49	13.10
-12	-1	-12	122.09	16.90
12	1	12	63.19	19.60
10	-2	-12	-19.60	10.90

10	-2	-12	4.30	27.70
-10	2	12	9.00	6.80
9	-2	-12	123.69	19.70
9	-2	-12	114.69	18.10
-9	2	12	124.89	13.60
8	-2	-12	39.10	11.80
8	-2	-12	47.80	16.60
-8	2	12	55.39	9.00
-8	2	12	42.30	13.00
7	-2	-12	129.79	18.80
7	-2	-12	74.79	16.10
-7	2	12	104.99	13.00
6	-2	-12	1334.37	69.29
6	-2	-12	1410.56	67.59
6	-2	-12	1417.96	68.59
-6	2	12	1399.86	65.79
5	-2	-12	182.78	22.30
5	-2	-12	194.08	24.50
5	-2	-12	206.58	23.50
-5	2	12	169.58	17.50
4	-2	-12	6.00	13.20
4	-2	-12	26.90	9.90
4	-2	-12	-1.60	13.10
4	-2	-12	19.90	11.00
4	-2	-12	18.20	9.40
-4	2	12	16.50	6.40
3	-2	-12	14.20	13.20
3	-2	-12	13.50	10.10
3	-2	-12	19.30	10.80
3	-2	-12	4.50	7.60
3	-2	-12	4.30	10.10
-3	2	12	5.30	5.00
2	-2	-12	-4.70	17.60
2	-2	-12	1.40	7.80
2	-2	-12	-5.00	9.10
-2	2	12	9.30	5.30
-2	2	12	-0.50	3.70
-2	2	12	-2.20	6.00
-1	2	12	186.38	12.70
-1	2	12	205.58	11.60
-1	2	12	185.48	13.20
0	2	12	1147.69	50.99
0	2	12	1065.89	51.89
1	2	12	579.84	26.20

1	2	12	520.45	26.30
1	2	12	566.14	27.80
2	2	12	508.35	28.80
2	2	12	591.04	29.30
2	2	12	634.14	27.00
-3	-2	-12	24.00	10.10
-3	-2	-12	5.30	9.20
-3	-2	-12	14.80	11.80
3	2	12	4.00	6.00
3	2	12	7.30	5.20
3	2	12	8.80	2.30
-4	-2	-12	403.16	21.20
-4	-2	-12	412.06	25.90
-4	-2	-12	376.36	27.60
-4	-2	-12	408.16	29.80
-4	-2	-12	406.56	23.50
4	2	12	450.45	19.70
4	2	12	376.16	23.90
-5	-2	-12	972.40	44.60
-5	-2	-12	860.71	49.99
-5	-2	-12	956.70	46.20
-5	-2	-12	932.81	47.80
-5	-2	-12	924.41	49.90
-5	-2	-12	918.11	45.40
5	2	12	979.40	43.20
-6	-2	-12	123.49	18.90
-6	-2	-12	131.69	11.00
-6	-2	-12	120.99	21.60
-6	-2	-12	154.48	14.10
-6	-2	-12	130.09	22.70
-6	-2	-12	133.89	13.10
6	2	12	136.69	8.90
-7	-2	-12	20.10	12.20
-7	-2	-12	8.70	5.50
-7	-2	-12	5.80	8.10
-7	-2	-12	18.70	10.90
-7	-2	-12	8.20	6.70
7	2	12	19.30	4.80
-8	-2	-12	389.56	27.70
-8	-2	-12	365.86	25.90
-8	-2	-12	345.67	23.50
-8	-2	-12	363.76	21.40
-8	-2	-12	359.96	22.00
-8	-2	-12	314.77	29.60

8	2	12	346.17	20.20
-9	-2	-12	11.80	7.40
-9	-2	-12	8.80	9.30
-9	-2	-12	32.70	12.20
-9	-2	-12	11.60	8.00
-9	-2	-12	13.00	13.70
-9	-2	-12	5.80	12.50
9	2	12	4.10	9.20
-10	-2	-12	93.39	16.80
-10	-2	-12	125.39	20.40
-10	-2	-12	92.59	14.20
-10	-2	-12	99.29	12.80
10	2	12	83.49	16.20
-11	-2	-12	25.30	8.90
-11	-2	-12	13.70	11.50
-11	-2	-12	50.29	14.20
-11	-2	-12	32.60	8.50
-11	-2	-12	7.00	18.20
-12	-2	-12	196.28	15.30
-12	-2	-12	178.68	22.40
-12	-2	-12	210.78	27.00
10	-3	-12	43.70	12.10
-10	3	12	48.90	8.80
9	-3	-12	30.20	12.50
9	-3	-12	63.89	18.50
-9	3	12	16.90	7.70
8	-3	-12	43.00	12.00
8	-3	-12	65.19	17.80
-8	3	12	56.29	13.90
-8	3	12	46.50	8.10
7	-3	-12	6.60	10.70
7	-3	-12	5.00	15.30
7	-3	-12	3.30	18.30
-7	3	12	7.10	6.30
6	-3	-12	260.17	28.80
6	-3	-12	299.57	22.50
6	-3	-12	267.77	25.20
-6	3	12	271.37	18.50
5	-3	-12	580.84	30.90
5	-3	-12	535.95	30.50
5	-3	-12	529.45	35.20
5	-3	-12	505.35	32.60
5	-3	-12	399.26	45.40
-5	3	12	491.55	28.30

4	-3	-12	25.20	9.80
4	-3	-12	43.50	13.10
4	-3	-12	23.90	9.90
4	-3	-12	25.30	14.90
4	-3	-12	25.90	9.40
-4	3	12	13.50	5.50
3	-3	-12	12.40	9.30
3	-3	-12	-7.60	11.10
3	-3	-12	21.60	14.20
3	-3	-12	3.40	9.20
3	-3	-12	2.30	7.90
2	-3	-12	449.76	26.30
2	-3	-12	454.45	28.70
2	-3	-12	450.85	29.00
2	-3	-12	504.75	30.40
-2	3	12	461.55	24.90
-2	3	12	460.95	24.80
-2	3	12	474.65	23.10
-1	3	12	233.98	14.50
-1	3	12	222.78	14.30
-1	3	12	236.88	12.70
0	3	12	1554.94	69.19
0	3	12	1491.65	70.39
1	3	12	40.50	5.20
1	3	12	51.69	7.40
1	3	12	42.50	4.60
1	3	12	44.80	5.00
2	3	12	970.80	46.60
2	3	12	1006.70	47.70
2	3	12	1036.20	45.40
-3	-3	-12	88.69	15.30
3	3	12	69.99	10.40
3	3	12	71.09	9.20
3	3	12	75.19	4.70
-4	-3	-12	235.88	14.40
-4	-3	-12	196.68	23.70
-4	-3	-12	287.07	26.70
-4	-3	-12	256.07	18.90
-4	-3	-12	233.28	17.70
4	3	12	272.47	12.20
4	3	12	223.08	17.80
-5	-3	-12	1529.95	68.69
-5	-3	-12	1477.45	69.29
-5	-3	-12	1431.96	73.69

-5	-3	-12	1455.55	73.79
-5	-3	-12	1452.65	69.79
-5	-3	-12	1488.35	71.29
5	3	12	1566.94	67.39
-6	-3	-12	53.69	14.80
-6	-3	-12	47.30	8.10
-6	-3	-12	48.80	11.10
-6	-3	-12	28.10	12.40
-6	-3	-12	39.60	8.70
-6	-3	-12	31.50	7.50
6	3	12	35.40	5.20
-7	-3	-12	427.86	24.10
-7	-3	-12	437.76	25.60
-7	-3	-12	459.75	28.60
-7	-3	-12	429.26	23.40
-7	-3	-12	376.26	30.90
7	3	12	440.36	21.80
-8	-3	-12	26.40	9.10
-8	-3	-12	10.00	11.70
-8	-3	-12	24.40	12.40
-8	-3	-12	28.40	7.50
-8	-3	-12	29.30	11.40
-8	-3	-12	18.90	6.90
8	3	12	26.30	6.10
-9	-3	-12	51.59	8.70
-9	-3	-12	42.90	8.50
-9	-3	-12	55.09	12.40
-9	-3	-12	34.50	14.90
-9	-3	-12	51.69	12.50
-9	-3	-12	55.59	10.70
9	3	12	41.00	9.10
-10	-3	-12	162.68	18.60
-10	-3	-12	132.19	14.20
-10	-3	-12	115.69	14.80
-10	-3	-12	146.49	16.00
-10	-3	-12	134.89	23.20
10	3	12	165.08	22.10
-11	-3	-12	30.60	8.00
-11	-3	-12	46.60	11.30
-11	-3	-12	48.70	13.50
-11	-3	-12	21.00	14.10
-11	-3	-12	37.40	8.70
-12	-3	-12	77.39	19.70
-12	-3	-12	45.90	14.00

-12	-3	-12	41.70	10.70
-12	-3	-12	34.40	8.60
12	3	12	71.59	23.80
10	-4	-12	54.09	12.10
-10	4	12	47.40	8.80
9	-4	-12	9.20	11.80
-9	4	12	13.90	11.90
-9	4	12	21.90	8.10
8	-4	-12	108.79	21.00
8	-4	-12	126.49	15.20
-8	4	12	99.69	13.50
-8	4	12	102.49	13.30
7	-4	-12	591.04	44.10
7	-4	-12	662.23	39.40
7	-4	-12	736.73	38.50
-7	4	12	701.73	35.70
-7	4	12	685.13	36.60
6	-4	-12	174.28	20.20
6	-4	-12	182.48	19.30
6	-4	-12	115.79	19.00
-6	4	12	146.59	14.20
5	-4	-12	42.90	15.80
5	-4	-12	36.00	9.90
5	-4	-12	26.80	16.50
5	-4	-12	16.80	9.20
5	-4	-12	25.00	10.50
-5	4	12	16.60	7.60
4	-4	-12	2.40	11.90
4	-4	-12	25.20	9.00
4	-4	-12	36.40	15.80
4	-4	-12	30.60	9.10
4	-4	-12	-4.70	8.70
-4	4	12	9.70	4.50
3	-4	-12	39.30	15.30
3	-4	-12	9.40	10.80
3	-4	-12	8.50	8.30
3	-4	-12	16.90	10.10
-3	4	12	9.60	4.00
2	-4	-12	19.10	8.70
2	-4	-12	0.80	8.10
2	-4	-12	12.40	7.10
2	-4	-12	21.30	10.40
-2	4	12	16.60	3.70
-1	4	12	1.30	4.10

-1	4	12	0.10	2.70
-1	4	12	5.90	4.90
0	4	12	1729.53	75.39
0	4	12	1649.13	76.69
0	4	12	1616.84	76.69
1	4	12	1774.82	81.09
1	4	12	1756.32	82.69
1	4	12	1820.42	80.89
1	4	12	1854.51	81.19
2	4	12	149.88	11.80
2	4	12	167.48	7.80
2	4	12	152.78	10.10
3	4	12	59.89	8.40
3	4	12	52.39	8.90
-4	-4	-12	90.79	15.90
-4	-4	-12	105.59	13.40
-4	-4	-12	93.89	11.30
-4	-4	-12	78.49	8.70
-4	-4	-12	82.79	16.50
4	4	12	74.29	10.20
4	4	12	91.49	13.10
-5	-4	-12	398.96	20.90
-5	-4	-12	381.56	32.20
-5	-4	-12	379.26	29.40
-5	-4	-12	357.06	22.50
-5	-4	-12	404.26	22.80
-5	-4	-12	384.26	25.20
5	4	12	442.96	19.00
-6	-4	-12	15.70	6.70
-6	-4	-12	-9.70	11.00
-6	-4	-12	-0.50	17.00
-6	-4	-12	17.00	5.30
-6	-4	-12	10.50	8.70
-6	-4	-12	22.80	7.20
6	4	12	10.80	3.20
-7	-4	-12	3.60	5.20
-7	-4	-12	4.90	6.20
-7	-4	-12	26.20	13.40
-7	-4	-12	16.70	8.00
-7	-4	-12	19.20	12.60
-7	-4	-12	7.40	9.00
-8	-4	-12	283.87	23.80
-8	-4	-12	309.97	23.90
-8	-4	-12	276.87	20.70

-8	-4	-12	284.77	18.70
-8	-4	-12	302.37	19.40
-8	-4	-12	284.07	27.90
8	4	12	320.97	17.30
-9	-4	-12	176.78	17.80
-9	-4	-12	197.98	15.60
-9	-4	-12	179.28	20.50
-9	-4	-12	201.38	21.80
-9	-4	-12	207.18	16.50
-9	-4	-12	187.58	25.50
9	4	12	180.88	15.80
-10	-4	-12	287.37	25.20
-10	-4	-12	221.08	17.30
-10	-4	-12	231.88	21.70
-10	-4	-12	260.47	18.50
-10	-4	-12	222.48	20.40
10	4	12	221.98	22.80
-11	-4	-12	279.67	19.00
-11	-4	-12	316.37	21.60
-11	-4	-12	253.27	24.80
-11	-4	-12	303.37	18.80
-11	-4	-12	318.57	25.10
-12	-4	-12	25.40	13.70
-12	-4	-12	27.30	20.00
-12	-4	-12	27.10	8.00
-12	-4	-12	26.90	9.60
12	4	12	41.90	16.60
10	-5	-12	15.90	11.40
-10	5	12	35.50	9.70
9	-5	-12	12.60	11.40
-9	5	12	14.50	7.80
-9	5	12	-2.10	10.90
8	-5	-12	15.80	11.40
8	-5	-12	-22.50	20.40
-8	5	12	7.70	7.50
-8	5	12	4.80	11.10
7	-5	-12	12.60	22.30
7	-5	-12	7.90	10.60
-7	5	12	-0.20	10.30
-7	5	12	11.00	7.10
6	-5	-12	88.59	14.20
6	-5	-12	87.49	19.80
6	-5	-12	75.09	11.70
6	-5	-12	114.29	18.30

-6	5	12	98.79	12.80
5	-5	-12	224.08	19.90
5	-5	-12	250.17	31.50
5	-5	-12	231.38	26.70
-5	5	12	246.08	16.40
4	-5	-12	366.56	25.50
4	-5	-12	431.76	25.00
4	-5	-12	405.16	27.50
4	-5	-12	384.66	23.90
4	-5	-12	333.47	34.50
-4	5	12	401.96	20.70
3	-5	-12	27.30	9.90
3	-5	-12	6.80	9.00
3	-5	-12	7.90	8.60
3	-5	-12	15.70	8.10
-3	5	12	5.90	3.60
2	-5	-12	335.57	22.40
2	-5	-12	340.67	24.80
2	-5	-12	313.97	20.90
2	-5	-12	325.57	20.90
2	-5	-12	320.77	20.70
-2	5	12	343.17	16.90
-1	5	12	1506.45	72.89
-1	5	12	1548.65	72.89
-1	5	12	1659.13	71.29
0	5	12	69.89	5.40
0	5	12	60.19	7.70
0	5	12	52.19	8.20
1	5	12	151.78	10.20
1	5	12	178.38	8.50
1	5	12	161.08	9.30
1	5	12	168.78	11.60
1	5	12	163.48	11.50
2	5	12	238.88	14.10
2	5	12	252.67	14.90
2	5	12	213.38	14.70
3	5	12	20.70	5.50
3	5	12	31.50	7.00
3	5	12	30.80	7.30
-4	-5	-12	29.00	15.10
-4	-5	-12	1.60	7.80
-4	-5	-12	6.10	4.70
-4	-5	-12	10.90	8.20
-4	-5	-12	30.60	14.10

4	5	12	-8.60	7.00
4	5	12	-10.40	7.90
-5	-5	-12	362.36	33.50
-5	-5	-12	371.76	21.80
-5	-5	-12	372.36	29.50
-5	-5	-12	355.46	22.30
-5	-5	-12	366.36	23.80
-5	-5	-12	400.06	20.00
-6	-5	-12	1071.49	50.69
-6	-5	-12	917.41	56.19
-6	-5	-12	1100.39	49.50
-6	-5	-12	1053.09	51.99
-6	-5	-12	1079.79	59.79
-6	-5	-12	1000.80	54.89
-6	-5	-12	1067.09	49.99
-7	-5	-12	109.99	14.10
-7	-5	-12	81.09	14.70
-7	-5	-12	101.49	17.10
-7	-5	-12	107.89	11.90
-7	-5	-12	41.50	34.50
-7	-5	-12	108.29	10.90
7	5	12	104.99	8.10
-8	-5	-12	149.09	15.80
-8	-5	-12	163.18	14.50
-8	-5	-12	181.08	13.70
-8	-5	-12	171.48	23.70
-8	-5	-12	152.98	19.00
-8	-5	-12	141.59	18.60
8	5	12	163.88	11.90
-9	-5	-12	355.66	23.50
-9	-5	-12	361.06	25.50
-9	-5	-12	362.66	25.10
-9	-5	-12	322.77	30.60
-9	-5	-12	361.66	22.10
-9	-5	-12	346.57	21.40
9	5	12	352.46	21.20
-10	-5	-12	183.78	21.90
-10	-5	-12	150.08	17.50
-10	-5	-12	121.49	19.50
-10	-5	-12	138.89	13.70
-10	-5	-12	163.68	15.80
-11	-5	-12	10.90	7.30
-11	-5	-12	-8.50	8.50
-11	-5	-12	-10.50	12.30

-11	-5	-12	32.10	11.70
-11	-5	-12	12.80	7.60
-12	-5	-12	95.59	11.90
-12	-5	-12	75.49	22.00
-12	-5	-12	107.09	13.40
-12	-5	-12	91.19	19.60
12	5	12	121.39	20.00
10	-6	-12	35.10	11.30
-10	6	12	10.40	15.70
-10	6	12	29.40	9.90
9	-6	-12	66.89	12.60
-9	6	12	59.09	10.00
-9	6	12	64.89	11.60
8	-6	-12	95.59	14.50
-8	6	12	85.29	14.50
7	-6	-12	181.68	19.80
7	-6	-12	185.98	20.70
-7	6	12	166.38	15.60
-7	6	12	147.29	19.00
6	-6	-12	1177.88	58.69
6	-6	-12	1156.28	60.09
6	-6	-12	1135.89	57.39
-6	6	12	1189.08	60.19
-6	6	12	1226.38	57.69
5	-6	-12	-0.30	8.60
5	-6	-12	3.60	13.20
5	-6	-12	25.00	10.40
5	-6	-12	2.70	13.30
-5	6	12	8.30	10.10
-5	6	12	9.50	6.80
4	-6	-12	49.99	11.50
4	-6	-12	15.70	10.30
4	-6	-12	33.70	8.40
4	-6	-12	38.40	9.90
-4	6	12	36.40	5.60
3	-6	-12	60.69	9.40
3	-6	-12	59.89	9.20
3	-6	-12	42.00	8.40
3	-6	-12	31.20	10.30
3	-6	-12	51.79	10.70
-3	6	12	58.69	6.20
2	-6	-12	62.19	9.10
2	-6	-12	50.69	8.20
2	-6	-12	81.89	12.90

2	-6	-12	71.59	10.00
2	-6	-12	79.99	13.30
-2	6	12	68.69	8.80
1	-6	-12	494.25	28.00
1	-6	-12	511.45	27.60
1	-6	-12	471.65	27.60
-1	6	12	541.55	24.10
0	6	12	291.27	14.10
0	6	12	284.07	16.40
0	6	12	280.67	17.10
1	6	12	1438.36	67.29
1	6	12	1448.06	67.59
1	6	12	1427.46	65.79
1	6	12	1497.95	66.69
2	6	12	277.77	17.40
2	6	12	307.47	17.10
2	6	12	300.47	16.00
2	6	12	292.97	17.00
-3	-6	-12	3.60	5.60
3	6	12	9.00	7.00
3	6	12	3.70	6.50
3	6	12	13.80	5.40
3	6	12	5.80	5.70
-4	-6	-12	833.42	42.50
-4	-6	-12	834.22	41.30
-4	-6	-12	904.51	39.60
-4	-6	-12	751.32	48.10
-4	-6	-12	831.62	48.90
4	6	12	912.91	41.60
4	6	12	811.42	41.90
4	6	12	758.22	42.10
-5	-6	-12	232.38	13.70
-5	-6	-12	210.88	18.50
-5	-6	-12	208.28	16.00
-5	-6	-12	209.28	15.90
-5	-6	-12	237.08	24.90
-5	-6	-12	190.28	24.20
5	6	12	214.28	11.80
-6	-6	-12	-17.80	11.50
-6	-6	-12	9.30	8.10
-6	-6	-12	14.30	11.10
-6	-6	-12	9.50	5.50
-6	-6	-12	-2.30	7.00
-6	-6	-12	-5.40	6.70

-6	-6	-12	7.60	20.00
6	6	12	2.60	2.90
-7	-6	-12	200.48	18.90
-7	-6	-12	192.08	23.70
-7	-6	-12	184.08	19.70
-7	-6	-12	188.98	16.50
-7	-6	-12	191.58	14.10
-7	-6	-12	206.98	15.10
-8	-6	-12	49.70	10.90
-8	-6	-12	73.29	10.90
-8	-6	-12	70.89	12.20
-8	-6	-12	69.29	13.40
-8	-6	-12	45.90	10.70
-8	-6	-12	44.80	9.60
8	6	12	61.79	8.70
-9	-6	-12	6.50	9.30
-9	-6	-12	8.30	13.90
-9	-6	-12	21.80	8.20
-9	-6	-12	14.40	7.50
-9	-6	-12	9.00	7.90
-9	-6	-12	16.70	10.60
9	6	12	10.90	7.70
-10	-6	-12	16.20	11.30
-10	-6	-12	-5.20	11.00
-10	-6	-12	5.20	7.50
-10	-6	-12	-1.00	8.00
-10	-6	-12	9.80	8.40
10	6	12	2.80	11.30
-11	-6	-12	156.98	13.60
-11	-6	-12	146.09	14.10
-11	-6	-12	158.08	15.60
-11	-6	-12	179.78	20.00
-11	-6	-12	163.68	19.50
-12	-6	-12	87.39	11.50
-12	-6	-12	112.49	13.30
-12	-6	-12	73.89	16.40
-12	-6	-12	102.89	17.20
12	6	12	77.69	17.00
10	-7	-12	29.30	11.40
-10	7	12	21.40	10.70
-10	7	12	44.80	12.30
9	-7	-12	-1.90	16.90
9	-7	-12	8.20	11.10
-9	7	12	3.30	10.10

-9	7	12	3.00	8.30
8	-7	-12	102.39	18.60
8	-7	-12	79.69	19.50
-8	7	12	97.69	14.70
-8	7	12	89.19	13.20
7	-7	-12	9.70	11.10
7	-7	-12	14.80	10.70
-7	7	12	5.30	7.50
-7	7	12	13.10	10.10
6	-7	-12	530.75	34.70
6	-7	-12	529.25	30.00
6	-7	-12	562.74	32.00
-6	7	12	448.66	31.40
-6	7	12	547.75	30.30
5	-7	-12	505.55	29.30
5	-7	-12	433.66	27.30
5	-7	-12	502.65	29.60
-5	7	12	438.86	25.20
-5	7	12	461.65	28.50
4	-7	-12	28.10	8.40
4	-7	-12	27.90	11.50
4	-7	-12	30.60	9.80
4	-7	-12	26.40	10.50
-4	7	12	15.00	4.50
-4	7	12	5.30	10.50
3	-7	-12	21.30	10.60
3	-7	-12	4.50	6.90
3	-7	-12	11.00	7.70
3	-7	-12	-7.20	8.90
3	-7	-12	-4.50	8.00
-3	7	12	1.60	2.90
2	-7	-12	12.60	8.00
2	-7	-12	23.10	8.90
2	-7	-12	19.00	7.00
2	-7	-12	26.10	8.20
2	-7	-12	30.10	12.90
-2	7	12	19.30	3.40
1	-7	-12	547.85	30.90
1	-7	-12	585.64	31.00
1	-7	-12	555.34	30.90
-1	7	12	643.64	27.50
0	-7	-12	1024.40	49.40
0	7	12	966.10	46.80
0	7	12	930.91	42.80

0	7	12	856.21	45.50
1	7	12	64.89	10.20
1	7	12	74.49	8.90
1	7	12	77.79	6.80
1	7	12	76.29	10.70
2	7	12	240.18	16.10
2	7	12	262.47	16.30
2	7	12	246.08	14.40
2	7	12	236.58	15.20
-3	-7	-12	379.76	20.20
3	7	12	368.26	22.30
3	7	12	389.76	21.10
3	7	12	365.06	21.00
3	7	12	347.07	22.00
-4	-7	-12	504.35	36.40
-4	-7	-12	514.05	24.40
-4	-7	-12	470.85	28.90
-4	-7	-12	489.85	39.10
-4	-7	-12	453.85	26.90
-4	-7	-12	455.75	26.50
4	7	12	467.45	29.20
4	7	12	495.35	26.70
-5	-7	-12	487.65	45.30
-5	-7	-12	534.15	35.50
-5	-7	-12	561.94	26.50
-5	-7	-12	544.95	28.20
-5	-7	-12	536.05	27.80
-5	-7	-12	552.74	29.50
-5	-7	-12	425.96	31.70
-6	-7	-12	11.40	5.20
-6	-7	-12	13.20	11.50
-6	-7	-12	11.70	6.60
-6	-7	-12	10.40	9.40
-6	-7	-12	-0.40	12.00
-6	-7	-12	16.30	11.10
6	7	12	21.00	3.50
-7	-7	-12	273.37	21.90
-7	-7	-12	268.07	25.50
-7	-7	-12	270.07	21.30
-7	-7	-12	290.77	17.30
-7	-7	-12	274.17	18.10
-7	-7	-12	280.77	19.50
-8	-7	-12	38.10	10.60
-8	-7	-12	38.60	9.30

-8	-7	-12	26.50	7.10
-8	-7	-12	43.80	13.60
-8	-7	-12	42.10	7.90
-8	-7	-12	33.50	9.90
8	7	12	32.00	6.10
-9	-7	-12	86.59	12.70
-9	-7	-12	49.60	11.60
-9	-7	-12	63.79	11.40
-9	-7	-12	98.19	14.10
-9	-7	-12	63.19	11.10
9	7	12	73.99	12.30
-10	-7	-12	173.08	17.10
-10	-7	-12	188.88	20.20
-10	-7	-12	176.88	14.80
-10	-7	-12	204.48	16.00
-10	-7	-12	185.88	19.90
10	7	12	152.98	19.60
-11	-7	-12	59.09	11.40
-11	-7	-12	46.90	11.80
-11	-7	-12	42.50	9.30
-11	-7	-12	45.20	8.50
-11	-7	-12	54.29	9.40
-12	-7	-12	57.59	14.70
-12	-7	-12	39.50	10.40
-12	-7	-12	45.70	9.40
12	7	12	42.70	18.80
10	-8	-12	42.70	11.70
-10	8	12	39.50	12.10
-10	8	12	25.30	11.70
9	-8	-12	20.10	11.10
9	-8	-12	31.50	15.20
-9	8	12	19.20	10.30
-9	8	12	13.30	9.60
8	-8	-12	152.18	26.20
-8	8	12	106.49	17.80
-8	8	12	102.59	14.70
7	-8	-12	381.96	26.10
7	-8	-12	393.16	27.20
-7	8	12	374.26	25.40
6	-8	-12	150.38	19.70
6	-8	-12	133.69	24.30
6	-8	-12	136.19	19.00
6	-8	-12	127.99	17.40
-6	8	12	112.79	17.00

5	-8	-12	30.60	11.20
5	-8	-12	55.29	10.00
5	-8	-12	62.29	12.20
5	-8	-12	29.80	12.90
-5	8	12	40.00	10.10
-5	8	12	54.79	8.00
4	-8	-12	-2.00	10.60
4	-8	-12	15.40	8.70
4	-8	-12	15.70	10.00
4	-8	-12	18.30	10.50
-4	8	12	1.00	4.30
-4	8	12	3.90	9.70
3	-8	-12	-5.20	8.40
3	-8	-12	10.90	8.70
3	-8	-12	19.90	11.20
3	-8	-12	1.60	7.10
3	-8	-12	17.70	8.50
-3	8	12	6.40	3.20
2	-8	-12	249.97	18.80
2	-8	-12	241.68	25.10
2	-8	-12	262.67	18.70
2	-8	-12	251.67	17.70
2	-8	-12	240.88	18.10
-2	8	12	280.57	13.60
1	-8	-12	83.89	10.00
1	-8	-12	52.19	8.60
1	-8	-12	76.29	10.00
1	-8	-12	72.69	13.30
1	-8	-12	72.39	15.70
-1	8	12	75.09	5.70
0	-8	-12	1212.98	60.29
0	-8	-12	1251.17	59.89
0	-8	-12	1305.17	59.79
0	8	12	1272.77	56.79
-1	-8	-12	208.98	17.30
1	8	12	239.68	21.10
1	8	12	216.98	16.00
1	8	12	212.18	16.70
1	8	12	203.58	12.60
-2	-8	-12	263.77	19.50
2	8	12	257.97	16.00
2	8	12	231.78	17.80
2	8	12	251.77	15.40
2	8	12	247.68	17.10

-3	-8	-12	89.79	10.30
3	8	12	86.69	15.00
3	8	12	82.39	12.10
3	8	12	79.69	13.30
3	8	12	60.59	11.10
-4	-8	-12	12.90	9.80
-4	-8	-12	38.00	14.00
-4	-8	-12	14.40	7.90
-4	-8	-12	-0.90	15.00
-4	-8	-12	4.50	8.10
-4	-8	-12	8.90	22.20
4	8	12	3.10	16.20
4	8	12	11.90	10.00
-5	-8	-12	139.29	13.30
-5	-8	-12	108.09	16.70
-5	-8	-12	137.39	14.30
-5	-8	-12	124.69	14.60
-5	-8	-12	136.09	10.70
-6	-8	-12	12.10	10.40
-6	-8	-12	2.80	7.30
-6	-8	-12	-22.10	12.30
-6	-8	-12	1.50	5.40
6	8	12	6.20	3.30
-7	-8	-12	198.58	23.70
-7	-8	-12	167.98	19.50
-7	-8	-12	200.38	19.10
-7	-8	-12	238.58	15.90
-7	-8	-12	197.38	17.00
-7	-8	-12	221.88	14.70
7	8	12	209.48	12.40
-8	-8	-12	350.26	22.00
-8	-8	-12	336.87	24.80
-8	-8	-12	403.86	25.20
-8	-8	-12	378.86	23.20
-8	-8	-12	373.96	29.70
-9	-8	-12	143.39	13.90
-9	-8	-12	134.59	12.60
-9	-8	-12	136.59	17.90
-9	-8	-12	162.78	18.80
-9	-8	-12	143.09	15.60
9	8	12	133.59	13.90
-10	-8	-12	96.59	14.20
-10	-8	-12	103.09	12.00
-10	-8	-12	85.69	12.60

-10	-8	-12	116.59	13.50
-10	-8	-12	89.09	16.80
10	8	12	102.69	18.00
-11	-8	-12	115.79	13.50
-11	-8	-12	85.49	11.40
-11	-8	-12	96.19	18.10
-11	-8	-12	117.49	13.90
10	-9	-12	53.39	11.90
-10	9	12	47.60	12.10
-10	9	12	59.39	15.70
9	-9	-12	0.70	10.90
9	-9	-12	15.30	14.40
-9	9	12	10.30	10.50
-9	9	12	12.60	9.70
8	-9	-12	-2.00	11.60
8	-9	-12	4.80	12.10
-8	9	12	21.50	9.90
-8	9	12	4.20	9.90
7	-9	-12	10.60	9.90
7	-9	-12	31.40	12.10
-7	9	12	26.20	9.80
6	-9	-12	16.50	11.20
6	-9	-12	-27.20	15.50
6	-9	-12	0.60	9.00
6	-9	-12	26.60	14.60
-6	9	12	24.30	12.00
-6	9	12	-10.00	9.40
5	-9	-12	213.28	21.20
5	-9	-12	194.08	17.60
5	-9	-12	202.48	22.70
5	-9	-12	191.48	23.40
-5	9	12	200.08	18.80
-5	9	12	182.68	15.20
4	-9	-12	332.77	22.40
4	-9	-12	332.17	28.10
4	-9	-12	321.37	21.50
-4	9	12	326.17	22.70
-4	9	12	338.17	18.20
3	-9	-12	-13.80	12.60
3	-9	-12	14.00	8.00
3	-9	-12	9.20	8.90
3	-9	-12	16.40	8.20
3	-9	-12	7.20	8.00
-3	9	12	7.50	3.40

2	-9	-12	700.63	41.30
2	-9	-12	709.23	36.70
2	-9	-12	722.93	36.30
2	-9	-12	709.13	36.50
2	-9	-12	670.43	36.10
-2	9	12	743.03	33.30
1	-9	-12	806.22	39.50
1	-9	-12	770.42	39.80
1	-9	-12	750.72	39.80
1	-9	-12	767.22	39.50
1	-9	-12	789.72	45.10
-1	9	12	856.21	36.60
0	-9	-12	338.17	21.40
0	-9	-12	306.27	21.60
0	-9	-12	345.67	21.00
0	-9	-12	327.57	22.60
0	9	12	362.86	16.30
-1	-9	-12	82.29	13.10
-1	-9	-12	87.19	14.80
-1	-9	-12	74.69	9.50
-2	-9	-12	475.25	32.70
-2	-9	-12	420.96	28.00
-2	-9	-12	458.05	26.70
2	9	12	498.45	28.40
2	9	12	490.65	25.20
-3	-9	-12	97.19	23.40
-3	-9	-12	84.19	14.40
-3	-9	-12	66.09	13.90
-3	-9	-12	62.19	10.10
3	9	12	52.09	9.80
3	9	12	32.20	11.60
-4	-9	-12	68.99	9.00
-4	-9	-12	81.99	10.00
-4	-9	-12	56.59	13.20
-4	-9	-12	51.89	16.10
-4	-9	-12	71.59	14.50
-4	-9	-12	69.79	10.80
-4	-9	-12	55.39	9.90
-5	-9	-12	362.96	21.20
-5	-9	-12	363.16	19.80
-5	-9	-12	320.47	24.70
-5	-9	-12	399.76	30.90
-5	-9	-12	397.36	22.60
-5	-9	-12	371.46	25.60

-6	-9	-12	580.64	28.50
-6	-9	-12	589.44	36.50
-6	-9	-12	532.45	29.70
-6	-9	-12	568.94	32.00
-6	-9	-12	541.25	29.10
-6	-9	-12	489.55	38.90
6	9	12	583.84	26.40
-7	-9	-12	58.59	10.70
-7	-9	-12	57.99	9.50
-7	-9	-12	92.19	12.60
-7	-9	-12	58.89	14.20
-7	-9	-12	58.39	11.00
-7	-9	-12	79.99	11.70
7	9	12	69.69	8.10
-8	-9	-12	127.29	17.30
-8	-9	-12	132.19	17.30
-8	-9	-12	132.19	14.60
-8	-9	-12	118.59	13.60
-8	-9	-12	135.39	13.20
-8	-9	-12	127.59	17.90
-9	-9	-12	398.36	26.20
-9	-9	-12	385.46	22.90
-9	-9	-12	433.76	23.90
-9	-9	-12	413.46	24.70
-9	-9	-12	412.46	26.30
9	9	12	370.96	23.10
-10	-9	-12	107.19	14.00
-10	-9	-12	92.29	12.90
-10	-9	-12	92.69	11.50
-10	-9	-12	127.49	17.20
-10	-9	-12	106.29	17.20
-11	-9	-12	-4.20	7.80
-11	-9	-12	15.60	10.60
-11	-9	-12	17.40	10.20
-11	-9	-12	9.80	7.30
9	-10	-12	58.09	14.00
9	-10	-12	41.60	12.20
-9	10	12	20.40	10.80
-9	10	12	46.30	10.60
8	-10	-12	8.60	12.50
8	-10	-12	5.20	11.40
-8	10	12	20.40	9.90
-8	10	12	8.20	10.00
7	-10	-12	526.55	31.20

7	-10	-12	492.15	29.70
-7	10	12	452.55	29.30
6	-10	-12	483.15	34.40
6	-10	-12	478.25	30.30
6	-10	-12	505.45	27.70
6	-10	-12	362.86	34.70
-6	10	12	446.86	27.80
-6	10	12	477.05	32.60
5	-10	-12	-3.40	11.70
5	-10	-12	9.20	8.40
5	-10	-12	-1.40	11.80
5	-10	-12	18.00	12.10
-5	10	12	-9.10	8.70
-5	10	12	3.50	7.20
4	-10	-12	69.79	14.00
4	-10	-12	66.69	11.80
4	-10	-12	98.39	16.80
4	-10	-12	58.09	10.30
4	-10	-12	85.39	14.90
-4	10	12	80.89	9.00
3	-10	-12	227.58	17.80
3	-10	-12	245.08	18.60
3	-10	-12	216.78	24.70
3	-10	-12	205.58	19.10
3	-10	-12	242.68	17.70
-3	10	12	240.28	13.10
2	-10	-12	172.88	24.00
2	-10	-12	156.68	15.40
2	-10	-12	178.88	16.50
2	-10	-12	158.88	15.90
2	-10	-12	181.08	16.10
-2	10	12	165.78	9.70
1	-10	-12	280.97	32.20
1	-10	-12	355.96	22.10
1	-10	-12	351.36	22.60
1	-10	-12	360.76	21.90
1	-10	-12	391.26	22.00
1	-10	-12	301.27	24.60
-1	10	12	390.96	17.50
0	-10	-12	11.20	8.20
0	-10	-12	1.70	7.00
0	-10	-12	-5.70	7.80
-1	-10	-12	695.83	35.70
-1	-10	-12	732.63	39.10

-1	-10	-12	720.23	36.30
-1	-10	-12	679.83	36.60
-2	-10	-12	20.90	9.00
-2	-10	-12	16.70	9.00
-2	-10	-12	24.00	17.00
-2	-10	-12	10.30	14.90
-2	-10	-12	18.30	9.30
-2	-10	-12	2.70	10.80
-3	-10	-12	38.80	22.40
-3	-10	-12	-8.30	12.40
-3	-10	-12	10.80	9.10
-3	-10	-12	36.60	11.10
-3	-10	-12	8.70	11.00
-3	-10	-12	-1.80	6.50
-4	-10	-12	940.11	42.10
-4	-10	-12	903.41	43.30
-4	-10	-12	838.02	45.70
-4	-10	-12	894.31	43.70
-4	-10	-12	913.21	44.00
-4	-10	-12	772.22	45.40
-5	-10	-12	70.19	11.80
-5	-10	-12	55.59	9.10
-5	-10	-12	80.49	11.90
-5	-10	-12	63.49	12.00
-5	-10	-12	56.89	11.00
-5	-10	-12	76.49	16.70
-5	-10	-12	52.59	8.90
-6	-10	-12	-1.10	9.30
-6	-10	-12	2.00	9.70
-6	-10	-12	-1.80	7.00
-6	-10	-12	10.60	14.90
-6	-10	-12	0.80	6.80
-6	-10	-12	6.60	5.70
6	10	12	3.00	4.10
-7	-10	-12	397.86	22.10
-7	-10	-12	361.16	23.50
-7	-10	-12	419.56	26.20
-7	-10	-12	373.06	32.10
-7	-10	-12	390.76	22.70
-7	-10	-12	389.86	25.30
7	10	12	434.26	20.70
-8	-10	-12	117.19	17.00
-8	-10	-12	124.89	13.20
-8	-10	-12	115.19	14.70

-8	-10	-12	105.09	17.30
-8	-10	-12	150.28	19.80
-8	-10	-12	116.69	12.40
8	10	12	111.89	11.50
-9	-10	-12	33.60	10.70
-9	-10	-12	12.80	8.50
-9	-10	-12	21.80	11.00
-9	-10	-12	10.80	6.70
-9	-10	-12	13.20	7.70
9	10	12	17.30	8.50
-10	-10	-12	6.10	9.80
-10	-10	-12	9.30	7.90
-10	-10	-12	9.40	7.30
-10	-10	-12	20.40	10.30
-10	-10	-12	4.40	7.20
-11	-10	-12	58.29	11.60
-11	-10	-12	80.39	11.90
-11	-10	-12	66.59	11.10
-11	-10	-12	66.59	9.60
9	-11	-12	29.30	12.70
9	-11	-12	24.00	12.30
-9	11	12	4.70	10.00
-9	11	12	17.70	11.10
8	-11	-12	224.98	21.20
8	-11	-12	186.58	20.10
-8	11	12	200.48	19.50
-8	11	12	204.68	19.90
7	-11	-12	39.20	16.70
7	-11	-12	38.40	10.80
-7	11	12	20.80	14.10
6	-11	-12	263.67	28.70
6	-11	-12	220.28	23.70
6	-11	-12	278.37	20.50
6	-11	-12	256.97	29.60
-6	11	12	236.18	20.40
5	-11	-12	6.10	9.00
5	-11	-12	15.90	11.60
5	-11	-12	0.40	12.90
5	-11	-12	-0.40	12.50
-5	11	12	-4.30	7.60
-5	11	12	4.30	9.00
4	-11	-12	175.88	18.50
4	-11	-12	195.98	19.00
4	-11	-12	173.78	17.20

4	-11	-12	137.49	23.10
4	-11	-12	187.08	18.30
-4	11	12	160.88	12.40
3	-11	-12	8.40	7.90
3	-11	-12	28.70	13.00
3	-11	-12	23.40	14.40
3	-11	-12	12.10	11.90
3	-11	-12	29.20	9.10
-3	11	12	23.30	4.40
2	-11	-12	16.10	8.00
2	-11	-12	9.60	8.20
2	-11	-12	6.80	7.40
2	-11	-12	-17.80	15.60
2	-11	-12	4.20	8.60
-2	11	12	13.60	3.10
1	-11	-12	585.24	30.70
1	-11	-12	580.14	31.80
1	-11	-12	590.84	31.00
1	-11	-12	589.94	31.20
1	-11	-12	522.65	32.40
1	-11	-12	583.64	39.40
-1	11	12	603.44	27.40
0	-11	-12	-0.90	6.90
0	-11	-12	10.80	10.50
0	-11	-12	2.50	10.70
0	-11	-12	22.60	7.80
0	-11	-12	13.30	8.10
0	11	12	15.70	2.70
-1	-11	-12	347.67	22.90
-1	-11	-12	394.46	23.70
-1	-11	-12	348.47	22.30
-1	-11	-12	377.16	28.80
1	11	12	410.56	17.80
-2	-11	-12	240.68	18.80
-2	-11	-12	296.27	18.70
-2	-11	-12	230.38	30.40
-2	-11	-12	261.07	19.70
-2	-11	-12	295.77	22.20
-2	-11	-12	243.68	20.90
-3	-11	-12	813.12	40.30
-3	-11	-12	792.82	40.70
-3	-11	-12	767.22	40.80
-3	-11	-12	765.62	42.50
-3	-11	-12	755.92	39.30

-3	-11	-12	836.72	38.80
-4	-11	-12	344.57	29.40
-4	-11	-12	436.56	26.70
-4	-11	-12	400.96	24.60
-4	-11	-12	416.96	22.10
-4	-11	-12	395.86	26.10
-4	-11	-12	396.36	24.10
-4	-11	-12	421.86	25.30
-5	-11	-12	192.88	24.10
-5	-11	-12	209.58	19.80
-5	-11	-12	224.38	17.60
-5	-11	-12	235.78	15.00
-5	-11	-12	210.78	18.90
-5	-11	-12	260.77	22.80
-6	-11	-12	27.30	10.50
-6	-11	-12	39.20	8.00
-6	-11	-12	43.50	10.90
-6	-11	-12	46.90	8.40
-6	-11	-12	26.10	9.80
-6	-11	-12	35.30	7.10
6	11	12	46.80	7.60
-7	-11	-12	142.09	17.00
-7	-11	-12	171.28	15.50
-7	-11	-12	142.19	14.50
-7	-11	-12	146.59	13.10
-7	-11	-12	165.98	18.30
7	11	12	156.38	11.80
-8	-11	-12	154.58	14.50
-8	-11	-12	144.59	13.50
-8	-11	-12	129.79	17.80
-8	-11	-12	142.19	17.90
-8	-11	-12	181.68	15.80
8	11	12	144.69	13.20
-9	-11	-12	3.30	11.00
-9	-11	-12	24.80	19.70
-9	-11	-12	14.40	12.00
-9	-11	-12	8.70	15.90
-9	-11	-12	-1.00	6.80
-10	-11	-12	312.97	23.20
-10	-11	-12	335.47	19.90
-10	-11	-12	297.37	19.60
-10	-11	-12	347.27	20.70
-10	-11	-12	334.37	23.10
-11	-11	-12	6.40	7.20

-11	-11	-12	3.40	8.50
-11	-11	-12	12.00	10.70
-11	-11	-12	6.40	7.70
9	-12	-12	7.20	13.20
9	-12	-12	21.50	12.10
-9	12	12	7.20	12.00
-9	12	12	15.80	11.90
8	-12	-12	171.48	20.40
8	-12	-12	165.88	18.60
-8	12	12	166.78	18.60
-8	12	12	154.98	19.20
7	-12	-12	99.19	16.30
7	-12	-12	149.79	18.90
-7	12	12	114.49	19.00
6	-12	-12	487.45	32.50
6	-12	-12	534.85	37.90
6	-12	-12	529.65	29.60
6	-12	-12	536.95	35.10
-6	12	12	448.56	29.90
5	-12	-12	203.18	24.90
5	-12	-12	267.67	23.40
5	-12	-12	240.18	18.80
5	-12	-12	222.98	25.00
-5	12	12	217.28	20.10
-5	12	12	242.08	17.90
4	-12	-12	13.60	9.20
4	-12	-12	11.00	10.70
4	-12	-12	34.20	15.00
4	-12	-12	8.90	8.10
4	-12	-12	8.60	9.80
-4	12	12	14.80	6.10
3	-12	-12	56.39	10.40
3	-12	-12	43.40	21.30
3	-12	-12	51.19	9.40
3	-12	-12	61.99	10.30
3	-12	-12	40.50	10.70
-3	12	12	43.30	7.00
2	-12	-12	549.35	36.80
2	-12	-12	412.86	36.30
-2	12	12	513.45	24.20
1	-12	-12	195.68	18.00
1	-12	-12	189.68	16.50
1	-12	-12	219.28	17.40
1	-12	-12	196.08	16.20

1	-12	-12	185.08	19.50
-1	12	12	186.08	10.60
0	-12	-12	535.15	31.30
0	-12	-12	587.24	30.60
0	-12	-12	578.14	30.40
0	-12	-12	500.15	33.20
0	-12	-12	613.24	31.30
0	12	12	598.14	26.70
-1	-12	-12	5.40	7.90
-1	-12	-12	7.90	14.00
-1	-12	-12	9.00	9.40
-1	-12	-12	14.70	8.00
-1	-12	-12	-2.20	9.30
1	12	12	13.60	2.60
-2	-12	-12	354.26	23.40
-2	-12	-12	366.16	21.70
-2	-12	-12	403.16	22.60
-2	-12	-12	344.77	23.70
-2	-12	-12	302.77	34.80
-2	-12	-12	402.66	25.70
-3	-12	-12	255.27	17.70
-3	-12	-12	235.98	17.10
-3	-12	-12	235.88	19.30
-3	-12	-12	279.97	19.10
-3	-12	-12	221.58	24.80
-3	-12	-12	220.98	21.90
-4	-12	-12	20.70	8.40
-4	-12	-12	6.30	9.10
-4	-12	-12	14.70	7.00
-4	-12	-12	0.40	9.90
-4	-12	-12	-7.60	9.00
-4	-12	-12	40.30	10.80
-5	-12	-12	23.60	8.50
-5	-12	-12	27.80	12.30
-5	-12	-12	5.40	13.00
-5	-12	-12	30.90	8.40
-5	-12	-12	23.90	6.90
-5	-12	-12	10.20	9.20
-6	-12	-12	61.89	11.10
-6	-12	-12	55.79	8.00
-6	-12	-12	62.39	12.30
-6	-12	-12	59.29	10.30
-6	-12	-12	58.69	9.00
-6	-12	-12	80.89	12.90

6	12	12	47.60	6.90
-7	-12	-12	27.00	11.20
-7	-12	-12	21.80	7.90
-7	-12	-12	21.90	7.80
-7	-12	-12	26.20	10.30
-7	-12	-12	10.90	10.60
7	12	12	19.60	6.60
-8	-12	-12	490.95	30.10
-8	-12	-12	503.85	27.50
-8	-12	-12	486.85	27.00
-8	-12	-12	513.55	28.10
-8	-12	-12	489.35	29.70
8	12	12	504.65	26.60
-9	-12	-12	68.19	9.80
-9	-12	-12	103.49	17.40
-9	-12	-12	89.99	14.10
-9	-12	-12	55.29	11.60
-9	-12	-12	94.89	13.10
-10	-12	-12	105.19	12.10
-10	-12	-12	86.99	12.60
-10	-12	-12	93.19	17.10
-10	-12	-12	86.89	12.10
-10	-12	-12	85.39	12.00
-11	-12	-12	40.50	8.30
-11	-12	-12	23.90	9.50
-11	-12	-12	61.99	9.80
9	-13	-12	4.30	15.10
9	-13	-12	30.80	15.30
-9	13	12	14.60	12.60
-9	13	12	-1.60	9.50
8	-13	-12	23.50	14.10
8	-13	-12	0.70	9.80
-8	13	12	6.10	11.20
-8	13	12	9.80	10.00
7	-13	-12	73.49	14.50
7	-13	-12	70.19	11.00
-7	13	12	91.59	12.70
6	-13	-12	24.20	14.80
6	-13	-12	29.20	15.40
6	-13	-12	30.00	9.30
6	-13	-12	5.80	19.10
-6	13	12	3.10	10.00
5	-13	-12	271.17	26.00
5	-13	-12	261.87	22.80

5	-13	-12	232.58	19.20
5	-13	-12	212.18	25.40
-5	13	12	168.28	20.00
-5	13	12	175.98	19.70
4	-13	-12	323.17	24.10
4	-13	-12	317.77	21.90
4	-13	-12	316.97	25.10
4	-13	-12	326.57	23.50
4	-13	-12	335.27	30.80
-4	13	12	322.07	18.90
3	-13	-12	66.99	11.00
3	-13	-12	91.29	19.40
3	-13	-12	70.19	11.30
3	-13	-12	96.19	15.60
-3	13	12	84.39	8.60
2	-13	-12	329.27	22.10
2	-13	-12	337.27	22.20
2	-13	-12	321.67	22.40
2	-13	-12	339.47	23.30
-2	13	12	345.77	17.50
1	-13	-12	214.18	20.10
1	-13	-12	270.47	20.30
-1	13	12	240.68	12.90
0	-13	-12	429.66	24.90
0	-13	-12	441.86	26.20
0	-13	-12	456.25	25.40
0	-13	-12	464.05	26.60
0	-13	-12	396.16	29.00
0	13	12	442.96	21.10
-1	-13	-12	157.18	16.00
-1	-13	-12	171.48	25.30
-1	-13	-12	149.88	14.80
-1	-13	-12	149.29	14.80
-1	-13	-12	155.88	17.30
1	13	12	168.08	8.80
-2	-13	-12	292.27	21.40
-2	-13	-12	310.97	20.00
-2	-13	-12	311.77	41.90
-2	-13	-12	357.66	23.90
-2	-13	-12	293.17	20.60
-2	-13	-12	321.97	20.50
-2	-13	-12	312.97	27.90
2	13	12	336.67	15.30
-3	-13	-12	398.76	23.40

-3	-13	-12	379.66	23.30
-3	-13	-12	396.76	24.00
-3	-13	-12	412.56	29.40
-3	-13	-12	410.86	26.90
-3	-13	-12	380.16	24.10
-4	-13	-12	125.19	15.20
-4	-13	-12	130.89	17.90
-4	-13	-12	140.19	17.50
-4	-13	-12	103.79	15.90
-4	-13	-12	114.49	13.00
-5	-13	-12	309.37	18.30
-5	-13	-12	319.17	19.90
-5	-13	-12	252.87	20.10
-5	-13	-12	267.27	30.50
-5	-13	-12	269.47	19.30
-5	-13	-12	253.77	20.90
-6	-13	-12	256.67	17.90
-6	-13	-12	264.97	19.80
-6	-13	-12	185.58	22.80
-6	-13	-12	201.28	18.90
-6	-13	-12	235.78	16.60
-6	-13	-12	230.98	18.20
-7	-13	-12	53.29	9.50
-7	-13	-12	68.39	11.60
-7	-13	-12	65.49	11.20
-7	-13	-12	63.09	9.50
-7	-13	-12	45.50	11.00
7	13	12	64.89	11.00
-8	-13	-12	10.50	7.50
-8	-13	-12	15.90	10.90
-8	-13	-12	21.80	8.50
-8	-13	-12	13.20	10.30
-8	-13	-12	21.80	8.20
8	13	12	16.80	8.60
-9	-13	-12	623.64	33.00
-9	-13	-12	596.94	30.70
-9	-13	-12	563.74	30.30
-9	-13	-12	551.64	30.20
-9	-13	-12	542.05	32.70
-10	-13	-12	57.79	10.30
-10	-13	-12	89.19	12.90
-10	-13	-12	67.29	11.30
-10	-13	-12	38.50	8.70
-11	-13	-12	6.70	9.10

-11	-13	-12	-9.10	7.80
-11	-13	-12	-6.40	9.50
-11	-13	-12	22.10	7.70
8	-14	-12	21.80	17.60
8	-14	-12	12.90	9.20
-8	14	12	12.40	11.30
-8	14	12	9.40	9.70
7	-14	-12	546.65	29.90
7	-14	-12	526.45	34.30
-7	14	12	495.25	30.30
6	-14	-12	299.87	35.30
6	-14	-12	306.07	27.40
6	-14	-12	308.77	29.90
6	-14	-12	298.27	31.20
5	-14	-12	174.68	21.40
5	-14	-12	142.39	27.00
5	-14	-12	166.68	24.50
5	-14	-12	143.79	16.20
-5	14	12	144.59	19.50
-5	14	12	120.99	17.50
4	-14	-12	87.19	14.00
4	-14	-12	66.59	24.20
4	-14	-12	105.29	16.10
4	-14	-12	91.09	12.70
4	-14	-12	75.59	12.70
-4	14	12	84.19	12.10
3	-14	-12	512.75	30.30
3	-14	-12	509.25	29.60
3	-14	-12	497.15	29.80
3	-14	-12	520.45	29.90
-3	14	12	510.95	25.90
2	-14	-12	65.09	10.20
2	-14	-12	63.09	10.90
2	-14	-12	72.99	11.10
2	-14	-12	85.79	11.60
-2	14	12	62.49	7.50
1	-14	-12	23.10	9.00
1	-14	-12	32.10	11.30
1	-14	-12	23.20	9.90
1	-14	-12	13.20	8.10
1	-14	-12	35.60	9.10
-1	14	12	23.60	4.10
0	-14	-12	26.20	9.90
0	-14	-12	22.10	8.30

0	-14	-12	4.20	12.60
0	-14	-12	20.70	7.90
0	14	12	23.50	3.70
-1	-14	-12	758.42	46.60
-1	-14	-12	841.02	41.40
-1	-14	-12	752.92	42.00
-1	-14	-12	793.02	40.50
1	14	12	849.91	37.00
-2	-14	-12	17.60	13.70
-2	-14	-12	3.10	8.90
-2	-14	-12	1.70	7.70
-2	-14	-12	-13.90	12.40
-2	-14	-12	11.60	7.60
2	14	12	10.30	2.90
-3	-14	-12	82.49	17.80
-3	-14	-12	81.39	12.40
-3	-14	-12	98.59	16.20
-3	-14	-12	83.09	14.50
-3	-14	-12	85.49	13.80
-3	-14	-12	72.49	10.60
3	14	12	69.29	5.70
-4	-14	-12	458.95	27.70
-4	-14	-12	389.96	31.30
-4	-14	-12	515.95	26.30
-4	-14	-12	462.15	26.30
-4	-14	-12	442.46	27.70
-4	-14	-12	492.25	27.10
4	14	12	482.05	22.00
-5	-14	-12	-12.70	8.80
-5	-14	-12	10.20	8.00
-5	-14	-12	-2.30	8.50
-5	-14	-12	11.40	9.20
-5	-14	-12	9.80	12.00
-5	-14	-12	3.80	8.00
5	14	12	2.50	5.30
-6	-14	-12	31.50	9.20
-6	-14	-12	66.59	9.30
-6	-14	-12	62.39	11.90
-6	-14	-12	63.59	10.80
-6	-14	-12	50.99	9.80
6	14	12	38.60	7.90
-7	-14	-12	267.57	20.40
-7	-14	-12	229.78	23.50
-7	-14	-12	291.37	19.70

-7	-14	-12	261.77	18.50
-7	-14	-12	307.57	19.20
-7	-14	-12	284.37	21.40
7	14	12	232.78	17.70
-8	-14	-12	25.60	14.60
-8	-14	-12	17.40	7.90
-8	-14	-12	33.40	12.20
-8	-14	-12	4.60	12.00
-8	-14	-12	10.50	14.60
8	14	12	-0.70	9.30
-9	-14	-12	213.18	16.10
-9	-14	-12	209.38	16.00
-9	-14	-12	197.58	16.60
-9	-14	-12	227.68	18.40
-10	-14	-12	9.00	7.70
-10	-14	-12	-12.90	8.90
-10	-14	-12	-7.40	7.60
-10	-14	-12	2.50	8.00
8	-15	-12	155.48	15.60
-8	15	12	166.88	18.30
-8	15	12	144.19	16.10
7	-15	-12	26.60	9.10
-7	15	12	32.80	10.30
6	-15	-12	194.38	33.70
6	-15	-12	176.68	18.20
6	-15	-12	190.58	25.40
-6	15	12	133.99	19.20
5	-15	-12	11.70	15.30
5	-15	-12	13.70	11.60
5	-15	-12	21.90	9.30
-5	15	12	35.00	13.40
-5	15	12	28.10	10.40
4	-15	-12	123.19	17.30
4	-15	-12	115.69	18.70
4	-15	-12	142.99	21.00
4	-15	-12	132.89	18.50
-4	15	12	154.68	14.20
3	-15	-12	-7.70	9.90
3	-15	-12	-11.70	9.50
3	-15	-12	-0.30	9.40
3	-15	-12	1.10	9.20
-3	15	12	7.70	5.90
2	-15	-12	101.79	15.90
2	-15	-12	84.39	12.20

2	-15	-12	94.29	16.80
2	-15	-12	73.39	11.90
-2	15	12	79.79	8.70
1	-15	-12	624.54	34.00
1	-15	-12	607.34	35.70
1	-15	-12	617.94	33.60
1	-15	-12	622.74	33.30
1	-15	-12	614.84	33.40
-1	15	12	618.54	29.70
0	-15	-12	-12.90	8.50
0	-15	-12	-10.80	13.20
0	-15	-12	1.20	8.80
0	-15	-12	-5.30	7.70
0	-15	-12	17.30	8.60
0	15	12	6.70	3.60
-1	-15	-12	99.49	13.60
-1	-15	-12	122.79	14.30
-1	-15	-12	79.59	13.30
-1	-15	-12	90.19	12.50
1	15	12	118.19	7.70
-2	-15	-12	1034.60	49.20
-2	-15	-12	982.40	49.40
-2	-15	-12	1020.00	49.20
-2	-15	-12	961.50	51.09
-2	-15	-12	1037.60	49.10
2	15	12	1013.80	46.10
-3	-15	-12	115.39	15.40
-3	-15	-12	113.39	14.10
-3	-15	-12	119.29	16.00
-3	-15	-12	92.69	13.20
-3	-15	-12	114.69	17.30
-3	-15	-12	122.69	14.50
3	15	12	110.29	7.50
-4	-15	-12	15.10	9.50
-4	-15	-12	15.30	10.10
-4	-15	-12	-3.30	8.50
-4	-15	-12	9.60	7.70
-4	-15	-12	14.10	8.20
4	15	12	13.00	4.20
-5	-15	-12	17.40	10.40
-5	-15	-12	12.70	9.20
-5	-15	-12	28.80	8.10
-5	-15	-12	27.80	8.50
-5	-15	-12	30.40	11.90

-5	-15	-12	10.20	8.80
5	15	12	17.00	6.40
-6	-15	-12	72.09	10.10
-6	-15	-12	72.69	10.70
-6	-15	-12	107.39	15.00
-6	-15	-12	68.79	10.90
-6	-15	-12	64.29	10.70
-7	-15	-12	-3.50	8.50
-7	-15	-12	1.60	8.20
-7	-15	-12	4.40	9.20
-7	-15	-12	7.40	8.90
-7	-15	-12	-4.30	9.00
7	15	12	2.20	8.70
-8	-15	-12	90.29	15.10
-8	-15	-12	99.99	13.70
-8	-15	-12	93.19	15.80
-8	-15	-12	118.89	14.90
8	15	12	72.29	11.80
-9	-15	-12	65.69	10.40
-9	-15	-12	89.59	12.90
-9	-15	-12	83.59	12.90
-9	-15	-12	90.29	13.20
-10	-15	-12	262.27	19.00
-10	-15	-12	282.27	18.20
-10	-15	-12	261.27	17.50
-10	-15	-12	266.67	17.90
8	-16	-12	114.09	12.60
-8	16	12	74.89	12.20
-8	16	12	102.89	14.40
7	-16	-12	-3.40	8.50
-7	16	12	4.10	9.90
6	-16	-12	364.76	39.70
6	-16	-12	322.57	21.40
6	-16	-12	335.47	27.70
-6	16	12	287.87	23.40
5	-16	-12	32.80	17.90
5	-16	-12	56.19	16.00
-5	16	12	38.10	15.40
-5	16	12	45.20	21.50
4	-16	-12	15.40	9.70
4	-16	-12	9.20	10.60
4	-16	-12	9.20	10.30
4	-16	-12	19.90	12.60
-4	16	12	8.70	9.30

3	-16	-12	231.28	20.70
3	-16	-12	193.18	19.60
3	-16	-12	207.58	20.30
3	-16	-12	217.18	19.90
-3	16	12	195.38	14.70
2	-16	-12	480.75	30.00
2	-16	-12	498.45	29.00
2	-16	-12	453.25	28.90
2	-16	-12	485.45	28.90
-2	16	12	471.45	24.60
1	-16	-12	202.98	18.40
1	-16	-12	225.98	19.80
1	-16	-12	239.18	18.50
1	-16	-12	199.68	18.20
1	-16	-12	217.28	22.70
-1	16	12	219.78	13.40
0	-16	-12	247.08	18.80
0	-16	-12	196.68	26.30
0	-16	-12	260.27	19.50
0	-16	-12	250.27	20.60
0	-16	-12	225.08	18.50
0	16	12	229.68	13.50
-1	-16	-12	30.30	13.80
-1	-16	-12	36.40	9.00
-1	-16	-12	60.89	19.10
-1	-16	-12	34.90	11.00
-1	-16	-12	45.40	9.60
-1	-16	-12	31.90	8.60
1	16	12	30.30	4.70
-2	-16	-12	332.87	20.80
-2	-16	-12	344.17	21.20
-2	-16	-12	296.37	23.20
-2	-16	-12	316.97	24.60
-2	-16	-12	308.57	21.10
2	16	12	310.27	16.20
-3	-16	-12	164.08	16.80
-3	-16	-12	142.29	15.20
-3	-16	-12	165.68	15.00
-3	-16	-12	153.58	16.10
-3	-16	-12	137.79	23.70
-3	-16	-12	156.08	17.80
3	16	12	160.78	9.90
-4	-16	-12	16.30	7.70
-4	-16	-12	21.90	9.70

-4	-16	-12	29.40	9.90
-4	-16	-12	3.20	7.70
-4	-16	-12	9.90	9.40
4	16	12	16.30	5.10
-5	-16	-12	55.49	9.00
-5	-16	-12	42.40	10.50
-5	-16	-12	46.30	12.40
-5	-16	-12	51.19	12.40
-5	-16	-12	41.40	10.80
-5	-16	-12	37.70	9.20
-6	-16	-12	12.30	8.30
-6	-16	-12	11.70	8.00
-6	-16	-12	-1.60	9.70
-6	-16	-12	37.10	9.40
-6	-16	-12	11.90	9.50
6	16	12	-2.10	8.50
-7	-16	-12	61.79	13.00
-7	-16	-12	42.80	9.40
-7	-16	-12	47.10	12.50
7	16	12	28.50	9.80
-8	-16	-12	518.55	28.00
-8	-16	-12	484.45	27.60
-8	-16	-12	479.85	27.40
-8	-16	-12	466.65	27.50
-8	-16	-12	515.45	28.40
-9	-16	-12	37.90	8.60
-9	-16	-12	25.60	12.90
-9	-16	-12	36.10	9.20
-9	-16	-12	31.50	9.00
-10	-16	-12	10.50	9.90
-10	-16	-12	14.40	8.30
-10	-16	-12	-1.90	7.70
-10	-16	-12	-2.80	7.20
7	-17	-12	39.70	8.80
-7	17	12	43.60	10.10
6	-17	-12	15.00	8.80
6	-17	-12	-4.00	13.60
-6	17	12	-4.40	9.50
5	-17	-12	330.27	22.10
5	-17	-12	365.76	26.00
5	-17	-12	310.87	30.00
-5	17	12	304.27	23.20
4	-17	-12	133.29	21.60
4	-17	-12	123.49	17.80

4	-17	-12	111.29	18.50
4	-17	-12	157.58	17.40
-4	17	12	150.78	18.50
3	-17	-12	129.59	18.60
3	-17	-12	118.39	17.30
3	-17	-12	125.79	17.40
3	-17	-12	119.59	17.30
-3	17	12	110.69	13.10
2	-17	-12	282.77	22.80
2	-17	-12	334.87	22.70
2	-17	-12	324.07	22.50
2	-17	-12	280.37	23.50
-2	17	12	295.77	17.80
1	-17	-12	16.00	9.20
1	-17	-12	-1.50	8.70
1	-17	-12	-4.20	8.30
1	-17	-12	-2.40	12.70
1	-17	-12	10.60	9.50
-1	17	12	5.10	5.20
0	-17	-12	319.97	21.70
0	-17	-12	326.77	21.30
0	-17	-12	347.67	23.40
0	-17	-12	315.47	30.40
0	-17	-12	300.07	22.00
0	17	12	313.17	17.30
-1	-17	-12	455.35	26.90
-1	-17	-12	514.25	28.10
-1	-17	-12	481.65	30.20
-1	-17	-12	461.15	27.00
1	17	12	496.05	23.90
-2	-17	-12	28.30	12.00
-2	-17	-12	13.10	8.40
-2	-17	-12	3.50	7.80
-2	-17	-12	11.70	7.90
-2	-17	-12	8.00	11.20
2	17	12	14.80	4.50
-3	-17	-12	1007.80	49.70
-3	-17	-12	970.50	48.40
-3	-17	-12	970.50	49.50
-3	-17	-12	1022.20	49.20
-3	-17	-12	1015.10	53.19
3	17	12	1008.60	46.30
-4	-17	-12	169.68	15.70
-4	-17	-12	171.88	18.20

-4	-17	-12	193.08	17.50
-4	-17	-12	151.58	17.20
-4	-17	-12	195.88	16.70
4	17	12	175.98	15.20
-5	-17	-12	167.98	16.30
-5	-17	-12	143.39	17.50
-5	-17	-12	171.18	20.50
-5	-17	-12	191.18	16.00
-5	-17	-12	144.39	21.60
-5	-17	-12	195.78	15.60
5	17	12	176.78	15.00
-6	-17	-12	48.90	10.40
-6	-17	-12	37.70	12.30
-6	-17	-12	44.00	10.00
-6	-17	-12	21.00	9.00
-6	-17	-12	49.90	9.10
6	17	12	18.30	9.80
-7	-17	-12	-1.70	7.80
-7	-17	-12	-0.30	8.80
-7	-17	-12	21.40	10.40
-7	-17	-12	20.80	9.40
7	17	12	5.80	10.60
-8	-17	-12	61.19	10.60
-8	-17	-12	72.69	10.50
-8	-17	-12	67.99	10.80
-8	-17	-12	64.39	9.50
-8	-17	-12	48.00	9.90
-9	-17	-12	349.37	20.40
-9	-17	-12	361.86	20.50
-9	-17	-12	332.27	20.10
-9	-17	-12	306.07	20.90
7	-18	-12	294.97	18.70
-7	18	12	271.17	28.90
-7	18	12	277.57	21.10
6	-18	-12	16.50	13.50
6	-18	-12	6.70	8.50
-6	18	12	0.70	9.70
5	-18	-12	183.88	20.40
5	-18	-12	154.48	16.30
5	-18	-12	189.68	26.80
-5	18	12	150.38	16.90
4	-18	-12	228.08	24.50
4	-18	-12	248.38	22.20
4	-18	-12	281.47	20.70

4	-18	-12	258.77	21.70
-4	18	12	180.58	26.60
3	-18	-12	338.57	24.40
3	-18	-12	298.27	22.70
3	-18	-12	313.67	23.00
3	-18	-12	305.47	22.80
-3	18	12	258.47	20.90
2	-18	-12	13.30	12.20
2	-18	-12	7.90	9.50
2	-18	-12	10.00	9.10
2	-18	-12	13.60	10.20
-2	18	12	6.50	7.20
1	-18	-12	12.40	13.50
1	-18	-12	2.90	8.60
1	-18	-12	4.60	9.10
1	-18	-12	4.40	9.60
1	-18	-12	6.10	8.60
-1	18	12	7.90	6.10
0	-18	-12	152.58	27.30
0	-18	-12	68.59	10.70
0	-18	-12	116.39	17.90
0	-18	-12	94.59	14.50
0	-18	-12	82.59	13.90
0	18	12	89.39	9.60
-1	-18	-12	423.66	25.20
-1	-18	-12	415.06	24.80
-1	-18	-12	402.46	27.50
-1	-18	-12	417.76	24.90
1	18	12	412.46	22.00
-2	-18	-12	66.59	13.40
-2	-18	-12	29.00	8.20
-2	-18	-12	38.30	9.20
-2	-18	-12	49.60	8.70
-2	-18	-12	52.59	11.50
2	18	12	29.40	5.80
-3	-18	-12	217.18	16.20
-3	-18	-12	192.28	18.50
-3	-18	-12	183.78	18.30
-3	-18	-12	184.38	26.70
-3	-18	-12	197.48	16.00
3	18	12	199.48	13.00
-4	-18	-12	177.68	18.00
-4	-18	-12	147.59	17.10
-4	-18	-12	145.79	15.40

-4	-18	-12	153.08	16.00
-4	-18	-12	160.78	14.60
4	18	12	175.38	13.50
-5	-18	-12	-1.40	7.60
-5	-18	-12	5.10	8.70
-5	-18	-12	18.60	11.60
-5	-18	-12	0.90	7.30
-5	-18	-12	5.60	9.20
5	18	12	6.60	8.60
-6	-18	-12	112.49	15.10
-6	-18	-12	117.79	18.20
-6	-18	-12	120.59	18.70
-6	-18	-12	119.79	16.10
-6	-18	-12	130.89	15.80
-6	-18	-12	120.69	14.60
6	18	12	122.89	17.30
-7	-18	-12	178.98	16.20
-7	-18	-12	177.08	17.40
-7	-18	-12	171.78	15.40
-7	-18	-12	176.88	16.20
-7	-18	-12	188.18	16.20
-8	-18	-12	33.80	9.60
-8	-18	-12	36.80	8.70
-8	-18	-12	39.80	8.40
-8	-18	-12	26.90	12.10
-8	-18	-12	19.50	8.80
-9	-18	-12	103.49	11.50
-9	-18	-12	109.79	11.20
-9	-18	-12	67.79	19.00
-9	-18	-12	108.89	12.60
6	-19	-12	40.50	9.10
6	-19	-12	60.49	17.90
-6	19	12	42.10	9.60
5	-19	-12	9.50	10.90
5	-19	-12	58.69	17.80
-5	19	12	21.10	9.00
4	-19	-12	61.49	14.30
4	-19	-12	56.99	11.00
4	-19	-12	63.39	11.40
4	-19	-12	52.49	11.00
3	-19	-12	31.80	11.50
3	-19	-12	23.10	10.60
3	-19	-12	50.49	12.00
3	-19	-12	52.69	11.20

-3	19	12	35.40	12.20
2	-19	-12	74.39	11.50
2	-19	-12	75.39	11.40
2	-19	-12	40.90	11.40
-2	19	12	70.09	10.00
1	-19	-12	137.39	24.50
1	-19	-12	142.19	15.50
1	-19	-12	170.78	16.80
1	-19	-12	127.19	15.70
1	-19	-12	161.98	18.50
-1	19	12	155.88	13.50
0	-19	-12	1.90	10.10
0	-19	-12	11.10	9.30
0	-19	-12	6.30	8.20
0	-19	-12	19.30	8.10
0	19	12	18.00	6.70
-1	-19	-12	-1.10	11.30
-1	-19	-12	-0.50	8.30
-1	-19	-12	5.40	7.80
-1	-19	-12	10.90	8.00
1	19	12	2.20	13.70
1	19	12	7.80	6.00
-2	-19	-12	802.92	39.80
-2	-19	-12	765.32	40.80
-2	-19	-12	792.32	39.90
-2	-19	-12	801.72	43.10
2	19	12	792.72	38.00
-3	-19	-12	79.69	12.80
-3	-19	-12	70.09	11.20
-3	-19	-12	75.59	17.30
-3	-19	-12	84.49	12.20
3	19	12	62.99	8.20
-4	-19	-12	5.80	7.60
-4	-19	-12	5.00	10.20
-4	-19	-12	7.50	9.50
-4	-19	-12	3.30	7.30
4	19	12	4.20	8.20
-5	-19	-12	6.10	9.00
-5	-19	-12	4.90	11.20
-5	-19	-12	-2.50	8.10
-5	-19	-12	6.80	9.80
5	19	12	10.10	9.60
-6	-19	-12	-28.30	14.30
-6	-19	-12	2.30	7.40

-6	-19	-12	-1.60	7.00
-6	-19	-12	15.00	8.70
-6	-19	-12	-2.30	8.50
-7	-19	-12	7.40	7.60
-7	-19	-12	19.60	8.30
-7	-19	-12	-0.70	8.60
-7	-19	-12	7.20	7.00
-8	-19	-12	15.50	6.90
-8	-19	-12	18.10	9.30
-8	-19	-12	18.70	8.90
-8	-19	-12	19.70	8.10
6	-20	-12	67.99	14.40
5	-20	-12	40.20	11.20
5	-20	-12	37.10	9.70
5	-20	-12	0.40	22.70
-5	20	12	3.00	8.80
4	-20	-12	23.60	13.00
4	-20	-12	21.00	9.50
4	-20	-12	12.20	10.20
-4	20	12	4.00	8.00
3	-20	-12	552.34	32.10
3	-20	-12	580.64	32.90
3	-20	-12	539.75	31.00
3	-20	-12	555.74	31.30
2	-20	-12	499.35	30.90
2	-20	-12	475.75	28.30
2	-20	-12	517.35	28.30
2	-20	-12	479.35	28.80
-2	20	12	395.96	28.00
1	-20	-12	33.20	18.80
1	-20	-12	45.30	16.80
-1	20	12	28.30	9.20
0	-20	-12	37.40	11.40
0	-20	-12	46.70	9.80
0	-20	-12	38.50	9.90
0	-20	-12	53.79	9.60
0	20	12	60.19	8.60
-1	-20	-12	0.20	11.40
-1	-20	-12	-13.40	7.80
-1	-20	-12	9.70	8.40
-1	-20	-12	-4.80	7.70
1	20	12	-17.20	13.50
1	20	12	-6.90	6.70
-2	-20	-12	15.40	20.00

-2	-20	-12	-7.50	7.40
-2	-20	-12	1.90	8.40
-2	-20	-12	-1.90	7.50
2	20	12	7.90	6.90
-3	-20	-12	116.79	15.60
-3	-20	-12	100.69	14.70
-3	-20	-12	125.19	19.60
-3	-20	-12	106.09	17.10
3	20	12	112.89	13.30
-4	-20	-12	0.60	9.50
-4	-20	-12	11.70	7.80
-4	-20	-12	3.90	7.50
-4	-20	-12	9.60	9.10
4	20	12	13.30	9.50
-5	-20	-12	47.50	9.30
-5	-20	-12	40.90	8.30
-5	-20	-12	50.09	8.50
-5	-20	-12	40.10	11.50
-6	-20	-12	19.40	6.90
-6	-20	-12	-4.60	14.20
-6	-20	-12	-2.40	7.90
-6	-20	-12	3.60	7.10
-7	-20	-12	204.28	15.20
-7	-20	-12	203.08	14.60
-7	-20	-12	197.08	15.00
-8	-20	-12	334.77	20.40
-8	-20	-12	330.57	18.60
-8	-20	-12	329.37	19.00
5	-21	-12	185.58	20.10
-5	21	12	170.58	15.70
4	-21	-12	37.60	11.00
4	-21	-12	62.79	14.60
4	-21	-12	44.70	9.90
-4	21	12	63.79	9.70
3	-21	-12	68.39	10.90
3	-21	-12	91.99	13.90
3	-21	-12	66.69	13.00
3	-21	-12	80.39	12.60
2	-21	-12	257.47	19.60
2	-21	-12	279.57	19.20
2	-21	-12	247.98	19.90
2	-21	-12	249.88	25.10
1	-21	-12	-6.00	8.30
1	-21	-12	-7.40	9.20

1	-21	-12	-6.30	9.60
0	-21	-12	-0.10	10.00
0	-21	-12	4.60	7.90
0	-21	-12	9.10	7.70
-1	-21	-12	247.98	18.80
-1	-21	-12	260.27	18.20
-1	-21	-12	241.78	21.40
-1	-21	-12	243.08	18.00
1	21	12	238.08	17.70
-2	-21	-12	43.00	8.60
-2	-21	-12	25.70	9.20
-2	-21	-12	61.29	13.80
-2	-21	-12	70.79	11.80
2	21	12	37.90	9.00
2	21	12	41.90	13.90
-3	-21	-12	620.64	32.50
-3	-21	-12	650.03	32.70
-3	-21	-12	652.93	40.00
-3	-21	-12	614.04	34.10
-4	-21	-12	16.10	7.00
-4	-21	-12	19.10	8.80
-4	-21	-12	12.30	6.80
-4	-21	-12	21.00	9.60
-5	-21	-12	74.59	10.80
-5	-21	-12	60.39	12.00
-5	-21	-12	60.79	9.40
-5	-21	-12	46.70	7.80
-6	-21	-12	-1.40	15.10
-6	-21	-12	30.60	7.10
-6	-21	-12	16.10	8.10
-6	-21	-12	25.30	6.90
-7	-21	-12	18.80	7.30
-7	-21	-12	18.40	6.20
-7	-21	-12	28.00	7.80
4	-22	-12	236.48	17.40
4	-22	-12	267.07	20.10
-4	22	12	250.77	17.00
3	-22	-12	83.09	23.00
3	-22	-12	83.19	13.20
3	-22	-12	75.99	12.30
3	-22	-12	95.39	13.80
2	-22	-12	41.10	9.60
2	-22	-12	41.30	9.00
2	-22	-12	37.40	10.30

1	-22	-12	24.30	8.10
1	-22	-12	10.20	8.50
1	-22	-12	-3.90	9.10
0	-22	-12	26.40	10.10
0	-22	-12	15.30	8.40
0	-22	-12	2.20	7.60
-1	-22	-12	266.57	21.00
-1	-22	-12	254.17	17.00
-1	-22	-12	246.88	18.00
-1	-22	-12	242.58	17.20
-2	-22	-12	-0.80	6.50
-2	-22	-12	-1.70	6.80
-2	-22	-12	-14.10	12.80
-2	-22	-12	15.00	8.40
-3	-22	-12	195.08	16.70
-3	-22	-12	174.78	14.10
-3	-22	-12	170.58	13.70
3	22	12	118.19	26.50
-4	-22	-12	241.88	16.50
-4	-22	-12	260.57	19.10
-4	-22	-12	266.17	17.00
-5	-22	-12	17.50	6.80
-5	-22	-12	18.50	10.40
-6	-22	-12	90.79	11.50
-6	-22	-12	90.89	9.60
-6	-22	-12	107.19	11.00
3	-23	-12	-7.70	11.10
3	-23	-12	-13.20	8.00
-3	23	12	2.70	7.10
2	-23	-12	70.89	11.00
2	-23	-12	72.19	12.60
2	-23	-12	53.59	9.70
1	-23	-12	16.80	8.60
1	-23	-12	28.10	7.80
1	-23	-12	31.40	9.20
0	-23	-12	22.00	9.90
0	-23	-12	19.50	8.20
0	-23	-12	10.60	7.70
-1	-23	-12	88.39	11.00
-1	-23	-12	56.19	8.60
-1	-23	-12	89.19	12.20
-1	-23	-12	66.19	12.40
-2	-23	-12	722.73	37.90
-2	-23	-12	801.12	41.50

-2	-23	-12	774.72	37.10
-2	-23	-12	723.13	37.00
-3	-23	-12	12.60	8.30
-3	-23	-12	-0.30	6.40
-3	-23	-12	4.10	6.20
3	23	12	-7.50	15.10
-4	-23	-12	8.40	8.60
-4	-23	-12	3.70	6.60
-4	-23	-12	15.70	6.40
4	23	12	21.80	14.70
-5	-23	-12	51.39	9.00
-5	-23	-12	49.30	7.10
-5	-23	-12	32.20	14.50
1	-24	-12	-5.40	8.30
1	-24	-12	-1.30	8.30
1	-24	-12	-2.60	7.40
0	-24	-12	66.99	11.10
0	-24	-12	68.09	11.40
0	-24	-12	72.09	12.10
-1	-24	-12	15.20	13.20
-1	-24	-12	5.60	7.40
-1	-24	-12	-4.30	6.60
-2	-24	-12	26.00	16.10
-2	-24	-12	18.90	6.40
-2	-24	-12	25.40	6.90
-2	-24	-12	3.80	7.70
-3	-24	-12	146.69	11.60
-3	-24	-12	126.99	13.60
-3	-24	-12	128.89	11.40
-4	-24	-12	67.99	9.10
-4	-24	-12	44.10	11.00
-4	-24	-12	55.99	9.00
1	18	-13	203.18	15.10
-1	-18	13	188.08	15.40
-1	-18	13	193.38	15.50
0	18	-13	-3.50	7.60
0	-18	13	2.10	10.40
0	-18	13	-20.80	17.40
1	-18	13	50.09	8.40
3	17	-13	41.50	7.80
-3	-17	13	32.10	6.50
2	17	-13	-1.30	6.90
-2	-17	13	2.00	6.70
-2	-17	13	-3.00	11.20

1	17	-13	98.89	12.40
-1	-17	13	85.19	12.40
-1	-17	13	112.69	13.10
0	17	-13	55.39	9.10
0	-17	13	55.29	14.40
0	-17	13	85.59	11.90
-1	17	-13	-2.10	15.30
-1	17	-13	-14.00	7.70
1	-17	13	12.50	7.30
-2	17	-13	304.17	24.60
-2	17	-13	314.17	20.70
2	-17	13	330.87	19.10
-3	17	-13	105.59	16.00
-3	17	-13	126.79	20.90
3	-17	13	101.09	12.00
4	16	-13	13.80	7.20
-4	-16	13	0.10	5.30
3	16	-13	396.46	21.90
-3	-16	13	365.46	20.80
2	16	-13	362.96	21.50
-2	-16	13	349.57	20.50
-2	-16	13	369.26	22.50
1	16	-13	130.89	13.80
-1	-16	13	126.89	14.30
-1	-16	13	131.79	13.90
0	16	-13	-3.70	7.20
0	-16	13	-3.10	7.20
0	-16	13	16.80	9.10
-1	16	-13	25.00	8.40
-1	16	-13	23.20	12.00
-2	16	-13	-1.80	8.30
-2	16	-13	18.60	14.60
-2	16	-13	0.60	10.60
-3	16	-13	24.30	10.50
-3	16	-13	26.10	9.80
-3	16	-13	1.20	13.70
3	-16	13	33.40	7.60
-4	16	-13	-6.30	10.00
-4	16	-13	-3.00	9.10
-4	16	-13	17.30	13.20
4	-16	13	7.90	7.20
-5	16	-13	164.48	15.00
-5	16	-13	143.99	19.50
5	-16	13	177.08	21.40

5	-16	13	173.48	14.90
5	15	-13	37.80	8.10
-5	-15	13	22.90	5.80
4	15	-13	97.09	12.50
-4	-15	13	97.29	10.50
3	15	-13	11.20	7.50
-3	-15	13	2.60	5.80
-3	-15	13	-11.80	11.60
2	15	-13	29.90	8.00
-2	-15	13	28.30	9.30
-2	-15	13	42.50	7.80
1	15	-13	3.40	7.70
-1	-15	13	20.40	7.50
-1	-15	13	24.80	8.20
0	15	-13	92.39	20.70
0	15	-13	84.39	13.90
0	-15	13	89.49	13.90
0	-15	13	80.49	14.00
-1	15	-13	18.10	15.00
-1	15	-13	14.70	8.10
-1	15	-13	-14.70	11.20
1	-15	13	-10.80	11.30
1	-15	13	9.40	7.40
-2	15	-13	80.19	11.30
-2	15	-13	53.59	9.60
-2	15	-13	63.89	14.70
2	-15	13	42.60	8.40
-3	15	-13	341.67	22.30
-3	15	-13	296.37	30.00
-3	15	-13	332.67	22.40
3	-15	13	360.76	21.00
-4	15	-13	18.60	12.00
-4	15	-13	13.90	10.20
-4	15	-13	23.10	9.30
4	-15	13	5.60	10.90
4	-15	13	12.40	8.10
-5	15	-13	96.69	13.80
-5	15	-13	87.09	13.10
-5	15	-13	81.29	13.40
5	-15	13	77.59	14.10
5	-15	13	117.59	13.70
-6	15	-13	134.29	16.30
-6	15	-13	180.38	21.60
-6	15	-13	187.48	15.70

6	-15	13	155.38	15.00
6	-15	13	143.69	16.40
6	14	-13	54.59	9.50
6	14	-13	56.79	14.00
-6	-14	13	67.99	9.20
5	14	-13	-4.20	7.50
5	14	-13	-12.20	13.20
-5	-14	13	-2.90	5.60
4	14	-13	206.48	16.60
4	14	-13	219.88	23.30
-4	-14	13	238.88	15.40
3	14	-13	73.79	15.30
3	14	-13	51.39	9.30
-3	-14	13	85.49	11.50
-3	-14	13	61.99	12.00
2	14	-13	261.07	19.60
-2	-14	13	276.57	20.20
-2	-14	13	289.27	18.30
1	14	-13	139.89	16.70
-1	-14	13	157.08	16.60
-1	-14	13	160.28	15.60
0	14	-13	13.80	13.40
0	14	-13	40.70	10.20
0	-14	13	37.80	9.40
0	-14	13	19.50	8.50
-1	14	-13	22.70	11.50
-1	14	-13	44.80	10.40
-1	14	-13	39.20	12.60
1	-14	13	41.50	9.40
1	-14	13	33.40	11.40
-2	14	-13	118.19	16.90
-2	14	-13	163.98	17.90
-2	14	-13	123.79	17.90
2	-14	13	117.49	15.30
-3	14	-13	62.79	12.40
-3	14	-13	62.39	10.90
-3	14	-13	36.90	10.30
3	-14	13	43.50	8.80
3	-14	13	76.99	13.00
-4	14	-13	199.68	19.00
-4	14	-13	216.88	18.40
-4	14	-13	215.38	19.30
4	-14	13	213.28	17.50
4	-14	13	207.38	19.60

-5	14	-13	28.60	11.30
-5	14	-13	16.50	11.20
-5	14	-13	21.80	9.40
5	-14	13	24.60	9.20
5	-14	13	13.40	8.90
-6	14	-13	58.89	14.00
-6	14	-13	69.09	12.00
-6	14	-13	43.90	9.70
6	-14	13	33.20	9.30
6	-14	13	72.19	12.00
7	13	-13	60.19	12.10
7	13	-13	50.09	10.80
-7	-13	13	67.49	9.10
6	13	-13	6.70	11.30
6	13	-13	6.70	8.20
-6	-13	13	7.20	6.00
5	13	-13	9.70	7.80
5	13	-13	-16.20	11.10
-5	-13	13	8.20	5.80
4	13	-13	45.20	12.30
4	13	-13	11.50	8.30
-4	-13	13	16.80	6.80
3	13	-13	32.30	9.20
3	13	-13	23.10	12.90
-3	-13	13	18.90	7.70
-3	-13	13	12.70	10.30
2	13	-13	4.90	14.90
2	13	-13	6.30	8.20
-2	-13	13	17.20	10.40
1	13	-13	149.79	16.80
-1	-13	13	164.18	16.00
-1	-13	13	196.08	17.20
0	13	-13	168.68	24.20
0	13	-13	183.08	17.60
0	-13	13	179.38	16.70
0	-13	13	197.78	17.00
-1	13	-13	46.40	9.90
-1	13	-13	49.00	13.30
-1	13	-13	52.99	12.00
1	-13	13	57.79	11.10
1	-13	13	46.90	8.90
-2	13	-13	272.37	20.90
-2	13	-13	253.87	20.70
2	-13	13	264.37	19.40

2	-13	13	253.17	28.10
-3	13	-13	-2.40	10.50
-3	13	-13	-16.70	11.70
-3	13	-13	-16.50	10.00
3	-13	13	3.10	10.30
3	-13	13	-17.70	11.10
-4	13	-13	230.18	19.50
-4	13	-13	239.78	19.40
-4	13	-13	263.87	21.00
4	-13	13	245.08	19.50
4	-13	13	236.78	19.20
-5	13	-13	42.50	11.80
-5	13	-13	49.10	11.10
-5	13	-13	30.80	10.10
5	-13	13	38.80	9.30
5	-13	13	20.50	9.20
-6	13	-13	41.30	9.70
-6	13	-13	61.89	12.00
-6	13	-13	30.70	13.10
6	-13	13	63.29	11.20
6	-13	13	31.20	8.60
-7	13	-13	101.19	15.80
-7	13	-13	78.49	13.90
-7	13	-13	71.39	10.50
7	-13	13	87.99	12.10
7	12	-13	16.90	9.70
-7	-12	13	-0.20	9.40
-7	-12	13	-1.30	6.40
6	12	-13	67.89	11.80
6	12	-13	86.39	13.80
-6	-12	13	63.99	10.40
-6	-12	13	83.39	10.40
5	12	-13	14.70	10.60
5	12	-13	9.60	8.40
-5	-12	13	32.00	15.80
-5	-12	13	4.00	6.50
4	12	-13	55.19	12.40
4	12	-13	41.50	10.00
-4	-12	13	31.60	8.40
3	12	-13	466.55	26.80
3	12	-13	452.05	31.40
-3	-12	13	454.25	34.80
-3	-12	13	466.95	26.00
2	12	-13	108.89	18.80

2	12	-13	117.09	14.60
-2	-12	13	96.79	15.40
-2	-12	13	89.69	13.30
1	12	-13	226.48	23.10
1	12	-13	223.38	18.50
-1	-12	13	229.68	16.80
-1	-12	13	200.68	17.90
0	12	-13	132.89	21.90
0	12	-13	189.88	23.20
0	12	-13	145.89	15.70
0	-12	13	150.38	15.50
0	-12	13	133.59	15.30
-1	12	-13	166.68	18.30
-1	12	-13	164.18	17.20
-1	12	-13	123.39	20.00
1	-12	13	197.88	16.60
1	-12	13	169.98	17.90
-2	12	-13	28.30	9.10
-2	12	-13	7.60	9.80
-2	12	-13	1.70	9.90
2	-12	13	-5.80	6.90
-3	12	-13	16.40	10.00
-3	12	-13	14.80	9.60
-3	12	-13	3.10	10.10
3	-12	13	11.70	7.80
-4	12	-13	29.60	10.40
-4	12	-13	26.60	17.40
-4	12	-13	45.10	16.40
4	-12	13	35.80	15.20
-5	12	-13	366.26	24.10
-5	12	-13	371.16	25.60
-5	12	-13	335.27	23.30
5	-12	13	327.47	22.70
5	-12	13	332.57	23.40
-6	12	-13	13.10	9.50
-6	12	-13	-1.30	10.30
-6	12	-13	4.50	12.50
6	-12	13	12.90	10.20
6	-12	13	12.70	8.20
-7	12	-13	40.60	11.10
-7	12	-13	41.40	9.70
-7	12	-13	58.69	13.10
7	-12	13	29.00	8.20
-8	12	-13	151.88	17.10

-8	12	-13	216.98	31.80
-8	12	-13	191.58	17.20
8	-12	13	176.78	14.20
8	11	-13	126.59	23.80
-8	-11	13	182.98	13.80
-8	-11	13	195.78	15.10
7	11	-13	147.99	16.20
7	11	-13	158.98	17.30
-7	-11	13	135.09	13.60
-7	-11	13	142.59	12.80
6	11	-13	2.60	9.90
6	11	-13	-2.50	8.60
-6	-11	13	7.00	6.20
-6	-11	13	-1.20	8.50
5	11	-13	82.29	14.50
5	11	-13	101.89	17.60
-5	-11	13	98.29	12.50
-5	-11	13	102.99	16.80
4	11	-13	24.30	9.10
-4	-11	13	37.70	8.30
3	11	-13	9.10	10.00
3	11	-13	11.70	8.90
-3	-11	13	1.10	9.50
-3	-11	13	1.30	6.80
2	11	-13	49.00	9.30
2	11	-13	63.09	12.10
-2	-11	13	41.30	8.30
-2	-11	13	63.59	10.20
1	11	-13	30.60	10.90
1	11	-13	17.30	9.00
-1	-11	13	21.30	8.10
-1	-11	13	22.80	7.40
0	11	-13	9.00	12.10
0	11	-13	17.40	8.60
0	-11	13	10.30	7.50
0	-11	13	10.40	7.50
-1	11	-13	-5.20	12.40
-1	11	-13	5.00	19.80
-1	11	-13	-3.50	8.30
-1	11	-13	16.50	11.40
1	-11	13	-1.50	7.10
1	-11	13	-15.80	8.70
-2	11	-13	164.18	17.50
-2	11	-13	115.19	18.00

-2	11	-13	121.79	18.40
-2	11	-13	135.19	17.20
2	-11	13	129.79	15.20
-3	11	-13	395.06	25.00
-3	11	-13	362.06	24.40
-3	11	-13	344.27	24.20
3	-11	13	395.46	24.10
-4	11	-13	12.10	9.80
-4	11	-13	-3.70	9.00
-4	11	-13	5.40	10.50
4	-11	13	-14.00	7.90
-5	11	-13	28.30	10.50
-6	11	-13	535.75	32.00
-6	11	-13	495.15	29.40
-6	11	-13	454.95	29.00
6	-11	13	482.75	28.20
6	-11	13	484.85	29.00
-7	11	-13	104.79	15.20
-7	11	-13	118.19	15.60
-7	11	-13	142.79	20.70
7	-11	13	97.19	17.00
7	-11	13	118.59	14.50
-8	11	-13	6.00	8.80
-8	11	-13	17.50	10.90
-8	11	-13	-6.90	20.00
8	-11	13	13.90	7.20
8	10	-13	12.20	9.40
-8	-10	13	20.70	10.80
-8	-10	13	7.10	6.60
7	10	-13	106.19	15.50
7	10	-13	113.39	17.30
-7	-10	13	101.99	12.10
6	10	-13	76.29	12.00
6	10	-13	63.09	10.60
-6	-10	13	68.29	9.50
-6	-10	13	80.69	11.60
5	10	-13	41.30	12.10
-5	-10	13	40.40	8.20
-5	-10	13	50.79	12.60
4	10	-13	138.69	15.90
4	10	-13	131.49	17.50
-4	-10	13	132.79	13.40
-4	-10	13	186.28	22.60
-4	-10	13	138.69	18.70

3	10	-13	3.20	8.00
3	10	-13	12.90	10.00
-3	-10	13	9.90	9.10
-3	-10	13	3.40	7.30
2	10	-13	642.24	33.90
2	10	-13	615.74	34.70
-2	-10	13	612.14	32.80
-2	-10	13	616.94	33.50
1	10	-13	41.50	8.70
1	10	-13	32.80	10.60
-1	-10	13	16.10	7.50
-1	-10	13	35.40	9.20
0	10	-13	8.50	10.60
0	10	-13	10.30	8.20
0	-10	13	16.50	7.70
0	-10	13	14.80	8.20
-1	10	-13	327.77	22.90
-1	10	-13	315.87	25.00
-1	10	-13	371.36	23.30
-1	10	-13	322.57	25.00
1	-10	13	347.67	22.10
1	-10	13	317.57	22.50
-2	10	-13	403.16	30.00
-2	10	-13	373.86	26.50
-2	10	-13	386.56	25.10
-2	10	-13	428.76	26.50
2	-10	13	454.15	25.10
-3	10	-13	39.90	15.20
-3	10	-13	34.10	10.70
-3	10	-13	13.00	9.80
3	-10	13	35.00	8.00
-4	10	-13	285.57	22.50
-4	10	-13	274.17	21.00
-4	10	-13	221.78	19.80
4	-10	13	249.38	19.40
-5	10	-13	14.10	11.40
-5	10	-13	20.50	9.60
5	-10	13	12.80	9.20
-6	10	-13	90.19	13.80
-6	10	-13	102.09	17.30
-6	10	-13	75.99	12.40
6	-10	13	84.29	12.60
-7	10	-13	148.49	21.90
-7	10	-13	106.89	15.80

-7	10	-13	98.69	16.00
7	-10	13	177.08	24.30
7	-10	13	132.99	14.70
-8	10	-13	50.49	11.40
-8	10	-13	61.99	13.70
-8	10	-13	71.69	11.00
8	-10	13	61.89	9.50
-9	10	-13	21.30	22.60
-9	10	-13	33.20	11.70
-9	10	-13	15.60	8.90
9	-10	13	10.00	7.50
8	9	-13	129.29	15.70
-8	-9	13	108.39	12.10
-8	-9	13	121.29	13.70
7	9	-13	223.58	19.00
7	9	-13	215.08	20.80
-7	-9	13	237.28	16.70
-7	-9	13	226.88	16.30
6	9	-13	12.60	11.50
6	9	-13	3.50	10.00
-6	-9	13	4.60	7.40
-6	-9	13	9.30	7.70
5	9	-13	-11.00	9.50
5	9	-13	4.40	9.10
-5	-9	13	11.90	6.90
-5	-9	13	1.30	7.80
4	9	-13	58.89	9.90
4	9	-13	91.19	12.50
-4	-9	13	61.69	12.60
-4	-9	13	48.10	9.80
-4	-9	13	68.99	11.80
3	9	-13	130.89	17.30
3	9	-13	126.49	15.50
-3	-9	13	110.49	13.30
-3	-9	13	141.59	16.90
2	9	-13	87.19	14.00
2	9	-13	103.29	16.00
-2	-9	13	67.29	9.50
1	9	-13	275.37	20.20
1	9	-13	253.37	21.20
-1	-9	13	296.87	18.60
0	9	-13	119.19	16.80
0	9	-13	113.59	15.20
0	-9	13	106.39	13.60

-1	9	-13	22.40	12.20
-1	9	-13	29.10	8.70
-1	9	-13	50.49	11.50
-1	9	-13	34.60	9.90
1	-9	13	25.10	9.00
-2	9	-13	243.38	19.10
-2	9	-13	234.28	21.50
-2	9	-13	252.17	20.50
-2	9	-13	288.37	23.90
2	-9	13	289.27	19.20
-3	9	-13	91.99	15.70
-3	9	-13	82.79	12.10
-3	9	-13	78.69	12.20
-3	9	-13	85.79	11.80
3	-9	13	103.29	14.40
-4	9	-13	149.09	21.80
-4	9	-13	172.38	18.00
-4	9	-13	224.18	30.10
-4	9	-13	157.08	20.70
4	-9	13	181.38	17.50
-5	9	-13	230.38	23.10
-5	9	-13	211.58	20.70
-5	9	-13	215.18	30.30
-5	9	-13	200.18	18.80
-5	9	-13	179.38	26.40
5	-9	13	224.78	19.50
-6	9	-13	6.90	20.80
-6	9	-13	8.10	10.20
-6	9	-13	6.10	12.70
-6	9	-13	26.20	10.00
6	-9	13	11.70	11.30
-7	9	-13	305.07	22.80
-7	9	-13	296.67	21.80
-7	9	-13	298.47	27.80
7	-9	13	349.77	21.80
7	-9	13	280.57	30.00
-8	9	-13	8.60	10.10
-8	9	-13	24.30	9.30
-8	9	-13	15.80	14.30
8	-9	13	7.10	7.80
-9	9	-13	128.19	14.90
-9	9	-13	119.49	17.00
-9	9	-13	149.69	19.00
9	-9	13	141.19	14.20

9	8	-13	94.99	14.70
-9	-8	13	98.29	13.40
-8	-8	13	43.30	9.00
-8	-8	13	66.49	11.00
7	8	-13	7.90	12.10
7	8	-13	2.20	10.30
-7	-8	13	11.20	7.00
-7	-8	13	11.50	7.20
6	8	-13	195.98	19.70
-6	-8	13	220.28	16.80
-6	-8	13	212.18	22.90
5	8	-13	-1.50	9.30
5	8	-13	9.60	9.50
-5	-8	13	4.60	6.50
-5	-8	13	13.60	8.00
4	8	-13	323.97	22.00
4	8	-13	308.97	23.40
-4	-8	13	319.57	25.20
-4	-8	13	305.67	21.20
-4	-8	13	316.37	20.20
3	8	-13	499.65	28.80
3	8	-13	494.25	29.80
-3	-8	13	523.25	27.70
2	8	-13	-4.60	8.00
2	8	-13	-6.20	8.40
-2	-8	13	0.00	5.80
1	8	-13	553.54	31.20
1	8	-13	541.85	30.50
-1	-8	13	565.24	29.60
0	8	-13	320.07	31.60
0	8	-13	253.77	20.60
0	8	-13	272.37	19.90
0	-8	13	259.77	18.30
-1	8	-13	151.48	18.40
-1	8	-13	151.48	15.60
-1	8	-13	153.18	22.80
-1	8	-13	123.19	15.80
1	-8	13	155.38	15.80
1	-8	13	141.89	16.50
-2	8	-13	15.60	11.10
-2	8	-13	-2.30	10.70
-2	8	-13	-6.10	7.70
-2	8	-13	-6.70	7.80
-2	8	-13	2.30	10.50

2	-8	13	9.80	9.20
-3	8	-13	-15.80	9.40
-3	8	-13	-5.50	7.80
-3	8	-13	16.60	15.30
-3	8	-13	15.20	9.90
-3	8	-13	15.30	12.50
3	-8	13	1.00	7.10
3	-8	13	4.90	10.30
-4	8	-13	153.18	19.20
-4	8	-13	129.19	17.30
-4	8	-13	122.59	15.30
-4	8	-13	110.39	16.10
-4	8	-13	137.89	17.90
4	-8	13	131.29	15.70
4	-8	13	93.79	15.00
-5	8	-13	340.67	32.50
-5	8	-13	345.17	28.90
-5	8	-13	361.46	28.00
-5	8	-13	368.66	26.20
-5	8	-13	373.26	25.30
5	-8	13	333.97	23.60
-6	8	-13	1.60	10.10
-6	8	-13	34.70	14.90
-6	8	-13	-3.00	9.60
-6	8	-13	-2.60	12.80
-6	8	-13	13.70	12.30
6	-8	13	8.50	10.60
-7	8	-13	96.99	30.90
-7	8	-13	73.79	11.50
-7	8	-13	56.99	20.10
-8	8	-13	154.88	24.80
-8	8	-13	154.98	16.70
-8	8	-13	143.59	16.50
8	-8	13	150.98	16.30
-9	8	-13	18.40	14.50
-9	8	-13	3.30	8.70
-9	8	-13	33.80	11.40
9	-8	13	9.90	8.40
9	7	-13	6.80	9.50
-9	-7	13	3.70	6.80
8	7	-13	500.65	28.00
-8	-7	13	467.65	25.50
-8	-7	13	460.75	27.00
7	7	-13	149.39	18.00

-7	-7	13	121.19	13.00
-7	-7	13	97.29	14.00
6	7	-13	-15.10	9.00
6	7	-13	19.90	11.50
-6	-7	13	0.60	6.60
-6	-7	13	6.80	7.00
5	7	-13	415.36	26.30
5	7	-13	365.86	25.90
-5	-7	13	401.66	23.10
-5	-7	13	391.76	23.30
4	7	-13	147.99	17.60
4	7	-13	100.69	16.40
-4	-7	13	123.99	15.60
-4	-7	13	133.49	13.00
3	7	-13	19.40	8.70
3	7	-13	21.30	9.40
-3	-7	13	19.40	7.00
2	7	-13	53.09	9.20
2	7	-13	69.29	11.00
-2	-7	13	55.89	8.20
1	7	-13	7.60	8.90
1	7	-13	13.50	7.90
-1	-7	13	3.40	6.10
0	7	-13	316.07	21.60
0	7	-13	323.07	28.70
0	-7	13	347.47	25.00
0	-7	13	314.07	20.60
-1	7	-13	22.10	9.10
-1	7	-13	28.50	11.50
-1	7	-13	33.20	9.10
1	-7	13	30.30	8.40
1	-7	13	40.90	7.70
-2	7	-13	248.28	18.90
-2	7	-13	241.78	21.40
-2	7	-13	212.18	28.70
-2	7	-13	248.98	22.00
-2	7	-13	257.17	17.70
-2	7	-13	192.78	26.70
2	-7	13	204.18	16.90
-3	7	-13	138.59	17.30
-3	7	-13	131.89	17.10
-3	7	-13	106.49	13.90
-3	7	-13	158.88	21.70
-3	7	-13	110.59	17.40

3	-7	13	132.19	15.90
-4	7	-13	169.38	19.70
-4	7	-13	163.58	21.10
-4	7	-13	172.78	25.00
-4	7	-13	133.29	17.70
-4	7	-13	135.79	15.00
4	-7	13	136.89	18.90
4	-7	13	146.29	15.90
-5	7	-13	7.00	11.40
-5	7	-13	27.40	12.50
-5	7	-13	14.20	14.00
-5	7	-13	-3.20	10.10
-5	7	-13	9.00	8.60
5	-7	13	-20.30	12.50
5	-7	13	9.80	8.60
-6	7	-13	1076.59	52.19
-6	7	-13	1060.79	56.29
-6	7	-13	1050.09	54.59
-6	7	-13	1056.89	52.49
-6	7	-13	1040.10	56.19
6	-7	13	1008.60	52.99
6	-7	13	1015.20	56.69
-7	7	-13	80.19	12.30
-7	7	-13	58.69	16.10
-7	7	-13	60.29	13.30
-7	7	-13	51.19	17.80
-7	7	-13	96.59	14.30
-8	7	-13	4.40	10.00
-8	7	-13	-18.40	14.80
-8	7	-13	6.30	26.40
-8	7	-13	12.10	9.50
8	-7	13	18.50	8.90
-9	7	-13	21.90	11.20
-9	7	-13	-4.80	8.80
-9	7	-13	10.90	14.40
9	-7	13	-1.60	8.80
-10	7	-13	62.89	16.30
-10	7	-13	1.70	8.60
-10	7	-13	15.00	11.70
10	-7	13	23.30	10.70
9	6	-13	148.49	16.80
-9	-6	13	153.38	12.60
8	6	-13	94.59	16.10
-8	-6	13	50.69	10.00

-8	-6	13	64.49	10.90
-7	-6	13	93.89	15.80
-7	-6	13	71.99	12.00
6	6	-13	101.09	14.00
6	6	-13	85.19	12.60
-6	-6	13	93.59	13.00
-6	-6	13	127.79	13.10
5	6	-13	62.79	12.00
5	6	-13	32.10	10.20
-5	-6	13	57.09	8.30
-5	-6	13	61.79	8.70
4	6	-13	12.70	9.10
4	6	-13	-0.70	9.60
-4	-6	13	8.70	7.70
-4	-6	13	-1.00	5.80
3	6	-13	94.79	11.90
3	6	-13	107.49	16.80
-3	-6	13	80.69	11.10
2	6	-13	602.24	33.40
-2	-6	13	612.74	31.30
1	6	-13	-2.30	14.50
1	6	-13	22.40	8.30
1	6	-13	15.40	8.80
-1	-6	13	5.90	6.40
-1	-6	13	23.40	7.20
0	6	-13	65.29	9.80
0	6	-13	66.49	13.20
0	6	-13	53.99	12.70
0	6	-13	64.89	10.30
0	-6	13	68.19	10.90
0	-6	13	66.09	11.50
-1	6	-13	922.61	48.00
-1	6	-13	982.60	47.20
-1	6	-13	933.21	46.70
-1	6	-13	957.40	46.10
-1	6	-13	846.72	48.10
1	-6	13	897.21	45.20
1	-6	13	922.91	44.20
-2	6	-13	81.09	11.70
-2	6	-13	92.29	13.20
-2	6	-13	117.49	17.70
-2	6	-13	109.49	14.90
-2	6	-13	81.99	13.20
2	-6	13	85.49	11.80

-3	6	-13	24.40	7.60
-3	6	-13	19.20	8.90
-3	6	-13	9.10	12.60
-3	6	-13	35.10	9.50
-3	6	-13	27.50	10.60
3	-6	13	14.30	7.70
-4	6	-13	371.26	25.50
-4	6	-13	368.86	28.90
-4	6	-13	357.06	25.00
-4	6	-13	328.17	24.90
-4	6	-13	375.16	26.80
-4	6	-13	342.37	21.50
4	-6	13	333.37	23.00
-5	6	-13	96.29	14.20
-5	6	-13	132.39	17.90
-5	6	-13	104.89	19.30
-5	6	-13	106.19	16.10
-5	6	-13	102.29	14.40
5	-6	13	87.69	11.90
5	-6	13	114.79	18.20
-6	6	-13	4.70	9.60
-6	6	-13	9.20	14.70
-6	6	-13	8.40	11.50
-6	6	-13	3.30	13.00
6	-6	13	1.50	13.70
6	-6	13	21.90	11.70
-7	6	-13	101.59	16.00
-7	6	-13	100.29	15.10
-7	6	-13	108.39	17.40
-7	6	-13	110.99	20.60
-7	6	-13	110.69	17.60
7	-6	13	136.39	20.80
-8	6	-13	46.00	16.70
-8	6	-13	49.40	11.80
-8	6	-13	32.40	10.20
-8	6	-13	38.50	17.30
-9	6	-13	-11.90	14.60
-9	6	-13	8.30	9.90
-9	6	-13	29.50	20.80
9	-6	13	2.10	9.60
-10	6	-13	3.60	8.80
-10	6	-13	31.00	19.30
-10	6	-13	15.60	11.20
10	-6	13	30.50	11.20

9	5	-13	196.08	18.60
-9	-5	13	183.08	13.70
8	5	-13	13.30	10.50
-8	-5	13	23.00	9.30
-8	-5	13	21.90	6.90
7	5	-13	234.68	22.20
-7	-5	13	253.77	19.10
-7	-5	13	250.37	17.30
6	5	-13	46.30	12.00
6	5	-13	58.49	15.00
-6	-5	13	49.30	8.40
-6	-5	13	78.39	11.80
5	5	-13	-12.00	11.70
5	5	-13	11.70	13.40
5	5	-13	12.90	10.60
-5	-5	13	-2.60	6.40
4	5	-13	148.09	17.10
4	5	-13	160.48	18.80
4	5	-13	147.29	19.40
-4	-5	13	152.18	15.00
-4	-5	13	175.38	13.70
3	5	-13	15.60	9.60
3	5	-13	-5.60	11.90
3	5	-13	20.60	12.40
-3	-5	13	2.00	5.80
-3	-5	13	7.40	8.50
2	5	-13	26.50	12.50
2	5	-13	44.70	10.00
2	5	-13	39.80	9.50
-2	-5	13	20.60	8.50
-2	-5	13	56.39	10.00
1	5	-13	470.95	35.30
1	5	-13	512.35	34.10
1	5	-13	555.54	30.20
1	5	-13	552.54	31.10
-1	-5	13	569.44	28.40
-1	-5	13	578.74	28.20
0	5	-13	111.19	19.90
0	5	-13	133.89	22.20
0	5	-13	140.99	16.00
0	5	-13	118.59	14.80
0	-5	13	112.09	11.20
0	-5	13	95.39	12.30
-1	5	-13	-8.00	9.40

-1	5	-13	-14.10	10.20
-1	5	-13	-4.70	7.10
-1	5	-13	6.90	9.30
1	-5	13	-3.20	5.00
1	-5	13	-10.70	7.10
-2	5	-13	221.68	20.30
-2	5	-13	189.88	15.90
-2	5	-13	221.78	21.00
-2	5	-13	209.48	16.90
-2	5	-13	158.28	20.70
2	-5	13	216.88	14.70
-3	5	-13	273.87	18.50
-3	5	-13	303.17	26.00
-3	5	-13	297.77	22.80
-3	5	-13	317.67	22.70
-3	5	-13	286.17	24.80
-3	5	-13	287.87	22.40
3	-5	13	289.47	18.60
-4	5	-13	4.10	8.40
-4	5	-13	3.90	14.70
-4	5	-13	-4.60	6.30
-4	5	-13	-9.40	8.80
-4	5	-13	2.60	12.80
4	-5	13	4.50	7.40
-5	5	-13	239.48	22.10
-5	5	-13	276.57	22.60
-5	5	-13	254.47	18.10
-5	5	-13	212.18	28.00
-5	5	-13	294.47	21.10
5	-5	13	228.38	19.20
5	-5	13	222.88	20.90
-6	5	-13	285.67	25.80
-6	5	-13	258.77	19.50
-6	5	-13	278.17	21.40
-6	5	-13	271.77	24.10
-6	5	-13	247.58	27.90
6	-5	13	202.38	24.30
6	-5	13	273.77	22.70
-7	5	-13	493.25	33.40
-7	5	-13	437.26	27.40
-7	5	-13	433.26	26.40
-7	5	-13	423.46	30.10
-7	5	-13	492.25	33.40
7	-5	13	419.46	32.70

-8	5	-13	30.30	10.00
-8	5	-13	26.80	9.60
-8	5	-13	32.70	15.40
-8	5	-13	16.70	12.90
8	-5	13	18.10	21.20
-9	5	-13	64.19	11.00
-9	5	-13	59.59	11.50
-9	5	-13	58.89	17.40
-9	5	-13	52.59	15.80
9	-5	13	63.19	12.10
-10	5	-13	39.80	14.60
-10	5	-13	25.20	11.10
-10	5	-13	24.40	9.60
10	-5	13	35.00	12.30
10	4	-13	59.79	19.90
-10	-4	13	32.30	7.20
9	4	-13	147.69	17.70
9	4	-13	156.78	27.30
-9	-4	13	151.68	12.70
8	4	-13	5.30	15.40
8	4	-13	21.00	11.30
-8	-4	13	9.10	6.60
-8	-4	13	1.60	8.70
7	4	-13	68.49	12.10
7	4	-13	65.69	15.80
-7	-4	13	75.79	10.30
-7	-4	13	73.99	11.60
6	4	-13	231.48	24.30
6	4	-13	212.18	22.30
-6	-4	13	210.88	15.90
-6	-4	13	217.88	16.90
5	4	-13	196.18	21.40
5	4	-13	223.38	27.00
5	4	-13	186.38	22.50
-5	-4	13	222.38	15.60
-5	-4	13	195.28	15.50
4	4	-13	299.87	23.80
4	4	-13	297.47	24.40
-4	-4	13	307.47	19.40
-4	-4	13	307.07	18.70
3	4	-13	121.79	21.40
3	4	-13	182.88	19.00
3	4	-13	197.78	22.80
3	4	-13	224.28	25.90

-3	-4	13	140.49	18.90
-3	-4	13	155.18	15.80
2	4	-13	232.98	19.20
2	4	-13	203.38	22.00
2	4	-13	206.18	18.60
2	4	-13	232.68	22.10
-2	-4	13	215.78	16.50
1	4	-13	117.29	21.20
1	4	-13	132.59	19.60
1	4	-13	138.39	15.90
1	4	-13	136.89	18.10
1	4	-13	141.99	16.80
-1	-4	13	149.49	12.30
0	4	-13	658.03	34.70
0	4	-13	617.24	37.30
0	4	-13	597.34	36.10
0	4	-13	652.03	36.10
0	4	-13	688.63	35.70
0	-4	13	737.03	32.60
-1	4	-13	180.38	19.30
-1	4	-13	217.98	18.40
-1	4	-13	196.38	20.40
-1	4	-13	212.88	17.10
1	-4	13	181.38	16.40
1	-4	13	203.48	13.20
-2	4	-13	134.19	18.20
-2	4	-13	174.48	19.60
-2	4	-13	166.88	15.20
-2	4	-13	170.28	14.30
-2	4	-13	150.58	18.90
2	-4	13	169.38	12.30
-3	4	-13	3.20	10.80
-3	4	-13	-9.00	10.60
-3	4	-13	7.60	6.40
-3	4	-13	-3.00	8.60
-3	4	-13	4.80	12.20
-3	4	-13	-7.10	7.30
3	-4	13	-0.80	4.90
-4	4	-13	193.38	24.30
-4	4	-13	192.08	20.00
-4	4	-13	201.38	21.20
-4	4	-13	164.18	14.30
4	-4	13	186.58	14.20
-5	4	-13	392.16	24.60

-5	4	-13	352.56	26.30
-5	4	-13	385.16	26.60
-5	4	-13	366.96	25.50
-5	4	-13	387.76	22.50
-5	4	-13	350.26	31.80
5	-4	13	372.16	22.30
-6	4	-13	74.79	12.80
-6	4	-13	69.89	15.10
-6	4	-13	102.29	13.00
-6	4	-13	91.29	15.20
-6	4	-13	113.79	19.40
6	-4	13	86.39	15.00
6	-4	13	76.69	13.90
-7	4	-13	107.09	20.70
-7	4	-13	171.98	22.50
-7	4	-13	163.28	24.90
-7	4	-13	137.69	16.10
-7	4	-13	125.49	16.50
-8	4	-13	61.09	10.80
-8	4	-13	45.30	10.10
-8	4	-13	44.50	12.70
-8	4	-13	35.70	14.10
-8	4	-13	26.20	14.00
8	-4	13	31.40	17.80
-9	4	-13	-11.50	9.20
-9	4	-13	2.70	15.40
-9	4	-13	-2.30	14.90
-9	4	-13	-0.10	8.80
9	-4	13	14.20	12.00
-10	4	-13	86.49	17.90
-10	4	-13	102.99	16.20
-10	4	-13	79.99	15.80
10	-4	13	91.59	15.30
-11	4	-13	158.98	23.30
-11	4	-13	82.69	13.40
-11	4	-13	110.69	15.50
11	-4	13	96.29	18.50
10	3	-13	15.60	14.20
-10	-3	13	20.10	6.30
9	3	-13	51.69	15.70
9	3	-13	56.39	12.20
-9	-3	13	59.89	10.10
8	3	-13	457.55	30.00
8	3	-13	440.76	31.40

-8	-3	13	459.45	25.30
-8	-3	13	478.15	27.80
7	3	-13	27.00	11.60
7	3	-13	26.20	12.50
6	3	-13	22.30	12.00
6	3	-13	17.30	11.60
-6	-3	13	2.40	6.50
-6	-3	13	7.20	6.10
5	3	-13	471.95	30.20
5	3	-13	467.85	29.60
-5	-3	13	441.76	24.90
4	3	-13	51.99	13.90
4	3	-13	51.09	15.20
4	3	-13	46.80	11.10
3	3	-13	60.99	12.20
3	3	-13	47.10	11.60
3	3	-13	59.09	13.30
3	3	-13	45.70	11.10
-3	-3	13	56.89	10.50
-3	-3	13	58.29	8.70
2	3	-13	15.80	12.20
2	3	-13	13.60	10.90
2	3	-13	-5.50	9.50
2	3	-13	9.00	9.80
2	3	-13	4.60	8.00
-2	-3	13	8.70	6.80
1	3	-13	13.50	9.70
1	3	-13	3.40	9.40
1	3	-13	4.10	9.70
1	3	-13	12.90	10.20
1	3	-13	11.00	8.40
-1	-3	13	7.10	5.20
0	3	-13	341.47	24.20
0	3	-13	348.97	23.50
0	3	-13	331.67	25.30
0	3	-13	380.46	23.20
0	3	-13	362.46	23.90
0	-3	13	379.46	19.20
-1	3	-13	45.40	9.80
-1	3	-13	29.20	9.20
-1	3	-13	59.99	11.10
-1	3	-13	33.90	11.40
-1	3	-13	53.09	9.80
1	-3	13	48.70	7.70

-2	3	-13	169.28	20.10
-2	3	-13	152.28	18.90
-2	3	-13	129.69	17.10
-2	3	-13	155.28	14.00
-2	3	-13	142.69	14.60
-2	3	-13	149.59	17.10
2	-3	13	159.58	11.10
-3	3	-13	69.59	14.10
-3	3	-13	62.79	11.70
-3	3	-13	65.49	9.50
-3	3	-13	62.29	8.20
-3	3	-13	75.49	13.10
-3	3	-13	61.09	10.70
3	-3	13	60.89	8.20
-4	3	-13	172.58	24.30
-4	3	-13	130.69	18.10
-4	3	-13	191.78	19.50
-4	3	-13	159.18	20.10
-4	3	-13	157.88	15.50
-4	3	-13	159.08	13.20
4	-3	13	162.58	12.10
-5	3	-13	294.27	23.20
-5	3	-13	288.87	24.30
-5	3	-13	327.17	22.80
-5	3	-13	294.67	19.00
-5	3	-13	295.37	29.30
5	-3	13	298.27	18.20
-6	3	-13	1564.24	74.29
-6	3	-13	1483.05	76.49
-6	3	-13	1531.65	72.19
-6	3	-13	1447.46	74.79
-6	3	-13	1558.84	71.79
6	-3	13	1524.05	71.49
-7	3	-13	67.29	13.60
-7	3	-13	74.89	16.90
-7	3	-13	65.39	10.80
-7	3	-13	81.59	14.30
7	-3	13	73.29	11.80
-8	3	-13	4.40	8.30
-8	3	-13	-21.10	13.50
-8	3	-13	9.60	11.90
-8	3	-13	3.30	12.10
-8	3	-13	10.60	7.90
8	-3	13	12.80	13.80

-9	3	-13	4.30	14.40
-9	3	-13	-10.90	8.30
-9	3	-13	-0.20	9.10
-9	3	-13	9.80	15.90
9	-3	13	22.80	12.80
9	-3	13	-4.00	22.80
-10	3	-13	37.90	14.90
-10	3	-13	48.70	10.60
-10	3	-13	62.09	16.80
-10	3	-13	45.00	10.00
10	-3	13	30.50	15.20
-11	3	-13	16.60	10.80
-11	3	-13	37.10	16.50
-11	3	-13	8.90	9.10
-11	3	-13	12.40	16.60
11	-3	13	10.50	19.40
10	2	-13	121.29	22.40
-10	-2	13	121.09	11.70
9	2	-13	71.69	13.20
9	2	-13	71.09	14.50
-9	-2	13	66.39	10.40
8	2	-13	68.59	14.40
8	2	-13	49.99	14.50
-8	-2	13	60.59	11.10
-8	-2	13	48.00	8.70
7	2	-13	391.76	27.40
7	2	-13	418.56	29.00
-7	-2	13	371.06	23.40
-7	-2	13	371.16	22.10
6	2	-13	259.47	23.60
6	2	-13	282.47	24.50
-6	-2	13	280.17	17.90
-6	-2	13	278.17	18.90
5	2	-13	16.70	10.60
5	2	-13	29.10	11.60
-5	-2	13	4.00	5.70
4	2	-13	30.20	14.00
4	2	-13	3.50	10.70
4	2	-13	22.60	11.00
-4	-2	13	18.50	6.60
-4	-2	13	2.70	12.40
3	2	-13	532.85	31.50
3	2	-13	562.34	33.30
3	2	-13	514.55	30.80

-3	-2	13	550.24	28.30
-3	-2	13	509.35	29.50
2	2	-13	447.46	30.00
2	2	-13	514.65	29.90
2	2	-13	437.96	29.50
2	2	-13	531.95	28.60
2	2	-13	469.85	29.90
-2	-2	13	532.15	26.50
1	2	-13	105.49	17.50
1	2	-13	69.69	11.30
1	2	-13	99.49	14.60
1	2	-13	64.49	11.10
1	2	-13	98.39	12.70
0	2	-13	145.49	18.30
0	2	-13	98.99	16.10
0	2	-13	107.09	15.60
0	2	-13	95.39	15.00
0	2	-13	118.99	16.00
-1	2	-13	1813.62	86.99
-1	2	-13	1858.81	86.29
-1	2	-13	1887.81	87.49
-1	2	-13	1891.51	87.79
-1	2	-13	1848.02	87.79
1	-2	13	1875.81	85.29
-2	2	-13	113.39	15.90
-2	2	-13	116.49	16.40
-2	2	-13	115.79	14.00
-2	2	-13	90.89	17.00
-2	2	-13	140.59	19.10
-2	2	-13	111.59	13.30
2	-2	13	114.39	9.10
-3	2	-13	-15.50	12.40
-3	2	-13	15.00	9.90
-3	2	-13	11.60	11.90
-3	2	-13	-8.10	9.00
-3	2	-13	13.50	6.50
-3	2	-13	4.80	6.70
3	-2	13	12.00	4.30
-4	2	-13	182.68	16.10
-4	2	-13	191.58	20.50
-4	2	-13	155.38	23.90
-4	2	-13	207.48	14.20
-4	2	-13	178.08	19.50
-4	2	-13	202.48	20.90

4	-2	13	182.38	12.30
-5	2	-13	82.19	13.60
-5	2	-13	89.29	14.00
-5	2	-13	79.99	10.80
-5	2	-13	65.59	10.70
5	-2	13	82.29	9.60
-6	2	-13	8.80	6.30
-6	2	-13	17.60	9.70
-6	2	-13	10.70	10.20
-6	2	-13	2.60	12.70
-6	2	-13	5.80	7.50
-6	2	-13	12.30	11.30
6	-2	13	7.00	6.20
-7	2	-13	28.80	10.60
-7	2	-13	21.80	14.10
-7	2	-13	14.20	11.20
-7	2	-13	24.70	8.70
-7	2	-13	34.90	8.10
7	-2	13	40.70	9.10
-8	2	-13	72.99	13.50
-8	2	-13	76.49	13.40
-8	2	-13	59.49	13.60
-8	2	-13	58.69	9.70
-8	2	-13	81.49	15.70
8	-2	13	71.89	13.00
-9	2	-13	8.20	16.90
-9	2	-13	4.70	20.10
-9	2	-13	10.30	18.60
-9	2	-13	0.50	8.50
9	-2	13	21.10	17.90
-10	2	-13	-6.50	8.90
-10	2	-13	18.90	13.70
-10	2	-13	10.50	8.90
-10	2	-13	-16.60	15.10
10	-2	13	-1.90	15.20
-11	2	-13	340.77	29.50
-11	2	-13	309.97	40.90
-11	2	-13	338.87	21.20
-11	2	-13	338.57	22.40
10	1	-13	68.49	15.40
-10	-1	13	57.59	7.90
9	1	-13	31.80	12.80
9	1	-13	11.80	13.40
-9	-1	13	38.30	7.60

8	1	-13	25.40	14.20
8	1	-13	31.90	15.70
-8	-1	13	24.00	10.80
7	1	-13	53.19	12.60
7	1	-13	31.50	13.30
-7	-1	13	36.40	7.10
-7	-1	13	50.89	9.30
6	1	-13	335.17	27.10
6	1	-13	352.56	25.80
-6	-1	13	330.47	20.40
5	1	-13	36.80	12.00
5	1	-13	40.80	11.60
-5	-1	13	45.30	7.50
4	1	-13	325.57	27.30
4	1	-13	255.87	21.80
-4	-1	13	278.57	17.40
-4	-1	13	264.87	22.70
3	1	-13	4.40	10.90
3	1	-13	2.70	10.00
3	1	-13	6.50	10.00
3	1	-13	6.40	10.50
-3	-1	13	-3.20	6.60
-3	-1	13	2.70	7.20
2	1	-13	89.59	12.10
2	1	-13	75.69	11.60
2	1	-13	87.79	12.10
2	1	-13	111.29	12.60
-2	-1	13	74.79	10.80
-2	-1	13	76.79	10.90
1	1	-13	1071.89	53.39
1	1	-13	1054.39	53.29
1	1	-13	1117.79	53.79
1	1	-13	1029.90	53.99
-1	-1	13	1171.48	52.69
0	1	-13	482.45	27.90
0	1	-13	445.36	27.60
0	1	-13	451.65	28.90
0	1	-13	460.15	28.50
0	-1	13	541.55	24.20
-1	1	-13	100.29	18.30
-1	1	-13	119.79	13.00
-1	1	-13	105.29	14.90
-1	1	-13	125.39	16.90
-1	1	-13	98.49	14.10

1	-1	13	120.39	10.80
-2	1	-13	74.49	11.80
-2	1	-13	85.19	15.80
-2	1	-13	111.39	16.50
-2	1	-13	94.29	12.90
-2	1	-13	81.69	15.20
2	-1	13	96.19	7.90
-3	1	-13	173.88	15.20
-3	1	-13	169.98	18.10
-3	1	-13	194.18	18.50
-3	1	-13	188.28	14.10
-3	1	-13	165.78	20.00
-3	1	-13	201.88	20.50
-3	1	-13	197.28	22.50
3	-1	13	190.68	11.30
3	-1	13	150.88	19.40
-4	1	-13	95.89	13.60
-4	1	-13	100.89	15.20
-4	1	-13	156.38	17.70
-4	1	-13	134.69	11.60
-4	1	-13	154.78	18.90
-4	1	-13	135.69	14.20
4	-1	13	120.59	9.20
-5	1	-13	119.09	15.00
-5	1	-13	101.19	14.80
-5	1	-13	118.89	11.50
-5	1	-13	123.09	18.30
-5	1	-13	86.79	13.80
5	-1	13	109.59	9.20
-6	1	-13	966.80	51.79
-6	1	-13	985.20	48.40
-6	1	-13	983.40	49.90
-6	1	-13	994.90	52.69
-6	1	-13	981.90	54.19
-6	1	-13	973.00	47.80
6	-1	13	1004.10	47.00
-7	1	-13	298.97	23.80
-7	1	-13	338.37	20.10
-7	1	-13	356.46	27.50
-7	1	-13	285.37	20.70
-7	1	-13	284.37	29.60
7	-1	13	297.87	19.50
-8	1	-13	-8.60	10.20
-8	1	-13	-5.20	13.80

-8	1	-13	8.50	7.20
-8	1	-13	15.00	12.30
-8	1	-13	-2.40	7.40
8	-1	13	-11.20	9.50
-9	1	-13	87.19	16.90
-9	1	-13	70.89	11.10
-9	1	-13	70.19	18.20
-9	1	-13	48.60	13.10
-9	1	-13	72.09	12.50
9	-1	13	91.59	16.50
-10	1	-13	52.59	16.00
-10	1	-13	16.30	8.90
-10	1	-13	33.40	13.30
-10	1	-13	33.90	8.90
10	-1	13	1.60	17.10
-11	1	-13	14.10	10.60
-11	1	-13	11.00	8.80
-11	1	-13	-3.30	14.70
-11	1	-13	51.79	15.50
10	0	-13	22.10	25.60
10	0	-13	3.40	12.00
-10	0	13	3.40	6.00
9	0	-13	216.68	21.40
9	0	-13	175.78	22.00
-9	0	13	174.78	14.20
-8	0	13	18.10	11.30
-8	0	13	18.20	8.70
7	0	-13	29.10	11.80
7	0	-13	33.80	13.80
-7	0	13	41.30	7.90
6	0	-13	409.96	30.00
6	0	-13	416.06	28.40
-6	0	13	424.16	23.80
5	0	-13	303.27	25.90
5	0	-13	286.87	23.50
-5	0	13	318.07	18.90
4	0	-13	312.27	27.10
4	0	-13	362.46	24.40
4	0	-13	348.07	25.40
4	0	-13	309.37	26.60
-4	0	13	344.37	19.80
-4	0	13	321.97	22.90
3	0	-13	13.30	11.80
3	0	-13	-5.60	11.40

3	0	-13	17.60	10.50
3	0	-13	4.80	9.40
-3	0	13	5.00	6.60
-3	0	13	15.30	6.80
2	0	-13	26.90	10.20
2	0	-13	49.70	11.00
2	0	-13	31.10	11.00
2	0	-13	39.20	10.90
-2	0	13	17.20	6.30
1	0	-13	28.40	10.60
1	0	-13	21.90	8.30
1	0	-13	26.20	8.70
1	0	-13	14.30	9.20
1	0	-13	13.80	9.50
-1	0	13	32.90	5.50
0	0	-13	928.71	51.99
0	0	-13	1045.40	51.69
0	0	-13	1035.00	51.89
0	0	-13	1032.50	51.29
0	0	-13	1085.89	52.59
0	0	13	1163.38	49.00
-1	0	-13	9.00	9.30
-1	0	-13	-6.20	8.10
-1	0	-13	-5.20	8.10
-1	0	-13	18.80	10.50
1	0	13	5.30	3.70
-2	0	-13	482.85	28.40
-2	0	-13	420.86	29.00
-2	0	-13	452.35	26.10
-2	0	-13	435.76	25.00
-2	0	-13	446.76	27.10
-2	0	-13	426.06	23.70
2	0	13	431.46	24.60
2	0	13	459.55	22.50
-3	0	-13	17.50	11.50
-3	0	-13	6.70	6.30
-3	0	-13	7.60	7.30
-3	0	-13	14.60	8.30
-3	0	-13	8.90	9.50
-3	0	-13	18.70	10.90
-3	0	-13	10.70	8.50
3	0	13	11.10	3.50
-4	0	-13	439.86	31.70
-4	0	-13	427.26	23.20

-4	0	-13	444.56	27.80
-4	0	-13	425.86	28.80
-4	0	-13	437.76	26.10
-4	0	-13	417.96	24.80
-4	0	-13	406.56	29.50
4	0	13	437.26	21.40
-5	0	-13	1005.50	52.69
-5	0	-13	959.90	51.99
-5	0	-13	960.40	49.40
-5	0	-13	1017.80	47.90
-5	0	-13	1036.10	53.09
-5	0	-13	1006.40	48.80
5	0	13	1025.30	46.90
-6	0	-13	14.90	6.20
-6	0	-13	-1.10	11.90
-6	0	-13	-5.90	10.90
-6	0	-13	3.80	7.20
-6	0	-13	8.30	8.80
-6	0	-13	13.70	9.90
6	0	13	6.10	4.70
-7	0	-13	73.09	14.80
-7	0	-13	75.29	15.10
-7	0	-13	93.29	15.70
-7	0	-13	74.09	11.70
-7	0	-13	83.79	12.90
7	0	13	83.39	10.80
-8	0	-13	111.39	13.50
-8	0	-13	131.09	18.50
-8	0	-13	112.69	14.10
-8	0	-13	116.39	14.00
-8	0	-13	103.99	16.70
8	0	13	97.89	12.00
-9	0	-13	7.30	8.50
-9	0	-13	-11.00	10.50
-9	0	-13	27.00	14.30
-9	0	-13	-5.30	13.80
-9	0	-13	6.10	8.30
9	0	13	6.80	12.70
-10	0	-13	204.38	26.20
-10	0	-13	190.58	15.70
-10	0	-13	161.58	15.70
-10	0	-13	176.38	20.90
10	0	13	130.19	22.40
-11	0	-13	13.80	8.50

-11	0	-13	14.60	14.10
-11	0	-13	9.90	9.90
-11	0	-13	31.10	16.30
10	-1	-13	41.20	12.30
-10	1	13	36.30	7.50
9	-1	-13	129.49	22.80
9	-1	-13	103.99	19.40
-9	1	13	112.49	12.40
8	-1	-13	277.47	32.60
-8	1	13	252.77	24.20
-8	1	13	321.27	19.70
7	-1	-13	94.19	15.90
7	-1	-13	80.39	13.60
-7	1	13	79.79	11.80
6	-1	-13	176.48	23.30
6	-1	-13	181.18	20.70
-6	1	13	173.98	14.60
5	-1	-13	538.15	32.00
5	-1	-13	492.85	30.20
5	-1	-13	473.15	32.40
-5	1	13	523.35	26.50
-5	1	13	405.16	30.80
4	-1	-13	4.40	11.60
4	-1	-13	1.30	12.50
4	-1	-13	-1.70	9.70
4	-1	-13	15.30	11.70
-4	1	13	8.40	6.00
-4	1	13	-0.40	7.80
3	-1	-13	5.40	7.90
3	-1	-13	-5.40	9.90
3	-1	-13	11.10	10.40
3	-1	-13	-3.90	9.80
3	-1	-13	-3.40	8.80
-3	1	13	0.60	6.10
-3	1	13	-2.70	5.60
2	-1	-13	108.69	14.50
2	-1	-13	110.49	16.00
2	-1	-13	89.39	16.40
2	-1	-13	117.79	13.40
2	-1	-13	112.09	17.60
-2	1	13	114.59	11.60
1	-1	-13	1.30	10.30
1	-1	-13	23.90	10.50
1	-1	-13	6.90	7.70

1	-1	-13	13.20	9.20
1	-1	-13	24.10	9.90
-1	1	13	9.70	4.30
0	-1	-13	1037.60	54.29
0	-1	-13	1057.79	52.79
0	-1	-13	1038.60	53.49
0	-1	-13	1063.69	53.29
0	1	13	1254.97	50.79
-1	-1	-13	97.49	14.30
-1	-1	-13	96.69	17.50
-1	-1	-13	93.69	11.90
-1	-1	-13	106.49	16.30
1	1	13	106.39	7.80
-2	-1	-13	80.19	10.30
-2	-1	-13	54.59	11.40
-2	-1	-13	73.89	12.40
-2	-1	-13	63.69	12.40
2	1	13	50.09	7.40
2	1	13	57.99	6.30
-3	-1	-13	40.10	12.10
-3	-1	-13	55.29	12.30
-3	-1	-13	72.09	11.40
-3	-1	-13	57.19	7.40
-3	-1	-13	31.60	7.50
-3	-1	-13	50.09	10.70
3	1	13	43.50	8.90
-4	-1	-13	4.70	9.00
-4	-1	-13	21.10	6.70
-4	-1	-13	10.80	11.70
-4	-1	-13	26.30	13.90
-4	-1	-13	27.40	7.90
-4	-1	-13	22.70	10.70
-4	-1	-13	28.60	10.30
4	1	13	14.80	3.30
-5	-1	-13	513.95	33.50
-5	-1	-13	507.15	28.10
-5	-1	-13	524.15	26.50
-5	-1	-13	515.35	28.60
-5	-1	-13	482.15	33.00
-5	-1	-13	513.15	31.90
5	1	13	526.05	25.00
-6	-1	-13	839.72	41.20
-6	-1	-13	869.71	43.00
-6	-1	-13	874.51	46.80

-6	-1	-13	828.92	47.80
-6	-1	-13	790.32	45.90
6	1	13	840.62	39.90
-7	-1	-13	184.08	14.60
-7	-1	-13	196.98	25.30
-7	-1	-13	178.58	18.20
-7	-1	-13	168.18	15.40
-7	-1	-13	229.08	24.50
7	1	13	186.48	13.20
-8	-1	-13	42.60	14.80
-8	-1	-13	16.40	7.50
-8	-1	-13	15.10	13.70
-8	-1	-13	13.90	7.70
-9	-1	-13	11.70	13.40
-9	-1	-13	25.30	14.70
-9	-1	-13	24.70	8.20
-9	-1	-13	15.00	8.30
-9	-1	-13	8.60	10.00
9	1	13	-0.50	11.00
-10	-1	-13	85.79	12.50
-10	-1	-13	93.49	14.50
-10	-1	-13	62.69	10.30
10	1	13	20.60	32.30
-11	-1	-13	40.30	13.90
-11	-1	-13	16.70	9.50
-11	-1	-13	31.80	15.30
-11	-1	-13	28.50	8.80
10	-2	-13	139.29	18.10
-10	2	13	112.49	12.30
9	-2	-13	13.40	10.70
9	-2	-13	18.60	15.40
-9	2	13	-2.70	6.30
8	-2	-13	130.69	20.40
-8	2	13	145.19	21.50
-8	2	13	160.78	14.80
7	-2	-13	311.17	29.30
7	-2	-13	348.07	25.90
-7	2	13	340.17	20.80
6	-2	-13	63.69	16.60
6	-2	-13	107.59	16.20
6	-2	-13	117.59	18.30
-6	2	13	94.19	12.40
5	-2	-13	92.69	16.80
5	-2	-13	94.89	15.10

5	-2	-13	88.09	17.10
5	-2	-13	83.29	25.30
-5	2	13	86.49	12.80
-5	2	13	104.49	11.90
4	-2	-13	14.90	12.50
4	-2	-13	14.20	8.60
4	-2	-13	11.00	10.60
4	-2	-13	-1.50	11.60
4	-2	-13	2.90	9.60
-4	2	13	-4.40	6.60
3	-2	-13	416.56	24.40
3	-2	-13	401.26	25.90
3	-2	-13	356.86	27.80
3	-2	-13	392.06	27.00
3	-2	-13	419.36	26.50
-3	2	13	412.86	21.90
2	-2	-13	33.80	10.80
2	-2	-13	31.80	7.90
2	-2	-13	49.70	11.00
2	-2	-13	50.69	11.30
-2	2	13	30.90	6.00
1	-2	-13	448.46	27.50
1	-2	-13	445.76	28.70
1	-2	-13	430.86	28.30
1	-2	-13	474.35	27.20
1	-2	-13	480.55	26.70
-1	2	13	516.95	23.60
0	-2	-13	730.13	37.10
0	-2	-13	678.13	38.40
0	-2	-13	687.33	37.10
0	2	13	747.13	33.70
-1	-2	-13	1716.43	81.89
-1	-2	-13	1708.73	83.09
-1	-2	-13	1686.33	81.19
1	2	13	1821.62	79.09
1	2	13	1772.62	80.29
-2	-2	-13	7.10	9.10
-2	-2	-13	3.80	7.90
-2	-2	-13	18.40	11.10
-2	-2	-13	0.00	10.70
2	2	13	7.00	6.00
2	2	13	9.30	2.90
-3	-2	-13	11.10	6.10
-3	-2	-13	-2.30	7.80

-3	-2	-13	8.20	8.40
-3	-2	-13	3.40	11.00
-3	-2	-13	11.60	10.60
-3	-2	-13	24.50	11.80
3	2	13	12.00	3.10
3	2	13	17.00	7.00
-4	-2	-13	468.95	32.10
-4	-2	-13	447.56	34.20
-4	-2	-13	427.76	25.80
-4	-2	-13	445.86	23.20
-4	-2	-13	439.56	28.00
-4	-2	-13	449.56	25.10
-4	-2	-13	408.26	28.80
4	2	13	464.65	21.40
-5	-2	-13	226.58	23.70
-5	-2	-13	238.48	24.80
-5	-2	-13	259.37	17.90
-5	-2	-13	241.48	15.10
-5	-2	-13	238.78	21.20
-5	-2	-13	240.98	18.40
5	2	13	256.37	13.10
-6	-2	-13	57.39	9.50
-6	-2	-13	38.70	9.60
-6	-2	-13	48.90	11.10
-6	-2	-13	40.20	8.30
-6	-2	-13	41.80	13.70
6	2	13	64.89	6.70
-7	-2	-13	87.29	15.00
-7	-2	-13	114.79	11.60
-7	-2	-13	100.09	17.30
-7	-2	-13	116.59	20.70
-7	-2	-13	90.59	12.70
7	2	13	80.79	9.20
-8	-2	-13	195.98	18.40
-8	-2	-13	160.38	22.70
-8	-2	-13	192.28	25.50
-8	-2	-13	167.88	15.90
8	2	13	173.88	14.30
-9	-2	-13	5.70	7.30
-9	-2	-13	25.90	14.70
-9	-2	-13	9.00	8.00
-9	-2	-13	16.80	13.70
-9	-2	-13	3.60	9.50
9	2	13	7.00	10.00

-10	-2	-13	179.98	15.80
-10	-2	-13	183.48	20.70
-10	-2	-13	159.68	15.00
-10	-2	-13	148.99	24.90
-11	-2	-13	427.06	23.80
-11	-2	-13	356.16	30.80
-11	-2	-13	405.56	24.70
-11	-2	-13	429.46	27.70
10	-3	-13	-4.60	11.50
-10	3	13	12.20	7.00
9	-3	-13	17.20	18.10
9	-3	-13	-4.40	10.80
-9	3	13	8.10	6.90
8	-3	-13	56.69	21.20
8	-3	-13	44.60	13.20
-8	3	13	46.60	8.80
-8	3	13	32.70	13.70
7	-3	-13	233.58	28.40
7	-3	-13	185.88	21.10
-7	3	13	183.28	15.60
6	-3	-13	273.67	24.60
6	-3	-13	246.88	21.60
6	-3	-13	228.08	25.90
-6	3	13	228.68	17.40
5	-3	-13	89.89	16.10
5	-3	-13	102.59	13.60
5	-3	-13	68.69	15.60
-5	3	13	87.69	15.20
-5	3	13	75.29	11.60
4	-3	-13	157.78	19.40
4	-3	-13	201.28	17.60
4	-3	-13	177.28	24.10
4	-3	-13	195.48	22.30
4	-3	-13	172.38	18.10
3	-3	-13	35.00	8.20
3	-3	-13	8.40	9.60
3	-3	-13	21.70	9.90
3	-3	-13	33.70	10.60
3	-3	-13	44.70	13.20
-3	3	13	33.60	5.50
2	-3	-13	226.38	20.90
2	-3	-13	248.08	17.50
2	-3	-13	219.88	18.90
2	-3	-13	237.38	19.60

2	-3	-13	216.88	22.10
-2	3	13	228.38	13.60
1	-3	-13	1864.61	88.89
1	-3	-13	1877.91	90.29
1	-3	-13	1850.21	88.99
1	-3	-13	1864.01	89.19
-1	3	13	2143.29	87.39
0	-3	-13	39.60	11.70
0	-3	-13	28.30	9.20
0	-3	-13	12.90	8.20
0	3	13	19.10	3.50
-1	-3	-13	59.29	12.20
-1	-3	-13	42.60	9.80
-1	-3	-13	23.30	15.30
1	3	13	38.70	5.20
1	3	13	40.70	7.00
1	3	13	29.60	4.80
-2	-3	-13	90.49	14.90
-2	-3	-13	85.69	13.50
-2	-3	-13	111.99	15.70
2	3	13	97.09	6.20
2	3	13	69.49	10.30
-3	-3	-13	23.30	12.50
-3	-3	-13	19.10	10.00
-3	-3	-13	9.40	5.50
-3	-3	-13	9.90	8.70
-3	-3	-13	-3.90	22.80
-3	-3	-13	12.90	8.40
3	3	13	17.40	2.80
3	3	13	16.30	6.30
-4	-3	-13	133.19	24.60
-4	-3	-13	142.39	14.40
-4	-3	-13	140.29	18.50
-4	-3	-13	119.79	10.70
-4	-3	-13	149.59	19.80
-4	-3	-13	104.59	16.30
-4	-3	-13	120.09	15.40
4	3	13	138.99	7.80
-5	-3	-13	281.97	26.80
-5	-3	-13	310.67	19.70
-5	-3	-13	300.87	22.40
-5	-3	-13	299.17	17.20
-5	-3	-13	300.67	19.50
-5	-3	-13	290.07	26.30

5	3	13	299.67	15.10
-6	-3	-13	2522.05	122.29
-6	-3	-13	2588.34	118.79
-6	-3	-13	2542.45	120.49
-6	-3	-13	2603.84	118.19
-6	-3	-13	2560.84	117.59
-6	-3	-13	2609.44	123.49
6	3	13	2641.54	116.49
-7	-3	-13	256.67	16.00
-7	-3	-13	222.88	27.60
-7	-3	-13	237.48	16.60
-7	-3	-13	254.57	29.40
-7	-3	-13	253.87	23.00
-7	-3	-13	233.98	19.20
7	3	13	215.88	13.90
-8	-3	-13	13.20	7.60
-8	-3	-13	7.40	8.20
-8	-3	-13	4.20	12.90
-8	-3	-13	-5.40	7.80
-8	-3	-13	19.90	11.10
8	3	13	-0.70	6.10
-9	-3	-13	87.59	12.20
-9	-3	-13	64.89	13.50
-9	-3	-13	74.09	17.10
-9	-3	-13	106.59	13.90
-9	-3	-13	87.39	11.70
9	3	13	95.49	15.40
-10	-3	-13	65.19	11.50
-10	-3	-13	79.59	12.40
-10	-3	-13	105.99	13.80
-10	-3	-13	65.59	13.90
-11	-3	-13	33.40	13.50
-11	-3	-13	38.90	10.80
-11	-3	-13	16.90	8.10
-11	-3	-13	15.60	13.30
10	-4	-13	22.20	12.00
-10	4	13	6.20	7.40
9	-4	-13	132.19	18.60
-9	4	13	131.89	14.00
8	-4	-13	54.69	15.90
8	-4	-13	54.79	19.80
-8	4	13	59.79	9.70
7	-4	-13	29.10	11.80
7	-4	-13	47.30	14.20

7	-4	-13	42.40	17.10
6	-4	-13	590.94	40.10
6	-4	-13	579.94	34.90
6	-4	-13	612.44	35.30
-6	4	13	626.84	32.10
5	-4	-13	128.09	18.20
5	-4	-13	84.59	12.10
5	-4	-13	101.09	16.60
5	-4	-13	65.09	12.00
-5	4	13	103.39	14.60
4	-4	-13	22.50	10.10
4	-4	-13	42.90	12.80
4	-4	-13	32.70	9.70
4	-4	-13	15.20	8.50
4	-4	-13	54.89	15.20
-4	4	13	27.90	6.10
3	-4	-13	9.00	7.30
3	-4	-13	4.50	8.40
3	-4	-13	-17.60	13.40
3	-4	-13	-1.70	10.30
3	-4	-13	20.10	10.00
-3	4	13	7.50	4.30
2	-4	-13	126.99	20.30
2	-4	-13	79.39	10.00
2	-4	-13	85.49	13.10
2	-4	-13	64.29	9.00
2	-4	-13	83.89	11.30
-2	4	13	80.99	7.50
1	-4	-13	171.28	16.30
1	-4	-13	141.99	15.80
1	-4	-13	187.28	16.80
1	-4	-13	160.18	21.20
-1	4	13	155.48	10.20
0	-4	-13	1127.89	55.69
0	-4	-13	1103.39	57.99
0	-4	-13	1148.39	55.69
0	4	13	1238.98	52.99
-1	-4	-13	282.07	27.60
-1	-4	-13	262.37	20.60
1	4	13	285.67	16.60
1	4	13	246.98	16.40
1	4	13	302.67	14.60
1	4	13	342.77	21.80
-2	-4	-13	329.67	23.80

-2	-4	-13	333.87	31.20
2	4	13	376.56	17.30
2	4	13	396.56	19.90
-3	-4	-13	-11.80	5.70
-3	-4	-13	2.00	10.00
-3	-4	-13	-4.60	8.60
-3	-4	-13	4.00	13.80
-3	-4	-13	-2.40	10.70
3	4	13	5.00	2.00
3	4	13	-0.40	6.20
-4	-4	-13	445.46	25.70
-4	-4	-13	412.26	26.40
-4	-4	-13	428.16	34.20
-4	-4	-13	440.96	23.20
-4	-4	-13	579.84	51.29
-4	-4	-13	466.45	30.70
-4	-4	-13	446.96	26.40
-5	-4	-13	396.86	23.70
-5	-4	-13	409.16	25.80
-5	-4	-13	408.46	31.70
-5	-4	-13	416.06	23.50
-5	-4	-13	373.26	31.00
-5	-4	-13	417.66	21.70
5	4	13	413.76	19.60
-6	-4	-13	29.30	7.30
-6	-4	-13	20.30	8.40
-6	-4	-13	11.20	17.80
-6	-4	-13	12.70	5.40
-6	-4	-13	33.40	9.10
-6	-4	-13	1.80	15.30
6	4	13	17.40	3.80
-7	-4	-13	328.97	29.10
-7	-4	-13	289.07	33.90
-7	-4	-13	295.07	19.50
-7	-4	-13	303.77	18.50
-7	-4	-13	296.57	24.30
-7	-4	-13	302.87	21.00
7	4	13	303.27	16.50
-8	-4	-13	347.07	23.30
-8	-4	-13	310.37	21.80
-8	-4	-13	244.28	30.70
-8	-4	-13	310.27	29.30
-8	-4	-13	332.17	25.50
-8	-4	-13	311.07	20.40

8	4	13	311.87	18.20
-9	-4	-13	62.09	13.00
-9	-4	-13	112.09	18.60
-9	-4	-13	74.79	14.20
-9	-4	-13	95.89	13.60
-9	-4	-13	86.19	15.40
-9	-4	-13	70.59	11.90
9	4	13	89.19	13.90
-10	-4	-13	139.09	13.90
-10	-4	-13	132.09	17.20
-10	-4	-13	152.98	21.70
-10	-4	-13	215.58	23.60
-10	-4	-13	157.88	14.70
10	4	13	151.18	22.10
-11	-4	-13	30.30	8.20
-11	-4	-13	15.10	10.60
-11	-4	-13	48.90	13.90
-11	-4	-13	54.19	11.00
10	-5	-13	139.79	17.60
-10	5	13	129.99	13.60
9	-5	-13	95.49	17.10
-9	5	13	119.09	13.50
8	-5	-13	119.79	19.90
7	-5	-13	6.50	12.70
7	-5	-13	16.30	11.60
7	-5	-13	29.30	20.40
-7	5	13	26.50	7.90
6	-5	-13	16.70	17.80
6	-5	-13	26.60	11.20
6	-5	-13	30.30	11.00
5	-5	-13	247.78	22.10
5	-5	-13	230.08	22.10
5	-5	-13	300.57	21.30
-5	5	13	261.97	18.40
4	-5	-13	133.09	18.40
4	-5	-13	144.99	18.10
4	-5	-13	115.39	15.90
4	-5	-13	139.79	21.00
4	-5	-13	152.58	16.00
-4	5	13	112.19	10.60
3	-5	-13	66.99	15.40
3	-5	-13	59.39	11.60
3	-5	-13	57.39	9.60
3	-5	-13	43.70	10.30

3	-5	-13	33.60	8.40
-3	5	13	49.99	6.70
2	-5	-13	293.87	20.70
2	-5	-13	259.37	23.10
2	-5	-13	291.67	20.10
2	-5	-13	282.57	19.50
-2	5	13	307.97	15.40
1	-5	-13	412.46	28.40
1	-5	-13	427.66	24.50
1	-5	-13	416.76	24.70
1	-5	-13	393.36	25.00
-1	5	13	458.45	21.00
0	-5	-13	781.02	40.80
0	-5	-13	806.92	44.30
0	-5	-13	783.92	40.90
0	5	13	870.41	37.60
-1	-5	-13	0.90	8.60
-1	-5	-13	-2.20	10.20
-1	-5	-13	-7.70	17.50
1	5	13	4.50	2.70
-2	-5	-13	21.10	9.60
-2	-5	-13	15.70	13.40
2	5	13	20.20	9.40
-3	-5	-13	-11.00	9.10
-3	-5	-13	-28.60	14.40
-3	-5	-13	0.40	6.10
-3	-5	-13	8.80	12.00
3	5	13	0.90	7.90
3	5	13	11.10	6.70
-4	-5	-13	236.48	18.40
-4	-5	-13	260.67	15.30
-4	-5	-13	268.27	29.90
-4	-5	-13	259.37	18.40
-4	-5	-13	312.17	25.30
-4	-5	-13	220.48	19.10
-5	-5	-13	1093.19	53.09
-5	-5	-13	1101.19	55.19
-5	-5	-13	1057.19	60.29
-5	-5	-13	1124.09	52.29
-5	-5	-13	1091.19	59.19
-5	-5	-13	1124.39	53.79
5	5	13	1209.18	50.79
-6	-5	-13	209.88	14.10
-6	-5	-13	191.18	15.80

-6	-5	-13	239.18	32.50
-6	-5	-13	209.08	18.20
-6	-5	-13	181.18	18.80
6	5	13	216.98	11.40
-7	-5	-13	351.26	20.30
-7	-5	-13	355.86	38.50
-7	-5	-13	356.96	20.90
-7	-5	-13	313.07	29.60
-7	-5	-13	339.47	22.30
-7	-5	-13	354.46	24.90
7	5	13	359.96	18.40
-8	-5	-13	2.50	9.90
-8	-5	-13	12.70	8.60
-8	-5	-13	7.00	7.60
-8	-5	-13	1.80	13.70
-8	-5	-13	-11.90	9.70
-8	-5	-13	-2.10	12.10
8	5	13	1.40	5.60
-9	-5	-13	24.00	7.40
-9	-5	-13	18.60	10.90
-9	-5	-13	38.60	8.40
-9	-5	-13	19.10	12.80
-9	-5	-13	35.50	9.80
-9	-5	-13	39.20	14.70
9	5	13	25.70	8.40
-10	-5	-13	174.78	20.60
-10	-5	-13	121.69	13.40
-10	-5	-13	166.38	17.10
-10	-5	-13	147.99	21.80
-10	-5	-13	145.89	14.30
10	5	13	118.89	20.10
-11	-5	-13	46.00	18.10
-11	-5	-13	56.09	12.60
-11	-5	-13	24.80	12.00
-11	-5	-13	42.40	10.20
-11	-5	-13	67.79	11.40
10	-6	-13	164.38	18.10
-10	6	13	166.08	16.10
9	-6	-13	-13.50	10.60
-9	6	13	0.30	7.20
8	-6	-13	37.80	14.30
8	-6	-13	32.20	14.90
-8	6	13	31.50	11.60
-8	6	13	15.70	11.90

7	-6	-13	600.84	35.30
7	-6	-13	649.64	36.00
-7	6	13	610.94	32.80
-7	6	13	585.44	34.60
6	-6	-13	33.70	10.40
6	-6	-13	20.10	11.70
6	-6	-13	17.80	11.10
-6	6	13	5.60	17.20
5	-6	-13	272.97	26.70
5	-6	-13	274.37	23.00
5	-6	-13	293.87	23.10
5	-6	-13	313.67	21.30
-5	6	13	263.97	18.60
4	-6	-13	2.40	8.60
4	-6	-13	-5.60	16.00
4	-6	-13	-3.00	8.00
4	-6	-13	13.60	10.90
-4	6	13	6.50	5.10
3	-6	-13	210.98	19.60
3	-6	-13	209.38	17.20
3	-6	-13	214.68	18.00
3	-6	-13	239.08	20.70
-3	6	13	213.08	12.80
2	-6	-13	-7.10	7.10
2	-6	-13	12.40	8.10
2	-6	-13	2.90	8.90
2	-6	-13	-6.10	6.70
2	-6	-13	-14.80	11.00
-2	6	13	3.40	3.00
1	-6	-13	9.40	11.00
1	-6	-13	44.50	12.90
1	-6	-13	12.80	8.50
1	-6	-13	9.60	8.00
1	-6	-13	14.20	7.70
-1	6	13	17.50	4.40
0	-6	-13	1468.05	69.79
0	-6	-13	1449.06	72.69
0	-6	-13	1511.85	69.59
0	-6	-13	1425.46	69.89
0	6	13	1548.84	66.99
-1	-6	-13	1539.65	74.89
-1	-6	-13	1389.66	70.09
-1	-6	-13	1438.36	69.89
1	6	13	1555.24	66.69

-2	-6	-13	201.98	21.40
-2	-6	-13	215.28	20.50
-2	-6	-13	223.18	29.70
2	6	13	177.88	12.80
2	6	13	210.78	14.40
-3	-6	-13	82.09	10.90
-3	-6	-13	87.39	17.50
-3	-6	-13	93.89	13.70
-3	-6	-13	108.69	18.70
-3	-6	-13	77.19	11.20
3	6	13	81.79	11.60
3	6	13	98.59	14.50
-4	-6	-13	102.69	15.10
-4	-6	-13	94.99	18.00
-4	-6	-13	106.69	10.40
-4	-6	-13	91.09	15.00
-4	-6	-13	101.69	15.50
-4	-6	-13	113.39	18.60
-5	-6	-13	58.49	9.50
-5	-6	-13	57.09	8.70
-5	-6	-13	39.90	14.70
-5	-6	-13	58.19	10.00
-5	-6	-13	79.69	19.70
-5	-6	-13	68.69	9.50
5	6	13	63.69	4.80
-6	-6	-13	142.69	11.80
-6	-6	-13	144.59	14.80
-6	-6	-13	151.48	17.00
-6	-6	-13	171.68	24.20
-7	-6	-13	120.69	11.80
-7	-6	-13	126.59	17.10
-7	-6	-13	132.99	22.60
-7	-6	-13	153.48	20.80
-7	-6	-13	109.99	14.50
-7	-6	-13	108.29	12.80
7	6	13	119.49	9.00
-8	-6	-13	233.98	25.60
-8	-6	-13	195.28	19.80
-8	-6	-13	226.78	18.30
-8	-6	-13	201.08	16.60
-8	-6	-13	220.38	22.70
8	6	13	206.68	14.10
-9	-6	-13	80.09	13.10
-9	-6	-13	49.10	8.50

-9	-6	-13	50.69	13.30
-9	-6	-13	59.59	11.70
-9	-6	-13	58.79	9.80
-9	-6	-13	58.89	15.40
9	6	13	74.69	12.60
-10	-6	-13	409.26	25.40
-10	-6	-13	435.86	24.20
-10	-6	-13	419.86	28.30
-10	-6	-13	417.06	23.80
-10	-6	-13	413.56	28.20
-11	-6	-13	363.26	25.40
-11	-6	-13	440.36	27.80
-11	-6	-13	325.67	28.60
-11	-6	-13	436.46	22.80
-11	-6	-13	401.56	24.30
10	-7	-13	-6.80	9.40
-10	7	13	10.70	9.10
9	-7	-13	5.50	11.10
-9	7	13	-14.30	12.90
-9	7	13	0.30	8.10
8	-7	-13	65.19	14.30
8	-7	-13	41.30	14.70
-8	7	13	49.80	10.10
-8	7	13	39.40	12.10
7	-7	-13	48.10	12.90
-7	7	13	60.89	11.70
-7	7	13	80.59	13.00
6	-7	-13	225.98	20.10
6	-7	-13	203.18	21.30
6	-7	-13	170.78	23.90
-6	7	13	171.68	20.50
-6	7	13	172.28	26.70
5	-7	-13	435.26	28.20
5	-7	-13	421.86	30.80
5	-7	-13	449.46	26.30
5	-7	-13	460.25	28.80
-5	7	13	430.86	24.70
-5	7	13	422.76	29.20
4	-7	-13	285.87	30.70
4	-7	-13	218.88	21.30
4	-7	-13	210.88	24.70
-4	7	13	230.98	14.40
3	-7	-13	21.40	8.20
3	-7	-13	28.90	8.70

3	-7	-13	11.50	7.30
3	-7	-13	17.90	9.90
3	-7	-13	2.60	10.30
-3	7	13	10.70	3.70
2	-7	-13	393.06	29.40
2	-7	-13	444.36	25.30
2	-7	-13	430.46	25.30
2	-7	-13	465.25	25.20
2	-7	-13	454.35	26.50
-2	7	13	479.75	22.00
1	-7	-13	589.64	32.00
1	-7	-13	604.44	36.50
1	-7	-13	578.84	31.60
1	-7	-13	577.64	31.80
1	-7	-13	620.44	32.30
-1	7	13	642.94	28.80
0	-7	-13	183.88	16.70
0	-7	-13	218.28	27.10
0	-7	-13	176.48	16.40
0	-7	-13	168.98	17.50
0	7	13	193.48	10.10
-1	-7	-13	94.29	16.10
-1	-7	-13	66.39	16.60
-1	-7	-13	80.69	15.20
1	7	13	78.79	5.70
-2	-7	-13	-5.70	16.20
-2	-7	-13	11.90	11.40
-2	-7	-13	23.40	11.50
-3	-7	-13	16.90	6.70
-3	-7	-13	17.30	13.00
-3	-7	-13	21.20	10.20
-3	-7	-13	10.60	16.60
-3	-7	-13	9.70	13.10
3	7	13	9.10	7.20
3	7	13	21.10	9.70
-4	-7	-13	58.89	10.10
-4	-7	-13	102.59	18.10
-4	-7	-13	76.69	18.40
-4	-7	-13	58.59	9.40
-5	-7	-13	64.49	9.50
-5	-7	-13	48.70	8.10
-5	-7	-13	78.49	14.70
-5	-7	-13	69.09	15.00
-5	-7	-13	55.69	7.40

-5	-7	-13	96.99	22.80
-6	-7	-13	1175.58	56.39
-6	-7	-13	1334.77	65.69
-6	-7	-13	1186.68	55.39
-6	-7	-13	1033.60	58.09
-6	-7	-13	1123.49	72.99
-6	-7	-13	1134.39	56.59
-6	-7	-13	1188.08	57.79
6	7	13	1228.48	53.49
-7	-7	-13	27.90	7.60
-7	-7	-13	34.90	12.80
-7	-7	-13	15.60	9.20
-7	-7	-13	23.50	11.80
-7	-7	-13	16.10	6.40
-7	-7	-13	14.30	7.70
-8	-7	-13	95.99	12.50
-8	-7	-13	72.99	12.80
-8	-7	-13	74.09	12.70
-8	-7	-13	65.19	12.30
-8	-7	-13	74.29	15.50
-8	-7	-13	69.79	10.80
-9	-7	-13	140.39	26.60
-9	-7	-13	178.98	21.60
-9	-7	-13	166.38	14.00
-9	-7	-13	177.48	15.10
-9	-7	-13	154.88	18.50
-9	-7	-13	184.98	16.80
9	7	13	174.48	15.30
-10	-7	-13	106.49	12.80
-10	-7	-13	113.49	18.10
-10	-7	-13	108.69	15.30
-10	-7	-13	107.89	12.30
-10	-7	-13	92.79	14.00
-11	-7	-13	70.09	11.60
-11	-7	-13	39.80	14.50
-11	-7	-13	51.79	11.70
-11	-7	-13	62.59	10.10
-11	-7	-13	79.89	12.20
10	-8	-13	42.00	13.80
-10	8	13	29.50	14.90
9	-8	-13	75.69	18.10
9	-8	-13	89.79	13.50
-9	8	13	91.09	11.90
-9	8	13	74.09	12.80

8	-8	-13	43.20	12.20
8	-8	-13	33.10	13.30
-8	8	13	30.00	9.40
-8	8	13	24.30	11.20
7	-8	-13	305.17	24.10
7	-8	-13	339.57	24.00
-7	8	13	288.97	20.80
-7	8	13	271.47	23.60
6	-8	-13	1215.08	59.09
6	-8	-13	1124.89	59.59
6	-8	-13	1208.18	56.79
6	-8	-13	1112.79	60.89
-6	8	13	1175.68	56.69
-6	8	13	1079.29	58.59
5	-8	-13	64.19	12.30
5	-8	-13	53.59	13.50
5	-8	-13	64.49	12.50
5	-8	-13	67.39	10.30
-5	8	13	55.79	8.80
-5	8	13	72.19	11.60
4	-8	-13	16.80	9.50
4	-8	-13	29.90	11.60
4	-8	-13	4.70	8.80
-4	8	13	12.90	5.10
-4	8	13	26.40	11.20
3	-8	-13	12.70	8.30
3	-8	-13	22.10	8.60
3	-8	-13	37.10	10.10
3	-8	-13	8.90	8.10
3	-8	-13	30.80	12.30
-3	8	13	24.90	4.20
2	-8	-13	73.59	13.20
2	-8	-13	59.29	13.90
2	-8	-13	71.59	9.70
2	-8	-13	61.79	9.00
2	-8	-13	74.69	11.10
-2	8	13	60.69	6.00
1	-8	-13	362.86	29.00
1	-8	-13	332.97	22.00
1	-8	-13	355.26	22.70
1	-8	-13	351.66	21.80
1	-8	-13	337.27	22.10
-1	8	13	358.26	17.20
0	-8	-13	331.47	31.20

0	-8	-13	300.97	21.80
0	-8	-13	344.27	22.50
0	-8	-13	308.97	21.10
0	-8	-13	328.47	21.20
0	8	13	355.86	16.20
-1	-8	-13	851.41	44.70
-1	-8	-13	868.51	50.39
-1	-8	-13	892.81	43.50
-1	-8	-13	874.21	43.50
-2	-8	-13	332.37	22.60
-2	-8	-13	286.77	23.20
-2	-8	-13	276.17	25.60
-2	-8	-13	349.96	33.10
-3	-8	-13	-2.50	13.20
-3	-8	-13	11.70	7.00
-3	-8	-13	4.50	11.10
-3	-8	-13	1.10	14.70
-3	-8	-13	-1.10	16.80
-4	-8	-13	384.26	26.20
-4	-8	-13	409.56	39.70
-4	-8	-13	394.56	33.70
-4	-8	-13	396.56	23.90
-4	-8	-13	395.66	21.10
-4	-8	-13	375.06	23.50
-5	-8	-13	247.88	20.20
-5	-8	-13	189.78	24.30
-5	-8	-13	288.17	15.80
-5	-8	-13	251.57	27.70
-5	-8	-13	270.37	35.80
-5	-8	-13	260.97	18.40
-5	-8	-13	258.17	17.70
-6	-8	-13	32.70	9.60
-6	-8	-13	22.00	6.60
-6	-8	-13	35.30	13.40
-6	-8	-13	10.80	7.40
-6	-8	-13	37.00	12.40
-6	-8	-13	16.60	7.40
6	8	13	29.30	4.10
-7	-8	-13	88.39	12.70
-7	-8	-13	89.09	15.70
-7	-8	-13	82.29	12.30
-7	-8	-13	91.19	14.40
-7	-8	-13	81.59	12.70
-7	-8	-13	97.49	11.20

-8	-8	-13	80.99	12.60
-8	-8	-13	91.79	11.80
-8	-8	-13	124.89	23.40
-8	-8	-13	78.09	12.90
-8	-8	-13	90.79	17.20
-8	-8	-13	91.39	14.50
8	8	13	70.49	10.00
-9	-8	-13	11.40	12.00
-9	-8	-13	24.20	9.90
-9	-8	-13	16.50	7.60
-9	-8	-13	14.10	7.20
-9	-8	-13	4.40	7.70
9	8	13	8.10	8.40
-10	-8	-13	8.00	7.80
-10	-8	-13	-5.40	8.50
-10	-8	-13	-15.60	10.90
-10	-8	-13	2.90	7.50
-10	-8	-13	6.70	10.10
-11	-8	-13	37.80	13.70
-11	-8	-13	19.50	7.50
-11	-8	-13	12.50	10.80
-11	-8	-13	17.80	8.40
9	-9	-13	41.60	12.00
9	-9	-13	54.09	16.00
-9	9	13	40.70	12.10
-9	9	13	41.40	10.30
8	-9	-13	179.68	19.90
8	-9	-13	170.48	20.30
-8	9	13	147.99	19.50
-8	9	13	149.99	17.50
7	-9	-13	22.60	12.40
7	-9	-13	9.70	10.80
-7	9	13	14.70	11.30
6	-9	-13	306.07	22.00
6	-9	-13	284.07	23.80
6	-9	-13	258.57	27.50
6	-9	-13	289.47	30.70
-6	9	13	224.48	29.90
-6	9	13	239.98	21.90
5	-9	-13	126.59	18.50
5	-9	-13	119.89	19.90
5	-9	-13	107.19	15.20
5	-9	-13	103.49	15.60
-5	9	13	95.49	16.80

-5	9	13	80.89	10.50
4	-9	-13	101.79	19.20
4	-9	-13	113.79	20.70
4	-9	-13	130.09	17.00
4	-9	-13	119.99	14.90
-4	9	13	124.29	10.60
-4	9	13	106.39	17.10
3	-9	-13	9.80	9.30
3	-9	-13	1.90	7.90
3	-9	-13	22.50	8.10
3	-9	-13	-2.00	12.10
-3	9	13	5.90	3.70
2	-9	-13	23.90	8.70
2	-9	-13	16.00	7.80
2	-9	-13	26.50	9.00
2	-9	-13	44.70	8.60
2	-9	-13	48.50	13.90
-2	9	13	38.70	5.00
1	-9	-13	339.07	20.90
1	-9	-13	315.47	29.20
1	-9	-13	310.17	21.00
1	-9	-13	324.27	21.70
1	-9	-13	327.37	21.10
-1	9	13	354.46	16.50
0	-9	-13	390.76	22.80
0	-9	-13	378.06	33.20
0	-9	-13	371.26	23.10
0	-9	-13	372.76	24.50
0	-9	-13	393.16	23.30
-1	-9	-13	56.09	12.20
-1	-9	-13	86.99	14.10
-1	-9	-13	86.49	13.50
-1	-9	-13	88.29	15.50
-1	-9	-13	39.90	17.90
-2	-9	-13	93.99	12.70
-2	-9	-13	79.99	15.40
-2	-9	-13	68.79	19.00
-2	-9	-13	78.59	12.00
-2	-9	-13	114.39	15.50
-3	-9	-13	136.19	13.30
-3	-9	-13	129.49	14.70
-3	-9	-13	126.09	19.20
-3	-9	-13	211.08	31.00
-3	-9	-13	165.18	19.60

-3	-9	-13	168.88	21.50
-3	-9	-13	133.89	20.10
-4	-9	-13	342.37	18.70
-4	-9	-13	342.27	31.40
-4	-9	-13	343.37	21.80
-4	-9	-13	323.57	22.00
-4	-9	-13	285.47	26.60
-4	-9	-13	324.07	23.40
-4	-9	-13	324.67	44.20
-5	-9	-13	1032.80	48.90
-5	-9	-13	1036.20	47.70
-5	-9	-13	1031.40	50.49
-5	-9	-13	913.81	51.19
-5	-9	-13	1007.00	54.49
-5	-9	-13	1012.90	48.40
-6	-9	-13	6.70	13.70
-6	-9	-13	8.20	5.90
-6	-9	-13	4.50	6.80
-6	-9	-13	-11.10	11.30
-6	-9	-13	11.70	8.00
-6	-9	-13	19.80	9.30
6	9	13	15.80	3.90
-7	-9	-13	481.45	26.50
-7	-9	-13	480.95	25.60
-7	-9	-13	475.05	25.00
-7	-9	-13	418.66	32.70
-7	-9	-13	420.46	28.10
-7	-9	-13	439.46	28.40
7	9	13	473.75	23.00
-8	-9	-13	88.99	13.00
-8	-9	-13	76.59	16.30
-8	-9	-13	78.49	11.50
-8	-9	-13	74.49	12.40
-8	-9	-13	74.29	13.40
-8	-9	-13	85.19	12.20
-9	-9	-13	12.30	10.30
-9	-9	-13	5.30	7.00
-9	-9	-13	8.60	16.00
-9	-9	-13	24.00	8.30
-9	-9	-13	6.40	7.90
9	9	13	10.30	8.30
-10	-9	-13	196.08	15.40
-10	-9	-13	158.68	14.50
-10	-9	-13	209.78	18.10

-10	-9	-13	178.28	19.70
-10	-9	-13	178.88	15.80
-11	-9	-13	17.90	8.30
-11	-9	-13	33.80	10.20
-11	-9	-13	23.80	8.60
-11	-9	-13	15.90	12.10
-11	-9	-13	19.50	7.80
9	-10	-13	-9.50	13.80
9	-10	-13	14.00	11.10
-9	10	13	1.20	10.00
-9	10	13	8.70	13.40
8	-10	-13	68.19	12.70
8	-10	-13	92.79	13.50
-8	10	13	53.59	11.50
-8	10	13	55.09	10.60
7	-10	-13	557.54	35.10
7	-10	-13	503.15	34.10
-7	10	13	595.44	42.00
6	-10	-13	87.99	15.70
6	-10	-13	97.19	17.50
6	-10	-13	66.69	14.00
6	-10	-13	53.29	15.60
-6	10	13	50.39	11.40
5	-10	-13	200.88	22.40
5	-10	-13	196.68	18.60
5	-10	-13	181.38	23.20
5	-10	-13	183.28	22.40
-5	10	13	158.18	16.20
-5	10	13	206.28	19.40
4	-10	-13	18.40	12.50
4	-10	-13	-7.80	8.60
4	-10	-13	10.30	9.80
4	-10	-13	-5.40	8.90
4	-10	-13	-14.80	10.70
-4	10	13	3.20	5.40
-4	10	13	-5.60	9.70
3	-10	-13	117.19	14.90
3	-10	-13	165.28	21.90
3	-10	-13	134.19	17.10
3	-10	-13	121.19	14.80
3	-10	-13	118.79	20.50
-3	10	13	141.29	9.60
2	-10	-13	10.10	9.00
2	-10	-13	0.80	8.50

2	-10	-13	7.60	8.40
2	-10	-13	-19.70	13.80
2	-10	-13	11.60	8.40
-2	10	13	6.00	3.00
1	-10	-13	159.68	20.70
1	-10	-13	206.78	16.40
1	-10	-13	187.98	17.40
1	-10	-13	203.18	16.40
1	-10	-13	206.08	27.30
1	-10	-13	183.18	17.20
-1	10	13	206.68	10.70
0	-10	-13	1317.57	65.19
0	-10	-13	1360.56	64.39
0	-10	-13	1332.57	64.69
0	-10	-13	1392.06	67.09
0	-10	-13	1371.06	69.49
0	-10	-13	1342.47	64.19
0	10	13	1421.36	61.59
-1	-10	-13	371.26	26.10
-1	-10	-13	327.97	22.20
-1	-10	-13	364.16	21.70
-1	-10	-13	254.57	33.40
-1	-10	-13	371.16	22.40
-2	-10	-13	126.69	17.00
-2	-10	-13	79.79	13.80
-2	-10	-13	119.19	14.80
-2	-10	-13	151.78	20.50
-2	-10	-13	122.09	16.30
-2	-10	-13	119.99	17.00
-3	-10	-13	63.89	11.30
-3	-10	-13	99.69	14.90
-3	-10	-13	54.99	18.70
-3	-10	-13	69.69	11.20
-3	-10	-13	65.69	12.50
-4	-10	-13	50.59	16.30
-4	-10	-13	-1.00	9.60
-4	-10	-13	17.60	6.50
-4	-10	-13	17.40	10.90
-4	-10	-13	16.40	12.00
-4	-10	-13	33.00	11.30
-4	-10	-13	20.90	10.50
-5	-10	-13	153.08	16.00
-5	-10	-13	152.98	14.70
-5	-10	-13	150.88	12.30

-5	-10	-13	130.39	19.10
-5	-10	-13	181.88	21.20
-5	-10	-13	163.58	19.20
-6	-10	-13	9.70	10.60
-6	-10	-13	7.00	7.40
-6	-10	-13	-4.60	14.30
-6	-10	-13	13.60	6.70
-6	-10	-13	5.30	10.00
-6	-10	-13	0.80	7.40
6	10	13	11.00	4.30
-7	-10	-13	49.50	15.50
-7	-10	-13	26.10	10.60
-7	-10	-13	44.20	8.00
-7	-10	-13	61.69	9.20
-7	-10	-13	52.59	8.40
-7	-10	-13	46.30	11.00
7	10	13	54.19	8.30
-8	-10	-13	250.47	16.90
-8	-10	-13	261.57	18.90
-8	-10	-13	217.88	21.10
-8	-10	-13	263.17	17.90
-8	-10	-13	240.18	21.50
-8	-10	-13	200.68	30.00
-9	-10	-13	123.79	20.70
-9	-10	-13	133.49	12.60
-9	-10	-13	172.78	24.40
9	10	13	120.99	14.70
-10	-10	-13	253.07	21.40
-10	-10	-13	227.38	16.70
-10	-10	-13	233.28	20.20
-10	-10	-13	226.98	16.50
-10	-10	-13	265.57	17.90
-11	-10	-13	124.09	16.90
-11	-10	-13	131.19	22.00
-11	-10	-13	102.29	12.90
-11	-10	-13	106.39	11.90
-11	-10	-13	102.99	12.90
9	-11	-13	-37.00	18.30
9	-11	-13	17.60	12.10
-9	11	13	10.20	10.80
-9	11	13	12.10	9.90
8	-11	-13	21.50	11.50
8	-11	-13	4.90	11.90
-8	11	13	4.00	10.30

-8	11	13	18.30	9.70
7	-11	-13	41.50	14.80
7	-11	-13	34.50	12.10
-7	11	13	52.09	11.50
6	-11	-13	40.80	15.60
6	-11	-13	10.20	12.70
6	-11	-13	8.80	9.30
6	-11	-13	15.60	15.10
-6	11	13	-9.90	9.90
5	-11	-13	578.14	33.50
5	-11	-13	519.15	33.80
5	-11	-13	533.05	30.20
5	-11	-13	528.75	34.10
-5	11	13	507.65	29.00
-5	11	13	525.85	31.10
4	-11	-13	303.57	25.30
4	-11	-13	311.67	22.20
4	-11	-13	296.97	21.30
4	-11	-13	270.67	22.30
4	-11	-13	274.97	22.30
-4	11	13	277.27	17.30
-4	11	13	267.67	22.10
3	-11	-13	30.40	9.70
3	-11	-13	39.80	10.30
3	-11	-13	32.70	8.90
3	-11	-13	25.80	20.20
3	-11	-13	25.90	8.80
-3	11	13	27.90	4.90
2	-11	-13	741.53	42.50
2	-11	-13	796.52	44.20
2	-11	-13	714.43	44.00
2	-11	-13	707.73	42.50
2	-11	-13	701.93	42.30
-2	11	13	714.03	34.30
1	-11	-13	274.67	20.40
1	-11	-13	276.47	20.20
1	-11	-13	287.27	23.10
1	-11	-13	274.77	19.20
1	-11	-13	264.97	19.50
1	-11	-13	325.77	30.80
-1	11	13	281.47	14.30
0	-11	-13	189.38	18.80
0	-11	-13	154.58	16.60
0	-11	-13	172.98	29.80

0	-11	-13	174.38	15.30
0	-11	-13	150.48	20.10
0	-11	-13	179.58	15.30
0	11	13	171.38	9.30
-1	-11	-13	36.90	12.80
-1	-11	-13	35.70	9.10
-1	-11	-13	7.20	11.20
-1	-11	-13	24.60	7.70
-1	-11	-13	30.20	10.00
1	11	13	27.40	4.10
-2	-11	-13	100.29	14.50
-2	-11	-13	106.59	13.20
-2	-11	-13	94.39	16.70
-2	-11	-13	151.88	21.70
-3	-11	-13	139.79	16.80
-3	-11	-13	185.18	17.80
-3	-11	-13	158.48	15.00
-3	-11	-13	179.18	22.20
-3	-11	-13	107.89	18.70
-3	-11	-13	152.38	20.60
-3	-11	-13	203.38	36.70
-4	-11	-13	88.19	11.70
-4	-11	-13	108.69	19.20
-4	-11	-13	112.29	18.00
-4	-11	-13	104.59	12.30
-4	-11	-13	138.89	22.20
-4	-11	-13	129.69	17.80
-4	-11	-13	98.59	13.50
-5	-11	-13	423.46	25.50
-5	-11	-13	419.96	26.60
-5	-11	-13	412.46	22.80
-5	-11	-13	403.86	24.10
-6	-11	-13	539.65	31.90
-6	-11	-13	563.34	28.10
-6	-11	-13	471.05	31.60
-6	-11	-13	537.45	29.20
-6	-11	-13	517.65	28.80
6	11	13	559.44	25.70
-7	-11	-13	-0.90	9.90
-7	-11	-13	19.30	7.80
-7	-11	-13	15.20	8.00
-7	-11	-13	-10.80	10.30
-7	-11	-13	17.40	10.70
7	11	13	10.20	5.90

-8	-11	-13	128.99	14.50
-8	-11	-13	131.69	13.30
-8	-11	-13	169.48	19.00
-8	-11	-13	115.19	18.30
-8	-11	-13	137.09	15.50
8	11	13	130.59	12.70
-9	-11	-13	327.97	20.80
-9	-11	-13	288.07	19.60
-9	-11	-13	317.77	21.50
-9	-11	-13	326.97	23.90
-9	-11	-13	288.77	24.00
9	11	13	313.17	20.60
-10	-11	-13	34.70	10.90
-10	-11	-13	26.50	7.70
-10	-11	-13	20.40	10.50
-10	-11	-13	23.90	8.20
-11	-11	-13	12.60	7.70
-11	-11	-13	11.30	9.30
-11	-11	-13	13.60	7.60
-11	-11	-13	25.50	12.80
9	-12	-13	-1.40	14.30
9	-12	-13	-8.00	12.70
-9	12	13	14.80	11.10
-9	12	13	-12.30	12.30
8	-12	-13	-9.70	12.40
8	-12	-13	-6.30	10.70
-8	12	13	-11.80	9.50
-8	12	13	10.40	9.40
7	-12	-13	415.96	25.30
7	-12	-13	367.46	27.40
-7	12	13	360.26	25.80
6	-12	-13	839.92	48.50
6	-12	-13	780.72	46.90
6	-12	-13	835.32	43.20
6	-12	-13	872.01	45.50
-6	12	13	843.22	43.40
5	-12	-13	55.49	14.40
5	-12	-13	41.40	12.80
5	-12	-13	60.19	13.80
5	-12	-13	39.70	10.00
-5	12	13	51.89	11.10
4	-12	-13	10.80	10.70
4	-12	-13	-6.70	9.90
4	-12	-13	9.20	9.00

4	-12	-13	10.10	9.50
4	-12	-13	24.10	14.60
-4	12	13	23.00	6.60
3	-12	-13	52.39	10.80
3	-12	-13	53.99	10.00
3	-12	-13	63.49	11.00
3	-12	-13	46.60	10.30
3	-12	-13	47.00	15.80
-3	12	13	70.69	7.80
2	-12	-13	224.78	18.60
2	-12	-13	222.68	18.90
2	-12	-13	230.98	30.20
2	-12	-13	216.98	18.70
2	-12	-13	215.78	18.70
-2	12	13	208.58	12.30
1	-12	-13	246.18	20.60
1	-12	-13	208.88	19.10
1	-12	-13	216.38	18.50
1	-12	-13	203.38	30.90
1	-12	-13	284.77	25.30
-1	12	13	201.48	11.80
0	-12	-13	-2.60	8.40
0	-12	-13	20.80	10.80
0	-12	-13	10.10	7.40
0	-12	-13	-1.90	7.90
0	12	13	4.60	2.70
-1	-12	-13	933.01	48.80
-1	-12	-13	868.61	44.60
-1	-12	-13	929.61	45.90
-1	-12	-13	901.31	44.80
-1	-12	-13	877.11	45.60
1	12	13	950.20	41.40
-2	-12	-13	153.68	18.90
-2	-12	-13	170.58	18.40
-2	-12	-13	168.08	16.50
-2	-12	-13	176.18	15.90
-2	-12	-13	205.98	29.20
-2	-12	-13	176.78	18.40
-3	-12	-13	-6.80	9.60
-3	-12	-13	-11.70	8.90
-3	-12	-13	3.90	15.30
-3	-12	-13	4.20	9.40
-3	-12	-13	-31.40	37.20
-3	-12	-13	8.30	8.50

-3	-12	-13	-15.50	12.60
-4	-12	-13	1101.69	52.19
-4	-12	-13	1143.29	54.29
-4	-12	-13	1099.79	52.89
-4	-12	-13	1053.09	53.19
-4	-12	-13	1093.19	53.49
-4	-12	-13	1068.69	53.69
-5	-12	-13	86.99	11.30
-5	-12	-13	78.19	11.70
-5	-12	-13	71.79	10.10
-5	-12	-13	78.49	11.40
-5	-12	-13	76.99	10.40
-5	-12	-13	97.39	15.30
-6	-12	-13	-0.70	10.80
-6	-12	-13	-7.20	11.20
-6	-12	-13	2.20	9.80
-6	-12	-13	2.70	8.10
-6	-12	-13	3.20	7.10
-6	-12	-13	-10.80	7.70
6	12	13	7.70	5.60
-7	-12	-13	468.45	26.00
-7	-12	-13	450.05	29.40
-7	-12	-13	462.45	27.10
-7	-12	-13	494.05	29.10
-7	-12	-13	464.95	26.20
7	12	13	483.35	25.90
-8	-12	-13	43.00	8.80
-8	-12	-13	41.00	8.30
-8	-12	-13	43.70	8.70
-8	-12	-13	36.80	11.40
-8	-12	-13	44.90	11.30
8	12	13	41.60	8.50
-9	-12	-13	53.89	9.20
-9	-12	-13	51.69	9.00
-9	-12	-13	54.59	11.30
-9	-12	-13	35.90	11.40
-9	-12	-13	38.20	8.60
-10	-12	-13	12.60	7.90
-10	-12	-13	9.30	7.80
-10	-12	-13	-2.40	10.80
-10	-12	-13	8.70	8.30
-10	-12	-13	0.00	10.20
-11	-12	-13	82.19	12.40
-11	-12	-13	66.29	12.20

-11	-12	-13	91.39	13.40
-11	-12	-13	100.79	11.80
8	-13	-13	96.49	15.70
8	-13	-13	83.99	15.40
-8	13	13	108.09	16.30
-8	13	13	85.59	11.90
7	-13	-13	17.00	9.80
7	-13	-13	5.40	13.80
-7	13	13	24.10	10.90
6	-13	-13	214.28	19.60
5	-13	-13	142.59	23.60
5	-13	-13	124.19	16.00
5	-13	-13	101.69	17.20
5	-13	-13	119.29	20.10
-5	13	13	91.69	12.60
-5	13	13	102.69	16.70
4	-13	-13	12.90	11.20
4	-13	-13	25.20	9.30
4	-13	-13	46.30	11.20
4	-13	-13	24.90	16.30
4	-13	-13	24.90	10.60
-4	13	13	31.10	7.30
3	-13	-13	24.00	9.70
3	-13	-13	13.80	9.80
3	-13	-13	27.30	9.60
3	-13	-13	36.70	17.70
3	-13	-13	29.10	10.40
-3	13	13	23.70	5.40
2	-13	-13	7.20	9.00
2	-13	-13	10.50	8.90
2	-13	-13	10.30	8.60
2	-13	-13	0.80	8.30
-2	13	13	4.60	3.70
1	-13	-13	754.22	42.70
1	-13	-13	795.92	40.40
1	-13	-13	787.02	40.10
1	-13	-13	782.32	41.30
1	-13	-13	806.02	40.80
-1	13	13	787.72	36.70
0	-13	-13	130.49	20.20
0	-13	-13	166.58	18.40
0	-13	-13	167.08	18.40
0	13	13	180.88	9.70
-1	-13	-13	74.79	10.50

-1	-13	-13	68.59	12.40
-1	-13	-13	96.59	15.60
-1	-13	-13	90.79	12.40
1	13	13	90.39	6.20
-2	-13	-13	501.95	27.80
-2	-13	-13	473.95	28.30
-2	-13	-13	562.24	39.40
-2	-13	-13	471.75	28.20
-2	-13	-13	475.75	28.70
-2	-13	-13	505.65	30.30
2	13	13	526.15	23.20
-3	-13	-13	685.43	39.50
-3	-13	-13	675.63	39.20
-3	-13	-13	724.63	36.50
-3	-13	-13	725.43	38.10
-4	-13	-13	364.96	25.50
-4	-13	-13	329.47	26.70
-4	-13	-13	375.56	23.30
-4	-13	-13	397.46	24.20
-4	-13	-13	416.16	22.80
-4	-13	-13	384.76	24.80
-5	-13	-13	524.05	29.40
-5	-13	-13	544.15	29.10
-5	-13	-13	509.45	29.80
-5	-13	-13	527.35	28.00
-5	-13	-13	480.75	28.80
-6	-13	-13	181.98	19.00
-6	-13	-13	146.79	16.10
-6	-13	-13	176.68	21.70
-6	-13	-13	174.48	14.90
-6	-13	-13	149.09	15.60
-6	-13	-13	147.19	18.20
6	13	13	132.19	12.20
-7	-13	-13	111.49	14.10
-7	-13	-13	105.39	13.30
-7	-13	-13	143.69	17.80
-7	-13	-13	120.79	14.40
-7	-13	-13	113.89	15.40
-7	-13	-13	126.49	14.90
7	13	13	103.29	12.20
-8	-13	-13	173.08	16.60
-8	-13	-13	150.08	14.80
-8	-13	-13	173.48	20.80
-8	-13	-13	170.78	15.70

8	13	13	125.59	14.90
-9	-13	-13	51.09	9.40
-9	-13	-13	65.29	11.80
-9	-13	-13	55.99	10.80
-9	-13	-13	49.90	9.50
-9	-13	-13	72.69	12.60
-10	-13	-13	249.38	19.00
-10	-13	-13	240.48	16.30
-10	-13	-13	219.28	16.60
-10	-13	-13	203.98	17.10
-11	-13	-13	14.30	12.60
-11	-13	-13	13.80	12.20
-11	-13	-13	12.80	10.50
8	-14	-13	122.79	14.30
8	-14	-13	134.69	22.90
-8	14	13	129.69	17.30
7	-14	-13	187.98	17.00
7	-14	-13	193.28	24.10
-7	14	13	168.98	19.20
6	-14	-13	289.87	28.60
6	-14	-13	301.97	21.90
6	-14	-13	258.67	33.00
6	-14	-13	281.37	28.20
-6	14	13	273.07	23.00
5	-14	-13	106.59	19.10
5	-14	-13	119.79	17.00
5	-14	-13	133.59	21.60
5	-14	-13	94.39	17.10
-5	14	13	103.89	17.70
-5	14	13	153.18	19.30
4	-14	-13	124.99	18.70
4	-14	-13	111.59	20.00
4	-14	-13	88.99	13.20
4	-14	-13	97.99	12.70
4	-14	-13	78.99	12.40
-4	14	13	82.49	13.40
3	-14	-13	66.59	11.30
3	-14	-13	32.00	10.00
3	-14	-13	42.60	10.20
3	-14	-13	43.20	10.70
-3	14	13	50.89	8.80
2	-14	-13	211.28	20.50
2	-14	-13	200.78	18.90
2	-14	-13	223.68	19.20

2	-14	-13	205.48	18.90
-2	14	13	211.88	12.90
1	-14	-13	210.58	20.30
1	-14	-13	196.48	17.20
1	-14	-13	166.48	18.30
1	-14	-13	183.38	17.70
1	-14	-13	202.68	17.70
-1	14	13	184.98	11.20
0	-14	-13	1158.68	55.99
0	-14	-13	1115.59	57.69
0	-14	-13	1108.79	55.49
0	-14	-13	1168.98	55.59
0	-14	-13	1183.18	56.29
0	14	13	1142.19	52.49
-1	-14	-13	83.39	14.30
-1	-14	-13	110.19	18.60
-1	-14	-13	77.89	14.40
-1	-14	-13	104.79	16.10
-1	-14	-13	91.99	21.10
1	14	13	89.49	6.70
-2	-14	-13	56.39	20.50
-2	-14	-13	110.89	14.80
-2	-14	-13	120.19	15.20
-2	-14	-13	75.39	10.60
[truncated: 441,896 more chars]
