# Supplementary material for: Structure-property relationships of photofunctional diiridium(II) complexes with tetracationic charge and an unsupported Ir–Ir bond
Source: Commun Chem. 2022 Nov 23;5:159. doi: 10.1038/s42004-022-00775-4 (PMC9814866; doi:10.1038/s42004-022-00775-4)
Supplement: Supplementary file 10 — Supplementary Data 7 [file 42004_2022_775_MOESM10_ESM.pdf]

```
data_Ir(III)(t-Bu-terpyr)(PPh3)(CO)Br[OTf]2
_audit_creation_date              2018-06-20
_audit_creation_method
;
Olex2 1.2
(compiled 2018.05.29 svn.r3508 for OlexSys, GUI svn.r5506)
;
_shelxl_version_number            2013-2
_audit_contact_author_address     ?
_audit_contact_author_email       ?
_audit_contact_author_name        ''
_audit_contact_author_phone       ?
_publ_contact_author_id_orcid     ?
_publ_section_references
;
Dolomanov, O.V., Bourhis, L.J., Gildea, R.J, Howard, J.A.K. & Puschmann,
H.
 (2009), J. Appl. Cryst. 42, 339-341.

Sheldrick, G.M. (2015). Acta Cryst. A71, 3-8.

Sheldrick, G.M. (2015). Acta Cryst. C71, 3-8.
;
_chemical_name_common             ?
_chemical_name_systematic
;
 ?
;
_chemical_formula_moiety          'C46 H49 Br Ir N3 O P, 2(C F3 O3 S),
C2 H3 N'
_chemical_formula_sum             'C50 H52 Br F6 Ir N4 O7 P S2'
_chemical_formula_weight          1302.15
_chemical_absolute_configuration  ?
_chemical_melting_point           ?
loop_
  _atom_type_symbol
  _atom_type_description
  _atom_type_scat_dispersion_real
  _atom_type_scat_dispersion_imag
  _atom_type_scat_source
 'C' 'C' 0.0033 0.0016 'International Tables Vol C Tables 4.2.6.8 and
6.1.1.4'
 'H' 'H' 0.0000 0.0000 'International Tables Vol C Tables 4.2.6.8 and
6.1.1.4'
```

```
 'Br' 'Br' -0.2901 2.4595
 'International Tables Vol C Tables 4.2.6.8 and 6.1.1.4'
 'F' 'F' 0.0171 0.0103 'International Tables Vol C Tables 4.2.6.8 and
6.1.1.4'
 'Ir' 'Ir' -1.4442 7.9887
 'International Tables Vol C Tables 4.2.6.8 and 6.1.1.4'
 'N' 'N' 0.0061 0.0033 'International Tables Vol C Tables 4.2.6.8 and
6.1.1.4'
 'O' 'O' 0.0106 0.0060 'International Tables Vol C Tables 4.2.6.8 and
6.1.1.4'
 'P' 'P' 0.1023 0.0942 'International Tables Vol C Tables 4.2.6.8 and
6.1.1.4'
 'S' 'S' 0.1246 0.1234 'International Tables Vol C Tables 4.2.6.8 and
6.1.1.4'

_shelx_space_group_comment
;
The symmetry employed for this shelxl refinement is uniquely defined
by the following loop, which should always be used as a source of
symmetry information in preference to the above space-group names.
They are only intended as comments.
;
_space_group_crystal_system        'orthorhombic'
_space_group_IT_number             29
_space_group_name_H-M_alt          'P c a 21'
_space_group_name_Hall             'P 2c -2ac'
loop_
  _space_group_symop_operation_xyz
 'x, y, z'
 '-x, -y, z+1/2'
 'x+1/2, -y, z'
 '-x+1/2, y, z+1/2'

_cell_length_a                     20.0061(10)
_cell_length_b                     10.4813(5)
_cell_length_c                     24.9756(13)
_cell_angle_alpha                  90
_cell_angle_beta                   90
_cell_angle_gamma                  90
_cell_volume                       5237.1(5)
_cell_formula_units_Z              4
_cell_measurement_reflns_used      9300
_cell_measurement_temperature      100(2)
_cell_measurement_theta_max        27.50
```

```
_cell_measurement_theta_min       2.34
_shelx_estimated_absorpt_T_max    ?
_shelx_estimated_absorpt_T_min    ?
_exptl_absorpt_coefficient_mu     3.498
_exptl_absorpt_correction_T_max   0.1512
_exptl_absorpt_correction_T_min   0.1097
_exptl_absorpt_correction_type    multi-scan
_exptl_absorpt_process_details
 'SADABS-2014/5 (Bruker,2014/5) was used for absorption correction.
wR2(int) was 0.1192 before and 0.0606 after correction. The Ratio of
minimum to maximum transmission is 0.7255. The \l/2 correction factor
is Not present.'
_exptl_crystal_colour             'light yellow'
_exptl_crystal_colour_modifier    light
_exptl_crystal_colour_primary     yellow
_exptl_crystal_density_diffrn     1.651
_exptl_crystal_density_meas       ?
_exptl_crystal_density_method     ?
_exptl_crystal_description        block
_exptl_crystal_F_000              2596
_exptl_crystal_size_max           0.21
_exptl_crystal_size_mid           0.19
_exptl_crystal_size_min           0.17
_exptl_special_details
;
 ?
;
_exptl_transmission_factor_max    ?
_exptl_transmission_factor_min    ?
_diffrn_reflns_av_R_equivalents   0.0494
_diffrn_reflns_av_unetI/netI      0.0210
_diffrn_reflns_Laue_measured_fraction_full  0.998
_diffrn_reflns_Laue_measured_fraction_max  0.997
_diffrn_reflns_limit_h_max        25
_diffrn_reflns_limit_h_min        -26
_diffrn_reflns_limit_k_max        13
_diffrn_reflns_limit_k_min        -13
_diffrn_reflns_limit_l_max        32
_diffrn_reflns_limit_l_min        -32
_diffrn_reflns_number             155835
_diffrn_reflns_point_group_measured_fraction_full  0.999
_diffrn_reflns_point_group_measured_fraction_max  0.997
_diffrn_reflns_theta_full         25.242
_diffrn_reflns_theta_max          27.550
```

```
_diffrn_reflns_theta_min          2.340
_diffrn_ambient_temperature       100.01
_diffrn_detector_area_resol_mean   ?
_diffrn_measured_fraction_theta_full  0.998
_diffrn_measured_fraction_theta_max  0.997
_diffrn_measurement_device_type    'Bruker APEX-II CCD'
_diffrn_measurement_method         '\f and \w scans'
_diffrn_radiation_type             MoK\a
_diffrn_radiation_wavelength       0.71073
_diffrn_source                     ?
_diffrn_standards_number           0
_reflns_Friedel_coverage           0.953
_reflns_Friedel_fraction_full      0.999
_reflns_Friedel_fraction_max       0.996
_reflns_number_gt                  11769
_reflns_number_total               12071
_reflns_special_details
;
 Reflections were merged by SHELXL according to the crystal
 class for the calculation of statistics and refinement.

 _reflns_Friedel_fraction is defined as the number of unique
 Friedel pairs measured divided by the number that would be
 possible theoretically, ignoring centric projections and
 systematic absences.
;
_reflns_threshold_expression       'I > 2\s(I)'
_computing_cell_refinement         'SAINT v8.37A (Bruker, 2015)'
_computing_data_collection         ?
_computing_data_reduction          'SAINT v8.37A (Bruker, 2015)'
_computing_molecular_graphics      'Olex2 (Dolomanov et al., 2009)'
_computing_publication_material    'Olex2 (Dolomanov et al., 2009)'
_computing_structure_refinement    'ShelXL (Sheldrick, 2015)'
_computing_structure_solution      'ShelXT (Sheldrick, 2015)'
_refine_diff_density_max           0.881
_refine_diff_density_min           -1.447
_refine_diff_density_rms           0.099
_refine_ls_abs_structure_details
;
 Flack x determined using 5576 quotients [(I+)-(I-)]/[(I+)+(I-)]
 (Parsons and Flack (2004), Acta Cryst. A60, s61).
;
_refine_ls_abs_structure_Flack     -0.0065(17)
_refine_ls_extinction_coef         .
```

```
_refine_ls_extinction_method      none
_refine_ls_goodness_of_fit_ref    1.176
_refine_ls_hydrogen_treatment     mixed
_refine_ls_matrix_type            full
_refine_ls_number_parameters      664
_refine_ls_number_reflns          12071
_refine_ls_number_restraints      1
_refine_ls_R_factor_all           0.0246
_refine_ls_R_factor_gt            0.0234
_refine_ls_restrained_S_all       1.176
_refine_ls_shift/su_max           0.001
_refine_ls_shift/su_mean          0.000
_refine_ls_structure_factor_coef  Fsqd
_refine_ls_weighting_details
;
 w=1/[\s^2^(Fo^2^)+(0.0180P)^2^+8.9831P]
 where P=(Fo^2^+2Fc^2^)/3
;
_refine_ls_weighting_scheme       calc
_refine_ls_wR_factor_gt           0.0549
_refine_ls_wR_factor_ref          0.0552
_refine_special_details
;
 ?
;
_olex2_refinement_description
;
1. Fixed Uiso
 At 1.2 times of:
  All C(H) groups
 At 1.5 times of:
  All C(H,H) groups, All C(H,H,H) groups
2.a Aromatic/amide H refined with riding coordinates:
 C13(H13), C10(H10), C8(H8), C33(H33), C32(H32), C46(H46), C3(H3),
C34(H34),
 C16(H16), C43(H43), C42(H42), C15(H15), C5(H5), C31(H31), C30(H30),
C45(H45),
 C36(H36), C40(H40), C2(H2), C39(H39), C38(H38), C44(H44), C37(H37)
2.b Idealised Me refined as rotating group:
 C23(H23A,H23B,H23C), C27(H27A,H27B,H27C), C24(H24A,H24B,H24C),
C20(H20A,H20B,
 H20C), C18(H18A,H18B,H18C), C26(H26A,H26B,H26C), C28(H28A,H28B,H28C),
C19(H19A,
 H19B,H19C), C50(H50A,H50B,H50C)
```

```
;
_atom_sites_solution_hydrogens     mixed
_atom_sites_solution_primary       dual
_atom_sites_solution_secondary     ?
loop_
  _atom_site_label
  _atom_site_type_symbol
  _atom_site_fract_x
  _atom_site_fract_y
  _atom_site_fract_z
  _atom_site_U_iso_or_equiv
  _atom_site_adp_type
  _atom_site_occupancy
  _atom_site_site_symmetry_order
  _atom_site_calc_flag
  _atom_site_refinement_flags_posn
  _atom_site_refinement_flags_adp
  _atom_site_refinement_flags_occupancy
  _atom_site_disorder_assembly
  _atom_site_disorder_group
 Ir1 Ir 0.53580(2) 0.43242(2) 0.38425(2) 0.01156(5) Uani 1 1 d . . . . .
 P1 P 0.50943(6) 0.27245(12) 0.44709(5) 0.0128(2) Uani 1 1 d . . . . .
 S1 S 0.30810(7) -0.09885(13) 0.33390(5) 0.0204(3) Uani 1 1 d . . . . .
 S2 S 0.39632(6) 0.21668(12) 0.08474(5) 0.0187(3) Uani 1 1 d . . . . .
 N2 N 0.4482(2) 0.4158(4) 0.34648(17) 0.0114(8) Uani 1 1 d . . . . .
 O4 O 0.27069(19) -0.2155(4) 0.34244(16) 0.0214(8) Uani 1 1 d . . . . .
 O1 O 0.67692(19) 0.4779(4) 0.42367(17) 0.0261(9) Uani 1 1 d . . . . .
 F2 F 0.3934(2) -0.2305(4) 0.2761(2) 0.0561(13) Uani 1 1 d . . . . .
 O2 O 0.3609(2) -0.0775(4) 0.37196(19) 0.0397(14) Uani 1 1 d . . . . .
 C13 C 0.3671(2) 0.6450(5) 0.43475(19) 0.0141(10) Uani 1 1 d . . . . .
 H13 H 0.3223 0.6475 0.4221 0.017 Uiso 1 1 calc R . . . .
 O7 O 0.4415(2) 0.1199(4) 0.06569(16) 0.0237(8) Uani 1 1 d . . . . .
 C7 C 0.4460(2) 0.3421(4) 0.30214(19) 0.0126(9) Uani 1 1 d . . . . .
 C10 C 0.3401(2) 0.4974(4) 0.33051(19) 0.0131(9) Uani 1 1 d . . . . .
 H10 H 0.3042 0.5513 0.3409 0.016 Uiso 1 1 calc R . . . .
 N1 N 0.5600(2) 0.2980(4) 0.32705(17) 0.0135(8) Uani 1 1 d . . . . .
 C9 C 0.3351(2) 0.4223(5) 0.2845(2) 0.0138(9) Uani 1 1 d . . . . .
 F6 F 0.26918(17) 0.2100(4) 0.11093(15) 0.0318(8) Uani 1 1 d . . . . .
 C8 C 0.3888(3) 0.3433(5) 0.2704(2) 0.0145(11) Uani 1 1 d . . . . .
 H8 H 0.3862 0.2909 0.2395 0.017 Uiso 1 1 calc R . . . .
 F1 F 0.3898(2) -0.0304(5) 0.2568(2) 0.0568(13) Uani 1 1 d . . . . .
 O6 O 0.3760(2) 0.3091(4) 0.04494(18) 0.0294(9) Uani 1 1 d . . . . .
 C6 C 0.5086(3) 0.2701(5) 0.2926(2) 0.0145(10) Uani 1 1 d . . . . .
 C12 C 0.4134(2) 0.5697(4) 0.4087(2) 0.0123(9) Uani 1 1 d . . . . .
```

C33 C 0.5341(3) -0.0895(4) 0.3856(4) 0.0230(9) Uani 1 1 d . . . . .
H33 H 0.5083 -0.1549 0.3693 0.028 Uiso 1 1 calc R . . . .
C32 C 0.6018(3) -0.1061(5) 0.3925(2) 0.0224(12) Uani 1 1 d . . . . .
H32 H 0.6223 -0.1831 0.3811 0.027 Uiso 1 1 calc R . . . .
C46 C 0.3939(3) 0.3156(6) 0.5054(3) 0.0232(13) Uani 1 1 d . . . . .
H46 H 0.4232 0.3450 0.5327 0.028 Uiso 1 1 calc R . . . .
C3 C 0.6280(2) 0.1460(5) 0.2824(2) 0.0165(10) Uani 1 1 d . . . . .
H3 H 0.6700 0.1041 0.2798 0.020 Uiso 1 1 calc R . . . .
O3 O 0.2678(2) 0.0104(4) 0.32062(18) 0.0294(9) Uani 1 1 d . . . . .
F5 F 0.2995(2) 0.0633(4) 0.05564(17) 0.0413(10) Uani 1 1 d . . . . .
F4 F 0.3260(2) 0.0495(4) 0.13885(17) 0.0434(11) Uani 1 1 d . . . . .
C14 C 0.3851(3) 0.7180(5) 0.4795(2) 0.0159(10) Uani 1 1 d . . . . .
C29 C 0.5418(2) 0.1192(5) 0.4256(2) 0.0145(9) Uani 1 1 d . . . . .
C34 C 0.5033(3) 0.0226(5) 0.4024(2) 0.0183(10) Uani 1 1 d . . . . .
H34 H 0.4565 0.0334 0.3982 0.022 Uiso 1 1 calc R . . . .
F3 F 0.3103(2) -0.1563(5) 0.23149(16) 0.0508(11) Uani 1 1 d . . . . .
C16 C 0.4983(3) 0.6429(5) 0.4659(2) 0.0160(10) Uani 1 1 d . . . . .
H16 H 0.5441 0.6463 0.4758 0.019 Uiso 1 1 calc R . . . .
C1 C 0.6232(2) 0.4600(5) 0.4116(2) 0.0154(10) Uani 1 1 d . . . . .
O5 O 0.4127(2) 0.2691(4) 0.13653(18) 0.0336(10) Uani 1 1 d . . . . .
C21 C 0.2736(2) 0.4267(5) 0.2481(2) 0.0147(10) Uani 1 1 d . . . . .
C43 C 0.3067(3) 0.2292(6) 0.4262(3) 0.0264(13) Uani 1 1 d . . . . .
H43 H 0.2770 0.1994 0.3993 0.032 Uiso 1 1 calc R . . . .
C42 C 0.3756(3) 0.2247(5) 0.4181(2) 0.0191(11) Uani 1 1 d . . . . .
H42 H 0.3928 0.1927 0.3853 0.023 Uiso 1 1 calc R . . . .
C15 C 0.4525(3) 0.7180(5) 0.4929(2) 0.0178(10) Uani 1 1 d . . . . .
H15 H 0.4675 0.7709 0.5213 0.021 Uiso 1 1 calc R . . . .
C5 C 0.5165(2) 0.1803(5) 0.2527(2) 0.0147(10) Uani 1 1 d . . . . .
H5 H 0.4806 0.1630 0.2289 0.018 Uiso 1 1 calc R . . . .
C4 C 0.5771(2) 0.1142(5) 0.2469(2) 0.0146(10) Uani 1 1 d . . . . .
C23 C 0.2437(3) 0.2925(5) 0.2442(2) 0.0235(12) Uani 1 1 d . . . . .
H23A H 0.2772 0.2337 0.2298 0.035 Uiso 1 1 calc GR . . . .
H23B H 0.2047 0.2941 0.2204 0.035 Uiso 1 1 calc GR . . . .
H23C H 0.2299 0.2638 0.2799 0.035 Uiso 1 1 calc GR . . . .
C31 C 0.6404(3) -0.0111(5) 0.4161(2) 0.0238(12) Uani 1 1 d . . . . .
H31 H 0.6871 -0.0234 0.4210 0.029 Uiso 1 1 calc R . . . .
C35 C 0.5478(3) 0.2883(5) 0.5124(2) 0.0198(11) Uani 1 1 d . . . . .
C41 C 0.4201(3) 0.2670(5) 0.4580(2) 0.0202(11) Uani 1 1 d . . . . .
C27 C 0.3583(3) 0.9332(5) 0.5165(3) 0.0251(12) Uani 1 1 d . . . . .
H27A H 0.4037 0.9361 0.5312 0.038 Uiso 1 1 calc GR . . . .
H27B H 0.3281 0.9812 0.5399 0.038 Uiso 1 1 calc GR . . . .
H27C H 0.3581 0.9712 0.4806 0.038 Uiso 1 1 calc GR . . . .
C24 C 0.2210(3) 0.5196(6) 0.2690(2) 0.0213(11) Uani 1 1 d . . . . .
H24A H 0.2060 0.4921 0.3045 0.032 Uiso 1 1 calc GR . . . .

H24B H 0.1829 0.5213 0.2444 0.032 Uiso 1 1 calc GR . . . .
H24C H 0.2405 0.6052 0.2715 0.032 Uiso 1 1 calc GR . . . .
C30 C 0.6106(3) 0.1011(5) 0.4324(2) 0.0201(11) Uani 1 1 d . . . . .
H30 H 0.6368 0.1664 0.4483 0.024 Uiso 1 1 calc R . . . .
C11 C 0.3985(2) 0.4926(5) 0.36123(19) 0.0117(9) Uani 1 1 d . . . . .
C22 C 0.2957(3) 0.4718(6) 0.1922(2) 0.0214(11) Uani 1 1 d . . . . .
H22A H 0.315(4) 0.542(7) 0.195(3) 0.032 Uiso 1 1 d R . . . .
H22B H 0.261(4) 0.474(7) 0.168(3) 0.032 Uiso 1 1 d R . . . .
C20 C 0.5695(4) -0.1180(6) 0.2340(3) 0.0350(15) Uani 1 1 d . . . . .
H20A H 0.6018 -0.1339 0.2627 0.053 Uiso 1 1 calc GR . . . .
H20B H 0.5714 -0.1880 0.2080 0.053 Uiso 1 1 calc GR . . . .
H20C H 0.5244 -0.1127 0.2492 0.053 Uiso 1 1 calc GR . . . .
C45 C 0.3250(3) 0.3213(6) 0.5132(3) 0.0354(15) Uani 1 1 d . . . . .
H45 H 0.3077 0.3553 0.5456 0.042 Uiso 1 1 calc R . . . .
C36 C 0.5458(3) 0.1798(5) 0.5453(2) 0.0242(12) Uani 1 1 d . . . . .
H36 H 0.5213 0.1065 0.5345 0.029 Uiso 1 1 calc R . . . .
C40 C 0.5828(3) 0.3951(5) 0.5295(2) 0.0254(12) Uani 1 1 d . . . . .
H40 H 0.5831 0.4699 0.5080 0.030 Uiso 1 1 calc R . . . .
C2 C 0.6186(2) 0.2374(5) 0.3214(2) 0.0174(10) Uani 1 1 d . . . . .
H2 H 0.6544 0.2578 0.3449 0.021 Uiso 1 1 calc R . . . .
C25 C 0.3348(3) 0.7930(5) 0.5130(2) 0.0195(11) Uani 1 1 d . . . . .
C47 C 0.3524(3) -0.1311(7) 0.2718(3) 0.0363(16) Uani 1 1 d . . . . .
C39 C 0.6175(4) 0.3941(6) 0.5778(3) 0.0434(19) Uani 1 1 d . . . . .
H39 H 0.6418 0.4673 0.5890 0.052 Uiso 1 1 calc R . . . .
C17 C 0.5867(3) 0.0083(5) 0.2059(2) 0.0190(11) Uani 1 1 d . . . . .
C18 C 0.5409(4) 0.0280(7) 0.1578(3) 0.0379(17) Uani 1 1 d . . . . .
H18A H 0.4942 0.0188 0.1691 0.057 Uiso 1 1 calc GR . . . .
H18B H 0.5512 -0.0357 0.1303 0.057 Uiso 1 1 calc GR . . . .
H18C H 0.5479 0.1138 0.1432 0.057 Uiso 1 1 calc GR . . . .
C38 C 0.6163(5) 0.2854(7) 0.6093(3) 0.051(2) Uani 1 1 d . . . . .
H38 H 0.6408 0.2834 0.6418 0.061 Uiso 1 1 calc R . . . .
C44 C 0.2819(3) 0.2777(6) 0.4741(3) 0.0333(15) Uani 1 1 d . . . . .
H44 H 0.2350 0.2809 0.4799 0.040 Uiso 1 1 calc R . . . .
C37 C 0.5797(4) 0.1800(6) 0.5938(2) 0.0365(16) Uani 1 1 d . . . . .
H37 H 0.5778 0.1072 0.6164 0.044 Uiso 1 1 calc R . . . .
C26 C 0.2642(3) 0.7885(6) 0.4891(2) 0.0272(13) Uani 1 1 d . . . . .
H26A H 0.2654 0.8195 0.4521 0.041 Uiso 1 1 calc GR . . . .
H26B H 0.2343 0.8428 0.5103 0.041 Uiso 1 1 calc GR . . . .
H26C H 0.2477 0.7005 0.4896 0.041 Uiso 1 1 calc GR . . . .
C48 C 0.3184(3) 0.1304(6) 0.0981(2) 0.0254(12) Uani 1 1 d . . . . .
C28 C 0.3330(3) 0.7321(6) 0.5688(2) 0.0279(13) Uani 1 1 d . . . . .
H28A H 0.3191 0.6428 0.5658 0.042 Uiso 1 1 calc GR . . . .
H28B H 0.3012 0.7784 0.5915 0.042 Uiso 1 1 calc GR . . . .
H28C H 0.3776 0.7362 0.5849 0.042 Uiso 1 1 calc GR . . . .

```
 C19 C 0.6591(3) 0.0026(6) 0.1863(2) 0.0250(12) Uani 1 1 d . . . . .
 H19A H 0.6719 0.0858 0.1716 0.037 Uiso 1 1 calc GR . . . .
 H19B H 0.6631 -0.0627 0.1584 0.037 Uiso 1 1 calc GR . . . .
 H19C H 0.6885 -0.0191 0.2163 0.037 Uiso 1 1 calc GR . . . .
 Br1 Br 0.55902(3) 0.60348(5) 0.31558(2) 0.01777(10) Uani 1 1 d . . . . .
 N3 N 0.4789(2) 0.5647(4) 0.42566(17) 0.0119(8) Uani 1 1 d . . . . .
 C49 C 0.5715(5) 0.5470(7) 0.1527(3) 0.0427(18) Uani 1 1 d . . . . .
 N4 N 0.6190(4) 0.5828(10) 0.1350(3) 0.078(3) Uani 1 1 d . . . . .
 C50 C 0.5086(6) 0.5067(13) 0.1758(4) 0.090(4) Uani 1 1 d . . . . .
 H50A H 0.4988 0.4190 0.1647 0.135 Uiso 1 1 calc GR . . . .
 H50B H 0.4728 0.5634 0.1635 0.135 Uiso 1 1 calc GR . . . .
 H50C H 0.5115 0.5106 0.2149 0.135 Uiso 1 1 calc GR . . . .

loop_
  _atom_site_aniso_label
  _atom_site_aniso_U_11
  _atom_site_aniso_U_22
  _atom_site_aniso_U_33
  _atom_site_aniso_U_23
  _atom_site_aniso_U_13
  _atom_site_aniso_U_12
 Ir1 0.01086(7) 0.00909(7) 0.01472(8) -0.00138(10) -0.00140(9)
0.00030(6)
 P1 0.0142(6) 0.0099(6) 0.0143(6) -0.0009(5) -0.0011(5) -0.0004(5)
 S1 0.0175(6) 0.0181(6) 0.0257(7) 0.0011(5) -0.0034(5) -0.0034(5)
 S2 0.0173(6) 0.0159(6) 0.0228(6) -0.0032(5) 0.0032(5) -0.0037(5)
 N2 0.0104(18) 0.0084(19) 0.0153(19) -0.0004(15) -0.0042(15) -0.0032(15)
 O4 0.0186(18) 0.0194(19) 0.026(2) 0.0038(16) -0.0019(15) -0.0037(15)
 O1 0.0158(19) 0.031(2) 0.032(2) -0.0017(18) -0.0057(16) -0.0039(17)
 F2 0.045(3) 0.040(3) 0.083(4) 0.014(2) 0.031(2) 0.020(2)
 O2 0.031(2) 0.037(2) 0.051(4) 0.006(2) -0.022(2) -0.0138(19)
 C13 0.013(2) 0.016(2) 0.013(2) -0.0015(19) -0.0004(18) -0.0004(19)
 O7 0.024(2) 0.019(2) 0.028(2) -0.0025(17) 0.0057(17) -0.0005(16)
 C7 0.013(2) 0.009(2) 0.016(2) 0.0000(17) 0.0011(18) -0.0005(18)
 C10 0.009(2) 0.009(2) 0.021(2) -0.0043(18) 0.0016(18) -0.0010(17)
 N1 0.0121(19) 0.0126(19) 0.016(2) -0.0049(16) 0.0023(16) -0.0005(16)
 C9 0.012(2) 0.013(2) 0.015(2) 0.0038(18) -0.0001(18) -0.0018(18)
 F6 0.0194(16) 0.037(2) 0.039(2) 0.0033(16) 0.0052(14) 0.0009(15)
 C8 0.017(3) 0.013(2) 0.014(2) -0.003(2) 0.0034(19) 0.000(2)
 F1 0.045(3) 0.044(3) 0.081(4) 0.020(2) 0.028(2) -0.008(2)
 O6 0.030(2) 0.018(2) 0.040(3) 0.0080(18) 0.0102(18) 0.0011(17)
 C6 0.017(2) 0.013(2) 0.013(2) 0.001(2) -0.002(2) 0.0029(19)
 C12 0.012(2) 0.009(2) 0.016(2) -0.0018(18) -0.0013(18) 0.0006(17)
 C33 0.028(2) 0.012(2) 0.029(2) -0.005(4) -0.004(3) -0.0031(19)
```

C32 0.028(3) 0.014(2) 0.025(3) -0.001(2) 0.001(2) 0.0030(19)
C46 0.026(3) 0.017(3) 0.026(3) 0.003(2) 0.007(2) 0.004(2)
C3 0.013(2) 0.017(2) 0.020(3) 0.0005(19) 0.0026(19) 0.0038(19)
O3 0.030(2) 0.0176(19) 0.041(2) 0.0045(19) 0.0003(19) 0.0047(16)
F5 0.035(2) 0.036(2) 0.053(2) -0.0155(19) -0.0041(18) -0.0152(17)
F4 0.037(2) 0.042(2) 0.051(3) 0.0304(19) 0.0141(19) 0.0032(17)
C14 0.023(3) 0.012(2) 0.013(2) -0.0032(19) -0.0006(19) 0.003(2)
C29 0.017(2) 0.012(2) 0.014(2) 0.0025(19) -0.0010(19) 0.0022(19)
C34 0.019(2) 0.015(2) 0.021(2) -0.0002(19) 0.0003(19) -0.001(2)
F3 0.070(3) 0.056(3) 0.026(2) -0.001(2) 0.007(2) 0.002(2)
C16 0.017(2) 0.012(2) 0.018(2) -0.0023(19) -0.0046(19) -0.0064(19)
C1 0.010(2) 0.009(2) 0.028(3) 0.0020(19) -0.0032(19) 0.0052(18)
O5 0.026(2) 0.041(3) 0.034(2) -0.019(2) -0.0041(18) 0.0020(19)
C21 0.011(2) 0.016(2) 0.017(2) -0.0031(19) -0.0027(18) 0.0022(18)
C43 0.020(3) 0.021(3) 0.038(3) 0.014(3) 0.000(2) -0.002(2)
C42 0.019(2) 0.012(2) 0.026(3) 0.008(2) 0.003(2) 0.0010(19)
C15 0.021(3) 0.016(2) 0.017(2) -0.0045(19) -0.003(2) 0.003(2)
C5 0.014(2) 0.013(2) 0.017(2) -0.0005(19) -0.001(2) 0.0009(18)
C4 0.015(2) 0.012(2) 0.017(2) 0.0031(19) 0.0027(19) 0.0007(19)
C23 0.023(3) 0.022(3) 0.026(3) 0.003(2) -0.007(2) -0.006(2)
C31 0.020(3) 0.020(3) 0.031(3) 0.000(2) -0.003(2) 0.005(2)
C35 0.027(3) 0.015(2) 0.017(3) 0.000(2) -0.005(2) 0.001(2)
C41 0.023(3) 0.014(2) 0.024(3) 0.005(2) 0.001(2) 0.002(2)
C27 0.026(3) 0.018(3) 0.030(3) -0.007(2) -0.002(2) 0.005(2)
C24 0.014(2) 0.029(3) 0.021(3) -0.006(2) -0.005(2) 0.005(2)
C30 0.017(2) 0.016(2) 0.027(3) -0.003(2) -0.003(2) 0.000(2)
C11 0.015(2) 0.010(2) 0.010(2) 0.0006(18) 0.0008(18) -0.0021(18)
C22 0.019(3) 0.027(3) 0.018(3) 0.005(2) -0.001(2) 0.000(2)
C20 0.044(4) 0.017(3) 0.044(4) -0.010(3) 0.018(3) -0.005(3)
C45 0.038(4) 0.029(3) 0.039(4) 0.008(3) 0.022(3) 0.010(3)
C36 0.035(3) 0.013(3) 0.025(3) 0.001(2) -0.005(2) -0.004(2)
C40 0.046(4) 0.013(3) 0.017(3) 0.001(2) -0.009(2) -0.001(2)
C2 0.013(2) 0.017(2) 0.023(3) 0.000(2) 0.001(2) 0.0031(18)
C25 0.020(3) 0.020(3) 0.019(3) -0.006(2) -0.001(2) 0.002(2)
C47 0.030(3) 0.030(4) 0.049(4) 0.012(3) 0.018(3) 0.000(3)
C39 0.082(6) 0.020(3) 0.028(3) 0.003(3) -0.025(4) -0.015(3)
C17 0.019(3) 0.015(2) 0.023(3) -0.003(2) 0.002(2) 0.004(2)
C18 0.044(4) 0.045(4) 0.024(3) -0.019(3) -0.009(3) 0.021(3)
C38 0.098(7) 0.025(3) 0.029(3) 0.005(3) -0.034(4) -0.013(4)
C44 0.019(3) 0.029(3) 0.052(4) 0.013(3) 0.013(3) 0.006(2)
C37 0.068(5) 0.017(3) 0.025(3) 0.009(2) -0.011(3) -0.004(3)
C26 0.020(3) 0.034(3) 0.028(3) -0.015(3) 0.001(2) 0.010(2)
C48 0.022(3) 0.026(3) 0.028(3) 0.002(2) 0.002(2) -0.006(2)
C28 0.032(3) 0.033(3) 0.019(3) -0.006(2) 0.003(2) 0.004(3)

```
 C19 0.021(3) 0.026(3) 0.028(3) -0.005(2) 0.005(2) 0.005(2)
 Br1 0.0203(2) 0.0123(2) 0.0207(2) 0.0013(2) 0.0022(2) -0.00063(19)
 N3 0.0120(19) 0.0092(19) 0.0145(19) -0.0009(15) -0.0017(15) 0.0010(15)
 C49 0.064(5) 0.032(4) 0.032(4) -0.001(3) -0.017(4) -0.006(4)
 N4 0.054(5) 0.131(9) 0.050(5) -0.005(5) 0.001(4) -0.009(5)
 C50 0.116(9) 0.110(9) 0.044(5) 0.016(6) -0.016(6) -0.080(8)

_geom_special_details
;
 All esds (except the esd in the dihedral angle between two l.s. planes)
 are estimated using the full covariance matrix.  The cell esds are
taken
 into account individually in the estimation of esds in distances,
angles
 and torsion angles; correlations between esds in cell parameters are
only
 used when they are defined by crystal symmetry.  An approximate
(isotropic)
 treatment of cell esds is used for estimating esds involving l.s.
planes.
;
loop_
  _geom_bond_atom_site_label_1
  _geom_bond_atom_site_label_2
  _geom_bond_distance
  _geom_bond_site_symmetry_2
  _geom_bond_publ_flag
 Ir1 P1 2.3564(13) . ?
 Ir1 N2 1.999(4) . ?
 Ir1 N1 2.064(4) . ?
 Ir1 C1 1.900(5) . ?
 Ir1 Br1 2.5242(6) . ?
 Ir1 N3 2.070(4) . ?
 P1 C29 1.813(5) . ?
 P1 C35 1.809(5) . ?
 P1 C41 1.809(6) . ?
 S1 O4 1.450(4) . ?
 S1 O2 1.439(4) . ?
 S1 O3 1.439(4) . ?
 S1 C47 1.819(7) . ?
 S2 O7 1.440(4) . ?
 S2 O6 1.446(4) . ?
 S2 O5 1.443(4) . ?
 S2 C48 1.832(6) . ?
```

N2 C7 1.351(6) . ?
N2 C11 1.331(6) . ?
O1 C1 1.131(6) . ?
F2 C47 1.330(8) . ?
C13 H13 0.9500 . ?
C13 C12 1.380(7) . ?
C13 C14 1.401(7) . ?
C7 C8 1.393(7) . ?
C7 C6 1.480(7) . ?
C10 H10 0.9500 . ?
C10 C9 1.396(7) . ?
C10 C11 1.398(6) . ?
N1 C6 1.372(7) . ?
N1 C2 1.341(6) . ?
C9 C8 1.401(7) . ?
C9 C21 1.531(7) . ?
F6 C48 1.330(7) . ?
C8 H8 0.9500 . ?
F1 C47 1.347(8) . ?
C6 C5 1.381(7) . ?
C12 C11 1.466(7) . ?
C12 N3 1.378(6) . ?
C33 H33 0.9500 . ?
C33 C32 1.375(7) . ?
C33 C34 1.392(7) . ?
C32 H32 0.9500 . ?
C32 C31 1.391(8) . ?
C46 H46 0.9500 . ?
C46 C41 1.392(8) . ?
C46 C45 1.392(9) . ?
C3 H3 0.9500 . ?
C3 C4 1.392(7) . ?
C3 C2 1.378(7) . ?
F5 C48 1.329(7) . ?
F4 C48 1.333(7) . ?
C14 C15 1.388(7) . ?
C14 C25 1.528(7) . ?
C29 C34 1.397(7) . ?
C29 C30 1.399(7) . ?
C34 H34 0.9500 . ?
F3 C47 1.339(9) . ?
C16 H16 0.9500 . ?
C16 C15 1.383(7) . ?
C16 N3 1.353(6) . ?

C21 C23 1.532(7) . ?
C21 C24 1.525(7) . ?
C21 C22 1.538(7) . ?
C43 H43 0.9500 . ?
C43 C42 1.394(8) . ?
C43 C44 1.391(9) . ?
C42 H42 0.9500 . ?
C42 C41 1.406(8) . ?
C15 H15 0.9500 . ?
C5 H5 0.9500 . ?
C5 C4 1.404(7) . ?
C4 C17 1.522(7) . ?
C23 H23A 0.9800 . ?
C23 H23B 0.9800 . ?
C23 H23C 0.9800 . ?
C31 H31 0.9500 . ?
C31 C30 1.380(7) . ?
C35 C36 1.404(8) . ?
C35 C40 1.389(8) . ?
C27 H27A 0.9800 . ?
C27 H27B 0.9800 . ?
C27 H27C 0.9800 . ?
C27 C25 1.545(8) . ?
C24 H24A 0.9800 . ?
C24 H24B 0.9800 . ?
C24 H24C 0.9800 . ?
C30 H30 0.9500 . ?
C22 H22A 0.83(8) . ?
C22 H22B 0.92(7) . ?
C20 H20A 0.9800 . ?
C20 H20B 0.9800 . ?
C20 H20C 0.9800 . ?
C20 C17 1.537(8) . ?
C45 H45 0.9500 . ?
C45 C44 1.383(10) . ?
C36 H36 0.9500 . ?
C36 C37 1.387(8) . ?
C40 H40 0.9500 . ?
C40 C39 1.391(8) . ?
C2 H2 0.9500 . ?
C25 C26 1.535(7) . ?
C25 C28 1.534(8) . ?
C39 H39 0.9500 . ?
C39 C38 1.385(9) . ?

```
 C17 C18 1.523(8) . ?
 C17 C19 1.530(7) . ?
 C18 H18A 0.9800 . ?
 C18 H18B 0.9800 . ?
 C18 H18C 0.9800 . ?
 C38 H38 0.9500 . ?
 C38 C37 1.381(9) . ?
 C44 H44 0.9500 . ?
 C37 H37 0.9500 . ?
 C26 H26A 0.9800 . ?
 C26 H26B 0.9800 . ?
 C26 H26C 0.9800 . ?
 C28 H28A 0.9800 . ?
 C28 H28B 0.9800 . ?
 C28 H28C 0.9800 . ?
 C19 H19A 0.9800 . ?
 C19 H19B 0.9800 . ?
 C19 H19C 0.9800 . ?
 C49 N4 1.112(11) . ?
 C49 C50 1.449(13) . ?
 C50 H50A 0.9800 . ?
 C50 H50B 0.9800 . ?
 C50 H50C 0.9800 . ?

loop_
  _geom_angle_atom_site_label_1
  _geom_angle_atom_site_label_2
  _geom_angle_atom_site_label_3
  _geom_angle
  _geom_angle_site_symmetry_1
  _geom_angle_site_symmetry_3
  _geom_angle_publ_flag
 P1 Ir1 Br1 177.59(3) . . ?
 N2 Ir1 P1 93.20(12) . . ?
 N2 Ir1 N1 79.60(16) . . ?
 N2 Ir1 Br1 84.42(12) . . ?
 N2 Ir1 N3 79.18(16) . . ?
 N1 Ir1 P1 91.59(12) . . ?
 N1 Ir1 Br1 88.36(12) . . ?
 N1 Ir1 N3 158.71(16) . . ?
 C1 Ir1 P1 94.30(16) . . ?
 C1 Ir1 N2 172.2(2) . . ?
 C1 Ir1 N1 97.88(19) . . ?
 C1 Ir1 Br1 88.09(15) . . ?
```

C1 Ir1 N3 102.95(19) . . ?
N3 Ir1 P1 91.19(12) . . ?
N3 Ir1 Br1 87.99(11) . . ?
C29 P1 Ir1 110.71(17) . . ?
C35 P1 Ir1 116.09(18) . . ?
C35 P1 C29 101.3(2) . . ?
C41 P1 Ir1 110.09(17) . . ?
C41 P1 C29 111.7(2) . . ?
C41 P1 C35 106.7(3) . . ?
O4 S1 C47 102.7(3) . . ?
O2 S1 O4 114.4(2) . . ?
O2 S1 O3 116.1(3) . . ?
O2 S1 C47 103.6(3) . . ?
O3 S1 O4 114.6(2) . . ?
O3 S1 C47 103.0(3) . . ?
O7 S2 O6 114.9(2) . . ?
O7 S2 O5 114.9(3) . . ?
O7 S2 C48 104.3(3) . . ?
O6 S2 C48 102.5(3) . . ?
O5 S2 O6 115.1(3) . . ?
O5 S2 C48 102.6(3) . . ?
C7 N2 Ir1 117.7(3) . . ?
C11 N2 Ir1 118.1(3) . . ?
C11 N2 C7 123.3(4) . . ?
C12 C13 H13 119.5 . . ?
C12 C13 C14 121.0(5) . . ?
C14 C13 H13 119.5 . . ?
N2 C7 C8 119.1(4) . . ?
N2 C7 C6 113.3(4) . . ?
C8 C7 C6 127.5(4) . . ?
C9 C10 H10 120.3 . . ?
C9 C10 C11 119.4(4) . . ?
C11 C10 H10 120.3 . . ?
C6 N1 Ir1 113.7(3) . . ?
C2 N1 Ir1 126.9(4) . . ?
C2 N1 C6 119.3(4) . . ?
C10 C9 C8 119.0(5) . . ?
C10 C9 C21 122.0(4) . . ?
C8 C9 C21 119.0(5) . . ?
C7 C8 C9 119.5(5) . . ?
C7 C8 H8 120.2 . . ?
C9 C8 H8 120.2 . . ?
N1 C6 C7 115.2(4) . . ?
N1 C6 C5 120.7(5) . . ?

C5 C6 C7 124.1(5) . . ?
C13 C12 C11 124.0(4) . . ?
N3 C12 C13 121.0(4) . . ?
N3 C12 C11 114.9(4) . . ?
C32 C33 H33 119.9 . . ?
C32 C33 C34 120.3(5) . . ?
C34 C33 H33 119.9 . . ?
C33 C32 H32 119.7 . . ?
C33 C32 C31 120.7(5) . . ?
C31 C32 H32 119.7 . . ?
C41 C46 H46 119.7 . . ?
C41 C46 C45 120.5(6) . . ?
C45 C46 H46 119.7 . . ?
C4 C3 H3 119.4 . . ?
C2 C3 H3 119.4 . . ?
C2 C3 C4 121.2(5) . . ?
C13 C14 C25 123.3(5) . . ?
C15 C14 C13 116.2(5) . . ?
C15 C14 C25 120.5(5) . . ?
C34 C29 P1 124.6(4) . . ?
C34 C29 C30 119.6(5) . . ?
C30 C29 P1 115.8(4) . . ?
C33 C34 C29 119.5(5) . . ?
C33 C34 H34 120.2 . . ?
C29 C34 H34 120.2 . . ?
C15 C16 H16 119.4 . . ?
N3 C16 H16 119.4 . . ?
N3 C16 C15 121.2(5) . . ?
O1 C1 Ir1 174.3(5) . . ?
C9 C21 C23 108.9(4) . . ?
C9 C21 C22 108.5(4) . . ?
C23 C21 C22 109.7(4) . . ?
C24 C21 C9 111.7(4) . . ?
C24 C21 C23 109.8(4) . . ?
C24 C21 C22 108.2(4) . . ?
C42 C43 H43 120.3 . . ?
C44 C43 H43 120.3 . . ?
C44 C43 C42 119.3(6) . . ?
C43 C42 H42 119.6 . . ?
C43 C42 C41 120.8(5) . . ?
C41 C42 H42 119.6 . . ?
C14 C15 H15 119.2 . . ?
C16 C15 C14 121.7(5) . . ?
C16 C15 H15 119.2 . . ?

C6 C5 H5 119.6 . . ?
C6 C5 C4 120.7(5) . . ?
C4 C5 H5 119.6 . . ?
C3 C4 C5 116.6(5) . . ?
C3 C4 C17 120.8(4) . . ?
C5 C4 C17 122.5(4) . . ?
C21 C23 H23A 109.5 . . ?
C21 C23 H23B 109.5 . . ?
C21 C23 H23C 109.5 . . ?
H23A C23 H23B 109.5 . . ?
H23A C23 H23C 109.5 . . ?
H23B C23 H23C 109.5 . . ?
C32 C31 H31 120.2 . . ?
C30 C31 C32 119.6(5) . . ?
C30 C31 H31 120.2 . . ?
C36 C35 P1 116.2(4) . . ?
C40 C35 P1 124.4(4) . . ?
C40 C35 C36 119.1(5) . . ?
C46 C41 P1 119.2(4) . . ?
C46 C41 C42 118.7(5) . . ?
C42 C41 P1 121.9(4) . . ?
H27A C27 H27B 109.5 . . ?
H27A C27 H27C 109.5 . . ?
H27B C27 H27C 109.5 . . ?
C25 C27 H27A 109.5 . . ?
C25 C27 H27B 109.5 . . ?
C25 C27 H27C 109.5 . . ?
C21 C24 H24A 109.5 . . ?
C21 C24 H24B 109.5 . . ?
C21 C24 H24C 109.5 . . ?
H24A C24 H24B 109.5 . . ?
H24A C24 H24C 109.5 . . ?
H24B C24 H24C 109.5 . . ?
C29 C30 H30 119.8 . . ?
C31 C30 C29 120.4(5) . . ?
C31 C30 H30 119.8 . . ?
N2 C11 C10 119.6(4) . . ?
N2 C11 C12 113.9(4) . . ?
C10 C11 C12 126.5(4) . . ?
C21 C22 H22A 110(5) . . ?
C21 C22 H22B 113(4) . . ?
H22A C22 H22B 112(6) . . ?
H20A C20 H20B 109.5 . . ?
H20A C20 H20C 109.5 . . ?

H20B C20 H20C 109.5 . . ?
C17 C20 H20A 109.5 . . ?
C17 C20 H20B 109.5 . . ?
C17 C20 H20C 109.5 . . ?
C46 C45 H45 119.9 . . ?
C44 C45 C46 120.3(6) . . ?
C44 C45 H45 119.9 . . ?
C35 C36 H36 120.1 . . ?
C37 C36 C35 119.7(5) . . ?
C37 C36 H36 120.1 . . ?
C35 C40 H40 119.6 . . ?
C35 C40 C39 120.8(5) . . ?
C39 C40 H40 119.6 . . ?
N1 C2 C3 121.5(5) . . ?
N1 C2 H2 119.2 . . ?
C3 C2 H2 119.2 . . ?
C14 C25 C27 108.6(5) . . ?
C14 C25 C26 112.2(4) . . ?
C14 C25 C28 107.4(4) . . ?
C26 C25 C27 109.4(5) . . ?
C28 C25 C27 110.6(5) . . ?
C28 C25 C26 108.6(5) . . ?
F2 C47 S1 112.2(5) . . ?
F2 C47 F1 107.1(5) . . ?
F2 C47 F3 107.1(6) . . ?
F1 C47 S1 111.2(5) . . ?
F3 C47 S1 111.8(4) . . ?
F3 C47 F1 107.2(6) . . ?
C40 C39 H39 120.3 . . ?
C38 C39 C40 119.4(6) . . ?
C38 C39 H39 120.3 . . ?
C4 C17 C20 107.0(4) . . ?
C4 C17 C18 110.8(4) . . ?
C4 C17 C19 111.3(4) . . ?
C18 C17 C20 110.0(5) . . ?
C18 C17 C19 108.8(5) . . ?
C19 C17 C20 108.9(5) . . ?
C17 C18 H18A 109.5 . . ?
C17 C18 H18B 109.5 . . ?
C17 C18 H18C 109.5 . . ?
H18A C18 H18B 109.5 . . ?
H18A C18 H18C 109.5 . . ?
H18B C18 H18C 109.5 . . ?
C39 C38 H38 119.7 . . ?

C37 C38 C39 120.5(6) . . ?
C37 C38 H38 119.7 . . ?
C43 C44 H44 119.8 . . ?
C45 C44 C43 120.4(6) . . ?
C45 C44 H44 119.8 . . ?
C36 C37 H37 119.8 . . ?
C38 C37 C36 120.3(6) . . ?
C38 C37 H37 119.8 . . ?
C25 C26 H26A 109.5 . . ?
C25 C26 H26B 109.5 . . ?
C25 C26 H26C 109.5 . . ?
H26A C26 H26B 109.5 . . ?
H26A C26 H26C 109.5 . . ?
H26B C26 H26C 109.5 . . ?
F6 C48 S2 111.4(4) . . ?
F6 C48 F4 107.4(5) . . ?
F5 C48 S2 111.0(4) . . ?
F5 C48 F6 108.2(5) . . ?
F5 C48 F4 107.7(5) . . ?
F4 C48 S2 110.9(4) . . ?
C25 C28 H28A 109.5 . . ?
C25 C28 H28B 109.5 . . ?
C25 C28 H28C 109.5 . . ?
H28A C28 H28B 109.5 . . ?
H28A C28 H28C 109.5 . . ?
H28B C28 H28C 109.5 . . ?
C17 C19 H19A 109.5 . . ?
C17 C19 H19B 109.5 . . ?
C17 C19 H19C 109.5 . . ?
H19A C19 H19B 109.5 . . ?
H19A C19 H19C 109.5 . . ?
H19B C19 H19C 109.5 . . ?
C12 N3 Ir1 113.3(3) . . ?
C16 N3 Ir1 128.3(3) . . ?
C16 N3 C12 118.5(4) . . ?
N4 C49 C50 177.2(11) . . ?
C49 C50 H50A 109.5 . . ?
C49 C50 H50B 109.5 . . ?
C49 C50 H50C 109.5 . . ?
H50A C50 H50B 109.5 . . ?
H50A C50 H50C 109.5 . . ?
H50B C50 H50C 109.5 . . ?

_shelx_res_file

```
;
TITL zfr190619a_b.res in Pca2(1)
REM Old TITL zfr190619a in Pca2(1)
REM SHELXT solution in Pca2(1)
REM R1 0.255, Rweak 0.039, Alpha 0.029, Orientation as input
REM Flack x = 0.292 ( 0.005 ) from Parsons' quotients
REM Formula found by SHELXT: C65 N12 O10 Ir
CELL 0.71073 20.0061 10.4813 24.9756 90 90 90
ZERR 4 0.001 0.0005 0.0013 0 0 0
LATT -1
SYMM -X,-Y,0.5+Z
SYMM 0.5+X,-Y,+Z
SYMM 0.5-X,+Y,0.5+Z
SFAC C H Br F Ir N O P S
UNIT 200 208 4 24 4 16 28 4 8

L.S. 50
PLAN  1
TEMP -173.14
BOND $H
LIST 6
fmap 2
ACTA
OMIT 0 1 -2
OMIT 2 0 -1
OMIT 2 0 2
OMIT 0 1 2
REM <olex2.extras>
REM <HklSrc "%.\\zfr190619a.hkl">
REM </olex2.extras>

WGHT    0.018000    8.983100
FVAR       0.06158
IR1   5    0.535803    0.432422    0.384251    11.00000    0.01086
0.00909 =
         0.01472   -0.00138   -0.00140    0.00030
P1    8    0.509434    0.272447    0.447087    11.00000    0.01418
0.00987 =
         0.01431   -0.00089   -0.00112   -0.00038
S1    9    0.308098   -0.098846    0.333903    11.00000    0.01746
0.01814 =
         0.02572    0.00109   -0.00340   -0.00336
S2    9    0.396322    0.216681    0.084736    11.00000    0.01729
0.01587 =
```

```
         0.02284   -0.00323    0.00318   -0.00374
N2     6     0.448162     0.415763     0.346479     11.00000     0.01039
0.00841 =
         0.01527   -0.00043   -0.00415   -0.00323
O4     7     0.270690    -0.215525     0.342442     11.00000     0.01863
0.01939 =
         0.02620    0.00377   -0.00191   -0.00367
O1     7     0.676917     0.477936     0.423667     11.00000     0.01576
0.03061 =
         0.03180   -0.00170   -0.00569   -0.00387
F2     4     0.393417    -0.230504     0.276079     11.00000     0.04547
0.03995 =
         0.08298    0.01433    0.03052    0.01975
O2     7     0.360902    -0.077536     0.371960     11.00000     0.03146
0.03699 =
         0.05062    0.00606   -0.02184   -0.01381
C13    1     0.367123     0.645031     0.434752     11.00000     0.01328
0.01569 =
         0.01345   -0.00149   -0.00038   -0.00045
AFIX  43
H13   2    0.322348    0.647494    0.422129    11.00000   -1.20000
AFIX   0

O7     7     0.441529     0.119862     0.065688     11.00000     0.02446
0.01858 =
         0.02819   -0.00253    0.00570   -0.00048
C7     1     0.446045     0.342108     0.302144     11.00000     0.01314
0.00886 =
         0.01590    0.00000    0.00107   -0.00046
C10    1     0.340101     0.497422     0.330514     11.00000     0.00905
0.00946 =
         0.02084   -0.00430    0.00160   -0.00101
AFIX  43
H10   2    0.304163    0.551293    0.340858    11.00000   -1.20000
AFIX   0
N1     6     0.560005     0.297978     0.327047     11.00000     0.01210
0.01260 =
         0.01568   -0.00490    0.00228   -0.00051
C9     1     0.335104     0.422345     0.284501     11.00000     0.01248
0.01347 =
         0.01535    0.00377   -0.00005   -0.00175

F6     4     0.269175     0.209976     0.110925     11.00000     0.01936
0.03661 =
```

```
         0.03931    0.00331    0.00521    0.00095
C8     1     0.388805     0.343326     0.270423     11.00000     0.01730
0.01262 =
         0.01351   -0.00253    0.00343   -0.00013
AFIX  43
H8    2    0.386178    0.290947    0.239452    11.00000   -1.20000
AFIX   0

F1     4     0.389843    -0.030407     0.256821     11.00000     0.04515
0.04417 =
         0.08097    0.02018    0.02843   -0.00843
O6     7     0.375996     0.309138     0.044940     11.00000     0.02993
0.01812 =
         0.04013    0.00802    0.01023    0.00105
C6     1     0.508565     0.270124     0.292648     11.00000     0.01722
0.01321 =
         0.01306    0.00140   -0.00164    0.00292
C12    1     0.413420     0.569655     0.408746     11.00000     0.01187
0.00852 =
         0.01649   -0.00177   -0.00134    0.00060
C33    1     0.534142    -0.089539     0.385603     11.00000     0.02824
0.01206 =
         0.02874   -0.00497   -0.00389   -0.00308
AFIX  43
H33    2    0.508332   -0.154916    0.369272    11.00000   -1.20000
AFIX   0
C32    1     0.601769    -0.106059     0.392511     11.00000     0.02823
0.01441 =
         0.02460   -0.00064    0.00136    0.00296
AFIX  43
H32    2    0.622317   -0.183080    0.381059    11.00000   -1.20000
AFIX   0
C46    1     0.393889     0.315581     0.505414     11.00000     0.02637
0.01708 =
         0.02611    0.00349    0.00715    0.00418
AFIX  43
H46    2    0.423208    0.345011    0.532668    11.00000   -1.20000
AFIX   0
C3     1     0.628012     0.146018     0.282443     11.00000     0.01254
0.01661 =
         0.02048    0.00051    0.00265    0.00382
AFIX  43
H3     2    0.669977    0.104072    0.279803    11.00000   -1.20000
AFIX   0
```

```
O3     7     0.267802     0.010399     0.320623     11.00000     0.02965
0.01765 =
         0.04081    0.00446    0.00032    0.00471
F5     4     0.299504     0.063260     0.055641     11.00000     0.03509
0.03594 =
         0.05299   -0.01554   -0.00414   -0.01517
F4     4     0.325959     0.049502     0.138854     11.00000     0.03654
0.04217 =
         0.05134    0.03038    0.01410    0.00324
C14    1     0.385127     0.717968     0.479474     11.00000     0.02311
0.01168 =
         0.01283   -0.00320   -0.00064    0.00328
C29    1     0.541771     0.119165     0.425613     11.00000     0.01731
0.01197 =
         0.01427    0.00247   -0.00096    0.00223
C34    1     0.503320     0.022612     0.402441     11.00000     0.01870
0.01550 =
         0.02066   -0.00025    0.00031   -0.00107
AFIX  43
H34   2    0.456470    0.033381    0.398198    11.00000   -1.20000
AFIX   0

F3     4     0.310313    -0.156256     0.231490     11.00000     0.06988
0.05619 =
         0.02641   -0.00143    0.00684    0.00205
C16    1     0.498261     0.642927     0.465901     11.00000     0.01740
0.01241 =
         0.01811   -0.00226   -0.00460   -0.00642
AFIX  43
H16   2    0.544083    0.646324    0.475781    11.00000   -1.20000
AFIX   0
C1     1     0.623240     0.460049     0.411619     11.00000     0.00965
0.00909 =
         0.02756    0.00202   -0.00321    0.00521

O5     7     0.412747     0.269107     0.136531     11.00000     0.02595
0.04056 =
         0.03420   -0.01893   -0.00412    0.00203
C21    1     0.273555     0.426729     0.248087     11.00000     0.01118
0.01610 =
         0.01688   -0.00312   -0.00267    0.00224
C43    1     0.306709     0.229179     0.426180     11.00000     0.01975
0.02111 =
```

```
         0.03822    0.01376    0.00022   -0.00198
AFIX  43
H43   2    0.277022    0.199368    0.399257    11.00000   -1.20000
AFIX   0
C42   1     0.375623     0.224745     0.418118     11.00000     0.01898
0.01184 =
         0.02646    0.00763    0.00267    0.00102
AFIX  43
H42   2    0.392772    0.192739    0.385289    11.00000   -1.20000
AFIX   0
C15   1     0.452480     0.717994     0.492884     11.00000     0.02076
0.01562 =
         0.01704   -0.00450   -0.00330    0.00273
AFIX  43
H15   2    0.467493    0.770932    0.521321    11.00000   -1.20000
AFIX   0
C5    1     0.516509     0.180268     0.252704     11.00000     0.01392
0.01282 =
         0.01734   -0.00054   -0.00122    0.00086
AFIX  43
H5    2    0.480579    0.162997    0.228914    11.00000   -1.20000
AFIX   0
C4    1     0.577121     0.114172     0.246882     11.00000     0.01514
0.01205 =
         0.01670    0.00307    0.00271    0.00074
C23   1     0.243658     0.292518     0.244169     11.00000     0.02253
0.02219 =
         0.02583    0.00253   -0.00747   -0.00559
AFIX 137
H23A  2    0.277234    0.233720    0.229767    11.00000   -1.50000
H23B  2    0.204710    0.294134    0.220381    11.00000   -1.50000
H23C  2    0.229902    0.263832    0.279852    11.00000   -1.50000
AFIX   0
C31   1     0.640413    -0.011080     0.416110     11.00000     0.02048
0.01980 =
         0.03113   -0.00035   -0.00334    0.00516
AFIX  43
H31   2    0.687063   -0.023378    0.420992    11.00000   -1.20000
AFIX   0
C35   1     0.547810     0.288253     0.512354     11.00000     0.02717
0.01534 =
         0.01683    0.00016   -0.00504    0.00094
C41   1     0.420082     0.267044     0.457961     11.00000     0.02325
0.01354 =
```

```
         0.02380    0.00516    0.00108    0.00225
C27    1     0.358343     0.933201     0.516490     11.00000     0.02649
0.01846 =
         0.03046   -0.00662   -0.00174    0.00459
AFIX 137
H27A  2    0.403744    0.936098    0.531173    11.00000   -1.50000
H27B  2    0.328096    0.981184    0.539881    11.00000   -1.50000
H27C  2    0.358130    0.971208    0.480640    11.00000   -1.50000
AFIX   0
C24    1     0.221035     0.519633     0.268973     11.00000     0.01367
0.02905 =
         0.02130   -0.00616   -0.00538    0.00486
AFIX 137
H24A  2    0.205984    0.492103    0.304481    11.00000   -1.50000
H24B  2    0.182888    0.521270    0.244368    11.00000   -1.50000
H24C  2    0.240474    0.605244    0.271496    11.00000   -1.50000
AFIX   0
C30    1     0.610554     0.101074     0.432378     11.00000     0.01716
0.01605 =
         0.02715   -0.00290   -0.00258   -0.00035
AFIX  43
H30   2    0.636841    0.166381    0.448262    11.00000   -1.20000
AFIX   0
C11    1     0.398488     0.492591     0.361230     11.00000     0.01494
0.00991 =
         0.01020    0.00062    0.00081   -0.00210
C22    1     0.295678     0.471767     0.192220     11.00000     0.01919
0.02682 =
         0.01806    0.00520   -0.00083    0.00027
H22A  2    0.314849    0.542243    0.194670    11.00000   -1.50000
H22B  2    0.261383    0.473965    0.167753    11.00000   -1.50000
C20    1     0.569506    -0.118004     0.233994     11.00000     0.04428
0.01651 =
         0.04431   -0.00974    0.01786   -0.00473
AFIX 137
H20A  2    0.601793   -0.133850    0.262743    11.00000   -1.50000
H20B  2    0.571365   -0.187978    0.208011    11.00000   -1.50000
H20C  2    0.524411   -0.112687    0.249154    11.00000   -1.50000
AFIX   0
C45    1     0.325042     0.321319     0.513235     11.00000     0.03808
0.02894 =
         0.03913    0.00807    0.02197    0.00992
AFIX  43
H45   2    0.307659    0.355340    0.545625    11.00000   -1.20000
```

```
AFIX   0
C36    1     0.545815     0.179774     0.545319     11.00000     0.03474
0.01283 =
         0.02505    0.00083   -0.00522   -0.00416
AFIX  43
H36    2    0.521332    0.106529    0.534483    11.00000   -1.20000
AFIX   0
C40    1     0.582807     0.395141     0.529471     11.00000     0.04581
0.01332 =
         0.01692    0.00105   -0.00873   -0.00055
AFIX  43
H40    2    0.583075    0.469881    0.507953    11.00000   -1.20000
AFIX   0
C2     1     0.618594     0.237360     0.321430     11.00000     0.01293
0.01681 =
         0.02250   -0.00040    0.00088    0.00305
AFIX  43
H2     2    0.654440    0.257821    0.344853    11.00000   -1.20000
AFIX   0
C25    1     0.334784     0.793030     0.512984     11.00000     0.01997
0.01984 =
         0.01860   -0.00579   -0.00077    0.00187

C47    1     0.352436    -0.131110     0.271790     11.00000     0.02996
0.03006 =
         0.04893    0.01191    0.01780   -0.00012
C39    1     0.617485     0.394097     0.577753     11.00000     0.08214
0.02006 =
         0.02796    0.00302   -0.02523   -0.01463
AFIX  43
H39    2    0.641754    0.467254    0.588994    11.00000   -1.20000
AFIX   0
C17    1     0.586681     0.008268     0.205875     11.00000     0.01926
0.01488 =
         0.02284   -0.00282    0.00155    0.00351
C18    1     0.540920     0.028036     0.157833     11.00000     0.04422
0.04528 =
         0.02434   -0.01938   -0.00931    0.02108
AFIX 137
H18A  2    0.494243    0.018767    0.169083    11.00000   -1.50000
H18B  2    0.551197   -0.035695    0.130327    11.00000   -1.50000
H18C  2    0.547881    0.113751    0.143183    11.00000   -1.50000
AFIX   0
C38    1     0.616298     0.285439     0.609272     11.00000     0.09758
```

```
0.02524 =
         0.02892    0.00501   -0.03427   -0.01251
AFIX  43
H38   2    0.640836    0.283424    0.641826    11.00000   -1.20000
AFIX   0
C44   1     0.281850     0.277710     0.474056     11.00000     0.01870
0.02931 =
         0.05179    0.01304    0.01265    0.00594
AFIX  43
H44   2    0.234953    0.280910    0.479868    11.00000   -1.20000
AFIX   0
C37   1     0.579730     0.179980     0.593767     11.00000     0.06757
0.01741 =
         0.02453    0.00852   -0.01071   -0.00359
AFIX  43
H37   2    0.577790    0.107209    0.616389    11.00000   -1.20000
AFIX   0
C26   1     0.264150     0.788545     0.489079     11.00000     0.02001
0.03361 =
         0.02811   -0.01513    0.00070    0.01013
AFIX 137
H26A  2    0.265399    0.819493    0.452066    11.00000   -1.50000
H26B  2    0.234287    0.842769    0.510283    11.00000   -1.50000
H26C  2    0.247663    0.700511    0.489624    11.00000   -1.50000
AFIX   0

C48   1     0.318440     0.130437     0.098137     11.00000     0.02206
0.02624 =
         0.02789    0.00225    0.00220   -0.00627
C28   1     0.333020     0.732111     0.568812     11.00000     0.03217
0.03305 =
         0.01852   -0.00630    0.00307    0.00354
AFIX 137
H28A  2    0.319069    0.642781    0.565751    11.00000   -1.50000
H28B  2    0.301213    0.778424    0.591456    11.00000   -1.50000
H28C  2    0.377650    0.736210    0.584894    11.00000   -1.50000
AFIX   0
C19   1     0.659076     0.002568     0.186277     11.00000     0.02066
0.02601 =
         0.02821   -0.00508    0.00514    0.00534
AFIX 137
H19A  2    0.671944    0.085762    0.171632    11.00000   -1.50000
H19B  2    0.663133   -0.062657    0.158351    11.00000   -1.50000
H19C  2    0.688522   -0.019051    0.216293    11.00000   -1.50000
```

```
AFIX   0
BR1    3     0.559022     0.603477     0.315585     11.00000     0.02029
0.01231 =
         0.02071    0.00129    0.00217   -0.00063
N3     6     0.478933     0.564695     0.425661     11.00000     0.01203
0.00923 =
         0.01453   -0.00091   -0.00173    0.00098

C49    1     0.571524     0.546971     0.152656     11.00000     0.06385
0.03209 =
         0.03206   -0.00089   -0.01695   -0.00594
N4     6     0.618993     0.582795     0.135005     11.00000     0.05392
0.13115 =
         0.04993   -0.00537    0.00098   -0.00927
C50    1     0.508564     0.506723     0.175808     11.00000     0.11626
0.10998 =
         0.04371    0.01575   -0.01622   -0.07990
AFIX 137
H50A  2    0.498799    0.419017    0.164686    11.00000   -1.50000
H50B  2    0.472768    0.563362    0.163458    11.00000   -1.50000
H50C  2    0.511537    0.510565    0.214941    11.00000   -1.50000
AFIX   0
HKLF 4

REM  zfr190619a_b.res in Pca2(1)
REM R1 =  0.0234 for   11769 Fo > 4sig(Fo)  and  0.0246 for all   12071
data
REM    664 parameters refined using      1 restraints

END

WGHT      0.0180      8.9835

REM Highest difference peak  0.881,  deepest hole -1.447,  1-sigma
level  0.099
Q1    1   0.5360  0.4326  0.4308  11.00000  0.05    0.88
;
_shelx_res_checksum                85826
_shelx_hkl_file
;
   0   2   0   65.46    3.14
   0   2   0   67.85    3.13
   0   2   0   68.04    3.11
   0   2   0   66.86    3.08
```

0	2	0	67.81	3.13
0	2	0	67.06	3.19
0	-2	0	70.50	3.19
0	-2	0	66.24	3.14
0	-2	0	65.42	3.08
0	-2	0	67.64	3.12
0	-2	0	68.85	3.11
0	-2	0	71.47	3.14
0	3	0	22.31	1.12
0	3	0	21.65	1.27
0	3	0	22.44	1.22
0	3	0	21.99	1.20
0	-3	0	21.55	1.20
0	3	0	23.22	1.20
0	3	0	23.62	1.17
0	-3	0	22.25	1.16
0	-3	0	23.57	1.29
0	-3	0	23.41	1.21
0	-3	0	22.36	1.12
0	-3	0	23.59	1.19
0	-4	0	19.12	1.20
0	4	0	19.23	0.99
0	4	0	17.81	1.18
0	4	0	17.99	1.11
0	4	0	16.94	1.03
0	-4	0	19.15	1.09
0	-4	0	18.32	1.10
0	-4	0	19.11	1.06
0	4	0	17.69	1.09
0	-4	0	18.90	1.07
0	-4	0	17.70	1.03
0	-4	0	17.05	0.97
0	5	0	78.20	3.53
0	5	0	76.98	3.61
0	5	0	75.61	3.82
0	-5	0	79.03	3.66
0	-5	0	77.79	3.62
0	-5	0	74.42	3.61
0	-5	0	69.98	3.92
0	-5	0	69.34	3.64
0	-5	0	79.09	3.65
0	5	0	79.17	3.70
0	5	0	75.62	3.65
0	-5	0	69.98	3.51

0	-6	0	410.76	18.21
0	-6	0	372.48	18.06
0	-6	0	388.35	18.18
0	6	0	410.19	18.18
0	6	0	398.65	18.21
0	6	0	408.10	18.29
0	-6	0	427.69	18.38
0	6	0	386.47	18.41
0	-6	0	435.79	18.25
0	-6	0	373.51	18.23
0	6	0	413.90	18.08
0	-7	0	270.04	12.13
0	7	0	254.31	11.94
0	-7	0	235.11	11.76
0	7	0	270.53	11.92
0	-7	0	269.25	11.90
0	-7	0	247.02	11.98
0	7	0	262.91	12.05
0	-7	0	268.91	11.95
0	-7	0	277.99	11.99
0	7	0	250.15	12.20
0	7	0	259.20	11.79
0	-7	0	256.37	11.92
0	-8	0	59.26	3.00
0	8	0	50.34	3.18
0	-8	0	60.81	3.22
0	8	0	51.03	3.20
0	-8	0	53.22	2.87
0	-8	0	52.56	2.69
0	8	0	56.81	2.74
0	8	0	54.02	2.88
0	-8	0	57.46	2.92
0	-8	0	55.34	2.90
0	8	0	51.18	2.91
0	-9	0	58.36	3.18
0	-9	0	56.69	3.06
0	-9	0	57.28	3.03
0	-9	0	60.20	3.08
0	-9	0	56.70	3.40
0	9	0	55.92	3.08
0	9	0	61.01	2.87
0	9	0	51.71	3.34
0	9	0	59.41	3.33
0	-9	0	52.92	2.80

0	-9	0	52.15	3.05
0	-10	0	15.20	1.14
0	10	0	18.17	1.98
0	10	0	15.47	1.98
0	-10	0	16.51	1.43
0	10	0	17.62	1.59
0	-10	0	15.92	1.49
0	-10	0	18.49	1.71
0	-10	0	16.59	1.76
0	-10	0	17.16	1.56
0	10	0	18.77	1.36
0	11	0	0.32	0.81
0	-11	0	0.32	0.78
0	-11	0	0.17	0.77
0	-11	0	-0.53	0.44
0	11	0	-1.71	1.20
0	11	0	-1.29	0.64
0	-11	0	0.39	0.70
0	-11	0	-1.75	0.91
0	11	0	-0.11	1.20
0	12	0	6.95	1.26
0	12	0	3.06	1.60
0	-12	0	5.61	1.20
0	-12	0	4.28	0.71
0	-13	0	47.32	3.14
0	13	0	40.37	3.90
0	-13	0	44.33	2.86
-1	-2	0	74.58	3.42
-1	-2	0	76.35	3.39
1	-2	0	77.24	3.42
-1	-2	0	73.90	3.48
1	-2	0	69.50	3.46
1	2	0	77.65	3.43
-1	-2	0	76.61	3.47
-1	2	0	75.32	3.43
-1	2	0	71.96	3.41
-1	2	0	73.77	3.51
-1	2	0	73.75	3.47
-1	-2	0	71.20	3.44
1	2	0	75.08	3.43
1	-2	0	77.94	3.42
1	-2	0	70.80	3.42
-1	-2	0	77.88	3.44
-1	2	0	76.48	3.46

1	-2	0	80.99	3.53
1	2	0	72.24	3.50
1	-2	0	72.49	3.40
1	-2	0	75.54	3.46
1	2	0	73.80	3.49
1	2	0	77.66	3.39
1	2	0	74.09	3.47
1	3	0	19.45	1.04
1	3	0	20.29	1.11
1	-3	0	20.77	1.04
-1	3	0	19.42	1.00
1	-3	0	20.49	1.04
1	-3	0	18.08	0.99
1	3	0	20.26	1.06
-1	3	0	19.11	1.17
-1	3	0	18.82	1.04
-1	3	0	19.33	1.10
1	-3	0	20.16	1.08
-1	-3	0	18.06	1.06
-1	-3	0	19.71	1.04
-1	-3	0	19.64	0.99
-1	3	0	19.82	1.10
1	-3	0	20.41	1.05
-1	-3	0	20.88	1.11
1	-3	0	18.38	1.08
1	-3	0	20.84	1.18
-1	-3	0	20.17	1.04
1	3	0	20.00	0.99
1	3	0	20.01	1.16
1	3	0	19.27	1.08
-1	-3	0	20.87	1.14
1	4	0	26.70	1.31
1	-4	0	25.94	1.39
1	4	0	24.92	1.43
1	-4	0	24.73	1.32
1	-4	0	25.70	1.44
-1	4	0	26.50	1.58
-1	4	0	25.39	1.44
1	4	0	26.32	1.42
-1	-4	0	27.72	1.47
1	4	0	25.21	1.52
1	4	0	27.24	1.39
-1	-4	0	26.06	1.39
1	-4	0	27.52	1.40

1	4	0	26.46	1.46
-1	4	0	26.33	1.33
-1	4	0	26.77	1.40
1	-4	0	27.75	1.41
1	-4	0	26.67	1.55
1	-4	0	26.42	1.43
-1	-4	0	24.82	1.42
-1	-4	0	26.74	1.38
-1	-4	0	25.21	1.32
-1	4	0	26.41	1.46
-1	-4	0	27.59	1.53
1	5	0	1.39	0.34
1	5	0	1.32	0.42
-1	5	0	1.40	0.32
-1	5	0	1.50	0.48
1	-5	0	1.22	0.27
1	5	0	2.53	0.59
-1	5	0	1.31	0.41
1	-5	0	1.17	0.32
-1	-5	0	0.96	0.31
-1	-5	0	1.32	0.30
1	-5	0	1.30	0.48
1	-5	0	1.14	0.41
1	5	0	1.29	0.30
-1	-5	0	1.52	0.44
-1	-5	0	1.79	0.46
1	-5	0	1.71	0.36
1	-5	0	1.61	0.43
-1	5	0	1.55	0.44
1	5	0	1.28	0.39
-1	-5	0	1.31	0.40
1	-5	0	1.77	0.43
-1	-5	0	1.41	0.42
-1	-5	0	1.97	0.55
-1	5	0	1.08	0.35
-1	6	0	21.77	1.45
1	-6	0	27.79	1.56
-1	-6	0	22.97	1.51
1	-6	0	22.29	1.32
1	6	0	25.33	1.34
1	6	0	24.70	1.68
-1	-6	0	24.59	1.32
1	-6	0	22.94	1.52
1	-6	0	25.27	1.47

-1	-6	0	25.99	1.68
1	6	0	24.58	1.45
-1	-6	0	23.78	1.43
1	-6	0	26.18	1.55
-1	-6	0	23.37	1.49
1	6	0	23.91	1.55
-1	6	0	25.24	1.60
-1	6	0	25.23	1.57
-1	6	0	24.99	1.46
1	6	0	24.77	1.48
-1	6	0	24.46	1.75
1	-6	0	26.22	1.69
-1	-6	0	25.72	1.47
1	-6	0	25.01	1.44
-1	-6	0	27.13	1.57
1	7	0	0.02	0.52
1	7	0	0.17	0.42
-1	-7	0	-0.01	0.43
1	7	0	0.15	0.55
1	-7	0	-0.14	0.39
-1	-7	0	0.11	0.44
-1	7	0	0.79	0.51
-1	-7	0	0.16	0.31
-1	7	0	-0.37	0.40
-1	7	0	0.67	0.56
1	-7	0	0.10	0.28
-1	-7	0	1.60	0.57
1	-7	0	0.92	0.43
-1	-7	0	-0.16	0.57
-1	7	0	0.28	0.45
-1	7	0	0.26	0.58
1	7	0	0.33	0.53
-1	-7	0	-0.11	0.34
-1	-7	0	0.54	0.58
1	-7	0	0.21	0.62
1	7	0	0.31	0.40
1	-7	0	0.86	0.51
1	-7	0	-0.25	0.32
1	-7	0	0.77	0.51
1	-8	0	2.73	0.96
-1	8	0	3.23	0.59
-1	-8	0	5.49	0.97
1	-8	0	2.76	0.66
1	8	0	3.39	0.90

-1	-8	0	3.65	0.69
-1	-8	0	3.09	0.78
-1	8	0	4.65	1.01
1	-8	0	4.22	0.76
1	8	0	3.67	0.55
1	8	0	3.05	0.65
-1	-8	0	2.77	0.59
1	8	0	3.42	0.86
1	-8	0	4.13	0.69
1	-8	0	4.11	0.79
-1	-8	0	2.66	0.46
-1	-8	0	4.11	0.94
-1	-8	0	1.90	0.68
1	-8	0	3.72	0.53
1	8	0	2.20	0.57
-1	8	0	2.84	0.65
1	-8	0	2.90	0.54
-1	8	0	4.00	0.72
-1	8	0	2.80	0.94
-1	9	0	1.36	0.77
1	9	0	-0.33	0.55
-1	9	0	0.24	0.50
1	9	0	0.67	0.90
1	-9	0	0.64	0.37
1	-9	0	0.93	0.74
-1	-9	0	0.45	0.65
-1	-9	0	0.13	0.47
-1	-9	0	0.71	0.62
-1	9	0	0.06	0.87
-1	-9	0	-0.07	0.64
-1	-9	0	2.10	0.85
1	-9	0	0.32	0.58
1	-9	0	0.44	1.00
1	-9	0	-0.09	0.56
1	-9	0	0.84	0.51
1	-9	0	-0.36	0.70
-1	9	0	0.35	0.61
1	9	0	0.10	0.48
-1	-9	0	-0.35	0.89
-1	-9	0	0.04	0.37
1	10	0	2.41	0.77
-1	10	0	1.29	1.05
-1	10	0	1.85	0.67
-1	-10	0	1.43	0.77

1	10	0	1.10	1.10
-1	10	0	1.12	0.82
1	10	0	-0.59	0.87
1	-10	0	0.55	0.65
-1	-10	0	1.35	0.66
-1	-10	0	0.12	0.73
1	-10	0	1.34	0.88
-1	-10	0	0.23	0.92
1	-10	0	1.14	0.48
1	-10	0	0.71	0.59
1	10	0	1.58	0.57
1	-10	0	1.69	0.96
1	-10	0	0.98	0.73
-1	-10	0	0.70	0.41
-1	-10	0	0.63	0.82
-1	10	0	2.81	1.12
-1	11	0	4.73	1.11
-1	-11	0	7.48	1.10
1	-11	0	6.77	1.25
1	11	0	5.51	1.02
1	11	0	4.55	1.04
1	-11	0	4.25	0.97
-1	-11	0	6.13	1.00
-1	11	0	5.89	0.90
1	-11	0	5.07	0.99
-1	11	0	5.85	1.48
-1	-11	0	5.53	1.09
-1	-11	0	5.29	0.94
-1	-11	0	4.40	0.81
-1	11	0	6.80	1.65
1	-11	0	6.48	0.85
1	-11	0	5.19	1.03
1	-11	0	5.36	1.17
1	11	0	5.74	1.60
-1	-12	0	15.69	1.76
1	12	0	16.11	1.81
1	12	0	12.82	2.44
1	-12	0	16.99	1.70
-1	12	0	14.37	1.89
1	-12	0	14.59	1.26
-1	-12	0	14.81	1.82
-1	12	0	20.78	3.76
-1	-12	0	15.06	1.21
1	-12	0	18.84	1.89

-1	-13	0	2.61	1.29
-1	-13	0	2.84	0.98
1	-13	0	1.73	0.91
-1	13	0	1.92	1.24
1	13	0	-1.53	1.76
1	-13	0	3.36	1.17
-1	13	0	0.18	1.85
2	1	0	221.53	9.89
2	1	0	218.35	9.84
-2	-2	0	58.39	2.66
2	2	0	55.60	2.66
2	-2	0	55.74	2.58
2	-2	0	48.33	2.54
-2	-2	0	56.65	2.55
2	-2	0	54.92	2.63
-2	2	0	58.91	2.60
-2	2	0	56.46	2.69
-2	2	0	58.08	2.64
2	-2	0	57.01	2.68
-2	2	0	55.79	2.65
-2	-2	0	53.63	2.59
-2	-2	0	54.39	2.60
2	2	0	56.88	2.59
2	2	0	55.72	2.55
2	-2	0	54.35	2.63
2	-2	0	56.13	2.59
2	2	0	56.73	2.57
-2	-2	0	55.76	2.62
2	2	0	56.55	2.64
-2	-2	0	56.14	2.57
-2	2	0	57.33	2.58
2	3	0	5.98	0.47
2	3	0	5.81	0.38
-2	3	0	5.73	0.52
2	3	0	6.00	0.48
-2	-3	0	5.15	0.46
2	-3	0	5.29	0.42
-2	3	0	5.70	0.56
2	-3	0	5.86	0.51
-2	-3	0	6.32	0.57
2	3	0	4.52	0.57
2	-3	0	5.68	0.44
-2	3	0	6.20	0.46
-2	-3	0	5.53	0.54

2	-3	0	5.63	0.43
-2	3	0	5.94	0.44
-2	-3	0	5.44	0.45
-2	3	0	5.58	0.65
-2	-3	0	5.93	0.40
2	-3	0	5.85	0.54
2	-3	0	6.07	0.44
2	3	0	5.06	0.57
2	-3	0	5.99	0.57
-2	-3	0	5.64	0.44
2	4	0	88.96	4.25
2	-4	0	95.53	4.24
-2	4	0	93.14	4.27
2	-4	0	87.24	4.32
2	4	0	90.21	4.35
2	4	0	94.11	4.28
-2	-4	0	92.06	4.43
-2	4	0	94.76	4.23
2	-4	0	95.82	4.25
-2	-4	0	91.56	4.28
-2	-4	0	87.19	4.29
-2	4	0	92.78	4.46
2	4	0	85.30	4.38
2	-4	0	95.65	4.44
-2	-4	0	93.98	4.35
-2	-4	0	87.96	4.23
-2	4	0	90.24	4.33
2	-4	0	93.30	4.31
2	4	0	92.73	4.18
-2	-4	0	92.48	4.19
-2	4	0	92.24	4.35
2	-4	0	88.75	4.21
2	4	0	89.12	4.31
2	-4	0	87.51	4.26
-2	5	0	76.93	3.78
-2	5	0	76.58	3.78
2	5	0	76.30	3.87
2	-5	0	80.95	3.89
-2	-5	0	77.03	3.81
2	5	0	77.30	3.69
-2	-5	0	75.64	3.65
-2	-5	0	71.02	3.70
2	5	0	77.36	3.77
-2	5	0	80.48	3.64

-2	5	0	77.94	3.92
2	5	0	74.86	3.78
-2	-5	0	80.55	3.79
-2	-5	0	77.28	3.70
-2	5	0	77.82	3.69
2	-5	0	72.81	3.61
2	-5	0	81.62	3.69
2	5	0	74.47	3.67
2	-5	0	78.91	3.66
2	-5	0	76.31	3.87
2	5	0	78.54	3.58
2	-5	0	75.97	3.68
-2	-6	0	220.16	10.08
2	-6	0	200.36	9.91
-2	6	0	218.96	10.09
2	-6	0	232.88	10.26
-2	-6	0	229.16	10.17
-2	-6	0	205.23	10.04
-2	-6	0	214.07	10.02
-2	-6	0	236.06	10.13
-2	-6	0	222.74	9.96
-2	6	0	213.56	10.11
-2	6	0	204.21	10.24
-2	-6	0	206.05	9.88
2	6	0	233.49	10.03
2	-6	0	208.99	10.00
-2	6	0	229.89	9.95
2	-6	0	220.65	10.12
2	-6	0	220.24	10.04
-2	6	0	226.58	10.02
2	6	0	220.51	10.25
2	6	0	218.60	10.10
2	-6	0	206.92	10.07
2	-6	0	217.04	9.97
2	6	0	217.03	9.99
2	6	0	218.57	9.88
-2	7	0	314.37	14.44
2	-7	0	315.47	14.41
2	7	0	306.73	14.42
-2	-7	0	324.10	14.53
-2	-7	0	331.47	14.63
2	-7	0	301.33	14.52
-2	7	0	300.75	14.73
2	7	0	314.52	14.29

-2	-7	0	295.72	14.48
2	-7	0	325.45	14.47
-2	-7	0	313.84	14.46
2	-7	0	300.81	14.32
2	7	0	324.47	14.45
2	7	0	324.55	14.57
-2	-7	0	300.13	14.29
2	7	0	294.43	14.68
2	-7	0	314.23	14.45
-2	7	0	319.02	14.55
2	-7	0	346.23	14.47
2	-7	0	334.92	14.72
-2	-7	0	338.28	14.57
-2	7	0	324.57	14.60
-2	7	0	326.98	14.35
2	8	0	52.21	2.96
-2	8	0	57.48	3.11
2	8	0	55.55	3.33
-2	8	0	59.39	3.24
-2	-8	0	61.87	3.18
2	8	0	60.41	3.20
-2	-8	0	59.41	3.25
2	8	0	54.52	2.97
-2	-8	0	55.95	2.81
2	-8	0	53.88	2.83
2	-8	0	56.53	2.98
-2	-8	0	50.19	3.02
-2	8	0	59.56	2.90
-2	8	0	56.24	2.99
2	-8	0	62.57	3.39
-2	-8	0	59.44	2.98
2	-8	0	58.47	2.97
2	-8	0	65.14	3.07
-2	-8	0	58.15	3.09
2	8	0	56.55	2.82
2	-8	0	60.64	3.09
-2	-8	0	58.86	3.02
2	-8	0	54.09	3.08
-2	8	0	54.37	3.31
-2	9	0	8.53	1.03
2	-9	0	8.19	1.34
-2	-9	0	8.90	0.78
-2	-9	0	7.66	1.12
2	-9	0	6.61	0.76

-2	-9	0	7.40	1.08
2	-9	0	8.00	1.60
2	-9	0	7.11	1.00
2	9	0	6.80	1.42
-2	-9	0	9.20	1.38
2	9	0	7.58	0.88
-2	9	0	8.51	1.26
2	9	0	8.00	1.40
2	-9	0	4.97	0.97
2	-9	0	7.37	0.95
2	9	0	7.80	1.04
-2	9	0	7.21	1.45
-2	9	0	7.50	1.34
2	-9	0	8.42	1.24
-2	-9	0	8.18	1.19
-2	-9	0	5.67	0.97
-2	-9	0	10.00	1.43
2	-10	0	5.49	1.02
-2	10	0	4.83	1.13
2	10	0	6.44	1.29
2	-10	0	4.38	0.76
2	-10	0	4.34	0.88
2	10	0	5.43	0.88
2	-10	0	6.09	1.31
-2	10	0	5.29	0.87
-2	-10	0	4.95	0.97
-2	10	0	4.26	1.25
2	-10	0	6.90	1.14
-2	10	0	6.45	1.29
2	10	0	5.08	1.18
-2	-10	0	3.82	0.93
2	10	0	6.00	0.91
-2	-10	0	4.81	0.99
2	-10	0	5.76	0.93
2	-10	0	7.13	1.13
-2	-10	0	4.29	0.72
-2	-10	0	5.98	1.02
-2	11	0	2.83	1.03
2	11	0	3.27	2.13
-2	-11	0	1.96	0.54
2	-11	0	2.78	0.91
2	-11	0	2.29	1.08
-2	11	0	2.72	1.23
2	-11	0	1.04	0.51

2	-11	0	1.72	0.79
-2	11	0	0.87	0.73
-2	-11	0	0.74	1.41
-2	-11	0	1.59	0.77
-2	-11	0	1.60	1.00
-2	11	0	1.49	1.25
2	-11	0	2.04	1.06
2	11	0	2.18	0.65
2	11	0	2.21	0.82
2	-11	0	0.91	0.72
-2	-12	0	4.71	0.88
2	12	0	8.12	1.90
2	12	0	7.40	1.16
-2	12	0	7.08	1.30
2	-12	0	5.12	0.82
-2	-12	0	5.19	1.14
2	-12	0	7.76	1.48
-2	-12	0	4.97	1.28
-2	12	0	7.54	1.87
2	-12	0	4.48	1.05
2	-13	0	45.69	2.87
-2	-13	0	46.86	3.19
-2	13	0	47.54	3.18
2	-13	0	44.60	2.82
-2	-13	0	42.06	2.84
-2	13	0	40.99	3.87
2	13	0	43.98	3.95
-3	0	0	0.03	0.05
3	0	0	0.11	0.08
-3	0	0	-0.07	0.10
3	0	0	0.04	0.05
3	0	0	0.02	0.09
-3	0	0	0.08	0.10
-3	0	0	0.07	0.07
-3	0	0	0.27	0.10
3	0	0	-0.11	0.10
3	0	0	0.13	0.09
-3	0	0	0.16	0.08
3	0	0	-0.07	0.08
3	-1	0	18.83	0.96
3	-1	0	20.53	0.97
-3	-1	0	19.94	1.03
-3	1	0	19.68	0.91
-3	-1	0	20.75	0.96

3	1	0	20.61	0.96
-3	-1	0	19.45	0.92
-3	-1	0	18.82	0.96
-3	1	0	20.57	0.97
-3	1	0	18.41	1.02
3	-1	0	18.72	1.01
3	1	0	20.07	0.96
3	1	0	19.53	0.93
3	1	0	19.29	1.03
3	1	0	19.31	0.95
-3	-1	0	19.70	0.91
-3	1	0	20.54	0.97
-3	1	0	20.21	0.97
3	1	0	19.37	0.93
-3	-1	0	19.22	0.95
3	-2	0	3.01	0.30
3	2	0	3.23	0.43
-3	2	0	3.11	0.31
3	-2	0	2.85	0.43
-3	2	0	3.08	0.44
-3	2	0	2.99	0.35
3	2	0	3.10	0.31
-3	-2	0	3.39	0.28
3	-2	0	2.98	0.30
3	-2	0	2.94	0.41
3	2	0	3.02	0.26
-3	-2	0	3.24	0.30
-3	-2	0	3.10	0.32
-3	2	0	3.03	0.30
3	-2	0	2.82	0.33
-3	-2	0	2.95	0.28
-3	2	0	2.59	0.44
3	2	0	3.11	0.28
3	2	0	3.15	0.35
-3	-2	0	2.84	0.41
-3	-2	0	3.76	0.33
-3	-3	0	31.54	1.55
-3	3	0	30.17	1.72
3	-3	0	31.81	1.51
3	3	0	32.53	1.51
-3	3	0	31.18	1.65
-3	-3	0	32.02	1.51
3	-3	0	31.36	1.51
-3	-3	0	31.45	1.57

3	3	0	32.78	1.54
-3	-3	0	29.68	1.56
-3	-3	0	32.26	1.68
-3	3	0	32.61	1.56
3	-3	0	31.97	1.64
3	-3	0	31.59	1.64
3	3	0	32.66	1.58
-3	3	0	32.58	1.64
-3	-3	0	32.08	1.61
3	-3	0	30.06	1.55
3	3	0	30.38	1.66
3	3	0	30.02	1.63
-3	3	0	32.01	1.57
3	-3	0	30.87	1.72
-3	-4	0	15.03	0.98
3	-4	0	13.56	0.82
3	4	0	14.92	1.02
-3	-4	0	14.31	0.89
-3	-4	0	14.03	0.87
3	4	0	12.34	1.00
-3	-4	0	15.14	0.82
-3	4	0	12.99	0.99
3	4	0	15.49	0.92
3	-4	0	12.81	0.98
-3	-4	0	13.94	0.89
-3	-4	0	15.61	1.03
3	4	0	14.73	0.80
-3	4	0	16.11	0.88
3	-4	0	14.86	0.99
3	-4	0	15.43	1.10
-3	4	0	13.52	1.05
-3	4	0	15.28	0.92
-3	4	0	14.43	0.96
3	-4	0	14.84	0.83
3	-4	0	15.26	0.86
3	4	0	15.79	0.87
3	-4	0	14.62	0.88
-3	5	0	178.79	8.15
-3	5	0	160.70	8.08
3	-5	0	165.43	8.03
-3	-5	0	185.66	8.17
-3	5	0	181.26	8.05
3	5	0	177.09	7.93
3	5	0	178.80	8.01

3	5	0	183.37	8.05
3	-5	0	180.00	8.10
3	-5	0	167.95	7.98
-3	5	0	171.58	8.28
3	5	0	180.35	8.12
3	-5	0	187.89	8.27
3	-5	0	181.35	8.01
3	5	0	169.22	8.20
-3	-5	0	171.39	8.06
-3	-5	0	167.59	8.05
3	-5	0	175.64	8.03
-3	-5	0	181.29	8.15
3	-5	0	169.79	8.11
3	5	0	168.78	8.16
-3	5	0	175.11	8.15
-3	-5	0	171.56	7.99
3	6	0	73.67	3.57
3	-6	0	77.17	3.66
-3	6	0	72.37	3.69
-3	-6	0	73.30	3.44
-3	-6	0	68.03	3.58
3	-6	0	69.36	3.64
-3	6	0	70.54	3.56
3	6	0	72.26	3.66
3	-6	0	69.94	3.55
3	-6	0	68.55	3.47
3	6	0	71.60	3.77
3	-6	0	79.32	3.55
-3	6	0	71.98	3.84
-3	-6	0	69.64	3.56
-3	6	0	76.09	3.53
3	-6	0	77.92	3.52
3	6	0	74.32	3.53
-3	-6	0	78.23	3.72
-3	6	0	71.84	3.68
-3	-6	0	71.65	3.50
3	6	0	75.15	3.43
-3	-6	0	74.55	3.69
3	-7	0	50.71	2.70
3	-7	0	48.78	2.80
-3	-7	0	50.97	2.66
-3	-7	0	56.28	2.92
-3	-7	0	53.54	2.83
3	-7	0	52.44	2.67

3	7	0	45.62	2.62
3	7	0	53.10	2.86
-3	7	0	54.20	2.91
-3	7	0	56.37	2.67
-3	7	0	51.87	2.86
-3	7	0	51.73	2.72
-3	-7	0	50.73	2.57
3	-7	0	60.57	2.79
3	-7	0	56.08	3.06
3	-7	0	48.99	2.59
3	-7	0	55.66	2.83
-3	-7	0	52.82	2.74
3	7	0	54.26	2.73
3	7	0	51.81	2.67
-3	-7	0	50.90	2.76
-3	7	0	49.64	3.07
3	7	0	50.22	2.94
-3	-7	0	54.65	2.89
3	8	0	21.16	1.47
-3	8	0	20.79	1.86
-3	-8	0	21.33	1.73
3	-8	0	22.61	1.56
-3	8	0	24.69	1.81
-3	-8	0	20.50	1.45
-3	8	0	21.43	1.50
3	-8	0	20.73	1.43
-3	8	0	22.71	1.42
-3	-8	0	20.54	1.53
3	-8	0	22.89	1.53
3	-8	0	21.58	1.89
-3	-8	0	21.00	1.59
3	8	0	20.37	1.44
3	-8	0	19.65	1.28
3	8	0	23.06	1.30
3	8	0	23.85	1.82
-3	-8	0	19.78	1.25
3	-8	0	21.82	1.42
3	8	0	21.80	1.64
-3	-8	0	23.32	1.74
-3	8	0	21.69	1.63
-3	-8	0	23.33	1.56
3	-8	0	20.90	1.65
3	-9	0	5.87	0.71
3	-9	0	7.49	1.36

-3	-9	0	4.98	0.72
3	9	0	5.20	0.92
-3	-9	0	6.20	0.86
-3	9	0	6.21	1.01
3	-9	0	6.50	1.30
-3	-9	0	5.19	0.93
-3	9	0	6.10	1.20
3	-9	0	6.74	0.97
-3	-9	0	5.32	0.95
-3	-9	0	7.55	1.19
-3	-9	0	5.36	0.84
3	-9	0	5.27	0.97
3	-9	0	5.18	0.92
3	-9	0	5.50	0.93
-3	9	0	7.91	1.12
3	9	0	5.53	0.79
3	9	0	4.26	1.05
-3	9	0	5.69	1.21
3	9	0	6.58	1.32
-3	10	0	16.42	1.95
3	-10	0	13.36	1.09
-3	10	0	17.51	1.40
3	10	0	13.24	1.30
3	-10	0	13.72	1.71
3	-10	0	17.03	1.59
-3	-10	0	13.44	1.50
3	-10	0	12.82	1.32
-3	-10	0	14.80	1.54
-3	-10	0	13.40	1.45
3	10	0	12.74	1.78
3	-10	0	14.91	2.09
-3	-10	0	13.42	1.03
-3	10	0	12.61	1.95
3	10	0	14.52	1.12
3	-10	0	13.84	1.31
3	-10	0	14.31	1.44
-3	10	0	14.21	1.67
-3	-10	0	14.53	1.36
3	10	0	15.54	1.79
3	-11	0	63.70	3.43
3	-11	0	67.28	3.50
3	11	0	62.45	3.31
3	-11	0	66.37	3.37
-3	-11	0	59.80	3.41

-3	-11	0	66.08	3.59
-3	11	0	63.24	3.65
3	-11	0	57.21	3.48
3	11	0	64.83	3.58
-3	11	0	62.93	3.29
3	11	0	59.55	3.92
-3	11	0	61.94	3.88
-3	11	0	61.85	4.13
3	-11	0	55.55	3.13
-3	-11	0	57.10	3.07
3	12	0	66.75	3.74
-3	12	0	69.74	4.57
3	12	0	66.39	4.57
-3	-12	0	81.40	4.20
-3	12	0	63.88	4.60
-3	-12	0	68.34	3.88
3	-12	0	75.39	3.95
-3	-12	0	70.53	3.93
3	-12	0	66.91	3.57
3	-12	0	78.00	3.89
3	-13	0	33.73	2.37
-3	13	0	34.11	2.90
-3	13	0	30.48	3.32
-3	-13	0	36.40	2.42
-3	-13	0	33.67	2.90
3	13	0	28.91	3.59
4	0	0	3.13	0.19
-4	0	0	3.07	0.26
4	0	0	3.08	0.32
-4	0	0	2.87	0.21
-4	0	0	2.11	0.32
-4	0	0	2.77	0.30
4	0	0	3.00	0.26
4	0	0	2.77	0.28
4	0	0	2.76	0.26
-4	0	0	2.43	0.25
4	0	0	2.93	0.32
-4	0	0	2.69	0.18
-4	-1	0	151.06	6.76
-4	1	0	156.53	6.77
4	-1	0	152.89	6.81
-4	1	0	153.24	6.75
-4	-1	0	144.95	6.68
-4	1	0	152.79	6.71

-4	-1	0	151.70	6.75
-4	1	0	152.63	6.76
4	1	0	141.57	6.70
4	1	0	149.89	6.74
4	-1	0	143.76	6.76
-4	-1	0	154.53	6.76
-4	-1	0	145.04	6.76
-4	1	0	148.66	6.82
4	-1	0	147.44	6.75
4	1	0	153.22	6.83
4	-1	0	150.06	6.76
-4	-1	0	143.31	6.67
-4	1	0	151.83	6.80
-4	-1	0	154.14	6.82
-4	-1	0	144.69	6.74
4	1	0	149.49	6.70
4	2	0	28.37	1.57
-4	-2	0	32.80	1.51
4	2	0	33.18	1.50
-4	-2	0	31.68	1.50
-4	2	0	32.39	1.54
4	2	0	30.97	1.49
4	-2	0	31.53	1.61
4	-2	0	30.99	1.53
4	-2	0	30.43	1.56
-4	2	0	32.43	1.54
4	2	0	31.48	1.53
4	-2	0	30.18	1.54
-4	2	0	31.53	1.56
4	2	0	30.86	1.51
4	2	0	29.08	1.72
-4	-2	0	33.30	1.65
-4	-2	0	32.49	1.50
-4	-2	0	30.82	1.49
-4	2	0	31.00	1.64
4	3	0	22.33	1.08
-4	3	0	20.84	1.22
4	-3	0	21.26	1.16
-4	-3	0	21.46	1.09
4	-3	0	21.79	1.29
4	3	0	19.84	1.23
-4	3	0	21.73	1.13
4	-3	0	21.37	1.11
4	-3	0	21.25	1.22

4	3	0	20.70	1.12
-4	-3	0	21.27	1.07
-4	3	0	20.51	1.29
4	3	0	19.42	1.16
-4	3	0	21.28	1.12
4	-3	0	20.00	1.10
-4	3	0	21.00	1.19
-4	-3	0	22.61	1.27
4	3	0	20.60	1.05
-4	-3	0	21.57	1.13
-4	-3	0	20.81	1.12
-4	-3	0	19.85	1.10
4	-4	0	0.19	0.21
-4	4	0	0.22	0.33
-4	4	0	0.62	0.29
4	-4	0	0.27	0.41
4	-4	0	0.07	0.20
-4	-4	0	0.50	0.23
4	-4	0	-0.03	0.22
4	4	0	0.23	0.21
4	4	0	0.69	0.26
-4	-4	0	-0.03	0.37
-4	4	0	0.30	0.27
-4	-4	0	0.41	0.26
-4	4	0	-0.36	0.36
4	4	0	0.06	0.33
-4	-4	0	0.41	0.28
4	-4	0	0.20	0.34
-4	-4	0	-0.03	0.29
-4	-4	0	0.27	0.25
4	-4	0	0.40	0.21
4	4	0	0.34	0.33
-4	4	0	-0.05	0.44
4	-4	0	-0.04	0.24
4	4	0	0.38	0.26
4	-5	0	40.50	1.93
4	5	0	36.83	2.15
4	-5	0	38.68	1.99
-4	-5	0	37.07	1.94
4	-5	0	36.60	2.10
4	-5	0	39.32	1.94
4	5	0	40.21	1.96
-4	5	0	41.15	2.14
-4	5	0	40.19	2.02

-4	5	0	38.86	2.30
4	5	0	37.89	2.15
-4	5	0	36.70	2.13
-4	-5	0	38.42	2.01
-4	-5	0	37.15	1.91
4	-5	0	36.24	2.07
-4	-5	0	37.08	2.00
-4	5	0	42.32	2.01
-4	-5	0	39.45	2.08
4	-5	0	36.75	1.95
4	-5	0	39.21	2.21
4	5	0	38.37	1.88
-4	-5	0	40.90	2.18
4	5	0	40.28	2.01
4	6	0	49.07	2.63
4	-6	0	47.92	2.51
-4	-6	0	48.83	2.63
-4	-6	0	48.59	2.39
-4	-6	0	52.16	2.70
-4	-6	0	50.88	2.54
-4	-6	0	45.29	2.51
4	-6	0	46.31	2.43
-4	6	0	47.58	2.81
-4	6	0	48.67	2.67
4	6	0	46.78	2.72
-4	6	0	50.89	2.49
4	6	0	50.38	2.53
4	-6	0	46.72	2.62
4	6	0	50.50	2.38
-4	6	0	51.51	2.55
4	6	0	46.76	2.67
-4	6	0	52.77	2.71
4	-6	0	52.52	2.84
4	-6	0	52.71	2.45
-4	-6	0	47.93	2.45
4	-6	0	47.07	2.63
4	-6	0	52.30	2.47
4	-7	0	119.08	5.26
-4	7	0	120.14	5.29
4	-7	0	107.81	5.31
4	-7	0	116.30	5.67
-4	-7	0	111.16	5.35
-4	-7	0	113.39	5.18
4	-7	0	104.87	5.41

-4	7	0	118.35	5.52
-4	-7	0	103.43	5.33
4	7	0	96.78	5.21
4	7	0	108.23	5.32
4	7	0	113.73	5.25
-4	-7	0	110.26	5.46
4	7	0	114.26	5.60
-4	-7	0	109.73	5.23
-4	7	0	103.62	5.62
4	-7	0	116.95	5.43
4	-7	0	105.62	5.22
-4	-7	0	117.01	5.50
-4	-7	0	115.32	5.46
-4	7	0	113.34	5.49
-4	7	0	109.89	5.33
4	7	0	116.72	5.47
4	-8	0	54.49	2.98
4	8	0	63.41	3.60
-4	8	0	58.61	3.21
4	-8	0	52.03	3.05
-4	-8	0	54.92	3.03
4	-8	0	62.17	3.01
-4	-8	0	61.06	3.24
-4	-8	0	60.14	3.17
-4	-8	0	59.05	3.24
-4	-8	0	56.07	2.82
-4	8	0	56.01	3.01
-4	8	0	62.99	3.28
4	-8	0	58.74	2.92
4	-8	0	54.44	2.86
-4	8	0	52.33	3.32
-4	8	0	60.17	2.96
4	8	0	59.52	3.21
4	8	0	56.83	2.81
4	-8	0	62.50	3.47
-4	-8	0	55.39	3.02
4	-8	0	56.53	3.15
4	8	0	56.76	2.94
4	8	0	55.16	3.29
-4	9	0	5.00	0.98
4	-9	0	4.65	0.84
4	-9	0	5.05	0.73
4	-9	0	3.75	0.76
4	9	0	3.88	1.02

4	-9	0	5.94	0.90
4	-9	0	3.62	1.22
4	9	0	4.44	0.78
-4	9	0	5.02	1.14
-4	-9	0	3.96	0.59
4	-9	0	5.19	0.88
-4	9	0	5.16	0.81
-4	9	0	3.35	1.13
4	-9	0	4.91	1.01
-4	-9	0	3.73	0.81
4	9	0	4.58	0.85
-4	-9	0	3.55	0.71
-4	-9	0	5.26	0.83
4	9	0	4.51	1.02
-4	-9	0	3.47	1.07
-4	-9	0	4.59	0.94
-4	10	0	9.23	1.59
4	10	0	8.32	1.57
4	-10	0	7.19	1.09
4	-10	0	7.55	0.87
4	-10	0	7.62	1.07
-4	-10	0	8.32	1.25
4	-10	0	7.57	1.19
4	-10	0	7.96	1.10
4	-10	0	7.67	1.33
4	10	0	8.76	1.08
-4	-10	0	7.37	0.83
-4	-10	0	6.93	1.14
4	10	0	8.47	1.63
-4	10	0	8.57	1.55
-4	-10	0	9.01	1.05
-4	-10	0	9.62	1.44
-4	10	0	7.73	1.24
4	10	0	7.22	0.93
-4	10	0	8.13	1.26
4	-10	0	7.83	1.53
4	11	0	14.09	1.39
4	-11	0	14.16	1.43
-4	-11	0	13.35	1.09
4	-11	0	10.81	1.46
-4	-11	0	15.94	1.76
-4	-11	0	14.63	1.47
-4	11	0	17.29	1.88
-4	11	0	12.28	1.78

4	11	0	12.23	2.08
4	-11	0	13.74	1.18
4	-11	0	13.79	1.67
-4	11	0	14.78	1.47
4	-11	0	12.92	1.40
-4	11	0	13.74	2.00
-4	-12	0	3.77	0.99
4	-12	0	3.99	1.02
-4	12	0	5.21	1.85
-4	-12	0	2.03	1.18
4	-12	0	2.75	0.90
4	12	0	1.70	1.58
-4	12	0	5.14	1.33
4	-12	0	3.04	0.72
-4	-13	0	31.63	2.92
-4	13	0	33.26	2.99
-4	-13	0	28.64	2.27
4	-13	0	29.45	2.24
4	13	0	24.44	3.79
-5	0	0	0.83	0.20
5	0	0	0.58	0.17
5	0	0	0.46	0.17
5	0	0	0.39	0.15
5	0	0	0.25	0.18
-5	0	0	0.29	0.08
-5	0	0	0.30	0.14
5	0	0	0.14	0.18
-5	0	0	0.60	0.17
5	0	0	0.41	0.10
-5	0	0	0.38	0.10
-5	0	0	0.16	0.19
-5	-1	0	0.03	0.14
-5	1	0	0.27	0.16
-5	1	0	0.14	0.14
-5	1	0	0.37	0.15
5	-1	0	0.50	0.15
5	1	0	0.39	0.15
5	-1	0	0.06	0.13
5	1	0	0.10	0.22
-5	-1	0	-0.05	0.16
-5	1	0	0.22	0.17
-5	-1	0	0.36	0.24
5	1	0	0.24	0.08
5	1	0	0.16	0.11

5	-1	0	0.47	0.22
-5	1	0	0.29	0.22
-5	1	0	0.05	0.23
-5	-1	0	0.26	0.16
5	1	0	0.26	0.15
5	-1	0	0.28	0.23
-5	-1	0	0.09	0.17
5	-2	0	65.24	3.03
5	2	0	65.70	3.00
5	-2	0	63.45	3.05
-5	2	0	65.47	3.04
-5	2	0	61.87	3.13
5	2	0	64.76	3.01
-5	-2	0	65.44	3.01
5	2	0	65.96	3.00
5	2	0	63.26	3.14
5	-2	0	66.33	3.12
-5	-2	0	64.54	2.96
5	-2	0	66.12	3.04
-5	-2	0	66.35	3.02
-5	2	0	62.38	3.12
5	2	0	64.52	3.00
-5	-2	0	65.68	3.14
-5	2	0	67.40	3.06
-5	-2	0	64.66	2.96
-5	-2	0	61.89	3.03
5	2	0	66.73	3.04
5	3	0	8.47	0.54
-5	-3	0	7.89	0.58
-5	3	0	7.39	0.77
-5	3	0	8.08	0.66
-5	3	0	7.70	0.58
5	3	0	7.45	0.61
5	3	0	7.91	0.52
-5	-3	0	7.02	0.60
-5	-3	0	7.98	0.77
-5	-3	0	8.09	0.57
5	-3	0	7.93	0.55
5	-3	0	7.92	0.76
-5	3	0	8.61	0.61
5	3	0	8.02	0.58
-5	-3	0	7.99	0.55
5	-3	0	7.24	0.59
5	3	0	8.07	0.74

-5	3	0	7.58	0.73
5	-3	0	7.93	0.56
-5	-3	0	7.80	0.53
5	-4	0	136.66	6.05
-5	-4	0	130.80	6.15
5	4	0	139.08	6.10
5	4	0	126.24	6.25
5	4	0	133.78	6.18
5	4	0	132.44	6.09
5	-4	0	126.95	6.15
-5	-4	0	131.62	6.12
-5	4	0	143.27	6.19
-5	4	0	129.87	6.38
5	-4	0	137.46	6.26
-5	-4	0	135.85	6.20
5	-4	0	141.68	6.40
-5	4	0	139.20	6.27
-5	-4	0	135.97	6.32
-5	4	0	136.21	6.19
-5	-4	0	136.73	6.19
5	4	0	126.33	6.31
-5	-4	0	127.96	6.17
5	-4	0	126.51	6.16
-5	4	0	136.98	6.32
5	-4	0	136.53	6.28
-5	5	0	129.53	5.60
5	5	0	123.30	5.52
5	5	0	119.69	5.60
-5	-5	0	118.07	5.53
-5	5	0	119.65	5.61
-5	-5	0	124.86	5.65
5	-5	0	125.06	5.48
5	-5	0	125.18	5.85
-5	-5	0	123.22	5.77
5	-5	0	123.22	5.50
-5	5	0	117.39	5.85
5	-5	0	115.23	5.67
5	5	0	116.94	5.77
5	5	0	120.72	5.49
5	-5	0	119.37	5.70
-5	5	0	130.59	5.74
5	5	0	111.12	5.71
5	-5	0	115.41	5.56
-5	5	0	116.36	5.74

-5	-5	0	118.67	5.50
-5	-5	0	114.26	5.58
5	-5	0	116.68	5.59
-5	-5	0	114.39	5.61
-5	6	0	78.59	3.71
5	-6	0	76.77	3.66
5	-6	0	75.29	3.81
5	-6	0	70.92	3.61
-5	6	0	79.55	3.53
-5	-6	0	75.87	3.73
-5	-6	0	69.13	3.44
5	6	0	67.33	3.70
5	6	0	67.55	3.69
-5	-6	0	69.98	3.55
5	-6	0	68.57	3.50
-5	-6	0	72.22	3.41
5	-6	0	65.12	3.48
5	-6	0	68.30	3.46
-5	6	0	74.44	3.55
-5	-6	0	73.57	3.62
5	6	0	72.20	3.53
5	6	0	72.53	3.39
-5	6	0	67.30	3.87
-5	6	0	71.08	3.70
-5	-6	0	69.27	3.52
5	6	0	74.40	3.44
5	-6	0	77.44	3.44
5	-7	0	85.38	4.36
5	7	0	89.14	4.14
-5	7	0	93.40	4.45
-5	-7	0	84.97	4.40
5	-7	0	92.48	4.67
5	7	0	84.86	4.07
-5	-7	0	86.12	4.11
-5	7	0	84.33	4.58
5	7	0	81.52	4.47
5	-7	0	94.32	4.17
5	-7	0	81.93	4.23
5	-7	0	85.61	4.37
-5	-7	0	88.67	4.36
5	7	0	87.54	4.26
-5	7	0	87.66	4.27
-5	-7	0	81.37	4.24
-5	7	0	94.97	4.24

-5	-7	0	82.53	4.13
5	7	0	86.66	4.37
-5	7	0	87.09	4.45
5	-7	0	82.06	4.15
-5	-7	0	91.90	4.45
-5	-7	0	86.26	4.27
5	-7	0	91.57	4.14
-5	-8	0	1.11	0.56
5	8	0	1.39	0.45
5	-8	0	0.94	0.47
5	-8	0	1.80	0.53
5	-8	0	0.80	0.95
-5	8	0	1.19	0.73
5	-8	0	1.44	0.74
-5	-8	0	1.11	0.66
5	8	0	0.77	0.72
-5	8	0	1.49	0.79
-5	-8	0	2.05	0.43
-5	8	0	0.48	0.57
-5	-8	0	1.93	0.59
-5	-8	0	1.21	0.71
-5	8	0	0.40	0.93
5	8	0	1.03	0.73
5	-8	0	0.92	0.55
-5	-8	0	1.80	0.64
-5	-8	0	1.73	0.85
-5	8	0	1.28	0.74
5	8	0	1.80	0.50
5	-8	0	1.15	0.71
5	-8	0	1.07	0.42
-5	-9	0	32.48	2.10
5	-9	0	35.10	2.38
-5	-9	0	36.74	2.32
-5	-9	0	31.75	1.86
5	9	0	33.58	2.01
-5	9	0	34.97	2.42
5	-9	0	36.87	1.98
5	-9	0	39.57	2.75
5	9	0	36.19	2.37
-5	9	0	36.10	2.12
5	-9	0	37.13	2.24
5	9	0	35.61	1.88
-5	9	0	32.66	2.60
5	-9	0	35.15	2.10

-5	-9	0	34.22	2.06
5	-9	0	36.96	2.08
5	-9	0	33.80	1.95
-5	9	0	36.47	2.47
5	9	0	32.40	2.44
-5	10	0	84.49	4.97
5	-10	0	83.49	4.54
5	-10	0	94.51	4.37
-5	-10	0	82.04	4.45
-5	-10	0	82.73	4.25
5	10	0	84.39	4.14
-5	10	0	90.65	4.38
-5	10	0	90.61	4.83
-5	-10	0	82.27	4.15
5	-10	0	80.30	4.23
5	10	0	86.82	4.71
5	-10	0	89.24	4.44
5	-10	0	93.07	4.30
5	10	0	87.52	4.25
-5	10	0	88.46	4.71
5	-10	0	93.78	5.00
-5	-10	0	83.90	4.46
-5	-11	0	78.11	4.23
5	-11	0	76.03	3.94
5	-11	0	82.95	4.19
-5	-11	0	76.61	3.84
5	11	0	80.24	4.76
-5	11	0	77.65	4.92
5	11	0	81.51	3.98
-5	11	0	72.83	4.80
-5	-11	0	78.80	4.41
5	-11	0	76.25	4.30
5	-11	0	89.82	4.06
-5	-12	0	82.60	4.49
-5	12	0	87.19	4.84
5	-12	0	78.99	4.17
5	12	0	76.75	5.04
-5	12	0	75.68	5.13
-5	-12	0	86.05	4.70
5	-12	0	82.31	4.54
5	-12	0	92.81	4.29
5	-12	0	85.83	4.46
-5	-13	0	49.04	3.49
-5	-13	0	43.06	2.93

5	-13	0	44.20	2.85
5	13	0	39.60	4.02
-5	13	0	50.62	3.64
-6	0	0	9.82	0.65
6	0	0	9.77	0.66
-6	0	0	9.35	0.68
-6	0	0	10.50	0.62
-6	0	0	10.71	0.62
6	0	0	10.18	0.51
-6	0	0	9.55	0.60
-6	0	0	9.43	0.48
6	0	0	9.68	0.67
-6	-1	0	6.58	0.51
6	-1	0	6.23	0.42
-6	1	0	7.07	0.47
-6	-1	0	6.02	0.47
-6	1	0	5.63	0.56
6	1	0	6.43	0.46
6	1	0	6.53	0.65
-6	-1	0	6.37	0.50
-6	1	0	6.64	0.49
6	1	0	6.74	0.39
-6	1	0	6.23	0.43
-6	1	0	6.08	0.44
-6	-1	0	5.98	0.58
6	-1	0	6.07	0.59
6	-1	0	7.04	0.46
-6	1	0	6.38	0.55
-6	-1	0	6.55	0.46
6	-1	0	5.33	0.44
6	-1	0	5.74	0.46
6	-1	0	6.03	0.56
6	1	0	6.58	0.34
6	-2	0	100.42	4.80
6	2	0	103.22	4.69
-6	2	0	108.59	4.73
-6	-2	0	99.81	4.61
-6	2	0	103.68	4.82
6	-2	0	97.22	4.72
-6	2	0	103.34	4.73
6	2	0	101.68	4.66
6	-2	0	100.60	4.73
6	2	0	102.40	4.84
-6	-2	0	98.65	4.72

6	2	0	102.61	4.67
-6	2	0	102.88	4.83
-6	-2	0	105.84	4.74
-6	-2	0	102.49	4.61
6	2	0	102.47	4.69
-6	-2	0	107.52	4.84
6	-2	0	98.42	4.71
-6	-2	0	105.08	4.72
6	-3	0	2.81	0.38
-6	3	0	2.46	0.50
6	3	0	2.77	0.36
6	3	0	2.18	0.45
6	3	0	2.64	0.44
6	3	0	2.55	0.41
-6	-3	0	2.00	0.45
-6	-3	0	2.68	0.41
-6	-3	0	3.03	0.42
6	-3	0	2.46	0.52
-6	-3	0	2.57	0.36
6	-3	0	1.99	0.45
6	-3	0	2.20	0.44
-6	3	0	2.40	0.50
-6	3	0	2.46	0.47
6	3	0	1.84	0.39
-6	3	0	2.66	0.42
-6	3	0	3.09	0.45
6	3	0	3.33	0.36
6	-3	0	2.66	0.40
-6	-3	0	2.27	0.31
-6	4	0	2.87	0.54
-6	-4	0	3.85	0.67
6	4	0	2.41	0.44
6	4	0	1.86	0.51
-6	-4	0	2.38	0.48
6	4	0	3.09	0.40
6	-4	0	2.38	0.50
-6	4	0	2.60	0.61
6	-4	0	3.00	0.66
-6	-4	0	2.32	0.46
6	-4	0	2.64	0.41
6	-4	0	2.50	0.50
-6	4	0	2.84	0.51
-6	-4	0	2.52	0.39
6	4	0	2.93	0.45

6	4	0	2.34	0.38
-6	-4	0	2.89	0.46
-6	4	0	2.98	0.53
6	-4	0	2.71	0.40
6	4	0	2.84	0.48
-6	4	0	2.85	0.43
-6	-4	0	2.16	0.40
6	-5	0	32.27	1.85
-6	-5	0	33.48	1.76
6	5	0	34.49	1.67
-6	-5	0	34.65	1.80
6	5	0	30.10	1.80
6	5	0	30.16	1.95
6	-5	0	34.48	1.63
6	-5	0	34.87	2.07
6	5	0	29.57	1.86
-6	5	0	34.61	1.91
6	-5	0	33.06	1.94
6	5	0	35.03	1.68
6	-5	0	32.85	1.74
-6	-5	0	32.73	1.97
-6	-5	0	32.79	1.72
-6	5	0	33.90	1.80
-6	-5	0	33.23	1.67
-6	5	0	33.02	2.11
-6	5	0	36.11	1.82
6	5	0	32.97	1.78
-6	-5	0	32.89	1.80
-6	5	0	32.90	1.97
6	-5	0	31.73	1.74
-6	6	0	1.00	0.45
6	6	0	1.61	0.42
6	-6	0	0.70	0.34
6	6	0	0.35	0.52
-6	6	0	1.12	0.45
6	6	0	0.59	0.53
6	-6	0	0.30	0.44
-6	-6	0	0.26	0.55
6	-6	0	0.60	0.34
-6	-6	0	-0.16	0.45
-6	-6	0	0.16	0.38
-6	6	0	0.34	0.54
6	6	0	1.26	0.44
6	-6	0	0.21	0.33

6	6	0	0.73	0.35
6	6	0	1.11	0.32
6	-6	0	0.08	0.55
-6	-6	0	0.59	0.31
6	-6	0	0.49	0.32
6	-6	0	0.84	0.74
-6	6	0	0.52	0.77
-6	6	0	0.12	0.51
-6	-6	0	0.66	0.39
-6	-6	0	1.81	0.47
-6	-7	0	5.01	0.78
-6	7	0	5.83	1.19
-6	-7	0	5.38	0.68
-6	-7	0	5.73	1.03
-6	7	0	4.51	0.94
6	-7	0	4.84	0.78
-6	7	0	6.41	0.98
6	-7	0	5.57	0.71
-6	-7	0	5.18	0.94
6	-7	0	4.85	0.68
6	-7	0	5.30	0.65
6	7	0	5.66	0.95
6	-7	0	6.11	0.62
6	-7	0	6.77	0.99
6	7	0	3.60	0.63
6	7	0	5.81	0.70
-6	-7	0	5.34	0.79
-6	-7	0	4.61	0.79
-6	7	0	5.77	0.89
6	7	0	4.88	1.01
-6	7	0	5.59	0.86
6	-8	0	8.43	0.84
6	-8	0	7.85	1.32
-6	8	0	7.91	1.38
-6	-8	0	6.57	0.99
-6	-8	0	6.75	0.93
6	8	0	8.34	0.77
-6	8	0	6.06	1.24
-6	-8	0	9.65	1.37
-6	8	0	6.10	1.24
6	8	0	7.95	0.79
-6	-8	0	5.95	0.91
6	-8	0	6.61	0.79
6	8	0	7.80	1.26

-6	8	0	9.11	1.12
6	8	0	7.96	1.18
-6	-8	0	8.60	0.76
6	-8	0	9.88	1.57
6	-8	0	6.87	0.91
-6	-8	0	6.92	1.20
6	-8	0	8.91	0.89
6	-8	0	7.86	0.88
6	-9	0	0.15	0.62
6	-9	0	0.44	1.10
6	9	0	0.04	0.52
6	-9	0	0.85	0.48
-6	-9	0	0.76	0.43
-6	-9	0	0.20	0.58
-6	9	0	1.08	0.97
6	9	0	1.13	0.86
6	-9	0	0.14	0.91
6	9	0	0.45	0.46
-6	9	0	0.51	0.85
-6	9	0	0.43	0.67
6	9	0	0.74	0.81
6	-9	0	0.74	0.64
-6	-9	0	0.77	0.54
-6	9	0	0.00	0.95
6	-9	0	0.01	0.56
-6	-9	0	-0.23	0.68
-6	-9	0	0.72	0.64
6	-9	0	0.83	0.45
6	-10	0	30.78	1.99
6	-10	0	28.77	1.82
-6	10	0	32.28	2.48
6	10	0	27.38	2.40
6	10	0	29.73	1.72
6	10	0	30.16	1.85
-6	10	0	31.09	2.39
-6	-10	0	29.94	2.23
-6	-10	0	29.08	1.71
-6	-10	0	28.76	2.04
6	-10	0	33.18	1.85
6	-10	0	29.18	2.12
6	-10	0	28.74	2.72
6	10	0	34.23	2.35
-6	10	0	28.20	2.45
6	-10	0	32.79	1.98

-6	-10	0	29.00	1.92
-6	-10	0	30.83	2.07
-6	10	0	33.53	2.04
6	-11	0	10.18	1.07
6	11	0	11.96	1.13
6	-11	0	8.73	1.93
6	-11	0	13.32	1.35
-6	-11	0	11.74	1.79
6	-11	0	12.65	1.25
-6	11	0	14.02	2.32
-6	11	0	10.09	1.84
6	-11	0	11.02	1.51
-6	-11	0	8.50	1.00
-6	-11	0	12.84	1.57
6	11	0	11.66	2.02
6	-12	0	5.23	1.19
-6	-12	0	7.93	1.52
6	12	0	4.11	1.84
-6	-12	0	3.70	0.99
6	-12	0	4.48	0.97
-6	12	0	5.47	1.61
6	-12	0	5.72	1.08
-6	-13	0	7.75	1.24
6	-13	0	7.80	1.47
-6	-13	0	15.54	2.35
-6	13	0	13.87	2.69
6	13	0	9.51	2.02
-7	0	0	0.09	0.22
-7	0	0	0.17	0.17
7	0	0	0.27	0.20
-7	0	0	0.03	0.19
-7	0	0	0.00	0.23
7	0	0	0.00	0.23
7	0	0	0.40	0.11
7	0	0	0.07	0.25
7	0	0	0.28	0.16
-7	0	0	0.51	0.18
-7	-1	0	132.58	5.94
-7	1	0	132.76	5.85
7	-1	0	129.52	5.95
-7	-1	0	131.91	5.99
-7	1	0	128.14	6.00
7	-1	0	123.84	5.91
7	-1	0	126.70	5.86

-7	-1	0	128.34	5.89
7	1	0	134.22	5.82
-7	1	0	130.92	5.87
7	-1	0	125.17	5.90
-7	-1	0	126.92	5.89
-7	-1	0	130.08	5.93
7	1	0	122.97	5.79
7	-1	0	128.38	5.88
-7	1	0	133.42	5.91
7	-1	0	127.45	5.98
-7	-1	0	131.03	5.91
7	1	0	128.33	5.92
7	1	0	125.53	5.98
-7	1	0	131.24	5.92
-7	-2	0	73.76	3.35
7	-2	0	70.89	3.45
-7	-2	0	68.99	3.36
7	2	0	72.31	3.27
7	-2	0	76.05	3.25
-7	2	0	73.56	3.38
7	2	0	73.56	3.31
-7	-2	0	64.54	3.53
-7	-2	0	70.38	3.35
-7	-2	0	70.86	3.32
-7	2	0	68.98	3.47
7	2	0	70.33	3.48
-7	2	0	70.43	3.24
-7	2	0	73.92	3.35
7	-2	0	69.87	3.34
7	2	0	69.87	3.26
-7	2	0	70.75	3.44
7	-2	0	71.08	3.34
7	-2	0	67.04	3.35
-7	-2	0	70.04	3.31
-7	3	0	16.91	1.22
-7	3	0	17.33	1.04
7	3	0	14.76	1.14
7	3	0	17.22	0.94
7	3	0	17.45	0.96
7	-3	0	16.20	0.97
7	-3	0	16.40	1.15
7	3	0	17.25	0.94
7	3	0	16.12	0.96
-7	-3	0	16.46	1.18

-7	3	0	17.20	1.02
-7	3	0	17.89	1.07
7	-3	0	17.01	1.00
7	-3	0	16.44	1.01
-7	-3	0	17.15	0.87
-7	-3	0	17.13	0.88
-7	-3	0	17.78	0.96
-7	3	0	15.70	1.16
7	3	0	17.26	1.01
-7	-3	0	16.93	1.01
-7	-3	0	17.14	0.99
-7	4	0	176.47	8.22
7	4	0	167.84	8.22
7	4	0	181.65	8.05
-7	-4	0	172.08	7.99
7	-4	0	167.95	8.04
-7	4	0	181.77	8.07
7	-4	0	179.53	8.26
-7	4	0	185.41	8.13
-7	-4	0	177.39	7.98
-7	4	0	190.08	8.08
7	-4	0	174.59	8.11
-7	-4	0	174.60	7.97
-7	4	0	173.95	8.25
7	4	0	182.97	7.96
7	-4	0	162.55	8.02
7	4	0	180.18	8.05
-7	-4	0	163.89	8.05
-7	-4	0	172.29	7.98
-7	-4	0	176.03	8.21
7	4	0	166.20	8.08
7	-4	0	174.40	8.16
7	4	0	178.99	7.97
7	-5	0	126.42	5.93
7	5	0	115.99	5.75
7	5	0	123.18	5.69
7	-5	0	118.85	5.78
-7	-5	0	120.02	5.57
-7	-5	0	116.95	5.59
-7	-5	0	124.02	5.63
-7	5	0	114.49	5.91
-7	-5	0	117.79	5.57
-7	-5	0	117.55	5.68
7	-5	0	117.02	5.63

-7	5	0	123.25	5.67
7	5	0	123.88	5.67
7	-5	0	117.42	5.63
-7	5	0	131.66	5.79
7	5	0	116.62	5.85
7	5	0	121.83	5.55
-7	5	0	127.77	5.67
-7	5	0	115.37	5.83
7	5	0	123.61	5.53
-7	-5	0	123.20	5.85
7	-5	0	119.01	5.73
7	-6	0	147.09	7.16
7	-6	0	164.30	7.54
7	6	0	161.33	7.07
-7	6	0	163.06	7.36
7	6	0	148.83	7.42
-7	-6	0	157.54	7.42
7	-6	0	151.19	7.33
7	6	0	164.44	7.28
-7	-6	0	153.75	7.10
7	6	0	154.09	7.20
-7	6	0	151.69	7.53
-7	6	0	164.63	7.22
-7	-6	0	149.28	7.23
-7	-6	0	158.46	7.21
7	6	0	144.45	7.33
-7	6	0	155.95	7.23
7	6	0	153.38	7.08
-7	6	0	157.38	7.44
-7	-6	0	150.75	7.09
7	-6	0	158.64	7.32
7	-6	0	141.57	7.17
-7	-6	0	151.08	7.16
7	7	0	20.73	1.67
-7	7	0	21.35	1.72
7	-7	0	23.59	1.24
7	-7	0	23.36	1.94
7	7	0	20.99	1.43
-7	7	0	23.99	1.70
7	7	0	22.88	1.26
-7	-7	0	21.44	1.45
7	-7	0	20.67	1.68
-7	-7	0	21.87	1.30
-7	7	0	22.09	1.51

-7	-7	0	20.45	1.39
7	-7	0	21.78	1.34
7	-7	0	20.45	1.38
7	-7	0	21.00	1.50
-7	-7	0	21.47	1.34
-7	7	0	21.79	1.97
-7	-7	0	21.06	1.77
-7	7	0	22.53	1.49
-7	-7	0	23.08	1.52
7	7	0	20.59	1.43
7	-8	0	-0.04	0.55
7	-8	0	0.45	0.48
-7	8	0	0.59	0.63
-7	8	0	0.50	0.80
-7	8	0	-0.79	0.78
-7	-8	0	0.86	0.51
7	-8	0	-0.11	0.36
-7	-8	0	0.14	0.51
7	8	0	-0.26	0.69
7	-8	0	0.72	1.12
7	-8	0	0.78	0.46
7	8	0	0.68	0.42
-7	-8	0	-0.05	0.36
-7	8	0	0.02	0.74
7	-8	0	0.29	0.74
-7	-8	0	0.78	0.52
7	-8	0	0.64	0.43
-7	-8	0	0.12	0.60
7	8	0	0.59	0.38
7	9	0	48.17	3.08
-7	-9	0	47.98	2.72
-7	9	0	47.09	3.33
7	9	0	52.05	2.50
-7	-9	0	46.77	2.99
7	9	0	52.97	3.02
7	-9	0	51.00	2.54
7	-9	0	58.22	3.43
7	9	0	49.14	2.50
7	-9	0	43.90	2.84
7	-9	0	49.63	2.61
-7	9	0	53.81	3.13
-7	-9	0	47.36	2.53
-7	9	0	49.44	2.78
-7	9	0	46.34	3.04

7	-9	0	47.31	2.85
-7	-9	0	49.33	2.52
-7	-9	0	49.94	2.79
-7	-10	0	53.44	2.82
7	10	0	54.63	3.52
7	-10	0	56.13	3.83
7	10	0	57.25	3.37
7	-10	0	53.12	3.22
7	-10	0	59.45	2.93
7	-10	0	59.67	3.12
-7	10	0	51.03	3.86
7	-10	0	54.56	2.93
-7	-10	0	56.86	3.39
-7	-10	0	55.84	3.18
7	10	0	54.98	2.81
7	10	0	57.56	2.85
-7	10	0	53.28	3.72
7	-10	0	59.89	2.87
7	-11	0	49.65	3.03
7	11	0	50.28	3.59
-7	11	0	54.95	3.52
7	-11	0	53.85	2.85
-7	-11	0	51.46	3.17
7	-11	0	50.88	3.17
7	-11	0	55.89	2.77
-7	-11	0	49.87	2.67
-7	-11	0	50.62	3.35
-7	11	0	57.09	3.81
7	-12	0	101.47	5.17
-7	-12	0	111.07	5.49
7	-12	0	96.83	5.50
7	12	0	100.93	6.10
-7	12	0	113.85	5.95
-7	-12	0	109.68	5.84
7	-12	0	105.95	5.39
7	-13	0	35.51	2.47
-7	-13	0	37.80	3.03
7	13	0	36.02	3.40
-7	13	0	40.97	3.78
-7	-13	0	40.63	3.58
7	-13	0	34.85	2.47
8	0	0	31.68	1.50
8	0	0	32.23	1.69
8	0	0	29.92	1.67

-8	0	0	31.54	1.69
8	0	0	34.09	1.62
-8	0	0	29.97	1.60
-8	0	0	32.94	1.67
8	0	0	31.34	1.61
8	-1	0	25.53	1.42
-8	-1	0	27.30	1.53
-8	-1	0	27.26	1.45
8	1	0	27.71	1.28
-8	1	0	27.93	1.35
8	1	0	26.59	1.43
8	-1	0	28.29	1.42
8	-1	0	27.91	1.54
8	1	0	26.71	1.52
8	-1	0	27.01	1.41
-8	-1	0	29.27	1.44
-8	1	0	26.53	1.39
8	-1	0	26.94	1.36
8	-1	0	27.01	1.48
-8	1	0	25.53	1.55
-8	1	0	27.62	1.44
-8	-1	0	28.20	1.48
-8	-1	0	26.09	1.41
-8	2	0	118.16	5.15
8	2	0	104.90	5.00
8	-2	0	104.73	5.10
8	2	0	115.22	5.09
8	-2	0	114.08	5.23
8	2	0	107.47	5.25
-8	-2	0	93.32	5.14
-8	2	0	117.80	5.13
-8	2	0	108.62	5.01
-8	-2	0	113.80	5.25
8	-2	0	111.76	5.10
8	-2	0	112.04	5.02
-8	2	0	108.87	5.20
8	-2	0	107.86	5.12
-8	-2	0	112.01	5.12
8	-2	0	108.65	5.21
-8	-2	0	110.97	5.12
-8	2	0	112.29	5.26
-8	-2	0	107.39	5.09
-8	-2	0	114.22	5.13
-8	2	0	113.19	5.10

8	-3	0	8.13	0.67
-8	-3	0	8.70	0.71
-8	-3	0	9.24	0.73
8	-3	0	9.80	0.89
-8	3	0	7.30	0.85
8	3	0	9.14	0.65
8	-3	0	8.84	0.65
8	3	0	8.63	0.63
-8	3	0	9.00	0.75
-8	-3	0	8.42	0.67
-8	3	0	10.02	0.72
-8	-3	0	7.91	0.90
-8	3	0	9.24	0.73
-8	-3	0	8.91	0.51
-8	3	0	8.51	0.92
-8	-3	0	9.00	0.50
-8	-3	0	8.32	0.65
8	-3	0	8.67	0.67
8	3	0	8.30	0.87
8	3	0	8.14	0.62
8	4	0	154.76	7.50
-8	4	0	171.85	7.57
-8	-4	0	160.87	7.44
8	-4	0	159.37	7.50
-8	4	0	167.97	7.51
-8	-4	0	155.21	7.51
-8	4	0	160.34	7.68
8	4	0	162.77	7.49
-8	-4	0	168.59	7.69
-8	-4	0	164.75	7.39
8	4	0	166.38	7.40
-8	-4	0	156.85	7.43
-8	4	0	159.16	7.69
-8	-4	0	161.80	7.38
8	-4	0	153.69	7.47
8	-4	0	158.65	7.54
8	4	0	167.41	7.47
-8	4	0	179.22	7.55
8	4	0	157.65	7.68
8	-4	0	162.33	7.62
8	-4	0	167.19	7.72
-8	5	0	4.48	0.67
8	-5	0	3.21	0.62
-8	-5	0	4.66	0.66

8	5	0	2.96	0.57
8	5	0	2.88	0.56
8	5	0	3.15	0.67
-8	5	0	3.28	0.56
-8	-5	0	2.47	0.50
-8	-5	0	3.80	0.73
8	5	0	3.41	0.62
-8	5	0	2.98	0.62
8	5	0	2.73	0.52
-8	5	0	4.72	0.89
-8	-5	0	4.12	0.60
8	-5	0	3.38	0.83
-8	-5	0	2.59	0.54
8	-5	0	3.61	0.53
8	-5	0	3.51	0.52
-8	5	0	2.66	0.78
8	5	0	4.76	0.52
8	-5	0	3.14	0.69
-8	-6	0	39.08	2.04
8	-6	0	39.11	2.08
-8	-6	0	39.63	2.16
8	6	0	40.95	2.13
-8	6	0	35.36	2.51
-8	6	0	41.40	2.42
-8	6	0	40.87	2.29
-8	-6	0	37.98	2.09
8	-6	0	39.51	2.21
8	6	0	34.60	2.33
8	-6	0	39.42	2.08
-8	6	0	41.22	2.17
8	-6	0	40.78	2.44
-8	-6	0	40.12	2.01
8	-6	0	37.61	2.31
8	6	0	34.98	2.23
-8	-6	0	35.80	2.03
8	6	0	38.47	2.21
8	6	0	39.74	1.96
-8	-6	0	36.18	2.37
8	6	0	41.05	1.99
-8	6	0	41.77	2.17
8	7	0	16.95	1.41
8	7	0	17.43	1.06
-8	-7	0	14.09	1.57
8	7	0	18.94	1.10

8	7	0	13.64	1.42
8	7	0	18.40	1.28
-8	-7	0	19.10	1.11
8	-7	0	17.96	1.20
-8	-7	0	15.74	1.17
8	7	0	14.28	1.51
8	-7	0	16.13	1.14
-8	-7	0	15.05	1.19
8	-7	0	16.56	1.79
-8	7	0	16.98	1.33
-8	7	0	20.05	1.66
-8	-7	0	17.49	1.28
-8	7	0	13.94	1.75
8	-7	0	17.07	1.33
-8	-7	0	13.79	1.23
-8	7	0	19.92	1.37
-8	7	0	17.07	1.53
8	-7	0	17.94	1.61
-8	-8	0	0.52	0.41
8	-8	0	-0.38	1.06
8	-8	0	1.31	0.48
-8	-8	0	1.10	0.52
8	8	0	1.58	0.45
-8	8	0	1.72	0.88
-8	8	0	1.07	0.68
8	-8	0	0.91	0.56
-8	8	0	1.57	0.89
-8	-8	0	1.20	0.74
-8	-8	0	1.25	0.57
-8	8	0	2.39	0.90
-8	-8	0	1.24	0.51
8	8	0	0.50	0.72
8	-8	0	1.48	0.51
-8	-9	0	1.32	0.54
8	-9	0	0.69	0.51
8	9	0	0.24	0.81
-8	9	0	-0.78	1.07
8	-9	0	0.90	0.53
8	9	0	1.59	0.44
-8	-9	0	0.70	0.63
8	-9	0	1.10	1.20
-8	9	0	-0.14	0.80
-8	-9	0	0.75	0.62
8	9	0	1.13	0.49

-8	-9	0	0.92	0.47
-8	9	0	-0.27	0.86
-8	-9	0	0.56	0.76
8	-9	0	1.49	0.71
8	-9	0	0.13	0.46
8	10	0	10.09	0.98
-8	10	0	9.88	2.03
-8	10	0	11.49	1.83
-8	-10	0	10.37	1.72
8	-10	0	10.55	1.02
8	-10	0	13.23	2.04
8	10	0	10.33	2.13
-8	-10	0	9.16	0.99
8	-10	0	9.52	1.03
8	-10	0	11.14	1.39
-8	-10	0	10.53	1.39
8	10	0	10.00	1.90
8	-10	0	11.73	1.23
8	11	0	8.06	1.54
-8	11	0	5.86	1.61
8	-11	0	8.66	1.39
8	-11	0	5.05	0.83
-8	11	0	4.02	1.62
-8	-11	0	5.59	0.90
-8	-11	0	7.18	1.36
-8	-11	0	5.57	1.23
8	-11	0	6.36	1.17
-8	12	0	2.52	1.54
8	-12	0	2.82	0.86
8	-12	0	2.80	0.85
-8	-12	0	4.30	0.98
8	12	0	0.55	1.49
-8	-12	0	0.99	1.24
8	-12	0	2.19	1.10
-9	0	0	-0.14	0.23
9	0	0	-0.04	0.31
-9	0	0	-0.05	0.31
-9	0	0	-0.06	0.23
9	0	0	-0.10	0.21
9	0	0	-0.10	0.11
-9	0	0	0.60	0.31
9	0	0	0.10	0.25
-9	0	0	-0.01	0.20
-9	1	0	162.42	7.10

9	-1	0	153.28	7.19
-9	-1	0	164.54	7.21
9	1	0	156.19	7.29
-9	1	0	151.27	7.30
9	-1	0	156.84	7.17
9	-1	0	152.92	7.20
-9	1	0	161.86	7.22
9	1	0	148.07	7.19
9	-1	0	159.70	7.28
9	-1	0	152.83	7.11
-9	-1	0	152.88	7.22
-9	-1	0	143.90	7.17
-9	-1	0	160.29	7.24
9	-1	0	161.13	7.23
-9	-1	0	156.57	7.18
-9	1	0	162.10	7.20
-9	1	0	158.38	7.16
9	1	0	160.74	7.10
-9	1	0	159.85	7.17
-9	-1	0	159.09	7.31
9	-2	0	99.47	4.73
9	2	0	101.30	4.52
9	-2	0	99.16	4.73
9	-2	0	96.77	4.58
-9	2	0	96.98	4.69
-9	-2	0	98.43	4.59
9	-2	0	102.45	4.54
-9	-2	0	100.01	4.76
-9	-2	0	94.70	4.60
-9	-2	0	96.08	4.62
9	2	0	96.86	4.76
9	2	0	94.00	4.48
-9	-2	0	102.44	4.65
-9	2	0	100.85	4.65
-9	-2	0	104.59	4.64
-9	-2	0	96.31	4.60
9	-2	0	95.03	4.62
-9	2	0	99.65	4.58
-9	2	0	102.81	4.63
-9	2	0	97.80	4.77
-9	2	0	97.19	4.52
9	-2	0	97.42	4.61
9	-3	0	84.34	4.27
9	-3	0	88.76	4.40

-9	-3	0	93.10	4.46
9	3	0	91.37	4.22
-9	-3	0	89.21	4.23
9	3	0	88.57	4.20
-9	3	0	88.24	4.45
-9	3	0	97.23	4.30
-9	3	0	94.30	4.31
-9	-3	0	95.23	4.27
-9	-3	0	91.31	4.29
-9	3	0	94.47	4.29
9	3	0	89.14	4.20
9	-3	0	86.52	4.25
9	3	0	88.90	4.45
-9	3	0	92.61	4.43
9	-3	0	89.27	4.27
-9	-3	0	83.00	4.27
9	4	0	118.47	5.43
-9	-4	0	109.01	5.37
9	-4	0	114.95	5.49
-9	4	0	116.68	5.73
9	-4	0	114.40	5.51
-9	-4	0	121.70	5.72
-9	-4	0	120.69	5.35
-9	-4	0	114.54	5.52
-9	-4	0	117.94	5.47
9	4	0	120.65	5.47
9	4	0	114.88	5.71
9	4	0	119.33	5.51
9	-4	0	112.77	5.54
-9	4	0	124.68	5.53
-9	4	0	125.49	5.55
9	-4	0	113.59	5.64
9	4	0	115.17	5.50
-9	4	0	122.33	5.57
-9	4	0	113.70	5.70
-9	-4	0	119.10	5.46
9	5	0	161.05	7.68
-9	-5	0	162.80	7.83
-9	5	0	163.74	7.89
9	-5	0	165.72	7.77
9	-5	0	158.26	7.61
9	5	0	161.04	7.61
-9	-5	0	159.84	7.54
9	5	0	167.01	7.51

9	5	0	171.30	7.63
9	-5	0	171.86	7.91
-9	5	0	174.67	7.73
9	5	0	169.04	7.48
9	-5	0	162.31	7.68
-9	-5	0	158.18	7.54
-9	-5	0	166.44	7.50
9	5	0	151.79	7.82
-9	-5	0	158.44	7.64
-9	5	0	169.76	7.65
-9	5	0	176.60	7.67
-9	-5	0	167.04	7.49
9	-5	0	155.54	7.59
-9	5	0	161.26	7.85
9	-6	0	28.71	1.77
-9	6	0	31.13	1.89
9	6	0	30.88	1.57
-9	6	0	30.95	1.78
-9	6	0	30.45	1.78
9	-6	0	29.42	1.97
-9	6	0	27.85	2.12
-9	6	0	29.54	1.99
9	-6	0	29.99	2.11
9	6	0	26.94	1.96
9	6	0	29.24	1.53
-9	-6	0	26.45	1.70
9	-6	0	26.39	1.64
9	6	0	28.70	1.82
-9	-6	0	29.45	1.60
-9	-6	0	29.26	2.02
9	-6	0	28.56	1.66
9	6	0	29.32	1.76
9	6	0	28.75	1.71
-9	-6	0	28.37	1.59
-9	-6	0	28.89	1.66
-9	-6	0	30.20	1.65
-9	7	0	1.82	1.00
9	7	0	2.54	0.44
9	7	0	2.86	0.81
9	-7	0	2.09	0.49
-9	7	0	3.33	0.68
-9	-7	0	2.56	0.56
9	-7	0	1.73	0.78
9	-7	0	3.18	1.05

-9	-7	0	3.49	0.63
-9	-7	0	3.50	0.62
-9	7	0	1.95	0.66
-9	-7	0	1.94	0.54
9	-7	0	1.83	0.59
-9	7	0	2.49	0.85
-9	7	0	2.48	0.86
9	7	0	3.47	0.82
9	7	0	3.63	0.63
9	-7	0	4.11	0.70
9	7	0	0.96	0.68
-9	8	0	13.67	2.02
9	-8	0	12.82	1.10
-9	-8	0	13.37	1.07
9	8	0	12.27	1.44
-9	8	0	15.88	1.72
-9	8	0	14.73	1.66
-9	-8	0	15.71	1.23
-9	-8	0	13.84	1.57
9	-8	0	13.01	2.20
-9	8	0	15.14	1.38
9	-8	0	15.43	1.33
9	8	0	14.80	1.14
9	-8	0	16.72	1.19
9	8	0	11.90	1.52
-9	-8	0	15.22	1.30
-9	-9	0	10.55	1.53
-9	9	0	9.81	1.77
9	-9	0	11.53	2.04
-9	-9	0	11.17	1.26
-9	-9	0	10.66	1.21
9	-9	0	8.92	1.25
9	9	0	10.32	1.55
9	9	0	10.57	0.95
9	-9	0	10.88	1.12
-9	-9	0	9.54	0.95
9	-9	0	10.00	1.01
9	-10	0	30.35	2.17
-9	-10	0	29.46	2.42
-9	10	0	27.22	2.87
-9	10	0	32.80	2.64
-9	-10	0	32.90	2.16
9	-10	0	31.25	2.03
9	10	0	25.31	2.42

-9	-10	0	29.68	1.75
9	-10	0	28.39	1.90
9	-10	0	35.87	3.04
9	-11	0	62.73	3.45
9	-11	0	59.03	3.24
9	-11	0	57.73	3.58
-9	11	0	60.72	4.05
-9	-11	0	63.19	3.94
9	11	0	59.05	4.03
-9	-11	0	64.88	3.62
-9	-12	0	27.93	2.68
9	-12	0	25.42	2.30
-9	12	0	29.36	3.25
9	12	0	25.96	2.72
9	-12	0	26.59	2.03
9	-12	0	28.88	1.95
-9	-12	0	28.02	2.20
-10	0	0	34.63	1.84
-10	0	0	35.96	1.95
-10	0	0	35.67	1.91
10	0	0	33.37	1.90
10	0	0	37.16	1.83
10	0	0	34.74	1.70
-10	0	0	37.75	1.99
10	0	0	36.31	1.85
-10	0	0	36.04	1.86
-10	0	0	38.36	1.87
10	1	0	118.70	5.68
10	-1	0	113.72	5.57
-10	1	0	128.60	5.59
-10	1	0	115.30	5.69
-10	-1	0	119.21	5.58
10	1	0	119.58	5.48
10	-1	0	120.89	5.56
10	1	0	115.21	5.58
-10	-1	0	121.52	5.67
-10	1	0	120.09	5.55
10	-1	0	116.47	5.48
10	-1	0	119.67	5.62
10	-1	0	123.92	5.67
-10	-1	0	123.08	5.64
10	1	0	118.40	5.41
-10	-1	0	117.32	5.61
-10	-1	0	122.13	5.57

-10	1	0	121.29	5.50
10	-1	0	117.27	5.57
-10	1	0	125.08	5.61
-10	-1	0	116.61	5.56
-10	1	0	125.17	5.46
-10	-2	0	86.20	4.10
10	-2	0	90.54	4.05
-10	2	0	84.44	4.18
10	-2	0	83.15	4.13
-10	2	0	90.66	4.17
10	-2	0	84.19	4.07
10	2	0	83.58	3.97
10	-2	0	87.91	4.24
-10	2	0	91.86	4.14
-10	2	0	88.59	4.30
-10	-2	0	88.04	4.16
10	-2	0	85.58	4.12
-10	2	0	86.47	4.03
10	2	0	91.50	4.02
-10	-2	0	85.32	4.13
-10	-2	0	84.33	4.11
-10	-2	0	83.64	4.10
10	-2	0	88.89	4.25
-10	-2	0	88.79	4.28
-10	-2	0	83.75	4.12
10	2	0	83.21	4.26
-10	2	0	92.82	4.08
-10	-3	0	1.47	0.38
-10	3	0	0.66	0.56
-10	-3	0	2.83	0.49
-10	-3	0	1.69	0.53
10	3	0	1.36	0.28
10	-3	0	1.47	0.57
10	-3	0	0.92	0.32
-10	-3	0	1.05	0.42
-10	3	0	1.32	0.38
10	-3	0	0.35	0.30
-10	3	0	1.74	0.53
10	3	0	0.61	0.48
10	3	0	0.97	0.27
-10	3	0	1.65	0.40
-10	-3	0	1.22	0.33
10	-3	0	1.19	0.48
10	-3	0	1.13	0.32

-10	3	0	1.70	0.40
-10	-4	0	67.18	3.02
-10	-4	0	67.67	3.03
10	4	0	67.71	3.18
-10	4	0	71.46	3.29
-10	-4	0	66.69	3.46
10	4	0	67.08	3.16
10	4	0	65.55	3.21
10	-4	0	61.99	3.39
-10	4	0	69.75	3.30
-10	4	0	69.35	3.30
-10	4	0	62.78	3.46
-10	-4	0	62.62	3.25
-10	-4	0	63.15	3.14
-10	-4	0	66.19	3.26
-10	4	0	63.70	3.46
-10	-4	0	64.76	3.18
-10	-4	0	66.93	3.23
10	-4	0	66.29	3.27
10	4	0	62.60	3.45
10	-4	0	64.46	3.22
10	-4	0	65.00	3.25
-10	5	0	77.40	4.19
-10	5	0	85.87	3.99
10	5	0	75.05	3.99
10	5	0	78.88	3.87
-10	5	0	83.14	3.93
10	-5	0	76.53	3.87
10	-5	0	73.64	3.84
-10	-5	0	79.86	3.92
-10	-5	0	80.37	3.71
10	5	0	81.70	3.77
-10	5	0	77.58	4.14
-10	-5	0	78.49	3.81
10	-5	0	83.31	4.20
10	-5	0	78.09	4.05
10	5	0	76.62	3.90
10	-5	0	78.28	3.93
-10	5	0	85.96	3.94
10	5	0	74.89	4.11
-10	-5	0	78.30	3.83
-10	-5	0	81.97	4.14
-10	-5	0	83.65	3.72
-10	-6	0	35.74	1.82

10	-6	0	33.54	2.31
-10	-6	0	32.75	1.80
10	6	0	36.16	1.79
10	-6	0	33.08	1.88
10	6	0	32.59	1.93
-10	6	0	29.35	2.30
10	-6	0	32.37	1.87
-10	6	0	34.39	2.09
-10	6	0	34.57	2.00
10	-6	0	32.66	1.97
-10	6	0	34.85	2.28
-10	-6	0	35.66	2.25
-10	-6	0	31.53	1.77
-10	-6	0	31.62	1.85
10	6	0	33.69	1.97
-10	6	0	37.34	2.00
10	6	0	31.60	2.02
-10	-6	0	34.95	1.97
10	-6	0	34.57	2.19
10	6	0	32.13	2.19
10	6	0	33.05	1.79
-10	-7	0	87.95	4.55
10	7	0	85.96	4.79
-10	7	0	91.91	4.68
10	7	0	93.43	4.28
-10	-7	0	92.70	4.38
-10	7	0	96.06	4.88
10	7	0	97.78	4.35
10	7	0	81.29	4.62
10	7	0	97.89	4.59
10	-7	0	87.31	4.46
-10	7	0	95.20	4.58
-10	-7	0	93.80	4.36
10	-7	0	93.92	4.63
-10	7	0	86.72	5.03
10	-7	0	88.57	4.47
10	-7	0	95.72	4.96
10	-8	0	35.55	2.11
10	-8	0	36.45	2.15
10	-8	0	35.44	2.28
10	8	0	42.02	2.02
-10	8	0	40.33	2.70
-10	-8	0	30.99	3.30
10	-8	0	39.68	2.82

10	8	0	39.82	2.40
-10	-8	0	31.68	2.48
-10	-8	0	39.41	2.03
-10	8	0	33.28	2.81
10	9	0	2.93	0.98
10	9	0	2.30	1.00
10	9	0	3.18	0.58
10	-9	0	2.53	0.80
-10	-9	0	3.74	0.77
-10	-9	0	2.48	0.98
10	-9	0	3.19	0.67
-10	9	0	2.57	1.43
-10	-9	0	3.94	0.63
10	-9	0	3.64	0.72
-10	9	0	3.07	1.13
10	-9	0	2.12	1.25
-10	-10	0	0.07	0.68
-10	10	0	-0.81	1.49
10	-10	0	0.65	0.58
-10	-10	0	0.70	0.54
10	-10	0	-0.16	0.82
10	10	0	0.19	1.10
-10	10	0	0.37	1.19
10	-10	0	0.58	0.65
-10	-10	0	1.84	1.08
10	-11	0	8.40	1.43
10	-11	0	4.21	1.03
10	-11	0	5.50	0.92
10	11	0	4.62	1.62
-10	11	0	2.83	1.40
-10	-11	0	2.57	1.04
-10	-11	0	6.58	1.57
10	12	0	3.27	1.40
-10	12	0	1.38	1.80
10	-12	0	3.07	1.21
-10	-12	0	2.11	1.00
10	-12	0	3.67	0.97
10	-12	0	4.03	0.90
-10	-12	0	4.00	1.39
-11	0	0	-0.21	0.29
11	0	0	0.08	0.15
-11	0	0	-0.40	0.32
-11	0	0	0.37	0.40
-11	0	0	0.16	0.25

-11	0	0	0.07	0.31
-11	0	0	0.69	0.39
11	0	0	-0.16	0.22
11	0	0	-0.21	0.37
11	0	0	0.28	0.30
11	-1	0	54.74	2.67
-11	1	0	53.66	2.82
-11	-1	0	51.86	2.73
11	-1	0	54.40	2.80
-11	-1	0	54.75	2.80
-11	1	0	55.54	2.52
-11	1	0	54.25	2.67
11	-1	0	52.50	2.56
-11	-1	0	58.68	2.71
11	-1	0	48.71	2.86
11	-1	0	51.23	2.67
-11	1	0	57.79	2.72
11	1	0	50.16	2.67
11	-1	0	55.00	2.68
-11	1	0	57.41	2.70
-11	-1	0	55.72	2.78
-11	-1	0	57.45	2.69
11	1	0	52.05	2.78
-11	-1	0	51.42	2.66
-11	2	0	107.54	4.97
11	-2	0	99.94	4.92
-11	-2	0	98.24	4.88
-11	-2	0	108.94	4.90
-11	-2	0	109.82	5.08
11	2	0	107.28	4.80
-11	2	0	109.14	4.93
-11	2	0	106.78	4.84
-11	2	0	100.50	4.81
11	2	0	98.64	4.74
11	-2	0	108.13	4.85
11	-2	0	102.83	4.85
-11	2	0	104.91	5.10
-11	-2	0	105.91	4.97
11	-2	0	102.91	5.03
-11	-2	0	104.27	4.92
11	-2	0	100.94	4.90
-11	2	0	105.27	4.95
11	-2	0	107.66	5.02
11	2	0	102.26	5.06

-11	-2	0	101.38	4.91
-11	-2	0	100.67	4.91
-11	-3	0	186.35	8.79
-11	3	0	198.66	8.82
11	-3	0	182.65	8.75
-11	-3	0	188.61	8.77
-11	-3	0	183.77	8.78
11	3	0	187.04	8.96
11	-3	0	199.71	8.96
-11	3	0	195.78	8.99
-11	3	0	194.59	8.75
11	-3	0	190.23	8.76
-11	-3	0	186.11	8.72
-11	-3	0	197.59	8.96
11	3	0	184.73	8.65
-11	-3	0	195.74	8.73
-11	3	0	189.80	8.89
11	3	0	189.74	8.66
-11	3	0	198.64	8.78
11	-3	0	188.74	8.91
-11	-3	0	191.12	8.74
11	-3	0	184.53	8.78
-11	4	0	90.88	4.52
-11	-4	0	87.26	4.27
-11	-4	0	91.34	4.50
11	-4	0	90.08	4.44
11	4	0	86.59	4.48
11	-4	0	81.99	4.25
-11	-4	0	88.74	4.25
-11	4	0	86.57	4.30
11	4	0	92.85	4.18
11	-4	0	84.32	4.25
11	4	0	89.12	4.17
-11	4	0	90.71	4.27
-11	-4	0	86.20	4.14
-11	4	0	93.22	4.31
-11	-4	0	88.75	4.27
11	-4	0	84.58	4.22
11	4	0	84.85	4.19
-11	-4	0	90.33	4.21
-11	4	0	88.41	4.49
-11	-5	0	55.97	2.55
-11	-5	0	53.80	2.61
-11	-5	0	53.23	2.54

11	5	0	51.61	3.02
11	-5	0	57.52	3.16
11	-5	0	51.42	2.99
11	-5	0	50.57	2.80
11	5	0	55.68	2.68
-11	5	0	57.23	2.85
-11	5	0	56.16	2.90
11	-5	0	52.54	2.77
-11	5	0	53.54	3.07
-11	-5	0	54.09	2.70
-11	-5	0	51.97	2.75
-11	-5	0	53.95	2.81
-11	5	0	52.18	3.08
11	5	0	52.22	2.78
11	-5	0	52.77	2.74
-11	-5	0	54.27	2.80
-11	-5	0	58.80	3.10
11	5	0	54.30	2.73
-11	5	0	54.85	2.82
11	6	0	20.94	1.58
-11	6	0	24.83	1.92
-11	6	0	24.61	1.64
-11	-6	0	23.16	1.31
11	-6	0	23.17	1.46
11	6	0	23.12	1.64
11	-6	0	24.01	1.97
11	-6	0	23.36	1.48
-11	-6	0	24.27	1.56
11	6	0	23.79	1.53
11	6	0	22.36	1.83
-11	6	0	24.48	1.68
11	6	0	26.33	1.42
-11	6	0	22.20	1.93
-11	-6	0	24.92	1.43
-11	-6	0	22.34	1.31
-11	-6	0	23.87	1.52
-11	6	0	25.41	1.62
11	-6	0	22.94	1.89
11	-6	0	23.22	1.57
11	7	0	17.03	1.10
11	-7	0	14.40	1.16
-11	-7	0	15.68	1.25
-11	7	0	13.75	1.83
11	7	0	12.35	1.57

11	-7	0	14.45	1.31
-11	7	0	14.40	1.64
-11	-7	0	13.70	1.09
11	-7	0	13.56	1.12
11	7	0	13.19	1.41
-11	-7	0	14.85	1.07
11	7	0	16.32	1.40
11	-7	0	16.56	1.81
-11	-8	0	46.61	2.66
11	-8	0	44.30	2.50
11	8	0	47.81	2.40
11	-8	0	43.79	2.66
11	-8	0	48.76	3.24
-11	8	0	49.45	3.45
-11	-8	0	45.50	2.42
11	8	0	50.23	2.76
11	-8	0	42.05	3.05
-11	-8	0	46.94	2.46
11	8	0	42.75	2.77
-11	8	0	45.62	3.39
-11	-8	0	46.72	2.91
-11	9	0	8.43	1.93
11	-9	0	9.28	1.33
-11	-9	0	9.93	1.30
-11	-9	0	8.02	1.15
11	-9	0	11.94	1.84
-11	-9	0	9.73	1.49
11	-9	0	10.72	1.18
-11	9	0	10.62	1.93
-11	-9	0	10.67	1.03
11	-9	0	9.56	1.21
11	9	0	9.83	1.58
11	9	0	9.15	1.55
11	9	0	11.34	1.03
11	10	0	9.52	1.99
-11	-10	0	11.57	1.11
-11	-10	0	15.41	2.05
11	-10	0	11.63	1.24
-11	-10	0	14.13	1.67
11	-10	0	12.39	1.28
-11	10	0	13.31	2.17
11	-10	0	9.42	1.42
11	-11	0	17.49	1.65
-11	-11	0	19.03	2.31

11	-11	0	15.64	1.53
11	11	0	16.68	2.25
-11	11	0	18.55	2.85
11	-11	0	12.92	1.82
-11	-11	0	18.02	1.82
11	-12	0	31.17	2.16
11	-12	0	29.06	2.13
-11	12	0	25.75	3.18
-11	-12	0	28.89	2.25
-11	-12	0	27.60	2.69
11	12	0	26.13	2.72
11	-12	0	24.87	2.44
12	0	0	175.97	7.92
-12	0	0	181.78	7.99
-12	0	0	174.70	7.97
-12	0	0	173.03	8.00
12	0	0	171.38	8.02
12	0	0	163.98	7.95
12	0	0	168.35	7.79
-12	0	0	174.16	8.07
-12	0	0	181.47	8.09
-12	0	0	162.33	7.94
12	-1	0	104.48	4.97
12	-1	0	105.62	5.09
-12	-1	0	110.71	5.19
-12	1	0	114.02	5.12
-12	-1	0	110.94	5.10
-12	1	0	99.81	5.07
-12	1	0	103.73	5.24
-12	1	0	111.96	5.09
-12	1	0	107.20	4.94
-12	-1	0	113.94	5.22
-12	1	0	112.59	4.91
12	1	0	102.15	5.20
12	-1	0	113.14	5.13
-12	-1	0	113.28	5.12
12	1	0	104.79	5.10
12	-1	0	108.53	5.10
-12	-1	0	99.64	5.07
-12	1	0	110.51	5.14
12	1	0	105.70	4.90
12	1	0	111.91	5.02
12	-1	0	109.70	5.21
-12	-1	0	104.70	5.15

-12	2	0	56.32	3.05
-12	2	0	59.13	2.88
12	-2	0	55.44	2.85
12	2	0	54.30	3.01
-12	-2	0	57.45	3.02
12	2	0	58.62	2.74
12	-2	0	58.53	2.81
-12	-2	0	57.64	2.88
-12	2	0	61.36	2.78
-12	-2	0	56.51	2.87
12	2	0	54.82	2.66
-12	2	0	56.52	2.77
12	-2	0	58.39	3.00
-12	-2	0	57.81	2.84
-12	-2	0	55.93	2.83
-12	-2	0	54.10	2.84
-12	2	0	60.67	2.93
12	-2	0	57.82	3.02
-12	2	0	59.20	2.91
12	-2	0	56.92	2.78
12	-2	0	55.27	2.87
-12	-2	0	58.62	2.95
-12	-3	0	8.40	0.82
12	3	0	8.46	1.05
12	-3	0	9.12	1.10
12	-3	0	9.95	1.14
-12	-3	0	8.15	0.76
-12	-3	0	8.42	0.80
-12	3	0	9.36	0.90
12	-3	0	7.92	0.78
12	-3	0	8.78	0.76
-12	-3	0	7.75	0.86
12	-3	0	8.31	0.73
-12	-3	0	8.88	0.78
12	3	0	8.65	0.64
-12	3	0	7.90	1.07
-12	-3	0	8.49	1.08
-12	3	0	7.65	1.09
-12	3	0	8.60	0.64
-12	3	0	8.93	0.83
-12	-3	0	7.41	1.01
12	3	0	8.98	0.70
-12	3	0	9.37	0.86
12	-3	0	9.21	0.70

-12	-4	0	28.13	2.36
-12	-4	0	33.71	1.87
-12	4	0	30.97	2.10
-12	-4	0	31.12	1.73
12	-4	0	35.60	2.12
12	-4	0	31.72	1.79
12	-4	0	32.13	2.06
12	4	0	31.04	2.06
12	4	0	31.99	1.73
-12	4	0	32.55	1.91
-12	-4	0	33.80	1.77
12	-4	0	32.21	1.81
12	-4	0	32.34	1.82
-12	4	0	32.70	2.11
-12	4	0	32.70	1.86
-12	-4	0	32.42	1.77
12	4	0	32.08	1.74
-12	-4	0	33.45	2.12
-12	4	0	35.01	1.88
-12	-4	0	31.21	1.83
12	5	0	20.66	1.68
12	-5	0	20.70	1.40
12	-5	0	21.12	1.38
12	5	0	22.02	1.36
12	5	0	22.27	1.32
-12	-5	0	22.92	1.77
-12	-5	0	21.61	1.43
12	-5	0	21.18	1.35
-12	-5	0	21.96	1.33
-12	5	0	22.64	1.48
-12	5	0	21.25	1.75
-12	-5	0	20.62	1.46
-12	-5	0	21.58	1.46
-12	5	0	21.78	1.78
12	-5	0	17.32	1.65
-12	-5	0	20.77	1.22
-12	5	0	22.82	1.54
-12	5	0	21.24	1.51
12	5	0	20.87	1.37
12	-6	0	86.11	4.39
-12	-6	0	86.69	4.37
12	6	0	85.20	4.62
-12	6	0	86.61	4.39
-12	-6	0	84.48	4.24

12	6	0	82.50	4.35
12	-6	0	82.20	4.28
-12	-6	0	91.59	4.08
12	-6	0	85.47	4.32
-12	6	0	88.63	4.72
-12	-6	0	84.84	4.23
-12	6	0	102.00	4.78
-12	6	0	87.40	4.66
-12	-6	0	85.52	4.10
12	-6	0	93.70	4.74
12	6	0	89.74	4.30
12	6	0	93.12	4.20
-12	7	0	49.73	3.36
12	-7	0	50.99	2.78
12	7	0	55.36	2.68
-12	-7	0	53.13	2.72
-12	7	0	47.82	3.26
12	-7	0	52.38	2.80
12	-7	0	57.12	3.42
12	7	0	55.31	2.86
-12	-7	0	50.89	2.89
-12	-7	0	53.84	2.61
12	-7	0	51.63	2.90
-12	-8	0	84.81	4.19
-12	-8	0	87.39	4.16
-12	8	0	88.00	4.83
-12	8	0	82.14	4.96
12	-8	0	86.26	4.31
12	8	0	81.86	4.52
12	-8	0	75.80	4.42
12	-8	0	93.68	4.98
12	-8	0	86.73	4.47
-12	-8	0	89.71	4.72
12	8	0	91.35	4.16
-12	-8	0	87.65	4.44
-12	-9	0	44.85	2.70
12	9	0	47.23	2.88
-12	-9	0	45.27	3.05
12	9	0	41.98	2.95
-12	9	0	48.90	3.29
12	-9	0	39.80	2.75
12	-9	0	42.93	2.51
12	-9	0	44.01	2.56
12	9	0	46.44	2.36

-12	-9	0	43.86	2.40
12	-10	0	10.16	1.49
-12	10	0	9.45	1.72
12	-10	0	8.00	1.19
-12	-10	0	10.27	1.10
-12	-10	0	8.40	1.57
12	10	0	9.00	1.61
-12	-10	0	13.02	2.13
12	-10	0	8.07	1.36
-12	-11	0	1.24	0.89
12	11	0	0.92	1.22
-12	-11	0	2.06	1.22
12	-11	0	-1.59	1.01
-12	11	0	2.95	1.72
12	-11	0	1.83	0.80
12	-11	0	0.72	0.70
12	-12	0	4.77	1.11
-12	-12	0	4.75	1.85
12	12	0	2.20	1.51
-12	-12	0	4.46	1.49
-12	12	0	5.61	1.72
12	-12	0	2.70	0.95
12	-12	0	4.16	1.38
-13	0	0	0.15	0.35
-13	0	0	0.04	0.32
-13	0	0	0.15	0.53
13	0	0	0.10	0.18
13	0	0	-0.33	0.26
-13	0	0	-0.20	0.37
-13	0	0	-0.26	0.42
-13	0	0	0.35	0.48
13	0	0	-0.57	0.37
13	0	0	0.13	0.42
13	-1	0	18.13	1.18
-13	-1	0	17.77	1.24
13	-1	0	17.89	1.19
13	-1	0	16.91	1.34
-13	1	0	17.26	1.18
-13	1	0	17.27	1.18
13	1	0	17.19	0.89
-13	-1	0	16.61	1.32
-13	1	0	17.36	1.32
-13	1	0	17.06	1.20
13	1	0	17.12	1.05

-13	-1	0	16.02	1.14
-13	-1	0	17.86	1.34
13	1	0	17.65	1.13
13	-1	0	16.52	0.97
-13	-1	0	16.02	1.09
13	1	0	19.19	1.30
-13	1	0	16.03	1.12
13	-1	0	17.19	1.11
-13	-1	0	18.90	1.18
-13	-2	0	70.52	3.55
13	-2	0	72.65	3.30
-13	-2	0	67.78	3.33
-13	-2	0	66.33	3.44
-13	-2	0	68.44	3.39
-13	-2	0	68.19	3.35
-13	2	0	69.55	3.38
13	2	0	68.59	3.23
13	-2	0	63.52	3.25
13	-2	0	63.47	3.36
13	-2	0	65.58	3.34
13	2	0	68.64	3.54
13	2	0	65.01	3.14
-13	2	0	67.80	3.41
-13	2	0	66.89	3.23
-13	-2	0	67.73	3.34
-13	2	0	72.49	3.62
-13	2	0	71.84	3.28
13	-2	0	70.77	3.70
-13	-2	0	66.44	3.37
13	-2	0	66.27	3.52
13	-3	0	52.07	2.87
13	-3	0	51.87	2.84
-13	-3	0	54.58	2.83
-13	-3	0	55.67	2.89
-13	3	0	53.42	2.82
13	3	0	58.58	2.71
13	3	0	59.24	3.12
13	3	0	55.22	2.74
-13	-3	0	57.54	2.86
-13	3	0	54.27	2.93
-13	3	0	59.31	3.06
-13	-3	0	56.63	2.85
-13	-3	0	56.38	2.95
13	-3	0	55.04	3.08

-13	-3	0	54.76	2.87
13	-3	0	62.48	2.76
-13	3	0	56.86	2.91
13	-3	0	61.10	3.10
-13	-3	0	59.26	3.12
-13	3	0	61.01	3.18
13	-3	0	51.86	2.80
-13	3	0	59.68	2.71
13	-4	0	50.00	2.92
-13	4	0	51.64	3.00
-13	4	0	59.39	2.99
-13	4	0	52.15	2.94
13	4	0	62.35	3.26
-13	4	0	59.81	3.26
13	-4	0	65.04	3.30
13	4	0	53.31	2.83
13	-4	0	59.97	3.22
13	-4	0	54.75	2.91
-13	-4	0	53.91	2.95
-13	-4	0	62.01	2.92
-13	-4	0	64.11	3.01
13	4	0	62.30	2.87
-13	-4	0	54.69	3.04
-13	-4	0	53.33	2.87
-13	-4	0	60.13	2.90
13	-4	0	51.93	2.91
-13	4	0	67.44	3.36
-13	-4	0	59.87	3.26
13	-5	0	11.03	1.06
13	5	0	12.21	1.02
13	5	0	11.94	1.01
-13	5	0	10.78	1.55
-13	5	0	12.15	1.20
-13	-5	0	13.06	0.99
13	-5	0	11.45	1.00
-13	-5	0	12.03	0.99
-13	-5	0	11.46	1.10
-13	5	0	9.58	1.35
-13	-5	0	13.34	0.95
13	-5	0	10.10	0.99
-13	5	0	13.63	1.48
-13	-5	0	13.90	1.10
13	5	0	13.17	1.42
-13	-6	0	0.28	0.64

13	6	0	1.59	0.78
13	-6	0	0.34	0.49
-13	-6	0	0.94	0.27
13	-6	0	0.21	0.41
13	6	0	1.84	0.53
-13	-6	0	2.11	0.58
13	-6	0	0.55	0.42
-13	6	0	0.30	1.01
13	6	0	1.63	0.58
-13	-6	0	0.67	0.46
-13	-6	0	1.15	0.37
13	-6	0	0.97	0.93
13	6	0	0.22	0.57
-13	6	0	0.38	0.82
-13	-7	0	14.93	1.17
-13	-7	0	15.08	1.32
-13	7	0	15.17	2.00
13	-7	0	14.24	1.33
13	7	0	15.26	1.35
13	-7	0	13.38	1.20
13	7	0	15.62	1.69
13	7	0	14.09	1.38
-13	7	0	15.79	1.81
-13	-7	0	15.16	0.99
-13	-7	0	16.21	1.32
13	7	0	14.91	1.17
13	-7	0	14.70	1.24
13	-7	0	16.37	2.05
-13	-8	0	23.01	1.37
13	-8	0	21.50	1.85
-13	8	0	20.79	2.22
13	8	0	22.80	1.44
-13	8	0	21.46	2.49
13	8	0	21.53	1.67
13	-8	0	21.22	1.52
-13	-8	0	20.62	1.47
-13	-8	0	20.78	2.04
13	8	0	17.63	1.81
13	-8	0	22.41	2.34
-13	-8	0	20.44	1.70
13	-8	0	21.07	1.53
-13	9	0	6.44	1.51
13	-9	0	3.11	0.85
13	-9	0	3.64	0.79

-13	-9	0	6.35	1.28
13	-9	0	4.43	0.84
-13	-9	0	3.86	0.76
13	9	0	3.17	0.95
13	9	0	4.02	1.12
-13	-9	0	4.18	0.87
13	-10	0	2.93	0.99
-13	-10	0	5.17	1.08
-13	10	0	7.30	1.87
13	10	0	5.42	1.37
-13	-10	0	6.41	1.06
-13	-10	0	6.40	1.42
13	-10	0	3.44	0.90
13	-11	0	0.66	1.00
-13	11	0	4.52	1.65
13	11	0	3.97	1.29
13	-11	0	2.01	0.77
13	-11	0	1.46	0.91
-13	-11	0	3.89	1.34
-13	-11	0	3.20	1.00
14	0	0	187.00	8.31
-14	0	0	180.32	8.49
-14	0	0	177.18	8.48
14	0	0	176.89	8.16
14	0	0	181.13	8.36
-14	0	0	182.55	8.41
-14	0	0	187.33	8.38
-14	0	0	169.62	8.34
14	0	0	182.55	8.43
-14	-1	0	71.14	3.55
-14	1	0	68.17	3.45
14	-1	0	65.46	3.39
14	1	0	67.89	3.42
-14	1	0	69.18	3.61
-14	1	0	69.06	3.61
-14	1	0	64.04	3.38
14	-1	0	70.14	3.44
-14	1	0	69.11	3.43
-14	-1	0	71.84	3.56
-14	-1	0	66.11	3.49
-14	-1	0	66.94	3.42
14	1	0	70.33	3.31
14	-1	0	70.14	3.43
14	-1	0	66.66	3.25

-14	-1	0	71.29	3.43
-14	-1	0	65.73	3.38
14	1	0	70.70	3.55
14	1	0	68.59	3.18
-14	1	0	72.47	3.42
-14	-2	0	32.20	2.05
14	-2	0	32.79	2.06
-14	2	0	31.88	1.87
-14	2	0	32.98	1.91
-14	2	0	32.25	2.11
14	-2	0	30.40	1.69
-14	2	0	33.97	1.80
-14	-2	0	32.59	1.84
-14	-2	0	35.40	2.00
-14	2	0	32.54	1.76
14	2	0	31.93	1.68
14	-2	0	32.28	1.82
-14	-2	0	32.40	1.89
-14	-2	0	32.77	1.86
14	-2	0	34.46	1.94
14	-2	0	33.57	1.79
-14	2	0	32.79	1.68
14	2	0	31.17	1.58
-14	-2	0	30.08	1.79
-14	-2	0	29.66	1.79
14	2	0	32.74	2.04
14	-3	0	29.08	1.91
14	-3	0	29.17	1.98
14	-3	0	29.00	1.64
-14	-3	0	28.04	1.64
-14	3	0	27.84	1.72
-14	3	0	28.84	1.62
-14	3	0	28.63	1.99
-14	-3	0	24.82	1.64
-14	3	0	24.94	1.48
-14	-3	0	27.24	1.69
14	-3	0	28.24	1.67
14	-3	0	27.41	1.58
14	-3	0	26.98	1.94
14	3	0	28.26	1.90
-14	-3	0	29.69	1.65
14	3	0	28.49	1.45
-14	-3	0	27.48	1.65
-14	-3	0	27.49	1.95

-14	3	0	25.61	2.19
-14	-3	0	31.09	1.84
14	3	0	27.98	1.52
-14	4	0	0.37	0.80
-14	-4	0	0.46	0.46
-14	-4	0	1.05	0.77
14	-4	0	0.26	0.40
14	-4	0	1.07	0.45
-14	-4	0	0.51	0.44
-14	-4	0	-0.01	0.53
-14	-4	0	0.73	0.45
14	-4	0	0.10	0.40
14	-4	0	1.33	0.81
14	4	0	0.72	0.37
-14	4	0	-0.56	0.66
14	4	0	-0.33	0.58
-14	-4	0	0.45	0.43
-14	-4	0	0.13	0.63
-14	4	0	0.29	0.56
14	4	0	0.32	0.38
14	-5	0	22.28	1.89
-14	5	0	21.00	1.78
-14	-5	0	19.62	1.33
14	-5	0	17.30	1.34
-14	-5	0	21.18	1.42
-14	-5	0	20.27	1.34
14	5	0	19.86	1.29
-14	5	0	18.06	1.89
14	5	0	19.44	1.70
14	-5	0	17.71	1.31
14	5	0	19.28	1.28
-14	-5	0	20.93	1.65
14	-5	0	19.11	1.36
-14	-5	0	19.43	1.29
-14	-6	0	54.97	2.69
-14	-6	0	49.38	2.70
14	6	0	54.70	2.73
-14	6	0	51.79	3.31
14	-6	0	54.84	2.86
14	-6	0	51.31	2.74
-14	-6	0	43.24	3.39
-14	-6	0	51.85	2.85
14	-6	0	50.47	2.78
-14	-6	0	49.82	2.57

14	-7	0	40.18	2.34
14	-7	0	41.24	2.37
14	7	0	41.79	2.49
-14	-7	0	38.93	2.29
-14	-7	0	45.93	2.07
14	-7	0	43.09	3.00
-14	-7	0	42.25	2.35
14	7	0	43.77	2.45
-14	7	0	37.66	3.07
-14	-7	0	43.20	2.28
-14	-7	0	42.04	2.47
14	-7	0	38.49	2.56
-14	7	0	40.67	2.92
-14	-7	0	40.83	2.10
14	-8	0	96.74	4.87
14	8	0	102.94	4.99
-14	-8	0	98.56	4.57
14	8	0	95.57	5.04
-14	-8	0	94.39	4.75
14	-8	0	100.41	5.00
-14	-8	0	93.66	4.97
-14	8	0	100.53	5.48
14	-8	0	94.99	4.86
-14	-8	0	99.85	5.31
14	-9	0	101.97	5.10
14	9	0	96.19	5.37
-14	9	0	107.94	5.83
14	-9	0	103.17	5.27
-14	-9	0	102.13	5.27
-14	-9	0	105.55	5.65
-14	-9	0	98.59	4.96
14	-9	0	101.65	5.10
14	-10	0	8.03	1.47
14	-10	0	8.87	1.35
-14	10	0	8.71	1.90
14	10	0	10.10	1.84
14	-10	0	7.48	1.32
-14	-10	0	7.39	1.26
-14	-10	0	7.53	1.43
14	11	0	3.26	1.37
14	-11	0	0.82	1.21
14	-11	0	2.03	0.83
-14	11	0	4.03	1.63
-14	-11	0	1.05	1.25

-14	-11	0	3.48	1.07
14	-11	0	1.96	0.91
-15	0	0	-0.36	0.34
-15	0	0	-0.78	0.53
-15	0	0	0.22	0.65
-15	0	0	-0.41	0.42
15	0	0	-0.34	0.39
-15	0	0	-0.36	0.44
15	0	0	-0.11	0.50
15	0	0	0.24	0.22
-15	0	0	0.04	0.55
15	0	0	-0.46	0.33
15	-1	0	4.09	0.68
-15	1	0	3.45	0.85
-15	-1	0	4.85	0.82
-15	-1	0	3.82	0.80
15	1	0	5.14	0.65
-15	-1	0	5.96	1.01
15	1	0	3.54	0.69
-15	1	0	5.73	0.86
15	1	0	4.98	0.70
15	-1	0	5.81	0.79
15	-1	0	4.91	0.87
-15	1	0	3.35	0.62
-15	-1	0	4.77	0.81
-15	1	0	4.98	0.85
-15	1	0	4.54	0.85
-15	1	0	5.01	0.71
-15	-1	0	3.93	0.67
15	1	0	4.03	0.40
15	-1	0	3.74	0.47
-15	-1	0	4.43	0.89
15	2	0	10.70	0.60
-15	2	0	12.43	1.34
-15	2	0	7.14	0.99
15	-2	0	7.42	0.70
15	-2	0	10.65	1.32
15	-2	0	8.99	0.85
-15	2	0	6.81	0.72
-15	-2	0	5.80	1.13
-15	2	0	8.99	1.00
-15	2	0	11.02	0.90
-15	-2	0	7.73	0.97
-15	-2	0	6.16	0.91

-15	-3	0	20.25	1.76
15	4	0	2.06	0.48
15	-4	0	1.87	0.51
-15	-4	0	2.45	0.78
15	-4	0	1.54	0.46
15	-4	0	0.81	0.43
-15	4	0	1.80	0.58
-15	-4	0	0.93	0.53
-15	-5	0	10.24	0.96
-15	-5	0	8.63	1.34
-15	-5	0	9.88	1.03
15	-5	0	9.64	0.99
-15	5	0	10.75	1.74
-15	-5	0	13.29	1.07
15	5	0	9.93	0.95
-15	5	0	9.84	1.70
15	5	0	12.44	1.02
15	5	0	12.51	1.52
15	-5	0	9.26	1.03
15	-5	0	9.76	1.06
-15	-5	0	10.65	1.07
15	-5	0	13.25	1.64
15	6	0	23.08	2.00
-15	6	0	21.08	2.26
-15	6	0	23.63	2.12
15	-6	0	21.94	1.53
-15	-6	0	21.54	1.42
15	-6	0	22.01	1.55
15	6	0	23.50	1.54
-15	-6	0	21.98	1.59
-15	-6	0	22.18	1.50
15	6	0	24.12	1.57
15	-6	0	19.52	1.55
-15	-6	0	21.87	1.83
-15	-6	0	21.47	1.51
-15	7	0	1.81	1.16
15	7	0	3.06	0.72
-15	-7	0	3.22	0.69
-15	-7	0	3.06	0.73
15	-7	0	4.34	1.32
-15	7	0	2.01	1.33
-15	-7	0	1.94	0.81
15	7	0	3.06	0.81
15	-7	0	3.23	0.78

-15	-7	0	2.74	1.10
-15	-7	0	3.68	0.53
15	-7	0	3.85	0.73
-15	-7	0	3.55	0.80
15	-7	0	1.96	0.64
15	-8	0	1.67	0.83
-15	-8	0	0.37	0.72
15	-8	0	1.61	0.67
-15	8	0	2.03	1.23
15	8	0	2.37	0.81
-15	-8	0	2.47	0.80
15	8	0	1.90	0.87
15	-8	0	1.91	0.73
-15	-8	0	1.20	0.64
-15	-8	0	2.25	1.05
-15	-9	0	1.11	0.68
15	-9	0	0.83	0.83
-15	-9	0	0.72	0.95
-15	9	0	1.35	1.53
15	-9	0	1.15	0.79
15	9	0	1.88	1.10
15	-9	0	0.52	0.65
-15	-9	0	3.17	1.34
-15	10	0	4.40	1.58
15	-10	0	-0.03	0.89
15	10	0	3.84	1.17
-15	-10	0	3.89	1.28
15	-10	0	1.12	0.79
-15	-10	0	1.57	0.93
15	-10	0	0.05	1.02
-15	11	0	5.87	1.85
-15	-11	0	5.12	1.71
15	-11	0	4.37	1.25
-15	-11	0	4.62	1.11
15	-11	0	1.91	0.87
15	-11	0	3.45	0.95
15	11	0	7.90	2.21
-16	0	0	8.64	1.31
16	0	0	8.79	0.62
-16	0	0	9.32	1.02
16	0	0	7.86	0.90
16	0	0	11.86	1.07
-16	0	0	7.11	1.00
-16	0	0	8.84	0.91

-16	0	0	9.72	1.12
-16	0	0	8.54	1.17
16	1	0	43.43	2.08
-16	1	0	42.35	2.62
16	-1	0	41.81	2.15
-16	1	0	40.64	2.63
16	-1	0	43.65	2.36
-16	-1	0	45.12	2.55
16	1	0	46.12	2.53
16	-1	0	46.47	2.37
-16	1	0	43.84	2.39
-16	-1	0	44.24	2.40
16	1	0	42.75	2.37
-16	-1	0	43.37	2.40
16	-1	0	45.33	2.38
-16	-1	0	40.12	2.49
16	1	0	46.93	2.25
-16	1	0	43.58	2.41
-16	1	0	42.14	2.35
-16	-1	0	39.70	2.32
-16	-1	0	42.25	2.58
16	-2	0	47.77	2.50
-16	-2	0	46.65	2.64
-16	2	0	51.93	3.00
-16	2	0	49.31	2.72
16	2	0	51.51	2.52
-16	2	0	50.13	3.03
-16	-2	0	47.81	2.69
-16	2	0	50.31	2.40
-16	-2	0	48.69	2.68
-16	2	0	49.86	2.62
-16	-2	0	47.61	2.64
16	-2	0	49.81	2.66
-16	2	0	50.58	2.44
16	-2	0	56.53	2.83
16	-2	0	54.66	2.65
-16	-2	0	55.11	2.95
-16	2	0	49.22	2.66
16	2	0	49.98	2.39
16	2	0	52.64	2.88
-16	-2	0	48.61	2.74
-16	-2	0	52.66	2.89
-16	3	0	34.05	2.39
16	3	0	34.34	2.28

-16	-3	0	34.98	2.04
16	-3	0	34.55	1.99
-16	3	0	39.19	2.34
16	-3	0	38.13	2.18
-16	-3	0	35.02	2.06
-16	-3	0	33.10	2.01
16	3	0	35.74	1.77
-16	-3	0	36.73	2.25
-16	3	0	32.75	1.99
16	3	0	35.55	1.85
-16	-3	0	33.63	1.99
-16	3	0	33.67	1.86
-16	-3	0	34.40	2.33
16	-3	0	33.72	1.89
-16	4	0	19.99	1.89
16	4	0	19.14	1.70
-16	-4	0	20.85	1.40
-16	-4	0	18.90	1.37
-16	4	0	18.91	1.83
16	4	0	19.58	1.18
16	4	0	17.10	1.21
-16	-4	0	19.14	1.64
-16	-4	0	18.46	1.40
16	-4	0	18.14	1.30
16	-4	0	22.61	1.96
16	-4	0	17.81	1.37
-16	-4	0	19.50	1.36
16	-4	0	18.27	1.33
16	-5	0	25.52	1.66
16	5	0	25.16	1.55
16	5	0	26.83	1.57
-16	-5	0	24.18	1.64
16	-5	0	24.42	1.64
16	-5	0	24.75	1.62
-16	-5	0	24.04	1.62
16	5	0	20.35	2.29
-16	-5	0	24.98	1.57
-16	5	0	21.23	2.09
-16	-5	0	25.31	1.92
16	-5	0	31.01	2.33
-16	6	0	18.85	2.09
-16	-6	0	18.74	1.50
-16	-6	0	16.95	1.28
16	-6	0	16.05	1.34

16	-6	0	16.26	1.40
-16	-6	0	16.23	1.71
16	-6	0	17.11	1.41
-16	-6	0	17.38	1.38
-16	-6	0	16.41	1.40
-16	6	0	22.16	2.22
16	6	0	19.02	1.93
16	6	0	20.68	1.44
16	6	0	17.63	1.37
16	7	0	36.81	2.31
-16	-7	0	35.29	2.29
-16	-7	0	34.73	2.14
-16	7	0	36.36	2.89
-16	-7	0	38.44	2.16
16	7	0	36.61	2.22
-16	-7	0	34.52	1.93
-16	-7	0	38.81	2.73
16	-7	0	34.97	2.19
16	-7	0	35.58	2.17
16	-7	0	33.45	2.27
16	8	0	82.07	4.26
-16	-8	0	85.27	4.80
16	-8	0	80.99	4.21
-16	-8	0	84.26	4.39
16	-8	0	80.50	4.33
-16	-8	0	81.99	4.15
-16	8	0	78.64	4.98
16	8	0	81.16	4.41
16	-8	0	82.77	4.23
16	-9	0	62.54	3.46
-16	-9	0	62.71	3.99
-16	9	0	64.31	4.28
16	-9	0	62.84	3.66
16	-9	0	62.79	3.46
-16	-9	0	62.20	3.57
-16	-9	0	58.22	3.27
16	9	0	60.82	3.64
-16	-10	0	14.49	1.75
16	-10	0	14.75	1.61
-16	10	0	12.91	2.13
16	-10	0	14.98	1.54
16	-10	0	15.12	1.83
-16	-10	0	17.86	2.25
16	10	0	16.61	2.07

17	0	0	-0.06	0.44
-17	0	0	-0.04	0.65
-17	0	0	0.42	0.67
17	0	0	0.20	0.45
17	0	0	0.08	0.23
-17	0	0	0.34	0.40
17	0	0	-0.46	0.53
-17	0	0	-0.12	0.79
-17	-1	0	0.88	0.80
17	-1	0	2.33	0.62
17	1	0	1.41	0.47
-17	1	0	0.21	0.80
-17	1	0	0.87	0.45
17	1	0	4.01	0.80
17	-1	0	1.33	0.33
17	1	0	0.73	0.51
17	-1	0	1.37	0.51
-17	-1	0	1.88	0.72
-17	-1	0	2.50	0.85
-17	1	0	1.29	0.74
17	1	0	1.55	0.29
-17	1	0	1.37	0.64
-17	-1	0	1.13	0.52
17	-1	0	2.02	0.58
17	2	0	24.88	1.31
-17	-2	0	25.14	1.62
-17	-2	0	26.56	1.63
17	-2	0	24.03	1.45
17	-2	0	29.45	1.84
-17	-2	0	24.66	1.96
17	2	0	28.09	1.93
-17	2	0	24.37	1.64
-17	-2	0	23.86	1.96
-17	2	0	22.92	1.60
17	-2	0	27.75	1.61
17	2	0	28.96	1.50
-17	2	0	25.71	2.02
-17	-2	0	26.80	1.82
17	-2	0	22.70	1.60
-17	3	0	15.69	1.16
-17	-3	0	15.48	1.64
17	3	0	17.14	1.13
-17	3	0	17.17	1.83
17	3	0	18.83	1.64

17	-3	0	19.23	1.53
-17	3	0	14.51	1.38
-17	-3	0	16.27	1.26
-17	-3	0	13.33	1.42
-17	-3	0	16.89	1.38
-17	-3	0	15.48	1.26
17	-3	0	14.81	1.24
17	3	0	16.94	0.97
17	-4	0	32.77	1.94
-17	-4	0	31.57	2.01
-17	4	0	37.80	2.42
17	4	0	34.17	1.88
17	4	0	38.77	2.48
17	-4	0	31.91	2.00
-17	-4	0	33.40	1.99
-17	-4	0	32.76	2.24
-17	-4	0	35.10	2.28
-17	4	0	35.28	2.51
17	-4	0	32.53	2.02
-17	-4	0	32.60	2.05
-17	5	0	25.29	2.33
-17	-5	0	25.20	1.65
-17	-5	0	25.11	1.73
-17	-5	0	24.14	2.02
17	-5	0	30.64	2.25
17	-5	0	25.14	1.74
17	5	0	25.84	1.61
17	5	0	24.84	2.15
17	5	0	25.74	1.60
17	-5	0	27.45	1.70
-17	5	0	23.73	2.35
17	-5	0	26.66	1.72
-17	-5	0	26.00	1.78
-17	-5	0	25.48	1.69
-17	-6	0	7.87	1.73
-17	-6	0	6.35	1.00
17	-6	0	5.06	1.01
-17	-6	0	6.31	0.95
17	-6	0	6.25	1.02
17	6	0	6.53	1.29
17	-6	0	5.52	0.97
-17	-6	0	5.65	1.03
17	6	0	5.00	0.82
-17	-6	0	6.08	1.04

17	-6	0	9.55	1.70
-17	6	0	6.12	1.42
17	6	0	7.02	1.05
17	-7	0	4.79	0.83
-17	7	0	4.83	1.41
-17	-7	0	6.43	1.35
17	7	0	6.56	1.17
17	7	0	5.53	0.95
-17	-7	0	6.13	1.06
-17	-7	0	5.36	0.90
17	-7	0	4.75	0.83
17	-7	0	6.68	1.12
-17	-7	0	4.66	0.94
-17	8	0	6.85	1.80
-17	-8	0	8.46	1.70
17	8	0	8.20	1.61
17	-8	0	7.41	1.08
17	-8	0	7.27	1.07
17	8	0	9.60	1.27
-17	-8	0	9.14	1.32
17	-8	0	8.12	1.46
-17	-8	0	6.35	1.15
17	-9	0	2.53	0.88
-17	9	0	3.27	1.48
-17	-9	0	3.50	1.34
-17	-9	0	2.95	0.90
-17	-9	0	3.56	0.99
17	-9	0	2.74	1.05
17	-9	0	2.29	0.83
17	9	0	4.90	1.10
-17	-10	0	18.86	2.32
-17	-10	0	15.64	1.89
17	10	0	17.33	2.01
17	-10	0	15.57	1.81
17	-10	0	16.75	1.64
-17	10	0	22.53	2.85
17	-10	0	16.67	1.64
18	0	0	3.29	0.49
-18	0	0	3.53	0.88
18	0	0	2.87	0.61
18	0	0	5.58	0.81
-18	0	0	3.21	0.69
-18	0	0	3.85	1.14
-18	0	0	3.85	0.95

18	0	0	2.59	0.77
18	-1	0	0.51	0.38
-18	1	0	1.02	0.63
18	-1	0	0.59	0.48
18	1	0	1.60	0.73
-18	-1	0	1.64	0.93
18	-1	0	0.66	0.58
-18	1	0	0.47	0.67
18	1	0	0.79	0.28
-18	1	0	1.11	0.79
-18	-1	0	-0.19	0.79
-18	-1	0	0.51	0.71
18	1	0	1.18	0.49
-18	-1	0	0.86	0.51
18	-1	0	2.27	0.62
-18	2	0	38.24	2.65
18	2	0	39.44	2.47
-18	-2	0	36.08	2.31
-18	2	0	39.47	2.18
-18	-2	0	37.19	2.52
-18	-2	0	38.55	2.20
-18	2	0	35.67	2.17
18	-2	0	42.41	2.19
18	2	0	39.49	2.01
18	2	0	36.95	1.86
-18	-2	0	36.87	2.52
18	-2	0	36.73	2.17
18	-2	0	35.17	1.99
18	-2	0	41.86	2.27
-18	-2	0	36.35	2.17
18	3	0	12.43	1.51
-18	-3	0	11.97	1.16
18	3	0	14.60	1.07
18	-3	0	12.64	1.19
-18	3	0	14.13	1.10
-18	-3	0	10.88	1.35
-18	3	0	13.03	1.22
-18	-3	0	13.49	1.67
18	-3	0	17.21	1.58
18	3	0	13.92	0.98
-18	3	0	15.27	1.26
-18	3	0	11.87	1.30
-18	3	0	13.40	1.70
18	-3	0	12.58	1.02

18	-4	0	6.81	1.31
18	-4	0	3.24	0.62
-18	-4	0	4.21	1.06
-18	-4	0	3.08	0.68
18	4	0	3.24	0.66
18	-4	0	3.94	0.72
-18	-4	0	3.84	0.74
18	4	0	3.94	0.63
-18	4	0	3.70	1.09
-18	4	0	3.16	0.69
18	4	0	3.21	1.00
-18	-4	0	3.60	0.78
18	-5	0	1.11	0.56
18	5	0	1.60	1.01
-18	-5	0	1.64	0.65
18	5	0	1.10	0.57
18	5	0	2.03	0.59
-18	-5	0	1.53	0.68
18	-5	0	2.00	1.23
-18	5	0	0.72	1.01
-18	-5	0	0.21	0.99
18	-5	0	0.43	0.57
18	-5	0	1.70	0.66
-18	5	0	2.56	1.17
-18	-5	0	1.59	0.62
-18	-6	0	11.03	1.23
18	-6	0	13.06	1.28
-18	6	0	13.21	2.07
-18	-6	0	14.04	1.88
-18	-6	0	13.35	1.28
18	-6	0	13.43	1.33
18	6	0	13.80	1.30
-18	-6	0	15.01	1.48
18	6	0	12.93	1.29
18	-6	0	14.64	1.35
18	-7	0	25.37	1.94
-18	7	0	23.76	2.88
-18	-7	0	22.91	2.00
18	7	0	22.94	1.91
-18	-7	0	27.50	2.54
18	-7	0	25.52	1.89
18	-7	0	22.28	1.74
-18	-7	0	25.71	1.77
18	7	0	24.69	1.83

-18	-8	0	33.56	2.19
18	-8	0	31.59	2.27
18	-8	0	33.86	2.46
-18	-8	0	37.28	2.94
-18	-8	0	33.28	2.43
-18	8	0	35.51	3.22
18	8	0	40.87	2.48
18	-8	0	33.38	2.32
-18	9	0	29.98	3.12
-18	-9	0	29.74	2.30
-18	-9	0	34.04	2.88
18	-9	0	28.56	2.27
18	-9	0	27.65	2.09
18	9	0	35.85	2.45
18	-9	0	25.75	2.06
-19	0	0	0.91	0.86
-19	0	0	-0.47	0.46
-19	0	0	0.78	0.78
19	0	0	-0.62	0.65
19	0	0	-0.06	0.51
19	0	0	-0.01	0.28
19	0	0	-0.17	0.52
19	1	0	12.51	0.87
-19	1	0	12.93	1.21
19	-1	0	11.56	1.26
19	1	0	11.66	1.03
-19	-1	0	15.22	1.56
19	-1	0	12.81	0.88
19	1	0	12.06	1.43
19	-1	0	10.64	1.23
-19	1	0	10.58	1.66
-19	-1	0	12.05	1.56
19	-1	0	14.39	1.21
-19	1	0	14.63	1.41
19	-2	0	52.39	2.72
-19	2	0	44.99	2.63
-19	-2	0	48.60	2.83
-19	-2	0	47.56	2.99
19	-2	0	47.00	2.43
-19	2	0	48.93	3.08
-19	-2	0	43.54	2.82
-19	2	0	45.65	2.60
19	2	0	48.72	2.42
-19	-2	0	50.02	2.66

19	2	0	43.17	2.31
19	2	0	50.21	2.90
19	-3	0	211.56	9.41
-19	-3	0	204.57	9.45
19	3	0	200.94	8.97
-19	-3	0	197.05	9.32
-19	3	0	186.17	9.08
-19	3	0	191.60	9.28
-19	-3	0	195.04	9.29
-19	3	0	187.31	9.07
19	-3	0	200.11	9.15
-19	-3	0	190.00	9.25
-19	3	0	207.21	9.69
19	3	0	198.79	9.66
19	3	0	201.53	9.05
19	-3	0	198.37	9.28
19	-4	0	75.51	4.02
-19	-4	0	72.17	3.97
19	-4	0	73.88	3.65
19	4	0	73.21	3.54
-19	4	0	67.80	3.75
19	-4	0	71.55	3.73
19	4	0	74.11	3.48
-19	-4	0	71.27	3.75
-19	4	0	68.17	3.75
-19	4	0	71.23	4.26
19	4	0	76.42	4.13
-19	-4	0	70.73	3.72
-19	-4	0	70.78	3.76
-19	-5	0	15.13	1.42
-19	5	0	14.84	1.42
19	5	0	16.07	1.29
-19	5	0	17.22	2.15
-19	-5	0	15.14	1.48
-19	-5	0	14.39	1.40
19	-5	0	15.14	1.28
19	5	0	16.57	1.24
19	-5	0	15.65	1.37
-19	-6	0	7.08	1.29
19	6	0	5.42	1.15
19	-6	0	6.73	1.03
-19	-6	0	7.15	1.12
19	-6	0	8.08	1.15
-19	6	0	7.03	1.69

19	6	0	8.34	1.20
-19	-6	0	5.05	1.27
-19	-6	0	5.61	0.93
19	-7	0	0.33	0.82
-19	-7	0	-0.48	0.85
-19	-7	0	0.51	1.24
-19	7	0	0.30	1.38
19	-7	0	-0.37	0.78
19	7	0	0.72	0.81
19	-7	0	-0.31	0.74
-19	-7	0	0.75	0.99
19	7	0	1.83	0.83
19	-8	0	6.21	1.24
-19	8	0	6.78	1.63
19	-8	0	5.17	0.96
-19	-8	0	6.81	1.43
19	8	0	6.14	1.06
19	-8	0	5.74	1.00
-19	-8	0	6.38	1.14
19	-9	0	31.11	2.26
-19	9	0	34.61	3.22
19	-9	0	32.90	2.26
19	9	0	31.23	2.37
19	-9	0	33.16	2.37
-19	-9	0	33.11	2.48
-19	-9	0	34.71	2.88
-20	0	0	0.66	0.95
20	0	0	0.61	0.33
20	0	0	1.56	0.59
-20	0	0	0.20	0.89
20	0	0	1.29	0.81
20	0	0	0.66	0.56
-20	0	0	-0.49	0.54
20	1	0	8.54	1.08
-20	-1	0	5.99	1.02
-20	-1	0	6.92	1.15
20	1	0	10.82	1.42
-20	1	0	5.19	1.17
-20	1	0	6.04	1.08
20	-1	0	6.37	1.00
20	-1	0	5.38	0.69
-20	1	0	7.10	1.11
-20	-1	0	7.18	1.03
20	1	0	7.03	0.62

-20	2	0	3.40	0.71
20	2	0	3.16	0.50
20	2	0	4.02	1.07
20	-2	0	2.42	0.49
20	-2	0	2.33	0.67
20	-2	0	5.10	0.89
-20	2	0	3.06	1.13
-20	-2	0	2.40	0.71
-20	2	0	2.61	0.74
-20	2	0	3.27	0.84
-20	-2	0	2.28	0.92
-20	-2	0	3.25	0.70
-20	3	0	5.38	0.94
20	-3	0	7.57	1.05
20	3	0	5.60	0.64
-20	3	0	4.71	1.26
-20	-3	0	4.75	0.84
20	-3	0	4.68	0.81
-20	-3	0	4.83	0.85
20	3	0	4.89	0.82
-20	-3	0	5.40	1.26
-20	-3	0	5.18	0.99
20	-3	0	7.19	1.30
20	3	0	5.72	1.13
-20	3	0	4.56	0.71
-20	-4	0	0.63	0.72
20	-4	0	0.00	0.50
20	-4	0	1.19	1.10
-20	4	0	0.57	0.62
20	-4	0	1.54	0.71
-20	-4	0	0.12	1.63
-20	4	0	0.18	1.10
-20	-4	0	0.07	0.69
20	4	0	1.35	0.44
-20	-4	0	1.20	0.64
20	5	0	1.99	0.58
-20	-5	0	0.89	0.78
-20	-5	0	0.52	0.69
-20	-5	0	1.46	1.11
20	5	0	2.15	0.65
20	-5	0	0.92	0.63
-20	-5	0	0.13	0.64
-20	5	0	0.51	0.74
20	-5	0	0.50	0.66

-20	5	0	0.48	1.32
-20	-6	0	0.21	0.80
20	-6	0	0.98	0.74
-20	6	0	0.29	0.74
20	6	0	0.28	0.75
-20	6	0	-0.01	1.34
-20	-6	0	0.07	1.21
20	6	0	1.16	0.80
-20	-6	0	0.13	0.70
20	-6	0	-0.15	0.66
20	7	0	8.42	1.39
20	-7	0	10.05	1.41
-20	-7	0	8.45	1.39
-20	-7	0	9.92	1.50
20	7	0	10.56	1.35
20	-7	0	5.71	1.24
-20	7	0	7.22	1.31
-20	-7	0	7.25	1.50
-20	7	0	13.11	2.37
-20	-8	0	5.96	1.10
-20	-8	0	6.85	1.12
-20	8	0	12.93	2.54
20	-8	0	6.74	1.35
20	8	0	9.36	1.56
20	-8	0	5.57	1.02
-21	0	0	-1.78	0.92
-21	0	0	-0.22	0.60
21	0	0	-0.08	0.32
-21	0	0	-0.36	0.73
-21	0	0	-1.43	0.96
21	0	0	-0.41	0.58
21	0	0	-0.80	0.58
-21	1	0	19.68	1.63
21	-1	0	21.76	1.57
21	1	0	19.07	1.33
-21	-1	0	20.91	1.62
-21	-1	0	21.27	1.61
-21	1	0	19.22	2.10
21	-1	0	21.09	1.27
-21	-1	0	20.82	2.04
21	-1	0	21.65	1.65
21	1	0	20.51	1.17
-21	1	0	22.13	1.77
-21	1	0	20.83	1.63

21	2	0	74.14	3.69
-21	-2	0	76.47	4.04
-21	2	0	77.42	4.07
21	-2	0	76.43	3.78
-21	2	0	83.70	4.55
-21	2	0	70.56	3.91
-21	-2	0	78.14	4.20
21	-2	0	82.20	4.08
21	-2	0	81.14	4.06
-21	-2	0	83.05	4.07
21	3	0	160.36	7.26
21	-3	0	158.34	7.41
-21	3	0	155.93	7.61
-21	-3	0	155.60	7.66
-21	-3	0	157.07	7.58
21	-3	0	161.31	7.68
21	-3	0	161.36	7.59
-21	3	0	162.29	8.08
-21	3	0	147.20	7.33
-21	-3	0	164.38	7.63
-21	4	0	135.39	6.66
21	4	0	134.21	6.25
-21	-4	0	130.08	6.58
21	-4	0	135.46	6.61
21	-4	0	140.58	7.25
-21	-4	0	131.36	6.62
-21	4	0	137.12	7.23
-21	-4	0	145.13	7.00
-21	-4	0	136.77	6.64
21	-4	0	135.45	6.49
-21	5	0	21.38	1.81
-21	-5	0	21.53	1.82
-21	-5	0	23.60	1.81
-21	5	0	24.36	2.79
-21	-5	0	22.51	2.44
-21	-5	0	23.75	1.94
21	-5	0	23.18	1.80
21	5	0	22.25	1.53
21	-5	0	22.90	1.65
-21	-6	0	1.15	1.27
-21	-6	0	2.13	0.97
21	-6	0	1.92	0.71
-21	-6	0	2.07	0.90
-21	6	0	2.93	0.83

21	6	0	2.76	0.76
21	-6	0	1.32	0.85
-21	6	0	1.74	1.32
-21	-7	0	0.06	0.89
-21	7	0	1.30	1.51
-21	-7	0	-0.10	1.15
-21	-7	0	-0.04	0.78
21	-7	0	-0.40	0.70
-21	7	0	-0.97	0.72
21	-7	0	0.69	0.78
21	7	0	0.33	0.78
-21	8	0	0.38	0.77
21	-8	0	-0.56	0.76
21	-8	0	0.46	0.80
-21	-8	0	2.76	1.00
-22	0	0	12.14	1.58
22	0	0	8.78	1.28
22	0	0	12.04	1.18
22	0	0	10.84	0.84
-22	0	0	11.94	1.93
-22	0	0	10.16	1.30
22	1	0	0.88	0.36
-22	-1	0	-0.14	0.64
-22	-1	0	0.75	0.73
-22	1	0	1.21	0.81
-22	-1	0	1.04	0.97
-22	1	0	2.29	1.08
-22	1	0	0.29	0.67
22	-1	0	1.83	0.76
22	1	0	1.75	0.54
22	-1	0	-0.10	0.66
-22	-1	0	1.21	0.98
22	-1	0	0.54	0.44
-22	-2	0	2.26	0.83
-22	-2	0	3.80	1.05
-22	-2	0	4.12	0.83
-22	2	0	1.51	0.87
-22	-2	0	1.46	1.34
22	-2	0	4.48	0.93
-22	2	0	2.40	1.25
-22	2	0	2.57	0.78
22	-2	0	1.72	0.55
22	-2	0	1.24	0.68
22	-3	0	0.50	0.67

-22	3	0	0.72	0.87
-22	-3	0	0.48	0.70
22	-3	0	-0.07	0.58
-22	3	0	-0.57	1.37
22	-3	0	2.47	0.91
-22	-3	0	0.02	0.74
-22	-3	0	0.81	0.90
-22	-3	0	1.68	1.11
-22	4	0	-0.26	1.50
22	-4	0	1.78	0.82
-22	4	0	0.73	0.80
-22	-4	0	0.35	0.99
-22	-4	0	-0.69	1.03
-22	-4	0	-0.33	0.74
22	-4	0	0.50	0.60
-22	-4	0	0.56	0.84
-22	5	0	1.45	0.82
22	5	0	2.06	0.70
22	-5	0	0.88	0.84
22	5	0	2.00	0.75
-22	-5	0	0.63	0.83
-22	-5	0	-1.08	1.26
-22	-5	0	0.58	0.81
-22	5	0	1.09	1.41
22	-5	0	1.80	0.64
22	6	0	1.93	0.68
22	6	0	1.64	0.85
22	-6	0	1.53	0.82
-22	-6	0	-1.98	1.15
-22	-6	0	1.86	0.93
-22	6	0	-0.32	1.42
-22	-6	0	0.13	0.87
-22	6	0	1.77	0.80
22	-6	0	0.58	0.70
-22	7	0	3.91	0.92
22	-7	0	4.92	0.94
-22	-7	0	4.92	1.46
-22	7	0	1.94	1.89
22	7	0	4.72	0.95
-22	-7	0	4.88	1.09
22	-7	0	3.18	0.82
-22	-7	0	2.78	0.99
23	0	0	0.22	0.60
23	0	0	-0.15	0.74

-23	0	0	-0.40	0.84
23	0	0	0.59	0.46
-23	0	0	0.15	1.17
-23	0	0	0.93	0.79
-23	1	0	8.80	1.48
-23	-1	0	9.33	1.38
-23	1	0	7.87	1.46
-23	-1	0	10.11	1.45
-23	-1	0	9.00	1.62
-23	-1	0	9.93	1.97
23	-1	0	8.76	0.96
23	1	0	8.66	0.79
-23	1	0	8.71	1.55
23	-1	0	11.85	1.32
23	-1	0	9.59	1.43
23	1	0	11.17	1.02
23	-2	0	47.98	2.71
-23	-2	0	43.38	2.76
23	-2	0	44.20	2.38
-23	-2	0	50.74	3.09
23	-2	0	44.80	2.74
-23	-2	0	41.62	3.00
-23	2	0	44.21	3.49
-23	-2	0	43.25	2.75
-23	2	0	45.03	2.75
23	-3	0	72.50	3.69
23	-3	0	72.20	3.98
-23	3	0	77.65	4.76
-23	-3	0	77.96	4.07
-23	-3	0	71.22	3.97
-23	-3	0	76.45	4.34
-23	3	0	71.70	3.98
-23	4	0	94.29	5.22
-23	-4	0	89.96	4.62
-23	-4	0	90.33	4.62
-23	-4	0	91.54	5.08
-23	4	0	84.71	4.56
23	-4	0	80.91	4.31
23	-4	0	90.93	4.60
-23	-4	0	79.18	4.52
-23	-5	0	17.60	1.80
-23	5	0	15.71	1.64
23	-5	0	17.53	1.66
-23	-5	0	15.98	1.70

-23	-5	0	15.02	2.38
23	-5	0	17.25	1.45
-23	5	0	18.53	2.59
-23	-6	0	0.76	1.02
23	6	0	0.22	0.67
-23	-6	0	1.85	0.93
-23	6	0	0.13	0.78
-23	6	0	-0.26	1.47
23	-6	0	0.39	0.75
23	-6	0	0.89	0.75
-23	-6	0	0.89	1.31
24	0	0	5.30	1.17
-24	0	0	3.77	0.99
24	0	0	4.21	0.70
-24	0	0	3.01	1.34
-24	0	0	5.37	1.26
24	0	0	4.36	0.83
24	-1	0	20.43	1.64
24	1	0	19.11	1.30
-24	-1	0	22.44	2.15
-24	-1	0	19.79	1.82
-24	-1	0	17.62	2.37
-24	-1	0	16.37	1.74
-24	1	0	18.37	1.71
24	-1	0	21.06	1.94
24	-1	0	19.36	1.27
24	1	0	20.00	1.16
-24	1	0	19.06	2.75
-24	-2	0	8.04	1.57
-24	-2	0	10.51	1.59
24	-2	0	9.81	0.95
-24	-2	0	8.31	1.55
24	-2	0	10.74	2.18
24	-2	0	8.35	1.24
-24	-2	0	10.62	1.48
-24	2	0	8.72	1.82
-24	2	0	10.97	1.37
-24	3	0	-0.17	0.76
24	-3	0	-0.04	0.83
-24	-3	0	1.45	1.38
-24	-3	0	1.08	0.82
-24	3	0	1.87	1.44
24	-3	0	0.54	0.52
-24	-3	0	-0.01	0.95

-24	-3	0	2.31	1.21
24	-4	0	2.97	0.88
-24	-4	0	1.52	1.12
24	-4	0	2.18	0.67
-24	4	0	3.34	0.98
-24	-4	0	3.47	0.93
-24	-4	0	5.81	1.45
-24	4	0	4.56	1.51
-24	5	0	0.77	1.55
-24	-5	0	0.13	1.26
-24	-5	0	1.07	0.89
24	-5	0	-1.19	0.73
-24	-5	0	1.31	1.13
-25	0	0	0.26	1.18
-25	0	0	-1.37	0.74
25	0	0	-0.36	0.68
-25	0	0	-1.75	0.70
-25	0	0	1.18	1.54
25	0	0	0.74	0.42
25	-1	0	0.54	0.45
-25	-1	0	0.26	0.77
25	-1	0	0.90	0.84
-25	1	0	5.20	1.57
-25	1	0	-0.03	0.83
-25	-1	0	0.93	0.87
-25	-1	0	-2.52	1.23
-25	-1	0	2.57	1.44
25	-1	0	-0.51	0.74
25	-2	0	5.21	0.88
-25	-2	0	9.15	1.55
-25	-2	0	6.37	1.10
-25	2	0	8.36	1.70
-25	2	0	5.32	1.08
25	-2	0	5.54	1.03
-25	-2	0	5.74	1.06
-25	-3	0	41.43	2.61
25	-3	0	36.83	2.51
-25	3	0	39.45	3.33
-25	-3	0	41.69	3.40
-25	-3	0	41.79	3.32
25	-3	0	39.03	2.19
-25	-3	0	37.82	2.58
-26	0	0	33.41	2.31
0	-2	-1	0.05	0.12

0	2	-1	0.12	0.13
0	2	1	-0.02	0.12
0	-2	1	0.06	0.09
0	2	1	-0.05	0.18
0	-2	1	0.30	0.19
0	2	1	0.11	0.18
0	2	1	-0.19	0.13
0	-2	-1	0.21	0.20
0	2	-1	-0.04	0.11
0	2	1	0.11	0.17
0	2	1	0.18	0.12
0	-2	-1	0.06	0.13
0	2	-1	0.15	0.10
0	-2	-1	0.03	0.14
0	2	-1	0.04	0.18
0	-2	1	0.24	0.18
0	2	-1	-0.07	0.18
0	-2	1	-0.07	0.12
0	-2	1	0.15	0.16
0	-2	-1	0.03	0.14
0	-2	1	-0.02	0.11
0	-2	-1	0.01	0.12
0	-3	1	0.41	0.23
0	-3	1	-0.21	0.13
0	-3	1	-0.01	0.12
0	-3	1	0.27	0.22
0	3	-1	0.12	0.16
0	-3	-1	0.06	0.20
0	-3	-1	-0.11	0.25
0	-3	1	-0.05	0.28
0	-3	-1	0.23	0.22
0	3	1	0.21	0.17
0	3	1	0.23	0.20
0	3	-1	0.34	0.23
0	-3	1	0.10	0.19
0	3	1	0.25	0.22
0	-3	-1	-0.10	0.13
0	3	1	-0.12	0.15
0	3	-1	0.20	0.24
0	3	-1	0.10	0.14
0	-3	-1	-0.06	0.15
0	3	-1	0.34	0.30
0	3	1	0.02	0.25
0	-3	-1	0.16	0.19

0	3	1	-0.15	0.21
0	-3	1	0.28	0.23
0	-4	1	0.29	0.30
0	-4	1	0.11	0.28
0	4	-1	0.33	0.28
0	4	1	0.22	0.25
0	-4	1	-0.17	0.18
0	4	1	0.02	0.25
0	-4	-1	-0.12	0.25
0	-4	-1	0.02	0.22
0	4	-1	0.25	0.24
0	4	1	0.07	0.30
0	4	1	-0.04	0.27
0	-4	-1	0.29	0.30
0	-4	-1	0.28	0.28
0	4	-1	0.13	0.34
0	4	-1	0.16	0.17
0	-4	1	0.11	0.17
0	4	1	0.00	0.20
0	-4	1	0.07	0.22
0	-4	1	0.14	0.37
0	-4	1	0.23	0.28
0	4	1	0.28	0.30
0	-4	-1	0.10	0.22
0	-4	-1	0.19	0.33
0	5	1	-0.26	0.26
0	-5	-1	0.39	0.35
0	5	-1	0.57	0.49
0	-5	1	-0.14	0.31
0	5	-1	-0.01	0.22
0	5	-1	-0.21	0.27
0	5	-1	0.36	0.34
0	-5	-1	-0.02	0.20
0	-5	1	-0.10	0.42
0	-5	1	0.08	0.23
0	-5	-1	0.31	0.29
0	-5	-1	0.06	0.32
0	-5	1	-0.35	0.35
0	5	1	0.19	0.42
0	-5	-1	0.06	0.23
0	5	1	0.35	0.34
0	5	1	-0.09	0.28
0	5	1	0.33	0.31
0	5	1	-0.13	0.27

0	5	-1	0.18	0.29
0	-5	1	0.13	0.33
0	-6	1	0.08	0.47
0	-6	-1	0.16	0.29
0	-6	-1	0.59	0.38
0	-6	1	0.16	0.39
0	6	1	-0.05	0.45
0	-6	-1	1.24	0.49
0	-6	1	0.21	0.37
0	-6	-1	0.12	0.38
0	6	-1	-0.18	0.44
0	6	-1	-0.04	0.29
0	-6	1	1.46	0.49
0	6	-1	0.22	0.46
0	-6	-1	0.73	0.58
0	6	-1	-0.06	0.34
0	6	-1	0.58	0.50
0	6	1	0.16	0.31
0	-6	1	0.02	0.22
0	6	1	0.20	0.36
0	-6	-1	0.15	0.42
0	6	1	0.67	0.54
0	-6	1	-0.22	0.28
0	6	1	0.43	0.44
0	-6	1	0.39	0.54
0	-6	-1	0.00	0.28
0	7	1	1.24	0.62
0	-7	1	0.13	0.28
0	7	-1	0.30	0.34
0	-7	-1	0.60	0.40
0	7	1	0.31	0.38
0	-7	1	0.19	0.60
0	-7	1	-0.24	0.48
0	-7	1	-0.42	0.53
0	7	-1	-0.29	0.40
0	7	-1	0.07	0.48
0	7	-1	0.28	0.59
0	-7	1	0.87	0.57
0	-7	1	-0.09	0.41
0	-7	-1	0.12	0.31
0	7	1	-0.43	0.38
0	7	-1	-0.46	0.51
0	7	1	0.34	0.57
0	7	1	-0.25	0.43

0	-7	-1	0.04	0.47
0	-7	-1	0.50	0.47
0	-7	1	-0.37	0.35
0	-7	-1	1.09	0.67
0	-7	-1	0.84	0.57
0	-7	-1	0.17	0.43
0	8	-1	0.14	0.74
0	8	1	-0.13	0.58
0	8	-1	0.50	0.58
0	8	-1	-1.30	0.60
0	8	-1	0.06	0.53
0	8	1	0.40	0.52
0	-8	1	-0.20	0.58
0	8	1	0.52	0.53
0	-8	-1	0.21	0.68
0	-8	1	0.77	0.76
0	-8	-1	0.90	0.63
0	-8	1	-0.55	0.39
0	-8	-1	0.34	0.34
0	-8	1	-0.19	0.31
0	-8	-1	-0.02	0.42
0	-8	1	-0.07	0.49
0	8	-1	0.34	0.40
0	-8	-1	0.55	0.53
0	8	1	-0.57	0.42
0	-8	-1	0.02	0.56
0	-8	-1	-0.51	0.48
0	-8	1	0.58	0.61
0	-8	1	0.53	0.66
0	9	-1	-0.24	0.62
0	9	-1	0.09	0.48
0	-9	1	1.89	0.80
0	-9	1	-0.18	0.34
0	-9	-1	-0.10	0.34
0	9	-1	0.02	0.76
0	-9	-1	0.49	0.54
0	-9	-1	0.83	0.49
0	-9	1	0.11	0.47
0	-9	-1	0.83	0.94
0	9	1	0.14	0.50
0	9	1	-0.13	0.60
0	9	1	-0.53	0.84
0	9	-1	-0.72	0.82
0	-9	-1	0.51	0.66

0	-9	-1	0.58	0.79
0	-9	1	-0.18	0.71
0	-9	1	-0.68	0.90
0	9	1	-0.71	0.88
0	-9	-1	-0.33	0.56
0	-9	1	-0.72	0.58
0	-9	1	0.22	0.51
0	10	-1	0.85	1.07
0	10	1	0.43	1.10
0	-10	-1	-0.57	0.88
0	-10	-1	0.03	0.61
0	10	1	0.16	0.72
0	10	1	0.30	0.58
0	10	1	1.12	1.10
0	-10	-1	-0.22	0.77
0	-10	1	0.66	0.85
0	-10	1	0.55	0.93
0	-10	1	-0.64	0.69
0	-10	-1	0.36	0.66
0	-10	-1	-0.34	0.65
0	10	-1	0.46	0.73
0	10	-1	-1.49	0.90
0	-10	1	0.39	0.55
0	10	-1	0.45	0.56
0	-10	1	0.33	0.60
0	-10	-1	-0.28	0.44
0	-10	1	0.30	0.40
0	11	-1	-0.18	0.61
0	-11	1	0.22	0.60
0	11	1	-0.26	0.67
0	-11	-1	0.27	0.78
0	-11	-1	0.56	0.88
0	-11	-1	0.76	0.53
0	11	1	1.32	1.27
0	-11	1	0.04	0.45
0	-11	1	-0.31	0.97
0	-11	-1	0.21	0.85
0	11	1	-0.39	1.20
0	11	-1	-1.85	1.15
0	-11	1	-0.45	0.75
0	11	-1	0.04	1.30
0	11	-1	0.02	0.84
0	11	1	-0.05	0.80
0	-11	-1	-0.10	0.69

0	-11	1	0.42	0.81
0	-12	1	0.03	0.79
0	-12	1	-0.44	0.49
0	-12	1	0.04	0.92
0	-12	-1	-0.71	0.55
0	-12	-1	-0.33	0.85
0	12	1	0.81	1.01
0	12	-1	1.20	1.08
0	12	-1	0.21	1.42
0	12	1	-0.04	1.34
0	-12	-1	-0.72	1.73
0	13	-1	1.66	1.89
0	-13	1	2.31	1.33
0	-13	-1	0.27	1.19
0	-13	-1	1.41	0.92
0	-13	1	0.16	0.88
0	13	-1	1.27	1.61
0	13	1	0.43	1.77
-1	1	-1	86.81	4.13
-1	1	-1	91.25	4.11
-1	1	-1	100.83	4.13
1	-1	-1	92.94	4.14
-1	-1	1	81.68	3.75
1	1	1	77.16	3.72
-1	-1	1	92.26	3.81
-1	1	-1	92.48	4.21
1	1	1	82.88	3.73
-1	1	-1	89.33	4.09
-1	-1	1	78.19	3.72
-1	-1	-1	87.50	4.13
-1	-1	-1	89.81	4.14
1	1	-1	89.46	4.11
-1	1	1	81.20	3.70
-1	1	1	85.50	3.76
1	1	-1	91.37	4.17
-1	-1	-1	90.84	4.15
1	-1	1	88.93	3.81
-1	1	1	77.64	3.72
-1	1	1	82.10	3.80
-1	1	1	80.42	3.73
1	-2	-1	197.79	8.76
-1	-2	1	189.95	8.93
1	2	1	208.43	8.93
-1	2	-1	192.89	8.73

1	2	1	205.94	8.97
1	2	1	192.45	8.98
1	2	1	197.28	8.96
-1	2	-1	198.21	8.68
1	-2	-1	185.85	8.67
1	2	1	191.35	8.90
1	-2	-1	202.14	8.69
-1	2	-1	193.14	8.65
-1	-2	1	199.77	8.87
1	-2	-1	184.54	8.71
-1	-2	1	210.66	8.96
-1	2	-1	209.74	8.68
-1	-2	1	201.23	8.97
1	-2	-1	177.88	8.67
-1	2	-1	201.17	8.78
-1	2	-1	189.83	8.71
-1	-2	1	203.21	8.97
-1	-2	1	195.63	8.98
-1	-2	1	189.13	8.93
1	-2	-1	197.89	8.66
-1	-2	-1	186.55	8.66
-1	-2	-1	201.04	8.72
-1	2	1	198.92	8.97
-1	2	1	191.00	9.00
1	-2	1	212.13	9.03
1	2	-1	202.32	8.71
1	-2	1	212.38	8.93
1	-2	1	197.35	8.93
1	-2	1	184.72	8.89
-1	-2	-1	203.91	8.74
-1	-2	-1	175.28	8.63
-1	2	1	183.20	8.92
-1	2	1	197.93	8.92
1	2	-1	193.34	8.62
-1	2	1	200.08	8.92
-1	2	1	209.67	8.95
1	2	-1	192.40	8.68
-1	-2	-1	193.56	8.68
1	-2	1	201.75	8.97
1	2	-1	185.48	8.74
1	-2	1	192.74	8.96
-1	-2	-1	181.80	8.70
1	-3	-1	18.02	1.05
1	3	1	45.02	2.10

1	-3	-1	21.80	1.07
1	-3	-1	19.44	1.04
-1	-3	1	45.74	2.16
1	3	1	40.64	2.07
-1	3	-1	21.08	1.11
1	3	1	45.99	2.16
-1	-3	1	42.47	2.18
-1	-3	1	41.65	2.11
1	3	1	43.06	2.05
-1	-3	1	43.91	2.14
1	-3	-1	19.84	1.17
-1	-3	1	46.15	2.13
1	-3	-1	19.04	1.12
1	-3	-1	19.78	1.08
-1	3	-1	21.10	1.07
-1	-3	1	40.88	2.02
-1	3	-1	21.08	1.02
1	3	1	44.20	2.21
1	3	1	44.74	2.15
-1	3	-1	20.42	1.16
-1	-3	1	40.44	2.08
-1	3	-1	21.20	1.23
1	-3	1	44.70	2.21
-1	3	1	43.24	2.22
1	-3	1	44.89	2.10
1	-3	1	42.54	2.14
-1	3	1	44.08	2.12
-1	3	1	43.38	2.07
-1	-3	-1	20.18	1.03
-1	3	1	43.73	2.09
-1	-3	-1	22.03	1.14
1	-3	1	41.85	2.04
-1	-3	-1	20.33	1.06
-1	3	1	42.35	2.09
1	3	-1	20.67	0.99
-1	3	1	41.51	2.14
1	3	-1	20.96	1.11
1	-3	1	45.10	2.11
-1	-3	-1	19.46	1.10
1	-3	1	47.85	2.11
-1	-3	-1	20.88	1.16
-1	-3	-1	18.66	1.02
1	3	-1	20.11	1.10
1	-3	1	44.09	2.15

1	3	-1	20.15	1.07
1	3	-1	20.25	1.19
1	-4	-1	193.73	8.54
1	-4	-1	183.53	8.38
1	-4	-1	194.57	8.39
1	4	1	208.45	9.33
-1	4	-1	187.01	8.45
1	4	1	209.88	9.39
-1	-4	1	214.40	9.40
1	-4	-1	163.68	8.32
-1	-4	1	206.68	9.44
-1	-4	1	205.84	9.34
-1	-4	1	209.91	9.38
-1	4	-1	183.82	8.59
-1	4	-1	181.17	8.31
1	-4	-1	186.62	8.38
-1	4	-1	180.55	8.44
-1	-4	1	194.34	9.23
-1	-4	1	210.12	9.35
-1	4	-1	188.64	8.39
1	4	1	203.94	9.31
1	-4	-1	171.47	8.43
1	4	1	209.67	9.27
1	4	1	200.78	9.39
1	4	1	204.39	9.47
-1	-4	1	192.22	9.35
1	-4	1	206.70	9.34
-1	4	1	207.83	9.41
1	-4	1	221.39	9.35
1	-4	1	219.91	9.50
1	4	-1	184.78	8.29
-1	4	1	180.15	9.38
1	-4	1	194.44	9.25
1	-4	1	197.20	9.37
-1	-4	-1	198.46	8.45
1	-4	1	218.44	9.38
-1	-4	-1	172.81	8.42
-1	4	1	203.35	9.49
1	4	-1	181.94	8.43
1	4	-1	180.63	8.52
-1	-4	-1	194.47	8.50
-1	-4	-1	187.31	8.39
-1	4	1	203.56	9.33
-1	-4	-1	180.40	8.33

1	4	-1	186.14	8.39
-1	-4	-1	178.90	8.33
1	4	-1	189.89	8.42
-1	4	1	206.95	9.29
1	-5	-1	210.50	9.29
-1	5	-1	203.87	9.38
1	-5	-1	197.36	9.29
1	5	1	192.45	8.76
-1	-5	1	177.78	8.86
-1	-5	1	204.99	8.86
1	5	1	168.23	8.88
1	-5	-1	192.14	9.36
-1	-5	1	193.95	8.89
-1	-5	1	194.67	8.98
-1	5	-1	203.27	9.51
1	5	1	195.90	8.93
-1	-5	1	201.61	8.92
-1	5	-1	212.33	9.31
1	5	1	198.73	8.84
1	-5	-1	215.51	9.48
1	-5	-1	223.33	9.32
-1	5	-1	204.74	9.21
-1	5	-1	204.71	9.38
-1	-5	1	188.91	8.84
-1	-5	1	181.66	8.71
1	-5	-1	205.62	9.29
1	5	1	190.25	8.90
1	-5	-1	189.53	9.22
-1	5	1	196.33	8.83
1	5	-1	200.90	9.18
1	-5	1	203.68	8.85
-1	5	1	194.44	8.93
1	-5	1	211.42	8.88
-1	-5	-1	203.53	9.24
1	5	-1	205.55	9.33
-1	-5	-1	216.33	9.37
1	5	-1	199.33	9.36
-1	-5	-1	195.68	9.22
1	-5	1	182.80	8.89
1	-5	1	183.21	8.73
1	5	-1	204.21	9.30
-1	-5	-1	183.69	9.33
-1	5	1	193.21	8.87
-1	-5	-1	199.14	9.30

-1	5	1	193.31	9.05
1	-5	1	195.04	8.90
1	-5	1	207.95	9.04
-1	-5	-1	210.92	9.45
-1	5	1	199.00	8.78
1	5	-1	199.75	9.49
1	-5	1	187.48	8.84
-1	6	-1	191.64	8.76
-1	-6	1	200.03	9.21
-1	-6	1	196.24	9.16
1	-6	-1	193.99	8.76
1	6	1	203.94	9.15
-1	6	-1	185.78	9.02
-1	-6	1	210.36	9.24
1	-6	-1	196.91	8.76
1	6	1	198.23	9.13
1	-6	-1	190.79	8.76
-1	6	-1	192.22	8.65
-1	6	-1	192.39	8.87
-1	-6	1	187.97	9.00
1	-6	-1	201.93	8.78
1	-6	-1	172.52	8.66
1	6	1	195.06	9.38
-1	-6	1	183.63	9.18
1	-6	-1	170.41	9.22
-1	6	-1	187.87	8.84
1	-6	-1	200.47	8.98
-1	-6	1	212.84	9.35
1	6	1	200.94	9.27
1	-6	1	210.13	9.21
1	-6	1	183.69	9.02
-1	-6	-1	191.69	8.70
1	-6	1	189.26	9.21
-1	6	1	194.24	9.19
-1	6	1	204.51	9.29
1	6	-1	188.21	8.62
-1	6	1	188.28	9.38
1	6	-1	185.29	8.79
1	-6	1	198.92	9.16
1	-6	1	203.56	9.16
1	6	-1	195.98	9.02
-1	-6	-1	190.06	8.77
-1	6	1	196.66	9.08
1	6	-1	192.56	8.86

1	6	-1	191.95	8.76
-1	-6	-1	182.68	8.66
-1	-6	-1	171.24	8.80
1	-6	1	212.16	9.20
-1	6	1	202.12	9.15
-1	-6	-1	200.67	8.94
-1	-6	-1	209.13	8.85
1	-6	1	207.12	9.38
1	7	1	59.11	2.81
1	-7	-1	58.86	3.01
1	7	1	56.05	3.08
-1	-7	1	52.74	2.91
-1	7	-1	56.29	3.10
-1	7	-1	61.72	3.18
-1	-7	1	58.88	2.97
1	-7	-1	60.87	3.01
-1	-7	1	58.82	3.00
1	-7	-1	54.64	2.88
-1	-7	1	54.52	2.97
1	7	1	57.52	3.21
-1	-7	1	61.96	3.05
1	7	1	57.93	2.92
1	7	1	54.44	2.93
-1	7	-1	59.35	2.90
-1	-7	1	63.79	3.20
-1	7	-1	63.15	3.37
1	-7	-1	62.62	3.03
-1	7	-1	60.52	3.02
1	-7	-1	63.76	3.30
1	-7	-1	65.01	3.07
1	-7	-1	55.06	3.06
-1	-7	1	54.28	2.75
1	7	-1	54.09	2.92
-1	7	1	57.34	2.98
-1	-7	-1	61.57	2.98
1	7	-1	56.80	3.01
1	7	-1	56.06	3.04
-1	-7	-1	58.64	3.01
1	-7	1	59.57	3.01
1	7	-1	57.42	3.11
1	-7	1	55.88	2.92
-1	-7	-1	65.71	3.14
1	-7	1	52.31	2.76
1	-7	1	61.63	2.96

-1	-7	-1	53.45	3.05
-1	-7	-1	54.84	2.87
-1	7	1	57.53	2.84
1	-7	1	49.31	3.09
-1	7	1	52.08	3.24
1	-7	1	63.94	3.02
1	-7	1	65.00	3.26
-1	7	1	54.39	3.20
1	7	-1	57.59	3.27
-1	-7	-1	61.26	3.07
-1	-7	-1	62.80	3.25
-1	7	1	56.66	2.92
1	-8	-1	21.88	1.80
-1	-8	1	17.77	1.13
1	-8	-1	19.76	1.24
1	8	1	18.97	1.63
-1	8	-1	21.91	1.77
-1	8	-1	17.37	1.51
-1	-8	1	18.81	1.68
-1	-8	1	19.10	1.43
-1	8	-1	19.77	1.27
1	8	1	19.38	1.59
1	8	1	18.74	1.38
1	8	1	19.38	1.25
1	-8	-1	20.29	1.39
1	-8	-1	19.09	1.36
-1	-8	1	20.48	1.59
1	-8	-1	19.17	1.47
1	-8	-1	22.94	1.55
-1	-8	1	20.65	1.50
-1	8	-1	17.44	1.75
-1	-8	1	16.37	1.40
1	8	1	17.56	1.36
1	-8	-1	22.15	1.52
-1	-8	1	19.03	1.34
-1	8	-1	21.27	1.44
1	-8	1	19.48	1.50
1	-8	1	17.98	1.13
1	-8	1	19.27	1.38
-1	8	1	19.15	1.57
1	8	-1	19.68	1.76
1	8	-1	19.85	1.54
1	-8	1	17.60	1.32
-1	8	1	17.02	1.68

-1	-8	-1	19.98	1.38
-1	8	1	18.59	1.40
1	-8	1	19.43	1.76
-1	8	1	19.14	1.44
1	8	-1	21.34	1.48
1	8	-1	20.75	1.42
1	-8	1	19.57	1.32
1	8	-1	20.72	1.25
-1	8	1	20.81	1.28
-1	-8	-1	19.21	1.55
-1	-8	-1	20.58	1.43
-1	-8	-1	19.62	1.24
-1	-8	-1	21.55	1.73
1	-8	1	21.34	1.53
-1	-8	-1	21.85	1.47
-1	-8	-1	20.31	1.59
-1	-9	1	15.85	1.51
1	9	1	18.35	1.71
-1	-9	1	15.75	1.07
-1	9	-1	11.55	1.72
-1	-9	1	16.12	1.43
1	9	1	17.17	1.36
-1	9	-1	14.57	1.62
1	-9	-1	14.13	1.04
-1	-9	1	19.32	1.54
1	-9	-1	15.90	1.43
-1	-9	1	16.45	1.34
1	-9	-1	14.23	1.26
-1	-9	1	15.34	1.76
-1	9	-1	14.05	1.13
1	9	1	14.14	1.73
-1	9	-1	13.26	1.41
1	-9	-1	14.80	1.20
1	-9	-1	16.19	1.48
1	-9	-1	13.12	1.34
1	-9	-1	15.82	1.79
1	9	1	17.32	1.23
-1	-9	1	18.50	1.68
1	-9	1	14.35	1.38
1	-9	1	20.89	1.63
1	-9	1	16.12	1.58
1	9	-1	12.06	1.70
1	-9	1	16.25	1.08
1	-9	1	17.83	1.40

-1	-9	-1	14.64	1.52
-1	9	1	16.82	1.29
-1	9	1	16.01	1.80
1	9	-1	13.45	1.32
-1	9	1	15.68	1.46
-1	-9	-1	16.88	1.38
-1	-9	-1	12.76	1.01
1	9	-1	16.22	1.08
-1	-9	-1	12.54	1.51
-1	-9	-1	14.77	1.71
-1	9	1	17.05	1.75
-1	-9	-1	14.33	1.24
1	-9	1	16.23	1.88
1	-9	1	16.41	1.31
1	9	-1	11.95	1.64
-1	-9	-1	14.90	1.35
-1	-10	1	61.23	3.31
1	-10	-1	63.86	3.51
1	10	1	57.97	3.17
-1	-10	1	62.73	3.28
-1	10	-1	76.96	4.11
1	-10	-1	65.03	3.82
-1	10	-1	71.71	3.87
-1	-10	1	54.14	2.89
1	-10	-1	75.34	3.77
-1	-10	1	58.91	3.39
1	-10	-1	76.64	3.88
1	10	1	60.58	3.02
-1	-10	1	61.30	3.22
1	10	1	62.47	3.60
-1	10	-1	73.24	3.56
1	-10	-1	73.09	3.78
-1	10	-1	70.30	4.27
1	-10	-1	68.59	3.74
1	10	1	55.09	3.67
1	10	-1	78.75	4.12
-1	10	1	55.62	3.28
-1	10	1	67.67	3.64
-1	-10	-1	65.38	3.49
1	-10	1	52.74	3.26
1	-10	1	53.99	2.90
1	10	-1	69.03	3.77
1	10	-1	72.02	3.50
-1	10	1	56.67	3.04

1	-10	1	58.06	3.33
-1	-10	-1	74.25	3.94
-1	-10	-1	67.63	3.78
-1	-10	-1	73.92	3.70
1	-10	1	65.60	3.24
1	-10	1	61.93	3.37
-1	-10	-1	77.05	3.85
-1	10	1	52.74	3.60
1	-10	1	58.66	3.19
1	10	-1	68.44	4.26
-1	-10	-1	72.19	3.76
-1	11	-1	99.87	6.06
1	-11	-1	97.56	5.25
-1	-11	1	113.16	5.42
1	-11	-1	119.44	5.62
1	-11	-1	106.15	5.49
-1	11	-1	111.95	5.64
1	-11	-1	116.37	5.57
1	-11	-1	121.06	5.56
1	-11	-1	100.54	5.57
-1	-11	1	100.53	5.06
-1	11	-1	121.33	5.94
1	11	1	103.58	5.95
-1	-11	1	106.73	5.41
1	11	1	108.15	5.16
-1	11	-1	110.90	5.30
1	11	1	110.69	5.38
-1	-11	1	98.81	5.39
-1	-11	1	115.42	5.52
-1	-11	-1	113.20	5.56
-1	-11	-1	115.66	5.78
1	-11	1	120.59	5.47
-1	-11	-1	104.88	5.26
1	-11	1	109.87	5.48
-1	11	1	107.84	5.46
1	11	-1	113.51	5.25
-1	-11	-1	104.86	5.56
-1	-11	-1	117.09	5.63
1	11	-1	104.00	6.10
-1	11	1	101.15	5.98
-1	11	1	104.69	5.20
1	11	-1	111.67	5.55
1	-11	1	100.89	5.45
-1	11	1	114.63	5.80

1	-11	1	99.56	5.09
1	-11	1	111.22	5.43
1	12	1	102.30	5.96
-1	12	-1	105.13	5.46
1	12	1	98.32	5.19
1	-12	-1	107.02	5.39
-1	-12	1	111.47	5.40
1	-12	-1	111.56	5.38
-1	-12	1	108.95	5.33
1	-12	-1	97.09	5.02
-1	-12	1	97.21	4.88
1	12	-1	100.25	6.01
-1	-12	-1	111.00	5.44
1	-12	1	91.12	4.88
-1	12	1	95.43	6.35
1	-12	1	103.67	5.30
1	-12	1	113.42	5.37
-1	-12	-1	94.36	4.99
-1	12	1	105.91	5.37
-1	-12	-1	107.39	5.39
1	12	-1	105.40	5.36
-1	13	-1	34.38	3.09
-1	-13	1	28.95	2.26
1	-13	-1	38.41	2.74
1	-13	-1	35.12	2.59
1	13	1	29.48	3.41
-1	13	-1	34.70	3.83
-1	-13	1	32.28	2.64
-1	-13	-1	47.35	2.98
-1	-13	-1	37.60	2.60
1	-13	1	30.11	2.26
-1	13	1	23.76	3.48
1	-13	1	34.00	2.54
1	13	-1	38.81	3.70
2	0	-1	0.03	0.07
2	-1	-1	54.97	2.47
-2	-1	1	43.97	2.00
2	-1	-1	54.40	2.45
-2	1	-1	55.46	2.45
-2	1	-1	59.97	2.42
-2	1	-1	52.88	2.43
-2	-1	1	46.95	2.05
2	1	1	41.96	2.03
2	1	1	45.10	2.03

-2	-1	1	45.28	2.00
-2	-1	1	43.31	2.03
2	1	1	37.81	2.06
-2	-1	1	44.57	2.08
-2	-1	1	44.50	2.02
-2	1	-1	53.22	2.50
2	1	1	47.73	2.04
-2	1	-1	47.43	2.47
2	-1	1	43.63	2.03
-2	-1	-1	53.33	2.43
2	-1	1	44.05	2.08
2	1	-1	44.57	2.46
-2	-1	-1	53.26	2.43
2	-1	1	41.33	2.04
-2	-1	-1	52.87	2.43
2	1	-1	54.08	2.43
-2	1	1	42.85	2.07
-2	1	1	47.61	2.05
-2	1	1	46.27	2.05
-2	1	1	43.75	2.02
-2	-1	-1	53.00	2.39
-2	-1	-1	54.25	2.48
2	1	-1	53.30	2.40
2	-2	-1	99.81	4.68
2	-2	-1	104.63	4.73
-2	2	-1	106.54	4.65
-2	2	-1	107.12	4.62
-2	-2	1	95.95	4.37
-2	-2	1	99.85	4.36
2	2	1	92.79	4.38
-2	2	-1	96.70	4.73
2	2	1	99.31	4.34
-2	-2	1	90.94	4.32
-2	2	-1	105.38	4.68
2	-2	-1	97.23	4.63
2	2	1	92.86	4.31
2	-2	-1	104.01	4.59
-2	-2	1	91.75	4.34
-2	2	-1	102.22	4.71
-2	-2	1	94.00	4.40
-2	2	-1	102.54	4.62
2	-2	-1	103.01	4.67
-2	-2	1	98.21	4.28
2	2	1	92.81	4.40

2	-2	-1	101.44	4.64
-2	-2	-1	105.60	4.65
2	-2	1	92.64	4.31
2	-2	1	94.93	4.39
2	-2	1	98.75	4.45
2	2	-1	106.16	4.58
2	-2	1	92.94	4.37
-2	-2	-1	103.22	4.70
2	2	-1	98.76	4.71
-2	2	1	96.91	4.38
2	-2	1	103.38	4.32
-2	-2	-1	93.26	4.57
2	2	-1	102.91	4.64
2	-2	1	95.62	4.34
2	2	-1	104.17	4.65
-2	-2	-1	101.55	4.67
-2	-2	-1	99.88	4.65
-2	2	1	93.23	4.29
-2	-2	-1	98.90	4.62
-2	2	1	91.79	4.33
-2	2	1	96.64	4.43
2	2	-1	99.81	4.69
-2	2	1	91.01	4.33
-2	2	1	96.66	4.38
-2	3	-1	37.06	1.76
-2	3	-1	36.45	1.92
2	3	1	36.83	1.74
2	-3	-1	36.15	1.72
2	3	1	35.31	1.74
2	3	1	33.52	1.95
-2	-3	1	36.71	1.79
2	3	1	37.67	1.84
-2	3	-1	36.76	1.72
2	-3	-1	34.66	1.74
2	-3	-1	33.81	1.73
2	3	1	39.82	1.80
2	3	1	30.76	1.92
-2	-3	1	39.53	1.82
2	-3	-1	36.35	1.79
-2	3	-1	36.65	1.73
-2	-3	1	37.63	1.71
-2	-3	1	35.99	1.85
-2	-3	1	35.71	1.86
2	-3	-1	33.58	1.80

-2	3	-1	36.75	1.82
-2	-3	1	35.76	1.79
2	-3	-1	36.23	1.88
-2	-3	-1	31.75	1.69
-2	3	1	35.14	1.78
2	-3	1	36.78	1.93
-2	3	1	35.42	1.93
-2	-3	-1	36.57	1.84
-2	-3	-1	38.58	1.77
2	-3	1	39.70	1.77
2	3	-1	36.34	1.76
-2	3	1	36.65	1.78
2	-3	1	36.85	1.86
2	-3	1	36.58	1.83
2	3	-1	37.05	1.78
-2	3	1	36.05	1.85
2	-3	1	36.39	1.74
-2	-3	-1	33.93	1.78
-2	3	1	38.12	1.85
2	3	-1	32.73	1.83
2	3	-1	37.43	1.67
-2	-3	-1	38.35	1.83
-2	-3	-1	36.92	1.72
2	-3	1	37.05	1.79
2	4	1	35.81	1.84
-2	-4	1	37.42	1.96
2	-4	-1	32.35	1.81
2	-4	-1	35.17	1.71
-2	-4	1	35.02	1.89
2	4	1	37.29	1.97
2	-4	-1	34.81	1.92
2	-4	-1	31.08	1.72
2	4	1	34.92	1.98
2	-4	-1	33.46	1.70
2	4	1	36.86	1.81
-2	4	-1	32.68	1.81
2	4	1	39.03	1.89
-2	-4	1	35.91	1.88
-2	4	-1	32.78	1.93
-2	4	-1	34.07	1.80
-2	4	-1	36.12	1.69
-2	-4	1	36.93	1.97
2	-4	-1	35.96	1.72
2	4	1	37.65	1.96

-2	-4	1	37.91	1.90
-2	4	-1	35.57	1.75
-2	-4	1	37.78	1.78
2	-4	-1	34.86	1.79
-2	4	1	38.77	2.09
-2	-4	-1	36.78	1.86
-2	-4	-1	36.20	1.70
2	-4	1	39.79	1.89
2	4	-1	35.11	1.64
2	4	-1	34.85	1.77
2	-4	1	38.81	2.05
-2	4	1	37.57	1.97
-2	-4	-1	32.08	1.67
-2	-4	-1	33.28	1.74
2	-4	1	40.23	1.88
2	-4	1	38.86	1.96
-2	-4	-1	31.06	1.76
2	-4	1	35.53	1.93
-2	4	1	36.77	1.89
-2	4	1	38.46	1.87
-2	4	1	37.54	1.94
2	4	-1	33.68	1.78
2	4	-1	34.87	1.75
2	4	-1	34.04	1.88
2	-4	1	38.01	1.88
-2	-4	-1	37.36	1.85
2	5	1	33.63	1.87
2	5	1	35.01	1.77
-2	5	-1	31.48	1.86
2	5	1	33.99	1.87
-2	-5	1	33.95	1.66
-2	-5	1	32.46	1.84
-2	-5	1	31.38	1.79
-2	-5	1	34.43	1.89
2	-5	-1	32.24	1.73
-2	5	-1	35.39	1.69
2	5	1	34.02	1.95
-2	-5	1	33.48	1.91
2	-5	-1	29.99	1.67
-2	5	-1	34.82	1.77
2	-5	-1	32.27	1.71
2	-5	-1	30.82	1.93
-2	5	-1	35.86	1.87
2	5	1	34.21	1.69

-2	5	-1	30.62	1.96
2	-5	-1	34.26	1.74
-2	-5	1	31.95	1.76
2	-5	-1	35.53	1.98
2	5	1	33.02	1.76
-2	-5	1	34.99	1.80
2	-5	1	36.01	1.76
2	-5	1	35.76	1.89
-2	-5	-1	33.64	1.76
-2	5	1	28.68	2.01
2	-5	1	36.61	2.02
-2	5	1	33.38	1.85
2	5	-1	32.24	1.92
2	-5	1	32.32	1.67
-2	-5	-1	31.63	1.68
-2	-5	-1	34.86	1.86
2	5	-1	33.70	1.79
2	5	-1	33.90	1.62
-2	-5	-1	35.08	1.89
2	-5	1	35.14	1.79
-2	-5	-1	29.84	1.77
2	5	-1	34.17	1.83
-2	-5	-1	32.56	1.66
2	-5	1	29.85	1.89
2	5	-1	32.56	1.75
-2	5	1	35.54	1.91
-2	5	1	31.70	1.96
-2	5	1	34.35	1.77
2	-5	1	32.06	1.75
2	-6	-1	30.03	1.86
2	6	1	31.20	1.82
-2	-6	1	31.10	1.69
-2	-6	1	28.99	1.71
2	-6	-1	27.21	1.57
2	-6	-1	25.59	1.56
2	-6	-1	31.31	1.62
2	6	1	30.60	1.68
-2	-6	1	32.29	1.88
-2	-6	1	31.31	1.78
2	-6	-1	29.18	1.68
-2	-6	1	29.57	1.52
2	6	1	27.70	1.88
-2	6	-1	27.86	1.72
-2	-6	1	31.63	1.83

2	-6	-1	27.20	1.50
-2	6	-1	28.12	1.61
-2	6	-1	27.85	1.53
-2	6	-1	28.27	1.90
-2	6	-1	28.70	1.71
2	6	1	29.06	1.65
2	6	1	29.27	1.78
2	6	1	29.70	1.64
-2	-6	1	31.69	1.73
2	-6	-1	28.74	1.68
-2	-6	-1	27.55	1.60
-2	6	1	31.59	1.85
-2	6	1	28.07	1.94
2	6	-1	28.58	1.44
2	-6	1	29.47	1.66
2	-6	1	28.56	1.56
2	6	-1	28.68	1.70
2	6	-1	27.26	1.81
-2	6	1	28.08	1.66
-2	-6	-1	27.37	1.50
2	-6	1	32.62	2.04
-2	6	1	29.52	1.77
2	-6	1	31.72	1.69
2	6	-1	28.59	1.60
2	-6	1	30.80	1.80
2	6	-1	28.16	1.62
-2	6	1	32.26	1.65
-2	-6	-1	29.80	1.74
-2	-6	-1	26.46	1.53
2	-6	1	30.34	1.74
-2	-6	-1	29.75	1.76
-2	-6	-1	25.41	1.64
2	-6	1	26.48	1.81
2	-7	-1	92.80	4.52
2	-7	-1	83.10	4.29
-2	-7	1	70.17	3.93
2	7	1	82.99	4.07
-2	7	-1	85.41	4.33
-2	-7	1	84.61	4.06
-2	7	-1	89.99	4.11
2	-7	-1	90.63	4.25
2	7	1	83.52	3.79
2	7	1	77.70	3.87
2	-7	-1	83.55	4.19

2	7	1	74.98	4.17
-2	-7	1	83.75	4.13
-2	-7	1	76.29	3.99
2	-7	-1	79.76	4.09
-2	7	-1	82.22	4.51
2	-7	-1	84.48	4.16
-2	7	-1	85.55	4.21
2	-7	-1	91.36	4.22
-2	7	-1	89.32	4.38
2	7	1	80.67	3.91
-2	-7	1	75.65	3.73
-2	-7	1	83.36	3.95
-2	-7	1	80.16	3.92
2	7	-1	84.55	4.23
-2	-7	-1	78.36	4.25
-2	7	1	77.06	4.22
2	-7	1	84.46	3.96
-2	7	1	77.81	3.90
2	-7	1	73.96	3.76
-2	-7	-1	94.68	4.42
2	7	-1	89.40	4.34
-2	-7	-1	86.27	4.29
2	-7	1	80.56	3.89
-2	-7	-1	91.97	4.34
2	-7	1	78.31	3.91
-2	7	1	85.46	3.86
2	7	-1	88.74	4.03
2	7	-1	85.51	4.21
-2	-7	-1	89.01	4.13
2	-7	1	72.93	3.96
-2	7	1	79.57	4.00
2	-7	1	84.18	4.01
2	7	-1	78.06	4.45
2	-7	1	81.97	4.18
-2	-7	-1	83.20	4.21
-2	7	1	82.98	4.14
-2	-7	-1	84.02	4.08
2	8	1	7.71	1.28
-2	-8	1	6.55	0.88
-2	-8	1	8.43	1.05
2	-8	-1	6.92	1.15
2	-8	-1	6.05	1.01
2	-8	-1	6.48	1.00
2	8	1	7.61	0.82

-2	-8	1	7.14	1.04
2	8	1	7.60	0.97
-2	8	-1	4.90	1.11
-2	-8	1	8.71	1.09
-2	-8	1	7.59	0.72
-2	8	-1	5.98	1.18
2	-8	-1	4.93	0.84
2	-8	-1	7.55	1.05
-2	-8	1	6.98	1.22
-2	8	-1	5.19	0.78
-2	8	-1	4.97	1.01
2	8	1	7.83	1.21
2	8	1	7.67	0.95
-2	8	-1	5.37	0.82
2	-8	-1	5.73	0.69
2	-8	-1	6.66	0.84
-2	-8	1	7.61	1.28
-2	8	1	7.21	0.91
-2	8	1	6.30	0.97
2	8	-1	5.39	0.75
2	-8	1	7.46	1.41
-2	-8	-1	7.63	1.01
-2	-8	-1	4.60	0.66
-2	-8	-1	6.53	0.81
-2	-8	-1	6.05	1.14
2	8	-1	5.68	1.06
-2	8	1	7.83	1.31
2	8	-1	5.61	0.92
-2	8	1	7.94	1.08
2	8	-1	6.51	0.88
2	-8	1	9.14	0.94
2	-8	1	9.61	1.12
-2	-8	-1	5.03	0.96
2	8	-1	6.17	0.90
2	-8	1	7.70	1.20
2	-8	1	7.87	0.88
2	-8	1	7.10	0.70
-2	8	1	6.60	1.22
-2	-8	-1	5.94	0.89
2	-8	1	7.28	0.92
-2	-8	-1	5.06	0.93
2	9	1	3.95	0.61
2	-9	-1	2.21	0.58
-2	-9	1	3.00	0.82

-2	9	-1	4.76	1.07
-2	9	-1	2.56	0.99
-2	-9	1	2.61	1.00
-2	-9	1	3.22	0.78
2	-9	-1	2.80	0.78
2	-9	-1	2.62	1.03
-2	9	-1	1.12	0.72
-2	-9	1	3.73	0.98
2	-9	-1	2.75	0.53
-2	-9	1	3.68	0.72
2	9	1	2.51	1.01
-2	-9	1	3.84	0.79
-2	-9	1	2.84	0.47
-2	9	-1	4.12	0.69
2	-9	-1	2.97	0.68
2	-9	-1	4.74	0.89
2	-9	-1	3.35	0.84
2	9	1	3.96	0.99
2	9	1	2.58	0.68
-2	-9	-1	3.49	0.79
2	-9	1	2.94	0.67
-2	-9	-1	1.72	0.88
2	9	-1	2.01	0.83
2	9	-1	3.14	0.54
-2	-9	-1	3.13	0.96
2	-9	1	2.93	0.67
-2	-9	-1	3.02	0.54
-2	-9	-1	3.19	0.73
-2	9	1	3.30	0.82
2	-9	1	4.30	0.95
2	-9	1	4.66	0.95
-2	9	1	4.20	1.02
2	-9	1	4.37	1.21
2	9	-1	2.65	0.75
-2	-9	-1	2.86	0.62
-2	9	1	4.04	1.12
-2	-9	-1	2.22	0.79
2	-9	1	2.87	0.78
2	9	-1	3.87	1.09
2	-9	1	3.32	0.60
-2	9	1	2.68	0.71
2	-10	-1	2.07	0.74
2	-10	-1	1.44	0.90
2	10	1	2.43	0.72

-2	-10	1	2.09	0.83
-2	-10	1	2.02	0.50
-2	-10	1	1.67	0.64
2	10	1	2.13	1.26
-2	10	-1	-0.50	1.11
2	10	1	0.30	1.03
2	10	1	2.56	0.63
2	-10	-1	1.28	0.62
2	-10	-1	2.50	0.89
-2	-10	1	2.82	0.91
-2	10	-1	1.07	0.86
2	-10	-1	2.53	1.25
-2	10	-1	0.04	0.91
-2	-10	1	1.41	0.77
2	-10	-1	1.78	0.74
-2	10	-1	0.75	0.67
2	-10	-1	1.86	0.52
2	10	-1	1.87	0.72
-2	-10	-1	1.34	0.50
-2	10	1	0.84	1.12
-2	10	1	1.77	0.72
2	-10	1	1.72	0.75
-2	10	1	1.02	0.80
2	-10	1	3.54	1.03
-2	-10	-1	1.64	0.68
2	-10	1	3.76	1.01
-2	-10	-1	1.34	0.78
2	-10	1	2.86	0.78
2	10	-1	1.07	1.01
2	-10	1	2.31	0.72
2	10	-1	1.39	0.56
2	-10	1	1.44	0.49
2	-10	1	2.47	1.18
-2	-10	-1	1.50	0.75
-2	10	1	3.09	1.16
-2	-10	-1	1.14	0.94
2	-11	-1	0.40	0.76
2	-11	-1	1.15	0.56
-2	-11	1	1.56	0.50
2	11	1	2.05	1.54
-2	11	-1	-0.24	1.17
-2	11	-1	0.85	1.22
2	-11	-1	0.69	1.00
-2	-11	1	0.22	0.68

2	11	1	2.95	0.71
-2	11	-1	2.58	0.77
-2	11	-1	1.86	0.98
2	11	1	1.59	0.81
2	-11	-1	2.31	0.86
2	-11	-1	1.88	0.78
-2	-11	1	2.50	1.14
-2	-11	1	0.79	1.33
2	-11	-1	0.82	0.98
-2	11	1	4.11	1.37
-2	-11	-1	0.48	0.90
-2	11	1	4.13	1.03
2	-11	1	2.80	1.05
-2	11	1	2.26	0.78
2	11	-1	2.51	0.88
2	-11	1	1.13	0.79
2	-11	1	2.00	0.79
-2	-11	-1	0.41	1.20
-2	-11	-1	1.23	0.76
2	-11	1	2.42	0.57
2	-11	1	2.98	0.80
-2	11	1	1.26	1.27
2	11	-1	-0.41	0.87
-2	-11	-1	1.22	0.55
-2	-12	1	0.30	1.05
2	12	1	0.36	0.87
-2	-12	1	0.65	0.79
2	-12	-1	0.56	0.91
-2	12	-1	2.80	1.22
2	-12	-1	3.06	0.93
2	12	1	-1.14	1.51
2	-12	-1	0.95	0.64
-2	12	-1	2.88	1.77
-2	-12	-1	2.07	0.89
2	-12	1	0.77	0.89
-2	12	1	0.63	1.10
-2	12	1	3.16	1.50
2	12	-1	1.50	1.60
-2	-12	-1	2.14	1.06
-2	-12	-1	1.46	0.56
2	-12	1	0.16	0.88
2	-12	1	0.63	0.58
2	12	-1	1.96	0.99
2	13	1	0.01	1.75

-2	13	-1	0.23	1.33
-2	-13	1	0.50	1.24
2	-13	-1	-0.07	1.18
-2	13	-1	-1.31	1.93
-2	-13	1	0.48	0.88
2	-13	-1	-0.62	0.84
2	-13	1	0.21	0.84
-2	13	1	0.67	1.86
2	-13	1	0.29	1.01
-2	-13	-1	0.64	0.87
2	13	-1	0.47	1.78
-2	-13	-1	0.51	1.26
-2	13	1	2.16	1.36
-3	0	-1	0.13	0.06
-3	0	1	0.08	0.08
3	0	1	0.03	0.11
3	0	-1	0.05	0.08
-3	0	-1	0.02	0.12
-3	0	1	0.15	0.12
3	0	1	-0.03	0.10
3	0	-1	0.05	0.07
3	0	1	0.10	0.10
-3	0	-1	0.02	0.10
3	0	1	-0.03	0.07
3	0	-1	-0.03	0.10
-3	0	-1	-0.04	0.11
-3	0	-1	0.12	0.09
-3	0	1	0.12	0.13
-3	0	1	-0.05	0.10
3	0	1	0.03	0.06
-3	0	-1	0.04	0.11
3	0	1	0.05	0.09
-3	0	1	0.12	0.07
3	-1	-1	156.99	6.84
3	1	1	152.66	6.90
3	-1	-1	152.16	6.80
-3	1	-1	150.39	6.85
-3	-1	1	145.55	6.84
3	1	1	147.53	6.83
-3	-1	1	146.03	6.78
-3	1	-1	158.40	6.81
-3	-1	1	147.00	6.81
-3	-1	1	150.79	6.82
3	-1	-1	145.79	6.78

-3	-1	1	164.25	6.87
-3	-1	1	149.55	6.78
-3	1	-1	152.97	6.86
-3	1	-1	150.04	6.77
-3	-1	1	155.35	6.90
3	1	1	144.00	6.82
-3	1	-1	154.41	6.80
3	-1	1	143.10	6.84
-3	-1	-1	150.11	6.79
3	1	-1	157.18	6.81
-3	1	1	159.47	6.85
-3	-1	-1	151.75	6.77
3	-1	1	154.37	6.85
-3	-1	-1	150.18	6.81
-3	1	1	155.03	6.88
-3	-1	-1	149.27	6.78
-3	-1	-1	153.85	6.85
-3	1	1	156.13	6.89
3	1	-1	150.88	6.85
3	1	-1	147.41	6.76
3	1	-1	146.14	6.74
-3	-1	-1	146.67	6.76
-3	-1	-1	152.00	6.80
-3	-1	-1	147.15	6.77
3	-1	1	155.83	6.89
3	-1	1	154.02	6.85
-3	1	1	158.55	6.86
-3	1	1	149.96	6.83
3	1	-1	147.46	6.79
3	-2	-1	5.96	0.41
3	2	1	3.29	0.35
-3	2	-1	6.02	0.46
-3	2	-1	5.75	0.57
3	-2	-1	5.86	0.47
-3	-2	1	2.97	0.27
-3	-2	1	4.06	0.36
-3	2	-1	6.03	0.38
3	2	1	3.22	0.42
3	2	1	3.05	0.29
-3	2	-1	6.52	0.44
3	2	1	3.24	0.31
-3	2	-1	6.84	0.42
-3	-2	1	2.77	0.31
-3	-2	1	2.87	0.42

-3	-2	1	3.33	0.32
-3	-2	1	3.17	0.26
3	-2	-1	6.05	0.51
3	-2	-1	6.18	0.44
3	-2	-1	6.81	0.54
-3	2	-1	6.46	0.57
-3	-2	-1	5.68	0.53
3	-2	1	3.08	0.44
-3	2	1	2.95	0.43
3	-2	1	3.19	0.29
-3	-2	-1	5.89	0.41
3	2	-1	6.41	0.55
3	-2	1	3.77	0.48
3	-2	1	3.84	0.35
3	2	-1	6.66	0.37
3	2	-1	6.26	0.43
-3	2	1	3.15	0.37
-3	2	1	3.20	0.43
-3	-2	-1	5.82	0.36
3	2	-1	6.68	0.46
-3	-2	-1	6.30	0.44
-3	-2	-1	6.62	0.45
-3	-2	-1	5.97	0.43
3	-2	1	3.01	0.30
3	2	-1	6.17	0.47
-3	2	1	3.13	0.29
3	-2	1	3.51	0.27
-3	2	1	2.78	0.33
3	-3	-1	71.40	3.25
3	3	1	72.10	3.27
-3	3	-1	72.89	3.30
-3	-3	1	72.53	3.44
-3	-3	1	68.12	3.33
3	3	1	69.62	3.29
3	-3	-1	74.32	3.47
3	-3	-1	74.33	3.25
3	-3	-1	70.27	3.38
-3	-3	1	69.41	3.35
-3	3	-1	71.23	3.38
3	-3	-1	68.25	3.31
-3	3	-1	71.48	3.28
3	3	1	72.20	3.44
3	3	1	70.46	3.41
3	-3	-1	65.57	3.30

3	3	1	70.58	3.34
-3	-3	1	74.56	3.39
-3	3	-1	71.38	3.49
-3	3	-1	70.52	3.33
-3	-3	1	73.09	3.35
-3	3	-1	70.23	3.42
3	-3	-1	71.00	3.37
-3	-3	1	72.13	3.27
-3	3	1	71.74	3.34
-3	-3	-1	68.79	3.33
-3	-3	-1	73.10	3.34
-3	3	1	73.68	3.42
3	-3	1	72.52	3.32
3	3	-1	69.03	3.42
3	-3	1	70.88	3.34
-3	3	1	70.88	3.48
3	-3	1	76.52	3.51
3	3	-1	74.05	3.33
3	3	-1	72.41	3.24
-3	3	1	69.90	3.34
3	-3	1	73.07	3.30
3	3	-1	69.35	3.39
3	3	-1	71.86	3.32
-3	3	1	71.62	3.41
-3	-3	-1	65.43	3.24
3	-3	1	70.23	3.42
3	-3	1	69.27	3.39
-3	-3	-1	73.20	3.29
-3	-3	-1	73.61	3.42
-3	-3	-1	73.58	3.37
3	-4	-1	14.60	0.96
3	-4	-1	15.94	1.02
3	4	1	12.28	0.93
-3	4	-1	14.67	1.16
3	-4	-1	15.00	0.92
-3	-4	1	12.46	0.94
-3	4	-1	16.96	1.03
3	4	1	12.27	0.87
-3	4	-1	13.89	1.05
-3	-4	1	11.75	0.81
3	4	1	12.35	0.80
-3	4	-1	15.22	0.95
-3	-4	1	12.19	0.70
-3	-4	1	11.61	0.80

3	-4	-1	15.33	1.08
3	4	1	12.07	0.94
3	-4	-1	14.60	0.89
3	-4	-1	15.40	0.86
3	4	1	12.54	0.73
-3	-4	1	12.46	0.83
-3	4	-1	16.62	0.91
3	4	1	12.69	0.76
-3	-4	1	12.56	0.94
3	-4	-1	15.87	0.88
-3	4	1	12.40	0.91
-3	4	1	12.68	0.81
-3	4	1	11.43	1.00
3	4	-1	15.91	0.95
3	-4	1	12.28	0.73
3	4	-1	14.89	1.06
3	-4	1	9.75	0.97
-3	-4	-1	16.54	1.03
3	-4	1	13.76	1.03
3	-4	1	12.61	0.79
3	-4	1	13.17	0.77
-3	-4	-1	16.26	1.08
3	4	-1	16.59	0.82
-3	-4	-1	14.30	0.86
-3	-4	-1	15.63	0.95
3	4	-1	15.85	0.96
-3	4	1	13.19	0.81
-3	4	1	12.30	0.89
3	-4	1	13.68	0.78
-3	-4	-1	15.88	0.95
-3	-4	-1	15.79	0.89
3	-5	-1	124.78	5.77
3	-5	-1	132.38	5.94
-3	-5	1	145.18	6.66
3	5	1	143.18	6.89
-3	5	-1	128.67	5.72
3	-5	-1	114.61	5.66
-3	5	-1	128.47	5.66
-3	-5	1	147.94	6.77
-3	-5	1	155.00	6.91
3	5	1	140.07	6.93
3	5	1	149.38	6.78
-3	-5	1	134.67	6.77
3	-5	-1	127.22	5.65

-3	-5	1	149.12	6.88
3	5	1	146.47	6.86
-3	-5	1	143.08	6.79
3	-5	-1	118.71	5.78
3	-5	-1	128.01	5.66
3	5	1	144.42	6.71
-3	5	-1	119.16	5.82
3	-5	-1	115.69	5.69
-3	-5	1	142.57	6.87
-3	5	-1	128.95	5.82
3	5	1	146.21	6.69
-3	5	1	147.86	6.78
-3	5	1	151.52	6.76
-3	-5	-1	120.32	5.72
3	5	-1	119.67	5.70
3	-5	1	145.08	6.85
-3	-5	-1	132.64	5.84
-3	-5	-1	127.69	5.81
-3	5	1	153.31	6.91
3	-5	1	159.74	6.77
3	-5	1	141.54	6.82
3	5	-1	116.23	5.86
3	-5	1	144.94	6.78
-3	-5	-1	119.40	5.74
-3	-5	-1	114.71	5.61
3	5	-1	123.98	5.77
-3	5	1	147.65	7.03
3	5	-1	124.40	5.58
-3	5	1	148.25	6.87
3	-5	1	161.16	7.03
3	5	-1	108.66	5.86
3	-5	1	140.78	6.70
3	-5	1	140.53	6.84
-3	-5	-1	124.00	5.63
3	6	1	46.30	2.53
-3	6	-1	60.66	2.91
-3	-6	1	46.55	2.55
3	6	1	49.34	2.68
-3	6	-1	57.40	3.03
-3	-6	1	48.78	2.40
-3	-6	1	49.56	2.65
-3	-6	1	54.49	2.72
3	6	1	50.06	2.76
-3	6	-1	60.40	2.83

3	-6	-1	60.18	2.96
3	-6	-1	58.73	2.82
-3	-6	1	50.03	2.56
3	-6	-1	53.82	2.97
-3	-6	1	53.09	2.71
3	-6	-1	63.08	2.86
-3	-6	1	50.96	2.56
-3	6	-1	57.16	3.18
3	6	1	48.14	2.44
3	-6	-1	55.76	2.87
3	-6	-1	53.73	3.43
3	-6	-1	53.15	2.81
3	6	1	50.78	2.67
3	6	1	51.03	2.55
-3	6	-1	59.96	3.03
-3	6	1	50.55	2.71
3	6	-1	57.11	2.72
3	-6	1	46.35	2.89
-3	6	1	50.49	2.51
3	6	-1	58.52	2.87
-3	-6	-1	63.82	3.06
-3	-6	-1	55.60	2.89
3	-6	1	46.13	2.44
-3	6	1	50.71	2.84
-3	-6	-1	52.66	2.78
3	-6	1	48.12	2.62
-3	-6	-1	60.71	3.03
-3	-6	-1	59.01	2.79
3	-6	1	49.60	2.54
3	-6	1	52.05	2.67
3	6	-1	55.01	3.09
-3	6	1	50.07	2.55
3	6	-1	57.12	2.97
3	6	-1	59.73	2.90
-3	6	1	50.23	2.66
3	-6	1	53.45	2.56
-3	-6	-1	53.74	2.92
3	-6	1	52.73	2.51
-3	7	-1	3.77	0.77
3	7	1	3.57	0.76
-3	-7	1	4.97	0.84
3	-7	-1	2.76	0.63
3	7	1	3.43	0.62
3	-7	-1	3.21	0.48

3	-7	-1	3.91	0.85
-3	-7	1	4.26	0.66
3	7	1	3.27	0.62
3	-7	-1	3.74	0.70
-3	-7	1	3.56	0.50
3	7	1	3.45	0.57
3	-7	-1	2.76	0.52
-3	7	-1	3.45	0.55
3	7	1	5.33	0.93
-3	-7	1	3.24	0.65
3	-7	-1	3.98	0.72
-3	7	-1	2.52	0.82
3	-7	-1	3.97	0.64
-3	7	-1	2.65	0.77
-3	7	-1	1.60	0.51
-3	-7	1	4.67	0.85
-3	-7	1	3.74	0.61
-3	7	1	3.58	0.63
-3	-7	-1	4.39	0.79
-3	-7	-1	2.67	0.59
3	-7	1	3.98	0.92
3	7	-1	3.44	0.63
-3	7	1	4.55	0.98
-3	7	1	4.03	0.77
3	7	-1	3.00	0.69
-3	7	1	3.92	0.60
3	-7	1	4.33	0.68
-3	-7	-1	2.73	0.55
3	-7	1	3.00	0.54
3	7	-1	2.54	0.53
-3	-7	-1	3.27	0.74
-3	7	1	4.03	0.74
3	-7	1	3.42	0.65
3	-7	1	3.92	0.77
3	7	-1	2.87	0.43
3	-7	1	3.74	0.61
3	-7	1	2.87	0.52
3	7	-1	2.10	0.63
-3	-7	-1	2.65	0.49
-3	-7	-1	3.45	0.55
3	-8	-1	10.96	1.13
3	-8	-1	11.09	0.90
3	-8	-1	11.59	1.02
3	-8	-1	10.66	1.02

-3	-8	1	10.70	1.33
-3	8	-1	9.44	1.10
-3	-8	1	10.54	1.13
-3	8	-1	10.40	1.26
-3	-8	1	10.63	1.00
-3	-8	1	9.95	1.35
3	-8	-1	12.55	1.13
-3	8	-1	13.06	1.42
-3	8	-1	14.58	1.06
-3	-8	1	11.33	1.18
-3	8	-1	10.05	1.35
-3	-8	1	11.22	1.19
3	8	1	9.69	1.02
3	8	1	10.25	1.30
3	-8	-1	12.88	1.52
3	8	1	9.94	1.05
3	8	1	11.72	0.90
-3	-8	1	10.15	0.83
3	-8	-1	10.77	1.26
3	8	1	10.31	1.32
3	8	-1	13.42	1.07
3	-8	1	10.21	1.00
3	-8	1	12.66	1.55
3	-8	1	11.66	1.01
3	8	-1	10.70	1.07
-3	8	1	9.67	1.28
-3	-8	-1	11.44	1.35
3	8	-1	12.33	1.26
3	-8	1	10.59	1.12
-3	8	1	13.21	1.08
-3	-8	-1	13.14	1.42
3	-8	1	10.59	0.83
-3	-8	-1	9.96	0.87
-3	8	1	10.14	1.10
-3	-8	-1	12.46	1.19
3	-8	1	11.01	1.31
3	-8	1	12.41	1.18
-3	-8	-1	11.44	1.17
-3	8	1	11.77	1.40
-3	8	1	9.95	1.19
-3	-8	-1	11.10	1.03
3	8	-1	11.82	0.88
3	8	-1	9.97	1.38
3	-9	-1	32.51	2.13

3	-9	-1	35.92	2.06
-3	9	-1	29.55	2.43
-3	-9	1	32.27	2.03
3	9	1	31.49	2.38
-3	9	-1	32.67	2.21
3	9	1	32.07	1.92
3	-9	-1	35.34	2.53
3	-9	-1	33.09	1.94
3	-9	-1	29.42	1.81
-3	9	-1	37.96	2.38
-3	-9	1	35.05	2.08
3	-9	-1	33.59	1.98
3	9	1	32.62	1.82
-3	-9	1	31.39	2.08
3	9	1	32.53	2.31
-3	-9	1	33.64	2.30
-3	9	-1	35.52	1.94
3	-9	-1	28.80	2.01
-3	-9	1	31.72	2.14
-3	-9	1	31.96	1.73
-3	-9	-1	31.26	2.13
3	9	-1	32.28	2.24
-3	9	1	30.55	2.17
-3	-9	-1	32.17	1.78
-3	9	1	30.17	2.45
-3	-9	-1	33.83	2.02
3	-9	1	34.08	2.04
3	-9	1	30.30	1.79
-3	-9	-1	31.44	1.93
-3	9	1	33.04	1.98
3	-9	1	32.16	2.23
3	9	-1	31.74	1.75
-3	-9	-1	33.47	2.28
-3	9	1	36.22	2.32
-3	-9	-1	29.39	2.10
3	-9	1	31.51	2.08
3	9	-1	33.14	1.99
3	-9	1	40.07	2.15
3	-9	1	34.12	1.97
3	9	-1	31.69	2.41
3	-9	1	36.19	2.60
-3	10	-1	33.83	2.45
3	10	1	28.77	1.96
3	-10	-1	36.74	2.76

3	10	1	35.97	2.50
-3	-10	1	27.77	2.19
3	-10	-1	32.65	2.21
3	-10	-1	31.11	2.51
-3	10	-1	34.27	2.40
-3	-10	1	31.35	1.76
3	10	1	31.35	2.56
-3	10	-1	30.02	2.68
3	-10	-1	35.78	2.23
3	-10	-1	37.58	2.10
-3	-10	1	32.84	2.22
3	-10	-1	31.61	2.12
-3	10	-1	34.66	2.06
3	10	1	32.85	1.87
3	-10	-1	29.81	1.88
-3	-10	1	34.42	2.17
-3	-10	1	31.60	2.08
-3	10	1	32.07	2.30
3	10	-1	33.26	2.55
3	-10	1	33.65	2.07
3	10	-1	34.33	1.85
-3	-10	-1	35.38	2.15
-3	10	1	33.91	2.06
3	-10	1	34.78	2.70
3	-10	1	30.88	2.17
3	-10	1	30.81	1.83
3	-10	1	38.45	2.28
-3	-10	-1	31.75	2.21
3	-10	1	31.98	2.05
3	10	-1	35.57	2.47
-3	10	1	33.89	2.71
3	10	-1	31.22	2.08
3	-10	1	32.66	2.38
-3	10	1	36.36	2.64
-3	-10	-1	33.46	1.87
-3	-10	-1	33.66	2.06
-3	-10	-1	37.17	2.33
3	11	1	44.31	2.42
3	11	1	43.25	3.22
3	-11	-1	49.22	2.82
-3	11	-1	44.39	3.02
3	-11	-1	49.48	2.71
-3	11	-1	50.25	3.30
-3	-11	1	43.82	2.69

-3	11	-1	42.59	3.46
-3	-11	1	46.12	2.88
-3	-11	1	42.11	2.30
3	-11	-1	42.40	2.48
-3	11	-1	46.29	2.62
3	11	1	42.27	2.54
3	-11	-1	49.70	2.77
3	-11	-1	41.62	2.81
3	11	-1	46.14	2.69
-3	11	1	44.75	3.40
3	-11	1	43.16	2.38
3	-11	1	41.11	2.75
3	-11	1	48.37	2.85
3	-11	1	42.70	2.65
3	-11	1	52.03	2.71
-3	11	1	46.96	2.94
-3	-11	-1	50.37	2.98
-3	11	1	50.76	3.30
-3	11	1	44.25	2.57
-3	-11	-1	42.32	2.44
3	11	-1	49.28	3.38
-3	-11	-1	46.88	2.77
-3	-12	1	98.01	5.17
-3	-12	1	96.67	4.99
3	-12	-1	102.01	5.11
3	-12	-1	90.75	4.78
3	-12	-1	112.78	5.08
-3	12	-1	92.60	5.78
-3	12	-1	91.72	5.61
3	12	1	91.87	5.61
3	-12	1	103.80	4.93
3	12	-1	96.97	4.97
-3	12	1	86.71	5.64
-3	12	1	102.43	5.27
-3	-12	-1	101.02	5.14
-3	-12	-1	93.57	4.73
3	-12	1	94.93	4.96
-3	-12	-1	108.36	5.34
3	-12	1	88.28	4.61
3	12	-1	92.90	5.74
3	13	1	22.74	3.46
-3	13	-1	18.70	3.11
3	-13	-1	23.23	1.95
-3	-13	1	23.68	2.61

-3	-13	1	23.05	2.06
-3	13	-1	26.60	2.64
-3	-13	-1	22.76	2.46
3	13	-1	21.04	3.37
-3	13	1	23.01	2.56
-3	13	1	20.83	3.15
-3	-13	-1	17.68	1.93
3	-13	1	24.23	2.00
-4	0	-1	1000.00	44.58
4	-1	-1	52.51	2.48
-4	1	-1	51.58	2.55
-4	1	-1	53.92	2.48
-4	-1	1	64.71	3.08
-4	-1	1	63.84	3.00
-4	1	-1	53.23	2.44
-4	-1	1	65.08	2.99
-4	-1	1	64.77	3.02
-4	1	-1	52.56	2.54
4	1	1	64.99	2.98
4	1	1	65.51	3.08
-4	1	-1	54.22	2.47
4	1	1	68.61	3.04
4	1	1	67.52	3.00
-4	-1	1	62.61	3.01
-4	-1	1	67.41	3.05
4	-1	-1	53.05	2.46
4	-1	-1	53.57	2.47
4	1	1	65.51	3.01
4	-1	-1	54.51	2.53
-4	-1	-1	53.56	2.47
-4	-1	-1	53.31	2.45
-4	1	1	67.25	2.97
4	-1	1	66.74	2.99
4	-1	1	64.12	3.08
-4	1	1	64.16	3.08
-4	1	1	66.81	3.01
4	1	-1	54.96	2.49
4	1	-1	50.60	2.54
-4	-1	-1	55.27	2.49
4	1	-1	51.35	2.39
-4	1	1	61.56	3.04
-4	-1	-1	53.86	2.43
-4	-1	-1	53.16	2.54
4	1	-1	52.41	2.39

4	-1	1	67.08	3.03
-4	-1	-1	52.28	2.45
-4	1	1	66.69	3.03
-4	1	1	65.69	3.01
-4	-1	-1	53.23	2.41
4	-1	1	66.46	3.02
4	-1	1	64.27	3.02
4	2	1	126.92	5.81
4	2	1	129.97	5.83
-4	2	-1	172.19	8.00
-4	-2	1	135.76	5.85
-4	-2	1	123.88	5.84
4	2	1	132.96	5.77
-4	2	-1	180.59	7.92
4	2	1	130.19	5.84
4	-2	-1	173.44	7.88
-4	-2	1	122.90	5.77
4	2	1	117.47	5.94
-4	2	-1	186.73	7.91
4	-2	-1	172.16	7.99
-4	-2	1	131.30	5.79
4	2	1	111.56	5.87
-4	2	-1	162.16	8.05
-4	-2	1	126.82	5.79
-4	-2	1	133.26	5.93
-4	2	-1	180.05	7.83
-4	2	-1	180.60	7.90
4	-2	1	129.50	5.91
-4	-2	-1	174.80	7.87
-4	2	1	123.23	5.82
4	2	-1	175.61	7.84
4	-2	1	126.71	5.77
-4	2	1	134.89	5.87
-4	2	1	133.32	5.85
-4	-2	-1	185.18	7.99
4	-2	1	131.10	5.86
4	2	-1	181.17	7.89
4	2	-1	177.65	7.86
-4	-2	-1	184.95	7.84
-4	-2	-1	173.55	7.87
-4	-2	-1	164.37	7.96
-4	2	1	130.32	5.92
4	2	-1	152.69	7.92
4	2	-1	175.04	7.89

4	-2	1	131.71	6.03
4	-2	1	129.57	5.83
4	2	-1	156.65	8.07
-4	-2	-1	180.74	7.87
-4	3	-1	32.52	1.54
4	-3	-1	30.41	1.67
4	-3	-1	30.16	1.59
-4	3	-1	29.12	1.44
4	-3	-1	28.84	1.50
-4	-3	1	28.01	1.33
-4	-3	1	27.81	1.53
-4	-3	1	26.95	1.37
4	3	1	26.23	1.40
4	-3	-1	30.60	1.57
4	3	1	28.18	1.31
-4	-3	1	27.82	1.41
-4	3	-1	30.03	1.63
-4	3	-1	30.94	1.57
4	-3	-1	29.77	1.49
-4	-3	1	27.54	1.41
4	3	1	27.73	1.52
-4	-3	1	27.49	1.41
4	3	1	26.31	1.35
-4	3	-1	28.86	1.68
4	3	1	26.78	1.45
-4	3	-1	31.95	1.51
4	-3	1	27.65	1.33
4	-3	1	26.64	1.44
-4	3	1	28.15	1.47
4	-3	1	27.49	1.38
-4	3	1	28.11	1.50
-4	-3	-1	29.10	1.44
4	3	-1	26.14	1.49
-4	-3	-1	30.42	1.48
4	3	-1	32.18	1.51
4	-3	1	26.97	1.58
4	-3	1	26.92	1.50
4	3	-1	29.39	1.57
-4	3	1	27.27	1.56
4	3	-1	28.65	1.63
-4	3	1	29.29	1.42
-4	-3	-1	29.33	1.51
4	3	-1	30.22	1.51
-4	-3	-1	30.52	1.53

-4	-3	-1	30.51	1.53
-4	-3	-1	30.72	1.64
4	-3	1	27.05	1.39
4	3	-1	30.41	1.43
-4	3	1	27.59	1.40
4	-4	-1	146.36	6.73
-4	-4	1	143.63	6.44
4	4	1	140.26	6.67
-4	-4	1	145.81	6.58
-4	-4	1	137.36	6.54
-4	-4	1	137.07	6.51
-4	4	-1	150.79	6.53
-4	4	-1	148.95	6.56
-4	4	-1	141.45	6.75
4	4	1	143.31	6.44
4	4	1	133.92	6.62
-4	4	-1	138.71	6.67
4	-4	-1	141.74	6.62
-4	4	-1	148.63	6.63
4	-4	-1	146.29	6.44
-4	-4	1	147.68	6.53
4	-4	-1	141.99	6.61
4	-4	-1	148.67	6.45
4	4	1	140.75	6.46
4	4	1	144.79	6.54
4	-4	-1	138.44	6.52
-4	-4	1	149.79	6.68
4	-4	-1	133.60	6.52
-4	4	1	140.15	6.53
-4	-4	-1	149.35	6.68
4	-4	1	141.32	6.50
4	4	-1	146.17	6.52
4	4	-1	146.87	6.55
-4	4	1	140.20	6.63
4	-4	1	149.23	6.74
4	4	-1	134.04	6.67
4	-4	1	149.47	6.46
-4	4	1	145.89	6.63
4	-4	1	144.72	6.63
4	4	-1	134.75	6.63
-4	4	1	133.25	6.70
-4	-4	-1	141.38	6.56
4	4	-1	140.36	6.43
-4	-4	-1	137.45	6.54

-4	-4	-1	131.14	6.44
4	-4	1	134.65	6.52
4	-4	1	142.90	6.60
-4	-4	-1	145.85	6.59
-4	-5	1	59.96	3.23
-4	5	-1	60.79	3.02
-4	5	-1	61.95	2.92
-4	-5	1	64.32	3.27
4	-5	-1	57.12	2.98
-4	-5	1	63.13	3.12
4	5	1	64.43	3.02
4	5	1	61.96	3.26
4	-5	-1	56.18	2.87
4	5	1	64.91	3.12
4	5	1	64.50	3.02
-4	5	-1	56.69	3.03
4	5	1	61.95	3.26
-4	5	-1	56.40	3.15
-4	-5	1	65.38	3.00
4	-5	-1	57.95	2.85
-4	-5	1	61.90	3.13
-4	-5	1	60.63	3.11
-4	5	-1	61.73	2.87
-4	-5	1	67.45	3.22
4	-5	-1	59.69	2.80
4	-5	-1	57.46	2.97
4	5	1	63.68	3.34
4	-5	-1	61.29	3.14
4	-5	-1	62.54	2.81
4	5	-1	60.03	2.76
4	5	-1	60.05	2.88
4	-5	1	66.33	3.05
4	5	-1	61.34	2.90
-4	5	1	65.77	3.13
-4	-5	-1	58.74	2.81
4	-5	1	68.04	3.08
-4	5	1	66.92	3.12
4	-5	1	61.71	3.10
-4	-5	-1	61.57	3.06
4	-5	1	62.56	3.06
-4	-5	-1	58.33	2.91
4	-5	1	64.26	3.23
-4	-5	-1	56.19	2.91
4	-5	1	64.01	3.20

4	-5	1	67.42	3.38
-4	5	1	63.74	3.23
-4	5	1	63.36	3.24
-4	5	1	63.80	3.39
-4	-5	-1	54.86	2.78
-4	-5	-1	61.99	2.98
4	5	-1	54.62	3.02
-4	6	-1	222.61	10.02
4	-6	-1	205.14	9.82
-4	-6	1	218.60	9.83
4	-6	-1	208.62	9.97
-4	6	-1	217.99	9.90
-4	-6	1	199.81	9.85
4	6	1	196.36	10.03
4	6	1	211.13	9.76
-4	-6	1	215.46	9.72
4	-6	-1	209.90	9.88
4	6	1	215.70	9.86
4	6	1	219.94	9.97
-4	-6	1	217.44	9.96
-4	6	-1	231.20	9.85
-4	-6	1	209.90	9.88
-4	-6	1	210.24	9.98
4	-6	-1	223.65	10.14
-4	6	-1	206.97	10.02
4	-6	-1	226.46	9.81
4	6	1	207.38	10.01
-4	6	-1	212.59	10.18
-4	-6	1	217.32	10.00
4	-6	-1	222.31	9.95
4	-6	-1	217.53	9.79
-4	6	1	212.15	9.99
4	-6	1	208.72	9.85
-4	6	1	230.14	10.03
-4	6	1	213.96	9.86
4	6	-1	213.29	9.72
4	-6	1	228.52	9.79
4	6	-1	218.95	9.89
-4	-6	-1	205.51	9.90
4	6	-1	213.81	9.84
-4	-6	-1	214.16	9.79
-4	-6	-1	225.64	10.00
-4	6	1	230.27	9.85
-4	6	1	211.45	10.14

4	-6	1	201.98	9.77
4	6	-1	202.06	10.06
4	-6	1	216.70	9.95
-4	-6	-1	209.30	9.90
4	-6	1	206.12	9.94
4	6	-1	219.89	9.96
4	-6	1	229.73	9.82
4	-6	1	228.46	10.15
-4	-6	-1	227.13	10.04
-4	-6	-1	201.71	9.75
4	-7	-1	104.84	5.09
4	-7	-1	110.99	5.02
-4	7	-1	101.72	5.26
-4	7	-1	114.75	5.06
4	-7	-1	109.30	5.17
-4	-7	1	98.36	4.89
4	-7	-1	101.96	5.19
4	7	1	101.27	4.86
-4	-7	1	98.29	4.74
4	7	1	96.88	4.56
4	7	1	95.57	4.97
4	-7	-1	116.34	5.04
-4	-7	1	97.70	4.86
4	-7	-1	110.64	5.40
4	7	1	98.25	4.60
-4	-7	1	91.39	4.71
4	7	1	94.46	4.87
-4	7	-1	111.62	5.13
-4	7	-1	99.01	5.41
-4	-7	1	97.21	4.54
-4	-7	1	98.77	4.68
4	-7	-1	101.82	5.01
-4	-7	1	97.32	4.85
-4	7	-1	108.35	5.27
4	7	1	99.49	4.72
4	-7	1	92.18	4.69
4	-7	1	96.30	4.82
4	7	-1	108.39	5.21
-4	-7	-1	104.39	5.12
-4	7	1	88.71	4.99
4	-7	1	93.37	4.59
4	-7	1	101.05	4.65
4	7	-1	105.63	5.06
-4	7	1	95.76	4.71

-4	7	1	102.89	4.93
4	-7	1	102.70	4.70
-4	-7	-1	104.49	4.97
4	-7	1	95.56	4.79
4	7	-1	106.31	4.91
-4	-7	-1	98.75	5.11
-4	-7	-1	99.57	4.95
-4	7	1	97.09	4.85
4	7	-1	97.68	5.32
-4	-7	-1	110.71	5.28
4	-7	1	103.88	5.08
-4	-7	-1	111.65	5.24
4	7	-1	107.75	5.10
-4	7	1	105.25	4.69
-4	-8	1	29.27	1.85
-4	-8	1	32.55	2.14
4	-8	-1	28.81	1.83
4	-8	-1	29.50	1.72
-4	8	-1	29.93	1.89
4	-8	-1	32.30	2.03
-4	-8	1	30.32	2.00
4	-8	-1	29.75	1.92
-4	-8	1	31.39	1.92
4	8	1	31.21	2.07
-4	-8	1	32.05	1.65
4	-8	-1	34.81	1.86
-4	8	-1	32.10	2.12
4	-8	-1	32.70	1.78
4	8	1	30.84	2.15
4	-8	-1	31.91	2.35
-4	8	-1	33.89	1.83
4	8	1	31.35	1.68
-4	8	-1	30.78	2.29
4	8	1	28.12	1.71
-4	-8	1	26.78	2.02
-4	-8	1	29.03	1.86
-4	8	-1	34.70	2.15
4	8	-1	32.84	2.07
-4	-8	-1	31.63	2.01
-4	-8	-1	29.35	1.90
-4	8	1	29.69	2.24
-4	8	1	32.21	1.84
4	-8	1	36.00	1.91
4	-8	1	28.91	2.05

4	8	-1	31.12	1.65
-4	8	1	30.09	2.05
4	-8	1	29.27	1.68
-4	-8	-1	27.51	1.74
-4	8	1	29.18	1.86
-4	-8	-1	32.71	2.14
4	-8	1	29.66	1.81
4	8	-1	29.80	2.17
4	8	-1	30.95	1.85
-4	8	1	33.28	2.15
-4	-8	-1	31.56	2.08
-4	-8	-1	30.39	1.69
4	-8	1	30.06	1.90
-4	-8	-1	29.62	1.86
4	-8	1	33.17	2.33
4	-8	1	31.84	1.77
-4	9	-1	31.29	2.23
4	-9	-1	27.22	1.91
4	9	1	26.03	1.72
4	-9	-1	28.18	1.81
-4	9	-1	29.88	2.12
4	9	1	28.04	1.63
4	9	1	30.38	2.18
-4	-9	1	25.17	1.86
4	-9	-1	25.91	1.64
-4	-9	1	28.23	1.84
-4	-9	1	29.05	2.01
4	9	1	29.67	2.22
4	-9	-1	24.49	1.98
-4	-9	1	26.99	1.81
4	-9	-1	30.18	1.89
4	-9	-1	29.43	2.41
-4	9	-1	26.23	2.30
-4	9	-1	31.17	1.79
-4	-9	1	26.34	1.53
4	-9	-1	28.30	1.72
-4	-9	-1	28.01	1.88
-4	-9	-1	27.79	1.60
-4	-9	-1	25.10	1.73
-4	9	1	28.92	1.81
4	-9	1	26.24	1.88
4	-9	1	30.26	2.12
-4	-9	-1	29.95	2.04
4	9	-1	26.82	1.56

4	-9	1	28.05	1.74
4	-9	1	31.45	2.45
4	9	-1	24.86	2.20
-4	-9	-1	28.08	1.83
-4	9	1	31.43	2.25
4	9	-1	29.57	1.76
4	-9	1	27.00	1.60
-4	-9	-1	30.16	2.15
-4	9	1	26.37	2.17
4	9	-1	29.33	2.04
4	-9	1	26.60	1.79
-4	9	1	26.63	2.01
4	-9	1	31.04	1.90
4	-10	-1	11.24	1.34
-4	10	-1	8.65	1.21
-4	10	-1	9.01	1.35
4	10	1	12.00	1.12
-4	-10	1	10.68	0.88
4	-10	-1	7.57	1.51
4	10	1	10.14	1.79
4	-10	-1	12.24	1.75
-4	10	-1	5.43	1.33
4	-10	-1	7.49	0.93
-4	-10	1	8.82	1.22
4	-10	-1	9.03	1.13
-4	10	-1	6.70	1.40
-4	-10	1	12.32	1.41
4	-10	-1	9.36	1.08
4	10	1	10.70	1.06
-4	-10	1	13.22	1.55
4	10	1	8.21	1.69
4	-10	-1	5.40	1.15
-4	-10	1	10.21	1.39
4	-10	1	10.66	1.73
4	10	-1	9.04	1.45
4	-10	1	11.16	0.98
4	-10	1	10.54	1.32
4	-10	1	10.82	1.23
-4	-10	-1	7.82	0.88
4	10	-1	9.47	1.58
-4	-10	-1	6.29	1.22
4	10	-1	8.73	0.91
4	-10	1	10.34	1.16
-4	-10	-1	5.67	1.16

-4	10	1	10.89	1.81
4	-10	1	11.69	1.47
-4	-10	-1	9.30	1.45
-4	-10	-1	8.82	1.18
-4	10	1	11.95	1.37
-4	10	1	9.75	1.84
4	10	-1	9.17	1.11
-4	10	1	9.94	1.56
4	11	1	0.17	1.19
4	-11	-1	0.86	1.18
-4	11	-1	2.02	1.21
-4	-11	1	0.99	0.68
4	11	1	0.90	0.71
4	-11	-1	1.62	0.83
-4	-11	1	1.36	0.48
-4	11	-1	1.99	0.83
-4	-11	1	-0.43	0.88
4	-11	-1	1.93	0.62
-4	11	-1	1.48	1.14
-4	11	-1	1.78	1.30
4	-11	-1	-0.33	0.66
4	-11	-1	1.36	0.75
-4	-11	-1	0.56	0.75
4	-11	1	1.65	0.76
-4	11	1	3.31	1.30
4	11	-1	2.61	1.26
-4	-11	-1	1.11	0.54
4	-11	1	0.10	0.78
-4	11	1	1.36	1.27
4	-11	1	0.44	0.56
4	11	-1	1.92	0.78
4	-11	1	0.01	0.93
-4	-11	-1	1.45	0.96
-4	11	1	-0.30	0.97
4	-11	1	0.57	0.65
4	12	1	3.62	1.66
4	-12	-1	2.18	1.36
-4	-12	1	2.17	0.96
-4	-12	1	4.85	1.33
4	-12	-1	1.95	0.76
-4	12	-1	1.08	1.57
-4	12	-1	2.88	1.27
4	-12	-1	2.07	0.88
4	-12	-1	3.33	0.98

4	-12	1	2.46	0.67
4	-12	1	2.25	0.94
-4	12	1	1.74	1.66
-4	12	1	3.25	1.28
4	12	-1	2.67	1.61
-4	-12	-1	1.73	1.16
4	-12	1	3.77	1.04
-4	-12	-1	2.56	0.68
-4	-12	-1	1.74	0.96
4	12	-1	3.03	1.39
4	-13	-1	7.42	1.20
4	13	1	8.37	2.29
-4	-13	1	5.71	1.17
4	-13	-1	8.66	1.57
-4	-13	1	10.32	1.89
-4	13	-1	8.47	1.82
4	-13	1	6.67	1.14
4	13	-1	6.65	2.37
-4	-13	-1	8.48	1.75
-4	13	1	9.28	1.78
-4	-13	-1	6.91	1.22
5	0	-1	0.04	0.13
-5	0	-1	0.00	0.15
-5	0	-1	0.17	0.10
-5	0	-1	0.10	0.15
5	0	-1	0.09	0.09
5	0	1	0.01	0.18
-5	0	1	0.08	0.17
-5	0	1	-0.08	0.11
5	0	1	0.07	0.13
-5	0	-1	0.33	0.15
-5	0	-1	0.01	0.15
5	0	-1	0.22	0.13
5	0	1	0.09	0.17
5	0	-1	0.15	0.14
-5	0	1	-0.16	0.18
-5	0	1	0.22	0.20
5	0	-1	0.05	0.19
-5	0	1	0.06	0.13
-5	0	-1	0.10	0.18
5	0	1	-0.11	0.20
-5	0	-1	-0.03	0.19
5	0	1	0.21	0.10
-5	1	-1	17.12	1.04

5	-1	-1	16.92	0.94
-5	1	-1	18.11	0.93
5	1	1	16.49	0.88
-5	1	-1	18.07	0.89
5	-1	-1	17.18	0.87
5	1	1	17.43	0.98
-5	-1	1	15.66	0.86
-5	-1	1	16.67	0.86
5	-1	-1	17.28	0.99
-5	-1	1	17.13	0.97
5	1	1	15.41	0.84
-5	-1	1	16.24	0.88
-5	1	-1	18.01	0.90
-5	1	-1	17.61	1.00
5	-1	-1	16.83	0.90
5	1	1	15.33	0.94
-5	-1	1	17.12	0.92
5	1	1	17.17	0.86
-5	-1	1	17.18	0.88
-5	-1	-1	17.72	0.83
5	-1	1	16.16	0.89
-5	-1	-1	16.97	0.84
-5	1	1	17.00	0.87
-5	1	1	17.38	0.86
5	1	-1	17.34	0.94
-5	1	1	17.41	0.90
-5	1	1	15.98	0.90
5	1	-1	17.39	1.02
5	-1	1	16.87	0.86
-5	-1	-1	17.88	0.93
-5	-1	-1	17.59	0.91
5	-1	1	16.23	0.96
-5	1	1	17.13	0.83
-5	-1	-1	18.37	1.01
-5	-1	-1	17.31	0.92
-5	-1	-1	16.52	0.91
-5	2	-1	96.91	4.37
5	-2	-1	94.61	4.37
5	2	1	90.53	4.30
-5	-2	1	90.13	4.16
5	2	1	90.28	4.20
-5	2	-1	101.18	4.29
-5	-2	1	88.64	4.11
-5	-2	1	86.04	4.20

5	2	1	89.16	4.18
5	2	1	91.33	4.18
-5	2	-1	95.20	4.47
5	-2	-1	96.53	4.43
-5	-2	1	96.95	4.23
-5	-2	1	95.64	4.32
-5	2	-1	91.40	4.45
5	-2	-1	85.50	4.39
-5	-2	1	91.79	4.12
-5	2	-1	98.91	4.37
5	-2	-1	93.14	4.36
-5	2	-1	98.38	4.37
5	2	1	90.78	4.19
-5	2	1	95.17	4.23
-5	-2	-1	91.00	4.29
5	2	-1	93.55	4.30
-5	-2	-1	95.18	4.29
5	2	-1	91.57	4.32
5	-2	1	94.30	4.13
-5	-2	-1	93.27	4.30
5	-2	1	87.50	4.20
5	2	-1	91.48	4.45
-5	-2	-1	89.82	4.36
5	-2	1	89.41	4.22
5	2	-1	97.27	4.35
5	2	-1	92.97	4.34
5	2	-1	94.79	4.30
-5	2	1	88.98	4.28
-5	-2	-1	99.06	4.31
5	-2	1	93.86	4.29
-5	2	1	93.28	4.20
-5	-2	-1	92.10	4.34
-5	2	1	90.56	4.29
-5	-2	-1	100.08	4.47
5	-2	1	89.32	4.20
5	3	1	58.97	2.71
5	3	1	54.29	2.70
-5	-3	1	54.21	2.70
-5	-3	1	55.91	2.69
5	3	1	58.78	2.60
-5	3	-1	81.92	4.07
5	-3	-1	85.59	3.96
-5	3	-1	80.90	4.04
5	3	1	54.51	2.72

5	3	1	54.59	2.82
-5	3	-1	84.42	3.81
-5	3	-1	87.01	3.97
-5	3	-1	91.63	3.92
-5	-3	1	58.49	2.63
5	-3	-1	86.06	4.06
5	-3	-1	84.30	3.92
5	3	1	57.80	2.65
-5	-3	1	51.27	2.70
5	-3	-1	81.51	3.89
-5	-3	1	56.38	2.63
-5	-3	1	55.09	2.84
5	3	-1	82.89	3.89
-5	-3	-1	82.34	4.05
-5	-3	-1	78.86	3.92
5	-3	1	57.70	2.87
-5	3	1	55.94	2.69
5	3	-1	80.51	4.04
-5	3	1	56.71	2.84
5	-3	1	55.88	2.73
5	-3	1	53.37	2.68
5	3	-1	83.86	3.83
-5	-3	-1	82.92	3.90
-5	-3	-1	84.32	3.89
5	3	-1	87.61	3.89
5	-3	1	57.58	2.59
-5	3	1	58.05	2.81
5	3	-1	82.33	3.94
-5	3	1	58.31	2.76
-5	3	1	59.41	2.72
-5	-3	-1	83.19	3.83
5	-3	1	56.25	2.68
-5	4	-1	56.35	2.75
5	4	1	44.82	2.17
5	4	1	42.02	2.38
5	4	1	46.40	2.12
-5	-4	1	44.41	2.20
-5	-4	1	43.04	2.24
5	-4	-1	51.80	2.64
5	-4	-1	57.45	2.88
-5	4	-1	58.12	2.66
-5	-4	1	47.86	2.27
5	-4	-1	52.80	2.73
-5	4	-1	55.03	2.56

-5	-4	1	45.70	2.16
5	-4	-1	51.79	2.75
5	-4	-1	51.53	2.63
5	4	1	44.07	2.24
-5	-4	1	44.85	2.24
-5	-4	1	48.18	2.42
-5	4	-1	57.02	2.93
-5	4	-1	57.82	2.68
-5	4	-1	52.22	2.80
5	4	1	42.28	2.32
5	-4	1	44.02	2.37
-5	4	1	44.86	2.37
-5	4	1	44.68	2.24
-5	-4	-1	55.46	2.68
5	-4	1	41.04	2.22
-5	-4	-1	58.24	2.83
-5	4	1	49.03	2.28
5	4	-1	55.78	2.62
-5	4	1	45.36	2.35
5	-4	1	47.94	2.46
5	4	-1	56.83	2.68
5	-4	1	44.02	2.30
-5	-4	-1	53.79	2.65
5	4	-1	56.06	2.67
5	-4	1	42.82	2.21
-5	-4	-1	53.34	2.60
-5	4	1	46.68	2.47
5	4	-1	52.19	2.74
5	4	-1	53.92	2.55
-5	-4	-1	52.99	2.67
5	4	-1	53.18	2.81
5	-4	1	48.92	2.14
-5	-4	-1	52.38	2.56
5	-5	-1	103.08	4.89
5	-5	-1	105.09	4.66
-5	5	-1	106.88	4.92
-5	-5	1	86.48	4.44
-5	5	-1	99.71	4.96
5	-5	-1	106.59	5.04
5	-5	-1	98.97	4.88
-5	-5	1	97.78	4.50
5	5	1	96.31	4.46
-5	-5	1	85.29	4.38
5	5	1	91.96	4.34

5	5	1	90.54	4.57
5	-5	-1	96.28	4.76
-5	5	-1	104.54	4.82
-5	5	-1	105.40	4.78
-5	-5	1	89.25	4.41
5	-5	-1	105.56	4.67
5	5	1	90.99	4.60
-5	-5	1	95.13	4.43
-5	-5	1	99.59	4.63
5	-5	-1	95.82	4.77
-5	5	-1	99.28	5.06
5	5	1	96.61	4.32
-5	-5	-1	98.53	4.80
5	5	-1	104.83	4.80
-5	-5	-1	96.98	4.67
5	5	-1	101.71	4.66
-5	5	1	101.03	4.45
-5	-5	-1	100.42	4.71
-5	-5	-1	106.91	4.97
5	-5	1	96.35	4.57
-5	-5	-1	105.29	4.85
5	-5	1	87.96	4.39
-5	5	1	95.06	4.45
5	5	-1	100.20	4.97
-5	-5	-1	100.21	4.81
5	-5	1	101.36	4.74
-5	5	1	92.88	4.58
5	5	-1	94.28	4.91
5	-5	1	84.30	4.42
5	-5	1	101.03	4.37
5	-5	1	102.60	4.35
-5	5	1	90.81	4.69
5	-5	1	88.56	4.52
-5	5	1	98.21	4.58
5	5	-1	105.20	4.75
5	-6	-1	13.17	1.17
-5	-6	1	18.41	1.02
5	6	1	15.99	1.13
5	-6	-1	12.65	0.90
-5	-6	1	17.98	1.29
5	-6	-1	14.04	0.91
-5	-6	1	16.43	1.15
-5	6	-1	15.00	1.01
5	-6	-1	13.13	0.92

5	-6	-1	12.78	0.95
5	-6	-1	13.67	1.08
-5	-6	1	17.92	1.20
5	6	1	19.14	1.06
5	6	1	18.26	1.19
-5	6	-1	14.78	1.20
-5	-6	1	18.77	1.41
5	-6	-1	14.06	1.30
5	6	1	17.39	1.34
-5	-6	1	15.77	1.30
-5	6	-1	14.37	1.05
5	6	1	17.68	1.31
5	6	1	17.10	1.34
-5	6	-1	13.41	1.21
-5	-6	1	15.75	1.16
5	6	-1	13.78	0.84
5	-6	1	18.23	1.25
-5	-6	-1	14.88	1.03
5	-6	1	16.96	1.08
-5	-6	-1	14.14	1.14
-5	-6	-1	15.29	0.94
5	-6	1	16.79	1.36
5	-6	1	18.17	1.13
-5	6	1	18.72	1.33
-5	6	1	19.26	1.21
-5	-6	-1	13.05	1.02
-5	6	1	16.34	1.34
-5	6	1	18.50	1.19
5	-6	1	18.42	1.14
5	6	-1	13.44	0.94
-5	6	1	17.10	1.57
-5	-6	-1	12.82	1.24
-5	-6	-1	12.30	0.87
5	-6	1	19.77	1.09
5	6	-1	13.78	1.02
5	-6	1	18.90	1.48
5	6	-1	13.03	1.16
-5	-7	1	1.80	0.60
5	-7	-1	3.64	0.76
5	7	1	2.82	0.45
-5	-7	1	2.16	0.54
-5	-7	1	2.26	0.66
5	-7	-1	2.03	0.45
-5	7	-1	2.38	0.72

-5	7	-1	3.15	0.78
-5	7	-1	3.46	0.84
-5	-7	1	1.74	0.59
5	7	1	2.50	0.67
5	-7	-1	3.27	0.53
5	7	1	2.98	0.73
5	-7	-1	2.90	0.48
-5	7	-1	2.80	0.63
-5	-7	1	2.41	0.52
5	7	1	1.37	0.66
-5	7	-1	2.81	0.55
5	-7	-1	3.09	0.90
-5	-7	1	2.81	0.75
5	-7	-1	2.71	0.63
-5	-7	1	2.70	0.41
5	7	1	2.44	0.50
5	7	1	2.37	0.49
5	-7	-1	2.20	0.47
-5	-7	-1	2.64	0.56
5	-7	1	2.14	0.56
-5	7	1	1.96	0.69
-5	-7	-1	4.68	0.85
5	7	-1	3.69	0.55
5	7	-1	2.91	0.51
-5	7	1	2.05	0.56
-5	7	1	2.59	0.66
5	-7	1	2.68	0.45
5	-7	1	1.45	0.68
5	-7	1	1.92	0.49
-5	-7	-1	3.15	0.71
-5	7	1	2.11	0.59
5	7	-1	2.64	0.57
-5	-7	-1	2.47	0.60
5	7	-1	2.46	0.68
-5	-7	-1	2.00	0.43
5	7	-1	2.59	0.73
5	-7	1	1.68	0.52
-5	-7	-1	2.65	0.50
5	-7	1	1.81	0.43
-5	7	1	2.20	0.74
5	-7	1	2.60	0.95
5	-8	-1	40.76	2.16
5	8	1	43.41	2.43
-5	-8	1	37.87	2.23

5	-8	-1	38.87	2.38
5	-8	-1	39.75	2.21
-5	-8	1	40.58	2.50
-5	-8	1	37.53	2.20
5	-8	-1	37.11	2.11
-5	8	-1	43.53	2.23
-5	8	-1	45.20	2.54
-5	-8	1	36.46	2.21
-5	-8	1	36.98	2.37
5	-8	-1	42.78	2.74
-5	8	-1	39.68	2.50
-5	-8	1	38.29	2.40
5	8	1	38.76	2.03
-5	8	-1	39.39	2.71
5	8	1	37.16	2.01
5	-8	-1	39.31	2.12
5	-8	-1	38.28	2.32
-5	-8	1	39.02	2.00
5	8	1	36.71	2.49
5	8	-1	39.92	2.39
-5	8	1	38.26	2.70
5	8	-1	39.72	2.01
-5	8	1	40.82	2.23
-5	-8	-1	37.38	2.07
-5	-8	-1	40.46	2.43
5	-8	1	36.79	2.18
5	8	-1	38.02	2.53
-5	-8	-1	39.10	2.51
5	-8	1	39.08	2.31
-5	-8	-1	38.54	2.38
5	8	-1	40.73	2.19
-5	-8	-1	37.95	2.06
-5	-8	-1	39.04	2.25
5	-8	1	42.87	2.76
-5	8	1	38.07	2.41
5	-8	1	40.05	2.10
5	-8	1	36.11	2.04
-5	-8	-1	37.38	2.23
5	-8	1	42.37	2.21
5	-8	1	39.13	2.43
-5	8	1	42.77	2.53
-5	8	1	36.53	2.24
-5	9	-1	1.96	0.90
5	9	1	3.94	0.62

-5	-9	1	2.93	0.75
5	-9	-1	3.02	0.66
-5	-9	1	3.47	0.62
5	-9	-1	3.05	0.57
-5	9	-1	3.57	0.78
-5	9	-1	3.33	1.10
5	-9	-1	4.62	0.79
-5	9	-1	3.85	1.01
5	9	1	4.12	0.65
-5	-9	1	5.16	0.98
-5	-9	1	2.38	1.00
5	9	1	4.85	1.09
5	-9	-1	3.91	1.24
5	9	1	2.14	0.97
5	-9	-1	2.47	0.90
5	-9	-1	4.19	0.67
5	-9	-1	2.30	0.76
-5	-9	1	4.63	0.88
-5	9	1	3.84	1.16
5	9	-1	1.79	0.95
5	-9	1	3.81	0.68
5	-9	1	4.30	0.86
5	-9	1	2.59	0.95
5	9	-1	4.10	0.58
5	-9	1	3.53	0.58
5	-9	1	3.01	1.14
-5	9	1	3.10	1.10
-5	9	1	4.61	0.82
-5	-9	-1	3.79	0.57
5	9	-1	3.50	0.93
-5	9	1	3.70	1.01
5	-9	1	3.13	0.66
-5	-9	-1	3.39	0.90
-5	-9	-1	3.57	0.65
5	-9	1	3.74	0.76
5	9	-1	4.43	0.68
-5	-9	-1	3.11	0.73
5	-10	-1	9.60	1.17
5	-10	-1	11.02	1.75
-5	10	-1	8.90	1.27
5	-10	-1	10.51	1.25
5	10	1	10.00	1.79
5	10	1	9.63	1.16
5	-10	-1	9.14	1.27

-5	-10	1	11.43	1.41
-5	-10	1	11.09	1.28
-5	10	-1	8.60	1.59
5	-10	-1	8.30	1.07
5	10	1	13.08	1.09
-5	10	-1	9.10	1.54
-5	-10	1	12.77	1.33
-5	-10	1	12.47	0.93
-5	10	-1	8.52	1.53
5	-10	-1	8.69	0.98
5	-10	1	10.42	1.00
5	-10	1	11.12	1.38
-5	-10	-1	7.23	1.15
5	-10	1	12.79	1.19
5	-10	1	11.74	2.16
-5	10	1	11.24	1.95
-5	10	1	14.00	1.42
-5	-10	-1	9.04	0.92
5	10	-1	9.12	0.91
-5	10	1	12.06	1.91
-5	10	1	10.46	1.63
5	10	-1	9.05	2.22
5	-10	1	10.64	1.35
-5	-10	-1	7.90	1.24
5	-10	1	11.87	1.26
5	10	-1	10.13	1.14
-5	-10	-1	8.92	1.20
5	10	-1	9.95	1.44
-5	-11	1	7.84	0.82
-5	-11	1	5.17	1.02
5	-11	-1	7.02	1.22
5	11	1	4.18	1.46
-5	11	-1	9.46	1.41
5	-11	-1	8.52	1.01
-5	11	-1	9.61	1.82
5	-11	-1	7.09	1.18
-5	-11	1	9.02	1.43
5	-11	-1	8.89	1.26
5	11	1	8.96	1.07
-5	11	1	7.44	1.40
-5	-11	-1	8.15	1.31
5	-11	1	5.02	1.05
5	11	-1	6.53	1.20
5	-11	1	6.01	0.94

-5	-11	-1	7.65	0.97
-5	-11	-1	8.46	1.43
5	11	-1	7.89	1.63
5	-11	1	8.34	1.09
-5	11	1	7.46	1.68
5	-11	1	7.22	1.16
5	-12	-1	11.37	1.55
-5	-12	1	12.30	1.95
-5	-12	1	12.39	1.54
5	-12	-1	13.28	2.10
-5	12	-1	14.27	2.66
5	-12	-1	9.20	1.61
-5	12	-1	15.55	2.28
5	12	1	10.93	1.91
5	-12	-1	13.44	1.46
-5	12	1	11.03	2.20
5	12	-1	14.02	2.56
-5	12	1	11.95	2.12
5	-12	1	9.88	1.39
5	-12	1	8.18	1.33
-5	-12	-1	9.94	1.57
-5	-12	-1	13.45	1.96
5	-12	1	9.26	1.46
5	-12	1	10.06	1.11
-5	-13	1	6.29	1.24
5	-13	-1	6.23	0.95
-5	-13	1	5.26	1.55
-5	13	-1	7.98	1.79
5	-13	-1	3.74	1.11
5	13	1	7.87	2.06
-5	13	1	8.55	1.84
5	-13	1	5.63	1.10
-5	-13	-1	8.40	1.29
5	13	-1	4.91	2.15
-5	-13	-1	10.09	1.84
-6	0	-1	428.70	18.60
-6	0	1	417.30	18.84
-6	0	-1	415.68	18.63
6	0	1	415.53	18.88
6	0	1	406.17	18.86
-6	0	1	434.82	18.91
-6	0	-1	418.00	18.54
-6	0	-1	423.20	18.61
-6	0	-1	429.33	18.59

6	0	1	425.44	18.86
6	0	1	425.65	18.88
6	0	-1	383.69	18.58
-6	0	1	417.99	18.86
6	0	-1	395.35	18.58
-6	0	-1	393.17	18.58
-6	0	-1	419.57	18.50
6	0	-1	429.24	18.53
6	0	-1	427.51	18.61
-6	0	1	424.14	18.85
-6	-1	1	260.98	13.09
-6	-1	1	284.10	13.09
-6	1	-1	326.06	14.40
6	-1	-1	304.28	14.27
-6	-1	1	299.52	13.19
-6	1	-1	325.43	14.36
6	-1	-1	305.05	14.32
6	-1	-1	324.52	14.28
6	1	1	283.04	13.12
6	1	1	301.22	13.08
6	1	1	294.86	13.19
-6	-1	1	298.96	13.15
-6	1	-1	316.89	14.29
-6	1	-1	333.03	14.33
-6	1	-1	314.93	14.28
6	-1	-1	319.74	14.33
-6	-1	1	283.79	13.10
6	1	1	292.12	13.10
6	1	1	278.26	13.04
-6	1	-1	327.55	14.33
6	-1	-1	336.00	14.39
-6	-1	1	299.99	13.13
6	-1	1	284.93	13.12
-6	1	1	300.15	13.12
-6	-1	-1	307.61	14.21
6	-1	1	281.82	13.08
6	-1	1	299.05	13.09
-6	-1	-1	342.35	14.40
6	-1	1	303.35	13.19
-6	1	1	302.09	13.07
-6	-1	-1	303.99	14.32
-6	-1	-1	312.50	14.21
-6	-1	-1	322.91	14.32
-6	-1	-1	315.61	14.31

-6	1	1	300.65	13.07
-6	1	1	292.72	13.19
-6	-1	-1	310.19	14.31
-6	1	1	299.51	13.11
6	-1	1	289.07	13.17
6	1	-1	307.64	14.33
-6	1	1	299.81	13.13
6	-1	1	278.61	13.11
-6	-2	1	147.44	6.60
6	2	1	146.87	6.56
-6	-2	1	152.13	6.61
-6	2	-1	128.93	6.05
-6	-2	1	143.62	6.55
6	2	1	137.78	6.68
-6	2	-1	133.02	5.95
-6	2	-1	129.24	5.86
-6	2	-1	134.70	5.96
-6	-2	1	142.59	6.69
-6	2	-1	129.96	6.06
6	-2	-1	128.86	5.93
6	2	1	136.09	6.53
6	-2	-1	125.04	5.95
-6	2	-1	133.84	5.95
6	-2	-1	133.74	6.03
6	2	1	144.30	6.55
-6	-2	1	140.75	6.58
6	-2	-1	129.44	5.95
-6	2	1	150.87	6.60
-6	2	1	143.75	6.69
6	-2	1	140.82	6.59
-6	-2	-1	133.84	5.91
-6	2	1	142.10	6.57
-6	-2	-1	131.57	5.85
6	2	-1	126.85	5.86
-6	2	1	151.87	6.62
-6	-2	-1	125.26	5.95
6	-2	1	143.16	6.59
6	2	-1	123.76	6.05
6	2	-1	132.24	5.90
-6	-2	-1	134.03	6.06
6	2	-1	128.25	5.88
-6	-2	-1	128.77	5.86
6	-2	1	144.61	6.59
6	2	-1	134.77	5.94

-6	-2	-1	127.12	5.88
6	-2	1	148.38	6.51
-6	2	1	141.67	6.66
-6	-2	-1	126.44	5.95
-6	-2	-1	126.38	5.92
-6	-3	1	20.85	1.09
6	3	1	20.22	1.32
-6	-3	1	21.72	1.19
-6	3	-1	15.96	0.94
-6	-3	1	19.82	1.17
-6	-3	1	21.31	1.33
6	-3	-1	13.68	1.04
6	3	1	21.18	1.17
6	-3	-1	14.82	0.93
6	3	1	20.27	1.16
6	-3	-1	14.56	0.86
6	-3	-1	14.81	0.90
-6	3	-1	13.49	1.07
-6	-3	1	20.45	1.06
6	3	1	21.63	1.12
-6	3	-1	15.37	0.91
6	3	1	22.14	1.08
-6	3	-1	14.34	1.09
6	3	1	21.72	1.17
-6	3	-1	16.13	0.96
-6	-3	1	20.89	1.10
-6	-3	-1	15.07	0.84
-6	-3	-1	15.75	0.84
6	-3	1	20.69	1.32
6	-3	1	20.53	1.19
-6	3	1	22.10	1.22
-6	3	1	22.40	1.26
6	3	-1	15.72	0.88
6	3	-1	14.78	0.82
-6	-3	-1	15.31	0.86
6	3	-1	13.30	1.05
-6	-3	-1	16.82	1.10
6	3	-1	15.10	0.86
6	-3	1	24.30	1.40
-6	3	1	21.08	1.33
-6	-3	-1	14.58	0.91
-6	-3	-1	15.32	0.84
6	-3	1	21.25	1.16
6	3	-1	14.61	0.90

6	3	-1	14.36	0.90
-6	3	1	20.36	1.31
6	-3	1	21.08	1.15
-6	-4	1	33.67	1.67
6	-4	-1	23.30	1.36
-6	4	-1	24.30	1.41
6	4	1	31.47	1.69
-6	-4	1	30.62	1.67
6	4	1	32.51	1.62
6	-4	-1	24.98	1.55
6	4	1	32.75	1.56
6	4	1	29.92	1.84
-6	4	-1	23.14	1.32
-6	4	-1	22.70	1.48
-6	4	-1	21.08	1.58
-6	-4	1	32.80	1.62
6	-4	-1	23.31	1.29
-6	4	-1	24.95	1.33
-6	-4	1	32.10	1.69
6	-4	-1	23.39	1.27
6	4	1	30.76	1.74
6	4	1	32.46	1.72
-6	-4	1	34.10	1.88
6	-4	-1	23.34	1.43
-6	-4	1	29.41	1.68
6	-4	1	32.54	1.75
6	4	-1	23.37	1.19
-6	4	1	33.61	1.70
6	-4	1	32.63	1.55
-6	-4	-1	22.12	1.26
-6	-4	-1	22.83	1.24
6	-4	1	32.41	1.66
-6	4	1	32.42	1.91
-6	-4	-1	24.42	1.30
6	-4	1	29.62	1.66
-6	-4	-1	24.05	1.50
-6	4	1	35.03	1.80
6	4	-1	24.30	1.32
6	4	-1	24.57	1.31
6	4	-1	25.31	1.28
6	-4	1	31.85	1.92
-6	-4	-1	22.28	1.21
-6	4	1	35.63	1.73
-6	4	1	30.74	1.84

6	4	-1	22.25	1.35
6	4	-1	22.83	1.48
6	-4	1	31.28	1.83
-6	-4	-1	22.01	1.31
6	-5	-1	45.86	2.38
-6	5	-1	46.62	2.72
6	-5	-1	44.96	2.37
-6	5	-1	50.37	2.44
6	-5	-1	49.42	2.68
-6	-5	1	45.78	2.40
6	5	1	42.19	2.18
-6	-5	1	43.53	2.19
-6	-5	1	41.43	2.17
-6	-5	1	41.03	2.13
6	5	1	39.00	2.35
6	5	1	42.03	2.29
-6	5	-1	51.94	2.55
6	5	1	43.52	2.02
-6	5	-1	52.36	2.42
6	-5	-1	47.25	2.54
6	-5	-1	46.66	2.49
6	5	1	42.09	2.07
-6	5	-1	45.14	2.59
-6	-5	1	42.67	2.06
-6	-5	1	43.28	2.20
6	5	-1	47.73	2.28
-6	-5	-1	49.61	2.62
-6	5	1	40.07	2.47
6	5	-1	44.83	2.52
-6	-5	-1	45.83	2.43
-6	-5	-1	46.36	2.39
6	-5	1	41.60	2.25
-6	5	1	43.38	2.19
6	5	-1	50.15	2.36
6	-5	1	40.69	2.13
-6	5	1	45.20	2.21
6	5	-1	50.06	2.45
6	-5	1	42.82	2.03
6	-5	1	43.71	2.47
-6	-5	-1	49.06	2.43
6	-5	1	36.85	2.13
-6	-5	-1	45.81	2.28
6	-5	1	43.50	2.06
6	5	-1	48.08	2.41

-6	5	1	42.09	2.35
6	-5	1	40.63	2.33
6	5	-1	44.20	2.59
-6	-5	-1	46.24	2.34
-6	5	1	44.80	2.32
6	-6	-1	119.99	5.89
6	6	1	123.53	6.17
6	-6	-1	120.30	5.88
6	6	1	128.89	5.81
6	6	1	126.71	5.85
-6	-6	1	126.83	5.83
-6	-6	1	121.88	5.99
-6	-6	1	122.36	6.13
-6	6	-1	122.25	6.11
-6	-6	1	130.98	6.02
6	-6	-1	129.24	5.74
-6	-6	1	124.56	5.94
6	-6	-1	126.95	6.03
6	6	1	125.95	5.97
-6	6	-1	127.90	5.94
6	-6	-1	133.64	5.76
6	6	1	119.59	6.12
6	-6	-1	135.17	6.24
-6	6	-1	135.25	6.08
-6	6	-1	135.19	5.92
-6	-6	1	130.91	6.17
-6	6	-1	116.76	6.22
6	-6	-1	125.12	6.02
-6	-6	1	127.04	5.93
6	6	-1	129.07	5.76
6	-6	1	126.83	5.92
6	6	-1	124.15	5.89
6	-6	1	133.79	6.30
6	6	-1	120.91	6.09
6	-6	1	133.16	5.86
6	6	-1	122.88	5.92
6	-6	1	125.44	6.10
-6	6	1	125.31	5.98
6	-6	1	120.03	5.95
-6	-6	-1	128.84	6.12
-6	6	1	135.19	5.99
-6	6	1	120.47	6.27
-6	-6	-1	121.53	5.95
-6	-6	-1	117.81	5.75

-6	-6	-1	126.52	5.96
-6	-6	-1	122.14	5.90
6	-6	1	133.12	5.84
-6	6	1	126.03	6.15
6	-6	1	123.22	6.05
-6	-6	-1	127.04	5.83
-6	6	1	136.15	6.14
6	-7	-1	96.98	4.82
6	7	1	100.47	5.20
-6	-7	1	103.28	5.21
-6	-7	1	103.67	4.82
6	-7	-1	102.15	5.42
-6	-7	1	99.71	5.02
-6	7	-1	109.64	4.88
-6	7	-1	100.82	5.26
-6	-7	1	98.92	4.96
-6	7	-1	110.41	5.10
6	7	1	108.10	4.82
-6	-7	1	103.94	4.93
-6	-7	1	100.99	5.16
6	-7	-1	99.25	5.00
6	-7	-1	109.64	4.74
6	7	1	108.73	5.35
6	7	1	99.65	4.98
-6	-7	1	102.54	5.07
6	-7	-1	96.63	4.86
-6	7	-1	105.08	4.93
6	7	1	99.23	5.20
6	7	1	104.43	4.84
6	-7	-1	99.78	5.01
-6	7	-1	100.98	5.12
-6	-7	-1	96.81	4.91
-6	7	1	108.94	5.00
6	7	-1	100.67	4.90
-6	7	1	106.46	5.02
-6	7	1	100.06	5.35
6	-7	1	110.73	4.90
6	7	-1	100.28	4.70
-6	-7	-1	101.76	4.95
-6	-7	-1	98.54	4.76
6	7	-1	95.50	5.08
6	-7	1	106.90	4.84
-6	-7	-1	107.96	5.13
6	-7	1	103.22	5.15

-6	-7	-1	96.82	4.69
6	-7	1	101.71	4.91
-6	7	1	101.69	5.20
-6	-7	-1	97.46	4.88
6	-7	1	101.01	5.09
6	-7	1	99.03	4.97
6	7	-1	104.69	4.79
-6	7	1	112.61	5.22
6	-7	1	107.19	5.56
-6	8	-1	80.56	3.82
-6	8	-1	79.91	4.08
-6	-8	1	75.93	3.97
6	-8	-1	82.56	3.67
6	8	1	78.89	4.27
6	-8	-1	71.43	3.93
-6	-8	1	80.59	4.22
-6	-8	1	78.09	4.03
6	-8	-1	84.03	3.69
6	-8	-1	77.53	3.99
6	-8	-1	81.91	4.30
-6	-8	1	82.38	4.22
6	-8	-1	76.25	3.75
-6	-8	1	79.25	3.79
-6	8	-1	69.49	4.22
-6	8	-1	77.96	4.12
6	8	1	79.57	3.81
6	8	1	84.32	4.18
-6	-8	1	77.62	4.01
6	-8	-1	73.94	3.82
-6	8	1	85.96	4.30
-6	-8	-1	72.71	3.85
6	-8	1	74.47	4.16
-6	-8	-1	76.74	4.08
6	-8	1	86.66	4.43
-6	-8	-1	71.16	3.61
6	8	-1	74.23	3.61
6	-8	1	75.82	3.87
6	8	-1	81.16	3.99
-6	8	1	77.45	4.43
-6	8	1	84.05	4.01
6	-8	1	79.99	3.98
6	8	-1	69.55	4.08
-6	-8	-1	75.53	3.68
-6	-8	-1	71.86	3.83

6	8	-1	76.55	3.72
6	-8	1	76.66	4.07
-6	8	1	77.16	4.20
6	-8	1	81.32	3.85
6	-8	1	79.83	4.11
6	-9	-1	4.92	0.86
-6	-9	1	4.98	0.93
6	9	1	3.88	0.72
6	9	1	4.61	0.80
-6	-9	1	5.12	0.97
-6	9	-1	3.92	1.11
6	-9	-1	4.52	1.32
6	-9	-1	4.26	0.77
6	-9	-1	4.42	0.71
-6	-9	1	4.81	0.68
-6	-9	1	5.60	0.98
6	9	1	4.08	1.04
-6	-9	1	6.44	1.09
6	-9	-1	3.84	0.85
6	-9	-1	5.59	1.03
6	9	1	5.42	1.06
-6	9	-1	3.90	1.13
-6	9	-1	4.76	0.83
6	-9	-1	3.79	0.69
-6	9	-1	3.86	1.03
6	-9	1	4.51	0.87
-6	-9	-1	3.46	0.63
6	-9	1	5.86	0.80
6	9	-1	3.61	1.04
-6	-9	-1	3.59	0.78
6	9	-1	4.08	0.73
6	-9	1	5.93	0.87
-6	9	1	5.89	0.90
6	9	-1	4.19	0.98
6	-9	1	4.92	0.71
-6	-9	-1	2.81	0.66
6	-9	1	4.57	1.30
-6	9	1	4.70	1.14
-6	-9	-1	4.18	0.89
-6	9	1	6.64	1.15
6	9	-1	4.65	0.59
-6	9	1	5.05	1.17
-6	-9	-1	4.77	0.76
-6	-10	1	11.16	0.92

6	10	1	11.86	1.65
-6	10	-1	7.81	1.40
6	-10	-1	5.72	1.19
-6	10	-1	8.53	1.32
6	-10	-1	8.31	1.16
-6	-10	1	10.33	1.32
-6	10	-1	10.27	1.73
-6	-10	1	7.88	1.31
6	10	1	9.45	1.61
6	10	1	11.17	1.05
-6	10	-1	6.37	1.45
6	10	1	9.63	1.03
6	-10	-1	9.64	1.17
6	-10	-1	10.58	1.75
6	-10	-1	7.53	0.97
-6	-10	1	10.06	1.30
-6	-10	1	11.97	1.53
6	-10	-1	7.80	0.99
6	-10	1	10.72	1.33
-6	-10	-1	6.90	1.26
-6	10	1	9.46	1.63
-6	10	1	12.39	1.71
-6	10	1	13.98	2.05
6	10	-1	9.26	0.88
6	-10	1	12.47	2.16
-6	-10	-1	6.43	0.95
-6	-10	-1	8.27	1.42
6	-10	1	8.87	1.20
6	10	-1	7.62	1.02
6	-10	1	11.39	1.12
6	10	-1	7.31	1.54
-6	-10	-1	8.38	1.22
6	-10	1	10.72	1.34
-6	-10	-1	7.67	0.91
6	10	-1	10.29	1.74
6	-10	1	10.50	1.02
-6	11	-1	2.84	1.22
6	-11	-1	3.09	0.97
6	11	1	1.97	1.33
6	-11	-1	3.34	0.86
6	-11	-1	6.46	1.73
6	-11	-1	4.28	0.75
6	-11	-1	4.65	0.83
-6	11	-1	5.75	1.69

6	11	1	3.53	0.83
-6	-11	1	3.84	1.01
-6	-11	1	4.81	1.17
-6	-11	1	3.24	0.57
6	-11	1	3.37	0.66
-6	-11	-1	4.48	1.15
6	-11	1	5.40	2.63
6	11	-1	4.78	1.42
-6	11	1	2.14	1.15
6	-11	1	2.75	0.79
6	-11	1	3.74	0.83
-6	11	1	2.39	1.43
-6	-11	-1	2.65	0.97
6	-11	1	3.84	0.98
-6	-11	-1	5.24	0.75
6	11	-1	5.22	0.86
-6	12	-1	6.54	1.49
-6	-12	1	6.66	1.14
-6	12	-1	6.28	3.01
6	-12	-1	4.39	1.02
6	12	1	6.15	1.91
6	-12	-1	4.86	1.21
6	-12	-1	5.20	0.85
-6	-12	1	7.67	1.62
-6	-12	-1	5.77	1.41
6	-12	1	7.48	1.00
6	-12	1	7.86	1.39
-6	12	1	8.64	1.64
-6	-12	-1	5.22	1.06
6	12	-1	2.19	1.64
6	-12	1	6.81	1.31
6	13	1	20.50	3.05
6	-13	-1	23.35	1.92
-6	-13	1	21.60	2.07
6	-13	-1	18.55	1.70
-6	13	-1	30.82	3.26
-6	-13	1	27.66	2.69
-6	13	1	27.70	2.91
6	-13	1	24.02	1.97
-6	-13	-1	22.46	2.58
6	13	-1	17.46	2.90
-6	-13	-1	22.32	2.00
-7	0	-1	0.00	0.18
7	0	1	0.10	0.10

-7	0	-1	0.14	0.16
-7	0	-1	-0.05	0.18
-7	0	1	0.04	0.18
7	0	-1	0.03	0.15
7	0	1	-0.08	0.15
-7	0	1	0.06	0.18
-7	0	1	0.19	0.25
7	0	-1	0.09	0.14
-7	0	-1	-0.06	0.10
-7	0	-1	-0.04	0.16
-7	0	1	0.06	0.21
7	0	-1	-0.01	0.09
7	0	-1	0.35	0.20
7	0	1	-0.16	0.22
-7	0	-1	0.03	0.20
7	0	-1	-0.04	0.24
-7	0	-1	0.09	0.23
7	0	1	-0.36	0.21
7	0	1	0.05	0.23
-7	0	1	0.31	0.27
7	1	1	16.43	0.94
7	-1	-1	18.89	0.96
-7	1	-1	16.55	0.96
7	-1	-1	16.55	1.06
7	1	1	16.46	0.83
7	1	1	17.77	0.91
-7	-1	1	16.26	0.92
7	-1	-1	16.97	0.97
-7	-1	1	18.14	0.94
-7	1	-1	16.09	1.07
7	1	1	16.09	0.94
-7	-1	1	14.84	0.90
-7	1	-1	17.95	0.95
-7	-1	1	16.72	1.01
7	-1	-1	17.05	0.91
7	-1	-1	17.28	1.00
7	1	1	16.09	1.01
-7	1	-1	17.90	1.04
7	-1	-1	18.32	0.95
-7	-1	1	17.22	1.05
-7	1	-1	16.05	1.04
-7	1	-1	18.10	0.98
7	-1	1	17.72	0.93
-7	1	1	17.66	0.95

7	-1	1	17.21	1.05
-7	-1	-1	17.03	0.99
-7	-1	-1	17.64	1.06
-7	-1	-1	17.87	1.00
7	-1	1	15.92	0.93
-7	1	1	16.09	1.03
7	-1	1	16.19	0.93
7	1	-1	16.82	1.06
-7	1	1	17.66	0.88
-7	-1	-1	18.47	0.98
7	-1	1	15.21	0.88
-7	-1	-1	16.17	0.95
-7	1	1	16.91	0.90
7	-1	1	16.34	1.04
7	1	-1	17.63	0.96
-7	1	1	18.35	0.94
7	2	1	3.94	0.61
7	2	1	5.13	0.44
-7	-2	1	5.06	0.43
7	-2	-1	9.99	0.62
-7	2	-1	9.30	0.83
-7	2	-1	8.15	0.82
-7	2	-1	9.15	0.66
7	-2	-1	9.17	0.76
7	2	1	4.69	0.39
7	-2	-1	8.87	0.64
7	2	1	4.67	0.45
-7	2	-1	9.24	0.68
-7	2	-1	9.44	0.56
-7	-2	1	4.64	0.47
-7	2	-1	9.40	0.66
-7	-2	1	4.71	0.42
7	-2	-1	9.42	0.64
7	-2	-1	8.64	0.83
-7	-2	1	5.47	0.54
-7	-2	1	4.79	0.73
7	-2	1	5.67	0.47
-7	-2	-1	9.49	0.64
7	2	-1	9.34	0.81
7	2	-1	9.44	0.67
7	-2	1	4.29	0.46
-7	-2	-1	9.50	0.51
-7	-2	-1	9.39	0.79
-7	-2	-1	9.02	0.65

-7	2	1	5.21	0.53
7	-2	1	4.04	0.68
-7	2	1	6.09	0.49
7	2	-1	9.03	0.53
-7	2	1	4.94	0.60
7	-2	1	4.84	0.41
-7	2	1	3.99	0.62
7	2	-1	9.00	0.54
7	-2	1	4.73	0.46
-7	2	1	5.58	0.55
-7	-2	-1	8.95	0.62
-7	-2	-1	8.86	0.62
-7	-2	-1	9.79	0.67
7	3	1	1.49	0.32
-7	3	-1	2.73	0.45
7	-3	-1	3.11	0.55
7	3	1	1.94	0.31
7	-3	-1	2.66	0.41
-7	-3	1	1.50	0.30
7	3	1	1.63	0.44
-7	-3	1	2.53	0.44
7	3	1	2.26	0.38
-7	-3	1	1.23	0.26
7	-3	-1	2.94	0.46
7	-3	-1	3.34	0.42
-7	-3	1	2.15	0.35
-7	-3	1	3.50	0.66
7	3	1	2.05	0.42
-7	3	-1	3.12	0.50
-7	-3	1	1.96	0.29
-7	3	-1	3.93	0.50
-7	3	-1	2.76	0.60
-7	3	-1	2.73	0.53
-7	3	1	3.00	0.50
-7	-3	-1	3.03	0.35
7	3	-1	3.28	0.61
-7	-3	-1	2.98	0.36
-7	3	1	1.95	0.47
7	-3	1	1.78	0.34
-7	-3	-1	2.58	0.43
7	3	-1	2.90	0.47
7	-3	1	2.35	0.51
7	3	-1	3.27	0.46
7	-3	1	2.16	0.38

-7	3	1	2.50	0.42
7	3	-1	3.02	0.38
-7	-3	-1	2.80	0.38
-7	-3	-1	2.46	0.41
7	3	-1	2.60	0.40
7	3	-1	2.41	0.40
-7	3	1	2.78	0.53
-7	3	1	2.93	0.47
-7	-3	-1	3.60	0.62
7	-3	1	2.90	0.38
-7	-4	1	22.45	1.28
-7	-4	1	20.96	1.14
-7	4	-1	20.06	1.49
7	4	1	20.84	1.27
-7	4	-1	19.64	1.39
7	-4	-1	22.75	1.47
-7	-4	1	23.87	1.49
7	4	1	22.71	1.31
-7	-4	1	23.41	1.16
7	4	1	21.87	1.17
7	4	1	19.69	1.41
7	4	1	21.46	1.10
-7	-4	1	22.45	1.18
-7	4	-1	19.80	1.28
-7	4	-1	20.74	1.22
-7	-4	1	21.46	1.25
7	-4	-1	18.54	1.35
7	-4	-1	20.70	1.20
7	4	1	21.52	1.25
7	-4	-1	21.80	1.18
-7	4	-1	21.48	1.24
7	-4	-1	21.97	1.28
-7	-4	-1	20.76	1.15
7	-4	1	19.81	1.21
-7	4	1	22.03	1.35
-7	-4	-1	19.59	1.21
-7	-4	-1	20.09	1.12
-7	4	1	22.69	1.50
7	4	-1	21.22	1.19
7	4	-1	21.63	1.21
7	4	-1	20.41	1.10
7	-4	1	19.64	1.42
7	4	-1	18.68	1.38
-7	4	1	24.91	1.28

7	-4	1	19.51	1.26
7	4	-1	20.50	1.24
7	-4	1	21.28	1.50
7	-4	1	19.51	1.20
7	4	-1	22.15	1.18
-7	-4	-1	20.83	1.16
-7	4	1	23.11	1.30
-7	4	1	19.19	1.41
-7	-4	-1	20.39	1.14
-7	-4	-1	21.32	1.45
-7	-5	1	7.13	0.66
7	-5	-1	8.43	0.98
-7	5	-1	10.33	1.20
-7	-5	1	8.36	0.75
7	5	1	6.70	0.94
-7	-5	1	8.44	0.83
7	-5	-1	8.15	0.99
7	-5	-1	8.25	0.80
-7	5	-1	8.63	0.89
-7	-5	1	9.92	0.64
-7	-5	1	8.07	0.75
-7	5	-1	8.23	0.81
7	5	1	8.32	0.66
7	5	1	8.91	0.77
7	5	1	8.37	0.58
-7	5	-1	8.97	0.81
7	5	1	6.74	0.77
-7	5	-1	7.60	0.99
7	-5	-1	8.72	0.70
-7	-5	1	8.50	1.04
7	-5	-1	7.39	0.69
7	5	1	7.94	0.84
7	5	-1	8.83	0.62
-7	-5	-1	7.85	0.70
-7	-5	-1	8.28	1.05
-7	-5	-1	8.98	0.72
-7	5	1	8.68	1.09
7	5	-1	10.00	0.72
7	-5	1	7.99	0.79
7	-5	1	7.72	1.12
7	5	-1	6.91	0.84
7	-5	1	8.81	0.61
7	5	-1	8.92	0.77
7	5	-1	7.98	0.98

-7	5	1	8.58	0.83
7	-5	1	7.50	0.66
-7	5	1	8.60	0.76
7	-5	1	7.31	0.68
-7	5	1	6.23	0.93
7	5	-1	7.26	0.82
7	-5	1	7.24	0.99
-7	5	1	9.82	0.94
-7	-5	-1	8.66	0.77
-7	-5	-1	8.72	0.75
7	-6	-1	4.57	0.78
-7	6	-1	6.50	0.97
-7	-6	1	3.79	0.75
-7	6	-1	6.35	0.83
-7	-6	1	5.33	0.83
-7	-6	1	6.68	0.58
-7	6	-1	6.26	1.07
-7	6	-1	5.93	1.18
-7	-6	1	5.53	1.05
7	6	1	5.01	0.80
7	6	1	6.83	0.64
7	-6	-1	6.59	1.09
7	6	1	5.20	0.75
-7	-6	1	4.98	0.69
-7	6	-1	7.68	0.87
-7	-6	1	4.20	0.65
7	-6	-1	7.41	0.70
7	-6	-1	6.03	1.03
7	-6	-1	6.98	0.69
7	6	1	4.53	0.78
7	6	1	5.36	0.88
7	-6	1	4.71	0.75
-7	6	1	5.72	0.78
-7	-6	-1	4.83	0.83
-7	6	1	5.08	1.01
-7	6	1	4.81	0.98
7	-6	1	6.06	0.92
-7	-6	-1	5.37	0.72
7	-6	1	5.01	0.59
-7	-6	-1	6.78	0.69
-7	6	1	5.20	0.95
-7	-6	-1	6.37	0.74
-7	-6	-1	6.18	0.81
-7	6	1	5.18	0.81

7	-6	1	4.45	0.88
7	-6	1	5.70	0.65
7	-6	1	5.45	0.62
7	6	-1	6.19	0.77
7	-6	1	6.35	0.57
7	6	-1	6.23	0.85
7	6	-1	4.59	0.81
7	6	-1	6.86	0.59
7	6	-1	6.32	0.72
-7	-7	1	24.97	1.86
-7	-7	1	26.57	1.69
7	-7	-1	27.26	2.06
-7	7	-1	28.73	1.63
7	-7	-1	24.95	1.50
-7	7	-1	28.31	1.83
7	7	1	28.26	1.51
-7	-7	1	27.18	1.62
7	7	1	26.50	1.90
-7	7	-1	25.98	1.89
7	7	1	26.85	1.68
7	-7	-1	26.56	1.81
-7	-7	1	27.41	1.99
7	-7	-1	23.73	1.51
-7	-7	1	25.85	1.73
-7	7	-1	24.27	1.64
7	7	1	25.02	1.83
-7	-7	1	25.88	1.49
-7	-7	1	26.50	1.65
7	-7	-1	24.15	1.61
7	7	-1	22.60	1.76
7	-7	1	31.14	2.17
7	7	-1	23.14	1.51
-7	-7	-1	24.41	1.89
7	-7	1	29.46	1.53
-7	-7	-1	23.65	1.43
7	-7	1	26.85	1.56
-7	-7	-1	24.93	1.60
-7	-7	-1	25.70	1.65
7	-7	1	27.54	1.64
7	7	-1	26.80	1.44
-7	7	1	27.55	2.20
7	7	-1	25.78	1.60
-7	7	1	28.31	1.91
7	-7	1	25.74	1.93

-7	7	1	28.01	1.96
-7	7	1	26.89	1.71
7	-7	1	27.04	1.75
-7	-7	-1	23.94	1.55
-7	7	1	30.45	1.76
-7	8	-1	12.88	1.21
7	-8	-1	11.26	1.19
7	-8	-1	10.46	1.43
-7	-8	1	10.28	0.89
7	-8	-1	11.47	1.02
-7	8	-1	12.88	1.48
7	-8	-1	12.07	0.92
-7	-8	1	10.76	1.12
-7	-8	1	10.43	1.07
7	8	1	12.71	1.55
-7	-8	1	11.12	1.30
7	-8	-1	12.25	0.87
-7	-8	1	10.33	1.04
7	8	1	11.31	0.88
7	-8	-1	12.60	1.00
7	8	1	9.74	0.92
7	-8	-1	13.93	1.83
-7	8	-1	10.83	1.44
7	8	1	8.06	1.29
-7	8	-1	11.90	1.50
7	-8	1	9.80	0.91
-7	8	1	10.53	1.42
-7	-8	-1	11.33	1.12
7	8	-1	12.60	0.98
-7	8	1	10.17	1.50
-7	8	1	12.77	1.24
7	-8	1	11.70	0.86
7	8	-1	10.90	1.39
7	-8	1	11.64	1.01
-7	-8	-1	10.97	0.95
7	-8	1	13.69	1.80
7	-8	1	11.86	1.02
-7	8	1	7.39	1.42
-7	-8	-1	11.21	1.11
7	8	-1	11.41	0.91
7	-8	1	12.93	1.49
7	-8	1	10.71	1.14
7	-9	-1	3.91	0.74
-7	9	-1	3.93	1.06

-7	-9	1	4.53	0.99
7	-9	-1	2.88	0.79
7	9	1	4.67	1.04
7	9	1	4.26	0.78
7	-9	-1	3.58	1.23
-7	9	-1	3.56	1.18
-7	9	-1	4.38	0.89
7	9	1	1.51	0.98
7	-9	-1	3.85	0.69
-7	-9	1	2.86	0.70
-7	9	-1	4.33	1.18
7	-9	-1	4.50	0.81
-7	-9	1	4.25	0.80
-7	-9	1	3.21	0.54
-7	-9	-1	3.17	0.77
7	9	-1	3.99	1.01
7	-9	1	3.45	0.61
-7	9	1	2.82	0.82
-7	-9	-1	4.76	0.82
7	-9	1	4.24	0.65
7	-9	1	2.26	0.59
7	9	-1	4.40	0.60
7	-9	1	5.31	0.88
-7	9	1	3.38	1.22
-7	9	1	3.76	1.02
7	9	-1	3.37	1.09
7	-9	1	3.18	0.74
-7	-9	-1	3.86	0.97
7	-9	1	3.91	1.36
7	9	-1	4.94	0.72
-7	-9	-1	3.98	0.62
-7	9	1	3.90	1.00
7	10	1	3.16	1.05
-7	-10	1	1.84	0.98
-7	-10	1	0.97	0.65
-7	10	-1	1.88	1.38
7	10	1	2.28	0.61
7	-10	-1	2.63	1.40
-7	-10	1	2.42	0.94
7	-10	-1	1.79	0.59
7	-10	-1	2.23	0.76
7	-10	-1	2.95	0.75
7	10	1	1.66	1.05
-7	-10	1	2.89	0.54

7	-10	-1	2.23	0.63
7	-10	-1	2.66	0.73
7	-10	1	3.30	0.78
7	10	-1	2.74	0.56
-7	10	1	0.07	1.16
7	10	-1	2.36	1.09
7	10	-1	3.55	0.67
7	-10	1	1.28	0.66
-7	-10	-1	3.08	0.59
7	-10	1	1.60	0.72
7	10	-1	2.79	1.02
-7	-10	-1	2.01	0.97
7	-10	1	2.32	0.61
-7	10	1	2.21	1.06
7	-10	1	0.97	1.45
7	-10	1	1.94	0.58
-7	-10	-1	1.40	0.73
-7	-11	1	6.22	0.83
-7	-11	1	5.45	1.05
7	-11	-1	4.51	0.76
-7	11	-1	5.58	1.44
7	11	1	4.38	1.48
-7	11	-1	3.45	1.53
-7	-11	1	6.88	1.35
7	-11	-1	3.27	0.98
7	-11	-1	3.72	0.91
7	-11	1	7.17	1.31
-7	-11	-1	4.65	0.74
-7	11	1	5.11	1.43
-7	-11	-1	6.20	1.36
7	11	-1	4.52	1.40
-7	-11	-1	4.28	1.09
7	-11	1	5.78	1.16
-7	11	1	5.76	1.43
7	11	-1	6.13	0.83
7	-11	1	7.27	1.06
7	-11	1	7.39	0.98
-7	12	-1	2.96	1.48
7	-12	-1	4.47	0.98
7	12	1	2.20	1.77
-7	-12	1	2.79	1.52
7	-12	-1	3.81	0.79
7	-12	-1	4.16	1.12
-7	-12	1	1.81	0.91

7	-12	1	3.84	0.94
-7	-12	-1	2.44	0.91
-7	12	1	2.52	1.50
7	-12	1	3.66	1.06
7	12	-1	2.42	1.74
-7	-12	-1	4.27	1.50
7	-12	1	3.75	0.76
7	-13	-1	3.98	0.95
-7	13	-1	4.44	2.02
-7	-13	1	5.06	1.45
-7	-13	1	4.85	1.41
7	-13	-1	4.41	1.00
-7	13	1	3.80	1.97
-7	-13	-1	5.89	1.60
7	13	-1	6.13	1.82
7	-13	1	4.34	1.01
-7	-13	-1	2.36	1.36
8	0	1	409.18	17.68
8	0	-1	424.86	19.69
8	0	-1	426.96	19.70
-8	0	1	380.16	17.74
-8	0	1	408.81	17.70
8	0	1	397.36	17.70
8	0	1	365.01	17.66
-8	0	1	392.54	17.66
-8	0	1	395.43	17.73
-8	0	-1	456.91	19.61
-8	0	1	408.32	17.75
-8	0	-1	462.17	19.74
8	0	1	368.30	17.57
8	0	1	406.77	17.71
-8	0	-1	421.37	19.70
8	0	-1	459.40	19.65
8	0	-1	429.72	19.58
-8	0	-1	423.61	19.70
-8	0	-1	436.33	19.73
-8	0	-1	448.86	19.77
8	1	1	147.65	7.06
8	-1	-1	143.56	6.42
8	-1	-1	139.30	6.52
8	1	1	147.01	6.94
-8	1	-1	145.54	6.47
-8	1	-1	136.40	6.54
8	-1	-1	138.23	6.45

-8	1	-1	143.05	6.38
8	1	1	165.36	7.02
8	-1	-1	138.89	6.45
-8	1	-1	148.07	6.46
-8	-1	1	151.64	7.07
8	-1	-1	135.78	6.37
-8	-1	1	154.88	7.04
8	1	1	151.09	7.13
-8	-1	1	157.05	7.14
-8	1	-1	143.93	6.46
-8	-1	1	143.08	7.02
-8	1	-1	145.55	6.42
8	-1	-1	139.59	6.43
-8	-1	1	161.25	7.12
-8	1	1	154.08	7.06
-8	1	1	156.93	7.02
-8	-1	-1	144.02	6.53
8	-1	1	157.51	7.04
-8	-1	-1	133.76	6.43
8	1	-1	137.36	6.53
8	1	-1	144.74	6.30
-8	-1	-1	138.42	6.46
8	-1	1	155.64	7.15
-8	1	1	151.19	7.15
8	-1	1	155.27	7.06
8	-1	1	150.83	7.00
8	-1	1	147.32	7.06
-8	1	1	158.20	7.02
-8	-1	-1	140.65	6.46
-8	-1	-1	140.73	6.46
8	-1	1	158.93	7.13
-8	1	1	158.21	7.08
8	1	-1	135.80	6.44
-8	-1	-1	135.92	6.45
-8	1	1	157.09	6.97
-8	2	-1	49.19	2.25
8	2	1	59.74	3.09
8	2	1	58.69	2.86
8	2	1	63.92	2.91
-8	2	-1	47.58	2.27
-8	2	-1	45.27	2.17
-8	-2	1	59.66	2.92
-8	-2	1	64.15	3.11
8	-2	-1	43.82	2.22

8	-2	-1	45.70	2.35
8	-2	-1	45.06	2.36
-8	2	-1	43.52	2.39
-8	-2	1	63.75	3.01
-8	2	-1	46.13	2.38
-8	2	-1	48.21	2.28
-8	-2	1	61.91	2.97
-8	-2	1	61.26	2.92
8	-2	-1	44.04	2.23
-8	-2	1	59.94	2.95
8	-2	-1	46.90	2.15
-8	-2	1	59.25	2.94
-8	2	1	61.34	2.84
-8	-2	-1	45.29	2.08
-8	-2	-1	44.85	2.24
8	-2	1	66.44	3.12
8	2	-1	46.27	2.12
-8	2	1	67.08	3.01
8	-2	1	64.52	2.89
-8	2	1	59.82	3.09
-8	-2	-1	44.75	2.26
8	-2	1	61.65	2.96
-8	-2	-1	45.14	2.07
-8	-2	-1	47.43	2.40
8	-2	1	59.72	2.94
-8	-2	-1	41.97	2.24
8	-2	1	59.98	3.07
8	2	-1	45.38	2.12
-8	-2	-1	47.85	2.25
-8	2	1	63.57	2.96
-8	2	1	62.81	2.94
8	-2	1	57.96	2.95
-8	2	1	60.64	3.03
-8	-2	-1	45.33	2.23
8	2	-1	43.74	2.39
8	3	1	6.65	0.57
-8	3	-1	6.38	0.62
8	-3	-1	6.48	0.80
8	-3	-1	5.32	0.55
-8	-3	1	5.77	0.81
-8	-3	1	5.81	0.52
8	3	1	7.13	0.60
-8	-3	1	6.11	0.37
-8	-3	1	6.97	0.70

-8	-3	1	5.79	0.56
-8	3	-1	5.35	0.79
-8	-3	1	6.24	0.36
8	-3	-1	5.83	0.59
8	-3	-1	6.56	0.54
-8	3	-1	6.65	0.63
-8	3	-1	6.83	0.83
-8	-3	1	6.81	0.61
8	3	1	5.63	0.52
8	3	1	6.10	0.77
-8	3	-1	7.60	0.67
8	-3	1	5.56	0.55
-8	-3	-1	6.99	0.59
-8	-3	-1	6.51	0.54
-8	3	1	5.07	0.76
-8	-3	-1	5.46	0.57
-8	-3	-1	6.86	0.44
8	-3	1	6.31	0.81
-8	3	1	6.60	0.82
-8	-3	-1	6.33	0.82
8	3	-1	5.55	0.76
-8	-3	-1	6.38	0.63
8	-3	1	6.43	0.59
8	-3	1	5.78	0.55
-8	-3	-1	6.29	0.45
-8	3	1	6.98	0.64
8	3	-1	5.97	0.53
-8	3	1	6.76	0.66
8	3	-1	6.51	0.52
-8	3	1	6.99	0.62
8	3	-1	6.74	0.54
-8	-3	-1	6.37	0.58
8	-4	-1	12.55	1.13
-8	4	-1	12.35	1.21
-8	-4	1	18.69	1.00
8	-4	-1	14.96	0.95
8	4	1	16.33	1.32
-8	-4	1	18.46	1.13
8	4	1	18.44	1.13
-8	4	-1	15.57	1.08
8	4	1	16.54	1.13
-8	4	-1	13.74	1.19
8	4	1	17.88	1.14
-8	-4	1	19.00	1.16

8	-4	-1	16.63	0.93
-8	-4	1	17.41	0.98
-8	-4	1	18.59	1.37
-8	4	-1	14.91	1.01
-8	-4	1	21.25	1.07
8	-4	-1	13.54	1.20
8	-4	-1	13.69	0.99
8	4	1	18.75	1.08
-8	4	-1	15.83	1.00
8	-4	1	18.38	1.16
8	4	-1	14.46	0.94
8	-4	1	17.55	1.09
-8	-4	-1	14.83	0.91
8	4	-1	14.77	0.86
8	-4	1	15.91	1.39
-8	4	1	19.31	1.26
-8	-4	-1	14.68	1.20
-8	-4	-1	13.83	0.84
-8	-4	-1	14.24	0.93
-8	-4	-1	13.31	0.96
8	4	-1	12.95	1.17
-8	4	1	19.37	1.20
-8	4	1	17.41	1.40
8	-4	1	17.87	1.10
8	-4	1	18.12	1.33
-8	4	1	20.55	1.37
-8	-4	-1	13.65	0.85
8	4	-1	12.61	0.97
8	4	-1	13.48	1.16
-8	4	1	21.52	1.18
-8	-4	-1	14.70	0.82
8	4	-1	16.70	0.98
-8	-5	1	70.04	3.49
-8	-5	1	71.46	3.36
8	5	1	72.17	3.47
-8	5	-1	99.32	4.25
8	5	1	71.31	3.37
-8	-5	1	70.16	3.38
-8	5	-1	85.05	4.43
-8	-5	1	70.96	3.68
-8	5	-1	87.32	4.53
8	-5	-1	82.52	4.19
8	5	1	74.40	3.51
-8	-5	1	69.26	3.44

8	5	1	69.63	3.55
8	-5	-1	89.39	4.48
-8	-5	1	69.24	3.36
8	5	1	68.68	3.68
-8	5	-1	94.23	4.25
8	5	1	72.12	3.28
-8	5	-1	94.33	4.33
8	-5	-1	85.07	4.34
8	-5	-1	80.81	4.16
8	-5	-1	88.49	4.29
-8	-5	-1	88.31	4.14
-8	5	1	75.68	3.60
8	-5	1	75.09	3.78
-8	-5	-1	87.81	4.15
8	5	-1	91.79	4.21
-8	-5	-1	82.20	4.23
8	5	-1	87.43	4.20
8	5	-1	80.88	4.41
-8	-5	-1	87.81	4.42
-8	-5	-1	88.97	4.15
8	-5	1	69.37	3.53
8	5	-1	88.98	4.08
8	5	-1	84.85	4.28
8	-5	1	63.67	3.42
-8	5	1	71.08	3.67
-8	-5	-1	85.67	4.08
8	-5	1	70.23	3.45
8	-5	1	71.76	3.64
-8	5	1	71.19	3.47
-8	5	1	64.89	3.71
-8	5	1	77.10	3.52
-8	6	-1	96.60	4.33
8	6	1	79.88	4.22
-8	-6	1	81.28	3.94
-8	6	-1	94.03	4.35
8	-6	-1	83.14	4.25
-8	-6	1	81.39	3.85
8	-6	-1	84.91	4.43
8	6	1	82.39	4.06
-8	-6	1	80.68	3.89
8	6	1	78.43	3.97
-8	-6	1	79.92	4.21
-8	-6	1	80.89	4.01
-8	6	-1	87.42	4.57

8	-6	-1	84.71	4.38
-8	-6	1	81.19	3.94
8	6	1	78.30	4.11
8	6	1	83.12	3.86
8	-6	-1	97.58	4.66
-8	6	-1	88.36	4.69
-8	6	-1	95.32	4.46
8	-6	-1	89.33	4.28
8	-6	1	86.32	4.38
-8	6	1	78.83	4.34
8	-6	1	77.87	3.93
-8	6	1	85.59	4.16
-8	-6	-1	89.74	4.55
8	6	-1	82.55	4.42
8	-6	1	80.75	4.15
-8	6	1	86.17	4.03
8	6	-1	79.56	4.24
8	6	-1	88.49	4.34
8	-6	1	79.90	3.96
8	6	-1	87.75	4.30
-8	6	1	82.95	4.00
-8	-6	-1	91.08	4.27
-8	-6	-1	85.16	4.33
-8	-6	-1	88.79	4.25
8	-6	1	80.62	4.07
-8	-6	-1	88.35	4.21
-8	6	1	83.64	4.22
8	6	-1	92.81	4.20
8	7	1	169.55	8.21
-8	-7	1	164.71	8.21
-8	-7	1	166.43	7.93
8	-7	-1	146.56	7.29
-8	-7	1	172.19	7.93
8	-7	-1	163.06	7.60
-8	7	-1	168.32	7.40
8	7	1	165.43	8.00
8	-7	-1	150.04	7.16
8	-7	-1	142.47	7.15
-8	-7	1	166.34	7.85
8	7	1	173.30	7.86
-8	-7	1	168.79	8.23
8	-7	-1	150.54	7.33
-8	7	-1	151.23	7.64
-8	7	-1	169.41	7.22

8	7	1	176.90	8.13
-8	-7	1	159.11	8.03
8	7	1	163.23	8.25
-8	7	-1	150.97	7.22
-8	-7	1	170.63	8.01
-8	7	-1	150.79	7.47
8	-7	1	167.92	7.96
8	-7	1	174.90	8.14
-8	-7	-1	150.60	7.14
8	7	-1	150.29	7.19
8	-7	1	165.75	8.17
8	7	-1	161.51	7.27
-8	7	1	172.42	8.27
-8	-7	-1	147.05	7.17
-8	-7	-1	148.28	7.23
8	-7	1	189.95	8.45
-8	-7	-1	148.92	7.42
8	7	-1	162.58	7.04
-8	-7	-1	147.73	7.07
8	-7	1	187.33	7.82
-8	7	1	185.67	8.06
-8	7	1	185.29	8.23
8	7	-1	153.55	7.00
-8	7	1	170.77	8.40
8	-7	1	164.51	7.99
-8	7	1	162.94	8.02
8	7	-1	146.81	7.38
-8	-8	1	70.33	3.91
8	-8	-1	77.21	3.88
-8	8	-1	82.59	4.31
8	8	1	73.47	3.49
-8	-8	1	70.96	3.47
-8	-8	1	72.94	3.72
-8	-8	1	72.32	3.62
-8	8	-1	76.17	4.38
-8	-8	1	69.81	3.56
8	-8	-1	81.71	4.40
8	-8	-1	79.24	3.91
8	8	1	71.68	3.95
-8	8	-1	82.96	4.22
8	8	1	70.22	3.91
-8	8	-1	79.92	3.96
8	-8	-1	74.98	4.05
8	-8	-1	82.04	4.51

8	-8	1	69.52	3.57
-8	8	1	77.18	3.74
-8	8	1	72.07	3.99
8	8	-1	67.12	3.99
8	-8	1	69.09	3.63
8	-8	1	75.64	3.43
8	-8	1	78.20	4.18
8	8	-1	80.17	3.71
-8	-8	-1	76.38	3.89
8	-8	1	69.64	3.78
-8	-8	-1	77.12	3.78
-8	8	1	65.00	4.33
8	8	-1	81.82	3.75
8	8	-1	76.94	4.19
-8	-8	-1	75.94	3.99
-8	8	1	75.77	3.92
-8	9	-1	45.05	2.95
8	-9	-1	42.32	2.44
8	-9	-1	41.96	2.37
-8	-9	1	37.90	2.19
-8	9	-1	41.77	3.17
8	9	1	36.76	2.65
8	-9	-1	42.53	2.60
-8	-9	1	39.64	2.58
-8	-9	1	36.23	2.26
-8	-9	1	37.62	2.33
8	9	1	38.72	2.08
-8	9	-1	44.77	2.52
8	-9	-1	51.24	3.25
-8	-9	1	37.86	2.03
8	9	-1	43.99	2.20
8	-9	1	41.80	2.04
8	9	-1	44.15	2.26
8	9	-1	39.36	2.76
8	-9	1	43.12	2.11
-8	-9	-1	41.52	2.46
-8	-9	-1	42.37	2.28
8	-9	1	38.25	2.41
8	-9	1	36.95	2.15
8	-9	1	41.39	3.04
-8	9	1	37.30	2.88
-8	-9	-1	42.48	2.53
-8	9	1	39.57	2.67
8	-9	1	37.39	2.25

-8	-9	-1	41.58	2.74
8	10	1	13.01	1.92
-8	10	-1	11.88	2.09
-8	-10	1	11.04	1.75
8	10	1	9.98	1.79
-8	-10	1	11.00	1.41
8	-10	-1	9.07	1.35
8	10	1	11.86	2.33
-8	10	-1	10.22	1.87
8	-10	-1	11.52	2.16
8	-10	-1	8.05	1.07
-8	-10	1	11.07	0.99
8	-10	-1	10.65	1.22
8	-10	1	11.19	1.23
8	10	-1	7.96	1.54
-8	-10	-1	10.13	1.32
-8	10	1	12.04	1.78
-8	-10	-1	8.67	1.02
8	10	-1	10.58	1.09
8	-10	1	11.78	1.11
8	-10	1	11.99	1.10
8	10	-1	11.77	0.95
8	-10	1	11.29	1.41
-8	10	1	9.63	2.01
8	10	-1	11.07	2.03
-8	-10	-1	10.29	1.72
8	-10	1	13.82	1.71
8	-10	1	12.83	2.36
-8	11	-1	2.98	1.59
8	-11	-1	3.54	0.73
-8	11	-1	2.53	1.28
8	-11	-1	2.69	0.92
8	11	1	1.53	1.20
8	-11	-1	1.83	0.94
-8	-11	1	4.38	1.17
-8	-11	1	3.10	1.01
8	-11	1	2.89	0.96
8	-11	1	3.12	0.88
-8	11	1	0.04	2.06
-8	11	1	2.47	1.35
-8	-11	-1	2.57	1.12
8	11	-1	2.66	1.32
-8	-11	-1	4.10	0.69
-8	-11	-1	2.32	0.99

8	-12	-1	8.27	1.26
8	-12	-1	8.17	1.40
-8	12	-1	6.55	1.75
-8	-12	1	8.35	1.58
-8	-12	1	9.18	1.49
8	12	1	8.91	1.82
8	-12	-1	7.88	2.15
8	-12	1	7.52	1.16
-8	12	1	11.47	2.29
8	-12	1	9.35	1.24
-8	-12	-1	7.02	1.16
-8	-12	-1	9.75	1.69
8	12	-1	13.11	2.60
8	-12	1	8.12	1.65
9	0	1	-0.15	0.21
9	0	1	-0.03	0.31
-9	0	1	0.37	0.28
-9	0	1	0.22	0.31
-9	0	-1	-0.10	0.31
-9	0	-1	0.14	0.30
-9	0	1	0.41	0.32
9	0	1	0.38	0.27
9	0	-1	0.13	0.12
9	0	1	0.09	0.13
9	0	-1	0.27	0.28
-9	0	-1	-0.01	0.25
-9	0	-1	0.07	0.27
-9	0	-1	0.08	0.20
9	0	-1	0.39	0.23
-9	0	-1	0.16	0.17
9	0	-1	-0.10	0.21
-9	0	1	0.28	0.38
9	0	-1	0.02	0.21
-9	0	1	-0.03	0.25
-9	0	-1	0.04	0.13
-9	1	-1	6.11	0.58
-9	1	-1	6.71	0.61
9	1	1	7.72	0.47
-9	-1	1	5.90	0.83
-9	1	-1	7.01	0.52
9	-1	-1	7.45	0.81
-9	-1	1	7.94	0.76
9	-1	-1	6.89	0.68
9	-1	-1	6.19	0.66

9	1	1	8.64	0.64
-9	1	-1	7.44	0.64
-9	-1	1	9.49	0.64
-9	1	-1	9.63	0.67
-9	1	-1	7.55	0.79
9	-1	-1	7.04	0.50
-9	-1	1	8.11	0.70
9	1	1	7.45	0.77
9	-1	-1	7.28	0.59
-9	-1	1	8.13	0.66
-9	-1	-1	6.77	0.66
9	-1	1	7.06	0.53
9	-1	1	7.10	0.80
-9	-1	-1	7.84	0.64
9	1	-1	8.41	0.63
-9	1	1	8.92	0.69
-9	-1	-1	7.00	0.69
9	1	-1	7.30	0.77
-9	1	1	9.07	0.79
9	1	-1	8.01	0.39
-9	1	1	7.80	0.54
-9	1	1	6.91	0.63
9	-1	1	7.91	0.61
-9	1	1	10.33	0.70
-9	1	1	7.97	0.65
-9	-1	-1	7.99	0.62
9	-1	1	8.67	0.77
-9	-1	-1	7.38	0.76
-9	-1	-1	7.67	0.62
-9	2	-1	7.08	0.69
-9	-2	1	8.37	0.63
9	2	1	9.50	0.65
-9	-2	1	6.63	0.63
-9	-2	1	8.02	0.75
-9	-2	1	9.40	0.63
9	2	1	7.27	0.49
-9	-2	1	7.21	0.84
9	-2	-1	9.29	0.64
9	2	1	6.38	0.64
9	-2	-1	7.70	0.85
-9	2	-1	9.62	0.60
-9	2	-1	7.63	0.81
9	-2	-1	7.27	0.59
9	-2	-1	7.60	0.60

9	-2	-1	7.57	0.85
-9	2	-1	7.13	0.64
-9	-2	1	9.10	0.66
-9	-2	1	7.61	0.61
9	2	1	6.50	0.76
-9	2	-1	9.12	0.90
9	-2	-1	8.22	0.65
-9	-2	-1	7.96	0.65
-9	2	1	6.68	0.77
9	2	-1	7.71	0.82
9	-2	1	7.18	0.63
9	2	-1	7.76	0.48
-9	2	1	7.49	0.64
-9	-2	-1	10.39	0.68
-9	2	1	7.17	0.52
-9	-2	-1	8.35	0.68
-9	2	1	7.04	0.82
9	-2	1	7.48	0.58
-9	2	1	7.62	0.69
9	2	-1	7.78	0.49
9	-2	1	7.47	0.59
-9	-2	-1	7.49	0.70
9	-2	1	8.20	0.62
9	-2	1	6.87	0.85
-9	-2	-1	7.69	0.84
-9	-3	1	4.55	0.57
-9	-3	1	4.36	0.47
9	3	1	2.50	0.55
9	-3	-1	7.19	0.63
-9	-3	1	4.31	0.67
9	3	1	3.90	0.53
9	-3	-1	7.29	0.88
9	-3	-1	8.04	0.61
9	3	1	2.96	0.43
-9	-3	1	2.59	0.59
-9	3	-1	6.63	0.68
9	-3	-1	6.36	0.61
-9	3	-1	9.01	0.91
-9	3	-1	7.00	0.71
-9	3	-1	5.73	0.92
9	-3	-1	4.46	0.74
-9	-3	-1	5.35	0.60
-9	-3	-1	6.31	0.88
9	-3	1	5.99	0.81

-9	-3	-1	6.05	0.46
9	-3	1	4.52	0.50
9	-3	1	3.67	0.49
-9	-3	-1	8.34	0.66
9	3	-1	5.73	0.51
-9	-3	-1	6.68	0.66
-9	-3	-1	7.34	0.67
-9	3	1	3.22	0.59
-9	3	1	3.24	0.59
-9	-3	-1	7.63	0.43
9	-3	1	2.94	0.47
-9	3	1	2.85	0.62
9	3	-1	6.20	0.82
9	3	-1	7.35	0.61
9	3	-1	5.83	0.53
9	-3	1	4.51	0.42
-9	-3	-1	9.18	0.66
-9	4	-1	45.08	2.30
-9	4	-1	47.36	2.54
9	-4	-1	44.11	2.31
-9	-4	1	52.95	2.68
-9	-4	1	53.89	2.48
-9	-4	1	51.63	2.86
-9	4	-1	50.33	2.33
9	4	1	58.54	2.61
9	-4	-1	44.50	2.45
-9	4	-1	44.19	2.34
9	-4	-1	43.20	2.23
9	-4	-1	45.29	2.28
-9	4	-1	39.70	2.50
-9	-4	1	52.94	2.68
9	4	1	54.68	2.66
9	4	1	49.35	2.85
9	4	1	48.79	2.64
-9	-4	1	50.84	2.50
-9	-4	1	56.24	2.60
-9	-4	-1	44.30	2.30
-9	4	1	53.06	2.88
9	4	-1	40.69	2.47
-9	-4	-1	43.12	2.13
9	-4	1	49.87	2.68
9	4	-1	38.42	2.24
9	4	-1	43.42	2.19
9	-4	1	50.89	2.65

-9	4	1	49.92	2.89
-9	-4	-1	43.89	2.20
-9	-4	-1	40.93	2.13
9	-4	1	55.94	2.86
-9	4	1	54.17	2.74
9	-4	1	51.10	2.91
9	4	-1	44.97	2.17
-9	-4	-1	42.74	2.48
-9	-4	-1	52.34	2.26
-9	-4	-1	44.13	2.12
-9	4	1	58.40	2.73
9	-4	1	51.79	2.64
-9	-4	-1	45.59	2.30
9	4	-1	46.41	2.27
-9	4	1	57.95	2.69
-9	-5	1	19.82	1.21
9	-5	-1	21.32	1.32
-9	5	-1	23.45	1.48
-9	-5	1	18.24	1.05
9	-5	-1	21.67	1.40
9	-5	-1	23.06	1.30
-9	-5	1	16.31	1.45
9	5	1	15.30	1.40
9	5	1	15.09	1.20
-9	-5	1	16.10	1.01
-9	5	-1	23.70	1.41
-9	5	-1	24.51	1.43
-9	5	-1	20.10	1.75
-9	5	-1	22.63	1.67
9	5	1	16.40	1.20
9	-5	-1	21.14	1.67
9	5	1	18.15	1.18
-9	-5	1	19.46	1.23
9	-5	-1	22.82	1.61
9	5	1	20.15	1.11
9	-5	1	16.82	1.59
9	5	-1	21.57	1.61
-9	-5	-1	20.33	1.14
-9	5	1	21.88	1.53
9	-5	1	17.69	1.45
-9	-5	-1	20.76	1.23
9	5	-1	23.70	1.24
9	5	-1	17.62	1.36
-9	-5	-1	21.95	1.24

-9	5	1	19.96	1.28
-9	5	1	15.38	1.48
-9	-5	-1	25.38	1.31
-9	5	1	18.65	1.32
9	-5	1	16.86	1.12
9	-5	1	18.49	1.13
-9	-5	-1	20.68	1.26
9	-5	1	17.25	1.21
-9	-5	-1	22.79	1.39
9	5	-1	21.22	1.38
-9	-5	-1	20.60	1.63
-9	-6	1	16.18	1.18
-9	-6	1	15.15	1.05
-9	6	-1	21.95	1.55
-9	-6	1	14.22	1.48
9	6	1	16.32	1.15
9	6	1	15.05	1.01
-9	6	-1	23.33	1.45
9	-6	-1	20.06	1.30
9	6	1	12.95	1.38
9	-6	-1	20.40	1.74
9	6	1	13.58	1.22
9	-6	-1	20.62	1.64
9	-6	-1	20.10	1.43
9	-6	-1	19.78	1.30
-9	-6	1	15.94	1.03
-9	6	-1	21.60	1.43
9	6	1	14.99	1.24
-9	6	-1	19.54	1.83
-9	-6	1	15.06	1.00
-9	-6	1	14.79	1.12
-9	6	-1	21.15	1.66
-9	-6	-1	20.99	1.70
9	-6	1	14.03	1.16
-9	-6	-1	21.86	1.27
-9	6	1	15.51	1.59
9	6	-1	18.16	1.62
9	6	-1	20.92	1.39
-9	-6	-1	21.47	1.28
-9	6	1	16.81	1.19
9	6	-1	18.27	1.45
-9	-6	-1	21.13	1.39
-9	6	1	16.66	1.45
9	6	-1	22.39	1.22

9	6	-1	20.66	1.19
9	-6	1	15.55	1.07
-9	-6	-1	20.27	1.30
9	-6	1	15.10	1.46
9	-6	1	17.75	1.61
-9	6	1	15.70	1.24
-9	6	1	15.71	1.32
9	-6	1	14.03	1.05
9	6	-1	20.95	1.36
9	7	1	36.36	2.16
9	-7	-1	29.37	1.89
9	7	1	32.57	2.16
-9	7	-1	30.07	1.88
9	7	1	32.08	2.26
9	7	1	33.96	1.83
-9	-7	1	34.63	1.95
-9	-7	1	32.52	2.24
9	-7	-1	31.79	2.32
9	-7	-1	28.82	1.73
-9	7	-1	30.50	2.33
9	-7	-1	28.56	2.07
-9	7	-1	27.95	2.13
-9	7	-1	30.66	2.02
-9	-7	1	32.44	2.01
9	-7	-1	27.28	1.73
-9	-7	1	34.80	1.87
-9	-7	1	32.60	1.79
-9	7	-1	33.55	1.90
-9	7	1	33.57	2.31
9	-7	1	33.93	2.07
-9	7	1	36.98	2.30
-9	-7	-1	29.94	1.84
-9	7	1	30.94	2.45
9	-7	1	35.59	2.42
9	-7	1	31.73	2.22
-9	-7	-1	28.41	1.67
9	-7	1	34.66	1.90
9	-7	1	33.04	1.93
9	7	-1	30.37	1.61
9	7	-1	28.69	1.98
-9	7	1	37.64	2.08
-9	-7	-1	27.95	1.71
9	7	-1	30.88	1.60
9	7	-1	32.18	1.93

-9	7	1	33.21	2.04
-9	-8	1	16.39	1.39
9	8	1	17.44	1.15
-9	-8	1	16.12	1.26
-9	-8	1	14.69	1.09
-9	-8	1	12.42	1.60
-9	-8	1	18.16	1.32
9	8	1	14.38	1.54
-9	8	-1	13.65	1.64
9	-8	-1	13.15	1.11
9	-8	-1	13.53	1.12
-9	8	-1	16.41	1.42
9	-8	-1	13.31	1.29
-9	8	-1	14.88	1.52
9	8	1	17.47	1.52
9	8	1	16.63	1.70
-9	8	-1	11.76	2.30
9	8	-1	12.56	1.53
-9	-8	-1	11.97	1.25
9	8	-1	13.94	1.38
-9	-8	-1	12.91	1.17
9	-8	1	15.96	1.20
9	-8	1	15.43	2.06
-9	-8	-1	14.12	1.07
-9	8	1	15.21	2.10
9	8	-1	14.45	0.97
-9	8	1	18.27	2.44
-9	8	1	16.42	1.45
9	-8	1	18.27	1.32
9	-8	1	16.32	1.39
9	8	-1	15.16	1.00
-9	8	1	17.72	1.76
-9	9	-1	11.55	1.95
9	-9	-1	11.94	1.10
9	-9	-1	12.67	1.17
9	9	1	12.46	1.64
-9	-9	1	12.03	1.21
9	-9	-1	15.60	2.15
-9	9	-1	9.24	2.15
9	-9	-1	10.48	1.29
-9	-9	1	13.09	1.01
-9	-9	1	12.10	1.26
-9	-9	1	14.82	1.64
9	9	1	13.74	1.08

-9	-9	1	12.60	1.30
-9	-9	-1	12.78	1.07
-9	-9	-1	13.09	1.35
9	-9	1	8.86	1.30
9	-9	1	14.60	1.22
9	9	-1	11.17	1.64
-9	-9	-1	12.50	1.25
9	9	-1	13.55	1.01
9	-9	1	10.99	2.20
-9	9	1	10.14	1.75
-9	9	1	10.49	2.09
9	-9	1	12.86	1.10
9	9	-1	13.12	1.45
-9	-9	-1	13.83	1.64
-9	-10	1	32.78	2.26
-9	10	-1	34.89	3.01
9	10	1	31.55	2.59
9	-10	-1	32.37	2.32
9	10	1	35.49	1.96
-9	-10	1	34.60	2.62
-9	10	-1	34.51	2.79
9	-10	-1	36.05	2.19
9	-10	-1	33.62	3.29
-9	-10	1	33.83	1.90
9	-10	1	33.01	2.05
9	10	-1	31.53	2.69
-9	10	1	38.98	2.77
-9	-10	-1	33.72	1.96
-9	-10	-1	35.85	2.29
9	-10	1	39.35	3.17
9	-10	1	31.56	2.30
-9	10	1	32.85	3.04
-9	-10	-1	35.44	2.59
9	-10	1	35.09	2.18
-9	-11	1	32.91	2.87
9	11	1	30.28	3.03
-9	-11	1	32.14	2.43
9	-11	-1	30.60	2.44
9	-11	-1	35.67	2.28
-9	11	-1	32.78	2.99
9	-11	-1	30.37	2.02
-9	-11	-1	32.07	1.92
9	-11	1	33.69	2.28
-9	-11	-1	32.85	2.84

-9	11	1	32.15	2.92
9	11	-1	31.52	2.90
9	-11	1	32.35	2.40
9	-11	1	31.54	1.99
-9	-11	-1	32.04	2.38
-9	-12	1	21.29	1.88
9	-12	-1	24.90	1.89
9	12	1	16.32	2.60
-9	12	-1	21.56	3.05
9	-12	-1	19.65	1.73
9	-12	-1	19.68	2.08
-9	-12	1	16.80	2.33
9	12	-1	19.74	2.74
-9	-12	-1	21.58	2.46
9	-12	1	17.98	1.61
9	-12	1	18.29	1.72
9	-12	1	20.14	1.97
-9	12	1	17.44	3.01
-9	-12	-1	21.33	1.96
-10	0	1	90.09	4.17
-10	0	1	89.94	4.21
-10	0	-1	84.15	3.96
-10	0	-1	80.97	3.98
-10	0	-1	79.72	3.89
-10	0	1	88.55	4.28
-10	0	-1	87.92	3.92
-10	0	1	81.85	4.15
-10	0	1	85.70	4.25
10	0	-1	77.40	3.73
10	0	-1	81.22	3.94
10	0	1	86.85	4.18
10	0	1	95.19	4.25
10	0	-1	79.37	3.87
10	0	1	86.01	4.05
10	0	1	89.71	4.23
-10	0	-1	84.08	3.91
-10	0	1	90.81	4.19
10	0	-1	83.87	3.82
10	-1	-1	69.59	3.30
-10	1	-1	66.79	3.15
-10	1	-1	67.04	3.17
10	1	1	65.48	3.15
-10	1	-1	69.69	3.21
10	1	1	62.05	2.98

-10	1	-1	64.96	3.17
10	-1	-1	62.89	3.18
-10	1	-1	67.46	3.33
-10	-1	1	61.93	3.23
10	1	1	68.58	3.09
10	-1	-1	61.80	3.07
-10	-1	1	61.77	3.18
-10	-1	1	64.45	3.23
-10	1	-1	65.76	3.07
-10	-1	1	62.25	3.10
-10	-1	1	68.08	3.14
-10	1	-1	72.27	3.21
-10	-1	1	63.02	3.12
10	-1	-1	61.63	3.17
10	1	1	62.12	3.22
10	-1	-1	65.44	3.16
10	1	-1	64.05	3.03
-10	1	1	62.43	3.10
-10	1	1	65.78	2.99
10	-1	1	64.28	3.12
-10	-1	-1	64.38	3.28
10	-1	1	69.40	3.24
-10	-1	-1	69.00	3.20
-10	-1	-1	67.47	3.18
-10	-1	-1	65.73	3.17
10	-1	1	68.25	3.25
10	-1	1	65.15	3.14
10	1	-1	62.42	2.98
10	1	-1	65.26	3.17
-10	1	1	67.68	3.28
-10	1	1	64.72	3.16
10	1	-1	61.68	3.28
-10	1	1	69.17	3.14
10	-1	1	60.49	3.04
-10	-1	-1	68.33	3.24
-10	-2	1	20.33	1.20
-10	2	-1	25.60	1.35
-10	-2	1	22.04	1.19
10	-2	-1	23.19	1.35
-10	-2	1	21.09	1.21
10	2	1	18.04	1.32
-10	2	-1	26.20	1.47
10	-2	-1	26.61	1.41
-10	2	-1	27.34	1.57

-10	2	-1	26.22	1.38
-10	-2	1	21.50	1.40
-10	2	-1	26.16	1.62
10	2	1	19.13	1.10
10	-2	-1	25.63	1.35
10	2	1	18.52	1.03
-10	-2	1	18.95	1.15
10	-2	-1	24.04	1.41
10	-2	-1	27.15	1.56
-10	2	-1	28.31	1.47
-10	-2	1	21.10	1.15
10	-2	-1	26.64	1.60
-10	2	1	19.21	1.13
-10	-2	-1	25.97	1.42
10	-2	1	20.38	1.38
10	2	-1	24.78	1.24
-10	-2	-1	25.81	1.40
-10	2	1	20.51	1.08
-10	2	1	21.72	1.38
-10	-2	-1	25.48	1.46
10	-2	1	19.82	1.13
10	2	-1	25.39	1.27
-10	-2	-1	24.81	1.60
-10	-2	-1	25.27	1.42
10	-2	1	19.46	1.16
-10	2	1	20.82	1.28
10	-2	1	19.09	1.17
10	2	-1	23.87	1.58
-10	2	1	20.35	1.23
-10	-2	-1	27.11	1.46
10	-2	1	19.74	1.39
10	-2	1	18.79	1.11
-10	-2	-1	25.07	1.41
-10	2	1	22.69	1.20
10	-3	-1	39.69	2.16
10	3	1	31.56	1.97
10	-3	-1	36.51	1.96
10	3	1	32.15	1.70
-10	-3	1	35.49	2.01
-10	3	-1	38.30	2.00
10	-3	-1	37.40	1.99
-10	3	-1	39.66	1.99
10	3	1	34.21	1.75
-10	-3	1	31.53	1.74

10	3	1	34.09	1.73
-10	-3	1	34.99	1.75
-10	-3	1	32.57	1.74
-10	-3	1	31.76	1.78
-10	-3	1	34.54	1.81
-10	3	-1	37.70	2.20
10	-3	-1	36.31	2.13
-10	3	-1	40.20	2.03
-10	-3	1	35.07	1.86
-10	3	-1	36.77	1.83
10	-3	-1	38.53	1.96
-10	3	-1	38.42	2.18
10	-3	1	32.57	1.77
10	-3	1	32.59	1.79
-10	3	1	35.41	1.82
-10	3	1	35.69	1.82
-10	-3	-1	36.84	1.99
-10	-3	-1	38.55	1.97
10	-3	1	32.84	1.78
-10	3	1	32.61	1.95
10	-3	1	34.51	1.97
-10	3	1	35.68	1.86
10	3	-1	36.43	2.16
10	3	-1	37.26	1.87
-10	3	1	32.93	1.99
10	-3	1	33.12	1.65
10	3	-1	38.17	1.85
-10	-3	-1	36.91	2.17
-10	-3	-1	37.92	2.00
-10	-3	-1	37.89	1.96
10	-3	1	34.73	2.03
-10	-3	-1	37.56	1.91
-10	4	-1	2.00	0.72
-10	-4	1	2.70	0.50
-10	-4	1	2.67	0.54
10	4	1	1.81	0.39
-10	4	-1	2.34	0.47
10	-4	-1	2.02	0.44
10	-4	-1	0.56	0.67
-10	4	-1	2.54	0.64
10	-4	-1	2.39	0.44
-10	4	-1	2.55	0.54
-10	-4	1	1.95	0.61
-10	4	-1	2.37	0.54

10	-4	-1	2.75	0.48
10	4	1	1.35	0.59
-10	-4	1	2.04	0.24
10	4	1	3.23	0.54
10	-4	-1	1.94	0.59
-10	-4	-1	2.28	0.47
-10	-4	-1	2.08	0.40
10	-4	1	1.49	0.58
10	-4	1	1.53	0.38
10	4	-1	1.97	0.57
-10	4	1	3.48	0.63
-10	4	1	2.53	0.56
-10	-4	-1	1.61	0.65
-10	-4	-1	2.12	0.42
10	4	-1	1.12	0.38
-10	-4	-1	1.84	0.39
-10	4	1	1.13	0.66
-10	-4	-1	1.72	0.41
10	4	-1	3.12	0.50
10	-4	1	2.24	0.43
-10	-4	-1	1.74	0.47
10	4	-1	2.40	0.46
-10	4	1	2.23	0.55
-10	-4	-1	1.82	0.33
10	-4	1	1.75	0.42
-10	5	-1	3.79	0.79
-10	5	-1	2.64	0.79
10	-5	-1	2.53	0.47
-10	-5	1	6.17	0.56
-10	-5	1	6.84	1.13
-10	5	-1	5.54	0.76
10	5	1	8.89	0.84
10	5	1	7.13	0.80
10	5	1	7.09	0.86
-10	5	-1	3.30	0.60
-10	-5	1	6.08	0.56
10	5	1	6.63	1.04
10	-5	-1	2.13	0.52
10	-5	-1	4.09	0.60
-10	-5	1	8.51	0.96
10	-5	-1	2.12	0.76
10	-5	1	7.06	0.72
-10	5	1	6.79	0.89
-10	5	1	9.33	1.13

-10	-5	-1	2.69	0.73
10	5	-1	3.79	0.51
-10	-5	-1	2.53	0.55
10	-5	1	6.95	0.79
-10	-5	-1	3.51	0.50
-10	-5	-1	2.79	0.51
-10	-5	-1	5.07	0.69
-10	5	1	4.00	0.87
10	5	-1	2.81	0.56
10	5	-1	1.30	0.49
-10	5	1	6.05	1.25
10	-5	1	7.73	0.72
10	-5	1	5.85	1.03
10	5	-1	1.29	0.61
-10	-6	1	48.72	2.95
-10	-6	1	50.52	2.66
-10	6	-1	51.38	2.81
10	-6	-1	46.96	2.87
10	6	1	53.87	2.73
-10	6	-1	55.68	2.74
-10	-6	1	50.59	2.52
-10	-6	1	50.42	2.51
10	-6	-1	50.73	2.75
10	6	1	48.55	2.75
10	-6	-1	48.52	2.62
-10	6	-1	51.43	3.01
-10	-6	1	51.29	2.71
10	-6	-1	53.56	3.06
10	6	1	54.07	2.56
10	6	1	49.64	2.66
-10	6	-1	49.98	3.09
-10	6	-1	54.25	2.74
10	6	1	48.79	2.94
10	-6	-1	46.36	2.60
-10	-6	1	50.44	2.53
-10	-6	-1	50.71	2.95
10	-6	1	49.38	2.73
10	-6	1	50.93	2.91
10	6	-1	48.14	2.94
-10	6	1	50.54	2.82
10	-6	1	49.95	2.63
10	6	-1	53.08	2.50
-10	-6	-1	52.10	2.58
10	6	-1	48.59	2.74

-10	6	1	50.68	2.70
10	6	-1	48.27	2.65
10	6	-1	50.67	2.50
-10	6	1	52.63	2.75
10	-6	1	54.90	3.11
-10	6	1	50.97	2.97
-10	6	1	48.91	3.04
-10	-6	-1	52.01	2.72
-10	-6	-1	49.64	2.53
10	-6	1	49.00	2.62
10	6	-1	53.24	2.69
-10	7	-1	81.40	4.13
10	-7	-1	80.54	4.06
10	-7	-1	76.30	3.90
-10	-7	1	80.05	4.00
10	7	1	75.47	4.23
-10	7	-1	81.21	4.01
10	7	1	82.78	4.05
10	7	1	72.97	4.07
-10	-7	1	79.72	3.79
10	7	1	80.32	3.79
-10	-7	1	75.56	4.19
10	-7	-1	73.80	3.90
-10	-7	1	78.66	3.82
10	-7	-1	88.96	4.47
-10	7	-1	78.66	4.30
-10	7	-1	70.94	4.42
-10	-7	1	80.34	3.88
-10	-7	-1	74.41	3.99
10	-7	1	87.70	4.46
10	-7	1	77.73	4.04
-10	7	1	76.40	4.43
-10	7	1	80.75	4.01
10	7	-1	75.57	4.09
10	7	-1	85.21	3.77
-10	-7	-1	79.09	3.84
10	7	-1	81.48	4.01
10	7	-1	80.66	3.75
-10	7	1	78.75	4.16
-10	7	1	84.32	4.31
10	-7	1	74.47	3.88
-10	-7	-1	76.66	3.88
10	-7	1	76.21	3.91
10	-8	-1	67.90	3.44

10	-8	-1	70.78	4.00
10	8	1	50.56	4.46
-10	-8	1	66.14	3.21
-10	8	-1	69.58	3.93
-10	8	-1	65.19	4.06
10	8	1	68.62	3.23
10	8	1	70.74	3.63
10	-8	-1	63.50	3.39
10	-8	-1	66.08	3.58
-10	-8	1	62.95	3.33
-10	-8	1	61.45	4.19
-10	-8	1	66.74	3.28
10	-8	1	64.64	3.45
10	8	-1	68.43	3.24
10	8	-1	68.85	3.21
-10	-8	-1	66.78	3.32
-10	-8	-1	65.15	3.37
10	-8	1	72.18	4.35
-10	8	1	68.12	3.85
10	-8	1	62.31	3.32
10	8	-1	53.25	4.08
10	8	-1	72.15	3.61
-10	-8	-1	67.09	3.61
10	-8	1	64.46	3.51
-10	8	1	64.11	3.95
10	9	1	46.38	2.81
-10	-9	1	40.64	2.47
10	9	1	45.85	2.31
-10	-9	1	43.43	2.32
10	9	1	43.78	2.87
-10	9	-1	31.76	2.72
-10	-9	1	45.78	2.92
10	-9	-1	31.63	2.20
10	-9	-1	33.94	2.07
10	-9	-1	37.80	2.97
-10	9	-1	33.64	2.65
10	-9	-1	31.44	2.01
-10	-9	1	41.98	2.28
-10	-9	1	44.03	2.63
10	-9	1	41.55	2.49
10	-9	1	46.64	3.34
10	-9	1	42.65	2.64
10	9	-1	35.25	1.79
10	-9	1	42.91	2.40

-10	-9	-1	33.78	2.20
-10	-9	-1	32.50	2.48
-10	9	1	41.76	2.96
10	9	-1	36.02	2.45
-10	-9	-1	34.19	1.93
10	9	-1	31.61	2.49
-10	-9	-1	33.27	2.10
10	9	-1	35.91	1.86
10	10	1	17.85	2.09
-10	10	-1	21.09	2.63
10	-10	-1	22.91	1.95
-10	-10	1	18.69	1.70
-10	-10	1	19.37	1.29
10	-10	-1	22.77	1.61
-10	10	-1	23.37	2.48
-10	-10	1	20.59	2.11
10	-10	-1	22.73	1.72
-10	10	1	19.31	2.34
-10	-10	-1	26.56	2.28
10	-10	1	17.59	1.44
-10	-10	-1	21.98	1.83
10	10	-1	20.53	2.21
-10	-10	-1	22.45	1.54
10	-10	1	20.23	1.79
-10	10	1	15.05	2.76
10	-10	1	20.34	1.59
10	-11	-1	4.63	1.02
-10	-11	1	5.41	1.11
-10	11	-1	4.04	1.56
10	11	1	4.30	1.60
-10	-11	1	6.00	1.61
10	-11	-1	4.68	1.04
10	-11	-1	4.82	0.91
10	-11	1	4.30	1.09
10	11	-1	4.03	1.63
-10	-11	-1	5.41	1.53
-10	11	1	6.56	1.53
10	-11	1	4.31	0.99
-10	-11	-1	6.27	1.13
10	-11	1	4.96	0.88
10	-12	-1	8.49	1.77
10	12	1	7.22	1.66
10	-12	-1	8.30	1.33
-10	-12	1	8.18	1.25

-10	-12	1	7.62	1.53
-10	12	-1	7.16	1.86
10	-12	-1	8.26	1.33
10	12	-1	8.37	1.79
-10	-12	-1	9.98	1.47
10	-12	1	7.72	1.33
10	-12	1	6.91	1.45
10	-12	1	8.10	1.41
-10	-12	-1	10.84	1.71
-10	12	1	7.98	2.22
-11	0	1	0.17	0.25
11	0	-1	0.04	0.14
-11	0	-1	0.00	0.30
11	0	1	0.22	0.15
11	0	-1	-0.40	0.23
-11	0	-1	-0.22	0.26
-11	0	-1	0.61	0.45
11	0	-1	-0.06	0.38
-11	0	1	0.18	0.31
-11	0	1	0.10	0.29
-11	0	1	0.33	0.36
-11	0	-1	-0.16	0.28
-11	0	1	-0.36	0.41
-11	0	-1	-0.14	0.35
11	0	1	-0.35	0.21
11	0	1	-0.18	0.32
-11	0	1	-0.08	0.37
11	0	-1	0.12	0.26
11	0	1	-0.10	0.39
11	0	1	0.38	0.35
-11	1	-1	5.79	0.46
11	1	1	4.00	0.67
-11	-1	1	3.24	0.55
-11	-1	1	4.59	0.59
-11	-1	1	3.48	0.62
11	-1	-1	3.46	0.57
-11	1	-1	5.36	0.61
-11	-1	1	4.10	0.77
-11	1	-1	6.54	0.66
11	-1	-1	4.48	0.53
11	1	1	4.83	0.55
11	-1	-1	4.82	0.43
-11	1	-1	5.96	0.62
11	-1	-1	4.18	0.59

11	1	1	4.30	0.35
11	-1	-1	4.09	0.54
-11	1	1	3.55	0.48
11	-1	1	5.04	0.53
-11	1	1	4.27	0.60
-11	1	1	3.93	0.59
11	1	-1	3.67	0.48
-11	-1	-1	5.28	0.60
11	-1	1	3.31	0.55
11	-1	1	4.62	0.76
11	-1	1	3.42	0.40
-11	-1	-1	4.33	0.74
-11	1	1	5.60	0.60
-11	-1	-1	4.62	0.75
-11	1	1	3.67	0.37
11	1	-1	4.61	0.31
11	1	-1	3.54	0.69
11	1	-1	5.30	0.56
-11	-2	1	22.47	1.32
-11	-2	1	24.15	1.25
11	2	1	20.49	1.19
-11	-2	1	20.35	1.45
-11	-2	1	24.77	1.28
11	-2	-1	19.21	1.45
-11	2	-1	23.14	1.28
11	-2	-1	22.97	1.31
11	2	1	19.74	1.41
11	-2	-1	21.34	1.29
11	2	1	20.14	1.09
-11	-2	1	21.79	1.28
-11	-2	1	22.03	1.42
-11	2	-1	23.22	1.28
-11	2	-1	26.68	1.40
11	-2	-1	26.33	1.55
-11	2	-1	22.19	1.39
-11	-2	1	21.19	1.25
11	-2	-1	22.61	1.34
-11	2	-1	21.52	1.45
-11	2	-1	27.12	1.60
11	-2	-1	21.53	1.26
-11	-2	-1	25.11	1.35
11	2	-1	22.00	1.17
-11	2	1	20.92	1.19
-11	2	1	20.90	1.32

-11	-2	-1	22.88	1.33
-11	-2	-1	23.15	1.36
11	2	-1	21.40	1.13
11	-2	1	19.25	1.17
11	2	-1	22.68	1.50
11	-2	1	19.67	1.24
-11	2	1	20.92	1.17
-11	2	1	19.38	1.35
11	-2	1	24.32	1.49
-11	-2	-1	22.57	1.54
-11	-2	-1	26.53	1.36
-11	2	1	24.55	1.30
-11	-2	-1	25.50	1.40
11	-2	1	21.12	1.45
11	-2	1	20.80	1.26
-11	3	-1	127.28	5.99
11	3	1	120.49	5.51
-11	3	-1	129.50	5.78
11	-3	-1	123.36	5.73
11	-3	-1	121.56	5.91
-11	-3	1	121.22	5.53
-11	-3	1	114.54	5.75
-11	3	-1	122.74	5.60
11	3	1	109.91	5.74
11	3	1	110.56	5.46
-11	-3	1	119.33	5.55
-11	-3	1	118.29	5.58
-11	3	-1	118.84	5.90
11	-3	-1	114.72	5.75
11	-3	-1	119.64	5.73
-11	3	-1	125.71	5.80
-11	-3	1	125.32	5.55
11	-3	-1	129.69	5.92
-11	3	-1	124.16	5.74
11	3	1	128.67	5.55
-11	-3	1	118.36	5.64
-11	-3	1	119.19	5.58
-11	3	1	127.55	5.82
11	-3	1	121.47	5.74
11	-3	1	117.75	5.77
-11	-3	-1	123.90	5.74
-11	3	1	127.89	5.59
11	-3	1	114.42	5.58
-11	-3	-1	121.97	5.93

11	-3	1	110.60	5.56
11	3	-1	118.15	5.61
-11	3	1	121.50	5.63
-11	-3	-1	120.81	5.77
11	-3	1	119.25	5.44
11	3	-1	122.99	5.61
-11	-3	-1	123.76	5.73
-11	-3	-1	123.96	5.77
-11	-3	-1	120.47	5.69
-11	3	1	122.66	5.57
11	3	-1	120.09	5.94
11	-3	1	115.38	5.56
-11	3	1	108.84	5.68
-11	4	-1	125.90	5.79
11	-4	-1	122.09	5.91
-11	4	-1	116.17	5.97
11	4	1	127.14	5.62
-11	4	-1	126.16	6.03
-11	-4	1	117.64	5.72
-11	-4	1	125.06	5.63
11	4	1	113.24	5.89
11	-4	-1	115.39	5.75
11	4	1	123.65	5.63
-11	4	-1	126.45	5.81
11	-4	-1	122.14	5.96
-11	-4	1	117.51	5.69
-11	-4	1	119.43	5.69
-11	-4	1	124.68	5.60
-11	4	-1	127.18	5.79
11	-4	-1	117.88	5.76
-11	-4	1	120.67	5.90
-11	-4	1	116.83	5.59
11	-4	-1	118.96	5.72
11	4	1	115.41	5.63
11	4	-1	117.11	5.98
11	-4	1	115.12	5.68
-11	-4	-1	123.02	5.64
-11	-4	-1	124.82	5.71
11	-4	1	122.08	5.85
-11	-4	-1	122.86	5.50
-11	-4	-1	122.23	5.79
-11	-4	-1	128.19	5.51
11	-4	1	117.31	5.70
-11	4	1	127.07	5.75

11	-4	1	117.68	5.67
-11	-4	-1	121.32	5.97
-11	4	1	126.98	5.74
-11	-4	-1	120.33	5.77
-11	4	1	115.80	5.87
-11	4	1	124.20	5.94
-11	4	1	121.47	5.68
11	4	-1	124.02	5.65
-11	-4	-1	126.35	5.72
11	4	-1	114.59	5.68
11	-5	-1	41.63	2.74
-11	5	-1	42.61	2.52
-11	5	-1	43.62	2.26
-11	-5	1	46.30	2.23
11	5	1	42.82	2.29
-11	-5	1	44.37	2.34
-11	-5	1	42.32	2.59
-11	5	-1	38.64	2.55
11	5	1	40.52	2.31
11	-5	-1	39.12	2.21
11	-5	-1	38.94	2.42
-11	5	-1	41.29	2.28
-11	-5	1	42.52	2.35
-11	-5	1	44.41	2.05
-11	-5	1	42.35	2.35
-11	5	-1	41.53	2.26
11	-5	-1	39.78	2.17
11	5	1	45.07	2.23
-11	-5	1	41.32	2.04
11	5	1	42.79	2.58
11	-5	-1	38.57	2.13
-11	-5	-1	41.49	2.23
-11	-5	-1	37.90	2.10
-11	5	1	43.91	2.43
11	-5	1	42.66	2.34
11	5	-1	37.13	2.17
-11	-5	-1	40.24	1.97
11	-5	1	44.56	2.57
-11	5	1	44.55	2.62
-11	-5	-1	43.14	2.14
11	-5	1	44.06	2.71
11	5	-1	38.38	2.45
11	-5	1	41.72	2.29
-11	-5	-1	38.04	2.21

-11	-5	-1	40.43	1.97
11	5	-1	41.39	2.07
-11	5	1	39.07	2.62
11	5	-1	43.71	2.08
-11	-5	-1	39.06	2.48
-11	5	1	46.68	2.35
11	5	-1	40.63	2.10
11	-5	1	41.75	2.28
-11	5	1	46.18	2.41
-11	-5	-1	39.83	2.01
11	-6	-1	50.21	2.70
11	-6	-1	52.27	2.79
-11	-6	1	48.61	2.36
-11	6	-1	52.22	2.77
11	-6	-1	51.28	2.67
11	6	1	51.58	2.47
11	-6	-1	55.24	3.13
-11	-6	1	44.82	2.36
11	6	1	45.60	2.86
-11	6	-1	54.10	2.80
11	6	1	47.93	2.67
-11	-6	1	47.17	2.62
-11	-6	1	48.57	2.59
-11	6	-1	51.30	3.09
11	6	1	49.63	2.74
-11	6	-1	52.84	2.85
-11	-6	1	49.21	2.47
11	-6	-1	50.12	2.97
-11	6	-1	48.56	3.15
11	6	1	48.82	2.77
-11	-6	-1	51.84	2.76
-11	-6	-1	54.60	2.55
-11	6	1	50.14	2.67
-11	6	1	50.45	2.74
11	6	-1	47.78	2.69
11	-6	1	49.30	3.03
-11	-6	-1	50.92	2.46
11	6	-1	46.97	3.00
11	6	-1	45.93	2.77
-11	-6	-1	50.54	2.54
11	-6	1	47.52	2.54
11	6	-1	54.93	2.70
-11	6	1	49.32	2.93
-11	-6	-1	51.37	2.63

11	6	-1	53.86	2.55
11	6	-1	53.41	2.56
-11	6	1	49.59	2.64
-11	6	1	46.41	2.94
11	-6	1	46.17	2.64
11	-6	1	51.76	3.08
11	-6	1	47.08	2.55
11	7	1	9.74	1.20
11	-7	-1	10.69	1.04
-11	-7	1	11.76	0.90
11	7	1	10.10	1.29
-11	7	-1	10.08	1.52
-11	-7	1	9.87	1.39
11	7	1	12.47	0.97
11	-7	-1	11.60	1.65
11	7	1	9.85	1.42
11	-7	-1	11.96	1.17
-11	-7	1	11.83	0.95
-11	-7	1	9.97	1.09
11	-7	-1	12.35	1.04
-11	-7	1	10.58	1.20
-11	7	-1	9.07	1.75
-11	7	-1	11.12	1.42
-11	-7	-1	12.74	1.02
11	-7	1	10.93	1.12
11	-7	1	11.33	1.79
-11	-7	-1	12.85	1.03
11	7	-1	11.67	1.30
-11	-7	-1	11.93	1.16
11	7	-1	12.04	1.39
11	7	-1	14.86	0.99
11	7	-1	13.60	0.88
-11	7	1	11.67	1.49
11	-7	1	8.91	0.97
-11	7	1	12.22	1.76
11	-7	1	9.99	0.96
-11	8	-1	3.12	1.38
11	-8	-1	3.30	1.30
11	-8	-1	2.11	0.61
11	-8	-1	2.44	0.72
11	8	1	2.29	0.60
-11	-8	1	3.16	0.61
-11	-8	1	4.63	0.75
-11	-8	1	4.14	0.75

11	8	1	2.42	0.82
11	8	1	2.47	0.86
-11	8	-1	2.46	0.99
-11	-8	1	2.81	0.91
11	8	1	2.50	0.88
-11	-8	1	5.56	1.06
-11	-8	-1	2.47	0.64
11	8	-1	3.10	0.61
11	-8	1	3.51	1.35
-11	8	1	2.70	1.52
11	8	-1	5.10	0.77
-11	-8	-1	1.27	0.62
11	8	-1	2.96	0.86
11	-8	1	2.60	0.64
11	-8	1	1.41	0.54
11	8	-1	1.35	0.81
11	-8	1	2.48	0.70
-11	-8	-1	2.27	0.58
-11	-9	1	18.49	1.46
-11	9	-1	20.50	2.25
11	-9	-1	19.22	1.54
11	-9	-1	19.43	2.54
-11	-9	1	18.39	1.99
11	9	1	19.01	1.91
11	9	1	21.15	2.00
11	9	1	19.05	1.38
-11	-9	1	19.44	1.32
11	-9	-1	20.15	1.72
-11	-9	1	18.19	1.60
11	-9	-1	20.11	1.47
-11	9	-1	18.66	2.58
-11	-9	-1	20.13	2.02
-11	9	1	22.97	2.25
11	-9	1	20.34	1.56
-11	-9	-1	22.89	1.71
-11	9	1	19.60	2.58
11	-9	1	18.33	2.03
11	9	-1	19.73	1.95
11	-9	1	22.63	2.32
-11	-9	-1	19.75	1.45
11	-9	1	20.50	1.70
11	9	-1	20.27	1.32
-11	-9	-1	19.54	1.69
11	9	-1	21.61	1.80

-11	-10	1	63.75	3.99
-11	-10	1	65.87	3.23
11	-10	-1	71.25	3.60
11	10	1	63.74	3.96
-11	10	-1	67.45	4.25
11	-10	-1	63.13	3.72
-11	-10	1	67.57	3.71
11	-10	-1	67.07	3.53
11	10	-1	62.72	4.04
11	-10	1	67.44	3.53
11	-10	1	65.33	3.41
11	-10	1	57.67	3.70
-11	-10	-1	71.31	4.11
-11	-10	-1	66.29	3.41
-11	-10	-1	66.03	3.78
-11	10	1	66.37	4.12
11	-11	-1	55.20	3.45
-11	-11	1	50.92	3.16
11	-11	-1	55.92	3.23
11	11	1	49.95	3.56
-11	11	-1	61.44	4.23
11	-11	-1	51.51	3.11
-11	-11	1	51.24	3.57
11	-11	1	54.02	2.93
-11	-11	-1	60.10	3.38
-11	-11	-1	59.65	3.78
11	-11	1	52.80	3.05
-11	11	1	52.68	3.99
11	11	-1	52.53	3.70
11	-11	1	52.21	3.27
-11	12	-1	42.67	3.62
11	-12	-1	40.42	2.98
11	-12	-1	41.30	2.65
11	12	1	41.27	3.39
-11	-12	1	44.55	3.27
11	-12	-1	41.86	2.63
-11	-12	1	43.95	2.85
11	-12	1	37.38	2.93
11	12	-1	41.08	3.38
-11	12	1	45.42	3.73
-11	-12	-1	39.53	2.79
11	-12	1	42.53	2.71
-11	-12	-1	39.92	3.17
11	-12	1	40.93	2.57

-12	0	-1	50.16	2.42
12	0	1	52.84	2.70
12	0	1	46.86	2.45
-12	0	1	58.65	2.78
12	0	-1	40.37	2.23
-12	0	-1	53.52	2.36
-12	0	1	51.10	2.64
-12	0	1	50.35	2.68
-12	0	-1	46.79	2.34
12	0	1	59.13	2.77
-12	0	1	49.72	2.62
-12	0	-1	45.18	2.33
-12	0	1	51.64	2.61
12	0	-1	43.24	2.29
-12	0	1	47.32	2.75
12	0	-1	39.85	2.10
12	0	-1	43.94	2.39
12	0	1	53.30	2.64
12	0	1	55.53	2.64
12	-1	-1	3.83	0.55
-12	1	-1	6.10	0.60
-12	1	-1	3.61	0.64
-12	1	-1	3.97	0.65
12	-1	-1	5.36	0.57
-12	-1	1	2.08	0.43
12	-1	-1	4.73	0.65
12	-1	-1	3.78	0.41
12	1	1	1.30	0.41
12	1	1	1.45	0.48
12	1	1	2.04	0.42
-12	1	-1	4.21	0.63
12	-1	-1	4.94	0.64
-12	-1	1	4.17	0.79
-12	1	-1	3.16	0.44
-12	-1	1	1.92	0.59
12	-1	1	1.52	0.73
12	1	-1	4.51	0.31
12	-1	1	0.85	0.39
12	-1	1	3.01	0.58
-12	1	1	2.49	0.60
-12	-1	-1	4.39	0.72
-12	1	1	3.73	0.59
-12	-1	-1	3.05	0.60
-12	-1	-1	3.13	0.60

12	1	-1	5.27	0.49
-12	1	1	1.26	0.40
-12	-1	-1	3.70	0.62
12	1	-1	6.14	0.81
12	1	-1	4.87	0.59
12	-2	-1	4.63	0.52
-12	2	-1	6.62	0.65
-12	2	-1	5.58	0.85
12	-2	-1	4.83	0.87
12	2	1	5.56	0.65
12	-2	-1	6.73	0.96
12	2	1	4.71	0.42
-12	2	-1	4.64	0.58
-12	-2	1	7.32	0.64
-12	2	-1	3.98	0.69
12	-2	-1	5.32	0.59
12	2	1	4.86	0.81
-12	-2	1	4.72	0.73
-12	-2	1	6.24	0.70
12	-2	-1	4.61	0.61
-12	-2	1	5.49	0.91
-12	2	-1	7.44	0.95
-12	-2	1	4.13	0.64
-12	2	-1	4.77	0.72
12	-2	1	5.20	0.94
12	2	-1	5.15	0.37
-12	2	1	7.56	0.93
-12	-2	-1	4.63	0.81
-12	2	1	4.82	0.59
12	2	-1	4.04	0.45
-12	2	1	4.73	0.73
-12	-2	-1	6.18	0.78
-12	-2	-1	6.88	0.65
12	-2	1	3.64	0.58
12	-2	1	5.09	0.60
-12	-2	-1	6.35	0.89
-12	-2	-1	6.58	0.65
12	-2	1	6.45	0.92
12	-2	1	4.64	0.53
-12	2	1	6.21	1.13
-12	2	1	6.28	0.60
-12	-2	-1	4.89	0.64
-12	2	1	6.02	0.68
-12	-2	-1	5.46	0.69

12	2	-1	4.36	0.82
12	-2	1	5.00	0.58
12	3	1	9.82	0.70
-12	3	-1	10.76	0.86
-12	3	-1	10.56	0.92
-12	-3	1	9.83	0.87
12	-3	-1	10.35	0.79
12	3	1	10.75	0.83
-12	3	-1	10.78	0.96
12	-3	-1	9.82	1.12
-12	3	-1	7.89	1.10
-12	-3	1	9.72	1.04
12	-3	-1	11.05	1.12
12	-3	-1	9.98	0.79
-12	-3	1	10.38	0.81
-12	-3	1	10.09	0.86
-12	3	-1	10.99	1.21
-12	-3	1	10.08	0.89
12	-3	-1	9.39	0.82
-12	-3	1	10.25	0.82
-12	-3	1	10.25	1.14
12	3	1	9.68	1.09
-12	3	-1	10.08	0.74
12	3	-1	9.52	0.67
-12	-3	-1	9.96	0.91
-12	3	1	9.27	0.94
-12	-3	-1	10.66	0.84
-12	3	1	9.59	1.12
-12	-3	-1	9.18	1.13
12	-3	1	10.70	0.79
12	-3	1	10.68	1.15
12	-3	1	9.42	1.25
12	-3	1	9.35	0.83
-12	-3	-1	10.14	0.86
-12	3	1	9.28	0.88
-12	-3	-1	10.21	0.84
-12	3	1	11.18	0.87
12	3	-1	8.97	1.06
12	3	-1	9.58	0.68
-12	-3	-1	9.42	0.86
-12	3	1	9.63	1.14
-12	-3	-1	9.44	1.11
12	-3	1	9.38	0.79
12	-3	1	10.71	0.86

12	-4	-1	14.98	1.11
-12	-4	1	19.52	1.21
-12	-4	1	21.63	1.22
-12	-4	1	19.01	1.59
-12	-4	1	20.62	1.31
-12	-4	1	19.56	1.47
-12	4	-1	16.01	1.21
12	-4	-1	16.75	1.43
-12	-4	1	20.15	1.27
-12	4	-1	16.76	1.48
-12	4	-1	16.36	1.24
12	-4	-1	16.92	1.12
12	4	1	19.43	1.22
12	4	1	18.48	1.22
-12	-4	1	20.49	1.23
12	-4	-1	17.86	1.50
12	4	1	16.91	1.15
-12	4	-1	16.49	1.23
-12	4	-1	14.25	1.48
12	4	1	16.88	1.48
12	-4	-1	16.40	1.13
-12	4	1	19.07	1.55
-12	-4	-1	15.90	1.04
-12	4	1	19.20	1.38
-12	-4	-1	16.48	1.10
12	4	-1	16.42	1.04
-12	4	1	17.46	1.59
12	-4	1	18.09	1.22
12	4	-1	15.71	1.04
12	-4	1	19.18	1.25
-12	-4	-1	15.27	1.19
12	4	-1	17.21	1.43
-12	4	1	20.32	1.32
12	-4	1	18.56	1.55
-12	-4	-1	17.55	1.17
12	-4	1	20.10	1.27
-12	-4	-1	16.13	1.19
-12	-4	-1	17.21	1.46
12	-4	1	19.22	1.56
-12	4	1	20.27	1.37
12	5	1	1.44	0.46
12	5	1	2.65	0.49
-12	5	-1	4.66	0.95
12	-5	-1	1.71	0.49

-12	5	-1	1.12	0.93
-12	-5	1	1.91	0.44
-12	5	-1	3.44	0.66
12	5	1	0.61	0.61
12	-5	-1	1.65	0.41
-12	5	-1	2.00	0.66
-12	-5	1	2.87	0.85
-12	-5	1	1.65	0.58
-12	-5	1	1.91	0.45
12	5	1	1.91	0.48
-12	-5	1	2.12	0.52
-12	5	-1	1.48	0.57
12	-5	-1	1.05	0.49
12	-5	-1	1.74	0.70
-12	-5	1	0.78	0.41
12	-5	1	0.92	0.45
12	-5	1	1.14	0.39
-12	5	1	0.76	0.60
-12	-5	-1	4.11	0.67
-12	-5	-1	2.95	0.57
-12	5	1	0.67	0.83
12	5	-1	1.24	0.47
-12	-5	-1	2.34	0.39
12	-5	1	1.03	0.42
-12	-5	-1	2.93	0.62
12	5	-1	2.04	0.50
12	5	-1	1.43	0.66
12	-5	1	2.52	0.76
-12	5	1	0.73	0.57
-12	-5	-1	1.63	0.56
-12	-5	-1	1.97	0.37
-12	5	1	2.88	0.82
-12	5	1	1.60	0.57
-12	-5	-1	2.60	0.78
-12	-6	1	22.07	1.25
12	-6	-1	15.30	1.15
-12	6	-1	21.40	1.77
12	-6	-1	16.02	1.73
-12	-6	1	18.70	1.44
-12	6	-1	18.11	1.91
12	6	1	20.05	1.24
12	-6	-1	14.10	1.19
12	-6	-1	13.58	1.22
-12	-6	1	15.83	0.99

-12	6	-1	13.57	1.34
12	6	1	17.51	1.34
-12	-6	1	19.86	1.13
-12	-6	1	16.91	1.06
12	6	1	14.67	1.27
12	6	1	19.14	1.64
-12	-6	1	20.93	1.39
12	6	-1	14.81	1.20
12	-6	1	17.21	1.26
-12	6	1	16.34	1.90
12	6	-1	17.71	1.60
12	-6	1	17.25	1.33
-12	6	1	21.67	1.79
-12	-6	-1	15.76	1.22
12	-6	1	16.11	1.85
12	-6	1	15.31	1.22
12	6	-1	16.40	1.30
-12	6	1	19.07	1.40
-12	-6	-1	16.01	1.00
-12	-6	-1	18.32	1.06
-12	-6	-1	20.12	1.32
-12	-7	1	13.76	1.24
-12	-7	1	12.97	1.19
-12	7	-1	14.52	1.92
-12	-7	1	9.16	0.85
-12	-7	1	13.62	1.02
-12	7	-1	12.37	1.80
12	-7	-1	16.82	1.88
12	-7	-1	13.37	1.29
12	7	1	9.43	1.24
12	-7	-1	13.32	1.11
12	-7	-1	13.87	1.15
12	7	1	13.36	1.06
-12	-7	-1	17.61	1.17
-12	7	1	12.69	1.70
-12	-7	-1	14.92	1.47
12	7	-1	14.01	1.21
12	-7	1	11.13	1.04
-12	7	1	11.20	1.79
12	-7	1	8.54	1.08
12	-7	1	11.58	1.78
12	-7	1	9.73	1.01
-12	-7	-1	13.41	1.04
12	7	-1	15.63	1.08

-12	-8	1	33.91	1.86
12	-8	-1	27.57	1.84
12	8	1	31.04	2.39
-12	8	-1	29.31	2.73
12	8	1	29.00	1.75
12	8	1	28.55	2.12
12	-8	-1	30.19	2.06
-12	-8	1	31.15	1.99
12	8	1	29.48	2.16
-12	-8	1	30.79	2.32
-12	8	-1	31.46	2.51
12	-8	-1	34.37	2.68
-12	-8	1	27.16	1.67
-12	-8	-1	30.96	1.83
12	8	-1	29.27	2.22
12	8	-1	32.50	2.25
-12	8	1	28.56	2.64
-12	8	1	33.46	2.51
-12	-8	-1	27.27	1.77
12	-8	1	25.39	1.80
12	-8	1	30.77	2.71
12	8	-1	34.68	1.74
12	-8	1	27.15	1.83
-12	-8	-1	30.45	2.03
12	-8	1	26.36	1.95
12	9	1	21.31	2.04
12	9	1	22.28	1.43
-12	-9	1	20.50	2.10
12	-9	-1	17.26	1.49
12	-9	-1	21.07	1.59
12	9	1	18.03	1.84
-12	9	-1	23.97	2.38
12	-9	-1	18.14	1.69
-12	-9	1	21.46	1.70
-12	-9	1	19.10	1.36
-12	-9	-1	22.45	1.50
-12	-9	-1	22.37	1.76
12	-9	1	17.88	1.54
-12	9	1	21.35	2.32
-12	-9	-1	23.59	2.17
12	9	-1	22.03	1.37
12	9	-1	21.09	2.03
12	-9	1	16.36	1.82
12	-9	1	17.01	1.44

12	9	-1	20.07	1.88
-12	10	-1	22.53	2.64
12	-10	-1	19.17	1.89
-12	-10	1	25.93	2.07
12	-10	-1	21.57	1.65
12	10	1	21.66	2.36
-12	-10	1	20.34	1.47
-12	-10	1	22.89	2.46
12	-10	-1	22.02	1.73
-12	10	1	23.41	2.49
-12	-10	-1	23.74	2.01
12	-10	1	22.39	1.93
12	-10	1	22.76	1.85
-12	-10	-1	20.92	1.57
12	-10	1	19.07	1.64
-12	-10	-1	23.70	2.47
12	10	-1	22.49	2.43
-12	11	-1	4.43	1.72
-12	-11	1	4.38	0.98
12	-11	-1	7.75	1.29
12	-11	-1	5.87	1.02
-12	-11	1	6.08	1.42
12	-11	-1	6.37	1.31
12	11	1	3.29	1.37
-12	-11	-1	6.37	1.23
12	11	-1	3.55	1.42
12	-11	1	6.69	1.00
12	-11	1	5.86	1.06
12	-11	1	5.14	1.27
-12	11	1	4.51	1.89
-12	-11	-1	5.56	1.43
-12	-12	1	4.28	1.45
12	12	1	1.96	1.46
12	-12	-1	2.21	1.36
12	-12	-1	3.32	0.98
12	-12	-1	2.91	0.93
-12	-12	1	1.73	1.33
-12	12	-1	3.23	1.63
12	12	-1	2.03	1.47
-12	-12	-1	2.56	1.31
12	-12	1	2.49	1.30
12	-12	1	1.56	0.84
-12	12	1	4.04	1.68
12	-12	1	3.26	1.04

-13	0	-1	-0.35	0.43
-13	0	-1	0.17	0.37
-13	0	-1	0.01	0.39
13	0	1	-0.08	0.18
-13	0	-1	0.32	0.31
13	0	1	-0.31	0.30
13	0	-1	-0.12	0.27
13	0	-1	0.43	0.36
-13	0	1	-0.19	0.35
-13	0	1	-0.02	0.29
-13	0	1	0.10	0.44
13	0	-1	0.39	0.40
-13	0	1	-0.06	0.40
-13	0	1	0.57	0.51
13	0	1	-0.14	0.43
13	0	1	0.30	0.39
13	0	-1	0.13	0.17
-13	0	1	-0.37	0.36
-13	0	-1	0.02	0.49
-13	0	-1	-0.10	0.43
-13	-1	1	23.10	1.36
-13	-1	1	22.42	1.43
-13	-1	1	22.09	1.50
13	-1	-1	20.11	1.23
-13	1	-1	19.33	1.27
13	-1	-1	18.25	1.18
13	-1	-1	18.26	1.21
13	1	1	20.50	1.30
13	1	1	21.07	1.33
-13	1	-1	19.91	1.02
13	1	1	20.34	1.45
13	1	1	22.49	1.12
-13	-1	1	21.71	1.35
13	-1	-1	18.71	1.05
-13	1	-1	19.06	1.21
-13	1	-1	20.11	1.07
-13	1	-1	19.62	1.44
13	-1	-1	18.65	1.26
-13	-1	1	20.96	1.29
-13	-1	1	21.85	1.52
-13	1	-1	20.05	1.31
13	1	-1	19.08	0.95
13	-1	1	21.75	1.50
-13	1	1	22.23	1.35

13	1	-1	19.20	1.21
13	1	-1	18.09	1.33
-13	-1	-1	19.33	1.25
13	-1	1	22.69	1.37
13	-1	1	20.84	1.17
-13	1	1	21.91	1.51
-13	-1	-1	20.43	1.34
-13	-1	-1	20.16	1.40
-13	1	1	19.49	1.44
13	1	-1	18.67	1.07
13	-1	1	24.36	1.42
-13	1	1	19.78	1.37
13	-1	1	21.41	1.32
-13	1	1	20.97	1.57
-13	-1	-1	20.07	1.22
-13	-1	-1	18.76	1.26
-13	-1	-1	19.62	1.40
-13	2	-1	111.13	5.15
13	-2	-1	106.08	5.31
-13	-2	1	103.91	5.10
-13	-2	1	102.02	4.92
-13	-2	1	99.22	4.89
13	2	1	100.02	5.08
13	2	1	105.81	4.85
-13	-2	1	100.89	4.93
-13	2	-1	113.73	5.14
-13	2	-1	110.55	4.99
-13	-2	1	105.56	5.04
13	-2	-1	109.03	5.26
-13	2	-1	102.75	5.08
13	-2	-1	108.00	5.08
13	-2	-1	111.38	5.02
-13	-2	1	101.65	4.96
13	-2	-1	106.44	5.00
13	2	1	100.17	4.75
-13	2	-1	104.84	5.01
-13	2	-1	107.21	5.33
-13	-2	1	106.95	4.92
13	-2	-1	102.27	5.10
-13	-2	-1	108.60	5.09
-13	2	1	103.31	4.79
13	-2	1	104.53	4.94
-13	-2	-1	102.62	5.11
-13	2	1	107.77	4.94

13	2	-1	107.84	4.92
-13	-2	-1	107.67	5.13
13	2	-1	106.69	5.27
-13	-2	-1	105.12	5.10
-13	-2	-1	105.86	5.08
13	-2	1	104.95	4.87
-13	2	1	105.08	4.98
-13	2	1	101.26	4.82
13	-2	1	103.27	5.08
13	2	-1	102.86	4.86
13	-2	1	107.07	5.09
-13	2	1	99.92	4.89
13	-2	1	102.56	4.85
-13	2	1	108.27	5.16
-13	-2	-1	111.69	5.28
-13	-2	-1	108.12	5.16
-13	3	-1	114.55	5.35
13	3	1	120.41	5.43
13	-3	-1	111.38	5.48
13	-3	-1	112.76	5.27
-13	-3	1	121.32	5.47
-13	-3	1	119.07	5.58
-13	3	-1	116.69	5.39
13	-3	-1	109.70	5.33
-13	3	-1	110.66	5.57
-13	3	-1	106.22	5.16
-13	3	-1	114.68	5.28
-13	-3	1	116.16	5.71
13	3	1	113.19	5.36
13	3	1	110.90	5.70
13	-3	-1	120.63	5.49
-13	3	-1	114.00	5.47
-13	-3	1	114.89	5.46
-13	-3	1	116.71	5.52
13	-3	-1	111.54	5.30
-13	-3	1	115.10	5.51
-13	-3	1	112.87	5.48
-13	-3	-1	111.91	5.53
13	3	-1	117.17	5.16
13	-3	1	112.37	5.50
-13	3	1	117.20	5.59
-13	-3	-1	103.04	5.31
13	-3	1	114.09	5.46
13	-3	1	120.87	5.72

13	3	-1	110.12	5.13
-13	-3	-1	108.91	5.33
13	-3	1	114.13	5.52
13	-3	1	112.93	5.67
13	-3	1	114.80	5.52
-13	-3	-1	117.61	5.30
-13	3	1	122.11	5.77
-13	-3	-1	113.68	5.34
-13	3	1	119.49	5.52
-13	3	1	116.44	5.57
-13	3	1	111.54	5.30
-13	-3	-1	106.89	5.28
-13	-3	-1	108.26	5.29
13	3	-1	108.28	5.52
-13	3	1	119.20	5.46
13	-4	-1	201.10	9.98
-13	4	-1	210.51	10.13
13	-4	-1	209.20	9.85
-13	-4	1	226.40	10.52
13	4	1	219.04	10.53
-13	-4	1	221.87	10.22
-13	4	-1	222.59	9.88
-13	-4	1	212.22	10.20
13	-4	-1	210.13	9.83
13	-4	-1	224.18	10.08
-13	-4	1	217.93	10.30
-13	4	-1	219.96	9.90
-13	-4	1	234.67	10.34
-13	4	-1	214.37	9.84
13	-4	-1	213.79	9.82
-13	4	-1	218.89	10.06
-13	-4	1	220.11	10.29
-13	-4	1	236.21	10.23
13	4	1	212.91	10.19
13	4	1	230.51	10.23
-13	-4	-1	214.71	9.81
-13	-4	-1	208.74	9.86
13	-4	1	218.92	10.30
13	4	-1	220.60	9.71
13	-4	1	238.47	10.57
-13	-4	-1	205.96	10.12
13	4	-1	213.38	10.09
13	4	-1	209.08	9.72
-13	4	1	224.39	10.35

-13	4	1	232.55	10.31
-13	-4	-1	212.01	10.07
-13	4	1	219.29	10.44
-13	4	1	225.44	10.28
-13	-4	-1	205.13	9.85
13	-4	1	206.08	10.43
-13	4	1	222.55	10.56
13	-4	1	222.99	10.28
-13	-4	-1	208.38	9.74
13	-4	1	224.26	10.29
-13	-4	-1	220.00	9.79
-13	-5	1	46.18	2.41
-13	5	-1	47.85	2.59
-13	-5	1	46.55	2.44
13	-5	-1	45.73	2.48
-13	-5	1	48.82	2.69
13	5	1	47.85	2.49
-13	-5	1	44.02	2.54
-13	-5	1	49.71	2.44
-13	5	-1	44.97	2.85
13	5	1	47.38	2.83
-13	5	-1	49.66	2.95
13	-5	-1	45.24	2.52
-13	5	-1	49.59	2.64
13	5	1	51.02	2.47
13	5	1	48.61	2.51
13	-5	-1	44.66	2.53
-13	-5	1	45.17	2.55
13	5	-1	45.39	2.82
13	-5	1	45.80	2.49
13	5	-1	46.69	2.48
-13	-5	-1	45.28	2.34
-13	-5	-1	51.04	2.49
-13	-5	-1	48.78	2.52
-13	-5	-1	45.79	2.57
-13	5	1	49.62	2.59
-13	5	1	42.93	2.83
13	5	-1	48.85	2.42
-13	5	1	42.84	2.83
13	-5	1	47.98	2.56
13	-5	1	47.41	2.52
13	6	1	11.95	1.11
-13	-6	1	8.37	0.90
13	-6	-1	10.76	1.08

-13	-6	1	10.47	1.55
-13	-6	1	8.47	1.02
13	6	1	10.40	1.43
13	6	1	10.99	0.99
13	-6	-1	13.43	1.61
13	6	1	10.83	1.08
-13	6	-1	12.01	1.83
-13	-6	1	9.78	0.94
-13	-6	1	9.68	0.87
13	-6	-1	11.69	1.04
-13	6	-1	13.35	1.66
13	-6	-1	10.62	1.00
13	6	-1	11.52	1.09
-13	-6	-1	12.92	1.08
13	-6	1	10.27	0.97
13	-6	1	10.61	1.07
-13	-6	-1	12.98	1.13
13	-6	1	11.69	1.68
-13	6	1	8.53	1.50
-13	-6	-1	11.92	0.77
13	6	-1	11.19	1.14
-13	6	1	9.05	1.39
13	-6	1	8.87	0.95
-13	-6	-1	12.54	1.14
-13	-7	1	10.53	1.47
13	-7	-1	8.72	1.17
-13	-7	1	6.90	1.10
13	7	1	9.07	1.17
-13	7	-1	9.38	1.98
-13	-7	1	8.98	0.95
-13	-7	1	10.19	0.81
13	-7	-1	8.25	1.72
-13	7	-1	11.38	1.70
13	7	1	10.35	1.15
-13	-7	1	9.41	0.73
13	7	1	8.85	1.44
13	-7	-1	7.66	0.99
13	7	1	9.69	1.01
-13	-7	1	11.33	0.97
-13	-7	1	7.54	1.26
13	-7	-1	9.73	1.07
-13	-7	-1	9.88	1.14
13	7	-1	9.59	1.15
-13	-7	-1	9.93	0.83

13	-7	1	8.68	1.12
-13	7	1	8.48	1.50
13	7	-1	9.05	1.18
-13	-7	-1	9.31	1.02
13	-7	1	8.80	1.76
13	-7	1	7.05	0.99
-13	7	1	10.31	1.46
-13	8	-1	1.26	1.33
-13	-8	1	1.80	0.67
13	8	1	2.08	1.01
-13	-8	1	1.61	1.02
13	-8	-1	0.47	0.53
13	8	1	0.13	0.56
-13	-8	1	1.94	0.56
13	8	1	0.82	0.82
13	-8	-1	1.08	1.30
-13	-8	1	0.43	0.48
13	8	1	-0.73	0.70
-13	-8	1	1.75	0.75
13	-8	-1	1.21	0.71
-13	-8	1	1.80	0.42
13	-8	-1	-0.03	0.54
-13	8	-1	0.88	1.05
13	8	-1	1.51	0.82
13	-8	1	-0.15	0.49
-13	-8	-1	0.13	0.52
-13	8	1	0.47	1.23
-13	-8	-1	1.56	0.75
-13	-8	-1	1.51	0.59
13	8	-1	0.75	0.75
-13	8	1	-0.37	1.06
13	-8	1	0.47	0.82
13	-8	1	1.33	1.29
13	-8	1	0.88	0.56
-13	-9	1	28.98	2.39
-13	9	-1	26.12	2.59
13	9	1	23.28	2.08
-13	-9	1	25.55	1.64
13	9	1	26.93	2.04
13	-9	-1	20.85	1.69
13	-9	-1	21.75	1.85
-13	-9	1	24.07	1.93
13	-9	-1	23.22	1.71
-13	-9	-1	22.01	1.62

13	-9	1	26.61	1.97
13	9	-1	24.25	2.14
13	9	-1	26.94	1.86
-13	-9	-1	21.54	1.86
-13	9	1	26.93	2.63
13	-9	1	26.40	1.83
13	-9	1	23.41	1.75
-13	-9	-1	22.46	2.26
13	10	1	77.19	4.41
13	-10	-1	72.41	4.04
-13	-10	1	77.03	4.16
13	-10	-1	72.16	3.87
-13	-10	1	71.91	4.48
-13	10	-1	72.13	4.70
13	-10	-1	73.24	3.82
-13	-10	-1	69.97	3.65
13	-10	1	71.92	4.13
-13	-10	-1	72.95	4.39
-13	-10	-1	73.61	4.04
13	10	-1	69.14	4.23
-13	10	1	73.96	4.78
13	-10	1	78.04	4.03
13	-10	1	71.92	3.90
13	-11	-1	54.40	3.11
-13	11	-1	55.94	4.04
13	11	1	44.80	3.49
-13	-11	1	50.69	3.19
13	-11	-1	54.10	3.37
13	-11	-1	51.62	3.13
-13	-11	1	53.22	3.61
13	-11	1	54.26	3.10
13	11	-1	47.90	3.47
13	-11	1	55.87	3.35
13	-11	1	57.75	3.12
-13	11	1	54.31	3.96
-13	-11	-1	57.42	3.66
-13	-11	-1	54.41	3.30
-14	0	-1	16.41	1.29
-14	0	-1	16.57	1.15
14	0	1	15.73	0.99
-14	0	-1	19.34	1.39
14	0	-1	15.44	0.90
14	0	-1	19.30	1.17
-14	0	-1	14.85	1.19

-14	0	1	18.25	1.27
-14	0	1	19.13	1.26
14	0	-1	20.20	1.11
-14	0	1	16.89	1.47
14	0	1	18.35	1.31
14	0	1	18.32	1.21
-14	0	1	23.14	1.41
-14	0	-1	15.13	1.38
-14	0	-1	14.59	1.18
14	0	1	19.56	1.30
14	1	1	32.86	1.81
14	-1	-1	29.28	1.80
14	-1	-1	30.08	1.66
14	-1	-1	31.85	1.87
14	1	1	33.72	1.75
14	1	1	32.91	1.93
14	1	1	31.17	1.56
-14	1	-1	34.96	1.90
-14	-1	1	29.60	1.79
14	-1	-1	34.56	1.90
-14	-1	1	31.00	1.76
-14	1	-1	38.11	2.13
-14	-1	1	31.21	1.99
-14	-1	1	31.24	1.88
-14	1	-1	37.70	1.92
-14	-1	1	31.07	1.93
-14	1	-1	33.44	1.91
-14	-1	1	30.22	1.80
-14	1	-1	36.16	1.87
-14	-1	-1	31.20	1.86
-14	1	1	29.15	1.74
14	-1	1	35.10	2.01
14	-1	1	36.35	1.90
14	1	-1	30.46	1.57
14	1	-1	29.91	1.68
-14	1	1	30.47	1.79
-14	-1	-1	34.29	1.85
-14	-1	-1	38.94	2.02
14	1	-1	30.90	1.83
14	-1	1	28.74	1.60
-14	-1	-1	35.14	2.03
-14	-1	-1	32.46	1.99
-14	-1	-1	33.55	1.88
-14	1	1	28.20	1.82

14	-1	1	35.08	1.83
-14	1	1	30.52	1.79
14	-1	1	31.98	1.78
-14	1	1	31.83	2.00
-14	1	1	28.52	2.01
14	1	-1	32.70	1.98
-14	2	-1	12.62	0.92
14	-2	-1	8.44	0.71
-14	-2	1	8.72	1.15
14	-2	-1	9.73	0.97
14	-2	-1	9.26	0.83
14	-2	-1	8.49	0.83
14	-2	-1	8.06	0.83
-14	-2	1	9.80	0.95
-14	2	-1	11.03	1.03
-14	2	-1	11.07	0.99
-14	2	-1	8.83	0.79
-14	-2	1	11.41	0.89
14	2	1	12.91	0.88
-14	2	-1	11.44	1.02
-14	-2	1	11.19	0.98
14	2	1	11.22	0.68
-14	-2	1	10.81	0.90
-14	-2	1	10.90	1.20
-14	-2	1	9.48	0.99
14	2	1	13.54	1.21
-14	2	1	9.50	0.96
-14	2	1	10.62	1.24
14	-2	1	13.33	0.96
-14	-2	-1	10.04	0.92
14	-2	1	14.07	1.31
14	2	-1	8.52	0.56
-14	-2	-1	8.13	1.10
14	-2	1	14.03	1.29
-14	2	1	8.27	0.89
14	2	-1	9.27	1.11
-14	-2	-1	11.67	0.97
14	-2	1	9.19	0.77
14	-2	1	11.56	0.93
-14	2	1	11.50	0.88
-14	2	1	8.91	0.77
-14	2	1	7.12	0.98
14	2	-1	9.48	0.67
-14	-2	-1	11.60	1.22

-14	-3	1	1.29	0.39
-14	3	-1	0.91	0.93
14	3	1	2.34	0.66
-14	3	-1	1.28	0.49
14	3	1	1.40	0.41
-14	-3	1	1.11	0.46
14	-3	-1	0.98	0.39
14	-3	-1	0.79	0.41
14	-3	-1	1.36	0.72
-14	-3	1	0.68	0.70
14	-3	-1	1.53	0.72
14	-3	-1	1.18	0.39
-14	3	-1	0.83	0.52
-14	-3	1	0.38	0.45
-14	-3	1	0.49	0.65
-14	3	-1	3.43	0.81
-14	3	-1	1.97	0.56
-14	-3	1	1.27	0.48
-14	-3	1	1.26	0.41
14	3	-1	1.89	0.69
14	-3	1	2.17	0.75
-14	3	1	0.25	0.45
-14	3	1	0.77	0.51
14	-3	1	0.58	0.36
-14	3	1	1.12	0.66
14	-3	1	0.95	0.40
14	-3	1	1.67	0.75
-14	-3	-1	0.44	0.47
-14	3	1	2.17	0.40
14	3	-1	0.67	0.32
-14	-3	-1	2.52	0.78
14	3	-1	2.29	0.42
-14	3	1	0.79	0.51
14	-3	1	0.69	0.40
-14	-3	-1	1.29	0.49
-14	-3	-1	2.86	0.53
-14	3	1	1.17	0.83
-14	-3	-1	1.21	0.59
14	-4	-1	1.99	0.49
-14	-4	1	3.49	0.77
14	-4	-1	2.14	0.82
-14	4	-1	1.95	0.60
-14	-4	1	2.08	0.54
14	4	1	1.82	0.74

14	4	1	3.38	0.59
-14	4	-1	2.44	1.03
-14	-4	1	2.49	0.55
14	-4	-1	2.17	0.51
-14	-4	1	3.25	0.58
-14	4	-1	1.90	0.88
-14	-4	1	2.87	0.84
14	4	1	3.29	0.54
-14	4	-1	3.13	0.86
-14	-4	1	2.83	0.66
14	-4	-1	1.97	0.52
-14	-4	1	2.59	0.61
-14	-4	-1	1.93	0.54
-14	4	1	2.40	0.61
14	-4	1	3.83	1.00
-14	-4	-1	2.25	0.57
-14	-4	-1	1.85	0.52
-14	4	1	2.92	0.87
-14	-4	-1	1.83	0.67
-14	-4	-1	1.17	0.75
14	-4	1	2.70	0.54
14	-4	1	2.66	0.57
-14	4	1	2.98	0.89
14	-4	1	3.76	0.62
14	4	-1	1.54	0.41
-14	-4	-1	2.50	0.56
14	4	-1	1.88	0.75
-14	-4	-1	2.18	0.61
14	4	-1	1.99	0.43
-14	-5	1	34.03	1.86
14	-5	-1	34.95	1.97
-14	5	-1	34.39	2.46
-14	-5	1	37.02	2.14
14	5	1	33.03	1.88
-14	-5	1	34.11	1.96
14	5	1	32.45	1.86
14	5	1	30.93	2.21
14	-5	-1	36.99	2.39
14	-5	-1	34.36	2.02
-14	-5	1	33.28	1.85
14	-5	-1	31.85	2.00
-14	-5	1	31.02	1.80
-14	5	-1	33.51	2.38
-14	-5	-1	37.19	1.98

-14	5	1	30.61	2.36
-14	-5	-1	33.63	1.89
-14	-5	-1	34.62	2.05
14	5	-1	36.95	2.38
-14	-5	-1	35.35	1.98
14	-5	1	32.33	1.92
14	-5	1	32.19	1.89
14	5	-1	34.12	1.93
-14	5	1	32.60	1.98
14	5	-1	36.25	1.90
-14	5	1	32.28	2.28
14	-5	1	32.48	1.92
-14	-6	1	11.38	0.97
14	-6	-1	8.44	0.93
14	-6	-1	8.06	1.08
14	6	1	8.09	1.04
14	-6	-1	7.13	0.97
-14	-6	1	8.96	0.90
-14	-6	1	7.22	1.44
-14	6	-1	13.21	2.36
-14	6	-1	9.76	1.88
14	6	1	8.78	0.97
-14	-6	1	9.58	1.07
-14	-6	1	6.41	0.90
-14	-6	-1	11.27	1.29
-14	6	1	5.82	1.71
14	-6	1	7.79	1.03
-14	6	1	6.68	1.38
14	-6	1	7.61	0.95
14	-6	1	9.86	1.74
-14	-6	-1	10.98	0.83
-14	-6	-1	7.52	1.15
14	-6	1	4.94	0.90
14	6	-1	7.44	0.93
-14	-6	-1	11.14	1.02
14	7	1	4.34	0.72
14	7	1	5.20	1.16
14	-7	-1	2.81	0.71
-14	-7	1	4.93	0.86
14	-7	-1	2.67	0.61
14	-7	-1	2.26	1.25
-14	7	-1	5.38	1.16
14	7	1	3.38	0.88
14	-7	-1	3.36	0.76

-14	-7	1	4.81	0.76
-14	-7	1	4.33	1.04
-14	-7	1	3.09	0.66
-14	-7	1	5.91	0.89
14	7	1	7.57	1.17
-14	7	-1	4.39	1.41
14	7	-1	2.93	0.80
-14	-7	-1	5.98	0.92
-14	-7	-1	3.01	0.59
-14	-7	-1	6.24	1.02
14	-7	1	5.27	0.89
14	-7	1	4.82	0.88
14	7	-1	2.60	0.70
-14	7	1	5.04	1.41
-14	7	1	3.16	1.11
14	-7	1	4.94	1.35
14	-7	1	2.95	0.83
14	8	1	3.45	0.71
14	8	1	1.47	0.85
14	-8	-1	1.99	0.73
14	-8	-1	2.53	0.64
-14	-8	1	1.76	0.44
-14	-8	1	1.79	0.64
14	-8	-1	2.93	0.72
-14	8	-1	4.04	1.29
14	-8	1	1.38	0.64
14	8	-1	2.19	0.88
14	-8	1	1.30	0.60
-14	-8	-1	1.44	0.51
14	-8	1	1.37	0.74
14	-9	-1	6.38	1.30
-14	-9	1	13.90	1.24
14	-9	-1	7.97	1.18
-14	9	-1	9.42	1.79
14	-9	-1	7.29	1.12
-14	-9	1	11.33	1.59
14	9	1	9.71	1.89
-14	-9	1	16.48	2.19
-14	-9	-1	9.92	1.66
-14	9	1	12.74	2.31
-14	-9	-1	10.73	1.53
-14	-9	-1	10.46	1.21
-14	-10	1	3.61	1.29
-14	-10	1	3.05	0.99

-14	10	-1	1.60	1.51
14	-10	-1	2.91	0.93
14	-10	-1	3.03	1.00
14	10	1	3.39	1.21
14	-10	-1	1.44	0.89
14	-10	1	1.91	0.82
-14	-10	-1	2.61	0.97
-14	-10	-1	3.37	1.33
-14	10	1	3.22	1.60
-14	-10	-1	1.22	0.93
14	-10	1	1.22	0.77
14	10	-1	1.60	1.17
14	-10	1	1.80	0.91
14	11	1	11.89	2.11
-14	-11	1	15.76	2.24
-14	11	-1	11.91	2.05
14	-11	-1	11.05	1.46
14	-11	-1	10.77	1.97
14	-11	-1	12.04	1.55
-14	-11	1	9.67	1.68
14	-11	1	13.91	2.01
-14	-11	-1	14.56	1.81
14	11	-1	13.57	2.24
14	-11	1	9.59	1.46
-14	-11	-1	14.74	2.24
-14	11	1	14.78	2.57
14	-11	1	9.20	1.56
-15	0	1	0.48	0.47
-15	0	1	0.71	0.56
-15	0	-1	-0.01	0.55
-15	0	1	0.55	0.56
-15	0	-1	0.23	0.45
15	0	-1	0.24	0.36
-15	0	-1	0.46	0.49
15	0	1	-0.33	0.43
-15	0	-1	0.35	0.40
15	0	-1	0.13	0.51
-15	0	-1	-0.17	0.52
15	0	1	0.47	0.23
-15	0	-1	-0.52	0.64
15	0	1	-0.02	0.44
-15	0	1	-0.12	0.34
-15	0	1	-0.43	0.40
-15	0	1	-0.43	0.62

15	0	-1	0.04	0.19
15	0	1	-0.22	0.33
15	0	-1	0.01	0.37
15	-1	-1	29.04	1.52
15	-1	-1	32.14	1.76
-15	-1	1	18.04	1.45
-15	-1	1	21.53	1.41
15	1	1	20.42	1.30
15	1	1	18.53	1.34
-15	1	-1	28.59	1.98
-15	-1	1	19.64	1.30
15	-1	-1	30.03	1.73
-15	1	-1	27.60	1.71
-15	-1	1	21.64	1.63
-15	1	-1	30.02	1.80
-15	1	-1	30.77	1.78
15	1	1	19.94	1.47
15	1	1	20.37	1.09
15	-1	-1	29.94	1.71
-15	-1	1	20.27	1.39
-15	-1	1	20.93	1.56
-15	1	-1	29.36	1.79
15	-1	1	20.88	1.33
-15	1	1	20.72	1.40
15	1	-1	29.62	1.56
-15	-1	-1	31.14	1.93
15	-1	1	19.72	1.15
-15	1	1	20.16	1.39
-15	-1	-1	28.90	1.75
-15	-1	-1	30.33	1.78
-15	1	1	19.68	1.32
-15	-1	-1	28.64	1.70
15	1	-1	29.08	1.86
-15	-1	-1	30.71	1.92
-15	1	1	22.22	1.69
15	-1	1	22.61	1.39
15	-1	1	22.32	1.62
15	1	-1	29.63	1.43
15	1	-1	28.55	1.72
-15	1	1	19.44	1.58
-15	1	1	21.93	1.46
-15	-1	-1	28.16	1.86
15	-1	1	20.42	1.47
-15	-2	1	106.53	5.48

-15	-2	1	115.40	5.55
-15	-2	1	122.58	5.68
15	-2	-1	112.12	5.36
-15	-2	1	118.41	5.53
15	-2	-1	113.63	5.40
-15	-2	1	108.50	5.56
15	-2	-1	111.01	5.24
15	2	1	112.37	5.31
15	2	1	115.70	5.72
-15	2	-1	110.53	5.42
-15	2	-1	117.54	5.30
-15	-2	1	117.15	5.72
-15	2	-1	112.90	5.31
-15	2	-1	104.81	5.35
15	-2	-1	115.17	5.43
-15	2	-1	113.13	5.41
-15	2	-1	112.70	5.19
-15	-2	1	109.85	5.50
-15	2	-1	114.55	5.66
-15	2	1	113.14	5.76
15	-2	1	117.87	5.43
-15	-2	-1	112.18	5.40
15	2	-1	113.19	5.17
-15	-2	-1	119.16	5.48
-15	-2	-1	108.87	5.37
-15	2	1	116.79	5.78
-15	-2	-1	108.35	5.41
15	-2	1	121.36	5.64
15	-2	1	114.21	5.69
-15	2	1	114.55	5.29
-15	2	1	120.16	5.34
-15	2	1	121.26	5.55
-15	-2	-1	122.90	5.59
-15	-2	-1	104.54	5.34
15	-2	1	114.03	5.54
15	2	-1	109.27	5.54
-15	-2	-1	113.97	5.38
-15	2	1	118.07	5.60
15	-2	1	121.49	5.50
15	2	-1	108.95	5.09
-15	3	-1	322.56	14.75
15	-3	-1	322.06	14.45
-15	3	-1	316.53	14.48
-15	-3	1	323.63	15.23

-15	-3	1	345.70	15.31
15	3	1	338.78	15.13
-15	-3	1	344.31	15.20
15	3	1	325.66	15.05
-15	-3	1	315.95	15.20
-15	3	-1	298.99	14.44
15	-3	-1	308.81	14.47
15	-3	-1	318.68	14.38
-15	3	-1	304.44	14.28
15	-3	-1	323.14	14.78
-15	-3	1	324.20	15.23
-15	-3	1	328.80	15.42
15	3	1	322.96	15.44
-15	-3	1	317.48	15.18
-15	3	-1	321.68	14.52
-15	-3	-1	326.12	14.44
-15	3	1	315.32	15.02
-15	3	1	343.53	15.24
-15	3	1	326.98	15.48
15	-3	1	335.61	15.24
15	-3	1	330.70	15.17
-15	-3	-1	322.89	14.66
-15	-3	-1	309.68	14.47
15	-3	1	329.40	15.44
15	-3	1	356.26	15.11
-15	-3	-1	308.76	14.47
15	3	-1	323.77	14.26
15	3	-1	316.61	14.23
-15	-3	-1	309.89	14.43
-15	3	1	365.11	15.24
15	-3	1	364.78	15.46
-15	3	1	344.40	15.25
-15	-3	-1	307.48	14.42
15	3	-1	320.05	14.69
-15	-3	-1	333.15	14.50
-15	3	1	317.09	15.07
-15	-4	1	83.27	4.23
15	-4	-1	111.22	5.30
15	4	1	88.26	4.16
-15	-4	1	87.24	4.32
15	4	1	92.96	4.22
-15	-4	1	85.07	4.24
15	-4	-1	109.48	5.32
15	-4	-1	117.99	5.60

-15	-4	1	90.93	4.44
-15	-4	1	88.16	4.25
-15	4	-1	110.04	5.69
-15	4	-1	105.55	5.62
15	-4	-1	111.73	5.37
15	4	1	85.64	4.57
15	-4	1	83.90	4.30
15	-4	1	97.23	4.64
-15	4	1	83.88	4.31
-15	-4	-1	106.63	5.26
-15	-4	-1	113.22	5.36
15	4	-1	115.74	5.19
-15	4	1	85.51	4.63
-15	-4	-1	109.22	5.37
-15	-4	-1	110.78	5.30
15	-4	1	86.76	4.27
-15	-4	-1	107.06	5.31
15	4	-1	111.10	5.64
15	4	-1	111.81	5.18
15	-4	1	83.62	4.29
-15	4	1	86.46	4.54
-15	-5	1	29.22	1.83
-15	-5	1	31.13	1.77
15	-5	-1	40.29	2.36
15	5	1	30.78	1.77
15	5	1	28.99	1.76
15	5	1	26.98	2.17
-15	-5	1	29.89	1.75
-15	5	-1	44.36	2.85
-15	5	-1	39.17	2.91
15	-5	-1	42.28	2.73
-15	-5	1	28.01	1.71
-15	-5	1	31.76	2.07
15	-5	-1	40.41	2.31
15	-5	-1	40.51	2.35
-15	-5	-1	45.18	2.33
15	-5	1	31.02	1.80
15	5	-1	41.41	2.26
-15	-5	-1	43.52	2.49
15	-5	1	30.72	2.36
15	5	-1	44.27	2.23
-15	5	1	31.30	2.30
-15	-5	-1	40.06	2.30
-15	-5	-1	41.03	2.41

-15	5	1	25.13	2.34
15	-5	1	30.03	1.84
15	-5	1	29.08	1.83
15	5	-1	46.70	2.79
-15	-5	-1	41.56	2.25
-15	6	-1	22.38	2.12
-15	-6	1	28.21	2.02
-15	-6	1	24.28	1.81
15	6	1	29.04	1.75
-15	-6	1	26.45	1.67
-15	-6	1	29.34	2.16
-15	-6	1	26.52	1.59
15	-6	-1	18.96	1.50
15	6	1	28.70	1.78
15	6	1	27.56	2.16
15	-6	-1	25.06	1.61
-15	-6	1	26.57	1.67
-15	6	-1	23.63	2.21
15	-6	-1	22.88	1.56
-15	6	1	25.93	2.40
-15	6	1	25.15	2.24
15	6	-1	22.91	1.48
-15	-6	-1	21.02	1.35
-15	-6	-1	22.18	1.52
15	-6	1	26.93	1.77
-15	-6	-1	22.22	1.65
-15	-6	-1	23.02	1.53
15	6	-1	22.69	1.57
15	-6	1	25.47	1.73
-15	-6	-1	22.13	1.62
-15	-7	1	2.76	0.81
15	-7	-1	3.81	1.31
15	-7	-1	2.89	0.74
15	7	1	2.23	1.05
15	7	1	2.42	0.77
15	-7	-1	3.99	0.71
-15	-7	1	3.28	0.68
-15	-7	1	4.08	1.13
15	-7	-1	2.46	0.67
-15	7	-1	3.64	1.23
-15	-7	1	2.66	0.62
-15	-7	1	4.31	0.75
15	7	1	2.40	0.74
-15	7	-1	1.85	1.40

-15	-7	1	1.90	1.05
-15	-7	-1	3.70	0.75
-15	-7	-1	3.81	0.76
-15	7	1	2.46	1.17
15	7	-1	2.37	0.81
15	7	-1	2.63	0.73
15	-7	1	2.35	1.25
-15	-7	-1	3.51	0.83
-15	7	1	2.50	1.38
15	-7	1	2.87	0.74
15	-7	1	2.59	0.63
15	-7	1	3.08	0.72
15	8	1	11.92	1.46
-15	-8	1	8.95	1.45
-15	8	-1	6.88	1.45
-15	-8	1	6.89	1.11
15	-8	-1	7.62	1.22
15	8	1	9.46	1.39
15	-8	-1	6.14	1.07
-15	-8	1	8.26	1.18
15	-8	-1	8.08	1.12
15	-8	1	10.06	1.33
-15	8	1	7.76	1.52
-15	-8	-1	7.81	1.26
-15	-8	-1	7.12	0.99
-15	-8	-1	9.78	1.44
15	8	-1	8.06	1.40
15	-8	1	8.01	1.18
15	-8	1	8.41	1.14
15	8	-1	7.71	1.22
15	-9	-1	36.47	2.33
-15	9	-1	35.03	3.27
15	-9	-1	31.00	2.16
-15	-9	1	34.95	2.86
15	9	1	29.75	2.55
15	-9	-1	31.17	2.13
-15	-9	1	29.26	1.96
-15	-9	1	32.58	2.36
-15	-9	-1	32.40	2.84
-15	-9	-1	36.21	2.46
15	-9	1	34.74	2.10
-15	9	1	30.19	3.14
-15	-9	-1	30.14	2.07
15	-9	1	32.90	2.27

15	-9	1	30.09	2.18
15	9	-1	32.56	2.58
-15	10	-1	43.48	3.43
-15	-10	1	27.57	2.70
15	10	1	32.07	2.50
15	-10	-1	34.46	2.64
-15	-10	1	31.52	2.34
15	-10	-1	34.23	2.42
15	-10	-1	35.41	2.36
15	10	-1	33.27	2.64
-15	-10	-1	38.61	2.96
15	-10	1	32.93	2.52
-15	10	1	35.06	3.19
15	-10	1	34.63	2.22
-15	-10	-1	37.37	2.52
15	-10	1	31.19	2.26
15	-11	-1	37.71	2.38
15	-11	-1	31.43	2.69
15	11	1	50.96	4.15
-15	-11	1	53.03	4.52
-15	11	-1	31.71	3.44
-15	-11	1	38.03	2.68
15	-11	-1	35.09	2.37
-15	-11	-1	41.29	3.32
15	-11	1	39.70	2.57
-15	11	1	39.62	3.62
15	-11	1	34.77	2.51
-15	-11	-1	33.99	2.83
15	-11	1	38.50	2.90
-16	0	-1	18.96	1.45
16	0	-1	18.36	1.38
-16	0	1	28.85	2.02
-16	0	1	27.87	1.72
16	0	-1	17.25	1.16
16	0	-1	17.06	0.96
-16	0	1	25.38	1.72
16	0	1	29.22	1.79
-16	0	-1	16.19	1.49
16	0	1	30.10	1.70
16	0	1	26.20	1.68
16	0	-1	17.76	1.26
-16	0	1	27.39	1.80
-16	0	-1	17.74	1.26
-16	0	-1	17.92	1.33

-16	0	-1	21.78	1.63
-16	0	1	27.91	1.87
16	0	1	27.25	1.44
-16	0	1	27.63	1.67
-16	0	-1	17.60	1.35
16	-1	-1	28.56	1.57
-16	-1	1	23.55	1.66
16	1	1	25.96	1.63
-16	1	-1	30.79	1.81
-16	-1	1	24.36	1.65
16	-1	-1	32.69	1.85
-16	-1	1	23.04	1.87
-16	-1	1	25.17	1.59
16	1	1	25.96	1.35
16	-1	-1	30.66	1.76
-16	-1	1	26.06	1.84
16	1	1	29.79	1.59
-16	1	-1	32.69	1.87
-16	1	-1	31.11	2.10
16	-1	-1	29.16	1.77
16	1	1	28.36	1.83
-16	1	-1	27.59	2.11
-16	1	-1	29.78	1.87
-16	-1	1	26.29	1.79
-16	-1	-1	29.73	1.78
16	-1	1	28.04	1.67
-16	1	1	26.86	1.90
-16	1	1	23.90	1.59
-16	-1	-1	28.13	1.83
16	1	-1	29.04	1.80
-16	-1	-1	29.49	1.84
-16	1	1	26.54	1.69
-16	-1	-1	32.72	2.00
16	-1	1	31.52	1.76
-16	-1	-1	29.21	2.04
16	-1	1	27.50	1.63
16	1	-1	29.90	1.48
-16	1	1	24.67	1.67
16	1	-1	30.62	1.60
16	1	-1	34.58	2.00
-16	-1	-1	31.04	2.04
-16	1	1	22.67	1.94
16	-1	1	25.46	1.41
16	-2	-1	26.28	1.62

16	2	1	29.21	1.61
-16	2	-1	25.81	1.53
-16	-2	1	25.11	1.62
-16	-2	1	29.01	1.80
-16	-2	1	26.90	1.96
16	-2	-1	25.52	1.46
-16	2	-1	27.07	1.73
-16	-2	1	25.26	1.69
-16	2	-1	24.06	1.40
-16	-2	1	27.32	1.72
-16	2	-1	26.12	1.64
-16	2	-1	24.74	1.62
-16	-2	1	26.02	1.93
16	-2	-1	29.17	1.79
-16	2	-1	28.28	2.03
16	2	1	25.36	1.39
16	2	1	29.87	1.92
-16	-2	1	26.64	1.65
-16	2	1	28.36	2.86
-16	-2	-1	25.48	1.64
16	2	-1	27.41	1.42
-16	-2	-1	27.27	1.69
-16	-2	-1	26.95	1.88
-16	2	1	26.63	1.66
-16	-2	-1	26.78	1.87
-16	2	1	29.01	1.63
-16	-2	-1	27.54	1.94
16	-2	1	24.59	1.49
-16	-2	-1	25.94	1.64
16	-2	1	24.96	1.64
16	-2	1	23.36	1.91
-16	-2	-1	25.96	1.75
16	2	-1	28.74	1.88
16	-2	1	30.77	1.89
-16	2	1	28.19	1.74
16	-2	1	30.24	1.66
16	2	-1	26.38	1.31
-16	2	1	27.14	2.02
-16	2	1	26.29	1.66
-16	-3	1	17.09	1.52
16	-3	-1	18.56	1.37
16	3	1	17.47	1.56
-16	3	-1	14.97	1.09
16	3	1	16.33	1.00

-16	3	-1	14.06	1.14
16	3	1	14.97	1.14
16	-3	-1	15.28	1.15
-16	-3	1	17.14	1.22
16	-3	-1	14.36	1.15
-16	-3	1	16.45	1.28
16	-3	-1	13.88	1.03
-16	-3	1	14.62	1.55
-16	-3	1	16.89	1.31
-16	-3	1	16.20	1.62
-16	3	-1	15.43	1.62
-16	-3	-1	13.96	1.25
-16	-3	-1	14.50	1.43
16	-3	1	15.65	1.10
-16	3	1	18.18	1.68
16	-3	1	20.42	1.69
-16	3	1	16.40	1.40
16	3	-1	14.99	0.98
-16	-3	-1	12.92	1.16
-16	3	1	17.31	1.05
16	-3	1	17.29	1.16
-16	3	1	14.01	1.32
-16	3	1	15.56	1.09
-16	-3	-1	13.67	1.18
-16	-3	-1	13.28	1.21
16	3	-1	16.74	1.53
16	-3	1	14.51	1.21
16	3	-1	17.04	0.91
-16	-3	-1	14.87	1.57
-16	-4	1	20.48	1.43
-16	4	-1	19.02	1.92
-16	-4	1	23.03	1.76
16	4	1	20.05	1.27
16	-4	-1	23.14	1.88
16	4	1	19.99	1.33
16	4	1	19.00	1.71
-16	-4	1	19.03	1.36
16	-4	-1	17.62	1.42
-16	-4	1	19.36	1.41
16	-4	-1	20.00	1.37
-16	4	-1	21.95	1.99
-16	-4	1	22.19	1.45
16	-4	-1	18.43	1.33
-16	-4	-1	19.39	1.63

-16	4	1	20.68	1.81
-16	-4	-1	19.57	1.41
16	4	-1	19.53	1.21
-16	-4	-1	20.20	1.44
-16	4	1	22.19	1.95
-16	-4	-1	19.47	1.39
16	-4	1	18.69	1.36
-16	-4	-1	19.66	1.45
16	4	-1	19.09	1.72
16	4	-1	21.67	1.20
16	-4	1	21.33	1.87
16	-4	1	19.61	1.37
16	5	1	5.40	0.89
16	-5	-1	3.09	0.67
-16	-5	1	7.82	1.13
-16	-5	1	7.22	1.16
16	5	1	5.82	1.25
-16	5	-1	2.69	0.96
-16	-5	1	5.43	0.99
-16	-5	1	7.73	0.92
16	5	1	5.63	0.83
16	-5	-1	3.35	0.61
16	-5	-1	4.68	1.13
-16	-5	1	7.66	0.91
16	-5	-1	3.47	0.72
-16	5	-1	2.24	1.21
-16	-5	-1	2.85	0.62
16	5	-1	4.27	0.85
-16	-5	-1	2.77	0.73
-16	-5	-1	2.91	0.80
-16	-5	-1	2.95	0.90
-16	-5	-1	2.83	0.67
16	-5	1	6.88	0.93
16	-5	1	5.75	0.90
-16	5	1	6.43	1.17
16	5	-1	2.77	0.58
16	-5	1	7.02	1.35
16	-5	1	6.11	0.92
-16	5	1	8.55	1.36
-16	-6	1	31.65	2.06
-16	-6	1	32.26	1.84
16	-6	-1	33.51	2.00
-16	6	-1	32.63	2.65
-16	-6	1	33.66	2.46

16	6	1	33.31	1.98
16	-6	-1	33.03	2.02
16	-6	-1	28.23	1.90
16	6	1	33.32	2.01
-16	-6	1	33.05	2.26
16	-6	-1	35.11	2.48
16	6	1	33.73	2.48
-16	-6	1	30.74	1.90
-16	-6	1	32.35	1.94
-16	6	-1	30.49	2.70
16	-6	1	31.12	2.02
16	-6	1	30.39	1.98
16	6	-1	29.89	1.95
-16	-6	-1	31.20	2.26
-16	6	1	29.69	2.54
-16	-6	-1	33.59	2.09
16	-6	1	30.53	1.96
-16	-6	-1	31.89	1.84
-16	-6	-1	31.67	1.97
-16	6	1	33.42	2.68
16	6	-1	31.95	1.88
-16	-6	-1	32.53	1.97
16	-7	-1	8.24	1.14
16	7	1	7.84	1.26
16	7	1	7.07	1.21
-16	7	-1	12.63	2.05
-16	-7	1	8.05	1.41
-16	-7	1	6.86	0.95
16	-7	-1	10.67	1.16
-16	-7	1	8.35	1.19
16	-7	-1	9.66	1.28
-16	-7	1	4.62	0.84
-16	-7	1	8.36	1.03
16	-7	1	6.48	1.09
16	7	-1	10.79	1.35
-16	-7	-1	9.31	1.21
16	7	-1	10.45	1.23
16	-7	1	6.24	1.05
-16	-7	-1	12.21	1.34
-16	7	1	7.06	1.43
-16	-7	-1	12.74	1.22
16	-7	1	5.84	1.18
16	8	1	15.47	1.56
-16	8	-1	17.62	2.59

-16	-8	1	17.14	1.62
-16	-8	1	22.86	2.34
16	-8	-1	13.79	1.40
16	8	1	16.00	1.79
16	-8	-1	15.21	1.59
-16	-8	1	16.88	1.41
16	-8	-1	14.73	1.43
16	-8	1	14.61	1.42
16	-8	1	13.93	1.39
-16	-8	-1	16.22	1.69
-16	8	1	15.66	2.56
-16	-8	-1	15.54	1.48
-16	-8	-1	21.45	2.34
16	-8	1	12.09	1.50
16	8	-1	19.96	1.89
16	8	-1	14.48	1.51
-16	-9	1	19.26	1.83
16	-9	-1	14.45	1.74
16	-9	-1	14.13	1.57
16	-9	-1	15.22	1.61
-16	-9	1	23.23	2.35
16	9	1	20.48	1.99
-16	9	-1	19.40	2.68
-16	-9	1	17.36	1.51
-16	-9	-1	15.09	2.20
16	-9	1	15.10	1.81
-16	-9	-1	15.15	1.77
16	-9	1	18.18	1.72
-16	-9	-1	13.00	1.49
16	-9	1	17.66	1.69
-16	9	1	18.34	2.67
16	-10	-1	0.21	0.79
-16	-10	1	2.73	0.95
16	-10	-1	1.46	0.77
16	-10	-1	2.78	1.08
-16	-10	1	1.27	1.21
16	10	1	2.18	1.19
-16	10	-1	4.31	1.53
-16	10	1	4.40	1.53
-16	-10	-1	1.72	1.30
16	10	-1	0.70	1.18
-16	-10	-1	1.90	0.94
16	-10	1	0.31	0.75
16	-10	1	1.77	0.88

16	-10	1	2.09	1.08
-17	0	-1	0.06	0.66
17	0	1	0.35	0.26
-17	0	1	0.14	0.81
-17	0	1	-0.31	0.38
17	0	-1	-0.48	0.54
17	0	-1	-0.08	0.22
17	0	1	0.14	0.49
-17	0	-1	0.51	0.68
17	0	-1	0.04	0.41
-17	0	-1	-0.32	0.76
-17	0	-1	0.04	0.44
-17	0	1	0.51	0.53
-17	0	1	0.17	0.67
-17	0	1	-0.44	0.65
17	0	-1	0.42	0.43
17	0	1	-0.23	0.42
17	0	1	-0.03	0.59
-17	1	-1	13.25	1.49
17	-1	-1	13.72	1.11
-17	1	-1	13.00	1.54
-17	-1	1	16.71	1.50
17	1	1	17.41	1.29
-17	-1	1	17.11	1.27
17	-1	-1	13.62	1.12
-17	1	-1	15.27	1.27
17	-1	-1	13.37	0.84
-17	1	-1	11.68	1.09
-17	-1	1	16.83	1.54
-17	-1	1	17.32	1.67
17	1	1	17.72	1.24
17	1	1	16.66	1.48
-17	-1	1	15.71	1.44
17	1	1	17.08	0.97
-17	-1	-1	10.18	1.05
17	-1	1	18.63	1.35
-17	-1	-1	12.03	1.41
17	-1	1	17.28	1.34
17	-1	1	16.65	1.02
-17	1	1	14.73	1.22
17	1	-1	13.13	1.13
-17	-1	-1	14.01	1.52
-17	1	1	16.81	1.32
17	1	-1	13.58	0.75

17	1	-1	12.40	1.29
17	1	-1	13.30	0.92
-17	1	1	16.31	1.62
17	-1	1	15.82	1.26
-17	-1	-1	13.02	1.35
-17	1	1	16.74	1.37
-17	2	-1	70.28	3.74
-17	-2	1	71.03	3.76
-17	-2	1	68.56	3.62
17	-2	-1	77.59	3.78
-17	2	-1	75.00	4.08
17	2	1	66.89	3.25
-17	-2	1	69.35	3.51
17	2	1	71.03	3.41
-17	2	-1	76.70	3.69
17	-2	-1	72.41	3.57
-17	-2	1	67.59	3.49
17	-2	-1	72.86	3.73
-17	-2	1	68.54	3.56
-17	-2	1	67.43	3.75
17	2	1	70.47	3.75
-17	2	1	67.65	3.42
17	-2	1	68.04	3.37
-17	2	1	70.16	3.83
-17	-2	-1	74.02	3.97
-17	-2	-1	69.86	3.73
-17	-2	-1	72.47	3.84
17	-2	1	73.75	3.68
-17	-2	-1	72.88	3.74
-17	2	1	66.79	3.44
17	-2	1	73.22	3.49
17	-2	1	71.26	3.53
-17	2	1	65.25	3.51
-17	-2	-1	76.83	3.94
17	2	-1	77.17	3.98
17	2	-1	76.70	3.50
-17	-3	1	67.79	3.56
-17	-3	1	60.07	3.35
-17	-3	1	63.48	3.32
17	3	1	63.50	3.08
17	3	1	68.56	3.61
17	3	1	63.42	3.17
-17	-3	1	59.65	3.27
17	-3	-1	76.49	3.71

-17	3	-1	69.45	3.54
-17	-3	1	63.24	3.32
17	-3	-1	71.09	3.53
-17	3	-1	66.17	3.93
-17	3	1	64.14	3.72
17	-3	1	70.94	3.53
17	3	-1	72.27	3.83
-17	-3	-1	64.18	3.51
-17	3	1	66.17	3.24
-17	-3	-1	73.41	3.70
-17	-3	-1	63.62	3.54
-17	3	1	61.54	3.10
17	-3	1	68.25	3.23
-17	3	1	60.97	3.12
-17	-3	-1	66.41	3.54
17	3	-1	69.18	3.24
17	-3	1	63.87	3.31
-17	-4	1	100.91	4.94
-17	-4	1	91.28	4.71
-17	-4	1	95.76	4.72
-17	-4	1	92.51	4.76
17	-4	-1	90.45	4.58
17	4	1	101.96	5.11
17	-4	-1	91.48	4.63
-17	4	-1	95.26	5.10
-17	-4	1	92.87	4.70
17	4	1	98.02	4.64
17	-4	-1	90.65	4.68
17	-4	-1	98.12	5.10
-17	4	-1	99.10	4.91
-17	4	1	93.32	4.88
17	4	-1	95.86	4.46
17	-4	1	109.43	5.14
-17	-4	-1	99.81	4.77
17	4	-1	96.01	4.47
17	-4	1	94.93	4.75
-17	-4	-1	90.63	4.62
17	4	-1	98.13	5.03
-17	4	1	94.68	5.14
-17	-4	-1	89.58	4.64
17	-4	1	93.77	4.69
-17	5	-1	20.67	2.15
17	-5	-1	24.52	2.00
17	5	1	15.43	1.77

-17	-5	1	14.43	1.29
17	-5	-1	19.31	1.43
-17	-5	1	13.64	1.32
17	-5	-1	18.56	1.48
-17	-5	1	15.59	1.75
-17	-5	1	19.44	1.78
17	5	1	15.38	1.20
-17	-5	1	16.07	1.28
-17	-5	1	13.79	1.26
-17	5	-1	20.34	2.19
17	5	1	16.51	1.25
17	5	-1	20.20	1.33
-17	5	1	17.98	1.99
17	-5	1	18.48	2.00
17	-5	1	14.07	1.26
-17	-5	-1	19.69	1.53
17	5	-1	17.49	1.31
-17	-5	-1	18.83	1.78
-17	5	1	15.56	1.98
-17	-5	-1	20.87	1.48
-17	-5	-1	17.79	1.40
17	-5	1	13.48	1.23
17	-6	-1	0.92	0.60
-17	-6	1	1.48	0.61
-17	-6	1	0.51	0.56
-17	-6	1	1.12	1.03
-17	6	-1	1.27	1.18
-17	-6	1	0.47	0.99
-17	-6	1	-0.22	0.65
17	-6	-1	1.17	0.65
17	6	1	1.43	0.96
-17	-6	1	2.14	0.74
17	-6	-1	0.03	0.54
17	-6	-1	2.52	1.15
17	6	1	1.22	0.63
17	6	1	0.83	0.67
-17	-6	-1	0.37	0.59
17	-6	1	0.19	0.58
17	-6	1	1.33	1.20
-17	-6	-1	1.73	0.65
-17	6	1	-1.32	0.97
17	6	-1	1.06	0.64
17	-6	1	0.27	0.61
-17	-6	-1	0.94	0.70

17	-6	1	0.44	0.62
17	6	-1	1.49	0.62
-17	7	-1	2.34	1.35
-17	-7	1	4.30	0.84
17	-7	-1	3.71	0.75
17	-7	-1	4.86	0.89
17	7	1	6.98	1.37
17	7	1	5.40	0.94
-17	-7	1	4.85	0.94
-17	-7	1	4.49	0.68
-17	-7	1	4.80	0.88
-17	-7	1	4.92	1.20
17	-7	-1	3.55	0.77
17	7	1	3.50	0.90
-17	-7	-1	4.01	0.82
17	7	-1	4.78	0.90
-17	-7	-1	4.01	0.89
17	-7	1	4.46	0.92
-17	7	1	3.66	1.37
17	-7	1	4.76	0.85
17	-7	1	3.11	0.77
17	7	-1	4.56	0.83
17	-8	-1	13.44	1.50
17	-8	-1	13.00	1.56
-17	-8	1	15.35	1.74
-17	-8	1	13.13	1.38
-17	-8	1	11.41	2.19
17	-8	-1	12.52	1.35
17	8	1	12.43	1.76
17	8	1	16.52	1.95
-17	8	-1	15.05	2.63
17	-8	1	13.71	1.52
17	8	-1	12.31	1.76
-17	-8	-1	14.15	1.78
-17	-8	-1	14.23	2.27
17	-8	1	13.87	1.50
-17	-8	-1	17.08	1.49
17	8	-1	12.26	1.45
-17	8	1	14.69	2.65
17	-8	1	12.84	1.58
17	-9	-1	17.08	1.83
-17	-9	1	17.74	2.22
17	-9	-1	19.66	1.73
17	9	1	19.92	1.87

-17	9	-1	18.51	2.65
17	-9	-1	20.76	1.74
-17	-9	1	16.96	1.79
17	-9	1	16.72	1.65
-17	-9	-1	18.20	2.27
-17	9	1	15.35	2.50
17	9	-1	16.28	1.88
-17	-9	-1	20.87	1.66
17	-9	1	17.47	1.95
17	-9	1	15.22	1.62
-17	-9	-1	19.48	1.85
-17	-10	1	19.29	1.97
17	10	1	23.81	2.20
17	-10	-1	15.53	1.63
-17	10	-1	23.69	2.92
-17	-10	1	20.57	2.45
17	-10	-1	17.17	1.85
17	-10	-1	19.05	1.68
17	10	-1	18.19	2.03
17	-10	1	24.77	1.89
-17	10	1	24.54	2.95
17	-10	1	22.00	2.03
-17	-10	-1	22.48	2.39
-17	-10	-1	16.64	1.92
17	-10	1	19.61	1.79
-18	0	-1	22.65	1.92
-18	0	1	28.40	2.09
18	0	1	29.08	1.54
18	0	-1	22.40	1.40
18	0	-1	23.50	1.24
-18	0	1	27.34	1.80
-18	0	-1	22.96	1.88
-18	0	1	28.08	1.97
-18	0	-1	23.08	1.58
18	0	1	34.08	2.00
-18	0	-1	25.81	1.82
18	0	1	28.25	1.77
18	0	-1	22.87	1.54
18	0	1	29.24	1.81
18	1	1	19.98	1.11
18	1	1	18.28	1.43
18	-1	-1	13.62	0.97
-18	1	-1	15.01	1.37
-18	1	-1	14.52	1.58

18	1	1	20.90	1.65
18	-1	-1	14.69	1.18
-18	-1	1	17.38	1.35
-18	-1	1	17.77	1.64
-18	-1	1	23.35	1.92
-18	-1	1	19.54	1.72
18	1	1	23.00	1.37
18	-1	-1	15.08	1.15
18	-1	1	18.88	1.40
-18	1	1	16.99	1.73
18	1	-1	12.85	1.38
-18	-1	-1	13.09	1.64
-18	1	1	17.56	1.42
18	1	-1	13.67	1.19
18	1	-1	14.45	0.97
-18	-1	-1	13.98	1.19
-18	-1	-1	12.94	1.54
18	1	-1	14.32	0.81
-18	1	1	18.23	1.50
-18	-1	-1	14.88	1.50
18	-1	1	19.90	1.48
-18	1	1	16.23	1.40
18	-1	1	22.33	1.51
-18	2	-1	29.26	1.86
-18	2	-1	28.97	2.30
18	2	1	27.41	2.00
18	2	1	25.45	1.39
18	2	1	26.18	1.61
-18	-2	1	26.36	1.66
18	-2	-1	29.06	1.81
-18	-2	1	27.40	2.14
-18	-2	1	27.35	2.08
-18	-2	1	24.13	1.65
-18	-2	1	25.28	1.84
-18	2	-1	26.64	1.82
18	-2	-1	29.01	1.64
18	-2	-1	31.98	1.92
18	-2	1	27.20	1.73
-18	2	1	24.81	1.60
18	-2	1	29.91	1.88
-18	-2	-1	30.66	1.86
-18	-2	-1	30.28	2.18
18	2	-1	31.32	1.56
-18	2	1	26.58	2.15

18	2	-1	30.66	1.46
18	-2	1	28.96	1.71
18	2	-1	33.08	2.16
-18	-2	-1	27.71	2.00
18	-2	1	25.08	1.49
-18	-2	-1	28.43	1.83
-18	2	1	25.99	1.68
-18	2	1	25.64	1.68
-18	3	-1	6.23	1.22
18	-3	-1	7.89	0.95
-18	3	-1	6.76	1.19
18	-3	-1	6.75	0.78
-18	3	-1	5.99	1.06
-18	-3	1	9.56	1.04
18	-3	-1	6.18	1.30
-18	-3	1	10.10	1.60
-18	-3	1	9.21	1.09
-18	3	-1	5.53	0.94
-18	-3	1	9.43	1.23
18	3	1	11.83	1.42
-18	3	1	11.62	1.62
-18	-3	-1	6.09	1.03
-18	-3	-1	7.94	0.97
-18	-3	-1	6.50	1.14
-18	3	1	9.61	1.02
-18	-3	-1	7.39	1.18
18	3	-1	6.42	0.61
18	-3	1	7.45	1.30
18	-3	1	10.16	0.93
18	3	-1	8.04	1.34
-18	3	1	11.03	0.92
-18	3	1	8.72	0.99
18	-3	1	11.22	1.47
18	3	-1	7.26	0.76
-18	3	1	9.83	1.09
18	-3	1	11.57	1.10
-18	-4	1	3.29	1.11
-18	-4	1	3.47	0.77
18	-4	-1	5.87	1.19
-18	-4	1	3.16	0.72
18	4	1	3.49	1.00
-18	4	-1	4.54	1.19
-18	-4	1	3.05	0.67
-18	4	-1	3.66	0.70

18	4	1	3.54	0.67
18	-4	-1	2.82	0.69
18	4	1	3.77	0.70
18	-4	-1	5.85	0.87
18	-4	1	2.82	0.63
-18	-4	-1	5.65	1.05
-18	-4	-1	3.91	0.74
-18	4	1	3.68	1.04
18	-4	1	3.71	0.66
18	-4	1	5.15	1.04
-18	-4	-1	3.68	0.81
-18	4	1	4.17	1.15
-18	-4	-1	4.78	1.07
18	4	-1	4.54	0.76
18	4	-1	4.50	0.66
-18	5	-1	22.37	1.65
-18	-5	1	22.54	1.60
-18	-5	1	23.60	1.64
18	-5	-1	20.18	1.63
-18	-5	1	22.86	2.11
-18	5	-1	21.65	2.28
-18	-5	1	22.42	1.71
18	5	1	25.66	1.59
18	5	1	25.26	2.17
18	5	1	23.79	1.55
18	-5	-1	22.93	1.60
18	-5	-1	25.84	2.26
-18	5	-1	27.17	2.37
18	-5	-1	22.86	1.58
-18	-5	1	21.24	2.10
-18	5	1	22.47	2.29
-18	-5	-1	22.90	1.61
-18	-5	-1	24.51	1.74
18	-5	1	21.82	1.59
18	5	-1	21.44	1.46
-18	5	1	22.37	2.15
-18	-5	-1	22.99	1.59
-18	-5	-1	22.89	1.93
18	5	-1	22.44	1.46
18	-5	1	26.11	2.31
18	-5	1	23.20	1.66
-18	-6	1	25.32	1.74
18	6	1	26.60	1.82
18	6	1	28.30	1.79

-18	-6	1	27.59	2.35
-18	-6	1	22.88	1.88
18	-6	-1	28.29	1.86
18	-6	-1	24.06	1.76
-18	-6	1	25.68	1.72
18	6	1	25.60	2.39
18	-6	-1	25.22	1.79
-18	6	-1	25.51	2.52
18	6	-1	24.26	1.74
-18	-6	-1	27.60	1.94
18	-6	1	26.33	1.80
18	6	-1	25.56	1.71
18	-6	1	25.97	1.79
-18	-6	-1	25.79	1.78
-18	-6	-1	27.43	1.67
-18	6	1	28.52	2.55
18	7	1	20.21	1.81
-18	-7	1	21.69	2.38
-18	-7	1	19.27	1.82
18	-7	-1	25.82	1.97
18	-7	-1	25.37	1.94
-18	-7	1	19.69	1.59
-18	7	-1	24.54	2.96
18	-7	-1	23.65	1.80
18	7	1	20.32	1.76
18	-7	1	18.74	1.76
18	7	-1	27.16	1.96
18	7	-1	26.84	1.85
18	-7	1	21.25	1.72
-18	7	1	25.36	2.79
-18	-7	-1	25.27	1.87
18	-7	1	20.72	1.60
-18	-7	-1	26.73	2.07
18	-8	-1	40.17	2.60
-18	8	-1	43.72	3.45
18	-8	-1	42.62	2.62
-18	-8	1	41.56	2.73
-18	-8	1	46.06	3.27
-18	-8	1	42.28	2.52
18	-8	-1	44.67	2.72
18	8	1	43.47	2.72
-18	-8	-1	41.99	3.18
18	-8	1	42.07	2.65
-18	-8	-1	41.27	2.58

18	8	-1	41.48	2.68
18	-8	1	42.63	2.67
18	-8	1	40.77	2.73
-18	8	1	44.97	3.50
-18	-8	-1	42.14	2.76
-18	-9	1	58.80	3.41
-18	-9	1	62.03	3.95
18	-9	-1	57.19	3.34
-18	9	-1	59.59	4.26
18	-9	-1	60.48	3.49
18	9	1	60.79	3.51
18	-9	-1	58.92	3.34
18	-9	1	60.03	3.29
18	-9	1	56.28	3.44
18	-9	1	55.76	3.28
18	9	-1	55.25	3.52
-18	9	1	53.89	4.10
-18	-9	-1	61.97	3.55
-18	-9	-1	65.21	3.99
-19	0	-1	0.44	0.77
19	0	1	0.18	0.29
19	0	-1	-0.34	0.25
19	0	-1	0.41	0.48
-19	0	-1	-0.73	0.80
19	0	1	-0.43	0.55
19	0	-1	-0.11	0.51
-19	0	1	-0.38	0.76
-19	0	1	-0.11	0.45
-19	0	-1	-0.66	0.46
-19	0	1	0.18	0.64
19	0	1	-0.68	0.67
-19	0	1	1.26	0.76
19	-1	-1	2.15	0.61
-19	-1	1	2.57	0.63
19	-1	-1	2.19	0.40
-19	1	-1	1.16	0.94
-19	-1	1	3.15	0.99
-19	-1	1	3.82	0.95
19	1	1	5.13	0.90
-19	1	-1	1.32	0.58
-19	1	-1	4.29	0.88
19	1	1	2.58	0.71
19	-1	-1	3.50	0.66
19	1	1	3.29	0.84

19	1	1	2.73	0.50
-19	1	1	2.43	0.66
19	-1	1	4.13	0.74
-19	-1	-1	2.01	1.04
-19	1	1	4.37	1.08
19	1	-1	3.22	0.62
-19	-1	-1	1.74	0.82
19	-1	1	3.51	0.77
19	1	-1	1.93	0.65
-19	1	1	2.64	0.86
19	-1	1	2.75	0.46
-19	1	1	4.64	0.77
-19	-1	-1	1.39	0.56
-19	-1	-1	3.83	0.97
19	-2	-1	15.24	1.31
19	2	1	20.48	1.31
19	2	1	18.44	1.04
-19	2	-1	16.32	1.37
-19	2	-1	17.25	1.85
-19	-2	1	15.59	1.61
-19	2	-1	17.52	1.42
-19	-2	1	15.43	1.35
19	-2	-1	20.64	1.47
19	2	1	21.24	1.73
19	-2	-1	15.01	1.09
-19	-2	1	17.42	1.92
-19	-2	-1	15.22	1.59
19	-2	1	16.74	1.17
-19	2	1	15.11	1.37
19	-2	1	17.17	1.36
19	-2	1	18.38	1.42
-19	2	1	15.36	1.85
-19	-2	-1	14.71	1.82
19	-2	1	20.75	1.60
-19	2	1	15.56	1.43
-19	2	1	19.28	1.38
-19	-2	-1	15.83	1.32
-19	3	-1	19.85	1.57
19	3	1	25.92	1.51
-19	-3	1	24.26	1.58
-19	3	-1	23.38	2.15
-19	-3	1	19.04	1.53
-19	3	-1	20.16	1.52
19	-3	-1	18.45	1.34

-19	3	-1	20.17	1.42
-19	-3	1	19.84	1.72
-19	-3	1	19.20	2.09
19	3	1	24.06	2.75
19	-3	-1	21.13	1.54
19	3	1	23.22	1.27
-19	-3	-1	22.94	1.59
-19	-3	-1	22.31	1.72
-19	3	1	18.67	1.59
19	-3	1	20.31	1.54
-19	-3	-1	17.39	1.96
19	-3	1	17.84	1.38
-19	3	1	25.61	2.20
-19	-4	1	36.23	2.30
-19	4	-1	40.68	2.90
19	4	1	42.00	2.13
19	-4	-1	34.87	2.13
-19	-4	1	38.57	2.37
19	-4	-1	37.52	2.22
-19	-4	1	47.31	2.81
19	-4	-1	43.99	2.67
19	4	1	43.60	2.23
-19	4	-1	38.10	2.45
-19	4	-1	34.53	2.26
-19	-4	1	36.34	2.38
-19	-4	1	37.00	2.73
19	4	1	44.47	2.79
-19	4	1	39.53	2.91
-19	-4	-1	35.02	2.57
-19	4	1	37.87	2.38
19	-4	1	39.78	2.35
19	4	-1	39.67	2.00
-19	4	1	38.59	2.40
19	4	-1	40.86	1.95
19	-4	1	37.89	2.25
-19	-4	-1	36.44	2.58
-19	-4	-1	33.68	2.29
-19	-4	-1	37.05	2.32
19	5	1	9.16	1.02
-19	-5	1	6.65	1.14
-19	-5	1	6.67	1.23
-19	5	-1	6.31	1.43
19	-5	-1	6.08	0.91
-19	-5	1	8.44	1.14

19	5	1	6.25	1.11
19	-5	-1	7.68	1.45
19	5	1	8.25	1.49
-19	5	-1	8.84	1.17
19	-5	-1	7.72	1.06
-19	-5	1	10.19	1.71
19	-5	1	9.48	1.80
-19	-5	-1	8.00	1.19
-19	5	1	6.89	1.39
19	5	-1	9.01	0.98
19	5	-1	7.54	0.95
-19	-5	-1	7.82	1.18
19	-5	1	6.59	0.98
19	-5	1	5.66	1.05
-19	6	-1	-0.89	1.41
19	-6	-1	0.09	0.71
-19	-6	1	-1.04	0.64
-19	-6	1	0.23	1.06
19	6	1	0.87	0.78
19	-6	-1	-0.38	0.68
-19	6	-1	1.47	0.77
19	-6	-1	-0.30	0.59
-19	-6	1	0.14	0.67
19	6	1	0.51	0.74
19	6	1	0.93	1.15
-19	-6	1	0.52	0.73
-19	-6	-1	1.61	0.84
19	-6	1	1.58	0.76
19	-6	1	0.03	0.61
-19	-6	-1	1.06	0.80
19	6	-1	0.18	0.64
-19	6	1	-1.06	1.36
19	6	-1	1.71	0.71
19	7	1	2.36	0.89
-19	-7	1	1.41	0.96
-19	-7	1	3.23	1.35
19	-7	-1	2.35	0.81
19	-7	-1	1.67	0.77
19	-7	-1	0.98	0.84
-19	7	-1	2.40	1.46
-19	7	-1	-0.02	0.81
19	7	1	3.00	0.87
-19	-7	1	2.07	0.82
19	-7	1	2.87	0.94

19	7	-1	1.93	0.92
-19	-7	-1	1.42	1.01
-19	7	1	0.81	1.42
-19	-7	-1	1.85	0.81
-19	-7	-1	1.68	1.28
19	7	-1	2.94	0.85
19	-7	1	1.04	0.87
19	-8	-1	9.85	1.35
19	-8	-1	9.02	1.40
19	-8	-1	10.53	1.43
-19	8	-1	11.09	1.45
-19	-8	1	7.98	1.09
-19	8	-1	12.57	2.40
-19	-8	1	8.81	1.31
-19	-8	1	8.37	1.55
19	8	1	6.93	1.11
-19	-8	-1	9.91	1.53
-19	-8	-1	13.79	2.05
-19	-8	-1	9.79	1.45
19	8	-1	7.37	1.45
-19	8	1	8.96	1.71
19	-8	1	7.22	1.09
19	-8	1	6.43	1.36
19	9	1	4.37	1.07
-19	-9	1	2.55	1.32
19	-9	-1	0.00	0.78
-19	9	-1	-1.44	1.38
-19	-9	1	4.06	1.07
19	-9	-1	1.17	0.90
19	-9	-1	1.16	0.76
19	-9	1	1.48	0.88
-19	-9	-1	4.32	1.31
19	-9	1	2.54	0.85
-19	9	1	-0.34	1.45
-19	-9	-1	1.85	0.97
-20	0	1	16.43	1.75
-20	0	1	15.65	1.52
-20	0	1	16.77	1.43
-20	0	-1	16.91	1.85
-20	0	-1	15.22	1.35
20	0	1	18.33	1.48
-20	0	1	19.10	1.91
20	0	-1	15.82	1.34
-20	0	-1	13.62	1.68

20	0	1	19.39	1.12
20	0	-1	15.39	1.14
20	0	1	19.34	1.40
20	0	-1	15.18	0.94
20	0	1	17.61	1.63
-20	1	-1	55.77	3.12
20	1	1	60.00	3.20
20	-1	-1	60.08	3.02
20	1	1	60.86	3.34
-20	1	-1	58.18	3.51
-20	-1	1	53.60	3.09
-20	-1	1	59.74	3.34
20	-1	-1	59.43	3.10
20	1	1	56.76	2.96
-20	-1	1	59.27	3.62
-20	-1	1	58.56	3.30
20	-1	-1	57.79	2.81
-20	-1	1	58.00	3.08
20	1	1	57.63	2.80
-20	1	-1	57.98	3.21
-20	-1	-1	57.86	3.35
-20	1	1	56.66	3.45
-20	1	1	55.59	3.16
-20	1	1	57.25	3.12
20	-1	1	57.57	3.14
20	-1	1	60.64	3.12
20	1	-1	55.22	2.80
20	-1	1	58.51	2.87
20	-1	1	59.83	3.18
20	1	-1	58.12	2.71
-20	-1	-1	56.15	3.10
-20	1	1	56.84	3.14
-20	-2	1	44.26	2.45
-20	2	-1	44.48	2.58
-20	-2	1	39.85	2.41
20	2	1	42.38	2.78
-20	-2	1	44.81	2.70
20	2	1	42.55	2.11
20	-2	-1	48.33	2.59
-20	-2	1	43.06	2.67
20	-2	-1	45.30	2.54
20	-2	-1	44.14	2.30
-20	2	-1	41.30	3.01
20	2	1	40.13	2.28

-20	2	-1	42.59	2.54
20	-2	1	47.07	2.48
20	-2	1	42.65	2.48
-20	-2	-1	44.82	2.57
-20	2	1	40.85	2.47
-20	2	1	43.11	2.94
-20	2	1	42.33	2.41
-20	-2	-1	49.68	3.36
-20	-2	-1	41.93	2.74
20	-2	1	44.18	2.57
-20	-2	-1	43.65	2.56
-20	2	1	38.74	2.34
20	-2	1	41.25	2.22
-20	-3	1	10.64	1.54
-20	3	-1	7.13	1.43
-20	-3	1	8.87	1.39
20	3	1	8.45	1.54
-20	-3	1	9.24	1.23
20	3	1	11.18	0.87
20	-3	-1	10.58	1.37
-20	3	-1	9.24	1.04
-20	-3	1	9.23	1.14
-20	3	-1	9.16	1.22
20	3	1	11.16	1.11
-20	-3	1	8.65	1.46
20	-3	-1	9.36	0.94
20	-3	-1	7.86	1.08
20	-3	1	10.60	1.22
-20	-3	-1	8.84	1.16
-20	-3	-1	8.05	1.18
-20	3	1	10.02	1.08
-20	3	1	10.88	1.24
20	-3	1	10.08	0.98
-20	3	1	9.82	1.84
-20	-3	-1	8.07	1.42
20	-3	1	11.34	1.48
-20	-3	-1	8.94	1.40
20	4	1	1.73	0.55
-20	4	-1	1.01	0.93
-20	4	-1	1.51	0.66
-20	-4	1	2.08	0.66
20	-4	-1	2.82	0.69
-20	-4	1	2.01	1.22
20	4	1	0.45	1.06

20	-4	-1	1.12	0.91
20	-4	-1	2.56	0.60
-20	-4	1	2.30	0.75
-20	-4	1	1.93	0.86
-20	-4	1	1.97	1.01
-20	4	-1	2.10	1.21
-20	4	1	1.60	0.65
-20	-4	-1	2.78	0.87
20	4	-1	2.07	0.47
20	-4	1	2.16	0.59
-20	4	1	2.97	1.21
20	-4	1	1.34	1.16
-20	-4	-1	1.35	0.74
20	-4	1	2.13	0.65
-20	-4	-1	0.75	0.66
20	-5	-1	10.35	1.26
20	-5	-1	10.11	1.25
-20	5	-1	11.78	1.42
-20	-5	1	11.73	1.25
20	5	1	13.63	1.32
-20	-5	1	11.03	1.36
-20	-5	1	11.24	1.82
20	5	1	11.80	1.12
-20	-5	1	8.30	1.37
20	5	1	13.16	1.89
-20	5	-1	10.64	1.86
-20	-5	-1	8.81	1.36
20	-5	1	10.01	1.23
-20	-5	-1	10.03	1.32
-20	5	1	10.80	1.44
-20	-5	-1	11.73	1.33
20	5	-1	10.53	1.07
20	-5	1	10.19	1.29
-20	5	1	11.32	1.86
20	5	-1	11.54	1.01
20	6	1	7.25	1.04
-20	-6	1	6.60	1.20
20	6	1	7.01	1.28
-20	6	-1	5.25	0.97
20	-6	-1	6.92	1.14
-20	-6	1	5.01	1.47
-20	6	-1	6.29	1.58
20	-6	-1	6.72	1.19
-20	-6	1	7.69	1.42

20	-6	1	7.08	1.10
-20	6	1	7.55	1.59
-20	-6	-1	8.15	1.28
-20	-6	-1	6.25	1.12
20	6	-1	5.19	0.92
20	-6	1	5.94	0.99
20	6	-1	7.66	1.11
20	7	1	47.62	2.80
20	-7	-1	45.71	2.79
-20	7	-1	44.97	3.55
-20	-7	1	51.28	3.48
20	7	1	54.74	2.87
-20	-7	1	49.24	3.01
20	-7	-1	44.75	2.74
-20	7	-1	46.51	2.77
-20	-7	1	45.45	2.80
20	-7	-1	46.94	2.74
20	7	-1	48.01	2.67
20	7	-1	43.31	2.65
-20	-7	-1	45.08	2.87
20	-7	1	47.54	2.88
20	-7	1	48.65	2.86
-20	7	1	49.24	3.65
-20	-7	-1	45.09	3.29
-20	-7	-1	44.64	2.75
-20	8	-1	58.51	3.33
20	-8	-1	55.29	3.30
-20	8	-1	57.38	4.16
-20	-8	1	63.60	3.97
-20	-8	1	58.05	3.44
20	8	1	57.79	3.33
20	-8	-1	60.50	3.28
20	-8	-1	55.63	3.32
20	-8	1	58.28	3.27
-20	-8	-1	65.25	3.97
-20	8	1	57.98	4.18
-20	-8	-1	57.93	3.30
20	-8	1	55.35	3.30
-20	-8	-1	59.27	3.47
20	8	-1	55.68	3.31
-21	0	1	-0.74	0.87
21	0	-1	0.30	0.31
21	0	1	0.30	0.62
21	0	1	0.38	0.66

21	0	-1	-0.04	0.61
-21	0	-1	-0.84	0.59
21	0	1	-0.25	0.36
-21	0	1	0.64	0.97
-21	0	1	0.79	0.81
21	0	-1	0.20	0.51
-21	0	1	-0.43	0.57
21	0	1	0.36	0.82
-21	0	-1	0.13	0.98
-21	0	-1	0.64	0.91
21	1	1	2.50	0.44
21	1	1	2.68	0.95
-21	1	-1	3.82	1.16
21	1	1	3.53	0.67
21	-1	-1	3.29	0.62
-21	-1	1	4.02	1.01
21	-1	-1	4.05	0.75
-21	-1	1	1.90	0.76
21	-1	-1	2.62	0.75
-21	-1	1	2.74	0.75
-21	-1	1	3.78	1.39
-21	1	-1	2.28	0.97
-21	1	-1	1.42	0.70
21	-1	1	4.02	0.92
-21	-1	-1	4.83	1.16
-21	1	1	2.58	1.09
-21	-1	-1	3.01	0.77
21	-1	1	3.51	0.67
-21	1	1	2.59	0.94
-21	1	1	2.19	0.71
21	-1	1	3.53	0.87
21	1	-1	3.04	0.49
21	1	-1	4.16	0.67
-21	-1	-1	3.34	0.76
-21	1	1	3.71	0.81
21	-1	1	1.37	0.71
21	2	1	5.98	1.19
-21	-2	1	7.11	1.13
-21	-2	1	7.99	1.84
-21	-2	1	2.82	0.76
21	2	1	4.90	0.63
21	-2	-1	3.92	0.81
21	-2	-1	3.84	0.71
-21	-2	1	7.90	1.46

-21	2	-1	2.72	0.72
-21	2	-1	4.03	1.26
21	-2	1	4.79	0.74
-21	-2	-1	4.82	0.88
-21	-2	-1	3.79	0.84
21	-2	1	3.54	0.78
-21	-2	-1	3.44	1.04
-21	2	1	3.08	1.19
-21	2	1	8.04	1.09
21	-2	1	7.56	1.31
-21	2	1	2.45	0.74
-21	3	-1	4.35	1.34
-21	3	-1	1.97	0.79
21	-3	-1	4.00	0.80
-21	-3	1	5.24	1.03
-21	-3	1	2.59	0.78
21	-3	-1	2.77	0.65
-21	3	1	6.15	1.38
-21	3	1	2.32	0.79
-21	-3	-1	4.47	1.05
21	-3	1	3.47	0.78
21	-3	1	2.69	0.64
-21	-3	-1	3.91	0.87
-21	-3	-1	6.41	1.15
-21	-4	1	3.30	0.93
21	-4	-1	1.01	0.71
-21	-4	1	3.52	0.81
-21	4	-1	0.69	1.37
21	4	1	5.65	1.34
21	-4	-1	0.97	0.61
-21	4	-1	0.48	0.73
-21	4	1	1.15	0.75
-21	-4	-1	1.46	0.73
21	-4	1	2.29	0.70
21	-4	1	1.38	0.66
-21	-4	-1	1.82	0.78
-21	4	1	1.96	1.41
-21	-4	-1	1.71	0.91
-21	5	-1	0.51	0.74
21	-5	-1	0.34	0.62
21	-5	-1	0.54	0.62
21	5	1	1.12	0.70
-21	-5	1	1.79	0.75
-21	-5	1	-0.41	1.15

-21	-5	1	-0.46	0.79
21	5	1	0.78	0.74
-21	5	-1	-0.51	1.40
-21	-5	1	0.96	0.86
-21	-5	-1	1.34	0.84
21	-5	1	0.30	0.61
-21	5	1	0.60	1.39
-21	-5	-1	1.18	0.83
21	-5	1	0.02	0.76
21	5	-1	1.12	0.60
21	5	-1	1.35	0.60
-21	-5	-1	0.98	0.84
-21	5	1	0.07	0.67
-21	-6	1	2.51	0.81
-21	6	-1	0.72	1.38
21	6	1	1.72	0.89
-21	-6	1	2.40	0.91
21	6	1	1.83	0.81
-21	-6	1	1.19	1.16
-21	6	-1	1.89	0.76
21	-6	-1	0.12	0.79
21	-6	-1	1.59	0.69
-21	-6	-1	2.55	1.31
-21	-6	-1	1.49	0.91
21	6	-1	1.76	0.73
21	-6	1	1.60	0.70
21	-6	1	0.71	0.82
21	6	-1	1.10	0.90
-21	6	1	2.49	0.83
-21	6	1	3.40	1.44
-21	-6	-1	0.15	0.88
21	-7	-1	2.58	0.84
21	-7	-1	0.32	0.73
-21	7	-1	2.64	1.60
-21	7	-1	-0.14	0.78
-21	-7	1	2.35	1.33
-21	-7	1	1.42	0.91
-21	-7	1	0.36	0.84
21	7	1	1.52	0.81
-21	7	1	0.57	0.75
21	-7	1	0.75	0.75
-21	-7	-1	1.49	0.91
-21	-7	-1	3.23	1.29
-21	-7	-1	1.60	0.91

21	7	-1	0.17	1.14
21	-7	1	-0.51	0.73
21	7	-1	1.19	0.79
-21	7	1	1.11	1.50
21	-8	-1	3.97	0.92
21	-8	-1	2.78	0.89
-21	-8	1	3.20	0.99
21	-8	1	4.51	0.93
21	-8	1	2.65	0.86
-21	-8	-1	4.00	1.03
22	0	1	42.58	2.57
-22	0	1	40.10	2.50
-22	0	-1	52.62	3.40
22	0	-1	51.62	3.01
-22	0	1	43.70	2.99
22	0	1	41.38	2.39
-22	0	-1	51.12	2.98
-22	0	1	38.88	2.62
22	0	1	46.40	2.72
22	0	-1	54.25	2.58
22	0	1	41.27	2.14
22	0	-1	55.12	2.72
22	-1	-1	23.95	1.42
-22	-1	1	28.66	2.06
-22	-1	1	31.16	2.44
22	1	1	31.08	1.85
-22	1	-1	24.16	2.45
22	-1	-1	25.43	1.84
-22	1	-1	25.12	1.90
22	1	1	34.40	2.45
22	1	1	31.83	1.66
-22	-1	1	32.03	2.32
-22	-1	1	33.04	2.08
22	-1	-1	24.77	1.68
-22	1	1	32.62	2.13
22	-1	1	31.92	2.12
22	-1	1	31.18	1.75
22	1	-1	24.28	1.29
-22	1	1	28.47	2.08
-22	-1	-1	23.94	2.18
-22	-1	-1	26.36	1.82
-22	1	1	29.91	2.59
22	-1	1	30.41	2.03
22	1	-1	25.27	1.65

-22	-1	-1	27.24	1.86
22	-2	-1	54.57	3.06
-22	-2	1	57.74	3.21
22	-2	-1	56.23	2.82
-22	2	-1	55.50	3.17
-22	-2	1	54.92	3.20
22	2	1	58.21	2.81
-22	2	-1	51.26	3.01
22	-2	-1	58.35	3.12
-22	-2	1	58.96	3.46
-22	2	-1	54.09	3.67
22	2	1	57.32	2.94
22	2	1	57.15	3.53
-22	2	1	58.08	3.30
22	-2	1	57.60	3.28
-22	2	1	57.83	3.08
-22	2	1	64.79	3.81
-22	-2	-1	55.37	3.39
-22	-2	-1	54.06	3.11
22	-2	1	57.42	2.97
-22	-2	-1	59.54	3.14
22	-2	1	60.84	3.25
-22	-3	1	21.45	1.80
22	-3	-1	24.48	1.91
22	-3	-1	23.04	1.60
22	-3	-1	24.81	1.83
-22	-3	1	22.26	1.81
22	3	1	24.01	1.35
-22	3	-1	26.01	2.79
22	3	1	24.18	2.26
-22	-3	1	22.38	2.11
-22	3	-1	25.68	1.99
-22	-3	1	27.66	2.32
-22	-3	-1	25.89	2.12
22	-3	1	23.76	1.95
-22	-3	-1	21.62	1.84
22	-3	1	23.42	1.60
22	-3	1	22.61	1.80
-22	-3	-1	25.81	1.94
-22	3	1	23.87	1.93
-22	3	1	25.42	2.70
22	-4	-1	4.77	0.96
-22	-4	1	2.53	1.00
22	4	1	3.38	0.64

-22	-4	1	3.45	0.97
-22	4	-1	4.03	1.71
22	-4	-1	4.74	0.91
-22	-4	1	2.83	0.86
-22	-4	1	3.94	1.27
-22	4	-1	3.09	0.92
22	4	1	3.19	0.73
22	-4	1	3.10	0.71
-22	4	1	3.78	1.50
22	-4	1	3.55	0.94
-22	4	1	1.56	0.84
-22	-4	-1	3.19	0.93
-22	-4	-1	4.34	1.03
-22	-4	-1	3.66	1.07
22	-5	-1	1.89	0.69
-22	5	-1	3.51	1.51
-22	5	-1	3.16	0.85
-22	-5	1	2.23	0.87
-22	-5	1	2.47	0.94
-22	-5	1	1.52	1.37
22	5	1	2.25	0.67
22	-5	-1	2.32	0.88
-22	-5	-1	2.38	0.89
22	-5	1	1.95	0.68
-22	5	1	0.96	1.33
-22	-5	-1	4.13	1.61
-22	5	1	2.34	0.79
22	-5	1	1.91	0.88
-22	-5	-1	3.34	0.92
22	6	1	13.02	1.30
22	-6	-1	13.02	1.34
-22	-6	1	11.91	1.57
-22	6	-1	16.98	2.66
-22	6	-1	14.43	1.54
-22	-6	1	14.92	1.64
22	6	1	15.30	1.56
22	-6	-1	11.38	1.49
-22	-6	1	11.04	2.18
22	6	-1	13.18	1.23
-22	-6	-1	11.17	2.15
-22	-6	-1	11.02	1.58
22	-6	1	14.80	1.43
-22	-6	-1	15.30	1.65
-22	6	1	8.69	1.82

22	-6	1	11.36	1.47
22	6	-1	13.13	1.41
-22	6	1	14.09	1.57
-22	-7	1	21.87	1.98
-22	-7	1	17.32	2.47
-22	7	-1	22.36	3.57
22	-7	-1	14.34	1.50
22	7	1	22.11	1.75
22	-7	-1	18.08	1.62
-22	-7	1	17.90	1.87
-22	7	-1	17.81	1.66
-22	-7	-1	20.02	1.84
-22	7	1	21.89	1.85
-22	7	1	29.77	4.60
22	7	-1	17.31	1.53
22	-7	1	21.10	1.72
22	7	-1	14.58	1.62
22	-7	1	20.30	1.78
-22	-7	-1	12.09	2.25
-22	-7	-1	14.57	1.80
-23	0	-1	0.83	0.87
23	0	-1	-0.27	0.39
-23	0	1	-0.34	0.76
-23	0	-1	0.97	1.14
23	0	1	-0.55	0.79
-23	0	1	0.17	0.91
-23	0	1	-0.38	1.15
23	0	1	0.25	0.46
-23	0	-1	-0.25	0.80
23	0	-1	-0.95	0.64
23	0	-1	-0.20	0.64
23	0	1	-0.81	0.69
23	1	1	14.57	1.06
23	-1	-1	13.39	1.07
23	-1	-1	16.21	1.51
-23	-1	1	14.90	1.46
-23	1	-1	12.65	1.58
23	-1	-1	14.79	1.34
-23	-1	1	13.11	2.45
-23	-1	1	16.59	2.16
23	1	1	14.44	1.50
-23	-1	1	14.74	1.62
-23	1	-1	14.76	2.22
-23	1	-1	12.08	1.58

23	-1	1	15.00	1.18
-23	1	1	15.99	1.61
-23	-1	-1	13.58	1.55
-23	-1	-1	14.33	1.62
-23	1	1	20.75	2.25
23	-1	1	14.73	1.53
-23	1	1	15.03	1.66
23	-1	1	15.02	1.67
-23	-1	-1	13.29	2.03
-23	2	-1	7.00	1.72
23	2	1	5.42	0.78
23	-2	-1	8.83	1.32
-23	-2	1	6.49	1.34
-23	-2	1	5.39	1.28
-23	2	-1	7.70	1.35
23	2	1	7.36	0.92
23	-2	-1	7.28	1.30
-23	-2	1	5.52	1.02
-23	-2	1	5.56	1.08
23	-2	-1	6.66	0.87
-23	2	1	3.56	0.90
23	-2	1	4.87	1.04
-23	-2	-1	9.07	1.78
23	-2	1	6.23	0.88
-23	2	1	5.76	1.58
-23	-2	-1	4.17	1.03
23	-2	1	7.75	1.41
-23	-2	-1	8.82	1.39
-23	3	-1	35.65	2.35
-23	-3	1	29.48	2.13
-23	-3	1	31.60	2.41
23	-3	-1	30.33	1.98
-23	-3	1	30.26	2.26
-23	-3	1	31.98	2.67
23	-3	-1	34.63	2.40
-23	3	-1	39.45	3.38
23	-3	1	28.04	1.81
-23	3	1	28.40	3.02
-23	-3	-1	34.89	2.42
23	-3	1	31.56	2.25
-23	-3	-1	36.25	2.80
-23	-3	-1	40.51	2.72
-23	3	1	31.70	2.17
-23	-3	-1	35.53	2.45

-23	-4	1	24.50	1.97
23	4	1	23.66	1.56
23	-4	-1	18.95	1.79
-23	4	-1	17.76	1.66
-23	4	-1	20.58	2.66
-23	-4	1	25.31	1.89
23	-4	-1	16.39	1.35
-23	-4	1	23.92	2.18
-23	-4	1	23.05	2.68
23	-4	1	19.70	1.54
-23	-4	-1	16.36	1.83
-23	-4	-1	18.75	2.09
23	-4	1	23.52	1.88
-23	-4	-1	19.76	2.52
-23	4	1	21.36	1.84
-23	-4	-1	17.45	1.76
-23	4	1	24.17	2.68
-23	-5	1	18.17	1.72
-23	-5	1	16.08	2.42
-23	5	-1	18.40	2.65
23	-5	-1	15.08	1.39
23	5	1	16.63	1.56
23	-5	-1	16.52	1.65
23	5	1	16.84	1.26
-23	-5	1	18.59	1.71
-23	5	-1	15.69	1.66
23	-5	1	15.36	1.44
-23	-5	-1	15.77	1.85
-23	-5	-1	15.17	1.66
23	-5	1	15.10	1.63
-23	5	1	20.37	2.60
-23	-5	-1	18.98	2.40
-23	5	1	16.25	1.65
23	-6	-1	4.35	0.95
23	6	1	4.13	0.79
23	-6	-1	6.08	1.00
-23	-6	1	5.46	1.48
-23	-6	1	4.62	1.07
-23	6	-1	3.58	1.59
-23	-6	1	3.86	1.08
-23	6	-1	5.99	1.07
-23	6	1	3.60	0.98
-23	-6	-1	4.60	1.20
23	-6	1	2.71	0.85

-23	-6	-1	4.28	1.02
-23	-6	-1	7.82	1.50
-23	6	1	6.23	1.78
23	-6	1	3.03	0.88
-24	0	1	11.92	2.17
24	0	-1	9.11	1.06
24	0	1	9.67	1.60
-24	0	1	10.10	1.32
-24	0	-1	11.96	1.46
-24	0	1	8.24	1.27
-24	0	-1	13.80	2.20
-24	0	-1	12.57	1.79
24	0	1	11.44	0.94
24	0	-1	8.76	1.62
-24	0	1	10.10	1.70
24	0	-1	9.39	0.84
24	-1	-1	24.28	2.03
-24	-1	1	25.97	2.00
-24	1	-1	23.27	1.87
-24	-1	1	23.09	2.24
24	-1	-1	22.60	1.39
24	-1	-1	22.15	1.77
-24	1	-1	23.84	2.90
-24	1	-1	22.75	2.07
-24	-1	1	20.76	1.96
24	1	1	23.32	1.59
-24	-1	1	27.61	2.55
24	1	1	24.76	1.40
-24	1	1	22.55	1.91
24	-1	1	26.36	1.88
24	-1	1	23.54	1.47
-24	-1	-1	21.25	1.93
-24	-1	-1	22.96	2.22
-24	-1	-1	25.75	1.92
-24	1	1	24.01	2.87
-24	-1	-1	22.88	2.51
24	-1	1	27.20	2.07
-24	2	-1	15.97	1.62
-24	-2	1	19.67	2.33
-24	2	-1	18.13	2.76
24	-2	-1	17.37	1.82
-24	-2	1	18.15	1.88
24	-2	-1	14.95	1.16
24	-2	-1	18.34	1.55

-24	-2	1	19.62	1.82
-24	-2	1	21.44	2.26
24	-2	1	18.89	1.30
-24	-2	-1	18.77	2.29
-24	-2	-1	14.58	1.67
-24	-2	-1	16.03	1.76
-24	-2	-1	17.18	2.19
24	-2	1	16.07	1.74
-24	2	1	23.10	2.68
-24	2	1	16.89	1.65
-24	-3	1	1.38	1.23
-24	-3	1	2.01	0.92
-24	-3	1	2.01	1.04
24	-3	-1	2.97	0.59
24	-3	-1	2.33	0.89
-24	-3	1	0.20	1.32
-24	3	-1	4.01	1.54
-24	3	-1	2.83	0.88
24	-3	1	3.15	0.89
24	-3	1	1.17	0.57
-24	3	1	0.77	0.81
-24	3	1	1.41	1.43
-24	-3	-1	2.69	1.49
-24	-3	-1	2.52	1.19
-24	-3	-1	2.28	0.87
-24	-3	-1	1.04	0.96
-24	-4	1	5.42	1.22
-24	4	-1	4.23	1.62
-24	4	-1	5.84	1.12
24	-4	-1	4.30	0.74
24	-4	-1	3.71	0.96
-24	-4	1	5.01	1.51
-24	-4	1	4.63	0.97
-24	-4	-1	6.55	1.56
24	-4	1	3.11	0.95
24	-4	1	3.91	0.74
-24	4	1	4.43	1.57
-24	4	1	4.45	1.04
-24	-4	-1	5.44	1.03
-24	-4	-1	2.73	1.02
-24	-5	1	9.83	1.44
24	-5	-1	7.13	1.32
-24	-5	1	6.83	1.30
-24	5	-1	7.64	1.74

-24	-5	1	7.18	1.60
24	-5	1	5.87	1.02
-24	5	1	8.20	1.69
-24	-5	-1	7.99	1.29
-24	-5	-1	6.72	1.18
-24	-5	-1	9.83	1.63
-25	0	-1	-0.57	1.02
-25	0	-1	0.02	0.79
-25	0	-1	-0.48	1.56
-25	0	1	0.89	1.61
-25	0	-1	-0.19	0.78
25	0	1	-0.18	0.42
-25	0	1	0.36	0.81
25	0	-1	-0.11	0.38
-25	0	1	-0.89	0.71
25	0	1	1.27	0.83
25	0	1	0.70	0.79
25	0	-1	0.06	0.59
-25	-1	1	15.22	1.59
-25	-1	1	14.02	1.58
25	-1	-1	13.00	1.64
25	-1	-1	12.58	0.97
25	-1	-1	12.39	1.36
25	1	1	15.22	1.26
-25	1	-1	13.87	2.64
-25	-1	1	15.92	2.11
-25	1	-1	12.43	1.55
25	1	1	14.26	0.99
-25	-1	1	18.59	2.57
-25	-1	-1	13.71	1.52
25	-1	1	13.61	1.10
-25	-1	-1	14.53	2.33
25	-1	1	14.12	1.63
-25	-1	-1	15.22	2.12
-25	1	1	12.86	2.57
-25	1	1	14.56	1.57
-25	-1	-1	12.88	1.55
25	-2	-1	27.16	2.05
-25	2	-1	23.71	2.87
25	-2	-1	23.89	1.52
-25	-2	1	21.37	1.88
-25	-2	1	22.68	2.49
-25	-2	1	20.71	2.39
-25	2	-1	22.24	1.96

-25	-2	1	23.11	1.92
-25	2	1	21.64	2.69
-25	-2	-1	26.81	2.01
25	-2	1	23.79	1.90
-25	-2	-1	27.30	1.98
-25	-2	-1	22.75	2.65
-25	-2	-1	23.37	2.45
25	-2	1	21.88	1.46
-25	2	1	18.63	1.85
-25	-3	1	53.88	3.18
25	-3	-1	51.56	2.76
-25	-3	1	54.71	3.53
-25	-3	1	54.38	3.16
-25	3	-1	53.92	3.92
25	-3	-1	51.73	3.09
-25	-3	1	55.48	3.89
-25	3	1	58.95	3.93
-25	-3	-1	53.28	3.16
-25	-3	-1	52.15	3.13
-25	3	1	51.18	3.15
-25	-3	-1	57.98	3.85
25	-3	1	50.17	2.81
25	-3	1	54.30	3.15
0	-1	2	189.12	8.46
0	-1	-2	-0.03	0.04
0	1	-2	0.01	0.02
0	-2	2	506.61	21.75
0	-2	2	460.81	21.73
0	-2	2	519.40	21.81
0	2	2	488.40	21.80
0	2	-2	540.42	25.60
0	2	-2	596.35	25.68
0	2	2	464.62	21.71
0	2	-2	593.00	25.70
0	-2	-2	544.09	25.60
0	2	2	463.79	21.71
0	2	-2	609.20	25.64
0	-2	-2	552.16	25.65
0	-2	2	510.26	21.79
0	2	2	492.50	21.77
0	-2	-2	581.43	25.67
0	-2	2	453.93	21.71
0	2	2	503.06	21.74
0	3	2	30.96	1.62

0	3	2	32.51	1.57
0	-3	2	29.38	1.55
0	-3	-2	44.71	2.17
0	3	2	30.06	1.52
0	-3	2	31.67	1.65
0	3	-2	41.76	2.06
0	3	2	30.15	1.52
0	3	-2	45.20	2.11
0	-3	-2	44.56	2.04
0	-3	-2	46.15	2.09
0	3	2	30.34	1.52
0	3	-2	43.08	2.15
0	-3	2	32.20	1.58
0	3	-2	42.35	2.10
0	-3	-2	40.40	2.07
0	3	-2	41.59	2.20
0	-3	2	30.54	1.53
0	-3	2	29.95	1.45
0	-3	2	32.82	1.62
0	3	2	30.72	1.65
0	-3	-2	42.04	2.05
0	3	-2	42.00	1.98
0	-3	-2	41.18	2.12
0	-4	2	67.84	3.36
0	-4	-2	80.39	3.77
0	-4	-2	78.17	3.60
0	-4	-2	85.57	3.68
0	4	-2	78.15	3.69
0	4	-2	77.07	3.80
0	-4	2	68.43	3.25
0	-4	-2	73.76	3.60
0	-4	2	75.10	3.41
0	-4	-2	72.74	3.69
0	4	-2	76.06	3.63
0	4	2	71.91	3.46
0	4	2	70.87	3.33
0	-4	2	75.60	3.43
0	-4	2	74.60	3.44
0	4	-2	75.63	3.53
0	-4	2	76.02	3.53
0	4	2	66.75	3.64
0	4	2	70.12	3.36
0	-4	2	65.06	3.39
0	4	-2	76.85	3.71

0	4	2	70.33	3.41
0	-4	-2	74.56	3.63
0	5	-2	94.67	4.32
0	5	2	65.42	3.36
0	5	2	67.15	3.27
0	-5	2	66.98	3.21
0	-5	2	64.09	3.03
0	-5	2	68.85	3.24
0	-5	-2	102.15	4.48
0	5	2	64.01	3.14
0	5	2	63.58	3.19
0	-5	-2	87.69	4.49
0	-5	2	69.06	3.35
0	5	-2	94.21	4.51
0	5	2	62.76	3.15
0	5	-2	98.16	4.45
0	-5	2	63.49	3.26
0	5	-2	93.50	4.65
0	5	2	66.66	3.11
0	5	-2	92.44	4.52
0	-5	2	62.87	3.20
0	-5	-2	89.95	4.38
0	-5	-2	91.25	4.43
0	-5	-2	96.10	4.40
0	-5	2	61.93	3.15
0	-6	-2	279.39	13.30
0	6	-2	292.93	13.41
0	6	2	330.19	14.71
0	6	-2	302.10	13.32
0	-6	2	337.32	14.62
0	6	-2	288.70	13.56
0	-6	2	342.12	14.64
0	-6	-2	301.44	13.26
0	-6	-2	281.69	13.39
0	-6	2	322.02	14.58
0	6	2	313.14	14.60
0	6	2	317.25	14.49
0	-6	-2	303.13	13.50
0	6	2	326.08	14.56
0	6	2	308.92	14.80
0	-6	2	301.25	14.41
0	-6	2	291.42	14.59
0	6	2	307.58	14.55
0	6	-2	294.42	13.17

0	-6	-2	274.52	13.23
0	-6	-2	316.49	13.36
0	6	-2	284.61	13.39
0	-6	2	345.46	14.79
0	-6	2	330.59	14.61
0	-7	2	161.46	7.92
0	-7	2	184.98	8.17
0	7	2	167.16	8.10
0	7	-2	193.87	8.77
0	-7	2	170.17	8.11
0	-7	2	166.17	8.16
0	7	2	173.99	8.10
0	-7	2	187.47	8.36
0	7	-2	191.94	8.88
0	-7	-2	198.47	8.99
0	7	-2	190.19	8.85
0	7	2	170.42	8.01
0	-7	-2	189.70	8.78
0	-7	-2	210.70	8.84
0	7	-2	192.06	8.60
0	7	-2	180.95	9.04
0	7	2	165.32	8.37
0	-7	-2	196.06	8.72
0	-7	-2	165.90	8.97
0	7	2	182.17	8.29
0	-7	2	178.20	8.15
0	-7	2	184.79	8.19
0	-7	-2	182.00	8.76
0	-7	-2	177.17	8.65
0	-8	2	90.79	4.37
0	-8	-2	65.35	3.48
0	8	2	82.21	4.60
0	8	-2	58.59	3.27
0	-8	2	79.93	4.10
0	8	2	85.39	4.31
0	-8	-2	58.06	3.24
0	-8	-2	62.12	3.19
0	8	-2	62.09	3.02
0	-8	2	86.30	4.40
0	-8	2	93.67	4.65
0	8	2	84.63	4.30
0	8	2	90.13	4.21
0	8	2	84.61	4.65
0	-8	-2	62.48	3.14

0	-8	2	96.75	4.42
0	-8	-2	59.02	3.05
0	8	-2	65.53	3.39
0	-8	-2	67.94	3.31
0	-8	2	91.98	4.44
0	-8	-2	61.24	3.20
0	8	-2	61.04	3.19
0	-8	2	84.74	4.31
0	8	-2	60.04	3.55
0	-9	2	51.95	2.92
0	-9	-2	66.23	3.45
0	-9	-2	59.91	3.45
0	-9	2	55.40	3.32
0	9	2	54.34	2.82
0	9	2	53.87	2.94
0	9	-2	66.33	3.87
0	9	2	52.67	3.29
0	9	-2	68.77	3.74
0	9	-2	66.04	3.51
0	-9	2	57.79	3.07
0	-9	-2	64.79	3.36
0	-9	-2	65.73	3.76
0	9	2	59.10	3.31
0	-9	2	56.06	3.00
0	-9	2	50.07	2.66
0	-9	-2	60.53	3.23
0	-9	2	57.65	3.12
0	-9	-2	64.35	3.40
0	9	-2	68.52	3.21
0	-9	2	52.45	3.00
0	-9	-2	69.91	3.55
0	-10	2	9.46	1.60
0	10	-2	5.30	0.73
0	-10	-2	5.93	1.14
0	10	2	9.81	1.49
0	10	-2	6.65	1.21
0	10	2	7.85	1.25
0	-10	-2	4.43	0.68
0	10	-2	2.12	1.20
0	10	-2	5.89	1.03
0	-10	2	8.14	0.78
0	-10	-2	6.75	1.19
0	-10	2	7.58	1.15
0	-10	-2	6.07	0.97

0	-10	2	9.77	1.29
0	-10	2	6.96	1.10
0	-10	-2	5.13	0.89
0	10	2	7.90	1.45
0	-10	2	7.80	1.25
0	10	2	9.28	1.15
0	-10	-2	5.04	0.99
0	-11	-2	8.96	1.25
0	11	2	7.85	1.40
0	11	2	8.25	1.23
0	-11	2	8.53	1.22
0	-11	2	8.64	1.24
0	-11	-2	9.53	1.42
0	11	-2	10.44	1.51
0	-11	2	7.53	0.84
0	11	-2	9.43	1.16
0	11	-2	8.78	1.56
0	11	2	8.56	1.65
0	11	2	8.49	1.63
0	-11	2	10.89	1.82
0	11	-2	14.19	2.62
0	-11	2	6.68	1.29
0	-11	-2	8.34	1.01
0	-11	-2	10.33	1.46
0	-11	-2	7.95	1.46
0	12	-2	43.38	3.54
0	-12	2	41.41	2.59
0	-12	-2	41.15	2.38
0	-12	2	36.82	2.48
0	-12	-2	40.78	2.71
0	12	2	39.70	2.55
0	-12	-2	46.16	3.12
0	-12	2	33.83	2.24
0	12	2	29.94	3.14
0	12	-2	40.77	2.83
0	-13	2	39.95	2.72
0	13	2	41.14	3.86
0	-13	2	45.63	3.07
0	13	-2	39.92	3.96
0	-13	-2	46.09	2.98
0	-13	-2	37.81	2.66
1	1	2	86.62	4.06
-1	-1	2	86.19	4.05
-1	-1	2	92.90	4.11

1	1	2	94.33	4.16
-1	1	-2	97.97	4.30
1	-1	-2	92.89	4.32
1	1	2	88.55	4.09
-1	1	-2	94.14	4.27
-1	-1	2	90.88	4.10
-1	1	-2	91.58	4.29
-1	-1	2	87.33	4.05
-1	1	-2	102.84	4.38
-1	1	-2	94.72	4.31
1	1	2	88.18	4.03
1	-1	-2	97.68	4.27
1	1	2	88.30	4.05
1	-1	-2	90.47	4.32
1	1	-2	93.95	4.28
-1	1	2	89.95	4.08
-1	1	2	88.74	4.07
1	-1	2	95.36	4.15
1	-1	2	93.76	4.13
-1	1	2	92.09	4.10
-1	1	2	88.54	4.10
1	1	-2	95.36	4.32
-1	1	2	88.08	4.04
-1	-1	-2	97.93	4.32
-1	-1	-2	93.96	4.25
1	-1	2	85.84	4.07
-1	-1	-2	94.72	4.33
-1	1	2	93.39	4.05
-1	-1	-2	92.71	4.32
1	-2	-2	157.95	7.17
1	2	2	168.23	7.58
1	2	2	163.39	7.53
-1	-2	2	182.49	7.60
-1	2	-2	152.47	7.11
-1	2	-2	154.28	7.09
-1	-2	2	159.88	7.53
-1	-2	2	154.54	7.53
-1	2	-2	168.03	7.22
1	-2	-2	152.71	7.14
-1	-2	2	162.16	7.59
1	-2	-2	152.06	7.09
1	-2	-2	160.21	7.09
-1	2	-2	169.36	7.11
1	2	2	165.70	7.59

-1	2	-2	154.58	7.04
-1	-2	2	164.18	7.58
1	-2	-2	163.85	7.07
1	-2	-2	150.08	7.08
-1	2	-2	156.81	7.17
1	2	2	168.47	7.54
-1	-2	-2	155.65	7.09
1	-2	2	178.36	7.65
-1	2	2	171.35	7.60
-1	2	2	169.07	7.62
-1	2	2	177.26	7.55
1	2	-2	161.41	7.14
1	-2	2	161.08	7.49
1	-2	2	180.78	7.56
1	2	-2	152.42	7.09
-1	-2	-2	148.41	7.08
1	2	-2	158.07	7.13
-1	2	2	163.41	7.53
-1	-2	-2	153.03	7.13
1	2	-2	150.67	7.16
-1	-2	-2	163.31	7.13
1	-2	2	162.81	7.54
-1	-2	-2	154.78	7.03
-1	-2	-2	167.31	7.15
1	-2	2	170.85	7.61
-1	2	2	168.49	7.52
-1	2	2	162.74	7.54
1	-2	2	157.11	7.56
1	3	2	63.85	3.06
1	3	2	65.13	3.07
1	-3	-2	64.13	3.11
1	-3	-2	65.86	2.99
1	3	2	64.64	3.01
-1	-3	2	69.31	3.08
1	-3	-2	55.34	3.03
1	3	2	63.12	3.11
-1	3	-2	63.58	2.93
1	-3	-2	69.35	2.99
-1	3	-2	68.32	3.01
-1	-3	2	64.77	3.10
-1	-3	2	62.55	3.01
-1	-3	2	66.18	3.08
-1	3	-2	63.21	3.09
1	3	2	63.40	2.98

1	-3	-2	62.53	3.05
1	3	2	57.36	2.95
1	-3	-2	61.54	2.98
-1	3	-2	69.13	3.02
-1	-3	2	61.27	3.02
-1	3	-2	62.91	3.04
-1	3	-2	64.25	3.14
-1	-3	2	61.60	2.91
1	3	-2	63.53	2.99
-1	3	2	64.48	3.14
1	-3	2	68.52	3.15
-1	3	2	68.90	3.03
-1	-3	-2	68.84	3.05
-1	-3	-2	62.64	3.04
-1	-3	-2	62.18	2.99
1	-3	2	61.79	2.94
1	-3	2	62.17	3.04
1	3	-2	64.22	3.02
-1	-3	-2	63.45	2.93
-1	3	2	62.67	3.00
1	-3	2	62.25	3.00
1	3	-2	64.06	2.90
1	-3	2	69.69	3.03
-1	-3	-2	62.34	2.98
-1	3	2	67.16	3.10
1	-3	2	63.36	3.08
-1	3	2	62.58	2.99
-1	3	2	64.32	3.00
1	3	-2	65.70	3.06
-1	-3	-2	65.73	3.08
1	3	-2	55.69	3.19
1	-4	-2	21.96	1.31
-1	4	-2	23.43	1.14
-1	4	-2	23.70	1.30
1	-4	-2	22.55	1.19
1	-4	-2	23.50	1.23
1	4	2	27.48	1.59
1	4	2	25.69	1.41
-1	-4	2	26.88	1.45
-1	4	-2	21.06	1.31
-1	-4	2	31.00	1.55
1	-4	-2	23.86	1.24
-1	-4	2	28.33	1.59
-1	4	-2	23.60	1.25

-1	-4	2	26.61	1.33
-1	-4	2	26.15	1.47
-1	-4	2	29.22	1.54
1	4	2	27.82	1.41
1	4	2	28.02	1.56
1	4	2	28.81	1.45
1	-4	-2	23.10	1.38
-1	-4	2	27.17	1.50
1	4	2	27.20	1.50
1	-4	-2	21.64	1.22
1	4	-2	22.21	1.11
-1	4	2	25.48	1.62
1	-4	2	29.35	1.56
-1	4	2	28.58	1.44
-1	-4	-2	22.09	1.17
-1	-4	-2	20.92	1.28
-1	-4	-2	23.73	1.36
1	-4	2	26.70	1.35
-1	4	2	25.34	1.58
-1	-4	-2	22.57	1.23
1	4	-2	22.78	1.23
1	-4	2	24.41	1.47
1	-4	2	28.20	1.46
-1	-4	-2	21.98	1.18
1	-4	2	29.22	1.64
1	4	-2	22.10	1.27
-1	4	2	29.83	1.58
1	4	-2	20.69	1.34
-1	4	2	27.07	1.46
-1	-4	-2	23.97	1.30
1	-4	2	28.49	1.47
1	-4	2	30.68	1.50
1	5	2	45.60	2.42
-1	5	-2	44.65	2.48
1	-5	-2	47.10	2.25
1	-5	-2	45.59	2.44
-1	-5	2	44.72	2.20
-1	5	-2	43.69	2.24
-1	-5	2	50.64	2.43
-1	-5	2	45.32	2.34
1	5	2	48.44	2.34
-1	-5	2	46.14	2.41
-1	5	-2	43.25	2.31
1	5	2	46.20	2.54

-1	5	-2	45.78	2.15
-1	5	-2	44.32	2.37
1	5	2	48.35	2.47
1	-5	-2	42.14	2.19
-1	-5	2	47.39	2.46
-1	-5	2	46.65	2.51
1	5	2	45.19	2.32
-1	-5	2	44.14	2.37
1	5	2	46.66	2.28
1	-5	-2	41.14	2.30
1	-5	-2	43.81	2.23
1	-5	-2	48.63	2.27
-1	-5	-2	41.81	2.29
-1	-5	-2	45.14	2.38
-1	-5	-2	42.36	2.18
1	5	-2	44.25	2.47
1	-5	2	45.19	2.33
1	-5	2	44.46	2.39
1	-5	2	49.60	2.39
1	5	-2	44.38	2.10
-1	5	2	43.30	2.51
-1	-5	-2	43.61	2.24
1	5	-2	43.62	2.30
-1	5	2	44.96	2.34
1	-5	2	47.72	2.46
-1	5	2	46.58	2.37
1	-5	2	48.15	2.37
-1	5	2	46.86	2.48
-1	5	2	48.08	2.34
-1	-5	-2	47.13	2.32
1	5	-2	45.43	2.26
1	-5	2	52.69	3.06
1	5	-2	44.57	2.31
-1	-5	-2	44.85	2.18
1	-6	-2	10.11	0.80
1	-6	-2	11.72	1.17
-1	6	-2	11.47	0.78
-1	-6	2	11.40	0.98
-1	-6	2	10.41	0.96
1	6	2	11.57	1.03
-1	6	-2	10.03	1.02
1	6	2	11.34	0.88
1	6	2	10.61	0.95
-1	-6	2	10.95	1.07

-1	6	-2	11.26	0.91
1	-6	-2	11.55	0.87
1	6	2	11.26	0.87
-1	6	-2	10.09	1.20
-1	-6	2	12.89	1.17
1	6	2	10.24	1.09
-1	6	-2	10.50	1.01
1	-6	-2	12.02	0.95
-1	-6	2	11.31	0.87
1	-6	-2	10.29	1.03
-1	-6	2	11.83	1.06
1	6	2	12.00	0.91
1	-6	-2	11.94	0.94
1	6	-2	12.02	1.14
-1	6	2	11.97	1.00
1	-6	2	11.77	1.03
-1	-6	-2	10.79	0.82
-1	-6	-2	11.54	0.87
-1	6	2	12.32	1.11
-1	6	2	11.00	1.11
-1	6	2	12.57	0.91
-1	-6	-2	12.31	1.13
-1	6	2	11.08	0.88
1	-6	2	12.71	1.21
1	6	-2	11.65	0.74
-1	-6	-2	11.17	0.98
1	6	-2	11.94	0.91
1	6	-2	10.18	1.01
-1	-6	-2	12.19	1.01
1	6	-2	10.90	1.00
-1	-6	-2	12.27	0.88
1	-6	2	12.78	1.10
1	-6	2	11.17	0.72
1	-6	2	10.53	0.86
1	-6	2	10.49	0.93
1	-6	2	9.87	0.92
1	-7	-2	3.51	0.64
1	-7	-2	2.95	0.53
1	-7	-2	2.29	0.60
1	-7	-2	4.47	0.69
1	-7	-2	2.65	0.47
1	7	2	3.23	0.57
-1	7	-2	2.28	0.63
1	-7	-2	3.98	0.62

-1	-7	2	4.25	0.81
-1	-7	2	3.96	0.68
-1	-7	2	3.34	0.72
-1	-7	2	2.86	0.52
-1	-7	2	4.37	0.74
1	7	2	3.42	0.64
1	7	2	2.41	0.71
-1	-7	2	3.71	0.75
1	-7	-2	4.78	0.90
-1	7	-2	3.38	0.49
1	7	2	3.95	0.55
-1	7	-2	3.68	0.72
-1	7	-2	2.36	0.54
-1	-7	2	2.59	0.43
1	7	2	4.11	0.77
-1	7	-2	3.38	0.81
1	7	2	3.69	0.83
1	-7	2	4.36	0.91
1	-7	2	4.90	0.77
1	-7	2	3.48	0.65
1	7	-2	2.90	0.65
1	-7	2	2.82	0.48
-1	-7	-2	3.40	0.68
-1	-7	-2	3.06	0.77
1	-7	2	4.13	0.65
-1	7	2	3.96	0.71
-1	-7	-2	2.70	0.45
-1	-7	-2	2.71	0.64
-1	7	2	3.37	0.60
1	7	-2	3.72	0.66
-1	7	2	4.18	0.66
1	7	-2	3.37	0.61
1	-7	2	2.85	0.70
1	7	-2	2.70	0.75
1	7	-2	3.08	0.45
-1	7	2	3.57	0.85
-1	-7	-2	2.49	0.49
1	8	2	34.64	2.23
1	-8	-2	31.79	1.85
-1	-8	2	34.40	1.97
-1	-8	2	34.75	2.11
-1	8	-2	30.86	2.11
-1	8	-2	29.70	2.23
1	-8	-2	29.34	1.72

1	-8	-2	29.70	1.83
1	-8	-2	32.14	2.24
-1	-8	2	35.15	2.07
-1	-8	2	31.24	1.73
1	8	2	35.09	1.88
-1	8	-2	28.83	1.86
1	8	2	32.16	2.28
-1	-8	2	35.44	2.17
1	8	2	32.67	1.95
1	-8	-2	33.61	1.91
-1	-8	2	31.18	2.01
1	-8	-2	36.01	1.97
-1	-8	2	34.73	2.32
-1	8	-2	30.80	1.72
1	-8	-2	29.70	1.93
1	8	2	30.85	1.97
-1	8	-2	30.93	2.00
-1	8	2	31.06	2.28
-1	-8	-2	28.67	1.70
-1	8	2	34.02	2.04
1	-8	2	38.05	2.13
1	-8	2	31.84	1.94
-1	-8	-2	34.23	2.04
-1	-8	-2	31.96	1.87
1	-8	2	31.13	2.00
-1	-8	-2	32.10	1.90
1	-8	2	37.63	2.40
-1	8	2	35.21	2.29
1	-8	2	37.40	2.16
-1	-8	-2	32.33	1.84
1	-8	2	31.58	1.74
1	8	-2	34.36	2.07
-1	8	2	35.84	1.91
1	8	-2	31.98	1.66
1	8	-2	30.26	1.92
1	8	-2	28.26	1.87
1	8	-2	31.49	2.24
1	-8	2	36.79	2.01
-1	-8	-2	28.52	1.96
-1	8	2	30.85	1.97
-1	-8	-2	32.09	2.16
1	9	2	5.50	1.14
1	-9	-2	6.33	0.92
-1	-9	2	5.57	1.15

-1	9	-2	5.91	1.12
1	-9	-2	4.97	0.79
1	-9	-2	4.53	0.75
1	-9	-2	5.72	1.09
1	9	2	6.25	1.01
-1	-9	2	7.18	1.14
-1	9	-2	6.73	1.05
1	9	2	7.32	0.96
1	-9	-2	7.60	1.06
1	-9	-2	6.62	0.97
1	9	2	7.86	1.30
-1	-9	2	5.69	0.88
-1	-9	2	5.72	0.63
-1	-9	2	7.03	1.44
-1	-9	2	7.32	1.06
-1	9	-2	5.60	0.87
1	-9	-2	8.26	1.47
-1	-9	2	6.05	0.94
-1	9	-2	4.58	1.16
1	9	-2	7.11	0.80
1	-9	2	6.88	1.37
1	-9	2	5.06	0.96
-1	-9	-2	6.03	0.93
1	-9	2	6.74	1.30
-1	9	2	8.10	1.35
-1	-9	-2	5.86	1.21
-1	9	2	6.39	1.00
1	9	-2	7.61	1.16
-1	9	2	6.16	1.26
-1	-9	-2	6.36	0.86
-1	-9	-2	6.80	1.28
1	-9	2	6.65	1.35
-1	-9	-2	7.07	1.01
1	-9	2	6.42	0.93
1	9	-2	5.14	1.06
-1	9	2	5.86	1.06
1	-9	2	4.49	0.66
-1	-9	-2	5.31	0.76
1	-9	2	5.53	1.02
1	9	-2	6.10	1.08
1	10	2	9.74	1.73
1	10	2	9.03	1.19
-1	10	-2	15.22	1.82
-1	10	-2	11.72	1.45

-1	-10	2	8.65	1.60
1	-10	-2	12.37	1.49
1	-10	-2	9.52	1.33
-1	-10	2	8.54	1.12
1	-10	-2	13.29	1.33
1	-10	-2	10.17	1.03
1	-10	-2	10.37	1.22
-1	-10	2	7.28	1.32
-1	10	-2	14.05	1.14
-1	-10	2	11.62	1.36
-1	10	-2	11.35	1.88
1	-10	-2	13.51	1.57
1	10	2	10.03	1.13
1	-10	-2	14.57	2.80
-1	-10	2	9.91	1.51
1	10	2	9.96	1.53
-1	-10	2	8.29	0.80
1	10	-2	11.97	1.06
-1	10	2	8.31	1.34
-1	10	2	8.75	1.18
1	10	-2	11.03	1.78
-1	10	2	9.32	1.73
1	-10	2	10.16	1.27
1	-10	2	10.19	1.36
-1	-10	-2	11.75	1.27
-1	-10	-2	12.81	1.62
-1	10	2	9.23	1.70
-1	-10	-2	11.62	1.29
-1	-10	-2	11.19	1.31
1	-10	2	7.67	1.27
-1	-10	-2	12.04	1.39
-1	-10	-2	10.24	1.02
1	-10	2	9.38	1.63
1	-10	2	8.83	0.83
1	10	-2	9.46	1.85
1	-10	2	8.97	1.14
1	10	-2	12.04	1.43
-1	-11	2	3.41	0.55
-1	11	-2	1.93	1.25
-1	-11	2	7.38	1.20
1	11	2	3.07	1.31
-1	11	-2	2.78	0.72
1	-11	-2	1.31	0.82
1	-11	-2	1.99	1.01

1	11	2	4.18	0.93
-1	-11	2	3.65	1.06
1	-11	-2	4.71	0.98
1	-11	-2	3.91	0.70
1	-11	-2	1.07	0.96
-1	11	-2	3.82	1.36
1	-11	-2	3.39	0.93
-1	-11	2	4.09	1.11
-1	11	-2	3.05	1.04
1	11	2	4.32	0.83
-1	-11	2	3.17	0.82
-1	11	2	1.31	1.34
-1	-11	-2	3.02	0.85
1	-11	2	2.66	0.90
1	11	-2	4.21	1.44
1	11	-2	3.99	0.99
-1	11	2	4.77	1.01
1	-11	2	4.09	0.72
-1	-11	-2	2.72	0.80
1	-11	2	3.00	0.79
1	-11	2	4.33	0.96
1	-11	2	1.96	1.15
-1	-11	-2	2.06	0.88
-1	11	2	3.72	1.47
-1	-11	-2	2.56	0.59
-1	11	2	3.59	0.86
1	-12	-2	4.08	1.13
1	12	2	7.20	1.15
-1	-12	2	4.45	1.01
-1	12	-2	5.05	1.20
1	-12	-2	3.42	0.74
1	-12	-2	2.14	0.98
-1	12	-2	1.83	1.78
-1	-12	2	5.62	1.26
1	12	2	5.19	1.78
1	-12	2	4.70	0.82
-1	-12	-2	3.04	0.94
1	-12	2	6.20	1.42
1	12	-2	3.13	1.58
-1	12	2	5.43	1.85
-1	12	2	7.46	1.28
-1	-12	-2	1.85	1.01
1	-12	2	6.83	1.12
-1	-12	-2	2.87	0.70

1	12	-2	3.92	1.16
1	-13	-2	9.20	1.38
-1	13	-2	5.16	2.12
1	13	2	8.97	2.29
-1	-13	2	8.60	1.51
-1	13	-2	11.54	2.00
1	-13	-2	7.50	1.20
-1	-13	2	11.46	2.04
1	-13	2	9.15	1.55
-1	-13	-2	10.68	2.00
1	-13	2	7.82	1.19
1	13	-2	8.30	2.08
-1	-13	-2	5.47	1.11
-1	13	2	9.34	2.15
2	0	2	128.44	5.77
-2	1	-2	331.12	15.10
-2	1	-2	329.68	15.10
-2	-1	2	331.54	16.02
-2	1	-2	336.87	15.12
-2	1	-2	339.71	15.17
-2	-1	2	374.88	16.08
-2	1	-2	352.31	15.11
-2	1	-2	338.90	15.11
2	1	2	370.89	16.04
-2	1	-2	346.41	15.20
-2	-1	2	355.39	16.03
2	-1	-2	344.90	15.14
2	1	2	344.65	16.03
2	1	2	356.09	16.08
-2	1	2	351.79	16.07
2	-1	2	383.81	16.13
2	1	-2	330.41	15.12
2	1	-2	346.54	15.15
-2	-1	-2	324.06	15.11
-2	1	2	364.32	16.05
-2	-1	-2	338.52	15.11
-2	-1	-2	337.46	15.14
-2	1	2	363.85	16.06
2	-1	2	348.80	16.03
-2	1	2	351.41	16.01
2	-1	2	343.22	16.04
2	1	-2	331.12	15.09
-2	1	2	373.44	16.07
-2	-1	-2	343.81	15.11

-2	-1	-2	339.26	15.15
-2	1	2	369.90	16.07
2	-1	2	353.35	16.04
2	-1	2	366.49	16.09
-2	-2	2	98.90	4.36
2	-2	-2	82.14	3.99
2	2	2	93.71	4.35
-2	2	-2	84.37	4.02
2	-2	-2	84.17	4.03
-2	2	-2	89.80	4.00
2	-2	-2	92.31	3.94
2	2	2	94.77	4.37
-2	2	-2	84.08	4.07
2	2	2	98.43	4.31
2	-2	-2	85.30	4.01
-2	-2	2	95.51	4.35
-2	-2	2	88.84	4.29
-2	-2	2	90.99	4.30
-2	-2	2	94.12	4.37
2	2	2	89.95	4.29
2	-2	-2	89.42	4.08
-2	2	-2	90.64	4.12
-2	-2	2	95.63	4.38
-2	2	-2	89.47	3.96
-2	2	-2	95.31	3.99
2	-2	-2	81.72	3.97
2	-2	2	101.85	4.30
-2	-2	-2	86.49	3.99
2	-2	2	96.21	4.39
-2	-2	-2	89.48	4.05
-2	2	2	95.15	4.34
2	2	-2	87.37	4.03
-2	2	2	98.78	4.37
-2	2	2	92.97	4.29
-2	2	2	91.78	4.31
2	2	-2	88.21	3.99
2	-2	2	99.27	4.43
2	-2	2	90.49	4.27
-2	2	2	94.29	4.26
-2	-2	-2	83.08	3.98
-2	-2	-2	84.48	4.02
-2	2	2	93.85	4.39
-2	-2	-2	92.57	4.03
2	2	-2	83.80	4.04

2	-2	2	89.61	4.33
2	-2	2	90.85	4.30
2	-3	-2	4.13	0.44
2	-3	-2	3.73	0.52
-2	-3	2	7.91	0.64
2	-3	-2	2.92	0.34
-2	3	-2	3.60	0.52
-2	3	-2	3.68	0.43
2	3	2	8.19	0.61
-2	-3	2	7.29	0.41
-2	-3	2	7.09	0.51
-2	3	-2	3.11	0.48
2	3	2	7.60	0.59
-2	-3	2	7.30	0.60
-2	-3	2	8.14	0.62
2	-3	-2	3.64	0.45
2	-3	-2	3.78	0.34
-2	-3	2	7.40	0.52
-2	-3	2	7.37	0.61
2	3	2	7.28	0.52
-2	3	-2	3.74	0.38
-2	3	-2	3.36	0.38
-2	3	-2	4.01	0.33
2	3	2	7.09	0.68
2	3	2	7.07	0.51
2	3	2	7.03	0.45
-2	-3	-2	3.33	0.34
2	-3	2	7.93	0.53
-2	3	2	7.10	0.51
-2	3	2	7.69	0.61
2	3	-2	3.75	0.46
-2	3	2	6.69	0.69
2	3	-2	3.42	0.25
2	-3	2	7.37	0.51
2	-3	2	7.01	0.64
2	3	-2	4.25	0.48
-2	3	2	7.60	0.55
-2	3	2	8.19	0.64
2	3	-2	3.29	0.38
-2	-3	-2	3.34	0.28
-2	-3	-2	3.11	0.40
-2	-3	-2	4.15	0.46
-2	-3	-2	3.35	0.38
2	-3	2	7.40	0.57

2	-3	2	7.34	0.46
-2	-3	-2	3.79	0.47
-2	3	2	7.53	0.51
2	-3	2	7.35	0.71
-2	-4	2	40.54	2.11
-2	4	-2	41.23	2.18
2	-4	-2	42.31	2.09
-2	4	-2	44.62	2.07
-2	-4	2	45.33	2.18
2	4	2	44.27	2.12
2	4	2	43.92	2.21
2	-4	-2	44.94	2.04
-2	-4	2	41.24	2.11
-2	4	-2	42.79	2.15
2	-4	-2	42.61	2.24
-2	-4	2	41.77	1.99
2	4	2	42.59	2.24
-2	4	-2	42.93	2.00
-2	-4	2	42.05	2.19
-2	4	-2	41.29	2.30
2	4	2	42.04	2.06
2	-4	-2	38.28	2.03
2	-4	-2	38.60	2.12
-2	4	-2	43.82	2.09
2	4	2	42.83	2.20
2	4	2	38.33	2.04
2	-4	-2	38.84	2.05
2	4	-2	38.44	2.20
-2	4	2	40.66	2.08
-2	4	2	41.53	2.29
2	4	-2	41.63	2.08
-2	-4	-2	41.48	2.03
-2	-4	-2	43.52	2.16
-2	-4	-2	39.27	2.11
2	-4	2	46.33	2.12
2	-4	2	42.85	2.04
-2	-4	-2	41.60	2.07
2	4	-2	42.89	2.13
-2	4	2	43.93	2.16
-2	-4	-2	38.05	1.97
2	-4	2	45.09	2.22
2	4	-2	42.55	2.11
-2	4	2	43.66	2.10
-2	-4	-2	44.78	2.19

2	-4	2	39.73	2.15
-2	4	2	44.89	2.23
2	-4	2	43.43	2.28
2	4	-2	42.10	1.94
2	-4	2	42.74	2.11
-2	5	-2	105.29	4.91
2	-5	-2	99.63	4.80
2	5	2	87.83	4.37
2	5	2	86.42	4.28
2	-5	-2	100.35	4.84
2	5	2	93.64	4.20
2	-5	-2	98.60	4.77
2	-5	-2	94.88	4.87
-2	-5	2	86.88	4.27
2	-5	-2	109.35	4.79
-2	-5	2	88.66	4.30
-2	-5	2	90.23	4.33
-2	-5	2	86.95	4.05
2	5	2	81.69	4.12
-2	-5	2	86.03	4.20
-2	5	-2	106.96	4.74
-2	-5	2	82.26	4.20
2	5	2	93.04	4.32
-2	5	-2	101.97	5.05
2	5	2	88.98	4.12
-2	5	-2	104.42	4.82
2	-5	-2	107.53	5.02
-2	5	-2	103.30	4.95
-2	-5	2	95.92	4.26
2	5	-2	96.14	4.98
2	5	-2	101.52	4.85
-2	-5	-2	98.59	4.86
2	5	-2	102.11	4.87
-2	-5	-2	97.52	4.71
2	-5	2	94.98	4.19
-2	5	2	90.01	4.25
2	-5	2	92.67	4.42
-2	5	2	91.89	4.33
-2	-5	-2	101.93	4.76
-2	5	2	76.09	4.52
2	5	-2	105.96	4.83
2	-5	2	93.08	4.21
2	-5	2	93.68	4.31
2	-5	2	84.73	4.25

2	-5	2	83.56	4.08
-2	-5	-2	108.37	4.91
2	5	-2	100.71	4.66
-2	-5	-2	108.81	4.95
-2	-5	-2	102.03	4.82
-2	5	2	85.97	4.16
2	-5	2	86.71	4.19
-2	5	2	89.84	4.19
2	-6	-2	216.79	9.53
2	-6	-2	192.12	9.59
-2	-6	2	222.47	9.94
-2	-6	2	207.03	9.83
2	-6	-2	220.50	9.76
-2	-6	2	218.91	9.99
2	-6	-2	225.62	9.51
-2	-6	2	214.93	9.91
2	-6	-2	190.76	9.44
2	6	2	210.28	10.04
-2	6	-2	211.89	9.43
2	6	2	214.83	9.73
2	6	2	222.56	9.83
2	-6	-2	198.83	9.50
-2	6	-2	211.76	9.66
-2	6	-2	203.07	9.80
-2	6	-2	219.76	9.65
2	6	2	217.64	9.96
2	6	2	211.48	9.92
-2	6	-2	211.81	9.53
-2	-6	2	198.26	9.83
2	-6	-2	210.10	9.48
-2	-6	2	200.39	9.65
2	-6	2	229.11	9.86
-2	6	2	214.84	9.78
-2	-6	-2	201.64	9.52
2	-6	2	220.66	9.82
2	-6	2	229.26	10.09
-2	6	2	212.43	10.08
2	-6	2	207.61	9.82
2	-6	2	226.16	9.92
2	-6	2	200.38	9.69
-2	6	2	216.59	9.88
2	6	-2	185.53	9.70
2	-6	2	198.62	9.87
2	6	-2	204.88	9.51

-2	-6	-2	196.76	9.57
-2	-6	-2	201.96	9.44
2	6	-2	204.33	9.34
-2	-6	-2	225.58	9.63
2	6	-2	208.59	9.60
-2	-6	-2	225.84	9.68
-2	-6	-2	200.05	9.40
2	6	-2	200.30	9.55
-2	6	2	218.79	9.82
-2	-7	2	227.44	10.42
2	-7	-2	185.93	9.18
2	7	2	220.36	10.32
-2	-7	2	233.82	10.59
-2	-7	2	240.63	10.43
2	-7	-2	196.35	9.28
-2	7	-2	208.51	9.17
2	7	2	228.31	10.55
2	7	2	226.87	10.28
-2	-7	2	206.67	10.40
2	7	2	214.05	10.48
2	7	2	224.06	10.38
2	-7	-2	208.67	9.28
-2	-7	2	214.07	10.19
-2	7	-2	200.73	9.62
2	7	2	211.22	10.64
2	-7	-2	223.22	9.29
-2	7	-2	196.57	9.41
-2	-7	2	232.19	10.52
2	-7	-2	205.71	9.25
-2	7	-2	200.12	9.43
-2	-7	2	219.71	10.47
2	-7	-2	182.50	9.35
-2	7	-2	200.56	9.29
2	-7	-2	221.30	9.58
-2	7	2	220.45	10.44
-2	-7	-2	198.56	9.16
2	7	-2	202.40	9.28
2	7	-2	198.33	9.09
-2	7	2	235.66	10.59
-2	-7	-2	190.22	9.34
-2	-7	-2	215.74	9.40
2	-7	2	206.78	10.23
2	7	-2	191.91	9.55
2	-7	2	241.55	10.49

2	-7	2	241.95	10.44
-2	-7	-2	196.01	9.18
-2	7	2	216.02	10.68
2	-7	2	225.41	10.40
-2	-7	-2	199.07	9.30
2	-7	2	240.67	10.40
-2	7	2	236.54	10.34
-2	-7	-2	205.80	9.45
2	7	-2	199.03	9.38
2	7	-2	192.66	9.31
-2	7	2	224.95	10.38
2	-7	2	207.86	10.44
2	-7	2	249.43	10.71
2	8	2	100.10	5.42
2	-8	-2	106.86	5.45
2	8	2	99.73	5.04
2	-8	-2	121.19	5.37
2	-8	-2	113.30	5.35
-2	-8	2	107.76	5.26
2	8	2	103.41	4.97
2	-8	-2	115.40	5.32
-2	-8	2	105.17	5.20
2	-8	-2	110.67	5.35
-2	-8	2	99.23	5.16
2	8	2	96.02	5.07
2	-8	-2	121.94	5.69
-2	-8	2	106.17	5.35
-2	-8	2	100.10	4.87
-2	8	-2	109.17	5.35
-2	8	-2	113.36	5.50
-2	-8	2	111.51	5.16
2	8	2	109.84	5.33
-2	8	-2	116.17	5.22
2	-8	-2	101.05	5.22
-2	8	-2	117.35	5.56
-2	-8	2	103.61	5.12
-2	8	-2	108.66	5.74
2	8	-2	112.06	5.48
-2	8	2	101.22	5.46
-2	-8	-2	109.12	5.22
-2	-8	-2	113.86	5.45
-2	-8	-2	108.33	5.36
-2	-8	-2	113.52	5.24
2	8	-2	100.84	5.35

-2	8	2	105.23	5.18
2	-8	2	115.33	5.49
2	8	-2	106.90	5.69
-2	8	2	100.13	5.09
2	-8	2	106.82	5.12
2	8	-2	102.15	5.33
2	-8	2	111.56	5.11
-2	-8	-2	118.64	5.50
-2	8	2	111.80	5.37
2	-8	2	118.03	5.19
-2	-8	-2	100.33	5.39
-2	8	2	106.42	5.03
-2	-8	-2	116.32	5.58
2	-8	2	99.03	4.92
2	-8	2	100.62	5.18
2	-8	2	113.15	5.23
2	8	-2	105.92	5.37
-2	9	-2	16.28	1.57
2	-9	-2	16.86	1.44
2	-9	-2	13.57	1.12
-2	-9	2	17.52	1.73
-2	9	-2	16.86	1.20
-2	-9	2	16.51	1.35
2	9	2	14.74	1.27
2	-9	-2	14.70	1.42
2	9	2	15.56	1.70
2	9	2	16.84	1.25
2	-9	-2	14.24	1.36
-2	-9	2	15.01	1.05
2	-9	-2	14.15	1.20
2	-9	-2	15.44	1.66
-2	-9	2	16.06	1.48
-2	9	-2	13.76	1.78
2	9	2	19.23	1.74
-2	9	-2	15.86	1.53
2	-9	-2	16.26	1.26
-2	-9	2	16.08	1.44
-2	-9	2	18.94	1.58
2	-9	2	20.95	1.64
2	9	-2	10.71	1.66
2	-9	2	18.33	1.34
2	9	-2	12.99	1.31
-2	-9	-2	13.24	1.27
-2	-9	-2	15.56	1.09

2	-9	2	15.07	1.44
-2	-9	-2	17.58	1.62
-2	9	2	16.53	1.34
-2	-9	-2	13.83	1.43
-2	-9	-2	15.32	1.40
2	-9	2	15.43	1.66
2	-9	2	20.80	2.03
2	9	-2	15.51	1.05
2	-9	2	15.59	1.07
-2	-9	-2	16.71	1.27
-2	9	2	16.84	1.48
-2	-9	-2	16.63	1.63
2	-9	2	18.31	1.37
2	9	-2	14.06	1.58
-2	9	2	16.80	1.78
-2	9	2	14.61	1.77
-2	10	-2	22.59	2.01
2	-10	-2	16.37	1.57
-2	-10	2	15.81	1.49
-2	10	-2	18.81	1.38
2	-10	-2	15.89	1.66
-2	-10	2	16.09	1.41
2	-10	-2	20.44	1.52
-2	-10	2	14.83	1.56
2	10	2	14.97	2.08
2	-10	-2	16.16	1.24
-2	10	-2	16.94	2.10
2	-10	-2	19.59	1.68
2	10	2	15.01	1.34
-2	-10	2	15.38	1.70
2	10	2	16.04	1.29
2	-10	-2	20.91	2.08
-2	-10	2	13.73	1.01
2	10	2	13.23	1.90
2	-10	-2	16.87	1.45
-2	10	-2	18.01	1.78
-2	-10	-2	14.94	1.44
-2	-10	-2	19.13	1.67
2	-10	2	14.71	1.82
-2	10	2	16.74	1.58
2	-10	2	13.22	1.42
2	10	-2	17.73	1.21
-2	-10	-2	17.81	1.68
-2	10	2	14.08	1.91

-2	-10	-2	17.16	1.27
-2	10	2	15.93	1.43
2	10	-2	17.73	1.55
2	-10	2	16.70	1.09
2	-10	2	16.70	1.44
2	-10	2	17.87	1.76
2	10	-2	19.22	2.04
2	-10	2	18.54	2.32
2	-10	2	16.14	1.38
-2	10	2	17.93	1.99
-2	-10	-2	17.14	1.48
-2	-11	2	7.63	1.25
-2	-11	2	6.98	0.79
2	-11	-2	9.35	1.30
-2	11	-2	9.93	1.21
2	-11	-2	8.25	1.30
2	-11	-2	8.46	1.29
2	-11	-2	5.51	0.98
2	11	2	6.56	1.61
-2	11	-2	10.68	1.65
2	11	2	8.48	1.25
2	-11	-2	8.46	1.35
2	-11	-2	9.67	1.59
2	11	2	6.56	1.13
-2	-11	2	8.35	1.68
-2	11	-2	8.23	1.60
-2	11	-2	8.21	1.72
2	-11	2	8.25	1.30
2	-11	2	5.88	0.86
-2	-11	-2	7.48	0.98
-2	-11	-2	9.06	1.27
-2	-11	-2	8.17	1.51
2	11	-2	7.68	2.44
2	-11	2	8.90	1.32
2	-11	2	6.19	1.08
-2	11	2	9.64	1.66
2	-11	2	7.69	1.22
-2	11	2	7.07	1.55
-2	11	2	9.29	1.55
2	11	-2	8.98	1.43
-2	11	2	8.98	1.25
-2	12	-2	9.00	1.81
2	-12	-2	6.60	0.92
-2	12	-2	9.51	2.06

2	-12	-2	7.27	1.35
2	12	2	5.49	1.82
2	12	2	7.51	1.11
2	-12	-2	9.87	1.49
-2	-12	2	5.88	1.30
-2	-12	2	7.51	1.37
2	-12	2	6.04	0.78
-2	12	2	6.18	1.32
2	-12	2	9.27	1.27
-2	-12	-2	9.36	1.45
2	12	-2	8.06	1.27
2	12	-2	7.26	1.99
2	-12	2	4.85	1.04
-2	-12	-2	6.86	1.07
-2	-12	-2	7.54	1.23
-2	12	2	8.78	1.85
-2	13	-2	31.39	2.84
-2	-13	2	36.15	2.53
2	13	2	31.34	3.50
-2	-13	2	36.67	2.81
2	-13	-2	33.21	2.34
-2	13	-2	33.38	3.68
-2	13	2	42.13	3.58
2	-13	2	39.05	2.66
2	13	-2	26.76	3.63
2	-13	2	32.54	2.47
-2	-13	-2	31.32	2.31
-2	-13	-2	36.06	2.78
3	0	2	0.00	0.08
-3	0	2	0.04	0.07
-3	0	-2	0.13	0.08
3	0	2	-0.06	0.13
3	0	-2	-0.02	0.09
3	0	-2	0.08	0.07
-3	0	-2	0.25	0.14
-3	0	-2	0.12	0.10
-3	0	2	0.18	0.11
3	0	2	-0.08	0.10
3	0	2	0.03	0.10
3	0	-2	0.03	0.09
3	0	2	0.22	0.13
-3	0	-2	0.04	0.15
-3	0	-2	0.06	0.11
3	0	2	0.07	0.10

3	0	-2	0.07	0.12
-3	0	2	0.02	0.14
3	0	-2	-0.01	0.07
3	0	-2	-0.01	0.13
-3	0	2	0.09	0.07
-3	0	2	0.02	0.12
-3	0	-2	0.00	0.15
3	-1	-2	15.88	0.78
-3	1	-2	15.15	0.89
3	1	2	16.82	0.84
-3	-1	2	16.24	0.79
3	-1	-2	15.50	0.75
3	1	2	17.34	0.85
-3	1	-2	15.64	0.81
3	1	2	16.38	0.90
-3	-1	2	16.12	0.91
-3	1	-2	15.81	0.80
-3	-1	2	15.98	0.90
-3	1	-2	16.34	0.80
-3	-1	2	17.74	0.81
-3	-1	2	16.45	0.83
3	-1	-2	14.94	0.81
-3	1	-2	15.20	0.90
-3	-1	2	16.98	0.90
3	1	2	16.27	0.80
3	-1	-2	16.26	0.82
-3	-1	2	17.16	0.78
-3	1	-2	17.13	0.82
-3	1	-2	15.87	0.80
-3	1	2	16.28	0.88
-3	-1	-2	17.21	0.82
3	1	-2	15.84	0.78
-3	1	2	17.25	0.87
3	-1	2	16.13	0.84
-3	1	2	17.53	0.90
-3	-1	-2	16.36	0.83
-3	-1	-2	16.46	0.79
-3	1	2	16.47	0.82
3	-1	2	16.31	0.85
3	1	-2	14.05	0.77
3	-1	2	16.84	0.93
-3	1	2	17.73	0.87
-3	-1	-2	15.61	0.81
-3	-1	-2	16.10	0.81

-3	1	2	16.65	0.90
3	1	-2	16.40	0.85
3	-1	2	16.37	0.85
-3	-1	-2	15.95	0.86
-3	-1	-2	13.84	0.77
3	1	-2	16.07	0.76
3	1	-2	15.63	0.74
3	-2	-2	106.06	4.85
-3	-2	2	112.73	5.25
3	2	2	112.45	5.25
-3	2	-2	110.68	4.87
-3	-2	2	111.20	5.17
3	2	2	110.22	5.16
3	-2	-2	109.48	4.88
3	-2	-2	105.06	4.87
3	-2	-2	108.17	4.92
-3	-2	2	114.31	5.10
-3	2	-2	102.83	4.82
3	2	2	110.85	5.17
-3	2	-2	115.75	4.82
-3	-2	2	118.23	5.22
3	2	2	111.97	5.20
-3	2	-2	109.19	4.85
-3	-2	2	111.98	5.25
-3	-2	2	110.06	5.13
-3	2	-2	102.51	4.93
3	-2	-2	100.40	4.81
-3	2	-2	99.83	4.95
-3	-2	2	115.48	5.18
3	-2	2	108.50	5.17
3	-2	2	119.61	5.32
-3	2	2	109.27	5.15
-3	2	2	120.44	5.23
-3	-2	-2	102.83	4.82
-3	-2	-2	103.88	4.85
3	2	-2	100.27	4.86
3	-2	2	110.29	5.20
-3	-2	-2	106.96	4.84
3	2	-2	107.39	4.83
-3	-2	-2	105.62	4.85
3	-2	2	112.78	5.16
-3	-2	-2	108.65	4.92
3	-2	2	113.55	5.26
3	2	-2	105.69	4.76

3	-2	2	119.22	5.15
-3	2	2	115.14	5.19
-3	2	2	117.92	5.23
-3	2	2	114.61	5.26
3	2	-2	108.97	4.86
3	-3	-2	99.90	4.63
-3	3	-2	105.93	4.65
3	-3	-2	102.06	4.50
-3	-3	2	122.58	5.76
3	3	2	123.85	5.75
3	3	2	123.54	5.85
3	-3	-2	86.66	4.71
-3	3	-2	102.90	4.60
3	-3	-2	94.72	4.56
-3	3	-2	103.90	4.56
3	-3	-2	96.24	4.58
-3	-3	2	130.76	5.83
3	-3	-2	99.22	4.70
-3	-3	2	122.05	5.86
-3	3	-2	106.75	4.56
-3	-3	2	123.72	5.78
-3	3	-2	96.06	4.69
-3	-3	2	126.60	5.88
-3	-3	2	130.32	5.83
-3	3	-2	95.25	4.75
3	3	2	123.87	5.87
3	3	2	127.77	5.79
-3	-3	2	126.30	5.68
-3	3	2	135.48	5.87
3	-3	2	143.17	5.76
-3	-3	-2	103.56	4.68
-3	-3	-2	98.49	4.56
-3	-3	-2	103.62	4.60
-3	-3	-2	101.23	4.63
3	3	-2	95.35	4.65
3	-3	2	126.92	5.94
-3	3	2	123.41	5.91
3	3	-2	99.96	4.58
3	3	-2	101.58	4.48
3	3	-2	98.84	4.60
3	-3	2	123.56	5.78
-3	3	2	133.45	5.80
-3	3	2	120.65	5.70
-3	3	2	121.00	5.77

3	-3	2	126.18	5.88
-3	-3	-2	95.60	4.60
3	-3	2	122.44	5.83
3	-3	2	124.02	5.75
-3	3	2	126.96	5.84
-3	-4	2	58.71	2.97
3	4	2	63.57	2.97
-3	-4	2	65.51	3.03
3	-4	-2	54.22	2.74
3	4	2	60.63	3.10
-3	4	-2	62.06	2.75
-3	-4	2	58.57	3.06
3	-4	-2	55.35	2.82
-3	4	-2	58.33	2.78
3	4	2	64.67	3.06
-3	-4	2	60.66	3.06
3	-4	-2	57.05	2.93
-3	-4	2	62.63	3.10
-3	4	-2	54.54	2.89
3	4	2	61.01	3.08
3	4	2	59.60	2.87
-3	4	-2	60.49	2.85
-3	4	-2	58.73	3.00
3	-4	-2	58.47	2.69
3	-4	-2	57.37	2.80
3	-4	-2	54.67	2.74
-3	-4	2	57.97	2.95
-3	-4	2	62.99	2.85
3	4	2	61.18	2.91
-3	4	-2	61.85	2.72
3	4	-2	59.59	2.63
-3	4	2	62.30	2.98
-3	-4	-2	59.56	2.84
3	4	-2	57.11	2.76
3	-4	2	64.32	3.19
-3	-4	-2	58.06	2.72
-3	4	2	61.65	2.97
-3	-4	-2	51.96	2.63
-3	4	2	59.77	3.15
3	-4	2	60.23	3.23
3	4	-2	56.42	2.79
3	-4	2	67.20	2.95
3	-4	2	62.10	2.91
-3	4	2	64.19	3.05

3	-4	2	59.71	2.97
-3	-4	-2	54.12	2.79
3	4	-2	53.28	2.87
-3	-4	-2	58.81	2.78
-3	-4	-2	57.55	2.87
-3	4	2	66.68	3.10
3	5	2	86.84	4.41
3	5	2	91.98	4.23
-3	5	-2	98.58	4.85
3	-5	-2	104.49	4.76
3	-5	-2	96.53	4.63
3	5	2	88.30	4.20
-3	-5	2	87.59	4.30
-3	-5	2	90.66	4.42
3	-5	-2	93.88	4.66
-3	-5	2	86.57	4.30
3	5	2	92.48	4.41
-3	5	-2	108.60	4.65
-3	-5	2	97.22	4.36
3	-5	-2	93.55	4.68
3	-5	-2	98.58	4.79
-3	5	-2	88.75	5.09
-3	-5	2	88.47	4.40
3	5	2	85.99	4.45
3	-5	-2	102.00	4.90
-3	-5	2	91.95	4.16
3	-5	-2	106.43	4.65
-3	5	-2	102.70	4.81
3	5	2	89.56	4.29
-3	5	-2	105.31	4.73
-3	-5	2	95.01	4.43
-3	-5	-2	94.54	4.74
3	5	-2	103.88	4.72
3	5	-2	101.90	4.72
3	-5	2	95.14	4.43
3	5	-2	102.14	4.55
3	-5	2	87.48	4.21
-3	5	2	93.42	4.29
3	-5	2	98.44	4.30
-3	5	2	86.15	4.52
3	5	-2	92.01	4.85
3	-5	2	92.42	4.38
3	-5	2	88.47	4.30
-3	-5	-2	104.39	4.81

-3	5	2	84.79	4.53
-3	-5	-2	92.43	4.57
3	-5	2	95.43	4.55
3	-5	2	96.69	4.27
-3	5	2	88.00	4.29
-3	5	2	91.78	4.38
-3	-5	-2	99.52	4.64
-3	-5	-2	98.80	4.71
3	5	-2	102.84	4.76
-3	-5	-2	106.91	4.84
3	6	2	106.28	5.06
3	-6	-2	110.63	5.23
-3	6	-2	99.22	5.15
3	6	2	105.39	5.27
3	-6	-2	99.23	5.02
3	-6	-2	99.67	4.88
-3	-6	2	107.86	5.27
-3	6	-2	101.90	5.10
3	-6	-2	103.45	4.86
3	6	2	108.59	5.15
3	6	2	107.10	5.07
-3	-6	2	105.06	5.17
-3	-6	2	112.09	5.31
3	-6	-2	105.56	4.97
-3	6	-2	104.46	4.95
-3	-6	2	104.35	5.17
-3	6	-2	112.74	4.87
3	6	2	111.16	5.30
3	-6	-2	98.22	4.91
-3	6	-2	101.91	5.22
-3	-6	2	112.02	5.30
-3	-6	2	109.50	4.99
3	6	2	109.23	5.37
3	-6	-2	114.26	4.90
-3	-6	2	114.21	5.20
-3	-6	-2	110.22	5.08
3	-6	2	113.86	5.29
3	-6	2	109.16	5.29
3	-6	2	114.90	5.14
3	6	-2	104.93	4.76
-3	6	2	110.23	5.25
3	-6	2	101.49	5.04
-3	-6	-2	102.81	4.86
3	6	-2	110.03	5.03

3	6	-2	105.74	4.94
3	6	-2	95.50	5.13
-3	6	2	117.14	5.36
-3	6	2	111.59	5.14
3	-6	2	102.78	5.23
3	6	-2	104.99	4.96
-3	6	2	106.21	5.41
-3	-6	-2	99.70	4.98
-3	6	2	110.25	5.16
3	-6	2	115.90	5.44
-3	-6	-2	110.42	5.08
-3	-6	-2	101.15	4.94
3	-6	2	102.25	5.15
-3	-6	-2	96.26	4.79
3	-7	-2	31.42	1.83
-3	7	-2	32.62	1.88
3	7	2	28.77	1.86
3	7	2	28.03	1.92
3	7	2	27.48	1.68
-3	-7	2	29.40	1.85
3	7	2	28.09	1.56
-3	-7	2	26.16	1.67
3	-7	-2	32.58	1.77
-3	7	-2	34.10	2.07
-3	7	-2	32.27	2.25
3	-7	-2	36.15	1.84
-3	-7	2	26.88	1.86
-3	-7	2	28.13	1.45
-3	-7	2	28.50	1.80
-3	7	-2	34.83	1.78
3	-7	-2	30.94	1.96
-3	-7	2	27.05	1.68
3	7	2	27.40	1.57
3	-7	-2	32.63	1.81
3	7	2	28.43	1.80
3	-7	-2	34.93	2.21
3	-7	-2	34.81	1.94
-3	-7	2	30.37	1.89
-3	7	-2	33.18	2.05
3	-7	2	27.29	1.66
-3	-7	-2	32.06	2.02
3	7	-2	30.47	2.10
3	-7	2	28.61	1.65
3	7	-2	32.20	1.96

3	-7	2	29.92	1.76
3	-7	2	27.71	1.86
3	7	-2	33.22	1.88
3	7	-2	33.66	1.91
-3	-7	-2	31.84	1.74
3	-7	2	28.12	1.76
3	-7	2	27.35	1.51
-3	7	2	29.06	1.66
-3	-7	-2	28.16	1.89
-3	-7	-2	31.13	1.72
-3	7	2	31.57	1.89
-3	-7	-2	33.84	2.03
-3	7	2	28.59	1.79
3	-7	2	32.63	2.11
3	7	-2	32.67	1.64
-3	7	2	27.69	2.06
-3	7	2	28.40	1.70
-3	-7	-2	30.70	1.84
-3	-8	2	9.49	1.14
-3	8	-2	6.43	0.97
-3	-8	2	9.69	1.14
3	8	2	7.97	0.89
3	-8	-2	10.09	1.42
3	-8	-2	9.92	1.13
-3	-8	2	7.90	1.29
3	8	2	10.29	0.89
3	-8	-2	7.88	1.02
-3	-8	2	10.32	0.98
-3	8	-2	9.82	1.43
3	8	2	7.53	0.96
3	8	2	10.78	1.32
3	-8	-2	9.23	0.81
-3	-8	2	9.46	0.70
3	-8	-2	8.98	0.90
3	-8	-2	7.70	0.90
-3	8	-2	9.08	1.21
-3	-8	2	8.91	1.30
-3	8	-2	9.36	1.30
-3	8	-2	8.52	0.92
3	8	2	5.42	1.08
-3	-8	2	7.91	1.05
3	-8	-2	9.17	1.02
-3	8	2	8.97	1.04
3	8	-2	9.34	0.72

3	-8	2	7.87	1.32
3	8	-2	8.22	0.97
3	-8	2	8.19	0.74
3	8	-2	10.35	1.32
3	8	-2	9.04	1.01
3	-8	2	7.42	0.89
3	8	-2	8.82	1.15
-3	-8	-2	10.10	1.28
-3	-8	-2	9.50	0.96
-3	8	2	9.65	1.15
-3	8	2	9.49	1.38
3	-8	2	9.31	1.14
-3	8	2	8.59	1.36
-3	-8	-2	9.70	0.83
3	-8	2	8.37	0.94
-3	8	2	9.29	1.03
3	-8	2	9.36	1.54
-3	-8	-2	7.54	1.11
3	-8	2	9.73	1.09
-3	-8	-2	9.42	1.27
-3	-8	-2	7.60	0.89
3	-9	-2	5.46	1.07
3	-9	-2	7.22	0.82
-3	-9	2	10.58	0.82
3	9	2	7.61	1.49
3	-9	-2	8.42	1.62
-3	9	-2	7.53	1.08
-3	-9	2	11.48	1.35
-3	9	-2	8.45	1.29
3	-9	-2	7.61	0.96
-3	-9	2	11.09	1.14
-3	9	-2	6.26	0.96
3	9	2	9.12	1.48
-3	9	-2	7.85	1.40
3	-9	-2	7.15	0.99
-3	-9	2	10.24	1.49
3	-9	-2	8.36	1.14
3	9	2	9.53	0.98
3	9	2	9.77	1.10
3	-9	-2	7.10	1.24
-3	-9	2	10.70	1.27
-3	-9	2	9.67	1.16
-3	-9	-2	7.53	1.16
-3	9	2	10.06	1.35

-3	-9	-2	7.15	1.06
-3	-9	-2	7.96	1.17
3	-9	2	10.03	1.09
-3	9	2	10.05	1.17
3	-9	2	10.56	0.85
-3	9	2	11.84	1.49
3	9	-2	6.46	0.81
-3	-9	-2	8.75	1.42
-3	-9	-2	7.99	1.02
3	9	-2	5.83	1.03
3	-9	2	11.74	1.79
3	-9	2	10.43	1.54
3	-9	2	9.01	1.14
-3	9	2	10.87	1.64
-3	-9	-2	6.98	0.82
3	9	-2	5.76	1.14
3	-9	2	10.42	1.10
3	-9	2	13.12	1.37
3	9	-2	7.79	1.31
-3	-10	2	62.45	3.37
3	-10	-2	78.58	3.85
-3	10	-2	69.68	4.36
-3	-10	2	56.02	3.32
3	-10	-2	66.58	3.89
-3	-10	2	63.83	3.29
3	-10	-2	69.50	3.92
3	10	2	55.71	3.62
3	-10	-2	75.61	3.74
-3	10	-2	77.66	3.68
-3	10	-2	80.32	4.21
-3	-10	2	55.58	2.85
3	-10	-2	75.56	4.30
3	-10	-2	71.61	3.81
3	10	2	64.21	3.92
-3	10	-2	76.36	4.11
-3	-10	2	58.71	3.22
3	10	2	56.68	3.05
3	10	2	57.70	3.00
3	-10	-2	68.16	3.64
-3	-10	-2	76.26	3.87
-3	10	2	55.20	3.70
3	-10	2	60.48	3.77
3	-10	2	57.81	3.20
3	-10	2	64.86	3.35

3	10	-2	73.40	3.79
-3	10	2	58.40	3.14
-3	10	2	67.37	3.71
3	10	-2	76.08	3.52
3	10	-2	70.16	4.25
-3	-10	-2	69.43	3.88
3	-10	2	56.21	2.94
-3	-10	-2	67.79	3.61
3	-10	2	55.78	3.40
3	-10	2	54.57	3.28
3	-10	2	62.25	3.18
-3	-10	-2	68.97	3.60
3	10	-2	77.04	4.11
-3	-10	-2	75.15	3.97
-3	10	2	56.46	3.35
-3	-11	2	55.20	2.79
3	-11	-2	59.18	3.49
-3	-11	2	61.42	3.26
3	-11	-2	49.06	2.83
3	-11	-2	54.55	3.09
3	11	2	55.57	2.95
-3	11	-2	55.09	3.33
-3	-11	2	58.04	3.38
3	-11	-2	47.66	3.06
3	-11	-2	52.68	3.06
-3	11	-2	55.87	3.53
-3	11	-2	49.27	3.77
-3	11	-2	59.18	2.91
3	11	2	53.38	3.71
3	11	2	54.19	3.03
3	-11	-2	54.76	3.00
3	-11	2	53.39	3.26
-3	11	2	59.28	3.45
3	-11	2	60.42	3.36
3	11	-2	53.60	3.61
3	-11	2	59.22	3.20
-3	-11	-2	57.93	3.12
-3	-11	-2	56.38	3.35
-3	-11	-2	49.26	2.78
3	-11	2	55.00	2.89
-3	11	2	49.10	3.79
3	-11	2	64.52	3.22
-3	11	2	57.78	3.74
3	11	-2	52.98	3.04

-3	-12	2	44.69	2.97
3	-12	-2	41.54	2.55
3	-12	-2	37.91	2.32
-3	12	-2	41.25	2.91
3	12	2	37.46	3.43
-3	-12	2	42.02	2.72
3	-12	-2	40.63	2.64
-3	12	-2	35.98	3.41
3	-12	2	46.54	2.73
-3	-12	-2	44.64	2.91
-3	12	2	46.08	3.07
-3	-12	-2	36.77	2.25
3	-12	2	41.09	2.70
-3	12	2	42.84	3.53
3	12	-2	39.23	3.40
3	-12	2	39.90	2.31
-3	-12	-2	42.17	2.67
3	12	-2	39.46	2.55
3	-13	-2	42.44	2.78
-3	-13	2	47.79	3.39
-3	13	-2	44.81	3.37
3	13	2	33.04	3.95
-3	-13	2	43.54	2.82
3	-13	-2	38.81	2.45
-3	13	-2	41.19	3.85
3	-13	2	44.44	2.86
-3	-13	-2	41.83	2.79
-3	13	2	46.29	3.19
-3	-13	-2	48.37	3.23
3	13	-2	44.17	4.11
-4	0	-2	230.39	10.60
-4	0	-2	244.68	10.63
4	0	2	218.80	9.83
-4	0	-2	228.21	10.62
-4	0	2	223.61	9.88
-4	0	2	220.18	9.84
4	0	-2	232.09	10.53
4	0	2	201.68	9.79
-4	0	2	220.41	9.80
4	0	-2	239.38	10.64
4	0	2	215.31	9.84
4	0	2	222.47	9.84
-4	0	2	224.96	9.84
4	0	-2	229.16	10.56

-4	0	-2	238.24	10.62
4	0	2	220.04	9.85
4	0	-2	233.89	10.58
4	0	-2	249.92	10.54
-4	0	2	220.27	9.82
-4	0	-2	233.40	10.56
-4	1	-2	123.02	5.47
4	-1	-2	119.61	5.44
-4	-1	2	94.36	4.47
4	1	2	95.71	4.42
4	1	2	94.74	4.40
-4	1	-2	122.42	5.47
-4	-1	2	95.54	4.48
-4	-1	2	95.35	4.40
4	-1	-2	120.20	5.46
-4	-1	2	95.84	4.39
-4	-1	2	92.21	4.40
4	1	2	94.64	4.38
4	1	2	95.72	4.43
-4	1	-2	130.03	5.49
-4	1	-2	122.65	5.56
-4	1	-2	122.14	5.46
-4	-1	2	102.24	4.47
4	1	2	99.85	4.49
-4	1	-2	115.52	5.46
-4	1	-2	114.51	5.53
4	1	-2	119.04	5.40
-4	-1	-2	122.60	5.46
-4	1	2	96.20	4.37
4	1	-2	114.73	5.38
-4	1	2	96.93	4.44
-4	1	2	98.64	4.42
4	1	-2	125.68	5.51
4	-1	2	97.81	4.41
4	1	-2	118.97	5.43
-4	-1	-2	123.34	5.48
-4	1	2	96.04	4.36
-4	1	2	97.18	4.43
4	-1	2	96.06	4.42
-4	1	2	97.26	4.48
-4	1	2	98.18	4.39
-4	-1	-2	125.48	5.53
-4	-1	-2	110.44	5.44
4	-1	2	98.25	4.49

-4	-1	-2	119.42	5.43
4	-1	2	98.96	4.43
-4	-1	-2	119.30	5.47
-4	1	2	100.50	4.49
4	2	2	1.86	0.35
-4	-2	2	1.13	0.18
4	2	2	1.33	0.23
4	-2	-2	2.14	0.34
-4	-2	2	1.26	0.24
-4	-2	2	1.19	0.19
-4	2	-2	1.92	0.35
-4	2	-2	2.67	0.32
-4	-2	2	1.11	0.30
4	-2	-2	2.01	0.29
-4	-2	2	1.37	0.29
-4	-2	2	1.37	0.31
4	-2	-2	2.05	0.28
-4	-2	2	0.92	0.21
-4	2	-2	2.75	0.50
-4	2	-2	2.27	0.29
4	2	2	1.51	0.27
-4	2	-2	2.20	0.32
-4	2	-2	2.80	0.29
4	2	2	1.77	0.27
-4	2	2	1.35	0.28
4	-2	2	2.18	0.41
-4	-2	-2	2.24	0.32
4	2	-2	2.45	0.34
-4	-2	-2	2.23	0.28
4	2	-2	2.42	0.29
4	-2	2	1.66	0.26
-4	2	2	1.24	0.24
4	-2	2	1.35	0.26
-4	2	2	1.32	0.32
-4	2	2	1.12	0.27
-4	-2	-2	2.73	0.30
4	-2	2	1.11	0.24
4	2	-2	2.29	0.25
4	2	-2	2.21	0.30
-4	2	2	1.11	0.21
4	-2	2	1.54	0.23
-4	-2	-2	2.60	0.42
-4	-2	-2	2.06	0.31
4	-2	2	1.38	0.32

4	-3	-2	15.48	0.90
4	3	2	18.13	1.07
4	-3	-2	16.75	0.99
4	3	2	19.49	1.03
-4	-3	2	18.09	0.92
-4	3	-2	17.51	0.96
4	-3	-2	16.18	1.06
-4	3	-2	18.13	0.93
-4	3	-2	16.90	0.90
4	-3	-2	17.07	0.94
-4	-3	2	19.34	1.05
-4	-3	2	18.54	1.15
-4	-3	2	17.53	1.12
4	3	2	18.50	1.13
-4	-3	2	18.72	0.97
4	-3	-2	17.56	1.03
-4	3	-2	17.71	1.01
-4	3	-2	15.55	1.07
-4	-3	2	18.68	1.07
-4	-3	2	17.76	1.01
4	3	2	18.13	0.99
-4	3	-2	17.02	1.15
-4	3	2	18.07	1.00
4	3	-2	16.63	0.84
4	3	-2	16.92	0.94
4	-3	2	18.41	1.01
4	3	-2	16.05	1.00
4	-3	2	17.70	1.05
-4	3	2	17.68	1.16
4	3	-2	16.50	0.96
-4	3	2	20.34	1.12
-4	-3	-2	17.26	1.07
4	-3	2	19.73	1.20
-4	-3	-2	16.47	0.95
-4	-3	-2	17.61	0.97
-4	3	2	19.02	1.05
4	-3	2	17.90	1.15
4	-3	2	18.47	0.98
-4	3	2	18.77	1.11
-4	-3	-2	16.44	0.96
4	-3	2	19.77	0.97
4	3	-2	17.16	0.93
-4	4	-2	0.47	0.30
4	-4	-2	0.50	0.22

-4	-4	2	0.87	0.36
-4	4	-2	0.00	0.39
-4	4	-2	0.45	0.30
4	-4	-2	0.10	0.38
4	4	2	0.65	0.26
-4	4	-2	0.59	0.25
4	-4	-2	0.41	0.22
-4	-4	2	0.95	0.38
4	-4	-2	0.57	0.31
-4	-4	2	1.15	0.23
4	4	2	1.17	0.37
-4	4	-2	0.83	0.27
4	-4	-2	0.48	0.25
4	-4	-2	0.62	0.32
-4	4	-2	0.40	0.49
4	4	2	0.72	0.36
4	4	2	1.04	0.32
-4	-4	2	0.59	0.34
-4	-4	2	1.09	0.45
4	-4	-2	0.41	0.20
-4	-4	2	0.68	0.27
-4	-4	2	0.77	0.29
-4	-4	-2	0.67	0.26
4	4	-2	0.51	0.30
4	4	-2	0.66	0.17
4	-4	2	0.55	0.25
4	4	-2	0.01	0.17
-4	4	2	0.60	0.29
4	4	-2	0.30	0.26
4	-4	2	0.47	0.42
4	-4	2	0.73	0.22
4	4	-2	0.56	0.33
-4	4	2	0.82	0.30
4	-4	2	0.59	0.29
4	-4	2	0.90	0.27
-4	4	2	1.36	0.41
-4	4	2	0.64	0.39
-4	-4	-2	0.43	0.32
-4	-4	-2	0.63	0.24
4	-4	2	0.71	0.46
-4	-4	-2	0.82	0.33
-4	4	2	0.84	0.50
-4	-4	-2	0.56	0.39
4	-5	-2	55.07	2.90

4	5	2	58.77	2.78
4	5	2	58.61	3.06
-4	-5	2	60.52	2.95
4	5	2	58.97	2.90
-4	-5	2	59.54	3.04
-4	-5	2	60.04	3.00
4	-5	-2	61.59	2.72
4	5	2	56.30	3.04
4	5	2	57.93	2.83
-4	-5	2	59.35	2.77
-4	-5	2	54.38	2.88
-4	5	-2	60.88	2.85
-4	-5	2	59.90	2.92
4	-5	-2	52.53	2.78
4	-5	-2	57.89	3.02
-4	5	-2	60.25	2.79
-4	5	-2	55.41	3.11
-4	5	-2	58.68	2.99
-4	5	-2	56.73	2.97
-4	-5	2	60.02	3.07
4	-5	-2	54.09	2.79
4	-5	-2	58.22	2.89
4	5	2	61.38	3.01
4	-5	-2	57.31	2.71
4	-5	2	61.36	3.15
4	5	-2	58.56	2.83
-4	-5	-2	62.35	2.92
-4	5	2	59.50	3.01
4	-5	2	57.89	2.98
-4	-5	-2	60.50	2.98
-4	5	2	63.02	2.92
-4	-5	-2	53.61	2.81
-4	-5	-2	52.55	2.65
4	5	-2	59.03	2.66
-4	5	2	57.50	3.15
4	-5	2	57.68	2.84
-4	5	2	61.17	3.07
4	5	-2	53.28	2.96
4	-5	2	62.51	3.06
4	-5	2	56.58	2.89
-4	-5	-2	56.15	2.75
4	5	-2	58.13	2.81
-4	5	2	60.26	2.91
-4	-5	-2	55.58	2.85

-4	-6	2	78.20	4.01
4	-6	-2	73.28	3.72
4	6	2	78.77	3.78
4	6	2	77.08	4.07
-4	6	-2	76.99	3.96
4	6	2	84.15	4.02
4	-6	-2	81.72	3.69
-4	6	-2	82.41	3.94
-4	6	-2	82.24	3.81
-4	-6	2	82.64	4.00
-4	6	-2	84.17	3.74
4	-6	-2	78.73	4.02
-4	6	-2	76.08	4.08
4	-6	-2	77.81	3.85
4	-6	-2	73.95	3.75
-4	-6	2	76.72	3.88
4	6	2	75.67	4.01
4	-6	-2	75.82	3.89
4	6	2	80.56	3.74
-4	-6	2	75.35	3.86
-4	-6	2	81.84	3.91
-4	-6	2	81.79	4.05
-4	-6	2	81.55	3.71
4	6	2	77.93	3.87
4	-6	-2	69.31	3.90
4	6	-2	81.26	3.86
4	-6	2	87.84	3.87
4	6	-2	79.65	3.78
-4	6	2	68.32	4.31
4	-6	2	80.71	4.35
4	-6	2	75.81	3.77
-4	-6	-2	84.78	3.97
4	-6	2	78.48	3.87
4	-6	2	84.92	3.82
-4	-6	-2	74.12	3.81
-4	6	2	85.58	4.07
4	6	-2	73.59	3.98
-4	6	2	78.92	4.00
4	6	-2	76.96	3.78
-4	-6	-2	72.47	3.60
4	-6	2	79.35	3.96
-4	-6	-2	81.37	3.89
-4	6	2	79.11	3.87
4	-6	2	82.16	4.02

-4	-6	-2	78.75	3.71
4	6	-2	76.81	3.59
-4	6	2	85.00	3.88
-4	-6	-2	77.79	3.80
-4	7	-2	131.00	6.01
-4	-7	2	125.21	5.70
4	-7	-2	114.09	5.74
4	7	2	113.95	6.12
4	-7	-2	123.50	5.82
-4	7	-2	121.91	6.02
-4	-7	2	120.52	6.04
-4	7	-2	117.52	6.18
4	-7	-2	129.97	6.14
4	7	2	123.87	5.77
4	-7	-2	123.14	5.72
-4	-7	2	116.77	5.89
-4	7	-2	131.89	5.76
-4	-7	2	122.75	5.92
4	-7	-2	132.22	5.75
-4	-7	2	124.65	6.04
4	7	2	121.36	5.89
-4	-7	2	129.44	5.92
4	-7	-2	116.10	5.91
4	7	2	119.28	6.05
4	7	2	124.76	5.77
-4	-7	2	127.02	6.08
4	-7	-2	123.68	5.87
4	7	2	127.13	6.06
-4	7	-2	120.27	5.84
4	7	-2	121.49	5.60
4	-7	2	135.41	5.92
-4	-7	-2	131.51	6.01
4	-7	2	123.58	5.90
-4	7	2	120.08	5.89
4	-7	2	134.65	6.24
4	7	-2	115.31	6.08
-4	-7	-2	120.88	5.84
-4	7	2	133.80	6.13
-4	7	2	124.15	6.03
4	-7	2	117.12	5.77
4	7	-2	117.41	5.80
-4	-7	-2	130.92	5.98
4	7	-2	124.07	5.83
4	-7	2	122.24	6.00

-4	-7	-2	116.45	5.86
-4	7	2	115.71	6.18
-4	7	2	130.05	5.88
-4	-7	-2	113.40	5.63
4	-7	2	129.34	6.04
4	-7	2	129.66	5.85
4	7	-2	127.36	5.93
-4	-7	-2	119.78	5.71
-4	-8	2	45.97	2.32
4	-8	-2	63.93	3.55
-4	8	-2	61.17	3.36
4	-8	-2	61.58	3.03
-4	-8	2	46.16	2.80
-4	-8	2	49.39	2.84
-4	8	-2	62.78	3.31
4	8	2	47.48	2.89
-4	8	-2	65.67	3.05
-4	-8	2	47.82	2.75
4	-8	-2	57.93	3.10
4	8	2	50.79	2.81
4	-8	-2	60.22	3.24
-4	8	-2	58.07	3.48
4	-8	-2	57.68	3.02
4	8	2	43.63	2.73
4	8	2	46.06	2.43
4	-8	-2	58.53	3.20
4	-8	-2	66.07	3.08
4	8	2	45.56	2.41
-4	-8	2	45.17	2.58
-4	-8	2	45.46	2.59
-4	-8	2	50.05	2.65
-4	8	2	48.70	3.00
4	8	-2	55.96	3.41
4	-8	2	49.23	2.78
4	-8	2	50.89	2.98
4	-8	2	47.14	2.42
4	8	-2	62.92	3.28
-4	8	2	46.10	2.60
-4	-8	-2	57.44	2.98
4	-8	2	52.20	2.63
-4	-8	-2	55.91	3.16
-4	-8	-2	55.59	2.93
4	8	-2	57.59	3.08
-4	-8	-2	58.80	3.14

-4	-8	-2	65.61	3.39
4	-8	2	48.49	2.52
4	-8	2	48.78	2.59
4	-8	2	44.32	2.62
-4	8	2	51.01	2.58
-4	-8	-2	63.29	3.32
-4	8	2	49.86	2.89
-4	8	2	47.63	2.75
4	8	-2	58.72	2.86
-4	9	-2	20.68	1.85
-4	9	-2	19.24	1.40
-4	9	-2	18.76	1.74
-4	-9	2	15.68	1.56
4	9	2	17.38	1.24
-4	-9	2	17.54	1.57
4	-9	-2	19.58	1.43
4	9	2	17.06	1.78
-4	-9	2	14.76	1.35
4	-9	-2	18.13	2.01
4	9	2	17.29	1.22
-4	9	-2	18.36	1.96
4	-9	-2	17.31	1.62
4	-9	-2	14.99	1.46
4	9	2	17.39	1.76
4	-9	-2	18.18	1.32
4	-9	-2	17.90	1.37
-4	-9	2	16.27	1.41
4	-9	-2	15.77	1.23
-4	-9	2	17.01	1.07
4	9	-2	17.08	1.13
4	-9	2	16.25	1.34
4	-9	2	20.16	1.52
4	-9	2	20.05	1.35
4	-9	2	15.13	1.44
4	-9	2	16.26	1.12
4	-9	2	16.67	1.77
-4	-9	-2	17.39	1.39
-4	-9	-2	18.08	1.23
-4	9	2	12.39	1.78
-4	-9	-2	17.52	1.47
-4	-9	-2	17.70	1.74
4	9	-2	18.46	1.65
4	9	-2	18.68	1.37
-4	9	2	20.66	1.92

4	9	-2	17.24	1.82
-4	-9	-2	17.24	1.23
-4	9	2	17.54	1.63
4	-9	2	20.38	2.09
-4	9	2	16.68	1.45
-4	-9	-2	19.88	1.63
4	10	2	12.33	1.80
4	-10	-2	8.09	1.23
4	10	2	9.29	1.56
4	10	2	10.80	1.17
-4	10	-2	8.34	1.31
-4	-10	2	10.71	1.31
-4	-10	2	12.89	1.53
4	-10	-2	6.07	0.91
4	-10	-2	7.42	1.22
-4	-10	2	10.72	1.52
-4	-10	2	10.93	0.87
4	-10	-2	5.42	1.13
-4	10	-2	7.45	1.18
4	-10	-2	7.86	1.54
-4	10	-2	9.58	1.60
-4	10	-2	9.37	1.72
4	-10	-2	7.51	1.05
4	10	2	11.15	1.14
-4	-10	2	12.51	1.31
4	-10	-2	7.17	1.07
4	-10	2	12.08	1.35
-4	-10	-2	5.68	1.11
4	-10	2	11.58	1.23
-4	10	2	10.35	1.57
-4	10	2	9.75	1.82
4	10	-2	7.12	1.12
4	-10	2	12.13	1.22
4	10	-2	6.89	1.43
-4	-10	-2	7.97	1.31
-4	-10	-2	7.23	1.16
4	-10	2	10.18	0.96
4	-10	2	14.20	1.54
4	10	-2	9.35	1.55
-4	10	2	12.98	1.38
-4	10	2	13.56	1.93
-4	-10	-2	7.31	0.89
4	10	-2	9.18	0.86
-4	-10	-2	7.18	1.20

4	-10	2	11.76	2.15
-4	11	-2	-1.09	1.21
4	11	2	-0.07	0.66
4	-11	-2	1.69	0.76
-4	11	-2	0.98	0.76
4	-11	-2	1.24	0.86
-4	-11	2	0.94	0.93
4	-11	-2	1.61	0.57
-4	11	-2	1.88	1.26
-4	-11	2	1.22	0.43
-4	11	-2	0.98	1.15
4	11	2	-0.91	1.13
-4	-11	2	0.25	0.64
4	11	2	0.52	0.63
4	-11	-2	1.00	0.68
4	-11	2	1.35	0.80
-4	-11	-2	1.42	0.70
4	-11	2	0.89	0.67
-4	-11	-2	1.83	0.98
4	-11	2	0.68	0.51
4	11	-2	0.67	1.20
-4	-11	-2	0.85	0.57
4	-11	2	1.01	0.79
-4	11	2	1.09	0.99
-4	11	2	2.30	1.32
4	11	-2	0.71	0.81
-4	11	2	1.62	1.34
4	-11	2	1.31	1.01
-4	12	-2	0.00	1.41
-4	-12	2	2.53	0.97
4	-12	-2	0.69	0.84
4	-12	-2	1.87	0.88
4	-12	-2	1.22	0.84
4	-12	-2	0.56	1.07
4	12	2	0.79	1.52
-4	-12	2	0.53	1.09
-4	12	-2	-0.18	1.15
-4	12	2	-1.32	1.56
-4	-12	-2	1.01	0.86
4	-12	2	1.51	0.84
4	-12	2	0.83	0.89
4	12	-2	3.97	1.58
-4	-12	-2	2.19	1.21
-4	-12	-2	2.07	0.66

4	12	-2	0.78	0.85
4	-12	2	1.19	0.58
-4	-13	2	18.89	2.60
-4	-13	2	12.58	1.69
4	-13	-2	12.30	1.66
4	-13	-2	10.66	1.36
-4	13	-2	15.51	2.54
4	13	2	15.89	2.70
-4	-13	-2	14.25	1.79
-4	13	2	21.39	2.58
4	13	-2	17.29	3.17
-4	-13	-2	17.46	2.51
4	-13	2	13.35	1.67
-5	0	2	0.05	0.12
5	0	2	-0.07	0.17
-5	0	2	0.14	0.15
-5	0	2	0.28	0.20
-5	0	2	-0.20	0.16
-5	0	-2	0.02	0.14
-5	0	2	-0.05	0.16
5	0	2	0.00	0.12
-5	0	-2	-0.26	0.19
5	0	-2	-0.01	0.15
5	0	-2	-0.04	0.11
-5	0	-2	0.01	0.20
-5	0	2	0.26	0.22
-5	0	-2	0.10	0.16
5	0	2	0.02	0.15
-5	0	-2	-0.05	0.17
-5	0	-2	0.08	0.10
5	0	2	0.11	0.14
5	0	-2	0.15	0.10
5	0	-2	0.06	0.10
5	0	2	0.02	0.18
-5	-1	2	107.46	4.89
-5	-1	2	108.64	4.88
5	1	2	104.80	4.99
5	1	2	98.32	4.90
5	-1	-2	95.76	4.36
-5	1	-2	88.18	4.53
5	-1	-2	97.80	4.33
5	1	2	114.24	4.93
5	-1	-2	87.59	4.36
-5	-1	2	113.44	4.97

5	1	2	110.38	4.91
-5	1	-2	96.81	4.34
5	1	2	107.49	4.89
-5	1	-2	96.53	4.33
5	1	2	98.19	4.98
-5	-1	2	106.81	4.98
-5	1	-2	93.04	4.43
-5	1	-2	99.11	4.36
-5	-1	2	105.00	4.89
-5	1	2	110.42	4.89
-5	-1	-2	92.64	4.36
5	-1	2	107.96	4.97
-5	1	2	106.97	5.06
-5	-1	-2	100.59	4.43
-5	1	2	105.00	4.85
-5	1	2	107.51	4.91
-5	1	2	114.72	5.00
-5	-1	-2	90.35	4.36
-5	1	2	114.28	4.89
-5	-1	-2	88.39	4.33
5	1	-2	98.18	4.39
5	1	-2	98.39	4.32
-5	-1	-2	96.24	4.32
-5	-1	-2	96.57	4.29
5	-1	2	109.55	4.90
5	-1	2	96.64	4.97
5	1	-2	95.35	4.42
-5	1	2	108.59	4.86
-5	-2	2	85.41	3.85
5	-2	-2	77.47	3.63
5	2	2	83.94	3.82
-5	2	-2	80.56	3.56
-5	-2	2	82.41	3.91
5	-2	-2	75.67	3.62
-5	2	-2	76.81	3.75
5	-2	-2	80.95	3.69
-5	-2	2	82.19	3.74
-5	-2	2	78.85	3.79
5	2	2	81.19	3.79
5	2	2	76.31	3.78
5	-2	-2	72.10	3.62
-5	2	-2	80.32	3.62
-5	2	-2	74.61	3.71
-5	-2	2	82.93	3.75

5	2	2	79.78	3.90
-5	-2	2	81.37	3.70
-5	-2	2	82.54	3.71
-5	2	-2	78.80	3.63
-5	2	-2	79.96	3.61
5	2	-2	78.64	3.54
5	-2	2	82.07	3.80
5	-2	2	80.95	3.75
-5	-2	-2	76.72	3.58
-5	-2	-2	80.10	3.72
-5	2	2	79.38	3.88
5	-2	2	82.89	3.90
-5	-2	-2	79.63	3.54
-5	2	2	86.57	3.84
-5	-2	-2	78.22	3.57
5	-2	2	80.84	3.82
-5	-2	-2	75.70	3.62
-5	2	2	84.73	3.84
5	2	-2	74.85	3.57
5	2	-2	74.22	3.71
-5	-2	-2	81.57	3.63
-5	-2	-2	72.67	3.52
5	-2	2	83.09	3.81
5	2	-2	78.71	3.63
-5	2	2	85.07	3.89
5	2	-2	79.57	3.59
-5	2	2	82.17	3.78
-5	-3	2	28.30	1.49
5	3	2	27.85	1.52
5	-3	-2	12.57	0.76
-5	3	-2	13.72	0.83
-5	3	-2	12.73	0.85
5	-3	-2	12.35	0.79
5	3	2	27.96	1.62
5	-3	-2	12.28	0.84
-5	-3	2	26.19	1.61
5	3	2	30.31	1.47
-5	3	-2	13.67	1.03
-5	-3	2	30.11	1.54
5	-3	-2	13.37	0.95
-5	3	-2	13.57	0.79
-5	-3	2	30.56	1.47
-5	-3	2	28.74	1.42
-5	-3	2	29.41	1.64

5	3	2	29.77	1.50
-5	3	-2	13.40	0.74
-5	-3	2	28.69	1.40
-5	3	-2	12.09	0.99
5	3	-2	14.32	0.84
-5	-3	-2	12.81	0.78
5	3	-2	13.02	0.79
-5	3	2	29.50	1.65
-5	3	2	28.99	1.48
5	3	-2	12.51	0.84
5	3	-2	13.51	0.78
5	-3	2	28.70	1.64
5	3	-2	13.11	0.70
5	-3	2	30.05	1.42
5	-3	2	29.25	1.70
-5	3	2	30.62	1.64
5	-3	2	28.27	1.47
-5	-3	-2	12.96	0.81
-5	-3	-2	13.00	0.79
-5	-3	-2	13.36	0.79
-5	-3	-2	12.70	0.95
5	-3	2	27.79	1.49
5	-3	2	30.63	1.54
-5	3	2	28.57	1.60
-5	4	-2	209.74	9.84
-5	-4	2	200.12	9.60
-5	4	-2	208.11	9.67
5	-4	-2	216.37	9.79
-5	4	-2	207.86	9.52
-5	-4	2	217.28	9.61
5	-4	-2	208.14	9.68
5	-4	-2	207.36	9.60
-5	-4	2	207.83	9.55
5	4	2	208.39	9.60
5	4	2	200.10	9.68
5	4	2	203.36	9.74
-5	4	-2	204.37	9.75
5	-4	-2	219.23	9.69
5	4	2	212.08	9.54
5	-4	-2	194.17	9.57
-5	4	-2	216.19	9.70
-5	-4	2	216.40	9.63
-5	-4	2	199.07	9.71
-5	-4	2	211.23	9.74

-5	-4	2	205.50	9.49
-5	4	-2	221.74	9.63
-5	4	2	223.15	9.63
-5	4	2	227.74	9.72
5	-4	2	195.27	9.58
5	-4	2	210.20	9.67
-5	-4	-2	205.07	9.60
5	4	-2	221.35	9.60
5	4	-2	215.95	9.61
-5	-4	-2	206.34	9.56
5	4	-2	213.92	9.60
5	-4	2	231.10	9.85
5	-4	2	213.59	9.72
-5	-4	-2	223.03	9.76
-5	4	2	211.63	9.71
5	-4	2	224.64	9.52
5	4	-2	202.34	9.68
-5	-4	-2	217.57	9.63
-5	4	2	211.67	9.59
5	-4	2	205.35	9.57
5	4	-2	208.90	9.48
-5	4	2	206.02	9.78
-5	-4	-2	204.73	9.62
-5	-5	2	130.39	6.24
-5	5	-2	138.15	6.82
-5	-5	2	131.55	5.94
5	5	2	130.49	6.08
5	-5	-2	133.02	6.51
5	5	2	127.57	6.24
-5	5	-2	139.16	6.72
5	-5	-2	144.55	6.39
-5	5	-2	142.69	6.56
5	-5	-2	140.62	6.61
-5	-5	2	132.69	6.11
5	-5	-2	146.41	6.77
-5	-5	2	136.58	6.13
5	-5	-2	143.79	6.39
-5	5	-2	146.32	6.65
5	5	2	125.33	6.21
-5	5	-2	151.70	6.52
-5	-5	2	123.90	6.21
-5	-5	2	125.80	6.04
5	-5	-2	140.86	6.64
5	-5	-2	131.15	6.50

-5	-5	2	124.05	6.08
5	5	-2	142.10	6.54
-5	-5	-2	144.92	6.59
5	-5	2	138.69	6.36
5	-5	2	132.23	6.21
-5	5	2	126.32	6.31
5	-5	2	124.01	6.07
5	5	-2	142.18	6.52
5	5	-2	128.59	6.64
-5	-5	-2	150.81	6.72
5	5	-2	139.61	6.38
-5	-5	-2	137.25	6.55
5	-5	2	126.80	6.02
-5	5	2	136.81	6.10
5	-5	2	143.84	6.01
-5	5	2	137.12	6.22
5	-5	2	127.76	6.16
-5	-5	-2	140.69	6.57
-5	5	2	127.33	6.19
-5	5	2	135.38	6.08
-5	-5	-2	137.48	6.47
5	6	2	113.71	5.09
5	-6	-2	98.83	4.86
-5	-6	2	113.63	5.34
5	-6	-2	103.98	5.14
5	-6	-2	104.74	4.76
5	-6	-2	104.81	4.98
-5	6	-2	109.91	4.86
-5	-6	2	108.64	5.12
5	-6	-2	98.97	4.85
-5	-6	2	105.62	5.27
5	6	2	102.42	5.42
-5	-6	2	113.07	5.44
-5	-6	2	108.58	5.23
-5	-6	2	114.75	5.28
-5	-6	2	108.74	5.41
-5	6	-2	104.11	4.92
5	6	2	101.80	5.40
5	6	2	114.43	5.38
5	-6	-2	98.11	4.98
5	6	2	109.66	5.15
5	6	2	106.60	5.25
-5	6	-2	103.42	5.09
-5	6	-2	90.77	5.37

-5	6	-2	112.95	5.06
5	-6	-2	107.25	4.75
5	-6	2	117.18	5.17
5	-6	2	107.64	5.25
-5	6	2	107.11	5.25
5	-6	2	119.21	5.22
5	6	-2	98.11	5.06
5	6	-2	103.96	4.89
-5	-6	-2	106.31	5.07
-5	6	2	109.31	5.39
5	6	-2	106.51	4.86
5	-6	2	105.86	5.33
-5	6	2	118.39	5.27
5	-6	2	108.28	5.18
5	-6	2	124.36	5.62
-5	6	2	120.12	5.44
5	6	-2	102.48	4.70
-5	-6	-2	101.00	4.91
-5	-6	-2	101.69	4.92
-5	-6	-2	106.86	4.97
-5	-6	-2	100.73	4.81
-5	-6	-2	95.01	4.68
5	-6	2	113.67	5.40
-5	6	2	104.42	5.52
-5	-7	2	21.82	1.59
-5	7	-2	24.22	1.55
5	-7	-2	24.20	1.46
5	7	2	21.46	1.26
-5	-7	2	22.67	1.20
-5	7	-2	24.46	1.90
5	7	2	21.39	1.43
5	-7	-2	23.98	1.34
-5	-7	2	21.06	1.41
5	-7	-2	23.40	1.63
5	-7	-2	23.57	1.89
5	7	2	21.25	1.64
5	7	2	22.81	1.61
-5	-7	2	23.04	1.68
-5	7	-2	26.37	1.48
5	-7	-2	25.35	1.38
5	-7	-2	22.12	1.40
-5	7	-2	27.43	1.68
-5	7	-2	24.28	1.77
-5	-7	2	23.26	1.58

5	7	2	20.67	1.59
5	7	2	21.63	1.30
-5	-7	2	21.44	1.40
5	-7	-2	21.97	1.58
5	7	-2	21.77	1.74
5	-7	2	24.47	1.37
5	-7	2	22.02	1.29
-5	-7	-2	25.39	1.75
-5	-7	-2	24.02	1.38
5	-7	2	19.41	1.67
-5	-7	-2	22.04	1.27
-5	7	2	22.68	1.62
-5	7	2	24.15	1.48
-5	7	2	22.65	1.45
5	7	-2	25.05	1.62
5	7	-2	23.77	1.52
5	-7	2	21.84	1.51
-5	-7	-2	22.98	1.49
5	7	-2	24.00	1.26
5	-7	2	21.28	1.38
-5	-7	-2	23.67	1.65
-5	7	2	24.96	1.71
-5	-7	-2	22.28	1.50
5	-7	2	22.71	1.91
5	7	-2	23.03	1.48
-5	7	2	23.09	1.77
-5	-8	2	20.23	1.55
5	8	2	20.87	1.68
5	8	2	18.78	1.64
-5	-8	2	20.69	1.14
5	-8	-2	20.26	1.73
5	-8	-2	18.13	1.32
-5	-8	2	19.55	1.42
-5	8	-2	20.14	1.38
5	-8	-2	17.51	1.54
5	8	2	18.52	1.70
-5	-8	2	21.13	1.76
-5	8	-2	15.94	1.80
-5	8	-2	20.38	1.69
-5	8	-2	20.48	1.68
5	-8	-2	20.30	1.27
5	-8	-2	19.42	1.25
5	8	2	20.79	1.28
5	-8	-2	18.68	1.46

-5	-8	2	20.50	1.64
-5	-8	2	22.83	1.85
-5	-8	2	18.65	1.42
5	-8	2	20.51	1.39
-5	-8	-2	18.11	1.39
-5	8	2	18.86	1.94
-5	8	2	20.24	1.47
-5	-8	-2	16.63	1.14
5	-8	2	18.20	1.46
5	-8	2	21.70	1.48
5	-8	2	20.90	2.03
-5	8	2	19.32	1.46
5	8	-2	19.46	1.32
5	8	-2	17.66	1.67
-5	-8	-2	20.27	1.72
5	8	-2	18.44	1.50
5	-8	2	19.65	1.72
-5	-8	-2	18.89	1.52
-5	8	2	20.35	1.77
5	-8	2	20.27	1.32
-5	8	2	20.08	1.64
-5	-8	-2	17.96	1.37
5	-8	2	18.11	1.21
5	8	-2	18.99	1.09
-5	-8	-2	18.04	1.20
5	9	2	27.85	1.64
-5	-9	2	27.46	2.02
5	9	2	27.42	2.15
5	-9	-2	22.91	1.93
-5	9	-2	26.69	1.72
-5	9	-2	25.22	2.27
-5	9	-2	25.71	2.11
5	-9	-2	28.69	1.68
-5	-9	2	27.44	1.90
5	-9	-2	23.20	1.67
5	-9	-2	31.35	2.37
5	9	2	28.84	1.58
-5	-9	2	27.27	1.84
-5	-9	2	26.50	1.52
5	9	2	27.28	2.09
-5	9	-2	28.74	2.09
5	-9	-2	25.61	1.84
5	-9	-2	25.38	1.55
5	-9	-2	24.49	1.59

-5	9	2	28.30	2.23
5	-9	2	26.73	1.60
-5	9	2	26.57	2.06
5	9	-2	27.43	1.99
-5	-9	-2	25.48	1.55
5	9	-2	26.69	1.47
5	-9	2	27.56	1.91
-5	9	2	26.00	2.29
-5	-9	-2	26.68	1.93
5	-9	2	29.75	1.71
5	-9	2	30.00	2.52
5	-9	2	25.33	2.12
5	-9	2	28.77	2.34
5	-9	2	30.12	2.36
-5	-9	-2	25.79	1.79
5	9	-2	22.42	2.06
5	9	-2	26.22	1.66
-5	9	2	29.61	1.87
-5	-9	-2	22.79	1.47
-5	-10	2	81.29	4.41
-5	-10	2	82.83	4.09
-5	-10	2	82.99	4.46
-5	10	-2	80.60	3.95
5	-10	-2	77.68	4.01
5	-10	-2	72.36	4.14
-5	-10	2	88.26	4.41
5	-10	-2	82.72	3.89
5	10	2	86.61	4.19
-5	10	-2	78.67	4.63
5	-10	-2	72.90	3.87
5	-10	-2	85.04	3.94
5	-10	-2	80.45	4.57
5	10	2	93.32	4.76
-5	10	-2	81.08	4.35
5	10	2	81.01	4.19
-5	10	-2	82.54	4.40
-5	-10	2	89.29	4.72
5	-10	2	88.52	4.44
-5	-10	-2	75.00	3.81
5	10	-2	80.03	3.94
-5	-10	-2	74.14	4.08
5	-10	2	83.56	4.22
-5	10	2	82.29	4.65
5	-10	2	80.98	4.50

5	-10	2	93.47	4.33
5	10	-2	79.79	4.26
-5	10	2	79.55	4.93
5	10	-2	78.67	3.70
5	-10	2	94.70	5.07
-5	10	2	88.72	4.76
5	10	-2	70.84	4.47
-5	-10	-2	68.57	3.71
5	-10	2	97.43	4.46
-5	-10	-2	80.23	4.10
-5	10	2	92.59	4.63
-5	-11	2	73.47	4.02
5	11	2	66.49	3.41
-5	-11	2	64.30	3.26
-5	11	-2	73.07	4.18
-5	-11	2	66.97	3.73
-5	11	-2	73.32	3.73
5	-11	-2	67.47	3.95
5	-11	-2	75.23	3.68
5	-11	-2	73.53	3.84
5	11	2	67.53	4.26
5	-11	-2	68.07	3.64
-5	11	-2	71.86	4.59
-5	11	2	74.20	4.08
5	-11	2	66.85	3.43
5	-11	2	76.28	3.61
-5	11	2	61.90	4.31
5	11	-2	71.18	3.73
-5	-11	-2	68.39	3.58
-5	-11	-2	66.85	3.86
5	-11	2	68.29	3.69
-5	-11	-2	77.08	4.13
5	-11	2	63.81	3.80
5	11	-2	75.33	4.49
5	-12	-2	73.31	3.83
5	-12	-2	69.19	4.16
-5	12	-2	70.32	4.92
5	12	2	71.62	4.71
-5	-12	2	77.14	4.38
-5	12	-2	83.53	4.62
-5	-12	2	76.06	4.13
5	-12	-2	72.97	4.06
-5	12	2	63.93	4.61
-5	-12	-2	81.53	4.38

5	-12	2	77.35	4.10
5	12	-2	76.02	4.81
-5	-12	-2	72.30	3.77
-5	-12	-2	75.77	4.14
5	-12	2	70.14	3.75
5	-12	2	83.54	3.99
-5	12	2	80.02	4.47
-5	-13	2	56.84	3.73
5	-13	-2	47.32	2.99
-5	13	-2	60.03	3.92
5	-13	-2	56.90	3.26
-5	-13	2	52.37	3.26
5	13	2	50.94	4.16
-5	-13	-2	57.68	3.82
-5	13	2	55.16	3.83
5	13	-2	54.01	4.28
-5	-13	-2	50.81	3.27
5	-13	2	52.16	3.19
6	0	-2	75.50	3.73
-6	0	2	76.00	3.82
6	0	2	82.52	3.81
6	0	2	80.40	3.71
-6	0	-2	81.75	3.77
6	0	-2	78.69	3.65
6	0	-2	78.94	3.64
-6	0	2	83.53	3.84
-6	0	-2	84.78	3.75
6	0	-2	78.67	3.71
6	0	-2	84.25	3.77
6	0	2	77.09	3.79
-6	0	2	84.12	3.75
-6	0	-2	82.07	3.67
-6	0	2	83.95	3.79
-6	0	2	80.23	3.76
6	0	2	80.43	3.78
6	0	2	85.44	3.84
-6	0	-2	81.10	3.75
-6	0	-2	83.43	3.74
-6	0	-2	78.23	3.79
-6	1	-2	11.74	0.80
-6	-1	2	9.98	0.62
6	-1	-2	9.95	0.71
6	-1	-2	11.08	0.64
6	1	2	9.51	0.64

6	1	2	11.18	0.58
-6	1	-2	9.88	0.77
-6	-1	2	10.19	0.73
-6	-1	2	10.34	0.75
-6	-1	2	10.70	0.62
-6	1	-2	11.92	0.64
6	1	2	11.21	0.66
-6	1	-2	11.42	0.67
6	-1	-2	10.06	0.65
-6	1	-2	11.22	0.66
6	-1	-2	9.54	0.60
6	1	2	10.19	0.66
6	1	2	10.82	0.73
6	1	2	11.62	0.70
-6	1	-2	9.58	0.65
6	-1	2	10.36	0.77
-6	1	2	11.35	0.63
-6	1	2	10.76	0.61
6	-1	2	9.67	0.60
-6	1	2	9.93	0.62
6	-1	2	11.45	0.65
-6	1	2	9.99	0.66
-6	1	2	9.24	0.72
-6	1	2	9.31	0.60
-6	1	2	9.92	0.66
6	-1	2	11.08	0.65
6	1	-2	9.78	0.72
-6	-1	-2	11.48	0.57
-6	-1	-2	11.76	0.77
-6	-1	-2	10.37	0.66
-6	-1	-2	10.00	0.68
-6	-1	-2	10.79	0.61
-6	1	2	9.85	0.75
6	1	-2	10.90	0.77
-6	-1	-2	11.79	0.68
-6	-2	2	7.51	0.63
6	2	2	7.99	0.58
6	2	2	7.73	0.54
6	2	2	6.66	0.48
6	-2	-2	4.03	0.66
-6	2	-2	5.03	0.68
-6	-2	2	6.63	0.49
6	-2	-2	4.41	0.45
-6	-2	2	7.25	0.48

-6	-2	2	6.89	0.68
-6	2	-2	5.12	0.62
-6	-2	2	7.60	0.51
6	-2	-2	5.05	0.45
-6	2	-2	5.57	0.46
-6	2	-2	5.11	0.46
-6	2	-2	5.69	0.48
-6	2	-2	4.74	0.41
6	2	2	7.56	0.66
6	-2	-2	5.02	0.40
6	2	-2	5.35	0.51
-6	2	2	5.81	0.63
-6	2	2	6.41	0.63
-6	2	2	7.25	0.52
-6	2	2	6.82	0.63
6	-2	2	7.65	0.53
6	2	-2	6.04	0.45
-6	2	2	7.03	0.66
6	-2	2	7.64	0.48
-6	-2	-2	5.93	0.47
6	2	-2	4.30	0.58
-6	-2	-2	5.59	0.41
-6	-2	-2	4.71	0.43
-6	-2	-2	5.54	0.42
6	-2	2	7.27	0.52
6	2	-2	5.32	0.35
6	-2	2	7.08	0.52
-6	-2	-2	5.45	0.38
6	2	-2	4.41	0.38
-6	-2	-2	5.59	0.60
-6	-2	-2	4.53	0.43
-6	-2	-2	4.62	0.52
6	-2	2	6.29	0.67
6	3	2	60.81	2.96
6	-3	-2	63.86	3.14
-6	3	-2	66.68	3.09
6	3	2	62.73	2.99
-6	-3	2	60.80	2.86
-6	3	-2	66.38	3.37
-6	3	-2	68.92	3.19
-6	3	-2	66.04	3.19
6	3	2	64.55	2.94
6	3	2	58.21	2.97
6	-3	-2	68.04	3.28

-6	-3	2	62.81	2.89
-6	-3	2	59.48	2.96
-6	3	-2	62.53	3.32
6	3	2	60.87	3.10
-6	3	-2	71.24	3.24
-6	-3	2	66.68	3.02
6	-3	-2	64.70	3.21
6	-3	-2	63.99	3.18
-6	-3	2	62.11	3.11
-6	-3	2	61.60	2.89
-6	3	2	65.43	3.01
-6	-3	-2	69.66	3.14
6	-3	2	60.05	2.95
6	-3	2	64.87	3.18
6	-3	2	61.81	2.87
-6	-3	-2	64.11	3.04
6	3	-2	63.61	3.32
6	3	-2	69.71	3.13
6	-3	2	57.49	2.95
-6	-3	-2	71.11	3.34
6	3	-2	67.30	3.16
-6	3	2	66.61	3.05
6	3	-2	63.59	3.17
6	3	-2	67.70	3.09
-6	3	2	62.80	3.11
6	3	-2	72.53	3.19
6	-3	2	60.58	3.00
-6	-3	-2	65.02	3.19
-6	3	2	59.47	2.96
-6	3	2	61.05	3.09
6	-3	2	65.72	3.11
-6	-3	-2	66.34	3.14
-6	-3	-2	66.38	3.15
-6	-4	2	10.58	0.69
6	4	2	11.86	0.74
-6	4	-2	6.68	0.86
-6	-4	2	10.63	0.69
6	-4	-2	7.39	0.69
-6	4	-2	7.65	0.57
-6	-4	2	10.42	0.99
6	-4	-2	7.25	0.88
-6	4	-2	8.15	0.76
-6	-4	2	10.23	0.76
6	-4	-2	6.67	0.57

6	4	2	11.90	0.79
-6	4	-2	7.61	0.65
6	4	2	10.82	0.84
6	4	2	11.03	0.84
-6	4	-2	8.11	0.97
-6	4	-2	8.29	0.70
-6	-4	2	9.25	0.90
-6	-4	2	10.47	0.77
6	-4	-2	7.65	0.62
6	4	2	10.17	0.94
6	-4	-2	7.21	0.80
-6	-4	2	9.98	0.85
-6	4	2	12.11	0.91
6	-4	2	11.91	0.67
6	4	-2	7.78	0.52
6	-4	2	10.77	0.73
-6	4	2	11.06	0.84
6	4	-2	6.95	0.67
6	-4	2	10.93	0.84
6	-4	2	11.77	1.11
6	4	-2	7.35	0.73
6	4	-2	8.45	0.66
-6	4	2	10.25	0.93
-6	-4	-2	8.63	0.88
-6	-4	-2	8.34	0.67
6	4	-2	7.21	0.72
-6	-4	-2	7.42	0.62
6	-4	2	11.24	1.00
-6	4	2	10.30	0.78
-6	4	2	9.89	1.00
-6	-4	-2	8.35	0.64
-6	-4	-2	7.87	0.66
6	-4	2	10.49	0.75
-6	5	-2	5.41	1.01
6	5	2	4.79	0.56
-6	-5	2	5.58	0.87
-6	-5	2	3.85	0.77
-6	-5	2	4.92	0.75
6	5	2	4.88	0.69
6	-5	-2	4.29	0.70
-6	-5	2	5.27	0.61
6	-5	-2	4.11	0.52
-6	-5	2	4.67	0.67
-6	5	-2	4.99	0.62

6	5	2	4.44	0.79
6	5	2	6.10	0.63
-6	5	-2	4.13	0.81
6	-5	-2	4.22	0.63
6	-5	-2	4.30	0.81
-6	-5	2	4.92	0.55
-6	5	-2	4.71	0.66
-6	5	-2	4.23	0.52
-6	5	-2	4.52	0.74
6	-5	-2	4.62	0.54
-6	-5	2	5.24	0.51
-6	-5	-2	4.29	0.64
6	5	-2	4.04	0.42
6	-5	2	5.55	0.66
-6	5	2	4.68	0.78
6	-5	2	4.79	0.52
-6	5	2	4.59	0.80
-6	5	2	5.26	0.71
6	-5	2	5.10	0.56
6	5	-2	4.49	0.57
6	-5	2	5.60	0.91
6	-5	2	4.89	0.54
-6	-5	-2	4.09	0.57
6	5	-2	3.62	0.61
-6	-5	-2	3.81	0.57
6	-5	2	5.54	0.88
-6	-5	-2	3.70	0.59
-6	-5	-2	3.90	0.72
6	5	-2	4.45	0.60
6	5	-2	4.76	0.62
-6	5	2	5.36	0.64
-6	5	2	4.98	0.89
-6	6	-2	7.78	0.96
-6	6	-2	6.95	1.31
6	-6	-2	6.04	0.73
6	6	2	4.70	0.87
6	6	2	4.76	0.63
-6	-6	2	4.22	0.65
6	-6	-2	6.07	0.63
-6	-6	2	4.34	0.85
-6	-6	2	4.09	0.55
-6	-6	2	5.68	0.82
-6	-6	2	4.82	0.81
6	-6	-2	6.35	1.11

-6	-6	2	7.05	0.99
-6	6	-2	6.41	0.81
6	6	2	4.32	0.71
-6	6	-2	6.97	0.78
6	6	2	5.00	0.92
-6	6	-2	5.34	0.97
-6	-6	2	5.09	0.68
6	-6	-2	7.42	0.92
6	-6	-2	5.38	0.63
-6	-6	-2	6.66	1.00
-6	-6	-2	5.95	0.76
6	6	-2	4.65	0.91
-6	-6	-2	6.88	0.85
-6	6	2	5.55	0.76
-6	6	2	5.49	0.83
6	6	-2	4.30	0.71
6	-6	2	4.47	0.64
-6	-6	-2	5.67	0.65
-6	6	2	5.86	1.00
6	-6	2	6.70	1.09
-6	6	2	4.49	0.77
6	6	-2	6.20	0.77
6	-6	2	5.17	0.57
6	-6	2	6.48	0.70
-6	-6	-2	6.25	0.72
6	6	-2	6.19	0.53
6	-6	2	5.10	0.75
6	-6	2	4.83	0.56
-6	6	2	5.64	0.85
6	-6	2	6.54	0.93
6	6	-2	6.43	0.67
6	7	2	3.50	0.78
-6	7	-2	3.07	0.82
6	-7	-2	3.04	0.50
6	-7	-2	2.85	0.53
-6	7	-2	4.12	0.65
-6	-7	2	2.64	0.72
6	-7	-2	2.75	0.53
6	7	2	3.57	0.63
-6	7	-2	2.82	0.77
-6	-7	2	4.39	0.89
-6	-7	2	4.11	0.80
6	-7	-2	3.98	0.95
-6	-7	2	3.79	0.76

-6	7	-2	4.02	0.68
-6	-7	2	3.26	0.61
6	-7	-2	2.16	0.75
-6	7	-2	3.21	0.86
-6	-7	2	2.98	0.48
6	7	2	2.81	0.74
-6	-7	2	2.97	0.57
6	7	2	2.25	0.75
6	7	2	2.83	0.56
6	-7	-2	3.16	0.57
6	-7	-2	2.52	0.58
-6	-7	-2	3.88	0.65
-6	-7	-2	3.45	0.61
6	-7	2	5.32	1.12
-6	-7	-2	3.27	0.72
6	7	-2	3.78	0.71
6	-7	2	3.22	0.50
6	-7	2	2.92	0.53
-6	7	2	3.05	0.64
-6	-7	-2	2.91	0.61
6	7	-2	1.75	0.68
6	7	-2	3.37	0.58
6	-7	2	3.21	0.60
-6	7	2	2.25	0.76
-6	7	2	2.98	0.85
-6	7	2	1.74	1.00
6	-7	2	3.35	0.67
-6	-7	-2	3.88	0.82
-6	7	2	3.24	0.60
6	7	-2	3.13	0.49
6	-7	2	4.09	0.76
6	-7	2	2.89	0.82
-6	-8	2	6.99	1.26
6	-8	-2	9.85	0.96
6	8	2	7.95	1.19
6	8	2	7.32	1.19
6	-8	-2	10.32	1.65
6	8	2	8.36	0.85
-6	-8	2	7.65	0.69
6	-8	-2	10.31	0.92
-6	8	-2	10.47	1.10
-6	8	-2	9.54	1.38
-6	8	-2	8.49	1.41
-6	-8	2	5.62	0.95

6	-8	-2	10.39	0.90
6	-8	-2	10.05	0.81
6	-8	-2	6.86	1.31
6	8	2	7.08	1.21
-6	-8	2	7.65	1.19
-6	-8	2	8.19	1.32
-6	8	-2	10.06	1.39
-6	-8	2	7.84	0.93
6	-8	-2	9.24	1.10
-6	-8	-2	9.16	1.05
6	-8	2	7.99	1.65
6	8	-2	9.05	1.23
-6	8	2	6.76	1.08
6	8	-2	9.48	1.42
-6	8	2	9.03	1.43
6	8	-2	8.90	1.00
-6	8	2	7.87	1.37
-6	-8	-2	11.01	1.05
6	-8	2	7.49	0.75
6	-8	2	7.22	1.40
-6	-8	-2	12.02	1.42
-6	8	2	6.52	1.22
6	8	-2	9.23	0.74
6	-8	2	6.10	0.85
-6	-8	-2	9.34	0.86
-6	8	2	8.50	1.20
6	-8	2	8.32	1.13
6	-8	2	5.56	0.97
6	-8	2	7.33	0.88
-6	9	-2	10.06	1.59
-6	9	-2	10.08	1.23
-6	9	-2	8.92	1.54
6	9	2	11.95	1.53
6	-9	-2	9.07	1.04
-6	-9	2	10.43	1.29
6	-9	-2	9.64	1.81
-6	-9	2	11.70	0.90
6	-9	-2	10.33	1.02
-6	-9	2	10.78	1.32
6	9	2	12.83	1.05
-6	-9	2	11.73	1.23
-6	9	-2	11.87	1.60
6	-9	-2	7.72	1.47
6	-9	-2	11.88	1.30

-6	-9	2	11.39	1.45
6	-9	-2	9.77	1.07
6	-9	-2	11.11	1.01
6	9	2	10.09	1.53
-6	9	2	10.67	1.51
-6	9	2	10.10	1.69
6	-9	2	12.58	1.27
6	-9	2	12.17	2.03
-6	9	2	11.45	1.33
-6	-9	-2	10.74	1.27
-6	-9	-2	11.81	1.18
6	9	-2	9.78	0.85
6	9	-2	11.49	1.04
6	9	-2	9.64	1.53
6	-9	2	11.70	1.03
-6	-9	-2	10.43	0.97
6	9	-2	11.10	1.38
6	-9	2	11.52	1.15
-6	-9	-2	12.14	1.38
6	-9	2	10.33	0.94
6	-9	2	9.48	1.24
-6	9	2	11.66	1.72
6	10	2	1.93	1.06
-6	10	-2	1.50	1.19
-6	10	-2	1.53	0.79
-6	-10	2	2.87	0.53
-6	-10	2	1.81	0.90
6	10	2	2.46	1.10
6	-10	-2	2.14	0.73
6	-10	-2	2.23	1.22
6	-10	-2	0.99	0.77
6	-10	-2	2.02	0.61
6	10	2	2.83	0.64
-6	-10	2	2.12	0.81
6	-10	-2	1.78	0.58
-6	10	-2	1.53	1.03
-6	-10	2	3.07	0.95
-6	-10	2	1.38	0.75
-6	10	-2	1.60	1.32
6	-10	-2	1.32	0.66
-6	10	2	1.44	1.17
-6	-10	-2	1.55	0.83
6	-10	2	3.31	0.80
6	-10	2	3.35	0.71

6	-10	2	2.61	1.33
-6	-10	-2	1.21	0.56
6	10	-2	2.01	1.05
6	10	-2	2.15	0.51
-6	-10	-2	2.28	0.79
6	10	-2	2.31	0.68
6	-10	2	2.13	0.80
6	10	-2	0.86	1.01
-6	10	2	1.74	1.01
-6	-10	-2	1.16	0.80
6	-10	2	2.22	0.86
6	-10	2	2.75	0.59
6	-11	-2	6.14	1.06
6	-11	-2	6.25	1.22
6	-11	-2	6.05	1.00
-6	11	-2	5.17	1.70
-6	-11	2	5.81	1.13
6	-11	-2	4.41	1.77
-6	11	-2	6.24	1.44
-6	-11	2	6.12	1.28
-6	-11	2	5.37	0.75
6	11	2	6.68	1.50
6	-11	-2	5.29	1.04
-6	11	2	3.45	1.46
6	-11	2	6.03	0.84
6	-11	2	5.07	1.01
-6	-11	-2	5.16	0.78
6	11	-2	7.01	1.58
6	-11	2	6.24	1.16
6	11	-2	6.16	1.17
-6	-11	-2	6.67	1.33
6	-11	2	7.86	1.13
-6	-11	-2	7.01	1.24
-6	11	2	4.47	1.41
6	12	2	-0.98	1.59
-6	12	-2	2.19	1.42
6	-12	-2	0.71	0.71
6	-12	-2	-1.27	0.76
-6	-12	2	-1.42	1.25
-6	12	-2	-0.28	1.82
-6	-12	2	-0.79	0.80
6	-12	-2	-0.37	0.97
6	-12	2	0.37	0.61
-6	-12	-2	-0.26	1.18

-6	-12	-2	1.60	0.89
-6	12	2	1.71	1.32
6	-12	2	0.37	0.76
6	-12	2	-0.27	0.96
6	12	-2	1.08	1.70
-6	13	-2	4.08	2.00
6	-13	-2	4.33	1.09
6	-13	-2	4.03	0.93
-6	-13	2	5.35	1.12
6	13	2	2.23	1.82
-6	-13	2	5.80	1.56
6	-13	2	5.18	1.01
6	13	-2	3.12	1.71
-6	13	2	8.34	1.74
-6	-13	-2	5.92	1.09
-6	-13	-2	3.35	1.50
-7	0	2	0.09	0.17
-7	0	-2	0.14	0.21
-7	0	2	-0.14	0.24
-7	0	-2	0.10	0.24
-7	0	-2	0.12	0.13
7	0	-2	0.04	0.15
7	0	2	0.34	0.25
-7	0	-2	-0.04	0.18
7	0	2	0.01	0.16
-7	0	2	-0.02	0.24
7	0	-2	-0.02	0.25
-7	0	-2	0.03	0.18
7	0	2	-0.03	0.28
-7	0	2	0.10	0.18
7	0	-2	-0.04	0.09
7	0	-2	-0.08	0.14
7	0	2	0.14	0.12
-7	0	-2	0.07	0.23
-7	0	2	0.07	0.21
7	0	-2	0.35	0.16
-7	0	-2	0.13	0.20
-7	-1	2	30.47	1.53
7	1	2	31.93	1.56
-7	-1	2	30.67	1.53
7	1	2	30.23	1.47
7	1	2	30.69	1.57
-7	-1	2	30.84	1.63
-7	1	-2	18.28	1.15

7	1	2	30.68	1.63
-7	-1	2	30.96	1.53
-7	1	-2	18.91	1.02
-7	1	-2	17.87	1.12
-7	1	-2	19.11	1.14
7	-1	-2	18.09	1.04
7	1	2	31.37	1.56
7	-1	-2	18.11	0.97
-7	-1	2	29.26	1.51
-7	1	-2	18.43	1.03
7	1	2	32.74	1.57
7	-1	-2	19.34	1.00
-7	-1	2	31.35	1.65
7	1	-2	19.08	1.12
-7	1	2	27.78	1.63
7	-1	2	28.91	1.50
7	1	-2	18.65	1.07
-7	1	2	31.99	1.52
7	-1	2	32.50	1.56
7	-1	2	29.85	1.64
-7	-1	-2	17.48	1.06
-7	1	2	28.94	1.51
-7	-1	-2	18.83	1.04
-7	1	2	31.11	1.53
-7	1	2	29.65	1.64
7	1	-2	18.38	1.01
7	-1	2	29.53	1.55
-7	1	2	32.04	1.50
-7	-1	-2	18.79	0.95
-7	-1	-2	19.67	0.91
-7	-1	-2	19.81	1.13
-7	1	2	31.25	1.53
7	-1	2	32.16	1.69
-7	1	2	29.36	1.77
7	2	2	65.20	3.19
-7	2	-2	56.54	2.63
7	-2	-2	56.00	2.73
-7	2	-2	54.23	2.56
7	-2	-2	53.09	2.63
-7	-2	2	68.58	3.24
-7	-2	2	71.00	3.31
7	-2	-2	55.99	2.70
-7	2	-2	55.93	2.63
7	2	2	67.35	3.37

-7	2	-2	55.27	2.77
-7	-2	2	64.44	3.23
7	-2	-2	53.51	2.61
-7	-2	2	68.64	3.39
-7	-2	2	64.90	3.20
-7	-2	2	70.72	3.20
7	2	2	71.94	3.25
-7	2	-2	58.44	2.65
7	-2	-2	54.66	2.59
7	2	2	68.76	3.23
-7	2	-2	52.87	2.76
7	2	-2	50.62	2.52
7	2	-2	53.19	2.75
-7	2	2	71.93	3.25
7	2	-2	49.29	2.71
7	-2	2	65.70	3.24
-7	-2	-2	53.86	2.61
-7	-2	-2	55.12	2.53
7	-2	2	71.02	3.19
-7	-2	-2	56.76	2.52
-7	2	2	67.30	3.31
-7	2	2	67.70	3.35
-7	2	2	71.49	3.29
7	-2	2	67.38	3.25
-7	-2	-2	54.44	2.64
-7	-2	-2	54.97	2.75
7	-2	2	68.75	3.37
7	-2	2	67.58	3.25
7	2	-2	52.61	2.52
-7	-2	-2	54.86	2.57
-7	2	2	71.18	3.23
-7	-2	-2	55.06	2.64
-7	-2	-2	53.15	2.56
-7	-3	2	287.56	13.26
7	-3	-2	330.14	15.03
-7	3	-2	357.84	15.03
-7	-3	2	309.58	13.56
7	3	2	267.77	13.42
7	-3	-2	316.42	14.96
-7	3	-2	344.82	15.01
-7	3	-2	320.66	14.90
-7	-3	2	292.55	13.27
7	3	2	308.58	13.42
-7	3	-2	334.75	15.18

7	-3	-2	351.05	15.11
-7	-3	2	300.12	13.33
7	3	2	282.20	13.54
-7	-3	2	275.87	13.40
7	3	2	281.43	13.39
-7	3	-2	330.38	15.15
-7	3	-2	349.40	15.04
7	-3	-2	325.38	15.01
-7	-3	2	311.83	13.45
-7	-3	-2	319.58	14.88
-7	3	2	297.57	13.53
-7	-3	-2	342.85	15.03
-7	-3	-2	326.23	14.92
7	-3	2	290.87	13.43
7	-3	2	282.97	13.40
-7	3	2	321.54	13.47
-7	-3	-2	327.90	14.93
7	-3	2	318.04	13.65
-7	3	2	297.97	13.53
7	3	-2	317.43	14.97
-7	-3	-2	329.79	14.98
7	3	-2	320.53	15.15
7	-3	2	296.26	13.30
-7	-3	-2	338.56	14.91
-7	3	2	328.94	13.46
-7	3	2	292.86	13.39
7	3	-2	331.17	14.98
-7	-3	-2	317.85	15.01
7	-3	2	309.41	13.53
7	3	-2	337.92	14.93
7	-3	2	279.34	13.40
-7	-3	-2	346.70	15.15
-7	-4	2	73.67	3.78
-7	4	-2	60.99	2.95
7	4	2	78.68	3.79
-7	4	-2	56.86	3.12
-7	-4	2	80.31	3.97
7	-4	-2	60.63	3.08
-7	4	-2	57.38	2.75
-7	-4	2	79.45	3.68
7	-4	-2	56.77	2.87
-7	4	-2	65.40	2.90
-7	-4	2	79.79	3.82
7	4	2	75.32	3.82

7	-4	-2	58.06	2.93
7	4	2	78.03	3.73
7	4	2	76.33	3.95
-7	4	-2	56.98	3.07
-7	-4	2	82.36	3.72
7	-4	-2	59.84	3.01
7	-4	-2	54.55	2.83
7	4	2	82.81	3.84
-7	4	-2	61.58	2.91
-7	-4	-2	56.77	2.89
-7	4	2	78.42	3.98
7	4	-2	58.36	2.75
7	-4	2	75.88	3.76
-7	4	2	79.35	3.94
7	4	-2	54.57	2.90
7	-4	2	73.19	3.76
-7	4	2	81.83	3.79
-7	-4	-2	56.97	2.84
-7	-4	-2	62.92	3.09
7	4	-2	55.84	2.94
-7	-4	-2	57.78	2.83
-7	-4	-2	60.25	2.83
7	4	-2	61.80	2.85
-7	4	2	85.64	3.90
7	-4	2	79.53	3.84
7	-4	2	84.76	4.07
-7	4	2	85.72	3.84
7	-4	2	79.15	3.94
7	4	-2	60.53	2.87
7	-4	2	78.70	3.65
-7	-5	2	126.53	6.01
-7	-5	2	131.97	6.29
-7	5	-2	116.31	5.05
-7	5	-2	103.79	5.35
-7	5	-2	104.12	4.90
7	-5	-2	100.26	5.03
-7	-5	2	126.29	6.11
-7	5	-2	110.09	5.08
7	-5	-2	104.12	5.16
-7	-5	2	132.72	6.07
-7	5	-2	102.86	5.26
7	5	2	132.22	6.10
7	5	2	123.84	6.28
-7	-5	2	128.39	5.97

7	-5	-2	112.15	5.31
-7	5	-2	117.67	5.18
7	5	2	122.36	6.18
-7	-5	2	124.10	6.26
-7	-5	2	128.55	6.11
7	-5	-2	99.98	5.00
7	5	2	136.24	6.17
7	-5	-2	101.35	5.11
7	5	2	131.64	6.02
7	5	-2	111.63	5.00
7	-5	2	131.67	6.18
7	5	-2	110.97	5.06
-7	5	2	129.53	6.27
7	-5	2	126.82	6.06
7	-5	2	141.45	6.42
7	5	-2	107.50	4.89
7	-5	2	134.19	5.95
-7	5	2	142.05	6.25
-7	5	2	133.00	6.10
-7	-5	-2	110.42	5.25
-7	-5	-2	105.30	5.07
7	5	-2	100.43	5.13
-7	-5	-2	101.07	4.98
7	5	-2	109.27	5.04
-7	5	2	137.22	6.14
-7	-5	-2	109.53	5.04
7	-5	2	124.69	6.08
7	-5	2	134.38	6.26
-7	5	2	124.51	6.33
-7	-5	-2	106.06	5.02
-7	-6	2	146.99	6.85
-7	6	-2	168.11	7.29
-7	-6	2	147.37	6.76
-7	-6	2	140.94	6.94
7	-6	-2	151.49	7.27
-7	-6	2	154.45	6.93
7	-6	-2	153.51	7.40
7	6	2	149.04	6.92
7	6	2	146.69	6.81
-7	6	-2	157.89	7.33
-7	-6	2	148.68	7.13
-7	6	-2	155.45	7.54
7	6	2	139.87	7.12
7	-6	-2	149.22	7.26

-7	-6	2	153.17	6.93
7	-6	-2	169.52	7.62
-7	-6	2	141.96	7.10
-7	6	-2	167.37	7.45
-7	6	-2	155.63	7.66
7	-6	-2	152.55	7.40
7	6	2	136.64	7.05
-7	-6	-2	153.04	7.33
-7	6	2	156.84	6.96
7	-6	2	147.45	7.08
-7	6	2	148.88	7.12
7	6	-2	153.75	7.11
-7	-6	-2	154.03	7.29
7	6	-2	152.60	7.29
-7	6	2	136.60	7.20
-7	-6	-2	151.03	7.21
7	-6	2	154.47	6.80
7	-6	2	161.11	7.30
-7	6	2	150.69	6.93
7	-6	2	142.60	6.86
7	6	-2	150.23	7.44
7	-6	2	156.02	6.77
7	6	-2	161.71	7.33
-7	6	2	160.18	7.11
7	-6	2	140.40	6.90
-7	-6	-2	158.11	7.51
7	-6	2	145.56	7.00
-7	-6	-2	155.08	7.28
7	6	-2	162.87	7.23
-7	-7	2	9.28	1.22
-7	-7	2	10.17	0.92
7	-7	-2	9.43	0.89
-7	7	-2	9.70	1.06
-7	-7	2	11.19	1.09
-7	7	-2	12.28	1.06
-7	7	-2	10.84	1.25
-7	-7	2	10.04	1.11
7	-7	-2	11.14	0.91
7	7	2	8.87	1.18
-7	-7	2	9.68	1.35
7	-7	-2	11.55	1.30
-7	-7	2	11.01	0.75
7	7	2	9.19	0.98
7	-7	-2	10.70	1.05

7	7	2	8.95	0.85
7	7	2	8.83	1.18
7	-7	-2	11.04	1.49
-7	7	-2	11.09	1.31
-7	-7	2	11.31	0.98
-7	7	-2	10.36	1.64
7	-7	2	11.22	0.78
-7	7	2	11.62	1.34
-7	-7	-2	10.67	1.00
7	7	-2	11.59	0.75
-7	7	2	9.86	1.08
7	-7	2	10.28	0.98
7	-7	2	10.14	0.91
-7	-7	-2	9.05	0.97
-7	-7	-2	11.64	1.36
-7	-7	-2	9.63	0.87
7	-7	2	11.22	0.96
7	7	-2	9.79	1.07
-7	-7	-2	9.05	1.07
-7	7	2	9.19	1.27
7	-7	2	9.75	0.82
-7	7	2	12.31	1.09
7	7	-2	11.87	0.94
7	7	-2	10.25	1.01
7	-7	2	13.52	2.28
7	-7	2	8.89	1.36
-7	7	2	10.31	1.56
7	7	-2	9.55	1.17
7	-8	-2	9.74	1.42
-7	-8	2	10.31	1.04
7	8	2	10.72	1.32
7	-8	-2	10.20	1.20
7	-8	-2	12.96	1.61
-7	8	-2	9.51	1.51
-7	8	-2	11.40	1.44
7	-8	-2	12.20	1.00
-7	-8	2	12.16	1.29
7	8	2	8.15	1.28
7	8	2	10.40	1.36
-7	-8	2	9.93	0.83
-7	8	-2	9.82	1.47
-7	-8	2	10.93	1.08
-7	8	-2	13.09	1.17
7	8	2	9.80	0.93

-7	-8	2	11.99	1.33
7	-8	-2	10.78	1.02
-7	-8	-2	11.60	1.15
7	8	-2	9.45	1.08
7	-8	2	11.55	1.75
7	-8	2	11.05	1.50
-7	8	2	9.17	1.39
7	-8	2	11.66	0.93
7	8	-2	10.20	1.35
-7	8	2	12.69	1.55
7	-8	2	11.06	1.13
7	-8	2	10.93	1.00
-7	8	2	11.57	1.26
-7	-8	-2	11.63	0.96
-7	-8	-2	11.49	1.07
7	8	-2	10.89	1.03
-7	8	2	11.07	1.47
7	-8	2	10.65	0.91
7	9	2	19.28	1.35
7	-9	-2	22.13	1.29
-7	-9	2	19.86	2.00
-7	9	-2	20.25	1.89
7	-9	-2	18.92	1.55
7	9	2	20.41	1.84
7	9	2	20.53	1.92
7	-9	-2	19.50	1.39
7	-9	-2	18.12	1.96
-7	-9	2	20.07	1.24
7	-9	-2	21.57	2.22
-7	9	-2	20.06	1.94
-7	9	-2	17.91	2.13
-7	-9	2	18.09	1.51
-7	9	-2	23.33	1.61
-7	-9	2	18.62	1.50
-7	-9	2	19.74	1.65
-7	-9	-2	17.08	1.49
7	-9	2	22.30	1.33
-7	-9	-2	19.07	1.52
7	-9	2	20.74	1.47
7	-9	2	22.07	2.32
-7	-9	-2	17.54	1.31
7	9	-2	15.56	2.45
7	-9	2	19.31	1.32
7	9	-2	21.06	1.23

7	-9	2	19.80	1.61
7	9	-2	20.29	1.36
-7	9	2	19.56	2.21
7	-9	2	21.65	1.53
-7	9	2	21.07	1.89
-7	9	2	19.36	1.96
7	9	-2	21.32	1.72
-7	9	2	22.46	1.85
-7	-9	-2	18.54	1.79
7	10	2	50.25	2.70
-7	-10	2	50.59	2.59
-7	10	-2	49.65	3.19
7	10	2	54.48	3.27
7	-10	-2	43.02	2.79
7	10	2	49.52	3.38
7	-10	-2	42.97	2.62
7	-10	-2	44.22	2.43
-7	-10	2	50.02	3.23
-7	10	-2	44.70	2.66
-7	-10	2	51.76	2.98
7	-10	-2	44.20	2.52
-7	10	-2	41.36	3.32
7	-10	-2	50.17	3.41
-7	-10	2	53.67	2.94
7	-10	-2	51.55	2.51
7	-10	2	57.51	2.88
7	10	-2	50.24	3.00
-7	-10	-2	45.06	2.73
7	-10	2	52.08	2.92
7	-10	2	50.95	3.04
7	-10	2	58.22	2.76
-7	-10	-2	44.11	2.48
7	-10	2	57.41	3.71
7	10	-2	43.16	3.08
-7	-10	-2	48.95	2.97
7	10	-2	45.64	2.30
7	-10	2	48.27	2.73
7	10	-2	47.57	2.51
-7	10	2	45.83	3.47
-7	10	2	53.59	3.38
7	-11	-2	76.21	3.50
-7	-11	2	71.81	4.02
-7	11	-2	68.30	4.52
-7	11	-2	66.65	4.26

7	-11	-2	68.03	3.88
-7	-11	2	66.91	3.78
7	-11	-2	71.04	3.76
7	-11	-2	66.98	3.59
7	11	2	65.38	4.17
-7	11	2	60.57	4.27
7	-11	2	63.11	3.41
7	-11	2	65.08	3.80
7	-11	2	67.11	3.68
7	-11	2	73.68	3.46
-7	11	2	70.56	4.12
7	11	-2	68.69	3.53
-7	-11	-2	71.50	3.90
-7	-11	-2	66.66	3.51
7	11	-2	67.75	4.38
-7	-11	-2	73.44	4.12
7	12	2	74.75	5.22
-7	12	-2	92.02	5.14
7	-12	-2	83.28	4.40
-7	-12	2	80.66	4.44
7	-12	-2	81.30	4.71
-7	-12	2	85.69	4.92
7	-12	-2	90.67	4.60
7	12	-2	88.97	5.50
7	-12	2	83.02	4.54
7	-12	2	82.23	4.39
-7	12	2	87.48	4.92
-7	-12	-2	86.40	5.02
-7	-12	-2	85.79	4.63
7	-13	-2	19.70	1.72
-7	13	-2	27.29	3.50
-7	-13	2	21.57	2.46
7	13	2	13.74	2.92
7	-13	-2	24.21	1.92
-7	-13	-2	20.71	2.47
7	13	-2	13.87	2.78
-7	13	2	24.08	3.28
7	-13	2	20.31	1.93
8	0	-2	41.97	2.09
8	0	2	25.76	1.44
-8	0	-2	44.41	2.01
8	0	-2	41.07	2.10
-8	0	-2	50.34	2.17
-8	0	-2	41.63	2.12

8	0	-2	38.77	1.98
8	0	2	24.14	1.28
8	0	2	30.54	1.47
-8	0	2	23.67	1.41
-8	0	-2	44.88	2.17
-8	0	2	23.62	1.44
-8	0	2	29.39	1.37
-8	0	-2	43.95	2.08
-8	0	2	25.01	1.45
-8	0	-2	40.23	2.18
8	0	2	25.30	1.41
-8	0	-2	43.63	2.14
8	0	2	27.69	1.40
-8	-1	2	195.55	8.90
8	-1	-2	191.11	8.57
-8	1	-2	184.98	8.70
8	-1	-2	187.36	8.52
8	1	2	189.17	8.83
-8	-1	2	196.82	8.90
-8	1	-2	197.08	8.54
-8	-1	2	192.83	8.94
8	-1	-2	188.42	8.60
-8	1	-2	181.87	8.59
8	1	2	187.74	8.93
-8	-1	2	203.15	9.00
-8	1	-2	200.24	8.62
-8	-1	2	179.28	8.88
-8	1	-2	191.57	8.58
-8	-1	2	200.21	9.00
8	1	2	192.34	9.00
8	1	2	199.79	8.92
8	1	2	199.64	8.91
8	1	2	204.59	8.93
8	-1	-2	188.63	8.58
-8	1	2	205.87	8.95
-8	1	2	193.13	8.86
8	-1	2	202.22	9.03
-8	1	2	197.45	9.03
-8	-1	-2	186.78	8.60
-8	1	2	202.92	8.83
-8	1	2	193.34	9.01
8	-1	2	193.44	8.91
-8	1	2	206.57	8.91
-8	-1	-2	193.52	8.48

8	1	-2	189.23	8.68
-8	-1	-2	195.14	8.46
8	-1	2	197.03	9.01
8	-1	2	191.61	8.93
-8	-1	-2	179.88	8.83
-8	-1	-2	179.31	8.67
8	-1	2	189.22	8.87
8	1	-2	184.82	8.58
-8	1	2	195.33	8.88
-8	2	-2	4.98	0.72
8	-2	-2	5.22	0.71
-8	-2	2	4.22	0.51
-8	-2	2	4.47	0.47
-8	-2	2	4.16	0.41
-8	-2	2	4.02	0.62
-8	2	-2	6.50	0.50
8	-2	-2	5.73	0.50
-8	2	-2	5.87	0.58
8	-2	-2	5.91	0.52
8	-2	-2	6.91	0.52
8	2	2	4.00	0.37
8	2	2	5.36	0.52
-8	-2	2	4.04	0.60
8	2	2	4.64	0.52
-8	2	-2	5.72	0.57
-8	2	-2	4.62	0.75
-8	2	-2	6.27	0.56
-8	-2	2	4.26	0.43
8	2	2	2.93	0.58
8	2	-2	5.51	0.55
-8	-2	-2	5.86	0.57
8	2	-2	4.90	0.39
-8	-2	-2	5.23	0.58
-8	-2	-2	6.64	0.54
-8	2	2	3.88	0.49
-8	-2	-2	6.00	0.56
-8	-2	-2	6.31	0.38
8	-2	2	4.21	0.71
8	-2	2	3.87	0.44
8	2	-2	3.71	0.69
8	-2	2	3.83	0.43
-8	2	2	3.22	0.60
-8	-2	-2	5.85	0.57
-8	2	2	4.48	0.53

8	-2	2	4.13	0.45
8	-2	2	3.74	0.43
-8	-2	-2	5.90	0.42
-8	2	2	3.10	0.51
8	2	-2	5.41	0.36
-8	-2	-2	5.59	0.73
-8	3	-2	6.02	0.86
-8	3	-2	7.59	0.69
-8	-3	2	6.80	0.53
-8	-3	2	6.49	0.59
8	3	2	5.96	0.55
8	-3	-2	7.31	0.62
-8	-3	2	7.84	0.75
-8	3	-2	7.12	0.53
8	-3	-2	7.42	0.58
8	-3	-2	7.16	0.83
-8	3	-2	8.11	0.67
8	3	2	6.67	0.64
-8	3	-2	7.03	0.66
8	-3	-2	6.83	0.63
8	3	2	6.79	0.63
-8	-3	2	6.32	0.53
-8	-3	2	7.35	0.84
-8	3	-2	6.43	0.84
8	3	2	5.30	0.75
8	-3	2	7.51	0.51
-8	-3	-2	7.19	0.59
8	-3	2	6.61	0.58
-8	-3	-2	7.58	0.50
-8	-3	-2	6.97	0.63
-8	-3	-2	7.30	0.63
-8	3	2	7.35	0.70
8	-3	2	6.75	0.60
8	3	-2	6.68	0.56
-8	-3	-2	6.05	0.84
-8	3	2	7.12	0.69
-8	3	2	6.86	0.82
-8	-3	-2	6.18	0.54
-8	3	2	6.12	0.78
8	3	-2	6.89	0.80
8	3	-2	6.26	0.60
8	-3	2	6.75	0.59
-8	3	2	7.12	0.62
-8	-3	-2	7.98	0.68

8	-3	2	7.85	0.88
8	3	-2	8.15	0.63
-8	-3	-2	6.79	0.53
-8	-4	2	58.68	3.01
8	-4	-2	66.57	3.33
8	4	2	60.50	3.01
8	4	2	62.01	3.03
-8	4	-2	69.61	3.19
8	-4	-2	60.13	3.14
-8	-4	2	64.33	3.06
-8	4	-2	69.06	3.13
-8	4	-2	66.00	3.14
-8	-4	2	64.05	2.88
8	4	2	58.61	3.02
8	-4	-2	65.29	3.25
-8	-4	2	61.82	2.85
-8	-4	2	61.19	2.91
8	-4	-2	62.52	3.06
-8	4	-2	60.48	3.34
-8	-4	2	62.65	3.20
8	4	2	59.75	3.20
8	4	2	60.99	2.95
8	-4	-2	61.80	3.11
-8	4	-2	64.33	3.33
-8	4	2	61.17	3.21
8	-4	2	61.31	3.18
-8	-4	-2	65.66	3.31
8	-4	2	57.26	2.98
8	-4	2	63.39	3.29
-8	4	2	58.86	3.17
-8	4	2	66.44	3.07
8	-4	2	62.20	2.85
-8	-4	-2	62.97	3.03
8	4	-2	63.99	2.99
-8	-4	-2	62.68	3.08
8	4	-2	60.20	3.30
8	-4	2	57.39	2.98
-8	4	2	65.81	3.11
8	4	-2	65.93	3.06
-8	4	2	63.06	3.01
8	4	-2	59.72	3.11
8	4	-2	64.38	3.09
-8	-4	-2	64.00	3.01
-8	-4	-2	60.74	2.94

-8	-4	-2	57.99	3.11
8	-4	2	59.31	3.04
8	4	-2	64.95	3.07
8	5	2	20.49	1.17
-8	-5	2	19.35	1.11
8	5	2	19.29	1.24
-8	-5	2	18.20	1.44
8	-5	-2	17.41	1.22
-8	-5	2	18.69	1.49
8	5	2	19.60	1.31
8	5	2	17.89	1.44
-8	-5	2	19.65	1.33
-8	5	-2	17.38	1.43
8	-5	-2	16.81	1.46
-8	-5	2	18.75	1.15
-8	5	-2	19.08	1.24
-8	-5	2	21.08	1.10
8	-5	-2	18.42	1.11
8	-5	-2	17.40	1.14
-8	5	-2	17.54	1.19
-8	5	-2	16.91	1.53
-8	-5	2	19.46	1.23
8	-5	-2	17.74	1.39
8	5	2	19.30	1.35
-8	5	-2	18.20	1.00
-8	5	-2	18.54	1.28
-8	5	2	18.57	1.53
8	-5	2	19.45	1.18
-8	-5	-2	17.73	1.18
8	-5	2	19.57	1.49
8	5	-2	18.88	1.02
8	5	-2	16.16	1.24
-8	-5	-2	18.36	1.11
-8	-5	-2	17.43	1.12
-8	-5	-2	18.65	1.11
-8	5	2	20.84	1.40
-8	5	2	20.82	1.32
8	5	-2	18.90	1.10
-8	5	2	18.07	1.43
8	5	-2	19.73	1.19
8	-5	2	22.04	1.63
-8	-5	-2	17.06	1.44
8	-5	2	19.21	1.25
8	-5	2	18.74	1.18

-8	5	2	19.97	1.24
8	5	-2	18.61	1.18
8	-6	-2	46.98	2.71
-8	-6	2	44.49	2.09
8	6	2	38.27	2.36
-8	-6	2	37.06	2.43
8	-6	-2	42.56	2.41
8	-6	-2	43.66	2.32
8	-6	-2	43.39	2.52
8	6	2	42.55	2.26
-8	-6	2	41.66	2.53
-8	-6	2	42.71	2.29
-8	-6	2	39.59	2.13
-8	6	-2	49.00	2.52
8	-6	-2	43.43	2.29
8	6	2	42.41	2.33
-8	6	-2	47.84	2.41
-8	-6	2	40.31	2.21
-8	6	-2	40.24	2.75
-8	-6	2	42.16	2.27
8	6	2	38.40	2.46
-8	6	-2	45.36	2.64
-8	6	-2	47.38	2.37
8	6	2	43.84	2.15
8	6	-2	40.30	2.46
-8	6	2	43.96	2.46
8	-6	2	40.30	2.22
-8	6	2	36.57	2.60
-8	-6	-2	42.61	2.27
8	-6	2	46.06	2.10
-8	-6	-2	43.62	2.29
-8	-6	-2	44.06	2.32
8	-6	2	40.72	2.31
8	-6	2	44.10	2.05
8	6	-2	45.69	2.25
8	-6	2	42.09	2.67
8	6	-2	44.78	2.35
-8	6	2	42.91	2.50
-8	-6	-2	40.77	2.36
8	6	-2	45.25	2.15
-8	-6	-2	42.34	2.60
-8	6	2	41.60	2.26
8	-6	2	41.79	2.18
8	6	-2	43.58	2.37

8	-6	2	43.35	2.50
-8	6	2	45.76	2.33
-8	-7	2	2.68	0.54
-8	7	-2	3.62	0.91
-8	-7	2	3.28	0.86
8	7	2	2.37	0.80
8	-7	-2	3.12	0.56
-8	7	-2	3.07	1.02
-8	-7	2	2.57	0.80
-8	7	-2	4.50	0.71
8	-7	-2	2.85	0.64
8	-7	-2	2.90	1.02
8	7	2	4.95	0.72
8	-7	-2	3.33	0.85
8	7	2	1.74	0.69
-8	7	-2	4.19	0.84
-8	7	-2	1.99	0.61
-8	-7	2	1.84	0.61
8	7	2	2.23	0.73
8	7	2	2.73	0.55
-8	-7	2	3.53	0.74
8	-7	2	4.47	0.70
8	7	-2	1.33	0.73
8	-7	2	3.45	0.55
8	-7	2	3.39	0.62
8	7	-2	3.29	0.58
8	-7	2	3.67	0.72
-8	7	2	1.88	0.82
8	-7	2	3.10	0.87
-8	-7	-2	4.06	0.94
-8	-7	-2	3.97	0.66
-8	7	2	3.74	0.69
-8	-7	-2	3.19	0.65
-8	-7	-2	1.92	0.61
8	-7	2	2.45	1.12
8	7	-2	4.91	0.70
-8	7	2	2.27	0.96
-8	7	2	3.38	0.68
-8	-7	-2	2.90	0.61
8	7	-2	3.26	0.73
-8	-8	2	6.33	1.38
-8	8	-2	7.54	1.42
8	-8	-2	7.10	1.41
-8	-8	2	6.11	0.89

8	8	2	4.94	0.99
-8	8	-2	7.77	1.43
8	-8	-2	7.60	1.05
-8	-8	2	4.69	0.93
-8	8	-2	7.52	1.36
8	-8	-2	7.89	0.87
-8	8	-2	6.04	1.12
8	8	2	8.13	0.86
-8	-8	2	8.19	0.95
8	-8	-2	4.77	1.01
-8	-8	2	7.66	0.75
8	8	2	8.47	1.24
8	-8	-2	8.79	0.89
8	8	-2	6.33	0.99
8	8	-2	6.48	1.16
8	-8	2	4.67	1.25
-8	-8	-2	7.48	0.83
8	-8	2	7.44	0.70
8	-8	2	5.03	0.87
8	-8	2	6.62	0.77
8	8	-2	9.73	0.75
-8	-8	-2	9.21	1.05
-8	8	2	8.17	1.43
-8	8	2	8.18	1.15
8	8	-2	7.83	0.92
-8	8	2	6.39	1.17
8	-8	2	7.05	0.86
8	-8	2	6.37	0.87
-8	-8	-2	6.60	0.99
8	-9	-2	2.65	1.28
8	-9	-2	1.13	0.54
-8	9	-2	1.35	0.96
-8	-9	2	1.65	0.84
-8	-9	2	2.26	0.89
8	9	2	0.47	0.79
-8	-9	2	1.56	0.49
-8	-9	2	1.17	0.67
-8	9	-2	0.62	0.76
8	-9	-2	1.94	0.58
-8	-9	2	2.22	0.67
-8	9	-2	0.42	1.20
8	9	2	1.44	0.55
8	9	2	1.68	0.91
8	-9	-2	1.52	0.74

-8	9	2	3.38	0.91
-8	-9	-2	2.60	0.73
8	-9	2	1.64	0.51
8	-9	2	0.35	1.21
8	-9	2	1.41	0.54
8	9	-2	2.05	0.50
8	-9	2	1.48	0.56
8	9	-2	2.04	0.63
-8	-9	-2	2.90	0.60
-8	9	2	1.18	1.11
8	-9	2	0.84	1.00
8	9	-2	0.77	0.93
-8	-9	-2	1.25	0.68
8	-9	2	1.24	0.66
-8	-10	2	26.97	2.31
-8	10	-2	23.81	2.40
8	-10	-2	24.10	1.93
-8	-10	2	27.80	1.99
8	10	2	24.76	2.38
8	-10	-2	26.43	1.80
8	-10	-2	22.85	3.02
-8	10	-2	21.82	2.50
8	-10	-2	22.67	1.68
8	10	2	25.84	1.68
-8	-10	2	25.39	1.52
8	-10	2	27.57	2.10
-8	-10	-2	25.94	1.92
-8	10	2	27.72	2.32
8	10	-2	29.52	1.77
-8	10	2	24.60	2.54
-8	-10	-2	24.07	1.61
8	-10	2	29.86	2.87
8	10	-2	23.63	2.39
-8	-10	-2	24.83	2.21
8	-10	2	26.68	1.88
8	-10	2	28.96	1.64
8	-10	2	28.17	2.02
8	10	-2	26.52	1.61
8	-10	2	26.99	1.69
8	-11	-2	6.33	0.95
8	-11	-2	6.41	1.07
-8	-11	2	8.77	1.51
-8	11	-2	5.60	1.56
8	11	2	8.63	1.59

-8	11	-2	4.99	1.68
-8	-11	2	8.75	1.47
8	-11	-2	7.64	1.40
-8	-11	-2	7.91	1.37
8	11	-2	7.14	1.56
-8	-11	-2	3.80	1.07
8	-11	2	7.18	1.43
-8	11	2	11.72	2.09
8	-11	2	11.10	1.46
-8	-11	-2	6.54	0.88
8	-11	2	7.18	1.11
8	-11	2	7.80	1.11
8	-12	-2	2.22	0.85
-8	12	-2	2.54	1.78
8	12	2	0.57	1.51
-8	-12	2	2.03	1.29
8	-12	-2	-0.03	0.78
-8	-12	2	1.97	0.86
-8	-12	-2	1.21	0.84
8	12	-2	2.09	1.60
8	-12	2	1.41	0.80
8	-12	2	0.35	1.02
8	-12	2	1.12	0.73
-8	-12	-2	3.20	1.40
-8	12	2	1.90	1.44
9	0	2	0.34	0.29
9	0	-2	0.04	0.20
-9	0	-2	-0.05	0.30
-9	0	-2	0.27	0.26
-9	0	-2	0.37	0.33
-9	0	2	-0.03	0.31
9	0	-2	0.06	0.26
-9	0	2	0.03	0.24
9	0	-2	0.24	0.18
9	0	2	-0.23	0.32
-9	0	2	0.35	0.37
-9	0	-2	0.00	0.16
-9	0	-2	0.06	0.21
-9	0	-2	0.01	0.24
9	0	2	0.07	0.20
-9	0	-2	-0.14	0.24
-9	0	2	0.18	0.22
9	0	2	0.07	0.14
9	0	-2	0.11	0.21

-9	0	2	0.00	0.30
9	0	-2	0.09	0.12
9	1	2	81.27	3.77
-9	-1	2	76.39	3.87
9	-1	-2	62.79	3.04
-9	1	-2	58.95	3.04
-9	1	-2	65.16	2.98
9	-1	-2	61.98	2.96
9	-1	-2	61.73	3.05
9	1	2	83.36	3.79
-9	-1	2	80.15	3.76
-9	1	-2	62.89	3.06
9	1	2	80.20	3.66
9	-1	-2	61.99	3.02
-9	1	-2	59.89	3.17
-9	1	-2	65.81	3.08
-9	-1	2	77.50	3.80
-9	1	-2	66.26	3.05
-9	1	-2	64.91	3.08
9	1	2	78.34	3.79
-9	-1	2	77.08	3.97
-9	-1	2	83.07	3.76
-9	-1	2	74.79	3.73
9	1	2	78.33	3.86
9	-1	2	79.45	3.85
-9	1	2	82.79	3.76
-9	1	2	74.84	3.86
9	-1	2	80.59	3.76
-9	1	2	82.02	3.69
9	-1	2	81.28	3.88
-9	-1	-2	63.95	2.88
-9	-1	-2	64.03	2.90
-9	-1	-2	61.01	3.05
-9	-1	-2	67.62	3.08
-9	-1	-2	61.25	3.07
-9	1	2	79.45	3.73
-9	1	2	82.47	3.65
-9	-1	-2	67.64	3.17
9	-1	2	75.35	3.70
-9	-1	-2	66.17	3.05
9	1	-2	63.45	3.04
9	-1	2	80.11	3.78
-9	1	2	83.10	3.91
9	1	-2	62.31	3.15

-9	2	-2	144.28	6.96
9	-2	-2	146.65	6.91
9	2	2	129.69	6.23
-9	2	-2	149.47	6.93
-9	2	-2	146.72	6.75
9	2	2	138.68	6.31
-9	-2	2	140.19	6.46
9	-2	-2	145.94	6.78
-9	-2	2	138.25	6.30
9	2	2	128.00	6.43
-9	-2	2	135.83	6.31
-9	-2	2	130.57	6.27
9	-2	-2	140.74	6.77
-9	2	-2	153.05	6.84
-9	-2	2	143.15	6.40
-9	2	-2	158.23	6.84
-9	-2	2	127.84	6.30
9	-2	-2	151.56	6.88
9	2	2	144.74	6.31
-9	2	-2	152.22	6.79
9	2	-2	146.25	6.64
-9	-2	-2	143.11	6.82
-9	-2	-2	151.69	6.64
-9	2	2	145.62	6.37
9	-2	2	141.07	6.27
9	-2	2	132.81	6.33
9	-2	2	131.07	6.29
-9	-2	-2	151.73	6.83
-9	2	2	144.45	6.31
-9	2	2	135.95	6.35
-9	-2	-2	144.95	6.65
9	2	-2	151.00	6.84
-9	-2	-2	152.32	6.94
9	2	-2	142.68	6.65
9	2	-2	140.89	6.94
-9	2	2	135.04	6.46
-9	2	2	129.38	6.20
-9	2	2	136.82	6.20
-9	-2	-2	151.13	6.79
-9	-2	-2	141.83	6.80
9	-2	2	133.21	6.33
9	-2	2	138.62	6.43
9	-2	2	145.39	6.50
-9	2	2	143.97	6.29

9	3	2	86.38	4.21
-9	3	-2	94.63	4.27
-9	3	-2	89.11	4.44
9	-3	-2	85.64	4.23
-9	3	-2	90.24	4.12
-9	-3	2	85.14	4.20
-9	3	-2	88.89	4.43
-9	-3	2	90.97	4.27
-9	3	-2	90.83	4.26
9	-3	-2	86.06	4.19
9	-3	-2	85.55	4.24
-9	-3	2	94.69	4.34
-9	-3	2	96.09	4.46
9	-3	-2	92.41	4.38
-9	3	-2	91.81	4.25
9	3	2	92.83	4.26
-9	-3	2	83.82	4.25
9	3	2	94.13	4.24
9	-3	-2	86.36	4.35
9	3	2	88.70	4.44
-9	-3	-2	89.43	4.10
-9	-3	-2	90.78	4.23
-9	3	2	95.52	4.33
-9	-3	-2	85.14	4.15
9	-3	2	90.93	4.41
-9	3	2	89.69	4.25
-9	-3	-2	93.19	4.26
-9	-3	-2	90.90	4.08
-9	3	2	94.57	4.30
-9	-3	-2	88.40	4.26
-9	3	2	88.09	4.42
9	-3	2	86.47	4.27
-9	-3	-2	90.25	4.17
9	3	-2	85.36	4.15
9	-3	2	84.85	4.26
-9	-3	-2	93.25	4.42
9	-3	2	92.34	4.15
9	-3	2	89.17	4.27
9	3	-2	87.75	4.10
9	3	-2	88.42	4.42
9	3	-2	90.96	4.23
-9	3	2	88.74	4.40
-9	4	-2	214.18	9.29
-9	4	-2	197.75	9.43

-9	4	-2	213.79	9.26
9	-4	-2	199.45	9.34
-9	-4	2	170.05	8.19
9	4	2	180.76	8.14
9	4	2	170.92	8.19
-9	-4	2	187.69	8.24
-9	-4	2	181.84	8.00
-9	4	-2	212.17	9.24
9	-4	-2	203.57	9.27
-9	4	-2	199.43	9.46
-9	-4	2	178.40	8.00
9	-4	-2	194.22	9.18
9	-4	-2	192.29	9.22
-9	-4	2	158.72	8.14
-9	4	-2	192.02	9.05
9	4	2	171.43	8.38
-9	-4	2	175.62	8.10
9	4	2	184.65	8.21
-9	-4	2	177.18	8.38
9	-4	2	188.80	8.47
9	-4	2	172.02	8.22
9	4	-2	196.12	9.20
-9	-4	-2	205.50	9.25
9	-4	2	183.32	8.03
-9	-4	-2	209.13	9.42
9	4	-2	195.47	9.42
-9	-4	-2	200.15	9.11
-9	-4	-2	202.61	9.10
-9	4	2	190.79	8.25
9	4	-2	193.38	9.20
-9	-4	-2	201.70	9.25
9	-4	2	172.95	8.18
-9	4	2	188.54	8.28
-9	4	2	176.95	8.37
-9	-4	-2	196.32	9.18
-9	4	2	186.23	8.20
-9	-4	-2	198.87	9.09
9	4	-2	211.60	9.18
-9	4	2	173.19	8.37
9	4	-2	200.06	9.19
9	-4	2	164.35	8.18
-9	-4	-2	192.63	9.05
9	-4	2	180.26	8.35
9	5	2	73.24	3.51

-9	-5	2	65.11	3.45
-9	-5	2	70.03	3.32
-9	-5	2	72.88	3.71
9	-5	-2	57.80	3.16
9	5	2	71.18	3.38
-9	5	-2	58.71	3.28
-9	5	-2	66.18	3.04
-9	-5	2	70.38	3.48
9	5	2	69.25	3.69
9	-5	-2	57.50	2.99
9	-5	-2	57.24	2.95
-9	-5	2	69.60	3.34
9	-5	-2	59.19	3.06
9	-5	-2	60.27	3.27
-9	-5	2	65.66	3.65
9	5	2	69.32	3.52
-9	5	-2	57.87	3.32
9	5	2	70.40	3.46
-9	5	-2	64.19	3.12
-9	5	-2	63.90	3.06
-9	-5	-2	61.77	2.91
-9	-5	-2	62.37	3.26
9	5	-2	60.23	2.99
9	5	-2	56.49	3.05
9	5	-2	61.65	2.98
-9	-5	-2	57.49	2.94
-9	-5	-2	57.23	3.02
9	-5	2	68.66	3.51
9	-5	2	65.60	3.42
9	-5	2	77.60	3.84
-9	5	2	70.50	3.69
-9	5	2	74.37	3.59
-9	5	2	72.17	3.47
-9	-5	-2	58.42	2.90
9	-5	2	71.12	3.44
9	5	-2	59.57	2.92
-9	5	2	65.72	3.69
-9	5	2	72.60	3.52
9	-5	2	69.50	3.66
-9	-6	2	31.20	2.07
-9	-6	2	32.50	1.68
9	-6	-2	27.05	1.64
9	6	2	30.89	2.09
-9	6	-2	31.19	1.75

-9	-6	2	30.06	1.72
9	-6	-2	28.43	1.77
-9	6	-2	29.54	1.85
-9	-6	2	29.71	1.81
9	6	2	30.52	1.82
9	6	2	32.57	1.71
9	-6	-2	29.19	1.64
9	6	2	30.99	1.94
-9	-6	2	33.50	1.89
-9	6	-2	30.28	1.77
-9	6	-2	25.78	2.21
-9	6	-2	28.84	2.00
9	6	2	30.88	1.92
9	-6	-2	29.98	2.06
-9	-6	2	30.71	1.66
-9	-6	2	30.77	2.13
9	-6	-2	26.48	1.90
9	6	-2	26.24	1.80
-9	-6	-2	28.91	1.62
-9	-6	-2	26.72	1.71
9	-6	2	33.62	2.12
9	6	-2	28.12	1.68
9	-6	2	30.34	1.87
9	-6	2	30.08	1.76
-9	6	2	31.89	2.08
-9	6	2	32.75	1.90
9	-6	2	29.90	1.75
9	-6	2	36.88	2.32
9	6	-2	29.87	1.71
-9	6	2	30.66	2.19
-9	-6	-2	28.82	1.61
-9	-6	-2	27.98	1.62
-9	6	2	32.06	2.01
-9	6	2	30.00	1.82
9	6	-2	29.78	1.59
-9	-6	-2	29.68	2.01
9	6	-2	26.10	1.61
-9	7	-2	2.62	0.68
9	7	2	1.43	0.76
-9	7	-2	1.36	0.79
9	-7	-2	1.70	0.48
9	7	2	1.13	0.63
9	-7	-2	2.53	0.82
-9	-7	2	3.08	0.71

9	7	2	2.31	0.71
-9	-7	2	2.69	0.77
-9	-7	2	2.66	0.50
-9	7	-2	2.53	0.80
-9	-7	2	2.16	0.60
-9	-7	2	1.21	0.47
9	7	2	1.61	0.47
9	-7	-2	3.14	0.60
-9	7	-2	1.35	0.99
9	-7	-2	2.20	0.67
9	-7	-2	3.50	0.98
9	7	-2	3.41	0.48
9	-7	2	1.59	0.60
-9	-7	-2	2.77	0.52
9	-7	2	1.50	1.10
-9	-7	-2	2.40	0.56
-9	7	2	2.30	0.77
9	-7	2	1.08	0.78
9	-7	2	2.06	0.52
9	-7	2	2.16	0.52
-9	7	2	1.54	1.03
-9	-7	-2	2.50	0.61
-9	7	2	1.62	0.62
-9	7	2	3.03	0.72
9	7	-2	4.51	0.69
9	7	-2	1.68	0.70
-9	7	2	1.81	0.85
9	7	-2	2.01	0.65
9	-8	-2	14.84	1.22
-9	-8	2	14.78	1.09
9	-8	-2	16.71	1.26
9	8	2	16.81	1.58
9	-8	-2	17.76	2.19
-9	8	-2	18.23	1.77
9	8	2	13.32	1.64
-9	-8	2	15.10	1.20
-9	-8	2	15.57	1.67
-9	-8	2	16.62	1.37
9	-8	-2	17.29	1.45
-9	8	-2	17.75	1.66
9	8	2	17.55	1.20
-9	8	-2	15.89	1.44
9	8	2	13.56	1.59
9	-8	2	18.87	2.08

9	8	-2	18.51	1.61
-9	8	2	15.53	2.05
9	-8	2	17.81	1.40
9	8	-2	18.85	1.11
9	8	-2	16.42	1.67
9	8	-2	17.48	1.21
9	-8	2	16.21	1.27
-9	8	2	17.97	1.77
-9	-8	-2	15.92	1.20
-9	-8	-2	15.72	1.29
9	-8	2	17.87	1.13
9	-8	2	15.38	1.18
-9	-8	-2	15.04	1.46
-9	-9	2	26.11	1.70
-9	-9	2	25.72	2.11
9	-9	-2	25.55	1.61
-9	-9	2	25.97	1.48
-9	9	-2	22.90	2.21
9	-9	-2	24.26	2.39
-9	-9	2	25.58	1.84
9	-9	-2	25.28	1.85
-9	9	-2	22.45	2.36
-9	-9	2	25.72	1.88
9	9	2	22.06	2.03
9	-9	-2	23.84	1.62
9	9	2	24.96	1.59
9	-9	2	26.57	1.74
9	9	-2	20.88	2.05
9	-9	2	24.92	1.87
9	9	-2	25.22	1.96
9	9	-2	24.57	1.38
-9	-9	-2	22.66	1.71
9	-9	2	23.62	2.57
-9	9	2	24.23	2.26
9	-9	2	27.49	1.56
9	9	-2	24.19	1.54
9	-9	2	27.02	1.64
-9	-9	-2	23.34	1.53
-9	-9	-2	22.67	1.75
-9	9	2	23.08	2.29
9	-10	-2	42.20	2.45
-9	10	-2	36.95	3.17
9	-10	-2	44.66	3.99
9	10	2	40.00	2.22

9	10	2	35.96	2.81
-9	10	-2	43.37	3.11
9	-10	-2	38.38	2.58
-9	-10	2	38.85	2.08
-9	-10	2	43.91	2.90
9	-10	-2	39.11	2.33
-9	-10	2	41.23	2.55
-9	-10	-2	40.33	2.56
-9	-10	-2	41.15	2.28
9	-10	2	40.15	2.58
9	-10	2	42.77	3.38
9	-10	2	38.98	2.28
9	-10	2	40.73	2.45
-9	10	2	37.34	3.19
-9	10	2	43.64	2.95
9	10	-2	40.27	2.92
-9	-10	-2	40.19	2.85
9	10	-2	41.82	2.23
-9	-11	2	63.03	3.95
9	11	2	56.39	3.95
9	-11	-2	57.47	3.92
-9	-11	2	62.23	3.59
9	-11	-2	66.08	3.50
9	-11	-2	61.70	3.33
-9	11	-2	67.11	4.12
9	11	-2	60.96	4.07
9	-11	2	62.40	3.46
9	-11	2	59.19	3.51
9	-11	2	57.03	3.18
-9	-11	-2	58.49	3.19
-9	-11	-2	60.90	3.57
-9	-11	-2	63.24	3.98
-9	11	2	66.70	4.01
9	-12	-2	32.50	2.51
9	-12	-2	34.41	2.26
9	12	2	34.82	3.33
-9	-12	2	40.52	2.67
9	-12	-2	29.18	2.12
-9	-12	2	40.44	3.09
-9	12	-2	30.81	3.47
9	-12	2	40.53	2.77
9	-12	2	37.63	2.39
-9	12	2	43.75	3.64
9	12	-2	30.86	2.95

-9	-12	-2	33.67	2.83
9	-12	2	42.34	2.59
-9	-12	-2	31.94	2.38
10	0	2	149.54	7.12
-10	0	2	156.11	7.12
10	0	2	146.83	6.98
-10	0	2	152.17	7.19
10	0	-2	153.76	6.86
-10	0	-2	134.27	7.16
-10	0	-2	157.78	6.91
-10	0	2	152.85	7.17
-10	0	-2	151.85	7.11
-10	0	-2	164.21	7.05
-10	0	-2	160.20	6.88
10	0	2	157.58	7.14
10	0	-2	144.86	6.91
10	0	-2	153.98	7.01
-10	0	-2	147.34	7.02
10	0	-2	151.36	7.06
-10	0	2	161.21	7.08
10	0	2	154.95	7.14
10	0	-2	148.88	7.00
-10	0	2	156.40	7.10
-10	0	-2	160.91	7.04
10	0	2	152.51	7.10
-10	-1	2	160.13	7.58
-10	1	-2	194.71	8.38
-10	-1	2	173.33	7.65
10	1	2	144.66	7.62
10	-1	-2	174.08	8.34
10	1	2	159.79	7.63
-10	1	-2	189.91	8.37
10	-1	-2	179.52	8.32
-10	1	-2	190.66	8.26
-10	-1	2	172.84	7.54
-10	1	-2	184.47	8.34
-10	-1	2	156.44	7.50
-10	-1	2	169.77	7.54
-10	-1	2	165.15	7.63
-10	1	-2	188.91	8.35
10	1	2	155.16	7.42
-10	1	-2	169.58	8.33
10	1	2	162.56	7.57
10	1	2	159.22	7.63

-10	1	-2	175.76	8.47
10	-1	-2	171.00	8.41
10	-1	-2	179.38	8.24
10	1	-2	180.44	8.34
-10	1	2	171.63	7.53
-10	1	2	169.06	7.69
10	-1	2	151.47	7.54
-10	1	2	172.52	7.39
-10	-1	-2	190.33	8.35
10	-1	2	166.91	7.54
-10	1	2	169.82	7.58
-10	-1	-2	180.83	8.38
-10	1	2	167.45	7.51
10	-1	2	168.86	7.64
-10	-1	-2	187.59	8.37
10	1	-2	179.97	8.45
-10	-1	-2	185.97	8.44
-10	1	2	165.29	7.66
10	-1	2	158.95	7.47
10	-1	2	171.99	7.65
-10	-1	-2	177.36	8.35
-10	-2	2	38.42	2.20
-10	-2	2	37.43	1.98
-10	2	-2	37.49	2.18
10	-2	-2	37.41	2.11
-10	2	-2	40.24	2.01
-10	-2	2	42.67	2.02
-10	2	-2	40.34	2.09
-10	2	-2	43.05	2.27
10	-2	-2	38.50	2.06
10	2	2	37.67	2.18
10	2	2	44.36	2.05
-10	2	-2	43.47	2.10
-10	2	-2	39.51	2.05
-10	-2	2	39.78	2.16
-10	-2	2	37.59	2.06
10	-2	-2	38.51	2.00
10	2	2	38.91	1.93
10	-2	-2	39.56	2.02
10	-2	-2	38.61	2.19
10	2	2	41.24	2.02
-10	-2	2	38.28	2.03
-10	-2	2	38.07	2.01
-10	2	-2	43.77	2.01

-10	2	2	35.16	1.92
-10	-2	-2	42.10	2.08
10	-2	2	47.46	2.24
10	2	-2	36.73	1.86
-10	-2	-2	40.98	2.24
10	-2	2	39.58	2.00
-10	-2	-2	39.70	2.06
-10	-2	-2	40.71	2.05
-10	2	2	38.53	2.20
-10	2	2	38.37	2.08
-10	2	2	40.99	1.99
-10	2	2	41.47	2.03
-10	2	2	41.53	2.09
-10	2	2	40.17	1.92
-10	-2	-2	43.08	2.10
10	-2	2	38.06	2.24
10	-2	2	39.22	2.04
-10	-2	-2	43.66	2.08
10	2	-2	35.39	2.19
10	3	2	22.65	1.29
10	-3	-2	18.79	1.17
-10	3	-2	21.24	1.24
-10	3	-2	19.55	1.24
10	-3	-2	19.90	1.17
-10	-3	2	26.67	1.41
-10	3	-2	19.74	1.10
-10	-3	2	24.39	1.60
-10	3	-2	19.38	1.46
10	-3	-2	19.78	1.41
-10	-3	2	24.94	1.32
10	3	2	25.07	1.37
-10	3	-2	19.99	1.46
-10	3	-2	21.93	1.26
-10	-3	2	23.95	1.37
10	-3	-2	19.95	1.40
-10	-3	2	24.69	1.33
10	-3	-2	18.20	1.20
10	3	2	21.13	1.53
10	-3	2	24.42	1.27
10	3	-2	19.84	1.06
10	3	-2	20.60	1.23
-10	-3	-2	20.84	1.00
-10	3	2	26.29	1.39
-10	-3	-2	21.28	1.24

-10	-3	-2	21.11	1.21
-10	3	2	25.41	1.40
10	-3	2	22.84	1.37
10	-3	2	25.51	1.60
10	-3	2	22.39	1.36
-10	-3	-2	20.20	1.02
-10	-3	-2	19.97	1.43
-10	3	2	25.34	1.46
-10	3	2	21.62	1.53
10	-3	2	22.49	1.36
10	3	-2	18.96	1.40
-10	-3	-2	19.64	1.19
-10	3	2	24.01	1.58
10	3	-2	18.58	1.08
-10	-3	-2	20.41	1.24
10	4	2	16.48	1.08
10	-4	-2	16.55	1.14
-10	4	-2	20.12	1.23
-10	-4	2	16.70	1.04
-10	-4	2	17.41	1.39
-10	4	-2	17.71	1.43
-10	-4	2	16.66	1.14
10	4	2	18.80	1.09
-10	-4	2	17.71	1.26
10	-4	-2	16.47	1.36
-10	-4	2	17.04	1.03
-10	-4	2	15.67	1.09
-10	4	-2	17.63	1.52
-10	4	-2	18.60	1.21
10	-4	-2	18.13	1.10
-10	4	-2	19.35	1.25
10	4	2	15.16	1.31
10	4	2	17.48	1.15
10	-4	-2	17.05	1.14
-10	4	2	16.91	1.19
10	4	-2	19.40	1.14
-10	-4	-2	17.88	1.13
-10	-4	-2	17.51	0.97
10	4	-2	17.73	1.03
-10	-4	-2	18.01	1.20
10	4	-2	16.57	1.38
-10	-4	-2	18.57	1.10
10	-4	2	15.02	1.09
-10	-4	-2	17.30	1.05

-10	4	2	16.29	1.34
-10	-4	-2	18.65	1.19
10	-4	2	17.65	1.39
10	4	-2	16.00	1.09
-10	4	2	15.37	1.39
-10	4	2	17.24	1.21
-10	4	2	17.45	1.14
-10	-4	-2	18.60	1.43
10	-4	2	16.74	1.08
10	-4	2	16.82	1.08
10	-4	2	17.15	0.92
10	-5	-2	86.93	4.46
-10	-5	2	88.66	4.38
10	5	2	88.12	4.60
-10	-5	2	87.03	4.57
-10	-5	2	90.58	4.14
10	-5	-2	89.57	4.37
10	5	2	84.74	4.38
10	-5	-2	90.31	4.57
-10	-5	2	91.71	4.60
10	5	2	95.65	4.29
-10	-5	2	89.53	4.37
10	5	2	90.85	4.39
10	-5	-2	86.69	4.31
-10	5	-2	85.70	4.60
-10	5	-2	91.80	4.40
-10	-5	2	87.42	4.24
-10	5	-2	89.98	4.60
10	-5	-2	85.89	4.26
-10	-5	2	89.14	4.15
-10	5	-2	93.99	4.35
-10	5	-2	95.22	4.37
10	-5	2	85.90	4.33
-10	5	2	86.84	4.60
-10	5	2	95.45	4.48
10	-5	2	88.09	4.40
10	5	-2	92.18	4.25
10	5	-2	84.46	4.33
10	-5	2	90.46	4.55
-10	5	2	92.52	4.36
10	5	-2	88.97	4.29
-10	-5	-2	92.13	4.36
-10	-5	-2	91.12	4.57
10	-5	2	96.71	4.72

-10	-5	-2	83.10	4.33
-10	-5	-2	88.44	4.17
10	-5	2	88.68	4.34
-10	5	2	89.85	4.60
-10	-5	-2	88.03	4.26
-10	-5	-2	88.12	4.21
10	5	-2	90.84	4.24
-10	5	2	94.68	4.42
-10	-6	2	47.76	2.30
-10	-6	2	42.27	2.67
-10	6	-2	56.75	2.97
10	-6	-2	54.23	2.91
10	6	2	42.80	2.71
10	6	2	46.12	2.50
-10	-6	2	41.97	2.70
10	-6	-2	51.28	2.76
10	-6	-2	54.82	3.06
-10	-6	2	42.72	2.23
10	-6	-2	56.07	3.23
-10	-6	2	42.65	2.26
-10	6	-2	58.69	2.91
10	6	2	42.48	2.52
10	6	2	45.81	2.43
-10	-6	2	44.40	2.48
-10	6	-2	46.31	3.25
-10	6	-2	58.82	2.89
10	-6	-2	51.05	2.80
-10	-6	2	45.64	2.46
10	6	2	49.04	2.34
-10	6	-2	57.76	3.21
10	6	-2	56.91	2.65
10	6	-2	54.77	2.83
-10	6	2	44.09	2.79
10	-6	2	50.91	2.92
10	6	-2	55.04	2.85
-10	6	2	46.11	2.52
10	-6	2	44.23	2.39
-10	-6	-2	57.65	2.78
-10	-6	-2	53.79	2.87
-10	-6	-2	56.15	2.73
-10	6	2	49.89	2.78
-10	-6	-2	53.33	3.11
-10	-6	-2	52.09	2.70
10	6	-2	54.25	2.71

-10	6	2	46.92	2.46
-10	6	2	45.75	2.60
10	-6	2	43.03	2.38
10	6	-2	49.79	2.91
10	-6	2	48.35	2.73
10	-6	2	44.56	2.48
-10	-7	2	29.95	2.20
10	-7	-2	35.61	2.60
-10	-7	2	32.38	1.81
-10	-7	2	35.11	1.78
10	7	2	36.70	1.84
-10	-7	2	33.82	1.98
-10	7	-2	40.36	2.22
10	-7	-2	35.28	2.20
10	-7	-2	34.30	2.07
-10	-7	2	32.61	2.14
10	7	2	32.46	2.12
-10	7	-2	39.53	2.55
10	-7	-2	37.72	2.07
-10	7	-2	36.91	2.37
10	7	2	28.69	2.19
10	7	2	30.03	2.08
-10	7	-2	31.84	2.72
10	-7	2	31.87	1.89
-10	7	2	37.07	2.35
10	7	-2	39.58	2.00
10	7	-2	39.11	2.22
10	-7	2	31.10	2.02
-10	7	2	28.51	2.39
10	-7	2	36.81	2.58
-10	-7	-2	38.17	2.05
10	7	-2	31.26	2.23
-10	7	2	34.34	2.06
10	7	-2	41.03	1.93
-10	-7	-2	36.69	2.04
10	-7	2	32.75	1.88
-10	-7	-2	37.85	2.19
-10	7	2	32.88	2.19
-10	7	2	33.53	2.01
-10	8	-2	50.20	2.77
10	-8	-2	42.83	2.42
10	8	2	43.49	2.65
-10	-8	2	41.62	2.87
10	-8	-2	48.71	3.09

10	-8	-2	41.92	2.41
10	-8	-2	44.99	2.62
-10	8	-2	43.18	2.94
10	8	2	44.28	2.27
-10	8	-2	40.71	3.23
-10	-8	2	43.23	2.34
-10	-8	2	42.62	2.19
10	8	-2	45.09	2.59
-10	-8	-2	40.83	2.39
10	8	-2	44.71	2.30
10	8	-2	37.94	2.69
10	-8	2	42.50	2.33
10	-8	2	39.21	2.50
-10	-8	-2	41.22	2.51
-10	-8	-2	42.83	2.35
-10	8	2	37.94	3.03
-10	8	2	48.17	2.90
10	8	-2	49.80	2.24
-10	-9	2	16.45	1.08
10	-9	-2	13.49	1.24
-10	-9	2	13.90	1.38
10	9	2	13.19	1.76
10	-9	-2	10.98	2.22
10	-9	-2	14.87	1.41
-10	9	-2	14.10	1.92
10	9	2	16.47	1.66
-10	9	-2	8.71	1.62
-10	-9	2	14.62	1.80
10	-9	-2	9.71	1.41
-10	-9	2	16.52	1.46
10	9	2	16.79	1.23
-10	9	2	11.36	1.81
10	9	-2	13.94	0.99
-10	-9	-2	11.37	1.14
-10	-9	-2	15.84	1.48
10	-9	2	15.08	1.47
-10	-9	-2	12.83	1.31
10	9	-2	13.25	1.14
10	9	-2	13.86	1.61
10	-9	2	12.07	2.27
10	-9	2	14.90	1.34
10	-9	2	13.46	1.17
10	9	-2	9.76	1.70
-10	9	2	12.89	2.47

10	-10	-2	2.52	0.93
-10	-10	2	4.58	0.92
10	-10	-2	3.65	0.75
-10	10	-2	2.54	1.56
10	-10	-2	4.44	0.86
-10	-10	2	5.45	1.29
-10	-10	2	4.31	0.78
10	10	2	3.56	1.22
-10	10	-2	5.55	1.41
-10	-10	-2	3.70	1.11
-10	-10	-2	3.92	0.74
10	10	-2	3.41	0.70
-10	10	2	3.48	1.33
10	-10	2	3.36	0.97
10	10	-2	0.99	1.11
10	-10	2	5.92	0.92
-10	-10	-2	4.94	1.02
-10	-11	2	15.13	2.35
10	11	2	12.99	2.45
-10	-11	2	12.42	1.66
-10	11	-2	11.90	2.02
10	-11	-2	10.05	1.64
10	-11	-2	13.13	1.53
10	-11	-2	11.04	1.32
-10	-11	-2	10.82	1.60
-10	11	2	16.58	2.35
-10	-11	-2	12.46	1.81
10	-11	2	15.67	1.37
10	11	-2	11.58	2.00
-10	-11	-2	12.08	1.21
10	-11	2	12.40	1.57
10	-11	2	14.53	1.71
10	-12	-2	0.94	0.77
-10	-12	2	1.83	1.22
10	12	2	3.21	1.53
-10	-12	2	2.51	1.00
-10	12	-2	-0.26	1.61
10	-12	-2	2.03	1.22
10	-12	-2	2.62	0.96
-10	12	2	6.06	1.98
10	12	-2	1.07	1.35
-10	-12	-2	1.96	0.96
10	-12	2	2.55	1.18
-10	-12	-2	1.59	1.25

10	-12	2	1.03	0.83
10	-12	2	0.92	0.81
11	0	-2	-0.02	0.24
-11	0	2	-0.12	0.42
-11	0	-2	0.25	0.37
11	0	-2	0.00	0.13
-11	0	-2	-0.02	0.17
11	0	-2	0.01	0.38
-11	0	2	-0.17	0.39
-11	0	2	0.32	0.37
-11	0	2	-0.20	0.26
-11	0	-2	0.07	0.23
-11	0	-2	-0.23	0.27
-11	0	-2	-0.35	0.30
11	0	-2	0.03	0.24
11	0	2	0.02	0.35
11	0	-2	0.39	0.25
-11	0	-2	0.42	0.41
-11	0	2	0.02	0.31
11	0	2	0.20	0.25
11	0	2	0.30	0.40
-11	0	-2	0.17	0.28
11	0	2	-0.03	0.16
-11	-1	2	78.47	3.96
11	-1	-2	96.23	4.49
11	1	2	85.39	3.99
-11	1	-2	97.81	4.50
-11	-1	2	85.96	4.11
11	-1	-2	91.91	4.39
11	1	2	81.77	4.10
11	1	2	80.79	3.87
-11	1	-2	96.25	4.39
-11	-1	2	84.03	4.12
-11	1	-2	98.01	4.54
-11	1	-2	93.94	4.66
-11	-1	2	78.78	4.04
-11	1	-2	97.79	4.51
-11	1	-2	85.82	4.49
-11	-1	2	87.50	4.00
11	1	2	80.82	4.02
-11	-1	2	85.03	3.99
-11	1	-2	98.12	4.54
11	-1	-2	97.17	4.49
11	-1	-2	96.55	4.49

11	-1	-2	92.59	4.51
-11	-1	-2	99.03	4.62
-11	-1	-2	97.38	4.52
-11	1	2	82.69	4.13
11	-1	2	81.60	3.91
11	-1	2	80.01	4.00
-11	-1	-2	98.74	4.54
-11	1	2	86.40	4.04
-11	-1	-2	92.40	4.51
11	-1	2	85.80	4.00
-11	1	2	88.04	4.17
11	1	-2	91.50	4.60
-11	1	2	85.43	3.97
11	1	-2	92.66	4.50
-11	-1	-2	96.81	4.57
11	-1	2	87.30	4.14
11	-1	2	85.00	4.11
-11	1	2	87.59	4.00
-11	-1	-2	95.40	4.57
11	-2	-2	77.62	4.05
11	-2	-2	85.21	4.13
11	2	2	94.74	4.67
-11	2	-2	87.43	4.18
-11	-2	2	89.08	4.47
-11	2	-2	91.99	4.11
-11	-2	2	90.35	4.50
-11	2	-2	89.94	4.01
-11	-2	2	101.29	4.69
-11	2	-2	88.53	4.01
-11	-2	2	96.70	4.63
-11	2	-2	80.87	4.05
-11	-2	2	91.16	4.53
11	-2	-2	86.15	4.04
-11	2	-2	84.68	4.09
-11	2	-2	85.83	4.26
11	-2	-2	84.16	4.19
-11	-2	2	98.13	4.50
-11	-2	2	100.73	4.53
11	-2	-2	80.36	3.98
11	2	2	91.68	4.40
11	2	2	101.57	4.51
-11	2	2	96.69	4.53
11	-2	2	89.76	4.52
-11	2	2	96.64	4.44

-11	-2	-2	88.83	4.05
-11	-2	-2	86.90	4.09
11	-2	2	99.51	4.72
-11	2	2	92.65	4.41
11	-2	2	98.85	4.47
11	-2	2	93.38	4.48
-11	-2	-2	90.43	4.23
-11	2	2	103.41	4.53
-11	-2	-2	84.68	4.06
-11	-2	-2	81.49	4.06
-11	2	2	93.09	4.69
-11	2	2	95.80	4.57
11	2	-2	83.98	4.21
11	2	-2	83.29	3.85
-11	-2	-2	84.70	4.06
11	-2	2	98.63	4.66
-11	-2	-2	76.52	4.17
-11	2	2	87.91	4.41
11	-3	-2	103.76	5.02
-11	3	-2	110.61	5.31
11	-3	-2	100.44	5.02
-11	-3	2	106.61	5.22
-11	3	-2	109.21	4.94
11	-3	-2	98.60	5.05
-11	3	-2	107.82	5.25
11	3	2	116.44	5.23
-11	-3	2	112.87	5.43
-11	3	-2	107.55	5.04
-11	-3	2	114.04	5.19
-11	-3	2	108.55	5.24
-11	-3	2	115.49	5.35
11	3	2	107.15	5.42
11	-3	-2	107.44	5.21
-11	3	-2	103.36	5.08
11	-3	-2	108.82	5.21
11	3	2	114.35	5.22
-11	3	-2	112.00	5.10
-11	-3	2	108.39	5.19
11	3	2	106.13	5.15
-11	-3	2	106.41	5.23
11	-3	2	108.27	5.23
11	3	-2	104.73	4.91
11	-3	2	104.69	5.25
-11	3	2	116.45	5.25

11	-3	2	107.96	5.25
-11	3	2	112.57	5.30
-11	3	2	112.86	5.24
-11	3	2	116.68	5.47
-11	-3	-2	113.27	5.06
-11	3	2	110.78	5.36
11	-3	2	114.87	5.49
-11	-3	-2	104.15	5.07
-11	-3	-2	109.74	5.03
-11	-3	-2	103.76	4.98
11	-3	2	110.74	5.41
11	3	-2	106.14	5.26
-11	-3	-2	110.67	5.25
11	3	-2	105.87	4.89
11	-3	2	117.33	5.14
-11	-3	-2	110.21	5.09
-11	4	-2	104.50	4.93
11	4	2	97.32	4.60
11	4	2	91.78	4.57
-11	4	-2	105.00	5.17
11	-4	-2	99.19	4.90
11	-4	-2	105.04	5.11
11	4	2	94.47	4.85
-11	-4	2	94.73	4.62
11	-4	-2	101.38	4.85
-11	-4	2	100.18	4.86
-11	4	-2	98.59	5.14
11	-4	-2	97.34	4.89
-11	-4	2	96.26	4.63
-11	4	-2	107.79	4.94
-11	-4	2	99.05	4.55
-11	-4	2	102.07	4.71
-11	-4	2	97.12	4.53
11	4	2	100.81	4.58
11	-4	-2	106.53	5.08
-11	4	-2	106.32	4.93
-11	-4	-2	100.57	4.81
11	-4	2	94.88	4.62
-11	-4	-2	101.82	4.91
-11	-4	-2	105.99	5.14
-11	4	2	99.92	4.69
11	4	-2	102.24	5.14
-11	4	2	98.30	4.67
11	4	-2	104.03	4.77

11	4	-2	101.21	4.82
-11	-4	-2	100.75	4.67
-11	-4	-2	101.02	4.92
11	-4	2	93.52	4.79
11	-4	2	86.97	4.60
-11	-4	-2	103.77	4.69
-11	4	2	102.37	4.64
11	-4	2	92.36	4.63
-11	4	2	93.25	4.80
-11	-4	-2	93.64	4.80
-11	-4	-2	105.07	4.87
11	4	-2	105.62	4.86
-11	4	2	99.77	4.87
-11	-5	2	55.78	2.94
11	-5	-2	62.48	3.29
11	5	2	54.81	3.18
-11	-5	2	55.07	3.15
-11	-5	2	59.68	2.81
-11	-5	2	56.57	2.64
-11	5	-2	66.76	3.64
-11	-5	2	58.65	2.97
11	5	2	59.19	2.86
11	-5	-2	65.46	3.35
-11	-5	2	57.72	2.64
-11	5	-2	66.88	3.70
11	5	2	55.62	2.94
11	-5	-2	65.50	3.26
11	-5	-2	61.33	3.49
-11	5	-2	69.56	3.40
-11	-5	2	57.62	2.82
-11	-5	2	59.67	3.21
-11	-5	2	61.25	3.23
-11	5	-2	69.18	3.36
-11	5	-2	69.36	3.36
-11	-5	-2	68.08	3.13
11	-5	2	53.96	2.95
11	5	-2	68.70	3.31
11	-5	2	54.79	2.90
-11	5	2	57.51	3.22
-11	5	2	58.80	3.26
11	5	-2	63.07	3.30
11	5	-2	68.54	3.23
-11	-5	-2	62.72	3.57
11	-5	2	54.58	2.91

-11	-5	-2	65.75	3.34
-11	-5	-2	67.83	3.28
11	-5	2	54.69	3.15
-11	5	2	60.12	3.06
-11	-5	-2	63.77	3.16
-11	-5	-2	62.56	3.33
-11	5	2	59.28	2.96
-11	-5	-2	63.66	3.10
-11	-5	-2	64.81	3.11
-11	5	2	60.60	3.03
11	-5	2	59.26	3.31
11	5	-2	67.04	3.23
11	6	2	32.26	2.23
-11	6	-2	31.26	2.17
11	6	2	34.86	2.05
11	-6	-2	24.60	1.64
-11	-6	2	33.56	1.77
-11	-6	2	34.22	1.79
11	-6	-2	29.82	2.15
11	6	2	33.66	2.04
-11	6	-2	25.56	2.17
-11	-6	2	31.36	1.69
-11	-6	2	32.62	1.69
11	-6	-2	27.76	1.76
11	6	2	35.17	1.85
-11	6	-2	28.84	1.77
11	-6	-2	27.57	1.99
-11	-6	2	33.78	2.25
-11	-6	2	33.61	1.96
-11	6	-2	29.66	1.86
-11	6	-2	29.23	1.81
11	-6	-2	27.53	1.63
11	6	-2	28.83	1.59
-11	6	2	34.86	2.06
11	6	-2	27.08	1.63
11	6	-2	26.48	1.68
-11	6	2	34.42	2.44
11	-6	2	30.73	1.88
-11	-6	-2	28.63	1.64
-11	-6	-2	29.20	1.76
11	6	-2	24.32	1.76
-11	-6	-2	27.97	1.56
11	-6	2	36.64	2.47
11	-6	2	34.54	1.91

-11	6	2	31.60	2.11
-11	6	2	32.81	1.97
-11	6	2	33.21	2.27
11	-6	2	32.73	1.99
-11	7	-2	4.64	1.06
-11	-7	2	6.41	0.75
-11	7	-2	4.85	1.25
11	-7	-2	4.77	0.78
11	-7	-2	5.58	0.81
-11	7	-2	5.52	1.01
-11	-7	2	7.38	0.78
11	-7	-2	4.81	0.91
11	7	2	6.56	1.18
11	-7	-2	6.69	1.21
11	7	2	8.75	0.91
-11	-7	2	6.29	0.90
-11	-7	2	7.40	1.30
11	7	2	7.92	1.26
11	7	2	7.82	1.17
-11	-7	-2	5.45	0.97
11	-7	2	7.12	0.83
-11	-7	-2	4.73	0.68
11	-7	2	5.53	0.80
11	-7	2	7.01	0.98
11	7	-2	5.44	0.77
11	-7	2	7.71	1.44
-11	-7	-2	4.59	0.70
11	7	-2	5.45	0.92
-11	7	2	6.84	1.32
11	7	-2	5.43	0.71
-11	7	2	6.16	1.31
-11	-8	2	6.53	1.20
11	-8	-2	6.82	1.48
-11	-8	2	7.50	0.84
11	8	2	9.73	1.38
11	-8	-2	7.65	0.95
11	-8	-2	5.96	0.93
-11	8	-2	6.45	1.44
-11	-8	2	6.37	0.91
11	8	2	8.97	0.97
-11	-8	2	6.68	1.05
-11	8	-2	7.35	1.34
11	8	2	8.87	1.29
11	8	2	7.37	1.41

11	-8	-2	5.78	1.04
-11	-8	-2	6.20	1.08
-11	8	2	7.38	1.61
11	-8	2	8.56	0.99
-11	-8	-2	7.18	0.95
11	-8	2	9.33	1.94
11	-8	2	7.34	1.11
-11	-8	-2	7.92	1.08
11	8	-2	6.41	1.34
11	-8	2	8.10	0.91
11	8	-2	7.92	0.89
11	8	-2	7.81	1.26
11	8	-2	7.01	0.81
11	-9	-2	23.62	1.70
-11	-9	2	21.02	2.05
-11	9	-2	26.95	3.18
11	-9	-2	25.66	1.75
11	-9	-2	22.03	1.92
-11	9	-2	25.28	2.48
-11	-9	2	21.11	1.35
11	9	2	22.90	1.48
-11	-9	2	20.34	1.49
11	-9	-2	33.95	2.94
-11	-9	2	20.19	1.70
11	9	2	20.38	2.01
11	9	2	18.75	1.95
11	9	-2	26.39	1.60
11	-9	2	23.22	1.62
11	9	-2	27.52	2.22
11	-9	2	27.56	2.64
11	-9	2	19.10	1.49
11	9	-2	26.00	1.51
-11	-9	-2	28.38	1.94
11	9	-2	26.37	2.03
-11	9	2	22.06	2.24
11	-9	2	20.91	1.71
-11	9	2	20.29	2.50
-11	-9	-2	26.66	1.71
-11	-10	2	19.64	2.25
-11	-10	2	18.04	1.28
-11	10	-2	22.69	2.54
11	10	2	16.11	2.12
11	-10	-2	21.03	1.85
11	-10	-2	21.87	1.69

11	-10	-2	20.55	1.63
-11	-10	2	19.02	1.82
-11	10	2	18.86	2.41
11	10	-2	20.03	2.21
-11	-10	-2	20.82	1.56
-11	-10	-2	21.86	2.31
-11	-10	-2	26.54	2.03
11	-10	2	19.78	1.65
11	-10	2	18.41	1.44
11	-10	2	15.04	1.89
11	-11	-2	18.63	1.61
-11	-11	2	22.08	2.43
-11	-11	2	22.28	1.90
11	11	2	21.59	2.46
11	-11	-2	19.16	1.96
-11	11	-2	21.66	2.99
11	-11	-2	20.17	1.79
11	-11	2	18.75	1.96
11	11	-2	14.47	2.35
11	-11	2	21.13	1.76
11	-11	2	16.66	1.61
-11	11	2	21.21	2.94
-11	-11	-2	18.33	2.39
-11	-11	-2	18.10	1.82
11	-12	-2	19.97	1.92
11	12	2	26.24	2.83
11	-12	-2	20.55	1.85
11	-12	-2	20.44	2.18
-11	-12	2	27.34	2.20
-11	-12	2	26.73	2.62
-11	12	-2	28.38	3.00
11	-12	2	22.89	1.86
11	-12	2	22.21	2.33
-11	12	2	33.62	3.19
11	-12	2	23.14	2.02
11	12	-2	20.80	2.66
-11	-12	-2	15.86	1.89
-11	-12	-2	21.76	2.50
12	0	-2	218.70	9.92
-12	0	-2	228.73	10.17
-12	0	-2	215.48	9.88
12	0	2	213.86	10.28
-12	0	2	237.05	10.44
-12	0	2	230.15	10.45

12	0	-2	219.10	10.16
-12	0	-2	236.35	10.15
-12	0	2	239.08	10.41
12	0	2	219.27	10.43
-12	0	2	212.71	10.38
-12	0	2	233.53	10.56
-12	0	-2	223.90	10.22
12	0	2	222.59	10.47
-12	0	2	223.64	10.51
-12	0	-2	211.65	10.11
12	0	2	236.93	10.46
12	0	-2	210.29	10.08
-12	0	-2	233.29	10.14
12	0	2	238.23	10.48
12	0	-2	213.04	9.99
12	1	2	61.16	3.18
12	1	2	67.03	3.10
-12	1	-2	79.33	3.73
-12	-1	2	61.94	3.06
12	1	2	58.24	3.08
-12	1	-2	74.86	3.72
-12	-1	2	63.56	3.07
-12	1	-2	77.70	3.75
12	-1	-2	76.85	3.70
-12	1	-2	78.30	3.58
12	-1	-2	75.33	3.68
-12	-1	2	64.17	3.23
-12	-1	2	55.77	3.01
12	-1	-2	75.63	3.58
-12	-1	2	59.95	3.14
-12	1	-2	76.66	3.90
12	-1	-2	75.43	3.72
-12	-1	2	63.28	3.19
12	1	2	61.80	2.92
12	-1	2	62.99	3.09
-12	-1	-2	76.80	3.79
12	-1	2	59.40	2.96
-12	1	2	63.14	3.22
12	1	-2	77.12	3.72
12	-1	2	66.33	3.22
12	-1	2	60.15	3.08
-12	-1	-2	80.19	3.80
12	1	-2	72.99	3.83
-12	-1	-2	78.13	3.73

-12	1	2	64.26	3.06
-12	-1	-2	80.43	3.86
-12	-1	-2	78.11	3.75
12	-1	2	62.61	3.19
-12	1	2	64.80	3.12
-12	-1	-2	75.82	3.73
-12	1	2	63.58	3.26
-12	1	2	62.81	3.07
12	-2	-2	47.22	2.51
12	-2	-2	47.06	2.33
12	2	2	46.06	2.64
-12	2	-2	43.93	2.40
-12	2	-2	45.62	2.35
-12	2	-2	49.62	2.47
-12	-2	2	50.26	2.50
-12	2	-2	47.00	2.54
-12	2	-2	48.97	2.35
-12	-2	2	47.05	2.51
-12	-2	2	46.49	2.46
-12	-2	2	46.24	2.44
-12	-2	2	51.42	2.48
-12	-2	2	53.26	2.64
-12	2	-2	46.00	2.62
12	-2	-2	46.19	2.38
-12	-2	2	50.36	2.67
12	2	2	47.87	2.35
-12	2	-2	48.53	2.47
12	2	2	52.24	2.49
12	-2	2	46.48	2.48
12	-2	2	48.32	2.43
12	2	-2	44.37	2.57
-12	-2	-2	47.10	2.42
-12	2	2	53.01	2.74
-12	2	2	45.59	2.39
-12	-2	-2	49.09	2.50
12	-2	2	48.53	2.66
12	-2	2	51.53	2.70
-12	-2	-2	47.84	2.46
-12	-2	-2	49.48	2.42
-12	-2	-2	45.38	2.42
-12	2	2	46.07	2.68
-12	2	2	49.21	2.69
12	-2	2	47.92	2.42
-12	2	2	51.29	2.56

-12	2	2	49.14	2.39
-12	2	2	51.20	2.50
-12	-2	-2	45.02	2.44
-12	-2	-2	50.13	2.61
12	2	-2	46.92	2.19
-12	2	2	47.69	2.38
-12	3	-2	13.23	0.97
12	3	2	9.82	0.76
-12	-3	2	11.48	0.95
12	3	2	11.28	1.13
-12	-3	2	12.42	1.13
12	-3	-2	11.73	0.93
12	-3	-2	12.47	0.90
-12	-3	2	10.84	0.83
12	-3	-2	12.49	0.90
-12	3	-2	14.39	1.06
-12	3	-2	13.66	1.28
12	3	2	11.54	0.91
12	-3	-2	12.08	1.22
-12	-3	2	10.59	0.89
-12	3	-2	13.60	1.33
-12	-3	2	10.45	0.87
-12	3	-2	12.63	0.86
12	-3	-2	12.35	1.14
-12	-3	2	12.34	1.21
-12	3	-2	13.75	1.06
-12	-3	2	11.67	0.88
12	3	2	11.48	0.90
-12	-3	-2	12.60	0.97
-12	3	2	10.77	1.15
-12	-3	-2	12.53	0.95
-12	3	2	11.77	0.91
12	-3	2	11.54	0.89
12	-3	2	12.69	1.32
-12	-3	-2	12.88	1.01
12	-3	2	11.06	0.85
-12	-3	-2	12.69	1.00
-12	-3	-2	12.82	0.94
-12	-3	-2	11.23	1.24
12	3	-2	11.98	0.74
-12	3	2	12.24	1.01
-12	3	2	11.54	1.16
12	3	-2	11.40	1.18
-12	3	2	11.54	0.93

12	-3	2	10.91	1.18
12	3	-2	11.28	0.76
12	-3	2	10.87	0.89
12	4	2	11.97	1.22
12	-4	-2	10.54	0.89
-12	-4	2	12.12	0.91
-12	-4	2	11.09	0.93
12	-4	-2	12.39	1.21
12	-4	-2	11.13	1.25
-12	4	-2	11.67	1.39
-12	-4	2	12.81	0.91
-12	4	-2	12.10	1.05
12	-4	-2	9.81	0.90
-12	4	-2	12.29	1.06
12	-4	-2	11.53	0.90
12	4	2	11.99	0.98
-12	4	-2	12.10	1.04
-12	-4	2	12.47	1.22
12	4	2	13.12	0.97
-12	-4	2	11.36	1.01
-12	4	-2	12.85	1.31
-12	-4	2	12.47	1.34
12	4	2	12.11	0.89
-12	-4	2	13.56	0.98
-12	-4	-2	11.71	0.94
-12	4	2	11.55	1.10
12	-4	2	11.00	0.94
12	-4	2	14.28	1.34
12	4	-2	11.09	0.79
-12	-4	-2	11.83	1.01
12	4	-2	11.24	0.85
-12	4	2	11.21	1.34
-12	4	2	12.46	1.06
-12	-4	-2	12.37	0.65
-12	-4	-2	11.68	0.86
-12	-4	-2	10.84	1.27
-12	-4	-2	11.67	0.97
12	-4	2	11.50	0.96
-12	-4	-2	11.66	0.63
-12	-4	-2	11.76	1.00
12	4	-2	14.37	1.29
-12	4	2	11.72	1.25
12	-4	2	11.81	0.95
-12	4	2	12.51	1.04

-12	-5	2	35.60	2.11
-12	5	-2	47.53	2.47
-12	-5	2	37.86	2.37
12	5	2	36.80	2.38
12	-5	-2	43.00	2.41
12	5	2	36.84	2.08
-12	-5	2	36.29	2.40
-12	-5	2	39.26	2.28
12	5	2	40.70	2.05
-12	-5	2	40.10	1.99
-12	5	-2	47.14	2.50
12	-5	-2	43.98	2.38
12	-5	-2	44.90	2.34
-12	-5	2	35.62	1.98
-12	5	-2	42.41	2.73
-12	-5	2	37.50	2.10
-12	5	-2	44.59	2.82
12	-5	-2	42.69	2.61
-12	5	-2	45.90	2.46
12	5	2	40.47	2.10
12	-5	2	40.82	2.52
-12	-5	-2	44.01	2.34
-12	5	2	37.90	2.21
-12	-5	-2	43.09	2.21
12	-5	2	34.35	2.32
-12	-5	-2	45.59	2.15
12	5	-2	46.09	2.32
12	-5	2	35.90	2.10
-12	5	2	39.11	2.46
-12	-5	-2	42.51	2.42
-12	-5	-2	42.29	2.67
-12	5	2	41.00	2.22
-12	5	2	36.65	2.12
12	-5	2	36.43	2.07
-12	-5	-2	45.59	2.44
12	5	-2	42.34	2.35
-12	5	2	36.88	2.40
12	5	-2	47.27	2.31
-12	-5	-2	42.17	2.28
-12	-5	-2	43.69	2.13
12	-5	2	34.19	2.04
12	-6	-2	90.41	4.66
12	-6	-2	84.21	4.27
-12	-6	2	82.45	4.00

-12	-6	2	85.13	4.34
-12	-6	2	90.93	4.16
12	6	2	90.26	4.22
-12	6	-2	85.92	4.66
12	6	2	87.04	4.37
-12	-6	2	89.43	4.39
-12	6	-2	91.78	4.34
12	-6	-2	84.25	4.32
12	6	2	90.65	4.34
-12	-6	2	87.69	4.18
12	-6	-2	82.08	4.21
-12	6	-2	84.24	4.73
-12	-6	2	90.06	4.00
-12	-6	2	88.86	4.60
12	6	2	86.36	4.61
12	-6	2	85.52	4.37
12	-6	2	83.21	4.28
-12	6	2	83.99	4.64
-12	6	2	89.98	4.65
12	6	-2	81.83	4.30
12	6	-2	89.97	4.23
-12	6	2	83.89	4.32
12	6	-2	87.73	4.15
12	-6	2	93.37	4.78
-12	-6	-2	84.54	4.32
-12	-6	-2	87.79	4.22
12	-6	2	84.47	4.28
-12	-6	-2	88.80	4.07
12	-7	-2	61.54	3.21
12	-7	-2	63.46	3.31
12	-7	-2	69.69	3.73
-12	-7	2	71.12	3.35
-12	7	-2	51.27	3.95
12	7	2	71.55	3.86
-12	-7	2	65.76	3.59
-12	-7	2	63.01	3.60
-12	-7	2	66.20	3.37
12	-7	-2	60.53	3.16
-12	7	-2	53.65	3.69
12	7	2	72.70	3.43
-12	-7	2	71.46	3.28
-12	-7	-2	60.13	3.14
-12	7	2	67.72	3.95
12	7	-2	62.19	3.05

12	-7	2	67.55	3.49
12	-7	2	64.19	3.50
12	-7	2	70.44	3.65
-12	-7	-2	64.86	3.06
-12	7	2	67.24	4.01
12	7	-2	63.95	3.20
12	-7	2	77.23	4.13
-12	8	-2	60.60	3.72
12	8	2	63.57	3.55
-12	-8	2	63.72	3.72
-12	-8	2	64.38	3.10
-12	-8	2	62.25	3.40
-12	-8	2	64.01	3.10
12	8	2	60.22	3.54
-12	-8	2	60.91	3.12
12	8	2	62.78	3.18
-12	8	-2	55.80	3.94
12	-8	-2	57.44	3.15
12	-8	-2	56.73	3.27
12	-8	-2	63.89	3.78
12	8	2	63.32	3.76
12	-8	-2	57.20	3.12
-12	8	2	59.53	3.98
12	-8	2	63.47	3.44
12	8	-2	61.49	2.95
-12	-8	-2	54.36	3.04
12	-8	2	69.57	4.08
12	8	-2	58.01	3.38
12	-8	2	61.36	3.27
-12	-8	-2	59.62	3.28
-12	8	2	64.49	3.78
-12	-8	-2	59.65	3.03
12	8	-2	65.27	3.22
12	-8	2	60.21	3.30
12	9	2	63.59	3.41
-12	-9	2	57.69	2.91
12	9	2	58.61	2.98
-12	-9	2	58.00	3.27
12	9	2	55.58	3.47
-12	-9	2	57.69	3.60
12	-9	-2	60.74	3.37
12	-9	-2	56.35	3.17
-12	9	-2	64.86	3.89
12	-9	-2	57.55	3.19

-12	-9	-2	58.88	3.10
12	9	-2	62.25	2.94
-12	-9	-2	57.56	3.35
-12	9	2	58.10	3.76
12	9	-2	54.57	3.53
12	-9	2	55.82	3.06
12	-9	2	57.65	3.16
12	-9	2	51.40	3.94
12	9	-2	59.59	4.11
12	-10	-2	10.18	1.28
12	10	2	5.74	1.41
-12	10	-2	11.06	1.85
-12	-10	2	8.15	1.58
-12	-10	2	9.38	1.51
12	-10	-2	9.99	1.27
12	-10	-2	9.42	1.51
-12	-10	-2	11.18	1.61
12	-10	2	4.55	1.01
-12	-10	-2	10.76	1.65
12	10	-2	9.06	1.68
-12	10	2	5.43	1.37
12	-10	2	6.29	1.07
-12	-10	-2	8.18	1.22
12	-10	2	5.93	0.96
12	11	2	0.65	1.23
12	-11	-2	0.28	0.82
-12	11	-2	-1.71	1.47
-12	-11	2	1.02	0.84
-12	-11	2	-0.38	1.18
12	-11	-2	-1.27	0.98
12	-11	-2	0.06	0.77
-12	-11	-2	0.54	1.16
12	-11	2	0.68	0.76
-12	-11	-2	0.95	0.81
12	-11	2	-0.09	1.02
-12	11	2	-0.14	1.71
12	11	-2	-0.86	1.24
12	-11	2	1.98	0.79
12	-12	-2	7.12	1.14
12	-12	-2	7.41	1.16
12	12	2	1.07	1.43
12	-12	-2	7.28	1.65
-12	-12	2	5.40	1.35
12	-12	2	4.91	1.35

12	12	-2	5.90	1.59
12	-12	2	6.05	1.15
12	-12	2	3.71	0.88
13	0	2	0.89	0.43
-13	0	2	0.19	0.45
13	0	2	0.01	0.31
-13	0	-2	-0.41	0.47
13	0	-2	-0.16	0.26
-13	0	-2	-0.15	0.32
-13	0	2	-0.01	0.55
13	0	-2	0.45	0.43
-13	0	2	-0.04	0.28
-13	0	2	0.48	0.39
-13	0	2	0.02	0.43
13	0	2	0.53	0.41
13	0	-2	0.13	0.16
13	0	2	-0.35	0.45
13	0	-2	0.08	0.29
-13	0	-2	-0.34	0.42
-13	0	-2	0.24	0.39
13	0	2	0.16	0.20
-13	0	2	0.18	0.35
-13	0	-2	0.37	0.41
13	-1	-2	26.27	1.40
-13	1	-2	27.38	1.51
-13	1	-2	30.46	1.64
-13	1	-2	28.43	1.63
13	1	2	30.07	1.50
13	-1	-2	27.85	1.54
-13	-1	2	30.38	1.83
-13	-1	2	27.64	1.62
-13	1	-2	26.38	1.40
-13	1	-2	26.76	1.62
13	1	2	28.37	1.69
13	-1	-2	28.36	1.56
-13	-1	2	29.18	1.76
13	1	2	30.44	1.81
13	1	2	30.59	1.72
-13	1	-2	28.71	1.80
-13	1	-2	26.31	1.57
-13	-1	2	31.12	1.69
13	-1	-2	26.35	1.56
-13	-1	2	29.33	1.67
-13	-1	2	32.96	1.89

-13	-1	-2	29.09	1.60
13	1	-2	25.91	1.36
-13	1	2	30.32	1.91
13	-1	2	30.28	1.69
-13	-1	-2	28.98	1.75
-13	-1	-2	28.53	1.70
13	-1	2	32.89	1.81
13	-1	2	27.01	1.52
13	-1	2	30.05	1.84
-13	-1	-2	29.16	1.64
-13	-1	-2	27.29	1.71
-13	1	2	29.73	1.86
13	-1	2	27.96	1.66
13	1	-2	27.93	1.72
-13	1	2	27.31	1.71
13	1	-2	25.10	1.55
-13	1	2	29.70	1.67
-13	-1	-2	26.73	1.59
-13	-2	2	47.07	2.40
-13	-2	2	46.16	2.41
-13	-2	2	47.98	2.39
-13	2	-2	44.86	2.33
-13	-2	2	47.93	2.48
-13	2	-2	46.34	2.49
13	-2	-2	47.83	2.48
13	-2	-2	44.75	2.32
-13	2	-2	46.41	2.48
13	-2	-2	44.18	2.43
13	2	2	47.19	2.59
-13	2	-2	47.22	2.49
13	2	2	48.29	2.43
-13	2	-2	49.70	2.71
13	-2	-2	48.19	2.40
-13	2	-2	48.29	2.40
-13	-2	2	47.81	2.60
-13	-2	2	51.89	2.65
-13	-2	2	47.36	2.44
13	2	2	44.04	2.26
-13	-2	-2	45.23	2.49
-13	2	2	44.21	2.28
-13	2	2	49.09	2.44
-13	-2	-2	43.13	2.41
-13	2	2	45.73	2.40
-13	-2	-2	48.97	2.45

-13	-2	-2	47.02	2.63
-13	2	2	44.88	2.66
-13	2	2	46.20	2.35
13	2	-2	49.67	2.64
-13	2	2	51.29	2.69
13	-2	2	45.36	2.62
13	-2	2	40.45	2.40
-13	-2	-2	46.83	2.44
-13	2	2	48.23	2.33
-13	-2	-2	45.51	2.46
13	-2	2	47.78	2.39
13	-2	2	42.53	2.33
13	-2	2	51.90	2.72
-13	-2	-2	50.75	2.49
-13	-3	2	18.35	1.27
-13	-3	2	17.06	1.48
13	-3	-2	20.68	1.31
-13	-3	2	16.72	1.26
13	-3	-2	22.43	1.66
13	3	2	21.47	1.53
13	-3	-2	19.20	1.34
-13	3	-2	26.85	1.77
-13	-3	2	15.82	1.19
-13	3	-2	18.66	1.42
-13	3	-2	19.71	1.34
-13	3	-2	21.91	1.69
13	-3	-2	20.15	1.29
13	-3	-2	23.53	1.61
-13	3	-2	23.85	1.46
-13	3	-2	22.77	1.37
13	3	2	18.32	1.25
13	3	2	19.57	1.11
-13	-3	2	22.51	1.62
-13	-3	2	21.95	1.22
-13	-3	2	22.47	1.29
-13	3	2	23.47	1.59
-13	-3	-2	20.26	1.35
-13	-3	-2	21.42	1.42
13	3	-2	23.04	1.17
13	-3	2	23.97	1.70
13	3	-2	23.26	1.66
13	3	-2	19.50	1.14
13	-3	2	21.85	1.62
-13	-3	-2	25.36	1.42

-13	3	2	16.78	1.24
-13	-3	-2	26.86	1.42
13	-3	2	20.84	1.15
-13	3	2	19.20	1.53
-13	3	2	17.17	1.34
-13	3	2	19.09	1.28
13	-3	2	17.05	1.23
-13	-3	-2	22.59	1.66
13	-3	2	16.57	1.17
-13	-3	-2	18.47	1.50
-13	-4	2	47.72	2.56
-13	-4	2	41.60	2.15
-13	4	-2	44.93	2.38
-13	-4	2	39.78	2.11
-13	-4	2	39.24	2.20
13	4	2	42.04	2.50
-13	4	-2	39.42	2.32
13	4	2	40.90	2.11
13	-4	-2	42.89	2.59
13	4	2	42.06	2.16
-13	-4	2	39.70	2.33
-13	4	-2	40.88	2.60
13	-4	-2	41.77	2.25
-13	-4	2	48.05	2.25
13	-4	-2	36.57	2.26
13	4	2	34.78	2.16
-13	4	-2	50.20	2.74
-13	4	-2	50.00	2.13
13	-4	-2	36.94	2.29
13	-4	-2	45.07	2.63
-13	4	-2	37.67	2.34
-13	-4	-2	47.39	2.37
13	-4	2	42.57	2.50
-13	4	2	44.15	2.24
13	4	-2	43.05	2.19
13	-4	2	34.56	2.14
13	-4	2	36.03	2.17
13	-4	2	46.33	2.64
-13	4	2	36.92	2.21
13	-4	2	34.94	2.15
13	4	-2	49.19	2.66
13	4	-2	38.49	2.12
-13	4	2	37.47	2.28
-13	-4	-2	48.40	2.24

-13	-4	-2	44.96	2.62
-13	-4	-2	37.25	2.26
-13	-4	-2	38.98	2.33
-13	-4	-2	47.94	2.33
-13	4	2	40.87	2.50
-13	-5	2	19.81	1.67
13	-5	-2	16.61	1.58
-13	-5	2	20.42	1.33
-13	-5	2	19.19	1.47
-13	5	-2	18.72	1.44
13	5	2	21.66	1.47
13	5	2	24.98	1.46
-13	5	-2	22.23	1.49
13	5	2	24.95	1.43
13	-5	-2	18.98	1.29
-13	-5	2	21.83	1.34
-13	5	-2	24.02	1.94
-13	-5	2	22.35	1.46
-13	-5	2	24.07	1.81
13	-5	-2	17.33	1.35
13	-5	-2	17.28	1.33
-13	-5	2	21.61	1.33
13	5	2	24.55	1.82
-13	-5	-2	24.64	1.45
13	5	-2	17.16	1.30
-13	-5	-2	20.81	1.02
13	-5	2	21.47	1.46
-13	-5	-2	19.83	1.01
13	5	-2	19.02	1.22
-13	5	2	21.61	1.83
-13	5	2	22.54	1.53
13	-5	2	19.52	1.40
-13	-5	-2	21.54	1.38
13	-5	2	20.40	1.41
-13	5	2	22.23	1.81
13	-6	-2	20.86	1.43
13	6	2	24.71	1.53
13	6	2	25.78	1.97
-13	-6	2	23.79	1.95
-13	-6	2	21.48	1.57
-13	-6	2	24.93	1.45
13	-6	-2	21.34	1.46
13	-6	-2	23.19	2.02
-13	-6	2	22.78	1.44

13	6	2	22.57	1.62
-13	-6	2	24.90	1.47
-13	6	-2	21.72	2.18
13	6	2	26.00	1.63
13	-6	-2	21.92	1.50
-13	6	-2	24.12	2.06
-13	-6	2	24.63	1.82
-13	-6	-2	21.43	1.56
13	-6	2	23.28	1.53
-13	6	2	22.36	2.01
13	-6	2	23.80	1.63
13	-6	2	24.69	2.16
13	6	-2	19.20	1.51
13	-6	2	25.54	1.57
-13	-6	-2	22.54	1.23
-13	6	2	22.74	2.11
-13	-6	-2	22.25	1.47
13	6	-2	23.18	1.45
13	7	2	8.32	0.97
-13	7	-2	6.52	1.25
-13	-7	2	7.77	1.43
-13	-7	2	8.67	1.52
-13	-7	2	8.51	0.90
13	7	2	6.63	1.08
-13	7	-2	5.42	1.36
-13	-7	2	7.85	0.87
13	7	2	7.39	1.09
13	-7	-2	5.04	0.86
13	-7	-2	5.99	1.31
-13	-7	2	7.40	0.63
13	-7	-2	5.88	1.03
13	7	2	5.35	1.09
-13	-7	-2	6.62	0.98
-13	-7	-2	6.17	1.02
-13	-7	-2	7.23	0.78
13	-7	2	6.89	1.04
13	-7	2	8.58	1.59
13	-7	2	8.53	0.97
13	-7	2	6.44	0.96
-13	7	2	8.86	1.57
-13	7	2	7.05	1.41
13	7	-2	7.19	1.10
13	7	-2	6.56	1.05
-13	-8	2	12.30	1.10

13	-8	-2	10.51	1.08
13	8	2	13.36	1.80
13	8	2	13.98	1.57
13	-8	-2	11.62	2.14
13	-8	-2	9.91	1.22
13	-8	-2	9.99	1.11
-13	-8	2	13.11	0.99
-13	8	-2	13.33	2.03
-13	-8	2	12.94	1.37
-13	-8	2	11.47	1.10
13	8	2	15.54	1.44
13	8	2	13.75	1.22
-13	8	-2	11.52	1.90
-13	-8	2	12.92	1.76
-13	-8	-2	12.27	1.15
13	-8	2	13.07	1.18
13	-8	2	11.92	1.24
13	8	-2	7.31	1.31
-13	-8	-2	10.94	1.04
-13	8	2	13.01	1.99
-13	-8	-2	11.73	1.38
13	-8	2	14.70	2.21
-13	8	2	11.90	1.97
13	8	-2	9.15	1.44
13	9	2	6.11	1.07
-13	-9	2	6.32	0.92
-13	-9	2	7.86	1.24
-13	9	-2	11.50	2.12
13	-9	-2	5.18	0.85
13	9	2	6.66	1.25
13	-9	-2	6.07	1.25
13	-9	-2	6.48	1.08
-13	-9	2	5.76	1.39
13	9	-2	7.23	1.24
-13	-9	-2	8.10	1.20
13	-9	2	7.21	1.10
-13	-9	-2	7.71	1.14
13	-9	2	5.35	1.02
13	-9	2	5.23	0.90
13	9	-2	6.60	1.28
-13	9	2	6.14	1.47
13	10	2	11.63	1.96
13	-10	-2	7.35	1.47
13	-10	-2	6.46	1.19

13	-10	-2	7.82	1.33
-13	-10	2	9.30	1.58
-13	10	-2	10.89	2.07
-13	-10	-2	7.78	1.24
13	10	-2	12.67	1.90
13	-10	2	5.60	1.07
13	-10	2	7.29	1.44
-13	-10	-2	13.06	2.10
-13	-10	-2	11.09	1.57
13	-10	2	4.69	0.95
-13	11	-2	5.24	1.64
13	11	2	3.30	1.32
-13	-11	2	7.82	1.43
13	-11	-2	4.69	0.95
13	-11	-2	3.13	1.04
-13	-11	2	3.92	1.30
13	-11	-2	3.06	1.16
13	-11	2	3.28	0.85
13	-11	2	3.20	1.11
13	-11	2	2.57	0.92
13	11	-2	4.84	1.40
-13	-11	-2	8.05	1.54
-13	-11	-2	6.05	1.18
-13	11	2	6.03	1.73
14	0	-2	79.03	3.62
-14	0	2	88.16	4.21
14	0	2	84.32	4.04
14	0	2	88.04	4.28
-14	0	2	85.12	4.33
-14	0	2	79.56	4.16
14	0	-2	77.22	3.83
-14	0	2	86.21	4.23
-14	0	-2	76.75	4.00
14	0	-2	76.55	3.71
-14	0	-2	75.34	3.85
14	0	-2	77.32	3.92
14	0	2	83.11	4.22
14	0	2	90.11	4.31
-14	0	2	92.01	4.28
14	0	2	90.07	4.26
-14	0	2	88.72	4.41
-14	0	-2	82.58	3.90
-14	0	-2	83.29	3.95
-14	0	-2	81.37	3.89

-14	-1	2	128.45	6.28
14	1	2	130.50	6.15
14	-1	-2	123.11	5.88
-14	1	-2	130.68	5.73
-14	1	-2	128.49	5.98
-14	1	-2	113.24	5.91
-14	-1	2	133.31	6.33
-14	1	-2	130.25	5.95
-14	-1	2	128.08	6.39
-14	-1	2	140.68	6.49
-14	1	-2	128.23	5.77
14	1	2	129.43	6.43
-14	1	-2	130.08	5.97
14	1	2	136.14	6.32
-14	-1	2	137.28	6.32
-14	1	-2	123.89	6.14
-14	-1	2	141.79	6.44
14	-1	-2	127.50	5.77
14	-1	-2	124.14	5.91
14	1	2	131.73	6.35
14	-1	-2	123.42	5.94
-14	-1	-2	122.84	5.95
-14	-1	-2	131.79	6.04
14	-1	2	129.07	6.19
14	-1	2	138.76	6.34
-14	1	2	137.07	6.37
-14	1	2	135.48	6.31
-14	1	2	130.16	6.49
-14	-1	-2	118.71	5.92
14	-1	2	131.10	6.43
14	-1	2	138.84	6.35
-14	1	2	134.72	6.52
-14	-1	-2	124.81	5.94
14	-1	2	143.69	6.42
14	1	-2	124.92	5.72
14	1	-2	125.01	5.94
14	1	-2	128.19	6.07
14	1	-2	127.07	5.66
-14	1	2	128.72	6.29
-14	-1	-2	123.86	6.02
-14	1	2	141.42	6.33
-14	-1	-2	130.86	6.07
-14	2	-2	98.66	4.70
-14	-2	2	98.60	4.60

-14	2	-2	99.36	4.80
-14	-2	2	93.20	4.58
-14	-2	2	95.38	4.61
-14	2	-2	102.63	4.80
-14	2	-2	100.33	4.77
14	-2	-2	96.27	4.63
14	-2	-2	100.62	4.73
14	2	2	94.59	4.43
14	2	2	93.31	4.79
14	-2	-2	103.12	4.79
-14	2	-2	101.47	5.02
-14	2	-2	99.05	4.62
-14	-2	2	97.61	4.76
-14	-2	2	91.60	4.64
-14	2	-2	88.12	4.72
-14	-2	2	87.63	4.55
-14	-2	2	98.60	4.79
14	-2	-2	98.50	4.77
14	2	2	96.46	4.56
14	-2	2	98.87	4.63
-14	2	2	97.39	4.83
-14	-2	-2	96.15	4.75
-14	2	2	97.00	4.41
-14	-2	-2	99.69	4.75
14	-2	2	92.19	4.76
-14	-2	-2	97.06	4.75
-14	2	2	91.39	4.49
-14	2	2	97.63	4.61
14	-2	2	101.04	4.84
-14	2	2	99.71	4.68
-14	2	2	96.82	4.50
14	2	-2	101.25	4.95
-14	-2	-2	93.71	4.77
-14	-2	-2	99.86	4.94
-14	-2	-2	100.43	4.79
14	-2	2	94.65	4.52
-14	-2	-2	102.68	4.82
14	-2	2	97.82	4.57
-14	2	2	93.77	4.84
-14	-3	2	60.92	3.19
-14	-3	2	55.66	3.01
14	3	2	58.49	3.24
-14	3	-2	55.22	2.97
-14	3	-2	50.56	2.66

-14	3	-2	54.05	2.82
-14	-3	2	59.71	3.04
-14	-3	2	56.20	2.95
14	-3	-2	53.55	2.76
14	-3	-2	51.12	2.65
-14	-3	2	60.71	3.28
-14	-3	2	57.56	3.02
-14	3	-2	50.54	2.61
14	-3	-2	53.54	2.70
-14	-3	2	58.45	2.98
-14	3	-2	53.38	3.08
-14	3	-2	49.78	2.72
-14	3	-2	55.93	2.81
14	-3	-2	58.03	2.92
14	3	2	60.95	2.98
14	3	2	57.60	2.87
-14	3	2	60.07	3.12
-14	-3	-2	51.90	2.73
14	-3	2	62.02	2.90
-14	-3	-2	52.21	2.72
-14	3	2	60.60	3.13
14	3	-2	50.90	2.50
14	3	-2	50.79	2.96
-14	3	2	59.07	2.95
-14	-3	-2	54.34	2.80
-14	-3	-2	49.13	2.76
-14	-3	-2	51.71	2.76
-14	3	2	62.11	3.05
-14	3	2	61.68	3.33
14	-3	2	63.73	3.31
14	3	-2	53.00	2.51
14	-3	2	56.41	3.01
-14	-3	-2	54.58	3.01
-14	-3	-2	55.74	2.74
14	-3	2	57.74	3.24
14	-3	2	58.28	2.96
14	-4	-2	7.34	0.86
-14	4	-2	8.22	1.33
-14	-4	2	7.54	1.29
14	-4	-2	8.79	0.85
-14	-4	2	7.36	0.79
14	-4	-2	8.75	1.22
-14	4	-2	9.50	1.02
-14	-4	2	8.95	0.89

-14	-4	2	8.34	0.98
-14	-4	2	9.06	1.26
14	-4	-2	7.28	0.81
-14	-4	2	7.62	0.88
-14	-4	2	7.61	0.87
-14	4	-2	9.62	1.45
14	4	2	8.62	0.87
14	4	2	7.99	0.78
14	4	2	7.12	1.18
-14	-4	-2	8.55	1.06
-14	-4	-2	8.30	0.91
-14	4	2	7.57	0.99
-14	-4	-2	7.73	0.93
14	-4	2	8.08	0.85
14	4	-2	8.36	0.70
-14	4	2	7.87	1.32
14	-4	2	8.26	1.46
-14	-4	-2	8.29	1.12
-14	4	2	7.75	1.27
14	-4	2	8.42	0.76
14	-4	2	6.94	0.82
-14	-4	-2	7.71	1.32
-14	-4	-2	8.91	0.95
14	4	-2	7.72	0.73
14	-4	2	8.42	0.88
-14	-4	-2	8.99	0.91
14	-5	-2	6.33	1.32
14	5	2	3.63	0.69
14	-5	-2	3.30	0.58
-14	-5	2	5.82	0.77
14	-5	-2	3.27	0.60
14	-5	-2	2.69	0.61
-14	-5	2	6.18	1.17
14	5	2	3.54	0.87
14	5	2	5.38	0.77
-14	5	-2	1.70	1.04
-14	-5	2	5.81	0.75
-14	-5	2	3.88	0.63
-14	-5	2	6.00	0.85
-14	-5	2	5.44	0.97
14	5	2	3.81	0.67
-14	5	-2	5.41	1.03
-14	5	2	6.48	1.26
-14	5	2	2.67	1.02

14	-5	2	4.11	0.73
-14	-5	-2	4.61	0.81
14	5	-2	4.95	0.77
14	5	-2	4.28	0.66
-14	-5	-2	3.49	0.63
14	-5	2	3.91	0.73
-14	5	2	3.63	0.73
14	-5	2	4.64	0.80
-14	-5	-2	5.22	0.70
-14	-5	-2	3.78	0.73
14	6	2	56.39	2.97
14	6	2	46.99	2.98
14	-6	-2	52.33	3.16
-14	-6	2	53.34	2.74
-14	6	-2	55.89	3.54
-14	-6	2	55.57	3.05
14	6	2	56.94	2.84
14	-6	-2	56.35	3.07
-14	-6	2	53.28	2.95
-14	-6	2	57.89	2.79
-14	6	-2	55.29	3.64
14	6	2	45.60	3.51
14	-6	-2	57.34	2.96
-14	-6	2	55.18	2.79
-14	-6	-2	56.56	2.98
-14	6	2	53.98	3.80
14	-6	2	55.23	2.97
-14	6	2	54.43	3.35
14	-6	2	61.81	3.48
-14	-6	-2	55.11	2.80
14	-6	2	54.39	2.91
14	6	-2	59.09	2.92
-14	-6	-2	61.52	2.66
14	-6	2	54.18	2.90
-14	-7	2	65.63	3.68
-14	-7	2	71.92	3.99
-14	-7	2	66.98	3.64
-14	-7	2	73.29	3.43
-14	-7	2	65.80	3.36
14	-7	-2	66.72	3.63
14	7	2	75.99	3.71
14	7	2	68.88	3.70
14	7	2	68.42	4.01
14	-7	-2	75.99	4.09

14	-7	-2	67.52	3.57
14	7	2	70.19	3.47
-14	-7	2	68.97	3.43
-14	7	-2	68.08	4.09
-14	7	-2	64.93	4.20
14	-7	2	77.17	4.33
14	-7	2	68.33	3.57
-14	7	2	68.34	4.13
14	-7	2	67.33	3.57
-14	-7	-2	69.03	3.66
14	7	-2	71.26	3.53
-14	7	2	67.20	4.05
-14	-7	-2	70.15	3.27
14	-7	2	60.05	4.18
14	7	-2	68.21	3.64
-14	-7	-2	65.28	3.49
14	8	2	71.11	4.21
-14	8	-2	74.64	4.41
14	-8	-2	72.03	3.70
-14	-8	2	71.54	3.55
14	8	2	79.44	3.88
14	-8	-2	69.55	3.74
-14	-8	2	71.75	3.53
-14	-8	2	73.61	4.19
-14	-8	2	70.54	3.80
14	-8	-2	70.69	3.86
-14	-8	2	67.22	3.44
14	8	2	68.33	3.58
14	8	2	71.71	3.92
-14	-8	-2	72.54	3.87
-14	8	2	70.75	4.28
14	-8	2	74.25	3.86
14	-8	2	69.96	3.73
-14	-8	-2	69.31	3.67
14	8	-2	67.94	3.91
-14	-8	-2	73.37	3.48
14	8	-2	74.01	3.77
14	-8	2	68.02	3.69
14	-9	-2	64.62	3.46
-14	9	-2	64.06	4.30
-14	-9	2	68.00	4.22
14	-9	-2	65.12	3.61
14	9	2	70.09	4.06
14	-9	-2	66.46	3.44

-14	-9	2	67.12	3.43
-14	-9	2	70.16	3.83
14	-9	2	67.99	3.68
14	-9	2	69.24	3.64
14	9	-2	57.78	3.79
-14	-9	-2	66.54	3.69
-14	-9	-2	63.69	4.04
14	-9	2	68.83	3.83
-14	9	2	68.83	4.39
-14	-9	-2	62.59	3.37
14	10	2	10.25	1.85
14	-10	-2	18.54	1.71
-14	-10	2	13.42	2.09
-14	10	-2	19.57	2.87
14	-10	-2	17.66	1.72
14	-10	-2	17.61	1.81
-14	-10	2	13.36	1.64
14	-10	2	11.36	1.35
-14	-10	-2	18.53	2.28
14	-10	2	12.63	1.65
14	-10	2	11.92	1.44
-14	-10	-2	15.63	1.58
14	10	-2	17.43	2.05
-14	10	2	15.82	2.71
-14	-10	-2	19.31	1.87
14	-11	-2	6.19	1.09
14	-11	-2	5.39	1.37
-14	11	-2	6.62	1.79
-14	-11	2	3.80	1.08
14	11	2	5.23	1.42
-14	-11	2	2.79	1.39
14	-11	-2	5.11	0.99
14	11	-2	5.86	1.48
-14	-11	-2	5.37	1.23
14	-11	2	3.75	1.31
-14	11	2	4.02	1.57
14	-11	2	6.38	1.15
-14	-11	-2	2.87	1.38
14	-11	2	3.09	0.90
15	0	-2	0.70	0.34
-15	0	-2	0.00	0.51
-15	0	-2	0.70	0.58
15	0	2	0.15	0.24
15	0	2	-0.83	0.42

-15	0	2	0.35	0.34
-15	0	-2	0.17	0.40
-15	0	2	-0.01	0.44
-15	0	-2	0.03	0.44
15	0	2	0.20	0.59
15	0	-2	-0.48	0.33
15	0	2	0.14	0.37
15	0	-2	0.15	0.19
-15	0	2	-0.42	0.54
-15	0	2	0.42	0.68
-15	0	2	0.18	0.50
-15	0	-2	0.17	0.56
-15	0	-2	-0.79	0.62
-15	0	2	0.15	0.48
-15	1	-2	1.18	0.33
-15	1	-2	0.39	0.51
-15	-1	2	1.15	0.55
-15	-1	2	1.04	0.48
-15	-1	2	1.09	0.44
15	-1	-2	0.92	0.29
15	1	2	2.00	0.51
-15	-1	2	1.15	0.51
-15	-1	2	0.42	0.70
-15	1	-2	0.35	0.48
-15	1	-2	0.83	0.44
15	1	2	1.29	0.29
15	-1	-2	2.37	0.50
15	1	2	1.34	0.59
-15	1	-2	1.31	0.64
-15	1	-2	1.53	0.60
15	-1	-2	1.69	0.44
15	1	2	1.48	0.49
-15	-1	2	2.56	0.72
15	-1	2	1.16	0.45
-15	-1	-2	0.31	0.48
-15	-1	-2	1.03	0.42
-15	1	2	0.48	0.46
-15	1	2	1.19	0.72
15	1	-2	1.68	0.46
-15	-1	-2	0.78	0.62
15	-1	2	0.99	0.30
-15	1	2	1.68	0.61
15	-1	2	2.24	0.64
-15	1	2	1.89	0.50

-15	-1	-2	1.65	0.53
-15	1	2	0.89	0.53
15	1	-2	2.60	0.64
15	-1	2	0.92	0.47
-15	-1	-2	2.68	0.72
15	-1	2	0.35	0.65
-15	-1	-2	0.96	0.56
-15	1	2	2.83	0.61
-15	1	2	1.24	0.42
-15	2	-2	16.30	1.25
-15	-2	2	20.37	1.40
15	2	2	17.18	1.07
15	-2	-2	13.86	1.11
-15	2	-2	13.81	1.10
-15	2	-2	13.65	1.00
15	2	2	20.38	1.55
-15	2	-2	13.13	1.23
15	-2	-2	16.26	1.10
-15	-2	2	17.33	1.58
-15	2	-2	16.23	1.53
-15	-2	2	21.52	1.28
15	-2	-2	13.91	0.95
-15	-2	2	18.96	1.35
-15	2	-2	17.51	1.17
-15	-2	2	21.83	1.30
-15	2	-2	12.33	1.19
-15	-2	2	22.12	1.61
-15	-2	-2	12.29	1.32
-15	-2	-2	14.71	1.13
-15	2	2	23.53	1.70
15	-2	2	15.70	1.26
-15	2	2	16.96	1.36
-15	-2	-2	12.26	1.14
-15	-2	-2	16.84	1.23
-15	-2	-2	15.99	1.14
-15	2	2	21.35	1.25
-15	2	2	20.24	1.35
-15	-2	-2	17.60	1.48
15	-2	2	16.11	1.14
15	-2	2	15.39	1.58
15	-2	2	19.58	1.29
15	-2	2	22.29	1.53
-15	-2	-2	16.49	1.28
-15	2	2	16.34	1.65

15	3	2	8.75	0.74
-15	3	-2	12.21	1.04
-15	-3	2	9.24	0.97
-15	3	-2	9.40	1.58
-15	3	-2	11.97	1.17
15	-3	-2	12.20	1.05
-15	-3	2	11.96	1.07
15	3	2	9.42	0.95
-15	-3	2	11.13	1.33
15	-3	-2	10.56	0.94
-15	3	-2	13.90	1.02
-15	-3	-2	9.93	1.20
15	-3	2	10.61	0.89
-15	-3	-2	15.23	1.11
-15	-3	-2	12.98	1.49
-15	-3	-2	13.96	1.07
15	-3	2	7.09	0.85
-15	-4	2	5.24	0.82
15	-4	-2	2.75	0.59
-15	-4	2	3.74	0.88
-15	-4	2	1.99	0.58
-15	-4	2	3.14	0.69
-15	4	-2	4.97	1.06
15	-4	-2	4.12	0.71
15	-4	-2	1.97	0.50
15	4	-2	2.84	0.58
-15	-4	-2	1.06	0.58
-15	4	2	2.03	0.66
-15	-4	-2	2.51	0.75
-15	4	2	3.87	1.06
-15	-5	2	2.46	0.64
-15	5	-2	4.83	1.37
-15	-5	2	4.91	0.79
15	-5	-2	3.68	0.77
15	5	2	5.21	0.82
-15	-5	2	1.77	0.56
-15	-5	2	3.12	0.94
15	-5	-2	4.04	0.74
15	-5	-2	4.83	1.15
-15	5	-2	9.73	1.74
-15	-5	2	1.41	0.59
15	-5	-2	4.91	0.84
-15	-5	2	3.76	0.94
15	5	2	4.73	1.00

15	5	2	3.37	0.63
15	5	-2	6.03	0.83
15	-5	2	3.04	1.12
-15	-5	-2	4.96	0.92
15	-5	2	2.33	0.63
15	-5	2	1.53	0.58
-15	5	2	4.18	1.26
15	-5	2	2.68	0.59
-15	5	2	2.95	1.04
-15	-5	-2	7.01	0.97
15	5	-2	2.65	0.55
-15	-6	2	13.97	1.17
15	-6	-2	10.37	1.13
15	6	2	12.52	1.20
-15	-6	2	10.73	1.05
15	-6	-2	9.70	1.07
-15	-6	2	13.85	1.64
-15	6	-2	12.86	1.85
-15	-6	2	13.97	1.12
15	6	2	11.09	1.60
-15	6	-2	11.81	1.94
15	6	2	13.60	1.24
-15	-6	2	14.78	1.62
15	6	2	12.40	1.23
15	-6	-2	11.81	1.09
15	-6	2	12.62	1.14
15	-6	2	11.09	1.15
-15	6	2	11.51	1.97
15	6	-2	10.15	1.12
-15	-6	-2	10.84	1.14
-15	6	2	15.79	1.78
15	-6	2	12.55	1.14
-15	-6	-2	11.63	1.21
-15	-6	-2	12.56	1.23
-15	-6	-2	12.58	1.07
15	6	-2	12.07	1.03
15	7	2	3.79	0.83
15	-7	-2	2.47	0.68
15	-7	-2	2.08	0.75
-15	-7	2	3.20	1.14
-15	-7	2	3.42	0.68
15	7	2	3.60	0.80
-15	-7	2	2.80	0.69
-15	7	-2	4.87	1.27

15	7	2	3.00	0.71
15	-7	-2	2.39	0.59
-15	-7	2	3.40	1.03
-15	7	-2	1.17	1.35
-15	-7	2	2.93	0.78
15	-7	-2	3.18	1.21
15	7	2	3.84	1.05
-15	-7	2	2.22	0.57
15	7	-2	1.89	0.78
15	-7	2	2.33	0.67
15	-7	2	3.68	0.77
15	-7	2	2.74	0.74
15	7	-2	1.74	0.63
-15	-7	-2	3.53	0.89
-15	7	2	2.87	1.40
-15	-7	-2	3.69	0.73
15	-7	2	3.29	1.39
-15	7	2	2.66	1.22
-15	8	-2	7.22	1.49
15	8	2	8.90	1.15
15	8	2	7.98	1.44
-15	-8	2	9.04	1.31
15	-8	-2	6.78	1.26
15	8	2	9.29	1.55
-15	-8	2	7.60	1.34
15	-8	-2	8.37	1.17
15	-8	-2	8.72	1.14
15	8	2	7.73	1.39
-15	-8	2	8.31	0.95
-15	-8	2	8.52	1.11
-15	-8	-2	7.54	1.29
-15	-8	-2	6.80	1.20
15	8	-2	9.25	1.27
15	-8	2	9.55	1.16
-15	8	2	11.09	2.01
15	8	-2	8.54	1.36
15	-8	2	6.49	1.13
15	-8	2	8.33	1.28
-15	9	-2	-0.55	1.49
-15	-9	2	1.43	0.90
15	-9	-2	0.41	0.83
15	-9	-2	0.27	0.74
-15	-9	2	2.08	0.66
15	-9	-2	0.25	0.64

-15	-9	2	1.23	1.19
15	9	2	1.41	1.11
-15	-9	-2	3.92	1.33
-15	9	2	3.27	1.52
15	-9	2	0.58	0.77
-15	-9	-2	1.12	0.79
-15	-9	-2	1.41	0.98
15	-9	2	1.30	0.68
15	-9	2	1.52	0.86
15	9	-2	2.05	1.14
15	10	2	10.61	1.80
-15	-10	2	7.76	1.45
15	-10	-2	12.05	1.79
15	-10	-2	11.68	1.50
-15	10	-2	12.18	2.04
-15	-10	2	11.03	1.54
15	-10	-2	9.30	1.44
15	-10	2	5.70	1.02
15	-10	2	7.55	1.18
-15	-10	-2	11.70	2.06
15	-10	2	6.72	1.34
-15	-10	-2	12.05	1.62
15	10	-2	14.65	1.91
-15	10	2	5.72	1.67
15	-11	-2	5.08	0.99
-15	11	-2	6.68	1.85
-15	-11	2	2.95	1.00
15	-11	-2	3.30	0.88
15	-11	-2	3.11	1.16
-15	-11	-2	7.24	1.98
15	-11	2	1.74	0.83
-15	-11	-2	5.05	1.55
15	-11	2	2.28	1.16
15	-11	2	2.30	0.92
-15	11	2	1.52	1.70
-16	0	-2	23.24	1.79
16	0	-2	25.66	1.38
-16	0	-2	26.61	1.74
16	0	2	30.60	1.79
16	0	-2	22.86	1.23
16	0	2	33.35	1.84
-16	0	2	31.84	1.99
-16	0	2	26.73	1.73
-16	0	2	27.42	1.84

-16	0	-2	24.81	1.78
-16	0	2	27.58	2.10
-16	0	2	30.56	1.81
-16	0	2	33.05	1.93
16	0	-2	24.27	1.50
16	0	2	31.94	1.90
-16	0	-2	25.64	1.82
-16	0	-2	22.75	1.54
-16	0	2	29.62	1.86
16	0	2	29.04	1.56
-16	0	-2	24.98	1.61
-16	1	-2	65.55	3.64
-16	-1	2	63.42	3.47
-16	1	-2	71.11	3.44
-16	-1	2	64.36	3.26
16	-1	-2	69.88	3.38
16	1	2	63.32	3.04
-16	-1	2	63.86	3.38
-16	-1	2	62.57	3.22
16	1	2	63.85	3.40
-16	1	-2	62.11	3.38
-16	-1	2	62.74	3.28
16	-1	-2	67.07	3.18
16	1	2	66.15	3.24
16	1	2	60.71	3.27
16	-1	-2	71.84	3.34
-16	1	-2	67.19	3.44
-16	-1	2	63.06	3.39
-16	1	2	62.97	3.22
-16	1	2	65.46	3.49
16	1	-2	68.28	3.07
-16	1	2	63.48	3.25
-16	-1	-2	65.24	3.53
16	-1	2	58.70	3.06
-16	-1	-2	69.94	3.58
16	-1	2	60.14	3.42
-16	1	2	59.63	3.22
16	-1	2	68.89	3.39
-16	-1	-2	62.17	3.37
-16	1	2	65.73	3.55
16	1	-2	65.34	3.13
-16	1	2	64.91	3.26
-16	-1	-2	69.71	3.56
16	-1	2	67.26	3.28

-16	1	2	73.56	4.05
16	-1	2	61.56	3.25
-16	-1	-2	66.59	3.43
16	1	-2	66.89	3.40
-16	-1	-2	63.35	3.40
-16	-2	2	61.28	3.33
-16	-2	2	60.28	3.11
-16	-2	2	62.18	3.34
16	2	2	59.33	2.87
16	2	2	61.47	3.07
-16	2	-2	68.40	3.37
-16	-2	2	56.86	3.03
16	2	2	61.42	3.30
16	-2	-2	62.98	3.40
-16	2	-2	68.95	3.74
16	-2	-2	66.44	3.45
-16	2	-2	68.46	3.33
-16	-2	2	57.59	3.16
-16	-2	2	61.44	3.10
16	-2	-2	69.03	3.39
-16	-2	2	57.02	3.05
-16	2	-2	64.88	3.41
16	-2	-2	66.71	3.25
16	-2	2	63.67	3.06
-16	2	2	61.62	3.11
-16	-2	-2	65.34	3.48
-16	2	2	58.66	3.00
16	-2	2	69.10	3.29
-16	2	2	60.29	3.37
-16	2	2	58.73	2.99
-16	-2	-2	66.54	3.42
-16	-2	-2	64.45	3.41
16	-2	2	57.41	3.10
-16	-2	-2	66.97	3.42
-16	-2	-2	70.76	3.66
16	-2	2	54.79	3.27
16	-2	2	57.62	2.95
-16	-2	-2	71.93	3.56
-16	-3	2	24.42	1.64
16	-3	-2	23.00	1.50
16	-3	-2	22.13	1.45
-16	-3	2	25.02	1.62
-16	-3	2	22.80	1.50
-16	3	-2	20.86	1.88

16	3	2	25.39	1.56
-16	3	-2	22.62	1.49
16	3	2	23.36	1.85
16	-3	-2	25.80	1.65
-16	-3	2	24.37	1.83
16	-3	-2	23.60	1.38
-16	-3	2	24.17	1.89
-16	3	-2	20.02	1.59
-16	-3	2	27.06	1.62
-16	3	-2	24.88	1.72
16	3	2	23.32	1.36
-16	-3	2	24.27	1.92
-16	-3	2	26.38	1.59
-16	3	2	23.88	1.93
-16	-3	-2	21.97	1.52
-16	-3	-2	23.37	1.55
16	3	-2	23.17	1.19
16	-3	2	26.09	1.53
16	-3	2	27.14	2.21
-16	-3	-2	24.17	1.89
-16	-3	-2	20.98	1.55
16	-3	2	23.61	1.56
16	3	-2	22.66	1.23
-16	3	2	22.63	1.38
-16	-3	-2	21.07	1.48
16	-3	2	24.43	1.46
-16	3	2	23.66	1.61
-16	-3	-2	25.79	1.73
-16	3	2	26.02	1.68
16	-4	-2	14.52	1.30
-16	-4	2	13.39	1.18
16	-4	-2	20.12	1.66
-16	4	-2	14.99	1.26
16	4	2	16.10	1.55
-16	-4	2	15.56	1.54
16	4	2	14.29	1.05
16	-4	-2	12.75	1.39
-16	4	-2	16.68	1.82
-16	-4	2	16.11	1.64
-16	-4	2	13.10	1.19
-16	-4	2	14.79	1.13
-16	4	-2	15.35	1.97
16	-4	-2	15.37	1.18
16	4	2	13.45	1.16

-16	-4	2	12.91	1.19
-16	-4	-2	15.60	1.29
16	4	-2	15.89	1.01
-16	4	2	12.36	1.68
16	-4	2	14.58	1.14
-16	4	2	16.62	1.60
16	-4	2	15.45	1.00
16	-4	2	13.49	1.13
-16	-4	-2	16.34	1.26
-16	-4	-2	16.55	1.48
16	4	-2	15.09	1.02
16	-4	2	15.76	1.72
-16	-4	-2	16.80	1.32
-16	-4	-2	15.39	1.25
16	5	2	8.74	1.04
16	-5	-2	5.96	0.87
-16	5	-2	5.36	0.89
16	-5	-2	5.57	0.79
16	5	2	7.15	1.33
-16	5	-2	5.32	1.33
-16	-5	2	8.56	1.23
-16	-5	2	6.26	1.04
-16	-5	2	7.73	0.94
16	5	2	7.64	0.91
-16	-5	2	7.91	0.95
16	-5	-2	5.46	1.14
16	-5	-2	5.52	0.82
-16	-5	2	7.70	1.02
-16	5	-2	4.68	1.17
16	5	-2	4.47	0.99
-16	5	2	8.30	1.52
16	5	-2	5.45	0.75
-16	-5	-2	4.93	0.84
-16	-5	-2	5.43	0.83
-16	-5	-2	5.56	0.98
-16	5	2	9.24	1.52
-16	-5	-2	4.80	0.95
16	-5	2	8.06	1.32
-16	-5	-2	7.49	1.27
16	-5	2	8.36	0.92
-16	-6	2	34.02	1.99
-16	-6	2	30.47	1.93
16	-6	-2	26.48	1.84
16	6	2	32.49	2.53

16	6	2	35.58	2.12
16	-6	-2	31.76	2.35
16	-6	-2	27.53	1.84
-16	6	-2	26.34	2.52
-16	-6	2	33.93	2.12
-16	-6	2	37.39	2.03
-16	-6	2	36.05	2.54
-16	6	-2	28.49	2.51
-16	-6	2	36.04	2.42
16	6	2	32.99	2.09
-16	-6	-2	26.98	1.84
-16	6	2	36.79	2.71
-16	-6	-2	25.65	1.59
16	-6	2	34.23	2.08
-16	-6	-2	28.01	1.95
16	-6	2	33.60	2.06
16	6	-2	29.68	1.70
-16	-6	-2	27.71	1.83
16	6	-2	29.44	1.81
16	-6	2	32.58	2.08
-16	6	2	33.00	2.67
-16	-6	-2	29.69	2.01
16	-7	-2	61.56	3.41
-16	-7	2	66.63	3.20
16	-7	-2	58.98	3.34
16	-7	-2	61.37	3.29
-16	-7	2	56.31	3.07
16	7	2	63.50	3.40
-16	-7	2	63.03	3.42
16	7	2	63.77	3.38
16	7	2	62.23	3.80
-16	7	-2	64.39	3.98
-16	-7	2	58.44	3.17
-16	-7	2	63.09	3.79
-16	7	2	59.67	3.88
16	-7	2	59.93	3.36
16	-7	2	63.09	3.32
-16	-7	-2	64.78	3.49
-16	-7	-2	61.54	3.31
16	7	-2	63.40	3.40
16	7	-2	63.92	3.27
16	-7	2	60.82	3.31
16	8	2	68.78	4.37
16	-8	-2	67.28	3.58

16	-8	-2	63.40	3.52
16	-8	-2	67.91	3.67
-16	8	-2	69.74	4.45
16	8	2	75.11	3.85
-16	-8	2	74.26	3.89
-16	-8	2	67.23	3.62
16	8	2	72.01	4.00
-16	-8	2	73.69	4.37
16	-8	2	68.19	3.75
16	-8	2	72.03	3.77
16	8	-2	62.18	3.74
16	8	-2	67.12	3.56
16	-8	2	68.12	3.87
-16	8	2	73.72	4.57
-16	-8	-2	68.40	3.79
-16	-8	-2	67.64	3.52
-16	9	-2	31.29	3.24
-16	-9	2	33.09	2.84
16	-9	-2	35.21	2.47
16	-9	-2	34.58	2.35
16	-9	-2	37.37	2.32
-16	-9	2	33.80	2.44
16	9	2	35.54	2.55
-16	-9	-2	33.43	2.20
-16	-9	-2	36.25	2.90
-16	-9	-2	32.69	2.46
-16	9	2	34.13	3.20
16	9	-2	35.40	2.58
16	-9	2	32.70	2.27
16	-9	2	32.20	2.27
16	-9	2	34.92	2.45
16	-10	-2	22.45	1.84
-16	-10	2	20.54	1.96
16	-10	-2	23.09	1.85
-16	10	-2	20.80	2.90
16	-10	-2	25.82	2.22
16	10	2	24.63	2.23
-16	-10	2	19.04	2.42
16	-10	2	22.29	1.82
16	-10	2	21.29	2.12
16	-10	2	20.91	1.84
-16	10	2	23.15	2.77
16	10	-2	19.98	2.23
-16	-10	-2	21.68	2.11

-16	-10	-2	25.17	2.53
17	0	2	0.00	0.57
-17	0	2	0.51	0.64
-17	0	-2	0.27	0.77
-17	0	2	-0.15	0.78
17	0	-2	-0.24	0.40
-17	0	-2	0.43	0.43
17	0	-2	0.44	0.37
-17	0	-2	-0.07	0.68
-17	0	2	-0.57	0.50
-17	0	2	0.00	0.43
17	0	2	-0.54	0.56
17	0	2	-0.25	0.40
-17	0	2	-0.37	0.52
-17	0	-2	-0.02	0.67
17	0	2	0.29	0.27
17	0	-2	-0.07	0.21
-17	0	2	0.04	0.67
-17	-1	2	5.33	0.95
17	1	2	5.23	0.96
17	1	2	5.41	0.56
-17	-1	2	7.02	1.06
-17	1	-2	2.77	0.58
-17	-1	2	5.37	0.96
-17	-1	2	5.94	1.09
-17	-1	2	4.89	1.03
-17	1	-2	4.44	0.92
17	-1	-2	5.08	0.74
-17	1	-2	4.85	0.80
17	1	2	6.07	0.91
-17	-1	2	6.38	0.84
17	-1	-2	3.50	0.48
17	1	2	6.43	0.87
17	-1	-2	3.88	0.63
-17	1	2	5.69	0.80
17	-1	2	5.18	0.59
-17	-1	-2	4.26	0.91
-17	1	2	5.49	0.94
17	1	-2	4.58	0.52
-17	-1	-2	3.62	0.62
17	-1	2	4.96	0.92
-17	-1	-2	3.63	0.83
17	1	-2	3.56	0.36
17	-1	2	5.19	0.98

-17	1	2	6.32	0.90
-17	1	2	6.45	1.03
17	-1	2	4.68	0.83
17	1	-2	3.18	0.61
-17	-1	-2	5.21	0.98
-17	1	2	4.44	0.92
17	2	2	17.11	1.29
-17	-2	2	16.27	1.25
17	2	2	17.30	1.57
-17	-2	2	17.75	1.72
-17	-2	2	22.29	1.62
-17	-2	2	18.16	1.45
-17	2	-2	21.20	1.52
-17	2	-2	21.32	1.36
17	-2	-2	20.63	1.29
17	-2	-2	26.31	1.55
-17	-2	2	16.51	1.36
17	2	2	16.05	0.99
17	-2	-2	20.93	1.43
-17	-2	2	15.54	1.22
-17	2	-2	20.59	1.50
-17	2	-2	21.79	1.90
-17	-2	2	16.33	1.67
17	-2	2	13.68	1.21
-17	-2	-2	21.77	1.66
-17	-2	-2	20.43	1.83
-17	2	2	18.76	1.27
17	-2	2	15.52	1.10
-17	-2	-2	23.05	1.48
-17	2	2	15.23	1.26
-17	2	2	15.49	1.28
-17	-2	-2	21.50	1.78
17	-2	2	18.02	1.29
-17	2	2	18.76	1.67
-17	2	2	16.99	1.31
17	-2	2	19.99	1.60
-17	-2	-2	23.20	1.52
17	-3	-2	13.73	1.33
-17	-3	2	15.76	1.42
-17	-3	2	15.75	1.27
17	3	2	21.87	1.73
-17	3	-2	10.73	1.14
17	3	2	18.07	1.12
17	-3	-2	8.97	0.98

-17	-3	2	17.44	1.65
-17	-3	2	18.19	1.33
17	3	2	19.33	1.31
-17	-3	2	15.54	1.38
-17	-3	2	15.64	1.76
-17	3	-2	10.45	1.32
-17	3	-2	9.44	1.61
-17	-3	-2	9.76	1.40
-17	-3	-2	10.77	1.20
-17	-3	-2	10.68	1.06
-17	3	2	19.96	1.14
17	-3	2	20.86	1.66
-17	3	2	17.97	1.29
17	-3	2	20.48	1.33
17	-3	2	16.56	1.32
-17	-3	-2	9.56	1.08
-17	3	2	18.58	1.83
-17	3	2	14.94	1.17
-17	-4	2	78.80	4.18
-17	4	-2	70.51	3.75
17	-4	-2	72.93	3.78
17	4	2	71.40	3.67
-17	-4	2	74.78	3.87
17	4	2	75.55	3.79
-17	-4	2	72.23	3.82
-17	-4	2	74.19	3.79
17	4	2	78.76	4.24
-17	4	-2	76.14	4.22
17	-4	-2	86.11	4.00
-17	-4	2	78.86	4.10
-17	4	-2	74.36	4.05
17	-4	-2	73.72	3.69
-17	-4	2	73.86	3.81
17	-4	-2	74.72	3.72
-17	4	-2	68.64	3.55
-17	-4	-2	72.71	3.84
17	4	-2	77.37	3.52
-17	-4	-2	70.87	3.74
17	-4	2	72.83	3.78
17	-4	2	80.91	3.64
-17	4	2	73.51	3.97
17	4	-2	74.84	3.52
17	-4	2	80.63	4.28
-17	-4	-2	69.72	3.71

-17	-4	-2	73.29	3.83
-17	4	2	77.79	4.26
-17	5	-2	23.85	1.67
17	5	2	19.00	1.37
-17	-5	2	19.48	1.86
17	5	2	21.47	1.93
-17	-5	2	20.03	1.51
-17	-5	2	17.22	1.41
-17	5	-2	22.74	2.48
17	-5	-2	24.46	1.70
-17	-5	2	19.14	1.43
-17	-5	2	21.67	2.01
-17	-5	2	19.06	1.50
17	-5	-2	24.00	2.12
17	-5	-2	21.50	1.59
17	-5	-2	23.28	1.63
17	5	2	19.38	1.46
-17	5	-2	25.76	2.33
-17	-5	-2	25.70	1.87
-17	-5	-2	24.56	1.58
17	-5	2	19.66	1.45
17	-5	2	24.94	2.34
-17	5	2	19.87	2.10
-17	-5	-2	25.40	1.66
17	-5	2	19.79	1.44
-17	-5	-2	23.59	1.71
17	5	-2	23.85	1.48
-17	5	2	19.98	2.03
17	5	-2	25.74	1.48
17	-6	-2	0.57	0.59
17	-6	-2	0.40	1.16
17	6	2	3.57	0.79
17	6	2	4.12	0.81
-17	-6	2	3.77	0.72
-17	-6	2	3.55	0.81
-17	6	-2	0.29	1.13
-17	-6	2	3.05	0.69
-17	-6	2	3.46	0.77
-17	-6	2	2.82	1.12
17	-6	-2	1.31	0.64
17	-6	-2	0.10	0.50
-17	-6	2	3.18	1.06
17	6	2	4.32	1.18
-17	6	-2	-0.62	0.52

-17	6	2	3.57	1.23
17	6	-2	0.79	0.59
-17	-6	-2	1.30	0.65
17	-6	2	3.41	0.76
17	-6	2	4.68	0.79
-17	-6	-2	0.79	0.61
17	-6	2	4.78	1.27
-17	-6	-2	1.08	0.63
17	6	-2	0.86	0.62
-17	-7	2	2.02	0.62
17	7	2	1.87	0.84
-17	-7	2	3.08	0.86
-17	-7	2	2.08	0.68
17	-7	-2	1.82	0.76
17	-7	-2	2.07	0.70
17	-7	-2	3.04	0.78
17	7	2	2.78	0.87
-17	7	-2	-0.14	1.20
-17	-7	2	1.73	1.10
17	7	2	1.39	1.10
17	7	-2	3.32	0.84
-17	-7	-2	1.59	0.68
17	-7	2	1.49	0.76
-17	7	2	1.57	1.21
-17	-7	-2	2.50	0.78
17	-7	2	2.28	0.73
17	7	-2	2.35	0.78
17	8	2	0.82	0.91
-17	8	-2	0.10	1.41
17	8	2	0.90	1.29
17	-8	-2	-0.66	0.59
17	-8	-2	0.11	0.81
-17	-8	2	0.99	0.72
-17	-8	2	2.25	1.34
17	-8	-2	0.03	0.75
-17	-8	2	0.82	0.83
-17	-8	-2	2.68	1.06
17	8	-2	2.01	1.03
-17	8	2	2.60	1.54
17	8	-2	0.27	0.77
17	-8	2	0.22	0.78
-17	-8	-2	1.84	0.80
17	-8	2	0.68	0.81
17	-8	2	0.33	0.84

17	9	2	12.56	1.69
17	-9	-2	8.57	1.46
-17	-9	2	13.34	1.64
17	-9	-2	9.75	1.52
17	-9	-2	7.97	1.38
-17	9	-2	9.10	1.78
-17	-9	2	11.54	2.07
17	9	-2	9.55	1.61
-17	-9	-2	11.14	2.01
-17	9	2	15.48	2.48
17	-9	2	12.15	1.69
-17	-9	-2	8.26	1.53
-17	-9	-2	10.63	1.50
17	-9	2	12.40	1.47
17	-9	2	8.49	1.43
17	-10	-2	29.42	2.26
-17	-10	2	29.74	2.77
17	10	2	31.62	2.56
17	-10	-2	29.02	2.08
-17	10	-2	30.56	3.19
-17	-10	2	29.34	2.30
17	-10	-2	28.39	2.10
17	-10	2	31.34	2.35
17	-10	2	26.26	2.14
17	10	-2	30.84	2.50
-17	-10	-2	26.97	2.26
-17	10	2	30.65	3.19
17	-10	2	31.78	2.14
-17	-10	-2	30.53	2.71
-18	0	-2	21.82	1.78
18	0	-2	22.65	1.21
-18	0	-2	22.56	1.58
-18	0	2	22.70	1.58
18	0	-2	23.73	1.56
18	0	2	26.70	1.67
-18	0	2	24.54	1.73
-18	0	-2	20.40	1.94
18	0	2	22.17	1.40
18	0	-2	26.30	1.39
-18	0	-2	24.86	1.92
-18	0	2	24.31	1.81
-18	0	2	22.68	1.87
18	0	2	28.09	1.81
18	-1	-2	14.26	1.19

-18	-1	2	24.07	1.61
-18	-1	2	25.83	1.62
-18	1	-2	14.76	1.41
-18	-1	2	24.39	2.10
-18	-1	2	23.66	1.87
-18	-1	2	25.68	1.95
18	1	2	28.03	1.67
18	1	2	27.18	1.90
18	-1	-2	17.16	1.18
-18	1	-2	13.12	1.62
18	1	2	24.37	1.39
18	1	2	25.97	1.73
-18	-1	2	26.27	1.83
18	-1	-2	14.61	0.95
18	-1	2	28.41	1.81
18	1	-2	14.67	0.90
18	-1	2	25.95	1.70
-18	1	2	24.63	1.74
18	-1	2	25.24	1.71
-18	1	2	23.29	1.65
-18	-1	-2	14.94	1.59
18	1	-2	16.26	0.82
-18	-1	-2	14.88	1.27
-18	-1	-2	17.66	1.70
18	1	-2	14.74	1.22
-18	-1	-2	13.65	1.50
-18	1	2	26.34	1.67
-18	1	2	24.02	1.96
18	-2	-2	24.43	1.54
18	2	2	30.24	2.10
18	2	2	29.21	1.78
-18	2	-2	22.34	1.61
-18	-2	2	27.97	1.97
-18	-2	2	28.41	2.25
18	-2	-2	26.21	1.61
-18	-2	2	28.84	1.76
-18	-2	2	31.01	2.04
-18	-2	2	28.39	1.77
18	-2	-2	20.76	1.31
-18	2	-2	21.31	2.03
-18	2	-2	20.31	1.55
18	2	2	25.56	1.66
-18	-2	2	26.38	2.12
-18	-2	-2	22.62	1.58

-18	2	2	29.14	1.70
-18	2	2	28.39	1.77
-18	2	2	28.82	2.23
18	-2	2	29.85	2.07
-18	-2	-2	23.41	1.90
18	-2	2	29.05	1.82
-18	-2	-2	22.91	1.77
-18	-2	-2	23.01	1.77
18	-2	2	27.30	1.60
18	-2	2	32.64	1.83
-18	2	2	28.02	1.79
-18	-3	2	13.98	1.50
-18	3	-2	16.09	1.31
18	3	2	14.03	1.61
-18	3	-2	14.62	1.49
18	-3	-2	15.90	1.12
-18	-3	2	13.04	1.15
18	3	2	13.35	0.95
-18	3	-2	14.94	1.77
-18	3	-2	14.52	1.29
-18	-3	2	13.76	1.35
18	-3	-2	16.40	1.50
-18	-3	2	13.90	1.75
18	-3	-2	12.96	1.24
18	-3	-2	16.92	1.23
-18	-3	2	12.91	1.22
-18	3	2	14.63	1.00
-18	-3	-2	14.33	1.26
18	-3	2	14.04	1.07
-18	-3	-2	14.35	1.44
18	-3	2	15.02	1.17
-18	-3	-2	17.80	1.69
-18	3	2	12.29	1.07
18	-3	2	14.59	1.59
-18	-3	-2	14.44	1.28
18	-3	2	12.85	1.34
-18	3	2	14.31	1.70
18	-4	-2	2.02	0.56
-18	4	-2	1.91	0.64
-18	-4	2	1.35	0.67
18	-4	-2	2.31	0.63
18	4	2	2.05	0.94
18	-4	-2	3.01	0.86
18	4	2	1.76	0.64

-18	-4	2	0.87	1.02
-18	-4	2	1.20	0.57
-18	4	-2	3.05	1.07
-18	-4	2	1.48	0.59
-18	-4	2	0.70	0.86
18	4	2	1.70	0.51
18	-4	-2	1.88	0.61
-18	-4	-2	2.31	0.71
-18	4	2	1.89	0.96
-18	4	2	0.17	0.96
-18	-4	-2	1.82	0.67
-18	-4	-2	1.52	0.94
18	4	-2	2.49	0.47
18	-4	2	1.41	0.56
18	-4	2	1.60	0.55
-18	-4	-2	3.14	0.71
18	4	-2	1.75	0.42
18	-5	-2	20.31	1.46
18	-5	-2	20.23	2.25
18	5	2	24.17	1.55
18	-5	-2	18.80	1.45
18	5	2	23.87	1.66
-18	5	-2	23.68	2.31
18	-5	-2	19.69	1.55
-18	-5	2	21.49	1.66
-18	-5	2	20.50	2.16
18	5	2	23.63	2.14
-18	5	-2	25.96	2.30
-18	-5	2	20.99	1.57
-18	5	-2	19.87	1.54
-18	-5	2	21.82	2.08
-18	-5	2	21.39	1.63
18	5	-2	20.30	1.35
18	-5	2	22.99	1.65
18	-5	2	23.87	2.27
-18	5	2	24.09	2.30
18	5	-2	20.94	1.33
18	-5	2	22.60	1.58
-18	-5	-2	18.88	1.76
-18	-5	-2	20.97	1.58
-18	-5	-2	17.29	1.50
-18	-5	-2	20.59	1.65
-18	5	2	23.16	2.18
-18	-6	2	29.70	2.39

-18	-6	2	23.65	1.72
18	6	2	25.83	1.76
-18	6	-2	29.60	2.62
-18	-6	2	23.74	1.70
18	-6	-2	25.02	1.77
18	6	2	30.47	2.44
-18	-6	2	24.42	1.88
18	-6	-2	23.36	1.79
-18	6	-2	25.89	1.82
18	-6	-2	23.40	1.72
18	6	2	28.67	1.90
-18	-6	-2	25.25	1.83
18	6	-2	24.64	1.64
-18	-6	-2	25.30	1.68
-18	6	2	26.34	2.50
18	6	-2	25.91	1.73
-18	-6	-2	25.35	1.88
18	-6	2	24.47	1.80
18	-6	2	25.22	1.81
-18	7	-2	15.64	2.65
-18	7	-2	13.17	1.53
18	-7	-2	12.80	1.47
18	7	2	14.34	1.59
18	-7	-2	15.58	1.53
18	7	2	14.26	1.52
-18	-7	2	11.98	1.46
-18	-7	2	8.80	1.61
-18	-7	2	10.95	1.82
18	7	2	18.18	2.31
18	-7	-2	13.75	1.36
-18	-7	2	11.85	1.32
-18	-7	-2	15.18	1.69
18	7	-2	16.31	1.41
18	-7	2	10.39	1.48
-18	-7	-2	15.84	1.68
18	7	-2	14.76	1.61
-18	7	2	10.61	1.88
18	-7	2	9.97	1.33
-18	8	-2	33.79	3.17
-18	8	-2	33.76	2.34
-18	-8	2	32.95	2.25
18	8	2	40.06	2.53
-18	-8	2	38.78	2.59
18	-8	-2	30.34	2.33

18	-8	-2	32.60	2.26
-18	-8	2	42.68	3.09
18	-8	-2	30.23	2.24
18	8	-2	37.08	2.54
-18	-8	-2	34.95	2.44
-18	-8	-2	28.72	2.48
18	8	-2	37.65	2.41
18	-8	2	36.37	2.58
-18	-8	-2	40.92	2.96
-18	8	2	37.68	3.23
18	-8	2	34.68	2.39
-18	-9	2	47.82	2.88
18	-9	-2	45.28	2.83
18	9	2	39.08	2.88
-18	9	-2	46.71	3.69
-18	-9	2	45.68	3.38
-18	9	-2	41.00	2.71
18	-9	-2	41.58	2.69
18	-9	-2	46.50	2.73
18	-9	2	47.09	2.74
-18	9	2	49.11	3.70
18	9	-2	46.36	2.96
-18	-9	-2	46.67	2.93
-18	-9	-2	44.00	2.68
-18	-9	-2	46.10	3.37
18	-9	2	45.88	2.93
19	0	2	0.58	0.62
-19	0	2	0.32	0.50
-19	0	-2	0.12	0.82
-19	0	-2	-0.17	0.77
19	0	2	0.29	0.32
19	0	2	0.18	0.64
-19	0	2	-0.04	0.62
-19	0	-2	0.51	0.51
-19	0	2	-0.26	0.77
19	0	2	-0.12	0.49
-19	0	2	-0.77	0.67
19	0	-2	0.32	0.28
19	0	-2	0.13	0.52
19	0	-2	0.01	0.42
19	1	2	9.84	0.76
-19	1	-2	9.57	1.61
19	1	2	10.17	1.13
-19	1	-2	12.66	1.17

19	-1	-2	10.94	1.10
-19	-1	2	7.01	1.32
-19	-1	2	10.91	1.51
-19	-1	2	10.05	1.05
-19	-1	2	12.87	1.35
19	1	2	7.73	1.08
19	-1	-2	12.08	1.01
19	1	2	10.47	1.38
19	-1	-2	10.68	0.77
-19	1	-2	8.77	1.35
-19	-1	2	8.77	1.25
-19	1	2	8.09	1.62
-19	1	2	9.53	1.06
-19	-1	-2	8.85	1.06
-19	1	2	9.28	1.08
-19	1	2	8.46	1.30
-19	-1	-2	10.34	1.46
19	-1	2	9.05	0.80
19	-1	2	10.59	1.09
-19	-1	-2	9.55	1.64
19	-1	2	10.32	1.25
19	-1	2	9.49	1.24
-19	-1	-2	10.22	1.55
19	1	-2	10.06	0.74
-19	-2	2	47.69	3.13
19	2	2	51.78	2.66
19	-2	-2	50.09	2.51
-19	2	-2	48.72	2.76
19	-2	-2	52.87	2.73
-19	-2	2	48.71	2.91
-19	-2	2	47.27	2.72
-19	-2	2	46.90	2.68
19	2	2	49.18	2.97
-19	2	-2	47.00	3.34
19	-2	-2	51.64	2.73
-19	-2	2	50.37	2.89
19	2	2	47.74	2.43
-19	2	-2	47.52	2.73
-19	2	2	45.96	2.66
-19	-2	-2	49.98	2.90
-19	2	2	50.49	2.74
-19	-2	-2	50.59	2.74
-19	-2	-2	46.63	3.20
19	-2	2	49.45	2.54

19	-2	2	49.13	2.73
-19	-2	-2	49.62	2.77
-19	2	2	46.98	3.08
-19	2	2	47.84	2.61
19	-2	2	53.81	2.73
19	-2	2	56.76	2.92
-19	3	-2	107.99	5.37
-19	-3	2	106.84	5.34
-19	-3	2	101.79	5.20
-19	3	-2	112.81	5.55
-19	3	-2	107.91	5.39
-19	-3	2	109.27	5.52
-19	-3	2	105.17	5.25
19	3	2	105.16	5.01
19	-3	-2	118.28	5.39
-19	3	-2	116.20	5.99
19	3	2	108.52	5.17
-19	-3	2	101.61	5.50
19	-3	-2	118.09	5.59
-19	-3	-2	118.37	5.57
-19	-3	-2	111.19	5.58
19	-3	2	114.35	5.16
19	-3	2	104.72	5.13
-19	-3	-2	115.58	5.68
-19	3	2	103.90	5.27
19	-3	2	112.71	5.54
-19	3	2	112.28	5.70
-19	-3	-2	103.67	5.57
19	-3	2	105.31	5.27
-19	-4	2	63.01	3.50
-19	-4	2	73.33	3.88
19	-4	-2	67.52	3.43
19	4	2	67.23	3.85
-19	4	-2	62.45	3.66
-19	-4	2	64.82	3.42
19	-4	-2	69.96	3.50
-19	4	-2	63.19	3.54
19	4	2	64.89	3.28
19	4	2	66.57	3.40
19	-4	-2	67.71	3.58
-19	-4	2	66.61	3.51
19	-4	-2	75.00	3.83
-19	4	-2	68.24	4.12
-19	-4	2	62.73	3.82

-19	-4	-2	63.03	3.58
-19	-4	-2	70.90	3.72
-19	-4	-2	64.70	3.52
19	-4	2	66.91	3.42
-19	4	2	63.95	3.99
19	-4	2	68.83	3.77
19	-4	2	68.85	3.53
-19	-4	-2	67.96	3.60
-19	-5	2	23.45	1.71
-19	5	-2	31.44	2.73
-19	5	-2	29.08	1.93
19	-5	-2	29.71	1.88
19	-5	-2	32.89	2.18
-19	-5	2	24.68	1.86
-19	-5	2	25.07	1.83
19	-5	-2	31.79	1.87
-19	-5	2	28.24	2.32
19	5	2	23.36	2.34
19	-5	-2	27.69	1.92
19	5	2	26.52	1.69
19	5	2	26.03	1.80
19	5	-2	27.66	1.65
19	-5	2	30.19	2.66
-19	-5	-2	27.64	2.01
19	-5	2	27.45	1.78
-19	5	2	27.60	2.46
-19	-5	-2	24.63	1.87
-19	-5	-2	26.79	1.90
19	-5	2	26.66	1.84
19	5	-2	28.79	1.67
-19	6	-2	8.95	1.39
19	6	2	13.60	1.38
19	-6	-2	10.32	1.38
-19	-6	2	10.15	1.36
-19	6	-2	10.34	1.92
19	6	2	11.55	2.10
-19	-6	2	10.83	1.95
-19	-6	2	10.04	1.29
19	-6	-2	9.35	1.31
-19	-6	2	9.88	1.18
19	6	2	10.28	1.35
19	-6	-2	9.21	1.18
19	-6	2	10.13	1.24
-19	6	2	9.94	1.88

-19	-6	-2	11.52	1.47
19	6	-2	10.90	1.15
19	6	-2	9.87	1.18
19	-6	2	10.19	1.30
-19	-6	-2	9.95	1.30
19	-7	-2	7.20	1.17
19	7	2	2.39	1.38
-19	-7	2	5.21	1.06
-19	7	-2	2.55	1.53
-19	-7	2	4.68	1.42
-19	-7	2	5.73	0.91
-19	7	-2	3.50	0.96
19	-7	-2	4.34	0.94
19	7	2	3.68	0.97
19	-7	-2	4.77	0.96
19	7	2	6.94	1.37
19	7	-2	4.32	0.91
19	-7	2	5.60	1.09
-19	7	2	4.16	1.51
-19	-7	-2	4.77	1.05
19	-7	2	4.41	0.99
-19	-7	-2	4.32	0.92
19	7	-2	4.97	1.01
-19	8	-2	4.70	0.96
19	-8	-2	7.07	1.11
-19	-8	2	4.24	1.04
-19	-8	2	5.54	1.44
-19	8	-2	4.51	1.63
19	-8	-2	5.57	1.03
19	8	2	3.35	1.02
19	-8	-2	3.03	0.88
-19	-8	2	5.47	1.35
-19	8	2	3.90	1.48
19	-8	2	6.01	1.23
19	8	-2	4.67	1.02
19	-8	2	4.41	0.97
-19	-8	-2	6.08	1.12
-19	-8	-2	5.49	1.06
-19	-8	-2	5.99	1.44
19	-9	-2	24.85	2.09
-19	9	-2	28.56	2.02
19	-9	-2	24.83	1.97
19	-9	-2	24.21	1.91
-19	-9	2	24.86	2.59

19	9	2	23.25	2.26
-19	-9	2	25.08	2.13
19	9	-2	22.69	2.12
-19	9	2	23.70	2.94
-19	-9	-2	23.93	2.12
-19	-9	-2	27.91	2.86
19	-9	2	28.56	2.02
19	-9	2	27.26	2.13
-20	0	-2	0.07	0.56
-20	0	-2	1.22	0.93
20	0	2	0.79	0.37
20	0	-2	-0.19	0.56
20	0	-2	0.88	0.31
-20	0	2	1.93	0.75
20	0	-2	1.82	0.48
-20	0	-2	1.29	0.98
20	0	2	3.41	0.86
20	0	2	3.06	0.74
20	0	2	0.50	0.62
-20	0	2	1.17	0.58
-20	0	2	0.35	0.91
-20	-1	2	12.74	1.48
20	1	2	9.96	0.83
-20	-1	2	10.41	1.08
20	-1	-2	11.19	1.02
20	1	2	9.93	1.22
-20	-1	2	11.40	1.54
20	-1	-2	7.88	0.75
-20	-1	2	7.43	1.09
-20	1	-2	9.52	1.60
-20	1	-2	8.59	1.17
-20	1	-2	8.90	1.41
20	-1	-2	8.19	1.09
-20	-1	2	9.05	1.61
20	1	2	13.48	1.22
20	-1	2	9.30	0.86
20	-1	2	8.15	1.15
-20	1	2	12.14	1.25
-20	1	2	9.96	1.57
-20	-1	-2	11.13	1.54
20	-1	2	13.71	1.33
-20	-1	-2	8.01	1.13
-20	1	2	8.74	1.17
20	-1	2	13.95	1.33

-20	1	2	7.86	1.40
-20	2	-2	0.17	0.58
20	2	2	1.59	0.94
-20	-2	2	2.12	0.62
-20	-2	2	0.39	0.56
20	-2	-2	0.45	0.55
20	-2	-2	1.75	0.73
-20	2	-2	0.30	1.00
20	2	2	1.01	0.38
-20	-2	2	1.34	0.85
-20	-2	2	3.16	0.86
-20	2	-2	0.27	0.80
20	-2	-2	0.28	0.38
20	-2	2	2.84	0.77
-20	-2	-2	0.92	0.83
-20	-2	-2	1.28	1.07
20	-2	2	1.50	0.69
20	-2	2	0.72	0.44
-20	2	2	0.70	0.70
20	-2	2	3.50	0.89
-20	-2	-2	0.69	0.60
-20	2	2	0.83	1.01
-20	2	2	2.17	0.68
-20	-2	-2	0.16	0.57
-20	2	2	0.60	0.61
-20	-3	2	5.78	1.12
20	-3	-2	6.76	1.23
20	-3	-2	5.28	0.83
-20	-3	2	5.66	0.88
20	-3	-2	4.99	0.81
-20	3	-2	7.90	1.50
-20	3	-2	4.69	0.83
20	3	2	6.17	0.77
-20	-3	2	6.32	1.09
-20	-3	2	6.48	1.07
20	3	2	9.31	1.51
20	3	2	6.53	1.12
-20	3	2	5.88	1.31
20	-3	2	7.17	1.10
-20	-3	-2	6.09	1.11
-20	-3	-2	6.56	1.27
-20	3	2	5.55	0.97
20	-3	2	7.41	1.10
20	-3	2	7.91	1.45

20	-3	2	5.19	0.84
-20	-3	-2	9.35	1.35
-20	-3	-2	7.10	1.11
-20	4	-2	3.46	0.80
20	-4	-2	3.55	0.67
-20	-4	2	2.25	0.85
-20	-4	2	3.64	1.31
-20	4	-2	4.37	1.33
-20	-4	2	4.66	1.25
20	-4	-2	2.68	0.97
20	-4	-2	3.80	0.74
-20	4	-2	2.82	1.05
-20	-4	2	5.40	1.19
20	4	2	5.09	0.85
20	4	2	2.18	1.17
-20	-4	2	5.85	0.95
20	-4	2	4.18	0.85
-20	-4	-2	4.61	0.83
-20	4	2	5.32	0.93
-20	-4	-2	3.98	0.87
-20	-4	-2	4.82	0.94
20	-4	2	5.05	0.85
20	-4	2	6.14	1.27
-20	4	2	3.56	1.28
-20	-5	2	2.08	0.83
20	5	2	2.78	1.16
20	-5	-2	5.29	1.05
-20	-5	2	3.95	1.22
20	5	2	2.46	0.79
20	5	2	2.64	0.71
-20	5	-2	3.22	0.89
20	-5	-2	2.18	0.85
-20	5	-2	4.31	1.56
20	-5	-2	2.32	0.70
-20	-5	2	0.46	0.74
-20	-5	-2	3.12	0.83
20	5	-2	3.29	0.60
20	-5	2	3.33	0.80
20	-5	2	3.70	0.78
-20	-5	-2	3.28	0.84
-20	-5	-2	4.02	0.89
20	5	-2	3.87	0.69
-20	5	2	3.57	1.41
20	-6	-2	1.20	0.79

-20	-6	2	0.04	0.85
-20	-6	2	1.59	0.77
-20	6	-2	1.87	0.81
20	-6	-2	0.58	0.70
-20	-6	2	1.69	1.44
20	-6	-2	0.54	0.63
-20	-6	2	3.09	1.38
-20	6	-2	3.69	1.51
20	6	2	1.84	0.89
20	6	2	2.44	1.50
20	6	2	-0.02	0.84
-20	6	2	-0.67	1.21
20	-6	2	0.71	0.71
20	-6	2	0.09	0.80
-20	-6	-2	0.66	0.79
20	6	-2	1.26	0.79
20	6	-2	1.46	0.68
-20	-6	-2	2.25	0.96
-20	7	-2	0.85	1.41
20	7	2	1.05	1.25
-20	-7	2	0.83	0.93
20	-7	-2	0.81	0.79
-20	7	-2	1.42	0.75
20	-7	-2	0.56	0.76
20	-7	-2	1.15	0.73
-20	-7	2	3.48	1.32
20	7	2	3.00	0.85
-20	-7	-2	1.00	0.83
20	-7	2	0.51	0.77
-20	-7	-2	1.25	0.89
20	-7	2	0.93	0.76
-20	7	2	0.60	1.34
20	7	-2	0.14	0.83
20	7	-2	3.34	0.88
20	8	2	7.53	1.54
20	-8	-2	7.37	1.29
20	-8	-2	4.38	1.03
-20	-8	2	6.93	1.58
-20	8	-2	5.89	0.98
-20	8	-2	2.16	1.63
-20	-8	2	5.97	1.11
20	-8	-2	7.22	1.33
-20	-8	-2	6.64	1.24
20	8	-2	9.18	1.51

-20	-8	-2	4.87	1.45
-20	8	2	4.60	1.67
20	-8	2	7.20	1.24
-20	-8	-2	6.23	1.14
20	-8	2	5.49	1.02
-21	0	-2	-0.51	0.59
21	0	2	0.19	0.40
-21	0	-2	0.11	1.12
21	0	2	-0.10	0.61
-21	0	2	-1.27	0.74
21	0	2	0.16	0.81
-21	0	2	0.95	0.97
21	0	-2	-0.22	0.30
-21	0	2	0.16	0.97
-21	0	-2	0.18	1.03
21	0	2	1.53	0.73
21	0	-2	-0.13	0.45
-21	0	2	0.18	0.61
21	0	-2	-0.64	0.55
21	-1	-2	15.68	1.39
-21	-1	2	14.42	1.30
21	-1	-2	15.62	1.03
-21	-1	2	14.18	1.60
-21	1	-2	16.26	1.45
21	1	2	13.58	1.62
-21	1	-2	16.75	1.70
21	-1	-2	15.20	1.26
21	1	2	13.60	0.99
21	1	2	13.59	1.42
21	1	2	13.80	1.34
-21	-1	2	13.24	1.92
-21	-1	2	15.19	1.38
-21	1	-2	19.49	2.04
-21	1	2	11.98	1.89
-21	1	2	14.56	1.39
-21	1	2	13.34	1.90
21	-1	2	15.77	1.50
-21	-1	-2	15.23	1.44
21	-1	2	13.52	1.05
21	-1	2	13.46	1.38
-21	1	2	16.68	1.43
21	-1	2	15.86	1.51
-21	-1	-2	15.66	1.89
21	-2	-2	89.32	4.37

21	2	2	78.09	3.91
-21	2	-2	86.44	4.88
21	-2	-2	89.98	4.32
-21	2	-2	83.76	4.38
21	-2	-2	85.69	4.10
-21	-2	2	77.63	4.61
21	2	2	79.03	3.73
21	2	2	75.97	4.28
-21	-2	2	80.18	4.27
-21	-2	2	75.46	4.02
-21	-2	2	79.60	4.04
21	-2	2	78.83	3.84
-21	-2	-2	84.30	4.39
-21	2	2	81.71	4.52
21	-2	2	81.24	4.05
-21	-2	-2	81.48	4.52
-21	-2	-2	83.23	4.38
21	-2	2	77.33	4.09
-21	2	2	77.53	4.06
-21	2	2	73.36	3.90
21	-2	2	80.76	4.18
-21	3	-2	111.97	5.91
21	3	2	126.32	5.85
-21	-3	2	123.82	6.41
21	-3	-2	127.64	5.94
-21	-3	2	124.88	6.11
21	-3	-2	121.24	5.69
21	3	2	123.28	6.45
21	3	2	127.91	5.98
-21	-3	2	126.07	6.16
-21	3	-2	122.84	6.40
21	-3	-2	123.78	5.88
-21	-3	2	121.90	6.22
21	-3	2	122.76	5.98
21	-3	2	127.22	6.17
-21	-3	-2	115.63	5.97
-21	-3	-2	118.15	5.92
-21	-3	-2	115.96	5.88
-21	3	2	124.06	6.17
-21	3	2	132.20	6.63
21	-3	2	127.94	6.31
-21	-4	2	102.54	5.21
-21	-4	2	97.50	5.08
21	-4	-2	99.63	5.01

21	4	2	104.25	5.04
21	-4	-2	100.15	4.75
-21	4	-2	92.79	4.92
-21	-4	2	107.08	5.55
21	-4	-2	97.42	4.85
21	4	2	101.98	5.56
-21	4	-2	99.44	5.60
-21	-4	2	101.38	5.18
21	4	2	102.64	4.88
21	-4	2	107.28	5.19
-21	-4	-2	91.16	4.86
-21	4	2	100.98	5.78
-21	-4	-2	92.65	4.87
21	-4	2	100.05	5.04
-21	4	2	105.77	5.24
-21	-4	-2	97.36	4.97
21	5	2	29.80	1.85
21	5	2	26.92	2.75
21	-5	-2	27.92	1.87
-21	-5	2	28.45	2.07
-21	-5	2	22.97	2.02
-21	-5	2	23.08	1.88
21	5	2	27.78	1.92
-21	5	-2	21.07	2.94
21	-5	-2	25.70	1.78
-21	-5	2	33.15	2.69
-21	5	-2	26.39	1.96
21	-5	2	29.37	2.06
-21	5	2	25.36	2.01
21	-5	2	29.49	1.90
-21	5	2	26.60	2.85
-21	-5	-2	25.79	1.95
-21	-5	-2	24.26	2.09
21	6	2	8.55	1.45
21	-6	-2	9.85	1.44
-21	-6	2	9.25	1.65
-21	6	-2	9.13	1.76
-21	6	-2	13.88	1.48
21	6	2	9.58	1.27
-21	-6	2	8.19	1.52
21	6	2	9.23	1.79
-21	-6	2	7.80	1.32
21	-6	-2	10.70	1.29
21	-6	2	7.55	1.32

21	6	-2	10.20	1.21
21	6	-2	11.12	1.27
21	-6	2	8.44	1.21
-21	-6	-2	12.79	1.54
-21	-6	-2	13.59	1.60
-21	6	2	7.75	1.68
-21	-7	2	5.06	1.42
-21	-7	2	3.15	0.93
-21	-7	2	3.28	0.94
21	-7	-2	2.25	0.80
21	-7	-2	3.93	0.91
-21	7	-2	2.82	0.86
-21	7	-2	3.09	1.64
21	-7	-2	2.54	0.94
21	7	2	2.49	0.87
-21	-7	-2	3.26	1.29
21	7	-2	3.75	0.82
21	7	-2	6.14	1.26
-21	7	2	3.80	1.53
21	-7	2	3.50	0.87
21	-7	2	3.66	0.90
-21	-7	-2	3.81	1.08
-21	-7	-2	2.24	0.91
21	-8	-2	8.01	1.38
-21	-8	2	5.32	1.15
-21	-8	-2	10.42	1.71
-22	0	2	13.11	1.39
22	0	-2	13.90	1.00
22	0	2	14.35	1.46
22	0	-2	11.26	0.83
-22	0	-2	13.70	1.97
22	0	2	12.70	1.43
22	0	2	14.14	1.04
22	0	2	16.23	1.71
-22	0	-2	13.62	1.40
-22	0	2	12.93	1.97
-22	0	2	13.91	1.59
22	0	-2	11.27	1.32
22	-1	-2	2.42	0.46
22	-1	-2	2.45	0.68
-22	-1	2	2.80	0.81
22	1	2	5.96	1.16
22	-1	-2	2.06	0.72
-22	1	-2	3.69	1.33

-22	1	-2	1.78	0.89
22	1	2	3.63	0.66
-22	-1	2	1.34	1.06
-22	-1	2	2.40	1.01
-22	-1	2	3.05	0.70
22	-1	2	4.57	0.97
-22	-1	-2	2.65	0.74
-22	-1	-2	1.10	0.70
22	-1	2	2.54	0.60
-22	1	2	2.90	0.88
-22	1	2	2.46	1.24
22	-1	2	2.09	0.73
-22	1	2	2.56	0.81
-22	-1	-2	3.00	1.10
-22	-2	2	3.19	1.05
-22	-2	2	5.02	1.14
-22	-2	2	3.24	0.85
-22	2	-2	3.64	0.88
22	-2	-2	4.76	0.82
-22	-2	2	3.51	0.81
22	2	2	3.30	0.68
22	2	2	3.97	1.17
-22	2	-2	1.71	1.22
22	2	2	6.80	1.08
22	-2	-2	6.11	1.17
22	-2	2	3.01	1.01
-22	-2	-2	4.93	1.22
-22	2	2	4.34	0.92
-22	2	2	3.58	1.25
-22	-2	-2	3.67	0.88
-22	-2	-2	4.64	0.89
22	-2	2	4.76	0.97
-22	2	2	4.77	0.82
22	-2	2	4.06	0.88
22	-2	2	4.63	0.87
-22	3	-2	0.95	1.41
-22	3	-2	0.98	0.82
22	-3	-2	1.56	0.86
-22	-3	2	-0.26	0.67
22	3	2	0.79	0.53
22	-3	-2	1.04	0.60
-22	-3	2	-0.12	1.02
-22	-3	2	0.36	1.20
-22	-3	2	0.65	0.72

22	-3	-2	2.04	0.78
22	3	2	1.00	0.78
22	3	2	1.13	1.16
-22	-3	-2	0.34	0.76
-22	3	2	0.23	1.33
22	-3	2	2.76	1.16
22	-3	2	1.62	0.81
-22	3	2	0.32	0.76
-22	-3	-2	0.28	0.71
22	-3	2	0.14	0.58
-22	-3	-2	0.36	0.94
22	4	2	-0.33	1.18
-22	-4	2	-1.22	1.05
-22	4	-2	1.09	0.80
22	-4	-2	0.25	0.63
22	4	2	-0.47	0.62
22	-4	-2	-1.13	0.54
-22	4	-2	-0.43	1.55
-22	-4	2	0.22	0.90
-22	-4	2	0.88	0.73
-22	-4	2	0.19	0.80
-22	-4	-2	-1.00	0.71
-22	4	2	1.48	1.27
22	-4	2	0.93	0.76
-22	-4	-2	-0.30	0.93
22	-4	2	0.95	0.62
-22	4	2	0.52	0.74
-22	-4	-2	0.72	0.97
22	5	2	3.21	1.48
22	5	2	2.14	0.70
-22	5	-2	1.62	0.76
-22	-5	2	0.72	0.95
22	-5	-2	0.56	0.78
22	-5	-2	0.32	0.58
-22	-5	2	2.62	1.47
-22	5	-2	1.25	1.39
-22	-5	2	-0.40	0.80
-22	-5	2	1.32	0.97
22	-5	2	-0.38	0.73
-22	5	2	0.32	0.69
-22	5	2	0.63	1.32
22	-5	2	1.22	0.66
-22	-5	-2	1.28	0.88
-22	-5	-2	1.38	0.85

22	6	2	1.52	1.41
-22	-6	2	1.53	1.30
-22	-6	2	0.37	0.85
22	6	2	1.49	0.72
22	-6	-2	1.25	0.81
-22	6	-2	0.85	0.83
22	-6	-2	2.00	0.72
-22	-6	2	0.72	0.87
-22	6	-2	4.92	1.63
22	-6	2	0.93	0.72
22	6	-2	2.46	0.67
-22	-6	-2	2.00	0.93
-22	6	2	1.47	1.47
22	-6	2	-0.12	0.69
-22	-6	-2	1.44	0.84
-22	-6	-2	2.83	1.30
22	6	-2	2.24	0.73
-22	6	2	0.65	0.80
-22	7	-2	1.55	0.83
-22	-7	2	2.45	0.96
-22	-7	2	1.51	1.38
22	7	2	3.43	0.89
22	-7	-2	1.94	0.88
22	-7	-2	3.01	0.82
-22	7	-2	2.41	2.63
-22	7	2	-0.49	0.91
-22	-7	-2	3.65	1.41
22	7	-2	2.02	0.83
-22	-7	-2	2.62	0.95
22	-7	2	3.23	0.88
22	-7	2	1.84	0.79
22	7	-2	3.22	0.91
-22	-7	-2	2.37	1.09
23	0	2	0.64	0.80
-23	0	2	-0.86	0.78
-23	0	2	0.07	1.16
-23	0	2	0.85	0.90
-23	0	-2	-1.23	1.14
-23	0	-2	0.41	0.78
23	0	-2	0.00	0.37
23	0	2	0.38	0.51
23	0	2	0.55	0.82
23	0	2	-0.69	1.05
23	0	-2	-0.21	0.70

23	1	2	1.89	0.52
23	1	2	0.83	0.74
23	-1	-2	1.16	0.75
23	1	2	-0.09	1.01
23	-1	-2	2.25	0.53
23	-1	-2	3.03	0.70
-23	-1	2	2.46	0.90
-23	1	-2	2.29	0.86
-23	-1	2	1.46	1.17
-23	-1	2	2.33	0.75
-23	1	-2	3.43	1.29
23	-1	2	1.31	0.56
23	-1	2	1.26	1.07
-23	1	2	1.90	1.31
-23	-1	-2	3.16	0.93
-23	-1	-2	2.21	1.15
23	-1	2	2.63	0.94
-23	1	2	3.07	0.93
23	-1	2	1.40	0.85
-23	-1	-2	2.19	0.86
-23	1	2	3.31	0.96
23	2	2	56.83	3.66
-23	2	-2	59.77	3.52
-23	-2	2	54.42	3.25
-23	2	-2	66.00	3.73
-23	-2	2	59.95	3.59
23	-2	-2	63.04	3.16
23	-2	-2	64.89	3.48
-23	-2	2	59.61	3.58
-23	-2	2	59.95	3.27
23	-2	-2	64.92	3.39
-23	2	-2	58.94	4.19
23	2	2	60.64	2.91
-23	-2	-2	67.40	3.62
-23	-2	-2	63.84	3.80
-23	2	2	57.09	4.00
23	-2	2	59.21	3.38
23	-2	2	57.58	3.00
23	-2	2	62.75	3.36
-23	-2	-2	64.07	3.59
-23	2	2	57.32	3.32
23	-3	-2	88.17	4.37
-23	-3	2	81.38	4.48
-23	-3	2	82.06	4.37

23	-3	-2	81.77	4.06
23	3	2	85.02	3.96
23	3	2	88.15	4.73
-23	-3	2	88.16	4.73
-23	-3	2	82.50	4.30
23	3	2	79.26	4.05
-23	3	-2	79.13	4.34
-23	3	-2	80.40	5.15
23	-3	2	79.89	4.38
-23	-3	-2	87.01	4.41
-23	-3	-2	84.07	4.56
23	-3	2	78.73	4.07
-23	-3	-2	78.88	4.39
-23	3	2	82.77	4.95
-23	3	2	79.93	4.33
23	4	2	59.11	3.98
-23	4	-2	47.27	2.97
-23	-4	2	59.58	3.40
-23	-4	2	59.60	3.26
23	-4	-2	48.77	2.72
-23	-4	2	60.29	3.92
-23	4	-2	57.96	3.88
23	-4	-2	53.79	3.06
23	4	2	56.01	3.09
23	4	2	58.87	2.94
-23	4	2	61.18	4.00
-23	-4	-2	54.72	3.08
23	-4	2	55.51	3.31
-23	4	2	55.96	3.27
-23	-4	-2	48.09	3.10
23	-4	2	58.24	3.08
-23	-5	2	20.96	1.86
-23	5	-2	20.31	1.85
23	5	2	19.89	1.83
-23	5	-2	22.51	2.84
-23	-5	2	20.73	2.52
-23	-5	2	16.13	1.75
23	-5	-2	23.62	1.89
23	5	2	21.18	1.44
23	-5	-2	20.16	1.61
-23	5	2	21.55	2.68
23	-5	2	18.93	1.75
23	-5	2	20.10	1.55
-23	5	2	19.65	1.77

-23	-5	-2	24.28	1.92
-23	-5	-2	21.97	2.06
-23	6	-2	10.32	1.50
23	-6	-2	6.41	1.05
23	-6	-2	8.45	1.53
23	6	2	9.97	1.27
-23	-6	2	9.47	1.72
23	6	2	8.28	1.19
-23	-6	2	8.54	1.29
-23	-6	2	8.34	1.51
-23	-6	-2	7.74	1.29
-23	6	2	10.28	1.62
-23	-6	-2	7.86	1.37
23	-6	2	8.19	1.37
-23	-6	-2	8.14	1.59
-23	6	2	7.46	1.83
23	-6	2	9.14	1.42
-24	0	-2	21.71	2.40
24	0	2	21.70	1.75
-24	0	2	16.90	1.73
-24	0	-2	16.95	1.62
-24	0	2	17.45	1.88
24	0	-2	13.64	0.96
24	0	2	22.69	1.88
-24	0	2	21.14	2.57
24	0	-2	13.55	1.09
24	0	2	19.95	1.31
-24	0	2	20.17	1.80
24	1	2	21.05	2.25
-24	1	-2	17.33	1.77
-24	-1	2	17.38	2.27
24	-1	-2	18.01	1.78
-24	-1	2	17.22	1.80
-24	-1	2	17.93	1.79
24	-1	-2	16.38	1.14
-24	-1	2	21.46	2.08
24	1	2	19.54	1.24
24	1	2	19.38	1.58
-24	1	-2	18.91	2.75
-24	1	-2	14.36	1.63
-24	1	2	19.00	2.73
24	-1	2	17.18	1.78
24	-1	2	18.00	1.70
-24	-1	-2	19.81	1.77

24	-1	2	18.16	1.28
-24	1	2	21.12	1.73
-24	-1	-2	19.41	2.39
-24	-1	-2	17.03	1.71
-24	-2	2	1.70	0.94
24	-2	-2	2.83	0.94
24	2	2	2.54	0.54
-24	2	-2	3.68	1.81
-24	2	-2	2.26	0.76
24	2	2	1.64	1.28
24	2	2	1.64	0.73
-24	-2	2	3.53	1.26
-24	-2	2	1.49	0.94
-24	-2	2	1.25	1.22
24	-2	-2	3.46	0.82
24	-2	-2	2.70	0.53
24	-2	2	1.78	0.57
-24	-2	-2	4.08	1.31
-24	2	2	1.53	0.79
-24	-2	-2	3.27	0.88
24	-2	2	1.71	0.86
-24	-2	-2	3.34	0.98
-24	2	2	2.66	1.43
24	3	2	10.78	1.22
24	-3	-2	11.31	1.61
24	3	2	8.88	0.92
-24	3	-2	8.59	1.41
-24	-3	2	9.17	1.53
-24	3	-2	14.43	2.50
-24	-3	2	11.41	1.73
24	-3	-2	10.66	1.01
-24	-3	2	9.45	1.53
-24	-3	2	7.77	1.28
-24	3	2	5.93	1.57
-24	-3	-2	9.44	1.84
24	-3	2	10.22	1.05
-24	-3	-2	8.64	1.53
-24	3	2	8.52	1.40
24	-3	2	7.76	1.39
-24	-3	-2	8.11	1.41
24	4	2	3.53	0.61
24	-4	-2	2.42	0.96
24	4	2	2.09	0.85
-24	-4	2	2.70	1.00

-24	4	-2	2.39	0.91
-24	-4	2	2.03	0.92
24	-4	-2	2.50	0.67
-24	4	-2	2.10	1.57
-24	-4	2	3.69	1.50
-24	-4	2	5.84	1.71
-24	-4	-2	-1.06	1.34
-24	-4	-2	2.88	0.91
24	-4	2	1.73	0.66
24	-4	2	2.69	0.84
-24	4	2	2.95	1.50
-24	-4	-2	1.83	1.03
-24	4	2	1.23	0.88
24	5	2	6.64	1.03
-24	-5	2	5.88	1.49
24	-5	-2	6.32	1.07
-24	-5	2	6.30	1.25
-24	-5	2	3.41	1.01
-24	5	-2	3.68	1.69
-24	-5	-2	9.71	1.57
-24	-5	-2	7.99	1.31
-24	-5	-2	5.88	1.12
24	-5	2	5.43	1.03
-24	5	2	5.05	1.62
-25	0	2	0.40	1.62
25	0	2	1.18	0.81
25	0	2	0.97	0.83
25	0	2	0.06	0.45
-25	0	-2	0.49	1.07
-25	0	2	0.48	0.83
-25	0	-2	-0.05	0.80
-25	0	-2	2.36	1.63
-25	0	2	-0.21	0.77
-25	1	-2	9.96	1.48
25	1	2	9.27	1.30
-25	1	-2	8.10	1.82
25	1	2	5.77	0.79
25	-1	-2	9.50	1.56
-25	-1	2	8.78	1.48
25	-1	-2	11.68	1.23
-25	-1	2	5.11	1.63
-25	-1	2	6.02	1.05
-25	-1	2	11.30	1.92
25	-1	-2	8.94	0.83

-25	1	2	6.50	1.68
-25	-1	-2	10.64	1.40
25	-1	2	7.31	0.90
-25	-1	-2	9.04	1.45
-25	1	2	5.98	1.06
25	-1	2	7.94	1.43
-25	-1	-2	8.93	1.64
-25	-1	-2	11.41	2.04
25	-2	-2	5.84	1.15
25	-2	-2	6.57	0.86
-25	2	-2	6.35	1.68
-25	-2	2	4.28	1.01
-25	2	-2	3.32	1.02
25	2	2	4.94	0.75
-25	-2	2	5.84	1.47
-25	-2	2	6.77	1.07
-25	-2	2	3.97	1.38
-25	-2	-2	6.00	1.07
25	-2	2	4.60	0.91
-25	-2	-2	8.79	1.62
-25	2	2	4.34	0.96
-25	-2	-2	4.06	0.99
-25	-2	-2	6.78	1.54
-25	2	2	5.72	1.51
25	-2	2	4.35	0.97
25	-3	-2	18.06	1.38
25	-3	-2	22.73	1.87
-25	-3	2	29.15	2.59
-25	3	-2	21.85	2.83
-25	-3	2	29.38	2.90
-25	-3	2	25.72	2.05
-25	-3	2	25.82	2.00
-25	3	-2	21.31	2.01
-25	-3	-2	20.70	1.90
-25	-3	-2	20.83	1.90
25	-3	2	19.56	1.58
25	-3	2	21.87	1.95
-25	3	2	30.86	2.87
-25	3	2	22.04	1.95
-25	-3	-2	26.18	2.75
0	0	-3	0.12	0.07
0	0	3	0.12	0.14
0	0	-3	0.10	0.09
0	0	3	0.06	0.07

0	0	-3	0.09	0.04
0	0	-3	0.14	0.12
0	0	-3	0.24	0.11
0	0	-3	-0.04	0.04
0	0	3	0.21	0.08
0	0	3	0.10	0.04
0	0	3	0.29	0.09
0	0	3	0.20	0.12
0	1	-3	0.07	0.04
0	1	-3	0.25	0.10
0	-1	-3	0.01	0.10
0	-1	3	0.04	0.07
0	1	-3	-0.13	0.14
0	1	3	0.19	0.11
0	-1	3	0.06	0.06
0	1	3	-0.01	0.10
0	-1	-3	-0.07	0.10
0	1	3	0.06	0.10
0	-1	3	0.14	0.16
0	1	-3	0.21	0.19
0	1	3	0.13	0.06
0	1	-3	0.03	0.07
0	-1	-3	0.09	0.11
0	1	3	0.04	0.15
0	1	-3	0.05	0.12
0	-1	3	0.16	0.09
0	1	3	0.10	0.12
0	-1	-3	0.09	0.06
0	-1	3	0.03	0.11
0	-1	3	0.12	0.18
0	-1	-3	-0.05	0.14
0	-1	-3	-0.03	0.11
0	-2	-3	0.16	0.22
0	2	3	0.14	0.17
0	2	3	0.09	0.16
0	-2	3	0.07	0.18
0	-2	-3	-0.05	0.15
0	2	3	-0.10	0.11
0	-2	3	-0.02	0.12
0	-2	3	0.00	0.14
0	2	-3	0.05	0.07
0	2	-3	0.29	0.23
0	2	-3	0.09	0.12
0	2	3	0.00	0.18

0	2	-3	-0.01	0.13
0	2	-3	0.01	0.18
0	2	-3	0.24	0.24
0	-2	-3	0.10	0.12
0	-2	3	-0.15	0.24
0	2	3	0.10	0.22
0	-2	-3	0.08	0.16
0	2	3	0.08	0.14
0	-2	-3	0.02	0.19
0	-2	3	0.10	0.24
0	-3	-3	0.04	0.22
0	3	3	0.01	0.19
0	-3	3	0.58	0.33
0	-3	3	0.11	0.10
0	3	3	0.04	0.16
0	-3	3	0.14	0.25
0	3	3	0.03	0.18
0	3	3	0.15	0.26
0	3	3	0.04	0.22
0	3	3	0.29	0.25
0	-3	3	0.17	0.29
0	3	-3	0.15	0.12
0	3	-3	0.17	0.21
0	3	-3	-0.06	0.18
0	3	-3	-0.01	0.22
0	3	-3	0.14	0.28
0	3	-3	0.19	0.32
0	-3	-3	0.32	0.27
0	-3	-3	-0.08	0.17
0	-3	3	-0.03	0.16
0	-3	3	0.04	0.18
0	-3	-3	0.02	0.20
0	-3	-3	0.06	0.17
0	4	-3	0.38	0.31
0	-4	-3	-0.15	0.20
0	4	3	-0.11	0.25
0	-4	-3	-0.30	0.31
0	-4	3	-0.12	0.33
0	4	-3	-0.12	0.27
0	-4	3	-0.03	0.24
0	4	-3	0.17	0.16
0	4	-3	-0.03	0.28
0	-4	-3	0.10	0.28
0	4	3	0.05	0.33

0	-4	3	0.07	0.26
0	-4	3	0.02	0.24
0	-4	-3	-0.10	0.19
0	-4	3	0.14	0.14
0	-4	-3	0.00	0.25
0	-4	3	-0.16	0.34
0	4	-3	-0.08	0.23
0	4	3	-0.10	0.23
0	4	3	0.28	0.25
0	4	-3	-0.19	0.23
0	-4	-3	-0.17	0.21
0	5	-3	0.48	0.49
0	5	3	0.45	0.31
0	-5	3	-0.21	0.29
0	5	3	0.09	0.40
0	-5	3	-0.04	0.56
0	-5	-3	-0.27	0.26
0	5	-3	-0.07	0.19
0	5	3	-0.22	0.26
0	-5	3	0.23	0.26
0	-5	3	-0.24	0.32
0	-5	3	-0.01	0.18
0	5	-3	0.20	0.33
0	-5	3	0.42	0.49
0	5	3	0.75	0.47
0	-5	3	0.28	0.42
0	-5	-3	0.19	0.34
0	-5	-3	-0.05	0.25
0	5	3	-0.10	0.31
0	5	-3	-0.17	0.29
0	5	-3	-0.12	0.37
0	-5	-3	0.17	0.30
0	-5	-3	0.02	0.33
0	6	-3	0.13	0.44
0	-6	3	-0.15	0.37
0	6	-3	-0.16	0.25
0	6	3	0.06	0.33
0	6	-3	0.39	0.45
0	6	3	-0.38	0.33
0	-6	3	0.30	0.39
0	6	-3	0.24	0.46
0	-6	3	-0.30	0.50
0	-6	3	-0.31	0.54
0	6	3	0.29	0.39

0	-6	-3	-0.25	0.27
0	-6	-3	0.69	0.54
0	-6	3	0.63	0.50
0	-6	3	0.53	0.32
0	-6	3	-0.56	0.31
0	-6	-3	-0.08	0.34
0	6	-3	-0.21	0.34
0	6	3	-0.30	0.51
0	-6	-3	-0.25	0.41
0	6	3	0.22	0.46
0	6	3	0.10	0.35
0	-6	-3	0.15	0.30
0	-6	-3	0.31	0.44
0	7	3	0.25	0.64
0	7	-3	-0.25	0.30
0	-7	-3	0.06	0.51
0	-7	3	-0.74	0.53
0	-7	3	0.21	0.23
0	7	3	0.27	0.63
0	7	-3	0.18	0.54
0	-7	3	-0.02	0.57
0	7	3	0.13	0.40
0	-7	-3	-0.10	0.63
0	-7	-3	0.22	0.33
0	-7	-3	-0.13	0.43
0	7	-3	-0.24	0.42
0	-7	3	-0.26	0.34
0	7	3	0.63	0.42
0	7	3	-0.08	0.46
0	7	-3	-0.10	0.48
0	-7	3	-0.50	0.41
0	-7	-3	0.08	0.37
0	7	-3	0.95	0.67
0	-7	3	-0.31	0.44
0	-7	3	0.11	0.70
0	7	3	0.25	0.45
0	-8	3	-0.07	0.28
0	8	-3	0.09	0.46
0	8	-3	0.54	0.70
0	-8	3	-0.01	0.53
0	-8	-3	0.00	0.52
0	-8	-3	0.34	0.37
0	8	3	-0.19	0.45
0	8	3	-0.26	0.64

0	-8	-3	-0.39	0.41
0	-8	-3	0.39	0.63
0	-8	3	-0.72	0.41
0	8	3	-0.40	0.48
0	8	-3	0.32	0.49
0	-8	-3	-0.05	0.73
0	8	3	-0.33	0.80
0	8	-3	0.38	0.65
0	-8	3	0.27	0.76
0	-8	-3	-0.24	0.46
0	-8	3	0.19	0.52
0	8	-3	0.31	0.55
0	-8	-3	-0.06	0.46
0	8	3	-0.34	0.51
0	-8	3	-0.10	0.66
0	-8	3	0.56	0.68
0	9	3	0.29	0.93
0	9	-3	-0.18	0.45
0	-9	-3	0.18	0.47
0	-9	-3	-0.50	0.60
0	-9	-3	0.56	0.44
0	-9	-3	-0.05	0.58
0	-9	-3	0.74	0.77
0	9	-3	-0.13	0.65
0	-9	-3	0.20	0.93
0	-9	3	0.05	0.81
0	9	3	-1.00	0.55
0	-9	3	0.26	0.34
0	9	-3	0.75	0.96
0	9	-3	-0.15	0.85
0	-9	3	0.75	0.55
0	9	3	0.57	0.57
0	-9	-3	-0.50	0.62
0	-9	3	-1.50	0.75
0	-9	3	0.37	0.99
0	9	3	0.42	0.88
0	-9	3	-0.04	0.62
0	-10	-3	0.67	0.60
0	10	-3	-0.38	0.70
0	10	3	0.09	0.99
0	-10	3	1.83	1.00
0	10	-3	-0.23	0.52
0	-10	3	-0.31	0.59
0	-10	3	-0.12	0.91

0	-10	-3	0.86	0.78
0	-10	3	0.48	0.69
0	10	3	-0.83	0.64
0	-10	-3	-0.12	0.90
0	-10	3	-0.07	0.55
0	-10	-3	-1.15	0.45
0	10	3	-0.02	0.68
0	10	-3	1.53	1.13
0	10	-3	-0.55	0.92
0	-10	3	-0.03	0.39
0	-10	-3	-0.93	0.71
0	10	3	-0.44	1.05
0	-10	-3	0.40	0.70
0	11	3	-0.29	0.69
0	11	-3	-0.02	0.85
0	11	3	-0.14	1.31
0	-11	-3	-0.49	0.82
0	-11	3	-0.21	0.79
0	-11	3	-0.18	0.72
0	-11	-3	-0.86	0.54
0	-11	-3	-0.21	0.76
0	-11	-3	0.00	0.66
0	11	3	0.99	0.83
0	11	-3	-0.25	1.23
0	11	3	-1.20	1.15
0	-11	3	0.15	0.68
0	-11	3	-0.05	0.42
0	-11	3	0.65	1.09
0	-12	-3	-0.32	1.04
0	12	-3	-1.40	1.49
0	-12	-3	-0.96	0.90
0	-12	-3	-0.74	0.62
0	-12	3	-0.35	0.97
0	12	-3	-0.40	0.99
0	12	3	-0.29	1.62
0	-12	3	0.15	0.83
0	12	3	0.49	0.92
0	-13	-3	0.57	0.84
0	13	3	-0.73	1.74
0	-13	-3	0.78	1.17
0	-13	3	0.05	0.88
0	13	-3	-1.57	1.93
0	13	-3	1.23	1.18
0	-13	3	-0.75	1.25

1	0	3	-0.01	0.07
1	0	-3	0.22	0.12
-1	0	-3	0.06	0.06
1	0	3	-0.10	0.07
-1	0	-3	0.05	0.06
-1	0	-3	-0.06	0.13
-1	0	3	0.00	0.08
1	0	-3	0.05	0.10
1	0	-3	-0.01	0.05
-1	0	-3	0.18	0.09
-1	0	-3	-0.01	0.10
-1	0	3	0.13	0.08
1	0	3	0.00	0.12
1	0	3	0.18	0.15
1	0	3	-0.01	0.05
-1	0	3	0.23	0.13
-1	0	3	0.06	0.12
-1	0	-3	0.28	0.13
-1	0	3	0.02	0.07
1	0	3	0.09	0.08
1	0	-3	0.01	0.11
1	1	3	114.29	5.48
-1	1	-3	116.26	5.42
-1	1	-3	116.93	5.44
1	-1	-3	123.77	5.47
-1	-1	3	120.28	5.44
-1	-1	3	126.39	5.57
-1	-1	3	121.75	5.55
-1	1	-3	120.07	5.40
-1	-1	3	116.44	5.53
-1	1	-3	133.95	5.55
1	-1	-3	123.17	5.49
1	1	3	134.42	5.58
-1	1	-3	121.85	5.51
1	-1	-3	119.20	5.45
1	1	3	116.88	5.53
1	1	3	123.54	5.49
1	1	3	121.95	5.53
-1	-1	3	119.75	5.48
-1	1	-3	124.37	5.46
-1	-1	3	116.29	5.49
1	-1	-3	118.49	5.42
1	-1	-3	117.41	5.44
1	-1	3	118.24	5.52

1	-1	3	136.67	5.61
-1	-1	-3	121.49	5.45
1	1	-3	118.81	5.40
1	1	-3	120.25	5.49
1	1	-3	118.55	5.49
-1	-1	-3	118.67	5.46
-1	-1	-3	115.77	5.41
-1	-1	-3	124.38	5.49
-1	1	3	128.44	5.56
1	-1	3	113.30	5.51
1	-1	3	127.17	5.58
-1	1	3	117.74	5.46
-1	1	3	125.96	5.53
1	-1	3	116.06	5.49
-1	1	3	121.00	5.54
1	-1	3	124.86	5.47
1	1	-3	116.57	5.46
-1	1	3	121.16	5.49
-1	-1	-3	119.77	5.41
-1	1	3	122.79	5.51
-1	-1	-3	117.75	5.44
1	2	3	54.17	2.59
-1	2	-3	91.39	4.32
1	2	3	53.33	2.51
1	-2	-3	90.82	4.28
-1	2	-3	98.67	4.25
1	-2	-3	100.91	4.21
-1	2	-3	98.85	4.37
1	2	3	54.15	2.57
-1	-2	3	51.68	2.52
-1	2	-3	90.91	4.24
1	2	3	54.05	2.59
1	2	3	55.77	2.47
1	2	3	51.59	2.52
1	-2	-3	94.56	4.30
-1	2	-3	90.84	4.21
-1	-2	3	52.59	2.51
1	-2	-3	86.99	4.19
-1	-2	3	56.67	2.62
-1	2	-3	93.37	4.16
1	-2	-3	88.08	4.23
-1	-2	3	53.09	2.58
-1	-2	3	54.46	2.60
-1	2	3	54.90	2.53

-1	-2	-3	90.64	4.22
1	2	-3	93.71	4.21
1	2	-3	94.59	4.30
-1	2	3	53.87	2.51
-1	2	3	50.53	2.59
1	-2	3	54.13	2.46
1	-2	3	57.42	2.64
1	-2	3	56.93	2.55
-1	2	3	56.11	2.53
1	-2	3	60.39	2.67
1	2	-3	90.04	4.27
-1	-2	-3	92.77	4.26
1	2	-3	89.13	4.31
-1	-2	-3	88.49	4.27
-1	2	3	52.82	2.50
1	-2	3	52.39	2.52
-1	2	3	53.96	2.59
-1	-2	-3	89.98	4.21
-1	2	3	52.04	2.53
1	-2	3	52.32	2.54
1	3	3	66.56	3.04
-1	-3	3	64.34	3.04
-1	3	-3	68.51	3.41
-1	3	-3	69.03	3.32
1	-3	-3	67.31	3.30
1	-3	-3	71.51	3.39
-1	-3	3	64.45	3.11
1	-3	-3	70.71	3.27
-1	-3	3	70.25	3.16
1	3	3	66.37	3.14
1	3	3	63.13	3.10
-1	3	-3	73.34	3.47
1	3	3	59.51	2.98
-1	-3	3	66.48	3.15
-1	-3	3	60.80	3.05
1	-3	-3	70.48	3.36
-1	3	-3	71.92	3.21
-1	3	-3	68.53	3.28
1	3	3	66.55	3.16
1	-3	-3	76.92	3.28
1	3	3	63.11	3.03
-1	3	-3	74.61	3.32
-1	3	3	64.41	3.05
1	-3	3	67.64	3.16

1	-3	3	63.03	3.08
-1	3	3	66.77	3.05
-1	3	3	64.89	3.04
-1	3	3	62.88	3.03
-1	-3	-3	70.42	3.28
1	-3	3	63.29	2.96
1	-3	3	68.14	3.21
-1	3	3	62.86	3.17
1	3	-3	70.43	3.28
1	3	-3	71.67	3.31
1	3	-3	70.25	3.34
-1	-3	-3	71.20	3.36
1	-3	3	68.48	3.08
-1	-3	-3	73.75	3.34
1	-3	3	63.44	3.04
1	3	-3	71.59	3.38
-1	-3	-3	72.56	3.21
-1	3	3	66.88	3.14
1	3	-3	62.16	3.49
-1	-3	-3	61.71	3.33
-1	-3	-3	67.49	3.34
1	-4	-3	188.76	9.06
1	4	3	182.47	8.30
-1	-4	3	171.18	8.15
1	-4	-3	182.11	8.97
-1	-4	3	193.08	8.32
1	-4	-3	202.04	9.13
1	4	3	180.24	8.25
-1	4	-3	198.74	9.05
-1	4	-3	215.76	9.03
-1	4	-3	198.02	9.11
1	4	3	176.41	8.20
-1	-4	3	184.69	8.23
-1	-4	3	169.40	8.01
1	-4	-3	210.09	8.99
1	-4	-3	204.18	8.98
-1	-4	3	195.70	8.26
-1	4	-3	196.44	8.89
1	4	3	176.73	8.14
-1	-4	3	172.79	8.16
1	4	3	175.34	8.12
-1	-4	3	177.44	8.21
1	4	3	165.72	8.08
-1	4	-3	201.12	9.00

1	-4	-3	189.12	8.98
-1	4	3	177.40	8.32
-1	4	3	180.30	8.26
-1	-4	-3	193.64	8.91
1	-4	3	174.24	8.15
-1	-4	-3	215.20	9.12
-1	-4	-3	186.22	9.05
-1	-4	-3	187.21	8.99
1	4	-3	192.91	9.15
1	-4	3	167.95	8.18
-1	4	3	173.25	8.14
1	-4	3	184.15	8.17
1	4	-3	195.49	8.99
-1	-4	-3	211.71	9.06
1	4	-3	200.48	9.04
-1	4	3	179.36	8.17
-1	-4	-3	187.60	8.96
1	-4	3	198.22	8.35
-1	4	3	176.24	8.14
1	4	-3	200.25	9.08
1	-4	3	173.27	8.04
1	4	-3	194.34	8.86
1	-4	3	186.10	8.25
-1	-5	3	299.35	13.09
-1	-5	3	276.18	12.97
1	5	3	271.42	12.91
-1	5	-3	270.68	11.97
-1	5	-3	271.22	12.09
1	5	3	249.22	12.98
1	-5	-3	248.48	12.15
-1	-5	3	304.35	13.15
1	5	3	287.34	13.04
1	5	3	293.71	12.97
1	-5	-3	286.19	12.27
-1	-5	3	274.11	12.82
1	-5	-3	247.85	12.06
-1	5	-3	267.84	12.32
1	-5	-3	267.60	12.05
1	-5	-3	289.07	12.09
-1	-5	3	267.83	12.99
-1	-5	3	302.84	13.06
1	5	3	284.54	13.17
-1	5	-3	260.89	12.19
-1	5	-3	274.69	12.17

1	-5	-3	241.99	12.03
-1	-5	3	303.28	13.07
1	5	3	297.31	13.11
1	-5	3	312.64	13.10
1	-5	3	255.65	13.31
-1	5	3	291.26	12.95
1	-5	3	303.68	13.01
-1	5	3	290.79	12.97
1	5	-3	265.24	12.16
-1	-5	-3	285.77	12.24
1	-5	3	271.38	13.01
1	5	-3	259.05	11.92
1	5	-3	265.20	12.29
-1	-5	-3	287.89	12.15
-1	5	3	287.43	12.98
-1	-5	-3	253.33	12.03
1	5	-3	260.33	12.07
-1	5	3	275.09	13.16
-1	5	3	291.45	12.96
-1	5	3	306.94	13.13
-1	-5	-3	267.60	12.09
-1	-5	-3	259.88	11.98
1	5	-3	278.05	12.15
1	-5	3	277.20	12.97
1	-5	3	280.24	12.85
-1	-5	-3	246.30	12.14
-1	-6	3	87.77	4.53
-1	6	-3	105.26	4.81
1	-6	-3	103.84	4.90
1	-6	-3	92.81	4.67
-1	6	-3	97.64	4.82
-1	-6	3	92.17	4.50
1	6	3	97.31	4.67
-1	6	-3	96.59	4.69
-1	-6	3	93.53	4.57
1	-6	-3	90.21	4.61
-1	-6	3	98.83	4.62
-1	-6	3	101.87	4.61
1	6	3	90.27	4.48
1	6	3	95.53	4.58
-1	6	-3	99.89	4.55
-1	-6	3	90.89	4.31
1	6	3	90.54	4.72
1	6	3	89.40	4.43

1	-6	-3	95.31	4.77
1	-6	-3	107.63	4.71
1	6	3	91.97	4.43
-1	-6	3	99.93	4.71
-1	6	-3	99.38	4.99
1	-6	3	89.05	4.33
-1	6	3	94.44	4.45
-1	6	3	93.79	4.65
-1	-6	-3	107.12	4.77
-1	6	3	90.30	4.50
-1	-6	-3	98.16	4.58
1	-6	3	86.72	4.55
1	6	-3	98.33	4.69
-1	-6	-3	91.36	4.62
1	6	-3	97.32	4.78
1	-6	3	106.88	4.79
1	6	-3	96.47	4.51
-1	6	3	94.20	4.49
1	-6	3	88.78	4.48
1	6	-3	97.82	4.77
1	6	-3	98.62	4.95
1	-6	3	102.05	4.58
-1	-6	-3	105.57	4.87
-1	6	3	90.29	4.50
1	-6	3	99.84	4.54
-1	6	3	90.57	4.74
-1	-6	-3	91.67	4.75
-1	-6	-3	96.38	4.69
1	-6	3	103.49	4.63
-1	-7	3	60.03	3.00
1	7	3	55.11	2.79
-1	7	-3	61.43	3.34
1	-7	-3	60.89	3.17
1	7	3	55.03	2.94
-1	7	-3	65.20	3.34
-1	-7	3	55.47	3.09
-1	7	-3	61.55	3.52
-1	-7	3	53.11	2.91
-1	-7	3	58.85	3.01
1	-7	-3	58.00	3.09
-1	-7	3	52.89	2.86
1	7	3	55.72	2.85
1	7	3	54.47	3.11
1	7	3	53.81	2.81

1	7	3	58.36	3.08
-1	7	-3	62.63	3.20
-1	-7	3	53.13	2.63
-1	-7	3	54.84	2.94
1	-7	-3	62.58	3.29
-1	7	-3	64.79	3.03
1	-7	-3	63.16	3.16
1	-7	-3	69.61	3.24
1	-7	-3	66.83	3.42
-1	-7	-3	63.12	3.19
1	-7	3	53.71	2.85
-1	-7	-3	68.33	3.39
-1	-7	-3	71.68	3.32
1	7	-3	61.78	3.28
-1	7	3	53.89	2.85
-1	7	3	53.28	2.87
-1	-7	-3	59.30	3.09
1	-7	3	53.04	2.67
1	7	-3	64.82	3.32
-1	7	3	56.02	2.81
1	7	-3	61.88	2.98
1	7	-3	59.11	3.46
1	7	-3	63.82	3.21
-1	7	3	56.10	3.18
-1	7	3	57.46	3.10
-1	-7	-3	63.58	3.08
-1	-7	-3	58.50	3.28
1	-7	3	58.77	2.97
1	-7	3	56.86	2.89
1	-7	3	62.19	3.04
1	-7	3	59.20	3.18
-1	8	-3	13.97	1.01
-1	8	-3	11.19	1.26
1	-8	-3	13.80	1.13
-1	-8	3	15.54	1.59
-1	8	-3	13.28	1.17
1	8	3	13.99	1.48
-1	-8	3	18.35	1.46
1	-8	-3	13.36	1.12
1	-8	-3	13.05	1.19
1	8	3	13.65	1.23
1	-8	-3	13.75	1.54
-1	8	-3	15.23	1.39
-1	8	-3	12.56	1.48

-1	-8	3	15.24	1.32
1	8	3	15.59	1.18
1	-8	-3	15.70	1.26
1	-8	-3	13.24	1.25
1	8	3	13.82	1.22
1	8	3	17.63	1.58
1	-8	-3	11.89	0.99
-1	-8	3	16.57	1.21
-1	-8	3	16.26	1.49
-1	-8	3	15.64	1.26
-1	-8	3	15.32	0.93
1	8	-3	13.84	1.33
1	8	-3	13.00	1.13
1	-8	3	15.60	1.51
-1	-8	-3	15.22	1.36
-1	-8	-3	12.81	1.00
-1	-8	-3	15.94	1.52
1	-8	3	17.13	1.46
-1	-8	-3	13.04	1.15
-1	8	3	15.61	1.59
1	8	-3	10.78	1.38
1	8	-3	12.46	1.21
1	-8	3	14.52	0.97
-1	-8	-3	13.50	1.11
1	-8	3	15.49	1.19
1	8	-3	12.44	0.90
-1	-8	-3	11.99	1.25
-1	8	3	15.33	1.29
-1	8	3	14.99	1.57
-1	8	3	17.31	1.23
-1	8	3	14.71	1.27
1	-8	3	15.15	1.23
1	-8	3	16.20	1.73
1	-8	3	17.67	1.28
-1	-9	3	12.26	1.20
-1	-9	3	15.29	1.57
-1	-9	3	11.03	1.54
-1	-9	3	12.02	0.90
1	9	3	14.01	1.82
-1	-9	3	12.37	1.63
1	-9	-3	15.59	1.30
-1	9	-3	17.43	1.56
1	-9	-3	16.94	1.80
-1	9	-3	15.26	1.82

1	-9	-3	15.27	1.44
1	-9	-3	16.26	1.28
1	-9	-3	15.26	1.18
1	9	3	14.57	1.21
1	9	3	13.41	1.23
1	-9	-3	17.61	1.50
-1	9	-3	15.46	1.51
-1	-9	3	16.14	1.39
1	9	3	12.47	1.63
-1	-9	3	12.95	1.35
-1	9	-3	17.50	1.15
1	-9	-3	16.02	1.39
-1	-9	-3	15.23	1.16
-1	-9	-3	17.07	1.60
-1	9	3	14.62	1.27
1	9	-3	17.17	1.61
-1	9	3	14.04	1.31
1	-9	3	13.13	0.91
1	-9	3	15.43	1.70
1	-9	3	12.75	1.20
1	-9	3	13.07	1.52
-1	-9	-3	15.90	1.29
1	9	-3	17.19	1.13
-1	9	3	11.54	1.48
-1	9	3	17.09	1.80
1	-9	3	15.55	1.29
1	9	-3	13.81	1.40
1	-9	3	15.87	1.59
-1	-9	-3	14.44	1.34
-1	-9	-3	13.53	1.26
1	9	-3	16.94	1.80
-1	-9	-3	14.97	1.38
1	-9	3	10.95	1.20
-1	-9	-3	14.57	1.74
-1	10	-3	109.31	5.60
-1	10	-3	104.81	5.98
1	-10	-3	109.27	5.39
1	-10	-3	115.42	5.46
-1	-10	3	113.88	5.57
-1	-10	3	117.62	5.66
1	10	3	103.49	5.96
-1	10	-3	113.76	5.21
1	10	3	122.30	5.94
-1	-10	3	104.91	5.21

-1	-10	3	104.45	5.61
-1	10	-3	120.40	5.77
1	-10	-3	102.82	5.54
1	-10	-3	112.41	6.20
1	-10	-3	124.19	5.55
-1	-10	3	120.87	5.66
1	-10	-3	103.53	5.27
1	10	3	110.24	5.39
1	10	3	111.71	5.48
1	-10	-3	108.57	5.45
-1	-10	-3	100.80	5.26
1	-10	3	113.67	5.56
1	10	-3	107.52	5.51
1	10	-3	111.75	5.15
-1	-10	-3	110.84	5.46
-1	-10	-3	112.48	5.52
1	-10	3	102.03	5.24
1	-10	3	105.14	5.61
1	10	-3	106.72	5.96
-1	10	3	112.80	5.44
-1	-10	-3	114.15	5.58
1	-10	3	117.55	5.60
1	-10	3	118.31	5.72
-1	10	3	107.00	6.02
-1	10	3	118.19	5.94
-1	-10	-3	109.82	5.30
-1	10	3	111.04	5.58
1	10	-3	116.43	5.70
1	-10	3	126.06	5.74
-1	-10	-3	101.44	5.50
1	11	3	62.60	4.16
-1	-11	3	61.71	3.23
1	11	3	63.75	3.45
-1	-11	3	73.23	3.86
-1	11	-3	92.84	5.30
-1	11	-3	90.47	4.78
1	11	3	66.24	3.57
1	-11	-3	83.90	4.55
1	-11	-3	95.84	4.61
-1	-11	3	71.77	3.82
1	-11	-3	91.04	4.65
1	-11	-3	87.25	4.40
1	-11	-3	88.22	4.61
-1	-11	3	66.45	3.64

1	-11	-3	80.90	4.67
-1	11	-3	93.95	5.45
1	-11	3	63.79	3.72
-1	11	3	67.92	3.53
1	-11	3	61.83	3.27
1	11	-3	87.34	4.68
-1	-11	-3	93.04	4.64
-1	11	3	76.78	4.20
-1	11	3	65.78	4.26
1	11	-3	92.16	5.35
1	-11	3	73.93	3.74
1	-11	3	67.37	3.64
-1	-11	-3	97.23	4.70
-1	-11	-3	86.96	4.70
-1	-11	-3	82.02	4.37
-1	11	3	69.24	3.72
1	-12	-3	57.19	3.27
1	12	3	46.28	2.80
-1	12	-3	50.09	3.96
-1	-12	3	50.18	3.07
-1	-12	3	44.88	2.87
1	-12	-3	52.20	2.91
1	-12	-3	61.02	3.24
1	12	3	41.99	3.53
-1	12	-3	53.19	3.48
1	-12	3	48.05	2.91
-1	12	3	50.07	3.00
-1	12	3	42.57	3.68
-1	-12	-3	48.90	2.90
1	12	-3	53.49	3.31
-1	-12	-3	57.78	3.28
-1	-12	-3	60.08	3.31
1	12	-3	49.79	3.97
-1	13	-3	54.01	4.53
1	-13	-3	55.93	3.40
-1	-13	3	55.93	3.39
-1	13	-3	54.78	3.74
1	-13	-3	65.02	3.55
1	-13	-3	56.18	3.18
1	13	3	52.15	4.38
-1	-13	3	62.50	3.72
-1	-13	-3	66.43	3.76
1	-13	3	63.03	3.63
-1	13	3	52.67	4.38

1	13	-3	53.74	4.58
-1	-13	-3	56.74	3.44
1	13	-3	67.65	4.49
1	-13	3	56.65	3.38
2	0	3	45.21	2.04
-2	0	3	42.92	2.05
-2	0	-3	69.75	3.25
-2	0	3	44.25	2.02
2	0	3	43.09	2.07
2	0	-3	72.29	3.30
-2	0	3	44.98	2.03
-2	0	-3	69.22	3.24
-2	0	-3	70.68	3.28
2	0	3	41.23	2.03
2	0	3	40.05	2.05
-2	0	3	44.52	1.98
-2	0	-3	74.77	3.33
2	0	-3	70.79	3.24
-2	0	-3	66.30	3.28
-2	0	3	48.39	2.07
2	0	-3	72.00	3.26
2	0	3	43.93	2.03
-2	0	3	44.93	2.09
2	0	-3	76.65	3.29
-2	0	-3	70.63	3.25
2	1	3	85.10	4.04
2	1	3	86.89	4.00
2	-1	-3	82.35	3.91
-2	-1	3	92.28	4.09
-2	1	-3	86.58	3.97
-2	-1	3	87.82	4.04
2	-1	-3	88.63	3.93
-2	-1	3	88.11	3.97
2	-1	-3	84.87	3.97
-2	1	-3	89.45	3.96
2	1	3	83.98	4.02
-2	1	-3	93.94	4.09
-2	-1	3	85.75	3.99
-2	-1	3	87.62	3.99
-2	1	-3	88.90	3.95
2	1	3	87.61	4.07
-2	1	-3	84.53	4.04
2	-1	-3	87.95	4.00
-2	1	-3	91.01	3.98

2	-1	-3	88.03	4.00
-2	-1	3	85.67	4.07
2	1	-3	88.83	4.00
2	1	-3	86.52	4.00
-2	1	3	87.70	4.00
-2	1	3	95.52	4.08
-2	-1	-3	84.95	3.93
2	1	-3	86.22	3.93
-2	1	3	93.22	4.04
2	-1	3	88.71	4.02
2	-1	3	92.30	4.11
2	1	-3	87.10	4.01
2	-1	3	90.55	4.01
-2	-1	-3	87.88	3.95
-2	-1	-3	87.48	4.02
-2	-1	-3	81.83	3.96
-2	1	3	88.42	4.06
-2	1	3	87.78	3.98
-2	1	3	90.88	4.07
2	-1	3	89.01	4.04
2	-1	3	90.14	4.13
-2	-1	-3	85.48	3.95
-2	-1	-3	87.21	3.97
2	-1	3	82.09	4.03
-2	-1	-3	85.36	4.00
2	2	3	240.71	11.01
-2	2	-3	219.12	9.64
2	-2	-3	192.19	9.49
-2	-2	3	245.43	11.01
-2	-2	3	243.38	11.03
2	2	3	245.06	11.02
-2	-2	3	241.06	10.95
2	2	3	239.51	10.95
2	-2	-3	207.34	9.57
2	-2	-3	225.58	9.48
2	-2	-3	202.30	9.53
2	2	3	246.29	11.03
2	2	3	239.50	10.96
-2	2	-3	205.33	9.48
2	-2	-3	208.65	9.59
-2	-2	3	231.48	10.94
-2	2	-3	229.16	9.68
-2	2	-3	211.83	9.52
-2	2	-3	233.47	9.54

-2	-2	3	256.52	11.04
-2	2	-3	208.89	9.55
-2	2	3	244.31	10.99
-2	-2	-3	202.75	9.52
-2	2	3	241.23	10.92
2	-2	3	252.86	11.06
2	-2	3	237.70	10.96
2	-2	3	245.63	10.97
-2	2	3	228.75	10.93
2	-2	3	234.44	10.99
-2	-2	-3	216.75	9.58
-2	-2	-3	207.90	9.57
2	-2	3	232.11	10.92
2	2	-3	217.28	9.58
2	2	-3	209.93	9.58
2	-2	3	267.59	11.12
2	2	-3	204.18	9.58
-2	2	3	243.16	10.99
-2	-2	-3	205.60	9.52
-2	2	3	250.14	11.03
-2	2	3	244.87	11.05
2	2	-3	210.78	9.51
-2	2	3	259.63	10.99
2	3	3	9.74	0.64
-2	-3	3	10.01	0.75
2	-3	-3	7.72	0.68
-2	3	-3	8.54	0.57
-2	-3	3	9.77	0.62
-2	3	-3	7.47	0.67
2	-3	-3	7.47	0.49
-2	-3	3	10.72	0.78
2	3	3	10.29	0.74
2	3	3	10.11	0.70
2	-3	-3	6.82	0.62
2	3	3	9.16	0.64
2	-3	-3	6.07	0.51
-2	3	-3	7.39	0.46
2	3	3	10.89	0.75
2	-3	-3	6.96	0.57
-2	3	-3	7.78	0.59
-2	-3	3	9.82	0.63
-2	3	-3	7.74	0.54
-2	3	-3	8.54	0.72
-2	-3	3	9.94	0.72

2	3	-3	6.99	0.54
2	-3	3	11.23	0.68
2	3	-3	6.47	0.63
-2	-3	-3	7.45	0.61
2	-3	3	10.98	0.82
2	3	-3	7.73	0.64
2	-3	3	11.94	0.87
-2	3	3	10.63	0.70
-2	3	3	9.28	0.61
-2	3	3	9.98	0.67
2	-3	3	9.72	0.68
-2	3	3	9.01	0.62
-2	-3	-3	7.02	0.58
2	3	-3	7.00	0.64
2	-3	3	10.89	0.58
-2	-3	-3	8.19	0.61
-2	3	3	9.37	0.74
-2	3	3	9.94	0.81
-2	-3	-3	6.99	0.51
2	-3	3	10.83	0.64
2	-4	-3	1.54	0.35
2	4	3	0.44	0.36
2	4	3	0.54	0.32
2	-4	-3	1.05	0.27
-2	-4	3	0.60	0.37
2	4	3	0.32	0.24
2	-4	-3	0.69	0.36
-2	-4	3	0.41	0.24
-2	4	-3	1.09	0.33
2	-4	-3	1.49	0.29
2	4	3	0.98	0.27
-2	4	-3	0.51	0.35
-2	-4	3	0.32	0.25
-2	4	-3	1.64	0.36
-2	4	-3	1.37	0.25
-2	-4	3	1.01	0.36
2	4	3	0.53	0.28
-2	4	-3	1.54	0.50
-2	4	-3	1.12	0.33
-2	-4	3	0.83	0.37
2	4	3	0.77	0.34
2	-4	-3	1.27	0.28
-2	-4	3	0.61	0.38
2	-4	3	0.49	0.25

2	4	-3	0.99	0.32
2	-4	3	0.49	0.58
-2	-4	-3	1.11	0.34
-2	4	3	0.72	0.29
2	4	-3	1.32	0.44
2	-4	3	0.60	0.28
2	4	-3	1.23	0.21
-2	4	3	0.50	0.28
-2	-4	-3	1.49	0.38
-2	-4	-3	1.39	0.28
2	4	-3	1.48	0.36
2	-4	3	1.32	0.49
2	-4	3	0.53	0.20
-2	4	3	0.64	0.27
-2	-4	-3	1.30	0.37
-2	4	3	0.50	0.41
2	4	-3	1.25	0.32
-2	-4	-3	1.47	0.32
-2	-4	-3	0.94	0.25
-2	4	3	0.51	0.34
2	-4	3	0.56	0.31
-2	-5	3	11.99	0.87
2	-5	-3	16.25	1.01
-2	-5	3	12.57	0.97
2	5	3	11.93	1.04
-2	5	-3	17.08	1.25
-2	-5	3	11.86	0.89
-2	5	-3	15.35	1.18
2	-5	-3	16.61	1.06
-2	-5	3	14.82	1.07
-2	-5	3	13.06	1.09
-2	-5	3	12.28	0.70
2	5	3	12.08	0.97
2	-5	-3	15.98	1.11
2	5	3	12.14	0.82
2	-5	-3	16.83	1.27
-2	5	-3	18.39	1.16
-2	-5	3	13.16	1.06
2	5	3	13.77	1.05
2	-5	-3	17.53	1.01
2	-5	-3	14.61	1.00
-2	5	-3	15.75	1.05
-2	5	-3	17.63	0.95
2	5	3	13.25	0.89

2	-5	3	15.17	1.20
2	5	-3	15.77	0.85
2	5	-3	16.32	1.05
-2	5	3	12.81	1.06
-2	-5	-3	18.40	1.21
-2	-5	-3	17.92	1.17
-2	-5	-3	14.47	1.10
2	5	-3	15.34	1.10
2	-5	3	12.50	0.87
-2	5	3	11.15	0.84
-2	5	3	13.15	0.95
2	5	-3	16.30	1.22
-2	5	3	12.11	0.88
-2	-5	-3	16.82	1.00
-2	5	3	11.67	0.86
2	5	-3	16.21	1.09
2	-5	3	13.83	0.75
2	-5	3	14.26	0.92
-2	-5	-3	16.64	1.03
-2	-5	-3	15.83	0.91
2	-5	3	12.69	1.10
2	-5	3	12.58	0.95
-2	-6	3	33.66	1.88
-2	6	-3	37.14	2.01
2	6	3	30.26	1.84
-2	6	-3	39.17	2.13
2	-6	-3	33.21	1.95
-2	-6	3	30.43	1.77
-2	-6	3	28.53	1.68
2	6	3	28.58	1.88
2	6	3	31.10	1.62
2	-6	-3	35.37	1.94
-2	-6	3	27.85	1.64
2	6	3	30.38	1.78
-2	-6	3	28.47	1.46
2	-6	-3	40.66	1.99
-2	6	-3	37.51	2.33
2	-6	-3	37.30	2.01
2	-6	-3	36.12	2.09
2	6	3	30.47	1.66
-2	-6	3	32.29	1.92
2	-6	-3	38.25	2.25
-2	-6	3	29.49	1.83
2	6	3	28.19	1.60

-2	6	-3	36.37	2.16
-2	6	-3	39.14	1.89
2	-6	3	28.72	1.51
2	-6	3	32.81	1.72
-2	6	3	25.80	1.99
-2	-6	-3	39.85	2.13
-2	-6	-3	40.34	2.19
2	6	-3	36.36	2.08
2	6	-3	38.48	2.25
2	-6	3	31.47	1.87
2	-6	3	28.99	1.65
-2	-6	-3	35.91	1.99
-2	-6	-3	33.94	2.07
-2	6	3	27.98	1.87
-2	-6	-3	36.68	1.93
-2	6	3	28.62	1.65
2	6	-3	38.00	2.08
-2	6	3	29.97	1.66
2	-6	3	31.75	1.76
2	-6	3	32.60	2.02
2	6	-3	38.14	2.01
2	-6	3	28.44	1.73
-2	6	3	28.54	1.74
-2	-6	-3	36.73	1.87
2	6	-3	37.47	1.80
-2	6	3	29.06	1.65
2	7	3	48.75	2.53
2	-7	-3	50.43	2.81
-2	-7	3	43.51	2.55
-2	7	-3	48.64	2.57
2	7	3	50.43	2.46
2	-7	-3	53.78	2.57
-2	-7	3	46.09	2.53
2	7	3	46.03	2.62
-2	-7	3	48.19	2.64
2	7	3	48.76	2.75
-2	7	-3	45.18	2.71
-2	7	-3	52.31	2.44
-2	-7	3	49.41	2.72
2	7	3	46.55	2.79
2	-7	-3	47.23	2.53
-2	-7	3	54.99	2.69
2	-7	-3	45.64	2.46
2	-7	-3	50.51	2.57

-2	7	-3	45.39	2.87
2	-7	-3	43.90	2.64
2	7	3	46.25	2.44
-2	-7	3	44.78	2.73
-2	7	-3	49.25	2.80
2	-7	-3	48.82	2.52
2	-7	3	48.78	2.53
-2	-7	-3	51.11	2.77
2	-7	3	53.27	2.65
-2	-7	-3	46.56	2.46
-2	-7	-3	46.97	2.64
2	-7	3	44.80	2.35
-2	-7	-3	47.31	2.42
-2	7	3	47.02	2.59
2	-7	3	52.79	2.73
2	7	-3	48.68	2.61
-2	-7	-3	48.27	2.56
2	7	-3	46.88	2.83
-2	7	3	50.28	2.80
-2	-7	-3	54.17	2.70
-2	7	3	49.86	2.51
2	7	-3	51.09	2.68
2	-7	3	45.79	2.56
2	7	-3	48.75	2.33
-2	7	3	46.62	2.52
-2	7	3	45.98	2.86
2	-7	3	50.00	2.56
2	7	-3	48.45	2.56
2	-7	3	51.33	2.84
2	-8	-3	24.40	1.51
2	-8	-3	25.66	1.61
-2	8	-3	27.16	1.91
2	8	3	21.16	1.63
2	-8	-3	28.39	2.03
2	8	3	24.16	1.86
-2	-8	3	20.64	1.47
2	8	3	23.83	1.85
2	-8	-3	23.44	1.70
-2	-8	3	22.38	1.84
2	8	3	22.45	1.49
-2	-8	3	21.92	1.18
-2	-8	3	21.83	1.61
-2	8	-3	27.57	1.50
-2	8	-3	26.43	1.67

-2	8	-3	22.72	2.00
-2	-8	3	22.76	1.76
-2	8	-3	27.50	1.88
2	8	3	23.00	1.40
2	-8	-3	28.85	1.69
-2	-8	3	21.66	1.59
2	-8	-3	25.85	1.69
2	8	3	19.49	1.43
2	-8	-3	24.64	1.60
-2	-8	3	21.93	1.69
2	8	-3	26.26	1.67
-2	8	3	21.49	1.57
2	8	-3	26.65	1.84
2	-8	3	21.52	1.25
2	-8	3	22.23	1.47
-2	-8	-3	23.99	1.50
-2	8	3	19.77	1.51
2	-8	3	21.17	1.55
-2	-8	-3	27.04	1.66
2	8	-3	25.35	1.38
2	-8	3	26.11	1.81
-2	8	3	24.21	1.77
2	-8	3	22.28	1.50
-2	-8	-3	27.16	1.86
2	-8	3	23.34	2.00
2	8	-3	25.45	2.02
-2	-8	-3	20.30	1.85
-2	-8	-3	25.23	1.75
2	-8	3	24.19	1.65
-2	-8	-3	27.74	1.93
-2	8	3	19.11	1.72
2	8	-3	26.65	1.74
-2	8	3	23.00	1.51
2	9	3	22.76	1.56
-2	-9	3	23.10	1.32
-2	-9	3	22.06	1.65
-2	-9	3	24.26	1.75
2	-9	-3	16.61	1.26
2	-9	-3	16.83	1.42
-2	9	-3	18.30	1.67
2	-9	-3	20.61	1.55
2	-9	-3	19.15	1.53
-2	-9	3	25.66	1.83
2	-9	-3	20.21	1.40

-2	9	-3	20.61	1.32
-2	9	-3	18.04	1.96
2	9	3	22.97	2.25
-2	9	-3	21.22	1.82
-2	-9	3	26.27	1.92
2	-9	-3	18.25	1.38
2	9	3	22.65	1.92
2	9	3	23.16	1.56
-2	-9	3	25.33	1.98
2	-9	-3	18.99	1.90
-2	-9	-3	17.18	1.33
2	-9	3	25.25	1.70
2	-9	3	24.93	1.67
-2	9	3	24.30	1.65
-2	-9	-3	16.65	1.23
2	-9	3	27.62	2.01
-2	9	3	23.66	1.75
-2	-9	-3	20.78	1.74
2	-9	3	25.56	2.11
2	-9	3	24.10	1.38
-2	-9	-3	18.87	1.61
2	9	-3	17.11	1.50
-2	9	3	23.25	2.07
-2	9	3	26.63	2.10
2	9	-3	18.40	1.92
-2	-9	-3	18.01	1.77
2	-9	3	22.10	1.74
2	-9	3	26.29	1.89
2	9	-3	20.30	1.73
-2	-9	-3	19.57	1.44
-2	-9	-3	20.58	1.56
2	-10	-3	17.67	2.10
-2	10	-3	20.75	1.38
2	-10	-3	18.72	1.68
-2	10	-3	18.72	1.97
-2	-10	3	14.68	1.01
-2	-10	3	15.51	1.58
2	10	3	15.74	1.92
2	-10	-3	17.13	1.35
-2	10	-3	18.36	1.84
-2	10	-3	22.30	2.02
2	-10	-3	20.05	1.72
2	10	3	16.16	1.31
2	10	3	15.07	1.34

-2	-10	3	18.44	1.85
-2	-10	3	14.37	1.46
2	10	3	20.52	2.04
-2	-10	3	14.34	1.39
2	-10	-3	18.38	1.51
2	-10	-3	17.90	1.66
2	-10	-3	21.34	1.54
-2	10	3	15.85	1.58
-2	-10	-3	18.47	1.35
2	-10	3	15.09	1.38
-2	10	3	16.35	1.47
-2	-10	-3	17.64	1.91
2	-10	3	16.55	1.44
2	-10	3	14.24	1.46
2	10	-3	21.10	2.13
-2	10	3	16.32	2.04
2	10	-3	18.84	2.01
2	10	-3	18.36	1.21
-2	-10	-3	18.30	1.43
-2	10	3	17.93	1.99
2	-10	3	15.01	1.06
-2	-10	-3	20.39	1.58
-2	-10	-3	19.29	1.71
2	10	-3	17.32	1.62
2	-10	3	15.85	1.76
2	-10	3	15.83	1.91
-2	11	-3	6.91	1.25
2	-11	-3	6.83	1.08
2	-11	-3	8.31	1.27
2	-11	-3	8.31	1.13
2	-11	-3	6.91	0.93
-2	-11	3	6.80	1.27
2	11	3	6.98	1.22
-2	11	-3	8.99	1.55
-2	11	-3	9.30	1.77
-2	-11	3	6.67	1.20
2	11	3	6.69	0.93
2	11	3	7.03	1.63
2	-11	-3	9.11	1.55
2	-11	-3	8.87	1.36
-2	11	-3	8.25	1.25
-2	-11	3	5.27	0.70
-2	-11	3	7.95	1.26
2	11	-3	6.91	1.16

-2	11	3	5.71	1.48
-2	-11	-3	5.08	0.97
-2	-11	-3	7.35	1.22
-2	11	3	8.25	1.50
2	-11	3	6.41	1.09
-2	-11	-3	6.68	1.20
2	-11	3	6.12	1.14
2	-11	3	7.18	1.14
2	11	-3	7.67	1.55
-2	11	3	7.84	1.55
-2	-11	-3	8.82	1.51
2	-11	3	5.86	0.78
2	-11	3	7.08	1.28
-2	12	-3	8.07	1.42
2	-12	-3	7.57	1.47
-2	-12	3	9.25	1.50
-2	12	-3	3.58	2.13
-2	-12	3	9.09	1.47
2	12	3	11.76	2.30
2	-12	-3	6.44	1.23
2	-12	-3	6.45	0.98
-2	-12	-3	6.24	1.16
2	-12	3	7.59	1.19
-2	12	3	8.21	1.37
2	-12	3	7.78	0.99
2	12	-3	7.95	1.94
-2	12	3	6.98	1.64
-2	-12	-3	6.60	1.12
2	12	-3	6.93	1.23
-2	-12	-3	6.74	1.28
2	-12	3	8.21	1.60
2	13	3	8.04	2.02
-2	13	-3	5.29	2.15
-2	13	-3	4.36	1.55
-2	-13	3	7.47	1.22
-2	-13	3	11.04	2.05
2	-13	-3	6.21	0.94
2	-13	-3	6.36	1.16
-2	13	3	8.08	2.08
-2	-13	-3	6.21	1.23
2	-13	3	5.78	1.08
2	-13	3	9.07	1.43
-2	-13	-3	8.48	1.53
2	13	-3	6.57	2.35

3	0	-3	0.06	0.15
-3	0	-3	0.09	0.10
-3	0	3	0.05	0.13
3	0	3	-0.10	0.12
-3	0	3	0.08	0.07
3	0	3	0.04	0.11
3	0	-3	0.04	0.07
-3	0	3	-0.01	0.15
-3	0	-3	0.13	0.15
3	0	-3	0.17	0.09
-3	0	-3	0.07	0.12
3	0	-3	0.04	0.13
3	0	3	0.08	0.13
-3	0	3	0.07	0.09
-3	0	-3	0.05	0.18
3	0	-3	0.03	0.16
-3	0	-3	0.23	0.13
3	0	3	0.06	0.10
-3	0	3	0.07	0.11
-3	0	3	-0.18	0.15
3	0	3	0.07	0.17
3	0	-3	0.04	0.11
-3	0	-3	0.15	0.18
3	1	3	6.66	0.43
-3	1	-3	13.88	0.73
3	-1	-3	13.46	0.69
3	-1	-3	14.30	0.77
-3	-1	3	6.37	0.51
-3	1	-3	15.00	0.89
3	-1	-3	14.11	0.75
3	1	3	6.67	0.42
-3	-1	3	5.98	0.48
-3	1	-3	13.96	0.74
-3	1	-3	14.44	0.76
3	-1	-3	13.44	0.68
3	1	3	6.12	0.48
-3	1	-3	15.07	0.74
-3	-1	3	5.96	0.39
-3	-1	3	6.42	0.38
3	1	3	6.04	0.42
3	1	3	5.43	0.39
3	-1	-3	14.04	0.81
-3	-1	3	6.28	0.34
-3	1	-3	13.11	0.85

-3	-1	3	6.58	0.36
-3	-1	-3	14.60	0.81
-3	-1	-3	14.69	0.74
-3	1	3	6.20	0.36
3	-1	3	7.08	0.59
-3	1	3	6.24	0.44
3	-1	3	6.67	0.53
3	1	-3	13.60	0.80
3	-1	3	5.94	0.41
-3	-1	-3	13.84	0.74
-3	-1	-3	13.29	0.75
3	-1	3	6.67	0.42
-3	1	3	6.77	0.45
3	-1	3	6.33	0.41
3	1	-3	13.08	0.79
-3	-1	-3	13.12	0.72
3	1	-3	13.78	0.70
3	1	-3	13.70	0.68
-3	1	3	6.10	0.45
-3	1	3	6.03	0.38
-3	1	3	6.48	0.50
3	-1	3	6.07	0.43
3	1	-3	13.18	0.74
-3	-1	-3	13.72	0.80
3	1	-3	13.51	0.68
3	2	3	249.99	11.24
3	2	3	243.12	11.23
3	-2	-3	235.19	10.68
-3	-2	3	258.25	11.31
-3	2	-3	261.46	10.76
-3	2	-3	253.35	10.92
-3	-2	3	241.91	11.23
-3	-2	3	253.82	11.25
3	2	3	246.78	11.28
3	-2	-3	219.62	10.72
-3	2	-3	239.27	10.79
-3	-2	3	242.20	11.15
-3	-2	3	242.74	11.15
-3	2	-3	244.32	10.73
3	-2	-3	240.20	10.81
-3	2	-3	244.30	10.76
3	-2	-3	236.39	10.84
3	-2	-3	246.55	10.80
-3	-2	3	235.40	11.20

3	-2	3	243.81	11.21
3	-2	3	267.67	11.40
-3	-2	-3	228.56	10.75
3	2	-3	235.73	10.82
-3	-2	-3	242.26	10.78
3	2	-3	234.96	10.74
-3	2	3	252.50	11.32
-3	2	3	233.67	11.20
-3	2	3	256.96	11.29
-3	2	3	261.74	11.30
3	-2	3	245.93	11.27
-3	2	3	244.68	11.17
-3	-2	-3	234.89	10.79
3	-2	3	257.60	11.24
-3	2	3	258.79	11.26
3	2	-3	241.72	10.81
3	-2	3	262.92	11.35
-3	-2	-3	225.79	10.77
-3	-3	3	266.54	11.37
-3	3	-3	268.35	11.61
3	3	3	256.07	11.29
3	-3	-3	239.63	11.54
3	-3	-3	226.89	11.67
3	3	3	242.73	11.37
3	3	3	243.24	11.27
-3	-3	3	255.56	11.40
-3	3	-3	263.36	11.51
3	-3	-3	261.55	11.67
-3	3	-3	250.94	11.67
-3	-3	3	255.44	11.34
-3	-3	3	239.42	11.27
3	-3	-3	264.71	11.47
3	3	3	253.86	11.37
-3	-3	3	241.95	11.38
-3	3	-3	265.92	11.74
3	3	3	249.22	11.41
-3	-3	3	242.97	11.29
3	-3	-3	263.00	11.58
-3	3	-3	258.98	11.56
-3	3	-3	267.84	11.54
3	-3	-3	238.96	11.51
-3	-3	-3	250.08	11.58
3	-3	3	241.93	11.33
-3	-3	-3	269.58	11.60

3	-3	3	271.30	11.49
3	3	-3	258.15	11.54
-3	3	3	268.40	11.39
3	-3	3	259.80	11.41
-3	3	3	237.34	11.27
-3	-3	-3	259.31	11.55
3	3	-3	263.27	11.60
-3	3	3	248.46	11.22
-3	-3	-3	231.32	11.54
-3	3	3	244.99	11.42
3	-3	3	240.38	11.24
3	-3	3	229.14	11.28
-3	3	3	249.50	11.34
3	3	-3	245.61	11.62
-3	3	3	251.33	11.31
3	-3	3	268.79	11.29
-3	-3	-3	272.47	11.64
3	4	3	60.05	2.97
-3	-4	3	54.29	2.93
3	-4	-3	57.29	2.87
3	-4	-3	58.58	2.62
-3	4	-3	58.16	2.72
-3	-4	3	63.84	2.94
-3	-4	3	61.59	2.94
-3	-4	3	59.17	2.97
-3	4	-3	57.26	2.96
3	4	3	58.27	2.80
-3	4	-3	58.20	2.69
-3	-4	3	57.77	2.84
-3	4	-3	57.68	2.63
3	-4	-3	53.02	2.76
3	-4	-3	54.98	2.73
-3	-4	3	55.62	2.81
3	-4	-3	52.83	2.66
3	4	3	58.29	2.95
-3	4	-3	53.70	2.85
3	4	3	60.66	2.94
3	4	3	58.51	2.83
-3	4	-3	57.43	2.78
3	-4	-3	51.15	2.68
3	-4	3	59.10	2.92
-3	4	3	52.77	2.88
-3	4	3	58.13	3.01
3	-4	3	66.02	3.10

-3	-4	-3	58.03	2.82
-3	4	3	62.26	2.97
-3	-4	-3	51.59	2.73
3	4	-3	50.03	2.82
3	-4	3	57.44	2.77
-3	4	3	57.18	2.77
-3	-4	-3	56.88	2.71
-3	4	3	58.70	2.86
-3	4	3	60.52	2.90
-3	-4	-3	60.50	2.79
-3	-4	-3	52.71	2.66
3	-4	3	59.23	2.98
3	4	-3	55.09	2.76
3	4	-3	57.72	2.71
3	-4	3	56.95	2.83
-3	5	-3	68.96	3.15
3	5	3	61.12	3.18
3	5	3	59.79	3.12
3	-5	-3	66.96	3.37
3	-5	-3	67.44	3.08
-3	-5	3	67.70	3.13
-3	5	-3	67.05	3.07
3	5	3	62.63	3.01
-3	-5	3	60.78	3.01
3	-5	-3	61.11	3.12
-3	5	-3	66.59	3.17
-3	-5	3	64.80	3.15
-3	-5	3	61.04	3.13
-3	5	-3	67.47	3.43
-3	-5	3	57.99	3.01
-3	5	-3	63.53	3.31
3	-5	-3	59.15	3.11
3	5	3	61.55	3.14
3	5	3	53.25	3.02
-3	5	-3	66.70	3.24
3	5	3	59.79	2.90
-3	-5	3	65.04	3.18
3	-5	-3	60.24	3.22
3	-5	-3	64.62	3.20
-3	-5	3	60.96	2.84
-3	5	3	58.84	2.96
3	5	-3	64.03	3.19
-3	-5	-3	63.52	3.10
3	5	-3	65.58	2.96

3	-5	3	69.68	3.32
-3	5	3	61.37	3.09
3	5	-3	69.21	3.17
3	5	-3	65.35	3.19
3	-5	3	59.71	3.07
3	5	-3	59.23	3.31
-3	5	3	61.38	3.28
-3	-5	-3	69.55	3.28
3	-5	3	60.71	2.92
-3	5	3	64.15	3.03
-3	-5	-3	63.82	3.15
3	-5	3	63.67	3.17
-3	5	3	61.18	3.01
-3	-5	-3	59.81	3.19
3	-5	3	68.66	3.01
3	-5	3	59.55	3.01
-3	-5	-3	70.86	3.27
-3	-5	-3	59.51	2.98
3	6	3	67.37	3.54
-3	-6	3	67.39	3.54
-3	-6	3	73.54	3.60
3	-6	-3	64.48	3.32
3	-6	-3	66.46	3.46
-3	6	-3	66.36	3.36
-3	-6	3	73.37	3.54
-3	-6	3	67.96	3.22
3	6	3	68.56	3.63
3	6	3	73.67	3.60
3	6	3	66.19	3.30
3	-6	-3	65.29	3.30
-3	6	-3	64.04	3.34
3	-6	-3	74.33	3.64
-3	6	-3	66.33	3.53
-3	-6	3	72.62	3.59
3	-6	-3	69.45	3.28
-3	6	-3	61.40	3.58
3	-6	-3	67.26	3.38
-3	-6	3	66.68	3.42
-3	-6	3	66.58	3.42
-3	6	-3	70.09	3.69
3	-6	-3	73.58	3.29
3	6	3	69.39	3.42
3	6	3	67.29	3.34
3	-6	3	74.43	3.43

-3	6	3	66.21	3.65
3	6	-3	65.96	3.57
3	6	-3	71.39	3.43
3	6	-3	71.01	3.42
3	-6	3	67.53	3.30
-3	-6	-3	73.59	3.49
3	-6	3	66.64	3.49
-3	-6	-3	74.45	3.50
-3	6	3	68.21	3.41
3	6	-3	68.66	3.14
-3	6	3	73.71	3.42
-3	6	3	73.73	3.63
3	6	-3	68.42	3.35
3	-6	3	65.25	3.41
3	-6	3	73.73	3.60
-3	-6	-3	65.00	3.40
-3	-6	-3	65.67	3.17
-3	6	3	67.71	3.48
3	-6	3	73.52	3.78
-3	-6	-3	65.96	3.28
3	-6	3	75.73	3.46
-3	-6	-3	66.51	3.36
-3	7	-3	9.30	0.98
3	-7	-3	10.07	1.31
-3	-7	3	4.71	0.72
-3	7	-3	10.12	1.29
3	-7	-3	8.63	0.87
3	7	3	7.48	1.03
-3	-7	3	5.58	0.52
-3	-7	3	6.85	1.08
3	-7	-3	8.59	1.01
-3	-7	3	5.19	0.90
3	-7	-3	7.86	0.98
-3	-7	3	5.51	1.06
-3	7	-3	9.41	1.15
3	7	3	4.49	0.78
3	-7	-3	8.49	0.78
3	7	3	5.15	0.72
3	7	3	4.94	0.98
-3	7	-3	9.79	0.86
3	-7	-3	9.68	0.88
3	-7	-3	8.79	0.84
-3	-7	3	6.12	1.06
-3	7	-3	8.15	1.11

-3	-7	3	5.85	0.78
3	7	3	6.28	0.88
3	-7	3	4.34	0.71
3	7	-3	7.76	0.92
-3	-7	-3	8.02	1.01
-3	-7	-3	7.45	0.72
3	-7	3	5.25	0.93
3	7	-3	8.73	0.92
-3	-7	-3	11.39	1.13
3	7	-3	8.93	0.99
-3	7	3	4.78	0.95
-3	-7	-3	9.73	1.16
3	-7	3	5.92	0.90
3	7	-3	9.43	1.17
-3	7	3	4.91	0.79
3	-7	3	5.29	0.82
3	-7	3	6.47	1.09
3	-7	3	6.04	0.76
3	-7	3	5.91	0.57
3	7	-3	8.69	0.62
-3	7	3	5.24	0.96
-3	-7	-3	8.18	0.78
-3	-7	-3	8.48	0.85
-3	7	3	5.00	0.88
-3	7	3	4.29	0.80
3	-8	-3	36.03	1.96
-3	8	-3	32.83	2.20
-3	-8	3	41.52	2.33
-3	8	-3	35.27	2.21
3	8	3	37.85	2.47
3	-8	-3	32.28	2.03
-3	-8	3	36.12	1.86
-3	8	-3	36.14	1.87
-3	-8	3	35.93	2.27
-3	8	-3	35.82	2.22
3	-8	-3	35.20	1.90
3	8	3	37.31	2.40
3	-8	-3	35.45	2.39
-3	-8	3	36.77	2.39
-3	-8	3	35.87	2.15
3	-8	-3	32.66	2.08
3	8	3	39.56	2.32
-3	-8	3	37.03	2.45
3	-8	-3	33.12	1.95

-3	8	-3	29.92	2.34
-3	-8	3	35.46	2.19
3	8	3	36.50	2.00
3	8	3	38.70	2.04
3	-8	-3	32.27	1.87
3	8	-3	29.85	2.27
-3	-8	-3	35.76	2.22
3	8	-3	34.01	2.03
-3	-8	-3	31.43	1.79
-3	8	3	38.24	2.15
3	-8	3	38.36	2.15
-3	8	3	34.54	2.24
-3	8	3	36.36	2.18
-3	-8	-3	35.36	2.19
-3	8	3	37.93	2.53
3	-8	3	39.68	2.15
-3	-8	-3	32.84	2.10
3	8	-3	35.35	2.14
-3	8	3	42.63	2.50
-3	-8	-3	32.48	1.97
3	-8	3	42.19	2.66
3	-8	3	37.28	2.25
3	-8	3	34.93	1.94
3	8	-3	32.51	1.69
3	-8	3	40.37	2.40
3	-8	3	40.68	2.27
-3	-8	-3	33.14	1.85
-3	9	-3	21.93	1.93
3	-9	-3	23.28	1.75
3	-9	-3	23.65	1.79
-3	9	-3	25.06	1.54
-3	9	-3	20.75	2.14
-3	-9	3	21.27	1.78
3	9	3	22.03	1.52
-3	-9	3	25.99	1.83
3	-9	-3	23.79	1.60
3	9	3	22.36	1.99
3	-9	-3	26.00	1.69
3	-9	-3	22.96	1.52
-3	-9	3	21.37	1.66
3	9	3	21.82	1.48
-3	-9	3	21.69	1.60
3	9	3	25.30	1.90
3	-9	-3	24.30	2.03

3	-9	-3	25.56	1.60
-3	-9	3	22.74	1.23
-3	9	-3	27.43	1.92
-3	-9	3	21.59	1.89
-3	9	3	21.57	1.63
3	-9	3	21.57	1.64
-3	-9	-3	25.60	1.99
3	-9	3	21.54	1.55
-3	-9	-3	23.58	1.79
-3	9	3	21.36	1.76
3	9	-3	24.96	1.35
3	-9	3	20.25	1.31
3	-9	3	26.36	1.82
3	-9	3	25.98	1.60
3	9	-3	27.93	1.98
-3	9	3	19.26	2.00
3	9	-3	24.17	1.68
3	9	-3	20.89	2.05
-3	9	3	24.97	1.97
-3	-9	-3	23.89	1.69
3	-9	3	24.07	2.00
3	-9	3	23.50	2.24
-3	-9	-3	22.90	1.73
-3	-9	-3	24.85	1.49
3	-10	-3	62.83	3.70
-3	10	-3	60.77	3.51
-3	10	-3	54.63	3.71
-3	-10	3	51.78	3.07
3	10	3	49.98	2.72
-3	-10	3	53.10	2.93
3	-10	-3	54.15	3.19
3	-10	-3	58.36	3.12
3	-10	-3	61.49	3.14
3	-10	-3	51.48	3.21
-3	10	-3	57.70	3.44
3	-10	-3	60.17	3.05
3	10	3	50.40	4.29
3	10	3	48.55	2.70
-3	-10	3	47.05	2.94
-3	10	-3	60.89	2.97
3	-10	-3	53.98	2.96
3	10	3	47.99	3.30
-3	-10	3	49.24	2.51
-3	-10	3	54.32	3.07

3	10	-3	62.67	3.45
-3	10	3	47.23	3.36
3	-10	3	57.70	2.89
3	-10	3	50.97	2.98
-3	-10	-3	57.99	3.27
3	10	-3	55.08	3.11
3	-10	3	55.06	3.49
3	-10	3	47.59	2.59
-3	10	3	52.06	3.04
-3	10	3	58.11	3.44
3	-10	3	54.18	3.07
-3	-10	-3	55.40	2.93
-3	10	3	52.43	2.85
-3	-10	-3	54.08	3.23
-3	-10	-3	55.23	2.90
3	10	-3	55.97	3.63
-3	-10	-3	54.81	3.14
3	10	-3	54.42	2.79
3	-10	3	52.84	2.91
3	-11	-3	45.23	2.68
-3	11	-3	46.27	3.13
-3	-11	3	45.25	2.84
3	11	3	38.97	3.13
3	-11	-3	39.11	2.41
3	-11	-3	42.71	2.75
-3	11	-3	43.87	3.05
-3	11	-3	43.98	2.50
-3	11	-3	41.68	3.40
3	-11	-3	50.62	2.63
3	-11	-3	45.85	2.67
3	11	3	40.26	2.41
-3	-11	3	40.26	2.14
3	11	3	41.00	2.41
3	-11	-3	40.64	2.75
-3	-11	3	44.15	2.63
-3	-11	-3	40.36	2.39
3	-11	3	44.47	2.61
-3	11	3	43.06	2.79
3	-11	3	40.91	2.27
-3	11	3	46.79	3.24
3	-11	3	38.47	2.67
-3	-11	-3	42.42	2.68
3	11	-3	44.25	2.67
-3	11	3	42.00	3.24

3	-11	3	48.09	2.84
3	-11	3	49.15	2.64
-3	-11	-3	46.12	2.88
3	11	-3	43.83	3.26
3	12	3	59.51	4.27
3	-12	-3	64.11	3.41
-3	12	-3	64.59	3.82
-3	-12	3	63.30	3.56
-3	12	-3	56.61	4.33
3	-12	-3	61.53	3.50
-3	-12	3	68.60	3.84
-3	-12	-3	58.65	3.51
3	12	-3	58.57	3.45
3	-12	3	69.57	3.55
3	-12	3	57.28	3.11
-3	12	3	53.13	4.22
3	-12	3	63.87	3.54
-3	-12	-3	55.20	3.16
-3	-12	-3	68.69	3.76
3	12	-3	59.22	4.28
-3	12	3	62.28	3.72
-3	13	-3	10.10	2.30
3	-13	-3	9.61	1.35
-3	13	-3	12.13	2.39
-3	-13	3	12.20	2.33
3	13	3	8.70	2.40
-3	-13	3	10.41	1.58
3	-13	-3	12.27	1.59
-3	13	3	10.40	1.57
-3	-13	-3	12.43	1.77
3	13	-3	12.88	2.64
3	-13	3	10.91	1.55
-3	-13	-3	9.20	1.43
3	-13	3	12.50	1.69
4	0	-3	131.23	5.83
4	0	3	87.90	4.07
-4	0	3	88.96	4.00
-4	0	3	88.04	4.07
-4	0	-3	134.59	5.91
4	0	3	87.85	4.10
4	0	-3	126.22	5.80
-4	0	3	92.36	4.14
-4	0	-3	126.04	5.87
4	0	3	83.60	4.09

4	0	-3	128.90	5.85
4	0	-3	134.04	5.82
-4	0	3	91.50	4.02
-4	0	-3	125.18	5.82
4	0	3	93.00	4.08
-4	0	-3	117.29	5.87
4	0	-3	131.96	5.79
4	0	-3	127.43	5.90
-4	0	-3	128.27	5.85
-4	0	-3	134.79	5.85
-4	0	3	90.66	4.06
4	0	3	84.72	4.05
-4	-1	3	62.85	2.95
4	1	3	64.34	3.06
-4	1	-3	83.32	3.73
-4	1	-3	76.05	3.81
-4	-1	3	65.98	3.06
-4	-1	3	62.08	3.04
-4	1	-3	81.36	3.73
4	1	3	64.25	2.99
-4	1	-3	87.36	3.74
-4	-1	3	66.54	2.95
4	-1	-3	86.04	3.68
4	-1	-3	80.55	3.68
4	1	3	64.53	2.98
-4	1	-3	82.99	3.73
-4	1	-3	81.40	3.72
-4	1	-3	81.45	3.84
4	-1	-3	84.06	3.74
4	1	3	61.30	2.95
-4	-1	3	64.47	2.89
4	1	-3	80.48	3.69
-4	1	3	67.48	3.05
-4	-1	-3	83.62	3.76
4	-1	3	64.64	2.99
-4	1	3	65.35	2.95
4	1	-3	79.66	3.78
4	1	-3	82.20	3.69
-4	1	3	68.28	3.01
-4	1	3	69.12	3.01
4	1	-3	80.20	3.68
4	1	-3	79.21	3.78
4	-1	3	63.43	3.00
4	-1	3	58.89	2.98

4	1	-3	79.21	3.65
-4	-1	-3	79.89	3.74
-4	-1	-3	81.07	3.79
-4	1	3	65.17	3.06
-4	-1	-3	81.60	3.73
-4	-1	-3	80.24	3.70
-4	1	3	62.56	2.94
-4	-1	-3	70.92	3.70
4	-1	3	64.64	3.07
-4	1	3	63.88	3.00
4	-2	-3	83.67	3.77
-4	-2	3	90.04	4.10
4	2	3	88.11	4.02
-4	-2	3	89.28	4.00
4	2	3	83.79	4.04
-4	2	-3	82.22	3.69
-4	2	-3	78.02	3.85
-4	2	-3	77.04	3.81
4	-2	-3	76.13	3.66
4	2	3	85.39	4.11
4	-2	-3	80.84	3.74
-4	2	-3	81.36	3.73
-4	-2	3	87.69	4.12
-4	2	-3	84.10	3.72
-4	-2	3	85.08	4.11
-4	-2	3	85.50	3.94
-4	-2	3	83.97	4.00
-4	2	-3	80.87	3.68
-4	-2	3	85.30	3.95
-4	-2	3	87.41	3.94
4	2	3	86.19	4.02
4	-2	3	87.04	4.03
4	2	-3	82.04	3.75
-4	2	3	89.90	4.09
-4	2	3	92.57	4.08
-4	-2	-3	77.30	3.72
-4	2	3	89.76	4.11
4	2	-3	74.62	3.70
-4	-2	-3	81.85	3.79
4	2	-3	80.12	3.65
-4	2	3	84.25	3.99
-4	-2	-3	81.23	3.71
4	-2	3	81.86	4.04
-4	-2	-3	79.14	3.69

4	-2	3	91.55	4.20
4	-2	3	88.22	4.00
4	-2	3	90.25	4.14
4	2	-3	78.44	3.67
4	-2	3	82.11	4.00
-4	2	3	92.74	4.06
-4	-3	3	9.45	0.50
-4	3	-3	6.36	0.81
-4	3	-3	7.46	0.58
-4	-3	3	9.10	0.60
4	-3	-3	6.58	0.56
-4	-3	3	8.76	0.56
4	3	3	9.35	0.61
4	3	3	9.52	0.69
4	-3	-3	6.90	0.62
4	-3	-3	5.97	0.47
-4	-3	3	10.03	0.75
4	-3	-3	6.53	0.54
4	3	3	9.44	0.75
-4	3	-3	7.30	0.51
-4	-3	3	8.86	0.74
4	-3	-3	6.95	0.70
4	3	3	8.77	0.68
-4	3	-3	7.10	0.52
-4	3	-3	7.25	0.72
4	3	-3	7.93	0.54
-4	3	3	8.96	0.70
4	-3	3	8.38	0.60
-4	-3	-3	6.93	0.52
4	-3	3	9.45	0.60
4	-3	3	9.33	0.64
4	3	-3	7.43	0.51
-4	-3	-3	6.22	0.52
-4	3	3	9.37	0.67
4	-3	3	9.59	0.58
-4	3	3	9.77	0.73
-4	-3	-3	6.94	0.57
4	3	-3	6.99	0.40
-4	3	3	8.38	0.77
4	3	-3	6.09	0.60
-4	-3	-3	7.55	0.56
4	-3	3	7.81	0.85
-4	3	3	8.38	0.60
4	-3	3	9.77	0.89

-4	-3	-3	7.22	0.67
4	3	-3	7.44	0.61
-4	-4	3	33.71	1.73
-4	-4	3	30.04	1.57
4	-4	-3	31.03	1.68
4	-4	-3	33.04	1.79
4	-4	-3	31.00	1.71
-4	4	-3	35.21	1.68
4	4	3	29.20	1.72
-4	4	-3	34.90	1.81
-4	-4	3	31.11	1.46
4	4	3	28.73	1.70
-4	-4	3	28.57	1.72
4	4	3	30.72	1.57
-4	4	-3	34.54	1.70
-4	4	-3	35.88	1.75
4	-4	-3	33.67	1.91
4	-4	-3	31.83	1.79
4	-4	-3	34.37	1.60
-4	-4	3	29.62	1.60
-4	4	-3	32.82	1.95
-4	4	-3	31.46	1.89
-4	-4	3	31.79	1.78
4	4	3	30.40	1.60
-4	-4	3	32.07	1.68
-4	4	3	31.01	1.65
4	-4	3	31.64	1.55
-4	-4	-3	33.56	1.73
4	-4	3	29.69	1.59
4	-4	3	34.94	1.87
-4	-4	-3	34.58	1.78
4	-4	3	30.42	1.65
4	4	-3	34.37	1.78
-4	4	3	30.26	1.59
-4	-4	-3	31.08	1.74
-4	4	3	28.98	1.82
-4	4	3	30.07	1.71
4	-4	3	34.93	1.59
-4	-4	-3	32.25	1.69
-4	-4	-3	34.80	1.87
4	4	-3	34.05	1.72
4	4	-3	33.92	1.56
4	4	-3	31.39	1.82
4	-4	3	31.68	1.79

-4	4	3	28.98	1.50
-4	-5	3	12.89	1.08
-4	-5	3	15.32	1.09
-4	5	-3	19.03	1.51
-4	-5	3	15.65	1.12
-4	5	-3	20.16	1.13
-4	5	-3	17.76	1.33
4	5	3	14.52	1.11
-4	-5	3	14.22	0.95
4	-5	-3	17.71	0.99
4	5	3	13.84	1.09
-4	5	-3	16.36	1.31
4	-5	-3	17.85	1.21
4	-5	-3	16.80	1.37
4	5	3	15.01	0.91
4	-5	-3	17.09	1.01
-4	-5	3	13.50	0.95
-4	-5	3	14.41	0.78
4	5	3	15.73	0.97
4	-5	-3	17.43	1.09
-4	-5	3	13.97	1.17
-4	5	-3	20.71	1.17
4	-5	-3	17.60	1.22
4	-5	-3	17.21	1.07
4	5	3	15.32	1.10
-4	5	3	15.86	1.02
4	-5	3	14.51	0.87
4	5	-3	18.40	1.14
-4	-5	-3	19.57	1.30
-4	-5	-3	18.78	1.10
-4	5	3	11.74	1.04
-4	-5	-3	19.73	1.25
-4	-5	-3	16.24	1.10
-4	5	3	12.59	1.16
4	-5	3	13.34	0.92
4	5	-3	19.07	0.92
4	-5	3	14.61	0.91
4	5	-3	19.11	1.13
-4	5	3	15.76	1.18
4	-5	3	15.52	1.04
4	5	-3	17.66	1.30
-4	-5	-3	17.32	1.16
-4	5	3	13.53	0.96
4	-5	3	16.37	1.31

4	-5	3	14.59	1.20
4	-6	-3	161.49	7.80
4	-6	-3	157.74	7.91
-4	-6	3	152.37	7.48
4	6	3	153.12	7.26
4	6	3	162.50	7.49
4	-6	-3	158.96	7.79
-4	-6	3	152.55	7.36
4	6	3	159.76	7.35
-4	6	-3	180.58	7.76
4	6	3	157.20	7.18
-4	6	-3	164.71	8.02
4	6	3	154.09	7.50
4	6	3	151.36	7.54
-4	-6	3	162.57	7.48
-4	-6	3	157.88	7.51
-4	-6	3	160.18	7.16
4	-6	-3	179.76	8.09
-4	6	-3	173.31	7.85
4	-6	-3	169.41	7.88
-4	6	-3	173.40	7.95
4	-6	-3	175.21	7.72
-4	6	-3	167.45	8.14
-4	-6	3	149.40	7.33
-4	6	3	167.05	7.36
-4	-6	-3	159.30	7.86
-4	6	3	156.58	7.45
4	6	-3	157.17	8.01
4	6	-3	170.07	7.83
-4	6	3	158.60	7.35
-4	-6	-3	164.36	7.76
-4	6	3	170.29	7.55
4	-6	3	148.97	7.42
4	-6	3	155.00	7.35
4	6	-3	172.60	7.89
-4	-6	-3	176.47	7.94
4	6	-3	169.26	7.62
4	6	-3	170.09	7.85
4	-6	3	167.60	7.32
4	-6	3	156.95	7.25
4	-6	3	165.74	7.37
-4	-6	-3	170.10	7.85
4	-6	3	164.20	7.51
-4	-6	-3	178.59	7.98

4	-7	-3	60.00	2.94
4	7	3	41.37	2.53
4	-7	-3	59.03	3.03
-4	7	-3	62.68	3.21
4	7	3	45.79	2.51
4	-7	-3	66.06	2.96
-4	-7	3	44.04	2.49
4	7	3	42.41	2.19
-4	7	-3	59.04	3.28
4	7	3	40.01	2.44
-4	-7	3	42.14	2.50
4	-7	-3	66.95	3.44
4	7	3	41.94	2.13
4	-7	-3	57.74	3.10
-4	-7	3	41.50	2.05
-4	7	-3	64.83	2.97
4	-7	-3	54.25	3.13
-4	-7	3	40.37	2.28
4	7	3	42.50	2.29
-4	7	-3	56.80	3.06
-4	-7	3	45.93	2.41
-4	7	-3	61.99	3.49
-4	-7	3	40.23	2.43
4	-7	-3	58.12	2.99
-4	-7	3	39.42	2.27
-4	-7	-3	62.18	3.23
-4	7	3	43.23	2.64
-4	-7	-3	53.94	2.81
4	-7	3	41.76	2.27
4	7	-3	59.41	3.05
4	-7	3	41.99	2.38
-4	-7	-3	58.16	2.95
-4	7	3	45.22	2.56
-4	-7	-3	57.47	3.10
-4	-7	-3	58.77	3.05
4	-7	3	44.60	2.24
-4	7	3	40.99	2.27
4	7	-3	54.11	3.29
4	7	-3	59.97	3.13
4	-7	3	41.16	2.50
-4	7	3	40.44	2.41
-4	7	3	43.74	2.30
4	-7	3	45.94	2.35
4	-7	3	40.31	2.13

4	7	-3	60.34	3.06
4	7	-3	59.96	2.79
4	-7	3	43.62	2.69
-4	-7	-3	64.16	3.20
4	-8	-3	62.89	3.31
4	-8	-3	65.08	3.63
4	8	3	73.62	3.76
4	8	3	68.34	3.70
-4	-8	3	71.73	3.78
4	-8	-3	62.83	3.14
4	-8	-3	60.94	3.16
-4	8	-3	67.64	3.16
-4	-8	3	65.93	3.69
-4	-8	3	68.09	3.55
-4	-8	3	68.83	3.55
4	8	3	62.60	3.78
-4	8	-3	61.79	3.49
4	-8	-3	71.85	3.20
-4	8	-3	61.82	3.68
-4	-8	3	67.00	3.68
-4	-8	3	66.62	3.25
4	-8	-3	58.55	3.34
-4	-8	3	73.73	3.63
-4	8	-3	65.39	3.47
4	-8	-3	60.09	3.21
4	8	3	67.58	3.39
-4	8	3	69.35	3.54
-4	-8	-3	55.68	2.99
4	8	-3	57.92	3.55
4	-8	3	74.67	3.93
4	-8	3	74.85	3.60
-4	-8	-3	64.03	3.43
4	8	-3	63.03	2.96
4	-8	3	71.73	3.74
4	-8	3	67.63	3.35
-4	8	3	63.60	3.91
-4	-8	-3	57.46	3.29
4	8	-3	64.60	3.40
4	8	-3	64.62	3.25
4	-8	3	73.39	3.49
4	-8	3	67.48	3.52
-4	8	3	76.02	3.85
-4	8	3	70.29	3.53
-4	-8	-3	64.48	3.14

4	-8	3	65.55	3.58
-4	8	3	70.46	3.68
-4	-8	-3	66.82	3.48
-4	-8	-3	63.11	3.27
4	-9	-3	38.17	2.13
4	9	3	38.94	2.17
4	-9	-3	41.76	2.20
-4	9	-3	36.93	2.79
-4	-9	3	38.20	2.36
4	-9	-3	44.00	2.83
4	-9	-3	35.32	2.35
4	-9	-3	36.58	2.34
-4	-9	3	37.63	2.50
4	-9	-3	37.07	2.20
-4	-9	3	37.73	1.97
4	-9	-3	37.79	2.12
-4	-9	3	38.21	2.35
4	9	3	39.58	2.09
4	9	3	40.18	2.64
-4	9	-3	38.18	2.55
-4	-9	3	42.35	2.46
-4	9	-3	42.61	2.16
-4	9	-3	42.10	2.59
4	9	3	38.97	2.71
-4	-9	-3	35.27	2.27
4	-9	3	38.67	2.08
-4	-9	-3	39.93	2.09
-4	9	3	40.83	2.35
4	-9	3	47.18	2.46
4	-9	3	46.37	2.89
4	9	-3	37.47	1.91
4	-9	3	38.17	2.40
4	9	-3	39.36	2.49
-4	9	3	38.79	2.79
-4	9	3	43.96	2.70
-4	-9	-3	34.97	2.34
4	9	-3	37.68	2.64
4	-9	3	38.79	2.64
-4	-9	-3	39.64	2.55
-4	-9	-3	32.97	1.96
4	-9	3	41.41	2.27
4	-9	3	39.61	2.40
4	9	-3	35.44	2.20
-4	9	3	36.82	2.49

4	-10	-3	38.49	2.14
4	10	3	40.29	2.22
-4	-10	3	37.84	2.42
4	-10	-3	34.90	2.35
4	10	3	38.70	2.86
-4	-10	3	37.95	2.01
4	10	3	36.97	2.23
-4	10	-3	35.73	2.46
4	-10	-3	33.90	2.12
-4	-10	3	37.61	2.42
4	-10	-3	32.35	1.98
-4	10	-3	37.37	2.07
4	-10	-3	34.09	2.03
-4	-10	3	39.40	2.71
4	-10	-3	35.25	2.73
-4	10	-3	36.19	2.52
4	10	3	41.43	2.83
-4	10	-3	31.34	2.76
-4	-10	3	40.46	2.57
4	-10	-3	31.70	2.22
4	-10	3	39.52	2.14
4	-10	3	39.23	2.50
-4	-10	-3	32.36	1.94
-4	-10	-3	34.39	2.20
-4	-10	-3	34.55	2.35
-4	10	3	33.36	2.86
-4	-10	-3	32.69	2.37
4	10	-3	35.85	2.48
4	10	-3	31.22	2.88
-4	10	3	42.85	2.97
4	10	-3	33.07	2.11
-4	-10	-3	27.40	2.21
4	-10	3	41.04	2.41
-4	10	3	41.73	2.65
4	-10	3	44.73	3.17
4	-10	3	45.66	2.40
4	-10	3	45.17	2.59
-4	10	3	41.13	2.42
-4	-11	3	-0.56	0.66
4	-11	-3	0.88	0.88
4	11	3	0.09	0.66
4	-11	-3	2.12	0.78
-4	11	-3	-0.88	1.21
-4	11	-3	0.29	0.79

4	-11	-3	0.57	0.55
4	11	3	0.22	1.12
-4	11	-3	0.13	1.38
-4	-11	3	0.12	0.91
4	11	3	-0.17	0.68
4	-11	-3	0.21	0.69
4	-11	3	-0.11	0.77
4	11	-3	0.53	1.29
4	-11	3	0.46	0.49
-4	11	3	0.75	1.30
4	-11	3	1.48	0.81
-4	-11	-3	0.90	0.93
4	-11	3	1.51	1.02
-4	-11	-3	1.69	0.77
-4	11	3	-1.21	0.90
4	-11	3	0.99	0.70
4	11	-3	2.04	0.84
-4	11	3	3.97	2.27
-4	-11	-3	0.73	0.57
-4	12	-3	10.74	1.71
4	-12	-3	8.27	1.63
-4	12	-3	8.03	1.96
4	12	3	5.48	1.70
4	-12	-3	8.19	1.26
-4	-12	3	6.34	1.39
4	-12	-3	10.27	1.36
-4	-12	3	5.78	1.18
4	-12	-3	6.48	1.13
-4	-12	-3	6.66	1.24
4	12	-3	9.17	1.49
-4	-12	-3	7.09	0.94
-4	12	3	9.69	1.93
4	-12	3	7.61	1.22
-4	-12	-3	7.05	1.41
-4	12	3	7.97	1.80
4	12	-3	9.31	1.89
4	-12	3	5.73	0.96
4	-12	3	7.45	1.36
-4	13	-3	10.38	1.93
-4	-13	3	8.30	1.33
-4	-13	3	9.79	1.98
4	-13	-3	8.70	1.55
4	13	3	9.69	2.32
4	-13	-3	8.96	1.18

-4	13	3	11.56	1.82
-4	-13	-3	10.37	1.57
4	-13	3	8.83	1.53
4	13	-3	8.01	2.24
-4	-13	-3	10.69	1.86
-5	0	3	0.20	0.19
5	0	3	-0.27	0.19
-5	0	3	0.09	0.11
-5	0	-3	-0.14	0.15
-5	0	3	0.17	0.21
5	0	-3	0.02	0.14
-5	0	3	0.06	0.16
-5	0	-3	0.02	0.20
-5	0	-3	0.17	0.23
-5	0	-3	0.15	0.23
-5	0	3	0.06	0.19
5	0	-3	0.00	0.11
-5	0	-3	0.24	0.19
-5	0	-3	-0.02	0.12
5	0	3	-0.03	0.15
5	0	-3	0.18	0.25
5	0	3	0.12	0.17
5	0	-3	0.00	0.15
5	0	3	0.03	0.21
5	0	-3	0.15	0.11
-5	-1	3	18.63	0.95
-5	1	-3	13.92	0.77
5	-1	-3	13.70	0.73
-5	1	-3	14.57	0.78
5	1	3	17.26	0.96
-5	-1	3	18.84	0.95
-5	1	-3	14.94	0.93
5	1	3	19.23	1.00
-5	1	-3	14.15	0.79
-5	1	-3	14.32	0.80
5	1	3	17.83	1.07
5	-1	-3	13.36	0.76
-5	1	-3	14.84	0.78
-5	-1	3	18.07	1.06
5	1	3	18.93	0.99
5	-1	-3	12.75	0.77
5	1	3	19.65	1.05
-5	-1	3	18.53	1.06
5	1	3	18.56	0.95

-5	-1	3	18.07	0.95
5	-1	-3	13.18	0.79
-5	-1	3	20.35	1.09
-5	-1	-3	13.27	0.85
5	1	-3	14.39	0.85
-5	1	3	18.32	1.05
5	1	-3	14.28	0.74
-5	1	3	18.73	1.08
5	-1	3	18.54	0.99
-5	-1	-3	14.43	0.74
-5	-1	-3	13.73	0.79
5	1	-3	14.08	0.66
-5	1	3	18.79	0.94
-5	1	3	17.52	0.98
-5	-1	-3	13.13	0.79
-5	-1	-3	13.83	0.80
-5	1	3	19.19	1.00
-5	1	3	17.32	0.93
5	-1	3	18.03	1.09
-5	1	3	19.99	0.99
5	-1	3	19.30	0.99
5	-1	3	18.99	0.98
-5	-2	3	49.76	2.21
-5	2	-3	56.32	2.54
-5	2	-3	53.18	2.68
-5	-2	3	44.55	2.32
5	2	3	46.03	2.29
-5	-2	3	49.81	2.37
-5	-2	3	45.54	2.38
-5	-2	3	48.46	2.19
-5	-2	3	48.32	2.40
-5	-2	3	48.94	2.22
-5	2	-3	50.39	2.64
5	-2	-3	53.81	2.55
-5	2	-3	55.49	2.55
5	-2	-3	51.47	2.48
-5	-2	3	47.05	2.22
-5	2	-3	54.98	2.52
5	-2	-3	54.51	2.60
5	-2	-3	50.75	2.53
-5	2	-3	54.59	2.50
5	2	3	46.66	2.29
5	2	-3	48.86	2.49
-5	2	3	49.44	2.33

-5	2	3	47.93	2.26
5	-2	3	49.15	2.26
-5	-2	-3	53.56	2.62
5	-2	3	46.72	2.31
-5	-2	-3	53.08	2.53
5	2	-3	53.51	2.44
5	2	-3	55.13	2.57
-5	-2	-3	53.68	2.54
5	-2	3	46.87	2.28
-5	2	3	47.91	2.37
-5	2	3	51.78	2.35
5	-2	3	50.01	2.48
-5	-2	-3	52.27	2.52
-5	-2	-3	46.81	2.43
5	2	-3	53.10	2.50
-5	-2	-3	52.62	2.53
5	-2	3	48.50	2.41
-5	2	3	47.27	2.36
-5	-2	-3	55.68	2.50
5	-2	3	45.11	2.29
5	-3	-3	71.45	3.51
5	3	3	80.40	3.77
-5	3	-3	73.42	3.68
-5	3	-3	75.01	3.54
-5	-3	3	79.25	3.79
5	-3	-3	67.40	3.44
-5	3	-3	76.08	3.48
-5	-3	3	80.73	3.68
-5	-3	3	83.18	3.78
5	3	3	82.41	3.80
-5	-3	3	82.68	3.86
5	-3	-3	72.23	3.54
5	-3	-3	71.61	3.67
-5	3	-3	78.49	3.52
5	-3	-3	79.95	3.65
-5	-3	3	80.97	3.69
-5	3	-3	72.61	3.65
-5	3	-3	78.47	3.46
-5	-3	3	77.59	3.71
5	3	3	78.55	3.84
-5	-3	3	81.80	3.94
-5	-3	3	79.09	3.92
5	-3	3	76.71	3.76
5	-3	3	85.68	3.95

5	3	-3	74.52	3.60
5	-3	3	80.21	3.84
5	3	-3	73.62	3.45
-5	-3	-3	74.27	3.51
5	-3	3	86.67	4.03
-5	-3	-3	75.44	3.49
5	3	-3	71.29	3.53
-5	-3	-3	72.32	3.48
-5	3	3	80.10	3.77
5	-3	3	75.35	3.79
-5	3	3	86.73	3.84
5	3	-3	76.55	3.48
5	-3	3	82.98	3.75
-5	-3	-3	70.88	3.50
-5	3	3	80.65	3.89
-5	3	3	81.67	3.93
-5	-3	-3	79.34	3.65
5	-4	-3	44.11	2.36
-5	-4	3	28.28	1.73
5	4	3	28.91	1.67
5	-4	-3	40.88	2.12
-5	-4	3	29.28	1.60
-5	-4	3	29.71	1.46
-5	4	-3	46.71	2.20
5	-4	-3	44.29	2.27
5	-4	-3	42.17	2.25
-5	-4	3	29.11	1.54
5	4	3	29.32	1.57
-5	-4	3	31.15	1.63
5	4	3	29.91	1.75
-5	4	-3	42.96	2.36
-5	4	-3	44.10	2.12
-5	4	-3	41.68	2.44
-5	-4	3	32.35	1.79
5	-4	-3	39.87	2.15
5	4	3	30.92	1.61
-5	4	-3	44.23	2.26
-5	-4	3	31.69	1.71
5	-4	3	32.11	1.89
5	4	-3	46.96	2.20
5	4	-3	44.37	2.16
5	4	-3	43.62	2.16
5	-4	3	29.03	1.55
5	-4	3	32.60	1.56

-5	4	3	28.95	1.80
5	-4	3	28.36	1.58
5	-4	3	30.42	1.79
-5	-4	-3	43.00	2.19
-5	4	3	31.39	1.61
5	-4	3	29.69	1.65
-5	4	3	29.79	1.71
-5	-4	-3	45.11	2.34
-5	-4	-3	42.79	2.18
5	4	-3	42.30	2.01
-5	-4	-3	43.45	2.15
-5	4	3	33.23	1.66
5	4	-3	40.29	2.25
-5	-4	-3	42.87	2.19
-5	4	3	34.25	1.77
5	-5	-3	6.25	0.64
5	5	3	5.92	0.75
-5	5	-3	7.27	0.83
5	-5	-3	7.61	0.89
-5	-5	3	6.37	0.64
-5	5	-3	7.04	0.69
-5	5	-3	8.07	0.93
5	-5	-3	6.80	0.74
-5	5	-3	9.05	0.76
5	5	3	6.74	0.83
-5	-5	3	6.93	0.78
-5	-5	3	6.68	0.85
5	-5	-3	7.05	0.86
-5	-5	3	6.99	0.48
-5	5	-3	7.73	1.16
5	5	3	5.81	0.66
-5	-5	3	6.30	0.82
5	-5	-3	7.58	0.65
5	5	3	6.34	0.66
-5	-5	3	7.14	0.91
-5	-5	3	6.22	0.61
-5	5	-3	7.71	0.69
-5	-5	-3	8.62	0.81
-5	5	3	7.24	0.83
5	-5	3	6.22	0.73
-5	5	3	5.31	0.96
-5	-5	-3	7.33	0.69
5	-5	3	7.27	0.59
-5	-5	-3	7.14	0.91

5	-5	3	6.75	0.63
5	-5	3	7.08	0.59
5	5	-3	8.04	0.70
5	5	-3	7.22	0.49
-5	5	3	6.01	0.68
5	-5	3	5.80	0.94
5	5	-3	7.75	0.87
5	5	-3	8.23	0.73
-5	-5	-3	6.71	0.74
5	-5	3	7.78	0.96
-5	5	3	6.05	0.84
-5	-5	-3	7.97	0.69
-5	5	3	7.24	0.76
5	6	3	29.52	1.94
5	-6	-3	25.29	1.58
-5	-6	3	32.97	2.03
-5	6	-3	27.65	1.49
5	-6	-3	26.55	1.49
5	6	3	30.38	1.78
5	-6	-3	28.81	1.86
5	6	3	31.10	1.71
-5	-6	3	30.54	2.03
5	-6	-3	25.29	1.48
5	-6	-3	23.76	1.39
-5	6	-3	27.21	1.68
-5	-6	3	32.75	1.81
-5	6	-3	28.70	1.91
-5	-6	3	31.29	1.77
5	-6	-3	26.96	1.37
5	6	3	31.41	1.96
5	-6	-3	25.56	1.64
-5	6	-3	24.08	1.75
-5	-6	3	32.24	1.91
5	6	3	33.82	1.99
-5	6	-3	26.48	1.57
-5	-6	3	31.17	1.95
-5	-6	3	33.33	1.60
5	-6	3	32.07	1.69
-5	6	3	31.83	2.19
-5	-6	-3	27.35	1.64
5	6	-3	24.86	1.70
5	6	-3	27.08	1.55
-5	-6	-3	24.89	1.45
-5	-6	-3	25.29	1.54

-5	6	3	32.34	1.81
-5	-6	-3	26.56	1.73
5	-6	3	30.92	1.77
-5	6	3	36.18	2.05
5	6	-3	25.27	1.53
5	-6	3	37.86	2.23
-5	6	3	31.52	1.94
-5	-6	-3	25.19	1.55
5	-6	3	32.80	2.03
5	6	-3	25.75	1.29
-5	6	3	34.42	1.85
5	-6	3	32.16	1.88
5	-6	3	34.18	1.73
-5	-7	3	0.88	0.65
5	-7	-3	-0.07	0.35
5	-7	-3	-0.33	0.36
-5	7	-3	0.01	0.61
5	-7	-3	0.74	0.38
5	7	3	0.16	0.40
5	-7	-3	-0.32	0.41
-5	7	-3	-0.02	0.44
-5	7	-3	0.37	0.44
-5	7	-3	0.04	0.67
-5	-7	3	-0.31	0.39
-5	-7	3	0.84	0.56
-5	-7	3	0.61	0.68
-5	-7	3	-0.24	0.36
5	-7	-3	0.39	0.35
-5	7	-3	0.55	0.60
5	7	3	0.50	0.52
5	7	3	0.63	0.54
-5	-7	3	0.08	0.63
5	-7	-3	-1.26	0.71
5	-7	-3	0.54	0.56
5	7	3	1.24	0.48
5	7	3	-0.56	0.59
-5	-7	3	0.12	0.29
5	7	-3	0.80	0.49
5	-7	3	0.25	0.42
5	7	-3	0.66	0.26
5	-7	3	-1.55	0.72
-5	7	3	0.59	0.59
5	-7	3	0.36	0.70
5	7	-3	0.53	0.46

-5	7	3	0.46	0.50
5	-7	3	0.46	0.32
-5	7	3	0.81	0.49
5	7	-3	0.87	0.61
-5	-7	-3	0.26	0.61
-5	-7	-3	-0.08	0.45
5	-7	3	0.28	0.40
5	7	-3	0.26	0.53
-5	-7	-3	-0.07	0.37
-5	-7	-3	0.09	0.55
-5	-7	-3	0.07	0.35
-5	7	3	0.17	0.56
-5	7	3	0.89	0.71
5	-7	3	0.41	0.62
5	-7	3	-0.24	0.37
-5	8	-3	29.95	1.97
-5	8	-3	27.51	2.08
5	-8	-3	28.50	1.93
-5	-8	3	31.79	1.55
-5	8	-3	26.84	2.23
5	8	3	28.71	2.13
-5	8	-3	30.83	1.72
-5	-8	3	29.22	1.82
5	8	3	30.90	2.10
5	-8	-3	32.76	2.27
5	-8	-3	27.11	1.67
-5	-8	3	30.61	1.87
5	8	3	28.68	2.06
5	-8	-3	29.47	1.64
5	8	3	30.45	1.72
-5	-8	3	33.27	1.98
5	-8	-3	27.53	1.83
-5	-8	3	26.60	2.03
-5	-8	3	27.38	2.09
5	-8	-3	29.72	1.74
5	-8	-3	26.60	1.80
-5	-8	-3	28.94	2.07
5	-8	3	30.88	1.67
5	-8	3	29.13	1.91
-5	8	3	33.97	2.22
-5	-8	-3	27.90	1.83
-5	-8	-3	26.99	1.91
-5	8	3	25.97	2.31
5	8	-3	26.64	2.11

5	8	-3	29.87	1.75
-5	-8	-3	27.70	1.79
5	8	-3	28.52	1.46
-5	8	3	31.91	1.90
-5	8	3	31.81	2.06
5	-8	3	34.29	1.92
-5	-8	-3	26.83	1.64
5	-8	3	28.82	1.81
5	8	-3	30.06	1.95
5	-8	3	30.95	2.18
-5	8	3	27.13	1.86
5	-8	3	31.12	1.76
5	-8	3	32.84	2.43
-5	9	-3	4.08	0.74
-5	-9	3	2.92	0.89
-5	9	-3	2.50	1.04
5	9	3	2.52	0.92
-5	9	-3	4.11	1.04
5	-9	-3	4.00	0.71
5	9	3	2.29	0.99
5	-9	-3	2.83	0.87
-5	-9	3	2.87	0.40
5	9	3	3.35	0.65
-5	9	-3	4.06	1.25
5	-9	-3	3.21	0.61
5	9	3	2.64	0.82
5	-9	-3	3.78	1.19
5	-9	-3	4.21	0.85
5	-9	-3	3.33	0.74
-5	-9	3	1.76	0.76
5	-9	-3	2.89	0.67
-5	-9	3	2.45	0.71
5	9	-3	3.12	0.46
-5	9	3	3.63	0.95
5	-9	3	2.56	0.63
5	-9	3	2.77	0.54
5	9	-3	3.56	0.72
-5	-9	-3	3.32	0.58
5	-9	3	2.13	0.75
-5	9	3	3.04	1.10
-5	9	3	2.75	1.11
5	9	-3	2.60	1.02
-5	9	3	4.13	0.84
5	-9	3	2.28	1.15

-5	-9	-3	3.00	0.78
5	-9	3	2.80	1.43
5	-9	3	3.40	0.84
5	9	-3	2.92	0.88
5	-10	-3	9.60	1.26
5	10	3	6.43	2.11
5	-10	-3	9.64	1.35
-5	10	-3	10.04	1.72
5	10	3	6.69	1.03
-5	-10	3	8.01	1.22
-5	10	-3	10.15	1.91
-5	10	-3	10.79	1.80
-5	-10	3	7.92	1.49
5	-10	-3	10.61	1.18
5	10	3	6.22	1.38
5	10	3	6.74	0.83
-5	-10	3	6.16	1.15
-5	-10	3	6.27	0.68
5	-10	-3	10.26	1.95
-5	10	-3	11.20	1.27
-5	-10	3	7.49	1.07
5	-10	-3	9.50	1.10
5	-10	-3	11.01	1.07
-5	-10	-3	8.41	1.32
5	10	-3	9.50	1.58
5	-10	3	6.38	1.15
5	-10	3	8.33	1.71
5	10	-3	11.37	1.21
-5	10	3	9.05	1.56
-5	-10	-3	10.61	1.03
5	-10	3	7.81	1.16
-5	-10	-3	9.11	1.24
5	10	-3	9.56	1.69
5	-10	3	5.86	0.81
-5	10	3	4.17	1.38
-5	10	3	6.44	1.39
5	10	-3	9.05	1.23
5	-10	3	7.63	1.06
5	-10	3	6.20	1.08
5	11	3	21.78	2.02
5	11	3	17.68	2.22
-5	11	-3	16.39	2.48
-5	-11	3	17.14	1.64
5	-11	-3	21.08	1.56

-5	-11	3	20.06	2.04
-5	11	-3	22.91	3.57
5	-11	-3	18.44	1.49
5	-11	-3	18.74	1.61
-5	11	-3	18.75	2.16
-5	11	-3	21.61	1.72
5	-11	-3	16.51	1.64
5	-11	3	17.00	1.29
5	-11	3	17.72	1.62
-5	-11	-3	19.80	1.76
-5	-11	-3	17.85	1.39
-5	-11	-3	18.62	2.01
-5	11	3	16.82	2.08
-5	11	3	18.02	2.43
5	11	-3	17.90	1.63
5	11	-3	21.52	2.42
5	-11	3	16.64	1.63
5	-11	3	18.16	1.56
5	-12	-3	14.18	1.40
5	-12	-3	16.45	1.70
-5	-12	3	15.51	1.72
5	-12	-3	13.71	1.72
5	12	3	16.32	2.50
-5	12	-3	12.60	2.21
-5	-12	3	17.97	2.18
-5	12	-3	12.23	2.29
-5	-12	-3	14.63	2.02
5	-12	3	16.94	1.32
5	-12	3	15.25	1.63
-5	12	3	13.64	2.18
-5	-12	-3	12.38	1.32
5	12	-3	14.90	2.65
-5	-12	-3	17.89	1.70
5	-12	3	17.30	1.66
-5	12	3	16.68	2.39
-5	13	-3	4.52	1.75
5	-13	-3	2.79	0.94
-5	-13	3	2.85	0.99
5	13	3	2.68	1.87
-5	-13	3	3.54	1.52
5	-13	-3	2.93	0.99
5	13	-3	3.12	1.92
-5	-13	-3	1.27	0.88
5	-13	3	4.11	0.99

-5	13	3	4.07	1.55
-5	-13	-3	2.36	1.60
-6	0	-3	172.55	7.68
-6	0	-3	167.44	7.59
-6	0	-3	173.23	7.69
-6	0	-3	163.00	7.69
6	0	3	142.66	7.05
6	0	3	148.24	6.99
6	0	-3	161.85	7.56
-6	0	3	149.77	6.98
-6	0	3	156.79	6.94
-6	0	3	162.74	7.07
-6	0	-3	171.09	7.66
6	0	3	164.24	7.08
6	0	-3	170.83	7.56
-6	0	3	153.51	6.99
6	0	-3	166.05	7.60
6	0	3	143.91	6.98
6	0	3	143.96	6.93
-6	0	3	166.22	6.98
6	0	-3	168.56	7.71
6	0	3	155.10	6.98
6	0	-3	169.80	7.63
-6	-1	3	464.86	20.77
-6	1	-3	491.38	21.31
6	1	3	463.27	20.87
6	1	3	465.28	20.77
-6	-1	3	484.13	20.88
6	-1	-3	481.04	21.30
6	-1	-3	478.15	21.33
6	1	3	487.53	20.85
-6	1	-3	480.48	21.34
6	1	3	439.82	20.76
-6	-1	3	443.90	20.76
6	1	3	455.34	20.80
-6	1	-3	468.07	21.32
-6	1	-3	500.10	21.44
6	1	3	440.36	20.81
-6	-1	3	443.82	20.76
6	-1	-3	467.25	21.29
-6	1	-3	489.51	21.33
-6	1	-3	480.18	21.42
6	-1	-3	458.56	21.33
-6	-1	3	472.40	20.86

-6	-1	3	480.15	20.87
-6	1	-3	466.80	21.33
-6	-1	-3	470.02	21.26
6	-1	3	452.12	20.78
-6	1	3	471.88	20.76
-6	-1	-3	477.39	21.34
6	-1	3	473.95	20.87
6	-1	3	441.95	20.80
-6	-1	-3	464.49	21.33
6	1	-3	469.32	21.29
-6	1	3	473.04	20.87
-6	1	3	479.01	20.82
-6	1	3	470.01	20.76
-6	1	3	459.74	20.77
6	1	-3	482.64	21.39
-6	-1	-3	491.13	21.32
-6	1	3	489.50	20.77
-6	-1	-3	445.13	21.31
-6	1	3	445.48	20.74
-6	1	3	478.55	20.89
6	-1	3	468.05	20.80
-6	-1	-3	486.50	21.40
6	2	3	49.18	2.48
-6	2	-3	68.64	3.05
6	2	3	55.44	2.51
-6	-2	3	50.45	2.44
6	-2	-3	66.57	3.07
-6	2	-3	66.15	3.02
-6	-2	3	49.89	2.61
6	2	3	51.42	2.61
6	2	3	54.51	2.55
-6	2	-3	66.82	3.07
-6	2	-3	65.81	3.22
6	-2	-3	63.41	3.01
-6	2	-3	63.26	3.19
-6	-2	3	49.29	2.48
6	-2	-3	64.09	3.05
-6	-2	3	50.48	2.61
-6	-2	3	52.79	2.44
-6	-2	3	55.48	2.60
6	-2	3	52.38	2.47
-6	-2	-3	64.34	3.06
-6	-2	-3	65.15	3.03
6	-2	3	53.82	2.52

-6	2	3	53.78	2.61
-6	2	3	52.92	2.57
6	2	-3	65.42	2.94
-6	-2	-3	63.70	3.05
6	2	-3	67.02	3.02
6	-2	3	50.23	2.51
6	2	-3	61.41	3.17
-6	-2	-3	58.21	2.97
6	2	-3	60.11	2.98
-6	2	3	53.75	2.47
-6	3	-3	13.37	0.89
6	-3	-3	13.25	0.97
6	3	3	8.82	0.82
-6	-3	3	10.73	0.61
-6	3	-3	13.17	0.79
-6	-3	3	10.95	0.65
6	3	3	11.13	0.74
6	-3	-3	12.31	0.86
-6	3	-3	11.91	1.00
6	3	3	9.54	0.72
-6	3	-3	13.40	1.11
-6	-3	3	10.97	0.63
6	-3	-3	13.03	0.77
-6	3	-3	13.21	0.83
6	-3	-3	12.68	0.83
-6	-3	3	11.16	0.85
-6	-3	3	8.42	0.85
-6	3	-3	13.74	0.86
-6	-3	3	9.95	0.71
6	3	3	11.65	0.72
-6	-3	3	10.88	0.60
-6	-3	3	10.55	0.92
6	3	3	10.62	0.79
6	-3	3	10.23	0.75
6	-3	3	11.42	1.00
6	3	-3	12.68	0.75
-6	3	3	11.81	0.85
-6	-3	-3	13.85	1.00
6	3	-3	13.35	0.82
6	-3	3	9.89	0.70
6	-3	3	10.88	0.65
6	-3	3	10.95	0.70
-6	3	3	11.21	0.79
6	3	-3	13.48	0.87

6	-3	3	10.59	0.93
-6	-3	-3	13.11	0.83
6	3	-3	11.97	0.82
-6	-3	-3	13.68	0.83
-6	3	3	11.35	0.70
-6	-3	-3	13.57	0.80
-6	-3	-3	13.66	0.84
-6	3	3	9.90	0.86
-6	3	3	10.03	0.92
6	4	3	7.67	0.63
-6	4	-3	7.10	0.59
6	4	3	7.97	0.68
-6	4	-3	8.74	1.04
6	4	3	7.34	0.69
6	-4	-3	6.79	0.86
6	-4	-3	6.53	0.79
6	-4	-3	6.45	0.56
-6	-4	3	7.67	0.63
-6	-4	3	8.70	0.83
6	-4	-3	7.13	0.68
-6	-4	3	7.21	0.55
-6	-4	3	8.47	0.68
-6	4	-3	8.04	0.70
6	4	3	7.59	0.81
-6	-4	3	7.70	0.83
-6	4	-3	5.50	0.84
-6	4	-3	8.66	0.66
-6	-4	3	7.61	0.91
6	-4	-3	7.16	0.63
-6	-4	3	8.34	0.55
-6	4	-3	7.04	0.74
-6	-4	-3	7.38	0.67
6	-4	3	10.64	1.05
6	-4	3	7.73	0.62
-6	4	3	7.51	0.81
6	-4	3	7.10	0.62
-6	4	3	8.12	0.75
-6	-4	-3	8.11	0.88
6	4	-3	7.50	0.48
-6	4	3	8.83	0.66
-6	4	3	7.65	0.79
6	-4	3	8.05	0.61
6	4	-3	7.52	0.63
6	-4	3	8.18	0.92

-6	-4	-3	7.36	0.65
6	4	-3	8.30	0.67
-6	4	3	7.84	0.90
6	4	-3	8.44	0.65
-6	-4	-3	7.46	0.64
6	-4	3	8.16	0.72
6	4	-3	6.50	0.71
-6	-4	-3	7.13	0.63
-6	-5	3	66.01	3.38
-6	-5	3	69.93	3.22
-6	-5	3	67.67	3.31
-6	5	-3	74.40	3.50
-6	5	-3	77.25	3.62
-6	-5	3	66.83	3.55
-6	5	-3	78.67	3.71
-6	5	-3	80.64	3.57
6	5	3	67.01	3.55
6	-5	-3	69.58	3.53
6	-5	-3	74.11	3.69
-6	-5	3	65.19	3.53
-6	5	-3	73.34	3.89
6	-5	-3	75.56	3.82
-6	-5	3	70.07	3.39
6	-5	-3	72.39	3.58
6	5	3	68.66	3.31
6	5	3	64.77	3.48
6	-5	-3	69.88	3.66
6	5	3	68.61	3.38
-6	5	-3	69.66	3.78
-6	-5	3	71.25	3.45
-6	-5	-3	77.91	3.62
-6	-5	-3	70.22	3.59
6	-5	3	72.15	3.40
6	5	-3	76.55	3.58
-6	5	3	70.19	3.53
-6	5	3	74.72	3.43
6	-5	3	77.38	3.74
6	-5	3	69.82	3.45
6	5	-3	69.24	3.69
6	-5	3	71.38	3.33
-6	-5	-3	76.85	3.78
6	5	-3	77.05	3.58
-6	5	3	64.45	3.62
6	5	-3	75.39	3.59

-6	5	3	69.51	3.37
-6	5	3	73.30	3.53
-6	-5	-3	72.47	3.59
6	-5	3	68.23	3.37
-6	-5	-3	70.05	3.53
6	5	-3	73.10	3.40
6	-5	3	70.12	3.55
6	-6	-3	91.47	4.38
-6	6	-3	99.72	4.37
6	6	3	89.14	4.39
6	-6	-3	86.99	4.48
-6	-6	3	89.75	4.34
-6	6	-3	85.72	4.80
6	6	3	86.80	4.55
-6	6	-3	90.73	4.64
6	6	3	88.71	4.59
-6	6	-3	91.15	4.43
-6	-6	3	93.32	4.61
6	-6	-3	94.25	4.69
-6	-6	3	93.36	4.21
-6	-6	3	94.77	4.45
6	-6	-3	90.78	4.50
-6	-6	3	87.40	4.56
6	-6	-3	84.70	4.35
-6	-6	3	89.46	4.40
-6	6	-3	101.61	4.57
-6	-6	3	89.50	4.52
6	6	3	91.55	4.30
6	-6	3	91.60	4.57
6	-6	3	85.97	4.37
-6	-6	-3	89.55	4.42
6	-6	3	88.51	4.47
6	6	-3	85.85	4.56
6	6	-3	93.06	4.43
-6	6	3	89.75	4.39
-6	6	3	95.80	4.43
6	6	-3	89.99	4.18
6	6	-3	92.56	4.40
6	-6	3	90.45	4.32
-6	-6	-3	95.40	4.61
-6	6	3	101.80	4.61
-6	-6	-3	94.00	4.46
6	6	-3	90.91	4.38
-6	-6	-3	89.21	4.41

-6	6	3	89.53	4.54
6	-6	3	94.80	4.29
-6	6	3	85.94	4.67
6	-6	3	100.40	4.78
-6	-6	-3	90.38	4.34
6	7	3	115.92	5.38
-6	-7	3	104.68	5.45
-6	-7	3	115.57	5.71
-6	7	-3	129.99	5.57
6	7	3	109.72	5.70
6	-7	-3	125.70	5.95
6	-7	-3	122.95	5.41
-6	-7	3	112.06	5.45
-6	7	-3	111.53	5.85
6	-7	-3	116.33	5.58
6	-7	-3	116.38	5.58
6	-7	-3	114.92	5.70
6	7	3	111.70	5.69
6	-7	-3	120.85	5.42
-6	7	-3	117.22	5.64
6	7	3	117.32	5.65
-6	7	-3	112.46	5.95
-6	-7	3	120.13	5.58
-6	-7	3	113.97	5.57
-6	-7	3	114.16	5.52
6	-7	-3	111.16	5.71
-6	-7	3	112.94	5.67
6	7	3	107.20	5.47
-6	7	-3	125.58	5.78
-6	-7	-3	124.63	5.85
6	-7	3	123.28	5.40
-6	-7	-3	118.82	5.69
6	7	-3	110.91	5.83
6	7	-3	114.00	5.60
6	-7	3	107.85	5.47
6	-7	3	108.72	5.57
6	-7	3	128.23	5.94
-6	-7	-3	114.99	5.52
-6	7	3	113.65	5.66
-6	7	3	116.86	5.50
-6	-7	-3	115.18	5.63
6	7	-3	115.22	5.35
-6	-7	-3	111.01	5.63
-6	7	3	128.11	5.76

6	-7	3	111.73	5.38
-6	7	3	120.39	5.53
6	-7	3	115.94	5.68
6	7	-3	118.31	5.58
6	-8	-3	87.12	4.64
6	8	3	93.58	4.72
-6	8	-3	103.46	4.51
6	8	3	90.82	4.35
6	-8	-3	98.67	4.97
-6	8	-3	92.64	5.06
-6	8	-3	95.49	4.73
-6	8	-3	90.84	4.84
6	8	3	85.42	4.76
6	-8	-3	89.99	4.65
6	-8	-3	89.37	4.50
-6	-8	3	85.85	4.45
6	-8	-3	86.77	4.45
-6	-8	3	88.73	4.71
-6	-8	3	94.35	4.55
-6	-8	3	93.17	4.23
6	-8	-3	94.50	4.34
-6	-8	3	91.61	4.54
6	8	3	87.18	4.72
6	-8	-3	98.06	4.34
6	8	-3	90.77	4.24
-6	-8	-3	88.38	4.55
6	-8	3	97.76	4.40
6	-8	3	88.71	4.71
6	-8	3	89.72	4.36
-6	-8	-3	89.37	4.75
-6	8	3	95.71	4.80
6	8	-3	92.06	4.48
-6	-8	-3	88.82	4.56
-6	8	3	92.78	4.53
-6	-8	-3	90.78	4.43
6	-8	3	88.42	4.60
6	8	-3	84.68	4.81
-6	8	3	83.05	4.48
6	8	-3	96.01	4.88
6	-8	3	98.76	4.98
-6	8	3	93.16	4.72
6	-8	3	87.76	4.49
-6	8	3	88.52	4.90
6	-8	3	101.28	4.50

-6	-9	3	48.01	2.91
-6	9	-3	44.33	2.76
6	9	3	48.48	2.60
6	-9	-3	45.64	2.95
-6	9	-3	43.81	2.79
6	-9	-3	42.77	2.22
-6	-9	3	50.25	3.02
6	-9	-3	39.46	2.53
-6	9	-3	39.07	2.97
6	9	3	49.69	3.01
-6	9	-3	42.93	2.38
6	-9	-3	41.25	2.29
-6	-9	3	46.89	2.75
6	-9	-3	40.12	2.37
-6	-9	3	51.31	2.87
-6	-9	3	50.66	2.41
6	-9	-3	39.79	2.58
6	9	3	52.20	3.06
6	9	3	43.59	3.02
-6	9	3	52.49	3.19
-6	9	3	45.43	3.20
6	-9	3	54.07	2.80
6	-9	3	56.06	2.66
-6	-9	-3	39.38	2.39
6	9	-3	44.94	2.64
-6	-9	-3	38.82	2.46
6	-9	3	47.25	2.84
6	9	-3	40.76	2.04
6	9	-3	38.00	2.78
6	9	-3	42.12	2.33
6	-9	3	59.18	3.48
6	-9	3	49.41	2.58
6	-9	3	46.47	2.72
-6	9	3	49.27	2.79
-6	-9	-3	41.36	2.24
-6	9	3	49.46	3.02
-6	-10	3	15.93	1.74
-6	10	-3	15.86	2.13
-6	10	-3	13.14	2.27
6	10	3	13.81	1.81
6	-10	-3	16.09	2.25
-6	-10	3	14.85	0.96
6	-10	-3	11.79	1.15
-6	-10	3	15.78	1.46

-6	-10	3	18.55	1.69
-6	10	-3	11.54	1.42
-6	-10	3	14.00	1.48
6	-10	-3	14.07	1.33
6	-10	-3	11.55	1.44
6	-10	-3	15.02	1.30
6	-10	-3	14.00	1.15
-6	10	-3	13.86	1.84
6	10	3	14.52	1.27
6	10	3	15.99	1.96
6	-10	3	15.15	1.31
6	10	-3	11.53	1.83
6	-10	3	18.69	1.61
6	-10	3	14.16	1.38
-6	-10	-3	14.60	1.18
-6	10	3	14.60	2.04
-6	-10	-3	14.60	1.45
6	10	-3	14.67	1.67
-6	-10	-3	13.81	1.44
-6	-10	-3	13.92	1.62
-6	10	3	16.55	1.84
6	-10	3	15.09	2.13
6	-10	3	13.01	1.50
6	10	-3	13.63	1.31
6	-10	3	13.68	1.13
6	-11	-3	5.01	0.94
-6	11	-3	4.95	1.34
6	-11	-3	3.60	1.00
6	-11	-3	4.11	0.76
-6	11	-3	4.78	1.09
-6	-11	3	5.77	1.31
-6	-11	3	3.54	0.99
6	-11	-3	3.92	2.41
6	11	3	6.46	1.45
-6	11	-3	3.98	1.58
6	-11	-3	4.17	0.90
6	-11	3	3.38	0.95
6	-11	3	4.07	0.73
-6	11	3	4.43	1.51
6	-11	3	5.57	1.12
-6	-11	-3	3.12	0.95
6	-11	3	5.72	0.94
-6	-11	-3	4.14	0.74
6	11	-3	4.64	0.91

-6	-11	-3	4.98	1.23
6	11	-3	1.95	1.30
6	12	3	7.30	1.96
-6	12	-3	6.45	2.15
-6	12	-3	5.88	1.68
-6	-12	3	7.82	1.23
6	-12	-3	6.42	1.13
-6	-12	3	7.39	1.64
6	-12	-3	5.40	1.15
6	-12	-3	7.14	1.02
-6	-12	-3	7.45	1.59
6	-12	3	7.38	1.31
-6	12	3	9.29	1.65
6	12	-3	7.45	1.95
-6	-12	-3	6.46	1.10
6	-12	3	7.68	1.84
-6	-12	-3	6.73	1.11
6	-12	3	8.15	1.03
6	-13	-3	13.54	1.69
-6	-13	3	13.07	1.68
-6	-13	3	16.71	2.31
6	-13	-3	12.63	1.54
-6	13	-3	15.95	2.48
6	13	3	15.25	2.73
-6	-13	-3	15.24	1.72
6	13	-3	18.02	2.78
-6	-13	-3	13.76	2.35
-6	13	3	16.74	2.71
6	-13	3	14.67	1.65
7	0	3	0.08	0.21
-7	0	-3	0.07	0.24
-7	0	3	0.17	0.21
-7	0	3	0.08	0.25
7	0	3	-0.02	0.24
-7	0	-3	-0.27	0.24
7	0	-3	0.06	0.12
-7	0	-3	-0.05	0.20
7	0	3	0.20	0.13
-7	0	3	0.18	0.28
-7	0	3	0.02	0.17
-7	0	3	-0.06	0.22
7	0	-3	0.12	0.15
-7	0	-3	0.18	0.24
7	0	3	0.06	0.17

-7	0	-3	0.12	0.38
7	0	3	-0.06	0.34
-7	0	3	-0.05	0.23
7	0	-3	0.00	0.14
7	-1	-3	71.94	3.32
-7	1	-3	73.24	3.35
-7	1	-3	72.16	3.37
7	1	3	68.94	3.31
7	1	3	69.01	3.27
-7	-1	3	67.65	3.35
7	1	3	75.00	3.36
-7	1	-3	74.23	3.41
-7	1	-3	68.51	3.48
7	1	3	69.41	3.37
7	1	3	70.42	3.24
-7	-1	3	68.72	3.25
-7	1	-3	69.05	3.38
7	-1	-3	73.00	3.38
-7	1	-3	71.82	3.40
-7	-1	3	71.11	3.26
-7	1	-3	73.17	3.49
-7	-1	3	71.60	3.38
-7	-1	3	71.29	3.27
-7	-1	3	72.09	3.38
7	-1	-3	70.93	3.34
7	-1	-3	72.35	3.37
-7	-1	-3	72.12	3.40
7	-1	3	66.76	3.25
7	-1	3	71.24	3.29
7	-1	3	69.66	3.37
-7	1	3	74.33	3.26
-7	-1	-3	65.91	3.32
-7	-1	-3	73.83	3.34
-7	-1	-3	73.04	3.39
-7	1	3	67.41	3.24
-7	1	3	69.79	3.25
7	1	-3	72.36	3.34
-7	-1	-3	67.00	3.38
-7	1	3	70.22	3.25
7	-1	3	64.77	3.29
7	1	-3	74.81	3.44
7	-1	3	69.46	3.41
-7	1	3	66.66	3.50
-7	-1	-3	73.05	3.46

-7	-2	3	34.63	1.68
7	2	3	31.58	1.85
-7	-2	3	31.23	1.85
7	-2	-3	29.30	1.45
7	-2	-3	30.24	1.58
7	2	3	34.45	1.79
-7	-2	3	32.84	1.71
-7	2	-3	25.74	1.66
-7	-2	3	36.99	1.85
-7	2	-3	29.28	1.52
-7	2	-3	29.53	1.46
-7	-2	3	34.47	1.88
7	2	3	32.75	1.70
-7	2	-3	30.84	1.52
-7	2	-3	28.58	1.49
-7	-2	3	36.24	1.73
-7	2	-3	29.95	1.70
7	-2	-3	27.51	1.49
-7	2	-3	31.06	1.54
7	-2	-3	27.59	1.51
-7	-2	-3	29.88	1.66
-7	-2	-3	29.86	1.52
7	-2	3	33.38	1.73
7	2	-3	29.07	1.54
7	-2	3	34.54	1.70
7	2	-3	26.21	1.40
-7	2	3	37.09	1.79
-7	2	3	35.38	1.75
-7	-2	-3	29.69	1.42
-7	-2	-3	26.44	1.45
7	-2	3	34.28	1.90
-7	2	3	34.96	1.87
-7	-2	-3	29.21	1.51
-7	2	3	34.80	1.81
-7	2	3	34.97	1.71
-7	-2	-3	28.72	1.51
7	-2	3	34.53	1.75
-7	-2	-3	29.11	1.45
-7	-2	-3	28.34	1.43
-7	-3	3	8.94	0.57
-7	-3	3	8.87	0.53
-7	3	-3	11.42	0.71
-7	3	-3	12.44	0.80
-7	-3	3	10.30	0.85

7	3	3	8.51	0.66
-7	-3	3	8.98	0.90
-7	-3	3	9.23	0.50
-7	-3	3	8.61	0.67
-7	3	-3	10.64	1.07
7	3	3	8.45	0.80
7	-3	-3	10.27	0.75
7	-3	-3	10.33	0.77
7	3	3	10.15	0.69
7	-3	-3	12.11	0.71
7	3	3	7.42	0.81
-7	-3	3	6.66	0.84
7	-3	-3	9.37	0.89
-7	-3	3	9.04	0.60
-7	3	-3	10.33	0.77
-7	3	-3	8.66	0.94
-7	3	-3	10.81	0.81
-7	3	3	10.55	0.76
7	3	-3	11.32	0.68
-7	-3	-3	10.70	0.78
7	3	-3	12.98	0.76
7	-3	3	8.93	0.97
7	-3	3	8.26	0.64
-7	3	3	8.48	0.88
7	3	-3	13.32	0.81
7	-3	3	8.41	0.90
-7	3	3	8.14	0.81
-7	-3	-3	9.35	0.71
-7	-3	-3	9.80	0.64
-7	-3	-3	8.83	0.64
-7	3	3	10.28	0.71
7	-3	3	9.77	0.61
-7	-3	-3	11.47	0.78
-7	-3	-3	11.01	0.77
-7	-3	-3	11.12	0.69
7	-3	3	8.05	0.64
7	-3	3	7.80	0.67
-7	3	3	10.41	0.80
7	3	-3	9.44	0.73
-7	-3	-3	10.88	0.94
-7	4	-3	10.04	0.69
-7	-4	3	9.34	0.97
7	4	3	10.01	0.83
-7	4	-3	8.50	0.96

7	-4	-3	9.35	0.74
7	4	3	11.15	0.80
7	4	3	11.41	0.78
-7	-4	3	10.46	0.79
-7	-4	3	10.43	0.74
-7	4	-3	11.55	0.83
7	-4	-3	10.13	0.70
-7	-4	3	10.23	0.67
-7	-4	3	11.47	1.07
7	-4	-3	9.19	0.98
-7	-4	3	11.00	0.65
-7	4	-3	8.67	1.09
-7	-4	3	11.25	0.69
-7	-4	3	11.93	0.96
7	4	3	10.05	0.95
7	4	3	12.03	0.91
7	-4	-3	7.82	0.94
7	-4	-3	9.31	0.80
-7	4	-3	10.06	0.76
-7	4	-3	9.47	0.85
-7	-4	-3	10.19	0.76
7	4	-3	9.88	0.73
7	-4	3	9.51	0.73
7	4	-3	8.60	0.80
7	4	-3	10.99	0.76
-7	4	3	10.05	0.98
7	-4	3	10.90	0.83
-7	-4	-3	10.86	1.03
7	-4	3	10.08	0.77
-7	4	3	9.63	1.00
7	-4	3	11.80	1.16
-7	4	3	11.79	0.95
-7	4	3	12.10	0.91
-7	-4	-3	9.76	0.76
-7	-4	-3	10.62	0.77
7	-4	3	10.99	1.07
7	-4	3	12.09	0.71
-7	4	3	11.41	0.81
-7	-4	-3	11.07	0.74
7	4	-3	9.98	0.76
-7	5	-3	4.97	0.70
-7	-5	3	3.58	0.64
7	5	3	3.46	0.70
-7	5	-3	5.22	0.66

-7	5	-3	4.09	0.77
7	5	3	4.90	0.59
-7	-5	3	4.21	0.81
7	-5	-3	5.07	0.75
-7	5	-3	5.22	0.58
7	-5	-3	4.85	0.67
-7	-5	3	3.88	0.59
-7	-5	3	5.52	0.95
-7	-5	3	3.68	0.51
7	5	3	4.76	0.64
7	-5	-3	5.74	0.97
-7	5	-3	5.43	0.99
7	-5	-3	5.43	0.56
7	5	3	5.09	0.80
-7	5	-3	4.67	0.91
-7	-5	3	4.67	0.49
-7	-5	3	4.19	0.81
7	5	3	3.24	0.68
7	-5	-3	4.66	0.59
7	-5	3	5.77	1.04
-7	5	3	5.40	0.88
-7	5	3	4.82	0.66
-7	-5	-3	4.14	0.60
-7	-5	-3	5.69	0.95
-7	-5	-3	5.55	0.61
7	-5	3	5.39	0.53
-7	5	3	4.52	0.81
7	-5	3	4.06	0.53
7	-5	3	3.92	0.64
7	5	-3	5.43	0.63
7	5	-3	3.80	0.43
-7	-5	-3	5.00	0.65
-7	5	3	4.19	0.72
7	5	-3	3.61	0.64
7	-5	3	4.17	0.54
7	5	-3	4.67	0.72
-7	5	3	4.15	0.70
-7	-5	-3	3.58	0.62
7	-5	3	5.31	0.83
7	5	-3	5.14	0.63
7	6	3	18.63	1.43
7	6	3	18.37	1.26
-7	-6	3	17.93	1.48
-7	6	-3	20.39	1.63

-7	6	-3	22.58	1.34
-7	-6	3	18.33	1.54
7	-6	-3	20.14	1.69
7	6	3	17.24	1.47
-7	6	-3	22.67	1.41
7	-6	-3	19.03	1.40
-7	-6	3	19.58	1.55
7	-6	-3	19.68	1.53
-7	-6	3	19.72	1.31
-7	-6	3	18.51	1.26
7	-6	-3	19.02	1.28
7	-6	-3	20.36	1.26
-7	6	-3	22.55	1.87
7	6	3	19.84	1.19
-7	6	-3	23.72	1.53
-7	-6	3	18.16	1.17
7	-6	3	18.16	1.31
7	-6	3	17.77	1.16
-7	6	3	18.39	1.46
7	6	-3	21.69	1.32
-7	-6	-3	20.79	1.27
7	6	-3	18.91	1.48
-7	-6	-3	19.26	1.32
7	-6	3	20.96	1.12
-7	6	3	19.71	1.48
7	6	-3	20.27	1.37
7	-6	3	19.94	1.59
7	-6	3	18.72	1.22
7	6	-3	23.18	1.35
-7	-6	-3	20.60	1.35
-7	-6	-3	19.73	1.33
-7	6	3	21.81	1.37
-7	-6	-3	20.77	1.60
-7	6	3	19.41	1.70
-7	6	3	20.44	1.31
7	6	-3	20.91	1.10
7	-6	3	22.50	1.77
7	-7	-3	27.02	1.61
-7	7	-3	27.51	1.88
-7	7	-3	26.12	1.72
7	-7	-3	30.00	2.12
-7	-7	3	27.35	1.83
7	-7	-3	26.31	1.85
-7	-7	3	25.03	1.53

-7	-7	3	25.57	1.66
7	-7	-3	27.27	1.76
-7	-7	3	26.27	1.35
-7	-7	3	25.13	1.91
7	7	3	26.32	1.49
7	-7	-3	26.23	1.59
-7	7	-3	25.33	2.06
7	7	3	24.62	1.59
-7	7	-3	28.83	1.67
7	7	3	23.52	1.76
-7	-7	3	23.35	1.82
-7	7	-3	25.92	1.98
7	7	3	23.74	1.83
-7	-7	3	25.19	1.62
7	-7	3	24.70	1.67
-7	7	3	26.99	1.69
7	7	-3	27.31	1.38
-7	7	3	22.28	2.08
-7	7	3	26.15	1.84
7	7	-3	26.91	1.63
-7	-7	-3	24.30	1.67
7	7	-3	28.35	1.72
7	-7	3	29.08	1.57
-7	-7	-3	26.85	1.69
-7	-7	-3	27.27	1.57
-7	-7	-3	27.11	1.73
-7	7	3	24.40	1.61
7	-7	3	27.42	1.44
7	7	-3	27.67	1.68
-7	7	3	26.79	1.90
7	-7	3	24.87	1.55
7	-7	3	25.50	1.47
7	-7	3	22.69	2.34
-7	-7	-3	26.78	1.96
7	7	-3	27.12	1.88
7	-7	3	24.37	1.91
7	8	3	3.58	0.89
7	-8	-3	1.87	0.57
7	-8	-3	1.51	0.82
-7	8	-3	0.91	0.98
-7	8	-3	2.60	0.70
7	8	3	1.85	0.81
-7	-8	3	2.83	0.64
-7	8	-3	1.94	0.92

7	-8	-3	2.64	0.56
-7	-8	3	2.78	0.77
-7	-8	3	2.84	0.63
7	8	3	2.77	0.57
-7	-8	3	3.10	0.51
-7	8	-3	1.73	0.84
7	8	3	2.82	0.85
7	-8	-3	1.73	1.06
7	-8	-3	2.48	0.66
-7	-8	3	2.83	0.89
7	-8	3	2.48	0.55
7	-8	3	3.23	0.71
-7	-8	-3	2.50	0.64
7	-8	3	2.66	0.53
-7	8	3	4.29	1.07
-7	-8	-3	1.73	0.52
7	8	-3	2.34	0.86
-7	-8	-3	2.51	0.57
7	-8	3	3.59	0.81
7	8	-3	2.62	0.41
-7	8	3	2.38	0.97
-7	8	3	3.86	0.93
7	8	-3	3.12	0.61
-7	8	3	3.03	0.77
7	-8	3	2.85	1.00
7	-8	3	2.41	0.57
7	-8	3	3.78	1.21
-7	-9	3	2.59	0.89
-7	-9	3	1.49	0.67
7	-9	-3	2.24	1.21
7	9	3	1.31	0.91
7	-9	-3	2.50	0.48
-7	9	-3	1.94	1.07
-7	-9	3	1.11	0.36
7	-9	-3	0.35	0.68
-7	9	-3	1.66	1.04
7	9	3	2.26	0.59
7	-9	-3	2.16	0.59
7	9	3	2.21	1.05
-7	9	-3	1.43	0.90
7	-9	-3	2.08	0.56
-7	-9	3	2.48	0.70
7	-9	-3	1.14	0.56
7	9	3	0.69	0.82

-7	9	-3	1.58	0.72
7	-9	3	2.15	0.52
7	-9	3	1.87	1.32
-7	9	3	1.82	0.86
7	-9	3	1.88	0.76
7	9	-3	1.70	0.41
-7	9	3	1.74	1.16
7	-9	3	0.89	0.66
-7	-9	-3	1.79	0.57
7	-9	3	1.64	0.58
7	9	-3	2.43	0.62
-7	-9	-3	1.53	0.73
7	-9	3	1.95	0.53
-7	9	3	1.33	1.04
-7	-9	-3	2.08	0.66
7	-10	-3	0.41	0.63
-7	10	-3	1.14	1.02
-7	-10	3	1.39	0.90
-7	-10	3	1.70	0.75
7	-10	-3	0.08	0.61
7	-10	-3	-0.36	1.32
-7	10	-3	0.63	0.78
7	-10	-3	0.85	0.62
-7	-10	3	0.40	0.94
7	10	3	-0.38	0.92
-7	10	-3	0.56	1.19
7	10	3	1.85	0.65
7	-10	-3	0.01	0.73
7	10	3	-0.19	1.08
-7	10	3	-0.10	0.99
-7	-10	-3	1.75	0.59
7	-10	3	0.20	1.51
7	-10	3	1.30	0.86
-7	-10	-3	0.68	0.86
7	-10	3	0.53	0.59
7	10	-3	1.20	0.67
7	-10	3	-0.07	0.72
-7	-10	-3	0.77	0.71
7	10	-3	0.55	1.13
7	-10	3	1.56	0.62
7	10	-3	1.77	0.48
-7	10	3	1.58	1.17
7	10	-3	0.33	0.92
7	-10	3	1.14	0.54

-7	-11	3	6.79	1.37
7	11	3	4.38	1.44
-7	11	-3	6.41	1.63
-7	11	-3	4.55	1.68
-7	-11	3	4.67	1.01
7	-11	-3	4.90	0.90
7	-11	-3	3.21	0.95
7	-11	-3	6.16	1.06
7	-11	3	5.16	0.92
7	11	-3	5.60	0.88
-7	11	3	4.37	1.54
-7	-11	-3	4.79	1.31
7	-11	3	7.09	1.11
-7	-11	-3	6.51	1.13
-7	11	3	6.42	1.38
7	-11	3	4.97	1.09
7	11	-3	4.25	1.43
7	-11	3	5.28	1.03
-7	-11	-3	6.54	0.86
-7	-12	3	5.58	1.65
7	12	3	5.73	1.90
7	-12	-3	5.61	1.21
-7	-12	3	4.73	1.04
7	-12	-3	7.91	1.41
7	-12	-3	8.03	1.27
-7	12	-3	3.90	1.49
-7	-12	-3	8.34	1.40
7	-12	3	4.21	0.82
-7	-12	-3	5.66	1.52
7	12	-3	6.07	1.92
7	-12	3	4.45	0.95
7	-12	3	5.07	1.15
-7	12	3	7.00	1.61
-7	-13	3	4.87	1.42
7	-13	-3	3.35	0.96
7	13	3	3.36	1.73
7	-13	-3	3.91	0.99
7	13	-3	4.09	1.78
-7	-13	-3	4.26	1.40
7	-13	3	4.02	1.54
-8	0	3	439.89	19.40
-8	0	-3	414.01	17.84
-8	0	-3	416.64	17.80
8	0	3	443.51	19.37

8	0	-3	351.79	17.71
-8	0	3	443.17	19.30
8	0	-3	392.45	17.66
8	0	3	401.26	19.26
8	0	3	409.24	19.34
8	0	3	456.30	19.43
-8	0	3	409.75	19.41
-8	0	3	455.77	19.45
-8	0	-3	414.28	17.81
-8	0	-3	403.49	17.70
8	0	-3	385.52	17.77
8	0	-3	384.72	17.75
-8	0	-3	386.53	17.84
-8	0	3	452.70	19.36
-8	0	-3	407.51	17.78
8	0	3	439.53	19.38
-8	0	3	384.16	19.42
-8	1	-3	136.32	6.07
8	1	3	135.50	6.76
-8	1	-3	122.83	6.17
-8	-1	3	147.76	6.72
-8	1	-3	126.27	6.06
8	-1	-3	129.00	6.06
-8	-1	3	139.82	6.71
-8	-1	3	145.81	6.83
-8	-1	3	148.06	6.87
8	-1	-3	124.17	5.98
8	1	3	145.55	6.84
8	-1	-3	129.32	6.04
-8	-1	3	143.15	6.82
-8	1	-3	134.69	6.06
8	1	3	144.91	6.69
8	1	3	147.00	6.74
-8	-1	3	154.96	6.74
8	1	3	149.08	6.76
-8	1	-3	138.06	6.08
8	1	3	157.50	6.81
8	-1	-3	133.04	6.02
-8	1	-3	137.08	6.01
8	-1	3	152.23	6.89
-8	1	3	150.43	6.78
-8	1	3	148.54	6.71
8	-1	3	138.58	6.70
-8	1	3	143.60	6.71

8	-1	3	144.58	6.77
-8	1	3	146.99	6.69
8	-1	3	148.97	6.84
-8	-1	-3	132.77	6.08
-8	-1	-3	133.55	5.95
-8	1	3	157.15	6.68
-8	-1	-3	133.16	6.07
8	1	-3	132.03	6.02
8	1	-3	130.17	6.12
8	-1	3	145.26	6.77
-8	1	3	149.34	6.72
-8	1	3	150.99	6.87
-8	-1	-3	132.14	6.17
-8	-1	-3	133.51	6.00
-8	2	-3	82.23	3.74
8	2	3	56.30	2.83
-8	2	-3	78.44	3.91
8	-2	-3	79.59	3.73
8	2	3	62.73	2.93
-8	-2	3	62.45	3.03
-8	-2	3	56.71	2.82
-8	2	-3	83.59	3.77
8	2	3	56.87	3.00
-8	-2	3	64.57	3.00
-8	2	-3	74.86	3.89
8	2	3	59.63	2.88
-8	-2	3	61.05	2.88
-8	2	-3	84.93	3.78
-8	-2	3	63.78	2.85
-8	-2	3	54.41	2.99
-8	2	-3	72.45	3.73
-8	2	-3	77.81	3.71
8	-2	-3	81.57	3.82
8	-2	-3	81.53	3.71
-8	-2	3	57.32	2.87
8	-2	3	57.77	2.89
-8	-2	-3	75.80	3.71
8	2	-3	80.06	3.59
8	2	-3	81.40	3.79
-8	2	3	58.92	2.87
8	2	-3	75.24	3.62
8	-2	3	59.13	3.02
-8	-2	-3	75.89	3.75
-8	-2	-3	79.82	3.70

-8	-2	-3	82.20	3.64
-8	-2	-3	78.61	3.76
-8	2	3	63.31	3.05
-8	2	3	62.21	2.88
8	-2	3	59.96	2.85
-8	2	3	59.53	3.01
-8	2	3	60.85	2.94
-8	-2	-3	81.70	3.88
-8	-2	-3	78.48	3.76
-8	-2	-3	78.61	3.67
8	-2	3	58.16	2.87
-8	2	3	64.60	2.95
-8	3	-3	31.78	1.66
-8	-3	3	36.23	1.99
-8	-3	3	31.98	1.93
-8	-3	3	34.03	1.70
8	-3	-3	29.51	1.64
8	-3	-3	29.53	1.63
8	3	3	31.57	1.75
-8	-3	3	35.64	1.89
8	-3	-3	28.30	1.57
-8	3	-3	31.16	1.56
-8	3	-3	30.90	1.87
8	3	3	34.83	1.77
-8	3	-3	32.88	1.65
8	3	3	32.51	1.93
8	-3	-3	30.49	1.77
8	3	3	35.64	1.84
-8	3	-3	29.75	1.83
-8	-3	3	32.12	1.76
-8	-3	3	34.97	1.70
-8	3	-3	33.28	1.67
8	3	-3	31.68	1.65
-8	3	3	32.95	1.92
8	-3	3	33.51	1.78
8	-3	3	33.92	1.77
-8	-3	-3	32.17	1.53
-8	-3	-3	29.97	1.56
-8	-3	-3	28.06	1.52
8	-3	3	34.50	1.97
-8	3	3	37.76	1.86
8	-3	3	34.94	1.70
-8	-3	-3	29.99	1.64
8	-3	3	33.73	1.80

-8	3	3	37.55	1.86
-8	-3	-3	31.30	1.52
-8	3	3	33.56	1.93
8	3	-3	30.86	1.60
8	3	-3	28.91	1.55
-8	-3	-3	32.43	1.82
-8	-3	-3	30.17	1.62
-8	3	3	35.48	1.77
8	3	-3	28.47	1.56
-8	-3	-3	33.19	1.68
8	4	3	14.15	1.04
-8	-4	3	13.62	0.80
-8	4	-3	19.27	1.14
8	4	3	14.00	0.90
8	-4	-3	16.25	1.10
8	-4	-3	16.74	1.15
-8	4	-3	16.77	1.43
-8	4	-3	16.99	1.17
-8	-4	3	13.65	0.80
8	-4	-3	16.13	1.03
-8	-4	3	14.24	1.08
8	4	3	13.60	1.12
-8	-4	3	12.76	0.79
-8	-4	3	14.17	1.18
-8	-4	3	9.69	1.07
-8	-4	3	13.17	0.74
-8	4	-3	19.98	1.17
-8	4	-3	17.82	1.00
8	4	3	12.94	0.93
8	-4	-3	17.05	1.52
-8	4	-3	16.31	1.35
8	-4	-3	17.53	1.29
8	4	3	13.59	0.94
-8	-4	3	12.15	0.89
-8	-4	-3	18.87	1.04
8	-4	3	12.99	0.88
-8	-4	-3	18.88	1.10
-8	-4	-3	18.00	1.14
8	-4	3	15.48	1.32
-8	-4	-3	18.43	1.34
8	4	-3	15.46	1.11
8	4	-3	18.25	1.10
8	-4	3	12.91	0.95
-8	4	3	14.93	1.02

8	-4	3	15.10	1.20
-8	4	3	13.90	1.13
-8	4	3	11.30	1.17
8	-4	3	14.13	0.81
8	-4	3	12.69	0.90
8	4	-3	18.21	1.06
-8	-4	-3	18.17	1.13
-8	4	3	13.27	0.93
8	4	-3	19.17	1.12
-8	4	3	14.14	1.04
-8	-4	-3	17.59	1.06
8	5	3	59.12	3.08
8	-5	-3	67.82	3.46
-8	5	-3	66.62	3.71
-8	5	-3	71.74	3.43
-8	5	-3	75.43	3.40
-8	-5	3	60.17	2.91
8	-5	-3	66.91	3.37
-8	5	-3	72.52	3.48
8	-5	-3	65.59	3.51
8	-5	-3	70.67	3.64
8	5	3	62.41	2.94
-8	5	-3	67.21	3.64
8	5	3	58.22	2.99
-8	-5	3	61.72	2.85
8	5	3	59.97	3.08
-8	-5	3	56.65	3.18
-8	-5	3	59.32	3.21
8	5	3	58.08	3.20
-8	-5	3	60.76	2.86
-8	-5	3	57.92	2.99
8	-5	-3	65.12	3.32
-8	5	-3	67.45	3.25
-8	-5	3	60.74	3.07
8	-5	3	61.14	2.96
-8	-5	-3	69.05	3.34
-8	-5	-3	71.20	3.62
-8	5	3	56.78	3.23
8	-5	3	61.91	2.85
-8	5	3	62.11	3.14
-8	-5	-3	69.56	3.33
8	-5	3	55.86	3.02
8	5	-3	71.92	3.36
-8	5	3	63.83	3.00

8	5	-3	66.15	3.44
8	5	-3	68.96	3.37
-8	5	3	62.16	3.06
-8	-5	-3	64.79	3.33
-8	-5	-3	65.05	3.40
-8	5	3	60.73	3.18
8	-5	3	57.83	3.19
8	-5	3	64.52	3.36
8	5	-3	72.34	3.36
8	-5	3	58.53	2.98
-8	6	-3	127.79	5.75
-8	6	-3	113.78	5.43
8	-6	-3	114.29	5.71
8	-6	-3	112.77	5.67
-8	6	-3	127.57	5.59
-8	6	-3	115.88	5.97
8	6	3	116.74	5.82
-8	-6	3	115.46	5.70
-8	-6	3	120.50	5.64
8	6	3	115.31	5.91
-8	6	-3	116.92	5.88
-8	-6	3	120.35	5.58
-8	-6	3	116.67	5.89
8	-6	-3	120.83	5.89
-8	-6	3	120.92	5.50
8	6	3	121.94	5.79
8	6	3	123.84	5.60
8	6	3	116.76	5.68
8	-6	-3	113.12	5.57
8	-6	-3	112.13	5.53
-8	-6	3	119.88	5.92
-8	6	-3	122.12	5.64
-8	6	3	115.47	5.87
8	6	-3	123.34	5.55
8	-6	3	115.16	5.62
-8	6	3	108.88	5.95
-8	6	3	121.90	5.86
-8	6	3	127.64	5.74
8	-6	3	137.62	6.12
8	6	-3	114.03	5.72
8	-6	3	120.99	5.78
8	-6	3	120.02	5.88
-8	-6	-3	111.35	5.62
8	6	-3	114.18	5.57

-8	-6	-3	116.85	5.56
-8	-6	-3	117.66	5.57
-8	-6	-3	113.86	5.53
8	6	-3	125.12	5.62
-8	6	3	126.11	5.70
-8	-6	-3	123.42	5.85
8	6	-3	116.47	5.38
8	-6	3	116.45	5.67
-8	-7	3	104.61	4.95
-8	-7	3	87.55	4.33
-8	7	-3	79.55	4.49
-8	7	-3	82.41	4.09
8	7	3	84.20	4.60
-8	7	-3	82.87	4.35
-8	-7	3	89.99	4.20
8	7	3	98.58	4.77
-8	7	-3	87.42	4.02
8	-7	-3	86.63	4.42
8	-7	-3	77.36	4.16
8	7	3	91.48	4.30
8	-7	-3	81.12	4.13
-8	-7	3	94.89	4.53
8	-7	-3	75.05	3.94
8	7	3	83.36	4.66
-8	-7	3	86.46	4.65
-8	7	-3	86.39	4.20
-8	-7	3	82.74	4.43
8	-7	-3	76.42	3.96
-8	-7	3	85.86	4.66
8	-7	3	84.96	4.64
-8	-7	-3	79.65	4.07
-8	7	3	83.84	4.77
8	-7	3	93.19	4.24
-8	7	3	90.41	4.44
8	7	-3	80.25	3.75
8	-7	3	84.98	4.33
8	-7	3	90.93	4.53
-8	7	3	96.37	4.50
8	7	-3	82.94	4.08
-8	-7	-3	80.86	4.06
8	7	-3	82.75	3.94
8	-7	3	99.50	4.94
8	-7	3	85.52	4.40
-8	-7	-3	79.86	3.93

8	-7	3	100.27	4.32
-8	-7	-3	81.99	4.28
-8	-7	-3	80.06	3.99
-8	7	3	91.56	4.66
8	7	-3	79.31	3.99
8	7	-3	75.24	4.18
-8	7	3	88.03	4.65
-8	-8	3	74.01	4.01
-8	-8	3	73.35	3.84
-8	-8	3	72.89	3.80
-8	-8	3	74.04	3.50
-8	-8	3	71.88	3.70
8	-8	-3	77.30	4.16
8	-8	-3	75.20	4.24
-8	8	-3	87.23	4.08
-8	8	-3	77.56	4.46
8	-8	-3	79.07	4.02
-8	8	-3	81.13	4.44
8	8	3	70.42	4.02
8	-8	-3	82.38	4.54
8	8	3	74.28	3.64
-8	8	-3	86.25	4.34
8	-8	-3	79.08	4.03
8	8	3	71.72	4.00
-8	8	3	73.55	3.84
-8	-8	-3	80.78	4.14
8	-8	3	72.46	3.67
-8	8	3	72.91	4.05
8	-8	3	72.80	3.89
8	-8	3	79.49	3.60
8	8	-3	88.41	4.19
8	8	-3	78.93	4.34
-8	8	3	72.36	4.62
8	8	-3	80.78	3.78
-8	-8	-3	80.46	4.07
-8	-8	-3	80.13	3.97
8	-8	3	82.23	4.40
8	8	-3	83.97	3.98
8	-8	3	79.88	3.79
-8	8	3	77.38	4.10
8	-8	3	74.31	3.77
-8	9	-3	60.21	3.95
8	-9	-3	71.94	3.95
-8	-9	3	54.19	3.02

-8	9	-3	62.18	3.91
8	-9	-3	58.36	3.20
-8	9	-3	65.42	3.31
8	9	3	56.01	2.92
8	-9	-3	62.82	3.46
-8	-9	3	53.90	3.35
-8	-9	3	56.72	2.76
8	9	3	60.99	3.44
-8	-9	3	58.74	3.16
8	9	3	53.39	3.36
8	9	3	52.63	3.40
-8	-9	3	56.44	3.14
8	9	-3	61.67	2.95
-8	-9	-3	61.09	3.17
8	-9	3	65.29	3.84
-8	-9	-3	62.25	3.38
8	9	-3	56.03	3.62
-8	-9	-3	59.16	3.31
8	-9	3	56.00	2.95
8	-9	3	61.43	2.90
-8	9	3	56.11	3.59
8	-9	3	64.66	3.05
8	9	-3	64.70	3.18
8	-9	3	55.63	3.08
-8	9	3	58.36	3.40
8	-9	3	56.31	3.21
8	-10	-3	6.35	1.15
8	10	3	3.01	1.13
-8	-10	3	5.57	1.22
8	-10	-3	7.69	1.12
-8	10	-3	11.56	1.85
8	10	3	5.20	0.76
8	-10	-3	11.88	2.33
-8	-10	3	5.30	0.98
-8	10	-3	6.54	1.55
8	-10	3	3.30	0.96
8	-10	3	4.40	0.73
-8	10	3	4.54	1.22
-8	10	3	1.34	1.17
8	-10	3	5.26	0.89
8	10	-3	5.81	1.05
-8	-10	-3	5.58	1.30
-8	-10	-3	4.97	0.96
8	-10	3	4.96	1.78

8	-10	3	4.39	0.91
-8	-10	-3	6.60	1.00
8	10	-3	5.85	1.35
8	-10	3	8.03	1.47
8	-11	-3	-0.23	0.64
8	-11	-3	0.93	0.71
-8	-11	3	-0.22	1.08
-8	11	-3	0.11	1.28
-8	-11	3	-0.32	0.86
-8	11	-3	-0.17	1.40
8	-11	-3	-0.27	0.81
8	11	3	-0.65	1.14
-8	-11	-3	0.29	0.64
8	11	-3	0.93	1.31
-8	-11	-3	0.43	0.88
8	-11	3	0.08	0.83
-8	-11	-3	-0.39	0.95
8	-11	3	-0.22	0.61
8	-11	3	0.88	0.91
8	-11	3	0.41	0.60
-8	11	3	-0.39	1.17
8	11	-3	-0.04	0.70
8	-12	-3	6.13	1.05
8	-12	-3	4.35	1.22
8	12	3	3.47	1.59
8	-12	-3	4.43	0.96
-8	-12	3	3.02	0.91
-8	-12	3	3.66	1.43
-8	12	-3	6.47	1.86
8	12	-3	3.06	1.69
8	-12	3	3.68	0.80
8	-12	3	4.58	1.11
-8	-12	-3	5.21	1.47
-8	-12	-3	4.04	0.96
-8	12	3	6.06	1.63
8	-12	3	1.54	0.81
-9	0	3	-0.19	0.26
-9	0	3	-0.04	0.25
-9	0	3	-0.04	0.31
-9	0	-3	0.12	0.18
9	0	-3	-0.08	0.12
-9	0	-3	0.12	0.26
-9	0	-3	0.18	0.33
-9	0	-3	0.26	0.29

-9	0	-3	-0.16	0.21
9	0	3	-0.03	0.20
9	0	-3	-0.15	0.18
-9	0	3	0.29	0.27
-9	0	-3	-0.19	0.24
9	0	3	-0.24	0.33
-9	0	-3	-0.10	0.31
-9	0	3	-0.33	0.36
9	0	-3	0.35	0.21
9	0	-3	0.12	0.15
9	0	3	0.34	0.16
9	0	3	0.09	0.30
-9	-1	3	1.49	0.29
9	1	3	1.67	0.37
-9	-1	3	1.30	0.33
9	1	3	1.24	0.23
-9	1	-3	2.48	0.47
-9	1	-3	3.37	0.48
-9	1	-3	2.67	0.49
-9	-1	3	0.64	0.38
9	1	3	1.30	0.42
-9	1	-3	2.89	0.46
-9	-1	3	1.06	0.39
9	-1	-3	1.97	0.39
9	-1	-3	2.70	0.41
-9	1	-3	1.95	0.48
9	1	3	1.63	0.43
9	1	3	1.40	0.29
-9	1	-3	3.05	0.38
9	-1	-3	2.82	0.40
-9	-1	3	1.50	0.37
9	-1	-3	2.10	0.33
-9	-1	3	1.13	0.49
9	1	3	1.61	0.36
-9	-1	3	1.53	0.30
-9	1	-3	2.46	0.43
-9	-1	-3	2.84	0.46
-9	-1	-3	2.46	0.39
9	1	-3	2.37	0.42
-9	-1	-3	2.23	0.41
9	-1	3	1.10	0.30
9	-1	3	1.92	0.57
-9	-1	-3	3.04	0.29
-9	1	3	0.81	0.41

-9	1	3	1.09	0.34
9	-1	3	1.48	0.31
-9	-1	-3	3.46	0.63
-9	1	3	0.36	0.37
-9	1	3	0.70	0.24
9	1	-3	2.76	0.41
9	-1	3	1.86	0.31
-9	-1	-3	2.51	0.45
9	-1	3	0.71	0.41
-9	1	3	1.15	0.38
-9	1	3	1.47	0.33
9	2	3	41.54	2.09
-9	2	-3	44.76	2.12
-9	-2	3	43.34	2.31
9	2	3	39.52	2.28
9	-2	-3	40.78	2.09
-9	2	-3	39.86	2.12
-9	2	-3	41.88	2.32
9	-2	-3	43.72	2.10
-9	2	-3	39.94	2.29
-9	-2	3	41.02	2.13
-9	-2	3	46.50	2.16
9	-2	-3	41.53	2.19
-9	-2	3	42.43	2.11
-9	2	-3	43.14	2.16
-9	-2	3	37.64	2.28
9	2	3	44.52	2.22
-9	-2	3	41.87	2.39
-9	2	-3	43.63	2.10
-9	2	-3	44.62	2.17
9	2	3	47.19	2.18
9	-2	-3	41.32	2.14
-9	2	3	43.62	2.31
-9	-2	-3	44.74	1.99
9	2	-3	42.45	1.94
9	-2	3	39.61	2.13
-9	2	3	48.44	2.17
9	-2	3	42.77	2.18
9	-2	3	44.56	2.33
-9	2	3	47.98	2.37
-9	-2	-3	43.30	2.28
9	2	-3	37.93	1.96
-9	-2	-3	37.06	2.14
-9	-2	-3	41.68	2.15

9	-2	3	43.56	2.37
9	2	-3	43.85	2.19
-9	2	3	42.36	2.22
-9	2	3	44.52	2.22
-9	2	3	42.62	2.05
-9	2	3	36.73	2.04
-9	-2	-3	43.94	2.16
-9	2	3	47.17	2.16
-9	-2	-3	42.47	2.10
-9	-2	-3	44.42	2.15
-9	-2	-3	42.24	2.02
9	3	3	165.62	8.19
-9	3	-3	163.13	7.88
9	-3	-3	159.38	7.63
-9	3	-3	180.33	7.72
-9	-3	3	181.91	8.16
-9	3	-3	178.92	7.71
-9	3	-3	162.93	7.59
9	-3	-3	162.88	7.69
9	-3	-3	165.23	7.79
-9	-3	3	184.32	8.40
9	-3	-3	169.92	7.80
-9	-3	3	169.75	8.22
-9	-3	3	168.76	8.37
-9	3	-3	162.10	7.87
9	3	3	183.15	8.22
-9	-3	3	179.54	8.23
-9	-3	3	174.55	8.16
9	3	3	173.07	8.39
9	3	3	190.05	8.27
-9	3	-3	180.73	7.72
-9	3	3	176.05	8.33
-9	-3	-3	155.19	7.57
-9	-3	-3	165.80	7.68
-9	-3	-3	173.73	7.72
-9	-3	-3	168.08	7.71
9	3	-3	154.49	7.59
-9	-3	-3	169.04	7.56
9	3	-3	172.13	7.71
-9	3	3	195.53	8.30
9	-3	3	175.44	8.24
9	-3	3	184.02	8.15
9	-3	3	174.17	8.25
9	-3	3	171.76	8.24

-9	-3	-3	165.41	7.84
-9	-3	-3	165.91	7.59
-9	3	3	191.39	8.28
-9	-3	-3	164.64	7.58
9	-3	3	186.61	8.39
-9	3	3	179.30	8.39
-9	3	3	185.47	8.22
9	3	-3	166.76	7.55
9	4	3	43.54	2.55
9	4	3	48.99	2.35
-9	-4	3	41.89	2.55
-9	4	-3	50.93	2.84
-9	-4	3	46.16	2.15
9	4	3	42.38	2.36
-9	-4	3	50.42	2.28
-9	-4	3	48.43	2.26
9	-4	-3	49.46	2.59
9	-4	-3	51.99	2.53
-9	-4	3	47.85	2.38
9	4	3	47.31	2.43
-9	-4	3	44.15	2.58
9	-4	-3	49.24	2.74
-9	4	-3	54.88	2.67
-9	-4	3	44.44	2.61
-9	4	-3	43.21	2.81
9	-4	-3	52.46	2.63
-9	4	-3	53.34	2.63
-9	4	-3	54.02	2.61
-9	-4	3	45.66	2.16
-9	4	-3	48.91	2.45
-9	-4	-3	50.82	2.61
9	4	-3	53.85	2.59
-9	-4	-3	53.89	2.59
9	-4	3	47.06	2.24
-9	4	3	48.76	2.48
-9	-4	-3	50.16	2.47
9	-4	3	45.28	2.37
-9	-4	-3	52.01	2.62
-9	-4	-3	47.68	2.43
-9	-4	-3	49.20	2.49
9	-4	3	44.48	2.36
9	-4	3	47.54	2.42
-9	4	3	47.64	2.44
9	4	-3	52.68	2.49

-9	4	3	50.96	2.38
-9	-4	-3	49.53	2.79
9	-4	3	47.78	2.60
-9	4	3	42.92	2.55
-9	4	3	49.62	2.60
9	4	-3	55.94	2.58
9	-4	3	47.14	2.70
-9	5	-3	21.59	1.43
9	-5	-3	19.44	1.23
9	5	3	10.65	0.97
-9	5	-3	21.60	1.63
9	-5	-3	19.87	1.34
-9	-5	3	11.66	1.24
-9	5	-3	23.20	1.41
-9	5	-3	21.28	1.17
9	5	3	11.55	1.07
-9	5	-3	20.42	1.72
-9	-5	3	10.69	0.74
-9	-5	3	13.80	0.80
9	-5	-3	20.70	1.31
9	-5	-3	19.69	1.61
-9	-5	3	11.57	0.76
-9	-5	3	9.43	1.14
9	-5	-3	19.51	1.55
-9	5	-3	23.74	1.36
9	5	3	12.89	0.91
-9	-5	3	12.37	0.94
-9	-5	3	10.86	0.79
9	5	3	10.92	1.14
9	5	3	10.63	0.93
9	5	-3	17.31	1.34
9	-5	3	10.24	0.93
-9	5	3	13.44	0.96
-9	-5	-3	20.25	1.24
9	-5	3	10.27	0.84
-9	5	3	12.72	1.07
-9	5	3	9.14	1.20
9	5	-3	22.16	1.31
9	-5	3	13.79	1.29
-9	-5	-3	20.12	1.60
-9	5	3	11.82	1.20
9	-5	3	11.27	1.39
9	-5	3	11.71	0.89
9	5	-3	22.62	1.32

-9	-5	-3	22.06	1.36
-9	-5	-3	21.92	1.29
-9	5	3	11.46	1.10
-9	-5	-3	19.32	1.22
9	-5	3	12.75	0.77
9	5	-3	22.46	1.29
-9	6	-3	44.63	3.08
-9	6	-3	52.49	2.76
9	6	3	52.24	2.80
9	-6	-3	48.83	2.79
9	6	3	52.25	3.07
-9	6	-3	53.20	2.63
-9	-6	3	55.88	3.12
9	-6	-3	52.03	2.95
-9	-6	3	49.29	2.97
-9	6	-3	48.90	2.93
-9	-6	3	54.43	2.83
-9	6	-3	49.77	2.66
9	6	3	54.37	2.94
-9	-6	3	52.76	2.72
-9	6	-3	46.69	2.42
9	-6	-3	47.65	2.68
9	6	3	56.74	2.73
9	-6	-3	47.01	2.53
9	-6	-3	47.81	2.58
-9	-6	3	49.66	3.03
9	6	3	52.11	2.93
-9	-6	3	56.08	2.64
9	-6	3	55.91	2.90
9	-6	3	50.52	2.78
-9	-6	-3	48.47	2.54
-9	-6	-3	50.95	2.56
9	6	-3	48.44	2.60
9	-6	3	58.35	3.30
9	6	-3	54.48	2.64
9	-6	3	56.56	3.10
9	6	-3	44.61	2.71
-9	-6	-3	50.07	2.55
-9	6	3	52.87	3.15
-9	6	3	57.43	2.83
-9	6	3	56.70	3.04
-9	-6	-3	50.30	2.65
9	6	-3	51.62	2.40
9	6	-3	53.75	2.57

9	-6	3	56.59	2.61
-9	6	3	56.23	2.90
9	-6	3	55.01	2.77
-9	6	3	56.17	3.07
-9	-6	-3	51.10	2.92
-9	-7	3	9.63	0.86
-9	7	-3	12.76	1.16
9	-7	-3	12.70	1.59
-9	-7	3	8.40	1.23
9	-7	-3	11.82	1.00
9	7	3	9.49	1.31
-9	-7	3	8.74	0.98
9	7	3	9.41	0.93
9	-7	-3	11.75	1.02
9	7	3	8.99	1.18
-9	7	-3	9.90	1.49
9	7	3	10.45	1.18
9	-7	-3	11.32	1.14
9	-7	-3	10.07	1.38
-9	7	-3	12.70	1.33
-9	7	-3	9.96	1.70
-9	-7	3	9.33	0.76
-9	-7	3	9.36	1.43
9	7	-3	12.44	1.03
-9	-7	-3	12.44	1.01
9	-7	3	9.60	0.76
-9	7	3	7.91	1.65
-9	7	3	9.16	1.33
-9	-7	-3	11.94	1.06
-9	7	3	10.25	1.11
9	-7	3	14.12	2.11
9	7	-3	11.91	1.24
-9	7	3	9.03	1.18
-9	7	3	9.17	1.34
9	-7	3	11.05	0.92
-9	-7	-3	11.81	1.09
9	7	-3	11.31	0.79
9	-7	3	8.32	0.91
9	7	-3	11.86	1.27
9	-7	3	8.86	0.86
9	-7	3	10.18	1.07
9	7	-3	13.30	1.14
9	-7	3	10.02	1.68
9	8	3	11.31	1.57

9	-8	-3	15.85	1.92
-9	8	-3	18.54	1.80
-9	8	-3	15.13	1.41
-9	-8	3	14.60	1.54
-9	-8	3	14.94	1.00
9	8	3	14.06	1.55
9	-8	-3	15.84	1.20
-9	-8	3	14.94	1.31
-9	8	-3	15.41	1.58
9	-8	-3	14.03	1.37
-9	8	-3	14.33	1.80
-9	-8	3	16.15	1.19
9	8	3	14.26	1.17
-9	-8	3	14.86	1.59
9	-8	-3	14.79	1.17
9	8	-3	14.49	1.53
9	8	-3	16.41	1.63
9	-8	3	19.17	2.20
-9	-8	-3	14.83	1.16
9	-8	3	15.65	1.18
-9	-8	-3	16.02	1.31
9	-8	3	14.45	1.12
9	-8	3	15.86	1.34
-9	8	3	10.20	1.91
9	8	-3	13.33	1.25
9	-8	3	14.14	1.23
9	-8	3	14.97	0.96
9	8	-3	14.64	1.14
-9	8	3	16.22	1.69
9	-9	-3	5.70	0.92
-9	9	-3	5.56	1.50
9	-9	-3	5.47	1.03
-9	-9	3	3.05	1.02
9	9	3	2.44	0.67
9	9	3	3.64	1.02
-9	-9	3	3.04	0.72
-9	-9	3	3.72	0.68
-9	-9	3	3.79	0.96
-9	9	-3	6.54	1.16
9	-9	-3	4.33	1.34
9	-9	-3	5.79	0.95
-9	-9	3	3.32	0.79
-9	9	-3	7.54	1.37
9	9	3	3.62	1.01

9	-9	3	4.27	0.77
9	9	-3	4.96	0.89
-9	-9	-3	5.26	0.79
9	-9	3	3.90	0.61
-9	9	3	2.89	1.19
-9	9	3	3.13	1.13
-9	-9	-3	5.18	0.91
9	-9	3	2.85	0.81
9	-9	3	2.34	0.62
-9	-9	-3	5.57	1.12
9	-9	3	4.32	1.45
9	9	-3	5.32	1.02
9	9	-3	5.17	1.16
9	10	3	16.49	1.92
9	-10	-3	15.57	1.33
9	10	3	13.52	1.27
9	-10	-3	15.23	1.55
-9	-10	3	13.10	1.92
-9	10	-3	11.48	2.27
-9	10	-3	17.46	2.27
9	-10	-3	15.67	1.41
-9	-10	3	15.39	1.56
9	-10	3	16.62	1.18
9	-10	3	14.32	1.56
9	10	-3	16.37	1.30
-9	10	3	13.49	2.28
9	10	-3	12.15	2.04
9	-10	3	15.44	1.39
-9	-10	-3	15.95	1.32
-9	-10	-3	15.83	1.54
-9	10	3	12.50	1.99
9	-10	3	18.68	2.63
9	-10	3	13.26	1.18
-9	-10	-3	14.80	1.93
9	-11	-3	24.46	1.91
-9	11	-3	21.99	2.58
-9	-11	3	23.51	2.56
9	11	3	19.57	2.65
9	-11	-3	20.73	1.70
-9	-11	3	22.92	2.09
9	11	-3	23.16	2.62
-9	-11	-3	23.71	2.00
-9	-11	-3	24.48	2.49
-9	11	3	22.39	2.52

9	-11	3	25.49	1.96
-9	-11	-3	19.92	1.64
9	-11	3	24.01	2.06
9	-12	-3	18.91	1.72
9	-12	-3	24.01	1.85
-9	12	-3	28.66	3.30
9	12	3	18.42	2.65
-9	-12	3	19.85	2.37
-9	-12	3	17.48	1.85
9	-12	-3	21.61	2.20
9	12	-3	22.91	2.74
-9	-12	-3	21.53	2.46
-9	12	3	23.60	3.01
9	-12	3	16.18	1.93
-9	-12	-3	19.18	1.92
9	-12	3	19.85	1.60
9	-12	3	23.56	1.86
10	0	-3	98.57	4.85
10	0	-3	102.11	4.96
-10	0	3	113.44	5.27
-10	0	-3	111.36	4.86
10	0	3	110.07	5.28
-10	0	3	113.94	5.21
-10	0	-3	98.32	5.07
-10	0	-3	109.60	5.05
10	0	3	104.97	5.13
-10	0	-3	112.37	5.02
-10	0	3	117.10	5.25
-10	0	-3	110.87	4.94
-10	0	3	111.64	5.32
10	0	-3	103.65	4.95
-10	0	3	106.65	5.25
-10	0	-3	115.07	5.01
10	0	-3	103.73	4.82
10	0	3	119.71	5.35
10	0	3	110.58	5.25
10	0	3	114.95	5.30
-10	0	3	111.15	5.36
-10	1	-3	46.98	2.36
10	-1	-3	43.76	2.22
10	1	3	31.57	1.65
-10	-1	3	30.69	1.57
-10	1	-3	44.15	2.49
10	-1	-3	47.69	2.30

-10	1	-3	47.79	2.36
10	-1	-3	44.76	2.33
-10	-1	3	27.72	1.71
-10	-1	3	31.15	1.75
10	1	3	32.06	1.72
-10	1	-3	44.53	2.33
-10	1	-3	48.94	2.26
10	1	3	29.02	1.71
10	1	3	29.30	1.63
10	1	3	27.65	1.65
-10	-1	3	30.52	1.61
-10	1	-3	47.94	2.37
-10	-1	3	28.54	1.76
-10	-1	3	32.99	1.61
-10	-1	3	30.65	1.68
10	1	3	29.75	1.51
-10	1	-3	46.69	2.40
-10	1	3	28.48	1.78
-10	1	3	30.72	1.66
-10	1	3	30.58	1.61
-10	-1	-3	47.24	2.36
-10	1	3	33.20	1.61
-10	-1	-3	46.98	2.21
10	-1	3	28.10	1.53
10	-1	3	31.63	1.81
10	-1	3	31.22	1.63
-10	-1	-3	46.90	2.45
-10	1	3	31.70	1.76
-10	-1	-3	47.77	2.36
-10	-1	-3	43.77	2.39
-10	1	3	30.59	1.44
-10	-1	-3	47.45	2.17
10	-1	3	28.44	1.61
10	-1	3	30.97	1.74
10	1	-3	49.60	2.33
-10	2	-3	122.73	6.03
-10	-2	3	118.87	5.91
10	2	3	122.37	5.86
10	-2	-3	122.46	5.87
-10	2	-3	118.58	5.84
-10	2	-3	126.95	5.89
-10	2	-3	130.77	5.89
-10	-2	3	132.21	6.06
10	-2	-3	126.28	5.82

-10	-2	3	127.86	5.93
10	2	3	124.57	6.08
-10	2	-3	124.99	5.81
10	2	3	136.52	5.99
10	2	3	127.53	5.94
-10	-2	3	125.14	6.07
10	-2	-3	119.31	5.80
-10	2	-3	131.46	5.83
-10	-2	3	128.57	5.91
10	-2	-3	123.85	5.90
-10	-2	3	131.38	6.09
-10	2	-3	126.76	6.00
-10	-2	3	117.93	5.89
10	-2	3	136.05	6.15
-10	-2	-3	130.68	5.88
-10	2	3	130.37	5.83
-10	-2	-3	126.57	5.83
-10	2	3	128.70	5.92
-10	2	3	134.80	6.01
-10	-2	-3	130.98	5.69
-10	-2	-3	132.68	6.01
-10	2	3	120.33	5.82
-10	-2	-3	121.86	5.83
10	-2	3	121.40	5.91
-10	2	3	131.86	5.90
10	-2	3	135.03	5.92
10	2	-3	125.06	5.90
-10	-2	-3	121.29	5.86
10	-2	3	127.66	6.07
-10	-2	-3	125.60	5.71
-10	2	3	127.39	6.10
-10	2	3	128.03	5.95
10	-2	3	125.50	5.96
-10	2	3	132.93	6.12
-10	-2	-3	128.22	5.87
-10	3	-3	34.13	2.06
10	-3	-3	32.99	1.79
-10	-3	3	30.28	1.76
10	-3	-3	33.89	1.82
-10	-3	3	25.47	1.69
10	3	3	28.45	1.53
10	-3	-3	35.12	1.99
-10	3	-3	36.30	1.86
10	3	3	27.99	1.59

-10	-3	3	28.25	1.48
10	3	3	27.07	1.71
-10	3	-3	36.00	1.87
-10	3	-3	32.67	2.06
10	3	3	23.68	1.44
10	-3	-3	34.66	1.99
-10	-3	3	29.18	1.75
-10	3	-3	33.01	1.73
-10	3	-3	35.36	1.84
-10	-3	3	28.01	1.46
-10	-3	3	27.56	1.53
-10	-3	3	26.15	1.50
10	-3	-3	34.73	1.77
10	-3	3	26.55	1.52
-10	-3	-3	34.86	1.85
-10	3	3	26.39	1.65
10	-3	3	26.12	1.51
-10	3	3	28.75	1.61
10	-3	3	28.38	1.75
-10	-3	-3	30.62	1.75
-10	-3	-3	33.23	1.67
10	3	-3	35.53	1.85
-10	3	3	27.61	1.72
-10	-3	-3	34.16	2.01
10	3	-3	32.64	1.69
10	3	-3	34.17	1.65
10	-3	3	26.77	1.44
-10	3	3	28.57	1.55
-10	-3	-3	34.78	1.64
-10	-3	-3	32.07	1.83
10	-3	3	25.95	1.51
-10	-3	-3	33.14	1.75
-10	3	3	29.53	1.53
-10	-3	-3	32.69	1.81
-10	4	-3	10.86	1.22
-10	4	-3	11.18	0.96
10	4	3	10.61	0.99
10	-4	-3	11.01	0.87
-10	-4	3	11.21	1.11
-10	-4	3	9.52	0.81
10	4	3	10.22	0.83
10	4	3	10.14	1.05
-10	4	-3	9.12	1.20
10	-4	-3	10.28	0.79

10	-4	-3	11.19	0.87
10	4	3	9.29	0.81
-10	-4	3	10.39	0.76
-10	-4	3	8.83	1.03
-10	4	-3	11.21	0.96
-10	-4	3	8.39	1.10
-10	4	-3	11.23	0.78
10	-4	-3	10.89	1.14
-10	4	-3	11.84	0.94
-10	-4	3	10.17	0.73
-10	-4	3	10.32	0.88
-10	4	3	10.19	1.13
-10	-4	-3	10.07	0.93
-10	-4	-3	11.78	0.76
-10	4	3	10.73	0.86
-10	-4	-3	10.58	0.75
10	-4	3	9.04	0.82
-10	4	3	10.26	0.96
-10	-4	-3	9.97	0.85
10	-4	3	10.13	0.79
-10	-4	-3	10.49	0.72
10	-4	3	9.17	0.80
-10	-4	-3	11.60	1.16
-10	4	3	9.96	0.98
10	4	-3	11.62	0.90
10	-4	3	9.55	0.72
10	-4	3	10.10	1.22
10	4	-3	12.05	0.75
-10	-4	-3	10.30	0.88
-10	-4	-3	10.96	0.79
10	-4	3	9.56	1.15
10	4	-3	9.73	0.83
-10	4	3	10.81	1.08
10	-5	-3	12.25	1.35
-10	5	-3	15.17	1.41
10	5	3	14.31	1.33
10	-5	-3	15.14	1.35
10	-5	-3	14.33	1.05
-10	5	-3	15.59	1.20
-10	-5	3	17.27	1.42
-10	-5	3	16.06	0.97
-10	5	-3	12.54	0.86
10	5	3	14.15	1.13
-10	5	-3	14.05	1.13

-10	5	-3	14.00	1.56
-10	-5	3	16.91	1.48
-10	-5	3	17.79	1.16
10	-5	-3	14.71	0.97
10	-5	-3	13.29	1.06
-10	5	-3	13.48	1.10
-10	-5	3	15.44	0.89
-10	-5	3	15.12	0.87
10	5	3	14.71	1.20
-10	-5	3	16.80	1.37
10	5	3	16.87	1.09
10	-5	3	16.20	1.17
-10	-5	-3	14.06	1.11
-10	5	3	19.04	1.46
-10	5	3	15.18	1.25
10	-5	3	16.61	1.60
10	5	-3	15.02	1.06
-10	-5	-3	13.37	0.91
-10	5	3	15.61	1.27
-10	5	3	15.19	1.50
10	-5	3	14.13	1.08
-10	-5	-3	12.58	1.01
-10	5	3	18.56	1.19
10	5	-3	14.83	1.04
10	5	-3	11.81	1.06
10	-5	3	17.38	0.95
-10	-5	-3	13.00	1.05
10	-5	3	15.48	1.46
10	-5	3	15.48	1.08
-10	-5	-3	14.51	1.39
10	5	-3	15.51	0.97
-10	-5	-3	13.45	0.94
-10	-6	3	77.80	3.79
-10	-6	3	77.50	4.21
10	-6	-3	75.19	4.07
-10	6	-3	81.53	4.04
-10	-6	3	81.13	3.76
10	-6	-3	82.86	4.22
10	-6	-3	76.73	3.82
-10	6	-3	73.76	4.20
-10	-6	3	77.69	3.97
10	6	3	75.04	3.94
10	-6	-3	79.60	3.90
10	6	3	75.63	4.21

-10	-6	3	83.38	4.08
10	6	3	83.52	3.87
10	6	3	76.37	4.04
10	6	3	82.73	4.06
-10	-6	3	80.32	4.22
-10	-6	3	81.68	3.80
-10	6	-3	83.59	3.92
-10	6	-3	80.24	3.95
-10	-6	3	81.66	3.76
10	-6	-3	75.81	3.95
-10	6	-3	75.09	4.30
-10	-6	-3	74.28	3.92
10	6	-3	80.54	3.88
-10	6	3	82.65	4.15
-10	-6	-3	76.59	3.78
-10	6	3	81.50	3.96
-10	-6	-3	83.93	4.20
-10	-6	-3	78.94	3.84
10	6	-3	74.75	3.98
10	6	-3	80.21	3.83
10	-6	3	76.96	3.90
-10	6	3	85.73	4.05
10	-6	3	77.03	3.92
10	-6	3	80.46	4.04
10	-6	3	83.50	4.37
-10	-6	-3	80.39	3.81
10	-6	3	79.88	4.20
-10	6	3	78.35	4.21
-10	6	3	80.55	4.31
10	-7	-3	96.57	4.76
-10	-7	3	105.07	5.21
-10	-7	3	103.82	5.18
10	7	3	109.70	5.31
10	-7	-3	107.78	5.25
-10	7	-3	97.93	5.20
-10	7	-3	100.50	4.99
-10	7	-3	94.13	5.31
-10	7	-3	104.49	4.85
-10	-7	3	108.88	4.99
10	7	3	101.80	5.30
10	-7	-3	94.98	4.91
10	7	3	109.29	5.03
-10	-7	3	101.66	5.39
-10	-7	3	106.41	4.95

10	-7	-3	92.79	4.80
10	7	3	103.49	5.42
-10	-7	-3	97.38	4.87
10	7	-3	91.86	4.97
-10	-7	-3	99.20	4.75
-10	-7	-3	97.92	4.75
10	7	-3	100.94	4.72
10	-7	3	116.23	5.69
10	-7	3	102.01	5.12
-10	7	3	103.75	5.42
-10	7	3	106.07	5.16
10	7	-3	104.79	4.87
10	-7	3	106.47	5.25
-10	7	3	105.53	5.55
10	-7	3	105.51	5.09
-10	8	-3	36.46	2.87
-10	-8	3	32.23	1.87
10	-8	-3	35.23	2.08
-10	8	-3	34.82	2.93
-10	-8	3	32.21	2.33
-10	8	-3	37.10	2.22
10	8	3	33.84	1.88
10	-8	-3	35.30	2.09
10	-8	-3	40.90	2.79
10	8	3	29.92	2.43
10	8	3	33.40	2.20
10	-8	-3	34.24	2.26
-10	-8	3	34.43	1.77
10	8	3	27.04	2.87
-10	-8	3	32.89	2.17
-10	8	3	35.74	2.45
10	-8	3	31.72	2.08
-10	-8	-3	36.18	2.20
10	-8	3	31.16	1.88
10	8	-3	37.06	1.99
10	-8	3	34.90	2.80
-10	8	3	34.33	2.68
10	8	-3	32.42	2.36
-10	-8	-3	35.98	2.13
10	8	-3	38.45	2.25
-10	-8	-3	37.76	2.09
10	-8	3	28.78	1.95
-10	-9	3	51.81	2.61
10	-9	-3	52.14	2.87

-10	9	-3	56.21	3.52
10	-9	-3	57.59	3.54
-10	-9	3	51.94	2.97
10	9	3	53.03	2.75
-10	-9	3	52.88	2.80
10	-9	-3	52.96	3.08
-10	9	-3	50.78	3.51
10	-9	-3	49.56	3.01
-10	-9	3	51.56	3.30
-10	-9	3	51.29	2.97
10	9	3	51.14	3.31
10	9	3	52.29	3.23
10	9	-3	49.43	3.29
-10	9	3	51.80	3.34
-10	-9	-3	54.09	2.92
-10	-9	-3	51.50	2.81
10	-9	3	51.38	2.79
10	-9	3	51.55	3.04
10	-9	3	52.03	2.90
-10	9	3	55.12	3.68
10	9	-3	55.06	3.15
-10	-9	-3	55.89	3.06
10	-9	3	61.03	3.66
10	9	-3	53.99	2.75
-10	-10	3	12.32	1.44
10	-10	-3	11.41	1.72
-10	10	-3	10.89	2.17
-10	-10	3	13.56	1.87
10	-10	-3	11.32	1.34
10	10	3	13.05	1.37
10	10	3	11.83	1.90
-10	10	-3	14.44	2.38
10	-10	-3	12.65	1.28
10	-10	3	12.70	1.23
-10	10	3	12.35	2.07
-10	-10	-3	14.41	1.64
10	10	-3	15.21	1.18
10	-10	3	12.86	1.35
10	10	-3	12.46	1.92
-10	-10	-3	13.00	1.88
-10	-10	-3	12.47	1.26
10	-10	3	10.99	1.45
10	-11	-3	3.69	0.97
-10	-11	3	4.98	1.53

10	-11	-3	2.38	0.81
-10	-11	3	3.93	1.00
10	-11	-3	1.63	1.03
10	11	3	5.12	1.57
-10	11	-3	1.69	1.68
-10	-11	-3	1.87	0.91
10	-11	3	3.63	0.82
10	11	-3	1.64	1.42
-10	-11	-3	2.49	0.79
10	-11	3	3.62	1.18
-10	11	3	0.89	1.35
10	-11	3	2.27	0.84
-10	-11	-3	3.57	1.43
-10	-12	3	4.47	1.46
-10	12	-3	6.70	1.96
10	12	3	3.45	1.49
10	-12	-3	5.65	1.11
10	-12	-3	5.70	1.32
10	-12	-3	4.78	0.95
-10	-12	3	5.01	1.16
10	-12	3	5.58	0.95
-10	-12	-3	6.29	1.47
10	-12	3	6.23	1.06
10	-12	3	5.65	1.29
-10	-12	-3	4.25	1.13
-10	12	3	7.86	2.07
10	12	-3	6.77	1.63
-11	0	-3	-0.15	0.19
-11	0	3	-0.08	0.40
-11	0	-3	-0.32	0.39
11	0	-3	0.52	0.26
11	0	3	0.45	0.45
-11	0	-3	0.32	0.31
11	0	3	0.08	0.40
-11	0	-3	0.23	0.29
11	0	3	0.35	0.19
11	0	3	0.45	0.35
11	0	-3	-0.01	0.23
-11	0	3	0.22	0.46
11	0	3	0.17	0.26
11	0	-3	-0.08	0.14
-11	0	-3	0.33	0.30
-11	0	3	0.19	0.29
-11	0	3	0.38	0.34

-11	0	-3	0.08	0.38
-11	0	3	0.05	0.35
11	0	-3	0.67	0.26
-11	0	-3	0.25	0.31
-11	0	3	0.46	0.37
-11	-1	3	15.32	1.11
-11	-1	3	18.46	1.12
-11	-1	3	15.60	1.18
-11	1	-3	20.61	1.48
11	-1	-3	23.25	1.29
-11	-1	3	15.77	1.02
-11	-1	3	16.83	1.24
11	1	3	14.20	1.00
-11	1	-3	23.87	1.36
11	1	3	14.52	0.87
-11	1	-3	21.60	1.33
11	-1	-3	25.51	1.35
-11	-1	3	17.01	0.98
-11	1	-3	23.62	1.41
-11	1	-3	23.40	1.37
-11	1	-3	23.98	1.24
11	-1	-3	24.11	1.21
11	1	3	12.88	1.09
11	1	3	14.96	1.12
11	-1	-3	22.34	1.29
11	1	3	14.62	1.18
-11	-1	3	16.62	1.01
-11	-1	-3	23.20	1.17
-11	1	3	15.44	1.22
11	-1	3	15.62	1.18
-11	1	3	16.48	1.07
11	-1	3	16.35	1.24
-11	-1	-3	21.39	1.43
-11	1	3	18.09	1.02
-11	-1	-3	22.38	1.35
11	1	-3	24.00	1.32
-11	-1	-3	24.02	1.50
-11	-1	-3	23.60	1.13
-11	1	3	13.80	0.98
11	-1	3	14.01	0.99
-11	-1	-3	23.09	1.34
11	-1	3	15.50	1.04
11	-1	3	14.32	0.90
-11	-1	-3	21.21	1.33

-11	1	3	16.32	1.03
-11	-2	3	72.73	3.50
-11	2	-3	72.89	3.77
11	-2	-3	74.16	3.53
-11	2	-3	78.41	3.62
-11	-2	3	73.79	3.52
-11	2	-3	73.43	3.60
-11	-2	3	73.02	3.56
11	-2	-3	72.20	3.50
-11	2	-3	66.71	3.54
-11	2	-3	78.19	3.53
11	2	3	75.60	3.56
-11	-2	3	75.43	3.67
11	-2	-3	70.98	3.62
-11	-2	3	75.66	3.50
11	2	3	73.96	3.53
-11	-2	3	73.47	3.68
-11	-2	3	71.13	3.66
-11	2	-3	70.12	3.70
11	2	3	68.81	3.42
11	-2	-3	74.74	3.59
11	2	3	69.14	3.67
-11	2	-3	74.61	3.53
-11	-2	3	71.02	3.48
-11	-2	-3	77.22	3.60
-11	-2	-3	74.20	3.73
-11	2	3	77.23	3.72
11	-2	3	72.85	3.67
-11	-2	-3	73.05	3.37
-11	-2	-3	72.45	3.58
-11	-2	-3	71.37	3.57
11	-2	3	75.15	3.50
-11	2	3	66.82	3.41
-11	2	3	77.78	3.60
11	-2	3	69.12	3.48
-11	2	3	73.14	3.41
-11	2	3	71.19	3.52
-11	2	3	74.54	3.46
-11	2	3	76.09	3.52
-11	-2	-3	74.89	3.60
-11	-2	-3	74.66	3.57
-11	2	3	74.20	3.73
11	-2	3	69.54	3.53
11	-2	3	74.34	3.77

-11	-2	-3	76.90	3.36
11	-3	-3	124.04	6.02
-11	3	-3	125.46	5.81
-11	-3	3	118.00	6.05
11	3	3	129.07	5.88
11	3	3	127.92	5.91
11	-3	-3	125.76	6.05
-11	-3	3	124.36	6.08
11	-3	-3	128.47	5.87
-11	-3	3	131.55	6.02
-11	-3	3	122.77	5.88
-11	-3	3	132.05	5.84
-11	3	-3	121.32	6.09
11	3	3	121.22	6.07
-11	-3	3	125.77	5.83
-11	3	-3	129.31	5.90
11	-3	-3	120.67	5.92
-11	3	-3	129.17	6.16
-11	-3	3	124.02	5.88
-11	3	-3	121.20	5.89
11	-3	-3	124.84	5.89
-11	3	-3	130.83	5.95
11	3	3	118.23	5.82
-11	3	-3	133.43	5.96
11	-3	3	124.58	5.89
11	-3	3	118.72	5.90
-11	3	3	136.00	5.99
-11	-3	-3	125.26	5.93
-11	-3	-3	131.22	5.92
-11	3	3	132.45	5.89
-11	-3	-3	121.80	5.92
-11	-3	-3	126.08	5.72
-11	-3	-3	127.50	5.85
11	-3	3	127.33	6.06
11	3	-3	130.63	5.93
11	3	-3	126.89	5.72
11	-3	3	127.88	6.14
-11	-3	-3	124.35	5.83
-11	-3	-3	127.10	6.10
-11	-3	-3	127.34	5.70
11	3	-3	116.10	5.75
-11	3	3	126.56	5.89
-11	3	3	122.25	5.97
11	-3	3	123.53	5.86

-11	3	3	131.11	6.12
-11	4	-3	72.50	3.44
11	-4	-3	70.15	3.40
-11	-4	3	86.08	4.19
-11	4	-3	72.62	3.42
11	4	3	87.05	4.15
11	4	3	81.96	4.15
-11	4	-3	65.07	3.65
-11	-4	3	88.68	4.09
11	-4	-3	68.79	3.39
-11	-4	3	88.78	4.10
11	-4	-3	69.76	3.60
-11	-4	3	84.97	4.19
-11	4	-3	64.23	3.22
-11	-4	3	92.82	4.34
-11	-4	3	83.54	4.39
-11	4	-3	65.45	3.67
-11	-4	3	88.22	4.43
11	-4	-3	68.03	3.32
11	4	3	82.25	4.39
11	-4	-3	69.07	3.56
-11	4	-3	69.62	3.41
-11	-4	-3	67.55	3.40
11	-4	3	88.28	4.04
11	4	-3	64.76	3.30
-11	-4	-3	63.15	3.24
11	-4	3	83.22	4.19
-11	-4	-3	67.17	3.18
-11	4	3	84.50	4.37
-11	-4	-3	67.33	3.40
-11	4	3	87.89	4.24
-11	-4	-3	67.73	3.35
11	-4	3	87.78	4.20
-11	-4	-3	67.02	3.27
-11	4	3	91.61	4.44
11	-4	3	83.99	4.38
-11	-4	-3	70.16	3.21
11	4	-3	70.00	3.24
11	-4	3	81.64	4.20
-11	-4	-3	67.73	3.61
11	4	-3	71.54	3.36
-11	4	3	90.32	4.28
-11	4	3	92.53	4.20
-11	5	-3	46.07	2.91

-11	-5	3	36.40	2.06
-11	5	-3	53.36	2.64
11	5	3	38.18	2.01
11	5	3	34.51	2.07
11	-5	-3	49.42	2.59
-11	-5	3	36.07	2.35
-11	5	-3	45.37	2.94
11	-5	-3	47.61	2.60
11	-5	-3	47.33	2.51
-11	-5	3	41.41	1.95
-11	-5	3	38.77	1.93
11	5	3	37.73	2.14
11	5	3	34.81	2.30
11	-5	-3	48.13	2.79
-11	5	-3	50.26	2.64
-11	-5	3	37.75	2.23
-11	-5	3	35.62	2.31
-11	5	-3	53.80	2.70
-11	-5	-3	47.91	2.40
11	5	-3	53.03	2.58
11	-5	3	35.66	2.02
-11	-5	-3	49.56	2.45
11	-5	3	37.79	2.35
-11	5	3	37.37	2.17
11	-5	3	35.51	2.02
-11	5	3	39.66	2.09
11	5	-3	51.76	2.49
-11	-5	-3	48.72	2.54
11	5	-3	51.18	2.54
-11	5	3	38.79	2.35
-11	5	3	37.02	2.17
-11	-5	-3	49.20	2.63
-11	-5	-3	47.05	2.84
11	-5	3	39.97	2.58
11	-5	3	36.30	2.08
-11	5	3	36.36	2.39
-11	-5	-3	49.49	2.62
11	5	-3	44.84	2.57
-11	6	-3	63.86	3.62
11	-6	-3	64.05	3.17
-11	6	-3	56.43	3.65
-11	-6	3	61.16	2.96
11	-6	-3	63.14	3.30
11	-6	-3	67.09	3.62

-11	6	-3	67.39	3.30
11	6	3	62.99	3.04
11	6	3	63.36	3.23
-11	-6	3	60.14	3.24
11	6	3	56.06	3.17
-11	-6	3	60.49	2.94
-11	6	-3	62.99	3.64
-11	-6	3	57.41	2.87
11	6	3	56.73	3.38
11	-6	-3	65.16	3.53
-11	6	-3	64.55	3.36
-11	-6	3	62.43	2.86
-11	-6	3	59.56	3.14
11	-6	-3	57.30	3.20
-11	-6	3	60.64	3.41
11	-6	3	58.39	3.08
-11	6	3	61.66	3.43
11	6	-3	64.62	3.15
-11	-6	-3	64.95	3.12
11	-6	3	64.34	3.60
-11	-6	-3	64.42	3.29
-11	6	3	63.19	3.21
11	6	-3	61.08	3.21
-11	6	3	63.35	3.14
-11	6	3	62.33	3.29
11	-6	3	59.60	3.10
-11	6	3	60.31	3.47
11	-6	3	58.91	3.17
11	6	-3	67.25	3.18
-11	-6	-3	61.13	3.17
11	6	-3	58.40	3.29
-11	-7	3	2.99	0.51
-11	-7	3	2.82	0.48
11	7	3	2.65	0.77
11	7	3	1.87	0.87
-11	-7	3	2.66	0.51
-11	-7	3	2.13	0.62
11	-7	-3	3.33	0.60
11	-7	-3	3.12	0.59
-11	7	-3	4.02	1.06
-11	7	-3	3.69	1.31
11	-7	-3	3.52	1.13
-11	-7	3	2.18	0.83
11	7	3	2.75	0.77

-11	7	-3	2.97	0.75
11	-7	-3	3.81	0.74
11	7	3	3.47	0.63
-11	-7	3	1.93	0.84
-11	-7	-3	2.80	0.68
11	-7	3	2.27	0.68
11	-7	3	1.88	0.52
-11	-7	-3	3.58	0.65
-11	7	3	2.15	1.08
11	-7	3	3.45	1.21
11	-7	3	2.66	0.61
-11	7	3	2.00	0.87
11	7	-3	3.11	0.84
11	7	-3	3.57	0.72
-11	-7	-3	4.92	0.85
11	7	-3	4.28	0.83
11	8	3	3.11	1.01
11	-8	-3	3.23	0.73
-11	-8	3	2.79	0.51
-11	-8	3	3.11	0.56
11	-8	-3	3.68	1.24
11	8	3	2.74	0.65
-11	-8	3	2.95	0.98
-11	-8	3	2.37	0.66
11	8	3	1.92	0.89
11	-8	-3	2.94	0.76
-11	8	-3	4.23	1.11
-11	-8	3	2.90	0.59
-11	-8	3	1.67	0.90
-11	8	-3	3.65	1.37
11	-8	-3	3.94	0.67
11	8	3	3.31	0.88
-11	-8	-3	3.03	0.68
11	-8	3	2.45	1.49
-11	-8	-3	3.06	0.72
11	8	-3	4.51	0.89
11	8	-3	3.05	0.61
11	-8	3	2.82	0.73
11	-8	3	3.38	0.64
11	-8	3	2.75	0.63
11	8	-3	3.34	0.94
-11	8	3	2.21	1.32
-11	-8	-3	2.95	0.82
11	9	3	35.61	2.05

11	-9	-3	32.14	2.32
11	-9	-3	32.78	2.09
-11	-9	3	35.55	2.06
11	9	3	31.51	2.58
-11	-9	3	33.01	1.87
11	9	3	35.39	2.54
-11	-9	3	35.55	2.62
11	-9	-3	41.43	3.09
11	9	3	33.71	2.52
-11	9	-3	33.36	2.83
11	-9	-3	33.78	2.11
-11	-9	3	36.88	2.29
11	9	-3	35.38	1.94
11	-9	3	32.92	2.07
11	-9	3	36.69	2.29
-11	-9	-3	32.29	2.28
-11	-9	-3	34.10	2.05
11	9	-3	33.07	2.51
11	-9	3	35.97	2.16
11	-9	3	37.29	3.03
-11	9	3	31.21	2.86
11	9	-3	39.04	2.41
-11	9	3	39.64	2.79
11	-10	-3	49.12	3.05
11	-10	-3	49.26	2.86
-11	-10	3	49.60	3.46
11	-10	-3	49.12	2.83
11	10	3	48.60	3.40
-11	-10	3	52.56	3.07
-11	10	-3	57.62	3.68
11	-10	3	49.38	2.78
-11	-10	-3	46.88	2.77
11	-10	3	50.98	2.96
-11	-10	-3	51.53	3.45
-11	10	3	55.20	3.60
-11	-10	-3	49.07	3.08
11	10	-3	48.47	3.45
11	10	-3	53.50	2.73
11	-11	-3	41.74	2.59
-11	-11	3	38.65	3.06
-11	11	-3	42.26	3.73
-11	-11	3	42.82	2.71
11	-11	-3	40.32	2.83
11	11	3	38.41	3.04

11	-11	-3	40.90	2.47
-11	-11	-3	39.10	2.37
-11	11	3	44.75	3.52
11	-11	3	39.19	2.39
-11	-11	-3	42.06	2.68
11	-11	3	37.32	2.50
11	11	-3	35.25	3.13
-11	-11	-3	40.09	3.15
11	-11	3	37.99	2.73
11	12	3	27.20	2.89
-11	-12	3	38.06	3.03
11	-12	-3	36.13	2.80
11	-12	-3	39.84	2.48
11	-12	-3	37.53	2.53
-11	12	-3	39.80	3.41
-11	-12	3	33.74	2.49
11	-12	3	35.11	2.69
11	12	-3	36.40	3.20
11	-12	3	31.12	2.33
-11	-12	-3	36.51	3.07
-11	12	3	36.54	3.42
-11	-12	-3	35.66	2.63
11	-12	3	34.01	2.22
-12	0	-3	75.92	3.71
-12	0	3	76.41	3.87
-12	0	-3	81.40	3.86
-12	0	-3	75.61	3.73
-12	0	3	81.83	3.84
12	0	3	75.03	3.76
12	0	-3	75.46	3.68
12	0	-3	74.02	3.50
12	0	-3	70.01	3.55
-12	0	-3	78.62	3.72
-12	0	3	75.57	3.75
-12	0	3	78.75	3.69
-12	0	-3	80.72	3.79
12	0	3	74.94	3.72
12	0	3	78.09	3.79
-12	0	3	77.35	3.76
12	0	-3	76.33	3.66
12	0	3	79.10	3.97
-12	0	-3	76.44	3.58
-12	0	3	75.14	3.72
12	0	3	70.71	3.57

-12	0	-3	77.63	3.52
-12	1	-3	15.94	1.13
12	1	3	18.36	1.02
-12	-1	3	16.59	1.29
-12	1	-3	17.28	1.17
-12	-1	3	17.87	1.11
12	-1	-3	14.70	1.04
12	-1	-3	14.80	1.06
-12	1	-3	20.11	1.24
-12	-1	3	17.82	1.16
-12	1	-3	16.39	0.98
-12	1	-3	21.29	1.38
12	1	3	18.52	1.18
-12	-1	3	18.40	1.18
12	1	3	20.29	1.31
12	-1	-3	14.55	0.93
-12	-1	3	19.64	1.27
-12	-1	3	19.08	1.34
-12	1	-3	18.47	1.17
12	1	3	19.87	1.28
12	-1	-3	17.03	1.05
-12	-1	3	16.36	1.37
-12	1	3	17.21	1.39
-12	1	3	16.19	1.20
-12	-1	-3	17.92	1.13
12	-1	3	17.86	1.06
12	1	-3	16.74	1.07
12	-1	3	21.65	1.49
-12	1	3	19.85	1.35
-12	1	3	17.65	1.15
-12	1	3	19.44	1.17
12	-1	3	18.41	1.17
12	-1	3	19.55	1.23
-12	-1	-3	16.05	1.14
-12	-1	-3	19.17	1.31
-12	-1	-3	20.72	1.26
12	-1	3	21.57	1.37
-12	1	3	18.19	1.17
-12	-2	3	11.52	0.83
-12	-2	3	10.67	0.86
-12	2	-3	11.41	0.90
12	-2	-3	10.92	0.80
12	-2	-3	11.22	0.84
12	-2	-3	10.68	0.88

12	2	3	10.66	0.72
12	2	3	9.82	1.00
-12	2	-3	11.47	0.86
-12	-2	3	10.20	1.03
-12	-2	3	10.55	0.86
-12	2	-3	11.16	0.96
12	2	3	11.93	0.92
-12	-2	3	10.55	1.09
12	2	3	11.56	0.91
-12	2	-3	11.87	0.97
-12	-2	3	10.23	1.12
-12	2	-3	15.42	1.24
-12	2	-3	12.54	0.91
-12	-2	3	11.87	0.81
-12	-2	3	11.67	0.93
-12	-2	-3	13.03	0.93
12	-2	3	10.93	1.12
-12	2	3	12.52	1.12
12	-2	3	11.68	0.85
12	-2	3	8.92	0.77
-12	-2	-3	10.09	0.93
-12	2	3	10.45	0.95
-12	2	3	11.96	0.88
-12	-2	-3	11.75	1.03
12	-2	3	12.16	1.26
12	-2	3	10.90	0.86
-12	2	3	11.55	0.98
-12	-2	-3	13.89	0.94
-12	-2	-3	12.42	1.16
-12	2	3	10.32	1.15
-12	-2	-3	12.89	1.04
-12	2	3	11.43	0.79
-12	2	3	9.63	0.81
-12	2	3	9.71	0.79
-12	3	-3	2.75	0.56
12	3	3	2.07	0.38
-12	3	-3	4.28	0.62
-12	3	-3	2.09	0.42
-12	3	-3	1.82	0.71
-12	-3	3	2.80	0.67
-12	-3	3	2.45	0.66
-12	-3	3	2.83	0.50
12	3	3	2.06	0.62
-12	-3	3	3.77	0.65

12	-3	-3	1.95	0.42
12	3	3	3.17	0.54
12	3	3	2.66	0.52
12	-3	-3	2.99	0.65
12	-3	-3	2.28	0.44
-12	-3	3	2.57	0.74
-12	-3	3	2.87	0.53
-12	3	-3	2.19	0.49
12	-3	-3	3.34	0.51
-12	-3	3	2.93	0.49
-12	-3	3	2.85	0.49
-12	3	-3	2.16	0.54
-12	3	-3	2.63	0.72
12	-3	3	2.47	0.69
-12	3	3	2.34	0.51
12	-3	3	3.20	0.51
-12	-3	-3	2.72	0.77
-12	-3	-3	2.33	0.51
-12	3	3	2.13	0.70
12	-3	3	3.30	0.77
12	3	-3	2.08	0.34
12	3	-3	1.90	0.40
-12	-3	-3	3.19	0.50
-12	-3	-3	2.46	0.31
12	-3	3	3.39	0.59
-12	3	3	2.61	0.64
-12	3	3	3.61	0.64
12	-3	3	3.02	0.53
-12	-3	-3	3.74	0.64
-12	-3	-3	2.69	0.52
-12	3	3	2.52	0.48
-12	-3	-3	2.83	0.51
-12	-3	-3	2.22	0.28
-12	3	3	3.41	0.69
12	-4	-3	19.16	1.56
-12	-4	3	18.76	1.44
-12	-4	3	18.93	1.13
-12	4	-3	17.44	1.64
12	4	3	16.12	1.12
12	-4	-3	17.39	1.23
-12	4	-3	17.58	1.10
-12	4	-3	21.62	1.37
-12	-4	3	17.12	1.20
12	-4	-3	19.03	1.19

-12	-4	3	16.74	1.45
-12	-4	3	17.87	1.23
12	4	3	18.97	1.21
-12	4	-3	19.49	1.34
12	4	3	17.89	1.21
-12	4	-3	19.28	1.31
-12	-4	3	17.74	1.10
-12	-4	3	17.49	1.55
12	-4	-3	17.47	1.21
-12	4	-3	19.73	1.63
12	4	3	16.99	1.42
12	-4	-3	18.29	1.47
-12	-4	-3	18.90	1.20
12	-4	3	15.92	1.15
-12	4	3	18.23	1.34
-12	-4	-3	17.57	1.21
12	-4	3	17.98	1.19
12	4	-3	18.00	1.06
-12	4	3	16.97	1.54
-12	-4	-3	19.59	1.56
-12	4	3	17.81	1.49
12	4	-3	19.41	1.23
-12	-4	-3	18.34	1.27
-12	4	3	19.27	1.30
12	-4	3	18.15	1.08
12	-4	3	15.58	1.49
-12	-4	-3	18.88	0.98
-12	-4	-3	19.28	1.01
12	4	-3	17.59	1.14
12	-4	3	16.63	1.19
-12	4	3	19.01	1.22
-12	-4	-3	17.89	1.30
-12	5	-3	2.10	0.82
-12	-5	3	-0.41	0.66
12	-5	-3	1.24	0.49
12	5	3	1.35	0.51
-12	-5	3	1.87	0.68
-12	-5	3	1.05	0.41
12	5	3	1.14	0.48
12	-5	-3	1.47	0.42
-12	5	-3	-0.11	0.84
-12	5	-3	0.45	0.56
-12	5	-3	0.25	0.51
-12	-5	3	2.63	0.54

-12	5	-3	0.93	0.58
12	-5	-3	0.34	0.41
-12	-5	3	2.09	0.71
12	-5	-3	2.49	0.79
12	-5	-3	0.47	0.62
-12	-5	3	0.79	0.49
12	5	3	0.48	0.43
12	5	3	1.10	0.68
12	5	-3	0.56	0.41
-12	-5	-3	0.75	0.72
12	-5	3	2.87	0.78
-12	-5	-3	1.73	0.40
12	-5	3	0.75	0.42
-12	-5	-3	0.61	0.53
-12	5	3	0.64	0.61
12	5	-3	1.20	0.44
12	-5	3	0.70	0.46
12	-5	3	0.11	0.39
-12	5	3	1.88	0.70
12	5	-3	0.62	0.47
12	-5	3	0.50	0.86
-12	5	3	0.79	0.62
-12	-5	-3	1.17	0.35
-12	5	3	2.01	0.54
-12	-5	-3	1.30	0.44
-12	-5	-3	0.73	0.30
-12	5	3	2.13	0.83
-12	-5	-3	1.27	0.46
12	-6	-3	18.53	1.74
12	-6	-3	15.75	1.24
12	-6	-3	16.80	1.20
12	-6	-3	16.70	1.33
-12	-6	3	19.20	1.12
12	6	3	22.57	1.54
-12	-6	3	25.37	1.86
12	6	3	20.63	1.78
-12	6	-3	12.05	1.87
-12	-6	3	26.38	1.37
-12	-6	3	20.29	1.11
-12	-6	3	25.57	1.55
-12	6	-3	16.05	1.37
-12	-6	3	21.77	1.77
12	6	3	24.48	1.47
-12	-6	3	21.48	1.32

-12	6	3	23.79	1.56
-12	-6	-3	17.39	1.12
12	-6	3	22.17	1.54
12	6	-3	16.89	1.23
12	-6	3	19.79	1.42
-12	6	3	21.39	2.03
-12	-6	-3	20.03	1.37
12	6	-3	16.44	1.35
12	-6	3	22.56	2.17
-12	-6	-3	20.66	1.32
12	6	-3	16.44	1.17
12	-6	3	21.05	1.45
-12	6	3	26.77	1.92
12	7	3	26.33	1.92
-12	-7	3	19.13	2.20
12	-7	-3	33.37	2.47
12	7	3	19.86	2.21
-12	-7	3	28.80	1.51
-12	-7	3	25.74	1.46
12	-7	-3	26.55	1.69
-12	7	-3	28.33	2.50
-12	-7	3	28.24	1.96
12	-7	-3	28.28	1.79
12	7	3	22.46	2.33
12	-7	-3	28.40	1.89
-12	-7	3	24.81	1.37
-12	-7	3	29.14	1.77
12	7	3	26.43	1.60
12	-7	3	23.54	1.59
12	-7	3	22.49	1.71
12	7	-3	28.34	1.78
-12	7	3	26.36	2.15
-12	7	3	23.80	2.21
12	-7	3	29.64	2.48
12	-7	3	24.27	1.74
12	7	-3	26.65	1.63
-12	-7	-3	30.05	1.78
12	7	-3	25.61	2.11
-12	-7	-3	34.76	2.56
-12	-7	-3	28.50	1.66
-12	-8	3	18.73	1.18
12	8	3	18.21	1.93
-12	-8	3	20.96	1.80
12	-8	-3	19.08	1.47

-12	8	-3	15.74	2.54
12	8	3	16.80	1.70
-12	8	-3	27.32	2.28
12	8	3	17.19	1.36
12	-8	-3	22.91	2.29
12	-8	-3	19.04	1.64
-12	-8	3	18.02	1.91
-12	-8	3	18.26	1.48
-12	-8	3	15.64	1.18
12	8	3	16.98	1.67
-12	-8	3	15.81	1.15
12	8	-3	19.89	1.35
12	8	-3	20.42	1.87
12	-8	3	16.70	1.33
12	8	-3	20.15	1.58
-12	-8	-3	24.53	1.72
-12	8	3	19.72	1.94
12	-8	3	16.61	1.46
-12	-8	-3	20.03	1.46
12	-8	3	16.38	1.34
-12	-8	-3	21.02	1.50
-12	8	3	15.30	2.12
12	-8	3	20.41	2.35
12	-9	-3	26.35	1.85
12	9	3	25.31	2.33
-12	-9	3	26.10	2.30
12	9	3	23.75	1.64
-12	-9	3	23.76	1.46
12	9	3	26.28	2.18
12	9	3	25.90	2.16
-12	9	-3	33.07	2.80
-12	-9	3	25.18	1.86
12	-9	-3	28.21	1.85
12	-9	-3	25.63	1.97
-12	-9	-3	28.42	2.03
12	9	-3	28.55	1.65
12	-9	3	22.02	1.74
-12	9	3	25.23	2.50
-12	-9	-3	27.17	1.83
12	-9	3	23.07	1.63
12	9	-3	29.04	2.27
12	9	-3	24.69	2.57
12	-10	-3	20.39	1.88
-12	-10	3	17.60	2.37

12	-10	-3	20.84	1.64
-12	10	-3	23.27	2.89
12	10	3	14.83	2.21
12	-10	-3	20.68	1.61
-12	-10	3	18.39	1.82
-12	-10	-3	20.42	1.97
12	-10	3	15.62	1.48
-12	-10	-3	17.79	1.60
-12	10	3	22.54	2.41
12	-10	3	17.48	1.77
12	-10	3	19.82	1.71
12	10	-3	19.48	2.39
-12	-10	-3	19.50	2.42
-12	11	-3	0.63	1.62
-12	-11	3	3.25	1.29
12	-11	-3	0.96	0.79
12	11	3	1.90	1.21
12	-11	-3	0.97	0.88
12	-11	-3	0.51	0.99
-12	-11	3	2.24	0.89
12	-11	3	1.41	0.86
-12	11	3	3.90	1.85
-12	-11	-3	1.34	0.95
12	-11	3	2.27	1.08
12	11	-3	2.48	1.26
12	-11	3	1.62	0.79
-12	-11	-3	0.99	1.18
12	-12	-3	2.30	0.91
12	-12	-3	1.33	0.85
-12	-12	3	1.96	1.10
12	-12	-3	2.14	1.34
12	-12	3	1.73	1.10
12	-12	3	-0.27	0.96
12	-12	3	-0.03	0.98
13	0	3	0.37	0.22
-13	0	-3	0.00	0.21
-13	0	3	-0.13	0.56
13	0	-3	0.36	0.32
-13	0	-3	0.37	0.39
13	0	3	0.27	0.46
-13	0	-3	-0.48	0.46
-13	0	3	0.69	0.46
13	0	3	-0.21	0.39
-13	0	3	0.48	0.40

-13	0	-3	0.02	0.35
-13	0	3	-0.23	0.33
13	0	-3	-0.23	0.29
-13	0	-3	-0.12	0.44
13	0	3	0.22	0.33
13	0	-3	0.19	0.24
13	0	-3	-0.13	0.16
-13	0	3	0.29	0.46
-13	0	3	-0.29	0.36
13	0	3	-0.10	0.42
-13	0	-3	-0.04	0.37
-13	0	-3	0.14	0.28
-13	-1	3	43.69	2.31
13	-1	-3	42.75	2.23
-13	-1	3	41.89	2.25
-13	1	-3	41.08	2.23
13	1	3	39.70	2.00
13	-1	-3	42.73	2.18
-13	-1	3	42.61	2.15
13	-1	-3	41.74	2.17
13	-1	-3	42.08	2.06
-13	-1	3	38.57	2.10
-13	-1	3	39.77	2.24
13	1	3	40.45	2.18
-13	1	-3	41.79	2.26
-13	1	-3	42.69	2.27
-13	1	-3	42.69	2.08
-13	1	-3	41.50	2.22
13	1	3	43.25	2.25
13	1	3	39.39	2.27
-13	-1	3	42.70	2.36
-13	1	-3	45.51	2.28
-13	1	-3	43.58	2.43
-13	-1	3	41.15	2.16
-13	1	3	41.57	2.33
-13	1	3	41.01	2.21
-13	1	3	40.42	2.39
-13	-1	-3	42.73	2.38
13	-1	3	40.00	2.04
13	-1	3	39.22	2.30
-13	-1	-3	38.60	2.31
13	-1	3	40.12	2.55
-13	1	3	40.54	2.13
-13	-1	-3	43.40	2.25

13	-1	3	38.96	2.15
13	1	-3	40.61	2.19
-13	1	3	39.85	2.13
13	-1	3	39.54	2.16
-13	-1	-3	44.42	2.28
-13	-1	-3	40.74	2.23
-13	-2	3	89.56	4.30
13	2	3	84.82	4.00
-13	2	-3	104.66	5.03
-13	2	-3	102.01	4.96
13	-2	-3	104.71	4.98
-13	2	-3	104.84	5.22
-13	2	-3	106.67	4.88
-13	-2	3	86.01	4.13
-13	-2	3	88.82	4.12
-13	-2	3	82.27	4.07
-13	-2	3	84.15	4.10
13	-2	-3	103.01	4.92
13	2	3	81.51	4.28
-13	2	-3	109.14	5.02
13	-2	-3	103.68	4.87
-13	-2	3	87.44	4.30
13	2	3	90.13	4.17
-13	-2	3	83.61	4.17
-13	-2	3	83.23	4.27
-13	2	-3	104.59	4.93
-13	2	3	85.26	4.02
-13	-2	-3	104.27	4.99
-13	2	3	85.81	3.98
-13	-2	-3	101.46	4.97
-13	-2	-3	104.94	5.14
-13	-2	-3	106.77	4.97
-13	-2	-3	101.03	4.98
-13	-2	-3	104.84	5.00
-13	-2	-3	100.27	4.98
-13	2	3	86.13	4.34
-13	2	3	85.80	4.04
-13	2	3	87.76	4.36
13	-2	3	85.12	4.30
13	-2	3	87.74	4.11
13	-2	3	84.27	4.06
-13	2	3	90.75	4.12
13	-2	3	90.71	4.37
-13	2	3	75.17	4.23

13	-2	3	79.01	4.13
-13	3	-3	126.64	6.14
13	-3	-3	124.46	5.82
-13	3	-3	128.30	5.92
-13	-3	3	152.69	7.32
-13	-3	3	146.97	7.11
13	-3	-3	121.87	5.81
-13	3	-3	129.43	5.92
-13	3	-3	121.65	5.75
-13	-3	3	146.89	7.05
13	-3	-3	127.84	5.99
13	3	3	155.58	7.11
-13	3	-3	124.04	5.82
-13	-3	3	148.85	7.31
-13	-3	3	149.27	7.12
13	3	3	148.88	7.02
-13	-3	3	145.25	7.11
-13	3	-3	127.18	6.07
13	3	3	147.52	7.32
-13	-3	3	160.08	7.27
13	3	3	160.46	7.14
-13	-3	3	156.71	7.08
13	-3	-3	121.03	5.88
-13	3	-3	117.32	5.84
13	-3	3	167.90	7.46
13	-3	3	157.29	7.03
-13	3	3	154.76	7.21
-13	-3	-3	129.10	6.08
13	3	-3	118.78	5.65
13	-3	3	148.16	7.09
-13	3	3	155.53	7.37
13	3	-3	121.48	5.63
-13	3	3	150.89	7.35
-13	-3	-3	122.89	5.87
-13	3	3	151.21	7.07
13	-3	3	146.64	7.29
-13	-3	-3	120.12	5.82
-13	3	3	156.03	7.11
-13	-3	-3	126.81	5.85
13	-3	3	152.91	7.15
-13	-3	-3	123.36	5.89
-13	-3	-3	125.56	5.90
-13	4	-3	179.19	8.54
13	-4	-3	175.86	8.31

-13	-4	3	179.24	8.47
-13	-4	3	174.90	8.50
13	4	3	178.52	8.80
-13	-4	3	178.46	8.55
13	-4	-3	178.99	8.28
-13	4	-3	171.78	8.12
13	4	3	193.61	8.58
13	-4	-3	166.09	8.43
-13	-4	3	182.26	8.55
13	-4	-3	184.05	8.48
-13	4	-3	176.48	8.60
-13	4	-3	183.07	8.34
13	-4	-3	179.83	8.25
-13	-4	3	181.63	8.77
13	4	3	185.04	8.51
13	4	3	177.12	8.49
-13	-4	3	196.19	8.69
-13	-4	3	194.61	8.48
-13	4	-3	165.13	8.26
-13	-4	3	186.11	8.79
-13	4	-3	187.35	8.35
-13	4	-3	183.44	8.30
-13	-4	-3	184.07	8.21
13	-4	3	198.29	8.90
-13	4	3	184.64	8.70
-13	-4	-3	181.46	8.33
-13	-4	-3	181.52	8.53
-13	4	3	185.31	8.82
-13	-4	-3	172.84	8.27
-13	4	3	183.24	8.56
13	-4	3	183.38	8.57
13	-4	3	175.17	8.72
13	-4	3	184.44	8.58
-13	4	3	184.44	8.54
13	4	-3	182.65	8.11
-13	-4	-3	173.09	8.16
-13	-4	-3	183.01	7.99
-13	4	3	185.02	8.63
13	4	-3	169.15	8.15
-13	-4	-3	178.57	8.33
-13	-4	-3	180.94	7.99
-13	-5	3	106.39	5.16
-13	-5	3	105.46	5.04
13	-5	-3	101.24	5.05

13	5	3	106.56	5.09
-13	5	-3	106.56	5.50
-13	-5	3	102.99	5.16
13	-5	-3	115.58	5.43
-13	-5	3	112.66	5.04
13	5	3	103.29	5.14
13	5	3	111.36	5.19
13	-5	-3	104.51	5.15
13	-5	-3	105.54	5.14
13	5	3	105.37	5.45
-13	5	-3	110.88	5.53
-13	-5	3	109.05	5.44
-13	5	-3	109.57	5.18
-13	-5	3	110.42	5.29
13	-5	3	101.87	5.14
-13	5	3	106.08	5.45
13	5	-3	108.59	4.98
-13	5	3	111.35	5.17
-13	-5	-3	103.19	5.17
-13	-5	-3	102.99	4.84
-13	-5	-3	104.26	5.10
13	5	-3	108.01	5.07
13	-5	3	106.96	5.19
-13	-5	-3	100.90	4.93
-13	5	3	107.18	5.41
-13	-5	-3	102.81	4.97
13	-5	3	102.69	5.14
-13	-5	-3	110.49	4.87
13	5	-3	102.85	5.06
-13	-6	3	15.37	1.64
13	-6	-3	15.21	1.25
13	6	3	17.19	1.65
-13	-6	3	16.15	1.58
-13	6	-3	20.86	1.90
13	6	3	14.76	1.22
-13	-6	3	14.97	1.09
13	-6	-3	15.68	1.28
-13	-6	3	15.52	1.10
-13	-6	3	15.61	1.25
13	-6	-3	17.79	2.59
13	6	3	14.23	1.31
-13	6	-3	18.08	2.02
13	-6	-3	16.50	1.20
13	6	3	16.12	1.29

-13	6	3	15.45	1.31
13	-6	3	12.53	1.15
-13	-6	-3	16.68	1.07
13	6	-3	16.75	1.18
-13	6	3	16.03	1.81
-13	6	3	15.02	1.73
-13	-6	-3	15.89	1.37
13	-6	3	15.41	1.30
-13	-6	-3	18.21	1.30
13	6	-3	17.29	1.19
13	6	-3	15.61	1.37
13	-6	3	15.96	1.20
13	-6	3	16.94	1.94
13	-7	-3	5.33	1.20
-13	-7	3	4.83	1.08
-13	-7	3	5.54	0.81
13	7	3	6.48	0.82
13	-7	-3	3.19	0.74
13	7	3	6.09	1.06
-13	7	-3	5.01	1.34
-13	-7	3	4.06	1.03
13	7	3	5.76	1.08
13	-7	-3	3.77	0.81
-13	7	-3	4.62	1.10
-13	-7	3	4.05	0.52
13	-7	-3	3.74	0.63
-13	-7	3	6.03	0.79
13	7	3	5.63	1.14
-13	-7	-3	4.18	0.71
13	-7	3	8.77	1.82
-13	7	3	5.58	1.13
13	-7	3	4.79	0.88
13	7	-3	4.65	0.84
13	-7	3	5.36	1.02
-13	-7	-3	3.95	0.77
13	-7	3	4.75	0.84
-13	7	3	5.76	1.31
-13	-7	-3	4.15	0.77
13	7	-3	5.01	0.79
13	7	-3	4.97	0.97
-13	-8	3	17.46	1.86
-13	-8	3	16.36	1.72
13	8	3	15.79	1.64
13	-8	-3	20.68	1.64

-13	8	-3	20.83	2.63
-13	-8	3	14.41	1.39
-13	8	-3	21.96	2.33
13	-8	-3	19.64	2.21
13	8	3	13.10	1.83
-13	-8	3	14.93	1.14
13	-8	-3	20.80	1.48
13	8	3	15.42	2.52
13	-8	-3	19.12	1.53
-13	-8	3	15.80	1.13
13	8	3	14.95	1.30
-13	-8	3	14.61	1.03
13	8	-3	21.19	1.66
-13	8	3	16.71	2.03
-13	8	3	11.92	2.09
13	8	-3	21.34	1.83
-13	-8	-3	20.83	1.43
13	-8	3	13.36	1.25
13	-8	3	16.64	2.29
13	-8	3	14.69	1.33
-13	-8	-3	20.22	1.65
-13	-8	-3	20.46	1.54
13	8	-3	22.56	1.38
13	9	3	24.76	1.64
-13	-9	3	20.84	2.30
13	-9	-3	23.92	1.68
-13	9	-3	24.15	2.56
-13	-9	3	23.47	1.48
13	9	3	22.56	2.11
13	9	3	23.23	2.34
-13	-9	3	23.38	1.84
13	-9	-3	22.61	1.72
13	-9	-3	22.79	1.88
13	-9	3	26.63	1.72
13	-9	3	24.65	1.74
-13	-9	-3	23.98	1.92
13	9	-3	23.33	1.83
13	9	-3	23.37	1.46
13	9	-3	22.62	2.12
-13	-9	-3	22.74	1.70
-13	9	3	21.54	2.49
13	-9	3	23.79	1.92
-13	10	-3	74.27	4.84
-13	-10	3	80.11	4.34

-13	-10	3	80.88	4.68
13	-10	-3	73.61	3.80
13	10	3	76.08	4.54
13	-10	-3	69.04	3.78
13	-10	-3	72.89	4.02
-13	-10	-3	72.10	3.97
13	-10	3	76.26	4.33
13	-10	3	77.92	4.06
13	-10	3	79.03	4.19
13	10	-3	65.45	4.16
-13	-10	-3	67.24	3.69
-13	-10	-3	71.52	4.33
-13	10	3	80.13	4.92
13	-11	-3	58.01	3.37
-13	-11	3	48.61	3.09
-13	-11	3	48.95	3.47
-13	11	-3	60.85	4.25
13	11	3	46.28	3.42
13	-11	-3	56.84	3.32
13	-11	-3	60.19	3.68
13	-11	3	51.09	3.24
-13	-11	-3	62.72	3.93
13	-11	3	50.62	2.99
13	-11	3	50.18	2.91
-13	-11	-3	60.20	3.57
13	11	-3	56.18	3.86
-13	11	3	59.49	3.98
-14	0	3	7.97	0.94
-14	0	3	6.09	0.85
-14	0	3	7.79	0.97
14	0	3	7.61	0.81
14	0	3	7.35	0.59
-14	0	3	6.74	0.80
14	0	-3	7.48	0.63
14	0	-3	6.22	0.48
-14	0	3	7.88	0.76
-14	0	3	9.50	1.04
-14	0	-3	8.13	0.87
14	0	3	10.27	1.09
-14	0	3	5.37	0.94
14	0	-3	8.42	0.73
-14	-1	3	22.04	1.45
-14	-1	3	21.49	1.48
-14	-1	3	26.53	1.65

-14	1	-3	20.84	1.34
14	-1	-3	20.02	1.19
-14	-1	3	25.18	1.42
14	1	3	24.62	1.61
14	1	3	24.99	1.60
-14	-1	3	29.26	1.65
-14	1	-3	21.20	1.43
-14	-1	3	26.39	1.59
14	-1	-3	23.06	1.34
14	-1	-3	18.85	1.35
-14	-1	3	19.89	1.69
-14	1	-3	19.36	1.20
-14	1	-3	21.49	1.45
14	1	3	21.89	1.27
14	1	3	22.51	1.47
-14	1	-3	25.59	1.49
-14	1	-3	26.11	1.72
14	-1	-3	23.14	1.36
14	-1	3	20.85	1.29
-14	-1	-3	23.31	1.42
-14	1	3	20.39	1.51
-14	1	3	20.95	1.71
14	-1	3	21.96	1.66
-14	1	3	26.06	1.69
-14	-1	-3	24.88	1.61
14	-1	3	23.94	1.46
-14	-1	-3	22.97	1.46
-14	-1	-3	26.33	1.60
14	-1	3	26.33	1.66
-14	1	3	27.70	1.58
-14	1	3	25.03	1.49
14	-1	3	24.94	1.49
-14	-1	-3	21.00	1.53
14	1	-3	24.39	1.39
-14	1	3	23.93	1.45
-14	2	-3	5.90	1.11
-14	-2	3	5.67	0.85
14	2	3	9.94	0.98
-14	-2	3	11.50	1.11
14	2	3	8.64	0.65
-14	2	-3	9.00	0.94
-14	-2	3	10.05	0.79
14	-2	-3	5.49	0.61
-14	-2	3	5.96	1.13

-14	-2	3	10.64	1.18
14	-2	-3	8.60	0.75
14	-2	-3	6.42	0.76
-14	2	-3	6.02	0.89
-14	-2	3	7.11	0.88
-14	2	-3	6.55	0.71
-14	2	3	6.81	0.63
14	-2	3	7.19	0.69
-14	2	3	8.40	0.86
-14	2	3	5.64	0.97
-14	-2	-3	6.38	0.98
-14	2	3	7.82	0.98
14	-2	3	8.33	0.81
-14	-2	-3	7.63	0.90
-14	2	3	8.35	0.79
-14	-2	-3	6.31	0.83
14	-2	3	9.68	0.87
14	-3	-3	9.19	0.82
-14	-3	3	9.77	1.19
-14	-3	3	8.59	0.91
-14	3	-3	8.98	0.86
-14	3	-3	9.24	1.30
14	-3	-3	8.01	0.78
-14	3	-3	8.38	1.33
-14	-3	3	10.26	1.16
-14	-3	3	7.23	0.84
-14	-3	3	9.23	0.86
14	3	3	7.50	0.70
14	3	3	6.30	0.91
-14	3	-3	7.79	0.86
14	-3	-3	7.71	0.84
-14	-3	3	9.02	1.29
-14	3	-3	7.94	0.98
14	3	3	7.44	1.11
-14	-3	3	7.84	0.90
-14	-3	3	8.13	0.76
-14	3	-3	10.60	1.06
-14	3	-3	9.34	0.88
-14	3	3	10.82	1.24
-14	-3	-3	10.03	0.98
14	-3	3	8.89	0.80
14	-3	3	9.14	1.35
-14	-3	-3	9.12	0.91
14	-3	3	8.44	1.26

-14	3	3	7.80	1.29
14	-3	3	6.98	0.74
-14	-3	-3	7.64	1.04
14	3	-3	8.06	0.58
-14	3	3	6.98	0.86
-14	3	3	6.82	0.99
-14	3	3	7.25	1.11
-14	-3	-3	7.32	0.89
-14	-3	-3	8.88	0.98
-14	3	3	9.51	0.94
-14	-3	-3	10.05	0.92
-14	-3	-3	9.17	1.28
14	-3	3	6.04	0.81
14	3	-3	9.38	0.61
14	-4	-3	4.74	0.78
-14	-4	3	2.70	0.57
-14	-4	3	3.38	0.59
-14	-4	3	3.61	0.91
-14	-4	3	2.26	0.62
-14	4	-3	5.73	0.80
-14	4	-3	7.57	1.36
14	-4	-3	5.14	0.69
-14	-4	3	2.46	0.51
-14	4	-3	8.10	0.96
14	-4	-3	4.07	0.71
-14	-4	3	2.71	0.56
14	4	3	2.83	0.63
-14	-4	3	2.18	0.73
14	-4	-3	5.39	0.92
14	4	3	3.32	0.64
-14	4	-3	6.66	1.15
14	4	3	3.85	0.84
14	4	3	3.42	0.67
-14	-4	3	2.39	0.81
14	-4	3	2.86	0.55
-14	-4	-3	6.71	0.90
14	-4	3	2.25	0.52
-14	-4	-3	6.82	0.85
14	-4	3	3.59	0.65
14	4	-3	4.78	0.56
-14	4	3	2.76	0.88
-14	4	3	2.22	0.80
-14	4	3	3.05	0.65
14	4	-3	5.18	0.64

14	-4	3	3.52	0.98
-14	-4	-3	5.51	0.97
-14	-5	3	20.44	1.46
-14	-5	3	21.70	1.33
14	-5	-3	17.21	1.22
14	5	3	21.41	1.45
14	-5	-3	19.71	1.73
-14	5	-3	19.21	1.83
-14	-5	3	21.48	1.36
-14	-5	3	22.42	1.82
14	5	3	20.62	1.73
14	5	3	23.10	1.58
14	-5	-3	16.02	1.28
14	-5	-3	19.25	1.33
-14	-5	3	20.35	1.37
-14	-5	3	20.13	1.74
-14	5	-3	18.86	1.92
14	5	3	21.37	1.41
14	-5	3	21.01	1.41
-14	-5	-3	18.22	1.16
14	5	-3	17.41	1.14
-14	-5	-3	19.48	1.37
-14	5	3	19.95	1.52
14	-5	3	20.12	1.42
-14	-5	-3	18.19	1.32
-14	5	3	20.30	1.83
14	5	-3	16.93	1.23
-14	-5	-3	19.50	0.94
-14	5	3	21.50	1.89
-14	-5	-3	17.69	1.32
14	6	3	6.15	1.12
14	-6	-3	8.78	0.91
-14	6	-3	13.22	1.99
-14	-6	3	8.01	1.01
-14	-6	3	5.11	0.82
14	-6	-3	7.68	1.05
-14	-6	3	6.69	1.12
-14	-6	3	7.37	0.83
-14	-6	3	5.63	0.84
14	6	3	8.57	1.00
14	6	-3	6.67	1.38
14	-6	3	10.99	1.87
-14	6	3	5.39	1.20
14	-6	3	4.77	0.85

14	6	-3	5.73	0.87
14	-6	3	3.58	0.80
-14	6	3	5.17	1.15
14	-6	3	4.70	0.96
-14	-6	-3	8.53	0.70
-14	-7	3	8.19	1.07
-14	-7	3	10.24	0.95
-14	-7	3	8.07	1.55
14	7	3	7.02	1.09
14	7	3	8.03	1.27
-14	-7	3	10.25	1.61
-14	7	-3	6.05	1.32
14	-7	-3	11.96	1.90
14	-7	-3	7.30	1.03
14	-7	-3	5.94	0.97
-14	-7	3	6.36	0.93
14	7	3	10.35	1.60
14	-7	-3	6.94	0.95
14	7	3	10.70	1.25
-14	7	-3	6.06	1.46
-14	7	3	9.97	1.94
-14	-7	-3	6.41	0.82
14	-7	3	5.00	1.30
14	7	-3	8.45	1.27
14	-7	3	7.31	0.99
-14	-7	-3	9.34	1.18
14	-7	3	9.48	1.93
-14	-7	-3	10.40	1.12
14	-7	3	6.03	0.95
-14	7	3	9.91	1.69
14	7	-3	4.45	0.91
14	8	3	14.81	1.91
-14	-8	3	15.49	1.46
14	-8	-3	12.63	1.20
14	-8	-3	12.44	1.30
-14	-8	3	15.25	1.88
-14	8	-3	14.00	2.17
14	-8	-3	11.46	1.36
14	8	3	12.09	1.21
14	8	3	11.95	1.43
14	8	3	15.48	1.63
14	8	-3	13.29	1.32
-14	8	3	13.19	2.01
14	8	-3	17.05	1.69

14	-8	3	10.91	1.19
14	-8	3	10.20	1.33
-14	-8	-3	14.09	1.52
14	-8	3	9.09	1.20
-14	-8	-3	13.45	1.32
-14	-8	-3	10.45	1.10
14	9	3	18.78	2.39
14	-9	-3	13.05	1.60
14	-9	-3	16.56	1.46
-14	9	-3	16.48	2.66
-14	-9	3	19.12	1.73
-14	-9	3	20.39	2.35
14	9	3	20.21	2.11
-14	-9	3	16.65	1.26
14	-9	-3	17.12	1.48
-14	-9	-3	15.67	1.79
14	9	-3	18.25	2.08
14	-9	3	15.00	1.59
-14	-9	-3	17.12	1.55
-14	9	3	20.28	2.50
14	-9	3	13.82	1.42
14	-9	3	14.77	1.73
-14	-10	3	3.96	1.36
14	-10	-3	5.02	1.04
-14	10	-3	2.44	1.64
-14	-10	3	5.84	1.18
14	-10	-3	3.53	0.91
14	10	3	3.55	1.28
14	-10	-3	4.51	1.11
14	-10	3	3.82	1.00
-14	-10	-3	3.41	1.33
14	10	-3	3.59	1.25
14	-10	3	4.61	0.90
-14	-10	-3	6.46	1.20
-14	10	3	3.14	1.55
14	-10	3	4.90	1.13
-14	-10	-3	2.61	0.90
14	-11	-3	6.35	1.46
-14	-11	3	4.45	1.12
-14	-11	3	4.96	1.41
14	-11	-3	5.12	1.05
-14	11	-3	6.76	1.81
14	-11	-3	7.43	1.14
14	11	3	1.75	1.38

14	11	-3	4.69	1.46
14	-11	3	5.01	0.99
-14	-11	-3	6.39	1.55
14	-11	3	4.24	1.09
14	-11	3	4.37	1.33
-14	-11	-3	5.63	1.14
-14	11	3	5.91	1.66
-15	0	3	-0.08	0.48
-15	0	3	-0.39	0.70
-15	0	-3	0.10	0.40
15	0	3	-0.04	0.44
15	0	3	-0.58	0.57
15	0	-3	0.16	0.37
-15	0	-3	0.27	0.51
15	0	3	-0.11	0.59
-15	0	-3	0.01	0.46
-15	0	-3	-0.17	0.54
-15	0	3	-0.28	0.42
15	0	-3	0.41	0.30
-15	0	3	-0.12	0.55
15	0	3	0.10	0.25
15	0	-3	-0.15	0.18
-15	0	-3	-0.29	0.56
-15	0	3	0.56	0.36
15	0	3	-0.43	0.38
-15	0	3	-0.52	0.49
-15	0	3	0.08	0.44
-15	-1	3	25.54	1.59
15	-1	-3	21.69	1.17
-15	-1	3	23.79	1.68
-15	-1	3	24.99	1.72
-15	1	-3	19.32	1.46
15	1	3	27.64	1.69
-15	-1	3	24.04	1.53
15	1	3	26.35	1.76
15	-1	-3	20.96	1.38
15	1	3	27.03	1.41
15	-1	-3	21.44	1.35
15	1	3	26.45	1.63
-15	1	-3	20.56	1.38
-15	-1	3	27.32	1.79
-15	-1	3	27.37	1.92
-15	1	-3	23.55	1.28
-15	1	-3	21.39	1.48

-15	-1	3	25.88	1.64
-15	1	-3	21.12	1.68
15	-1	-3	20.32	1.30
-15	1	-3	20.92	1.17
15	-1	3	30.04	1.77
-15	-1	-3	20.48	1.42
15	-1	3	26.85	1.44
15	1	-3	23.03	1.39
-15	1	3	24.19	1.55
-15	1	3	26.30	1.82
-15	1	3	24.25	1.58
-15	1	3	27.53	1.70
15	-1	3	26.89	1.62
-15	-1	-3	21.36	1.61
-15	-1	-3	22.55	1.42
-15	-1	-3	22.34	1.59
-15	-1	-3	20.38	1.53
-15	1	3	25.55	1.93
15	-1	3	25.16	1.81
-15	1	3	29.04	1.66
-15	-1	-3	22.86	1.48
15	-1	3	25.96	1.61
15	2	3	63.61	3.11
-15	2	-3	69.20	3.40
-15	2	-3	71.69	3.64
15	-2	-3	68.01	3.55
-15	-2	3	63.84	3.35
15	-2	-3	70.58	3.41
-15	2	-3	71.49	3.84
15	2	3	65.21	3.48
-15	-2	3	66.36	3.28
-15	2	-3	69.29	3.51
-15	2	-3	66.85	3.54
-15	-2	3	64.16	3.28
-15	2	-3	72.98	3.59
-15	-2	3	63.03	3.24
-15	2	-3	72.00	3.61
-15	-2	3	68.18	3.50
-15	-2	3	62.51	3.22
15	-2	-3	70.98	3.56
15	-2	-3	74.34	3.52
-15	-2	3	65.47	3.51
15	2	3	66.73	3.28
-15	-2	3	64.28	3.44

-15	2	3	67.84	3.37
-15	2	3	69.14	3.28
-15	-2	-3	67.85	3.57
-15	-2	-3	70.45	3.56
-15	-2	-3	69.02	3.61
-15	2	3	61.69	3.16
15	-2	3	66.71	3.32
-15	-2	-3	70.45	3.56
-15	2	3	66.68	3.18
-15	-2	-3	74.33	3.64
-15	-2	-3	72.67	3.77
15	-2	3	62.87	3.49
15	-2	3	63.87	3.18
15	-2	3	66.61	3.25
-15	2	3	61.57	3.54
-15	2	3	63.90	3.53
-15	-2	-3	74.31	3.58
15	3	3	96.67	4.54
-15	-3	3	87.21	4.46
-15	-3	3	94.69	4.51
15	-3	-3	109.66	5.15
15	3	3	90.54	4.77
-15	-3	3	91.97	4.78
-15	3	-3	100.64	5.17
-15	-3	3	91.90	4.75
-15	-3	3	86.55	4.54
-15	3	-3	108.92	5.53
-15	3	-3	124.87	5.52
-15	-3	3	96.78	4.73
15	-3	-3	107.49	5.21
15	3	3	89.85	4.39
-15	-3	3	85.62	4.51
15	-3	-3	112.09	5.28
-15	3	-3	102.91	5.10
15	-3	-3	103.73	5.10
-15	-3	3	92.01	4.55
15	-3	3	96.82	4.93
-15	-3	-3	109.43	5.21
-15	3	3	93.95	4.53
15	-3	3	96.66	4.46
15	-3	3	92.42	4.48
-15	-3	-3	110.43	5.45
15	-3	3	98.28	4.85
-15	3	3	95.24	4.55

15	-3	3	93.70	4.56
-15	-3	-3	105.11	5.20
-15	-3	-3	101.72	5.17
-15	-3	-3	107.09	5.22
-15	3	3	102.73	4.55
-15	-3	-3	108.76	5.19
-15	3	3	89.97	4.81
-15	-4	3	130.20	6.41
-15	4	-3	128.50	6.29
-15	4	-3	136.30	6.84
-15	4	-3	130.82	6.70
15	-4	-3	134.37	6.43
15	4	3	138.47	6.74
15	4	3	137.29	6.44
-15	-4	3	130.58	6.37
-15	-4	3	139.54	6.72
15	-4	-3	132.11	6.49
-15	-4	3	131.97	6.45
15	4	3	127.12	6.34
15	-4	-3	148.17	6.72
-15	-4	3	144.64	6.64
-15	4	-3	130.35	6.45
-15	-4	3	142.73	6.40
15	-4	-3	141.27	6.44
-15	-4	3	132.78	6.45
15	-4	3	146.26	6.88
15	-4	3	132.09	6.42
-15	4	3	137.08	6.59
-15	-4	-3	140.26	6.45
-15	4	3	133.42	6.74
-15	-4	-3	139.33	6.53
-15	-4	-3	136.44	6.48
-15	4	3	133.99	6.45
-15	-4	-3	131.35	6.39
15	4	-3	142.03	6.25
15	-4	3	131.55	6.46
15	4	-3	130.96	6.27
-15	-4	-3	142.29	6.45
15	-4	3	142.98	6.28
15	-5	-3	64.29	3.24
15	5	3	61.77	3.19
15	-5	-3	52.34	3.78
-15	-5	3	63.58	3.09
-15	-5	3	57.73	3.08

-15	5	-3	64.58	3.29
-15	5	-3	65.35	3.76
-15	-5	3	65.39	3.41
15	-5	-3	68.12	3.70
-15	-5	3	58.68	3.06
15	5	3	61.28	3.14
15	-5	-3	61.23	3.34
-15	-5	3	61.32	3.51
-15	-5	3	59.11	3.17
15	5	3	58.86	3.51
15	-5	3	62.29	3.20
-15	-5	-3	66.37	3.22
15	-5	3	58.54	3.17
-15	-5	-3	58.37	3.37
-15	5	3	61.52	3.51
-15	-5	-3	63.99	3.28
15	5	-3	68.57	3.11
-15	-5	-3	62.21	3.12
15	5	-3	62.22	3.17
-15	-6	3	9.39	0.96
-15	-6	3	10.83	1.53
-15	-6	3	8.76	1.00
15	6	3	7.68	1.07
-15	6	-3	14.12	1.85
-15	-6	3	8.74	0.94
-15	-6	3	9.82	1.60
15	6	3	8.46	1.53
-15	6	-3	11.34	2.00
15	-6	-3	12.71	1.20
15	6	3	9.76	1.20
15	-6	-3	10.74	1.17
15	6	3	9.37	1.12
15	-6	-3	12.57	1.09
-15	-6	-3	12.77	1.28
-15	6	3	8.65	1.81
15	-6	3	10.01	1.95
15	-6	3	6.49	1.02
-15	6	3	11.30	1.66
15	-6	3	9.22	0.99
15	6	-3	14.27	1.08
15	6	-3	14.77	1.24
-15	-6	-3	13.77	1.23
-15	-7	3	14.54	1.17
-15	-7	3	13.10	1.78

-15	7	-3	9.70	1.93
15	-7	-3	11.91	1.29
-15	-7	3	16.11	1.39
15	7	3	16.15	1.60
-15	7	-3	11.18	2.24
15	7	3	10.58	1.71
-15	-7	3	16.12	1.23
15	-7	-3	10.45	1.08
15	-7	-3	12.84	1.92
15	7	3	16.15	1.41
15	-7	-3	12.60	1.25
15	7	3	15.19	1.43
-15	-7	3	14.57	1.83
-15	-7	3	10.89	1.13
-15	-7	-3	11.45	1.31
15	-7	3	14.39	2.27
15	7	-3	8.07	1.29
15	-7	3	14.05	1.35
15	7	-3	13.83	1.18
-15	7	3	15.79	2.90
-15	-7	-3	10.70	0.85
15	-7	3	13.34	1.31
-15	-7	-3	11.30	1.25
-15	7	3	16.02	2.06
15	-7	3	13.15	1.24
-15	-8	3	5.73	0.88
-15	-8	3	6.31	0.79
-15	-8	3	5.46	0.96
15	8	3	6.86	1.22
-15	-8	3	6.07	1.38
-15	8	-3	11.37	2.04
15	-8	-3	9.78	1.20
15	-8	-3	10.80	1.27
15	-8	-3	9.13	1.29
15	8	3	6.41	1.35
15	8	3	6.49	1.14
-15	-8	-3	8.31	0.93
-15	8	3	8.13	1.47
15	-8	3	6.24	1.29
15	8	-3	10.74	1.44
15	-8	3	6.45	0.99
15	8	-3	11.02	1.26
15	-8	3	5.92	1.08
-15	-8	-3	8.71	1.25

-15	-8	-3	10.11	1.37
-15	9	-3	26.51	3.15
15	-9	-3	26.62	1.99
-15	-9	3	22.22	2.03
15	-9	-3	26.55	1.85
15	-9	-3	25.79	2.01
-15	-9	3	23.88	2.44
15	9	3	23.19	2.23
15	9	3	19.29	2.48
-15	-9	-3	28.54	2.20
15	9	-3	26.91	2.29
-15	-9	-3	23.62	1.88
-15	9	3	19.06	2.86
15	-9	3	23.60	1.74
15	-9	3	23.47	1.86
15	-9	3	22.98	1.95
15	10	3	35.13	2.71
-15	-10	3	37.07	2.90
-15	10	-3	34.84	3.20
15	-10	-3	30.25	2.26
-15	-10	3	36.20	2.49
15	-10	-3	33.21	2.28
15	-10	-3	32.58	2.55
15	-10	3	36.15	2.67
-15	-10	-3	32.91	2.41
-15	-10	-3	31.58	2.80
-15	10	3	31.73	3.15
15	-10	3	35.02	2.32
-15	-10	-3	35.32	2.20
15	-10	3	33.96	2.35
15	10	-3	33.90	2.63
15	-11	-3	25.81	1.99
-15	-11	3	27.65	2.21
15	-11	-3	24.27	2.25
15	-11	-3	27.43	2.00
15	-11	3	26.58	2.04
15	-11	3	26.92	2.49
-16	0	-3	51.19	2.67
-16	0	3	46.62	2.68
-16	0	3	46.33	2.68
16	0	-3	45.28	2.20
16	0	3	50.58	2.46
-16	0	3	48.74	2.71
-16	0	-3	48.92	2.74

-16	0	-3	48.53	2.73
16	0	3	47.47	2.65
16	0	-3	43.78	2.28
-16	0	3	49.89	2.82
16	0	3	51.14	2.81
-16	0	-3	45.65	2.52
16	0	3	58.08	2.80
-16	0	3	48.82	2.62
-16	0	3	45.69	2.58
-16	0	3	50.53	2.96
16	0	-3	45.38	2.46
16	0	3	53.80	2.75
16	-1	-3	18.69	1.17
-16	1	-3	19.23	1.94
-16	-1	3	16.99	1.28
-16	-1	3	20.28	1.59
-16	-1	3	17.50	1.33
-16	1	-3	17.76	1.35
-16	1	-3	17.48	1.58
-16	-1	3	16.19	1.68
-16	-1	3	19.41	1.26
16	1	3	21.37	1.52
-16	1	-3	16.04	1.21
16	1	3	18.67	1.10
-16	-1	3	18.48	1.49
-16	-1	3	18.48	1.40
-16	1	-3	15.36	0.99
16	1	3	20.85	1.43
16	1	3	17.68	1.34
16	-1	-3	14.64	0.96
-16	1	-3	15.27	0.93
16	-1	-3	16.85	1.17
-16	-1	3	21.70	1.51
16	-1	3	18.64	1.65
-16	-1	-3	15.98	1.50
-16	1	3	16.79	1.72
16	-1	3	19.24	1.34
-16	-1	-3	15.71	1.23
16	-1	3	22.56	1.62
-16	-1	-3	15.11	1.49
-16	-1	-3	18.94	1.48
-16	1	3	19.39	1.36
16	1	-3	19.23	1.23
-16	-1	-3	16.17	1.47

-16	1	3	18.56	1.38
16	-1	3	16.94	1.12
-16	1	3	18.54	1.59
16	-1	3	19.73	1.38
-16	1	3	20.81	1.34
16	-2	-3	27.22	1.63
-16	-2	3	31.40	1.92
-16	2	-3	24.90	1.67
16	-2	-3	29.91	1.68
-16	2	-3	23.85	1.61
-16	-2	3	30.96	1.84
-16	-2	3	34.35	2.20
-16	-2	3	31.68	2.20
-16	-2	3	32.90	1.84
16	2	3	32.22	1.69
16	2	3	31.93	2.12
16	2	3	32.22	1.94
16	-2	-3	25.93	1.44
-16	-2	3	34.54	2.02
-16	-2	3	33.07	1.93
-16	2	-3	25.37	1.65
16	-2	-3	28.17	1.58
-16	2	-3	25.91	2.00
-16	-2	3	35.23	2.12
16	-2	3	32.19	1.91
-16	-2	-3	26.34	1.93
16	-2	3	31.97	2.07
-16	-2	-3	25.71	1.64
-16	-2	-3	25.02	1.63
-16	-2	-3	24.77	1.65
-16	-2	-3	23.52	1.72
-16	2	3	32.04	2.20
-16	2	3	35.44	1.82
16	-2	3	33.17	1.87
-16	2	3	32.66	1.89
-16	-2	-3	25.62	1.80
-16	2	3	28.45	1.88
16	-2	3	28.99	2.13
16	-2	3	29.96	1.74
16	-3	-3	1.09	0.42
-16	-3	3	1.34	0.57
-16	3	-3	1.32	0.79
-16	3	-3	1.13	0.57
-16	-3	3	2.07	0.72

-16	-3	3	0.49	0.41
-16	-3	3	0.51	0.55
16	3	3	1.13	0.74
16	3	3	1.03	0.36
-16	-3	3	1.17	0.48
16	-3	-3	0.55	0.41
16	-3	-3	0.13	0.41
-16	-3	3	0.81	0.81
-16	-3	3	0.83	0.53
-16	-3	3	0.50	0.85
16	-3	-3	2.04	0.63
-16	3	-3	0.74	0.73
-16	3	-3	1.23	0.48
16	3	3	0.92	0.55
16	-3	3	0.64	0.50
-16	3	3	1.10	0.59
16	-3	3	0.78	0.91
-16	-3	-3	0.59	0.59
-16	-3	-3	1.00	0.50
-16	3	3	0.48	0.64
-16	3	3	1.16	0.70
16	-3	3	0.52	0.40
16	-3	3	0.30	0.43
-16	-3	-3	0.31	0.54
-16	-3	-3	1.33	0.73
-16	-3	-3	1.46	0.54
16	4	3	12.45	1.48
-16	4	-3	10.10	0.98
-16	-4	3	10.05	1.09
16	4	3	12.10	1.13
16	-4	-3	9.68	0.97
-16	-4	3	10.83	1.07
-16	-4	3	12.37	1.59
-16	4	-3	10.22	1.08
-16	-4	3	11.03	1.42
-16	-4	3	12.23	1.04
-16	-4	3	10.99	1.12
16	4	3	12.48	0.99
-16	4	-3	12.19	1.68
16	-4	3	10.69	1.07
-16	4	3	9.11	1.60
16	4	-3	9.55	0.78
16	4	-3	11.40	0.81
16	-4	3	15.58	1.80

-16	-4	-3	11.11	1.07
-16	4	3	12.18	1.20
-16	-4	-3	11.51	1.28
-16	-4	-3	9.77	1.10
-16	4	3	11.81	1.51
16	-4	3	11.37	1.08
-16	-4	-3	9.88	1.24
-16	-4	-3	10.80	1.08
16	-4	3	14.41	0.95
16	5	3	1.44	0.87
-16	-5	3	0.32	0.48
-16	5	-3	1.77	1.14
16	-5	-3	0.68	0.46
16	5	3	1.49	0.62
-16	5	-3	1.17	0.53
-16	-5	3	-0.52	0.92
16	5	3	0.86	0.50
16	-5	-3	0.33	0.49
-16	-5	3	0.13	0.55
16	-5	-3	0.99	0.86
16	-5	-3	0.69	0.54
-16	5	-3	1.13	0.95
-16	-5	3	0.59	0.56
-16	-5	3	1.38	0.96
-16	-5	3	0.62	0.53
-16	-5	-3	1.51	0.60
16	-5	3	0.60	1.06
-16	5	3	0.36	0.82
16	-5	3	0.72	0.52
-16	-5	-3	1.07	0.81
-16	-5	-3	1.46	0.62
16	-5	3	0.62	0.50
16	5	-3	0.79	0.44
-16	5	3	0.97	1.05
-16	-5	-3	0.44	0.53
16	5	-3	1.08	0.83
-16	-6	3	28.05	1.68
16	-6	-3	27.29	1.83
16	6	3	29.87	2.26
16	-6	-3	30.63	2.33
-16	-6	3	23.97	1.76
16	-6	-3	26.12	1.88
-16	-6	3	23.40	1.63
16	-6	-3	24.51	1.80

-16	-6	3	28.04	2.19
16	6	3	25.33	1.82
-16	6	-3	26.11	2.57
-16	-6	3	27.14	2.23
16	6	3	29.27	1.83
-16	-6	3	24.95	1.62
-16	6	-3	29.08	1.82
-16	6	-3	31.29	2.57
-16	-6	-3	27.92	1.93
-16	6	3	24.29	2.57
-16	6	3	25.55	2.33
16	-6	3	23.43	1.73
-16	-6	-3	27.18	1.82
16	-6	3	25.98	1.78
16	6	-3	26.61	1.65
16	6	-3	26.64	1.78
16	-7	-3	14.81	1.42
-16	7	-3	15.49	2.23
16	-7	-3	14.48	1.30
-16	-7	3	18.84	1.36
16	-7	-3	14.45	1.40
16	7	3	19.36	2.06
-16	-7	3	16.00	1.30
-16	7	-3	17.11	1.46
-16	-7	3	15.86	1.35
16	7	3	21.89	1.66
16	7	3	15.60	1.59
-16	-7	3	19.87	2.09
-16	-7	3	17.19	1.56
16	7	-3	14.92	1.31
-16	-7	-3	16.83	1.55
16	7	-3	19.57	1.55
16	-7	3	15.47	1.49
-16	7	3	16.33	2.16
16	-7	3	18.11	1.49
-16	-7	-3	15.89	1.42
-16	-8	3	21.31	2.38
16	8	3	14.92	2.27
16	-8	-3	19.71	1.52
16	-8	-3	16.34	1.59
16	8	3	20.09	1.93
-16	-8	3	17.04	1.41
-16	-8	3	19.62	1.85
16	8	3	18.09	1.88

-16	8	-3	18.48	2.71
16	-8	-3	17.47	1.59
-16	-8	3	16.03	1.60
-16	-8	-3	19.01	1.85
16	-8	3	15.64	1.55
-16	-8	-3	17.78	1.56
16	8	-3	22.89	1.98
16	-8	3	14.27	1.57
16	8	-3	16.85	1.56
-16	8	3	15.73	2.51
-16	-9	3	11.92	2.09
-16	9	-3	10.07	1.94
16	9	3	13.40	1.78
16	-9	-3	10.75	1.50
16	9	3	12.02	2.11
-16	-9	3	11.28	1.71
16	-9	-3	12.99	1.66
16	-9	-3	11.96	1.46
16	-9	3	12.76	1.65
-16	9	3	12.45	2.01
-16	-9	-3	13.15	1.70
16	9	-3	10.43	1.67
16	-9	3	12.19	1.41
-16	-9	-3	9.47	1.50
16	-10	-3	4.50	1.16
16	10	3	6.26	1.40
-16	-10	3	8.92	1.44
-16	10	-3	5.97	1.84
16	-10	-3	5.10	0.95
-16	-10	3	7.60	1.55
16	-10	-3	6.18	1.01
16	-10	3	8.64	1.70
-16	-10	-3	3.76	1.10
-16	-10	-3	7.09	1.59
16	10	-3	5.16	1.31
-16	10	3	6.85	1.74
16	-10	3	6.09	1.06
-17	0	3	0.09	0.52
17	0	3	0.62	0.60
-17	0	3	0.67	0.69
-17	0	3	-0.29	0.50
-17	0	-3	0.60	0.79
-17	0	3	0.48	0.84
17	0	3	-0.19	0.43

17	0	3	-0.08	0.58
-17	0	3	0.09	0.43
-17	0	-3	0.77	0.72
17	0	-3	0.27	0.33
17	0	3	0.03	0.28
-17	0	-3	-0.41	0.38
17	0	-3	-0.07	0.43
17	0	-3	0.48	0.22
-17	0	3	0.05	0.62
-17	0	-3	1.09	0.74
17	1	3	3.40	0.66
-17	1	-3	4.08	0.84
17	-1	-3	4.01	0.73
17	-1	-3	5.06	0.72
-17	-1	3	3.20	0.97
-17	1	-3	3.69	0.66
17	-1	-3	4.84	0.50
-17	-1	3	2.56	0.56
-17	-1	3	3.81	0.89
-17	1	-3	4.63	0.99
-17	-1	3	3.47	0.81
17	1	3	2.73	0.48
-17	-1	3	2.07	0.52
-17	-1	3	3.23	0.69
17	1	3	2.81	0.71
-17	-1	3	4.30	0.80
17	1	3	2.16	0.70
-17	-1	3	2.93	0.95
-17	1	3	3.98	0.73
-17	1	3	4.83	0.76
17	-1	3	3.45	0.90
-17	1	3	2.95	0.64
17	-1	3	3.56	0.72
17	-1	3	1.76	0.83
-17	-1	-3	3.88	0.84
-17	1	3	2.26	0.80
-17	1	3	3.31	0.76
-17	-1	-3	4.35	0.96
17	-1	3	3.77	0.68
-17	-1	-3	3.33	0.85
17	-1	3	2.62	0.53
-17	-1	-3	4.40	0.80
-17	-2	3	55.76	3.20
17	2	3	56.87	2.90

-17	-2	3	52.60	2.81
17	2	3	57.94	3.12
-17	-2	3	51.75	3.04
-17	2	-3	49.20	2.50
-17	-2	3	52.99	2.94
-17	2	-3	48.32	2.59
17	-2	-3	47.80	2.60
-17	2	-3	44.45	2.58
-17	2	-3	45.98	2.94
-17	-2	3	54.26	2.89
17	-2	-3	50.75	2.57
-17	-2	3	53.73	3.10
17	2	3	52.42	2.65
17	-2	-3	46.49	2.39
17	-2	-3	48.27	2.53
-17	-2	3	51.14	2.80
-17	-2	3	55.27	2.90
17	-2	3	52.61	2.85
17	-2	3	63.97	3.14
-17	2	3	52.48	3.13
17	-2	3	57.51	2.85
-17	-2	-3	45.65	2.57
-17	-2	-3	48.98	2.70
17	-2	3	52.02	2.72
-17	2	3	51.98	2.79
-17	2	3	50.86	2.85
-17	-2	-3	46.79	2.85
-17	-2	-3	45.93	2.59
-17	2	3	51.22	2.75
-17	-2	-3	46.30	2.74
17	-3	-3	73.16	3.65
17	-3	-3	55.07	3.92
-17	3	-3	70.73	3.45
17	-3	-3	63.79	3.61
17	3	3	66.00	3.59
-17	-3	3	60.90	3.26
17	-3	-3	72.41	3.50
-17	3	-3	60.29	3.77
-17	3	-3	70.37	3.97
17	3	3	64.20	3.12
-17	-3	3	59.43	3.31
17	3	3	67.34	3.26
-17	-3	3	64.10	3.54
-17	3	-3	75.88	3.67

-17	-3	3	66.17	3.59
-17	-3	3	59.79	3.20
-17	-3	3	61.73	3.27
17	-3	3	55.26	3.39
-17	-3	-3	66.96	3.55
-17	-3	-3	69.70	3.60
-17	-3	-3	73.23	3.56
17	-3	3	70.96	3.87
-17	-3	-3	70.27	3.62
17	-3	3	62.26	3.30
-17	3	3	66.43	3.65
-17	3	3	59.38	3.83
17	-3	3	66.67	3.18
17	-4	-3	69.74	3.39
-17	4	-3	60.11	3.67
-17	-4	3	59.64	3.37
-17	-4	3	58.98	3.07
17	4	3	60.04	4.21
-17	-4	3	56.62	3.03
-17	-4	3	53.17	2.96
-17	-4	3	53.87	3.05
17	4	3	60.23	3.06
17	4	3	57.58	2.91
17	-4	-3	61.31	3.13
-17	4	-3	57.69	3.17
17	-4	-3	57.93	3.20
17	-4	-3	59.10	3.10
-17	4	-3	64.60	3.60
-17	-4	3	62.49	3.43
-17	4	-3	56.34	3.03
-17	4	3	54.79	3.17
17	4	-3	61.83	2.89
-17	4	3	54.07	3.41
-17	-4	-3	56.03	3.14
17	-4	3	51.67	3.07
-17	-4	-3	59.64	3.19
17	4	-3	64.48	2.92
17	-4	3	54.75	2.98
17	-4	3	62.43	2.90
-17	-4	-3	59.52	3.22
17	-4	3	66.16	3.55
-17	-4	-3	60.55	3.27
17	-5	-3	20.87	2.03
-17	-5	3	13.86	1.24

-17	-5	3	16.36	1.36
17	-5	-3	18.36	1.39
-17	-5	3	17.43	1.79
17	5	3	16.63	1.83
-17	-5	3	14.13	1.31
17	5	3	14.12	1.24
-17	-5	3	16.93	1.33
-17	5	-3	18.53	1.44
17	5	3	16.80	1.35
-17	5	-3	20.07	3.42
-17	5	-3	19.63	2.21
-17	-5	3	16.59	1.96
17	-5	-3	20.99	1.51
17	-5	-3	18.32	1.43
-17	5	3	18.50	1.99
-17	-5	-3	17.87	1.44
17	-5	3	16.80	2.20
-17	-5	-3	20.52	1.66
-17	-5	-3	19.08	1.43
-17	-5	-3	19.33	1.54
17	-5	3	15.99	1.32
17	5	-3	18.41	1.23
17	-5	3	15.65	1.31
17	5	-3	19.52	1.29
-17	5	3	15.76	1.97
-17	6	-3	6.58	1.49
17	6	3	10.72	1.22
-17	-6	3	10.14	1.21
-17	6	-3	7.85	1.11
17	6	3	10.65	1.33
17	-6	-3	7.81	1.52
-17	-6	3	10.65	1.14
-17	-6	3	10.43	1.84
17	6	3	10.54	1.72
17	-6	-3	7.26	0.98
-17	-6	3	12.41	1.74
17	-6	-3	6.61	1.09
17	-6	-3	6.33	1.00
-17	-6	3	10.61	1.20
-17	-6	3	7.72	1.04
-17	6	3	10.27	1.63
17	-6	3	10.36	1.20
-17	-6	-3	7.18	1.10
17	-6	3	10.41	1.15

-17	-6	-3	6.71	0.90
17	6	-3	8.20	0.97
17	6	-3	6.30	1.06
-17	-6	-3	6.83	1.12
-17	-7	3	5.39	0.98
-17	7	-3	4.60	0.86
17	7	3	3.77	1.23
17	-7	-3	6.42	1.06
17	7	3	5.07	1.02
17	-7	-3	5.15	0.82
-17	-7	3	4.91	0.80
-17	7	-3	2.92	1.53
17	7	3	6.05	1.27
-17	-7	3	6.90	1.35
17	-7	-3	4.39	0.80
-17	-7	3	4.84	0.93
17	-7	3	5.40	0.96
17	7	-3	5.11	1.06
-17	-7	-3	5.36	0.95
17	7	-3	4.50	0.88
17	-7	3	4.23	0.84
-17	-7	-3	3.83	0.88
-17	7	3	4.67	1.39
17	8	3	11.54	2.08
17	-8	-3	7.69	1.47
17	8	3	9.29	1.59
17	-8	-3	8.89	1.38
17	-8	-3	10.64	1.22
-17	8	-3	9.98	1.40
-17	8	-3	8.38	1.78
-17	-8	3	8.61	1.28
-17	-8	3	6.95	1.13
-17	-8	3	7.74	1.66
-17	-8	-3	8.29	1.40
17	-8	3	7.90	1.37
17	-8	3	8.51	1.43
-17	-8	-3	5.74	1.17
17	8	-3	8.35	1.59
-17	8	3	8.67	1.80
17	8	-3	9.46	1.30
17	-9	-3	19.57	1.74
-17	-9	3	19.03	1.87
-17	9	-3	23.42	1.90
17	9	3	19.10	1.91

-17	-9	3	20.84	2.31
17	-9	-3	22.02	1.93
-17	9	-3	22.16	2.74
17	-9	-3	18.87	1.83
17	9	-3	22.13	2.02
-17	9	3	18.36	2.57
17	-9	3	18.68	1.69
17	-9	3	17.50	1.93
-17	-9	-3	25.78	1.98
-17	-9	-3	17.74	1.82
-17	-10	3	16.62	1.88
-17	10	-3	17.97	2.84
17	-10	-3	16.55	1.63
17	-10	-3	16.84	1.65
17	10	3	17.10	2.16
-17	-10	3	18.88	2.51
17	-10	-3	17.92	1.90
-17	10	-3	16.07	1.68
17	10	-3	21.61	2.61
-17	-10	-3	18.56	2.41
-17	10	3	16.37	2.79
17	-10	3	16.39	1.85
17	-10	3	17.88	1.75
-17	-10	-3	19.35	1.88
-18	0	-3	54.14	3.40
18	0	3	73.01	3.69
-18	0	3	68.26	3.65
-18	0	3	66.16	3.76
-18	0	-3	60.68	3.40
-18	0	-3	63.45	3.30
18	0	-3	61.38	3.12
18	0	-3	58.93	2.79
18	0	-3	58.56	2.89
-18	0	3	67.68	3.61
18	0	3	76.73	3.65
-18	0	-3	60.71	3.40
18	0	3	68.62	3.60
-18	0	3	65.61	3.52
-18	-1	3	25.23	1.97
-18	1	-3	33.62	2.31
18	1	3	32.75	1.85
18	1	3	29.95	2.00
18	1	3	25.85	1.88
18	-1	-3	31.57	1.86

-18	-1	3	31.53	2.57
-18	-1	3	28.04	1.71
18	-1	-3	28.26	1.62
18	-1	-3	31.27	1.76
-18	1	-3	32.79	2.04
-18	-1	3	25.81	1.92
-18	-1	3	25.29	1.72
-18	-1	3	29.76	2.09
-18	1	-3	30.07	2.03
18	-1	3	33.84	1.98
-18	-1	-3	31.84	2.14
18	-1	3	27.61	1.81
-18	-1	-3	32.22	2.20
-18	1	3	28.01	2.09
-18	1	3	26.98	1.77
-18	-1	-3	30.52	2.19
-18	1	3	29.14	1.85
-18	-1	-3	30.54	1.93
-18	1	3	27.48	1.76
18	-1	3	30.71	1.88
18	-1	3	24.52	1.61
18	-2	-3	17.80	1.29
-18	2	-3	15.64	1.33
-18	-2	3	16.93	1.84
18	2	3	15.41	1.57
18	-2	-3	18.84	1.34
18	-2	-3	18.49	1.12
-18	-2	3	16.75	1.49
-18	2	-3	19.19	1.91
18	2	3	17.70	1.38
-18	-2	3	14.43	1.22
-18	-2	3	17.40	1.28
-18	-2	3	14.87	1.71
-18	2	-3	17.94	1.22
-18	2	-3	15.01	1.41
-18	-2	3	16.92	1.56
18	2	3	15.63	1.22
-18	2	3	16.33	1.32
18	-2	3	16.02	1.11
-18	-2	-3	15.92	1.81
-18	2	3	18.11	1.25
-18	2	3	16.72	1.31
-18	-2	-3	15.47	1.56
-18	2	3	15.89	2.09

-18	-2	-3	16.64	1.41
-18	-2	-3	19.67	1.70
18	-2	3	17.69	1.35
18	-2	3	16.64	1.71
18	-3	-3	9.90	1.14
-18	3	-3	7.07	1.36
-18	-3	3	8.52	1.32
-18	-3	3	6.73	1.13
18	3	3	7.91	1.42
18	-3	-3	6.17	0.96
18	-3	-3	8.08	0.91
-18	3	-3	7.76	1.02
18	3	3	7.03	0.73
-18	-3	3	7.43	1.00
18	-3	-3	6.67	0.80
-18	-3	3	6.62	0.93
-18	3	-3	6.92	1.24
-18	3	-3	6.93	1.07
-18	-3	3	8.52	1.65
18	-3	3	9.40	1.38
18	-3	3	6.60	0.94
18	-3	3	6.24	0.94
-18	-3	-3	6.18	0.97
-18	3	3	7.32	1.22
-18	-3	-3	6.16	1.05
18	-3	3	7.23	0.84
-18	-3	-3	7.04	1.41
-18	-3	-3	6.46	0.92
-18	3	3	6.97	0.86
-18	4	-3	18.53	1.84
18	-4	-3	19.18	1.44
18	-4	-3	16.77	1.32
-18	-4	3	15.27	1.42
18	4	3	15.47	1.18
-18	4	-3	15.41	1.99
18	4	3	17.68	1.40
18	-4	-3	16.28	1.26
18	-4	-3	18.31	1.71
-18	-4	3	14.71	1.93
18	4	3	17.87	1.78
-18	-4	3	15.03	1.30
-18	-4	3	16.84	1.72
-18	4	-3	16.37	1.40
18	-4	3	17.76	1.29

-18	-4	-3	16.59	1.43
18	-4	3	16.79	2.10
-18	4	3	16.05	1.65
18	-4	3	16.80	1.37
-18	-4	-3	16.50	1.65
-18	-4	-3	16.58	1.44
-18	-4	-3	16.14	1.43
-18	4	3	17.44	1.94
18	-4	3	17.31	1.29
18	-5	-3	5.28	0.89
-18	-5	3	7.75	1.45
-18	5	-3	7.99	1.08
18	-5	-3	7.03	0.95
-18	-5	3	8.44	1.17
18	5	3	9.89	1.71
-18	-5	3	10.91	1.72
18	5	3	11.50	1.08
-18	5	-3	5.37	1.40
18	5	3	11.53	1.23
-18	-5	3	7.00	1.07
18	-5	-3	6.86	1.01
-18	-5	3	8.60	1.21
-18	-5	3	9.40	1.21
18	-5	3	10.06	1.13
-18	-5	-3	6.37	1.07
-18	-5	-3	5.92	0.92
18	5	-3	4.63	0.79
-18	-5	-3	8.42	1.65
-18	-5	-3	5.57	0.92
-18	5	3	9.64	1.78
18	-5	3	8.76	1.15
18	-5	3	15.43	2.06
18	5	-3	6.95	0.89
-18	5	3	9.83	1.90
18	-6	-3	32.20	2.06
18	-6	-3	31.78	2.00
18	6	3	29.14	2.48
-18	-6	3	29.12	2.02
-18	-6	3	28.27	1.92
-18	6	-3	31.66	2.08
-18	-6	3	29.90	1.95
18	-6	-3	30.10	2.07
-18	-6	3	31.70	2.53
18	6	3	30.98	1.95

-18	6	-3	31.20	2.80
-18	-6	3	29.61	1.88
18	6	3	32.61	2.07
18	6	-3	34.99	1.92
-18	-6	-3	33.72	1.98
18	-6	3	28.75	1.97
18	6	-3	31.42	1.98
-18	6	3	29.33	2.64
-18	-6	-3	33.26	2.17
18	-6	3	30.47	2.00
-18	7	-3	46.76	3.69
18	-7	-3	45.60	2.73
18	7	3	48.02	3.37
18	-7	-3	44.84	2.59
18	-7	-3	44.77	2.68
-18	7	-3	43.46	2.68
-18	-7	3	43.45	2.43
-18	-7	3	43.22	3.28
-18	-7	3	43.09	2.69
18	7	3	47.10	2.73
18	7	3	45.33	2.79
-18	-7	3	43.23	2.51
18	7	-3	46.23	2.54
-18	7	3	42.26	3.50
18	-7	3	46.37	2.74
-18	-7	-3	41.07	2.62
18	7	-3	41.21	2.66
-18	-7	-3	45.93	2.87
18	-7	3	40.94	2.66
-18	-8	3	26.77	1.93
18	-8	-3	26.61	2.17
18	-8	-3	29.66	2.14
-18	-8	3	30.50	2.69
-18	8	-3	29.73	2.20
18	8	3	29.12	2.21
18	-8	-3	32.51	2.12
18	8	3	30.89	2.71
-18	8	-3	35.43	3.08
-18	-8	3	30.72	2.32
-18	-8	-3	31.21	2.16
18	8	-3	30.77	2.09
18	-8	3	27.13	2.07
-18	-8	-3	29.89	2.30
-18	8	3	29.03	2.91

18	-8	3	28.05	2.19
18	8	-3	28.95	2.21
-18	9	-3	34.14	2.31
18	-9	-3	33.31	2.28
-18	-9	3	34.99	2.42
18	-9	-3	32.56	2.34
-18	-9	3	35.40	2.93
-18	9	-3	36.59	3.41
18	9	3	31.16	2.47
18	-9	-3	33.28	2.26
-18	-9	-3	31.92	2.48
18	9	-3	35.19	2.52
-18	9	3	28.43	3.16
18	-9	3	33.99	2.35
-18	-9	-3	34.56	2.94
18	-9	3	31.53	2.21
-18	-9	-3	33.39	2.35
19	0	-3	1.16	0.49
-19	0	-3	-1.05	0.74
-19	0	-3	1.14	0.89
-19	0	3	-0.64	0.74
19	0	3	-0.06	0.32
-19	0	-3	0.56	0.56
19	0	3	-0.12	0.65
19	0	-3	0.47	0.37
19	0	3	0.59	0.50
-19	0	3	0.22	0.81
-19	0	3	-0.68	0.59
-19	0	3	0.47	0.54
19	0	3	0.19	0.70
-19	-1	3	1.76	0.91
-19	-1	3	-0.04	0.52
19	1	3	1.37	0.68
19	-1	-3	1.80	0.38
19	-1	-3	3.11	0.60
19	1	3	0.79	0.55
-19	1	-3	1.14	0.56
-19	1	-3	2.56	0.90
-19	-1	3	0.77	0.75
19	1	3	0.21	0.79
19	1	3	0.95	0.39
-19	-1	3	0.56	0.71
-19	-1	3	0.74	0.50
-19	1	-3	2.61	0.91

19	-1	-3	3.29	0.62
-19	1	3	0.01	0.48
-19	-1	-3	2.71	0.90
-19	-1	-3	2.54	0.98
-19	-1	-3	3.17	1.14
19	-1	3	1.41	0.66
-19	1	3	0.73	0.80
19	-1	3	0.00	0.55
19	-1	3	0.30	0.37
-19	-1	-3	1.60	0.64
19	-1	3	1.15	0.77
-19	1	3	0.89	0.60
-19	2	-3	22.74	2.32
19	-2	-3	27.11	1.61
-19	2	-3	23.26	1.71
-19	-2	3	24.17	1.69
19	2	3	29.19	1.81
-19	-2	3	26.32	1.71
-19	2	-3	19.59	1.61
-19	-2	3	29.84	2.03
19	2	3	28.49	2.02
19	2	3	24.93	1.46
-19	-2	3	22.94	1.90
19	-2	-3	22.28	1.34
19	-2	-3	22.77	1.55
19	-2	3	27.79	1.76
-19	-2	-3	25.00	2.01
-19	-2	-3	20.02	1.60
-19	2	3	25.86	1.72
19	-2	3	28.03	1.97
-19	2	3	26.30	1.64
-19	-2	-3	21.98	1.60
19	-2	3	24.92	1.74
-19	-2	-3	21.07	1.80
19	-2	3	24.93	1.54
-19	2	3	25.27	2.15
-19	2	3	24.28	1.73
19	3	3	11.35	0.97
-19	3	-3	13.97	1.47
-19	3	-3	10.42	1.27
19	3	3	15.33	1.35
19	-3	-3	13.64	1.27
19	-3	-3	10.51	1.04
-19	3	-3	14.88	1.36

19	-3	-3	15.39	1.23
-19	-3	3	10.76	1.12
-19	-3	3	12.45	1.89
19	3	3	13.99	1.71
-19	-3	3	10.57	1.33
-19	3	-3	14.39	1.97
-19	-3	3	11.04	1.40
-19	-3	-3	11.68	1.70
-19	3	3	14.91	1.87
19	-3	3	15.12	1.24
-19	-3	-3	13.99	1.31
19	-3	3	11.58	1.21
-19	-3	-3	11.14	1.44
-19	-3	-3	11.12	1.23
19	-3	3	11.18	1.05
-19	3	3	10.28	1.05
-19	-4	3	13.45	1.50
-19	4	-3	17.91	1.50
19	-4	-3	16.73	1.48
19	4	3	18.80	1.49
19	4	3	17.09	1.25
-19	-4	3	18.64	1.89
-19	-4	3	16.92	2.13
19	4	3	19.27	1.94
-19	-4	3	15.68	1.42
19	-4	-3	22.22	1.86
-19	4	-3	17.53	1.87
-19	4	-3	19.48	2.25
19	-4	-3	15.46	1.31
19	-4	-3	18.96	1.37
-19	-4	3	14.52	1.32
-19	-4	-3	16.41	1.46
19	-4	3	21.36	1.90
19	-4	3	18.52	1.39
19	-4	3	14.54	1.27
19	-4	3	15.77	1.43
-19	4	3	18.92	2.13
-19	-4	-3	18.19	1.52
-19	-4	-3	15.70	1.78
-19	-4	-3	18.96	1.56
19	5	3	3.86	1.28
19	-5	-3	3.68	0.70
19	-5	-3	4.41	0.75
-19	-5	3	4.59	1.22

-19	-5	3	4.29	0.87
19	5	3	7.33	1.00
19	-5	-3	3.91	0.77
-19	-5	3	5.26	2.03
-19	5	-3	5.54	1.39
19	-5	-3	5.35	0.99
19	5	3	5.50	0.90
-19	-5	3	3.56	0.74
-19	5	-3	3.83	0.83
-19	-5	3	3.95	0.83
19	-5	3	3.51	0.76
-19	-5	-3	6.16	1.02
19	-5	3	4.83	0.96
-19	-5	-3	4.63	0.88
-19	5	3	1.54	1.20
-19	-5	-3	6.97	1.16
19	5	-3	3.74	0.72
19	5	-3	6.48	0.82
-19	-6	3	8.17	1.29
19	-6	-3	10.87	1.41
19	6	3	10.17	1.81
19	-6	-3	10.38	1.23
19	-6	-3	9.57	1.31
-19	-6	3	10.02	1.68
-19	6	-3	12.00	1.43
-19	6	-3	9.33	1.79
19	6	3	9.81	1.44
-19	-6	3	8.57	1.28
19	6	3	8.74	1.34
-19	-6	3	11.12	1.18
19	-6	3	8.86	1.28
-19	-6	-3	11.55	1.52
-19	-6	-3	9.29	1.35
19	6	-3	8.09	1.07
-19	6	3	9.89	1.74
19	-6	3	9.40	1.34
19	6	-3	10.68	1.16
19	7	3	4.01	1.42
19	-7	-3	4.11	0.91
-19	7	-3	3.57	0.97
19	7	3	2.83	0.92
19	-7	-3	5.07	0.83
-19	7	-3	5.07	1.67
19	7	3	3.25	1.00

19	-7	-3	4.03	0.97
-19	-7	3	2.87	1.07
-19	-7	3	1.84	1.32
-19	-7	3	3.38	0.80
19	7	-3	4.01	0.85
19	7	-3	3.80	0.98
19	-7	3	2.96	0.90
-19	-7	-3	2.12	0.93
-19	-7	-3	4.22	0.95
-19	7	3	3.37	1.43
19	-7	3	2.11	0.93
-19	-8	3	6.30	1.12
-19	-8	3	9.75	1.60
-19	8	-3	9.17	1.37
-19	8	-3	6.74	1.75
19	-8	-3	9.62	1.45
19	8	3	5.18	1.10
19	-8	-3	5.89	1.04
19	-8	-3	6.50	1.30
19	8	3	8.45	1.54
-19	8	3	2.92	1.52
-19	-8	-3	8.67	1.23
-19	-8	-3	5.53	1.04
19	-8	3	6.60	1.37
19	-8	3	5.48	0.97
19	8	-3	7.79	1.44
19	-9	-3	2.45	0.98
-19	-9	3	1.18	1.41
-19	9	-3	2.50	0.93
19	-9	-3	2.58	0.87
19	-9	-3	1.94	0.86
-19	9	-3	1.71	2.10
19	9	3	4.85	1.61
-19	-9	3	2.77	1.00
-19	9	3	1.03	1.55
-19	-9	-3	3.07	0.96
19	-9	3	0.72	0.87
-19	-9	-3	2.53	1.74
19	-9	3	2.22	0.87
-19	-9	-3	4.67	1.73
19	9	-3	2.00	1.04
-20	0	3	37.94	2.38
20	0	-3	36.98	1.81
-20	0	-3	38.69	2.50

-20	0	3	35.85	2.52
20	0	3	41.21	2.09
-20	0	3	35.56	2.81
-20	0	3	39.98	2.34
20	0	3	42.71	2.41
20	0	3	42.84	2.43
20	0	3	39.75	2.50
20	0	-3	35.70	1.91
-20	0	-3	36.47	2.26
-20	0	-3	39.19	2.62
-20	-1	3	53.64	3.23
20	1	3	53.24	2.69
-20	1	-3	57.28	3.19
20	1	3	50.51	3.00
-20	1	-3	57.93	4.36
20	-1	-3	58.60	2.85
-20	-1	3	51.38	2.90
-20	1	-3	57.82	3.30
20	-1	-3	60.87	3.00
-20	-1	3	57.81	3.26
20	1	3	54.78	2.95
20	1	3	55.92	3.17
-20	-1	3	52.59	3.10
20	-1	3	55.65	3.04
-20	1	3	54.47	2.95
-20	-1	-3	60.09	3.21
20	-1	3	55.27	2.74
20	-1	3	58.06	3.08
-20	1	3	54.81	3.32
-20	1	3	50.46	2.89
-20	-1	-3	60.14	3.44
20	-1	3	53.83	2.99
-20	1	3	50.75	2.99
-20	2	-3	36.25	2.36
-20	-2	3	40.49	2.38
-20	-2	3	38.35	2.37
20	2	3	43.43	2.44
-20	2	-3	43.42	2.89
20	2	3	40.93	2.16
20	-2	-3	36.59	2.43
-20	-2	3	42.19	2.61
20	-2	-3	39.62	2.05
20	-2	-3	42.08	2.28
-20	2	-3	38.45	2.35

-20	-2	3	42.12	2.64
20	2	3	42.30	2.85
20	-2	3	43.06	2.47
-20	-2	-3	41.42	2.36
-20	2	3	39.06	2.32
-20	2	3	45.15	2.95
20	-2	3	42.10	2.25
-20	2	3	40.13	2.39
20	-2	3	46.36	2.69
20	-2	3	42.35	2.49
-20	-2	-3	36.82	2.34
-20	-2	-3	37.17	2.68
-20	-2	-3	37.48	2.54
-20	2	3	41.78	2.45
20	-3	-3	19.99	1.39
20	-3	-3	16.47	1.21
20	-3	-3	16.00	1.53
20	3	3	17.68	1.20
20	3	3	18.05	1.48
-20	-3	3	18.62	1.43
-20	3	-3	14.30	1.43
20	3	3	15.14	1.84
-20	-3	3	16.39	1.66
-20	3	-3	15.77	2.20
-20	-3	3	18.48	1.79
-20	-3	3	14.37	1.51
-20	-3	-3	18.99	1.94
-20	3	3	17.80	1.51
-20	-3	-3	15.93	1.46
-20	3	3	17.06	2.06
20	-3	3	18.37	1.29
-20	-3	-3	16.67	1.48
20	-3	3	17.24	1.45
20	-3	3	17.79	1.88
-20	-3	-3	14.29	1.62
20	-3	3	17.82	1.44
20	4	3	8.04	0.96
-20	-4	3	6.34	0.98
20	-4	-3	8.30	0.99
-20	-4	3	7.62	1.38
-20	4	-3	6.03	1.48
20	-4	-3	8.92	1.13
20	-4	-3	8.29	1.16
-20	4	-3	7.46	1.18

-20	-4	3	6.83	1.73
-20	-4	3	9.43	1.70
20	-4	-3	12.22	1.42
20	4	3	10.81	1.73
20	-4	3	7.02	1.01
20	-4	3	7.70	1.10
-20	-4	-3	8.16	1.25
-20	-4	-3	8.42	1.39
20	-4	3	7.35	1.18
20	-4	3	12.56	1.61
-20	-4	-3	7.32	1.08
-20	4	3	8.05	1.45
-20	-5	3	18.41	1.70
20	-5	-3	17.82	1.50
-20	-5	3	22.01	2.17
-20	-5	3	18.93	1.66
20	-5	-3	18.32	1.51
20	5	3	19.02	2.11
20	-5	-3	16.06	1.61
20	5	3	20.86	1.71
20	5	3	19.86	1.54
-20	5	-3	17.81	2.68
-20	5	-3	21.20	1.70
-20	-5	3	16.49	1.56
-20	-5	-3	14.85	1.67
-20	5	3	14.88	2.45
-20	-5	-3	16.08	1.53
20	-5	3	19.62	1.58
-20	-5	-3	19.28	1.64
20	-5	3	20.83	1.63
-20	-6	3	26.01	1.93
-20	-6	3	27.60	2.15
20	-6	-3	28.94	1.91
-20	-6	3	26.79	1.93
-20	-6	3	30.88	2.77
20	6	3	28.84	2.00
-20	6	-3	29.66	2.90
20	6	3	28.59	2.09
20	-6	-3	25.03	1.83
20	6	3	31.04	2.87
20	-6	-3	25.08	1.95
-20	6	-3	26.21	2.00
-20	6	3	29.91	2.89
20	-6	3	29.34	2.00

20	6	-3	24.39	1.76
-20	-6	-3	23.81	1.97
-20	-6	-3	27.31	2.13
20	-6	3	25.54	2.04
-20	-7	3	37.51	2.43
20	7	3	37.67	3.09
20	-7	-3	40.50	2.53
-20	7	-3	42.95	2.56
-20	-7	3	43.10	3.11
-20	-7	3	39.20	2.64
20	-7	-3	35.07	2.48
20	7	3	37.44	2.46
-20	7	-3	41.70	3.39
20	-7	-3	41.01	2.50
20	-7	3	40.16	2.54
20	-7	3	43.54	2.54
20	7	-3	36.64	2.41
-20	-7	-3	37.91	2.60
-20	7	3	39.32	3.19
20	7	-3	43.23	2.47
-20	-7	-3	42.01	2.67
-20	-8	3	41.82	2.69
-20	-8	3	39.19	3.22
20	-8	-3	43.93	2.69
-20	8	-3	41.73	2.65
-20	8	-3	42.50	3.62
20	-8	-3	45.52	2.72
20	-8	-3	45.56	2.66
20	8	3	40.19	2.65
20	8	-3	39.79	2.67
-20	8	3	42.66	3.49
20	-8	3	39.71	2.56
-20	-8	-3	40.51	2.76
-20	-8	-3	41.35	2.81
20	-8	3	43.10	2.68
-21	0	-3	0.44	0.64
21	0	3	0.00	0.65
-21	0	3	0.17	0.77
21	0	-3	0.17	0.60
21	0	3	-0.24	0.39
21	0	-3	0.18	0.41
-21	0	3	-0.06	0.96
-21	0	3	0.24	0.58
21	0	3	-0.16	0.79

21	0	3	-0.26	0.83
21	0	-3	0.05	0.29
-21	0	3	0.36	0.90
-21	0	-3	2.86	1.74
-21	1	-3	6.69	1.15
-21	-1	3	4.84	1.07
-21	-1	3	6.21	1.00
21	-1	-3	4.28	0.61
-21	-1	3	3.47	0.81
21	-1	-3	6.59	0.90
-21	1	-3	5.47	1.29
21	1	3	7.11	1.20
21	1	3	4.81	0.74
21	1	3	3.95	1.15
21	-1	-3	5.65	1.02
21	1	3	4.30	0.92
-21	1	-3	2.66	0.76
-21	-1	3	5.35	0.98
21	-1	3	5.13	0.75
21	-1	3	7.13	1.33
21	-1	3	7.92	1.33
-21	1	3	3.44	0.86
-21	-1	-3	4.13	1.11
-21	1	3	4.35	0.87
-21	-1	-3	4.72	0.88
21	-1	3	4.85	0.91
-21	1	3	4.57	1.21
21	-2	-3	2.56	0.51
-21	2	-3	2.38	0.71
21	2	3	4.72	0.90
-21	-2	3	3.12	0.79
21	2	3	4.89	1.07
21	-2	-3	3.35	0.76
-21	-2	3	5.64	1.33
-21	2	-3	3.67	1.32
-21	-2	3	3.28	0.75
21	2	3	2.99	0.55
-21	-2	3	4.13	1.23
-21	-2	-3	2.39	0.71
21	-2	3	4.09	1.01
-21	2	3	5.76	0.82
-21	2	3	2.76	0.80
21	-2	3	6.84	1.25
21	-2	3	3.94	0.72

-21	-2	-3	2.51	0.98
21	-2	3	3.31	0.84
-21	-2	-3	3.48	0.88
-21	2	3	4.45	1.23
21	3	3	0.29	0.45
21	-3	-3	0.42	0.53
21	3	3	3.00	0.83
-21	-3	3	-0.65	0.59
21	3	3	1.04	1.00
-21	3	-3	0.65	0.77
21	-3	-3	2.13	0.72
-21	-3	3	2.40	0.91
-21	-3	3	0.95	0.70
-21	-3	3	4.60	1.12
-21	3	-3	-0.01	1.18
-21	3	3	1.26	1.07
21	-3	3	1.52	0.68
21	-3	3	2.59	1.02
-21	3	3	0.48	0.78
-21	-3	-3	1.70	0.67
21	-3	3	3.02	0.80
-21	-3	-3	-0.03	0.69
-21	-3	-3	0.70	0.91
21	-3	3	0.12	0.55
-21	-4	3	1.54	0.75
-21	-4	3	5.95	1.30
21	4	3	3.40	0.72
-21	4	-3	1.47	0.83
21	-4	-3	3.69	0.75
21	-4	-3	6.60	1.37
-21	-4	3	3.44	0.92
-21	4	-3	3.40	1.62
21	-4	-3	1.42	0.68
-21	-4	3	2.52	0.76
21	-4	3	1.94	0.73
21	-4	3	2.97	0.82
-21	4	3	3.99	1.52
-21	-4	-3	1.40	0.86
-21	-4	-3	2.53	0.94
-21	-4	-3	2.54	0.84
-21	4	3	2.21	0.89
21	5	3	1.76	0.87
-21	-5	3	0.32	0.68
-21	5	-3	4.83	1.62

21	-5	-3	0.20	0.71
-21	-5	3	0.64	1.30
-21	-5	3	1.91	0.92
-21	5	-3	0.13	0.72
-21	-5	3	0.42	0.76
21	5	3	1.85	0.80
21	-5	-3	0.96	0.68
21	5	3	-0.13	1.37
21	-5	-3	1.34	0.69
21	-5	3	0.56	0.67
-21	-5	-3	-0.11	0.77
-21	5	3	1.05	1.32
21	-5	3	1.30	0.81
-21	-5	-3	1.72	0.91
21	-6	-3	0.89	0.74
-21	6	-3	3.22	1.50
-21	6	-3	1.61	0.80
-21	-6	3	1.50	1.00
21	-6	-3	0.91	0.69
21	6	3	0.76	0.90
-21	-6	3	-0.42	0.72
21	-6	-3	0.23	0.81
21	6	3	2.08	0.78
21	6	3	2.35	1.36
-21	-6	3	0.25	1.26
21	-6	3	1.48	0.69
21	6	-3	1.00	0.64
-21	-6	-3	0.72	0.77
21	-6	3	0.22	0.75
21	6	-3	1.95	0.79
-21	6	3	1.75	1.31
-21	-6	-3	1.88	0.92
-21	7	-3	0.21	1.53
-21	-7	3	1.86	1.28
21	7	3	1.20	0.86
21	7	3	-0.49	1.32
-21	-7	3	0.49	0.79
-21	7	-3	-0.29	0.75
21	-7	-3	0.70	0.86
21	-7	-3	2.79	0.87
21	-7	-3	0.48	0.75
-21	-7	3	1.95	0.91
21	7	-3	3.40	0.85
21	7	-3	1.48	0.79

21	-7	3	1.65	0.78
21	-7	3	1.14	0.76
-21	-7	-3	1.43	0.96
-21	7	3	0.83	1.44
-21	-7	-3	1.50	0.92
22	0	-3	79.24	4.03
22	0	3	65.47	3.24
-22	0	-3	71.97	4.03
22	0	-3	79.26	3.63
22	0	3	67.28	3.52
22	0	-3	78.03	3.55
22	0	3	63.73	3.56
-22	0	3	64.78	3.51
-22	0	-3	72.25	4.36
-22	0	3	65.74	3.91
22	0	3	67.57	3.66
-22	0	3	62.26	3.57
-22	-1	3	54.45	3.26
-22	1	-3	46.34	2.92
22	1	3	60.16	3.10
-22	1	-3	52.97	3.47
22	1	3	56.97	2.82
22	-1	-3	47.07	2.44
-22	-1	3	54.54	3.07
22	-1	-3	53.63	2.88
22	1	3	50.83	3.61
-22	-1	3	57.89	3.45
22	1	3	57.12	3.23
22	-1	-3	49.42	2.62
-22	-1	3	52.60	3.09
-22	1	3	56.18	3.15
-22	-1	-3	51.24	2.89
-22	-1	-3	51.56	4.15
22	-1	3	57.58	2.88
-22	1	3	51.41	3.54
-22	-1	-3	48.87	2.89
22	-1	3	56.96	3.24
22	-1	3	57.42	3.17
22	-1	3	60.69	3.25
-22	1	3	54.79	3.10
-22	-2	3	24.21	2.14
-22	-2	3	22.06	1.82
22	2	3	25.48	1.85
-22	2	-3	22.56	1.91

22	-2	-3	23.24	1.49
-22	-2	3	25.36	1.81
22	2	3	22.30	2.23
22	-2	-3	26.46	1.77
22	-2	-3	24.97	1.80
-22	-2	3	30.18	2.19
-22	2	-3	26.39	2.62
22	2	3	24.87	1.51
-22	2	3	28.01	2.45
-22	2	3	25.62	1.95
22	-2	3	24.37	1.63
22	-2	3	28.01	1.97
-22	2	3	23.43	1.69
-22	-2	-3	27.60	2.20
-22	-2	-3	24.73	1.93
-22	-2	-3	20.36	1.81
22	-2	3	27.57	1.97
22	-2	3	24.61	2.07
22	-3	-3	17.80	1.55
22	3	3	18.07	2.13
-22	3	-3	16.18	1.64
-22	-3	3	18.61	1.64
22	3	3	17.63	1.32
22	3	3	18.93	1.61
22	-3	-3	17.88	1.64
22	-3	-3	16.77	1.31
-22	3	-3	16.03	2.22
-22	-3	3	17.49	2.14
-22	-3	3	16.14	1.58
22	-3	3	18.24	1.39
-22	-3	-3	15.76	1.57
22	-3	3	16.85	1.53
-22	-3	-3	18.62	1.93
-22	3	3	20.43	1.77
-22	-3	-3	14.96	1.69
22	-3	3	20.85	1.78
-22	3	3	21.02	2.58
22	4	3	3.36	0.78
-22	-4	3	4.50	1.15
22	4	3	4.00	0.99
22	-4	-3	3.23	0.71
22	4	3	3.89	1.45
22	-4	-3	1.86	0.75
-22	-4	3	2.14	0.87

-22	4	-3	2.29	0.88
-22	4	-3	0.79	1.65
-22	-4	3	2.93	0.94
-22	-4	3	3.53	1.21
-22	-4	-3	0.98	0.98
-22	-4	-3	2.66	0.92
-22	-4	-3	2.86	0.93
22	-4	3	3.41	0.97
-22	4	3	3.50	1.38
-22	4	3	1.82	0.82
22	-4	3	3.49	0.75
22	-5	-3	24.53	1.92
22	-5	-3	23.96	1.69
-22	-5	3	26.59	2.07
-22	5	-3	29.61	2.93
22	5	3	32.38	1.91
22	5	3	34.55	2.88
-22	5	-3	23.56	1.88
-22	-5	3	28.23	2.25
-22	-5	3	31.39	2.91
-22	-5	3	26.53	2.15
-22	5	3	26.15	2.05
22	-5	3	31.73	1.94
-22	5	3	27.35	2.84
22	-5	3	29.14	2.13
-22	-5	-3	23.07	1.99
-22	-5	-3	25.77	1.99
22	-6	-3	40.31	2.46
-22	-6	3	34.90	2.37
22	6	3	36.96	2.97
-22	-6	3	39.00	2.99
-22	6	-3	44.34	2.60
22	6	3	40.91	3.03
-22	6	-3	35.12	3.43
22	-6	-3	40.44	2.59
-22	-6	3	35.05	2.46
22	6	3	34.75	2.22
-22	-6	-3	42.23	2.68
-22	6	3	37.77	3.24
22	-6	3	33.52	2.32
22	-6	3	37.84	2.33
-22	-6	-3	37.72	2.71
-22	7	-3	25.27	1.95
-22	-7	3	20.66	2.52

-22	-7	3	21.67	1.96
22	7	3	21.47	1.74
22	-7	-3	28.00	2.56
22	-7	-3	27.02	1.95
22	-7	-3	30.04	2.93
22	-7	3	20.94	1.76
22	7	-3	23.75	1.76
22	7	-3	21.08	1.70
22	-7	3	22.19	1.85
-22	-7	-3	20.84	2.01
-22	-7	-3	26.52	2.35
-23	0	3	-0.17	0.63
-23	0	3	0.26	1.09
23	0	3	0.95	0.57
23	0	-3	0.81	0.42
-23	0	-3	-0.27	0.84
-23	0	3	-0.60	0.76
23	0	3	-0.11	0.81
23	0	3	-0.56	1.06
-23	0	-3	-0.19	1.25
23	0	3	1.20	0.86
-23	0	3	-0.43	0.89
-23	-1	3	0.00	1.87
-23	-1	3	1.96	0.76
23	1	3	2.34	0.59
-23	1	-3	1.63	0.82
-23	1	-3	3.22	1.39
23	1	3	2.17	1.16
23	-1	-3	3.50	0.87
23	-1	-3	3.49	0.68
-23	-1	3	2.29	0.88
-23	-1	3	4.37	1.24
23	-1	-3	3.52	0.70
23	1	3	1.25	0.82
23	-1	3	1.65	0.54
-23	1	3	2.41	0.92
23	-1	3	2.46	0.96
-23	1	3	0.65	1.21
23	-1	3	2.92	0.97
23	-1	3	2.66	1.01
-23	-1	-3	3.24	1.01
-23	-1	-3	2.86	0.91
-23	1	3	1.96	0.87
-23	-1	-3	5.37	1.32

23	-2	-3	9.00	1.31
-23	2	-3	9.30	1.87
23	-2	-3	12.11	1.34
23	2	3	10.58	1.09
-23	-2	3	11.51	1.39
-23	2	-3	8.19	1.43
23	2	3	11.18	1.62
-23	-2	3	11.91	1.41
23	2	3	11.77	1.48
23	-2	-3	9.86	0.95
-23	-2	3	14.34	1.91
-23	-2	3	12.40	1.92
-23	-2	-3	10.81	1.59
23	-2	3	11.76	1.60
-23	2	3	11.88	1.91
23	-2	3	11.37	1.11
-23	2	3	11.81	1.47
23	-2	3	13.17	1.59
-23	-2	-3	9.97	1.53
-23	3	-3	25.31	3.17
23	-3	-3	24.31	1.56
-23	3	-3	22.32	1.90
-23	-3	3	22.40	1.82
23	3	3	27.53	2.43
-23	-3	3	25.24	2.21
-23	-3	3	24.49	2.39
-23	-3	3	22.66	1.97
23	3	3	23.12	1.48
23	-3	-3	26.45	1.90
23	-3	3	20.12	1.53
-23	-3	-3	23.13	2.07
-23	3	3	27.02	2.74
-23	-3	-3	25.29	2.00
-23	3	3	21.52	1.85
23	-3	3	23.59	1.92
-23	-3	-3	24.65	2.21
-23	-4	3	8.34	1.36
-23	-4	3	13.47	2.32
-23	-4	3	9.64	1.43
23	4	3	10.94	1.88
23	4	3	11.96	1.54
23	4	3	10.70	1.10
-23	-4	3	10.34	1.68
-23	4	-3	14.00	2.60

23	-4	-3	12.75	1.60
23	-4	-3	12.10	1.17
-23	4	-3	12.38	1.44
23	-4	3	9.50	1.09
23	-4	3	10.70	1.43
-23	4	3	10.40	1.39
-23	-4	-3	11.91	1.73
-23	-4	-3	14.00	1.60
-23	4	3	13.00	2.27
23	5	3	8.20	1.11
23	-5	-3	4.64	0.86
23	5	3	9.09	1.70
-23	-5	3	10.04	1.72
-23	-5	3	8.68	1.54
-23	5	-3	5.08	0.99
-23	-5	3	7.28	1.16
23	-5	-3	6.19	1.10
-23	5	-3	7.55	1.70
23	5	3	13.52	1.67
-23	-5	-3	6.44	1.25
-23	5	3	8.30	1.73
23	-5	3	9.43	1.35
-23	5	3	7.95	1.23
-23	-5	-3	4.90	1.06
23	-5	3	6.94	1.19
23	-6	-3	8.65	1.42
-23	-6	3	7.45	1.21
-23	-6	3	9.35	1.33
-23	-6	3	8.52	1.61
23	6	3	9.19	1.80
-23	6	-3	8.68	1.82
23	6	3	8.75	1.27
-23	6	-3	9.10	1.52
-23	6	3	15.37	2.93
23	-6	3	8.33	1.44
-23	6	3	13.86	2.29
-23	-6	-3	10.99	1.56
23	-6	3	9.03	1.82
-23	-6	-3	7.49	1.43
24	0	3	30.70	1.76
-24	0	3	29.82	2.17
24	0	3	33.76	2.47
-24	0	3	28.96	2.91
-24	0	-3	35.28	2.95

-24	0	-3	29.73	2.20
-24	0	3	27.02	2.22
-24	0	3	27.87	2.17
24	0	3	31.72	2.24
24	0	3	33.66	2.17
24	-1	-3	32.04	1.87
24	1	3	33.77	2.93
-24	-1	3	31.88	2.24
24	1	3	30.99	2.64
24	1	3	29.29	1.71
24	-1	-3	33.06	2.30
24	-1	-3	31.88	1.71
-24	-1	3	32.26	2.22
-24	1	-3	35.04	3.22
-24	-1	3	30.16	2.41
-24	-1	3	32.52	2.65
-24	1	-3	31.17	2.19
-24	-1	-3	29.32	2.26
-24	-1	-3	34.64	2.91
24	-1	3	24.11	2.46
24	-1	3	30.10	2.31
-24	1	3	28.50	2.12
24	-1	3	27.39	1.74
24	-1	3	30.04	2.22
-24	-1	-3	31.49	2.24
-24	1	3	30.05	3.02
24	2	3	19.10	1.65
24	-2	-3	13.92	1.42
24	2	3	21.14	2.30
24	-2	-3	13.25	1.06
24	2	3	19.43	1.31
-24	2	-3	11.06	1.48
-24	-2	3	23.16	1.90
-24	-2	3	22.48	2.34
24	-2	-3	15.31	1.68
-24	-2	3	20.78	1.88
-24	-2	3	14.77	2.19
-24	2	-3	14.39	2.25
24	-2	3	19.86	1.76
-24	-2	-3	17.67	2.24
-24	-2	-3	17.09	1.60
-24	2	3	18.88	1.72
24	-2	3	18.11	1.35
-24	2	3	19.30	2.63

-24	-2	-3	16.44	1.70
-24	-3	3	6.87	1.19
24	3	3	6.17	0.90
-24	-3	3	7.30	1.59
-24	3	-3	3.83	1.02
24	3	3	5.23	1.64
24	-3	-3	6.09	0.88
-24	-3	3	7.47	1.51
-24	-3	3	8.28	1.52
24	-3	-3	8.96	1.49
24	3	3	5.22	1.02
-24	3	-3	2.31	1.62
-24	-3	-3	8.57	1.21
-24	-3	-3	4.02	1.07
-24	3	3	6.50	1.12
-24	3	3	6.33	1.49
24	-3	3	8.67	1.35
24	-3	3	6.11	0.99
-24	-4	3	6.09	1.22
24	-4	-3	6.84	1.09
24	-4	-3	6.27	0.99
-24	-4	3	7.55	1.36
-24	-4	3	5.09	1.06
24	4	3	5.44	1.08
-24	4	-3	5.84	1.06
-24	-4	3	7.45	1.58
-24	4	-3	3.23	1.69
24	4	3	6.97	1.67
24	4	3	5.89	0.96
24	-4	3	6.84	1.13
-24	-4	-3	5.69	1.09
-24	4	3	7.39	1.32
-24	4	3	4.85	1.57
24	-4	3	4.93	0.82
-24	-4	-3	11.27	1.63
-24	-5	3	9.62	1.32
-24	-5	3	12.14	1.80
24	-5	-3	10.49	1.48
24	5	3	13.02	2.28
24	5	3	10.93	1.21
24	-5	3	9.84	1.48
-24	-5	-3	10.06	1.66
-24	-5	-3	13.69	1.58
-25	0	-3	0.78	0.82

-25	0	3	0.71	0.83
-25	0	3	1.32	1.46
25	0	3	0.00	0.49
-25	0	3	-0.31	0.77
25	0	3	0.11	0.78
-25	0	-3	0.39	1.62
25	0	3	-2.59	1.17
25	-1	-3	12.96	1.21
25	1	3	9.11	1.71
-25	-1	3	9.69	1.48
-25	1	-3	19.04	2.90
25	-1	-3	12.68	1.67
-25	-1	3	9.03	1.49
25	1	3	8.17	1.46
-25	1	-3	9.65	1.51
-25	-1	3	11.01	1.53
25	-1	-3	11.59	0.89
25	1	3	9.89	0.94
-25	-1	3	9.49	1.82
25	-1	3	10.71	1.49
-25	-1	-3	12.77	1.49
-25	-1	-3	11.10	1.53
-25	-1	-3	15.48	2.45
-25	1	3	9.40	1.45
25	-1	3	9.24	1.01
-25	1	3	16.65	2.41
-25	2	-3	25.24	3.05
-25	-2	3	29.71	2.64
25	2	3	28.53	1.62
-25	-2	3	27.51	2.16
-25	-2	3	27.72	2.78
25	2	3	29.30	1.98
25	-2	-3	26.36	2.12
25	2	3	31.43	2.97
-25	-2	3	28.00	2.17
-25	2	-3	24.57	2.05
25	-2	-3	27.15	1.57
-25	2	3	30.88	2.17
-25	-2	-3	27.30	2.05
25	-2	3	28.09	1.76
-25	2	3	30.83	2.82
-25	-2	-3	32.70	2.80
-25	-2	-3	25.96	2.05
25	-2	3	24.25	2.09

25	3	3	27.22	2.89
-25	3	-3	26.82	3.07
25	3	3	27.56	1.68
-25	-3	3	28.04	3.02
25	-3	-3	26.68	1.69
-25	-3	3	30.49	2.25
25	-3	-3	32.71	2.17
-25	-3	3	32.05	2.20
-25	-3	3	33.32	2.68
25	3	3	28.30	1.96
-25	3	-3	33.82	3.09
-25	-3	-3	23.01	2.09
25	-3	3	30.47	2.23
-25	3	3	30.06	3.01
-25	3	3	29.89	2.15
25	-3	3	29.39	2.32
-25	-3	-3	29.56	2.20
0	0	4	249.98	11.67
0	0	-4	327.60	14.08
0	0	-4	310.80	14.00
0	0	4	267.34	11.65
0	0	4	264.09	11.72
0	0	-4	304.78	13.97
0	0	-4	311.98	14.01
0	0	-4	308.17	14.03
0	1	-4	174.19	7.95
0	1	-4	169.44	7.96
0	-1	4	190.98	8.39
0	1	-4	176.09	8.08
0	1	4	198.95	8.46
0	1	-4	179.25	8.03
0	-1	-4	178.93	8.03
0	1	-4	199.34	8.13
0	-1	-4	169.56	8.00
0	1	4	185.87	8.47
0	-1	4	178.26	8.40
0	1	4	172.03	8.39
0	-1	-4	172.98	7.95
0	-1	4	201.47	8.53
0	-1	-4	176.81	8.01
0	-1	-4	184.40	8.08
0	-1	4	178.68	8.43
0	1	4	203.98	8.53
0	-1	-4	174.77	8.01

0	-1	4	186.88	8.47
0	1	4	185.70	8.44
0	1	4	181.68	8.45
0	-2	4	642.09	28.92
0	2	-4	613.11	27.46
0	2	4	635.43	28.89
0	2	-4	596.97	27.33
0	2	-4	615.77	27.40
0	2	4	663.58	28.97
0	-2	4	678.19	28.99
0	-2	-4	599.09	27.41
0	2	4	620.31	28.94
0	-2	-4	598.66	27.36
0	-2	-4	602.23	27.42
0	-2	4	715.07	29.01
0	2	-4	655.48	27.49
0	-2	-4	604.76	27.33
0	-2	4	621.19	28.86
0	2	4	612.86	28.84
0	-2	-4	627.02	27.37
0	2	4	633.45	28.88
0	3	4	107.91	5.04
0	-3	4	114.44	5.19
0	3	4	109.83	4.99
0	3	4	111.39	5.00
0	-3	4	116.76	5.17
0	3	4	108.16	5.01
0	3	4	113.02	5.14
0	3	-4	119.74	5.58
0	3	-4	121.52	5.35
0	-3	-4	102.49	5.49
0	3	-4	124.37	5.52
0	-3	-4	128.01	5.49
0	-3	4	110.39	5.09
0	-3	-4	113.85	5.46
0	3	4	109.76	5.06
0	-3	-4	115.71	5.54
0	3	4	105.91	5.15
0	3	-4	118.12	5.46
0	-3	4	102.21	5.04
0	-3	-4	125.61	5.57
0	3	-4	122.79	5.79
0	-3	4	105.75	5.02
0	3	-4	118.99	5.49

0	-4	4	110.66	5.09
0	4	-4	130.09	5.79
0	4	-4	119.69	5.72
0	4	-4	126.09	5.60
0	-4	4	100.69	5.05
0	-4	-4	131.39	5.75
0	-4	4	104.25	5.03
0	-4	4	119.35	5.19
0	4	4	102.27	5.32
0	-4	-4	116.66	5.71
0	-4	4	113.93	5.21
0	-4	-4	117.53	5.72
0	-4	-4	126.34	5.67
0	4	-4	121.20	5.84
0	4	4	109.97	5.06
0	4	-4	125.41	5.92
0	4	4	112.31	5.16
0	4	4	106.58	4.99
0	-4	4	103.82	4.89
0	-4	-4	130.75	5.86
0	4	4	110.29	5.03
0	-4	-4	115.34	5.80
0	4	-4	128.94	5.78
0	5	4	135.32	6.38
0	-5	-4	135.51	6.64
0	-5	-4	148.39	6.49
0	-5	4	147.49	6.50
0	5	4	136.03	6.32
0	-5	-4	133.83	6.53
0	-5	-4	137.14	6.68
0	5	-4	150.06	6.64
0	5	4	139.38	6.49
0	-5	4	148.97	6.57
0	5	4	136.92	6.32
0	5	-4	145.13	6.55
0	5	-4	146.24	6.81
0	-5	4	131.34	6.18
0	-5	4	127.42	6.37
0	5	4	132.92	6.54
0	-5	4	130.56	6.33
0	5	-4	138.29	6.67
0	5	-4	146.30	6.40
0	-5	-4	133.82	6.53
0	-5	4	139.88	6.41

0	6	-4	251.31	11.65
0	-6	4	231.52	11.02
0	-6	-4	279.22	11.70
0	6	4	234.08	10.97
0	-6	4	252.38	11.12
0	6	-4	246.70	11.92
0	6	4	237.52	11.00
0	-6	4	261.63	11.28
0	6	-4	254.21	11.78
0	6	4	232.46	11.25
0	-6	4	231.01	11.06
0	6	4	239.29	10.98
0	6	4	234.68	11.06
0	-6	-4	248.32	11.65
0	-6	4	260.69	11.16
0	6	4	246.43	11.19
0	-6	-4	257.65	11.57
0	6	-4	262.64	11.74
0	-6	4	228.65	10.83
0	-6	-4	237.83	11.76
0	-6	-4	239.13	11.60
0	6	-4	258.67	11.48
0	-6	4	245.24	11.09
0	-6	-4	271.98	11.85
0	7	4	114.51	5.62
0	7	-4	82.21	4.05
0	-7	4	125.41	5.70
0	7	4	118.15	5.52
0	-7	4	109.35	5.61
0	-7	4	109.15	5.56
0	7	4	112.71	5.84
0	-7	-4	78.64	3.87
0	7	-4	76.43	4.26
0	-7	-4	74.57	3.87
0	-7	-4	75.02	4.02
0	7	4	120.53	5.82
0	7	-4	80.23	3.74
0	7	-4	79.13	3.94
0	-7	-4	89.71	4.22
0	7	4	113.44	5.54
0	-7	4	111.62	5.35
0	-7	-4	81.11	3.95
0	7	-4	79.38	4.08
0	-7	4	130.75	5.75

0	-7	4	119.70	5.65
0	-7	-4	88.97	4.02
0	7	4	116.05	5.56
0	-7	4	124.00	5.86
0	-8	4	113.53	5.52
0	8	-4	125.08	6.32
0	-8	-4	134.12	5.98
0	8	4	114.35	5.44
0	8	4	109.77	5.47
0	-8	4	116.67	5.65
0	8	4	110.23	5.56
0	-8	-4	115.58	5.81
0	8	-4	125.36	6.05
0	-8	4	118.48	5.59
0	-8	4	123.76	5.68
0	-8	-4	132.03	6.20
0	8	-4	113.31	5.89
0	-8	-4	114.03	5.99
0	-8	4	124.05	5.86
0	8	4	121.08	5.82
0	8	-4	129.64	6.09
0	-8	4	104.81	5.56
0	-8	-4	123.20	5.81
0	8	4	112.38	5.83
0	8	-4	126.43	5.68
0	-8	-4	120.71	5.91
0	-8	4	107.61	5.26
0	9	4	128.72	6.09
0	-9	4	130.25	6.53
0	-9	-4	147.41	6.57
0	9	4	121.75	6.12
0	9	-4	133.73	6.62
0	9	-4	138.05	6.23
0	-9	-4	131.54	6.36
0	-9	-4	123.66	6.56
0	-9	-4	130.04	6.46
0	-9	4	120.08	5.89
0	-9	4	131.31	6.34
0	-9	4	133.81	6.33
0	-9	-4	124.99	6.35
0	-9	-4	143.00	6.52
0	9	4	135.32	6.51
0	9	-4	139.80	6.71
0	-9	4	134.00	6.26

0	9	4	122.23	6.58
0	-9	4	126.24	6.19
0	9	-4	133.24	6.94
0	-9	-4	139.78	6.78
0	10	4	78.19	4.27
0	10	4	70.81	3.72
0	-10	4	70.78	3.85
0	10	-4	57.49	3.36
0	10	-4	58.18	3.78
0	-10	4	66.12	4.55
0	10	-4	64.15	3.52
0	-10	-4	56.88	3.08
0	-10	-4	56.14	3.18
0	10	-4	60.76	2.90
0	10	4	71.18	4.30
0	-10	-4	60.47	3.24
0	-10	4	75.49	3.84
0	-10	4	75.26	4.04
0	10	4	73.16	3.78
0	-10	-4	63.30	3.35
0	-10	4	68.31	3.46
0	-10	-4	57.67	3.03
0	-10	4	79.92	3.94
0	-10	-4	50.83	3.27
0	-11	-4	35.84	2.49
0	11	4	40.62	3.63
0	-11	4	37.90	2.07
0	-11	4	43.99	2.70
0	11	-4	37.28	2.63
0	-11	-4	39.83	2.45
0	-11	4	40.59	2.49
0	11	4	41.58	2.53
0	11	-4	37.15	3.18
0	11	4	40.37	3.12
0	-11	-4	43.59	2.52
0	-11	-4	39.69	2.26
0	-11	4	40.02	2.51
0	-12	4	9.28	1.47
0	-12	-4	11.86	1.62
0	-12	-4	11.76	1.32
0	12	-4	13.69	1.98
0	-12	-4	9.83	1.71
0	12	4	9.14	2.13
0	12	-4	9.77	1.95

0	12	4	10.03	1.55
0	-12	4	8.92	1.67
0	-13	4	33.24	2.49
0	13	-4	28.66	2.63
0	13	-4	22.85	3.44
0	-13	-4	29.83	2.24
0	-13	4	38.04	2.83
0	-13	-4	26.98	1.90
0	13	4	34.49	3.61
0	-13	-4	32.59	2.55
1	0	-4	-0.08	0.15
-1	0	-4	0.03	0.10
-1	0	4	0.05	0.10
-1	0	-4	-0.19	0.16
1	0	-4	-0.03	0.10
-1	0	4	-0.11	0.15
1	0	4	0.23	0.19
1	0	4	0.06	0.11
1	0	4	-0.03	0.13
1	0	-4	0.12	0.18
1	0	4	-0.07	0.16
-1	0	-4	0.17	0.12
1	0	-4	0.01	0.13
-1	0	-4	0.08	0.18
1	0	4	0.04	0.07
1	0	4	0.01	0.10
-1	0	4	0.09	0.18
-1	0	-4	0.03	0.08
-1	0	4	-0.05	0.13
-1	0	-4	-0.06	0.11
-1	0	4	0.08	0.10
1	1	4	8.12	0.48
1	-1	-4	6.71	0.48
-1	1	-4	8.19	0.64
1	-1	-4	7.06	0.44
1	1	4	7.28	0.49
-1	-1	4	7.73	0.57
-1	-1	4	7.39	0.39
-1	-1	4	6.76	0.50
-1	1	-4	7.51	0.45
-1	1	-4	7.22	0.41
-1	1	-4	7.44	0.49
-1	-1	4	7.38	0.55
-1	-1	4	7.08	0.40

1	1	4	7.54	0.57
1	1	4	7.25	0.49
-1	1	-4	6.68	0.55
1	-1	-4	6.83	0.53
-1	-1	4	7.18	0.43
1	1	4	7.08	0.46
1	-1	-4	6.51	0.45
1	-1	-4	7.21	0.48
-1	1	-4	7.41	0.41
-1	-1	-4	6.99	0.41
-1	1	4	7.48	0.48
1	-1	4	8.65	0.63
1	-1	4	7.52	0.42
1	-1	4	7.69	0.50
-1	-1	-4	7.54	0.45
1	1	-4	7.35	0.55
-1	-1	-4	7.14	0.48
1	-1	4	7.23	0.58
-1	1	4	7.33	0.49
1	1	-4	7.06	0.39
-1	1	4	8.03	0.55
-1	-1	-4	6.76	0.45
-1	-1	-4	7.33	0.47
-1	-1	-4	7.30	0.53
1	1	-4	6.72	0.53
-1	1	4	6.86	0.46
1	-1	4	7.55	0.46
-1	1	4	7.40	0.38
1	1	-4	6.59	0.49
-1	1	4	7.93	0.51
1	-1	4	7.73	0.42
-1	2	-4	207.14	8.84
1	2	4	177.91	8.41
-1	-2	4	180.83	8.36
1	2	4	175.39	8.44
1	2	4	187.42	8.34
1	-2	-4	188.85	8.69
1	-2	-4	184.58	8.64
-1	2	-4	188.45	8.60
-1	2	-4	190.47	8.61
-1	-2	4	192.53	8.48
1	-2	-4	196.58	8.75
-1	-2	4	178.43	8.38
1	-2	-4	199.06	8.67

-1	-2	4	184.66	8.44
1	2	4	174.75	8.35
-1	2	-4	187.38	8.66
1	-2	-4	191.81	8.75
-1	2	-4	191.82	8.70
1	2	4	193.18	8.48
-1	2	-4	181.41	8.69
-1	-2	4	194.32	8.49
1	2	4	184.97	8.39
-1	2	-4	196.03	8.79
-1	-2	-4	193.75	8.71
1	2	-4	192.13	8.73
1	2	-4	199.66	8.79
-1	-2	-4	191.45	8.75
-1	-2	-4	190.88	8.65
-1	2	4	184.91	8.37
-1	2	4	179.64	8.34
1	-2	4	169.20	8.39
1	-2	4	191.32	8.42
-1	-2	-4	187.15	8.62
-1	2	4	183.74	8.46
1	-2	4	204.83	8.54
1	2	-4	197.21	8.77
1	2	-4	192.94	8.65
-1	2	4	195.96	8.47
-1	-2	-4	184.74	8.68
1	-2	4	194.44	8.50
-1	2	4	189.06	8.40
1	-2	4	177.83	8.37
-1	2	4	191.70	8.40
-1	-2	-4	197.25	8.73
-1	3	-4	66.77	3.14
-1	-3	4	77.23	3.60
-1	-3	4	82.69	3.62
-1	-3	4	71.60	3.46
1	-3	-4	66.04	3.18
1	3	4	73.46	3.47
1	3	4	72.85	3.40
-1	3	-4	68.34	3.14
1	-3	-4	63.22	3.10
-1	3	-4	64.31	3.09
-1	-3	4	70.46	3.46
1	3	4	73.54	3.56
1	3	4	74.37	3.58

1	3	4	71.33	3.39
-1	3	-4	65.61	3.28
1	-3	-4	70.33	3.22
1	3	4	72.20	3.44
-1	3	-4	64.71	3.22
-1	3	-4	67.20	3.00
1	-3	-4	72.14	3.09
-1	-3	4	74.16	3.53
1	-3	4	76.16	3.61
-1	-3	-4	60.84	3.09
1	3	-4	67.07	3.17
1	3	-4	66.43	3.09
1	-3	4	81.96	3.66
-1	-3	-4	68.55	3.19
-1	3	4	75.68	3.49
1	3	-4	65.23	3.23
-1	3	4	75.72	3.46
1	-3	4	70.00	3.49
-1	3	4	75.95	3.47
-1	3	4	73.54	3.44
-1	-3	-4	62.45	3.17
-1	-3	-4	65.95	3.08
1	-3	4	71.79	3.46
1	3	-4	62.60	3.30
1	3	-4	64.05	3.11
1	-3	4	68.82	3.37
-1	3	4	75.91	3.46
-1	3	4	71.04	3.58
-1	-3	-4	64.49	3.13
-1	3	4	78.28	3.58
-1	4	-4	5.69	0.57
1	-4	-4	4.99	0.49
-1	-4	4	10.45	0.80
1	4	4	9.68	0.66
1	-4	-4	6.46	0.55
-1	4	-4	6.91	0.63
-1	4	-4	5.49	0.75
-1	4	-4	5.82	0.42
-1	4	-4	5.33	0.56
1	4	4	9.78	0.69
1	-4	-4	5.12	0.53
1	4	4	10.39	0.83
-1	-4	4	11.45	0.92
-1	-4	4	10.08	0.72

-1	-4	4	10.42	0.72
1	-4	-4	6.27	0.68
1	-4	-4	5.67	0.69
1	-4	-4	5.61	0.54
1	4	4	10.60	0.84
1	4	4	10.41	0.76
1	4	4	10.37	0.72
-1	4	-4	5.60	0.72
1	4	-4	4.64	0.70
1	-4	4	10.43	0.58
-1	-4	-4	6.17	0.61
-1	-4	-4	5.62	0.53
1	4	-4	5.28	0.56
1	-4	4	9.90	0.71
-1	4	4	9.98	0.93
1	-4	4	12.16	1.00
1	-4	4	9.37	0.75
-1	-4	-4	5.05	0.64
1	-4	4	9.38	1.02
-1	-4	-4	5.78	0.53
-1	-4	-4	6.08	0.63
1	4	-4	5.58	0.65
1	4	-4	5.76	0.58
1	-4	4	10.60	0.75
-1	4	4	9.59	0.84
-1	4	4	11.72	0.88
-1	4	4	9.11	0.81
-1	-5	4	5.29	0.58
-1	-5	4	5.99	0.80
-1	-5	4	5.47	0.69
1	5	4	6.30	0.70
-1	-5	4	6.76	0.87
-1	5	-4	11.11	0.91
1	5	4	5.60	0.67
-1	-5	4	6.33	0.65
1	-5	-4	10.33	0.79
-1	5	-4	10.77	0.65
-1	5	-4	10.91	1.09
-1	-5	4	5.88	0.41
1	5	4	4.88	0.64
-1	5	-4	10.02	0.95
1	-5	-4	8.90	0.73
-1	-5	4	4.39	0.84
1	5	4	6.18	0.61

1	5	4	6.28	0.81
1	-5	-4	11.12	0.95
1	-5	-4	9.82	0.78
-1	5	-4	10.10	0.80
1	-5	-4	9.31	0.74
1	5	4	5.49	0.82
-1	5	-4	11.87	0.92
1	-5	4	6.04	0.90
1	5	-4	10.08	0.95
1	5	-4	11.24	0.81
1	5	-4	11.33	0.88
1	-5	4	6.38	0.46
-1	5	4	6.51	0.77
-1	-5	-4	9.32	0.69
-1	5	4	6.96	0.63
1	5	-4	10.37	0.98
-1	-5	-4	10.13	0.95
-1	5	4	6.97	0.62
-1	-5	-4	10.08	0.92
1	-5	4	5.98	0.71
1	-5	4	5.06	0.59
-1	-5	-4	9.37	0.77
1	-5	4	7.02	0.98
1	5	-4	10.29	0.59
-1	-5	-4	9.45	0.75
1	-5	4	5.92	0.67
-1	5	4	5.45	0.79
-1	-5	-4	9.60	0.87
-1	5	4	5.82	0.66
-1	5	4	6.26	0.62
1	-6	-4	45.13	2.23
1	-6	-4	41.20	2.16
-1	6	-4	44.22	2.32
-1	6	-4	42.29	2.05
-1	6	-4	42.17	2.21
-1	-6	4	46.04	2.43
1	-6	-4	38.45	2.17
1	6	4	42.83	2.32
-1	6	-4	41.65	2.50
1	6	4	42.34	2.48
1	6	4	41.76	2.20
1	6	4	40.65	2.18
-1	-6	4	47.08	2.50
-1	-6	4	40.44	2.23

-1	-6	4	39.29	2.27
-1	-6	4	40.24	2.03
1	-6	-4	45.29	2.45
1	6	4	42.76	2.23
1	6	4	44.60	2.45
-1	6	-4	42.40	2.39
-1	-6	4	42.18	2.32
-1	-6	4	45.65	2.40
1	-6	-4	40.73	2.28
1	6	-4	41.92	2.00
-1	6	4	45.56	2.56
-1	6	4	40.43	2.25
1	-6	4	42.79	2.24
-1	6	4	43.71	2.42
1	-6	4	43.55	2.29
1	6	-4	43.82	2.29
-1	6	4	41.24	2.27
-1	-6	-4	44.49	2.40
-1	-6	-4	40.24	2.28
-1	-6	-4	41.85	2.19
1	6	-4	40.96	2.20
-1	-6	-4	41.71	2.09
-1	6	4	41.02	2.23
-1	-6	-4	44.12	2.28
1	-6	4	39.58	2.30
1	-6	4	48.86	2.48
1	6	-4	39.93	2.46
1	-6	4	45.57	2.35
1	6	-4	40.47	2.31
-1	-6	-4	39.85	2.15
1	-6	4	47.21	2.52
-1	6	4	44.72	2.25
1	7	4	4.52	0.88
-1	7	-4	4.48	0.92
1	-7	-4	3.79	0.55
-1	7	-4	4.15	0.87
-1	-7	4	4.24	0.69
1	-7	-4	2.69	0.51
1	7	4	4.57	0.77
1	-7	-4	3.45	0.69
-1	-7	4	5.37	0.87
-1	7	-4	5.06	0.85
-1	-7	4	3.56	0.46
1	-7	-4	4.38	0.67

-1	7	-4	4.14	0.66
-1	-7	4	5.68	1.09
1	-7	-4	3.51	0.84
-1	-7	4	4.60	0.85
-1	-7	4	5.37	1.08
-1	7	-4	4.99	0.57
1	7	4	4.88	0.88
1	-7	-4	5.12	0.80
-1	-7	4	4.01	0.80
1	7	4	4.81	0.74
1	7	4	5.03	0.75
1	7	4	4.55	0.70
-1	7	4	3.73	0.60
1	-7	4	5.52	0.79
1	7	-4	4.25	0.76
1	7	-4	3.38	0.76
-1	7	4	3.37	0.82
-1	-7	-4	4.50	0.88
1	-7	4	5.32	0.71
-1	7	4	4.40	0.78
-1	7	4	4.48	0.75
1	-7	4	4.76	0.82
-1	7	4	4.83	1.03
-1	7	4	4.02	0.72
-1	-7	-4	3.68	0.73
-1	-7	-4	5.81	1.02
-1	-7	-4	3.93	0.65
1	7	-4	5.18	0.82
-1	-7	-4	3.61	0.66
1	-7	4	5.36	1.02
-1	-7	-4	4.15	0.59
1	7	-4	3.43	0.69
1	-7	4	4.52	0.46
1	-7	4	5.26	0.87
1	-7	4	4.51	0.89
1	7	-4	3.94	0.49
-1	8	-4	2.69	0.85
1	-8	-4	3.34	0.59
-1	8	-4	2.50	0.77
1	-8	-4	3.88	0.69
-1	-8	4	2.79	0.96
-1	8	-4	2.86	0.49
1	8	4	3.19	0.65
1	8	4	3.48	0.88

-1	8	-4	2.89	0.76
1	8	4	3.10	0.64
-1	-8	4	4.80	0.93
-1	-8	4	2.78	0.61
-1	-8	4	2.50	0.43
1	-8	-4	2.50	0.72
1	8	4	3.00	0.65
1	-8	-4	2.45	0.89
-1	-8	4	4.08	0.74
1	-8	-4	2.34	0.58
-1	8	-4	2.73	0.67
1	-8	-4	4.19	0.77
1	8	4	3.62	0.68
-1	-8	4	4.46	0.88
-1	-8	4	4.00	0.70
1	8	-4	3.43	0.56
1	-8	4	4.57	1.10
1	-8	4	4.04	0.80
-1	8	4	3.17	0.69
1	8	-4	2.65	0.73
-1	-8	-4	2.60	0.55
1	-8	4	4.89	0.73
-1	-8	-4	2.72	0.55
1	8	-4	3.08	0.73
1	-8	4	2.86	0.51
1	-8	4	4.69	0.93
1	-8	4	2.28	0.55
-1	-8	-4	2.72	0.61
-1	-8	-4	1.83	0.80
-1	8	4	3.74	0.96
-1	8	4	5.91	1.06
1	8	-4	2.74	0.89
-1	8	4	4.10	0.72
1	8	-4	1.12	0.55
-1	-8	-4	3.03	0.82
1	-8	4	3.33	0.83
-1	8	4	4.65	0.73
-1	9	-4	15.80	1.10
-1	-9	4	15.03	1.90
1	9	4	12.87	1.75
-1	-9	4	13.00	1.35
-1	9	-4	15.51	1.50
1	-9	-4	15.28	1.33
1	9	4	12.17	1.61

-1	-9	4	17.64	1.67
1	-9	-4	14.86	1.44
-1	-9	4	13.39	0.86
1	-9	-4	14.15	1.26
1	9	4	14.02	1.19
-1	-9	4	12.84	1.21
1	-9	-4	16.54	1.46
1	-9	-4	13.10	1.71
1	9	4	12.38	1.22
1	-9	-4	13.52	1.14
-1	9	-4	14.46	1.81
-1	-9	4	15.93	1.66
1	-9	-4	13.85	1.20
-1	-9	4	12.14	1.31
-1	9	-4	16.43	1.54
-1	-9	-4	13.67	1.23
1	-9	4	13.68	1.88
-1	9	4	13.99	1.31
-1	9	4	16.96	1.78
1	-9	4	13.31	1.66
-1	9	4	12.08	1.59
-1	-9	-4	14.57	1.34
1	9	-4	15.15	1.56
1	9	-4	14.21	1.44
1	-9	4	12.36	0.90
-1	-9	-4	12.52	1.15
-1	9	4	13.23	1.28
1	-9	4	13.55	1.24
1	9	-4	16.70	1.80
1	9	-4	14.16	1.04
-1	-9	-4	15.89	1.55
-1	-9	-4	14.11	1.73
1	-9	4	15.21	1.33
-1	-9	-4	15.62	1.23
1	-9	4	15.49	1.57
1	-9	4	10.78	1.18
-1	10	-4	2.68	0.94
-1	-10	4	7.21	1.26
-1	10	-4	3.64	0.62
1	-10	-4	2.66	0.84
1	-10	-4	3.40	0.70
-1	-10	4	4.26	1.05
1	-10	-4	1.72	0.73
-1	10	-4	1.43	1.24

1	-10	-4	3.16	0.95
-1	-10	4	3.77	0.78
1	-10	-4	2.94	0.73
1	10	4	4.95	1.27
1	10	4	5.92	1.37
1	-10	-4	2.23	0.83
-1	-10	4	4.50	0.54
-1	10	-4	2.47	1.09
-1	-10	4	4.09	0.96
1	10	4	5.65	0.87
1	10	4	5.43	0.85
1	-10	-4	2.78	1.22
-1	-10	-4	2.00	0.63
1	-10	4	4.23	0.89
1	-10	4	5.28	1.21
-1	10	4	4.09	1.25
-1	-10	-4	3.89	0.78
-1	10	4	6.06	0.93
-1	10	4	5.88	1.42
1	10	-4	4.66	1.35
-1	-10	-4	3.27	0.89
1	-10	4	5.82	0.96
-1	-10	-4	2.77	0.73
1	-10	4	4.48	0.64
1	-10	4	3.89	0.80
1	10	-4	4.76	0.93
-1	-10	-4	3.76	1.02
1	10	-4	3.07	1.20
1	10	-4	2.85	0.62
-1	10	4	4.81	0.95
-1	-10	-4	2.61	0.76
1	-10	4	3.65	1.12
-1	-11	4	10.73	1.35
1	11	4	9.45	1.91
-1	11	-4	10.37	1.67
1	-11	-4	9.94	1.37
-1	11	-4	9.76	1.79
1	-11	-4	9.91	1.36
-1	-11	4	12.01	1.94
1	-11	-4	8.68	1.41
-1	-11	4	9.52	0.82
1	11	4	12.11	1.32
1	-11	-4	9.96	1.31
1	-11	-4	10.10	1.10

1	11	4	12.71	1.37
-1	-11	-4	9.21	1.11
1	-11	4	8.70	1.44
1	-11	4	12.10	1.50
-1	-11	-4	11.62	1.47
-1	-11	-4	9.15	1.31
-1	11	4	9.80	1.51
-1	11	4	13.92	2.17
-1	-11	-4	11.09	1.46
1	11	-4	12.13	1.56
1	-11	4	11.30	1.33
-1	11	4	9.34	1.74
1	-11	4	10.78	0.89
-1	12	-4	10.31	1.97
1	12	4	8.49	1.51
-1	-12	4	8.99	1.44
1	-12	-4	8.18	1.53
1	-12	-4	7.55	1.18
1	12	4	8.69	1.81
-1	-12	4	9.32	1.50
1	-12	-4	9.89	1.53
-1	-12	-4	9.63	1.53
1	-12	4	7.43	1.15
1	12	-4	10.18	2.10
1	12	-4	8.34	1.36
-1	-12	-4	7.65	1.12
1	-12	4	8.32	1.55
-1	12	4	8.65	2.00
-1	12	4	9.87	1.68
-1	-12	-4	9.82	1.59
-1	13	-4	-0.60	2.05
1	13	4	-1.47	1.85
1	-13	-4	0.90	1.06
-1	13	-4	1.06	1.38
1	-13	-4	0.51	0.78
-1	-13	4	-1.08	1.19
-1	-13	4	-0.24	0.83
1	-13	-4	1.29	0.88
-1	-13	-4	0.09	0.88
-1	13	4	-1.64	1.85
-1	-13	-4	1.00	0.95
1	-13	4	0.01	0.87
1	13	-4	-0.18	1.07
1	-13	4	3.17	1.32

-1	-13	-4	0.93	1.24
1	13	-4	-2.92	1.83
-2	0	-4	312.08	14.01
2	0	-4	314.25	13.95
-2	0	-4	297.76	13.95
2	0	4	380.38	15.75
-2	0	-4	307.73	13.97
2	0	4	328.29	15.64
-2	0	-4	302.58	13.99
-2	0	4	347.11	15.62
-2	0	4	333.10	15.65
-2	0	-4	311.51	13.94
2	0	4	358.47	15.63
2	0	4	357.26	15.69
-2	0	4	334.04	15.62
2	0	4	343.68	15.62
2	0	-4	307.32	14.02
2	0	4	337.80	15.66
2	0	-4	311.50	13.98
2	0	-4	315.99	14.02
-2	0	4	375.79	15.69
-2	0	-4	338.32	14.07
-2	-1	4	345.58	15.48
-2	1	-4	391.68	17.13
-2	-1	4	366.64	15.52
2	-1	-4	380.11	17.08
-2	-1	4	353.34	15.48
2	1	4	323.54	15.43
-2	1	-4	420.28	17.18
-2	1	-4	370.75	17.04
2	1	4	350.80	15.42
2	1	4	336.66	15.47
-2	-1	4	327.90	15.40
2	-1	-4	377.96	17.04
2	1	4	336.44	15.45
2	-1	-4	374.84	17.01
-2	-1	4	333.73	15.38
-2	1	-4	375.12	17.01
-2	1	-4	377.19	17.02
2	-1	-4	383.58	17.08
2	1	4	359.87	15.53
-2	1	-4	390.12	17.06
-2	-1	4	349.83	15.38
2	-1	4	343.26	15.42

2	1	-4	373.20	17.07
2	-1	4	365.94	15.53
-2	-1	-4	377.69	17.01
-2	-1	-4	385.97	17.11
2	-1	4	348.28	15.46
2	1	-4	389.57	17.10
-2	1	4	357.09	15.49
2	1	-4	371.49	16.98
2	-1	4	353.02	15.41
-2	-1	-4	374.40	17.01
-2	1	4	343.79	15.44
-2	1	4	346.91	15.48
-2	1	4	336.85	15.46
-2	1	4	319.85	15.36
2	-1	4	377.76	15.58
-2	1	4	332.93	15.42
2	1	-4	383.49	17.09
-2	-1	-4	377.53	17.02
2	-1	4	320.47	15.44
2	1	-4	376.21	16.97
-2	-1	-4	384.17	17.08
-2	-1	-4	373.88	17.04
-2	-1	-4	366.50	17.03
2	-2	-4	252.97	11.42
-2	2	-4	246.19	11.38
2	2	4	270.57	12.27
-2	2	-4	278.26	11.54
2	2	4	261.34	12.25
2	2	4	273.98	12.32
-2	-2	4	276.98	12.33
-2	-2	4	250.33	12.24
2	2	4	258.37	12.31
2	-2	-4	238.79	11.31
2	2	4	286.95	12.36
-2	2	-4	249.91	11.31
2	-2	-4	255.26	11.43
-2	2	-4	267.32	11.38
-2	2	-4	252.97	11.31
-2	-2	4	265.15	12.24
2	-2	-4	253.15	11.32
2	2	4	277.54	12.20
-2	-2	4	287.99	12.37
-2	2	-4	253.13	11.35
-2	2	4	274.45	12.34

-2	2	4	267.78	12.23
2	2	-4	251.33	11.42
2	-2	4	261.66	12.25
2	-2	4	313.74	12.46
-2	-2	-4	257.31	11.41
-2	-2	-4	250.26	11.42
-2	-2	-4	241.56	11.34
-2	2	4	254.08	12.22
2	2	-4	258.58	11.33
2	-2	4	256.39	12.28
-2	-2	-4	246.90	11.28
-2	2	4	284.88	12.29
2	-2	4	277.45	12.29
2	2	-4	252.65	11.43
2	-2	4	289.94	12.39
-2	-2	-4	255.32	11.40
-2	2	4	272.40	12.28
-2	2	4	283.09	12.31
-2	2	4	262.67	12.34
2	2	-4	243.69	11.45
-2	-2	-4	245.27	11.36
-2	3	-4	24.91	1.20
-2	3	-4	23.62	1.41
2	3	4	21.10	1.18
-2	3	-4	24.62	1.46
-2	-3	4	21.50	1.22
-2	-3	4	19.53	1.19
2	-3	-4	27.83	1.24
2	3	4	19.77	1.09
2	3	4	22.88	1.24
2	-3	-4	22.39	1.27
2	-3	-4	24.59	1.39
-2	3	-4	26.28	1.31
2	3	4	22.49	1.01
-2	-3	4	22.16	1.27
2	3	4	22.77	1.23
-2	-3	4	19.23	1.10
-2	-3	4	20.54	1.10
2	3	4	19.92	1.12
2	-3	-4	22.70	1.33
-2	3	-4	24.95	1.32
-2	3	-4	23.87	1.27
-2	-3	-4	23.72	1.27
2	-3	4	20.04	1.10

-2	-3	-4	24.15	1.34
-2	3	4	21.38	1.15
-2	3	4	20.21	1.14
2	-3	4	23.85	1.35
-2	3	4	21.36	1.21
-2	3	4	21.55	1.09
-2	3	4	20.62	1.08
2	-3	4	20.98	1.14
-2	-3	-4	23.71	1.26
-2	3	4	20.02	1.27
2	-3	4	20.47	1.27
2	-3	4	19.47	1.14
2	3	-4	23.02	1.36
-2	-3	-4	26.38	1.36
-2	-3	-4	25.45	1.35
-2	3	4	20.60	1.16
2	3	-4	24.38	1.27
2	3	-4	22.27	1.38
2	-3	4	20.25	1.03
-2	-4	4	32.32	1.81
2	-4	-4	32.86	1.86
2	-4	-4	32.07	1.74
2	-4	-4	34.51	1.93
2	4	4	31.45	1.71
2	4	4	31.49	1.76
2	4	4	30.22	1.62
2	4	4	31.27	1.61
2	-4	-4	33.41	1.78
2	4	4	28.83	1.52
-2	4	-4	34.73	1.83
-2	-4	4	29.61	1.62
2	-4	-4	34.76	1.78
2	4	4	33.62	1.77
-2	4	-4	33.61	1.95
-2	-4	4	29.37	1.72
-2	4	-4	38.52	1.82
-2	4	-4	36.76	1.68
2	4	4	32.51	1.51
-2	4	-4	35.49	1.78
-2	-4	4	34.22	1.79
-2	-4	4	30.42	1.62
2	-4	-4	37.48	1.74
2	4	-4	36.16	1.81
-2	4	4	32.83	1.66

-2	-4	-4	32.72	1.84
2	-4	4	29.67	1.52
-2	4	4	27.49	1.67
2	-4	4	34.50	1.82
-2	4	4	29.35	1.81
-2	-4	-4	34.47	1.76
2	-4	4	35.12	1.90
-2	-4	-4	35.74	1.78
2	4	-4	31.07	1.93
-2	4	4	31.43	1.65
-2	-4	-4	34.46	1.98
2	4	-4	34.92	1.86
2	-4	4	31.53	1.68
-2	4	4	31.51	1.62
-2	4	4	34.17	1.80
2	4	-4	36.08	1.79
2	-4	4	30.51	1.62
2	-4	4	32.55	1.64
2	5	4	65.04	3.18
2	-5	-4	76.81	3.45
2	-5	-4	73.39	3.49
-2	-5	4	60.85	3.03
-2	-5	4	65.91	3.18
-2	5	-4	75.90	3.38
-2	5	-4	74.76	3.77
-2	-5	4	63.99	3.20
-2	5	-4	74.06	3.57
2	-5	-4	76.29	3.69
2	-5	-4	67.18	3.56
2	-5	-4	67.57	3.46
2	5	4	61.49	3.02
2	5	4	57.19	2.92
-2	5	-4	70.79	3.65
2	5	4	63.24	3.23
2	-5	-4	72.56	3.47
2	5	4	60.27	3.01
-2	-5	4	60.75	3.04
-2	-5	4	59.91	3.13
-2	5	-4	74.62	3.50
2	5	4	59.06	3.11
2	5	-4	68.86	3.68
-2	5	4	60.02	3.01
2	5	-4	71.80	3.54
-2	5	4	63.15	3.07

-2	-5	-4	76.22	3.59
-2	5	4	65.36	3.19
-2	5	4	61.53	3.01
-2	-5	-4	68.86	3.48
-2	-5	-4	78.27	3.64
-2	5	4	62.94	3.04
2	-5	4	65.54	3.22
2	-5	4	62.59	3.04
2	-5	4	63.12	3.06
2	-5	4	66.14	3.49
-2	-5	-4	71.86	3.35
2	5	-4	72.29	3.49
2	5	-4	70.51	3.58
2	-5	4	60.54	3.10
-2	-5	-4	70.41	3.46
-2	-5	-4	68.14	3.56
2	-6	-4	101.88	5.10
2	-6	-4	100.27	4.90
2	-6	-4	95.75	4.87
2	6	4	110.58	5.37
-2	6	-4	102.91	4.93
-2	-6	4	122.53	5.64
-2	6	-4	101.36	5.08
2	6	4	107.15	5.33
2	-6	-4	109.78	4.88
-2	-6	4	110.21	5.44
-2	-6	4	123.68	5.58
2	-6	-4	105.76	4.91
2	-6	-4	96.10	5.00
-2	-6	4	108.16	5.44
2	6	4	118.41	5.62
2	6	4	112.44	5.54
-2	6	-4	103.83	5.25
-2	-6	4	115.67	5.57
2	6	4	116.71	5.43
-2	6	-4	112.01	4.80
-2	-6	4	112.73	5.53
2	6	4	112.99	5.67
-2	6	-4	113.69	5.05
-2	6	4	115.23	5.43
-2	-6	-4	100.17	4.76
-2	-6	-4	114.39	5.10
-2	6	4	113.98	5.42
2	-6	4	124.11	5.51

2	-6	4	128.34	5.76
-2	-6	-4	97.38	4.99
2	-6	4	112.17	5.28
-2	-6	-4	100.78	4.87
-2	6	4	111.41	5.41
-2	-6	-4	99.28	4.91
2	-6	4	112.48	5.44
2	-6	4	104.36	5.60
-2	-6	-4	112.83	5.03
2	6	-4	104.65	4.99
-2	6	4	112.32	5.70
2	-6	4	120.16	5.46
-2	6	4	113.39	5.47
2	6	-4	99.50	5.00
2	6	-4	99.00	5.15
2	6	-4	102.97	4.92
2	-6	4	125.09	5.62
-2	6	4	120.94	5.65
-2	-7	4	183.56	8.47
-2	7	-4	168.76	8.27
-2	-7	4	165.75	8.25
-2	7	-4	167.78	8.40
2	-7	-4	192.79	8.08
2	7	4	168.28	8.12
2	-7	-4	190.28	8.37
2	-7	-4	161.49	8.19
2	7	4	173.26	8.22
-2	-7	4	170.81	8.25
2	7	4	167.91	8.49
2	7	4	183.34	8.45
2	-7	-4	166.71	8.04
-2	7	-4	177.07	8.11
2	-7	-4	177.82	8.06
2	7	4	175.77	8.35
2	-7	-4	169.76	8.08
-2	-7	4	194.18	8.38
2	7	4	177.00	8.16
-2	-7	4	181.44	8.39
-2	-7	4	167.20	7.99
-2	-7	4	176.93	8.35
-2	7	-4	172.13	8.45
-2	7	4	180.48	8.21
-2	-7	-4	186.54	8.29
2	-7	4	197.58	8.59

2	-7	4	185.40	8.26
-2	-7	-4	169.94	7.92
2	7	-4	174.16	8.09
2	-7	4	192.08	8.32
-2	7	4	172.99	8.22
-2	-7	-4	187.80	8.20
-2	7	4	168.65	8.21
-2	7	4	166.10	8.51
2	-7	4	167.26	8.29
-2	-7	-4	169.73	8.10
-2	-7	-4	166.26	8.17
2	-7	4	172.27	8.06
2	-7	4	190.24	8.41
2	7	-4	172.81	8.40
-2	7	4	188.10	8.48
2	7	-4	168.84	7.84
2	7	-4	164.12	8.16
-2	7	4	174.88	8.27
-2	-7	-4	166.60	8.02
2	-7	4	170.61	8.24
2	7	-4	181.26	8.20
2	-8	-4	151.10	6.44
-2	-8	4	119.57	5.96
-2	-8	4	129.10	6.24
2	-8	-4	133.96	6.40
2	8	4	117.31	5.93
2	8	4	121.93	6.09
-2	-8	4	113.89	5.98
-2	-8	4	126.17	6.09
-2	8	-4	142.48	6.64
-2	8	-4	131.04	6.57
-2	8	-4	143.30	6.28
2	8	4	131.86	6.24
2	-8	-4	128.00	6.36
-2	8	-4	136.85	6.88
2	-8	-4	131.10	6.43
2	-8	-4	141.22	6.74
2	8	4	120.39	5.85
2	-8	-4	136.84	6.40
-2	8	-4	136.51	6.66
2	8	4	118.63	6.28
-2	-8	4	129.72	6.15
2	-8	-4	129.35	6.56
2	8	4	125.72	5.87

-2	-8	4	137.97	6.11
-2	-8	-4	129.78	6.35
-2	8	4	117.02	5.94
2	-8	4	115.93	6.01
-2	-8	-4	135.13	6.46
2	8	-4	131.64	6.17
2	8	-4	137.01	6.58
-2	8	4	111.86	6.25
-2	-8	-4	126.26	6.54
2	-8	4	135.19	6.37
-2	8	4	130.78	6.23
-2	8	4	128.38	5.93
2	-8	4	133.83	6.16
-2	-8	-4	144.59	6.68
2	-8	4	120.37	5.75
2	-8	4	129.04	5.99
2	-8	4	135.90	6.08
2	8	-4	129.99	6.50
-2	8	4	123.84	6.01
2	8	-4	130.78	6.80
-2	-8	-4	144.78	6.58
2	-8	4	122.14	5.97
-2	-8	-4	131.41	6.26
-2	-9	4	77.35	4.24
-2	-9	4	86.05	4.21
2	9	4	74.35	3.92
2	-9	-4	76.72	3.75
2	9	4	75.38	3.92
2	-9	-4	76.90	4.16
-2	9	-4	78.68	4.08
2	-9	-4	71.72	3.68
-2	-9	4	73.81	4.08
2	-9	-4	73.12	3.77
-2	9	-4	73.40	4.24
-2	9	-4	75.89	4.01
2	-9	-4	66.23	3.84
2	-9	-4	74.19	3.77
-2	9	-4	75.63	3.59
2	9	4	79.24	4.66
2	9	4	76.01	4.40
-2	-9	4	81.08	4.17
2	9	4	76.39	4.15
-2	-9	4	78.45	4.05
2	-9	-4	78.18	3.79

-2	-9	4	77.50	3.69
2	9	-4	70.63	3.84
2	9	-4	77.57	4.00
-2	-9	-4	78.29	4.06
-2	9	4	75.45	4.42
2	9	-4	72.83	3.47
2	-9	4	85.78	4.08
-2	-9	-4	72.00	3.62
-2	-9	-4	74.50	3.81
2	-9	4	78.93	4.04
-2	-9	-4	68.84	3.66
-2	9	4	82.35	4.10
2	-9	4	88.95	4.21
-2	-9	-4	70.74	3.91
2	-9	4	73.36	4.11
2	9	-4	70.05	4.20
2	-9	4	73.41	3.76
2	-9	4	85.16	4.29
2	-9	4	82.55	4.54
-2	9	4	88.02	4.43
-2	-9	-4	77.72	3.98
-2	9	4	82.05	4.00
2	-10	-4	22.05	1.66
-2	10	-4	21.26	1.98
-2	10	-4	25.30	1.51
-2	-10	4	23.10	1.82
2	-10	-4	23.24	1.66
-2	-10	4	23.67	1.34
2	-10	-4	26.12	1.86
2	-10	-4	20.93	1.82
2	-10	-4	21.00	1.78
-2	10	-4	24.85	2.11
2	10	4	24.30	2.17
-2	-10	4	23.77	1.78
2	10	4	25.44	1.69
-2	-10	4	25.69	1.92
2	-10	-4	23.00	1.55
2	-10	-4	20.61	2.23
2	10	4	26.93	2.26
2	10	4	23.13	1.64
-2	10	-4	24.78	2.40
-2	-10	4	27.55	2.14
2	-10	4	23.28	1.41
-2	10	4	22.92	2.26

-2	10	4	26.12	2.39
2	10	-4	26.02	2.08
2	-10	4	29.14	1.87
-2	-10	-4	20.78	1.50
-2	-10	-4	25.69	1.86
-2	-10	-4	23.07	1.68
2	-10	4	23.94	1.85
-2	-10	-4	17.83	2.28
-2	-10	-4	22.96	1.55
2	10	-4	24.53	2.26
2	-10	4	28.16	2.42
-2	10	4	24.87	1.78
2	-10	4	27.06	2.11
2	10	-4	22.90	1.82
2	-10	4	22.66	1.74
-2	10	4	23.58	1.89
2	-11	-4	12.90	1.67
2	11	4	11.95	2.12
-2	11	-4	16.17	1.90
-2	11	-4	17.99	2.62
2	-11	-4	15.37	1.51
2	-11	-4	12.38	1.22
2	-11	-4	13.76	1.46
-2	11	-4	16.08	2.32
-2	-11	4	16.77	1.71
2	11	4	13.01	1.37
2	-11	-4	12.53	1.63
-2	-11	4	13.05	1.96
2	-11	-4	10.88	1.51
-2	-11	4	14.01	0.97
2	11	4	13.41	1.40
-2	-11	4	14.06	1.44
-2	11	4	14.62	2.23
2	-11	4	15.17	1.43
2	11	-4	14.78	2.14
-2	-11	-4	15.51	1.52
-2	11	4	15.12	1.71
-2	-11	-4	12.88	1.27
-2	11	4	14.97	2.28
-2	-11	-4	16.31	1.74
2	-11	4	14.98	1.55
2	-11	4	14.30	1.51
-2	-11	-4	14.93	1.49
2	-11	4	14.91	1.05

2	11	-4	11.79	1.58
-2	12	-4	11.95	1.66
2	-12	-4	11.95	1.60
-2	-12	4	10.37	1.51
-2	-12	4	7.66	1.46
2	-12	-4	6.61	1.59
2	-12	-4	9.65	1.57
2	12	4	10.17	2.06
2	-12	4	9.92	1.68
-2	12	4	9.20	1.83
2	-12	4	10.57	1.47
-2	-12	-4	10.13	1.56
2	12	-4	9.20	1.89
-2	-12	-4	9.97	1.21
-2	12	4	12.23	2.13
-2	-12	-4	11.52	1.72
2	12	-4	10.30	1.68
2	13	4	29.24	3.35
2	-13	-4	26.18	2.04
-2	-13	4	30.54	2.36
-2	-13	4	31.96	2.90
-2	13	-4	25.83	2.73
-2	13	-4	24.39	3.57
2	-13	-4	23.16	1.82
2	-13	4	29.28	2.31
-2	13	4	33.15	3.39
2	-13	4	37.24	2.63
-2	-13	-4	20.04	2.00
-2	-13	-4	26.52	2.51
2	13	-4	24.03	3.65
-3	0	-4	0.15	0.17
3	0	-4	-0.03	0.12
-3	0	-4	-0.05	0.11
3	0	-4	0.17	0.17
3	0	-4	-0.06	0.08
3	0	4	-0.10	0.11
-3	0	-4	0.19	0.12
-3	0	4	-0.04	0.16
3	0	4	0.13	0.21
3	0	4	0.05	0.13
3	0	-4	0.08	0.21
-3	0	4	0.08	0.20
-3	0	4	0.01	0.12
3	0	-4	0.02	0.06

-3	0	4	0.00	0.07
-3	0	4	-0.05	0.13
-3	0	-4	0.19	0.15
3	0	-4	0.04	0.18
-3	0	-4	0.17	0.23
-3	0	-4	-0.06	0.22
3	0	4	-0.03	0.15
3	0	4	0.18	0.15
-3	0	4	0.19	0.17
3	1	4	46.19	2.28
-3	1	-4	34.82	1.64
3	1	4	48.79	2.28
3	-1	-4	34.77	1.62
-3	-1	4	48.75	2.23
-3	1	-4	34.79	1.66
3	-1	-4	34.20	1.58
-3	1	-4	34.27	1.68
3	-1	-4	33.99	1.70
-3	-1	4	45.90	2.33
-3	-1	4	50.96	2.37
-3	-1	4	49.41	2.29
-3	1	-4	34.89	1.65
-3	1	-4	34.08	1.77
-3	-1	4	46.42	2.23
3	1	4	45.11	2.26
-3	-1	4	48.40	2.23
3	-1	-4	34.48	1.66
-3	1	-4	37.49	1.82
3	-1	-4	32.50	1.71
3	-1	4	50.54	2.27
3	1	-4	33.93	1.69
-3	1	4	49.30	2.30
-3	1	4	51.83	2.34
3	1	-4	34.28	1.71
-3	-1	-4	34.16	1.65
-3	-1	-4	33.71	1.73
-3	1	4	47.32	2.21
-3	-1	-4	31.58	1.64
3	1	-4	33.16	1.59
-3	-1	-4	33.65	1.66
-3	-1	-4	35.50	1.72
3	1	-4	33.63	1.73
3	-1	4	47.80	2.27
3	1	-4	34.77	1.59

-3	1	4	48.38	2.35
3	1	-4	34.66	1.61
-3	-1	-4	35.58	1.64
3	-1	4	48.52	2.38
3	-1	4	45.55	2.28
-3	1	4	48.38	2.26
3	-1	4	53.28	2.45
3	-1	4	48.66	2.30
-3	1	4	49.69	2.31
-3	2	-4	161.90	7.16
3	2	4	187.18	7.90
-3	2	-4	157.49	7.29
-3	2	-4	165.54	7.18
-3	2	-4	157.98	7.12
-3	-2	4	179.82	7.78
3	-2	-4	144.66	7.10
3	2	4	168.15	7.68
3	-2	-4	152.29	7.09
3	-2	-4	151.19	7.23
3	2	4	149.49	7.80
-3	2	-4	159.88	7.12
-3	-2	4	166.85	7.76
-3	2	-4	165.70	7.34
3	2	4	171.77	7.67
-3	-2	4	165.63	7.65
3	-2	-4	151.04	7.17
-3	-2	4	159.54	7.64
3	-2	-4	164.37	7.24
-3	-2	4	172.34	7.71
-3	2	-4	163.25	7.19
-3	2	4	178.02	7.75
3	2	-4	162.49	7.24
-3	2	4	181.71	7.76
3	2	-4	156.92	7.23
3	-2	4	164.39	7.71
3	2	-4	157.24	7.13
3	-2	4	190.45	7.88
-3	-2	-4	151.61	7.21
3	-2	4	174.48	7.80
-3	2	4	169.75	7.76
-3	2	4	162.23	7.63
3	2	-4	151.39	7.20
-3	2	4	174.93	7.71
-3	-2	-4	163.56	7.24

-3	2	4	166.87	7.62
3	-2	4	150.94	7.68
-3	-2	-4	161.12	7.18
-3	2	4	155.53	7.76
3	-2	4	174.39	7.69
-3	3	-4	185.69	8.13
3	3	4	199.84	9.17
3	-3	-4	168.56	8.18
3	-3	-4	177.72	8.25
3	-3	-4	187.21	8.30
3	3	4	204.74	9.18
-3	3	-4	179.88	8.33
-3	3	-4	188.67	8.40
3	3	4	194.83	9.26
-3	-3	4	210.72	9.25
-3	-3	4	210.08	9.29
3	-3	-4	158.50	8.18
-3	-3	4	191.81	9.15
-3	3	-4	184.16	8.19
-3	-3	4	193.14	9.27
3	3	4	206.46	9.28
3	-3	-4	187.44	8.10
-3	3	-4	192.71	8.19
-3	3	-4	180.51	8.23
-3	-3	4	195.06	9.17
3	3	4	196.71	9.26
-3	3	4	199.11	9.31
3	-3	4	194.76	9.18
3	3	-4	167.64	8.26
-3	-3	-4	175.50	8.23
3	3	-4	186.51	8.17
-3	3	4	192.83	9.15
-3	-3	-4	190.04	8.27
3	-3	4	220.93	9.41
3	-3	4	199.88	9.23
-3	3	4	208.53	9.21
-3	3	4	195.75	9.12
3	-3	4	212.56	9.33
3	-3	4	200.38	9.13
-3	-3	-4	187.44	8.23
-3	-3	-4	182.81	8.18
3	3	-4	177.52	8.26
-3	3	4	210.71	9.23
3	3	-4	170.97	8.38

3	-3	4	208.98	9.19
-3	3	4	212.71	9.29
-3	-3	-4	170.94	8.17
3	-4	-4	98.78	4.85
-3	-4	4	100.87	4.95
3	4	4	102.74	5.06
-3	4	-4	100.72	5.06
3	4	4	107.73	5.08
-3	4	-4	107.01	4.90
3	4	4	101.97	4.95
-3	-4	4	108.91	5.07
-3	4	-4	106.94	5.15
3	-4	-4	112.11	5.07
3	-4	-4	112.14	4.82
3	-4	-4	99.46	4.89
-3	4	-4	110.97	4.82
3	-4	-4	101.77	4.97
-3	-4	4	115.89	5.10
3	4	4	108.35	4.96
-3	-4	4	100.99	5.06
-3	4	-4	105.90	4.96
3	-4	-4	105.80	4.91
-3	-4	4	103.68	4.97
-3	-4	4	108.86	5.10
-3	4	-4	113.93	4.91
3	4	4	105.20	5.09
3	-4	4	106.36	5.12
-3	4	4	109.49	5.02
3	-4	4	113.87	5.22
3	4	-4	98.53	5.02
3	-4	4	103.93	4.97
-3	4	4	99.45	5.12
-3	-4	-4	96.14	4.93
-3	4	4	110.76	5.00
-3	4	4	112.24	5.10
-3	4	4	105.94	4.90
-3	-4	-4	100.77	4.87
-3	-4	-4	100.89	4.89
3	-4	4	110.47	4.97
-3	-4	-4	105.86	5.00
-3	-4	-4	109.35	4.98
3	4	-4	108.73	4.89
3	4	-4	104.78	4.96
-3	5	-4	5.91	0.54

3	5	4	6.02	0.81
-3	5	-4	6.30	0.89
3	-5	-4	5.18	0.59
3	-5	-4	6.06	0.56
-3	5	-4	5.22	0.82
-3	-5	4	5.41	0.84
3	-5	-4	4.62	0.78
3	-5	-4	5.09	0.76
-3	-5	4	6.00	0.62
3	-5	-4	5.00	0.56
-3	-5	4	6.67	1.00
3	5	4	6.50	0.75
-3	5	-4	6.13	0.68
-3	5	-4	6.15	0.67
-3	5	-4	5.00	0.72
-3	-5	4	6.22	0.76
3	-5	-4	5.66	0.71
3	5	4	6.53	0.65
3	5	4	5.63	0.74
-3	-5	4	5.95	0.61
-3	-5	4	6.67	0.82
3	5	4	6.91	0.64
-3	5	4	6.04	0.97
-3	-5	-4	4.96	0.60
-3	5	4	6.47	0.64
3	5	-4	6.41	0.65
3	-5	4	5.96	0.47
3	-5	4	6.19	0.61
-3	-5	-4	5.55	0.76
3	-5	4	7.68	0.67
-3	5	4	6.17	0.68
3	5	-4	5.19	0.79
-3	5	4	5.84	0.62
3	5	-4	5.99	0.73
3	-5	4	6.64	0.91
-3	5	4	5.46	0.74
3	-5	4	7.75	1.00
3	5	-4	6.46	0.67
-3	-5	-4	6.75	0.76
-3	5	4	6.27	0.85
-3	-5	-4	5.01	0.58
3	-5	4	5.98	0.61
-3	-5	-4	5.01	0.69
-3	-6	4	19.45	1.21

-3	6	-4	16.55	1.02
3	6	4	20.54	1.44
3	6	4	18.01	1.20
3	-6	-4	15.13	1.06
-3	6	-4	14.73	1.34
3	-6	-4	15.42	1.06
3	-6	-4	16.21	1.32
-3	-6	4	18.27	1.41
3	6	4	17.80	1.15
-3	-6	4	18.06	1.21
3	-6	-4	15.68	1.23
-3	-6	4	22.04	1.48
-3	-6	4	16.39	1.33
3	-6	-4	18.83	1.07
3	-6	-4	17.31	1.18
3	6	4	18.68	1.08
3	6	4	17.20	1.38
3	6	4	18.12	1.32
-3	6	-4	16.32	1.16
-3	-6	4	19.68	1.47
-3	6	-4	15.26	1.48
-3	6	-4	16.54	1.33
-3	-6	-4	15.41	1.20
3	6	-4	18.03	1.22
3	-6	4	19.13	1.48
-3	-6	-4	15.86	1.09
-3	-6	-4	17.82	1.26
-3	6	4	18.91	1.20
3	6	-4	15.87	1.32
-3	-6	-4	18.17	1.26
3	-6	4	18.49	1.26
3	-6	4	20.57	1.60
-3	-6	-4	14.61	0.91
3	-6	4	20.95	1.26
-3	6	4	17.50	1.46
-3	6	4	18.78	1.14
-3	6	4	22.30	1.46
3	6	-4	18.22	1.23
3	-6	4	17.05	1.17
3	6	-4	16.81	1.13
-3	6	4	20.02	1.31
3	-6	4	20.11	1.07
-3	6	4	19.41	1.25
-3	-6	-4	16.27	1.05

3	7	4	38.98	2.40
3	-7	-4	35.69	2.10
-3	-7	4	38.96	1.87
-3	-7	4	38.14	2.29
-3	7	-4	35.42	2.32
3	7	4	38.13	2.06
3	-7	-4	38.57	2.34
-3	-7	4	36.50	2.13
-3	-7	4	42.72	2.39
-3	-7	4	37.61	2.14
3	7	4	40.16	2.15
-3	-7	4	38.76	2.36
3	-7	-4	40.30	1.96
3	-7	-4	32.81	1.95
-3	7	-4	35.10	2.02
3	-7	-4	34.02	1.93
-3	7	-4	37.77	1.90
3	7	4	36.65	2.02
3	7	4	41.45	2.39
-3	-7	4	40.45	2.32
3	7	4	36.21	2.25
-3	7	-4	36.91	2.18
3	-7	-4	35.55	2.03
-3	7	-4	34.81	2.21
-3	-7	-4	39.16	2.17
3	-7	4	37.95	2.19
3	7	-4	34.36	2.01
3	-7	4	43.07	2.60
-3	-7	-4	33.84	2.10
3	-7	4	41.13	2.41
-3	7	4	38.71	2.22
-3	-7	-4	32.10	1.90
-3	7	4	37.61	2.14
-3	7	4	37.00	2.49
3	-7	4	36.87	1.96
-3	-7	-4	37.46	2.18
3	-7	4	42.43	2.24
-3	-7	-4	33.56	1.78
3	7	-4	36.34	2.06
3	7	-4	36.68	2.31
-3	7	4	41.26	2.38
3	7	-4	36.00	2.08
3	-7	4	36.31	2.11
-3	7	4	40.53	2.16

3	-7	4	42.58	2.15
-3	-7	-4	33.10	1.98
3	-8	-4	0.33	0.44
-3	-8	4	0.94	0.85
3	-8	-4	1.84	0.87
3	-8	-4	0.44	0.52
-3	8	-4	0.67	0.46
3	8	4	0.47	0.48
3	8	4	-0.02	0.48
3	-8	-4	0.95	0.62
3	8	4	0.47	0.42
-3	-8	4	0.68	0.74
3	-8	-4	1.03	0.58
3	8	4	-0.17	0.55
-3	8	-4	0.76	0.74
-3	-8	4	0.38	0.43
-3	-8	4	-0.23	0.50
3	-8	-4	0.03	0.46
3	8	4	-0.35	0.80
-3	8	-4	0.81	0.68
3	8	4	0.24	0.66
-3	-8	4	-0.48	0.71
-3	8	-4	0.61	0.82
3	-8	-4	0.26	0.46
-3	-8	4	0.32	0.59
3	-8	4	0.29	0.30
-3	-8	-4	2.72	0.82
3	8	-4	0.67	0.66
-3	8	4	0.89	0.61
-3	8	4	0.93	0.76
3	8	-4	1.12	0.31
3	8	-4	0.27	0.54
-3	8	4	1.16	0.61
3	8	-4	0.10	0.65
3	-8	4	0.62	0.52
-3	-8	-4	1.08	0.75
-3	8	4	0.08	0.79
3	-8	4	0.35	0.47
-3	8	4	0.61	0.57
3	-8	4	0.65	0.51
-3	-8	-4	0.81	0.41
-3	-8	-4	0.65	0.48
-3	-8	-4	-0.18	0.40
-3	-8	-4	0.42	0.59

3	-8	4	0.49	0.68
3	8	-4	1.48	0.58
3	-8	4	0.68	0.92
3	-8	4	-1.28	0.73
3	-9	-4	18.02	1.54
-3	-9	4	19.44	1.53
3	-9	-4	15.55	1.32
-3	-9	4	20.05	1.09
-3	9	-4	18.25	1.63
-3	-9	4	21.62	1.83
3	-9	-4	16.07	1.48
-3	-9	4	18.96	1.50
-3	9	-4	18.65	1.30
3	9	4	20.08	1.43
3	-9	-4	19.51	1.83
3	-9	-4	18.32	1.33
-3	-9	4	18.55	1.68
-3	9	-4	19.24	1.96
3	9	4	19.28	1.91
3	9	4	18.14	1.67
-3	9	-4	18.25	1.76
3	9	4	19.84	1.94
-3	-9	4	23.68	2.28
3	9	4	20.57	1.35
3	-9	-4	15.25	1.25
3	-9	-4	19.63	1.45
-3	9	4	21.94	1.68
3	9	-4	18.24	1.89
-3	9	4	17.43	1.57
-3	-9	-4	16.66	1.18
-3	-9	-4	15.99	1.73
3	-9	4	22.47	1.97
-3	-9	-4	17.20	1.51
3	-9	4	22.20	1.77
-3	-9	-4	15.52	1.27
3	-9	4	19.45	1.56
3	-9	4	19.26	1.20
3	-9	4	22.64	1.53
-3	9	4	17.32	1.78
3	9	-4	17.17	1.03
3	9	-4	19.72	1.68
3	-9	4	23.05	2.18
-3	-9	-4	18.80	1.43
3	9	-4	19.31	1.47

-3	9	4	22.88	2.05
3	-9	4	19.24	1.51
-3	10	-4	29.97	2.70
3	-10	-4	31.95	2.22
3	-10	-4	30.00	2.21
3	-10	-4	31.25	2.65
3	10	4	40.21	2.26
3	-10	-4	32.87	2.08
3	10	4	42.67	3.03
3	-10	-4	33.74	2.15
-3	-10	4	37.61	2.43
-3	-10	4	39.78	2.61
3	-10	-4	33.40	1.99
-3	-10	4	40.49	2.43
-3	10	-4	34.62	1.94
3	10	4	37.26	2.73
-3	-10	4	41.89	2.70
-3	10	-4	34.43	2.44
3	-10	-4	30.39	1.94
3	10	4	37.50	2.24
-3	10	-4	34.07	2.53
-3	-10	-4	33.82	2.25
-3	-10	-4	30.55	2.28
3	-10	4	40.61	2.62
3	-10	4	43.36	3.22
3	-10	4	37.51	2.07
3	10	-4	36.47	2.47
-3	10	4	41.13	2.96
3	-10	4	39.26	2.46
3	10	-4	31.78	2.15
-3	10	4	41.87	2.60
3	-10	4	40.56	2.41
3	-10	4	41.98	2.39
-3	10	4	33.86	2.75
-3	-10	-4	32.79	1.94
3	10	-4	32.97	2.69
-3	-10	-4	33.53	2.12
-3	10	4	38.76	2.53
-3	-11	4	46.69	2.97
-3	-11	4	44.13	2.74
-3	11	-4	57.36	3.40
3	-11	-4	56.12	3.06
3	-11	-4	44.19	3.01
3	-11	-4	47.83	3.05

3	-11	-4	53.89	2.98
3	11	4	43.99	2.55
3	-11	-4	55.11	2.89
-3	11	-4	45.21	3.65
3	11	4	45.23	2.52
3	11	4	39.61	3.22
-3	11	-4	47.95	3.50
3	-11	-4	49.98	2.76
-3	-11	4	58.31	3.95
3	-11	4	49.13	2.78
3	-11	4	46.04	2.39
3	11	-4	51.52	3.61
-3	-11	-4	52.75	3.16
-3	11	4	45.60	3.35
-3	11	4	40.50	3.25
-3	11	4	48.77	2.95
3	-11	4	43.08	2.81
3	11	-4	48.00	2.97
3	-11	4	52.15	2.79
3	-11	4	48.07	2.96
-3	-11	-4	52.02	2.97
-3	-11	-4	48.74	2.75
-3	-12	4	16.34	2.12
-3	12	-4	22.23	3.03
3	-12	-4	18.77	1.83
-3	-12	4	23.66	1.90
3	-12	-4	19.75	1.75
3	-12	-4	16.83	1.48
3	12	4	17.49	2.67
-3	12	-4	16.78	2.23
-3	12	4	17.58	2.41
3	-12	4	19.48	1.89
3	-12	4	22.56	1.85
3	12	-4	18.41	1.91
-3	-12	-4	15.49	1.45
-3	-12	-4	21.88	2.12
-3	12	4	21.28	2.13
-3	-12	-4	19.77	1.81
3	12	-4	14.38	2.68
-3	13	-4	12.84	2.44
3	13	4	9.05	2.39
3	-13	-4	11.00	1.54
3	-13	-4	7.38	1.36
-3	13	-4	2.52	2.08

-3	-13	4	11.09	1.77
-3	-13	4	8.63	1.56
3	-13	4	8.63	1.50
3	-13	4	8.92	1.69
3	13	-4	4.51	2.38
-3	-13	-4	10.64	1.67
-3	-13	-4	8.21	1.52
-4	0	4	123.08	5.81
4	0	-4	95.14	4.44
-4	0	-4	88.03	4.41
4	0	4	126.55	5.80
4	0	-4	95.84	4.33
-4	0	-4	95.33	4.36
4	0	-4	92.95	4.35
4	0	-4	97.93	4.38
-4	0	-4	97.06	4.41
-4	0	4	127.82	5.75
4	0	4	122.22	5.79
-4	0	4	131.46	5.79
4	0	4	128.50	5.81
-4	0	-4	96.95	4.46
4	0	-4	94.21	4.30
4	0	4	126.95	5.84
4	0	-4	96.98	4.39
-4	0	4	128.90	5.74
-4	0	-4	98.88	4.37
4	0	4	129.00	5.84
-4	0	4	132.36	5.89
4	1	4	67.88	3.46
-4	-1	4	76.14	3.50
-4	-1	4	77.03	3.36
-4	1	-4	46.26	2.14
-4	1	-4	46.97	2.30
4	-1	-4	45.67	2.07
-4	-1	4	70.99	3.46
-4	-1	4	74.03	3.32
-4	1	-4	43.94	2.13
-4	-1	4	76.27	3.36
4	-1	-4	45.56	2.10
-4	1	-4	44.99	2.16
-4	1	-4	48.20	2.15
4	1	4	66.11	3.45
4	-1	-4	40.42	2.19
-4	-1	4	71.54	3.35

4	1	4	68.06	3.37
-4	1	-4	42.26	2.24
4	1	4	74.39	3.41
4	1	-4	44.30	2.21
4	-1	4	71.99	3.41
4	1	-4	45.53	2.20
-4	1	4	79.12	3.44
4	-1	4	77.01	3.40
-4	1	4	78.90	3.50
4	1	-4	44.69	2.08
-4	1	4	72.78	3.34
-4	-1	-4	46.51	2.13
4	-1	4	73.05	3.42
-4	1	4	75.05	3.42
4	-1	4	73.72	3.62
-4	1	4	70.96	3.36
4	1	-4	43.82	2.08
4	1	-4	45.90	2.07
-4	-1	-4	45.63	2.14
-4	-1	-4	44.29	2.20
-4	-1	-4	41.15	2.13
-4	1	4	74.59	3.42
4	-1	4	73.51	3.50
-4	-1	-4	47.16	2.21
-4	2	-4	107.66	4.82
4	-2	-4	102.21	4.80
4	-2	-4	104.40	4.70
-4	2	-4	107.22	4.97
-4	-2	4	86.62	3.85
-4	-2	4	84.65	3.91
-4	-2	4	89.35	4.03
4	2	4	84.43	3.94
-4	2	-4	98.59	4.81
-4	2	-4	111.76	4.80
4	-2	-4	101.75	4.73
-4	-2	4	82.32	3.86
-4	-2	4	84.20	4.03
-4	2	-4	105.00	4.76
-4	2	-4	107.74	4.80
4	-2	-4	104.49	4.83
-4	2	-4	98.23	4.90
4	2	4	85.12	4.03
-4	-2	4	86.01	3.92
4	2	4	80.72	3.96

-4	2	4	83.45	3.85
-4	-2	-4	104.60	4.79
-4	2	4	83.77	3.89
4	2	-4	98.62	4.80
-4	2	4	89.79	4.03
4	-2	4	85.16	3.94
4	-2	4	80.64	3.93
4	2	-4	106.22	4.88
4	-2	4	84.84	4.06
4	2	-4	101.94	4.86
-4	2	4	83.21	4.01
-4	2	4	88.38	4.00
-4	2	4	91.84	4.01
-4	-2	-4	102.19	4.79
4	-2	4	73.72	4.01
-4	2	4	89.22	3.97
4	2	-4	106.52	4.75
-4	-2	-4	103.88	4.79
-4	-2	-4	104.88	4.84
4	-2	4	89.96	4.14
4	2	-4	103.27	4.73
-4	-2	-4	106.42	4.79
4	-3	-4	3.90	0.47
-4	-3	4	7.83	0.73
4	-3	-4	3.33	0.47
-4	3	-4	4.46	0.47
4	-3	-4	3.72	0.58
4	3	4	7.14	0.61
-4	3	-4	3.93	0.70
4	3	4	8.46	0.59
-4	3	-4	3.52	0.64
4	3	4	7.03	0.67
-4	3	-4	4.87	0.52
4	-3	-4	4.44	0.37
-4	-3	4	6.87	0.70
-4	-3	4	7.67	0.51
-4	3	-4	4.56	0.40
4	-3	-4	4.11	0.32
-4	-3	4	7.09	0.54
-4	-3	4	8.00	0.73
-4	-3	4	6.80	0.60
-4	3	-4	4.88	0.49
-4	-3	-4	4.57	0.44
-4	-3	-4	3.03	0.71

4	-3	4	7.79	0.59
4	3	-4	4.05	0.50
4	3	-4	4.09	0.42
-4	3	4	6.72	0.71
-4	-3	-4	4.24	0.44
4	-3	4	7.52	0.57
-4	3	4	7.65	0.69
4	-3	4	7.50	0.91
4	-3	4	8.06	0.60
4	3	-4	4.60	0.41
-4	3	4	6.76	0.76
-4	3	4	7.57	0.54
-4	-3	-4	4.62	0.52
4	3	-4	4.34	0.55
-4	3	4	8.07	0.64
4	-3	4	7.62	0.51
4	-3	4	8.12	0.79
-4	-4	4	11.90	0.88
-4	4	-4	12.87	0.75
4	4	4	10.77	0.78
4	4	4	11.23	0.90
-4	4	-4	10.14	0.99
4	-4	-4	12.26	1.01
-4	-4	4	10.12	0.88
-4	4	-4	12.61	0.83
4	-4	-4	10.32	0.79
-4	-4	4	11.66	0.97
-4	4	-4	12.26	1.13
-4	4	-4	11.07	0.81
-4	-4	4	10.78	0.77
4	-4	-4	13.04	0.92
4	-4	-4	13.26	0.69
4	4	4	10.80	0.89
4	-4	-4	11.48	0.76
-4	-4	4	11.26	0.73
-4	-4	4	11.56	0.96
4	4	4	12.46	0.92
4	-4	-4	11.63	0.89
-4	4	-4	12.40	0.90
-4	-4	-4	11.76	0.80
4	-4	4	11.92	0.83
4	-4	4	10.98	0.69
-4	-4	-4	11.49	0.85
-4	4	4	12.34	0.87

-4	-4	-4	11.54	0.80
-4	4	4	12.35	0.95
-4	-4	-4	12.62	0.98
4	-4	4	10.54	0.75
4	4	-4	11.89	0.80
4	-4	4	12.81	1.14
4	-4	4	11.55	1.02
-4	4	4	11.20	0.77
-4	4	4	11.81	0.91
-4	-4	-4	13.98	0.92
4	-4	4	12.21	0.78
4	4	-4	12.17	0.86
-4	4	4	11.43	1.05
4	4	-4	10.86	0.91
-4	4	4	11.18	0.70
-4	5	-4	51.25	2.51
4	-5	-4	49.96	2.68
4	-5	-4	46.81	2.46
4	-5	-4	46.08	2.43
4	-5	-4	52.79	2.36
4	5	4	41.59	2.34
-4	5	-4	50.44	2.65
4	5	4	41.83	2.32
4	5	4	45.98	2.35
-4	-5	4	41.78	2.17
-4	-5	4	44.35	2.32
-4	-5	4	39.56	2.18
-4	5	-4	52.63	2.42
-4	5	-4	49.61	2.69
4	-5	-4	47.44	2.58
4	-5	-4	51.13	2.55
-4	-5	4	42.67	2.35
-4	-5	4	41.06	2.32
4	5	4	41.88	2.18
4	5	4	43.21	2.15
-4	-5	4	42.36	2.38
-4	5	-4	50.07	2.47
-4	5	4	42.55	2.27
-4	-5	-4	50.99	2.63
4	-5	4	44.13	2.27
4	5	-4	45.43	2.62
-4	5	4	40.98	2.09
4	5	-4	48.50	2.47
-4	5	4	44.72	2.24

4	-5	4	45.07	2.17
-4	5	4	42.19	2.17
-4	5	4	43.85	2.46
-4	-5	-4	46.96	2.52
-4	-5	-4	52.69	2.58
-4	-5	-4	48.40	2.48
-4	-5	-4	46.45	2.44
4	-5	4	40.74	2.09
4	-5	4	47.31	2.55
-4	5	4	46.26	2.39
4	5	-4	48.62	2.54
4	-5	4	42.65	2.40
4	-5	4	40.58	2.17
4	6	4	30.51	1.90
4	6	4	29.79	1.94
-4	-6	4	30.85	1.91
-4	-6	4	32.77	1.94
-4	-6	4	30.49	1.74
4	-6	-4	27.38	1.38
4	-6	-4	22.58	1.55
-4	6	-4	27.53	1.45
-4	-6	4	34.58	2.00
-4	6	-4	23.61	1.69
-4	6	-4	26.85	1.53
4	-6	-4	24.33	1.42
4	6	4	31.81	1.70
-4	6	-4	27.07	1.51
-4	6	-4	25.48	1.59
-4	-6	4	29.88	1.74
4	6	4	32.42	1.95
4	-6	-4	23.46	1.43
-4	-6	4	31.88	1.98
-4	6	-4	24.27	1.82
4	-6	-4	26.12	1.58
4	-6	-4	24.47	1.76
4	6	4	30.12	1.74
-4	-6	-4	24.40	1.57
-4	-6	-4	26.27	1.63
-4	6	4	33.04	1.79
4	-6	4	29.64	1.63
-4	-6	-4	23.34	1.46
4	-6	4	35.46	2.19
-4	-6	-4	26.93	1.66
-4	-6	-4	24.67	1.42

4	-6	4	31.92	2.00
4	6	-4	24.00	1.55
4	6	-4	24.53	1.48
4	-6	4	31.76	1.75
-4	6	4	31.59	1.87
4	-6	4	31.95	1.86
4	6	-4	22.28	1.66
4	6	-4	24.53	1.54
-4	6	4	32.68	1.76
-4	6	4	32.23	1.99
4	-6	4	30.10	1.73
4	-7	-4	58.49	2.75
4	-7	-4	54.55	2.90
-4	7	-4	60.58	3.05
-4	7	-4	60.35	2.77
-4	-7	4	60.00	3.03
4	7	4	55.00	3.13
4	-7	-4	52.74	2.80
4	-7	-4	55.47	2.74
4	7	4	53.01	3.03
-4	-7	4	52.62	2.85
-4	7	-4	56.97	3.13
-4	7	-4	56.31	3.31
4	-7	-4	52.78	2.96
-4	-7	4	53.52	2.87
-4	7	-4	52.68	2.87
-4	-7	4	57.42	3.09
4	-7	-4	52.64	2.81
4	7	4	55.98	3.08
-4	-7	4	50.72	3.00
4	7	4	55.32	2.87
-4	-7	4	55.61	3.05
4	-7	-4	56.04	3.22
4	7	4	54.00	2.78
4	-7	4	59.27	2.93
4	-7	4	54.54	3.11
4	-7	4	52.94	2.95
-4	7	4	52.95	2.86
-4	7	4	51.53	3.16
4	-7	4	61.99	2.86
4	7	-4	53.53	3.12
4	7	-4	53.93	2.86
4	7	-4	57.97	2.95
-4	7	4	54.66	2.97

4	-7	4	64.98	3.36
4	-7	4	55.88	2.86
4	7	-4	54.30	2.89
4	-7	4	54.23	2.71
-4	-7	-4	51.17	2.91
-4	7	4	58.52	2.89
-4	-7	-4	53.19	2.78
-4	-7	-4	57.11	3.00
-4	7	4	57.90	3.09
-4	-7	-4	54.22	2.87
-4	-7	-4	56.83	3.03
-4	-8	4	42.78	2.70
4	-8	-4	45.65	2.62
4	-8	-4	43.32	2.64
4	8	4	44.72	2.31
-4	-8	4	42.50	2.47
4	-8	-4	43.50	2.42
-4	-8	4	46.05	2.78
-4	8	-4	47.24	2.83
4	8	4	44.55	2.66
4	-8	-4	45.38	2.51
-4	8	-4	46.53	2.95
4	-8	-4	44.05	2.46
-4	8	-4	50.34	2.46
4	8	4	45.43	2.80
4	-8	-4	48.78	2.95
-4	-8	4	48.46	2.68
-4	-8	4	43.04	2.66
4	8	4	48.68	2.80
-4	8	-4	51.48	2.80
-4	-8	4	44.13	2.50
4	-8	-4	50.46	2.49
4	8	4	43.46	2.39
-4	8	4	46.97	2.53
-4	-8	-4	48.57	2.79
-4	-8	-4	46.90	2.56
4	8	-4	47.43	2.66
-4	8	4	42.37	2.49
4	-8	4	50.64	2.95
-4	8	4	41.76	2.88
-4	-8	-4	45.00	2.64
-4	8	4	45.94	2.66
-4	-8	-4	45.87	2.44
4	-8	4	47.61	2.47

4	8	-4	43.93	2.88
4	-8	4	49.14	2.81
4	-8	4	47.25	2.50
4	-8	4	43.13	2.29
-4	-8	-4	48.64	2.77
-4	8	4	50.45	2.87
4	-8	4	52.18	2.64
4	8	-4	44.09	2.55
4	-8	4	43.10	2.54
4	-9	-4	55.27	2.74
4	-9	-4	50.88	2.79
-4	-9	4	38.80	2.46
4	9	4	42.51	2.27
-4	9	-4	55.24	2.69
4	-9	-4	45.82	2.87
4	9	4	39.39	2.63
-4	9	-4	56.42	3.14
4	-9	-4	45.67	2.91
4	9	4	40.78	2.33
-4	-9	4	47.96	2.68
-4	-9	4	43.31	2.48
-4	9	-4	51.59	3.12
4	9	4	45.30	2.84
4	9	4	41.62	2.83
4	-9	-4	45.67	2.69
4	-9	-4	51.87	2.69
-4	-9	4	44.79	2.67
-4	9	-4	51.08	3.36
4	-9	-4	57.16	3.32
-4	9	4	42.45	2.64
-4	9	4	40.64	2.93
-4	-9	-4	56.44	3.30
-4	-9	-4	48.23	2.94
4	-9	4	41.25	2.21
4	-9	4	45.39	3.11
-4	-9	-4	50.86	2.86
-4	9	4	41.81	2.49
4	-9	4	47.13	2.44
4	-9	4	44.57	2.60
4	9	-4	52.12	2.99
4	-9	4	45.23	2.80
-4	-9	-4	48.97	2.68
4	9	-4	50.72	2.82
4	-9	4	42.42	2.56

4	9	-4	49.76	3.25
-4	9	4	45.21	2.92
-4	-10	4	12.96	1.76
-4	10	-4	10.68	1.78
4	-10	-4	9.48	1.34
4	-10	-4	11.75	1.16
4	10	4	10.48	1.70
4	10	4	9.40	1.20
4	-10	-4	9.82	1.59
-4	-10	4	10.95	1.32
-4	10	-4	9.20	1.20
-4	-10	4	9.06	1.34
4	10	4	12.41	1.05
4	-10	-4	10.43	1.23
4	10	4	13.64	1.89
4	-10	-4	9.76	1.07
-4	10	-4	10.12	1.75
4	-10	-4	14.22	2.04
-4	10	-4	10.20	1.66
4	-10	-4	13.02	1.30
-4	-10	-4	12.40	1.55
-4	-10	-4	9.93	1.09
4	-10	4	13.01	1.64
-4	10	4	11.19	1.86
4	-10	4	14.51	1.33
-4	10	4	12.85	2.05
4	-10	4	12.23	1.37
4	-10	4	13.70	2.26
4	-10	4	14.15	1.33
-4	10	4	13.46	1.62
4	10	-4	10.28	1.32
-4	-10	-4	11.26	1.30
4	10	-4	12.04	1.67
-4	-10	-4	11.89	1.57
4	-10	4	12.00	0.96
4	-11	-4	6.90	1.18
-4	-11	4	5.89	1.23
-4	11	-4	5.42	1.74
4	-11	-4	4.61	0.86
-4	-11	4	5.71	0.99
4	11	4	5.71	1.47
4	11	4	4.18	0.82
4	11	4	6.14	0.79
-4	11	-4	4.63	1.39

4	-11	-4	3.90	1.03
-4	11	-4	6.52	1.33
4	-11	-4	4.45	0.92
4	-11	4	4.73	0.89
4	-11	4	4.64	0.70
4	-11	4	6.79	1.29
4	-11	4	4.66	1.25
-4	-11	-4	5.17	0.93
4	11	-4	5.45	1.53
-4	-11	-4	4.99	1.15
4	-11	4	6.36	1.05
-4	11	4	5.98	1.17
-4	11	4	5.38	1.53
4	11	-4	5.11	0.99
-4	-11	-4	4.67	0.82
-4	12	-4	3.54	1.79
-4	12	-4	6.37	1.47
4	-12	-4	4.79	0.91
4	-12	-4	6.26	1.28
4	-12	-4	6.58	0.97
4	-12	-4	4.27	1.11
4	12	4	3.27	1.67
-4	-12	4	2.37	0.96
-4	-12	4	4.62	1.29
4	-12	4	2.81	0.88
4	-12	4	4.20	0.70
-4	-12	-4	5.56	1.31
-4	-12	-4	4.79	0.90
-4	12	4	2.61	1.66
4	12	-4	5.54	1.69
4	-12	4	5.37	1.04
-4	12	4	3.77	1.36
4	12	-4	6.79	1.14
-4	-12	-4	4.21	1.13
-4	-13	4	7.58	1.71
-4	-13	4	11.28	1.56
4	-13	-4	7.67	1.15
-4	13	-4	8.07	1.69
4	13	4	11.03	2.53
4	-13	-4	7.03	1.13
4	13	-4	5.42	2.16
-4	-13	-4	10.14	1.88
-4	13	4	12.42	2.20
4	-13	4	8.88	1.27

-4	-13	-4	10.05	1.59
5	0	4	0.03	0.12
-5	0	4	-0.05	0.15
-5	0	-4	-0.23	0.22
-5	0	-4	0.00	0.25
5	0	-4	0.36	0.20
-5	0	4	-0.03	0.10
5	0	4	-0.15	0.16
5	0	4	0.17	0.20
5	0	-4	0.09	0.20
-5	0	4	0.00	0.16
-5	0	-4	-0.20	0.12
-5	0	-4	-0.01	0.16
5	0	4	-0.02	0.23
5	0	-4	0.27	0.16
5	0	-4	0.06	0.13
-5	0	4	0.14	0.11
5	0	4	0.27	0.22
5	0	-4	0.11	0.12
5	0	-4	-0.03	0.13
-5	0	4	0.03	0.19
-5	0	-4	-0.04	0.14
5	0	4	0.13	0.13
-5	0	4	0.37	0.26
-5	0	-4	-0.11	0.19
-5	-1	4	59.45	2.72
5	1	4	58.52	2.77
-5	1	-4	51.74	2.41
5	-1	-4	48.06	2.46
-5	-1	4	61.46	2.86
5	-1	-4	50.54	2.35
5	-1	-4	50.81	2.40
-5	-1	4	57.52	2.71
-5	1	-4	47.93	2.49
5	-1	-4	51.21	2.40
-5	-1	4	57.83	2.71
5	1	4	54.86	2.73
5	1	4	56.78	2.82
-5	1	-4	53.56	2.56
5	-1	-4	46.65	2.39
-5	1	-4	51.91	2.40
-5	1	-4	49.67	2.41
5	1	4	56.31	2.75
-5	1	-4	52.51	2.39

5	-1	-4	51.20	2.33
5	1	4	58.98	2.77
-5	1	4	58.61	2.76
-5	-1	-4	51.42	2.37
5	1	-4	48.90	2.36
5	-1	4	57.73	2.76
5	1	-4	46.98	2.56
-5	-1	-4	45.74	2.39
5	-1	4	57.52	2.75
5	-1	4	58.07	2.77
5	1	-4	50.65	2.35
-5	-1	-4	51.04	2.45
-5	1	4	61.78	2.78
-5	1	4	55.81	2.71
5	1	-4	51.49	2.30
-5	1	4	61.93	2.87
-5	1	4	61.67	2.77
5	-1	4	58.15	2.85
-5	1	4	59.42	2.70
-5	-1	-4	50.71	2.41
-5	-1	-4	52.02	2.48
-5	-1	-4	52.18	2.40
5	-1	4	60.04	2.76
5	1	-4	50.31	2.45
5	2	4	58.40	2.88
-5	2	-4	57.68	2.67
-5	2	-4	58.08	2.69
-5	2	-4	57.25	2.65
5	2	4	56.87	2.79
-5	-2	4	60.23	2.71
5	-2	-4	54.63	2.70
-5	-2	4	59.14	2.70
-5	2	-4	59.74	2.70
-5	2	-4	59.04	2.66
5	-2	-4	55.29	2.61
-5	-2	4	62.47	2.89
-5	-2	4	57.18	2.67
-5	-2	4	56.84	2.87
5	2	4	59.03	2.78
-5	2	-4	54.44	2.81
5	-2	-4	53.76	2.68
-5	-2	4	57.36	2.88
5	2	4	57.26	2.68
-5	2	-4	56.39	2.85

-5	-2	4	55.30	2.68
5	-2	4	59.55	2.77
5	-2	4	60.03	2.98
-5	2	4	58.27	2.73
5	2	-4	56.15	2.63
-5	2	4	58.67	2.85
5	2	-4	54.77	2.59
5	2	-4	54.16	2.74
-5	-2	-4	53.54	2.67
-5	2	4	58.95	2.85
5	-2	4	60.53	2.91
-5	-2	-4	58.07	2.68
-5	2	4	62.42	2.84
5	2	-4	51.51	2.65
-5	-2	-4	57.70	2.66
5	-2	4	56.07	2.78
-5	2	4	60.23	2.88
-5	-2	-4	59.87	2.68
-5	2	4	59.49	2.81
-5	-2	-4	52.94	2.59
5	-2	4	55.15	2.79
-5	-2	-4	56.67	2.70
-5	-3	4	310.59	14.55
-5	-3	4	316.93	14.32
5	3	4	311.83	14.56
5	-3	-4	276.86	13.40
5	3	4	300.20	14.48
5	-3	-4	290.77	13.51
-5	3	-4	293.51	13.62
5	-3	-4	288.44	13.48
-5	3	-4	311.84	13.48
5	3	4	320.03	14.43
5	-3	-4	311.43	13.54
-5	-3	4	313.58	14.32
-5	3	-4	313.24	13.44
5	3	4	315.03	14.44
5	-3	-4	310.84	13.59
-5	-3	4	305.71	14.36
-5	-3	4	340.32	14.54
-5	3	-4	316.81	13.44
-5	3	-4	315.40	13.51
-5	-3	4	329.18	14.43
-5	3	-4	307.73	13.69
-5	-3	4	313.80	14.43

-5	-3	4	327.63	14.58
5	3	-4	290.38	13.41
5	3	-4	280.65	13.49
-5	3	4	339.57	14.49
-5	3	4	307.38	14.40
5	-3	4	298.39	14.43
5	3	-4	299.06	13.43
5	-3	4	312.56	14.48
-5	-3	-4	283.94	13.45
-5	-3	-4	283.66	13.46
-5	3	4	342.20	14.54
-5	-3	-4	289.82	13.48
5	3	-4	304.93	13.51
-5	3	4	317.68	14.56
5	-3	4	350.77	14.69
5	-3	4	328.55	14.41
5	-3	4	333.17	14.59
5	-3	4	309.31	14.41
-5	-3	-4	287.45	13.45
-5	3	4	328.63	14.53
-5	-3	-4	310.54	13.58
-5	4	-4	193.19	8.52
5	4	4	175.84	8.59
-5	-4	4	182.37	8.40
-5	4	-4	179.72	8.74
-5	-4	4	177.06	8.59
5	4	4	171.80	8.54
-5	4	-4	190.69	8.46
5	-4	-4	198.28	8.68
5	-4	-4	189.40	8.57
5	-4	-4	177.69	8.50
-5	-4	4	194.67	8.49
-5	-4	4	180.98	8.46
-5	4	-4	199.64	8.58
-5	-4	4	187.87	8.62
5	4	4	183.63	8.43
5	4	4	188.38	8.46
-5	-4	4	180.36	8.29
5	-4	-4	182.50	8.57
5	-4	-4	175.41	8.44
-5	-4	4	190.76	8.56
-5	4	-4	176.61	8.67
5	-4	4	169.00	8.44
-5	-4	-4	192.68	8.64

-5	4	4	202.80	8.61
-5	-4	-4	180.54	8.52
5	-4	4	195.92	8.63
-5	4	4	186.64	8.44
5	4	-4	190.74	8.48
5	4	-4	171.10	8.57
-5	-4	-4	176.35	8.47
-5	4	4	178.00	8.63
-5	-4	-4	182.23	8.51
5	4	-4	189.44	8.54
5	-4	4	194.67	8.73
-5	4	4	199.64	8.51
5	-4	4	175.30	8.50
-5	4	4	189.96	8.56
5	-4	4	182.64	8.41
-5	-4	-4	193.20	8.53
5	4	-4	191.25	8.48
-5	5	-4	96.22	4.45
-5	5	-4	100.58	4.61
5	-5	-4	97.32	4.32
-5	-5	4	93.23	4.84
-5	-5	4	96.38	4.82
-5	-5	4	103.42	4.77
5	5	4	92.63	4.83
-5	5	-4	105.88	4.46
-5	5	-4	87.09	4.68
5	-5	-4	96.26	4.56
-5	-5	4	96.09	4.71
-5	-5	4	97.91	4.65
5	-5	-4	90.14	4.44
-5	-5	4	102.17	4.89
5	5	4	97.34	4.69
5	-5	-4	97.50	4.71
5	5	4	104.94	4.84
5	5	4	96.14	4.65
-5	5	-4	98.83	4.52
5	5	4	95.82	4.83
5	-5	-4	90.19	4.48
-5	5	-4	93.85	4.82
5	-5	-4	89.42	4.57
5	-5	4	107.61	4.66
5	-5	4	96.19	4.70
-5	5	4	93.15	4.91
-5	5	4	101.48	4.69

5	5	-4	95.72	4.53
-5	-5	-4	89.75	4.49
5	-5	4	103.26	5.00
-5	5	4	107.96	4.88
-5	5	4	105.54	4.75
-5	-5	-4	91.91	4.45
5	-5	4	96.68	4.77
5	-5	4	105.22	4.90
5	-5	4	98.14	4.63
-5	5	4	97.36	4.80
5	5	-4	87.13	4.62
5	5	-4	95.54	4.48
-5	-5	-4	98.43	4.65
-5	-5	-4	100.92	4.55
-5	-5	-4	88.49	4.51
-5	6	-4	42.17	2.16
5	6	4	28.17	1.72
-5	-6	4	32.77	1.88
-5	-6	4	29.12	1.74
5	6	4	28.05	1.87
5	-6	-4	42.95	2.42
5	6	4	29.56	1.90
-5	-6	4	35.94	2.02
-5	-6	4	32.19	2.00
5	-6	-4	39.01	2.09
-5	6	-4	40.01	2.37
5	-6	-4	36.11	2.19
-5	6	-4	42.28	2.27
-5	-6	4	30.23	1.71
-5	6	-4	41.81	2.05
5	-6	-4	40.45	2.21
5	-6	-4	35.85	2.05
5	6	4	32.29	1.93
5	-6	-4	37.71	1.97
5	-6	-4	43.00	1.96
5	6	4	31.10	1.69
-5	-6	4	28.06	1.89
-5	6	-4	39.57	2.46
-5	6	4	34.14	1.82
5	6	-4	40.98	2.13
5	-6	4	32.29	2.34
5	-6	4	31.70	2.02
5	6	-4	37.30	2.30
5	-6	4	29.05	1.72

5	-6	4	33.00	1.70
5	-6	4	31.16	1.83
-5	-6	-4	37.39	2.12
-5	6	4	29.80	2.12
-5	6	4	34.16	2.00
-5	-6	-4	39.23	2.29
5	-6	4	30.88	1.63
-5	-6	-4	35.69	2.04
5	6	-4	40.27	2.17
-5	-6	-4	41.57	2.22
-5	6	4	29.92	1.87
-5	6	4	32.58	1.76
-5	-6	-4	39.04	2.16
-5	-7	4	49.10	2.86
5	7	4	52.02	2.60
-5	-7	4	56.16	2.86
-5	-7	4	55.46	2.97
5	-7	-4	52.45	2.80
5	-7	-4	52.35	3.35
5	7	4	47.13	2.67
5	-7	-4	53.33	2.95
5	-7	-4	57.26	2.69
-5	7	-4	52.53	3.12
5	7	4	54.27	2.91
5	-7	-4	61.72	2.71
5	-7	-4	54.45	2.82
-5	-7	4	47.77	2.64
-5	7	-4	52.47	2.87
5	7	4	47.33	2.85
5	7	4	45.41	2.88
-5	7	-4	58.74	2.78
5	-7	-4	55.48	2.94
-5	-7	4	53.24	2.71
-5	7	-4	52.95	3.23
-5	7	-4	59.37	3.04
-5	-7	4	52.58	2.85
5	7	-4	58.14	3.13
-5	7	4	56.08	2.99
5	-7	4	46.55	2.65
-5	-7	-4	55.59	2.79
-5	7	4	50.67	2.68
-5	7	4	50.51	2.82
-5	7	4	56.18	2.75
5	7	-4	50.60	3.08

5	-7	4	57.74	3.19
-5	-7	-4	57.03	2.97
-5	-7	-4	56.42	3.05
5	-7	4	49.78	2.77
5	-7	4	49.68	2.55
-5	-7	-4	53.04	2.86
5	-7	4	52.16	2.63
5	7	-4	54.54	2.86
5	7	-4	52.91	2.84
-5	7	4	50.58	3.01
5	-7	4	52.73	2.72
-5	-7	-4	53.23	2.89
5	-7	4	53.41	2.96
-5	-8	4	31.77	2.17
-5	-8	4	31.69	1.94
-5	-8	4	33.97	2.16
5	8	4	32.20	2.17
-5	8	-4	29.72	2.41
-5	-8	4	31.25	2.23
-5	-8	4	31.35	1.96
5	-8	-4	34.44	2.42
-5	8	-4	36.50	2.24
-5	8	-4	35.61	1.89
5	-8	-4	29.78	1.85
5	-8	-4	31.03	1.91
5	-8	-4	33.27	2.10
5	-8	-4	33.31	1.89
5	8	4	35.61	2.25
5	8	4	32.09	2.24
-5	8	-4	31.84	2.27
5	8	4	32.46	1.86
5	-8	-4	32.63	1.80
5	-8	-4	32.94	2.08
-5	-8	-4	31.56	1.85
5	-8	4	30.87	1.91
-5	8	4	30.45	1.96
5	-8	4	34.75	1.90
5	-8	4	34.21	2.41
5	-8	4	32.38	1.76
-5	-8	-4	33.13	2.13
-5	8	4	35.51	2.35
-5	8	4	31.95	2.44
5	-8	4	32.78	2.32
5	8	-4	29.37	2.23

5	8	-4	35.60	1.99
-5	-8	-4	32.82	2.21
5	-8	4	36.17	2.08
-5	-8	-4	30.64	2.00
5	-8	4	31.39	2.03
-5	8	4	35.15	2.18
-5	8	4	31.43	2.01
5	8	-4	35.34	2.09
-5	-8	-4	33.56	1.98
5	9	4	17.96	1.87
-5	9	-4	19.54	1.41
5	-9	-4	16.14	1.50
5	9	4	17.16	1.31
5	-9	-4	17.91	1.96
-5	-9	4	17.32	1.59
-5	-9	4	15.44	1.40
5	-9	-4	17.68	1.31
5	-9	-4	15.22	1.64
5	9	4	18.26	1.63
5	-9	-4	18.80	1.35
-5	9	-4	17.42	1.81
-5	9	-4	16.29	2.02
5	-9	-4	16.96	1.37
5	-9	-4	17.54	1.22
-5	-9	4	17.08	1.72
-5	9	-4	18.47	1.70
5	9	4	15.62	1.72
5	-9	4	16.70	1.13
-5	9	4	20.87	1.96
-5	9	4	16.51	1.63
5	9	-4	16.72	1.41
5	9	-4	18.90	1.63
5	-9	4	16.85	1.31
5	-9	4	16.63	1.40
5	9	-4	17.67	1.78
-5	9	4	15.12	1.50
-5	-9	-4	17.20	1.30
5	-9	4	16.43	1.37
-5	9	4	16.49	1.83
5	-9	4	20.33	1.60
5	-9	4	17.91	2.19
-5	-9	-4	16.84	1.51
5	-10	-4	84.95	4.38
5	10	4	95.00	5.16

5	-10	-4	96.12	4.82
5	-10	-4	89.79	4.92
5	10	4	88.00	4.56
-5	-10	4	93.68	4.75
5	-10	-4	80.41	4.28
-5	10	-4	83.25	5.02
-5	-10	4	89.16	4.94
5	-10	-4	93.02	4.29
-5	10	-4	94.52	4.31
5	-10	-4	89.29	4.24
-5	10	-4	83.15	4.64
5	-10	-4	79.75	4.53
-5	-10	4	93.18	4.80
-5	-10	4	96.07	4.86
-5	10	-4	89.42	4.83
5	-10	4	101.49	4.70
5	-10	4	93.32	4.75
5	-10	4	90.85	4.83
-5	10	4	87.67	5.25
-5	-10	-4	81.04	4.25
5	-10	4	90.93	4.51
5	-10	4	103.08	4.85
5	10	-4	91.52	4.62
5	-10	4	99.74	5.43
-5	-10	-4	84.76	4.45
-5	-10	-4	82.53	4.48
-5	10	4	93.94	4.97
-5	10	4	98.02	5.20
5	10	-4	81.95	4.35
5	10	-4	82.02	4.87
-5	11	-4	76.94	5.21
-5	11	-4	56.38	4.17
-5	11	-4	64.78	3.99
5	11	4	59.97	3.27
5	-11	-4	64.83	3.33
-5	-11	4	63.78	3.75
5	-11	-4	58.77	3.63
5	-11	-4	74.11	5.45
5	11	4	60.69	3.99
5	-11	-4	63.24	3.49
-5	-11	4	64.08	3.53
5	-11	4	62.40	3.58
-5	11	4	54.77	4.05
5	11	-4	61.63	4.11

-5	-11	-4	67.42	3.81
5	-11	4	70.48	3.37
5	-11	4	66.41	3.49
-5	-11	-4	64.10	3.58
5	11	-4	64.80	3.49
5	-11	4	57.80	3.12
-5	11	4	61.25	3.70
-5	-11	-4	61.73	3.32
-5	12	-4	31.09	3.38
-5	12	-4	39.59	3.10
5	12	4	34.95	3.11
-5	-12	4	38.54	2.49
5	-12	-4	34.55	2.25
5	-12	-4	30.61	2.52
-5	-12	4	38.03	2.95
5	-12	-4	37.03	2.50
-5	12	4	32.36	3.85
5	-12	4	33.19	2.38
5	12	-4	38.21	2.42
5	-12	4	38.77	2.42
-5	-12	-4	34.84	2.18
-5	-12	-4	38.30	2.50
-5	-12	-4	39.82	2.83
5	-12	4	34.24	2.04
-5	12	4	37.77	2.85
5	12	-4	35.49	3.60
5	-13	-4	32.12	2.23
5	-13	-4	32.03	2.37
5	13	4	27.51	3.32
-5	13	-4	40.41	3.54
-5	-13	4	27.90	2.75
-5	-13	4	26.87	2.15
-5	-13	-4	33.11	2.41
5	-13	4	29.10	2.17
-5	13	4	29.55	2.99
-5	-13	-4	33.02	3.05
5	13	-4	28.08	3.59
6	0	4	170.88	8.12
-6	0	-4	173.02	7.65
6	0	4	183.05	8.17
6	0	-4	170.88	7.52
6	0	-4	169.80	7.54
6	0	4	177.67	8.19
-6	0	4	185.87	8.15

6	0	-4	160.97	7.53
-6	0	4	176.79	8.10
-6	0	-4	167.29	7.67
6	0	4	181.53	8.16
-6	0	-4	164.67	7.61
6	0	-4	163.99	7.58
-6	0	-4	176.77	7.61
-6	0	4	172.39	8.16
-6	0	-4	167.46	7.56
6	0	-4	167.53	7.68
-6	0	-4	155.92	7.66
6	0	4	177.98	8.20
-6	0	4	179.99	8.18
-6	0	4	192.13	8.27
6	1	4	27.09	1.41
-6	-1	4	27.01	1.47
-6	1	-4	18.77	1.07
6	-1	-4	19.98	1.02
-6	1	-4	21.57	1.11
6	-1	-4	19.72	1.04
-6	1	-4	21.90	1.26
-6	-1	4	26.57	1.35
-6	1	-4	20.72	1.07
6	1	4	28.64	1.41
-6	-1	4	26.30	1.35
-6	-1	4	28.85	1.52
-6	1	-4	17.89	1.18
6	-1	-4	19.15	1.05
6	1	4	28.00	1.48
6	1	4	24.57	1.38
6	-1	-4	19.52	1.09
6	1	4	27.49	1.37
6	-1	-4	20.72	1.09
6	1	4	30.13	1.52
-6	-1	4	27.05	1.36
-6	-1	4	26.78	1.49
6	-1	4	26.76	1.38
6	-1	4	28.16	1.42
-6	1	4	27.85	1.39
6	1	-4	19.73	1.03
-6	-1	-4	21.08	1.04
-6	1	4	27.60	1.42
-6	-1	-4	20.25	1.13
6	-1	4	27.84	1.51

-6	-1	-4	17.85	1.09
6	1	-4	20.28	0.95
-6	1	4	25.49	1.35
-6	-1	-4	19.65	1.09
6	-1	4	28.07	1.41
6	1	-4	19.24	1.14
6	1	-4	20.10	1.01
-6	1	4	26.71	1.38
-6	1	4	28.43	1.53
-6	1	4	27.54	1.34
-6	-1	-4	21.12	1.10
-6	1	4	26.64	1.47
-6	-1	-4	21.76	1.18
-6	2	-4	69.83	3.23
-6	-2	4	62.25	3.02
6	2	4	62.43	3.11
6	-2	-4	67.91	3.25
-6	-2	4	62.56	3.20
-6	-2	4	67.71	2.95
6	2	4	68.63	3.18
-6	2	-4	70.04	3.43
-6	2	-4	63.64	3.38
6	2	4	64.39	3.21
-6	-2	4	62.77	3.06
6	-2	-4	64.61	3.24
6	2	4	65.59	3.01
-6	-2	4	70.37	3.22
-6	-2	4	67.62	3.03
6	2	4	62.55	3.09
-6	2	-4	73.65	3.26
-6	2	-4	66.75	3.22
-6	2	4	66.30	3.22
6	-2	4	64.20	3.11
-6	-2	-4	70.81	3.20
-6	-2	-4	68.74	3.25
6	2	-4	67.69	3.20
6	-2	4	69.67	3.36
-6	2	4	65.11	3.20
-6	2	4	65.76	3.14
6	2	-4	68.06	3.19
6	-2	4	64.39	3.11
-6	-2	-4	61.92	3.17
-6	2	4	67.01	3.16
-6	-2	-4	68.47	3.25

6	-2	4	66.26	3.08
-6	-2	-4	72.55	3.36
6	2	-4	68.05	3.13
-6	2	4	66.83	3.14
6	2	-4	70.58	3.30
-6	-2	-4	69.28	3.25
6	-2	4	64.41	3.25
-6	2	4	64.60	3.05
-6	-2	-4	70.24	3.25
-6	-3	4	19.29	1.30
6	3	4	20.52	1.12
6	-3	-4	21.35	1.29
-6	-3	4	20.13	1.00
6	-3	-4	22.22	1.18
-6	3	-4	25.13	1.29
-6	3	-4	23.69	1.24
-6	-3	4	18.88	1.25
-6	-3	4	19.03	1.10
6	3	4	17.86	1.13
6	3	4	18.13	1.24
6	-3	-4	21.47	1.25
-6	3	-4	23.40	1.31
-6	3	-4	20.90	1.43
-6	-3	4	19.24	1.25
-6	-3	4	19.94	1.01
-6	-3	4	20.60	1.00
-6	3	-4	21.99	1.52
-6	3	-4	23.83	1.24
-6	-3	4	19.84	1.06
-6	3	4	21.12	1.09
-6	-3	-4	22.41	1.28
6	3	-4	23.64	1.30
-6	-3	-4	21.68	1.25
-6	3	4	20.28	1.29
6	-3	4	19.52	1.16
6	-3	4	20.57	1.33
-6	-3	-4	23.26	1.23
-6	3	4	19.82	1.18
6	3	-4	23.70	1.17
-6	-3	-4	23.96	1.28
-6	-3	-4	22.15	1.39
6	-3	4	18.09	1.10
6	-3	4	21.05	1.43
6	3	-4	20.76	1.25

-6	3	4	21.08	1.24
6	3	-4	21.20	1.29
-6	3	4	21.23	1.24
6	-3	4	20.12	1.10
6	-3	4	19.73	1.08
-6	-3	-4	22.29	1.25
-6	4	-4	1.56	0.40
-6	4	-4	1.60	0.51
6	-4	-4	1.77	0.32
6	4	4	1.38	0.36
6	-4	-4	2.03	0.49
-6	-4	4	1.66	0.35
-6	-4	4	1.95	0.51
-6	4	-4	2.00	0.72
-6	-4	4	1.36	0.22
-6	-4	4	1.09	0.44
-6	-4	4	1.21	0.49
-6	-4	4	1.27	0.23
6	-4	-4	1.46	0.53
6	4	4	1.38	0.46
-6	-4	4	1.35	0.36
6	-4	-4	0.92	0.31
6	4	4	1.44	0.36
-6	4	-4	1.99	0.37
-6	4	-4	2.10	0.44
-6	-4	4	1.13	0.26
6	-4	-4	1.52	0.37
-6	4	-4	1.28	0.34
6	4	4	1.12	0.37
6	-4	4	0.90	0.29
-6	4	4	1.33	0.50
-6	4	4	1.40	0.40
-6	-4	-4	1.77	0.38
6	4	-4	2.03	0.40
6	-4	4	0.97	0.34
6	-4	4	1.15	0.32
6	-4	4	1.57	0.62
6	-4	4	1.35	0.38
-6	-4	-4	1.49	0.38
6	-4	4	1.40	0.53
-6	-4	-4	1.69	0.47
6	4	-4	1.28	0.35
-6	4	4	1.12	0.40
6	4	-4	1.34	0.34

6	4	-4	1.35	0.41
-6	-4	-4	1.56	0.37
-6	-4	-4	1.27	0.35
-6	4	4	1.38	0.33
-6	4	4	1.74	0.48
-6	-5	4	72.42	3.48
-6	-5	4	69.89	3.71
-6	5	-4	67.63	3.23
6	-5	-4	61.61	3.13
-6	5	-4	65.49	3.14
6	5	4	73.13	3.55
6	-5	-4	63.78	3.20
-6	-5	4	72.63	3.76
-6	5	-4	60.62	3.41
6	5	4	75.42	3.51
-6	-5	4	71.47	3.56
6	-5	-4	64.22	3.29
6	-5	-4	67.29	3.31
-6	-5	4	75.44	3.58
-6	-5	4	65.08	3.78
-6	5	-4	71.06	3.17
-6	5	-4	63.20	3.53
-6	5	-4	71.87	3.33
6	-5	-4	64.95	3.42
6	5	4	72.34	3.68
-6	-5	4	73.26	3.37
6	5	4	69.39	3.71
6	-5	4	73.10	3.55
-6	-5	-4	63.63	3.21
6	5	-4	67.81	3.19
-6	-5	-4	65.86	3.23
6	-5	4	76.06	3.77
6	-5	4	78.07	3.91
6	5	-4	65.50	3.19
6	5	-4	61.60	3.32
-6	5	4	78.71	3.73
6	-5	4	68.41	3.60
6	-5	4	73.41	3.49
-6	5	4	80.24	3.62
-6	-5	-4	61.85	3.21
-6	5	4	75.52	3.55
6	5	-4	66.65	3.23
6	-5	4	76.63	3.49
-6	-5	-4	66.46	3.38

-6	-5	-4	61.64	3.16
-6	5	4	72.66	3.68
-6	5	4	70.48	3.80
6	6	4	3.77	0.72
6	-6	-4	6.04	1.00
-6	-6	4	4.94	0.91
-6	6	-4	8.56	0.88
-6	6	-4	7.44	0.98
6	6	4	3.68	0.71
6	6	4	3.60	0.65
-6	-6	4	3.00	0.72
-6	-6	4	3.40	0.58
-6	6	-4	5.45	1.08
-6	-6	4	4.26	0.93
6	-6	-4	6.55	0.70
6	6	4	4.24	0.85
-6	-6	4	4.63	0.83
-6	6	-4	8.17	0.79
6	-6	-4	5.81	1.09
6	-6	-4	7.33	0.69
-6	-6	4	4.03	0.65
6	-6	-4	7.45	0.87
-6	6	-4	7.99	0.79
6	6	4	4.21	0.68
6	-6	4	3.85	0.64
6	-6	4	4.12	0.59
-6	-6	-4	7.24	0.77
6	6	-4	7.81	0.85
-6	6	4	3.36	0.69
-6	6	4	5.54	0.96
-6	6	4	3.89	0.71
-6	-6	-4	5.95	1.02
-6	-6	-4	6.26	0.83
6	-6	4	5.51	0.92
-6	-6	-4	8.10	0.76
6	-6	4	5.24	1.00
-6	6	4	4.33	0.90
6	6	-4	7.07	0.83
-6	6	4	4.72	0.82
6	6	-4	7.26	0.49
6	6	-4	6.86	0.80
6	-6	4	4.24	0.59
-6	-6	-4	7.06	0.88
6	-6	4	3.12	0.52

6	6	-4	6.32	0.98
-6	7	-4	12.16	1.10
6	-7	-4	11.16	0.78
-6	-7	4	7.02	0.85
6	-7	-4	10.10	1.00
-6	-7	4	8.45	1.17
6	7	4	8.54	1.12
-6	7	-4	12.17	1.49
6	7	4	8.76	0.84
6	-7	-4	9.67	0.82
-6	-7	4	9.50	1.14
-6	7	-4	11.90	0.99
-6	-7	4	8.42	1.23
6	-7	-4	9.32	0.89
-6	-7	4	8.79	0.92
6	7	4	7.75	1.14
6	-7	-4	9.69	1.41
-6	-7	4	7.05	1.10
6	7	4	9.24	1.11
6	7	4	7.99	0.92
6	-7	-4	10.56	1.23
6	-7	-4	8.53	0.89
-6	7	-4	10.62	1.34
-6	7	-4	12.35	1.14
-6	7	4	10.07	1.06
6	-7	4	8.96	0.87
6	-7	4	8.13	0.95
6	-7	4	6.84	1.34
-6	-7	-4	11.23	1.28
-6	7	4	10.17	0.98
6	-7	4	8.93	0.86
-6	7	4	6.67	1.14
6	7	-4	9.62	1.27
6	7	-4	9.77	0.99
6	-7	4	11.18	1.13
6	-7	4	9.27	1.39
-6	-7	-4	9.94	1.07
-6	-7	-4	10.91	1.14
-6	7	4	7.73	1.74
-6	7	4	7.68	1.13
6	-7	4	8.66	0.73
-6	-7	-4	10.16	0.91
6	7	-4	9.83	0.96
-6	-7	-4	10.55	1.00

6	-8	-4	2.25	0.68
-6	8	-4	3.53	0.91
-6	-8	4	1.74	0.55
-6	-8	4	2.74	0.82
6	8	4	1.21	0.85
-6	-8	4	1.23	0.56
6	-8	-4	2.57	0.88
6	-8	-4	3.29	0.56
6	-8	-4	3.57	0.52
6	8	4	2.09	0.55
-6	8	-4	3.41	1.11
6	8	4	1.39	0.79
6	-8	-4	3.02	1.06
6	-8	-4	2.83	0.60
-6	8	-4	4.11	0.66
6	-8	-4	2.65	0.57
-6	8	-4	2.92	0.89
-6	-8	4	2.45	0.84
6	8	4	0.98	0.68
6	-8	4	1.24	0.43
6	8	-4	2.12	0.92
-6	8	4	0.52	0.64
6	-8	4	1.02	0.86
-6	8	4	1.22	0.86
-6	8	4	1.94	0.96
6	-8	4	1.39	0.47
6	-8	4	1.22	0.61
-6	8	4	1.58	0.74
-6	8	4	1.87	0.62
6	-8	4	1.24	1.07
-6	-8	-4	2.81	0.71
6	8	-4	3.70	0.69
-6	-8	-4	2.72	0.89
-6	-8	-4	2.60	0.60
6	-8	4	1.58	0.71
6	-8	4	1.62	0.49
-6	-8	-4	3.10	0.60
-6	-9	4	6.02	1.08
6	9	4	5.65	0.96
6	9	4	6.34	1.13
-6	9	-4	5.63	1.12
-6	-9	4	4.87	0.83
-6	9	-4	7.79	1.48
-6	-9	4	6.86	1.13

-6	-9	4	5.52	0.86
6	9	4	7.34	1.15
6	9	4	5.31	1.19
6	-9	-4	4.94	1.04
6	-9	-4	4.60	0.77
-6	9	-4	5.15	1.35
6	-9	-4	6.94	1.35
-6	9	-4	5.63	0.87
6	-9	-4	4.90	0.86
6	-9	-4	5.21	0.88
6	-9	-4	5.28	1.09
6	-9	4	5.04	1.03
-6	9	4	6.06	1.30
6	-9	4	6.51	1.41
-6	-9	-4	6.18	1.00
-6	9	4	2.98	1.15
6	-9	4	5.78	0.89
-6	-9	-4	7.02	0.92
6	9	-4	5.06	1.19
6	-9	4	8.07	1.07
-6	9	4	4.79	1.01
6	-9	4	4.90	0.91
6	9	-4	6.00	1.02
6	-9	4	5.22	0.75
-6	-9	-4	4.97	0.89
6	9	-4	8.20	1.28
-6	-10	4	4.27	1.13
6	-10	-4	3.91	1.45
-6	10	-4	6.91	1.23
6	-10	-4	3.34	0.93
6	-10	-4	5.57	0.90
-6	-10	4	2.93	0.83
6	-10	-4	4.67	0.81
6	-10	-4	6.61	1.04
-6	10	-4	5.96	1.42
-6	10	-4	4.74	1.25
-6	-10	4	5.29	1.12
6	10	4	5.76	0.84
6	10	4	2.74	1.07
6	10	4	5.05	1.26
6	-10	-4	5.39	0.86
-6	-10	4	5.05	1.14
-6	10	-4	5.21	1.52
6	10	-4	5.82	1.13

-6	10	4	3.09	1.29
6	10	-4	4.69	1.32
-6	10	4	4.66	1.08
6	-10	4	6.45	1.22
6	10	-4	5.82	1.11
6	-10	4	4.48	0.68
6	-10	4	3.96	0.78
-6	-10	-4	5.17	0.95
-6	-10	-4	5.18	0.78
6	-10	4	5.66	1.05
-6	-10	-4	4.91	0.96
6	-10	4	3.06	1.46
6	-10	4	5.68	1.01
-6	11	-4	3.60	1.62
6	-11	-4	2.25	0.70
-6	-11	4	2.44	0.93
6	-11	-4	2.32	0.94
6	11	4	1.62	1.25
6	-11	-4	3.65	0.89
-6	-11	4	1.63	1.13
-6	11	-4	1.32	1.26
6	-11	4	2.83	0.64
6	-11	4	3.21	0.84
6	11	-4	5.02	0.97
6	-11	4	2.84	0.95
-6	-11	-4	2.06	1.03
6	11	-4	4.24	1.50
-6	-11	-4	2.07	0.94
6	-11	4	2.91	0.93
-6	11	4	1.43	1.29
-6	-11	-4	2.69	0.73
-6	-12	4	0.44	0.85
-6	12	-4	0.68	1.38
-6	12	-4	-0.93	2.13
-6	-12	4	0.28	1.32
6	-12	-4	2.61	0.90
6	12	4	1.21	1.58
6	-12	-4	1.73	0.84
6	-12	-4	1.37	0.97
-6	-12	-4	0.40	0.82
-6	-12	-4	-0.32	0.69
6	12	-4	1.13	1.76
-6	-12	-4	1.97	1.37
6	-12	4	1.29	0.98

6	-12	4	-0.43	0.72
6	-12	4	-0.27	0.55
-6	12	4	3.49	1.39
-6	-13	4	2.24	0.95
6	13	4	4.96	1.91
6	-13	-4	1.74	0.93
-6	13	-4	3.53	2.01
6	-13	-4	3.34	0.94
-6	-13	4	4.42	1.43
-6	-13	-4	4.33	1.53
6	-13	4	0.98	0.91
-6	13	4	0.05	1.58
-6	-13	-4	3.59	1.04
6	13	-4	3.98	1.77
7	0	-4	0.14	0.16
-7	0	4	0.17	0.16
7	0	4	0.31	0.23
-7	0	4	-0.04	0.25
7	0	-4	0.16	0.16
-7	0	4	0.02	0.25
-7	0	4	0.10	0.35
7	0	-4	0.25	0.28
-7	0	-4	-0.08	0.26
-7	0	-4	0.00	0.22
7	0	-4	0.03	0.16
-7	0	4	-0.08	0.20
7	0	4	0.15	0.18
-7	0	-4	-0.26	0.24
7	0	4	-0.02	0.28
7	0	4	0.20	0.16
7	0	4	0.01	0.21
-7	0	-4	-0.10	0.28
7	0	-4	0.23	0.16
-7	0	-4	0.03	0.19
7	0	4	0.17	0.34
-7	0	-4	0.08	0.16
-7	-1	4	40.30	1.92
-7	-1	4	39.68	2.05
-7	-1	4	37.93	2.02
-7	1	-4	36.61	1.80
-7	1	-4	38.30	1.83
7	1	4	38.11	1.95
-7	-1	4	39.83	2.08
7	-1	-4	34.57	1.75

7	-1	-4	37.11	1.79
-7	-1	4	41.52	1.94
7	1	4	42.50	2.09
7	1	4	38.38	1.92
-7	1	-4	35.45	1.80
-7	-1	4	37.53	1.91
-7	1	-4	37.12	1.78
7	1	4	41.10	1.98
7	1	4	37.76	1.97
7	-1	-4	31.20	1.80
-7	1	-4	33.33	1.92
-7	1	-4	37.00	1.82
7	1	4	42.31	2.08
7	-1	-4	34.88	1.80
7	-1	4	39.16	1.94
-7	1	4	41.36	2.01
-7	1	4	38.57	1.92
-7	1	4	39.07	2.05
-7	1	4	38.82	1.93
7	-1	4	39.98	1.99
-7	1	4	36.86	1.91
7	1	-4	36.00	1.87
-7	1	4	40.08	2.10
7	1	-4	36.51	1.64
-7	-1	-4	37.48	1.83
7	-1	4	39.37	1.97
-7	-1	-4	36.76	1.82
-7	-1	-4	37.94	1.73
7	1	-4	36.46	1.75
-7	-1	-4	32.83	1.82
-7	-1	-4	35.41	1.81
-7	1	4	41.24	1.93
-7	1	4	41.67	1.91
7	1	-4	35.44	1.69
7	-1	4	38.91	2.06
7	2	4	161.65	6.92
-7	-2	4	142.53	6.93
-7	2	-4	135.88	6.07
-7	2	-4	127.40	6.05
7	2	4	139.66	6.80
7	-2	-4	127.72	6.08
7	2	4	146.61	6.95
7	2	4	150.62	6.75
-7	-2	4	129.42	6.79

7	-2	-4	131.35	6.12
-7	-2	4	150.78	6.81
-7	-2	4	153.38	6.77
7	-2	-4	131.08	6.01
-7	2	-4	123.91	6.21
-7	-2	4	138.44	6.79
-7	-2	4	161.63	6.96
7	2	4	152.54	6.83
-7	2	-4	138.12	6.09
-7	-2	4	152.17	6.96
-7	2	-4	141.07	6.08
7	-2	-4	134.15	6.07
-7	2	-4	133.66	6.25
-7	2	-4	132.78	6.05
-7	2	4	159.90	6.88
7	2	-4	122.37	5.98
-7	-2	-4	141.99	6.20
-7	-2	-4	126.59	6.07
-7	-2	-4	134.54	6.00
-7	2	4	141.98	6.71
-7	2	4	151.28	6.96
7	-2	4	151.26	6.81
-7	-2	-4	133.07	6.08
-7	2	4	148.51	6.72
7	-2	4	149.63	6.95
-7	-2	-4	132.05	6.08
-7	-2	-4	129.58	6.05
-7	2	4	148.36	6.78
-7	-2	-4	121.78	6.00
-7	2	4	159.05	6.86
7	-2	4	152.48	6.85
-7	2	4	144.79	6.87
7	2	-4	136.64	6.13
7	-2	4	143.84	6.84
-7	3	-4	245.66	10.70
7	-3	-4	219.49	10.64
-7	3	-4	228.48	10.88
-7	3	-4	250.21	10.74
7	3	4	265.94	11.79
-7	3	-4	247.16	10.75
7	-3	-4	230.59	10.74
7	3	4	252.96	11.93
7	3	4	277.09	11.88
-7	-3	4	251.85	11.69

-7	-3	4	259.97	11.70
-7	-3	4	248.16	11.92
-7	-3	4	245.09	11.78
-7	-3	4	276.52	11.91
7	3	4	247.53	11.81
7	-3	-4	228.86	10.72
-7	3	-4	231.32	10.68
-7	-3	4	263.83	11.67
-7	3	-4	237.48	10.93
-7	-3	4	269.43	11.96
-7	3	-4	235.01	10.66
-7	-3	4	254.04	11.64
-7	3	4	285.75	11.86
-7	3	4	218.89	11.85
-7	3	4	277.89	11.87
-7	3	4	257.29	11.91
7	3	-4	242.26	10.61
-7	-3	-4	243.94	10.73
-7	-3	-4	228.40	10.72
-7	-3	-4	234.30	10.66
-7	-3	-4	232.43	10.71
7	3	-4	243.12	10.76
7	-3	4	246.17	11.82
-7	-3	-4	232.08	10.69
-7	3	4	280.05	11.95
7	-3	4	266.98	11.95
-7	-3	-4	249.92	10.86
-7	3	4	258.45	11.90
7	-3	4	263.35	11.74
7	-3	4	280.59	12.07
7	3	-4	231.07	10.66
7	-3	4	242.00	11.80
7	3	-4	222.19	10.68
-7	-4	4	287.04	12.55
-7	-4	4	271.61	12.33
7	4	4	260.71	12.51
-7	-4	4	279.88	12.64
-7	-4	4	270.80	12.32
-7	4	-4	255.83	12.05
-7	-4	4	263.91	12.45
7	4	4	260.01	12.61
-7	-4	4	281.55	12.38
7	-4	-4	253.85	11.90
7	-4	-4	249.95	11.85

7	-4	-4	238.85	11.77
7	4	4	276.34	12.53
7	-4	-4	271.66	12.02
7	4	4	277.58	12.42
-7	4	-4	262.88	12.11
7	4	4	269.84	12.45
-7	4	-4	253.66	11.76
-7	4	-4	277.18	11.92
7	-4	-4	264.49	11.93
-7	-4	4	268.94	12.31
-7	-4	4	264.75	12.61
-7	4	-4	279.11	11.83
-7	4	-4	268.06	11.87
7	-4	4	261.69	12.45
-7	4	4	301.30	12.59
7	-4	4	265.24	12.42
7	-4	4	277.77	12.62
-7	4	4	272.52	12.42
7	-4	4	274.85	12.52
7	4	-4	269.79	11.87
-7	-4	-4	254.54	11.82
-7	4	4	273.05	12.62
-7	4	4	282.47	12.60
7	4	-4	267.62	11.78
-7	-4	-4	245.14	11.85
-7	-4	-4	259.83	11.81
7	4	-4	247.21	11.87
7	-4	4	256.47	12.42
-7	4	4	290.93	12.51
-7	-4	-4	249.09	11.82
-7	-4	-4	271.62	12.01
7	-4	4	299.43	12.77
7	4	-4	268.56	11.82
7	5	4	161.41	7.92
7	5	4	170.42	7.69
7	-5	-4	162.11	7.74
-7	5	-4	171.10	7.83
-7	-5	4	162.99	7.91
7	-5	-4	175.70	8.03
-7	-5	4	164.67	7.55
-7	5	-4	163.12	8.11
7	-5	-4	168.67	7.89
7	-5	-4	162.27	7.80
-7	-5	4	164.35	7.63

-7	5	-4	182.85	7.92
7	5	4	161.68	7.73
-7	-5	4	164.26	7.75
-7	5	-4	184.84	7.79
7	-5	-4	162.97	7.88
-7	5	-4	164.72	8.03
-7	-5	4	173.55	7.72
-7	-5	4	165.01	7.57
7	5	4	157.40	7.82
-7	-5	4	165.29	7.93
-7	5	-4	161.75	7.70
-7	5	4	166.89	7.71
7	-5	4	180.36	8.10
-7	-5	-4	169.29	7.79
-7	-5	-4	163.07	7.82
7	-5	4	166.29	7.81
7	5	-4	171.11	7.81
-7	-5	-4	165.38	7.80
7	-5	4	159.82	7.73
-7	-5	-4	174.25	8.00
7	5	-4	176.16	7.79
7	5	-4	173.28	7.78
-7	5	4	185.58	7.81
-7	5	4	169.16	7.89
-7	-5	-4	165.90	7.77
-7	5	4	177.53	7.90
7	5	-4	155.89	7.88
-7	5	4	165.39	7.97
7	-5	4	170.80	7.64
7	-5	4	167.86	7.91
7	-5	4	163.89	7.69
7	6	4	20.59	1.58
-7	6	-4	19.77	1.32
-7	6	-4	18.41	1.57
-7	6	-4	20.48	1.44
-7	6	-4	17.39	1.69
-7	6	-4	20.86	1.25
-7	-6	4	20.66	1.64
-7	-6	4	21.82	1.15
-7	-6	4	21.56	1.37
-7	-6	4	23.37	1.68
7	-6	-4	18.15	1.59
-7	-6	4	21.25	1.29
7	6	4	20.84	1.31

7	-6	-4	17.72	1.22
-7	-6	4	24.37	1.43
7	-6	-4	18.61	1.17
7	6	4	22.13	1.37
7	-6	-4	18.43	1.46
-7	6	-4	19.29	1.20
7	6	4	19.35	1.47
7	-6	-4	17.54	1.33
-7	-6	4	19.55	1.58
7	-6	4	20.95	1.66
7	-6	4	22.87	1.87
7	-6	4	21.48	1.34
-7	6	4	18.75	1.76
-7	-6	-4	19.40	1.29
-7	6	4	20.66	1.38
7	6	-4	19.16	1.29
7	6	-4	19.37	1.00
-7	6	4	20.96	1.57
-7	-6	-4	18.08	1.21
7	-6	4	20.69	1.26
7	6	-4	17.20	1.39
7	-6	4	21.42	1.44
-7	-6	-4	19.47	1.30
7	6	-4	20.43	1.32
-7	6	4	23.02	1.62
7	-6	4	25.03	1.26
-7	-6	-4	18.07	1.50
-7	-6	-4	19.69	1.29
-7	6	4	23.29	1.48
7	6	-4	20.58	1.28
7	-7	-4	7.34	1.19
-7	7	-4	8.75	1.20
-7	7	-4	7.71	0.99
-7	7	-4	9.50	0.96
7	-7	-4	6.86	0.90
7	-7	-4	8.12	0.80
-7	-7	4	4.95	0.77
7	-7	-4	7.11	0.81
-7	-7	4	5.27	0.91
-7	-7	4	6.87	1.23
-7	-7	4	6.10	1.04
-7	-7	4	6.82	0.83
7	7	4	5.25	0.97
-7	7	-4	7.59	1.31

7	-7	-4	9.35	1.31
7	7	4	6.74	1.06
7	7	4	6.26	1.06
-7	-7	4	5.65	0.94
7	7	4	4.69	0.77
-7	7	-4	7.41	1.34
7	7	4	5.57	0.84
7	7	-4	10.32	1.21
-7	-7	-4	7.46	0.98
-7	7	4	6.79	1.06
7	-7	4	5.80	1.00
7	-7	4	6.68	1.31
7	-7	4	5.85	0.87
-7	7	4	5.26	0.98
7	-7	4	4.66	0.73
7	-7	4	6.27	0.75
-7	-7	-4	7.80	0.97
7	-7	4	6.69	1.18
7	7	-4	8.15	0.59
-7	-7	-4	7.47	0.90
7	-7	4	5.83	0.65
-7	7	4	5.30	0.75
-7	7	4	6.06	1.23
7	7	-4	8.40	0.90
7	7	-4	10.23	1.04
-7	-7	-4	8.03	1.25
-7	-7	-4	7.70	0.85
7	7	-4	8.71	0.97
-7	7	4	6.23	1.11
7	8	4	34.61	2.27
-7	-8	4	32.75	2.01
7	8	4	34.30	1.90
7	8	4	32.01	2.22
7	-8	-4	22.56	1.87
-7	-8	4	31.64	1.92
-7	-8	4	33.10	2.21
-7	-8	4	33.24	2.24
-7	8	-4	22.36	2.21
-7	8	-4	28.30	1.64
7	-8	-4	20.80	1.50
7	-8	-4	23.10	1.71
7	-8	-4	27.12	2.25
-7	8	-4	25.99	1.91
-7	8	-4	24.00	2.06

7	8	4	30.75	2.23
7	-8	-4	23.48	1.55
7	-8	4	33.79	1.85
-7	-8	-4	23.20	1.62
-7	8	4	34.39	2.28
-7	8	4	34.51	2.40
-7	8	4	33.34	2.09
7	8	-4	23.71	1.57
-7	-8	-4	24.93	1.68
-7	8	4	35.17	2.43
7	-8	4	33.16	2.68
-7	8	4	30.14	2.37
7	8	-4	23.89	1.89
7	-8	4	35.84	1.86
7	-8	4	34.09	1.97
7	-8	4	30.94	2.06
7	-8	4	35.66	2.05
-7	-8	-4	25.05	1.52
-7	9	-4	28.68	1.80
7	9	4	21.55	1.93
7	-9	-4	25.68	1.68
-7	-9	4	19.47	1.50
-7	-9	4	19.98	1.54
-7	9	-4	27.77	2.17
-7	9	-4	24.95	2.36
-7	-9	4	18.23	2.10
7	-9	-4	24.79	1.72
7	9	4	20.31	1.42
7	-9	-4	25.46	1.87
-7	-9	4	20.63	1.84
7	-9	-4	27.83	2.46
-7	9	-4	26.24	2.25
7	9	4	19.20	1.89
7	9	4	17.64	1.85
7	-9	4	21.30	1.67
7	9	-4	20.65	2.36
7	-9	4	19.07	1.31
7	9	-4	26.08	1.70
-7	-9	-4	24.91	1.83
-7	9	4	18.58	2.15
-7	9	4	24.33	2.70
-7	-9	-4	27.35	1.84
-7	9	4	18.98	1.86
7	-9	4	20.96	1.50

7	-9	4	22.75	1.69
-7	-9	-4	27.34	1.67
7	-9	4	22.57	2.43
7	-9	4	21.94	1.39
-7	10	-4	98.55	4.66
-7	-10	4	88.33	4.61
7	10	4	89.72	5.00
7	-10	-4	98.11	5.25
-7	-10	4	87.94	4.86
7	-10	-4	94.76	4.68
-7	-10	4	90.43	4.62
-7	10	-4	94.93	5.15
7	-10	-4	87.55	4.81
7	10	4	83.78	4.37
-7	10	-4	85.45	5.28
7	-10	-4	88.83	4.69
7	10	4	95.21	5.40
7	10	4	85.54	4.86
7	-10	-4	89.55	4.86
7	-10	4	101.80	4.56
7	-10	4	91.25	4.58
-7	-10	-4	91.76	4.77
7	-10	4	90.02	5.28
-7	-10	-4	91.27	4.54
-7	10	4	77.96	5.04
7	10	-4	100.42	4.98
-7	10	4	91.16	4.91
7	10	-4	88.62	5.14
7	10	-4	93.40	4.60
7	-10	4	87.12	4.69
7	-10	4	86.44	4.36
7	-10	4	96.73	4.42
7	11	4	49.34	3.59
7	-11	-4	47.91	2.77
7	-11	-4	46.54	2.70
-7	11	-4	50.54	3.47
-7	-11	4	58.69	3.47
-7	-11	4	54.16	3.22
-7	11	-4	44.26	3.65
7	-11	-4	43.93	2.95
-7	11	4	54.87	3.50
7	-11	4	56.75	2.93
-7	-11	-4	45.73	2.66
7	-11	4	53.56	3.26

-7	-11	-4	50.20	3.22
7	11	-4	49.21	2.73
-7	-11	-4	51.90	3.04
7	11	-4	47.03	3.47
-7	11	4	47.80	3.68
7	-11	4	55.92	3.13
7	-11	4	52.00	2.83
7	-12	-4	45.79	2.70
7	-12	-4	44.89	2.82
7	-12	-4	40.11	2.99
7	12	4	38.64	3.46
-7	-12	4	38.26	2.61
-7	12	-4	51.46	3.77
-7	-12	4	40.92	3.19
7	-12	4	40.95	2.55
7	-12	4	37.57	2.24
-7	12	4	44.50	3.27
-7	-12	-4	46.33	2.89
7	-12	4	39.10	3.18
-7	-12	-4	45.61	3.41
7	12	-4	44.09	3.77
-7	-12	-4	44.80	2.57
7	-13	-4	14.03	1.80
7	-13	-4	17.50	1.77
-8	0	4	117.39	5.50
-8	0	-4	124.62	5.62
8	0	-4	121.78	5.58
-8	0	4	111.55	5.38
8	0	-4	119.86	5.48
-8	0	4	116.75	5.39
-8	0	-4	113.28	5.65
-8	0	-4	126.54	5.64
-8	0	4	115.04	5.40
8	0	4	118.02	5.43
-8	0	4	117.79	5.33
-8	0	-4	118.54	5.60
-8	0	-4	127.08	5.66
-8	0	-4	125.58	5.53
8	0	-4	119.33	5.54
8	0	4	118.81	5.39
8	0	4	111.42	5.32
8	0	4	115.38	5.39
8	0	-4	114.39	5.52
8	0	4	114.32	5.41

8	0	4	123.55	5.53
-8	0	4	114.50	5.45
-8	-1	4	18.41	1.26
8	1	4	20.42	1.15
8	-1	-4	21.74	1.25
-8	1	-4	25.94	1.31
8	1	4	19.28	1.06
8	1	4	20.23	1.14
-8	-1	4	18.91	1.05
8	1	4	20.70	1.24
8	-1	-4	21.65	1.19
-8	1	-4	24.37	1.23
-8	-1	4	20.47	1.10
-8	1	-4	20.70	1.42
8	1	4	22.78	1.29
-8	-1	4	20.66	1.08
-8	1	-4	23.05	1.29
-8	1	-4	22.69	1.39
8	-1	-4	22.75	1.19
8	1	4	21.85	1.16
-8	1	-4	24.05	1.28
-8	-1	4	18.00	1.20
-8	1	-4	20.73	1.25
8	-1	-4	21.81	1.24
-8	-1	4	17.81	1.23
-8	-1	-4	24.69	1.41
8	-1	4	20.53	1.13
-8	1	4	19.23	1.10
-8	1	4	18.61	1.07
-8	-1	-4	23.19	1.29
8	-1	4	20.42	1.28
-8	1	4	20.22	1.07
-8	-1	-4	25.61	1.30
-8	1	4	19.75	1.29
8	-1	4	20.38	1.08
8	-1	4	18.53	1.11
-8	1	4	17.20	1.09
-8	1	4	19.24	1.21
8	-1	4	22.30	1.36
8	1	-4	22.30	1.21
-8	1	4	21.70	1.17
-8	1	4	20.26	1.06
-8	-1	-4	23.95	1.17
8	1	-4	23.17	1.35

-8	-1	-4	23.44	1.26
8	2	4	7.48	0.78
-8	2	-4	9.32	0.94
8	2	4	7.98	0.58
-8	2	-4	9.17	0.69
-8	2	-4	10.41	0.70
8	-2	-4	9.49	0.67
-8	-2	4	6.90	0.56
-8	-2	4	7.19	0.84
-8	-2	4	6.46	0.52
-8	-2	4	7.63	0.57
8	2	4	8.82	0.66
-8	2	-4	11.42	0.76
8	2	4	6.79	0.73
-8	2	-4	8.00	0.97
8	2	4	8.12	0.57
-8	2	-4	9.65	0.72
-8	2	-4	11.27	0.74
8	-2	-4	9.71	0.63
8	-2	-4	8.97	0.83
8	-2	-4	9.04	0.69
-8	-2	4	6.58	0.76
-8	-2	-4	9.32	0.73
-8	2	4	7.89	0.82
-8	-2	-4	10.06	0.61
-8	2	4	6.67	0.69
-8	-2	-4	10.77	0.73
-8	2	4	7.17	0.51
-8	-2	-4	9.32	0.68
-8	2	4	7.16	0.76
8	-2	4	7.37	0.60
-8	-2	-4	8.12	0.73
-8	2	4	7.79	0.67
-8	2	4	6.33	0.52
8	-2	4	7.63	0.67
-8	-2	-4	11.72	0.75
8	2	-4	7.55	0.57
8	2	-4	9.22	0.78
-8	2	4	7.96	0.58
8	-2	4	8.38	0.63
-8	-2	-4	10.62	0.90
8	-2	4	7.90	0.85
8	2	-4	9.03	0.51
-8	2	4	7.94	0.69

-8	3	-4	19.96	1.21
-8	-3	4	21.91	1.28
8	-3	-4	17.75	1.15
-8	3	-4	19.91	1.17
8	3	4	23.88	1.43
-8	3	-4	19.66	1.13
8	3	4	23.45	1.46
-8	-3	4	22.71	1.21
8	3	4	21.78	1.29
-8	3	-4	21.18	1.15
-8	3	-4	17.73	1.36
-8	-3	4	22.91	1.21
8	-3	-4	18.60	1.15
8	3	4	24.39	1.32
-8	3	-4	23.86	1.25
-8	3	-4	20.14	1.43
-8	-3	4	22.31	1.48
-8	-3	4	23.42	1.53
8	-3	-4	18.91	1.10
-8	-3	-4	18.81	1.17
8	-3	4	23.65	1.34
8	-3	4	22.33	1.30
8	-3	4	23.18	1.33
-8	-3	-4	20.24	1.17
8	3	-4	21.00	1.23
-8	3	4	23.38	1.30
8	-3	4	24.49	1.54
-8	-3	-4	16.78	1.07
-8	-3	-4	20.83	1.09
-8	3	4	22.58	1.45
8	-3	4	26.34	1.65
-8	3	4	24.77	1.40
-8	3	4	23.35	1.47
8	3	-4	18.79	1.12
-8	3	4	25.34	1.40
8	3	-4	20.41	1.13
-8	-3	-4	20.76	1.21
-8	-3	-4	19.09	1.03
-8	3	4	24.37	1.53
-8	-3	-4	19.41	1.13
-8	-3	-4	22.19	1.37
8	3	-4	19.98	1.03
-8	-4	4	74.31	3.38
-8	4	-4	73.62	3.53

-8	4	-4	79.56	3.67
-8	4	-4	70.92	3.84
-8	-4	4	71.40	3.51
-8	-4	4	66.73	3.67
-8	-4	4	73.16	3.38
8	4	4	75.05	3.49
8	-4	-4	72.40	3.55
8	4	4	75.21	3.61
8	4	4	68.68	3.54
8	-4	-4	71.44	3.62
8	4	4	67.12	3.67
-8	4	-4	73.04	3.90
-8	-4	4	76.18	3.74
8	-4	-4	74.97	3.74
8	-4	-4	71.90	3.67
-8	4	-4	80.88	3.63
-8	4	-4	80.97	3.71
-8	-4	4	73.03	3.35
-8	-4	-4	74.72	3.61
8	-4	4	77.05	3.72
8	4	-4	69.15	3.62
-8	-4	-4	70.08	3.63
8	-4	4	69.11	3.55
-8	4	4	71.59	3.67
8	-4	4	72.56	3.50
-8	-4	-4	77.60	3.82
8	-4	4	76.62	3.85
8	4	-4	78.47	3.66
8	4	-4	75.18	3.59
8	4	-4	78.57	3.55
-8	-4	-4	74.06	3.58
-8	-4	-4	75.25	3.65
-8	4	4	74.06	3.58
-8	4	4	78.42	3.64
8	-4	4	72.56	3.49
8	-4	4	72.04	3.52
-8	4	4	69.65	3.69
-8	4	4	73.60	3.49
-8	-4	-4	75.90	3.56
8	-5	-4	1.26	0.69
8	5	4	0.13	0.51
8	5	4	0.29	0.36
8	-5	-4	1.22	0.37
-8	-5	4	0.39	0.27

8	-5	-4	1.27	0.59
-8	-5	4	0.19	0.58
-8	-5	4	0.68	0.25
-8	5	-4	2.25	0.70
-8	5	-4	1.78	0.54
-8	-5	4	0.64	0.49
8	5	4	0.94	0.43
-8	5	-4	2.02	0.85
8	-5	-4	1.20	0.45
-8	5	-4	1.40	0.41
-8	5	-4	2.05	0.49
-8	-5	4	0.31	0.31
-8	-5	4	0.48	0.37
-8	5	-4	2.75	0.58
8	5	4	0.94	0.37
8	5	4	0.83	0.52
-8	-5	4	0.65	0.56
-8	-5	4	0.12	0.35
8	-5	-4	1.46	0.35
8	5	-4	1.32	0.50
8	-5	4	0.84	0.33
-8	-5	-4	1.83	0.43
-8	5	4	-0.23	0.45
8	-5	4	0.26	0.28
8	-5	4	0.77	0.38
8	-5	4	-0.03	0.63
8	5	-4	1.23	0.41
8	5	-4	2.37	0.52
8	-5	4	0.35	0.34
-8	5	4	0.85	0.39
-8	-5	-4	2.36	0.74
-8	5	4	-0.36	0.53
-8	5	4	0.41	0.62
8	-5	4	0.83	0.71
8	5	-4	1.30	0.39
-8	-5	-4	2.29	0.48
-8	5	4	0.72	0.46
-8	-5	-4	1.65	0.47
-8	-5	-4	2.06	0.45
8	6	4	25.32	1.60
-8	6	-4	33.98	1.94
8	6	4	28.17	1.55
-8	6	-4	33.07	1.76
-8	-6	4	26.99	1.58

-8	-6	4	28.76	1.63
-8	-6	4	28.04	1.50
-8	6	-4	29.79	1.80
-8	-6	4	26.38	1.86
-8	-6	4	25.33	1.82
-8	6	-4	31.06	2.08
-8	6	-4	30.07	1.65
-8	6	-4	30.17	2.25
8	-6	-4	29.46	1.67
-8	-6	4	25.37	1.53
8	-6	-4	26.94	1.81
-8	-6	4	28.06	1.94
8	6	4	24.86	1.74
8	-6	-4	29.72	1.73
8	-6	-4	29.09	2.06
-8	-6	4	26.84	1.36
8	6	4	25.22	1.81
8	-6	-4	26.37	1.92
-8	-6	-4	29.53	1.77
-8	6	4	27.11	1.71
8	-6	4	26.69	1.67
-8	6	4	28.19	1.64
-8	-6	-4	30.33	1.75
8	6	-4	26.33	1.90
-8	-6	-4	30.60	1.75
-8	6	4	26.35	1.97
8	-6	4	27.20	1.94
-8	-6	-4	30.43	1.70
-8	6	4	27.77	1.86
8	-6	4	26.07	1.57
8	6	-4	31.58	1.76
8	6	-4	32.16	1.76
-8	6	4	28.95	1.86
8	-6	4	28.46	1.45
-8	-6	-4	31.04	2.05
8	6	-4	31.55	1.75
8	-6	4	28.32	2.11
8	-6	4	27.05	1.53
-8	-7	4	12.96	1.41
8	7	4	12.11	0.99
8	-7	-4	10.61	1.41
-8	-7	4	10.53	0.93
-8	-7	4	8.54	1.28
-8	-7	4	11.19	1.05

8	-7	-4	12.58	1.57
-8	7	-4	14.93	1.18
8	7	4	10.76	1.32
8	-7	-4	13.53	1.21
8	-7	-4	13.37	1.02
8	7	4	10.50	1.22
-8	7	-4	13.68	1.53
8	-7	-4	13.05	1.07
-8	-7	4	10.63	1.57
-8	7	-4	12.91	1.40
-8	-7	-4	10.61	1.44
8	-7	4	12.66	0.90
-8	7	4	12.43	1.42
-8	7	4	8.96	1.48
-8	7	4	13.29	1.39
8	-7	4	10.88	0.91
8	7	-4	15.37	1.29
-8	7	4	13.96	1.14
-8	-7	-4	13.36	1.64
8	-7	4	10.82	1.04
8	-7	4	14.27	1.56
-8	-7	-4	13.98	1.07
8	7	-4	14.08	1.14
-8	-7	-4	14.05	1.16
8	7	-4	11.21	1.36
8	-7	4	11.74	1.00
-8	7	4	11.47	1.24
-8	-7	-4	13.11	1.16
8	7	-4	13.34	1.07
-8	-8	4	9.80	1.04
-8	-8	4	11.06	1.40
-8	8	-4	11.47	1.21
8	8	4	10.56	1.03
-8	8	-4	9.72	1.66
8	8	4	10.12	1.48
8	8	4	8.14	1.31
8	-8	-4	9.00	1.62
8	-8	-4	11.52	1.02
8	8	4	9.92	1.35
-8	-8	4	11.43	1.47
-8	8	-4	11.81	1.50
-8	8	-4	13.86	1.61
-8	-8	4	9.39	1.03
8	-8	-4	11.48	1.05

8	-8	-4	9.52	1.17
8	-8	-4	9.54	1.47
8	8	-4	11.49	1.06
-8	8	4	9.35	1.74
-8	-8	-4	10.97	1.19
-8	8	4	10.80	1.47
-8	8	4	12.66	1.56
8	-8	4	9.57	0.91
8	-8	4	11.31	1.09
-8	-8	-4	11.55	1.18
8	-8	4	12.19	1.76
8	-8	4	11.07	0.94
8	8	-4	12.11	1.30
8	-8	4	11.95	1.30
8	8	-4	11.16	0.74
8	8	-4	9.72	1.47
8	-8	4	10.52	1.19
-8	-8	-4	11.58	1.06
8	9	4	18.10	1.33
8	-9	-4	14.56	1.21
8	-9	-4	15.84	1.47
-8	-9	4	16.34	1.37
-8	-9	4	15.64	1.43
-8	9	-4	13.40	2.06
8	9	4	15.60	1.73
-8	-9	4	17.94	1.79
8	9	4	13.68	1.68
-8	-9	4	18.22	1.76
8	9	4	17.22	1.83
-8	9	-4	14.06	1.44
8	-9	-4	15.28	1.87
8	-9	4	19.72	2.37
8	-9	4	17.11	1.23
-8	-9	-4	14.30	1.37
8	-9	4	16.74	1.51
8	-9	4	18.43	1.23
-8	9	4	17.82	1.91
8	9	-4	15.95	1.25
8	-9	4	16.32	1.37
-8	9	4	18.15	1.83
-8	-9	-4	12.96	1.36
-8	-9	-4	12.42	1.19
8	9	-4	13.23	1.65
8	-9	4	19.63	1.51

8	-10	-4	19.15	1.46
8	-10	-4	19.22	1.56
8	-10	-4	18.72	1.74
-8	-10	4	20.90	2.01
-8	-10	4	17.63	1.64
8	-10	-4	19.56	2.34
-8	10	-4	16.33	2.41
-8	10	-4	24.40	1.95
8	10	4	18.92	2.02
8	10	4	19.19	1.46
-8	10	-4	19.65	2.26
8	10	4	18.01	2.06
8	-10	4	17.72	1.34
-8	-10	-4	19.21	1.67
8	-10	4	17.61	1.55
8	-10	4	20.52	1.99
8	10	-4	15.02	2.13
8	-10	4	19.42	1.72
-8	10	4	13.15	2.22
-8	-10	-4	18.44	1.45
8	10	-4	19.60	1.52
-8	10	4	18.60	2.19
8	-10	4	17.90	2.66
8	-10	4	18.92	1.37
8	-11	-4	2.92	0.83
-8	11	-4	0.77	1.33
-8	11	-4	0.52	1.46
8	-11	-4	1.60	0.75
-8	-11	4	0.58	1.22
-8	-11	4	1.01	0.96
8	11	4	0.92	1.16
8	-11	-4	1.89	1.04
8	-11	4	0.93	0.63
-8	-11	-4	0.12	0.93
8	-11	4	1.78	0.82
-8	-11	-4	1.91	1.07
8	-11	4	0.74	0.57
-8	11	4	0.43	1.23
8	-11	4	1.08	0.99
8	11	-4	1.45	0.74
8	11	-4	3.13	1.55
-8	-11	-4	1.46	0.76
-8	-12	4	-0.33	0.78
8	-12	-4	2.51	1.11

8	-12	-4	1.12	0.80
8	12	4	2.23	1.49
-8	12	-4	0.21	1.67
-8	-12	4	-0.23	1.26
8	-12	-4	0.74	0.84
8	-12	4	1.72	1.00
8	-12	4	1.13	0.82
8	12	-4	3.41	1.57
-8	12	4	-0.65	1.46
-8	-12	-4	0.97	0.89
-8	-12	-4	0.81	1.26
9	0	-4	0.01	0.15
9	0	-4	0.11	0.19
-9	0	4	0.17	0.32
-9	0	-4	0.31	0.33
-9	0	-4	0.08	0.29
9	0	4	0.00	0.23
9	0	-4	0.03	0.21
-9	0	-4	-0.02	0.31
-9	0	4	0.18	0.29
-9	0	4	0.31	0.40
9	0	4	0.04	0.30
9	0	4	-0.10	0.44
-9	0	-4	-0.15	0.21
-9	0	-4	-0.10	0.18
9	0	-4	-0.05	0.15
-9	0	4	0.02	0.21
-9	0	4	0.29	0.27
-9	0	4	0.02	0.26
9	0	4	-0.01	0.17
-9	0	-4	0.21	0.35
-9	-1	4	28.57	1.56
9	-1	-4	23.99	1.29
9	1	4	28.42	1.70
-9	1	-4	22.71	1.31
9	-1	-4	23.04	1.25
9	1	4	30.10	1.64
9	-1	-4	22.13	1.22
9	-1	-4	21.81	1.29
-9	1	-4	24.60	1.35
-9	1	-4	20.48	1.44
9	1	4	30.84	1.63
-9	-1	4	32.22	1.62
-9	-1	4	28.17	1.71

-9	1	-4	25.29	1.36
9	1	4	30.47	1.55
-9	-1	4	31.83	1.75
9	1	4	32.91	1.65
-9	1	-4	25.26	1.34
-9	1	-4	23.38	1.43
-9	-1	4	29.98	1.77
-9	1	-4	25.37	1.27
9	1	4	34.18	1.77
9	-1	4	31.02	1.76
-9	1	4	30.44	1.60
-9	-1	-4	24.69	1.45
-9	1	4	30.99	1.51
-9	1	4	30.73	1.74
-9	-1	-4	24.43	1.29
9	1	-4	23.16	1.39
9	1	-4	23.20	1.26
-9	-1	-4	24.16	1.34
-9	1	4	31.16	1.79
-9	-1	-4	23.79	1.19
-9	1	4	29.85	1.60
-9	1	4	30.31	1.53
9	-1	4	30.49	1.82
-9	-1	-4	25.33	1.35
-9	-1	-4	21.53	1.34
9	-1	4	31.35	1.64
-9	1	4	32.85	1.67
9	-1	4	31.95	1.64
-9	1	4	31.76	1.58
9	-1	4	29.92	1.57
-9	-2	4	132.07	5.88
-9	-2	4	109.63	5.75
9	-2	-4	117.82	5.39
-9	2	-4	123.55	5.38
-9	2	-4	107.44	5.50
-9	-2	4	117.16	5.70
-9	2	-4	116.90	5.53
-9	2	-4	110.23	5.33
9	2	4	126.11	5.68
9	2	4	128.62	5.75
-9	2	-4	118.52	5.33
9	2	4	122.10	5.87
-9	-2	4	119.60	5.86
-9	-2	4	128.84	5.88

9	2	4	119.16	5.69
9	-2	-4	111.44	5.30
9	2	4	129.33	5.81
-9	-2	4	116.66	5.66
-9	2	-4	115.15	5.31
9	-2	-4	111.91	5.28
-9	-2	4	121.72	5.71
-9	2	-4	117.23	5.35
-9	-2	-4	114.02	5.35
-9	-2	-4	114.62	5.27
9	-2	4	117.60	5.72
-9	-2	-4	121.40	5.37
-9	2	4	129.15	5.72
-9	-2	-4	120.89	5.49
-9	2	4	126.53	5.70
9	2	-4	107.47	5.19
-9	-2	-4	112.14	5.35
-9	2	4	123.94	5.76
-9	2	4	129.44	5.91
9	-2	4	125.18	5.87
-9	-2	-4	115.95	5.22
9	2	-4	112.52	5.15
9	-2	4	130.44	5.72
9	-2	4	122.01	5.76
-9	2	4	124.61	5.63
-9	2	4	130.25	5.79
9	2	-4	112.70	5.40
-9	2	4	115.60	5.61
-9	-2	-4	108.12	5.31
-9	-3	4	127.43	6.04
-9	3	-4	137.65	6.17
9	3	4	125.62	6.29
-9	-3	4	125.89	6.11
-9	-3	4	135.90	6.06
-9	-3	4	136.80	6.13
-9	-3	4	125.42	6.29
9	-3	-4	128.98	6.09
-9	3	-4	126.39	6.12
9	3	4	141.30	6.22
-9	3	-4	127.17	6.37
9	-3	-4	127.66	6.15
9	3	4	133.04	6.13
9	-3	-4	138.15	6.27
9	3	4	125.91	6.12

-9	3	-4	139.76	6.19
-9	3	-4	133.70	6.38
-9	-3	4	141.71	6.29
-9	3	-4	132.47	6.09
9	-3	-4	130.89	6.17
-9	-3	4	137.25	6.32
-9	3	4	142.42	6.19
-9	3	4	132.06	6.29
9	-3	4	117.58	6.13
-9	-3	-4	133.34	6.08
-9	-3	-4	143.56	6.34
9	3	-4	138.70	6.21
-9	-3	-4	136.57	6.05
-9	3	4	143.74	6.22
-9	-3	-4	129.34	6.16
9	3	-4	123.83	6.06
9	-3	4	131.30	6.16
9	-3	4	126.84	6.15
9	-3	4	128.14	6.28
9	3	-4	134.63	6.00
-9	-3	-4	132.58	6.18
-9	3	4	129.95	6.23
-9	-3	-4	133.31	6.04
-9	3	4	134.22	6.11
-9	-3	-4	128.31	6.15
-9	3	4	134.63	6.31
-9	-3	-4	122.13	6.04
-9	4	-4	140.49	6.10
-9	-4	4	124.33	5.60
9	4	4	116.30	5.90
-9	4	-4	136.45	6.11
9	-4	-4	129.14	6.09
-9	4	-4	129.76	6.34
-9	4	-4	124.34	5.94
-9	4	-4	137.44	6.06
-9	-4	4	116.10	5.90
-9	-4	4	126.65	5.86
-9	4	-4	124.68	6.28
9	4	4	126.55	5.80
-9	-4	4	124.06	5.51
9	-4	-4	121.49	5.97
-9	-4	4	113.83	5.70
-9	-4	4	123.25	5.93
-9	-4	4	125.03	5.50

-9	-4	4	120.67	5.60
9	4	4	121.37	5.69
9	4	4	117.32	5.74
9	-4	-4	125.16	6.16
9	-4	-4	125.77	6.06
-9	4	4	127.08	5.70
-9	-4	-4	126.29	6.07
-9	4	4	119.76	5.89
9	-4	4	114.35	5.75
9	-4	4	119.37	5.71
9	4	-4	131.57	6.07
9	4	-4	129.88	6.01
-9	-4	-4	130.39	5.96
9	4	-4	134.59	5.95
-9	4	4	121.82	5.89
-9	-4	-4	129.16	6.04
9	-4	4	133.30	6.08
9	-4	4	123.23	5.91
-9	-4	-4	125.47	6.06
-9	-4	-4	137.48	6.27
-9	-4	-4	126.54	5.97
-9	4	4	131.50	5.80
-9	4	4	127.91	5.82
9	-4	4	113.86	5.71
9	4	-4	120.00	6.02
-9	5	-4	102.93	5.27
-9	-5	4	110.37	5.33
-9	-5	4	107.35	4.90
-9	-5	4	108.12	5.22
9	-5	-4	96.23	4.90
9	5	4	111.00	5.20
-9	5	-4	99.25	4.77
-9	-5	4	102.89	5.28
9	5	4	103.60	5.08
-9	-5	4	109.02	4.91
-9	5	-4	110.77	4.91
-9	5	-4	108.77	5.00
9	5	4	104.52	5.30
9	5	4	109.65	5.04
-9	5	-4	99.95	5.18
9	-5	-4	99.34	5.03
9	-5	-4	100.46	4.96
9	-5	-4	94.40	4.82
-9	5	-4	106.57	4.95

-9	-5	4	110.17	4.94
9	-5	-4	110.93	5.17
-9	-5	4	101.58	5.07
-9	-5	4	106.34	4.92
9	5	4	99.87	5.14
-9	5	4	108.58	5.06
9	-5	4	105.26	5.05
9	5	-4	105.81	4.88
9	-5	4	105.63	5.15
-9	5	4	101.93	5.31
-9	-5	-4	102.31	4.85
-9	5	4	109.59	5.15
-9	5	4	105.51	5.27
-9	-5	-4	106.59	4.95
-9	-5	-4	104.03	5.13
9	5	-4	105.46	4.90
9	-5	4	107.01	5.29
9	-5	4	112.03	5.46
9	-5	4	104.20	5.08
9	5	-4	99.08	4.95
-9	-5	-4	103.40	4.94
9	5	-4	97.13	4.95
-9	-5	-4	103.99	4.83
-9	5	4	113.06	5.24
-9	-5	-4	99.27	4.87
-9	-6	4	54.97	2.91
-9	6	-4	43.01	2.70
-9	6	-4	45.72	2.50
9	-6	-4	42.49	2.28
9	6	4	48.31	2.82
9	6	4	54.97	2.65
-9	6	-4	42.59	2.21
9	-6	-4	42.26	2.34
9	6	4	51.69	2.71
9	6	4	50.89	2.82
-9	-6	4	47.30	2.75
9	6	4	50.58	2.96
9	-6	-4	45.32	2.71
9	-6	-4	43.49	2.55
-9	6	-4	42.64	2.85
-9	-6	4	51.98	2.62
-9	-6	4	53.82	2.49
-9	6	-4	47.20	2.38
9	-6	-4	43.62	2.45

-9	-6	4	54.71	2.51
-9	-6	4	51.78	3.00
-9	-6	4	51.75	2.72
-9	6	-4	45.67	2.44
-9	-6	4	49.49	2.95
-9	6	4	51.22	3.07
-9	6	4	55.06	2.94
-9	6	4	53.50	2.80
9	-6	4	55.80	3.19
9	6	-4	45.80	2.36
9	6	-4	41.41	2.49
-9	6	4	52.34	2.72
9	6	-4	45.15	2.33
-9	-6	-4	42.88	2.39
9	-6	4	51.29	2.78
9	6	-4	47.11	2.37
-9	-6	-4	41.16	2.31
9	-6	4	53.89	2.53
9	-6	4	49.39	2.69
9	-6	4	51.81	2.65
-9	-6	-4	43.03	2.34
-9	-6	-4	43.47	2.31
-9	-6	-4	45.02	2.68
9	-6	4	52.96	3.02
-9	6	4	49.75	2.92
-9	7	-4	3.11	0.83
-9	-7	4	3.07	0.65
9	-7	-4	3.21	0.58
-9	-7	4	1.93	0.38
-9	7	-4	5.34	0.92
9	-7	-4	2.06	0.79
9	7	4	2.67	0.77
9	-7	-4	3.12	0.56
9	-7	-4	1.92	0.93
-9	-7	4	1.88	0.74
-9	7	-4	2.18	1.20
-9	7	-4	4.45	1.02
9	7	4	2.92	0.59
-9	-7	4	3.55	0.91
9	-7	-4	2.67	0.65
-9	-7	4	2.67	0.50
9	7	4	1.29	0.67
9	7	4	1.58	0.75
9	7	-4	3.21	0.67

9	-7	4	3.72	1.15
9	-7	4	2.69	0.43
-9	7	4	2.81	0.71
-9	-7	-4	3.16	0.59
-9	-7	-4	3.49	0.67
-9	7	4	1.10	0.61
-9	7	4	2.21	1.02
9	7	-4	3.23	0.81
-9	7	4	1.95	0.78
9	7	-4	4.02	0.74
9	-7	4	2.86	0.68
-9	-7	-4	4.63	0.82
9	-7	4	2.69	0.52
9	7	-4	1.87	0.61
-9	7	4	2.72	0.90
9	-8	-4	8.84	0.92
9	8	4	7.36	1.34
-9	8	-4	7.17	1.30
9	8	4	6.12	0.94
9	-8	-4	7.35	0.90
-9	8	-4	5.97	1.06
-9	-8	4	6.39	1.29
-9	8	-4	7.26	1.35
-9	8	-4	8.85	1.17
-9	-8	4	7.94	1.39
-9	-8	4	8.91	1.52
-9	-8	4	6.48	1.03
9	-8	-4	6.88	1.31
9	8	4	5.71	1.04
-9	-8	4	7.30	0.64
9	-8	-4	6.67	0.98
-9	-8	-4	6.89	1.03
9	-8	4	6.38	0.77
-9	8	4	4.69	1.34
9	-8	4	6.06	0.81
-9	8	4	4.18	1.00
9	-8	4	8.80	1.63
9	-8	4	5.90	0.98
9	8	-4	6.81	0.91
9	8	-4	7.79	1.23
9	8	-4	7.57	1.47
-9	-8	-4	6.89	0.94
9	9	4	19.08	2.23
9	-9	-4	15.84	1.32

-9	-9	4	16.64	1.84
-9	9	-4	20.08	1.53
-9	9	-4	14.11	1.92
9	9	4	13.94	1.77
9	-9	-4	15.58	1.52
9	9	4	17.66	1.36
-9	-9	4	16.19	1.67
-9	9	-4	17.53	2.34
9	9	4	14.08	1.71
-9	-9	4	17.12	1.83
9	-9	-4	18.35	2.17
-9	-9	4	19.07	1.52
9	-9	-4	14.64	1.50
9	9	-4	14.91	1.84
-9	-9	-4	16.13	1.43
9	-9	4	17.33	1.28
9	-9	4	18.00	1.60
-9	-9	-4	15.47	1.48
-9	-9	-4	14.93	1.31
9	9	-4	15.70	1.28
9	9	-4	18.34	1.64
9	-9	4	21.12	2.52
-9	9	4	17.83	1.97
9	-9	4	18.66	1.23
-9	9	4	14.84	1.96
9	-9	4	20.38	1.57
9	-9	4	17.29	1.44
9	10	4	51.78	3.40
-9	10	-4	57.43	3.95
9	10	4	49.74	3.42
-9	-10	4	50.95	3.02
9	-10	-4	55.06	3.30
9	-10	-4	57.43	3.13
9	10	4	49.55	2.78
-9	-10	4	51.57	3.40
-9	10	-4	59.66	3.85
9	-10	-4	59.58	3.17
9	10	-4	51.41	3.65
9	-10	4	52.60	3.13
-9	10	4	56.22	3.46
9	-10	4	53.39	4.22
-9	-10	-4	62.27	3.30
9	10	-4	60.32	3.06
-9	10	4	51.38	3.62

9	-10	4	56.68	2.71
-9	-10	-4	57.42	3.09
9	-10	4	53.55	2.97
9	-10	4	51.59	2.79
9	11	4	36.15	3.15
9	-11	-4	41.71	2.51
9	-11	-4	41.39	2.62
-9	-11	4	40.36	3.17
-9	11	-4	44.46	3.35
9	-11	-4	36.95	2.96
-9	-11	4	40.59	2.72
9	11	-4	43.76	3.47
-9	11	4	38.60	3.09
9	-11	4	41.30	2.73
-9	-11	-4	46.93	3.22
9	-11	4	38.77	2.50
-9	-11	-4	42.46	2.75
-9	-11	-4	39.77	2.44
9	-11	4	40.93	2.57
9	12	4	43.04	3.43
-9	-12	4	38.77	2.72
9	-12	-4	33.43	2.33
9	-12	-4	34.40	2.67
9	-12	-4	31.46	2.31
-9	-12	4	42.87	3.17
-9	12	-4	36.24	3.46
9	-12	4	43.23	2.69
-9	-12	-4	36.32	2.48
-9	12	4	45.01	3.64
9	12	-4	33.04	3.11
-9	-12	-4	32.48	2.88
9	-12	4	42.41	2.46
9	-12	4	41.78	2.85
-10	0	4	82.07	4.18
-10	0	-4	108.34	5.09
10	0	-4	105.48	5.05
10	0	4	85.82	4.09
-10	0	4	87.96	4.21
-10	0	-4	116.04	5.14
-10	0	-4	106.21	5.20
-10	0	4	89.40	4.27
10	0	4	83.38	4.18
-10	0	4	86.97	4.13
10	0	-4	104.51	5.07

10	0	-4	103.90	4.96
10	0	4	90.93	4.23
-10	0	-4	112.07	5.11
-10	0	4	92.36	4.19
10	0	4	87.06	4.23
-10	0	4	92.03	4.34
10	0	4	91.41	4.32
-10	0	-4	115.26	5.00
10	0	-4	106.71	4.94
-10	0	-4	111.70	5.17
-10	1	-4	101.76	4.98
-10	-1	4	74.20	3.83
-10	-1	4	84.92	3.93
10	-1	-4	98.33	4.72
-10	1	-4	102.15	4.90
10	1	4	83.04	3.87
-10	-1	4	79.75	3.73
10	1	4	78.26	3.86
-10	1	-4	107.37	4.76
-10	1	-4	99.19	4.83
10	-1	-4	99.57	4.77
10	1	4	76.25	3.68
-10	-1	4	80.87	3.75
10	-1	-4	99.09	4.82
10	1	4	81.79	3.78
-10	-1	4	75.80	3.82
10	-1	-4	93.66	4.84
-10	-1	4	76.87	3.86
10	1	4	71.71	3.78
-10	1	-4	107.75	4.85
-10	-1	4	75.27	3.71
10	1	4	81.84	3.78
-10	1	-4	110.91	4.87
-10	1	4	77.69	3.88
10	-1	4	79.47	3.79
-10	-1	-4	96.09	4.86
10	-1	4	82.23	3.88
-10	1	4	84.79	3.82
-10	-1	-4	104.67	4.86
10	-1	4	81.60	3.98
10	-1	4	74.77	3.77
-10	-1	-4	105.49	4.76
-10	1	4	80.11	3.94
-10	1	4	79.60	3.60

-10	-1	-4	106.90	4.94
-10	1	4	77.20	3.73
10	1	-4	102.46	4.78
-10	-1	-4	107.53	4.70
-10	-1	-4	104.13	4.85
-10	1	4	79.97	3.73
10	-1	4	75.39	3.70
-10	2	-4	51.05	2.73
10	2	4	53.97	2.54
-10	-2	4	50.42	2.53
-10	2	-4	49.98	2.74
-10	-2	4	46.43	2.70
-10	-2	4	51.38	2.74
-10	2	-4	54.20	2.58
10	-2	-4	49.73	2.58
10	-2	-4	49.55	2.47
10	2	4	53.02	2.59
10	2	4	47.09	2.69
-10	2	-4	51.02	2.54
-10	-2	4	50.55	2.55
-10	-2	4	54.00	2.74
-10	-2	4	49.60	2.51
-10	2	-4	46.45	2.51
-10	2	-4	53.47	2.51
10	-2	-4	50.76	2.47
-10	-2	4	53.79	2.54
-10	2	-4	49.01	2.50
10	2	4	54.39	2.68
10	2	4	50.26	2.51
10	-2	-4	49.29	2.53
10	-2	4	50.82	2.59
-10	-2	-4	52.48	2.56
10	2	-4	50.33	2.59
-10	-2	-4	51.63	2.55
10	-2	4	54.85	2.75
-10	-2	-4	52.11	2.56
-10	2	4	47.36	2.45
-10	-2	-4	47.41	2.51
-10	2	4	50.34	2.58
-10	-2	-4	50.25	2.69
10	-2	4	50.82	2.55
-10	2	4	49.91	2.46
-10	2	4	55.97	2.65
-10	-2	-4	51.80	2.38

10	-2	4	52.80	2.55
-10	2	4	53.92	2.54
-10	-2	-4	49.87	2.42
-10	2	4	52.42	2.77
-10	2	4	54.25	2.55
10	-2	4	55.09	2.85
10	-3	-4	3.19	0.53
10	-3	-4	3.93	0.54
-10	-3	4	5.74	0.92
10	3	4	6.05	0.68
-10	-3	4	7.59	0.88
-10	-3	4	6.27	0.69
-10	3	-4	4.07	0.61
-10	3	-4	3.69	0.72
-10	3	-4	3.11	0.52
10	3	4	5.85	0.83
10	-3	-4	3.53	0.47
-10	-3	4	7.64	0.62
-10	3	-4	2.74	0.75
-10	3	-4	3.92	0.58
10	3	4	6.27	0.61
-10	3	-4	4.32	0.63
-10	-3	4	7.12	0.98
-10	-3	4	7.33	0.63
-10	3	-4	3.85	0.57
10	-3	-4	3.23	0.65
10	3	4	6.66	0.83
-10	-3	4	6.67	0.63
10	3	-4	3.80	0.38
-10	-3	-4	3.25	0.55
10	-3	4	6.83	1.00
-10	3	4	6.58	0.65
-10	-3	-4	2.95	0.50
10	3	-4	4.99	0.64
-10	3	4	6.92	0.86
10	-3	4	6.69	0.64
-10	-3	-4	2.83	0.49
-10	-3	-4	3.62	0.74
-10	3	4	6.94	0.98
10	3	-4	3.99	0.47
-10	3	4	7.35	0.77
-10	3	4	7.09	0.88
-10	-3	-4	3.20	0.54
-10	-3	-4	2.79	0.40

10	-3	4	5.81	0.63
10	-3	4	6.55	0.58
-10	-3	-4	3.42	0.62
-10	-3	-4	3.24	0.64
-10	3	4	6.81	0.79
10	4	4	6.70	0.93
-10	4	-4	7.26	0.82
-10	-4	4	7.00	0.61
-10	4	-4	6.41	1.07
-10	-4	4	5.48	1.03
-10	-4	4	7.38	1.05
10	-4	-4	7.19	0.64
-10	-4	4	6.28	0.95
10	4	4	6.05	0.68
-10	4	-4	7.07	1.26
10	4	4	7.21	0.85
-10	-4	4	6.56	0.67
10	-4	-4	6.37	0.72
10	-4	-4	6.49	1.01
10	4	4	7.26	0.72
-10	4	-4	6.89	0.81
-10	4	-4	7.18	0.79
10	-4	-4	6.50	0.74
-10	4	-4	6.99	0.68
10	4	-4	7.25	0.76
-10	-4	-4	7.37	1.04
-10	4	4	6.97	0.73
-10	-4	-4	7.34	0.66
-10	-4	-4	6.90	0.73
10	-4	4	7.20	1.16
-10	-4	-4	7.25	0.60
10	-4	4	6.29	0.67
10	-4	4	5.86	1.06
10	4	-4	6.37	0.71
10	-4	4	6.64	0.70
-10	-4	-4	5.79	0.80
-10	4	4	7.04	0.96
-10	-4	-4	6.18	0.73
-10	4	4	6.51	1.00
-10	4	4	6.02	0.88
-10	4	4	6.78	0.88
10	4	-4	7.26	0.58
10	4	-4	7.16	0.73
10	-4	4	6.16	0.68

-10	-5	4	32.20	1.93
-10	5	-4	28.21	1.63
-10	-5	4	27.58	1.98
-10	5	-4	28.91	1.71
10	5	4	25.66	1.88
-10	-5	4	30.53	1.70
10	-5	-4	27.73	1.88
10	5	4	31.13	1.67
-10	5	-4	27.87	1.95
10	-5	-4	26.78	1.51
-10	5	-4	26.32	2.04
10	-5	-4	25.82	1.63
10	5	4	27.38	1.71
-10	5	-4	26.78	1.47
10	5	4	30.90	1.83
-10	-5	4	25.59	1.56
-10	-5	4	28.57	1.93
-10	-5	4	29.10	1.45
-10	5	-4	28.43	1.70
-10	-5	4	30.15	1.54
10	-5	-4	25.84	1.59
10	-5	-4	27.03	1.84
-10	-5	4	30.28	1.44
-10	-5	-4	27.97	1.54
10	5	-4	22.88	1.59
-10	-5	-4	26.29	1.48
10	-5	4	28.84	1.65
-10	-5	-4	25.99	1.64
10	-5	4	30.69	2.02
-10	5	4	27.61	1.96
-10	-5	-4	26.08	1.61
10	5	-4	28.65	1.51
10	-5	4	26.78	1.65
10	-5	4	32.74	2.25
10	-5	4	29.40	1.75
-10	5	4	32.19	1.98
10	5	-4	28.80	1.60
-10	5	4	28.88	1.82
-10	5	4	29.39	1.85
10	5	-4	27.03	1.59
-10	-5	-4	27.28	1.90
-10	-5	-4	27.45	1.58
-10	5	4	30.26	1.70
-10	6	-4	23.34	1.51

-10	6	-4	21.73	1.63
10	6	4	22.34	1.66
-10	6	-4	20.11	2.14
10	6	4	19.69	1.52
10	-6	-4	24.15	1.85
-10	-6	4	20.84	1.75
10	-6	-4	20.75	1.48
-10	-6	4	18.86	1.25
-10	-6	4	21.39	1.72
-10	6	-4	24.01	1.88
10	-6	-4	20.57	1.74
10	6	4	20.92	1.72
10	-6	-4	21.45	1.35
10	6	4	22.75	1.39
-10	-6	4	24.52	1.25
-10	-6	4	23.82	1.74
10	6	4	21.11	1.45
-10	-6	4	23.72	1.46
10	-6	-4	22.20	1.43
-10	-6	4	22.10	1.21
-10	6	-4	24.51	1.56
-10	-6	4	22.51	1.22
-10	6	-4	21.34	1.28
10	-6	4	21.23	1.40
-10	6	4	21.69	1.60
-10	-6	-4	24.32	1.52
-10	6	4	23.68	1.70
10	-6	4	24.06	1.83
-10	6	4	17.04	1.73
-10	-6	-4	21.91	1.40
10	6	-4	20.75	1.48
10	6	-4	18.05	1.54
10	-6	4	21.26	1.37
10	6	-4	22.97	1.47
10	-6	4	22.39	1.99
-10	-6	-4	21.71	1.35
10	-6	4	22.04	1.50
-10	-6	-4	22.28	1.78
-10	-6	-4	22.53	1.41
10	-6	4	22.87	1.24
-10	6	4	22.84	1.76
10	6	-4	22.80	1.40
-10	-7	4	41.63	2.17
-10	-7	4	44.44	2.12

-10	7	-4	61.37	3.06
-10	-7	4	41.96	2.63
-10	-7	4	41.55	2.08
-10	-7	4	42.67	2.54
10	-7	-4	58.18	3.14
-10	-7	4	41.88	2.38
-10	7	-4	58.60	3.21
10	-7	-4	55.36	3.37
10	-7	-4	57.13	3.01
-10	7	-4	54.26	3.54
10	7	4	44.25	2.29
10	7	4	38.45	2.60
10	7	4	42.19	2.57
10	-7	-4	54.08	2.93
10	7	4	39.26	2.50
-10	7	-4	61.56	3.47
-10	-7	-4	54.45	2.96
10	-7	4	41.29	2.43
10	-7	4	42.14	2.28
10	7	-4	59.72	2.95
10	-7	4	43.56	2.10
10	7	-4	60.64	3.07
-10	-7	-4	58.70	2.98
10	-7	4	42.14	2.33
10	7	-4	53.93	3.18
-10	7	4	45.40	2.71
10	-7	4	47.23	2.92
-10	7	4	43.71	2.39
-10	7	4	37.51	2.79
-10	-7	-4	57.79	3.08
-10	-8	4	45.33	2.18
-10	-8	4	35.83	2.84
-10	-8	4	44.33	2.83
-10	8	-4	45.54	2.46
-10	-8	4	46.13	2.70
10	8	4	40.34	2.79
10	8	4	45.50	2.73
10	-8	-4	45.45	2.95
-10	-8	4	45.10	2.20
-10	8	-4	37.71	3.39
10	-8	-4	41.17	2.51
10	-8	-4	41.67	2.31
10	8	4	46.97	2.40
-10	-8	4	44.65	2.36

10	-8	-4	39.34	2.36
-10	-8	-4	41.94	2.37
10	8	-4	34.63	2.58
10	-8	4	46.67	2.22
-10	-8	-4	40.83	2.45
10	8	-4	46.43	2.31
-10	8	4	37.41	3.13
10	-8	4	42.67	2.60
10	-8	4	44.06	2.47
-10	-8	-4	40.86	2.33
10	-8	4	44.43	2.39
-10	8	4	42.67	2.86
10	-8	4	47.60	2.37
10	-8	4	46.78	3.44
10	8	-4	45.12	2.49
-10	-9	4	45.91	2.73
-10	-9	4	50.49	3.13
10	9	4	43.63	2.54
10	9	4	46.59	3.11
-10	-9	4	46.05	2.56
-10	9	-4	38.89	2.97
10	-9	-4	37.18	2.45
10	-9	-4	44.77	3.11
-10	9	-4	38.34	3.03
10	9	4	43.43	2.96
10	-9	-4	36.55	2.26
-10	-9	4	50.52	2.89
10	-9	-4	36.94	2.25
-10	9	4	42.60	3.42
10	9	-4	37.00	2.76
10	-9	4	52.30	3.63
-10	-9	-4	41.39	2.46
-10	-9	-4	36.84	2.23
-10	-9	-4	39.43	2.39
10	-9	4	47.71	2.83
10	-9	4	48.66	2.68
-10	9	4	51.70	3.15
10	-9	4	49.90	2.42
10	9	-4	42.49	2.55
10	9	-4	41.80	2.23
10	-9	4	43.46	2.53
-10	10	-4	17.61	2.48
-10	-10	4	16.52	1.56
10	-10	-4	16.55	1.51

10	10	4	15.25	2.00
-10	-10	4	17.78	1.99
-10	10	-4	16.44	2.39
10	10	4	13.28	1.35
10	-10	-4	17.79	1.50
10	10	4	13.37	1.95
10	-10	4	14.06	1.58
-10	10	4	15.11	2.15
10	10	-4	16.54	2.09
10	-10	4	14.60	1.31
10	10	-4	19.97	1.44
10	-10	4	14.60	1.49
-10	-10	-4	18.39	1.78
-10	-10	-4	17.15	1.49
10	11	4	-0.21	1.38
-10	-11	4	2.46	1.36
-10	11	-4	-0.23	1.59
10	-11	-4	1.33	1.00
-10	-11	4	-0.46	0.75
10	-11	-4	0.50	0.79
10	-11	-4	-0.74	0.73
-10	-11	-4	0.43	0.76
10	11	-4	-1.63	1.26
10	-11	4	-0.27	0.77
10	-11	4	-0.12	1.01
-10	-11	-4	3.03	1.47
-10	-11	-4	1.04	0.87
-10	11	4	1.02	1.29
10	-11	4	-0.56	0.60
10	12	4	6.07	1.61
10	-12	-4	7.56	1.48
-10	-12	4	7.11	1.49
-10	-12	4	6.85	1.23
10	-12	-4	9.70	1.35
-10	12	-4	6.92	1.87
10	-12	-4	6.39	1.21
10	12	-4	7.03	1.74
10	-12	4	6.89	1.02
10	-12	4	5.90	1.28
-10	-12	-4	7.31	1.30
-10	-12	-4	8.84	1.56
10	-12	4	10.04	1.40
-10	12	4	8.93	2.14
-11	0	4	-0.04	0.35

11	0	4	0.03	0.33
-11	0	4	0.19	0.28
11	0	-4	0.17	0.23
11	0	-4	-0.14	0.15
-11	0	4	0.58	0.49
-11	0	4	0.42	0.40
-11	0	-4	0.01	0.22
-11	0	4	-0.47	0.32
11	0	4	-0.09	0.27
11	0	-4	0.09	0.26
11	0	-4	-0.02	0.22
-11	0	-4	-0.22	0.28
-11	0	4	-0.21	0.32
11	0	4	-0.10	0.39
-11	0	-4	-0.05	0.39
11	0	4	0.16	0.19
-11	0	-4	0.06	0.41
-11	0	-4	0.58	0.36
-11	0	-4	-0.16	0.29
-11	0	-4	0.18	0.30
11	0	4	-0.51	0.44
-11	1	-4	3.23	0.48
11	1	4	5.86	0.50
-11	1	-4	5.02	0.65
11	-1	-4	3.54	0.47
-11	1	-4	5.76	0.81
11	1	4	6.42	0.81
11	-1	-4	2.71	0.51
11	1	4	7.19	0.65
11	1	4	5.61	0.75
-11	-1	4	5.61	0.71
-11	-1	4	4.72	0.64
11	1	4	6.43	0.84
-11	1	-4	4.13	0.59
-11	-1	4	5.93	0.55
11	-1	-4	3.53	0.51
-11	-1	4	4.75	0.71
-11	1	-4	4.82	0.75
11	1	4	6.15	0.67
-11	-1	4	5.52	0.91
-11	-1	4	5.74	0.60
-11	1	-4	5.98	0.77
11	-1	-4	3.70	0.41
-11	-1	4	6.55	0.81

-11	1	-4	5.29	0.59
11	-1	4	7.32	0.87
-11	1	4	4.68	0.68
-11	-1	-4	4.46	0.61
-11	-1	-4	4.15	0.51
-11	1	4	5.19	0.76
-11	1	4	4.62	0.86
-11	1	4	5.67	0.61
-11	1	4	4.90	0.59
11	-1	4	7.10	0.95
-11	-1	-4	4.70	0.78
-11	1	4	5.75	0.68
-11	-1	-4	4.26	0.75
11	-1	4	6.83	0.71
11	1	-4	3.80	0.50
11	-1	4	5.36	0.50
11	-1	4	5.68	0.60
-11	-1	-4	4.83	0.60
-11	-1	-4	3.87	0.39
-11	-2	4	57.98	2.94
11	2	4	53.28	2.90
-11	2	-4	54.06	2.97
-11	2	-4	55.71	2.71
-11	-2	4	52.44	2.89
11	-2	-4	55.52	2.81
11	-2	-4	51.53	2.67
-11	-2	4	55.16	2.74
-11	2	-4	53.82	2.93
11	-2	-4	55.17	2.69
-11	-2	4	54.43	2.72
11	2	4	53.45	2.68
11	2	4	58.95	3.03
-11	-2	4	58.72	2.95
-11	-2	4	56.79	2.73
11	2	4	56.86	2.77
-11	2	-4	58.37	2.81
-11	-2	4	51.42	2.68
-11	2	-4	54.86	2.73
-11	2	-4	54.75	2.75
-11	2	-4	55.45	2.77
11	-2	-4	53.37	2.75
-11	-2	-4	53.85	2.76
11	-2	4	54.48	2.77
-11	2	4	57.32	2.65

11	-2	4	57.41	2.75
-11	2	4	56.44	2.75
-11	-2	-4	56.33	2.57
11	-2	4	58.71	3.04
11	2	-4	55.49	2.82
-11	2	4	55.89	2.93
11	-2	4	49.51	2.70
-11	2	4	58.61	2.84
-11	2	4	58.24	2.73
-11	-2	-4	54.74	2.73
-11	2	4	51.84	2.65
-11	-2	-4	53.85	2.77
-11	2	4	54.85	2.69
-11	-2	-4	56.95	2.78
-11	2	4	52.96	2.95
-11	-2	-4	55.79	2.77
11	-2	4	55.83	2.92
-11	-2	-4	55.97	2.92
11	-3	-4	106.21	5.11
-11	-3	4	102.41	4.70
-11	-3	4	108.31	4.94
-11	-3	4	93.91	4.93
-11	3	-4	109.76	5.40
-11	3	-4	108.46	5.06
-11	3	-4	103.31	5.11
-11	-3	4	104.69	4.77
-11	3	-4	118.84	5.20
-11	-3	4	96.85	4.68
-11	-3	4	103.01	4.97
11	3	4	95.96	4.72
11	-3	-4	114.51	5.27
-11	3	-4	111.08	5.16
11	3	4	103.79	4.87
11	-3	-4	109.54	5.08
11	3	4	97.63	4.95
-11	3	-4	108.17	5.13
-11	-3	4	97.32	4.75
11	3	4	100.28	4.76
11	-3	-4	105.63	5.15
-11	3	-4	108.53	5.37
11	-3	4	93.79	4.75
-11	-3	-4	112.84	5.34
11	3	-4	102.40	4.98
-11	-3	-4	108.26	5.14

-11	3	4	101.02	4.99
-11	3	4	102.82	4.98
-11	-3	-4	101.74	5.18
11	-3	4	103.67	4.69
-11	-3	-4	108.91	4.95
11	3	-4	109.52	4.93
-11	-3	-4	108.61	5.16
-11	3	4	101.30	4.84
-11	3	4	103.10	4.87
11	-3	4	92.60	4.77
-11	-3	-4	107.56	5.16
-11	3	4	107.99	4.75
11	3	-4	109.48	5.17
-11	-3	-4	104.00	5.07
-11	3	4	98.96	4.77
11	-3	4	100.88	4.96
-11	-3	-4	108.39	5.05
-11	-4	4	36.27	1.92
-11	4	-4	44.92	2.79
-11	-4	4	34.78	2.26
-11	-4	4	38.99	2.24
-11	-4	4	37.50	2.03
-11	4	-4	46.63	2.46
11	-4	-4	45.58	2.45
-11	-4	4	34.64	2.00
-11	4	-4	44.30	2.40
11	4	4	33.45	2.23
-11	4	-4	50.24	2.50
-11	4	-4	46.07	2.33
-11	4	-4	50.40	2.51
11	-4	-4	46.46	2.36
11	4	4	38.96	2.16
11	-4	-4	44.27	2.43
-11	-4	4	38.73	1.93
-11	4	-4	48.34	2.78
-11	-4	4	39.77	2.31
11	4	4	36.27	2.01
11	4	4	38.27	2.02
11	-4	-4	48.65	2.66
-11	4	4	36.60	2.31
11	-4	4	33.90	2.02
-11	4	4	39.73	2.29
-11	-4	-4	46.13	2.47
11	-4	4	40.50	2.74

-11	-4	-4	44.98	2.55
-11	4	4	36.97	2.26
-11	4	4	40.00	2.14
-11	4	4	40.23	2.03
-11	-4	-4	45.33	2.28
-11	-4	-4	48.09	2.30
11	4	-4	47.71	2.44
11	-4	4	39.94	1.90
-11	-4	-4	46.08	2.26
-11	-4	-4	49.03	2.48
11	4	-4	45.42	2.27
11	-4	4	36.26	2.02
-11	4	4	35.52	2.11
11	-4	4	41.00	2.34
-11	-4	-4	48.20	2.70
-11	-4	-4	47.69	2.44
11	4	-4	43.49	2.36
11	5	4	35.35	2.10
11	-5	-4	31.02	1.78
-11	5	-4	30.92	2.27
-11	-5	4	36.55	2.35
11	5	4	38.84	2.23
11	-5	-4	31.00	1.86
-11	5	-4	34.97	1.97
-11	5	-4	32.19	1.71
11	5	4	40.76	2.07
-11	-5	4	37.86	1.95
11	-5	-4	29.80	2.10
-11	-5	4	37.91	2.30
-11	-5	4	36.44	2.07
-11	-5	4	37.32	1.94
11	5	4	35.95	2.35
-11	5	-4	34.37	2.25
11	-5	-4	31.86	1.88
-11	-5	4	39.08	2.41
-11	5	-4	34.51	1.91
-11	5	-4	33.32	1.97
11	5	-4	33.33	1.84
-11	5	4	36.98	2.24
11	5	-4	33.45	1.75
11	5	-4	29.44	1.87
11	-5	4	42.46	2.60
-11	-5	-4	33.45	2.18
11	5	-4	33.13	1.84

11	-5	4	38.47	2.07
-11	5	4	39.73	2.10
-11	5	4	38.18	2.37
-11	5	4	38.74	2.22
-11	5	4	37.50	2.41
11	-5	4	38.71	2.40
11	-5	4	37.36	2.14
-11	-5	-4	33.39	1.91
11	-5	4	36.83	2.08
-11	-5	-4	34.69	1.86
-11	-5	-4	33.26	1.71
-11	-5	-4	32.31	1.92
-11	6	-4	32.21	2.00
-11	-6	4	30.84	1.64
11	6	4	32.44	2.04
11	6	4	32.19	1.99
11	6	4	30.57	2.17
11	-6	-4	33.56	2.24
-11	-6	4	31.24	1.91
11	6	4	32.87	1.85
11	-6	-4	29.60	1.83
-11	-6	4	33.41	1.61
-11	-6	4	30.88	2.11
-11	6	-4	32.36	1.91
11	-6	-4	29.16	1.73
-11	6	-4	28.76	1.63
11	-6	-4	29.28	1.91
-11	-6	4	32.45	2.20
11	-6	-4	35.89	2.51
-11	-6	4	32.68	1.73
-11	6	-4	30.24	2.47
-11	-6	4	33.61	1.74
-11	6	-4	29.27	2.24
11	-6	4	32.06	1.96
11	6	-4	34.10	1.85
-11	6	4	35.14	2.27
11	-6	4	29.74	1.85
11	6	-4	29.02	1.91
11	6	-4	31.41	1.75
-11	6	4	33.54	1.92
11	6	-4	31.22	1.77
-11	-6	-4	30.19	1.88
-11	6	4	32.31	2.22
-11	6	4	36.96	2.79

11	-6	4	30.64	1.83
-11	6	4	31.83	2.31
11	-6	4	35.58	2.49
-11	-6	-4	30.34	1.82
11	-7	-4	21.14	1.46
-11	-7	4	24.89	1.34
11	-7	-4	23.99	1.63
11	7	4	24.39	1.83
-11	7	-4	21.17	2.37
-11	-7	4	23.24	1.37
11	-7	-4	21.97	2.00
11	7	4	22.16	1.73
-11	-7	4	22.97	1.33
11	-7	-4	19.99	1.42
11	7	4	21.64	1.92
11	7	4	25.01	1.52
-11	-7	4	21.81	1.88
-11	7	-4	22.71	1.60
-11	-7	4	23.39	1.85
-11	-7	4	22.26	1.56
-11	7	-4	23.56	2.11
11	7	-4	22.99	1.48
-11	-7	-4	21.54	1.50
-11	-7	-4	21.34	1.60
-11	7	4	23.82	2.09
11	-7	4	24.77	2.33
11	7	-4	22.98	1.44
11	-7	4	23.74	1.65
-11	7	4	21.28	1.90
-11	7	4	22.17	1.68
11	-7	4	23.04	1.48
11	7	-4	20.50	1.71
11	-7	4	22.58	1.56
-11	8	-4	2.39	1.37
11	-8	-4	1.91	0.59
-11	-8	4	1.72	0.51
-11	-8	4	1.79	0.47
11	-8	-4	2.04	1.14
11	8	4	1.93	0.97
-11	-8	4	0.28	0.85
11	8	4	1.86	0.64
11	-8	-4	0.90	0.62
-11	-8	4	1.15	0.62
-11	-8	4	1.46	0.52

-11	8	-4	3.39	1.13
11	8	4	1.15	0.81
-11	-8	4	0.74	0.96
11	8	4	2.11	1.00
11	8	-4	2.13	1.19
-11	8	4	2.03	1.15
11	8	-4	2.73	0.60
11	-8	4	0.97	0.60
11	8	-4	0.83	0.82
-11	-8	-4	1.89	0.68
11	-8	4	0.90	0.50
11	-8	4	2.27	0.62
11	-8	4	0.70	1.59
-11	-8	-4	1.60	0.60
11	9	4	13.49	1.75
11	-9	-4	15.13	1.28
-11	-9	4	14.80	1.50
11	9	4	14.81	1.32
11	9	4	16.82	1.76
11	9	4	14.82	1.87
11	-9	-4	13.61	1.31
-11	9	-4	18.77	2.15
-11	-9	4	16.77	1.92
-11	-9	4	15.14	1.30
-11	9	-4	14.78	2.20
11	-9	-4	17.93	2.42
-11	9	4	16.29	2.20
11	9	-4	14.44	1.70
-11	-9	-4	13.30	1.33
11	9	-4	14.66	1.25
11	-9	4	14.06	1.48
11	-9	4	16.31	1.39
11	9	-4	11.34	1.83
11	-9	4	12.70	1.25
-11	9	4	13.90	1.98
-11	-9	-4	13.31	1.50
11	-9	4	12.92	2.45
-11	-10	4	22.34	2.43
11	-10	-4	24.83	1.82
11	-10	-4	25.08	1.80
-11	10	-4	26.55	2.66
-11	-10	4	22.14	1.88
11	10	4	21.25	2.33
11	-10	-4	24.34	2.04

11	10	4	24.48	2.44
11	10	-4	27.64	2.60
-11	10	4	25.88	2.50
-11	-10	-4	22.73	1.79
11	-10	4	20.94	1.57
11	-10	4	22.18	1.80
11	10	-4	24.76	1.64
-11	-10	-4	26.62	2.12
-11	-11	4	30.35	2.67
11	-11	-4	24.56	2.22
-11	11	-4	30.70	3.26
11	-11	-4	26.58	2.06
11	-11	-4	23.62	1.95
-11	-11	4	25.86	2.13
11	11	4	25.14	2.65
11	11	-4	21.22	2.53
-11	-11	-4	24.49	1.82
11	-11	4	24.30	1.82
11	-11	4	25.82	1.95
-11	11	4	31.12	3.11
11	-11	4	23.76	2.17
-11	-11	-4	24.87	2.59
-11	-11	-4	27.21	2.13
-11	12	-4	24.87	3.01
-11	-12	4	26.59	2.18
11	-12	-4	26.01	2.30
11	-12	-4	18.96	1.86
-11	-12	4	22.38	2.66
11	12	4	24.11	2.66
11	-12	-4	24.71	1.92
-11	-12	-4	22.77	2.61
11	12	-4	22.83	2.57
-11	-12	-4	21.29	2.02
11	-12	4	23.98	1.95
11	-12	4	26.06	2.34
-11	12	4	28.13	3.13
11	-12	4	24.76	1.88
12	0	-4	150.28	7.06
12	0	4	135.06	6.19
12	0	4	130.11	6.10
12	0	4	124.31	5.93
12	0	-4	152.49	7.03
12	0	4	125.20	6.10
-12	0	-4	152.34	7.24

-12	0	4	122.97	6.06
-12	0	-4	161.15	7.27
-12	0	4	132.23	6.22
-12	0	-4	154.41	7.35
12	0	-4	158.41	7.22
-12	0	-4	164.03	7.08
12	0	-4	150.52	7.18
-12	0	4	130.60	6.07
-12	0	4	134.58	6.06
-12	0	4	131.86	6.01
-12	0	-4	159.30	7.30
12	0	4	123.39	6.05
-12	0	4	130.12	6.15
-12	1	-4	104.31	4.86
12	-1	-4	97.98	4.76
-12	1	-4	97.60	4.83
12	1	4	108.28	4.93
12	1	4	100.64	5.00
-12	1	-4	101.55	4.85
12	-1	-4	97.17	4.81
-12	1	-4	105.12	4.85
12	-1	-4	99.86	4.69
-12	-1	4	98.65	4.84
-12	-1	4	101.42	4.97
-12	-1	4	104.45	4.87
-12	-1	4	104.23	5.00
-12	-1	4	98.23	4.85
-12	-1	4	99.79	4.98
-12	1	-4	100.38	5.00
-12	1	-4	102.57	4.72
-12	-1	4	111.31	5.10
-12	1	-4	100.15	4.85
12	1	4	102.21	4.80
-12	-1	4	106.50	4.90
12	-1	-4	100.43	4.75
12	1	4	101.86	4.94
-12	1	4	99.54	4.85
12	-1	4	98.33	4.81
-12	-1	-4	101.83	4.67
-12	1	4	105.72	4.95
12	-1	4	105.02	5.00
12	-1	4	102.52	4.93
-12	-1	-4	98.09	4.89
-12	1	4	105.06	4.87

-12	-1	-4	102.52	4.85
-12	-1	-4	101.68	4.86
-12	-1	-4	104.03	4.63
12	-1	4	107.09	4.93
12	-1	4	107.18	5.10
-12	-1	-4	106.92	4.85
-12	1	4	105.29	5.10
-12	1	4	107.43	4.87
-12	1	4	99.57	5.03
-12	-1	-4	107.46	4.97
-12	2	-4	54.18	2.58
12	2	4	48.67	2.77
-12	-2	4	54.27	2.62
-12	-2	4	52.43	2.81
-12	2	-4	51.70	2.66
12	-2	-4	52.48	2.57
12	-2	-4	48.43	2.76
-12	2	-4	52.72	2.78
-12	-2	4	53.84	2.62
12	-2	-4	51.37	2.64
12	2	4	55.48	2.72
-12	-2	4	50.95	2.68
-12	-2	4	52.59	2.82
-12	2	-4	49.65	2.62
-12	2	-4	50.68	2.85
-12	-2	4	51.61	2.79
-12	2	-4	55.29	2.70
-12	-2	4	48.94	2.56
12	2	4	51.10	2.54
-12	2	-4	49.15	2.59
12	2	4	53.13	2.65
-12	-2	4	49.33	2.59
12	-2	-4	49.98	2.53
-12	-2	-4	51.85	2.81
-12	2	4	51.97	2.83
-12	-2	-4	53.70	2.41
-12	-2	-4	53.69	2.44
12	-2	4	54.62	2.96
-12	-2	-4	52.68	2.65
12	-2	4	52.67	2.63
-12	2	4	52.30	2.61
-12	2	4	51.11	2.70
-12	-2	-4	53.38	2.64
12	-2	4	51.87	2.59

12	-2	4	51.34	2.80
-12	2	4	55.58	2.62
-12	-2	-4	52.00	2.67
12	-2	4	51.68	2.66
-12	-2	-4	47.46	2.65
-12	2	4	53.02	2.88
-12	2	4	51.68	2.53
-12	2	4	48.63	2.52
-12	-2	-4	50.69	2.64
-12	2	4	54.08	2.54
12	-3	-4	19.97	1.28
-12	-3	4	28.51	1.77
-12	3	-4	24.18	1.45
-12	3	-4	23.36	1.42
-12	-3	4	25.93	1.51
12	3	4	23.96	1.41
12	3	4	26.84	1.56
-12	3	-4	21.03	1.33
12	-3	-4	22.33	1.28
-12	-3	4	24.01	1.40
-12	3	-4	21.16	1.30
12	3	4	25.63	1.51
-12	3	-4	22.39	1.68
12	3	4	24.60	1.70
-12	-3	4	24.33	1.75
-12	3	-4	21.00	1.67
12	-3	-4	22.78	1.51
-12	-3	4	27.62	1.47
-12	3	-4	22.17	1.37
-12	-3	4	24.19	1.46
-12	-3	4	27.14	1.74
12	3	-4	22.86	1.41
-12	3	4	26.11	1.64
-12	-3	-4	22.45	1.15
-12	-3	-4	22.51	1.37
-12	-3	-4	20.00	1.29
-12	3	4	25.02	1.73
-12	3	4	26.34	1.49
-12	3	4	24.64	1.78
12	-3	4	24.49	1.50
12	-3	4	24.96	1.46
-12	-3	-4	20.17	1.36
-12	3	4	25.26	1.61
-12	3	4	26.34	1.52

-12	-3	-4	21.80	1.11
12	-3	4	26.46	1.43
-12	-3	-4	22.86	1.33
-12	-3	-4	22.33	1.63
12	3	-4	21.83	1.10
12	3	-4	20.59	1.16
12	-3	4	26.20	1.87
12	-3	4	23.63	1.74
-12	-3	-4	21.73	1.40
12	-4	-4	14.48	0.98
12	-4	-4	12.36	1.29
12	4	4	15.84	1.15
12	4	4	14.04	1.04
-12	-4	4	15.25	1.13
-12	4	-4	13.56	1.56
-12	4	-4	14.84	1.01
-12	-4	4	15.08	1.07
12	-4	-4	14.32	1.08
-12	4	-4	15.24	1.05
-12	4	-4	13.45	1.14
12	-4	-4	13.86	1.06
12	4	4	14.12	1.34
-12	-4	4	14.31	0.97
-12	-4	4	12.58	1.33
-12	4	-4	15.42	1.13
-12	4	-4	14.39	1.48
-12	-4	4	15.63	1.46
-12	-4	4	15.02	1.00
12	4	4	14.14	1.09
-12	4	-4	14.99	1.19
-12	-4	4	18.01	1.45
-12	-4	-4	13.59	1.02
12	-4	4	14.14	1.06
-12	4	4	13.77	1.25
12	-4	4	14.60	1.08
-12	-4	-4	14.58	1.00
12	4	-4	12.23	0.97
-12	4	4	14.26	1.21
-12	-4	-4	14.36	0.86
-12	-4	-4	13.59	1.15
-12	4	4	14.58	1.38
-12	-4	-4	15.41	1.13
12	4	-4	14.43	0.87
-12	-4	-4	13.53	1.08

-12	4	4	14.78	1.10
-12	-4	-4	14.53	1.42
12	-4	4	16.26	1.47
-12	-4	-4	15.60	0.91
12	-4	4	15.57	0.94
-12	4	4	14.33	1.48
-12	4	4	15.84	1.40
12	4	-4	15.36	1.11
-12	5	-4	40.67	2.28
-12	-5	4	40.70	2.56
-12	-5	4	39.83	2.51
-12	5	-4	37.66	2.21
12	-5	-4	36.35	2.36
12	5	4	40.79	2.53
12	-5	-4	37.47	2.06
-12	5	-4	38.91	2.64
-12	-5	4	42.08	2.27
-12	-5	4	37.34	2.21
-12	-5	4	40.71	2.09
12	5	4	38.19	2.23
12	-5	-4	40.73	2.19
-12	5	-4	39.73	2.21
-12	-5	4	43.63	2.48
-12	-5	4	43.14	2.10
12	5	4	41.14	2.21
-12	5	-4	38.09	2.54
-12	5	-4	37.69	1.97
12	-5	-4	38.48	2.16
12	5	-4	35.19	2.11
-12	-5	-4	36.13	2.43
12	-5	4	41.52	2.28
12	5	-4	40.84	2.13
-12	5	4	38.84	2.51
12	-5	4	39.78	2.61
-12	-5	-4	40.98	2.22
-12	5	4	40.73	2.37
-12	-5	-4	39.09	2.22
12	-5	4	46.59	2.78
-12	-5	-4	37.54	1.93
12	5	-4	38.47	2.01
-12	5	4	40.09	2.35
-12	5	4	41.84	2.25
-12	-5	-4	39.43	2.15
-12	-5	-4	41.12	2.00

-12	5	4	40.82	2.54
12	-5	4	40.70	2.23
-12	6	-4	89.72	5.02
12	6	4	93.57	4.52
-12	-6	4	86.31	4.40
12	-6	-4	97.35	4.93
12	6	4	88.78	4.30
12	-6	-4	90.63	4.60
12	6	4	85.91	4.65
-12	-6	4	87.58	4.64
12	-6	-4	93.25	4.66
12	6	4	84.67	4.42
-12	-6	4	92.46	4.18
-12	6	-4	96.75	4.64
12	-6	-4	90.33	4.52
-12	-6	4	84.70	4.50
-12	-6	4	88.56	4.20
-12	6	-4	93.74	4.99
-12	6	4	88.02	4.72
12	6	-4	100.76	4.75
-12	6	4	88.13	4.36
-12	-6	-4	92.36	4.65
-12	-6	-4	93.40	4.58
12	-6	4	89.59	4.35
12	6	-4	97.75	4.53
12	-6	4	91.30	4.45
12	-6	4	94.36	4.89
12	6	-4	85.52	4.61
12	6	-4	95.19	4.54
-12	6	4	89.66	4.68
-12	-6	-4	96.08	4.46
12	-6	4	82.24	4.33
-12	-7	4	60.79	3.32
12	7	4	59.26	3.03
-12	-7	4	59.10	2.88
-12	-7	4	60.68	2.81
-12	7	-4	62.13	4.15
12	-7	-4	67.32	3.77
12	7	4	54.50	3.43
12	-7	-4	61.23	3.31
12	-7	-4	65.06	3.40
-12	-7	4	54.42	3.12
-12	-7	4	56.60	2.89
12	-7	-4	63.50	3.24

-12	7	-4	58.38	3.93
-12	-7	4	58.05	3.44
12	7	4	63.66	3.33
12	7	-4	58.45	3.43
-12	-7	-4	62.49	3.39
-12	7	4	55.41	3.55
12	7	-4	68.22	3.29
12	7	-4	65.57	3.22
12	-7	4	57.07	3.18
-12	-7	-4	67.07	3.22
12	-7	4	61.22	3.07
-12	-7	-4	60.43	3.27
12	-7	4	63.03	3.78
-12	7	4	60.38	3.53
12	-8	-4	62.48	3.67
-12	-8	4	60.76	2.86
-12	-8	4	58.03	3.52
12	8	4	59.80	3.35
12	8	4	52.35	3.31
-12	8	-4	57.75	3.61
-12	-8	4	57.55	3.15
-12	8	-4	57.97	3.89
12	8	4	57.42	3.01
-12	-8	4	56.34	2.85
12	-8	-4	53.90	3.03
12	-8	-4	48.14	3.20
12	-8	-4	52.85	3.39
-12	-8	4	59.65	2.83
12	8	4	54.62	3.50
-12	-8	4	57.12	3.33
-12	8	4	56.97	3.67
12	-8	4	64.28	3.94
12	8	-4	52.13	3.30
12	-8	4	53.30	3.01
12	8	-4	58.52	2.93
-12	8	4	60.31	3.56
12	-8	4	56.86	3.20
12	-8	4	58.86	3.11
12	8	-4	60.14	3.08
-12	-8	-4	59.18	2.98
-12	-8	-4	56.41	3.01
-12	-8	-4	57.87	3.18
12	9	4	65.51	3.84
-12	-9	4	69.75	3.64

-12	-9	4	65.74	3.95
-12	-9	4	64.52	3.19
12	-9	-4	67.01	3.50
-12	9	-4	67.00	4.25
12	9	4	64.17	3.96
12	-9	-4	65.45	3.68
12	-9	-4	65.46	3.54
12	9	4	64.35	3.34
-12	-9	-4	66.90	3.66
12	-9	4	65.56	3.52
12	9	-4	68.99	3.37
-12	9	4	69.86	4.03
-12	-9	-4	62.04	3.47
12	9	-4	61.92	3.82
12	-9	4	62.12	3.41
12	9	-4	73.12	3.61
12	-10	-4	6.30	1.14
-12	-10	4	6.78	1.19
12	-10	-4	3.99	1.05
-12	10	-4	6.37	1.77
-12	-10	4	6.60	1.53
12	10	4	6.10	1.47
12	-10	-4	5.50	0.90
12	10	4	10.04	1.78
12	-10	4	7.23	1.41
-12	-10	-4	5.92	1.17
12	-10	4	5.60	0.92
-12	-10	-4	6.04	0.99
12	-10	4	5.30	0.99
12	10	-4	6.06	1.56
-12	10	4	7.30	1.50
-12	-11	4	1.22	1.20
12	-11	-4	-0.17	0.86
12	-11	-4	0.01	1.02
-12	-11	4	2.05	0.94
12	-11	-4	1.15	0.80
-12	11	-4	1.06	1.57
12	11	4	1.20	1.26
12	-11	4	0.39	0.78
-12	-11	-4	2.11	0.96
-12	-11	-4	0.30	0.76
-12	-11	-4	-0.71	1.17
-12	11	4	-1.95	1.68
12	-11	4	1.70	0.79

12	-11	4	0.26	1.02
12	11	-4	-0.62	1.21
13	0	4	0.13	0.49
13	0	-4	0.03	0.17
13	0	-4	-0.08	0.25
13	0	4	0.10	0.23
13	0	-4	-0.25	0.29
13	0	4	0.50	0.42
-13	0	-4	0.59	0.51
13	0	4	-0.31	0.27
-13	0	-4	0.24	0.32
-13	0	4	-0.58	0.55
-13	0	-4	0.14	0.26
-13	0	-4	0.12	0.41
13	0	-4	0.20	0.22
-13	0	-4	-0.23	0.37
-13	0	4	0.34	0.47
-13	0	-4	-0.17	0.46
-13	0	4	0.61	0.36
-13	0	4	0.16	0.39
13	0	4	-0.02	0.50
-13	0	4	-0.09	0.41
-13	0	-4	-0.38	0.37
-13	0	4	-0.23	0.36
-13	1	-4	4.13	0.75
-13	-1	4	1.53	0.66
-13	-1	4	1.35	0.44
13	-1	-4	3.70	0.55
-13	1	-4	3.32	0.60
13	-1	-4	2.96	0.51
-13	1	-4	3.96	0.72
-13	-1	4	1.41	0.40
13	1	4	0.98	0.40
-13	-1	4	2.85	0.51
13	-1	-4	3.44	0.58
13	-1	-4	2.69	0.41
13	1	4	1.41	0.51
-13	-1	4	1.43	0.49
13	1	4	1.31	0.29
-13	1	-4	5.00	0.90
-13	1	-4	3.88	0.62
-13	1	-4	3.44	0.52
13	1	4	1.10	0.52
-13	-1	4	1.26	0.50

13	1	4	1.24	0.54
-13	-1	4	2.00	0.59
-13	-1	4	2.86	0.50
-13	1	4	1.29	0.41
13	-1	4	1.16	0.31
13	-1	4	0.73	0.56
13	-1	4	0.95	0.37
13	-1	4	1.74	0.81
13	-1	4	1.49	0.46
-13	-1	-4	3.46	0.71
-13	-1	-4	4.08	0.82
-13	1	4	1.82	0.59
-13	-1	-4	3.25	0.57
-13	-1	-4	3.06	0.43
-13	1	4	1.60	0.68
-13	-1	-4	3.09	0.36
-13	1	4	2.09	0.46
-13	1	4	1.90	0.54
-13	-1	-4	3.55	0.60
-13	-1	-4	2.63	0.51
-13	2	-4	42.81	2.13
13	-2	-4	37.43	2.11
-13	-2	4	39.32	2.29
-13	2	-4	36.82	2.10
-13	2	-4	42.81	2.43
13	2	4	43.19	2.45
-13	-2	4	41.87	2.21
13	2	4	43.03	2.15
13	-2	-4	37.97	2.00
-13	-2	4	43.26	2.24
-13	-2	4	44.50	2.26
13	-2	-4	42.66	2.14
13	-2	-4	38.26	2.04
-13	2	-4	39.91	2.29
-13	-2	4	39.89	2.18
13	2	4	45.14	2.34
-13	2	-4	39.65	2.16
13	2	4	45.38	2.30
-13	-2	4	45.68	2.48
-13	2	-4	37.59	2.03
-13	-2	4	39.77	2.45
-13	2	-4	36.66	2.14
-13	-2	4	44.16	2.44
13	-2	4	39.86	2.26

-13	-2	-4	40.54	2.18
13	-2	4	45.99	2.26
-13	-2	-4	36.37	1.83
-13	-2	-4	40.30	2.34
-13	2	4	42.02	2.11
-13	-2	-4	38.38	2.16
-13	-2	-4	41.37	2.14
-13	2	4	40.58	2.50
-13	2	4	45.61	2.25
-13	2	4	44.64	2.48
13	-2	4	41.59	2.45
-13	-2	-4	37.77	1.82
-13	-2	-4	40.26	2.16
-13	2	4	44.68	2.18
-13	2	4	42.53	2.34
13	-2	4	49.50	2.60
-13	-2	-4	41.70	2.16
-13	2	4	35.27	2.36
-13	2	4	44.51	2.16
13	-2	4	38.91	2.17
-13	3	-4	41.19	2.24
13	3	4	38.39	2.33
-13	-3	4	42.06	2.39
-13	3	-4	40.59	2.17
13	3	4	36.84	2.09
13	3	4	39.55	2.15
-13	-3	4	41.40	2.37
13	-3	-4	40.30	2.21
13	3	4	35.92	1.99
-13	-3	4	38.47	2.05
-13	-3	4	39.58	2.14
-13	-3	4	40.66	2.33
-13	3	-4	38.51	2.50
-13	-3	4	40.52	2.03
13	-3	-4	40.88	2.12
-13	-3	4	41.16	2.10
13	-3	-4	39.93	2.13
-13	-3	4	37.76	2.10
-13	3	-4	43.66	2.55
13	-3	-4	46.72	2.41
-13	3	-4	38.23	2.16
-13	3	-4	39.85	2.52
-13	3	-4	41.93	2.27
-13	-3	-4	40.57	2.24

-13	-3	-4	39.39	2.21
13	3	-4	38.93	1.91
13	-3	4	38.15	2.35
-13	-3	-4	42.80	2.23
13	-3	4	41.17	2.51
-13	-3	-4	40.57	2.45
-13	3	4	41.44	2.10
-13	-3	-4	40.00	1.92
-13	-3	-4	40.42	1.90
-13	3	4	35.67	2.32
13	-3	4	39.51	2.04
-13	3	4	38.42	2.08
-13	3	4	38.31	2.21
-13	-3	-4	40.66	2.19
13	-3	4	35.09	2.04
13	-3	4	36.10	2.09
13	3	-4	39.04	1.97
-13	3	4	40.86	2.40
-13	3	4	38.13	2.42
-13	4	-4	12.24	0.96
-13	4	-4	8.79	1.04
13	-4	-4	8.51	0.91
13	-4	-4	12.58	0.89
13	4	4	14.19	1.11
-13	-4	4	15.44	1.46
-13	4	-4	9.94	1.02
-13	-4	4	16.91	1.57
-13	4	-4	12.24	1.11
13	4	4	14.98	1.04
-13	4	-4	12.52	1.52
13	-4	-4	9.72	0.93
13	4	4	11.55	1.12
13	4	4	15.12	1.38
-13	-4	4	13.55	1.02
-13	-4	4	13.89	1.12
13	4	-4	9.16	0.72
-13	4	4	17.78	1.16
13	4	-4	11.71	0.99
-13	-4	-4	8.19	0.63
-13	-4	-4	8.36	0.96
13	-4	4	16.68	1.53
13	-4	4	15.03	0.94
13	-4	4	11.54	1.02
13	-4	4	12.37	1.06

-13	-4	-4	9.16	0.67
-13	-4	-4	10.02	1.02
-13	4	4	12.74	1.22
-13	-4	-4	13.21	1.41
13	-5	-4	9.91	1.04
13	-5	-4	13.89	1.43
13	5	4	15.22	1.28
13	5	4	17.00	1.24
13	-5	-4	9.93	1.00
13	5	4	17.85	1.57
-13	-5	4	19.78	1.62
-13	-5	4	20.25	1.14
-13	5	-4	13.90	1.78
13	-5	-4	11.89	0.94
-13	-5	4	16.79	1.26
-13	-5	4	15.03	1.27
-13	5	-4	10.79	1.14
-13	-5	4	16.57	1.62
-13	-5	4	14.68	1.11
13	5	4	17.63	1.27
-13	5	4	16.79	1.61
-13	-5	-4	12.57	0.85
-13	5	4	16.89	1.71
13	5	-4	10.39	0.87
13	-5	4	13.80	1.17
13	5	-4	12.79	1.04
13	-5	4	13.29	1.20
-13	5	4	18.12	1.31
13	-5	4	18.91	1.88
13	5	-4	12.20	1.05
13	-5	4	18.89	1.34
13	-6	-4	19.02	1.50
13	6	4	22.75	1.49
-13	-6	4	22.49	1.35
13	6	4	21.80	1.89
-13	-6	4	21.85	1.89
-13	6	-4	23.43	2.10
-13	-6	4	22.27	1.89
-13	-6	4	20.95	1.49
-13	6	-4	23.96	2.30
13	6	4	22.74	1.59
-13	-6	4	20.88	1.33
13	6	4	22.62	1.60
13	-6	-4	22.34	1.54

13	-6	-4	21.46	1.43
13	6	-4	22.22	1.45
-13	6	4	21.14	1.95
-13	-6	-4	24.76	1.55
-13	6	4	21.03	1.56
13	6	-4	21.91	1.41
-13	6	4	20.07	2.01
13	-6	4	19.64	1.47
-13	-6	-4	27.37	1.68
13	6	-4	21.90	1.62
13	-6	4	28.31	2.26
13	-6	4	20.68	1.54
-13	-6	-4	24.17	1.37
-13	7	-4	19.62	2.09
13	-7	-4	21.01	2.07
13	7	4	17.15	1.53
-13	-7	4	17.53	1.21
-13	7	-4	19.10	2.35
-13	-7	4	16.83	2.05
13	7	4	16.77	1.81
13	-7	-4	17.59	1.33
-13	-7	4	18.35	1.90
13	7	4	18.39	1.39
-13	-7	4	19.52	1.23
-13	-7	4	15.01	1.79
13	-7	-4	20.05	1.45
13	-7	-4	17.85	1.66
13	7	-4	21.01	1.37
-13	7	4	19.64	2.15
13	7	-4	19.82	1.59
13	7	-4	19.95	1.45
-13	-7	-4	21.31	1.58
-13	-7	-4	21.36	1.34
-13	-7	-4	17.77	1.48
13	-7	4	19.17	1.36
13	-7	4	19.39	1.37
13	-7	4	18.03	1.48
13	-7	4	22.66	2.23
-13	7	4	17.22	1.87
-13	-8	4	10.06	0.86
-13	-8	4	9.87	0.95
13	8	4	10.57	1.67
13	8	4	10.10	1.67
13	-8	-4	11.40	1.17

13	8	4	12.44	1.53
13	-8	-4	10.07	1.83
13	8	4	10.80	1.18
-13	8	-4	10.09	1.80
13	-8	-4	7.64	1.01
13	-8	-4	7.92	1.21
-13	-8	4	10.01	0.93
-13	8	-4	10.64	1.93
-13	-8	4	9.24	1.70
-13	-8	4	10.78	1.26
-13	-8	4	12.81	1.71
-13	-8	-4	12.25	1.33
13	8	-4	10.64	1.48
-13	8	4	12.69	1.93
13	-8	4	8.16	1.15
-13	-8	-4	9.51	1.09
-13	8	4	13.90	2.20
13	8	-4	8.08	1.30
13	8	-4	10.21	1.04
13	-8	4	15.47	2.37
-13	-8	-4	9.39	1.19
13	-8	4	9.96	1.18
-13	-9	4	3.05	1.21
13	9	4	3.25	1.18
13	-9	-4	4.21	0.84
13	-9	-4	2.29	0.79
-13	-9	4	3.05	0.84
-13	-9	4	3.16	0.56
-13	9	-4	3.07	1.30
13	9	4	3.98	1.14
13	-9	-4	4.37	0.99
13	-9	4	3.46	0.79
13	9	-4	3.80	0.70
13	9	-4	2.40	1.16
-13	-9	-4	3.33	1.01
13	-9	4	2.99	0.74
-13	-9	-4	3.14	0.85
13	-9	4	2.41	0.91
-13	9	4	4.24	1.39
13	9	-4	4.36	0.98
-13	10	-4	5.20	1.91
13	-10	-4	8.21	1.33
13	-10	-4	8.25	1.31
13	-10	-4	7.38	1.49

13	10	4	11.72	2.05
13	10	4	4.64	1.31
-13	10	4	6.25	1.76
13	-10	4	5.03	1.20
13	-10	4	4.19	0.86
-13	-10	-4	5.20	0.97
-13	-10	-4	5.77	1.20
13	-10	4	5.26	0.95
13	10	-4	9.53	1.89
13	-11	-4	11.14	1.48
-13	11	-4	11.38	2.03
-13	-11	4	11.22	2.11
13	-11	-4	9.78	1.93
13	-11	-4	9.88	1.48
13	11	4	5.20	1.43
-13	-11	4	7.87	1.44
-13	-11	-4	13.65	2.15
13	-11	4	8.37	1.61
-13	11	4	6.53	1.79
13	11	-4	7.79	1.64
-13	-11	-4	10.35	1.70
13	-11	4	6.48	1.07
13	-11	4	5.43	0.95
-14	0	-4	181.53	8.10
-14	0	-4	171.91	8.07
-14	0	4	141.11	7.16
-14	0	-4	172.43	8.20
14	0	-4	169.42	8.01
14	0	-4	173.75	8.04
14	0	4	152.46	7.04
-14	0	4	159.33	7.19
14	0	4	151.35	7.23
-14	0	4	151.34	7.12
14	0	-4	169.82	7.81
-14	0	-4	180.40	8.14
-14	0	-4	175.32	7.83
14	0	4	154.32	7.20
-14	0	-4	176.79	7.88
14	0	4	157.62	7.24
-14	0	4	147.45	7.27
-14	0	4	155.62	7.20
14	0	-4	171.43	7.86
-14	0	-4	164.46	8.06
-14	0	4	154.01	7.36

14	0	4	161.25	7.28
14	1	4	105.39	5.03
-14	-1	4	103.36	5.08
-14	-1	4	107.75	5.21
-14	1	-4	104.28	5.00
-14	1	-4	108.38	4.82
14	1	4	98.55	5.02
14	-1	-4	104.13	4.79
-14	1	-4	100.39	5.17
-14	1	-4	105.49	5.01
-14	-1	4	103.76	5.09
-14	-1	4	101.92	4.93
-14	-1	4	107.49	4.97
-14	-1	4	101.71	4.94
14	-1	-4	104.25	4.97
14	1	4	102.73	5.11
-14	1	-4	95.43	4.96
14	-1	-4	105.61	4.88
14	1	4	101.01	4.86
-14	-1	4	110.31	5.01
-14	1	-4	104.99	4.93
14	1	4	106.95	5.10
-14	1	-4	105.49	5.02
14	-1	-4	103.88	4.92
-14	-1	4	110.13	5.11
-14	1	4	104.25	5.04
-14	-1	-4	106.48	5.11
-14	1	4	107.94	5.00
14	-1	4	100.51	5.01
-14	1	4	102.00	5.16
-14	-1	-4	98.77	4.98
-14	-1	-4	97.15	5.05
14	-1	4	100.71	5.10
14	-1	4	103.19	5.01
-14	-1	-4	108.80	5.00
-14	1	4	109.08	5.24
-14	1	4	101.99	4.95
-14	-1	-4	104.88	5.01
14	-1	4	97.64	4.87
-14	1	4	112.07	4.97
-14	-2	4	111.91	5.67
-14	-2	4	112.58	5.51
14	-2	-4	110.47	5.07
-14	2	-4	110.78	5.12

-14	-2	4	111.04	5.49
-14	2	-4	103.10	5.31
14	-2	-4	100.82	5.05
-14	2	-4	103.32	5.02
-14	-2	4	121.12	5.73
-14	-2	4	120.44	5.53
-14	2	-4	109.47	4.96
-14	-2	4	109.98	5.46
14	2	4	121.59	5.60
14	2	4	113.28	5.42
14	-2	-4	107.49	4.99
-14	2	-4	100.80	5.05
-14	-2	4	110.39	5.57
-14	2	-4	102.84	5.43
14	-2	-4	103.29	4.93
-14	-2	4	119.94	5.71
14	2	4	117.19	5.72
-14	-2	-4	105.95	5.05
14	-2	4	116.51	5.47
-14	-2	-4	106.98	5.07
-14	2	4	114.81	5.41
14	-2	4	113.58	5.69
-14	-2	-4	106.26	5.08
-14	-2	-4	107.20	5.08
-14	2	4	116.83	5.76
-14	-2	-4	107.28	5.24
14	-2	4	115.05	5.56
-14	-2	-4	99.75	5.07
14	-2	4	119.80	5.52
-14	2	4	119.41	5.51
14	-2	4	127.62	5.84
-14	2	4	117.08	5.61
-14	2	4	117.84	5.79
-14	2	4	116.50	5.42
-14	-2	-4	105.13	5.09
-14	3	-4	36.10	2.12
-14	3	-4	36.50	2.38
-14	3	-4	37.04	2.11
14	-3	-4	35.33	1.95
-14	-3	4	40.91	2.40
-14	-3	4	36.99	2.04
14	-3	-4	36.87	2.06
-14	-3	4	38.49	2.16
-14	-3	4	40.26	2.11

14	-3	-4	36.58	1.97
-14	-3	4	37.75	2.41
14	3	4	38.06	2.23
-14	-3	4	41.72	2.15
-14	3	-4	36.32	2.01
14	3	4	40.07	2.15
-14	-3	4	37.65	2.35
-14	3	-4	35.81	2.03
-14	3	-4	37.40	2.01
-14	-3	4	35.29	2.08
-14	3	-4	36.47	2.41
14	3	4	37.51	2.02
14	3	4	39.34	2.38
-14	3	4	37.63	2.44
-14	3	4	40.45	2.21
14	-3	4	42.16	2.09
-14	3	4	41.06	2.26
-14	-3	-4	37.84	2.08
-14	3	4	40.47	2.14
14	-3	4	40.36	2.60
-14	-3	-4	39.22	2.37
14	-3	4	35.73	2.38
14	-3	4	37.14	2.14
-14	-3	-4	35.19	2.05
-14	-3	-4	36.06	2.09
-14	-3	-4	38.34	2.08
-14	3	4	38.90	2.07
-14	-3	-4	35.38	2.07
14	3	-4	35.94	1.73
14	-3	4	37.49	2.08
-14	3	4	38.21	2.41
14	-4	-4	25.60	1.51
-14	-4	4	27.60	1.55
-14	4	-4	25.41	1.57
-14	4	-4	25.55	1.50
-14	4	-4	26.63	2.07
-14	-4	4	28.20	1.94
-14	-4	4	25.37	1.56
-14	-4	4	24.26	1.48
14	-4	-4	26.08	1.84
14	-4	-4	26.05	1.55
14	4	4	24.79	1.59
14	4	4	25.85	1.53
14	-4	-4	24.48	1.59

14	4	4	26.12	1.92
-14	-4	4	25.02	1.63
-14	4	-4	28.23	2.15
-14	-4	4	24.96	1.88
-14	-4	4	26.86	1.97
-14	-4	4	23.86	1.57
14	4	4	25.21	1.68
14	-4	4	26.95	1.45
-14	-4	-4	25.02	1.60
14	-4	4	25.33	1.57
-14	-4	-4	24.88	1.22
14	4	-4	23.67	1.42
-14	-4	-4	25.28	1.51
14	-4	4	25.58	1.60
14	4	-4	24.48	1.33
-14	4	4	26.46	1.62
-14	-4	-4	25.85	1.57
-14	-4	-4	26.82	1.65
14	-4	4	26.43	2.13
-14	4	4	23.67	1.89
14	5	4	14.84	1.52
14	5	4	14.49	1.17
-14	-5	4	14.36	1.17
14	-5	-4	20.74	1.74
-14	5	-4	18.43	1.29
-14	-5	4	14.34	1.05
14	-5	-4	17.89	1.30
14	5	4	14.16	1.30
-14	5	-4	16.87	1.95
-14	-5	4	12.83	1.59
14	-5	-4	15.79	1.19
-14	-5	4	15.78	1.53
-14	5	-4	17.77	1.82
14	5	4	14.74	1.23
-14	-5	4	14.72	1.08
-14	-5	4	14.87	1.41
14	-5	-4	16.67	1.25
14	-5	4	13.95	1.17
-14	5	4	14.89	1.28
14	5	-4	17.62	1.22
-14	5	4	15.28	1.69
14	5	-4	16.25	1.08
-14	-5	-4	17.69	1.35
-14	-5	-4	16.35	1.22

14	-5	4	14.46	0.96
-14	-5	-4	16.22	1.24
-14	5	4	14.09	1.58
-14	-5	-4	18.56	1.35
14	-5	4	12.61	1.10
-14	-5	-4	16.84	0.99
14	-6	-4	96.29	4.66
-14	-6	4	98.73	4.58
14	6	4	101.43	4.84
-14	-6	4	94.42	4.76
-14	-6	4	92.15	4.58
-14	-6	4	99.77	5.17
-14	6	-4	91.35	5.15
-14	-6	4	98.34	4.93
-14	6	-4	94.94	5.25
-14	6	-4	97.92	4.71
14	6	4	90.80	5.05
14	-6	-4	95.11	4.99
14	6	4	92.51	4.67
14	-6	-4	93.19	4.89
14	6	4	90.79	4.77
-14	6	4	95.86	5.16
14	6	-4	100.85	4.60
-14	-6	-4	102.12	4.47
-14	-6	-4	96.63	4.72
-14	6	4	94.76	5.09
-14	-6	-4	95.00	4.79
14	6	-4	84.30	4.78
14	6	-4	98.23	4.66
14	-6	4	95.71	4.80
14	-6	4	107.51	5.36
14	-7	-4	64.97	3.42
14	7	4	64.02	3.95
-14	7	-4	69.33	4.10
14	-7	-4	66.65	3.56
-14	-7	4	68.30	3.39
-14	-7	4	70.06	3.81
-14	-7	4	64.60	3.59
-14	7	-4	62.95	4.21
-14	-7	4	71.62	3.37
14	-7	-4	63.57	3.49
14	-7	-4	73.00	3.99
14	7	4	65.03	3.47
14	7	4	65.66	3.69

-14	-7	4	71.55	4.00
14	7	4	74.25	3.72
14	7	-4	67.35	3.41
-14	7	4	68.36	4.00
14	7	-4	62.57	3.57
-14	-7	-4	67.23	3.48
-14	-7	-4	64.05	3.59
-14	7	4	67.95	4.08
-14	-7	-4	68.41	3.27
14	-7	4	66.48	3.56
14	-7	4	75.96	4.29
14	7	-4	70.52	3.39
14	8	4	49.38	2.92
14	-8	-4	43.45	2.44
14	-8	-4	41.48	2.52
14	8	4	47.19	2.95
-14	-8	4	52.29	3.25
-14	-8	4	45.88	2.80
14	8	4	44.74	3.19
-14	8	-4	38.78	3.19
-14	-8	4	47.48	2.49
14	-8	-4	41.18	2.61
14	8	-4	40.90	2.69
-14	8	4	51.58	3.37
-14	-8	-4	47.78	2.71
14	8	-4	42.49	2.35
14	8	-4	48.02	2.52
-14	-8	-4	41.17	2.51
14	-8	4	48.46	2.84
-14	-8	-4	42.87	2.34
14	-8	4	47.37	2.67
14	-8	4	47.28	2.73
-14	9	-4	46.83	3.68
14	-9	-4	43.73	2.61
-14	-9	4	40.82	2.52
14	9	4	37.52	2.82
-14	-9	4	39.66	3.10
14	-9	-4	44.39	2.82
14	9	4	38.11	3.11
14	-9	-4	44.57	2.67
-14	9	4	42.15	3.22
14	-9	4	38.17	2.57
-14	-9	-4	44.13	2.65
14	9	-4	45.54	3.10

14	-9	4	39.22	2.48
-14	-9	-4	47.30	2.96
14	9	-4	48.00	2.45
14	-9	4	37.66	2.34
14	-10	-4	9.03	1.61
14	-10	-4	7.69	1.42
14	10	4	10.25	1.81
14	10	4	10.42	1.71
14	-10	-4	10.00	1.52
-14	-10	4	6.65	1.19
-14	-10	4	9.72	1.65
-14	10	-4	9.64	1.97
-14	10	4	6.40	1.73
14	-10	4	7.76	1.54
14	-10	4	6.80	1.27
14	10	-4	11.16	1.91
-14	-10	-4	10.47	1.32
-14	-10	-4	10.20	1.59
14	-11	-4	1.60	1.19
14	-11	-4	1.90	0.84
14	-11	-4	1.11	0.81
-14	11	-4	4.06	1.81
-14	-11	4	1.89	0.98
14	11	4	3.53	1.41
-14	-11	4	1.70	1.32
-14	-11	-4	1.61	1.33
14	-11	4	1.89	0.86
-14	-11	-4	2.14	0.94
-14	11	4	2.10	1.50
14	-11	4	0.52	1.15
14	11	-4	1.37	1.28
-15	0	4	0.40	0.56
15	0	-4	-0.28	0.31
-15	0	4	0.33	0.74
15	0	4	-0.18	0.37
15	0	-4	0.00	0.26
15	0	-4	0.22	0.19
15	0	4	0.52	0.62
-15	0	4	0.72	0.58
15	0	4	-0.73	0.42
-15	0	-4	0.00	0.37
-15	0	4	0.90	0.48
15	0	4	0.51	0.59
-15	0	-4	0.70	0.60

-15	0	-4	0.33	0.45
15	0	4	0.32	0.29
-15	0	-4	0.32	0.49
-15	0	-4	0.06	0.25
-15	0	-4	0.39	0.60
-15	0	-4	-0.05	0.30
-15	0	4	0.46	0.40
-15	0	4	0.15	0.36
-15	0	4	-0.48	0.46
15	-1	-4	4.84	0.68
-15	1	-4	3.86	0.60
-15	1	-4	5.63	1.11
-15	1	-4	4.75	0.84
-15	1	-4	5.75	0.92
15	1	4	5.69	1.10
-15	-1	4	6.36	0.89
-15	1	-4	4.33	0.82
-15	-1	4	7.42	0.94
15	-1	-4	5.82	0.68
-15	-1	4	6.63	0.95
-15	-1	4	6.19	0.72
15	-1	-4	4.72	0.51
-15	-1	4	6.72	0.72
-15	1	-4	5.13	0.80
15	1	4	5.23	0.57
15	-1	-4	4.96	0.72
-15	-1	4	4.42	0.80
-15	-1	4	6.53	1.07
15	1	4	4.38	0.77
15	1	4	5.02	0.93
-15	1	-4	3.96	0.74
-15	-1	-4	5.73	0.89
15	-1	4	7.29	1.02
-15	1	4	6.29	0.95
-15	1	4	4.49	0.94
-15	-1	-4	5.03	0.82
-15	1	4	8.05	0.83
-15	-1	-4	5.63	0.94
15	-1	4	5.86	0.92
-15	1	4	5.21	1.03
-15	-1	-4	3.76	0.64
-15	-1	-4	5.75	0.79
15	-1	4	5.36	0.59
-15	1	4	5.39	0.89

15	-1	4	4.70	0.75
-15	1	4	4.79	0.80
15	-1	4	5.54	0.83
-15	-1	-4	5.56	0.98
15	2	4	13.98	1.22
15	-2	-4	14.10	0.92
-15	2	-4	11.95	0.94
15	2	4	15.87	1.34
-15	2	-4	12.22	1.10
-15	2	-4	12.98	1.48
15	-2	-4	14.67	1.01
-15	2	-4	12.34	1.35
-15	-2	4	15.19	1.05
15	-2	-4	14.09	1.06
-15	2	-4	13.15	1.17
-15	-2	4	14.47	1.15
-15	-2	4	14.25	1.51
-15	-2	4	15.92	1.42
-15	-2	4	12.82	1.12
-15	2	-4	12.59	1.71
-15	2	-4	11.89	1.06
-15	-2	4	14.32	1.06
-15	-2	4	15.16	1.25
15	2	4	12.74	0.94
-15	-2	4	16.22	1.36
-15	2	4	13.56	1.24
-15	-2	-4	12.96	1.06
15	-2	4	12.87	1.10
-15	-2	-4	11.14	1.17
15	-2	4	13.68	1.45
-15	-2	-4	12.87	1.24
-15	2	4	14.93	1.08
-15	-2	-4	13.43	1.15
15	-2	4	14.24	1.10
-15	2	4	17.24	1.05
-15	-2	-4	13.51	1.12
-15	2	4	14.73	1.43
15	-2	4	13.95	1.00
-15	-2	-4	12.04	1.13
-15	2	4	14.84	1.52
15	-2	4	14.31	1.62
-15	-2	-4	13.34	1.41
-15	2	4	15.03	1.15
15	-3	-4	18.47	1.21

15	-3	-4	17.06	1.17
-15	-3	4	20.34	1.38
15	3	4	18.77	1.21
15	-3	-4	16.69	1.27
-15	-3	4	18.96	1.26
-15	-3	4	19.86	1.73
-15	-3	4	20.24	1.32
15	3	4	20.98	1.64
-15	-3	4	20.69	1.75
-15	3	-4	18.58	1.30
15	3	4	19.61	1.43
-15	3	-4	20.19	1.32
-15	-3	4	19.82	1.40
-15	-3	4	21.81	1.69
-15	-3	4	18.87	1.37
-15	3	-4	19.39	1.75
-15	-3	-4	19.79	1.36
-15	-3	-4	16.35	1.32
-15	3	4	20.99	1.70
-15	3	4	20.70	1.38
15	-3	4	18.10	1.33
15	-3	4	19.83	2.32
15	-3	4	17.87	1.25
-15	-3	-4	16.18	1.64
-15	-3	-4	15.28	1.36
-15	-3	-4	19.58	1.32
-15	-3	-4	18.33	1.35
-15	3	4	19.32	1.44
15	-3	4	20.37	1.29
15	-3	4	20.19	1.84
-15	4	-4	2.69	1.04
-15	-4	4	8.02	1.42
-15	-4	4	5.91	0.89
-15	4	-4	5.19	0.79
-15	-4	4	9.05	1.02
-15	-4	4	8.94	0.95
15	4	4	7.94	0.81
-15	4	-4	4.40	0.85
-15	4	-4	4.70	1.00
15	-4	-4	4.61	0.80
15	4	4	8.78	1.28
15	4	4	6.48	0.98
15	-4	-4	3.32	0.71
15	4	4	5.62	0.97

-15	4	4	7.98	1.02
15	-4	4	5.66	0.82
15	-4	4	5.95	0.81
-15	-4	-4	3.08	0.79
-15	-4	-4	6.73	0.91
15	4	-4	3.23	0.53
-15	-4	-4	5.86	0.83
15	-4	4	7.95	0.82
-15	-4	-4	5.97	0.95
15	-4	4	10.22	1.62
-15	5	-4	2.50	1.17
15	-5	-4	3.55	0.63
-15	-5	4	1.38	0.54
-15	-5	4	3.24	0.91
15	5	4	1.46	0.58
15	5	4	2.36	0.94
-15	-5	4	1.88	0.55
15	-5	-4	7.95	1.50
-15	-5	4	1.37	0.56
-15	-5	4	1.11	0.44
15	5	4	1.04	0.70
-15	5	-4	6.13	0.88
-15	-5	4	2.13	0.98
15	-5	4	1.50	0.57
15	-5	4	-0.96	0.91
-15	-5	-4	5.16	0.93
-15	-5	-4	5.58	0.98
15	-5	4	1.37	0.55
-15	5	4	1.54	0.65
15	5	-4	4.65	0.86
-15	6	-4	6.26	1.50
15	6	4	10.61	1.64
15	-6	-4	6.59	1.18
15	6	4	7.39	1.30
-15	-6	4	5.70	0.84
15	-6	-4	5.62	0.96
-15	-6	4	7.36	0.95
-15	-6	4	6.45	0.91
-15	-6	4	5.44	1.18
-15	-6	4	9.37	1.68
15	-6	-4	6.51	0.84
15	-6	-4	5.67	0.92
-15	6	-4	7.05	0.93
-15	6	-4	7.28	1.32

-15	-6	4	6.50	1.11
15	6	4	10.08	1.53
15	6	4	8.51	1.07
15	-6	4	5.66	0.94
-15	-6	-4	7.95	1.08
-15	-6	-4	8.34	1.09
-15	6	4	8.15	1.59
-15	6	4	6.16	1.30
15	-6	4	12.45	2.03
-15	-6	-4	6.55	0.65
15	6	-4	5.59	1.02
15	-6	4	6.42	0.95
15	6	-4	5.03	0.82
15	-7	-4	1.10	0.69
-15	-7	4	1.75	0.62
-15	-7	4	1.72	0.97
-15	7	-4	0.80	0.60
-15	-7	4	1.98	0.56
15	7	4	2.03	1.00
-15	-7	4	2.21	1.12
15	-7	-4	1.66	1.11
-15	-7	4	2.86	0.65
15	-7	-4	1.17	0.64
-15	-7	4	1.80	0.66
15	7	4	1.40	0.76
-15	7	-4	2.25	1.13
15	-7	-4	0.66	0.63
15	7	4	1.96	0.92
15	-7	4	2.07	0.67
-15	7	4	1.25	1.06
15	-7	4	1.99	1.32
15	-7	4	0.94	0.69
-15	-7	-4	1.71	0.49
-15	-7	-4	2.37	0.70
15	7	-4	1.10	0.73
-15	-7	-4	2.49	0.75
15	7	-4	0.76	0.58
-15	8	-4	3.04	1.35
-15	8	-4	1.64	0.71
-15	-8	4	3.40	0.66
15	8	4	4.36	1.30
15	-8	-4	0.94	0.78
-15	-8	4	3.69	0.94
-15	-8	4	1.65	1.15

15	-8	-4	1.99	0.67
15	-8	-4	2.64	0.73
15	8	4	4.56	1.07
15	8	4	3.17	1.02
15	8	-4	4.70	1.02
15	-8	4	2.84	0.90
15	-8	4	5.51	1.01
-15	-8	-4	3.16	0.77
-15	8	4	4.95	1.27
15	8	-4	1.39	0.73
-15	-8	-4	1.19	0.84
15	-9	-4	1.13	0.70
-15	9	-4	3.47	1.63
15	-9	-4	2.44	0.88
-15	9	-4	0.36	0.72
15	-9	-4	1.42	0.83
-15	-9	4	1.95	1.17
15	9	4	2.06	1.17
-15	-9	4	-0.73	0.85
15	9	4	0.48	1.01
15	9	-4	3.07	1.09
15	-9	4	0.67	0.82
-15	-9	-4	1.48	0.88
-15	9	4	0.98	1.49
15	-9	4	1.09	0.86
-15	-9	-4	1.37	0.88
15	10	4	1.73	1.13
15	-10	-4	1.42	0.89
-15	-10	4	0.58	0.90
15	-10	-4	0.22	0.79
-15	10	-4	4.42	1.55
15	-10	-4	0.58	1.10
-15	-10	4	5.08	1.33
-15	10	-4	0.68	0.89
-15	-10	-4	-0.08	0.86
15	-10	4	1.06	0.79
-15	10	4	3.13	1.56
15	-10	4	1.21	1.15
15	10	-4	2.41	1.19
-15	-11	4	3.62	1.07
15	-11	-4	4.42	0.94
15	-11	4	0.18	0.86
16	0	4	117.43	5.86
-16	0	-4	120.96	5.69

16	0	4	129.89	5.91
-16	0	4	113.91	5.72
-16	0	4	121.10	5.79
16	0	4	121.61	5.85
-16	0	4	117.51	5.92
-16	0	-4	112.34	5.76
-16	0	-4	114.09	5.58
-16	0	4	117.27	5.79
16	0	-4	119.88	5.35
-16	0	4	125.85	5.83
-16	0	4	120.21	6.02
16	0	4	121.72	5.80
16	0	4	119.44	5.61
16	0	-4	116.77	5.53
16	0	-4	113.20	5.28
-16	0	4	121.89	5.74
16	-1	-4	71.32	3.63
-16	-1	4	61.80	3.19
-16	-1	4	62.48	3.21
16	-1	-4	74.29	3.60
-16	-1	4	63.17	3.30
16	1	4	64.82	3.11
-16	1	-4	65.37	3.62
-16	-1	4	63.48	3.37
-16	1	-4	75.49	3.70
-16	1	-4	73.42	3.48
16	1	4	66.44	3.43
16	1	4	65.51	3.37
-16	-1	4	65.66	3.26
16	-1	-4	73.80	3.52
16	-1	-4	72.59	3.41
-16	-1	4	61.19	3.37
-16	1	-4	70.97	3.41
-16	1	-4	72.22	3.90
-16	-1	4	64.86	3.42
-16	-1	4	68.12	3.58
16	1	4	62.28	3.31
-16	1	4	62.40	3.21
-16	-1	-4	76.00	3.84
-16	-1	-4	72.93	3.77
16	-1	4	57.36	3.42
-16	-1	-4	75.32	3.80
16	-1	4	72.08	3.49
-16	1	4	61.15	3.47

-16	-1	-4	68.42	3.65
-16	1	4	65.46	3.26
16	-1	4	61.55	3.28
16	-1	4	64.38	3.30
-16	1	4	67.65	3.25
16	-1	4	62.43	3.12
-16	1	4	64.12	3.59
-16	2	-4	22.23	1.86
16	-2	-4	21.58	1.36
-16	-2	4	28.96	1.88
-16	-2	4	27.75	1.68
-16	-2	4	30.64	1.81
-16	-2	4	30.35	1.78
16	2	4	27.39	1.57
-16	-2	4	30.52	2.07
-16	-2	4	31.42	1.96
16	-2	-4	20.19	1.25
16	-2	-4	25.50	1.46
-16	2	-4	22.23	1.51
16	-2	-4	21.09	1.43
-16	-2	4	30.45	2.14
-16	2	-4	19.69	1.42
-16	2	-4	22.69	1.62
-16	-2	4	28.01	1.70
16	2	4	29.05	2.00
16	-2	4	26.77	2.02
16	-2	4	30.30	1.74
-16	-2	-4	21.37	1.61
16	-2	4	28.11	1.62
16	-2	4	28.80	1.77
-16	2	4	27.18	2.04
-16	-2	-4	21.23	1.77
-16	-2	-4	20.54	1.58
-16	2	4	29.24	1.77
-16	2	4	29.18	1.67
16	-2	4	29.86	2.02
-16	-2	-4	19.02	1.42
-16	-2	-4	22.81	1.51
16	-3	-4	14.19	1.26
-16	-3	4	7.90	0.92
-16	-3	4	8.11	1.38
-16	3	-4	12.54	1.56
-16	-3	4	9.01	1.05
-16	3	-4	14.86	1.63

16	-3	-4	11.96	1.02
-16	-3	4	8.90	1.07
16	3	4	9.55	1.30
16	3	4	8.90	1.09
-16	-3	4	8.83	1.26
16	-3	-4	12.99	1.12
-16	-3	4	7.26	1.44
-16	3	-4	12.95	1.13
-16	-3	4	10.07	1.04
-16	-3	4	8.05	0.87
16	3	4	8.38	0.79
16	-3	-4	11.62	0.96
-16	3	-4	12.39	1.15
-16	3	4	9.15	1.35
-16	3	4	9.59	1.05
16	-3	4	11.13	1.61
16	-3	4	9.79	0.90
16	-3	4	8.41	0.85
-16	-3	-4	12.86	1.22
16	-3	4	7.47	0.90
-16	3	4	9.11	1.08
-16	-3	-4	12.23	1.10
-16	-3	-4	13.81	1.32
-16	-3	-4	11.49	1.16
16	4	4	41.02	2.62
-16	-4	4	38.37	2.15
-16	4	-4	36.70	2.68
-16	-4	4	40.83	2.64
-16	-4	4	37.16	2.22
-16	4	-4	31.49	2.21
-16	-4	4	39.00	2.21
16	4	4	41.57	2.31
-16	-4	4	40.06	2.27
16	-4	-4	37.63	2.45
-16	4	-4	34.05	2.73
16	-4	-4	35.84	2.00
-16	-4	4	39.28	2.53
16	4	-4	34.86	1.81
-16	-4	-4	36.57	2.11
16	-4	4	42.06	2.89
-16	4	4	37.83	2.26
16	4	-4	37.98	1.78
-16	-4	-4	33.70	2.02
-16	4	4	39.41	2.44

16	-4	4	35.57	2.33
16	-4	4	40.40	2.27
-16	-4	-4	29.21	2.39
-16	-4	-4	39.88	2.14
16	-4	4	42.22	2.13
16	-5	-4	17.04	1.37
-16	5	-4	17.07	1.97
-16	5	-4	18.47	2.09
16	5	4	18.19	1.31
-16	-5	4	16.29	1.73
16	-5	-4	21.23	1.79
16	-5	-4	17.66	1.32
-16	-5	4	21.34	1.94
-16	-5	4	15.45	1.22
16	-5	-4	18.00	1.27
16	5	4	16.74	1.41
16	5	4	16.68	1.76
-16	-5	4	15.96	1.35
-16	-5	4	16.27	1.31
-16	5	-4	17.88	1.35
-16	-5	4	17.32	1.31
16	5	-4	17.60	1.13
-16	-5	-4	16.71	1.49
-16	-5	-4	19.59	1.46
-16	5	4	17.10	1.96
-16	5	4	15.80	1.83
16	-5	4	18.88	1.11
16	-5	4	16.26	1.33
16	-5	4	16.46	1.28
16	5	-4	13.72	1.30
-16	-5	-4	16.86	1.26
-16	-5	-4	18.32	1.39
16	-5	4	17.20	2.09
16	-6	-4	55.39	3.18
-16	6	-4	50.24	3.36
-16	-6	4	58.78	2.90
-16	-6	4	51.97	2.99
-16	-6	4	56.99	3.38
16	-6	-4	45.42	2.70
-16	6	-4	46.39	3.33
-16	6	-4	47.13	2.66
-16	-6	4	52.88	2.87
16	-6	-4	45.96	2.69
-16	-6	4	53.54	2.84

16	-6	-4	48.35	2.61
16	6	4	55.94	3.47
16	6	4	55.67	3.02
16	6	4	58.20	3.10
-16	-6	4	59.08	3.44
-16	-6	-4	47.96	2.78
-16	-6	-4	45.90	2.67
16	-6	4	53.91	3.00
16	6	-4	48.40	2.49
16	-6	4	52.55	2.99
-16	6	4	54.13	3.52
16	6	-4	46.97	2.63
-16	7	-4	69.72	4.16
16	7	4	78.16	4.47
16	-7	-4	63.79	3.49
-16	7	-4	65.69	3.45
-16	-7	4	75.18	3.85
16	-7	-4	65.66	3.49
-16	-7	4	77.80	4.46
-16	-7	4	72.90	3.84
-16	-7	4	76.43	4.27
16	-7	-4	63.23	3.36
16	7	4	77.48	4.11
16	7	4	85.37	4.17
-16	-7	4	76.66	4.09
16	7	-4	61.74	3.47
-16	-7	-4	65.32	3.56
16	-7	4	79.76	4.10
16	-7	4	80.08	4.04
-16	7	4	77.38	4.56
-16	-7	-4	61.19	3.41
16	7	-4	66.49	3.31
16	8	4	43.51	2.83
-16	8	-4	45.45	3.68
-16	-8	4	37.81	2.28
16	-8	-4	44.37	2.72
-16	-8	4	45.38	3.25
-16	-8	4	39.88	2.31
-16	-8	4	44.07	2.61
16	-8	-4	45.39	2.60
16	8	4	44.95	3.28
-16	8	-4	46.80	2.65
16	-8	-4	45.78	2.73
16	8	-4	44.71	2.91

16	8	-4	45.88	2.60
-16	-8	-4	42.62	2.69
-16	8	4	40.65	3.36
-16	-8	-4	44.78	2.90
16	-8	4	42.51	2.58
16	-8	4	43.02	2.64
16	-9	-4	20.65	2.07
16	-9	-4	20.73	1.73
16	9	4	24.95	2.10
16	-9	-4	20.09	1.80
-16	9	-4	22.96	1.86
-16	-9	4	21.04	2.43
-16	9	-4	25.24	2.88
16	9	4	22.95	2.47
-16	-9	4	24.00	2.08
-16	9	4	22.79	2.77
16	9	-4	19.71	2.04
16	-9	4	21.78	2.02
-16	-9	-4	20.25	1.93
-16	-9	-4	17.14	2.04
16	-9	4	23.65	1.86
16	-10	-4	16.75	1.80
-16	10	-4	14.88	2.79
-16	-10	4	12.03	1.62
-16	-10	4	13.12	2.20
16	-10	-4	14.06	1.57
-16	10	-4	14.21	1.67
16	-10	-4	16.45	1.56
16	10	4	13.32	1.96
-16	-10	-4	15.08	2.25
-16	-10	-4	12.98	1.82
-16	-10	-4	9.67	1.64
16	-10	4	12.84	1.83
-16	10	4	12.49	2.52
16	10	-4	12.68	2.00
16	-10	4	13.57	1.53
-17	0	4	-0.38	0.61
-17	0	-4	0.00	0.44
17	0	-4	0.08	0.21
17	0	4	-0.34	0.53
17	0	4	0.41	0.67
-17	0	4	-0.15	0.50
17	0	4	0.28	0.33
17	0	4	-0.07	0.75

17	0	4	-0.30	0.40
17	0	-4	0.62	0.32
-17	0	-4	-0.56	0.70
-17	0	4	0.36	0.71
17	0	-4	-0.19	0.39
-17	0	4	-1.89	1.35
-17	0	4	0.06	0.41
-17	0	4	0.42	0.53
-17	0	-4	0.07	0.66
-17	-1	4	5.97	1.01
-17	-1	4	4.79	0.78
-17	-1	4	7.37	1.07
-17	1	-4	3.22	0.66
-17	-1	4	5.07	0.82
17	1	4	6.48	1.00
-17	1	-4	3.73	0.72
17	-1	-4	3.76	0.49
-17	1	-4	4.59	0.88
-17	-1	4	5.64	1.12
-17	1	-4	4.46	0.93
17	1	4	5.52	1.07
17	1	4	5.38	0.88
17	-1	-4	4.86	0.75
17	-1	-4	4.61	0.68
17	-1	-4	4.81	0.74
-17	-1	4	5.61	0.95
17	1	4	6.27	0.64
-17	-1	4	5.78	0.96
17	-1	4	7.16	1.29
-17	1	4	6.42	0.91
17	-1	4	5.73	0.67
-17	-1	-4	3.97	0.89
17	-1	4	5.19	0.85
-17	-1	-4	3.32	0.86
-17	1	4	5.64	0.95
-17	-1	-4	6.16	1.03
-17	1	4	3.76	0.91
-17	1	4	7.22	1.06
17	-1	4	6.28	0.92
-17	-1	-4	4.35	0.72
17	-1	4	4.82	0.95
-17	2	-4	41.29	2.36
-17	-2	4	31.73	2.02
-17	-2	4	36.76	2.45

-17	-2	4	33.88	1.97
-17	-2	4	33.44	2.32
-17	-2	4	36.91	2.26
17	-2	-4	41.42	2.36
-17	-2	4	34.37	2.07
-17	-2	4	32.84	1.95
-17	2	-4	43.74	2.76
17	2	4	34.11	2.25
17	-2	-4	41.64	2.28
-17	2	-4	42.21	2.36
17	-2	-4	42.97	2.16
-17	-2	4	34.49	2.15
17	2	4	34.88	1.84
17	2	4	33.66	2.11
17	-2	-4	45.62	2.33
-17	2	-4	37.30	2.37
17	-2	4	36.11	2.28
-17	2	4	33.90	2.04
-17	-2	-4	43.47	2.53
-17	2	4	35.96	2.36
-17	-2	-4	41.70	2.37
17	-2	4	32.77	1.93
17	-2	4	33.70	2.05
-17	-2	-4	39.94	2.64
-17	-2	-4	42.93	2.47
-17	2	4	36.41	1.94
-17	2	4	33.96	1.99
17	-2	4	35.78	2.02
-17	-2	-4	40.35	2.37
-17	-3	4	39.59	2.32
-17	-3	4	41.88	2.77
17	3	4	46.09	2.71
17	-3	-4	38.40	2.10
-17	-3	4	41.66	2.38
-17	-3	4	43.73	2.68
17	3	4	45.89	2.46
17	-3	-4	43.02	2.37
-17	3	-4	37.80	2.26
17	-3	-4	41.29	2.18
17	-3	-4	39.27	2.37
-17	-3	4	44.42	2.44
-17	3	-4	40.32	2.76
-17	-3	4	41.91	2.48
-17	3	-4	39.73	2.47

-17	3	-4	35.84	2.18
17	-3	4	44.59	2.33
17	-3	4	42.27	2.33
17	-3	4	45.66	2.89
-17	3	4	42.79	2.43
-17	-3	-4	37.21	2.25
-17	-3	-4	39.53	2.35
-17	-3	-4	37.53	2.30
17	-3	4	41.76	2.62
17	-4	-4	40.41	2.35
-17	-4	4	42.65	2.36
-17	4	-4	38.42	2.31
17	4	4	45.78	2.52
17	-4	-4	40.76	2.24
-17	-4	4	44.96	2.82
-17	-4	4	43.14	2.42
-17	-4	4	39.07	2.45
17	4	4	42.14	2.32
-17	4	-4	40.61	2.28
-17	-4	4	46.05	2.90
-17	-4	4	42.42	2.44
17	-4	-4	40.26	2.24
-17	4	-4	42.40	2.89
17	-4	4	46.46	2.34
-17	-4	-4	41.66	2.41
-17	-4	-4	40.34	2.38
17	4	-4	39.47	1.98
-17	4	4	41.67	2.60
-17	-4	-4	39.43	2.32
17	-4	4	42.19	2.45
17	-4	4	51.55	3.02
17	-4	4	41.11	2.39
-17	4	4	45.70	2.91
-17	-4	-4	42.02	2.31
-17	-5	4	9.57	1.04
17	-5	-4	10.04	1.10
-17	-5	4	10.46	1.18
17	-5	-4	14.05	1.75
-17	5	-4	14.33	1.96
-17	-5	4	12.19	1.66
17	5	4	10.52	1.30
-17	-5	4	11.54	1.22
-17	5	-4	12.40	1.21
17	-5	-4	10.13	1.07

17	-5	-4	11.71	1.21
-17	-5	4	14.02	1.89
17	5	4	11.97	1.12
17	5	4	11.24	1.65
-17	-5	-4	12.14	1.49
-17	-5	-4	12.58	1.26
-17	5	4	11.47	1.81
17	5	-4	11.10	1.01
17	-5	4	8.80	1.12
-17	-5	-4	11.80	1.25
17	-5	4	11.66	1.17
-17	5	4	10.85	1.79
17	-5	4	16.58	2.06
17	5	-4	11.15	0.89
-17	-5	-4	12.11	1.22
-17	-6	4	9.59	1.05
17	6	4	12.04	1.81
-17	6	-4	12.87	1.28
-17	-6	4	11.90	1.74
-17	-6	4	9.46	1.21
17	-6	-4	13.16	1.96
-17	-6	4	10.25	1.21
17	6	4	12.27	1.37
17	-6	-4	11.87	1.12
17	-6	-4	9.66	1.24
-17	-6	4	10.18	1.94
17	-6	-4	13.33	1.28
17	6	4	10.64	1.26
-17	-6	4	8.91	1.16
-17	6	-4	14.90	2.19
-17	-6	-4	13.37	1.41
17	6	-4	13.19	1.24
-17	-6	-4	10.54	1.34
17	6	-4	10.56	1.05
17	-6	4	12.30	1.26
-17	6	4	9.75	1.62
17	-6	4	8.42	1.16
-17	7	-4	3.66	1.61
-17	-7	4	2.55	0.85
-17	-7	4	2.88	1.19
17	-7	-4	4.80	0.82
-17	-7	4	2.57	0.70
17	7	4	4.19	1.30
-17	-7	4	3.30	0.70

17	-7	-4	3.84	0.92
17	7	4	2.73	0.95
17	-7	-4	3.76	0.84
-17	7	-4	2.78	0.79
17	7	4	3.48	1.00
-17	7	4	3.37	1.46
17	7	-4	3.91	0.88
-17	-7	-4	3.35	0.97
17	-7	4	3.82	0.85
17	-7	4	2.20	0.74
17	7	-4	4.82	1.01
-17	-8	4	10.93	2.08
-17	-8	4	8.19	1.15
-17	-8	4	12.24	1.63
17	8	4	10.59	1.66
17	8	4	9.10	1.57
17	-8	-4	12.13	1.62
17	-8	-4	13.66	1.35
17	-8	-4	12.32	1.51
-17	8	-4	12.94	1.53
-17	8	-4	8.46	1.81
-17	8	4	12.96	2.38
17	-8	4	12.60	1.48
-17	-8	-4	13.66	1.47
17	-8	4	9.14	1.50
17	8	-4	11.56	1.66
17	8	-4	13.66	1.36
-17	-8	-4	10.21	1.62
-17	9	-4	3.39	1.01
-17	-9	4	8.40	1.50
-17	-9	4	8.78	1.30
17	9	4	6.51	1.50
17	9	4	4.78	1.18
-17	9	-4	3.10	1.54
17	-9	-4	4.16	1.01
17	-9	-4	4.94	1.13
17	-9	-4	5.81	1.07
17	-9	4	6.91	1.28
17	9	-4	6.24	1.16
-17	-9	-4	4.36	0.98
-17	-9	-4	7.03	1.15
-17	9	4	3.26	1.53
17	-9	4	6.84	1.06
17	-10	-4	22.06	1.89

-17	10	-4	29.14	3.05
17	-10	-4	20.88	2.00
17	-10	-4	22.39	1.85
-17	10	-4	24.72	1.95
-17	-10	4	27.51	2.26
17	10	4	28.50	3.29
-17	10	4	28.73	3.12
17	-10	4	26.21	2.20
17	-10	4	26.66	2.04
-17	-10	-4	23.18	2.49
-18	0	4	106.87	5.20
-18	0	-4	87.55	4.76
18	0	4	114.75	5.26
18	0	4	114.70	5.24
18	0	4	92.16	5.01
-18	0	4	106.43	5.12
-18	0	4	107.99	5.17
18	0	-4	97.28	4.55
-18	0	4	94.69	5.06
-18	0	-4	77.70	4.96
18	0	-4	98.00	4.30
-18	0	-4	95.69	4.71
-18	0	4	101.65	5.30
-18	0	-4	96.10	4.83
18	0	-4	90.34	4.22
-18	1	-4	60.84	3.37
-18	-1	4	68.44	3.67
18	1	4	72.66	3.89
18	-1	-4	68.27	3.34
-18	-1	4	73.79	3.67
18	-1	-4	62.71	3.17
-18	1	-4	69.08	3.74
18	1	4	80.30	3.83
-18	1	-4	66.66	3.47
-18	-1	4	71.44	3.91
18	-1	-4	70.06	3.23
18	1	4	66.63	3.62
-18	-1	4	72.15	3.89
-18	-1	4	75.12	3.85
-18	1	4	75.63	3.76
18	-1	4	78.10	3.81
-18	-1	-4	67.35	3.62
-18	1	4	66.58	3.72
-18	-1	-4	61.23	3.53

-18	-1	-4	53.99	3.89
-18	1	4	71.68	3.68
18	-1	4	69.07	3.55
-18	-1	-4	68.42	3.60
-18	1	4	75.33	3.73
-18	1	4	71.26	4.01
18	-1	4	84.88	3.95
-18	-2	4	7.89	0.95
-18	2	-4	13.03	1.21
18	2	4	9.82	1.32
-18	-2	4	6.90	1.20
-18	-2	4	9.22	0.96
-18	-2	4	8.05	1.44
-18	-2	4	7.65	1.28
-18	2	-4	12.85	1.17
18	-2	-4	12.17	0.91
-18	2	-4	14.66	1.33
18	-2	-4	13.95	1.15
-18	2	-4	13.48	1.76
18	-2	-4	12.76	1.11
18	2	4	8.61	0.78
18	2	4	10.17	1.23
18	-2	-4	12.28	1.14
-18	-2	4	9.75	1.32
-18	2	4	8.65	0.95
-18	-2	-4	12.43	1.17
-18	-2	-4	11.94	1.30
18	-2	4	9.56	1.08
-18	2	4	9.72	1.10
18	-2	4	8.29	1.00
-18	-2	-4	12.32	1.52
18	-2	4	10.63	1.53
18	-2	4	8.49	0.83
-18	-2	-4	12.36	1.44
-18	-3	4	0.43	0.47
-18	-3	4	-0.12	0.70
-18	3	-4	1.04	0.70
18	-3	-4	0.53	0.43
-18	-3	4	0.85	0.66
18	-3	-4	0.22	0.44
18	3	4	1.76	0.88
-18	-3	4	0.20	0.50
-18	3	-4	0.70	0.97
18	3	4	0.63	0.41

18	-3	-4	1.12	0.66
18	-3	-4	-0.20	0.52
-18	3	-4	0.51	0.87
-18	-3	4	-0.20	0.99
-18	3	-4	0.60	0.53
-18	-3	-4	0.58	0.84
18	-3	4	0.21	0.43
18	-3	4	1.27	0.55
18	-3	4	0.48	0.86
-18	-3	-4	0.91	0.70
18	-3	4	-0.05	0.56
-18	-3	-4	0.53	0.56
-18	3	4	0.20	0.87
-18	-3	-4	0.76	0.70
-18	-4	4	-0.29	0.67
18	-4	-4	1.84	0.85
-18	-4	4	1.06	0.82
-18	-4	4	1.03	0.94
18	-4	-4	0.61	0.52
-18	-4	4	0.22	0.64
-18	-4	4	0.65	0.54
18	4	4	0.81	0.50
18	4	4	0.14	0.81
-18	-4	4	-1.07	1.09
18	-4	-4	1.08	0.51
18	-4	-4	1.26	0.59
-18	4	-4	1.07	0.62
-18	4	-4	1.74	1.08
-18	4	-4	0.50	0.67
-18	4	-4	-0.54	1.04
18	4	4	0.37	0.68
-18	4	4	1.29	0.90
-18	4	4	0.92	0.94
18	-4	4	1.32	1.16
18	-4	4	0.59	0.52
-18	-4	-4	1.33	0.70
18	-4	4	0.34	0.52
-18	-4	-4	0.29	0.59
-18	-4	-4	1.31	0.84
18	-4	4	0.66	0.49
-18	-5	4	44.61	2.91
18	-5	-4	34.74	2.04
-18	-5	4	44.04	2.99
18	-5	-4	31.95	2.10

18	-5	-4	33.67	2.35
-18	-5	4	43.27	2.44
-18	-5	4	40.36	2.46
-18	5	-4	35.20	2.84
-18	-5	4	39.59	2.43
-18	-5	4	40.93	2.34
18	5	4	41.33	2.51
-18	5	-4	31.21	2.06
18	5	4	42.20	2.37
18	5	4	45.02	2.97
-18	-5	-4	32.34	1.99
-18	5	4	41.01	3.02
-18	-5	-4	35.85	2.18
18	-5	4	46.83	3.27
18	-5	4	42.74	2.48
18	-5	4	42.71	2.42
-18	-5	-4	28.06	2.33
18	5	-4	33.50	1.75
-18	5	4	38.43	2.78
18	5	-4	29.76	1.80
18	-6	-4	25.56	1.76
18	6	4	30.49	1.88
-18	-6	4	23.87	1.76
-18	-6	4	28.22	2.38
-18	-6	4	24.06	1.71
18	6	4	26.86	2.33
-18	-6	4	23.50	1.80
-18	6	-4	27.72	2.68
-18	6	-4	25.84	1.86
18	6	4	27.17	1.95
18	-6	-4	29.25	1.91
18	-6	-4	24.60	1.80
-18	-6	4	24.23	1.88
18	-6	4	27.35	1.87
18	6	-4	27.62	1.62
-18	6	4	27.67	2.53
18	6	-4	26.82	1.77
18	-6	4	24.19	1.80
-18	-6	-4	23.60	1.85
-18	-6	-4	26.50	1.94
18	7	4	31.76	2.20
18	-7	-4	27.91	2.11
-18	7	-4	26.32	3.15
-18	-7	4	28.88	1.95

-18	7	-4	31.30	2.13
18	-7	-4	27.43	1.92
18	-7	-4	28.47	2.12
-18	-7	4	27.94	1.92
18	7	4	32.04	2.28
-18	-7	4	31.32	2.14
18	7	4	30.59	2.82
-18	-7	4	35.17	2.85
18	-7	4	28.67	2.11
-18	-7	-4	30.52	2.08
-18	-7	-4	30.71	2.28
18	-7	4	32.94	2.17
18	7	-4	31.20	2.18
18	7	-4	31.19	1.91
-18	7	4	28.00	3.01
18	8	4	24.73	2.02
-18	-8	4	25.36	2.17
-18	8	-4	26.87	2.91
18	-8	-4	26.18	2.01
18	-8	-4	25.40	1.95
18	-8	-4	25.69	1.90
-18	-8	4	27.74	2.59
18	8	4	26.85	2.53
-18	8	-4	25.91	2.05
18	-8	4	24.71	2.14
18	8	-4	27.21	2.09
18	-8	4	24.61	1.91
-18	-8	-4	25.95	2.11
-18	8	4	26.42	2.74
18	8	-4	27.70	1.88
-18	-8	-4	25.87	2.05
18	-9	-4	13.63	1.54
18	9	4	12.92	1.86
18	-9	-4	14.04	1.64
18	-9	-4	13.02	1.48
-18	-9	4	19.69	2.41
-18	-9	4	14.33	1.77
18	9	4	14.94	2.35
-18	9	-4	12.27	2.14
-18	9	-4	11.50	1.54
18	-9	4	14.56	1.69
-18	-9	-4	13.36	1.78
18	9	-4	11.18	1.81
18	-9	4	18.51	1.62

-18	9	4	13.12	2.64
-18	-9	-4	15.89	1.80
-19	0	-4	0.45	0.83
-19	0	4	0.58	0.82
19	0	4	-0.78	0.68
-19	0	4	-0.02	0.54
-19	0	-4	0.34	0.54
19	0	4	-0.56	0.73
19	0	-4	-0.13	0.34
19	0	4	-0.71	0.50
-19	0	-4	-0.45	0.84
-19	0	4	0.60	0.65
-19	0	4	-0.42	0.80
19	0	-4	-0.41	0.46
19	0	4	-0.28	0.33
19	-1	-4	13.92	1.22
19	1	4	12.74	0.94
19	-1	-4	15.82	0.95
-19	1	-4	16.82	1.55
-19	1	-4	13.37	1.31
-19	-1	4	11.30	1.15
-19	-1	4	12.11	1.07
-19	-1	4	11.78	1.37
19	1	4	10.54	1.33
-19	-1	4	13.83	1.60
-19	1	-4	12.94	1.78
19	1	4	10.80	1.23
19	-1	-4	17.68	1.15
-19	-1	4	12.42	1.41
19	1	4	10.95	1.46
-19	1	4	10.12	1.55
-19	-1	-4	15.17	1.35
-19	1	4	13.16	1.39
-19	-1	-4	16.25	1.76
-19	-1	-4	13.15	1.63
-19	1	4	13.08	1.18
19	-1	4	11.81	1.30
19	-1	4	13.16	1.43
19	-1	4	12.46	1.23
19	-1	4	12.58	0.97
-19	1	4	11.43	1.19
-19	-2	4	24.81	1.81
19	2	4	21.75	1.71
19	-2	-4	26.47	1.67

-19	-2	4	22.43	1.55
19	2	4	22.96	1.38
-19	2	-4	24.53	1.76
-19	2	-4	25.83	2.31
19	-2	-4	27.50	1.66
19	2	4	26.02	1.97
19	-2	-4	26.71	1.47
-19	-2	4	23.20	1.86
-19	-2	4	21.29	1.55
-19	2	-4	24.13	1.84
-19	2	-4	23.92	1.52
19	-2	4	24.83	2.03
-19	2	4	22.15	1.63
-19	2	4	21.12	2.03
-19	2	4	22.35	1.49
-19	2	4	21.96	1.60
19	-2	4	23.75	1.65
-19	-2	-4	25.78	2.06
19	-2	4	23.46	1.45
-19	-2	-4	23.24	1.93
19	-2	4	24.38	1.63
-19	-2	-4	25.10	1.75
-19	-2	-4	26.60	1.77
-19	3	-4	67.28	3.47
19	-3	-4	69.92	3.47
19	-3	-4	67.63	3.35
-19	-3	4	60.61	3.46
-19	-3	4	54.62	3.05
19	-3	-4	71.70	3.57
-19	3	-4	66.62	3.58
-19	3	-4	65.71	4.04
-19	-3	4	55.87	3.25
19	-3	-4	69.66	3.56
19	3	4	58.68	3.48
19	3	4	56.70	2.95
-19	3	-4	64.17	3.53
19	3	4	59.77	3.22
19	-3	4	58.52	3.04
-19	3	4	59.65	3.61
19	-3	4	66.42	3.53
-19	-3	-4	66.45	3.57
-19	-3	-4	65.24	3.54
19	-3	4	59.66	3.20
-19	-3	-4	66.57	3.69

-19	-3	-4	63.11	3.57
19	-3	4	59.61	3.09
-19	-4	4	60.29	3.61
19	-4	-4	58.28	3.19
19	4	4	59.57	3.66
-19	4	-4	54.77	3.38
19	4	4	62.58	3.31
-19	-4	4	58.67	3.16
-19	-4	4	60.99	3.73
-19	4	-4	59.01	3.21
19	-4	-4	58.45	3.04
-19	-4	4	61.00	3.23
19	4	4	59.79	3.09
19	-4	-4	62.10	3.32
-19	-4	4	59.07	3.29
-19	4	-4	58.80	3.80
-19	-4	-4	58.70	3.23
19	-4	4	58.90	3.18
-19	-4	-4	57.81	3.18
19	-4	4	61.51	3.29
-19	4	4	60.48	3.76
19	-4	4	65.20	3.11
-19	-4	-4	63.16	3.37
-19	-4	-4	53.49	3.12
19	-5	-4	48.65	2.62
19	5	4	44.18	3.13
-19	5	-4	53.91	3.44
-19	-5	4	42.86	2.65
19	-5	-4	48.65	2.88
19	-5	-4	46.92	2.59
19	5	4	45.45	2.72
19	5	4	47.36	2.53
19	-5	-4	48.51	2.73
-19	5	-4	44.93	2.68
-19	-5	4	43.69	2.50
-19	-5	4	46.01	3.07
-19	-5	4	51.44	3.40
-19	-5	4	45.03	2.64
19	5	-4	45.90	2.30
19	5	-4	43.33	2.34
-19	-5	-4	45.51	2.70
19	-5	4	46.49	2.59
19	-5	4	43.37	2.64
-19	-5	-4	42.34	2.57

-19	-5	-4	47.22	2.74
-19	5	4	44.93	3.21
-19	-6	4	10.46	2.00
-19	6	-4	12.94	1.51
19	6	4	10.43	1.53
19	6	4	10.75	1.40
-19	-6	4	9.10	1.35
19	-6	-4	11.53	1.42
-19	-6	4	7.59	1.26
19	-6	-4	11.86	1.31
-19	-6	4	10.88	1.19
19	-6	-4	14.60	1.53
19	6	4	9.66	1.73
-19	6	-4	12.70	2.03
-19	-6	-4	12.84	1.63
19	-6	4	8.12	1.32
19	6	-4	11.80	1.12
19	-6	4	9.86	1.37
19	6	-4	12.66	1.22
-19	-6	-4	12.53	1.48
-19	6	4	7.94	1.81
19	7	4	5.58	1.09
-19	-7	4	7.00	1.13
-19	7	-4	4.61	1.05
19	-7	-4	6.40	1.13
19	-7	-4	5.39	1.05
-19	-7	4	6.12	1.44
19	7	4	5.78	1.54
-19	-7	4	6.82	0.99
19	-7	-4	5.50	0.99
-19	7	-4	4.88	1.59
19	-7	4	6.04	0.98
-19	-7	-4	5.62	1.10
19	7	-4	6.28	1.12
19	-7	4	5.21	1.09
19	7	-4	4.11	0.97
-19	7	4	8.16	1.77
-19	-7	-4	6.80	1.11
19	-8	-4	2.93	0.87
-19	8	-4	2.58	1.52
19	-8	-4	2.39	0.82
-19	8	-4	2.72	0.90
19	8	4	3.28	1.34
19	8	4	2.59	0.95

-19	-8	4	4.49	1.37
-19	-8	4	4.34	1.01
19	-8	-4	2.44	0.90
-19	-8	-4	4.09	1.01
19	8	-4	2.28	0.93
19	-8	4	2.46	0.82
-19	-8	-4	4.15	1.03
-19	8	4	2.54	1.43
19	-8	4	2.25	0.94
-19	-9	4	11.18	1.69
19	-9	-4	12.42	1.50
19	-9	-4	13.32	1.67
19	-9	-4	12.31	1.50
-19	-9	4	10.38	2.20
-19	9	-4	11.44	1.55
-19	-9	-4	12.25	1.98
19	-9	4	14.05	1.57
-19	9	4	13.56	2.50
-19	-9	-4	12.16	1.85
19	-9	4	11.34	1.57
19	9	-4	17.57	2.02
20	0	-4	12.02	0.75
20	0	-4	13.90	0.88
20	0	-4	12.45	1.22
-20	0	4	15.46	1.73
-20	0	4	17.08	1.49
-20	0	4	14.88	1.74
-20	0	-4	14.07	1.75
-20	0	4	13.07	1.32
-20	0	-4	10.83	1.30
20	0	4	16.97	1.45
-20	0	-4	11.60	1.66
20	0	4	15.88	1.11
20	0	4	19.68	1.65
-20	1	-4	6.32	2.04
-20	1	-4	8.52	1.45
-20	-1	4	6.28	1.04
-20	-1	4	7.25	1.28
-20	1	-4	6.78	1.09
20	1	4	7.09	1.20
20	-1	-4	6.08	0.65
-20	-1	4	9.19	1.19
-20	-1	4	6.65	1.41
20	1	4	11.29	1.43

20	1	4	8.56	0.85
20	-1	-4	6.41	1.00
-20	-1	4	10.77	1.39
20	-1	4	11.75	1.46
-20	1	4	7.14	1.34
20	-1	4	6.84	0.84
20	-1	4	10.01	1.25
20	-1	4	7.90	1.17
-20	-1	-4	6.34	1.17
-20	1	4	7.55	1.53
-20	1	4	8.98	1.17
-20	1	4	6.21	1.07
-20	-1	-4	6.38	1.10
20	-2	-4	1.10	0.42
-20	-2	4	1.39	0.85
-20	-2	4	1.88	0.84
-20	-2	4	1.82	0.61
-20	2	-4	0.48	1.05
20	-2	-4	0.33	0.92
20	2	4	1.57	0.45
20	-2	-4	2.65	0.70
-20	-2	4	1.15	0.55
-20	2	-4	1.11	0.62
-20	2	-4	-1.59	0.80
-20	2	4	0.51	0.60
-20	-2	-4	1.43	0.89
-20	2	4	2.84	1.25
20	-2	4	2.60	0.70
-20	-2	-4	1.85	0.66
-20	2	4	2.97	0.70
20	-2	4	2.53	0.77
-20	2	4	2.29	1.11
-20	-2	-4	-0.36	1.04
20	-2	4	1.98	0.95
20	-2	4	1.58	0.51
-20	-2	-4	0.99	0.66
-20	3	-4	0.28	0.82
-20	-3	4	1.40	0.63
20	-3	-4	0.28	0.55
20	3	4	2.10	0.84
20	-3	-4	0.15	0.72
20	3	4	1.21	0.93
20	-3	-4	1.01	0.64
20	-3	-4	1.77	0.51

20	3	4	1.33	0.52
-20	-3	4	0.79	0.78
-20	3	-4	1.49	1.20
-20	-3	4	2.40	0.94
-20	3	-4	0.06	0.63
20	-3	4	1.35	0.55
-20	-3	-4	-0.02	0.73
20	-3	4	1.02	0.69
-20	-3	-4	2.05	0.88
-20	-3	-4	-0.87	1.01
-20	3	4	-0.82	0.98
-20	-3	-4	0.98	0.69
20	-3	4	1.11	1.21
20	-3	4	0.88	0.65
20	4	4	2.66	0.61
-20	4	-4	1.17	1.20
-20	-4	4	0.86	0.80
20	-4	-4	1.56	0.58
20	4	4	2.18	0.82
-20	-4	4	3.18	0.80
-20	4	-4	1.95	0.76
20	4	4	2.31	1.12
20	-4	-4	2.41	0.69
-20	-4	4	3.36	1.10
20	-4	-4	0.48	0.84
-20	-4	4	2.36	0.72
20	-4	-4	1.72	0.76
20	-4	4	3.71	1.14
20	-4	4	1.67	0.74
-20	4	4	2.31	1.10
-20	-4	-4	0.76	0.83
-20	-4	-4	2.29	0.79
20	-4	4	2.07	0.64
-20	-4	-4	0.19	0.67
20	-4	4	2.26	0.69
-20	-5	4	3.04	1.15
20	-5	-4	2.86	0.78
20	-5	-4	2.95	0.92
-20	-5	4	2.42	0.84
-20	5	-4	3.15	1.50
20	5	4	2.52	0.90
20	-5	-4	1.59	0.67
-20	-5	4	1.71	0.72
20	5	4	2.76	0.79

-20	5	-4	1.64	0.80
20	5	4	1.78	1.10
-20	-5	4	1.93	0.78
-20	-5	-4	3.47	0.91
-20	5	4	3.74	1.41
20	-5	4	2.28	0.80
-20	-5	-4	3.10	1.11
20	-5	4	2.49	0.86
-20	-6	4	8.54	1.36
20	-6	-4	4.99	1.03
-20	6	-4	5.05	1.03
-20	-6	4	5.81	1.09
-20	-6	4	12.91	2.19
-20	-6	4	8.11	1.21
20	6	4	6.41	1.11
-20	6	-4	6.70	1.69
20	6	4	8.66	1.30
20	-6	-4	5.10	0.93
20	6	4	7.58	1.70
20	-6	-4	6.87	1.14
20	6	-4	5.98	1.18
-20	6	4	5.27	1.57
20	-6	4	7.58	1.25
-20	-6	-4	6.45	1.10
20	-6	4	5.72	1.07
-20	-6	-4	5.45	1.01
-20	-7	4	1.71	0.76
20	7	4	4.92	1.40
-20	-7	4	3.45	1.33
20	-7	-4	4.16	0.99
-20	7	-4	3.26	0.97
20	-7	-4	4.54	0.93
20	-7	-4	3.21	0.86
-20	7	-4	5.31	1.57
-20	-7	4	1.81	0.95
20	-7	4	3.39	0.85
-20	7	4	0.86	1.28
20	-7	4	2.73	0.85
20	7	-4	3.86	0.92
-20	-7	-4	3.05	1.01
-20	-7	-4	4.58	1.06
-20	8	-4	4.30	1.67
-20	8	-4	4.80	1.00
-20	-8	4	9.93	1.76

20	-8	-4	3.58	0.98
20	8	4	8.21	1.50
20	8	4	4.99	1.53
-20	-8	4	6.39	1.13
20	-8	-4	4.13	0.96
20	-8	-4	4.55	0.97
20	-8	4	5.34	1.06
-20	-8	-4	3.60	1.02
-20	-8	-4	5.55	1.14
20	-8	4	5.00	0.97
-20	8	4	4.91	1.64
20	8	-4	8.91	1.53
21	0	4	1.00	0.87
-21	0	4	0.33	0.96
-21	0	4	-0.51	0.91
21	0	-4	-0.19	0.56
-21	0	-4	0.84	0.66
-21	0	4	0.52	0.61
21	0	-4	0.11	0.27
21	0	4	1.05	0.86
21	0	4	0.05	0.66
21	0	-4	0.38	0.36
21	0	4	0.24	0.43
-21	0	4	-0.98	0.72
21	-1	-4	28.19	1.51
21	1	4	22.32	1.39
-21	-1	4	19.54	1.55
-21	-1	4	24.97	2.03
21	-1	-4	30.37	1.88
21	-1	-4	28.13	1.66
-21	-1	4	21.50	1.66
-21	1	-4	23.32	2.11
21	1	4	22.33	2.03
21	1	4	22.03	1.79
-21	1	-4	28.65	2.47
-21	-1	4	25.47	1.87
-21	1	-4	26.18	1.92
21	1	4	23.29	1.80
21	-1	4	23.12	1.42
21	-1	4	25.45	1.94
-21	1	4	18.63	2.12
21	-1	4	23.43	1.85
21	-1	4	21.44	1.75
-21	-1	-4	30.14	2.29

-21	-1	-4	30.32	2.00
-21	1	4	21.69	1.69
-21	1	4	20.31	1.70
21	2	4	42.67	2.59
21	-2	-4	44.72	2.23
21	-2	-4	44.14	2.48
-21	2	-4	39.08	2.55
-21	-2	4	44.67	3.17
-21	-2	4	35.20	2.95
21	-2	-4	47.43	2.44
21	2	4	42.90	2.27
-21	-2	4	43.86	2.51
21	2	4	46.80	2.90
-21	-2	4	42.91	2.51
-21	2	-4	43.76	3.20
-21	2	4	43.90	2.55
21	-2	4	42.45	2.34
-21	-2	-4	41.65	2.58
-21	2	4	40.68	2.39
21	-2	4	45.52	2.63
21	-2	4	46.62	2.84
-21	2	4	44.81	3.09
-21	-2	-4	40.60	2.80
-21	-2	-4	41.26	2.55
21	-2	4	45.47	2.59
-21	3	-4	43.90	3.40
-21	-3	4	48.50	2.79
-21	-3	4	48.80	3.17
21	3	4	52.14	2.92
-21	-3	4	52.18	3.07
21	3	4	49.75	2.60
21	-3	-4	47.43	2.70
21	-3	-4	51.75	2.68
-21	3	-4	46.10	2.80
21	-3	-4	46.16	2.48
-21	-3	4	48.37	2.84
21	3	4	50.64	3.29
21	-3	4	48.21	2.71
-21	-3	-4	44.82	2.77
-21	-3	-4	46.78	2.75
-21	3	4	50.40	2.94
-21	3	4	55.13	3.40
21	-3	4	55.45	2.83
21	-3	4	47.20	2.87

21	-3	4	48.52	3.10
-21	-3	-4	43.49	2.87
21	4	4	82.90	4.28
21	4	4	80.08	4.04
-21	-4	4	79.59	4.14
21	-4	-4	86.76	4.23
-21	4	-4	81.30	4.42
-21	-4	4	82.26	4.24
21	-4	-4	83.65	4.43
21	4	4	86.06	4.67
21	-4	-4	87.49	4.46
-21	4	-4	87.96	5.21
21	-4	-4	87.71	4.32
-21	-4	4	82.64	4.62
-21	-4	4	76.11	4.27
21	-4	4	82.88	4.31
-21	-4	-4	87.22	4.47
-21	-4	-4	76.70	4.36
21	-4	4	80.70	4.15
-21	-4	-4	81.75	4.39
-21	4	4	83.58	4.88
21	5	4	50.85	2.95
-21	-5	4	48.91	2.79
-21	-5	4	51.10	3.49
21	-5	-4	57.57	3.12
-21	5	-4	58.11	4.09
21	5	4	49.64	2.77
21	-5	-4	56.12	3.04
-21	-5	4	48.59	2.94
21	-5	-4	57.03	3.23
21	5	4	55.56	3.63
-21	5	-4	53.57	3.19
-21	-5	4	49.68	2.92
21	-5	4	46.59	2.97
-21	-5	-4	54.36	3.25
-21	-5	-4	55.96	3.20
-21	5	4	49.88	3.61
21	-5	4	50.60	2.83
21	-6	-4	21.09	1.67
-21	-6	4	20.17	2.56
21	6	4	27.78	2.10
21	6	4	28.93	2.69
-21	6	-4	22.10	2.77
21	-6	-4	22.71	1.76

-21	-6	4	25.38	2.15
21	6	4	23.92	1.84
21	-6	-4	20.52	1.77
-21	6	-4	23.32	1.85
-21	-6	4	22.49	1.80
-21	-6	-4	23.48	1.93
-21	-6	-4	21.63	1.89
-21	6	4	28.30	2.76
21	-6	4	29.85	1.98
21	-6	4	24.62	1.85
-21	7	-4	14.09	2.16
21	-7	-4	12.62	1.57
-21	-7	4	16.17	1.70
21	-7	-4	13.75	1.53
21	7	4	14.96	2.28
21	-7	-4	10.02	1.40
-21	-7	4	16.27	2.27
-21	7	-4	10.91	1.49
21	7	4	18.16	1.66
-21	-7	-4	13.07	1.64
21	-7	4	13.29	1.52
21	-7	4	13.27	1.52
-21	-7	-4	14.41	1.73
21	7	-4	13.15	1.38
-21	7	4	9.62	1.87
21	7	-4	9.32	1.35
-22	0	4	16.06	1.67
22	0	4	15.62	1.70
-22	0	4	14.34	1.37
22	0	4	14.00	1.76
-22	0	-4	12.53	1.57
-22	0	4	18.79	2.02
22	0	-4	16.49	1.49
22	0	4	14.32	1.19
-22	0	4	14.97	1.45
22	0	4	15.83	1.59
-22	0	-4	16.42	2.10
22	-1	-4	0.81	0.57
22	-1	-4	1.51	0.40
-22	1	-4	3.46	1.36
22	1	4	8.88	1.59
22	1	4	3.40	0.66
22	1	4	4.46	0.99
-22	-1	4	4.16	0.79

-22	-1	4	4.10	0.99
-22	1	-4	1.41	0.87
-22	-1	4	2.86	1.14
-22	-1	4	2.95	0.83
22	1	4	8.22	1.44
22	-1	-4	2.54	0.75
-22	1	4	3.49	0.88
22	-1	4	4.55	1.07
-22	1	4	3.00	0.85
22	-1	4	3.62	0.68
22	-1	4	4.29	0.92
-22	1	4	3.51	1.23
-22	-1	-4	1.53	0.79
22	-2	-4	0.69	0.67
-22	2	-4	0.44	0.79
22	-2	-4	1.25	0.73
22	2	4	4.30	0.97
-22	-2	4	1.37	0.97
22	2	4	1.52	1.12
22	2	4	1.37	0.57
22	-2	-4	1.65	0.53
-22	2	-4	1.52	1.33
-22	-2	4	1.77	0.75
-22	-2	4	1.47	0.68
-22	-2	4	1.20	1.02
22	-2	4	1.44	0.62
-22	2	4	1.91	0.87
22	-2	4	4.47	1.08
-22	-2	-4	2.11	1.00
-22	2	4	1.67	0.87
22	-2	4	3.26	0.97
-22	2	4	0.97	1.19
-22	-2	-4	2.22	0.79
-22	-2	-4	2.28	0.91
22	-2	4	1.98	0.87
-22	3	-4	1.62	1.60
-22	-3	4	0.67	0.72
22	3	4	2.10	0.95
22	-3	-4	2.39	0.76
22	3	4	1.50	0.64
-22	3	-4	0.78	0.74
-22	-3	4	0.87	0.95
22	-3	-4	1.43	0.60
22	3	4	2.05	1.20

-22	-3	4	0.89	0.71
22	-3	-4	3.21	0.87
-22	-3	4	2.62	1.21
-22	-3	-4	2.97	0.91
22	-3	4	1.55	0.88
-22	3	4	2.38	1.44
-22	-3	-4	0.57	1.07
22	-3	4	2.73	0.84
22	-3	4	1.10	0.59
-22	3	4	1.14	0.85
-22	-3	-4	2.81	0.85
22	4	4	0.52	0.97
-22	4	-4	1.37	1.67
22	4	4	-0.42	0.60
22	4	4	-1.23	1.25
-22	-4	4	0.37	0.75
22	-4	-4	0.06	0.58
-22	4	-4	0.13	0.71
-22	-4	4	1.18	0.74
-22	-4	4	-1.11	1.00
22	-4	-4	0.53	0.67
-22	-4	-4	0.55	1.20
-22	-4	-4	-0.43	0.68
-22	4	4	-0.10	1.28
-22	-4	-4	1.11	0.87
22	-4	4	-0.47	0.53
-22	4	4	-0.58	0.71
22	-4	4	0.41	0.81
22	-5	-4	3.09	0.83
-22	5	-4	1.05	1.46
22	-5	-4	1.69	0.76
-22	5	-4	0.25	0.71
22	-5	-4	2.77	0.70
-22	-5	4	3.31	1.09
22	5	4	2.43	0.77
-22	-5	4	1.05	0.92
-22	-5	4	0.77	0.80
22	5	4	0.77	1.28
-22	-5	4	1.25	1.46
22	-5	4	1.57	0.77
-22	-5	-4	1.62	1.07
-22	5	4	0.99	1.33
22	-5	4	1.78	0.69
-22	-5	-4	1.86	0.81

-22	6	-4	2.60	1.62
22	6	4	1.45	1.31
22	-6	-4	2.77	0.95
-22	-6	4	3.47	1.00
22	-6	-4	1.22	0.85
22	6	4	6.06	1.19
22	-6	-4	2.27	0.78
-22	-6	4	2.86	0.89
-22	6	-4	0.77	0.89
-22	-6	4	3.70	1.38
-22	-6	-4	1.28	0.88
-22	-6	-4	3.71	1.14
-22	6	4	1.68	1.50
22	-6	4	1.75	0.79
22	-6	4	1.24	0.76
22	7	4	3.50	1.41
22	-7	-4	1.79	0.84
-22	7	-4	2.84	0.90
22	7	4	3.05	1.16
-22	-7	4	1.47	0.93
22	7	-4	3.79	0.84
22	-7	4	1.33	0.80
-22	-7	-4	1.38	1.05
22	7	-4	3.00	0.80
22	-7	4	0.91	0.76
-22	-7	-4	4.59	1.29
23	0	4	-0.41	0.49
-23	0	-4	-0.29	0.80
-23	0	4	-0.11	0.66
-23	0	4	-0.05	0.79
-23	0	-4	0.41	1.36
23	0	4	0.38	0.82
-23	0	4	-1.48	1.08
-23	0	4	0.41	0.89
23	0	4	2.22	0.98
-23	-1	4	17.01	2.16
23	1	4	20.16	2.06
-23	-1	4	15.75	2.76
23	-1	-4	22.73	1.51
-23	1	-4	24.43	2.65
23	-1	-4	22.32	1.30
-23	1	-4	19.80	1.82
23	1	4	17.30	1.32
-23	-1	4	18.77	1.60

23	1	4	17.96	1.76
23	-1	-4	22.08	1.77
-23	-1	4	16.31	1.66
23	-1	4	18.36	1.32
-23	1	4	16.92	1.59
-23	-1	-4	22.51	2.41
-23	-1	-4	25.89	1.92
23	-1	4	19.62	1.90
23	-1	4	19.16	1.81
-23	-1	-4	19.23	1.93
-23	1	4	18.83	2.41
-23	1	4	15.19	1.74
23	-1	4	19.49	1.85
23	-2	-4	34.41	2.13
-23	-2	4	25.43	2.37
23	-2	-4	31.03	1.95
-23	-2	4	27.48	1.92
-23	-2	4	25.66	2.33
-23	2	-4	28.64	3.09
-23	-2	4	28.65	1.93
23	2	4	25.15	1.60
23	2	4	27.43	2.43
23	-2	-4	29.45	1.70
23	2	4	23.25	1.97
-23	2	-4	30.50	2.17
-23	2	4	29.90	2.86
-23	-2	-4	30.00	2.29
23	-2	4	27.58	2.06
23	-2	4	24.28	1.66
-23	-2	-4	25.07	4.18
23	-2	4	26.52	2.05
-23	-2	-4	29.07	2.17
-23	2	4	25.71	1.97
-23	3	-4	26.67	3.20
-23	-3	4	34.44	2.26
23	3	4	31.91	1.94
-23	-3	4	35.61	2.76
-23	-3	4	36.83	2.32
23	3	4	31.71	2.78
23	3	4	33.06	2.34
23	-3	-4	30.64	1.99
-23	3	-4	27.05	2.03
23	-3	-4	33.09	2.76
-23	-3	4	35.65	2.61

23	-3	-4	28.18	1.70
-23	-3	-4	26.31	2.20
23	-3	4	32.75	2.35
-23	3	4	33.92	3.00
23	-3	4	33.28	2.01
-23	3	4	34.49	2.29
-23	-3	-4	27.65	2.12
-23	-3	-4	25.23	2.34
-23	-4	4	44.16	2.97
23	-4	-4	50.11	2.99
-23	-4	4	44.62	2.72
-23	-4	4	46.32	2.84
23	4	4	46.75	3.47
23	4	4	46.28	2.46
-23	4	-4	53.85	3.86
-23	4	-4	49.76	2.98
23	-4	-4	49.16	2.70
-23	-4	4	47.53	3.41
-23	-4	-4	53.75	3.07
-23	4	4	40.49	2.70
23	-4	4	43.33	2.53
-23	4	4	45.28	3.41
-23	-4	-4	50.45	3.16
23	-4	4	47.52	2.78
23	5	4	23.19	2.19
-23	-5	4	31.69	2.80
23	5	4	27.38	1.81
-23	5	-4	29.06	3.09
-23	-5	4	25.95	2.16
23	-5	-4	29.80	1.94
23	-5	-4	34.74	2.27
23	5	4	32.08	2.73
-23	5	-4	28.97	2.16
-23	-5	4	24.22	2.05
23	-5	4	26.39	1.86
-23	5	4	28.84	2.90
-23	-5	-4	28.21	2.34
-23	-5	-4	29.81	2.23
-23	5	4	30.06	2.11
23	-5	4	27.05	2.02
-23	-6	4	15.18	1.74
-23	-6	4	17.99	1.78
23	6	4	16.96	1.52
-23	-6	4	16.56	2.39

-23	6	-4	18.59	3.25
23	6	4	14.42	2.45
-23	6	-4	17.23	1.84
23	-6	-4	13.03	1.60
-23	-6	-4	15.50	1.71
-23	-6	-4	12.16	1.90
23	-6	4	18.24	1.70
-24	0	4	47.06	2.98
-24	0	4	48.89	3.00
-24	0	4	50.52	3.61
-24	0	-4	38.48	2.49
-24	0	-4	36.45	3.16
-24	0	4	48.76	2.91
24	0	4	56.24	3.29
24	0	4	49.41	2.63
24	0	4	52.59	2.97
24	0	4	51.27	3.07
24	1	4	19.02	1.89
-24	-1	4	16.46	2.07
24	1	4	21.41	2.32
24	-1	-4	21.92	1.46
24	-1	-4	23.09	1.90
-24	-1	4	20.83	1.81
-24	1	-4	21.22	2.95
24	1	4	20.17	1.38
24	-1	-4	21.42	1.27
-24	-1	4	23.48	2.43
-24	1	-4	22.37	1.83
-24	-1	4	20.79	1.90
-24	1	4	21.28	1.81
-24	1	4	24.28	2.67
-24	-1	-4	23.81	2.65
-24	-1	-4	19.72	1.84
24	-1	4	22.70	2.19
-24	-1	-4	23.92	1.82
24	-1	4	20.41	1.40
24	-1	4	19.82	1.82
24	-2	-4	18.00	1.78
-24	2	-4	14.62	1.59
-24	2	-4	15.89	2.42
24	2	4	15.29	1.18
24	-2	-4	16.76	1.14
-24	-2	4	12.80	2.13
-24	-2	4	14.91	1.75

24	-2	-4	17.06	1.47
-24	-2	4	12.80	1.67
24	2	4	19.35	2.19
-24	-2	4	14.01	2.10
-24	-2	-4	15.43	1.72
-24	-2	-4	15.19	1.63
-24	2	4	12.70	2.40
-24	-2	-4	18.66	2.35
24	-2	4	15.68	1.24
-24	2	4	17.08	1.61
24	-2	4	14.18	1.59
-24	3	-4	0.72	1.51
24	3	4	0.56	0.56
-24	-3	4	2.06	1.03
24	-3	-4	2.31	0.92
-24	3	-4	0.26	0.82
-24	-3	4	0.94	1.31
24	3	4	-2.18	1.45
-24	-3	4	2.82	1.27
24	3	4	-0.53	0.96
-24	-3	4	1.77	0.98
24	-3	-4	0.23	0.50
-24	-3	-4	-0.20	0.79
24	-3	4	0.06	0.58
-24	-3	-4	0.65	0.87
24	-3	4	0.59	0.76
-24	3	4	-0.17	0.74
-24	3	4	1.39	1.28
24	-4	-4	1.67	0.61
-24	-4	4	0.25	0.97
-24	-4	4	3.08	0.98
-24	4	-4	-2.67	1.46
24	4	4	1.20	1.05
-24	-4	4	1.71	1.37
24	4	4	-0.79	1.27
24	4	4	1.06	0.64
-24	4	-4	1.45	0.80
24	-4	-4	1.24	0.98
-24	-4	4	0.87	1.27
-24	-4	-4	1.06	0.96
24	-4	4	0.23	0.63
-24	-4	-4	1.42	0.87
24	-4	4	2.46	0.88
-24	4	4	1.22	1.51

-24	4	4	0.93	0.77
25	0	4	-0.21	0.52
-25	0	4	0.16	0.90
-25	0	-4	-0.29	0.80
25	0	4	-1.95	1.28
-25	0	-4	0.56	1.83
-25	0	4	0.70	1.42
-25	0	4	-0.31	0.77
25	0	4	-0.10	0.77
25	-1	-4	9.40	1.00
25	1	4	5.21	0.87
25	1	4	4.57	1.51
-25	-1	4	4.08	0.98
-25	1	-4	4.19	1.05
-25	-1	4	6.19	1.37
25	-1	-4	7.08	0.69
-25	-1	4	6.83	1.71
-25	1	-4	7.89	1.96
25	1	4	4.66	1.03
-25	-1	4	4.94	1.08
25	-1	-4	7.44	1.27
-25	1	4	5.67	1.02
-25	-1	-4	3.40	1.62
25	-1	4	4.16	0.74
-25	1	4	6.96	1.66
25	-1	4	5.37	1.04
-25	-1	-4	6.38	1.14
-25	-1	-4	6.57	1.21
-25	2	-4	15.78	2.74
-25	-2	4	9.08	1.74
25	2	4	10.78	1.03
25	2	4	12.36	1.96
-25	2	-4	12.01	1.49
-25	-2	4	9.86	1.55
25	-2	-4	11.04	0.96
25	-2	-4	12.55	1.66
-25	-2	4	9.88	1.80
25	2	4	9.52	1.57
-25	-2	4	9.93	1.54
-25	-2	-4	13.74	1.57
-25	2	4	8.55	1.70
25	-2	4	10.10	1.55
25	-2	4	9.85	1.06
-25	-2	-4	10.01	1.79

-25	2	4	8.11	1.25
-25	-2	-4	8.61	1.55
-25	-3	4	11.75	1.60
-25	-3	4	12.96	1.73
25	3	4	12.45	2.41
25	3	4	13.10	1.66
-25	-3	4	16.72	2.21
-25	-3	4	13.65	2.60
25	3	4	13.76	1.19
25	-3	-4	17.51	1.25
-25	3	-4	18.07	2.83
25	-3	-4	19.21	1.79
25	-3	4	12.20	1.64
-25	-3	-4	15.16	1.74
-25	3	4	14.00	1.57
-25	-3	-4	14.99	1.74
-25	3	4	16.65	2.50
0	0	5	0.63	0.15
0	0	5	0.52	0.23
0	0	-5	1.35	0.23
0	0	-5	0.73	0.12
0	0	5	0.48	0.14
0	0	-5	1.13	0.29
0	0	5	0.96	0.30
0	0	-5	0.88	0.22
0	0	-5	0.60	0.16
0	0	5	0.86	0.12
0	0	5	0.85	0.20
0	0	-5	0.66	0.19
0	1	5	0.18	0.17
0	-1	-5	-0.01	0.15
0	1	-5	0.09	0.23
0	1	-5	-0.01	0.24
0	-1	5	0.20	0.26
0	-1	-5	0.14	0.09
0	-1	5	0.23	0.11
0	1	5	0.15	0.09
0	-1	5	-0.05	0.24
0	1	-5	0.33	0.15
0	1	-5	0.23	0.20
0	-1	5	0.21	0.11
0	1	5	0.16	0.20
0	-1	5	0.20	0.20
0	1	5	0.02	0.15

0	1	5	0.26	0.28
0	-1	-5	0.15	0.15
0	-1	-5	0.06	0.18
0	-1	-5	0.18	0.24
0	-1	5	0.22	0.14
0	1	5	0.00	0.16
0	1	-5	0.20	0.12
0	-1	-5	0.24	0.18
0	1	-5	0.00	0.10
0	-2	-5	-0.10	0.22
0	-2	5	-0.05	0.27
0	2	5	0.15	0.12
0	2	5	-0.01	0.18
0	-2	-5	0.02	0.19
0	2	5	0.08	0.22
0	-2	5	0.16	0.22
0	2	5	0.24	0.25
0	-2	-5	-0.06	0.21
0	2	-5	0.00	0.31
0	-2	5	0.23	0.10
0	2	-5	0.24	0.20
0	2	-5	0.14	0.15
0	-2	5	0.14	0.17
0	2	-5	-0.09	0.15
0	-2	-5	0.14	0.13
0	-2	-5	-0.05	0.17
0	-2	5	0.08	0.31
0	-2	5	-0.05	0.14
0	2	5	0.35	0.20
0	2	5	0.09	0.19
0	2	-5	0.10	0.10
0	3	5	-0.03	0.26
0	3	-5	0.08	0.34
0	-3	5	-0.07	0.20
0	3	-5	-0.29	0.17
0	3	-5	-0.10	0.24
0	3	-5	-0.08	0.25
0	3	5	0.40	0.20
0	3	5	-0.03	0.17
0	3	-5	-0.11	0.22
0	3	5	-0.05	0.19
0	-3	5	-0.12	0.33
0	3	5	-0.09	0.26
0	-3	5	0.13	0.17

0	-3	5	0.02	0.34
0	3	5	0.40	0.26
0	-3	-5	-0.14	0.19
0	3	5	-0.02	0.23
0	3	-5	0.14	0.10
0	-3	-5	0.22	0.22
0	-3	-5	0.16	0.27
0	-3	-5	0.10	0.17
0	-3	-5	-0.02	0.26
0	-3	5	-0.28	0.19
0	-4	-5	0.43	0.29
0	4	-5	0.46	0.36
0	-4	5	-0.17	0.38
0	4	-5	0.12	0.35
0	-4	5	0.02	0.28
0	-4	-5	0.11	0.27
0	-4	5	-0.02	0.24
0	-4	-5	-0.05	0.35
0	4	5	0.01	0.38
0	4	5	0.44	0.25
0	4	5	0.06	0.27
0	4	-5	0.35	0.40
0	4	5	-0.01	0.23
0	4	5	0.31	0.42
0	4	5	-0.09	0.25
0	4	-5	-0.08	0.13
0	-4	5	0.10	0.29
0	-4	-5	-0.01	0.22
0	-4	-5	-0.12	0.35
0	4	-5	0.01	0.27
0	4	-5	0.30	0.29
0	-5	5	0.31	0.32
0	5	-5	0.83	0.21
0	-5	-5	0.24	0.27
0	5	5	0.17	0.41
0	-5	-5	0.12	0.40
0	-5	5	0.09	0.52
0	5	-5	0.14	0.39
0	-5	-5	0.10	0.25
0	-5	5	0.27	0.16
0	5	-5	0.39	0.37
0	5	5	0.29	0.34
0	-5	5	-0.25	0.54
0	5	5	0.03	0.47

0	-5	5	0.16	0.26
0	5	5	0.42	0.31
0	5	-5	0.30	0.34
0	5	-5	-0.01	0.37
0	5	5	0.39	0.29
0	5	-5	0.60	0.53
0	5	5	0.20	0.34
0	-5	5	1.02	0.39
0	-6	-5	-0.01	0.42
0	-6	-5	0.31	0.32
0	-6	5	-0.49	0.47
0	-6	-5	0.45	0.55
0	6	5	-0.06	0.34
0	6	-5	0.16	0.21
0	-6	5	0.10	0.30
0	6	5	0.43	0.37
0	-6	5	-0.06	0.62
0	-6	-5	-0.03	0.34
0	6	5	-0.24	0.47
0	6	-5	-0.23	0.51
0	-6	5	-0.37	0.58
0	6	5	-0.22	0.30
0	-6	5	0.19	0.36
0	6	-5	0.03	0.44
0	-6	5	-0.11	0.37
0	-6	-5	-0.09	0.29
0	-6	5	-0.08	0.19
0	6	-5	-0.10	0.49
0	6	5	0.71	0.39
0	6	5	0.30	0.50
0	6	-5	-0.27	0.32
0	-7	5	-0.30	0.69
0	-7	-5	-0.20	0.46
0	7	5	0.48	0.45
0	-7	5	-0.18	0.35
0	-7	-5	0.89	0.68
0	7	5	0.12	0.45
0	-7	-5	0.22	0.51
0	-7	-5	0.26	0.35
0	-7	5	0.19	0.48
0	7	-5	0.65	0.57
0	-7	5	0.42	0.68
0	-7	-5	-0.23	0.32
0	7	5	-0.68	0.40

0	7	5	0.95	0.65
0	7	5	0.11	0.44
0	-7	5	0.05	0.58
0	7	-5	0.26	0.53
0	7	-5	0.06	0.28
0	7	-5	-0.46	0.64
0	-7	5	-0.07	0.45
0	7	-5	-0.12	0.41
0	-7	5	-0.19	0.24
0	-7	-5	0.13	0.46
0	8	5	-0.33	0.52
0	-8	5	0.29	0.44
0	-8	5	-0.42	0.63
0	-8	-5	0.12	0.44
0	8	5	0.72	0.55
0	8	-5	-0.17	0.60
0	8	-5	0.29	0.35
0	8	5	0.64	0.68
0	8	5	-0.14	0.51
0	-8	-5	-1.32	0.73
0	-8	5	0.32	0.25
0	-8	5	0.95	0.82
0	8	-5	-0.20	0.59
0	8	-5	-0.03	0.78
0	-8	5	-0.12	0.76
0	-8	-5	-0.10	0.54
0	-8	-5	0.04	0.43
0	8	5	-0.01	0.50
0	8	5	-0.54	0.84
0	-8	-5	-0.78	0.61
0	-8	5	0.29	0.87
0	-8	5	0.40	0.53
0	-8	-5	-0.30	0.52
0	8	-5	-0.59	0.53
0	-9	5	0.88	0.90
0	9	5	-0.50	0.58
0	-9	-5	0.27	0.97
0	-9	5	-0.84	0.98
0	9	-5	0.46	0.41
0	-9	5	0.08	0.76
0	9	-5	0.61	0.90
0	-9	5	-0.08	0.28
0	-9	-5	-0.60	0.46
0	9	-5	0.43	0.68

0	-9	-5	-0.45	0.60
0	-9	-5	0.75	0.50
0	-9	-5	-0.40	0.64
0	9	5	-0.28	0.61
0	9	5	0.84	0.90
0	9	5	0.51	1.07
0	-9	5	-0.76	0.47
0	9	-5	-0.03	0.83
0	-9	5	-1.02	0.87
0	-9	5	-1.18	0.58
0	-9	-5	0.90	0.80
0	10	-5	0.59	1.19
0	-10	-5	0.14	0.53
0	-10	-5	-0.11	0.68
0	-10	5	0.34	0.39
0	10	5	-0.28	0.66
0	10	-5	-0.26	0.94
0	10	-5	0.35	0.55
0	-10	5	0.29	0.62
0	-10	-5	-0.19	0.60
0	10	5	-1.41	0.97
0	10	5	1.07	0.70
0	-10	5	0.37	1.06
0	10	-5	-0.31	0.77
0	-10	5	-0.14	0.61
0	-10	5	0.09	0.74
0	-10	-5	-0.74	0.87
0	10	5	0.15	0.99
0	-10	-5	0.80	0.84
0	-11	-5	-0.46	0.78
0	11	-5	-0.06	1.40
0	-11	-5	0.38	0.61
0	-11	5	-0.59	0.60
0	-11	5	0.01	0.36
0	11	5	-0.90	1.13
0	11	5	-0.37	0.78
0	-11	-5	0.94	0.81
0	-11	5	-0.82	0.84
0	-11	-5	0.25	0.76
0	11	-5	-0.08	0.92
0	-11	5	-0.39	0.69
0	-12	5	0.91	0.85
0	-12	5	-0.11	0.92
0	-12	-5	-0.80	0.95

0	12	5	-0.04	0.86
0	-12	-5	-0.63	1.13
0	12	-5	0.24	1.61
0	12	5	-1.90	1.79
0	13	5	-3.04	1.82
0	-13	-5	0.64	0.87
0	13	-5	0.00	1.35
0	-13	5	0.58	0.87
0	13	-5	1.29	1.81
0	-13	-5	-0.64	0.75
0	-13	5	-0.55	1.23
0	-13	-5	0.90	1.24
-1	0	5	0.04	0.13
1	0	-5	-0.04	0.13
1	0	5	0.07	0.17
-1	0	-5	0.11	0.19
1	0	5	0.09	0.14
1	0	5	0.34	0.22
1	0	-5	0.09	0.05
-1	0	-5	-0.04	0.09
1	0	5	-0.02	0.09
-1	0	5	-0.18	0.19
-1	0	-5	-0.09	0.12
-1	0	5	-0.01	0.13
1	0	-5	-0.01	0.18
-1	0	-5	-0.03	0.13
-1	0	5	0.08	0.15
1	0	-5	0.18	0.16
-1	0	5	0.08	0.05
1	0	5	0.08	0.13
1	0	5	0.02	0.26
-1	0	5	0.14	0.23
-1	0	-5	0.32	0.28
-1	0	-5	0.04	0.16
1	0	-5	-0.03	0.21
1	0	-5	-0.11	0.15
-1	1	-5	17.64	0.94
1	1	5	34.05	1.65
1	-1	-5	17.13	0.93
-1	-1	5	29.49	1.55
-1	-1	5	29.19	1.44
-1	1	-5	17.60	1.06
-1	-1	5	31.75	1.62
1	1	5	30.02	1.51

1	1	5	28.75	1.53
-1	-1	5	29.82	1.49
1	1	5	32.11	1.54
-1	-1	5	30.87	1.45
1	1	5	29.35	1.54
1	-1	-5	17.71	0.96
-1	1	-5	17.66	0.97
-1	1	-5	18.54	0.89
1	-1	-5	18.78	1.03
-1	1	-5	18.84	1.10
1	-1	-5	17.07	0.94
-1	1	-5	18.22	0.89
1	-1	-5	17.30	0.97
-1	-1	-5	17.88	0.88
1	-1	5	32.70	1.63
-1	-1	-5	16.54	0.93
1	-1	5	31.14	1.46
1	-1	5	34.14	1.68
1	1	-5	18.16	0.98
1	1	-5	17.40	0.86
-1	1	5	33.78	1.63
-1	-1	-5	18.12	0.88
1	-1	5	30.65	1.55
1	1	-5	17.94	0.86
-1	1	5	32.45	1.56
-1	-1	-5	19.22	1.06
1	1	-5	17.75	1.05
1	-1	5	30.20	1.46
-1	1	5	30.07	1.54
1	-1	5	29.97	1.51
-1	1	5	30.87	1.42
-1	-1	-5	17.68	0.96
-1	1	5	29.68	1.51
1	1	-5	16.30	1.02
-1	-1	-5	17.69	0.95
-1	1	5	32.91	1.53
-1	-1	-5	17.19	0.94
1	2	5	254.38	12.01
1	-2	-5	287.01	13.41
-1	2	-5	294.38	13.36
1	2	5	260.10	11.99
1	-2	-5	303.32	13.45
-1	2	-5	302.94	13.31
-1	2	-5	327.31	13.57

-1	2	-5	276.76	13.61
-1	-2	5	256.14	11.92
-1	-2	5	286.46	12.09
1	-2	-5	294.16	13.39
-1	2	-5	292.69	13.40
-1	-2	5	244.24	11.94
-1	-2	5	267.51	12.29
-1	-2	5	280.61	12.07
1	-2	-5	303.29	13.47
1	2	5	269.47	12.06
1	-2	-5	299.71	13.38
1	2	5	268.66	11.97
1	2	5	250.45	11.91
1	-2	-5	289.88	13.33
-1	2	-5	306.95	13.43
1	2	5	285.87	11.93
1	-2	5	298.22	12.14
-1	2	5	274.35	11.98
-1	2	5	277.65	12.06
-1	2	5	266.68	11.97
1	-2	5	248.83	11.97
1	2	-5	307.94	13.45
1	2	-5	295.87	13.40
-1	-2	-5	260.88	13.48
-1	-2	-5	289.81	13.35
-1	-2	-5	293.19	13.35
-1	2	5	249.54	11.90
1	-2	5	251.98	11.93
1	-2	5	279.43	12.09
1	2	-5	300.01	13.35
-1	-2	-5	297.83	13.43
-1	-2	-5	312.27	13.43
1	-2	5	264.29	12.00
-1	2	5	259.21	11.95
-1	-2	-5	299.85	13.48
1	2	-5	316.48	13.53
-1	2	5	251.83	12.02
1	-2	5	268.84	11.88
-1	3	-5	343.70	15.55
1	3	5	338.99	15.46
1	3	5	346.71	15.42
-1	3	-5	342.27	15.41
1	-3	-5	343.15	15.61
-1	3	-5	371.78	15.73

1	3	5	340.32	15.34
1	-3	-5	351.46	15.62
-1	3	-5	343.90	15.50
-1	-3	5	355.63	15.55
1	3	5	323.94	15.38
1	-3	-5	331.02	15.53
-1	-3	5	332.51	15.48
1	3	5	357.70	15.54
-1	3	-5	342.77	15.41
-1	3	-5	342.43	15.66
-1	-3	5	332.59	15.39
-1	-3	5	317.41	15.41
1	-3	-5	357.16	15.51
-1	-3	5	366.68	15.56
-1	3	-5	355.45	15.57
1	3	5	352.45	15.53
1	3	5	348.42	15.36
1	3	-5	343.51	15.58
-1	3	5	332.69	15.37
-1	3	5	347.51	15.53
-1	3	5	337.62	15.54
1	3	-5	349.91	15.64
-1	-3	-5	330.04	15.49
1	3	-5	341.05	15.53
-1	-3	-5	326.93	15.52
1	-3	5	321.13	15.39
-1	-3	-5	333.63	15.60
1	-3	5	317.09	15.43
1	3	-5	342.09	15.50
1	-3	5	371.33	15.58
1	3	-5	342.19	15.67
-1	-3	-5	354.87	15.56
1	-3	5	341.63	15.45
-1	3	5	339.76	15.41
-1	3	5	342.44	15.43
-1	3	5	348.12	15.40
-1	-3	-5	359.03	15.61
1	-3	5	377.54	15.62
-1	3	5	339.95	15.41
-1	4	-5	330.73	14.81
-1	4	-5	319.51	14.58
1	4	5	328.89	14.39
1	4	5	308.51	14.13
-1	-4	5	308.30	14.21

1	-4	-5	335.93	14.73
1	-4	-5	341.65	14.58
-1	-4	5	300.56	14.48
1	-4	-5	312.80	14.68
-1	-4	5	294.16	14.22
-1	-4	5	310.96	14.30
-1	4	-5	320.83	14.74
-1	4	-5	335.33	14.64
1	4	5	315.34	14.14
-1	-4	5	341.09	14.40
1	4	5	322.19	14.22
-1	4	-5	323.64	14.46
1	-4	-5	300.89	14.55
1	4	5	324.52	14.36
-1	4	-5	330.11	14.64
1	-4	-5	310.04	14.59
1	4	5	316.55	14.27
1	4	5	304.91	14.19
-1	4	5	324.74	14.36
-1	-4	-5	308.95	14.57
-1	4	5	303.68	14.37
1	-4	5	336.49	14.39
1	4	-5	317.97	14.57
1	4	-5	328.93	14.67
-1	4	5	271.70	14.24
-1	-4	-5	315.10	14.68
-1	4	5	303.09	14.23
-1	4	5	319.65	14.23
-1	4	5	323.67	14.21
1	-4	5	352.71	14.45
-1	-4	-5	341.27	14.64
1	-4	5	304.89	14.21
-1	-4	-5	328.50	14.69
1	-4	5	303.23	14.25
1	4	-5	325.77	14.72
1	4	-5	324.35	14.78
1	-4	5	326.58	14.27
1	4	-5	330.24	14.62
-1	-4	-5	307.04	14.58
-1	5	-5	145.90	6.59
-1	-5	5	149.26	6.98
1	-5	-5	131.67	6.52
-1	5	-5	144.78	6.62
-1	5	-5	142.84	6.82

1	5	5	138.97	6.81
1	-5	-5	153.59	6.54
1	5	5	153.87	6.80
-1	5	-5	140.33	6.70
-1	-5	5	142.57	6.91
1	5	5	154.87	7.06
-1	-5	5	139.93	6.88
1	5	5	146.31	6.87
-1	-5	5	158.54	7.11
1	5	5	143.86	6.95
-1	-5	5	165.73	7.09
1	-5	-5	138.66	6.68
1	-5	-5	134.72	6.51
-1	5	-5	144.88	6.39
-1	5	-5	140.96	6.53
1	5	5	148.82	6.88
1	-5	-5	138.04	6.64
1	5	5	147.95	7.10
1	-5	5	141.64	6.73
1	5	-5	137.53	6.67
-1	5	5	145.00	6.87
-1	-5	-5	134.02	6.62
1	-5	5	141.97	6.93
-1	5	5	144.27	7.09
1	5	-5	140.78	6.59
1	-5	5	156.12	6.96
1	5	-5	147.82	6.63
-1	-5	-5	131.94	6.51
1	5	-5	139.13	6.53
-1	5	5	148.50	6.90
-1	-5	-5	138.21	6.52
-1	5	5	146.87	6.90
1	-5	5	160.07	7.16
1	5	-5	143.40	6.78
-1	-5	-5	146.97	6.60
1	-5	5	143.14	6.89
-1	5	5	156.29	7.07
-1	-5	-5	146.39	6.69
-1	5	5	147.75	6.87
1	-5	5	161.05	7.08
-1	-6	5	5.78	0.74
1	-6	-5	2.41	0.52
-1	-6	5	6.09	0.65
-1	6	-5	3.29	0.66

1	6	5	5.60	0.73
1	6	5	6.08	0.91
1	-6	-5	2.60	0.47
1	6	5	4.90	0.70
-1	-6	5	6.11	1.01
-1	-6	5	5.33	0.79
-1	-6	5	6.00	0.98
1	-6	-5	2.89	0.74
-1	-6	5	5.19	0.89
1	6	5	5.93	0.69
-1	6	-5	2.71	0.52
-1	6	-5	1.77	0.69
-1	6	-5	2.24	0.58
1	-6	-5	3.10	0.63
1	6	5	5.36	0.97
1	-6	-5	2.47	0.44
1	6	5	6.50	0.75
-1	6	-5	2.47	0.44
-1	6	5	5.48	0.68
1	-6	5	5.36	0.72
-1	6	5	6.71	0.75
1	-6	5	5.36	0.47
1	6	-5	3.36	0.70
1	6	-5	2.91	0.62
-1	-6	-5	3.30	0.60
-1	-6	-5	2.05	0.58
-1	-6	-5	2.93	0.48
-1	6	5	5.70	0.76
1	-6	5	6.06	0.75
1	6	-5	3.65	0.64
-1	-6	-5	3.13	0.54
-1	-6	-5	2.01	0.64
1	6	-5	2.56	0.35
1	6	-5	3.53	0.73
1	-6	5	6.14	1.05
-1	6	5	5.75	0.99
-1	6	5	5.50	0.74
1	-6	5	4.57	0.62
1	-6	5	7.94	1.13
1	7	5	33.42	2.24
1	7	5	35.35	2.24
1	7	5	33.88	1.88
1	-7	-5	34.16	1.89
-1	-7	5	34.96	2.17

1	-7	-5	37.12	2.24
1	7	5	36.61	1.96
1	-7	-5	34.13	1.88
-1	7	-5	33.04	2.12
-1	-7	5	38.23	2.22
-1	-7	5	33.71	1.96
-1	-7	5	35.80	2.09
-1	7	-5	33.20	1.93
1	-7	-5	33.66	1.91
1	-7	-5	36.79	1.95
-1	-7	5	33.79	2.03
-1	7	-5	32.66	2.28
1	-7	-5	31.10	2.01
-1	7	-5	38.25	2.10
-1	7	-5	36.17	1.74
-1	-7	5	37.86	2.28
1	7	5	37.44	2.09
1	7	5	34.50	1.97
1	-7	5	37.52	2.35
-1	7	5	34.15	1.99
-1	7	5	33.19	1.98
-1	-7	-5	34.88	1.91
-1	7	5	33.86	1.97
-1	-7	-5	36.07	2.02
1	-7	5	34.54	1.97
-1	7	5	36.32	2.31
1	7	-5	29.94	2.15
-1	-7	-5	30.74	2.05
1	7	-5	33.85	1.66
1	7	-5	33.22	1.94
-1	-7	-5	31.96	1.86
1	7	-5	31.39	2.04
1	-7	5	37.16	2.25
-1	7	5	34.23	2.28
-1	-7	-5	35.88	2.16
1	-7	5	34.74	1.74
1	-7	5	37.00	2.05
1	-7	5	35.18	2.08
-1	7	5	36.63	1.99
1	7	-5	35.87	2.05
1	-7	5	39.00	2.15
1	8	5	39.01	2.59
-1	-8	5	40.70	2.41
-1	-8	5	45.21	2.61

1	8	5	43.13	2.36
-1	8	-5	51.54	2.89
1	-8	-5	52.31	2.61
1	8	5	40.96	2.30
-1	-8	5	46.79	2.77
-1	-8	5	43.90	2.48
1	-8	-5	57.77	3.04
-1	-8	5	38.80	2.33
1	-8	-5	49.11	2.80
-1	-8	5	46.78	2.65
-1	8	-5	49.31	2.90
-1	8	-5	51.05	2.43
1	-8	-5	45.26	2.58
-1	8	-5	46.69	3.00
1	8	5	46.19	2.74
-1	-8	5	38.70	2.13
1	8	5	40.87	2.43
1	-8	-5	51.99	2.69
1	-8	-5	44.72	2.61
-1	8	-5	50.83	2.88
1	8	5	41.20	2.32
-1	8	5	42.13	2.37
1	-8	5	41.05	2.34
1	-8	5	44.50	2.65
1	-8	5	45.56	2.57
1	-8	5	45.75	2.44
-1	-8	-5	46.95	2.57
-1	8	5	46.82	2.74
1	8	-5	47.63	3.05
-1	8	5	41.34	2.36
-1	-8	-5	51.26	2.77
-1	8	5	39.26	2.36
-1	-8	-5	53.55	2.96
1	8	-5	51.67	2.83
1	8	-5	44.51	2.65
-1	-8	-5	49.91	2.66
1	8	-5	47.52	2.77
-1	8	5	41.30	2.35
-1	8	5	41.57	2.72
1	8	-5	48.93	2.36
1	-8	5	46.43	2.82
-1	-8	-5	47.51	2.74
1	-8	5	41.72	2.08
1	-8	5	42.71	2.41

-1	9	-5	37.01	1.86
1	-9	-5	34.29	2.05
1	-9	-5	31.32	1.95
-1	9	-5	29.83	2.55
-1	-9	5	34.27	1.74
-1	-9	5	31.97	2.17
1	-9	-5	34.28	2.06
1	9	5	33.51	2.06
1	-9	-5	31.55	2.18
1	9	5	40.64	2.65
1	-9	-5	38.13	2.42
1	-9	-5	32.35	2.03
1	-9	-5	36.59	2.18
-1	-9	5	36.92	2.25
1	9	5	32.67	2.50
-1	9	-5	36.24	2.30
1	9	5	35.20	2.03
-1	-9	5	36.99	2.44
1	9	5	32.49	2.20
-1	-9	5	35.59	2.43
-1	9	-5	35.50	2.30
-1	-9	5	36.05	2.14
-1	-9	-5	33.82	2.05
-1	9	5	40.34	2.64
-1	9	5	35.14	2.12
-1	-9	-5	32.07	1.96
1	9	-5	33.63	2.59
1	9	-5	33.63	2.23
1	9	-5	35.64	2.32
-1	-9	-5	35.15	2.44
1	-9	5	34.43	2.55
-1	9	5	34.47	2.14
1	-9	5	33.69	1.80
1	-9	5	35.41	2.17
1	-9	5	36.26	2.50
-1	9	5	35.04	2.49
-1	-9	-5	31.30	2.12
-1	-9	-5	36.76	2.26
1	-9	5	33.94	2.09
1	-9	5	36.99	2.39
1	9	-5	32.74	1.74
1	-9	5	35.82	2.17
-1	10	-5	67.84	3.75
1	10	5	48.97	2.79

-1	10	-5	63.07	3.63
1	-10	-5	59.59	3.26
1	10	5	45.72	2.78
1	-10	-5	67.54	3.43
-1	-10	5	51.68	2.90
-1	-10	5	54.13	3.03
-1	10	-5	66.81	3.12
-1	10	-5	63.90	4.04
1	-10	-5	70.42	4.37
1	-10	-5	64.48	3.40
1	-10	-5	60.65	3.33
1	-10	-5	70.47	3.51
-1	-10	5	48.75	3.02
1	-10	-5	58.45	3.50
1	10	5	54.38	3.43
-1	-10	5	50.33	2.47
-1	-10	5	55.98	3.33
1	10	5	47.04	3.34
-1	10	5	57.98	3.49
-1	10	5	53.96	2.90
1	-10	5	55.20	3.28
1	-10	5	50.21	2.90
-1	10	5	50.60	2.81
1	10	-5	60.27	3.05
-1	-10	-5	57.81	3.26
1	10	-5	62.82	3.97
1	-10	5	53.00	2.97
1	-10	5	48.87	2.51
-1	10	5	47.54	3.35
1	-10	5	51.45	2.98
-1	-10	-5	63.07	3.47
-1	-10	-5	58.78	3.50
-1	-10	-5	63.54	3.54
-1	-10	-5	61.02	3.40
1	-10	5	55.44	3.13
1	10	-5	70.80	3.81
1	10	-5	60.60	3.54
-1	-11	5	39.97	3.00
1	-11	-5	56.86	3.10
1	-11	-5	56.63	3.16
1	-11	-5	48.18	3.15
1	11	5	42.12	3.18
1	-11	-5	49.26	2.87
1	-11	-5	57.95	3.10

-1	11	-5	52.82	3.77
1	11	5	43.15	2.55
-1	11	-5	58.43	3.36
-1	-11	5	44.63	2.67
-1	-11	-5	51.48	2.88
1	-11	5	47.52	2.73
1	11	-5	41.07	4.28
-1	11	5	45.39	2.69
-1	11	5	39.44	3.24
1	-11	5	44.10	2.64
-1	11	5	52.00	3.36
1	-11	5	41.13	2.20
-1	-11	-5	57.47	3.11
1	11	-5	56.29	3.26
-1	-11	-5	49.05	3.17
1	-11	5	43.48	2.75
-1	-11	-5	59.42	3.20
1	-12	-5	32.23	2.19
1	-12	-5	27.54	2.18
-1	-12	5	30.44	2.46
-1	12	-5	26.88	3.15
1	12	5	26.71	2.95
1	-12	-5	25.64	1.91
-1	12	-5	30.50	2.47
-1	-12	5	30.68	2.24
1	-12	5	28.64	2.17
1	-12	5	30.10	2.31
-1	-12	-5	30.18	2.20
1	12	-5	25.70	2.43
1	12	-5	23.30	3.15
-1	12	5	31.20	2.32
-1	12	5	30.82	3.10
-1	-12	-5	27.25	1.95
-1	-12	-5	30.42	2.28
1	-13	-5	14.24	1.69
1	-13	-5	15.04	1.56
-1	-13	5	14.30	1.67
-1	13	-5	15.86	3.34
-1	13	-5	15.63	2.43
1	13	5	11.00	2.39
-1	-13	5	13.66	2.14
1	-13	-5	13.72	1.85
1	-13	5	12.69	1.65
1	-13	5	15.60	2.09

-1	-13	-5	12.56	1.71
-1	-13	-5	15.32	2.15
-1	-13	-5	13.59	1.49
-1	13	5	8.65	2.32
1	13	-5	15.39	2.13
1	13	-5	13.55	2.70
2	0	5	495.22	22.86
2	0	-5	466.72	21.43
-2	0	5	512.77	22.90
-2	0	-5	480.35	21.41
2	0	5	546.76	22.97
-2	0	5	536.01	22.90
-2	0	-5	477.55	21.34
-2	0	5	494.74	22.84
2	0	5	522.35	22.88
2	0	-5	485.94	21.45
2	0	-5	482.40	21.39
-2	0	-5	476.72	21.43
-2	0	-5	456.15	21.36
-2	0	5	518.01	22.81
2	0	5	521.75	22.82
-2	0	-5	466.52	21.38
2	0	5	450.33	22.83
-2	0	-5	495.81	21.50
2	0	-5	492.92	21.35
2	-1	-5	101.89	4.71
-2	1	-5	106.96	4.86
-2	1	-5	102.62	4.74
2	1	5	74.77	3.66
-2	-1	5	76.63	3.59
-2	1	-5	104.47	4.67
-2	-1	5	76.05	3.66
-2	-1	5	80.31	3.56
2	-1	-5	105.68	4.78
-2	-1	5	81.09	3.69
-2	-1	5	80.89	3.72
2	1	5	79.90	3.63
2	-1	-5	102.20	4.75
2	1	5	84.84	3.75
-2	1	-5	102.85	4.81
2	-1	-5	102.98	4.69
-2	1	-5	100.72	4.71
2	1	5	75.16	3.62
-2	1	-5	103.97	4.67

-2	-1	5	75.85	3.55
-2	1	5	75.31	3.53
-2	1	5	79.80	3.63
-2	-1	-5	100.55	4.71
2	-1	5	77.69	3.65
2	-1	5	77.00	3.58
-2	1	5	81.68	3.68
-2	-1	-5	100.60	4.70
-2	-1	-5	98.06	4.70
-2	1	5	77.61	3.61
2	-1	5	76.07	3.59
2	-1	5	80.01	3.73
2	-1	5	70.27	3.62
-2	1	5	77.55	3.65
-2	-1	-5	99.65	4.74
2	1	-5	99.65	4.78
-2	-1	-5	105.46	4.80
2	1	-5	103.43	4.79
-2	1	5	81.53	3.70
2	1	-5	106.18	4.63
-2	-1	-5	102.04	4.67
2	1	-5	102.60	4.64
2	-1	5	88.19	3.81
-2	-1	-5	105.20	4.67
2	1	-5	104.13	4.75
2	-2	-5	25.02	1.38
-2	-2	5	37.52	1.83
-2	2	-5	28.20	1.40
2	-2	-5	25.88	1.34
-2	2	-5	28.99	1.42
2	2	5	36.90	1.83
-2	-2	5	36.65	1.93
-2	2	-5	28.69	1.32
-2	-2	5	35.59	1.83
2	-2	-5	25.98	1.44
2	2	5	38.82	1.82
-2	-2	5	39.66	2.00
2	2	5	36.49	1.92
-2	2	-5	28.17	1.36
-2	-2	5	40.00	1.96
-2	2	-5	27.68	1.52
2	2	5	41.08	1.99
2	-2	-5	25.98	1.41
2	-2	-5	27.79	1.47

-2	2	-5	30.20	1.61
2	2	5	34.17	1.99
2	-2	-5	26.42	1.30
-2	2	5	37.12	1.80
2	2	-5	27.36	1.33
-2	2	5	39.88	1.97
-2	-2	-5	26.47	1.31
2	2	-5	26.12	1.44
-2	-2	-5	26.70	1.38
2	-2	5	43.36	2.08
-2	-2	-5	27.77	1.42
-2	-2	-5	26.29	1.34
-2	2	5	39.05	1.89
2	-2	5	41.25	2.04
-2	2	5	38.91	1.92
2	-2	5	41.11	1.91
2	2	-5	28.44	1.51
-2	2	5	36.29	1.94
-2	-2	-5	25.52	1.45
-2	-2	-5	29.26	1.45
-2	2	5	37.79	1.84
2	-2	5	41.25	1.81
2	-2	5	36.55	1.88
-2	-3	5	22.38	1.22
-2	-3	5	24.25	1.41
2	-3	-5	16.87	0.91
-2	3	-5	15.99	1.13
2	-3	-5	15.03	0.96
2	3	5	25.13	1.39
2	3	5	24.34	1.15
-2	-3	5	24.63	1.37
2	-3	-5	14.39	0.95
-2	-3	5	22.76	1.21
-2	3	-5	17.32	0.85
-2	3	-5	16.49	0.97
-2	3	-5	16.87	0.86
-2	3	-5	18.21	1.01
2	3	5	23.34	1.33
2	-3	-5	17.20	1.08
2	3	5	23.15	1.25
2	3	5	22.19	1.21
2	3	5	23.16	1.12
2	3	5	22.99	1.30
-2	3	-5	17.85	1.23

-2	-3	5	22.78	1.33
-2	3	-5	17.26	0.95
-2	3	5	24.18	1.38
-2	-3	-5	17.63	1.03
2	-3	5	26.09	1.50
-2	-3	-5	18.59	1.04
2	-3	5	24.93	1.28
2	-3	5	25.05	1.44
-2	3	5	22.45	1.20
-2	-3	-5	15.95	0.92
-2	3	5	23.68	1.40
2	3	-5	16.54	1.05
2	3	-5	15.63	1.07
2	-3	5	22.58	1.27
2	3	-5	15.78	1.05
2	-3	5	22.16	1.21
-2	3	5	22.92	1.27
-2	3	5	22.06	1.19
2	3	-5	16.38	0.92
-2	-3	-5	16.76	1.05
-2	-3	-5	15.82	0.95
-2	3	5	22.87	1.27
2	-4	-5	32.14	1.54
2	4	5	28.28	1.54
-2	-4	5	28.53	1.65
-2	-4	5	24.72	1.54
2	4	5	26.18	1.33
-2	-4	5	26.47	1.43
-2	4	-5	30.41	1.57
-2	4	-5	30.36	1.62
-2	-4	5	27.04	1.60
2	4	5	27.76	1.33
2	4	5	26.37	1.44
-2	-4	5	25.94	1.43
2	-4	-5	27.98	1.52
-2	4	-5	31.22	1.80
2	4	5	26.83	1.45
2	-4	-5	28.33	1.65
-2	4	-5	31.51	1.46
2	4	5	28.66	1.61
-2	4	-5	31.37	1.62
-2	4	-5	28.26	1.76
2	4	5	26.88	1.58
2	-4	-5	26.72	1.57

2	-4	-5	27.19	1.68
2	-4	5	26.94	1.33
-2	4	5	27.21	1.58
2	-4	5	30.81	1.72
-2	4	5	25.19	1.42
2	-4	5	29.30	1.69
-2	4	5	25.57	1.48
2	4	-5	29.46	1.57
2	-4	5	28.61	1.49
-2	4	5	27.65	1.48
-2	4	5	26.75	1.64
2	4	-5	29.38	1.68
2	4	-5	29.72	1.68
-2	4	5	25.81	1.42
2	-4	5	26.71	1.50
-2	-4	-5	30.39	1.56
2	4	-5	31.13	1.59
-2	-4	-5	29.24	1.68
-2	4	5	27.92	1.47
-2	-4	-5	31.63	1.66
2	-4	5	26.90	1.44
2	4	-5	27.85	1.76
-2	-4	-5	28.96	1.57
-2	5	-5	103.87	4.48
-2	5	-5	99.33	4.61
-2	-5	5	90.04	4.46
-2	5	-5	95.93	4.87
2	5	5	89.95	4.44
2	5	5	90.24	4.54
-2	5	-5	102.44	4.69
2	-5	-5	91.83	4.59
-2	-5	5	87.23	4.34
-2	-5	5	86.52	4.35
-2	-5	5	95.69	4.50
2	5	5	85.02	4.23
2	5	5	92.54	4.50
2	-5	-5	99.06	4.77
2	-5	-5	104.18	4.57
-2	-5	5	98.25	4.55
2	-5	-5	92.92	4.69
2	5	5	92.80	4.34
-2	5	-5	96.56	4.80
-2	5	-5	103.95	4.67
2	-5	-5	95.90	4.57

2	5	5	93.10	4.33
2	5	5	99.00	4.23
-2	-5	5	99.95	4.56
2	-5	-5	88.42	4.67
2	5	-5	100.25	4.65
-2	-5	-5	107.78	4.76
-2	5	5	91.75	4.37
2	5	-5	92.92	4.71
-2	5	5	93.96	4.37
-2	5	5	89.56	4.32
-2	-5	-5	94.36	4.59
-2	-5	-5	102.00	4.69
2	-5	5	90.10	4.22
-2	-5	-5	91.55	4.58
2	-5	5	98.85	4.55
2	5	-5	90.58	4.79
-2	5	5	88.42	4.67
-2	-5	-5	92.77	4.68
-2	5	5	91.66	4.33
2	5	-5	98.80	4.60
2	-5	5	92.15	4.38
2	-5	5	85.81	4.73
2	-5	5	89.77	4.41
2	-5	5	92.00	4.35
-2	-6	5	7.21	0.90
-2	-6	5	9.61	1.12
-2	6	-5	13.67	1.06
-2	-6	5	8.62	0.82
2	-6	-5	12.84	1.02
-2	6	-5	11.37	0.96
2	-6	-5	11.38	1.06
-2	-6	5	8.08	0.75
2	-6	-5	12.89	1.09
2	6	5	9.71	1.09
-2	6	-5	11.67	1.17
2	6	5	8.90	0.82
-2	6	-5	13.08	1.26
2	-6	-5	11.22	0.91
2	6	5	8.10	0.78
2	-6	-5	12.11	1.20
-2	6	-5	14.53	1.11
-2	-6	5	9.10	1.02
-2	-6	5	9.37	1.08
2	6	5	9.24	0.89

-2	6	-5	14.02	0.83
2	-6	-5	12.02	0.90
2	6	5	8.07	1.01
2	6	5	7.31	0.75
2	6	5	9.78	0.67
-2	6	5	8.81	0.82
-2	-6	-5	11.99	1.05
2	6	-5	11.82	1.19
-2	6	5	8.20	0.88
2	6	-5	13.20	0.99
-2	-6	-5	12.73	1.15
2	-6	5	9.66	0.97
2	-6	5	9.32	0.79
2	-6	5	9.02	1.14
-2	6	5	8.30	0.81
-2	6	5	8.69	1.07
2	-6	5	9.49	1.16
-2	-6	-5	12.15	0.92
2	6	-5	13.72	1.05
-2	-6	-5	12.98	1.10
2	-6	5	9.38	0.60
-2	6	5	8.05	0.85
-2	-6	-5	12.69	0.93
2	-6	5	9.89	0.86
-2	6	5	8.16	1.15
2	6	-5	12.86	1.08
-2	7	-5	29.61	1.80
-2	7	-5	32.26	2.02
2	7	5	22.56	1.51
-2	-7	5	24.97	1.54
2	-7	-5	33.02	1.77
-2	-7	5	25.45	1.68
2	7	5	27.42	1.86
2	7	5	24.78	1.65
2	-7	-5	28.24	1.83
2	7	5	22.66	1.79
-2	7	-5	31.54	1.59
-2	-7	5	25.68	1.58
-2	7	-5	30.17	2.14
2	7	5	24.18	1.50
-2	7	-5	29.97	2.09
-2	-7	5	28.62	1.85
2	-7	-5	29.24	1.76
2	7	5	23.28	1.52

2	-7	-5	27.19	1.71
2	-7	-5	30.47	2.05
-2	-7	5	24.88	1.78
-2	-7	5	27.92	1.92
2	-7	-5	27.87	1.69
-2	-7	-5	31.16	1.89
-2	-7	-5	27.51	1.68
-2	7	5	24.42	1.89
2	-7	5	26.58	1.70
-2	-7	-5	32.00	1.99
2	-7	5	28.43	2.01
2	-7	5	24.16	1.58
-2	-7	-5	28.25	1.87
2	-7	5	27.31	1.59
-2	7	5	26.80	1.81
-2	-7	-5	30.92	1.76
2	-7	5	25.05	1.85
-2	7	5	24.30	1.57
2	7	-5	31.10	1.77
2	-7	5	25.50	1.54
2	7	-5	26.96	2.03
2	7	-5	31.00	1.88
-2	7	5	24.95	1.52
-2	7	5	24.20	1.58
-2	7	5	25.64	1.56
2	-7	5	24.29	1.32
2	7	-5	30.97	1.86
2	-8	-5	31.62	1.95
2	-8	-5	31.83	1.92
-2	8	-5	31.03	2.17
2	-8	-5	30.24	1.86
-2	-8	5	25.30	2.07
2	8	5	26.93	1.58
-2	8	-5	35.74	2.20
-2	-8	5	28.44	2.04
2	8	5	24.86	1.84
2	-8	-5	36.26	1.98
2	-8	-5	33.76	2.23
2	8	5	24.86	1.97
-2	-8	5	30.42	2.04
-2	-8	5	28.10	1.74
2	8	5	28.38	1.71
-2	8	-5	32.22	2.40
-2	-8	5	23.65	1.74

-2	8	-5	36.39	1.80
2	8	5	29.41	2.11
2	8	5	26.17	1.71
2	-8	-5	30.40	1.99
-2	-8	5	27.88	1.86
2	-8	5	30.97	2.30
-2	8	5	30.31	1.78
-2	8	5	29.12	2.16
2	8	-5	30.81	2.32
-2	-8	-5	33.09	1.93
-2	8	5	28.02	1.73
2	-8	5	30.44	2.08
2	8	-5	31.34	2.05
2	-8	5	32.38	1.98
2	-8	5	27.32	1.79
2	-8	5	26.81	1.47
2	8	-5	34.09	2.12
2	-8	5	29.98	1.77
-2	-8	-5	29.84	2.09
-2	8	5	24.98	2.08
-2	8	5	25.56	1.74
2	8	-5	32.50	1.98
-2	-8	-5	32.81	1.86
-2	-8	-5	34.27	2.13
2	-8	5	28.41	1.74
-2	8	5	29.38	1.81
-2	-8	-5	32.68	2.20
2	-9	-5	67.86	3.59
-2	-9	5	71.11	3.80
2	9	5	71.56	4.05
2	-9	-5	70.40	3.58
2	-9	-5	78.15	3.65
-2	9	-5	76.84	3.42
-2	-9	5	77.40	3.91
2	-9	-5	70.08	3.60
2	-9	-5	69.16	3.54
-2	9	-5	73.91	3.86
-2	-9	5	72.84	3.67
2	9	5	68.99	3.55
2	9	5	67.95	3.76
2	9	5	74.13	4.12
2	-9	-5	65.57	3.71
-2	-9	5	70.11	3.87
2	-9	-5	71.91	4.01

-2	9	-5	67.99	3.87
2	9	5	68.59	3.53
-2	-9	5	62.41	3.67
-2	9	-5	68.51	4.15
2	-9	5	73.33	3.82
2	-9	5	68.73	3.67
-2	-9	-5	70.06	3.64
-2	9	5	71.61	3.64
2	9	-5	68.10	3.27
2	-9	5	73.94	3.96
2	9	-5	66.90	4.07
2	9	-5	71.42	3.82
-2	9	5	69.10	3.68
2	-9	5	68.56	3.36
-2	9	5	77.07	4.08
2	-9	5	74.09	3.69
2	-9	5	71.21	4.14
2	9	-5	67.95	3.74
-2	-9	-5	67.44	3.77
2	-9	5	66.94	3.72
-2	9	5	62.88	4.00
-2	-9	-5	69.50	3.87
-2	-9	-5	68.48	3.53
2	10	5	2.33	0.74
-2	-10	5	0.98	0.68
2	-10	-5	5.89	1.03
-2	-10	5	0.78	0.78
-2	-10	5	1.95	1.09
2	-10	-5	3.84	0.96
2	-10	-5	4.95	0.82
2	-10	-5	5.50	1.34
2	-10	-5	3.87	0.83
2	10	5	2.83	1.20
-2	10	-5	5.21	1.45
2	10	5	2.60	0.72
-2	-10	5	2.47	0.83
-2	10	-5	5.32	1.09
-2	10	-5	5.84	1.24
2	-10	-5	6.75	0.95
2	-10	-5	5.55	1.02
-2	10	-5	4.85	0.90
2	10	5	1.19	0.95
2	10	5	1.89	0.63
-2	10	5	1.74	0.85

2	-10	5	2.64	0.47
2	-10	5	1.30	0.81
2	-10	5	2.10	0.83
2	-10	5	0.85	0.71
2	-10	5	2.37	1.04
-2	-10	-5	5.00	1.00
-2	-10	-5	4.83	0.80
-2	-10	-5	4.11	0.85
-2	10	5	2.07	1.05
2	10	-5	4.77	0.96
2	10	-5	5.65	1.21
2	10	-5	4.07	1.18
-2	10	5	3.08	1.28
-2	-10	-5	4.51	0.95
-2	-11	5	8.18	1.45
2	11	5	8.19	1.17
-2	11	-5	8.82	1.73
2	-11	-5	8.11	1.49
-2	11	-5	6.02	1.35
-2	-11	5	7.48	1.21
2	11	5	8.71	1.68
2	-11	-5	6.14	1.06
2	-11	-5	6.90	1.07
2	-11	-5	5.54	1.08
2	-11	-5	6.15	0.91
-2	-11	5	7.31	1.08
-2	-11	-5	6.86	1.21
-2	11	5	7.31	1.20
2	-11	5	7.29	1.18
2	-11	5	6.62	0.77
-2	-11	-5	6.60	1.22
-2	-11	-5	7.13	1.50
-2	11	5	6.23	1.65
-2	-11	-5	5.92	0.89
2	11	-5	6.50	1.14
2	11	-5	6.17	1.62
-2	11	5	5.72	1.45
2	-11	5	6.22	1.21
2	-11	5	8.61	1.35
2	12	5	-1.71	1.34
-2	12	-5	-0.53	1.76
-2	-12	5	0.88	1.09
-2	12	-5	0.94	1.18
2	-12	-5	-0.21	0.66

2	-12	-5	0.27	0.95
-2	-12	5	0.34	0.84
2	-12	-5	1.39	0.95
2	12	-5	0.92	0.98
-2	-12	-5	-0.52	0.77
-2	-12	-5	-0.73	0.68
2	-12	5	2.69	1.21
-2	12	5	-2.55	1.42
-2	12	5	2.25	1.06
-2	-12	-5	-1.20	0.95
2	-12	5	-0.26	0.82
2	12	-5	0.61	1.65
-2	-13	5	16.96	1.72
-2	-13	5	13.09	2.44
2	-13	-5	12.44	1.58
-2	13	-5	13.39	1.97
2	-13	-5	12.64	1.50
2	13	5	12.28	2.22
-2	13	-5	6.25	2.20
2	13	-5	11.25	2.63
-2	13	5	15.41	3.23
-2	-13	-5	10.97	1.78
2	-13	5	16.35	2.13
-2	-13	-5	13.27	1.67
-2	-13	-5	11.33	2.15
2	13	-5	12.05	2.40
2	-13	5	13.56	1.67
-3	0	-5	-0.28	0.21
-3	0	5	0.03	0.21
3	0	-5	0.00	0.08
-3	0	5	-0.03	0.23
3	0	-5	0.11	0.14
3	0	5	0.02	0.12
3	0	5	0.03	0.14
-3	0	5	0.02	0.15
3	0	5	-0.08	0.18
-3	0	-5	0.14	0.15
3	0	5	-0.06	0.22
-3	0	5	0.15	0.06
3	0	5	0.38	0.31
-3	0	5	0.19	0.06
3	0	-5	0.07	0.22
3	0	5	0.22	0.17
-3	0	-5	0.08	0.21

-3	0	-5	0.04	0.26
-3	0	-5	0.19	0.16
-3	0	-5	-0.02	0.12
3	0	-5	0.00	0.21
-3	0	5	-0.08	0.17
-3	0	5	0.15	0.13
3	0	-5	0.14	0.19
-3	-1	5	52.99	2.63
-3	1	-5	62.68	2.78
3	1	5	59.08	2.72
3	1	5	55.46	2.59
-3	-1	5	54.11	2.54
3	-1	-5	56.80	2.87
-3	-1	5	54.49	2.52
-3	1	-5	59.74	2.83
3	-1	-5	57.30	2.80
3	1	5	53.08	2.58
3	-1	-5	58.00	2.70
-3	-1	5	54.78	2.62
3	1	5	56.58	2.61
-3	1	-5	57.37	2.91
3	-1	-5	60.16	2.87
-3	-1	5	58.60	2.70
-3	-1	5	51.74	2.51
3	1	5	53.13	2.57
-3	1	-5	64.67	2.99
-3	1	-5	60.29	2.80
-3	1	-5	62.92	2.78
3	-1	-5	59.81	2.78
3	-1	5	52.42	2.58
3	-1	5	55.02	2.56
3	1	-5	58.75	2.73
3	1	-5	59.77	2.72
-3	1	5	55.03	2.61
-3	1	5	58.71	2.66
-3	1	5	55.78	2.66
-3	-1	-5	59.19	2.81
3	-1	5	54.66	2.57
3	1	-5	60.95	2.85
-3	-1	-5	58.29	2.89
-3	1	5	51.04	2.56
3	-1	5	61.19	2.79
-3	1	5	54.03	2.59
3	1	-5	58.65	2.90

-3	-1	-5	57.01	2.80
3	-1	5	55.81	2.70
-3	1	5	51.89	2.48
3	-1	5	54.45	2.61
-3	-1	-5	62.17	2.78
-3	-1	-5	59.34	2.85
-3	-2	5	119.12	5.69
-3	2	-5	139.78	6.25
-3	2	-5	137.24	6.00
3	-2	-5	127.08	6.04
3	2	5	123.99	5.74
-3	-2	5	123.50	5.70
-3	2	-5	132.14	5.99
3	2	5	125.34	5.81
3	2	5	113.28	5.85
3	-2	-5	127.52	5.96
-3	2	-5	139.37	6.07
3	-2	-5	129.92	6.12
-3	-2	5	124.87	5.78
3	2	5	134.24	5.86
3	2	5	124.58	5.67
3	-2	-5	138.59	6.11
-3	-2	5	129.28	5.86
3	2	5	125.36	5.72
-3	2	-5	134.40	6.18
3	-2	-5	131.68	5.98
-3	2	-5	139.77	6.03
3	-2	-5	129.57	6.10
-3	-2	5	119.65	5.81
-3	2	-5	130.95	6.06
3	-2	5	128.97	5.88
-3	-2	-5	128.54	6.03
-3	2	5	127.35	5.80
-3	-2	-5	131.49	5.96
-3	-2	-5	129.80	6.11
-3	2	5	125.08	5.76
3	-2	5	126.64	5.73
-3	-2	-5	127.65	6.04
-3	2	5	129.68	5.83
-3	-2	-5	126.87	6.05
3	2	-5	132.33	6.11
3	2	-5	133.10	5.99
-3	-2	-5	138.79	6.11
3	2	-5	129.10	6.11

-3	2	5	123.19	5.67
3	-2	5	134.96	5.94
-3	2	5	125.19	5.81
-3	2	5	123.43	5.69
3	2	-5	124.47	6.17
3	-2	5	124.89	5.77
3	-2	5	130.53	5.77
-3	-3	5	230.05	10.70
3	3	5	230.75	10.69
3	3	5	228.89	10.69
3	-3	-5	255.39	11.87
3	3	5	225.48	10.58
3	-3	-5	243.48	11.80
3	-3	-5	269.64	11.91
-3	3	-5	265.88	11.73
-3	3	-5	255.64	11.95
-3	-3	5	249.26	10.74
-3	3	-5	282.87	11.82
-3	3	-5	273.73	11.73
-3	3	-5	281.03	12.04
3	3	5	233.43	10.60
-3	-3	5	230.53	10.59
-3	-3	5	223.21	10.57
-3	3	-5	270.87	11.84
3	3	5	243.24	10.73
3	-3	-5	263.25	11.72
-3	-3	-5	267.59	11.90
3	3	-5	261.46	11.88
-3	3	5	229.04	10.73
3	-3	5	232.56	10.65
3	3	-5	245.77	11.89
-3	-3	-5	247.65	11.79
3	-3	5	237.09	10.64
-3	3	5	234.75	10.64
-3	3	5	244.58	10.66
-3	-3	-5	270.13	11.85
-3	3	5	225.59	10.54
-3	3	5	222.30	10.55
3	3	-5	264.03	11.77
3	-3	5	264.80	10.86
-3	-3	-5	246.05	11.78
-3	-3	-5	255.58	11.87
3	3	-5	240.10	11.98
-3	3	5	247.41	10.72

3	-3	5	222.90	10.59
-3	4	-5	126.80	5.54
3	4	5	111.56	5.28
-3	4	-5	121.26	5.63
-3	-4	5	109.22	5.27
-3	4	-5	119.12	5.80
3	4	5	112.31	5.40
-3	-4	5	110.24	5.41
3	-4	-5	113.58	5.57
-3	4	-5	132.58	5.93
-3	-4	5	118.79	5.42
-3	-4	5	119.75	5.45
-3	4	-5	130.16	5.66
3	-4	-5	118.32	5.72
3	4	5	108.73	5.41
-3	4	-5	130.05	5.71
3	-4	-5	127.86	5.55
3	4	5	117.10	5.44
-3	-4	5	112.11	5.29
3	4	5	112.22	5.29
3	-4	-5	123.37	5.78
3	-4	-5	112.93	5.62
3	-4	5	106.38	5.28
3	4	-5	123.37	5.61
-3	4	5	108.09	5.51
-3	-4	-5	128.39	5.73
3	4	-5	122.97	5.74
-3	4	5	119.32	5.36
3	-4	5	128.39	5.59
-3	-4	-5	114.97	5.62
-3	-4	-5	113.28	5.69
3	4	-5	109.09	5.75
-3	4	5	113.28	5.49
-3	-4	-5	116.35	5.61
-3	-4	-5	123.50	5.71
-3	4	5	100.17	5.32
-3	4	5	117.53	5.34
-3	4	5	120.84	5.34
3	-4	5	118.82	5.49
-3	4	5	113.38	5.24
3	4	-5	120.00	5.72
3	-4	5	119.79	5.32
3	5	5	59.12	3.14
-3	-5	5	57.52	2.96

3	5	5	59.83	2.95
-3	5	-5	80.88	3.81
3	-5	-5	78.93	3.82
3	-5	-5	76.00	3.79
-3	5	-5	81.44	3.88
-3	5	-5	75.77	3.98
3	-5	-5	75.47	3.89
-3	-5	5	60.13	3.14
3	-5	-5	81.49	4.00
3	-5	-5	77.94	3.75
-3	-5	5	58.29	3.10
-3	5	-5	76.21	3.82
-3	-5	5	65.87	3.21
-3	5	-5	85.97	3.70
-3	-5	5	65.01	3.16
3	5	5	59.34	3.09
3	-5	-5	84.64	3.72
3	5	5	63.92	3.16
-3	5	-5	80.64	4.08
3	5	5	63.88	2.98
-3	-5	5	60.48	2.98
-3	5	5	63.25	3.20
-3	-5	-5	74.98	3.76
-3	-5	-5	85.08	3.91
3	-5	5	55.43	2.96
-3	5	5	61.65	3.01
3	-5	5	61.89	3.19
-3	5	5	54.92	3.01
3	5	-5	74.23	3.97
-3	5	5	58.68	2.95
-3	-5	-5	77.55	3.79
-3	-5	-5	81.28	3.90
3	5	-5	79.67	3.79
-3	-5	-5	74.38	3.85
-3	5	5	58.33	3.02
3	-5	5	59.59	3.03
-3	5	5	57.00	3.21
3	-5	5	71.29	3.34
3	5	-5	76.89	3.89
3	5	-5	78.82	3.82
3	-5	5	64.71	3.01
3	-5	5	59.77	2.85
-3	6	-5	17.77	1.00
3	6	5	10.33	0.65

3	6	5	9.54	1.07
-3	-6	5	11.30	1.09
3	6	5	10.62	1.06
-3	-6	5	9.28	0.99
3	-6	-5	15.51	1.05
3	6	5	8.43	0.93
3	-6	-5	15.55	1.08
-3	-6	5	10.06	1.15
3	6	5	8.51	0.70
-3	6	-5	15.54	1.54
-3	-6	5	9.26	0.81
-3	6	-5	16.25	1.15
-3	-6	5	10.20	0.83
-3	6	-5	15.82	1.38
3	6	5	10.33	0.86
3	-6	-5	17.59	1.40
3	-6	-5	17.66	1.05
-3	6	-5	17.23	1.20
-3	6	-5	14.94	1.32
3	-6	-5	15.06	1.23
3	6	5	9.94	0.84
3	-6	-5	16.30	1.17
-3	-6	5	8.98	1.09
3	6	-5	16.70	1.13
3	6	-5	17.41	1.19
-3	6	5	10.79	1.07
-3	6	5	8.92	0.92
-3	6	5	9.81	0.88
-3	-6	-5	14.84	1.23
3	-6	5	11.41	1.22
-3	6	5	10.99	0.87
-3	-6	-5	15.71	1.08
-3	-6	-5	15.47	1.07
3	-6	5	11.01	0.88
-3	-6	-5	17.13	1.26
3	-6	5	10.56	1.19
-3	6	5	9.74	1.11
-3	-6	-5	17.82	1.28
3	-6	5	9.57	0.66
3	-6	5	9.48	0.82
-3	6	5	9.15	0.77
3	6	-5	15.26	1.31
3	6	-5	14.60	1.19
3	-6	5	10.15	0.84

3	7	5	3.10	0.53
-3	7	-5	2.72	0.47
-3	-7	5	4.48	0.90
-3	-7	5	3.15	0.58
-3	-7	5	3.70	0.84
3	-7	-5	1.66	0.74
3	-7	-5	1.50	0.61
3	-7	-5	2.58	0.50
-3	-7	5	1.67	0.64
-3	7	-5	2.13	0.54
3	7	5	3.37	0.51
3	7	5	3.03	0.49
3	-7	-5	1.90	0.51
3	7	5	2.93	0.72
3	7	5	3.31	0.86
-3	7	-5	1.78	0.73
3	7	5	2.01	0.50
-3	7	-5	2.64	0.71
3	-7	-5	1.76	0.56
3	-7	-5	1.48	0.45
3	7	5	1.84	0.68
-3	-7	5	3.79	0.85
-3	-7	5	2.81	0.58
-3	7	-5	2.43	0.82
-3	7	5	2.78	0.82
-3	-7	-5	2.03	0.47
-3	7	5	1.79	0.58
-3	-7	-5	2.35	0.68
3	7	-5	3.01	0.83
3	7	-5	1.32	0.55
-3	7	5	2.85	0.60
3	-7	5	2.76	0.87
3	-7	5	2.64	0.88
3	7	-5	1.73	0.57
-3	7	5	2.56	0.57
3	7	-5	1.92	0.51
3	-7	5	2.53	0.42
3	-7	5	3.07	0.58
3	-7	5	2.93	0.70
-3	-7	-5	1.98	0.66
3	-7	5	1.63	0.53
-3	7	5	2.12	0.75
-3	7	5	2.50	0.59
-3	-7	-5	2.65	0.67

3	-7	5	2.42	0.50
-3	-7	-5	1.52	0.46
-3	8	-5	46.36	2.59
3	8	5	50.65	2.70
-3	8	-5	43.35	2.71
3	-8	-5	40.89	2.43
-3	8	-5	46.80	2.92
-3	-8	5	50.72	2.78
3	-8	-5	41.27	2.52
-3	-8	5	54.34	3.04
3	8	5	50.17	2.92
-3	-8	5	45.76	2.77
3	8	5	53.52	2.55
3	-8	-5	44.43	2.76
3	8	5	55.12	3.10
-3	-8	5	57.01	3.00
3	-8	-5	42.53	2.39
3	-8	-5	47.68	2.37
-3	8	-5	46.12	2.27
3	-8	-5	40.95	2.31
3	8	5	49.69	2.76
-3	-8	5	50.89	2.94
3	8	5	50.61	3.08
3	-8	-5	42.29	2.35
-3	-8	5	51.73	3.07
3	-8	5	56.12	2.79
3	8	-5	41.82	2.48
3	-8	5	49.56	2.54
-3	8	5	51.59	2.79
3	-8	5	50.32	2.83
-3	-8	-5	41.55	2.32
-3	8	5	53.63	2.87
-3	-8	-5	43.07	2.42
3	-8	5	57.06	3.09
3	-8	5	58.73	3.31
-3	-8	-5	41.20	2.49
-3	8	5	53.98	3.06
-3	8	5	49.12	2.77
3	-8	5	49.82	2.75
-3	-8	-5	46.59	2.66
3	8	-5	45.91	2.57
3	8	-5	37.40	2.72
3	8	-5	46.30	2.78
3	-8	5	56.01	2.91

-3	8	5	44.53	3.03
-3	-8	-5	48.04	2.62
-3	9	-5	47.96	2.39
-3	-9	5	32.30	2.08
3	9	5	32.55	1.97
3	-9	-5	48.97	2.54
-3	-9	5	31.60	2.05
3	-9	-5	46.76	2.53
3	9	5	33.12	2.45
3	-9	-5	44.26	2.60
-3	9	-5	43.76	3.09
3	-9	-5	45.69	2.46
-3	9	-5	49.55	2.85
-3	-9	5	35.86	2.37
3	-9	-5	42.26	2.46
3	9	5	35.04	2.49
3	-9	-5	44.39	2.89
3	-9	-5	42.63	2.66
3	9	5	31.88	2.23
3	9	5	34.98	1.84
-3	9	-5	47.75	2.87
3	9	5	32.08	1.83
-3	-9	5	33.21	2.23
-3	-9	-5	43.01	2.66
3	9	-5	42.65	2.99
3	-9	5	30.53	2.05
-3	-9	-5	44.50	2.57
3	-9	5	33.48	2.48
-3	-9	-5	46.40	2.78
3	-9	5	37.44	2.31
3	-9	5	30.66	2.54
-3	9	5	31.49	2.48
-3	-9	-5	42.22	2.44
3	-9	5	32.55	1.77
-3	9	5	32.80	2.18
3	9	-5	40.98	2.60
3	-9	5	39.20	2.76
3	9	-5	50.11	2.78
3	-9	5	37.63	2.11
-3	9	5	32.34	2.10
-3	9	5	35.98	2.46
-3	-10	5	70.21	3.96
3	-10	-5	76.73	3.99
3	-10	-5	85.08	3.99

3	-10	-5	72.34	4.12
3	10	5	74.04	3.71
3	-10	-5	79.40	3.90
3	-10	-5	76.17	4.46
-3	-10	5	79.64	4.17
-3	10	-5	83.31	3.79
3	10	5	71.77	3.78
3	-10	-5	70.71	3.86
-3	-10	5	76.24	4.12
3	10	5	73.89	4.36
-3	10	-5	73.91	4.62
3	10	5	79.05	3.87
-3	-10	5	73.83	3.93
3	10	5	81.72	4.49
-3	10	-5	81.33	4.32
3	-10	-5	69.82	3.99
-3	10	-5	82.58	4.35
-3	10	5	71.24	4.40
-3	-10	-5	71.51	4.14
-3	-10	-5	72.30	3.86
3	10	-5	76.08	4.07
3	-10	5	78.88	4.09
3	-10	5	77.67	3.91
3	-10	5	73.42	3.93
3	-10	5	72.54	4.00
3	-10	5	72.55	3.61
3	10	-5	76.77	4.57
-3	10	5	76.05	4.41
-3	10	5	75.63	4.03
-3	-10	-5	76.83	4.02
3	10	-5	84.42	4.28
-3	-11	5	47.41	3.26
3	-11	-5	47.25	2.64
3	-11	-5	47.38	2.74
-3	11	-5	41.64	3.51
-3	-11	5	43.80	2.90
3	11	5	41.17	3.20
-3	11	-5	51.84	4.48
-3	11	-5	44.17	3.34
-3	-11	5	46.16	2.72
3	11	5	45.14	2.46
3	-11	-5	42.22	2.86
3	-11	-5	43.55	2.56
3	-11	5	50.35	2.77

-3	-11	-5	44.76	2.71
-3	11	5	41.54	3.29
-3	-11	-5	42.91	2.54
3	-11	5	42.19	2.29
3	11	-5	48.44	2.81
3	11	-5	45.75	3.41
3	-11	5	41.75	2.73
-3	11	5	47.58	2.88
-3	11	5	44.95	3.36
-3	-11	-5	47.25	2.90
3	-11	5	43.19	2.68
3	-12	-5	22.77	1.97
-3	-12	5	22.15	2.23
-3	-12	5	20.45	1.88
3	-12	-5	20.05	1.67
3	-12	-5	24.08	1.87
-3	12	-5	20.26	3.06
-3	12	-5	21.49	2.46
3	12	5	21.56	2.71
-3	-12	-5	21.36	1.71
-3	-12	-5	23.16	2.21
3	-12	5	24.94	2.06
3	-12	5	19.38	1.82
-3	12	5	20.19	2.16
-3	12	5	19.26	2.76
3	12	-5	20.66	2.02
-3	-12	-5	21.41	1.91
3	12	-5	20.94	2.86
3	-13	-5	9.16	1.49
3	13	5	11.41	2.57
-3	-13	5	11.85	1.94
3	-13	-5	10.15	1.46
-3	13	-5	12.24	1.92
-3	-13	5	13.77	1.66
-3	13	-5	12.36	2.29
3	-13	5	10.20	1.46
-3	-13	-5	10.87	1.73
3	13	-5	9.26	2.48
-3	-13	-5	9.86	1.64
3	-13	5	10.61	1.54
4	0	5	329.49	14.93
-4	0	5	367.62	15.05
-4	0	5	355.64	14.95
4	0	-5	380.19	16.62

4	0	-5	366.30	16.60
-4	0	-5	342.42	16.70
4	0	5	307.82	15.04
-4	0	5	328.07	14.84
4	0	-5	364.47	16.55
4	0	-5	375.96	16.75
-4	0	-5	382.82	16.69
4	0	5	345.55	15.25
4	0	5	304.82	14.94
-4	0	5	318.75	14.94
4	0	-5	365.83	16.68
4	0	5	310.60	14.99
-4	0	-5	371.00	16.70
4	0	5	332.01	14.98
-4	0	-5	371.47	16.64
-4	0	5	339.55	14.90
-4	0	5	353.23	14.92
-4	0	-5	382.34	16.76
4	0	-5	376.31	16.66
-4	0	-5	374.65	16.64
4	1	5	77.48	3.77
-4	-1	5	81.96	3.66
4	-1	-5	98.27	4.49
-4	-1	5	85.86	3.83
4	1	5	82.05	3.72
4	1	5	75.83	3.77
-4	1	-5	96.44	4.44
-4	1	-5	99.01	4.42
-4	1	-5	80.51	4.59
-4	-1	5	77.53	3.64
-4	1	-5	99.25	4.58
4	1	5	73.63	3.78
4	-1	-5	96.90	4.32
-4	1	-5	103.22	4.41
4	-1	-5	96.40	4.45
4	-1	-5	97.47	4.38
4	1	5	74.71	3.69
-4	-1	5	83.67	3.70
4	-1	-5	98.47	4.42
-4	-1	5	80.52	3.64
4	1	-5	96.71	4.35
-4	1	5	80.16	3.62
-4	-1	-5	96.16	4.42
-4	-1	-5	88.42	4.41

-4	1	5	78.21	3.68
4	1	-5	95.20	4.33
4	1	-5	95.57	4.46
-4	-1	-5	94.11	4.40
4	1	-5	95.65	4.44
-4	1	5	83.21	3.73
4	1	-5	95.65	4.35
-4	-1	-5	99.08	4.41
4	-1	5	77.83	3.70
4	-1	5	74.70	3.71
-4	-1	-5	95.13	4.48
4	-1	5	80.40	3.74
4	-1	5	83.94	3.71
4	-1	5	81.03	3.89
-4	1	5	86.51	3.77
-4	1	5	83.00	3.73
4	-1	5	78.03	3.81
-4	1	5	81.60	3.80
4	1	-5	93.17	4.51
-4	-1	-5	95.14	4.49
4	2	5	41.04	2.14
-4	2	-5	59.01	3.10
-4	-2	5	45.51	2.32
4	2	5	45.65	2.24
4	-2	-5	62.54	3.00
4	-2	-5	64.90	3.10
-4	2	-5	64.17	3.27
-4	-2	5	50.32	2.37
4	-2	-5	65.12	3.14
4	2	5	43.60	2.25
-4	2	-5	65.99	3.05
-4	-2	5	46.30	2.13
-4	2	-5	65.38	3.11
-4	2	-5	68.39	3.12
-4	2	-5	65.09	3.09
-4	-2	5	47.21	2.24
-4	-2	5	44.82	2.19
-4	2	-5	64.91	3.24
4	-2	-5	67.77	3.02
-4	-2	5	47.07	2.16
4	2	5	44.77	2.31
4	2	-5	68.57	3.18
-4	-2	-5	67.48	3.18
-4	2	5	48.48	2.32

-4	2	5	46.76	2.17
4	2	-5	65.37	3.03
-4	-2	-5	64.84	3.16
4	-2	5	45.94	2.24
-4	-2	-5	66.64	3.09
-4	-2	-5	66.45	3.08
4	2	-5	67.99	3.15
-4	-2	-5	60.96	3.01
-4	2	5	49.75	2.32
-4	2	5	47.74	2.29
-4	2	5	46.90	2.27
4	-2	5	44.20	2.22
4	2	-5	64.28	3.17
-4	2	5	46.08	2.16
4	2	-5	68.01	2.98
-4	2	5	49.10	2.33
-4	-2	-5	65.91	3.10
-4	-2	-5	66.47	3.10
4	2	-5	66.48	3.03
4	-2	5	45.45	2.44
4	-2	5	46.38	2.38
-4	-3	5	46.10	2.29
4	3	5	50.91	2.48
4	3	5	46.68	2.35
-4	-3	5	47.70	2.33
4	3	5	48.00	2.42
-4	3	-5	46.77	2.21
-4	3	-5	44.78	2.13
4	-3	-5	45.24	2.09
4	-3	-5	41.31	2.13
-4	3	-5	43.04	2.38
4	-3	-5	46.19	2.32
-4	3	-5	48.38	2.23
-4	-3	5	46.20	2.47
4	-3	-5	44.05	2.22
-4	-3	5	51.97	2.51
4	-3	-5	44.78	2.27
-4	-3	5	48.28	2.40
-4	3	-5	46.45	2.47
4	3	5	42.39	2.40
-4	3	-5	48.85	2.15
-4	3	-5	47.24	2.25
4	3	5	46.42	2.46
-4	3	5	46.26	2.56

-4	3	5	50.30	2.42
4	3	-5	46.38	2.17
-4	3	5	48.28	2.49
-4	3	5	49.10	2.42
4	-3	5	48.68	2.40
4	3	-5	40.60	2.27
-4	-3	-5	42.25	2.18
4	3	-5	43.57	2.32
-4	-3	-5	43.84	2.26
-4	-3	-5	42.75	2.20
-4	-3	-5	41.86	2.30
4	3	-5	44.61	2.16
4	3	-5	44.93	2.27
-4	3	5	51.41	2.45
4	-3	5	49.63	2.45
4	-3	5	51.54	2.58
4	-3	5	46.92	2.35
4	-3	5	54.01	2.64
-4	3	5	47.71	2.30
-4	3	5	47.08	2.26
4	4	5	3.31	0.55
-4	4	-5	6.08	0.81
4	4	5	3.26	0.48
4	4	5	3.27	0.57
4	-4	-5	7.39	0.81
-4	4	-5	7.13	0.66
4	-4	-5	5.16	0.60
-4	4	-5	6.90	0.62
-4	4	-5	6.24	0.53
4	-4	-5	6.11	0.70
-4	4	-5	7.42	0.71
4	-4	-5	5.26	0.53
-4	-4	5	3.09	0.59
-4	-4	5	2.50	0.41
-4	-4	5	2.80	0.45
4	4	5	2.54	0.48
-4	-4	5	3.47	0.62
4	-4	-5	6.97	0.49
-4	-4	5	3.18	0.60
-4	4	-5	7.17	1.02
4	4	5	2.66	0.53
-4	-4	5	3.95	0.75
4	4	-5	5.16	0.72
4	-4	5	2.95	0.35

4	4	-5	7.26	0.72
-4	4	5	3.83	0.42
-4	4	5	3.62	0.75
-4	4	5	2.36	0.45
4	-4	5	2.98	0.43
4	-4	5	3.11	0.48
-4	-4	-5	7.60	0.69
-4	-4	-5	6.56	0.76
-4	-4	-5	6.78	0.59
-4	-4	-5	7.18	0.64
4	4	-5	7.49	0.61
4	-4	5	3.45	0.46
4	-4	5	3.05	0.66
-4	-4	-5	6.20	0.69
-4	4	5	3.59	0.69
-4	4	5	3.47	0.57
4	-4	5	3.35	0.63
-4	4	5	2.95	0.60
-4	5	-5	17.39	1.35
4	-5	-5	18.22	1.35
4	-5	-5	18.63	0.98
4	5	5	13.04	1.10
-4	5	-5	16.63	1.32
4	-5	-5	16.46	1.16
4	-5	-5	16.49	1.08
4	-5	-5	16.09	1.03
-4	-5	5	14.14	1.15
-4	5	-5	17.27	1.11
4	5	5	12.59	0.90
-4	-5	5	11.66	1.05
-4	-5	5	12.19	0.87
4	-5	-5	18.40	1.20
-4	5	-5	19.12	1.02
-4	5	-5	18.44	1.22
4	5	5	12.78	0.89
-4	5	-5	15.08	1.24
-4	-5	5	14.64	1.26
4	5	5	13.41	1.07
-4	-5	5	13.98	1.08
4	5	5	11.54	1.00
-4	-5	5	12.36	0.88
4	-5	5	12.31	0.88
-4	-5	-5	17.99	1.09
-4	-5	-5	19.15	1.22

4	-5	5	12.27	0.92
4	-5	5	12.89	0.78
4	-5	5	12.88	1.22
-4	-5	-5	15.79	1.16
-4	5	5	11.53	0.85
-4	5	5	13.48	1.01
4	5	-5	16.93	1.21
-4	5	5	12.92	0.89
-4	5	5	11.58	1.16
4	5	-5	17.50	1.10
4	5	-5	15.29	1.24
4	-5	5	14.48	1.33
4	-5	5	13.69	0.93
-4	-5	-5	15.97	1.07
-4	5	5	12.67	1.14
-4	5	5	12.92	0.99
-4	-5	-5	17.67	1.27
4	-6	-5	43.56	2.44
4	6	5	39.27	2.13
4	6	5	41.59	2.11
4	-6	-5	40.22	2.26
-4	6	-5	41.38	2.55
-4	6	-5	45.13	2.35
-4	-6	5	39.35	2.29
-4	-6	5	39.20	2.14
4	-6	-5	48.20	2.22
-4	6	-5	47.40	2.24
-4	6	-5	44.23	2.35
-4	-6	5	41.45	2.33
4	-6	-5	50.02	2.63
-4	-6	5	39.96	2.12
4	-6	-5	45.14	2.40
-4	6	-5	42.41	2.67
4	6	5	39.69	2.28
-4	6	-5	48.66	2.50
-4	-6	5	42.63	2.37
4	6	5	39.25	2.33
4	-6	-5	43.65	2.31
-4	-6	5	40.60	2.37
4	6	5	37.99	2.31
4	-6	5	42.40	2.41
4	6	-5	39.25	2.50
-4	6	5	40.60	2.18
4	-6	5	39.98	2.13

4	6	-5	42.93	2.39
-4	6	5	39.89	2.24
-4	6	5	40.32	2.08
4	-6	5	43.50	2.62
-4	-6	-5	42.73	2.28
-4	-6	-5	43.71	2.33
-4	-6	-5	42.30	2.40
4	6	-5	45.79	2.54
4	-6	5	39.72	2.01
4	-6	5	40.64	2.14
-4	6	5	42.31	2.40
-4	-6	-5	47.68	2.46
-4	6	5	39.46	2.12
-4	-6	-5	45.46	2.48
4	-6	5	39.79	2.22
4	6	-5	43.82	2.32
4	-7	-5	42.59	2.09
4	7	5	43.42	2.57
-4	7	-5	42.38	2.09
4	7	5	43.50	2.39
4	-7	-5	36.56	2.28
-4	7	-5	40.55	2.24
-4	7	-5	38.44	2.47
-4	-7	5	45.55	2.65
-4	-7	5	46.20	2.64
4	-7	-5	40.07	2.17
4	-7	-5	37.56	2.14
4	7	5	45.26	2.34
-4	-7	5	42.93	2.56
4	7	5	46.96	2.65
-4	7	-5	41.13	2.34
4	-7	-5	38.87	2.26
-4	7	-5	38.68	2.61
-4	-7	5	47.31	2.62
-4	-7	5	43.89	2.41
-4	-7	5	42.89	2.38
4	7	5	44.10	2.65
4	-7	-5	42.71	2.52
-4	-7	-5	41.42	2.37
-4	7	5	50.91	2.74
4	-7	5	44.48	2.69
-4	-7	-5	39.00	2.19
-4	7	5	45.15	2.41
4	-7	5	46.62	2.39

-4	-7	-5	40.02	2.40
-4	7	5	45.21	2.74
-4	-7	-5	38.24	2.27
4	-7	5	42.32	2.38
-4	-7	-5	40.79	2.15
-4	7	5	47.26	2.46
4	7	-5	41.46	2.29
4	7	-5	36.34	2.44
-4	7	5	43.52	2.50
4	-7	5	42.47	2.22
4	-7	5	49.47	2.92
4	7	-5	40.94	2.21
4	7	-5	41.29	2.28
4	-7	5	41.70	2.45
-4	-8	5	79.82	3.93
-4	8	-5	87.78	4.18
4	-8	-5	76.41	3.88
-4	8	-5	72.83	4.37
4	8	5	71.54	3.91
4	-8	-5	84.89	3.85
4	8	5	69.66	4.02
-4	-8	5	73.42	3.76
-4	8	-5	78.59	4.24
-4	8	-5	87.44	3.82
4	-8	-5	76.98	3.98
4	-8	-5	83.94	4.28
4	-8	-5	77.06	3.82
4	8	5	71.79	3.64
4	-8	-5	71.64	4.01
-4	-8	5	71.45	3.91
-4	-8	5	68.81	3.71
4	-8	-5	79.84	3.92
-4	-8	5	78.81	4.11
4	8	5	77.27	4.04
-4	-8	5	71.03	3.98
4	-8	5	78.47	3.73
4	-8	5	80.98	3.83
4	8	-5	75.17	4.30
4	8	-5	81.81	4.08
-4	-8	-5	78.12	4.10
4	-8	5	73.75	4.02
4	-8	5	73.50	3.54
-4	8	5	67.73	4.06
-4	-8	-5	73.55	4.05

-4	-8	-5	75.08	3.94
4	-8	5	69.15	3.80
4	-8	5	86.44	4.31
4	-8	5	70.24	3.72
-4	-8	-5	71.04	3.83
-4	8	5	81.06	4.07
-4	8	5	67.68	3.72
-4	8	5	71.84	3.84
-4	8	5	77.47	3.78
-4	-8	-5	82.96	4.15
4	8	-5	75.08	3.98
4	9	5	89.11	4.09
-4	9	-5	78.94	4.84
4	9	5	84.01	4.26
4	-9	-5	86.51	4.32
-4	9	-5	95.11	4.64
-4	9	-5	89.32	4.70
4	-9	-5	83.67	4.48
-4	-9	5	90.34	4.59
4	9	5	85.02	4.75
4	9	5	80.25	4.56
4	-9	-5	83.35	4.21
4	-9	-5	95.40	4.26
4	-9	-5	90.35	4.77
-4	-9	5	82.88	4.37
4	-9	-5	83.51	4.38
4	9	5	91.37	4.74
-4	9	-5	96.20	4.21
-4	-9	5	88.69	4.60
4	-9	-5	82.09	4.26
-4	-9	5	83.31	4.50
4	-9	5	84.38	4.46
4	-9	5	96.59	4.96
4	9	-5	91.47	4.52
4	9	-5	83.76	4.78
4	-9	5	85.17	4.16
-4	9	5	83.32	4.37
4	9	-5	83.93	4.37
-4	-9	-5	80.84	4.44
4	-9	5	99.82	4.54
-4	9	5	79.24	4.78
-4	9	5	92.49	4.74
4	-9	5	92.27	4.37
-4	-9	-5	84.22	4.25

-4	-9	-5	77.26	4.49
-4	9	5	88.05	4.52
4	-10	-5	46.08	3.19
4	-10	-5	39.47	2.69
-4	-10	5	47.11	3.07
4	-10	-5	41.73	2.55
4	10	5	53.63	3.94
-4	10	-5	47.93	2.44
4	10	5	48.51	3.08
4	10	5	46.05	2.70
4	10	5	53.74	2.51
4	-10	-5	41.34	2.71
4	-10	-5	42.99	2.46
-4	10	-5	46.58	3.02
-4	-10	5	47.99	3.11
-4	-10	5	45.61	2.84
4	-10	-5	49.08	2.53
-4	10	-5	42.16	3.21
4	-10	-5	47.57	2.46
4	10	5	48.84	3.31
-4	10	-5	48.73	2.99
-4	-10	5	48.57	2.86
4	-10	5	53.26	3.04
4	-10	5	49.99	2.84
4	10	-5	35.29	3.30
-4	-10	-5	40.71	2.67
-4	10	5	50.84	3.03
4	-10	5	54.75	3.57
-4	10	5	54.63	3.45
4	-10	5	54.99	2.83
4	10	-5	47.84	2.89
4	10	-5	45.64	2.64
-4	-10	-5	42.51	2.46
4	-10	5	48.21	2.53
4	-10	5	49.74	2.94
-4	10	5	47.15	3.36
-4	-10	-5	42.71	2.62
-4	-11	5	6.35	1.09
-4	11	-5	5.86	1.36
-4	-11	5	3.30	1.12
-4	11	-5	5.04	1.68
4	-11	-5	5.35	0.98
4	-11	-5	6.42	1.17
4	11	5	5.57	1.41

-4	11	-5	9.36	1.74
4	-11	-5	8.20	1.21
4	11	-5	7.57	1.58
-4	-11	-5	8.52	1.58
4	-11	5	4.64	0.80
4	-11	5	4.72	0.95
4	-11	5	5.65	1.02
4	-11	5	6.59	1.26
-4	-11	-5	7.15	0.99
-4	-11	-5	8.67	1.33
4	11	-5	8.04	1.18
-4	11	5	6.32	1.59
4	-11	5	7.17	1.09
-4	11	5	6.14	1.22
4	-12	-5	2.48	1.00
4	-12	-5	3.85	0.88
-4	12	-5	2.24	1.68
4	-12	-5	2.77	0.88
-4	-12	5	6.04	1.32
-4	12	-5	3.35	1.33
-4	-12	5	2.66	0.95
4	-12	-5	2.02	1.17
4	12	5	2.42	1.55
-4	-12	-5	3.01	1.03
4	-12	5	2.98	0.90
-4	-12	-5	4.06	0.90
-4	12	5	-1.05	1.55
4	-12	5	3.59	0.98
-4	-12	-5	3.06	1.27
4	12	-5	2.78	1.68
4	12	-5	5.26	1.15
-4	12	5	3.66	1.35
4	-13	-5	19.06	1.91
4	-13	-5	22.02	1.82
4	13	5	22.67	3.50
-4	13	-5	20.14	2.98
-4	-13	5	24.89	2.65
-4	-13	5	25.29	2.07
4	13	-5	18.99	3.23
4	-13	5	25.51	2.03
-4	13	5	25.35	3.84
-4	-13	-5	24.41	2.74
-4	-13	-5	20.10	1.95
5	0	5	0.31	0.15

-5	0	-5	-0.46	0.25
5	0	-5	0.47	0.23
-5	0	5	0.23	0.14
-5	0	-5	0.18	0.22
5	0	-5	0.31	0.12
5	0	-5	0.29	0.20
-5	0	-5	0.22	0.30
5	0	5	0.28	0.19
5	0	-5	0.09	0.23
5	0	5	0.21	0.27
-5	0	5	0.24	0.12
-5	0	-5	0.06	0.16
-5	0	5	0.46	0.28
5	0	-5	0.36	0.18
-5	0	5	-0.07	0.17
-5	0	-5	-0.04	0.18
5	0	-5	0.22	0.16
5	0	5	0.22	0.15
-5	0	-5	0.27	0.17
5	0	5	0.38	0.20
-5	0	5	0.17	0.24
-5	1	-5	8.41	0.56
5	-1	-5	7.95	0.54
-5	-1	5	11.48	0.63
5	1	5	12.25	0.73
5	-1	-5	7.86	0.56
-5	1	-5	8.16	0.78
5	-1	-5	7.99	0.54
5	-1	-5	8.11	0.57
-5	1	-5	8.02	0.56
5	1	5	12.19	0.71
5	-1	-5	7.36	0.63
5	1	5	10.49	0.72
-5	-1	5	11.91	0.66
-5	-1	5	11.46	0.64
-5	-1	5	10.86	0.77
-5	1	-5	8.49	0.61
-5	-1	5	13.27	0.88
-5	-1	5	12.26	0.61
-5	1	-5	6.93	0.68
5	1	5	11.23	0.77
5	-1	-5	7.56	0.45
-5	-1	5	12.05	0.67
-5	1	-5	8.01	0.54

-5	-1	-5	8.44	0.56
-5	-1	-5	6.73	0.58
-5	1	5	11.40	0.73
5	1	-5	8.01	0.63
-5	-1	-5	7.99	0.65
-5	-1	-5	8.34	0.54
5	1	-5	8.15	0.50
-5	1	5	11.24	0.69
-5	-1	-5	7.87	0.56
5	-1	5	12.14	0.87
-5	1	5	11.34	0.62
5	1	-5	7.72	0.56
5	-1	5	13.47	0.97
5	1	-5	7.94	0.46
-5	-1	-5	7.73	0.64
5	-1	5	11.66	0.70
5	-1	5	11.98	0.72
-5	1	5	12.19	0.74
-5	1	5	11.63	0.84
-5	1	5	12.13	0.77
5	-1	5	11.81	0.71
5	1	-5	7.63	0.64
-5	-2	5	20.63	0.98
-5	-2	5	20.40	0.99
5	-2	-5	20.80	1.23
-5	-2	5	19.67	1.08
-5	2	-5	24.02	1.26
5	-2	-5	23.68	1.15
5	2	5	20.74	1.11
-5	-2	5	20.97	1.24
5	2	5	20.15	1.09
5	-2	-5	21.63	1.14
5	-2	-5	22.48	1.28
-5	-2	5	19.81	1.20
5	2	5	17.84	1.00
-5	-2	5	18.42	1.04
-5	2	-5	23.20	1.46
-5	2	-5	24.99	1.27
-5	2	-5	21.42	1.40
-5	2	-5	23.48	1.21
-5	2	-5	24.33	1.21
5	-2	5	24.27	1.39
5	2	-5	21.47	1.34
5	2	-5	23.16	1.17

-5	-2	-5	23.03	1.18
5	2	-5	22.86	1.30
5	-2	5	18.43	1.08
5	-2	5	19.58	1.16
-5	-2	-5	25.65	1.26
-5	-2	-5	25.10	1.31
-5	2	5	20.09	1.01
-5	-2	-5	22.59	1.25
5	-2	5	15.10	1.52
-5	2	5	19.68	1.16
-5	-2	-5	23.58	1.24
5	2	-5	23.01	1.15
-5	-2	-5	23.45	1.30
5	2	-5	23.03	1.12
-5	2	5	19.78	1.22
-5	2	5	21.20	1.17
5	2	-5	23.36	1.27
5	-2	5	20.98	1.09
-5	2	5	19.90	1.01
-5	-3	5	51.25	2.61
5	3	5	48.84	2.56
5	-3	-5	54.03	2.68
-5	3	-5	60.81	2.73
5	3	5	47.50	2.49
5	-3	-5	52.56	2.76
-5	3	-5	53.63	2.93
-5	-3	5	53.24	2.59
-5	3	-5	56.87	2.79
-5	3	-5	61.27	2.78
-5	-3	5	48.74	2.36
-5	3	-5	57.34	2.71
-5	-3	5	48.82	2.30
-5	-3	5	50.87	2.30
-5	-3	5	47.04	2.56
5	-3	-5	57.35	2.87
-5	3	-5	57.67	3.04
5	3	5	50.50	2.44
-5	3	-5	61.36	2.77
-5	-3	5	53.58	2.46
-5	-3	5	48.01	2.41
5	-3	-5	54.31	2.82
5	3	5	50.16	2.43
-5	3	5	52.70	2.50
-5	-3	-5	61.72	2.90

5	-3	5	47.19	2.43
5	-3	5	52.16	2.65
5	-3	5	52.71	2.70
-5	3	5	48.51	2.57
-5	-3	-5	54.16	2.76
-5	-3	-5	60.33	2.78
-5	3	5	54.14	2.57
5	3	-5	53.27	2.81
5	3	-5	57.77	2.83
5	-3	5	48.67	2.48
-5	3	5	49.80	2.53
-5	3	5	52.71	2.58
5	-3	5	47.31	2.50
5	3	-5	57.46	2.71
-5	3	5	48.93	2.39
-5	-3	-5	56.82	2.81
-5	-3	-5	55.18	2.76
5	3	-5	57.70	2.72
-5	4	-5	44.01	2.53
-5	-4	5	40.13	2.33
5	4	5	44.54	2.19
5	4	5	43.73	2.18
-5	-4	5	42.52	2.38
5	4	5	41.40	2.29
-5	-4	5	43.19	2.24
-5	4	-5	50.33	2.66
5	-4	-5	43.01	2.32
5	-4	-5	47.36	2.50
-5	4	-5	46.62	2.38
5	-4	-5	45.49	2.39
-5	-4	5	42.66	2.36
-5	4	-5	49.11	2.32
-5	-4	5	40.77	2.12
5	4	5	41.83	2.33
5	4	5	45.78	2.52
-5	4	-5	49.26	2.35
5	-4	-5	42.39	2.24
-5	-4	5	42.53	2.18
5	-4	5	46.17	2.18
5	4	-5	46.00	2.29
5	-4	5	49.35	2.55
5	-4	5	41.49	2.18
-5	4	5	41.87	2.28
-5	-4	-5	50.44	2.49

5	4	-5	44.03	2.42
5	4	-5	45.40	2.39
5	-4	5	47.03	2.44
-5	4	5	44.60	2.40
-5	-4	-5	50.04	2.37
-5	-4	-5	44.56	2.36
-5	4	5	46.48	2.27
-5	-4	-5	44.79	2.31
-5	4	5	43.50	2.16
-5	-4	-5	44.44	2.31
5	-4	5	41.77	2.13
-5	4	5	47.41	2.37
-5	4	5	45.61	2.33
5	-4	5	42.41	2.25
5	4	-5	46.30	2.29
5	5	5	28.47	1.76
5	5	5	27.67	1.77
5	5	5	29.73	1.61
-5	5	-5	18.90	1.14
-5	5	-5	17.92	1.09
5	-5	-5	16.02	1.09
5	-5	-5	17.61	1.05
5	-5	-5	18.09	1.26
-5	5	-5	19.39	1.27
5	-5	-5	19.03	1.38
-5	-5	5	30.64	1.74
-5	-5	5	31.11	1.87
-5	5	-5	17.99	1.16
5	-5	-5	17.81	0.95
5	5	5	27.60	1.62
-5	-5	5	28.92	1.58
-5	5	-5	17.61	1.53
-5	-5	5	31.14	1.90
-5	-5	5	26.30	1.77
-5	-5	5	30.63	1.64
5	-5	-5	16.11	1.19
-5	5	-5	15.30	1.36
5	5	5	33.07	1.83
-5	-5	-5	16.85	1.11
-5	-5	-5	17.79	1.11
5	-5	5	29.56	1.55
-5	5	5	28.19	1.91
-5	-5	-5	18.87	1.22
-5	5	5	33.02	1.72

5	-5	5	29.55	1.63
5	-5	5	28.77	1.70
5	5	-5	16.33	1.21
-5	5	5	31.39	1.63
-5	5	5	28.99	1.50
-5	5	5	31.00	1.85
5	-5	5	33.86	2.08
-5	5	5	27.99	1.74
5	-5	5	31.19	1.62
5	5	-5	16.49	1.11
5	-5	5	28.94	1.90
5	5	-5	16.74	1.28
-5	-5	-5	16.99	1.16
-5	-5	-5	17.94	1.31
5	-6	-5	3.83	0.75
5	6	5	3.89	0.80
-5	6	-5	3.75	0.81
-5	6	-5	4.25	0.64
-5	-6	5	5.46	1.07
-5	-6	5	4.72	0.88
-5	6	-5	4.60	0.76
5	-6	-5	4.98	0.89
-5	-6	5	5.75	0.95
-5	-6	5	5.35	0.90
-5	-6	5	4.65	0.65
5	6	5	4.52	0.81
-5	6	-5	5.59	0.78
-5	6	-5	4.46	0.84
5	-6	-5	3.76	0.60
5	6	5	4.89	0.68
5	6	5	5.14	0.73
-5	-6	5	4.52	0.64
5	-6	-5	3.78	0.58
5	6	5	4.58	0.83
5	-6	-5	4.35	0.54
5	-6	-5	4.39	0.69
-5	6	-5	4.69	0.91
5	-6	5	4.64	0.85
-5	6	5	4.64	0.97
5	-6	5	4.72	0.52
5	-6	5	5.09	1.19
-5	6	5	5.11	0.71
-5	-6	-5	3.88	0.61
5	6	-5	4.45	0.74

-5	6	5	3.62	0.69
5	6	-5	5.00	0.93
5	-6	5	6.03	0.70
-5	6	5	4.45	0.92
5	6	-5	4.30	0.67
-5	-6	-5	3.68	0.73
5	-6	5	4.41	0.72
-5	-6	-5	3.75	0.65
-5	6	5	4.30	0.84
-5	-6	-5	4.49	0.80
-5	-6	-5	5.52	0.96
5	-6	5	4.79	0.63
5	7	5	7.30	0.81
-5	7	-5	11.01	0.92
5	-7	-5	9.34	0.77
5	-7	-5	9.37	0.88
5	7	5	6.06	0.85
-5	-7	5	7.02	1.22
-5	7	-5	9.68	1.05
5	7	5	5.91	1.00
5	-7	-5	8.95	1.48
-5	-7	5	6.96	0.79
-5	-7	5	6.01	1.00
5	7	5	7.69	1.14
5	7	5	7.06	1.03
5	-7	-5	10.55	0.81
-5	-7	5	6.32	1.12
-5	-7	5	5.86	0.80
5	-7	-5	9.91	1.03
5	-7	-5	9.84	1.14
5	-7	-5	9.02	0.86
-5	7	-5	7.94	1.30
-5	-7	5	6.25	1.22
-5	7	-5	8.43	1.25
-5	7	-5	9.19	1.07
-5	7	5	5.69	0.95
5	7	-5	9.28	0.96
-5	-7	-5	8.75	0.87
5	-7	5	6.04	0.86
-5	7	5	5.78	0.86
5	7	-5	8.86	0.99
-5	-7	-5	8.90	1.10
-5	7	5	6.70	1.00
5	7	-5	8.54	1.13

-5	7	5	5.51	1.13
5	-7	5	6.65	1.03
-5	7	5	7.38	1.22
-5	-7	-5	10.19	0.97
5	-7	5	6.83	0.77
5	-7	5	6.58	0.63
5	-7	5	7.40	1.43
5	-7	5	6.43	0.78
-5	-7	-5	7.61	1.21
-5	-7	-5	10.16	1.02
-5	-8	5	11.32	1.12
-5	8	-5	15.79	1.54
5	8	5	11.67	1.09
5	8	5	10.96	1.35
5	-8	-5	15.44	1.33
-5	-8	5	12.12	1.51
5	-8	-5	13.37	1.14
5	-8	-5	15.24	1.71
5	-8	-5	13.59	1.14
-5	-8	5	13.10	1.13
-5	8	-5	12.35	1.69
5	8	5	14.10	1.47
-5	8	-5	16.86	1.17
5	8	5	11.94	1.41
5	-8	-5	14.82	1.42
-5	-8	5	12.61	1.48
-5	8	-5	12.96	1.60
5	-8	-5	12.60	1.23
-5	-8	5	11.58	1.38
5	-8	-5	13.06	1.06
-5	8	5	8.79	1.16
-5	8	5	13.66	1.30
5	-8	5	11.14	1.05
-5	-8	-5	13.46	1.14
5	8	-5	12.64	1.52
5	-8	5	14.16	1.34
5	-8	5	15.25	1.86
-5	-8	-5	12.66	1.24
5	8	-5	15.05	1.34
5	-8	5	11.48	1.64
-5	-8	-5	13.86	1.54
5	8	-5	15.29	1.26
-5	-8	-5	16.28	1.23
-5	8	5	13.75	1.65

-5	8	5	11.40	1.35
5	-8	5	12.26	0.89
5	-8	5	12.90	1.09
5	-8	5	12.35	1.20
-5	8	5	12.60	1.58
-5	-8	-5	14.74	1.44
-5	9	-5	8.33	1.30
-5	9	-5	9.62	1.10
-5	-9	5	7.45	1.21
5	-9	-5	4.58	0.98
5	-9	-5	6.79	0.84
5	-9	-5	10.60	1.72
5	9	5	6.52	1.34
-5	9	-5	4.59	1.31
5	9	5	6.38	1.01
5	-9	-5	4.89	0.89
5	-9	-5	7.84	1.00
5	-9	-5	5.91	0.95
5	9	5	9.56	1.56
-5	9	-5	7.20	1.50
5	-9	-5	6.49	1.07
-5	-9	5	6.82	1.14
5	9	5	6.66	1.25
-5	-9	5	7.35	1.09
5	-9	5	5.71	0.90
5	9	-5	6.19	1.19
-5	-9	-5	5.32	1.08
5	-9	5	7.53	1.11
5	-9	5	6.67	0.73
5	-9	5	7.60	1.62
5	9	-5	7.02	1.07
-5	-9	-5	5.21	0.93
-5	9	5	7.06	1.34
-5	9	5	4.30	1.17
-5	9	5	7.60	1.13
5	-9	5	9.39	1.03
-5	-9	-5	7.76	1.23
5	-9	5	6.99	1.35
5	9	-5	8.37	1.34
-5	-10	5	40.30	2.42
-5	10	-5	49.19	2.50
5	10	5	36.79	2.30
5	10	5	45.83	2.93
-5	-10	5	38.26	2.67

-5	10	-5	41.08	3.99
5	10	5	37.51	4.07
-5	10	-5	44.94	3.00
-5	-10	5	43.30	2.79
5	-10	-5	49.69	2.50
5	-10	-5	46.81	3.18
5	-10	-5	41.16	2.70
5	-10	-5	36.85	2.72
5	-10	-5	40.36	2.47
5	-10	-5	45.45	2.56
-5	10	-5	46.70	3.33
-5	-10	5	39.08	2.48
5	-10	-5	46.06	2.43
5	10	-5	47.39	2.83
5	-10	5	41.20	3.22
5	-10	5	39.28	2.15
-5	-10	-5	39.76	2.65
-5	10	5	37.85	2.67
5	10	-5	44.45	2.60
-5	-10	-5	42.29	2.45
5	-10	5	40.17	2.43
5	10	-5	41.47	3.05
5	-10	5	43.88	2.40
-5	10	5	39.78	2.99
-5	-10	-5	43.84	2.64
-5	10	5	42.87	3.02
5	-10	5	44.81	2.66
5	-10	5	36.19	2.46
-5	-11	5	10.98	1.78
5	-11	-5	12.47	1.26
-5	11	-5	11.34	2.11
5	-11	-5	15.52	3.13
5	-11	-5	11.92	1.50
5	-11	-5	12.85	1.43
5	-11	-5	13.58	1.31
5	11	5	9.56	1.79
-5	11	-5	13.87	2.08
-5	11	-5	15.98	2.84
-5	-11	5	10.49	1.46
5	-11	5	10.53	1.39
5	-11	5	11.39	1.46
-5	11	5	8.71	1.72
-5	-11	-5	10.95	1.81
-5	-11	-5	12.42	1.56

5	-11	5	8.63	0.96
-5	11	5	13.01	2.23
-5	-11	-5	12.07	1.33
5	11	-5	13.56	1.51
5	-11	5	9.40	1.45
5	11	-5	12.06	2.17
-5	12	-5	7.98	2.00
5	-12	-5	10.79	1.48
-5	-12	5	12.09	2.22
-5	12	-5	8.41	1.74
5	-12	-5	7.83	1.37
5	12	5	10.80	1.95
5	-12	-5	8.92	1.35
-5	-12	5	10.67	1.52
5	-12	5	8.67	1.41
5	12	-5	10.01	1.57
-5	-12	-5	9.13	1.31
-5	-12	-5	8.52	1.47
-5	12	5	13.55	2.05
5	-12	5	13.36	1.55
-5	-12	-5	8.52	1.49
5	12	-5	8.21	2.11
-5	13	-5	10.28	2.17
5	-13	-5	8.59	1.47
-5	-13	5	10.55	1.75
5	13	5	5.75	1.98
5	-13	-5	10.15	1.56
-5	-13	5	5.85	1.14
-5	-13	-5	9.47	1.51
5	-13	5	7.10	1.14
-5	13	5	8.28	1.73
-5	-13	-5	12.44	2.39
5	13	-5	9.98	2.14
6	0	-5	78.47	3.75
6	0	-5	82.49	3.67
-6	0	5	91.49	4.12
-6	0	-5	77.43	3.75
6	0	5	89.61	4.13
-6	0	5	86.91	4.02
-6	0	5	94.65	4.25
6	0	5	88.92	4.14
-6	0	5	89.32	4.04
-6	0	-5	79.70	3.71
6	0	-5	78.70	3.83

-6	0	-5	84.56	3.75
6	0	5	85.92	4.16
-6	0	-5	81.15	3.80
6	0	5	84.11	4.10
-6	0	5	85.26	4.13
6	0	-5	76.82	3.71
6	0	-5	81.81	3.71
6	0	5	86.73	4.16
6	0	-5	79.62	3.66
-6	0	5	92.69	4.06
-6	0	-5	78.81	3.84
6	1	5	42.58	2.22
-6	1	-5	57.22	2.62
6	-1	-5	56.23	2.52
6	-1	-5	56.57	2.61
-6	-1	5	43.71	2.21
-6	-1	5	44.30	2.08
-6	1	-5	54.91	2.63
-6	-1	5	47.73	2.29
6	-1	-5	56.34	2.57
6	1	5	41.26	2.13
6	-1	-5	53.01	2.60
-6	1	-5	54.65	2.60
-6	-1	5	43.35	2.08
6	-1	-5	52.86	2.60
6	-1	-5	54.32	2.71
-6	1	-5	49.57	2.71
6	1	5	44.24	2.14
-6	-1	5	47.29	2.12
-6	1	5	44.43	2.13
-6	-1	-5	48.91	2.62
-6	1	5	41.90	2.10
-6	1	5	44.04	2.06
6	-1	5	44.72	2.17
6	-1	5	46.16	2.17
-6	-1	-5	51.58	2.61
6	1	-5	55.98	2.67
-6	-1	-5	55.41	2.70
6	1	-5	54.96	2.69
-6	1	5	47.46	2.18
6	-1	5	40.72	2.13
6	1	-5	56.40	2.50
6	1	-5	54.59	2.55
6	1	-5	55.42	2.58

6	-1	5	41.80	2.15
6	-1	5	44.29	2.26
-6	-1	-5	52.67	2.66
6	2	5	117.76	5.52
-6	-2	5	120.91	5.56
6	-2	-5	140.68	6.27
-6	-2	5	121.89	5.72
-6	-2	5	104.35	5.55
-6	-2	5	122.83	5.47
6	-2	-5	140.18	6.30
-6	-2	5	126.22	5.53
6	-2	-5	141.21	6.40
6	2	5	123.97	5.62
-6	2	-5	140.05	6.38
-6	2	-5	138.59	6.36
-6	2	-5	138.91	6.57
-6	-2	5	132.95	5.75
-6	2	-5	144.07	6.39
6	2	5	114.04	5.61
-6	2	-5	133.27	6.52
-6	-2	5	123.43	5.73
-6	2	-5	133.21	6.46
6	2	5	121.08	5.73
-6	2	5	123.14	5.70
-6	-2	-5	130.07	6.31
-6	-2	-5	139.63	6.35
6	2	-5	135.20	6.35
6	-2	5	125.39	5.76
6	-2	5	111.95	5.67
6	2	-5	141.69	6.28
6	-2	5	119.25	5.62
6	-2	5	110.41	5.61
-6	-2	-5	137.49	6.38
-6	-2	-5	147.38	6.50
6	2	-5	136.37	6.27
6	2	-5	142.48	6.45
-6	2	5	131.62	5.67
-6	2	5	128.87	5.66
-6	2	5	129.42	5.75
-6	-2	-5	139.60	6.38
-6	2	5	126.04	5.67
-6	-2	-5	139.67	6.41
6	-2	5	127.86	5.85
-6	-2	-5	133.02	6.37

-6	2	5	118.23	5.51
6	2	-5	141.97	6.31
-6	2	5	121.05	5.54
-6	3	-5	44.51	2.54
-6	-3	5	44.54	2.27
-6	-3	5	37.78	2.04
6	3	5	39.82	2.11
6	-3	-5	45.89	2.33
6	3	5	42.18	2.08
-6	3	-5	47.76	2.28
-6	-3	5	40.77	1.95
-6	3	-5	45.21	2.29
-6	-3	5	44.02	2.03
-6	3	-5	47.04	2.34
6	-3	-5	44.98	2.20
-6	-3	5	42.47	1.96
-6	-3	5	39.18	2.20
-6	3	-5	41.57	2.45
-6	3	-5	44.93	2.24
6	3	5	37.57	2.19
-6	-3	5	45.56	2.25
-6	3	-5	47.75	2.26
-6	-3	5	43.07	1.95
-6	3	5	43.59	2.15
-6	-3	-5	45.87	2.29
6	-3	5	38.98	2.11
6	3	-5	47.20	2.35
-6	-3	-5	49.03	2.28
-6	3	5	43.67	2.18
-6	-3	-5	44.87	2.29
6	3	-5	43.44	2.30
-6	3	5	41.26	2.18
-6	-3	-5	47.20	2.30
6	-3	5	42.17	2.37
-6	-3	-5	48.03	2.43
6	-3	5	42.02	2.28
6	-3	5	40.26	2.06
6	3	-5	46.24	2.20
-6	3	5	44.96	2.24
6	-3	5	39.89	2.07
-6	3	5	41.57	2.21
-6	3	5	39.85	2.02
-6	-3	-5	44.40	2.31
6	4	5	16.36	1.02

6	-4	-5	12.34	1.09
6	-4	-5	12.86	0.83
6	4	5	15.84	1.09
-6	-4	5	17.27	1.06
-6	-4	5	16.50	1.81
-6	4	-5	12.54	1.13
-6	-4	5	15.41	1.00
6	4	5	16.46	1.03
-6	4	-5	14.45	0.96
6	-4	-5	11.82	0.95
-6	-4	5	16.78	0.86
-6	-4	5	16.40	0.93
-6	4	-5	14.78	0.88
-6	4	-5	14.41	1.00
-6	-4	5	16.08	1.19
-6	4	-5	14.14	0.93
-6	4	-5	13.45	1.31
-6	-4	5	18.48	1.28
-6	-4	5	16.30	0.84
6	-4	-5	13.03	0.91
6	4	5	15.86	1.17
-6	-4	-5	12.10	0.90
-6	4	5	16.00	1.02
6	4	-5	13.92	0.89
-6	-4	-5	12.76	0.89
6	4	-5	12.92	0.99
-6	-4	-5	14.22	1.13
6	4	-5	13.65	0.87
-6	4	5	15.85	1.12
-6	-4	-5	13.82	0.94
6	-4	5	17.43	0.99
6	-4	5	18.76	1.43
-6	4	5	16.09	1.15
6	4	-5	13.51	1.00
-6	4	5	17.23	1.21
6	-4	5	17.36	1.33
6	-4	5	15.85	1.00
-6	4	5	15.14	1.23
-6	4	5	16.87	1.20
6	-4	5	17.69	1.10
-6	-4	-5	14.21	0.98
-6	5	-5	13.37	0.96
-6	5	-5	13.82	1.03
-6	-5	5	11.58	0.85

-6	-5	5	11.21	0.78
6	-5	-5	13.29	1.22
-6	5	-5	15.63	0.94
-6	-5	5	10.90	1.25
-6	-5	5	9.72	1.13
-6	5	-5	14.65	1.11
6	5	5	11.12	0.99
-6	5	-5	10.43	1.21
6	-5	-5	11.75	0.94
6	5	5	11.77	0.87
6	-5	-5	12.27	1.02
-6	-5	5	12.37	0.96
-6	-5	5	9.51	1.02
6	-5	-5	13.34	1.11
6	5	5	10.47	1.02
6	-5	-5	12.74	0.87
6	5	5	11.86	0.89
-6	5	-5	12.96	1.45
6	-5	5	11.32	0.77
-6	-5	-5	13.42	0.95
6	5	-5	12.35	1.03
6	5	-5	12.78	1.10
6	-5	5	10.81	1.20
-6	5	5	10.84	1.18
-6	5	5	11.73	0.97
-6	5	5	11.92	1.09
6	-5	5	12.62	0.83
-6	-5	-5	13.96	1.04
6	5	-5	14.01	0.97
-6	5	5	10.41	1.00
-6	-5	-5	14.86	1.04
6	-5	5	14.76	1.30
-6	-5	-5	13.62	0.97
6	-5	5	10.75	0.83
6	5	-5	13.48	0.94
-6	-5	-5	15.03	1.21
6	-5	5	10.70	0.87
-6	5	5	10.53	0.86
6	-6	-5	75.04	3.83
6	-6	-5	72.74	3.67
-6	6	-5	83.40	3.69
-6	-6	5	72.11	3.89
-6	-6	5	79.71	3.80
-6	6	-5	74.08	3.69

-6	-6	5	71.99	3.92
-6	6	-5	74.20	4.03
6	-6	-5	80.59	4.03
-6	6	-5	85.55	3.92
6	6	5	80.07	3.90
-6	-6	5	76.92	3.88
-6	-6	5	73.18	3.71
6	6	5	69.24	3.85
6	-6	-5	74.47	3.85
-6	6	-5	72.13	4.06
6	6	5	75.52	3.70
-6	6	-5	78.42	3.78
-6	-6	5	75.61	3.64
6	6	5	75.34	3.65
6	-6	-5	75.81	3.75
6	6	5	71.13	3.89
6	6	-5	78.96	3.76
-6	6	5	78.50	3.77
-6	-6	-5	79.46	3.96
-6	-6	-5	74.41	3.71
-6	6	5	81.49	3.95
-6	-6	-5	73.58	3.79
6	-6	5	75.56	3.95
6	-6	5	71.50	3.69
6	-6	5	78.31	3.66
6	6	-5	73.95	3.77
6	6	-5	69.77	3.90
-6	6	5	74.98	3.69
6	-6	5	81.85	4.15
6	-6	5	74.25	3.61
-6	6	5	78.63	3.86
-6	-6	-5	73.73	3.76
-6	-6	-5	77.32	3.80
6	-6	5	77.30	3.81
6	6	-5	77.84	3.74
-6	6	5	69.56	3.94
6	7	5	111.76	5.40
-6	7	-5	114.99	6.03
-6	7	-5	131.51	5.95
6	7	5	108.57	5.11
6	7	5	100.17	5.37
-6	-7	5	107.29	5.21
6	7	5	100.39	5.17
-6	7	-5	119.43	5.70

-6	7	-5	117.57	5.78
6	-7	-5	125.94	5.54
6	-7	-5	116.07	5.73
-6	-7	5	101.90	5.12
-6	-7	5	107.71	5.29
6	-7	-5	116.58	5.87
6	7	5	104.97	5.42
-6	7	-5	114.76	6.17
-6	-7	5	113.74	5.36
-6	-7	5	102.83	5.39
6	-7	-5	118.97	5.83
6	-7	-5	127.91	6.09
-6	7	-5	131.11	5.69
6	-7	-5	122.49	5.55
-6	-7	5	108.01	5.45
6	-7	-5	118.32	5.71
-6	-7	-5	119.05	5.78
6	7	-5	121.07	5.77
-6	7	5	109.73	5.18
-6	7	5	106.53	5.59
6	7	-5	123.28	5.75
6	-7	5	106.14	5.19
6	-7	5	104.52	5.06
6	-7	5	105.36	5.27
6	-7	5	111.88	5.12
-6	-7	-5	122.13	5.84
6	-7	5	108.96	5.44
-6	7	5	108.53	5.35
-6	7	5	114.97	5.26
-6	7	5	114.36	5.47
6	7	-5	110.18	5.98
-6	-7	-5	121.05	5.71
-6	-7	-5	115.31	5.79
6	-7	5	118.79	5.69
-6	-7	-5	122.73	5.96
6	-8	-5	137.63	6.42
-6	8	-5	134.79	5.94
6	-8	-5	130.40	5.80
6	8	5	116.27	5.68
6	8	5	104.67	5.68
-6	-8	5	114.37	5.57
-6	8	-5	118.16	6.47
6	-8	-5	134.48	5.81
6	-8	-5	117.37	6.13

6	8	5	108.07	5.67
-6	-8	5	109.22	5.37
6	-8	-5	119.75	6.09
6	8	5	110.48	5.31
-6	8	-5	126.67	6.36
-6	-8	5	108.92	5.62
-6	8	-5	141.62	6.29
6	-8	-5	116.55	5.96
-6	-8	5	107.16	5.43
6	-8	-5	118.02	5.96
-6	8	5	114.49	5.59
6	8	-5	124.72	6.02
-6	-8	-5	133.33	6.37
6	-8	5	112.72	5.73
-6	-8	-5	120.05	6.05
-6	8	5	122.40	5.79
6	-8	5	105.12	5.40
6	-8	5	122.47	5.46
6	-8	5	115.86	5.35
6	-8	5	106.21	5.50
6	-8	5	128.00	6.02
-6	8	5	103.93	5.40
-6	-8	-5	120.95	6.04
-6	-8	-5	120.62	5.95
6	-8	5	109.72	5.27
6	8	-5	116.69	6.30
-6	8	5	111.56	5.46
-6	8	5	111.42	5.82
-6	9	-5	86.80	4.44
6	-9	-5	88.75	3.94
-6	-9	5	103.12	5.04
-6	-9	5	93.22	4.83
6	-9	-5	77.40	4.28
6	-9	-5	78.20	4.24
-6	9	-5	88.57	4.06
6	9	5	92.77	4.72
-6	-9	5	93.62	4.92
6	-9	-5	89.39	4.63
-6	9	-5	78.55	4.68
-6	-9	5	89.54	5.17
6	9	5	93.65	5.10
6	9	5	94.62	5.20
6	9	5	106.57	5.22
6	-9	-5	80.29	4.08

-6	9	-5	78.55	4.48
6	-9	-5	77.82	4.10
6	-9	5	109.12	5.55
-6	9	5	100.88	5.28
6	9	-5	81.04	4.13
-6	-9	-5	75.25	4.02
6	-9	5	92.74	4.86
6	-9	5	114.68	4.97
6	9	-5	77.87	4.61
6	-9	5	106.31	4.81
6	-9	5	95.64	4.68
-6	9	5	94.52	5.36
6	-9	5	95.13	4.98
-6	9	5	96.54	5.09
-6	-9	-5	78.29	4.19
6	9	-5	86.11	4.29
-6	-9	-5	78.49	4.17
6	10	5	36.86	2.23
-6	10	-5	35.70	3.11
-6	10	-5	44.62	2.90
-6	10	-5	37.98	2.92
-6	10	-5	45.20	2.44
6	-10	-5	41.72	3.14
-6	-10	5	41.93	2.73
6	-10	-5	45.68	2.31
6	10	5	37.62	2.64
-6	-10	5	38.48	2.40
6	-10	-5	44.54	2.27
6	-10	-5	40.18	2.45
6	-10	-5	38.76	2.42
6	10	5	34.80	2.75
6	-10	-5	40.18	2.63
-6	-10	5	40.11	2.71
6	10	5	36.59	2.76
-6	10	5	40.73	2.67
-6	-10	-5	41.50	2.62
-6	-10	-5	38.06	2.53
6	-10	5	44.67	2.32
6	-10	5	38.55	2.37
-6	-10	-5	41.84	2.38
6	-10	5	43.73	2.55
6	-10	5	35.89	2.09
-6	10	5	34.36	2.92
6	10	-5	42.46	2.50

6	10	-5	39.16	2.96
6	-10	5	43.14	3.31
6	-10	5	36.29	2.49
6	10	-5	44.41	2.72
6	-11	-5	2.57	0.76
-6	11	-5	4.03	1.48
6	-11	-5	1.94	0.94
6	-11	-5	4.61	0.73
6	11	5	2.37	1.24
-6	11	-5	3.58	1.70
6	-11	-5	2.48	0.81
-6	-11	5	2.72	1.15
-6	-11	5	3.03	0.97
-6	-11	-5	2.04	0.98
6	-11	5	2.50	0.87
6	-11	5	3.04	0.66
6	11	-5	3.83	1.47
-6	11	5	1.49	1.45
-6	-11	-5	3.25	0.79
6	-11	5	2.55	0.95
6	11	-5	2.94	0.95
-6	-11	-5	1.97	1.03
6	-11	5	4.25	0.90
-6	12	-5	11.75	2.88
-6	-12	5	11.85	1.62
6	-12	-5	16.79	1.68
6	12	5	13.18	2.62
-6	12	-5	14.28	2.41
6	-12	-5	12.84	1.80
6	-12	-5	12.45	1.49
-6	-12	5	14.33	2.07
6	-12	5	15.18	1.72
6	12	-5	17.04	3.06
-6	-12	-5	17.76	1.71
6	-12	5	16.56	1.61
-6	-12	-5	13.76	1.46
6	-12	5	13.35	1.50
-6	-12	-5	15.22	2.02
-6	12	5	14.75	1.95
-6	-13	5	41.87	3.10
6	-13	-5	35.83	2.48
6	13	5	36.85	3.54
-6	-13	5	35.93	2.77
-6	13	-5	44.09	3.61

6	-13	-5	34.39	2.51
-6	-13	-5	35.31	3.05
-6	-13	-5	38.37	2.92
6	13	-5	30.53	3.43
-6	13	5	42.76	3.39
7	0	-5	0.04	0.21
-7	0	5	-0.18	0.22
-7	0	-5	0.04	0.31
7	0	-5	0.11	0.18
7	0	5	0.14	0.32
-7	0	-5	0.11	0.28
-7	0	-5	-0.14	0.26
7	0	-5	0.26	0.18
-7	0	-5	0.30	0.23
7	0	5	-0.21	0.30
7	0	-5	-0.07	0.15
-7	0	-5	0.06	0.28
7	0	-5	-0.13	0.29
-7	0	5	0.05	0.33
-7	0	5	0.11	0.16
7	0	5	0.12	0.27
7	0	5	-0.07	0.21
7	0	5	-0.04	0.18
-7	0	-5	-0.28	0.20
-7	0	5	0.06	0.28
7	0	5	0.04	0.17
-7	1	-5	1.69	0.43
-7	-1	5	1.20	0.38
7	-1	-5	2.28	0.38
7	1	5	1.04	0.37
-7	1	-5	2.37	0.37
7	1	5	1.28	0.27
7	1	5	0.66	0.26
7	-1	-5	1.69	0.31
7	-1	-5	2.40	0.40
7	-1	-5	2.07	0.37
-7	1	-5	2.48	0.37
7	1	5	1.17	0.31
-7	-1	5	1.05	0.33
-7	1	-5	2.34	0.45
-7	1	-5	2.58	0.42
-7	-1	5	0.88	0.27
-7	1	-5	1.70	0.46
7	1	5	1.77	0.45

7	1	5	1.20	0.28
-7	-1	5	0.97	0.22
7	-1	-5	1.89	0.29
7	-1	5	0.67	0.66
7	-1	5	0.92	0.26
7	-1	5	1.18	0.29
7	-1	5	1.17	0.25
-7	1	5	0.79	0.34
-7	-1	-5	1.74	0.38
-7	-1	-5	2.47	0.47
-7	1	5	1.08	0.22
7	1	-5	1.84	0.41
-7	1	5	0.99	0.30
-7	-1	-5	2.30	0.42
-7	-1	-5	1.95	0.35
7	1	-5	1.83	0.30
-7	-1	-5	2.43	0.34
-7	1	5	0.57	0.26
-7	1	5	0.98	0.33
-7	-1	-5	1.80	0.32
-7	1	5	1.04	0.28
7	-1	5	1.15	0.32
-7	1	5	1.07	0.41
-7	-2	5	8.73	0.59
7	-2	-5	10.39	0.60
7	2	5	8.22	0.65
-7	2	-5	10.31	0.73
7	2	5	8.25	0.80
-7	2	-5	8.58	0.68
-7	-2	5	8.03	0.55
-7	2	-5	9.37	0.69
-7	2	-5	9.73	0.72
7	2	5	7.29	0.56
-7	2	-5	7.33	0.90
7	-2	-5	8.82	0.67
-7	2	-5	9.41	0.68
-7	2	-5	8.10	0.88
7	2	5	7.53	0.59
-7	-2	5	8.09	0.55
-7	-2	5	7.12	0.77
7	2	5	7.42	0.73
7	-2	-5	8.76	0.68
-7	-2	5	8.59	0.84
7	-2	-5	9.02	0.76

7	-2	-5	9.63	0.58
-7	2	5	8.70	0.83
7	2	-5	9.64	0.80
7	2	-5	9.35	0.65
-7	-2	-5	7.93	0.67
-7	2	5	7.94	0.73
-7	2	5	8.12	0.55
-7	-2	-5	9.16	0.68
7	2	-5	9.93	0.56
7	-2	5	7.52	0.59
7	-2	5	7.22	1.05
-7	2	5	8.28	0.70
-7	-2	-5	10.70	0.72
-7	2	5	8.45	0.72
7	-2	5	6.77	0.58
7	2	-5	8.70	0.64
-7	2	5	9.16	0.72
7	-2	5	7.25	0.86
7	-2	5	8.00	0.62
-7	2	5	7.38	0.53
-7	-2	-5	9.17	0.63
-7	-2	-5	9.91	0.85
-7	-2	-5	9.24	0.71
-7	-2	-5	9.63	0.73
7	3	5	65.38	3.45
7	3	5	63.29	3.32
-7	3	-5	65.14	3.06
-7	-3	5	70.29	3.49
7	-3	-5	60.94	3.05
-7	3	-5	65.38	3.05
7	3	5	69.84	3.32
-7	3	-5	59.21	3.22
-7	-3	5	70.43	3.20
7	-3	-5	64.27	3.05
-7	3	-5	58.38	2.98
-7	-3	5	70.44	3.19
-7	-3	5	67.52	3.20
7	-3	-5	60.94	2.94
-7	3	-5	55.78	3.24
-7	3	-5	62.62	3.00
7	3	5	63.89	3.40
-7	3	-5	65.20	3.01
-7	-3	5	67.49	3.46
-7	-3	5	71.78	3.16

-7	-3	5	67.16	3.28
-7	3	5	75.86	3.50
-7	3	5	72.29	3.38
7	-3	5	70.29	3.28
-7	-3	-5	65.20	3.19
-7	-3	-5	64.29	3.04
-7	-3	-5	60.71	3.03
7	3	-5	65.07	3.10
7	3	-5	64.63	2.92
7	-3	5	70.57	3.61
-7	3	5	69.12	3.43
7	3	-5	62.35	2.97
-7	-3	-5	63.56	2.99
7	3	-5	58.44	3.01
-7	3	5	69.37	3.26
7	-3	5	70.54	3.51
-7	-3	-5	61.83	3.03
-7	3	5	69.78	3.44
7	-3	5	67.29	3.34
-7	-3	-5	61.04	3.03
7	-3	5	65.06	3.31
-7	3	5	74.55	3.42
-7	4	-5	11.77	0.94
7	4	5	11.57	0.83
-7	-4	5	10.70	0.80
-7	-4	5	11.05	0.68
-7	-4	5	10.76	0.66
-7	-4	5	9.85	1.01
-7	-4	5	10.22	1.09
-7	4	-5	9.01	1.26
7	4	5	10.11	0.85
7	-4	-5	10.47	0.87
7	-4	-5	12.57	0.76
-7	4	-5	12.25	0.90
-7	-4	5	10.44	0.78
-7	-4	5	13.34	1.12
-7	4	-5	10.89	1.11
7	-4	-5	10.60	1.03
-7	4	-5	10.47	0.77
-7	4	-5	11.40	0.86
7	4	5	12.64	0.83
-7	-4	5	11.71	0.64
7	-4	-5	12.21	0.86
7	4	5	10.21	0.98

-7	4	-5	10.67	0.80
-7	-4	-5	11.39	0.89
-7	-4	-5	10.25	0.83
-7	-4	-5	11.03	0.84
-7	4	5	8.18	1.00
7	4	-5	11.24	0.77
-7	4	5	10.87	0.98
7	-4	5	9.26	0.82
-7	4	5	12.28	0.94
7	4	-5	12.10	0.82
7	-4	5	11.38	0.75
-7	4	5	11.09	0.80
7	4	-5	12.45	0.95
7	4	-5	9.15	0.86
-7	4	5	12.67	1.04
7	-4	5	10.03	0.78
7	-4	5	10.42	1.12
-7	-4	-5	12.07	0.83
7	-4	5	10.39	1.22
-7	-4	-5	10.20	1.06
-7	4	5	12.56	1.07
-7	-5	5	0.49	0.40
-7	5	-5	0.90	0.41
-7	5	-5	0.94	0.67
-7	-5	5	0.91	0.54
-7	-5	5	0.74	0.61
-7	-5	5	0.06	0.55
7	-5	-5	0.92	0.52
7	5	5	-0.03	0.36
7	-5	-5	0.31	0.58
-7	5	-5	0.00	0.53
7	5	5	0.84	0.42
7	5	5	0.76	0.37
7	-5	-5	0.86	0.29
-7	5	-5	0.86	0.40
-7	-5	5	0.81	0.34
7	-5	-5	0.52	0.32
-7	5	-5	0.76	0.50
-7	-5	5	0.69	0.28
7	-5	-5	0.63	0.42
-7	5	-5	0.98	0.43
7	5	5	0.09	0.43
-7	5	5	0.49	0.59
-7	-5	-5	0.70	0.42

-7	5	5	0.10	0.53
-7	5	5	0.82	0.37
7	-5	5	1.16	0.79
-7	5	5	0.27	0.47
-7	-5	-5	0.69	0.40
-7	-5	-5	1.01	0.39
7	-5	5	0.88	0.38
7	5	-5	0.68	0.47
7	-5	5	0.89	0.31
7	-5	5	0.16	0.29
-7	5	5	0.75	0.46
7	5	-5	0.63	0.37
7	-5	5	0.97	0.61
7	-5	5	0.73	0.33
-7	-5	-5	0.77	0.38
-7	-5	-5	0.91	0.57
7	5	-5	0.74	0.44
7	5	-5	0.58	0.35
-7	-6	5	11.93	1.06
7	-6	-5	10.49	0.90
-7	6	-5	10.63	1.48
7	6	5	11.81	1.00
-7	6	-5	10.80	1.11
-7	-6	5	11.61	0.88
7	6	5	10.97	1.12
-7	6	-5	10.85	1.03
-7	-6	5	12.58	1.34
7	-6	-5	8.78	1.24
7	-6	-5	9.97	1.15
-7	-6	5	11.46	0.96
-7	6	-5	9.35	1.27
-7	-6	5	9.17	1.18
7	6	5	13.02	0.97
7	-6	-5	9.72	1.00
7	6	5	11.98	1.19
-7	-6	5	13.85	1.32
7	-6	-5	10.63	0.83
-7	6	-5	12.59	0.94
-7	-6	-5	10.64	0.92
-7	6	5	13.08	1.28
7	-6	5	14.36	1.57
7	6	-5	11.23	0.98
-7	-6	-5	12.98	1.27
7	-6	5	13.51	1.06

7	-6	5	12.21	0.90
-7	6	5	11.55	1.10
-7	6	5	12.13	1.44
-7	6	5	10.07	1.16
-7	-6	-5	10.04	0.93
7	-6	5	11.56	1.37
7	-6	5	11.34	0.92
7	6	-5	11.70	0.97
7	-6	5	11.13	0.85
-7	-6	-5	11.16	1.01
7	6	-5	11.36	1.01
-7	-6	-5	9.90	0.99
-7	6	5	13.07	1.00
7	6	-5	10.01	1.11
7	7	5	20.92	1.47
-7	-7	5	21.47	1.84
7	7	5	21.45	1.73
7	-7	-5	21.09	1.38
-7	-7	5	22.31	1.58
-7	7	-5	20.58	1.47
7	7	5	21.98	1.42
7	-7	-5	21.89	1.87
-7	-7	5	23.00	1.78
-7	-7	5	22.13	1.72
-7	7	-5	22.58	1.40
7	-7	-5	19.25	1.51
7	-7	-5	24.05	1.67
-7	-7	5	21.65	1.48
7	7	5	21.89	1.67
-7	-7	5	22.98	1.42
-7	7	-5	21.37	1.41
-7	7	-5	18.99	1.78
-7	7	-5	23.70	1.66
7	7	5	25.25	1.76
-7	7	-5	20.42	1.86
7	-7	-5	18.58	1.31
7	-7	5	23.11	1.53
7	7	-5	21.29	1.49
-7	-7	-5	20.08	1.48
7	-7	5	24.03	1.39
7	7	-5	21.73	1.44
-7	7	5	20.73	1.97
-7	7	5	24.78	1.63
-7	7	5	26.32	1.88

7	-7	5	22.57	2.20
7	7	-5	21.59	1.44
-7	-7	-5	21.39	1.45
7	7	-5	20.66	1.68
-7	7	5	21.98	1.48
7	-7	5	21.88	1.45
-7	-7	-5	20.27	1.73
7	-7	5	22.55	1.33
-7	-7	-5	19.70	1.49
-7	-7	-5	19.77	1.35
-7	7	5	22.88	1.71
7	-7	5	19.75	1.85
-7	-8	5	9.04	1.00
-7	-8	5	11.23	1.52
-7	-8	5	8.65	0.97
7	8	5	7.73	1.27
7	-8	-5	14.02	1.30
7	-8	-5	12.70	1.52
7	8	5	7.88	0.99
7	8	5	9.05	1.28
-7	8	-5	15.60	1.24
-7	8	-5	12.33	1.89
7	-8	-5	12.18	1.09
7	8	5	11.21	1.42
-7	-8	5	7.63	1.27
-7	8	-5	14.02	1.54
-7	8	-5	13.60	1.64
7	-8	-5	15.23	1.92
7	-8	-5	13.46	1.14
-7	-8	-5	11.05	1.12
-7	8	5	8.12	1.29
7	-8	5	10.35	1.19
7	-8	5	8.15	0.97
7	-8	5	9.65	1.61
7	-8	5	9.25	0.92
7	-8	5	9.26	1.09
-7	8	5	9.94	1.11
-7	8	5	8.79	1.34
7	-8	5	9.91	0.84
-7	8	5	8.93	1.20
7	8	-5	12.94	1.19
-7	8	5	8.78	1.58
-7	-8	-5	11.59	1.22
-7	-8	-5	13.56	1.22

7	8	-5	13.25	1.51
-7	9	-5	1.60	1.13
-7	9	-5	3.23	1.09
-7	9	-5	3.32	0.77
-7	-9	5	0.67	0.83
7	9	5	1.69	1.06
7	-9	-5	2.11	0.55
7	-9	-5	3.52	1.27
-7	-9	5	1.51	0.61
7	-9	-5	1.46	0.73
-7	-9	5	1.18	0.63
7	9	5	2.82	1.02
7	-9	-5	1.50	0.61
-7	-9	5	1.90	1.03
-7	9	-5	2.59	1.23
7	9	5	-0.03	0.86
7	9	5	2.47	0.66
-7	-9	-5	2.77	0.78
-7	9	5	2.29	1.18
-7	-9	-5	2.54	0.76
7	-9	5	1.98	0.62
7	-9	5	3.60	1.48
7	-9	5	1.17	0.48
-7	-9	-5	2.72	0.67
7	9	-5	0.93	0.94
-7	9	5	1.16	0.93
7	-9	5	1.92	0.61
7	9	-5	3.13	0.73
7	-9	5	1.51	0.78
7	-9	5	1.89	0.83
7	10	5	2.85	1.16
-7	10	-5	3.17	1.42
7	-10	-5	5.34	0.70
7	-10	-5	6.42	1.54
-7	-10	5	3.02	1.11
7	10	5	3.76	1.03
7	10	5	3.29	0.76
7	-10	-5	4.61	0.98
-7	-10	5	4.27	1.20
-7	10	-5	4.95	1.29
-7	10	-5	4.41	0.93
7	-10	-5	4.45	0.82
7	-10	-5	4.52	0.84
-7	-10	5	5.52	0.94

-7	-10	-5	4.56	0.90
7	-10	5	5.57	1.16
-7	10	5	5.52	1.42
7	10	-5	5.60	0.94
7	10	-5	3.96	1.29
7	-10	5	3.59	0.85
7	-10	5	3.80	0.66
-7	10	5	5.20	1.25
7	-10	5	5.76	0.84
7	-10	5	4.88	1.50
7	10	-5	5.37	1.52
-7	-10	-5	4.29	0.80
7	-10	5	3.24	0.80
-7	-11	5	2.93	1.07
-7	-11	5	1.00	0.96
-7	11	-5	4.03	1.41
7	11	5	1.19	1.24
-7	11	-5	2.92	1.59
7	-11	-5	0.09	0.71
7	-11	-5	1.63	0.89
7	-11	-5	0.70	0.68
-7	-11	-5	1.96	0.99
-7	-11	-5	2.14	0.73
7	11	-5	1.24	1.22
-7	-11	-5	0.94	1.08
7	-11	5	2.57	0.81
7	-11	5	0.70	0.80
-7	11	5	1.10	1.17
7	-11	5	0.44	0.79
7	11	-5	2.17	0.83
-7	11	5	1.37	1.66
7	-11	5	1.80	0.63
-7	-12	5	3.82	1.40
-7	-12	5	4.67	1.02
-7	12	-5	6.02	1.86
7	-12	-5	3.72	1.23
7	-12	-5	3.47	0.91
7	-12	-5	4.14	1.06
7	12	5	4.68	1.89
-7	-12	-5	5.37	0.95
7	-12	5	3.19	0.83
-7	-12	-5	3.43	0.96
7	-12	5	3.98	0.75
-7	-12	-5	3.97	1.57

7	12	-5	2.89	1.67
-7	12	5	2.67	1.47
8	0	-5	94.20	4.21
8	0	-5	89.28	4.37
-8	0	-5	86.66	4.44
-8	0	5	103.34	4.61
8	0	-5	89.92	4.25
8	0	5	95.36	4.68
-8	0	-5	83.96	4.34
-8	0	5	97.63	4.68
-8	0	5	104.68	4.68
8	0	5	99.20	4.73
-8	0	-5	91.47	4.22
-8	0	5	100.69	4.71
-8	0	-5	91.63	4.30
8	0	5	105.68	4.87
8	0	-5	93.50	4.19
-8	0	5	105.60	4.83
8	0	5	97.39	4.71
8	0	5	93.28	4.63
-8	0	-5	97.21	4.30
-8	0	-5	93.18	4.36
-8	1	-5	212.72	9.93
8	1	5	238.15	10.64
8	-1	-5	215.34	9.93
8	-1	-5	227.29	9.91
-8	-1	5	240.61	10.61
-8	-1	5	227.01	10.57
8	1	5	250.22	10.78
8	-1	-5	221.50	9.86
-8	-1	5	226.36	10.57
-8	1	-5	202.99	10.04
8	1	5	228.68	10.66
8	-1	-5	220.46	9.88
-8	1	-5	215.96	10.04
-8	1	-5	230.27	9.96
-8	1	-5	223.31	9.90
-8	-1	5	231.04	10.70
-8	1	-5	234.28	9.95
8	1	5	220.08	10.60
8	1	5	229.83	10.72
-8	1	-5	220.94	9.96
-8	-1	5	241.36	10.77
8	1	5	233.58	10.62

-8	-1	-5	219.98	9.94
-8	1	5	235.83	10.57
-8	1	5	239.44	10.61
-8	-1	-5	222.66	9.86
-8	1	5	238.97	10.73
-8	1	5	247.81	10.58
-8	1	5	239.57	10.78
8	-1	5	217.21	10.61
8	1	-5	216.73	9.77
-8	-1	-5	194.34	9.95
8	-1	5	238.49	10.73
8	-1	5	228.47	10.65
8	1	-5	221.40	9.88
-8	-1	-5	222.73	9.96
8	1	-5	217.20	10.03
8	1	-5	215.11	9.82
8	-1	5	230.79	10.65
-8	1	5	222.89	10.59
-8	1	5	245.68	10.67
-8	1	5	238.86	10.57
-8	-1	-5	208.37	9.94
-8	-1	-5	222.49	10.02
8	2	5	287.01	12.26
-8	2	-5	264.86	11.65
-8	2	-5	261.19	11.62
8	2	5	268.59	12.06
8	-2	-5	256.50	11.62
-8	2	-5	244.92	11.62
-8	-2	5	262.52	12.25
8	2	5	261.06	12.27
8	-2	-5	270.82	11.69
-8	-2	5	242.92	12.16
-8	-2	5	292.27	12.31
8	-2	-5	247.73	11.64
-8	2	-5	242.22	11.78
8	2	5	274.48	12.15
-8	-2	5	249.66	12.09
-8	2	-5	272.99	11.64
-8	2	-5	260.08	11.83
8	-2	-5	259.99	11.56
-8	-2	5	280.88	12.09
8	2	5	246.98	12.12
-8	-2	5	278.69	12.28
-8	2	5	248.72	12.02

8	2	-5	256.16	11.55
-8	2	5	268.36	12.26
-8	-2	-5	267.00	11.76
-8	2	5	270.94	12.17
8	-2	5	273.27	12.27
-8	2	5	287.42	12.16
-8	2	5	266.01	12.08
8	2	-5	259.28	11.55
-8	-2	-5	259.59	11.62
8	-2	5	272.16	12.13
-8	-2	-5	260.37	11.65
8	2	-5	250.21	11.47
-8	2	5	283.73	12.19
8	-2	5	257.68	12.16
-8	-2	-5	263.86	11.56
-8	-2	-5	250.57	11.64
-8	2	5	271.27	12.04
-8	-2	-5	232.71	11.57
-8	2	5	271.99	12.29
8	-2	5	249.56	12.14
-8	-2	-5	246.92	11.63
8	2	-5	255.09	11.71
-8	-3	5	33.36	2.04
8	-3	-5	36.15	1.96
-8	-3	5	38.66	2.12
-8	3	-5	40.80	1.96
-8	3	-5	37.27	1.97
-8	3	-5	37.07	2.23
8	-3	-5	37.31	1.87
-8	-3	5	37.45	1.80
-8	-3	5	38.71	2.13
-8	3	-5	42.00	2.01
8	-3	-5	36.74	1.96
8	3	5	34.09	1.89
-8	3	-5	38.05	1.94
-8	3	-5	36.84	2.18
8	3	5	38.08	2.03
-8	-3	5	32.63	1.82
8	3	5	36.33	1.90
-8	-3	5	35.17	1.85
-8	3	-5	36.16	1.91
8	3	5	36.70	2.05
8	3	-5	38.38	1.81
-8	-3	-5	39.24	2.14

8	-3	5	34.18	1.91
-8	-3	-5	36.45	1.96
8	3	-5	35.48	1.91
-8	3	5	38.08	2.09
-8	3	5	37.25	1.85
8	3	-5	38.39	1.90
-8	-3	-5	37.72	1.95
8	-3	5	36.09	1.90
-8	-3	-5	37.81	1.97
-8	3	5	35.98	2.02
-8	-3	-5	34.87	1.84
8	-3	5	36.91	2.11
8	-3	5	36.79	1.84
-8	-3	-5	36.77	1.79
-8	3	5	40.94	1.99
-8	-3	-5	37.17	1.96
-8	3	5	35.24	2.02
8	3	-5	38.12	2.04
-8	3	5	39.36	1.99
8	-3	5	38.24	2.23
-8	-3	-5	38.29	1.88
-8	4	-5	13.07	0.86
8	4	5	14.15	1.11
8	4	5	12.23	1.09
-8	-4	5	12.50	0.78
-8	-4	5	13.49	0.76
-8	-4	5	11.21	0.87
-8	-4	5	13.03	1.21
-8	-4	5	13.47	0.79
-8	4	-5	13.92	1.00
-8	4	-5	14.21	1.00
-8	4	-5	11.58	1.32
-8	4	-5	15.40	0.95
8	4	5	12.54	0.95
-8	4	-5	12.94	0.92
8	-4	-5	11.93	0.94
-8	-4	5	13.54	1.23
-8	-4	5	12.36	1.13
8	4	5	14.60	0.95
-8	4	-5	11.77	1.19
8	-4	-5	12.41	0.82
8	-4	-5	11.17	0.90
-8	-4	5	12.47	0.89
8	-4	5	12.77	0.91

-8	4	5	12.89	1.14
-8	-4	-5	12.63	0.93
8	4	-5	13.47	0.84
8	4	-5	14.06	1.02
8	4	-5	12.36	0.96
8	-4	5	13.36	1.25
-8	-4	-5	13.45	0.98
-8	4	5	13.60	1.09
-8	4	5	11.34	1.07
-8	-4	-5	12.44	1.16
8	-4	5	13.07	0.94
8	-4	5	14.01	1.35
8	4	-5	13.46	0.90
-8	4	5	12.89	1.16
-8	-4	-5	12.21	0.95
-8	-4	-5	13.31	0.88
-8	4	5	12.46	0.89
-8	4	5	13.37	1.04
-8	-4	-5	11.21	0.91
8	-4	5	13.58	0.84
-8	-5	5	13.19	1.29
-8	5	-5	14.06	1.09
-8	5	-5	13.87	0.98
-8	-5	5	12.70	0.80
8	5	5	14.92	1.19
-8	5	-5	14.05	1.13
8	5	5	12.30	0.97
8	5	5	12.43	0.93
-8	5	-5	14.07	1.33
-8	5	-5	13.63	0.96
-8	-5	5	12.18	1.26
-8	5	-5	10.57	1.53
-8	-5	5	11.35	1.17
-8	-5	5	10.69	0.89
8	-5	-5	13.31	0.98
-8	-5	5	12.82	0.79
8	-5	-5	12.03	1.04
-8	-5	5	12.78	0.90
-8	-5	5	13.07	0.75
8	-5	-5	12.58	0.89
8	-5	-5	13.33	1.28
8	-5	-5	14.09	1.21
8	5	5	10.79	1.13
8	5	5	11.80	1.04

8	5	-5	14.44	0.98
-8	5	5	12.43	1.30
8	-5	5	13.68	1.33
-8	-5	-5	12.96	0.99
8	5	-5	11.05	1.07
8	-5	5	11.83	0.99
8	-5	5	12.16	0.83
-8	5	5	13.45	1.19
-8	5	5	11.84	0.94
8	-5	5	13.20	0.94
8	5	-5	13.75	1.04
-8	-5	-5	11.68	0.97
8	-5	5	12.90	0.88
-8	-5	-5	14.03	1.29
-8	5	5	12.32	1.15
8	-5	5	16.20	1.48
-8	-5	-5	14.17	0.98
-8	-5	-5	12.78	1.00
-8	5	5	11.64	1.08
8	5	-5	14.71	0.99
8	-6	-5	42.93	2.49
-8	-6	5	52.53	2.56
-8	6	-5	50.40	2.58
-8	-6	5	50.69	2.97
8	-6	-5	42.14	2.38
-8	-6	5	52.64	2.43
-8	6	-5	46.96	2.48
8	-6	-5	42.98	2.31
-8	6	-5	45.31	2.90
-8	-6	5	53.62	2.89
8	6	5	47.28	2.87
-8	-6	5	53.13	2.66
8	6	5	52.29	2.63
8	-6	-5	45.58	2.70
-8	6	-5	44.23	2.72
8	6	5	47.32	2.67
-8	-6	5	49.25	2.91
-8	6	-5	50.51	2.40
-8	-6	5	52.43	2.69
8	6	5	50.51	2.83
8	-6	-5	43.22	2.56
-8	6	-5	45.75	2.32
8	-6	5	54.28	2.57
8	-6	5	50.27	2.60

8	-6	5	50.34	2.67
-8	-6	-5	45.71	2.40
8	-6	5	52.32	2.99
8	6	-5	46.14	2.39
8	-6	5	52.15	2.77
8	6	-5	40.55	2.55
-8	6	5	52.80	2.68
8	6	-5	46.52	2.38
-8	-6	-5	43.76	2.36
-8	-6	-5	42.89	2.42
-8	-6	-5	45.55	2.67
-8	6	5	53.39	2.92
8	-6	5	59.77	3.25
8	6	-5	46.61	2.41
-8	6	5	52.43	2.89
-8	-6	-5	43.80	2.41
-8	6	5	47.38	3.01
-8	6	5	52.96	2.78
8	-7	-5	125.18	6.08
-8	-7	5	126.24	6.19
8	-7	-5	138.36	6.51
-8	7	-5	143.11	6.13
-8	7	-5	137.92	6.34
8	-7	-5	127.86	6.15
-8	-7	5	124.29	6.30
-8	7	-5	123.44	6.61
-8	7	-5	126.78	6.51
8	-7	-5	128.06	6.28
-8	7	-5	121.40	6.04
8	7	5	125.49	5.96
-8	-7	5	126.09	6.07
-8	-7	5	125.37	5.95
8	7	5	125.64	6.31
8	7	5	120.49	6.25
8	-7	-5	123.29	6.26
8	7	-5	121.68	6.34
-8	7	5	133.14	6.14
-8	-7	-5	126.94	6.17
-8	-7	-5	124.88	6.10
8	-7	5	126.87	6.16
8	-7	5	135.54	6.61
8	7	-5	128.56	6.14
-8	7	5	126.10	6.04
-8	-7	-5	127.86	6.20

-8	-7	-5	129.85	6.43
8	7	-5	132.99	6.15
-8	7	5	121.88	6.40
-8	7	5	131.85	6.31
8	7	-5	137.04	6.20
8	-7	5	124.42	6.05
8	-7	5	127.18	5.96
-8	7	5	126.99	6.26
8	-7	5	128.06	5.92
8	-7	5	130.88	6.32
-8	-8	5	71.91	3.72
8	-8	-5	70.64	3.61
8	8	5	70.62	4.05
8	8	5	70.88	3.69
-8	8	-5	78.35	3.63
-8	8	-5	75.52	4.07
8	8	5	71.34	4.06
8	8	5	77.50	4.10
8	-8	-5	66.44	3.74
-8	-8	5	77.21	4.00
-8	8	-5	76.05	3.91
-8	-8	5	70.34	4.07
-8	-8	5	72.55	3.82
-8	8	-5	68.37	4.17
8	-8	-5	68.99	3.57
8	-8	-5	66.75	4.65
8	-8	-5	68.47	3.83
-8	8	5	74.71	4.07
8	-8	5	72.61	3.68
-8	-8	-5	70.22	3.67
-8	-8	-5	67.81	3.57
8	-8	5	71.27	3.92
8	-8	5	87.32	3.83
8	-8	5	78.77	3.66
-8	-8	-5	72.26	3.72
8	-8	5	85.99	4.51
8	8	-5	75.07	3.73
-8	8	5	73.25	4.32
-8	8	5	77.40	4.17
8	-8	5	72.50	3.80
8	8	-5	71.31	3.61
8	8	-5	66.63	3.94
-8	9	-5	66.80	3.29
8	-9	-5	69.80	3.86

8	9	5	48.62	2.71
8	-9	-5	58.91	3.44
-8	9	-5	62.73	3.93
8	9	5	54.12	3.28
-8	-9	5	50.54	2.78
8	9	5	47.35	3.14
-8	9	-5	64.89	3.84
8	9	5	48.64	3.13
-8	-9	5	50.85	2.89
-8	-9	5	51.26	3.07
-8	-9	5	50.71	3.15
8	-9	-5	60.05	3.25
8	-9	-5	55.45	3.36
-8	9	5	46.78	3.25
8	-9	5	60.82	2.94
8	-9	5	59.41	3.70
8	9	-5	63.19	3.24
-8	9	5	52.25	3.14
8	-9	5	50.96	2.98
8	9	-5	57.89	3.66
8	-9	5	52.89	2.69
-8	-9	-5	62.64	3.34
8	-9	5	49.56	2.67
-8	-9	-5	59.11	3.34
8	-9	5	51.27	2.84
-8	-9	-5	59.65	3.20
-8	-10	5	20.31	2.14
8	-10	-5	17.46	1.51
-8	10	-5	20.52	2.34
8	10	5	20.26	2.19
8	10	5	21.22	2.14
8	10	5	21.67	1.56
8	-10	-5	17.93	1.72
8	-10	-5	19.02	1.49
-8	-10	5	21.21	1.77
8	-10	-5	18.52	2.36
-8	10	-5	17.89	1.62
-8	10	-5	18.27	2.44
-8	-10	-5	16.97	1.66
8	-10	5	27.41	2.80
8	-10	5	23.63	2.58
-8	-10	-5	16.84	1.44
-8	10	5	21.75	2.49
-8	10	5	20.03	2.25

8	-10	5	22.62	1.68
8	10	-5	17.95	1.51
8	10	-5	20.26	2.24
8	-10	5	21.15	1.47
8	-10	5	25.27	1.58
8	-10	5	23.63	1.87
8	-11	-5	-0.17	0.98
8	-11	-5	1.83	0.72
8	11	5	1.03	1.20
-8	11	-5	2.23	1.61
-8	-11	5	-0.75	0.83
-8	-11	5	0.55	1.26
8	-11	-5	0.18	0.67
-8	11	-5	-2.12	1.22
-8	-11	-5	0.92	0.74
-8	-11	-5	0.55	0.93
8	11	-5	-1.44	1.40
8	-11	5	0.13	0.69
8	11	-5	-0.78	0.80
8	-11	5	0.69	0.94
8	-11	5	-0.10	0.58
8	-11	5	2.14	0.95
-8	11	5	0.32	1.23
-8	-11	-5	0.83	1.14
8	-12	-5	11.79	1.60
-8	12	-5	16.46	2.47
8	12	5	18.71	2.69
-8	-12	5	16.07	1.74
-8	-12	5	15.18	2.25
8	-12	-5	12.44	1.80
8	-12	-5	13.71	1.55
-8	12	5	12.60	1.98
-8	-12	-5	12.76	1.54
8	-12	5	13.45	1.56
-8	-12	-5	11.64	1.63
-8	-12	-5	16.17	2.28
8	-12	5	14.06	1.31
8	12	-5	13.03	2.41
9	0	-5	-0.03	0.30
9	0	5	0.25	0.28
9	0	5	-0.33	0.34
9	0	-5	0.14	0.17
-9	0	-5	0.15	0.27
-9	0	5	-0.49	0.36

-9	0	5	0.03	0.29
-9	0	-5	-0.03	0.37
-9	0	-5	-0.06	0.27
-9	0	-5	0.62	0.40
9	0	-5	0.03	0.20
9	0	-5	0.16	0.16
-9	0	-5	0.07	0.21
9	0	5	-0.20	0.47
9	0	-5	-0.02	0.18
9	0	5	-0.19	0.31
9	0	5	-0.18	0.23
-9	0	5	0.40	0.22
9	0	5	-0.03	0.18
-9	0	5	0.45	0.31
-9	0	5	-0.07	0.34
-9	0	-5	0.05	0.35
9	1	5	40.10	2.04
9	-1	-5	46.61	2.44
9	-1	-5	48.94	2.38
-9	1	-5	51.49	2.48
9	1	5	41.26	2.06
9	-1	-5	48.07	2.38
-9	-1	5	40.78	2.18
-9	1	-5	51.66	2.60
9	1	5	37.87	1.98
-9	-1	5	39.22	2.12
9	1	5	44.00	2.24
-9	-1	5	41.05	2.02
-9	1	-5	52.52	2.43
9	1	5	38.80	2.13
-9	-1	5	37.78	1.96
9	-1	-5	51.45	2.44
-9	-1	5	42.96	2.22
-9	1	-5	48.13	2.61
9	1	5	39.86	2.06
-9	-1	5	39.86	1.98
-9	1	-5	49.63	2.47
-9	1	5	38.37	2.01
-9	1	5	40.69	2.22
9	-1	5	39.44	2.06
9	-1	5	40.94	2.16
-9	-1	-5	50.47	2.47
9	1	-5	49.36	2.32
-9	-1	-5	46.24	2.49

9	-1	5	41.25	2.26
-9	1	5	40.24	2.01
-9	1	5	41.60	2.07
9	-1	5	38.22	1.99
-9	-1	-5	52.09	2.37
-9	1	5	39.15	1.93
-9	-1	-5	47.48	2.48
-9	1	5	40.82	1.97
-9	-1	-5	49.09	2.57
9	1	-5	47.37	2.54
-9	1	5	37.40	2.13
9	1	-5	49.82	2.27
9	1	-5	49.51	2.41
-9	1	5	40.60	1.94
-9	-1	-5	52.62	2.50
-9	2	-5	38.34	1.88
-9	-2	5	33.04	1.74
-9	-2	5	34.09	1.77
9	-2	-5	36.73	1.81
-9	2	-5	37.12	1.90
9	-2	-5	34.69	1.88
-9	2	-5	35.07	2.10
-9	2	-5	35.64	2.13
-9	-2	5	34.98	1.98
-9	-2	5	33.37	1.71
-9	-2	5	31.10	1.91
9	2	5	35.64	1.82
9	-2	-5	36.34	1.86
-9	2	-5	39.64	1.93
9	2	5	34.05	1.94
9	-2	-5	36.29	1.93
9	2	5	35.42	1.95
-9	-2	5	38.05	2.01
9	2	5	33.05	1.76
-9	2	-5	34.11	1.88
-9	-2	5	34.42	1.76
-9	2	-5	35.00	1.88
9	2	5	34.44	1.75
-9	-2	-5	38.36	2.06
-9	2	5	34.43	1.69
-9	2	5	35.45	2.00
-9	2	5	33.13	1.83
9	-2	5	34.47	1.81
-9	2	5	36.24	1.81

9	-2	5	33.55	1.78
9	2	-5	35.45	1.77
-9	2	5	32.33	1.68
-9	-2	-5	37.23	1.79
-9	-2	-5	36.28	1.90
-9	-2	-5	32.00	1.88
9	-2	5	34.67	1.99
-9	2	5	37.90	1.88
9	2	-5	36.61	1.69
-9	-2	-5	36.10	1.86
9	2	-5	38.15	1.98
-9	-2	-5	35.71	1.89
9	-2	5	34.26	1.78
-9	-2	-5	35.89	1.91
-9	-3	5	46.06	2.29
9	-3	-5	39.36	2.19
-9	3	-5	42.42	2.13
-9	-3	5	50.89	2.61
-9	3	-5	37.55	2.39
-9	-3	5	48.91	2.31
-9	3	-5	43.49	2.11
9	3	5	49.21	2.41
-9	3	-5	38.81	2.06
9	3	5	44.39	2.39
9	3	5	43.06	2.54
-9	3	-5	40.91	2.06
-9	3	-5	40.81	2.13
-9	-3	5	45.03	2.36
9	-3	-5	41.43	2.01
9	3	5	51.06	2.55
-9	-3	5	48.99	2.38
9	-3	-5	41.31	2.11
9	-3	-5	40.84	2.10
-9	-3	5	42.69	2.55
-9	3	-5	39.58	2.33
-9	-3	5	48.40	2.61
-9	3	5	50.11	2.62
-9	-3	-5	37.24	1.99
9	3	-5	35.86	2.01
-9	-3	-5	37.34	2.09
-9	-3	-5	40.11	2.11
-9	-3	-5	39.25	1.95
-9	-3	-5	41.38	2.00
-9	3	5	46.53	2.50

9	3	-5	42.25	1.93
-9	3	5	48.58	2.36
-9	3	5	49.66	2.46
9	-3	5	49.94	2.74
9	-3	5	46.53	2.34
-9	-3	-5	40.05	2.06
9	-3	5	49.19	2.62
-9	-3	-5	42.19	2.11
9	-3	5	44.66	2.40
-9	3	5	51.42	2.49
-9	-3	-5	42.09	2.29
9	3	-5	42.99	2.17
9	-3	5	47.07	2.42
-9	4	-5	81.11	4.33
-9	4	-5	81.72	4.29
-9	4	-5	81.15	3.95
9	-4	-5	84.61	4.07
-9	-4	5	86.49	4.49
-9	-4	5	91.19	4.16
-9	-4	5	90.40	4.08
9	4	5	92.05	4.43
-9	4	-5	89.97	4.08
9	4	5	92.53	4.29
-9	-4	5	87.75	4.28
-9	4	-5	78.89	3.97
-9	-4	5	88.94	4.51
-9	-4	5	93.67	4.48
-9	-4	5	90.45	4.16
-9	4	-5	88.51	4.02
-9	4	-5	90.00	4.10
9	4	5	85.74	4.32
9	4	5	86.47	4.49
9	-4	-5	80.86	3.94
-9	-4	5	91.99	4.07
9	-4	-5	81.01	4.04
9	-4	5	83.38	4.32
-9	4	5	91.71	4.37
9	4	-5	88.77	3.91
9	4	-5	75.38	4.00
9	-4	5	86.24	4.30
-9	4	5	90.68	4.26
9	-4	5	89.00	4.20
-9	4	5	83.79	4.44
-9	-4	-5	81.31	4.02

-9	4	5	96.22	4.52
-9	4	5	96.67	4.42
9	-4	5	90.78	4.52
-9	-4	-5	83.63	3.94
-9	-4	-5	81.98	4.05
-9	-4	-5	82.28	4.22
9	4	-5	85.59	4.07
-9	4	5	89.83	4.47
9	-4	5	92.45	4.66
-9	-4	-5	83.65	4.05
-9	-4	-5	81.66	3.98
9	4	-5	86.02	3.98
-9	-5	5	27.05	1.53
9	5	5	26.76	1.68
9	-5	-5	26.76	1.56
9	-5	-5	25.80	1.89
-9	-5	5	27.05	1.34
9	-5	-5	27.79	1.66
-9	-5	5	24.34	1.83
-9	5	-5	29.08	1.59
-9	-5	5	22.60	1.75
-9	5	-5	31.08	1.68
-9	-5	5	25.64	1.39
9	5	5	25.23	1.59
-9	5	-5	27.47	2.11
-9	-5	5	28.06	1.81
-9	-5	5	26.25	1.36
9	5	5	25.15	1.50
-9	5	-5	28.94	2.01
-9	5	-5	31.62	1.75
-9	-5	5	25.35	1.34
-9	5	-5	29.29	1.77
9	5	5	25.79	1.77
9	-5	-5	28.02	1.72
-9	5	5	24.34	1.86
-9	-5	-5	28.41	1.60
-9	5	5	25.75	1.77
-9	5	5	26.75	1.72
-9	-5	-5	29.84	1.66
-9	-5	-5	29.10	1.72
9	5	-5	30.22	1.65
-9	5	5	26.01	1.81
9	5	-5	29.74	1.69
-9	-5	-5	30.01	1.65

-9	-5	-5	30.27	1.71
9	5	-5	25.58	1.72
9	-5	5	26.82	1.40
9	-5	5	24.41	1.51
-9	5	5	26.58	1.52
9	-5	5	28.16	1.89
-9	-5	-5	27.62	1.92
9	-5	5	25.05	1.57
-9	5	5	28.03	1.67
9	-5	5	26.93	2.03
-9	6	-5	17.94	1.22
9	-6	-5	14.53	1.45
-9	-6	5	9.13	1.28
9	-6	-5	15.60	1.26
-9	-6	5	12.35	1.00
-9	6	-5	17.18	1.38
-9	-6	5	11.51	1.45
-9	6	-5	16.95	1.13
9	-6	-5	15.45	1.08
9	6	5	13.33	1.04
-9	-6	5	12.71	0.79
9	6	5	11.85	1.28
9	6	5	11.20	1.07
-9	6	-5	13.42	1.72
-9	6	-5	17.84	1.63
9	-6	-5	15.70	1.55
9	6	5	11.76	1.19
-9	6	-5	17.11	1.32
9	-6	-5	16.01	1.17
-9	-6	5	13.81	1.42
9	6	5	14.13	1.33
-9	-6	5	13.76	0.91
-9	-6	5	12.43	1.04
-9	6	5	15.24	1.26
9	6	-5	14.18	1.31
-9	-6	-5	16.83	1.21
-9	-6	-5	14.41	1.51
-9	6	5	10.97	1.37
-9	-6	-5	14.72	1.20
9	6	-5	16.88	1.21
-9	6	5	13.22	1.32
-9	6	5	14.19	1.12
-9	6	5	13.23	1.47
9	-6	5	11.82	0.94

9	-6	5	13.78	1.02
9	6	-5	17.27	1.20
-9	-6	-5	16.95	1.17
9	-6	5	14.01	1.55
9	-6	5	13.50	1.71
9	-6	5	12.88	1.09
-9	-6	-5	16.05	1.17
9	6	-5	18.10	1.22
9	-6	5	13.61	0.90
-9	7	-5	21.64	1.62
-9	-7	5	17.78	1.05
9	7	5	19.90	1.66
9	-7	-5	16.74	1.22
9	7	5	17.58	1.53
9	-7	-5	16.94	1.30
9	7	5	19.00	1.30
-9	-7	5	17.00	1.61
-9	-7	5	17.80	1.33
9	-7	-5	17.26	1.42
-9	7	-5	17.05	2.10
9	-7	-5	16.20	1.60
-9	7	-5	17.24	1.77
-9	-7	5	18.20	1.71
9	-7	-5	19.05	1.80
-9	7	-5	20.16	1.39
-9	-7	-5	18.47	1.33
-9	7	5	18.57	1.36
9	7	-5	18.30	1.34
-9	7	5	16.33	1.65
9	7	-5	19.74	1.36
-9	7	5	20.39	1.65
9	-7	5	20.16	1.18
9	-7	5	17.64	1.20
9	-7	5	18.84	2.03
9	7	-5	16.11	1.52
9	-7	5	17.70	1.27
-9	-7	-5	17.49	1.31
-9	-7	-5	20.39	1.39
-9	7	5	16.97	1.63
9	7	-5	17.50	1.31
9	-7	5	18.42	1.41
-9	7	5	15.27	1.84
-9	-8	5	3.18	0.73
9	8	5	2.89	0.93

-9	8	-5	5.81	1.27
-9	8	-5	5.08	0.85
9	8	5	4.13	0.70
9	-8	-5	5.49	1.31
9	8	5	3.56	0.92
9	-8	-5	4.82	0.82
-9	-8	5	4.15	1.06
-9	8	-5	5.22	1.21
-9	8	-5	4.95	0.96
9	-8	-5	4.47	0.86
9	-8	-5	4.25	0.79
-9	-8	-5	6.68	1.01
-9	-8	-5	3.50	0.86
9	-8	5	2.89	0.71
-9	8	5	3.69	0.96
9	-8	5	5.32	1.45
9	-8	5	3.62	0.59
-9	-8	-5	5.94	0.82
9	8	-5	5.69	1.13
9	-8	5	2.91	0.68
9	-8	5	5.89	0.91
9	-8	5	3.88	0.69
9	8	-5	5.35	0.79
-9	8	5	3.76	1.24
-9	-9	5	15.94	1.88
-9	-9	5	19.11	1.93
-9	-9	5	18.50	1.54
9	-9	-5	19.99	1.71
9	-9	-5	18.83	1.56
9	-9	-5	22.53	2.34
9	9	5	17.53	1.40
-9	9	-5	22.00	2.24
-9	9	-5	23.83	1.68
9	9	5	16.64	1.77
9	9	5	14.76	1.78
9	9	5	19.18	1.90
9	-9	-5	18.97	1.63
-9	9	-5	21.50	2.51
-9	-9	-5	19.74	1.54
9	-9	5	17.29	1.58
9	-9	5	16.27	1.44
9	-9	5	19.92	2.48
9	-9	5	18.83	1.25
-9	9	5	18.15	1.97

9	9	-5	21.22	2.06
-9	9	5	13.19	2.78
9	-9	5	20.04	1.57
-9	-9	-5	22.56	1.71
-9	-9	-5	21.46	1.66
9	9	-5	19.83	1.52
9	-9	5	18.32	1.31
9	9	-5	23.90	1.83
-9	10	-5	10.29	1.99
9	10	5	9.58	1.79
-9	10	-5	5.62	1.66
9	10	5	11.41	1.21
-9	-10	5	10.56	1.57
9	-10	-5	9.60	1.20
9	-10	-5	7.57	1.42
-9	-10	5	9.74	1.36
9	-10	-5	9.37	1.18
9	10	5	8.09	1.72
9	-10	5	10.31	1.08
9	-10	5	8.87	1.01
-9	10	5	10.71	1.89
-9	-10	-5	8.51	1.20
9	-10	5	12.38	1.29
-9	10	5	9.94	1.91
-9	-10	-5	10.39	1.37
9	-10	5	7.10	2.88
9	10	-5	9.57	1.23
9	10	-5	7.19	1.48
9	-10	5	8.26	1.37
9	-11	-5	8.26	1.56
9	11	5	10.71	2.20
-9	-11	5	9.13	1.81
9	-11	-5	10.77	1.36
9	-11	-5	8.35	1.42
-9	-11	5	8.95	1.55
-9	11	-5	13.43	2.10
9	-11	5	7.56	1.33
9	11	-5	12.46	2.53
9	11	-5	11.34	1.87
-9	-11	-5	11.10	1.34
-9	-11	-5	9.84	1.56
9	-11	5	10.10	1.16
-9	11	5	9.73	1.71
9	-11	5	7.97	1.31

-9	12	-5	14.56	2.80
9	12	5	6.05	1.61
-9	-12	5	6.43	1.12
9	-12	-5	12.32	1.59
9	-12	-5	9.38	1.84
-9	-12	5	7.53	1.53
9	-12	-5	9.47	1.39
-9	12	5	7.50	1.85
9	-12	5	5.99	1.25
9	-12	5	6.14	1.05
-9	-12	-5	8.84	1.63
-9	-12	-5	9.72	1.64
9	-12	5	4.47	1.04
9	12	-5	6.28	1.73
-10	0	5	137.73	7.09
10	0	5	144.14	6.98
10	0	5	148.97	7.03
10	0	5	157.61	7.00
-10	0	5	143.92	6.98
10	0	5	153.62	7.02
-10	0	5	157.98	7.14
10	0	-5	171.19	7.82
10	0	-5	169.89	7.94
10	0	5	146.28	6.90
-10	0	5	162.59	6.99
-10	0	-5	181.34	8.05
-10	0	5	149.96	7.05
10	0	-5	170.59	7.90
-10	0	-5	159.62	8.05
-10	0	-5	172.16	7.99
10	0	-5	175.66	7.83
10	0	5	159.18	7.17
-10	0	-5	180.24	8.00
-10	0	5	149.13	6.92
-10	0	-5	179.52	7.88
10	-1	-5	117.16	5.53
-10	-1	5	117.71	5.52
10	1	5	117.49	5.43
10	1	5	120.00	5.55
-10	-1	5	114.41	5.36
-10	-1	5	110.42	5.34
10	1	5	114.09	5.42
10	-1	-5	111.95	5.61
-10	1	-5	118.20	5.79

10	1	5	112.90	5.52
-10	1	-5	112.10	5.62
10	-1	-5	121.96	5.59
10	-1	-5	120.14	5.55
-10	1	-5	124.82	5.74
-10	-1	5	117.78	5.58
-10	1	-5	121.33	5.66
-10	1	-5	127.38	5.65
-10	1	-5	125.39	5.57
10	1	5	112.88	5.36
10	1	5	105.74	5.43
-10	1	-5	127.01	5.66
-10	-1	5	111.34	5.48
-10	-1	5	117.68	5.39
-10	-1	-5	124.96	5.51
-10	1	5	116.19	5.53
-10	1	5	123.17	5.47
-10	1	5	122.16	5.61
10	-1	5	114.03	5.44
10	-1	5	109.68	5.36
10	1	-5	119.77	5.45
-10	-1	-5	110.37	5.66
10	1	-5	119.52	5.41
10	1	-5	120.19	5.72
10	1	-5	117.20	5.57
-10	1	5	114.43	5.37
10	-1	5	122.43	5.64
-10	-1	-5	124.00	5.65
10	-1	5	115.77	5.51
-10	-1	-5	120.49	5.60
-10	1	5	120.87	5.26
10	-1	5	111.82	5.43
-10	1	5	119.31	5.26
-10	-1	-5	125.43	5.75
-10	-1	-5	122.34	5.66
-10	1	5	118.28	5.36
-10	1	5	117.14	5.39
-10	2	-5	44.53	2.57
-10	2	-5	48.86	2.34
10	2	5	36.96	1.98
-10	2	-5	49.98	2.41
10	2	5	39.96	2.17
10	2	5	36.07	2.06
-10	-2	5	36.21	1.94

10	2	5	38.54	2.04
10	2	5	39.36	2.18
-10	-2	5	38.97	1.98
10	2	5	37.04	1.98
-10	2	-5	45.29	2.56
-10	-2	5	37.08	2.17
-10	-2	5	42.00	2.23
10	-2	-5	46.44	2.28
10	-2	-5	47.67	2.40
-10	-2	5	41.08	2.01
10	-2	-5	44.64	2.30
-10	-2	5	42.20	2.24
-10	-2	5	41.33	2.01
-10	2	-5	46.90	2.40
10	-2	-5	44.53	2.35
-10	2	-5	47.66	2.37
-10	2	-5	44.50	2.35
10	2	-5	46.58	2.13
10	-2	5	36.76	2.00
-10	-2	-5	42.09	2.33
-10	2	5	41.77	2.02
10	-2	5	39.56	2.03
-10	-2	-5	49.74	2.23
-10	2	5	39.77	2.05
-10	2	5	40.56	1.99
-10	-2	-5	46.34	2.30
10	-2	5	37.95	2.04
-10	-2	-5	45.72	2.38
10	2	-5	47.34	2.44
-10	-2	-5	47.46	2.52
-10	-2	-5	43.61	2.36
-10	2	5	37.93	1.92
-10	-2	-5	46.57	2.38
10	-2	5	40.01	2.21
-10	2	5	42.38	2.11
-10	2	5	38.88	1.93
10	2	-5	46.93	2.20
-10	2	5	40.82	2.25
-10	2	5	40.30	2.20
-10	3	-5	0.47	0.34
-10	-3	5	0.41	0.53
-10	3	-5	0.73	0.40
-10	3	-5	0.17	0.31
10	3	5	0.25	0.45

-10	3	-5	-0.27	0.59
10	-3	-5	0.46	0.29
-10	-3	5	1.06	0.36
10	-3	-5	0.41	0.30
10	-3	-5	0.61	0.26
-10	3	-5	-0.01	0.49
-10	-3	5	0.44	0.27
-10	3	-5	0.26	0.37
-10	3	-5	0.09	0.37
-10	-3	5	0.36	0.29
-10	-3	5	0.63	0.29
-10	-3	5	0.34	0.52
10	3	5	0.54	0.28
10	3	5	0.72	0.37
10	-3	-5	-0.06	0.47
-10	-3	5	0.24	0.45
10	3	5	0.42	0.45
-10	-3	-5	0.30	0.34
-10	3	5	0.46	0.49
10	-3	5	0.41	0.27
10	3	-5	0.38	0.20
-10	-3	-5	0.38	0.39
10	-3	5	0.55	0.31
-10	-3	-5	0.65	0.34
-10	-3	-5	0.44	0.33
10	3	-5	0.30	0.38
-10	3	5	0.83	0.42
10	3	-5	0.38	0.28
-10	-3	-5	0.10	0.50
10	-3	5	0.35	0.56
-10	3	5	0.22	0.43
10	-3	5	0.99	0.32
-10	-3	-5	1.03	0.41
-10	-3	-5	0.29	0.26
-10	-3	-5	0.60	0.34
-10	3	5	0.37	0.53
-10	3	5	0.53	0.41
-10	3	5	0.44	0.33
-10	4	-5	2.96	0.54
-10	-4	5	3.84	0.75
10	4	5	3.15	0.58
-10	4	-5	3.63	0.79
-10	-4	5	2.86	0.41
10	4	5	3.27	0.65

-10	4	-5	3.50	0.56
-10	4	-5	2.57	0.52
-10	-4	5	3.05	0.77
10	4	5	3.53	0.55
-10	4	-5	3.59	0.61
-10	-4	5	2.88	0.52
10	-4	-5	3.44	0.49
-10	-4	5	2.80	0.70
-10	4	-5	3.32	0.63
10	4	5	2.17	0.58
10	-4	-5	2.97	0.55
10	-4	-5	3.96	0.63
10	-4	-5	2.79	0.64
-10	-4	5	2.61	0.48
-10	4	-5	2.72	0.83
-10	-4	-5	3.40	0.63
-10	-4	-5	3.51	0.62
10	-4	5	3.44	0.46
-10	-4	-5	5.02	0.85
-10	4	5	2.97	0.71
10	-4	5	3.09	0.54
10	-4	5	4.04	0.88
-10	-4	-5	3.47	0.50
10	4	-5	4.06	0.60
10	-4	5	3.13	0.57
10	4	-5	3.35	0.63
-10	4	5	2.18	0.62
10	4	-5	3.34	0.64
-10	4	5	3.38	0.62
10	-4	5	2.00	0.70
-10	4	5	2.28	0.69
-10	4	5	2.93	0.65
10	4	-5	3.91	0.47
-10	4	5	2.36	0.57
-10	-4	-5	4.12	0.69
-10	-4	-5	3.91	0.60
10	-5	-5	50.90	2.75
10	5	5	59.73	2.99
10	-5	-5	56.83	3.01
-10	5	-5	49.90	3.11
10	-5	-5	50.77	2.71
-10	5	-5	57.43	2.82
-10	-5	5	62.02	3.22
-10	-5	5	57.61	3.01

-10	-5	5	61.43	2.77
-10	-5	5	61.12	2.84
-10	5	-5	49.77	2.59
10	-5	-5	51.38	2.61
10	5	5	62.04	3.19
10	5	5	58.02	3.25
-10	-5	5	58.42	2.78
-10	5	-5	57.28	2.73
-10	5	-5	53.71	2.77
-10	-5	5	59.86	2.85
-10	5	-5	48.55	2.64
-10	-5	5	56.92	3.28
-10	-5	5	56.03	3.23
10	5	5	57.06	3.07
-10	5	-5	53.21	3.05
-10	5	5	58.78	2.99
10	-5	5	59.11	3.08
10	5	-5	51.20	2.71
-10	5	5	56.68	3.27
10	-5	5	59.45	2.87
10	-5	5	63.90	3.49
-10	5	5	62.97	3.14
-10	-5	-5	52.74	2.96
10	-5	5	56.30	3.01
-10	-5	-5	51.18	2.69
10	5	-5	53.98	2.68
-10	-5	-5	52.66	2.61
-10	-5	-5	52.53	2.75
10	5	-5	48.47	2.73
-10	5	5	62.66	3.30
10	-5	5	58.66	3.29
-10	-5	-5	52.25	2.65
-10	5	5	63.85	3.20
-10	5	5	60.87	3.25
10	5	-5	53.95	2.60
-10	-5	-5	50.83	2.74
-10	6	-5	88.38	4.69
-10	-6	5	84.71	3.99
-10	6	-5	94.20	4.32
-10	6	-5	85.72	4.15
10	-6	-5	95.46	4.64
10	6	5	89.31	4.40
-10	-6	5	88.13	3.99
-10	6	-5	91.72	4.44

10	6	5	88.00	4.16
-10	-6	5	83.93	4.57
10	-6	-5	83.78	4.47
-10	6	-5	85.36	4.37
10	-6	-5	86.00	4.30
-10	-6	5	87.98	3.98
10	-6	-5	82.04	4.19
10	6	5	84.68	4.48
-10	-6	5	84.53	4.21
-10	-6	5	82.41	4.46
-10	6	-5	84.01	4.82
10	6	5	83.35	4.33
-10	-6	5	84.82	4.38
10	-6	-5	84.75	4.37
-10	-6	5	82.60	4.03
-10	6	5	83.75	4.44
10	-6	5	85.42	4.29
10	6	-5	87.90	4.28
-10	-6	-5	84.99	4.24
-10	6	5	86.97	4.50
-10	6	5	82.87	4.17
-10	6	5	87.23	4.31
10	6	-5	81.39	4.38
-10	-6	-5	91.90	4.69
10	-6	5	82.04	4.14
-10	-6	-5	93.90	4.45
-10	6	5	85.97	4.41
-10	-6	-5	90.67	4.36
10	6	-5	91.23	4.24
10	-6	5	87.39	4.03
-10	-6	-5	84.62	4.27
-10	-6	-5	85.68	4.34
10	-6	5	80.75	4.18
10	-6	5	97.66	4.80
10	-6	5	85.15	4.50
10	6	-5	85.62	4.26
-10	7	-5	54.78	3.28
-10	-7	5	58.79	2.80
-10	7	-5	57.66	2.89
-10	-7	5	58.08	2.89
10	7	5	56.03	3.35
10	-7	-5	52.09	2.77
-10	7	-5	52.33	2.70
10	7	5	57.56	3.25

-10	-7	5	58.27	2.79
10	-7	-5	51.88	2.83
-10	-7	5	58.56	3.10
-10	-7	5	62.25	3.33
10	7	5	58.21	3.00
10	7	5	61.99	3.30
-10	7	-5	53.08	3.48
10	-7	-5	55.07	3.24
-10	-7	5	59.93	3.38
10	-7	-5	51.71	2.94
-10	7	-5	57.57	3.06
10	-7	5	68.08	3.77
-10	-7	-5	52.19	2.91
10	7	-5	57.58	2.87
-10	-7	-5	51.62	2.81
10	-7	5	55.21	3.04
10	7	-5	48.86	3.03
10	7	-5	56.98	2.83
10	-7	5	56.74	2.99
-10	7	5	57.06	3.09
-10	7	5	59.41	3.40
10	-7	5	56.95	3.14
-10	-7	-5	51.23	2.82
-10	7	5	56.88	3.49
10	-7	5	62.56	2.88
10	-8	-5	64.34	3.56
-10	8	-5	53.22	3.52
10	-8	-5	53.46	3.09
-10	-8	5	54.20	3.25
-10	8	-5	44.17	4.46
10	8	5	48.25	3.13
10	-8	-5	54.06	2.88
10	-8	-5	51.99	2.95
-10	-8	5	59.99	3.22
-10	-8	5	53.07	3.18
10	8	5	52.36	2.83
10	8	5	52.22	3.26
-10	8	-5	57.91	2.99
-10	-8	5	54.55	2.77
10	-8	5	53.37	2.91
10	8	-5	49.92	3.20
-10	-8	-5	56.75	2.97
-10	-8	-5	53.94	2.92
-10	-8	-5	55.24	3.04

-10	8	5	53.16	3.56
-10	8	5	54.28	3.33
10	-8	5	52.60	3.41
10	-8	5	56.78	2.72
10	-8	5	55.65	3.07
10	-8	5	52.91	2.82
10	-8	5	61.08	3.64
10	8	-5	59.19	3.06
10	8	-5	58.05	2.91
-10	-9	5	31.67	2.34
10	9	5	25.81	2.38
10	9	5	28.58	1.88
10	9	5	28.11	2.33
-10	-9	5	32.63	1.86
10	-9	-5	41.81	3.07
10	-9	-5	36.27	2.23
-10	-9	5	30.71	2.06
-10	-9	5	25.04	2.36
10	-9	-5	37.23	2.43
-10	9	-5	38.57	2.97
-10	9	-5	36.34	3.06
10	-9	-5	37.39	2.19
-10	-9	-5	37.57	2.24
10	-9	5	29.36	1.94
10	9	-5	36.00	2.18
10	9	-5	33.59	2.66
10	-9	5	27.57	1.80
10	-9	5	32.14	1.73
10	-9	5	34.77	2.82
10	-9	5	36.14	2.06
-10	9	5	31.32	2.42
-10	9	5	29.95	2.98
10	9	-5	40.16	2.44
-10	-9	-5	39.22	2.39
10	-9	5	29.43	2.05
-10	10	-5	8.29	1.73
-10	-10	5	5.18	1.39
10	-10	-5	6.33	1.13
10	10	5	4.63	1.31
-10	10	-5	4.23	1.46
10	10	5	4.03	1.22
10	-10	-5	5.18	1.06
10	-10	-5	5.01	0.90
-10	-10	5	5.08	0.97

-10	10	5	3.91	1.32
10	-10	5	5.21	1.01
-10	-10	-5	6.45	1.18
10	-10	5	4.08	0.93
-10	-10	-5	6.23	0.95
10	-10	5	5.57	0.84
10	10	-5	6.27	1.15
10	10	-5	6.15	1.45
10	-10	5	3.70	0.79
10	-11	-5	0.89	1.00
10	11	5	1.68	1.53
10	-11	-5	1.06	0.82
-10	-11	5	-0.48	0.83
-10	11	-5	2.20	1.68
10	-11	-5	0.08	0.71
10	11	-5	0.65	1.40
-10	-11	-5	1.37	0.76
10	-11	5	0.92	1.13
-10	-11	-5	0.68	0.89
10	-11	5	0.47	0.79
10	-11	5	-0.68	0.59
-10	11	5	0.19	1.29
10	-12	-5	5.26	1.00
10	12	5	1.90	1.38
-10	-12	5	1.15	0.96
-10	-12	5	2.65	1.30
10	-12	-5	3.49	1.09
-10	12	-5	5.82	1.70
10	-12	-5	3.48	1.28
-10	-12	-5	3.13	1.07
10	-12	5	2.12	0.89
10	12	-5	3.96	1.67
10	-12	5	3.14	1.08
-10	-12	-5	4.09	1.33
-10	12	5	3.32	1.89
11	0	-5	0.13	0.23
-11	0	5	-1.04	0.46
-11	0	-5	0.03	0.32
-11	0	5	0.23	0.34
-11	0	5	0.17	0.43
-11	0	-5	0.12	0.24
11	0	5	0.42	0.41
11	0	5	0.04	0.20
11	0	-5	0.20	0.26

-11	0	-5	0.14	0.28
11	0	5	0.70	0.53
-11	0	5	0.19	0.29
-11	0	-5	-0.08	0.39
11	0	5	0.02	0.33
11	0	5	-0.07	0.24
-11	0	5	0.24	0.38
11	0	-5	-0.16	0.17
-11	0	-5	0.46	0.44
11	0	-5	-0.27	0.21
-11	0	5	-0.29	0.38
-11	0	-5	0.06	0.33
-11	0	-5	0.06	0.43
-11	1	-5	51.83	2.41
-11	1	-5	48.94	2.59
-11	-1	5	48.82	2.56
11	1	5	46.72	2.40
-11	-1	5	42.04	2.26
11	1	5	44.38	2.37
11	1	5	45.17	2.46
-11	1	-5	51.09	2.52
11	-1	-5	46.40	2.35
-11	1	-5	44.95	2.63
11	1	5	45.86	2.27
-11	1	-5	46.10	2.47
11	1	5	47.27	2.37
11	-1	-5	49.08	2.40
-11	-1	5	46.58	2.46
-11	-1	5	44.98	2.42
-11	1	-5	48.87	2.52
-11	-1	5	48.37	2.31
-11	-1	5	44.71	2.43
-11	1	-5	49.09	2.49
11	-1	-5	49.71	2.41
11	-1	-5	46.51	2.45
11	1	5	47.87	2.51
-11	-1	5	45.76	2.34
11	-1	5	44.07	2.28
-11	1	5	44.35	2.30
11	1	-5	49.74	2.43
-11	1	5	48.86	2.30
-11	1	5	46.46	2.33
-11	-1	-5	51.09	2.62
-11	1	5	47.45	2.57

-11	-1	-5	49.33	2.50
11	-1	5	47.37	2.49
-11	-1	-5	50.04	2.43
-11	1	5	51.88	2.42
11	-1	5	48.16	2.64
11	-1	5	44.62	2.38
11	-1	5	42.32	2.35
-11	-1	-5	48.46	2.51
-11	1	5	43.92	2.47
-11	-1	-5	47.25	2.55
-11	-1	-5	50.05	2.33
-11	-2	5	116.46	5.14
11	2	5	112.76	5.18
-11	2	-5	105.14	5.06
11	-2	-5	106.27	5.09
-11	-2	5	110.26	5.32
11	-2	-5	109.65	5.02
11	2	5	106.44	5.22
11	-2	-5	112.01	5.12
-11	-2	5	104.51	5.29
-11	2	-5	112.11	5.05
-11	-2	5	103.14	5.08
11	-2	-5	107.14	5.02
-11	2	-5	115.89	5.14
11	2	5	112.08	5.12
-11	-2	5	102.94	5.11
11	2	5	116.57	5.32
-11	-2	5	115.11	5.35
-11	2	-5	102.47	5.08
11	2	5	104.05	5.30
-11	2	-5	101.99	5.29
-11	-2	5	112.25	5.14
11	2	5	107.07	5.12
-11	-2	-5	105.51	5.11
-11	-2	-5	109.68	4.93
-11	-2	-5	106.71	5.11
11	-2	5	105.33	5.14
-11	2	5	112.68	5.23
11	-2	5	104.67	5.18
-11	-2	-5	96.87	5.06
-11	2	5	111.72	5.34
-11	2	5	101.66	5.05
11	2	-5	108.32	5.17
-11	-2	-5	105.56	5.11

-11	-2	-5	110.41	4.99
-11	2	5	110.12	5.37
-11	2	5	113.71	5.10
-11	2	5	116.46	5.12
-11	2	5	111.12	5.07
-11	2	5	110.26	5.17
11	-2	5	110.73	5.16
11	-2	5	108.58	5.32
11	-2	5	113.25	5.45
-11	-2	-5	113.22	5.26
-11	3	-5	83.22	4.16
11	3	5	92.05	4.71
11	3	5	106.44	4.89
-11	-3	5	96.16	4.66
11	-3	-5	91.28	4.30
11	-3	-5	87.17	4.11
11	3	5	102.59	4.76
-11	3	-5	87.88	4.19
-11	-3	5	107.43	4.97
-11	3	-5	85.10	4.45
11	3	5	94.36	4.93
11	-3	-5	88.78	4.21
-11	3	-5	85.40	4.45
-11	-3	5	95.30	4.72
-11	-3	5	94.94	4.92
-11	-3	5	103.17	4.69
-11	3	-5	88.89	4.19
-11	3	-5	84.08	4.13
-11	-3	5	103.99	4.74
11	-3	-5	87.82	4.17
-11	3	-5	89.21	4.23
-11	-3	5	99.62	4.95
-11	3	5	100.70	4.83
-11	-3	-5	86.67	4.20
-11	-3	-5	83.90	4.19
11	3	-5	81.99	4.04
-11	-3	-5	85.97	4.08
-11	3	5	101.29	4.77
11	-3	5	96.98	4.94
-11	3	5	106.31	5.01
11	-3	5	98.42	4.76
-11	3	5	102.56	4.96
-11	-3	-5	84.80	4.20
-11	-3	-5	86.77	4.02

11	3	-5	89.09	3.97
-11	-3	-5	88.44	4.08
11	3	-5	87.13	4.24
-11	-3	-5	87.39	4.38
11	-3	5	99.87	4.70
-11	3	5	102.77	4.72
-11	3	5	101.52	4.84
11	-3	5	95.28	4.77
-11	4	-5	116.55	5.98
-11	4	-5	118.24	5.63
11	4	5	126.21	5.97
11	4	5	132.68	6.11
-11	-4	5	124.56	5.97
11	-4	-5	127.96	5.85
11	-4	-5	121.06	5.70
-11	-4	5	123.15	5.86
-11	4	-5	127.73	5.72
-11	4	-5	122.69	5.72
-11	-4	5	118.19	5.95
-11	-4	5	136.75	6.19
11	4	5	117.95	5.97
-11	4	-5	114.68	5.95
-11	4	-5	127.85	5.69
-11	-4	5	130.90	6.23
-11	-4	5	122.07	6.19
-11	4	-5	117.53	5.57
11	-4	-5	115.75	5.58
11	4	5	117.56	6.18
-11	-4	5	131.34	5.88
11	-4	-5	118.44	5.68
-11	4	5	125.67	6.13
-11	4	5	131.53	6.22
11	4	-5	109.16	5.58
-11	-4	-5	117.37	5.51
-11	-4	-5	122.31	5.56
11	-4	5	126.45	6.01
-11	-4	-5	119.05	5.69
11	-4	5	123.43	6.01
-11	-4	-5	122.64	5.90
11	-4	5	126.16	6.20
-11	4	5	132.13	5.96
11	4	-5	123.58	5.71
-11	-4	-5	116.02	5.69
-11	-4	-5	115.67	5.48

-11	4	5	133.46	6.09
-11	4	5	125.44	6.04
-11	-4	-5	118.46	5.67
-11	4	5	132.36	6.24
11	-4	5	134.21	6.38
11	-4	5	128.14	5.88
11	4	-5	120.81	5.51
-11	-5	5	71.68	3.43
11	-5	-5	74.98	3.75
11	5	5	75.83	3.72
-11	5	-5	71.32	3.56
-11	5	-5	75.14	3.76
11	5	5	71.18	3.54
-11	-5	5	70.61	3.57
-11	5	-5	70.66	4.03
-11	5	-5	79.41	3.80
11	5	5	66.92	3.60
11	5	5	67.05	3.82
11	-5	-5	73.10	3.61
-11	-5	5	70.30	3.85
-11	-5	5	68.32	3.82
-11	5	-5	72.99	3.65
-11	5	-5	75.19	4.14
-11	-5	5	73.97	3.42
11	-5	-5	74.15	3.72
-11	-5	5	74.19	3.80
-11	5	-5	79.51	3.73
11	5	-5	74.67	3.66
-11	-5	-5	74.05	3.56
11	-5	5	84.98	4.11
-11	-5	-5	75.68	3.98
11	-5	5	64.67	3.55
-11	5	5	71.89	3.83
-11	-5	-5	70.13	3.72
11	5	-5	70.40	3.70
-11	-5	-5	77.95	3.62
-11	5	5	75.37	3.71
11	-5	5	70.89	3.63
-11	5	5	73.61	3.56
-11	5	5	68.59	3.81
11	5	-5	78.02	3.72
11	-5	5	73.28	3.42
-11	-5	-5	73.37	3.70
-11	5	5	74.34	3.72

-11	-5	-5	72.37	3.73
11	5	-5	76.74	3.59
-11	5	5	74.78	3.87
11	-5	5	69.47	3.83
11	-6	-5	27.70	1.81
11	-6	-5	28.96	1.71
-11	-6	5	29.73	1.46
11	-6	-5	31.15	1.93
-11	6	-5	29.70	1.95
-11	-6	5	27.47	1.49
-11	-6	5	27.94	2.09
11	-6	-5	33.43	2.23
-11	-6	5	27.87	2.05
11	6	5	31.21	1.98
-11	6	-5	32.54	1.90
11	6	5	26.70	1.83
-11	-6	5	29.89	1.57
11	6	5	29.67	2.08
11	6	5	28.83	1.73
-11	6	-5	30.29	1.69
-11	6	-5	31.08	2.42
-11	6	-5	29.58	2.25
-11	-6	5	29.07	1.76
11	-6	5	30.30	2.39
11	6	-5	33.18	1.83
-11	-6	-5	30.47	1.76
-11	6	5	27.80	2.06
11	-6	5	31.06	1.54
11	-6	5	29.06	1.74
-11	6	5	29.91	1.79
-11	-6	-5	29.56	1.87
11	-6	5	28.95	1.82
-11	-6	-5	30.40	1.83
11	6	-5	30.20	1.83
11	6	-5	32.63	1.75
11	6	-5	28.26	1.91
-11	6	5	27.15	2.12
-11	-7	5	2.37	0.85
-11	-7	5	2.86	1.00
11	-7	-5	4.46	0.82
11	-7	-5	5.24	1.16
11	-7	-5	3.29	0.59
-11	-7	5	2.36	0.42
-11	7	-5	5.66	0.80

11	7	5	2.09	0.98
-11	-7	5	2.71	0.46
-11	-7	5	3.60	0.60
11	7	5	3.31	0.66
11	-7	-5	3.26	0.70
11	7	5	3.11	0.93
-11	7	-5	4.02	1.37
-11	7	-5	2.84	1.05
-11	7	-5	4.28	0.80
11	7	5	2.08	0.74
-11	-7	5	2.37	0.63
-11	-7	-5	4.23	0.87
-11	7	5	0.55	0.97
11	7	-5	4.29	0.80
11	-7	5	2.51	0.60
-11	-7	-5	3.66	0.71
-11	-7	-5	3.39	0.74
11	7	-5	4.42	0.68
11	7	-5	3.75	0.98
11	-7	5	2.48	0.44
11	-7	5	1.66	0.53
11	-7	5	1.99	0.62
-11	7	5	2.45	0.78
11	-7	5	1.88	1.25
-11	7	5	3.27	0.72
-11	-8	5	13.58	0.90
-11	-8	5	14.06	1.06
11	-8	-5	13.51	1.22
11	8	5	16.08	1.72
-11	-8	5	11.36	1.77
11	8	5	13.06	1.24
-11	8	-5	14.65	1.95
11	-8	-5	19.23	2.03
-11	-8	5	12.53	1.24
11	-8	-5	14.10	1.16
-11	-8	5	11.67	1.64
-11	8	-5	11.98	2.43
11	8	5	11.70	1.66
11	8	5	11.92	1.50
11	-8	-5	11.04	1.31
-11	8	5	12.46	2.16
-11	-8	-5	16.95	1.44
-11	8	5	11.37	2.02
11	-8	5	14.51	0.98

-11	-8	-5	14.95	1.32
11	-8	5	15.03	1.38
11	-8	5	11.57	1.09
11	8	-5	12.72	1.54
-11	-8	-5	14.41	1.29
11	-8	5	16.25	2.55
11	-8	5	14.87	1.22
11	-8	5	11.29	1.18
11	8	-5	14.39	1.23
11	9	5	28.57	2.42
11	-9	-5	27.91	1.87
11	9	5	28.66	2.34
11	9	5	29.71	2.39
-11	9	-5	23.72	2.57
-11	-9	5	32.46	2.50
11	-9	-5	27.86	1.81
-11	-9	5	31.17	2.07
-11	9	-5	27.65	2.63
11	-9	-5	25.60	2.76
11	9	5	30.36	1.88
11	-9	5	30.86	3.01
11	9	-5	29.69	1.81
11	9	-5	27.96	2.37
11	-9	5	29.84	2.12
-11	9	5	31.25	2.51
11	-9	5	30.32	2.00
-11	-9	-5	26.78	2.00
-11	-9	-5	24.44	1.82
-11	9	5	27.63	2.97
11	-9	5	28.31	1.84
11	-9	5	30.95	1.70
11	9	-5	31.60	2.14
-11	-10	5	36.21	2.90
-11	-10	5	33.63	2.40
11	10	5	36.55	2.89
-11	10	-5	36.13	3.00
11	-10	-5	30.61	2.01
11	-10	-5	29.02	2.26
11	-10	-5	30.88	2.07
11	10	5	34.62	2.76
11	-10	5	33.74	2.08
-11	-10	-5	26.33	1.99
11	10	-5	28.52	2.82
11	-10	5	34.16	2.33

-11	-10	-5	30.33	2.29
-11	10	5	35.56	2.96
11	10	-5	32.78	1.95
11	-11	-5	24.58	2.20
-11	-11	5	28.90	2.15
-11	-11	5	25.45	2.59
-11	11	-5	26.52	3.05
11	-11	-5	24.70	1.98
11	11	5	27.13	2.73
11	-11	-5	24.97	2.01
-11	11	5	27.09	3.03
11	11	-5	22.38	2.54
-11	-11	-5	25.00	2.03
11	-11	5	27.44	2.03
-11	-11	-5	25.91	1.93
11	-11	5	27.21	2.26
-11	-12	5	29.85	2.77
11	12	5	24.10	2.86
11	-12	-5	35.98	2.82
11	-12	-5	37.12	2.37
11	-12	-5	33.59	2.38
-11	-12	5	27.53	2.23
-11	12	-5	34.81	3.42
11	-12	5	26.60	2.38
-11	-12	-5	32.67	2.59
-11	12	5	27.30	3.17
11	12	-5	27.95	2.94
-11	-12	-5	39.44	3.09
11	-12	5	26.40	2.05
-12	0	-5	110.64	5.42
-12	0	5	109.57	5.37
12	0	5	111.30	5.39
-12	0	-5	111.93	5.53
-12	0	-5	116.55	5.38
-12	0	5	119.97	5.39
-12	0	-5	115.86	5.27
12	0	5	111.45	5.43
12	0	5	116.60	5.57
-12	0	5	112.50	5.55
-12	0	-5	117.42	5.42
12	0	-5	111.19	5.33
12	0	-5	108.00	5.20
-12	0	-5	120.79	5.50
-12	0	-5	119.53	5.45

12	0	-5	111.78	5.37
12	0	5	108.05	5.27
12	0	5	119.08	5.44
-12	0	5	115.42	5.31
-12	0	5	110.15	5.46
12	0	-5	116.44	5.23
-12	0	5	117.05	5.41
12	-1	-5	48.79	2.52
-12	-1	5	52.89	2.82
12	-1	-5	51.59	2.47
12	1	5	55.78	2.87
12	1	5	54.55	2.77
-12	1	-5	51.45	2.64
-12	1	-5	48.57	2.55
12	1	5	53.56	2.78
-12	-1	5	55.58	2.74
-12	1	-5	49.00	2.55
12	-1	-5	49.23	2.46
-12	-1	5	56.94	2.88
12	1	5	53.42	2.78
-12	-1	5	54.61	2.70
12	1	5	53.61	2.66
-12	-1	5	52.25	2.66
-12	1	-5	48.54	2.43
-12	1	-5	50.41	2.75
-12	-1	5	54.78	2.95
12	-1	-5	46.34	2.38
-12	1	-5	53.05	2.59
-12	-1	5	54.77	2.84
-12	1	-5	52.84	2.60
-12	1	5	52.66	2.89
-12	-1	-5	52.49	2.69
-12	-1	-5	49.68	2.36
12	-1	5	52.62	2.66
-12	1	5	54.99	2.71
12	-1	5	55.90	2.78
-12	1	5	54.53	2.68
-12	1	5	52.03	2.96
-12	1	5	54.65	2.79
-12	-1	-5	46.81	2.61
12	-1	5	53.47	2.76
-12	-1	-5	47.39	2.56
12	-1	5	56.55	2.99
-12	-1	-5	50.10	2.57

-12	1	5	57.06	2.73
12	-1	5	55.28	2.87
12	1	-5	48.04	2.47
12	2	5	10.59	0.93
12	2	5	10.05	0.92
-12	-2	5	11.04	0.88
-12	2	-5	12.72	0.99
-12	-2	5	12.09	1.16
-12	2	-5	10.56	0.90
12	-2	-5	12.27	0.82
-12	2	-5	13.00	1.01
-12	2	-5	11.86	0.96
12	-2	-5	11.46	0.91
-12	2	-5	11.54	1.18
-12	2	-5	10.98	0.88
-12	-2	5	11.55	1.06
12	-2	-5	12.11	0.96
-12	-2	5	11.05	0.89
-12	-2	5	12.52	1.20
12	2	5	10.19	1.01
12	2	5	11.34	0.80
12	2	5	9.77	0.91
12	-2	-5	11.13	0.82
-12	-2	5	10.51	0.78
-12	2	-5	10.88	1.22
-12	-2	5	11.43	0.82
-12	-2	-5	10.82	0.91
-12	2	5	9.72	1.20
-12	-2	-5	11.80	0.98
-12	2	5	12.00	0.83
-12	-2	-5	11.96	1.04
-12	2	5	9.84	0.84
12	-2	5	12.82	1.32
-12	2	5	11.70	0.93
12	-2	5	10.03	0.82
12	-2	5	11.85	1.19
-12	2	5	10.60	1.00
-12	2	5	13.04	1.13
-12	-2	-5	11.71	0.95
12	-2	5	12.09	0.92
-12	-2	-5	10.99	0.84
-12	-2	-5	11.94	1.18
12	2	-5	9.88	0.98
-12	2	5	11.99	0.82

12	-2	5	8.99	0.85
-12	-2	-5	11.79	0.72
-12	2	5	12.68	0.87
-12	3	-5	2.89	0.53
-12	-3	5	2.59	0.73
12	-3	-5	2.66	0.49
12	-3	-5	3.43	0.68
-12	3	-5	2.52	0.53
12	-3	-5	3.35	0.60
-12	3	-5	2.29	0.70
12	3	5	1.41	0.38
-12	3	-5	2.88	0.82
-12	3	-5	2.95	0.64
-12	-3	5	2.42	0.66
12	-3	-5	3.17	0.57
-12	-3	5	1.79	0.72
-12	-3	5	1.56	0.45
-12	-3	5	1.68	0.44
-12	-3	5	2.63	0.46
12	3	5	1.98	0.52
-12	3	-5	3.66	0.61
12	3	5	1.38	0.60
-12	3	-5	2.91	0.53
12	3	-5	3.30	0.34
12	3	-5	3.27	0.59
-12	3	5	2.09	0.73
12	-3	5	2.22	0.47
-12	-3	-5	3.36	0.57
-12	3	5	2.28	0.61
-12	3	5	1.39	0.51
-12	-3	-5	2.53	0.53
12	-3	5	2.17	0.68
12	-3	5	1.71	0.76
-12	-3	-5	3.84	0.67
-12	3	5	1.02	0.50
-12	-3	-5	3.23	0.73
-12	-3	-5	2.66	0.54
12	-3	5	1.34	0.41
12	3	-5	3.10	0.49
-12	3	5	2.29	0.45
-12	-3	-5	2.36	0.42
-12	-3	-5	2.89	0.56
12	-3	5	1.46	0.43
-12	-3	-5	2.56	0.50

-12	3	5	1.36	0.57
-12	4	-5	25.10	1.97
12	-4	-5	27.75	1.89
-12	-4	5	29.96	1.60
12	4	5	29.30	1.71
12	-4	-5	27.63	1.51
-12	-4	5	28.27	1.66
-12	-4	5	26.24	1.90
12	4	5	31.84	1.90
-12	4	-5	28.18	1.66
12	-4	-5	25.49	1.59
-12	4	-5	27.58	1.54
-12	-4	5	29.67	1.71
-12	-4	5	29.33	1.57
-12	-4	5	32.10	2.02
12	4	5	27.15	1.66
-12	4	-5	28.29	1.65
-12	4	-5	25.40	2.02
12	4	5	27.18	1.93
-12	4	-5	25.84	1.56
-12	-4	5	28.14	2.01
-12	4	-5	29.54	1.72
12	-4	-5	26.41	1.62
-12	-4	-5	27.65	1.68
-12	4	5	29.45	1.84
12	-4	5	29.10	1.69
-12	4	5	29.49	1.92
12	-4	5	28.79	1.70
12	4	-5	24.59	1.49
-12	4	5	29.57	1.69
12	4	-5	27.44	1.39
-12	-4	-5	27.18	1.42
12	4	-5	26.88	1.64
12	-4	5	28.67	2.02
12	-4	5	31.34	1.59
-12	4	5	29.83	2.05
-12	-4	-5	26.07	1.53
-12	-4	-5	26.65	1.48
-12	4	5	27.57	1.95
-12	-4	-5	26.63	1.64
-12	-4	-5	25.51	1.62
-12	4	5	28.56	1.79
-12	-4	-5	27.20	1.93
-12	-4	-5	26.45	1.48

-12	-5	5	17.61	1.33
-12	5	-5	31.24	1.88
-12	-5	5	18.62	1.27
-12	-5	5	19.60	1.18
-12	5	-5	28.65	1.69
12	-5	-5	30.37	1.82
-12	5	-5	28.68	2.28
-12	-5	5	19.03	1.15
12	-5	-5	28.22	1.69
12	-5	-5	30.10	1.85
12	5	5	18.98	1.61
-12	5	-5	34.17	2.15
-12	-5	5	19.33	1.69
-12	5	-5	29.20	2.24
12	5	5	19.25	1.33
-12	5	-5	30.04	1.91
12	5	5	17.38	1.32
-12	-5	5	18.95	1.67
-12	5	-5	28.78	1.76
-12	-5	5	17.84	1.59
12	5	5	21.19	1.57
-12	-5	-5	38.62	3.06
-12	5	5	17.59	1.74
12	5	-5	29.59	1.81
-12	-5	-5	31.24	1.63
-12	5	5	17.29	1.65
-12	5	5	21.66	1.66
-12	-5	-5	32.39	1.70
-12	5	5	22.00	2.08
12	-5	5	18.58	1.32
12	-5	5	20.96	1.90
-12	-5	-5	33.21	1.88
-12	5	5	19.84	1.33
-12	-5	-5	29.51	1.82
12	5	-5	31.90	1.66
-12	5	5	19.46	1.70
12	-5	5	19.68	1.12
12	-5	5	16.78	1.28
12	5	-5	31.54	1.82
-12	-6	5	48.93	2.41
12	-6	-5	49.94	2.88
12	-6	-5	43.41	2.46
-12	6	-5	48.41	2.94
-12	6	-5	46.41	2.51

12	-6	-5	43.78	2.51
12	6	5	45.58	2.64
-12	-6	5	49.79	2.38
-12	-6	5	47.50	2.87
-12	6	-5	41.89	2.29
12	6	5	48.31	2.79
-12	6	-5	41.84	2.40
-12	-6	5	47.50	2.60
12	-6	-5	41.94	2.35
12	6	5	47.64	2.55
-12	-6	5	45.00	2.83
12	6	5	48.53	2.90
-12	6	-5	43.86	3.04
-12	-6	-5	43.69	2.46
12	6	-5	45.70	2.44
-12	6	5	49.48	2.91
-12	-6	-5	49.74	2.56
12	6	-5	45.56	2.39
-12	6	5	46.21	2.94
-12	-6	-5	46.10	2.38
12	-6	5	51.39	3.23
-12	6	5	45.51	2.58
12	-6	5	44.95	2.54
12	6	-5	43.06	2.52
12	6	-5	45.61	2.45
12	-6	5	45.91	2.64
12	-6	5	50.77	2.35
12	7	5	12.81	1.53
12	-7	-5	9.27	1.57
-12	7	-5	7.14	1.48
12	7	5	12.13	1.18
-12	-7	5	12.37	1.17
-12	-7	5	9.20	0.79
-12	7	-5	8.67	0.87
12	-7	-5	5.94	1.01
-12	-7	5	13.20	1.66
-12	-7	5	11.58	1.50
-12	-7	5	11.85	0.96
12	-7	-5	5.61	0.84
12	7	5	12.91	1.50
-12	-7	5	12.31	0.97
12	7	5	12.69	1.27
12	7	-5	6.96	0.94
12	-7	5	13.02	2.07

12	-7	5	12.75	0.83
-12	-7	-5	9.79	1.11
-12	-7	-5	8.70	1.05
-12	7	5	10.02	1.71
-12	7	5	9.78	1.80
12	7	-5	6.32	1.16
12	7	-5	5.10	0.95
12	-7	5	10.68	1.06
12	-7	5	10.62	1.17
-12	-7	-5	5.41	1.01
-12	-8	5	13.94	1.87
12	8	5	19.23	1.92
12	-8	-5	14.48	1.28
12	-8	-5	16.71	1.39
-12	8	-5	13.39	2.31
12	-8	-5	20.87	2.27
12	8	5	14.76	1.81
-12	8	-5	20.21	2.21
12	8	5	14.79	1.33
-12	-8	5	17.58	1.09
-12	-8	5	17.35	1.43
-12	-8	5	14.27	1.09
12	8	5	17.41	1.68
-12	-8	5	16.91	1.88
-12	8	5	15.83	1.90
12	8	-5	16.03	1.31
-12	8	5	15.79	2.05
12	8	-5	16.63	1.39
-12	-8	-5	19.79	1.59
12	8	-5	16.85	1.79
12	-8	5	13.68	1.38
-12	-8	-5	15.58	1.42
12	-8	5	13.08	1.29
-12	-8	-5	17.22	1.42
-12	9	-5	19.70	2.46
-12	-9	5	22.79	1.67
-12	-9	5	16.66	2.00
12	-9	-5	20.14	1.52
12	-9	-5	20.22	1.58
12	9	5	17.02	1.89
12	9	5	16.48	2.01
12	-9	-5	19.78	1.77
12	9	-5	16.93	1.94
12	9	-5	23.35	1.62

12	-9	5	17.55	1.50
12	-9	5	15.27	1.50
-12	-9	-5	21.12	1.76
12	9	-5	19.85	1.51
-12	9	5	20.07	2.19
-12	-9	-5	20.40	1.63
12	-10	-5	12.78	1.34
12	10	5	9.91	2.09
12	-10	-5	12.65	1.36
-12	10	-5	12.08	2.13
-12	-10	5	9.00	1.60
12	-10	-5	11.91	1.64
12	10	5	11.04	1.78
-12	-10	5	9.75	1.78
-12	-10	-5	12.29	1.41
-12	-10	-5	11.88	1.68
-12	10	5	13.85	2.22
12	10	-5	12.63	1.61
12	-10	5	13.34	1.54
12	10	-5	11.01	2.12
12	-10	5	10.27	1.47
-12	11	-5	6.54	1.78
12	-11	-5	6.58	1.32
12	11	5	5.59	1.43
12	-11	-5	5.49	1.16
12	-11	-5	9.09	1.43
-12	-11	5	5.41	1.12
-12	-11	5	3.23	1.27
12	-11	5	3.15	0.87
12	-11	5	4.26	1.16
-12	-11	-5	7.22	1.49
-12	11	5	3.30	1.75
12	11	-5	5.23	1.39
-12	-11	-5	6.93	1.26
-12	-11	-5	8.65	1.58
-13	0	5	-0.09	0.46
-13	0	5	-0.44	0.38
-13	0	5	0.70	0.64
-13	0	5	-0.28	0.40
-13	0	-5	0.39	0.27
13	0	5	0.17	0.32
-13	0	-5	-0.29	0.49
13	0	-5	0.38	0.29
13	0	5	0.12	0.24

13	0	-5	-0.24	0.29
-13	0	-5	-0.12	0.43
-13	0	-5	0.28	0.35
-13	0	-5	-0.26	0.48
13	0	5	-0.14	0.49
13	0	5	0.40	0.39
-13	0	-5	0.38	0.40
-13	0	-5	0.66	0.45
-13	0	5	0.43	0.46
13	0	5	0.27	0.64
13	0	-5	0.20	0.19
13	0	-5	0.04	0.21
-13	0	5	-0.19	0.32
-13	-1	5	25.21	1.62
13	-1	-5	26.53	1.48
13	1	5	28.45	1.74
13	1	5	25.65	1.57
-13	1	-5	26.25	1.62
-13	1	-5	24.94	1.56
-13	1	-5	27.40	1.60
-13	1	-5	24.49	1.77
13	1	5	26.93	1.44
-13	-1	5	24.91	1.64
-13	1	-5	27.37	1.65
-13	-1	5	29.08	1.87
13	1	5	26.72	1.64
13	-1	-5	27.71	1.49
-13	-1	5	26.25	1.47
13	-1	-5	25.72	1.55
-13	-1	5	27.28	1.71
-13	1	-5	26.70	1.46
13	-1	-5	26.03	1.39
13	1	5	24.62	1.66
-13	1	-5	28.75	1.69
-13	-1	5	26.74	1.57
-13	-1	5	27.35	1.51
-13	-1	-5	24.53	1.68
-13	1	5	25.46	1.84
13	-1	5	24.88	1.56
-13	-1	-5	27.35	1.60
13	-1	5	25.30	1.44
-13	-1	-5	27.57	1.62
-13	-1	-5	25.78	1.59
-13	1	5	25.78	1.62

13	1	-5	26.33	1.51
-13	-1	-5	29.00	1.77
13	-1	5	28.82	1.92
13	-1	5	27.63	1.74
13	-1	5	29.41	1.62
-13	1	5	28.19	1.52
-13	1	5	25.24	1.52
-13	1	5	25.44	1.71
-13	-1	-5	27.43	1.45
-13	1	5	25.19	1.67
-13	-1	-5	28.18	1.36
13	-2	-5	111.72	5.29
-13	2	-5	113.61	5.38
-13	-2	5	108.51	5.17
-13	2	-5	112.07	5.51
-13	-2	5	114.72	5.39
-13	-2	5	101.03	5.12
13	2	5	107.12	5.38
-13	-2	5	102.08	5.14
13	2	5	104.73	5.12
-13	-2	5	118.13	5.43
13	2	5	119.00	5.34
-13	2	-5	103.33	5.37
13	2	5	110.46	5.22
-13	-2	5	105.69	5.25
-13	-2	5	108.44	5.34
-13	2	-5	120.61	5.46
-13	-2	5	118.52	5.20
13	-2	-5	115.03	5.39
13	-2	-5	112.94	5.40
-13	2	-5	109.75	5.64
13	-2	-5	114.67	5.32
-13	2	-5	119.01	5.34
13	2	5	105.41	5.26
-13	2	5	112.58	5.16
-13	2	5	110.18	5.46
-13	2	5	106.59	5.04
13	-2	5	113.62	5.21
-13	-2	-5	111.06	5.41
13	-2	5	115.59	5.52
-13	-2	-5	115.68	5.21
-13	2	5	109.98	5.28
13	-2	5	108.43	5.36
13	-2	5	107.86	5.16

-13	2	5	106.60	5.39
-13	-2	-5	119.22	5.18
-13	-2	-5	114.17	5.43
-13	-2	-5	108.07	5.40
-13	-2	-5	113.55	5.43
-13	-2	-5	114.19	5.57
-13	2	5	112.22	5.08
-13	2	5	103.24	5.10
13	-2	5	107.94	5.24
-13	2	5	111.02	5.10
-13	3	-5	92.81	4.76
-13	3	-5	96.83	4.50
13	-3	-5	92.89	4.37
13	-3	-5	103.69	4.60
13	3	5	104.32	5.19
13	-3	-5	91.11	4.47
-13	-3	5	103.81	5.44
-13	-3	5	113.46	5.49
-13	-3	5	117.06	5.22
-13	-3	5	119.24	5.50
-13	3	-5	88.09	4.68
-13	3	-5	86.70	4.43
13	3	5	112.92	5.27
-13	3	-5	95.51	4.44
13	3	5	119.28	5.37
-13	-3	5	103.23	5.16
-13	-3	5	108.65	5.23
-13	-3	5	104.20	5.21
13	3	5	111.96	5.48
-13	3	-5	91.68	4.50
13	-3	-5	85.49	4.38
-13	3	-5	90.41	4.41
-13	-3	-5	95.36	4.25
-13	3	5	111.56	5.53
13	3	-5	90.60	4.24
-13	-3	-5	96.07	4.22
-13	-3	-5	92.01	4.48
-13	-3	-5	86.13	4.40
-13	3	5	114.28	5.22
-13	-3	-5	80.78	4.60
13	-3	5	104.23	5.44
13	-3	5	106.55	5.24
13	-3	5	112.49	5.21
-13	3	5	111.72	5.35

-13	-3	-5	94.48	4.68
13	3	-5	91.10	4.17
13	-3	5	110.05	5.29
-13	3	5	106.82	5.48
-13	-3	-5	88.78	4.47
13	3	-5	93.77	4.51
-13	3	5	110.28	5.31
-13	-3	-5	93.60	4.48
-13	3	5	108.88	5.22
13	-3	5	115.49	5.63
-13	4	-5	146.58	6.62
13	4	5	139.43	6.27
-13	-4	5	127.35	6.39
-13	4	-5	137.57	6.93
13	4	5	126.65	6.40
-13	-4	5	129.29	6.41
13	-4	-5	146.62	6.77
-13	-4	5	121.18	6.12
-13	-4	5	137.82	6.16
13	4	5	125.42	6.13
13	-4	-5	141.06	6.59
-13	-4	5	142.17	6.39
-13	4	-5	133.88	6.56
-13	-4	5	135.19	6.07
13	4	5	128.56	6.14
13	-4	-5	139.99	6.52
-13	-4	5	124.37	6.04
-13	4	-5	138.78	6.64
13	-4	-5	137.52	6.62
-13	4	-5	144.47	6.64
-13	4	-5	139.99	6.91
13	-4	5	134.34	6.05
-13	4	5	128.18	6.19
13	-4	5	126.71	6.36
-13	4	5	128.84	6.44
-13	4	5	131.68	6.43
-13	-4	-5	132.22	6.72
13	4	-5	131.35	6.45
13	-4	5	131.81	6.19
-13	-4	-5	137.70	6.83
13	4	-5	143.33	6.38
-13	4	5	130.51	6.12
-13	-4	-5	135.55	6.62
13	4	-5	145.83	6.63

-13	-4	-5	140.13	6.45
13	-4	5	126.62	6.18
-13	-4	-5	138.77	6.36
-13	-4	-5	140.90	6.64
-13	-4	-5	143.04	6.40
-13	4	5	126.90	6.28
-13	4	5	130.44	6.25
-13	-4	-5	136.88	6.60
13	5	5	135.68	6.79
-13	-5	5	137.47	6.77
-13	5	-5	146.38	7.39
-13	-5	5	150.10	6.66
-13	5	-5	144.67	6.96
13	5	5	135.51	7.04
13	-5	-5	146.10	7.02
13	5	5	143.71	6.76
13	-5	-5	149.04	6.92
-13	5	-5	141.46	6.82
-13	-5	5	138.69	6.64
-13	5	-5	152.44	7.40
-13	-5	5	141.56	7.05
-13	-5	5	135.29	6.77
13	-5	-5	150.62	7.03
-13	5	-5	150.66	7.06
-13	-5	5	153.57	7.00
13	-5	-5	152.00	7.23
13	5	5	153.30	6.91
-13	-5	-5	147.94	7.00
-13	5	5	140.58	7.06
13	5	-5	153.10	6.83
13	5	-5	139.74	6.92
-13	-5	-5	144.94	7.05
13	-5	5	150.82	6.63
13	-5	5	140.92	6.83
13	5	-5	154.95	7.00
-13	-5	-5	147.93	6.81
13	-5	5	139.57	6.81
-13	5	5	144.77	6.77
-13	5	5	143.30	7.02
13	-5	5	160.64	7.33
13	6	5	58.36	3.44
13	-6	-5	57.31	3.06
-13	6	-5	60.32	3.59
13	-6	-5	56.75	3.03

13	-6	-5	56.63	2.90
-13	-6	5	59.44	2.99
-13	-6	5	59.52	3.52
-13	6	-5	57.11	2.96
13	6	5	57.84	3.79
-13	-6	5	61.74	3.47
-13	6	-5	51.78	2.78
-13	6	-5	54.03	3.79
-13	-6	5	56.98	3.17
-13	-6	5	65.84	3.01
13	6	5	61.82	3.15
-13	6	5	58.83	3.19
-13	-6	-5	60.50	2.85
13	-6	5	59.07	3.18
13	6	-5	59.04	2.97
13	-6	5	67.37	3.86
13	6	-5	53.24	3.04
-13	6	5	59.60	3.54
-13	-6	-5	56.95	3.01
13	-6	5	66.41	2.95
-13	6	5	60.75	3.55
-13	-6	-5	53.71	3.25
13	-6	5	61.13	3.25
13	6	-5	58.70	2.87
13	-7	-5	4.69	1.20
-13	7	-5	3.13	1.35
13	7	5	0.81	0.89
-13	7	-5	3.07	0.65
-13	-7	5	1.57	0.51
-13	-7	5	0.19	0.87
13	7	5	2.28	0.74
-13	7	-5	3.57	1.12
13	7	5	0.86	0.83
-13	-7	5	2.32	0.72
13	-7	-5	1.94	0.56
13	-7	-5	2.17	0.66
13	7	5	1.22	0.63
-13	-7	5	1.27	0.46
-13	-7	5	2.78	1.06
13	-7	5	1.83	0.57
13	7	-5	2.46	0.62
-13	-7	-5	3.38	0.68
-13	7	5	2.94	1.20
-13	-7	-5	2.98	0.78

13	-7	5	2.17	1.39
13	7	-5	2.98	0.65
13	7	-5	2.34	0.77
13	-7	5	1.57	0.64
-13	-7	-5	3.66	0.77
-13	7	5	2.09	0.92
13	-8	-5	27.64	1.89
13	8	5	21.96	1.90
13	-8	-5	35.19	2.50
-13	-8	5	21.53	1.40
-13	8	-5	23.68	2.89
13	-8	-5	26.24	1.75
13	-8	-5	28.34	2.03
-13	-8	5	26.77	2.16
13	8	5	19.90	2.12
-13	-8	5	22.62	1.30
-13	-8	5	21.49	1.71
13	8	5	24.62	2.12
-13	8	-5	28.88	2.60
-13	-8	5	23.75	2.19
-13	8	-5	23.39	1.78
13	8	-5	28.89	1.77
-13	-8	-5	28.13	1.89
-13	-8	-5	28.27	1.78
13	-8	5	24.63	1.68
-13	8	5	26.94	2.31
13	8	-5	30.44	1.96
13	-8	5	23.46	2.62
-13	-8	-5	27.83	2.00
13	8	-5	24.39	2.08
13	-9	-5	19.32	1.82
13	-9	-5	19.35	1.56
-13	-9	5	22.50	1.81
13	9	5	19.74	2.38
13	-9	-5	18.68	1.60
-13	9	-5	20.82	2.55
-13	-9	5	25.29	2.42
13	9	5	23.45	2.17
-13	-9	-5	21.96	1.95
13	9	-5	22.12	1.51
13	-9	5	23.48	1.76
13	9	-5	18.35	2.17
13	-9	5	20.82	1.82
-13	-9	-5	19.04	1.63

-13	9	5	20.40	2.36
13	9	-5	21.11	1.97
-13	10	-5	26.52	1.83
13	10	5	26.36	2.65
-13	-10	5	27.78	2.22
13	-10	-5	25.26	1.85
13	-10	-5	21.78	1.91
13	-10	-5	23.21	2.08
-13	-10	5	29.49	2.69
13	10	5	24.25	2.44
-13	10	-5	27.05	3.09
13	10	-5	22.27	2.43
-13	-10	-5	25.20	1.96
13	-10	5	28.75	2.25
-13	-10	-5	24.53	2.11
-13	10	5	31.26	3.07
13	-10	5	26.64	2.00
13	-11	-5	49.83	2.87
13	-11	-5	47.46	2.89
-13	-11	5	43.21	3.27
-13	11	-5	52.45	2.82
13	11	5	43.34	3.23
-13	11	-5	52.75	3.82
13	-11	-5	44.46	3.20
-13	-11	5	44.59	2.82
-13	-11	-5	39.07	3.43
-13	11	5	43.92	3.55
-13	-11	-5	47.90	3.46
13	-11	5	38.53	2.87
13	-11	5	43.90	2.72
-13	-11	-5	49.60	3.08
13	11	-5	44.20	3.39
14	0	5	103.78	4.89
14	0	-5	95.88	4.55
-14	0	-5	97.88	4.72
-14	0	-5	98.65	4.64
-14	0	5	97.75	4.73
14	0	-5	98.47	4.41
-14	0	5	97.47	4.64
14	0	5	93.00	4.58
-14	0	5	94.15	4.72
-14	0	5	95.64	4.93
-14	0	5	96.05	4.83
-14	0	-5	95.35	4.50

14	0	5	92.19	4.72
14	0	5	99.55	4.79
14	0	5	103.11	4.80
-14	0	-5	95.21	4.64
-14	0	-5	95.91	4.78
-14	0	5	101.68	4.76
-14	0	-5	100.05	4.67
14	0	-5	88.66	4.36
14	0	-5	90.58	4.57
-14	0	-5	94.86	4.42
-14	-1	5	2.20	0.53
-14	-1	5	2.71	0.67
14	-1	-5	0.72	0.29
-14	-1	5	1.63	0.46
14	-1	-5	1.27	0.41
-14	-1	5	1.72	0.76
-14	1	-5	1.24	0.48
-14	1	-5	1.60	0.41
14	1	5	2.33	0.62
-14	1	-5	2.03	0.54
-14	-1	5	1.85	0.43
-14	1	-5	1.88	0.63
-14	-1	-5	1.43	0.53
-14	-1	-5	2.13	0.54
-14	-1	-5	2.12	0.40
14	-1	5	2.87	0.50
-14	1	5	1.97	0.45
14	-1	5	2.11	0.70
-14	1	5	1.93	0.81
-14	1	5	2.24	0.59
-14	-1	-5	2.47	0.70
14	-1	5	1.66	0.46
-14	1	5	1.94	0.59
-14	-1	-5	1.52	0.36
-14	1	5	2.49	0.60
-14	-2	5	16.01	1.17
-14	2	-5	15.41	1.22
-14	-2	5	15.70	1.54
-14	2	-5	16.49	1.12
14	2	5	16.55	1.31
-14	2	-5	14.40	1.22
-14	-2	5	18.50	1.13
-14	-2	5	17.79	1.10
14	2	5	14.62	1.36

14	-2	-5	15.56	1.04
14	2	5	15.36	1.23
-14	-2	5	17.98	1.49
-14	2	-5	16.62	1.28
-14	2	-5	16.36	1.18
-14	-2	5	16.77	1.20
-14	-2	5	17.55	1.28
14	-2	-5	17.86	1.20
-14	2	-5	15.96	1.52
-14	2	-5	17.23	1.48
14	-2	-5	19.85	1.12
14	2	5	14.57	1.04
14	-2	5	16.07	1.56
-14	-2	-5	18.04	1.50
-14	2	5	16.84	1.45
-14	-2	-5	16.99	1.22
-14	2	5	17.91	1.17
-14	-2	-5	17.27	0.89
14	-2	5	15.80	1.48
-14	2	5	17.33	1.60
14	-2	5	16.02	1.19
-14	-2	-5	15.70	1.28
14	-2	5	16.42	1.17
-14	2	5	15.70	1.33
-14	2	5	17.97	1.12
-14	-2	-5	16.03	0.91
14	-2	5	14.73	1.08
-14	2	5	20.37	1.09
-14	-2	-5	17.52	1.23
-14	-2	-5	14.20	1.23
-14	-2	-5	17.26	1.29
-14	3	-5	4.61	0.85
-14	3	-5	4.69	0.94
-14	3	-5	4.13	0.74
-14	-3	5	5.64	0.65
14	-3	-5	3.77	0.64
14	-3	-5	4.70	0.84
-14	-3	5	6.23	0.81
-14	-3	5	6.07	0.73
-14	-3	5	6.00	0.99
-14	3	-5	3.13	0.65
-14	-3	5	4.81	0.78
-14	-3	5	4.46	0.81
-14	-3	5	6.55	1.04

14	-3	-5	4.94	0.63
14	3	5	5.39	1.04
-14	3	-5	3.71	0.78
14	3	5	4.36	0.64
14	-3	-5	4.09	0.71
-14	3	-5	2.94	0.56
-14	-3	5	4.96	1.01
14	3	5	4.92	0.80
14	3	5	5.01	0.85
-14	-3	-5	4.15	0.42
-14	3	5	4.02	0.80
-14	-3	-5	4.20	0.46
-14	3	5	5.40	0.99
-14	3	5	5.91	0.98
14	-3	5	4.40	0.73
-14	3	5	4.49	0.92
-14	-3	-5	4.16	0.69
-14	-3	-5	3.58	0.67
-14	3	5	4.04	0.82
14	-3	5	5.84	1.19
14	-3	5	5.22	0.96
-14	-3	-5	3.81	0.72
14	-3	5	5.90	0.73
-14	-3	-5	3.72	0.67
14	-3	5	3.58	0.66
-14	-3	-5	2.57	0.81
-14	3	5	6.01	0.81
-14	-3	-5	4.53	0.74
14	4	5	10.58	1.10
-14	-4	5	8.33	0.86
-14	-4	5	11.36	1.29
-14	-4	5	7.75	1.42
14	-4	-5	12.21	1.02
-14	4	-5	11.64	1.63
14	4	5	8.55	1.00
-14	-4	5	6.64	0.84
-14	4	-5	11.84	1.08
-14	-4	5	8.82	0.96
-14	-4	5	8.91	0.80
-14	4	-5	12.97	1.54
-14	-4	5	8.45	1.35
14	-4	-5	12.33	0.95
14	-4	-5	14.36	1.41
14	4	5	7.97	0.84

14	-4	-5	11.61	1.05
-14	4	-5	11.97	1.06
14	4	5	9.28	1.25
-14	-4	-5	13.71	1.13
14	4	-5	12.19	1.12
-14	4	5	10.60	1.30
-14	-4	-5	12.35	1.00
-14	4	5	7.30	0.96
14	-4	5	9.55	0.92
14	4	-5	12.54	0.93
14	4	-5	12.60	0.78
-14	-4	-5	13.35	0.81
-14	-4	-5	11.78	0.75
14	-4	5	8.49	0.87
-14	-4	-5	11.31	1.09
-14	-4	-5	12.68	1.07
-14	4	5	8.65	1.40
14	-4	5	9.13	0.92
-14	-5	5	33.28	2.08
14	5	5	36.16	2.46
-14	-5	5	36.89	2.38
-14	5	-5	38.93	2.09
14	-5	-5	41.69	2.60
-14	-5	5	33.79	1.93
14	-5	-5	38.10	2.26
-14	5	-5	38.74	2.20
-14	-5	5	36.75	1.98
14	5	5	36.63	2.10
-14	5	-5	41.63	2.85
-14	-5	5	34.51	2.07
14	5	5	34.91	2.09
14	-5	-5	38.15	2.25
-14	5	-5	40.81	2.76
-14	-5	5	37.27	2.47
14	5	5	37.32	2.22
14	-5	-5	39.33	2.14
14	5	-5	38.62	2.23
-14	5	5	38.45	2.47
-14	-5	-5	41.55	2.33
14	-5	5	35.58	2.10
-14	5	5	36.29	2.48
14	5	-5	39.50	2.03
14	-5	5	37.32	2.11
14	-5	5	40.41	2.82

14	5	-5	37.11	2.14
-14	-5	-5	41.10	2.28
14	-5	5	37.83	1.91
-14	5	5	37.50	2.13
-14	-5	-5	40.88	2.01
14	-6	-5	2.28	0.55
-14	6	-5	2.47	0.60
14	6	5	1.88	0.81
14	6	5	1.10	0.61
-14	-6	5	1.11	0.51
-14	-6	5	1.20	0.77
14	-6	-5	1.52	0.54
14	6	5	1.20	0.68
-14	-6	5	-0.17	0.98
-14	6	-5	2.48	1.28
14	-6	5	1.55	0.41
14	6	-5	1.72	0.50
-14	-6	-5	1.34	0.49
14	-6	5	0.68	1.15
14	-6	5	1.03	0.59
14	-6	5	1.17	0.52
-14	-6	-5	2.42	0.69
-14	6	5	1.27	0.83
-14	6	5	1.97	1.12
14	6	-5	2.04	0.58
14	-7	-5	1.89	0.55
14	-7	-5	1.82	0.62
-14	-7	5	1.83	0.61
14	7	5	2.25	0.99
-14	-7	5	1.66	1.16
14	-7	-5	1.22	0.69
14	-7	-5	2.49	1.14
14	7	5	1.96	0.77
-14	7	-5	3.37	1.13
-14	7	-5	0.82	1.29
-14	-7	5	3.15	1.06
-14	-7	5	1.23	0.55
14	7	-5	1.26	0.60
14	-7	5	1.39	1.25
-14	-7	-5	2.20	0.71
-14	-7	-5	1.53	0.56
-14	7	5	2.64	1.21
14	7	-5	2.16	0.79
14	-7	5	0.69	0.61

-14	7	5	2.03	1.05
14	7	-5	0.92	0.58
14	-7	5	1.35	0.73
-14	-8	5	5.02	0.96
-14	8	-5	4.63	0.79
14	-8	-5	4.13	0.83
-14	8	-5	5.01	1.39
14	-8	-5	4.64	0.74
-14	-8	5	5.12	1.26
14	8	5	3.92	0.97
14	-8	-5	4.60	0.93
14	8	5	3.31	1.16
-14	-8	5	6.07	0.82
14	-8	5	3.67	0.87
-14	-8	-5	3.57	0.87
14	8	-5	4.10	1.03
14	8	-5	5.40	1.01
14	8	-5	3.90	0.80
-14	8	5	4.25	1.29
-14	-8	-5	3.27	0.81
14	-8	5	3.07	0.75
-14	-9	5	15.35	2.25
14	9	5	10.48	1.82
-14	9	-5	9.62	1.27
14	-9	-5	11.07	1.24
14	9	5	13.77	1.96
-14	-9	5	15.13	1.53
14	-9	-5	10.49	1.54
-14	9	-5	9.42	1.87
14	-9	-5	9.42	1.29
14	9	-5	10.12	1.92
14	9	-5	10.15	1.14
-14	9	5	16.16	2.35
14	-9	5	10.04	1.45
-14	-9	-5	10.72	1.43
-14	-9	-5	8.86	1.56
14	-9	5	13.73	1.64
-14	10	-5	11.19	1.41
-14	-10	5	9.47	1.60
14	10	5	11.30	1.76
14	10	5	10.08	1.82
14	-10	-5	15.26	1.92
-14	-10	5	11.35	1.75
14	-10	-5	9.84	1.49

14	-10	-5	12.31	1.54
-14	10	-5	13.24	2.12
14	10	-5	11.76	1.86
14	-10	5	11.12	1.40
14	-10	5	11.20	1.64
-14	-10	-5	13.72	1.69
-14	10	5	14.09	2.10
-14	-11	5	6.93	1.54
14	-11	-5	7.76	1.34
-14	11	-5	6.63	1.18
14	11	5	3.88	1.40
-14	-11	5	6.35	1.20
14	-11	-5	6.40	1.03
-14	11	-5	4.21	1.73
14	-11	-5	8.48	1.75
14	11	-5	6.22	1.50
-14	-11	-5	8.93	1.57
-14	11	5	5.18	1.65
14	-11	5	5.66	1.39
14	-11	5	5.35	1.04
-14	-11	-5	3.98	1.40
-15	0	-5	0.60	0.52
-15	0	-5	0.23	0.42
-15	0	-5	0.63	0.64
-15	0	5	0.49	0.54
15	0	-5	0.55	0.36
15	0	-5	-0.35	0.32
15	0	5	-0.37	0.38
15	0	5	-0.59	0.59
15	0	5	0.18	0.57
-15	0	-5	0.18	0.60
-15	0	5	0.05	0.48
-15	0	5	-0.67	0.74
-15	0	-5	0.33	0.48
15	0	5	0.59	0.48
-15	0	5	0.38	0.42
-15	0	5	-0.86	0.46
15	0	-5	0.06	0.21
15	0	5	0.28	0.28
-15	0	5	-0.09	0.54
-15	0	-5	0.47	0.45
-15	0	-5	-0.12	0.30
15	0	-5	0.26	0.26
-15	1	-5	21.85	1.75

-15	1	-5	23.68	1.49
-15	1	-5	21.49	1.51
15	1	5	17.08	1.23
15	-1	-5	22.30	1.36
-15	1	-5	21.16	1.45
15	1	5	14.75	1.32
15	-1	-5	22.20	1.21
15	-1	-5	22.94	1.43
15	1	5	16.08	1.04
-15	-1	5	17.06	1.18
15	-1	-5	23.02	1.32
-15	-1	5	15.15	1.25
-15	-1	5	17.89	1.63
-15	-1	5	16.21	1.12
-15	-1	5	16.23	1.31
-15	-1	5	18.91	1.45
-15	1	-5	22.24	1.54
15	1	5	16.65	1.37
-15	-1	5	16.00	1.10
-15	1	-5	22.84	1.28
-15	-1	5	15.14	1.33
15	1	5	17.39	1.45
-15	-1	-5	22.57	1.16
-15	1	5	16.97	1.25
15	-1	5	15.64	1.05
-15	-1	-5	23.96	1.69
-15	1	5	16.81	1.67
-15	1	5	17.25	1.18
-15	-1	-5	21.55	1.47
-15	-1	-5	21.10	1.50
-15	1	5	16.09	1.42
15	-1	5	14.79	1.18
-15	1	5	17.63	1.33
-15	1	5	13.73	1.15
-15	-1	-5	21.46	1.61
15	-1	5	17.09	2.11
15	-1	5	16.17	1.24
-15	-1	-5	22.17	1.13
-15	-1	-5	22.47	1.49
15	-1	5	15.19	1.46
-15	-2	5	27.69	1.87
-15	-2	5	25.60	1.60
-15	-2	5	27.76	1.69
-15	2	-5	24.80	1.85

15	2	5	27.44	1.89
-15	-2	5	26.12	1.61
-15	-2	5	29.41	1.94
-15	-2	5	27.90	1.71
-15	-2	5	27.45	1.79
-15	2	-5	26.27	1.96
-15	-2	5	28.00	2.05
15	-2	-5	23.81	1.44
15	-2	-5	24.23	1.52
15	-2	-5	23.94	1.39
15	2	5	28.64	1.57
-15	2	-5	24.87	1.60
15	-2	-5	23.54	1.54
15	2	5	28.97	1.87
-15	2	-5	23.64	1.54
15	-2	5	27.01	1.59
-15	2	5	27.34	1.84
-15	2	5	28.83	1.57
-15	2	5	28.43	1.62
15	-2	5	29.84	1.70
-15	-2	-5	24.62	1.56
-15	2	5	26.92	1.95
15	-2	5	32.75	2.17
-15	-2	-5	27.71	1.88
-15	-2	-5	21.53	1.55
-15	2	5	30.04	2.30
-15	-2	-5	25.06	1.67
15	-2	5	28.77	1.73
15	-2	5	28.53	2.00
-15	2	5	28.43	1.68
-15	-2	-5	23.07	1.64
-15	-2	-5	24.26	1.56
-15	-3	5	73.80	3.96
15	-3	-5	89.25	4.19
15	-3	-5	82.78	4.14
15	3	5	72.08	3.62
-15	3	-5	79.84	4.12
-15	-3	5	72.80	3.97
-15	-3	5	70.27	3.62
-15	-3	5	73.19	3.77
-15	3	-5	86.36	4.33
-15	-3	5	77.41	3.70
-15	-3	5	69.25	3.66
15	3	5	75.69	3.98

-15	3	-5	82.24	4.51
15	3	5	80.26	3.88
-15	-3	5	74.33	4.00
-15	-3	5	76.19	3.72
15	-3	-5	83.02	4.03
15	-3	-5	85.31	4.05
-15	3	-5	80.40	4.07
15	3	5	74.30	3.74
-15	3	5	74.70	3.68
15	-3	5	78.05	3.66
-15	-3	-5	83.40	4.16
15	-3	5	72.90	3.74
-15	3	5	73.96	3.69
15	-3	5	70.73	3.67
-15	-3	-5	81.57	4.14
-15	-3	-5	85.99	4.12
-15	-3	-5	83.78	4.38
-15	3	5	73.78	4.00
-15	-3	-5	79.28	4.10
15	-3	5	83.91	4.18
-15	-4	5	58.62	3.37
-15	-4	5	62.31	3.41
-15	4	-5	51.90	3.15
-15	4	-5	49.26	2.66
-15	-4	5	64.64	3.05
15	4	5	56.96	3.35
15	-4	-5	50.31	2.65
15	-4	-5	49.38	2.70
15	4	5	57.90	3.03
-15	4	-5	50.60	3.10
-15	-4	5	57.50	2.97
-15	-4	5	59.49	3.09
-15	4	-5	47.35	2.64
15	4	5	54.02	3.06
-15	-4	5	55.44	3.06
15	4	5	62.71	3.23
15	-4	-5	51.85	2.60
-15	-4	5	56.08	3.08
15	-4	-5	54.09	2.93
15	-4	5	65.49	3.59
15	4	-5	48.97	2.47
-15	-4	-5	51.10	2.63
15	-4	5	63.94	3.00
-15	4	5	58.94	3.43

15	-4	5	61.08	3.12
-15	4	5	58.27	3.24
15	-4	5	59.59	3.08
-15	-4	-5	49.19	2.71
15	4	-5	50.70	2.39
-15	4	5	61.64	3.09
-15	-4	-5	45.01	2.99
-15	-4	-5	50.05	2.74
-15	5	-5	75.09	3.72
-15	5	-5	78.05	3.89
-15	-5	5	65.39	3.18
-15	-5	5	61.33	3.27
-15	5	-5	76.21	4.35
15	5	5	65.22	3.43
-15	-5	5	59.46	3.23
15	5	5	60.57	3.27
-15	-5	5	60.15	3.13
15	-5	-5	78.89	3.83
15	-5	-5	75.27	3.96
-15	5	-5	67.55	4.65
-15	-5	5	71.21	3.67
-15	-5	5	62.10	3.61
15	-5	-5	85.84	4.22
-15	-5	-5	80.49	3.59
15	5	-5	72.81	3.78
15	-5	5	62.61	3.32
-15	5	5	60.44	3.27
-15	-5	-5	75.02	4.00
-15	-5	-5	76.65	3.93
-15	5	5	63.31	3.69
-15	-5	-5	77.89	3.73
15	-5	5	67.67	3.10
15	5	-5	81.03	3.68
15	-6	-5	53.99	2.81
-15	6	-5	47.98	3.44
-15	-6	5	53.07	3.21
-15	-6	5	51.59	2.83
-15	-6	5	52.23	2.65
15	6	5	46.95	2.81
15	-6	-5	50.86	2.91
-15	6	-5	52.07	2.86
15	-6	-5	58.90	3.33
-15	6	-5	50.22	3.58
15	6	5	56.70	3.01

15	6	5	49.40	3.18
15	-6	-5	56.42	2.97
-15	-6	5	47.73	3.16
-15	-6	5	48.55	2.60
15	6	-5	56.31	2.87
-15	6	5	50.07	3.22
-15	-6	-5	51.72	2.90
15	6	-5	56.01	2.73
15	6	-5	51.63	2.88
15	-6	5	47.68	2.77
-15	-6	-5	54.01	3.00
15	-6	5	51.86	2.83
-15	6	5	50.70	3.23
-15	-6	-5	53.64	2.61
15	-6	5	57.81	3.52
-15	-7	5	10.41	1.00
15	7	5	10.99	1.37
-15	-7	5	10.55	1.73
15	-7	-5	11.86	1.33
-15	-7	5	11.67	1.93
-15	-7	5	8.83	1.24
15	-7	-5	9.97	1.19
15	7	5	10.64	1.58
15	7	5	8.80	1.47
-15	-7	5	9.96	1.04
-15	7	-5	11.06	2.05
-15	7	-5	11.16	1.16
15	7	-5	10.44	1.16
-15	-7	-5	8.41	1.32
15	-7	5	10.39	1.19
15	7	-5	10.93	1.07
-15	7	5	8.46	1.40
15	-7	5	11.00	1.84
15	-7	5	10.38	1.17
15	7	-5	9.90	1.34
-15	-7	-5	9.16	0.96
-15	-7	-5	10.99	1.24
-15	8	-5	13.38	2.38
15	-8	-5	10.65	1.27
-15	-8	5	10.78	1.41
-15	-8	5	12.31	2.10
15	8	5	12.58	2.03
15	-8	-5	10.48	1.39
-15	8	-5	11.37	1.31

15	8	5	13.31	1.66
15	8	5	14.85	2.13
-15	-8	5	11.48	1.08
15	-8	-5	11.91	1.23
15	8	-5	11.27	1.67
15	8	-5	11.69	1.22
-15	8	5	15.44	2.08
15	8	-5	12.04	1.30
-15	-8	-5	11.68	1.54
15	-8	5	13.25	1.34
-15	-8	-5	12.16	1.45
15	-8	5	11.02	1.41
15	9	5	12.18	1.79
-15	-9	5	9.90	1.63
-15	9	-5	16.22	1.48
-15	9	-5	13.85	2.58
-15	-9	5	14.35	1.69
15	-9	-5	14.48	1.72
15	9	5	13.43	2.16
15	-9	-5	15.65	1.60
15	-9	-5	14.89	1.42
15	9	-5	13.15	1.90
-15	-9	-5	12.46	2.19
15	-9	5	11.70	1.51
15	-9	5	10.42	1.45
-15	9	5	12.65	2.54
-15	-9	-5	12.34	1.74
-15	10	-5	27.93	3.01
15	10	5	24.93	2.69
-15	-10	5	27.40	2.24
15	10	5	27.65	2.52
15	-10	-5	25.56	2.02
-15	10	-5	26.19	2.04
-15	-10	5	28.05	2.67
15	-10	-5	25.55	1.93
15	-10	-5	22.26	2.21
15	-10	5	32.81	2.46
-15	-10	-5	25.28	2.15
-15	10	5	29.58	2.98
15	10	-5	18.35	2.27
15	-10	5	29.80	2.07
-15	-10	-5	24.81	2.00
-16	0	5	95.43	4.63
-16	0	5	91.90	4.95

16	0	-5	94.54	4.22
16	0	5	91.37	4.70
-16	0	-5	94.31	4.70
16	0	-5	87.64	4.16
-16	0	-5	91.03	4.58
16	0	5	92.87	4.54
-16	0	5	96.72	4.73
-16	0	5	96.46	4.85
16	0	5	95.12	4.79
-16	0	-5	91.12	4.22
16	0	5	103.44	4.90
-16	0	5	99.74	4.74
16	0	-5	93.66	4.41
16	0	5	97.91	4.79
-16	0	5	94.83	4.70
-16	0	-5	85.36	4.17
16	1	5	20.15	1.58
16	1	5	19.09	1.18
16	1	5	17.64	1.37
16	-1	-5	15.86	1.04
-16	-1	5	18.96	1.26
16	-1	-5	12.45	0.88
16	-1	-5	12.67	1.11
-16	1	-5	14.94	1.19
-16	-1	5	17.27	1.80
16	1	5	19.43	1.51
-16	-1	5	20.48	1.61
16	-1	-5	13.73	1.05
-16	-1	5	17.23	1.27
16	1	5	23.89	1.68
-16	1	-5	14.15	1.18
-16	-1	5	21.42	1.52
-16	-1	5	19.27	1.53
-16	-1	5	18.86	1.34
-16	1	-5	16.26	1.57
-16	1	-5	14.94	1.32
-16	-1	5	19.08	1.43
-16	-1	-5	17.71	1.53
-16	1	5	19.73	1.43
-16	-1	-5	15.23	1.42
16	-1	5	17.01	1.17
-16	1	5	18.41	1.32
16	-1	5	18.17	1.67
-16	1	5	23.08	1.37

16	-1	5	18.98	1.37
16	-1	5	21.83	1.44
-16	-1	-5	15.15	1.21
-16	1	5	17.94	1.61
-16	-2	5	3.58	0.69
-16	2	-5	0.92	0.45
16	-2	-5	1.94	0.54
-16	-2	5	3.42	0.72
-16	-2	5	1.96	0.82
-16	2	-5	2.31	0.87
-16	-2	5	3.09	0.54
-16	-2	5	4.04	0.69
-16	-2	5	3.30	0.71
-16	-2	5	3.59	0.64
-16	-2	5	1.76	0.93
-16	2	-5	-0.26	0.69
16	2	5	3.68	0.86
16	-2	-5	0.29	0.41
16	-2	-5	1.63	0.45
16	-2	-5	0.29	0.34
16	2	5	2.70	0.57
-16	2	-5	1.09	0.61
-16	-2	-5	0.84	0.51
16	-2	5	2.25	0.80
-16	2	5	2.69	0.94
16	-2	5	3.08	0.62
-16	2	5	3.04	0.63
-16	2	5	4.35	0.76
-16	-2	-5	1.17	0.63
-16	2	5	3.11	0.81
16	-2	5	2.33	0.98
16	-2	5	2.88	0.61
-16	-2	-5	1.02	0.43
-16	-2	-5	0.87	0.68
-16	-2	-5	1.67	0.83
16	-2	5	3.06	0.57
16	3	5	9.23	1.15
-16	-3	5	11.15	1.08
-16	-3	5	9.80	1.09
-16	-3	5	11.32	1.31
-16	-3	5	10.19	1.56
16	3	5	8.06	0.85
-16	-3	5	9.92	0.95
16	-3	-5	8.38	0.95

16	-3	-5	8.03	0.82
-16	3	-5	8.91	1.05
-16	3	-5	5.90	0.93
16	3	5	9.07	1.28
-16	3	-5	8.64	1.46
-16	3	-5	8.34	1.58
16	-3	-5	7.47	1.09
-16	-3	5	8.79	1.06
16	-3	-5	8.04	0.84
-16	-3	5	11.00	1.44
-16	-3	5	9.71	0.91
-16	3	5	11.07	1.44
-16	3	5	7.33	1.04
-16	-3	-5	6.66	1.08
16	-3	5	9.12	0.93
-16	-3	-5	7.70	0.97
16	-3	5	10.41	1.57
16	-3	5	9.46	1.01
16	-3	5	8.83	0.95
-16	3	5	10.10	1.07
-16	-3	-5	7.41	0.98
-16	-3	-5	7.52	1.15
16	-4	-5	3.19	0.56
-16	-4	5	2.07	0.55
-16	-4	5	1.23	0.59
-16	4	-5	1.70	1.13
16	-4	-5	0.89	0.57
16	-4	-5	2.11	0.56
16	4	5	2.11	0.65
-16	-4	5	1.46	0.98
16	-4	-5	3.49	0.81
16	4	5	2.18	0.70
16	4	5	1.88	0.84
-16	-4	5	1.12	0.74
-16	4	-5	2.02	0.66
-16	-4	5	2.25	0.64
-16	4	-5	2.65	1.09
-16	-4	5	2.37	0.52
16	-4	5	1.87	0.51
-16	4	5	1.84	0.65
-16	-4	-5	2.63	0.91
-16	-4	-5	2.69	0.71
-16	-4	-5	2.10	0.61
-16	4	5	2.65	0.88

16	4	-5	1.35	0.34
16	4	-5	3.63	0.60
16	-4	5	1.14	1.12
-16	-4	-5	2.24	0.75
-16	4	5	1.07	0.92
-16	-5	5	5.36	0.99
16	5	5	8.41	1.15
-16	-5	5	4.86	0.96
16	5	5	6.07	1.13
-16	5	-5	6.38	0.92
-16	-5	5	4.72	0.81
-16	5	-5	4.74	0.77
16	5	5	6.88	0.92
-16	-5	5	5.46	0.80
16	-5	-5	3.87	0.75
16	-5	-5	4.21	0.86
16	-5	-5	5.21	0.74
-16	5	-5	5.51	1.40
-16	5	-5	5.79	1.27
-16	-5	5	7.76	1.32
16	-5	-5	4.82	1.03
-16	-5	5	6.89	1.33
-16	-5	-5	6.47	0.87
16	-5	5	7.04	0.84
16	5	-5	5.99	0.68
-16	-5	-5	8.17	1.06
16	-5	5	5.25	0.92
-16	-5	-5	5.85	1.06
-16	5	5	5.04	1.21
16	5	-5	3.86	0.80
16	-5	5	6.48	0.91
-16	-5	-5	5.62	0.99
-16	5	5	4.41	1.10
-16	6	-5	14.81	2.25
-16	-6	5	15.98	1.22
-16	-6	5	15.37	1.30
-16	-6	5	17.42	1.40
-16	-6	5	17.02	1.88
-16	6	-5	22.54	2.30
16	-6	-5	18.07	1.46
-16	-6	5	16.53	2.11
-16	6	-5	16.55	1.39
-16	-6	5	15.20	1.33
16	6	5	17.73	1.58

16	6	5	18.72	1.45
16	6	5	17.92	1.97
16	-6	-5	21.77	1.97
16	-6	-5	15.87	1.44
16	-6	-5	15.66	1.32
16	6	-5	15.13	1.21
-16	-6	-5	16.86	1.48
-16	6	5	16.13	2.02
-16	6	5	10.67	1.78
16	-6	5	13.35	1.37
16	6	-5	15.92	1.43
16	-6	5	16.65	1.45
-16	-6	-5	19.59	1.56
16	-6	5	22.48	2.31
16	-7	-5	12.66	1.44
-16	-7	5	9.55	1.17
16	7	5	13.50	1.87
-16	7	-5	15.89	1.39
-16	-7	5	12.40	1.38
16	7	5	13.86	1.64
16	7	5	11.88	1.37
16	-7	-5	14.54	1.40
16	-7	-5	15.24	1.25
-16	7	-5	19.52	2.27
-16	-7	5	11.68	1.10
-16	-7	5	14.77	1.89
16	7	-5	15.41	1.53
16	7	-5	12.14	1.23
-16	-7	-5	14.51	1.49
-16	-7	-5	16.41	1.53
16	-7	5	9.96	1.23
16	-7	5	9.56	1.30
-16	7	5	12.87	1.95
-16	8	-5	17.68	1.54
16	8	5	20.07	2.01
-16	-8	5	16.17	1.29
-16	-8	5	16.50	1.37
16	-8	-5	17.23	1.45
-16	-8	5	20.32	2.40
-16	8	-5	23.24	2.87
16	-8	-5	15.12	1.62
-16	-8	5	16.56	1.63
16	8	5	18.55	2.36
16	-8	-5	15.57	1.60

-16	-8	-5	13.60	1.77
16	-8	5	17.56	1.64
16	-8	5	16.90	1.69
-16	8	5	20.09	2.54
16	8	-5	16.64	1.92
16	8	-5	17.95	1.51
-16	-8	-5	17.94	1.83
16	-9	-5	26.71	1.99
-16	-9	5	26.06	2.30
16	9	5	30.14	2.39
-16	9	-5	28.81	2.05
16	-9	-5	25.13	2.27
16	-9	-5	27.75	1.92
-16	9	-5	24.93	2.99
16	9	5	30.25	2.71
-16	-9	5	33.05	2.70
-16	-9	-5	23.07	2.02
-16	-9	-5	27.12	2.15
16	9	-5	28.72	2.26
16	-9	5	28.89	2.07
16	-9	5	28.21	2.29
-16	9	5	28.62	2.96
-16	10	-5	2.82	0.94
16	10	5	3.63	1.41
16	-10	-5	0.63	0.80
16	-10	-5	3.12	0.92
-16	10	-5	2.86	1.77
-16	-10	5	1.69	0.90
16	10	5	2.12	1.22
16	-10	-5	2.76	1.04
-16	-10	5	0.77	1.37
-16	-10	-5	1.91	1.06
16	-10	5	2.18	0.89
-16	-10	-5	1.07	0.99
-16	10	5	6.61	1.66
16	10	-5	-0.10	1.17
16	-10	5	1.03	1.02
17	0	5	-0.37	0.75
17	0	5	-0.14	0.57
-17	0	-5	0.61	0.70
-17	0	5	-0.13	0.52
17	0	5	0.30	0.46
-17	0	-5	-0.20	0.44
17	0	-5	-0.60	0.37

17	0	-5	0.09	0.21
-17	0	5	-0.20	0.62
-17	0	5	0.58	0.45
17	0	5	0.20	0.33
17	0	-5	0.14	0.29
-17	0	5	-0.22	0.69
-17	0	5	0.09	0.51
17	0	5	-0.58	0.69
-17	0	-5	0.09	0.72
17	1	5	11.32	1.30
17	1	5	11.01	1.13
-17	-1	5	10.84	0.97
-17	-1	5	8.72	1.17
-17	-1	5	10.80	1.23
17	-1	-5	11.51	1.00
-17	-1	5	10.25	1.60
-17	1	-5	12.81	1.15
-17	-1	5	11.86	1.30
17	-1	-5	11.71	0.92
-17	-1	5	13.23	1.14
17	-1	-5	10.02	1.02
-17	-1	5	12.92	1.40
-17	1	-5	11.63	1.51
-17	-1	5	11.78	1.03
17	1	5	12.60	1.34
-17	1	-5	15.60	1.34
17	1	5	12.98	0.92
17	-1	-5	11.71	0.79
-17	1	-5	12.94	1.05
17	-1	5	9.41	1.43
-17	-1	-5	11.30	1.14
-17	-1	-5	11.31	1.41
-17	1	5	11.40	1.37
-17	-1	-5	11.44	1.40
17	-1	5	14.55	1.50
17	-1	5	12.69	1.19
17	-1	5	10.95	0.90
-17	1	5	11.81	1.23
-17	-1	-5	12.76	1.49
-17	1	5	10.11	1.10
-17	1	5	11.62	1.10
17	-1	5	11.53	1.12
17	-2	-5	34.23	2.01
-17	-2	5	25.20	1.59

17	-2	-5	35.87	1.93
-17	-2	5	26.38	1.74
-17	-2	5	26.66	1.90
17	2	5	28.08	1.97
-17	-2	5	26.00	1.70
17	2	5	28.83	1.87
17	-2	-5	31.57	1.79
-17	-2	5	25.47	2.01
-17	-2	5	23.92	1.81
17	-2	-5	35.89	1.96
17	2	5	25.57	1.51
-17	2	-5	34.48	2.43
-17	-2	5	22.50	2.13
-17	2	-5	33.43	2.09
-17	2	-5	34.88	2.06
-17	-2	5	25.71	1.60
-17	2	5	26.78	1.59
-17	2	5	24.13	1.67
-17	-2	-5	31.80	2.01
-17	-2	-5	31.37	2.02
17	-2	5	30.22	2.04
-17	-2	-5	29.03	2.08
-17	-2	-5	36.00	2.18
-17	2	5	27.10	2.05
17	-2	5	23.38	1.67
-17	2	5	24.96	1.67
17	-2	5	26.48	1.68
-17	3	-5	29.84	2.39
-17	-3	5	25.50	1.62
-17	-3	5	26.22	1.98
17	3	5	29.77	1.86
-17	3	-5	25.76	1.78
17	-3	-5	34.14	1.94
17	-3	-5	30.34	1.73
-17	-3	5	26.13	1.76
17	3	5	25.03	1.56
-17	3	-5	25.32	1.82
17	-3	-5	27.23	1.79
-17	3	-5	30.81	2.16
17	3	5	26.18	1.99
-17	-3	5	25.63	1.80
-17	-3	5	26.53	2.20
17	-3	-5	26.15	1.63
-17	-3	5	27.36	1.68

-17	-3	-5	30.87	1.94
-17	3	5	26.70	1.72
17	-3	5	29.74	1.67
-17	3	5	23.54	2.10
-17	-3	-5	29.73	1.85
-17	-3	-5	26.48	1.88
-17	-3	-5	30.02	1.87
17	-3	5	25.49	2.03
17	-3	5	25.67	1.60
17	-3	5	29.57	2.37
17	-4	-5	57.56	3.03
-17	-4	5	51.50	3.12
17	4	5	46.23	3.00
17	-4	-5	65.00	3.26
-17	-4	5	48.64	3.01
-17	4	-5	59.02	3.61
-17	4	-5	57.94	3.05
-17	-4	5	48.53	2.66
-17	-4	5	47.20	2.56
17	-4	-5	59.04	3.14
-17	4	-5	56.76	3.12
-17	-4	5	47.59	2.64
17	4	5	52.22	2.81
-17	-4	5	46.30	2.69
17	4	5	48.26	2.58
17	-4	-5	60.79	3.04
17	-4	5	51.73	2.55
-17	4	5	45.91	3.07
-17	4	5	46.82	2.80
17	-4	5	50.14	2.71
17	-4	5	46.57	2.63
-17	-4	-5	56.81	3.15
-17	-4	-5	56.47	3.07
-17	-4	-5	57.02	3.15
-17	-4	-5	58.00	3.12
17	-4	5	57.87	3.37
-17	-5	5	24.19	2.06
17	-5	-5	22.48	1.70
-17	-5	5	22.00	1.60
-17	5	-5	21.42	1.68
17	-5	-5	25.80	1.67
17	5	5	23.52	2.07
-17	5	-5	26.91	2.49
-17	-5	5	23.80	2.27

-17	-5	5	21.05	1.59
17	-5	-5	29.21	2.09
-17	-5	5	19.36	1.44
-17	5	-5	22.53	2.39
17	5	5	21.69	1.56
17	-5	-5	25.58	1.59
17	5	5	24.87	1.78
17	-5	5	24.34	1.45
-17	-5	-5	26.13	1.80
17	5	-5	23.09	1.50
17	-5	5	22.09	2.41
-17	5	5	19.42	2.02
17	-5	5	22.29	1.56
17	-5	5	21.49	1.60
-17	-5	-5	24.26	1.76
-17	-5	-5	23.90	1.62
17	5	-5	23.87	1.38
-17	5	5	19.78	2.12
17	-6	-5	39.99	2.74
17	6	5	37.11	2.32
-17	-6	5	30.46	2.01
-17	6	-5	39.18	2.29
17	-6	-5	36.28	2.28
-17	6	-5	37.60	3.06
-17	-6	5	32.14	2.01
-17	-6	5	33.59	2.68
17	6	5	35.24	2.65
-17	-6	5	31.57	1.92
17	6	5	34.41	2.12
-17	-6	5	32.01	2.10
17	-6	-5	38.94	2.34
-17	-6	5	35.71	2.58
17	6	-5	36.29	2.20
17	-6	5	33.93	2.14
-17	-6	-5	36.19	2.37
-17	6	5	30.68	2.64
17	-6	5	32.65	2.12
17	6	-5	37.22	2.03
17	-7	-5	13.20	1.28
17	7	5	15.18	1.70
17	7	5	15.03	1.65
17	-7	-5	14.74	1.51
-17	-7	5	14.88	1.49
-17	-7	5	13.33	1.33

-17	7	-5	13.28	1.40
-17	-7	5	14.37	1.25
-17	-7	5	13.91	2.10
-17	7	-5	18.45	2.61
17	-7	-5	14.80	1.45
17	7	5	11.78	2.12
17	7	-5	13.55	1.54
17	-7	5	14.48	1.45
17	7	-5	15.74	1.26
17	-7	5	13.24	1.44
-17	-7	-5	11.85	1.64
-17	7	5	12.40	1.92
-17	-7	-5	12.39	1.45
17	-8	-5	3.08	0.91
-17	8	-5	3.76	0.97
-17	-8	5	3.53	1.04
17	-8	-5	4.08	1.05
-17	8	-5	4.27	1.59
17	8	5	0.78	1.24
17	-8	-5	4.08	0.85
17	8	5	1.86	0.98
-17	-8	5	3.36	1.28
-17	-8	-5	3.67	1.06
-17	8	5	3.11	1.42
17	8	-5	3.56	0.83
17	-8	5	2.84	1.02
-17	-8	-5	2.58	0.93
17	-8	5	2.98	0.81
17	8	-5	3.46	1.03
-17	9	-5	2.34	1.56
17	9	5	4.09	1.12
17	-9	-5	5.30	1.01
17	-9	-5	4.94	1.11
-17	-9	5	3.77	1.34
-17	-9	5	3.67	1.04
17	9	5	7.24	1.44
17	-9	-5	5.11	0.98
-17	9	-5	4.82	1.08
17	-9	5	5.63	1.21
-17	-9	-5	5.36	1.13
17	9	-5	3.08	1.10
-17	-9	-5	3.39	0.98
-17	9	5	5.21	1.52
17	-9	5	2.99	0.81

17	-10	-5	32.66	2.33
17	-10	-5	31.64	2.25
17	-10	-5	34.28	2.48
-17	-10	5	32.70	2.43
-17	10	-5	48.13	5.02
-17	10	-5	33.75	2.32
17	-10	5	29.45	2.23
17	-10	5	30.77	2.47
-17	10	5	36.10	3.23
-18	0	-5	104.10	5.36
-18	0	5	97.82	5.02
18	0	-5	111.91	4.95
18	0	-5	108.57	5.19
18	0	5	109.22	5.03
18	0	-5	106.86	4.89
18	0	5	96.87	4.92
-18	0	5	97.66	4.93
-18	0	-5	109.08	5.50
-18	0	-5	102.75	5.26
18	0	5	102.72	4.97
18	0	5	90.90	4.96
-18	0	5	97.54	4.87
-18	0	5	94.14	4.79
-18	0	5	97.33	4.80
18	0	5	98.96	4.68
-18	1	-5	28.20	2.25
-18	-1	5	40.48	2.61
18	-1	-5	32.29	1.80
-18	-1	5	41.80	2.59
-18	-1	5	41.18	2.41
18	1	5	42.56	2.23
-18	-1	5	41.22	2.35
18	1	5	45.68	2.58
18	-1	-5	27.07	2.01
-18	1	-5	30.69	2.01
18	-1	-5	33.81	1.69
18	1	5	44.52	2.62
18	1	5	41.56	2.45
-18	-1	5	45.09	2.55
-18	-1	5	42.36	2.32
-18	1	-5	28.07	1.86
-18	1	-5	28.69	1.60
18	-1	5	45.06	2.77
-18	1	5	42.03	2.72

18	-1	5	41.32	2.24
18	-1	5	40.51	2.43
18	-1	5	45.60	2.50
-18	1	5	41.92	2.39
-18	-1	-5	25.11	2.01
-18	1	5	42.55	2.44
-18	-1	-5	28.26	2.09
-18	-1	-5	30.33	2.16
-18	1	5	45.09	2.39
-18	-1	-5	31.92	2.15
-18	-2	5	9.65	0.98
-18	-2	5	8.57	1.26
18	-2	-5	7.91	0.98
18	-2	-5	8.33	0.96
18	-2	-5	9.52	0.93
-18	-2	5	8.33	1.46
-18	2	-5	7.82	1.18
-18	2	-5	7.50	1.31
-18	2	-5	7.97	1.23
-18	-2	5	9.46	0.97
18	2	5	10.90	1.31
18	2	5	9.40	1.32
-18	2	-5	6.23	0.99
-18	-2	5	8.10	1.31
18	-2	-5	8.06	0.75
18	2	5	9.04	0.84
-18	2	5	8.48	1.10
-18	2	5	8.46	1.09
-18	-2	-5	7.89	1.31
18	-2	5	10.18	1.06
-18	-2	-5	7.96	1.00
18	-2	5	11.84	1.50
-18	-2	-5	7.55	1.12
18	-2	5	10.13	1.10
-18	-2	-5	8.92	1.41
-18	2	5	8.44	1.42
18	-2	5	7.03	0.84
-18	2	5	8.74	0.96
18	-3	-5	4.64	0.68
-18	-3	5	2.60	0.78
-18	3	-5	2.95	0.83
18	3	5	2.86	1.06
-18	3	-5	6.02	1.21
-18	-3	5	3.41	0.64

-18	3	-5	4.04	0.76
-18	-3	5	2.74	0.62
-18	-3	5	2.64	1.14
-18	3	-5	3.92	1.03
-18	-3	5	3.13	0.86
18	3	5	3.19	0.57
18	-3	-5	4.28	0.78
18	-3	-5	3.77	0.72
18	-3	-5	3.45	0.71
-18	-3	-5	3.28	0.69
-18	-3	-5	4.65	0.98
18	-3	5	3.39	0.61
18	-3	5	3.68	0.69
-18	-3	-5	3.70	0.90
-18	-3	-5	3.78	0.85
18	-3	5	4.70	0.78
-18	3	5	3.10	0.99
18	-4	-5	2.67	0.65
18	-4	-5	4.03	0.67
18	4	5	3.86	0.68
-18	-4	5	5.63	0.91
-18	4	-5	2.59	1.16
-18	-4	5	2.78	1.21
18	4	5	4.63	1.13
-18	-4	5	3.96	1.07
18	-4	-5	4.90	1.01
-18	-4	5	7.43	1.16
18	4	5	5.76	1.18
-18	4	-5	2.17	0.67
-18	4	-5	4.03	1.23
18	-4	-5	4.72	0.87
-18	-4	5	4.29	0.75
-18	-4	5	5.30	0.89
-18	4	-5	3.08	0.80
-18	4	5	4.09	0.95
18	-4	5	4.19	1.28
-18	-4	-5	2.69	0.73
-18	-4	-5	3.93	0.77
18	-4	5	3.39	0.73
18	-4	5	5.27	0.88
-18	-4	-5	4.47	0.81
18	-4	5	5.07	0.87
-18	4	5	5.22	1.24
-18	-4	-5	3.32	0.93

-18	-5	5	22.86	2.18
18	-5	-5	21.64	1.67
-18	-5	5	25.35	2.48
-18	-5	5	23.51	1.76
-18	-5	5	23.93	1.71
18	5	5	25.20	1.68
18	5	5	25.47	2.25
-18	-5	5	23.89	1.60
-18	5	-5	20.10	2.45
18	-5	-5	24.36	1.72
-18	5	-5	23.55	1.70
18	-5	-5	23.54	1.64
18	-5	-5	28.84	2.00
18	5	5	26.91	1.92
-18	-5	5	24.31	1.76
18	5	-5	23.15	1.45
18	5	-5	22.12	1.33
-18	-5	-5	20.74	1.67
-18	5	5	24.07	2.13
18	-5	5	24.49	1.57
18	-5	5	24.49	1.75
-18	-5	-5	23.37	1.72
18	-5	5	23.45	1.69
-18	5	5	26.49	2.37
18	-5	5	28.53	2.70
-18	-5	-5	23.65	1.77
18	-6	-5	45.89	2.77
-18	-6	5	46.76	2.58
-18	-6	5	52.33	3.25
-18	6	-5	49.70	3.56
18	6	5	47.73	2.77
-18	6	-5	46.33	2.74
18	-6	-5	48.18	2.72
-18	-6	5	45.77	2.65
18	6	5	49.72	3.23
-18	-6	5	47.31	2.77
18	6	5	49.06	2.90
18	-6	-5	48.72	2.64
18	6	-5	43.67	2.59
-18	6	5	45.75	3.39
18	-6	5	46.55	2.74
-18	-6	-5	50.92	2.89
18	6	-5	48.97	2.47
-18	-6	-5	45.98	2.76

18	-6	5	44.94	2.71
18	7	5	41.21	3.20
-18	-7	5	37.28	2.26
-18	7	-5	39.72	3.60
-18	7	-5	42.05	2.59
18	-7	-5	41.55	2.63
-18	-7	5	39.16	2.49
18	-7	-5	40.97	2.42
-18	-7	5	41.10	3.17
18	-7	-5	43.50	2.64
18	7	5	39.35	2.63
18	7	5	44.02	3.32
-18	-7	5	37.98	2.33
18	-7	5	40.78	2.58
-18	-7	-5	41.72	2.57
-18	-7	-5	41.49	2.74
18	7	-5	36.90	2.59
-18	7	5	40.96	3.35
18	-7	5	38.73	2.52
18	7	-5	42.55	2.34
-18	-8	5	32.31	2.70
18	8	5	28.71	2.62
18	-8	-5	29.40	2.22
-18	-8	5	27.56	2.26
-18	8	-5	32.47	3.02
-18	8	-5	30.22	2.21
18	-8	-5	29.82	2.01
18	8	5	26.12	2.15
18	-8	-5	30.15	2.13
18	-8	5	25.00	2.01
-18	8	5	25.91	2.82
18	8	-5	29.85	2.21
18	-8	5	28.25	2.20
-18	-8	-5	30.84	2.28
18	8	-5	30.55	2.02
-18	-8	-5	29.52	2.24
18	-9	-5	17.95	1.66
18	9	5	15.81	2.38
-18	9	-5	19.61	2.88
-18	-9	5	20.10	1.82
18	-9	-5	17.34	1.78
18	9	5	18.70	1.98
-18	-9	5	20.91	2.47
18	-9	-5	17.96	1.67

-18	9	-5	16.36	1.64
-18	-9	-5	19.21	1.88
-18	9	5	16.92	2.72
18	-9	5	18.29	1.73
18	9	-5	15.50	1.92
18	-9	5	17.20	1.85
-18	-9	-5	13.90	1.87
-19	0	-5	-0.13	0.55
-19	0	-5	-0.86	0.86
-19	0	5	-0.25	0.93
-19	0	-5	-0.15	0.85
-19	0	5	-0.51	0.81
19	0	-5	0.16	0.26
-19	0	5	0.17	0.66
-19	0	5	0.34	0.52
19	0	-5	-0.22	0.47
19	0	5	0.01	0.39
19	0	5	-1.22	0.62
19	0	5	0.15	0.53
19	0	5	0.48	0.80
19	0	-5	0.15	0.31
-19	-1	5	4.48	0.75
-19	-1	5	3.85	0.96
19	-1	-5	3.25	0.62
-19	-1	5	3.44	1.06
19	1	5	4.84	0.80
-19	-1	5	3.72	0.64
19	1	5	3.67	0.91
-19	-1	5	5.02	0.97
-19	1	-5	3.78	1.08
-19	1	-5	4.77	1.02
19	1	5	4.80	0.71
-19	1	-5	1.82	0.64
19	1	5	3.57	0.88
19	-1	-5	3.17	0.52
19	-1	5	5.12	1.01
-19	1	5	3.01	0.65
-19	-1	-5	4.22	1.01
19	-1	5	5.24	0.74
-19	1	5	3.35	1.00
-19	-1	-5	3.49	0.72
-19	-1	-5	3.35	0.98
19	-1	5	5.05	0.91
19	-1	5	4.29	0.77

-19	1	5	4.03	0.94
-19	1	5	5.97	0.99
19	-2	-5	9.24	0.84
19	2	5	8.44	0.87
-19	-2	5	9.69	1.01
-19	-2	5	9.84	1.38
-19	-2	5	9.22	1.31
19	-2	-5	9.72	1.04
19	2	5	9.14	1.53
19	-2	-5	11.08	1.13
-19	-2	5	8.05	1.03
-19	2	-5	8.59	1.15
-19	2	-5	8.16	1.43
19	2	5	11.74	1.38
-19	2	-5	10.45	1.20
-19	2	-5	8.57	1.39
19	-2	-5	10.83	1.05
-19	-2	-5	12.36	1.51
19	-2	5	8.77	0.93
-19	-2	-5	8.04	1.56
-19	2	5	8.49	1.59
19	-2	5	9.80	1.18
19	-2	5	8.98	1.15
-19	2	5	9.77	1.14
-19	2	5	8.78	1.00
-19	-2	-5	9.71	1.15
19	-2	5	10.70	1.74
-19	-2	-5	8.64	1.16
-19	3	-5	2.78	0.74
-19	-3	5	1.34	0.73
19	-3	-5	2.94	0.58
19	3	5	1.68	0.54
19	3	5	2.40	0.84
19	-3	-5	3.78	0.65
-19	3	-5	3.40	0.88
-19	-3	5	3.02	1.00
-19	3	-5	5.31	1.06
-19	-3	5	2.25	0.59
19	-3	-5	2.96	0.78
-19	-3	5	2.04	0.64
-19	3	-5	3.47	1.20
19	3	5	1.39	0.88
19	-3	5	3.49	1.02
-19	-3	-5	3.89	0.84

19	-3	5	1.75	0.67
19	-3	5	1.39	0.66
-19	-3	-5	3.28	0.94
-19	-3	-5	3.25	1.03
-19	-3	-5	3.88	0.82
19	-3	5	1.77	0.54
-19	3	5	1.31	1.05
-19	-4	5	10.80	1.13
-19	4	-5	8.52	1.21
19	-4	-5	14.46	1.44
19	-4	-5	10.73	1.23
-19	4	-5	11.53	2.03
19	-4	-5	10.25	1.17
19	-4	-5	9.06	1.05
19	4	5	13.98	1.86
-19	-4	5	9.35	1.37
19	4	5	10.86	1.40
-19	-4	5	12.94	1.74
-19	-4	5	11.37	1.16
19	4	5	11.37	1.12
19	-4	5	10.03	1.11
-19	-4	-5	12.29	1.37
19	-4	5	12.17	1.17
-19	-4	-5	11.05	1.37
19	-4	5	15.14	2.11
-19	-4	-5	9.91	1.27
19	-4	5	11.56	1.25
-19	4	5	11.04	1.89
19	5	5	13.97	1.57
19	-5	-5	16.28	1.60
19	5	5	14.77	1.94
-19	-5	5	12.13	2.19
19	-5	-5	12.23	1.17
19	5	5	13.82	1.23
-19	5	-5	10.76	1.30
-19	-5	5	11.07	1.16
-19	5	-5	10.84	2.13
-19	-5	5	11.59	1.43
19	-5	-5	11.36	1.35
19	-5	-5	11.18	1.21
-19	-5	5	13.53	1.87
-19	-5	5	10.95	1.29
19	-5	5	11.33	1.17
-19	-5	-5	13.85	1.42

-19	-5	-5	11.28	1.30
-19	5	5	11.69	2.01
19	-5	5	10.60	1.26
-19	-5	-5	11.50	1.40
19	-6	-5	6.33	1.10
19	6	5	10.60	1.82
-19	-6	5	7.33	1.28
19	6	5	7.64	1.48
19	-6	-5	8.06	1.34
19	-6	-5	6.34	1.23
-19	6	-5	5.12	1.01
-19	-6	5	6.18	1.56
19	6	5	7.11	1.13
-19	-6	5	7.03	1.14
-19	6	-5	8.60	1.89
-19	-6	5	6.91	1.27
19	6	-5	5.04	0.90
19	-6	5	7.33	1.30
19	6	-5	6.01	1.09
-19	-6	-5	7.95	1.42
-19	-6	-5	6.33	1.09
19	-6	5	7.19	1.20
-19	6	5	7.70	1.64
-19	7	-5	2.84	1.48
19	7	5	4.41	0.98
-19	-7	5	3.98	1.11
-19	-7	5	4.58	1.36
-19	-7	5	4.20	1.43
19	7	5	1.87	1.34
-19	-7	5	3.46	0.84
19	-7	-5	3.52	0.79
-19	7	-5	4.19	1.01
19	-7	-5	4.04	0.93
19	-7	-5	3.49	0.91
-19	-7	-5	5.44	1.03
19	7	-5	4.52	0.79
-19	-7	-5	3.52	0.95
-19	7	5	2.81	1.44
19	-7	5	4.16	0.91
19	-7	5	3.03	0.94
19	7	-5	2.19	0.90
-19	8	-5	6.89	1.19
-19	-8	5	8.88	1.55
-19	8	-5	9.84	1.86

19	-8	-5	8.96	1.44
19	8	5	5.39	1.43
19	-8	-5	9.39	1.33
19	8	5	5.54	1.13
19	-8	-5	9.43	1.37
-19	-8	5	5.23	1.49
-19	8	5	7.05	1.68
19	-8	5	5.59	0.95
-19	-8	-5	8.19	1.33
-19	-8	-5	10.21	1.68
19	-8	5	6.16	1.29
19	8	-5	6.72	1.11
19	-9	-5	4.66	0.94
-19	9	-5	3.75	0.98
-19	-9	5	5.10	1.13
19	-9	-5	2.52	0.88
19	-9	-5	6.52	1.35
19	9	-5	5.40	1.80
19	-9	5	5.92	1.16
-19	-9	-5	4.05	1.12
19	-9	5	5.08	1.01
20	0	-5	97.90	4.76
-20	0	-5	91.84	5.07
20	0	5	106.81	5.36
20	0	5	111.25	5.37
-20	0	-5	93.60	4.82
20	0	5	104.81	5.28
20	0	-5	94.25	4.36
-20	0	5	102.11	5.44
20	0	5	104.22	5.02
20	0	-5	99.89	4.42
-20	0	5	106.23	5.19
-20	0	5	102.62	5.30
-20	0	5	105.76	5.23
20	1	5	65.71	3.51
-20	1	-5	64.73	4.94
20	-1	-5	76.03	3.50
-20	-1	5	59.75	3.83
-20	-1	5	57.61	3.45
20	-1	-5	72.14	3.39
20	1	5	61.99	3.15
20	1	5	64.17	3.46
-20	1	-5	69.40	3.77
-20	1	-5	66.67	3.86

20	-1	-5	75.21	3.70
20	1	5	64.93	3.59
-20	-1	5	65.65	3.31
-20	-1	5	63.69	3.65
-20	-1	5	63.93	3.33
-20	1	5	60.53	3.70
-20	-1	-5	74.33	3.82
-20	1	5	59.78	3.29
-20	1	5	60.47	3.39
20	-1	5	65.70	3.43
20	-1	5	66.44	3.46
-20	1	5	62.13	3.36
20	-1	5	63.19	3.19
-20	-1	-5	71.50	4.03
20	-1	5	69.45	3.65
-20	2	-5	33.23	2.29
20	-2	-5	35.17	2.05
-20	-2	5	40.12	2.30
20	-2	-5	36.78	2.10
20	-2	-5	37.68	1.88
20	2	5	35.85	2.63
-20	2	-5	35.71	2.79
-20	-2	5	37.54	2.55
-20	2	-5	33.97	2.21
20	2	5	40.18	2.13
-20	-2	5	39.53	2.56
20	2	5	40.74	2.52
-20	-2	-5	32.42	2.50
20	-2	5	38.87	2.18
20	-2	5	40.70	2.41
-20	-2	-5	35.07	2.19
-20	2	5	39.39	2.83
-20	-2	-5	33.12	2.23
-20	2	5	38.29	2.36
20	-2	5	41.30	2.69
-20	-2	-5	33.38	2.42
-20	2	5	36.37	2.20
20	-2	5	43.12	2.40
20	-3	-5	6.44	1.14
-20	-3	5	7.07	1.02
-20	-3	5	4.69	1.01
20	-3	-5	6.89	0.85
-20	3	-5	7.00	1.12
20	3	5	6.25	0.89

-20	3	-5	7.41	1.47
20	3	5	9.36	1.43
20	3	5	7.42	1.36
-20	-3	5	8.49	1.34
-20	3	-5	7.97	1.44
-20	-3	5	5.05	1.13
20	-3	-5	7.66	1.12
20	-3	5	6.33	1.36
20	-3	5	6.34	0.93
-20	-3	-5	7.54	1.21
20	-3	5	6.96	1.14
20	-3	5	6.09	0.88
-20	-3	-5	6.05	1.07
-20	-3	-5	6.51	1.01
-20	3	5	4.80	1.25
-20	-3	-5	7.19	1.12
-20	-4	5	7.30	1.14
20	-4	-5	5.97	0.96
20	-4	-5	6.58	1.00
20	4	5	8.29	1.41
-20	-4	5	9.38	1.19
-20	4	-5	4.57	0.92
20	-4	-5	6.64	1.20
-20	4	-5	7.77	1.56
20	-4	-5	7.48	1.33
20	4	5	7.75	1.02
-20	-4	5	10.50	1.76
20	4	5	7.37	1.37
-20	-4	5	7.41	1.32
-20	-4	-5	6.60	1.08
20	-4	5	7.55	1.13
-20	-4	-5	4.76	0.96
-20	-4	-5	5.47	0.98
-20	4	5	8.07	1.48
20	-4	5	7.09	1.16
20	-4	5	8.48	1.08
-20	-5	5	24.42	1.93
-20	-5	5	25.01	2.33
-20	-5	5	23.47	1.88
20	5	5	27.09	1.83
20	5	5	24.58	2.55
20	-5	-5	25.57	1.99
20	-5	-5	25.90	1.84
-20	5	-5	25.76	1.98

20	5	5	26.82	2.08
-20	-5	5	26.02	1.76
20	-5	-5	29.17	1.83
-20	5	-5	22.16	2.93
-20	-5	-5	26.95	2.09
-20	-5	-5	24.33	1.94
20	-5	5	24.16	1.94
20	-5	5	27.97	1.88
-20	5	5	24.86	2.77
-20	-6	5	54.85	3.65
20	6	5	51.72	2.93
-20	6	-5	46.07	3.71
-20	-6	5	46.43	2.89
-20	6	-5	49.96	2.85
20	-6	-5	44.18	2.79
-20	-6	5	51.44	2.99
-20	-6	5	49.01	2.78
20	6	5	46.31	3.52
20	6	5	54.00	3.08
20	-6	-5	47.85	2.67
20	-6	-5	46.96	2.72
20	-6	5	52.80	3.01
20	6	-5	47.82	2.39
-20	6	5	49.43	3.64
-20	-6	-5	45.94	2.88
20	6	-5	42.25	2.53
20	-6	5	51.70	2.95
-20	-6	-5	45.97	2.86
20	-7	-5	57.46	3.23
20	-7	-5	57.40	3.17
20	7	5	55.29	3.77
-20	-7	5	63.62	3.83
20	-7	-5	57.26	3.15
-20	7	-5	56.94	3.23
-20	-7	5	58.61	3.40
20	7	5	54.32	3.20
-20	7	-5	51.73	4.03
-20	7	5	55.54	3.87
20	-7	5	54.64	3.20
-20	-7	-5	56.80	3.30
20	7	-5	55.58	3.14
-20	-7	-5	57.88	3.32
20	-7	5	56.58	3.21
20	7	-5	53.56	3.06

20	8	5	26.57	2.14
-20	-8	5	26.27	2.14
20	-8	-5	31.13	2.23
20	-8	-5	30.26	2.20
20	8	5	24.34	2.64
20	-8	-5	29.56	2.18
-20	8	-5	32.02	3.18
-20	-8	5	31.21	2.76
-20	8	-5	29.51	2.20
20	-8	5	28.19	2.06
20	-8	5	26.96	2.06
-20	8	5	25.75	2.87
-20	-8	-5	31.34	2.42
-20	-8	-5	34.64	2.40
20	8	-5	31.68	2.25
21	0	5	0.38	0.44
-21	0	5	1.17	0.98
-21	0	-5	-0.08	0.64
-21	0	5	0.09	0.61
21	0	5	-0.96	0.79
-21	0	5	0.70	0.96
-21	0	5	-0.66	0.77
21	0	5	-0.31	0.65
-21	0	5	-0.70	0.49
21	0	5	-0.87	0.90
21	0	-5	0.23	0.31
21	1	5	0.06	0.93
21	-1	-5	1.10	0.50
-21	-1	5	0.38	0.63
21	-1	-5	0.56	0.57
21	1	5	-0.10	0.43
-21	1	-5	0.09	0.97
-21	1	-5	0.55	0.60
21	-1	-5	0.96	0.37
21	1	5	0.51	0.67
-21	-1	5	0.39	0.52
-21	-1	5	-0.87	0.87
-21	1	-5	-0.52	1.14
-21	-1	5	0.09	0.80
21	-1	5	-0.76	0.81
-21	-1	-5	2.15	1.01
-21	1	5	0.74	0.95
-21	-1	-5	1.13	0.68
21	-1	5	0.06	0.98

21	-1	5	0.72	0.70
-21	1	5	0.43	0.61
21	-1	5	0.68	0.46
-21	1	5	1.50	0.77
21	-2	-5	3.79	0.73
-21	-2	5	4.70	1.02
21	2	5	3.90	0.76
-21	-2	5	2.76	0.73
-21	2	-5	2.19	1.28
21	-2	-5	3.09	0.52
-21	-2	5	6.18	1.72
21	2	5	4.32	0.99
21	2	5	2.90	1.08
-21	-2	5	2.46	0.69
-21	2	-5	4.00	0.94
21	-2	-5	3.32	0.69
-21	2	5	2.65	0.76
-21	-2	-5	3.31	0.75
-21	2	5	2.96	0.71
-21	2	5	4.28	1.21
-21	-2	-5	4.68	1.17
-21	-2	-5	3.98	0.85
21	-2	5	2.57	1.09
21	-2	5	3.56	0.81
21	-2	5	2.83	0.62
21	-2	5	3.09	0.82
21	-3	-5	1.44	0.60
-21	3	-5	0.45	1.27
21	3	5	3.51	1.08
21	3	5	1.01	0.61
21	-3	-5	1.75	0.77
21	-3	-5	2.13	0.68
-21	-3	5	1.48	0.69
21	-3	-5	3.64	0.84
-21	3	-5	1.37	0.84
21	3	5	3.02	0.93
-21	-3	5	4.00	1.11
-21	-3	5	1.97	0.66
-21	-3	-5	1.29	0.80
21	-3	5	2.99	0.82
21	-3	5	3.48	1.50
-21	3	5	1.45	1.16
-21	-3	-5	0.92	0.74
21	-3	5	2.36	0.77

21	-3	5	1.58	0.63
-21	-3	-5	1.59	1.01
21	-4	-5	4.01	0.93
-21	-4	5	2.77	0.78
21	4	5	3.41	1.16
-21	-4	5	1.00	0.66
21	-4	-5	2.82	0.71
-21	-4	5	2.50	1.00
21	4	5	3.01	1.04
21	-4	-5	1.25	0.71
-21	4	-5	2.16	0.93
-21	4	-5	3.78	1.61
21	4	5	3.01	0.75
21	-4	-5	2.33	0.89
-21	-4	5	3.49	1.22
21	-4	5	1.54	0.87
-21	-4	-5	2.71	0.86
-21	-4	-5	1.58	0.90
21	-4	5	2.14	0.75
-21	-4	-5	1.87	0.98
21	-4	5	2.97	0.94
-21	4	5	0.16	1.33
21	-5	-5	1.38	0.67
-21	-5	5	1.29	0.72
-21	5	-5	3.13	1.52
21	-5	-5	0.32	0.67
-21	-5	5	1.50	0.70
-21	-5	5	0.45	0.87
-21	-5	5	2.66	1.34
21	5	5	2.51	1.49
21	-5	-5	1.82	0.82
21	5	5	1.55	0.99
-21	5	-5	2.63	0.86
21	5	5	2.73	0.76
-21	5	5	1.22	1.38
21	-5	5	1.68	0.76
21	-5	5	0.30	0.69
-21	-5	-5	1.37	0.87
-21	-5	-5	1.90	0.85
21	6	5	1.22	0.78
-21	6	-5	3.96	1.47
21	-6	-5	0.84	0.68
-21	-6	5	0.53	1.30
-21	-6	5	0.09	0.90

21	-6	-5	-0.45	0.65
21	6	5	1.94	1.35
-21	-6	5	0.61	0.98
-21	6	-5	-0.36	0.75
21	-6	-5	1.00	0.70
-21	-6	-5	2.05	0.91
21	-6	5	0.37	0.67
21	-6	5	2.12	0.79
-21	-6	-5	-0.52	0.88
-21	6	5	2.59	1.33
21	-7	-5	3.32	0.89
21	7	5	3.09	1.52
-21	7	-5	1.99	1.66
21	-7	-5	3.43	0.91
-21	7	-5	1.90	0.86
21	-7	-5	2.72	0.99
21	7	5	1.51	0.92
-21	-7	5	2.15	0.95
-21	-7	5	2.94	1.37
21	7	-5	2.67	0.82
21	-7	5	0.72	0.81
-21	-7	-5	3.89	1.22
-21	7	5	4.59	1.56
-21	-7	-5	1.75	0.92
21	-7	5	1.51	0.82
21	7	-5	2.65	0.83
22	0	5	60.52	3.07
22	0	5	60.52	3.43
-22	0	5	58.16	3.36
-22	0	-5	69.55	3.85
22	0	5	62.29	3.47
-22	0	5	60.37	3.29
22	0	5	61.69	3.55
-22	0	5	57.52	3.65
-22	0	5	57.01	3.18
-22	0	-5	72.22	4.25
-22	-1	5	88.58	4.81
22	1	5	91.70	4.77
-22	1	-5	85.43	4.74
-22	-1	5	91.03	4.99
22	1	5	92.12	4.93
22	1	5	90.40	4.47
-22	-1	5	93.72	4.64
-22	1	-5	95.49	5.26

22	-1	-5	95.58	4.38
22	1	5	93.49	4.86
22	-1	-5	92.82	4.28
-22	-1	5	90.98	4.67
22	-1	-5	92.53	4.64
22	-1	5	96.12	4.82
-22	1	5	88.42	5.05
22	-1	5	90.65	4.50
-22	1	5	92.58	4.71
-22	-1	-5	89.14	4.77
22	-1	5	100.68	4.89
22	-1	5	96.14	4.82
-22	1	5	87.02	4.61
22	-2	-5	38.92	2.27
22	-2	-5	35.77	1.95
-22	-2	5	37.46	2.65
-22	-2	5	42.47	2.79
-22	2	-5	33.66	2.41
22	-2	-5	38.28	2.14
22	2	5	39.41	2.21
22	2	5	37.83	2.59
-22	2	-5	37.35	3.04
-22	-2	5	37.47	2.39
-22	-2	5	40.25	2.42
22	2	5	43.27	2.90
22	-2	5	40.95	2.56
-22	-2	-5	31.92	2.60
-22	-2	-5	35.30	2.42
22	-2	5	40.49	2.58
-22	2	5	39.80	2.50
-22	2	5	38.04	3.09
-22	-2	-5	35.50	2.38
22	-2	5	38.23	2.25
22	-2	5	38.77	2.80
22	-3	-5	-0.53	0.50
-22	-3	5	3.00	1.20
-22	-3	5	0.53	0.74
22	3	5	0.79	1.17
-22	3	-5	-1.20	1.49
-22	-3	5	1.72	0.98
22	3	5	0.48	0.99
-22	-3	5	0.76	0.73
-22	3	-5	-0.03	0.70
22	3	5	0.92	0.67

22	-3	-5	0.21	0.75
22	-3	-5	-0.14	0.67
-22	3	5	-0.35	0.75
-22	3	5	1.81	1.42
22	-3	5	1.42	0.87
-22	-3	-5	-1.41	1.00
22	-3	5	1.37	0.60
22	-3	5	1.29	0.86
-22	-3	-5	-0.67	0.86
-22	-3	-5	0.13	0.83
22	4	5	6.86	1.60
22	4	5	7.36	1.11
-22	-4	5	7.56	1.43
-22	-4	5	5.88	1.02
22	4	5	8.18	1.66
22	-4	-5	5.01	0.92
-22	4	-5	4.91	1.02
22	-4	-5	4.38	0.86
22	-4	-5	5.45	0.96
-22	4	-5	7.89	1.85
-22	-4	5	4.91	1.14
-22	-4	5	6.94	1.06
22	-4	5	6.28	1.07
-22	-4	-5	3.02	1.02
-22	4	5	5.01	1.54
-22	-4	-5	3.92	0.96
22	-4	5	6.19	1.04
-22	5	-5	15.50	1.53
-22	-5	5	22.23	2.60
-22	5	-5	17.22	2.68
22	-5	-5	15.26	1.33
22	5	5	19.36	2.45
22	-5	-5	14.11	1.49
22	-5	-5	15.54	1.47
22	5	5	19.06	1.55
-22	-5	5	18.36	1.68
-22	-5	5	17.55	1.88
22	-5	5	22.68	1.59
-22	-5	-5	15.52	1.64
-22	5	5	20.05	2.48
22	-5	5	18.51	1.74
-22	-5	-5	15.76	1.88
-22	6	-5	50.75	3.94
22	-6	-5	50.00	3.00

22	-6	-5	53.00	2.99
22	-6	-5	49.97	2.84
-22	-6	5	42.12	2.79
-22	6	-5	48.42	2.98
22	6	5	43.26	3.23
-22	-6	5	43.28	2.58
22	6	5	41.99	2.60
-22	-6	5	43.66	3.21
-22	-6	-5	48.87	3.05
22	-6	5	43.14	2.69
-22	-6	-5	48.07	3.19
-22	6	5	44.22	3.47
22	-6	5	44.25	2.64
-23	0	5	1.37	0.85
23	0	5	-0.62	0.96
-23	0	-5	0.33	0.76
23	0	5	0.86	1.04
-23	0	5	-0.19	0.64
-23	0	5	0.93	0.91
-23	0	-5	-0.02	1.36
23	0	5	0.06	0.82
-23	0	5	1.17	1.15
-23	1	-5	2.71	0.95
-23	-1	5	3.08	0.82
23	1	5	3.57	0.65
23	-1	-5	3.33	0.84
23	1	5	5.36	1.30
-23	1	-5	3.16	1.43
-23	-1	5	1.85	0.85
23	-1	-5	3.34	0.66
-23	-1	5	3.03	1.20
23	1	5	2.45	1.06
23	-1	5	2.89	1.03
-23	-1	-5	4.02	0.92
-23	1	5	2.65	0.89
-23	1	5	2.70	0.94
-23	-1	-5	2.91	1.32
-23	-1	-5	4.49	1.05
23	-1	5	2.43	1.34
-23	1	5	2.37	1.42
23	-1	5	4.02	0.92
23	-1	5	2.97	0.67
-23	2	-5	3.05	1.62
23	-2	-5	1.93	0.71

23	2	5	2.81	0.65
23	2	5	3.79	1.11
-23	-2	5	2.98	1.21
-23	-2	5	2.59	0.80
23	2	5	1.23	1.23
23	-2	-5	1.94	0.80
-23	-2	5	1.62	1.21
23	-2	-5	2.37	0.53
-23	2	-5	1.61	0.83
-23	-2	5	4.41	0.88
23	-2	5	2.97	0.87
23	-2	5	2.47	1.02
-23	-2	-5	4.20	1.56
23	-2	5	2.93	0.66
-23	-2	-5	2.70	0.90
-23	-2	-5	1.53	0.91
-23	2	5	1.42	0.78
-23	2	5	3.99	1.51
23	-3	-5	12.75	1.08
23	3	5	10.61	1.13
-23	-3	5	11.40	1.42
-23	3	-5	16.02	2.86
-23	3	-5	11.46	1.46
-23	-3	5	10.26	1.59
23	3	5	14.78	2.30
-23	-3	5	14.28	1.99
-23	-3	5	8.28	1.39
23	3	5	12.03	1.71
23	-3	5	11.90	1.54
-23	-3	-5	14.25	1.72
-23	-3	-5	11.58	1.91
-23	-3	-5	13.04	1.50
-23	3	5	10.27	1.41
23	-3	5	9.84	1.11
-23	3	5	12.50	2.24
-23	4	-5	7.95	1.85
23	4	5	7.63	1.28
23	-4	-5	7.77	1.03
-23	-4	5	13.98	2.34
23	-4	-5	10.66	1.38
23	4	5	8.27	1.12
23	4	5	7.77	1.63
-23	-4	5	8.50	1.44
-23	-4	5	7.84	1.23

-23	-4	5	9.96	1.86
-23	4	-5	7.26	1.32
-23	-4	-5	10.05	1.63
23	-4	5	9.65	1.37
-23	4	5	8.92	1.58
-23	-4	-5	9.24	1.41
-23	4	5	8.07	1.33
23	-4	5	7.16	1.09
23	-5	-5	6.91	1.12
23	-5	-5	6.93	1.09
23	5	5	7.04	1.53
-23	-5	5	9.97	1.70
-23	-5	5	7.39	1.62
23	5	5	6.65	1.22
-23	5	-5	4.03	1.62
-23	5	-5	7.71	1.38
-23	-5	5	8.75	1.46
-23	-5	5	8.83	1.57
-23	-5	-5	4.27	1.15
-23	5	5	7.95	1.65
-23	-5	-5	8.18	1.46
23	-5	5	6.10	1.09
23	-5	5	7.42	1.19
23	-6	-5	2.44	0.91
-23	-6	5	4.85	1.03
23	-6	5	3.57	0.87
-23	-6	-5	4.20	1.10
24	0	5	56.97	3.44
-24	0	5	54.96	3.00
-24	0	5	51.57	3.73
-24	0	5	54.44	3.12
-24	0	-5	57.12	3.40
24	0	5	50.43	3.15
-24	0	-5	64.77	4.02
-24	0	5	53.37	3.12
24	0	5	51.81	2.79
-24	1	-5	33.10	3.33
-24	-1	5	34.13	2.94
24	1	5	35.39	2.33
-24	-1	5	32.58	2.22
-24	-1	5	32.33	2.50
24	1	5	38.12	2.74
24	-1	-5	34.94	1.77
24	1	5	31.43	1.89

-24	1	-5	33.19	2.31
24	-1	-5	36.32	2.29
-24	-1	5	33.11	2.35
24	-1	-5	34.82	1.89
-24	-1	-5	35.17	2.31
24	-1	5	30.66	2.30
-24	-1	-5	31.73	2.32
-24	1	5	31.93	2.25
-24	-1	-5	34.17	3.02
24	-1	5	30.14	1.91
24	-1	5	34.82	2.88
-24	1	5	32.70	2.95
-24	-2	5	15.63	1.75
24	-2	-5	15.66	1.56
-24	2	-5	9.75	1.46
24	2	5	14.68	1.19
24	2	5	14.26	1.81
24	2	5	17.85	2.20
-24	-2	5	13.34	1.69
24	-2	-5	10.76	0.95
-24	2	-5	10.66	2.18
-24	-2	5	12.41	2.11
-24	-2	5	18.72	2.11
24	-2	5	15.45	1.58
24	-2	5	14.30	1.25
-24	-2	-5	14.42	1.53
-24	2	5	13.79	1.56
-24	-2	-5	10.65	1.63
-24	2	5	14.49	2.26
-24	-2	-5	10.82	1.53
-24	3	-5	0.88	1.54
24	-3	-5	1.78	0.56
24	3	5	1.80	0.63
24	-3	-5	2.44	0.93
-24	-3	5	2.07	1.00
-24	-3	5	1.97	1.42
24	3	5	2.37	1.58
-24	-3	5	0.98	1.26
-24	-3	5	2.15	0.98
-24	3	-5	0.30	0.75
-24	-3	-5	1.78	0.88
24	-3	5	1.11	0.63
-24	3	5	1.61	0.82
-24	-3	-5	1.03	0.89

24	-3	5	2.00	0.86
-24	3	5	2.45	1.35
-24	-4	5	9.23	1.60
-24	-4	5	8.16	1.63
24	4	5	7.32	1.15
-24	-4	5	8.32	1.49
-24	4	-5	7.86	1.79
-24	-4	5	9.92	1.96
24	-4	-5	5.55	0.95
24	-4	-5	8.72	1.58
24	4	5	8.26	1.62
-24	4	-5	4.77	1.02
-24	-4	-5	5.77	1.07
-24	4	5	8.23	1.41
24	-4	5	8.38	1.48
-24	-4	-5	5.57	1.11
24	-4	5	7.40	1.08
-24	4	5	9.71	1.81
-25	0	-5	1.58	1.75
25	0	5	0.31	0.57
-25	0	5	1.01	0.86
-25	0	5	2.65	1.45
25	0	5	-0.85	0.90
-25	0	5	-0.30	0.81
-25	0	-5	1.91	0.90
25	0	5	0.60	1.43
-25	-1	5	15.55	2.49
-25	1	-5	8.43	1.49
25	-1	-5	12.06	1.74
25	-1	-5	9.86	0.76
25	1	5	15.08	2.20
-25	-1	5	11.22	1.93
-25	-1	5	12.67	1.58
-25	1	-5	10.08	2.14
-25	-1	5	12.75	1.71
25	1	5	11.17	1.14
25	-1	-5	10.78	0.97
-25	-1	-5	7.18	1.20
25	-1	5	11.15	1.13
-25	1	5	10.88	1.61
-25	1	5	12.63	1.53
-25	1	5	14.00	2.28
-25	-1	-5	8.18	1.49
-25	-1	-5	12.98	2.55

25	-1	5	12.45	1.66
-25	2	-5	10.72	1.47
-25	-2	5	10.83	1.57
25	-2	-5	11.45	0.94
25	-2	-5	10.86	1.60
25	2	5	9.30	1.08
25	-2	-5	9.63	1.73
-25	-2	5	10.11	1.77
25	2	5	10.11	1.87
-25	-2	5	12.02	1.56
-25	-2	5	6.92	1.54
-25	2	-5	15.48	2.71
-25	2	5	6.66	1.56
25	-2	5	8.25	1.02
25	-2	5	7.67	1.25
-25	-2	-5	9.62	1.53
-25	-2	-5	9.66	1.62
-25	2	5	9.59	1.47
-25	-2	-5	10.67	2.10
25	-3	-5	11.05	1.07
-25	-3	5	14.41	1.68
25	3	5	14.32	2.44
25	3	5	13.36	1.22
25	-3	-5	12.61	1.67
-25	-3	5	13.05	1.73
25	-3	5	12.29	1.60
-25	-3	-5	15.15	1.72
-25	3	5	15.92	1.79
-25	-3	-5	11.04	1.55
0	0	6	148.59	6.94
0	0	6	168.20	7.17
0	0	6	161.59	7.10
0	0	6	150.83	7.06
0	0	6	150.52	7.03
0	0	-6	98.73	4.57
0	0	-6	95.52	4.50
0	0	-6	101.65	4.63
0	0	-6	98.06	4.41
0	0	-6	98.75	4.53
0	0	6	150.55	6.99
0	0	-6	95.87	4.47
0	-1	-6	52.14	2.44
0	-1	6	24.05	1.20
0	-1	6	24.01	1.25

0	-1	-6	54.59	2.45
0	-1	-6	55.48	2.56
0	-1	-6	52.24	2.56
0	1	6	22.47	1.28
0	-1	-6	52.87	2.53
0	-1	-6	56.38	2.66
0	1	-6	52.39	2.47
0	1	-6	49.71	2.64
0	1	-6	52.51	2.46
0	1	-6	56.17	2.70
0	-1	6	26.24	1.39
0	-1	6	27.12	1.44
0	1	6	24.86	1.26
0	-1	-6	52.73	2.55
0	-1	6	24.23	1.18
0	1	-6	55.34	2.53
0	1	6	26.33	1.31
0	1	6	26.52	1.39
0	-1	6	24.08	1.31
0	1	-6	54.17	2.58
0	1	6	24.22	1.29
0	-2	6	379.00	18.35
0	2	6	429.60	18.37
0	-2	-6	426.70	19.36
0	2	6	388.29	18.37
0	-2	-6	424.42	19.30
0	2	-6	428.63	19.31
0	2	-6	471.22	19.50
0	-2	-6	407.18	19.24
0	2	6	388.80	18.42
0	2	-6	425.72	19.26
0	2	6	436.26	18.50
0	2	-6	426.75	19.20
0	-2	-6	418.03	19.31
0	-2	-6	439.46	19.32
0	-2	6	405.61	18.26
0	2	6	372.56	18.28
0	-2	6	439.70	18.52
0	2	-6	436.35	19.43
0	-2	6	410.35	18.41
0	2	-6	436.46	19.36
0	-2	6	388.30	18.31
0	-2	-6	427.35	19.41
0	2	6	431.27	18.38

0	-2	6	444.53	18.51
0	-3	6	267.88	11.70
0	3	6	258.82	11.50
0	3	-6	247.83	10.88
0	3	6	259.86	11.56
0	-3	6	245.02	11.49
0	-3	6	240.49	11.53
0	-3	-6	227.22	10.63
0	3	-6	237.52	10.64
0	3	6	243.26	11.62
0	3	6	264.40	11.66
0	3	-6	233.47	10.68
0	3	-6	231.22	10.53
0	3	-6	240.98	10.73
0	-3	-6	245.48	10.69
0	-3	6	255.76	11.59
0	3	6	251.55	11.54
0	-3	-6	227.16	10.76
0	-3	-6	225.36	10.67
0	3	-6	239.53	10.82
0	-3	-6	236.77	10.75
0	3	6	236.68	11.46
0	-3	6	282.26	11.72
0	-4	6	21.11	1.47
0	-4	-6	17.25	1.18
0	-4	-6	15.61	1.03
0	-4	-6	16.14	1.17
0	4	-6	18.32	1.24
0	4	6	19.71	1.35
0	4	6	21.78	1.26
0	4	6	20.88	1.24
0	4	-6	17.86	1.05
0	-4	-6	15.89	0.98
0	4	6	22.39	1.20
0	-4	-6	16.42	1.04
0	-4	6	22.64	1.32
0	-4	6	20.99	1.25
0	4	-6	16.41	1.26
0	-4	6	21.22	1.21
0	-4	6	24.02	1.54
0	4	-6	18.15	1.18
0	4	6	22.09	1.18
0	4	6	22.16	1.41
0	4	-6	16.72	1.02

0	5	-6	11.83	1.15
0	5	6	18.28	1.14
0	5	6	20.64	1.14
0	-5	6	18.78	1.15
0	5	-6	13.87	0.98
0	5	-6	12.37	1.02
0	-5	6	19.62	1.26
0	-5	-6	11.72	1.20
0	5	-6	11.39	1.05
0	5	6	17.68	1.35
0	-5	6	20.45	1.46
0	5	6	18.79	1.13
0	5	6	18.42	1.20
0	-5	6	18.98	1.20
0	5	-6	13.79	0.92
0	-5	-6	11.24	0.84
0	-5	-6	12.51	0.95
0	5	-6	13.13	0.68
0	5	6	17.82	1.20
0	-5	-6	12.33	1.18
0	-5	-6	12.56	0.90
0	-5	6	20.87	1.47
0	5	6	21.24	1.37
0	-6	-6	30.56	1.89
0	-6	6	36.52	1.94
0	-6	6	33.89	2.14
0	-6	6	31.42	1.86
0	6	6	33.17	1.86
0	6	-6	33.45	1.58
0	6	-6	30.58	1.80
0	6	6	31.05	1.82
0	6	-6	33.95	1.91
0	-6	6	31.56	1.57
0	-6	6	33.56	1.81
0	6	6	32.85	1.77
0	6	6	31.32	2.07
0	-6	-6	30.88	1.79
0	6	6	31.58	1.79
0	-6	-6	36.63	1.87
0	6	6	35.94	1.78
0	-6	6	35.09	2.11
0	6	-6	34.30	1.94
0	-6	-6	31.97	1.77
0	-6	-6	31.87	2.00

0	6	-6	31.99	1.99
0	6	6	35.07	2.10
0	6	-6	32.38	2.14
0	7	-6	83.73	3.85
0	-7	6	93.78	4.57
0	-7	-6	80.51	4.08
0	7	6	89.82	4.51
0	-7	-6	83.27	4.07
0	-7	-6	87.71	4.33
0	-7	6	99.77	4.73
0	-7	6	100.97	4.90
0	-7	6	89.92	4.61
0	7	-6	82.85	4.11
0	-7	-6	79.63	4.20
0	7	-6	78.43	4.26
0	7	-6	84.97	4.22
0	7	-6	82.73	4.46
0	7	6	91.86	4.53
0	7	6	91.24	4.61
0	-7	-6	92.59	4.17
0	7	6	99.88	4.86
0	-7	6	89.69	4.30
0	7	6	92.90	4.53
0	7	6	89.29	4.84
0	-7	6	100.83	4.81
0	-7	6	97.11	4.66
0	8	-6	222.11	10.91
0	-8	6	218.96	9.70
0	8	6	195.35	9.52
0	8	6	217.49	9.79
0	8	6	216.69	9.40
0	-8	6	187.58	9.52
0	8	6	196.74	9.81
0	-8	-6	231.70	10.73
0	-8	-6	212.01	10.43
0	8	6	190.57	9.39
0	8	-6	207.95	10.63
0	8	-6	261.18	11.18
0	8	-6	223.31	10.21
0	-8	-6	215.38	10.47
0	-8	6	212.00	9.57
0	-8	-6	211.39	10.59
0	-8	6	216.60	9.81
0	-8	6	207.80	9.62

0	-8	-6	240.52	10.53
0	-8	6	196.55	9.47
0	8	6	186.95	9.43
0	8	6	207.11	9.43
0	8	-6	244.25	10.66
0	-9	6	181.16	9.09
0	9	6	190.26	8.95
0	9	6	179.85	9.40
0	-9	6	188.39	9.03
0	9	6	178.84	9.05
0	9	-6	179.00	8.17
0	-9	-6	189.75	8.78
0	9	-6	199.32	8.73
0	9	-6	175.45	8.96
0	-9	-6	175.59	8.47
0	-9	-6	189.92	8.54
0	-9	6	212.53	9.62
0	-9	-6	166.19	8.59
0	-9	-6	169.21	8.40
0	9	6	185.50	8.94
0	9	6	204.82	8.93
0	-9	6	205.43	9.23
0	-9	6	182.07	8.68
0	9	-6	176.74	8.66
0	-9	6	196.71	9.11
0	-9	6	203.29	9.22
0	10	6	55.95	3.06
0	-10	6	59.39	3.61
0	-10	-6	36.26	2.24
0	10	-6	36.94	2.59
0	10	-6	38.54	3.01
0	-10	-6	37.57	2.46
0	-10	6	62.01	3.28
0	10	6	59.93	3.57
0	10	6	59.52	3.07
0	10	6	51.67	3.59
0	10	-6	45.40	2.72
0	-10	6	53.23	3.18
0	10	-6	35.88	1.97
0	-10	6	56.17	3.15
0	-10	-6	39.88	2.35
0	-10	-6	38.43	2.52
0	-10	-6	40.83	2.43
0	11	6	1.57	1.23

0	-11	-6	1.14	0.67
0	-11	-6	0.97	0.81
0	-11	6	0.60	0.67
0	11	6	0.42	0.83
0	11	-6	0.28	1.61
0	11	-6	1.45	0.94
0	-11	-6	0.85	0.76
0	-11	-6	0.84	0.81
0	12	-6	14.76	2.82
0	12	6	8.95	3.13
0	-12	6	13.94	1.87
0	-12	-6	17.29	1.77
0	-12	-6	14.32	1.45
0	-12	-6	15.89	2.16
0	-12	6	12.91	1.55
0	12	-6	16.44	2.05
0	13	-6	14.46	2.63
0	-13	6	35.19	2.69
0	-13	-6	20.11	2.23
0	-13	6	33.14	2.34
0	13	-6	19.03	2.49
0	-13	-6	22.80	1.91
0	-13	-6	19.21	1.78
0	13	6	28.53	3.53
1	0	-6	0.09	0.24
-1	0	-6	0.09	0.29
1	0	-6	0.00	0.06
-1	0	6	0.13	0.06
1	0	6	0.32	0.29
-1	0	6	0.01	0.25
-1	0	-6	-0.03	0.19
-1	0	6	0.27	0.27
-1	0	-6	0.06	0.23
1	0	-6	0.01	0.15
-1	0	6	0.07	0.16
1	0	-6	-0.09	0.15
1	0	6	-0.03	0.19
-1	0	-6	0.04	0.10
1	0	6	-0.01	0.15
-1	0	-6	0.07	0.15
1	0	6	0.06	0.10
-1	0	6	0.05	0.20
1	0	6	-0.02	0.17
1	0	-6	0.04	0.20

-1	0	6	-0.13	0.14
-1	0	-6	0.02	0.16
1	0	6	-0.06	0.23
1	0	-6	-0.14	0.22
1	-1	-6	35.18	1.77
-1	1	-6	35.32	1.67
1	-1	-6	34.93	1.83
-1	-1	6	31.55	1.58
-1	-1	6	29.12	1.55
1	-1	-6	34.59	1.74
1	-1	-6	36.53	1.74
-1	1	-6	34.85	1.86
-1	-1	6	28.39	1.35
-1	1	-6	36.04	1.68
1	1	6	26.57	1.41
1	1	6	29.81	1.58
1	1	6	28.63	1.46
1	-1	-6	34.80	1.73
-1	-1	6	26.71	1.48
1	-1	-6	34.56	1.62
1	1	6	27.75	1.46
-1	-1	6	27.31	1.40
-1	-1	6	26.59	1.33
-1	1	-6	37.36	1.74
1	1	6	28.61	1.46
-1	1	-6	35.30	1.77
-1	1	-6	37.41	1.91
-1	1	6	28.91	1.44
1	-1	6	28.09	1.47
1	-1	6	32.58	1.64
1	1	-6	33.84	1.66
-1	-1	-6	33.24	1.74
-1	1	6	27.95	1.45
1	1	-6	34.78	1.64
-1	-1	-6	35.27	1.75
1	1	-6	37.96	1.89
1	-1	6	27.90	1.43
-1	1	6	27.19	1.31
1	1	-6	33.54	1.92
-1	1	6	29.05	1.48
-1	1	6	28.47	1.42
-1	-1	-6	36.06	1.86
1	-1	6	29.49	1.37
1	-1	6	29.82	1.56

1	1	-6	36.24	1.73
-1	-1	-6	34.91	1.65
-1	1	6	29.07	1.54
1	1	-6	35.61	1.78
-1	-1	-6	32.59	1.73
1	-1	6	27.88	1.36
-1	-1	-6	36.49	1.66
1	-2	-6	115.70	5.37
1	2	6	132.20	5.88
-1	2	-6	114.46	5.39
-1	-2	6	125.19	5.84
-1	2	-6	115.28	5.34
-1	-2	6	136.69	6.03
-1	2	-6	113.34	5.51
1	-2	-6	120.80	5.44
-1	-2	6	121.10	5.88
-1	2	-6	117.19	5.29
-1	-2	6	137.23	6.05
1	-2	-6	115.83	5.30
-1	-2	6	130.74	5.78
1	2	6	122.22	5.83
1	2	6	117.31	6.01
1	2	6	123.64	5.95
-1	-2	6	126.49	5.95
-1	2	-6	121.88	5.43
1	-2	-6	118.03	5.40
1	-2	-6	116.14	5.39
1	-2	-6	120.81	5.49
1	2	6	136.56	6.04
1	2	6	128.65	5.90
-1	2	-6	123.84	5.58
1	-2	6	129.75	5.94
1	2	-6	118.17	5.37
-1	2	6	134.71	5.92
-1	-2	-6	111.53	5.38
-1	-2	-6	112.47	5.32
1	-2	6	134.78	6.06
1	2	-6	120.59	5.45
1	2	-6	117.66	5.32
1	-2	6	141.13	6.10
-1	2	6	120.75	5.81
-1	-2	-6	116.75	5.41
-1	2	6	139.30	6.04
-1	-2	-6	118.66	5.49

-1	2	6	122.74	5.89
1	-2	6	123.88	5.85
1	-2	6	129.10	5.81
1	-2	6	123.76	5.90
1	2	-6	114.09	5.66
-1	2	6	133.70	5.93
-1	-2	-6	113.50	5.35
1	2	-6	118.42	5.49
-1	-2	-6	117.19	5.42
-1	2	6	125.48	5.97
1	2	-6	120.45	5.26
1	3	6	82.33	4.22
1	3	6	83.10	4.14
1	-3	-6	78.60	3.75
1	-3	-6	74.15	3.65
-1	-3	6	87.12	4.08
1	3	6	91.01	4.25
-1	3	-6	78.23	3.61
-1	-3	6	85.60	4.11
1	3	6	88.68	4.07
-1	3	-6	74.95	3.79
-1	3	-6	78.21	3.51
-1	3	-6	80.37	3.70
-1	-3	6	85.87	4.18
-1	-3	6	94.55	4.28
1	-3	-6	78.53	3.73
1	3	6	89.17	4.06
1	3	6	84.23	4.13
1	-3	-6	78.91	3.62
-1	3	-6	75.64	3.65
-1	-3	6	93.01	4.28
-1	3	-6	78.43	3.51
-1	-3	-6	80.32	3.68
-1	3	6	86.80	4.05
-1	-3	-6	75.47	3.59
-1	3	6	96.40	4.27
1	3	-6	74.23	3.79
1	-3	6	97.18	4.34
1	3	-6	78.57	3.77
1	-3	6	82.14	4.40
-1	-3	-6	74.06	3.64
1	-3	6	85.51	4.08
-1	-3	-6	81.96	3.72
1	-3	6	82.26	4.12

-1	3	6	93.62	4.10
-1	3	6	91.20	4.12
-1	3	6	87.78	4.11
1	-3	6	86.64	4.15
-1	3	6	83.95	4.22
1	3	-6	79.55	3.60
-1	-3	-6	74.37	3.73
1	3	-6	72.27	3.72
1	3	-6	76.79	3.64
-1	-4	6	38.43	1.96
1	-4	-6	38.75	2.07
-1	4	-6	40.03	2.24
1	4	6	36.25	2.09
1	-4	-6	40.37	2.02
1	-4	-6	44.46	2.07
-1	4	-6	40.56	2.05
1	4	6	40.02	1.90
1	4	6	37.17	1.88
1	4	6	38.30	1.99
-1	4	-6	41.21	2.14
-1	-4	6	41.10	2.21
1	4	6	37.39	1.93
1	-4	-6	41.60	2.19
-1	4	-6	40.46	2.31
1	4	6	40.91	2.15
-1	4	-6	41.22	1.91
-1	-4	6	41.66	2.23
1	4	6	35.16	2.01
-1	-4	6	40.18	2.07
-1	-4	6	38.32	1.98
-1	4	-6	41.67	1.91
-1	4	-6	41.10	2.10
1	-4	6	37.38	2.00
-1	4	6	41.08	2.02
1	-4	6	39.42	2.05
1	4	-6	41.60	2.17
-1	4	6	41.64	2.14
1	-4	6	36.69	1.95
-1	4	6	38.48	2.16
-1	4	6	38.64	1.97
1	4	-6	40.14	2.22
-1	-4	-6	39.35	2.03
1	4	-6	42.40	2.06
1	-4	6	40.62	2.23

-1	-4	-6	38.82	2.08
1	4	-6	42.35	2.10
1	4	-6	38.48	2.28
-1	4	6	41.04	1.98
1	-4	6	38.82	2.19
-1	-4	-6	42.46	2.19
-1	4	6	40.91	1.96
-1	4	6	32.90	1.97
-1	-4	-6	38.97	2.15
-1	5	-6	21.72	1.52
-1	-5	6	18.63	1.29
1	-5	-6	24.71	1.51
1	5	6	18.30	1.23
1	5	6	19.16	1.42
-1	5	-6	21.46	1.65
1	-5	-6	21.43	1.40
1	-5	-6	23.17	1.31
-1	5	-6	22.61	1.36
1	5	6	19.90	1.42
1	-5	-6	25.85	1.36
-1	-5	6	21.06	1.46
-1	-5	6	19.01	1.21
1	5	6	19.55	1.15
-1	5	-6	23.89	1.44
1	5	6	19.80	1.17
1	5	6	18.93	1.22
-1	5	-6	22.41	1.44
1	5	6	20.78	1.13
-1	-5	6	23.27	1.51
1	-5	-6	22.73	1.35
-1	-5	6	19.70	1.19
-1	5	-6	23.70	1.17
-1	-5	-6	23.29	1.35
1	-5	6	18.97	1.24
1	5	-6	21.74	1.58
-1	5	6	20.96	1.25
-1	-5	-6	22.70	1.32
-1	5	6	20.36	1.18
1	5	-6	24.10	1.36
-1	5	6	22.60	1.46
1	-5	6	20.27	1.53
-1	5	6	19.90	1.21
-1	5	6	20.98	1.20
-1	5	6	19.84	1.39

-1	-5	-6	22.98	1.46
-1	5	6	19.95	1.18
1	5	-6	23.32	1.51
1	-5	6	19.83	1.26
1	-5	6	18.61	1.17
1	5	-6	24.03	1.41
1	5	-6	23.24	1.49
-1	-5	-6	24.63	1.43
-1	-5	-6	24.06	1.53
1	-5	6	22.46	1.51
1	6	6	14.09	1.09
1	6	6	15.99	1.13
1	6	6	15.28	1.29
-1	-6	6	17.34	1.39
-1	6	-6	13.32	1.33
1	-6	-6	14.08	1.02
-1	6	-6	15.08	0.86
1	-6	-6	13.17	0.98
-1	-6	6	14.77	1.13
-1	6	-6	13.62	1.24
-1	-6	6	18.65	1.46
-1	-6	6	15.93	1.20
1	6	6	17.31	1.01
1	-6	-6	14.49	1.15
-1	-6	6	14.01	1.04
1	6	6	15.96	1.31
-1	6	-6	15.51	1.16
1	6	6	15.99	1.07
1	6	6	15.36	1.15
-1	6	-6	15.25	1.08
-1	6	-6	16.04	1.20
1	-6	-6	14.80	1.31
1	-6	-6	16.15	1.09
1	6	-6	14.42	1.22
1	6	-6	14.29	1.07
1	6	-6	13.52	1.32
-1	-6	-6	13.99	1.19
-1	-6	-6	15.09	1.14
-1	-6	-6	14.73	1.02
-1	6	6	14.73	1.10
-1	6	6	15.08	1.12
-1	-6	-6	16.03	1.29
-1	6	6	13.73	1.36
1	-6	6	18.43	1.45

1	-6	6	16.23	0.87
-1	6	6	15.07	1.06
1	-6	6	17.07	1.08
1	-6	6	16.23	1.15
1	6	-6	15.40	1.14
1	-6	6	14.54	1.12
-1	6	6	17.60	1.40
1	-6	6	19.28	1.53
-1	6	6	15.39	1.11
-1	-6	-6	14.40	1.03
1	-7	-6	8.18	1.22
-1	-7	6	7.47	1.14
1	7	6	6.55	0.90
-1	-7	6	7.83	1.24
1	-7	-6	7.47	0.81
-1	-7	6	7.04	0.93
1	7	6	8.78	1.23
-1	7	-6	8.78	0.95
1	-7	-6	7.31	0.99
-1	7	-6	7.59	1.08
-1	-7	6	7.19	0.80
1	7	6	7.87	0.76
1	7	6	7.29	1.11
1	7	6	8.27	0.87
-1	-7	6	8.59	1.25
-1	7	-6	8.48	0.66
-1	7	-6	8.29	1.25
-1	7	-6	9.93	1.07
1	-7	-6	7.30	0.81
1	-7	-6	9.42	0.95
1	7	6	6.21	0.90
-1	-7	6	6.86	0.96
1	7	6	7.89	0.77
-1	7	6	7.11	0.85
1	-7	6	7.14	1.29
1	-7	6	8.25	1.21
-1	7	6	7.04	0.86
-1	-7	-6	7.67	0.84
-1	7	6	7.44	0.91
1	7	-6	6.74	0.92
1	-7	6	6.46	0.90
-1	7	6	7.74	1.27
-1	7	6	8.66	0.89
-1	-7	-6	9.74	1.03

-1	-7	-6	10.99	1.20
-1	7	6	7.44	0.86
1	-7	6	8.42	0.82
1	7	-6	8.86	1.01
-1	7	6	5.22	1.05
1	7	-6	8.78	1.06
-1	-7	-6	8.90	0.85
1	-7	6	6.48	0.91
1	-7	6	8.09	1.10
1	7	-6	7.64	1.08
-1	-8	6	27.53	1.76
1	8	6	28.53	1.71
1	-8	-6	28.29	2.06
1	-8	-6	26.69	1.66
1	8	6	25.59	1.74
1	8	6	29.24	2.28
-1	-8	6	28.14	2.05
1	8	6	26.54	2.06
-1	8	-6	26.01	2.06
1	8	6	26.98	1.77
-1	-8	6	32.21	2.22
-1	8	-6	29.71	1.98
-1	-8	6	31.86	2.14
-1	-8	6	29.23	1.91
1	-8	-6	29.98	1.77
1	-8	-6	26.80	1.69
1	8	6	26.57	1.85
-1	-8	6	28.96	1.84
-1	8	-6	26.55	2.04
-1	8	-6	29.10	1.47
1	-8	-6	24.75	1.78
1	-8	6	29.37	2.03
-1	-8	-6	27.52	2.02
-1	8	6	27.97	1.80
-1	-8	-6	23.42	1.63
-1	8	6	27.96	1.76
1	-8	6	30.74	2.16
1	8	-6	28.39	1.91
-1	8	6	32.17	1.78
1	-8	6	26.87	1.81
-1	-8	-6	26.59	1.79
1	-8	6	30.39	2.29
1	-8	6	28.54	1.45
-1	8	6	28.34	1.78

1	-8	6	29.75	1.86
-1	-8	-6	24.80	1.67
-1	-8	-6	28.25	1.87
1	8	-6	28.11	1.94
1	8	-6	28.51	1.89
1	8	-6	26.16	2.15
-1	8	6	27.18	1.76
-1	8	6	30.95	2.23
1	-8	6	27.79	1.76
-1	8	6	27.43	2.05
1	-9	-6	12.86	1.56
1	9	6	8.35	1.45
-1	9	-6	11.60	1.52
-1	9	-6	12.84	1.81
-1	9	-6	16.57	1.67
1	9	6	7.91	1.06
-1	-9	6	9.16	1.16
1	9	6	7.33	1.14
1	-9	-6	10.35	1.15
1	9	6	6.75	1.17
-1	-9	6	7.33	1.04
1	9	6	8.18	1.47
1	-9	-6	14.22	1.39
1	9	6	8.26	0.99
-1	-9	6	6.84	1.20
1	-9	-6	13.29	1.31
-1	-9	6	6.18	1.19
-1	-9	6	11.09	1.66
1	-9	-6	11.43	1.14
1	-9	6	8.90	1.78
-1	-9	-6	11.54	1.20
-1	9	6	6.91	1.28
-1	-9	-6	11.68	1.65
1	-9	6	8.86	0.69
1	-9	6	9.65	1.42
-1	-9	-6	10.72	1.27
-1	9	6	9.38	1.12
1	9	-6	12.83	1.45
-1	9	6	9.69	1.14
-1	-9	-6	15.26	1.51
1	-9	6	10.62	1.17
-1	9	6	8.02	1.66
1	9	-6	11.78	1.75
-1	9	6	9.00	1.17

-1	9	6	7.38	1.19
1	-9	6	8.57	1.10
1	9	-6	14.52	1.53
1	-9	6	9.86	1.52
-1	-9	-6	11.87	1.14
1	-9	6	8.23	1.06
1	-10	-6	8.66	1.15
-1	10	-6	11.20	1.74
-1	10	-6	11.26	1.57
1	-10	-6	8.14	1.08
1	-10	-6	10.41	1.26
1	10	6	6.90	1.44
1	10	6	7.32	1.11
1	-10	-6	10.15	1.28
-1	-10	6	7.40	1.15
1	-10	-6	13.77	2.72
-1	10	-6	9.78	1.66
1	-10	-6	9.01	1.20
1	10	6	8.56	1.70
1	10	6	6.26	0.92
-1	10	-6	10.17	1.02
1	10	6	6.05	1.10
-1	-10	6	7.79	1.27
-1	-10	6	6.40	1.12
-1	-10	6	12.46	1.88
1	-10	6	5.95	0.69
-1	-10	-6	10.89	1.22
1	10	-6	9.20	1.46
1	-10	6	8.27	1.07
-1	10	6	6.19	1.49
1	-10	6	7.04	1.28
-1	-10	-6	11.96	1.16
1	10	-6	12.97	1.87
-1	-10	-6	8.05	1.37
1	10	-6	9.61	1.84
-1	10	6	7.15	1.05
-1	10	6	6.61	1.21
-1	-10	-6	12.62	1.56
-1	10	6	6.62	1.46
1	-10	6	5.75	1.16
1	-11	-6	7.80	1.22
1	-11	-6	4.73	0.92
1	11	6	7.49	1.41
-1	-11	6	7.10	1.19

-1	11	-6	5.31	1.72
1	-11	-6	5.75	0.89
1	-11	-6	6.35	1.11
1	11	6	6.46	1.25
-1	11	-6	4.77	1.26
1	-11	-6	3.97	1.04
-1	-11	6	8.33	1.27
1	-11	6	7.00	1.18
1	-11	6	5.11	0.68
1	-11	6	5.73	0.95
1	11	-6	4.97	1.58
1	11	-6	5.45	1.14
-1	-11	-6	6.80	1.12
-1	11	6	7.46	1.42
-1	-11	-6	6.79	1.18
1	-11	6	7.30	1.34
-1	-11	-6	5.36	0.84
-1	11	6	6.73	1.57
-1	-11	-6	6.44	1.03
-1	11	6	11.82	2.13
1	-12	-6	2.68	0.86
1	12	6	2.97	1.64
1	-12	-6	4.04	1.03
-1	-12	6	3.86	1.16
-1	-12	6	3.14	0.94
-1	12	-6	2.35	1.26
1	-12	-6	3.30	1.13
-1	12	-6	3.93	1.81
-1	-12	-6	1.65	1.06
1	-12	6	2.38	1.12
-1	-12	-6	3.33	1.01
-1	12	6	1.92	1.70
1	-12	6	3.34	0.96
1	12	-6	0.24	1.62
-1	-12	-6	2.76	0.81
-1	12	6	3.08	1.03
-1	-13	6	2.38	0.97
-1	13	-6	4.15	2.20
1	-13	-6	3.82	1.24
1	-13	-6	2.88	0.93
-1	13	-6	3.90	1.53
-1	-13	6	3.21	1.51
1	-13	-6	5.80	1.07
1	13	6	2.55	2.00

-1	13	6	1.38	1.89
-1	-13	-6	3.39	1.02
1	13	-6	4.70	2.40
1	13	-6	5.16	1.43
-1	-13	-6	3.99	0.98
-1	-13	-6	5.03	1.34
1	-13	6	1.65	1.33
1	-13	6	3.12	0.97
-2	0	-6	102.55	4.56
-2	0	-6	103.92	4.76
2	0	-6	105.70	4.71
2	0	6	113.87	5.27
2	0	-6	99.21	4.58
2	0	6	124.47	5.38
-2	0	-6	100.70	4.62
-2	0	6	106.81	5.22
-2	0	6	118.44	5.30
2	0	6	109.50	5.15
2	0	-6	100.03	4.69
2	0	6	108.93	5.18
-2	0	-6	100.40	4.59
-2	0	6	111.78	5.19
2	0	-6	97.49	4.62
-2	0	6	119.34	5.30
2	0	6	109.72	5.24
-2	0	-6	92.82	4.66
-2	0	6	115.82	5.18
-2	1	-6	169.98	7.29
-2	-1	6	104.81	4.88
-2	1	-6	164.06	7.37
2	1	6	108.70	4.96
-2	-1	6	111.28	4.90
2	-1	-6	165.01	7.39
2	-1	-6	167.14	7.35
-2	-1	6	104.62	4.94
-2	-1	6	109.76	5.06
2	1	6	102.94	4.92
2	1	6	101.10	4.99
-2	1	-6	161.85	7.33
2	1	6	110.89	4.99
-2	1	-6	169.95	7.51
2	-1	-6	143.07	7.37
-2	1	-6	168.24	7.29
2	-1	-6	162.20	7.32

2	1	6	114.93	5.12
2	-1	-6	170.92	7.43
-2	-1	6	112.46	5.08
2	1	-6	163.01	7.23
2	-1	6	118.50	5.17
-2	1	6	117.49	5.08
-2	-1	-6	156.70	7.33
2	1	-6	161.27	7.26
-2	-1	-6	165.02	7.35
2	-1	6	113.18	5.09
2	1	-6	165.90	7.38
-2	-1	-6	137.76	7.37
-2	1	6	111.15	4.97
-2	1	6	113.35	5.04
-2	1	6	105.49	4.97
2	-1	6	95.28	5.07
2	1	-6	142.12	7.57
2	-1	6	104.87	4.97
-2	-1	-6	163.50	7.28
2	-1	6	113.13	4.93
-2	1	6	99.76	4.85
-2	-1	-6	161.19	7.37
2	-1	6	106.44	4.93
-2	1	6	104.93	4.99
-2	-1	-6	166.76	7.45
-2	-2	6	197.35	9.09
-2	2	-6	200.81	9.29
2	-2	-6	194.09	9.43
2	2	6	205.17	8.99
-2	2	-6	209.14	9.25
-2	2	-6	213.93	9.33
-2	2	-6	204.13	9.21
2	2	6	209.58	9.16
-2	-2	6	194.21	8.97
2	-2	-6	189.85	9.46
-2	-2	6	188.13	8.99
-2	2	-6	208.53	9.43
2	-2	-6	197.04	9.19
2	-2	-6	194.62	9.29
2	2	6	185.59	8.97
2	2	6	199.80	9.02
2	-2	-6	217.10	9.35
-2	2	-6	218.08	9.49
2	-2	-6	203.43	9.25

-2	-2	6	208.14	9.12
-2	-2	6	212.59	9.17
2	2	6	197.69	9.08
-2	2	6	198.03	9.04
2	2	-6	212.56	9.35
-2	-2	-6	209.48	9.33
2	-2	6	212.81	9.18
-2	2	6	185.78	8.93
2	-2	6	201.49	8.96
-2	-2	-6	196.48	9.28
-2	-2	-6	195.39	9.23
2	-2	6	203.64	9.07
2	2	-6	209.70	9.41
2	2	-6	210.70	9.22
-2	-2	-6	203.95	9.38
-2	2	6	195.31	9.09
-2	-2	-6	210.76	9.32
-2	2	6	207.40	9.08
-2	2	6	213.71	9.15
-2	2	6	189.08	9.00
2	-2	6	180.86	8.99
-2	-2	-6	199.08	9.23
2	2	-6	209.14	9.39
2	-3	-6	117.52	5.68
2	3	6	130.10	5.68
-2	3	-6	126.43	5.58
-2	3	-6	129.78	5.72
2	3	6	123.31	5.82
-2	-3	6	131.89	5.92
-2	-3	6	120.25	5.73
2	-3	-6	129.36	5.78
2	-3	-6	124.15	5.63
2	-3	-6	116.79	5.61
-2	3	-6	125.35	5.66
2	3	6	125.54	5.85
-2	-3	6	119.98	5.84
-2	3	-6	122.77	5.70
2	3	6	128.46	5.90
2	-3	-6	117.79	5.76
-2	3	-6	119.85	5.84
-2	-3	6	121.56	5.72
-2	3	-6	132.69	5.93
-2	3	-6	124.43	5.57
2	3	6	119.64	5.72

2	-3	6	120.61	5.78
-2	3	6	120.49	5.69
-2	3	6	131.48	5.90
2	-3	6	131.84	6.12
-2	3	6	118.16	5.87
2	3	-6	120.27	5.82
-2	3	6	131.54	5.77
2	3	-6	121.58	5.77
-2	-3	-6	119.74	5.63
-2	-3	-6	131.71	5.75
-2	3	6	123.31	5.73
2	3	-6	122.58	5.79
2	-3	6	122.86	5.73
-2	-3	-6	127.40	5.73
2	3	-6	127.49	5.64
-2	-3	-6	113.98	5.76
2	-3	6	131.27	5.98
-2	-3	-6	117.24	5.57
-2	-3	-6	118.79	5.68
-2	3	6	118.91	5.79
-2	3	6	130.68	5.80
2	-3	6	126.72	5.80
2	4	6	1.67	0.40
2	4	6	1.31	0.26
2	-4	-6	0.81	0.30
2	-4	-6	0.65	0.43
2	4	6	1.05	0.35
2	-4	-6	0.80	0.26
-2	4	-6	0.90	0.22
-2	-4	6	1.48	0.34
-2	-4	6	1.27	0.31
-2	-4	6	2.19	0.58
2	4	6	1.72	0.33
-2	4	-6	1.10	0.22
-2	-4	6	2.03	0.55
2	-4	-6	1.00	0.31
-2	4	-6	0.97	0.33
2	4	6	1.80	0.35
2	-4	-6	0.66	0.32
-2	-4	6	1.23	0.39
-2	4	-6	0.64	0.45
-2	4	-6	1.32	0.52
-2	4	-6	0.83	0.37
2	4	6	1.38	0.43

2	4	6	1.46	0.41
-2	4	6	1.91	0.33
-2	-4	-6	0.56	0.40
-2	4	6	1.27	0.40
2	-4	6	1.93	0.40
-2	4	6	1.59	0.36
2	4	-6	0.85	0.30
-2	4	6	1.48	0.45
2	4	-6	0.74	0.33
-2	-4	-6	0.85	0.31
-2	4	6	1.50	0.49
-2	4	6	2.19	0.41
2	-4	6	1.34	0.36
2	-4	6	2.10	0.64
2	-4	6	1.43	0.30
2	4	-6	1.08	0.45
2	4	-6	0.91	0.46
2	4	-6	0.85	0.43
2	-4	6	1.69	0.55
-2	-4	-6	1.26	0.49
-2	-4	-6	0.80	0.30
-2	-4	-6	1.17	0.44
-2	-5	6	44.89	2.47
-2	5	-6	43.42	2.50
2	5	6	44.13	2.47
-2	5	-6	45.77	2.36
2	5	6	48.11	2.50
2	-5	-6	44.43	2.25
-2	-5	6	43.30	2.27
2	-5	-6	47.71	2.26
-2	5	-6	46.84	2.31
2	-5	-6	42.84	2.29
2	5	6	42.58	2.16
-2	5	-6	47.18	2.39
2	-5	-6	43.07	2.40
2	5	6	46.01	2.16
-2	-5	6	44.13	2.40
2	5	6	44.76	2.28
2	-5	-6	46.23	2.47
-2	-5	6	49.90	2.55
-2	5	-6	46.17	2.15
-2	-5	6	42.94	2.28
2	5	6	40.28	2.33
-2	5	-6	44.41	2.57

2	5	6	44.16	2.26
-2	-5	-6	46.64	2.43
2	-5	6	44.96	2.35
-2	5	6	45.56	2.33
-2	5	6	44.80	2.25
-2	5	6	45.99	2.31
2	-5	6	44.50	2.33
-2	5	6	45.70	2.49
-2	5	6	43.07	2.25
2	-5	6	44.55	2.28
-2	-5	-6	42.79	2.28
-2	-5	-6	44.64	2.37
2	5	-6	46.13	2.29
-2	-5	-6	43.51	2.41
2	5	-6	42.58	2.51
-2	-5	-6	42.05	2.26
2	-5	6	49.15	2.67
2	5	-6	47.60	2.35
2	5	-6	42.37	2.41
2	-5	6	47.84	2.55
2	5	-6	45.80	2.43
-2	6	-6	36.10	2.33
-2	-6	6	42.47	2.38
-2	-6	6	40.42	2.18
2	-6	-6	33.64	2.08
-2	6	-6	38.86	1.83
2	6	6	39.19	2.27
2	-6	-6	38.81	2.09
2	6	6	37.52	2.07
-2	6	-6	38.10	2.11
-2	-6	6	39.50	2.19
-2	6	-6	40.14	2.11
2	6	6	45.06	2.44
2	6	6	40.72	2.42
2	-6	-6	39.00	2.22
-2	-6	6	45.88	2.48
2	6	6	39.59	2.16
-2	-6	6	41.60	2.32
2	-6	-6	34.91	1.95
-2	6	-6	36.68	2.22
2	-6	-6	34.23	1.97
-2	6	-6	36.28	2.02
2	6	6	42.16	2.04
-2	-6	6	42.44	2.45

2	6	6	41.56	2.18
2	6	-6	36.87	2.14
-2	6	6	39.93	2.18
2	6	-6	36.32	2.05
2	6	-6	36.52	2.28
2	-6	6	35.06	2.43
2	-6	6	41.30	2.00
2	-6	6	46.15	2.56
-2	-6	-6	37.43	1.99
-2	6	6	41.74	2.22
-2	6	6	41.80	2.17
-2	-6	-6	37.89	2.13
-2	-6	-6	36.45	1.98
2	-6	6	44.34	2.48
-2	6	6	41.43	2.46
-2	-6	-6	39.63	2.13
2	-6	6	43.60	2.25
-2	6	6	42.23	2.41
-2	-6	-6	36.37	2.11
2	-6	6	40.80	2.18
-2	6	6	41.22	2.21
2	6	-6	35.59	1.99
-2	6	6	45.21	2.24
-2	-7	6	81.37	4.08
2	7	6	74.98	3.92
2	7	6	82.45	4.11
-2	7	-6	85.54	4.53
2	-7	-6	89.90	4.41
2	-7	-6	77.47	4.23
-2	-7	6	70.72	3.82
2	7	6	73.79	3.77
-2	-7	6	77.55	3.96
-2	7	-6	86.82	4.15
-2	7	-6	89.24	3.94
2	7	6	77.06	3.80
2	-7	-6	87.23	4.09
2	-7	-6	80.53	4.09
-2	7	-6	83.83	4.36
-2	-7	6	81.06	4.04
-2	7	-6	80.90	4.40
2	-7	-6	80.67	4.09
2	7	6	79.49	3.65
-2	-7	6	72.44	3.80
2	7	6	71.95	4.07

-2	-7	6	80.52	4.02
2	-7	6	81.76	4.10
-2	7	6	81.53	4.12
-2	-7	-6	88.15	4.32
-2	-7	-6	87.89	4.24
2	7	-6	89.85	4.24
2	-7	6	75.12	3.81
2	-7	6	87.49	4.25
-2	-7	-6	82.18	4.26
2	7	-6	79.14	4.24
-2	-7	-6	84.44	4.13
2	-7	6	79.36	3.86
-2	-7	-6	79.63	4.08
-2	7	6	77.53	3.82
-2	7	6	77.92	3.81
-2	7	6	75.18	3.83
2	7	-6	79.47	4.44
2	7	-6	83.84	4.13
-2	7	6	74.08	3.79
2	-7	6	74.04	3.87
2	-7	6	74.76	3.59
-2	7	6	71.07	4.11
-2	7	6	81.09	3.84
2	-8	-6	108.44	5.64
2	8	6	116.88	5.25
-2	8	-6	117.75	5.95
2	8	6	107.37	5.25
2	-8	-6	115.54	5.69
2	8	6	107.99	5.75
-2	-8	6	108.19	5.42
2	-8	-6	107.49	5.80
2	8	6	118.65	5.77
-2	-8	6	109.98	5.62
2	8	6	107.54	5.36
-2	-8	6	114.00	5.72
-2	8	-6	130.97	5.94
2	-8	-6	130.90	5.70
-2	8	-6	113.42	6.14
2	8	6	106.87	5.39
2	-8	-6	128.22	6.03
-2	-8	6	104.43	5.44
2	8	6	109.78	5.54
-2	-8	6	110.14	5.55
-2	-8	6	119.48	5.65

2	8	-6	130.08	5.87
-2	8	6	110.03	5.44
-2	-8	-6	114.75	5.66
-2	-8	-6	121.22	5.92
2	-8	6	126.84	5.91
-2	-8	-6	114.05	5.70
-2	8	6	124.59	5.79
2	8	-6	121.37	5.91
2	-8	6	105.09	5.17
2	-8	6	121.16	5.57
-2	8	6	104.90	5.38
2	-8	6	121.55	5.68
2	-8	6	122.06	5.49
-2	8	6	111.24	5.41
2	8	-6	114.34	6.08
2	-8	6	109.35	5.42
-2	8	6	106.56	5.39
-2	8	6	106.77	5.77
-2	-8	-6	109.40	5.81
2	8	-6	113.83	5.82
2	-8	6	111.55	5.50
-2	-8	-6	126.10	5.84
2	9	6	182.49	8.38
-2	-9	6	163.97	7.99
-2	-9	6	156.92	8.02
2	-9	-6	151.29	7.42
-2	9	-6	150.32	7.67
2	-9	-6	149.05	7.58
2	-9	-6	170.50	7.44
2	9	6	149.61	7.94
-2	9	-6	169.46	7.23
-2	9	-6	171.88	7.72
2	9	6	165.59	7.89
2	-9	-6	161.11	7.79
2	9	6	163.30	7.81
-2	-9	6	176.22	8.15
-2	-9	6	180.74	8.20
2	-9	-6	148.63	7.38
2	9	6	164.48	8.10
-2	9	-6	148.16	7.94
2	9	6	164.73	8.36
-2	-9	-6	165.57	7.60
2	-9	6	181.16	8.04
2	9	-6	156.97	7.94

-2	-9	-6	152.22	7.46
-2	9	6	180.38	7.98
-2	-9	-6	153.43	7.37
2	9	-6	149.92	7.56
2	-9	6	167.28	8.21
-2	-9	-6	158.55	7.71
2	-9	6	159.40	7.69
2	9	-6	165.94	7.65
-2	9	6	160.12	7.93
-2	9	6	167.69	8.00
-2	-9	-6	142.11	7.56
2	-9	6	180.89	8.14
2	-9	6	177.65	8.43
2	-9	6	158.77	8.05
-2	9	6	183.28	8.38
-2	9	6	161.56	8.37
2	-10	-6	10.18	1.27
2	10	6	11.26	1.23
2	10	6	9.51	1.57
2	-10	-6	10.37	1.21
-2	10	-6	9.52	1.62
2	-10	-6	11.46	1.40
-2	10	-6	16.20	1.79
2	-10	-6	11.56	1.91
-2	10	-6	12.38	1.04
2	10	6	11.16	1.26
-2	-10	6	11.04	1.91
2	10	6	10.05	1.18
-2	10	-6	10.17	2.04
-2	-10	6	8.92	1.26
2	-10	-6	10.64	1.15
-2	-10	6	12.02	1.54
2	10	6	11.98	1.98
-2	-10	6	12.03	1.49
2	10	6	8.39	1.42
2	-10	-6	14.22	1.49
-2	10	6	10.62	1.81
2	-10	6	10.18	0.81
2	10	-6	11.26	1.91
2	-10	6	11.65	1.38
-2	-10	-6	10.31	1.27
2	-10	6	10.09	1.23
-2	10	6	10.99	1.46
-2	-10	-6	10.76	1.32

2	-10	6	9.61	1.74
-2	10	6	14.97	2.07
2	10	-6	13.09	1.65
2	10	-6	11.60	1.48
-2	-10	-6	10.55	1.18
-2	10	6	12.67	1.44
2	-10	6	10.30	1.34
2	-11	-6	6.95	1.15
2	-11	-6	7.72	1.33
2	-11	-6	7.16	1.11
2	-11	-6	7.86	1.36
-2	-11	6	8.01	1.25
2	-11	-6	8.19	1.24
2	11	6	9.34	1.17
2	11	6	8.56	1.63
-2	-11	6	10.24	1.47
-2	11	-6	7.87	1.72
-2	-11	-6	5.91	1.08
-2	11	6	12.46	2.04
2	-11	6	11.00	1.36
-2	11	6	11.73	2.30
-2	-11	-6	7.42	1.26
-2	11	6	8.80	1.49
2	-11	6	7.89	0.80
2	11	-6	7.81	1.48
2	11	-6	7.60	1.69
2	-11	6	10.03	1.40
2	-11	6	9.95	1.31
-2	11	6	5.57	1.61
-2	-11	-6	5.83	1.03
2	-12	-6	8.15	1.19
-2	12	-6	9.66	1.65
-2	12	-6	8.54	1.97
2	-12	-6	6.88	1.24
2	-12	-6	9.55	1.53
-2	-12	6	7.41	1.22
2	12	6	9.26	1.94
-2	-12	6	8.26	1.50
-2	12	6	7.16	1.72
-2	12	6	8.96	1.72
-2	-12	-6	6.71	1.22
2	12	-6	8.40	2.03
-2	-12	-6	8.35	1.43
2	12	-6	8.21	1.43

-2	-12	-6	6.06	1.12
2	-12	6	10.62	1.62
2	-12	6	7.45	1.15
-2	-13	6	30.34	2.35
2	-13	-6	24.95	2.10
2	-13	-6	23.78	1.96
-2	13	-6	27.86	3.82
2	13	6	29.26	3.33
-2	13	-6	23.46	2.78
-2	-13	6	40.91	3.06
-2	-13	-6	26.99	2.55
-2	-13	-6	25.65	1.94
2	-13	6	33.45	2.62
2	13	-6	20.69	2.36
2	13	-6	24.02	3.83
-2	-13	-6	28.15	2.19
2	-13	6	27.67	2.29
3	0	-6	-0.07	0.10
3	0	6	0.10	0.23
-3	0	6	0.16	0.23
-3	0	-6	0.11	0.23
-3	0	6	-0.09	0.14
-3	0	6	-0.05	0.25
3	0	6	0.16	0.15
3	0	6	0.14	0.16
3	0	6	-0.01	0.26
3	0	-6	-0.12	0.21
3	0	6	0.39	0.33
3	0	-6	0.12	0.17
-3	0	-6	0.01	0.17
-3	0	-6	0.28	0.33
3	0	-6	0.03	0.21
-3	0	6	0.05	0.26
-3	0	6	0.02	0.17
-3	0	-6	0.05	0.27
3	0	-6	-0.12	0.23
-3	0	-6	0.08	0.14
3	0	6	0.22	0.17
3	1	6	66.96	3.25
-3	1	-6	52.17	2.45
3	1	6	68.37	3.34
3	-1	-6	54.05	2.57
-3	-1	6	75.39	3.42
-3	1	-6	53.21	2.68

-3	-1	6	70.83	3.35
3	-1	-6	50.68	2.54
-3	-1	6	70.04	3.22
3	-1	-6	52.09	2.47
-3	1	-6	51.78	2.48
-3	-1	6	68.17	3.19
-3	1	-6	51.00	2.62
-3	-1	6	69.78	3.25
3	-1	-6	50.77	2.49
3	1	6	76.54	3.46
3	1	6	70.03	3.28
-3	-1	6	67.07	3.34
-3	1	-6	53.85	2.45
-3	1	-6	52.72	2.53
3	1	6	69.02	3.30
-3	-1	-6	49.38	2.59
-3	1	6	70.84	3.29
-3	-1	-6	48.94	2.49
-3	1	6	67.53	3.16
3	-1	6	67.79	3.29
-3	-1	-6	55.68	2.50
-3	-1	-6	50.89	2.48
3	1	-6	51.24	2.53
-3	1	6	68.78	3.28
-3	-1	-6	54.40	2.45
-3	1	6	75.33	3.38
3	1	-6	52.46	2.57
-3	1	6	69.43	3.31
3	-1	6	66.55	3.27
3	-1	6	72.46	3.26
-3	1	6	73.76	3.38
3	-1	6	73.65	3.42
3	1	-6	52.86	2.41
-3	-1	-6	51.62	2.54
3	1	-6	52.07	2.38
3	1	-6	51.75	2.56
3	-2	-6	316.42	15.06
-3	-2	6	315.48	14.73
-3	2	-6	337.59	15.14
-3	-2	6	334.56	14.67
-3	-2	6	355.90	14.93
-3	2	-6	350.54	15.20
3	-2	-6	331.47	15.23
3	2	6	336.00	14.74

3	-2	-6	329.10	15.16
3	2	6	307.89	14.83
3	-2	-6	347.04	15.22
-3	-2	6	326.09	14.86
-3	2	-6	341.32	15.11
3	-2	-6	334.64	15.11
-3	-2	6	340.68	14.85
3	2	6	346.91	14.93
3	2	6	314.42	14.85
-3	2	-6	333.42	15.17
3	-2	-6	342.09	15.21
-3	-2	6	304.81	14.74
-3	2	-6	339.19	15.31
-3	2	-6	353.61	15.38
3	2	6	313.50	14.78
-3	2	6	342.29	14.86
-3	-2	-6	334.59	15.25
-3	-2	-6	350.57	15.23
3	2	-6	336.59	15.25
3	2	-6	343.76	15.23
-3	-2	-6	336.68	15.13
-3	-2	-6	333.85	15.18
3	-2	6	355.98	14.95
3	-2	6	348.48	15.01
-3	2	6	318.81	14.70
-3	2	6	340.71	14.81
-3	-2	-6	321.89	15.09
3	2	-6	341.79	15.09
-3	2	6	316.38	14.85
-3	-2	-6	332.65	15.13
-3	-2	-6	323.15	15.16
-3	2	6	349.96	14.90
3	-2	6	312.14	14.76
3	2	-6	336.82	15.25
3	-2	6	336.61	14.74
3	2	-6	338.79	15.03
3	-2	6	306.10	14.80
3	-2	6	328.75	14.82
-3	2	6	311.83	14.74
3	-3	-6	353.58	15.82
-3	3	-6	366.96	16.14
3	3	6	387.55	16.94
3	3	6	363.96	16.87
-3	-3	6	397.97	16.95

3	-3	-6	361.07	15.99
-3	3	-6	364.25	15.81
-3	3	-6	358.10	15.81
-3	3	-6	348.30	15.89
-3	3	-6	370.23	15.92
-3	-3	6	347.43	16.75
-3	3	-6	361.78	15.93
3	3	6	355.01	16.86
3	-3	-6	354.46	15.98
-3	-3	6	366.31	16.76
3	-3	-6	310.59	15.86
-3	3	-6	352.27	16.07
3	3	6	333.93	16.85
-3	-3	6	363.39	16.89
3	-3	-6	345.32	15.90
-3	-3	6	385.91	16.88
3	3	6	361.80	16.77
-3	3	6	389.90	16.83
-3	-3	-6	343.53	15.86
3	3	-6	369.64	15.85
-3	3	6	358.22	16.90
-3	-3	-6	357.88	15.99
-3	3	6	367.56	16.73
3	-3	6	407.81	17.05
-3	-3	-6	361.31	15.94
3	-3	6	399.92	16.98
3	-3	6	363.01	16.82
-3	-3	-6	344.23	15.75
3	-3	6	344.65	16.77
3	3	-6	360.77	15.99
-3	3	6	400.77	16.93
-3	3	6	360.94	16.74
3	3	-6	359.29	16.00
3	-3	6	380.58	16.82
3	3	-6	338.61	16.00
-3	-3	-6	344.33	15.97
-3	3	6	381.60	16.82
-3	-3	-6	335.68	15.88
-3	3	6	396.21	16.86
-3	4	-6	174.96	8.25
-3	-4	6	194.27	9.22
3	4	6	201.99	9.23
-3	4	-6	189.85	8.28
3	-4	-6	181.31	8.34

-3	4	-6	186.08	8.11
3	-4	-6	175.40	8.22
3	-4	-6	178.73	8.15
3	-4	-6	181.06	8.32
3	4	6	208.81	9.40
-3	-4	6	216.46	9.42
-3	4	-6	171.06	8.39
-3	-4	6	204.34	9.35
-3	-4	6	194.91	9.35
-3	4	-6	183.03	8.20
3	4	6	206.39	9.22
3	-4	-6	187.53	8.13
-3	-4	6	194.25	9.21
3	4	6	193.56	9.33
3	4	6	197.14	9.36
3	-4	6	210.56	9.44
-3	-4	-6	185.45	8.29
-3	4	6	206.32	9.27
-3	4	6	201.26	9.49
-3	4	6	200.52	9.19
3	-4	6	220.56	9.53
-3	-4	-6	172.75	8.20
-3	4	6	194.31	9.40
-3	-4	-6	179.00	8.29
-3	4	6	214.27	9.28
-3	4	6	201.88	9.21
3	4	-6	170.20	8.35
3	-4	6	204.68	9.26
3	4	-6	184.48	8.18
3	-4	6	186.96	9.22
3	4	-6	178.58	8.31
-3	-4	-6	170.35	8.18
-3	-4	-6	171.58	8.28
3	4	-6	181.07	8.32
-3	5	-6	65.01	3.49
3	-5	-6	67.35	3.40
-3	-5	6	72.60	3.58
3	-5	-6	64.68	3.28
-3	-5	6	68.70	3.34
3	5	6	64.96	3.51
3	5	6	72.16	3.55
-3	5	-6	70.73	3.16
3	5	6	64.91	3.45
-3	-5	6	64.96	3.47

3	-5	-6	64.36	3.22
-3	5	-6	66.51	3.32
3	5	6	69.08	3.34
3	-5	-6	70.65	3.49
-3	5	-6	67.82	3.59
-3	-5	6	65.86	3.33
-3	5	-6	67.60	3.35
3	-5	-6	72.11	3.21
-3	5	-6	67.53	3.28
3	5	6	66.75	3.33
-3	-5	6	71.77	3.52
-3	5	6	75.15	3.43
-3	-5	-6	65.60	3.26
3	-5	6	67.77	3.41
-3	5	6	67.58	3.40
-3	-5	-6	71.48	3.40
-3	-5	-6	65.31	3.27
3	5	-6	69.78	3.27
-3	5	6	66.83	3.60
-3	5	6	69.72	3.32
-3	5	6	72.92	3.40
-3	-5	-6	67.94	3.38
3	5	-6	60.41	3.45
3	-5	6	64.34	3.33
3	-5	6	75.16	3.62
3	-5	6	70.69	3.39
-3	-5	-6	63.32	3.38
-3	5	6	76.29	3.59
3	5	-6	67.42	3.30
3	5	-6	65.93	3.39
-3	5	6	63.62	3.30
-3	-6	6	1.19	0.67
-3	-6	6	0.10	0.43
-3	-6	6	0.83	0.33
-3	-6	6	1.33	0.61
3	6	6	0.99	0.40
-3	6	-6	1.02	0.61
3	6	6	0.96	0.47
3	-6	-6	2.46	0.44
3	6	6	0.81	0.33
3	6	6	0.28	0.49
-3	6	-6	2.58	0.61
-3	6	-6	2.42	0.85
-3	-6	6	0.85	0.60

3	-6	-6	1.44	0.64
3	-6	-6	2.92	0.48
-3	6	-6	2.31	0.38
3	-6	-6	2.49	0.49
-3	-6	6	0.16	0.35
3	6	6	0.53	0.38
-3	6	-6	2.25	0.57
3	6	6	0.17	0.44
3	-6	-6	2.25	0.58
-3	6	-6	2.66	0.51
-3	-6	-6	1.69	0.55
3	-6	6	0.71	0.75
-3	6	6	0.69	0.63
-3	6	6	0.54	0.43
-3	6	6	0.62	0.37
-3	6	6	0.50	0.54
-3	6	6	0.50	0.38
-3	6	6	1.24	0.50
3	-6	6	0.55	0.35
-3	-6	-6	2.12	0.57
3	6	-6	2.36	0.57
-3	6	6	0.53	0.43
-3	-6	-6	1.37	0.41
3	6	-6	1.39	0.43
3	-6	6	0.91	0.41
3	-6	6	1.15	0.40
3	-6	6	0.34	0.24
3	6	-6	1.61	0.63
-3	-6	-6	3.00	0.65
3	6	-6	2.06	0.51
-3	-6	-6	1.95	0.41
3	-6	6	0.41	0.64
3	-7	-6	7.44	0.87
-3	7	-6	8.54	1.02
-3	7	-6	8.85	1.31
-3	-7	6	11.22	1.27
3	7	6	9.60	0.93
3	7	6	9.91	0.70
-3	-7	6	9.22	0.89
3	7	6	9.96	1.07
-3	-7	6	9.73	1.10
-3	7	-6	8.11	0.86
-3	7	-6	7.12	0.95
-3	7	-6	9.42	1.25

3	-7	-6	8.09	0.83
-3	-7	6	8.91	0.90
-3	-7	6	11.23	1.28
3	-7	-6	9.80	0.95
-3	-7	6	9.38	1.31
3	7	6	9.37	1.06
3	7	6	8.86	0.92
-3	7	-6	8.55	1.14
3	7	6	9.25	1.22
3	-7	-6	10.11	1.03
3	-7	-6	8.17	1.27
3	7	6	8.60	0.78
3	7	-6	9.00	0.96
-3	-7	-6	9.58	0.91
-3	-7	-6	8.14	1.03
3	7	-6	10.09	1.03
-3	-7	-6	11.23	1.17
-3	7	6	9.28	0.92
-3	-7	-6	9.26	1.13
3	-7	6	9.50	0.69
3	7	-6	7.66	1.17
-3	7	6	9.42	1.00
3	-7	6	8.81	0.92
3	7	-6	8.70	1.02
-3	7	6	8.17	1.29
-3	7	6	9.36	0.95
-3	7	6	11.17	1.36
-3	-7	-6	9.38	0.87
3	-7	6	11.85	1.36
3	-7	6	9.37	0.87
3	-7	6	8.14	0.97
3	-7	6	8.51	1.39
-3	7	6	10.42	1.05
3	-8	-6	25.33	2.00
3	-8	-6	22.89	1.69
3	8	6	24.02	1.72
-3	-8	6	23.46	1.61
-3	8	-6	22.30	1.87
-3	8	-6	21.37	1.92
-3	-8	6	22.09	1.55
-3	-8	6	25.48	1.78
3	8	6	21.12	1.87
-3	-8	6	23.93	1.93
3	8	6	23.46	1.95

3	8	6	21.75	1.44
3	-8	-6	21.97	1.50
-3	-8	6	25.30	1.96
3	8	6	20.98	1.54
3	8	6	22.07	1.73
-3	8	-6	25.42	1.77
3	8	6	23.84	1.33
3	-8	-6	25.31	1.55
-3	8	-6	25.44	1.44
-3	-8	6	24.34	1.94
3	-8	-6	23.64	1.55
-3	8	6	24.74	1.67
-3	-8	-6	23.32	1.53
-3	-8	-6	24.21	1.81
3	-8	6	23.42	1.67
3	-8	6	24.61	1.56
3	-8	6	24.40	1.79
-3	-8	-6	24.96	1.59
3	-8	6	22.80	1.56
3	8	-6	24.76	1.71
-3	8	6	26.99	1.94
-3	8	6	23.27	1.50
3	8	-6	23.15	1.67
-3	-8	-6	21.97	1.69
-3	8	6	22.16	1.59
3	-8	6	23.48	1.30
3	8	-6	22.95	1.95
-3	8	6	22.77	1.64
-3	8	6	21.01	1.92
3	-8	6	25.39	2.00
3	-8	6	24.59	2.13
-3	-8	-6	23.62	1.83
-3	9	-6	37.93	2.42
3	-9	-6	32.96	1.96
3	9	6	38.94	2.02
-3	9	-6	31.03	2.39
3	-9	-6	33.75	2.42
-3	9	-6	36.28	1.87
-3	9	-6	30.88	2.62
-3	-9	6	39.95	2.62
3	-9	-6	32.34	2.18
3	9	6	37.59	2.42
3	9	6	38.82	2.01
-3	-9	6	38.96	2.45

3	-9	-6	31.10	1.98
-3	-9	6	35.43	2.29
3	-9	-6	32.33	1.99
-3	-9	6	36.40	2.28
3	9	6	36.38	2.62
3	9	6	37.89	2.21
3	-9	-6	37.64	2.05
3	9	-6	29.76	2.48
-3	9	6	38.25	2.35
-3	-9	-6	30.12	2.25
3	-9	6	43.28	2.92
-3	9	6	37.37	2.70
-3	-9	-6	32.15	2.29
3	-9	6	37.81	2.91
3	-9	6	36.29	1.95
3	-9	6	40.52	2.30
-3	9	6	43.58	2.84
-3	9	6	36.71	2.27
-3	-9	-6	33.88	2.09
3	9	-6	36.73	2.29
3	9	-6	33.16	2.19
-3	-9	-6	33.56	1.96
3	-9	6	36.34	2.32
3	-9	6	37.95	2.48
3	-10	-6	9.25	1.10
3	10	6	12.74	2.08
3	-10	-6	11.93	1.36
-3	10	-6	10.84	1.76
3	10	6	9.02	1.70
-3	-10	6	9.65	1.31
-3	10	-6	9.30	1.65
3	10	6	12.20	1.06
3	10	6	10.58	1.07
-3	10	-6	10.61	1.11
3	10	6	11.30	1.26
3	-10	-6	10.77	1.36
-3	10	-6	10.65	1.75
3	-10	-6	12.34	1.99
-3	-10	6	11.32	1.95
3	10	6	10.23	1.43
3	-10	-6	10.39	1.20
-3	-10	6	10.39	1.28
3	-10	-6	12.01	1.20
3	10	-6	9.33	1.58

-3	10	6	12.84	1.52
-3	-10	-6	8.74	1.14
3	10	-6	9.55	1.36
3	-10	6	12.38	1.35
-3	10	6	9.89	2.04
-3	-10	-6	9.09	1.44
3	-10	6	9.81	0.85
-3	10	6	8.68	1.45
3	-10	6	11.96	1.28
3	10	-6	11.38	2.02
3	-10	6	9.40	1.74
-3	10	6	11.57	1.81
3	-10	6	12.87	1.34
-3	-10	-6	9.12	1.25
3	-11	-6	27.68	1.88
-3	11	-6	27.37	2.38
-3	-11	6	31.72	2.09
-3	-11	6	28.42	2.33
3	-11	-6	27.18	2.13
-3	-11	6	33.70	2.98
3	-11	-6	22.12	1.81
3	11	6	25.87	2.56
-3	11	-6	27.93	2.86
3	11	6	29.88	1.81
3	-11	-6	23.40	1.87
-3	-11	-6	25.46	1.75
-3	11	6	28.92	2.30
-3	11	6	36.72	3.26
-3	-11	-6	28.78	1.97
-3	11	6	26.06	2.52
3	-11	6	29.65	2.08
3	11	-6	27.05	2.69
-3	-11	-6	26.16	2.12
-3	11	6	28.98	2.27
3	-11	6	29.10	2.08
3	-11	6	27.36	1.61
3	11	-6	25.14	2.07
3	-11	6	27.20	2.10
-3	12	-6	6.55	1.52
3	12	6	12.52	2.27
3	-12	-6	8.17	1.18
-3	-12	6	7.84	1.43
3	-12	-6	8.78	1.58
-3	-12	6	11.04	1.88

3	-12	-6	5.72	1.00
3	-12	6	7.91	1.43
-3	12	6	8.88	2.02
-3	12	6	9.11	1.41
-3	-12	-6	8.40	1.28
3	-12	6	9.87	2.28
3	12	-6	7.61	1.30
-3	-12	-6	6.05	1.40
3	12	-6	7.15	2.00
-3	-13	6	2.79	1.54
-3	13	-6	7.44	1.79
3	13	6	2.70	2.23
-3	-13	6	1.23	0.89
-3	13	-6	1.68	2.16
3	-13	-6	5.85	1.10
3	-13	-6	4.31	1.07
-3	-13	-6	5.35	1.08
-3	-13	-6	3.85	1.11
-3	-13	-6	4.72	1.49
3	13	-6	6.33	2.13
3	-13	6	3.03	0.91
3	-13	6	2.31	1.23
-4	0	6	51.01	2.52
-4	0	6	59.87	2.78
4	0	6	58.54	2.83
4	0	-6	86.97	3.97
-4	0	6	53.07	2.66
-4	0	-6	86.48	3.95
-4	0	-6	80.58	4.02
4	0	-6	86.68	3.91
4	0	-6	85.65	3.92
-4	0	-6	83.80	3.99
-4	0	-6	88.59	3.93
-4	0	-6	84.62	3.94
-4	0	6	56.95	2.62
4	0	6	52.82	2.63
4	0	-6	84.27	3.99
-4	0	6	57.73	2.53
-4	0	6	59.25	2.70
-4	0	-6	85.16	4.09
4	0	6	53.11	2.72
-4	0	6	57.42	2.63
4	0	6	54.84	2.69
4	0	-6	81.92	4.03

4	1	6	88.65	4.25
-4	1	-6	78.31	3.91
-4	-1	6	96.22	4.27
-4	1	-6	84.94	3.84
4	1	6	88.14	4.30
4	1	6	87.55	4.22
-4	-1	6	99.27	4.38
4	-1	-6	78.43	3.87
-4	-1	6	87.58	4.15
4	-1	-6	76.69	3.79
4	-1	-6	81.77	3.85
-4	-1	6	91.20	4.18
-4	1	-6	79.51	3.80
-4	-1	6	88.37	4.29
4	1	6	88.05	4.26
4	1	6	87.46	4.24
-4	-1	6	91.60	4.18
-4	1	-6	83.79	3.79
-4	1	-6	85.04	3.79
4	-1	-6	82.51	3.68
4	-1	-6	81.34	3.78
4	1	6	96.49	4.41
4	-1	6	92.96	4.36
-4	1	6	85.93	4.22
-4	1	6	88.54	4.12
4	1	-6	81.17	3.75
-4	1	6	94.12	4.31
-4	1	6	94.80	4.35
4	1	-6	81.84	3.86
-4	-1	-6	81.10	3.80
-4	1	6	93.42	4.25
-4	-1	-6	80.87	3.82
4	-1	6	89.50	4.27
4	1	-6	81.08	3.86
-4	-1	-6	83.60	3.92
4	1	-6	82.16	3.70
4	-1	6	101.15	4.48
-4	1	6	91.83	4.26
4	-1	6	92.40	4.23
4	1	-6	81.17	3.73
4	-1	6	93.70	4.23
4	1	-6	80.56	3.88
-4	-1	-6	82.77	3.78
-4	-1	-6	80.88	3.81

-4	-1	-6	85.40	3.88
-4	2	-6	50.19	2.41
-4	2	-6	53.36	2.48
4	-2	-6	47.19	2.46
-4	-2	6	56.87	2.66
4	2	6	52.20	2.54
-4	-2	6	52.34	2.61
-4	-2	6	49.92	2.59
4	-2	-6	46.90	2.34
4	-2	-6	51.40	2.39
4	2	6	55.62	2.66
-4	-2	6	51.44	2.43
4	2	6	49.16	2.49
-4	2	-6	54.32	2.46
-4	2	-6	47.51	2.61
-4	-2	6	51.06	2.44
-4	-2	6	51.96	2.39
4	-2	-6	50.15	2.52
-4	2	-6	54.26	2.50
4	2	6	48.08	2.43
4	2	6	52.45	2.59
4	-2	-6	49.45	2.51
-4	2	-6	55.80	2.74
4	2	-6	51.05	2.38
4	-2	6	53.10	2.68
-4	-2	-6	47.73	2.64
-4	2	6	52.04	2.57
-4	-2	-6	51.10	2.44
-4	2	6	54.04	2.60
-4	2	6	50.01	2.44
4	-2	6	59.09	2.78
4	-2	6	51.79	2.52
-4	-2	-6	47.60	2.39
-4	2	6	48.71	2.61
-4	-2	-6	49.57	2.55
4	2	-6	48.80	2.55
4	2	-6	50.37	2.40
4	2	-6	50.08	2.53
4	-2	6	44.52	2.57
-4	2	6	52.62	2.41
-4	2	6	52.70	2.60
-4	-2	-6	50.41	2.47
4	2	-6	51.95	2.55
-4	-2	-6	55.75	2.45

4	2	-6	50.57	2.33
-4	-2	-6	54.68	2.48
4	-2	6	54.06	2.47
4	-2	6	53.05	2.49
-4	3	-6	38.79	2.17
-4	3	-6	38.37	1.91
-4	3	-6	33.80	2.05
-4	-3	6	36.99	1.94
-4	-3	6	36.66	1.84
4	3	6	33.54	1.83
-4	-3	6	32.84	1.69
4	-3	-6	39.00	2.00
4	3	6	34.39	1.78
4	-3	-6	35.62	1.89
-4	-3	6	33.85	1.88
4	3	6	34.97	1.74
-4	3	-6	37.86	1.91
-4	3	-6	38.35	1.89
4	3	6	35.91	1.92
-4	3	-6	37.68	1.81
4	3	6	32.80	1.86
4	-3	-6	35.57	1.77
4	-3	-6	35.74	1.95
-4	-3	6	34.53	1.72
-4	3	-6	38.81	1.81
-4	3	6	35.30	1.90
-4	-3	-6	36.72	1.97
4	3	-6	35.61	1.99
4	-3	6	37.42	2.06
-4	-3	-6	38.34	1.91
-4	3	6	32.87	1.69
4	3	-6	35.95	1.97
-4	-3	-6	36.45	1.85
4	3	-6	36.27	1.82
4	-3	6	35.21	1.98
4	-3	6	35.89	1.78
-4	3	6	34.99	1.81
-4	-3	-6	37.84	1.99
-4	3	6	33.96	1.81
-4	3	6	33.33	1.67
4	-3	6	33.10	1.74
4	3	-6	37.53	1.82
-4	3	6	34.92	1.91
4	3	-6	36.93	1.99

-4	-3	-6	34.81	1.88
4	-3	6	33.01	1.78
4	-4	-6	12.25	0.74
-4	-4	6	13.97	1.14
-4	4	-6	13.10	0.94
4	4	6	13.62	1.06
4	4	6	12.88	0.89
4	-4	-6	13.17	1.00
4	4	6	10.70	0.94
-4	4	-6	13.72	0.90
-4	4	-6	11.08	1.18
-4	4	-6	14.22	0.76
-4	4	-6	13.31	0.77
-4	-4	6	11.79	0.83
4	-4	-6	11.86	0.87
4	-4	-6	12.32	0.79
-4	-4	6	11.38	0.98
4	-4	-6	12.47	0.97
4	4	6	12.46	0.83
-4	-4	6	12.73	0.98
4	4	6	11.48	0.94
-4	4	-6	13.02	0.92
-4	-4	6	12.49	0.81
-4	4	-6	11.81	1.09
-4	-4	-6	11.06	0.85
4	-4	6	13.38	0.88
-4	4	6	11.76	0.89
-4	-4	-6	11.99	0.91
4	-4	6	14.70	1.18
-4	4	6	12.40	0.78
-4	4	6	13.00	1.12
-4	4	6	12.94	1.01
-4	-4	-6	13.57	1.02
-4	4	6	14.04	1.08
4	4	-6	13.65	0.85
-4	4	6	13.12	0.95
-4	4	6	12.96	0.95
4	-4	6	12.78	0.83
-4	-4	-6	11.92	0.83
4	-4	6	12.88	1.24
4	4	-6	11.63	0.99
4	-4	6	12.03	0.88
-4	-4	-6	11.87	0.94
4	4	-6	10.32	1.00

4	4	-6	11.84	1.00
4	-5	-6	33.73	1.89
-4	-5	6	26.58	1.53
-4	5	-6	36.96	2.22
-4	5	-6	39.60	2.08
-4	5	-6	39.75	2.00
4	-5	-6	35.36	1.97
4	-5	-6	37.73	2.19
4	5	6	28.92	1.58
4	5	6	25.98	1.70
-4	5	-6	39.24	2.05
-4	-5	6	27.00	1.72
-4	-5	6	28.20	1.56
-4	-5	6	28.94	1.73
4	5	6	27.60	1.70
4	-5	-6	37.28	1.86
4	-5	-6	34.13	2.03
-4	5	-6	40.38	1.88
-4	-5	6	30.20	1.86
4	5	6	27.90	1.56
4	5	6	28.35	1.76
-4	-5	6	29.29	1.80
-4	5	-6	37.66	2.30
4	5	-6	38.61	1.96
4	5	-6	36.93	2.07
-4	5	6	28.72	1.54
-4	5	6	29.98	1.68
4	-5	6	29.48	1.87
-4	5	6	27.76	1.50
-4	5	6	26.55	1.81
4	5	-6	34.47	2.15
-4	-5	-6	35.29	1.95
4	-5	6	31.62	2.00
-4	5	6	30.95	1.81
-4	5	6	28.40	1.64
-4	-5	-6	39.75	2.14
-4	-5	-6	36.90	1.97
4	-5	6	29.18	1.61
-4	-5	-6	36.98	2.07
4	-5	6	27.32	1.63
-4	-5	-6	39.06	2.08
4	-5	6	25.81	1.54
-4	5	6	30.20	1.65
-4	6	-6	44.14	2.27

4	-6	-6	40.38	2.16
-4	-6	6	48.23	2.56
-4	6	-6	40.51	2.47
4	-6	-6	39.94	2.33
-4	6	-6	42.14	2.63
-4	-6	6	43.88	2.52
4	-6	-6	42.80	2.49
-4	-6	6	41.19	2.26
4	6	6	43.93	2.27
-4	6	-6	44.98	2.11
-4	-6	6	44.36	2.47
4	6	6	44.34	2.28
-4	-6	6	42.75	2.28
4	-6	-6	36.76	2.18
-4	6	-6	41.71	2.26
4	6	6	47.77	2.56
-4	-6	6	44.31	2.47
-4	6	-6	46.71	2.40
4	-6	-6	44.81	2.12
4	6	6	40.76	2.48
4	6	6	39.18	2.41
4	6	-6	40.84	2.46
-4	-6	-6	37.98	2.18
4	-6	6	47.53	2.70
4	-6	6	44.86	2.30
-4	-6	-6	42.71	2.34
-4	-6	-6	43.71	2.40
4	6	-6	40.79	2.32
-4	6	6	42.62	2.27
-4	6	6	43.66	2.35
4	-6	6	43.15	2.15
-4	-6	-6	40.92	2.22
4	-6	6	43.17	2.38
4	-6	6	45.91	2.61
-4	6	6	48.22	2.59
4	6	-6	41.75	2.22
-4	6	6	45.28	2.39
-4	6	6	42.86	2.20
4	-6	6	41.62	2.28
-4	-6	-6	39.26	2.30
4	6	-6	45.41	2.45
-4	6	6	37.63	2.94
-4	6	6	45.01	2.40
4	-7	-6	63.13	3.37

-4	7	-6	63.77	3.23
4	7	6	64.15	3.32
-4	-7	6	64.85	3.32
4	-7	-6	55.82	3.12
-4	7	-6	56.77	3.45
-4	-7	6	64.49	3.51
-4	7	-6	60.19	3.07
-4	-7	6	66.13	3.35
-4	7	-6	59.07	3.34
4	-7	-6	54.61	2.98
-4	-7	6	72.74	3.61
4	7	6	64.15	3.51
-4	7	-6	61.60	3.07
-4	-7	6	65.06	3.53
4	-7	-6	57.67	2.97
4	7	6	63.89	3.28
-4	7	-6	63.28	2.91
-4	-7	6	68.37	3.60
4	7	6	70.49	3.62
4	7	6	64.76	3.60
4	-7	-6	62.85	2.92
-4	7	6	67.41	3.39
-4	-7	-6	57.76	3.02
4	7	-6	60.07	3.09
-4	-7	-6	57.33	2.98
-4	7	6	61.22	3.30
-4	7	6	62.68	3.66
4	7	-6	53.02	3.29
4	7	-6	55.10	3.09
4	-7	6	64.97	3.44
-4	-7	-6	58.60	3.20
4	-7	6	69.11	3.65
-4	7	6	72.60	3.67
-4	7	6	63.30	3.25
4	-7	6	77.28	3.87
4	-7	6	68.87	3.35
-4	7	6	65.84	3.43
4	-7	6	63.47	3.33
-4	-7	-6	61.58	3.19
-4	-7	-6	55.70	3.12
4	7	-6	59.10	3.02
4	-7	6	64.72	3.17
-4	8	-6	53.12	3.42
4	-8	-6	57.21	3.38

4	-8	-6	53.05	3.08
-4	-8	6	66.85	3.54
4	-8	-6	54.93	2.94
-4	8	-6	62.06	2.85
-4	-8	6	62.57	3.48
-4	-8	6	59.47	3.26
4	8	6	65.40	3.24
4	-8	-6	54.30	2.95
-4	8	-6	64.15	3.26
-4	8	-6	55.23	3.33
4	-8	-6	60.22	2.90
4	8	6	58.52	3.53
-4	-8	6	62.66	3.55
-4	-8	6	63.68	3.30
4	8	6	62.14	3.48
4	8	6	67.14	3.62
4	-8	6	60.56	3.36
4	-8	6	69.00	3.86
4	-8	6	70.48	3.43
4	8	-6	54.16	3.06
-4	-8	-6	55.84	2.93
4	-8	6	66.86	3.29
-4	8	6	64.12	3.38
4	8	-6	52.11	3.33
-4	-8	-6	55.96	3.16
4	8	-6	56.87	3.09
-4	-8	-6	59.66	3.22
-4	8	6	63.62	3.59
4	-8	6	63.86	3.29
4	-8	6	64.30	3.62
4	-8	6	60.49	3.06
-4	-8	-6	54.41	3.12
-4	8	6	62.86	3.72
-4	-8	-6	58.84	3.02
-4	8	6	64.22	3.34
-4	8	6	59.74	3.27
-4	-9	6	29.86	2.10
4	-9	-6	47.38	2.39
-4	9	-6	40.45	2.79
-4	9	-6	39.01	2.98
-4	-9	6	27.73	2.23
4	9	6	31.30	2.33
-4	9	-6	47.10	2.31
4	-9	-6	38.85	2.30

4	-9	-6	44.79	2.97
4	-9	-6	40.73	2.38
4	-9	-6	40.51	2.37
-4	9	-6	45.48	2.71
4	-9	-6	38.56	2.54
-4	-9	6	28.57	2.33
4	9	6	27.42	2.10
-4	-9	6	27.53	1.90
4	9	6	31.29	2.39
4	9	6	29.91	1.87
4	9	-6	44.42	2.62
4	9	-6	42.82	2.54
4	9	-6	40.02	2.86
-4	-9	-6	43.58	2.36
-4	-9	-6	39.25	2.82
4	-9	6	32.53	2.20
-4	9	6	35.87	2.54
4	-9	6	35.28	2.70
4	-9	6	31.64	1.93
4	-9	6	29.74	1.90
4	-9	6	27.59	1.99
-4	-9	-6	42.13	2.57
-4	9	6	29.93	2.08
4	-9	6	29.33	1.62
-4	9	6	26.14	2.32
-4	-10	6	15.84	1.49
4	10	6	14.75	1.40
-4	-10	6	16.31	1.75
4	-10	-6	16.09	1.38
4	-10	-6	16.38	1.35
4	-10	-6	15.66	1.40
-4	10	-6	17.69	1.34
-4	10	-6	16.92	2.03
-4	-10	6	19.95	2.11
4	-10	-6	16.44	2.14
-4	-10	6	14.94	1.47
4	10	6	16.10	1.95
-4	10	-6	13.56	2.15
4	-10	-6	18.17	1.47
4	-10	-6	14.25	1.59
4	10	6	16.32	1.78
4	10	6	18.10	2.12
-4	10	-6	18.20	1.81
-4	10	6	14.69	2.05

4	10	-6	18.41	1.57
4	-10	6	17.25	1.55
4	-10	6	16.85	1.47
4	-10	6	18.10	1.48
-4	10	6	17.39	1.79
4	-10	6	15.73	1.10
-4	-10	-6	14.05	1.48
-4	-10	-6	16.32	1.40
-4	-10	-6	16.06	1.63
-4	10	6	15.02	2.22
4	-10	6	17.17	1.86
4	10	-6	19.36	1.84
4	-10	6	20.11	3.08
4	10	-6	15.06	2.00
-4	10	6	15.95	1.73
4	11	6	2.78	1.35
4	-11	-6	1.23	0.75
-4	-11	6	1.30	0.79
-4	11	-6	0.22	1.10
4	-11	-6	2.71	0.83
4	-11	-6	1.26	0.67
-4	-11	6	2.37	1.12
4	-11	-6	2.03	0.95
-4	11	-6	2.81	1.46
4	11	-6	0.17	1.36
-4	-11	-6	0.75	0.94
-4	11	6	1.85	1.35
-4	-11	-6	1.14	0.78
4	-11	6	2.26	0.51
4	-11	6	1.68	0.83
4	-11	6	1.74	0.89
-4	11	6	2.69	1.07
-4	-11	-6	-0.07	0.62
4	11	-6	0.55	0.87
4	-11	6	1.38	0.83
4	-11	6	2.31	1.22
4	-12	-6	6.18	1.04
-4	12	-6	6.34	2.07
-4	-12	6	6.02	1.49
4	-12	-6	5.68	1.20
-4	-12	6	7.46	1.39
4	12	6	7.96	1.90
-4	12	-6	4.69	1.54
4	-12	-6	5.89	2.07

4	-12	6	10.00	1.37
-4	12	6	8.64	1.64
4	-12	6	9.44	1.38
4	12	-6	6.59	1.94
-4	-12	-6	5.71	1.41
4	12	-6	5.57	1.19
-4	-12	-6	4.24	1.10
-4	12	6	3.93	1.69
4	-13	-6	8.60	1.54
-4	-13	6	14.29	1.62
-4	-13	6	14.11	2.31
-4	13	-6	11.09	2.15
4	13	6	8.03	2.35
4	-13	-6	8.44	1.47
-4	-13	-6	8.28	1.77
4	13	-6	7.58	2.20
-4	-13	-6	9.55	1.38
4	-13	6	12.31	1.52
-5	0	6	-0.08	0.16
-5	0	6	-0.04	0.10
5	0	-6	-0.10	0.21
-5	0	6	0.18	0.19
-5	0	-6	0.03	0.18
-5	0	6	-0.06	0.11
-5	0	-6	0.19	0.19
-5	0	-6	-0.04	0.31
5	0	6	-0.22	0.19
-5	0	-6	0.22	0.25
5	0	-6	-0.09	0.20
5	0	6	-0.09	0.15
5	0	-6	-0.10	0.14
-5	0	-6	0.00	0.17
5	0	6	-0.04	0.27
5	0	-6	0.07	0.16
5	0	6	0.21	0.20
5	0	6	0.04	0.41
5	0	-6	-0.02	0.23
-5	0	6	0.35	0.24
-5	0	6	0.34	0.32
5	0	6	0.08	0.15
-5	1	-6	98.86	4.58
5	-1	-6	100.83	4.69
5	1	6	79.92	3.79
-5	-1	6	82.20	3.75

-5	1	-6	100.78	4.59
5	1	6	73.56	3.76
-5	1	-6	101.51	4.64
-5	-1	6	87.92	3.93
5	-1	-6	98.91	4.48
-5	-1	6	79.68	3.84
5	1	6	75.32	3.76
-5	1	-6	100.02	4.59
5	-1	-6	100.64	4.57
5	1	6	77.89	3.84
-5	-1	6	77.70	3.68
5	-1	-6	102.07	4.60
5	-1	-6	92.10	4.58
-5	1	-6	98.46	4.75
-5	1	-6	96.24	4.71
5	1	6	82.07	3.79
-5	-1	6	80.10	3.72
-5	-1	6	80.01	3.69
5	-1	-6	100.39	4.63
-5	-1	6	80.40	3.69
5	-1	6	81.18	3.77
-5	1	6	79.75	3.78
-5	-1	-6	100.52	4.59
5	1	-6	98.22	4.62
5	-1	6	88.25	4.03
5	-1	6	79.96	3.78
5	-1	6	81.98	3.77
-5	-1	-6	93.92	4.61
5	-1	6	80.24	3.89
-5	1	6	78.13	3.75
-5	-1	-6	103.84	4.59
5	1	-6	99.72	4.52
-5	-1	-6	96.44	4.68
-5	1	6	79.26	3.66
5	-1	6	78.96	3.80
5	1	-6	98.89	4.65
5	1	-6	98.33	4.53
-5	1	6	86.35	3.80
5	1	-6	101.06	4.69
-5	1	6	86.26	3.85
-5	1	6	85.25	3.91
-5	2	-6	319.59	13.83
-5	-2	6	282.02	13.34
-5	-2	6	293.09	13.50

5	-2	-6	309.51	13.92
-5	2	-6	309.76	13.83
5	2	6	279.74	13.33
-5	2	-6	306.84	14.03
-5	2	-6	321.07	14.09
-5	-2	6	286.53	13.32
-5	2	-6	319.82	13.89
5	-2	-6	295.93	13.76
5	-2	-6	291.02	13.86
-5	-2	6	300.07	13.31
-5	-2	6	303.22	13.38
-5	2	-6	310.55	13.87
-5	2	-6	327.11	13.90
5	2	6	286.05	13.44
-5	-2	6	332.71	13.58
5	-2	-6	305.07	13.78
5	2	6	296.92	13.41
5	2	6	288.47	13.50
5	2	-6	306.19	13.91
5	-2	6	313.98	13.57
5	-2	6	276.44	13.42
-5	-2	-6	298.64	13.80
-5	2	6	316.07	13.45
-5	2	6	295.71	13.47
5	-2	6	322.01	13.67
5	2	-6	293.37	13.78
5	2	-6	309.45	13.75
5	2	-6	307.36	13.78
-5	-2	-6	302.94	13.87
-5	2	6	276.51	13.31
5	-2	6	278.47	13.40
-5	-2	-6	322.69	13.97
-5	2	6	317.96	13.50
5	2	-6	310.20	13.95
-5	2	6	283.70	13.34
5	2	-6	313.83	13.97
-5	-2	-6	314.43	13.86
-5	2	6	307.08	13.54
5	-2	6	301.79	13.43
-5	-2	-6	260.09	13.90
-5	2	6	305.28	13.48
-5	-2	-6	314.20	13.94
-5	3	-6	182.65	8.04
-5	-3	6	216.08	10.04

5	-3	-6	166.90	7.99
5	-3	-6	169.30	8.10
-5	3	-6	172.52	8.23
5	3	6	236.11	10.44
5	3	6	212.72	10.18
5	-3	-6	176.11	8.16
-5	3	-6	168.43	8.27
-5	3	-6	176.83	8.04
5	-3	-6	175.17	7.97
5	3	6	221.91	10.12
-5	-3	6	216.81	10.08
-5	-3	6	208.42	9.96
-5	-3	6	219.74	10.24
5	3	6	215.21	10.24
-5	3	-6	182.82	8.10
-5	3	-6	185.77	8.11
-5	-3	6	225.83	10.13
-5	3	-6	184.49	8.37
5	-3	-6	188.25	8.22
5	-3	6	216.03	10.16
5	-3	6	228.36	10.13
-5	3	6	221.94	10.24
5	-3	6	210.55	10.11
-5	3	6	229.31	10.23
5	-3	6	232.90	10.31
5	3	-6	177.04	8.03
5	3	-6	179.63	8.03
5	3	-6	181.20	8.21
-5	3	6	213.02	10.01
-5	-3	-6	179.46	8.11
-5	3	6	226.12	10.20
-5	-3	-6	171.39	8.09
-5	-3	-6	176.89	8.09
-5	3	6	222.83	10.05
5	-3	6	231.89	10.38
-5	-3	-6	169.08	8.15
-5	3	6	240.19	10.25
-5	-3	-6	180.20	8.20
-5	-3	-6	180.88	8.10
5	3	-6	166.48	8.15
5	3	-6	178.35	8.19
-5	3	6	235.27	10.17
-5	-4	6	290.91	13.48
5	-4	-6	261.89	12.38

-5	4	-6	280.90	12.37
-5	4	-6	285.37	12.45
5	4	6	279.39	13.46
5	4	6	293.74	13.32
-5	-4	6	277.61	13.26
-5	4	-6	275.38	12.67
5	-4	-6	272.90	12.47
-5	-4	6	288.00	13.31
-5	4	-6	266.04	12.58
5	-4	-6	258.08	12.29
-5	-4	6	304.80	13.51
-5	-4	6	309.66	13.50
-5	4	-6	288.97	12.40
5	-4	-6	268.99	12.22
5	4	6	273.80	13.42
5	4	6	290.82	13.32
5	4	6	305.45	13.48
-5	4	-6	273.33	12.29
-5	-4	6	311.60	13.39
5	-4	-6	275.50	12.52
-5	-4	-6	287.19	12.51
-5	4	6	310.57	13.49
5	-4	6	280.11	13.38
-5	4	6	306.34	13.44
-5	4	6	273.27	13.35
5	-4	6	317.09	13.66
5	-4	6	282.79	13.33
-5	-4	-6	263.29	12.37
5	-4	6	293.26	13.32
-5	4	6	312.06	13.40
5	-4	6	314.62	13.54
-5	4	6	279.94	13.21
5	4	-6	258.08	12.48
5	4	-6	279.49	12.34
5	4	-6	273.82	12.34
-5	4	6	286.76	13.50
-5	-4	-6	266.46	12.37
5	4	-6	272.73	12.46
-5	-4	-6	276.71	12.41
-5	-4	-6	266.52	12.43
-5	4	6	291.54	13.41
5	-5	-6	50.30	2.57
-5	5	-6	55.89	2.58
5	5	6	54.24	3.02

5	-5	-6	52.41	2.48
-5	5	-6	49.46	2.89
-5	5	-6	55.31	2.68
-5	-5	6	56.71	3.05
5	-5	-6	49.74	2.74
-5	5	-6	53.64	2.66
-5	-5	6	55.23	2.82
5	-5	-6	50.44	2.65
5	5	6	60.65	3.08
5	5	6	57.20	2.87
-5	-5	6	58.24	3.10
5	5	6	58.35	2.87
-5	-5	6	59.14	2.98
-5	-5	6	56.42	2.87
-5	-5	6	60.83	3.11
5	5	6	54.61	3.01
5	-5	-6	54.10	2.86
-5	5	-6	56.18	2.78
5	5	-6	54.94	2.63
5	5	-6	53.47	2.74
5	-5	6	60.25	3.26
5	-5	6	58.73	2.88
-5	-5	-6	53.47	2.81
-5	-5	-6	54.18	2.72
-5	5	6	58.77	2.85
-5	5	6	64.80	3.11
-5	-5	-6	49.88	2.71
-5	5	6	60.38	2.95
-5	5	6	57.97	2.98
5	5	-6	50.32	2.82
5	-5	6	57.27	2.96
-5	-5	-6	51.08	2.64
-5	5	6	61.26	3.02
-5	5	6	56.87	3.13
-5	-5	-6	48.78	2.63
5	-5	6	55.17	2.87
-5	5	6	56.19	2.75
5	-5	6	57.69	3.13
5	5	-6	54.22	2.65
5	6	6	15.21	1.09
-5	-6	6	14.27	1.03
-5	6	-6	21.16	1.76
5	6	6	14.01	1.23
5	6	6	15.66	1.33

-5	6	-6	22.53	1.44
5	6	6	13.45	1.20
-5	6	-6	24.59	1.31
-5	-6	6	14.08	1.06
-5	6	-6	24.38	1.57
-5	6	-6	22.74	1.43
5	-6	-6	23.76	1.71
5	-6	-6	20.56	1.36
-5	-6	6	16.37	1.43
5	6	6	14.35	1.05
-5	-6	6	16.67	1.29
-5	-6	6	13.73	1.27
5	-6	-6	20.75	1.30
5	-6	-6	21.37	1.47
-5	6	-6	17.73	1.64
5	-6	-6	22.88	1.21
-5	-6	6	15.69	1.40
5	-6	6	15.27	1.06
-5	-6	-6	22.10	1.48
-5	6	6	15.79	0.99
5	6	-6	23.44	1.65
5	6	-6	20.86	1.37
-5	-6	-6	23.40	1.59
5	-6	6	14.74	1.48
-5	6	6	15.22	1.06
-5	-6	-6	20.83	1.35
5	6	-6	22.08	1.47
-5	-6	-6	21.93	1.38
-5	-6	-6	21.66	1.47
5	-6	6	16.78	1.16
-5	6	6	13.92	1.20
5	-6	6	14.20	1.04
5	-6	6	15.37	1.60
-5	6	6	17.03	1.39
5	-6	6	13.17	0.90
-5	6	6	15.59	1.25
-5	6	6	16.11	1.19
-5	6	6	13.58	1.48
5	-7	-6	12.80	1.21
5	7	6	12.92	1.29
-5	7	-6	14.25	1.20
5	7	6	17.54	1.47
5	-7	-6	12.84	1.03
5	7	6	13.52	1.34

5	-7	-6	14.87	1.73
-5	-7	6	16.32	1.42
-5	7	-6	11.25	1.41
-5	-7	6	14.44	1.10
5	7	6	13.89	1.13
5	-7	-6	12.08	1.06
-5	7	-6	13.53	1.20
-5	7	-6	14.67	1.36
-5	-7	6	14.71	1.37
-5	-7	6	14.77	1.50
-5	7	-6	14.37	1.04
5	-7	-6	16.14	0.98
-5	-7	6	14.43	1.12
5	7	6	13.91	1.15
-5	-7	6	14.53	1.50
-5	7	-6	12.78	1.64
-5	7	6	12.98	1.23
-5	7	6	14.31	1.26
5	7	-6	13.91	1.19
-5	7	6	12.72	1.12
5	7	-6	11.96	1.38
-5	-7	-6	13.82	1.24
-5	7	6	16.49	1.55
5	-7	6	15.48	1.60
5	-7	6	15.25	1.77
-5	-7	-6	14.01	1.26
5	7	-6	13.05	1.12
5	-7	6	14.35	1.09
5	-7	6	14.38	1.15
-5	-7	-6	12.82	1.09
-5	7	6	14.28	1.44
5	-7	6	13.66	1.09
5	-7	6	13.57	0.93
-5	-7	-6	16.38	1.43
-5	-7	-6	13.09	1.05
-5	-8	6	28.94	2.06
5	-8	-6	25.40	1.82
-5	8	-6	30.68	1.65
-5	-8	6	23.97	1.88
5	-8	-6	27.57	2.05
5	8	6	29.65	2.03
-5	-8	6	24.24	1.61
-5	8	-6	29.99	2.02
5	8	6	23.19	1.86

5	8	6	25.96	1.62
-5	8	-6	25.14	2.08
5	8	6	25.24	1.97
-5	8	-6	24.34	2.22
5	-8	-6	23.61	1.64
-5	-8	6	24.45	1.65
5	-8	-6	25.46	1.65
5	-8	-6	27.25	1.57
5	-8	6	25.41	1.43
-5	-8	-6	26.79	2.04
-5	-8	-6	26.21	1.82
-5	-8	-6	26.36	1.99
5	8	-6	27.22	1.85
-5	8	6	22.78	1.65
5	8	-6	26.89	2.09
5	-8	6	29.01	1.83
-5	8	6	25.04	2.09
5	-8	6	28.44	2.22
5	-8	6	22.66	1.71
5	8	-6	26.41	1.77
5	-8	6	25.08	2.12
5	-8	6	26.90	1.63
-5	8	6	24.17	1.77
-5	-8	-6	26.04	1.65
5	-8	6	23.19	1.60
-5	-8	-6	27.79	1.73
-5	8	6	26.49	2.15
-5	8	6	25.37	1.84
5	-9	-6	34.63	2.15
-5	-9	6	39.14	2.38
-5	-9	6	42.15	2.73
5	9	6	37.97	2.29
5	-9	-6	37.20	2.03
5	-9	-6	36.43	2.38
5	9	6	42.61	2.82
5	9	6	37.52	2.58
5	-9	-6	40.64	2.76
-5	9	-6	34.06	2.58
-5	9	-6	30.47	2.73
-5	-9	6	41.16	2.66
5	-9	-6	35.48	2.15
-5	9	-6	42.35	2.13
-5	9	-6	41.34	2.62
5	-9	-6	40.20	2.10

5	9	6	39.74	2.73
-5	9	6	44.52	2.95
5	-9	6	43.41	3.10
5	9	-6	39.72	2.44
-5	-9	-6	34.73	2.32
5	-9	6	41.76	2.49
-5	9	6	38.47	2.80
5	9	-6	36.57	2.74
-5	-9	-6	36.43	2.13
-5	-9	-6	33.68	2.23
-5	9	6	40.11	2.57
5	-9	6	44.19	2.61
5	-9	6	39.27	2.35
5	-9	6	43.69	2.35
5	-9	6	40.46	2.13
5	9	-6	36.42	2.27
5	-10	-6	49.80	2.88
5	-10	-6	49.81	3.46
-5	-10	6	57.11	3.33
-5	-10	6	54.37	3.08
-5	10	-6	53.70	3.33
5	-10	-6	51.79	2.74
5	10	6	51.54	2.93
-5	-10	6	56.64	3.43
-5	10	-6	53.93	3.44
5	-10	-6	50.66	2.86
5	-10	-6	48.27	3.02
5	10	6	61.27	3.55
5	10	6	43.78	3.50
-5	10	-6	59.21	2.81
5	-10	-6	57.88	2.82
-5	10	-6	49.68	3.66
-5	-10	6	53.58	3.32
-5	-10	-6	47.63	3.09
-5	-10	-6	51.82	2.99
5	-10	6	54.74	2.76
-5	10	6	56.52	3.29
-5	10	6	52.71	3.50
5	10	-6	46.74	3.41
5	10	-6	54.69	3.13
-5	-10	-6	50.07	2.84
-5	10	6	55.46	3.23
5	-10	6	52.42	3.04
5	10	-6	52.99	2.98

5	-10	6	54.03	3.17
5	-10	6	60.23	3.04
5	-10	6	58.40	3.26
5	-10	6	57.37	3.81
5	-11	-6	30.30	2.02
-5	-11	6	35.45	2.73
5	11	6	34.99	3.04
-5	11	-6	33.78	2.79
5	-11	-6	32.06	2.13
5	-11	-6	29.96	2.32
-5	11	-6	25.87	3.00
-5	-11	6	35.27	2.40
5	-11	-6	34.81	1.98
5	-11	6	35.64	2.47
-5	-11	-6	30.77	2.25
5	-11	6	36.00	2.40
5	11	-6	31.63	2.24
5	-11	6	34.03	1.98
5	-11	6	41.95	2.37
5	11	-6	26.65	2.84
-5	11	6	35.36	2.90
-5	11	6	37.68	2.62
-5	-11	-6	30.37	1.99
-5	12	-6	18.90	2.95
-5	-12	6	17.62	2.42
5	-12	-6	18.59	1.63
5	12	6	18.17	2.52
5	-12	-6	19.08	1.94
-5	12	-6	18.81	2.44
-5	-12	6	18.95	1.78
5	-12	-6	16.80	1.80
5	-12	6	21.49	1.73
-5	12	6	16.66	2.23
5	-12	6	20.50	1.84
-5	-12	-6	17.73	1.77
-5	-12	-6	17.74	2.11
5	12	-6	17.38	1.89
5	12	-6	14.79	2.92
-5	13	-6	10.93	2.36
5	-13	-6	9.87	1.59
-5	-13	6	6.16	1.14
-5	-13	6	8.81	1.59
5	13	6	6.55	1.93
5	-13	-6	10.43	1.68

-5	-13	-6	12.73	2.35
5	-13	6	5.77	1.56
5	13	-6	7.75	2.14
-5	-13	-6	11.50	1.68
-5	13	6	9.34	2.13
-6	0	6	40.83	2.39
-6	0	6	47.11	2.08
6	0	-6	46.35	2.44
6	0	6	45.07	2.18
6	0	-6	50.68	2.28
-6	0	6	42.22	2.28
6	0	6	45.17	2.23
-6	0	-6	46.10	2.35
-6	0	-6	49.71	2.50
6	0	6	42.80	2.19
6	0	-6	47.32	2.34
6	0	-6	49.07	2.36
6	0	6	42.94	2.16
-6	0	6	48.41	2.18
-6	0	-6	43.56	2.41
6	0	6	42.82	2.24
6	0	-6	49.05	2.24
-6	0	6	46.63	2.10
-6	0	-6	50.90	2.42
6	0	-6	47.98	2.31
6	-1	-6	47.85	2.27
6	1	6	57.18	2.76
6	1	6	59.05	2.77
6	-1	-6	45.34	2.40
-6	-1	6	59.90	2.63
-6	1	-6	48.80	2.30
-6	-1	6	55.89	2.66
6	-1	-6	47.08	2.31
-6	-1	6	56.74	2.82
6	-1	-6	48.82	2.19
6	1	6	55.10	2.76
6	-1	-6	45.77	2.29
-6	-1	6	57.30	2.67
-6	1	-6	47.58	2.47
6	1	6	56.27	2.84
-6	-1	6	61.00	2.71
-6	1	-6	45.95	2.44
-6	1	-6	47.16	2.30
-6	1	-6	45.86	2.33

6	-1	-6	48.24	2.29
6	1	6	53.71	2.74
-6	-1	-6	46.10	2.31
-6	1	6	53.90	2.72
6	1	-6	47.76	2.20
6	-1	6	57.47	2.87
6	-1	6	61.59	3.04
-6	-1	-6	47.50	2.27
6	-1	6	56.68	2.78
-6	-1	-6	47.41	2.40
6	1	-6	46.00	2.40
6	1	-6	48.14	2.37
-6	1	6	61.57	2.80
6	-1	6	58.39	2.76
6	-1	6	51.65	2.81
-6	1	6	57.15	2.65
-6	-1	-6	42.71	2.33
-6	1	6	62.18	2.92
-6	1	6	56.90	2.74
-6	-1	-6	47.07	2.31
6	-1	6	56.68	2.75
-6	-2	6	-0.30	0.31
-6	-2	6	0.19	0.23
-6	2	-6	0.60	0.24
-6	2	-6	0.75	0.32
-6	-2	6	0.36	0.14
-6	-2	6	0.15	0.16
-6	2	-6	0.26	0.41
6	-2	-6	0.76	0.20
6	-2	-6	0.65	0.27
-6	2	-6	1.07	0.51
6	2	6	0.50	0.26
-6	2	-6	0.65	0.29
-6	2	-6	0.56	0.25
6	2	6	0.13	0.28
6	-2	-6	0.07	0.20
6	2	6	0.15	0.23
6	-2	-6	0.52	0.29
6	2	6	0.51	0.27
-6	-2	6	0.05	0.18
-6	2	-6	0.67	0.28
6	-2	-6	0.58	0.22
-6	-2	-6	0.40	0.33
6	2	-6	0.66	0.42

6	-2	6	0.08	0.20
6	2	-6	0.61	0.29
6	-2	6	-0.53	0.39
-6	2	6	-0.14	0.17
-6	2	6	-0.16	0.29
-6	2	6	-0.15	0.33
-6	-2	-6	0.43	0.24
-6	2	6	0.16	0.35
-6	-2	-6	0.17	0.34
-6	2	6	0.23	0.29
-6	-2	-6	0.36	0.27
-6	-2	-6	0.62	0.25
6	2	-6	0.51	0.19
-6	2	6	0.26	0.27
6	-2	6	0.54	0.25
6	2	-6	0.58	0.18
6	2	-6	0.45	0.33
6	-2	6	0.83	0.53
6	2	-6	0.65	0.21
-6	2	6	-0.01	0.20
-6	-2	-6	0.28	0.29
-6	-2	-6	0.61	0.27
6	-2	6	0.25	0.22
6	3	6	10.13	0.76
-6	3	-6	10.47	0.72
6	3	6	9.95	0.76
-6	-3	6	10.20	0.60
6	3	6	8.59	0.84
-6	-3	6	9.21	0.89
-6	3	-6	10.14	1.00
-6	-3	6	9.67	0.58
6	-3	-6	10.11	0.77
-6	3	-6	11.10	0.81
-6	-3	6	10.53	0.62
-6	3	-6	9.69	0.77
6	-3	-6	9.75	0.64
-6	-3	6	11.13	0.73
-6	-3	6	11.41	1.00
-6	-3	6	9.98	0.70
-6	3	-6	9.72	1.12
-6	-3	6	10.55	0.96
6	-3	-6	10.47	0.82
-6	3	-6	12.30	0.85
-6	3	-6	11.00	0.75

-6	3	6	9.79	0.88
6	-3	6	9.74	1.02
-6	-3	-6	10.52	0.78
-6	3	6	11.47	0.93
6	3	-6	10.75	0.67
-6	-3	-6	10.88	0.76
6	-3	6	10.34	0.73
-6	3	6	11.84	0.92
-6	3	6	9.68	0.84
6	3	-6	9.54	0.81
-6	-3	-6	10.43	0.86
6	-3	6	10.63	1.11
-6	3	6	10.52	0.83
-6	3	6	11.03	0.68
-6	-3	-6	10.78	0.77
-6	-3	-6	10.18	0.75
6	-3	6	10.03	0.78
-6	-3	-6	10.61	0.92
6	-3	6	10.00	0.72
6	3	-6	10.97	0.89
-6	4	-6	2.04	0.73
-6	4	-6	1.72	0.57
6	4	6	1.37	0.36
6	-4	-6	2.05	0.45
-6	4	-6	3.06	0.43
-6	4	-6	3.02	0.59
6	4	6	1.66	0.50
-6	4	-6	2.67	0.47
-6	-4	6	2.85	0.57
6	-4	-6	1.48	0.37
6	-4	-6	1.78	0.33
-6	4	-6	2.58	0.41
6	-4	-6	2.19	0.54
6	4	6	1.64	0.42
-6	-4	6	2.06	0.35
-6	-4	6	1.51	0.47
-6	4	-6	2.55	0.48
-6	-4	6	1.76	0.37
-6	-4	6	2.56	0.66
6	4	6	1.65	0.39
-6	-4	6	1.51	0.56
6	-4	6	1.90	0.70
6	4	-6	2.82	0.55
-6	-4	-6	2.72	0.52

6	-4	6	2.06	0.41
6	4	-6	2.32	0.48
6	-4	6	1.59	0.34
-6	4	6	1.89	0.43
-6	-4	-6	2.54	0.47
6	4	-6	2.54	0.40
-6	-4	-6	2.62	0.61
6	-4	6	1.59	0.38
-6	4	6	2.06	0.37
-6	-4	-6	2.38	0.50
-6	4	6	2.23	0.58
-6	4	6	2.40	0.52
6	-4	6	1.65	0.59
-6	4	6	2.63	0.62
6	4	-6	2.47	0.40
-6	-4	-6	1.61	0.35
-6	4	6	2.97	0.57
6	-5	-6	33.53	1.90
6	-5	-6	33.53	1.80
-6	5	-6	33.57	2.34
-6	-5	6	35.09	1.91
-6	-5	6	35.52	2.17
-6	5	-6	33.50	2.15
-6	5	-6	33.13	2.00
6	5	6	35.09	2.09
6	-5	-6	34.98	1.99
-6	-5	6	35.06	1.84
-6	5	-6	36.50	1.80
-6	5	-6	37.73	1.85
-6	-5	6	34.83	2.11
-6	-5	6	37.74	2.00
-6	-5	6	39.27	2.21
-6	5	-6	37.73	1.95
6	5	6	34.10	2.03
6	5	6	34.21	1.91
-6	5	-6	36.96	2.02
6	5	6	38.15	2.13
6	5	6	36.00	1.91
6	-5	-6	36.63	2.13
-6	5	6	35.41	2.04
-6	5	6	34.86	2.18
-6	5	6	38.00	2.13
6	-5	6	34.19	1.97
6	5	-6	32.65	2.03

6	5	-6	35.08	1.97
-6	-5	-6	33.36	1.95
-6	5	6	36.17	2.03
6	-5	6	37.73	1.91
-6	-5	-6	35.43	1.91
6	-5	6	36.94	2.34
-6	-5	-6	36.58	1.95
6	-5	6	37.12	2.23
6	5	-6	35.74	1.87
6	5	-6	36.29	1.89
-6	-5	-6	32.78	1.88
-6	5	6	36.55	2.08
-6	5	6	37.29	1.90
-6	-5	-6	37.30	2.12
6	-5	6	34.67	1.91
-6	-6	6	5.24	0.70
6	6	6	6.41	0.74
-6	-6	6	6.75	1.04
6	-6	-6	6.57	0.75
-6	6	-6	6.59	0.76
-6	6	-6	6.27	0.97
-6	6	-6	7.23	0.89
-6	-6	6	4.75	0.64
-6	6	-6	8.23	0.88
-6	-6	6	5.23	0.86
6	6	6	6.38	0.89
6	-6	-6	6.55	0.66
-6	-6	6	5.52	0.89
-6	6	-6	5.24	1.11
-6	-6	6	6.19	0.97
6	-6	-6	7.22	1.11
6	6	6	4.86	0.91
6	6	6	5.47	0.71
6	-6	-6	5.62	0.84
6	-6	-6	6.24	0.55
6	6	6	5.19	0.98
-6	6	-6	5.87	1.10
-6	-6	-6	5.55	0.80
-6	-6	-6	7.11	0.91
-6	-6	-6	6.50	0.76
6	6	-6	5.89	1.00
-6	6	6	5.15	0.91
6	-6	6	6.81	1.01
-6	6	6	6.56	1.09

-6	6	6	4.67	0.91
6	-6	6	4.99	0.66
6	-6	6	5.83	0.70
6	-6	6	4.28	0.74
-6	-6	-6	6.22	0.74
6	6	-6	6.93	0.81
6	6	-6	5.98	0.84
6	6	-6	7.21	0.79
-6	6	6	5.10	0.87
-6	6	6	4.50	0.73
-6	6	6	5.87	0.85
-6	-6	-6	8.01	1.09
6	-6	6	4.63	0.56
6	7	6	25.77	1.67
6	-7	-6	20.62	1.32
-6	-7	6	27.98	1.69
-6	7	-6	22.21	1.49
-6	-7	6	25.45	1.90
-6	7	-6	20.89	1.98
6	7	6	27.58	1.90
-6	-7	6	33.01	2.06
6	7	6	27.70	1.64
-6	-7	6	27.90	2.04
6	-7	-6	20.99	1.17
-6	7	-6	22.29	1.62
6	7	6	26.68	1.89
6	-7	-6	23.26	1.82
-6	-7	6	27.18	1.62
-6	7	-6	18.74	1.74
6	7	6	28.64	1.96
6	-7	-6	19.24	1.47
6	-7	-6	18.57	1.21
-6	7	-6	21.37	1.43
-6	-7	6	26.06	1.81
-6	7	-6	23.10	1.34
6	-7	-6	17.76	1.33
-6	7	6	27.91	1.69
-6	-7	-6	19.70	1.40
-6	7	6	29.06	1.81
6	-7	6	29.90	2.28
6	7	-6	20.88	1.45
-6	-7	-6	21.50	1.52
6	7	-6	18.44	1.70
6	-7	6	29.26	1.64

-6	7	6	24.59	2.09
-6	7	6	29.27	2.06
6	-7	6	26.15	1.51
-6	7	6	27.56	1.87
6	7	-6	21.13	1.42
6	-7	6	29.28	2.11
-6	-7	-6	20.41	1.36
-6	-7	-6	19.13	1.45
6	-7	6	26.43	1.76
6	-7	6	26.04	1.65
-6	-7	-6	20.37	1.71
-6	8	-6	8.22	1.48
-6	8	-6	10.73	1.02
6	-8	-6	8.42	0.84
6	8	6	9.38	1.41
-6	-8	6	8.52	0.94
-6	-8	6	7.94	1.25
6	-8	-6	7.48	0.94
-6	-8	6	10.01	1.54
6	-8	-6	9.63	1.63
6	8	6	7.48	0.98
-6	-8	6	6.41	0.95
6	-8	-6	8.06	0.77
-6	8	-6	7.83	1.36
6	-8	-6	9.13	0.94
6	8	6	8.79	1.29
-6	8	-6	9.99	1.49
6	8	6	6.72	1.27
6	-8	-6	8.00	1.03
6	-8	6	8.29	0.92
-6	8	6	8.31	1.33
-6	-8	-6	8.29	0.97
6	8	-6	7.55	1.24
6	-8	6	10.34	1.28
6	-8	6	7.04	1.03
-6	8	6	7.98	1.33
6	-8	6	8.85	1.85
6	-8	6	7.22	0.88
6	8	-6	9.90	1.11
6	-8	6	6.66	0.73
-6	8	6	8.10	1.15
-6	8	6	7.78	1.09
-6	-8	-6	8.33	0.99
-6	8	6	7.61	1.05

-6	-8	-6	7.20	1.08
6	9	6	11.81	1.24
6	-9	-6	15.59	1.18
-6	9	-6	9.79	1.70
-6	-9	6	14.34	1.27
6	-9	-6	15.18	1.26
6	-9	-6	14.66	1.23
-6	9	-6	16.06	1.90
6	9	6	14.08	1.93
-6	-9	6	12.40	1.29
-6	9	-6	16.33	1.80
6	9	6	15.21	1.60
6	9	6	14.89	1.62
-6	-9	6	14.00	1.83
6	-9	-6	13.72	1.90
6	-9	-6	14.71	1.48
-6	9	-6	16.19	1.34
-6	-9	-6	14.11	1.53
6	-9	6	13.28	1.01
6	-9	6	14.64	1.22
6	-9	6	13.43	1.24
-6	-9	-6	17.17	1.36
6	9	-6	15.73	1.40
6	9	-6	15.62	1.58
6	-9	6	14.79	1.59
6	-9	6	12.25	1.33
-6	9	6	14.16	1.61
6	-9	6	12.11	2.21
-6	9	6	12.44	1.95
-6	-9	-6	13.97	1.27
-6	9	6	13.48	1.86
6	-10	-6	2.34	0.60
6	-10	-6	-0.27	1.25
-6	-10	6	0.38	1.14
-6	10	-6	0.39	0.71
6	-10	-6	1.44	0.68
6	10	6	-0.14	0.90
-6	10	-6	0.63	1.25
6	-10	-6	-0.02	0.55
6	-10	-6	0.80	0.64
6	10	6	0.13	0.92
6	10	6	2.41	1.41
-6	-10	6	-0.48	0.64
-6	-10	6	-1.11	0.88

6	10	6	0.64	0.66
-6	10	-6	1.38	1.10
6	-10	-6	-0.43	0.76
-6	10	-6	1.22	1.06
6	10	-6	1.39	0.77
-6	-10	-6	0.98	1.49
6	10	-6	0.93	1.06
-6	-10	-6	0.81	0.67
-6	-10	-6	0.84	0.63
-6	10	6	0.69	0.91
6	10	-6	0.83	1.06
6	-10	6	0.11	0.48
6	-10	6	0.46	0.69
-6	10	6	-0.63	1.19
6	-10	6	1.70	1.52
6	-10	6	0.85	0.76
6	-10	6	1.88	1.01
6	-10	6	1.29	0.68
6	-11	-6	1.46	0.64
-6	-11	6	-0.09	1.01
6	11	6	-0.08	1.02
6	-11	-6	0.52	0.72
-6	11	-6	1.23	1.49
6	-11	-6	-0.22	0.66
6	-11	-6	-0.30	0.91
6	11	6	0.23	1.18
-6	-11	6	0.90	0.87
-6	11	-6	-0.07	1.19
-6	-11	-6	1.56	0.71
-6	-11	-6	0.07	0.88
-6	11	6	-0.26	1.27
6	11	-6	1.53	1.36
6	-11	6	0.09	0.48
6	-11	6	0.40	0.75
6	-11	6	1.71	0.88
6	-11	6	0.52	0.77
-6	11	6	0.83	1.29
6	11	-6	0.95	0.87
6	-12	-6	1.35	0.96
-6	12	-6	-0.74	1.49
-6	-12	6	1.59	0.86
6	12	6	0.64	1.68
6	-12	-6	0.64	0.84
-6	-12	6	0.03	1.41

6	-12	-6	1.66	0.81
-6	12	-6	-1.89	2.80
-6	12	6	0.75	1.29
6	12	-6	-0.53	1.80
6	-12	6	1.39	0.76
6	-12	6	1.92	0.90
6	12	-6	2.17	1.09
-6	-12	-6	0.58	1.37
-6	-12	-6	2.16	0.90
6	-13	-6	2.15	1.10
6	-13	-6	0.48	0.90
-6	-13	6	1.90	1.18
-6	-13	-6	2.32	1.22
-7	0	-6	0.09	0.24
7	0	6	0.11	0.22
-7	0	6	0.08	0.38
7	0	-6	0.08	0.30
7	0	6	0.26	0.20
-7	0	6	0.35	0.16
7	0	6	-0.20	0.24
-7	0	-6	-0.01	0.20
7	0	6	-0.25	0.35
-7	0	6	0.02	0.24
-7	0	-6	0.32	0.37
-7	0	-6	0.10	0.29
7	0	6	0.20	0.24
-7	0	6	0.11	0.16
7	0	6	-0.29	0.32
-7	0	6	0.17	0.23
7	0	-6	0.17	0.18
7	0	-6	-0.01	0.30
7	0	-6	0.09	0.20
7	0	-6	0.02	0.16
-7	0	6	0.02	0.23
-7	0	-6	0.06	0.22
7	0	-6	0.24	0.23
-7	1	-6	122.10	5.51
-7	-1	6	122.76	5.77
7	1	6	127.51	5.71
-7	1	-6	122.85	5.69
7	1	6	120.64	5.69
7	1	6	118.69	5.78
-7	1	-6	128.18	5.55
-7	1	-6	122.39	5.57

7	-1	-6	120.14	5.49
7	1	6	116.65	5.71
7	1	6	117.25	5.69
-7	-1	6	117.12	5.61
7	-1	-6	123.46	5.51
-7	-1	6	121.57	5.62
7	-1	-6	113.53	5.63
7	-1	-6	116.72	5.51
7	-1	-6	118.95	5.52
-7	-1	6	132.99	5.67
-7	1	-6	110.49	5.65
-7	-1	6	127.86	5.86
7	-1	-6	122.40	5.43
7	1	-6	118.51	5.46
7	1	-6	116.26	5.48
-7	1	6	122.05	5.66
-7	1	6	129.30	5.70
-7	1	6	120.86	5.60
7	-1	6	124.06	5.80
-7	1	6	134.41	5.87
7	-1	6	115.35	5.68
-7	1	6	131.28	5.73
7	1	-6	119.76	5.41
-7	-1	-6	123.27	5.49
7	-1	6	119.99	5.70
7	1	-6	119.08	5.63
7	-1	6	124.46	5.73
-7	-1	-6	113.75	5.57
-7	-1	-6	117.57	5.55
7	-1	6	132.77	5.71
-7	-1	-6	118.79	5.60
-7	1	6	115.56	5.66
-7	2	-6	165.09	7.08
7	-2	-6	149.53	7.07
7	-2	-6	158.08	7.11
7	2	6	162.88	7.81
-7	-2	6	171.06	7.52
-7	2	-6	159.38	7.08
-7	2	-6	144.07	7.22
7	-2	-6	153.36	6.96
-7	2	-6	160.22	7.05
7	2	6	168.69	7.71
-7	2	-6	151.58	7.05
-7	2	-6	156.50	7.08

-7	-2	6	182.87	7.88
7	2	6	161.25	7.61
-7	-2	6	175.43	7.84
7	2	6	157.44	7.70
-7	-2	6	162.53	7.63
-7	-2	6	157.76	7.79
-7	2	-6	157.74	7.31
-7	-2	6	172.40	7.63
7	-2	-6	145.12	7.06
7	2	-6	150.90	7.15
-7	2	6	165.75	7.61
-7	2	6	168.95	7.79
-7	2	6	170.71	7.74
7	2	-6	150.32	6.93
-7	-2	-6	158.53	7.12
7	-2	6	175.17	7.96
7	-2	6	161.23	7.71
-7	-2	-6	147.59	7.06
-7	-2	-6	162.89	7.19
7	-2	6	170.20	7.70
7	-2	6	173.20	7.85
7	2	-6	149.58	7.04
-7	2	6	169.07	7.74
7	2	-6	153.25	6.98
-7	-2	-6	153.01	7.02
-7	-2	-6	148.61	7.07
-7	2	6	181.17	7.76
-7	-2	-6	159.44	7.07
-7	-2	-6	145.45	7.01
7	-2	6	158.59	7.70
-7	2	6	159.98	7.60
-7	2	6	175.62	7.85
7	-3	-6	167.31	8.12
-7	3	-6	192.40	8.14
-7	-3	6	150.49	6.73
-7	3	-6	168.54	8.30
-7	3	-6	186.63	8.15
7	3	6	142.01	6.91
-7	-3	6	139.12	6.74
7	-3	-6	174.02	8.15
-7	-3	6	156.19	6.78
-7	-3	6	148.70	6.75
-7	-3	6	153.71	7.05
-7	3	-6	172.29	8.07

-7	-3	6	138.95	6.83
7	3	6	149.58	6.87
7	3	6	141.54	7.01
-7	-3	6	158.88	7.06
-7	3	-6	179.27	8.38
-7	3	-6	183.18	8.09
-7	-3	6	144.21	7.01
-7	3	-6	177.66	8.10
7	-3	-6	167.59	8.01
-7	3	6	149.59	6.80
-7	-3	-6	166.30	8.10
-7	3	6	152.41	7.00
-7	-3	-6	176.92	8.15
-7	-3	-6	181.47	8.09
7	3	-6	178.68	8.04
7	-3	6	146.23	6.85
-7	3	6	145.60	6.98
-7	-3	-6	184.66	8.26
7	3	-6	178.00	8.19
-7	3	6	154.38	6.94
-7	3	6	157.73	7.04
-7	-3	-6	184.49	8.13
7	3	-6	166.92	8.12
-7	3	6	159.62	6.98
7	-3	6	146.62	6.88
7	-3	6	153.47	7.07
-7	-3	-6	170.97	8.12
7	-3	6	140.95	6.90
7	-3	6	153.67	7.17
7	3	-6	177.32	8.01
-7	4	-6	286.54	12.70
7	-4	-6	260.30	12.73
-7	4	-6	272.75	12.66
-7	4	-6	304.08	12.70
-7	-4	6	307.56	14.32
-7	4	-6	269.77	13.01
7	-4	-6	289.61	12.88
-7	4	-6	292.67	12.75
7	-4	-6	271.47	12.78
7	4	6	298.93	14.56
-7	-4	6	307.19	14.36
7	-4	-6	262.97	12.63
-7	4	-6	304.14	12.81
-7	-4	6	303.02	14.46

-7	-4	6	332.32	14.69
-7	-4	6	335.09	14.66
-7	4	-6	271.56	12.96
-7	-4	6	333.96	14.45
-7	-4	6	316.91	14.32
7	4	6	305.78	14.65
7	4	6	327.80	14.48
-7	-4	6	308.67	14.65
7	-4	6	315.86	14.45
7	-4	6	294.78	14.48
7	-4	6	345.12	14.84
-7	-4	-6	277.13	12.74
7	-4	6	333.87	14.70
7	4	-6	287.91	12.65
-7	4	6	327.41	14.62
-7	-4	-6	281.69	12.71
7	-4	6	309.29	14.55
7	4	-6	287.95	12.67
7	4	-6	260.42	12.77
-7	4	6	337.42	14.63
-7	4	6	314.32	14.65
-7	4	6	333.83	14.65
-7	-4	-6	264.99	12.71
-7	-4	-6	278.10	12.74
-7	4	6	331.31	14.56
-7	-4	-6	292.47	12.89
7	4	-6	288.42	12.80
-7	-4	-6	272.88	12.75
-7	4	6	321.56	14.44
7	-5	-6	85.18	4.22
7	5	6	84.22	4.29
-7	-5	6	88.63	4.42
-7	5	-6	91.37	4.23
-7	5	-6	79.30	4.39
-7	5	-6	82.99	4.04
7	5	6	85.38	4.36
-7	-5	6	84.00	4.37
7	5	6	81.92	4.15
7	-5	-6	81.73	4.13
7	5	6	85.95	4.14
-7	-5	6	85.43	4.16
-7	5	-6	94.94	4.10
7	-5	-6	88.95	4.35
-7	5	-6	84.28	4.51

-7	-5	6	90.20	4.17
7	-5	-6	79.78	4.03
-7	-5	6	89.25	4.37
-7	-5	6	84.70	4.06
-7	5	-6	84.30	4.16
-7	-5	-6	87.81	4.33
7	5	-6	90.03	4.10
-7	5	6	85.19	4.30
7	5	-6	87.83	4.09
-7	-5	-6	88.22	4.16
7	5	-6	87.00	4.20
7	5	-6	79.67	4.24
-7	5	6	88.76	4.14
-7	-5	-6	82.55	4.15
7	-5	6	82.50	4.16
7	-5	6	90.20	4.57
-7	5	6	90.71	4.26
-7	5	6	91.72	4.36
7	-5	6	88.05	4.43
7	-5	6	88.59	4.12
-7	-5	-6	83.28	4.12
-7	5	6	88.75	4.34
7	-5	6	85.08	4.24
-7	5	6	82.53	4.39
-7	-5	-6	85.24	4.13
-7	-6	6	24.94	1.52
7	-6	-6	34.76	2.14
-7	-6	6	21.84	1.80
-7	6	-6	33.85	1.91
-7	-6	6	23.25	1.35
-7	6	-6	30.49	2.17
-7	6	-6	36.11	1.79
-7	6	-6	30.60	1.85
-7	-6	6	22.36	1.67
7	6	6	23.97	1.44
-7	6	-6	35.34	2.01
7	6	6	21.98	1.59
7	-6	-6	31.55	1.73
-7	-6	6	23.37	1.44
7	-6	-6	31.59	1.93
7	6	6	26.12	1.71
7	-6	-6	29.84	1.81
7	6	6	21.15	1.44
7	6	6	20.82	1.65

-7	-6	6	25.88	1.82
7	6	-6	29.21	1.99
7	-6	6	24.27	2.02
-7	6	6	24.75	1.60
-7	6	6	24.12	1.69
-7	-6	-6	29.93	1.80
-7	6	6	23.26	1.66
7	-6	6	23.67	1.40
7	-6	6	22.85	1.86
-7	6	6	24.98	1.46
7	6	-6	33.85	1.84
-7	6	6	23.75	1.74
-7	6	6	25.60	1.90
-7	-6	-6	32.66	1.89
-7	-6	-6	30.21	1.86
7	-6	6	22.05	1.48
7	6	-6	34.23	1.87
7	-6	6	22.92	1.42
7	6	-6	32.40	1.90
-7	-6	-6	30.26	1.84
-7	-6	-6	31.64	2.07
7	7	6	3.46	0.78
-7	7	-6	2.18	0.90
-7	-7	6	2.04	0.89
7	7	6	3.32	0.79
7	7	6	3.58	0.63
7	7	6	2.74	0.84
7	-7	-6	2.01	0.61
-7	-7	6	2.95	0.93
7	-7	-6	2.14	0.49
-7	7	-6	4.84	0.88
-7	-7	6	4.22	0.68
7	-7	-6	2.81	0.61
7	-7	-6	2.75	0.90
-7	7	-6	3.39	0.66
-7	-7	6	3.52	0.71
7	7	6	3.72	0.64
-7	-7	6	2.48	0.76
-7	7	-6	3.40	1.00
-7	7	-6	3.47	0.61
7	7	-6	3.16	0.66
-7	7	6	3.86	0.86
-7	-7	-6	3.80	0.65
7	-7	6	3.91	0.57

-7	-7	-6	2.45	0.84
7	7	-6	1.78	0.76
-7	-7	-6	3.34	0.71
7	-7	6	3.84	0.60
-7	7	6	2.03	0.72
-7	-7	-6	2.97	0.62
7	7	-6	4.01	0.69
7	-7	6	3.36	0.65
7	-7	6	3.38	1.18
-7	7	6	3.83	0.83
-7	7	6	4.20	0.98
-7	7	6	3.47	1.02
7	7	-6	2.69	0.60
-7	-7	-6	3.62	0.70
7	-7	6	3.09	0.58
7	-7	6	2.90	0.93
-7	-8	6	11.40	1.43
-7	-8	6	13.96	1.65
7	-8	-6	14.15	1.21
7	-8	-6	13.10	1.28
7	-8	-6	14.75	1.17
7	-8	-6	16.30	1.97
7	8	6	11.58	1.39
-7	-8	6	12.93	1.15
7	8	6	12.72	1.43
7	8	6	12.57	1.56
-7	-8	6	13.20	1.15
-7	8	-6	13.93	1.67
-7	8	-6	17.76	1.51
7	8	6	11.86	1.15
-7	8	-6	18.46	1.32
-7	8	-6	15.57	1.98
7	-8	6	14.39	1.17
-7	-8	-6	16.01	1.36
-7	8	6	15.46	1.75
-7	8	6	10.76	1.56
7	-8	6	12.46	0.96
7	-8	6	11.28	1.19
-7	8	6	11.40	1.21
-7	-8	-6	13.24	1.20
7	-8	6	13.57	2.04
7	8	-6	13.64	1.58
7	-8	6	13.74	1.09
7	8	-6	12.98	1.25

-7	-8	-6	16.32	1.35
-7	8	6	12.58	1.46
-7	9	-6	52.68	2.56
7	-9	-6	43.36	2.49
-7	-9	6	46.16	2.59
-7	-9	6	46.84	2.66
7	9	6	49.11	3.06
7	-9	-6	49.37	3.17
7	9	6	43.42	3.00
7	-9	-6	44.17	2.54
-7	-9	6	45.32	2.91
7	9	6	45.21	2.54
7	-9	-6	45.34	2.74
-7	-9	6	47.15	2.97
-7	9	-6	38.24	3.04
-7	9	-6	51.78	2.94
7	9	-6	39.14	2.88
-7	9	6	46.06	3.18
7	-9	6	49.78	2.54
-7	9	6	46.96	2.92
-7	-9	-6	42.87	2.67
7	-9	6	46.38	2.63
7	9	-6	46.47	2.58
7	-9	6	44.09	2.42
7	-9	6	49.33	2.75
-7	-9	-6	40.84	2.48
-7	-9	-6	45.34	2.63
7	-9	6	46.15	2.77
7	-9	6	48.04	3.49
7	-10	-6	71.18	3.69
7	-10	-6	68.65	3.65
7	-10	-6	69.37	3.92
7	10	6	69.02	4.27
-7	-10	6	72.16	3.91
-7	10	-6	80.67	3.69
-7	-10	6	73.90	4.14
-7	10	-6	66.87	4.42
-7	10	-6	71.55	4.28
7	-10	-6	75.26	4.32
-7	-10	6	70.98	4.20
7	10	6	70.78	4.19
7	-10	6	81.45	3.98
7	-10	6	80.05	3.79
-7	10	6	71.28	4.41

-7	-10	-6	69.65	3.80
7	-10	6	76.27	4.66
-7	-10	-6	65.22	3.63
7	10	-6	66.11	4.21
-7	10	6	73.18	4.17
7	-10	6	70.44	3.62
7	-10	6	69.82	3.86
7	-10	6	75.45	4.03
7	10	-6	68.77	3.72
-7	-11	6	59.30	3.57
7	-11	-6	46.80	3.08
7	11	6	50.76	3.67
-7	11	-6	50.18	3.60
-7	-11	6	55.03	3.26
7	-11	-6	53.72	2.89
7	11	6	48.60	3.46
7	-11	-6	49.91	2.85
-7	11	-6	47.35	3.81
7	11	-6	50.98	2.90
7	-11	6	53.64	2.83
-7	11	6	54.93	3.48
-7	-11	-6	46.45	2.76
7	-11	6	55.73	3.31
-7	-11	-6	51.90	3.09
7	-11	6	54.73	3.20
7	-11	6	57.16	3.04
7	11	-6	45.13	3.57
-7	-12	6	18.74	1.84
7	-12	-6	26.21	2.05
-7	-12	6	21.17	2.48
-7	12	-6	31.42	3.28
7	-12	-6	24.50	1.93
7	12	6	17.10	3.02
7	12	-6	23.97	2.94
-7	-12	-6	30.57	2.85
-7	-12	-6	23.74	2.14
-7	12	6	25.68	2.68
7	-12	6	21.77	1.78
-7	-12	-6	25.02	2.13
8	0	6	160.82	7.55
8	0	6	156.11	7.46
-8	0	-6	163.63	7.20
-8	0	-6	144.57	7.31
8	0	6	163.86	7.54

8	0	-6	154.44	7.35
-8	0	6	169.68	7.65
-8	0	6	167.35	7.49
8	0	-6	157.68	7.15
-8	0	6	163.28	7.40
8	0	-6	160.41	7.18
8	0	6	169.09	7.50
-8	0	-6	157.78	7.26
-8	0	-6	156.35	7.34
-8	0	6	155.89	7.49
8	0	-6	150.11	7.21
8	0	6	158.20	7.50
-8	0	6	169.88	7.53
-8	0	-6	169.71	7.27
-8	0	-6	162.26	7.32
8	1	6	16.60	0.99
-8	-1	6	15.74	0.95
8	1	6	14.23	1.04
8	-1	-6	17.17	1.02
-8	-1	6	16.29	0.89
8	1	6	15.61	1.01
8	1	6	15.80	0.97
-8	1	-6	18.46	1.28
8	-1	-6	18.40	1.04
8	-1	-6	16.28	1.03
-8	1	-6	20.08	1.06
8	-1	-6	18.43	1.05
-8	-1	6	15.27	1.18
8	1	6	18.52	1.24
-8	-1	6	15.21	1.11
-8	1	-6	20.33	1.11
-8	1	-6	17.02	1.25
-8	1	-6	16.28	1.06
-8	-1	6	14.05	1.06
8	1	6	16.05	0.94
-8	-1	6	14.89	0.86
8	-1	6	16.52	0.98
-8	1	6	14.82	1.15
8	-1	6	16.38	1.01
-8	1	6	15.94	1.01
-8	-1	-6	19.09	1.01
8	1	-6	17.88	0.90
-8	1	6	15.97	0.87
8	1	-6	16.39	1.15

-8	-1	-6	16.05	1.10
8	-1	6	14.78	0.92
-8	1	6	14.36	1.06
-8	-1	-6	18.91	1.21
-8	1	6	15.71	0.96
-8	1	6	16.46	0.96
-8	-1	-6	18.45	1.08
8	1	-6	17.26	0.99
-8	-1	-6	17.23	1.08
8	1	-6	16.86	0.99
8	-1	6	16.21	0.99
8	-1	6	14.97	1.14
-8	1	6	15.33	0.91
-8	-1	-6	17.94	1.16
8	-2	-6	8.57	0.60
-8	-2	6	9.00	0.63
8	2	6	7.33	0.65
-8	-2	6	8.58	0.90
8	2	6	7.41	0.63
-8	-2	6	7.54	0.82
-8	2	-6	7.63	0.91
-8	-2	6	9.77	0.64
-8	2	-6	8.35	0.74
8	-2	-6	8.22	0.68
8	2	6	8.63	0.72
-8	2	-6	7.86	1.03
-8	-2	6	8.85	0.60
8	-2	-6	7.85	0.67
-8	2	-6	8.36	0.69
8	-2	-6	8.74	0.56
-8	2	-6	9.06	0.74
-8	-2	6	10.42	0.99
8	2	6	7.83	0.80
8	2	6	8.59	0.90
-8	2	-6	7.70	0.66
8	-2	-6	9.12	0.80
8	2	-6	8.17	0.49
-8	-2	-6	8.46	0.63
-8	2	6	9.10	0.94
-8	2	6	8.98	0.61
-8	-2	-6	7.21	0.69
-8	-2	-6	6.70	0.75
8	-2	6	9.04	0.97
-8	-2	-6	8.36	0.71

8	2	-6	8.32	0.66
8	-2	6	7.71	0.67
8	-2	6	7.50	0.65
-8	2	6	9.28	0.79
-8	-2	-6	9.65	0.90
8	2	-6	8.93	0.79
-8	-2	-6	7.62	0.67
-8	2	6	9.54	0.79
-8	2	6	7.46	0.73
8	-2	6	7.77	0.67
8	-2	6	8.51	1.23
8	2	-6	8.19	0.56
-8	2	6	8.98	0.60
-8	2	6	7.07	0.78
-8	-2	-6	8.02	0.76
8	-3	-6	43.62	2.30
-8	3	-6	44.74	2.61
-8	3	-6	45.33	2.29
8	3	6	50.37	2.63
8	3	6	52.53	2.49
8	-3	-6	44.05	2.19
-8	3	-6	48.37	2.34
-8	3	-6	44.01	2.53
-8	3	-6	43.58	2.26
-8	-3	6	46.92	2.42
8	3	6	46.00	2.61
-8	3	-6	47.18	2.32
-8	-3	6	53.58	2.70
-8	3	-6	46.52	2.27
8	3	6	48.48	2.31
-8	-3	6	50.82	2.26
8	3	6	46.88	2.49
8	-3	-6	42.91	2.29
-8	-3	6	52.89	2.37
-8	-3	6	47.33	2.63
-8	-3	6	46.97	2.65
8	3	-6	46.22	2.22
8	-3	6	48.91	2.79
-8	-3	-6	45.08	2.30
-8	-3	-6	45.00	2.30
-8	3	6	51.70	2.66
8	-3	6	47.11	2.43
-8	3	6	46.75	2.57
-8	3	6	51.98	2.57

8	3	-6	45.14	2.16
8	-3	6	48.32	2.49
-8	3	6	50.30	2.61
-8	-3	-6	44.97	2.31
8	-3	6	48.97	2.47
-8	3	6	51.44	2.55
-8	-3	-6	43.82	2.19
-8	-3	-6	43.78	2.31
8	3	-6	46.57	2.37
-8	-3	-6	45.19	2.23
8	-3	6	50.49	2.70
8	3	-6	43.47	2.27
-8	4	-6	8.00	0.82
-8	4	-6	9.06	0.76
8	-4	-6	8.15	0.78
-8	-4	6	6.10	0.90
8	-4	-6	8.65	0.77
-8	4	-6	9.21	0.79
-8	-4	6	7.86	1.05
8	4	6	6.54	0.74
-8	-4	6	8.02	0.56
-8	-4	6	7.28	0.58
-8	-4	6	8.44	1.05
8	4	6	7.99	0.74
-8	-4	6	8.42	0.70
-8	4	-6	8.81	0.70
-8	4	-6	9.12	0.85
8	-4	-6	7.88	0.64
-8	4	-6	5.72	0.99
8	4	6	8.04	0.87
8	4	6	7.87	0.89
-8	4	-6	8.52	1.19
8	4	-6	9.20	0.75
8	-4	6	7.13	0.72
8	-4	6	7.96	0.67
-8	4	6	7.17	0.69
8	-4	6	8.46	1.20
8	4	-6	8.33	0.84
-8	-4	-6	9.04	0.80
-8	-4	-6	7.99	0.76
-8	-4	-6	8.79	0.74
-8	-4	-6	9.45	1.05
8	4	-6	8.60	0.81
-8	4	6	7.55	0.98

-8	-4	-6	9.75	0.83
8	-4	6	7.83	0.68
-8	4	6	6.43	0.85
8	-4	6	9.39	1.15
-8	-4	-6	9.42	0.84
-8	4	6	7.67	0.97
-8	4	6	8.09	0.90
-8	4	6	7.82	0.86
8	4	-6	9.30	0.68
-8	-5	6	10.40	0.62
8	5	6	10.31	0.94
-8	-5	6	9.44	0.65
-8	-5	6	10.47	0.84
8	-5	-6	7.60	0.87
-8	5	-6	8.72	0.81
8	-5	-6	9.06	0.82
-8	5	-6	10.42	0.95
-8	5	-6	9.09	0.75
8	5	6	9.13	1.05
8	-5	-6	8.37	0.69
8	5	6	9.70	0.89
-8	-5	6	10.46	1.18
8	-5	-6	8.11	1.12
-8	-5	6	8.36	0.81
-8	-5	6	10.22	0.70
8	5	6	10.97	0.87
-8	5	-6	9.92	0.98
-8	5	-6	10.45	1.43
-8	5	-6	7.74	1.17
-8	-5	6	8.49	1.19
-8	-5	6	7.43	1.05
8	-5	6	9.92	1.27
-8	5	6	11.71	0.87
-8	5	6	8.97	1.12
-8	-5	-6	9.40	0.83
8	-5	6	9.31	0.81
-8	-5	-6	7.81	0.83
-8	-5	-6	9.14	1.13
8	5	-6	8.86	0.88
-8	5	6	10.75	1.02
8	-5	6	10.57	0.90
-8	-5	-6	8.33	0.88
8	5	-6	8.61	0.79
8	5	-6	8.85	0.80

-8	5	6	10.82	1.08
-8	5	6	9.84	1.16
8	-5	6	12.85	1.49
8	-5	6	10.69	0.80
-8	5	6	8.80	1.12
-8	-5	-6	8.67	0.82
8	5	-6	7.83	0.94
-8	-6	6	14.18	0.95
-8	-6	6	12.64	1.36
8	6	6	13.48	1.10
-8	-6	6	15.77	1.54
8	6	6	14.82	1.09
8	6	6	12.36	1.16
-8	-6	6	15.85	1.52
-8	-6	6	13.70	1.07
8	-6	-6	13.92	1.11
-8	6	-6	12.73	1.81
8	-6	-6	14.32	1.48
-8	6	-6	15.86	1.58
-8	6	-6	16.28	1.34
8	6	6	14.05	1.33
-8	6	-6	19.01	1.18
8	-6	-6	14.92	1.02
-8	6	-6	15.42	1.22
-8	6	-6	16.33	1.25
8	-6	-6	14.41	1.23
-8	-6	6	12.82	1.08
-8	-6	-6	16.16	1.18
-8	6	6	11.74	1.44
8	6	-6	15.92	1.13
8	-6	6	16.62	1.79
-8	6	6	14.72	1.35
-8	6	6	15.48	1.11
-8	-6	-6	15.10	1.48
8	6	-6	15.46	1.20
-8	6	6	14.40	1.25
8	6	-6	13.54	1.32
8	-6	6	17.17	1.61
8	-6	6	15.05	1.07
-8	-6	-6	15.69	1.13
8	-6	6	14.41	1.03
-8	6	6	15.08	1.43
-8	6	6	14.96	1.40
8	6	-6	16.79	1.16

8	-6	6	13.82	1.14
-8	-6	-6	15.11	1.18
-8	-6	-6	16.13	1.19
8	7	6	11.87	1.39
8	-7	-6	12.42	1.12
8	7	6	12.53	1.39
8	-7	-6	13.98	1.29
-8	7	-6	14.41	1.72
-8	-7	6	10.78	1.34
8	7	6	11.67	1.26
-8	-7	6	13.23	1.01
-8	-7	6	10.46	1.50
-8	7	-6	15.32	1.22
8	7	6	12.71	1.10
-8	7	-6	12.87	1.61
-8	7	-6	14.67	1.43
-8	-7	6	12.59	1.08
8	-7	-6	15.30	1.06
-8	7	-6	14.88	1.18
8	-7	-6	14.69	1.49
-8	7	6	13.45	1.16
-8	-7	-6	13.36	1.25
8	-7	6	12.09	0.93
8	7	-6	15.92	1.22
-8	7	6	11.16	1.62
-8	-7	-6	14.25	1.17
8	-7	6	12.48	1.14
-8	7	6	13.94	1.29
8	7	-6	11.64	1.41
8	7	-6	13.45	1.25
-8	7	6	13.25	1.53
-8	-7	-6	12.76	1.16
8	-7	6	11.78	1.03
-8	7	6	14.15	1.42
8	-7	6	14.56	1.88
8	-7	6	12.09	0.96
-8	7	6	10.18	1.47
8	7	-6	14.08	1.19
8	8	6	2.42	0.96
8	-8	-6	2.13	0.55
-8	-8	6	1.76	0.89
-8	-8	6	1.66	0.53
-8	-8	6	0.59	0.99
8	8	6	1.70	0.82

8	-8	-6	2.58	0.59
-8	8	-6	3.06	0.96
8	-8	-6	2.11	0.70
-8	8	-6	4.30	1.11
-8	-8	6	2.24	0.61
8	8	6	1.09	0.69
8	8	6	2.95	0.64
-8	8	-6	1.43	1.18
-8	8	-6	3.06	0.73
8	-8	-6	1.29	1.83
-8	8	6	-0.30	1.03
8	-8	6	1.98	0.50
-8	-8	-6	2.97	0.76
8	-8	6	3.71	1.39
8	-8	6	1.93	0.54
-8	-8	-6	2.64	0.75
8	8	-6	2.02	0.71
8	-8	6	1.75	0.70
8	8	-6	2.64	0.68
8	8	-6	2.18	0.93
-8	8	6	2.00	0.92
-8	8	6	2.12	0.84
-8	-8	-6	2.32	0.64
-8	-9	6	13.09	1.91
8	9	6	10.53	1.53
8	-9	-6	11.40	1.39
8	9	6	12.78	1.62
8	-9	-6	11.58	1.17
-8	-9	6	12.20	1.64
8	9	6	12.98	1.28
-8	-9	6	12.42	1.26
-8	-9	6	12.97	1.27
8	9	6	13.69	1.80
-8	9	-6	15.26	1.37
8	-9	-6	13.85	2.00
-8	9	-6	12.52	1.83
-8	9	-6	11.35	1.99
8	-9	6	12.06	1.37
8	-9	6	12.32	2.37
-8	-9	-6	11.35	1.33
8	9	-6	11.03	1.74
8	9	-6	12.40	1.27
-8	-9	-6	11.19	1.38
-8	-9	-6	11.94	1.24

-8	9	6	13.64	1.64
-8	9	6	11.26	1.73
8	-9	6	15.59	1.54
8	-9	6	15.96	1.16
8	-9	6	11.85	1.03
8	9	-6	12.26	1.42
8	-9	6	14.11	1.28
-8	-10	6	25.44	1.97
8	-10	-6	25.67	1.78
-8	-10	6	23.83	2.33
8	10	6	27.77	2.38
8	-10	-6	24.23	1.75
-8	10	-6	24.04	2.56
8	-10	-6	24.20	1.98
-8	10	-6	23.59	2.67
8	10	6	26.27	2.40
-8	10	-6	27.78	1.87
8	-10	6	28.42	2.10
8	-10	6	31.05	1.81
8	10	-6	25.28	2.40
8	-10	6	27.86	1.70
8	-10	6	27.23	1.92
-8	-10	-6	24.19	1.77
-8	10	6	30.85	2.41
-8	-10	-6	23.38	1.88
8	-10	6	28.84	3.03
8	10	-6	25.93	1.86
-8	10	6	24.34	2.61
-8	11	-6	13.43	2.77
8	11	6	6.14	1.55
8	-11	-6	10.15	1.33
-8	11	-6	11.30	1.78
-8	-11	6	6.94	1.22
8	-11	-6	10.24	1.86
8	11	6	5.15	1.40
-8	-11	6	6.61	1.62
8	-11	-6	8.94	1.31
-8	11	6	8.96	1.58
8	11	-6	10.39	1.91
8	-11	6	6.72	1.06
-8	-11	-6	8.84	1.31
8	-11	6	7.89	1.20
8	-11	6	5.68	1.18
-8	-11	-6	7.62	1.34

8	11	-6	10.08	1.45
8	12	6	0.16	1.54
-8	-12	6	0.40	1.24
8	-12	-6	-0.72	1.08
-8	-12	6	1.47	0.86
-8	12	-6	0.65	1.63
8	-12	-6	0.77	0.88
8	-12	-6	1.14	0.91
8	-12	6	2.91	0.95
8	12	-6	-0.30	1.46
-8	-12	-6	0.56	1.29
-8	12	6	3.20	1.49
-8	-12	-6	-1.40	0.88
-9	0	-6	-0.29	0.36
9	0	-6	0.03	0.32
9	0	6	0.04	0.38
9	0	-6	0.03	0.21
-9	0	-6	-0.24	0.39
9	0	6	0.33	0.30
-9	0	-6	0.12	0.31
-9	0	6	-0.16	0.35
9	0	-6	0.30	0.18
-9	0	6	0.12	0.29
-9	0	-6	-0.02	0.23
-9	0	6	0.40	0.46
9	0	-6	0.33	0.22
9	0	6	0.30	0.51
-9	0	-6	0.46	0.43
9	0	6	0.05	0.20
-9	0	6	-0.52	0.29
9	0	6	0.09	0.25
9	0	6	-0.08	0.29
-9	0	6	0.10	0.21
-9	0	-6	0.54	0.30
9	0	-6	0.21	0.19
-9	1	-6	0.75	0.32
9	1	6	4.00	0.43
-9	1	-6	0.48	0.29
9	1	6	2.70	0.52
9	-1	-6	0.70	0.25
-9	1	-6	0.67	0.43
-9	1	-6	1.24	0.48
9	-1	-6	0.77	0.21
-9	1	-6	0.52	0.33

-9	-1	6	3.20	0.56
-9	1	-6	0.77	0.51
-9	-1	6	2.75	0.53
-9	-1	6	3.12	0.42
9	1	6	3.04	0.47
-9	-1	6	3.03	0.38
-9	-1	6	3.18	0.55
9	1	6	3.61	0.69
9	1	6	2.95	0.54
9	-1	-6	0.77	0.27
9	1	6	2.55	0.47
-9	1	-6	0.50	0.28
9	-1	-6	0.73	0.31
-9	-1	6	3.53	0.63
-9	1	6	3.56	0.51
9	1	-6	0.57	0.26
-9	-1	-6	1.00	0.40
-9	1	6	3.51	0.40
9	1	-6	0.84	0.16
-9	-1	-6	0.12	0.35
9	1	-6	0.89	0.38
9	1	-6	1.05	0.25
-9	1	6	3.12	0.58
-9	1	6	2.99	0.55
-9	1	6	2.87	0.45
9	-1	6	3.45	0.56
9	-1	6	3.23	0.41
9	-1	6	3.70	0.61
-9	1	6	2.30	0.48
-9	-1	-6	0.85	0.26
-9	-1	-6	0.86	0.34
-9	-1	-6	0.67	0.41
9	-1	6	3.32	0.46
-9	-1	-6	1.14	0.36
-9	1	6	2.90	0.55
-9	1	6	3.47	0.61
-9	2	-6	40.80	2.01
-9	-2	6	32.27	1.70
9	-2	-6	38.81	1.98
-9	-2	6	31.24	1.89
-9	2	-6	40.85	2.04
-9	-2	6	32.48	1.71
-9	2	-6	39.94	2.00
-9	2	-6	36.80	2.21

9	2	6	33.44	1.90
-9	-2	6	32.96	1.94
-9	-2	6	30.96	1.68
-9	2	-6	37.17	1.99
9	2	6	34.41	1.74
9	2	6	36.47	1.98
9	-2	-6	38.49	1.90
9	2	6	34.44	1.78
9	2	6	32.81	1.70
9	-2	-6	40.90	2.06
-9	2	-6	39.28	2.28
9	-2	-6	35.84	1.99
-9	-2	6	35.08	1.98
9	-2	6	33.91	1.76
9	2	-6	39.36	1.91
-9	2	6	32.26	1.87
-9	-2	-6	38.44	2.01
9	-2	6	34.33	1.96
-9	-2	-6	37.77	2.02
9	2	-6	37.86	1.92
9	2	-6	38.82	1.81
-9	2	6	35.00	1.77
-9	-2	-6	41.28	2.03
9	2	-6	37.15	2.09
-9	2	6	30.15	1.64
-9	2	6	34.48	1.83
-9	2	6	33.50	1.79
-9	2	6	32.64	1.66
-9	-2	-6	40.13	2.17
-9	-2	-6	36.94	2.02
-9	-2	-6	32.36	2.10
-9	2	6	34.43	1.68
9	-2	6	31.17	1.73
9	-2	6	30.80	1.74
-9	-2	-6	40.57	1.93
-9	2	6	35.04	1.97
-9	-3	6	188.55	9.18
-9	-3	6	191.99	9.37
-9	-3	6	205.51	9.20
9	3	6	193.91	9.24
-9	3	-6	199.56	8.74
-9	-3	6	219.74	9.43
-9	3	-6	189.23	8.96
-9	3	-6	188.92	8.69

-9	-3	6	208.51	9.13
9	-3	-6	187.23	8.61
-9	3	-6	202.67	8.71
-9	-3	6	186.93	9.10
9	-3	-6	179.28	8.71
-9	3	-6	190.68	8.69
-9	3	-6	199.48	8.74
9	3	6	211.55	9.38
9	3	6	196.53	9.39
9	-3	-6	199.86	8.82
-9	3	-6	182.32	8.92
-9	-3	6	208.46	9.42
9	3	6	205.09	9.23
9	-3	-6	177.17	8.71
9	3	-6	191.25	8.78
9	3	-6	180.95	8.66
-9	-3	-6	190.99	8.70
9	3	-6	191.42	8.55
-9	-3	-6	178.52	8.71
-9	-3	-6	191.70	8.63
-9	3	6	209.09	9.30
-9	-3	-6	191.52	8.72
-9	3	6	198.06	9.40
9	-3	6	206.51	9.41
-9	3	6	197.26	9.36
9	-3	6	212.62	9.55
9	-3	6	200.07	9.25
-9	3	6	207.76	9.29
9	-3	6	182.88	9.23
-9	3	6	200.85	9.31
9	-3	6	201.48	9.19
-9	-3	-6	177.48	8.59
-9	3	6	200.34	9.16
-9	-3	-6	197.25	8.74
9	-4	-6	120.49	5.68
-9	4	-6	132.36	5.86
-9	4	-6	118.55	5.72
-9	-4	6	117.05	5.48
9	4	6	126.35	5.80
-9	-4	6	114.69	5.61
-9	4	-6	115.48	6.03
9	4	6	112.50	5.81
-9	-4	6	114.33	5.82
-9	-4	6	127.24	5.84

-9	4	-6	134.47	5.78
9	-4	-6	119.26	5.81
9	4	6	115.18	5.68
-9	4	-6	125.83	5.81
-9	4	-6	120.41	5.73
9	-4	-6	119.94	5.79
-9	-4	6	123.14	5.43
-9	-4	6	120.60	5.49
-9	-4	6	123.14	5.42
9	4	6	118.34	5.62
-9	-4	6	120.45	5.86
-9	4	-6	119.39	6.09
9	4	-6	127.03	5.66
9	-4	6	127.03	6.03
-9	4	6	120.97	5.58
-9	4	6	117.07	5.80
-9	-4	-6	123.01	5.76
-9	4	6	126.56	5.73
9	-4	6	116.52	5.68
9	-4	6	119.43	5.57
-9	4	6	123.83	5.75
9	4	-6	124.13	5.71
-9	-4	-6	120.38	5.78
-9	-4	-6	121.61	5.80
9	4	-6	127.26	5.84
-9	-4	-6	121.83	5.71
-9	4	6	124.42	5.85
9	4	-6	117.34	5.78
-9	-4	-6	122.99	5.80
-9	4	6	120.53	5.81
9	-4	6	118.26	5.85
9	-4	6	112.81	5.64
-9	5	-6	88.55	4.02
9	5	6	77.26	3.70
-9	-5	6	77.39	3.53
-9	-5	6	79.12	4.01
-9	-5	6	75.12	3.51
9	-5	-6	86.97	4.27
9	5	6	78.52	3.91
-9	5	-6	68.49	4.12
9	5	6	72.38	3.80
-9	5	-6	80.10	3.96
9	-5	-6	82.67	4.10
-9	-5	6	76.33	3.58

9	-5	-6	81.54	4.05
9	-5	-6	76.33	3.91
9	5	6	71.30	3.93
-9	5	-6	89.29	4.15
-9	-5	6	72.01	3.55
-9	5	-6	80.28	4.34
-9	-5	6	73.26	3.70
-9	5	-6	88.09	4.10
-9	-5	6	81.77	3.97
-9	-5	6	71.62	3.93
-9	5	-6	85.06	4.49
-9	5	6	73.01	3.96
-9	-5	-6	82.18	4.06
9	-5	6	76.46	3.63
-9	-5	-6	86.74	3.99
-9	5	6	76.27	3.94
9	-5	6	76.95	3.99
-9	5	6	79.14	3.91
9	-5	6	75.84	3.78
-9	-5	-6	82.03	4.03
9	5	-6	81.38	3.98
9	5	-6	84.23	4.07
9	5	-6	77.33	4.09
9	-5	6	72.99	3.71
-9	-5	-6	82.70	4.06
9	-5	6	80.53	4.16
-9	-5	-6	87.76	4.29
-9	5	6	76.82	3.92
-9	5	6	74.61	3.67
-9	-5	-6	80.26	4.02
-9	5	6	77.06	3.81
9	6	6	20.78	1.48
9	-6	-6	14.46	1.22
-9	-6	6	17.00	1.61
9	6	6	20.24	1.62
9	-6	-6	14.39	1.03
-9	-6	6	18.05	1.74
-9	-6	6	19.99	1.12
9	-6	-6	13.80	1.13
9	6	6	21.44	1.36
-9	6	-6	15.22	1.06
-9	6	-6	14.08	1.57
-9	6	-6	15.36	1.27
-9	-6	6	20.37	1.30

-9	6	-6	14.64	1.75
9	-6	-6	15.34	1.53
9	6	6	18.19	1.35
-9	6	-6	14.98	1.14
-9	6	-6	16.45	1.35
-9	-6	6	19.91	1.34
-9	-6	6	20.79	1.22
-9	6	-6	15.87	1.17
-9	-6	6	19.91	1.74
-9	6	6	17.04	1.69
-9	6	6	23.67	1.58
-9	6	6	22.66	1.66
9	-6	6	19.21	1.32
-9	-6	-6	14.33	1.17
-9	6	6	21.31	1.70
9	-6	6	23.72	1.91
-9	-6	-6	14.87	1.18
9	-6	6	19.71	1.77
9	6	-6	13.05	1.32
9	6	-6	15.08	1.16
-9	6	6	21.51	1.37
9	6	-6	15.81	1.18
9	-6	6	21.69	1.27
9	-6	6	20.23	1.43
9	6	-6	16.12	1.19
-9	-6	-6	14.48	1.13
-9	-6	-6	17.85	1.55
-9	-6	-6	15.04	1.19
-9	6	6	20.71	1.64
-9	7	-6	16.40	1.70
9	7	6	10.51	1.27
-9	7	-6	15.99	1.25
-9	7	-6	15.81	1.50
-9	-7	6	10.94	1.05
-9	7	-6	14.03	1.22
-9	-7	6	12.63	1.55
-9	-7	6	9.13	1.34
9	-7	-6	15.94	1.32
9	-7	-6	13.67	1.18
-9	7	-6	12.44	2.31
-9	-7	6	11.43	0.95
9	-7	-6	17.32	1.73
9	7	6	9.90	1.35
9	-7	-6	14.10	1.07

9	7	6	10.99	1.07
9	7	-6	14.26	1.21
-9	7	6	13.89	1.40
9	7	-6	15.34	1.24
9	-7	6	10.87	2.65
-9	7	6	11.92	1.45
-9	-7	-6	14.99	1.22
9	7	-6	10.94	1.37
9	-7	6	12.27	0.93
-9	7	6	10.53	1.45
-9	-7	-6	15.46	1.26
-9	7	6	11.20	1.13
-9	-7	-6	16.22	1.26
9	7	-6	15.36	1.23
-9	7	6	11.66	1.63
9	-7	6	12.00	1.15
9	-7	6	10.47	1.00
-9	8	-6	3.84	0.99
-9	8	-6	4.14	1.46
9	-8	-6	3.53	0.73
9	8	6	3.06	0.70
-9	8	-6	3.21	1.23
-9	-8	6	3.22	0.75
9	8	6	1.23	1.11
9	-8	-6	3.34	1.12
-9	8	-6	6.10	1.08
9	-8	-6	3.06	0.82
-9	-8	6	2.19	0.60
-9	-8	6	3.95	1.08
-9	-8	6	2.13	0.89
9	8	6	1.97	0.86
9	-8	-6	3.20	0.59
9	-8	6	1.49	0.47
-9	-8	-6	3.25	0.71
9	-8	6	1.94	0.55
9	-8	6	1.79	0.67
-9	8	6	2.47	0.87
9	8	-6	3.94	0.74
9	-8	6	3.50	1.36
-9	-8	-6	3.61	0.71
9	8	-6	3.45	0.81
9	-8	6	1.87	0.51
9	8	-6	1.91	0.87
-9	8	6	0.69	1.07

-9	-8	-6	3.07	0.76
-9	-9	6	34.38	2.23
-9	-9	6	34.78	2.61
9	-9	-6	42.06	3.00
9	-9	-6	37.91	2.46
-9	9	-6	37.84	2.88
9	9	6	32.97	2.53
-9	9	-6	35.71	3.11
-9	-9	6	33.09	2.18
-9	-9	6	36.46	2.65
-9	9	-6	40.34	2.33
9	9	6	33.83	2.10
9	9	6	37.76	2.64
9	-9	-6	37.21	2.23
9	9	6	34.99	2.60
-9	9	6	28.87	2.58
9	-9	6	40.04	2.05
9	-9	6	39.63	3.16
9	-9	6	41.51	2.34
9	9	-6	36.45	2.72
9	9	-6	39.82	2.50
9	-9	6	38.43	2.38
-9	-9	-6	34.80	2.24
-9	-9	-6	37.83	2.39
9	-9	6	36.98	2.22
-9	-9	-6	35.84	2.33
9	-9	6	36.33	2.04
9	9	-6	39.21	2.27
-9	10	-6	50.92	3.60
9	-10	-6	47.75	2.82
9	-10	-6	47.78	2.98
9	10	6	53.24	3.44
-9	10	-6	48.11	3.64
9	10	6	48.03	3.37
-9	-10	6	52.24	3.05
9	-10	-6	52.88	2.83
-9	-10	6	50.15	3.38
9	10	-6	53.15	2.85
9	-10	6	52.88	3.16
9	10	-6	47.17	3.36
9	-10	6	54.67	2.80
-9	10	6	53.32	3.45
-9	-10	-6	50.22	2.97
9	-10	6	55.46	3.02

-9	-10	-6	47.37	2.76
-9	11	-6	44.72	3.80
9	-11	-6	45.32	2.77
9	-11	-6	46.47	3.08
9	-11	-6	47.97	2.82
9	11	6	42.97	3.38
-9	-11	6	45.08	2.93
9	11	6	39.98	3.37
-9	-11	6	48.80	3.46
9	11	-6	48.48	3.63
-9	11	6	48.91	3.34
-9	-11	-6	46.60	2.80
-9	-11	-6	51.75	3.04
9	-11	6	43.79	2.84
9	11	-6	49.45	2.77
9	-11	6	48.53	2.81
-9	-12	6	37.10	2.94
-9	12	-6	28.10	3.27
9	-12	-6	30.73	2.57
9	-12	-6	29.94	2.20
9	12	6	33.87	3.08
-9	-12	6	34.84	2.54
9	-12	-6	34.31	2.36
-9	12	-6	33.03	1.97
-9	-12	-6	30.97	2.76
-9	-12	-6	31.13	2.40
-9	12	6	40.05	3.39
9	-12	6	35.55	2.42
9	12	-6	25.05	2.87
-9	-12	-6	31.29	2.36
10	0	6	108.08	4.95
10	0	-6	132.93	6.34
-10	0	-6	140.40	6.30
-10	0	6	107.84	5.13
-10	0	6	100.95	4.99
-10	0	-6	139.58	6.25
-10	0	6	100.80	4.95
10	0	6	105.54	5.01
10	0	-6	128.03	6.18
-10	0	-6	120.02	6.29
-10	0	6	113.80	4.97
10	0	6	107.45	5.00
10	0	-6	133.71	6.13
-10	0	6	101.16	4.93

-10	0	-6	132.02	6.28
-10	0	-6	141.94	6.14
-10	0	-6	129.91	6.24
10	0	-6	133.62	6.09
10	0	6	97.45	4.94
10	0	6	107.79	5.19
10	0	-6	137.41	6.08
10	0	6	103.33	4.89
-10	-1	6	24.29	1.59
10	-1	-6	22.07	1.32
10	1	6	23.54	1.37
10	1	6	25.51	1.39
-10	-1	6	23.58	1.27
-10	-1	6	21.89	1.25
-10	-1	6	21.26	1.42
10	-1	-6	21.73	1.36
-10	-1	6	21.37	1.47
-10	-1	6	25.41	1.35
-10	1	-6	22.92	1.55
-10	1	-6	23.59	1.43
10	1	6	22.49	1.28
-10	1	-6	23.16	1.54
10	1	6	21.86	1.44
-10	1	-6	24.21	1.30
-10	1	-6	23.75	1.39
10	-1	-6	23.65	1.25
10	1	6	24.77	1.60
-10	1	-6	23.62	1.35
10	-1	-6	23.73	1.26
10	-1	6	23.46	1.52
-10	-1	-6	23.67	1.24
10	1	-6	23.05	1.28
10	1	-6	23.65	1.13
-10	1	6	22.46	1.35
10	1	-6	23.63	1.46
-10	1	6	24.88	1.23
-10	1	6	26.39	1.42
-10	-1	-6	21.42	1.44
-10	1	6	22.25	1.30
10	-1	6	22.39	1.35
-10	-1	-6	23.82	1.38
-10	1	6	24.15	1.48
-10	-1	-6	22.86	1.40
-10	1	6	25.19	1.59

-10	-1	-6	23.67	1.50
-10	-1	-6	23.77	1.37
10	-1	6	22.59	1.66
10	1	-6	23.67	1.19
10	-1	6	21.98	1.37
10	-1	6	23.56	1.30
-10	1	6	23.24	1.22
10	2	6	15.98	1.25
10	2	6	13.64	1.01
10	2	6	14.90	1.03
-10	2	-6	6.48	0.66
-10	2	-6	7.57	0.70
10	2	6	14.17	0.98
-10	-2	6	16.20	1.30
-10	-2	6	15.23	1.24
-10	-2	6	17.27	0.98
10	-2	-6	6.86	0.75
10	-2	-6	7.06	0.66
-10	-2	6	15.03	0.92
-10	-2	6	16.54	1.00
10	2	6	13.18	1.11
-10	2	-6	5.87	0.79
10	2	6	14.51	0.96
10	-2	-6	6.50	0.62
-10	-2	6	14.73	1.14
-10	2	-6	6.44	0.68
-10	2	-6	6.71	0.74
-10	2	-6	6.86	0.97
10	-2	-6	6.65	0.57
-10	2	6	15.70	0.93
-10	2	6	16.13	0.95
10	2	-6	6.64	0.43
-10	-2	-6	7.88	0.91
-10	2	6	15.71	1.16
10	-2	6	14.83	0.97
-10	-2	-6	6.59	0.57
10	2	-6	7.73	0.78
-10	2	6	14.17	1.01
-10	2	6	15.36	1.05
10	2	-6	6.77	0.59
-10	2	6	15.12	1.29
-10	-2	-6	6.71	0.72
10	-2	6	14.02	1.24
10	2	-6	7.45	0.58

-10	-2	-6	5.46	0.69
-10	2	6	13.78	0.91
10	-2	6	14.38	0.99
-10	2	6	14.90	1.08
-10	-2	-6	6.85	0.72
10	-2	6	14.50	1.01
-10	-2	-6	6.45	0.70
10	-3	-6	8.14	0.77
-10	-3	6	12.05	1.21
-10	3	-6	10.03	0.83
-10	3	-6	8.66	0.77
10	-3	-6	8.36	0.78
-10	3	-6	9.10	0.86
10	3	6	11.53	0.92
10	3	6	12.52	1.14
-10	3	-6	7.22	1.05
-10	-3	6	10.90	0.82
-10	-3	6	11.69	0.88
10	-3	-6	9.15	0.67
10	-3	-6	9.30	1.01
10	3	6	11.78	1.05
-10	-3	6	11.87	1.09
-10	-3	6	11.34	0.77
-10	-3	6	11.68	1.09
10	3	6	11.41	0.86
-10	-3	6	11.55	1.23
-10	3	-6	9.77	1.18
-10	3	-6	10.51	0.85
-10	-3	-6	8.78	0.84
10	3	-6	9.82	0.89
-10	3	6	12.07	0.85
-10	-3	-6	8.99	0.71
-10	-3	-6	10.07	0.81
-10	3	6	12.77	1.04
-10	-3	-6	9.16	0.85
-10	-3	-6	10.08	1.07
-10	-3	-6	9.41	0.69
10	-3	6	12.38	1.31
10	-3	6	12.42	0.90
-10	3	6	12.02	1.01
-10	3	6	10.96	1.03
10	-3	6	10.43	1.21
-10	-3	-6	8.87	0.81
-10	3	6	11.70	1.18

10	3	-6	9.07	0.59
10	3	-6	8.51	0.72
10	-3	6	12.32	0.88
10	-3	6	12.11	0.86
-10	-3	-6	8.79	0.78
-10	3	6	12.52	1.08
-10	4	-6	17.31	1.27
-10	4	-6	19.34	1.31
10	4	6	15.71	1.32
-10	-4	6	13.63	1.38
10	-4	-6	17.91	1.09
10	4	6	15.40	1.29
-10	-4	6	13.65	0.99
-10	4	-6	20.93	1.25
10	-4	-6	17.94	1.22
-10	-4	6	14.67	0.89
-10	-4	6	15.67	0.93
10	-4	-6	19.57	1.23
10	-4	-6	17.75	1.38
10	4	6	14.81	1.08
-10	-4	6	14.12	1.30
-10	4	-6	17.56	1.55
-10	4	-6	15.55	1.36
-10	4	-6	18.21	1.16
10	4	6	13.01	1.05
-10	-4	6	15.85	1.42
-10	4	-6	20.07	1.72
-10	4	6	15.83	1.23
10	4	-6	17.41	1.21
-10	4	6	15.40	1.04
10	4	-6	18.13	1.28
-10	-4	-6	18.96	1.47
-10	4	6	17.03	1.23
-10	4	6	16.76	1.30
10	-4	6	16.84	1.59
10	-4	6	14.91	1.07
-10	-4	-6	19.22	1.24
-10	-4	-6	18.51	1.26
-10	-4	-6	19.00	1.24
-10	4	6	14.67	1.31
10	-4	6	15.24	1.41
10	-4	6	13.99	1.04
10	4	-6	18.48	1.06
10	-4	6	15.60	0.99

-10	-4	-6	19.18	1.12
-10	4	6	16.23	1.38
-10	-4	-6	19.50	1.21
10	4	-6	17.87	1.15
-10	-5	6	33.80	2.26
10	-5	-6	37.97	2.11
-10	-5	6	34.26	1.90
-10	5	-6	43.59	2.34
-10	-5	6	33.84	1.70
-10	-5	6	34.42	1.63
10	5	6	30.66	1.95
10	-5	-6	41.85	2.60
-10	-5	6	35.17	1.67
-10	5	-6	44.32	2.35
10	5	6	32.49	1.88
10	-5	-6	40.05	2.29
-10	5	-6	39.91	2.17
-10	-5	6	31.50	2.15
-10	5	-6	44.59	2.26
10	5	6	31.10	2.11
-10	5	-6	39.09	2.68
10	5	6	35.54	2.14
-10	5	-6	42.05	2.61
10	-5	-6	38.63	2.23
-10	-5	6	36.64	2.22
-10	-5	6	34.38	1.71
-10	-5	-6	39.25	2.28
10	5	-6	44.16	2.13
10	-5	6	35.67	2.42
-10	-5	-6	41.15	2.53
10	5	-6	37.98	2.27
-10	5	6	34.33	2.11
-10	-5	-6	39.97	2.20
10	-5	6	34.95	1.96
-10	5	6	34.39	1.87
10	5	-6	42.78	2.29
10	-5	6	33.40	1.90
10	5	-6	40.91	2.19
-10	-5	-6	42.37	2.17
-10	-5	-6	43.83	2.27
-10	-5	-6	44.06	2.29
-10	5	6	34.20	2.03
-10	5	6	32.58	2.16
10	-5	6	37.11	2.28

-10	5	6	34.56	2.13
10	-5	6	33.34	1.78
-10	5	6	31.36	2.17
10	-6	-6	47.79	2.87
-10	6	-6	46.71	2.45
-10	6	-6	41.83	3.09
10	6	6	40.43	2.64
10	6	6	43.85	2.35
-10	6	-6	47.57	2.62
-10	-6	6	46.00	2.69
10	6	6	40.89	2.49
-10	-6	6	43.66	2.23
-10	6	-6	51.55	2.71
-10	-6	6	44.29	2.38
10	-6	-6	44.47	2.53
10	-6	-6	47.85	2.63
-10	-6	6	42.86	2.21
-10	6	-6	51.66	2.56
-10	-6	6	44.58	2.12
-10	-6	6	42.71	2.14
10	6	6	47.12	2.59
10	-6	-6	45.97	2.42
-10	6	-6	45.92	2.94
-10	-6	6	43.37	2.68
-10	-6	-6	45.98	2.53
10	6	-6	48.37	2.56
-10	6	6	44.56	2.64
-10	6	6	44.00	2.67
10	-6	6	43.34	2.23
10	6	-6	42.73	2.64
10	-6	6	41.81	2.43
-10	6	6	43.76	2.36
-10	-6	-6	46.75	2.58
-10	-6	-6	45.45	2.52
-10	6	6	45.19	2.51
10	-6	6	52.05	3.03
-10	6	6	38.67	2.64
10	-6	6	43.73	2.37
10	6	-6	47.63	2.50
-10	6	6	43.56	2.58
10	6	-6	51.75	2.47
10	7	6	52.68	3.03
10	-7	-6	49.46	2.65
10	-7	-6	56.04	3.19

-10	-7	6	52.65	2.58
10	-7	-6	51.47	2.75
-10	7	-6	57.48	2.80
10	7	6	52.23	2.73
10	7	6	48.62	2.93
-10	7	-6	47.83	3.19
-10	7	-6	49.37	2.69
10	7	6	48.77	3.07
-10	-7	6	54.08	3.08
-10	7	-6	51.88	2.94
-10	-7	6	52.58	3.11
-10	-7	6	52.93	2.79
-10	7	-6	51.93	2.66
-10	7	-6	45.02	3.43
10	-7	-6	48.82	2.84
10	-7	6	57.12	3.37
10	7	-6	53.94	2.78
10	-7	6	49.38	2.75
-10	7	6	51.75	2.78
10	7	-6	45.33	2.95
-10	7	6	46.83	3.21
10	7	-6	55.05	2.76
-10	7	6	52.25	3.07
-10	-7	-6	50.61	2.75
10	-7	6	51.68	2.87
-10	-7	-6	51.14	2.83
-10	-7	-6	50.83	2.73
10	-7	6	51.92	2.60
10	-8	-6	40.12	2.43
-10	8	-6	38.84	2.82
-10	-8	6	32.52	2.42
10	8	6	29.11	2.26
10	-8	-6	40.21	2.20
-10	-8	6	31.65	2.25
-10	8	-6	34.56	2.88
10	8	6	33.68	2.01
-10	-8	6	35.41	1.91
-10	8	-6	40.71	2.34
10	8	6	31.15	2.37
-10	-8	6	35.11	2.50
10	-8	-6	43.55	2.87
-10	8	6	35.51	2.81
-10	-8	-6	37.52	2.29
-10	-8	-6	39.05	2.34

10	-8	6	40.67	3.00
10	8	-6	32.66	2.54
-10	8	6	32.33	2.45
-10	-8	-6	41.66	2.41
10	-8	6	35.55	1.84
10	8	-6	40.33	2.27
10	-8	6	32.25	2.15
10	8	-6	41.43	2.40
10	-8	6	31.89	2.01
-10	9	-6	28.07	2.88
-10	-9	6	37.88	2.67
10	-9	-6	31.06	2.75
-10	-9	6	33.11	2.62
10	-9	-6	30.47	1.88
10	9	6	33.45	2.59
10	-9	-6	29.98	2.18
10	-9	-6	30.87	1.95
10	9	6	32.97	2.62
-10	-9	6	34.81	2.10
-10	9	-6	27.86	2.67
-10	-9	6	34.65	2.28
10	9	6	38.39	2.30
10	9	-6	31.44	1.95
-10	9	6	36.75	2.59
-10	9	6	29.60	2.86
10	-9	6	32.07	2.30
10	-9	6	41.22	2.24
10	-9	6	40.61	3.23
-10	-9	-6	29.99	1.98
-10	-9	-6	31.78	2.14
10	9	-6	31.54	2.11
10	9	-6	25.67	2.36
10	-9	6	37.18	1.98
10	-9	6	34.80	2.20
10	10	6	23.54	2.16
-10	10	-6	18.58	2.48
-10	-10	6	21.78	1.77
10	-10	-6	21.94	1.88
-10	10	-6	24.44	3.62
10	10	6	16.09	2.14
-10	-10	6	15.85	2.23
10	-10	-6	20.16	1.69
10	-10	-6	21.66	1.63
-10	-10	-6	20.91	1.67

10	-10	6	22.80	1.44
-10	10	6	20.89	2.40
10	10	-6	23.44	1.73
-10	-10	-6	22.15	1.99
10	-10	6	20.93	1.78
10	10	-6	20.49	2.43
10	-10	6	21.71	1.85
-10	-11	6	14.90	2.23
-10	11	-6	16.71	2.47
10	-11	-6	18.19	1.69
10	-11	-6	11.95	1.87
10	11	6	15.86	2.55
-10	-11	6	14.59	1.65
10	11	6	12.90	2.34
10	-11	-6	16.45	1.65
-10	11	-6	17.25	1.42
-10	-11	-6	17.66	1.79
10	-11	6	14.73	1.56
-10	11	6	17.15	2.34
-10	-11	-6	15.30	1.65
10	11	-6	12.65	2.41
10	-11	6	12.10	2.28
10	-12	-6	1.81	0.90
10	-12	-6	4.79	1.03
-10	12	-6	2.44	0.85
10	-12	-6	4.22	1.34
10	12	6	1.85	1.46
-10	12	-6	2.42	1.64
-10	-12	6	7.13	1.51
-10	-12	6	3.02	1.00
10	-12	6	5.67	1.67
-10	12	6	5.78	1.99
10	-12	6	1.81	0.81
-10	-12	-6	3.82	1.07
-10	-12	-6	2.77	1.44
10	12	-6	1.83	1.61
11	0	6	0.07	0.26
-11	0	-6	0.00	0.33
-11	0	-6	0.55	0.47
-11	0	-6	-0.71	0.41
-11	0	-6	-0.07	0.26
11	0	6	0.04	0.37
-11	0	-6	0.40	0.43
-11	0	6	0.06	0.28

11	0	6	0.15	0.42
11	0	-6	0.11	0.20
-11	0	6	-0.65	0.37
11	0	6	0.36	0.34
11	0	-6	0.05	0.22
-11	0	-6	0.61	0.34
11	0	6	0.04	0.60
-11	0	6	-0.40	0.48
11	0	6	0.10	0.24
-11	0	6	-0.05	0.32
-11	0	6	0.10	0.35
11	0	-6	-0.20	0.25
-11	0	6	0.43	0.52
11	0	-6	-0.15	0.25
11	-1	-6	11.70	0.77
11	1	6	9.59	0.78
-11	-1	6	8.69	0.66
-11	1	-6	11.36	1.08
-11	1	-6	11.86	1.11
-11	1	-6	10.67	0.89
11	-1	-6	10.29	0.81
-11	-1	6	9.88	1.06
11	1	6	9.02	0.94
-11	-1	6	10.01	0.74
11	1	6	9.79	0.83
11	1	6	7.60	0.86
-11	1	-6	11.06	0.80
-11	-1	6	9.77	0.81
-11	-1	6	10.65	1.01
11	1	6	8.44	0.67
11	1	6	9.43	1.04
-11	1	-6	10.93	0.85
-11	-1	6	9.53	0.90
11	-1	-6	10.76	0.76
11	-1	-6	10.63	0.73
-11	1	-6	11.77	0.93
-11	1	-6	11.04	0.96
-11	1	6	10.47	1.09
-11	1	6	8.69	0.86
-11	1	6	9.53	0.73
11	-1	6	8.44	1.17
11	-1	6	9.06	0.77
11	1	-6	11.29	0.79
11	-1	6	9.06	0.99

11	1	-6	11.27	0.99
-11	-1	-6	10.94	0.74
-11	-1	-6	10.89	0.91
11	-1	6	8.76	0.69
11	-1	6	8.12	0.79
-11	1	6	10.04	0.83
11	1	-6	11.28	0.59
-11	-1	-6	11.05	0.90
11	1	-6	11.20	0.65
-11	-1	-6	10.10	1.01
-11	-1	-6	10.46	1.03
-11	1	6	9.72	0.92
-11	-1	-6	11.40	0.89
-11	1	6	7.94	0.73
-11	-2	6	9.61	0.81
11	-2	-6	9.33	0.73
-11	-2	6	11.75	1.11
-11	-2	6	11.51	0.80
11	2	6	10.29	1.08
11	-2	-6	7.62	0.75
-11	-2	6	10.21	1.00
-11	2	-6	9.64	0.89
11	2	6	10.28	0.86
-11	-2	6	9.55	0.72
-11	2	-6	8.38	1.10
-11	-2	6	9.98	0.75
-11	2	-6	9.10	0.83
-11	2	-6	9.12	0.78
11	-2	-6	10.26	0.90
-11	2	-6	10.82	0.90
-11	2	-6	8.80	1.07
11	2	6	10.05	0.89
-11	2	-6	10.10	0.81
11	2	6	11.59	1.01
-11	-2	6	9.83	1.12
11	2	6	10.44	0.87
11	-2	-6	8.80	0.66
11	2	6	10.72	0.78
11	-2	6	9.67	0.82
-11	2	6	10.72	0.85
-11	2	6	11.20	0.79
11	-2	6	8.65	0.75
11	-2	6	8.61	1.10
-11	-2	-6	8.47	0.65

-11	-2	-6	8.58	1.03
-11	2	6	10.38	1.17
-11	-2	-6	10.55	0.88
-11	-2	-6	11.83	0.87
-11	2	6	9.96	0.78
11	2	-6	8.88	0.50
-11	-2	-6	8.91	0.81
-11	2	6	10.51	1.04
11	-2	6	9.67	0.84
-11	-2	-6	9.63	0.86
11	2	-6	7.67	0.64
11	2	-6	8.67	0.89
11	-2	6	12.95	1.26
-11	2	6	11.51	0.79
-11	2	6	11.13	0.97
11	-3	-6	34.25	1.96
11	3	6	28.54	1.71
11	-3	-6	32.30	1.72
11	-3	-6	31.60	1.83
-11	3	-6	33.22	1.84
-11	-3	6	29.62	1.96
-11	3	-6	35.15	1.90
-11	3	-6	33.23	1.86
-11	-3	6	30.30	1.91
-11	-3	6	33.92	2.01
-11	3	-6	31.33	2.13
-11	-3	6	33.39	1.65
11	-3	-6	33.32	1.82
11	3	6	28.81	1.67
-11	-3	6	30.45	1.67
-11	3	-6	33.16	1.85
11	3	6	33.71	1.92
-11	-3	6	29.24	1.64
11	3	6	31.04	1.92
-11	-3	6	28.64	1.58
-11	3	-6	29.08	1.80
-11	3	-6	33.78	2.22
11	-3	6	29.22	1.98
11	3	-6	34.39	1.92
-11	-3	-6	35.30	2.10
-11	-3	-6	32.69	1.73
-11	3	6	30.25	1.82
-11	3	6	32.81	1.82
-11	-3	-6	35.20	1.86

11	3	-6	32.55	1.60
-11	-3	-6	34.71	1.87
11	3	-6	31.91	1.73
11	-3	6	27.20	1.69
-11	3	6	31.75	2.00
-11	-3	-6	31.80	1.77
-11	-3	-6	33.72	1.87
11	-3	6	29.45	1.70
-11	-3	-6	33.53	1.69
-11	3	6	30.70	1.77
11	-3	6	30.85	1.67
-11	3	6	30.57	1.91
-11	3	6	32.19	1.66
-11	-4	6	55.54	3.12
-11	4	-6	61.89	3.05
11	4	6	54.68	2.89
11	4	6	59.37	3.06
11	-4	-6	58.38	2.92
-11	4	-6	60.61	3.09
-11	-4	6	56.77	3.18
-11	-4	6	60.44	2.79
-11	4	-6	61.39	3.47
11	4	6	53.96	3.10
-11	4	-6	60.05	3.10
-11	-4	6	61.01	3.17
-11	-4	6	57.57	2.87
-11	-4	6	53.05	2.84
-11	4	-6	58.89	3.17
-11	-4	6	54.37	2.75
11	-4	-6	61.83	3.22
11	-4	-6	56.27	3.03
11	4	6	55.43	2.89
-11	4	-6	56.73	2.99
-11	4	-6	61.42	3.36
11	-4	-6	57.24	3.04
11	4	-6	57.32	2.98
-11	-4	-6	61.91	3.08
-11	-4	-6	61.71	3.07
11	4	-6	62.79	3.10
-11	-4	-6	58.44	2.89
-11	4	6	58.41	3.00
-11	4	6	58.48	3.12
-11	-4	-6	62.94	3.31
-11	4	6	58.48	3.18

11	-4	6	57.60	2.81
11	-4	6	64.15	3.38
-11	4	6	60.21	3.10
-11	4	6	58.35	3.01
11	-4	6	56.13	3.17
11	-4	6	51.46	2.88
11	-4	6	54.34	2.91
11	4	-6	59.91	2.86
-11	-4	-6	60.61	2.97
-11	4	6	57.86	2.85
-11	-4	-6	58.83	3.06
-11	5	-6	5.42	1.14
11	5	6	7.88	0.92
11	-5	-6	5.02	0.76
11	5	6	10.38	1.21
-11	5	-6	6.86	0.94
-11	5	-6	7.06	1.27
-11	5	-6	5.53	0.89
-11	-5	6	8.97	1.36
-11	-5	6	9.12	1.23
-11	-5	6	9.38	0.75
11	-5	-6	5.97	0.64
11	5	6	8.94	0.94
11	5	6	8.57	1.17
-11	-5	6	8.06	0.84
-11	-5	6	7.49	1.32
11	-5	-6	5.75	0.85
-11	5	-6	5.57	0.72
-11	5	-6	5.55	0.99
11	-5	6	8.22	0.91
-11	5	6	8.55	1.10
-11	-5	-6	7.05	1.21
11	5	-6	6.47	0.79
11	-5	6	9.16	0.87
-11	5	6	10.93	1.25
-11	-5	-6	6.78	0.84
-11	5	6	8.40	1.34
-11	-5	-6	5.79	0.69
-11	5	6	8.57	1.29
11	5	-6	4.49	0.85
-11	-5	-6	4.95	0.83
11	-5	6	9.44	0.79
11	5	-6	6.15	0.84
11	5	-6	5.86	0.67

-11	5	6	9.30	1.18
-11	-5	-6	5.66	0.79
-11	5	6	10.21	0.92
11	-5	6	12.68	1.61
-11	-5	-6	4.83	0.88
11	-5	6	9.88	1.41
-11	-6	6	43.26	2.02
11	6	6	42.68	2.61
-11	6	-6	33.98	2.63
11	-6	-6	34.12	1.92
11	-6	-6	33.55	2.12
11	6	6	42.70	2.31
-11	-6	6	43.48	2.09
-11	-6	6	43.35	2.09
11	6	6	37.58	2.58
-11	6	-6	35.80	1.98
-11	6	-6	37.08	2.20
-11	6	-6	38.45	2.10
-11	6	-6	34.32	2.48
-11	-6	6	41.19	2.31
11	6	6	42.25	2.44
-11	-6	6	41.27	2.63
11	-6	-6	38.94	2.42
-11	-6	6	42.75	2.08
-11	-6	6	41.43	2.63
11	-6	-6	34.16	2.04
-11	-6	-6	33.78	2.06
-11	6	6	42.45	2.32
11	-6	6	48.05	3.04
11	-6	6	39.96	2.30
11	6	-6	32.13	2.13
-11	6	6	38.76	2.59
11	6	-6	38.82	1.98
11	-6	6	43.30	2.16
11	6	-6	33.93	2.01
11	6	-6	35.83	2.05
-11	-6	-6	35.95	2.01
-11	6	6	41.24	2.71
-11	-6	-6	35.61	2.09
11	-6	6	40.65	2.37
-11	7	-6	27.09	1.81
-11	7	-6	26.25	1.64
11	-7	-6	28.97	1.88
-11	7	-6	24.99	3.18

11	-7	-6	29.28	2.24
11	-7	-6	25.35	1.74
-11	7	-6	23.55	2.40
11	-7	-6	25.82	1.61
-11	-7	6	29.27	1.51
11	7	6	31.09	2.13
11	7	6	27.52	1.80
11	7	6	27.29	2.02
-11	7	-6	25.56	1.71
-11	-7	6	27.84	2.23
-11	-7	6	27.96	1.82
-11	-7	6	28.85	1.51
-11	-7	6	28.92	1.55
-11	7	6	28.67	2.32
11	-7	6	34.81	2.67
-11	-7	-6	27.20	1.71
11	-7	6	27.68	1.90
-11	7	6	31.05	2.18
-11	7	6	29.12	1.87
-11	-7	-6	27.38	1.77
11	-7	6	29.28	1.63
11	7	-6	28.25	1.74
11	7	-6	29.74	1.69
11	-8	-6	3.76	0.63
11	8	6	3.70	1.10
11	-8	-6	3.88	0.73
-11	8	-6	5.37	1.25
11	8	6	3.49	1.01
11	-8	-6	3.29	0.89
-11	-8	6	3.06	0.73
11	-8	-6	3.54	1.23
-11	8	-6	4.28	1.50
-11	8	-6	4.81	0.84
11	8	6	3.28	0.90
-11	-8	6	5.27	1.27
-11	-8	6	4.35	1.17
11	8	6	4.44	0.75
-11	-8	6	3.96	0.61
-11	-8	-6	4.41	0.83
11	-8	6	4.55	1.57
11	-8	6	4.10	0.85
11	8	-6	2.73	0.96
11	-8	6	3.95	0.79
-11	8	6	4.97	1.09

11	-8	6	3.93	0.76
11	8	-6	3.85	0.82
-11	-8	-6	4.20	0.83
-11	8	6	4.23	1.34
-11	-8	-6	4.50	0.81
11	8	-6	5.20	0.96
11	-9	-6	5.45	0.96
-11	-9	6	6.46	1.34
11	9	6	5.59	1.22
11	9	6	5.27	1.25
-11	9	-6	4.99	1.43
11	-9	-6	6.93	1.12
-11	-9	6	7.39	1.19
-11	9	-6	8.25	1.02
-11	-9	6	7.79	2.10
-11	9	-6	7.74	1.60
11	-9	-6	5.37	1.02
11	-9	6	6.44	0.84
11	9	-6	5.82	1.29
11	9	-6	5.08	1.01
-11	9	6	6.41	1.30
11	-9	6	8.48	2.04
-11	-9	-6	6.19	0.93
11	9	-6	7.81	1.11
11	-9	6	6.24	1.07
-11	-9	-6	4.83	0.93
11	-9	6	6.06	1.18
11	10	6	29.82	2.78
11	-10	-6	38.68	2.36
11	10	6	28.94	2.72
-11	-10	6	29.41	2.24
-11	-10	6	29.25	2.75
11	-10	-6	36.17	2.59
11	-10	-6	37.34	2.31
-11	10	-6	37.54	2.22
-11	10	-6	38.58	3.33
11	10	-6	37.68	3.06
11	-10	6	33.83	2.20
-11	-10	-6	37.67	2.64
11	10	-6	38.57	2.32
-11	10	6	30.69	2.75
-11	-10	-6	36.11	2.36
-11	-11	6	29.65	2.18
11	-11	-6	30.51	2.25

11	11	6	25.14	2.69
-11	-11	6	25.12	2.58
-11	11	-6	36.63	2.07
11	11	6	24.28	2.62
11	-11	-6	31.51	2.22
-11	11	-6	31.00	3.24
11	-11	-6	28.19	2.44
11	11	-6	24.66	2.75
-11	11	6	28.99	3.08
-11	-11	-6	33.79	2.31
11	-11	6	26.87	2.01
-11	-11	-6	24.50	2.31
11	-11	6	28.28	2.28
-11	-12	6	28.62	2.76
11	-12	-6	22.81	1.95
11	-12	-6	24.71	2.55
11	12	6	24.80	2.92
-11	12	-6	28.18	3.19
-11	-12	6	32.40	2.32
-11	12	-6	26.98	1.93
11	-12	-6	26.19	1.97
-11	-12	-6	25.66	2.72
-11	12	6	30.80	3.24
11	12	-6	20.02	2.70
11	-12	6	30.18	2.57
12	0	6	161.96	7.55
-12	0	6	156.80	7.59
-12	0	-6	174.69	7.76
-12	0	6	167.94	7.43
-12	0	-6	169.98	7.90
-12	0	-6	169.89	7.89
12	0	-6	165.86	7.69
12	0	-6	170.56	7.80
-12	0	6	172.32	7.53
12	0	-6	165.40	7.84
-12	0	-6	168.48	7.91
12	0	6	155.02	7.57
-12	0	-6	173.51	7.96
-12	0	6	165.56	7.71
-12	0	6	165.17	7.54
12	0	-6	177.93	7.71
-12	0	-6	163.13	7.99
-12	0	6	154.34	7.50
12	0	6	153.07	7.52

12	0	6	169.14	7.73
12	0	6	156.97	7.43
-12	1	-6	82.75	4.15
-12	1	-6	89.70	4.06
12	1	6	76.75	4.00
12	-1	-6	83.38	4.00
12	1	6	79.28	3.83
-12	1	-6	81.20	4.31
-12	1	-6	85.69	4.15
12	1	6	82.32	3.91
-12	1	-6	85.48	4.19
12	-1	-6	82.97	4.12
12	-1	-6	91.01	4.06
-12	-1	6	82.72	4.12
-12	-1	6	84.11	4.02
12	-1	-6	83.83	4.04
-12	1	-6	85.08	4.25
-12	-1	6	78.23	3.97
-12	-1	6	79.02	3.98
-12	1	-6	91.30	4.19
12	1	6	76.18	3.92
12	1	6	82.17	3.92
-12	-1	6	82.17	3.88
-12	-1	6	82.47	3.83
-12	-1	6	75.80	3.79
12	1	-6	86.40	3.88
-12	1	6	81.47	4.04
-12	1	6	79.98	3.89
-12	-1	-6	86.60	3.99
12	-1	6	76.81	3.83
-12	-1	-6	84.98	4.27
-12	1	6	84.44	3.82
-12	-1	-6	88.98	4.18
-12	-1	-6	87.02	4.09
12	-1	6	82.79	4.02
12	1	-6	85.27	4.08
12	-1	6	83.25	4.21
-12	-1	-6	84.03	4.17
-12	1	6	77.23	3.84
12	1	-6	86.24	3.90
12	-1	6	77.99	3.92
-12	1	6	84.84	3.95
12	-1	6	75.84	3.90
-12	1	6	81.22	4.13

-12	-1	-6	80.03	4.20
12	2	6	42.64	2.28
12	2	6	43.31	2.47
12	2	6	44.96	2.33
-12	-2	6	40.93	2.20
12	-2	-6	36.18	2.01
12	-2	-6	37.24	1.91
-12	-2	6	42.97	2.23
-12	-2	6	46.07	2.56
-12	2	-6	36.60	2.22
-12	2	-6	37.59	2.29
-12	2	-6	38.85	1.99
12	-2	-6	35.63	1.92
-12	2	-6	38.18	2.05
12	-2	-6	38.50	2.03
-12	-2	6	45.75	2.51
-12	2	-6	37.58	2.02
12	2	6	41.63	2.23
-12	-2	6	46.07	2.28
-12	2	-6	40.67	2.09
-12	-2	6	43.72	2.25
-12	2	-6	38.90	2.05
12	2	6	42.36	2.35
-12	-2	6	40.88	2.43
12	2	6	46.32	2.54
12	2	-6	37.92	1.72
-12	-2	-6	33.49	2.02
12	-2	6	44.36	2.51
-12	-2	-6	34.45	2.06
12	-2	6	45.74	2.68
-12	-2	-6	33.20	2.18
12	2	-6	39.24	2.13
12	-2	6	44.93	2.29
-12	2	6	47.88	2.41
-12	-2	-6	36.51	2.05
-12	2	6	43.49	2.21
12	-2	6	43.05	2.32
-12	2	6	43.11	2.48
-12	2	6	43.44	2.22
-12	2	6	45.64	2.24
12	-2	6	42.54	2.25
-12	2	6	47.04	2.60
-12	2	6	44.63	2.25
-12	-2	-6	38.23	2.23

-12	-2	-6	37.52	1.84
12	3	6	14.14	0.99
-12	3	-6	14.11	1.04
-12	-3	6	15.16	1.44
-12	3	-6	14.83	1.09
-12	3	-6	12.46	1.39
-12	3	-6	13.94	1.16
12	-3	-6	14.37	1.19
12	3	6	14.93	1.11
12	3	6	14.81	1.37
-12	-3	6	14.43	1.40
12	-3	-6	13.01	1.03
12	3	6	13.80	1.25
-12	-3	6	14.65	0.95
-12	-3	6	15.59	1.02
-12	-3	6	14.19	0.99
-12	-3	6	14.88	1.31
-12	3	-6	14.86	1.12
-12	-3	6	15.72	1.06
-12	3	-6	14.81	1.52
12	-3	-6	14.14	0.94
12	-3	-6	13.20	0.98
-12	3	-6	15.61	1.12
-12	-3	-6	13.85	1.02
-12	-3	-6	14.66	0.88
-12	3	6	14.77	1.42
-12	3	6	16.33	1.23
-12	3	6	15.30	1.23
12	-3	6	14.76	1.04
12	3	-6	14.69	1.14
12	-3	6	15.39	1.04
-12	-3	-6	14.41	1.01
-12	3	6	14.48	1.03
12	3	-6	14.75	0.78
-12	3	6	14.37	1.27
-12	-3	-6	15.21	1.13
-12	-3	-6	14.46	1.35
12	3	-6	13.91	0.93
-12	-3	-6	13.76	1.09
12	-3	6	15.48	1.42
-12	-3	-6	14.70	1.07
12	-3	6	12.59	1.03
-12	3	6	15.49	1.15
-12	-4	6	-1.28	0.65

-12	4	-6	0.70	0.37
-12	4	-6	0.43	0.53
12	-4	-6	0.36	0.40
-12	-4	6	0.89	0.62
-12	4	-6	0.26	0.47
12	4	6	0.65	0.60
-12	4	-6	0.29	0.51
-12	-4	6	0.27	0.40
-12	-4	6	0.34	0.36
12	-4	-6	0.36	0.29
-12	-4	6	0.22	0.31
-12	4	-6	0.51	0.72
12	4	6	0.52	0.69
-12	4	-6	-0.12	0.83
12	4	6	0.03	0.45
12	-4	-6	0.56	0.69
-12	4	-6	0.91	0.51
-12	-4	6	0.90	0.43
-12	-4	6	0.62	0.75
12	4	6	0.37	0.40
12	-4	-6	0.27	0.40
-12	4	6	-0.06	0.54
12	4	-6	0.71	0.47
12	-4	6	0.08	0.36
-12	-4	-6	0.35	0.72
12	-4	6	0.18	0.70
-12	-4	-6	0.81	0.51
-12	4	6	0.58	0.69
-12	-4	-6	0.68	0.46
12	-4	6	0.94	0.86
12	-4	6	0.25	0.36
-12	4	6	1.13	0.67
-12	-4	-6	0.55	0.34
-12	4	6	0.19	0.41
-12	-4	-6	0.58	0.53
-12	4	6	0.50	0.58
12	-4	6	-0.33	0.37
-12	4	6	0.60	0.55
12	4	-6	0.47	0.26
-12	-4	-6	0.55	0.38
-12	-4	-6	0.80	0.47
-12	-4	-6	0.16	0.41
12	4	-6	0.35	0.42
-12	-5	6	12.82	0.94

12	-5	-6	9.55	0.98
12	5	6	12.59	1.14
-12	5	-6	6.24	1.21
-12	-5	6	15.23	0.99
12	-5	-6	8.86	0.79
-12	5	-6	9.40	1.05
-12	5	-6	9.20	1.44
-12	-5	6	13.89	1.45
12	5	6	14.60	1.17
-12	5	-6	9.01	0.89
12	5	6	12.98	1.36
-12	-5	6	15.66	1.17
12	5	6	12.62	1.37
-12	5	-6	8.95	1.01
-12	-5	6	13.95	1.57
12	-5	-6	8.65	0.91
-12	-5	6	13.71	1.08
-12	5	6	13.37	1.47
12	-5	6	12.37	1.07
-12	5	6	15.39	1.49
12	-5	6	15.11	1.00
12	5	-6	6.75	0.95
12	-5	6	13.08	1.74
-12	5	6	13.77	1.12
-12	-5	-6	7.87	0.95
-12	-5	-6	8.31	0.95
12	-5	6	14.31	1.14
-12	-5	-6	7.74	0.92
12	5	-6	8.04	0.73
-12	-6	6	76.76	4.20
-12	-6	6	75.34	3.69
-12	6	-6	92.74	4.45
12	6	6	73.65	3.97
12	-6	-6	97.19	4.77
12	6	6	79.59	4.08
-12	6	-6	89.76	4.88
12	-6	-6	87.58	4.33
-12	6	-6	90.13	4.38
12	-6	-6	89.17	4.52
12	6	6	73.01	4.19
-12	-6	6	78.67	3.69
-12	-6	6	74.88	3.88
-12	6	-6	84.12	4.98
12	6	6	77.10	3.86

12	-6	-6	89.38	4.46
-12	-6	6	76.09	4.14
12	6	-6	91.94	4.33
12	6	-6	84.24	4.49
-12	6	6	74.36	4.22
12	6	-6	89.51	4.38
-12	-6	-6	87.20	4.49
12	-6	6	87.04	4.52
-12	6	6	81.33	3.88
-12	-6	-6	90.26	4.45
12	-6	6	76.58	3.95
-12	-6	-6	90.41	4.36
12	-6	6	81.28	3.72
12	-6	6	76.08	3.89
12	6	-6	92.30	4.44
-12	6	6	79.97	4.19
-12	-7	6	53.91	2.70
-12	-7	6	54.21	2.96
-12	7	-6	69.17	4.46
-12	-7	6	56.35	3.33
12	-7	-6	70.12	3.80
12	7	6	52.57	3.11
-12	-7	6	56.71	3.34
-12	7	-6	73.77	3.69
12	7	6	58.19	3.27
12	7	6	53.32	3.31
12	-7	-6	72.38	3.66
12	-7	-6	74.70	3.88
12	-7	-6	79.72	4.20
-12	-7	6	48.74	2.77
12	7	6	57.18	2.93
-12	-7	6	56.26	2.64
-12	7	-6	70.29	4.23
12	-7	6	56.95	2.74
12	-7	6	55.41	2.97
12	7	-6	75.87	3.74
-12	-7	-6	73.54	3.83
-12	-7	-6	74.72	3.72
12	-7	6	54.95	3.05
12	-7	6	64.50	3.76
-12	7	6	55.96	3.47
12	7	-6	76.63	3.72
-12	-7	-6	75.07	3.78
12	7	-6	68.38	3.87

-12	8	-6	94.10	4.52
12	8	6	86.34	4.67
-12	-8	6	83.49	4.69
12	8	6	82.36	4.52
12	-8	-6	90.59	4.61
-12	-8	6	84.43	4.01
-12	8	-6	80.69	5.22
-12	8	-6	87.48	5.16
12	-8	-6	90.37	4.76
12	8	6	78.86	4.68
12	-8	-6	89.48	4.50
-12	-8	6	83.32	4.32
12	-8	-6	99.72	5.14
-12	-8	6	86.67	4.59
-12	8	6	79.52	4.80
-12	-8	-6	89.81	4.58
-12	-8	-6	92.40	4.71
12	8	-6	96.40	4.55
12	-8	6	88.15	4.05
12	-8	6	98.27	5.22
12	8	-6	97.39	4.60
-12	8	6	85.58	4.68
12	-8	6	81.67	4.30
-12	-8	-6	94.85	4.58
12	-8	6	84.93	4.41
12	8	-6	84.07	4.78
-12	-9	6	34.13	2.29
-12	9	-6	30.76	2.75
-12	9	-6	24.67	1.92
12	-9	-6	25.89	1.89
12	-9	-6	23.33	2.01
-12	-9	6	35.00	2.72
12	9	6	35.67	2.70
12	9	6	34.02	2.60
12	-9	-6	26.76	1.80
12	-9	6	35.75	2.23
12	9	-6	28.46	1.86
12	9	-6	28.79	2.34
-12	9	6	38.06	2.84
-12	-9	-6	29.02	2.04
-12	-9	-6	28.22	1.93
12	9	-6	29.49	1.90
12	-9	6	37.20	1.95
12	10	6	15.68	2.42

12	-10	-6	18.55	1.55
-12	10	-6	16.11	1.48
12	-10	-6	18.98	1.69
12	10	6	16.46	2.35
-12	-10	6	19.85	1.89
-12	-10	6	18.21	2.36
-12	10	-6	21.51	2.88
12	-10	-6	15.64	1.81
-12	-10	-6	18.12	1.81
12	10	-6	18.14	1.51
12	-10	6	19.73	1.70
12	10	-6	17.60	2.34
-12	10	6	17.79	2.36
-12	-10	-6	17.85	1.68
12	-10	6	19.36	1.82
-12	-11	6	3.85	1.36
12	11	6	4.65	1.42
12	-11	-6	5.37	1.02
12	-11	-6	2.74	1.15
-12	11	-6	6.08	1.73
12	-11	-6	5.10	1.09
-12	11	-6	5.24	0.96
12	11	6	3.22	1.32
-12	-11	6	3.89	0.96
-12	-11	-6	2.57	0.97
-12	-11	-6	4.83	0.95
12	11	-6	3.74	1.36
-12	11	6	5.08	1.84
12	-11	6	4.39	1.15
12	-11	6	2.49	0.84
-13	0	6	0.07	0.32
-13	0	6	0.12	0.44
-13	0	-6	0.05	0.51
13	0	6	-0.58	0.47
-13	0	-6	0.08	0.50
13	0	-6	0.30	0.22
-13	0	6	-0.10	0.47
-13	0	-6	0.67	0.44
-13	0	6	0.00	0.43
13	0	6	0.06	0.26
13	0	-6	0.04	0.20
13	0	6	0.22	0.38
-13	0	6	0.34	0.65
-13	0	-6	0.08	0.29

-13	0	6	-0.13	0.40
-13	0	-6	0.08	0.43
13	0	-6	-0.47	0.28
13	0	6	0.00	0.42
13	0	-6	-0.04	0.26
-13	0	-6	-0.07	0.33
-13	0	-6	-0.37	0.46
13	0	6	-0.49	0.67
-13	-1	6	1.39	0.56
-13	1	-6	2.27	0.61
-13	1	-6	1.12	0.44
13	1	6	1.26	0.44
13	1	6	1.02	0.68
13	1	6	0.85	0.54
-13	-1	6	0.52	0.46
-13	1	-6	2.76	0.63
13	1	6	1.99	0.50
-13	-1	6	0.99	0.39
-13	-1	6	1.08	0.50
-13	-1	6	1.32	0.34
-13	1	-6	2.04	0.42
13	-1	-6	1.96	0.39
-13	-1	6	1.12	0.69
-13	-1	6	0.60	0.53
13	-1	-6	2.13	0.47
-13	1	-6	1.57	0.56
-13	1	-6	1.91	0.49
13	-1	-6	1.93	0.41
13	-1	-6	1.66	0.33
13	1	6	1.22	0.35
-13	1	-6	1.77	0.67
13	1	6	1.23	0.51
-13	-1	-6	1.21	0.54
-13	1	6	1.53	0.40
-13	-1	-6	2.95	0.71
13	-1	6	0.55	0.58
-13	-1	-6	1.80	0.49
13	-1	6	1.39	0.47
-13	-1	-6	1.68	0.50
13	-1	6	1.18	0.40
13	1	-6	2.06	0.43
-13	1	6	2.40	0.74
-13	1	6	1.24	0.50
13	-1	6	1.02	0.34

-13	-1	-6	1.92	0.35
-13	1	6	0.92	0.52
-13	-1	-6	1.92	0.51
-13	1	6	1.28	0.50
13	1	-6	2.47	0.23
13	-1	6	0.46	0.65
-13	1	6	1.00	0.48
-13	2	-6	10.62	1.03
-13	-2	6	9.83	0.87
-13	2	-6	11.50	1.27
13	-2	-6	12.64	0.94
-13	2	-6	10.44	1.29
13	2	6	9.69	0.96
-13	2	-6	10.06	0.92
-13	2	-6	9.79	0.97
13	-2	-6	10.93	0.91
-13	2	-6	12.25	1.03
-13	2	-6	9.63	0.89
13	2	6	8.23	0.75
-13	-2	6	8.47	0.74
13	2	6	9.14	0.89
13	-2	-6	10.62	0.81
13	2	6	9.08	0.93
-13	-2	6	10.62	1.20
13	-2	-6	11.90	0.82
13	2	6	7.95	1.04
-13	-2	6	9.44	1.26
-13	-2	6	10.90	1.07
-13	-2	6	9.01	0.83
-13	-2	6	9.70	0.78
13	-2	6	8.85	1.19
-13	2	6	8.19	0.76
-13	2	6	11.12	0.77
13	-2	6	9.54	0.88
13	-2	6	8.36	0.75
-13	-2	-6	9.89	0.72
13	2	-6	11.04	1.04
13	-2	6	7.59	1.42
-13	-2	-6	11.70	1.08
-13	2	6	10.51	0.83
-13	2	6	8.41	1.02
-13	-2	-6	10.25	0.85
-13	2	6	9.65	0.85
-13	-2	-6	9.34	0.98

-13	-2	-6	11.18	1.00
13	-2	6	8.67	0.83
-13	2	6	9.51	1.27
-13	2	6	10.32	1.10
-13	2	6	9.39	0.85
-13	-2	-6	11.23	1.23
13	-3	-6	8.47	1.03
13	-3	-6	6.53	0.64
-13	-3	6	7.60	0.81
-13	-3	6	7.29	0.79
13	-3	-6	6.12	0.76
-13	-3	6	7.26	1.24
-13	-3	6	7.02	1.19
13	-3	-6	5.44	0.70
-13	3	-6	5.60	0.89
-13	-3	6	6.34	0.66
-13	3	-6	6.27	1.19
-13	3	-6	6.31	0.87
13	3	6	6.32	0.71
-13	3	-6	5.15	0.88
-13	3	-6	8.34	1.36
-13	-3	6	9.23	1.11
-13	3	-6	4.93	0.80
-13	-3	6	7.70	0.72
-13	3	-6	4.87	0.75
13	3	6	7.14	0.90
13	3	6	6.54	1.02
13	3	6	7.29	1.12
13	3	-6	5.29	0.43
13	-3	6	5.12	0.95
-13	-3	-6	6.44	1.16
-13	3	6	5.92	0.91
-13	-3	-6	4.06	0.69
13	3	-6	6.92	0.64
13	-3	6	5.76	0.73
-13	-3	-6	4.85	0.78
13	-3	6	7.21	0.74
-13	-3	-6	7.13	0.81
-13	-3	-6	6.01	0.71
-13	3	6	5.28	1.00
-13	3	6	8.35	1.12
-13	-3	-6	5.56	0.87
13	3	-6	5.84	0.85
-13	3	6	5.82	0.98

-13	3	6	7.09	0.78
13	-3	6	6.58	1.27
-13	3	6	7.49	1.10
13	-3	6	5.91	0.74
-13	-3	-6	7.16	0.84
-13	-3	-6	5.93	0.57
13	4	6	11.39	1.30
-13	-4	6	16.03	1.58
13	-4	-6	17.10	1.05
-13	4	-6	14.96	1.24
13	-4	-6	15.11	1.18
-13	-4	6	13.79	1.39
-13	-4	6	13.06	1.49
13	-4	-6	13.95	1.16
-13	4	-6	14.29	1.23
-13	-4	6	14.85	1.09
-13	-4	6	13.06	1.02
-13	-4	6	14.72	1.05
13	4	6	12.42	1.12
13	4	6	11.77	1.02
-13	4	-6	16.36	1.27
-13	4	-6	16.19	1.63
-13	4	-6	16.68	1.17
-13	4	-6	16.24	1.65
-13	-4	6	14.03	0.94
-13	4	-6	12.75	1.71
-13	4	6	15.54	1.46
13	4	-6	15.16	1.08
-13	4	6	14.19	1.08
-13	-4	-6	14.05	1.06
13	-4	6	12.36	1.06
-13	-4	-6	16.39	1.04
13	-4	6	11.78	0.99
13	4	-6	16.46	1.24
-13	4	6	17.42	1.44
13	-4	6	13.47	1.52
-13	-4	-6	16.82	1.24
-13	-4	-6	13.82	1.50
-13	-4	-6	16.49	1.22
13	-4	6	10.26	1.02
-13	-4	-6	13.55	0.93
13	4	-6	15.12	0.90
13	5	6	10.22	1.61
-13	5	-6	10.01	1.07

13	5	6	12.95	1.41
-13	-5	6	16.67	1.51
-13	5	-6	11.65	1.54
13	5	6	12.89	1.18
-13	-5	6	11.89	0.98
13	-5	-6	8.14	0.95
-13	-5	6	14.36	0.96
13	-5	-6	10.56	0.89
13	-5	-6	10.50	1.03
-13	-5	6	14.31	1.11
-13	5	-6	8.62	1.46
-13	-5	6	12.60	1.62
-13	5	-6	11.21	1.00
-13	5	-6	12.29	1.32
13	5	6	11.23	1.10
-13	-5	6	13.27	1.17
13	5	-6	9.99	1.04
-13	5	6	16.51	1.53
13	-5	6	12.45	1.16
-13	-5	-6	9.78	1.07
-13	5	6	13.23	1.14
13	-5	6	11.02	1.04
-13	-5	-6	9.61	0.90
13	5	-6	10.87	1.04
13	-5	6	13.11	1.00
13	5	-6	9.38	0.80
13	-5	6	15.34	1.86
-13	-5	-6	11.54	1.07
-13	5	6	13.65	1.53
13	6	6	22.08	1.57
13	-6	-6	17.05	1.68
-13	-6	6	25.42	2.13
-13	-6	6	23.43	2.24
13	6	6	23.59	1.95
-13	6	-6	17.69	1.56
13	-6	-6	18.81	1.50
13	-6	-6	19.63	1.31
-13	-6	6	22.17	1.39
-13	6	-6	21.94	1.40
-13	6	-6	20.91	2.20
-13	-6	6	22.99	1.56
-13	-6	6	21.99	1.35
13	-6	6	24.99	1.38
13	-6	6	22.48	1.60

13	6	-6	20.16	1.27
-13	-6	-6	18.24	1.31
-13	6	6	23.82	2.00
-13	-6	-6	19.86	1.49
13	6	-6	19.86	1.42
-13	6	6	23.64	2.08
-13	6	6	22.55	1.63
13	-6	6	29.77	2.42
-13	7	-6	20.71	2.51
13	-7	-6	21.30	1.54
13	-7	-6	19.86	1.38
-13	-7	6	21.63	2.15
-13	7	-6	18.47	1.35
-13	7	-6	20.70	1.46
13	-7	-6	22.84	2.08
13	7	6	20.59	1.95
13	-7	-6	19.88	1.71
-13	-7	6	19.66	1.29
13	7	6	23.39	1.93
-13	-7	6	23.72	1.33
-13	-7	6	19.89	1.56
-13	7	-6	17.83	2.13
13	7	6	18.33	1.65
-13	-7	6	19.88	1.96
13	7	-6	21.33	1.49
-13	7	6	22.05	2.00
-13	-7	-6	21.12	1.62
13	-7	6	25.15	2.47
13	7	-6	19.91	1.66
13	7	-6	21.79	1.42
13	-7	6	22.19	1.28
13	-7	6	19.37	1.49
-13	7	6	20.95	2.11
-13	-7	-6	19.31	1.56
-13	-7	-6	21.74	1.47
13	-7	6	18.30	1.55
13	8	6	0.91	0.99
13	-8	-6	1.69	1.81
13	8	6	0.29	0.96
-13	-8	6	-0.07	0.47
-13	-8	6	-0.45	0.91
-13	-8	6	-0.75	0.59
13	-8	-6	1.67	0.70
13	8	6	0.65	0.86

-13	8	-6	1.41	1.65
-13	8	-6	1.55	0.69
13	-8	-6	1.41	0.77
-13	-8	6	1.64	1.19
-13	8	-6	0.29	1.20
13	8	-6	0.85	0.67
13	8	-6	1.20	0.91
13	8	-6	1.17	0.77
-13	8	6	-0.27	0.98
13	-8	6	-0.15	1.70
13	-8	6	0.28	0.61
-13	-8	-6	2.06	0.71
-13	-8	-6	1.27	0.71
-13	9	-6	-0.28	1.42
-13	9	-6	1.23	0.65
13	-9	-6	0.22	0.81
-13	-9	6	-0.58	1.11
13	-9	-6	1.03	0.64
13	9	6	0.27	1.07
13	-9	-6	1.63	0.71
-13	-9	6	0.53	0.74
13	9	6	1.04	1.27
13	9	-6	1.16	1.16
13	-9	6	-0.35	0.77
-13	9	6	-0.19	1.08
-13	-9	-6	0.31	0.77
13	9	-6	-0.08	0.69
13	-9	6	0.05	0.64
-13	-9	-6	0.38	0.81
13	10	6	7.42	1.60
-13	-10	6	9.11	1.66
13	-10	-6	11.96	1.35
-13	10	-6	11.64	1.37
-13	-10	6	11.58	2.11
13	-10	-6	8.98	1.51
13	-10	-6	10.23	1.62
-13	10	-6	12.20	2.10
13	10	6	8.12	1.55
13	-10	6	8.34	1.32
-13	-10	-6	11.25	1.54
-13	-10	-6	11.70	1.66
13	-10	6	10.09	1.53
13	10	-6	14.88	2.00
-13	10	6	12.83	2.03

-13	-11	6	14.42	1.68
13	11	6	13.62	2.19
-13	11	-6	15.11	1.51
-13	11	-6	13.13	2.11
13	11	6	17.04	2.35
13	-11	-6	13.50	1.52
13	-11	-6	11.79	2.01
13	-11	-6	13.80	1.56
-13	-11	6	16.01	2.29
13	-11	6	14.65	1.62
-13	11	6	18.35	2.66
13	-11	6	14.76	1.85
-13	-11	-6	10.01	1.68
-13	-11	-6	10.44	1.58
13	11	-6	13.74	2.36
-14	0	-6	188.08	8.67
-14	0	-6	176.57	8.73
-14	0	-6	184.80	8.62
-14	0	6	197.56	9.19
-14	0	6	185.46	8.96
-14	0	-6	185.52	8.51
14	0	-6	179.75	8.54
-14	0	6	195.38	8.88
14	0	6	193.99	9.01
14	0	-6	191.48	8.37
-14	0	6	203.17	8.99
-14	0	6	203.47	9.01
14	0	6	182.61	8.86
14	0	-6	181.91	8.34
14	0	6	181.49	8.97
14	0	6	197.19	9.04
14	0	-6	187.61	8.51
14	0	6	198.13	9.24
-14	0	-6	185.93	8.59
-14	0	6	189.34	9.08
-14	0	-6	185.64	8.41
-14	-1	6	194.38	8.94
14	1	6	183.70	8.87
-14	-1	6	192.60	8.77
14	-1	-6	188.51	8.67
14	-1	-6	189.41	8.83
14	1	6	205.90	9.04
-14	1	-6	190.13	9.32
-14	-1	6	177.90	8.73

-14	1	-6	188.60	8.86
-14	-1	6	191.33	8.83
14	-1	-6	192.56	8.76
-14	1	-6	190.76	8.88
14	1	6	197.16	8.85
-14	-1	6	191.87	9.06
-14	1	-6	194.26	8.72
14	-1	-6	196.72	8.73
-14	1	-6	183.80	9.05
14	1	6	191.59	8.97
14	1	6	186.19	8.74
-14	-1	6	187.13	8.91
-14	1	-6	192.71	8.88
-14	-1	6	185.73	8.92
-14	-1	-6	195.20	8.86
14	1	-6	197.79	8.80
-14	-1	-6	190.76	8.98
14	-1	6	182.10	8.75
14	-1	6	182.72	8.86
-14	-1	-6	183.65	8.87
-14	1	6	194.83	8.81
-14	1	6	200.35	8.90
-14	-1	-6	183.79	8.93
-14	-1	-6	194.56	8.64
-14	1	6	194.56	9.01
-14	1	6	189.97	9.08
14	-1	6	187.84	8.93
-14	-1	-6	192.01	8.70
-14	1	6	201.96	8.77
-14	-1	-6	185.28	8.86
14	-1	6	191.88	8.88
-14	1	6	179.32	8.77
14	-1	6	193.91	9.08
14	2	6	19.68	1.45
14	2	6	20.74	1.46
-14	-2	6	22.12	1.31
-14	2	-6	21.39	1.47
14	2	6	20.93	1.31
-14	-2	6	23.47	1.53
14	2	6	20.95	1.58
14	-2	-6	20.76	1.26
-14	-2	6	21.22	1.31
-14	-2	6	19.98	1.59
-14	-2	6	21.85	1.69

14	2	6	21.65	1.55
14	-2	-6	22.94	1.42
-14	2	-6	22.09	1.34
-14	-2	6	22.47	1.42
-14	2	-6	21.29	1.49
14	2	6	22.95	1.46
-14	-2	6	25.30	1.84
-14	-2	6	21.81	1.39
-14	2	-6	20.79	1.44
-14	2	-6	22.10	1.71
14	-2	-6	23.50	1.30
14	-2	-6	20.95	1.38
-14	2	-6	19.67	1.38
-14	2	-6	21.46	1.71
14	-2	6	22.52	1.72
14	-2	6	25.14	1.94
-14	-2	-6	20.80	1.69
-14	-2	-6	20.62	1.44
-14	2	6	22.83	1.82
-14	-2	-6	21.96	1.16
14	-2	6	23.16	1.43
-14	2	6	23.17	1.35
-14	2	6	23.24	1.57
-14	2	6	21.69	1.28
-14	-2	-6	21.72	1.48
-14	-2	-6	20.24	1.48
-14	2	6	21.72	1.65
14	-2	6	22.31	1.44
-14	2	6	21.34	1.36
-14	-2	-6	22.47	1.24
14	-2	6	21.84	1.34
14	-3	-6	10.97	0.98
14	-3	-6	11.85	0.92
14	3	6	12.84	1.22
-14	3	-6	11.90	1.06
-14	-3	6	13.38	1.47
14	3	6	11.53	1.30
14	-3	-6	10.58	1.04
-14	-3	6	13.20	1.03
-14	3	-6	9.07	1.04
14	3	6	12.84	1.13
-14	3	-6	10.43	1.67
-14	3	-6	11.44	1.44
-14	3	-6	9.53	0.96

-14	-3	6	12.06	1.31
-14	-3	6	12.15	1.55
-14	-3	6	13.65	1.09
14	3	6	13.25	1.00
-14	-3	6	13.56	0.95
-14	-3	6	14.05	1.07
14	-3	-6	11.38	0.85
-14	-3	-6	10.27	0.71
14	-3	6	13.35	1.09
-14	-3	-6	10.48	1.04
-14	-3	-6	10.39	1.11
14	-3	6	13.78	1.66
14	-3	6	12.41	1.45
-14	-3	-6	10.52	0.81
-14	3	6	14.63	1.25
-14	-3	-6	11.10	1.02
-14	3	6	12.70	1.08
-14	3	6	13.06	1.38
14	-3	6	12.64	1.02
-14	3	6	13.54	1.06
-14	-3	-6	12.19	1.14
14	3	-6	11.82	1.12
14	3	-6	9.92	0.73
14	-3	6	13.23	1.03
-14	3	6	16.79	1.60
-14	-3	-6	11.78	1.38
-14	4	-6	0.83	0.97
14	-4	-6	2.53	0.60
-14	-4	6	2.57	0.54
-14	-4	6	1.71	0.48
-14	-4	6	2.24	0.93
14	4	6	2.80	0.54
14	-4	-6	2.65	0.79
-14	-4	6	2.70	1.02
14	4	6	2.78	0.68
-14	-4	6	2.41	0.47
14	4	6	2.74	0.64
14	-4	-6	2.70	0.55
-14	4	-6	3.82	0.71
-14	4	-6	1.96	0.90
-14	-4	6	2.60	0.59
14	4	6	1.50	0.71
-14	-4	6	1.55	0.74
14	-4	-6	2.16	0.48

-14	4	-6	2.70	0.56
-14	-4	-6	3.30	0.67
14	4	-6	1.85	0.49
14	4	-6	3.01	0.44
14	-4	6	1.77	0.52
-14	4	6	0.71	0.85
-14	4	6	1.28	0.71
-14	-4	-6	2.89	0.58
14	-4	6	1.95	0.51
-14	-4	-6	2.77	0.59
-14	4	6	2.58	0.62
14	-4	6	2.05	0.49
14	4	-6	2.27	0.63
-14	-4	-6	2.26	0.46
-14	-4	-6	2.67	0.53
-14	-5	6	41.00	2.59
14	5	6	41.24	2.65
14	-5	-6	33.73	1.88
-14	-5	6	39.37	2.11
14	5	6	38.24	2.29
14	-5	-6	38.65	2.43
-14	-5	6	42.89	2.17
-14	5	-6	34.83	1.98
-14	-5	6	41.71	2.70
14	-5	-6	32.38	2.03
-14	-5	6	39.03	2.24
14	-5	-6	33.89	2.01
-14	-5	6	40.89	2.29
-14	5	-6	32.55	1.94
-14	5	-6	32.56	2.62
14	5	6	45.33	2.46
-14	5	-6	32.74	2.55
14	5	6	38.27	2.27
14	-5	6	42.81	2.92
14	-5	6	38.80	2.28
-14	5	6	41.53	2.59
14	5	-6	35.02	2.05
14	5	-6	32.70	1.95
-14	-5	-6	35.38	1.83
-14	-5	-6	34.73	2.09
14	5	-6	34.23	1.78
14	-5	6	40.80	2.30
-14	-5	-6	32.83	2.05
14	-5	6	41.28	2.14

-14	5	6	40.31	2.28
-14	5	6	38.49	2.64
14	-6	-6	72.27	3.67
-14	-6	6	74.13	3.90
-14	6	-6	71.93	4.16
-14	-6	6	73.30	3.71
-14	-6	6	80.49	3.75
-14	6	-6	59.22	3.49
-14	6	-6	71.86	4.29
14	6	6	83.01	4.11
14	6	6	72.13	3.96
14	6	6	72.65	4.24
-14	6	-6	70.99	3.61
14	-6	-6	65.83	3.75
-14	-6	6	75.55	4.25
14	-6	-6	72.11	3.54
-14	-6	6	80.08	4.28
-14	-6	-6	75.10	3.47
14	6	-6	75.84	3.65
14	6	-6	67.37	3.62
14	-6	6	76.48	3.93
-14	-6	-6	71.76	3.72
-14	6	6	77.54	4.28
14	-6	6	78.50	4.02
14	6	-6	74.76	3.48
-14	6	6	75.54	3.90
-14	-6	-6	70.88	3.67
-14	6	6	75.98	4.29
14	-6	6	83.53	4.59
14	-6	6	82.07	3.72
-14	-7	6	80.54	4.03
-14	-7	6	79.62	4.27
14	-7	-6	98.74	5.01
14	-7	-6	99.81	5.05
14	7	6	80.14	4.70
-14	7	-6	100.99	5.57
-14	7	-6	95.71	4.93
-14	7	-6	94.22	5.54
14	-7	-6	110.01	5.41
-14	-7	6	89.64	4.04
-14	-7	6	86.38	4.70
14	-7	-6	94.82	4.85
14	7	6	79.76	4.41
-14	-7	6	82.46	4.62

14	-7	6	95.04	5.06
14	7	-6	92.57	5.04
14	-7	6	82.70	4.28
14	-7	6	91.73	4.01
-14	-7	-6	98.35	4.98
-14	7	6	83.32	4.71
14	-7	6	79.93	4.50
14	7	-6	106.51	4.94
14	7	-6	107.45	4.88
-14	-7	-6	100.59	4.80
-14	7	6	88.77	4.76
-14	-7	-6	102.94	5.10
14	8	6	50.73	3.09
-14	-8	6	52.68	2.95
14	-8	-6	47.82	2.68
-14	-8	6	53.38	3.35
14	8	6	53.06	3.24
14	-8	-6	47.62	2.81
-14	8	-6	50.94	2.75
14	8	6	49.53	3.33
-14	8	-6	52.02	3.57
-14	-8	6	49.10	2.57
14	-8	-6	49.81	2.94
14	8	-6	44.79	2.95
-14	-8	-6	51.40	2.97
-14	8	6	50.38	3.43
14	-8	6	50.09	2.87
14	8	-6	52.07	2.78
14	8	-6	50.40	2.74
14	-8	6	52.25	3.02
-14	-8	-6	48.19	2.84
14	-9	-6	11.88	1.33
-14	-9	6	19.49	2.40
14	9	6	18.57	2.41
14	-9	-6	15.11	1.54
14	9	6	15.02	2.16
-14	9	-6	18.32	2.72
-14	9	-6	13.12	1.39
14	-9	-6	14.56	1.68
-14	-9	6	17.31	1.70
14	-9	6	16.06	1.71
-14	9	6	16.87	2.52
14	9	-6	12.46	2.07
14	9	-6	17.63	1.43

14	-9	6	16.31	1.67
-14	-9	-6	13.77	1.79
14	-10	-6	7.05	1.14
14	-10	-6	5.60	1.31
14	-10	-6	8.87	1.39
14	10	6	5.81	1.34
-14	10	-6	5.10	0.99
-14	-10	6	7.20	1.52
-14	10	-6	8.80	1.82
14	10	6	6.81	1.46
-14	-10	6	7.56	1.24
14	-10	6	5.58	1.19
-14	-10	-6	7.40	1.16
-14	10	6	7.26	1.70
-14	-10	-6	8.31	1.49
14	-10	6	6.01	1.04
14	10	-6	3.84	1.27
-14	11	-6	5.67	1.16
14	11	6	3.36	1.40
14	-11	-6	3.80	0.98
-14	-11	6	3.38	1.05
-14	11	-6	1.54	1.70
14	-11	-6	2.73	0.94
-14	-11	6	1.91	1.29
14	-11	-6	4.28	1.25
14	11	6	3.83	1.39
14	-11	6	5.04	1.36
14	-11	6	4.99	0.97
-14	11	6	5.53	1.62
-14	-11	-6	4.85	1.22
14	11	-6	3.24	1.53
-15	0	-6	0.88	0.67
-15	0	-6	0.82	0.65
15	0	6	-0.27	0.30
-15	0	6	-0.09	0.56
15	0	-6	-0.18	0.36
15	0	6	-0.12	0.50
15	0	-6	-0.07	0.22
-15	0	-6	0.15	0.51
-15	0	-6	0.39	0.34
15	0	6	0.90	0.66
-15	0	-6	0.19	0.39
15	0	6	-0.41	0.61
-15	0	-6	-0.11	0.49

15	0	-6	0.37	0.32
-15	0	6	0.22	0.54
-15	0	6	0.05	0.52
-15	0	6	0.15	0.40
-15	0	-6	0.81	0.52
-15	0	6	-0.79	0.78
15	0	-6	0.08	0.26
-15	0	6	0.34	0.54
15	0	6	0.33	0.40
-15	-1	6	5.31	0.75
-15	1	-6	8.19	1.10
15	1	6	4.81	0.78
15	-1	-6	8.45	0.75
15	-1	-6	8.21	0.65
-15	1	-6	9.53	0.94
-15	-1	6	3.99	0.97
-15	1	-6	8.22	0.79
15	1	6	5.73	1.10
-15	-1	6	5.55	0.92
15	1	6	5.28	0.95
-15	-1	6	5.44	0.91
15	1	6	5.07	1.01
15	-1	-6	8.46	0.75
-15	-1	6	4.74	0.68
-15	1	-6	6.50	1.23
15	-1	-6	8.70	0.86
-15	1	-6	7.74	1.08
15	1	6	5.16	0.63
-15	-1	6	4.73	0.89
-15	-1	6	4.40	0.63
15	-1	6	5.05	0.89
15	-1	6	4.79	0.84
-15	-1	-6	6.82	0.92
-15	-1	-6	8.38	0.64
-15	-1	-6	7.64	0.94
-15	1	6	4.11	0.74
-15	-1	-6	7.88	1.13
-15	1	6	5.40	1.00
-15	1	6	3.78	0.80
-15	-1	-6	8.91	0.77
-15	1	6	4.56	0.87
15	-1	6	4.28	0.77
-15	1	6	4.57	0.77
-15	1	6	3.39	0.90

15	-1	6	6.05	1.09
15	-1	6	4.95	0.64
-15	2	-6	26.49	1.65
15	-2	-6	28.89	1.57
-15	-2	6	27.21	1.65
-15	2	-6	25.90	2.05
-15	-2	6	30.03	1.76
-15	-2	6	27.23	1.98
15	-2	-6	26.63	1.64
-15	-2	6	29.26	1.66
15	-2	-6	26.15	1.50
15	2	6	30.05	1.95
15	2	6	29.55	1.97
15	2	6	29.08	1.80
-15	-2	6	28.05	1.70
15	-2	-6	24.93	1.64
-15	2	-6	27.96	1.72
-15	-2	6	31.04	2.13
-15	-2	6	27.94	1.92
15	2	6	28.16	1.62
-15	-2	6	28.69	1.84
15	-2	6	28.19	1.75
-15	-2	-6	28.32	1.78
15	-2	6	32.10	2.19
-15	-2	-6	26.58	1.75
-15	-2	-6	26.65	1.36
-15	-2	-6	26.91	1.70
-15	-2	-6	26.43	1.70
-15	2	6	31.01	1.68
-15	2	6	30.37	1.71
-15	-2	-6	27.91	1.41
-15	2	6	29.76	2.02
-15	2	6	28.69	2.48
15	-2	6	27.88	1.64
-15	-2	-6	26.62	1.68
-15	2	6	29.70	1.62
15	-2	6	27.06	2.03
-15	-2	-6	28.08	1.98
15	-2	6	29.38	1.75
-15	3	-6	33.45	2.00
-15	-3	6	38.48	2.12
15	-3	-6	34.95	1.98
-15	-3	6	37.69	2.49
-15	-3	6	39.65	2.16

-15	3	-6	32.40	2.38
15	-3	-6	36.26	1.88
15	3	6	46.67	2.55
-15	-3	6	39.95	2.43
15	-3	-6	38.74	2.04
-15	-3	6	37.99	2.07
15	3	6	37.10	2.42
15	-3	-6	33.16	1.86
15	3	6	36.58	2.07
-15	3	-6	30.91	2.30
-15	3	-6	32.92	1.96
-15	-3	6	41.83	2.59
-15	-3	6	35.26	2.20
15	3	6	39.68	2.36
-15	-3	6	39.98	2.15
-15	-3	-6	29.21	1.97
-15	-3	-6	34.02	2.02
-15	3	6	38.28	2.15
-15	3	6	39.89	2.14
15	-3	6	37.07	2.10
-15	-3	-6	34.94	2.00
-15	-3	-6	33.21	1.68
15	-3	6	37.99	2.75
15	-3	6	37.63	2.11
15	-3	6	37.65	2.18
-15	3	6	40.00	2.48
-15	-3	-6	34.18	2.00
-15	4	-6	18.98	1.40
-15	-4	6	20.92	1.76
15	-4	-6	19.67	1.43
15	4	6	21.95	1.70
15	-4	-6	23.71	1.73
15	4	6	19.59	1.38
-15	-4	6	20.40	1.29
-15	-4	6	20.38	1.47
-15	4	-6	20.88	2.08
-15	4	-6	19.11	1.44
-15	-4	6	24.10	1.43
-15	-4	6	21.74	1.43
15	-4	-6	19.47	1.36
-15	4	-6	20.60	1.94
-15	-4	6	23.26	1.97
15	-4	-6	21.17	1.30
15	4	6	20.87	1.73

15	-4	6	18.50	1.45
15	-4	6	19.08	1.38
15	4	-6	19.60	1.24
-15	-4	-6	20.28	1.47
15	-4	6	23.09	1.99
-15	4	6	21.12	1.87
15	4	-6	18.28	1.08
15	-4	6	19.64	1.32
-15	-4	-6	20.51	1.13
-15	-4	-6	19.51	1.40
-15	-4	-6	18.36	1.69
-15	4	6	21.16	1.46
-15	4	6	21.43	1.71
-15	-4	-6	22.43	1.52
-15	5	-6	1.73	0.62
15	-5	-6	1.14	0.46
-15	5	-6	1.83	1.17
15	-5	-6	0.93	0.55
-15	-5	6	0.89	1.01
-15	5	-6	2.63	0.80
-15	5	-6	2.08	0.98
-15	-5	6	0.89	0.43
15	5	6	0.27	0.73
15	5	6	1.13	0.53
-15	-5	6	0.87	0.49
15	-5	-6	0.81	0.51
-15	-5	6	1.07	0.56
15	-5	6	1.55	0.58
-15	5	6	-0.31	1.00
-15	-5	-6	2.26	0.62
15	5	-6	1.31	0.62
-15	-5	-6	2.07	0.59
-15	5	6	0.96	0.60
15	-5	6	1.16	0.53
-15	5	6	-0.41	0.75
15	5	-6	1.13	0.40
-15	-5	-6	1.67	0.45
15	-5	6	0.48	0.50
-15	6	-6	8.03	1.69
15	-6	-6	14.07	1.62
15	6	6	9.26	1.12
15	6	6	5.72	1.17
15	-6	-6	9.64	0.95
-15	6	-6	8.72	1.07

15	-6	-6	9.07	1.11
-15	6	-6	8.97	1.05
-15	-6	6	8.78	0.87
15	-6	-6	6.94	1.08
-15	-6	6	7.07	1.08
-15	-6	6	9.78	1.59
-15	-6	6	9.51	1.89
-15	-6	6	8.10	0.98
-15	6	-6	10.38	1.94
15	6	6	7.72	1.12
15	-6	6	8.66	0.88
-15	6	6	8.01	1.61
15	-6	6	7.50	1.03
15	6	-6	10.16	1.20
-15	-6	-6	9.60	1.17
15	6	-6	10.62	1.17
-15	-6	-6	10.62	1.22
15	6	-6	8.99	0.90
15	-6	6	7.98	1.70
-15	6	6	10.68	1.75
-15	-6	-6	9.26	0.91
15	-6	6	8.45	1.04
-15	-7	6	0.66	0.73
-15	7	-6	1.15	1.17
-15	7	-6	-0.12	0.53
15	7	6	1.00	0.83
-15	-7	6	-0.82	1.24
15	7	6	-0.72	0.73
15	-7	-6	-0.12	0.60
-15	-7	6	0.95	0.63
15	-7	-6	0.32	0.72
-15	-7	6	0.63	0.48
-15	-7	6	-0.61	0.95
15	7	6	0.13	1.02
15	-7	-6	-0.06	0.51
15	7	-6	0.08	0.76
-15	-7	-6	-0.11	0.66
15	7	-6	0.16	0.55
-15	-7	-6	0.63	0.56
15	-7	6	0.83	0.59
15	7	-6	0.27	0.62
15	-7	6	0.76	0.67
-15	7	6	-1.30	0.94
-15	-7	-6	-1.20	0.64

15	-8	-6	4.28	0.88
15	8	6	2.63	1.28
15	-8	-6	2.20	0.87
15	-8	-6	3.87	0.74
-15	-8	6	3.90	1.44
-15	8	-6	3.83	0.83
-15	-8	6	5.26	0.83
15	8	6	3.33	1.16
-15	-8	6	4.62	0.91
-15	8	-6	0.71	1.48
15	-8	6	4.09	0.95
-15	8	6	4.85	1.31
15	8	-6	3.49	0.80
15	-8	6	3.06	0.81
15	8	-6	2.94	1.15
-15	-8	-6	2.74	0.94
15	8	-6	3.93	0.83
-15	-9	6	2.89	1.28
15	9	6	0.73	1.06
15	-9	-6	0.10	0.66
15	-9	-6	0.20	0.84
15	9	6	-0.83	1.17
-15	9	-6	1.70	0.78
-15	9	-6	0.98	1.43
15	-9	-6	0.26	0.96
-15	-9	6	1.22	0.93
-15	-9	-6	0.04	0.79
-15	-9	-6	2.57	0.95
-15	9	6	2.61	1.61
15	-9	6	0.89	0.90
15	-9	6	0.11	0.70
15	9	-6	-0.10	0.86
15	9	-6	0.15	1.10
15	-10	-6	-0.02	0.95
-15	-10	6	0.10	0.90
15	10	6	0.83	1.13
-15	-10	6	2.73	1.28
15	10	6	-1.08	1.23
15	-10	-6	-0.16	0.84
-15	10	-6	2.43	1.55
15	-10	-6	0.26	0.77
-15	10	-6	0.28	0.91
15	10	-6	1.23	1.20
-15	-10	-6	-0.48	0.83

15	-10	6	1.12	0.80
15	-10	6	0.20	1.07
-15	-10	-6	0.36	0.95
-15	10	6	1.65	1.41
-16	0	-6	182.67	8.21
16	0	6	177.21	8.32
-16	0	6	180.40	8.17
-16	0	6	171.70	8.25
-16	0	6	183.96	8.28
16	0	6	171.45	8.11
-16	0	-6	171.01	8.57
16	0	-6	179.91	8.09
16	0	6	172.65	8.29
16	0	6	185.03	8.43
-16	0	6	173.09	8.51
16	0	6	174.98	8.27
16	0	-6	181.58	8.36
16	0	-6	182.02	8.32
-16	0	-6	176.57	8.47
-16	0	6	174.01	8.38
-16	0	-6	170.38	8.40
-16	0	6	170.63	8.23
16	0	-6	188.62	8.13
16	1	6	130.05	6.13
16	1	6	128.47	5.97
-16	1	-6	113.15	5.83
-16	-1	6	119.12	6.02
-16	-1	6	116.78	5.84
16	-1	-6	118.81	5.61
-16	1	-6	119.66	5.89
16	1	6	120.38	5.96
16	-1	-6	120.64	5.75
-16	-1	6	131.61	5.95
-16	-1	6	124.64	5.85
16	1	6	120.61	5.79
-16	-1	6	124.06	6.03
-16	-1	6	122.38	6.20
16	1	6	128.58	6.07
-16	1	-6	121.30	6.12
16	-1	-6	117.84	5.82
-16	-1	6	117.98	5.81
-16	-1	6	124.41	6.01
-16	1	-6	121.21	5.79
16	-1	-6	128.31	5.69

16	-1	6	130.76	6.22
-16	-1	-6	117.79	5.97
-16	-1	-6	121.60	5.88
16	-1	6	120.74	5.95
-16	1	6	117.49	5.85
16	-1	6	119.17	5.80
-16	1	6	129.59	5.90
-16	-1	-6	121.03	5.56
-16	-1	-6	138.35	5.66
-16	-1	-6	121.94	6.01
-16	1	6	124.77	5.85
16	-1	6	118.54	6.04
16	-1	6	129.39	5.97
-16	1	6	121.88	6.11
-16	-2	6	41.03	2.46
16	-2	-6	41.53	2.21
-16	2	-6	41.62	2.49
-16	2	-6	40.81	2.67
-16	-2	6	42.15	2.30
-16	-2	6	38.51	2.19
-16	2	-6	38.02	2.25
16	-2	-6	41.17	2.26
-16	2	-6	39.62	2.32
-16	-2	6	45.38	2.73
-16	-2	6	43.87	2.56
16	-2	-6	41.43	2.17
-16	-2	6	41.75	2.26
-16	-2	6	41.20	2.40
-16	-2	6	37.65	2.17
16	2	6	41.80	2.51
16	2	6	40.48	2.16
16	-2	-6	39.49	2.09
-16	2	6	38.62	2.51
-16	2	6	43.87	2.25
-16	2	6	37.06	2.14
-16	-2	-6	38.59	2.37
-16	-2	-6	39.93	2.27
-16	2	6	38.95	2.23
-16	-2	-6	42.11	2.36
-16	-2	-6	41.17	2.32
16	-2	6	40.83	2.31
16	-2	6	40.35	2.27
16	-2	6	39.04	2.17
16	-2	6	46.08	2.82

16	-2	6	43.68	2.81
-16	-2	-6	40.99	2.55
-16	3	-6	0.86	0.66
-16	-3	6	0.38	0.44
-16	-3	6	0.65	0.73
-16	-3	6	1.21	0.48
-16	-3	6	0.84	0.54
16	3	6	1.31	0.80
16	-3	-6	0.69	0.62
16	3	6	0.96	0.71
16	-3	-6	0.76	0.45
-16	-3	6	1.64	0.60
16	-3	-6	1.27	0.45
16	3	6	1.57	0.47
-16	-3	6	-0.41	0.95
-16	-3	6	1.99	0.61
-16	-3	6	0.83	0.91
16	-3	-6	0.35	0.48
-16	3	-6	0.34	0.76
-16	3	-6	0.06	0.48
-16	3	6	0.95	0.76
-16	-3	-6	0.67	0.63
16	-3	6	1.26	0.59
16	-3	6	1.61	0.50
-16	-3	-6	1.14	0.54
-16	-3	-6	0.29	0.67
16	-3	6	1.33	1.08
-16	3	6	0.84	0.56
16	-3	6	1.68	0.51
-16	3	6	1.71	0.62
-16	-3	-6	0.77	0.53
-16	-4	6	33.95	2.39
16	-4	-6	30.49	1.89
-16	-4	6	28.87	1.88
16	4	6	31.41	2.10
16	-4	-6	25.07	1.69
16	-4	-6	28.42	1.62
-16	-4	6	26.06	1.77
16	-4	-6	25.35	1.61
16	4	6	28.96	2.22
-16	-4	6	32.21	1.83
-16	-4	6	28.50	1.71
-16	-4	6	25.77	2.11
-16	4	-6	26.24	1.72

16	4	6	29.34	1.76
-16	4	6	29.97	2.22
-16	-4	-6	24.43	1.74
16	4	-6	26.43	1.45
16	-4	6	26.35	1.84
-16	4	6	31.11	2.04
16	-4	6	31.59	2.42
-16	-4	-6	25.75	1.74
-16	-4	-6	28.07	1.80
16	4	-6	25.70	1.33
16	-4	6	32.30	1.74
-16	4	6	30.43	1.86
-16	-4	-6	24.95	1.74
-16	5	-6	47.79	3.22
16	5	6	45.45	2.93
16	-5	-6	52.92	2.90
16	5	6	43.87	2.53
16	-5	-6	47.23	2.54
16	-5	-6	43.46	2.57
-16	-5	6	47.90	2.91
-16	-5	6	49.27	2.49
-16	-5	6	48.05	3.07
-16	-5	6	42.55	2.53
-16	5	-6	43.14	2.53
16	-5	-6	47.19	2.47
-16	5	-6	47.12	3.16
-16	-5	6	45.58	2.40
-16	-5	6	43.33	2.50
16	5	6	45.17	2.75
-16	5	-6	43.55	2.49
-16	5	6	42.14	2.86
-16	-5	-6	39.27	2.81
-16	-5	-6	41.77	2.58
16	-5	6	49.48	2.40
16	-5	6	44.32	2.55
-16	-5	-6	49.13	2.26
-16	5	6	46.94	2.99
16	-5	6	43.96	2.54
-16	-5	-6	45.94	2.41
16	5	-6	43.36	2.40
16	5	-6	47.42	2.28
-16	-5	-6	46.89	2.66
-16	-6	6	105.99	5.56
16	6	6	109.56	5.37

-16	-6	6	107.09	4.99
-16	-6	6	97.18	4.94
16	6	6	104.12	5.56
-16	-6	6	99.02	5.16
16	-6	-6	89.20	4.57
-16	6	-6	79.31	4.41
16	-6	-6	101.51	4.98
16	-6	-6	90.75	4.58
16	-6	-6	92.78	4.44
16	6	6	99.35	5.17
-16	6	-6	90.78	4.53
-16	6	-6	91.28	5.13
-16	-6	6	106.53	5.52
-16	-6	-6	90.84	4.63
16	-6	6	110.18	4.91
16	6	-6	85.60	4.46
16	-6	6	97.19	5.15
16	-6	6	103.24	5.19
-16	-6	-6	88.93	4.57
16	-6	6	112.89	5.81
-16	-6	-6	89.28	4.20
-16	6	6	104.24	5.57
-16	6	6	102.11	5.55
16	6	-6	92.84	4.32
-16	7	-6	56.54	3.07
16	-7	-6	53.06	2.94
-16	7	-6	57.73	3.80
16	-7	-6	55.72	3.11
-16	-7	6	52.38	2.81
-16	-7	6	55.05	3.51
-16	-7	6	54.47	3.09
16	7	6	57.68	3.38
16	-7	-6	55.51	3.16
16	7	6	54.87	3.15
16	7	6	56.80	3.53
-16	-7	-6	49.75	3.31
16	7	-6	58.27	3.05
-16	-7	-6	59.41	3.23
-16	7	6	56.14	3.59
-16	-7	-6	55.72	2.76
16	7	-6	53.45	3.11
16	7	-6	57.80	2.93
16	-7	6	56.38	3.13
16	-7	6	52.98	3.03

16	-8	-6	25.18	2.03
-16	8	-6	29.10	1.96
-16	-8	6	37.04	3.03
16	-8	-6	27.95	2.10
-16	-8	6	32.75	2.32
-16	8	-6	29.16	3.18
16	-8	-6	29.89	1.90
16	8	6	34.54	2.63
16	8	6	36.12	3.03
-16	-8	-6	26.76	2.24
16	8	-6	27.46	2.31
16	8	-6	28.74	1.89
16	8	-6	27.91	1.91
-16	8	6	33.72	3.16
16	-8	6	39.14	2.43
-16	-8	-6	27.47	2.05
16	-8	6	34.82	2.35
-16	-9	6	15.18	1.78
-16	-9	6	15.06	2.19
-16	9	-6	10.78	1.91
16	9	6	12.04	2.13
16	-9	-6	10.47	1.45
-16	9	-6	12.00	1.55
16	-9	-6	12.53	1.81
16	9	6	14.64	1.81
16	-9	-6	13.69	1.36
-16	-9	-6	11.71	1.64
16	-9	6	14.36	1.80
-16	-9	-6	8.63	1.57
-16	9	6	15.63	2.51
16	-9	6	15.08	1.54
16	9	-6	13.89	1.78
16	10	6	5.23	1.48
-16	10	-6	7.73	1.87
-16	-10	6	6.88	1.16
16	10	6	4.61	1.29
16	-10	-6	5.30	1.19
16	-10	-6	6.08	1.05
-16	-10	6	2.78	1.41
16	-10	-6	5.69	1.09
-16	10	-6	6.71	1.14
-16	-10	-6	6.74	1.20
16	-10	6	4.75	0.98
16	-10	6	3.22	1.16

-16	-10	-6	6.48	1.19
-16	10	6	4.98	1.64
16	10	-6	4.15	1.28
-17	0	-6	0.25	0.46
17	0	6	0.81	0.58
-17	0	6	-0.65	0.50
17	0	-6	0.01	0.23
17	0	6	0.67	0.75
17	0	6	0.36	0.50
17	0	6	-0.02	0.36
-17	0	-6	1.21	0.78
-17	0	6	0.30	0.70
-17	0	-6	0.24	0.50
17	0	-6	-0.07	0.40
-17	0	6	0.36	0.55
-17	0	6	-0.14	0.60
17	0	-6	-0.07	0.42
-17	0	-6	-0.43	0.73
17	0	6	0.02	0.82
17	0	-6	0.02	0.28
-17	1	-6	23.11	1.56
-17	-1	6	18.58	1.56
17	-1	-6	24.25	1.37
-17	-1	6	20.82	1.72
17	-1	-6	21.36	1.50
17	1	6	19.32	1.26
17	-1	-6	23.29	1.45
17	1	6	19.52	1.65
17	-1	-6	23.92	1.29
-17	-1	6	19.43	1.34
17	1	6	20.39	1.74
-17	1	-6	23.53	1.76
-17	-1	6	20.06	1.30
-17	1	-6	23.71	1.98
-17	-1	6	20.45	1.56
17	1	6	19.53	1.60
17	1	6	19.08	1.47
-17	1	-6	19.84	1.62
-17	-1	6	19.96	1.42
-17	-1	6	20.62	1.63
17	-1	6	18.52	1.45
17	-1	6	21.73	1.74
-17	1	6	19.81	1.40
17	-1	6	19.20	1.49

-17	1	6	18.87	1.51
-17	-1	-6	24.56	1.86
17	-1	6	20.11	1.75
17	-1	6	18.52	1.38
-17	-1	-6	23.97	1.65
-17	1	6	20.55	1.40
-17	-1	-6	25.30	1.90
-17	1	6	18.05	1.64
17	2	6	29.86	2.01
-17	2	-6	34.14	2.20
17	-2	-6	37.29	1.95
-17	2	-6	33.56	2.11
-17	2	-6	30.97	2.40
-17	-2	6	28.65	1.78
-17	-2	6	28.39	1.98
17	-2	-6	33.21	1.90
17	2	6	28.29	1.98
17	-2	-6	34.91	2.02
-17	-2	6	27.55	1.83
-17	-2	6	28.19	1.91
-17	-2	6	26.67	1.67
-17	-2	6	28.25	2.28
-17	-2	6	27.24	1.68
-17	2	-6	29.96	2.52
-17	-2	6	28.03	2.11
-17	-2	-6	30.14	2.12
-17	-2	-6	32.33	2.06
-17	-2	-6	38.62	2.20
-17	2	6	26.92	2.10
-17	2	6	27.51	1.66
17	-2	6	28.03	1.77
-17	2	6	28.06	1.75
-17	2	6	29.05	1.75
-17	3	-6	24.06	2.21
17	-3	-6	26.14	1.55
17	3	6	37.84	2.28
-17	3	-6	26.35	2.12
17	3	6	34.60	1.98
-17	-3	6	35.64	2.14
17	-3	-6	25.34	1.63
17	3	6	35.67	2.44
17	-3	-6	25.75	1.71
-17	-3	6	31.84	2.60
-17	-3	6	39.09	2.42

-17	-3	6	34.89	2.22
-17	3	-6	24.02	1.64
17	-3	-6	24.22	1.48
-17	-3	6	39.41	2.18
-17	-3	6	34.57	2.01
-17	-3	6	38.48	2.08
-17	3	-6	22.43	1.69
17	-3	6	36.74	2.08
-17	-3	-6	22.13	1.70
-17	-3	-6	23.33	1.64
-17	-3	-6	24.38	1.76
-17	3	6	35.92	2.12
-17	-3	-6	23.91	1.72
17	-3	6	40.24	2.70
17	-3	6	36.88	2.16
17	-3	6	34.53	2.02
-17	3	6	34.97	2.46
-17	-4	6	21.74	1.64
17	-4	-6	32.08	1.91
17	-4	-6	31.61	2.01
17	4	6	21.18	1.87
17	4	6	21.28	1.46
-17	-4	6	23.30	1.52
-17	-4	6	20.03	1.39
17	-4	-6	36.29	2.17
-17	4	-6	30.55	2.02
17	-4	-6	33.42	1.89
17	4	6	22.79	1.74
-17	-4	6	21.81	1.56
-17	-4	6	22.23	2.21
-17	4	-6	28.47	2.54
-17	4	-6	33.10	2.01
-17	-4	6	22.76	2.11
17	-4	6	22.14	1.45
-17	4	6	19.95	1.97
-17	-4	-6	31.04	1.97
-17	4	6	21.79	1.75
17	-4	6	24.46	2.48
17	-4	6	21.03	1.51
-17	-4	-6	32.01	2.10
-17	-4	-6	31.17	2.03
-17	-4	-6	34.52	2.03
17	-4	6	20.99	1.55
-17	4	6	21.36	1.57

-17	5	-6	20.98	1.56
17	-5	-6	19.81	1.43
-17	5	-6	21.41	1.61
-17	5	-6	23.82	2.40
17	5	6	19.86	1.49
-17	-5	6	19.87	1.51
17	-5	-6	21.86	1.57
17	-5	-6	22.25	1.63
-17	-5	6	19.60	2.31
-17	-5	6	19.71	1.96
17	5	6	20.49	1.71
17	5	6	20.74	2.00
-17	-5	6	18.98	1.52
-17	5	-6	20.37	2.35
-17	-5	6	20.21	1.52
17	-5	-6	25.19	2.32
-17	-5	6	18.93	1.35
-17	-5	-6	20.92	1.53
17	-5	6	13.78	2.18
17	5	-6	19.26	1.43
17	-5	6	17.10	1.50
-17	5	6	21.68	2.00
17	5	-6	21.50	1.25
17	-5	6	18.98	1.49
-17	5	6	19.85	2.08
-17	-5	-6	23.75	1.72
-17	-5	-6	21.73	1.46
17	-5	6	21.74	1.36
17	-6	-6	16.35	1.88
-17	-6	6	16.42	1.44
-17	-6	6	16.19	1.26
17	-6	-6	12.71	1.34
17	6	6	13.91	1.45
-17	-6	6	22.07	2.06
-17	-6	6	18.26	1.37
-17	-6	6	15.22	1.41
17	-6	-6	11.67	1.34
-17	6	-6	14.84	1.38
17	6	6	19.46	1.68
17	-6	-6	13.37	1.36
-17	-6	6	19.13	2.29
-17	6	-6	15.05	2.25
17	6	6	17.52	2.02
17	-6	6	14.81	1.44

17	6	-6	14.45	1.09
17	-6	6	14.33	1.42
-17	-6	-6	12.78	1.42
-17	6	6	14.94	2.02
-17	-6	-6	15.15	1.45
17	6	-6	15.26	1.37
17	7	6	2.39	0.98
17	7	6	2.89	1.34
-17	-7	6	1.68	1.23
17	-7	-6	3.03	0.85
-17	-7	6	3.41	0.83
17	-7	-6	3.15	0.88
17	-7	-6	3.61	0.73
-17	7	-6	4.15	1.67
-17	7	-6	3.43	0.84
17	7	6	2.65	1.01
-17	-7	6	2.12	0.75
-17	-7	6	2.85	0.68
17	7	-6	3.48	0.99
-17	7	6	3.88	1.38
17	-7	6	2.18	0.79
-17	-7	-6	4.64	0.95
17	-7	6	2.32	0.82
-17	-7	-6	1.95	0.95
17	7	-6	4.85	0.95
-17	8	-6	7.84	1.39
17	8	6	7.36	1.46
-17	-8	6	7.17	1.19
-17	-8	6	6.44	1.31
17	-8	-6	5.58	1.02
-17	8	-6	6.49	1.79
17	-8	-6	8.66	1.54
17	8	6	6.49	1.25
17	-8	-6	6.91	1.15
-17	-8	6	6.06	1.55
17	-8	6	8.25	1.50
17	8	-6	7.05	1.21
17	-8	6	3.87	0.88
-17	-8	-6	4.79	1.17
-17	8	6	5.73	1.57
-17	-8	-6	7.30	1.20
17	8	-6	9.11	1.19
-17	9	-6	4.07	1.73
-17	-9	6	7.74	1.58

-17	9	-6	7.65	1.21
17	9	6	8.36	1.64
-17	-9	6	7.43	1.23
17	9	6	5.88	1.21
17	-9	-6	6.90	1.09
17	-9	-6	5.91	1.17
17	-9	-6	7.50	1.12
-17	-9	-6	5.35	1.12
17	9	-6	7.21	1.27
-17	-9	-6	7.46	1.19
17	-9	6	8.02	1.28
-17	9	6	8.23	1.59
17	-9	6	5.75	1.24
-17	-10	6	13.92	1.76
17	-10	-6	10.01	1.43
18	0	6	131.41	6.13
-18	0	-6	128.78	6.55
-18	0	6	119.24	6.07
18	0	6	118.45	5.72
18	0	6	118.68	5.92
-18	0	6	123.27	5.82
18	0	-6	126.91	5.94
18	0	-6	137.81	5.99
-18	0	6	124.30	5.95
18	0	6	110.40	5.96
-18	0	6	118.44	5.83
18	0	-6	134.65	6.24
-18	0	6	122.24	5.90
18	0	6	129.84	6.03
-18	0	-6	126.43	6.42
18	1	6	77.33	3.91
18	-1	-6	66.28	3.28
-18	1	-6	66.07	3.20
-18	-1	6	74.25	3.90
18	-1	-6	69.14	3.16
18	1	6	66.73	3.76
18	1	6	77.71	3.94
-18	1	-6	64.08	3.38
-18	1	-6	62.36	3.46
18	-1	-6	60.47	3.29
-18	-1	6	66.97	3.65
-18	1	-6	65.53	3.72
-18	-1	6	71.70	3.82
-18	-1	6	71.98	3.89

-18	-1	6	73.42	3.64
-18	-1	6	70.03	3.68
18	1	6	72.14	3.58
-18	1	6	74.00	3.72
-18	-1	-6	62.05	3.55
18	-1	6	72.56	3.59
18	-1	6	75.80	3.79
-18	1	6	72.35	3.68
-18	1	6	70.31	3.66
-18	1	6	60.26	4.64
-18	-1	-6	62.38	3.39
18	-1	6	84.40	4.10
18	-1	6	76.74	3.82
-18	-1	-6	61.83	3.53
-18	-2	6	19.63	1.39
18	2	6	22.77	1.78
-18	2	-6	21.77	1.62
18	2	6	21.10	1.73
-18	-2	6	20.00	1.73
-18	2	-6	24.23	2.18
-18	-2	6	20.56	1.42
18	-2	-6	24.80	1.50
-18	-2	6	20.65	1.66
18	2	6	19.58	1.31
-18	2	-6	22.34	1.75
-18	-2	6	25.04	2.71
18	-2	-6	22.36	1.34
-18	2	-6	23.67	1.77
18	-2	-6	24.44	1.51
18	-2	-6	21.07	1.55
18	-2	6	21.20	1.54
-18	2	6	18.90	1.50
-18	-2	-6	20.67	1.61
-18	2	6	19.13	1.83
18	-2	6	19.94	1.34
-18	2	6	20.42	1.49
18	-2	6	20.72	1.51
-18	-2	-6	22.53	1.59
-18	2	6	18.19	1.35
-18	-2	-6	21.90	1.83
-18	-2	-6	23.52	1.84
18	-2	6	22.40	1.94
-18	-3	6	1.99	0.55
-18	3	-6	1.59	1.04

18	-3	-6	1.82	0.61
-18	3	-6	4.11	0.89
-18	-3	6	1.84	0.78
-18	-3	6	1.26	0.53
18	3	6	1.00	0.94
18	-3	-6	2.65	0.61
18	3	6	2.11	0.53
18	-3	-6	2.61	0.70
18	-3	-6	2.32	0.55
-18	3	-6	3.40	1.10
-18	-3	6	1.51	0.73
-18	-3	6	1.16	0.81
-18	3	-6	2.54	0.67
18	3	6	1.89	0.78
-18	-3	-6	2.16	0.93
18	-3	6	1.66	0.55
-18	-3	-6	2.88	0.72
-18	-3	-6	2.76	0.71
18	-3	6	2.04	0.64
18	-3	6	3.06	0.69
-18	3	6	2.14	0.96
-18	-3	-6	2.21	0.80
18	-3	6	0.14	1.11
-18	-4	6	4.95	1.06
-18	4	-6	5.74	1.04
-18	4	-6	6.27	1.00
18	-4	-6	6.24	0.90
-18	-4	6	6.39	1.15
-18	4	-6	5.51	1.36
-18	-4	6	6.04	1.37
18	4	6	5.08	0.91
-18	-4	6	5.18	0.89
-18	-4	6	6.55	1.19
-18	4	-6	4.68	1.24
18	-4	-6	4.92	0.86
18	-4	-6	6.97	1.35
-18	-4	6	4.49	0.77
18	-4	-6	8.21	1.06
18	4	6	5.15	1.00
18	4	6	5.83	1.12
18	-4	6	6.02	1.02
-18	4	6	5.19	0.94
-18	4	6	5.50	1.22
-18	-4	-6	4.98	0.90

-18	-4	-6	6.04	0.93
18	-4	6	3.89	1.34
-18	-4	-6	6.42	1.01
18	-4	6	6.07	0.94
18	-4	6	5.83	0.96
-18	-4	-6	5.61	0.90
-18	-5	6	28.40	1.86
-18	-5	6	30.74	2.03
18	-5	-6	25.33	1.86
-18	5	-6	25.06	1.86
18	-5	-6	26.00	1.73
18	-5	-6	32.35	2.08
18	5	6	31.66	1.99
18	5	6	34.00	2.27
18	-5	-6	27.01	1.81
-18	-5	6	30.77	2.02
-18	-5	6	31.72	2.77
-18	-5	6	31.97	2.50
18	5	6	30.66	2.51
-18	-5	6	29.58	1.96
-18	5	-6	29.34	2.67
-18	5	6	29.16	2.54
-18	5	6	31.73	2.39
18	-5	6	33.62	1.83
18	-5	6	30.54	2.00
18	-5	6	32.35	2.06
18	-5	6	33.80	2.76
-18	-5	-6	24.53	1.90
18	5	-6	26.87	1.61
18	5	-6	27.86	1.46
-18	-5	-6	28.84	1.96
-18	-5	-6	25.86	1.81
-18	6	-6	38.36	2.38
18	6	6	33.38	2.70
-18	6	-6	39.12	3.35
-18	-6	6	30.65	1.94
-18	-6	6	38.28	2.69
18	6	6	36.06	2.39
18	6	6	33.25	2.23
18	-6	-6	40.25	2.25
18	-6	-6	36.25	2.43
-18	-6	6	31.64	2.11
-18	-6	6	34.44	2.19
18	-6	-6	40.53	2.40

18	6	-6	39.34	2.09
-18	-6	-6	36.74	2.44
-18	-6	-6	40.41	2.56
18	6	-6	37.08	2.25
-18	6	6	32.50	2.86
18	-6	6	32.23	2.14
18	-6	6	31.30	2.16
-18	-7	6	32.54	2.12
-18	-7	6	31.00	2.00
-18	-7	6	32.15	2.20
-18	-7	6	36.50	2.97
18	-7	-6	24.37	1.98
18	-7	-6	27.39	1.82
-18	7	-6	32.68	3.12
18	7	6	36.59	2.41
18	-7	-6	25.53	2.02
18	7	6	36.77	2.98
-18	7	-6	26.32	2.01
18	7	-6	25.97	2.10
18	-7	6	32.20	2.34
18	-7	6	33.73	2.28
-18	7	6	31.30	3.05
18	7	-6	24.87	1.71
-18	-7	-6	25.02	2.06
-18	-7	-6	29.53	2.17
18	8	6	18.71	1.84
-18	-8	6	22.81	2.39
-18	-8	6	15.79	1.88
18	-8	-6	16.74	1.67
-18	8	-6	13.70	1.70
18	-8	-6	15.56	1.55
-18	8	-6	14.53	2.54
18	-8	-6	15.35	1.46
18	8	6	18.90	2.22
18	8	-6	15.65	1.51
18	-8	6	17.68	1.80
-18	-8	-6	15.69	1.79
18	-8	6	16.23	1.62
-18	8	6	17.73	2.46
18	8	-6	15.63	1.75
-18	-8	-6	15.65	1.70
-18	9	-6	6.46	1.79
18	9	6	2.40	1.31
18	-9	-6	7.71	1.41

18	-9	-6	4.51	1.03
-18	-9	6	3.00	1.01
-18	9	-6	4.20	1.01
-18	-9	6	4.61	1.45
18	9	6	2.56	1.13
18	-9	-6	5.71	1.11
-18	9	6	2.18	1.52
-18	-9	-6	6.02	1.23
18	9	-6	5.43	1.20
18	-9	6	3.14	0.89
-18	-9	-6	5.78	1.23
18	-9	6	3.00	1.02
19	0	-6	-0.14	0.29
-19	0	6	0.77	0.66
-19	0	-6	0.45	0.59
-19	0	-6	-0.47	0.88
19	0	6	-0.11	0.68
-19	0	6	1.25	0.91
-19	0	6	0.37	0.85
-19	0	6	0.63	0.50
19	0	-6	0.05	0.22
19	0	6	0.57	0.60
19	0	6	-0.03	0.41
19	0	-6	-0.50	0.47
-19	0	-6	0.71	0.84
19	0	6	-0.03	0.85
19	-1	-6	18.16	1.07
-19	-1	6	14.85	1.63
-19	1	-6	17.46	1.19
19	1	6	14.71	1.40
19	1	6	18.73	1.69
-19	1	-6	16.59	1.47
-19	-1	6	17.65	1.25
-19	-1	6	14.62	1.72
19	-1	-6	21.03	1.37
19	1	6	16.73	1.60
19	-1	-6	17.69	1.38
19	1	6	17.63	1.19
-19	-1	6	14.24	1.28
-19	1	-6	18.25	1.96
-19	-1	6	15.42	1.57
-19	1	-6	16.51	1.72
19	-1	-6	18.46	1.18
-19	-1	-6	19.49	1.86

19	-1	6	16.28	1.19
-19	1	6	15.60	1.73
19	-1	6	16.25	1.41
-19	1	6	12.85	1.30
19	-1	6	17.63	1.49
19	-1	6	18.19	1.85
-19	-1	-6	14.77	1.79
-19	-1	-6	17.92	1.50
-19	1	6	16.09	1.52
19	2	6	25.94	1.99
-19	2	-6	24.66	1.80
-19	-2	6	24.63	1.63
19	-2	-6	27.75	1.62
-19	-2	6	25.09	1.96
-19	2	-6	23.31	2.25
-19	2	-6	24.28	1.78
19	-2	-6	26.30	1.64
19	2	6	23.59	1.53
-19	2	-6	23.87	1.91
-19	-2	6	24.49	1.90
19	-2	-6	25.69	1.47
19	2	6	27.46	2.16
-19	-2	6	24.90	1.61
19	-2	-6	26.08	1.76
19	-2	6	23.73	1.56
-19	-2	-6	23.96	1.99
19	-2	6	24.60	1.76
-19	-2	-6	22.91	2.02
-19	2	6	25.83	1.67
19	-2	6	26.62	1.76
-19	2	6	25.51	2.16
-19	-2	-6	26.91	1.82
-19	2	6	22.56	1.55
-19	-2	-6	26.34	1.78
19	-2	6	26.66	2.25
19	-3	-6	17.62	1.30
19	3	6	14.96	1.71
-19	3	-6	18.56	1.68
19	3	6	14.90	1.64
19	-3	-6	20.69	1.40
-19	-3	6	14.65	1.48
-19	3	-6	22.71	2.25
-19	-3	6	12.54	2.46
19	-3	-6	21.80	1.57

-19	-3	6	13.27	1.26
-19	3	-6	21.50	1.96
19	3	6	13.63	1.14
19	-3	-6	21.59	1.54
-19	-3	6	14.25	1.20
-19	3	-6	17.93	1.53
19	-3	6	14.40	1.29
-19	-3	-6	20.22	1.75
-19	-3	-6	20.61	1.82
19	-3	6	14.34	1.35
-19	3	6	14.68	1.84
-19	-3	-6	17.48	1.58
-19	-3	-6	18.58	1.58
19	-3	6	16.38	1.83
19	-3	6	13.08	1.15
19	-4	-6	43.58	2.52
19	-4	-6	43.56	2.33
-19	4	-6	40.23	2.43
-19	4	-6	38.64	3.07
-19	-4	6	49.14	2.71
-19	-4	6	48.04	2.89
19	-4	-6	39.78	2.27
19	4	6	49.64	2.72
-19	-4	6	51.04	3.19
-19	-4	6	49.18	2.78
19	4	6	50.11	3.27
19	-4	-6	40.54	2.44
19	4	6	50.46	2.98
19	-4	6	52.52	2.73
-19	-4	-6	40.75	2.43
-19	-4	-6	41.61	2.50
19	-4	6	57.17	3.56
19	-4	6	51.38	2.89
-19	4	6	50.61	3.32
-19	-4	-6	35.28	2.44
19	-4	6	50.29	2.77
19	5	6	36.67	2.27
19	5	6	41.47	2.56
-19	-5	6	35.93	2.37
19	-5	-6	48.74	2.83
-19	-5	6	36.58	2.29
19	5	6	35.56	2.78
19	-5	-6	52.64	3.08
-19	-5	6	38.76	2.80

-19	5	-6	52.24	3.54
19	-5	-6	49.18	2.72
-19	5	-6	47.93	2.77
19	-5	-6	45.96	2.61
-19	-5	6	33.86	2.16
-19	-5	-6	43.14	2.80
19	-5	6	41.18	2.10
19	-5	6	35.36	2.31
-19	5	6	33.89	2.95
19	-5	6	39.81	2.27
-19	-5	-6	45.67	2.81
-19	-5	-6	47.52	2.88
19	-6	-6	43.30	2.59
-19	-6	6	37.50	2.39
-19	-6	6	34.93	2.35
19	6	6	38.19	2.44
19	-6	-6	42.87	2.66
19	6	6	35.53	3.07
-19	-6	6	37.33	2.97
-19	-6	6	35.62	2.18
19	6	6	37.60	2.54
-19	6	-6	43.16	3.70
19	-6	-6	43.77	2.47
-19	6	-6	42.92	2.65
19	6	-6	39.35	2.39
19	6	-6	43.68	2.25
19	-6	6	37.72	2.37
19	-6	6	37.97	2.44
-19	-6	-6	44.30	2.76
-19	6	6	43.10	3.30
-19	-6	-6	39.93	2.65
19	-7	-6	2.62	0.89
-19	-7	6	3.88	0.89
-19	7	-6	6.20	1.64
-19	-7	6	2.98	0.94
-19	7	-6	2.66	0.92
19	7	6	3.51	0.98
19	-7	-6	3.94	0.90
19	7	6	4.54	1.43
19	-7	-6	3.15	0.82
-19	-7	6	2.72	1.03
-19	-7	6	5.26	1.52
-19	7	6	5.77	1.44
-19	-7	-6	4.06	0.96

19	-7	6	4.51	0.92
19	7	-6	3.34	0.95
19	-7	6	3.81	0.93
-19	-7	-6	5.81	1.10
19	7	-6	4.96	1.02
-19	-8	6	3.41	1.04
-19	8	-6	3.07	0.95
19	-8	-6	3.62	0.87
19	-8	-6	2.66	0.91
19	8	6	3.00	1.02
-19	8	-6	3.08	1.63
-19	-8	6	3.40	1.41
19	-8	-6	5.75	1.00
19	8	6	3.49	1.46
-19	-8	-6	4.45	1.17
19	-8	6	4.30	0.96
-19	-8	-6	3.50	1.06
-19	8	6	4.40	1.56
19	-8	6	4.57	1.02
19	8	-6	3.22	0.99
-20	0	6	13.29	1.71
-20	0	-6	12.89	1.77
20	0	-6	11.48	0.71
-20	0	6	14.42	1.19
20	0	6	19.98	1.67
-20	0	6	17.40	1.49
-20	0	-6	10.52	1.28
-20	0	6	12.28	1.29
-20	0	6	14.31	1.73
20	0	6	13.81	1.13
20	0	6	15.64	1.44
20	0	-6	8.33	0.62
20	-1	-6	18.75	1.21
20	1	6	26.88	2.07
20	-1	-6	23.68	1.53
-20	1	-6	20.33	1.94
-20	-1	6	23.26	1.93
20	1	6	24.61	1.81
-20	1	-6	20.14	1.66
20	-1	-6	23.99	1.36
20	1	6	23.21	1.52
20	1	6	30.65	2.07
-20	1	-6	19.32	2.62
-20	-1	6	21.82	1.62

-20	-1	6	25.49	1.99
-20	-1	6	21.12	1.63
20	-1	6	27.09	1.89
-20	1	6	25.78	1.88
-20	1	6	23.12	1.72
20	-1	6	27.82	2.10
20	-1	6	24.10	1.76
20	-1	6	22.29	1.52
-20	-1	-6	23.33	2.07
-20	1	6	22.72	2.14
-20	1	6	23.57	1.69
-20	-1	-6	21.95	1.69
-20	-2	6	8.18	1.11
-20	-2	6	12.27	1.58
20	-2	-6	8.89	1.05
20	2	6	9.71	0.97
20	-2	-6	7.02	1.11
-20	2	-6	7.86	1.21
20	2	6	10.39	1.54
-20	2	-6	8.14	1.27
20	2	6	12.42	1.56
-20	2	-6	6.41	1.16
20	-2	-6	11.17	1.08
-20	2	-6	6.77	1.43
-20	-2	6	9.04	1.11
20	-2	-6	8.02	0.82
20	-2	6	10.77	1.93
-20	-2	-6	6.61	1.17
-20	-2	-6	8.49	1.56
20	-2	6	11.39	1.29
-20	-2	-6	8.09	1.23
-20	2	6	9.13	1.72
-20	-2	-6	7.47	1.30
-20	2	6	8.97	1.11
20	-2	6	8.84	0.98
20	-2	6	10.71	1.28
20	3	6	3.47	1.00
-20	-3	6	2.58	0.97
20	-3	-6	2.58	0.74
-20	3	-6	1.67	0.69
-20	3	-6	3.04	1.70
-20	-3	6	2.38	0.90
-20	-3	6	1.70	0.67
20	-3	-6	2.26	0.68

-20	-3	6	0.98	0.60
20	-3	-6	1.51	0.67
20	-3	-6	1.88	0.54
-20	3	-6	1.69	1.16
20	3	6	2.16	0.57
20	3	6	1.44	1.05
20	-3	6	1.89	0.56
20	-3	6	1.74	0.78
-20	-3	-6	2.46	0.74
20	-3	6	2.45	1.13
-20	3	6	1.31	1.05
-20	-3	-6	2.91	0.93
-20	-3	-6	1.96	0.76
20	-3	6	2.68	0.72
20	-4	-6	7.07	1.00
-20	-4	6	7.38	1.37
-20	-4	6	9.83	1.55
-20	4	-6	5.05	0.97
20	4	6	6.18	1.15
20	-4	-6	7.80	1.31
20	4	6	7.51	1.41
-20	-4	6	7.61	1.13
20	-4	-6	6.93	1.27
-20	-4	6	6.74	1.08
20	4	6	6.30	1.07
-20	4	-6	4.80	1.47
20	-4	-6	7.73	1.04
20	-4	6	6.44	1.17
-20	-4	-6	6.52	1.09
-20	-4	-6	8.78	1.36
20	-4	6	5.87	1.06
20	-4	6	6.35	1.13
-20	-4	-6	7.97	1.16
-20	4	6	6.02	1.55
-20	-5	6	1.87	0.77
-20	5	-6	3.02	0.92
20	-5	-6	5.60	1.04
-20	5	-6	6.55	1.73
20	5	6	3.21	0.85
20	-5	-6	4.25	0.89
-20	-5	6	1.64	0.85
20	-5	-6	6.46	1.13
20	5	6	2.37	1.06
-20	-5	6	3.46	1.20

20	5	6	3.21	1.40
-20	-5	6	3.04	0.67
20	-5	6	1.81	0.80
20	-5	6	-0.07	0.73
-20	5	6	5.81	1.56
-20	-5	-6	5.74	1.07
-20	-5	-6	4.69	1.02
20	-6	-6	4.81	0.91
20	-6	-6	6.34	1.11
-20	6	-6	4.45	0.91
20	-6	-6	4.81	1.02
-20	-6	6	6.39	1.59
-20	-6	6	4.16	0.88
-20	-6	6	4.45	0.82
-20	-6	6	5.18	1.01
-20	6	-6	4.73	1.68
20	6	6	6.37	1.56
-20	-6	-6	4.55	1.07
-20	6	6	5.93	1.46
20	6	-6	3.85	0.66
20	6	-6	4.14	0.90
20	-6	6	6.96	1.27
20	-6	6	4.36	0.94
-20	-6	-6	8.17	1.38
20	7	6	7.61	1.42
20	-7	-6	4.30	0.88
-20	-7	6	8.25	1.57
-20	7	-6	8.78	1.77
20	-7	-6	6.34	1.16
-20	7	-6	2.54	0.85
20	-7	-6	3.71	0.89
20	7	6	4.72	1.34
-20	-7	6	6.02	1.19
20	7	-6	3.26	0.78
-20	7	6	8.93	1.65
-20	-7	-6	5.14	1.05
-20	-7	-6	5.38	1.09
20	-7	6	4.25	0.92
20	-7	6	5.35	0.98
-20	8	-6	-0.57	0.78
20	8	6	1.49	1.01
20	-8	-6	1.84	0.94
20	-8	-6	0.34	0.78
20	8	6	2.38	1.41

-20	-8	6	1.18	0.91
-20	8	-6	-0.02	1.56
-20	-8	6	1.06	1.35
20	-8	-6	1.72	0.85
-20	8	6	2.04	1.37
-20	-8	-6	1.95	1.19
20	-8	6	0.69	0.85
-20	-8	-6	1.60	0.90
20	8	-6	1.53	1.01
20	-8	6	1.20	0.77
-21	0	6	0.06	0.60
-21	0	6	0.22	0.83
-21	0	6	0.67	1.02
-21	0	-6	0.79	0.67
21	0	6	0.78	0.67
21	0	6	0.05	0.80
-21	0	6	-0.06	1.03
21	0	6	-0.24	0.45
21	1	6	22.15	1.77
21	1	6	22.37	1.97
-21	-1	6	23.78	1.68
-21	1	-6	18.40	1.69
-21	-1	6	21.28	2.01
-21	-1	6	22.15	1.57
21	-1	-6	20.67	1.21
21	-1	-6	21.96	2.16
21	-1	-6	23.04	1.36
-21	1	-6	20.76	2.29
21	1	6	21.05	1.99
21	1	6	21.94	1.44
-21	-1	6	22.58	1.86
21	-1	6	21.88	1.46
21	-1	6	25.58	1.90
-21	1	6	20.03	1.68
-21	1	6	19.69	2.16
21	-1	6	23.64	2.05
-21	1	6	21.82	1.68
21	-1	6	20.16	1.68
-21	-1	-6	22.14	2.06
-21	-1	-6	21.33	1.72
21	-2	-6	14.23	1.07
21	-2	-6	14.88	1.29
21	2	6	14.69	1.76
-21	2	-6	14.91	1.55

21	2	6	13.06	1.15
21	2	6	12.54	1.78
21	-2	-6	16.12	1.26
-21	2	-6	16.50	2.26
-21	-2	6	13.12	1.67
-21	-2	6	13.73	1.32
-21	-2	6	13.74	1.28
21	-2	6	13.55	1.22
21	-2	6	16.59	1.83
21	-2	6	13.75	1.40
21	-2	6	14.23	1.53
-21	2	6	10.38	1.21
-21	-2	-6	14.85	1.51
-21	2	6	12.53	1.95
-21	-2	-6	15.26	1.51
-21	-2	-6	14.16	1.88
-21	2	6	12.49	1.36
-21	-3	6	19.43	1.58
-21	-3	6	18.10	1.81
21	-3	-6	24.34	1.71
21	-3	-6	22.41	1.60
21	-3	-6	22.36	1.47
-21	-3	6	20.03	2.08
21	3	6	17.89	2.09
21	3	6	20.21	1.50
-21	3	-6	21.60	2.54
-21	-3	6	18.92	1.54
21	3	6	22.17	2.00
21	-3	-6	22.43	1.80
-21	3	-6	21.78	1.84
-21	-3	-6	20.89	1.95
21	-3	6	20.81	1.67
-21	-3	-6	16.47	1.73
-21	3	6	23.62	2.47
21	-3	6	20.48	1.52
-21	-3	-6	21.18	1.86
21	-3	6	20.98	1.77
21	4	6	34.49	2.78
-21	-4	6	36.49	2.75
21	4	6	33.35	2.48
-21	-4	6	34.33	2.20
21	-4	-6	36.65	2.23
21	4	6	34.49	2.14
-21	4	-6	36.03	2.42

21	-4	-6	36.96	2.18
-21	-4	6	31.60	2.08
21	-4	-6	42.92	2.86
21	-4	-6	37.69	2.44
-21	4	-6	36.33	3.36
21	-4	6	32.57	2.13
21	-4	6	36.55	2.16
21	-4	6	32.56	2.32
-21	4	6	36.78	3.09
-21	-4	-6	33.85	2.41
-21	-4	-6	37.51	2.53
-21	-4	-6	34.56	2.40
21	-5	-6	83.99	4.35
-21	5	-6	82.63	4.35
-21	-5	6	74.92	4.00
21	-5	-6	83.91	4.19
-21	5	-6	81.35	5.11
21	5	6	75.33	4.08
21	5	6	79.58	4.68
-21	-5	6	79.14	4.60
21	5	6	76.29	3.92
21	-5	-6	83.83	4.21
-21	-5	6	72.98	4.13
21	-5	6	72.69	3.95
-21	5	6	76.04	4.63
-21	-5	-6	82.08	4.40
-21	-5	-6	77.26	4.36
21	-5	6	76.00	4.07
-21	6	-6	43.59	2.60
-21	-6	6	40.23	2.71
-21	6	-6	38.51	3.47
-21	-6	6	40.41	2.49
21	-6	-6	41.10	2.48
21	6	6	42.40	2.53
21	6	6	43.98	3.21
21	-6	-6	41.44	2.47
-21	-6	6	44.11	3.26
-21	-6	6	42.86	2.63
21	-6	-6	39.86	2.57
-21	-6	-6	44.44	2.69
21	-6	6	39.12	2.56
-21	6	6	41.65	3.25
-21	-6	-6	40.27	2.82
21	-6	6	40.66	2.53

-21	7	-6	5.78	1.71
21	-7	-6	3.45	0.91
-21	-7	6	5.26	1.55
21	7	6	5.77	1.07
21	-7	-6	3.32	0.86
-21	7	-6	3.69	0.98
-21	-7	6	7.84	1.22
21	7	6	5.28	1.54
21	-7	-6	2.65	0.91
21	-7	6	6.23	1.06
21	7	-6	3.67	0.85
-21	-7	-6	5.27	1.28
21	-7	6	6.22	1.28
21	7	-6	4.60	0.79
-21	7	6	4.73	1.48
22	0	6	4.60	0.94
-22	0	6	3.47	0.84
-22	0	6	2.09	0.94
-22	0	6	3.42	0.73
22	0	6	3.30	0.97
-22	0	6	3.38	1.21
-22	0	-6	4.28	1.29
-22	0	-6	3.79	0.99
-22	-1	6	10.57	1.35
-22	-1	6	13.77	1.72
-22	1	-6	9.74	1.48
-22	1	-6	12.89	2.27
22	1	6	14.98	1.78
22	1	6	11.24	1.60
-22	-1	6	8.80	1.80
22	-1	-6	8.88	0.92
-22	-1	6	9.59	1.24
22	1	6	15.49	1.83
22	-1	-6	8.61	0.76
22	-1	-6	10.22	1.22
-22	1	6	14.97	1.98
-22	1	6	10.33	1.39
-22	1	6	11.16	1.41
22	-1	6	11.02	1.53
22	-1	6	13.33	1.82
22	-1	6	14.17	1.65
-22	-1	-6	9.51	1.49
22	-1	6	10.88	1.18
22	-2	-6	6.57	1.07

22	-2	-6	8.26	1.15
-22	-2	6	8.09	1.24
-22	-2	6	7.67	1.32
22	-2	-6	6.47	0.87
-22	2	-6	7.64	1.58
-22	-2	6	8.05	1.33
22	2	6	7.92	1.32
-22	2	-6	5.62	0.98
-22	-2	6	8.33	1.21
22	2	6	7.28	1.22
22	2	6	7.51	1.08
-22	-2	-6	5.79	1.01
22	-2	6	8.04	1.47
22	-2	6	7.90	1.48
-22	-2	-6	5.49	1.11
-22	2	6	7.56	1.51
22	-2	6	6.72	1.06
-22	2	6	8.39	1.39
-22	-2	-6	6.08	1.26
22	-3	-6	1.00	0.55
22	3	6	1.32	1.07
-22	3	-6	-0.06	0.75
-22	-3	6	1.42	0.73
-22	-3	6	1.57	0.77
-22	-3	6	2.95	1.10
22	3	6	0.05	1.09
22	3	6	0.53	0.61
22	-3	-6	-0.34	0.72
-22	3	-6	0.44	1.53
22	-3	-6	-0.78	0.61
-22	-3	6	-0.14	0.96
-22	-3	-6	0.01	1.03
22	-3	6	-0.67	0.77
22	-3	6	0.27	0.85
-22	-3	-6	0.62	0.96
22	-3	6	0.36	0.59
-22	3	6	0.51	0.75
-22	-3	-6	0.06	0.69
-22	3	6	-0.33	1.32
-22	-4	6	0.82	0.76
22	4	6	1.99	1.37
22	-4	-6	1.06	0.60
-22	-4	6	0.51	1.19
-22	-4	6	1.11	0.75

22	-4	-6	0.84	0.75
22	-4	-6	1.01	0.71
-22	4	-6	0.01	0.75
22	4	6	1.13	1.06
22	4	6	0.33	0.63
-22	-4	6	0.69	1.00
-22	4	6	1.31	1.27
22	-4	6	0.29	0.81
-22	-4	-6	-0.91	0.90
-22	-4	-6	1.80	0.83
22	-4	6	0.27	0.61
22	-5	-6	4.39	0.91
22	-5	-6	4.10	0.78
22	5	6	2.64	0.78
22	5	6	4.88	1.49
-22	5	-6	3.84	1.60
22	-5	-6	5.46	0.89
-22	5	-6	4.45	0.89
-22	-5	6	3.88	1.05
-22	-5	6	2.30	1.51
-22	-5	6	1.90	1.00
-22	-5	6	5.03	0.91
-22	-5	-6	3.76	0.88
-22	-5	-6	3.47	1.12
22	-5	6	2.87	0.76
22	-5	6	2.70	0.89
-22	5	6	4.28	1.41
22	-6	-6	6.01	1.03
22	-6	-6	5.26	1.03
22	6	6	4.02	0.93
-22	-6	6	5.88	1.44
-22	6	-6	5.28	1.73
22	6	6	5.85	1.47
-22	-6	6	3.11	1.02
22	-6	-6	4.96	0.91
-22	6	-6	4.65	1.08
22	-6	6	6.71	1.25
22	-6	6	4.37	0.91
-22	-6	-6	5.88	1.26
-22	-6	-6	9.50	1.47
-22	6	6	4.28	1.57
23	0	6	0.83	1.07
23	0	6	-0.49	0.75
-23	0	6	0.58	0.67

23	0	6	0.24	1.06
-23	0	6	2.12	1.29
-23	0	-6	-0.97	0.75
-23	0	6	-0.52	0.92
-23	0	6	0.27	0.90
23	0	6	0.21	0.55
-23	0	-6	0.46	1.37
23	1	6	8.11	1.29
-23	-1	6	10.78	1.40
23	-1	-6	6.64	1.23
23	-1	-6	7.13	0.73
23	1	6	7.69	1.04
23	1	6	7.75	1.48
-23	-1	6	7.09	1.01
-23	1	-6	9.33	1.71
-23	1	-6	5.04	0.99
23	-1	-6	7.47	0.98
-23	-1	6	10.38	1.86
23	1	6	8.41	1.69
-23	1	6	7.61	1.63
23	-1	6	8.12	1.45
-23	1	6	8.60	1.49
23	-1	6	7.72	1.38
23	-1	6	6.63	1.01
-23	-1	-6	5.62	1.10
-23	-1	-6	6.39	1.06
-23	-1	-6	12.73	2.06
23	2	6	21.45	1.46
23	2	6	23.67	2.06
23	-2	-6	28.77	1.74
-23	2	-6	27.40	3.10
-23	2	-6	23.12	1.96
23	-2	-6	30.07	1.94
23	-2	-6	26.38	1.56
-23	-2	6	21.59	1.74
23	2	6	20.23	2.27
-23	-2	6	20.40	2.23
-23	-2	6	19.95	1.70
-23	-2	6	17.00	2.17
-23	-2	-6	27.55	2.02
23	-2	6	19.61	1.47
-23	2	6	21.85	2.51
-23	-2	-6	23.52	2.53
-23	-2	-6	24.47	2.06

23	-2	6	22.46	1.96
23	-2	6	19.87	1.83
-23	2	6	19.70	1.77
23	3	6	21.58	1.54
23	3	6	22.65	2.05
-23	-3	6	21.46	1.78
-23	-3	6	24.78	2.36
23	3	6	27.13	2.67
23	-3	-6	17.40	1.29
-23	3	-6	20.63	2.95
-23	-3	6	25.62	2.26
-23	3	-6	18.25	1.68
23	-3	-6	19.67	1.63
-23	-3	6	20.44	1.77
23	-3	6	20.93	1.56
-23	3	6	21.68	2.49
-23	-3	-6	19.04	1.69
23	-3	6	22.87	1.89
-23	-3	-6	17.24	1.83
-23	3	6	21.67	1.81
-23	-3	-6	18.39	2.50
-23	-4	6	34.23	2.74
-23	-4	6	38.44	2.53
23	4	6	36.67	2.18
23	-4	-6	38.66	2.32
-23	-4	6	41.07	3.19
23	4	6	41.27	3.14
-23	-4	6	32.57	2.36
-23	4	-6	38.73	3.36
-23	4	-6	37.55	2.45
23	-4	-6	38.08	2.13
-23	4	6	36.23	3.04
-23	-4	-6	30.78	2.40
23	-4	6	37.07	2.41
23	-4	6	37.47	2.24
-23	4	6	37.42	2.45
-23	-4	-6	34.80	2.55
23	5	6	34.78	2.89
-23	-5	6	30.33	2.29
23	-5	-6	36.12	2.20
-23	5	-6	35.57	3.43
-23	-5	6	37.94	3.06
-23	5	-6	34.38	2.42
23	-5	-6	40.70	2.48

23	-5	-6	36.66	2.43
23	5	6	38.36	2.24
-23	-5	6	35.64	2.48
-23	-5	6	33.27	2.60
-23	-5	-6	35.28	2.49
-23	5	6	39.82	3.21
23	-5	6	36.05	2.27
23	-5	6	36.59	2.44
-23	-5	-6	36.19	2.57
-24	0	6	36.32	2.31
-24	0	-6	40.16	2.52
-24	0	6	43.85	2.52
24	0	6	35.51	2.83
24	0	6	35.24	2.46
24	0	6	36.00	2.12
-24	0	6	40.24	3.25
-24	0	-6	34.84	3.23
-24	0	6	33.32	2.59
-24	1	-6	25.69	2.01
-24	-1	6	23.92	2.66
-24	1	-6	23.83	3.09
24	1	6	22.17	1.58
24	1	6	22.04	2.47
24	-1	-6	25.92	1.52
-24	-1	6	26.14	2.23
24	-1	-6	28.69	2.00
-24	-1	6	26.63	2.08
-24	-1	6	24.53	1.85
24	-1	-6	25.23	1.39
24	-1	6	22.37	1.93
24	-1	6	26.90	2.27
-24	1	6	30.54	2.69
-24	-1	-6	27.12	1.98
-24	-1	-6	27.22	2.81
-24	-1	-6	22.80	1.98
24	-1	6	23.44	1.61
-24	1	6	27.17	2.00
-24	2	-6	14.35	1.59
24	2	6	15.03	1.22
-24	-2	6	14.16	1.72
-24	-2	6	16.78	2.32
24	-2	-6	13.80	1.01
-24	2	-6	12.31	2.21
24	-2	-6	14.14	1.52

-24	-2	6	13.71	1.66
24	2	6	14.80	2.44
-24	-2	6	15.90	2.13
-24	2	6	13.88	2.26
-24	-2	-6	15.98	1.54
24	-2	6	12.15	1.27
-24	-2	-6	13.04	1.58
-24	2	6	15.10	1.55
-24	-2	-6	11.09	2.15
24	-2	6	15.13	1.60
24	3	6	1.67	1.43
24	3	6	0.12	0.62
24	-3	-6	-0.22	0.86
-24	-3	6	0.24	1.13
-24	-3	6	0.37	0.88
-24	-3	6	-0.99	1.41
-24	3	-6	2.37	0.90
24	-3	-6	1.88	0.57
-24	-3	6	1.93	0.92
-24	3	-6	2.08	1.65
24	-3	6	0.63	0.64
24	-3	6	1.65	0.89
-24	3	6	0.76	1.30
-24	-3	-6	1.23	0.85
-24	3	6	1.48	0.85
-24	-3	-6	1.86	0.90
-24	4	-6	5.88	1.73
24	4	6	6.55	1.15
24	-4	-6	7.76	1.18
24	-4	-6	4.48	0.94
24	4	6	7.55	1.61
-24	4	-6	6.14	1.05
-24	-4	6	8.40	1.20
-24	-4	6	5.50	1.14
-24	-4	6	11.68	2.01
-24	-4	6	9.47	1.70
-24	-4	-6	3.40	0.95
-24	4	6	7.77	1.65
24	-4	6	5.60	1.11
-24	4	6	4.43	0.96
24	-4	6	5.49	1.04
-24	-4	-6	4.58	1.09
-25	0	-6	2.57	1.77
-25	0	6	-0.26	1.33

25	0	6	0.91	0.59
-25	0	6	-0.10	0.89
-25	0	-6	0.75	0.85
25	0	6	-0.16	0.84
-25	0	6	-0.86	0.72
25	0	6	-1.02	1.33
-25	-1	6	8.12	1.63
-25	-1	6	9.16	1.45
25	1	6	8.13	1.04
-25	-1	6	5.94	1.58
25	-1	-6	11.39	0.90
-25	1	-6	8.46	2.14
25	1	6	7.31	1.64
-25	1	-6	10.39	1.51
-25	-1	6	7.77	1.23
-25	-1	-6	8.69	1.55
-25	1	6	7.17	1.50
25	-1	6	8.86	1.52
25	-1	6	7.08	1.02
-25	-1	-6	8.32	1.84
-25	1	6	6.89	1.17
-25	-1	-6	10.95	1.55
-25	1	6	7.92	1.19
-25	2	-6	9.16	1.61
-25	-2	6	10.22	1.55
-25	-2	6	12.44	1.73
-25	-2	6	11.02	2.07
25	-2	-6	12.57	1.45
-25	2	-6	12.38	2.08
25	-2	-6	12.08	1.72
25	-2	-6	10.48	0.94
-25	-2	6	11.61	1.97
25	2	6	9.73	1.13
25	2	6	10.60	2.04
-25	2	6	10.28	1.50
-25	-2	-6	10.51	1.61
-25	2	6	9.26	1.68
25	-2	6	10.86	1.41
-25	-2	-6	12.45	1.57
25	-2	6	7.51	1.22
0	0	-7	1.03	0.27
0	0	7	0.38	0.19
0	0	-7	0.56	0.21
0	0	-7	0.27	0.29

0	0	7	0.38	0.19
0	0	-7	0.48	0.28
0	0	7	0.73	0.29
0	0	7	0.52	0.31
0	0	7	0.32	0.10
0	0	-7	0.39	0.20
0	0	-7	0.44	0.11
0	1	7	0.27	0.27
0	-1	-7	0.30	0.27
0	1	7	0.28	0.31
0	1	-7	0.31	0.24
0	1	-7	0.43	0.26
0	-1	-7	-0.01	0.19
0	-1	-7	-0.21	0.29
0	-1	7	0.16	0.17
0	1	-7	0.18	0.12
0	-1	7	-0.29	0.32
0	1	-7	0.21	0.18
0	-1	-7	0.15	0.10
0	1	7	-0.16	0.21
0	-1	7	0.31	0.12
0	-1	-7	0.34	0.23
0	1	-7	0.44	0.38
0	-1	7	-0.27	0.27
0	-1	-7	-0.06	0.16
0	1	-7	0.04	0.15
0	-1	-7	0.15	0.11
0	-1	7	-0.11	0.17
0	-1	7	0.14	0.15
0	1	7	-0.07	0.18
0	2	7	-0.04	0.22
0	2	7	0.04	0.13
0	-2	7	0.40	0.25
0	2	7	-0.05	0.24
0	-2	-7	-0.04	0.13
0	-2	7	0.66	0.36
0	-2	-7	0.35	0.25
0	2	-7	-0.03	0.14
0	-2	-7	0.17	0.26
0	2	7	0.10	0.33
0	-2	7	0.16	0.19
0	2	7	0.16	0.19
0	2	7	0.36	0.24
0	-2	-7	-0.03	0.21

0	2	-7	0.36	0.27
0	-2	-7	0.03	0.34
0	-2	7	0.04	0.15
0	-2	7	0.14	0.14
0	-2	-7	0.27	0.21
0	2	-7	0.16	0.19
0	-2	7	-0.11	0.33
0	2	-7	-0.34	0.24
0	2	-7	0.01	0.16
0	3	-7	0.04	0.36
0	3	-7	0.06	0.35
0	3	-7	0.03	0.14
0	-3	-7	-0.07	0.25
0	3	-7	-0.08	0.20
0	3	7	-0.09	0.23
0	-3	-7	-0.02	0.40
0	3	-7	0.12	0.35
0	-3	7	-0.38	0.25
0	-3	7	-0.03	0.17
0	-3	-7	0.17	0.27
0	3	7	-0.05	0.26
0	3	-7	0.16	0.24
0	3	7	0.05	0.17
0	3	7	0.49	0.36
0	-3	-7	0.06	0.27
0	-3	-7	0.10	0.18
0	-3	7	0.07	0.22
0	-3	-7	0.39	0.30
0	-3	7	0.03	0.39
0	3	7	0.07	0.35
0	3	7	-0.53	0.24
0	-3	7	0.11	0.40
0	4	7	-0.02	0.28
0	-4	7	-0.05	0.29
0	-4	7	0.20	0.24
0	-4	-7	-0.02	0.37
0	4	-7	0.32	0.42
0	4	7	0.51	0.46
0	-4	-7	0.16	0.25
0	4	-7	-0.01	0.48
0	4	-7	0.34	0.39
0	4	-7	-0.13	0.24
0	4	7	-0.21	0.26
0	4	7	0.18	0.29

0	-4	7	0.70	0.53
0	-4	-7	-0.19	0.25
0	-4	-7	0.39	0.43
0	4	7	0.59	0.30
0	-4	7	0.00	0.31
0	-4	7	0.42	0.61
0	4	-7	0.12	0.28
0	4	7	-0.33	0.33
0	4	7	-0.01	0.31
0	-4	-7	0.25	0.30
0	-5	7	0.17	0.25
0	-5	7	-0.28	0.51
0	5	-7	-0.41	0.44
0	-5	-7	0.08	0.30
0	-5	-7	0.23	0.26
0	-5	-7	0.23	0.39
0	-5	7	0.50	0.58
0	-5	7	0.00	0.36
0	5	7	0.42	0.37
0	-5	-7	0.56	0.38
0	-5	7	0.15	0.36
0	5	7	0.29	0.32
0	-5	-7	-0.11	0.41
0	5	7	-0.32	0.42
0	5	7	0.24	0.51
0	5	-7	-0.16	0.43
0	5	-7	-0.21	0.31
0	5	7	-0.07	0.37
0	5	7	0.42	0.29
0	5	-7	0.44	0.35
0	5	7	-0.04	0.26
0	5	-7	-0.35	0.43
0	6	7	-0.04	0.35
0	6	-7	0.11	0.52
0	-6	-7	-0.08	0.40
0	6	7	-0.33	0.45
0	-6	7	0.19	0.42
0	6	7	0.46	0.35
0	-6	7	1.19	0.65
0	6	-7	-0.45	0.50
0	-6	7	0.15	0.29
0	-6	7	0.01	0.66
0	6	-7	0.35	0.54
0	6	-7	0.49	0.40

0	-6	-7	-0.17	0.41
0	6	7	0.05	0.37
0	6	7	0.49	0.62
0	6	7	-0.39	0.36
0	-6	-7	-0.20	0.33
0	-6	7	0.10	0.38
0	6	7	0.20	0.33
0	-6	-7	0.26	0.29
0	-6	-7	-0.19	0.50
0	6	-7	-0.15	0.46
0	-7	-7	0.81	0.40
0	7	7	0.61	0.48
0	7	-7	0.53	0.52
0	7	7	0.52	0.65
0	7	7	-0.05	0.45
0	7	-7	0.18	0.58
0	7	-7	0.16	0.53
0	7	7	-0.14	0.43
0	7	7	0.41	0.42
0	7	7	-0.04	0.73
0	-7	7	1.30	0.79
0	-7	7	0.18	0.43
0	-7	7	0.06	0.48
0	-7	7	0.62	0.74
0	7	-7	0.46	0.61
0	-7	-7	0.59	0.41
0	-7	-7	0.46	0.51
0	-7	-7	0.28	0.50
0	-7	7	0.12	0.35
0	-7	-7	0.74	0.67
0	8	7	0.70	0.68
0	8	7	0.17	0.53
0	-8	7	0.03	0.49
0	-8	-7	-0.30	0.64
0	-8	-7	-0.38	0.45
0	-8	-7	-0.10	0.44
0	8	-7	0.73	0.64
0	8	7	0.33	0.50
0	-8	7	0.09	0.58
0	8	7	0.19	0.57
0	-8	7	1.42	0.80
0	8	-7	-0.90	0.81
0	8	7	-0.02	0.56
0	8	7	0.52	0.52

0	-8	7	1.53	0.90
0	8	-7	0.41	0.71
0	-8	-7	-0.56	0.70
0	-8	7	-0.83	0.93
0	-8	7	-0.36	0.56
0	8	7	-0.24	0.89
0	-8	-7	-0.11	0.64
0	-9	-7	-0.56	0.74
0	-9	7	0.39	0.66
0	9	7	-0.22	0.66
0	9	7	-0.25	0.77
0	-9	-7	0.05	0.47
0	9	-7	-0.67	0.71
0	9	-7	-0.05	0.72
0	-9	7	0.58	0.93
0	9	7	-0.28	0.61
0	9	7	0.39	0.61
0	-9	-7	0.06	0.49
0	-9	7	-0.16	0.61
0	9	7	-0.95	1.07
0	-9	-7	0.29	0.95
0	-9	7	-0.73	0.98
0	9	7	0.42	0.64
0	-9	-7	0.05	0.74
0	-9	7	0.15	0.64
0	9	-7	-1.53	0.95
0	-10	7	0.42	0.76
0	10	7	-0.34	0.72
0	10	-7	-0.66	0.95
0	10	7	0.30	0.75
0	10	-7	-0.50	1.15
0	10	-7	0.02	0.83
0	-10	-7	-1.28	0.87
0	-10	-7	1.48	0.78
0	-10	-7	-0.31	0.64
0	-10	7	0.08	0.65
0	10	7	0.03	1.08
0	10	7	0.43	0.70
0	-10	-7	-0.56	0.58
0	-10	7	0.95	1.13
0	10	7	0.86	1.01
0	-11	-7	-0.43	0.79
0	11	-7	-0.05	1.00
0	11	7	1.26	1.10

0	11	7	1.11	0.80
0	-11	-7	0.74	0.82
0	-11	-7	0.23	0.70
0	-11	7	-1.05	0.82
0	-11	-7	1.48	0.81
0	-11	7	0.20	0.74
0	-12	7	-1.06	1.07
0	-12	-7	1.15	0.78
0	-12	-7	1.00	0.95
0	12	7	0.18	1.69
0	12	-7	-0.77	1.11
0	-12	7	0.76	0.87
0	12	-7	0.22	1.76
0	-13	-7	-0.24	0.86
0	-13	-7	-0.43	1.21
0	13	-7	-0.86	2.09
0	-13	7	1.61	0.87
0	13	-7	1.60	1.40
0	13	7	1.17	1.83
0	-13	-7	-0.96	0.82
0	-13	7	2.09	1.35
-1	0	7	-0.07	0.15
-1	0	-7	-0.10	0.30
1	0	-7	0.18	0.22
1	0	7	0.04	0.18
1	0	-7	0.00	0.07
-1	0	-7	0.04	0.19
-1	0	-7	-0.17	0.25
-1	0	7	0.01	0.26
-1	0	7	0.18	0.31
1	0	7	0.22	0.21
-1	0	-7	-0.13	0.16
1	0	7	0.12	0.11
-1	0	-7	0.07	0.23
1	0	7	0.03	0.27
1	0	-7	0.14	0.19
1	0	-7	0.32	0.30
-1	0	-7	-0.02	0.11
-1	0	7	0.08	0.07
1	0	-7	0.00	0.15
1	0	7	0.01	0.34
-1	0	7	-0.02	0.18
1	0	7	0.14	0.20
-1	-1	7	47.32	2.37

-1	-1	7	52.73	2.56
1	-1	-7	36.11	1.85
1	1	7	48.75	2.42
1	-1	-7	37.40	1.98
1	1	7	47.19	2.42
-1	-1	7	47.97	2.43
1	-1	-7	38.00	1.88
-1	1	-7	38.57	1.81
1	-1	-7	38.31	1.74
1	1	7	49.11	2.43
1	-1	-7	36.40	1.93
-1	1	-7	37.86	1.98
-1	1	-7	36.89	2.04
1	-1	-7	37.57	1.88
-1	-1	7	46.89	2.29
1	1	7	48.67	2.38
-1	1	-7	38.05	1.87
-1	-1	7	52.01	2.54
-1	-1	7	49.95	2.32
1	1	7	54.00	2.58
1	-1	-7	38.61	1.74
-1	1	-7	38.65	1.79
-1	1	7	47.93	2.42
-1	1	7	53.39	2.55
1	-1	7	48.63	2.40
-1	1	7	51.79	2.39
1	-1	7	50.85	2.33
1	1	-7	37.76	1.85
-1	-1	-7	37.64	1.90
1	1	-7	38.37	1.79
-1	-1	-7	39.44	1.89
1	1	-7	36.88	1.96
1	-1	7	49.84	2.32
-1	-1	-7	38.99	2.02
1	1	-7	34.88	2.14
1	-1	7	50.63	2.44
-1	1	7	48.34	2.41
-1	-1	-7	40.20	1.78
1	1	-7	37.63	1.76
1	1	-7	36.76	1.91
-1	-1	-7	38.56	1.86
-1	1	7	50.05	2.46
1	-1	7	52.31	2.54
-1	-1	-7	38.93	1.78

1	-1	7	50.83	2.59
1	2	7	391.33	18.45
1	2	7	442.51	18.56
1	2	7	417.87	18.39
-1	-2	7	397.18	18.45
1	-2	-7	437.81	19.81
-1	-2	7	432.05	18.54
-1	-2	7	407.05	18.28
1	2	7	392.47	18.43
1	2	7	374.51	18.31
1	-2	-7	448.11	19.88
1	-2	-7	447.87	19.76
-1	2	-7	451.39	19.91
-1	-2	7	449.93	18.56
1	-2	-7	433.31	19.66
1	2	7	426.22	18.40
1	-2	-7	435.98	19.80
1	-2	-7	439.62	19.77
-1	2	-7	455.10	19.82
-1	2	-7	440.15	19.71
-1	2	-7	438.26	19.67
-1	-2	7	385.49	18.37
-1	-2	7	394.84	18.32
-1	2	-7	436.06	19.77
-1	-2	-7	436.51	19.80
-1	-2	-7	447.35	19.80
-1	2	7	388.57	18.45
1	2	-7	437.79	19.69
1	-2	7	405.67	18.44
-1	2	7	382.53	18.29
-1	-2	-7	427.72	19.76
-1	-2	-7	444.42	19.88
-1	-2	-7	434.70	19.74
1	-2	7	448.26	18.57
1	-2	7	411.43	18.31
-1	-2	-7	421.58	19.69
1	-2	7	458.07	18.62
1	2	-7	447.85	19.89
-1	2	7	346.71	18.45
-1	2	7	444.19	18.54
1	2	-7	434.48	19.75
1	2	-7	452.35	19.82
-1	2	7	433.70	18.43
-1	2	7	418.14	18.41

1	-2	7	377.79	18.40
1	2	-7	439.77	19.64
-1	3	-7	406.81	18.40
-1	-3	7	382.71	17.83
-1	-3	7	393.74	17.95
1	3	7	407.84	17.89
1	3	7	378.85	17.91
-1	3	-7	399.60	18.46
1	3	7	369.86	17.81
1	3	7	409.99	18.04
-1	3	-7	402.13	18.60
1	-3	-7	424.13	18.44
1	-3	-7	430.90	18.54
1	3	7	401.52	17.84
-1	3	-7	405.52	18.31
1	-3	-7	407.85	18.46
-1	3	-7	430.85	18.68
-1	-3	7	363.96	17.86
-1	-3	7	419.08	18.23
-1	3	-7	402.37	18.31
1	3	-7	412.72	18.39
1	-3	7	374.74	17.89
-1	3	7	389.23	17.99
1	3	-7	399.85	18.44
-1	3	7	384.33	17.80
-1	-3	-7	396.87	18.38
1	-3	7	435.44	18.11
-1	3	7	403.71	17.90
1	-3	7	396.65	17.93
-1	3	7	420.00	17.90
-1	3	7	419.13	18.04
-1	-3	-7	394.10	18.38
-1	-3	-7	421.77	18.52
1	-3	7	377.16	17.83
-1	-3	-7	401.85	18.55
-1	-3	-7	401.29	18.45
-1	3	7	387.37	17.87
1	3	-7	412.41	18.52
1	3	-7	412.71	18.59
1	3	-7	417.93	18.64
1	-3	7	437.60	18.08
-1	-3	-7	420.92	18.48
1	4	7	524.42	22.28
-1	4	-7	510.72	22.99

1	-4	-7	501.63	23.16
1	4	7	476.88	22.14
1	-4	-7	494.76	23.09
-1	-4	7	462.98	22.08
1	4	7	471.67	22.03
1	-4	-7	537.38	23.28
-1	4	-7	522.44	23.21
1	4	7	472.47	22.22
1	4	7	508.40	22.10
-1	4	-7	519.50	23.12
-1	-4	7	527.04	22.29
-1	-4	7	536.79	22.31
-1	-4	7	474.49	22.06
1	4	7	492.34	21.99
-1	4	-7	529.50	23.42
1	4	7	500.19	22.01
-1	-4	7	486.05	22.17
-1	4	-7	504.64	22.99
-1	4	-7	516.99	23.34
-1	4	-7	515.03	23.18
1	-4	-7	543.62	23.14
1	-4	-7	497.78	23.26
1	-4	7	502.33	22.15
-1	-4	-7	495.41	23.15
1	4	-7	519.39	23.31
1	4	-7	520.48	23.23
-1	4	7	483.64	22.09
1	-4	7	446.46	22.83
-1	-4	-7	528.46	23.19
-1	4	7	431.17	22.08
-1	-4	-7	505.29	23.26
1	-4	7	550.45	22.32
-1	-4	-7	504.15	23.11
1	4	-7	513.45	23.36
1	-4	7	469.21	22.06
-1	-4	-7	526.26	23.25
1	4	-7	514.13	23.16
-1	4	7	488.94	22.05
1	-4	7	451.95	22.10
1	4	-7	514.24	23.11
-1	4	7	478.30	22.24
-1	4	7	514.58	22.11
-1	4	7	529.28	22.28
-1	4	7	515.83	22.08

-1	5	-7	54.67	2.83
1	-5	-7	59.18	2.97
1	5	7	48.62	2.46
1	5	7	49.02	2.40
-1	5	-7	55.95	2.59
-1	5	-7	55.27	2.87
-1	-5	7	49.21	2.70
-1	5	-7	53.89	3.09
-1	-5	7	47.86	2.54
1	-5	-7	54.24	2.90
-1	5	-7	56.98	2.78
-1	5	-7	57.13	2.60
1	5	7	47.54	2.49
1	5	7	48.35	2.65
1	-5	-7	58.00	2.75
-1	5	-7	53.90	2.98
-1	-5	7	51.40	2.71
-1	-5	7	44.20	2.43
1	5	7	48.31	2.35
1	-5	-7	54.00	2.91
-1	-5	7	46.69	2.41
1	5	7	47.21	2.34
1	-5	-7	54.61	2.80
1	5	7	50.74	2.64
-1	-5	-7	54.03	2.93
1	5	-7	53.46	3.03
-1	-5	-7	59.28	2.95
-1	-5	-7	53.89	2.75
1	-5	7	52.27	2.73
-1	5	7	49.45	2.43
-1	5	7	49.32	2.66
1	5	-7	55.07	2.95
-1	5	7	48.32	2.47
-1	-5	-7	54.91	2.79
1	5	-7	56.63	2.91
1	-5	7	50.50	2.72
-1	5	7	44.76	2.37
1	-5	7	45.46	2.46
1	-5	7	46.78	2.41
-1	5	7	43.90	2.58
1	5	-7	56.25	2.77
1	-5	7	48.05	2.50
-1	5	7	48.57	2.45
1	5	-7	58.66	2.84

-1	-5	-7	59.18	2.85
-1	5	7	48.46	2.40
1	-6	-7	2.24	0.60
1	-6	-7	2.36	0.44
-1	6	-7	1.60	0.57
-1	6	-7	2.24	0.31
1	-6	-7	2.64	0.54
1	6	7	0.96	0.57
-1	6	-7	2.26	0.40
1	6	7	0.58	0.44
-1	-6	7	0.81	0.34
-1	6	-7	3.03	0.69
1	6	7	0.64	0.38
1	-6	-7	2.79	0.51
1	6	7	0.35	0.37
-1	-6	7	1.08	0.47
1	6	7	0.11	0.33
-1	6	-7	2.11	0.60
1	-6	-7	2.19	0.64
1	6	7	0.89	0.45
-1	-6	7	0.58	0.67
-1	-6	7	0.27	0.38
1	6	7	0.64	0.65
-1	-6	7	0.48	0.63
-1	6	-7	2.77	0.54
-1	6	-7	2.90	0.75
1	-6	7	0.00	0.67
1	-6	7	1.93	0.71
1	6	-7	1.86	0.67
-1	-6	-7	2.51	0.46
1	6	-7	2.15	0.49
-1	6	7	0.09	0.40
-1	6	7	0.39	0.36
-1	-6	-7	2.67	0.61
1	6	-7	2.27	0.60
1	6	-7	1.59	0.55
-1	6	7	1.06	0.47
1	6	-7	1.50	0.60
-1	-6	-7	2.34	0.45
-1	-6	-7	2.15	0.65
1	-6	7	0.40	0.42
1	-6	7	0.65	0.33
-1	6	7	0.68	0.40
1	-6	7	0.52	0.43

-1	6	7	0.86	0.70
-1	-6	-7	2.02	0.64
-1	6	7	0.48	0.57
1	-7	-7	2.68	0.85
-1	-7	7	2.00	0.82
-1	-7	7	2.89	0.87
1	7	7	3.01	0.63
-1	7	-7	1.81	0.52
1	-7	-7	2.41	0.60
1	7	7	3.61	0.60
1	-7	-7	1.39	0.59
-1	7	-7	1.64	0.83
-1	-7	7	3.87	0.82
1	7	7	2.94	0.52
-1	7	-7	3.05	0.76
1	7	7	2.88	0.73
-1	7	-7	3.75	0.74
-1	-7	7	3.89	0.74
-1	7	-7	1.64	0.69
-1	-7	7	2.53	0.51
-1	7	-7	2.15	0.36
-1	-7	7	2.30	0.64
1	-7	-7	1.83	0.46
1	7	7	3.69	0.53
1	7	7	2.95	0.60
1	-7	-7	1.26	0.48
1	7	-7	1.81	0.67
-1	7	7	3.02	0.56
-1	7	7	2.35	0.61
1	7	-7	0.49	0.70
-1	7	7	2.33	0.77
-1	-7	-7	2.61	0.52
-1	-7	-7	1.39	0.59
-1	-7	-7	2.75	0.67
-1	7	7	1.97	0.56
1	7	-7	3.20	0.69
1	-7	7	3.93	0.59
-1	-7	-7	2.38	0.74
-1	7	7	3.54	0.62
-1	7	7	3.00	0.88
1	7	-7	2.28	0.59
-1	-7	-7	2.01	0.52
1	-7	7	3.27	0.64
1	-7	7	4.07	0.97

-1	7	7	2.63	0.56
1	-7	7	2.45	0.62
1	-7	7	1.60	0.90
-1	8	-7	9.27	1.22
1	8	7	10.13	1.07
1	8	7	10.17	1.16
1	8	7	10.63	1.24
-1	8	-7	9.44	1.48
-1	-8	7	10.33	1.43
1	-8	-7	7.05	0.94
-1	8	-7	7.71	1.25
-1	-8	7	10.17	1.04
-1	-8	7	11.80	1.60
1	8	7	10.41	1.02
1	-8	-7	8.72	1.08
-1	-8	7	10.98	1.18
1	8	7	11.08	1.14
-1	-8	7	9.20	1.08
1	-8	-7	8.64	1.43
1	8	7	10.05	1.57
-1	8	-7	9.36	0.74
1	-8	-7	7.16	1.02
-1	-8	7	10.55	1.55
1	-8	-7	7.34	0.91
-1	-8	-7	8.30	1.06
1	-8	7	9.26	1.62
-1	-8	-7	10.74	1.23
-1	8	7	11.11	1.11
1	8	-7	8.43	1.36
-1	-8	-7	8.68	0.96
1	8	-7	10.06	1.24
1	-8	7	10.47	1.22
1	-8	7	12.53	1.61
-1	8	7	11.02	1.09
1	8	-7	8.78	1.17
-1	8	7	9.52	1.41
-1	8	7	9.77	1.07
-1	-8	-7	8.63	1.23
1	-8	7	10.96	1.03
-1	8	7	10.28	1.08
1	-8	7	11.49	1.17
-1	-8	-7	8.73	0.97
1	-8	7	10.54	1.41
-1	8	7	9.86	1.17

-1	8	7	11.39	1.61
1	9	7	56.08	3.10
1	9	7	53.97	3.19
-1	-9	7	59.58	3.29
-1	9	-7	68.03	3.15
-1	-9	7	61.64	3.53
1	-9	-7	60.99	3.40
1	-9	-7	63.78	3.44
-1	-9	7	56.82	3.16
-1	-9	7	62.35	3.64
1	9	7	61.31	3.61
-1	9	-7	69.10	4.03
1	-9	-7	71.04	3.52
1	9	7	63.56	3.05
-1	9	-7	63.54	3.71
1	9	7	58.00	3.02
1	-9	-7	60.95	3.58
-1	9	-7	76.66	3.77
1	-9	-7	69.27	3.85
-1	-9	7	58.06	3.18
1	9	7	57.40	3.53
-1	9	7	57.13	3.16
1	-9	7	60.03	3.41
-1	9	7	54.13	3.08
1	9	-7	59.45	3.94
1	-9	7	58.35	3.19
-1	9	7	63.34	3.13
-1	-9	-7	66.78	3.46
1	-9	7	57.80	3.51
1	9	-7	72.82	3.70
1	-9	7	58.51	3.23
-1	-9	-7	63.12	3.60
1	-9	7	62.30	3.63
-1	9	7	57.15	3.12
1	9	-7	62.17	3.64
-1	-9	-7	64.74	3.41
-1	-9	-7	65.79	3.71
-1	9	7	57.17	3.48
-1	-9	-7	68.66	3.61
-1	9	7	65.13	3.66
1	-9	7	50.38	3.59
1	10	7	83.41	4.21
-1	10	-7	90.31	5.56
1	-10	-7	97.61	5.24

1	10	7	96.40	4.90
-1	-10	7	85.21	4.38
-1	10	-7	114.93	5.44
1	-10	-7	103.62	5.12
1	10	7	78.48	4.83
1	10	7	88.61	4.21
-1	-10	7	92.11	4.56
-1	-10	7	79.79	4.43
1	-10	-7	105.00	5.09
1	-10	-7	97.68	5.01
-1	10	-7	100.05	5.36
1	10	7	79.49	4.44
-1	-10	-7	106.55	5.21
-1	-10	-7	96.77	5.26
1	-10	7	84.23	4.38
-1	-10	-7	102.48	5.10
-1	10	7	90.14	4.91
-1	10	7	82.64	4.85
1	10	-7	110.18	5.43
1	-10	7	78.35	4.39
-1	10	7	87.21	4.31
-1	10	7	82.12	4.32
-1	-10	-7	102.44	5.02
1	10	-7	95.94	5.63
1	-10	7	92.94	4.48
1	10	-7	103.03	5.31
1	11	7	24.28	1.78
-1	-11	7	27.86	2.12
-1	-11	7	22.58	1.82
1	-11	-7	29.63	2.14
-1	11	-7	25.05	2.85
1	-11	-7	24.49	2.02
1	-11	-7	25.00	1.89
1	-11	-7	25.25	1.94
1	-11	-7	20.57	2.02
1	11	7	26.22	2.50
-1	11	7	25.43	1.96
-1	-11	-7	28.57	2.05
-1	-11	-7	23.79	1.79
-1	-11	-7	26.65	1.91
1	11	-7	25.10	2.67
1	-11	7	25.72	1.90
1	11	-7	22.44	2.17
-1	11	7	20.61	2.51

1	-11	7	24.07	2.33
-1	-11	-7	22.46	1.98
1	12	7	14.88	2.48
-1	12	-7	7.82	2.03
1	-12	-7	12.92	1.64
-1	-12	7	17.04	1.72
-1	12	-7	8.77	1.59
1	-12	-7	11.41	1.65
1	-12	-7	11.62	1.42
-1	-12	7	12.73	1.95
-1	-12	-7	8.12	1.35
1	12	-7	8.95	2.05
1	-12	7	17.07	1.96
-1	-12	-7	8.87	1.60
-1	12	7	13.33	2.38
1	12	-7	9.72	1.48
1	-12	7	14.59	1.64
-1	-12	-7	11.92	1.80
-1	12	7	16.10	2.05
1	-13	-7	10.43	1.59
-1	13	-7	16.69	2.45
-1	-13	7	11.54	1.88
-1	13	-7	12.11	2.44
1	13	7	10.54	2.34
-1	-13	7	12.13	1.57
1	-13	-7	13.87	1.64
1	13	-7	14.87	2.30
-1	-13	-7	13.62	2.13
1	-13	7	13.17	2.08
1	-13	7	10.56	1.59
-1	-13	-7	11.66	1.59
-1	-13	-7	10.07	1.57
-1	13	7	13.30	2.65
1	13	-7	13.54	2.79
2	0	-7	68.39	3.19
-2	0	-7	69.82	3.21
-2	0	7	76.72	3.84
2	0	-7	65.49	3.23
2	0	7	75.76	3.86
2	0	-7	65.49	3.12
-2	0	-7	64.69	3.23
-2	0	7	86.61	3.98
2	0	7	81.39	3.80
-2	0	7	86.33	3.97

-2	0	-7	70.52	3.09
2	0	7	89.44	4.06
2	0	7	85.02	3.95
2	0	-7	62.88	3.37
2	0	7	81.35	3.83
-2	0	7	85.67	3.84
-2	0	-7	66.64	3.16
-2	0	-7	69.70	3.34
-2	-1	7	40.38	2.02
-2	-1	7	45.12	2.19
2	1	7	40.24	2.08
-2	-1	7	44.55	1.99
-2	1	-7	38.21	2.07
2	-1	-7	40.96	2.02
-2	1	-7	41.32	1.90
-2	1	-7	40.82	1.96
2	1	7	44.21	2.24
2	-1	-7	40.11	1.95
-2	-1	7	41.40	2.10
-2	1	-7	40.07	1.91
2	1	7	41.12	2.09
2	-1	-7	40.55	1.97
-2	-1	7	45.23	2.20
2	-1	-7	36.68	2.15
-2	-1	7	40.84	1.94
2	-1	-7	38.38	1.96
2	1	7	39.61	2.04
2	1	7	39.79	2.08
2	1	7	40.78	1.99
-2	1	-7	40.08	2.01
2	-1	7	40.70	2.09
-2	-1	-7	39.91	1.89
-2	-1	-7	37.18	1.94
-2	-1	-7	39.80	2.00
2	1	-7	40.97	1.84
2	-1	7	44.37	2.21
-2	-1	-7	37.97	1.98
-2	1	7	39.29	1.90
2	1	-7	40.46	1.88
-2	1	7	44.28	2.19
-2	-1	-7	40.10	2.11
-2	1	7	40.44	2.07
-2	1	7	44.43	2.06
2	1	-7	40.75	2.01

-2	-1	-7	40.42	1.98
2	1	-7	39.83	2.05
2	1	-7	41.52	2.09
2	-1	7	40.79	1.99
-2	1	7	42.72	2.14
-2	1	7	42.28	2.07
2	-1	7	41.50	2.06
2	-1	7	45.84	2.29
2	-1	7	42.95	2.01
-2	2	-7	51.00	2.39
-2	-2	7	58.45	2.92
2	2	7	68.61	3.15
2	-2	-7	48.35	2.44
2	-2	-7	50.76	2.58
-2	2	-7	52.47	2.43
2	2	7	60.76	3.03
2	2	7	59.99	3.00
-2	2	-7	51.43	2.64
-2	-2	7	64.33	3.08
-2	-2	7	60.01	2.88
2	-2	-7	51.08	2.51
-2	2	-7	53.85	2.54
-2	-2	7	61.05	3.03
-2	2	-7	51.24	2.48
2	-2	-7	50.92	2.53
-2	-2	7	62.82	2.83
-2	-2	7	64.63	3.12
2	-2	-7	49.90	2.36
-2	2	-7	54.59	2.73
2	2	7	64.55	2.95
2	-2	-7	48.63	2.47
2	2	-7	52.67	2.39
-2	2	7	61.44	2.97
2	2	-7	51.81	2.55
-2	2	7	58.91	2.94
-2	2	7	63.39	3.02
2	2	-7	49.70	2.58
-2	-2	-7	49.11	2.47
-2	-2	-7	54.23	2.43
2	-2	7	61.59	2.91
2	2	-7	50.63	2.61
-2	2	7	68.15	3.11
-2	2	7	59.08	2.84
-2	2	7	60.33	3.02

2	-2	7	66.54	3.21
-2	-2	-7	49.50	2.43
2	-2	7	63.21	2.88
2	-2	7	62.81	3.13
-2	-2	-7	51.69	2.51
2	-2	7	61.82	3.00
2	2	-7	50.77	2.34
2	-2	7	57.82	2.96
-2	-2	-7	49.84	2.60
2	3	7	75.56	3.43
-2	-3	7	69.54	3.43
-2	-3	7	70.03	3.57
2	3	7	72.95	3.43
2	-3	-7	61.92	2.90
-2	3	-7	62.37	3.02
-2	3	-7	57.31	3.11
-2	-3	7	71.43	3.46
2	-3	-7	59.30	2.86
-2	3	-7	63.61	2.83
2	-3	-7	61.20	3.02
-2	3	-7	61.25	2.96
2	-3	-7	59.93	2.96
-2	-3	7	74.04	3.84
2	3	7	72.96	3.50
-2	-3	7	74.13	3.63
-2	3	-7	63.91	2.82
2	3	7	77.31	3.66
2	-3	-7	59.29	3.03
2	3	7	72.20	3.58
2	3	7	71.84	3.55
-2	3	7	70.95	3.40
2	-3	7	79.32	3.73
2	-3	7	71.81	3.51
-2	3	7	67.93	3.47
2	-3	7	71.52	3.45
-2	-3	-7	67.81	3.02
2	-3	7	79.02	3.73
-2	-3	-7	59.21	3.06
-2	3	7	75.37	3.53
2	3	-7	59.45	3.12
2	3	-7	59.68	3.07
-2	3	7	76.02	3.53
-2	3	7	80.39	3.66
2	3	-7	58.66	3.03

-2	-3	-7	61.10	2.85
2	3	-7	63.98	2.89
2	-3	7	75.33	3.54
-2	-3	-7	56.50	2.94
-2	-3	-7	60.59	2.89
-2	3	7	70.17	3.61
2	4	7	7.99	0.54
-2	4	-7	7.92	0.93
-2	-4	7	7.96	1.00
-2	-4	7	8.11	0.77
2	4	7	7.84	0.58
-2	4	-7	10.71	1.05
-2	4	-7	9.94	0.75
2	4	7	6.66	0.71
-2	4	-7	9.71	0.61
-2	4	-7	10.77	0.61
-2	4	-7	9.19	0.80
2	4	7	7.08	0.72
2	-4	-7	10.79	0.74
-2	-4	7	7.64	0.88
2	-4	-7	10.68	0.90
2	4	7	7.44	0.80
2	-4	-7	10.05	0.68
-2	-4	7	7.74	0.65
2	-4	-7	9.11	0.76
-2	-4	7	7.23	0.62
-2	4	-7	10.92	0.93
2	-4	-7	10.10	0.87
2	4	7	8.05	0.62
2	4	7	7.53	0.81
2	-4	7	7.92	0.73
2	4	-7	10.12	0.96
-2	4	7	7.23	0.60
-2	4	7	7.24	0.73
2	4	-7	10.22	0.75
-2	-4	-7	8.44	0.75
-2	4	7	7.66	0.73
2	4	-7	10.82	0.77
2	4	-7	10.12	0.92
-2	-4	-7	10.17	0.88
2	-4	7	7.51	0.93
-2	4	7	7.38	0.62
2	-4	7	7.15	0.60
2	4	-7	9.11	0.89

-2	-4	-7	10.72	0.86
-2	4	7	7.57	0.99
2	-4	7	8.29	0.75
-2	-4	-7	8.37	0.89
-2	4	7	7.13	0.81
-2	4	7	9.03	0.68
-2	-4	-7	9.63	0.72
2	-4	7	8.08	0.98
-2	-5	7	60.19	3.02
2	5	7	52.49	2.90
2	5	7	54.88	2.92
2	5	7	54.34	2.82
2	-5	-7	55.97	2.79
2	-5	-7	56.26	2.86
-2	-5	7	58.47	2.95
2	-5	-7	53.82	2.95
2	5	7	51.84	2.69
-2	-5	7	53.87	2.85
-2	5	-7	59.62	2.68
-2	5	-7	54.42	3.06
-2	-5	7	51.20	2.72
-2	5	-7	57.70	3.19
-2	5	-7	60.31	2.93
2	-5	-7	56.44	2.81
-2	-5	7	52.03	2.70
-2	5	-7	58.32	2.85
2	-5	-7	59.93	3.05
-2	5	-7	57.95	2.91
2	5	7	55.86	2.60
2	5	7	54.54	2.74
2	5	7	52.35	2.59
-2	5	-7	60.61	2.69
-2	5	7	53.86	2.74
2	-5	7	54.76	2.78
2	5	-7	58.43	2.99
-2	5	7	52.91	2.85
-2	-5	-7	54.55	2.85
2	-5	7	60.80	3.10
-2	5	7	53.44	2.70
-2	5	7	57.23	2.78
-2	-5	-7	55.48	2.97
-2	5	7	55.50	2.69
-2	-5	-7	59.57	2.98
-2	5	7	55.56	2.99

-2	-5	-7	55.35	2.82
2	5	-7	59.57	2.89
2	-5	7	58.39	3.01
2	5	-7	58.25	2.84
2	5	-7	54.12	2.99
-2	-5	-7	58.88	2.93
2	5	-7	53.73	3.07
2	-5	7	53.92	2.71
2	-5	7	53.96	2.79
-2	6	-7	8.58	1.23
-2	-6	7	10.79	1.21
-2	-6	7	10.78	1.00
2	6	7	9.95	0.74
2	-6	-7	9.98	1.07
2	-6	-7	10.73	0.86
-2	-6	7	10.37	1.27
-2	6	-7	11.92	1.09
2	6	7	10.32	0.91
-2	6	-7	11.98	0.71
-2	6	-7	12.08	0.76
-2	-6	7	10.53	0.89
2	6	7	10.70	1.09
2	6	7	10.04	0.86
-2	6	-7	11.60	1.18
2	6	7	10.60	1.20
2	-6	-7	11.89	0.96
-2	6	-7	12.35	1.08
2	6	7	9.49	0.94
2	-6	-7	11.91	1.27
2	6	7	9.38	0.81
-2	6	-7	9.45	0.94
2	-6	-7	11.87	0.92
-2	-6	7	9.99	0.82
-2	-6	-7	10.74	0.88
2	-6	7	10.48	1.29
-2	6	7	10.40	0.85
-2	6	7	10.00	1.21
2	6	-7	11.29	1.11
-2	-6	-7	10.91	0.91
2	-6	7	9.27	0.82
-2	-6	-7	10.12	1.09
-2	6	7	10.90	0.95
-2	-6	-7	12.12	1.06
2	6	-7	11.56	1.21

2	-6	7	9.28	1.27
-2	6	7	10.56	1.14
2	6	-7	12.41	1.15
2	6	-7	12.06	1.02
-2	6	7	10.55	0.91
2	-6	7	11.71	1.40
-2	6	7	9.61	0.89
-2	6	7	10.59	0.97
-2	-6	-7	12.34	1.13
2	-6	7	9.41	0.92
2	6	-7	11.01	0.94
-2	-7	7	31.53	1.85
-2	7	-7	38.52	1.81
-2	7	-7	33.58	2.02
2	-7	-7	35.34	1.98
-2	-7	7	32.42	2.01
-2	7	-7	33.88	2.27
2	-7	-7	33.89	1.99
2	7	7	31.71	2.13
2	7	7	30.77	1.85
-2	-7	7	33.67	2.22
-2	7	-7	35.31	2.27
2	-7	-7	32.59	2.08
-2	7	-7	35.07	2.42
-2	7	-7	36.07	2.13
-2	-7	7	30.86	1.87
2	7	7	30.14	1.94
-2	-7	7	29.18	2.07
2	-7	-7	38.14	2.33
-2	-7	7	34.31	2.20
2	-7	-7	37.93	2.00
2	7	7	31.03	1.82
2	7	7	33.14	1.69
2	7	7	34.81	2.22
2	-7	7	35.87	2.35
-2	7	7	32.90	1.91
-2	-7	-7	38.83	2.20
-2	7	7	30.86	1.83
2	-7	7	31.34	1.83
-2	-7	-7	35.74	2.14
-2	7	7	29.14	2.16
-2	7	7	29.77	1.83
-2	-7	-7	36.75	2.00
-2	7	7	36.25	2.27

2	-7	7	31.43	1.87
-2	-7	-7	33.07	1.97
-2	7	7	34.46	1.90
2	-7	7	33.30	2.27
-2	-7	-7	33.66	2.17
2	7	-7	34.30	2.01
2	7	-7	35.52	2.17
2	7	-7	38.73	2.14
-2	7	7	30.38	1.87
2	-7	7	35.80	1.95
2	7	-7	34.96	2.38
-2	8	-7	14.61	1.72
2	-8	-7	15.92	1.44
-2	8	-7	16.08	1.61
-2	-8	7	16.85	1.74
2	-8	-7	18.48	1.36
2	8	7	17.76	1.38
2	8	7	16.07	1.33
2	8	7	17.90	1.18
2	8	7	17.80	1.50
-2	-8	7	18.05	1.54
2	8	7	16.50	1.58
2	8	7	17.84	2.04
2	-8	-7	14.49	1.25
-2	-8	7	18.91	1.37
-2	-8	7	17.47	1.75
2	-8	-7	18.33	1.73
-2	-8	7	17.64	1.82
-2	8	-7	19.50	1.62
-2	-8	7	18.36	1.38
2	-8	-7	16.33	1.27
-2	8	-7	18.97	1.09
2	8	7	18.76	1.22
-2	-8	-7	16.27	1.28
2	8	-7	16.47	1.51
-2	-8	-7	17.30	1.29
2	8	-7	18.66	1.50
-2	8	7	17.68	1.40
-2	8	7	18.35	1.35
2	-8	7	18.08	1.40
-2	8	7	19.22	1.86
-2	-8	-7	16.44	1.64
2	8	-7	17.02	1.74
2	-8	7	20.44	1.70

2	-8	7	20.99	1.89
-2	-8	-7	17.62	1.54
-2	8	7	17.94	1.72
2	-8	7	18.65	1.33
2	-8	7	19.20	1.98
-2	8	7	20.02	1.46
-2	8	7	19.41	1.46
2	-8	7	19.79	1.47
-2	8	7	16.60	1.37
-2	9	-7	28.39	2.44
-2	9	-7	28.06	2.17
2	9	7	26.82	1.59
2	-9	-7	32.16	2.34
2	-9	-7	25.17	1.94
2	9	7	24.07	1.59
-2	-9	7	23.08	1.81
2	-9	-7	25.32	1.74
2	-9	-7	30.12	1.90
2	9	7	25.30	2.15
-2	9	-7	29.96	2.16
-2	-9	7	27.81	1.98
-2	-9	7	26.21	2.27
-2	9	-7	31.84	1.61
2	9	7	25.41	1.75
2	-9	-7	27.54	1.79
-2	-9	7	24.75	1.83
2	9	7	21.90	1.88
2	9	7	28.23	2.37
2	9	-7	29.22	2.00
-2	9	7	25.67	1.81
-2	-9	-7	24.49	1.75
2	9	-7	28.86	2.05
-2	9	7	28.61	2.36
2	-9	7	25.38	2.11
-2	9	7	24.22	1.80
2	-9	7	26.51	1.83
2	9	-7	25.82	2.37
2	-9	7	30.70	2.50
2	-9	7	31.25	2.53
2	-9	7	26.79	1.87
-2	9	7	27.26	1.76
-2	-9	-7	27.78	2.27
-2	-9	-7	27.90	2.07
-2	-9	-7	24.80	1.90

-2	9	7	28.70	1.86
-2	-9	-7	26.42	1.83
-2	9	7	25.45	2.17
2	10	7	18.63	2.24
-2	-10	7	16.79	1.63
2	10	7	14.59	1.63
2	-10	-7	23.06	1.95
-2	-10	7	17.59	1.93
2	-10	-7	24.69	1.74
2	10	7	19.85	1.53
2	-10	-7	27.19	1.91
2	-10	-7	27.40	1.79
-2	10	-7	29.52	2.25
2	-10	-7	22.12	1.67
-2	10	-7	27.96	2.19
2	10	7	16.94	1.98
-2	-10	7	16.88	1.48
2	-10	-7	25.06	2.38
-2	10	-7	28.08	1.54
-2	10	-7	22.36	2.34
-2	-10	7	17.38	2.17
2	10	7	15.88	1.40
2	10	-7	22.35	1.96
2	-10	7	15.56	1.45
2	-10	7	19.87	1.65
2	-10	7	18.45	2.19
-2	-10	-7	23.50	1.81
2	10	-7	26.22	2.55
-2	10	7	17.22	1.61
-2	-10	-7	24.00	1.70
2	-10	7	14.26	1.57
-2	10	7	15.79	2.06
-2	10	7	17.38	2.24
-2	10	7	16.50	1.63
-2	-10	-7	24.52	1.90
2	10	-7	25.65	2.10
-2	-11	7	11.21	1.52
-2	-11	7	11.42	1.42
2	11	7	10.55	1.88
2	11	7	10.65	1.19
-2	11	-7	9.22	1.71
2	-11	-7	11.35	1.39
2	-11	-7	10.70	1.52
2	-11	-7	10.97	1.24

-2	11	-7	8.85	1.80
2	-11	-7	13.03	1.47
-2	-11	-7	11.52	1.38
2	11	-7	11.44	1.61
-2	11	7	9.10	1.53
2	11	-7	8.14	1.77
2	-11	7	11.46	1.57
-2	11	7	9.44	1.88
-2	-11	-7	11.71	1.34
2	-11	7	12.40	1.40
-2	11	7	10.37	1.87
2	-11	7	10.56	1.45
-2	12	-7	5.19	1.49
2	12	7	4.09	1.74
2	-12	-7	5.19	1.05
-2	12	-7	2.17	1.73
2	-12	-7	3.51	0.95
-2	-12	7	3.48	1.25
-2	-12	7	3.03	0.91
2	-12	-7	4.28	1.11
-2	12	7	2.50	1.57
2	12	-7	4.73	1.31
2	12	-7	2.42	1.78
-2	-12	-7	5.07	1.05
-2	12	7	4.89	1.15
2	-12	7	4.31	1.15
2	-12	7	2.91	0.94
-2	-12	-7	5.51	0.98
-2	-12	-7	2.91	1.25
-2	-13	7	19.14	2.61
-2	-13	7	16.42	1.80
2	-13	-7	21.30	1.93
2	-13	-7	18.46	1.78
-2	13	-7	17.44	2.87
-2	13	-7	22.37	2.78
2	13	7	13.87	2.33
2	13	-7	15.99	3.02
2	-13	7	18.40	2.24
2	13	-7	17.52	2.32
-2	-13	-7	23.41	2.41
2	-13	7	16.63	1.73
-2	-13	-7	22.17	1.82
-2	-13	-7	20.83	2.00
3	0	7	0.06	0.20

-3	0	-7	0.05	0.17
-3	0	-7	-0.06	0.13
3	0	-7	0.10	0.23
3	0	7	-0.66	0.31
-3	0	-7	-0.05	0.28
-3	0	7	0.00	0.28
3	0	-7	-0.04	0.11
-3	0	7	-0.13	0.29
3	0	7	-0.04	0.19
3	0	7	-0.11	0.28
3	0	-7	0.28	0.22
3	0	-7	0.08	0.19
-3	0	-7	0.01	0.22
-3	0	7	0.06	0.16
3	0	7	0.05	0.15
-3	0	7	-0.01	0.18
3	0	-7	0.25	0.26
-3	0	-7	-0.11	0.18
3	0	7	0.01	0.18
-3	0	-7	-0.21	0.27
3	1	7	33.37	2.00
3	-1	-7	36.28	1.78
-3	-1	7	37.71	1.96
-3	-1	7	41.30	2.02
3	-1	-7	35.00	1.86
-3	-1	7	39.16	1.88
-3	-1	7	39.90	1.85
3	-1	-7	36.85	1.79
3	1	7	34.39	1.92
3	1	7	37.27	1.95
-3	1	-7	33.25	1.80
3	1	7	36.19	1.96
-3	1	-7	36.53	1.85
3	-1	-7	38.87	1.89
-3	-1	7	36.97	1.80
3	1	7	41.05	2.13
-3	1	-7	35.03	1.79
3	1	7	39.17	1.91
-3	-1	7	41.30	2.08
-3	1	-7	38.24	1.75
-3	1	-7	34.90	1.91
3	1	-7	36.94	1.67
3	-1	7	36.91	1.95
-3	-1	-7	35.11	1.73

-3	-1	-7	35.27	1.93
-3	1	7	41.70	1.93
3	1	-7	35.49	1.71
-3	-1	-7	35.17	1.85
-3	1	7	38.74	1.93
-3	1	7	38.52	1.76
-3	1	7	41.69	2.05
3	-1	7	35.76	1.91
3	-1	7	41.67	2.09
-3	1	7	40.63	2.02
3	1	-7	36.88	1.86
3	-1	7	43.52	2.19
3	-1	7	40.68	1.89
-3	1	7	40.29	1.94
-3	-1	-7	34.56	1.82
3	1	-7	36.28	1.89
-3	2	-7	84.38	4.12
-3	2	-7	92.98	4.10
3	-2	-7	91.67	4.18
-3	-2	7	109.62	4.97
-3	2	-7	86.00	4.28
3	-2	-7	85.17	4.17
3	2	7	110.77	4.99
3	-2	-7	81.43	3.99
-3	-2	7	100.05	4.73
3	-2	-7	86.77	4.08
-3	2	-7	95.16	4.19
-3	2	-7	89.74	4.14
-3	-2	7	106.24	4.69
3	2	7	98.01	4.85
-3	-2	7	98.90	4.76
-3	2	-7	91.76	4.35
3	2	7	107.02	4.78
3	-2	-7	82.20	4.12
3	2	7	101.47	4.87
3	-2	-7	87.24	4.21
-3	-2	7	97.42	4.87
-3	-2	-7	88.75	4.21
3	-2	7	104.30	4.85
3	2	-7	90.33	4.26
3	-2	7	112.62	5.05
3	-2	7	99.56	4.77
-3	2	7	98.58	4.87
-3	2	7	108.34	4.89

-3	-2	-7	90.80	4.25
-3	-2	-7	82.79	4.12
3	2	-7	90.76	4.04
-3	2	7	109.57	4.93
3	2	-7	88.27	3.99
3	-2	7	105.44	4.75
-3	2	7	100.32	4.69
-3	-2	-7	90.28	4.08
-3	-2	-7	92.66	4.16
3	2	-7	84.59	4.22
-3	-2	-7	89.65	4.08
-3	-2	-7	85.23	4.07
-3	2	7	100.94	4.78
3	-3	-7	348.74	15.79
-3	-3	7	326.36	14.18
-3	-3	7	290.00	14.03
3	-3	-7	348.98	15.96
-3	-3	7	323.72	14.27
3	-3	-7	339.63	15.87
-3	3	-7	356.99	15.84
-3	3	-7	356.27	15.89
-3	3	-7	372.09	15.91
-3	-3	7	298.39	14.16
3	3	7	295.31	14.15
3	3	7	299.90	14.16
3	3	7	326.22	13.99
3	-3	-7	335.43	15.76
3	3	7	333.58	14.25
-3	3	-7	348.11	16.04
-3	3	-7	361.59	15.76
-3	3	-7	375.76	16.14
3	3	7	308.09	14.08
3	-3	-7	361.55	15.96
-3	-3	7	303.93	14.02
3	-3	7	296.61	14.05
-3	3	7	327.90	14.11
3	-3	7	304.06	14.11
3	3	-7	344.27	16.00
-3	3	7	308.64	14.20
-3	3	7	288.11	14.54
-3	3	7	314.13	13.99
-3	-3	-7	337.54	16.00
-3	-3	-7	344.71	15.74
3	-3	7	317.36	14.12

3	3	-7	358.12	15.79
-3	3	7	324.04	14.14
-3	-3	-7	334.45	15.85
-3	-3	-7	348.28	15.96
-3	3	7	295.16	14.03
-3	-3	-7	369.31	15.94
-3	-3	-7	333.61	15.81
3	3	-7	357.67	15.95
3	3	-7	351.91	15.97
3	-3	7	335.20	14.28
-3	3	7	332.29	14.12
3	-4	-7	240.22	10.73
3	4	7	200.74	9.10
-3	4	-7	241.89	10.58
3	-4	-7	220.80	10.60
3	-4	-7	237.96	10.52
-3	4	-7	238.41	10.88
3	-4	-7	223.21	10.69
-3	-4	7	210.02	9.27
-3	-4	7	191.87	9.10
3	4	7	197.07	8.97
-3	-4	7	212.91	9.34
-3	4	-7	223.78	10.48
-3	4	-7	240.00	10.64
-3	4	-7	229.83	10.80
-3	-4	7	191.66	9.25
3	4	7	205.64	9.31
3	4	7	181.98	9.23
-3	4	-7	239.39	10.48
3	-4	-7	226.01	10.51
-3	-4	7	191.81	9.10
-3	4	-7	243.48	10.66
3	4	7	193.17	9.22
3	4	7	202.20	9.13
3	4	7	207.72	9.00
-3	-4	-7	220.05	10.59
-3	4	7	191.74	9.06
3	-4	7	203.79	9.18
-3	4	7	207.82	9.21
-3	4	7	203.99	9.16
3	4	-7	235.28	10.55
-3	4	7	168.61	9.13
-3	-4	-7	239.58	10.67
3	4	-7	235.08	10.70

3	-4	7	221.93	9.45
-3	-4	-7	232.50	10.56
-3	4	7	186.78	9.28
3	-4	7	220.05	9.38
3	-4	7	187.95	9.11
-3	-4	-7	244.89	10.69
3	4	-7	228.29	10.71
3	4	-7	223.07	10.76
-3	4	7	211.72	9.32
-3	-4	-7	218.53	10.68
-3	4	7	209.01	9.18
-3	5	-7	78.75	3.78
3	-5	-7	80.85	3.92
3	5	7	67.49	3.35
3	-5	-7	71.95	3.72
3	5	7	65.95	3.45
-3	-5	7	73.98	3.60
3	-5	-7	80.05	3.66
-3	5	-7	81.06	3.59
3	-5	-7	76.39	3.66
3	-5	-7	74.26	3.83
-3	-5	7	63.36	3.32
-3	5	-7	78.27	3.72
-3	5	-7	77.78	3.57
-3	-5	7	65.40	3.33
3	5	7	73.08	3.58
3	5	7	67.08	3.31
-3	5	-7	81.27	3.82
-3	5	-7	72.92	3.95
-3	-5	7	65.38	3.48
3	5	7	67.74	3.52
-3	5	-7	76.25	4.03
-3	-5	7	72.21	3.54
-3	5	7	67.86	3.30
-3	5	7	72.06	3.42
-3	-5	-7	78.07	3.83
3	-5	7	67.29	3.40
-3	-5	-7	78.31	3.71
3	5	-7	72.65	3.92
-3	-5	-7	73.60	3.84
-3	-5	-7	74.83	3.72
3	5	-7	77.15	3.74
-3	5	7	67.71	3.30
3	-5	7	64.09	3.32

-3	5	7	69.76	3.40
-3	-5	-7	77.22	3.83
3	5	-7	78.71	3.70
-3	5	7	71.69	3.58
3	5	-7	77.93	3.86
3	-5	7	72.94	3.63
3	-5	7	71.51	3.69
-3	5	7	69.65	3.62
3	-5	7	69.85	3.39
3	5	-7	72.75	3.84
-3	5	7	68.70	3.38
3	6	7	1.03	0.71
-3	6	-7	2.67	0.60
-3	6	-7	2.77	0.40
-3	6	-7	2.79	0.50
-3	-6	7	1.38	0.62
-3	-6	7	1.60	0.56
-3	6	-7	3.00	0.56
3	-6	-7	2.49	0.48
3	-6	-7	2.56	0.46
3	6	7	1.29	0.42
3	-6	-7	3.20	0.64
3	6	7	0.84	0.52
-3	-6	7	1.64	0.43
3	-6	-7	3.33	0.52
-3	6	-7	3.98	0.72
3	-6	-7	2.13	0.70
3	6	7	1.38	0.51
-3	-6	7	1.90	0.78
-3	-6	7	1.47	0.43
-3	-6	7	1.52	0.69
-3	6	-7	2.23	0.70
3	6	7	1.27	0.46
-3	6	-7	3.32	0.82
3	6	7	1.75	0.32
3	6	-7	2.79	0.54
-3	-6	-7	2.96	0.51
-3	6	7	1.64	0.52
-3	-6	-7	3.20	0.64
3	-6	7	1.77	0.72
-3	-6	-7	2.98	0.66
3	6	-7	1.99	0.65
-3	6	7	1.76	0.43
-3	6	7	1.56	0.50

-3	6	7	1.49	0.64
-3	6	7	0.92	0.61
3	6	-7	3.40	0.64
3	6	-7	2.94	0.58
-3	6	7	0.84	0.45
-3	-6	-7	2.88	0.63
-3	-6	-7	3.33	0.54
3	-6	7	2.11	0.52
3	-6	7	0.57	0.35
3	-6	7	1.20	0.38
-3	6	7	1.89	0.45
3	-6	7	0.69	0.70
3	-7	-7	4.79	0.90
3	7	7	5.62	0.95
3	-7	-7	5.89	0.79
3	7	7	6.28	0.87
3	7	7	6.99	0.85
3	7	7	8.44	1.28
3	-7	-7	7.91	1.25
-3	-7	7	8.28	1.33
3	7	7	6.20	0.67
-3	-7	7	7.07	1.02
-3	-7	7	8.24	1.20
-3	-7	7	6.41	0.81
-3	-7	7	6.71	1.19
-3	7	-7	7.13	1.00
-3	-7	7	6.21	0.80
3	7	7	5.85	1.02
-3	7	-7	7.36	1.19
3	-7	-7	5.15	0.74
-3	7	-7	6.63	1.31
-3	7	-7	6.92	1.08
3	7	7	7.00	0.62
3	-7	-7	8.09	0.86
-3	7	-7	5.15	0.92
-3	7	7	7.17	1.25
3	7	-7	6.18	0.86
-3	-7	-7	7.12	1.07
-3	-7	-7	5.99	0.78
-3	7	7	6.14	0.93
-3	-7	-7	6.83	1.08
3	-7	7	6.00	0.85
-3	7	7	5.39	1.07
-3	7	7	6.72	0.99

-3	7	7	6.04	0.88
3	7	-7	5.46	0.95
3	7	-7	6.01	0.92
3	7	-7	6.68	1.16
-3	-7	-7	5.26	0.79
3	-7	7	6.88	1.08
-3	7	7	6.40	0.95
-3	7	7	5.57	0.82
3	-7	7	5.71	0.88
-3	-7	-7	6.80	0.99
3	-7	7	7.97	1.41
3	-7	7	5.44	0.74
-3	-8	7	27.66	2.14
3	8	7	27.02	1.93
-3	-8	7	28.47	1.83
3	8	7	29.69	1.56
3	-8	-7	26.39	1.60
-3	-8	7	28.01	1.81
3	8	7	28.73	1.80
3	-8	-7	27.29	2.06
-3	8	-7	25.45	2.00
-3	-8	7	28.85	1.98
3	-8	-7	22.89	1.57
3	-8	-7	24.13	1.74
-3	8	-7	21.69	2.09
-3	8	-7	27.78	1.50
-3	-8	7	31.13	2.24
-3	-8	7	33.36	2.44
3	8	7	27.97	2.13
-3	8	-7	26.54	1.87
3	-8	-7	23.12	1.59
3	8	7	30.10	2.21
-3	8	7	30.05	1.91
-3	8	7	28.80	1.86
-3	8	7	28.57	1.75
3	-8	7	29.93	1.85
-3	-8	-7	23.94	1.88
-3	-8	-7	23.32	1.58
-3	-8	-7	22.48	1.61
3	8	-7	22.84	1.76
-3	8	7	27.18	2.09
3	-8	7	27.64	1.89
3	-8	7	30.99	2.05
-3	8	7	30.71	1.92

3	-8	7	32.79	2.45
3	8	-7	28.05	1.78
3	8	-7	22.30	2.04
-3	8	7	25.82	1.81
-3	-8	-7	24.58	1.84
-3	8	7	29.92	2.17
3	-8	7	28.96	1.78
3	-8	7	29.76	2.28
-3	-8	-7	25.26	1.85
3	9	7	31.88	1.67
-3	-9	7	33.27	2.42
-3	9	-7	39.49	2.64
3	9	7	29.30	1.91
3	-9	-7	37.73	2.23
-3	-9	7	26.09	2.06
3	-9	-7	42.25	2.25
3	-9	-7	35.15	2.40
-3	9	-7	41.39	2.57
-3	9	-7	37.67	2.92
3	9	7	28.67	2.10
-3	-9	7	28.44	1.97
3	9	7	30.12	2.51
-3	9	-7	41.87	2.08
3	-9	-7	39.13	2.64
3	9	7	28.45	2.28
-3	-9	7	28.68	2.10
3	9	7	29.87	2.04
3	-9	-7	37.54	2.22
3	9	-7	36.66	2.81
-3	9	7	28.43	2.37
-3	9	7	32.58	2.08
3	-9	7	32.40	2.22
-3	-9	-7	36.45	2.46
-3	-9	-7	38.57	2.29
3	9	-7	39.00	2.46
3	9	-7	36.50	2.41
3	-9	7	31.08	1.98
3	-9	7	27.88	1.97
3	-9	7	32.33	2.67
-3	-9	-7	36.30	2.23
-3	9	7	29.21	2.04
-3	9	7	32.33	2.53
3	-9	7	32.13	1.95
-3	-9	-7	39.82	2.51

3	-10	-7	38.48	2.34
3	-10	-7	41.50	2.33
-3	10	-7	37.45	3.10
-3	10	-7	43.35	2.82
3	10	7	34.55	1.90
-3	10	-7	42.87	2.17
3	-10	-7	40.16	2.37
3	-10	-7	35.02	2.45
-3	-10	7	35.90	2.75
-3	-10	7	32.69	2.17
-3	-10	7	32.95	2.37
3	-10	-7	36.13	2.92
3	10	7	36.67	1.91
3	10	7	33.59	2.36
3	10	7	39.82	2.91
3	-10	-7	39.81	2.39
3	10	7	30.28	2.59
-3	-10	7	35.35	2.24
-3	10	-7	38.46	2.83
-3	10	7	28.94	2.66
3	10	-7	39.81	2.55
-3	10	7	36.57	2.33
3	-10	7	34.47	2.19
-3	10	7	37.77	2.92
3	-10	7	35.49	2.24
3	10	-7	44.45	2.68
-3	10	7	33.55	2.32
-3	-10	-7	37.53	2.30
3	-10	7	41.14	2.61
3	-10	7	31.55	2.23
-3	-10	-7	38.66	2.44
3	-11	-7	34.30	2.19
-3	-11	7	35.19	2.60
-3	-11	7	34.56	2.28
3	-11	-7	37.70	2.30
3	11	7	32.32	2.80
-3	11	-7	35.70	3.00
3	-11	-7	40.98	2.30
3	-11	-7	31.99	2.48
-3	11	-7	35.34	2.77
-3	-11	-7	34.73	2.23
-3	-11	-7	32.22	2.32
-3	11	7	37.01	2.47
3	-11	7	33.26	2.33

-3	11	7	30.77	2.89
3	11	-7	36.02	2.48
3	-11	7	34.07	2.29
3	-11	7	36.47	2.37
3	11	-7	35.49	3.12
-3	-12	7	9.84	1.52
-3	-12	7	12.24	1.66
3	-12	-7	6.13	1.26
-3	12	-7	5.75	2.28
-3	12	-7	7.89	1.63
3	-12	-7	7.94	1.17
3	12	7	9.39	1.99
3	-12	-7	8.96	1.31
-3	-12	-7	9.26	1.31
-3	-12	-7	8.20	1.36
3	12	-7	8.78	1.68
3	-12	7	9.59	1.47
-3	-12	-7	10.27	1.36
3	-12	7	11.29	1.63
-3	12	7	13.20	2.50
-3	12	7	13.44	1.87
3	12	-7	7.21	1.89
3	-13	-7	7.16	1.18
-3	13	-7	5.67	2.26
-3	-13	7	5.86	1.06
3	-13	-7	6.80	1.13
-3	13	-7	9.94	2.07
-3	-13	7	9.35	1.68
3	13	7	8.57	2.37
3	-13	7	6.78	1.47
3	13	-7	8.48	2.28
-3	-13	-7	7.17	1.22
-3	-13	-7	4.96	1.03
3	-13	7	5.13	1.02
-3	-13	-7	10.02	1.86
-4	0	-7	53.47	2.66
4	0	7	42.75	2.15
-4	0	-7	56.56	2.85
4	0	7	43.42	2.19
-4	0	7	46.01	2.03
-4	0	-7	60.09	2.65
-4	0	7	44.69	2.16
4	0	7	47.84	2.41
-4	0	7	43.60	2.03

4	0	7	44.19	2.17
-4	0	-7	57.06	2.68
-4	0	-7	53.04	2.72
-4	0	7	49.71	2.28
4	0	-7	54.53	2.69
4	0	-7	56.60	2.77
-4	0	7	41.46	2.28
-4	0	7	45.47	2.16
4	0	7	45.40	2.28
4	0	-7	55.24	2.74
-4	1	-7	20.23	1.17
-4	-1	7	12.01	0.66
4	-1	-7	17.90	1.09
-4	-1	7	13.46	0.88
4	-1	-7	21.32	0.95
-4	-1	7	12.91	0.73
4	1	7	13.18	0.75
4	-1	-7	21.53	1.21
4	1	7	12.58	0.84
-4	1	-7	20.19	1.09
4	1	7	11.10	0.83
4	1	7	13.95	1.03
4	1	7	12.55	0.79
-4	1	-7	19.11	1.25
-4	-1	7	12.67	0.73
-4	-1	7	13.82	0.99
4	-1	-7	20.05	1.20
-4	1	-7	20.83	1.09
-4	1	-7	19.51	1.31
-4	-1	7	12.38	0.84
-4	1	-7	20.61	1.12
4	-1	-7	21.37	1.12
4	1	7	12.67	0.79
4	-1	7	13.72	0.77
-4	1	7	12.54	0.80
4	-1	7	13.44	1.03
-4	1	7	14.37	0.92
-4	-1	-7	19.62	1.17
4	1	-7	21.34	1.20
-4	1	7	12.38	0.80
4	-1	7	12.95	0.82
4	1	-7	20.95	0.98
4	1	-7	19.99	1.04
-4	-1	-7	20.21	1.08

-4	-1	-7	20.64	1.13
4	1	-7	20.53	1.06
-4	-1	-7	19.64	1.19
-4	-1	-7	20.26	1.27
4	1	-7	20.29	1.22
-4	1	7	13.14	0.63
-4	-1	-7	20.36	1.12
-4	1	7	14.33	0.94
4	-1	7	11.99	0.79
-4	1	7	14.12	0.82
4	-1	7	12.82	0.98
4	-1	7	12.46	0.75
-4	2	-7	27.41	1.74
4	2	7	17.90	1.17
-4	2	-7	31.05	1.55
-4	-2	7	19.38	1.02
-4	-2	7	20.28	1.18
-4	2	-7	30.98	1.61
4	-2	-7	30.69	1.63
-4	2	-7	29.95	1.83
-4	2	-7	29.68	1.50
4	-2	-7	28.49	1.41
4	-2	-7	30.24	1.64
4	2	7	20.71	1.31
4	2	7	21.29	1.12
-4	-2	7	18.51	1.19
-4	-2	7	19.90	1.02
4	-2	-7	28.64	1.56
-4	2	-7	29.66	1.56
4	2	7	18.74	1.06
4	2	7	18.18	1.12
4	-2	-7	30.51	1.50
-4	-2	7	19.97	0.98
-4	2	7	19.74	1.13
4	2	-7	30.76	1.68
4	-2	7	19.02	1.10
4	-2	7	20.31	1.05
-4	-2	-7	32.23	1.53
4	-2	7	20.40	1.39
-4	-2	-7	29.09	1.51
-4	2	7	19.21	0.97
4	2	-7	28.55	1.64
4	2	-7	30.63	1.46
-4	-2	-7	29.20	1.57

-4	2	7	18.32	1.32
-4	-2	-7	30.51	1.68
-4	-2	-7	28.18	1.50
4	-2	7	19.52	1.12
4	2	-7	29.82	1.42
4	2	-7	30.22	1.49
-4	2	7	19.21	1.06
4	2	-7	28.76	1.66
4	-2	7	19.81	1.31
4	-2	7	19.09	1.06
-4	-2	-7	29.23	1.64
-4	2	7	19.82	1.15
-4	-2	-7	30.99	1.57
-4	3	-7	49.14	2.40
-4	3	-7	51.08	2.28
-4	3	-7	49.70	2.28
-4	3	-7	50.15	2.36
4	-3	-7	50.86	2.48
4	-3	-7	47.06	2.45
4	3	7	46.27	2.35
4	3	7	50.07	2.57
-4	-3	7	51.13	2.60
4	3	7	44.82	2.45
4	3	7	48.24	2.27
-4	-3	7	48.05	2.33
4	-3	-7	40.67	2.28
4	3	7	44.23	2.46
-4	-3	7	48.75	2.46
-4	-3	7	48.27	2.32
-4	-3	7	45.39	2.48
4	-3	-7	47.33	2.27
-4	3	-7	45.36	2.55
4	-3	-7	44.61	2.36
-4	3	-7	48.44	2.66
-4	3	-7	52.42	2.41
4	3	7	47.17	2.39
-4	3	7	47.47	2.29
-4	3	7	50.34	2.44
-4	3	7	47.30	2.31
4	-3	7	44.89	2.35
4	-3	7	51.58	2.71
-4	-3	-7	46.96	2.46
4	3	-7	49.88	2.29
4	3	-7	46.55	2.49

-4	3	7	51.72	2.50
4	3	-7	46.34	2.48
-4	3	7	48.96	2.52
4	3	-7	39.94	2.28
-4	-3	-7	49.52	2.47
-4	-3	-7	48.90	2.40
-4	-3	-7	47.26	2.33
-4	3	7	53.41	2.57
4	3	-7	46.88	2.47
-4	-3	-7	45.72	2.36
-4	3	7	51.12	2.45
4	-3	7	46.74	2.41
4	-3	7	52.12	2.63
4	-3	7	45.29	2.40
4	4	7	7.05	0.88
4	-4	-7	8.91	0.76
-4	4	-7	9.98	0.64
4	4	7	5.54	0.72
-4	4	-7	9.17	0.76
-4	-4	7	6.78	0.59
4	-4	-7	9.42	0.66
-4	4	-7	10.08	0.63
-4	-4	7	6.29	0.76
4	4	7	6.65	0.68
-4	4	-7	9.88	0.82
4	4	7	5.93	0.70
4	4	7	7.97	0.63
-4	-4	7	6.62	0.60
-4	-4	7	7.62	0.82
-4	4	-7	9.04	1.35
4	-4	-7	9.89	0.87
4	-4	-7	9.65	0.91
-4	4	-7	7.08	0.95
-4	-4	7	7.12	0.99
-4	4	-7	9.37	0.85
4	-4	-7	9.21	0.64
-4	4	7	7.36	0.92
-4	4	7	6.82	0.76
-4	-4	-7	9.43	0.81
-4	4	7	6.89	0.58
4	-4	7	7.35	0.71
-4	4	7	7.42	0.61
4	4	-7	8.83	0.88
4	-4	7	5.87	0.98

4	4	-7	9.43	0.93
-4	-4	-7	9.45	0.91
-4	4	7	7.45	0.72
4	-4	7	5.95	0.60
4	4	-7	8.93	0.71
-4	-4	-7	8.96	0.71
4	4	-7	9.35	0.95
-4	4	7	8.35	0.91
4	-4	7	6.87	0.66
-4	-4	-7	8.37	0.75
-4	-4	-7	8.65	0.86
-4	4	7	8.12	0.82
4	-4	7	7.45	1.07
-4	-5	7	8.70	1.10
4	5	7	8.13	0.79
-4	5	-7	5.33	0.72
4	5	7	9.41	1.02
-4	-5	7	7.47	0.68
-4	5	-7	5.89	0.80
4	5	7	7.07	0.84
4	-5	-7	6.39	0.77
4	5	7	7.20	0.85
-4	5	-7	4.84	0.54
-4	-5	7	7.41	0.90
-4	-5	7	7.92	0.69
-4	5	-7	5.17	0.50
4	-5	-7	5.72	0.56
4	-5	-7	6.00	0.57
4	5	7	8.28	0.73
-4	5	-7	4.46	0.70
4	-5	-7	6.13	0.67
-4	5	-7	4.61	0.92
4	-5	-7	6.16	0.94
-4	5	-7	5.78	1.02
-4	-5	7	7.90	0.97
-4	5	7	8.44	0.71
-4	-5	-7	5.23	0.62
-4	-5	-7	5.44	0.80
-4	5	7	8.80	0.88
-4	-5	-7	5.06	0.77
4	-5	7	8.00	0.77
-4	-5	-7	3.98	0.64
4	5	-7	5.12	0.88
-4	5	7	8.46	1.04

4	5	-7	6.56	0.78
-4	5	7	8.76	1.03
-4	5	7	8.36	0.81
-4	-5	-7	5.82	0.84
-4	5	7	8.07	0.84
4	-5	7	7.15	1.09
4	-5	7	7.75	0.69
4	-5	7	7.10	0.76
4	5	-7	5.38	0.65
4	5	-7	5.33	0.87
-4	-6	7	18.16	1.17
-4	6	-7	13.55	1.35
4	6	7	18.30	1.46
4	6	7	17.33	1.38
-4	-6	7	20.17	1.60
-4	-6	7	17.91	1.49
4	-6	-7	12.92	0.93
4	-6	-7	12.60	1.13
-4	6	-7	12.79	1.50
4	6	7	16.21	1.18
4	6	7	17.65	1.18
-4	-6	7	17.55	1.38
-4	6	-7	14.70	1.22
-4	6	-7	13.51	0.85
-4	-6	7	17.54	1.39
4	-6	-7	13.06	0.91
-4	6	-7	14.76	1.08
-4	-6	7	17.48	1.15
4	6	7	15.63	1.27
-4	6	-7	14.03	1.14
4	-6	-7	12.25	1.01
4	-6	-7	14.93	1.35
-4	6	-7	14.41	0.90
4	-6	7	18.32	1.21
4	6	-7	13.44	1.01
-4	6	7	18.05	1.50
4	6	-7	13.85	1.19
-4	6	7	18.76	1.28
-4	-6	-7	11.55	0.98
-4	-6	-7	13.05	1.21
-4	6	7	16.61	1.08
-4	-6	-7	11.36	1.19
-4	6	7	18.45	1.29
4	6	-7	11.95	1.26

4	-6	7	15.94	1.14
4	-6	7	16.05	1.23
-4	6	7	17.81	1.17
-4	6	7	17.55	1.32
4	-6	7	17.07	1.62
4	6	-7	11.91	1.12
-4	6	7	17.42	1.76
-4	-6	-7	11.75	0.99
4	-6	7	20.02	1.78
-4	-6	-7	13.71	1.17
4	7	7	89.84	4.49
4	7	7	88.07	4.79
-4	7	-7	83.74	4.47
-4	-7	7	92.45	4.77
4	7	7	89.20	4.69
4	-7	-7	80.73	4.28
-4	-7	7	93.67	4.73
-4	7	-7	85.39	4.18
-4	7	-7	94.25	4.02
4	7	7	95.59	4.82
4	7	7	91.24	4.51
-4	-7	7	91.06	4.52
4	-7	-7	82.83	4.10
4	-7	-7	87.62	4.46
-4	7	-7	85.57	4.20
-4	7	-7	90.46	4.33
-4	-7	7	94.50	4.74
4	-7	-7	82.31	4.15
-4	-7	7	94.72	4.55
-4	7	-7	78.10	4.55
-4	-7	7	99.49	4.81
-4	-7	-7	80.49	4.27
4	-7	7	91.61	4.83
4	-7	7	101.67	4.97
-4	7	7	90.89	4.60
-4	7	7	87.81	4.45
-4	-7	-7	88.46	4.31
-4	7	7	101.10	4.86
-4	7	7	90.16	4.85
4	-7	7	95.90	4.56
4	-7	7	89.41	4.62
-4	7	7	98.56	4.61
-4	-7	-7	83.84	4.31
-4	7	7	93.62	4.52

4	7	-7	84.20	4.26
4	7	-7	87.25	4.24
4	7	-7	82.85	4.14
-4	-7	-7	81.49	4.15
4	7	-7	81.53	4.48
-4	7	7	98.25	4.63
4	-7	7	89.69	4.53
-4	-7	-7	80.71	4.12
4	8	7	92.97	4.69
-4	8	-7	91.55	4.85
4	8	7	79.46	4.48
4	-8	-7	101.00	4.43
4	8	7	82.52	4.62
-4	-8	7	85.85	4.33
-4	8	-7	101.56	4.74
4	-8	-7	87.73	4.48
-4	-8	7	94.35	4.64
4	-8	-7	84.07	4.63
-4	-8	7	87.06	4.34
-4	-8	7	93.81	4.90
-4	-8	7	85.39	4.53
-4	8	-7	98.68	4.36
4	8	7	87.03	4.28
4	-8	-7	89.01	4.47
4	-8	-7	95.68	4.83
-4	8	-7	84.62	4.96
-4	8	7	82.40	4.22
-4	-8	-7	88.41	4.52
-4	-8	-7	87.90	4.47
-4	-8	-7	87.95	4.65
4	8	-7	89.86	4.62
4	-8	7	89.07	4.67
4	-8	7	91.20	4.35
4	-8	7	82.02	4.38
-4	8	7	89.33	4.66
4	-8	7	82.69	4.31
-4	8	7	85.62	4.41
-4	8	7	92.64	4.44
-4	-8	-7	88.83	4.66
4	8	-7	84.73	4.87
-4	-8	-7	91.12	4.69
-4	8	7	80.60	4.70
4	-8	7	95.77	4.91
4	8	-7	97.15	4.64

-4	8	7	86.30	4.31
-4	8	7	88.38	4.38
-4	9	-7	97.86	4.40
-4	9	-7	82.59	4.86
4	-9	-7	88.05	4.53
-4	9	-7	98.65	4.85
4	-9	-7	90.39	4.73
4	-9	-7	90.45	4.44
-4	-9	7	94.15	4.75
4	-9	-7	91.44	4.53
4	9	7	82.30	4.60
4	9	7	82.20	4.35
-4	-9	7	84.10	4.42
-4	-9	7	87.10	4.65
4	-9	-7	98.59	5.02
4	9	7	92.86	4.81
4	9	7	82.44	4.80
4	-9	-7	101.75	4.49
-4	9	-7	90.10	5.13
4	-9	7	86.30	4.51
4	-9	7	89.57	4.45
-4	9	7	93.40	4.54
4	9	-7	88.77	4.66
-4	9	7	89.31	4.55
-4	9	7	79.40	4.82
-4	-9	-7	84.95	4.50
4	-9	7	90.82	4.46
4	-9	7	92.88	5.06
-4	-9	-7	83.92	4.67
4	9	-7	96.50	4.74
4	-9	7	95.08	4.59
-4	9	7	93.71	4.91
4	9	-7	83.41	4.96
4	-10	-7	51.05	2.93
-4	-10	7	51.41	3.23
4	-10	-7	51.60	2.90
-4	10	-7	55.51	3.23
4	-10	-7	53.54	3.51
4	-10	-7	55.69	2.87
4	10	7	53.37	3.50
-4	-10	7	54.76	3.07
4	10	7	60.77	3.74
4	-10	-7	53.58	2.83
-4	-10	7	53.69	3.48

-4	10	-7	46.42	3.58
4	10	7	50.24	3.24
-4	10	-7	52.43	4.00
-4	10	-7	59.13	2.76
4	-10	-7	46.39	3.08
-4	-10	7	50.55	3.01
-4	-10	-7	51.52	3.17
4	10	-7	53.61	3.09
-4	-10	-7	49.79	2.86
4	-10	7	49.32	3.08
4	10	-7	47.14	3.44
4	-10	7	59.79	3.08
-4	-10	-7	53.61	3.02
4	-10	7	55.18	3.03
-4	10	7	54.18	3.18
-4	10	7	59.50	3.66
-4	10	7	45.92	3.36
-4	10	7	57.77	3.21
4	10	-7	56.39	3.19
4	-10	7	58.74	3.32
4	-11	-7	7.07	1.21
-4	11	-7	5.96	1.46
-4	-11	7	3.33	0.97
4	-11	-7	5.63	1.16
4	-11	-7	6.43	1.19
-4	-11	7	3.98	1.13
-4	11	-7	2.88	1.54
4	11	7	2.93	1.09
4	11	7	4.64	1.43
4	-11	-7	5.82	0.97
4	11	-7	8.71	1.51
4	11	-7	5.66	1.50
-4	11	7	6.30	1.23
4	-11	7	5.62	1.03
-4	-11	-7	5.45	0.96
4	-11	7	6.63	1.25
4	-11	7	4.64	0.93
-4	-11	-7	5.06	1.01
-4	11	7	3.13	1.50
4	-12	-7	6.38	1.21
4	-12	-7	5.71	1.05
-4	12	-7	3.90	2.05
-4	-12	7	4.95	1.58
-4	-12	7	6.03	1.05

-4	12	-7	7.82	1.63
4	12	7	4.28	1.68
4	12	-7	3.75	2.00
4	-12	7	4.80	1.03
4	12	-7	7.13	1.38
-4	-12	-7	7.38	1.20
-4	-12	-7	6.20	1.05
4	-12	7	7.99	1.50
-4	12	7	2.78	1.38
-4	-12	-7	5.94	1.38
-4	13	-7	31.24	3.55
4	13	7	37.29	3.82
4	-13	-7	28.90	2.25
4	-13	-7	31.19	2.33
-4	-13	7	33.83	2.94
-4	-13	7	32.44	2.40
4	13	-7	30.25	3.40
-4	-13	-7	29.20	2.31
4	-13	7	36.89	2.89
-4	-13	-7	33.72	3.08
-4	-13	-7	26.92	2.57
-5	0	-7	-0.12	0.19
5	0	7	-0.02	0.24
-5	0	-7	0.11	0.20
5	0	-7	-0.03	0.18
5	0	7	-0.24	0.35
-5	0	-7	-0.05	0.35
-5	0	7	-0.03	0.21
5	0	7	-0.13	0.29
-5	0	-7	0.16	0.29
5	0	-7	-0.13	0.16
-5	0	-7	0.18	0.27
5	0	-7	-0.19	0.26
-5	0	7	0.03	0.18
5	0	7	-0.14	0.15
-5	0	-7	-0.06	0.20
-5	0	7	-0.46	0.56
-5	0	7	0.12	0.11
5	0	-7	-0.01	0.25
5	0	-7	-0.02	0.26
-5	0	7	0.06	0.10
5	0	7	0.07	0.18
-5	0	7	0.25	0.22
-5	1	-7	51.06	2.53

-5	-1	7	56.33	2.64
-5	1	-7	52.58	2.68
-5	-1	7	56.83	2.62
5	-1	-7	49.64	2.47
-5	-1	7	55.40	2.72
-5	1	-7	53.33	2.49
5	-1	-7	49.87	2.53
5	1	7	54.92	2.77
-5	1	-7	48.13	2.61
5	1	7	55.09	2.68
5	1	7	56.61	2.72
5	-1	-7	49.60	2.58
5	1	7	52.92	2.71
-5	-1	7	52.40	2.58
5	-1	-7	49.51	2.46
-5	1	-7	54.48	2.49
-5	1	-7	49.80	2.46
5	-1	-7	50.67	2.33
5	1	7	63.63	2.94
-5	-1	7	55.69	2.59
-5	-1	7	51.43	2.74
-5	1	7	59.27	2.72
5	1	-7	50.52	2.52
-5	-1	-7	55.48	2.47
5	1	-7	51.43	2.55
-5	1	7	55.13	2.83
-5	-1	-7	52.65	2.59
-5	1	7	53.88	2.68
5	-1	7	54.75	2.70
-5	-1	-7	46.80	2.52
-5	1	7	53.83	2.55
-5	-1	-7	52.50	2.49
5	-1	7	61.52	2.99
5	-1	7	56.52	2.84
-5	1	7	57.69	2.71
-5	-1	-7	51.14	2.49
5	1	-7	52.67	2.41
5	-1	7	56.69	2.74
5	1	-7	48.31	2.58
-5	-1	-7	50.65	2.61
5	1	-7	52.08	2.42
5	-1	7	54.53	2.68
-5	1	7	58.45	2.78
5	-1	7	58.49	2.69

-5	-2	7	5.15	0.65
-5	2	-7	10.30	1.07
-5	-2	7	5.09	0.42
5	2	7	5.20	0.73
-5	-2	7	5.56	0.42
5	-2	-7	9.24	0.72
-5	2	-7	10.44	0.73
-5	-2	7	5.22	0.59
-5	2	-7	11.27	0.79
5	2	7	5.45	0.55
5	2	7	4.83	0.60
-5	-2	7	5.58	0.76
5	-2	-7	10.13	0.83
5	2	7	5.09	0.51
-5	2	-7	9.05	0.66
-5	2	-7	11.12	0.74
5	2	7	4.96	0.54
5	-2	-7	9.81	0.65
5	-2	-7	10.22	0.56
-5	-2	7	5.37	0.43
-5	2	-7	9.23	0.94
5	-2	-7	9.93	0.81
5	-2	7	5.80	0.86
5	2	-7	9.74	0.64
5	2	-7	9.55	0.60
5	2	-7	10.34	0.58
5	-2	7	5.24	0.53
5	2	-7	9.41	0.84
-5	-2	-7	10.61	0.70
5	-2	7	5.66	0.51
-5	2	7	4.89	0.71
5	-2	7	5.68	0.50
-5	2	7	5.53	0.48
-5	2	7	5.96	0.60
-5	-2	-7	10.65	0.73
-5	-2	-7	10.92	0.69
-5	-2	-7	9.41	0.83
5	2	-7	9.83	0.81
5	-2	7	6.00	0.89
-5	-2	-7	10.27	0.84
5	2	-7	9.61	0.90
-5	2	7	5.33	0.59
5	-2	7	5.44	0.50
-5	2	7	4.73	0.38

-5	-2	-7	9.53	0.71
-5	-2	-7	9.13	0.74
-5	2	7	5.72	0.77
5	-3	-7	74.45	3.94
5	-3	-7	83.67	3.90
-5	3	-7	85.27	3.95
-5	3	-7	86.46	4.05
-5	-3	7	79.18	3.81
5	3	7	82.82	3.90
5	-3	-7	82.48	4.08
-5	-3	7	77.62	4.01
-5	-3	7	79.58	3.84
-5	3	-7	79.31	4.20
5	-3	-7	80.18	4.02
5	3	7	87.91	4.09
-5	-3	7	83.82	4.11
5	3	7	76.79	3.95
-5	3	-7	86.51	4.01
5	3	7	81.22	4.01
-5	3	-7	84.51	3.95
5	-3	-7	90.79	4.13
-5	3	-7	88.56	4.33
-5	-3	7	83.02	3.93
5	-3	7	76.21	3.87
-5	-3	-7	88.05	4.01
-5	3	7	87.94	3.96
-5	3	7	79.25	3.80
-5	3	7	88.35	4.05
-5	-3	-7	91.45	4.14
-5	3	7	82.88	3.81
-5	-3	-7	91.96	4.05
5	3	-7	82.86	3.95
-5	-3	-7	83.97	4.02
5	-3	7	80.65	3.94
-5	3	7	82.45	3.97
5	-3	7	84.08	3.92
5	-3	7	88.64	4.22
-5	-3	-7	82.14	4.09
-5	-3	-7	85.34	3.99
5	3	-7	87.20	3.94
-5	3	7	79.49	4.01
-5	3	7	83.70	4.02
5	3	-7	82.88	4.11
5	3	-7	82.72	4.12

5	3	-7	81.39	4.11
5	-3	7	86.11	4.12
-5	4	-7	19.90	1.22
-5	4	-7	17.28	1.40
5	4	7	25.96	1.46
5	-4	-7	16.23	1.15
-5	-4	7	25.03	1.60
-5	4	-7	19.02	1.64
5	-4	-7	20.25	1.33
5	4	7	28.57	1.69
-5	-4	7	27.80	1.74
-5	-4	7	24.07	1.39
-5	-4	7	26.57	1.55
5	-4	-7	17.77	1.04
5	-4	-7	17.24	1.22
-5	-4	7	25.03	1.37
-5	4	-7	20.89	1.20
5	4	7	25.48	1.43
5	4	7	25.31	1.54
-5	4	-7	17.65	1.14
-5	4	-7	21.66	1.30
-5	4	-7	18.90	1.07
5	-4	-7	19.32	1.02
5	4	7	25.95	1.58
5	4	-7	19.29	1.27
-5	4	7	27.60	1.65
-5	4	7	25.49	1.65
-5	4	7	26.16	1.58
5	-4	7	24.58	1.48
-5	-4	-7	20.81	1.22
5	4	-7	18.08	1.11
-5	4	7	27.07	1.53
-5	-4	-7	18.01	1.25
5	-4	7	24.76	1.42
5	-4	7	25.50	1.47
5	-4	7	28.00	1.78
5	-4	7	30.38	1.84
-5	-4	-7	20.49	1.35
-5	-4	-7	18.11	1.17
-5	4	7	25.27	1.33
5	4	-7	19.84	1.35
-5	4	7	24.61	1.52
-5	-4	-7	19.48	1.14
5	4	-7	16.72	1.28

5	4	-7	18.99	1.11
-5	-5	7	18.89	1.40
-5	-5	7	19.13	1.19
-5	5	-7	13.27	0.85
-5	5	-7	14.44	1.07
-5	5	-7	14.35	1.30
-5	-5	7	19.30	1.21
-5	-5	7	20.79	1.54
-5	5	-7	12.70	1.06
-5	5	-7	14.06	1.01
5	5	7	22.84	1.54
5	5	7	20.16	1.39
5	-5	-7	11.30	0.95
5	-5	-7	13.08	0.82
5	5	7	19.29	1.39
5	5	7	21.02	1.26
5	-5	-7	12.91	1.18
-5	5	-7	14.78	0.93
5	5	7	20.75	1.24
-5	5	-7	13.53	1.27
5	-5	-7	12.78	1.06
-5	-5	7	21.63	1.42
-5	-5	7	20.91	1.56
5	-5	-7	12.49	0.85
-5	-5	-7	14.38	1.18
5	-5	7	22.60	1.28
-5	-5	-7	13.12	1.09
5	5	-7	12.90	1.13
5	5	-7	13.93	0.96
-5	5	7	20.07	1.34
-5	5	7	20.42	1.41
5	-5	7	18.88	1.29
-5	5	7	19.48	1.20
-5	5	7	19.09	1.52
5	-5	7	24.23	1.76
-5	-5	-7	13.77	1.07
5	5	-7	13.34	0.96
-5	-5	-7	13.04	0.98
-5	5	7	20.67	1.36
5	-5	7	20.36	1.23
-5	5	7	20.11	1.16
5	-5	7	18.72	1.57
-5	5	7	21.27	1.45
-5	-5	-7	13.31	0.95

5	5	-7	13.14	1.08
-5	-6	7	11.67	0.97
-5	6	-7	15.48	1.49
5	6	7	16.84	1.37
5	-6	-7	19.20	1.54
5	-6	-7	16.63	1.11
-5	-6	7	15.29	1.25
5	-6	-7	16.43	1.30
5	6	7	12.90	1.05
-5	-6	7	13.53	1.35
-5	6	-7	19.18	1.38
-5	6	-7	17.71	1.04
5	6	7	13.15	1.19
5	6	7	12.99	1.20
-5	-6	7	14.63	1.46
-5	6	-7	18.63	1.29
-5	6	-7	16.98	1.66
-5	6	-7	17.85	1.26
-5	-6	7	13.51	1.23
-5	6	-7	19.29	1.13
-5	-6	7	12.65	0.98
5	6	7	12.34	1.02
5	-6	-7	16.95	1.20
5	-6	-7	18.03	1.05
-5	6	7	14.61	1.17
-5	6	7	12.78	1.41
-5	6	7	12.59	1.16
-5	6	7	16.60	1.39
-5	-6	-7	18.58	1.33
-5	6	7	12.03	1.19
5	6	-7	16.34	1.30
5	-6	7	15.40	1.04
-5	6	7	13.06	1.01
5	-6	7	13.93	1.46
5	-6	7	15.86	1.55
-5	-6	-7	17.37	1.32
-5	-6	-7	19.12	1.44
5	6	-7	18.55	1.21
-5	-6	-7	16.27	1.18
5	-6	7	13.31	1.00
5	-6	7	13.31	1.07
-5	-6	-7	17.52	1.21
5	6	-7	16.73	1.47
5	7	7	4.43	0.68

5	-7	-7	4.32	0.67
5	7	7	5.49	0.86
-5	-7	7	3.97	0.82
5	7	7	5.98	1.03
-5	7	-7	4.37	0.72
-5	-7	7	6.46	1.09
-5	-7	7	5.14	1.05
-5	7	-7	5.23	0.90
-5	-7	7	4.97	0.79
5	7	7	4.84	1.00
5	-7	-7	4.34	0.73
-5	7	-7	4.79	0.99
5	-7	-7	4.82	0.81
-5	-7	7	4.50	0.73
5	7	7	5.02	0.71
-5	-7	7	4.84	0.92
-5	7	-7	4.89	0.70
5	-7	-7	5.53	1.15
-5	7	-7	5.38	1.11
-5	7	-7	4.95	0.95
5	-7	-7	4.27	0.70
5	7	-7	5.14	0.92
-5	7	7	4.40	0.70
5	-7	7	4.56	0.72
5	7	-7	5.67	0.86
-5	-7	-7	5.30	0.93
-5	-7	-7	3.86	0.67
-5	7	7	5.54	0.65
-5	-7	-7	4.40	0.67
-5	-7	-7	5.89	1.07
-5	7	7	5.62	0.88
-5	7	7	4.86	1.05
5	-7	7	4.94	1.03
5	-7	7	5.15	0.82
-5	7	7	6.84	0.90
-5	-7	-7	3.83	0.78
5	-7	7	5.14	0.78
-5	7	7	5.50	0.83
5	-7	7	4.79	1.16
-5	7	7	5.49	1.12
5	7	-7	2.57	0.86
-5	8	-7	4.95	0.86
-5	8	-7	2.86	1.08
5	8	7	3.88	1.05

5	8	7	3.67	0.93
5	8	7	3.80	0.74
5	-8	-7	5.20	1.17
5	-8	-7	5.80	0.80
5	-8	-7	5.55	0.98
5	-8	-7	3.70	0.64
-5	-8	7	6.17	1.17
-5	-8	7	4.65	0.74
-5	-8	7	3.85	0.92
-5	8	-7	5.47	1.03
-5	8	-7	2.99	1.02
5	8	7	5.39	0.96
5	-8	-7	3.61	0.68
-5	-8	7	3.85	0.70
-5	8	7	4.68	0.89
-5	8	7	5.51	0.94
5	-8	7	4.45	0.91
-5	-8	-7	3.94	0.75
5	-8	7	4.74	0.74
5	-8	7	4.84	0.72
5	8	-7	4.60	1.05
5	8	-7	6.08	1.05
-5	8	7	3.89	0.87
5	8	-7	4.95	0.99
-5	-8	-7	3.86	0.84
-5	8	7	4.91	1.09
-5	8	7	4.27	1.12
-5	8	7	2.94	0.74
5	-8	7	3.86	1.17
-5	-8	-7	4.33	1.00
-5	-8	-7	4.66	0.75
-5	-9	7	8.06	1.39
-5	-9	7	7.65	1.09
5	-9	-7	8.37	1.01
-5	-9	7	8.02	1.43
-5	9	-7	10.04	1.63
5	9	7	8.18	1.71
5	-9	-7	10.15	1.58
5	9	7	7.23	1.31
5	9	7	7.04	1.15
5	-9	-7	9.77	1.07
5	-9	-7	8.00	0.94
-5	9	-7	9.55	1.07
-5	-9	7	10.28	1.44

-5	9	-7	8.37	1.62
5	-9	-7	7.68	1.01
5	-9	-7	9.65	1.17
5	9	7	8.95	1.47
-5	9	-7	9.04	1.56
5	-9	7	9.64	1.14
5	-9	7	9.17	1.06
5	9	-7	6.26	1.38
-5	-9	-7	9.37	1.10
5	9	-7	10.44	1.45
-5	-9	-7	8.53	1.11
-5	9	7	8.52	1.37
-5	9	7	10.80	1.67
5	-9	7	10.83	1.52
5	9	-7	9.21	1.25
5	-9	7	7.50	1.11
-5	-9	-7	7.57	1.38
5	-9	7	10.73	2.07
-5	9	7	7.30	1.27
-5	9	7	10.63	1.45
-5	-10	7	30.91	2.32
5	10	7	35.28	2.93
-5	-10	7	29.78	2.60
-5	10	-7	23.95	2.64
5	-10	-7	23.97	1.76
5	-10	-7	24.01	1.86
5	-10	-7	23.01	1.85
5	10	7	30.07	2.25
-5	10	-7	26.02	2.30
-5	10	-7	27.57	1.73
-5	-10	7	29.81	2.03
5	-10	-7	27.29	1.72
5	-10	-7	27.73	1.66
5	-10	-7	28.09	2.53
-5	10	-7	26.60	2.37
-5	-10	-7	25.69	1.77
-5	10	7	28.29	2.23
5	-10	7	30.68	2.37
-5	10	7	24.06	2.53
-5	-10	-7	24.70	1.89
5	-10	7	30.64	2.00
5	-10	7	30.80	1.97
5	10	-7	19.87	2.34
5	-10	7	32.79	3.13

-5	10	7	31.58	2.28
5	-10	7	25.79	2.06
5	10	-7	24.17	1.91
-5	-10	-7	23.65	2.10
5	10	-7	23.82	2.08
5	-11	-7	24.15	1.88
5	11	7	20.88	2.42
-5	11	-7	30.38	1.62
5	-11	-7	26.71	1.89
5	-11	-7	27.00	1.80
-5	11	-7	23.47	2.96
5	11	7	16.88	2.11
-5	11	-7	26.87	2.61
-5	-11	7	18.35	2.12
-5	-11	7	19.00	1.80
5	-11	-7	26.10	2.20
5	-11	7	20.41	1.83
5	-11	7	25.65	1.81
5	-11	7	20.98	1.82
5	11	-7	28.12	2.12
-5	11	7	19.90	2.03
-5	-11	-7	24.36	2.08
-5	-11	-7	23.53	1.88
-5	11	7	23.18	2.53
5	11	-7	23.97	2.76
-5	-12	7	19.71	2.56
-5	-12	7	22.60	1.90
-5	12	-7	22.43	2.65
5	-12	-7	20.39	1.74
5	-12	-7	18.10	2.01
-5	12	-7	17.34	3.03
5	-12	-7	18.39	1.87
5	12	7	22.49	2.83
-5	-12	-7	25.30	2.27
5	12	-7	17.02	3.08
5	12	-7	19.09	2.01
5	-12	7	19.97	1.82
-5	-12	-7	19.10	1.71
-5	-12	-7	20.39	1.87
5	-12	7	23.77	1.99
-5	12	7	21.38	2.32
5	-13	-7	2.41	1.12
-5	-13	7	0.68	0.96
5	-13	-7	0.58	0.92

5	13	7	2.98	1.89
-5	-13	-7	2.24	0.92
6	0	7	279.17	13.56
-6	0	-7	281.38	12.65
6	0	7	287.45	13.62
-6	0	-7	282.19	12.49
-6	0	-7	274.78	12.59
-6	0	-7	270.19	12.58
-6	0	7	300.56	13.50
6	0	-7	276.21	12.43
6	0	7	314.26	13.64
6	0	7	309.37	13.57
-6	0	7	322.18	13.59
-6	0	-7	272.07	12.50
-6	0	7	290.36	13.58
-6	0	7	278.90	13.44
6	0	-7	280.96	12.51
6	0	-7	281.07	12.46
-6	0	7	321.62	13.73
6	0	-7	268.52	12.51
-6	0	7	317.13	13.56
-6	0	7	294.04	13.44
6	0	-7	270.15	12.60
-6	0	-7	281.63	12.48
6	0	7	294.12	13.57
6	-1	-7	55.08	2.70
-6	-1	7	33.56	1.66
6	-1	-7	52.63	2.77
-6	-1	7	34.06	1.60
-6	1	-7	58.16	2.68
6	-1	-7	57.73	2.67
6	1	7	32.29	1.70
-6	-1	7	32.26	1.59
-6	-1	7	31.97	1.77
-6	-1	7	34.90	1.69
-6	1	-7	52.10	2.81
6	1	7	33.17	1.72
-6	-1	7	34.36	1.60
6	1	7	30.26	1.72
-6	1	-7	51.34	2.72
6	1	7	31.88	1.72
-6	-1	7	35.49	1.94
-6	1	-7	55.72	2.66
-6	1	-7	51.44	2.66

6	1	7	31.13	1.78
6	-1	-7	57.19	2.65
6	-1	-7	55.04	2.66
-6	1	-7	55.78	2.86
6	-1	-7	58.14	2.53
6	-1	7	34.87	2.02
-6	-1	-7	57.27	2.66
6	1	-7	57.57	2.58
-6	-1	-7	51.49	2.67
-6	1	7	33.17	1.57
6	-1	7	31.45	1.74
-6	1	7	33.46	1.70
-6	1	7	35.64	1.78
-6	-1	-7	54.02	2.72
6	-1	7	32.89	1.70
-6	1	7	32.47	1.69
-6	-1	-7	56.30	2.78
6	1	-7	52.18	2.68
-6	-1	-7	53.19	2.65
6	1	-7	57.10	2.61
6	-1	7	31.00	1.70
-6	1	7	36.99	1.90
-6	-1	-7	53.52	2.79
-6	1	7	35.30	1.74
6	1	-7	54.89	2.78
6	-1	7	36.01	1.73
-6	-2	7	80.82	4.15
6	2	7	81.09	4.09
-6	2	-7	87.86	4.39
-6	2	-7	88.56	4.14
6	2	7	84.75	4.07
6	-2	-7	88.50	4.00
-6	2	-7	91.53	4.13
6	-2	-7	86.35	4.23
6	2	7	80.24	3.99
-6	-2	7	87.77	3.97
-6	2	-7	80.68	4.29
6	-2	-7	89.90	4.05
-6	2	-7	91.96	4.11
6	-2	-7	87.61	4.13
6	-2	-7	85.43	4.17
-6	-2	7	92.57	4.27
-6	-2	7	81.96	4.08
-6	-2	7	89.89	4.02

-6	2	-7	86.35	4.16
-6	-2	7	87.84	3.95
-6	2	7	92.23	4.12
-6	-2	-7	81.61	4.13
6	-2	7	78.47	4.07
-6	-2	-7	85.34	4.11
6	2	-7	88.48	4.18
6	-2	7	84.21	4.08
6	2	-7	90.14	4.03
6	2	-7	90.59	4.03
-6	2	7	83.09	4.00
6	2	-7	91.14	4.01
6	-2	7	90.76	4.27
-6	-2	-7	85.88	4.11
-6	2	7	87.11	4.13
6	-2	7	90.07	4.06
-6	2	7	91.92	4.16
6	-2	7	91.16	4.37
6	2	-7	90.15	4.24
-6	2	7	91.76	4.23
-6	-2	-7	81.88	4.08
-6	2	7	82.43	3.95
6	2	-7	87.61	4.23
-6	-2	-7	87.67	4.11
6	-2	7	86.41	4.07
-6	-2	-7	86.13	4.21
-6	-3	7	42.90	2.33
-6	3	-7	36.98	1.90
6	3	7	40.31	2.15
6	-3	-7	35.55	1.73
-6	-3	7	40.89	1.95
6	-3	-7	34.20	1.72
6	3	7	41.68	2.11
-6	3	-7	32.49	1.99
-6	-3	7	41.51	1.99
6	3	7	39.72	2.23
6	-3	-7	32.83	1.86
6	-3	-7	34.31	1.91
-6	3	-7	38.81	1.88
-6	-3	7	37.36	2.22
-6	-3	7	39.10	2.04
-6	3	-7	33.04	2.08
-6	-3	7	42.96	2.09
-6	3	-7	34.52	1.80

-6	3	-7	36.54	2.24
-6	3	-7	37.77	1.82
-6	-3	7	38.27	1.93
-6	-3	-7	34.65	1.87
-6	3	7	43.07	2.24
6	-3	7	40.15	2.14
-6	3	7	43.92	2.18
6	-3	7	41.90	2.35
-6	3	7	41.95	2.19
-6	-3	-7	37.91	2.02
-6	3	7	42.22	2.01
6	3	-7	35.18	1.95
6	3	-7	35.31	1.78
-6	-3	-7	35.54	1.85
6	3	-7	36.54	1.77
6	-3	7	41.35	2.11
6	3	-7	37.23	2.02
6	3	-7	35.00	1.94
-6	-3	-7	34.35	1.95
6	-3	7	41.56	2.10
6	-3	7	45.28	2.47
-6	-3	-7	37.59	1.86
-6	3	7	43.37	2.25
-6	3	7	40.00	1.98
-6	3	7	43.88	2.27
-6	-3	-7	35.00	1.85
-6	4	-7	6.43	0.70
-6	-4	7	5.68	0.53
-6	-4	7	5.17	0.78
6	4	7	6.15	0.68
-6	4	-7	6.51	0.75
-6	4	-7	6.08	0.57
-6	4	-7	6.93	1.12
-6	4	-7	6.34	0.60
6	-4	-7	4.79	0.64
-6	-4	7	6.78	0.71
6	-4	-7	5.95	0.83
-6	4	-7	5.82	0.72
6	-4	-7	5.33	0.70
6	4	7	6.15	0.79
-6	4	-7	4.53	0.90
6	4	7	5.95	0.70
-6	-4	7	5.66	0.97
6	4	7	5.47	0.62

-6	-4	7	6.06	0.57
-6	-4	7	6.33	0.91
6	-4	-7	5.81	0.48
6	-4	-7	6.06	0.53
6	4	7	5.61	0.85
6	-4	7	4.66	0.56
-6	4	7	6.77	0.93
-6	4	7	4.55	0.75
-6	4	7	6.18	0.50
6	4	-7	6.30	0.75
-6	-4	-7	5.13	0.76
-6	4	7	7.17	0.87
-6	-4	-7	5.98	0.83
-6	-4	-7	6.94	0.69
-6	-4	-7	5.73	0.66
6	-4	7	6.00	0.89
6	4	-7	6.35	0.61
-6	-4	-7	5.29	0.58
6	4	-7	6.29	0.61
6	-4	7	5.85	0.65
-6	4	7	5.57	0.72
-6	4	7	6.02	0.56
6	-4	7	7.29	0.68
6	4	-7	6.57	0.80
-6	4	7	5.21	0.81
6	-4	7	6.98	1.05
-6	5	-7	8.32	0.64
6	5	7	6.01	0.70
6	-5	-7	7.53	0.76
-6	5	-7	6.84	0.82
-6	5	-7	9.23	0.72
-6	-5	7	5.92	0.59
-6	5	-7	7.52	0.90
6	5	7	6.40	0.78
-6	-5	7	6.23	0.93
6	5	7	4.96	0.81
-6	-5	7	5.92	0.65
-6	-5	7	6.03	0.88
6	-5	-7	7.20	0.62
6	5	7	5.51	0.96
-6	5	-7	6.37	1.30
6	-5	-7	8.50	0.84
-6	5	-7	7.17	0.86
6	5	7	7.03	0.72

6	-5	-7	7.88	0.57
-6	-5	7	6.58	0.84
-6	-5	7	6.83	1.12
6	-5	-7	7.11	1.01
-6	5	-7	6.95	1.06
6	5	-7	8.56	0.77
-6	5	7	6.06	0.87
-6	-5	-7	8.09	0.85
-6	5	7	4.69	0.83
6	5	-7	8.11	0.87
6	-5	7	6.21	1.12
-6	5	7	5.81	0.66
6	-5	7	6.52	0.72
6	-5	7	5.87	0.65
6	-5	7	6.40	0.73
-6	-5	-7	7.74	1.02
6	-5	7	7.96	1.15
-6	-5	-7	7.55	0.77
-6	5	7	6.90	0.91
-6	-5	-7	7.16	0.71
-6	5	7	5.46	0.95
-6	5	7	5.90	0.83
6	5	-7	7.54	0.73
-6	-5	-7	7.33	0.84
6	5	-7	6.17	0.93
-6	-6	7	87.60	4.30
6	6	7	77.05	4.19
-6	6	-7	80.29	3.89
-6	-6	7	78.73	3.94
6	6	7	80.22	4.21
-6	6	-7	80.22	3.86
6	6	7	81.35	3.99
6	-6	-7	83.27	4.11
-6	-6	7	82.30	4.27
-6	-6	7	84.98	4.14
6	-6	-7	76.18	3.76
6	6	7	81.63	4.01
-6	6	-7	77.25	3.72
6	-6	-7	74.35	3.95
-6	-6	7	83.53	4.03
-6	6	-7	76.85	4.17
6	-6	-7	73.31	3.85
-6	6	-7	82.95	3.99
-6	-6	7	79.97	4.23

-6	6	-7	84.69	3.77
6	6	7	85.24	4.28
6	-6	-7	78.24	3.64
-6	6	-7	76.05	4.23
-6	-6	-7	77.48	4.03
6	6	-7	78.23	3.82
-6	6	7	86.08	4.29
-6	6	7	78.09	4.31
-6	-6	-7	82.12	3.88
-6	6	7	86.89	4.18
-6	-6	-7	79.77	3.91
-6	6	7	86.23	4.11
-6	-6	-7	76.97	3.93
6	-6	7	85.69	4.01
-6	6	7	80.95	3.98
6	6	-7	77.72	3.93
6	6	-7	82.11	3.87
-6	6	7	80.14	4.14
6	-6	7	84.01	4.34
6	-6	7	77.23	4.01
-6	-6	-7	73.27	3.83
6	6	-7	71.30	4.05
6	-6	7	80.71	4.09
6	-6	7	88.54	4.54
-6	-7	7	49.99	2.91
-6	-7	7	51.18	2.77
-6	-7	7	47.69	2.83
-6	7	-7	59.81	2.75
6	-7	-7	56.73	3.12
-6	7	-7	53.35	3.19
6	7	7	50.50	2.90
-6	7	-7	55.63	2.66
6	-7	-7	49.84	2.80
-6	7	-7	59.33	3.05
-6	-7	7	47.54	2.91
-6	7	-7	47.71	3.30
6	-7	-7	52.39	2.95
-6	-7	7	48.25	2.53
6	7	7	45.55	2.55
6	7	7	46.58	2.80
6	7	7	46.08	2.80
-6	7	-7	55.61	2.90
6	-7	-7	54.15	2.60
6	-7	-7	52.60	2.74

-6	-7	7	49.28	2.60
-6	7	-7	55.90	2.86
6	7	7	44.28	2.57
-6	7	7	48.06	2.69
-6	-7	-7	52.51	3.07
6	-7	7	48.35	2.59
-6	-7	-7	53.18	2.84
6	-7	7	49.93	2.99
6	-7	7	53.08	3.19
6	-7	7	52.20	2.58
-6	-7	-7	54.96	2.93
6	-7	7	47.97	2.68
6	7	-7	54.56	2.82
-6	7	7	52.02	2.94
6	7	-7	55.49	2.91
-6	7	7	47.79	2.74
6	7	-7	49.00	3.10
-6	7	7	51.52	2.80
-6	7	7	48.66	2.57
-6	7	7	46.96	2.99
-6	-7	-7	49.99	2.79
-6	-7	-7	50.27	2.89
6	-8	-7	142.36	6.32
-6	8	-7	135.29	6.91
-6	8	-7	146.53	6.77
6	-8	-7	142.51	6.32
6	8	7	114.44	6.16
6	8	7	120.55	5.82
6	-8	-7	135.99	6.54
-6	-8	7	130.16	6.16
6	-8	-7	146.16	6.93
6	-8	-7	136.84	6.48
-6	-8	7	118.13	5.83
-6	-8	7	115.56	6.10
-6	-8	7	121.20	5.91
6	8	7	115.76	6.10
-6	8	-7	149.98	6.44
6	8	7	131.23	6.23
-6	8	-7	127.62	7.00
6	-8	-7	130.51	6.68
-6	8	-7	136.22	6.52
6	-8	7	123.94	6.02
-6	-8	-7	133.03	6.63
-6	-8	-7	128.76	6.49

-6	8	7	133.49	6.29
6	-8	7	127.58	5.86
-6	-8	-7	139.86	6.58
6	8	-7	126.57	6.84
-6	8	7	113.90	5.86
-6	8	7	119.12	6.26
6	-8	7	139.23	6.53
-6	8	7	120.63	6.03
6	8	-7	129.75	6.57
-6	8	7	123.99	6.05
6	-8	7	116.21	5.88
-6	-9	7	117.26	6.17
-6	-9	7	123.51	6.17
6	-9	-7	117.27	5.74
6	-9	-7	126.65	6.19
-6	9	-7	135.44	5.70
-6	9	-7	111.51	6.34
-6	9	-7	114.82	6.29
-6	9	-7	129.16	6.09
6	9	7	114.41	5.81
6	9	7	111.34	6.25
6	-9	-7	116.25	5.95
-6	-9	7	121.48	5.95
6	9	7	114.65	6.17
-6	-9	7	120.47	5.90
6	-9	-7	123.06	5.59
6	9	7	133.04	6.34
6	-9	-7	114.29	5.77
6	9	-7	126.17	5.95
6	-9	7	131.83	6.58
6	9	-7	114.48	5.84
-6	9	7	124.06	6.11
6	-9	7	116.16	5.92
-6	9	7	126.00	7.25
-6	9	7	128.01	6.09
6	-9	7	115.82	6.03
-6	-9	-7	115.82	5.74
6	-9	7	132.16	6.03
6	9	-7	102.45	6.36
-6	-9	-7	113.50	5.89
6	-9	7	129.41	5.89
-6	-9	-7	119.13	5.84
-6	9	7	115.90	6.39
-6	-10	7	25.99	1.94

-6	-10	7	26.81	2.47
6	-10	-7	34.12	2.20
6	-10	-7	24.39	1.83
-6	10	-7	31.66	1.91
6	-10	-7	29.36	1.69
6	-10	-7	26.56	1.84
6	-10	-7	33.68	2.72
6	10	7	23.78	2.29
6	10	7	24.41	2.20
-6	10	-7	26.09	2.50
-6	10	-7	28.58	2.36
-6	10	-7	22.03	2.69
6	-10	-7	25.28	2.07
-6	-10	7	27.59	2.33
6	10	-7	25.06	1.96
6	-10	7	26.93	1.91
-6	10	7	29.24	2.21
6	-10	7	31.28	2.23
6	10	-7	22.69	2.49
-6	10	7	26.86	2.56
-6	-10	-7	28.38	1.98
6	-10	7	31.47	2.94
6	-10	7	26.39	2.01
6	-10	7	29.43	1.87
-6	-10	-7	27.33	1.84
6	-11	-7	3.96	1.12
-6	11	-7	4.36	1.84
6	-11	-7	4.54	0.95
-6	-11	7	6.20	1.34
-6	-11	7	6.19	1.10
-6	11	-7	6.66	1.56
6	11	7	6.19	1.39
-6	11	-7	6.36	0.89
6	-11	-7	5.38	0.97
6	11	7	7.66	1.53
6	-11	-7	6.36	1.29
-6	-11	-7	5.69	1.16
6	-11	7	7.19	1.30
-6	11	7	4.67	1.49
6	11	-7	5.54	1.09
-6	11	7	5.08	1.52
-6	-11	-7	5.44	0.94
6	-11	7	7.16	1.39
6	-11	7	4.50	1.02

6	11	-7	1.69	1.45
-6	12	-7	8.14	1.95
6	-12	-7	8.72	1.54
6	12	7	13.45	2.63
6	-12	-7	9.93	1.78
-6	-12	7	11.37	1.79
-6	-12	7	8.05	1.44
6	-12	-7	10.56	1.41
-6	12	7	15.09	2.22
6	-12	7	8.95	1.46
6	12	-7	10.59	1.66
-6	-12	-7	12.06	2.30
6	12	-7	8.43	2.04
-6	-12	-7	10.85	1.47
-6	-12	-7	14.13	1.60
6	-12	7	8.09	1.33
7	0	7	-0.05	0.35
7	0	-7	0.13	0.20
-7	0	-7	0.60	0.39
-7	0	-7	0.46	0.26
-7	0	-7	-0.15	0.32
-7	0	7	0.02	0.25
-7	0	7	0.42	0.21
7	0	-7	0.32	0.19
-7	0	7	0.19	0.15
-7	0	7	0.13	0.31
-7	0	-7	-0.64	0.36
7	0	7	0.07	0.24
7	0	-7	0.14	0.29
7	0	7	0.10	0.20
7	0	7	0.22	0.31
-7	0	-7	0.34	0.29
-7	0	7	0.10	0.38
7	0	7	-0.09	0.22
-7	0	-7	0.17	0.24
7	0	-7	0.52	0.26
7	0	-7	0.09	0.30
7	0	-7	0.05	0.16
-7	1	-7	34.47	1.99
-7	1	-7	31.70	1.95
7	-1	-7	35.05	1.80
-7	-1	7	35.94	1.66
7	-1	-7	36.62	1.78
7	1	7	32.31	1.85

7	-1	-7	33.61	1.90
7	-1	-7	35.99	1.66
-7	-1	7	34.51	1.66
7	-1	-7	33.71	1.77
-7	1	-7	34.97	1.87
7	1	7	31.68	1.78
-7	-1	7	36.71	1.99
7	1	7	34.35	1.77
7	1	7	33.86	1.77
-7	-1	7	35.83	1.73
-7	1	-7	35.61	1.80
7	-1	-7	36.05	1.77
-7	-1	7	32.10	1.85
7	1	7	35.84	1.79
-7	1	-7	33.67	1.78
-7	-1	7	33.52	1.66
-7	1	-7	34.82	1.79
-7	-1	7	34.79	1.62
-7	1	7	36.56	1.81
7	-1	7	33.86	1.76
-7	1	7	27.73	1.97
-7	1	7	36.20	1.97
7	-1	7	32.89	1.76
7	1	-7	36.23	1.68
-7	-1	-7	29.59	1.92
-7	-1	-7	34.46	1.80
7	1	-7	35.43	1.72
-7	-1	-7	36.63	1.76
-7	1	7	34.77	1.64
7	1	-7	34.22	1.78
7	-1	7	33.38	1.81
7	-1	7	36.70	1.78
-7	-1	-7	33.06	1.89
7	1	-7	35.26	1.91
7	1	-7	33.13	1.87
-7	1	7	37.72	1.82
7	-1	7	36.06	2.08
7	-1	7	33.19	1.95
-7	-1	-7	35.79	1.91
-7	1	7	33.12	1.74
-7	2	-7	9.31	0.70
-7	2	-7	6.52	0.88
-7	-2	7	7.37	0.52
7	2	7	7.14	0.66

-7	-2	7	7.74	0.57
7	-2	-7	7.56	0.52
7	2	7	7.36	0.74
-7	-2	7	7.57	0.77
-7	2	-7	7.66	0.64
-7	-2	7	9.26	0.97
7	2	7	6.51	0.61
7	-2	-7	7.76	0.60
-7	-2	7	7.61	0.53
-7	2	-7	8.36	0.69
7	-2	-7	7.06	0.68
-7	2	-7	7.98	0.64
-7	2	-7	5.70	0.96
-7	-2	7	7.56	0.45
7	-2	-7	7.66	0.70
7	-2	-7	7.61	0.65
7	2	7	7.55	0.64
-7	-2	-7	7.59	0.69
7	-2	7	6.91	0.65
7	2	-7	7.72	0.77
7	-2	7	8.07	0.93
7	-2	7	7.17	0.61
-7	2	7	6.81	0.76
-7	2	7	6.92	0.85
-7	-2	-7	9.17	0.64
-7	-2	-7	7.02	0.78
-7	2	7	7.83	0.72
7	2	-7	7.03	0.81
7	2	-7	8.47	0.56
-7	-2	-7	7.51	0.65
7	-2	7	7.16	0.61
7	2	-7	7.49	0.70
-7	2	7	6.08	0.71
-7	-2	-7	7.81	0.63
-7	-2	-7	7.85	0.82
-7	2	7	7.32	0.58
-7	2	7	7.38	0.74
-7	2	7	7.47	0.51
-7	-2	-7	6.82	0.71
7	2	-7	8.64	0.54
7	-2	7	6.40	1.02
-7	3	-7	25.38	1.80
-7	3	-7	25.41	1.40
7	3	7	20.64	1.25

-7	3	-7	23.84	1.68
-7	-3	7	19.90	1.05
-7	3	-7	26.70	1.40
7	-3	-7	26.00	1.30
7	3	7	20.02	1.05
-7	-3	7	21.82	1.08
-7	-3	7	21.01	1.15
-7	3	-7	28.20	1.50
7	3	7	21.62	1.24
7	-3	-7	24.85	1.30
-7	3	-7	27.69	1.47
7	3	7	18.33	1.34
-7	-3	7	18.30	1.35
-7	-3	7	20.84	1.44
-7	-3	7	21.11	1.06
-7	-3	7	22.52	1.50
7	-3	-7	25.41	1.49
7	-3	-7	24.76	1.45
-7	-3	7	20.17	1.16
-7	3	7	19.97	1.35
-7	3	7	22.57	1.39
-7	-3	-7	24.89	1.52
7	-3	7	21.08	1.24
7	3	-7	26.76	1.34
7	-3	7	21.45	1.62
-7	-3	-7	25.98	1.43
7	3	-7	26.41	1.36
-7	3	7	22.80	1.33
-7	3	7	21.81	1.13
7	3	-7	23.60	1.48
7	-3	7	19.38	1.20
-7	-3	-7	24.74	1.46
-7	3	7	19.63	1.07
-7	3	7	22.06	1.43
-7	3	7	20.91	1.33
7	-3	7	19.82	1.48
-7	-3	-7	26.22	1.61
7	3	-7	26.20	1.54
-7	-3	-7	24.36	1.43
7	-3	7	20.64	1.21
-7	-3	-7	25.88	1.45
-7	4	-7	7.74	0.69
-7	-4	7	10.34	1.13
7	4	7	8.77	0.83

-7	4	-7	7.77	1.26
7	-4	-7	7.84	0.98
7	-4	-7	7.71	0.76
-7	-4	7	9.90	0.59
-7	-4	7	8.90	1.08
-7	-4	7	9.66	0.65
-7	4	-7	9.13	0.86
-7	4	-7	8.26	1.06
7	4	7	10.65	0.83
7	-4	-7	8.64	0.64
-7	4	-7	8.46	0.71
7	-4	-7	9.10	0.82
-7	4	-7	9.67	0.82
-7	-4	7	7.60	0.96
-7	4	-7	8.11	0.82
7	4	7	8.02	0.93
-7	-4	7	9.09	0.79
-7	-4	7	10.30	0.75
-7	-4	-7	7.99	0.77
-7	-4	-7	9.18	0.86
-7	4	7	11.06	0.96
-7	4	7	10.21	0.94
-7	4	7	10.27	0.78
-7	-4	-7	8.32	1.00
7	4	-7	9.08	0.69
-7	-4	-7	8.84	0.78
7	-4	7	10.03	1.18
7	-4	7	8.96	1.26
-7	4	7	10.94	1.06
-7	4	7	10.75	1.04
7	4	-7	9.37	0.87
-7	-4	-7	8.70	0.78
7	-4	7	9.47	0.79
7	4	-7	6.47	0.85
-7	4	7	9.28	0.98
7	-4	7	9.47	0.76
7	4	-7	10.20	0.72
7	-4	7	8.78	0.81
-7	-4	-7	8.06	0.74
7	5	7	14.06	1.03
7	5	7	15.77	1.30
-7	5	-7	14.02	0.92
-7	5	-7	15.70	1.13
-7	5	-7	14.62	1.14

-7	-5	7	14.35	1.09
-7	-5	7	13.76	1.35
7	5	7	14.46	1.13
-7	-5	7	11.96	1.21
-7	-5	7	14.67	1.00
7	-5	-7	12.16	1.01
7	-5	-7	12.33	0.87
-7	5	-7	14.93	1.12
7	-5	-7	12.47	1.24
-7	5	-7	15.04	0.99
7	-5	-7	12.87	1.06
7	5	7	15.24	1.05
-7	5	-7	13.74	1.61
-7	5	-7	12.60	1.33
7	5	7	13.63	1.21
-7	-5	7	13.54	0.89
-7	-5	7	13.21	1.40
-7	-5	-7	13.67	1.12
7	5	-7	14.22	1.00
7	-5	7	14.05	1.45
7	-5	7	14.00	1.42
7	-5	7	13.61	1.02
-7	-5	-7	14.73	1.04
-7	-5	-7	11.70	1.27
-7	5	7	15.47	1.27
-7	-5	-7	13.68	1.02
-7	5	7	15.18	1.29
7	5	-7	12.53	1.18
-7	5	7	14.39	1.20
-7	-5	-7	14.41	1.04
7	5	-7	13.38	0.98
7	-5	7	13.35	1.00
-7	5	7	12.86	1.28
7	5	-7	14.33	1.13
7	-5	7	14.73	1.01
-7	5	7	14.60	1.00
-7	5	7	14.41	1.18
-7	-6	7	6.37	0.76
7	-6	-7	5.73	0.96
7	6	7	6.73	1.09
7	-6	-7	5.38	0.62
7	6	7	5.17	0.80
7	-6	-7	5.90	0.76
-7	-6	7	5.05	0.99

-7	6	-7	5.29	0.87
-7	-6	7	6.94	1.07
-7	6	-7	5.62	0.97
-7	-6	7	5.19	0.67
-7	6	-7	6.07	1.23
-7	6	-7	6.66	1.00
-7	-6	7	6.89	1.23
-7	-6	7	5.27	0.92
7	-6	-7	4.96	0.83
-7	6	-7	5.14	0.90
7	6	7	5.50	0.93
-7	6	-7	5.62	0.72
-7	6	-7	5.39	0.61
7	6	7	5.18	0.86
7	6	7	6.48	0.83
7	6	-7	4.77	0.82
-7	-6	-7	5.29	0.77
-7	-6	-7	4.79	0.73
7	-6	7	6.21	0.74
-7	6	7	4.64	0.81
7	-6	7	7.10	1.76
-7	6	7	5.59	1.10
7	-6	7	6.48	1.06
7	-6	7	4.82	0.77
-7	6	7	7.07	0.99
-7	6	7	6.04	0.78
-7	6	7	5.69	0.97
-7	6	7	5.84	1.08
7	6	-7	4.66	0.79
7	-6	7	6.16	0.72
-7	-6	-7	7.29	1.12
7	6	-7	4.73	0.97
-7	-6	-7	5.75	0.86
-7	-6	-7	5.08	0.86
7	6	-7	5.75	0.85
7	-7	-7	33.06	2.16
-7	-7	7	33.42	2.33
7	-7	-7	34.46	1.93
-7	7	-7	38.94	2.30
7	7	7	33.84	2.03
7	-7	-7	31.72	2.01
-7	7	-7	35.47	2.45
7	7	7	33.38	2.29
-7	7	-7	36.22	1.89

7	7	7	39.67	2.42
7	-7	-7	36.17	2.40
-7	7	-7	40.83	2.00
-7	7	-7	35.42	2.07
-7	7	-7	33.86	2.60
-7	-7	7	37.02	2.07
-7	-7	7	38.95	2.46
7	7	7	41.18	2.60
-7	-7	7	35.02	1.98
7	7	7	33.66	2.29
7	7	-7	36.38	2.08
7	-7	7	38.60	2.74
-7	7	7	38.81	2.20
-7	7	7	36.83	2.06
-7	-7	-7	32.03	2.02
7	-7	7	35.17	2.48
7	-7	7	34.21	2.13
7	-7	7	33.46	2.03
-7	-7	-7	36.66	2.33
7	-7	7	38.97	2.03
-7	-7	-7	34.38	2.14
-7	-7	-7	35.26	2.09
-7	7	7	37.19	2.32
7	7	-7	35.65	2.03
-7	-7	-7	35.40	2.07
7	7	-7	33.15	2.29
-7	7	7	33.76	2.24
-7	7	7	35.00	2.49
7	7	-7	37.65	2.12
-7	7	7	39.41	2.45
-7	-8	7	9.42	1.66
-7	-8	7	8.93	1.03
-7	8	-7	12.47	0.94
-7	8	-7	11.44	1.57
-7	8	-7	10.87	1.78
-7	-8	7	9.24	1.02
7	8	7	7.94	1.25
-7	8	-7	11.49	1.49
7	-8	-7	12.98	1.86
-7	8	-7	13.17	1.19
7	-8	-7	11.47	1.02
7	-8	-7	10.52	1.09
7	8	7	8.80	1.28
-7	8	-7	9.56	1.24

7	8	7	9.60	1.52
7	8	7	6.89	1.07
7	-8	-7	10.12	1.23
-7	-8	7	8.21	1.36
7	-8	7	9.48	1.00
-7	8	7	8.60	1.43
7	-8	7	8.40	1.01
-7	-8	-7	11.09	1.12
-7	8	7	9.59	1.61
7	-8	7	8.80	0.99
-7	-8	-7	11.99	1.20
-7	-8	-7	12.22	1.24
7	8	-7	11.56	1.19
-7	8	7	6.71	1.30
7	8	-7	10.32	1.55
-7	8	7	8.26	1.11
7	-8	7	13.55	1.99
-7	8	7	10.08	1.46
-7	9	-7	7.53	1.59
7	-9	-7	7.29	1.14
7	-9	-7	5.84	1.09
-7	-9	7	7.10	1.09
-7	-9	7	8.64	1.46
-7	9	-7	8.14	1.19
7	9	7	9.16	1.76
-7	-9	7	8.14	1.45
-7	-9	7	7.71	1.07
7	9	7	6.92	0.93
-7	9	-7	9.06	1.37
-7	9	-7	10.57	1.72
7	9	7	8.41	1.56
7	-9	-7	6.54	1.47
7	9	7	7.19	1.32
7	-9	-7	7.67	1.02
7	-9	7	6.94	1.04
7	-9	7	7.87	1.71
-7	9	7	7.11	1.41
7	9	-7	6.14	1.27
-7	9	7	7.07	1.40
-7	-9	-7	7.62	1.15
7	-9	7	5.97	0.98
-7	-9	-7	7.53	1.08
7	9	-7	9.83	1.22
-7	-9	-7	6.54	1.15

7	-9	7	5.07	1.03
7	-9	7	9.20	1.45
-7	10	-7	2.60	0.55
-7	10	-7	1.56	0.82
-7	10	-7	1.13	1.25
7	-10	-7	2.58	0.74
7	-10	-7	1.98	0.69
7	10	7	1.34	1.08
-7	-10	7	0.81	0.70
-7	10	-7	3.91	1.32
7	10	7	2.92	1.04
-7	-10	7	1.94	1.02
7	-10	-7	0.83	0.87
-7	-10	7	2.84	1.30
7	-10	-7	1.48	1.33
7	-10	7	2.50	0.76
7	-10	7	1.99	1.07
7	-10	7	2.21	0.69
7	-10	7	2.01	1.44
7	10	-7	0.32	1.15
-7	10	7	3.04	1.13
7	-10	7	3.05	0.90
-7	-10	-7	3.23	0.92
7	10	-7	2.92	0.82
-7	10	7	3.35	1.33
-7	-10	-7	1.01	0.70
7	11	7	6.01	1.32
7	-11	-7	4.56	0.90
7	-11	-7	3.91	1.05
-7	-11	7	6.17	1.58
-7	11	-7	3.86	1.46
-7	-11	7	5.64	1.14
-7	11	-7	6.90	1.83
7	11	7	3.93	1.43
7	-11	-7	5.73	0.98
-7	11	-7	6.63	0.99
7	-11	7	5.12	0.97
7	11	-7	7.44	1.16
-7	-11	-7	6.33	1.24
-7	11	7	6.88	1.35
-7	-11	-7	6.38	1.09
7	-11	7	5.24	1.08
7	-11	7	5.59	1.03
7	11	-7	5.58	1.82

7	-12	-7	7.67	1.20
7	12	7	7.60	2.04
-7	-12	7	4.67	1.42
7	-12	-7	9.29	1.30
-7	12	-7	7.60	2.05
-7	-12	7	5.83	1.06
7	12	-7	5.52	1.85
-7	-12	-7	6.87	1.13
7	-12	7	6.67	1.10
-7	-12	-7	6.12	1.82
-7	12	7	8.30	1.68
-7	-12	-7	8.19	1.43
-8	0	7	151.55	6.80
-8	0	7	161.12	6.95
8	0	7	153.19	6.96
-8	0	-7	151.81	7.37
8	0	7	135.54	6.91
-8	0	-7	162.89	7.33
8	0	7	153.16	6.95
-8	0	-7	145.94	7.34
-8	0	7	157.28	6.83
8	0	7	143.66	7.00
-8	0	7	158.90	7.12
-8	0	7	143.54	6.95
8	0	-7	159.81	7.27
8	0	-7	152.65	7.36
8	0	-7	158.88	7.14
8	0	-7	158.10	7.22
8	0	-7	160.41	7.15
-8	0	7	149.64	6.83
-8	0	-7	162.43	7.22
-8	0	-7	168.40	7.27
8	0	-7	158.42	7.21
-8	0	-7	149.82	7.26
8	1	7	207.37	9.90
-8	1	-7	247.59	10.59
-8	1	-7	222.05	10.74
-8	1	-7	233.80	10.65
-8	1	-7	246.61	10.62
8	-1	-7	231.38	10.59
-8	-1	7	204.72	9.72
8	1	7	208.26	9.81
8	-1	-7	233.19	10.48
-8	-1	7	231.08	10.00

-8	1	-7	232.26	10.77
-8	-1	7	209.93	9.88
8	1	7	212.65	9.83
8	-1	-7	242.69	10.57
8	1	7	224.73	9.83
8	-1	-7	227.56	10.58
8	1	7	216.56	9.81
-8	-1	7	212.46	9.71
8	-1	-7	227.66	10.72
-8	-1	7	199.24	9.84
-8	1	-7	232.56	10.61
8	-1	-7	238.17	10.56
8	1	-7	231.77	10.53
-8	-1	-7	239.71	10.56
8	-1	7	223.88	9.93
-8	1	7	222.75	9.76
-8	-1	-7	233.96	10.68
8	1	-7	228.76	10.47
8	-1	7	222.79	9.82
8	-1	7	211.62	9.85
8	1	-7	235.48	10.72
-8	-1	-7	223.94	10.62
-8	1	7	228.07	9.99
-8	1	7	217.18	9.70
-8	1	7	195.77	9.78
8	1	-7	234.44	10.66
-8	-1	-7	216.30	10.62
8	1	-7	223.46	10.55
-8	-1	-7	236.99	10.60
8	-1	7	209.98	9.82
-8	1	7	235.76	9.85
-8	1	7	231.72	9.81
8	-1	7	196.75	9.79
-8	-1	-7	234.66	10.70
-8	-2	7	48.36	2.41
-8	2	-7	56.29	2.80
-8	-2	7	51.13	2.42
-8	2	-7	59.08	2.80
8	-2	-7	54.27	2.77
8	-2	-7	56.56	2.78
8	2	7	49.77	2.51
-8	-2	7	53.33	2.72
-8	-2	7	48.49	2.61
8	-2	-7	58.92	2.83

8	-2	-7	55.83	2.67
-8	-2	7	51.97	2.67
8	2	7	46.64	2.60
8	2	7	48.28	2.50
-8	2	-7	53.05	2.75
8	-2	-7	56.82	2.65
-8	2	-7	55.53	3.02
-8	2	-7	58.98	2.78
-8	-2	7	54.61	2.44
8	2	7	48.30	2.43
-8	2	-7	53.65	2.98
-8	2	-7	60.26	2.76
8	2	-7	56.96	2.66
-8	2	7	49.49	2.38
-8	-2	-7	57.10	2.78
8	-2	7	50.97	2.71
-8	2	7	48.09	2.41
8	2	-7	53.64	2.75
8	-2	7	45.33	2.48
-8	2	7	54.13	2.71
8	2	-7	59.18	2.63
-8	-2	-7	58.83	2.73
8	-2	7	52.72	2.84
-8	-2	-7	54.61	2.83
-8	-2	-7	57.56	2.92
8	-2	7	50.17	2.51
-8	2	7	51.17	2.53
-8	2	7	49.67	2.60
-8	-2	-7	53.93	2.78
8	2	-7	55.46	2.87
-8	-2	-7	53.83	2.76
8	-2	7	47.10	2.49
-8	2	7	53.66	2.57
-8	-2	-7	50.28	2.76
-8	2	7	53.16	2.56
-8	3	-7	51.42	2.59
-8	3	-7	50.69	2.54
-8	3	-7	49.96	2.61
8	-3	-7	51.18	2.45
-8	3	-7	46.73	2.80
8	-3	-7	49.98	2.61
-8	-3	7	48.82	2.65
-8	-3	7	42.87	2.58
8	3	7	49.61	2.46

-8	3	-7	56.06	2.57
-8	-3	7	50.37	2.25
-8	-3	7	51.02	2.69
8	-3	-7	47.52	2.57
-8	3	-7	50.43	2.88
8	3	7	46.67	2.47
-8	-3	7	50.95	2.28
8	3	7	46.65	2.60
-8	-3	7	46.78	2.39
-8	3	-7	55.39	2.63
-8	-3	7	49.55	2.33
8	3	7	47.81	2.29
8	3	-7	49.80	2.59
8	-3	7	48.14	2.44
8	3	-7	51.67	2.49
-8	-3	-7	55.55	2.55
8	3	-7	52.14	2.69
8	-3	7	53.49	2.84
-8	-3	-7	54.65	2.60
8	-3	7	46.53	2.46
-8	3	7	46.92	2.57
-8	3	7	47.88	2.35
-8	-3	-7	49.64	2.62
-8	-3	-7	47.71	2.46
-8	3	7	50.71	2.65
-8	3	7	51.55	2.53
-8	-3	-7	53.86	2.76
-8	3	7	50.76	2.56
-8	-3	-7	50.56	2.58
-8	3	7	45.70	2.53
8	-3	7	47.60	2.45
-8	-3	-7	48.87	2.59
8	-3	7	48.83	2.69
-8	-4	7	2.21	0.31
-8	4	-7	2.17	0.83
8	4	7	2.55	0.46
-8	4	-7	2.16	0.69
-8	4	-7	2.76	0.48
-8	-4	7	1.59	0.37
8	-4	-7	1.66	0.44
-8	-4	7	2.19	0.72
-8	-4	7	1.85	0.57
-8	-4	7	2.27	0.39
-8	4	-7	2.37	0.52

8	-4	-7	2.24	0.49
-8	-4	7	2.42	0.71
8	-4	-7	2.29	0.35
-8	4	-7	2.85	0.52
8	4	7	2.20	0.57
-8	4	-7	2.99	0.54
-8	4	-7	2.15	0.41
-8	-4	7	2.15	0.38
8	4	7	1.81	0.46
8	4	-7	3.17	0.55
-8	-4	-7	2.84	0.56
8	-4	7	1.21	0.77
-8	4	7	1.76	0.41
8	4	-7	2.40	0.53
-8	-4	-7	2.53	0.46
8	-4	7	1.95	0.44
8	4	-7	3.10	0.47
8	4	-7	2.54	0.52
-8	4	7	0.88	0.49
-8	4	7	2.07	0.62
-8	4	7	1.94	0.50
8	-4	7	1.62	0.37
-8	4	7	2.07	0.59
-8	-4	-7	2.83	0.51
8	-4	7	2.11	0.45
8	-4	7	2.05	0.69
-8	4	7	1.68	0.62
-8	-4	-7	1.69	0.39
-8	-4	-7	3.37	0.55
-8	-4	-7	2.40	0.64
8	-5	-7	65.93	3.46
8	5	7	69.91	3.54
-8	5	-7	71.27	3.49
-8	5	-7	66.42	3.44
-8	-5	7	69.74	3.54
8	-5	-7	74.08	3.69
8	-5	-7	69.86	3.54
-8	-5	7	73.24	3.42
8	-5	-7	64.73	3.32
-8	5	-7	69.39	3.37
-8	-5	7	69.63	3.78
-8	-5	7	73.89	3.85
-8	-5	7	75.35	3.83
-8	-5	7	75.42	3.52

-8	5	-7	67.85	3.86
-8	5	-7	65.72	3.76
-8	5	-7	72.78	3.56
8	5	7	67.13	3.66
8	5	7	68.67	3.76
-8	5	-7	72.85	3.41
-8	-5	-7	66.07	3.48
8	-5	7	70.72	3.51
-8	5	7	67.20	3.78
-8	5	7	76.12	3.77
-8	-5	-7	73.17	3.49
-8	5	7	71.66	3.76
-8	-5	-7	68.69	3.47
8	-5	7	71.17	3.64
-8	-5	-7	71.22	3.67
8	-5	7	80.46	4.06
8	5	-7	65.90	3.56
8	5	-7	74.44	3.40
-8	5	7	78.09	3.53
-8	-5	-7	69.01	3.45
-8	5	7	73.73	3.74
8	5	-7	69.30	3.52
8	5	-7	70.51	3.40
8	-5	7	69.53	3.56
-8	-5	-7	71.57	3.44
-8	5	7	76.63	3.68
8	-5	7	75.34	3.88
-8	6	-7	71.38	3.25
-8	-6	7	55.46	3.33
8	6	7	55.47	3.02
-8	-6	7	61.63	2.91
8	-6	-7	63.13	3.30
8	-6	-7	61.29	3.16
8	-6	-7	64.12	3.40
-8	-6	7	59.05	3.02
8	-6	-7	68.43	3.58
8	6	7	61.64	3.02
-8	6	-7	61.23	3.79
-8	-6	7	59.18	3.01
-8	6	-7	64.99	3.34
-8	-6	7	62.68	3.33
-8	6	-7	63.19	3.26
-8	-6	7	58.19	3.28
-8	6	-7	58.48	3.61

-8	6	-7	69.31	3.45
-8	6	-7	64.01	3.17
8	6	7	55.01	3.17
8	6	7	54.69	3.24
8	-6	7	58.76	3.38
8	-6	7	66.97	3.63
-8	6	7	59.36	3.25
-8	-6	-7	63.34	3.31
-8	-6	-7	65.25	3.35
-8	6	7	60.15	3.23
-8	6	7	62.29	3.01
8	-6	7	58.04	3.02
-8	6	7	64.67	3.35
8	-6	7	60.05	2.96
8	6	-7	61.34	3.47
-8	6	7	55.88	3.35
-8	-6	-7	64.16	3.53
8	-6	7	57.80	3.10
-8	-6	-7	64.59	3.29
8	6	-7	68.35	3.27
8	6	-7	67.31	3.36
8	6	-7	65.38	3.24
-8	6	7	61.75	3.17
-8	-6	-7	66.59	3.31
8	7	7	60.65	3.50
-8	-7	7	63.62	3.16
-8	-7	7	66.13	3.30
-8	7	-7	52.80	3.34
-8	7	-7	56.49	2.94
8	7	7	67.73	3.61
-8	7	-7	60.27	2.93
-8	7	-7	59.23	2.85
-8	7	-7	62.43	3.21
-8	7	-7	53.59	3.44
8	-7	-7	56.35	3.09
8	-7	-7	54.17	2.86
-8	-7	7	60.48	3.55
-8	-7	7	60.41	3.53
8	7	7	60.35	3.52
8	-7	-7	52.77	2.96
8	-7	-7	62.52	3.36
8	7	7	63.44	3.24
-8	-7	-7	55.79	2.97
8	7	-7	54.96	2.93

-8	7	7	62.06	3.48
-8	-7	-7	55.71	3.00
-8	7	7	66.61	3.54
8	-7	7	62.39	3.27
8	-7	7	70.42	3.98
-8	-7	-7	55.87	3.03
-8	7	7	64.09	3.26
8	-7	7	61.37	3.34
8	-7	7	64.72	3.19
-8	7	7	66.55	3.60
8	7	-7	60.42	3.02
8	7	-7	56.58	3.02
8	7	-7	50.56	3.17
-8	7	7	66.41	3.75
-8	-8	7	154.55	7.07
8	8	7	152.95	7.14
-8	8	-7	156.73	7.06
-8	8	-7	148.25	7.62
-8	-8	7	143.84	6.70
-8	8	-7	145.35	7.12
8	8	7	133.98	7.04
8	-8	-7	146.48	7.35
-8	8	-7	162.17	7.44
8	-8	-7	147.84	7.12
8	8	7	141.02	7.10
-8	8	-7	165.19	7.14
8	-8	-7	155.12	7.22
8	8	7	141.00	6.72
-8	8	-7	144.01	7.78
-8	-8	7	142.37	6.82
-8	-8	-7	144.90	7.23
-8	8	7	145.14	7.03
-8	8	7	135.80	7.24
8	8	-7	152.07	7.22
-8	8	7	139.78	6.80
8	8	-7	141.17	8.01
-8	-8	-7	146.46	7.17
8	-8	7	147.00	6.94
8	-8	7	152.14	6.72
8	8	-7	161.45	7.27
8	-8	7	123.37	6.89
8	-9	-7	67.00	3.95
8	9	7	67.20	4.01
8	9	7	72.61	3.73

8	-9	-7	74.20	3.74
-8	-9	7	67.99	3.73
-8	9	-7	79.08	3.63
-8	-9	7	71.64	4.09
-8	-9	7	64.99	4.00
8	9	7	68.13	4.05
8	9	7	73.56	4.11
-8	9	-7	70.29	4.46
-8	9	-7	80.04	3.77
8	-9	-7	71.87	3.79
-8	9	-7	70.74	4.31
8	-9	-7	80.73	4.36
-8	-9	7	70.12	3.62
8	-9	7	84.04	4.63
-8	-9	-7	70.19	3.88
-8	9	7	68.76	3.93
8	9	-7	68.12	4.21
8	-9	7	73.14	3.59
8	9	-7	77.95	3.93
-8	9	7	65.93	4.11
8	-9	7	70.99	3.71
8	9	-7	73.67	3.84
-8	-9	-7	69.19	3.76
8	-9	7	78.87	3.80
8	-9	7	67.54	3.81
-8	-9	-7	72.08	3.88
-8	10	-7	17.32	1.27
8	-10	-7	17.48	1.45
-8	-10	7	16.31	1.98
-8	10	-7	17.87	1.57
8	10	7	19.23	2.06
8	-10	-7	15.17	1.67
8	-10	-7	16.68	1.49
-8	10	-7	12.97	2.36
8	10	7	13.88	1.90
-8	-10	7	16.02	1.60
-8	10	-7	18.38	2.32
8	10	-7	16.84	2.12
-8	-10	-7	16.06	1.47
-8	10	7	15.83	1.95
8	10	-7	15.79	1.59
-8	-10	-7	18.85	1.61
8	-10	7	17.10	2.38
8	-10	7	16.80	1.67

-8	10	7	15.77	2.35
8	-10	7	16.07	1.46
-8	11	-7	-3.12	2.56
-8	-11	7	0.98	0.91
8	-11	-7	0.95	0.80
-8	11	-7	1.62	0.63
8	-11	-7	1.01	0.71
8	11	7	-0.99	1.22
-8	-11	7	2.08	1.41
8	11	7	1.83	1.26
-8	11	-7	1.06	1.53
-8	11	7	2.10	1.22
8	11	-7	0.62	0.91
-8	-11	-7	0.38	0.87
8	-11	7	2.50	0.81
8	-11	7	2.36	1.01
8	-11	7	1.12	0.78
8	11	-7	1.22	1.57
-8	-12	7	8.60	1.46
8	12	7	8.83	1.92
8	-12	-7	9.67	1.63
-8	-12	7	11.13	2.02
8	-12	-7	11.23	1.54
8	-12	-7	11.85	1.85
-8	12	-7	10.87	2.03
8	-12	7	9.20	1.49
-8	-12	-7	8.40	1.77
-8	-12	-7	10.18	1.59
-8	-12	-7	9.32	1.27
-8	12	7	12.21	1.95
9	0	7	0.29	0.31
-9	0	-7	0.04	0.25
9	0	7	0.29	0.50
9	0	-7	0.00	0.18
9	0	-7	-0.18	0.17
9	0	-7	-0.14	0.32
-9	0	7	0.09	0.22
-9	0	7	-0.30	0.30
-9	0	-7	0.08	0.42
9	0	-7	-0.07	0.22
-9	0	-7	0.16	0.29
-9	0	7	-0.08	0.73
9	0	7	-0.03	0.21
-9	0	-7	-0.09	0.36

9	0	7	0.34	0.26
9	0	-7	0.11	0.22
-9	0	-7	-0.67	0.42
-9	0	7	0.11	0.32
9	0	7	0.14	0.29
-9	0	-7	0.18	0.31
-9	0	7	-1.06	0.42
9	0	7	-0.12	0.44
-9	-1	7	20.84	1.18
-9	-1	7	19.78	1.06
-9	1	-7	24.24	1.35
-9	1	-7	22.46	1.40
9	1	7	17.84	1.24
9	1	7	18.98	1.17
9	-1	-7	23.91	1.30
-9	1	-7	23.09	1.34
-9	1	-7	22.98	1.53
9	1	7	18.35	1.20
-9	1	-7	23.99	1.30
-9	-1	7	20.29	1.32
9	-1	-7	21.95	1.44
-9	-1	7	22.54	1.45
-9	1	-7	20.63	1.48
9	-1	-7	23.06	1.27
9	-1	-7	22.73	1.30
9	1	7	20.17	1.19
-9	-1	7	17.70	1.22
9	1	7	18.90	1.12
9	-1	-7	23.66	1.18
-9	-1	7	18.17	1.03
9	1	7	19.76	1.45
-9	-1	-7	20.40	1.38
9	1	-7	20.98	1.23
-9	1	7	20.36	1.40
-9	1	7	19.99	1.15
9	1	-7	23.01	1.14
9	-1	7	18.22	1.12
9	1	-7	23.63	1.26
9	1	-7	23.65	1.45
-9	1	7	21.29	1.23
-9	-1	-7	22.66	1.32
9	-1	7	19.93	1.17
9	-1	7	20.19	1.19
-9	1	7	18.38	1.11

-9	-1	-7	23.07	1.25
9	-1	7	19.54	1.19
9	-1	7	17.46	1.31
-9	-1	-7	22.26	1.34
-9	-1	-7	23.10	1.44
-9	1	7	18.64	1.04
-9	-1	-7	23.91	1.48
-9	1	7	18.85	1.08
-9	-2	7	87.56	4.07
9	2	7	75.75	3.81
-9	-2	7	72.48	3.82
-9	-2	7	82.94	3.99
-9	2	-7	78.31	4.23
-9	-2	7	78.81	3.94
9	-2	-7	82.56	4.04
9	2	7	80.76	3.76
-9	2	-7	85.30	4.03
9	2	7	83.87	4.03
9	-2	-7	85.37	4.06
9	2	7	76.37	3.96
9	2	7	81.84	3.84
-9	-2	7	79.00	3.76
-9	2	-7	84.96	4.08
9	-2	-7	86.50	3.92
9	2	7	78.61	3.86
-9	2	-7	89.29	4.05
-9	2	-7	86.32	4.06
-9	2	-7	85.97	4.04
9	-2	-7	81.88	4.02
-9	-2	7	74.55	3.73
9	-2	7	79.98	3.84
-9	2	7	83.58	4.03
-9	2	7	79.24	3.83
-9	2	7	83.64	3.85
-9	2	7	79.38	3.94
-9	2	7	75.07	3.72
9	-2	7	81.38	4.20
-9	-2	-7	83.74	4.07
-9	-2	-7	82.08	4.04
9	2	-7	86.04	3.87
9	2	-7	82.59	4.15
9	2	-7	82.90	3.93
-9	-2	-7	85.24	3.97
9	-2	7	83.31	4.01

9	-2	7	77.58	3.83
-9	-2	-7	87.94	4.19
9	-2	7	74.94	3.81
9	2	-7	82.56	3.99
-9	2	7	84.10	3.88
-9	-2	-7	82.49	4.08
-9	-2	-7	86.70	4.06
-9	2	7	80.59	3.71
-9	3	-7	89.93	4.06
-9	3	-7	90.81	4.11
-9	3	-7	81.66	4.08
-9	-3	7	89.49	4.52
-9	3	-7	90.19	4.11
-9	-3	7	96.06	4.25
-9	-3	7	94.40	4.31
-9	-3	7	86.51	4.21
9	3	7	92.00	4.36
9	-3	-7	85.98	4.16
9	3	7	84.84	4.50
-9	3	-7	79.45	4.30
-9	3	-7	85.70	4.05
-9	3	-7	79.81	4.34
9	-3	-7	80.71	4.07
9	-3	-7	83.76	4.09
-9	-3	7	90.68	4.55
9	3	7	83.77	4.36
9	3	7	91.39	4.20
-9	-3	7	98.80	4.60
9	-3	-7	84.48	3.96
9	3	7	95.37	4.54
-9	-3	7	87.55	4.29
-9	3	7	94.17	4.44
9	3	-7	86.55	3.91
-9	-3	-7	85.70	4.09
-9	-3	-7	84.52	4.08
-9	-3	-7	80.51	3.97
9	-3	7	87.09	4.33
-9	3	7	89.45	4.43
-9	3	7	92.31	4.27
-9	-3	-7	87.05	4.25
9	-3	7	95.70	4.73
-9	-3	-7	83.64	4.09
-9	-3	-7	85.93	4.01
-9	-3	-7	83.70	4.09

9	3	-7	86.32	4.17
9	-3	7	88.36	4.38
9	-3	7	85.18	4.35
9	-3	7	93.57	4.59
-9	3	7	97.24	4.58
-9	3	7	97.84	4.44
9	3	-7	79.81	4.05
-9	-4	7	12.32	1.29
-9	4	-7	14.21	1.10
-9	-4	7	11.75	0.80
9	-4	-7	13.78	1.05
9	-4	-7	14.02	1.02
-9	-4	7	12.03	0.72
-9	4	-7	13.85	1.12
9	4	7	12.71	0.96
-9	-4	7	12.96	0.77
-9	-4	7	11.95	0.66
-9	-4	7	10.77	0.85
9	4	7	11.08	0.96
9	4	7	11.40	1.12
9	4	7	11.40	1.18
-9	4	-7	13.13	1.50
-9	4	-7	16.95	1.12
-9	4	-7	14.20	0.98
9	-4	-7	13.58	0.87
-9	-4	7	14.05	1.33
-9	4	-7	15.21	1.02
-9	4	-7	12.73	1.36
-9	-4	7	9.85	1.14
9	-4	7	11.15	0.90
-9	4	7	11.72	1.12
-9	4	7	12.56	0.87
-9	-4	-7	14.89	1.08
-9	-4	-7	14.03	1.04
-9	-4	-7	13.97	0.97
9	-4	7	11.37	0.92
9	4	-7	11.71	1.07
-9	-4	-7	13.59	1.29
-9	-4	-7	13.70	1.07
-9	4	7	11.05	1.16
-9	4	7	11.89	1.08
9	-4	7	11.43	1.34
9	4	-7	14.97	0.88
9	4	-7	13.11	1.11

9	-4	7	11.44	1.43
-9	4	7	11.45	1.21
-9	4	7	12.18	1.15
-9	-4	-7	13.76	1.04
9	4	-7	14.92	0.97
9	-4	7	11.48	0.88
-9	5	-7	19.77	1.99
-9	-5	7	26.43	1.34
9	5	7	23.72	1.59
-9	5	-7	25.30	1.58
9	5	7	25.47	1.76
-9	-5	7	25.82	1.50
-9	-5	7	26.81	1.42
-9	5	-7	19.54	1.74
-9	5	-7	24.58	1.43
-9	5	-7	24.67	1.53
9	-5	-7	22.00	1.28
-9	5	-7	21.61	1.49
-9	5	-7	21.71	1.33
9	5	7	25.29	1.54
-9	-5	7	23.19	1.85
-9	-5	7	25.70	1.33
9	-5	-7	22.95	1.49
-9	-5	7	26.46	1.32
9	-5	-7	22.71	1.42
9	-5	-7	24.53	1.70
-9	-5	7	23.79	1.77
-9	-5	7	29.21	1.93
-9	-5	-7	22.89	1.44
9	-5	7	24.55	1.58
-9	-5	-7	21.95	1.47
-9	5	7	24.81	1.49
-9	5	7	23.78	1.78
-9	-5	-7	21.70	1.46
-9	-5	-7	23.09	1.39
-9	-5	-7	22.88	1.73
9	-5	7	24.00	1.50
9	5	-7	20.43	1.51
-9	5	7	30.14	1.88
-9	5	7	27.75	1.82
-9	5	7	26.12	1.76
9	-5	7	27.15	2.12
9	5	-7	22.94	1.38
9	5	-7	24.39	1.51

9	-5	7	24.59	1.92
-9	-5	-7	21.35	1.44
9	-5	7	25.13	1.47
-9	5	7	25.44	1.67
-9	-6	7	17.13	1.62
-9	6	-7	19.03	2.02
-9	-6	7	19.37	1.76
9	-6	-7	20.15	1.23
9	-6	-7	18.74	1.36
-9	6	-7	20.01	1.80
-9	6	-7	21.46	1.36
9	-6	-7	21.25	1.73
-9	6	-7	22.23	1.58
9	6	7	21.77	1.37
-9	6	-7	20.43	1.26
-9	-6	7	19.05	1.28
-9	-6	7	19.99	1.19
-9	-6	7	21.15	1.34
-9	-6	7	20.73	1.76
-9	6	-7	21.35	1.49
9	6	7	17.53	1.44
9	6	7	19.91	1.60
-9	6	-7	18.73	1.40
9	-6	-7	19.70	1.45
-9	-6	-7	20.22	1.42
9	-6	7	20.40	1.42
9	-6	7	20.36	1.24
9	6	-7	16.76	1.50
-9	6	7	20.80	1.63
9	6	-7	22.54	1.45
-9	-6	-7	17.25	1.36
-9	-6	-7	19.57	1.35
9	-6	7	21.63	1.81
-9	6	7	20.60	1.51
9	6	-7	20.45	1.36
-9	6	7	19.68	1.71
-9	6	7	19.24	1.66
-9	-6	-7	18.76	1.41
-9	-6	-7	19.25	1.67
-9	6	7	18.52	1.32
-9	6	7	18.65	1.71
9	-6	7	20.05	2.06
9	-6	7	18.36	1.30
9	6	-7	21.62	1.36

-9	-6	-7	18.81	1.39
-9	-7	7	29.81	2.17
-9	7	-7	30.22	1.71
-9	-7	7	26.59	1.57
-9	-7	7	25.79	1.68
-9	-7	7	24.66	1.96
9	7	7	26.34	1.68
9	-7	-7	29.28	1.70
-9	7	-7	28.27	2.23
9	7	7	26.57	1.97
9	7	7	26.56	1.87
9	-7	-7	33.94	2.32
-9	7	-7	35.02	2.12
9	-7	-7	29.19	1.82
-9	7	-7	32.61	1.85
-9	7	-7	27.91	1.81
-9	7	-7	25.88	2.52
9	-7	-7	29.06	1.95
9	-7	7	26.94	1.66
-9	7	7	24.49	1.93
-9	-7	-7	30.52	1.83
9	-7	7	26.48	1.76
9	-7	7	28.15	1.57
-9	7	7	25.21	2.01
-9	7	7	28.46	2.21
9	7	-7	29.67	1.84
9	7	-7	32.08	1.90
-9	-7	-7	29.26	1.90
-9	7	7	26.42	1.71
-9	-7	-7	29.78	1.86
9	7	-7	28.36	1.80
9	-7	7	26.87	2.60
9	7	-7	27.85	2.03
-9	-8	7	53.96	3.30
9	-8	-7	62.67	3.57
-9	-8	7	51.49	3.21
9	8	7	50.95	3.19
-9	8	-7	54.33	3.69
-9	8	-7	63.38	3.49
-9	8	-7	59.02	2.93
9	8	7	51.75	2.84
-9	8	-7	52.71	3.01
9	8	7	44.38	3.47
-9	-8	7	51.81	2.93

-9	8	-7	63.11	3.07
-9	-8	7	54.77	2.81
9	-8	-7	54.95	3.09
9	-8	-7	55.86	2.97
-9	8	-7	57.32	3.81
9	-8	-7	56.00	3.22
9	8	7	58.92	3.33
-9	-8	-7	52.33	3.03
-9	-8	-7	58.93	3.15
-9	8	7	49.60	3.35
-9	8	7	52.68	3.16
9	8	-7	58.08	3.09
9	-8	7	58.43	2.78
9	-8	7	53.07	3.00
9	-8	7	55.20	2.90
9	8	-7	61.48	3.15
9	8	-7	51.66	3.34
-9	-8	-7	56.57	3.13
9	-8	7	64.57	3.81
9	9	7	8.74	1.59
-9	9	-7	6.52	1.57
9	-9	-7	9.09	1.56
9	9	7	12.54	1.90
-9	9	-7	9.62	0.97
-9	-9	7	11.37	1.61
9	-9	-7	8.53	1.25
-9	9	-7	9.44	1.67
-9	-9	7	9.26	1.21
9	-9	-7	9.81	2.00
-9	-9	7	11.67	1.61
-9	9	-7	8.27	1.32
-9	-9	7	8.67	1.21
9	9	7	6.27	1.26
9	-9	-7	6.75	1.08
9	-9	7	9.37	1.44
9	9	-7	6.79	1.40
-9	-9	-7	8.00	1.43
-9	-9	-7	7.04	1.23
9	-9	7	12.05	2.39
-9	-9	-7	7.94	1.16
9	9	-7	10.88	1.25
9	-9	7	8.75	1.15
9	-9	7	10.11	1.05
-9	9	7	7.59	1.64

9	9	-7	8.61	1.48
9	-9	7	9.56	1.30
-9	-10	7	3.21	0.86
-9	10	-7	2.46	1.46
-9	-10	7	2.56	1.11
9	10	7	1.90	1.08
9	-10	-7	-0.02	1.08
-9	10	-7	1.04	1.29
-9	10	-7	0.73	0.55
9	-10	-7	0.10	0.62
9	10	7	0.74	1.09
9	-10	-7	1.67	0.67
-9	-10	-7	0.51	0.71
-9	10	7	2.07	1.18
9	-10	7	2.25	0.69
9	10	-7	0.94	0.79
9	10	-7	1.56	1.18
9	-10	7	1.59	0.86
-9	-10	-7	0.74	0.79
9	-10	7	2.82	0.92
-9	11	-7	2.66	0.72
9	-11	-7	3.39	1.09
-9	11	-7	3.51	1.76
9	11	7	3.62	1.46
9	11	7	1.32	1.52
9	-11	-7	1.91	0.83
-9	-11	7	4.76	1.36
-9	-11	7	2.42	1.01
9	-11	-7	2.96	0.87
9	-11	7	3.01	0.98
9	-11	7	1.91	0.83
9	11	-7	0.40	1.43
-9	-11	-7	4.16	1.13
9	11	-7	3.39	0.95
-9	-11	-7	2.57	0.99
-9	11	7	2.29	1.36
-9	12	-7	6.71	1.79
9	-12	-7	4.42	1.02
9	-12	-7	3.95	0.95
9	12	7	5.04	1.64
-9	-12	7	3.62	1.02
-9	-12	7	4.87	1.45
9	-12	-7	5.70	1.37
-9	12	7	4.32	1.75

-9	-12	-7	5.65	1.22
9	-12	7	2.96	0.92
9	12	-7	1.84	1.60
-9	-12	-7	5.18	1.05
-9	-12	-7	5.34	1.50
-10	0	-7	152.86	6.89
10	0	-7	144.33	6.69
-10	0	-7	146.63	6.89
10	0	-7	144.84	6.76
10	0	7	190.68	8.56
-10	0	-7	138.55	6.81
-10	0	-7	151.03	6.73
-10	0	7	201.16	8.56
10	0	7	170.74	8.55
10	0	-7	144.70	6.93
10	0	7	181.03	8.61
-10	0	-7	135.41	6.89
10	0	-7	145.75	6.69
-10	0	7	192.55	8.74
-10	0	-7	153.96	6.83
-10	0	7	186.93	8.44
-10	0	7	174.45	8.55
10	0	-7	152.32	6.67
10	0	7	177.38	8.51
-10	0	7	188.08	8.59
10	0	7	185.83	8.59
10	0	7	197.84	8.83
-10	-1	7	126.79	6.21
10	1	7	133.04	6.35
10	1	7	127.52	6.12
10	1	7	124.82	6.25
-10	-1	7	135.04	6.25
-10	1	-7	141.59	6.88
-10	1	-7	141.92	6.74
10	1	7	133.71	6.16
10	1	7	122.36	6.16
-10	1	-7	152.29	6.76
-10	-1	7	142.43	6.37
-10	1	-7	150.43	6.69
10	-1	-7	147.87	6.62
-10	1	-7	152.04	6.73
10	-1	-7	137.23	6.70
10	1	7	129.71	6.26
-10	1	-7	131.25	6.86

10	-1	-7	141.40	6.65
-10	-1	7	143.20	6.13
-10	1	-7	147.24	6.78
-10	-1	7	123.98	6.04
-10	1	7	140.60	6.04
10	-1	7	129.99	6.18
-10	1	7	135.49	6.13
-10	-1	-7	146.62	6.78
10	1	-7	143.39	6.84
-10	1	7	137.09	6.36
10	-1	7	131.40	6.25
10	1	-7	147.49	6.67
10	1	-7	141.52	6.61
-10	-1	-7	134.36	6.77
-10	-1	-7	148.94	6.75
-10	1	7	136.55	6.04
-10	1	7	140.78	6.19
-10	-1	-7	141.70	6.76
-10	1	7	134.74	6.02
10	-1	7	122.07	6.11
10	1	-7	146.87	6.55
-10	1	7	127.16	6.25
-10	-1	-7	149.87	6.86
10	-1	7	129.36	6.17
-10	1	7	126.52	6.09
-10	-1	-7	151.57	6.65
-10	-2	7	65.45	3.31
10	2	7	62.71	3.17
10	2	7	62.53	3.07
10	2	7	60.64	3.26
-10	-2	7	67.93	3.38
10	-2	-7	64.81	3.30
10	-2	-7	66.31	3.26
-10	2	-7	61.98	3.51
-10	-2	7	61.90	3.05
-10	2	-7	70.26	3.30
-10	2	-7	66.81	3.53
10	2	7	66.14	3.38
-10	-2	7	64.32	3.06
10	-2	-7	69.92	3.19
-10	2	-7	63.29	3.29
-10	-2	7	62.49	3.26
-10	2	-7	68.61	3.30
-10	-2	7	68.34	3.10

-10	2	-7	67.27	3.33
-10	2	-7	68.14	3.33
10	-2	-7	71.54	3.33
10	2	7	62.92	3.14
10	2	7	62.17	3.12
10	-2	7	62.53	3.13
-10	-2	-7	64.45	3.29
10	-2	7	62.70	3.14
10	2	-7	67.41	3.42
10	2	-7	64.85	3.21
-10	2	7	57.86	3.21
-10	2	7	65.47	3.13
-10	2	7	66.59	3.20
-10	2	7	59.25	3.02
-10	-2	-7	66.25	3.21
-10	2	7	62.36	3.04
-10	-2	-7	67.73	3.32
10	2	-7	69.97	3.21
-10	2	7	69.33	3.39
10	-2	7	64.95	3.31
-10	2	7	63.77	3.27
-10	-2	-7	70.55	3.48
10	-2	7	61.29	3.11
10	2	-7	70.31	3.10
-10	2	7	64.86	3.13
-10	-2	-7	65.78	3.31
-10	-2	-7	66.43	3.33
-10	3	-7	10.81	1.31
10	3	7	7.18	0.65
10	3	7	7.94	1.09
10	-3	-7	12.19	1.08
-10	-3	7	7.52	0.60
10	-3	-7	10.09	0.73
-10	-3	7	5.86	0.92
-10	-3	7	7.92	0.66
-10	3	-7	11.88	0.97
-10	3	-7	11.19	0.93
-10	3	-7	10.49	0.84
-10	3	-7	10.48	0.88
-10	3	-7	10.55	0.97
10	-3	-7	10.95	0.88
-10	-3	7	7.49	0.70
-10	-3	7	6.31	1.07
10	3	7	6.17	0.89

10	-3	-7	11.06	0.89
10	3	7	7.88	0.73
-10	-3	7	6.39	1.12
-10	3	-7	9.93	1.19
10	3	7	8.83	0.82
-10	3	7	7.80	0.95
-10	-3	-7	11.48	0.81
-10	-3	-7	11.02	0.93
10	-3	7	7.53	1.13
10	-3	7	7.22	0.74
-10	3	7	8.47	0.91
10	3	-7	10.43	0.85
-10	-3	-7	10.72	0.91
10	3	-7	11.12	0.99
-10	-3	-7	14.08	1.20
10	-3	7	7.49	0.70
-10	3	7	8.27	0.91
-10	-3	-7	10.39	0.89
-10	-3	-7	10.33	0.94
-10	3	7	7.67	0.68
-10	3	7	8.41	0.94
-10	3	7	8.53	1.10
10	-3	7	8.26	0.74
10	3	-7	11.48	0.68
-10	-3	-7	9.74	0.91
10	-3	7	7.32	1.23
-10	4	-7	23.60	1.45
10	4	7	18.94	1.42
-10	4	-7	22.57	1.75
10	-4	-7	18.74	1.49
-10	4	-7	23.95	1.38
-10	4	-7	22.89	1.86
10	4	7	17.80	1.22
-10	-4	7	17.17	1.12
-10	-4	7	18.57	1.04
-10	4	-7	22.60	1.46
-10	-4	7	18.79	1.55
10	4	7	18.45	1.47
-10	-4	7	16.51	1.43
10	-4	-7	21.56	1.23
10	-4	-7	21.95	1.41
10	-4	-7	22.40	1.38
-10	4	-7	19.60	1.42
-10	-4	7	17.34	0.99

-10	4	-7	21.86	1.33
-10	-4	7	19.19	1.54
10	4	7	16.54	1.21
10	-4	7	17.20	1.19
-10	4	7	19.34	1.52
-10	-4	-7	21.27	1.28
10	4	-7	20.79	1.44
-10	4	7	17.21	1.38
-10	-4	-7	22.06	1.41
10	4	-7	19.70	1.38
-10	-4	-7	22.03	1.41
-10	-4	-7	21.10	1.63
-10	-4	-7	22.17	1.40
10	-4	7	18.64	1.58
10	-4	7	18.36	1.14
-10	4	7	18.05	1.33
10	-4	7	16.91	1.68
10	4	-7	22.70	1.20
-10	4	7	16.03	1.37
10	-4	7	17.23	1.18
-10	-4	-7	22.19	1.40
-10	4	7	17.96	1.37
-10	4	7	18.47	1.14
10	4	-7	21.74	1.29
-10	-5	7	54.99	3.19
-10	-5	7	56.74	3.17
-10	5	-7	54.35	3.25
-10	5	-7	54.04	3.33
-10	-5	7	53.46	3.12
10	-5	-7	54.61	2.94
10	5	7	57.95	3.11
-10	5	-7	59.80	3.00
-10	-5	7	56.48	2.65
10	5	7	55.06	2.87
-10	-5	7	56.29	2.86
-10	-5	7	54.34	2.66
-10	-5	7	58.22	2.62
10	-5	-7	53.98	2.91
10	5	7	52.01	3.10
-10	5	-7	56.29	2.88
-10	5	-7	57.72	2.97
-10	5	-7	61.39	2.91
10	-5	-7	58.78	3.40
-10	5	-7	54.65	2.83

10	5	7	53.99	2.95
-10	-5	7	57.75	2.68
10	-5	-7	53.82	2.76
10	-5	7	54.03	3.19
10	-5	7	58.42	2.96
-10	5	7	55.87	3.09
10	-5	7	54.79	2.79
-10	5	7	54.69	3.10
-10	-5	-7	57.30	2.90
-10	5	7	58.96	3.17
-10	-5	-7	58.33	2.94
-10	-5	-7	53.83	2.90
-10	5	7	56.90	2.83
-10	-5	-7	55.03	2.82
10	5	-7	51.79	2.94
10	-5	7	63.04	3.43
10	-5	7	52.69	2.87
-10	5	7	57.26	3.08
-10	-5	-7	57.47	3.18
-10	-5	-7	53.23	2.91
10	5	-7	57.74	2.84
10	5	-7	58.01	2.77
10	5	-7	56.37	2.95
-10	5	7	57.71	3.01
10	6	7	22.73	1.50
-10	6	-7	19.22	1.36
-10	-6	7	19.24	1.75
10	6	7	21.12	1.73
10	-6	-7	18.15	1.43
10	-6	-7	19.28	1.20
-10	6	-7	20.67	1.55
-10	6	-7	18.11	1.35
-10	6	-7	18.55	1.24
10	6	7	24.09	1.81
-10	-6	7	25.58	1.43
-10	-6	7	20.46	1.42
-10	6	-7	17.86	1.76
-10	-6	7	20.68	1.89
10	-6	-7	19.51	1.70
10	-6	-7	18.15	1.34
-10	6	-7	18.13	2.01
-10	-6	7	22.58	1.24
10	6	7	20.97	1.60
-10	6	7	22.78	1.82

10	-6	7	22.93	1.33
-10	6	7	22.70	1.73
-10	6	7	23.78	1.93
-10	6	7	22.10	1.46
10	-6	7	21.30	1.43
10	6	-7	18.99	1.22
10	6	-7	15.52	1.46
10	6	-7	20.45	1.39
10	-6	7	25.78	2.23
-10	-6	-7	18.87	1.37
10	6	-7	18.62	1.31
10	-6	7	20.65	1.51
-10	6	7	20.69	1.74
-10	-6	-7	21.11	1.41
-10	-6	-7	16.67	1.36
10	-7	-7	76.68	3.90
10	7	7	87.00	4.35
-10	7	-7	90.09	4.03
-10	7	-7	79.89	4.51
10	-7	-7	86.48	4.40
10	-7	-7	75.44	4.01
-10	7	-7	81.82	3.92
10	-7	-7	75.99	4.12
-10	7	-7	77.48	3.92
-10	-7	7	92.31	4.75
-10	7	-7	74.36	4.65
-10	-7	7	88.39	4.42
-10	-7	7	92.62	4.21
10	7	7	85.69	4.71
-10	-7	7	90.87	4.72
-10	7	-7	84.77	4.22
10	7	7	88.13	4.60
-10	-7	-7	76.06	4.00
10	-7	7	90.83	4.25
-10	7	7	89.34	4.66
10	7	-7	78.71	4.48
10	-7	7	84.41	4.39
10	7	-7	83.24	4.05
-10	-7	-7	78.63	4.09
-10	-7	-7	79.00	4.02
10	7	-7	78.23	4.23
-10	7	7	91.07	4.39
10	-7	7	88.55	4.50
-10	7	7	84.76	4.76

-10	8	-7	70.37	4.21
10	8	7	67.17	3.52
-10	-8	7	70.01	3.44
-10	-8	7	67.11	3.95
10	-8	-7	65.95	3.58
10	8	7	70.53	3.98
-10	-8	7	66.03	3.77
10	8	7	66.21	3.83
10	-8	-7	69.46	3.50
10	-8	-7	74.52	4.11
-10	-8	7	69.08	3.97
-10	8	-7	65.85	3.50
-10	8	-7	69.68	4.13
-10	8	-7	69.49	3.47
-10	8	-7	72.18	3.60
10	-8	-7	64.11	3.72
-10	-8	-7	72.58	3.62
10	8	-7	64.68	3.86
10	8	-7	73.72	3.68
-10	8	7	67.64	4.19
-10	-8	-7	70.04	3.71
10	-8	7	72.20	3.74
10	8	-7	72.11	3.62
-10	-8	-7	65.10	3.61
10	-8	7	71.52	3.62
-10	8	7	68.62	3.93
10	-8	7	73.04	4.39
10	-8	7	74.84	3.46
10	9	7	17.98	1.95
-10	-9	7	22.13	2.25
-10	9	-7	21.01	2.70
10	-9	-7	19.44	1.86
10	9	7	16.65	1.97
10	9	7	21.07	2.19
10	-9	-7	26.42	2.67
-10	-9	7	17.06	1.56
-10	9	-7	23.83	1.54
10	-9	-7	22.06	1.68
10	-9	-7	23.23	1.60
-10	-9	7	14.27	1.94
-10	9	-7	23.61	2.43
10	-9	7	16.94	1.48
10	-9	7	17.49	1.61
-10	9	7	12.71	2.23

-10	9	7	16.07	1.94
10	9	-7	21.28	2.19
10	9	-7	24.44	1.83
10	-9	7	23.58	2.73
-10	-9	-7	23.02	1.72
10	-9	7	19.17	1.33
10	-9	7	20.71	1.70
10	9	-7	24.53	1.76
-10	-9	-7	23.86	1.86
-10	10	-7	3.14	1.39
10	-10	-7	2.15	0.90
-10	-10	7	2.83	0.77
10	-10	-7	2.25	0.79
10	10	7	3.01	1.15
-10	10	-7	2.79	0.67
10	10	7	2.52	1.07
-10	-10	7	-0.48	1.21
10	-10	-7	1.85	0.72
10	-10	7	1.50	0.81
10	10	-7	4.10	0.92
-10	-10	-7	2.16	0.92
-10	10	7	2.07	1.29
10	10	-7	0.62	1.33
10	-10	7	2.15	0.64
10	-10	7	1.40	0.80
-10	-10	-7	3.73	0.91
10	-11	-7	-0.48	0.88
10	-11	-7	-1.19	1.04
10	11	7	0.48	1.23
-10	-11	7	0.20	1.15
-10	11	-7	1.93	0.78
-10	-11	7	-0.25	0.85
-10	11	-7	-0.28	1.60
10	11	7	1.76	1.45
10	-11	-7	0.54	0.83
10	11	-7	0.78	1.43
10	-11	7	1.56	0.75
10	-11	7	-0.49	1.47
-10	11	7	2.35	1.27
-10	-11	-7	2.59	1.01
-10	-11	-7	-0.51	0.81
10	12	7	2.70	1.55
10	-12	-7	3.06	0.95
-10	-12	7	5.16	1.15

10	-12	-7	5.52	0.98
-10	12	-7	2.61	1.71
-10	-12	7	4.98	1.48
10	-12	-7	4.21	1.33
10	12	-7	0.41	1.57
-10	-12	-7	3.45	1.11
-10	-12	-7	3.06	1.36
10	-12	7	2.69	0.93
-10	12	7	5.38	1.96
11	0	-7	0.16	0.25
-11	0	-7	0.25	0.37
-11	0	7	-0.25	0.46
-11	0	7	0.02	0.29
-11	0	7	-0.15	0.36
-11	0	7	-0.82	0.49
11	0	7	0.23	0.26
-11	0	7	-0.10	0.38
11	0	7	-0.08	0.28
11	0	-7	0.28	0.27
11	0	-7	-0.07	0.25
11	0	-7	-0.06	0.23
11	0	7	0.02	0.69
11	0	7	-0.02	0.34
-11	0	-7	0.33	0.51
11	0	7	0.05	0.38
-11	0	-7	0.49	0.41
-11	0	-7	0.21	0.49
11	0	-7	-0.56	0.37
-11	0	-7	0.39	0.29
11	0	7	-0.21	0.44
-11	0	-7	0.55	0.47
11	1	7	34.81	1.94
11	1	7	33.39	1.83
11	-1	-7	31.54	1.69
11	1	7	36.34	2.12
11	-1	-7	32.39	1.68
-11	-1	7	34.54	2.07
-11	1	-7	32.64	1.84
-11	-1	7	34.49	1.72
-11	1	-7	30.60	1.80
-11	-1	7	32.69	1.90
-11	1	-7	33.84	1.75
-11	1	-7	34.11	1.81
-11	-1	7	30.67	1.69

11	1	7	31.23	1.84
-11	1	-7	35.08	1.90
11	1	7	32.45	1.75
-11	-1	7	34.70	1.98
11	-1	-7	30.82	1.76
-11	1	-7	32.94	1.99
11	1	7	30.55	1.81
11	-1	-7	34.61	1.72
-11	-1	7	36.19	1.83
-11	1	-7	30.58	1.99
-11	-1	-7	34.40	1.96
-11	-1	-7	31.61	1.83
11	-1	7	30.91	1.74
11	-1	7	30.55	1.80
-11	1	7	33.88	1.81
-11	1	7	32.04	1.76
11	1	-7	33.24	1.93
11	-1	7	34.58	2.18
11	1	-7	33.10	1.64
11	1	-7	33.56	1.73
-11	-1	-7	28.67	1.89
-11	-1	-7	32.74	1.69
-11	-1	-7	33.35	1.87
-11	1	7	34.17	1.71
-11	1	7	29.79	1.90
-11	1	7	32.56	2.06
-11	-1	-7	33.74	1.84
11	-1	7	32.44	1.84
11	1	-7	33.98	1.57
-11	1	7	36.39	1.88
11	-1	7	32.74	1.96
11	-2	-7	113.42	5.58
-11	2	-7	115.60	5.59
-11	-2	7	119.44	5.46
11	2	7	109.96	5.22
-11	2	-7	112.94	5.80
-11	2	-7	117.36	5.60
11	-2	-7	127.06	5.61
11	2	7	107.79	5.36
11	2	7	112.48	5.15
11	-2	-7	119.48	5.47
11	2	7	106.41	5.19
-11	-2	7	102.82	5.12
11	2	7	119.95	5.45

-11	-2	7	113.88	5.38
-11	2	-7	115.83	5.78
-11	2	-7	119.25	5.62
-11	-2	7	106.77	5.34
-11	-2	7	111.42	5.14
11	-2	-7	113.22	5.52
11	2	7	107.69	5.26
-11	2	-7	125.49	5.57
-11	-2	7	119.16	5.18
11	-2	7	107.13	5.21
11	2	-7	120.28	5.70
-11	2	7	113.17	5.28
11	-2	7	109.62	5.37
11	-2	7	102.84	5.22
-11	2	7	111.02	5.37
-11	2	7	111.11	5.12
-11	2	7	107.03	5.43
-11	-2	-7	117.35	5.57
11	2	-7	117.20	5.35
-11	-2	-7	119.03	5.62
-11	-2	-7	118.88	5.47
-11	-2	-7	121.68	5.76
-11	-2	-7	109.95	5.57
-11	-2	-7	123.36	5.62
11	2	-7	121.55	5.49
11	-2	7	105.43	5.19
-11	2	7	111.62	5.11
-11	2	7	108.35	5.15
11	3	7	62.17	3.26
-11	-3	7	62.24	3.20
-11	3	-7	82.40	3.81
11	-3	-7	74.82	3.76
-11	3	-7	76.62	4.12
11	3	7	64.72	3.28
-11	-3	7	70.78	3.55
11	3	7	67.16	3.48
-11	3	-7	78.86	3.79
11	3	7	65.24	3.14
-11	3	-7	73.68	3.76
11	-3	-7	81.84	3.89
-11	-3	7	67.84	3.52
-11	-3	7	62.34	3.14
-11	3	-7	75.94	3.79
11	-3	-7	71.03	3.78

11	3	7	70.87	3.51
-11	3	-7	83.56	3.86
-11	-3	7	61.11	3.44
-11	-3	7	68.94	3.20
11	-3	-7	76.56	3.67
-11	3	-7	72.90	4.06
-11	-3	7	66.25	3.22
-11	-3	-7	77.29	3.82
11	-3	7	63.20	3.28
-11	-3	-7	79.15	4.00
11	-3	7	62.60	3.28
-11	3	7	70.47	3.39
-11	-3	-7	77.22	3.76
11	-3	7	65.09	3.24
-11	-3	-7	79.41	3.82
-11	3	7	68.28	3.33
11	3	-7	74.49	3.70
11	-3	7	66.29	3.71
-11	-3	-7	77.22	3.67
11	-3	7	67.87	3.52
-11	3	7	67.02	3.19
-11	3	7	70.99	3.57
-11	3	7	65.66	3.34
11	3	-7	78.32	3.57
-11	-3	-7	77.58	3.81
11	3	-7	78.51	3.89
-11	4	-7	186.10	8.23
11	4	7	185.17	9.21
-11	-4	7	183.96	8.96
11	-4	-7	167.84	8.23
-11	-4	7	208.73	9.26
-11	-4	7	195.76	8.97
11	4	7	201.55	9.20
-11	4	-7	187.46	8.28
-11	-4	7	189.83	9.22
11	4	7	202.87	9.01
-11	4	-7	175.08	8.56
-11	-4	7	184.00	8.85
11	-4	-7	177.14	8.25
-11	4	-7	170.26	8.17
-11	4	-7	186.33	8.28
-11	-4	7	205.05	8.89
-11	4	-7	173.85	8.54
-11	4	-7	167.29	8.18

-11	-4	7	193.62	9.25
11	4	7	187.93	9.04
11	-4	-7	179.20	8.11
11	-4	-7	185.90	8.37
-11	4	7	197.63	8.94
-11	-4	-7	174.09	8.10
11	-4	7	209.81	9.45
-11	4	7	186.91	9.12
11	4	-7	183.47	8.06
11	-4	7	191.27	9.05
-11	-4	-7	173.49	8.24
-11	-4	-7	177.95	8.18
11	-4	7	193.55	9.04
-11	4	7	196.30	9.21
11	-4	7	189.51	8.94
-11	-4	-7	176.61	8.44
-11	-4	-7	178.57	8.26
-11	-4	-7	170.74	8.24
-11	4	7	203.63	9.11
11	-4	7	194.12	9.23
-11	4	7	196.48	9.09
11	4	-7	163.43	8.17
11	4	-7	182.45	8.31
-11	4	7	194.14	9.24
-11	5	-7	67.29	3.44
-11	-5	7	75.60	3.81
11	5	7	69.64	3.48
-11	-5	7	66.11	3.46
11	-5	-7	71.64	3.57
-11	5	-7	69.01	3.89
11	5	7	66.69	3.55
-11	-5	7	68.61	3.29
11	5	7	68.19	3.75
11	-5	-7	68.12	3.37
-11	5	-7	63.96	3.93
-11	-5	7	66.54	3.79
-11	-5	7	72.08	3.30
-11	5	-7	74.91	3.63
-11	5	-7	71.10	3.57
11	5	7	70.64	3.74
11	-5	-7	66.22	3.50
-11	5	-7	70.60	3.45
-11	-5	7	68.85	3.77
-11	5	-7	74.54	3.52

-11	5	7	69.31	3.78
-11	-5	-7	71.60	3.79
11	5	-7	72.41	3.36
11	-5	7	67.94	3.38
11	5	-7	61.94	3.50
11	-5	7	63.69	3.48
11	5	-7	71.88	3.44
-11	5	7	68.68	3.73
-11	-5	-7	66.96	3.51
11	-5	7	70.59	3.81
-11	-5	-7	68.53	3.45
-11	-5	-7	70.58	3.54
-11	5	7	71.28	3.67
11	5	-7	72.51	3.56
-11	5	7	73.19	3.44
-11	-5	-7	68.93	3.52
-11	5	7	69.90	3.74
-11	-5	-7	65.15	3.38
11	-5	7	75.20	4.04
-11	5	7	72.40	3.61
11	-5	7	69.07	3.55
-11	6	-7	26.59	1.69
-11	-6	7	19.03	1.71
11	6	7	21.12	1.77
-11	6	-7	21.21	2.37
11	-6	-7	25.91	1.73
11	6	7	16.56	1.46
-11	-6	7	19.93	1.06
-11	-6	7	20.79	1.13
-11	-6	7	20.07	1.11
-11	6	-7	25.44	1.56
-11	6	-7	25.42	2.10
-11	6	-7	25.81	1.63
-11	-6	7	20.27	1.37
-11	-6	7	17.44	1.85
-11	6	-7	26.39	1.84
11	6	7	18.34	1.40
11	-6	-7	25.16	1.50
11	6	7	19.99	1.68
11	-6	-7	24.00	1.63
11	-6	-7	26.65	1.98
-11	6	7	18.29	1.84
-11	6	7	18.59	1.65
11	-6	7	24.06	2.20

-11	6	7	21.05	1.39
11	6	-7	24.80	1.77
-11	-6	-7	24.91	1.64
-11	-6	-7	24.34	1.68
11	-6	7	20.46	1.46
11	-6	7	20.00	1.23
-11	-6	-7	26.55	1.70
11	6	-7	26.54	1.63
11	6	-7	26.84	1.71
11	-6	7	18.67	1.35
11	6	-7	27.94	1.57
11	7	7	2.52	0.99
-11	-7	7	3.04	1.11
11	7	7	2.30	0.69
-11	-7	7	3.18	0.49
-11	7	-7	2.85	0.72
-11	7	-7	3.98	0.78
11	7	7	3.12	1.12
11	-7	-7	3.48	0.82
-11	-7	7	2.38	0.62
11	7	7	1.60	1.22
11	-7	-7	3.95	1.08
11	-7	-7	3.45	0.58
11	-7	-7	2.27	0.69
-11	7	-7	2.78	0.61
-11	-7	7	3.53	1.03
-11	7	-7	3.41	1.38
-11	7	-7	4.36	1.26
11	-7	7	3.35	0.65
11	-7	7	3.92	1.41
11	7	-7	4.05	0.72
-11	-7	-7	3.72	0.71
11	-7	7	2.36	0.67
11	-7	7	3.43	0.67
11	7	-7	1.39	0.77
-11	-7	-7	3.83	0.75
-11	7	7	3.13	0.94
-11	7	7	2.44	1.05
-11	7	7	2.69	0.69
-11	-7	-7	4.11	0.88
11	7	-7	3.63	0.63
-11	8	-7	34.85	2.81
11	-8	-7	39.11	2.80
11	-8	-7	34.92	2.05

11	-8	-7	35.81	2.20
-11	8	-7	37.86	2.08
-11	-8	7	28.69	2.41
11	8	7	35.15	2.49
-11	8	-7	33.07	2.10
-11	-8	7	29.74	2.03
-11	-8	7	33.12	1.78
11	-8	-7	35.46	2.37
-11	-8	7	32.58	2.57
11	8	7	28.19	2.20
11	8	-7	39.15	2.22
-11	8	7	29.40	2.59
11	-8	7	38.76	3.05
-11	-8	-7	35.41	2.31
-11	-8	-7	36.58	2.23
11	8	-7	33.34	2.51
11	8	-7	38.90	2.18
11	-8	7	32.07	2.11
-11	-8	-7	36.15	2.21
11	-8	7	31.03	2.02
11	-8	7	33.28	1.79
-11	8	7	32.41	2.39
-11	-9	7	20.61	2.20
11	9	7	19.54	2.02
11	-9	-7	21.99	1.66
11	-9	-7	20.46	1.80
11	9	7	22.76	2.21
-11	9	-7	21.00	2.48
-11	9	-7	21.36	1.51
-11	-9	7	24.40	1.77
-11	-9	7	25.67	3.41
11	-9	-7	20.20	1.53
-11	9	-7	22.72	2.57
11	9	-7	24.19	1.72
11	9	-7	19.43	2.07
11	-9	7	23.27	1.46
11	-9	7	21.20	1.76
-11	-9	-7	22.18	1.84
-11	-9	-7	21.93	1.65
11	-9	7	20.50	1.65
-11	9	7	25.84	2.29
11	9	-7	21.46	1.91
11	-10	-7	11.66	1.31
11	10	7	14.17	2.23

11	-10	-7	12.56	1.66
-11	10	-7	13.73	2.19
-11	10	-7	10.89	1.37
-11	-10	7	15.63	1.63
-11	-10	7	12.62	2.28
11	-10	-7	10.82	1.37
11	10	7	13.58	2.20
-11	10	7	17.57	2.32
11	10	-7	8.93	1.83
11	-10	7	17.34	1.64
-11	-10	-7	13.48	1.77
11	10	-7	14.56	1.49
-11	11	-7	38.15	2.18
11	11	7	30.72	2.79
11	11	7	29.55	2.96
11	-11	-7	32.36	2.61
-11	-11	7	30.68	2.76
-11	11	-7	31.65	3.29
11	-11	-7	33.73	2.33
11	-11	-7	34.98	2.39
-11	-11	7	34.78	2.39
-11	-11	-7	31.35	2.33
-11	11	7	37.76	3.32
-11	-11	-7	35.56	2.44
11	11	-7	28.81	2.91
11	-11	7	35.83	2.27
11	-11	7	32.91	2.53
-11	-12	7	17.63	1.89
11	-12	-7	30.13	2.14
-12	0	7	50.94	2.86
12	0	7	53.90	2.86
-12	0	-7	51.40	2.54
12	0	-7	50.31	2.47
-12	0	-7	49.40	2.67
12	0	7	52.82	2.70
12	0	-7	55.63	2.47
12	0	-7	51.61	2.54
-12	0	7	56.23	2.79
-12	0	-7	48.22	2.78
-12	0	-7	52.17	2.73
12	0	7	52.57	2.78
-12	0	-7	50.86	2.67
-12	0	7	54.68	2.79
-12	0	7	59.56	2.85

12	0	7	56.24	3.10
-12	0	7	54.12	2.66
12	0	-7	50.01	2.59
12	0	7	54.43	2.79
12	0	7	55.30	2.85
-12	0	-7	56.40	2.79
-12	0	7	52.60	3.02
12	1	7	49.31	2.62
12	-1	-7	55.69	2.54
-12	1	-7	51.97	2.54
-12	-1	7	55.83	2.63
12	-1	-7	48.41	2.48
12	1	7	51.20	2.65
12	1	7	52.75	2.75
12	-1	-7	48.22	2.49
-12	1	-7	50.69	2.79
-12	1	-7	51.92	2.66
-12	1	-7	52.82	2.64
-12	-1	7	54.12	2.90
-12	-1	7	49.43	2.50
-12	1	-7	51.35	2.70
-12	-1	7	52.27	2.53
-12	1	-7	49.13	2.83
12	-1	-7	49.99	2.59
-12	-1	7	51.70	2.71
12	1	7	49.04	2.56
-12	-1	7	53.82	2.77
-12	1	-7	48.25	2.63
12	1	7	46.72	2.63
12	1	-7	51.22	2.41
12	1	-7	50.72	2.54
-12	-1	-7	54.03	2.80
-12	1	7	50.61	2.76
12	-1	7	52.61	2.65
-12	-1	-7	51.19	2.63
-12	-1	-7	51.28	2.48
-12	-1	-7	52.61	2.65
12	1	-7	51.38	2.36
12	1	-7	49.58	2.74
-12	-1	-7	48.24	2.70
-12	1	7	48.83	2.57
-12	1	7	52.30	2.52
12	-1	7	50.76	2.77
-12	1	7	50.16	2.87

12	-1	7	48.36	2.55
-12	1	7	48.40	2.72
12	-1	7	53.65	2.99
12	-1	7	51.09	2.65
-12	1	7	51.91	2.67
-12	2	-7	54.24	2.80
12	-2	-7	51.14	2.70
-12	2	-7	52.42	3.06
-12	-2	7	55.04	2.60
-12	-2	7	54.00	2.85
-12	-2	7	57.17	2.94
12	2	7	49.97	2.67
-12	2	-7	55.18	2.83
12	2	7	51.30	2.59
12	2	7	53.58	2.66
-12	2	-7	53.66	3.00
-12	2	-7	58.13	2.82
12	-2	-7	55.03	2.79
12	2	7	49.89	2.79
-12	-2	7	53.07	2.56
-12	-2	7	48.63	2.76
12	2	7	53.03	2.88
12	-2	-7	58.37	2.79
-12	2	-7	57.66	2.85
-12	-2	7	49.57	2.52
-12	2	-7	54.59	2.75
12	-2	-7	55.90	2.67
12	2	7	47.76	2.57
12	-2	7	51.01	2.99
-12	2	7	51.31	2.90
-12	2	7	46.81	2.48
-12	2	7	51.17	2.54
-12	2	7	51.42	2.70
-12	-2	-7	53.47	2.81
-12	-2	-7	55.59	2.99
12	-2	7	48.53	2.61
12	2	-7	55.42	2.90
-12	-2	-7	55.39	2.64
-12	-2	-7	52.79	2.82
-12	2	7	52.61	2.54
-12	-2	-7	54.66	2.75
-12	2	7	48.40	2.78
12	2	-7	55.00	2.51
-12	2	7	52.58	2.53

12	-2	7	50.30	2.64
12	2	-7	54.55	2.68
12	-2	7	49.40	2.58
12	-2	7	48.72	2.82
-12	-2	-7	50.51	2.83
-12	2	7	51.33	2.56
12	2	-7	52.81	2.61
12	-3	-7	0.84	0.56
-12	-3	7	1.48	0.33
12	3	7	1.76	0.69
-12	-3	7	0.24	0.56
-12	3	-7	0.46	0.47
-12	3	-7	0.31	0.83
-12	-3	7	0.72	0.72
-12	-3	7	1.69	0.44
-12	-3	7	1.42	0.38
-12	3	-7	0.70	0.39
-12	-3	7	0.85	0.41
-12	3	-7	1.41	0.73
12	-3	-7	1.42	0.41
-12	3	-7	1.41	0.54
-12	3	-7	0.53	0.46
-12	-3	7	1.48	0.72
12	3	7	0.95	0.41
12	-3	-7	0.64	0.30
12	3	7	1.43	0.49
12	3	7	0.78	0.54
-12	3	-7	1.14	0.48
12	-3	-7	0.99	0.39
12	-3	7	1.01	0.41
12	-3	7	1.25	0.73
-12	3	7	1.48	0.42
-12	-3	-7	1.16	0.35
12	-3	7	1.28	0.42
-12	-3	-7	1.77	0.50
12	-3	7	1.43	0.43
-12	3	7	1.14	0.73
12	3	-7	0.94	0.22
-12	-3	-7	1.29	0.47
-12	-3	-7	0.46	0.41
12	3	-7	1.21	0.51
-12	-3	-7	1.39	0.47
-12	-3	-7	0.31	0.63
-12	3	7	0.85	0.51

-12	-3	-7	1.15	0.46
12	3	-7	1.31	0.41
-12	3	7	1.13	0.55
-12	3	7	1.94	0.62
-12	3	7	1.75	0.58
-12	4	-7	51.13	3.16
12	-4	-7	58.00	2.96
-12	4	-7	50.37	2.71
12	4	7	57.33	2.86
-12	-4	7	61.15	3.20
-12	4	-7	57.28	2.77
-12	-4	7	56.09	3.17
-12	-4	7	56.01	2.81
12	4	7	53.68	3.08
-12	4	-7	50.65	2.70
-12	4	-7	54.38	2.78
12	-4	-7	51.73	2.73
-12	-4	7	52.00	2.78
-12	4	-7	51.36	2.79
-12	4	-7	51.29	3.11
12	4	7	52.28	2.84
-12	-4	7	51.72	2.67
12	-4	-7	49.34	2.73
12	4	7	56.45	3.07
12	-4	-7	50.85	2.60
-12	-4	7	54.94	3.11
-12	-4	7	57.81	2.73
-12	-4	-7	53.17	2.68
12	4	-7	49.37	2.67
12	4	-7	53.32	2.51
-12	4	7	56.86	2.98
-12	-4	-7	51.11	2.76
-12	4	7	55.14	2.94
-12	-4	-7	51.01	2.57
-12	-4	-7	52.98	3.01
-12	-4	-7	53.16	2.78
-12	-4	-7	51.02	2.76
12	4	-7	53.70	2.81
12	-4	7	55.58	2.76
12	-4	7	59.56	3.36
-12	4	7	55.81	3.16
12	-4	7	55.90	3.16
-12	4	7	54.76	3.07
12	-4	7	53.76	2.86

-12	-4	-7	51.52	2.59
-12	4	7	53.04	3.00
12	-4	7	51.66	2.84
-12	4	7	54.84	2.77
12	5	7	29.67	2.16
-12	5	-7	41.83	2.35
-12	-5	7	32.45	1.75
12	5	7	32.58	1.99
-12	5	-7	43.81	2.97
-12	-5	7	34.37	1.81
-12	5	-7	43.56	2.84
-12	-5	7	33.06	2.34
12	-5	-7	43.81	2.28
-12	5	-7	44.86	2.44
-12	-5	7	32.36	2.26
-12	-5	7	30.38	1.89
12	5	7	35.29	2.20
12	-5	-7	43.72	2.46
-12	5	-7	46.80	2.51
12	-5	-7	39.66	2.39
12	5	7	32.50	1.95
-12	5	-7	42.37	2.36
-12	-5	7	34.00	1.94
12	-5	7	33.41	1.94
12	5	-7	43.41	2.42
12	5	-7	46.04	2.46
-12	-5	-7	43.33	2.52
-12	5	7	37.74	2.28
12	-5	7	33.60	1.82
12	-5	7	40.12	2.64
12	5	-7	46.83	2.23
12	-5	7	33.53	1.99
-12	5	7	34.65	1.92
-12	-5	-7	46.43	2.47
-12	5	7	34.80	2.23
-12	-5	-7	44.09	2.44
12	6	7	63.79	3.50
-12	6	-7	64.57	3.36
-12	6	-7	66.08	3.92
-12	-6	7	65.77	3.74
12	-6	-7	71.05	3.74
-12	-6	7	63.60	3.16
-12	6	-7	65.28	3.34
12	6	7	71.48	3.71

12	6	7	65.81	3.74
12	6	7	64.50	3.37
-12	-6	7	66.28	3.75
12	-6	-7	64.68	3.44
-12	-6	7	65.49	3.39
-12	6	-7	69.55	3.45
12	-6	-7	65.69	3.49
12	-6	-7	65.06	3.30
-12	6	-7	69.50	4.04
12	6	-7	62.41	3.35
12	-6	7	68.23	3.24
-12	6	7	66.11	3.75
12	-6	7	76.39	4.12
-12	6	7	64.74	3.66
12	6	-7	69.23	3.32
-12	-6	-7	66.38	3.50
-12	-6	-7	67.19	3.45
12	-6	7	61.60	3.39
12	6	-7	64.17	3.52
12	-6	7	65.76	3.48
12	6	-7	68.34	3.46
-12	6	7	65.61	3.37
-12	7	-7	16.80	1.29
12	7	7	16.38	1.70
12	7	7	14.64	1.45
-12	7	-7	17.62	2.46
12	-7	-7	17.67	1.23
12	-7	-7	17.19	1.38
-12	-7	7	15.13	1.74
-12	7	-7	18.93	2.02
-12	-7	7	14.63	0.93
-12	-7	7	15.28	0.98
12	-7	-7	17.92	1.51
-12	-7	7	13.76	1.25
12	7	7	16.28	1.88
-12	-7	7	15.68	0.96
-12	7	-7	18.52	1.41
12	-7	-7	18.30	1.87
-12	-7	7	13.70	1.99
12	-7	7	17.36	1.10
-12	7	7	15.67	1.36
-12	-7	-7	16.92	1.46
-12	7	7	17.93	1.99
12	-7	7	13.48	1.33

-12	-7	-7	16.50	1.45
12	7	-7	17.46	1.43
12	-7	7	14.33	1.26
12	7	-7	16.94	1.36
12	-7	7	16.57	2.27
12	7	-7	17.60	1.61
-12	-8	7	19.75	1.55
12	-8	-7	19.73	1.69
12	8	7	21.72	2.09
-12	8	-7	20.76	1.48
12	8	7	23.88	2.11
-12	-8	7	18.89	1.26
-12	-8	7	23.35	2.31
-12	8	-7	18.10	1.49
-12	8	-7	20.35	2.74
12	-8	-7	24.06	2.26
12	-8	-7	19.05	1.44
12	-8	-7	21.33	1.58
12	8	7	21.13	1.90
-12	-8	7	20.73	2.06
-12	8	-7	18.15	2.31
12	8	-7	20.00	1.60
12	-8	7	19.76	1.65
12	-8	7	19.81	1.53
12	8	-7	20.00	1.86
-12	8	7	19.60	2.07
-12	8	7	19.97	2.28
12	-8	7	20.51	1.30
-12	-8	-7	23.29	1.72
12	8	-7	21.62	1.61
12	-8	7	23.24	2.86
-12	-8	-7	19.02	1.59
-12	9	-7	0.72	1.16
12	-9	-7	-0.18	0.57
12	-9	-7	0.31	0.65
12	9	7	1.25	1.03
-12	-9	7	-0.32	1.06
12	-9	-7	1.69	0.87
-12	-9	7	0.61	0.75
-12	9	-7	0.17	0.68
12	9	7	1.44	0.96
-12	9	7	0.34	1.03
12	9	-7	1.11	0.75
12	-9	7	0.32	0.51

12	9	-7	0.51	1.08
-12	-9	-7	0.27	0.78
12	-9	7	0.49	0.64
12	9	-7	-0.13	0.77
-12	-9	-7	0.53	0.78
12	-10	-7	5.40	0.89
12	10	7	4.87	1.49
12	10	7	4.46	1.45
-12	10	-7	7.25	1.12
-12	-10	7	4.76	1.15
12	-10	-7	6.18	1.05
12	-10	-7	5.84	1.23
-12	10	-7	4.82	1.90
-12	-10	7	5.41	1.45
12	-10	7	4.58	1.12
12	-10	7	5.03	0.97
12	10	-7	3.89	1.39
-12	-10	-7	3.63	0.97
-12	-10	-7	5.16	1.13
12	10	-7	5.57	1.00
-12	10	7	3.67	1.41
12	-11	-7	0.93	0.91
-12	11	-7	1.99	0.81
-12	-11	7	0.08	1.16
-12	-11	7	1.78	0.92
12	-11	-7	1.82	0.96
12	11	7	0.84	1.18
12	11	7	0.51	1.25
-12	11	-7	-0.86	1.52
12	-11	-7	0.58	1.14
12	-11	7	2.42	1.07
-12	-11	-7	1.82	0.97
-12	11	7	-0.91	1.64
12	11	-7	1.36	1.32
12	-11	7	0.19	0.79
-12	-11	-7	-0.08	0.91
-13	0	7	0.09	0.32
13	0	-7	-0.16	0.27
-13	0	7	-0.37	0.55
13	0	-7	-0.02	0.32
13	0	7	0.09	0.55
13	0	7	0.15	0.36
-13	0	7	-0.45	0.64
-13	0	-7	0.28	0.33

13	0	-7	0.22	0.22
-13	0	7	0.19	0.45
-13	0	-7	-0.53	0.51
-13	0	-7	0.39	0.46
-13	0	-7	0.12	0.36
-13	0	7	-0.51	0.42
-13	0	-7	-0.50	0.51
-13	0	7	-0.01	0.42
13	0	7	0.03	0.44
13	0	7	0.18	0.27
-13	0	-7	0.06	0.40
13	0	7	0.92	0.71
13	0	-7	0.06	0.24
-13	0	-7	-0.25	0.48
-13	1	-7	36.89	2.00
-13	1	-7	33.71	1.94
-13	1	-7	32.36	2.16
-13	-1	7	28.31	1.83
13	-1	-7	34.05	1.81
13	1	7	31.93	1.82
-13	-1	7	31.51	1.91
13	1	7	29.48	1.87
13	1	7	29.75	1.77
-13	-1	7	30.60	1.66
13	1	7	31.79	2.17
13	1	7	29.38	1.65
-13	1	-7	37.47	1.85
13	1	7	29.30	1.76
13	-1	-7	34.73	1.81
13	-1	-7	32.89	1.88
-13	-1	7	28.90	1.85
-13	-1	7	35.05	1.79
-13	1	-7	34.04	2.01
13	-1	-7	34.03	1.77
-13	1	-7	33.42	2.10
-13	-1	7	27.03	1.60
-13	1	-7	35.92	1.96
-13	-1	7	29.90	2.05
-13	-1	-7	33.81	2.12
-13	-1	-7	33.05	1.95
13	-1	7	29.63	1.75
-13	1	7	29.34	2.07
13	-1	7	31.69	1.80
-13	-1	-7	36.53	1.78

-13	1	7	30.93	1.81
13	-1	7	33.74	2.22
-13	-1	-7	33.36	2.15
-13	-1	-7	31.26	2.04
13	-1	7	29.99	1.66
-13	-1	-7	36.18	1.98
-13	1	7	29.43	1.90
13	1	-7	35.25	1.67
13	-1	7	31.20	1.94
13	1	-7	34.61	1.85
13	1	-7	34.52	1.63
-13	1	7	32.60	1.82
-13	1	7	27.96	1.71
-13	1	7	30.26	1.65
13	2	7	56.56	2.93
-13	2	-7	64.73	3.35
-13	2	-7	62.88	3.58
13	2	7	56.44	2.86
-13	2	-7	64.98	3.37
-13	-2	7	52.31	2.80
13	-2	-7	68.81	3.21
-13	2	-7	68.43	3.28
13	2	7	61.31	3.19
13	2	7	56.87	2.88
-13	2	-7	66.75	3.54
-13	-2	7	51.90	2.79
-13	-2	7	57.49	2.84
13	2	7	56.17	3.08
-13	-2	7	56.22	3.07
13	2	7	54.42	2.95
-13	2	-7	65.90	3.34
-13	-2	7	56.88	3.19
-13	2	-7	62.57	3.32
13	-2	-7	69.36	3.29
-13	-2	7	61.28	2.89
-13	-2	7	58.91	3.11
13	-2	-7	62.66	3.21
-13	-2	-7	61.76	3.35
13	-2	7	58.97	2.91
13	-2	7	57.97	3.13
-13	2	7	59.02	3.12
-13	-2	-7	65.52	3.26
-13	-2	-7	69.30	3.54
13	2	-7	66.85	3.08

-13	-2	-7	67.85	3.37
-13	2	7	58.06	2.82
-13	-2	-7	65.81	3.36
-13	2	7	57.40	2.77
13	-2	7	59.21	3.31
-13	-2	-7	67.25	3.15
13	-2	7	56.39	2.93
13	2	-7	66.89	3.01
-13	2	7	57.49	3.22
13	2	-7	67.58	3.45
-13	2	7	58.10	2.78
13	-2	7	55.03	2.86
-13	2	7	52.20	2.76
-13	2	7	58.74	3.00
-13	2	7	57.70	2.82
-13	-3	7	68.24	3.60
-13	3	-7	59.95	3.03
13	-3	-7	56.19	2.97
-13	-3	7	72.18	3.89
13	-3	-7	61.09	3.05
13	3	7	71.73	3.65
-13	-3	7	76.63	3.95
-13	3	-7	57.79	3.32
-13	-3	7	79.83	4.00
13	-3	-7	60.67	2.85
13	3	7	71.54	3.89
-13	-3	7	75.63	3.64
13	-3	-7	56.80	2.90
-13	-3	7	79.65	3.64
-13	3	-7	57.59	2.96
-13	3	-7	56.48	2.96
13	3	7	75.56	3.72
-13	3	-7	54.22	2.94
-13	-3	7	70.85	3.56
13	3	7	83.56	3.97
13	-3	7	76.75	3.66
13	-3	7	69.04	3.69
13	3	-7	57.68	2.80
-13	-3	-7	55.88	2.77
-13	-3	-7	53.15	2.98
-13	3	7	74.71	3.73
-13	3	7	73.89	3.90
-13	-3	-7	57.39	2.98
-13	3	7	71.96	3.98

-13	-3	-7	58.02	2.88
-13	3	7	74.43	3.67
-13	-3	-7	56.91	3.21
-13	3	7	77.59	3.81
-13	-3	-7	54.89	2.93
13	-3	7	70.71	3.92
13	3	-7	58.53	2.67
-13	3	7	75.44	3.61
-13	-3	-7	60.50	2.89
13	3	-7	59.45	3.07
-13	-3	-7	57.99	3.01
13	-3	7	74.83	3.69
13	-4	-7	81.11	4.01
13	-4	-7	79.59	3.89
-13	4	-7	82.77	4.44
-13	-4	7	75.05	3.65
-13	4	-7	79.06	3.98
13	-4	-7	88.23	4.18
-13	-4	7	70.38	3.63
-13	-4	7	72.25	3.97
-13	-4	7	78.11	4.02
13	4	7	72.55	3.94
-13	-4	7	69.00	3.52
13	-4	-7	77.99	4.03
-13	4	-7	77.67	4.40
-13	-4	7	77.14	3.58
-13	4	-7	81.17	4.03
-13	4	-7	78.86	3.99
-13	-4	7	69.41	3.91
13	4	7	69.95	3.67
13	4	7	78.98	3.92
13	4	7	74.45	3.70
-13	-4	-7	81.77	4.07
-13	-4	-7	81.18	3.90
-13	-4	-7	81.03	3.92
-13	4	7	74.61	3.62
13	-4	7	79.83	4.19
-13	4	7	74.22	3.94
-13	-4	-7	81.16	4.06
13	4	-7	84.10	3.78
-13	-4	-7	80.19	3.83
13	4	-7	82.39	4.10
13	-4	7	69.08	3.68
-13	4	7	75.30	3.83

13	-4	7	75.73	3.60
13	-4	7	73.45	3.70
13	4	-7	78.95	3.92
-13	5	-7	97.46	5.37
13	-5	-7	106.99	5.12
-13	-5	7	89.48	4.22
13	5	7	83.30	4.40
-13	-5	7	93.38	4.71
13	-5	-7	99.69	4.92
13	5	7	87.00	4.36
-13	5	-7	95.78	4.81
-13	-5	7	83.79	4.18
-13	5	-7	99.32	4.87
-13	-5	7	87.96	4.67
-13	-5	7	85.01	4.34
-13	-5	7	86.94	4.35
13	5	7	86.05	4.65
-13	5	-7	100.03	5.30
13	5	7	97.03	4.65
13	-5	-7	99.92	4.92
13	-5	-7	98.58	4.78
-13	5	7	89.14	4.33
13	-5	7	95.97	4.97
-13	-5	-7	100.74	4.95
13	-5	7	91.80	4.45
13	5	-7	97.69	4.85
-13	-5	-7	99.79	4.78
13	5	-7	103.99	4.71
-13	-5	-7	101.11	4.93
-13	5	7	91.12	4.63
13	-5	7	89.94	4.25
-13	5	7	89.48	4.66
13	5	-7	102.58	4.95
13	-5	7	86.43	4.39
-13	-6	7	72.78	4.00
-13	6	-7	61.45	3.75
-13	-6	7	64.33	3.55
-13	-6	7	67.04	3.36
13	-6	-7	54.48	3.34
-13	6	-7	57.56	3.64
-13	6	-7	59.73	3.05
13	-6	-7	59.74	3.02
13	-6	-7	57.78	3.19
-13	-6	7	74.26	3.38

13	6	7	74.82	3.88
-13	6	-7	60.01	3.09
13	6	7	64.93	3.89
-13	-6	-7	58.96	3.23
13	-6	7	80.94	4.31
-13	6	7	66.40	3.91
13	-6	7	65.21	3.60
13	6	-7	63.05	3.00
13	-6	7	71.24	3.41
13	-6	7	70.93	3.66
-13	-6	-7	57.14	3.16
-13	6	7	70.11	3.57
13	6	-7	64.27	3.20
-13	-6	-7	59.28	3.05
13	7	7	1.47	0.76
-13	-7	7	2.85	0.50
13	-7	-7	3.36	0.77
-13	-7	7	1.80	0.66
-13	7	-7	0.92	1.15
-13	7	-7	2.23	1.42
13	7	7	3.13	0.99
13	-7	-7	2.92	0.58
-13	7	-7	3.24	0.80
13	-7	-7	2.63	1.08
13	-7	-7	2.18	0.68
-13	-7	7	2.45	0.98
-13	7	-7	3.16	0.70
13	7	7	1.57	1.00
-13	-7	7	3.66	1.29
13	-7	7	2.13	1.41
-13	7	7	1.34	0.92
13	-7	7	1.13	0.54
-13	7	7	2.09	1.18
13	7	-7	4.22	0.80
13	-7	7	1.49	0.66
-13	-7	-7	2.47	0.73
13	7	-7	2.04	0.84
13	7	-7	3.62	0.66
-13	-7	-7	2.98	0.79
-13	-7	-7	4.25	0.75
13	-7	7	1.02	0.73
-13	-8	7	6.68	1.45
13	-8	-7	6.56	1.10
-13	8	-7	8.93	1.70

-13	8	-7	7.27	1.14
13	8	7	6.85	1.41
-13	8	-7	12.23	3.07
-13	-8	7	9.20	1.17
13	8	7	7.83	1.47
13	-8	-7	5.87	1.09
13	-8	-7	7.50	1.32
13	8	7	7.26	1.33
-13	-8	7	7.28	1.41
-13	8	7	7.96	1.51
13	8	-7	7.87	1.13
-13	-8	-7	7.74	1.07
-13	-8	-7	7.25	1.29
13	-8	7	6.88	1.13
13	-8	7	6.10	0.87
13	8	-7	8.80	1.19
13	-8	7	6.83	1.32
13	8	-7	6.63	1.22
-13	9	-7	28.43	3.06
-13	-9	7	28.37	2.67
13	-9	-7	30.39	2.19
13	9	7	27.38	2.69
-13	-9	7	29.23	2.03
13	-9	-7	30.17	2.04
-13	9	-7	31.84	1.95
13	9	7	24.71	2.42
13	-9	-7	27.32	1.89
13	-9	7	28.05	2.03
-13	9	7	31.62	2.60
-13	-9	-7	31.95	2.28
13	-9	7	27.47	2.09
13	9	-7	24.80	2.50
13	9	-7	29.42	2.00
-13	10	-7	29.03	1.99
-13	10	-7	29.16	3.09
13	10	7	25.41	2.49
13	-10	-7	27.06	2.15
-13	-10	7	26.42	2.54
13	-10	-7	30.98	2.33
13	10	7	24.07	2.57
13	-10	-7	29.59	1.98
-13	-10	7	27.62	2.25
-13	-10	-7	28.54	2.17
13	-10	7	24.80	1.94

-13	-10	-7	30.52	2.32
13	-10	7	27.35	2.21
-13	10	7	25.63	2.97
13	10	-7	27.09	2.58
13	11	7	39.37	3.24
13	11	7	44.32	3.36
13	-11	-7	46.99	2.86
13	-11	-7	48.30	2.85
-13	11	-7	47.28	3.88
-13	11	-7	50.83	2.81
-13	-11	7	46.16	3.36
13	-11	-7	46.88	3.29
-13	-11	7	43.87	2.84
-13	-11	-7	43.02	2.94
-13	-11	-7	48.57	3.06
13	-11	7	44.39	3.02
-13	11	7	48.99	3.65
13	-11	7	43.07	2.74
13	11	-7	44.42	3.44
14	0	-7	20.57	1.38
-14	0	7	17.03	1.28
-14	0	7	20.13	1.38
-14	0	7	16.22	1.62
-14	0	-7	22.69	1.49
-14	0	-7	25.07	1.55
14	0	7	14.52	1.16
-14	0	7	16.33	1.09
14	0	-7	26.13	1.22
14	0	7	18.45	1.72
-14	0	-7	20.27	1.70
14	0	7	14.30	1.36
14	0	7	15.12	1.06
-14	0	7	15.13	1.31
14	0	-7	24.75	1.35
-14	0	-7	22.86	1.69
-14	0	-7	23.84	1.64
14	0	7	17.33	1.32
14	0	-7	20.84	1.18
-14	0	-7	20.01	1.28
-14	1	-7	7.09	0.86
14	1	7	8.21	0.98
-14	1	-7	6.41	0.93
14	1	7	8.23	1.08
-14	-1	7	7.57	1.03

-14	-1	7	8.65	0.97
-14	1	-7	8.11	0.92
-14	1	-7	8.93	1.04
-14	1	-7	6.83	1.19
14	-1	-7	5.64	0.75
14	-1	-7	5.82	0.63
-14	-1	7	9.50	0.76
14	1	7	9.25	1.07
-14	-1	7	7.28	1.35
14	1	7	7.97	0.89
-14	-1	7	12.29	1.20
-14	1	-7	5.59	0.71
-14	1	-7	7.99	1.11
14	1	7	8.91	0.77
14	1	7	10.29	1.48
-14	-1	7	7.99	0.80
14	1	-7	5.74	0.47
-14	1	7	7.15	1.09
14	-1	7	8.29	0.88
14	1	-7	8.02	0.45
-14	1	7	9.79	1.03
-14	1	7	9.48	0.82
-14	-1	-7	7.50	0.89
14	-1	7	8.71	0.93
-14	-1	-7	7.55	0.91
-14	1	7	7.39	1.03
14	1	-7	7.17	0.76
-14	-1	-7	7.32	0.93
-14	-1	-7	6.52	1.11
14	-1	7	6.64	1.21
14	-1	7	10.83	1.40
-14	1	7	7.19	1.37
-14	-1	-7	9.72	1.11
-14	-1	-7	7.35	0.67
14	-1	7	8.01	0.76
-14	2	-7	23.57	1.83
14	2	7	20.99	1.30
14	-2	-7	21.86	1.46
-14	2	-7	22.93	1.50
14	2	7	19.07	1.43
-14	2	-7	20.96	1.51
-14	-2	7	20.78	1.48
-14	-2	7	19.28	1.57
14	2	7	21.54	1.44

-14	-2	7	21.47	1.28
14	2	7	22.17	1.42
-14	-2	7	22.56	1.34
-14	-2	7	20.68	1.65
-14	2	-7	24.63	1.60
14	-2	-7	25.44	1.48
14	-2	-7	24.68	1.36
14	2	7	20.07	1.80
14	-2	-7	23.05	1.35
-14	2	-7	23.27	1.44
-14	2	-7	23.97	1.85
-14	-2	7	20.94	1.39
-14	-2	7	20.81	1.26
-14	2	-7	25.07	1.59
14	2	7	22.97	1.60
-14	-2	-7	21.48	1.58
-14	2	7	21.85	1.54
14	-2	7	20.28	1.39
-14	2	7	21.51	1.62
-14	2	7	21.30	1.33
-14	2	7	20.69	1.25
14	-2	7	21.16	1.70
-14	2	7	22.12	1.80
14	-2	7	20.32	2.03
-14	-2	-7	23.48	1.40
-14	-2	-7	23.84	1.56
14	-2	7	19.72	1.29
-14	-2	-7	22.66	1.27
-14	-2	-7	26.03	1.81
14	-2	7	19.68	1.39
-14	-2	-7	21.70	1.51
-14	2	7	21.44	1.31
-14	-3	7	0.53	0.64
14	3	7	1.51	0.43
14	3	7	2.69	0.70
14	3	7	1.26	0.56
-14	3	-7	2.23	1.02
-14	3	-7	1.01	0.45
-14	3	-7	3.07	0.92
-14	-3	7	1.17	0.38
14	-3	-7	1.30	0.45
-14	-3	7	1.37	0.50
-14	-3	7	1.22	0.84
-14	-3	7	1.91	0.46

14	-3	-7	1.24	0.38
14	-3	-7	1.69	0.54
-14	3	-7	2.06	0.88
-14	-3	7	2.01	0.90
-14	3	-7	1.05	0.63
14	-3	-7	1.57	0.55
-14	-3	7	1.39	0.52
14	3	7	1.56	0.67
-14	3	7	0.99	0.48
-14	-3	-7	2.29	0.50
-14	-3	-7	1.58	0.41
-14	-3	-7	1.30	0.57
14	-3	7	2.22	0.55
-14	3	7	0.78	0.65
-14	-3	-7	2.11	0.85
-14	3	7	1.84	0.67
-14	-3	-7	2.09	0.70
14	3	-7	2.10	0.30
14	-3	7	1.48	0.49
14	3	-7	1.63	0.42
-14	-3	-7	2.10	0.68
14	-3	7	0.15	0.74
14	-3	7	2.00	0.50
-14	-3	-7	1.96	0.54
-14	3	7	2.06	0.72
14	3	-7	1.47	0.61
14	-3	7	1.96	0.95
-14	-4	7	17.08	1.15
-14	-4	7	15.90	1.22
14	4	7	15.11	1.25
-14	-4	7	18.73	1.84
-14	-4	7	13.89	1.52
14	-4	-7	19.34	1.35
-14	4	-7	19.69	1.42
-14	4	-7	17.80	1.34
14	4	7	15.99	1.40
-14	4	-7	18.73	1.87
-14	-4	7	14.91	1.14
14	-4	-7	20.13	1.25
14	-4	-7	19.38	1.32
-14	-4	7	14.96	1.03
-14	4	-7	20.63	2.02
14	4	7	15.99	1.48
14	4	7	14.40	1.14

14	-4	-7	21.80	1.61
14	4	-7	18.84	1.06
14	-4	7	15.53	1.19
-14	-4	-7	18.77	1.27
14	4	-7	20.86	1.48
14	4	-7	18.55	1.26
-14	-4	-7	18.66	1.28
14	-4	7	15.66	1.12
-14	-4	-7	19.43	1.44
-14	4	7	15.51	1.20
-14	-4	-7	18.43	1.37
-14	4	7	14.18	1.49
-14	4	7	15.67	1.62
-14	-4	-7	20.67	1.27
14	-4	7	15.43	1.17
14	-5	-7	16.17	1.32
-14	-5	7	18.00	1.29
-14	-5	7	16.61	1.95
-14	5	-7	16.88	1.27
14	-5	-7	16.20	1.15
-14	-5	7	17.48	1.72
14	-5	-7	21.46	1.80
-14	5	-7	16.18	1.35
14	5	7	16.19	1.75
-14	-5	7	17.60	1.14
-14	5	-7	18.65	2.09
-14	-5	7	18.15	1.23
-14	5	-7	17.03	2.10
14	-5	-7	15.60	1.49
14	5	7	16.02	1.71
-14	-5	7	16.96	1.35
-14	-5	-7	16.55	1.19
-14	5	7	17.83	1.78
14	-5	7	16.99	1.30
14	5	-7	15.71	1.31
14	-5	7	20.32	2.20
14	5	-7	17.77	1.39
14	-5	7	17.31	1.50
14	5	-7	16.99	1.05
-14	-5	-7	16.15	1.37
14	-5	7	18.22	1.18
-14	5	7	17.16	1.74
-14	5	7	18.58	1.35
-14	-5	-7	15.50	1.88

-14	6	-7	6.96	1.53
-14	-6	7	4.34	0.81
-14	-6	7	6.55	1.29
-14	-6	7	6.47	0.93
14	-6	-7	7.95	1.03
14	6	7	5.11	1.71
-14	-6	7	5.05	1.11
-14	6	-7	7.39	1.03
14	-6	-7	7.76	1.00
-14	-6	7	5.94	0.74
-14	6	-7	7.22	1.39
14	6	7	5.10	1.00
-14	6	-7	7.23	1.15
14	6	7	5.10	1.05
14	-6	-7	6.50	0.85
14	-6	7	5.88	1.46
14	6	-7	5.95	1.06
14	-6	7	4.70	0.96
-14	-6	-7	7.85	1.11
-14	-6	-7	9.11	0.98
14	-6	7	5.93	0.79
14	6	-7	9.09	1.11
14	6	-7	6.93	0.80
-14	6	7	4.48	0.82
-14	6	7	3.97	1.14
-14	6	7	4.20	1.06
14	-6	7	5.17	0.89
-14	-6	-7	8.54	1.12
14	7	7	13.26	1.47
-14	7	-7	12.78	1.24
-14	7	-7	13.72	2.28
14	-7	-7	15.73	1.16
-14	-7	7	12.29	1.02
14	7	7	13.92	1.80
-14	-7	7	12.98	1.02
-14	7	-7	15.62	2.11
14	7	7	13.33	1.86
-14	-7	7	13.20	1.28
-14	7	-7	13.85	1.31
14	-7	-7	13.81	1.34
-14	-7	7	15.27	1.83
14	-7	-7	17.26	1.85
-14	-7	7	11.20	2.10
14	-7	-7	12.24	1.37

-14	-7	-7	14.93	1.46
14	-7	7	15.33	2.28
14	7	-7	12.65	1.30
14	-7	7	10.62	1.27
-14	-7	-7	14.85	1.43
-14	-7	-7	12.74	1.25
14	7	-7	13.85	1.18
14	-7	7	14.06	1.08
14	7	-7	16.50	1.57
-14	7	7	10.38	1.99
14	-7	7	11.51	1.22
-14	7	7	13.78	1.86
-14	-8	7	6.18	1.00
14	8	7	8.31	1.45
14	-8	-7	7.62	1.13
-14	8	-7	7.22	1.49
14	8	7	6.22	1.32
14	-8	-7	6.46	0.95
-14	8	-7	7.35	1.08
14	-8	-7	6.77	1.21
-14	-8	7	6.97	1.31
14	8	-7	5.66	1.14
14	8	-7	8.69	1.18
14	-8	7	7.53	1.25
-14	-8	-7	6.21	1.55
14	-8	7	6.07	0.89
-14	8	7	4.91	1.33
14	-8	7	8.08	1.13
-14	-8	-7	4.70	0.96
14	8	-7	5.66	1.10
14	-9	-7	7.42	1.46
-14	-9	7	8.54	1.69
-14	9	-7	8.92	1.27
14	-9	-7	7.32	1.36
-14	9	-7	8.37	2.02
14	9	7	6.41	1.45
-14	-9	7	10.06	1.40
14	-9	-7	8.08	1.14
14	9	7	6.54	1.63
-14	-9	-7	8.66	1.16
14	-9	7	8.17	1.57
-14	-9	-7	9.52	1.58
-14	9	7	10.30	1.87
14	9	-7	7.29	1.43

14	9	-7	8.53	1.31
14	-9	7	10.62	1.39
14	-10	-7	4.91	1.11
14	-10	-7	5.78	1.29
-14	10	-7	4.61	0.96
-14	-10	7	4.27	1.36
-14	-10	7	3.62	1.06
14	10	7	4.78	1.40
14	-10	-7	5.00	1.02
-14	10	-7	5.50	1.71
14	10	7	2.85	1.20
14	-10	7	3.11	0.83
-14	10	7	3.89	1.59
-14	-10	-7	2.97	0.99
-14	-10	-7	3.89	1.14
14	-10	7	2.92	1.08
14	10	-7	4.17	1.26
14	-11	-7	7.43	1.36
-14	11	-7	6.79	1.20
14	-11	-7	4.83	0.99
14	11	7	7.99	1.48
-14	-11	7	5.12	1.80
-14	11	-7	6.61	1.91
14	11	7	5.31	1.44
14	-11	-7	5.39	1.34
-14	-11	7	8.23	1.50
14	-11	7	5.16	1.52
-14	11	7	4.94	1.73
14	-11	7	4.96	1.54
-14	-11	-7	7.57	1.37
15	0	-7	0.07	0.24
-15	0	7	-0.28	0.55
-15	0	-7	-0.45	0.57
-15	0	7	0.17	0.57
-15	0	7	-0.42	0.59
-15	0	-7	0.33	0.43
15	0	7	-0.58	0.51
-15	0	7	-1.00	0.46
15	0	-7	-0.07	0.34
15	0	-7	0.02	0.39
15	0	7	0.50	0.35
-15	0	-7	0.00	0.65
-15	0	7	-0.05	0.41
-15	0	-7	0.02	0.58

15	0	7	-0.34	0.36
-15	0	7	-0.04	0.80
15	0	-7	0.31	0.25
15	0	7	0.43	0.95
15	0	7	0.21	0.70
-15	0	-7	-0.14	0.63
-15	1	-7	33.16	2.30
15	-1	-7	34.79	1.88
15	-1	-7	36.12	1.85
15	1	7	31.69	1.95
15	1	7	40.14	2.26
-15	-1	7	32.39	1.79
15	1	7	33.31	1.82
-15	-1	7	33.77	2.08
-15	1	-7	34.51	2.19
15	1	7	35.40	2.10
-15	-1	7	33.26	2.12
-15	1	-7	35.47	2.14
-15	-1	7	32.48	1.86
15	-1	-7	35.50	1.98
15	1	7	35.98	2.05
-15	-1	7	31.55	2.03
15	-1	-7	33.74	1.79
-15	-1	7	34.04	1.97
-15	1	-7	34.31	2.03
-15	-1	7	34.05	2.34
15	-1	7	30.68	2.14
15	1	-7	34.15	1.92
-15	-1	-7	38.30	1.88
-15	1	7	33.17	1.95
-15	-1	-7	33.46	2.05
-15	-1	-7	36.00	1.91
-15	1	7	31.59	2.02
15	-1	7	37.77	2.43
15	-1	7	32.19	1.82
-15	1	7	30.98	2.32
-15	1	7	33.62	2.15
15	-1	7	32.19	1.93
15	-1	7	33.93	1.98
-15	-1	-7	34.03	2.17
-15	1	7	30.31	1.86
-15	1	7	34.05	1.85
-15	-1	-7	35.09	2.24
-15	2	-7	27.85	1.71

-15	-2	7	27.48	1.65
15	2	7	28.66	1.82
15	2	7	32.27	1.98
15	-2	-7	25.74	1.67
-15	-2	7	28.89	2.04
-15	-2	7	30.92	1.73
-15	-2	7	27.41	1.84
15	-2	-7	29.43	1.56
15	2	7	29.65	1.83
-15	-2	7	27.16	1.65
15	-2	-7	28.69	1.66
-15	-2	7	28.54	1.96
-15	-2	7	29.51	1.78
-15	-2	7	30.12	2.18
15	2	7	29.08	1.96
-15	2	-7	30.32	2.03
-15	2	-7	24.43	2.05
15	-2	-7	26.36	1.52
15	2	7	27.75	1.66
-15	2	-7	25.67	1.76
-15	-2	-7	28.22	2.02
-15	-2	-7	26.88	1.75
15	-2	7	27.82	2.04
-15	2	7	31.26	1.71
-15	2	7	28.39	1.64
-15	-2	-7	25.78	1.79
-15	2	7	30.06	2.02
15	-2	7	28.90	1.68
-15	-2	-7	28.39	1.53
15	-2	7	31.65	2.31
15	-2	7	30.04	1.79
15	-2	7	31.24	1.79
-15	-3	7	11.19	1.09
-15	3	-7	7.35	0.89
15	3	7	12.64	1.35
-15	3	-7	7.84	1.51
15	-3	-7	10.02	1.01
15	3	7	14.48	1.33
15	-3	-7	8.33	0.79
-15	-3	7	10.69	1.47
-15	3	-7	7.34	1.38
-15	3	-7	8.45	1.08
15	3	7	10.85	0.95
-15	-3	7	12.14	0.93

15	-3	-7	6.57	0.85
-15	-3	7	12.89	1.07
-15	-3	7	10.57	1.64
-15	-3	7	10.25	1.31
-15	-3	7	11.27	0.97
15	-3	-7	7.52	0.80
-15	-3	-7	6.91	1.03
-15	-3	-7	7.83	1.04
15	-3	7	11.26	1.02
-15	3	7	13.02	1.06
15	-3	7	11.12	0.97
-15	3	7	12.40	1.37
15	-3	7	11.83	1.03
-15	-3	-7	7.80	0.98
-15	-3	-7	8.35	0.77
-15	-3	-7	6.71	0.89
15	-3	7	12.36	1.65
-15	3	7	12.47	1.16
-15	4	-7	82.55	4.14
15	-4	-7	81.35	4.15
-15	-4	7	89.08	4.27
15	-4	-7	84.81	4.25
-15	4	-7	84.27	4.61
-15	-4	7	87.43	4.56
15	-4	-7	92.52	4.39
15	4	7	77.94	4.33
-15	-4	7	84.04	4.13
15	4	7	84.83	4.57
-15	-4	7	81.90	4.23
15	-4	-7	84.49	4.06
-15	4	-7	82.56	4.16
-15	4	-7	80.26	4.62
-15	-4	7	90.57	4.21
15	4	7	94.24	4.49
-15	-4	7	91.27	4.67
-15	-4	-7	84.30	4.04
15	4	-7	83.06	4.25
15	-4	7	83.66	4.33
15	4	-7	85.54	3.87
-15	4	7	78.78	4.62
15	-4	7	85.72	4.29
15	-4	7	90.41	4.21
-15	4	7	89.66	4.40
-15	-4	-7	85.52	3.96

-15	4	7	86.40	4.23
-15	-4	-7	84.28	4.22
-15	-4	-7	76.90	4.23
15	4	-7	82.81	3.98
-15	-4	-7	81.30	4.07
15	-5	-7	87.17	4.23
15	-5	-7	81.92	4.24
-15	-5	7	77.63	3.91
15	5	7	79.04	4.41
-15	5	-7	86.06	4.74
-15	5	-7	83.89	4.77
15	5	7	85.99	4.26
15	-5	-7	96.11	4.49
-15	-5	7	86.87	3.98
15	5	7	77.40	4.09
15	-5	-7	80.76	4.09
-15	-5	7	85.17	4.50
-15	-5	7	79.64	4.07
-15	5	-7	75.24	4.37
-15	5	-7	78.65	4.12
15	5	-7	88.02	4.29
-15	5	7	81.35	4.39
-15	5	7	79.06	4.06
-15	-5	-7	84.06	4.00
-15	-5	-7	81.08	4.24
15	-5	7	78.61	4.10
-15	-5	-7	85.01	4.29
15	-5	7	86.69	3.96
15	-5	7	71.98	4.27
15	5	-7	85.10	3.97
-15	5	7	78.41	4.40
15	-5	7	95.44	4.76
15	-6	-7	43.58	2.53
15	-6	-7	43.74	2.54
-15	-6	7	42.12	2.24
-15	-6	7	42.89	2.87
-15	6	-7	42.76	2.41
-15	-6	7	41.63	3.00
15	6	7	41.05	2.82
15	-6	-7	49.15	2.90
15	6	7	46.50	2.67
15	6	7	39.26	2.49
-15	6	-7	45.60	3.20
15	-6	-7	41.81	2.35

-15	-6	7	37.22	2.38
-15	6	-7	41.24	2.44
-15	-6	7	39.19	2.19
-15	6	-7	44.83	3.17
-15	-6	-7	43.88	2.60
-15	6	7	40.81	2.86
15	6	-7	40.67	2.48
-15	6	7	43.94	2.94
15	-6	7	45.18	2.49
15	6	-7	43.71	2.27
15	6	-7	45.01	2.53
-15	-6	-7	43.99	2.31
-15	-6	-7	41.65	2.55
15	-6	7	39.43	2.41
15	-6	7	43.70	2.21
15	-6	7	45.80	3.24
15	-7	-7	17.22	1.30
-15	-7	7	17.77	1.48
-15	-7	7	20.66	2.38
15	7	7	14.27	1.63
15	7	7	19.04	1.79
15	-7	-7	15.08	1.46
15	-7	-7	16.51	1.46
15	7	7	16.21	2.00
-15	-7	7	17.63	2.09
-15	-7	7	16.69	1.22
-15	7	-7	14.49	1.36
-15	7	-7	15.45	2.23
15	7	-7	16.89	1.27
15	-7	7	19.22	1.27
-15	-7	-7	14.83	1.54
15	7	-7	14.78	1.56
15	-7	7	19.84	1.57
-15	-7	-7	16.91	1.28
15	7	-7	16.39	1.44
-15	7	7	14.53	2.05
15	-7	7	18.45	1.51
15	8	7	-0.70	1.24
15	8	7	0.95	0.97
-15	8	-7	0.62	0.72
15	-8	-7	1.17	0.60
15	-8	-7	0.57	0.82
-15	8	-7	1.43	1.53
-15	-8	7	0.81	0.74

15	-8	-7	0.64	0.78
-15	-8	7	0.70	1.24
15	8	-7	0.01	1.05
15	-8	7	0.25	0.74
-15	-8	-7	1.92	0.89
-15	8	7	0.97	1.17
-15	-8	-7	-0.28	0.73
15	-8	7	1.47	0.72
15	8	-7	-0.93	0.66
15	8	-7	1.26	0.75
-15	9	-7	21.35	1.74
15	9	7	18.63	2.20
-15	-9	7	19.38	2.41
15	-9	-7	22.17	1.65
15	9	7	20.34	2.41
-15	-9	7	21.58	1.92
15	-9	-7	21.18	1.82
15	-9	-7	21.90	2.08
-15	9	-7	20.23	2.86
15	-9	7	24.63	2.02
15	-9	7	21.38	1.77
-15	-9	-7	20.30	1.92
15	9	-7	19.35	2.11
15	9	-7	22.90	1.68
-15	-9	-7	20.37	1.90
-15	9	7	19.31	2.82
-15	10	-7	39.28	3.52
15	-10	-7	38.88	2.49
-15	-10	7	37.25	2.57
15	10	7	37.44	2.81
15	-10	-7	38.42	2.77
-15	10	-7	44.12	2.59
-15	-10	7	36.42	3.02
15	-10	-7	38.24	2.47
15	10	7	31.70	3.05
15	-10	7	39.55	2.49
-15	-10	-7	36.18	2.69
-15	10	7	37.28	3.27
15	-10	7	39.57	2.81
15	10	-7	35.67	2.87
-15	-10	-7	36.57	2.66
16	0	7	3.45	0.60
16	0	-7	2.14	0.51
16	0	7	4.95	0.78

-16	0	-7	1.88	0.51
16	0	7	3.47	0.90
16	0	7	5.93	0.99
-16	0	7	3.47	0.81
-16	0	-7	3.19	0.86
-16	0	-7	2.26	0.62
-16	0	7	3.83	0.77
-16	0	7	3.04	0.60
16	0	-7	1.18	0.31
16	1	7	19.87	1.63
-16	-1	7	19.58	1.34
-16	-1	7	20.52	1.71
-16	-1	7	20.50	1.29
-16	-1	7	20.76	1.60
-16	1	-7	17.51	1.72
16	1	7	20.76	1.28
16	-1	-7	16.65	1.24
16	1	7	18.43	1.43
-16	1	-7	15.45	1.48
16	-1	-7	17.64	1.17
-16	-1	7	22.01	1.40
16	-1	-7	18.79	1.12
-16	1	-7	18.10	1.31
16	1	7	20.35	1.60
16	-1	-7	15.02	1.00
-16	1	-7	15.71	1.42
-16	-1	7	24.99	1.63
-16	-1	7	20.64	1.52
-16	-1	7	20.23	1.91
16	1	7	22.45	1.76
-16	1	7	20.84	1.65
-16	1	7	22.70	1.44
16	-1	7	19.00	1.28
-16	-1	-7	16.95	1.63
16	-1	7	19.78	1.76
-16	-1	-7	16.52	1.14
16	-1	7	16.41	1.37
-16	-1	-7	16.13	1.53
16	-1	7	19.84	1.51
16	-1	7	21.14	1.94
-16	-1	-7	17.49	1.33
-16	1	7	19.65	1.36
-16	1	7	19.35	1.50
16	-2	-7	11.67	0.96

16	-2	-7	9.54	0.83
16	2	7	7.43	1.05
-16	-2	7	9.40	1.25
-16	2	-7	12.08	1.59
-16	-2	7	8.04	1.37
-16	-2	7	7.90	1.28
16	-2	-7	9.78	0.96
-16	2	-7	9.61	1.22
16	-2	-7	10.12	0.89
-16	-2	7	8.24	0.97
-16	2	-7	9.55	1.04
-16	2	-7	10.13	1.56
-16	-2	7	7.29	0.83
-16	-2	7	9.06	0.87
-16	-2	7	7.78	1.12
16	2	7	8.24	0.84
16	2	7	8.89	1.24
-16	-2	7	8.86	1.08
-16	2	7	9.15	0.93
16	-2	7	8.56	1.00
16	-2	7	8.58	0.85
-16	2	7	9.05	1.28
-16	2	7	9.40	0.99
-16	2	7	7.27	1.05
16	-2	7	8.04	0.97
-16	-2	-7	10.47	0.82
-16	-2	-7	10.22	1.06
16	-2	7	8.99	1.66
-16	-2	-7	10.09	1.24
-16	-2	-7	7.79	1.01
16	3	7	6.46	1.30
-16	3	-7	3.83	0.99
-16	-3	7	6.51	1.13
-16	-3	7	5.79	0.99
-16	-3	7	6.54	0.81
-16	-3	7	6.14	0.78
-16	3	-7	4.14	0.81
-16	3	-7	3.78	1.04
16	-3	-7	4.84	0.77
16	-3	-7	4.25	0.81
16	-3	-7	4.68	0.71
-16	-3	7	7.87	1.28
-16	-3	7	5.57	0.94
-16	-3	7	5.24	1.02

16	3	7	5.73	0.96
-16	3	-7	5.11	0.88
-16	3	7	5.45	1.28
-16	3	7	6.12	0.94
16	-3	7	4.48	0.87
-16	-3	-7	3.81	0.80
-16	-3	-7	5.45	0.64
-16	3	7	6.62	0.94
-16	-3	-7	5.45	1.07
-16	-3	-7	4.47	0.79
16	-3	7	6.79	1.47
16	-3	7	5.14	0.86
16	-3	7	6.00	0.80
-16	4	-7	3.37	1.19
-16	4	-7	0.87	0.57
-16	4	-7	0.59	0.72
-16	-4	7	1.29	0.51
-16	-4	7	0.51	0.86
16	-4	-7	1.50	0.51
-16	-4	7	1.15	0.47
-16	-4	7	1.82	0.63
16	4	7	2.74	0.92
-16	-4	7	1.55	1.15
-16	-4	7	1.00	0.62
16	4	7	1.25	0.50
16	-4	-7	1.16	0.46
-16	-4	7	1.27	0.64
16	-4	-7	0.80	0.53
16	-4	-7	2.63	0.72
-16	-4	-7	1.95	0.70
16	-4	7	2.32	0.58
-16	-4	-7	1.12	0.75
16	-4	7	1.82	0.60
16	4	-7	2.97	0.53
16	-4	7	0.68	1.11
16	-4	7	1.09	0.57
-16	4	7	0.91	0.62
16	4	-7	1.67	0.30
-16	4	7	1.64	0.82
-16	4	7	1.12	0.86
-16	-4	-7	2.41	0.59
-16	-4	-7	2.34	0.77
-16	-4	-7	1.17	0.41
16	-5	-7	1.27	0.90

-16	5	-7	1.39	0.70
16	5	7	2.54	0.92
-16	5	-7	2.00	0.60
-16	-5	7	1.47	1.25
-16	-5	7	0.97	0.48
-16	-5	7	2.37	0.59
16	-5	-7	0.46	0.44
16	-5	-7	0.75	0.56
16	5	7	1.87	0.66
16	5	7	1.46	0.90
-16	-5	7	2.02	0.89
16	-5	-7	0.47	0.57
-16	5	-7	-0.36	1.02
-16	5	-7	0.53	1.24
-16	-5	7	1.41	0.63
-16	-5	-7	0.63	0.43
-16	5	7	0.90	0.66
16	5	-7	1.39	0.39
-16	-5	-7	-0.06	0.56
-16	-5	-7	1.37	0.71
-16	5	7	1.43	0.96
16	-5	7	1.88	0.53
16	-5	7	0.95	0.56
16	5	-7	1.11	0.69
16	5	-7	1.39	0.57
-16	5	7	1.91	1.08
16	-5	7	0.68	0.54
-16	6	-7	9.43	1.14
-16	-6	7	7.88	0.90
16	6	7	5.49	1.24
16	6	7	8.63	1.19
16	-6	-7	11.08	1.72
-16	-6	7	6.57	1.46
-16	6	-7	13.68	2.11
16	-6	-7	6.27	1.08
-16	-6	7	10.20	1.74
16	-6	-7	4.73	1.02
16	-6	-7	8.51	0.94
16	6	7	7.35	1.25
-16	-6	7	6.73	1.22
-16	-6	7	6.14	1.02
-16	-6	-7	6.89	0.89
-16	6	7	7.64	1.36
16	6	-7	9.19	1.21

-16	-6	-7	11.07	1.26
16	6	-7	8.30	1.24
16	-6	7	7.62	0.92
16	-6	7	5.90	1.56
16	-6	7	6.27	1.05
16	-6	7	8.15	1.10
-16	-6	-7	7.42	1.33
16	6	-7	7.21	0.87
16	7	7	32.97	2.61
16	-7	-7	28.62	2.03
-16	7	-7	29.45	1.99
16	7	7	32.02	2.36
16	-7	-7	33.57	2.13
16	-7	-7	27.79	2.00
-16	-7	7	31.59	2.53
-16	7	-7	35.45	2.94
16	7	7	30.63	2.18
-16	-7	7	28.96	2.03
-16	-7	7	27.11	1.72
16	-7	7	28.24	2.04
16	-7	7	29.86	1.99
16	7	-7	32.03	2.05
-16	-7	-7	30.21	2.15
16	7	-7	31.21	1.86
16	7	-7	27.04	2.07
-16	7	7	31.26	2.57
-16	-7	-7	29.14	2.07
16	8	7	17.43	2.33
-16	-8	7	18.65	2.46
16	-8	-7	18.57	1.75
-16	-8	7	18.56	1.66
-16	8	-7	18.34	1.65
16	-8	-7	21.55	1.85
16	8	7	17.18	1.96
-16	8	-7	17.51	2.87
16	-8	-7	21.10	1.53
16	-8	7	17.97	1.66
-16	8	7	15.87	2.56
-16	-8	-7	17.85	1.91
-16	-8	-7	19.39	1.79
16	8	-7	18.14	1.65
16	8	-7	20.16	1.57
16	-8	7	16.43	1.67
16	8	-7	20.28	2.09

-16	9	-7	1.20	1.52
16	9	7	5.87	1.40
16	9	7	5.24	1.21
-16	-9	7	6.29	1.23
16	-9	-7	2.39	0.75
16	-9	-7	0.85	0.94
-16	9	-7	1.74	0.95
16	-9	-7	0.74	0.84
-16	-9	7	6.83	1.55
-16	9	7	5.12	1.53
-16	-9	-7	0.63	1.06
16	-9	7	4.52	1.17
16	9	-7	-0.34	0.96
-16	-9	-7	2.22	0.89
16	-9	7	4.21	0.89
-16	-10	7	5.07	1.50
-16	10	-7	14.28	2.72
16	-10	-7	10.83	1.50
16	10	7	6.20	1.39
16	-10	-7	10.84	1.49
-16	-10	7	11.38	1.58
16	-10	-7	11.03	1.63
16	10	7	11.03	2.12
-16	10	-7	11.93	1.65
-16	-10	-7	13.15	1.88
-16	10	7	7.81	1.83
16	-10	7	7.93	1.65
-16	-10	-7	10.00	1.62
16	10	-7	9.21	1.95
16	-10	7	8.02	1.32
17	0	7	-0.38	0.57
-17	0	-7	-0.02	0.54
17	0	7	-1.01	0.71
17	0	-7	-0.16	0.40
-17	0	7	0.09	0.51
17	0	7	-0.14	0.62
-17	0	7	-1.23	0.70
-17	0	-7	0.51	0.75
17	0	-7	-0.33	0.41
17	0	-7	-0.12	0.24
-17	0	-7	0.10	0.67
17	0	7	-0.06	0.81
-17	0	7	-0.85	0.52
17	0	-7	-0.24	0.26

-17	0	7	-0.06	0.61
-17	0	-7	-0.63	0.75
17	-1	-7	4.98	0.67
17	1	7	2.99	0.78
-17	1	-7	5.61	1.09
-17	-1	7	3.75	0.64
17	-1	-7	4.13	0.77
-17	-1	7	4.13	0.67
17	1	7	1.41	0.82
17	1	7	4.13	1.02
-17	-1	7	0.93	0.79
-17	1	-7	4.62	1.06
-17	-1	7	2.53	0.78
-17	-1	7	2.28	0.75
17	-1	-7	4.23	0.57
17	-1	-7	4.05	0.74
-17	-1	7	2.24	0.55
-17	1	-7	4.60	0.95
-17	-1	7	4.91	1.01
17	-1	7	3.13	0.92
-17	1	7	3.66	0.66
-17	1	7	2.50	0.61
-17	1	7	2.38	0.75
-17	1	7	2.33	0.81
-17	-1	-7	3.93	1.03
-17	-1	-7	4.01	0.69
-17	-1	-7	3.15	0.74
17	-1	7	2.78	0.73
17	-1	7	2.44	0.86
-17	-1	-7	5.59	0.97
17	-2	-7	10.45	1.21
-17	-2	7	9.18	0.98
-17	-2	7	11.49	1.23
-17	2	-7	12.83	1.36
17	-2	-7	8.89	1.06
-17	-2	7	9.78	1.13
-17	2	-7	10.18	1.61
-17	-2	7	12.75	1.57
17	2	7	11.23	0.96
-17	2	-7	13.31	1.51
17	-2	-7	11.78	0.98
17	2	7	11.68	1.59
-17	2	-7	11.30	1.10
-17	-2	7	10.77	1.27

17	2	7	10.37	1.31
-17	-2	7	11.48	1.37
17	-2	-7	13.51	1.05
-17	-2	7	10.65	1.00
-17	-2	-7	12.01	1.39
17	-2	7	11.14	1.10
-17	-2	-7	11.20	0.74
-17	2	7	10.98	1.11
17	-2	7	9.61	0.93
-17	2	7	9.59	1.02
-17	-2	-7	9.78	1.34
-17	-2	-7	10.98	1.09
17	-2	7	14.53	1.20
-17	-2	-7	11.67	1.34
17	-2	7	14.93	1.94
-17	2	7	12.74	1.16
-17	3	-7	15.53	1.92
-17	-3	7	5.60	1.04
-17	-3	7	7.38	0.88
-17	-3	7	7.27	1.32
17	-3	-7	11.80	1.09
17	3	7	7.03	1.17
17	3	7	8.49	1.31
-17	3	-7	11.06	1.13
17	-3	-7	12.37	1.20
17	-3	-7	11.07	0.96
-17	3	-7	11.15	1.98
-17	-3	7	7.05	1.12
17	3	7	8.53	0.87
17	-3	-7	12.05	1.01
-17	-3	7	7.31	1.30
-17	-3	7	6.67	1.13
-17	-3	7	6.11	0.89
-17	3	-7	12.02	1.32
17	-3	7	7.76	0.97
-17	-3	-7	11.25	1.16
17	-3	7	7.43	0.99
-17	-3	-7	9.68	1.27
17	-3	7	8.56	1.75
17	-3	7	7.03	0.86
-17	3	7	7.54	1.19
-17	-3	-7	10.47	1.32
-17	3	7	7.15	1.02
-17	-4	7	19.01	2.16

17	-4	-7	27.51	1.57
17	4	7	20.51	1.86
17	4	7	17.81	1.39
-17	-4	7	17.57	1.54
17	-4	-7	24.84	1.62
-17	4	-7	24.56	2.35
-17	-4	7	19.58	1.31
-17	4	-7	23.67	1.77
17	-4	-7	25.50	1.73
17	4	7	21.39	1.74
-17	-4	7	19.61	1.40
-17	4	-7	22.82	2.42
-17	4	-7	22.85	1.68
17	-4	-7	29.73	1.85
-17	-4	7	19.78	1.52
-17	4	7	17.79	1.48
17	-4	7	19.44	1.43
-17	-4	-7	24.16	1.71
-17	-4	-7	23.35	1.78
17	-4	7	21.75	2.30
-17	-4	-7	23.11	1.74
17	-4	7	20.89	1.40
17	-4	7	20.05	1.49
-17	-4	-7	24.06	1.77
-17	4	7	18.70	1.95
-17	4	7	19.82	1.70
-17	5	-7	41.91	2.49
17	-5	-7	41.66	2.44
-17	-5	7	34.33	2.19
17	5	7	38.72	2.23
-17	-5	7	38.81	2.68
-17	-5	7	33.83	2.92
-17	-5	7	36.88	2.17
17	-5	-7	44.33	2.36
-17	5	-7	41.66	2.42
17	5	7	36.55	2.68
-17	-5	7	36.06	2.24
17	-5	-7	41.65	2.51
-17	-5	7	33.70	2.03
17	5	7	36.99	2.45
-17	5	-7	44.14	3.23
17	5	-7	42.32	2.32
-17	5	7	33.27	2.66
-17	-5	-7	46.99	2.37

-17	5	7	38.99	2.59
-17	-5	-7	42.27	2.54
17	-5	7	40.83	2.08
-17	-5	-7	43.90	2.57
17	-5	7	37.36	2.23
17	5	-7	42.23	2.14
17	-5	7	37.55	2.24
-17	-6	7	37.62	3.00
17	6	7	36.51	2.52
17	-6	-7	37.67	2.11
17	-6	-7	37.05	2.26
17	-6	-7	38.26	2.27
17	-6	-7	34.04	3.52
-17	-6	7	37.95	2.28
17	6	7	36.38	2.30
17	6	7	38.22	2.77
-17	6	-7	38.64	3.01
-17	-6	7	34.34	2.26
-17	6	-7	34.74	2.22
-17	-6	7	35.84	2.08
-17	-6	7	38.60	2.84
17	6	-7	36.04	1.95
17	6	-7	32.07	2.18
17	-6	7	41.06	2.07
-17	-6	-7	35.07	2.31
17	-6	7	36.88	2.28
-17	6	7	38.30	2.81
-17	-6	-7	34.85	2.30
17	-6	7	36.26	2.33
-17	7	-7	10.95	2.02
17	7	7	8.68	1.59
-17	-7	7	8.63	1.67
17	-7	-7	14.54	1.47
17	-7	-7	11.61	1.45
-17	7	-7	9.17	1.31
-17	-7	7	8.19	1.01
17	7	7	8.60	1.56
17	-7	-7	11.64	1.19
-17	-7	7	7.79	1.07
17	-7	7	9.63	1.34
17	7	-7	11.84	1.16
-17	-7	-7	12.18	1.53
17	-7	7	10.66	1.30
-17	-7	-7	11.30	1.60

-17	7	7	11.07	2.20
17	7	-7	12.31	1.66
17	-8	-7	0.39	0.90
-17	8	-7	1.80	1.41
17	-8	-7	0.27	0.76
17	8	7	-0.14	0.95
-17	-8	7	0.87	1.36
17	-8	-7	0.74	0.63
-17	-8	7	1.08	0.88
-17	8	-7	-0.54	0.84
17	8	7	2.46	1.25
17	-8	7	1.09	0.75
-17	-8	-7	1.05	0.92
-17	-8	-7	1.41	0.84
17	8	-7	0.51	1.02
17	8	-7	0.91	0.66
-17	8	7	0.85	1.36
17	-8	7	1.11	0.95
-17	-9	7	8.50	1.71
17	9	7	6.13	1.26
17	-9	-7	6.44	1.16
-17	9	-7	8.99	1.54
-17	-9	7	8.50	1.33
-17	9	-7	4.33	1.71
17	9	7	8.48	1.64
17	-9	-7	8.20	1.31
17	-9	-7	7.68	1.11
17	-9	7	8.12	1.61
17	9	-7	5.98	1.30
-17	-9	-7	5.28	1.15
-17	9	7	6.90	1.65
17	-9	7	8.19	1.34
-17	-9	-7	7.70	1.26
18	0	7	70.92	3.85
-18	0	7	76.48	3.71
-18	0	7	76.82	3.94
18	0	-7	88.16	4.08
-18	0	-7	78.84	4.42
18	0	-7	86.36	3.80
-18	0	7	74.38	4.05
-18	0	-7	81.28	4.18
-18	0	7	74.36	3.75
18	0	7	82.14	4.17
18	0	7	73.15	3.97

-18	0	-7	78.71	3.88
-18	0	7	75.95	3.87
18	0	7	71.65	3.68
18	0	7	72.90	3.94
18	0	-7	81.15	3.77
-18	0	-7	77.67	4.30
-18	-1	7	74.96	4.13
-18	-1	7	77.29	3.93
18	1	7	76.98	4.05
-18	-1	7	75.24	3.87
18	1	7	80.01	4.18
18	1	7	81.06	4.25
-18	-1	7	77.44	4.08
18	-1	-7	83.16	3.93
-18	1	-7	76.25	4.34
-18	1	-7	73.19	4.11
-18	1	-7	78.92	3.92
-18	-1	7	76.37	3.90
18	-1	-7	80.33	3.74
-18	-1	7	75.17	4.15
18	-1	-7	85.88	3.82
18	1	7	79.09	4.09
18	1	7	79.46	3.87
-18	-1	-7	78.34	4.06
18	-1	7	81.59	4.05
18	-1	7	79.38	4.04
-18	-1	-7	75.02	4.20
-18	1	7	80.11	4.00
18	-1	7	77.40	3.87
-18	1	7	77.32	4.28
-18	1	7	78.98	3.92
-18	1	7	78.61	3.92
18	-1	7	86.99	4.33
18	-2	-7	14.49	1.12
18	2	7	15.68	1.15
18	-2	-7	13.92	1.00
-18	2	-7	13.93	1.58
18	2	7	15.50	1.60
-18	-2	7	12.95	1.18
18	2	7	17.19	1.68
-18	2	-7	12.37	1.26
18	-2	-7	13.85	1.12
-18	-2	7	13.58	1.44
-18	2	-7	13.78	1.88

-18	2	-7	13.93	1.49
-18	-2	7	14.80	1.20
-18	-2	7	15.39	1.30
18	-2	-7	13.36	1.20
-18	-2	7	13.80	1.56
-18	2	7	12.72	1.14
-18	-2	-7	12.59	1.52
-18	2	7	15.10	1.31
18	-2	7	15.95	1.34
-18	-2	-7	13.05	1.24
18	-2	7	14.52	1.34
-18	2	7	16.20	1.32
-18	2	7	15.38	1.67
18	-2	7	14.87	1.14
-18	-2	-7	14.76	1.59
-18	-2	-7	12.84	1.29
18	-2	7	15.97	1.97
18	-3	-7	3.51	0.74
-18	-3	7	3.52	0.66
-18	-3	7	3.47	0.64
-18	3	-7	2.02	1.04
-18	3	-7	3.12	1.15
-18	3	-7	4.32	0.97
18	3	7	3.14	0.62
18	-3	-7	3.17	0.61
-18	-3	7	3.56	0.84
-18	3	-7	2.69	0.73
18	-3	-7	4.60	0.95
18	3	7	3.95	1.02
18	-3	-7	3.83	0.79
-18	-3	7	3.88	0.84
-18	-3	7	2.43	0.97
18	3	7	4.74	0.97
18	-3	7	3.71	0.67
-18	-3	-7	4.65	0.85
-18	-3	-7	3.64	0.92
-18	-3	-7	3.48	0.79
-18	-3	-7	2.92	0.83
-18	3	7	5.15	0.79
18	-3	7	3.91	0.74
18	-3	7	3.96	0.73
18	-3	7	3.46	1.32
-18	3	7	3.64	1.04
18	-4	-7	1.39	0.61

-18	-4	7	2.62	0.78
18	-4	-7	2.24	0.83
-18	-4	7	2.85	0.82
18	4	7	2.42	1.02
-18	4	-7	1.43	0.70
-18	4	-7	2.17	0.85
18	4	7	2.61	0.92
-18	4	-7	0.72	1.11
-18	-4	7	0.46	0.87
18	-4	-7	1.84	0.59
18	4	7	1.25	0.57
-18	-4	7	2.31	0.60
-18	-4	7	1.45	0.63
-18	4	-7	2.79	1.29
18	-4	-7	0.79	0.49
18	-4	7	0.79	1.37
18	-4	7	2.49	0.64
-18	-4	-7	2.59	0.84
18	-4	7	1.45	0.66
-18	-4	-7	1.72	0.64
-18	-4	-7	1.75	0.74
-18	4	7	1.02	0.83
-18	4	7	0.69	0.92
-18	-4	-7	1.48	0.77
18	-4	7	0.96	0.57
-18	5	-7	10.37	1.72
18	5	7	10.26	1.83
-18	-5	7	10.23	1.15
-18	-5	7	10.92	1.33
-18	-5	7	9.53	1.31
18	-5	-7	8.93	1.09
18	-5	-7	10.80	1.35
-18	5	-7	10.41	1.26
18	-5	-7	8.72	1.02
18	5	7	10.35	1.17
-18	-5	7	8.25	1.04
18	-5	-7	10.44	1.21
18	5	7	11.33	1.62
-18	-5	7	9.22	1.78
-18	-5	-7	8.73	1.14
-18	5	7	9.02	1.80
18	5	-7	7.79	0.74
18	-5	7	12.04	1.28
18	-5	7	11.49	1.22

-18	-5	-7	10.19	1.35
18	-5	7	11.56	1.12
-18	-5	-7	10.71	1.29
18	5	-7	9.03	1.03
-18	5	7	10.48	1.84
18	6	7	53.31	3.56
18	-6	-7	51.79	3.01
18	-6	-7	50.32	2.75
-18	6	-7	52.67	3.90
-18	-6	7	47.24	2.90
18	-6	-7	51.37	2.95
18	6	7	55.20	3.19
-18	6	-7	51.45	2.92
-18	-6	7	51.63	2.75
-18	-6	7	51.04	2.96
18	6	7	51.63	3.03
-18	-6	7	54.59	3.46
18	6	-7	47.86	2.76
18	6	-7	53.47	2.61
18	-6	7	53.22	3.01
-18	-6	-7	49.79	3.01
-18	-6	-7	52.37	3.09
-18	6	7	54.49	3.63
18	-6	7	52.84	2.99
-18	7	-7	54.89	4.11
18	7	7	46.67	3.42
-18	-7	7	46.22	2.64
18	-7	-7	58.95	3.14
18	-7	-7	60.14	3.37
18	7	7	49.33	2.97
-18	-7	7	48.28	3.53
18	-7	-7	56.59	3.30
-18	-7	7	47.89	2.75
-18	-7	7	49.43	2.87
-18	7	-7	61.96	3.34
18	7	-7	62.28	3.05
-18	-7	-7	54.86	3.32
18	-7	7	48.89	2.94
18	7	-7	54.92	3.32
-18	-7	-7	60.39	3.44
18	-7	7	48.12	2.87
-18	7	7	49.21	3.55
-18	-8	7	22.49	2.37
18	-8	-7	21.47	1.70

18	-8	-7	23.45	1.80
-18	-8	7	20.48	1.96
18	8	7	16.22	2.25
-18	8	-7	23.23	1.95
-18	8	-7	22.89	2.84
18	-8	-7	24.95	1.91
18	8	7	17.72	1.89
18	-8	7	22.02	1.75
-18	8	7	22.28	2.53
-18	-8	-7	19.32	2.02
18	8	-7	17.66	1.93
18	8	-7	24.80	2.38
18	-8	7	23.62	1.98
-18	-8	-7	21.01	1.94
18	-9	-7	4.96	1.09
-18	-9	7	5.47	1.45
18	-9	-7	3.93	1.03
18	9	7	0.29	1.21
18	9	7	3.96	1.16
-18	9	-7	6.20	1.75
18	-9	-7	2.76	0.97
-18	-9	7	2.98	1.01
-18	9	-7	3.43	0.97
18	-9	7	6.81	1.41
-18	-9	-7	2.74	1.00
18	-9	7	3.67	0.91
-18	9	7	5.31	1.60
18	9	-7	4.92	1.20
-18	-9	-7	6.71	1.27
-19	0	7	0.36	0.47
-19	0	7	0.89	0.73
19	0	-7	-0.05	0.22
-19	0	7	0.87	0.87
19	0	7	0.03	0.43
19	0	-7	0.22	0.28
-19	0	-7	-0.43	0.55
-19	0	7	-0.10	0.69
19	0	7	0.03	0.73
-19	0	7	0.17	0.52
-19	0	7	0.15	0.74
19	0	7	0.72	0.59
-19	0	-7	0.47	0.86
19	0	7	0.27	0.90
-19	-1	7	6.48	1.03

19	1	7	5.66	0.82
-19	-1	7	6.21	1.19
-19	-1	7	5.55	0.95
-19	1	-7	4.07	0.79
19	1	7	6.50	1.13
19	1	7	5.09	0.94
-19	1	-7	5.41	1.08
-19	-1	7	5.14	0.85
19	-1	-7	4.95	0.82
-19	1	-7	5.27	1.23
19	-1	-7	5.34	0.73
19	-1	-7	4.53	0.72
19	-1	-7	3.80	0.59
19	1	7	5.00	0.91
-19	1	-7	4.01	0.75
-19	-1	-7	4.16	0.88
19	-1	7	5.98	1.07
-19	1	7	5.66	1.00
19	-1	7	6.12	0.99
19	-1	7	4.86	0.83
-19	-1	-7	5.53	1.12
19	-1	7	9.90	1.81
-19	1	7	5.30	1.13
-19	1	7	7.98	1.29
-19	-1	-7	3.20	1.00
-19	1	7	5.37	0.84
19	2	7	9.63	1.69
19	-2	-7	8.01	1.10
19	2	7	10.05	1.53
-19	2	-7	5.99	1.28
-19	2	-7	10.39	1.45
-19	2	-7	8.93	1.47
-19	-2	7	5.65	1.05
19	-2	-7	8.24	0.84
-19	-2	7	7.49	0.95
-19	-2	7	4.53	1.00
19	-2	-7	10.23	1.00
-19	-2	7	7.43	0.94
-19	2	-7	8.77	1.14
19	2	7	6.31	0.88
19	-2	-7	9.72	0.96
-19	2	7	4.36	1.12
-19	-2	-7	10.02	1.53
-19	-2	-7	8.37	1.20

19	-2	7	7.63	1.57
19	-2	7	6.84	1.12
19	-2	7	6.57	1.08
19	-2	7	5.75	0.84
-19	-2	-7	6.49	1.15
-19	-2	-7	7.78	1.14
-19	2	7	7.47	1.11
-19	2	7	5.96	0.95
19	-3	-7	9.41	1.19
19	-3	-7	11.87	1.20
-19	3	-7	13.09	1.50
-19	3	-7	11.36	1.30
19	3	7	11.37	1.61
-19	-3	7	6.86	1.33
19	-3	-7	10.98	1.07
19	3	7	10.19	1.59
19	3	7	9.37	1.03
-19	-3	7	9.89	1.10
-19	-3	7	8.89	1.04
-19	3	-7	9.52	1.61
-19	3	-7	10.94	1.77
19	-3	-7	11.34	1.02
-19	-3	-7	10.82	1.34
19	-3	7	9.71	1.19
19	-3	7	10.08	1.05
-19	-3	-7	11.92	1.30
19	-3	7	11.27	2.44
19	-3	7	11.85	1.25
-19	-3	-7	10.27	1.46
-19	3	7	10.18	1.72
-19	-3	-7	12.36	1.54
19	-4	-7	8.36	1.28
-19	4	-7	4.88	1.46
19	4	7	8.59	1.50
19	-4	-7	6.68	0.95
19	-4	-7	4.76	0.90
-19	-4	7	8.47	1.27
19	4	7	8.89	1.03
19	4	7	8.70	1.67
-19	-4	7	6.11	0.97
-19	4	-7	4.88	0.91
19	-4	-7	6.35	1.08
-19	-4	7	8.56	1.64
-19	-4	7	6.40	1.01

19	-4	7	8.48	1.18
19	-4	7	9.99	1.83
19	-4	7	6.87	1.02
-19	-4	-7	5.88	0.96
-19	4	7	5.26	1.30
-19	-4	-7	5.08	0.99
-19	-4	-7	7.02	1.06
19	-4	7	9.36	1.13
19	5	7	11.14	1.85
19	-5	-7	7.74	1.16
-19	-5	7	9.91	1.25
-19	5	-7	9.13	1.69
19	-5	-7	8.14	1.23
-19	-5	7	10.19	1.14
19	5	7	12.02	1.40
-19	-5	7	13.91	1.93
-19	-5	7	9.74	1.39
19	5	7	12.73	1.69
-19	5	-7	9.79	1.28
-19	5	7	9.01	1.68
19	-5	7	9.80	1.25
19	-5	7	10.68	1.36
-19	-5	-7	8.54	1.39
19	-5	7	13.41	1.19
-19	-5	-7	9.16	1.43
-19	6	-7	19.04	1.73
-19	6	-7	14.19	2.88
19	-6	-7	20.18	1.65
-19	-6	7	16.61	1.61
-19	-6	7	16.94	1.62
19	-6	-7	17.42	1.66
-19	-6	7	16.35	1.39
19	6	7	17.31	2.44
19	-6	-7	20.77	1.49
19	6	7	16.70	1.66
-19	-6	7	19.56	2.38
-19	-6	-7	16.84	1.79
-19	-6	-7	19.10	1.81
19	6	-7	17.20	1.55
19	-6	7	18.20	1.69
19	-6	7	18.66	1.68
19	6	-7	16.39	1.42
-19	6	7	17.58	2.45
-19	-7	7	1.45	1.32

19	7	7	2.37	0.91
-19	7	-7	4.06	1.53
-19	-7	7	1.43	0.92
19	-7	-7	1.00	0.78
19	7	7	2.18	1.28
19	-7	-7	3.81	0.88
19	-7	-7	2.53	0.76
-19	7	-7	1.73	0.84
-19	-7	7	3.48	0.86
19	7	-7	2.11	0.97
-19	-7	-7	2.45	0.98
-19	7	7	-0.91	1.22
19	-7	7	1.04	0.76
19	-7	7	1.70	0.82
-19	-7	-7	3.26	0.92
19	7	-7	2.64	0.76
-19	8	-7	3.45	1.72
19	-8	-7	2.05	0.86
19	-8	-7	3.05	0.89
-19	8	-7	3.22	0.96
19	-8	-7	4.09	0.92
19	8	7	-0.45	1.45
-19	-8	7	3.04	1.02
-19	-8	7	2.55	1.43
19	8	7	3.04	1.08
-19	-8	-7	2.85	1.14
19	-8	7	1.85	0.89
19	8	-7	2.61	1.02
-19	-8	-7	4.43	1.07
19	-8	7	1.64	0.81
-19	8	7	6.26	1.68
-20	0	-7	176.72	8.63
20	0	-7	190.13	8.18
20	0	7	176.31	8.42
-20	0	-7	173.18	8.84
-20	0	7	171.52	8.40
-20	0	7	166.39	8.32
20	0	7	180.90	8.57
-20	0	7	175.72	8.56
20	0	7	185.66	8.49
20	0	-7	183.05	8.16
20	0	7	175.92	8.20
-20	0	7	175.51	8.22
-20	0	7	176.06	8.30

20	-1	-7	94.53	4.48
20	1	7	78.10	4.24
20	-1	-7	98.51	4.30
20	-1	-7	91.47	4.22
-20	-1	7	72.52	4.10
20	1	7	77.90	4.52
20	1	7	79.33	4.10
-20	1	-7	98.75	5.02
-20	-1	7	75.00	3.92
-20	-1	7	74.61	3.86
20	1	7	77.58	3.84
-20	1	-7	79.35	5.01
-20	1	-7	82.40	4.58
-20	-1	7	79.27	4.22
20	-1	7	82.42	4.33
-20	1	7	79.56	4.00
20	-1	7	75.16	4.04
-20	1	7	77.89	4.01
20	-1	7	78.27	3.86
-20	1	7	71.77	3.90
20	-1	7	79.35	4.09
-20	-1	-7	86.96	4.61
-20	-1	-7	87.09	4.81
-20	1	7	79.74	4.34
-20	-2	7	19.01	1.50
20	2	7	19.64	1.39
20	-2	-7	15.34	1.10
-20	2	-7	16.20	2.12
-20	-2	7	21.02	1.81
20	-2	-7	16.92	1.27
20	-2	-7	17.06	1.45
20	-2	-7	17.01	1.28
-20	2	-7	12.31	1.71
-20	2	-7	15.16	1.54
-20	2	-7	13.89	1.43
-20	-2	7	18.61	1.60
20	2	7	19.53	1.91
-20	-2	7	18.93	1.89
20	2	7	21.12	1.97
20	-2	7	22.57	1.66
20	-2	7	19.80	1.42
20	-2	7	21.29	1.68
-20	-2	-7	14.19	1.50
-20	2	7	21.31	2.09

-20	-2	-7	17.32	1.85
-20	-2	-7	13.52	1.78
-20	2	7	17.56	1.42
20	-2	7	20.84	2.22
-20	-2	-7	17.46	1.48
-20	-3	7	-0.26	0.58
20	3	7	0.38	1.01
20	-3	-7	1.03	0.70
-20	-3	7	-0.92	0.89
20	3	7	0.17	0.51
-20	3	-7	0.51	0.71
-20	3	-7	0.44	1.22
-20	-3	7	0.53	0.61
20	-3	-7	0.32	0.47
20	3	7	0.81	1.01
20	-3	-7	0.09	0.56
20	-3	-7	0.11	0.62
-20	-3	-7	1.68	0.67
20	-3	7	-1.42	1.01
20	-3	7	0.85	0.55
-20	3	7	-0.99	0.92
-20	-3	-7	1.51	0.94
20	-3	7	-0.37	0.66
-20	-3	-7	0.50	0.68
20	-3	7	0.12	0.61
-20	4	-7	1.42	1.26
20	4	7	5.19	0.89
20	4	7	7.31	1.15
-20	-4	7	5.60	1.03
-20	4	-7	3.05	0.86
-20	-4	7	4.68	1.27
20	-4	-7	2.56	0.85
20	-4	-7	3.83	0.97
-20	-4	7	5.63	0.92
20	-4	-7	3.32	0.73
20	-4	-7	3.01	0.89
-20	-4	7	5.66	1.08
20	4	7	6.12	1.32
-20	-4	-7	2.94	0.95
-20	4	7	6.67	1.48
20	-4	7	5.11	0.96
20	-4	7	6.71	1.12
-20	-4	-7	3.08	0.85
20	-4	7	5.85	0.87

-20	-4	-7	6.08	0.98
20	5	7	25.72	2.73
-20	5	-7	24.37	3.09
-20	-5	7	30.79	2.20
20	5	7	31.39	2.31
20	-5	-7	32.00	2.14
20	-5	-7	30.81	1.98
20	5	7	29.48	2.04
-20	-5	7	27.62	2.03
-20	-5	7	26.98	1.86
-20	5	-7	27.36	2.14
20	-5	-7	31.25	1.92
-20	-5	7	30.16	2.56
20	-5	7	31.52	2.07
-20	5	7	30.14	2.85
-20	-5	-7	27.70	2.14
20	-5	7	31.57	2.17
-20	-5	-7	29.52	2.21
-20	-6	7	71.25	4.02
20	-6	-7	80.73	4.10
20	6	7	75.69	4.02
-20	-6	7	78.50	4.66
-20	6	-7	79.07	4.19
-20	-6	7	73.91	3.83
20	-6	-7	79.51	4.18
20	6	7	75.66	4.60
-20	-6	7	77.65	4.06
20	-6	-7	80.01	4.02
-20	6	-7	78.83	4.94
-20	-6	-7	79.34	4.25
20	6	-7	71.35	3.83
20	-6	7	76.03	4.07
20	-6	7	79.39	4.08
-20	-6	-7	77.90	4.23
-20	6	7	76.25	4.61
20	6	-7	78.73	3.72
20	7	7	51.80	3.20
20	-7	-7	57.61	3.18
20	7	7	57.28	3.73
-20	-7	7	52.87	3.32
-20	-7	7	56.34	3.73
20	-7	-7	58.35	3.11
20	-7	-7	54.41	3.22
-20	7	-7	53.85	3.21

-20	7	-7	57.47	4.17
20	-7	7	55.80	3.16
20	-7	7	55.16	3.16
-20	7	7	58.41	3.87
20	7	-7	58.02	2.92
-20	-7	-7	55.43	3.35
20	7	-7	55.23	3.05
-20	-7	-7	53.29	3.42
-20	-8	7	18.68	1.95
20	8	7	21.94	3.01
-20	8	-7	18.75	1.76
20	-8	-7	20.81	1.85
20	-8	-7	18.01	1.73
-20	-8	7	23.00	2.49
20	-8	-7	13.95	1.68
-20	8	-7	24.57	4.40
-20	-8	-7	19.50	2.33
20	-8	7	18.40	1.80
20	-8	7	24.34	1.85
20	8	-7	17.23	1.83
-20	8	7	25.28	3.07
-20	-8	-7	25.71	2.97
-21	0	7	-0.53	0.86
-21	0	7	0.26	0.57
21	0	7	-0.04	0.65
-21	0	-7	-0.27	0.67
21	0	7	0.69	1.02
-21	0	7	-0.64	0.81
21	0	7	0.24	0.52
21	0	7	0.32	0.90
-21	0	7	0.71	0.59
-21	1	-7	3.87	1.30
21	1	7	2.50	0.65
-21	-1	7	1.97	0.71
-21	1	-7	2.93	0.92
21	1	7	3.29	1.08
-21	-1	7	2.27	0.90
21	1	7	2.77	1.00
21	1	7	2.96	0.88
-21	-1	7	3.19	1.03
21	-1	-7	2.50	0.44
21	-1	-7	3.03	0.55
21	-1	-7	2.36	0.79
-21	-1	-7	2.77	1.07

21	-1	7	2.07	0.65
21	-1	7	1.55	0.72
-21	1	7	2.94	0.75
21	-1	7	3.31	0.92
21	-1	7	3.77	1.13
-21	-1	-7	1.67	0.75
-21	1	7	3.83	0.88
-21	1	7	2.88	1.13
21	2	7	1.88	1.10
21	-2	-7	0.85	0.59
-21	-2	7	1.17	0.95
21	-2	-7	0.55	0.69
21	2	7	1.54	0.63
21	2	7	0.74	0.99
-21	-2	7	2.76	1.32
-21	2	-7	1.03	0.76
-21	2	-7	1.90	1.29
-21	-2	7	1.01	0.64
-21	-2	7	1.05	0.61
21	-2	-7	1.26	0.48
-21	-2	-7	1.35	0.77
-21	2	7	0.15	1.03
-21	-2	-7	1.25	0.78
21	-2	7	1.06	0.59
21	-2	7	3.03	1.14
21	-2	7	1.04	0.80
-21	2	7	0.57	0.63
-21	-2	-7	0.83	1.02
21	-2	7	0.26	0.82
-21	3	-7	0.09	0.78
21	-3	-7	0.91	0.57
21	-3	-7	0.73	0.57
-21	3	-7	-0.53	1.18
21	-3	-7	1.07	0.78
21	3	7	1.14	1.11
21	3	7	0.78	1.10
21	-3	-7	1.92	0.76
-21	-3	7	-0.29	1.01
21	3	7	1.15	0.65
-21	-3	7	-0.02	0.61
-21	-3	7	0.97	0.64
-21	-3	7	1.49	0.93
-21	3	7	1.51	1.29
-21	-3	-7	1.84	0.81

21	-3	7	-0.30	0.62
21	-3	7	0.07	0.71
-21	-3	-7	0.27	0.76
-21	-3	-7	1.70	0.99
21	-3	7	0.42	0.78
21	4	7	2.44	1.35
21	4	7	1.90	0.74
21	-4	-7	2.15	0.66
21	4	7	2.98	1.16
-21	4	-7	2.33	1.64
-21	-4	7	1.83	0.91
21	-4	-7	2.10	0.84
-21	-4	7	4.02	0.88
-21	-4	7	3.13	0.78
-21	-4	7	2.65	1.22
21	-4	-7	3.07	0.74
-21	4	-7	2.74	0.82
-21	-4	-7	1.09	0.86
-21	-4	-7	2.62	0.91
21	-4	7	2.10	0.70
21	-4	7	2.15	0.80
-21	4	7	2.44	1.39
21	-4	7	2.25	0.86
21	-5	-7	0.51	0.69
-21	-5	7	0.61	0.92
-21	5	-7	0.79	1.57
21	5	7	0.69	1.27
-21	5	-7	0.17	0.75
21	-5	-7	1.27	0.77
-21	-5	7	1.36	0.79
21	5	7	0.86	0.74
21	-5	-7	-0.17	0.63
-21	-5	7	1.66	1.46
-21	5	7	2.30	1.30
-21	-5	-7	0.89	0.78
21	-5	7	0.68	0.74
21	-5	7	-0.28	0.63
-21	-5	-7	0.45	0.95
21	6	7	1.91	0.78
21	6	7	2.73	1.31
-21	-6	7	2.32	0.80
21	-6	-7	1.90	0.81
-21	6	-7	0.37	0.73
-21	-6	7	1.11	1.28

21	-6	-7	3.31	0.80
-21	-6	7	0.25	0.90
21	-6	-7	1.18	0.72
-21	6	-7	1.92	1.47
-21	-6	-7	1.88	0.89
-21	6	7	1.51	1.33
-21	-6	-7	0.61	1.07
21	-6	7	1.33	0.73
21	-6	7	0.72	0.75
21	-7	-7	-0.02	0.79
-21	-7	7	2.59	1.05
-21	7	-7	2.09	1.65
21	7	7	0.08	0.86
21	-7	-7	1.48	0.78
21	-7	-7	0.65	0.88
21	7	7	2.21	1.39
-21	-7	7	2.56	1.33
-21	7	-7	1.16	0.89
21	7	-7	2.05	0.72
21	-7	7	0.18	0.80
21	7	-7	0.40	0.78
-21	7	7	0.86	1.36
-21	-7	-7	2.25	0.88
-21	-7	-7	0.83	1.12
21	-7	7	0.40	0.75
-22	0	-7	141.62	7.28
-22	0	7	120.78	6.41
22	0	7	136.84	6.60
22	0	7	134.55	6.59
22	0	7	133.74	6.29
22	0	7	133.77	6.56
-22	0	-7	142.75	7.02
-22	0	7	133.74	6.74
-22	0	7	130.36	6.30
-22	0	7	127.61	6.39
22	-1	-7	86.44	4.05
22	1	7	97.97	5.04
22	1	7	98.41	5.09
-22	1	-7	85.82	5.02
-22	-1	7	91.76	4.97
-22	-1	7	100.61	5.16
22	-1	-7	87.13	4.39
-22	-1	7	92.75	4.82
22	-1	-7	90.90	4.12

22	1	7	96.94	5.12
-22	-1	7	92.96	4.74
22	1	7	93.48	4.69
-22	1	-7	83.44	4.55
-22	-1	-7	88.09	5.51
22	-1	7	92.37	4.96
-22	-1	-7	83.10	4.56
22	-1	7	101.01	5.00
22	-1	7	93.62	4.70
-22	1	7	100.79	5.31
-22	1	7	98.00	4.87
-22	1	7	88.29	4.75
22	2	7	15.07	1.94
22	-2	-7	14.85	1.37
-22	2	-7	12.47	1.51
22	-2	-7	13.67	1.10
-22	-2	7	13.27	1.43
-22	-2	7	15.36	1.42
22	-2	-7	14.31	1.28
-22	-2	7	13.00	1.84
22	2	7	15.62	1.32
-22	2	-7	11.64	2.37
-22	-2	7	12.80	1.86
22	2	7	17.66	2.02
-22	-2	-7	12.38	1.92
22	-2	7	12.66	1.63
-22	-2	-7	14.84	1.61
-22	-2	-7	11.65	1.66
22	-2	7	14.73	1.29
-22	2	7	14.35	2.31
-22	2	7	15.08	1.61
22	-2	7	13.72	1.69
-22	-3	7	1.03	0.77
22	-3	-7	1.65	0.74
-22	-3	7	1.18	1.11
22	-3	-7	0.98	0.75
22	3	7	0.77	1.08
22	-3	-7	2.47	0.75
22	3	7	1.85	1.18
-22	-3	7	2.05	0.78
-22	3	-7	2.97	1.61
-22	3	-7	1.08	0.75
22	-3	-7	1.37	0.58
-22	-3	7	3.30	1.11

22	3	7	1.77	0.68
-22	3	7	3.16	1.29
-22	-3	-7	1.77	1.00
-22	-3	-7	1.06	0.92
22	-3	7	1.71	0.86
22	-3	7	1.74	0.89
22	-3	7	2.32	0.72
-22	-3	-7	1.03	1.10
22	-4	-7	1.64	0.79
-22	-4	7	3.74	1.13
22	-4	-7	1.02	0.59
-22	-4	7	1.64	0.79
22	4	7	2.32	1.44
-22	-4	7	2.11	0.82
22	4	7	2.40	0.73
22	-4	-7	1.17	0.76
-22	4	-7	3.14	1.59
-22	4	-7	1.06	0.71
-22	-4	7	1.78	1.30
-22	-4	-7	2.54	0.91
22	-4	7	1.89	0.82
-22	4	7	1.15	1.27
22	-4	7	3.15	0.74
-22	-4	-7	1.34	1.06
-22	-5	7	18.88	2.02
22	-5	-7	23.04	1.78
-22	5	-7	22.50	2.86
-22	-5	7	22.01	1.92
-22	5	-7	22.34	1.87
22	5	7	17.26	1.55
22	-5	-7	23.47	1.65
-22	-5	7	21.42	2.55
22	5	7	22.39	2.39
-22	-5	7	17.68	1.64
22	-5	-7	23.64	1.86
-22	-5	-7	21.04	2.11
-22	5	7	17.51	2.41
22	-5	7	19.03	1.69
-22	-5	-7	24.90	1.94
22	-5	7	19.79	1.58
22	-6	-7	66.18	3.68
-22	6	-7	70.04	3.84
22	6	7	57.02	3.28
-22	-6	7	62.26	3.47

22	-6	-7	76.26	3.80
-22	-6	7	61.97	3.89
22	6	7	60.30	3.98
-22	6	-7	76.68	4.77
22	-6	-7	67.82	3.82
22	-6	7	57.73	3.38
-22	-6	-7	70.79	3.91
-22	-6	-7	60.85	3.91
22	-6	7	58.14	3.31
-22	6	7	57.64	4.01
-23	0	7	-1.74	1.20
-23	0	7	-0.47	0.85
23	0	7	0.46	0.54
-23	0	7	0.18	0.67
23	0	7	-1.15	1.02
-23	0	7	0.97	0.90
-23	0	-7	-1.22	1.41
23	0	7	0.84	0.85
-23	0	-7	0.85	0.85
-23	-1	7	-0.06	1.15
-23	-1	7	0.39	0.80
23	1	7	0.25	1.09
-23	-1	7	-0.23	1.18
23	1	7	0.01	0.56
23	-1	-7	0.55	0.45
23	-1	-7	0.21	0.74
23	1	7	0.85	0.81
-23	1	-7	0.66	1.44
-23	-1	7	0.61	0.72
-23	1	-7	-0.62	0.75
23	-1	-7	0.59	0.40
23	-1	7	0.68	0.80
-23	1	7	-0.80	1.31
-23	-1	-7	0.13	0.78
23	-1	7	0.80	0.56
-23	1	7	-0.02	0.87
-23	-1	-7	1.90	1.29
-23	-1	-7	0.28	0.77
-23	-2	7	3.18	0.85
23	-2	-7	4.82	0.77
-23	-2	7	3.18	1.26
23	2	7	2.94	1.24
-23	-2	7	3.76	0.92
-23	-2	7	4.40	1.25

-23	2	-7	4.80	0.97
23	2	7	3.03	0.69
-23	2	-7	2.67	1.76
23	-2	-7	3.94	0.76
23	-2	-7	4.73	0.93
-23	-2	-7	4.28	0.99
-23	-2	-7	5.04	1.38
23	-2	7	4.15	1.11
-23	2	7	2.08	0.87
23	-2	7	4.35	0.74
-23	2	7	3.52	1.44
-23	-2	-7	4.42	0.89
23	-2	7	1.85	0.81
-23	-3	7	6.02	1.01
23	3	7	7.50	1.07
23	3	7	7.77	1.71
23	-3	-7	6.73	0.93
-23	-3	7	7.63	1.47
-23	-3	7	7.80	1.39
23	-3	-7	7.31	1.28
-23	3	-7	7.95	1.29
-23	-3	7	7.76	1.33
-23	3	-7	8.41	1.91
23	-3	7	6.40	1.05
-23	-3	-7	10.25	1.56
23	-3	7	6.58	1.06
-23	3	7	6.74	1.10
-23	-3	-7	6.60	1.08
-23	3	7	5.28	1.46
23	-4	-7	4.07	0.90
23	4	7	5.09	1.54
-23	-4	7	2.71	1.02
-23	4	-7	5.33	1.62
-23	-4	7	5.98	1.68
23	-4	-7	4.12	0.93
23	4	7	3.97	0.81
-23	-4	7	3.98	1.29
-23	-4	7	4.85	0.99
23	-4	-7	3.12	0.69
-23	4	-7	1.73	0.83
-23	4	7	4.25	1.44
-23	-4	-7	3.88	1.03
-23	-4	-7	2.17	0.95
23	-4	7	3.90	0.82

23	-4	7	3.14	0.95
-23	-5	7	6.50	1.33
23	5	7	4.96	0.93
23	-5	-7	2.30	0.68
23	5	7	4.83	1.49
-23	-5	7	6.06	1.00
23	-5	-7	5.63	1.04
-23	5	-7	3.87	0.90
-23	-5	7	5.21	1.57
-23	-5	7	6.21	1.22
-23	5	-7	4.55	1.77
23	-5	-7	2.88	0.89
23	-5	7	5.26	1.11
-23	-5	-7	3.91	0.93
-23	-5	-7	2.55	1.02
23	-5	7	3.77	0.98
-23	5	7	1.48	1.40
24	0	7	62.26	3.35
-24	0	7	64.15	3.62
24	0	7	70.65	4.02
-24	0	7	63.41	3.50
24	0	7	64.58	3.67
-24	0	-7	68.90	4.28
-24	0	-7	60.26	3.56
-24	0	7	67.03	4.11
-24	-1	7	27.10	2.25
-24	1	-7	35.01	3.42
-24	-1	7	27.58	2.45
-24	-1	7	32.13	2.13
24	-1	-7	34.31	1.71
24	1	7	31.80	2.73
24	-1	-7	35.63	1.80
-24	1	-7	31.93	2.34
24	1	7	31.83	1.90
-24	-1	7	33.75	2.98
-24	1	7	32.03	2.20
-24	-1	-7	31.62	3.06
-24	1	7	31.51	2.80
-24	-1	-7	33.39	2.34
24	-1	7	36.47	2.56
24	-1	7	34.03	2.27
-24	1	7	28.55	2.06
-24	-1	-7	32.60	2.29
24	-1	7	28.53	1.90

24	-2	-7	11.65	1.17
-24	2	-7	14.26	1.55
24	-2	-7	13.79	1.49
24	-2	-7	11.24	0.91
24	2	7	13.00	2.00
-24	-2	7	13.45	2.11
-24	-2	7	14.86	1.55
24	2	7	13.43	1.24
-24	-2	7	15.27	2.36
-24	2	-7	7.23	2.02
-24	-2	7	11.27	1.68
24	-2	7	13.80	1.61
-24	2	7	11.22	1.77
24	-2	7	11.43	1.21
-24	2	7	12.03	1.47
-24	-2	-7	9.24	1.53
-24	-2	-7	8.05	1.52
-24	-2	-7	12.34	1.55
-24	3	-7	3.86	1.66
24	-3	-7	2.87	0.62
-24	-3	7	3.25	1.60
24	-3	-7	2.27	0.84
-24	-3	7	2.92	1.33
24	3	7	2.39	1.48
-24	3	-7	3.19	0.94
24	3	7	2.60	0.73
-24	-3	7	1.93	1.01
-24	-3	7	2.16	0.98
24	-3	7	1.73	0.66
-24	-3	-7	2.54	0.94
-24	-3	-7	1.98	0.85
-24	3	7	1.67	0.85
24	-3	7	4.57	1.00
-24	3	7	2.82	1.42
-24	-4	7	2.05	0.99
24	-4	-7	2.35	0.74
-24	-4	7	3.54	1.06
24	-4	-7	2.55	1.02
-24	-4	7	2.96	1.28
24	4	7	3.62	1.44
-24	4	-7	3.70	1.77
24	4	7	3.23	0.77
-24	-4	7	3.28	1.53
-24	4	-7	1.03	1.52

-24	4	7	2.82	0.94
24	-4	7	2.38	0.82
-24	4	7	1.30	1.32
-24	-4	-7	1.86	0.97
-24	-4	-7	1.81	0.96
25	0	7	-0.61	0.87
-25	0	-7	-1.31	0.75
-25	0	-7	-0.45	1.79
-25	0	7	1.23	1.35
25	0	7	1.29	1.34
-25	0	7	-0.28	0.83
25	0	7	0.34	0.58
-25	0	7	-0.06	0.88
25	1	7	4.48	0.78
-25	1	-7	4.13	1.88
25	1	7	5.00	1.81
-25	-1	7	5.07	1.39
-25	-1	7	4.94	1.04
-25	-1	7	6.64	1.15
-25	1	-7	2.43	0.90
25	-1	-7	3.02	0.50
25	-1	7	3.98	0.75
-25	1	7	3.44	1.41
-25	-1	-7	6.50	1.73
-25	1	7	5.88	1.05
-25	1	7	4.70	1.06
-25	-1	-7	2.79	0.89
-25	-1	-7	2.15	0.93
25	-1	7	5.09	1.49
25	-1	7	3.58	0.97
25	-2	-7	6.26	1.23
-25	-2	7	5.12	1.03
-25	-2	7	8.24	1.24
25	2	7	6.05	1.69
25	-2	-7	7.33	0.81
25	-2	-7	7.73	1.53
25	2	7	3.50	0.74
-25	-2	-7	8.30	1.51
-25	2	7	4.83	0.98
-25	-2	-7	9.04	1.47
25	-2	7	3.69	0.95
0	0	8	133.11	6.20
0	0	-8	109.69	4.99
0	0	8	136.13	6.12

0	0	-8	111.04	5.15
0	0	8	126.00	6.04
0	0	8	138.96	6.26
0	0	-8	101.18	5.04
0	0	8	124.88	6.16
0	0	-8	108.89	4.85
0	0	8	147.93	6.35
0	-1	8	88.70	4.34
0	-1	8	91.70	4.40
0	1	-8	71.23	3.45
0	1	-8	68.48	3.22
0	1	-8	74.41	3.34
0	-1	8	99.71	4.56
0	-1	-8	72.31	3.49
0	-1	8	88.20	4.24
0	1	8	87.07	4.37
0	-1	-8	71.52	3.21
0	1	-8	68.32	3.62
0	-1	8	91.99	4.28
0	1	8	97.91	4.34
0	1	-8	69.35	3.26
0	-1	8	97.99	4.49
0	1	8	89.14	4.37
0	1	-8	71.19	3.38
0	-1	-8	68.76	3.31
0	1	8	103.60	4.55
0	-1	-8	68.40	3.20
0	-1	-8	71.00	3.36
0	1	8	94.30	4.41
0	2	-8	220.91	10.23
0	2	8	260.97	12.38
0	-2	-8	213.01	9.98
0	-2	-8	220.52	10.22
0	-2	-8	226.00	10.13
0	-2	8	298.57	12.57
0	-2	8	266.80	12.42
0	-2	8	272.20	12.25
0	2	-8	219.25	9.97
0	2	8	264.08	12.42
0	2	8	296.29	12.54
0	2	8	295.84	12.40
0	2	-8	224.36	10.08
0	-2	8	266.42	12.28
0	-2	-8	211.78	10.10

0	2	-8	233.34	10.30
0	2	-8	226.21	10.00
0	-2	-8	228.53	10.10
0	-2	8	259.42	12.35
0	-2	-8	219.89	10.08
0	2	8	256.11	12.25
0	-3	-8	65.17	2.91
0	3	-8	61.87	3.00
0	3	8	54.51	2.50
0	3	-8	61.71	2.83
0	3	8	48.23	2.39
0	3	-8	62.79	3.09
0	-3	-8	64.42	3.01
0	-3	8	54.62	2.73
0	-3	-8	54.88	3.03
0	-3	-8	57.37	2.99
0	3	-8	62.61	2.93
0	3	8	52.07	2.67
0	-3	-8	61.43	3.14
0	-3	-8	64.42	3.08
0	-3	8	47.64	2.48
0	3	-8	65.19	3.24
0	3	8	51.62	2.52
0	-3	8	49.12	2.93
0	-3	8	50.47	2.57
0	3	8	50.34	2.51
0	-3	8	48.16	2.42
0	-3	8	51.88	2.33
0	3	-8	59.56	3.16
0	3	8	49.05	2.59
0	4	-8	1.59	0.37
0	-4	-8	2.02	0.43
0	4	8	0.82	0.40
0	4	8	0.77	0.37
0	-4	8	0.79	0.38
0	4	-8	1.99	0.65
0	4	-8	1.89	0.53
0	4	8	0.94	0.36
0	4	-8	2.37	0.45
0	-4	-8	1.41	0.38
0	4	8	0.78	0.29
0	-4	8	0.66	0.26
0	4	-8	1.94	0.50
0	4	8	0.89	0.31

0	4	8	0.88	0.55
0	-4	8	0.99	0.35
0	4	8	0.58	0.36
0	-4	8	0.87	0.54
0	-4	8	1.04	0.59
0	-4	-8	1.83	0.33
0	-4	-8	2.25	0.59
0	-4	-8	1.93	0.50
0	-5	-8	5.22	0.66
0	5	-8	6.01	0.67
0	-5	8	8.87	0.84
0	5	8	9.97	0.75
0	5	-8	5.91	0.72
0	-5	8	11.23	1.20
0	5	8	9.91	1.13
0	-5	-8	6.27	0.91
0	5	8	9.16	0.86
0	5	8	10.31	0.73
0	5	-8	6.38	0.86
0	-5	8	9.16	0.89
0	5	-8	5.05	0.87
0	5	8	8.95	0.81
0	-5	-8	5.59	0.58
0	-5	-8	6.40	0.74
0	5	8	9.82	0.81
0	5	-8	5.79	0.96
0	-5	8	9.22	0.74
0	-5	8	9.31	1.20
0	5	8	9.90	1.02
0	-5	-8	5.57	0.73
0	6	8	31.26	2.14
0	6	-8	40.34	2.17
0	6	8	37.06	1.92
0	6	-8	35.53	2.38
0	6	8	37.71	2.31
0	6	8	33.43	1.98
0	6	-8	38.20	2.04
0	-6	8	36.44	1.94
0	6	8	33.74	1.88
0	6	8	33.68	1.89
0	-6	8	35.92	2.06
0	-6	-8	36.26	2.19
0	-6	8	40.05	2.35
0	-6	-8	40.50	2.11

0	-6	8	35.76	2.01
0	6	-8	36.00	2.26
0	-6	-8	38.27	2.01
0	-6	8	40.44	2.33
0	6	8	36.97	2.01
0	6	-8	37.75	2.20
0	-6	-8	36.16	2.14
0	-6	-8	36.87	2.04
0	7	8	119.38	5.73
0	7	8	124.98	5.85
0	-7	-8	113.43	5.64
0	7	-8	118.94	5.65
0	-7	8	124.24	5.90
0	-7	8	127.24	6.09
0	7	8	113.72	6.05
0	7	-8	128.75	5.79
0	7	8	125.07	5.79
0	-7	-8	126.72	5.89
0	7	8	131.61	6.13
0	-7	-8	116.65	5.62
0	-7	-8	124.57	5.68
0	7	-8	110.33	6.00
0	7	-8	115.82	5.87
0	-7	8	119.40	5.77
0	7	8	118.28	5.71
0	-7	-8	111.19	5.76
0	7	8	112.19	5.81
0	-7	8	113.01	5.81
0	-7	8	129.90	6.15
0	7	-8	115.43	5.76
0	-8	8	287.12	13.46
0	8	8	272.14	13.77
0	-8	-8	314.35	13.70
0	8	8	289.98	13.43
0	8	8	313.56	13.37
0	8	-8	285.92	14.09
0	-8	8	277.49	13.50
0	-8	8	298.62	13.57
0	8	8	277.14	13.49
0	-8	-8	295.74	13.65
0	-8	8	301.95	13.80
0	-8	-8	282.02	13.63
0	-8	-8	311.19	13.91
0	8	8	313.27	13.82

0	-8	8	301.67	13.63
0	8	-8	292.77	13.87
0	-8	-8	280.73	13.80
0	8	8	269.96	13.39
0	-8	8	316.67	13.74
0	8	-8	318.41	13.82
0	9	8	114.63	5.58
0	9	8	94.39	4.83
0	9	8	106.99	4.86
0	9	-8	84.40	4.64
0	9	8	105.71	5.41
0	-9	-8	79.07	4.52
0	-9	8	100.25	5.29
0	-9	8	101.25	5.07
0	-9	-8	86.48	4.71
0	9	8	93.76	4.99
0	9	-8	85.89	4.90
0	-9	8	91.98	5.01
0	9	8	93.46	5.34
0	-9	-8	86.37	4.38
0	9	-8	97.71	4.68
0	-9	-8	92.78	4.49
0	-9	-8	88.28	4.39
0	-9	8	98.99	5.18
0	10	8	77.52	4.34
0	10	-8	73.74	3.94
0	-10	8	86.40	4.43
0	-10	-8	67.99	3.56
0	10	8	89.46	4.79
0	10	8	79.56	4.18
0	-10	-8	70.65	3.73
0	-10	-8	62.61	3.73
0	10	8	89.22	4.21
0	10	8	77.38	4.76
0	-10	-8	65.21	3.57
0	10	-8	69.44	4.27
0	10	-8	66.33	3.85
0	-10	8	84.11	4.31
0	11	8	1.06	0.81
0	11	-8	3.62	1.20
0	-11	-8	1.02	0.71
0	11	8	2.65	1.26
0	-11	8	0.55	0.91
0	11	-8	1.82	1.47

0	-11	-8	1.49	0.76
0	-11	-8	1.54	0.83
0	-11	8	-0.10	0.70
0	-11	-8	3.00	0.94
0	-12	-8	5.35	1.09
0	12	-8	3.85	1.33
0	12	8	7.08	2.05
0	-12	8	8.91	1.47
0	-12	-8	4.51	1.02
0	12	-8	5.81	1.97
0	-12	-8	5.12	1.42
0	-12	8	7.96	1.38
0	13	-8	42.29	3.21
0	-13	-8	42.34	2.71
0	-13	-8	42.05	2.93
0	-13	8	32.95	2.38
0	-13	-8	38.44	2.59
0	13	8	28.30	3.46
0	13	-8	31.79	4.28
0	-13	8	39.55	3.06
-1	0	8	0.00	0.18
-1	0	-8	0.08	0.12
1	0	-8	-0.14	0.21
-1	0	-8	0.37	0.33
-1	0	-8	-0.20	0.22
1	0	-8	0.01	0.08
1	0	8	0.09	0.18
-1	0	-8	0.04	0.18
-1	0	8	0.11	0.22
1	0	-8	0.00	0.18
1	0	8	0.07	0.11
1	0	-8	0.10	0.25
1	0	8	0.10	0.29
1	0	8	-0.31	0.31
-1	0	8	0.24	0.35
-1	0	-8	-0.29	0.15
-1	0	8	0.05	0.30
1	0	8	0.19	0.20
-1	0	8	0.03	0.08
1	0	-8	0.25	0.24
1	0	8	0.03	0.20
[truncated: 518,590 more chars]
