# Supplementary material for: Structure-property relationships of photofunctional diiridium(II) complexes with tetracationic charge and an unsupported Ir–Ir bond
Source: Commun Chem. 2022 Nov 23;5:159. doi: 10.1038/s42004-022-00775-4 (PMC9814866; doi:10.1038/s42004-022-00775-4)
Supplement: Supplementary file 11 — Supplementary Data 8 [file 42004_2022_775_MOESM11_ESM.pdf]

```
data_Ir(III)(t-Bu-terpyr)(PPh3)(NN)I[OTf]2
_audit_creation_date              2017-08-10
_audit_creation_method
;
Olex2 1.2
(compiled 2017.07.20 svn.r3457 for OlexSys, GUI svn.r5370)
;
_shelxl_version_number            2013-2
_publ_contact_author_address      ?
_publ_contact_author_email        ?
_publ_contact_author_id_orcid     ?
_publ_contact_author_name         ''
_publ_contact_author_phone        ?
_publ_section_references
;
Dolomanov, O.V., Bourhis, L.J., Gildea, R.J, Howard, J.A.K. & Puschmann,
H.
 (2009), J. Appl. Cryst. 42, 339-341.

Sheldrick, G.M. (2015). Acta Cryst. A71, 3-8.

Sheldrick, G.M. (2015). Acta Cryst. C71, 3-8.
;
_chemical_name_common             ?
_chemical_name_systematic
;
 ?
;
_chemical_formula_moiety          'C51 H58 Ir N6 P, 2(C F3 O3 S), C2 H3
N'
_chemical_formula_sum             'C55 H61 F6 Ir N7 O6 P S2'
_chemical_formula_weight          1317.39
_chemical_melting_point           ?
loop_
  _atom_type_symbol
  _atom_type_description
  _atom_type_scat_dispersion_real
  _atom_type_scat_dispersion_imag
  _atom_type_scat_source
 'C' 'C' 0.0033 0.0016 'International Tables Vol C Tables 4.2.6.8 and
6.1.1.4'
 'H' 'H' 0.0000 0.0000 'International Tables Vol C Tables 4.2.6.8 and
6.1.1.4'
 'N' 'N' 0.0061 0.0033 'International Tables Vol C Tables 4.2.6.8 and
```

```
6.1.1.4'
 'O' 'O' 0.0106 0.0060 'International Tables Vol C Tables 4.2.6.8 and
6.1.1.4'
 'F' 'F' 0.0171 0.0103 'International Tables Vol C Tables 4.2.6.8 and
6.1.1.4'
 'P' 'P' 0.1023 0.0942 'International Tables Vol C Tables 4.2.6.8 and
6.1.1.4'
 'S' 'S' 0.1246 0.1234 'International Tables Vol C Tables 4.2.6.8 and
6.1.1.4'
 'Ir' 'Ir' -1.4442 7.9887
 'International Tables Vol C Tables 4.2.6.8 and 6.1.1.4'

_shelx_space_group_comment
;
The symmetry employed for this shelxl refinement is uniquely defined
by the following loop, which should always be used as a source of
symmetry information in preference to the above space-group names.
They are only intended as comments.
;
_space_group_crystal_system       'monoclinic'
_space_group_IT_number            14
_space_group_name_H-M_alt         'P 1 21/c 1'
_space_group_name_Hall            '-P 2ybc'
loop_
  _space_group_symop_operation_xyz
 'x, y, z'
 '-x, y+1/2, -z+1/2'
 '-x, -y, -z'
 'x, -y-1/2, z-1/2'

_cell_length_a                    12.5751(6)
_cell_length_b                    21.4466(10)
_cell_length_c                    20.6816(9)
_cell_angle_alpha                 90
_cell_angle_beta                  90.2280(10)
_cell_angle_gamma                 90
_cell_volume                      5577.6(4)
_cell_formula_units_Z             4
_cell_measurement_reflns_used     9258
_cell_measurement_temperature     100(2)
_cell_measurement_theta_max       27.54
_cell_measurement_theta_min       2.72
_shelx_estimated_absorpt_T_max    0.669
_shelx_estimated_absorpt_T_min    0.614
```

```
_exptl_absorpt_coefficient_mu     2.574
_exptl_absorpt_correction_T_max   0.7456
_exptl_absorpt_correction_T_min   0.4113
_exptl_absorpt_correction_type    multi-scan
_exptl_absorpt_process_details
'SADABS-2014/5 (Bruker,2014/5) was used for absorption correction.
wR2(int) was 0.1185 before and 0.0554 after correction. The Ratio of
minimum to maximum transmission is 0.5516. The \l/2 correction factor
is Not present.'
_exptl_crystal_colour             yellow
_exptl_crystal_colour_primary     yellow
_exptl_crystal_density_diffrn     1.569
_exptl_crystal_density_meas       ?
_exptl_crystal_density_method     ?
_exptl_crystal_description        block
_exptl_crystal_F_000              2664
_exptl_crystal_size_max           0.21
_exptl_crystal_size_mid           0.19
_exptl_crystal_size_min           0.17
_exptl_special_details
;
 ?
;
_exptl_transmission_factor_max    ?
_exptl_transmission_factor_min    ?
_diffrn_reflns_av_R_equivalents   0.0420
_diffrn_reflns_av_unetI/netI      0.0364
_diffrn_reflns_Laue_measured_fraction_full 0.998
_diffrn_reflns_Laue_measured_fraction_max 0.996
_diffrn_reflns_limit_h_max        13
_diffrn_reflns_limit_h_min        -16
_diffrn_reflns_limit_k_max        27
_diffrn_reflns_limit_k_min        -23
_diffrn_reflns_limit_l_max        26
_diffrn_reflns_limit_l_min        -26
_diffrn_reflns_number             57582
_diffrn_reflns_point_group_measured_fraction_full 0.998
_diffrn_reflns_point_group_measured_fraction_max 0.996
_diffrn_reflns_theta_full         25.242
_diffrn_reflns_theta_max          27.549
_diffrn_reflns_theta_min          2.123
_diffrn_ambient_temperature       100.01
_diffrn_detector_area_resol_mean  ?
_diffrn_measured_fraction_theta_full 0.998
```

```
_diffrn_measured_fraction_theta_max 0.996
_diffrn_measurement_device_type   'Bruker APEX-II CCD'
_diffrn_measurement_method        '\f and \w scans'
_diffrn_radiation_type            MoK\a
_diffrn_radiation_wavelength      0.71073
_diffrn_source                    ?
_diffrn_standards_number          0
_reflns_Friedel_coverage          0.000
_reflns_Friedel_fraction_full     .
_reflns_Friedel_fraction_max      .
_reflns_number_gt                 11271
_reflns_number_total              12822
_reflns_special_details
;
 Reflections were merged by SHELXL according to the crystal
 class for the calculation of statistics and refinement.

 _reflns_Friedel_fraction is defined as the number of unique
 Friedel pairs measured divided by the number that would be
 possible theoretically, ignoring centric projections and
 systematic absences.
;
_reflns_threshold_expression      'I > 2\s(I)'
_computing_cell_refinement        'SAINT v8.37A (Bruker, 2015)'
_computing_data_collection        ?
_computing_data_reduction         'SAINT v8.37A (Bruker, 2015)'
_computing_molecular_graphics     'Olex2 (Dolomanov et al., 2009)'
_computing_publication_material   'Olex2 (Dolomanov et al., 2009)'
_computing_structure_refinement   'ShelXL (Sheldrick, 2015)'
_computing_structure_solution     'ShelXT (Sheldrick, 2015)'
_refine_diff_density_max          2.634
_refine_diff_density_min          -1.995
_refine_diff_density_rms          0.244
_refine_ls_extinction_coef        .
_refine_ls_extinction_method      none
_refine_ls_goodness_of_fit_ref    1.101
_refine_ls_hydrogen_treatment     constr
_refine_ls_matrix_type            full
_refine_ls_number_parameters      715
_refine_ls_number_reflns          12822
_refine_ls_number_restraints      36
_refine_ls_R_factor_all           0.0557
_refine_ls_R_factor_gt            0.0460
_refine_ls_restrained_S_all       1.114
```

```
_refine_ls_shift/su_max           0.003
_refine_ls_shift/su_mean          0.000
_refine_ls_structure_factor_coef  Fsqd
_refine_ls_weighting_details
;
 w=1/[\s^2^(Fo^2^)+(0.0590P)^2^+31.6050P]
 where P=(Fo^2^+2Fc^2^)/3
;
_refine_ls_weighting_scheme       calc
_refine_ls_wR_factor_gt           0.1194
_refine_ls_wR_factor_ref          0.1301
_refine_special_details
;
 ?
;
_olex2_refinement_description
;
1. Fixed Uiso
 At 1.2 times of:
  All C(H) groups, All N(H) groups
 At 1.5 times of:
  All C(H,H,H) groups
2. Uiso/Uaniso restraints and constraints
Uanis(C53) \\sim Ueq, Uanis(N7) \\sim Ueq, Uanis(C54) \\sim Ueq: with
sigma of
0.01 and sigma for terminal atoms of 0.02
Uanis(F4) \\sim Ueq, Uanis(F5) \\sim Ueq, Uanis(F6) \\sim Ueq: with
sigma of
0.005 and sigma for terminal atoms of 0.01
3.a Aromatic/amide H refined with riding coordinates:
 N4(H4), N5(H5), C1(H1), C2(H2), C4(H4A), C7(H7), C9(H9), C12(H12),
C14(H14),
 C15(H15), C29(H29), C30(H30), C31(H31), C32(H32), C33(H33), C35(H35),
C36(H36),
  C37(H37), C38(H38), C39(H39), C41(H41), C42(H42), C43(H43), C44(H44),
C45(H45)
3.b Idealised Me refined as rotating group:
 C17(H17A,H17B,H17C),     C18(H18A,H18B,H18C),     C19(H19A,H19B,H19C),
C21(H21A,H21B,
 H21C), C22(H22A,H22B,H22C), C23(H23A,H23B,H23C), C25(H25A,H25B,H25C),
C26(H26A,
 H26B,H26C),         C27(H27A,H27B,H27C),         C46(H46A,H46B,H46C),
C50(H50A,H50B,H50C),
 C55(H55A,H55B,H55C)
```

```
;
_atom_sites_solution_hydrogens    geom
_atom_sites_solution_primary      dual
_atom_sites_solution_secondary    ?
loop_
  _atom_site_label
  _atom_site_type_symbol
  _atom_site_fract_x
  _atom_site_fract_y
  _atom_site_fract_z
  _atom_site_U_iso_or_equiv
  _atom_site_adp_type
  _atom_site_occupancy
  _atom_site_site_symmetry_order
  _atom_site_calc_flag
  _atom_site_refinement_flags_posn
  _atom_site_refinement_flags_adp
  _atom_site_refinement_flags_occupancy
  _atom_site_disorder_assembly
  _atom_site_disorder_group
 Ir1 Ir 0.73551(2) 0.55109(2) 0.72623(2) 0.01550(7) Uani 1 1 d . . . . .
 P1 P 0.92207(10) 0.54667(6) 0.72027(6) 0.0168(2) Uani 1 1 d . . . . .
 N1 N 0.7284(3) 0.64563(19) 0.71403(18) 0.0164(8) Uani 1 1 d . . . . .
 N2 N 0.7252(3) 0.55540(18) 0.63091(19) 0.0154(8) Uani 1 1 d . . . . .
 N3 N 0.7213(3) 0.45774(19) 0.7049(2) 0.0187(8) Uani 1 1 d . . . . .
 N4 N 0.7335(4) 0.54928(19) 0.8249(2) 0.0192(8) Uani 1 1 d . . . . .
 H4 H 0.7958 0.5529 0.8441 0.023 Uiso 1 1 calc R . . . .
 N5 N 0.5725(3) 0.55324(18) 0.72847(19) 0.0166(8) Uani 1 1 d . . . . .
 H5 H 0.5413 0.5600 0.6910 0.020 Uiso 1 1 calc R . . . .
 N6 N 0.3966(4) 0.5210(3) 0.9247(2) 0.0379(12) Uani 1 1 d . . . . .
 C1 C 0.7168(4) 0.6891(2) 0.7602(2) 0.0186(10) Uani 1 1 d . . . . .
 H1 H 0.7158 0.6765 0.8042 0.022 Uiso 1 1 calc R . . . .
 C2 C 0.7062(4) 0.7513(2) 0.7454(2) 0.0210(10) Uani 1 1 d . . . . .
 H2 H 0.6987 0.7808 0.7793 0.025 Uiso 1 1 calc R . . . .
 C3 C 0.7064(4) 0.7714(2) 0.6816(2) 0.0183(9) Uani 1 1 d . . . . .
 C4 C 0.7178(4) 0.7259(2) 0.6338(2) 0.0178(9) Uani 1 1 d . . . . .
 H4A H 0.7189 0.7376 0.5895 0.021 Uiso 1 1 calc R . . . .
 C5 C 0.7274(4) 0.6641(2) 0.6507(2) 0.0152(9) Uani 1 1 d . . . . .
 C6 C 0.7255(4) 0.6120(2) 0.6031(2) 0.0149(9) Uani 1 1 d . . . . .
 C7 C 0.7158(4) 0.6167(2) 0.5366(2) 0.0154(9) Uani 1 1 d . . . . .
 H7 H 0.7211 0.6562 0.5161 0.018 Uiso 1 1 calc R . . . .
 C8 C 0.6980(4) 0.5628(2) 0.5000(2) 0.0154(9) Uani 1 1 d . . . . .
 C9 C 0.6943(4) 0.5050(2) 0.5318(2) 0.0151(9) Uani 1 1 d . . . . .
 H9 H 0.6810 0.4680 0.5079 0.018 Uiso 1 1 calc R . . . .
```

C10 C 0.7099(4) 0.5018(2) 0.5981(2) 0.0161(9) Uani 1 1 d . . . . .
C11 C 0.7114(4) 0.4458(2) 0.6402(2) 0.0183(9) Uani 1 1 d . . . . .
C12 C 0.7046(4) 0.3853(2) 0.6171(2) 0.0201(10) Uani 1 1 d . . . . .
H12 H 0.6979 0.3783 0.5719 0.024 Uiso 1 1 calc R . . . .
C13 C 0.7073(4) 0.3344(2) 0.6591(2) 0.0227(10) Uani 1 1 d . . . . .
C14 C 0.7098(4) 0.3489(3) 0.7251(2) 0.0266(11) Uani 1 1 d . . . . .
H14 H 0.7046 0.3162 0.7559 0.032 Uiso 1 1 calc R . . . .
C15 C 0.7197(4) 0.4093(2) 0.7460(2) 0.0220(10) Uani 1 1 d . . . . .
H15 H 0.7255 0.4172 0.7911 0.026 Uiso 1 1 calc R . . . .
C16 C 0.6929(4) 0.8408(2) 0.6662(2) 0.0215(10) Uani 1 1 d . . . . .
C17 C 0.5856(5) 0.8626(3) 0.6932(3) 0.0283(12) Uani 1 1 d . . . . .
H17A H 0.5856 0.8578 0.7403 0.042 Uiso 1 1 calc GR . . . .
H17B H 0.5283 0.8375 0.6744 0.042 Uiso 1 1 calc GR . . . .
H17C H 0.5745 0.9066 0.6821 0.042 Uiso 1 1 calc GR . . . .
C18 C 0.7837(5) 0.8771(3) 0.6987(3) 0.0323(13) Uani 1 1 d . . . . .
H18A H 0.7787 0.9212 0.6869 0.048 Uiso 1 1 calc GR . . . .
H18B H 0.8521 0.8603 0.6843 0.048 Uiso 1 1 calc GR . . . .
H18C H 0.7782 0.8728 0.7458 0.048 Uiso 1 1 calc GR . . . .
C19 C 0.6961(6) 0.8531(3) 0.5933(3) 0.0342(14) Uani 1 1 d . . . . .
H19A H 0.6862 0.8978 0.5852 0.051 Uiso 1 1 calc GR . . . .
H19B H 0.6391 0.8296 0.5719 0.051 Uiso 1 1 calc GR . . . .
H19C H 0.7650 0.8399 0.5762 0.051 Uiso 1 1 calc GR . . . .
C20 C 0.6760(4) 0.5658(2) 0.4275(2) 0.0160(9) Uani 1 1 d . . . . .
C21 C 0.6945(5) 0.6308(2) 0.4002(2) 0.0225(10) Uani 1 1 d . . . . .
H21A H 0.6799 0.6307 0.3536 0.034 Uiso 1 1 calc GR . . . .
H21B H 0.7685 0.6432 0.4078 0.034 Uiso 1 1 calc GR . . . .
H21C H 0.6469 0.6605 0.4215 0.034 Uiso 1 1 calc GR . . . .
C22 C 0.5579(4) 0.5487(3) 0.4170(3) 0.0243(11) Uani 1 1 d . . . . .
H22A H 0.5448 0.5066 0.4336 0.036 Uiso 1 1 calc GR . . . .
H22B H 0.5413 0.5499 0.3706 0.036 Uiso 1 1 calc GR . . . .
H22C H 0.5128 0.5786 0.4399 0.036 Uiso 1 1 calc GR . . . .
C23 C 0.7474(4) 0.5188(2) 0.3917(2) 0.0201(10) Uani 1 1 d . . . . .
H23A H 0.7297 0.4764 0.4055 0.030 Uiso 1 1 calc GR . . . .
H23B H 0.8222 0.5275 0.4019 0.030 Uiso 1 1 calc GR . . . .
H23C H 0.7359 0.5227 0.3450 0.030 Uiso 1 1 calc GR . . . .
C24 C 0.7033(5) 0.2675(3) 0.6353(3) 0.0305(12) Uani 1 1 d . . . . .
C25 C 0.5909(7) 0.2438(4) 0.6429(5) 0.072(3) Uani 1 1 d . . . . .
H25A H 0.5668 0.2515 0.6872 0.108 Uiso 1 1 calc GR . . . .
H25B H 0.5890 0.1989 0.6340 0.108 Uiso 1 1 calc GR . . . .
H25C H 0.5441 0.2656 0.6124 0.108 Uiso 1 1 calc GR . . . .
C26 C 0.7401(7) 0.2614(3) 0.5654(3) 0.0480(19) Uani 1 1 d . . . . .
H26A H 0.6898 0.2830 0.5368 0.072 Uiso 1 1 calc GR . . . .
H26B H 0.7433 0.2172 0.5535 0.072 Uiso 1 1 calc GR . . . .
H26C H 0.8108 0.2800 0.5609 0.072 Uiso 1 1 calc GR . . . .

C27 C 0.7816(8) 0.2272(3) 0.6750(4) 0.056(2) Uani 1 1 d . . . . .
H27A H 0.8515 0.2474 0.6759 0.084 Uiso 1 1 calc GR . . . .
H27B H 0.7878 0.1859 0.6551 0.084 Uiso 1 1 calc GR . . . .
H27C H 0.7552 0.2228 0.7193 0.084 Uiso 1 1 calc GR . . . .
C28 C 0.9600(4) 0.4852(2) 0.6641(2) 0.0190(10) Uani 1 1 d . . . . .
C29 C 0.9420(4) 0.4911(2) 0.5973(2) 0.0203(10) Uani 1 1 d . . . . .
H29 H 0.9179 0.5298 0.5803 0.024 Uiso 1 1 calc R . . . .
C30 C 0.9589(4) 0.4414(2) 0.5557(3) 0.0250(11) Uani 1 1 d . . . . .
H30 H 0.9456 0.4460 0.5107 0.030 Uiso 1 1 calc R . . . .
C31 C 0.9950(4) 0.3849(3) 0.5798(3) 0.0279(12) Uani 1 1 d . . . . .
H31 H 1.0070 0.3509 0.5513 0.033 Uiso 1 1 calc R . . . .
C32 C 1.0134(5) 0.3780(3) 0.6456(3) 0.0286(12) Uani 1 1 d . . . . .
H32 H 1.0379 0.3392 0.6620 0.034 Uiso 1 1 calc R . . . .
C33 C 0.9962(4) 0.4276(2) 0.6878(3) 0.0217(10) Uani 1 1 d . . . . .
H33 H 1.0090 0.4225 0.7327 0.026 Uiso 1 1 calc R . . . .
C34 C 0.9923(4) 0.5279(2) 0.7950(2) 0.0204(10) Uani 1 1 d . . . . .
C35 C 0.9506(5) 0.4834(3) 0.8374(3) 0.0258(11) Uani 1 1 d . . . . .
H35 H 0.8860 0.4628 0.8270 0.031 Uiso 1 1 calc R . . . .
C36 C 1.0034(5) 0.4694(3) 0.8947(3) 0.0328(13) Uani 1 1 d . . . . .
H36 H 0.9742 0.4396 0.9236 0.039 Uiso 1 1 calc R . . . .
C37 C 1.0990(5) 0.4988(3) 0.9096(3) 0.0327(13) Uani 1 1 d . . . . .
H37 H 1.1352 0.4891 0.9487 0.039 Uiso 1 1 calc R . . . .
C38 C 1.1409(5) 0.5420(3) 0.8678(3) 0.0302(12) Uani 1 1 d . . . . .
H38 H 1.2062 0.5620 0.8781 0.036 Uiso 1 1 calc R . . . .
C39 C 1.0888(4) 0.5568(2) 0.8106(3) 0.0251(11) Uani 1 1 d . . . . .
H39 H 1.1187 0.5865 0.7819 0.030 Uiso 1 1 calc R . . . .
C40 C 0.9822(4) 0.6190(2) 0.6925(2) 0.0193(10) Uani 1 1 d . . . . .
C41 C 0.9640(4) 0.6723(2) 0.7306(3) 0.0220(10) Uani 1 1 d . . . . .
H41 H 0.9259 0.6690 0.7700 0.026 Uiso 1 1 calc R . . . .
C42 C 1.0021(5) 0.7297(3) 0.7102(3) 0.0293(12) Uani 1 1 d . . . . .
H42 H 0.9890 0.7658 0.7355 0.035 Uiso 1 1 calc R . . . .
C43 C 1.0590(5) 0.7348(3) 0.6532(3) 0.0360(14) Uani 1 1 d . . . . .
H43 H 1.0829 0.7745 0.6388 0.043 Uiso 1 1 calc R . . . .
C44 C 1.0806(6) 0.6819(3) 0.6175(3) 0.0380(14) Uani 1 1 d . . . . .
H44 H 1.1215 0.6853 0.5791 0.046 Uiso 1 1 calc R . . . .
C45 C 1.0439(5) 0.6242(3) 0.6367(3) 0.0285(12) Uani 1 1 d . . . . .
H45 H 1.0604 0.5882 0.6121 0.034 Uiso 1 1 calc R . . . .
C46 C 0.6726(5) 0.5439(3) 0.9353(2) 0.0289(12) Uani 1 1 d . . . . .
H46A H 0.7483 0.5364 0.9435 0.043 Uiso 1 1 calc GR . . . .
H46B H 0.6304 0.5112 0.9560 0.043 Uiso 1 1 calc GR . . . .
H46C H 0.6526 0.5846 0.9532 0.043 Uiso 1 1 calc GR . . . .
C47 C 0.6519(4) 0.5435(2) 0.8635(2) 0.0222(10) Uani 1 1 d . . . . .
C48 C 0.5455(4) 0.5379(2) 0.8411(2) 0.0210(10) Uani 1 1 d . . . . .
C49 C 0.5090(4) 0.5462(2) 0.7768(2) 0.0188(10) Uani 1 1 d . . . . .

```
 C50 C 0.3916(4) 0.5482(2) 0.7635(3) 0.0239(11) Uani 1 1 d . . . . .
 H50A H 0.3794 0.5536 0.7170 0.036 Uiso 1 1 calc GR . . . .
 H50B H 0.3600 0.5833 0.7870 0.036 Uiso 1 1 calc GR . . . .
 H50C H 0.3589 0.5092 0.7779 0.036 Uiso 1 1 calc GR . . . .
 C51 C 0.4647(5) 0.5289(3) 0.8882(2) 0.0259(11) Uani 1 1 d . . . . .
 S1 S 0.43036(10) 0.65216(6) 0.57570(6) 0.0206(2) Uani 1 1 d . . . . .
 F1 F 0.4578(4) 0.7129(3) 0.6840(2) 0.0718(15) Uani 1 1 d . . . . .
 F2 F 0.3116(3) 0.66125(18) 0.67885(18) 0.0432(9) Uani 1 1 d . . . . .
 F3 F 0.3277(4) 0.74634(17) 0.6260(2) 0.0532(11) Uani 1 1 d . . . . .
 O1 O 0.3365(3) 0.63100(18) 0.54258(18) 0.0289(8) Uani 1 1 d . . . . .
 O2 O 0.4936(3) 0.6970(2) 0.5417(2) 0.0378(10) Uani 1 1 d . . . . .
 O3 O 0.4900(3) 0.60275(19) 0.60692(18) 0.0285(8) Uani 1 1 d . . . . .
 C52 C 0.3791(5) 0.6957(3) 0.6443(3) 0.0328(13) Uani 1 1 d . . . . .
 S2 S 0.84114(16) 0.71659(8) 0.92776(8) 0.0468(5) Uani 1 1 d . . . . .
 F4 F 0.9193(6) 0.6369(3) 1.0078(3) 0.119(3) Uani 1 1 d . U . . .
 F5 F 0.9229(7) 0.6202(3) 0.8984(3) 0.111(2) Uani 1 1 d . U . . .
 F6 F 1.0307(5) 0.6817(3) 0.9528(4) 0.111(2) Uani 1 1 d . U . . .
 O4 O 0.7371(5) 0.6876(3) 0.9250(3) 0.0780(19) Uani 1 1 d . . . . .
 O5 O 0.8314(7) 0.7577(3) 0.9828(3) 0.089(3) Uani 1 1 d . . . . .
 O6 O 0.8815(4) 0.7469(2) 0.8714(2) 0.0433(12) Uani 1 1 d . . . . .
 C53 C 0.9277(8) 0.6631(5) 0.9481(5) 0.075(3) Uani 1 1 d . U . . .
 N7 N 0.2339(7) 0.8835(3) 0.5963(3) 0.0612(19) Uani 1 1 d . U . . .
 C54 C 0.3095(7) 0.8749(3) 0.5683(3) 0.0482(18) Uani 1 1 d . U . . .
 C55 C 0.4081(7) 0.8631(4) 0.5326(4) 0.056(2) Uani 1 1 d . . . . .
 H55A H 0.3986 0.8757 0.4873 0.084 Uiso 1 1 calc GR . . . .
 H55B H 0.4252 0.8186 0.5345 0.084 Uiso 1 1 calc GR . . . .
 H55C H 0.4662 0.8872 0.5520 0.084 Uiso 1 1 calc GR . . . .

loop_
  _atom_site_aniso_label
  _atom_site_aniso_U_11
  _atom_site_aniso_U_22
  _atom_site_aniso_U_33
  _atom_site_aniso_U_23
  _atom_site_aniso_U_13
  _atom_site_aniso_U_12
 Ir1  0.02054(11)  0.01823(10)  0.00772(10)  0.00157(6)  0.00009(6)
0.00000(7)
 P1 0.0218(6) 0.0160(6) 0.0125(5) 0.0029(4) -0.0007(5) 0.0010(5)
 N1 0.0169(19) 0.021(2) 0.0117(18) -0.0012(15) 0.0014(15) -0.0003(16)
 N2 0.0173(19) 0.020(2) 0.0085(17) 0.0008(15) 0.0018(14) 0.0024(15)
 N3 0.025(2) 0.020(2) 0.0110(18) 0.0015(15) 0.0002(16) -0.0013(16)
 N4 0.026(2) 0.021(2) 0.0110(18) 0.0018(15) -0.0003(16) -0.0023(17)
 N5 0.023(2) 0.0187(19) 0.0087(18) -0.0018(15) -0.0007(15) -0.0012(16)
```

N6 0.038(3) 0.056(3) 0.019(2) 0.007(2) 0.003(2) -0.007(3)

C1 0.021(2) 0.026(2) 0.009(2) -0.0034(18) 0.0013(17) 0.0034(19)

C2 0.031(3) 0.018(2) 0.014(2) -0.0058(18) -0.0019(19) 0.005(2)

C3 0.022(2) 0.019(2) 0.014(2) -0.0028(18) 0.0012(18) 0.0024(19)

C4 0.018(2) 0.022(2) 0.013(2) 0.0008(18) -0.0011(17) 0.0008(18)

C5 0.015(2) 0.021(2) 0.009(2) -0.0025(17) 0.0006(16) 0.0045(18)

C6 0.014(2) 0.018(2) 0.013(2) 0.0003(17) 0.0026(16) 0.0032(17)

C7 0.017(2) 0.017(2) 0.012(2) -0.0010(17) 0.0008(17) 0.0014(18)

C8 0.015(2) 0.019(2) 0.012(2) 0.0003(17) 0.0011(17) 0.0006(17)

C9 0.015(2) 0.017(2) 0.012(2) 0.0015(17) -0.0022(17) 0.0013(17)

C10 0.018(2) 0.018(2) 0.012(2) -0.0001(17) 0.0024(17) 0.0037(18)

C11 0.020(2) 0.022(2) 0.013(2) 0.0007(18) -0.0003(18) 0.0015(19)

C12 0.022(2) 0.023(2) 0.015(2) 0.0037(19) -0.0019(18) -0.001(2)

C13 0.026(3) 0.021(2) 0.022(2) 0.006(2) -0.005(2) -0.004(2)

C14 0.024(3) 0.035(3) 0.020(3) 0.009(2) 0.000(2) 0.000(2)

C15 0.027(3) 0.027(3) 0.013(2) 0.008(2) -0.0023(19) -0.005(2)

C16 0.029(3) 0.016(2) 0.020(2) -0.0010(19) 0.002(2) 0.003(2)

C17 0.030(3) 0.026(3) 0.029(3) 0.001(2) 0.002(2) 0.010(2)

C18 0.037(3) 0.023(3) 0.037(3) -0.005(2) 0.004(3) -0.004(2)

C19 0.061(4) 0.019(3) 0.022(3) 0.000(2) 0.007(3) 0.006(3)

C20 0.019(2) 0.018(2) 0.011(2) 0.0010(17) -0.0017(17) -0.0009(18)

C21 0.037(3) 0.021(2) 0.010(2) 0.0020(18) -0.001(2) 0.000(2)

C22 0.025(3) 0.031(3) 0.017(2) -0.001(2) -0.004(2) 0.003(2)

C23 0.029(3) 0.020(2) 0.012(2) -0.0032(18) 0.0016(19) 0.000(2)

C24 0.045(3) 0.019(2) 0.028(3) 0.006(2) -0.007(2) -0.003(2)

C25 0.055(5) 0.052(5) 0.110(8) -0.027(5) 0.005(5) -0.023(4)

C26 0.094(6) 0.022(3) 0.029(3) -0.002(2) -0.006(3) 0.002(3)

C27 0.097(7) 0.033(4) 0.038(4) 0.006(3) -0.016(4) 0.018(4)

C28 0.020(2) 0.019(2) 0.018(2) 0.0004(19) -0.0008(18) 0.0011(19)

C29 0.018(2) 0.023(2) 0.020(2) 0.0034(19) -0.0010(19) 0.0017(19)

C30 0.024(3) 0.029(3) 0.022(3) -0.004(2) 0.002(2) -0.006(2)

C31 0.029(3) 0.021(3) 0.033(3) -0.006(2) 0.005(2) -0.003(2)

C32 0.031(3) 0.022(3) 0.033(3) 0.004(2) 0.005(2) 0.002(2)

C33 0.018(2) 0.022(2) 0.025(3) 0.005(2) 0.000(2) 0.0031(19)

C34 0.020(2) 0.022(2) 0.019(2) -0.0007(19) -0.0013(19) 0.0036(19)

C35 0.029(3) 0.026(3) 0.023(3) 0.004(2) -0.003(2) 0.001(2)

C36 0.047(4) 0.030(3) 0.022(3) 0.009(2) -0.007(2) 0.003(3)

C37 0.037(3) 0.038(3) 0.022(3) 0.003(2) -0.013(2) 0.007(3)

C38 0.029(3) 0.034(3) 0.028(3) -0.002(2) -0.010(2) 0.004(2)

C39 0.026(3) 0.026(3) 0.023(3) 0.003(2) -0.003(2) 0.000(2)

C40 0.017(2) 0.020(2) 0.022(2) 0.0026(19) -0.0029(18) 0.0040(19)

C41 0.018(2) 0.025(2) 0.024(2) -0.002(2) 0.0009(19) 0.002(2)

C42 0.028(3) 0.020(3) 0.040(3) -0.005(2) -0.006(2) 0.004(2)

C43 0.042(3) 0.025(3) 0.041(3) 0.009(3) -0.002(3) -0.007(3)

```
 C44 0.050(4) 0.037(3) 0.028(3) 0.007(3) 0.009(3) -0.010(3)
 C45 0.037(3) 0.027(3) 0.022(3) -0.001(2) 0.005(2) -0.003(2)
 C46 0.038(3) 0.036(3) 0.013(2) 0.002(2) -0.002(2) -0.011(2)
 C47 0.033(3) 0.020(2) 0.014(2) 0.0024(18) 0.002(2) -0.005(2)
 C48 0.030(3) 0.019(2) 0.014(2) -0.0006(18) 0.003(2) -0.001(2)
 C49 0.025(3) 0.017(2) 0.014(2) -0.0007(17) 0.0034(19) -0.0027(19)
 C50 0.024(3) 0.028(3) 0.020(2) 0.004(2) 0.002(2) -0.004(2)
 C51 0.033(3) 0.030(3) 0.015(2) 0.001(2) 0.000(2) -0.006(2)
 S1 0.0234(6) 0.0231(6) 0.0154(5) 0.0015(5) 0.0004(4) 0.0032(5)
 F1 0.065(3) 0.092(4) 0.059(3) -0.050(3) -0.012(2) -0.008(3)
 F2 0.050(2) 0.046(2) 0.0343(19) 0.0044(16) 0.0186(17) 0.0118(18)
 F3 0.066(3) 0.0234(18) 0.070(3) 0.0000(18) 0.024(2) 0.0100(18)
 O1 0.037(2) 0.0246(19) 0.0254(19) -0.0038(16) -0.0089(17) 0.0008(16)
 O2 0.030(2) 0.038(2) 0.045(3) 0.017(2) 0.0058(19) 0.0022(18)
 O3 0.031(2) 0.034(2) 0.0210(18) 0.0056(16) 0.0007(15) 0.0108(17)
 C52 0.035(3) 0.032(3) 0.032(3) -0.008(2) 0.003(2) -0.002(2)
 S2 0.0706(12) 0.0448(9) 0.0247(7) 0.0152(7) -0.0130(8) -0.0308(9)
 F4 0.129(5) 0.126(5) 0.101(4) 0.087(4) 0.050(4) 0.077(4)
 F5 0.148(6) 0.096(4) 0.089(4) -0.032(4) 0.023(4) -0.030(4)
 F6 0.083(4) 0.124(5) 0.125(5) 0.066(4) -0.046(4) -0.036(4)
 O4 0.067(4) 0.083(5) 0.085(5) 0.033(4) 0.008(3) 0.000(3)
 O5 0.143(7) 0.082(4) 0.042(3) 0.017(3) 0.034(4) 0.069(5)
 O6 0.079(4) 0.027(2) 0.024(2) 0.0043(17) 0.012(2) -0.003(2)
 C53 0.073(5) 0.079(6) 0.075(5) 0.013(5) 0.025(5) 0.008(5)
 N7 0.091(5) 0.055(4) 0.038(3) -0.011(3) -0.009(4) 0.026(4)
 C54 0.079(5) 0.032(3) 0.034(3) 0.000(3) -0.016(3) 0.008(3)
 C55 0.072(5) 0.041(4) 0.054(5) 0.014(3) -0.010(4) -0.008(4)

_geom_special_details
;
 All esds (except the esd in the dihedral angle between two l.s. planes)
 are estimated using the full covariance matrix.  The cell esds are
taken
 into account individually in the estimation of esds in distances,
angles
 and torsion angles; correlations between esds in cell parameters are
only
 used when they are defined by crystal symmetry.  An approximate
(isotropic)
 treatment of cell esds is used for estimating esds involving l.s.
planes.
;
loop_
  _geom_bond_atom_site_label_1
```

```
 _geom_bond_atom_site_label_2
 _geom_bond_distance
 _geom_bond_site_symmetry_2
 _geom_bond_publ_flag
Ir1 P1 2.3517(13) . ?
Ir1 N1 2.045(4) . ?
Ir1 N2 1.977(4) . ?
Ir1 N3 2.058(4) . ?
Ir1 N4 2.042(4) . ?
Ir1 N5 2.051(4) . ?
P1 C28 1.821(5) . ?
P1 C34 1.822(5) . ?
P1 C40 1.819(5) . ?
N1 C1 1.342(6) . ?
N1 C5 1.368(6) . ?
N2 C6 1.343(6) . ?
N2 C10 1.349(6) . ?
N3 C11 1.368(6) . ?
N3 C15 1.343(6) . ?
N4 H4 0.8800 . ?
N4 C47 1.308(7) . ?
N5 H5 0.8800 . ?
N5 C49 1.291(6) . ?
N6 C51 1.156(7) . ?
C1 H1 0.9500 . ?
C1 C2 1.375(7) . ?
C2 H2 0.9500 . ?
C2 C3 1.389(7) . ?
C3 C4 1.397(7) . ?
C3 C16 1.531(7) . ?
C4 H4A 0.9500 . ?
C4 C5 1.376(7) . ?
C5 C6 1.490(6) . ?
C6 C7 1.383(6) . ?
C7 H7 0.9500 . ?
C7 C8 1.400(6) . ?
C8 C9 1.405(6) . ?
C8 C20 1.524(6) . ?
C9 H9 0.9500 . ?
C9 C10 1.385(6) . ?
C10 C11 1.483(6) . ?
C11 C12 1.386(7) . ?
C12 H12 0.9500 . ?
C12 C13 1.395(7) . ?
```

C13 C14 1.398(7) . ?
C13 C24 1.519(7) . ?
C14 H14 0.9500 . ?
C14 C15 1.373(8) . ?
C15 H15 0.9500 . ?
C16 C17 1.535(7) . ?
C16 C18 1.535(8) . ?
C16 C19 1.530(7) . ?
C17 H17A 0.9800 . ?
C17 H17B 0.9800 . ?
C17 H17C 0.9800 . ?
C18 H18A 0.9800 . ?
C18 H18B 0.9800 . ?
C18 H18C 0.9800 . ?
C19 H19A 0.9800 . ?
C19 H19B 0.9800 . ?
C19 H19C 0.9800 . ?
C20 C21 1.523(7) . ?
C20 C22 1.545(7) . ?
C20 C23 1.541(7) . ?
C21 H21A 0.9800 . ?
C21 H21B 0.9800 . ?
C21 H21C 0.9800 . ?
C22 H22A 0.9800 . ?
C22 H22B 0.9800 . ?
C22 H22C 0.9800 . ?
C23 H23A 0.9800 . ?
C23 H23B 0.9800 . ?
C23 H23C 0.9800 . ?
C24 C25 1.510(10) . ?
C24 C26 1.527(9) . ?
C24 C27 1.543(9) . ?
C25 H25A 0.9800 . ?
C25 H25B 0.9800 . ?
C25 H25C 0.9800 . ?
C26 H26A 0.9800 . ?
C26 H26B 0.9800 . ?
C26 H26C 0.9800 . ?
C27 H27A 0.9800 . ?
C27 H27B 0.9800 . ?
C27 H27C 0.9800 . ?
C28 C29 1.405(7) . ?
C28 C33 1.403(7) . ?
C29 H29 0.9500 . ?

C29 C30 1.388(7) . ?
C30 H30 0.9500 . ?
C30 C31 1.386(8) . ?
C31 H31 0.9500 . ?
C31 C32 1.386(8) . ?
C32 H32 0.9500 . ?
C32 C33 1.394(8) . ?
C33 H33 0.9500 . ?
C34 C35 1.399(7) . ?
C34 C39 1.398(8) . ?
C35 H35 0.9500 . ?
C35 C36 1.387(8) . ?
C36 H36 0.9500 . ?
C36 C37 1.392(9) . ?
C37 H37 0.9500 . ?
C37 C38 1.374(9) . ?
C38 H38 0.9500 . ?
C38 C39 1.386(8) . ?
C39 H39 0.9500 . ?
C40 C41 1.408(7) . ?
C40 C45 1.397(7) . ?
C41 H41 0.9500 . ?
C41 C42 1.386(8) . ?
C42 H42 0.9500 . ?
C42 C43 1.385(9) . ?
C43 H43 0.9500 . ?
C43 C44 1.382(9) . ?
C44 H44 0.9500 . ?
C44 C45 1.381(8) . ?
C45 H45 0.9500 . ?
C46 H46A 0.9800 . ?
C46 H46B 0.9800 . ?
C46 H46C 0.9800 . ?
C46 C47 1.508(7) . ?
C47 C48 1.419(8) . ?
C48 C49 1.417(7) . ?
C48 C51 1.423(7) . ?
C49 C50 1.500(7) . ?
C50 H50A 0.9800 . ?
C50 H50B 0.9800 . ?
C50 H50C 0.9800 . ?
S1 O1 1.436(4) . ?
S1 O2 1.434(4) . ?
S1 O3 1.448(4) . ?

```
 S1 C52 1.819(6) . ?
 F1 C52 1.336(7) . ?
 F2 C52 1.335(7) . ?
 F3 C52 1.318(7) . ?
 S2 O4 1.449(7) . ?
 S2 O5 1.446(7) . ?
 S2 O6 1.430(4) . ?
 S2 C53 1.635(11) . ?
 F4 C53 1.362(11) . ?
 F5 C53 1.380(12) . ?
 F6 C53 1.358(12) . ?
 N7 C54 1.130(11) . ?
 C54 C55 1.467(12) . ?
 C55 H55A 0.9800 . ?
 C55 H55B 0.9800 . ?
 C55 H55C 0.9800 . ?

loop_
  _geom_angle_atom_site_label_1
  _geom_angle_atom_site_label_2
  _geom_angle_atom_site_label_3
  _geom_angle
  _geom_angle_site_symmetry_1
  _geom_angle_site_symmetry_3
  _geom_angle_publ_flag
 N1 Ir1 P1 94.40(11) . . ?
 N1 Ir1 N3 159.13(17) . . ?
 N1 Ir1 N4 98.14(15) . . ?
 N1 Ir1 N5 86.40(15) . . ?
 N2 Ir1 P1 90.63(12) . . ?
 N2 Ir1 N1 80.10(16) . . ?
 N2 Ir1 N3 80.00(16) . . ?
 N2 Ir1 N4 175.27(17) . . ?
 N2 Ir1 N5 87.71(16) . . ?
 N3 Ir1 P1 92.02(13) . . ?
 N4 Ir1 P1 93.89(13) . . ?
 N4 Ir1 N3 101.21(16) . . ?
 N4 Ir1 N5 87.80(17) . . ?
 N5 Ir1 P1 178.01(11) . . ?
 N5 Ir1 N3 86.61(16) . . ?
 C28 P1 Ir1 109.06(17) . . ?
 C28 P1 C34 104.7(2) . . ?
 C28 P1 C40 107.8(2) . . ?
 C34 P1 Ir1 116.41(17) . . ?
```

C40 P1 Ir1 113.47(16) . . ?

C40 P1 C34 104.8(2) . . ?

C1 N1 Ir1 127.2(3) . . ?

C1 N1 C5 118.6(4) . . ?

C5 N1 Ir1 113.9(3) . . ?

C6 N2 Ir1 118.0(3) . . ?

C6 N2 C10 123.7(4) . . ?

C10 N2 Ir1 118.1(3) . . ?

C11 N3 Ir1 113.5(3) . . ?

C15 N3 Ir1 128.2(4) . . ?

C15 N3 C11 118.3(4) . . ?

Ir1 N4 H4 115.7 . . ?

C47 N4 Ir1 128.6(4) . . ?

C47 N4 H4 115.7 . . ?

Ir1 N5 H5 115.2 . . ?

C49 N5 Ir1 129.6(4) . . ?

C49 N5 H5 115.2 . . ?

N1 C1 H1 119.1 . . ?

N1 C1 C2 121.7(4) . . ?

C2 C1 H1 119.1 . . ?

C1 C2 H2 119.6 . . ?

C1 C2 C3 120.8(4) . . ?

C3 C2 H2 119.6 . . ?

C2 C3 C4 117.1(4) . . ?

C2 C3 C16 120.0(4) . . ?

C4 C3 C16 123.0(4) . . ?

C3 C4 H4A 119.9 . . ?

C5 C4 C3 120.2(4) . . ?

C5 C4 H4A 119.9 . . ?

N1 C5 C4 121.5(4) . . ?

N1 C5 C6 114.6(4) . . ?

C4 C5 C6 123.6(4) . . ?

N2 C6 C5 113.2(4) . . ?

N2 C6 C7 119.4(4) . . ?

C7 C6 C5 127.1(4) . . ?

C6 C7 H7 120.3 . . ?

C6 C7 C8 119.4(4) . . ?

C8 C7 H7 120.3 . . ?

C7 C8 C9 118.7(4) . . ?

C7 C8 C20 121.7(4) . . ?

C9 C8 C20 119.5(4) . . ?

C8 C9 H9 119.9 . . ?

C10 C9 C8 120.2(4) . . ?

C10 C9 H9 119.9 . . ?

N2 C10 C9 118.4(4) . . ?
N2 C10 C11 113.1(4) . . ?
C9 C10 C11 128.5(4) . . ?
N3 C11 C10 115.1(4) . . ?
N3 C11 C12 121.1(4) . . ?
C12 C11 C10 123.7(4) . . ?
C11 C12 H12 119.4 . . ?
C11 C12 C13 121.1(5) . . ?
C13 C12 H12 119.4 . . ?
C12 C13 C14 115.8(5) . . ?
C12 C13 C24 122.5(5) . . ?
C14 C13 C24 121.7(5) . . ?
C13 C14 H14 119.4 . . ?
C15 C14 C13 121.3(5) . . ?
C15 C14 H14 119.4 . . ?
N3 C15 C14 122.1(5) . . ?
N3 C15 H15 118.9 . . ?
C14 C15 H15 118.9 . . ?
C17 C16 C3 108.5(4) . . ?
C17 C16 C18 109.8(4) . . ?
C17 C16 C19 109.4(5) . . ?
C18 C16 C3 108.6(4) . . ?
C19 C16 C3 111.7(4) . . ?
C19 C16 C18 108.7(5) . . ?
C16 C17 H17A 109.5 . . ?
C16 C17 H17B 109.5 . . ?
C16 C17 H17C 109.5 . . ?
H17A C17 H17B 109.5 . . ?
H17A C17 H17C 109.5 . . ?
H17B C17 H17C 109.5 . . ?
C16 C18 H18A 109.5 . . ?
C16 C18 H18B 109.5 . . ?
C16 C18 H18C 109.5 . . ?
H18A C18 H18B 109.5 . . ?
H18A C18 H18C 109.5 . . ?
H18B C18 H18C 109.5 . . ?
C16 C19 H19A 109.5 . . ?
C16 C19 H19B 109.5 . . ?
C16 C19 H19C 109.5 . . ?
H19A C19 H19B 109.5 . . ?
H19A C19 H19C 109.5 . . ?
H19B C19 H19C 109.5 . . ?
C8 C20 C21 112.1(4) . . ?
C8 C20 C22 107.4(4) . . ?

C8 C20 C23 110.0(4) . . ?
C21 C20 C22 108.3(4) . . ?
C21 C20 C23 109.3(4) . . ?
C22 C20 C23 109.7(4) . . ?
C20 C21 H21A 109.5 . . ?
C20 C21 H21B 109.5 . . ?
C20 C21 H21C 109.5 . . ?
H21A C21 H21B 109.5 . . ?
H21A C21 H21C 109.5 . . ?
H21B C21 H21C 109.5 . . ?
C20 C22 H22A 109.5 . . ?
C20 C22 H22B 109.5 . . ?
C20 C22 H22C 109.5 . . ?
H22A C22 H22B 109.5 . . ?
H22A C22 H22C 109.5 . . ?
H22B C22 H22C 109.5 . . ?
C20 C23 H23A 109.5 . . ?
C20 C23 H23B 109.5 . . ?
C20 C23 H23C 109.5 . . ?
H23A C23 H23B 109.5 . . ?
H23A C23 H23C 109.5 . . ?
H23B C23 H23C 109.5 . . ?
C13 C24 C25 108.3(6) . . ?
C13 C24 C26 112.2(5) . . ?
C13 C24 C27 109.6(5) . . ?
C25 C24 C27 110.6(7) . . ?
C26 C24 C25 110.9(6) . . ?
C26 C24 C27 105.1(6) . . ?
C24 C25 H25A 109.5 . . ?
C24 C25 H25B 109.5 . . ?
C24 C25 H25C 109.5 . . ?
H25A C25 H25B 109.5 . . ?
H25A C25 H25C 109.5 . . ?
H25B C25 H25C 109.5 . . ?
C24 C26 H26A 109.5 . . ?
C24 C26 H26B 109.5 . . ?
C24 C26 H26C 109.5 . . ?
H26A C26 H26B 109.5 . . ?
H26A C26 H26C 109.5 . . ?
H26B C26 H26C 109.5 . . ?
C24 C27 H27A 109.5 . . ?
C24 C27 H27B 109.5 . . ?
C24 C27 H27C 109.5 . . ?
H27A C27 H27B 109.5 . . ?

H27A C27 H27C 109.5 . . ?
H27B C27 H27C 109.5 . . ?
C29 C28 P1 121.3(4) . . ?
C33 C28 P1 120.0(4) . . ?
C33 C28 C29 118.2(5) . . ?
C28 C29 H29 119.5 . . ?
C30 C29 C28 121.1(5) . . ?
C30 C29 H29 119.5 . . ?
C29 C30 H30 120.0 . . ?
C31 C30 C29 120.0(5) . . ?
C31 C30 H30 120.0 . . ?
C30 C31 H31 120.0 . . ?
C30 C31 C32 120.0(5) . . ?
C32 C31 H31 120.0 . . ?
C31 C32 H32 119.8 . . ?
C31 C32 C33 120.4(5) . . ?
C33 C32 H32 119.8 . . ?
C28 C33 H33 119.8 . . ?
C32 C33 C28 120.3(5) . . ?
C32 C33 H33 119.8 . . ?
C35 C34 P1 120.1(4) . . ?
C39 C34 P1 121.0(4) . . ?
C39 C34 C35 118.9(5) . . ?
C34 C35 H35 119.9 . . ?
C36 C35 C34 120.3(5) . . ?
C36 C35 H35 119.9 . . ?
C35 C36 H36 120.0 . . ?
C35 C36 C37 120.1(6) . . ?
C37 C36 H36 120.0 . . ?
C36 C37 H37 120.1 . . ?
C38 C37 C36 119.9(5) . . ?
C38 C37 H37 120.1 . . ?
C37 C38 H38 119.7 . . ?
C37 C38 C39 120.7(6) . . ?
C39 C38 H38 119.7 . . ?
C34 C39 H39 119.9 . . ?
C38 C39 C34 120.1(5) . . ?
C38 C39 H39 119.9 . . ?
C41 C40 P1 116.6(4) . . ?
C45 C40 P1 124.2(4) . . ?
C45 C40 C41 119.2(5) . . ?
C40 C41 H41 120.2 . . ?
C42 C41 C40 119.6(5) . . ?
C42 C41 H41 120.2 . . ?

C41 C42 H42 119.7 . . ?
C41 C42 C43 120.7(5) . . ?
C43 C42 H42 119.7 . . ?
C42 C43 H43 120.2 . . ?
C44 C43 C42 119.5(5) . . ?
C44 C43 H43 120.2 . . ?
C43 C44 H44 119.5 . . ?
C43 C44 C45 121.0(6) . . ?
C45 C44 H44 119.5 . . ?
C40 C45 H45 120.0 . . ?
C44 C45 C40 119.9(5) . . ?
C44 C45 H45 120.0 . . ?
H46A C46 H46B 109.5 . . ?
H46A C46 H46C 109.5 . . ?
H46B C46 H46C 109.5 . . ?
C47 C46 H46A 109.5 . . ?
C47 C46 H46B 109.5 . . ?
C47 C46 H46C 109.5 . . ?
N4 C47 C46 117.8(5) . . ?
N4 C47 C48 123.5(5) . . ?
C48 C47 C46 118.7(5) . . ?
C47 C48 C51 117.6(5) . . ?
C49 C48 C47 126.6(5) . . ?
C49 C48 C51 115.5(5) . . ?
N5 C49 C48 122.8(5) . . ?
N5 C49 C50 117.8(4) . . ?
C48 C49 C50 119.3(4) . . ?
C49 C50 H50A 109.5 . . ?
C49 C50 H50B 109.5 . . ?
C49 C50 H50C 109.5 . . ?
H50A C50 H50B 109.5 . . ?
H50A C50 H50C 109.5 . . ?
H50B C50 H50C 109.5 . . ?
N6 C51 C48 177.5(6) . . ?
O1 S1 O3 113.8(2) . . ?
O1 S1 C52 104.0(3) . . ?
O2 S1 O1 115.8(3) . . ?
O2 S1 O3 115.0(3) . . ?
O2 S1 C52 103.7(3) . . ?
O3 S1 C52 102.3(3) . . ?
F1 C52 S1 110.9(4) . . ?
F1 C52 F2 107.2(5) . . ?
F2 C52 S1 111.2(4) . . ?
F3 C52 S1 112.0(4) . . ?

```
 F3 C52 F1 108.1(5) . . ?
 F3 C52 F2 107.3(5) . . ?
 O4 S2 C53 108.0(5) . . ?
 O5 S2 O4 102.4(5) . . ?
 O5 S2 C53 106.5(5) . . ?
 O6 S2 O4 119.1(4) . . ?
 O6 S2 O5 113.3(3) . . ?
 O6 S2 C53 106.9(4) . . ?
 F4 C53 S2 117.9(7) . . ?
 F5 C53 S2 104.4(8) . . ?
 F5 C53 F4 113.4(9) . . ?
 F6 C53 S2 116.6(8) . . ?
 F6 C53 F4 97.6(9) . . ?
 F6 C53 F5 106.8(8) . . ?
 N7 C54 C55 179.3(9) . . ?
 C54 C55 H55A 109.5 . . ?
 C54 C55 H55B 109.5 . . ?
 C54 C55 H55C 109.5 . . ?
 H55A C55 H55B 109.5 . . ?
 H55A C55 H55C 109.5 . . ?
 H55B C55 H55C 109.5 . . ?

_shelx_res_file
;
TITL z_a.res in P2(1)/c
REM Old TITL z in P2(1)/c
REM SHELXT solution in P2(1)/c
REM R1 0.116, Rweak 0.005, Alpha 0.046, Orientation as input
REM Formula found by SHELXT: C59 N3 O9 F4 P S2 Ir
CELL 0.71073 12.5751 21.4466 20.6816 90 90.228 90
ZERR 4 0.0006 0.001 0.0009 0 0.001 0
LATT 1
SYMM -X,0.5+Y,0.5-Z
SFAC C H N O F P S Ir
UNIT 220 244 28 24 24 4 8 4
ISOR 0.01 0.02 C53 N7 C54
ISOR 0.005 0.01 F4 F5 F6

L.S. 20
PLAN  1
SIZE 0.21 0.19 0.17
TEMP -173.14
BOND $H
list 4
```

```
fmap 2
acta
REM <olex2.extras>
REM <HklSrc "%.\\.hkl">
REM </olex2.extras>

WGHT    0.059000   31.605000
FVAR       0.20303
IR1   8    0.735508    0.551093    0.726227    11.00000    0.02054
0.01823 =
         0.00772    0.00157    0.00009    0.00000
P1    6    0.922074    0.546674    0.720272    11.00000    0.02182
0.01599 =
         0.01247    0.00293   -0.00067    0.00105
N1    3    0.728395    0.645626    0.714027    11.00000    0.01691
0.02065 =
         0.01174   -0.00118    0.00142   -0.00031
N2    3    0.725210    0.555402    0.630905    11.00000    0.01731
0.02024 =
         0.00854    0.00083    0.00176    0.00240
N3    3    0.721324    0.457742    0.704887    11.00000    0.02512
0.01990 =
         0.01098    0.00155    0.00024   -0.00126
N4    3    0.733513    0.549279    0.824946    11.00000    0.02589
0.02068 =
         0.01100    0.00178   -0.00032   -0.00233
AFIX  43
H4    2    0.795821    0.552887    0.844103    11.00000   -1.20000
AFIX   0
N5    3    0.572515    0.553244    0.728470    11.00000    0.02256
0.01871 =
         0.00866   -0.00179   -0.00071   -0.00119
AFIX  43
H5    2    0.541296    0.559974    0.690958    11.00000   -1.20000
AFIX   0
N6    3    0.396640    0.521022    0.924727    11.00000    0.03787
0.05634 =
         0.01944    0.00692    0.00314   -0.00729
C1    1    0.716764    0.689058    0.760198    11.00000    0.02075
0.02626 =
         0.00881   -0.00345    0.00127    0.00342
AFIX  43
H1    2    0.715816    0.676492    0.804239    11.00000   -1.20000
AFIX   0
```

```
C2     1     0.706186     0.751262     0.745405     11.00000     0.03100
0.01843 =
         0.01351   -0.00578   -0.00186    0.00457
AFIX  43
H2    2    0.698657    0.780842    0.779271    11.00000   -1.20000
AFIX   0
C3     1     0.706371     0.771401     0.681556     11.00000     0.02200
0.01877 =
         0.01420   -0.00281    0.00119    0.00237
C4     1     0.717769     0.725870     0.633802     11.00000     0.01845
0.02163 =
         0.01339    0.00078   -0.00114    0.00077
AFIX  43
H4A   2    0.718866    0.737594    0.589513    11.00000   -1.20000
AFIX   0
C5     1     0.727411     0.664097     0.650728     11.00000     0.01493
0.02141 =
         0.00940   -0.00249    0.00056    0.00448
C6     1     0.725514     0.612007     0.603088     11.00000     0.01379
0.01805 =
         0.01300    0.00035    0.00261    0.00315
C7     1     0.715796     0.616744     0.536640     11.00000     0.01669
0.01727 =
         0.01220   -0.00103    0.00081    0.00145
AFIX  43
H7    2    0.721138    0.656215    0.516061    11.00000   -1.20000
AFIX   0
C8     1     0.698017     0.562850     0.499982     11.00000     0.01465
0.01918 =
         0.01250    0.00025    0.00112    0.00064
C9     1     0.694285     0.505020     0.531838     11.00000     0.01547
0.01749 =
         0.01239    0.00151   -0.00216    0.00128
AFIX  43
H9    2    0.681018    0.468027    0.507862    11.00000   -1.20000
AFIX   0
C10    1     0.709887     0.501792     0.598064     11.00000     0.01816
0.01821 =
         0.01186   -0.00007    0.00236    0.00365
C11    1     0.711413     0.445837     0.640170     11.00000     0.01975
0.02241 =
         0.01288    0.00067   -0.00032    0.00148
C12    1     0.704596     0.385292     0.617111     11.00000     0.02237
0.02312 =
```

```
         0.01485    0.00373   -0.00185   -0.00101
AFIX  43
H12   2    0.697937    0.378306    0.571916    11.00000   -1.20000
AFIX   0
C13   1     0.707345     0.334427     0.659130     11.00000     0.02595
0.02053 =
         0.02165    0.00637   -0.00517   -0.00433
C14   1     0.709814     0.348859     0.725055     11.00000     0.02443
0.03516 =
         0.02027    0.00915    0.00030   -0.00043
AFIX  43
H14   2    0.704566    0.316201    0.755911    11.00000   -1.20000
AFIX   0
C15   1     0.719677     0.409326     0.746041     11.00000     0.02667
0.02661 =
         0.01275    0.00801   -0.00228   -0.00507
AFIX  43
H15   2    0.725524    0.417164    0.791125    11.00000   -1.20000
AFIX   0
C16   1     0.692854     0.840769     0.666158     11.00000     0.02858
0.01626 =
         0.01960   -0.00100    0.00202    0.00267
C17   1     0.585631     0.862620     0.693190     11.00000     0.03033
0.02558 =
         0.02909    0.00077    0.00222    0.01021
AFIX 137
H17A  2    0.585627    0.857778    0.740308    11.00000   -1.50000
H17B  2    0.528287    0.837453    0.674383    11.00000   -1.50000
H17C  2    0.574525    0.906581    0.682136    11.00000   -1.50000
AFIX   0
C18   1     0.783667     0.877057     0.698723     11.00000     0.03730
0.02279 =
         0.03685   -0.00478    0.00392   -0.00448
AFIX 137
H18A  2    0.778686    0.921206    0.686870    11.00000   -1.50000
H18B  2    0.852100    0.860311    0.684313    11.00000   -1.50000
H18C  2    0.778179    0.872817    0.745772    11.00000   -1.50000
AFIX   0
C19   1     0.696100     0.853130     0.593318     11.00000     0.06084
0.01943 =
         0.02247   -0.00024    0.00707    0.00580
AFIX 137
H19A  2    0.686223    0.897786    0.585236    11.00000   -1.50000
H19B  2    0.639130    0.829644    0.571912    11.00000   -1.50000
```

```
H19C  2    0.765024    0.839878    0.576158    11.00000   -1.50000
AFIX   0
C20    1     0.676025     0.565827     0.427525     11.00000     0.01939
0.01773 =
         0.01093    0.00099   -0.00165   -0.00095
C21    1     0.694501     0.630849     0.400160     11.00000     0.03652
0.02115 =
         0.00993    0.00200   -0.00056    0.00000
AFIX 137
H21A  2    0.679918    0.630663    0.353577    11.00000   -1.50000
H21B  2    0.768537    0.643174    0.407790    11.00000   -1.50000
H21C  2    0.646934    0.660545    0.421505    11.00000   -1.50000
AFIX   0
C22    1     0.557949     0.548652     0.416958     11.00000     0.02477
0.03083 =
         0.01732   -0.00122   -0.00402    0.00349
AFIX 137
H22A  2    0.544789    0.506591    0.433627    11.00000   -1.50000
H22B  2    0.541296    0.549904    0.370645    11.00000   -1.50000
H22C  2    0.512843    0.578562    0.439896    11.00000   -1.50000
AFIX   0
C23    1     0.747446     0.518837     0.391710     11.00000     0.02867
0.02003 =
         0.01164   -0.00324    0.00156    0.00028
AFIX 137
H23A  2    0.729676    0.476396    0.405509    11.00000   -1.50000
H23B  2    0.822190    0.527500    0.401855    11.00000   -1.50000
H23C  2    0.735878    0.522743    0.344997    11.00000   -1.50000
AFIX   0
C24    1     0.703256     0.267458     0.635340     11.00000     0.04485
0.01917 =
         0.02753    0.00581   -0.00688   -0.00343
C25    1     0.590907     0.243787     0.642905     11.00000     0.05524
0.05167 =
         0.10983   -0.02664    0.00521   -0.02269
AFIX 137
H25A  2    0.566784    0.251497    0.687189    11.00000   -1.50000
H25B  2    0.588967    0.198916    0.634021    11.00000   -1.50000
H25C  2    0.544053    0.265585    0.612414    11.00000   -1.50000
AFIX   0
C26    1     0.740101     0.261365     0.565371     11.00000     0.09354
0.02171 =
         0.02885   -0.00208   -0.00604    0.00166
AFIX 137
```

```
H26A  2    0.689837    0.282984    0.536811    11.00000   -1.50000
H26B  2    0.743289    0.217172    0.553487    11.00000   -1.50000
H26C  2    0.810826    0.280029    0.560903    11.00000   -1.50000
AFIX   0
C27    1     0.781574     0.227201     0.675009     11.00000     0.09689
0.03335 =
         0.03771    0.00599   -0.01600    0.01844
AFIX 137
H27A  2    0.851502    0.247362    0.675855    11.00000   -1.50000
H27B  2    0.787766    0.185916    0.655068    11.00000   -1.50000
H27C  2    0.755182    0.222765    0.719292    11.00000   -1.50000
AFIX   0
C28    1     0.960033     0.485231     0.664129     11.00000     0.01956
0.01925 =
         0.01813    0.00036   -0.00076    0.00113
C29    1     0.942024     0.491148     0.597296     11.00000     0.01804
0.02343 =
         0.01952    0.00343   -0.00101    0.00172
AFIX  43
H29   2    0.917940    0.529839    0.580332    11.00000   -1.20000
AFIX   0
C30    1     0.958854     0.441390     0.555696     11.00000     0.02448
0.02878 =
         0.02185   -0.00366    0.00201   -0.00635
AFIX  43
H30   2    0.945650    0.446043    0.510694    11.00000   -1.20000
AFIX   0
C31    1     0.994950     0.384877     0.579830     11.00000     0.02944
0.02137 =
         0.03292   -0.00617    0.00548   -0.00272
AFIX  43
H31   2    1.007044    0.350895    0.551327    11.00000   -1.20000
AFIX   0
C32    1     1.013385     0.377984     0.645571     11.00000     0.03125
0.02174 =
         0.03270    0.00364    0.00461    0.00246
AFIX  43
H32   2    1.037897    0.339165    0.661983    11.00000   -1.20000
AFIX   0
C33    1     0.996195     0.427634     0.687760     11.00000     0.01825
0.02156 =
         0.02517    0.00487    0.00012    0.00307
AFIX  43
H33   2    1.009025    0.422455    0.732739    11.00000   -1.20000
```

```
AFIX   0
C34   1    0.992325    0.527885    0.795002    11.00000    0.02033
0.02209 =
         0.01879  -0.00069  -0.00134   0.00365
C35   1    0.950637    0.483421    0.837445    11.00000    0.02895
0.02566 =
         0.02290   0.00436  -0.00295   0.00148
AFIX  43
H35   2   0.885963   0.462771   0.827037   11.00000  -1.20000
AFIX   0
C36   1    1.003372    0.469381    0.894650    11.00000    0.04658
0.02956 =
         0.02214   0.00933  -0.00736   0.00260
AFIX  43
H36   2   0.974210   0.439635   0.923633   11.00000  -1.20000
AFIX   0
C37   1    1.098970    0.498831    0.909633    11.00000    0.03723
0.03833 =
         0.02246   0.00256  -0.01301   0.00664
AFIX  43
H37   2   1.135205   0.489096   0.948742   11.00000  -1.20000
AFIX   0
C38   1    1.140861    0.542007    0.867771    11.00000    0.02889
0.03422 =
         0.02750  -0.00191  -0.00998   0.00424
AFIX  43
H38   2   1.206195   0.561955   0.878130   11.00000  -1.20000
AFIX   0
C39   1    1.088789    0.556764    0.810581    11.00000    0.02589
0.02591 =
         0.02340   0.00308  -0.00334  -0.00045
AFIX  43
H39   2   1.118687   0.586547   0.781920   11.00000  -1.20000
AFIX   0
C40   1    0.982219    0.618969    0.692523    11.00000    0.01663
0.01971 =
         0.02161   0.00264  -0.00289   0.00397
C41   1    0.964043    0.672327    0.730570    11.00000    0.01772
0.02475 =
         0.02351  -0.00154   0.00090   0.00157
AFIX  43
H41   2   0.925937   0.669044   0.769995   11.00000  -1.20000
AFIX   0
C42   1    1.002088    0.729689    0.710156    11.00000    0.02826
```

```
0.01985 =
         0.03985   -0.00478   -0.00648    0.00352
AFIX  43
H42   2    0.989019    0.765815    0.735490    11.00000   -1.20000
AFIX   0
C43   1     1.058972     0.734847     0.653181     11.00000     0.04184
0.02510 =
         0.04118    0.00944   -0.00178   -0.00735
AFIX  43
H43   2    1.082907    0.774477    0.638772    11.00000   -1.20000
AFIX   0
C44   1     1.080644     0.681944     0.617464     11.00000     0.04951
0.03687 =
         0.02763    0.00690    0.00920   -0.00960
AFIX  43
H44   2    1.121460    0.685323    0.579069    11.00000   -1.20000
AFIX   0
C45   1     1.043859     0.624239     0.636750     11.00000     0.03655
0.02725 =
         0.02160   -0.00125    0.00500   -0.00275
AFIX  43
H45   2    1.060448    0.588162    0.612120    11.00000   -1.20000
AFIX   0
C46   1     0.672604     0.543944     0.935338     11.00000     0.03798
0.03591 =
         0.01274    0.00229   -0.00224   -0.01104
AFIX 137
H46A  2    0.748318    0.536358    0.943529    11.00000   -1.50000
H46B  2    0.630372    0.511183    0.956017    11.00000   -1.50000
H46C  2    0.652599    0.584602    0.953170    11.00000   -1.50000
AFIX   0
C47   1     0.651861     0.543464     0.863460     11.00000     0.03251
0.01996 =
         0.01417    0.00242    0.00173   -0.00545
C48   1     0.545456     0.537854     0.841118     11.00000     0.03002
0.01938 =
         0.01372   -0.00059    0.00338   -0.00141
C49   1     0.508968     0.546186     0.776765     11.00000     0.02535
0.01699 =
         0.01400   -0.00071    0.00341   -0.00266
C50   1     0.391631     0.548246     0.763503     11.00000     0.02425
0.02770 =
         0.01988    0.00411    0.00160   -0.00436
AFIX 137
```

```
H50A  2    0.379392    0.553606    0.717007    11.00000   -1.50000
H50B  2    0.359964    0.583289    0.786964    11.00000   -1.50000
H50C  2    0.358898    0.509189    0.777874    11.00000   -1.50000
AFIX   0
C51    1     0.464666     0.528924     0.888236     11.00000     0.03293
0.02953 =
         0.01524    0.00146   -0.00040   -0.00570

S1     7     0.430365     0.652160     0.575701     11.00000     0.02343
0.02305 =
         0.01540    0.00145    0.00041    0.00322
F1     5     0.457842     0.712874     0.684029     11.00000     0.06452
0.09215 =
         0.05878   -0.05032   -0.01178   -0.00759
F2     5     0.311552     0.661249     0.678854     11.00000     0.04980
0.04553 =
         0.03431    0.00440    0.01861    0.01183
F3     5     0.327733     0.746341     0.626026     11.00000     0.06623
0.02342 =
         0.07009   -0.00004    0.02431    0.01002
O1     4     0.336505     0.630996     0.542580     11.00000     0.03652
0.02464 =
         0.02536   -0.00376   -0.00892    0.00083
O2     4     0.493569     0.697044     0.541732     11.00000     0.03037
0.03813 =
         0.04494    0.01717    0.00581    0.00215
O3     4     0.489973     0.602748     0.606916     11.00000     0.03076
0.03379 =
         0.02098    0.00555    0.00066    0.01085
C52    1     0.379125     0.695699     0.644298     11.00000     0.03486
0.03153 =
         0.03204   -0.00795    0.00275   -0.00239
S2     7     0.841137     0.716594     0.927763     11.00000     0.07060
0.04484 =
         0.02473    0.01517   -0.01297   -0.03083
F4     5     0.919270     0.636930     1.007812     11.00000     0.12931
0.12609 =
         0.10111    0.08689    0.05027    0.07653
F5     5     0.922879     0.620247     0.898391     11.00000     0.14846
0.09635 =
         0.08881   -0.03203    0.02337   -0.02982
F6     5     1.030699     0.681694     0.952849     11.00000     0.08269
0.12449 =
         0.12478    0.06580   -0.04556   -0.03642
```

```
O4     4     0.737133     0.687573     0.924987     11.00000     0.06652
0.08276 =
         0.08493    0.03295    0.00815    0.00006
O5     4     0.831427     0.757734     0.982790     11.00000     0.14268
0.08192 =
         0.04212    0.01749    0.03374    0.06905
O6     4     0.881523     0.746940     0.871376     11.00000     0.07905
0.02675 =
         0.02418    0.00426    0.01173   -0.00330
C53    1     0.927716     0.663137     0.948077     11.00000     0.07257
0.07858 =
         0.07505    0.01271    0.02520    0.00816
N7     3     0.233896     0.883542     0.596265     11.00000     0.09067
0.05525 =
         0.03759   -0.01074   -0.00910    0.02572
C54    1     0.309463     0.874901     0.568262     11.00000     0.07864
0.03170 =
         0.03425    0.00009   -0.01645    0.00800
C55    1     0.408058     0.863146     0.532552     11.00000     0.07177
0.04121 =
         0.05410    0.01439   -0.01024   -0.00794
AFIX 137
H55A  2    0.398608    0.875739    0.487343    11.00000   -1.50000
H55B  2    0.425226    0.818611    0.534483    11.00000   -1.50000
H55C  2    0.466184    0.887239    0.551982    11.00000   -1.50000
AFIX   0
HKLF 4

REM  z_a.res in P2(1)/c
REM R1 =  0.0460 for   11271 Fo > 4sig(Fo)  and  0.0557 for all   12822
data
REM    715 parameters refined using     36 restraints

END

WGHT      0.0533     29.9608

REM Highest difference peak   2.634,   deepest hole -1.995,   1-sigma
level  0.244
Q1    1   0.8270  0.6472  0.9020  11.00000  0.05    2.63
;
_shelx_res_checksum               70385
_shelx_hkl_file
;
```

3	0	0	0.10	0.70
4	0	0	1.00	0.90
-5	0	0	5698.33	273.87
5	0	0	5891.61	273.97
-5	0	0	5726.03	274.57
-6	0	0	2077.69	104.49
-6	0	0	2052.49	103.59
-6	0	0	2363.66	103.99
7	0	0	3931.11	215.08
-7	0	0	5138.59	215.18
-7	0	0	4414.96	214.68
-7	0	0	4557.84	215.78
-8	0	0	69.19	7.10
-8	0	0	56.69	8.30
-8	0	0	68.09	6.40
8	0	0	68.79	7.50
9	0	0	1903.81	91.79
-9	0	0	2074.09	91.49
-9	0	0	1827.22	90.79
-9	0	0	1708.73	92.09
10	0	0	44.80	8.40
-10	0	0	37.20	6.30
-10	0	0	45.20	7.80
-10	0	0	42.40	6.70
-11	0	0	860.81	41.90
11	0	0	842.92	43.00
-11	0	0	761.22	43.90
-12	0	0	118.09	14.70
-12	0	0	135.19	11.20
12	0	0	126.49	12.70
13	0	0	377.56	24.40
-13	0	0	418.36	23.20
-14	0	0	68.89	10.80
-15	0	0	292.27	19.90
-16	0	0	-2.00	8.20
-2	-1	0	1293.57	59.79
2	-1	0	1277.57	59.89
-2	-1	0	1213.58	59.79
-2	-1	0	1223.38	60.09
-2	1	0	1296.47	59.99
-2	1	0	1232.48	59.79
-3	-1	0	1986.30	91.09
-3	-1	0	1814.32	90.99
3	-1	0	1936.41	91.19

-3	-1	0	1886.01	91.49
-3	1	0	1965.70	91.49
-3	1	0	1892.51	91.09
-4	-1	0	289.77	13.10
-4	-1	0	243.38	12.90
-4	-1	0	245.28	13.60
4	-1	0	249.88	13.10
-4	1	0	218.68	13.30
-4	1	0	265.17	13.60
-5	-1	0	32.50	4.30
5	-1	0	38.30	4.10
-5	-1	0	22.20	3.70
5	1	0	27.30	4.30
-5	1	0	34.20	3.90
-6	-1	0	365.16	20.20
-6	-1	0	378.96	19.10
-6	-1	0	408.76	19.40
6	-1	0	377.46	19.40
-6	1	0	356.96	19.10
-6	1	0	347.57	20.00
6	1	0	369.06	19.90
-7	-1	0	314.47	17.30
-7	-1	0	300.67	18.70
7	-1	0	347.77	17.80
-7	-1	0	339.07	17.70
-7	1	0	336.17	17.40
7	1	0	322.67	18.40
-7	1	0	327.17	18.60
-8	-1	0	373.06	20.10
8	-1	0	394.26	20.90
-8	-1	0	342.67	21.50
-8	-1	0	414.06	20.60
-8	1	0	412.96	20.80
-8	1	0	358.66	20.10
-8	1	0	368.06	21.60
8	1	0	382.16	21.20
-9	-1	0	84.09	8.00
-9	-1	0	56.09	9.80
9	-1	0	80.59	8.50
-9	-1	0	82.29	7.50
-9	1	0	76.99	6.90
9	1	0	64.49	8.90
-9	1	0	83.49	10.00
-9	1	0	84.29	8.50

-10	-1	0	322.47	17.70
10	-1	0	314.37	18.70
-10	-1	0	285.27	20.10
-10	-1	0	342.57	18.70
-10	1	0	292.87	17.60
-10	1	0	289.67	19.80
-10	1	0	326.07	18.80
10	1	0	314.57	19.10
-11	-1	0	23.50	8.10
11	-1	0	45.20	8.90
11	1	0	24.00	6.30
-11	1	0	23.30	7.40
-12	-1	0	87.29	14.10
12	-1	0	106.89	11.60
-12	-1	0	107.09	10.20
-12	1	0	105.39	10.30
-12	1	0	84.39	13.90
12	1	0	89.09	11.80
13	-1	0	7.60	6.30
-13	-1	0	17.10	5.90
13	1	0	6.60	6.40
-13	1	0	12.70	6.20
-14	-1	0	29.10	7.40
-14	1	0	35.50	7.40
-15	-1	0	14.50	8.00
-15	1	0	14.70	7.20
-16	-1	0	63.69	10.30
-16	1	0	63.69	10.40
-1	-2	0	4.60	0.70
-1	-2	0	7.00	1.20
1	-2	0	4.40	0.90
-1	2	0	6.50	0.90
-2	-2	0	328.87	15.90
2	-2	0	327.57	16.10
-2	-2	0	346.47	16.40
-2	2	0	324.47	16.00
2	2	0	315.57	15.90
-2	2	0	324.77	16.20
-3	-2	0	733.43	37.30
3	-2	0	791.12	37.00
-3	-2	0	827.42	36.90
-3	2	0	798.62	36.90
-3	2	0	784.42	37.20
3	2	0	773.62	36.80

3	2	0	644.74	37.10
-4	-2	0	1722.23	85.79
-4	-2	0	1742.43	86.49
-4	-2	0	1886.11	85.89
4	-2	0	1819.72	86.09
4	2	0	1814.32	86.49
-4	2	0	1884.41	86.39
-4	2	0	1721.43	85.89
5	-2	0	418.46	20.10
-5	-2	0	401.56	19.80
-5	-2	0	355.76	20.60
5	2	0	372.16	20.50
-5	2	0	409.76	19.90
-5	2	0	391.86	20.50
-6	-2	0	1718.93	89.39
-6	-2	0	2055.99	88.79
6	-2	0	1906.01	88.89
-6	-2	0	1787.22	88.49
6	2	0	1831.92	89.29
-6	2	0	1757.12	88.49
-6	2	0	1857.81	89.39
7	-2	0	106.89	7.20
-7	-2	0	96.69	7.30
-7	-2	0	79.39	8.60
-7	-2	0	101.09	7.00
-7	2	0	104.29	7.20
7	2	0	83.79	8.20
-7	2	0	98.59	8.40
-8	-2	0	652.83	28.90
-8	-2	0	557.34	28.30
8	-2	0	561.44	28.80
-8	-2	0	506.95	29.80
-8	2	0	543.65	28.40
8	2	0	545.65	29.40
-8	2	0	535.55	29.60
-9	-2	0	392.36	19.50
9	-2	0	361.26	19.40
-9	-2	0	337.27	18.70
-9	-2	0	306.37	20.70
-9	2	0	343.77	18.90
-9	2	0	318.17	20.50
9	2	0	342.57	20.30
-10	-2	0	255.67	14.40
-10	-2	0	199.58	16.70

-10	-2	0	273.77	15.50
-10	2	0	237.38	14.50
-10	2	0	219.48	16.70
-10	2	0	238.28	15.70
10	2	0	213.88	15.80
-11	-2	0	463.75	29.20
-11	-2	0	533.25	26.80
-11	2	0	464.95	29.00
-11	2	0	523.85	26.90
11	2	0	500.95	28.30
-12	-2	0	109.89	14.90
-12	-2	0	144.19	11.00
-12	2	0	126.69	11.20
-12	2	0	125.79	15.00
12	2	0	118.09	13.20
-13	-2	0	205.38	14.90
13	2	0	178.58	16.40
-13	2	0	211.88	14.80
-14	-2	0	26.40	7.20
-14	2	0	28.10	7.40
-15	-2	0	348.57	23.00
-15	2	0	386.66	23.20
-16	-2	0	29.50	8.90
-16	2	0	12.30	8.00
0	-3	0	2.00	0.60
0	-3	0	0.40	0.60
0	3	0	1.40	0.50
-1	-3	0	3521.85	163.48
1	3	0	3363.46	163.48
-2	-3	0	1139.59	54.59
2	-3	0	1151.48	54.89
-2	-3	0	1173.28	55.19
-2	3	0	1141.89	54.79
2	3	0	1088.39	54.59
-2	3	0	1116.39	54.89
2	3	0	1214.18	55.19
-3	-3	0	1410.96	66.19
3	-3	0	1385.76	66.39
-3	-3	0	1364.06	66.79
-3	3	0	1373.46	66.39
3	3	0	1297.27	66.09
3	3	0	1495.85	66.89
-3	3	0	1382.26	66.49
-4	-3	0	445.76	24.10

-4	-3	0	524.15	23.50
4	3	0	458.45	23.30
-4	3	0	473.95	23.60
-4	3	0	471.55	23.90
4	3	0	479.65	24.10
-5	-3	0	2985.30	148.89
-5	-3	0	3156.58	148.49
5	-3	0	3176.98	148.39
-5	-3	0	3014.60	147.99
-5	3	0	3065.79	148.19
5	3	0	3214.28	148.89
-5	3	0	3158.38	148.79
6	-3	0	254.67	14.10
-6	-3	0	285.47	13.80
-6	-3	0	238.98	13.40
-6	-3	0	230.88	14.70
-6	3	0	244.98	14.50
-6	3	0	251.27	13.80
6	3	0	259.67	14.70
-7	-3	0	546.45	30.40
-7	-3	0	645.04	29.50
7	-3	0	597.84	29.70
-7	-3	0	570.14	29.10
-7	3	0	553.04	30.20
7	3	0	587.94	30.40
-7	3	0	575.34	29.30
8	-3	0	35.70	6.10
-8	-3	0	39.90	6.60
-8	-3	0	43.90	5.80
-8	3	0	37.00	7.00
-8	3	0	39.80	5.70
8	3	0	30.50	5.50
-9	-3	0	215.38	12.60
-9	-3	0	187.38	11.80
-9	-3	0	163.48	14.00
9	-3	0	195.78	12.50
-9	3	0	199.28	12.20
-9	3	0	177.48	13.80
9	3	0	196.58	13.70
-10	-3	0	288.27	20.70
-10	-3	0	334.67	18.40
-10	-3	0	377.16	19.30
10	3	0	311.57	20.20
-10	3	0	332.87	18.50

-10	3	0	305.77	20.40
-11	-3	0	287.47	21.20
-11	-3	0	314.57	18.40
11	3	0	310.07	20.40
-11	3	0	311.87	21.20
-11	3	0	350.16	18.80
-12	-3	0	369.56	20.70
-12	-3	0	312.97	23.90
12	3	0	377.66	22.80
-12	3	0	336.37	23.90
-13	-3	0	103.19	16.30
13	3	0	113.49	13.80
-13	3	0	115.59	11.50
-14	-3	0	246.78	17.50
-14	3	0	246.98	17.40
-15	-3	0	15.60	7.70
-15	3	0	16.40	7.60
-16	-3	0	366.36	24.80
-16	3	0	400.06	24.50
0	-4	0	1136.79	56.19
0	-4	0	1215.58	56.59
0	4	0	1196.58	56.59
0	4	0	1173.48	56.19
1	-4	0	193.78	10.50
-1	-4	0	213.98	10.30
-1	-4	0	215.18	11.00
1	4	0	205.38	10.30
1	4	0	233.78	11.10
-1	4	0	209.58	10.60
-1	4	0	193.38	10.40
-2	-4	0	80.09	5.50
2	-4	0	83.39	5.00
-2	-4	0	84.99	4.50
2	4	0	84.99	4.50
-2	4	0	77.79	4.90
2	4	0	77.09	5.30
-2	4	0	80.79	4.80
3	-4	0	201.68	10.40
-3	-4	0	197.68	11.00
-3	-4	0	202.28	10.00
3	4	0	202.28	10.80
3	4	0	183.28	9.90
-3	4	0	187.88	10.40
-4	-4	0	2.40	2.30

4	-4	0	4.10	1.90
-4	-4	0	5.10	1.60
-4	4	0	2.60	1.80
-4	4	0	3.60	1.70
4	4	0	4.70	1.50
4	4	0	1.30	2.00
-5	-4	0	77.49	5.30
5	-4	0	76.89	5.90
-5	-4	0	76.19	6.70
5	4	0	69.49	5.20
5	4	0	72.39	6.40
-5	4	0	68.29	5.90
-5	4	0	73.09	5.30
-6	-4	0	71.79	5.40
6	-4	0	56.59	6.10
-6	-4	0	70.89	6.90
-6	-4	0	60.29	5.20
-6	4	0	65.29	6.50
6	4	0	82.19	7.30
-6	4	0	53.29	5.30
-7	-4	0	22.80	4.10
-7	-4	0	22.60	3.50
7	-4	0	20.00	3.80
-7	-4	0	17.80	4.50
-7	4	0	21.00	3.70
7	4	0	19.10	4.30
-7	4	0	14.20	3.90
-8	-4	0	86.99	9.60
-8	-4	0	90.59	7.10
8	-4	0	87.49	7.70
-8	-4	0	100.79	7.70
-8	4	0	92.89	9.00
-8	4	0	92.09	7.30
8	4	0	97.99	9.10
-9	-4	0	115.49	12.40
9	-4	0	146.39	11.00
-9	-4	0	160.98	10.50
-9	-4	0	147.69	9.80
9	4	0	142.89	12.00
-9	4	0	150.58	10.20
-9	4	0	133.39	11.90
-10	-4	0	123.39	9.40
-10	-4	0	98.19	12.20
-10	-4	0	139.19	10.60

10	4	0	102.99	11.80
-10	4	0	112.59	12.00
-10	4	0	130.59	9.90
-11	-4	0	71.89	8.50
-11	-4	0	44.50	9.00
-11	4	0	74.39	11.80
-11	4	0	87.89	9.00
11	4	0	68.89	11.40
-12	-4	0	25.00	8.40
-12	-4	0	10.10	5.30
-12	4	0	5.30	7.90
12	4	0	-1.00	6.10
-12	4	0	13.00	5.70
13	4	0	-0.90	7.40
-13	4	0	2.30	5.50
-14	-4	0	58.49	10.40
-14	4	0	59.99	10.80
-15	-4	0	65.49	12.10
-15	4	0	54.69	9.50
-16	-4	0	15.90	8.80
-16	4	0	17.50	8.40
0	-5	0	4.90	1.20
0	5	0	1.60	1.00
0	5	0	2.00	1.40
1	-5	0	3540.25	177.58
-1	-5	0	3742.63	177.98
-1	5	0	3902.21	177.78
1	5	0	3983.30	178.08
-1	5	0	3543.95	177.48
2	-5	0	660.63	31.50
-2	-5	0	628.24	31.10
-2	-5	0	655.73	32.00
2	5	0	659.13	32.00
-2	5	0	650.03	31.30
-2	5	0	646.64	31.50
2	5	0	626.04	31.10
3	-5	0	2132.59	100.69
-3	-5	0	2107.99	100.19
-3	-5	0	2096.69	101.19
3	5	0	2001.90	100.19
-3	5	0	2127.39	100.79
3	5	0	2208.58	101.19
-3	5	0	2074.99	100.49
-4	-5	0	52.89	5.30

4	-5	0	52.19	4.40
-4	-5	0	54.89	3.80
4	5	0	56.89	5.20
-4	5	0	50.39	4.50
4	5	0	45.90	3.70
-4	5	0	51.49	4.00
5	-5	0	1182.48	54.49
-5	-5	0	1083.99	54.29
-5	-5	0	1052.79	54.99
5	5	0	1059.09	53.79
-5	5	0	1151.88	54.69
5	5	0	1199.18	54.99
-5	5	0	1087.49	54.09
6	-5	0	1601.04	79.19
-6	-5	0	1787.72	78.79
-6	-5	0	1485.65	79.79
-6	5	0	1589.04	78.89
6	5	0	1739.93	79.89
-6	5	0	1613.64	79.59
-7	-5	0	678.13	31.20
7	-5	0	594.94	31.60
-7	-5	0	575.84	32.40
-7	-5	0	591.44	30.90
-7	5	0	616.74	32.20
7	5	0	679.63	32.60
-7	5	0	608.64	31.20
-8	-5	0	225.18	16.80
-8	-5	0	313.87	15.60
8	-5	0	279.27	15.90
-8	-5	0	274.67	15.10
-8	5	0	287.47	15.60
-8	5	0	258.67	16.60
8	5	0	269.97	17.00
-9	-5	0	154.28	10.10
-9	-5	0	160.88	10.80
-9	-5	0	136.89	12.80
9	-5	0	140.39	10.80
9	5	0	158.58	12.70
-9	5	0	146.19	12.30
-9	5	0	155.98	10.60
-10	-5	0	417.86	26.80
-10	-5	0	450.25	24.30
-10	-5	0	512.05	25.10
-10	5	0	425.96	26.50

10	5	0	467.45	26.60
-10	5	0	473.15	24.70
-11	-5	0	666.23	34.50
-11	-5	0	761.32	37.00
-11	-5	0	602.04	37.40
11	5	0	715.33	36.90
-11	5	0	609.14	36.70
-11	5	0	662.03	34.80
-12	-5	0	499.15	34.00
-12	-5	0	616.14	30.70
-12	5	0	620.74	31.00
12	5	0	601.44	33.20
-12	5	0	517.45	33.10
-13	-5	0	39.80	12.10
-13	5	0	58.49	9.60
13	5	0	63.19	10.50
-14	-5	0	219.28	15.90
-14	5	0	213.08	16.20
-15	-5	0	10.90	7.80
-15	5	0	15.40	7.90
-16	-5	0	257.27	19.50
-16	5	0	219.78	18.50
0	-6	0	169.08	9.50
0	6	0	175.58	9.40
0	6	0	208.68	10.20
1	-6	0	10.00	1.70
-1	-6	0	4.80	1.90
-1	-6	0	4.80	1.20
1	6	0	3.40	1.10
-1	6	0	2.60	1.30
1	6	0	1.80	1.90
-1	6	0	5.00	1.40
-2	-6	0	852.81	41.00
-2	-6	0	900.21	42.10
2	-6	0	872.11	41.50
-2	6	0	886.61	41.60
2	6	0	821.42	41.00
-2	6	0	749.03	41.60
2	6	0	909.11	42.10
-3	-6	0	71.29	4.30
3	-6	0	69.59	5.10
-3	-6	0	63.59	5.70
-3	6	0	69.19	4.80
3	6	0	65.19	5.60

3	6	0	67.89	4.30
-3	6	0	68.89	4.90
4	-6	0	275.87	14.10
-4	-6	0	264.07	14.80
-4	-6	0	265.87	13.40
-4	6	0	250.87	13.70
4	6	0	292.77	14.80
-4	6	0	258.17	14.10
4	6	0	228.98	13.40
5	-6	0	46.10	5.20
-5	-6	0	44.90	5.90
-5	6	0	43.40	4.60
-5	6	0	44.90	5.10
5	6	0	62.69	6.30
5	6	0	36.90	4.00
-6	-6	0	4.90	2.30
-6	-6	0	13.10	4.10
6	-6	0	7.00	3.00
6	6	0	14.10	3.90
6	6	0	9.40	2.50
-6	6	0	3.90	3.20
-6	6	0	8.30	2.80
-7	-6	0	45.60	5.20
-7	-6	0	54.59	7.60
7	-6	0	40.50	5.80
-7	-6	0	54.19	5.20
-7	6	0	45.80	5.60
-7	6	0	46.80	6.70
7	6	0	66.89	7.70
-8	-6	0	270.27	15.50
-8	-6	0	258.67	17.70
-8	-6	0	310.37	15.90
8	-6	0	281.17	16.50
-8	6	0	279.57	15.90
-8	6	0	264.57	17.00
8	6	0	300.27	17.80
-9	-6	0	34.70	5.90
-9	-6	0	25.70	6.40
-9	-6	0	38.80	5.50
9	-6	0	35.30	7.30
9	6	0	47.00	9.20
-9	6	0	33.70	6.30
-9	6	0	45.10	6.50
-10	-6	0	5.30	4.00

-10	-6	0	6.00	6.00
-10	-6	0	3.80	4.40
-10	6	0	0.40	5.40
-10	6	0	7.70	4.70
10	6	0	14.90	6.10
-11	-6	0	137.19	10.70
-11	-6	0	123.69	14.60
-11	6	0	127.99	14.20
-11	6	0	134.99	10.60
-12	-6	0	49.70	12.20
-12	-6	0	80.59	9.50
-12	6	0	65.89	13.10
-12	6	0	79.49	9.00
-13	-6	0	6.90	6.80
-13	6	0	10.50	6.10
13	6	0	10.10	9.70
-14	-6	0	17.70	6.90
-14	6	0	20.80	7.20
-15	-6	0	89.09	13.10
-15	6	0	90.39	13.00
0	-7	0	2.00	1.40
0	7	0	0.70	1.20
1	-7	0	2461.45	121.59
-1	-7	0	2585.64	121.29
-1	-7	0	2685.03	122.19
1	7	0	2524.85	121.29
-1	7	0	2225.28	121.69
-1	7	0	2705.33	121.89
1	7	0	2676.43	122.29
-2	-7	0	661.43	33.10
-2	-7	0	675.13	32.00
2	-7	0	590.94	32.90
-2	7	0	668.63	32.20
2	7	0	658.13	31.90
-2	7	0	691.13	32.60
2	7	0	677.43	33.10
3	-7	0	2408.86	111.89
-3	-7	0	2338.87	112.49
-3	-7	0	2313.37	111.19
3	7	0	2453.45	112.39
-3	7	0	2254.57	111.49
3	7	0	2246.48	111.19
-3	7	0	2343.57	111.89
-4	-7	0	484.55	23.30

-4	-7	0	426.46	24.60
4	-7	0	493.05	24.10
4	7	0	494.45	24.70
-4	7	0	451.65	24.00
-4	7	0	477.75	23.70
5	-7	0	1652.13	78.19
-5	-7	0	1565.44	78.89
-5	-7	0	1667.43	77.49
-5	7	0	1612.74	78.29
5	7	0	1717.23	78.79
5	7	0	1485.15	77.29
-5	7	0	1601.44	77.69
-6	-7	0	326.57	20.40
6	-7	0	381.56	19.60
-6	-7	0	385.36	18.60
6	7	0	362.16	20.20
-6	7	0	378.06	19.00
6	7	0	323.37	18.30
-6	7	0	349.57	19.50
-7	-7	0	462.85	21.80
-7	-7	0	388.36	23.50
7	-7	0	410.76	22.60
7	7	0	398.96	21.50
-7	7	0	425.26	22.00
-7	7	0	421.06	23.00
7	7	0	425.66	24.90
-8	-7	0	51.29	9.20
-8	-7	0	70.59	7.00
8	-7	0	78.79	7.80
-8	-7	0	76.79	6.80
8	-7	0	66.59	9.00
-8	7	0	68.39	8.70
-8	7	0	78.29	6.80
-9	-7	0	372.46	19.20
-9	-7	0	331.17	21.60
-9	-7	0	339.47	18.90
9	-7	0	347.07	20.60
9	7	0	337.27	21.70
-9	7	0	336.97	19.10
-9	7	0	343.67	20.90
-10	-7	0	271.57	21.90
-10	-7	0	365.66	19.30
-10	-7	0	333.07	18.60
10	-7	0	331.87	19.50

-10	7	0	346.77	18.80
-10	7	0	316.47	21.00
10	7	0	342.77	21.40
-11	-7	0	447.26	23.50
-11	-7	0	376.56	27.30
-11	-7	0	483.75	24.40
11	-7	0	419.46	24.10
-11	7	0	342.17	25.50
11	7	0	463.05	26.90
12	-7	0	271.77	18.00
-12	-7	0	244.58	21.20
-12	-7	0	264.27	17.10
-12	7	0	303.97	17.80
12	7	0	309.37	21.40
-12	7	0	243.78	20.50
-13	-7	0	95.09	11.10
-13	7	0	106.59	11.80
13	7	0	134.09	16.60
-14	-7	0	319.27	20.60
-14	7	0	316.77	20.70
-15	-7	0	23.40	8.40
-15	7	0	22.40	7.90
0	-8	0	230.38	12.20
0	8	0	235.08	12.90
0	8	0	238.88	12.60
-1	-8	0	273.37	14.60
-1	-8	0	273.27	13.60
1	-8	0	252.57	13.90
1	8	0	284.87	13.60
-1	8	0	256.77	13.70
-1	8	0	250.97	14.30
-1	8	0	273.37	14.20
1	8	0	279.07	14.80
-2	-8	0	816.32	39.90
-2	-8	0	797.82	38.60
2	-8	0	795.32	39.20
2	8	0	762.42	38.60
-2	8	0	767.82	38.80
2	8	0	865.71	40.00
-2	8	0	796.22	39.30
3	-8	0	14.10	2.90
-3	-8	0	21.20	2.60
-3	-8	0	21.40	3.90
-3	8	0	16.90	2.90

3	8	0	16.60	2.60
-3	8	0	15.50	2.70
3	8	0	13.50	3.70
-4	-8	0	1139.49	54.39
-4	-8	0	1094.69	55.99
4	-8	0	1165.08	55.29
-4	8	0	1125.59	55.29
-4	8	0	1104.29	54.79
4	8	0	1097.39	54.39
4	8	0	1193.38	56.29
-5	-8	0	451.75	22.20
5	-8	0	451.65	23.30
-5	-8	0	444.76	24.20
5	8	0	425.86	22.10
5	8	0	477.95	24.00
-5	8	0	416.56	23.10
-5	8	0	431.16	22.60
6	-8	0	1057.39	53.89
-6	-8	0	1064.09	54.89
6	-8	0	1052.89	54.89
-6	-8	0	1152.38	52.99
6	8	0	1009.60	52.89
-6	8	0	1095.29	54.09
6	8	0	1237.88	54.89
-6	8	0	1066.69	53.29
-7	-8	0	136.99	11.60
7	-8	0	124.79	9.20
7	-8	0	130.99	10.10
-7	-8	0	143.19	8.80
7	8	0	148.59	11.40
-7	8	0	121.29	10.00
-7	8	0	136.29	9.20
7	8	0	125.79	8.50
8	-8	0	677.13	35.50
-8	-8	0	752.82	35.10
-8	-8	0	700.63	35.00
-8	-8	0	646.24	37.30
8	-8	0	634.54	35.90
8	8	0	771.32	37.40
-8	8	0	700.23	35.30
-8	8	0	687.13	36.50
9	-8	0	548.45	29.50
-9	-8	0	568.04	29.00
-9	-8	0	624.14	29.30

-9	8	0	547.05	29.30
9	8	0	588.24	31.40
-9	8	0	493.15	30.60
-10	-8	0	400.96	22.00
-10	-8	0	433.86	22.30
10	-8	0	393.26	22.60
-10	-8	0	344.97	25.10
10	8	0	432.46	24.80
-10	8	0	408.06	22.30
-10	8	0	388.36	24.20
11	-8	0	559.64	30.60
-11	-8	0	629.74	30.60
-11	-8	0	570.74	30.00
-11	-8	0	492.75	33.10
-11	8	0	557.64	30.10
-11	8	0	518.25	32.30
11	8	0	620.54	33.00
-12	-8	0	122.59	11.60
-12	-8	0	146.09	23.40
12	-8	0	131.19	12.40
12	8	0	170.78	16.00
-12	8	0	142.19	12.20
-13	-8	0	254.57	17.90
-13	8	0	279.97	18.00
-14	-8	0	12.60	7.30
-14	8	0	21.60	7.00
-15	-8	0	285.87	20.30
-15	8	0	273.27	19.40
0	-9	0	1.00	1.80
0	9	0	0.50	1.70
0	9	0	1.30	2.40
0	9	0	1.60	2.10
1	-9	0	195.78	11.80
-1	-9	0	230.88	11.50
-1	-9	0	220.58	12.70
1	9	0	221.68	12.80
-1	9	0	201.18	12.30
1	9	0	213.68	11.60
-1	9	0	256.57	12.40
-1	9	0	217.58	11.70
-2	-9	0	257.77	13.10
2	-9	0	237.78	13.70
-2	-9	0	261.27	14.70
-2	9	0	234.98	13.30

2	9	0	272.77	14.80
-2	9	0	264.47	14.00
-3	-9	0	139.49	9.80
-3	-9	0	136.09	7.80
3	-9	0	145.59	9.00
-3	9	0	148.69	8.90
3	9	0	137.19	9.80
-3	9	0	133.09	8.30
3	9	0	143.29	7.90
-4	-9	0	52.29	7.00
4	-9	0	53.59	5.60
4	9	0	62.69	7.30
4	9	0	55.79	4.60
-4	9	0	53.99	5.50
-4	9	0	57.29	5.10
-5	-9	0	209.08	10.50
5	-9	0	177.38	11.90
-5	-9	0	178.18	12.70
-5	9	0	185.08	11.60
5	9	0	190.58	10.50
-5	9	0	190.88	11.20
5	9	0	190.78	12.70
-6	-9	0	82.29	6.10
-6	-9	0	72.79	8.90
6	-9	0	61.79	7.40
6	-9	0	77.39	7.70
6	9	0	70.79	6.00
-6	9	0	92.59	6.90
-6	9	0	61.69	7.40
6	9	0	81.89	8.90
7	-9	0	116.99	8.70
-7	-9	0	130.89	8.30
7	-9	0	125.19	9.90
7	9	0	102.69	7.90
-7	9	0	122.29	8.80
-7	9	0	100.79	9.50
7	9	0	122.39	10.70
8	-9	0	7.20	3.60
-8	-9	0	2.60	3.50
-8	-9	0	-1.30	5.40
8	-9	0	1.10	7.20
8	9	0	5.40	5.10
-8	9	0	4.20	3.70
8	9	0	4.50	3.40

-8	9	0	6.70	4.70
-9	-9	0	45.50	6.80
-9	-9	0	28.50	7.60
9	-9	0	41.80	7.10
-9	-9	0	41.00	6.60
9	9	0	57.69	10.10
-9	9	0	55.49	7.30
-9	9	0	35.80	7.00
-10	-9	0	245.78	14.30
-10	-9	0	189.68	17.40
10	-9	0	211.48	14.50
-10	-9	0	220.78	13.90
10	9	0	221.08	16.90
-10	9	0	223.88	14.40
-10	9	0	199.28	16.40
-11	-9	0	15.40	5.90
11	-9	0	4.50	5.10
-11	-9	0	7.00	9.00
-11	-9	0	18.10	5.40
11	9	0	21.80	7.30
-11	9	0	12.40	6.60
-11	9	0	21.40	5.80
-12	-9	0	58.19	9.40
-12	9	0	67.19	9.70
12	9	0	51.29	10.80
-13	-9	0	34.40	7.40
-14	-9	0	-0.30	6.90
-14	9	0	2.30	6.60
-15	-9	0	18.60	8.90
-15	9	0	33.40	8.10
0	-10	0	9275.97	461.45
0	10	0	9999.00	462.35
0	10	0	9945.50	462.05
1	-10	0	226.08	12.70
-1	-10	0	224.98	13.50
1	10	0	234.28	13.80
1	10	0	236.88	12.40
-1	10	0	231.28	13.40
-1	10	0	247.88	13.20
-1	10	0	233.18	12.40
-2	-10	0	2852.21	141.19
-2	-10	0	2696.43	138.99
2	-10	0	2875.41	139.19
-2	10	0	3141.39	139.59

2	10	0	2860.41	138.59
2	10	0	3149.88	140.19
-2	10	0	2809.62	138.79
-3	-10	0	792.02	47.60
3	-10	0	1036.60	47.70
-3	10	0	969.90	47.00
3	10	0	955.00	46.70
3	10	0	1032.60	48.50
-3	10	0	1011.00	47.70
-4	-10	0	936.61	45.70
4	-10	0	959.10	46.90
-4	-10	0	895.61	49.00
4	10	0	1007.40	47.60
4	10	0	917.91	45.70
-4	10	0	942.71	46.20
-4	10	0	945.41	46.70
-5	-10	0	693.83	33.80
-5	-10	0	667.93	36.30
5	-10	0	679.83	35.00
-5	10	0	685.03	35.10
5	10	0	751.72	36.00
-5	10	0	686.23	34.30
5	10	0	662.33	33.90
6	-10	0	1929.61	92.79
6	-10	0	1861.11	92.49
-6	-10	0	2009.20	91.49
-6	-10	0	1818.82	93.59
6	10	0	2050.39	93.49
-6	10	0	1862.31	92.69
6	10	0	1834.42	91.49
-6	10	0	1886.61	91.89
-7	-10	0	1079.69	58.49
7	-10	0	1124.69	57.39
-7	-10	0	1244.48	56.09
7	-10	0	1169.18	56.79
7	10	0	1079.19	55.89
-7	10	0	1097.09	57.39
7	10	0	1250.77	58.29
-7	10	0	1154.48	56.49
-8	-10	0	36.10	7.50
8	-10	0	33.20	6.50
-8	-10	0	48.80	6.10
-8	10	0	34.70	5.30
8	10	0	32.90	4.70

-8	10	0	40.80	8.00
8	10	0	35.70	6.70
-9	-10	0	297.77	17.00
-9	-10	0	330.07	17.10
9	-10	0	311.27	17.70
-9	-10	0	270.47	20.60
-9	10	0	276.57	19.00
9	10	0	293.57	20.00
-9	10	0	277.37	17.30
-10	-10	0	245.88	20.20
-10	-10	0	241.28	15.60
10	-10	0	249.38	16.30
-10	-10	0	286.77	16.00
10	10	0	260.77	19.20
-10	10	0	251.97	18.30
-11	-10	0	617.64	31.40
11	-10	0	546.05	30.20
-11	-10	0	590.94	29.60
-11	-10	0	499.95	34.00
-11	10	0	558.54	29.90
11	10	0	577.84	33.00
-11	10	0	480.65	31.90
-12	-10	0	237.68	17.10
12	10	0	278.87	21.30
-12	10	0	282.87	17.80
-13	-10	0	385.06	23.40
-13	10	0	389.26	23.30
-14	-10	0	7.90	7.40
-14	10	0	4.50	6.50
-15	-10	0	297.27	21.50
-15	10	0	288.37	20.30
0	-11	0	-1.60	2.10
0	11	0	2.50	2.80
0	11	0	1.90	2.10
0	11	0	-4.50	2.10
-1	-11	0	305.97	16.50
1	-11	0	258.37	15.40
-1	-11	0	277.87	15.00
1	11	0	324.37	16.80
-1	11	0	283.97	15.80
-1	11	0	279.47	15.10
-1	11	0	298.47	16.20
1	11	0	287.77	15.00
-2	-11	0	17.30	2.60

2	-11	0	13.30	3.20
-2	-11	0	18.10	4.00
2	11	0	13.70	4.10
2	11	0	16.10	2.50
-2	11	0	13.80	2.80
-2	11	0	13.40	3.10
3	-11	0	181.58	12.00
-3	-11	0	202.88	10.90
-3	-11	0	214.08	13.40
3	11	0	227.88	13.30
-3	11	0	194.88	11.40
3	11	0	197.58	10.90
-3	11	0	195.68	12.10
-4	-11	0	82.39	8.80
4	-11	0	71.39	7.50
-4	-11	0	82.09	5.50
-4	11	0	80.39	7.10
4	11	0	77.19	5.70
4	11	0	96.19	9.00
-4	11	0	66.89	6.10
-5	-11	0	183.18	10.10
5	-11	0	162.48	11.80
-5	-11	0	182.28	13.00
-5	11	0	184.28	11.70
5	11	0	216.08	13.40
-5	11	0	158.98	10.80
5	11	0	163.38	10.10
6	-11	0	21.50	4.20
-6	-11	0	26.50	4.50
-6	-11	0	32.40	6.20
6	-11	0	22.50	5.30
6	11	0	26.60	4.70
-6	11	0	20.50	4.40
6	11	0	32.80	6.30
-6	11	0	33.70	5.50
-7	-11	0	55.09	10.40
7	-11	0	57.19	8.80
7	-11	0	57.49	7.00
-7	-11	0	63.39	6.30
-7	11	0	74.39	6.70
7	11	0	53.59	6.00
-7	11	0	57.59	8.40
7	11	0	79.99	9.60
-8	-11	0	20.50	4.30

8	-11	0	14.30	4.00
-8	-11	0	13.80	6.70
8	11	0	15.80	6.10
-8	11	0	19.50	4.80
-8	11	0	14.50	5.20
8	11	0	19.70	4.00
9	-11	0	111.49	10.30
-9	-11	0	126.89	9.80
-9	-11	0	114.49	14.10
9	11	0	130.09	13.60
-9	11	0	128.39	12.40
-9	11	0	114.99	10.10
-10	-11	0	186.98	18.60
-10	-11	0	223.28	13.50
10	-11	0	191.98	14.00
-10	-11	0	174.68	13.10
-10	11	0	179.98	13.60
-10	11	0	178.78	15.90
10	11	0	234.68	17.50
-11	-11	0	19.20	5.70
-11	-11	0	25.50	9.90
11	-11	0	13.90	5.40
11	11	0	18.10	8.70
-11	11	0	16.60	6.10
-11	11	0	6.10	7.50
-12	-11	0	8.80	6.20
-12	11	0	22.00	6.50
12	11	0	13.40	8.20
-13	-11	0	11.20	8.10
-13	11	0	29.00	7.30
-14	-11	0	102.79	13.50
-14	11	0	101.39	12.70
0	-12	0	2975.80	141.49
0	12	0	2923.71	142.39
0	12	0	2900.81	141.19
0	12	0	3044.80	142.09
-1	-12	0	29.10	4.90
-1	-12	0	24.90	3.90
1	-12	0	28.30	4.00
1	12	0	20.50	4.40
-1	12	0	23.60	4.50
-1	12	0	20.20	3.90
1	12	0	28.00	3.90
-1	12	0	29.90	4.30

-2	-12	0	476.25	23.00
-2	-12	0	465.55	24.80
2	-12	0	443.36	23.80
2	12	0	461.55	24.90
-2	12	0	430.56	23.20
2	12	0	446.56	22.90
-2	12	0	446.46	24.30
-2	12	0	496.75	24.20
-3	-12	0	6.20	2.20
3	-12	0	-0.40	3.40
-3	-12	0	0.00	3.90
3	12	0	5.70	2.30
-3	12	0	1.70	2.50
3	12	0	-2.20	3.60
-3	12	0	2.30	2.90
-4	-12	0	891.21	42.40
-4	-12	0	841.92	44.60
4	-12	0	828.92	43.60
-4	12	0	912.81	43.80
-4	12	0	852.61	42.90
4	12	0	924.71	44.70
4	12	0	841.72	42.40
-5	-12	0	91.69	6.30
-5	-12	0	70.99	9.70
5	-12	0	80.79	8.20
5	12	0	84.19	6.40
-5	12	0	91.49	8.00
5	12	0	93.69	9.40
-5	12	0	83.79	7.20
6	-12	0	444.86	25.60
-6	-12	0	427.56	26.60
-6	-12	0	508.45	23.70
6	-12	0	470.65	24.80
6	12	0	446.16	23.70
-6	12	0	466.35	24.30
6	12	0	482.85	26.30
-6	12	0	458.25	25.30
-7	-12	0	112.09	12.20
-7	-12	0	112.39	8.20
7	-12	0	109.79	9.20
7	12	0	123.69	11.40
-7	12	0	91.89	10.10
7	12	0	95.49	8.00
-7	12	0	120.59	8.60

-8	-12	0	584.54	28.00
8	-12	0	545.45	28.90
-8	-12	0	526.35	31.80
-8	12	0	525.35	30.00
8	12	0	510.25	27.70
-8	12	0	534.35	28.50
8	12	0	584.44	30.80
9	-12	0	243.68	15.50
-9	-12	0	256.47	14.70
-9	-12	0	203.88	19.30
-9	12	0	246.08	14.90
-9	12	0	236.58	17.20
9	12	0	237.18	14.30
9	12	0	240.88	18.00
-10	-12	0	155.48	18.30
-10	-12	0	194.08	13.10
-10	-12	0	193.88	13.20
10	-12	0	182.48	13.70
-10	12	0	158.78	15.50
-10	12	0	181.38	13.50
10	12	0	216.98	17.60
-11	-12	0	457.35	24.60
-11	-12	0	373.36	31.00
11	12	0	460.95	28.60
-11	12	0	441.26	24.50
-12	-12	0	183.38	14.60
-12	12	0	179.78	14.50
12	12	0	181.58	18.50
-13	12	0	112.39	12.60
-14	-12	0	19.50	8.40
-14	12	0	8.50	7.10
0	-13	0	-4.90	2.50
0	13	0	-3.30	2.70
0	13	0	1.50	3.90
0	13	0	-2.50	2.30
-1	-13	0	2305.77	108.99
1	-13	0	2225.58	109.29
-1	13	0	2218.48	108.99
1	13	0	2255.67	108.79
-1	13	0	2246.68	109.79
1	13	0	2364.36	110.49
-1	13	0	2309.07	110.19
2	-13	0	47.80	6.20
-2	-13	0	62.49	7.50

-2	-13	0	57.19	5.10
-2	13	0	56.29	6.50
2	13	0	53.29	7.60
2	13	0	55.39	5.00
-2	13	0	47.30	7.00
-2	13	0	59.99	5.50
-3	-13	0	748.83	36.30
-3	-13	0	760.32	38.70
3	-13	0	694.43	37.40
-3	13	0	709.63	36.70
3	13	0	720.63	36.30
-3	13	0	766.42	37.70
3	13	0	788.22	38.70
-4	-13	0	275.27	14.00
-4	-13	0	257.57	17.10
4	-13	0	250.57	15.70
-4	13	0	256.17	15.40
-4	13	0	253.57	14.60
4	13	0	288.27	17.00
4	13	0	247.08	14.00
5	-13	0	891.71	46.00
-5	-13	0	900.31	44.20
-5	-13	0	877.41	47.00
-5	13	0	905.71	44.80
5	13	0	981.70	47.00
5	13	0	865.51	44.30
-5	13	0	918.91	45.80
-6	-13	0	342.57	17.90
6	-13	0	342.87	19.20
6	-13	0	303.07	20.20
-6	-13	0	325.27	21.40
-6	13	0	345.67	19.90
6	13	0	389.36	21.30
-6	13	0	340.67	18.80
6	13	0	327.37	18.00
7	-13	0	286.87	16.90
-7	-13	0	300.87	15.80
-7	-13	0	283.57	20.00
7	13	0	254.77	15.70
-7	13	0	282.17	16.60
-7	13	0	280.97	17.90
7	13	0	316.27	19.40
-8	-13	0	343.67	17.90
-8	-13	0	306.57	22.20

8	-13	0	329.47	19.00
-8	13	0	297.67	20.00
8	13	0	304.37	17.70
8	13	0	356.16	21.50
-8	13	0	315.27	18.20
9	-13	0	134.69	10.90
-9	-13	0	98.89	15.80
-9	-13	0	126.79	9.80
-9	13	0	115.09	12.70
9	13	0	146.89	14.50
-9	13	0	119.89	11.10
9	13	0	101.99	9.30
-10	-13	0	356.46	20.30
10	-13	0	350.06	21.00
-10	-13	0	276.37	25.00
-10	-13	0	384.06	20.20
-10	13	0	338.67	22.90
10	13	0	398.56	24.30
-10	13	0	321.07	20.10
-11	-13	0	193.38	15.10
11	13	0	261.57	20.60
-11	13	0	213.48	15.50
-12	-13	0	182.48	14.90
12	13	0	186.98	20.40
-12	13	0	209.28	15.40
-13	-13	0	45.10	14.90
-13	13	0	38.50	8.20
-14	-13	0	226.88	18.50
-14	13	0	226.38	17.80
0	-14	0	351.36	18.20
0	14	0	343.17	19.00
0	14	0	337.37	17.90
0	14	0	320.37	19.50
-1	-14	0	21.50	3.60
1	-14	0	22.50	4.30
1	14	0	21.70	5.00
-1	14	0	16.20	3.40
-1	14	0	16.90	4.60
-1	14	0	20.80	5.20
1	14	0	25.70	4.50
-2	-14	0	11.00	4.60
-2	-14	0	11.40	3.00
2	-14	0	12.30	4.10
2	14	0	7.80	2.80

-2	14	0	14.60	4.30
-2	14	0	14.90	4.10
2	14	0	7.30	4.30
-2	14	0	13.60	3.40
3	-14	0	42.60	6.00
-3	-14	0	42.10	8.40
-3	-14	0	55.79	5.10
3	14	0	50.09	5.00
3	14	0	39.70	8.00
-3	14	0	44.30	6.70
-3	14	0	47.50	5.70
4	-14	0	210.58	14.20
-4	-14	0	204.38	15.50
-4	-14	0	236.78	12.10
-4	14	0	218.88	13.80
4	14	0	211.38	12.10
4	14	0	210.88	15.30
-4	14	0	210.08	12.80
5	-14	0	101.99	10.20
-5	-14	0	89.49	11.60
-5	-14	0	103.19	7.20
5	14	0	89.29	11.30
-5	14	0	106.09	8.30
5	14	0	96.49	7.40
-5	14	0	94.19	9.10
-6	-14	0	55.69	10.70
-6	-14	0	51.79	5.90
6	-14	0	49.40	7.10
6	14	0	40.50	6.10
6	14	0	47.10	9.70
-6	14	0	51.99	8.30
-6	14	0	50.99	7.00
7	-14	0	5.50	4.10
-7	-14	0	19.40	7.20
-7	-14	0	13.50	3.80
7	14	0	3.60	6.00
7	14	0	8.50	3.50
-7	14	0	1.80	4.80
-7	14	0	4.50	4.50
-8	-14	0	155.48	16.30
-8	-14	0	186.58	11.60
8	-14	0	176.98	12.80
8	14	0	183.58	15.50
-8	14	0	165.68	14.10

8	14	0	160.78	11.40
-8	14	0	195.68	12.70
-9	-14	0	20.40	9.10
9	-14	0	31.20	6.10
-9	-14	0	38.10	7.10
9	14	0	17.80	8.00
9	14	0	40.00	6.90
-9	14	0	36.70	6.50
-10	-14	0	17.50	5.60
-10	-14	0	19.20	9.80
-10	-14	0	18.70	5.90
10	-14	0	16.40	6.10
10	14	0	18.10	8.30
-10	14	0	22.90	6.40
-11	-14	0	86.09	10.90
-11	14	0	73.29	9.90
11	14	0	81.39	15.40
-12	-14	0	17.50	7.10
-12	14	0	14.30	6.70
-13	-14	0	6.40	8.90
-13	14	0	10.00	6.80
-14	-14	0	19.60	8.50
-14	14	0	7.80	8.00
0	-15	0	0.60	3.20
0	15	0	-2.60	2.80
0	15	0	6.70	4.00
0	15	0	-1.20	4.10
-1	-15	0	1513.65	71.79
1	-15	0	1450.05	72.19
1	15	0	1450.65	72.69
1	15	0	1488.45	73.49
-1	15	0	1458.65	71.69
-1	15	0	1516.65	72.89
-1	15	0	1467.55	73.29
1	15	0	1500.05	71.59
-2	-15	0	88.69	9.40
-2	-15	0	94.29	7.00
2	-15	0	97.79	8.40
-2	15	0	89.39	9.10
-2	15	0	81.79	8.40
-2	15	0	86.29	7.20
2	15	0	85.29	6.70
2	15	0	88.29	10.00
-3	-15	0	424.66	25.20

-3	-15	0	452.15	22.60
3	-15	0	434.86	24.20
3	15	0	453.95	25.30
3	15	0	436.96	22.60
-3	15	0	426.86	23.10
-3	15	0	454.75	24.70
-3	15	0	452.45	24.30
-4	-15	0	116.79	12.20
4	-15	0	129.49	11.30
-4	-15	0	139.39	8.50
-4	15	0	118.99	9.10
4	15	0	144.79	12.20
4	15	0	128.69	8.50
-4	15	0	131.29	10.40
-5	-15	0	1901.71	98.19
-5	-15	0	2053.69	95.29
5	15	0	2094.09	98.19
5	15	0	1868.91	95.29
-5	15	0	2008.50	95.99
-5	15	0	1982.90	97.09
-6	-15	0	388.26	19.70
6	-15	0	379.86	21.20
-6	-15	0	330.87	23.70
-6	15	0	369.26	21.80
6	15	0	380.06	23.10
6	15	0	376.56	19.90
-6	15	0	382.36	20.60
-7	-15	0	431.96	29.60
-7	-15	0	529.55	25.70
7	-15	0	498.85	27.10
-7	15	0	490.65	28.00
-7	15	0	513.15	26.60
7	15	0	530.95	29.20
7	15	0	485.55	25.80
8	-15	0	217.48	14.40
-8	-15	0	204.38	12.70
-8	-15	0	183.78	18.10
8	15	0	222.78	17.40
8	15	0	199.58	12.80
-8	15	0	198.08	15.50
-8	15	0	212.78	15.20
9	-15	0	175.98	13.30
-9	-15	0	183.28	11.80
-9	-15	0	152.38	18.10

-9	15	0	155.28	14.90
9	15	0	182.98	16.80
-9	15	0	159.78	12.30
9	15	0	158.28	11.60
-10	-15	0	255.87	24.80
-10	-15	0	309.27	17.60
-10	15	0	288.67	18.20
10	15	0	324.17	23.20
-11	-15	0	192.58	15.30
-11	15	0	204.48	15.60
11	15	0	216.78	21.20
-12	-15	0	501.95	28.00
-12	15	0	466.95	27.30
-13	-15	0	1.40	7.80
-13	15	0	11.30	7.20
0	-16	0	568.44	30.30
0	16	0	645.24	32.40
0	16	0	583.74	30.00
0	16	0	575.24	31.10
1	-16	0	59.69	7.40
-1	-16	0	54.79	6.30
-1	16	0	53.29	7.70
-1	16	0	64.09	6.40
1	16	0	54.29	7.60
-1	16	0	51.79	8.80
1	16	0	59.39	6.20
1	16	0	58.99	9.40
-2	-16	0	254.97	14.50
2	-16	0	240.58	15.70
2	16	0	282.77	17.50
-2	16	0	257.67	15.90
-2	16	0	268.57	16.80
-2	16	0	255.47	14.80
2	16	0	255.37	14.40
-3	-16	0	282.57	19.20
-3	-16	0	323.37	16.50
3	-16	0	290.07	18.30
-3	16	0	319.77	18.30
-3	16	0	281.47	16.90
-3	16	0	310.07	18.90
3	16	0	308.37	16.50
3	16	0	325.57	19.70
-4	-16	0	155.28	9.00
-4	-16	0	149.49	13.10

4	-16	0	109.89	11.50
-4	16	0	136.69	11.10
4	16	0	119.79	8.90
4	16	0	154.18	13.30
-4	16	0	120.19	9.80
-5	-16	0	133.49	13.90
-5	-16	0	127.09	8.80
5	16	0	126.69	9.00
5	16	0	144.09	13.00
-5	16	0	132.49	11.50
-5	16	0	122.29	9.80
-6	-16	0	114.09	8.30
6	-16	0	110.69	10.10
-6	-16	0	130.39	14.00
6	16	0	104.29	8.50
6	16	0	109.39	12.60
-6	16	0	102.09	9.00
-6	16	0	103.29	10.90
-7	-16	0	-1.90	7.10
7	-16	0	3.80	4.40
-7	-16	0	6.00	3.70
-7	16	0	-1.10	4.80
7	16	0	-2.10	6.40
7	16	0	2.30	3.80
-7	16	0	5.50	4.80
-8	-16	0	84.19	8.10
-8	-16	0	103.29	15.20
8	-16	0	83.99	10.00
8	16	0	68.49	13.50
-8	16	0	76.59	11.30
-8	16	0	87.69	8.80
8	16	0	69.09	8.10
-9	-16	0	43.10	10.90
-9	-16	0	57.19	8.00
9	16	0	56.49	10.60
-9	16	0	39.20	7.30
9	16	0	40.20	6.00
-10	16	0	17.80	6.60
10	16	0	11.70	9.50
10	16	0	10.80	5.10
-11	-16	0	21.10	7.30
11	16	0	39.80	11.30
-11	16	0	28.90	7.40
-12	-16	0	18.30	7.70

-12	16	0	33.60	8.10
-13	-16	0	51.69	10.30
-13	16	0	44.70	9.30
0	-17	0	2.30	4.10
0	17	0	2.90	5.10
0	17	0	1.70	3.90
0	17	0	2.10	3.50
1	-17	0	660.93	34.90
-1	-17	0	689.63	34.40
1	17	0	674.53	36.50
-1	17	0	646.54	36.20
-1	17	0	689.93	34.40
1	17	0	701.33	34.20
1	17	0	678.13	35.60
-1	17	0	689.53	35.60
-2	-17	0	4.50	3.60
2	-17	0	-0.50	4.70
-2	17	0	5.90	5.20
-2	17	0	-3.80	4.30
-2	17	0	2.90	3.70
2	17	0	0.10	5.60
2	17	0	8.90	3.50
-3	-17	0	152.08	9.00
3	-17	0	98.19	11.00
-3	-17	0	115.79	12.20
3	17	0	114.99	12.40
3	17	0	129.19	8.80
-3	17	0	121.79	10.80
-3	17	0	120.29	11.50
-3	17	0	121.29	9.50
-4	-17	0	157.88	15.20
-4	-17	0	191.08	11.30
4	17	0	189.78	11.40
-4	17	0	187.08	13.60
4	17	0	181.48	15.50
-4	17	0	200.28	14.40
-4	17	0	187.18	12.30
-5	-17	0	349.47	24.60
-5	-17	0	420.06	20.70
5	17	0	393.06	20.90
-5	17	0	387.76	21.60
5	17	0	426.16	24.90
-5	17	0	377.76	22.90
-6	-17	0	15.80	7.70

-6	-17	0	7.40	3.60
-6	17	0	-0.30	4.70
6	17	0	1.10	3.70
6	17	0	-2.20	6.70
-6	17	0	6.90	5.60
-7	-17	0	85.79	7.80
-7	-17	0	72.29	11.60
7	-17	0	91.29	10.20
7	17	0	67.69	8.10
7	17	0	101.89	13.00
-7	17	0	88.69	10.90
-7	17	0	92.59	9.40
-8	-17	0	48.40	11.10
-8	-17	0	71.19	7.90
8	-17	0	94.39	10.30
-8	17	0	71.29	12.80
-8	17	0	72.09	9.70
8	17	0	78.49	14.20
8	17	0	75.19	8.30
-9	-17	0	167.58	21.40
-9	-17	0	187.08	12.20
9	17	0	145.69	12.00
9	17	0	195.58	18.80
-9	17	0	174.18	13.00
-10	17	0	319.17	19.20
10	17	0	299.97	24.00
10	17	0	272.77	17.60
-11	-17	0	259.77	18.20
-11	17	0	238.48	17.00
11	17	0	270.17	27.70
-12	-17	0	138.09	14.80
-12	17	0	135.79	14.10
0	-18	0	1713.23	85.89
0	18	0	1800.42	87.79
0	18	0	1784.72	85.59
0	18	0	1770.42	86.99
-1	-18	0	9.50	4.60
1	-18	0	2.00	4.90
-1	18	0	14.70	6.30
1	18	0	15.50	5.10
-1	18	0	15.20	5.10
1	18	0	22.40	7.00
1	18	0	18.90	4.50
-1	18	0	17.20	4.60

2	-18	0	1009.40	51.99
-2	-18	0	1021.70	50.79
-2	18	0	1033.70	51.09
2	18	0	1070.29	53.49
2	18	0	990.70	52.19
2	18	0	1031.70	50.69
-2	18	0	1061.39	53.29
-2	18	0	1037.30	52.49
-3	-18	0	120.29	13.10
-3	-18	0	117.29	8.90
-3	18	0	130.29	12.60
3	18	0	114.19	8.80
-3	18	0	108.39	9.60
-3	18	0	121.99	11.30
3	18	0	128.19	13.10
-4	-18	0	822.82	46.60
-4	-18	0	885.51	43.20
4	18	0	936.81	47.00
-4	18	0	899.61	46.00
4	18	0	845.92	43.30
-4	18	0	860.41	43.90
-4	18	0	874.11	45.30
-5	-18	0	395.36	20.70
-5	-18	0	383.66	25.40
-5	18	0	390.86	21.80
5	18	0	418.66	25.00
-5	18	0	378.46	23.20
5	18	0	387.16	20.90
-6	-18	0	458.65	23.40
-6	-18	0	403.66	28.50
6	18	0	497.05	28.00
6	18	0	439.36	23.70
-6	18	0	440.86	26.20
-7	-18	0	188.88	20.60
-7	-18	0	222.38	13.00
-7	18	0	195.98	16.00
-7	18	0	218.08	14.50
7	18	0	230.48	18.80
7	18	0	201.18	13.30
-8	-18	0	37.10	11.60
-8	-18	0	44.60	5.90
-8	18	0	53.49	9.30
8	18	0	42.10	6.10
8	18	0	63.09	11.10

9	18	0	383.36	23.50
9	18	0	492.25	29.80
-9	18	0	452.15	25.10
10	18	0	55.19	9.20
10	18	0	71.19	13.50
-10	18	0	88.19	11.60
-11	-18	0	313.27	20.40
-11	18	0	288.77	20.10
-12	18	0	19.80	7.90
0	-19	0	-0.20	4.70
0	19	0	-2.00	6.40
0	19	0	7.00	4.20
0	19	0	-3.20	4.80
-1	-19	0	16.50	4.70
1	-19	0	16.30	5.30
-1	19	0	16.60	4.70
-1	19	0	20.00	6.70
-1	19	0	24.80	5.50
1	19	0	20.50	7.20
1	19	0	21.80	5.40
1	19	0	16.80	4.50
-2	-19	0	31.10	5.20
-2	19	0	35.80	8.20
2	19	0	30.80	4.90
2	19	0	42.90	8.90
-2	19	0	37.30	6.10
-2	19	0	28.90	6.40
-3	-19	0	12.90	4.30
-3	19	0	15.00	6.10
3	19	0	14.30	7.40
-3	19	0	12.10	5.00
-3	19	0	11.90	5.30
3	19	0	12.60	4.00
-4	-19	0	74.19	7.20
-4	-19	0	49.30	9.70
4	19	0	70.49	7.30
-4	19	0	59.29	9.70
-4	19	0	59.79	9.40
-4	19	0	55.59	8.30
4	19	0	50.89	10.00
-5	-19	0	88.29	7.50
-5	-19	0	42.50	9.80
5	19	0	69.49	7.90
-5	19	0	77.79	9.00

-5	19	0	66.29	10.60
-5	19	0	70.59	11.90
-6	-19	0	14.50	4.20
-6	-19	0	34.50	13.20
-6	19	0	9.50	5.20
6	19	0	5.50	4.30
-6	19	0	1.60	5.60
6	19	0	12.80	8.30
-7	-19	0	19.90	4.80
-7	19	0	9.30	5.40
7	19	0	12.00	8.40
7	19	0	19.40	4.90
8	19	0	10.30	5.10
8	19	0	22.70	9.30
-8	19	0	18.60	6.50
9	19	0	13.30	5.30
9	19	0	18.20	10.10
-9	19	0	28.20	6.70
-10	19	0	16.30	7.20
10	19	0	13.50	10.40
10	19	0	8.70	5.60
-11	-19	0	12.00	13.00
-11	19	0	23.10	7.70
0	-20	0	1292.27	67.69
0	20	0	1460.15	70.29
0	20	0	1324.07	68.89
0	20	0	1430.06	67.49
-1	-20	0	244.68	15.40
1	20	0	243.28	18.90
-1	20	0	259.67	15.40
1	20	0	236.08	16.90
-1	20	0	252.57	17.00
-1	20	0	242.38	18.30
1	20	0	245.88	15.10
-2	-20	0	689.63	35.20
2	20	0	708.43	38.60
2	20	0	698.73	35.10
-2	20	0	706.23	38.30
-2	20	0	689.13	35.60
-2	20	0	685.43	37.10
2	20	0	657.33	36.80
-3	-20	0	4.80	4.10
-3	20	0	9.00	6.70
3	20	0	-2.70	6.70

-3	20	0	18.60	5.90
-3	20	0	5.30	4.90
3	20	0	13.60	4.50
-4	-20	0	291.17	15.70
-4	20	0	270.97	16.90
-4	20	0	267.97	19.60
4	20	0	254.97	15.60
4	20	0	274.17	20.80
-4	20	0	259.17	18.10
-5	-20	0	529.05	27.50
-5	20	0	525.15	28.70
5	20	0	577.14	32.30
5	20	0	519.25	27.80
-5	20	0	509.15	33.10
-5	20	0	548.45	31.60
-6	-20	0	909.71	46.20
6	20	0	865.81	46.50
6	20	0	1032.30	51.29
-6	20	0	928.31	47.50
-6	20	0	952.10	50.19
7	20	0	191.28	19.70
-7	20	0	221.58	15.10
7	20	0	201.58	13.60
8	20	0	113.59	10.60
-8	20	0	114.19	12.20
8	20	0	140.29	18.50
9	20	0	330.87	26.30
9	20	0	298.57	18.90
-9	20	0	348.17	20.80
10	20	0	130.89	27.30
-10	20	0	137.69	13.50
-11	20	0	176.98	15.70
0	21	0	3.80	4.80
0	21	0	-0.90	6.50
0	21	0	-2.40	6.20
-1	21	0	201.38	16.90
-1	21	0	162.78	14.40
1	21	0	177.28	16.20
1	21	0	166.18	12.30
1	21	0	179.28	16.70
-1	21	0	171.58	12.70
-2	-21	0	-7.20	4.40
2	21	0	-3.20	5.60
-2	21	0	2.10	5.80

2	21	0	-5.80	4.30
2	21	0	-7.90	6.70
-2	21	0	-2.20	4.90
-3	-21	0	322.67	18.90
-3	21	0	344.47	22.00
3	21	0	388.66	24.20
3	21	0	314.37	21.30
-3	21	0	323.87	19.80
3	21	0	330.97	19.00
-3	21	0	367.76	23.30
-4	-21	0	63.39	8.10
4	21	0	63.39	8.00
-4	21	0	53.09	8.10
4	21	0	62.09	10.60
-4	21	0	64.59	10.80
-5	-21	0	47.40	7.30
-5	21	0	26.30	6.60
5	21	0	36.40	9.50
5	21	0	19.30	5.20
-5	21	0	29.70	8.60
6	21	0	92.29	9.50
-6	21	0	133.29	15.60
6	21	0	98.39	16.50
-6	21	0	87.59	10.80
-7	21	0	141.39	12.90
7	21	0	115.69	18.30
7	21	0	126.59	10.90
8	21	0	22.10	5.60
-8	21	0	14.40	7.10
8	21	0	22.80	11.10
9	21	0	50.29	12.30
9	21	0	34.30	7.90
-9	21	0	38.50	8.30
-10	21	0	43.00	8.90
0	22	0	417.86	22.10
0	22	0	321.27	34.40
1	22	0	0.80	4.90
-1	22	0	-7.40	7.30
1	22	0	-10.60	7.60
-1	22	0	-4.00	5.00
-2	22	0	357.76	20.90
2	22	0	344.67	24.50
-2	22	0	346.57	24.20
2	22	0	371.26	20.30

3	22	0	-0.30	7.90
3	22	0	2.10	4.60
-3	22	0	4.20	6.70
-3	22	0	-1.80	8.00
4	22	0	127.69	10.40
-4	22	0	130.19	11.70
-4	22	0	126.29	15.80
4	22	0	121.09	16.90
5	22	0	119.59	17.10
-5	22	0	113.79	16.10
5	22	0	116.89	10.20
-5	22	0	112.29	11.80
-6	22	0	332.07	20.00
6	22	0	327.17	25.20
-6	22	0	320.87	24.10
6	22	0	293.27	18.40
7	22	0	15.60	10.50
7	22	0	25.20	5.80
-7	22	0	34.10	7.60
-7	22	0	21.20	9.50
8	22	0	70.79	9.60
-8	22	0	63.69	11.30
8	22	0	46.00	14.40
-9	22	0	192.88	16.10
-10	22	0	36.80	9.30
0	23	0	-4.20	5.10
0	23	0	2.40	7.90
-1	23	0	526.65	32.40
1	23	0	545.85	32.90
-1	23	0	520.85	28.80
1	23	0	537.95	28.60
2	23	0	75.49	15.30
2	23	0	105.09	10.20
-2	23	0	112.79	15.90
-2	23	0	99.39	12.10
3	23	0	578.54	30.80
-3	23	0	576.64	31.70
3	23	0	592.04	35.50
-3	23	0	607.54	35.40
4	23	0	26.10	10.00
-4	23	0	21.60	6.80
4	23	0	30.40	6.00
-4	23	0	26.40	9.20
-5	23	0	198.88	19.80

5	23	0	187.08	20.70
5	23	0	194.98	13.80
-5	23	0	204.68	15.10
-6	23	0	236.58	17.00
-6	23	0	262.07	22.10
6	23	0	242.88	22.80
6	23	0	220.48	15.30
7	23	0	152.08	12.70
7	23	0	168.48	21.40
-7	23	0	198.68	20.20
-7	23	0	165.78	14.60
-8	23	0	131.09	19.60
-8	23	0	117.79	13.50
8	23	0	108.99	20.00
-9	23	0	34.50	9.30
0	24	0	83.69	10.50
0	24	0	81.59	16.00
1	24	0	-5.20	8.30
1	24	0	6.40	5.50
-1	24	0	10.60	9.80
-1	24	0	1.90	5.80
-2	24	0	13.70	6.40
2	24	0	15.20	9.20
-2	24	0	14.50	9.00
2	24	0	25.50	6.10
-3	24	0	0.50	9.70
3	24	0	-11.40	9.50
-3	24	0	4.30	6.30
3	24	0	15.00	5.50
-4	24	0	11.20	9.70
-4	24	0	-0.80	6.40
4	24	0	19.20	11.00
4	24	0	6.80	5.50
-5	24	0	49.10	8.60
5	24	0	44.60	7.00
5	24	0	29.30	11.40
-5	24	0	37.40	11.70
-6	24	0	13.70	10.30
6	24	0	5.20	5.70
-6	24	0	11.80	7.20
6	24	0	-2.20	10.60
-7	24	0	9.80	7.60
-7	24	0	-4.50	10.50
7	24	0	11.10	12.30

-8	24	0	-1.70	7.40
-8	24	0	8.50	11.80
8	24	0	1.40	11.90
0	25	0	-1.30	10.20
0	25	0	-4.80	5.90
-1	25	0	435.16	29.50
-1	25	0	438.76	24.80
1	25	0	405.16	29.50
2	25	0	-0.40	5.70
2	25	0	-4.90	9.30
-2	25	0	6.50	6.70
-2	25	0	-3.20	9.50
3	25	0	135.59	12.10
3	25	0	136.19	19.80
-3	25	0	134.59	13.10
-3	25	0	146.19	19.60
4	25	0	-13.20	9.80
-4	25	0	7.80	7.00
-4	25	0	-8.90	10.10
4	25	0	2.60	5.60
-5	25	0	375.36	28.50
-5	25	0	393.06	23.70
5	25	0	367.66	21.80
5	25	0	380.46	29.80
-6	25	0	69.29	12.70
6	25	0	103.09	20.50
-6	25	0	102.89	18.90
-7	25	0	213.38	17.90
7	25	0	233.38	25.70
-7	25	0	238.18	24.50
0	26	0	6.10	10.30
0	26	0	16.00	6.80
-1	26	0	-3.60	6.60
-1	26	0	-2.30	10.20
1	26	0	-3.90	6.00
1	26	0	-6.10	10.70
2	26	0	64.39	12.70
-2	26	0	55.79	13.20
-2	26	0	63.99	11.40
2	26	0	62.19	10.30
-3	26	0	-4.10	7.20
3	26	0	4.20	6.90
-3	26	0	5.50	11.30
3	26	0	-0.90	10.60

-4	26	0	6.20	10.80
-4	26	0	13.50	7.50
4	26	0	3.60	11.60
-5	26	0	-7.50	7.20
5	26	0	3.10	12.20
-5	26	0	20.70	11.20
0	27	0	0.20	11.70
0	27	0	4.80	6.70
-1	27	0	159.68	15.00
1	27	0	171.28	13.90
1	27	0	141.59	21.90
-1	27	0	121.69	21.40
2	27	0	15.80	11.20
-2	27	0	7.30	7.50
2	27	0	-2.70	6.20
-2	27	0	-5.00	11.60
-3	27	0	117.19	21.00
3	27	0	131.99	21.90
-3	27	0	112.09	14.00
4	27	0	26.70	14.70
-4	27	0	53.19	9.40
-4	27	0	50.49	13.40
-16	0	1	20.70	8.80
-15	0	1	8.90	7.20
13	0	-1	2.80	6.30
-13	0	1	-2.70	5.60
12	0	-1	12.90	6.60
-12	0	1	3.10	8.10
-11	0	1	-3.40	6.10
11	0	-1	-10.00	4.80
-11	0	1	-1.40	4.20
10	0	-1	8.80	5.30
-10	0	1	10.80	7.00
-10	0	1	7.40	5.20
-10	0	1	14.90	4.30
-9	0	1	5.80	4.20
-9	0	1	7.10	3.50
9	0	-1	1.90	4.60
-9	0	1	-2.40	5.10
-8	0	1	6.90	5.30
-8	0	1	17.70	4.30
8	0	-1	6.80	4.10
-7	0	1	9.10	3.00
-7	0	1	6.40	4.20

7	0	-1	7.70	3.60
6	0	-1	2.80	2.50
-6	0	1	2.20	3.30
6	0	-1	-0.50	2.90
-6	0	1	-2.20	2.20
-5	0	1	13.00	3.30
-5	0	1	7.40	2.30
5	0	-1	12.40	3.00
4	0	-1	-2.50	1.50
-4	0	1	3.30	1.90
-4	0	1	-1.80	1.50
4	0	-1	3.30	1.90
3	0	-1	1.60	1.60
3	0	-1	3.00	1.50
-3	0	1	3.40	1.50
-3	0	1	3.90	2.00
2	0	-1	-0.30	0.50
-2	0	1	1.60	0.70
-2	0	1	0.40	1.00
2	0	-1	1.40	0.70
-2	0	-1	-0.30	0.80
-2	0	-1	0.80	0.80
-2	0	-1	0.90	0.80
-3	0	-1	2.40	1.30
-3	0	-1	0.70	1.30
-3	0	-1	4.50	1.60
-4	0	-1	8.00	2.00
-4	0	-1	1.00	2.20
-4	0	-1	1.50	2.10
-5	0	-1	2.20	2.60
-5	0	-1	-1.70	2.00
-5	0	-1	1.50	2.30
-6	0	-1	-1.70	3.00
-6	0	-1	1.30	2.60
-6	0	-1	-0.20	2.30
-7	0	-1	-2.10	3.20
7	0	1	-1.10	1.80
-7	0	-1	1.70	3.40
-7	0	-1	-5.00	2.40
-8	0	-1	-2.80	3.90
8	0	1	-1.00	1.80
-8	0	-1	7.60	3.10
-8	0	-1	7.40	3.40
-9	0	-1	-6.00	3.40

-9	0	-1	-5.90	3.70
9	0	1	-0.10	2.30
-9	0	-1	-6.70	4.30
10	0	1	0.60	2.90
-10	0	-1	18.20	4.70
-10	0	-1	4.90	6.30
-10	0	-1	19.60	5.20
-11	0	-1	-9.10	5.60
11	0	1	-4.30	3.00
-11	0	-1	-3.10	4.00
-12	0	-1	4.40	7.10
12	0	1	6.40	4.30
13	0	1	4.60	4.40
-13	0	-1	7.90	6.00
-14	0	-1	13.60	6.30
-15	0	-1	-8.30	7.10
-16	0	-1	14.50	8.30
-16	-1	1	225.88	18.00
-16	1	1	175.88	17.40
-15	-1	1	24.60	8.10
-15	1	1	28.80	8.00
-14	-1	1	660.23	35.00
-14	1	1	640.04	34.80
-13	-1	1	88.79	11.50
13	-1	-1	102.29	12.60
-13	1	1	95.19	10.70
13	1	-1	86.49	12.50
-12	-1	1	656.03	41.10
-12	-1	1	795.72	38.60
12	-1	-1	776.92	40.00
-12	1	1	662.43	41.00
12	1	-1	779.42	40.40
-12	1	1	790.42	38.60
-11	-1	1	305.37	22.50
-11	-1	1	354.86	19.70
11	-1	-1	360.96	21.30
-11	1	1	325.67	22.50
11	1	-1	350.26	21.40
-11	1	1	358.36	19.80
-10	-1	1	1203.38	59.69
-10	-1	1	1339.07	60.59
10	-1	-1	1224.98	60.99
-10	-1	1	1118.39	61.79
-10	1	1	1183.08	59.79

10	1	-1	1215.18	61.09
-10	1	1	1139.49	61.79
-10	1	1	1303.27	60.79
9	-1	-1	926.51	46.80
-9	-1	1	861.61	47.50
-9	-1	1	909.81	45.70
-9	-1	1	985.40	46.30
9	-1	-1	900.31	46.10
9	1	-1	941.91	47.10
-9	1	1	876.11	47.60
-9	1	1	981.70	46.60
-9	1	1	928.51	45.80
8	-1	-1	1787.52	88.09
-8	-1	1	1680.33	88.69
-8	-1	1	1811.32	87.29
8	-1	-1	1837.82	87.69
-8	-1	1	2041.90	87.89
-8	1	1	1697.63	88.59
-8	1	1	1781.22	87.29
8	1	-1	1839.92	88.39
-7	-1	1	1961.60	96.09
7	-1	-1	1999.90	96.49
-7	-1	1	1902.81	97.39
-7	-1	1	2248.88	96.59
7	-1	-1	2035.60	96.99
-7	1	1	1884.81	97.29
-7	1	1	1972.00	96.19
7	1	-1	2019.50	97.09
6	-1	-1	937.31	44.90
6	-1	-1	926.11	44.40
-6	-1	1	890.51	44.10
-6	-1	1	850.21	45.20
-6	-1	1	982.80	44.40
-6	1	1	868.81	45.10
6	1	-1	901.21	45.00
-6	1	1	883.01	44.10
-5	-1	1	7061.29	336.17
-5	-1	1	7121.49	337.17
5	-1	-1	7132.89	336.37
5	-1	-1	7286.17	336.87
-5	1	1	7122.59	337.07
-5	1	1	6855.41	336.27
5	1	-1	7288.37	336.97
5	1	-1	6871.41	336.37

-4	-1	1	331.07	17.90
4	-1	-1	359.96	17.40
-4	-1	1	340.57	17.10
4	1	-1	335.27	17.80
-4	1	1	354.06	17.90
4	1	-1	342.77	17.20
-3	-1	1	8223.78	389.86
2	-1	-1	262.27	13.30
-2	-1	1	265.77	13.10
-2	-1	1	258.27	13.60
-2	1	1	273.27	13.30
2	1	-1	289.17	13.20
-2	1	1	270.17	13.60
-1	1	-1	1447.06	68.79
2	-1	1	532.25	25.30
-2	-1	-1	535.85	25.30
-2	-1	-1	503.55	25.40
2	-1	1	510.95	25.30
2	1	1	513.15	25.20
-2	1	-1	524.55	25.30
-2	1	-1	544.35	25.30
3	-1	1	2007.80	91.89
3	-1	1	1936.01	91.39
-3	-1	-1	1850.81	91.59
-3	-1	-1	1981.40	91.39
-3	1	-1	1868.41	91.39
3	1	1	1838.62	91.29
-3	1	-1	1941.51	91.59
-4	-1	-1	6937.71	336.27
-4	1	-1	7237.48	336.27
-5	-1	-1	1993.60	98.09
-5	-1	-1	1974.80	98.59
5	-1	1	2127.29	98.29
-5	-1	-1	2243.78	98.29
-5	1	-1	2002.60	98.59
-5	1	-1	1965.00	98.09
-6	-1	-1	1185.68	52.99
-6	-1	-1	1000.70	52.59
-6	-1	-1	1005.90	53.39
6	-1	1	1143.49	52.99
-6	1	-1	1033.10	52.69
-6	1	-1	1181.28	53.09
-6	1	-1	1042.90	53.29
-7	-1	-1	141.29	10.10

-7	-1	-1	143.99	9.10
-7	-1	-1	130.19	8.60
-7	1	-1	120.79	8.60
-7	1	-1	132.69	9.30
-7	1	-1	130.29	9.70
-8	-1	-1	971.00	48.20
-8	-1	-1	916.71	49.30
-8	-1	-1	1109.09	48.80
-8	1	-1	1099.29	49.00
-8	1	-1	949.01	49.40
8	1	1	856.91	48.70
-8	1	-1	962.40	48.30
-9	-1	-1	128.59	11.70
-9	-1	-1	148.59	10.50
-9	-1	-1	146.09	9.90
-9	1	-1	148.49	12.00
-9	1	-1	144.89	10.50
-9	1	-1	132.19	9.40
9	1	1	129.79	10.80
-10	-1	-1	1226.08	60.59
-10	-1	-1	1128.49	62.09
-10	-1	-1	1352.56	61.39
10	1	1	1228.58	61.59
-10	1	-1	1139.59	61.99
-10	1	-1	1203.88	60.59
-10	1	-1	1333.97	61.79
-11	-1	-1	114.29	13.40
-11	-1	-1	123.59	10.60
11	1	1	120.29	11.90
-11	1	-1	139.09	10.70
-11	1	-1	133.99	13.20
-12	-1	-1	672.83	33.80
-12	-1	-1	595.34	36.20
-12	1	-1	579.44	36.00
12	1	1	660.53	35.10
-12	1	-1	689.93	33.90
-13	-1	-1	8.30	5.80
13	1	1	3.40	6.10
-13	1	-1	11.60	6.30
-14	-1	-1	298.37	19.80
-14	1	-1	315.97	19.80
-15	-1	-1	7.90	7.60
-15	1	-1	9.00	7.70
-16	-1	-1	376.06	24.20

-16	1	-1	359.96	24.30
-16	-2	1	34.60	9.30
-16	2	1	41.30	9.10
-15	-2	1	136.49	14.20
-15	2	1	160.78	14.40
-14	-2	1	18.20	6.50
-14	2	1	28.60	7.00
13	-2	-1	185.88	15.90
-13	2	1	230.28	15.00
13	2	-1	189.88	17.00
-12	-2	1	214.08	14.20
12	-2	-1	203.58	15.80
-12	-2	1	169.68	18.00
-12	2	1	227.68	14.50
-12	2	1	206.98	18.20
12	2	-1	210.18	16.60
11	-2	-1	473.85	26.50
-11	-2	1	489.65	25.20
-11	-2	1	424.66	28.00
-11	2	1	486.75	25.30
11	2	-1	469.15	27.00
-11	2	1	445.36	28.10
-10	-2	1	518.65	27.10
-10	-2	1	464.85	28.70
10	-2	-1	522.85	27.50
10	-2	-1	516.95	27.10
-10	-2	1	469.65	26.20
-10	2	1	476.95	28.60
-10	2	1	507.75	26.50
10	2	-1	527.55	28.20
-9	-2	1	270.37	14.70
9	-2	-1	248.48	15.30
-9	-2	1	241.68	17.10
-9	-2	1	291.37	15.50
9	-2	-1	243.78	15.70
-9	2	1	252.37	14.90
9	2	-1	248.58	16.40
-9	2	1	246.98	17.10
-8	-2	1	630.54	31.60
-8	-2	1	579.24	33.30
-8	-2	1	661.93	32.00
8	-2	-1	647.04	32.60
8	-2	-1	633.24	32.10
-8	2	1	641.54	31.80

8	2	-1	630.14	33.00
-8	2	1	608.24	33.20
-7	-2	1	12.10	3.20
-7	-2	1	25.80	4.70
7	-2	-1	16.70	3.90
-7	-2	1	17.40	3.00
7	-2	-1	12.80	3.70
-7	2	1	12.90	3.20
7	2	-1	11.60	3.80
-7	2	1	9.50	3.80
6	-2	-1	1140.89	55.99
-6	-2	1	1246.28	55.89
6	-2	-1	1144.69	56.29
-6	-2	1	1092.79	56.69
-6	-2	1	1110.79	55.59
-6	2	1	1118.39	55.69
-6	2	1	1173.78	56.79
6	2	-1	1175.28	56.69
-5	-2	1	337.57	15.30
-5	-2	1	291.17	15.00
5	-2	-1	287.87	15.30
-5	-2	1	261.17	16.00
-5	2	1	295.07	16.00
-5	2	1	292.97	15.20
5	2	-1	283.37	16.00
-4	-2	1	441.96	20.40
-4	-2	1	393.36	20.30
-4	-2	1	405.66	21.20
4	-2	-1	400.86	20.60
-4	2	1	417.96	21.10
4	2	-1	423.86	21.20
-4	2	1	402.86	20.50
-3	-2	1	857.81	42.70
3	-2	-1	903.31	43.00
-3	-2	1	921.41	42.80
-3	-2	1	871.11	43.40
-3	2	1	901.41	43.30
-3	2	1	901.81	42.90
3	2	-1	887.71	43.40
2	-2	-1	152.78	7.90
-2	-2	1	160.88	7.70
-2	-2	1	149.29	8.30
2	2	-1	151.08	8.20
-2	2	1	149.09	8.10

2	2	-1	148.99	7.60
-2	2	1	161.98	7.90
-1	-2	1	82.49	4.00
1	-2	-1	80.69	4.40
-1	-2	1	77.49	4.50
1	2	-1	84.99	4.00
-1	2	1	84.19	4.40
1	-2	1	29.00	1.80
1	-2	1	31.20	1.90
-1	-2	-1	30.20	1.90
-1	-2	-1	30.00	2.20
-1	2	-1	31.20	1.80
-2	-2	-1	23.70	2.40
-2	-2	-1	17.40	1.80
2	-2	1	18.70	2.00
2	-2	1	21.80	1.80
-2	2	-1	20.50	1.90
2	2	1	19.70	1.80
-3	-2	-1	617.04	31.40
-3	-2	-1	666.23	31.10
3	-2	1	644.34	31.10
3	-2	1	632.44	31.20
-3	2	-1	636.94	31.10
3	2	1	619.04	31.00
-3	2	-1	672.93	31.30
-4	-2	-1	662.33	33.50
-4	-2	-1	710.33	33.00
4	-2	1	665.13	33.20
4	-2	1	702.03	33.30
4	2	1	595.34	33.20
-4	2	-1	725.33	33.40
-4	2	-1	675.73	33.10
-5	-2	-1	629.84	33.20
-5	-2	-1	743.63	32.70
-5	-2	-1	640.24	32.50
5	-2	1	682.33	32.80
-5	2	-1	635.84	32.60
-5	2	-1	645.94	33.00
6	-2	1	1055.49	48.50
-6	-2	-1	955.00	48.10
-6	-2	-1	901.91	48.80
-6	-2	-1	1075.69	48.30
-6	2	-1	965.00	48.10
-6	2	-1	971.10	48.80

-7	-2	-1	265.17	16.30
-7	-2	-1	321.17	15.50
-7	-2	-1	278.27	15.00
7	-2	1	293.07	15.60
-7	2	-1	277.07	16.10
7	2	1	239.18	15.70
-7	2	-1	269.47	15.10
-8	-2	-1	86.09	7.80
8	-2	1	104.89	7.60
-8	-2	-1	76.29	7.20
-8	-2	-1	86.59	9.00
-8	2	-1	100.29	8.20
8	2	1	88.59	8.70
-8	2	-1	98.19	8.80
-8	2	-1	102.79	7.60
-9	-2	-1	670.33	34.10
-9	-2	-1	584.84	35.40
-9	-2	-1	775.52	34.80
-9	2	-1	744.33	35.10
9	2	1	636.74	35.10
-9	2	-1	679.53	34.20
-9	2	-1	624.54	35.50
-10	-2	-1	168.48	14.90
-10	-2	-1	199.78	13.00
-10	-2	-1	226.38	14.00
-10	2	-1	205.18	15.30
-10	2	-1	214.28	13.10
10	2	1	191.08	14.20
-11	-2	-1	196.38	12.70
-11	-2	-1	146.39	15.40
11	2	1	171.58	14.10
-11	2	-1	194.78	12.80
-11	2	-1	169.48	15.10
-12	-2	-1	94.39	10.10
-12	-2	-1	66.39	13.20
-12	2	-1	93.99	13.10
12	2	1	82.79	11.90
-13	-2	-1	304.47	19.40
13	2	1	300.77	20.80
-13	2	-1	328.77	19.50
-14	-2	-1	11.30	6.50
-14	2	-1	22.50	6.90
-15	-2	-1	175.98	16.60
-15	2	-1	210.38	16.50

-16	-2	-1	22.20	8.60
-16	2	-1	20.80	8.60
-16	-3	1	37.60	9.20
-16	3	1	50.89	9.80
-15	-3	1	36.20	8.10
-15	3	1	13.00	7.60
-14	-3	1	36.60	7.30
-14	3	1	32.10	7.60
13	-3	-1	9.50	6.10
-13	-3	1	17.20	6.90
13	3	-1	5.90	7.80
-13	3	1	21.90	6.40
-12	-3	1	115.89	15.50
12	-3	-1	125.29	12.30
-12	-3	1	126.19	10.70
12	3	-1	130.09	14.00
-12	3	1	109.09	14.80
-12	3	1	113.49	12.10
-11	-3	1	132.09	10.20
11	-3	-1	118.59	11.50
-11	-3	1	99.49	13.80
-11	-3	1	123.69	11.40
-11	3	1	114.29	13.80
-11	3	1	139.79	10.70
11	3	-1	110.79	12.80
-10	-3	1	18.30	4.60
-10	-3	1	9.40	6.30
-10	-3	1	12.10	4.90
10	-3	-1	17.40	4.90
-10	3	1	16.50	5.80
-10	3	1	22.10	4.90
10	3	-1	15.20	6.10
-9	-3	1	247.18	13.70
-9	-3	1	215.58	16.20
9	-3	-1	230.08	14.60
-9	-3	1	262.07	14.40
9	-3	-1	235.28	14.80
-9	3	1	237.38	14.00
9	3	-1	217.88	15.60
-9	3	1	220.08	15.80
8	-3	-1	105.09	8.80
-8	-3	1	120.39	8.30
-8	-3	1	110.69	7.90
-8	-3	1	77.49	9.70

8	-3	-1	130.39	8.90
-8	3	1	102.49	9.70
8	3	-1	88.89	9.80
-8	3	1	118.29	8.10
-7	-3	1	17.00	3.30
-7	-3	1	15.50	4.60
7	-3	-1	15.90	3.80
7	-3	-1	13.30	3.30
-7	-3	1	20.60	3.10
-7	3	1	9.30	3.20
-7	3	1	7.50	3.60
7	3	-1	13.90	4.30
6	-3	-1	29.10	4.70
-6	-3	1	25.70	4.00
-6	-3	1	16.20	4.30
-6	-3	1	21.30	3.10
6	3	-1	24.20	4.30
-6	3	1	27.80	4.20
-6	3	1	26.40	4.30
-5	-3	1	1430.56	63.39
5	-3	-1	1320.67	63.69
-5	-3	1	1283.07	63.19
-5	-3	1	1207.08	64.19
-5	3	1	1305.07	64.09
-5	3	1	1345.57	63.49
5	3	-1	1329.17	64.19
-4	-3	1	165.08	8.50
-4	-3	1	148.49	9.40
-4	-3	1	169.48	8.60
-4	3	1	152.78	8.80
-4	3	1	163.38	9.30
4	3	-1	161.88	9.60
-3	-3	1	38.50	4.00
3	-3	-1	41.40	3.40
-3	-3	1	41.10	2.90
-3	3	1	48.80	3.70
3	3	-1	43.70	2.90
-3	3	1	43.70	3.20
3	3	-1	47.70	4.00
-2	-3	1	38.10	3.40
-2	-3	1	35.80	2.20
2	-3	-1	37.30	2.80
-2	3	1	40.30	2.60
2	3	-1	31.00	2.20

2	3	-1	39.30	3.30
-2	3	1	35.60	2.90
-1	-3	1	205.98	10.40
-1	-3	1	201.78	9.80
1	-3	-1	194.88	10.10
1	3	-1	204.18	10.40
-1	3	1	194.38	10.10
1	3	-1	210.08	9.80
-1	3	1	205.38	10.20
0	-3	1	6.40	1.00
0	-3	-1	10.10	1.10
0	-3	1	7.20	0.90
0	3	-1	9.40	0.90
0	3	-1	5.50	1.00
0	3	1	10.80	1.10
0	3	1	5.80	1.20
0	3	1	8.40	1.30
-2	-3	-1	1513.75	72.19
2	-3	1	1484.15	72.19
-2	-3	-1	1538.85	72.69
2	-3	1	1538.05	72.29
-2	3	-1	1517.75	72.29
2	3	1	1474.55	72.19
-2	3	-1	1545.35	72.29
-3	-3	-1	24.20	3.20
-3	-3	-1	26.00	2.30
3	-3	1	20.60	2.30
3	-3	1	22.60	2.70
3	3	1	22.40	2.20
-3	3	-1	23.80	2.50
-3	3	-1	16.70	2.30
4	-3	1	440.66	22.50
4	-3	1	454.65	21.90
-4	-3	-1	428.06	22.40
-4	-3	-1	486.85	21.80
4	3	1	376.16	22.10
-4	3	-1	451.55	22.10
-4	3	-1	436.36	21.90
5	-3	1	1061.89	51.69
-5	-3	-1	1011.60	52.19
5	-3	1	1044.90	51.69
-5	-3	-1	1157.78	51.59
5	3	1	1023.90	51.39
5	3	1	1109.09	52.19

-5	3	-1	1032.70	51.59
-5	3	-1	1070.29	51.99
6	-3	1	320.07	17.00
-6	-3	-1	343.17	16.70
6	-3	1	319.97	17.00
-6	-3	-1	294.77	17.50
-6	-3	-1	304.87	16.50
6	3	1	305.27	17.40
-6	3	-1	318.97	16.70
-6	3	-1	316.97	17.30
7	-3	1	31.80	5.20
7	-3	1	30.20	5.00
-7	-3	-1	28.80	4.50
-7	-3	-1	34.50	4.90
-7	-3	-1	30.80	6.10
7	3	1	34.20	5.00
-7	3	-1	30.70	4.40
-7	3	-1	33.10	5.80
8	-3	1	15.20	4.40
-8	-3	-1	11.40	3.60
8	-3	1	14.90	3.90
-8	-3	-1	22.00	3.90
8	3	1	3.20	3.90
-8	3	-1	7.80	3.90
-8	3	-1	14.30	3.80
-9	-3	-1	25.90	4.80
-9	-3	-1	12.60	5.60
-9	-3	-1	21.40	4.30
9	-3	1	30.20	5.30
9	-3	1	9.40	5.90
-9	3	-1	18.80	5.00
9	3	1	12.20	5.30
-9	3	-1	16.00	4.40
-9	3	-1	17.40	5.10
-10	-3	-1	151.68	13.80
10	-3	1	177.48	15.80
-10	-3	-1	167.38	11.50
-10	-3	-1	183.58	12.70
-10	3	-1	159.38	11.60
10	3	1	171.88	13.30
-10	3	-1	165.78	13.70
-11	-3	-1	199.58	13.20
-11	-3	-1	169.28	15.90
11	-3	1	196.08	15.10

-11	3	-1	188.38	12.90
11	3	1	206.88	15.10
-11	3	-1	174.88	15.70
12	-3	1	42.60	7.60
-12	-3	-1	38.60	9.80
-12	-3	-1	48.20	8.50
12	3	1	39.20	7.90
-12	3	-1	35.90	8.30
-12	3	-1	45.20	9.40
-13	-3	-1	57.49	10.10
-13	3	-1	52.89	9.50
-14	-3	-1	201.28	15.20
-14	3	-1	179.38	14.90
-15	-3	-1	16.20	7.90
-15	3	-1	27.00	7.90
-16	-3	-1	83.29	14.20
-16	3	-1	60.29	10.30
-16	-4	1	24.90	8.90
-16	4	1	16.20	8.00
-15	-4	1	309.07	20.40
-15	4	1	309.67	20.40
-14	-4	1	15.70	6.80
-14	4	1	22.10	7.20
-13	-4	1	239.18	15.50
13	4	-1	185.08	21.50
-13	4	1	238.08	15.70
-12	-4	1	259.57	16.00
12	-4	-1	255.77	17.30
-12	4	1	234.08	19.70
-12	4	1	260.57	18.00
11	-4	-1	269.47	17.10
-11	-4	1	262.27	16.00
-11	-4	1	248.58	19.30
-11	-4	1	297.87	17.20
-11	4	1	275.07	16.20
-11	4	1	224.38	18.70
11	4	-1	255.97	19.00
-10	-4	1	365.16	22.60
-10	-4	1	375.16	19.70
10	-4	-1	354.26	20.80
-10	-4	1	399.26	20.50
-10	4	1	342.67	20.00
-10	4	1	318.57	21.90
10	4	-1	361.36	22.00

-9	-4	1	721.43	36.40
-9	-4	1	665.33	38.50
9	-4	-1	713.03	37.30
9	-4	-1	718.13	37.30
-9	-4	1	820.42	37.00
-9	4	1	685.73	38.30
-9	4	1	739.43	36.70
9	4	-1	738.03	38.20
-8	-4	1	658.83	38.70
-8	-4	1	756.82	37.10
8	-4	-1	728.03	37.90
8	-4	-1	775.92	37.90
-8	-4	1	834.82	37.50
-8	4	1	701.33	38.60
8	4	-1	778.92	38.80
-8	4	1	742.03	37.30
-7	-4	1	30.20	4.40
7	-4	-1	32.90	5.50
-7	-4	1	42.10	4.80
7	-4	-1	27.30	4.80
-7	-4	1	28.30	5.20
-7	4	1	37.50	4.90
-7	4	1	43.20	6.60
7	4	-1	39.90	6.60
6	-4	-1	3669.63	172.68
-6	-4	1	3457.65	171.98
-6	-4	1	3937.91	172.28
-6	-4	1	3369.36	173.18
-6	4	1	3457.25	172.28
6	4	-1	3747.73	173.28
-6	4	1	3669.93	173.18
5	-4	-1	529.05	26.20
-5	-4	1	494.25	26.80
-5	-4	1	480.15	25.60
-5	-4	1	565.04	25.70
-5	4	1	504.95	26.50
5	4	-1	537.65	26.80
-5	4	1	517.95	25.90
-4	-4	1	558.64	29.10
-4	-4	1	599.94	28.10
4	-4	-1	590.74	28.60
4	4	-1	605.94	29.10
-4	4	1	582.44	28.80
-4	4	1	557.34	28.30

4	4	-1	545.75	28.00
-3	-4	1	30.00	3.60
3	-4	-1	27.10	2.80
-3	-4	1	25.60	2.20
-3	4	1	27.90	3.10
3	4	-1	26.10	2.20
3	4	-1	33.50	3.60
-3	4	1	25.80	3.00
-2	-4	1	2565.34	121.09
-2	-4	1	2474.65	120.29
2	-4	-1	2544.15	120.79
-2	4	1	2488.25	120.59
-2	4	1	2493.65	120.79
2	4	-1	2659.73	121.09
-1	-4	1	337.47	15.70
1	-4	-1	310.87	16.10
-1	-4	1	336.47	16.50
1	4	-1	334.37	16.40
-1	4	1	327.87	16.20
1	4	-1	316.57	15.70
-1	4	1	317.37	16.00
0	-4	1	2174.38	102.69
0	-4	-1	2129.79	102.49
0	-4	1	2088.69	102.39
0	4	1	2158.38	102.79
0	4	1	2169.38	102.59
0	4	-1	2073.39	102.39
0	4	1	2276.37	102.79
0	4	-1	2198.68	102.79
-1	-4	-1	3462.35	156.28
-1	-4	-1	3186.98	155.78
1	4	1	3126.99	155.78
1	4	1	3423.06	156.28
-1	4	-1	3231.98	155.78
-2	-4	-1	1768.32	80.89
-2	-4	-1	1658.83	80.19
2	-4	1	1648.34	80.29
2	-4	1	1716.23	80.39
2	4	1	1808.32	80.89
2	4	1	1569.04	80.19
-2	4	-1	1674.93	80.29
-2	4	-1	1636.94	80.29
3	-4	1	10.10	1.90
3	-4	1	8.10	1.80

-3	-4	-1	10.40	1.60
-3	-4	-1	9.40	2.10
3	4	1	8.10	2.00
3	4	1	11.20	2.00
-3	4	-1	10.10	2.10
-3	4	-1	7.80	2.50
4	-4	1	1007.90	47.10
-4	-4	-1	986.10	46.80
-4	-4	-1	924.71	47.50
4	-4	1	951.60	46.90
-4	4	-1	950.40	47.10
-4	4	-1	968.50	46.90
4	4	1	996.40	47.50
-5	-4	-1	1120.79	58.99
-5	-4	-1	1308.87	58.29
5	-4	1	1214.28	58.39
5	-4	1	1239.68	58.59
-5	4	-1	1236.58	58.69
5	4	1	1243.88	58.99
-5	4	-1	1174.78	58.29
5	4	1	1113.29	58.09
-6	-4	-1	443.36	23.20
-6	-4	-1	447.36	24.40
-6	-4	-1	491.75	23.40
6	-4	1	450.95	23.70
6	-4	1	467.15	23.50
-6	4	-1	443.66	23.30
-6	4	-1	489.15	24.10
6	4	1	484.15	24.30
6	4	1	417.56	23.10
7	-4	1	243.98	14.40
-7	-4	-1	235.68	15.20
-7	-4	-1	236.98	13.80
7	-4	1	257.07	14.20
-7	-4	-1	282.97	14.20
-7	4	-1	246.68	13.90
-7	4	-1	248.88	14.90
7	4	1	255.47	15.00
8	-4	1	413.06	22.70
-8	-4	-1	420.56	22.20
-8	-4	-1	468.25	22.60
-8	-4	-1	382.96	23.60
8	-4	1	437.36	22.70
-8	4	-1	419.16	22.30

8	4	1	432.56	23.60
-8	4	-1	426.26	23.50
-9	-4	-1	389.16	20.20
9	-4	1	371.96	20.50
-9	-4	-1	334.57	21.80
-9	-4	-1	418.76	20.70
9	-4	1	354.36	20.80
9	4	1	364.86	21.50
-9	4	-1	363.86	20.10
-9	4	-1	367.66	21.70
-10	-4	-1	69.09	9.10
-10	-4	-1	69.99	7.90
-10	-4	-1	68.19	10.80
10	-4	1	84.39	8.40
10	4	1	70.19	10.10
-10	4	-1	84.99	10.40
-10	4	-1	87.39	8.30
-11	-4	-1	844.72	40.30
-11	-4	-1	719.33	42.30
11	-4	1	808.12	40.80
11	4	1	810.42	41.80
-11	4	-1	802.52	40.40
-11	4	-1	724.63	42.00
12	-4	1	229.28	15.70
-12	-4	-1	240.38	15.10
-12	-4	-1	193.48	18.50
-12	4	-1	197.38	17.90
-12	4	-1	233.88	15.40
12	4	1	220.98	17.10
-13	-4	-1	215.48	15.70
-13	4	-1	223.78	15.70
13	4	1	227.38	18.00
-14	-4	-1	22.40	7.50
-14	4	-1	24.30	7.10
-15	-4	-1	381.96	23.70
-15	4	-1	367.16	23.30
-16	-4	-1	6.50	8.50
-16	4	-1	0.40	7.30
-16	-5	1	9.50	9.00
-16	5	1	25.60	8.70
-15	-5	1	35.20	8.10
-15	5	1	22.00	7.20
-14	-5	1	7.70	6.60
-14	5	1	6.70	6.60

-13	-5	1	11.50	5.60
-13	5	1	12.90	5.80
13	5	-1	3.60	8.00
12	-5	-1	3.30	4.60
-12	-5	1	4.90	5.20
-12	-5	1	10.30	9.00
12	5	-1	0.40	7.60
-12	5	1	14.40	7.60
-12	5	1	11.50	5.60
-11	-5	1	1.20	5.10
11	-5	-1	8.00	5.00
-11	-5	1	12.60	4.70
-11	5	1	14.10	5.30
11	5	-1	6.40	6.60
-11	5	1	3.30	6.80
-10	-5	1	-3.70	3.60
10	-5	-1	1.00	3.60
-10	-5	1	-0.30	5.70
-10	-5	1	1.50	4.40
-10	5	1	-2.50	5.20
10	5	-1	-1.50	6.70
-10	5	1	3.40	3.90
-9	-5	1	3.40	5.60
9	-5	-1	2.80	3.40
-9	-5	1	4.70	3.90
-9	-5	1	7.50	3.30
9	-5	-1	5.80	4.30
-9	5	1	13.10	4.10
-9	5	1	6.30	5.50
-8	-5	1	89.89	7.30
-8	-5	1	77.99	6.70
8	-5	-1	98.89	8.30
-8	-5	1	75.69	9.40
8	5	-1	76.19	9.30
-8	5	1	90.89	7.30
-8	5	1	75.29	8.70
7	-5	-1	273.87	15.70
-7	-5	1	269.27	14.80
-7	-5	1	269.57	16.90
-7	-5	1	297.17	15.10
7	5	-1	290.67	16.80
-7	5	1	270.87	16.40
-7	5	1	269.47	15.00
6	-5	-1	299.97	16.30

-6	-5	1	288.17	15.40
-6	-5	1	271.67	17.00
-6	-5	1	322.07	15.60
-6	5	1	293.27	16.80
-6	5	1	295.07	15.80
6	5	-1	298.17	17.00
-5	-5	1	49.40	4.00
-5	-5	1	46.30	5.90
5	-5	-1	41.20	4.60
-5	5	1	43.70	4.40
5	5	-1	45.40	5.90
5	5	-1	42.10	4.40
-5	5	1	46.80	5.10
-4	-5	1	83.39	5.00
-4	-5	1	76.19	6.50
4	-5	-1	82.09	5.80
4	5	-1	84.89	6.40
-4	5	1	79.69	5.90
-4	5	1	83.49	5.40
4	5	-1	75.39	4.90
-3	-5	1	121.19	6.30
-3	-5	1	129.29	7.80
3	-5	-1	106.89	7.60
-3	5	1	112.89	7.00
3	5	-1	107.99	6.20
3	5	-1	136.69	7.60
-3	5	1	114.19	6.70
-2	-5	1	360.26	18.00
-2	-5	1	396.96	19.10
2	-5	-1	360.56	18.50
-2	5	1	361.26	18.40
-2	5	1	373.36	18.70
2	5	-1	399.86	19.10
2	5	-1	354.06	18.00
-1	-5	1	141.09	7.90
1	-5	-1	134.89	7.50
-1	-5	1	138.89	7.00
-1	5	1	127.99	7.30
1	5	-1	136.39	7.00
-1	5	1	143.29	7.70
1	5	-1	147.19	8.00
0	-5	-1	264.47	13.30
0	-5	1	265.17	13.60
0	-5	1	243.58	13.20

0	5	-1	249.68	13.10
0	5	1	274.27	13.80
0	5	-1	271.77	13.70
0	5	1	287.07	13.70
0	5	1	286.27	13.40
-1	-5	-1	370.66	18.50
-1	-5	-1	405.66	19.20
1	-5	1	373.46	18.60
-1	5	-1	373.66	18.70
-1	5	-1	363.96	18.50
1	5	1	402.96	19.20
1	5	1	359.16	18.50
-2	-5	-1	16.30	1.90
2	-5	1	17.50	2.40
2	-5	1	16.20	2.10
-2	-5	-1	17.40	3.10
2	5	1	16.60	1.90
-2	5	-1	14.90	2.20
2	5	1	15.80	3.20
-2	5	-1	16.20	2.20
3	-5	1	39.80	3.60
3	-5	1	41.90	3.30
-3	-5	-1	47.40	3.20
-3	-5	-1	39.80	4.40
3	5	1	38.10	3.20
3	5	1	39.70	4.30
-3	5	-1	42.00	3.60
-3	5	-1	41.30	3.50
4	-5	1	8.50	1.90
-4	-5	-1	7.40	1.90
-4	-5	-1	5.10	2.50
4	-5	1	10.40	2.30
-4	5	-1	5.20	1.80
4	5	1	3.50	2.40
4	5	1	5.80	1.80
-4	5	-1	10.90	2.30
-5	-5	-1	104.79	8.00
-5	-5	-1	108.19	6.60
5	-5	1	103.19	7.30
5	-5	1	105.69	6.70
-5	5	-1	96.69	6.80
-5	5	-1	101.99	7.20
5	5	1	110.39	7.80
5	5	1	98.99	6.50

6	-5	1	54.79	5.20
-6	-5	-1	48.70	6.60
-6	-5	-1	70.59	5.30
6	-5	1	55.99	5.50
6	5	1	51.99	5.00
-6	5	-1	59.79	5.50
-6	5	-1	55.09	5.90
6	5	1	49.10	6.60
7	-5	1	12.20	3.30
7	-5	1	14.70	3.60
-7	-5	-1	15.80	3.20
-7	-5	-1	24.00	3.80
-7	-5	-1	9.40	3.90
-7	5	-1	11.70	3.30
7	5	1	17.10	4.20
-7	5	-1	6.50	3.50
-8	-5	-1	3.30	3.30
-8	-5	-1	4.60	3.20
-8	-5	-1	3.50	4.20
8	-5	1	3.60	3.00
8	-5	1	9.00	3.80
8	5	1	8.40	4.30
-8	5	-1	4.10	3.50
-8	5	-1	5.40	4.00
9	-5	1	3.40	3.20
-9	-5	-1	8.10	4.00
-9	-5	-1	2.40	4.90
-9	-5	-1	5.90	3.70
9	-5	1	7.50	4.50
9	5	1	3.80	4.60
-9	5	-1	0.30	3.50
-9	5	-1	4.60	4.80
-10	-5	-1	-1.30	4.50
-10	-5	-1	2.20	4.10
10	-5	1	4.40	3.80
-10	-5	-1	5.20	5.90
10	5	1	-0.80	5.60
-10	5	-1	3.50	5.30
-10	5	-1	3.90	4.40
11	-5	1	7.70	4.90
-11	-5	-1	13.60	7.60
-11	-5	-1	20.30	5.40
-11	5	-1	16.30	5.50
-11	5	-1	7.70	6.30

11	5	1	15.80	6.30
12	-5	1	10.20	5.30
-12	-5	-1	2.60	5.30
-12	-5	-1	28.70	8.30
-12	5	-1	4.80	5.50
-12	5	-1	3.30	7.00
12	5	1	3.80	6.20
-13	-5	-1	7.40	5.80
-13	5	-1	14.80	6.30
13	5	1	1.00	7.00
-14	-5	-1	-0.50	6.80
-14	5	-1	-2.70	6.20
-15	-5	-1	24.60	8.20
-15	5	-1	29.50	7.80
-16	-5	-1	0.30	8.90
-16	5	-1	-0.30	7.60
-15	-6	1	363.76	22.90
-15	6	1	347.27	22.60
-14	-6	1	18.40	7.00
-14	6	1	27.10	7.30
-13	-6	1	106.99	11.30
13	6	-1	97.19	14.60
-13	6	1	130.89	11.50
-12	-6	1	324.27	23.50
-12	-6	1	312.47	18.90
12	6	-1	345.87	22.50
-12	6	1	315.27	22.80
-12	6	1	303.87	18.80
-11	-6	1	419.86	22.30
-11	-6	1	388.56	21.60
-11	-6	1	337.97	25.00
-11	6	1	335.37	24.30
-11	6	1	421.16	22.00
11	6	-1	410.06	24.80
-10	-6	1	146.29	10.90
-10	-6	1	122.89	9.90
-10	6	1	119.39	10.30
10	6	-1	145.69	13.70
-10	6	1	126.89	13.10
9	-6	-1	603.74	32.90
-9	-6	1	609.34	31.40
-9	-6	1	562.44	33.80
-9	-6	1	700.53	32.00
-9	6	1	627.54	31.90

-9	6	1	578.64	33.40
9	6	-1	658.03	33.80
-8	-6	1	482.45	28.20
-8	-6	1	501.45	25.90
8	-6	-1	487.75	27.00
-8	-6	1	563.04	26.30
-8	6	1	480.85	27.60
-8	6	1	491.85	26.30
8	6	-1	561.54	28.30
-7	-6	1	168.78	10.40
-7	-6	1	187.38	10.70
-7	-6	1	185.28	13.00
7	-6	-1	174.88	11.40
-7	6	1	182.98	12.20
-7	6	1	189.38	11.10
-6	-6	1	1609.04	78.29
-6	-6	1	1561.74	79.89
-6	-6	1	1776.02	78.49
6	-6	-1	1663.83	79.19
-6	6	1	1604.94	78.69
-6	6	1	1613.44	79.49
6	6	-1	1588.24	80.79
-5	-6	1	88.19	5.60
5	-6	-1	94.89	6.60
-5	-6	1	85.49	7.70
5	6	-1	88.99	7.60
-5	6	1	80.49	6.70
5	6	-1	83.49	5.60
-5	6	1	82.09	5.90
4	-6	-1	1446.86	67.19
-4	-6	1	1418.26	66.39
-4	-6	1	1326.67	67.69
4	6	-1	1495.85	67.69
-4	6	1	1395.16	67.29
4	6	-1	1292.57	66.29
-4	6	1	1345.77	66.79
-3	-6	1	301.07	15.30
-3	-6	1	301.67	16.70
3	-6	-1	315.27	16.10
3	6	-1	310.47	15.40
-3	6	1	309.77	15.80
3	6	-1	326.37	16.70
-3	6	1	312.07	16.20
-2	-6	1	1946.01	95.89

2	-6	-1	2025.40	96.49
-2	-6	1	2121.69	97.09
-2	6	1	2074.59	96.69
-2	6	1	1943.51	96.29
2	6	-1	2110.69	96.99
2	6	-1	1914.31	95.89
1	-6	-1	209.88	12.60
1	-6	-1	239.78	12.60
-1	-6	1	241.38	12.10
-1	-6	1	258.57	13.10
-1	6	1	257.27	12.80
1	6	-1	260.37	13.10
1	6	-1	242.38	12.10
-1	6	1	248.58	12.50
-1	-6	-1	265.67	15.10
-1	-6	-1	293.07	14.50
1	-6	1	302.77	14.60
-1	6	-1	305.27	14.80
-1	6	-1	299.87	14.50
1	6	1	280.37	15.30
1	6	1	285.57	14.50
-2	-6	-1	1998.00	94.49
-2	-6	-1	1928.91	93.59
2	-6	1	1998.50	93.89
2	-6	1	1920.61	93.79
2	6	1	1879.21	93.59
-2	6	-1	1999.00	93.89
2	6	1	2037.80	94.49
-3	-6	-1	38.20	4.70
3	-6	1	34.80	3.80
-3	-6	-1	46.40	3.30
3	-6	1	38.60	3.40
-3	6	-1	40.90	3.60
-3	6	-1	38.20	3.60
3	6	1	42.50	3.30
3	6	1	45.50	4.70
4	-6	1	1170.68	56.09
4	-6	1	1172.38	56.39
-4	-6	-1	1108.39	56.99
-4	-6	-1	1220.78	55.99
-4	6	-1	1166.38	56.39
-4	6	-1	1137.29	56.09
4	6	1	1122.59	55.89
4	6	1	1197.48	56.89

5	-6	1	781.02	38.10
5	-6	1	777.42	38.50
-5	-6	-1	726.23	39.10
-5	-6	-1	821.82	38.00
5	6	1	821.72	39.10
-5	6	-1	763.22	38.10
5	6	1	741.93	37.80
-5	6	-1	773.22	38.50
6	-6	1	1319.67	64.39
-6	-6	-1	1427.76	63.89
-6	-6	-1	1236.78	64.99
6	-6	1	1319.97	63.99
-6	6	-1	1349.07	64.59
-6	6	-1	1313.97	63.99
6	6	1	1402.06	64.99
6	6	1	1182.18	63.59
7	-6	1	535.05	27.20
-7	-6	-1	454.95	27.90
-7	-6	-1	582.44	26.70
7	-6	1	531.65	26.80
-7	6	-1	517.15	27.50
7	6	1	507.75	26.40
-7	6	-1	517.25	26.70
7	6	1	532.25	28.00
-8	-6	-1	753.12	37.70
8	-6	1	779.12	38.10
-8	-6	-1	833.32	37.90
-8	-6	-1	680.53	39.30
8	-6	1	705.13	38.20
-8	6	-1	739.93	37.80
-8	6	-1	732.83	38.80
8	6	1	807.32	39.40
-9	-6	-1	269.97	18.70
9	-6	1	280.27	16.60
-9	-6	-1	278.37	16.10
-9	-6	-1	314.97	16.70
9	6	1	297.07	18.10
-9	6	-1	254.47	17.60
-9	6	-1	290.57	16.50
-10	-6	-1	183.28	12.60
-10	-6	-1	180.18	12.00
-10	-6	-1	168.88	15.00
10	-6	1	172.58	12.40
10	6	1	204.98	14.70

-10	6	-1	166.88	11.90
-10	6	-1	165.88	14.20
-11	-6	-1	822.82	40.50
11	-6	1	833.72	41.00
-11	-6	-1	713.63	42.90
-11	6	-1	776.92	40.40
11	6	1	863.91	42.70
-11	6	-1	718.13	42.10
-12	-6	-1	155.98	12.80
-12	-6	-1	153.58	17.70
12	-6	1	153.28	13.10
-12	6	-1	162.68	12.90
12	6	1	177.08	15.80
-12	6	-1	145.19	15.50
-13	-6	-1	134.09	14.20
-13	6	-1	139.79	12.90
13	6	1	167.78	23.40
-14	-6	-1	3.60	6.80
-14	6	-1	-0.60	6.50
-15	-6	-1	469.75	27.10
-15	6	-1	432.86	26.70
-15	-7	1	6.00	7.60
-15	7	1	29.70	8.20
-14	-7	1	88.39	11.60
-14	7	1	100.59	12.30
-13	-7	1	20.30	6.40
13	7	-1	33.20	9.00
-13	7	1	31.40	6.70
-12	-7	1	237.78	15.30
-12	-7	1	206.68	20.60
12	7	-1	257.97	18.70
-12	7	1	237.18	15.70
-12	7	1	209.18	20.00
-11	-7	1	235.98	19.50
-11	-7	1	229.48	14.90
-11	-7	1	252.77	15.80
-11	7	1	228.18	18.30
-11	7	1	228.48	19.00
-10	-7	1	276.97	15.90
-10	-7	1	239.28	19.10
-10	-7	1	250.67	15.20
-10	7	1	257.97	15.80
10	7	-1	287.07	18.50
-10	7	1	238.58	18.00

-9	-7	1	145.59	15.90
-9	-7	1	157.98	10.60
-9	-7	1	159.68	11.20
-9	7	1	162.78	13.40
9	7	-1	180.68	13.80
-9	7	1	176.88	11.30
-8	-7	1	152.38	9.50
-8	-7	1	117.49	12.10
8	-7	-1	130.69	10.60
-8	-7	1	136.29	9.10
-8	7	1	141.29	9.60
8	7	-1	156.48	12.20
-8	7	1	134.29	11.50
-7	-7	1	177.68	12.80
-7	-7	1	160.08	10.00
7	-7	-1	176.38	11.40
-7	-7	1	161.58	10.10
-7	7	1	154.88	10.20
-7	7	1	166.88	11.70
7	7	-1	207.68	13.20
-6	-7	1	208.98	10.80
-6	-7	1	193.08	13.20
6	-7	-1	182.28	11.70
6	7	-1	229.18	13.40
6	7	-1	176.08	10.60
-6	7	1	188.58	12.20
-6	7	1	181.28	11.00
5	-7	-1	1010.20	48.50
-5	-7	1	1026.30	47.50
-5	-7	1	950.40	49.20
-5	7	1	953.10	48.60
5	7	-1	925.71	47.40
-5	7	1	935.11	47.90
5	7	-1	1086.39	49.20
4	-7	-1	10.70	2.90
-4	-7	1	17.30	2.40
-4	-7	1	10.60	3.40
-4	7	1	11.00	2.50
-4	7	1	8.00	2.60
4	7	-1	9.20	1.80
4	7	-1	12.10	3.50
-3	-7	1	914.11	45.70
3	-7	-1	946.61	46.50
-3	-7	1	989.20	47.20

3	7	-1	978.10	48.00
-3	7	1	960.40	46.20
3	7	-1	904.01	45.70
-3	7	1	984.40	46.70
-2	-7	1	487.05	24.20
2	-7	-1	441.16	24.10
-2	-7	1	473.25	22.90
-2	7	1	465.25	23.30
-2	7	1	474.95	23.70
2	7	-1	467.55	22.90
2	7	-1	490.45	25.10
1	-7	-1	2088.29	97.69
-1	-7	1	1982.90	97.09
-1	-7	1	2195.98	98.09
1	-7	-1	1936.31	97.39
-1	7	1	1780.02	97.39
-1	7	1	2146.89	97.79
1	7	-1	1976.20	96.99
1	7	-1	2223.68	98.19
0	-7	1	47.90	4.20
0	-7	1	48.20	3.60
0	-7	-1	55.09	3.80
0	7	1	50.09	4.60
0	7	1	58.29	4.30
0	7	-1	56.29	4.60
0	7	1	56.59	3.80
0	7	-1	50.29	3.50
-1	-7	-1	1598.64	75.79
1	-7	1	1553.04	75.89
-1	-7	-1	1633.64	76.59
-1	7	-1	1506.65	75.69
1	7	1	1673.43	76.69
-1	7	-1	1625.44	76.19
1	7	1	1549.94	75.79
-1	7	-1	1552.34	76.19
2	-7	1	5.00	1.70
2	-7	1	9.80	2.00
-2	-7	-1	5.20	1.40
-2	-7	-1	7.50	2.40
-2	7	-1	6.10	1.70
-2	7	-1	4.00	1.60
2	7	1	5.60	1.50
2	7	1	5.50	2.60
-3	-7	-1	1108.79	54.79

-3	-7	-1	1103.89	53.59
3	-7	1	1114.49	54.09
3	-7	1	1073.69	53.79
-3	7	-1	1083.89	53.79
3	7	1	1197.28	54.89
-3	7	-1	1127.59	54.09
4	-7	1	116.19	7.20
4	-7	1	122.49	7.60
-4	-7	-1	123.19	8.80
-4	7	-1	103.29	7.10
4	7	1	112.99	7.10
4	7	1	135.19	8.70
-4	7	-1	119.59	7.60
5	-7	1	192.38	11.10
5	-7	1	196.78	11.60
-5	-7	-1	203.88	12.80
-5	-7	-1	187.88	10.80
-5	7	-1	196.28	11.20
5	7	1	227.58	12.80
-5	7	-1	200.48	11.60
5	7	1	183.18	10.70
-6	-7	-1	815.12	36.80
6	-7	1	732.33	37.40
-6	-7	-1	693.93	38.30
6	-7	1	725.13	36.90
-6	7	-1	740.83	37.00
-6	7	-1	716.33	37.50
6	7	1	705.73	36.60
6	7	1	826.22	38.30
-7	-7	-1	793.82	43.40
-7	-7	-1	922.51	41.90
7	-7	1	856.01	42.60
7	-7	1	841.32	42.00
-7	7	-1	833.42	42.00
7	7	1	895.21	43.30
-7	7	-1	861.91	42.80
7	7	1	760.22	41.50
8	-7	1	415.06	22.50
-8	-7	-1	373.16	24.30
8	-7	1	409.16	23.30
-8	-7	-1	477.65	22.50
-8	-7	-1	428.36	22.40
-8	7	-1	406.96	23.50
-8	7	-1	432.96	22.60

8	7	1	437.46	24.20
9	-7	1	22.40	4.40
-9	-7	-1	19.50	6.80
-9	-7	-1	21.40	4.60
-9	-7	-1	24.30	4.80
9	7	1	25.40	6.40
-9	7	-1	15.30	5.00
-9	7	-1	26.60	5.10
-10	-7	-1	47.90	8.60
10	-7	1	57.39	7.90
-10	-7	-1	53.59	7.90
-10	-7	-1	61.99	7.40
10	7	1	65.09	10.90
-10	7	-1	48.00	9.80
-10	7	-1	49.90	7.30
11	-7	1	167.88	12.90
-11	-7	-1	163.68	12.40
-11	-7	-1	153.68	16.60
-11	7	-1	196.68	19.40
11	7	1	181.28	15.90
-11	7	-1	148.99	14.80
12	-7	1	220.88	15.60
-12	-7	-1	224.88	15.40
-12	-7	-1	239.18	28.40
-12	7	-1	211.28	17.80
12	7	1	214.58	18.70
-12	7	-1	220.48	15.40
-13	-7	-1	55.29	9.20
-13	7	-1	67.89	10.20
-14	-7	-1	174.58	15.80
-14	7	-1	207.98	15.80
-15	-7	-1	26.10	8.50
-15	7	-1	30.30	7.90
-15	-8	1	101.39	14.30
-15	8	1	124.99	13.80
-14	-8	1	14.50	6.90
-14	8	1	13.30	7.20
-13	-8	1	33.30	7.00
-13	8	1	55.39	10.10
13	8	-1	29.90	9.40
-12	-8	1	87.69	17.30
-12	-8	1	70.99	9.80
12	8	-1	83.09	13.60
-12	8	1	94.99	10.40

-11	-8	1	290.97	16.70
-11	-8	1	290.67	17.30
-11	-8	1	226.28	20.90
-11	8	1	252.77	19.80
11	8	-1	310.67	20.20
-11	8	1	264.27	16.60
-10	-8	1	276.47	21.40
-10	-8	1	343.87	18.40
-10	-8	1	315.37	17.90
-10	8	1	322.87	20.90
-10	8	1	293.67	17.80
10	8	-1	323.77	20.90
-9	-8	1	8.30	4.20
-9	-8	1	5.60	6.00
-9	-8	1	14.70	3.90
9	8	-1	18.30	5.90
-9	8	1	11.60	5.20
-9	8	1	15.20	4.30
8	-8	-1	189.08	13.30
-8	-8	1	178.88	14.50
-8	-8	1	199.78	11.60
-8	-8	1	213.28	11.90
8	8	-1	203.68	14.60
-8	8	1	194.98	11.90
-8	8	1	177.28	13.80
7	-8	-1	61.69	7.60
-7	-8	1	70.69	6.00
-7	8	1	73.49	6.60
7	8	-1	70.49	8.60
-7	8	1	58.29	7.80
6	-8	-1	662.03	34.30
-6	-8	1	638.54	35.30
-6	-8	1	710.93	33.20
-6	8	1	666.33	33.70
6	8	-1	723.33	35.10
-6	8	1	677.53	34.60
6	8	-1	641.24	33.10
-5	-8	1	198.68	13.80
-5	-8	1	218.88	11.50
5	-8	-1	219.68	12.90
5	8	-1	223.88	13.70
-5	8	1	218.08	12.90
5	8	-1	209.48	11.50
-5	8	1	218.78	12.10

-4	-8	1	115.29	9.30
4	-8	-1	130.29	8.70
-4	-8	1	130.79	7.10
-4	8	1	131.69	8.50
4	8	-1	128.69	7.20
4	8	-1	123.39	9.30
-4	8	1	123.09	7.70
-3	-8	1	114.29	8.60
3	-8	-1	120.89	7.80
-3	-8	1	125.69	6.80
3	8	-1	119.29	6.80
-3	8	1	136.49	8.10
3	8	-1	112.09	8.60
-3	8	1	130.89	7.50
-2	-8	1	200.38	12.10
2	-8	-1	210.98	11.40
-2	-8	1	214.08	10.60
-2	8	1	199.68	11.10
-2	8	1	224.08	11.70
2	8	-1	201.08	10.50
2	8	-1	202.28	12.10
-1	-8	1	17.60	2.40
-1	-8	1	26.10	3.90
1	8	-1	21.60	3.90
1	8	-1	21.60	2.40
-1	8	1	19.40	3.00
-1	8	1	28.50	3.50
0	-8	-1	1311.07	63.09
0	-8	1	1230.38	62.99
0	8	1	1331.97	63.79
0	8	1	1396.76	63.69
0	8	1	1322.67	63.19
0	8	-1	1303.87	63.69
0	8	-1	1306.57	62.89
1	-8	1	57.39	4.40
-1	-8	-1	66.29	4.20
-1	-8	-1	50.39	5.20
-1	8	-1	64.99	4.70
1	8	1	58.09	4.70
-1	8	-1	54.39	4.10
-1	8	-1	53.69	4.80
-2	-8	-1	1201.98	58.59
-2	-8	-1	1220.88	57.39
2	-8	1	1207.28	57.79

2	8	1	1087.29	57.69
-2	8	-1	1194.78	57.49
2	8	1	1194.58	58.59
-2	8	-1	1262.97	57.99
-3	-8	-1	215.08	11.50
3	-8	1	223.48	12.00
3	-8	1	227.78	12.30
-3	-8	-1	218.08	13.10
-3	8	-1	219.88	12.20
3	8	1	239.98	13.30
-3	8	-1	227.78	11.80
3	8	1	205.18	11.60
-4	-8	-1	712.63	37.60
-4	-8	-1	753.92	36.10
4	-8	1	743.63	36.80
4	-8	1	748.73	36.50
4	8	1	709.43	36.10
-4	8	-1	758.12	36.40
4	8	1	768.62	37.50
-4	8	-1	726.33	36.70
5	-8	1	185.68	10.60
-5	-8	-1	161.98	12.20
5	-8	1	197.58	11.40
-5	-8	-1	197.28	10.20
5	8	1	176.28	11.90
5	8	1	178.38	10.30
-5	8	-1	186.68	10.50
-5	8	-1	168.58	10.90
-6	-8	-1	628.24	28.10
6	-8	1	554.44	28.90
-6	-8	-1	503.85	29.70
6	-8	1	556.54	28.30
6	8	1	531.35	27.90
-6	8	-1	555.34	28.30
-6	8	-1	542.95	28.80
6	8	1	588.64	29.60
7	-8	1	291.97	16.50
-7	-8	-1	299.17	15.50
7	-8	1	278.97	15.70
-7	-8	-1	245.88	17.30
7	8	1	252.17	15.10
-7	8	-1	302.07	15.80
-7	8	-1	274.67	16.50
7	8	1	296.17	17.80

-8	-8	-1	79.29	10.70
8	-8	1	84.89	7.80
-8	-8	-1	106.09	7.90
8	-8	1	89.99	9.10
8	8	1	76.09	11.70
-8	8	-1	95.19	8.00
8	8	1	85.69	7.30
-8	8	-1	87.49	9.30
-9	-8	-1	275.97	20.40
-9	-8	-1	354.26	18.10
-9	-8	-1	318.37	17.70
9	-8	1	309.17	17.90
-9	8	-1	283.17	19.10
-9	8	-1	328.07	18.00
-10	-8	-1	15.20	5.20
10	-8	1	2.60	4.00
-10	-8	-1	3.70	7.40
-10	-8	-1	9.50	4.40
-10	8	-1	6.20	5.50
-10	8	-1	9.30	4.80
10	8	1	6.30	8.80
11	-8	1	183.38	14.10
-11	-8	-1	208.88	14.10
-11	8	-1	214.98	14.30
11	8	1	213.68	18.30
-11	8	-1	183.88	16.20
-12	-8	-1	208.78	15.00
12	-8	1	199.58	15.40
-12	8	-1	218.28	14.90
12	8	1	204.98	18.80
-13	-8	-1	175.28	15.30
-13	8	-1	177.68	14.30
-14	-8	-1	18.20	7.70
-14	8	-1	33.60	7.80
-15	-8	-1	174.18	16.70
-15	8	-1	166.08	15.40
-15	-9	1	-4.00	7.90
-15	9	1	12.70	7.80
-14	-9	1	360.36	23.20
-14	9	1	402.16	23.50
-13	-9	1	93.29	11.50
-13	9	1	129.69	12.20
-12	-9	1	320.17	19.70
-12	9	1	338.57	20.10

12	9	-1	338.77	23.70
-11	-9	1	253.87	16.30
-11	-9	1	223.38	21.60
-11	-9	1	304.37	17.20
-11	9	1	278.37	17.10
11	9	-1	279.87	20.30
-11	9	1	263.47	20.10
-10	-9	1	498.75	32.50
-10	-9	1	569.34	29.20
-10	-9	1	611.34	29.60
-10	9	1	537.45	31.90
10	9	-1	581.64	32.20
-10	9	1	562.94	29.80
-9	-9	1	405.26	26.90
-9	-9	1	449.46	23.60
-9	-9	1	496.15	24.00
-9	9	1	435.06	26.10
-9	9	1	423.16	23.90
9	9	-1	481.45	26.60
-8	-9	1	400.06	25.80
-8	-9	1	485.05	23.20
-8	-9	1	456.65	23.10
-8	9	1	406.96	24.90
8	9	-1	471.65	25.70
-8	9	1	436.16	23.50
-7	-9	1	1358.56	61.59
-7	-9	1	1211.58	63.89
7	-9	-1	1250.97	62.79
-7	9	1	1302.17	61.99
7	9	-1	1375.06	63.59
7	9	-1	1135.29	61.29
-7	9	1	1249.47	63.09
6	-9	-1	38.10	5.90
-6	-9	1	46.60	4.50
-6	-9	1	40.20	7.70
6	9	-1	37.30	4.40
-6	9	1	32.60	6.20
-6	9	1	38.70	5.40
6	9	-1	47.50	7.40
5	-9	-1	2037.10	97.69
-5	-9	1	1806.52	99.59
5	9	-1	2184.68	98.29
-5	9	1	2081.59	97.79
-5	9	1	2054.99	96.99

5	9	-1	1944.91	96.49
-4	-9	1	676.13	33.20
4	-9	-1	700.53	34.40
4	9	-1	645.94	34.20
4	9	-1	732.53	35.10
-4	9	1	679.33	34.40
-4	9	1	662.53	33.70
3	-9	-1	1126.09	57.39
-3	-9	1	1215.78	60.09
-3	-9	1	1150.28	56.49
-3	9	1	1207.28	57.59
3	9	-1	1128.29	56.49
-3	9	1	1174.18	56.99
3	9	-1	1258.17	58.19
-2	-9	1	2.70	3.10
2	-9	-1	4.20	2.50
-2	-9	1	6.60	1.60
2	-9	-1	5.00	2.00
2	9	-1	0.50	2.60
-2	9	1	3.70	2.40
2	9	-1	3.10	1.50
-2	9	1	2.60	2.10
-1	-9	1	2896.61	142.99
1	-9	-1	2943.61	143.39
1	9	-1	3172.38	144.09
-1	9	1	2878.71	143.19
1	9	-1	2946.21	142.89
-1	9	1	3076.69	143.89
-1	9	1	3138.19	143.79
0	-9	-1	18.40	2.70
0	-9	1	10.40	2.10
0	9	1	14.30	2.50
0	9	1	8.40	2.70
0	9	1	12.20	2.60
0	9	-1	10.80	2.40
0	9	-1	8.70	2.60
-1	-9	-1	2891.31	135.29
-1	9	-1	2781.32	134.89
-1	9	-1	2878.21	134.79
1	9	1	2940.11	135.39
1	9	1	2728.73	134.29
-1	9	-1	2701.03	134.19
2	-9	1	360.16	18.20
-2	-9	-1	363.76	19.10

-2	9	-1	339.97	18.20
-2	9	-1	342.67	17.70
2	9	1	358.96	19.20
2	9	1	336.17	17.60
-3	-9	-1	3337.87	154.08
-3	-9	-1	3027.50	152.58
3	-9	1	3307.47	153.18
3	-9	1	3215.88	152.98
3	9	1	3412.06	154.08
-3	9	-1	3254.17	153.18
-3	9	-1	3079.39	152.68
3	9	1	3025.10	152.48
4	-9	1	91.19	6.40
-4	-9	-1	92.99	8.30
-4	-9	-1	90.29	5.90
4	-9	1	93.99	7.30
-4	9	-1	85.09	6.20
-4	9	-1	92.59	6.80
4	9	1	89.59	6.00
4	9	1	101.39	8.60
-5	-9	-1	2934.71	136.89
5	-9	1	2982.30	137.89
5	-9	1	2858.51	137.29
-5	-9	-1	2796.62	138.69
-5	9	-1	2847.71	137.69
5	9	1	2703.03	136.89
5	9	1	3087.19	138.69
-5	9	-1	2757.02	137.19
-6	-9	-1	1408.56	64.19
-6	-9	-1	1285.47	66.09
6	-9	1	1275.07	64.49
6	-9	1	1329.97	65.19
-6	9	-1	1354.16	64.49
6	9	1	1265.57	64.09
-6	9	-1	1293.97	65.09
6	9	1	1390.36	65.79
7	-9	1	672.53	34.30
-7	-9	-1	734.23	34.00
-7	-9	-1	654.23	36.10
7	-9	1	671.33	35.00
7	9	1	632.74	33.80
-7	9	-1	653.33	34.10
7	9	1	748.73	36.10
-7	9	-1	657.03	35.00

8	-9	1	249.88	15.20
-8	-9	-1	244.98	17.90
-8	-9	-1	292.67	15.20
8	9	1	273.57	17.50
-8	9	-1	268.57	16.60
8	9	1	244.28	14.70
-8	9	-1	260.17	15.10
-9	-9	-1	447.76	24.20
-9	-9	-1	473.45	24.20
9	-9	1	445.76	24.40
9	9	1	479.85	27.10
-9	9	-1	426.56	25.70
-9	9	-1	444.66	24.40
10	-9	1	999.30	51.39
-10	-9	-1	854.01	53.49
-10	-9	-1	1030.30	51.19
-10	-9	-1	1143.49	51.39
-10	9	-1	1047.50	51.39
-10	9	-1	989.60	52.89
10	9	1	1091.19	53.59
11	-9	1	103.79	10.70
-11	-9	-1	74.99	15.40
-11	-9	-1	101.19	10.40
-11	9	-1	113.89	10.10
-11	9	-1	114.89	13.30
11	9	1	122.59	14.90
-12	-9	-1	671.63	35.80
-12	9	-1	673.53	36.00
12	9	1	688.03	38.90
-13	-9	-1	20.60	7.00
-13	9	-1	14.00	7.10
-14	-9	-1	512.85	29.80
-14	9	-1	519.45	29.50
-15	-9	-1	2.20	8.60
-15	9	-1	16.40	7.80
-15	-10	1	19.60	8.70
-15	10	1	25.50	8.60
-14	-10	1	11.70	7.40
-14	10	1	17.80	7.60
-13	-10	1	7.70	6.50
-13	10	1	13.80	6.90
-12	-10	1	16.70	5.90
-12	10	1	23.80	6.10
12	10	-1	18.20	8.60

-11	-10	1	48.10	8.90
-11	-10	1	23.20	9.50
-11	-10	1	35.30	6.20
-11	10	1	56.19	8.20
11	10	-1	36.20	8.40
-11	10	1	24.10	8.00
-10	-10	1	0.80	8.00
-10	-10	1	19.90	5.20
-10	10	1	10.80	7.00
-10	10	1	29.20	9.40
10	10	-1	21.40	7.20
-9	-10	1	14.50	4.50
-9	-10	1	22.80	7.80
-9	-10	1	8.80	4.00
-9	10	1	13.60	4.80
9	10	-1	13.80	6.90
-9	10	1	15.40	6.30
-8	-10	1	19.10	4.10
-8	-10	1	23.40	6.60
-8	10	1	19.20	4.50
-8	10	1	10.70	5.00
8	10	-1	19.70	6.00
7	-10	-1	82.99	8.80
-7	-10	1	97.19	11.20
-7	-10	1	94.69	7.10
-7	10	1	102.29	8.00
7	10	-1	80.19	6.90
7	10	-1	111.59	10.80
-7	10	1	96.69	9.60
-6	-10	1	89.79	9.70
6	-10	-1	67.79	8.10
-6	-10	1	67.39	5.60
6	10	-1	66.69	5.70
-6	10	1	66.89	6.60
6	10	-1	92.89	9.10
-6	10	1	80.59	7.80
5	-10	-1	88.89	8.30
-5	-10	1	103.79	9.70
-5	-10	1	97.79	6.20
-5	10	1	94.09	8.00
5	10	-1	90.59	6.30
5	10	-1	104.09	9.30
-5	10	1	83.49	7.10
4	-10	-1	77.59	7.20

-4	-10	1	92.59	8.30
-4	-10	1	90.89	5.50
4	10	-1	81.49	5.80
4	10	-1	102.09	8.70
-4	10	1	86.19	7.20
-4	10	1	78.49	6.20
-3	-10	1	92.49	8.20
-3	-10	1	97.49	5.80
3	-10	-1	87.69	7.30
-3	10	1	95.79	7.30
-3	10	1	91.89	6.40
3	10	-1	110.99	8.60
3	10	-1	97.19	6.40
-2	-10	1	101.39	7.80
2	-10	-1	74.79	6.50
2	-10	-1	80.79	6.40
-2	-10	1	81.79	5.20
-2	10	1	82.09	6.50
-2	10	1	73.79	5.60
-1	-10	1	1.20	1.80
1	-10	-1	1.00	2.50
-1	-10	1	2.20	2.80
-1	10	1	-2.10	2.00
1	10	-1	1.20	2.90
1	10	-1	0.20	1.80
-1	10	1	-3.50	2.60
-1	10	1	-0.70	2.40
0	-10	-1	27.40	3.90
0	10	-1	20.30	3.50
0	10	-1	36.60	5.00
0	10	1	20.70	3.70
0	10	1	37.60	5.40
0	10	1	31.80	4.40
-1	-10	-1	1.50	3.30
1	-10	1	-2.30	2.00
-1	-10	-1	0.40	1.90
-1	10	-1	-0.40	2.00
1	10	1	0.60	1.90
-1	10	-1	-3.40	2.20
-1	10	-1	-3.00	1.70
1	10	1	0.10	2.80
-2	-10	-1	485.85	24.80
2	-10	1	464.35	23.80
-2	-10	-1	460.55	23.20

2	10	1	456.55	23.20
-2	10	-1	440.26	23.30
-2	10	-1	469.85	23.90
2	10	1	492.35	24.90
-3	-10	-1	5.30	3.40
3	-10	1	2.60	2.20
-3	-10	-1	0.90	1.80
3	-10	1	6.50	3.00
-3	10	-1	1.50	2.20
3	10	1	4.20	3.50
3	10	1	1.20	1.90
-3	10	-1	4.50	2.30
-4	-10	-1	5.60	3.90
4	-10	1	5.20	2.40
4	-10	1	0.00	3.10
-4	-10	-1	6.30	2.20
-4	10	-1	-0.10	2.30
4	10	1	3.20	2.20
-4	10	-1	5.50	2.80
4	10	1	8.10	4.40
5	-10	1	11.30	2.90
-5	-10	-1	14.70	2.70
5	-10	1	13.10	3.90
-5	-10	-1	9.50	4.40
-5	10	-1	9.40	3.10
-5	10	-1	11.00	3.10
5	10	1	7.20	4.50
5	10	1	10.90	2.90
6	-10	1	146.49	9.70
-6	-10	-1	144.39	12.50
6	-10	1	151.18	10.90
-6	-10	-1	149.09	9.30
6	10	1	138.49	9.30
-6	10	-1	144.09	10.40
-6	10	-1	155.48	9.90
6	10	1	175.38	12.40
7	-10	1	53.29	8.10
-7	-10	-1	64.69	6.20
7	-10	1	41.10	6.20
7	10	1	59.29	9.10
-7	10	-1	56.69	6.60
7	10	1	48.60	5.90
-7	10	-1	44.80	7.40
-8	-10	-1	-5.10	6.10

8	-10	1	5.60	3.40
-8	-10	-1	1.10	3.60
8	10	1	3.80	3.50
-8	10	-1	3.80	4.10
8	10	1	4.00	5.60
-8	10	-1	0.40	3.50
-9	-10	-1	16.80	4.80
-9	-10	-1	19.90	7.50
9	-10	1	16.90	4.30
9	10	1	26.70	6.50
-9	10	-1	17.20	5.50
-9	10	-1	21.10	5.10
9	10	1	20.80	4.60
10	-10	1	16.00	4.80
-10	-10	-1	25.90	8.60
-10	-10	-1	21.70	5.70
-10	-10	-1	21.30	5.50
10	10	1	21.20	7.70
-10	10	-1	19.80	6.60
-11	-10	-1	7.30	9.10
11	-10	1	7.00	5.00
-11	-10	-1	-3.00	5.00
-11	10	-1	6.30	6.70
-11	10	-1	4.90	5.60
11	10	1	6.90	7.70
-12	-10	-1	16.70	6.40
12	10	1	30.60	8.50
-13	-10	-1	27.70	7.50
-13	10	-1	23.40	7.30
-14	-10	-1	23.10	8.10
-14	10	-1	23.30	7.80
-15	-10	-1	10.90	9.00
-15	10	-1	11.90	7.70
-14	-11	1	289.07	19.60
-14	11	1	282.77	19.60
-13	-11	1	269.47	18.10
-13	11	1	259.47	18.10
-12	-11	1	401.86	22.90
-12	11	1	403.66	23.50
12	11	-1	384.46	26.90
11	-11	-1	450.55	26.80
-11	-11	1	449.36	31.40
-11	-11	1	510.05	26.10
-11	-11	1	478.25	25.80

-11	11	1	479.75	26.40
11	11	-1	484.65	29.40
-11	11	1	450.55	32.80
-10	-11	1	566.94	28.60
-10	-11	1	484.95	32.70
-10	-11	1	609.34	28.80
-10	11	1	566.94	29.20
10	11	-1	556.14	31.90
-10	11	1	463.05	31.00
-9	-11	1	257.67	20.20
-9	-11	1	299.97	16.30
-9	-11	1	295.87	16.20
-9	11	1	265.57	18.80
-9	11	1	268.57	16.70
9	11	-1	285.37	19.30
-8	-11	1	142.59	14.30
-8	-11	1	163.48	9.90
8	11	-1	154.68	13.10
-8	11	1	135.79	12.30
-8	11	1	146.69	10.70
8	11	-1	137.79	9.60
-7	-11	1	1447.76	66.79
-7	-11	1	1284.97	69.49
7	-11	-1	1324.07	68.69
7	11	-1	1304.87	66.59
-7	11	1	1356.66	68.59
-7	11	1	1418.06	67.49
7	11	-1	1518.25	69.29
-6	-11	1	147.69	8.80
6	-11	-1	153.38	11.40
-6	-11	1	133.39	12.60
-6	11	1	144.59	11.00
-6	11	1	148.09	9.60
6	11	-1	140.99	8.90
6	11	-1	152.78	11.80
5	-11	-1	1095.19	54.09
-5	-11	1	1095.19	52.39
-5	-11	1	1033.20	54.79
-5	11	1	1114.29	53.99
5	11	-1	1112.89	54.49
-5	11	1	1084.59	53.09
5	11	-1	1058.39	52.49
-4	-11	1	65.99	4.90
-4	-11	1	67.39	7.90

4	-11	-1	54.99	5.80
4	-11	-1	64.59	7.10
-4	11	1	66.79	6.60
4	11	-1	65.59	8.10
4	11	-1	72.19	5.00
-4	11	1	66.59	5.60
-3	-11	1	2319.17	109.79
3	-11	-1	2238.68	108.89
3	-11	-1	2029.30	108.59
-3	11	1	2418.56	109.19
3	11	-1	2201.48	107.79
-3	11	1	2234.18	108.29
3	11	-1	2387.26	109.69
2	-11	-1	25.80	4.00
-2	-11	1	22.20	3.20
-2	-11	1	30.00	5.50
2	11	-1	17.30	4.10
-2	11	1	20.70	3.40
-2	11	1	19.80	3.50
2	11	-1	22.70	3.20
1	-11	-1	1919.61	91.99
-1	-11	1	1829.52	91.89
-1	-11	1	1908.11	92.59
-1	11	1	1915.41	92.59
1	11	-1	1885.41	91.29
-1	11	1	1996.20	92.49
-1	11	1	1900.51	91.69
1	11	-1	1939.01	92.79
0	-11	1	9.00	2.60
0	-11	-1	19.00	3.20
0	11	1	14.40	2.90
0	11	1	15.70	3.20
0	11	-1	19.90	3.20
0	11	1	11.70	3.50
0	11	-1	13.40	3.40
-1	-11	-1	1839.02	89.89
-1	-11	-1	1951.40	88.89
1	-11	1	1840.72	89.09
1	11	1	1826.02	88.79
-1	11	-1	1933.81	89.59
-1	11	-1	1793.82	88.69
-1	11	-1	1774.62	89.59
1	11	1	1868.51	90.09
-2	-11	-1	69.39	7.40

-2	-11	-1	88.19	5.60
2	-11	1	82.49	6.50
2	11	1	69.09	7.70
2	11	1	87.89	5.70
-2	11	-1	77.69	6.70
-2	11	-1	70.09	5.60
-2	11	-1	79.69	6.60
-3	-11	-1	575.74	28.20
3	-11	1	556.24	29.00
-3	-11	-1	543.05	29.90
3	11	1	562.94	28.20
-3	11	-1	569.14	28.40
3	11	1	594.94	30.10
-3	11	-1	586.14	29.10
-4	-11	-1	794.62	38.90
-4	-11	-1	775.32	41.00
4	-11	1	811.72	40.10
4	-11	1	825.52	39.60
4	11	1	816.52	40.80
-4	11	-1	795.12	39.90
-4	11	-1	787.82	39.30
4	11	1	766.72	39.00
-5	-11	-1	891.41	42.10
-5	-11	-1	826.92	44.30
5	-11	1	870.71	43.40
5	-11	1	863.71	42.70
5	11	1	888.71	44.10
-5	11	-1	843.62	42.50
5	11	1	845.42	42.20
-5	11	-1	856.61	43.20
-6	-11	-1	1011.80	54.19
6	-11	1	1047.40	52.49
-6	-11	-1	1130.19	51.99
6	-11	1	1075.39	53.19
-6	11	-1	1035.10	52.99
6	11	1	1014.20	51.89
-6	11	-1	1067.59	52.39
6	11	1	1133.09	54.09
7	-11	1	338.97	21.00
7	-11	1	371.56	20.30
-7	-11	-1	400.46	19.80
-7	-11	-1	360.46	22.90
7	11	1	352.06	19.60
-7	11	-1	373.26	21.20

-7	11	-1	368.76	20.20
7	11	1	404.66	22.20
-8	-11	-1	201.68	12.20
-8	-11	-1	157.28	15.20
8	-11	1	196.08	12.60
-8	11	-1	198.28	12.60
-8	11	-1	189.58	13.90
8	11	1	211.88	15.20
8	11	1	187.78	12.00
-9	-11	-1	327.77	16.90
-9	-11	-1	244.48	20.00
9	-11	1	281.27	17.10
9	11	1	262.57	16.40
-9	11	-1	278.77	17.10
9	11	1	328.27	19.90
-9	11	-1	284.87	18.70
-10	-11	-1	1128.09	52.59
-10	-11	-1	1122.19	52.79
10	-11	1	1015.70	52.89
-10	-11	-1	940.81	55.79
-10	11	-1	982.00	54.39
10	11	1	1099.59	55.29
-10	11	-1	1061.59	52.69
-11	-11	-1	108.49	17.50
11	-11	1	145.29	12.20
-11	-11	-1	146.99	12.20
-11	11	-1	132.09	11.40
11	11	1	156.18	16.10
-11	11	-1	135.49	14.80
-12	-11	-1	435.56	24.70
12	11	1	395.56	27.90
-12	11	-1	447.36	25.00
-13	-11	-1	18.50	7.40
-13	11	-1	18.70	6.60
-14	-11	-1	273.37	19.70
-14	11	-1	259.27	18.70
-14	-12	1	21.00	8.10
-14	12	1	35.20	8.10
-13	-12	1	167.08	14.70
-13	12	1	176.98	14.50
-12	-12	1	91.29	11.30
-12	12	1	112.59	12.10
12	12	-1	130.19	16.80
-11	-12	1	272.07	16.80

-11	-12	1	268.87	16.70
-11	-12	1	246.98	23.60
11	12	-1	293.67	21.40
-11	12	1	249.78	17.30
-10	-12	1	393.16	21.90
10	-12	-1	379.66	23.20
-10	-12	1	409.26	22.00
-10	-12	1	364.26	27.20
-10	12	1	392.96	25.30
-10	12	1	398.56	22.30
10	12	-1	449.26	26.10
-9	-12	1	93.29	8.60
-9	-12	1	99.59	8.80
-9	-12	1	76.49	12.00
9	12	-1	119.49	13.10
-9	12	1	101.99	11.90
-9	12	1	97.79	8.90
-8	-12	1	93.99	7.70
-8	-12	1	89.69	12.90
-8	12	1	76.89	10.30
8	12	-1	76.59	7.30
-8	12	1	87.69	8.60
8	12	-1	106.39	11.80
-7	-12	1	24.30	6.80
-7	-12	1	16.40	3.70
7	12	-1	18.00	6.00
7	12	-1	14.30	3.70
-7	12	1	13.10	5.00
-7	12	1	17.80	4.30
6	-12	-1	795.82	42.10
-6	-12	1	773.72	43.00
-6	-12	1	841.12	40.10
-6	12	1	846.72	40.90
6	12	-1	887.71	42.80
-6	12	1	798.92	41.90
6	12	-1	784.72	40.10
-5	-12	1	36.30	4.10
-5	-12	1	47.70	8.70
5	-12	-1	22.60	5.20
-5	12	1	33.50	6.30
-5	12	1	32.90	5.40
5	12	-1	41.10	8.00
5	12	-1	36.20	4.40
4	-12	-1	729.13	38.40

-4	-12	1	760.62	36.80
-4	-12	1	757.02	39.40
4	-12	-1	736.23	38.00
4	12	-1	740.33	36.90
-4	12	1	734.23	37.40
4	12	-1	829.12	39.30
-4	12	1	760.92	38.40
3	-12	-1	1.10	2.80
-3	-12	1	9.10	2.20
3	-12	-1	0.40	3.50
-3	-12	1	5.80	3.90
3	12	-1	6.00	2.10
-3	12	1	3.50	2.70
-3	12	1	6.60	3.20
3	12	-1	6.30	3.80
-2	-12	1	539.45	28.60
2	-12	-1	510.75	27.60
-2	-12	1	546.05	26.80
2	12	-1	523.25	26.60
-2	12	1	513.95	27.10
2	12	-1	583.64	28.80
-2	12	1	568.14	28.10
-1	-12	1	-2.60	3.10
1	-12	-1	1.10	2.80
-1	-12	1	-0.50	2.10
-1	12	1	-2.80	3.00
-1	12	1	0.20	2.40
-1	12	1	3.20	3.00
1	12	-1	1.60	2.00
1	12	-1	1.70	3.20
0	-12	1	1196.88	58.79
0	-12	-1	1213.48	58.89
0	12	1	1236.98	58.79
0	12	-1	1207.78	59.39
0	12	1	1207.88	59.59
0	12	1	1235.78	60.09
0	12	-1	1208.68	59.89
0	12	-1	1197.08	58.49
-1	-12	-1	71.89	5.60
1	-12	1	64.29	5.90
-1	12	-1	70.19	6.90
-1	12	-1	76.69	6.50
1	12	1	65.29	5.60
1	12	1	66.09	7.40

-1	12	-1	74.79	5.40
2	-12	1	845.62	41.70
-2	-12	-1	862.81	41.00
-2	-12	-1	816.82	42.60
-2	12	-1	870.31	42.00
2	12	1	867.91	42.90
-2	12	-1	832.52	42.10
2	12	1	813.12	41.00
-2	12	-1	809.12	41.00
-3	-12	-1	70.89	5.40
-3	-12	-1	77.59	8.10
3	-12	1	59.79	6.80
-3	12	-1	68.09	6.60
3	12	1	68.09	8.40
-3	12	-1	73.59	5.80
3	12	1	69.49	5.60
4	-12	1	283.97	16.50
-4	-12	-1	283.57	17.80
4	-12	1	290.77	15.90
-4	-12	-1	295.27	15.10
4	12	1	265.37	15.10
4	12	1	308.17	17.80
-4	12	-1	277.07	16.10
-4	12	-1	279.77	15.50
5	-12	1	36.90	5.80
-5	-12	-1	55.59	8.40
-5	-12	-1	53.59	5.00
5	-12	1	37.00	5.60
5	12	1	45.50	8.60
5	12	1	38.10	5.00
-5	12	-1	30.40	5.40
-5	12	-1	40.40	6.20
6	-12	1	666.93	34.30
-6	-12	-1	707.93	33.70
-6	-12	-1	660.63	36.40
6	-12	1	675.43	35.30
6	12	1	752.82	36.40
-6	12	-1	666.23	34.10
-6	12	-1	637.34	34.90
6	12	1	642.04	33.70
7	-12	1	248.58	14.90
-7	-12	-1	247.58	17.80
-7	-12	-1	264.37	14.40
-7	12	-1	234.38	15.90

7	12	1	226.08	14.20
7	12	1	267.77	17.30
-7	12	-1	264.57	15.00
8	-12	1	214.98	13.50
-8	-12	-1	222.98	13.00
-8	-12	-1	207.58	17.10
8	12	1	188.78	12.70
-8	12	-1	218.08	13.60
-8	12	-1	188.38	14.70
8	12	1	233.38	16.40
-9	-12	-1	275.87	15.40
-9	-12	-1	244.48	19.90
9	-12	1	249.68	15.80
-9	12	-1	234.78	17.30
9	12	1	233.28	14.90
-9	12	-1	249.58	15.70
9	12	1	274.97	18.60
-10	-12	-1	134.39	10.80
10	-12	1	116.29	10.90
-10	-12	-1	102.49	11.10
-10	-12	-1	105.89	16.20
-10	12	-1	119.39	11.20
10	12	1	143.89	14.90
-10	12	-1	125.49	13.40
-11	-12	-1	166.08	13.30
11	12	1	183.68	17.20
-11	12	-1	143.99	13.10
-12	-12	-1	133.59	13.30
-12	12	-1	149.19	13.20
12	12	1	149.59	17.20
-13	-12	-1	234.58	17.90
-13	12	-1	238.98	17.40
-14	-12	-1	-5.40	8.20
-14	12	-1	15.50	7.10
-14	-13	1	54.89	9.20
-14	13	1	36.80	9.10
-13	-13	1	31.30	7.60
-13	13	1	28.60	7.30
-12	-13	1	85.79	11.50
12	13	-1	108.59	17.20
-12	13	1	103.49	11.80
-11	-13	1	229.18	14.50
-11	-13	1	213.08	14.70
11	13	-1	212.48	19.50

-11	13	1	201.48	14.70
-10	-13	1	133.89	18.70
-10	-13	1	149.69	11.80
10	-13	-1	159.68	13.10
-10	-13	1	161.18	11.60
10	13	-1	150.48	16.60
-10	13	1	141.49	15.10
-10	13	1	164.68	12.60
-9	-13	1	114.99	15.80
-9	-13	1	132.69	9.70
9	-13	-1	108.39	13.40
9	13	-1	129.29	14.20
-9	13	1	120.29	10.70
-9	13	1	110.09	13.10
-8	-13	1	4.70	7.20
-8	-13	1	8.00	3.70
-8	13	1	5.00	5.10
8	13	-1	5.90	6.20
8	13	-1	5.70	3.60
-8	13	1	8.90	4.30
-7	-13	1	48.90	5.60
-7	-13	1	35.20	8.50
7	13	-1	29.40	5.40
7	13	-1	38.70	7.60
-7	13	1	33.80	6.70
-7	13	1	39.80	6.10
-6	-13	1	268.77	14.40
-6	-13	1	230.18	17.90
6	-13	-1	310.97	19.00
-6	13	1	281.87	15.50
6	13	-1	246.48	14.40
6	13	-1	276.47	18.00
-6	13	1	262.37	16.80
-5	-13	1	478.55	28.40
-5	-13	1	526.75	25.50
5	-13	-1	501.15	27.70
-5	13	1	527.75	26.40
5	13	-1	503.55	25.50
5	13	-1	498.55	28.10
-5	13	1	520.05	27.40
-4	-13	1	151.58	8.40
4	-13	-1	140.79	9.80
4	-13	-1	128.59	10.70
-4	-13	1	127.09	11.70

-4	13	1	136.19	9.30
4	13	-1	126.69	11.40
-4	13	1	154.68	10.50
4	13	-1	140.79	8.50
-3	-13	1	111.09	6.70
3	-13	-1	93.19	8.50
-3	-13	1	96.99	9.80
-3	13	1	106.49	8.60
3	13	-1	105.69	6.70
3	13	-1	92.09	9.50
-3	13	1	100.29	7.60
-2	-13	1	77.59	8.00
-2	-13	1	76.99	5.60
2	-13	-1	70.09	7.10
2	13	-1	68.19	8.00
-2	13	1	61.79	7.10
-2	13	1	73.09	7.90
2	13	-1	72.59	5.50
-2	13	1	73.79	6.20
-1	-13	1	570.64	30.30
1	-13	-1	580.84	29.40
-1	-13	1	588.94	28.90
-1	13	1	620.54	30.10
1	13	-1	586.44	28.70
1	13	-1	554.04	30.40
-1	13	1	573.04	29.10
-1	13	1	548.55	30.20
0	-13	-1	47.20	5.20
0	-13	1	41.00	5.00
0	13	1	51.69	5.00
0	13	1	41.10	6.00
0	13	-1	48.80	4.80
0	13	-1	46.70	5.90
0	13	1	39.40	6.90
0	13	-1	34.90	6.70
1	-13	1	1234.78	59.69
-1	-13	-1	1242.88	59.39
1	13	1	1246.78	59.39
1	13	1	1260.77	60.29
-1	13	-1	1270.67	60.19
-1	13	-1	1216.68	59.19
-1	13	-1	1128.39	60.29
1	13	1	1186.38	60.79
-2	-13	-1	33.80	7.10

2	-13	1	38.30	5.70
-2	-13	-1	45.10	4.70
2	13	1	41.30	6.20
-2	13	-1	46.70	5.80
-2	13	-1	49.70	4.90
-2	13	-1	34.10	6.30
2	13	1	38.20	4.70
-3	-13	-1	383.06	18.90
-3	-13	-1	343.07	21.00
3	-13	1	357.46	20.00
-3	13	-1	353.86	19.10
-3	13	-1	396.56	20.20
3	13	1	357.16	21.00
3	13	1	364.86	18.90
-4	-13	-1	238.58	16.50
4	-13	1	236.58	15.00
-4	-13	-1	261.37	13.60
4	13	1	250.27	13.60
4	13	1	250.87	16.20
-4	13	-1	261.17	14.10
-4	13	-1	238.28	14.70
5	-13	1	439.46	24.50
-5	-13	-1	454.75	22.80
-5	-13	-1	426.46	25.80
5	-13	1	427.76	23.50
5	13	1	445.36	25.40
-5	13	-1	449.66	23.30
-5	13	-1	458.05	24.20
5	13	1	456.45	22.90
6	-13	1	153.48	12.50
6	-13	1	170.68	11.10
-6	-13	-1	167.58	10.20
-6	-13	-1	163.28	13.90
-6	13	-1	143.99	11.60
6	13	1	154.88	10.30
-6	13	-1	166.78	10.90
6	13	1	171.38	13.50
7	-13	1	303.17	17.30
-7	-13	-1	310.47	16.60
-7	-13	-1	294.37	20.50
7	13	1	293.37	19.60
-7	13	-1	279.57	18.20
-7	13	-1	316.17	17.30
7	13	1	290.27	16.60

-8	-13	-1	148.89	10.20
-8	-13	-1	127.89	14.80
8	-13	1	139.19	10.80
8	13	1	137.39	10.00
-8	13	-1	147.99	10.70
8	13	1	147.79	13.80
-8	13	-1	124.39	12.00
-9	-13	-1	30.50	9.90
-9	-13	-1	23.20	5.20
9	-13	1	28.20	5.90
-9	13	-1	27.60	5.70
-9	13	-1	29.80	7.30
9	13	1	28.70	5.20
9	13	1	26.90	7.80
-10	-13	-1	34.50	10.20
10	-13	1	31.30	5.90
10	13	1	36.00	9.80
-10	13	-1	50.69	8.90
-10	13	-1	28.70	7.50
10	13	1	33.10	5.90
-11	-13	-1	77.69	10.70
-11	13	-1	56.29	9.40
11	13	1	100.29	15.90
-12	-13	-1	157.98	14.40
-12	13	-1	154.98	13.90
-13	-13	-1	19.00	8.00
-13	13	-1	35.60	8.00
-14	-13	-1	112.79	15.20
-14	13	-1	88.79	13.30
-14	14	1	5.90	8.00
-13	-14	1	318.97	20.50
-13	14	1	302.97	20.50
-12	-14	1	154.88	13.90
-12	14	1	159.98	13.70
12	14	-1	169.58	19.80
-11	-14	1	275.77	17.80
-11	14	1	272.07	18.30
11	14	-1	303.67	23.40
-10	-14	1	252.87	15.10
10	-14	-1	243.38	16.50
-10	-14	1	185.48	22.10
-10	-14	1	228.68	15.40
-10	14	1	249.18	15.70
10	14	-1	261.57	20.40

-9	-14	1	883.91	51.79
-9	-14	1	1027.30	47.60
9	-14	-1	912.11	49.50
-9	14	1	905.01	50.39
-9	14	1	995.50	48.40
9	14	-1	1065.89	51.39
9	14	-1	918.61	47.40
-8	-14	1	548.75	34.80
-8	-14	1	639.94	30.50
8	14	-1	544.35	30.30
8	14	-1	676.33	34.40
-8	14	1	613.34	31.50
-8	14	1	578.04	33.10
-7	-14	1	230.68	12.40
-7	-14	1	201.88	17.20
-7	14	1	209.28	13.50
7	14	-1	195.58	12.30
7	14	-1	230.88	16.30
-7	14	1	202.88	15.30
-6	-14	1	1521.15	81.39
-6	-14	1	1648.34	78.19
6	14	-1	1767.32	81.19
-6	14	1	1619.24	80.39
-6	14	1	1641.34	79.09
6	14	-1	1559.04	78.29
5	-14	-1	282.47	18.50
-5	-14	1	317.77	16.10
-5	-14	1	294.97	19.90
-5	14	1	315.27	18.30
-5	14	1	289.67	16.80
5	14	-1	304.37	16.10
5	14	-1	322.97	19.60
4	-14	-1	897.91	47.90
-4	-14	1	933.61	49.10
-4	-14	1	986.90	46.20
4	-14	-1	956.20	47.70
4	14	-1	1020.90	48.90
-4	14	1	933.41	47.90
4	14	-1	928.41	46.20
-4	14	1	943.31	47.00
-3	-14	1	59.49	8.80
3	-14	-1	57.19	7.30
-3	-14	1	59.69	5.20
3	14	-1	58.39	5.20

-3	14	1	51.09	6.00
-3	14	1	60.09	7.50
3	14	-1	58.59	8.50
-2	-14	1	1511.25	71.99
-2	-14	1	1483.95	73.79
2	-14	-1	1468.35	72.89
-2	14	1	1447.96	72.29
2	14	-1	1458.35	71.79
-2	14	1	1506.75	73.59
-2	14	1	1559.84	73.39
2	14	-1	1509.85	73.89
1	-14	-1	71.19	7.00
-1	-14	1	85.99	6.50
-1	14	1	75.29	7.70
1	14	-1	85.39	6.20
-1	14	1	66.29	8.30
1	14	-1	90.89	8.80
-1	14	1	79.39	6.80
0	-14	-1	2329.07	112.89
0	-14	1	2331.27	112.79
0	14	1	2298.17	113.59
0	14	1	2399.16	112.79
0	14	-1	2348.86	113.49
0	14	-1	2367.16	114.09
0	14	-1	2336.67	112.49
0	14	1	2388.36	114.19
-1	-14	-1	102.79	7.60
1	-14	1	115.39	8.20
1	14	1	104.89	7.70
-1	14	-1	100.59	7.50
-1	14	-1	114.99	8.60
1	14	1	118.29	9.00
1	14	1	108.09	10.00
-1	14	-1	103.69	9.20
-2	-14	-1	823.02	39.90
-2	-14	-1	823.22	41.80
2	-14	1	808.12	40.60
2	14	1	791.22	39.80
-2	14	-1	797.22	41.20
2	14	1	841.32	42.00
-2	14	-1	817.42	41.00
-2	14	-1	778.02	39.90
-3	-14	-1	309.07	19.60
3	-14	1	325.17	18.40

-3	-14	-1	338.37	17.00
-3	14	-1	309.97	17.30
-3	14	-1	325.57	18.30
3	14	1	314.07	17.00
-3	14	-1	322.47	18.70
3	14	1	342.47	19.70
4	-14	1	849.12	43.60
-4	-14	-1	893.81	42.20
-4	-14	-1	847.22	44.80
4	14	1	827.02	42.20
-4	14	-1	835.82	42.50
4	14	1	907.41	44.80
-4	14	-1	855.51	43.50
5	-14	1	1130.39	55.59
-5	-14	-1	1158.38	54.69
5	-14	1	1099.09	56.29
-5	-14	-1	1104.49	57.49
-5	14	-1	1096.29	55.99
5	14	1	1110.19	54.79
5	14	1	1196.68	57.39
-5	14	-1	1087.99	55.09
-6	-14	-1	616.84	30.50
-6	-14	-1	571.74	33.80
6	-14	1	631.44	31.50
6	14	1	645.14	33.40
6	14	1	570.94	30.50
-6	14	-1	582.84	32.00
-6	14	-1	610.74	31.10
-7	-14	-1	129.99	14.30
-7	-14	-1	132.59	9.40
7	-14	1	127.09	10.10
-7	14	-1	112.19	11.10
-7	14	-1	129.49	10.10
7	14	1	148.59	13.30
7	14	1	123.59	9.40
8	-14	1	217.38	14.00
-8	-14	-1	192.78	17.80
-8	-14	-1	221.68	13.10
8	14	1	190.18	12.90
-8	14	-1	214.38	13.50
8	14	1	230.18	16.70
-8	14	-1	186.68	15.10
9	-14	1	441.16	24.40
-9	-14	-1	465.75	23.60

-9	-14	-1	392.26	28.40
-9	14	-1	464.15	24.30
-9	14	-1	392.76	25.60
9	14	1	397.66	23.30
9	14	1	476.25	27.50
-10	-14	-1	213.68	21.10
10	-14	1	219.08	18.50
-10	14	-1	229.18	15.20
-10	14	-1	239.88	25.60
10	14	1	265.27	19.80
10	14	1	205.48	14.60
-11	-14	-1	559.24	29.70
11	14	1	505.45	33.10
-11	14	-1	525.55	29.40
-12	-14	-1	37.90	8.10
-12	14	-1	26.80	7.90
-13	-14	-1	267.97	19.40
-13	14	-1	257.97	18.60
-14	-14	-1	20.50	9.40
-14	14	-1	25.40	8.40
-13	-15	1	16.10	7.60
-13	15	1	25.60	7.80
-12	-15	1	8.40	6.90
-12	15	1	21.20	7.30
-11	-15	1	9.40	5.90
-11	15	1	15.40	6.70
11	15	-1	12.30	9.80
-10	-15	1	-0.70	10.40
-10	-15	1	16.50	5.00
-10	-15	1	4.50	5.10
-10	15	1	6.00	5.90
10	15	-1	4.10	8.60
-9	-15	1	14.10	4.50
-9	-15	1	27.10	9.60
9	-15	-1	10.30	5.40
9	15	-1	5.10	4.30
-9	15	1	20.40	7.10
-9	15	1	22.20	6.00
9	15	-1	16.40	8.50
-8	-15	1	44.90	6.40
-8	-15	1	43.10	9.50
-8	15	1	25.50	5.50
-8	15	1	24.70	6.70
8	15	-1	42.20	8.40

8	15	-1	23.40	4.70
-7	-15	1	0.30	6.90
-7	-15	1	2.30	3.40
-7	15	1	-0.80	4.80
7	15	-1	6.00	3.60
7	15	-1	0.30	6.10
-7	15	1	0.60	4.40
-6	-15	1	134.39	14.20
-6	-15	1	118.89	8.20
-6	15	1	107.59	9.00
6	15	-1	111.69	8.30
6	15	-1	120.19	12.30
-6	15	1	125.89	11.40
-5	-15	1	11.80	3.10
5	-15	-1	2.60	3.10
-5	-15	1	4.50	6.20
-5	15	1	8.80	4.40
5	15	-1	8.70	3.00
5	15	-1	10.00	5.70
-5	15	1	5.30	4.70
-4	-15	1	26.10	6.80
-4	-15	1	23.40	3.30
4	-15	-1	7.80	5.80
-4	15	1	7.30	3.90
-4	15	1	21.70	5.20
4	15	-1	20.80	5.80
4	15	-1	15.00	3.20
3	-15	-1	142.19	11.40
-3	-15	1	160.08	12.90
-3	-15	1	159.58	9.40
3	15	-1	152.38	9.30
-3	15	1	147.49	10.30
3	15	-1	157.38	12.70
-3	15	1	163.08	12.30
-3	15	1	162.38	11.60
-2	-15	1	7.20	3.00
-2	-15	1	10.40	4.90
2	-15	-1	6.50	4.00
-2	15	1	11.20	4.70
-2	15	1	9.30	3.60
-2	15	1	9.40	4.10
2	15	-1	12.50	3.00
2	15	-1	6.30	4.80
-1	-15	1	23.90	4.00

1	-15	-1	16.30	4.30
-1	15	1	20.50	4.50
-1	15	1	29.80	5.40
-1	15	1	44.50	7.70
1	15	-1	33.20	6.10
1	15	-1	31.50	4.70
0	-15	-1	27.80	4.30
0	-15	1	18.70	4.10
0	15	-1	24.70	3.80
0	15	1	16.40	5.10
0	15	1	32.30	5.30
0	15	-1	16.40	4.30
0	15	1	13.70	4.30
0	15	-1	30.00	6.00
-1	-15	-1	15.20	3.60
1	-15	1	8.90	3.90
1	15	1	6.50	3.20
-1	15	-1	15.40	4.60
-1	15	-1	7.30	3.30
1	15	1	15.30	4.00
1	15	1	16.50	5.20
-2	-15	-1	3.60	2.90
-2	-15	-1	2.10	4.60
2	-15	1	-3.60	3.70
-2	15	-1	-3.40	2.80
2	15	1	2.00	2.90
-2	15	-1	-1.40	3.40
2	15	1	-4.70	4.30
-2	15	-1	-3.30	3.80
-3	-15	-1	11.10	3.20
3	-15	1	10.60	4.90
-3	-15	-1	11.00	5.10
-3	15	-1	15.00	3.80
-3	15	-1	8.90	4.80
-3	15	-1	14.60	4.20
3	15	1	12.40	3.20
3	15	1	17.40	5.60
-4	-15	-1	9.00	3.10
-4	-15	-1	10.70	5.80
4	-15	1	2.40	4.90
-4	15	-1	10.10	3.60
4	15	1	11.90	5.70
-4	15	-1	5.90	3.70
4	15	1	8.20	3.20

5	-15	1	15.40	6.00
-5	-15	-1	19.00	6.60
-5	15	-1	17.60	4.40
5	15	1	31.70	5.50
-5	15	-1	15.70	4.70
5	15	1	26.50	6.90
-6	-15	-1	47.80	8.60
-6	-15	-1	51.99	6.30
6	-15	1	69.89	7.80
6	15	1	52.99	6.70
-6	15	-1	49.00	8.30
6	15	1	40.60	7.80
-6	15	-1	56.19	7.40
7	-15	1	10.30	4.30
-7	-15	-1	-2.60	7.60
-7	-15	-1	14.80	4.00
-7	15	-1	13.40	5.30
7	15	1	8.50	4.10
7	15	1	12.80	6.80
-7	15	-1	17.20	4.90
-8	-15	-1	5.20	4.20
-8	-15	-1	1.10	7.70
8	-15	1	1.50	4.20
8	15	1	1.90	4.00
-8	15	-1	3.70	5.80
8	15	1	-5.30	6.40
-8	15	-1	1.10	5.00
-9	-15	-1	25.70	10.30
-9	-15	-1	13.10	5.10
9	-15	1	3.50	4.80
-9	15	-1	14.60	6.50
9	15	1	16.10	7.80
-9	15	-1	23.40	6.30
9	15	1	11.90	4.80
10	15	1	-1.60	8.80
10	15	1	5.70	5.00
-10	15	-1	9.70	6.20
-11	-15	-1	17.40	7.10
11	15	1	40.10	10.60
-11	15	-1	19.20	6.40
-12	-15	-1	12.10	7.80
-12	15	-1	12.70	6.90
-13	-15	-1	6.50	8.10
-13	15	-1	11.40	7.00

-13	16	1	240.78	18.40
-12	-16	1	352.56	21.80
-12	16	1	354.46	22.30
-11	-16	1	131.09	12.60
-11	16	1	127.29	12.00
11	16	-1	118.89	19.20
-10	-16	1	218.18	15.10
-10	16	1	216.08	15.70
10	16	-1	224.28	20.30
-9	-16	1	215.38	21.20
9	-16	-1	200.58	16.30
-9	-16	1	228.38	13.40
-9	16	1	218.28	14.40
9	16	-1	226.58	18.90
9	16	-1	183.18	13.20
-8	-16	1	364.76	18.90
-8	-16	1	315.97	24.80
8	-16	-1	339.87	23.70
-8	16	1	331.57	22.40
8	16	-1	371.66	23.20
-8	16	1	359.56	20.40
8	16	-1	331.57	18.90
-7	-16	1	10.20	3.80
-7	-16	1	-3.30	7.40
-7	16	1	13.70	6.20
7	16	-1	14.30	7.20
-7	16	1	8.70	5.60
7	16	-1	14.60	3.90
-6	-16	1	811.62	38.90
-6	-16	1	761.12	43.10
6	16	-1	833.82	42.40
-6	16	1	752.52	41.30
-6	16	1	797.62	40.00
6	16	-1	766.62	39.00
-5	-16	1	83.89	7.00
-5	-16	1	96.59	12.60
-5	16	1	97.09	8.60
-5	16	1	86.49	10.20
5	16	-1	90.69	11.40
5	16	-1	83.69	7.10
-4	-16	1	460.05	29.90
-4	-16	1	546.55	26.80
4	-16	-1	593.04	34.60
-4	16	1	550.94	29.10

4	16	-1	513.15	26.80
-4	16	1	535.55	27.80
4	16	-1	553.14	30.20
3	-16	-1	182.28	13.40
-3	-16	1	199.88	11.30
-3	-16	1	169.08	14.20
-3	16	1	198.48	14.40
3	16	-1	195.38	14.60
-3	16	1	211.38	13.60
-3	16	1	187.38	12.10
3	16	-1	191.68	11.20
-2	-16	1	239.08	13.20
-2	-16	1	231.38	15.80
2	-16	-1	227.18	14.60
-2	16	1	218.78	13.70
-2	16	1	222.18	15.60
2	16	-1	239.28	13.00
-2	16	1	240.38	15.00
2	16	-1	230.98	16.00
-1	-16	1	87.79	7.40
1	-16	-1	76.39	8.20
1	16	-1	83.49	6.90
1	16	-1	86.59	8.70
-1	16	1	86.89	9.10
1	16	-1	95.39	10.00
-1	16	1	86.99	7.60
-1	16	1	78.89	9.60
0	-16	1	2550.84	121.59
0	-16	-1	2526.85	121.69
0	16	1	2558.84	122.59
0	16	-1	2464.85	121.19
0	16	1	2518.25	123.29
0	16	-1	2489.25	122.99
0	16	-1	2553.84	122.49
0	16	1	2580.84	121.59
1	-16	1	0.50	4.00
-1	-16	-1	11.60	4.00
-1	16	-1	8.50	4.10
1	16	1	12.40	5.90
-1	16	-1	9.80	5.10
1	16	1	9.80	3.70
-1	16	-1	17.20	3.80
1	16	1	17.20	5.10
2	-16	1	569.34	29.90

-2	-16	-1	572.74	28.90
-2	16	-1	572.24	30.80
-2	16	-1	563.44	29.00
2	16	1	563.44	29.00
2	16	1	586.74	31.50
-2	16	-1	569.44	30.20
-3	-16	-1	91.49	10.90
3	-16	1	86.19	9.50
-3	-16	-1	103.49	7.50
-3	16	-1	97.89	7.90
3	16	1	92.79	10.80
-3	16	-1	98.29	9.10
3	16	1	92.79	7.50
-3	16	-1	95.69	9.90
4	-16	1	871.91	47.20
-4	-16	-1	968.50	45.70
-4	-16	-1	869.21	48.30
4	16	1	928.61	45.80
-4	16	-1	931.51	47.20
-4	16	-1	962.40	47.70
-4	16	-1	901.01	46.10
4	16	1	999.90	48.80
-5	-16	-1	359.26	18.10
-5	-16	-1	314.37	21.80
5	16	1	347.77	21.40
5	16	1	328.57	18.20
-5	16	-1	328.37	18.60
-5	16	-1	324.07	19.80
-6	-16	-1	603.64	35.10
-6	-16	-1	639.34	31.30
6	16	1	661.43	34.80
-6	16	-1	566.64	33.00
6	16	1	602.54	31.50
-6	16	-1	637.94	32.10
7	-16	1	148.89	11.30
-7	-16	-1	135.49	9.70
-7	-16	-1	99.89	15.50
7	16	1	145.09	14.20
7	16	1	124.09	9.90
-7	16	-1	121.79	11.90
-7	16	-1	139.99	10.90
-8	-16	-1	123.59	9.50
-8	-16	-1	99.89	16.20
8	-16	1	113.09	11.00

-8	16	-1	100.19	12.10
8	16	1	99.69	9.40
-8	16	-1	124.69	10.70
8	16	1	127.39	14.70
-9	-16	-1	531.05	27.50
-9	-16	-1	481.05	33.40
9	16	1	546.35	31.80
-9	16	-1	543.55	28.50
9	16	1	492.25	27.60
-10	16	-1	122.89	12.00
10	16	1	130.99	17.30
10	16	1	116.19	11.00
-11	-16	-1	386.06	22.90
11	16	1	338.27	26.80
-11	16	-1	363.86	22.50
-12	16	-1	75.39	12.00
-13	-16	-1	202.38	17.50
-13	16	-1	176.68	16.70
-12	17	1	234.98	17.70
-11	-17	1	105.19	12.20
11	17	-1	111.89	19.40
-11	17	1	107.19	12.50
-10	17	1	158.48	13.70
10	17	-1	173.78	18.70
-9	-17	1	206.08	22.00
-9	-17	1	191.88	17.50
-9	17	1	247.48	16.00
9	17	-1	203.48	13.90
9	17	-1	261.87	20.00
-8	-17	1	211.98	12.60
-8	-17	1	174.68	20.80
8	-17	-1	186.48	16.10
-8	17	1	204.08	23.50
8	17	-1	235.08	18.00
-8	17	1	205.38	14.20
8	17	-1	192.58	12.80
-7	-17	1	440.46	31.90
-7	-17	1	500.55	25.20
7	17	-1	473.25	25.40
7	17	-1	516.55	29.60
-7	17	1	490.05	28.40
-7	17	1	503.65	26.60
-6	-17	1	326.57	23.30
-6	-17	1	336.47	17.90

-6	17	1	339.07	19.30
6	17	-1	373.96	22.70
6	17	-1	327.57	18.10
-6	17	1	309.37	20.80
-5	-17	1	946.21	51.29
-5	-17	1	966.50	47.40
-5	17	1	942.01	49.80
-5	17	1	970.00	48.50
5	17	-1	903.41	47.50
5	17	-1	1101.09	51.39
-4	-17	1	0.40	3.30
-4	-17	1	7.20	5.90
4	17	-1	0.10	3.10
4	17	-1	2.50	6.40
-4	17	1	2.10	4.00
-4	17	1	-0.90	4.70
-4	17	1	0.40	5.50
-3	-17	1	960.70	51.39
3	-17	-1	959.60	50.69
-3	-17	1	1003.20	48.70
3	17	-1	1039.10	51.59
-3	17	1	1007.70	50.69
3	17	-1	966.00	48.60
-3	17	1	1004.90	49.50
-3	17	1	1012.10	51.19
2	-17	-1	1.00	4.80
-2	-17	1	-7.50	3.10
2	17	-1	-3.20	5.10
-2	17	1	1.40	4.50
2	17	-1	-0.10	2.90
-2	17	1	-2.70	4.00
-2	17	1	-4.70	5.10
-1	-17	1	459.15	23.90
1	-17	-1	438.96	24.50
1	17	-1	486.55	26.30
1	17	-1	441.16	25.00
-1	17	1	452.95	25.30
-1	17	1	454.35	26.10
1	17	-1	440.06	23.50
-1	17	1	468.65	24.10
0	-17	-1	277.47	16.80
0	-17	1	288.97	16.80
0	17	-1	303.07	18.80
0	17	1	292.77	16.60

0	17	-1	285.47	16.10
0	17	1	316.47	19.10
0	17	-1	281.97	17.50
0	17	1	288.97	17.80
-1	-17	-1	866.41	44.40
1	-17	1	871.01	44.90
-1	17	-1	874.21	45.50
-1	17	-1	886.01	44.30
-1	17	-1	914.41	46.40
1	17	1	963.10	47.00
1	17	1	874.31	45.60
1	17	1	913.61	44.40
2	-17	1	296.17	18.00
-2	-17	-1	303.07	16.80
-2	17	-1	291.37	18.90
-2	17	-1	309.47	18.20
-2	17	-1	304.67	16.90
2	17	1	309.87	16.70
2	17	1	305.17	20.00
-3	-17	-1	57.49	6.50
-3	-17	-1	58.99	10.10
3	-17	1	51.99	9.20
-3	17	-1	45.10	6.90
-3	17	-1	60.29	8.20
3	17	1	56.59	6.40
-3	17	-1	63.19	9.00
3	17	1	57.89	9.90
-4	-17	-1	226.38	17.70
-4	-17	-1	244.88	13.60
-4	17	-1	239.68	15.40
4	17	1	258.57	17.90
-4	17	-1	217.48	14.10
4	17	1	222.38	13.70
-4	17	-1	235.58	16.30
-5	-17	-1	248.08	13.70
-5	-17	-1	238.98	18.30
-5	17	-1	222.68	15.60
5	17	1	218.88	13.90
5	17	1	252.47	18.10
-5	17	-1	230.78	14.60
-6	-17	-1	72.99	7.50
-6	-17	-1	83.09	13.40
-6	17	-1	81.69	9.90
6	17	1	95.79	12.90

6	17	1	71.29	7.80
-6	17	-1	73.29	8.40
-7	-17	-1	19.90	8.80
-7	-17	-1	3.70	4.00
-7	17	-1	7.80	5.80
-7	17	-1	11.40	6.20
7	17	1	20.90	8.50
7	17	1	8.40	4.40
8	-17	1	210.48	15.00
-8	-17	-1	191.48	20.50
-8	-17	-1	213.68	13.40
-8	17	-1	208.18	14.50
-8	17	-1	187.78	16.10
8	17	1	253.47	18.90
8	17	1	195.78	13.50
-9	-17	-1	5.60	10.00
-9	17	-1	6.70	6.00
9	17	1	4.50	5.00
9	17	1	14.00	8.90
10	17	1	97.09	10.70
10	17	1	117.89	17.30
-10	17	-1	113.19	12.10
-11	17	-1	23.50	7.00
-12	-17	-1	86.19	13.40
-12	17	-1	57.49	9.40
-12	18	1	113.09	13.60
-11	-18	1	62.99	11.40
-11	18	1	71.69	11.90
-10	18	1	201.58	15.40
10	18	-1	170.98	19.90
9	18	-1	12.30	9.10
-9	18	1	28.30	7.00
9	18	-1	14.20	5.10
-8	-18	1	25.70	4.80
8	18	-1	23.80	5.00
8	18	-1	23.50	9.00
-8	18	1	25.70	6.80
-7	-18	1	15.20	4.00
-7	-18	1	18.50	9.30
7	18	-1	6.70	7.40
-7	18	1	2.50	5.90
7	18	-1	6.00	4.20
-7	18	1	14.90	5.60
-6	-18	1	60.69	10.70

-6	-18	1	94.39	7.60
6	18	-1	83.19	7.70
-6	18	1	56.19	10.90
-6	18	1	77.99	11.90
6	18	-1	77.09	13.20
-5	-18	1	14.70	7.80
-5	-18	1	31.70	5.60
5	18	-1	24.80	4.40
5	18	-1	17.40	7.20
-5	18	1	21.80	5.40
-5	18	1	14.20	6.00
-4	-18	1	341.77	17.20
-4	-18	1	259.57	21.00
-4	18	1	303.77	19.70
-4	18	1	318.57	18.40
-4	18	1	334.77	20.90
4	18	-1	330.67	21.50
4	18	-1	307.87	17.10
-3	-18	1	4.30	3.60
-3	-18	1	1.80	6.00
-3	18	1	1.50	5.60
-3	18	1	0.20	4.70
-3	18	1	-0.30	4.50
3	18	-1	7.80	3.40
3	18	-1	3.00	6.10
-2	-18	1	16.60	4.10
2	-18	-1	7.90	5.50
2	18	-1	15.70	3.90
2	18	-1	6.30	5.70
-2	18	1	14.10	6.50
-2	18	1	15.90	5.30
-2	18	1	15.20	4.80
-1	-18	1	75.29	7.80
1	-18	-1	62.39	9.10
-1	18	1	75.99	7.90
-1	18	1	71.29	10.80
-1	18	1	55.19	9.00
1	18	-1	64.39	8.80
1	18	-1	69.59	10.90
1	18	-1	73.19	7.10
0	-18	1	785.72	38.20
0	-18	-1	752.22	38.20
0	18	1	719.03	40.00
0	18	-1	761.72	37.60

0	18	1	774.42	39.30
0	18	1	745.93	37.90
0	18	-1	711.73	39.80
0	18	-1	745.63	39.00
-1	-18	-1	4.80	4.00
1	-18	1	3.90	4.70
-1	18	-1	5.30	4.80
-1	18	-1	-2.20	5.40
1	18	1	1.30	5.00
1	18	1	11.60	6.70
1	18	1	6.10	4.10
-1	18	-1	7.10	3.90
2	-18	1	325.27	19.00
-2	-18	-1	328.77	17.60
2	18	1	324.67	17.60
2	18	1	302.17	18.90
2	18	1	297.67	20.40
-2	18	-1	289.97	19.80
-2	18	-1	308.87	17.60
-2	18	-1	314.77	19.10
3	-18	1	4.30	9.60
-3	-18	-1	5.30	3.80
-3	18	-1	6.00	4.20
-3	18	-1	5.90	4.50
3	18	1	0.60	3.70
-3	18	-1	0.60	5.10
3	18	1	-5.80	5.90
-4	-18	-1	76.29	7.10
-4	-18	-1	74.79	12.10
4	18	1	59.19	9.80
-4	18	-1	52.19	7.80
-4	18	-1	51.69	8.80
4	18	1	50.99	7.00
-4	18	-1	69.09	10.30
-5	-18	-1	106.99	8.40
-5	-18	-1	76.59	13.50
-5	18	-1	103.99	12.10
-5	18	-1	103.39	10.90
-5	18	-1	97.59	9.40
5	18	1	102.59	13.60
5	18	1	104.69	8.80
-6	-18	-1	282.77	15.80
-6	-18	-1	277.97	21.40
6	18	1	268.87	16.00

6	18	1	306.97	20.60
-6	18	-1	264.87	17.40
-6	18	-1	257.37	18.00
-7	-18	-1	82.89	15.20
-7	-18	-1	92.49	9.00
7	18	1	109.09	14.30
-7	18	-1	107.29	10.00
-7	18	-1	82.79	11.40
7	18	1	96.89	9.30
-8	-18	-1	96.59	8.70
-8	-18	-1	87.19	17.90
8	18	1	83.19	14.70
8	18	1	80.29	9.20
-8	18	-1	83.89	9.40
9	18	1	82.59	9.70
9	18	1	83.49	16.30
-9	18	-1	107.69	11.40
10	18	1	9.20	10.10
-10	18	-1	10.50	6.70
10	18	1	12.70	5.90
-11	18	-1	58.39	9.30
-12	18	-1	29.30	8.00
-11	19	1	211.68	16.70
-10	19	1	501.35	28.50
10	19	-1	495.45	33.40
9	19	-1	230.28	21.50
9	19	-1	186.28	13.40
-9	19	1	210.88	15.50
-8	19	1	267.27	17.60
8	19	-1	256.57	16.00
8	19	-1	301.17	22.60
-7	-19	1	746.03	37.70
-7	19	1	771.72	39.30
7	19	-1	814.82	42.90
7	19	-1	704.43	37.90
-6	-19	1	22.90	4.20
-6	19	1	23.60	5.90
6	19	-1	17.90	4.70
6	19	-1	34.10	9.40
-6	19	1	17.70	6.90
-5	-19	1	730.33	36.30
5	19	-1	693.33	36.40
-5	19	1	718.43	39.10
5	19	-1	773.42	40.60

-4	-19	1	31.50	4.70
-4	19	1	24.60	6.40
4	19	-1	35.40	8.50
-4	19	1	22.10	7.20
4	19	-1	35.50	5.90
-4	19	1	20.10	5.60
-3	-19	1	905.01	44.60
-3	19	1	878.81	46.80
3	19	-1	946.31	48.10
-3	19	1	873.31	45.40
-3	19	1	952.30	47.90
3	19	-1	864.71	44.50
-2	-19	1	5.80	4.20
-2	19	1	15.40	6.50
2	19	-1	19.00	6.80
-2	19	1	9.30	5.10
-2	19	1	16.30	5.80
2	19	-1	9.30	5.40
2	19	-1	7.80	4.00
1	-19	-1	2107.19	96.49
-1	-19	1	1862.61	91.49
-1	19	1	1892.71	93.89
1	19	-1	1750.62	92.59
-1	19	1	1912.01	91.69
1	19	-1	1868.01	91.19
-1	19	1	1804.72	92.89
1	19	-1	1929.81	93.89
0	-19	1	80.79	9.00
0	-19	-1	75.19	9.10
0	19	1	94.99	12.30
0	19	-1	80.79	9.50
0	19	1	93.09	8.70
0	19	1	73.79	9.90
0	19	-1	88.29	11.80
0	19	-1	91.39	8.10
1	-19	1	750.72	41.10
-1	-19	-1	796.32	40.60
-1	19	-1	827.82	40.50
1	19	1	860.11	43.60
-1	19	-1	758.72	41.80
-1	19	-1	822.72	43.10
1	19	1	821.02	40.60
-2	-19	-1	2.80	4.10
2	-19	1	0.30	5.80

-2	19	-1	5.70	4.90
2	19	1	-1.10	6.20
2	19	1	-1.30	4.50
2	19	1	11.50	4.40
-2	19	-1	6.40	6.50
-2	19	-1	4.00	4.40
-3	-19	-1	584.94	29.50
-3	19	-1	567.94	31.40
-3	19	-1	562.54	30.00
3	19	1	556.64	29.60
-3	19	-1	598.74	32.50
3	19	1	589.24	33.20
-4	-19	-1	523.25	32.30
-4	-19	-1	564.64	28.30
-4	19	-1	508.25	34.40
4	19	1	558.14	28.50
-4	19	-1	560.14	29.00
4	19	1	589.54	32.50
-4	19	-1	537.35	30.30
-5	-19	-1	556.84	36.30
-5	-19	-1	635.34	32.10
-5	19	-1	628.14	32.90
-5	19	-1	662.13	35.40
-5	19	-1	604.24	34.20
5	19	1	631.34	32.40
5	19	1	715.03	36.80
-6	-19	-1	392.36	21.50
-6	-19	-1	387.76	26.70
6	19	1	400.76	21.80
-6	19	-1	383.96	23.90
6	19	1	420.86	26.10
-6	19	-1	397.66	22.40
-7	-19	-1	105.39	9.20
-7	-19	-1	119.69	18.20
7	19	1	119.79	16.10
-7	19	-1	115.79	11.00
7	19	1	109.09	10.10
-8	-19	-1	263.77	34.70
8	19	1	247.18	15.70
-8	19	-1	250.57	16.90
8	19	1	258.87	21.70
9	19	1	55.59	9.30
9	19	1	70.69	12.40
-9	19	-1	72.49	10.60

10	19	1	282.57	18.50
10	19	1	303.97	26.60
-10	19	-1	311.87	19.80
-11	19	-1	69.69	11.90
11	19	1	39.00	7.70
-11	20	1	15.60	7.80
-10	20	1	20.00	7.20
10	20	-1	8.60	11.90
9	20	-1	9.80	5.00
9	20	-1	16.00	10.70
-9	20	1	20.80	7.20
-8	20	1	9.30	6.60
8	20	-1	11.40	9.70
8	20	-1	1.40	4.50
7	20	-1	17.70	4.90
-7	20	1	22.80	6.80
7	20	-1	16.30	9.30
-6	-20	1	62.19	7.40
6	20	-1	56.29	7.90
6	20	-1	80.19	14.60
-6	20	1	75.29	9.90
-5	-20	1	10.50	4.00
-5	20	1	1.80	5.00
-5	20	1	6.70	9.20
5	20	-1	6.80	7.60
-5	20	1	7.60	7.10
5	20	-1	2.10	4.00
-4	-20	1	70.49	7.80
-4	20	1	78.09	11.20
4	20	-1	89.49	13.80
-4	20	1	65.89	9.50
4	20	-1	74.79	7.60
-3	-20	1	80.29	8.00
3	20	-1	77.59	12.80
3	20	-1	92.69	8.10
-3	20	1	75.09	12.90
-3	20	1	85.29	11.60
-3	20	1	79.99	9.70
-2	-20	1	201.08	13.70
2	20	-1	265.27	18.30
-2	20	1	236.88	17.80
-2	20	1	232.68	14.60
2	20	-1	209.58	13.40
-2	20	1	197.28	15.70

2	20	-1	213.48	15.80
-1	-20	1	-0.20	4.50
-1	20	1	-10.60	6.20
-1	20	1	-3.80	5.10
1	20	-1	-2.60	5.20
1	20	-1	3.80	4.00
1	20	-1	-7.20	6.90
-1	20	1	2.70	4.70
0	-20	1	19.00	5.60
0	20	-1	13.00	6.50
0	20	1	6.40	5.20
0	20	-1	13.90	4.60
0	20	1	8.30	4.80
0	20	1	6.50	6.70
0	20	-1	17.10	5.60
1	-20	1	78.89	11.00
-1	-20	-1	109.19	9.60
1	20	1	93.29	9.20
1	20	1	100.09	11.10
1	20	1	84.69	13.00
-1	20	-1	89.29	8.70
-1	20	-1	85.29	12.40
-1	20	-1	70.99	10.70
-2	-20	-1	7.00	4.30
-2	20	-1	4.70	4.70
-2	20	-1	11.90	6.60
-2	20	-1	8.10	5.10
2	20	1	12.90	4.70
2	20	1	15.70	7.60
2	20	1	12.10	6.20
-3	-20	-1	42.50	7.10
3	20	1	37.10	6.90
-3	20	-1	28.50	5.60
-3	20	-1	20.10	5.90
3	20	1	25.50	8.30
3	20	1	23.70	6.50
-4	-20	-1	14.50	4.60
4	20	1	11.40	7.70
4	20	1	9.10	4.60
-4	20	-1	8.30	5.00
-4	20	-1	7.80	6.90
-4	20	-1	12.10	5.50
-5	-20	-1	20.00	4.50
5	20	1	14.30	4.80

5	20	1	28.40	9.30
-5	20	-1	15.00	6.40
-5	20	-1	31.20	7.10
-5	20	-1	15.20	7.10
-6	-20	-1	15.90	4.70
-6	20	-1	14.10	7.70
6	20	1	3.70	8.10
6	20	1	3.40	4.60
-6	20	-1	19.50	5.80
7	20	1	86.79	9.50
-7	20	-1	85.49	10.70
7	20	1	102.69	15.60
8	20	1	6.30	9.60
8	20	1	5.30	5.40
-8	20	-1	15.90	6.50
9	20	1	13.40	5.90
9	20	1	5.20	10.00
-9	20	-1	11.70	7.20
10	20	1	10.40	5.80
-10	20	-1	11.70	7.10
-11	20	-1	24.10	8.20
-10	21	1	203.28	16.60
9	21	-1	234.08	24.90
-9	21	1	196.88	16.50
8	21	-1	112.99	18.80
8	21	-1	92.39	9.90
-8	21	1	127.29	12.80
7	21	-1	256.37	22.20
7	21	-1	245.48	15.70
-7	21	1	279.97	17.50
-6	21	1	124.49	12.00
6	21	-1	120.49	10.10
6	21	-1	119.99	16.40
-6	21	1	135.39	16.40
-5	-21	1	572.44	29.40
5	21	-1	554.04	29.60
-5	21	1	592.14	31.10
-5	21	1	579.84	34.30
5	21	-1	576.24	34.20
-4	-21	1	134.39	10.10
4	21	-1	135.89	10.10
-4	21	1	142.89	15.60
4	21	-1	136.89	15.90
-4	21	1	120.09	11.90

-3	-21	1	412.66	23.10
3	21	-1	434.76	27.50
3	21	-1	428.86	23.00
-3	21	1	438.56	27.00
-3	21	1	455.95	27.90
-3	21	1	423.16	24.40
-2	-21	1	2.10	4.80
-2	21	1	5.40	5.80
-2	21	1	2.90	7.20
2	21	-1	5.20	4.50
-2	21	1	9.30	5.70
2	21	-1	-1.10	7.00
-1	-21	1	775.92	42.00
1	21	-1	813.72	43.60
-1	21	1	794.82	43.80
-1	21	1	857.61	42.50
1	21	-1	874.21	45.40
-1	21	1	845.42	45.20
1	21	-1	858.31	41.80
0	21	-1	34.40	7.00
0	21	-1	51.19	7.90
0	21	1	51.79	6.80
0	21	1	26.90	10.50
0	21	1	43.80	9.30
0	21	-1	34.10	8.30
1	21	1	707.73	35.20
1	21	1	701.63	38.50
-1	21	-1	699.33	35.10
1	21	1	632.24	36.60
-1	21	-1	660.23	36.70
2	21	1	47.30	7.60
2	21	1	39.40	7.40
-2	21	-1	39.30	8.40
-2	21	-1	39.10	7.20
-2	21	-1	34.60	6.30
2	21	1	48.10	9.90
3	21	1	923.41	46.00
3	21	1	966.10	49.90
3	21	1	876.51	47.90
-3	21	-1	918.61	49.00
-3	21	-1	913.81	46.30
-3	21	-1	912.71	48.40
-4	21	-1	176.18	16.50
4	21	1	169.58	17.80

4	21	1	172.88	12.30
5	21	1	414.06	23.00
-5	21	-1	427.56	27.00
-5	21	-1	417.96	23.70
5	21	1	421.66	28.20
-6	21	-1	405.76	25.80
6	21	1	356.86	21.20
6	21	1	392.36	26.90
-6	21	-1	374.86	22.10
7	21	1	287.67	23.70
-7	21	-1	325.27	19.20
-7	21	-1	290.47	22.50
7	21	1	273.97	17.80
8	21	1	41.10	6.90
8	21	1	38.60	11.80
-8	21	-1	44.20	7.90
-9	21	-1	27.30	7.50
9	21	1	14.40	6.10
9	21	1	30.00	11.60
-10	21	-1	405.86	24.90
9	22	-1	46.00	12.80
-9	22	1	46.20	8.60
-8	22	1	149.39	14.30
8	22	-1	151.58	21.20
-7	22	1	67.19	12.60
7	22	-1	50.99	8.60
7	22	-1	52.59	17.60
-7	22	1	61.69	11.00
6	22	-1	240.08	15.90
-6	22	1	294.07	22.60
-6	22	1	277.77	18.10
-5	22	1	86.99	11.20
5	22	-1	78.59	17.50
5	22	-1	93.79	9.00
-5	22	1	78.39	15.00
-4	-22	1	169.28	13.00
4	22	-1	205.48	21.80
-4	22	1	209.38	18.60
-4	22	1	183.48	14.20
4	22	-1	186.28	12.50
-3	-22	1	-7.20	4.30
-3	22	1	-4.50	5.50
3	22	-1	-7.90	12.00
-3	22	1	-13.30	6.90

3	22	-1	0.40	4.20
-2	-22	1	216.98	14.80
-2	22	1	245.88	19.70
-2	22	1	218.18	16.40
2	22	-1	226.98	14.30
1	22	-1	4.80	4.80
-1	22	1	-0.60	10.00
1	22	-1	3.10	8.50
-1	22	1	1.00	5.60
0	22	1	393.06	22.80
0	22	-1	423.36	26.70
0	22	-1	388.36	22.50
0	22	1	425.46	27.10
-1	22	-1	49.10	8.60
1	22	1	38.60	9.50
-1	22	-1	46.50	9.00
1	22	1	51.99	7.10
2	22	1	446.86	28.30
-2	22	-1	432.96	23.90
2	22	1	411.96	23.60
-2	22	-1	441.76	27.50
3	22	1	60.29	8.80
3	22	1	78.39	12.20
-3	22	-1	69.39	13.50
-3	22	-1	67.49	12.40
-4	22	-1	186.38	14.50
4	22	1	199.38	19.40
4	22	1	207.18	13.80
-4	22	-1	224.88	18.50
5	22	1	8.30	5.30
5	22	1	12.40	8.90
-5	22	-1	16.10	6.00
-5	22	-1	12.60	7.90
-6	22	-1	220.68	15.60
-6	22	-1	232.18	19.80
6	22	1	224.58	20.80
6	22	1	189.08	14.40
7	22	1	91.49	11.00
7	22	1	124.39	18.60
-7	22	-1	113.49	12.20
-7	22	-1	118.29	16.90
-8	22	-1	133.59	13.20
8	22	1	110.59	11.70
-8	22	-1	129.49	18.40

8	22	1	133.69	20.40
-9	22	-1	89.89	12.80
9	23	-1	21.00	19.70
-9	23	1	24.10	8.70
8	23	-1	1.80	12.40
-8	23	1	1.20	7.30
-8	23	1	2.80	11.10
-7	23	1	6.90	7.40
-7	23	1	-7.60	10.00
7	23	-1	21.70	10.80
-6	23	1	14.90	9.50
6	23	-1	18.30	5.40
-6	23	1	17.50	7.40
6	23	-1	7.70	10.00
5	23	-1	145.99	19.40
-5	23	1	178.18	19.40
5	23	-1	164.28	12.10
-5	23	1	170.78	14.50
4	23	-1	8.30	9.70
-4	23	1	3.60	6.20
4	23	-1	17.20	5.00
-4	23	1	12.10	9.40
-3	23	1	100.89	15.80
3	23	-1	85.79	16.30
-3	23	1	111.59	11.50
3	23	-1	105.79	9.70
-2	23	1	1.30	6.00
2	23	-1	-1.30	8.70
2	23	-1	16.80	5.20
-2	23	1	0.60	9.00
1	23	-1	145.09	17.20
-1	23	1	150.68	17.70
1	23	-1	171.68	12.10
0	23	1	47.80	10.20
0	23	1	58.39	9.60
0	23	-1	58.99	10.70
0	23	-1	53.79	9.10
1	23	1	307.87	18.50
-1	23	-1	309.57	18.50
-1	23	-1	289.97	22.40
1	23	1	296.67	23.00
2	23	1	4.60	13.90
-2	23	-1	16.30	7.20
2	23	1	8.50	5.50

-2	23	-1	2.70	8.10
-3	23	-1	8.80	6.10
-3	23	-1	8.10	8.00
3	23	1	11.10	5.30
4	23	1	33.10	6.60
4	23	1	23.40	12.90
-4	23	-1	45.10	7.80
-4	23	-1	54.89	10.40
5	23	1	53.39	13.90
-5	23	-1	81.29	15.60
-5	23	-1	66.59	10.60
5	23	1	74.49	9.80
6	23	1	54.49	18.30
6	23	1	56.79	9.60
-6	23	-1	68.89	11.40
-6	23	-1	85.89	16.20
-7	23	-1	71.19	11.10
7	23	1	70.19	9.90
-7	23	-1	46.10	11.30
-8	23	-1	45.60	11.00
8	23	1	24.20	15.90
-8	23	-1	31.80	8.40
-9	23	-1	-3.40	10.00
-9	23	-1	3.20	7.60
-8	24	1	197.88	17.20
8	24	-1	187.18	24.60
-8	24	1	233.18	24.10
-7	24	1	22.40	7.90
-7	24	1	20.40	10.90
7	24	-1	21.60	12.00
-6	24	1	314.87	22.10
-6	24	1	373.86	27.70
6	24	-1	385.66	28.50
-5	24	1	17.30	9.80
5	24	-1	19.80	10.90
5	24	-1	15.60	5.40
-5	24	1	15.30	7.50
4	24	-1	409.36	22.90
-4	24	1	421.76	29.30
4	24	-1	437.46	29.30
-4	24	1	403.16	24.70
-3	24	1	19.90	7.20
3	24	-1	10.60	9.90
3	24	-1	19.50	5.60

-3	24	1	13.00	9.60
2	24	-1	803.62	44.70
-2	24	1	766.52	40.80
2	24	-1	758.82	39.80
-2	24	1	798.92	44.90
-1	24	1	6.60	8.90
-1	24	1	23.40	6.80
1	24	-1	29.20	10.40
1	24	-1	17.30	5.80
0	24	1	548.55	30.30
0	24	1	550.44	34.50
0	24	-1	567.74	34.20
0	24	-1	540.05	29.50
1	24	1	19.50	7.50
1	24	1	8.40	10.20
-1	24	-1	5.00	8.50
2	24	1	296.37	18.80
-2	24	-1	313.17	19.30
-2	24	-1	311.67	23.80
2	24	1	320.17	24.50
-3	24	-1	49.90	8.00
-3	24	-1	35.20	10.20
3	24	1	27.70	10.20
3	24	1	41.60	7.00
4	24	1	278.37	17.90
-4	24	-1	290.37	19.10
4	24	1	304.47	24.50
-4	24	-1	294.67	23.50
5	24	1	257.07	17.30
-5	24	-1	287.77	18.80
5	24	1	275.47	24.40
-5	24	-1	276.77	23.30
6	24	1	92.39	11.00
6	24	1	107.59	20.00
-6	24	-1	105.09	17.60
-6	24	-1	97.29	12.60
7	24	1	115.09	20.50
-7	24	-1	157.48	19.30
-7	24	-1	122.79	13.80
-8	24	-1	123.59	20.30
-8	24	-1	123.39	14.00
-7	25	1	23.30	12.20
7	25	-1	25.90	12.70
-7	25	1	22.40	8.60

6	25	-1	-5.90	12.00
-6	25	1	6.80	7.50
-6	25	1	-8.80	9.60
-5	25	1	15.10	7.90
-5	25	1	23.90	11.70
5	25	-1	16.40	11.30
4	25	-1	-13.10	10.30
-4	25	1	-3.70	9.50
-4	25	1	2.00	7.20
4	25	-1	-0.50	5.30
-3	25	1	0.40	9.40
3	25	-1	5.30	10.00
3	25	-1	8.00	5.30
-3	25	1	-3.10	6.80
-2	25	1	5.90	9.80
-2	25	1	-2.00	6.60
2	25	-1	-1.70	10.40
2	25	-1	10.00	5.60
-1	25	1	11.50	6.70
-1	25	1	-2.50	10.00
1	25	-1	8.70	9.40
1	25	-1	14.90	6.00
0	25	1	9.30	6.50
0	25	1	-9.60	9.20
0	25	-1	15.80	10.40
1	25	1	7.10	6.30
1	25	1	9.30	10.20
-1	25	-1	13.90	6.20
-1	25	-1	8.80	9.20
-2	25	-1	-1.50	6.10
-2	25	-1	4.40	10.20
2	25	1	-1.40	9.70
2	25	1	6.80	6.10
-3	25	-1	12.20	6.50
3	25	1	6.10	10.10
-3	25	-1	15.40	9.90
4	25	1	20.70	12.20
-4	25	-1	16.50	10.00
4	25	1	11.40	11.00
-4	25	-1	13.00	7.20
5	25	1	23.40	11.70
-5	25	-1	27.20	7.80
5	25	1	-7.40	10.70
-5	25	-1	14.30	10.20

-6	25	-1	-0.20	9.70
6	25	1	9.60	12.00
-6	25	-1	-7.20	6.70
-7	25	-1	17.80	12.40
7	25	1	-12.90	11.80
-7	25	-1	25.30	8.00
-5	26	1	48.40	9.40
-5	26	1	39.40	12.80
5	26	-1	39.10	13.70
-4	26	1	27.50	12.30
4	26	-1	41.30	12.40
-4	26	1	57.89	9.50
3	26	-1	16.80	6.00
3	26	-1	1.10	10.70
-3	26	1	16.40	7.70
-3	26	1	11.90	10.50
2	26	-1	113.39	16.20
-2	26	1	140.49	19.90
-2	26	1	114.79	13.50
1	26	-1	9.50	10.30
-1	26	1	-5.80	6.70
-1	26	1	5.10	10.70
1	26	-1	2.20	6.20
0	26	1	329.07	21.20
0	26	-1	325.67	26.40
0	26	-1	333.57	20.50
0	26	1	344.17	27.30
1	26	1	-1.50	10.40
-1	26	-1	8.20	6.70
1	26	1	13.90	7.10
-1	26	-1	11.50	10.80
-2	26	-1	168.48	14.90
2	26	1	183.18	22.40
2	26	1	195.28	14.80
-2	26	-1	199.28	20.80
-3	26	-1	43.10	8.40
3	26	1	33.50	7.20
3	26	1	34.90	12.60
-3	26	-1	30.10	11.10
4	26	1	188.68	15.00
-4	26	-1	192.68	16.40
-4	26	-1	198.68	21.90
4	26	1	233.78	24.30
-5	26	-1	91.99	13.00

-5	26	-1	110.99	19.70
5	26	1	75.19	15.50
-4	27	1	-6.60	7.80
4	27	-1	-5.60	11.30
-4	27	1	-21.80	11.60
-3	27	1	71.59	13.30
3	27	-1	100.19	20.60
-3	27	1	76.29	14.40
-2	27	1	9.60	11.80
-2	27	1	20.20	7.90
2	27	-1	24.30	13.10
2	27	-1	11.60	6.50
1	27	-1	97.69	11.70
1	27	-1	106.49	19.80
-1	27	1	79.79	14.40
-1	27	1	84.49	13.20
0	27	1	30.60	8.20
0	27	1	8.10	11.60
0	27	-1	34.50	7.50
0	27	-1	12.70	11.10
-1	27	-1	143.59	13.80
-1	27	-1	125.19	20.60
1	27	1	155.68	14.10
1	27	1	155.48	21.90
2	27	1	38.20	7.60
2	27	1	26.60	13.80
-2	27	-1	25.70	11.50
-2	27	-1	34.40	8.40
-3	27	-1	100.09	19.30
-3	27	-1	87.19	12.90
3	27	1	83.59	11.80
3	27	1	85.49	15.00
4	27	1	47.20	13.90
-4	27	-1	32.00	8.30
-4	27	-1	37.80	13.20
-16	0	2	13.50	8.30
-15	0	2	855.21	44.80
-14	0	2	31.90	7.30
13	0	-2	431.56	26.90
-13	0	2	446.46	25.30
-12	0	2	210.38	19.60
12	0	-2	256.07	18.00
-12	0	2	269.37	15.80
-11	0	2	1804.02	95.99

-11	0	2	1981.20	93.69
11	0	-2	1998.00	95.19
-10	0	2	752.52	43.20
-10	0	2	871.41	41.80
10	0	-2	815.92	42.40
-10	0	2	816.92	40.80
-9	0	2	1033.60	47.80
-9	0	2	935.41	47.00
9	0	-2	959.20	48.40
-9	0	2	876.81	49.00
-8	0	2	3615.34	176.88
8	0	-2	3727.73	177.18
8	0	-2	3872.61	178.08
-8	0	2	3617.54	178.58
-7	0	2	80.49	8.00
7	0	-2	79.89	6.50
7	0	-2	71.79	7.50
-7	0	2	66.59	5.90
-6	0	2	4158.28	200.18
6	0	-2	4193.28	200.38
-6	0	2	4107.29	201.28
6	0	-2	4382.26	201.18
5	0	-2	50.79	5.30
-5	0	2	37.20	3.90
-5	0	2	47.30	5.40
5	0	-2	29.30	4.60
-4	0	2	8669.73	411.36
3	0	-2	450.15	22.70
3	0	-2	485.55	23.30
-3	0	2	477.65	23.40
-3	0	2	454.65	22.60
-2	0	2	3370.56	166.58
2	0	-2	3597.64	167.08
-2	0	2	3586.04	167.18
-1	0	2	772.62	35.40
1	0	-2	703.53	35.10
-1	0	-2	590.04	30.50
-1	0	-2	673.63	30.60
1	0	2	656.53	30.50
-2	0	-2	2312.07	111.09
2	0	2	2282.87	111.09
-2	0	-2	2377.26	111.09
2	0	2	2382.96	111.09
-3	0	-2	5776.52	273.77

-4	0	-2	1464.15	73.29
-4	0	-2	1627.74	73.39
4	0	2	1532.65	73.39
-4	0	-2	1493.25	73.49
5	0	2	2598.44	125.69
-5	0	-2	2533.55	125.39
-5	0	-2	2510.75	125.79
-5	0	-2	2872.11	125.69
-6	0	-2	1293.47	65.79
6	0	2	1502.05	67.29
-6	0	-2	1181.88	65.09
-6	0	-2	1431.66	65.59
-7	0	-2	2774.12	138.29
-7	0	-2	3222.88	138.79
-7	0	-2	2678.03	138.89
-8	0	-2	6.80	3.20
-8	0	-2	10.80	4.00
-9	0	-2	642.34	33.30
-9	0	-2	576.14	34.40
-9	0	-2	750.72	34.20
-10	0	-2	52.69	6.60
-10	0	-2	30.20	6.60
-10	0	-2	53.69	9.70
-11	0	-2	403.76	21.90
-11	0	-2	373.66	23.90
-12	0	-2	-7.20	6.40
-12	0	-2	6.00	5.60
-13	0	-2	186.08	14.30
-14	0	-2	17.50	7.30
-15	0	-2	248.88	18.40
-16	0	-2	16.90	8.60
-16	-1	2	18.20	8.20
-16	1	2	11.30	8.10
-15	-1	2	30.00	8.10
-14	-1	2	96.89	11.90
-14	1	2	111.49	11.80
13	-1	-2	102.99	12.80
13	1	-2	72.79	13.00
-13	1	2	99.09	10.50
12	-1	-2	75.29	11.40
-12	-1	2	90.39	9.50
-12	-1	2	87.29	14.40
12	1	-2	80.99	12.40
-12	1	2	79.89	9.70

-12	1	2	79.59	14.50
11	-1	-2	67.69	10.20
-11	-1	2	66.99	8.40
11	1	-2	60.79	11.30
-11	1	2	82.89	13.00
-11	1	2	84.49	8.70
10	-1	-2	9.50	5.10
-10	-1	2	4.10	5.00
-10	-1	2	19.00	4.50
-10	-1	2	2.10	5.90
-10	1	2	11.00	4.20
10	1	-2	8.00	5.30
-10	1	2	6.00	6.30
-10	1	2	-2.80	5.20
-9	-1	2	95.29	11.50
9	-1	-2	117.49	10.20
-9	-1	2	101.19	8.30
-9	-1	2	101.09	9.10
9	-1	-2	111.89	9.00
-9	1	2	111.09	11.60
9	1	-2	95.19	10.50
-9	1	2	125.39	8.70
8	-1	-2	293.17	16.70
8	-1	-2	265.67	15.70
-8	-1	2	280.07	15.30
-8	-1	2	318.37	16.00
-8	-1	2	264.87	17.60
-8	1	2	275.57	17.60
-8	1	2	263.87	15.20
8	1	-2	260.17	16.80
7	-1	-2	37.40	5.30
-7	-1	2	35.90	7.00
7	-1	-2	47.10	6.10
-7	-1	2	48.10	4.60
-7	1	2	39.80	6.40
7	1	-2	44.00	6.50
-7	1	2	32.00	4.80
7	1	-2	36.80	4.90
6	-1	-2	49.20	4.90
-6	-1	2	28.90	6.10
-6	-1	2	35.30	4.10
6	-1	-2	39.90	5.50
-6	1	2	35.80	6.20
6	1	-2	39.10	4.40

-6	1	2	38.20	4.40
6	1	-2	34.50	5.80
-5	-1	2	1744.33	85.39
-5	-1	2	1788.12	86.59
5	-1	-2	1865.91	86.29
5	-1	-2	1765.12	85.69
-5	1	2	1793.12	86.49
5	1	-2	1795.12	85.59
5	1	-2	1844.12	86.39
-5	1	2	1708.53	85.49
-4	-1	2	38.60	3.00
4	-1	-2	34.90	4.10
4	-1	-2	41.20	3.50
-4	-1	2	30.80	4.50
4	1	-2	33.90	4.30
4	1	-2	31.50	3.20
-4	1	2	37.70	3.70
-4	1	2	32.30	4.20
3	-1	-2	769.32	38.40
-3	-1	2	775.32	38.20
-3	-1	2	809.52	39.00
3	-1	-2	790.52	38.80
-3	1	2	791.42	38.30
3	1	-2	819.12	39.00
-3	1	2	833.72	39.00
3	1	-2	791.02	38.30
2	-1	-2	628.54	30.60
-2	-1	2	647.74	30.40
2	-1	-2	654.33	30.90
-2	-1	2	624.54	31.00
2	1	-2	633.94	30.40
-2	1	2	650.63	31.00
2	1	-2	637.24	31.00
-2	1	2	617.84	30.60
0	-1	-2	338.97	16.10
0	1	2	342.07	16.40
0	1	-2	313.37	15.80
-1	-1	-2	359.06	16.90
-1	-1	-2	344.67	16.90
-1	1	-2	361.86	16.90
1	1	2	327.07	17.10
-2	-1	-2	318.07	15.90
2	-1	2	332.37	15.70
-2	-1	-2	341.77	15.90

-2	1	-2	314.27	15.90
2	1	2	313.47	15.80
-2	1	-2	308.97	15.90
2	1	2	322.17	16.00
-2	1	-2	328.27	15.70
-3	-1	-2	32.50	3.10
-3	-1	-2	39.60	3.10
3	-1	2	36.70	2.90
-3	1	-2	37.00	3.00
-3	1	-2	41.40	3.20
-3	1	-2	35.10	3.00
3	1	2	36.20	3.00
4	-1	2	505.75	24.50
-4	-1	-2	539.35	24.60
-4	-1	-2	469.65	24.70
-4	1	-2	512.85	24.70
-4	1	-2	456.95	24.40
4	1	2	469.15	24.40
-4	1	-2	505.65	24.70
-5	-1	-2	281.97	16.40
-5	-1	-2	322.27	16.10
-5	-1	-2	289.47	16.00
5	-1	2	321.97	16.20
-5	1	-2	290.07	15.90
-5	1	-2	338.77	16.20
-5	1	-2	312.67	16.40
-6	-1	-2	103.29	8.40
-6	-1	-2	142.79	8.20
6	-1	2	119.29	8.00
-6	-1	-2	115.59	7.70
-6	1	-2	126.99	8.20
-6	1	-2	118.29	8.50
-6	1	-2	109.89	7.70
7	-1	2	138.99	9.60
-7	-1	-2	138.49	10.20
-7	-1	-2	160.38	9.80
-7	-1	-2	129.79	9.10
-7	1	-2	166.18	9.90
-7	1	-2	129.49	10.00
-7	1	-2	146.49	9.00
8	-1	2	41.20	6.10
-8	-1	-2	33.10	5.50
-8	-1	-2	39.60	7.00
-8	-1	-2	54.99	6.30

-8	1	-2	44.20	7.00
-8	1	-2	37.20	5.70
-8	1	-2	58.69	6.60
-9	-1	-2	58.69	7.40
9	-1	2	43.20	7.00
-9	-1	-2	48.20	6.70
-9	1	-2	44.20	8.30
-9	1	-2	69.79	7.00
-9	1	-2	54.59	7.60
-10	-1	-2	-2.80	4.90
10	-1	2	11.20	4.20
-10	-1	-2	6.10	4.10
-10	-1	-2	7.70	5.10
-10	1	-2	8.90	5.40
-10	1	-2	5.90	3.80
-11	-1	-2	15.40	5.10
11	-1	2	11.60	5.40
-11	-1	-2	4.40	6.00
-11	1	-2	15.90	5.40
-11	1	-2	-1.40	5.60
12	-1	2	139.59	12.40
-12	-1	-2	108.19	14.50
-12	-1	-2	129.19	11.70
-12	1	-2	149.29	14.70
-12	1	-2	162.58	17.70
-13	-1	-2	10.60	6.10
-13	1	-2	19.50	6.40
-14	-1	-2	27.00	7.40
-14	1	-2	29.80	7.70
-15	-1	-2	28.80	8.20
-15	1	-2	15.90	7.50
-16	-1	-2	32.20	9.30
-16	1	-2	29.70	9.30
-16	-2	2	48.30	9.60
-16	2	2	46.30	9.40
-15	-2	2	236.18	17.90
-15	2	2	242.38	17.50
-14	2	2	27.20	7.10
13	-2	-2	260.27	19.00
-13	-2	2	257.97	17.30
13	2	-2	244.08	23.10
-13	2	2	293.37	17.30
-12	-2	2	405.86	34.60
-12	-2	2	428.56	23.00

12	-2	-2	424.36	24.70
-12	2	2	424.36	23.10
-12	2	2	364.66	26.50
-11	-2	2	312.77	16.90
-11	-2	2	233.78	19.90
-11	-2	2	279.17	17.80
11	-2	-2	295.57	18.30
11	2	-2	258.07	19.60
-11	2	2	271.57	19.90
-11	2	2	286.67	16.80
-10	-2	2	719.03	41.10
-10	-2	2	855.81	39.80
10	-2	-2	781.22	40.30
10	-2	-2	755.52	39.60
-10	-2	2	796.92	38.90
-10	2	2	712.93	41.30
10	2	-2	777.32	40.60
-10	2	2	786.72	39.10
-9	-2	2	374.96	25.60
9	-2	-2	460.55	24.70
-9	-2	2	508.65	24.10
-9	-2	2	440.36	23.30
9	-2	-2	469.05	24.10
-9	2	2	405.76	25.40
9	2	-2	430.16	25.10
-9	2	2	472.95	23.60
-8	-2	2	1092.99	58.19
8	-2	-2	1166.18	56.99
-8	-2	2	1141.29	56.39
-8	-2	2	1310.77	56.99
8	-2	-2	1178.78	57.59
8	2	-2	1121.99	57.69
-8	2	2	1149.98	56.59
-8	2	2	1109.89	58.19
-7	-2	2	10.10	4.70
7	-2	-2	19.50	4.00
-7	-2	2	17.60	3.00
7	-2	-2	12.80	3.50
-7	-2	2	19.60	3.30
7	2	-2	12.60	4.20
-7	2	2	9.10	4.10
-7	2	2	17.30	3.40
-6	-2	2	1905.31	85.69
6	-2	-2	1768.62	85.89

-6	-2	2	1700.23	85.39
6	-2	-2	1832.82	86.39
-6	-2	2	1719.23	86.79
-6	2	2	1756.82	85.59
-6	2	2	1779.12	86.69
6	2	-2	1808.32	86.59
5	-2	-2	20.10	3.00
-5	-2	2	14.60	2.70
-5	-2	2	14.60	2.40
-5	-2	2	5.90	3.20
5	-2	-2	18.00	3.10
-5	2	2	16.30	2.50
-5	2	2	15.40	3.30
5	2	-2	12.40	3.30
4	-2	-2	2037.40	96.39
-4	-2	2	1952.90	95.59
4	-2	-2	2041.00	95.99
-4	-2	2	1938.41	96.59
-4	2	2	2074.59	96.59
4	2	-2	1998.20	96.59
-4	2	2	2023.00	95.89
-3	-2	2	126.09	6.90
3	-2	-2	144.89	7.80
3	-2	-2	131.09	7.30
-3	-2	2	137.89	8.00
3	2	-2	132.09	7.00
-3	2	2	132.79	7.30
3	2	-2	144.59	8.10
-3	2	2	138.89	7.80
2	-2	-2	86.29	5.00
-2	-2	2	94.79	5.50
-2	-2	2	78.99	4.60
2	-2	-2	92.79	5.30
-2	2	2	96.69	5.40
2	2	-2	98.89	5.60
-2	2	2	90.69	5.00
1	-2	-2	1415.56	68.89
-1	-2	2	1515.35	68.89
1	-2	-2	1480.95	68.69
-1	2	2	1559.74	68.89
-1	2	2	1308.07	68.69
1	2	-2	1387.16	68.79
1	-2	2	173.98	8.60
-1	-2	-2	160.98	8.80

-1	-2	-2	169.88	8.60
-1	2	-2	179.28	8.60
-1	2	-2	181.68	8.30
1	2	2	162.88	8.60
2	-2	2	2109.99	98.69
-2	-2	-2	2089.89	98.89
-2	-2	-2	2097.59	98.69
2	-2	2	2050.29	98.39
2	2	2	2008.40	98.59
-2	2	-2	2069.39	98.69
-2	2	-2	2097.19	98.49
3	-2	2	1365.96	63.49
-3	-2	-2	1294.17	63.69
-3	-2	-2	1367.26	63.39
3	-2	2	1308.17	63.29
-3	2	-2	1364.26	63.39
3	2	2	1253.07	63.39
-3	2	-2	1320.07	63.49
-4	-2	-2	580.44	27.20
-4	-2	-2	523.15	27.50
4	-2	2	554.14	27.10
-4	2	-2	538.05	27.20
4	2	2	529.25	27.10
-4	2	-2	585.04	27.30
-5	-2	-2	90.39	7.40
-5	-2	-2	122.89	7.10
5	-2	2	112.79	7.00
5	-2	2	111.99	7.20
5	2	2	101.49	6.60
-5	2	-2	113.69	7.00
-5	2	-2	108.89	7.30
-6	-2	-2	751.92	33.90
6	-2	2	692.43	33.90
6	-2	2	703.03	33.90
-6	-2	-2	635.04	34.30
-6	-2	-2	660.23	33.70
-6	2	-2	640.24	33.70
-6	2	-2	656.73	34.10
-6	2	-2	698.93	34.00
-7	-2	-2	293.57	16.90
-7	-2	-2	348.07	17.30
7	-2	2	315.57	17.20
-7	-2	-2	288.97	17.70
-7	2	-2	345.37	17.60

-7	2	-2	310.47	17.70
-7	2	-2	310.97	17.00
-8	-2	-2	351.96	21.50
-8	-2	-2	417.66	20.80
-8	-2	-2	379.76	20.40
8	-2	2	399.26	20.90
-8	2	-2	375.26	20.50
-8	2	-2	351.16	21.30
-8	2	-2	417.26	21.20
9	-2	2	713.63	37.60
-9	-2	-2	677.23	38.50
-9	-2	-2	834.62	37.90
-9	-2	-2	733.53	37.30
-9	2	-2	736.93	37.30
-9	2	-2	682.03	38.40
-9	2	-2	795.52	38.10
-10	-2	-2	93.99	8.80
-10	-2	-2	92.89	9.70
10	-2	2	108.49	9.40
-10	-2	-2	90.79	11.50
-10	2	-2	96.59	8.40
-10	2	-2	96.79	10.80
11	-2	2	657.73	33.60
-11	-2	-2	583.84	34.80
-11	-2	-2	670.93	33.20
-11	2	-2	647.64	33.20
-11	2	-2	590.34	34.70
12	-2	2	263.67	16.70
-12	-2	-2	215.78	18.70
-12	-2	-2	267.37	15.90
-12	2	-2	199.08	18.00
-12	2	-2	239.08	18.80
-13	-2	-2	335.37	20.70
-13	2	-2	346.07	20.70
-14	-2	-2	10.60	6.60
-14	2	-2	14.90	7.00
-15	-2	-2	241.18	18.20
-15	2	-2	230.18	18.00
-16	-2	-2	11.10	9.00
-16	2	-2	12.10	8.40
-16	-3	2	237.88	18.90
-16	3	2	226.08	18.50
-15	-3	2	9.30	7.30
-15	3	2	16.90	7.10

-14	-3	2	246.68	16.80
-14	3	2	246.28	17.00
13	-3	-2	366.76	23.60
-13	-3	2	371.46	22.10
13	3	-2	391.86	25.30
-13	3	2	392.86	22.20
-12	-3	2	97.29	14.80
-12	-3	2	126.79	10.40
12	-3	-2	135.79	12.60
12	3	-2	113.39	14.10
-12	3	2	109.99	10.50
-12	3	2	90.49	14.80
-11	-3	2	427.06	30.50
11	-3	-2	536.25	28.30
-11	-3	2	506.05	26.50
-11	-3	2	528.25	27.40
-11	3	2	465.35	29.50
11	3	-2	520.55	29.50
-11	3	2	517.95	26.90
-10	-3	2	97.99	12.30
-10	-3	2	133.39	9.60
10	-3	-2	132.59	12.60
-10	-3	2	160.18	10.70
10	-3	-2	118.59	11.30
-10	3	2	112.99	12.90
-10	3	2	131.69	10.00
-9	-3	2	1091.89	47.90
-9	-3	2	924.71	47.10
9	-3	-2	976.70	48.40
9	-3	-2	955.90	48.00
-9	-3	2	893.21	49.40
-9	3	2	964.60	47.40
9	3	-2	927.31	49.60
-9	3	2	925.61	49.30
-8	-3	2	23.00	4.00
8	-3	-2	19.80	4.60
8	-3	-2	18.30	4.10
-8	-3	2	23.10	3.70
-8	-3	2	11.50	4.90
-8	3	2	10.50	5.10
-8	3	2	19.40	4.00
8	3	-2	10.60	4.70
7	-3	-2	842.22	42.70
7	-3	-2	882.51	43.10

-7	-3	2	932.71	42.40
-7	-3	2	860.11	42.10
-7	-3	2	799.02	43.70
-7	3	2	831.12	42.20
-7	3	2	839.22	43.60
7	3	-2	897.31	43.70
6	-3	-2	236.18	12.40
6	-3	-2	214.68	12.60
-6	-3	2	200.58	11.50
-6	-3	2	214.18	11.80
-6	-3	2	197.78	13.10
-6	3	2	223.48	13.20
-6	3	2	213.08	11.80
6	3	-2	212.88	13.20
-5	-3	2	1255.27	62.49
5	-3	-2	1298.77	62.09
5	-3	-2	1268.17	61.69
-5	-3	2	1377.66	61.39
-5	-3	2	1132.39	61.49
-5	3	2	1301.27	62.39
5	3	-2	1306.07	62.39
-5	3	2	1270.17	61.49
-4	-3	2	607.84	27.20
-4	-3	2	546.15	27.00
-4	-3	2	511.85	28.10
4	-3	-2	573.34	27.80
-4	3	2	553.74	28.00
4	3	-2	545.85	28.10
-4	3	2	560.54	27.40
-3	-3	2	64.49	5.30
3	-3	-2	72.09	4.90
-3	-3	2	72.79	4.20
3	-3	-2	76.09	4.90
-3	-3	2	78.29	4.20
-3	3	2	75.79	5.20
3	3	-2	81.49	5.50
-3	3	2	85.29	4.70
-2	-3	2	1130.09	53.79
-2	-3	2	1102.29	54.59
2	-3	-2	1106.29	54.39
2	-3	-2	1136.09	54.29
-2	3	2	1145.79	54.49
2	3	-2	1162.48	54.59
-2	3	2	1146.09	54.19

1	-3	-2	1664.33	81.29
-1	-3	2	1758.82	81.59
1	-3	-2	1741.73	81.49
1	3	-2	1800.62	81.49
-1	3	2	1717.13	81.49
-1	3	2	1571.34	81.29
0	-3	-2	36.40	2.40
0	-3	2	30.70	2.40
0	-3	-2	37.30	2.60
0	-3	2	32.20	2.00
0	3	2	40.70	2.60
0	3	-2	34.50	2.40
0	3	-2	39.60	2.00
-1	-3	-2	2002.00	93.09
1	-3	2	1944.01	92.79
-1	-3	-2	1923.41	92.79
-1	3	-2	1947.41	92.69
1	3	2	1886.21	92.79
-1	3	-2	2014.70	92.69
2	-3	2	478.75	23.50
2	-3	2	502.75	23.80
-2	-3	-2	487.75	24.20
-2	-3	-2	514.05	23.80
-2	3	-2	480.75	23.60
-2	3	-2	466.45	23.70
2	3	2	489.45	23.80
3	-3	2	1734.13	80.39
-3	-3	-2	1699.13	80.69
3	-3	2	1665.13	80.09
-3	-3	-2	1769.62	80.29
-3	3	-2	1544.95	80.49
-3	3	-2	1717.63	80.29
3	3	2	1636.74	80.29
4	-3	2	52.39	3.70
-4	-3	-2	50.59	4.50
-4	-3	-2	45.70	3.80
4	-3	2	43.60	3.90
4	3	2	44.80	3.80
-4	3	-2	43.30	4.00
-4	3	-2	49.40	3.90
-5	-3	-2	1778.12	78.49
-5	-3	-2	1517.05	78.89
5	-3	2	1687.23	78.59
5	-3	2	1666.93	78.39

-5	3	-2	1594.54	78.39
5	3	2	1521.05	78.19
-5	3	-2	1651.43	78.59
-6	-3	-2	593.24	30.20
-6	-3	-2	574.54	31.00
-6	-3	-2	654.93	30.40
6	-3	2	605.14	30.30
6	-3	2	612.84	30.60
-6	3	-2	626.04	30.80
-6	3	-2	582.04	30.30
-7	-3	-2	237.28	13.70
-7	-3	-2	245.88	15.00
7	-3	2	251.87	13.90
7	-3	2	248.08	14.10
-7	-3	-2	253.47	14.00
-7	3	-2	228.28	13.80
-7	3	-2	259.47	14.60
-8	-3	-2	547.15	27.70
8	-3	2	531.75	28.00
-8	-3	-2	594.14	28.00
-8	-3	-2	488.95	28.70
8	-3	2	539.95	27.90
8	3	2	519.45	28.40
-8	3	-2	526.75	28.60
-8	3	-2	581.74	28.50
-8	3	-2	527.45	27.80
9	-3	2	96.29	8.30
-9	-3	-2	80.59	9.90
-9	-3	-2	104.09	8.40
-9	-3	-2	85.19	7.70
-9	3	-2	93.49	9.60
9	3	2	84.49	9.10
-9	3	-2	95.19	9.40
-9	3	-2	86.49	7.60
-10	-3	-2	550.94	26.00
10	-3	2	462.95	25.50
-10	-3	-2	412.96	26.80
-10	-3	-2	478.95	25.20
10	3	2	484.75	26.40
-10	3	-2	462.55	25.00
-10	3	-2	436.36	26.60
-11	-3	-2	354.36	19.90
11	-3	2	354.86	20.30
-11	-3	-2	312.07	22.10

-11	3	-2	318.47	21.80
11	3	2	371.56	21.30
-11	3	-2	327.37	19.50
12	-3	2	430.16	25.90
-12	-3	-2	443.86	28.30
-12	-3	-2	494.45	25.70
12	3	2	456.95	26.80
-12	3	-2	487.25	25.80
-12	3	-2	435.66	27.80
-13	-3	-2	5.10	5.70
-13	3	-2	3.00	5.90
13	3	2	-1.80	8.20
-14	-3	-2	276.27	18.80
-14	3	-2	273.07	18.80
-15	-3	-2	10.60	7.40
-15	3	-2	12.20	7.90
-16	-3	-2	217.98	18.70
-16	3	-2	181.28	17.00
-16	-4	2	17.30	8.60
-16	4	2	14.30	8.30
-15	-4	2	31.60	8.20
-15	4	2	31.80	7.90
-14	-4	2	5.30	6.40
-14	4	2	20.30	6.70
13	-4	-2	35.10	9.40
-13	-4	2	38.00	6.70
13	4	-2	32.10	8.60
-13	4	2	53.99	9.20
-12	-4	2	23.90	5.60
-12	-4	2	15.70	9.00
12	-4	-2	24.50	6.20
-12	4	2	9.20	8.20
12	4	-2	18.30	7.70
-11	-4	2	73.59	8.00
-11	-4	2	57.59	10.10
11	-4	-2	80.89	9.90
-11	-4	2	91.29	10.10
11	4	-2	84.39	12.60
-11	4	2	80.69	8.80
-11	4	2	72.99	12.40
-10	-4	2	23.10	5.30
-10	-4	2	14.10	6.50
10	-4	-2	14.10	5.20
-10	-4	2	23.20	4.50

-10	4	2	16.50	6.30
-10	4	2	16.90	4.40
10	4	-2	19.80	6.30
-9	-4	2	72.69	11.00
-9	-4	2	87.59	7.40
9	-4	-2	102.89	9.10
-9	-4	2	102.29	8.50
9	-4	-2	88.29	8.80
-9	4	2	77.49	10.70
9	4	-2	91.49	10.90
-9	4	2	100.19	8.10
-8	-4	2	39.20	5.30
-8	-4	2	31.10	6.00
8	-4	-2	32.70	6.10
-8	-4	2	26.30	4.80
8	-4	-2	32.20	6.00
-8	4	2	32.80	5.30
-8	4	2	31.40	5.60
7	-4	-2	1.60	2.90
-7	-4	2	0.80	3.80
-7	-4	2	3.90	2.60
7	-4	-2	9.00	3.40
-7	-4	2	4.30	2.50
-7	4	2	4.80	3.90
-7	4	2	10.40	3.00
7	4	-2	0.10	4.40
-6	-4	2	262.07	13.00
6	-4	-2	235.88	13.50
6	-4	-2	235.28	13.60
-6	-4	2	210.68	14.30
-6	-4	2	248.08	12.70
6	4	-2	245.48	14.40
-6	4	2	246.88	14.30
-6	4	2	225.98	12.90
-5	-4	2	18.40	3.70
-5	-4	2	21.60	3.10
5	-4	-2	28.50	3.70
-5	-4	2	12.80	2.30
5	-4	-2	22.50	4.00
-5	4	2	22.70	3.50
-5	4	2	13.70	3.10
5	4	-2	24.20	4.30
4	-4	-2	286.87	15.40
-4	-4	2	270.27	15.90

4	-4	-2	303.77	15.30
-4	-4	2	318.67	14.70
-4	-4	2	285.17	14.60
4	4	-2	289.57	15.80
-4	4	2	295.67	15.60
-4	4	2	295.97	15.00
-3	-4	2	622.14	32.00
3	-4	-2	645.54	31.50
-3	-4	2	674.53	30.90
3	-4	-2	648.74	31.60
3	4	-2	631.24	31.90
3	4	-2	627.94	30.90
-3	4	2	645.14	31.40
-3	4	2	656.23	31.80
-2	-4	2	13.90	2.70
2	-4	-2	10.30	2.20
-2	-4	2	12.90	1.10
2	-4	-2	10.90	1.80
2	4	-2	10.20	2.00
-2	4	2	12.80	1.80
2	4	-2	12.70	1.10
-2	4	2	9.00	1.70
1	-4	-2	10.50	1.80
1	-4	-2	13.50	2.10
-1	-4	2	11.60	2.40
1	4	-2	15.50	1.10
-1	4	2	13.20	1.80
1	4	-2	13.40	2.50
-1	4	2	15.20	2.20
0	-4	2	897.21	44.60
0	-4	-2	962.10	44.80
0	-4	2	911.11	44.20
0	-4	-2	944.81	44.50
0	4	-2	889.11	44.20
0	4	2	971.70	44.80
0	4	2	923.71	44.50
0	4	-2	929.61	44.60
-1	-4	-2	353.66	18.10
-1	-4	-2	363.96	17.60
1	-4	2	362.36	17.60
-1	4	-2	368.66	17.60
-1	4	-2	378.66	17.60
1	4	2	337.07	18.10
1	4	2	356.36	17.70

-2	-4	-2	138.89	7.20
2	-4	2	139.79	7.40
-2	-4	-2	141.09	8.00
2	-4	2	137.49	7.10
2	4	2	126.19	7.20
-2	4	-2	137.59	7.10
-2	4	-2	133.89	7.30
-3	-4	-2	44.50	4.30
3	-4	2	47.70	3.20
-3	-4	-2	48.70	3.40
3	-4	2	46.80	3.60
-3	4	-2	46.50	3.70
3	4	2	46.30	3.50
-3	4	-2	44.20	3.30
4	-4	2	26.60	3.60
-4	-4	-2	36.10	3.30
4	-4	2	27.70	2.90
-4	-4	-2	27.70	4.20
-4	4	-2	25.00	3.10
-4	4	-2	30.10	3.30
4	4	2	31.20	3.20
-5	-4	-2	740.63	33.50
-5	-4	-2	606.94	34.10
5	-4	2	722.83	33.80
5	-4	2	682.83	33.40
-5	4	-2	684.73	33.70
5	4	2	628.54	33.30
-5	4	-2	689.23	33.60
6	-4	2	285.27	14.90
-6	-4	-2	266.67	15.90
6	-4	2	280.47	15.30
-6	4	-2	281.57	15.40
6	4	2	282.27	15.70
-6	4	-2	264.87	14.80
6	4	2	271.27	14.70
-7	-4	-2	33.90	6.40
-7	-4	-2	39.10	5.10
7	-4	2	41.50	5.70
7	-4	2	29.70	4.90
-7	-4	-2	39.90	5.30
7	4	2	35.30	6.20
-7	4	-2	37.50	4.80
-7	4	-2	34.00	5.80
8	-4	2	-1.30	2.80

8	-4	2	9.40	3.80
-8	-4	-2	9.30	3.50
-8	-4	-2	6.80	3.30
-8	-4	-2	8.70	4.30
8	4	2	7.30	3.90
-8	4	-2	6.00	3.30
-8	4	-2	4.20	3.70
9	-4	2	14.20	4.10
-9	-4	-2	9.60	5.50
-9	-4	-2	13.50	4.00
9	-4	2	17.30	4.70
-9	-4	-2	17.80	4.80
-9	4	-2	16.30	4.50
-9	4	-2	16.20	5.20
9	4	2	11.60	4.80
-9	4	-2	5.30	4.80
10	-4	2	13.40	4.40
-10	-4	-2	20.70	5.40
-10	-4	-2	31.50	5.30
-10	-4	-2	17.50	6.40
-10	4	-2	19.80	6.30
-10	4	-2	18.80	4.80
10	4	2	15.00	5.60
11	-4	2	57.39	8.70
-11	-4	-2	72.79	8.80
-11	-4	-2	58.09	11.70
11	4	2	76.89	10.70
-11	4	-2	60.69	8.80
-11	4	-2	56.09	9.20
12	-4	2	47.60	9.30
-12	-4	-2	67.89	9.10
-12	-4	-2	41.60	8.90
-12	4	-2	33.10	8.00
-12	4	-2	49.60	9.20
12	4	2	57.29	11.10
-13	-4	-2	13.60	6.30
-13	4	-2	19.40	6.80
-14	-4	-2	16.20	7.40
-14	4	-2	34.10	7.60
-15	-4	-2	29.30	8.20
-15	4	-2	25.20	8.40
-16	-4	-2	26.10	10.00
-16	4	-2	40.00	8.90
-16	-5	2	222.58	17.90

-16	5	2	212.98	17.60
-15	-5	2	89.19	12.90
-15	5	2	103.29	12.90
-14	-5	2	290.37	18.70
-14	5	2	289.97	19.00
-13	-5	2	65.39	9.60
-13	5	2	71.19	9.80
13	5	-2	64.29	13.00
-12	-5	2	175.88	18.50
12	-5	-2	208.08	15.20
-12	-5	2	208.58	13.60
-12	5	2	170.88	18.10
12	5	-2	202.18	17.00
-12	5	2	204.78	13.90
-11	-5	2	521.35	27.90
-11	-5	2	468.05	31.00
-11	-5	2	558.94	28.60
11	-5	-2	564.04	29.40
-11	5	2	533.85	28.10
-11	5	2	501.35	31.10
11	5	-2	554.14	30.50
-10	-5	2	812.42	46.60
10	-5	-2	873.31	45.20
-10	-5	2	993.30	44.70
-10	-5	2	869.61	43.90
10	5	-2	918.41	46.30
-10	5	2	786.52	46.20
-10	5	2	918.21	44.40
9	-5	-2	808.82	42.10
-9	-5	2	920.71	41.50
-9	-5	2	798.32	40.90
9	-5	-2	857.91	42.20
-9	-5	2	677.83	44.90
-9	5	2	847.72	41.40
9	5	-2	881.61	43.30
-9	5	2	795.32	43.20
8	-5	-2	52.49	7.00
8	-5	-2	46.60	6.60
-8	-5	2	42.20	8.70
-8	-5	2	55.79	6.10
-8	-5	2	59.49	5.60
8	5	-2	57.59	8.90
-8	5	2	50.59	8.00
-8	5	2	52.99	5.90

-7	-5	2	838.22	42.10
7	-5	-2	848.02	43.00
7	-5	-2	893.11	43.10
-7	-5	2	776.62	43.80
-7	-5	2	946.31	42.40
7	5	-2	908.71	43.90
-7	5	2	841.42	43.70
-7	5	2	835.62	42.40
-6	-5	2	318.57	16.70
-6	-5	2	304.47	18.50
6	-5	-2	336.97	17.70
6	-5	-2	328.97	17.70
6	5	-2	335.17	20.00
-6	5	2	326.07	17.20
-6	5	2	330.27	18.30
-5	-5	2	2851.51	138.49
5	-5	-2	2905.11	137.79
5	-5	-2	2968.80	137.79
-5	-5	2	3096.29	137.09
-5	-5	2	2797.12	136.99
-5	5	2	2940.71	138.19
5	5	-2	2673.53	138.99
-5	5	2	2803.82	137.39
-4	-5	2	322.57	15.40
4	-5	-2	311.77	16.20
4	-5	-2	320.87	16.30
-4	-5	2	297.27	16.80
-4	5	2	307.77	16.50
4	5	-2	327.97	16.90
-4	5	2	303.97	15.90
4	5	-2	292.67	15.40
-3	-5	2	3701.33	174.08
-3	-5	2	3605.14	175.28
3	-5	-2	3655.93	174.88
-3	5	2	3721.13	174.68
-3	5	2	3736.33	175.08
3	5	-2	3446.86	174.08
3	5	-2	3857.81	175.28
2	-5	-2	88.69	5.60
-2	-5	2	100.49	6.30
2	-5	-2	89.99	5.70
-2	-5	2	93.89	4.90
2	5	-2	91.89	4.90
2	5	-2	106.49	6.30

-2	5	2	94.89	5.90
-1	-5	2	139.19	7.60
-1	-5	2	155.88	8.60
1	-5	-2	150.78	8.20
1	-5	-2	166.08	8.50
1	5	-2	165.38	8.70
-1	5	2	149.49	8.20
-1	5	2	161.48	8.50
1	5	-2	148.79	7.60
0	-5	2	2339.07	111.49
0	-5	2	2333.07	111.09
0	-5	-2	2413.86	111.29
0	5	2	2428.76	111.69
0	5	-2	2232.98	110.99
0	5	2	2263.27	111.39
0	5	-2	2385.86	111.59
0	5	2	2329.67	111.69
-1	-5	-2	4003.20	191.48
-1	-5	-2	4041.60	190.98
1	5	2	4178.18	191.58
1	5	2	3881.31	190.98
-2	-5	-2	16.40	2.00
2	-5	2	11.50	1.80
2	-5	2	13.20	2.20
-2	-5	-2	13.10	2.30
-2	5	-2	13.80	2.10
-2	5	-2	13.10	1.90
2	5	2	8.80	1.60
2	5	2	10.60	2.40
-3	-5	-2	1325.17	64.39
-3	-5	-2	1355.06	65.19
3	-5	2	1296.87	64.29
3	-5	2	1384.76	64.69
3	5	2	1386.46	65.09
-3	5	-2	1338.27	64.49
-3	5	-2	1322.67	64.39
4	-5	2	2597.14	125.69
-4	-5	-2	2624.64	126.59
4	-5	2	2734.73	126.09
4	5	2	2516.05	125.69
-4	5	-2	2615.34	125.89
4	5	2	2789.22	126.49
-4	5	-2	2593.14	125.89
-5	-5	-2	1571.44	79.49

5	-5	2	1626.84	78.59
5	-5	2	1654.93	78.99
-5	-5	-2	1723.23	78.69
-5	5	-2	1607.04	78.69
5	5	2	1533.45	78.49
5	5	2	1711.73	79.39
-5	5	-2	1662.23	78.89
-6	-5	-2	803.72	44.70
-6	-5	-2	995.30	44.00
6	-5	2	891.51	44.20
6	-5	2	909.61	43.90
6	5	2	914.71	44.70
6	5	2	852.51	43.70
-6	5	-2	894.71	44.00
-6	5	-2	893.81	44.30
7	-5	2	1398.46	66.89
-7	-5	-2	1533.45	66.59
-7	-5	-2	1235.78	67.39
7	-5	2	1369.66	66.39
-7	5	-2	1334.17	66.39
-7	5	-2	1343.77	66.99
7	5	2	1283.37	66.19
7	5	2	1430.46	67.39
8	-5	2	751.62	37.80
8	-5	2	748.73	38.30
-8	-5	-2	683.93	39.00
-8	-5	-2	855.51	38.00
-8	-5	-2	743.63	37.70
-8	5	-2	719.43	38.50
-8	5	-2	736.73	37.80
8	5	2	773.02	38.80
-9	-5	-2	68.39	7.60
-9	-5	-2	51.99	9.00
-9	-5	-2	62.99	7.10
9	-5	2	57.09	6.80
9	-5	2	61.09	8.00
9	5	2	66.39	8.80
-9	5	-2	56.69	8.40
-9	5	-2	64.39	7.40
-10	-5	-2	935.21	51.99
-10	-5	-2	1104.79	50.69
-10	-5	-2	1021.80	50.19
10	-5	2	1012.80	50.39
-10	5	-2	968.50	50.09

10	5	2	1050.69	51.69
-10	5	-2	940.71	51.39
11	-5	2	2.90	4.60
-11	-5	-2	16.60	6.90
-11	-5	-2	9.80	5.00
-11	5	-2	12.20	5.70
-11	5	-2	12.10	6.40
11	5	2	14.30	6.10
-12	-5	-2	372.46	26.00
-12	-5	-2	414.26	22.70
12	-5	2	400.26	23.10
-12	5	-2	357.06	24.90
12	5	2	409.46	24.50
-12	5	-2	409.06	22.80
-13	-5	-2	32.20	6.90
-13	5	-2	23.10	6.80
-14	-5	-2	373.26	22.80
-14	5	-2	345.97	22.40
-15	-5	-2	29.40	8.90
-15	5	-2	29.00	8.50
-16	-5	-2	340.77	23.30
-16	5	-2	295.77	22.10
-15	-6	2	36.00	8.40
-15	6	2	70.29	11.70
-14	-6	2	16.80	6.60
-14	6	2	16.30	6.50
-13	-6	2	32.40	6.70
13	6	-2	26.00	9.00
-13	6	2	54.89	9.10
-12	-6	2	118.79	18.00
-12	-6	2	166.78	12.40
12	-6	-2	158.58	14.00
12	6	-2	194.28	16.50
-12	6	2	161.78	17.00
-12	6	2	189.58	12.80
-11	-6	2	25.80	6.30
11	-6	-2	14.30	5.30
-11	-6	2	34.90	9.20
-11	-6	2	18.30	5.00
11	6	-2	27.30	7.20
-11	6	2	20.30	5.60
-11	6	2	18.50	7.40
-10	-6	2	26.10	5.30
-10	-6	2	24.20	7.10

10	-6	-2	18.20	5.10
-10	-6	2	44.90	6.70
10	6	-2	52.19	9.70
-10	6	2	34.80	7.80
-9	-6	2	19.90	6.50
9	-6	-2	35.50	7.10
-9	-6	2	34.80	6.40
9	-6	-2	25.50	5.60
-9	-6	2	31.00	5.60
9	6	-2	49.50	8.80
-9	6	2	26.20	4.70
-9	6	2	25.40	6.40
8	-6	-2	214.48	13.70
-8	-6	2	196.08	12.20
8	-6	-2	215.18	13.60
-8	-6	2	220.18	12.70
8	6	-2	236.68	15.20
-8	6	2	214.68	12.70
-8	6	2	223.48	14.70
-7	-6	2	14.00	4.80
7	-6	-2	20.50	4.10
-7	-6	2	14.90	3.10
-7	-6	2	17.20	3.10
7	-6	-2	17.30	4.10
7	6	-2	17.00	5.10
-7	6	2	18.20	4.70
-7	6	2	25.90	4.70
-6	-6	2	564.54	29.80
-6	-6	2	573.74	31.70
6	-6	-2	587.24	30.80
6	-6	-2	613.14	30.90
6	6	-2	676.03	32.00
-6	6	2	620.44	30.40
-6	6	2	621.34	31.50
-5	-6	2	80.79	7.90
5	-6	-2	79.89	6.70
5	-6	-2	87.49	6.70
-5	-6	2	93.29	5.60
5	6	-2	94.79	7.80
-5	6	2	93.79	6.40
-5	6	2	90.59	7.20
4	-6	-2	4.00	2.40
-4	-6	2	11.50	2.00
-4	-6	2	5.30	2.90

4	-6	-2	9.90	2.20
4	6	-2	14.60	3.50
-4	6	2	12.20	2.70
4	6	-2	7.60	1.50
-4	6	2	5.50	2.20
-3	-6	2	43.50	4.90
-3	-6	2	42.80	2.80
3	-6	-2	38.60	3.80
3	-6	-2	40.50	3.90
3	6	-2	39.70	5.60
-3	6	2	44.60	4.30
3	6	-2	43.00	2.80
-3	6	2	45.40	3.80
-2	-6	2	135.59	6.80
-2	-6	2	144.99	8.30
2	-6	-2	124.09	7.70
2	-6	-2	136.59	7.80
2	6	-2	131.69	6.80
-2	6	2	139.19	7.90
-2	6	2	127.49	7.60
-1	-6	2	2.90	1.60
-1	-6	2	1.40	0.90
1	-6	-2	1.90	1.40
1	-6	-2	5.00	1.60
-1	6	2	2.60	1.60
1	6	-2	2.30	0.80
0	-6	-2	1106.99	52.39
0	-6	2	1122.49	52.69
0	-6	2	1069.39	52.19
0	6	2	1118.39	52.79
0	6	2	1083.39	52.89
0	6	-2	1030.70	52.09
0	6	-2	1095.99	52.89
0	6	2	1112.79	52.49
-1	-6	-2	99.59	6.50
-1	-6	-2	106.99	5.70
1	-6	2	94.09	5.70
1	6	2	101.89	6.50
-1	6	-2	108.39	5.90
-1	6	-2	94.19	5.90
-1	6	-2	96.99	5.50
1	6	2	102.09	5.80
-2	-6	-2	82.99	6.10
2	-6	2	89.29	5.10

2	-6	2	88.39	5.30
-2	-6	-2	90.09	5.10
2	6	2	78.69	6.20
2	6	2	85.59	5.20
-2	6	-2	75.49	5.40
-2	6	-2	90.69	5.30
-3	-6	-2	288.47	14.20
3	-6	2	271.17	14.10
3	-6	2	261.77	14.40
-3	-6	-2	282.27	15.20
-3	6	-2	271.27	14.30
3	6	2	286.37	15.20
3	6	2	276.47	14.20
-3	6	-2	283.47	14.30
-4	-6	-2	551.24	27.70
4	-6	2	521.15	26.40
-4	-6	-2	543.75	26.50
4	-6	2	540.05	26.90
4	6	2	509.45	26.50
-4	6	-2	533.35	26.70
4	6	2	595.44	27.70
-4	6	-2	504.55	26.60
-5	-6	-2	288.97	14.00
5	-6	2	247.28	14.40
5	-6	2	262.97	13.80
-5	-6	-2	255.67	15.30
5	6	2	243.38	13.80
-5	6	-2	251.87	14.00
5	6	2	280.37	15.20
-5	6	-2	261.37	14.30
6	-6	2	333.07	17.70
-6	-6	-2	357.86	17.80
-6	-6	-2	328.77	19.10
6	-6	2	328.37	18.40
6	6	2	300.47	17.60
-6	6	-2	317.97	17.90
-6	6	-2	349.57	18.40
6	6	2	395.26	19.20
-7	-6	-2	354.86	21.40
7	-6	2	358.76	20.50
-7	-6	-2	417.96	20.10
7	-6	2	379.26	19.90
-7	6	-2	383.66	20.80
-7	6	-2	369.66	20.00

7	6	2	410.86	21.40
7	6	2	351.96	19.70
8	-6	2	15.80	4.50
-8	-6	-2	10.90	3.70
-8	-6	-2	15.70	5.30
-8	-6	-2	16.10	3.70
8	-6	2	12.00	3.60
-8	6	-2	16.00	4.20
-8	6	-2	15.90	4.50
8	6	2	15.50	4.70
-9	-6	-2	10.30	4.10
9	-6	2	7.30	3.40
-9	-6	-2	5.30	5.20
-9	-6	-2	10.40	4.50
-9	6	-2	0.90	3.60
-9	6	-2	5.90	4.30
9	6	2	12.10	5.00
10	-6	2	13.20	4.40
-10	-6	-2	11.60	6.70
-10	-6	-2	14.80	4.80
-10	-6	-2	16.20	5.20
-10	6	-2	10.60	4.70
-10	6	-2	12.10	5.80
10	6	2	15.10	5.80
11	-6	2	190.48	13.50
-11	-6	-2	196.08	13.50
-11	-6	-2	184.78	16.60
-11	6	-2	173.38	15.30
-11	6	-2	153.98	15.50
11	6	2	223.78	16.20
-12	-6	-2	11.60	5.50
-12	-6	-2	20.90	8.60
12	-6	2	5.90	5.30
12	6	2	9.00	6.80
-12	6	-2	12.60	6.10
-12	6	-2	5.30	6.80
-13	-6	-2	18.70	6.80
-13	6	-2	12.00	6.30
-14	-6	-2	28.10	7.60
-14	6	-2	17.60	7.20
-15	-6	-2	38.60	9.00
-15	6	-2	7.70	8.00
-15	-7	2	11.10	7.80
-15	7	2	11.60	7.30

-14	-7	2	247.28	17.50
-14	7	2	259.37	17.90
-13	-7	2	59.79	9.50
-13	7	2	54.89	9.00
13	7	-2	69.79	13.90
-12	-7	2	148.09	11.70
-12	-7	2	138.09	18.20
12	-7	-2	146.19	13.40
-12	7	2	109.89	18.40
-12	7	2	171.18	12.40
12	7	-2	129.89	15.30
-11	-7	2	427.06	28.90
-11	-7	2	521.15	25.80
11	-7	-2	473.15	26.50
-11	-7	2	490.75	25.00
11	7	-2	479.65	28.00
-11	7	2	448.36	28.40
-11	7	2	406.96	26.50
-10	-7	2	655.53	33.90
10	-7	-2	663.43	35.20
-10	-7	2	752.72	34.60
-10	-7	2	614.24	37.00
-10	7	2	615.04	36.50
10	7	-2	691.93	36.60
-10	7	2	676.53	34.40
-9	-7	2	275.17	19.50
-9	-7	2	350.06	17.20
-9	-7	2	302.37	16.50
9	-7	-2	292.27	17.90
-9	7	2	268.27	19.00
9	7	-2	319.57	19.40
-9	7	2	289.47	16.80
8	-7	-2	212.28	13.40
8	-7	-2	197.08	13.10
-8	-7	2	205.68	11.90
-8	-7	2	187.78	15.00
-8	-7	2	224.38	12.30
8	7	-2	212.58	14.40
-8	7	2	185.08	14.20
-8	7	2	229.78	12.70
7	-7	-2	856.31	42.80
7	-7	-2	867.31	43.00
-7	-7	2	735.03	45.10
-7	-7	2	929.71	41.90

-7	-7	2	830.22	41.70
-7	7	2	858.81	42.30
7	7	-2	913.71	43.90
-7	7	2	832.52	43.50
6	-7	-2	9.90	3.20
-6	-7	2	10.00	2.50
6	-7	-2	7.80	3.60
-6	-7	2	6.80	4.10
-6	7	2	4.40	3.20
6	7	-2	9.90	4.10
-6	7	2	10.30	3.00
5	-7	-2	1571.74	76.09
5	-7	-2	1610.24	76.29
-5	-7	2	1477.95	76.89
-5	-7	2	1654.73	75.19
-5	7	2	1535.85	75.69
5	7	-2	1655.13	76.79
-5	7	2	1583.94	76.49
5	7	-2	1485.05	75.09
4	-7	-2	169.88	9.90
4	-7	-2	167.08	9.90
-4	-7	2	162.88	10.70
-4	-7	2	171.28	8.70
-4	7	2	165.68	9.40
4	7	-2	167.88	10.60
-4	7	2	163.88	10.10
4	7	-2	156.88	8.60
-3	-7	2	319.07	17.50
3	-7	-2	340.47	17.00
3	-7	-2	307.27	16.80
-3	-7	2	318.17	15.90
-3	7	2	324.47	17.10
3	7	-2	345.57	17.50
-3	7	2	320.87	16.60
3	7	-2	318.27	15.90
2	-7	-2	166.08	9.40
-2	-7	2	171.28	10.00
2	-7	-2	162.78	9.30
-2	-7	2	170.18	8.40
2	7	-2	175.78	10.00
-2	7	2	168.78	9.60
-2	7	2	152.78	9.10
2	7	-2	158.38	8.30
-1	-7	2	386.66	20.20

1	-7	-2	406.26	20.80
1	-7	-2	431.56	21.10
-1	-7	2	425.76	21.20
1	7	-2	412.36	21.20
-1	7	2	447.76	21.10
-1	7	2	398.66	20.70
1	7	-2	410.96	20.10
0	-7	2	113.59	7.30
0	-7	2	123.89	6.80
0	-7	-2	132.59	7.00
0	7	-2	121.89	6.60
0	7	2	123.49	7.80
0	7	-2	126.49	7.60
0	7	2	138.19	7.70
0	7	2	125.29	7.30
-1	-7	-2	2212.28	108.09
-1	-7	-2	2308.47	107.39
1	-7	2	2307.07	107.39
1	7	2	2267.07	108.19
-1	7	-2	2204.08	107.29
-1	7	-2	2169.38	107.69
-1	7	-2	2313.47	107.59
-2	-7	-2	39.80	4.70
2	-7	2	37.60	3.70
-2	-7	-2	43.10	3.40
-2	7	-2	43.60	3.60
2	7	2	46.30	4.80
2	7	2	41.60	3.50
-2	7	-2	37.60	3.50
3	-7	2	353.16	17.60
-3	-7	-2	332.27	18.30
3	-7	2	336.37	17.20
3	7	2	347.67	18.40
-3	7	-2	342.27	17.40
-3	7	-2	332.27	17.30
3	7	2	339.77	17.20
-4	-7	-2	33.40	3.50
4	-7	2	32.10	3.40
4	-7	2	29.30	4.30
-4	-7	-2	27.10	5.30
4	7	2	24.90	3.40
-4	7	-2	32.50	3.80
4	7	2	29.80	5.10
-4	7	-2	26.90	3.80

-5	-7	-2	1724.43	77.99
5	-7	2	1625.24	77.99
5	-7	2	1647.14	78.49
-5	-7	-2	1584.84	79.29
5	7	2	1527.05	77.89
-5	7	-2	1587.94	78.09
-5	7	-2	1612.24	78.39
5	7	2	1667.73	79.09
6	-7	2	747.83	37.10
6	-7	2	761.12	37.80
-6	-7	-2	815.22	37.10
-6	-7	-2	663.43	38.30
-6	7	-2	746.53	37.60
-6	7	-2	763.62	37.30
6	7	2	786.32	38.30
6	7	2	707.43	36.90
7	-7	2	215.98	12.80
-7	-7	-2	226.38	15.00
7	-7	2	223.28	13.70
-7	-7	-2	246.48	13.00
7	7	2	215.08	12.80
-7	7	-2	201.38	13.60
7	7	2	244.88	14.60
-7	7	-2	211.58	12.90
-8	-7	-2	457.75	22.10
8	-7	2	404.06	21.90
8	-7	2	419.66	22.80
-8	-7	-2	375.86	23.90
-8	7	-2	415.26	22.10
-8	7	-2	412.26	23.00
8	7	2	389.06	21.60
8	7	2	422.96	23.60
-9	-7	-2	166.18	10.60
9	-7	2	138.49	10.00
-9	-7	-2	136.29	10.10
-9	-7	-2	108.29	12.50
-9	7	-2	133.29	10.20
9	7	2	140.59	12.30
-9	7	-2	124.59	11.40
10	-7	2	360.76	21.10
-10	-7	-2	407.76	21.30
-10	-7	-2	319.17	23.50
-10	-7	-2	426.56	21.60
-10	7	-2	370.16	21.00

10	7	2	396.46	23.30
-10	7	-2	358.86	22.70
-11	-7	-2	113.89	10.20
11	-7	2	97.79	10.10
-11	-7	-2	92.59	13.80
-11	7	-2	77.69	12.30
-11	7	-2	84.99	9.50
11	7	2	116.39	13.10
-12	-7	-2	540.05	39.60
12	-7	2	569.94	31.00
-12	-7	-2	603.44	31.30
-12	7	-2	509.05	32.80
-12	7	-2	614.94	31.00
12	7	2	588.84	33.30
-13	-7	-2	32.60	7.40
-13	7	-2	30.00	7.30
-14	-7	-2	205.08	16.30
-14	7	-2	195.38	15.10
-15	-7	-2	5.40	8.20
-15	7	-2	0.90	7.70
-15	-8	2	176.48	16.30
-15	8	2	176.28	15.80
-14	-8	2	36.80	7.90
-13	-8	2	106.59	11.20
-13	8	2	120.19	11.60
13	8	-2	112.89	15.30
-12	-8	2	295.97	17.90
-12	-8	2	258.27	23.10
12	-8	-2	271.77	19.10
12	8	-2	344.27	22.20
-12	8	2	328.77	18.80
11	-8	-2	280.97	18.40
-11	-8	2	277.17	22.30
-11	-8	2	303.67	17.80
-11	-8	2	295.57	17.10
-11	8	2	288.87	17.70
11	8	-2	309.57	20.70
-11	8	2	270.57	20.80
-10	-8	2	197.98	13.00
10	-8	-2	186.68	13.80
-10	-8	2	180.48	12.20
-10	-8	2	185.48	17.20
-10	8	2	198.68	13.10
-10	8	2	200.28	16.00

10	8	-2	206.48	15.80
-9	-8	2	213.58	13.00
-9	-8	2	246.48	13.60
-9	-8	2	207.28	16.60
9	-8	-2	216.98	14.50
9	8	-2	239.28	16.50
-9	8	2	227.38	13.80
-9	8	2	202.18	16.00
-8	-8	2	443.66	21.20
-8	-8	2	363.66	23.60
8	-8	-2	389.46	22.10
8	-8	-2	365.36	22.10
-8	-8	2	405.86	20.90
-8	8	2	414.56	21.60
8	8	-2	441.86	23.80
-8	8	2	391.96	23.20
7	-8	-2	213.48	14.50
-7	-8	2	258.37	13.10
7	-8	-2	232.18	14.10
-7	-8	2	217.98	15.80
-7	-8	2	226.28	12.90
-7	8	2	236.18	15.10
-7	8	2	229.28	13.60
7	8	-2	271.67	15.90
-6	-8	2	1312.97	63.49
6	-8	-2	1299.17	64.19
6	-8	-2	1334.37	63.99
-6	-8	2	1209.48	64.89
-6	8	2	1313.37	64.49
-6	8	2	1323.67	63.49
6	8	-2	1208.88	62.79
6	8	-2	1460.55	64.99
5	-8	-2	254.77	14.60
5	-8	-2	266.67	14.50
-5	-8	2	236.68	16.80
-5	-8	2	271.57	13.20
5	8	-2	224.28	13.80
-5	8	2	256.67	14.90
5	8	-2	285.47	15.40
-5	8	2	251.07	14.00
-4	-8	2	1214.68	59.19
4	-8	-2	1210.68	60.19
4	-8	-2	1260.87	60.39
-4	8	2	1220.48	60.49

-4	8	2	1231.28	59.89
4	8	-2	1359.56	60.99
4	8	-2	1166.58	59.19
-3	-8	2	63.79	4.10
3	-8	-2	72.39	5.80
3	-8	-2	70.69	5.50
-3	-8	2	73.49	6.90
3	8	-2	76.69	6.60
-3	8	2	70.29	5.70
-3	8	2	61.89	4.90
3	8	-2	68.79	4.20
2	-8	-2	1068.59	54.19
-2	-8	2	1084.79	53.39
2	-8	-2	1113.79	54.39
-2	-8	2	1136.79	54.89
-2	8	2	1055.79	53.99
-2	8	2	1183.38	54.59
2	8	-2	1064.59	53.29
2	8	-2	1235.28	54.89
-1	-8	2	0.50	2.00
1	-8	-2	7.80	2.20
1	-8	-2	1.60	1.80
-1	-8	2	-2.20	1.20
1	8	-2	2.80	1.20
-1	8	2	-1.80	1.70
1	8	-2	0.50	2.00
-1	8	2	2.20	2.10
0	-8	-2	3572.64	194.68
0	-8	2	4081.59	193.48
0	8	2	4175.68	193.78
0	8	-2	4035.10	193.28
0	8	2	4325.57	194.28
0	8	-2	4263.67	194.18
-1	-8	-2	251.47	14.10
-1	-8	-2	258.07	14.50
1	-8	2	277.17	13.70
1	8	2	264.87	14.70
1	8	2	267.47	13.80
-1	8	-2	280.97	14.00
-1	8	-2	258.77	14.10
2	-8	2	3165.78	152.18
-2	-8	-2	3186.88	152.48
2	8	2	3411.56	152.68
2	8	2	3097.39	151.48

-2	8	-2	3009.90	151.38
-2	8	-2	3240.58	151.78
-3	-8	-2	349.77	18.20
3	-8	2	368.86	18.70
-3	-8	-2	369.66	19.60
3	-8	2	351.36	18.30
-3	8	-2	350.36	18.30
3	8	2	395.86	19.80
3	8	2	345.37	18.20
-3	8	-2	362.36	18.60
-4	-8	-2	277.87	14.10
4	-8	2	267.37	14.20
4	-8	2	277.57	14.70
-4	-8	-2	266.77	15.60
-4	8	-2	263.17	14.30
4	8	2	247.08	14.10
4	8	2	296.97	15.70
-4	8	-2	258.37	14.40
-5	-8	-2	1144.89	59.29
-5	-8	-2	1265.57	57.89
5	-8	2	1221.98	58.49
5	-8	2	1172.88	57.89
-5	8	-2	1153.08	58.29
5	8	2	1328.47	59.39
5	8	2	1108.79	57.79
-5	8	-2	1165.68	57.99
6	-8	2	1479.15	71.79
-6	-8	-2	1384.56	72.59
6	-8	2	1449.76	71.09
-6	-8	-2	1577.94	71.19
-6	8	-2	1439.46	71.69
6	8	2	1354.16	70.99
-6	8	-2	1475.45	71.29
6	8	2	1606.34	72.59
7	-8	2	447.96	24.50
7	-8	2	441.86	23.50
-7	-8	-2	493.35	23.70
-7	-8	-2	439.16	25.50
7	8	2	498.65	25.40
-7	8	-2	441.06	23.80
-7	8	-2	446.56	24.40
7	8	2	432.06	23.40
8	-8	2	147.39	11.20
-8	-8	-2	170.58	10.90

-8	-8	-2	160.78	13.60
8	-8	2	162.78	10.50
8	8	2	147.89	10.50
-8	8	-2	174.08	11.00
8	8	2	181.08	13.10
-8	8	-2	153.08	11.90
-9	-8	-2	865.41	39.40
-9	-8	-2	700.83	41.20
9	-8	2	760.62	39.10
-9	-8	-2	754.52	39.10
-9	8	-2	787.32	39.30
-9	8	-2	736.43	40.20
9	8	2	799.02	41.00
-10	-8	-2	56.09	9.20
-10	-8	-2	69.09	8.70
-10	-8	-2	73.59	8.90
10	-8	2	56.89	8.00
-10	8	-2	63.59	10.30
-10	8	-2	66.09	8.40
10	8	2	62.19	11.50
11	-8	2	916.31	47.20
-11	-8	-2	840.32	49.99
-11	-8	-2	990.10	47.40
11	8	2	1015.60	49.70
-11	8	-2	936.71	47.40
-11	8	-2	880.81	48.90
-12	-8	-2	74.89	10.90
12	-8	2	81.69	14.00
-12	8	-2	71.69	14.80
-12	8	-2	106.59	11.20
12	8	2	81.49	14.30
-13	-8	-2	276.87	18.60
-13	8	-2	285.47	18.70
-14	-8	-2	5.70	7.30
-14	8	-2	7.00	6.70
-15	-8	-2	228.68	19.60
-15	8	-2	250.57	18.60
-15	-9	2	24.30	8.30
-15	9	2	14.50	7.30
-14	-9	2	56.39	9.00
-14	9	2	66.49	11.30
-13	-9	2	48.90	7.60
13	9	-2	42.70	9.90
-13	9	2	62.59	10.10

-12	-9	2	6.70	5.30
12	-9	-2	3.20	8.20
12	9	-2	16.20	8.20
-12	9	2	15.80	6.20
11	-9	-2	57.59	9.30
-11	-9	2	81.49	9.50
-11	-9	2	66.49	8.50
-11	9	2	85.69	9.60
11	9	-2	94.49	13.20
-10	-9	2	18.60	5.00
-10	-9	2	14.80	4.30
10	-9	-2	9.20	4.70
-10	-9	2	11.50	7.80
-10	9	2	15.80	5.20
10	9	-2	19.70	7.10
-10	9	2	14.40	6.60
-9	-9	2	2.70	3.80
-9	-9	2	11.70	3.60
9	-9	-2	7.70	4.50
-9	-9	2	9.10	6.60
-9	9	2	4.00	4.30
9	9	-2	12.60	5.80
-9	9	2	6.00	5.30
-8	-9	2	128.29	8.70
8	-9	-2	115.29	9.70
-8	-9	2	111.69	12.40
-8	-9	2	114.39	8.40
8	9	-2	117.99	11.90
-8	9	2	108.19	11.20
-8	9	2	140.89	9.40
-7	-9	2	240.78	17.60
-7	-9	2	290.87	15.00
-7	-9	2	312.07	15.00
7	-9	-2	275.47	16.50
7	-9	-2	271.77	16.10
-7	9	2	281.37	15.70
7	9	-2	295.67	17.90
-7	9	2	252.47	17.00
-6	-9	2	58.99	4.90
6	-9	-2	65.09	7.20
6	-9	-2	61.49	6.50
-6	-9	2	57.39	8.40
6	9	-2	61.29	8.40
-6	9	2	49.40	7.00

6	9	-2	47.50	4.90
-6	9	2	66.09	6.00
5	-9	-2	110.39	7.90
-5	-9	2	115.49	6.50
5	-9	-2	104.69	8.50
-5	-9	2	100.09	9.40
5	9	-2	104.89	7.00
5	9	-2	97.69	9.20
-5	9	2	108.09	7.60
-5	9	2	104.19	8.40
4	-9	-2	18.10	3.50
-4	-9	2	12.90	4.10
4	-9	-2	17.40	3.80
-4	-9	2	21.60	2.60
4	9	-2	16.90	4.10
-4	9	2	19.40	3.20
-4	9	2	17.10	3.60
4	9	-2	20.50	2.80
-3	-9	2	521.65	28.70
3	-9	-2	563.44	28.10
-3	-9	2	570.44	27.00
3	-9	-2	539.35	28.10
3	9	-2	558.64	28.70
-3	9	2	549.74	27.60
3	9	-2	543.65	26.90
-3	9	2	588.04	28.40
-2	-9	2	39.70	5.30
-2	-9	2	31.10	3.00
2	-9	-2	38.50	4.50
2	-9	-2	42.30	4.50
-2	9	2	33.20	4.00
2	9	-2	30.50	2.80
2	9	-2	36.30	5.30
-2	9	2	32.30	4.60
-1	-9	2	247.38	13.40
1	-9	-2	241.08	12.80
-1	-9	2	231.38	12.10
1	9	-2	224.68	13.40
-1	9	2	264.57	13.40
1	9	-2	225.98	11.90
-1	9	2	216.38	12.60
0	-9	2	2.20	1.70
0	-9	-2	4.60	2.00
0	9	2	3.60	2.10

0	9	-2	2.30	2.30
0	9	-2	2.70	1.50
0	9	2	0.80	2.30
0	9	2	-1.70	2.60
1	-9	2	796.02	38.00
-1	-9	-2	825.42	38.00
-1	-9	-2	735.03	38.70
-1	9	-2	761.22	37.70
1	9	2	782.52	39.10
1	9	2	761.52	38.00
-1	9	-2	730.23	38.40
-1	9	-2	837.72	38.40
-2	-9	-2	719.13	34.90
-2	-9	-2	659.13	36.00
2	-9	2	747.43	35.30
-2	9	-2	722.23	34.90
2	9	2	699.83	35.00
-2	9	-2	743.53	35.40
-3	-9	-2	121.09	8.90
3	-9	2	106.19	7.00
-3	-9	-2	109.99	6.80
3	-9	2	101.79	7.30
-3	9	-2	109.39	7.30
-3	9	-2	109.69	6.90
3	9	2	100.19	6.80
3	9	2	126.99	9.10
4	-9	2	19.20	3.50
-4	-9	-2	22.30	3.50
4	-9	2	20.40	3.60
-4	-9	-2	16.00	4.00
4	9	2	9.10	4.00
-4	9	-2	19.20	4.00
4	9	2	22.20	3.70
-4	9	-2	22.50	3.90
-5	-9	-2	191.38	10.00
5	-9	2	162.78	10.10
5	-9	2	166.98	11.00
-5	-9	-2	153.68	11.90
5	9	2	169.28	12.10
5	9	2	178.38	10.00
-5	9	-2	172.28	10.60
-5	9	-2	161.08	10.20
-6	-9	-2	130.69	11.30
6	-9	2	138.99	9.60

6	-9	2	134.49	8.80
-6	-9	-2	135.79	8.80
-6	9	-2	120.69	9.50
-6	9	-2	149.99	9.30
6	9	2	123.29	8.60
6	9	2	140.29	11.10
7	-9	2	9.10	4.20
-7	-9	-2	11.70	5.20
-7	-9	-2	10.80	3.50
7	-9	2	12.20	3.40
-7	9	-2	18.30	4.00
7	9	2	11.40	3.50
-7	9	-2	12.10	4.10
7	9	2	14.40	5.00
-8	-9	-2	6.50	4.00
-8	-9	-2	7.70	5.40
8	-9	2	2.50	3.40
8	9	2	10.60	5.30
8	9	2	4.50	3.50
-8	9	-2	4.90	3.80
-8	9	-2	2.40	4.10
-9	-9	-2	40.20	8.30
-9	-9	-2	45.60	7.30
9	-9	2	46.90	7.10
-9	9	-2	67.79	9.40
9	9	2	52.19	7.40
9	9	2	70.19	12.10
-9	9	-2	58.29	7.80
-10	-9	-2	22.00	7.60
-10	-9	-2	21.10	5.60
10	-9	2	22.60	5.10
-10	-9	-2	17.80	5.20
10	9	2	32.70	14.20
-10	9	-2	16.20	5.60
-10	9	-2	17.80	5.80
-11	-9	-2	75.49	10.00
-11	-9	-2	42.20	18.40
11	-9	2	63.49	9.30
-11	9	-2	85.79	9.80
-11	9	-2	64.19	11.80
-12	-9	-2	115.19	11.90
-12	9	-2	96.29	10.50
-13	-9	-2	17.70	7.10
-13	9	-2	19.40	6.50

-14	-9	-2	9.40	7.40
-14	9	-2	13.50	7.60
-15	-9	-2	1.50	8.60
-15	9	-2	19.90	8.40
-15	-10	2	380.16	24.00
-15	10	2	379.96	24.10
-14	-10	2	41.80	8.30
-14	10	2	64.09	11.40
-13	-10	2	146.29	13.00
-13	10	2	157.18	13.60
13	10	-2	158.38	26.70
-12	-10	2	210.98	14.80
12	10	-2	215.88	19.80
-12	10	2	239.78	15.70
-11	-10	2	535.55	27.00
-11	-10	2	412.26	31.70
-11	-10	2	503.05	26.50
11	10	-2	534.95	30.40
-11	10	2	454.35	30.20
-11	10	2	517.55	27.30
-10	-10	2	537.65	35.30
-10	-10	2	657.43	31.50
-10	-10	2	618.44	31.30
-10	10	2	575.64	34.30
10	10	-2	639.64	34.40
-9	-10	2	461.75	21.90
-9	-10	2	415.76	21.50
-9	-10	2	366.16	25.40
9	-10	-2	388.56	22.60
-9	10	2	385.66	24.30
-9	10	2	404.96	22.20
9	10	-2	403.56	24.70
8	-10	-2	1400.46	70.99
-8	-10	2	1537.55	69.89
-8	-10	2	1447.96	69.89
-8	-10	2	1284.57	72.49
8	10	-2	1554.04	72.49
-8	10	2	1439.06	72.09
-8	10	2	1426.46	70.39
-7	-10	2	26.40	3.60
7	-10	-2	15.60	4.40
-7	-10	2	20.90	6.30
7	-10	-2	28.60	5.60
-7	10	2	17.30	3.90

-7	10	2	21.90	5.80
7	10	-2	28.70	6.00
7	10	-2	15.80	3.40
-6	-10	2	1601.14	82.99
6	-10	-2	1619.24	82.09
6	-10	-2	1689.53	81.79
-6	-10	2	1703.43	80.49
6	10	-2	1813.32	82.79
6	10	-2	1577.54	80.49
-6	10	2	1734.63	81.39
-6	10	2	1703.53	82.39
5	-10	-2	296.77	16.30
-5	-10	2	284.67	14.80
5	-10	-2	290.57	16.80
-5	-10	2	290.77	17.80
5	10	-2	282.17	14.90
-5	10	2	274.97	16.60
5	10	-2	313.17	17.40
-5	10	2	287.67	15.80
4	-10	-2	2190.28	107.09
-4	-10	2	2177.68	105.69
-4	-10	2	2214.98	107.89
4	-10	-2	2280.27	106.99
4	10	-2	2347.17	107.69
-4	10	2	2290.07	107.29
-4	10	2	2247.48	106.49
4	10	-2	2015.00	105.79
-3	-10	2	72.29	4.20
3	-10	-2	57.29	6.10
3	-10	-2	57.99	5.70
-3	-10	2	55.29	6.90
-3	10	2	59.29	5.40
3	10	-2	53.59	6.90
3	10	-2	59.69	4.20
-3	10	2	60.59	6.10
2	-10	-2	1452.25	72.19
-2	-10	2	1496.95	72.89
-2	-10	2	1473.75	71.29
2	-10	-2	1537.25	72.49
-2	10	2	1440.76	71.89
2	10	-2	1559.64	72.89
2	10	-2	1426.76	71.19
-2	10	2	1545.45	72.59
-1	-10	2	97.99	7.40

-1	-10	2	86.59	5.80
1	-10	-2	97.59	6.70
-1	10	2	88.39	7.50
1	10	-2	97.89	5.70
1	10	-2	94.99	7.40
-1	10	2	84.69	6.50
-1	10	2	103.79	7.20
0	-10	2	4323.57	208.08
0	-10	-2	4492.75	208.38
0	10	-2	4318.07	208.98
0	10	2	4477.65	208.98
0	10	-2	4284.67	207.88
0	10	2	4412.76	209.18
0	10	2	4355.96	208.38
1	-10	2	410.56	20.80
-1	-10	-2	411.26	20.70
1	10	2	413.26	22.10
1	10	2	410.46	21.50
-1	10	-2	400.46	20.50
-1	10	-2	395.96	21.40
1	10	2	418.86	20.90
-1	10	-2	427.76	21.30
-2	-10	-2	2550.64	120.89
-2	-10	-2	2569.14	122.19
2	-10	2	2585.44	121.19
-2	10	-2	2394.76	120.79
2	10	2	2663.13	122.29
-2	10	-2	2492.95	121.49
-2	10	-2	2588.94	121.39
2	10	2	2426.96	120.89
-3	-10	-2	1059.59	50.89
3	-10	2	1087.69	51.49
-3	-10	-2	1011.00	52.29
-3	10	-2	1050.09	51.39
3	10	2	1114.39	52.59
3	10	2	1009.20	50.89
-3	10	-2	1026.10	50.89
-4	-10	-2	1475.95	70.09
4	-10	2	1478.85	70.89
-4	-10	-2	1404.96	71.79
4	-10	2	1457.55	70.39
4	10	2	1407.66	70.19
-4	10	-2	1449.26	70.69
4	10	2	1517.15	71.79

-4	10	-2	1460.25	70.29
5	-10	2	2470.15	115.29
5	-10	2	2363.76	114.69
-5	-10	-2	2260.17	116.09
-5	-10	-2	2485.25	114.49
-5	10	-2	2369.36	115.09
5	10	2	2283.57	114.39
5	10	2	2551.44	116.19
-5	10	-2	2345.67	114.59
-6	-10	-2	798.22	38.60
6	-10	2	766.22	38.80
6	-10	2	774.72	39.60
-6	-10	-2	748.33	40.70
-6	10	-2	766.72	39.40
6	10	2	736.03	38.60
6	10	2	855.71	40.70
-6	10	-2	799.92	39.00
7	-10	2	1252.87	60.99
-7	-10	-2	1216.88	63.09
7	-10	2	1205.18	61.79
-7	-10	-2	1339.27	60.89
7	10	2	1159.68	60.59
-7	10	-2	1207.68	60.89
-7	10	-2	1194.28	61.59
7	10	2	1387.36	62.89
-8	-10	-2	244.38	14.00
8	-10	2	220.58	13.80
-8	-10	-2	225.08	16.80
8	10	2	266.47	16.90
-8	10	-2	212.38	13.70
-8	10	-2	233.08	15.20
8	10	2	220.88	13.60
9	-10	2	1467.45	72.89
-9	-10	-2	1629.64	72.99
-9	-10	-2	1320.57	80.29
9	10	2	1606.94	75.19
-9	10	-2	1441.16	74.19
9	10	2	1393.56	72.59
-9	10	-2	1544.85	73.19
-10	-10	-2	78.79	14.30
10	-10	2	94.19	9.40
-10	-10	-2	76.19	9.80
-10	-10	-2	109.39	10.00
10	10	2	85.59	13.50

-10	10	-2	85.19	11.30
-11	-10	-2	481.15	32.50
-11	-10	-2	553.34	29.10
11	-10	2	534.35	28.80
-11	10	-2	536.05	28.60
-11	10	-2	473.55	30.50
11	10	2	588.24	32.10
-12	-10	-2	118.89	12.10
12	10	2	118.89	16.10
-12	10	-2	103.19	11.80
-13	-10	-2	343.27	22.20
-13	10	-2	361.86	22.10
-14	-10	-2	27.30	8.50
-14	10	-2	15.90	7.90
-15	-10	-2	252.07	20.20
-15	10	-2	231.68	18.60
-14	-11	2	61.19	10.30
-14	11	2	38.40	8.40
-13	-11	2	-0.50	6.00
-13	11	2	1.80	6.30
-12	-11	2	75.29	9.70
12	11	-2	102.99	15.90
-12	11	2	59.49	9.50
-11	-11	2	167.18	19.50
-11	-11	2	156.68	12.10
-11	-11	2	143.29	11.50
-11	11	2	144.99	11.90
11	11	-2	159.68	16.60
-10	-11	2	5.80	4.30
10	-11	-2	1.40	3.50
-10	-11	2	19.90	5.00
-10	-11	2	7.90	8.10
10	11	-2	4.60	7.00
-10	11	2	13.50	7.10
-10	11	2	10.10	5.30
-9	-11	2	78.49	13.70
-9	-11	2	73.39	7.40
-9	-11	2	86.99	7.80
9	-11	-2	55.29	8.10
9	11	-2	81.39	11.70
-9	11	2	86.39	8.60
-9	11	2	69.69	10.90
-8	-11	2	336.97	16.90
-8	-11	2	279.87	20.60

-8	-11	2	316.77	16.90
8	-11	-2	292.97	18.10
-8	11	2	286.47	19.50
8	11	-2	345.87	20.30
-8	11	2	305.07	17.70
-7	-11	2	610.64	29.40
-7	-11	2	589.04	33.00
7	-11	-2	561.54	30.70
7	11	-2	689.13	32.70
-7	11	2	582.74	31.80
7	11	-2	527.25	29.30
-7	11	2	583.34	30.30
-6	-11	2	59.89	9.50
6	-11	-2	36.20	7.00
-6	-11	2	45.70	4.60
6	-11	-2	39.50	6.50
-6	11	2	44.50	7.40
6	11	-2	61.59	8.70
6	11	-2	50.29	4.80
-6	11	2	52.19	6.30
5	-11	-2	480.25	26.00
-5	-11	2	484.95	27.50
5	-11	-2	468.85	26.50
-5	11	2	500.05	25.60
5	11	-2	557.94	27.30
-5	11	2	518.85	26.70
5	11	-2	470.85	24.60
-4	-11	2	2.10	4.00
4	-11	-2	2.60	2.80
4	-11	-2	0.30	3.10
-4	-11	2	4.40	1.80
-4	11	2	2.50	2.70
-4	11	2	1.60	3.20
4	11	-2	0.90	1.90
4	11	-2	-1.60	4.00
3	-11	-2	337.57	18.30
-3	-11	2	332.37	19.30
-3	-11	2	338.47	16.90
3	-11	-2	301.07	18.30
-3	11	2	340.87	18.60
-3	11	2	323.47	17.70
3	11	-2	384.66	19.40
-2	-11	2	74.99	5.00
2	-11	-2	66.89	6.00

-2	-11	2	80.39	7.50
2	-11	-2	80.89	6.60
-2	11	2	73.99	6.80
-2	11	2	56.39	5.70
2	11	-2	75.39	7.30
-1	-11	2	466.45	24.20
1	-11	-2	471.15	23.50
-1	-11	2	425.86	22.80
1	11	-2	488.45	24.40
1	11	-2	457.45	22.70
-1	11	2	467.85	24.10
-1	11	2	440.46	23.30
-1	11	2	458.75	24.30
0	-11	-2	141.29	8.40
0	-11	2	132.69	8.20
0	11	-2	135.39	9.30
0	11	2	129.19	8.30
0	11	2	137.09	9.10
0	11	2	136.29	9.90
0	11	-2	138.39	7.80
-1	-11	-2	131.39	7.60
1	-11	2	113.39	7.80
1	11	2	126.39	9.30
1	11	2	119.69	7.70
1	11	2	129.29	8.60
-1	11	-2	128.69	8.20
-1	11	-2	120.89	8.60
-1	11	-2	119.89	7.40
2	-11	2	74.69	6.30
-2	-11	-2	83.89	5.60
-2	11	-2	75.79	6.60
2	11	2	77.69	7.60
2	11	2	79.09	5.80
-2	11	-2	87.39	6.50
-2	11	-2	71.09	5.50
3	-11	2	15.60	3.50
-3	-11	-2	8.70	4.30
-3	-11	-2	13.10	2.50
3	11	2	12.00	2.70
-3	11	-2	7.70	2.50
3	11	2	13.70	4.50
-3	11	-2	14.60	3.30
4	-11	2	12.70	3.80
4	-11	2	9.80	2.90

-4	-11	-2	21.00	3.70
-4	-11	-2	14.30	4.70
-4	11	-2	11.50	3.10
4	11	2	15.10	5.00
4	11	2	11.90	2.80
-4	11	-2	13.40	3.10
5	-11	2	372.86	20.20
-5	-11	-2	338.37	22.70
-5	-11	-2	393.76	19.90
5	-11	2	387.36	21.00
5	11	2	444.66	22.60
-5	11	-2	388.66	20.30
5	11	2	381.46	20.00
-5	11	-2	378.16	20.70
6	-11	2	271.07	16.10
-6	-11	-2	277.17	14.70
-6	-11	-2	216.08	23.20
6	-11	2	254.57	14.80
6	11	2	247.98	14.60
6	11	2	309.77	17.50
-6	11	-2	250.37	14.90
-6	11	-2	261.07	15.60
-7	-11	-2	7.60	9.00
-7	-11	-2	3.70	3.50
7	-11	2	3.40	3.20
7	-11	2	11.10	4.70
7	11	2	13.60	5.50
-7	11	-2	2.30	3.90
-7	11	-2	12.40	4.20
7	11	2	3.40	3.40
-8	-11	-2	86.19	8.10
8	-11	2	77.79	7.90
-8	-11	-2	82.39	11.90
8	11	2	78.89	7.90
-8	11	-2	83.69	8.20
-8	11	-2	77.99	9.30
9	-11	2	91.99	9.10
-9	-11	-2	107.29	9.60
-9	-11	-2	95.19	13.20
9	11	2	129.19	13.00
-9	11	-2	110.09	11.30
-9	11	-2	108.29	9.70
9	11	2	78.79	8.90
-10	-11	-2	191.88	18.20

10	-11	2	185.68	13.50
-10	11	-2	191.68	13.80
10	11	2	225.88	17.30
-10	11	-2	188.58	15.50
-11	-11	-2	58.89	10.10
11	-11	2	53.19	9.10
-11	-11	-2	47.50	10.90
11	11	2	75.49	13.40
-11	11	-2	76.19	9.90
-11	11	-2	36.40	8.20
-12	-11	-2	173.28	14.50
12	11	2	179.88	17.70
-12	11	-2	161.18	13.30
-13	-11	-2	10.30	7.30
-13	11	-2	12.50	6.80
-14	-11	-2	74.99	13.80
-14	11	-2	84.89	12.50
-14	-12	2	36.60	9.70
-14	12	2	69.29	11.90
-13	-12	2	81.19	11.60
-13	12	2	95.99	12.00
-12	-12	2	270.47	16.90
-12	12	2	248.88	17.40
12	12	-2	267.87	22.30
-11	-12	2	184.28	13.30
-11	-12	2	153.18	20.40
-11	-12	2	179.08	12.90
11	12	-2	180.88	18.10
-11	12	2	194.48	14.00
-10	-12	2	232.88	21.40
-10	-12	2	251.97	15.00
-10	-12	2	249.68	15.00
10	12	-2	251.27	19.50
-10	12	2	237.28	15.30
-10	12	2	225.08	18.70
-9	-12	2	196.88	18.70
-9	-12	2	233.88	13.50
-9	-12	2	226.28	13.30
-9	12	2	224.88	16.90
-9	12	2	209.28	15.30
9	12	-2	221.18	17.20
-8	-12	2	316.47	16.30
8	-12	-2	279.97	17.60
-8	-12	2	268.07	21.00

-8	12	2	313.87	17.40
8	12	-2	313.07	19.90
-8	12	2	287.07	19.20
-7	-12	2	15.60	6.40
7	-12	-2	16.40	4.50
-7	-12	2	13.80	3.30
-7	12	2	19.50	4.40
-7	12	2	8.80	4.90
7	12	-2	14.60	3.30
7	12	-2	16.30	6.00
-6	-12	2	1406.56	66.09
6	-12	-2	1382.26	67.59
-6	-12	2	1281.47	68.99
6	-12	-2	1318.27	67.99
6	12	-2	1302.17	66.09
-6	12	2	1408.26	68.29
-6	12	2	1413.36	66.99
6	12	-2	1464.85	68.79
5	-12	-2	6.30	4.30
5	-12	-2	6.20	3.40
-5	-12	2	9.40	2.20
-5	-12	2	4.60	5.10
-5	12	2	4.80	3.30
5	12	-2	7.60	2.50
-5	12	2	2.00	3.50
5	12	-2	-3.40	4.20
4	-12	-2	708.23	37.40
4	-12	-2	753.82	37.20
-4	-12	2	707.13	38.30
-4	12	2	770.62	37.60
4	12	-2	739.13	38.00
-4	12	2	724.73	36.60
4	12	-2	730.03	35.70
-3	-12	2	58.49	4.50
-3	-12	2	48.50	7.40
3	-12	-2	43.30	6.50
3	-12	-2	57.89	6.20
3	12	-2	56.29	4.50
-3	12	2	59.29	6.50
-3	12	2	54.59	5.70
3	12	-2	56.69	7.50
-2	-12	2	272.67	16.00
2	-12	-2	272.17	15.10
-2	-12	2	241.08	14.80

-2	12	2	258.37	14.50
2	12	-2	279.47	16.10
2	12	-2	268.27	13.80
-2	12	2	284.37	15.60
1	-12	-2	5.80	2.70
-1	-12	2	2.40	2.10
-1	-12	2	6.10	3.50
-1	12	2	2.00	2.90
-1	12	2	-1.00	3.10
1	12	-2	6.90	3.70
1	12	-2	5.40	2.10
-1	12	2	1.20	2.70
0	-12	-2	1131.49	54.49
0	-12	2	1128.69	54.39
0	12	2	1099.59	55.59
0	12	2	1103.09	54.49
0	12	-2	1108.49	55.39
0	12	2	1161.18	55.29
0	12	-2	1107.29	54.09
-1	-12	-2	28.00	4.00
1	-12	2	25.60	3.60
1	12	2	18.70	4.70
1	12	2	23.70	4.10
-1	12	-2	23.00	4.60
-1	12	-2	25.70	3.70
1	12	2	26.80	3.60
-1	12	-2	24.80	4.40
-2	-12	-2	433.96	23.50
-2	-12	-2	444.56	21.80
2	-12	2	456.65	22.40
-2	12	-2	404.56	21.60
-2	12	-2	449.06	22.60
2	12	2	432.56	21.90
-2	12	-2	394.96	22.60
2	12	2	413.86	23.50
-3	-12	-2	78.89	5.70
3	-12	2	75.29	6.80
-3	-12	-2	73.49	8.30
3	12	2	75.59	6.00
3	12	2	54.09	7.90
-3	12	-2	76.09	6.00
-3	12	-2	79.09	6.70
-4	-12	-2	576.94	31.10
4	-12	2	587.84	29.90

-4	-12	-2	608.44	28.90
-4	12	-2	558.74	29.00
4	12	2	551.14	28.90
-4	12	-2	573.74	29.70
4	12	2	586.54	31.00
-5	-12	-2	173.08	14.10
5	-12	2	194.58	11.70
5	-12	2	198.58	12.80
-5	-12	-2	202.88	11.20
5	12	2	185.38	13.80
5	12	2	185.78	11.30
-5	12	-2	188.68	11.60
-5	12	-2	194.18	12.20
6	-12	2	687.23	35.10
-6	-12	-2	757.22	34.90
6	-12	2	675.53	36.10
-6	-12	-2	660.83	37.20
6	12	2	751.92	37.30
-6	12	-2	643.44	35.70
-6	12	-2	705.03	35.20
6	12	2	685.53	34.80
-7	-12	-2	393.86	20.10
-7	-12	-2	331.47	22.90
7	-12	2	372.86	20.30
-7	12	-2	341.67	21.10
7	12	2	377.36	20.00
-7	12	-2	401.56	20.60
7	12	2	383.46	22.60
-8	-12	-2	159.68	10.60
-8	-12	-2	129.09	14.40
8	-12	2	156.98	10.90
8	12	2	149.09	10.60
8	12	2	163.18	14.00
-8	12	-2	151.78	11.10
-8	12	-2	136.19	12.10
-9	-12	-2	194.68	18.40
-9	-12	-2	237.48	14.50
9	-12	2	235.18	14.50
-9	12	-2	212.28	16.00
9	12	2	220.68	17.60
-9	12	-2	230.88	14.80
9	12	2	222.28	14.10
-10	-12	-2	25.60	9.60
10	-12	2	60.79	8.90

-10	12	-2	55.29	10.90
10	12	2	47.30	8.30
10	12	2	41.50	9.00
-10	12	-2	71.89	9.00
-11	-12	-2	483.85	26.90
11	12	2	491.85	30.10
-11	12	-2	456.15	26.60
-12	-12	-2	70.19	11.70
-12	12	-2	76.19	11.10
-13	-12	-2	150.28	15.00
-13	12	-2	156.38	14.00
-14	-12	-2	13.90	8.90
-14	12	-2	24.10	7.30
-14	13	2	238.78	18.20
-13	-13	2	110.89	12.40
-12	-13	2	243.08	16.60
12	13	-2	242.08	22.20
-12	13	2	270.57	17.70
-11	-13	2	403.26	22.20
-11	-13	2	332.17	34.80
-11	-13	2	383.46	22.00
-11	13	2	392.96	23.00
11	13	-2	430.26	27.50
-10	-13	2	138.39	10.90
-10	-13	2	126.09	17.70
-10	-13	2	145.79	10.70
-10	13	2	143.19	11.90
10	13	-2	151.18	16.10
-10	13	2	120.09	15.00
-9	-13	2	291.17	23.70
-9	-13	2	354.86	18.60
-9	-13	2	332.87	18.70
9	13	-2	372.96	22.80
-9	13	2	348.17	19.70
-9	13	2	315.57	22.20
8	-13	-2	37.70	7.10
-8	-13	2	57.89	6.30
-8	-13	2	36.20	8.80
8	13	-2	45.70	5.90
8	13	-2	60.29	10.80
-8	13	2	49.40	9.70
-8	13	2	48.70	6.80
-7	-13	2	765.52	36.00
-7	-13	2	654.63	39.60

7	-13	-2	724.23	37.70
-7	13	2	721.43	36.90
-7	13	2	719.73	38.60
7	13	-2	820.12	39.50
7	13	-2	697.33	36.00
-6	-13	2	66.29	5.20
6	-13	-2	56.79	7.40
-6	-13	2	51.89	9.80
6	13	-2	45.10	5.30
6	13	-2	57.89	9.70
-6	13	2	56.29	6.40
-6	13	2	52.49	8.30
-5	-13	2	755.42	36.70
-5	-13	2	733.83	39.80
5	-13	-2	750.62	38.40
5	-13	-2	707.33	38.90
-5	13	2	749.33	37.80
5	13	-2	833.32	39.80
-5	13	2	759.52	38.90
5	13	-2	731.53	36.80
-4	-13	2	7.80	2.20
4	-13	-2	-2.90	4.10
4	-13	-2	4.90	3.40
-4	-13	2	-0.40	4.30
4	13	-2	0.80	4.70
-4	13	2	-0.90	3.40
-4	13	2	0.40	3.20
4	13	-2	1.60	2.10
-3	-13	2	872.81	44.60
3	-13	-2	903.21	43.70
3	-13	-2	818.02	43.50
-3	-13	2	883.41	42.10
3	13	-2	849.32	42.10
-3	13	2	819.32	42.80
-3	13	2	886.91	43.90
3	13	-2	909.91	44.50
-2	-13	2	10.10	4.00
-2	-13	2	12.70	2.70
2	-13	-2	7.10	3.70
-2	13	2	5.80	3.00
-2	13	2	5.10	3.90
-2	13	2	9.70	3.70
2	13	-2	13.30	2.40
2	13	-2	8.30	3.90

1	-13	-2	1589.14	77.29
-1	-13	2	1555.24	77.99
-1	-13	2	1568.54	76.69
-1	13	2	1577.34	77.09
1	13	-2	1579.04	76.49
-1	13	2	1658.23	77.99
-1	13	2	1573.64	78.19
1	13	-2	1655.53	78.29
0	-13	-2	68.89	6.00
0	-13	2	58.69	5.70
0	13	-2	59.19	5.20
0	13	-2	62.29	7.30
0	13	2	59.19	5.80
0	13	2	66.69	6.60
0	13	2	58.39	7.40
1	-13	2	1015.80	49.99
-1	-13	-2	1059.89	49.90
1	13	2	1031.50	49.90
1	13	2	1037.90	51.49
-1	13	-2	1022.80	50.89
-1	13	-2	1041.30	50.49
-1	13	-2	970.70	49.50
1	13	2	987.20	50.59
-2	-13	-2	226.28	12.20
-2	-13	-2	219.28	14.30
2	-13	2	221.68	13.00
-2	13	-2	211.78	12.10
-2	13	-2	215.48	13.40
2	13	2	225.98	14.60
-2	13	-2	225.58	13.10
2	13	2	211.78	12.40
-3	-13	-2	845.72	40.10
3	-13	2	830.82	41.00
-3	-13	-2	801.62	42.10
-3	13	-2	794.72	41.10
3	13	2	796.92	40.20
-3	13	-2	817.22	41.00
3	13	2	845.42	42.30
-3	13	-2	800.92	40.20
-4	-13	-2	458.75	22.90
-4	-13	-2	447.16	25.30
4	-13	2	451.85	24.10
4	13	2	429.76	23.00
-4	13	-2	451.75	23.20

4	13	2	459.95	25.40
-4	13	-2	446.26	23.90
-5	-13	-2	1006.40	48.40
5	-13	2	995.00	48.90
-5	-13	-2	990.40	50.89
5	-13	2	976.30	49.60
-5	13	-2	953.30	49.30
5	13	2	1072.89	50.89
-5	13	-2	970.40	48.70
5	13	2	943.01	48.40
6	-13	2	848.02	43.60
6	-13	2	838.32	44.50
-6	-13	-2	861.81	46.00
-6	-13	-2	923.91	43.20
6	13	2	946.41	45.80
6	13	2	855.01	43.30
-6	13	-2	852.11	43.50
-6	13	-2	860.91	44.30
7	-13	2	281.27	16.50
-7	-13	-2	268.47	19.70
-7	-13	-2	306.17	16.20
-7	13	-2	276.47	17.40
7	13	2	311.57	19.30
-7	13	-2	283.37	16.40
7	13	2	262.07	16.00
-8	-13	-2	402.86	27.50
-8	-13	-2	493.05	24.30
8	-13	2	473.25	24.70
-8	13	-2	442.36	25.80
-8	13	-2	471.75	24.80
8	13	2	473.35	27.10
8	13	2	429.86	24.20
-9	-13	-2	43.30	7.80
-9	-13	-2	39.90	10.30
9	-13	2	41.80	8.00
-9	13	-2	40.00	7.30
9	13	2	42.00	8.70
9	13	2	23.90	5.40
-9	13	-2	33.20	6.60
-10	-13	-2	358.56	26.90
10	-13	2	392.36	22.30
10	13	2	341.87	21.40
10	13	2	436.96	25.70
-10	13	-2	384.06	22.10

-10	13	-2	393.76	24.20
-11	-13	-2	90.19	11.80
11	13	2	86.49	15.50
-11	13	-2	112.79	11.60
-12	13	-2	265.57	17.60
-13	-13	-2	-8.60	7.40
-13	13	-2	-2.30	7.10
-14	-13	-2	345.47	23.70
-14	13	-2	322.17	22.00
-14	14	2	13.50	8.00
-13	-14	2	2.30	6.70
-13	14	2	-0.70	7.00
-12	-14	2	39.60	7.10
-12	14	2	58.19	10.70
12	14	-2	31.90	10.40
-11	-14	2	6.10	5.30
-11	14	2	9.50	6.00
11	14	-2	10.40	9.40
-10	-14	2	21.10	5.30
-10	-14	2	22.80	10.10
-10	-14	2	22.20	5.20
-10	14	2	13.20	5.90
10	14	-2	13.30	8.80
-9	-14	2	53.39	7.40
-9	-14	2	25.30	10.80
-9	-14	2	43.60	7.10
-9	14	2	29.50	8.50
9	14	-2	46.70	10.00
-9	14	2	51.39	8.60
-8	-14	2	31.20	4.40
-8	-14	2	31.00	9.30
-8	14	2	21.70	6.50
8	14	-2	28.60	8.00
-8	14	2	19.60	6.50
8	14	-2	27.00	4.30
-7	-14	2	16.10	3.40
7	-14	-2	8.40	4.30
-7	-14	2	6.90	7.00
7	14	-2	6.90	3.10
-7	14	2	3.10	5.20
-7	14	2	9.80	4.60
7	14	-2	6.50	6.00
-6	-14	2	92.89	11.80
6	-14	-2	62.69	8.20

-6	-14	2	77.69	6.00
6	14	-2	61.09	10.30
-6	14	2	70.59	7.80
6	14	-2	66.49	6.20
-6	14	2	68.99	9.40
5	-14	-2	48.30	8.90
-5	-14	2	56.29	5.10
5	-14	-2	55.59	7.40
-5	-14	2	58.69	10.20
5	14	-2	53.79	9.20
-5	14	2	52.99	6.90
5	14	-2	56.79	5.40
-5	14	2	57.69	8.00
-4	-14	2	91.89	10.70
4	-14	-2	78.59	9.30
-4	-14	2	92.79	6.20
4	-14	-2	88.59	8.40
4	14	-2	109.09	10.50
-4	14	2	79.59	7.70
-4	14	2	85.19	9.00
4	14	-2	90.69	6.30
-3	-14	2	13.20	5.00
3	-14	-2	8.80	4.40
-3	-14	2	19.50	2.90
3	14	-2	16.00	2.70
-3	14	2	15.80	4.00
3	14	-2	9.30	5.10
-3	14	2	12.60	4.50
-2	-14	2	66.79	8.10
2	-14	-2	64.99	7.10
-2	-14	2	67.89	5.40
-2	14	2	73.69	7.40
-2	14	2	58.69	6.20
2	14	-2	60.69	8.20
-2	14	2	65.09	8.30
2	14	-2	59.99	5.20
-1	-14	2	-2.50	2.60
1	-14	-2	0.40	3.00
-1	14	2	-3.10	3.90
1	14	-2	-0.40	3.70
-1	14	2	-0.90	2.80
1	14	-2	-0.70	2.30
-1	14	2	1.60	3.60
0	-14	-2	382.16	20.80

0	-14	2	403.86	20.70
0	14	-2	400.96	21.40
0	14	2	381.76	22.40
0	14	2	388.56	20.80
0	14	-2	414.86	20.30
0	14	-2	377.26	22.00
0	14	2	406.66	21.80
1	-14	2	1.00	3.00
-1	-14	-2	0.70	2.70
1	14	2	0.60	2.80
-1	14	-2	-1.60	3.50
1	14	2	-2.10	4.40
-1	14	-2	0.00	3.00
1	14	2	-0.60	3.50
-1	14	-2	0.60	2.60
2	-14	2	42.90	6.10
-2	-14	-2	56.59	5.10
-2	-14	-2	43.30	7.90
-2	14	-2	34.10	6.60
-2	14	-2	39.20	5.10
-2	14	-2	41.90	5.90
2	14	2	38.30	6.30
2	14	2	38.90	5.20
3	-14	2	24.30	4.90
-3	-14	-2	26.60	3.70
-3	-14	-2	15.10	5.30
-3	14	-2	15.40	4.10
3	14	2	20.80	3.70
-3	14	-2	18.50	3.50
-3	14	-2	21.80	4.20
3	14	2	12.00	5.40
-4	-14	-2	132.89	8.50
-4	-14	-2	113.39	11.70
4	-14	2	125.09	10.10
4	14	2	114.99	11.40
-4	14	-2	125.59	8.80
4	14	2	128.49	8.70
-4	14	-2	131.89	9.70
-5	-14	-2	89.89	7.30
5	-14	2	72.09	9.40
-5	-14	-2	84.09	11.30
5	14	2	95.29	7.40
-5	14	-2	98.49	8.00
-5	14	-2	91.59	8.60

5	14	2	89.69	11.00
-6	-14	-2	26.90	7.20
6	-14	2	24.70	4.60
-6	-14	-2	35.60	5.60
-6	14	-2	30.60	5.70
-6	14	-2	35.00	6.50
6	14	2	18.50	6.30
6	14	2	22.60	4.50
7	-14	2	19.00	4.40
-7	-14	-2	5.50	6.80
-7	-14	-2	19.80	4.40
-7	14	-2	15.90	4.40
7	14	2	22.10	4.60
7	14	2	17.60	7.00
-7	14	-2	12.00	5.10
-8	-14	-2	99.59	8.80
-8	-14	-2	66.39	13.50
8	-14	2	93.99	9.30
-8	14	-2	106.79	9.60
8	14	2	89.09	8.80
-8	14	-2	92.49	10.80
8	14	2	99.69	12.70
-9	-14	-2	67.29	8.30
9	-14	2	52.99	8.60
-9	-14	-2	50.89	11.10
-9	14	-2	35.80	7.10
9	14	2	49.60	7.70
-9	14	-2	66.99	8.90
9	14	2	41.20	8.70
-10	-14	-2	27.40	10.00
-10	14	-2	15.80	7.40
10	14	2	13.60	5.50
-10	14	-2	20.80	6.40
10	14	2	35.30	9.30
-11	-14	-2	23.30	7.30
11	14	2	26.90	9.80
-11	14	-2	21.70	6.90
-12	-14	-2	1.40	8.80
-12	14	-2	13.00	7.10
-13	-14	-2	9.90	8.10
-13	14	-2	11.20	7.20
-14	-14	-2	18.10	9.80
-14	14	-2	18.30	8.40
-13	15	2	99.49	13.00

-12	-15	2	73.09	11.00
12	15	-2	86.89	17.60
-12	15	2	95.59	12.00
-11	-15	2	195.98	14.70
-11	15	2	207.78	15.10
11	15	-2	256.57	21.60
-10	-15	2	177.88	12.50
-10	-15	2	132.89	20.70
-10	-15	2	159.08	14.70
-10	15	2	183.68	13.50
10	15	-2	170.58	18.00
-9	-15	2	569.44	39.30
-9	-15	2	707.23	34.10
9	15	-2	761.02	38.50
-9	15	2	640.54	37.70
-9	15	2	683.93	35.20
-8	-15	2	41.70	9.90
-8	-15	2	51.79	6.40
-8	15	2	38.50	7.70
8	15	-2	42.50	6.40
8	15	-2	48.10	8.90
-8	15	2	52.49	8.20
-7	-15	2	92.29	6.90
7	-15	-2	77.89	9.10
-7	-15	2	83.19	13.50
-7	15	2	85.09	8.10
7	15	-2	102.19	12.40
7	15	-2	65.39	6.80
-7	15	2	65.59	10.70
-6	-15	2	82.49	6.40
6	-15	-2	72.09	8.50
-6	-15	2	59.39	11.80
-6	15	2	77.29	7.70
6	15	-2	81.59	11.30
-6	15	2	70.99	9.80
6	15	-2	75.09	6.40
-5	-15	2	1004.80	48.60
-5	-15	2	948.81	51.89
5	-15	-2	1016.10	50.59
-5	15	2	1013.40	49.80
5	15	-2	1051.99	51.79
5	15	-2	956.00	48.70
-5	15	2	1018.10	51.09
-4	-15	2	152.48	9.00

4	-15	-2	130.79	11.20
4	-15	-2	149.59	11.30
-4	-15	2	155.38	13.20
4	15	-2	165.68	13.10
-4	15	2	137.19	10.40
-4	15	2	159.28	11.70
4	15	-2	153.78	9.10
-3	-15	2	896.31	42.20
-3	-15	2	811.72	44.70
3	-15	-2	829.42	43.80
3	15	-2	856.61	42.10
3	15	-2	897.31	44.80
-3	15	2	855.11	43.00
-3	15	2	891.91	44.20
2	-15	-2	248.18	15.60
-2	-15	2	268.87	14.20
-2	-15	2	253.67	16.70
-2	15	2	248.08	14.90
-2	15	2	268.67	16.10
2	15	-2	285.87	17.00
2	15	-2	250.47	13.90
-2	15	2	267.37	16.70
-1	-15	2	1136.69	55.89
1	-15	-2	1129.89	56.49
1	15	-2	1157.78	55.69
-1	15	2	1156.48	57.29
-1	15	2	1148.49	57.79
1	15	-2	1167.28	57.79
-1	15	2	1139.39	56.29
0	-15	-2	11.90	3.60
0	-15	2	11.20	3.70
0	15	2	8.50	4.70
0	15	2	16.10	4.30
0	15	2	10.10	3.60
0	15	-2	11.30	3.10
0	15	-2	14.20	5.00
0	15	-2	22.90	4.50
1	-15	2	2288.47	108.39
-1	-15	-2	2276.97	108.09
-1	15	-2	2238.18	109.39
-1	15	-2	2220.78	108.89
1	15	2	2265.87	109.19
1	15	2	2291.57	109.99
-1	15	-2	2178.28	107.79

1	15	2	2234.38	108.09
-2	-15	-2	129.69	8.00
2	-15	2	107.09	8.90
-2	15	-2	106.49	7.80
-2	15	-2	116.19	9.90
2	15	2	92.89	10.60
2	15	2	110.39	8.10
2	15	2	93.09	10.40
-2	15	-2	103.29	8.90
-3	-15	-2	1047.10	54.09
3	-15	2	1058.69	52.79
-3	-15	-2	1093.09	51.89
-3	15	-2	1055.39	52.89
-3	15	-2	1017.00	53.09
-3	15	-2	1039.20	51.89
3	15	2	1112.89	54.39
3	15	2	1060.79	51.99
-4	-15	-2	741.73	40.70
-4	-15	-2	799.52	38.10
4	-15	2	767.92	39.40
4	15	2	779.32	40.70
-4	15	-2	739.63	38.20
4	15	2	767.42	38.20
-4	15	-2	768.72	39.20
5	-15	2	847.92	45.10
-5	-15	-2	855.11	46.60
-5	-15	-2	907.71	43.60
-5	15	-2	878.01	43.90
5	15	2	945.21	46.60
5	15	2	875.31	43.70
-5	15	-2	865.31	44.80
-6	-15	-2	297.97	16.40
-6	-15	-2	307.97	20.40
6	15	2	306.27	19.90
-6	15	-2	275.87	17.80
-6	15	-2	285.27	16.90
6	15	2	292.37	16.60
-7	-15	-2	470.25	23.50
-7	-15	-2	394.66	27.30
7	-15	2	443.06	24.30
-7	15	-2	458.65	24.00
7	15	2	503.85	27.30
-7	15	-2	413.36	25.00
7	15	2	416.96	23.50

8	-15	2	603.84	31.90
-8	-15	-2	620.74	31.20
-8	-15	-2	541.65	35.20
-8	15	-2	653.23	35.60
8	15	2	577.54	31.20
-8	15	-2	575.54	32.90
8	15	2	657.23	34.80
9	-15	2	46.90	8.50
-9	-15	-2	65.39	12.00
9	15	2	63.79	12.90
-9	15	-2	30.70	7.20
-9	15	-2	54.99	8.10
9	15	2	44.40	8.20
10	15	2	709.13	38.90
10	15	2	613.74	34.70
-10	15	-2	691.33	35.70
-11	15	-2	36.40	7.50
11	15	2	42.20	10.50
-12	-15	-2	366.46	23.40
-12	15	-2	348.47	22.20
-13	-15	-2	31.60	9.30
-13	15	-2	21.00	7.70
-13	16	2	33.40	8.70
-12	-16	2	124.89	14.60
-12	16	2	133.89	13.70
-11	-16	2	-3.50	5.50
11	16	-2	17.10	10.10
-11	16	2	7.90	6.80
-10	-16	2	119.89	11.00
-10	16	2	119.59	12.00
10	16	-2	132.29	17.80
-9	-16	2	25.60	10.30
-9	-16	2	27.80	4.90
-9	16	2	35.20	7.10
9	16	-2	29.20	9.20
-8	-16	2	128.59	16.90
-8	-16	2	139.79	9.40
8	16	-2	160.98	15.30
-8	16	2	113.59	13.80
-8	16	2	142.39	11.50
8	16	-2	113.39	9.30
-7	-16	2	7.10	3.40
7	-16	-2	7.50	4.80
-7	-16	2	4.00	7.90

-7	16	2	7.90	5.80
7	16	-2	10.70	3.60
7	16	-2	7.40	7.30
6	-16	-2	267.27	16.10
-6	-16	2	243.18	13.30
-6	-16	2	225.98	18.60
-6	16	2	234.38	14.90
6	16	-2	264.27	17.90
-6	16	2	224.38	16.50
6	16	-2	213.08	13.30
-5	-16	2	170.98	15.70
5	-16	-2	193.78	13.60
-5	-16	2	203.28	11.10
5	16	-2	183.98	11.10
-5	16	2	202.28	12.50
5	16	-2	202.08	15.80
-5	16	2	191.88	14.30
-4	-16	2	28.50	4.80
-4	-16	2	24.90	6.50
-4	16	2	19.90	5.10
4	16	-2	24.30	4.40
-4	16	2	24.80	5.50
4	16	-2	19.40	6.20
-3	-16	2	26.50	6.90
-3	-16	2	30.60	4.80
3	-16	-2	24.10	6.00
3	16	-2	22.40	6.10
-3	16	2	39.30	6.40
-3	16	2	26.10	5.90
-3	16	2	21.30	4.90
3	16	-2	23.90	4.30
-2	-16	2	29.30	5.90
2	-16	-2	31.50	5.70
-2	-16	2	32.80	5.10
2	16	-2	35.20	6.50
-2	16	2	26.50	4.70
-2	16	2	41.30	7.10
-2	16	2	27.30	5.30
2	16	-2	31.30	4.70
-1	-16	2	3.30	3.30
1	-16	-2	-2.10	3.70
1	16	-2	-1.80	2.60
-1	16	2	-4.80	4.70
-1	16	2	-6.50	4.00

-1	16	2	-2.10	3.50
1	16	-2	-10.40	4.40
0	-16	2	55.79	6.90
0	-16	-2	53.29	7.00
0	16	2	60.69	8.20
0	16	-2	53.89	6.10
0	16	2	82.19	9.90
0	16	-2	68.69	7.60
0	16	2	59.59	7.00
0	16	-2	77.59	9.20
-1	-16	-2	4.00	3.50
1	-16	2	9.10	4.10
-1	16	-2	-0.70	4.30
1	16	2	4.50	4.20
-1	16	-2	4.70	3.70
1	16	2	8.00	5.50
-1	16	-2	7.00	3.30
1	16	2	3.60	3.60
-2	-16	-2	92.99	7.30
2	-16	2	71.49	8.50
-2	16	-2	83.09	9.20
-2	16	-2	88.99	8.30
2	16	2	77.29	7.30
2	16	2	65.79	8.30
-2	16	-2	71.49	6.90
2	16	2	89.49	10.40
-3	-16	-2	63.29	6.30
-3	-16	-2	61.39	8.50
3	-16	2	41.90	8.00
-3	16	-2	53.49	6.30
-3	16	-2	56.79	8.40
3	16	2	47.60	8.00
-3	16	-2	53.89	7.40
3	16	2	57.49	6.50
-4	-16	-2	233.18	17.60
-4	-16	-2	271.77	14.50
4	-16	2	224.68	16.30
4	16	2	274.67	18.10
-4	16	-2	276.67	16.50
-4	16	-2	251.47	14.70
4	16	2	242.68	14.50
-4	16	-2	247.98	15.70
-5	-16	-2	4.60	6.30
-5	-16	-2	10.40	3.80

-5	16	-2	8.00	4.10
-5	16	-2	2.30	3.90
5	16	2	11.30	6.20
5	16	2	7.30	3.90
-6	-16	-2	137.49	9.70
-6	-16	-2	128.89	14.40
6	16	2	170.48	14.50
6	16	2	126.59	9.90
-6	16	-2	123.99	10.40
-6	16	-2	134.29	11.50
-7	-16	-2	67.19	10.90
-7	-16	-2	64.39	7.50
7	16	2	53.29	7.60
-7	16	-2	54.19	7.90
7	16	2	71.49	12.20
-7	16	-2	47.00	9.10
-8	-16	-2	2.00	9.20
8	-16	2	4.20	4.90
-8	-16	-2	14.60	4.40
-8	16	-2	10.90	5.20
8	16	2	7.20	7.30
8	16	2	8.20	4.60
-8	16	-2	10.50	5.90
-9	-16	-2	49.60	10.90
-9	16	-2	30.60	6.60
9	16	2	67.09	13.80
9	16	2	29.00	5.90
10	16	2	33.30	9.80
-10	16	-2	56.49	9.80
10	16	2	37.30	6.50
11	16	2	120.69	12.00
-11	16	-2	117.09	12.90
-12	-16	-2	6.30	8.30
-12	16	-2	11.20	7.20
-13	-16	-2	50.19	10.60
-13	16	-2	55.69	9.60
-12	17	2	91.39	12.40
-11	-17	2	219.58	15.40
11	17	-2	205.88	22.20
-11	17	2	206.28	16.40
-10	-17	2	173.68	12.90
-10	17	2	162.58	14.00
10	17	-2	146.29	19.80
9	17	-2	139.19	11.10

-9	17	2	182.58	13.60
9	17	-2	182.78	18.20
-8	-17	2	6.40	3.80
8	-17	-2	0.70	4.10
-8	-17	2	21.20	11.20
8	17	-2	4.70	4.00
-8	17	2	11.90	6.10
8	17	-2	18.90	8.00
-7	-17	2	532.25	25.80
-7	-17	2	416.96	31.10
7	-17	-2	521.45	28.40
-7	17	2	558.34	27.70
7	17	-2	495.05	26.00
-7	17	2	485.15	29.30
7	17	-2	528.35	30.50
-6	-17	2	13.80	7.80
6	-17	-2	4.50	4.80
-6	-17	2	13.10	3.40
6	17	-2	9.60	3.60
6	17	-2	13.30	6.90
-6	17	2	2.20	5.30
-6	17	2	7.60	5.00
-5	-17	2	877.91	41.00
-5	-17	2	753.32	44.60
-5	17	2	841.42	42.30
5	17	-2	812.42	41.00
5	17	-2	903.21	44.90
-5	17	2	814.92	43.70
-4	-17	2	5.50	3.20
-4	-17	2	3.30	5.90
4	17	-2	4.10	5.80
-4	17	2	-4.20	4.30
-4	17	2	-3.20	4.80
4	17	-2	3.90	2.90
-3	-17	2	338.17	16.90
-3	-17	2	269.97	19.80
3	17	-2	323.77	20.30
-3	17	2	313.97	20.00
3	17	-2	320.37	16.60
-3	17	2	305.17	19.30
-3	17	2	310.47	17.90
-2	-17	2	17.40	6.30
-2	-17	2	14.00	3.80
2	-17	-2	20.30	5.50

-2	17	2	13.20	4.90
-2	17	2	7.10	4.20
2	17	-2	7.10	5.30
-2	17	2	12.90	5.60
2	17	-2	16.20	3.40
-1	-17	2	317.77	16.70
1	-17	-2	287.37	17.40
-1	17	2	316.27	18.40
1	17	-2	303.07	16.10
-1	17	2	287.17	16.90
-1	17	2	279.47	18.90
1	17	-2	275.37	18.90
0	-17	-2	72.49	7.60
0	-17	2	61.79	7.50
0	17	2	57.19	8.60
0	17	2	64.59	7.30
0	17	-2	64.49	8.00
0	17	2	51.69	9.70
0	17	-2	77.29	6.60
0	17	-2	64.99	9.70
-1	-17	-2	390.76	20.30
1	-17	2	354.66	20.70
1	17	2	371.56	22.90
1	17	2	401.16	20.50
-1	17	-2	373.76	19.90
-1	17	-2	365.36	22.10
-1	17	-2	360.66	21.10
2	-17	2	7.40	4.80
-2	-17	-2	0.40	3.50
-2	17	-2	1.30	3.30
2	17	2	3.50	4.20
-2	17	-2	2.70	3.80
2	17	2	-5.10	5.50
2	17	2	3.70	3.80
-2	17	-2	2.10	4.70
-3	-17	-2	83.79	11.80
-3	-17	-2	108.19	8.10
3	-17	2	84.79	10.10
3	17	2	84.09	8.20
-3	17	-2	112.59	10.50
3	17	2	101.89	11.70
-3	17	-2	92.39	8.10
-3	17	-2	109.19	9.50
-4	-17	-2	539.95	26.90

-4	-17	-2	498.05	30.10
-4	17	-2	524.05	27.20
-4	17	-2	518.45	28.30
4	17	2	527.55	27.10
4	17	2	518.65	30.10
-4	17	-2	529.55	28.90
-5	-17	-2	354.26	18.30
-5	-17	-2	336.67	22.60
-5	17	-2	332.37	20.00
5	17	2	342.07	18.70
5	17	2	324.87	21.80
-5	17	-2	317.57	18.80
-6	-17	-2	418.16	21.60
-6	-17	-2	386.46	26.20
-6	17	-2	424.96	22.40
-6	17	-2	386.46	23.40
6	17	2	390.66	21.80
6	17	2	410.66	25.20
-7	-17	-2	78.19	7.90
-7	-17	-2	49.30	10.10
7	17	2	48.70	7.90
-7	17	-2	59.09	9.70
-7	17	-2	57.49	10.10
7	17	2	53.49	9.50
-8	-17	-2	134.79	18.00
-8	17	-2	144.19	13.50
8	17	2	142.59	11.40
8	17	2	153.88	16.40
-8	17	-2	156.18	11.50
9	17	2	47.60	10.20
9	17	2	38.70	6.70
-9	17	-2	53.99	9.50
-10	17	-2	156.98	13.50
10	17	2	142.09	12.40
10	17	2	147.19	18.30
11	17	2	31.70	7.40
-11	17	-2	38.50	7.60
-12	-17	-2	238.88	19.10
-12	17	-2	222.78	17.40
-12	18	2	236.18	17.50
-11	18	2	224.18	17.20
11	18	-2	224.88	23.30
-10	-18	2	251.37	16.40
-10	18	2	242.88	17.60

10	18	-2	244.18	23.30
9	18	-2	85.69	8.90
-9	18	2	99.29	10.60
9	18	-2	117.19	16.90
-8	18	2	622.04	32.00
8	18	-2	538.15	30.20
8	18	-2	636.54	35.30
7	-18	-2	-2.10	5.30
-7	-18	2	5.70	3.60
7	18	-2	2.30	3.70
7	18	-2	-0.50	7.30
-7	18	2	3.60	5.50
-7	18	2	5.40	7.20
-6	-18	2	981.40	47.50
-6	18	2	965.30	49.10
6	18	-2	1098.29	52.09
-6	18	2	888.51	50.49
6	18	-2	938.81	47.70
-5	-18	2	-1.10	3.30
-5	-18	2	1.10	8.20
-5	18	2	0.80	5.50
5	18	-2	-0.90	3.30
-5	18	2	0.30	4.80
5	18	-2	3.40	6.50
-4	-18	2	1253.57	70.29
-4	-18	2	1413.66	67.09
4	18	-2	1323.17	66.99
-4	18	2	1373.86	68.29
-4	18	2	1466.85	70.39
-4	18	2	1385.16	69.59
4	18	-2	1494.75	70.69
-3	-18	2	42.50	6.10
-3	-18	2	57.39	10.60
-3	18	2	32.70	5.80
-3	18	2	39.10	7.40
3	18	-2	51.19	10.50
3	18	-2	36.70	5.50
-3	18	2	35.10	7.50
-2	-18	2	551.14	27.20
-2	18	2	516.95	30.00
2	18	-2	520.65	26.80
2	18	-2	532.85	30.10
-2	18	2	527.95	28.00
-2	18	2	520.05	29.20

1	-18	-2	238.88	16.00
-1	-18	2	277.97	15.40
-1	18	2	278.47	16.00
1	18	-2	261.17	14.70
-1	18	2	274.07	18.30
-1	18	2	271.57	17.20
1	18	-2	236.58	16.40
1	18	-2	263.77	18.00
0	-18	-2	758.82	40.70
0	-18	2	812.32	40.70
0	18	2	809.42	40.80
0	18	-2	840.22	42.70
0	18	2	799.62	42.00
0	18	-2	794.82	41.60
0	18	2	824.12	43.20
0	18	-2	819.52	40.10
-1	-18	-2	542.65	29.20
1	-18	2	559.94	29.70
1	18	2	604.14	32.10
-1	18	-2	539.35	30.30
1	18	2	588.34	29.50
-1	18	-2	567.64	31.20
1	18	2	556.94	30.60
-1	18	-2	563.44	28.90
-2	-18	-2	239.48	13.90
2	-18	2	218.78	15.20
2	18	2	224.38	15.60
-2	18	-2	237.78	16.40
-2	18	-2	225.18	13.80
-2	18	-2	237.38	15.30
2	18	2	235.38	14.20
2	18	2	241.08	17.50
-3	-18	-2	342.77	18.00
3	-18	2	301.07	19.50
-3	18	-2	325.47	18.20
3	18	2	310.77	19.90
3	18	2	334.17	21.90
-3	18	-2	343.57	20.60
-3	18	-2	331.87	19.60
3	18	2	321.87	18.30
-4	-18	-2	33.90	6.10
-4	-18	-2	34.40	8.60
-4	18	-2	25.60	5.70
4	18	2	33.80	5.30

4	18	2	30.40	7.50
-4	18	-2	39.80	7.40
-4	18	-2	26.30	5.10
-5	-18	-2	377.06	24.70
-5	-18	-2	392.86	20.60
5	18	2	374.46	20.90
-5	18	-2	331.47	27.40
-5	18	-2	371.46	21.20
-5	18	-2	385.66	22.40
5	18	2	429.46	24.90
-6	-18	-2	113.19	9.20
-6	-18	-2	125.89	15.20
6	18	2	102.39	9.60
-6	18	-2	112.89	10.10
6	18	2	120.69	14.80
-6	18	-2	100.79	11.10
-7	-18	-2	184.38	19.20
-7	-18	-2	185.98	12.60
7	18	2	195.08	18.00
7	18	2	191.48	13.10
-7	18	-2	185.18	14.80
-7	18	-2	193.58	13.50
-8	-18	-2	42.60	10.90
8	18	2	31.40	6.20
8	18	2	48.60	10.20
-8	18	-2	43.70	7.10
-9	18	-2	154.38	12.80
9	18	2	124.39	11.80
9	18	2	131.99	17.20
-10	18	-2	15.00	6.70
10	18	2	5.30	9.60
10	18	2	13.30	6.10
-11	18	-2	90.09	12.40
-12	-18	-2	2.70	9.10
-12	18	-2	8.80	7.50
-11	19	2	70.19	12.30
-10	19	2	88.99	12.00
10	19	-2	66.29	13.50
9	19	-2	9.00	9.70
9	19	-2	-0.60	4.30
-9	19	2	12.90	7.00
8	19	-2	12.50	4.40
8	19	-2	21.50	9.90
-8	19	2	25.40	6.60

-7	-19	2	19.00	5.00
7	19	-2	11.50	4.20
-7	19	2	9.90	6.20
7	19	-2	11.90	8.70
-6	-19	2	45.00	6.30
-6	19	2	52.29	10.50
6	19	-2	40.10	9.10
-6	19	2	56.49	9.10
6	19	-2	42.60	6.70
-5	-19	2	13.70	3.90
5	19	-2	22.70	7.50
-5	19	2	12.70	5.90
-5	19	2	3.60	6.00
5	19	-2	14.00	3.90
-4	-19	2	4.90	3.50
-4	19	2	11.70	7.20
4	19	-2	16.90	7.70
4	19	-2	4.20	3.40
-4	19	2	0.30	5.00
-4	19	2	3.80	5.60
-3	-19	2	32.50	4.80
-3	19	2	21.80	5.50
-3	19	2	20.00	7.30
-3	19	2	43.30	7.80
3	19	-2	20.10	7.00
3	19	-2	34.00	5.60
-2	-19	2	6.70	4.20
-2	19	2	12.80	5.30
2	19	-2	17.40	3.90
-2	19	2	4.30	6.30
2	19	-2	8.60	6.20
-2	19	2	3.30	5.50
-1	-19	2	100.29	9.00
-1	19	2	92.29	10.60
1	19	-2	87.09	9.80
-1	19	2	101.79	12.30
-1	19	2	94.09	9.20
1	19	-2	93.59	11.90
1	19	-2	96.49	8.20
0	-19	2	29.60	5.90
0	-19	-2	34.50	7.40
0	19	-2	28.90	7.40
0	19	-2	28.50	5.70
0	19	2	17.90	7.20

0	19	2	25.90	5.70
0	19	-2	43.80	6.40
-1	-19	-2	118.89	9.80
1	-19	2	118.39	10.40
1	19	2	126.59	10.20
-1	19	-2	124.89	9.30
1	19	2	132.99	11.30
-1	19	-2	116.49	10.80
-1	19	-2	102.29	12.30
1	19	2	98.59	12.80
-2	-19	-2	17.50	4.50
2	-19	2	3.30	5.30
2	19	2	8.20	5.30
2	19	2	5.40	4.50
2	19	2	7.80	6.80
-2	19	-2	6.10	4.90
-2	19	-2	4.60	4.20
-2	19	-2	12.60	6.10
-3	-19	-2	14.90	4.50
3	19	2	4.60	4.20
-3	19	-2	6.40	5.70
-3	19	-2	-1.20	4.40
3	19	2	-0.20	6.50
-3	19	-2	3.30	4.20
3	19	2	2.20	5.00
-4	-19	-2	16.80	4.30
-4	-19	-2	0.20	6.80
-4	19	-2	7.50	6.70
4	19	2	-2.80	6.70
4	19	2	10.70	4.70
-4	19	-2	9.40	4.60
-4	19	-2	7.40	5.00
-5	-19	-2	87.69	14.80
-5	-19	-2	118.39	8.70
5	19	2	104.19	9.20
-5	19	-2	98.99	12.30
-5	19	-2	82.19	10.60
-5	19	-2	79.29	9.30
5	19	2	87.39	13.70
-6	-19	-2	41.10	11.00
-6	-19	-2	57.69	8.80
-6	19	-2	48.10	8.40
6	19	2	40.40	6.20
-6	19	-2	38.80	7.80

-6	19	-2	38.00	6.90
6	19	2	46.00	10.40
-7	-19	-2	17.10	10.40
7	19	2	14.40	8.50
7	19	2	14.90	5.30
-7	19	-2	22.20	6.10
8	19	2	6.20	8.70
-8	19	-2	9.10	6.30
8	19	2	6.20	5.40
-9	19	-2	20.60	6.60
9	19	2	18.80	6.10
9	19	2	8.90	10.40
10	19	2	12.50	6.40
-10	19	-2	23.60	7.60
11	19	2	33.80	7.40
-11	19	-2	33.30	7.70
-11	20	2	174.58	15.70
-10	20	2	327.37	20.80
10	20	-2	296.67	30.40
-9	20	2	360.96	22.70
9	20	-2	375.26	28.00
8	20	-2	363.66	21.10
8	20	-2	424.86	27.80
-8	20	2	395.86	23.40
7	20	-2	26.50	4.90
7	20	-2	30.70	9.60
-7	20	2	31.40	7.00
-6	-20	2	643.34	33.40
-6	20	2	663.33	34.60
6	20	-2	616.34	32.90
6	20	-2	690.53	37.70
-5	-20	2	119.69	9.10
5	20	-2	145.69	15.30
5	20	-2	124.59	9.20
-5	20	2	108.19	15.00
-5	20	2	114.39	11.60
-4	-20	2	820.42	40.70
-4	20	2	818.42	43.70
4	20	-2	769.82	40.60
4	20	-2	876.21	45.00
-4	20	2	824.52	45.40
-3	-20	2	10.80	4.40
3	20	-2	7.70	3.80
-3	20	2	-0.20	5.60

-3	20	2	3.90	5.20
3	20	-2	7.30	6.70
-3	20	2	4.70	6.50
-2	-20	2	665.73	33.70
2	20	-2	661.13	33.40
2	20	-2	621.34	35.40
2	20	-2	681.13	37.20
-2	20	2	666.93	36.10
-2	20	2	662.43	37.20
-2	20	2	673.43	34.60
-1	-20	2	29.40	5.50
-1	20	2	40.70	7.50
1	20	-2	19.10	5.80
-1	20	2	22.20	5.70
1	20	-2	31.40	7.60
-1	20	2	25.80	7.80
1	20	-2	22.00	4.60
0	-20	2	858.71	43.80
0	20	2	854.81	45.30
0	20	2	891.41	46.80
0	20	-2	849.12	46.00
0	20	-2	854.81	44.90
0	20	2	885.81	44.00
0	20	-2	873.81	43.30
1	-20	2	144.29	12.80
-1	20	-2	174.48	13.60
-1	20	-2	188.68	15.40
-1	20	-2	180.58	11.90
1	20	2	190.28	14.40
1	20	2	172.48	12.60
1	20	2	172.78	15.80
2	20	2	1330.47	66.39
-2	20	-2	1297.77	63.09
2	20	2	1318.57	63.29
-2	20	-2	1292.97	64.59
-2	20	-2	1190.68	72.19
2	20	2	1254.07	64.79
3	20	2	253.87	19.80
-3	20	-2	250.37	15.40
3	20	2	256.77	15.30
-3	20	-2	253.07	16.80
-3	20	-2	228.88	17.90
3	20	2	234.88	17.00
4	20	2	44.30	7.60

-4	20	-2	39.60	6.90
4	20	2	43.00	9.70
-4	20	-2	39.90	6.40
-4	20	-2	47.90	8.60
-5	-20	-2	560.34	36.00
5	20	2	610.64	31.60
-5	20	-2	650.23	34.50
-5	20	-2	564.64	33.80
5	20	2	639.94	35.60
-6	-20	-2	385.16	31.30
-6	20	-2	469.95	27.70
6	20	2	505.35	29.60
6	20	2	428.86	24.20
-6	20	-2	434.26	24.70
-7	20	-2	221.68	15.30
7	20	2	211.18	20.50
7	20	2	218.18	14.70
-8	20	-2	42.50	7.60
8	20	2	34.80	6.80
8	20	2	44.40	11.30
9	20	2	451.55	30.00
-9	20	-2	442.86	25.20
9	20	2	396.36	24.10
-10	20	-2	16.90	7.60
-11	20	-2	451.45	27.20
10	21	-2	14.50	12.60
-10	21	2	8.60	7.70
-9	21	2	31.30	8.30
9	21	-2	34.40	12.20
-8	21	2	48.90	8.50
-7	21	2	166.08	14.00
7	21	-2	158.58	11.70
7	21	-2	208.68	21.30
-6	21	2	32.40	9.60
6	21	-2	34.60	9.70
6	21	-2	31.00	5.20
-6	21	2	21.60	6.70
-5	21	2	164.48	13.50
-5	21	2	153.38	17.30
5	21	-2	153.48	11.00
5	21	-2	196.38	17.80
-4	-21	2	46.70	5.70
-4	21	2	54.39	10.30
4	21	-2	40.10	9.60

-4	21	2	57.49	8.20
4	21	-2	37.80	6.80
-3	-21	2	75.59	8.30
3	21	-2	99.19	14.30
-3	21	2	66.49	13.10
-3	21	2	83.79	13.20
3	21	-2	82.59	8.00
-2	-21	2	20.90	5.40
-2	21	2	12.40	7.70
2	21	-2	16.20	4.60
-2	21	2	23.60	7.10
2	21	-2	6.30	7.00
2	21	-2	14.60	6.40
-2	21	2	22.90	6.60
-1	-21	2	363.26	21.50
-1	21	2	333.57	26.70
-1	21	2	395.96	22.20
1	21	-2	373.76	23.10
1	21	-2	393.46	21.00
-1	21	2	428.46	25.90
1	21	-2	413.26	25.70
0	-21	2	42.20	6.70
0	21	-2	46.60	7.50
0	21	-2	50.39	7.60
0	21	2	42.00	7.90
0	21	2	40.10	9.30
0	21	2	52.19	8.90
1	21	2	21.50	5.70
-1	21	-2	11.20	7.30
-1	21	-2	20.10	5.90
1	21	2	12.90	7.80
-1	21	-2	10.50	4.90
1	21	2	15.00	6.20
2	21	2	50.49	8.50
2	21	2	41.70	7.80
-2	21	-2	44.90	7.70
-2	21	-2	45.10	8.70
-2	21	-2	60.09	8.10
2	21	2	50.49	9.90
-3	21	-2	120.59	12.60
3	21	2	133.49	15.90
-3	21	-2	122.39	14.10
3	21	2	108.99	13.10
-3	21	-2	108.69	10.20

3	21	2	121.69	10.50
-4	21	-2	11.30	7.10
4	21	2	-2.80	7.80
4	21	2	7.00	6.50
4	21	2	9.00	5.00
-4	21	-2	12.30	5.70
5	21	2	91.69	15.40
5	21	2	62.29	9.20
-5	21	-2	81.39	9.70
-5	21	-2	87.49	13.40
6	21	2	27.70	6.00
-6	21	-2	21.30	6.30
-6	21	-2	30.90	8.40
6	21	2	50.69	11.40
-7	21	-2	17.00	6.30
7	21	2	17.90	10.60
7	21	2	0.40	5.50
-7	21	-2	6.90	7.90
-8	21	-2	32.20	7.40
8	21	2	46.40	12.40
8	21	2	36.00	6.80
-9	21	-2	20.90	7.30
9	21	2	22.30	6.80
-10	21	-2	64.89	12.00
10	21	2	31.90	7.80
-9	22	2	156.28	14.50
9	22	-2	146.69	22.50
8	22	-2	115.39	19.30
-8	22	2	131.09	13.40
7	22	-2	-3.40	10.40
-7	22	2	12.20	7.20
-7	22	2	-5.90	9.90
-6	22	2	508.55	32.40
6	22	-2	527.45	32.20
6	22	-2	467.55	26.10
-6	22	2	504.25	28.30
5	22	-2	29.20	5.20
-5	22	2	18.40	9.10
5	22	-2	25.20	9.30
-5	22	2	34.10	7.60
4	22	-2	351.16	19.30
4	22	-2	327.27	24.40
-4	22	2	346.87	24.90
-4	22	2	374.96	21.50

-3	-22	2	-5.00	4.50
-3	22	2	-0.90	8.50
3	22	-2	-11.60	7.20
-3	22	2	-3.30	5.50
3	22	-2	-0.80	4.10
-2	-22	2	151.38	12.20
-2	22	2	162.88	17.10
2	22	-2	153.08	16.50
-2	22	2	135.89	13.90
2	22	-2	175.88	11.30
-1	-22	2	37.00	6.30
1	22	-2	42.20	10.00
-1	22	2	39.10	9.60
-1	22	2	32.20	6.80
1	22	-2	39.70	6.00
0	22	2	615.24	32.00
0	22	2	599.04	35.50
0	22	-2	582.74	34.80
0	22	-2	601.14	31.20
-1	22	-2	95.09	13.70
1	22	2	97.19	10.20
1	22	2	86.79	15.20
-1	22	-2	108.39	9.90
2	22	2	238.68	20.30
2	22	2	248.78	15.90
-2	22	-2	250.57	19.50
-2	22	-2	240.48	15.60
-3	22	-2	28.30	6.10
3	22	2	24.40	6.00
3	22	2	31.40	9.30
-3	22	-2	19.50	8.00
4	22	2	156.78	18.10
-4	22	-2	160.08	16.50
4	22	2	154.88	12.20
-4	22	-2	145.69	12.50
-5	22	-2	210.58	18.70
5	22	2	176.48	20.90
-5	22	-2	212.28	15.10
5	22	2	223.18	14.40
6	22	2	251.07	16.40
-6	22	-2	260.67	17.00
-6	22	-2	230.78	20.50
6	22	2	261.97	23.40
7	22	2	156.08	20.70

-7	22	-2	165.88	13.80
7	22	2	157.38	13.20
-7	22	-2	164.38	18.30
8	22	2	51.29	7.90
8	22	2	74.59	14.40
-8	22	-2	60.99	12.20
-8	22	-2	43.10	8.70
-9	22	-2	162.08	14.70
9	22	2	133.29	13.00
-9	23	2	89.99	13.80
9	23	-2	119.89	22.00
8	23	-2	40.30	12.90
-8	23	2	38.70	8.50
-8	23	2	46.20	13.10
-7	23	2	412.06	25.80
7	23	-2	434.76	30.90
-7	23	2	450.75	31.00
-6	23	2	26.10	10.80
6	23	-2	20.90	10.50
-6	23	2	20.30	7.70
5	23	-2	484.95	30.90
5	23	-2	425.06	24.60
-5	23	2	521.85	31.70
-5	23	2	454.25	26.80
4	23	-2	19.50	5.00
-4	23	2	2.00	9.30
4	23	-2	10.10	9.50
-4	23	2	0.40	6.50
3	23	-2	244.18	14.80
3	23	-2	246.08	21.20
-3	23	2	233.08	21.50
-3	23	2	244.18	16.80
2	23	-2	4.30	8.80
-2	23	2	2.00	6.00
2	23	-2	8.30	4.80
-2	23	2	4.90	8.50
1	23	-2	387.26	26.40
-1	23	2	390.36	22.90
-1	23	2	385.36	26.70
1	23	-2	391.36	21.60
0	23	-2	49.20	8.50
0	23	2	27.80	10.10
0	23	-2	51.59	10.70
-1	23	-2	395.06	22.10

1	23	2	393.66	22.70
-1	23	-2	387.86	26.30
2	23	2	22.80	6.10
2	23	2	8.50	9.30
-2	23	-2	10.00	7.90
-2	23	-2	22.30	6.00
3	23	2	222.48	20.80
-3	23	-2	236.48	15.30
-3	23	-2	220.08	19.80
3	23	2	224.68	15.30
4	23	2	104.09	17.30
-4	23	-2	91.09	11.20
-4	23	-2	124.39	15.90
4	23	2	108.89	10.70
-5	23	-2	183.48	14.30
-5	23	-2	187.48	18.80
5	23	2	167.78	13.60
5	23	2	220.18	21.00
-6	23	-2	249.58	21.60
6	23	2	265.07	23.50
-6	23	-2	231.88	16.90
6	23	2	243.18	16.50
7	23	2	77.69	14.70
7	23	2	67.79	10.90
-7	23	-2	106.29	17.00
-7	23	-2	88.39	11.80
-8	23	-2	267.77	22.70
-8	23	-2	253.07	18.10
8	23	2	213.28	16.60
-9	23	-2	19.30	8.20
-9	23	-2	23.40	12.00
8	24	-2	3.90	12.30
-8	24	2	7.30	8.50
-8	24	2	-6.10	11.10
7	24	-2	36.10	13.10
-7	24	2	9.30	7.90
-7	24	2	2.20	10.20
-6	24	2	32.20	8.30
6	24	-2	14.10	10.70
-6	24	2	17.90	11.10
5	24	-2	15.10	11.20
-5	24	2	-2.00	6.80
-5	24	2	3.90	9.60
-4	24	2	6.00	10.60

-4	24	2	12.80	7.10
4	24	-2	9.00	9.50
4	24	-2	10.40	4.90
3	24	-2	-8.30	9.10
-3	24	2	-3.40	6.50
-3	24	2	13.00	9.40
3	24	-2	1.60	4.80
2	24	-2	5.30	8.90
2	24	-2	7.20	5.00
-2	24	2	-2.10	6.20
-2	24	2	16.50	9.40
-1	24	2	3.40	10.10
-1	24	2	3.20	6.30
1	24	-2	3.10	5.20
1	24	-2	9.20	9.20
0	24	2	70.49	10.70
0	24	-2	81.89	9.60
0	24	-2	73.89	15.00
0	24	2	48.50	11.20
1	24	2	6.10	6.10
-1	24	-2	-7.80	7.70
1	24	2	-5.90	8.90
-1	24	-2	2.80	5.50
-2	24	-2	19.90	9.70
2	24	2	9.40	9.70
2	24	2	23.40	10.30
-2	24	-2	19.60	6.40
-3	24	-2	9.70	6.10
3	24	2	2.40	10.00
-3	24	-2	0.80	8.30
4	24	2	47.90	12.90
-4	24	-2	37.30	10.20
-4	24	-2	37.80	7.60
-5	24	-2	20.60	9.80
5	24	2	23.30	11.80
-5	24	-2	30.80	7.50
5	24	2	29.90	11.90
6	24	2	12.20	11.40
-6	24	-2	21.80	9.60
-6	24	-2	32.30	7.40
6	24	2	18.20	7.50
-7	24	-2	20.30	7.30
-7	24	-2	20.70	11.50
7	24	2	30.40	12.50

7	24	2	17.20	6.60
-8	24	-2	4.00	7.50
-8	24	-2	6.80	10.70
7	25	-2	87.49	16.40
-7	25	2	88.99	14.30
-7	25	2	134.39	21.90
-6	25	2	12.20	8.00
-6	25	2	34.70	12.20
6	25	-2	4.30	11.00
5	25	-2	312.47	26.70
-5	25	2	330.37	21.50
-5	25	2	339.87	27.20
4	25	-2	16.40	10.70
-4	25	2	12.70	10.70
-4	25	2	29.20	7.90
-3	25	2	377.86	22.70
-3	25	2	347.97	27.70
3	25	-2	378.16	27.30
3	25	-2	340.77	20.40
-2	25	2	22.50	10.70
-2	25	2	24.70	7.50
2	25	-2	23.60	6.10
2	25	-2	37.80	11.00
1	25	-2	376.86	27.50
-1	25	2	394.56	23.10
1	25	-2	370.46	21.70
-1	25	2	374.66	28.30
0	25	2	-3.30	9.80
0	25	-2	5.60	5.80
0	25	-2	0.30	8.80
0	25	2	10.10	6.80
-1	25	-2	421.76	25.30
-1	25	-2	430.86	29.80
1	25	2	443.46	25.70
1	25	2	504.25	31.70
2	25	2	45.10	11.70
-2	25	-2	44.00	7.50
-2	25	-2	54.89	11.60
2	25	2	47.10	8.00
-3	25	-2	214.98	21.20
3	25	2	217.28	15.80
-3	25	-2	210.18	16.00
3	25	2	231.58	23.20
4	25	2	250.77	24.20

-4	25	-2	233.18	17.20
4	25	2	218.78	16.30
-4	25	-2	259.57	22.40
5	25	2	182.68	14.70
-5	25	-2	180.68	15.70
-5	25	-2	198.28	20.80
5	25	2	196.28	22.60
6	25	2	59.69	11.00
6	25	2	65.69	15.40
-6	25	-2	65.79	13.80
-6	25	-2	72.39	12.00
-7	25	-2	138.59	20.90
-7	25	-2	167.48	15.20
-5	26	2	13.10	8.00
-5	26	2	-14.70	12.20
5	26	-2	14.00	12.00
-4	26	2	9.40	7.60
4	26	-2	1.80	10.50
-4	26	2	-7.50	10.70
-3	26	2	10.20	7.90
3	26	-2	17.40	12.30
-3	26	2	19.20	11.50
2	26	-2	52.79	12.40
-2	26	2	79.49	12.50
-2	26	2	68.59	13.40
2	26	-2	60.29	9.60
-1	26	2	21.10	7.80
1	26	-2	30.40	7.00
1	26	-2	9.20	10.10
-1	26	2	19.40	11.10
0	26	2	75.49	12.20
0	26	-2	79.29	13.80
0	26	-2	82.59	10.80
0	26	2	67.39	13.20
-1	26	-2	4.60	6.40
1	26	2	-4.80	6.70
-1	26	-2	-11.50	8.90
1	26	2	3.50	11.80
-2	26	-2	15.20	9.90
2	26	2	35.80	7.70
2	26	2	24.70	11.50
-2	26	-2	32.20	7.70
3	26	2	4.80	12.30
-3	26	-2	3.70	9.50

3	26	2	8.50	6.60
-3	26	-2	7.10	7.00
-4	26	-2	145.09	13.70
4	26	2	129.29	13.20
4	26	2	140.89	22.20
-4	26	-2	148.79	20.00
5	26	2	59.79	8.80
5	26	2	86.29	16.40
-5	26	-2	79.79	12.10
-5	26	-2	58.69	13.30
-3	27	2	104.39	14.30
3	27	-2	109.19	16.50
-3	27	2	110.49	21.80
2	27	-2	13.00	11.40
-2	27	2	17.90	12.60
-2	27	2	14.60	8.10
1	27	-2	172.98	13.50
1	27	-2	151.48	21.20
-1	27	2	132.39	15.10
-1	27	2	148.49	22.70
0	27	2	42.10	8.80
0	27	-2	38.80	7.40
0	27	-2	34.90	11.70
0	27	2	21.30	13.00
1	27	2	160.78	15.10
-1	27	-2	155.98	20.90
-1	27	-2	180.08	14.60
1	27	2	158.28	22.60
-2	27	-2	4.50	10.90
2	27	2	13.10	7.10
-2	27	-2	9.30	7.00
2	27	2	-0.10	11.70
-3	27	-2	104.09	12.20
3	27	2	90.99	17.10
3	27	2	66.59	11.90
-3	27	-2	63.29	12.70
-16	0	3	19.40	8.20
-15	0	3	-1.80	6.90
-14	0	3	10.00	6.10
13	0	-3	11.40	7.40
12	0	-3	0.30	7.80
-12	0	3	4.90	5.00
-11	0	3	14.40	7.60
-11	0	3	11.60	4.70

10	0	-3	11.90	5.60
-10	0	3	-2.10	5.00
-10	0	3	0.60	6.10
-10	0	3	-3.90	3.70
9	0	-3	13.80	4.10
-9	0	3	6.00	3.50
9	0	-3	8.80	5.10
-9	0	3	4.10	5.60
-8	0	3	2.60	4.30
8	0	-3	6.80	3.90
8	0	-3	4.20	3.40
-8	0	3	-4.90	2.80
7	0	-3	-0.60	3.50
7	0	-3	8.20	3.00
-7	0	3	2.70	4.00
-7	0	3	9.10	2.60
-6	0	3	-2.00	2.20
-6	0	3	-0.30	3.10
6	0	-3	-1.70	2.30
6	0	-3	-0.30	2.70
5	0	-3	3.20	2.10
-5	0	3	2.60	2.00
-5	0	3	-1.60	2.80
5	0	-3	-1.20	2.50
-4	0	3	0.60	2.20
-4	0	3	3.80	1.50
4	0	-3	0.70	1.90
4	0	-3	-2.00	1.30
3	0	-3	3.50	1.80
-3	0	3	0.70	1.80
3	0	-3	2.00	1.10
-3	0	3	1.80	1.10
-2	0	3	2.30	1.50
2	0	-3	3.70	1.40
-2	0	3	0.20	0.70
2	0	-3	1.30	0.80
1	0	-3	0.60	0.90
1	0	-3	2.00	0.60
-1	0	3	2.70	1.00
-1	0	3	2.30	0.60
0	0	3	1.40	0.50
0	0	3	1.00	0.40
0	0	-3	1.10	0.40
-1	0	-3	0.70	0.70

1	0	3	0.60	0.50
1	0	3	0.80	0.40
1	0	3	0.80	0.70
-2	0	-3	1.50	0.90
2	0	3	1.20	1.00
2	0	3	1.50	0.90
-2	0	-3	9.10	1.60
-3	0	-3	2.30	1.30
-3	0	-3	1.90	1.20
3	0	3	0.80	1.10
3	0	3	1.80	1.40
-4	0	-3	-0.90	1.60
4	0	3	2.60	1.60
-4	0	-3	0.00	1.80
-5	0	-3	0.90	2.30
-5	0	-3	2.80	2.20
-5	0	-3	0.40	2.10
5	0	3	0.50	1.90
-6	0	-3	2.10	2.80
6	0	3	-1.10	2.30
-6	0	-3	3.80	2.90
-6	0	-3	-2.00	2.90
7	0	3	-0.10	2.70
-7	0	-3	-1.90	3.00
-7	0	-3	2.00	3.20
-7	0	-3	0.40	2.90
-8	0	-3	7.00	3.40
-8	0	-3	0.20	4.00
-8	0	-3	4.40	3.50
8	0	3	-2.00	3.20
-9	0	-3	2.20	3.70
-9	0	-3	-4.80	3.80
-9	0	-3	-2.70	4.10
9	0	3	-2.10	3.50
-10	0	-3	10.90	4.30
10	0	3	-0.70	4.40
-10	0	-3	-0.60	4.70
-11	0	-3	-4.00	5.80
-11	0	-3	4.70	4.50
11	0	3	-6.40	4.60
-12	0	-3	-5.30	6.90
12	0	3	-1.10	5.30
-13	0	-3	3.50	5.50
-14	0	-3	7.30	6.70

-15	0	-3	7.90	7.80
-16	0	-3	15.00	8.80
-16	-1	3	268.47	20.40
-16	1	3	303.37	20.30
-15	-1	3	94.79	12.30
-15	1	3	92.09	12.20
-14	-1	3	220.18	15.60
-14	1	3	226.88	16.20
-13	-1	3	404.16	24.70
-13	1	3	455.15	24.80
13	1	-3	479.45	28.10
-12	-1	3	267.87	16.00
-12	-1	3	194.68	19.80
12	-1	-3	279.17	19.00
-12	1	3	270.37	16.00
-12	1	3	233.48	20.20
12	1	-3	262.67	19.00
-11	-1	3	983.70	48.20
11	-1	-3	1002.40	50.19
-11	-1	3	946.51	55.59
-11	-1	3	994.60	49.40
11	1	-3	1009.10	50.39
-11	1	3	962.20	48.30
-11	1	3	865.31	51.19
-10	-1	3	168.08	11.30
10	-1	-3	179.48	12.20
-10	-1	3	153.68	14.70
-10	-1	3	181.98	12.50
10	-1	-3	180.58	13.40
-10	1	3	171.08	11.40
-10	1	3	176.18	14.90
10	1	-3	153.38	15.50
-9	-1	3	1369.36	66.49
9	-1	-3	1343.27	67.09
9	-1	-3	1420.46	68.09
-9	-1	3	1297.27	68.69
-9	-1	3	1497.15	67.29
-9	1	3	1277.67	68.59
9	1	-3	1359.36	71.59
-9	1	3	1382.86	66.69
8	-1	-3	33.10	4.60
-8	-1	3	25.40	5.80
8	-1	-3	42.20	6.80
-8	-1	3	35.00	4.90

-8	1	3	28.70	5.50
8	1	-3	30.60	5.50
8	1	-3	32.10	5.20
-8	1	3	26.80	5.00
-7	-1	3	1616.24	83.69
7	-1	-3	1761.62	82.49
7	-1	-3	1733.73	83.19
-7	-1	3	1666.93	81.99
7	1	-3	1728.03	82.29
-7	1	3	1632.74	83.59
-7	1	3	1718.63	82.09
7	1	-3	1800.12	83.49
-6	-1	3	21.80	4.40
-6	-1	3	19.30	3.70
6	-1	-3	29.00	4.20
6	-1	-3	27.40	5.20
-6	1	3	32.60	5.70
-6	1	3	22.80	3.80
6	1	-3	28.60	4.40
6	1	-3	18.80	3.10
-5	-1	3	3606.24	171.88
-5	-1	3	3391.06	170.68
5	-1	-3	3676.53	171.58
5	-1	-3	3620.54	170.98
-5	1	3	3511.75	170.68
-5	1	3	3696.33	171.88
5	1	-3	3610.24	170.78
5	1	-3	3623.64	171.68
4	-1	-3	636.74	30.60
-4	-1	3	612.64	29.70
-4	-1	3	589.34	30.80
4	-1	-3	601.34	30.00
-4	1	3	573.24	30.20
-4	1	3	642.64	30.90
4	1	-3	618.64	29.80
4	1	-3	636.54	30.70
-3	-1	3	2134.29	99.69
3	-1	-3	2042.60	98.99
3	-1	-3	2106.09	99.49
-3	-1	3	1996.10	98.69
-3	1	3	2150.38	99.59
3	1	-3	2024.70	98.79
-3	1	3	2053.39	98.89
3	1	-3	2127.99	99.59

2	-1	-3	57.89	4.10
-2	-1	3	60.69	3.30
-2	-1	3	74.89	4.50
2	-1	-3	57.19	3.60
2	1	-3	59.69	3.30
-2	1	3	57.09	3.60
2	1	-3	64.89	4.40
-2	1	3	61.89	4.30
-1	-1	3	1564.34	72.19
1	-1	-3	1519.45	71.89
1	-1	-3	1479.45	72.19
1	1	-3	1581.54	72.19
-1	1	3	1378.86	71.99
-1	1	3	1573.24	72.19
1	1	-3	1498.95	71.69
0	-1	-3	913.71	48.60
0	-1	-3	1089.09	48.60
0	-1	3	1099.19	48.50
0	-1	3	890.81	48.40
0	1	3	1083.39	48.60
0	1	-3	1034.00	48.50
0	1	-3	1103.59	48.50
0	1	3	942.11	48.60
-2	-1	-3	1054.79	49.60
2	-1	3	1046.50	49.30
-2	-1	-3	1018.50	49.60
2	-1	3	1032.80	49.60
-2	1	-3	1059.99	49.70
2	1	3	1018.80	49.60
2	1	3	1049.79	49.60
-2	1	-3	1005.10	49.30
-3	-1	-3	33.60	3.00
-3	-1	-3	38.50	3.10
3	-1	3	37.80	2.70
-3	1	-3	31.50	2.80
-3	1	-3	29.90	3.00
3	1	3	32.90	3.00
3	1	3	30.40	2.90
-3	1	-3	41.30	3.20
-4	-1	-3	1373.96	68.79
4	-1	3	1453.25	68.59
-4	1	-3	1439.76	68.69
-4	1	-3	1508.55	68.99
-4	1	-3	1435.66	68.69

4	1	3	1424.66	68.79
4	1	3	1374.06	68.59
5	-1	3	1600.14	75.59
-5	-1	-3	1487.25	75.49
-5	-1	-3	1492.25	75.79
-5	-1	-3	1715.93	75.69
-5	1	-3	1475.55	75.49
-5	1	-3	1693.33	75.79
5	1	3	1577.94	75.69
-5	1	-3	1524.55	75.69
-6	-1	-3	2923.91	129.19
-6	-1	-3	2563.94	128.89
-6	-1	-3	2514.95	129.29
6	-1	3	2740.63	129.09
6	1	3	2672.03	129.19
-6	1	-3	2924.41	129.29
-6	1	-3	2602.04	129.29
-6	1	-3	2633.24	128.99
-7	-1	-3	117.89	9.00
7	-1	3	127.49	8.50
-7	-1	-3	137.69	8.90
-7	-1	-3	103.29	8.30
-7	1	-3	132.19	8.70
-7	1	-3	114.29	9.00
-7	1	-3	122.99	8.20
7	1	3	115.39	8.80
-8	-1	-3	894.21	40.10
8	-1	3	778.12	39.80
-8	-1	-3	786.52	39.60
-8	-1	-3	713.03	40.20
-8	1	-3	777.12	39.50
-8	1	-3	739.93	40.20
8	1	3	781.52	40.00
-8	1	-3	868.31	40.10
-9	-1	-3	-0.70	3.70
-9	-1	-3	5.40	4.40
9	-1	3	2.20	3.50
-9	-1	-3	3.50	4.20
-9	1	-3	1.90	3.50
9	1	3	3.10	3.60
-9	1	-3	-0.80	3.90
-9	1	-3	-3.50	4.40
-10	-1	-3	554.24	32.10
-10	-1	-3	592.94	30.80

10	-1	3	610.14	31.30
-10	1	-3	602.34	30.90
-10	1	-3	569.54	32.00
10	1	3	615.64	31.50
-11	-1	-3	5.40	6.40
-11	-1	-3	10.40	5.20
11	-1	3	6.70	5.30
11	1	3	8.10	5.60
-11	1	-3	2.50	5.20
-11	1	-3	11.60	6.70
-12	-1	-3	685.63	39.90
-12	-1	-3	648.84	37.90
12	-1	3	734.03	36.70
-12	1	-3	623.74	37.60
12	1	3	716.23	36.90
-12	1	-3	703.03	37.40
-13	-1	-3	-2.00	6.00
-13	1	-3	1.00	5.60
-14	-1	-3	233.48	17.30
-14	1	-3	243.88	17.30
-15	-1	-3	32.50	8.60
-15	1	-3	70.19	12.50
-16	-1	-3	274.87	21.40
-16	1	-3	287.17	20.90
-16	-2	3	147.09	15.60
-16	2	3	200.08	16.20
-15	-2	3	115.69	13.30
-15	2	3	103.79	12.70
-14	-2	3	215.38	15.40
-14	2	3	194.88	15.10
13	-2	-3	10.80	7.10
-13	-2	3	23.80	6.00
13	2	-3	6.00	7.20
-13	2	3	19.10	5.80
-12	-2	3	234.88	15.50
-12	-2	3	222.78	20.50
12	-2	-3	265.57	18.20
12	2	-3	247.88	19.00
-12	2	3	256.37	20.50
-12	2	3	266.17	15.80
-11	-2	3	3.30	5.30
-11	-2	3	-2.40	7.00
-11	-2	3	10.40	4.70
11	-2	-3	-0.50	5.20

-11	2	3	-2.00	7.00
11	2	-3	11.50	6.20
-11	2	3	10.00	4.80
10	-2	-3	122.89	11.40
10	-2	-3	136.79	10.50
-10	-2	3	128.69	9.10
-10	-2	3	122.09	10.40
-10	-2	3	85.39	12.90
-10	2	3	122.99	9.30
10	2	-3	122.49	12.10
-10	2	3	111.59	12.70
9	-2	-3	22.00	5.50
-9	-2	3	23.50	4.90
9	-2	-3	23.10	5.00
-9	-2	3	19.00	4.00
-9	-2	3	22.70	6.60
-9	2	3	10.20	5.60
9	2	-3	27.20	6.40
-9	2	3	18.50	4.30
-8	-2	3	224.28	15.60
8	-2	-3	260.07	15.10
-8	-2	3	275.27	14.10
8	-2	-3	234.28	14.00
-8	-2	3	234.58	13.40
-8	2	3	234.48	15.70
-8	2	3	231.68	13.60
-7	-2	3	11.60	3.10
7	-2	-3	19.10	3.50
-7	-2	3	15.70	5.20
-7	-2	3	13.20	2.90
7	-2	-3	20.20	4.10
7	2	-3	8.40	4.20
-7	2	3	10.80	4.20
6	-2	-3	145.59	8.90
-6	-2	3	116.19	7.30
6	-2	-3	114.59	7.90
-6	-2	3	104.99	9.40
-6	2	3	128.59	9.30
6	2	-3	121.69	9.00
6	2	-3	118.99	7.40
-6	2	3	126.79	7.60
5	-2	-3	18.40	2.90
-5	-2	3	6.70	2.40
-5	-2	3	8.80	3.70

5	-2	-3	15.50	3.20
-5	2	3	14.60	2.50
5	2	-3	10.80	3.30
-5	2	3	9.70	3.10
5	2	-3	17.50	2.40
4	-2	-3	1191.98	58.49
-4	-2	3	1172.98	57.69
4	-2	-3	1231.78	58.19
-4	-2	3	1182.48	58.89
4	2	-3	1188.48	57.79
-4	2	3	1248.38	58.89
4	2	-3	1228.68	58.79
-4	2	3	1216.48	57.99
3	-2	-3	731.53	35.10
3	-2	-3	720.83	34.70
-3	-2	3	703.33	34.30
-3	-2	3	693.23	35.30
3	2	-3	701.43	34.30
-3	2	3	744.23	35.30
-3	2	3	729.03	34.60
3	2	-3	705.03	35.20
-2	-2	3	58.69	4.30
2	-2	-3	59.29	3.60
2	-2	-3	60.29	4.00
-2	2	3	61.69	4.20
2	2	-3	61.49	4.30
-2	2	3	47.70	3.60
2	2	-3	60.89	3.10
-1	-2	3	44.10	3.20
1	-2	-3	51.79	3.00
1	-2	-3	47.70	3.30
-1	2	3	51.09	3.30
-1	2	3	47.10	3.10
1	2	-3	50.09	2.40
1	2	-3	50.29	3.30
0	-2	-3	664.03	32.60
0	-2	3	697.63	32.50
0	-2	-3	675.83	32.70
0	2	-3	679.03	32.30
0	2	3	675.73	32.70
0	2	3	647.94	32.60
0	2	-3	712.13	32.50
-1	-2	-3	150.78	7.80
-1	-2	-3	153.48	8.00

1	2	3	136.79	7.80
1	2	3	155.58	7.80
-1	2	-3	158.98	7.80
-1	2	-3	158.78	7.80
-2	-2	-3	2733.83	130.29
-2	-2	-3	2827.32	130.49
2	2	3	2656.83	130.29
-2	2	-3	2855.51	130.29
2	2	3	2730.73	130.79
-2	2	-3	2646.94	130.29
-3	-2	-3	1308.77	59.09
-3	-2	-3	1231.78	59.29
3	-2	3	1235.78	58.69
3	2	3	1279.77	59.29
3	2	3	1144.59	59.09
-3	2	-3	1263.17	58.89
-3	2	-3	1332.17	59.19
-3	2	-3	1047.60	59.29
4	-2	3	878.91	41.70
-4	-2	-3	862.01	42.20
-4	2	-3	931.51	42.20
4	2	3	895.31	42.60
-4	2	-3	805.42	41.90
4	2	3	813.62	41.90
-4	2	-3	857.21	41.90
-5	-2	-3	406.76	21.90
5	-2	3	433.66	21.40
-5	-2	-3	469.65	21.70
-5	2	-3	433.76	21.80
-5	2	-3	390.66	21.50
-5	2	-3	430.86	21.70
5	2	3	411.36	21.50
-6	-2	-3	332.07	19.60
-6	-2	-3	384.46	19.30
6	-2	3	375.46	19.10
-6	-2	-3	337.07	19.10
-6	2	-3	362.86	19.50
-6	2	-3	407.66	19.50
-6	2	-3	368.76	19.20
-7	-2	-3	936.41	40.90
-7	-2	-3	788.72	40.50
-7	-2	-3	746.43	41.00
7	-2	3	824.42	40.60
-7	2	-3	791.42	40.50

-7	2	-3	898.81	41.00
-7	2	-3	736.63	40.90
8	-2	3	189.68	11.90
-8	-2	-3	172.98	12.70
-8	-2	-3	195.48	11.90
-8	-2	-3	218.98	12.30
-8	2	-3	204.18	12.60
-8	2	-3	172.48	12.60
-8	2	-3	187.18	11.70
9	-2	3	395.36	22.00
-9	-2	-3	376.16	23.10
-9	-2	-3	389.46	21.70
-9	-2	-3	453.35	22.60
-9	2	-3	377.56	22.90
-9	2	-3	415.66	22.00
-9	2	-3	434.46	22.50
10	-2	3	4.50	4.20
-10	-2	-3	4.20	5.40
-10	-2	-3	10.90	8.10
-10	-2	-3	14.70	4.60
-10	2	-3	10.20	4.10
-10	2	-3	8.40	5.20
11	-2	3	527.85	27.70
-11	-2	-3	470.45	29.10
-11	-2	-3	519.05	27.40
-11	2	-3	528.05	27.50
-11	2	-3	473.85	28.70
-12	-2	-3	45.30	9.50
12	-2	3	45.10	7.60
-12	-2	-3	59.29	9.50
-12	2	-3	65.69	8.50
-12	2	-3	29.90	8.20
-13	-2	-3	83.99	11.20
-13	2	-3	118.09	11.70
-14	-2	-3	-3.00	6.50
-14	2	-3	-1.60	6.10
-15	-2	-3	96.49	13.30
-15	2	-3	94.99	13.60
-16	-2	-3	52.79	10.40
-16	2	-3	37.60	9.10
-16	-3	3	176.88	17.00
-16	3	3	207.88	16.70
-15	-3	3	37.80	8.10
-15	3	3	31.60	7.60

-14	-3	3	102.49	11.80
-14	3	3	108.19	11.70
13	-3	-3	248.78	18.50
-13	-3	3	260.87	16.30
13	3	-3	246.98	19.30
-13	3	3	248.28	16.30
12	-3	-3	11.40	6.10
-12	-3	3	4.90	5.00
-12	-3	3	1.90	8.10
-12	3	3	16.80	8.60
12	3	-3	3.90	6.70
-12	3	3	8.60	5.00
-11	-3	3	132.79	14.30
-11	-3	3	153.08	10.80
11	-3	-3	147.09	12.70
-11	-3	3	161.38	12.30
-11	3	3	138.89	11.00
-11	3	3	121.99	14.40
11	3	-3	145.49	13.50
-10	-3	3	209.18	12.20
-10	-3	3	165.08	16.40
10	-3	-3	188.58	13.90
-10	-3	3	199.98	13.20
10	-3	-3	196.38	13.70
-10	3	3	206.78	12.50
-10	3	3	164.58	15.40
10	3	-3	186.68	14.80
-9	-3	3	310.07	17.60
-9	-3	3	302.77	20.50
9	-3	-3	314.67	18.60
9	-3	-3	336.97	19.50
-9	-3	3	361.46	18.50
-9	3	3	331.77	18.00
9	3	-3	353.36	20.30
-9	3	3	321.07	20.60
-8	-3	3	-8.40	4.20
8	-3	-3	3.20	3.70
8	-3	-3	1.60	3.50
-8	-3	3	0.10	3.10
-8	-3	3	1.60	2.50
-8	3	3	0.90	3.10
-8	3	3	4.50	4.50
8	3	-3	-0.20	4.30
-7	-3	3	809.52	37.50

-7	-3	3	732.63	37.10
-7	-3	3	705.63	38.90
7	-3	-3	778.42	38.40
7	-3	-3	752.72	37.80
7	3	-3	775.82	39.20
-7	3	3	758.32	37.40
-7	3	3	748.33	39.00
-6	-3	3	16.70	2.60
-6	-3	3	10.60	4.70
6	-3	-3	20.10	3.50
6	-3	-3	19.90	3.80
-6	-3	3	12.00	4.10
-6	3	3	10.00	3.80
-6	3	3	20.20	3.60
5	-3	-3	359.56	18.10
-5	-3	3	316.17	18.80
-5	-3	3	381.46	17.70
-5	-3	3	336.07	17.40
5	-3	-3	352.36	18.50
-5	3	3	344.97	18.80
5	3	-3	346.37	18.90
-5	3	3	355.56	17.80
-4	-3	3	245.58	14.10
4	-3	-3	251.27	13.80
-4	-3	3	275.77	13.00
-4	-3	3	246.88	12.80
4	3	-3	271.77	14.30
-4	3	3	239.48	13.20
-4	3	3	267.37	14.20
-3	-3	3	177.28	8.60
3	-3	-3	170.48	9.50
3	-3	-3	167.08	9.20
-3	-3	3	167.78	9.80
-3	-3	3	183.68	8.60
-3	3	3	166.08	9.60
3	3	-3	165.98	9.80
2	-3	-3	1853.41	89.59
-2	-3	3	1827.72	89.69
2	-3	-3	1844.82	89.39
-2	3	3	1823.92	89.39
-2	3	3	1939.71	89.69
2	3	-3	1965.40	89.79
1	-3	-3	2322.67	108.79
-1	-3	3	2257.27	108.79

1	-3	-3	2343.57	108.59
-1	3	3	2319.27	108.89
-1	3	3	2144.09	108.69
1	3	-3	2324.67	108.79
0	-3	3	758.12	37.10
0	-3	3	791.52	37.30
0	-3	-3	811.92	37.60
0	-3	-3	756.02	37.40
0	3	-3	791.32	37.30
0	3	3	781.02	37.60
0	3	-3	757.42	37.10
0	3	3	784.02	37.50
1	-3	3	360.26	16.80
-1	-3	-3	314.27	17.10
-1	-3	-3	354.56	16.90
1	3	3	352.66	17.00
1	3	3	333.87	16.90
-1	3	-3	347.87	16.80
2	-3	3	270.57	13.30
-2	-3	-3	264.97	13.60
2	-3	3	260.97	12.70
-2	-3	-3	266.67	13.20
-2	3	-3	261.97	13.20
-2	3	-3	262.87	12.80
2	3	3	253.77	13.20
3	-3	3	2212.38	104.99
3	-3	3	2087.09	104.49
-3	-3	-3	2188.08	105.29
-3	-3	-3	2306.77	104.99
-3	3	-3	2213.48	104.69
4	-3	3	110.09	6.10
-4	-3	-3	105.59	7.00
4	-3	3	105.09	6.70
-4	-3	-3	110.39	6.60
-4	3	-3	117.19	6.50
-4	3	-3	98.69	6.40
4	3	3	102.49	6.50
-5	-3	-3	386.56	17.70
-5	-3	-3	316.77	18.10
5	-3	3	345.57	17.30
5	-3	3	353.36	17.80
-5	3	-3	322.57	17.60
-5	3	-3	326.97	17.60
5	3	3	326.97	17.50

6	-3	3	1121.79	54.49
6	-3	3	1115.09	54.19
-6	-3	-3	1032.80	54.89
-6	-3	-3	1221.58	54.49
-6	3	-3	1073.89	54.59
6	3	3	1004.80	54.09
-6	3	-3	1106.69	54.39
-6	3	-3	1253.77	54.89
-7	-3	-3	758.42	42.00
7	-3	3	837.62	41.30
-7	-3	-3	903.91	41.60
-7	-3	-3	842.12	41.40
-7	3	-3	826.02	41.40
-7	3	-3	918.41	42.00
-7	3	-3	759.82	41.70
-8	-3	-3	170.28	12.30
-8	-3	-3	174.18	11.00
-8	-3	-3	201.18	11.50
8	-3	3	162.78	11.00
-8	3	-3	176.88	11.20
-8	3	-3	191.78	12.10
-8	3	-3	162.38	11.80
-9	-3	-3	60.39	8.70
9	-3	3	57.19	6.90
-9	-3	-3	72.29	7.60
-9	-3	-3	61.79	7.10
-9	3	-3	42.50	7.90
-9	3	-3	59.19	7.10
-9	3	-3	58.69	8.40
10	-3	3	553.84	28.70
-10	-3	-3	508.35	30.20
-10	-3	-3	568.44	28.70
-10	3	-3	539.75	28.50
-10	3	-3	530.85	29.90
-11	-3	-3	76.79	9.00
11	-3	3	63.09	9.10
-11	-3	-3	73.39	11.60
-11	3	-3	60.59	11.00
-11	3	-3	70.89	8.90
-12	-3	-3	149.29	16.40
12	-3	3	190.78	14.00
-12	-3	-3	188.68	13.80
-12	3	-3	204.98	16.20
-12	3	-3	190.58	14.00

-13	-3	-3	14.20	6.50
-13	3	-3	8.10	6.20
-14	-3	-3	86.09	12.20
-14	3	-3	81.79	11.60
-15	-3	-3	17.50	8.40
-15	3	-3	0.60	7.90
-16	-3	-3	197.38	18.20
-16	3	-3	193.88	17.30
-16	-4	3	206.88	18.40
-16	4	3	245.18	18.10
-15	-4	3	89.49	12.70
-15	4	3	109.69	12.70
-14	-4	3	41.80	7.60
-14	4	3	42.20	7.80
13	-4	-3	143.19	14.30
-13	-4	3	151.78	12.40
-13	4	3	160.28	12.40
13	4	-3	141.19	16.00
-12	-4	3	539.95	27.10
-12	-4	3	469.55	31.30
12	-4	-3	530.25	29.10
-12	4	3	465.75	31.20
12	4	-3	522.45	30.00
11	-4	-3	140.49	12.00
-11	-4	3	129.79	10.00
-11	-4	3	132.59	11.40
-11	-4	3	113.89	14.30
-11	4	3	106.49	14.00
11	4	-3	107.99	12.60
-11	4	3	131.59	10.40
-10	-4	3	667.73	38.70
10	-4	-3	729.23	37.30
-10	-4	3	689.33	35.60
-10	-4	3	719.03	36.30
10	4	-3	754.92	38.10
-10	4	3	676.93	38.50
-10	4	3	714.63	35.90
9	-4	-3	309.17	17.70
9	-4	-3	276.37	17.00
-9	-4	3	237.38	20.40
-9	-4	3	324.47	16.60
-9	-4	3	283.67	15.90
-9	4	3	296.17	16.30
-9	4	3	280.37	18.90

9	4	-3	291.77	18.40
-8	-4	3	686.23	31.20
-8	-4	3	566.84	32.80
8	-4	-3	594.84	31.50
8	-4	-3	619.34	32.00
-8	-4	3	595.14	30.60
8	4	-3	639.94	32.90
-8	4	3	595.34	32.80
-8	4	3	617.84	31.10
7	-4	-3	51.79	6.50
-7	-4	3	42.90	7.60
-7	-4	3	59.09	5.20
-7	-4	3	53.19	4.80
7	-4	-3	46.80	6.10
-7	4	3	49.90	7.30
7	4	-3	51.79	7.80
-7	4	3	58.79	5.40
6	-4	-3	2489.45	117.79
-6	-4	3	2339.87	116.99
-6	-4	3	2515.55	117.29
-6	-4	3	2343.67	118.59
6	-4	-3	2493.45	118.09
-6	4	3	2381.86	117.39
6	4	-3	2572.94	118.69
-6	4	3	2506.55	118.59
5	-4	-3	8.30	2.80
-5	-4	3	9.70	2.00
-5	-4	3	6.70	3.20
-5	-4	3	8.40	1.80
5	-4	-3	8.30	2.50
5	4	-3	6.30	3.90
-5	4	3	7.90	2.40
-5	4	3	12.70	3.20
-4	-4	3	2046.90	103.09
4	-4	-3	2172.78	102.79
-4	-4	3	2047.70	101.79
-4	-4	3	2249.87	101.89
4	-4	-3	2221.38	102.59
-4	4	3	2193.78	102.99
-4	4	3	2062.29	102.29
3	-4	-3	15.70	2.80
-3	-4	3	18.50	1.60
3	-4	-3	18.80	2.90
-3	-4	3	18.70	3.00

-3	-4	3	16.60	1.60
3	4	-3	19.80	3.50
-3	4	3	14.10	2.40
-3	4	3	16.70	3.00
-2	-4	3	365.06	18.70
-2	-4	3	363.76	17.50
2	-4	-3	365.86	18.30
2	-4	-3	368.86	18.50
-2	4	3	372.96	18.60
2	4	-3	371.26	18.60
-2	4	3	366.66	18.30
-1	-4	3	1079.99	51.89
1	-4	-3	1125.29	51.69
1	-4	-3	1085.69	51.89
-1	-4	3	1047.40	50.99
1	4	-3	1019.10	50.99
-1	4	3	1100.79	51.89
-1	4	3	1051.29	51.69
1	4	-3	1110.29	51.79
0	4	3	6656.73	315.37
-1	-4	-3	584.24	28.70
-1	-4	-3	580.84	29.10
1	-4	3	614.14	28.60
1	4	3	558.14	28.70
1	4	3	609.84	28.90
-1	4	-3	583.24	28.50
-1	4	-3	617.54	28.60
-2	-4	-3	402.46	20.20
2	-4	3	397.76	19.70
2	-4	3	397.76	19.30
-2	-4	-3	413.86	19.70
-2	4	-3	400.06	19.40
2	4	3	381.26	19.70
-2	4	-3	396.56	19.60
3	-4	3	22.00	2.00
-3	-4	-3	30.40	2.80
-3	-4	-3	23.20	3.50
3	-4	3	19.30	2.70
-3	4	-3	19.80	2.90
-3	4	-3	25.70	2.30
3	4	3	20.60	2.70
4	-4	3	359.16	18.00
4	-4	3	376.16	18.60
-4	-4	-3	388.36	18.40

-4	-4	-3	337.87	18.90
4	4	3	356.46	18.40
-4	4	-3	374.06	18.30
-4	4	-3	349.57	18.40
5	-4	3	867.11	41.70
5	-4	3	878.61	42.30
-5	-4	-3	837.52	42.70
-5	-4	-3	934.71	42.10
5	4	3	815.72	41.90
-5	4	-3	838.72	42.00
-5	4	-3	838.52	42.00
-6	-4	-3	867.21	44.20
6	-4	3	884.01	43.20
-6	-4	-3	948.51	43.50
6	-4	3	906.91	43.70
-6	4	-3	864.41	43.50
6	4	3	807.22	43.20
-6	4	-3	906.01	43.70
-7	-4	-3	902.51	47.30
7	-4	3	992.10	46.40
7	-4	3	950.10	46.70
-7	4	-3	925.51	46.40
7	4	3	923.51	46.30
-7	4	-3	954.60	46.90
8	-4	3	85.69	7.00
-8	-4	-3	87.19	7.60
-8	-4	-3	85.99	8.80
-8	-4	-3	83.79	7.50
-8	4	-3	81.09	7.40
-8	4	-3	80.99	8.10
-8	4	-3	80.79	8.20
-9	-4	-3	710.43	32.30
9	-4	3	630.74	31.70
-9	-4	-3	606.54	31.80
-9	-4	-3	562.24	33.10
-9	4	-3	558.84	32.50
-9	4	-3	632.44	32.60
-9	4	-3	620.84	31.80
10	-4	3	60.29	7.50
-10	-4	-3	51.29	10.20
-10	-4	-3	54.19	7.90
-10	-4	-3	65.59	8.90
-10	4	-3	50.99	7.30
-10	4	-3	45.20	9.30

-11	-4	-3	699.33	34.30
11	-4	3	683.23	34.10
-11	-4	-3	599.04	35.90
-11	4	-3	645.24	33.80
-11	4	-3	607.14	35.40
-12	-4	-3	148.79	12.50
12	-4	3	141.89	12.30
-12	-4	-3	148.29	15.70
-12	4	-3	157.18	11.90
-12	4	-3	140.19	14.50
-13	-4	-3	406.06	23.40
-13	4	-3	376.66	23.10
-14	-4	-3	4.30	6.90
-14	4	-3	8.50	7.00
-15	-4	-3	229.68	18.20
-15	4	-3	221.78	18.00
-16	-4	-3	2.20	8.70
-16	4	-3	12.00	8.60
-15	-5	3	10.00	7.20
-15	5	3	17.40	7.40
-14	-5	3	4.30	6.20
-14	5	3	3.00	6.10
-13	-5	3	7.90	5.50
-13	5	3	6.40	5.40
13	5	-3	1.90	7.30
12	-5	-3	20.30	6.60
-12	-5	3	17.90	10.00
-12	-5	3	18.70	5.40
-12	5	3	24.60	9.40
-12	5	3	26.20	5.60
12	5	-3	14.00	7.30
-11	-5	3	8.40	4.40
11	-5	-3	15.30	5.80
-11	-5	3	-1.30	5.20
-11	-5	3	6.20	8.10
-11	5	3	12.30	4.90
11	5	-3	10.80	7.20
-11	5	3	20.50	8.20
-10	-5	3	27.60	5.70
10	-5	-3	33.60	6.20
-10	-5	3	30.50	8.10
-10	-5	3	34.90	6.10
-10	5	3	17.20	6.90
10	5	-3	26.90	6.70

-10	5	3	49.90	7.00
-9	-5	3	1.80	3.80
-9	-5	3	3.30	3.20
-9	-5	3	5.90	5.50
9	-5	-3	7.20	4.30
9	-5	-3	5.10	4.50
-9	5	3	8.40	3.90
9	5	-3	4.30	5.10
-9	5	3	3.40	5.80
8	-5	-3	7.40	3.80
-8	-5	3	10.40	3.40
-8	-5	3	9.50	2.90
-8	-5	3	8.00	5.20
8	-5	-3	4.70	3.70
-8	5	3	4.80	4.90
8	5	-3	11.80	5.30
-8	5	3	13.50	3.60
-7	-5	3	43.90	4.80
-7	-5	3	41.50	4.40
7	-5	-3	44.10	6.00
-7	-5	3	28.70	5.60
7	-5	-3	41.00	6.00
-7	5	3	32.60	5.50
-7	5	3	47.10	5.10
7	5	-3	42.30	7.30
-6	-5	3	237.98	15.90
-6	-5	3	284.77	14.10
6	-5	-3	262.07	15.20
-6	-5	3	251.77	13.90
6	-5	-3	270.17	14.90
-6	5	3	272.17	14.40
-6	5	3	258.27	15.70
6	5	-3	296.67	16.30
-5	-5	3	69.49	4.60
-5	-5	3	59.59	6.80
-5	-5	3	77.79	4.70
5	-5	-3	71.89	6.10
5	-5	-3	73.99	6.00
-5	5	3	69.69	6.50
5	5	-3	67.29	7.10
-5	5	3	69.79	5.20
-4	-5	3	38.90	5.00
4	-5	-3	40.60	4.30
-4	-5	3	40.90	2.90

4	-5	-3	38.30	4.30
-4	-5	3	34.40	2.80
4	5	-3	33.30	4.90
-4	5	3	34.00	3.80
-4	5	3	37.30	4.60
-3	-5	3	36.00	4.30
-3	-5	3	40.40	2.40
3	-5	-3	40.70	3.80
3	-5	-3	30.70	3.60
3	5	-3	39.40	4.40
-3	5	3	30.90	3.20
-3	5	3	35.40	3.80
2	-5	-3	167.48	9.50
2	-5	-3	187.98	9.90
-2	-5	3	178.18	10.10
-2	-5	3	184.08	8.70
-2	5	3	188.48	9.90
-2	5	3	165.78	9.50
2	5	-3	180.28	8.70
1	-5	-3	6.00	1.50
-1	-5	3	4.80	0.80
-1	-5	3	8.10	1.80
1	-5	-3	5.30	1.60
1	5	-3	5.70	0.90
-1	5	3	4.70	1.60
-1	5	3	5.60	1.80
0	-5	3	12.40	1.50
0	-5	-3	9.70	1.90
0	-5	-3	13.60	2.10
0	-5	3	14.20	2.20
0	5	3	15.40	2.30
0	5	-3	12.90	1.40
0	5	3	15.60	2.50
-1	-5	-3	1.60	1.50
-1	-5	-3	2.60	1.10
1	-5	3	3.70	1.10
1	5	3	1.40	1.30
-1	5	-3	1.70	1.00
1	5	3	3.00	1.40
-2	-5	-3	59.59	4.90
2	-5	3	67.09	4.20
-2	-5	-3	67.29	4.20
2	5	3	58.09	4.80
-2	5	-3	65.09	4.20

-2	5	-3	65.89	3.90
3	-5	3	203.98	12.70
3	-5	3	237.68	11.80
-3	-5	-3	254.17	12.20
-3	-5	-3	245.38	13.00
3	5	3	223.78	12.10
-3	5	-3	229.88	12.10
-3	5	-3	234.18	11.90
4	-5	3	47.80	4.60
-4	-5	-3	49.40	5.20
-4	-5	-3	54.69	4.20
4	-5	3	44.20	3.60
4	5	3	35.50	4.40
-4	5	-3	53.89	4.00
-4	5	-3	50.39	4.40
4	5	3	51.39	4.20
5	-5	3	137.69	7.80
5	-5	3	125.69	8.40
-5	-5	-3	143.89	8.30
-5	-5	-3	122.99	9.30
5	5	3	138.09	8.20
-5	5	-3	137.59	8.30
-5	5	-3	129.69	8.20
-6	-5	-3	9.00	3.60
-6	-5	-3	8.30	2.70
6	-5	3	5.10	2.40
6	-5	3	7.70	3.10
6	5	3	8.90	2.60
-6	5	-3	3.00	3.00
6	5	3	6.00	2.90
-6	5	-3	4.70	2.90
7	-5	3	123.39	9.50
7	-5	3	127.19	8.50
-7	-5	-3	116.09	10.10
-7	-5	-3	157.68	9.10
-7	5	-3	124.29	8.70
7	5	3	135.89	8.80
-7	5	-3	107.89	9.30
7	5	3	150.18	10.20
-8	-5	-3	73.89	6.80
8	-5	3	65.59	6.30
-8	-5	-3	55.79	8.30
-8	-5	-3	65.29	6.90
8	-5	3	63.29	7.30

8	5	3	67.89	8.10
-8	5	-3	60.69	6.80
-8	5	-3	67.49	7.70
-9	-5	-3	19.20	4.60
-9	-5	-3	18.60	5.50
-9	-5	-3	22.20	4.80
9	-5	3	9.90	3.70
-9	5	-3	21.40	4.80
-9	5	-3	7.50	4.60
9	5	3	20.20	4.90
-10	-5	-3	15.30	5.60
10	-5	3	15.30	4.60
-10	-5	-3	18.70	6.50
-10	-5	-3	19.30	5.40
10	5	3	15.60	5.70
-10	5	-3	13.30	5.70
-10	5	-3	4.50	4.40
11	-5	3	17.40	5.20
-11	-5	-3	20.90	5.60
-11	-5	-3	20.30	7.30
-11	5	-3	45.70	8.40
11	5	3	15.60	6.00
-11	5	-3	12.30	6.30
12	-5	3	-4.30	4.80
-12	-5	-3	-2.50	7.30
-12	-5	-3	3.30	5.60
12	5	3	3.80	6.30
-12	5	-3	-11.30	6.20
-12	5	-3	2.60	5.90
-13	-5	-3	14.50	6.60
-13	5	-3	17.80	6.80
-14	-5	-3	8.10	7.10
-14	5	-3	13.00	7.40
-15	-5	-3	8.50	7.80
-15	5	-3	14.60	8.40
-15	-6	3	190.58	15.90
-15	6	3	190.08	15.80
-14	-6	3	70.59	12.60
-14	6	3	119.59	11.90
-13	-6	3	271.97	17.20
-13	6	3	279.47	17.50
13	6	-3	265.07	21.00
-12	-6	3	229.98	21.80
-12	-6	3	263.97	16.10

12	-6	-3	282.27	18.50
12	6	-3	269.27	19.90
-12	6	3	293.37	16.90
-12	6	3	236.38	21.00
-11	-6	3	116.79	9.30
11	-6	-3	105.39	11.20
-11	-6	3	89.49	10.40
-11	-6	3	103.49	14.40
11	6	-3	122.59	13.60
-11	6	3	86.19	13.50
-11	6	3	133.39	10.20
-10	-6	3	658.63	34.00
10	-6	-3	671.33	35.60
-10	-6	3	735.23	34.70
-10	-6	3	615.84	37.30
-10	6	3	697.03	34.60
-10	6	3	658.33	37.20
10	6	-3	674.43	36.80
9	-6	-3	225.58	15.00
9	-6	-3	239.48	15.20
-9	-6	3	258.07	14.20
-9	-6	3	216.88	16.80
-9	-6	3	229.38	13.40
-9	6	3	222.48	16.60
-9	6	3	247.58	14.20
9	6	-3	243.38	16.50
8	-6	-3	481.95	26.00
-8	-6	3	479.55	24.50
8	-6	-3	475.15	25.90
-8	-6	3	538.45	25.00
-8	-6	3	459.45	27.20
8	6	-3	494.25	26.90
-8	6	3	464.45	25.00
-8	6	3	481.15	27.00
7	-6	-3	50.09	6.20
-7	-6	3	57.69	5.30
-7	-6	3	53.99	7.90
7	-6	-3	57.59	6.60
-7	-6	3	54.79	5.00
-7	6	3	61.29	7.70
7	6	-3	59.99	7.70
-7	6	3	59.59	5.80
-6	-6	3	2820.62	125.49
6	-6	-3	2573.24	126.39

6	-6	-3	2620.94	126.39
-6	-6	3	2534.55	125.29
-6	6	3	2550.34	125.79
-6	6	3	2622.34	126.99
6	6	-3	2697.93	127.09
5	-6	-3	5.10	3.00
5	-6	-3	5.00	2.60
-5	-6	3	11.20	3.60
-5	-6	3	7.80	1.80
-5	-6	3	9.20	1.80
-5	6	3	10.50	3.40
-5	6	3	4.40	2.40
5	6	-3	8.20	3.60
-4	-6	3	1337.17	63.69
4	-6	-3	1323.47	64.79
-4	-6	3	1248.18	65.19
4	-6	-3	1348.97	64.69
-4	6	3	1339.97	64.39
4	6	-3	1419.56	65.29
-4	6	3	1327.67	64.99
-3	-6	3	37.20	2.30
3	-6	-3	34.50	3.70
-3	-6	3	34.90	4.60
3	-6	-3	35.80	3.90
3	6	-3	33.70	2.30
3	6	-3	39.30	4.80
-3	6	3	34.90	3.40
-3	6	3	39.30	4.20
2	-6	-3	64.89	5.10
2	-6	-3	69.19	4.90
-2	-6	3	63.39	3.50
-2	-6	3	68.19	5.20
-2	6	3	64.39	4.70
2	6	-3	61.99	3.40
2	6	-3	62.69	5.10
-2	6	3	66.79	5.00
1	-6	-3	267.97	13.70
-1	-6	3	244.88	13.70
-1	-6	3	264.97	12.70
1	-6	-3	271.37	13.40
1	6	-3	263.97	13.80
-1	6	3	270.97	13.80
-1	6	3	238.78	13.50
1	6	-3	260.67	12.60

0	-6	3	2598.04	121.49
0	-6	3	2596.14	120.99
0	-6	-3	2630.24	121.29
0	6	3	2600.94	121.69
0	6	-3	2575.24	121.49
0	6	3	2586.54	121.79
0	6	-3	2440.36	120.89
0	6	3	2360.26	121.89
1	-6	3	825.92	40.20
-1	-6	-3	872.91	41.00
-1	-6	-3	853.21	40.40
1	6	3	867.01	40.80
-1	6	-3	815.42	40.40
1	6	3	740.63	40.70
-1	6	-3	822.02	40.10
1	6	3	884.31	41.10
-2	-6	-3	648.34	30.80
2	-6	3	643.54	30.80
-2	-6	-3	627.14	31.50
-2	6	-3	645.84	30.70
-2	6	-3	559.64	31.00
2	6	3	622.54	30.80
2	6	3	671.63	31.70
-3	-6	-3	601.94	32.30
3	-6	3	669.13	31.70
3	-6	3	654.13	31.30
3	6	3	631.54	31.50
-3	6	-3	652.33	31.60
-3	6	-3	630.34	31.40
3	6	3	667.73	32.40
4	-6	3	1594.24	79.59
-4	-6	-3	1749.03	79.89
-4	-6	-3	1645.94	80.79
4	-6	3	1709.03	80.19
-4	6	-3	1649.73	79.99
4	6	3	1779.72	80.79
4	6	3	1575.34	79.79
-4	6	-3	1614.24	79.79
5	-6	3	926.11	45.60
5	-6	3	941.21	46.20
-5	-6	-3	1025.90	46.00
-5	-6	-3	900.21	46.90
-5	6	-3	921.21	46.00
5	6	3	913.31	45.90

5	6	3	948.01	46.70
-5	6	-3	947.11	46.00
-6	-6	-3	1625.44	84.49
-6	-6	-3	1851.41	83.59
6	-6	3	1774.62	83.99
6	-6	3	1720.93	83.29
-6	6	-3	1674.03	83.59
6	6	3	1634.24	83.39
-6	6	-3	1751.92	83.79
6	6	3	1829.02	84.49
7	-6	3	921.21	45.40
-7	-6	-3	874.91	46.90
7	-6	3	941.61	46.20
-7	-6	-3	1030.20	45.80
-7	6	-3	893.01	46.00
7	6	3	949.60	46.60
-7	6	-3	911.91	45.70
7	6	3	870.31	45.50
8	-6	3	22.40	3.90
-8	-6	-3	22.60	5.50
-8	-6	-3	21.50	4.10
8	-6	3	20.80	5.00
8	6	3	17.60	4.00
-8	6	-3	25.20	4.90
-8	6	-3	21.80	4.20
8	6	3	25.00	5.70
9	-6	3	390.46	20.80
-9	-6	-3	338.77	22.60
-9	-6	-3	444.46	21.40
-9	-6	-3	395.26	21.20
9	6	3	397.86	22.40
-9	6	-3	359.16	20.80
-9	6	-3	354.26	21.90
10	-6	3	36.70	5.50
-10	-6	-3	34.50	7.30
-10	-6	-3	26.10	5.40
-10	-6	-3	24.90	6.70
-10	6	-3	17.80	5.20
10	6	3	34.50	7.00
-10	6	-3	27.10	6.90
-11	-6	-3	949.51	45.00
11	-6	3	894.01	44.70
-11	-6	-3	805.92	47.00
11	6	3	947.61	46.60

-11	6	-3	793.12	46.00
12	-6	3	102.19	11.00
-12	-6	-3	119.69	11.40
-12	-6	-3	132.49	15.60
12	6	3	113.09	13.40
-12	6	-3	114.09	13.70
-12	6	-3	113.49	10.70
-13	-6	-3	489.55	27.30
-13	6	-3	464.75	27.20
-14	-6	-3	78.79	12.40
-14	6	-3	73.39	12.10
-15	-6	-3	261.67	19.60
-15	6	-3	242.68	18.90
-15	-7	3	70.09	12.90
-15	7	3	104.49	12.50
-14	-7	3	133.49	20.90
-14	7	3	143.19	13.50
-13	-7	3	22.20	6.20
13	7	-3	32.60	8.80
-13	7	3	52.29	9.20
12	-7	-3	14.90	6.50
-12	-7	3	14.40	9.60
-12	-7	3	10.70	7.10
-12	-7	3	4.90	4.80
-12	7	3	11.90	5.80
12	7	-3	2.60	7.00
11	-7	-3	220.98	15.50
-11	-7	3	192.98	13.40
-11	-7	3	239.88	14.50
-11	-7	3	200.18	19.30
-11	7	3	199.58	16.10
-11	7	3	213.68	18.00
11	7	-3	233.78	17.60
-10	-7	3	129.49	14.50
-10	-7	3	121.69	10.20
10	-7	-3	130.09	11.40
-10	-7	3	116.19	9.20
-10	7	3	118.49	10.10
10	7	-3	148.59	13.40
-10	7	3	116.79	13.40
-9	-7	3	76.09	11.50
-9	-7	3	75.19	7.00
9	-7	-3	76.89	8.90
-9	-7	3	87.79	7.80

-9	7	3	87.39	7.90
-9	7	3	75.99	10.50
9	7	-3	103.39	11.00
-8	-7	3	8.80	5.20
-8	-7	3	2.70	2.70
-8	-7	3	0.70	2.90
8	-7	-3	-0.70	4.20
8	-7	-3	5.30	4.00
-8	7	3	1.40	3.20
8	7	-3	7.80	4.90
-8	7	3	-1.30	4.60
-7	-7	3	710.33	32.70
-7	-7	3	647.94	32.50
-7	-7	3	622.14	34.90
7	-7	-3	653.43	33.80
7	-7	-3	635.34	33.70
-7	7	3	649.24	34.60
-7	7	3	659.83	33.20
7	7	-3	702.33	35.00
6	-7	-3	5.70	3.50
-6	-7	3	4.40	2.30
-6	-7	3	5.00	2.10
6	-7	-3	3.00	3.00
-6	-7	3	9.70	4.10
-6	7	3	1.20	2.70
-6	7	3	-1.40	3.30
6	7	-3	5.20	3.90
-5	-7	3	442.56	24.50
5	-7	-3	436.46	23.40
-5	-7	3	438.96	22.10
5	-7	-3	454.25	23.50
-5	-7	3	466.45	22.10
-5	7	3	445.26	23.80
-5	7	3	452.05	22.90
5	7	-3	460.65	24.00
4	-7	-3	27.50	4.20
4	-7	-3	27.20	4.40
-4	-7	3	30.20	2.60
-4	7	3	32.10	3.90
-4	7	3	29.90	4.80
4	7	-3	38.40	5.10
4	7	-3	30.40	2.60
-3	-7	3	872.61	42.70
3	-7	-3	889.21	43.90

3	-7	-3	880.31	43.80
-3	-7	3	911.11	44.50
-3	7	3	898.01	44.10
-3	7	3	902.61	43.60
3	7	-3	973.60	44.40
3	7	-3	834.32	42.70
-2	-7	3	5.70	1.10
2	-7	-3	8.40	2.20
-2	-7	3	2.70	2.10
2	-7	-3	5.10	2.00
-2	7	3	5.40	2.00
2	7	-3	1.20	2.20
-2	7	3	4.90	2.00
2	7	-3	3.70	0.90
-1	-7	3	530.75	26.40
-1	-7	3	564.44	27.60
1	-7	-3	556.64	27.10
1	-7	-3	548.15	27.50
-1	7	3	543.35	27.50
1	7	-3	586.74	27.60
1	7	-3	543.65	26.40
-1	7	3	523.25	27.20
0	-7	3	97.29	6.10
0	-7	3	98.59	5.40
0	7	3	96.09	6.30
0	7	3	93.59	6.70
0	7	-3	95.39	5.30
0	7	3	94.69	6.10
0	7	-3	100.69	6.20
-1	-7	-3	45.10	4.40
-1	-7	-3	41.00	4.00
1	-7	3	49.90	3.40
1	7	3	47.20	4.70
-1	7	-3	44.50	3.80
1	7	3	46.40	4.10
1	7	3	42.00	3.80
-1	7	-3	39.30	3.10
-2	-7	-3	100.09	6.90
-2	-7	-3	86.89	6.10
2	-7	3	94.89	5.80
2	7	3	89.19	5.80
2	7	3	94.59	6.90
-2	7	-3	97.09	5.70
-2	7	-3	88.29	5.60

3	-7	3	630.74	31.90
3	-7	3	650.73	31.50
-3	-7	-3	634.94	31.70
-3	-7	-3	670.83	32.90
-3	7	-3	636.54	31.70
-3	7	-3	634.14	31.70
3	7	3	697.03	32.90
3	7	3	622.84	31.70
4	-7	3	574.04	28.10
-4	-7	-3	561.64	29.50
-4	-7	-3	591.44	28.30
4	-7	3	589.64	28.70
-4	7	-3	555.44	28.40
-4	7	-3	567.14	28.40
4	7	3	561.14	28.40
4	7	3	574.04	29.40
-5	-7	-3	698.33	32.40
5	-7	3	661.13	32.80
5	-7	3	654.93	32.10
-5	-7	-3	639.24	33.70
-5	7	-3	627.44	32.50
5	7	3	620.14	32.30
5	7	3	667.43	33.40
-5	7	-3	653.23	32.50
-6	-7	-3	205.18	13.40
6	-7	3	202.78	11.20
6	-7	3	191.88	12.30
-6	-7	-3	208.68	11.60
-6	7	-3	202.08	12.00
6	7	3	183.68	11.40
-6	7	-3	180.28	11.70
6	7	3	219.68	13.00
7	-7	3	68.89	6.20
-7	-7	-3	78.49	6.70
-7	-7	-3	67.09	8.50
7	-7	3	74.49	7.10
7	7	3	83.29	8.30
-7	7	-3	70.99	7.30
7	7	3	66.99	6.50
-7	7	-3	72.79	6.90
8	-7	3	116.59	8.40
-8	-7	-3	118.59	11.30
-8	-7	-3	122.29	9.00
8	-7	3	105.79	9.20

8	7	3	105.29	8.60
-8	7	-3	120.79	9.70
-8	7	-3	110.89	8.90
8	7	3	127.89	10.80
9	-7	3	40.70	6.60
-9	-7	-3	48.10	7.40
-9	-7	-3	40.00	7.50
-9	-7	-3	52.49	7.30
9	7	3	58.99	9.50
-9	7	-3	53.59	7.30
-9	7	-3	41.00	8.30
10	-7	3	515.15	26.70
-10	-7	-3	511.55	27.10
-10	-7	-3	458.15	29.30
-10	7	-3	481.15	26.70
10	7	3	541.45	28.90
-10	7	-3	496.25	28.30
-11	-7	-3	19.00	6.00
11	-7	3	17.60	5.10
-11	-7	-3	9.20	7.30
-11	7	-3	24.20	6.10
-11	7	-3	9.10	6.50
11	7	3	17.20	6.80
12	-7	3	114.19	10.80
-12	-7	-3	121.49	11.40
-12	7	-3	111.69	11.80
12	7	3	121.09	14.10
-12	7	-3	96.79	13.50
-13	-7	-3	17.70	7.00
-13	7	-3	5.50	6.50
-14	-7	-3	116.99	13.90
-14	7	-3	122.99	13.30
-15	-7	-3	20.80	8.90
-15	7	-3	33.90	8.70
-15	-8	3	62.69	9.60
-15	8	3	79.69	12.40
-14	8	3	103.39	12.40
-13	-8	3	27.50	6.90
-13	8	3	32.10	6.80
13	8	-3	41.30	10.00
-12	-8	3	115.59	10.70
-12	-8	3	138.79	18.90
-12	-8	3	145.39	12.30
12	-8	-3	125.59	12.90

-12	8	3	140.59	11.90
12	8	-3	145.09	16.00
-11	-8	3	27.50	5.30
11	-8	-3	50.69	9.60
-11	-8	3	21.60	6.10
-11	-8	3	11.90	8.80
-11	8	3	34.00	8.50
-11	8	3	49.90	8.20
11	8	-3	24.90	8.00
-10	-8	3	227.08	18.80
-10	-8	3	243.38	14.20
10	-8	-3	238.88	16.00
-10	-8	3	269.17	15.10
-10	8	3	230.58	15.00
-10	8	3	220.48	17.90
10	8	-3	268.07	18.00
-9	-8	3	261.27	14.80
-9	-8	3	300.47	15.30
-9	-8	3	238.18	19.00
9	-8	-3	245.78	16.30
-9	8	3	276.87	15.70
9	8	-3	268.27	18.20
-9	8	3	245.78	18.00
-8	-8	3	370.56	17.60
-8	-8	3	284.77	20.30
8	-8	-3	320.87	18.80
8	-8	-3	310.57	19.00
-8	-8	3	315.67	17.20
-8	8	3	332.67	18.10
8	8	-3	341.37	20.10
-8	8	3	325.37	20.10
7	-8	-3	14.30	3.90
-7	-8	3	12.50	2.90
7	-8	-3	21.20	4.40
-7	-8	3	17.80	5.60
-7	-8	3	14.90	3.00
-7	8	3	17.10	3.70
-7	8	3	14.70	4.70
7	8	-3	20.70	5.40
-6	-8	3	183.18	13.90
-6	-8	3	236.98	11.40
6	-8	-3	215.08	13.00
-6	-8	3	220.68	11.40
6	-8	-3	213.28	12.90

-6	8	3	199.68	13.40
-6	8	3	213.88	12.30
6	8	-3	203.88	13.90
5	-8	-3	18.50	3.90
-5	-8	3	12.60	4.30
5	-8	-3	13.40	3.30
-5	-8	3	12.10	2.10
5	8	-3	13.20	2.10
5	8	-3	15.20	4.20
-5	8	3	12.70	3.60
-5	8	3	15.90	3.00
-4	-8	3	912.11	48.60
-4	-8	3	958.80	46.70
4	-8	-3	985.80	48.00
4	-8	-3	1010.60	48.10
4	8	-3	924.11	46.70
-4	8	3	994.10	47.60
4	8	-3	1001.50	48.50
-4	8	3	1013.50	48.40
3	-8	-3	72.39	5.70
-3	-8	3	63.69	6.50
3	-8	-3	66.09	5.60
-3	-8	3	61.89	3.80
3	8	-3	58.19	6.30
3	8	-3	58.49	3.70
-3	8	3	66.19	5.10
-3	8	3	59.19	5.60
2	-8	-3	152.08	9.20
-2	-8	3	147.79	9.60
2	-8	-3	162.98	8.90
-2	-8	3	148.39	7.80
-2	8	3	146.79	8.80
2	8	-3	151.78	7.70
-2	8	3	157.68	9.40
2	8	-3	150.68	9.50
-1	-8	3	372.96	19.40
-1	-8	3	366.66	18.20
1	-8	-3	380.86	19.40
1	-8	-3	375.06	18.90
1	8	-3	369.86	18.10
-1	8	3	348.77	18.90
1	8	-3	368.86	19.40
-1	8	3	392.16	19.50
0	-8	-3	1609.24	74.79

0	-8	3	1566.34	74.49
0	-8	3	1529.45	75.09
0	8	-3	1467.45	74.29
0	8	3	1609.74	75.39
0	8	3	1574.24	75.59
0	8	-3	1543.35	75.19
0	8	3	1582.24	74.99
1	-8	3	37.40	3.50
-1	-8	-3	46.50	3.70
1	8	3	35.20	4.40
-1	8	-3	33.20	4.00
-1	8	-3	40.80	3.20
1	8	3	42.60	3.80
1	8	3	45.80	5.20
-2	-8	-3	1023.80	46.30
-2	-8	-3	900.71	47.10
-2	8	-3	983.10	46.40
2	8	3	957.60	47.30
2	8	3	943.91	46.30
-2	8	-3	919.51	46.10
3	-8	3	12.00	2.60
-3	-8	-3	7.40	2.10
-3	-8	-3	2.60	2.80
3	8	3	9.80	3.50
3	8	3	7.10	2.20
-3	8	-3	7.00	2.00
-3	8	-3	6.50	2.30
-4	-8	-3	473.85	27.40
4	-8	3	551.24	26.70
4	-8	3	527.05	26.10
-4	-8	-3	552.64	26.20
-4	8	-3	525.25	26.30
4	8	3	532.95	27.50
-4	8	-3	522.45	26.40
4	8	3	515.35	26.30
5	-8	3	1076.49	51.99
-5	-8	-3	1013.80	52.89
5	-8	3	1059.19	51.29
-5	-8	-3	1125.19	51.59
-5	8	-3	1039.60	51.69
5	8	3	1069.59	52.69
-5	8	-3	1063.99	51.69
5	8	3	1012.40	51.49
-6	-8	-3	77.69	6.30

6	-8	3	61.69	5.70
-6	-8	-3	82.19	8.60
6	-8	3	57.79	6.60
6	8	3	79.59	8.30
6	8	3	70.39	6.20
-6	8	-3	62.89	6.50
-6	8	-3	65.89	6.70
7	-8	3	164.68	10.20
-7	-8	-3	145.69	12.40
-7	-8	-3	189.88	10.70
7	-8	3	184.18	11.70
-7	8	-3	171.38	10.60
7	8	3	160.38	12.20
7	8	3	160.38	10.40
-7	8	-3	156.58	11.10
8	-8	3	216.28	12.70
-8	-8	-3	191.48	15.30
-8	-8	-3	245.28	13.30
8	-8	3	199.28	13.90
8	8	3	234.78	15.10
8	8	3	207.78	13.00
-8	8	-3	207.48	13.00
-8	8	-3	205.68	14.00
9	-8	3	834.72	41.60
-9	-8	-3	901.71	42.10
-9	-8	-3	744.63	43.80
9	8	3	887.51	43.70
-9	8	-3	832.02	42.00
-9	8	-3	790.22	42.70
9	8	3	803.52	41.60
-10	-8	-3	10.20	5.30
-10	-8	-3	22.80	6.90
10	-8	3	8.10	4.20
-10	8	-3	7.20	5.90
-10	8	-3	41.10	7.00
10	8	3	19.50	6.50
11	-8	3	370.06	20.80
-11	-8	-3	417.76	21.60
-11	-8	-3	295.47	24.10
-11	8	-3	384.06	21.00
-11	8	-3	310.77	22.50
11	8	3	377.16	23.60
-12	-8	-3	25.10	6.90
-12	8	-3	23.40	6.60

-12	8	-3	19.60	8.30
12	8	3	13.50	7.70
-13	-8	-3	52.69	8.70
-13	8	-3	77.49	11.20
-14	-8	-3	12.60	7.80
-14	8	-3	14.40	6.90
-15	-8	-3	110.89	15.20
-15	8	-3	113.69	14.20
-15	-9	3	53.69	9.40
-15	9	3	72.39	12.40
-14	-9	3	153.28	16.10
-14	9	3	167.88	14.60
-13	-9	3	65.79	10.00
-13	9	3	73.49	10.40
13	9	-3	75.89	14.70
-12	-9	3	216.48	15.20
-12	-9	3	198.58	14.20
12	-9	-3	227.58	19.50
-12	9	3	215.68	15.20
12	9	-3	232.38	19.00
-11	-9	3	743.03	38.00
11	-9	-3	742.13	39.80
-11	-9	3	687.13	42.80
-11	-9	3	832.92	38.70
-11	9	3	665.03	41.60
-11	9	3	755.42	38.80
11	9	-3	824.12	41.80
-10	-9	3	236.58	20.10
10	-9	-3	254.97	16.80
-10	-9	3	276.47	15.50
-10	-9	3	267.67	14.90
10	9	-3	259.17	18.50
-10	9	3	235.08	15.30
-10	9	3	246.98	19.00
-9	-9	3	395.36	27.00
9	-9	-3	450.55	25.00
-9	-9	3	489.15	23.60
-9	-9	3	437.16	23.20
9	9	-3	471.85	26.50
-9	9	3	433.86	26.50
-9	9	3	453.45	24.10
8	-9	-3	376.86	21.60
-8	-9	3	363.76	23.70
-8	-9	3	384.16	20.00

-8	-9	3	417.56	20.30
-8	9	3	373.96	22.90
-8	9	3	373.36	20.70
8	9	-3	412.46	23.20
7	-9	-3	790.92	39.80
7	-9	-3	744.23	40.00
-7	-9	3	776.42	38.30
-7	-9	3	820.02	38.40
-7	-9	3	740.63	41.10
-7	9	3	786.72	39.10
-7	9	3	748.73	40.50
7	9	-3	854.61	41.00
-6	-9	3	45.50	4.20
-6	-9	3	50.79	8.40
6	-9	-3	44.60	6.10
6	-9	-3	40.40	6.80
6	9	-3	53.69	7.70
-6	9	3	40.30	6.10
-6	9	3	44.90	5.20
5	-9	-3	3239.98	153.78
-5	-9	3	3120.29	154.68
-5	-9	3	3128.09	152.38
5	-9	-3	3221.58	153.98
5	9	-3	2932.01	152.38
-5	9	3	3211.98	153.28
5	9	-3	3486.75	154.58
-5	9	3	3340.47	154.28
-4	-9	3	8.10	1.50
4	-9	-3	5.10	2.80
-4	-9	3	2.40	3.50
4	-9	-3	7.10	3.10
-4	9	3	3.40	2.90
4	9	-3	6.00	3.50
4	9	-3	7.10	1.60
-4	9	3	2.00	2.40
3	-9	-3	1889.31	91.29
-3	-9	3	1883.61	91.89
-3	-9	3	1839.72	89.99
3	-9	-3	1907.91	91.39
-3	9	3	1855.31	90.89
3	9	-3	2028.80	91.89
3	9	-3	1814.82	89.99
-3	9	3	1928.31	91.49
-2	-9	3	999.90	49.99

2	-9	-3	1062.89	49.80
-2	-9	3	970.10	48.50
2	-9	-3	1021.40	49.50
2	9	-3	964.90	48.40
2	9	-3	1055.19	49.99
-2	9	3	968.00	49.30
-2	9	3	1060.69	49.90
-1	-9	3	4784.12	237.48
-1	-9	3	5065.49	238.68
1	-9	-3	5015.60	238.58
1	-9	-3	5061.69	238.08
-1	9	3	5138.49	238.58
-1	9	3	4773.42	238.08
1	9	-3	5232.68	238.68
0	-9	-3	319.47	15.60
0	-9	3	314.77	15.30
0	-9	3	284.27	15.90
0	9	-3	305.67	15.10
0	9	3	305.87	15.90
0	9	3	308.37	16.30
0	9	3	285.57	16.60
0	9	-3	285.17	16.10
-1	-9	-3	4638.44	217.28
1	-9	3	4662.33	217.18
1	9	3	4426.46	217.38
-1	9	-3	4378.16	216.88
-1	9	-3	4458.25	217.58
-1	9	-3	4687.13	217.48
1	9	3	4586.84	218.48
1	9	3	4745.83	217.88
2	-9	3	1800.72	84.09
-2	-9	-3	1763.52	85.09
-2	-9	-3	1789.42	83.99
-2	9	-3	1788.72	84.29
-2	9	-3	1663.73	84.19
2	9	3	1703.23	84.09
2	9	3	1802.62	85.19
-2	9	-3	1710.53	83.79
3	-9	3	688.73	34.50
-3	-9	-3	722.43	34.30
-3	-9	-3	705.23	35.60
3	9	3	707.33	35.60
-3	9	-3	702.23	34.50
3	9	3	669.33	34.30

-3	9	-3	686.53	34.20
4	-9	3	966.10	48.20
-4	-9	-3	1034.20	48.30
4	-9	3	984.50	48.70
-4	-9	-3	986.40	49.80
4	9	3	1051.29	49.80
-4	9	-3	994.00	48.40
-4	9	-3	986.70	48.50
4	9	3	934.51	48.30
5	-9	3	1180.88	57.29
5	-9	3	1132.79	56.49
-5	-9	-3	1184.78	56.69
-5	-9	-3	1133.79	58.19
-5	9	-3	1160.68	56.99
-5	9	-3	1139.89	56.79
5	9	3	1277.07	58.29
5	9	3	1125.79	56.69
-6	-9	-3	2351.36	119.79
6	-9	3	2403.36	118.09
6	-9	3	2519.55	118.99
-6	-9	-3	2645.24	118.29
6	9	3	2348.36	118.19
-6	9	-3	2405.06	118.39
6	9	3	2655.53	119.79
-6	9	-3	2403.06	118.69
-7	-9	-3	106.39	8.00
7	-9	3	88.69	9.00
-7	-9	-3	96.99	10.60
7	-9	3	98.69	7.50
-7	9	-3	107.09	8.80
-7	9	-3	91.39	7.80
7	9	3	131.29	10.30
7	9	3	90.09	7.60
8	-9	3	1096.09	53.49
-8	-9	-3	973.10	55.39
-8	-9	-3	1213.48	53.79
8	9	3	1014.40	53.39
-8	9	-3	1048.80	54.39
-8	9	-3	1068.69	53.79
8	9	3	1189.68	55.49
-9	-9	-3	5.40	4.50
-9	-9	-3	13.30	6.20
9	-9	3	9.80	3.70
9	9	3	8.20	4.20

9	9	3	10.20	6.00
-9	9	-3	10.80	4.90
-9	9	-3	5.40	5.20
-10	-9	-3	672.83	33.80
10	-9	3	652.33	33.20
-10	-9	-3	574.14	36.20
-10	9	-3	638.04	34.90
-10	9	-3	603.04	33.50
10	9	3	700.13	36.00
-11	-9	-3	49.40	10.40
11	-9	3	58.09	8.60
-11	-9	-3	49.90	7.70
11	9	3	62.49	12.80
-11	9	-3	55.09	11.20
-11	9	-3	58.69	9.50
-12	-9	-3	515.25	31.70
12	9	3	564.84	32.80
-12	9	-3	559.34	30.20
-13	-9	-3	66.79	11.70
-13	9	-3	29.30	7.40
-14	-9	-3	130.79	15.20
-14	9	-3	132.89	13.90
-15	-9	-3	91.39	15.70
-15	9	-3	82.09	13.60
-15	-10	3	10.30	7.60
-15	10	3	1.40	7.40
-14	-10	3	51.89	8.40
-14	10	3	41.40	8.40
-13	-10	3	37.80	7.10
-13	10	3	32.60	7.30
13	10	-3	42.40	10.40
-12	-10	3	18.70	5.60
-12	-10	3	11.50	5.40
-12	10	3	7.40	6.00
12	10	-3	15.20	8.50
-11	-10	3	17.80	5.00
-11	-10	3	28.20	5.80
11	-10	-3	22.70	6.20
-11	-10	3	-0.70	9.50
-11	10	3	30.10	6.10
-11	10	3	15.50	8.10
11	10	-3	21.50	7.80
-10	-10	3	62.59	8.00
-10	-10	3	54.19	10.60

10	-10	-3	63.69	9.30
-10	-10	3	66.09	7.20
-10	10	3	47.40	9.50
10	10	-3	87.79	12.80
-9	-10	3	21.70	7.90
9	-10	-3	39.80	7.90
-9	-10	3	30.40	4.60
-9	-10	3	33.00	4.90
-9	10	3	43.30	7.20
-9	10	3	37.20	7.40
9	10	-3	28.20	7.30
-8	-10	3	9.10	3.30
-8	-10	3	8.40	3.10
8	-10	-3	12.00	4.60
-8	-10	3	8.30	6.40
-8	10	3	10.90	4.10
-8	10	3	13.80	5.80
8	10	-3	18.60	6.20
-7	-10	3	12.40	5.60
7	-10	-3	4.60	3.70
-7	-10	3	10.00	2.80
-7	-10	3	8.10	2.80
7	-10	-3	12.40	5.10
-7	10	3	20.20	4.00
7	10	-3	11.40	5.30
-7	10	3	9.30	5.00
-6	-10	3	20.40	5.50
6	-10	-3	9.90	3.60
6	-10	-3	10.60	4.60
-6	-10	3	8.30	2.30
-6	10	3	14.20	4.40
-6	10	3	8.10	3.60
6	10	-3	16.40	5.20
6	10	-3	9.10	2.50
5	-10	-3	18.80	4.20
5	-10	-3	11.90	3.40
-5	-10	3	19.00	3.00
-5	-10	3	11.90	4.80
-5	10	3	28.50	4.90
5	10	-3	11.80	2.30
5	10	-3	14.10	4.60
-5	10	3	11.80	3.80
4	-10	-3	269.37	15.70
-4	-10	3	253.77	16.40

4	-10	-3	293.27	15.70
-4	-10	3	280.47	13.80
4	10	-3	252.47	16.00
-4	10	3	277.27	15.00
4	10	-3	273.87	13.80
-4	10	3	284.37	15.90
-3	-10	3	4.50	1.50
-3	-10	3	1.40	3.30
3	-10	-3	7.10	3.10
3	-10	-3	1.20	3.00
-3	10	3	6.40	2.90
3	10	-3	4.90	1.50
3	10	-3	2.20	3.20
-3	10	3	2.10	2.50
2	-10	-3	108.89	7.40
2	-10	-3	105.19	7.70
-2	-10	3	102.99	6.00
-2	-10	3	112.39	8.40
2	10	-3	108.49	8.00
-2	10	3	110.89	7.90
2	10	-3	98.79	5.80
-2	10	3	91.09	6.90
-1	-10	3	159.88	10.10
-1	-10	3	155.28	8.70
1	-10	-3	162.48	9.50
1	10	-3	163.08	10.30
-1	10	3	153.68	9.50
1	10	-3	156.98	8.40
-1	10	3	163.98	10.10
0	-10	-3	22.20	3.70
0	-10	3	9.30	2.20
0	10	-3	23.30	4.60
0	10	3	26.80	5.40
0	10	3	13.40	3.10
0	10	3	16.20	3.00
-1	-10	-3	69.39	5.00
-1	10	-3	53.59	4.50
1	10	3	60.19	5.80
-1	10	-3	56.49	5.20
1	10	3	65.69	7.00
1	10	3	58.19	5.00
-1	10	-3	55.39	5.60
-2	-10	-3	90.59	5.80
2	-10	3	81.39	6.00

-2	-10	-3	90.19	7.60
2	10	3	85.49	8.10
2	10	3	81.49	5.90
-2	10	-3	81.09	6.40
-2	10	-3	87.99	6.20
-2	10	-3	71.89	5.60
-3	-10	-3	161.88	11.60
-3	-10	-3	181.18	10.00
3	-10	3	181.18	10.50
-3	10	-3	169.28	9.90
3	10	3	177.38	10.10
-3	10	-3	180.78	10.30
4	-10	3	69.09	6.50
4	-10	3	66.29	5.70
-4	-10	-3	57.39	7.80
-4	-10	-3	78.99	5.80
-4	10	-3	69.09	5.90
4	10	3	78.99	5.90
-4	10	-3	77.79	6.10
4	10	3	71.69	8.50
5	-10	3	186.28	12.70
-5	-10	-3	226.68	12.00
-5	-10	-3	227.88	14.30
5	-10	3	202.38	11.80
-5	10	-3	191.18	11.90
-5	10	-3	205.88	12.40
5	10	3	246.88	14.60
5	10	3	205.28	12.00
-6	-10	-3	11.90	4.70
-6	-10	-3	9.40	3.30
6	-10	3	12.40	4.30
6	-10	3	6.60	2.70
6	10	3	9.40	3.30
-6	10	-3	7.00	3.30
6	10	3	8.90	4.60
-6	10	-3	5.20	3.20
-7	-10	-3	12.70	3.90
7	-10	3	11.70	5.10
7	-10	3	11.20	3.30
-7	-10	-3	14.30	5.50
-7	10	-3	7.20	3.90
7	10	3	8.00	3.50
-7	10	-3	13.20	4.10
7	10	3	8.70	5.60

8	-10	3	71.19	7.30
-8	-10	-3	75.59	11.30
-8	-10	-3	88.69	8.00
8	10	3	77.59	10.50
8	10	3	69.49	7.70
-8	10	-3	78.79	8.90
-8	10	-3	78.79	7.40
-9	-10	-3	98.59	9.10
-9	-10	-3	76.09	12.20
9	-10	3	83.29	8.10
9	10	3	82.49	8.70
9	10	3	91.79	11.90
-9	10	-3	89.29	10.20
-9	10	-3	87.39	8.90
-10	-10	-3	16.30	7.80
10	-10	3	9.00	4.60
10	10	3	9.10	7.20
-10	10	-3	7.30	6.30
10	10	3	12.50	5.00
-10	10	-3	15.30	5.80
-11	-10	-3	6.90	6.00
11	-10	3	6.80	4.90
-11	-10	-3	6.00	13.70
-11	10	-3	1.60	6.70
-11	10	-3	17.70	6.10
11	10	3	5.50	7.60
12	10	3	30.00	10.50
-13	-10	-3	-1.40	7.00
-13	10	-3	4.50	6.60
-14	-10	-3	9.20	8.40
-14	10	-3	16.90	7.70
-15	-10	-3	12.50	9.40
-15	10	-3	-5.60	7.20
-14	-11	3	190.98	15.60
-14	11	3	200.18	16.20
-13	-11	3	97.89	11.10
-13	11	3	92.49	11.80
-12	-11	3	125.39	11.10
12	11	-3	111.59	16.70
-12	11	3	125.59	12.10
-11	-11	3	645.94	43.70
-11	-11	3	845.72	39.10
11	-11	-3	736.23	40.50
-11	-11	3	778.32	38.70

11	11	-3	795.52	42.70
-11	11	3	768.52	39.40
-10	-11	3	154.28	10.70
-10	-11	3	133.19	10.10
10	-11	-3	142.89	12.50
-10	-11	3	114.79	16.70
10	11	-3	142.59	15.10
-10	11	3	156.08	11.80
-10	11	3	134.69	14.80
-9	-11	3	599.94	28.40
9	-11	-3	548.55	30.10
-9	-11	3	501.95	32.60
-9	-11	3	563.54	28.30
-9	11	3	562.14	29.30
-9	11	3	508.15	31.30
9	11	-3	586.64	31.90
-8	-11	3	71.69	6.50
-8	-11	3	89.79	6.80
8	-11	-3	71.49	8.70
-8	-11	3	57.19	9.60
-8	11	3	56.49	9.90
8	11	-3	68.69	10.50
-8	11	3	85.59	8.00
-7	-11	3	891.11	50.59
-7	-11	3	997.10	47.40
7	-11	-3	973.60	49.10
7	11	-3	1049.79	50.39
-7	11	3	937.61	49.80
-7	11	3	1005.00	48.50
6	-11	-3	27.90	5.90
-6	-11	3	22.50	6.50
6	-11	-3	35.10	6.20
-6	11	3	43.00	7.60
6	11	-3	25.00	5.60
-6	11	3	33.60	5.80
6	11	-3	27.80	4.20
5	-11	-3	2146.09	108.49
5	-11	-3	2306.87	108.49
-5	-11	3	2195.48	106.79
-5	-11	3	2158.98	109.49
5	11	-3	2352.16	109.19
-5	11	3	2299.67	108.89
-5	11	3	2235.98	107.79
4	-11	-3	9.90	3.80

-4	-11	3	22.90	5.50
4	-11	-3	14.40	3.60
-4	11	3	15.40	3.80
4	11	-3	19.50	2.90
-4	11	3	15.10	3.40
4	11	-3	14.30	4.50
-3	-11	3	1404.46	66.69
3	-11	-3	1437.96	68.29
3	-11	-3	1417.86	68.19
-3	-11	3	1326.17	68.69
-3	11	3	1362.36	67.59
-3	11	3	1452.95	68.39
3	11	-3	1408.96	68.59
3	11	-3	1350.66	66.59
2	-11	-3	18.80	3.50
-2	-11	3	19.50	2.90
2	-11	-3	24.00	4.10
-2	-11	3	20.30	4.40
-2	11	3	25.40	5.00
2	11	-3	24.50	2.70
2	11	-3	24.40	4.60
-2	11	3	15.60	3.40
-1	-11	3	912.61	44.50
-1	-11	3	909.31	45.90
1	-11	-3	948.91	45.30
-1	11	3	876.41	45.20
-1	11	3	907.71	46.10
1	11	-3	911.61	45.90
-1	11	3	981.30	45.90
0	-11	-3	234.38	12.50
0	-11	3	217.18	12.10
0	11	3	226.58	12.60
0	11	-3	225.58	13.30
0	11	-3	218.78	11.80
0	11	3	230.18	13.80
0	11	3	230.78	13.20
1	-11	3	1129.99	52.99
-1	-11	-3	1119.79	52.99
1	11	3	1118.99	54.29
-1	11	-3	1029.80	53.49
-1	11	-3	1039.60	52.49
1	11	3	1120.49	53.79
1	11	3	1098.79	53.09
-1	11	-3	1072.49	53.29

2	-11	3	806.22	38.60
-2	-11	-3	732.73	39.50
-2	-11	-3	820.92	38.30
2	11	3	783.82	38.40
-2	11	-3	759.42	38.10
2	11	3	806.72	39.90
-2	11	-3	792.22	38.70
-2	11	-3	743.13	38.80
-3	-11	-3	410.06	20.10
-3	-11	-3	368.46	21.70
3	-11	3	394.86	20.50
3	11	3	389.96	20.20
-3	11	-3	403.36	20.60
-3	11	-3	381.96	20.00
3	11	3	389.46	22.00
4	-11	3	1169.68	57.29
-4	-11	-3	1117.49	58.29
-4	-11	-3	1194.38	56.59
4	11	3	1226.08	58.59
-4	11	-3	1153.98	57.09
4	11	3	1136.59	56.69
-4	11	-3	1171.38	56.79
-5	-11	-3	438.26	24.80
5	-11	3	429.46	23.40
-5	-11	-3	462.95	22.60
5	-11	3	433.06	22.70
5	11	3	471.65	24.90
5	11	3	411.26	22.60
-5	11	-3	428.06	23.10
-5	11	-3	432.76	22.70
-6	-11	-3	1256.37	67.39
6	-11	3	1339.67	66.39
-6	-11	-3	1426.66	65.49
6	-11	3	1363.86	65.49
-6	11	-3	1310.97	66.09
-6	11	-3	1355.16	65.69
6	11	3	1272.27	65.49
6	11	3	1447.96	67.49
-7	-11	-3	128.59	8.90
7	-11	3	102.79	10.30
7	-11	3	112.89	8.50
-7	-11	-3	96.99	11.60
-7	11	-3	124.89	9.90
7	11	3	117.89	11.90

-7	11	-3	120.29	9.20
7	11	3	117.49	8.80
-8	-11	-3	361.06	18.70
-8	-11	-3	277.57	21.10
8	-11	3	334.47	18.50
8	11	3	344.87	21.20
-8	11	-3	350.96	19.00
-8	11	-3	326.27	19.70
8	11	3	323.97	18.50
-9	-11	-3	-0.90	13.00
-9	-11	-3	7.20	4.70
9	-11	3	4.00	3.80
9	11	3	3.10	6.60
-9	11	-3	5.20	5.00
-9	11	-3	10.90	4.60
9	11	3	0.80	4.20
10	-11	3	369.86	20.50
-10	-11	-3	310.87	24.40
10	11	3	386.76	24.40
-10	11	-3	345.87	22.30
-10	11	-3	381.86	21.00
10	11	3	347.27	20.50
11	-11	3	32.00	6.10
-11	-11	-3	36.30	7.50
-11	11	-3	36.70	8.40
-11	11	-3	28.70	6.70
11	11	3	39.70	9.80
-12	-11	-3	402.86	28.20
-12	11	-3	414.36	24.20
-13	-11	-3	-0.20	7.20
-13	11	-3	10.40	6.90
-14	-11	-3	241.48	19.20
-14	11	-3	200.18	17.00
-14	-12	3	115.29	15.90
-14	12	3	118.99	13.30
-13	-12	3	36.60	7.20
-13	12	3	58.69	10.70
-12	-12	3	128.79	11.90
-12	12	3	160.18	13.20
12	12	-3	153.18	18.00
-11	-12	3	44.80	7.70
-11	-12	3	25.70	5.60
-11	-12	3	30.20	12.30
11	-12	-3	15.90	7.00

-11	12	3	17.70	5.90
11	12	-3	18.70	8.80
-10	-12	3	179.18	12.40
-10	-12	3	188.88	19.40
10	-12	-3	182.88	14.70
-10	-12	3	202.68	12.70
10	12	-3	202.08	17.90
-10	12	3	194.88	17.70
-10	12	3	213.68	14.00
-9	-12	3	92.39	15.30
-9	-12	3	115.99	8.70
-9	-12	3	110.59	8.60
9	-12	-3	118.09	10.90
9	12	-3	129.99	14.00
-9	12	3	111.69	13.20
-9	12	3	101.79	12.70
8	-12	-3	374.96	21.30
-8	-12	3	324.27	23.60
-8	-12	3	384.06	19.20
-8	-12	3	365.16	19.20
-8	12	3	366.36	20.40
8	12	-3	403.66	23.10
-8	12	3	333.47	22.40
7	-12	-3	55.69	8.00
-7	-12	3	57.49	5.40
-7	-12	3	65.59	11.10
-7	12	3	63.39	7.10
-7	12	3	53.49	9.40
7	12	-3	80.19	10.50
7	12	-3	42.20	5.30
6	-12	-3	346.47	19.20
-6	-12	3	351.06	21.40
-6	-12	3	338.67	17.20
6	-12	-3	289.17	19.40
-6	12	3	337.27	20.00
-6	12	3	333.17	18.50
6	12	-3	383.36	20.60
6	12	-3	312.67	17.20
-5	-12	3	75.39	9.70
-5	-12	3	77.89	5.20
5	-12	-3	79.69	7.70
5	-12	-3	62.69	8.30
5	12	-3	77.19	9.20
5	12	-3	73.59	5.50

-5	12	3	68.39	8.20
-5	12	3	73.59	7.10
4	-12	-3	660.33	33.20
4	-12	-3	597.34	33.10
-4	-12	3	638.54	34.20
-4	-12	3	650.93	31.40
-4	12	3	632.04	32.50
4	12	-3	683.33	34.00
-4	12	3	651.13	33.50
3	-12	-3	-3.20	3.20
3	-12	-3	2.20	3.20
-3	-12	3	2.60	3.70
-3	-12	3	3.30	1.90
3	12	-3	4.00	2.10
-3	12	3	-0.20	3.20
3	12	-3	1.90	4.10
-3	12	3	-2.00	3.00
-2	-12	3	327.87	19.00
2	-12	-3	329.57	18.60
2	-12	-3	334.07	18.20
-2	-12	3	326.67	17.00
2	12	-3	358.46	19.20
-2	12	3	317.47	17.90
-2	12	3	355.76	18.80
-1	-12	3	4.20	3.50
-1	-12	3	-0.40	2.10
1	-12	-3	3.40	2.80
-1	12	3	2.50	2.80
-1	12	3	1.90	3.40
-1	12	3	0.40	2.90
1	12	-3	5.30	3.40
1	12	-3	2.10	2.00
0	-12	3	1044.60	52.39
0	-12	-3	1109.29	52.79
0	12	3	1066.59	52.89
0	12	3	1114.59	53.69
0	12	-3	1097.69	53.59
0	12	-3	1060.79	52.19
0	12	3	1086.39	54.09
1	-12	3	367.96	19.10
-1	-12	-3	363.96	19.00
-1	12	-3	364.56	18.70
1	12	3	369.66	19.30
1	12	3	382.76	20.90

-1	12	-3	366.26	19.60
-1	12	-3	367.56	20.00
1	12	3	369.76	20.10
-2	-12	-3	564.74	30.00
2	-12	3	600.44	28.90
-2	-12	-3	586.14	28.60
-2	12	-3	566.84	29.30
-2	12	-3	549.35	28.30
2	12	3	548.75	28.70
-2	12	-3	574.94	29.10
2	12	3	580.74	30.30
-3	-12	-3	187.98	10.50
3	-12	3	191.28	11.30
-3	-12	-3	163.38	14.00
-3	12	-3	177.08	11.30
-3	12	-3	183.48	11.10
-3	12	-3	171.18	10.50
3	12	3	183.68	10.70
3	12	3	199.48	12.90
-4	-12	-3	460.95	23.10
4	-12	3	457.15	24.00
4	12	3	441.36	23.20
-4	12	-3	434.06	23.70
4	12	3	494.65	25.50
-4	12	-3	433.46	23.20
-5	-12	-3	1140.69	55.29
5	-12	3	1119.49	55.49
5	-12	3	1146.59	56.29
5	12	3	1088.49	55.39
-5	12	-3	1090.39	55.89
-5	12	-3	1102.29	55.49
5	12	3	1264.17	57.69
6	-12	3	410.36	23.50
-6	-12	-3	432.96	22.20
6	-12	3	430.36	22.30
-6	-12	-3	355.26	27.50
-6	12	-3	443.56	22.60
6	12	3	478.25	24.90
-6	12	-3	421.56	23.00
6	12	3	402.26	22.20
-7	-12	-3	212.48	16.10
-7	-12	-3	199.88	12.80
7	-12	3	210.38	12.70
7	12	3	206.08	12.80

7	12	3	241.08	16.00
-7	12	-3	189.78	13.60
-7	12	-3	203.18	13.10
8	-12	3	179.08	11.80
-8	-12	-3	183.68	11.90
-8	-12	-3	168.08	15.30
-8	12	-3	198.88	12.50
-8	12	-3	165.08	13.10
8	12	3	185.28	14.80
8	12	3	163.78	11.70
-9	-12	-3	425.56	28.20
-9	-12	-3	494.25	24.80
9	-12	3	445.06	24.70
-9	12	-3	412.56	24.80
9	12	3	527.25	27.90
9	12	3	443.86	24.60
-9	12	-3	450.25	26.20
-10	-12	-3	4.90	7.80
10	-12	3	11.80	4.80
-10	12	-3	8.60	6.30
10	12	3	8.50	5.10
-10	12	-3	12.90	5.60
10	12	3	4.00	7.70
-11	-12	-3	257.57	17.70
-11	12	-3	250.27	17.10
11	12	3	268.17	21.00
-12	-12	-3	23.30	7.80
-12	12	-3	20.10	6.90
-13	-12	-3	121.49	14.80
-13	12	-3	129.29	12.60
-14	-12	-3	21.90	9.30
-14	12	-3	9.00	7.00
-14	13	3	18.80	7.70
-13	-13	3	59.79	10.70
-13	13	3	74.99	11.60
-12	-13	3	10.60	5.80
12	13	-3	7.00	9.40
-12	13	3	29.40	7.00
-11	-13	3	48.90	8.30
-11	-13	3	54.99	13.30
11	13	-3	43.60	10.10
-11	13	3	67.09	10.00
-10	-13	3	269.17	15.20
10	-13	-3	254.87	17.40

-10	-13	3	256.67	15.10
-10	-13	3	234.38	22.60
-10	13	3	240.58	21.60
10	13	-3	261.57	20.60
-10	13	3	244.08	15.90
-9	-13	3	213.18	12.30
9	-13	-3	184.48	14.50
-9	-13	3	198.28	12.40
9	13	-3	197.58	17.00
-9	13	3	201.18	16.90
-9	13	3	203.48	13.70
8	-13	-3	44.70	6.90
-8	-13	3	46.10	5.60
-8	-13	3	38.10	5.70
-8	-13	3	30.80	9.00
-8	13	3	32.40	6.90
-8	13	3	43.70	6.70
8	13	-3	28.70	7.50
-7	-13	3	105.69	13.00
-7	-13	3	116.79	7.60
7	-13	-3	123.89	10.60
-7	13	3	121.69	9.60
7	13	-3	113.09	12.10
7	13	-3	105.09	7.60
-7	13	3	101.19	11.30
6	-13	-3	30.70	5.50
-6	-13	3	32.30	4.10
-6	-13	3	23.70	6.30
6	13	-3	23.30	3.30
6	13	-3	26.70	6.20
-6	13	3	29.90	5.80
-6	13	3	27.00	4.80
5	-13	-3	230.78	15.70
5	-13	-3	246.78	15.00
-5	-13	3	221.88	16.60
5	13	-3	246.68	13.00
5	13	-3	230.48	15.90
-5	13	3	257.27	15.90
-5	13	3	256.17	14.20
4	-13	-3	69.39	8.00
4	-13	-3	75.29	7.60
-4	-13	3	65.79	9.40
-4	-13	3	77.39	5.10
4	13	-3	56.29	8.80

-4	13	3	77.29	8.30
-4	13	3	73.99	7.00
4	13	-3	72.09	5.30
-3	-13	3	247.58	16.60
-3	-13	3	267.87	13.70
3	-13	-3	264.17	15.60
3	-13	-3	262.27	15.60
3	13	-3	257.87	13.60
-3	13	3	273.37	15.90
-3	13	3	268.97	15.00
3	13	-3	261.67	16.40
2	-13	-3	265.87	14.80
-2	-13	3	250.97	15.70
-2	13	3	253.37	15.30
2	13	-3	265.87	15.80
2	13	-3	253.57	13.20
-2	13	3	238.78	14.40
1	-13	-3	185.48	11.30
-1	-13	3	187.68	10.50
-1	-13	3	172.98	12.10
-1	13	3	189.38	11.40
1	13	-3	180.08	12.30
-1	13	3	190.98	12.90
-1	13	3	198.68	12.20
1	13	-3	187.68	10.20
0	-13	-3	29.60	4.70
0	-13	3	23.30	4.20
0	13	3	16.00	4.50
0	13	-3	22.00	4.30
0	13	-3	24.30	3.70
0	13	3	22.20	4.20
0	13	3	28.60	4.30
-1	-13	-3	291.47	15.10
1	-13	3	280.97	15.10
-1	13	-3	253.97	16.00
-1	13	-3	259.87	14.50
1	13	3	284.47	16.20
1	13	3	285.47	15.30
1	13	3	282.67	17.10
-1	13	-3	277.47	15.60
-2	-13	-3	13.60	3.20
2	-13	3	4.90	3.10
-2	-13	-3	-0.60	3.80
-2	13	-3	10.20	3.30

2	13	3	6.60	3.00
-2	13	-3	7.10	2.60
-2	13	-3	4.30	3.20
2	13	3	3.10	4.40
2	13	3	9.30	3.50
-3	-13	-3	135.19	8.40
-3	-13	-3	122.79	10.70
3	-13	3	141.59	9.40
3	13	3	121.09	8.60
-3	13	-3	123.69	8.30
-3	13	-3	113.49	9.20
-3	13	-3	133.19	9.00
3	13	3	142.89	11.30
4	-13	3	70.19	7.70
-4	-13	-3	64.49	9.40
-4	-13	-3	94.39	6.60
4	13	3	66.49	9.40
-4	13	-3	88.29	6.80
4	13	3	75.29	6.80
-4	13	-3	83.59	7.20
-5	-13	-3	123.09	8.30
-5	-13	-3	114.09	11.50
5	-13	3	110.89	9.50
-5	13	-3	109.49	9.00
5	13	3	112.29	8.40
5	13	3	110.09	11.50
-5	13	-3	117.79	8.50
-6	-13	-3	207.98	16.80
-6	-13	-3	253.57	13.90
6	-13	3	245.28	14.00
6	-13	3	215.48	15.20
6	13	3	239.08	13.90
6	13	3	238.88	16.70
-6	13	-3	236.78	14.70
-6	13	-3	246.68	14.10
-7	-13	-3	27.50	4.70
7	-13	3	16.60	4.00
-7	-13	-3	10.00	6.10
7	13	3	23.10	6.40
7	13	3	19.50	4.60
-7	13	-3	15.50	4.50
-7	13	-3	16.30	4.30
8	-13	3	118.19	9.60
-8	-13	-3	104.09	13.30

-8	-13	-3	115.59	9.80
8	13	3	112.79	13.10
8	13	3	117.99	9.70
-8	13	-3	142.59	10.30
-8	13	-3	114.59	11.10
-9	-13	-3	35.10	9.30
9	-13	3	38.00	7.60
9	13	3	30.50	5.70
9	13	3	43.50	9.00
-9	13	-3	37.60	7.20
-9	13	-3	43.90	8.10
-10	-13	-3	263.67	22.30
10	-13	3	294.77	17.80
-10	13	-3	305.47	18.30
10	13	3	295.37	21.20
-10	13	-3	291.57	19.90
10	13	3	260.97	17.20
-11	13	-3	8.30	6.40
11	13	3	10.60	8.90
-12	-13	-3	83.59	12.90
-12	13	-3	80.49	11.50
-13	-13	-3	14.80	8.60
-13	13	-3	14.50	7.80
-14	-13	-3	93.89	15.70
-14	13	-3	78.59	13.40
-13	14	3	41.10	8.10
-12	-14	3	240.88	16.40
12	14	-3	276.77	23.40
-12	14	3	251.57	17.40
-11	-14	3	197.98	13.70
11	14	-3	209.58	20.40
-11	14	3	199.08	15.10
10	-14	-3	151.08	13.70
-10	-14	3	182.68	11.40
-10	-14	3	164.18	11.50
-10	-14	3	129.19	20.40
10	14	-3	157.98	17.00
-10	14	3	176.88	13.20
-9	-14	3	194.78	11.70
-9	-14	3	178.98	18.40
9	-14	-3	169.08	14.20
-9	-14	3	195.78	12.00
-9	14	3	173.68	16.70
-9	14	3	177.48	13.40

9	14	-3	205.18	17.30
-8	-14	3	212.28	11.80
-8	-14	3	170.58	17.50
8	-14	-3	203.68	14.60
-8	14	3	193.78	16.00
8	14	-3	214.68	17.00
7	-14	-3	287.37	17.80
-7	-14	3	257.27	20.50
-7	-14	3	284.87	15.10
-7	14	3	300.07	16.90
-7	14	3	275.27	18.80
7	14	-3	314.97	19.60
7	14	-3	266.07	15.20
-6	-14	3	1614.84	77.09
-6	-14	3	1436.06	80.29
6	-14	-3	1643.44	79.19
-6	14	3	1628.24	79.89
6	14	-3	1516.35	77.09
6	14	-3	1749.22	80.39
-6	14	3	1670.83	78.49
5	-14	-3	15.90	4.40
-5	-14	3	12.80	5.80
-5	-14	3	19.00	2.80
-5	14	3	13.10	4.40
5	14	-3	16.10	5.90
5	14	-3	19.30	3.00
-5	14	3	13.30	4.70
-4	-14	3	1564.44	82.79
4	-14	-3	1590.04	82.09
4	-14	-3	1720.43	82.09
-4	-14	3	1691.53	80.19
4	14	-3	1648.04	80.09
-4	14	3	1646.44	81.19
-4	14	3	1725.73	82.39
4	14	-3	1800.12	82.89
3	-14	-3	1.10	3.90
-3	-14	3	0.00	4.30
-3	-14	3	3.50	2.30
3	-14	-3	-7.60	3.70
-3	14	3	0.40	3.80
3	14	-3	2.40	2.20
-3	14	3	-1.40	3.60
3	14	-3	0.60	4.80
-2	-14	3	639.14	30.60

-2	-14	3	590.14	32.60
2	-14	-3	615.24	31.80
2	14	-3	617.14	32.70
-2	14	3	616.54	31.50
2	14	-3	601.84	30.30
-2	14	3	627.94	32.40
-2	14	3	641.34	32.90
-1	-14	3	31.80	4.60
-1	-14	3	30.90	5.10
1	-14	-3	36.60	5.60
-1	14	3	27.10	4.50
1	14	-3	37.30	7.00
1	14	-3	32.60	4.10
-1	14	3	38.20	6.50
-1	14	3	37.90	6.00
0	-14	-3	2143.89	102.19
0	-14	3	2134.09	101.89
0	14	-3	2107.39	103.09
0	14	3	2193.58	103.19
0	14	3	2116.19	102.29
0	14	-3	2045.20	101.49
0	14	3	2118.19	103.59
1	-14	3	881.61	43.00
-1	-14	-3	900.31	42.90
1	14	3	878.11	44.30
1	14	3	860.21	43.10
-1	14	-3	855.61	43.50
1	14	3	888.81	44.80
-1	14	-3	841.72	43.90
-1	14	-3	854.11	42.40
2	-14	3	735.33	36.50
-2	-14	-3	780.12	36.10
-2	14	-3	689.43	37.00
-2	14	-3	684.43	35.70
2	14	3	704.33	36.20
-2	14	-3	738.13	36.80
2	14	3	694.83	37.00
2	14	3	780.02	38.30
3	-14	3	213.98	13.10
-3	-14	-3	224.28	12.30
-3	-14	-3	199.38	14.50
3	14	3	214.98	12.40
-3	14	-3	215.38	13.50
-3	14	-3	204.98	13.00

3	14	3	224.48	15.20
-3	14	-3	205.68	12.10
4	-14	3	110.49	9.50
-4	-14	-3	121.69	11.20
-4	-14	-3	112.99	8.00
4	14	3	113.19	11.20
4	14	3	110.39	8.20
-4	14	-3	106.79	8.20
-4	14	-3	106.59	8.80
-5	-14	-3	575.24	28.70
-5	-14	-3	549.45	31.40
5	-14	3	545.25	30.00
-5	14	-3	553.94	29.00
5	14	3	595.34	31.50
5	14	3	557.94	28.90
-5	14	-3	575.44	29.70
-6	-14	-3	400.76	24.20
-6	-14	-3	401.76	20.60
-6	14	-3	378.66	20.90
6	14	3	405.96	23.70
-6	14	-3	373.66	21.60
6	14	3	355.46	20.70
7	-14	3	615.64	31.40
-7	-14	-3	544.05	34.10
-7	-14	-3	631.74	31.00
-7	14	-3	626.84	31.50
7	14	3	641.64	33.80
-7	14	-3	596.84	32.20
7	14	3	572.94	31.10
8	-14	3	9.50	4.30
-8	-14	-3	6.00	4.50
-8	-14	-3	8.30	7.20
8	14	3	7.50	6.80
8	14	3	4.30	4.40
-8	14	-3	-0.50	4.60
-8	14	-3	3.10	4.70
-9	-14	-3	380.56	28.60
9	-14	3	442.26	24.30
9	14	3	442.56	24.20
9	14	3	497.35	27.80
-9	14	-3	422.96	25.90
-9	14	-3	453.35	24.60
10	14	3	9.50	5.50
10	14	3	17.10	8.70

-10	14	-3	14.40	6.50
-10	14	-3	9.30	6.30
11	14	3	545.45	32.40
11	14	3	469.75	27.70
-11	14	-3	519.35	28.60
-12	-14	-3	6.50	7.60
-12	14	-3	6.30	7.00
-13	-14	-3	346.17	23.50
-13	14	-3	340.17	22.20
-13	15	3	4.20	7.30
-12	-15	3	13.00	6.10
-12	15	3	9.20	6.50
12	15	-3	24.70	10.50
-11	-15	3	24.00	5.80
-11	15	3	33.20	7.70
11	15	-3	14.30	9.90
-10	-15	3	-13.50	12.00
-10	-15	3	8.40	4.90
10	15	-3	-8.10	9.00
-10	15	3	7.10	6.20
-9	-15	3	10.10	10.00
-9	-15	3	17.80	4.10
9	-15	-3	17.40	6.00
-9	-15	3	20.90	4.70
-9	15	3	17.40	8.90
-9	15	3	14.70	5.80
9	15	-3	11.40	8.00
-8	-15	3	36.20	9.80
-8	-15	3	39.80	5.70
8	-15	-3	37.10	7.10
-8	15	3	46.90	8.70
-8	15	3	50.49	8.30
8	15	-3	40.90	8.40
8	15	-3	30.70	5.80
-7	-15	3	29.30	4.90
-7	-15	3	28.10	7.80
7	-15	-3	37.70	6.70
7	15	-3	22.90	3.80
7	15	-3	38.00	7.60
-7	15	3	23.90	5.10
-7	15	3	15.10	6.20
-6	-15	3	5.20	6.40
6	-15	-3	3.60	4.40
-6	-15	3	12.50	2.90

6	15	-3	6.80	6.30
-6	15	3	14.10	4.30
6	15	-3	7.30	2.90
-6	15	3	9.80	5.30
5	-15	-3	138.79	11.40
-5	-15	3	155.78	8.60
-5	-15	3	120.69	13.20
5	15	-3	140.99	13.10
-5	15	3	128.49	11.90
5	15	-3	149.88	8.70
-5	15	3	147.19	10.70
-4	-15	3	24.50	3.20
4	-15	-3	15.70	6.60
4	-15	-3	21.80	5.00
-4	-15	3	23.20	6.30
-4	15	3	17.60	4.60
4	15	-3	23.20	3.20
4	15	-3	23.90	6.30
-4	15	3	25.10	5.40
-3	-15	3	65.69	8.90
3	-15	-3	54.99	8.20
-3	-15	3	43.40	4.80
-3	15	3	43.70	6.90
3	15	-3	69.49	9.10
-3	15	3	51.19	8.00
3	15	-3	44.70	4.60
-2	-15	3	87.79	6.60
2	-15	-3	83.99	8.20
-2	-15	3	86.49	9.30
2	15	-3	89.29	9.70
-2	15	3	90.29	9.80
2	15	-3	87.29	6.20
-2	15	3	74.29	7.60
-2	15	3	95.09	9.00
1	-15	-3	12.10	3.80
-1	-15	3	17.00	3.50
1	15	-3	14.30	4.80
-1	15	3	12.50	4.90
-1	15	3	17.10	4.30
-1	15	3	13.70	4.60
1	15	-3	12.30	2.90
0	-15	3	1.40	3.00
0	-15	-3	0.00	3.10
0	15	3	-3.50	4.40

0	15	3	-8.20	3.60
0	15	-3	-1.80	4.30
0	15	3	4.40	3.70
0	15	-3	-0.70	2.60
0	15	-3	-2.70	3.00
1	-15	3	50.29	6.50
-1	-15	-3	61.59	6.20
-1	15	-3	47.50	5.50
-1	15	-3	79.69	8.40
1	15	3	58.89	6.50
1	15	3	70.29	9.20
1	15	3	49.90	8.50
-1	15	-3	54.39	6.80
-2	-15	-3	7.00	3.10
2	-15	3	1.40	3.80
-2	15	-3	-1.20	3.30
2	15	3	-5.00	4.60
2	15	3	-1.20	3.30
-2	15	-3	3.70	2.80
-2	15	-3	-2.30	4.00
2	15	3	-3.10	3.70
-3	-15	-3	219.38	12.20
3	-15	3	203.08	13.20
-3	-15	-3	208.08	15.00
-3	15	-3	212.58	13.60
3	15	3	195.58	12.30
3	15	3	206.68	15.10
-3	15	-3	189.28	11.90
-3	15	-3	206.68	13.10
4	-15	3	5.20	4.50
-4	-15	-3	11.60	6.40
-4	-15	-3	12.40	3.50
4	15	3	11.40	5.80
-4	15	-3	12.40	4.20
4	15	3	15.60	4.00
-4	15	-3	10.50	3.70
-4	15	-3	17.00	4.40
-5	-15	-3	41.80	5.80
5	-15	3	25.20	6.00
-5	-15	-3	34.10	7.70
5	15	3	30.90	6.90
-5	15	-3	29.60	5.30
5	15	3	37.10	6.30
-5	15	-3	24.30	4.70

-6	-15	-3	25.40	7.30
-6	-15	-3	17.70	4.10
-6	15	-3	14.40	4.50
6	15	3	14.30	4.40
-6	15	-3	16.70	4.70
6	15	3	24.00	7.00
-7	-15	-3	8.80	7.20
-7	15	-3	9.80	4.80
7	15	3	8.40	4.40
7	15	3	4.00	6.30
-7	15	-3	6.40	4.90
-8	-15	-3	21.70	8.40
-8	-15	-3	41.30	7.60
8	-15	3	28.90	5.50
-8	15	-3	22.90	5.90
8	15	3	19.90	5.30
8	15	3	29.60	7.90
9	-15	3	7.00	4.90
-9	-15	-3	17.00	9.30
9	15	3	1.40	7.70
-9	15	-3	-0.70	5.90
-9	15	-3	12.50	5.70
9	15	3	2.00	4.90
10	15	3	18.60	5.60
10	15	3	9.00	8.50
-10	15	-3	10.60	5.90
-11	15	-3	9.80	6.50
11	15	3	7.50	6.10
-12	-15	-3	13.60	8.50
-12	15	-3	13.90	7.10
-13	-15	-3	20.70	9.80
-13	15	-3	32.60	8.10
-13	16	3	30.70	8.70
-12	16	3	267.77	18.00
-11	-16	3	49.70	9.10
-11	16	3	75.09	11.10
11	16	-3	51.69	13.00
-10	-16	3	167.88	12.50
10	16	-3	177.78	19.00
-10	16	3	192.98	14.40
9	-16	-3	43.20	7.80
-9	-16	3	25.00	5.10
-9	-16	3	33.00	5.30
-9	16	3	28.00	6.30

9	16	-3	21.30	9.80
-8	-16	3	360.26	28.90
8	-16	-3	453.95	26.00
-8	-16	3	470.75	23.00
8	16	-3	420.76	23.00
-8	16	3	418.96	27.20
8	16	-3	499.05	28.20
-8	16	3	494.25	25.00
-7	-16	3	10.60	3.20
-7	-16	3	7.90	8.20
7	-16	-3	4.80	4.70
-7	16	3	8.10	6.60
-7	16	3	11.30	5.90
7	16	-3	3.30	6.80
7	16	-3	1.20	3.20
6	-16	-3	458.85	25.50
-6	-16	3	482.05	22.80
-6	-16	3	408.76	27.70
-6	16	3	468.75	24.50
6	16	-3	454.05	27.00
-6	16	3	428.36	26.10
6	16	-3	436.06	22.90
5	-16	-3	42.50	6.60
-5	-16	3	50.49	5.00
-5	-16	3	35.70	8.50
5	16	-3	37.30	5.10
-5	16	3	38.50	6.90
-5	16	3	49.10	7.30
5	16	-3	44.50	7.90
-4	-16	3	377.76	23.60
-4	-16	3	396.46	19.80
4	16	-3	405.06	23.50
-4	16	3	379.56	21.30
-4	16	3	396.46	22.80
4	16	-3	369.86	19.70
3	-16	-3	-2.30	4.90
-3	-16	3	2.20	2.70
-3	-16	3	1.30	4.80
3	16	-3	2.00	5.20
-3	16	3	-2.90	4.60
-3	16	3	-3.80	3.90
3	16	-3	0.60	2.50
-3	16	3	6.40	4.60
-2	-16	3	172.98	14.00

-2	-16	3	217.28	11.50
2	-16	-3	190.98	13.00
2	16	-3	188.48	10.90
2	16	-3	176.68	14.20
-2	16	3	207.88	13.70
-2	16	3	202.48	14.60
-2	16	3	196.88	12.70
-1	-16	3	31.20	4.40
1	-16	-3	42.00	5.60
-1	16	3	25.40	5.00
1	16	-3	22.70	5.90
1	16	-3	25.00	4.50
-1	16	3	25.20	5.30
-1	16	3	22.30	6.10
0	-16	3	1450.15	68.19
0	-16	-3	1417.76	68.39
0	16	3	1388.06	70.19
0	16	-3	1382.16	67.69
0	16	-3	1395.46	69.69
0	16	-3	1357.86	68.89
0	16	3	1351.96	69.79
0	16	3	1465.25	68.59
1	-16	3	126.49	9.50
-1	-16	-3	127.99	9.20
1	16	3	123.89	10.40
1	16	3	120.99	11.70
-1	16	-3	121.39	9.60
-1	16	-3	128.19	8.50
1	16	3	125.69	9.50
-1	16	-3	124.29	10.90
2	-16	3	294.27	17.40
-2	-16	-3	321.67	16.60
-2	16	-3	305.17	17.50
2	16	3	286.57	19.20
2	16	3	303.47	16.90
-2	16	-3	291.07	16.20
2	16	3	298.37	18.00
-2	16	-3	293.17	18.10
-3	-16	-3	79.69	11.40
-3	-16	-3	116.39	8.20
3	-16	3	102.09	9.90
3	16	3	115.49	8.60
-3	16	-3	121.59	9.40
-3	16	-3	118.69	10.20

-3	16	-3	111.49	8.20
3	16	3	87.29	11.40
-4	-16	-3	513.55	25.10
-4	-16	-3	464.15	27.90
4	-16	3	503.15	26.70
-4	16	-3	481.35	26.60
-4	16	-3	484.05	25.20
-4	16	-3	471.85	26.10
4	16	3	468.95	25.30
4	16	3	480.25	27.90
-5	-16	-3	295.57	16.40
-5	-16	-3	286.27	19.90
-5	16	-3	284.77	16.70
5	16	3	305.97	20.20
-5	16	-3	289.87	17.60
5	16	3	297.07	16.70
-6	-16	-3	482.75	31.00
-6	-16	-3	552.44	27.60
-6	16	-3	517.65	27.90
-6	16	-3	519.55	28.80
6	16	3	530.05	27.80
6	16	3	589.84	31.10
-7	-16	-3	736.13	36.60
-7	-16	-3	692.43	40.70
-7	16	-3	770.42	37.30
-7	16	-3	643.04	37.90
7	16	3	777.52	40.10
7	16	3	719.33	36.90
-8	-16	-3	56.69	11.80
8	-16	3	59.79	8.90
8	16	3	64.49	8.70
8	16	3	38.70	8.80
-8	16	-3	65.59	8.80
-9	-16	-3	217.08	24.20
-9	16	-3	290.07	17.50
9	16	3	257.97	21.30
-9	16	-3	280.27	25.40
9	16	3	265.87	16.70
10	16	3	16.20	9.40
10	16	3	7.70	5.70
-10	16	-3	5.30	6.20
-11	16	-3	335.77	20.60
11	16	3	281.17	24.20
-12	-16	-3	14.60	8.80

-12	16	-3	19.70	7.60
-13	-16	-3	242.98	20.70
-13	16	-3	222.48	18.20
-12	17	3	14.40	7.20
-11	17	3	258.47	18.60
11	17	-3	291.57	25.10
-10	-17	3	9.00	5.30
10	17	-3	-4.90	9.20
-10	17	3	9.80	6.60
-9	-17	3	216.58	13.90
9	17	-3	222.28	20.10
-9	17	3	219.78	15.70
8	-17	-3	11.20	6.20
-8	-17	3	2.70	3.40
8	17	-3	2.20	3.60
-8	17	3	8.10	5.40
8	17	-3	6.10	8.20
7	-17	-3	367.66	22.40
-7	-17	3	378.06	19.20
-7	17	3	341.67	23.50
7	17	-3	328.37	19.30
-7	17	3	375.46	21.40
7	17	-3	436.96	24.90
6	-17	-3	-0.40	4.90
-6	-17	3	4.40	3.00
-6	-17	3	2.00	8.00
-6	17	3	1.30	5.50
6	17	-3	-7.20	6.70
6	17	-3	2.80	3.00
-5	-17	3	70.69	5.90
5	-17	-3	61.39	9.30
-5	-17	3	49.50	9.50
-5	17	3	55.89	7.60
5	17	-3	55.69	11.00
5	17	-3	63.09	5.90
-5	17	3	55.29	9.70
-4	-17	3	77.49	12.10
-4	-17	3	102.89	7.10
-4	17	3	95.49	10.50
4	17	-3	90.99	6.80
-4	17	3	82.89	9.40
4	17	-3	101.49	11.80
-3	-17	3	272.67	14.40
-3	-17	3	238.78	17.80

3	17	-3	287.17	18.20
-3	17	3	249.48	17.00
-3	17	3	263.57	15.90
3	17	-3	254.47	14.10
-3	17	3	272.47	18.30
2	-17	-3	21.70	6.70
-2	-17	3	37.40	4.60
-2	-17	3	26.60	6.30
-2	17	3	27.10	5.80
-2	17	3	37.40	7.60
2	17	-3	26.90	6.40
-2	17	3	33.00	5.80
2	17	-3	34.50	4.70
1	-17	-3	28.10	5.10
-1	-17	3	29.90	4.60
-1	17	3	25.90	6.50
-1	17	3	26.80	5.10
1	17	-3	23.40	5.90
-1	17	3	30.40	6.20
1	17	-3	24.10	3.80
0	-17	3	183.78	12.30
0	-17	-3	190.48	12.60
0	17	-3	210.38	14.50
0	17	-3	188.18	13.00
0	17	3	211.98	15.50
0	17	3	201.48	12.80
0	17	-3	199.48	11.60
0	17	3	187.28	15.10
-1	-17	-3	87.59	7.60
1	-17	3	62.99	8.00
-1	17	-3	77.19	8.20
-1	17	-3	70.19	6.80
-1	17	-3	83.69	9.70
1	17	3	94.19	10.80
1	17	3	70.09	7.70
1	17	3	81.09	8.80
2	-17	3	49.20	6.60
-2	-17	-3	52.69	6.50
2	17	3	47.70	7.90
-2	17	-3	49.70	7.30
-2	17	-3	48.70	7.40
-2	17	-3	47.90	5.90
2	17	3	46.20	8.20
2	17	3	49.99	6.80

-3	-17	-3	7.90	3.80
3	-17	3	-6.10	4.60
-3	17	-3	8.60	3.70
-3	17	-3	3.60	4.90
3	17	3	8.50	4.40
-3	17	-3	1.60	3.70
3	17	3	5.20	6.70
3	17	3	2.80	3.90
4	-17	3	226.38	15.40
-4	-17	-3	209.18	16.90
-4	-17	-3	234.98	13.20
-4	17	-3	232.48	15.30
4	17	3	231.88	16.90
4	17	3	211.98	13.50
-4	17	-3	199.68	13.30
-4	17	-3	228.78	14.60
-5	-17	-3	25.00	4.60
-5	-17	-3	14.10	8.10
5	17	3	29.70	7.80
-5	17	-3	17.60	5.10
-5	17	-3	24.30	5.30
5	17	3	23.10	4.90
-5	17	-3	20.80	5.50
-6	-17	-3	39.00	6.50
-6	-17	-3	41.20	9.60
-6	17	-3	36.00	6.20
6	17	3	37.10	9.00
-6	17	-3	28.50	5.50
6	17	3	35.90	5.60
-7	-17	-3	14.60	8.30
-7	17	-3	15.50	6.10
7	17	3	2.80	8.00
7	17	3	7.50	4.80
-8	-17	-3	53.39	11.00
-8	17	-3	66.89	9.20
8	17	3	62.99	10.20
-8	17	-3	62.99	9.00
8	17	3	52.89	8.80
9	17	3	77.79	12.20
-9	17	-3	80.59	10.40
9	17	3	67.59	9.70
-10	17	-3	90.39	11.60
10	17	3	80.09	10.60
10	17	3	100.59	16.10

-11	17	-3	12.10	6.60
11	17	3	2.30	6.50
-12	-17	-3	118.89	16.50
-12	17	-3	113.99	13.40
-12	18	3	21.80	8.00
11	18	-3	44.80	13.50
-11	18	3	71.99	11.90
-10	-18	3	67.09	11.60
10	18	-3	50.49	12.20
-10	18	3	62.39	10.80
9	18	-3	110.39	18.10
-9	18	3	129.79	12.50
8	18	-3	192.68	18.80
-8	18	3	208.68	14.90
8	18	-3	187.78	12.00
-7	-18	3	54.29	5.90
7	-18	-3	51.79	10.10
-7	18	3	31.30	6.80
7	18	-3	38.50	9.10
7	18	-3	37.50	6.10
-6	-18	3	229.68	12.80
6	-18	-3	229.48	16.30
6	18	-3	241.08	18.30
6	18	-3	219.28	13.00
-6	18	3	237.88	15.30
-6	18	3	204.78	17.10
-5	-18	3	2.30	3.10
5	18	-3	1.10	3.00
-5	18	3	-4.50	4.90
5	18	-3	-3.90	6.90
-5	18	3	0.90	5.70
-4	-18	3	247.98	13.40
-4	18	3	245.88	17.00
-4	18	3	238.48	15.40
4	18	-3	227.58	17.60
4	18	-3	239.88	13.30
-4	18	3	228.78	17.90
-3	-18	3	9.70	3.60
3	18	-3	16.70	3.30
-3	18	3	7.10	5.20
-3	18	3	1.80	5.80
-3	18	3	5.20	5.30
3	18	-3	11.20	6.20
-2	-18	3	40.30	4.90

2	18	-3	19.20	6.50
2	18	-3	30.30	4.10
-2	18	3	28.90	7.10
-2	18	3	18.40	5.20
-2	18	3	32.50	6.70
-1	-18	3	138.69	9.70
-1	18	3	116.39	16.50
-1	18	3	139.09	10.60
1	18	-3	128.69	8.70
1	18	-3	121.19	12.40
-1	18	3	113.69	12.90
0	-18	3	45.00	7.20
0	-18	-3	50.19	7.30
0	18	3	55.39	8.80
0	18	-3	42.60	6.00
0	18	-3	51.79	9.40
0	18	-3	44.20	8.00
0	18	3	41.30	7.90
0	18	3	43.80	7.50
1	-18	3	36.40	5.70
-1	-18	-3	23.10	4.90
-1	18	-3	31.20	5.80
1	18	3	20.50	5.50
1	18	3	18.10	6.70
1	18	3	34.00	5.60
-1	18	-3	26.00	6.50
-1	18	-3	24.10	5.40
-2	-18	-3	245.38	13.90
2	-18	3	229.38	14.70
-2	18	-3	229.88	15.00
-2	18	-3	214.08	15.80
2	18	3	226.08	15.50
-2	18	-3	240.88	13.50
2	18	3	232.98	14.30
2	18	3	190.98	17.10
3	-18	3	27.80	6.10
-3	-18	-3	40.80	5.30
3	18	3	25.30	7.40
-3	18	-3	38.90	7.60
-3	18	-3	28.70	4.80
3	18	3	29.10	6.10
-3	18	-3	21.30	5.90
3	18	3	32.20	5.50
-4	-18	-3	14.30	4.10

-4	-18	-3	3.70	6.70
4	18	3	8.50	4.40
-4	18	-3	10.70	4.70
-4	18	-3	5.10	5.20
4	18	3	17.40	7.10
-4	18	-3	3.60	4.30
-5	-18	-3	183.88	11.30
-5	-18	-3	143.59	15.80
-5	18	-3	172.58	14.00
5	18	3	171.88	12.00
-5	18	-3	162.08	13.00
5	18	3	157.18	15.90
-5	18	-3	178.18	12.00
-6	-18	-3	124.69	15.60
-6	18	-3	143.99	11.10
-6	18	-3	132.29	12.00
6	18	3	144.39	10.90
6	18	3	138.99	15.40
-7	-18	-3	14.00	9.70
7	18	3	-7.40	7.00
-7	18	-3	14.90	5.60
7	18	3	6.00	4.90
-7	18	-3	8.20	5.90
-8	-18	-3	21.00	12.70
8	18	3	-0.10	5.20
8	18	3	4.60	9.70
-8	18	-3	13.90	5.90
9	18	3	56.19	9.40
-9	18	-3	70.19	10.50
9	18	3	63.19	11.50
-10	18	-3	2.50	6.30
10	18	3	-2.80	7.10
11	18	3	147.79	14.00
-11	18	-3	180.88	15.10
-12	-18	-3	8.10	9.70
-12	18	-3	9.70	7.90
-11	19	3	470.65	27.70
10	19	-3	29.20	11.60
-10	19	3	28.80	7.50
-9	19	3	316.37	20.40
9	19	-3	307.57	25.00
-8	19	3	15.70	6.80
8	19	-3	14.20	4.20
8	19	-3	25.40	9.20

7	-19	-3	768.62	45.10
-7	19	3	755.22	38.20
7	19	-3	762.42	41.30
7	19	-3	649.74	36.10
-6	-19	3	5.20	3.50
-6	19	3	8.50	5.70
6	19	-3	4.00	3.50
6	19	-3	7.00	7.50
-5	-19	3	1107.59	53.29
5	19	-3	1033.00	53.29
-5	19	3	1112.79	55.09
-5	19	3	1067.99	56.59
5	19	-3	1167.48	57.49
-4	-19	3	79.29	7.10
-4	19	3	77.09	11.40
4	19	-3	75.79	12.60
-4	19	3	71.29	12.90
4	19	-3	77.39	6.80
-4	19	3	74.59	9.40
-3	-19	3	331.37	17.90
3	19	-3	312.97	17.60
3	19	-3	356.56	22.10
-3	19	3	328.37	19.60
-3	19	3	323.77	20.90
-3	19	3	346.57	22.40
-2	-19	3	74.49	8.00
2	19	-3	87.79	7.10
-2	19	3	76.09	9.30
-2	19	3	77.49	10.60
-2	19	3	76.29	11.90
2	19	-3	92.19	11.80
-1	-19	3	681.53	34.70
-1	19	3	693.03	38.00
-1	19	3	685.13	35.50
1	19	-3	660.03	35.90
-1	19	3	674.73	36.70
1	19	-3	701.03	37.40
1	19	-3	682.83	34.20
0	-19	3	219.58	14.60
0	19	3	245.98	16.30
0	19	-3	221.78	15.40
0	19	3	243.88	18.30
0	19	-3	241.28	17.10
0	19	-3	243.28	13.80

0	19	3	234.08	15.10
1	-19	3	315.67	18.80
-1	19	-3	314.87	19.40
1	19	3	317.07	18.70
1	19	3	302.67	19.90
-1	19	-3	339.77	20.70
1	19	3	333.77	22.00
-1	19	-3	332.87	17.90
2	-19	3	427.16	23.70
-2	19	-3	425.16	23.90
-2	19	-3	416.36	22.40
-2	19	-3	402.16	24.90
2	19	3	417.76	26.20
2	19	3	422.46	22.90
2	19	3	419.36	24.30
-3	-19	-3	350.46	19.00
-3	19	-3	238.78	25.30
3	19	3	342.37	21.80
-3	19	-3	287.97	17.30
3	19	3	302.37	17.70
-3	19	-3	286.17	18.50
3	19	3	301.97	19.40
4	19	3	173.58	16.20
-4	19	-3	169.88	14.20
-4	19	-3	145.69	12.60
4	19	3	154.98	11.60
-4	19	-3	159.38	11.50
4	19	3	158.28	13.70
-5	-19	-3	290.67	22.50
-5	19	-3	325.87	21.20
-5	19	-3	321.37	18.80
5	19	3	327.97	18.80
5	19	3	375.06	23.40
-5	19	-3	314.87	20.00
-6	-19	-3	467.85	31.70
-6	19	-3	469.65	28.70
6	19	3	553.64	31.60
6	19	3	523.65	27.60
-6	19	-3	507.95	28.70
-6	19	-3	568.24	30.20
-7	-19	-3	132.39	17.90
-7	19	-3	164.58	12.50
7	19	3	137.49	12.00
7	19	3	181.88	18.10

8	19	3	217.48	19.80
8	19	3	185.08	14.40
-8	19	-3	222.98	15.10
-9	19	-3	-2.20	5.80
9	19	3	-6.90	5.40
9	19	3	-10.40	9.00
10	19	3	222.18	17.00
-10	19	-3	275.07	17.90
-11	19	-3	27.50	7.70
11	19	3	12.00	7.10
-11	20	3	34.70	8.30
-10	20	3	31.10	8.00
10	20	-3	19.60	12.70
9	20	-3	49.30	18.90
-9	20	3	87.19	12.00
8	20	-3	25.30	11.80
-8	20	3	14.50	7.20
7	20	-3	20.00	9.10
-7	20	3	12.90	6.20
7	20	-3	14.80	4.20
6	20	-3	10.90	4.00
-6	20	3	6.50	5.70
6	20	-3	14.60	8.20
-5	-20	3	14.30	4.00
-5	20	3	15.80	6.50
5	20	-3	14.40	7.90
-5	20	3	7.90	8.50
5	20	-3	14.00	3.90
-4	-20	3	10.00	3.90
4	20	-3	6.20	3.60
4	20	-3	17.30	7.60
-4	20	3	5.80	7.10
-4	20	3	15.80	6.10
-4	20	3	4.10	6.70
-3	-20	3	71.89	7.60
-3	20	3	62.79	9.20
-3	20	3	62.49	9.40
3	20	-3	84.39	12.70
-3	20	3	65.99	12.60
3	20	-3	67.69	6.80
-2	-20	3	8.90	4.50
2	20	-3	3.50	3.50
-2	20	3	1.90	5.40
-2	20	3	8.60	9.20

2	20	-3	7.20	6.20
-2	20	3	0.40	6.50
-1	-20	3	1.20	4.30
1	20	-3	0.00	4.70
1	20	-3	-6.70	5.90
1	20	-3	2.40	3.60
-1	20	3	-4.20	5.90
-1	20	3	4.60	7.10
-1	20	3	-5.20	5.00
0	-20	3	8.70	5.10
0	20	-3	16.60	5.10
0	20	-3	17.40	4.40
0	20	3	16.70	6.10
0	20	3	20.00	7.50
0	20	3	19.50	5.70
0	20	-3	33.20	7.50
1	-20	3	0.30	5.40
1	20	3	-0.70	4.90
1	20	3	-6.80	6.00
-1	20	-3	-7.80	4.80
-1	20	-3	6.90	8.80
1	20	3	-11.20	6.70
-1	20	-3	2.70	4.00
2	-20	3	18.50	10.30
-2	20	-3	11.20	5.30
-2	20	-3	11.60	6.50
2	20	3	15.40	5.10
2	20	3	25.90	8.80
2	20	3	12.20	5.90
-2	20	-3	17.30	4.70
-3	20	-3	4.20	4.60
3	20	3	9.10	4.90
3	20	3	3.60	5.70
3	20	3	7.00	7.30
-3	20	-3	4.00	4.80
-3	20	-3	8.70	6.30
-4	20	-3	31.40	5.80
4	20	3	31.80	6.00
-4	20	-3	31.00	7.30
4	20	3	32.40	8.80
-4	20	-3	32.70	6.30
4	20	3	54.09	10.20
-5	-20	-3	39.10	9.80
-5	20	-3	19.20	6.60

-5	20	-3	29.40	7.70
5	20	3	39.80	10.10
-5	20	-3	14.70	6.10
5	20	3	27.40	5.80
-6	-20	-3	21.80	10.70
-6	20	-3	12.70	5.50
-6	20	-3	12.80	7.10
6	20	3	5.30	8.20
6	20	3	9.00	5.30
7	20	3	5.80	5.30
-7	20	-3	-3.30	5.70
-7	20	-3	8.60	7.00
7	20	3	9.00	10.10
8	20	3	0.00	9.80
8	20	3	-0.60	5.60
-8	20	-3	9.70	6.20
-9	20	-3	25.00	7.60
10	20	3	7.10	6.50
-10	20	-3	15.20	7.70
11	20	3	14.70	7.20
-11	20	-3	22.90	8.60
10	21	-3	159.78	22.20
-10	21	3	176.88	16.00
9	21	-3	145.09	21.20
-9	21	3	152.88	14.40
-8	21	3	66.19	11.10
8	21	-3	44.20	11.70
7	21	-3	244.38	21.50
7	21	-3	212.88	15.10
-7	21	3	246.98	17.00
6	21	-3	11.80	10.90
-6	21	3	10.90	9.50
-6	21	3	5.80	6.40
6	21	-3	9.70	4.20
5	21	-3	622.84	33.10
-5	21	3	669.13	35.30
-5	21	3	695.73	38.70
-4	-21	3	7.70	4.10
-4	21	3	-6.70	5.80
-4	21	3	-6.10	8.30
4	21	-3	0.20	3.60
-3	-21	3	441.16	22.80
-3	21	3	432.16	27.50
3	21	-3	404.66	22.40

-3	21	3	436.06	24.50
-2	-21	3	118.09	9.60
2	21	-3	107.49	8.80
2	21	-3	100.49	11.40
-2	21	3	95.49	14.40
-2	21	3	117.09	12.90
-2	21	3	96.19	12.40
-1	-21	3	695.33	35.70
-1	21	3	713.03	36.50
-1	21	3	694.73	39.30
1	21	-3	699.73	35.10
1	21	-3	660.63	37.10
1	21	-3	656.23	40.10
-1	21	3	742.43	38.20
0	-21	3	56.09	7.30
0	21	-3	60.79	10.00
0	21	-3	48.30	9.20
0	21	3	53.79	7.70
0	21	3	48.40	10.40
0	21	-3	41.70	7.20
0	21	3	43.40	8.20
1	-21	3	698.03	36.20
-1	21	-3	668.93	38.00
-1	21	-3	695.63	35.00
1	21	3	712.53	35.90
-1	21	-3	673.63	36.70
1	21	3	689.53	38.90
1	21	3	665.53	37.30
2	21	3	85.79	10.10
2	21	3	83.69	11.80
-2	21	-3	105.69	10.90
-2	21	-3	105.79	9.30
-2	21	-3	91.89	13.00
2	21	3	79.09	14.90
3	21	3	162.38	14.30
-3	21	-3	151.78	15.00
-3	21	-3	149.99	11.70
3	21	3	170.68	16.90
-3	21	-3	142.89	13.80
3	21	3	145.49	11.80
4	21	3	243.18	20.30
-4	21	-3	241.88	18.50
4	21	3	237.08	15.20
4	21	3	210.98	17.20

-4	21	-3	238.38	15.10
5	21	3	143.39	15.40
5	21	3	180.28	13.20
-5	21	-3	185.68	13.20
-5	21	-3	190.08	16.80
5	21	3	205.58	19.00
-6	21	-3	646.04	36.60
6	21	3	655.63	33.70
6	21	3	660.03	38.30
-6	21	-3	606.74	33.80
-7	21	-3	146.39	12.80
7	21	3	130.69	12.40
7	21	3	155.28	19.10
-7	21	-3	154.48	16.80
-8	21	-3	213.38	18.80
-8	21	-3	172.58	14.70
8	21	3	173.48	16.20
8	21	3	192.78	21.30
9	21	3	8.80	6.60
-9	21	-3	-1.30	6.90
10	21	3	331.77	22.30
-10	21	-3	400.86	23.50
9	22	-3	57.59	13.30
-9	22	3	68.99	11.70
-8	22	3	94.59	12.60
8	22	-3	93.99	19.10
7	22	-3	-6.00	9.20
-7	22	3	8.90	7.40
6	22	-3	172.88	13.20
-6	22	3	222.18	16.10
6	22	-3	223.08	20.60
-6	22	3	240.08	21.30
-5	22	3	-0.20	6.80
5	22	-3	1.10	3.80
5	22	-3	-1.30	8.40
-5	22	3	-7.60	8.50
4	22	-3	130.69	9.70
-4	22	3	132.09	13.00
-4	22	3	131.59	16.80
4	22	-3	126.99	16.20
-3	-22	3	2.90	7.80
3	22	-3	4.90	7.80
-3	22	3	-1.10	8.30
-3	22	3	-2.80	5.80

3	22	-3	16.20	4.40
-2	-22	3	397.86	22.70
2	22	-3	424.06	22.20
2	22	-3	433.96	27.00
-2	22	3	409.36	24.20
-1	-22	3	35.80	6.90
-1	22	3	48.50	10.00
1	22	-3	55.09	9.60
1	22	-3	49.99	7.40
0	-22	3	296.97	18.70
0	22	-3	308.77	21.90
0	22	3	307.57	19.00
0	22	-3	302.47	17.70
0	22	3	308.37	22.70
-1	22	-3	1.50	7.50
1	22	3	14.80	6.10
-1	22	-3	3.60	4.70
1	22	3	-0.90	7.70
2	22	3	277.37	18.20
2	22	3	293.67	23.30
-2	22	-3	304.07	17.80
-2	22	-3	313.17	21.60
-3	22	-3	73.69	9.00
-3	22	-3	72.59	13.20
3	22	3	74.69	9.70
3	22	3	82.49	12.50
4	22	3	141.39	17.70
-4	22	-3	165.08	16.30
4	22	3	147.79	12.20
-4	22	-3	145.99	12.00
5	22	3	308.37	23.80
-5	22	-3	285.67	18.00
-5	22	-3	306.37	21.80
5	22	3	274.47	18.00
6	22	3	98.69	10.90
6	22	3	126.99	18.60
-6	22	-3	97.29	15.40
-6	22	-3	96.29	11.10
7	22	3	139.59	20.10
-7	22	-3	125.59	13.20
-7	22	-3	150.78	17.40
-8	22	-3	16.00	9.50
-8	22	-3	23.90	7.70
8	22	3	34.10	16.20

8	22	3	25.10	7.00
-9	22	-3	219.48	21.30
9	22	3	181.98	15.50
-9	22	-3	207.18	16.70
9	23	-3	124.49	21.90
-9	23	3	92.49	13.70
-8	23	3	4.30	7.20
8	23	-3	9.80	12.70
7	23	-3	150.68	20.10
-7	23	3	148.89	21.20
-7	23	3	161.98	15.10
-6	23	3	12.00	10.20
6	23	-3	9.70	10.30
-6	23	3	-0.60	7.10
-5	23	3	253.47	17.50
5	23	-3	223.48	14.50
5	23	-3	235.28	21.30
-5	23	3	224.28	22.20
4	23	-3	42.70	10.90
-4	23	3	63.39	9.30
4	23	-3	52.19	7.50
-4	23	3	44.10	11.00
-3	23	3	74.49	12.80
3	23	-3	79.19	12.70
3	23	-3	78.79	8.20
-3	23	3	83.39	11.60
2	23	-3	65.39	7.90
-2	23	3	54.29	10.90
-2	23	3	67.69	10.50
2	23	-3	72.39	14.70
-1	-23	3	19.60	8.20
1	23	-3	14.60	8.40
1	23	-3	20.90	5.30
-1	23	3	17.60	6.70
-1	23	3	27.90	10.40
0	23	-3	56.69	8.20
0	23	3	50.49	7.80
0	23	-3	33.90	9.40
1	23	3	80.89	14.30
-1	23	-3	115.69	10.40
-1	23	-3	100.89	14.80
1	23	3	106.99	16.80
2	23	3	21.30	10.20
-2	23	-3	-3.20	7.20

-2	23	-3	6.20	5.30
-3	23	-3	61.79	10.40
3	23	3	85.29	13.30
3	23	3	63.39	11.10
-4	23	-3	202.78	14.60
4	23	3	168.88	20.50
4	23	3	205.88	16.00
-4	23	-3	206.98	18.60
5	23	3	43.30	12.70
-5	23	-3	53.49	11.20
-5	23	-3	69.69	10.50
5	23	3	55.59	13.30
6	23	3	61.69	9.80
-6	23	-3	92.79	11.10
6	23	3	84.99	14.80
-6	23	-3	80.19	12.30
-7	23	-3	34.80	7.90
-7	23	-3	33.20	10.20
7	23	3	43.00	7.70
7	23	3	41.20	13.20
8	23	3	-0.40	6.30
-8	23	-3	-4.20	7.10
-8	23	-3	-2.90	9.40
-9	23	-3	-5.10	7.80
-9	23	-3	-4.60	10.60
8	24	-3	117.19	22.30
-8	24	3	87.19	15.70
-8	24	3	120.99	14.70
-7	24	3	14.20	11.60
7	24	-3	5.10	11.50
-7	24	3	-1.40	8.10
6	24	-3	840.32	47.00
-6	24	3	773.32	42.80
-6	24	3	796.52	47.30
-5	24	3	32.20	11.60
5	24	-3	17.50	10.50
-5	24	3	8.30	8.00
4	24	-3	496.55	27.10
-4	24	3	506.85	29.20
4	24	-3	528.45	33.30
-4	24	3	510.15	34.00
3	24	-3	9.70	8.90
-3	24	3	12.60	7.30
3	24	-3	20.30	5.10

-3	24	3	17.20	11.30
-2	24	3	151.58	19.40
-2	24	3	145.49	14.00
2	24	-3	166.18	18.40
2	24	-3	162.68	11.60
1	24	-3	62.59	8.70
1	24	-3	65.19	11.60
-1	24	3	76.79	12.80
-1	24	3	56.99	8.70
0	24	3	580.74	31.90
0	24	3	564.34	35.80
0	24	-3	588.44	30.70
0	24	-3	589.54	35.10
-1	24	-3	339.77	20.40
-1	24	-3	364.76	25.30
1	24	3	350.16	21.60
1	24	3	354.86	26.60
-2	24	-3	135.99	16.60
2	24	3	111.39	20.00
2	24	3	134.09	12.70
-3	24	-3	134.99	12.40
3	24	3	152.38	13.00
-3	24	-3	139.69	17.40
-4	24	-3	81.39	15.80
-4	24	-3	54.79	8.00
4	24	3	20.90	17.30
4	24	3	55.59	8.10
-5	24	-3	271.87	22.00
5	24	3	235.78	17.30
5	24	3	306.47	35.10
-5	24	-3	240.38	17.50
-6	24	-3	296.97	23.60
6	24	3	260.07	18.40
-6	24	-3	279.67	18.80
6	24	3	346.97	37.70
-7	24	-3	108.99	18.40
7	24	3	114.39	12.90
-7	24	-3	145.69	13.50
-8	24	-3	8.70	10.20
-8	24	-3	0.50	7.30
8	24	3	6.10	8.30
-7	25	3	7.10	8.60
-7	25	3	9.90	12.60
7	25	-3	0.60	12.20

-6	25	3	8.40	8.40
-6	25	3	12.90	11.80
6	25	-3	17.40	11.30
5	25	-3	30.70	12.20
-5	25	3	19.20	11.40
-5	25	3	-0.50	7.60
-4	25	3	10.10	11.40
4	25	-3	32.00	10.90
-4	25	3	20.30	8.30
-3	25	3	-0.70	10.80
3	25	-3	-2.20	9.90
3	25	-3	7.40	6.20
-3	25	3	11.30	7.80
-2	25	3	6.80	7.40
-2	25	3	3.90	10.80
2	25	-3	-5.60	9.20
2	25	-3	-5.50	4.60
-1	25	3	125.59	18.60
-1	25	3	106.09	12.70
1	25	-3	85.69	10.50
1	25	-3	131.39	17.60
0	25	-3	-5.70	8.50
0	25	3	0.80	6.50
0	25	-3	11.70	5.60
0	25	3	-9.90	11.00
-1	25	-3	7.40	9.20
-1	25	-3	16.00	9.90
1	25	3	2.50	10.20
1	25	3	16.60	7.20
-2	25	-3	62.19	11.80
-2	25	-3	61.39	10.30
2	25	3	72.79	11.10
2	25	3	41.20	12.80
-3	25	-3	179.08	18.90
3	25	3	149.49	21.20
3	25	3	180.68	14.40
-3	25	-3	163.28	13.90
4	25	3	33.70	11.90
-4	25	-3	17.90	9.50
4	25	3	23.80	7.20
-4	25	-3	30.10	7.40
5	25	3	4.20	6.60
-5	25	-3	12.80	7.30
-5	25	-3	7.60	9.20

5	25	3	13.80	11.90
6	25	3	19.60	12.80
6	25	3	18.30	7.00
-6	25	-3	7.00	7.10
-6	25	-3	21.30	10.90
-7	25	-3	0.10	9.80
7	25	3	7.00	7.50
-5	26	3	-18.90	12.60
5	26	-3	-18.40	10.90
-5	26	3	-9.70	7.80
-4	26	3	206.88	17.10
4	26	-3	172.98	22.30
-4	26	3	199.78	23.60
-3	26	3	23.50	8.20
3	26	-3	1.60	10.90
-3	26	3	-4.40	12.10
-2	26	3	137.29	21.70
-2	26	3	143.79	14.30
2	26	-3	102.89	19.10
-1	26	3	15.60	11.80
-1	26	3	14.40	7.70
1	26	-3	4.40	9.70
1	26	-3	13.90	5.90
0	26	3	421.76	25.10
0	26	-3	411.66	29.10
0	26	3	388.46	30.50
-1	26	-3	30.40	7.00
1	26	3	10.90	11.80
-1	26	-3	19.60	10.00
1	26	3	31.30	8.00
-2	26	-3	144.29	12.50
2	26	3	128.69	13.60
-2	26	-3	105.29	18.10
2	26	3	77.79	15.20
3	26	3	6.40	6.90
-3	26	-3	4.70	9.50
-3	26	-3	1.30	6.70
3	26	3	7.10	12.10
4	26	3	110.09	12.60
-4	26	-3	89.89	18.00
4	26	3	111.49	21.90
-4	26	-3	106.39	13.00
5	26	3	170.98	24.10
5	26	3	144.49	14.30

-5	26	-3	166.88	20.90
-5	26	-3	142.69	14.70
-3	27	3	250.97	27.20
-3	27	3	247.78	19.40
3	27	-3	273.17	25.10
-2	27	3	8.40	7.80
2	27	-3	17.30	11.60
-2	27	3	5.00	13.30
-1	27	3	217.18	18.50
-1	27	3	249.38	26.20
1	27	-3	229.48	22.90
0	27	3	68.49	15.50
0	27	3	54.69	9.50
0	27	-3	48.90	12.80
0	27	-3	63.19	10.50
1	27	3	170.08	23.10
-1	27	-3	112.19	12.30
1	27	3	101.99	13.90
-1	27	-3	121.99	19.40
2	27	3	47.80	14.10
-2	27	-3	72.89	13.80
-2	27	-3	46.10	8.40
2	27	3	66.59	9.70
3	27	3	101.49	16.70
-3	27	-3	110.39	12.90
-3	27	-3	116.29	19.70
3	27	3	93.09	13.30
-16	0	4	124.39	14.20
-15	0	4	298.47	19.60
-14	0	4	187.98	14.10
-13	0	4	76.19	13.00
13	0	-4	112.79	13.60
-12	0	4	731.73	44.10
12	0	-4	809.22	42.60
-12	0	4	825.82	40.30
-11	0	4	11.50	7.30
11	0	-4	8.20	6.60
-11	0	4	-9.70	7.50
-11	0	4	-0.80	4.40
10	0	-4	414.16	24.10
10	0	-4	401.96	22.40
-10	0	4	416.76	21.80
9	0	-4	409.56	23.50
9	0	-4	407.36	22.10

-9	0	4	425.56	21.70
-9	0	4	399.66	24.60
-8	0	4	1387.56	67.99
8	0	-4	1423.66	69.89
8	0	-4	1403.56	68.39
-8	0	4	1409.66	70.19
-7	0	4	75.99	5.70
7	0	-4	72.99	6.40
-7	0	4	79.59	8.50
6	0	-4	3047.30	140.59
6	0	-4	2938.01	139.49
-6	0	4	2858.81	140.79
-6	0	4	2850.11	139.19
5	0	-4	391.66	20.00
-5	0	4	381.96	18.80
-5	0	4	360.36	20.20
5	0	-4	365.56	18.90
4	0	-4	959.70	46.40
-4	0	4	965.10	45.50
-4	0	4	920.11	46.60
4	0	-4	937.21	45.50
3	0	-4	448.16	21.60
3	0	-4	448.26	22.40
-3	0	4	439.56	22.50
-3	0	4	439.26	21.60
2	0	-4	2074.09	94.69
2	0	-4	1984.50	94.09
-2	0	4	1772.22	94.09
-2	0	4	2098.89	94.79
0	0	4	123.09	6.20
0	0	-4	123.09	6.20
0	0	4	119.99	6.20
0	0	-4	113.59	6.10
2	0	4	374.66	18.70
2	0	4	389.16	19.10
-2	0	-4	404.46	19.10
-2	0	-4	382.86	18.80
3	0	4	1286.37	67.39
-3	0	-4	1496.95	67.09
-3	0	-4	1411.16	66.89
3	0	4	1398.56	66.79
4	0	4	1427.16	66.99
-4	0	-4	1363.56	67.09
-4	0	-4	1494.85	67.29

4	0	4	1301.77	67.09
5	0	4	662.83	32.90
-5	0	-4	605.14	32.90
-5	0	-4	735.33	33.10
-5	0	-4	662.33	33.00
-6	0	-4	69.19	6.30
6	0	4	81.99	6.20
-6	0	-4	76.99	6.40
-6	0	-4	71.29	6.20
-7	0	-4	923.61	49.70
7	0	4	1026.10	49.60
-7	0	-4	950.50	49.40
-7	0	-4	1129.39	49.90
-8	0	-4	42.40	6.70
8	0	4	52.39	6.40
-8	0	-4	49.60	6.80
-8	0	-4	55.29	5.90
-9	0	-4	505.85	26.80
-9	0	-4	483.65	27.50
-9	0	-4	542.45	27.40
9	0	4	500.15	26.90
10	0	4	-7.80	3.80
-10	0	-4	-4.20	4.70
-10	0	-4	1.10	3.80
-11	0	-4	1540.75	71.99
11	0	4	1487.45	72.09
-11	0	-4	1331.57	72.79
-12	0	-4	0.10	6.30
-12	0	-4	0.00	5.60
12	0	4	-2.20	5.30
-13	0	-4	428.76	25.00
-14	0	-4	24.00	7.60
-15	0	-4	93.69	13.60
-16	0	-4	105.69	15.10
-16	-1	4	3.40	7.90
-16	1	4	10.80	7.80
-15	-1	4	34.30	7.60
-14	-1	4	-0.10	5.80
-14	1	4	5.60	6.20
-13	-1	4	0.90	5.20
13	-1	-4	4.40	6.90
-13	1	4	-2.80	5.20
13	1	-4	-3.90	6.90
-12	-1	4	21.80	5.40

12	-1	-4	14.60	7.30
-12	-1	4	-0.60	8.30
-12	1	4	9.40	8.40
12	1	-4	20.70	6.80
-12	1	4	19.20	5.40
-11	-1	4	77.99	12.80
11	-1	-4	98.89	11.80
-11	-1	4	73.09	8.00
-11	-1	4	74.49	10.00
-11	1	4	65.29	8.20
-11	1	4	79.19	15.10
11	1	-4	84.59	11.00
-10	-1	4	14.30	6.80
10	-1	-4	-3.40	4.80
10	-1	-4	4.90	4.80
-10	-1	4	4.30	3.70
-10	-1	4	0.60	4.50
-10	1	4	5.90	3.80
10	1	-4	3.90	5.40
-10	1	4	0.50	6.00
-9	-1	4	48.30	9.20
9	-1	-4	59.79	7.20
-9	-1	4	40.10	5.80
9	-1	-4	48.40	10.20
-9	1	4	58.59	9.70
9	1	-4	74.79	9.40
-9	1	4	59.49	6.30
9	1	-4	63.39	6.90
-8	-1	4	14.60	3.20
-8	-1	4	11.60	5.50
8	-1	-4	13.90	3.80
8	1	-4	7.30	4.80
-8	1	4	8.10	4.80
-8	1	4	10.20	3.00
8	1	-4	16.50	3.50
-7	-1	4	117.19	7.60
7	-1	-4	131.79	9.60
7	-1	-4	117.79	8.20
-7	-1	4	105.89	10.20
7	1	-4	127.69	9.90
-7	1	4	111.39	7.60
7	1	-4	121.19	7.90
-7	1	4	115.49	10.10
6	-1	-4	1.30	3.10

-6	-1	4	4.40	2.20
-6	-1	4	1.90	3.60
6	-1	-4	7.30	2.60
6	1	-4	13.90	2.70
-6	1	4	3.60	3.80
-6	1	4	5.50	2.20
5	-1	-4	425.36	21.20
-5	-1	4	379.56	21.20
-5	-1	4	410.46	22.30
5	-1	-4	455.55	22.10
5	1	-4	422.96	21.00
5	1	-4	407.26	22.30
-5	1	4	431.36	22.40
-5	1	4	431.96	21.00
-4	-1	4	2.90	1.30
4	-1	-4	2.60	2.10
-4	-1	4	4.10	2.40
4	-1	-4	6.70	1.80
4	1	-4	3.60	1.40
4	1	-4	1.90	2.50
-4	1	4	5.00	2.50
3	-1	-4	132.79	7.20
3	-1	-4	143.29	8.00
-3	-1	4	130.69	6.90
-3	-1	4	126.39	8.00
3	1	-4	130.49	6.90
3	1	-4	135.49	8.00
-3	1	4	135.19	8.10
-3	1	4	135.19	7.20
-2	-1	4	43.70	2.80
-2	-1	4	41.00	3.70
2	-1	-4	49.30	3.70
2	-1	-4	50.29	3.00
2	1	-4	47.40	3.80
2	1	-4	48.20	2.70
-2	1	4	43.00	3.70
-2	1	4	45.90	3.10
-1	-1	4	8.40	1.00
-1	-1	4	4.80	1.40
1	-1	-4	6.20	1.40
1	-1	-4	5.10	1.00
1	1	-4	4.60	1.20
-1	1	4	7.60	1.40
1	1	-4	5.60	0.90

-1	1	4	5.30	1.10
0	-1	-4	1659.03	78.19
0	-1	4	1640.14	78.09
0	-1	4	1641.64	78.09
0	-1	-4	1648.14	78.29
0	1	-4	1661.43	78.09
0	1	4	1556.14	78.19
0	1	4	1644.04	78.19
0	1	-4	1704.13	78.09
1	-1	4	3297.47	155.48
-1	-1	-4	3290.27	155.58
1	1	4	3207.38	155.58
1	1	4	3320.67	155.48
-2	-1	-4	211.68	10.70
2	-1	4	205.38	10.70
-2	-1	-4	206.68	10.70
2	-1	4	212.78	10.10
-2	1	-4	199.38	10.20
2	1	4	209.98	10.60
2	1	4	211.38	10.80
-2	1	-4	218.08	10.80
-3	-1	-4	85.89	5.40
3	-1	4	88.59	6.10
3	-1	4	89.99	5.00
-3	-1	-4	101.99	5.60
-3	1	-4	101.59	5.70
-3	1	-4	83.59	5.10
3	1	4	92.59	5.70
3	1	4	89.69	5.40
-4	-1	-4	475.65	24.90
-4	-1	-4	539.25	25.00
4	-1	4	500.75	24.60
-4	1	-4	463.45	24.70
4	1	4	500.45	24.90
-4	1	-4	497.35	25.00
4	1	4	471.65	24.90
-4	1	-4	565.24	25.20
-5	-1	-4	470.15	24.70
-5	-1	-4	527.55	24.70
5	-1	4	499.65	24.40
-5	1	-4	536.05	24.80
-5	1	-4	459.35	24.50
5	1	4	446.36	24.50
5	1	4	496.05	24.70

-5	1	-4	477.45	24.60
6	-1	4	6.00	2.40
-6	-1	-4	5.60	2.80
-6	-1	-4	7.90	3.00
-6	-1	-4	7.90	2.90
-6	1	-4	8.00	2.90
-6	1	-4	3.70	2.90
-6	1	-4	5.70	3.00
6	1	4	6.90	2.80
7	-1	4	28.00	5.10
-7	-1	-4	38.90	5.30
-7	-1	-4	27.80	5.60
7	1	4	32.50	5.40
-7	1	-4	28.60	5.70
-7	1	-4	33.10	5.20
-7	1	-4	36.00	5.30
8	-1	4	59.79	6.50
-8	-1	-4	53.69	7.30
-8	-1	-4	62.09	6.70
-8	-1	-4	82.99	6.90
-8	1	-4	52.59	6.60
-8	1	-4	62.29	7.40
-8	1	-4	65.29	7.30
8	1	4	61.89	6.90
-9	-1	-4	130.39	10.10
9	-1	4	99.99	8.90
-9	-1	-4	94.39	10.00
-9	-1	-4	128.89	9.30
-9	1	-4	118.99	10.00
-9	1	-4	105.29	8.60
9	1	4	108.99	9.30
-10	-1	-4	189.88	12.40
-10	-1	-4	174.88	14.00
10	-1	4	190.88	12.80
-10	1	-4	172.78	13.70
10	1	4	197.68	13.30
-10	1	-4	186.58	12.30
-11	-1	-4	15.90	5.40
11	-1	4	1.30	5.30
-11	-1	-4	9.50	6.10
-11	1	-4	-0.80	6.50
-11	1	-4	10.20	4.90
11	1	4	11.20	5.50
-12	-1	-4	8.80	5.80

12	-1	4	14.00	5.40
-12	-1	-4	6.10	6.60
-12	1	-4	17.20	7.10
12	1	4	2.90	5.60
-12	1	-4	16.10	5.60
-13	-1	-4	13.10	6.60
-13	1	-4	24.90	6.70
-14	-1	-4	63.49	11.80
-14	1	-4	76.69	11.70
-15	-1	-4	41.20	9.20
-15	1	-4	35.00	9.10
-16	-1	-4	-0.50	8.50
-16	1	-4	7.70	8.60
-16	-2	4	234.88	19.30
-16	2	4	283.77	19.30
-15	-2	4	82.09	12.10
-15	2	4	91.89	12.10
-14	-2	4	91.09	10.70
-14	2	4	96.79	11.70
-13	-2	4	17.60	5.80
13	-2	-4	20.60	8.60
13	2	-4	8.40	7.70
-13	2	4	19.40	5.90
-12	-2	4	428.16	29.00
12	-2	-4	463.95	27.30
-12	-2	4	480.65	24.70
-12	2	4	462.25	24.80
-12	2	4	401.16	28.70
12	2	-4	471.05	27.50
-11	-2	4	4.50	5.80
-11	-2	4	6.00	4.30
11	-2	-4	18.40	7.00
-11	-2	4	2.50	7.20
11	2	-4	13.70	6.10
-11	2	4	7.40	8.70
-11	2	4	9.00	4.70
-10	-2	4	277.07	15.70
10	-2	-4	239.68	15.60
-10	-2	4	262.87	14.50
-10	-2	4	240.88	18.10
10	2	-4	250.27	17.00
-10	2	4	247.28	14.70
-10	2	4	230.48	18.00
9	-2	-4	26.80	6.60

-9	-2	4	33.00	5.20
-9	-2	4	21.30	6.90
9	-2	-4	40.20	5.50
-9	-2	4	26.30	4.20
9	2	-4	15.70	5.50
-9	2	4	15.60	6.00
-9	2	4	31.90	4.60
-8	-2	4	141.49	8.90
-8	-2	4	146.79	9.60
-8	-2	4	152.88	12.10
8	-2	-4	146.69	9.90
8	-2	-4	146.09	11.10
-8	2	4	132.49	9.10
8	2	-4	148.59	11.60
-8	2	4	134.39	11.60
-7	-2	4	7.10	4.40
7	-2	-4	13.00	4.10
7	-2	-4	6.30	3.10
-7	-2	4	7.50	2.60
7	2	-4	10.20	2.80
-7	2	4	8.10	4.10
7	2	-4	12.50	4.30
-7	2	4	6.20	2.60
6	-2	-4	1331.67	65.39
-6	-2	4	1328.57	66.69
-6	-2	4	1328.27	64.89
6	-2	-4	1353.06	66.19
-6	2	4	1357.56	65.19
-6	2	4	1366.36	66.69
6	2	-4	1389.06	66.49
6	2	-4	1362.86	65.19
-5	-2	4	8.40	1.90
5	-2	-4	10.30	2.50
-5	-2	4	10.60	3.70
5	-2	-4	7.90	2.80
5	2	-4	4.80	3.90
5	2	-4	7.10	2.00
-5	2	4	14.00	3.50
-5	2	4	10.90	2.30
4	-2	-4	656.13	31.50
-4	-2	4	629.54	31.80
-4	-2	4	633.14	30.40
4	-2	-4	645.14	31.00
-4	2	4	631.24	30.80

4	2	-4	624.84	30.50
-4	2	4	635.04	31.70
4	2	-4	588.14	32.40
3	-2	-4	19.10	2.50
3	-2	-4	18.80	2.90
-3	-2	4	24.10	3.50
-3	-2	4	15.50	1.70
3	2	-4	16.00	1.80
-3	2	4	18.40	2.40
-3	2	4	21.20	3.30
3	2	-4	21.70	3.50
-2	-2	4	2831.72	133.79
2	-2	-4	2870.61	133.79
2	-2	-4	2833.92	133.39
2	2	-4	2891.21	133.79
-2	2	4	2558.44	133.39
-2	2	4	2859.91	133.79
-1	-2	4	60.69	3.80
1	-2	-4	56.89	3.80
1	-2	-4	57.49	3.50
1	2	-4	55.99	2.90
-1	2	4	51.69	3.60
-1	2	4	56.69	3.80
1	2	-4	59.49	3.80
0	-2	-4	1232.88	58.29
0	-2	4	1238.98	57.89
0	-2	-4	1213.58	58.09
0	-2	4	1186.48	57.99
0	2	4	1226.68	58.19
0	2	-4	1228.18	57.89
0	2	-4	1200.18	57.99
1	-2	4	797.72	38.30
-1	-2	-4	819.82	38.40
-1	2	-4	804.62	38.30
1	2	4	761.02	38.40
1	2	4	805.62	38.50
1	2	4	803.72	38.40
-2	-2	-4	1201.48	57.49
-2	-2	-4	1199.28	57.59
2	2	4	1169.78	57.49
-2	2	-4	1135.69	57.39
-2	2	-4	1232.28	57.49
-2	2	-4	1211.58	56.89
2	2	4	1242.18	57.59

-3	-2	-4	871.51	43.00
-3	-2	-4	933.31	42.90
3	-2	4	884.51	42.30
3	2	4	832.72	42.90
3	2	4	901.71	43.00
-3	2	-4	955.80	43.00
-3	2	-4	865.31	42.50
-3	2	-4	850.91	43.00
4	-2	4	134.29	7.20
-4	-2	-4	142.99	7.80
-4	-2	-4	130.79	7.90
-4	2	-4	131.49	7.50
4	2	4	134.79	7.80
-4	2	-4	130.19	7.80
-4	2	-4	127.19	7.70
4	2	4	124.99	7.70
-5	-2	-4	1964.60	99.19
-5	-2	-4	2199.08	99.09
5	-2	4	2078.69	98.69
-5	2	-4	1973.70	98.89
-5	2	-4	2271.97	99.29
-5	2	-4	1997.30	98.99
5	2	4	2102.39	99.19
5	2	4	1939.31	98.99
-6	-2	-4	608.84	32.40
6	-2	4	663.53	31.90
-6	-2	-4	706.63	32.30
-6	2	-4	614.94	32.10
-6	2	-4	616.04	32.20
6	2	4	594.34	31.90
6	2	4	656.53	32.30
-6	2	-4	670.13	32.30
-7	-2	-4	676.23	37.40
7	-2	4	718.93	36.80
-7	-2	-4	729.83	37.00
-7	-2	-4	818.92	37.20
-7	2	-4	746.13	37.00
7	2	4	721.53	37.20
-7	2	-4	689.43	37.20
-7	2	-4	821.42	37.50
-8	-2	-4	151.98	11.30
8	-2	4	149.39	10.30
-8	-2	-4	164.68	10.60
-8	-2	-4	185.58	11.10

-8	2	-4	150.28	11.10
-8	2	-4	150.88	10.60
-8	2	-4	159.28	10.90
8	2	4	169.18	11.10
-9	-2	-4	561.64	32.40
9	-2	4	592.94	31.40
-9	-2	-4	667.63	32.10
-9	-2	-4	609.94	31.50
-9	2	-4	545.95	32.10
9	2	4	625.64	32.10
-9	2	-4	619.34	31.70
-9	2	-4	680.23	32.40
-10	-2	-4	17.00	5.80
10	-2	4	9.10	4.20
-10	-2	-4	20.70	4.80
10	2	4	13.40	4.80
-10	2	-4	11.10	4.50
-10	2	-4	8.40	5.20
-11	-2	-4	527.75	27.20
11	-2	4	508.45	27.40
-11	-2	-4	467.65	28.80
-11	2	-4	451.15	28.30
11	2	4	532.15	28.10
-11	2	-4	512.25	27.30
-12	-2	-4	-12.90	6.40
-12	-2	-4	4.90	6.60
12	-2	4	0.60	4.90
-12	2	-4	0.00	5.90
-12	2	-4	8.60	5.80
12	2	4	-4.60	5.50
-13	-2	-4	246.38	16.70
-13	2	-4	219.88	16.30
-14	-2	-4	41.60	8.90
-14	2	-4	44.60	8.30
-15	-2	-4	210.08	17.50
-15	2	-4	212.08	17.70
-16	-2	-4	51.89	10.10
-16	2	-4	37.50	9.50
-16	-3	4	35.60	8.90
-16	3	4	50.69	9.40
-15	-3	4	76.89	12.10
-15	3	4	108.39	12.90
-14	-3	4	66.29	10.20
-14	3	4	44.50	7.90

13	-3	-4	194.68	16.90
-13	-3	4	191.28	13.80
-13	3	4	213.58	14.10
13	3	-4	176.38	17.00
12	-3	-4	48.80	11.10
-12	-3	4	33.40	10.80
-12	-3	4	44.00	8.00
-12	3	4	59.39	8.30
12	3	-4	40.30	8.40
-12	3	4	38.50	16.40
-11	-3	4	109.19	15.10
-11	-3	4	149.59	12.10
-11	-3	4	145.89	10.60
11	3	-4	145.69	14.00
-11	3	4	162.18	11.00
-11	3	4	125.79	14.80
-10	-3	4	48.50	6.80
-10	-3	4	55.39	10.80
10	-3	-4	56.59	8.30
-10	-3	4	49.60	8.00
10	-3	-4	56.99	9.30
-10	3	4	51.89	7.00
-10	3	4	52.99	10.90
10	3	-4	46.90	9.60
9	-3	-4	495.85	26.90
-9	-3	4	488.15	26.00
9	-3	-4	535.15	27.90
-9	-3	4	561.64	26.80
-9	-3	4	476.65	28.90
9	3	-4	522.05	28.30
-9	3	4	496.15	29.00
-9	3	4	510.35	26.30
8	-3	-4	59.99	7.70
8	-3	-4	70.29	7.00
-8	-3	4	69.69	5.70
-8	-3	4	59.89	10.70
-8	-3	4	70.39	6.50
8	3	-4	64.19	8.10
-8	3	4	59.69	6.10
-8	3	4	47.40	8.70
7	-3	-4	420.86	22.30
-7	-3	4	394.56	23.10
-7	-3	4	448.76	21.20
7	-3	-4	404.46	21.60

-7	-3	4	396.56	20.70
-7	3	4	394.56	22.80
-7	3	4	418.76	21.10
7	3	-4	401.86	22.70
-6	-3	4	28.20	3.90
6	-3	-4	42.20	4.70
-6	-3	4	22.90	4.00
-6	-3	4	31.90	6.40
6	-3	-4	46.10	5.70
-6	3	4	37.00	6.10
-6	3	4	40.30	4.20
6	3	-4	33.50	6.50
5	-3	-4	1664.33	79.29
-5	-3	4	1577.94	78.49
5	-3	-4	1679.93	79.79
-5	-3	4	1609.64	80.19
-5	3	4	1593.34	78.89
5	3	-4	1721.13	80.09
-5	3	4	1683.03	80.09
4	-3	-4	16.60	2.80
-4	-3	4	12.30	2.00
-4	-3	4	27.60	4.40
4	-3	-4	19.70	3.60
-4	3	4	22.50	4.10
4	3	-4	17.20	3.50
4	3	-4	19.20	2.20
-4	3	4	18.50	2.90
3	-3	-4	940.41	45.20
3	-3	-4	961.70	44.90
-3	-3	4	931.41	44.20
-3	-3	4	917.71	45.40
3	3	-4	913.61	44.20
-3	3	4	927.11	45.40
-3	3	4	903.81	44.90
2	-3	-4	1234.48	58.49
2	-3	-4	1216.28	58.19
-2	-3	4	1223.78	58.59
-2	3	4	1114.79	58.19
2	3	-4	1265.67	58.59
2	3	-4	1162.08	57.49
-2	3	4	1282.07	58.59
1	-3	-4	733.73	34.20
-1	-3	4	722.63	34.40
1	-3	-4	692.03	34.40

-1	3	4	716.33	34.50
1	3	-4	755.22	34.40
-1	3	4	668.93	34.30
1	3	-4	676.93	33.60
0	-3	-4	14.80	2.20
0	-3	4	13.10	1.70
0	-3	-4	17.50	1.90
0	3	-4	12.20	1.70
0	3	4	14.00	1.90
0	3	-4	14.90	1.40
0	3	-4	14.70	1.30
0	3	4	10.40	2.00
-1	-3	-4	311.27	15.90
-1	3	-4	316.27	15.70
1	3	4	340.67	16.00
1	3	4	309.17	15.90
-1	3	-4	317.37	15.50
-1	3	-4	328.87	15.80
-2	-3	-4	47.30	3.30
-2	-3	-4	40.50	3.60
-2	3	-4	51.79	3.40
-2	3	-4	47.10	2.60
2	3	4	40.50	3.40
-2	3	-4	45.80	3.30
-3	-3	-4	389.36	18.70
3	-3	4	353.96	17.80
-3	-3	-4	387.76	18.90
3	3	4	330.57	18.50
-3	3	-4	353.46	18.50
-3	3	-4	386.16	18.60
3	3	4	378.46	18.90
-3	3	-4	375.26	18.10
-4	-3	-4	307.17	15.50
4	-3	4	293.07	14.80
-4	-3	-4	298.27	15.80
4	3	4	314.37	15.80
-4	3	-4	281.67	15.40
-4	3	-4	313.47	15.20
-4	3	-4	306.07	15.60
4	3	4	272.97	15.40
5	-3	4	865.01	42.30
-5	-3	-4	934.61	42.90
-5	-3	-4	833.42	43.20
5	3	4	810.72	42.70

-5	3	-4	878.11	42.70
-5	3	-4	818.42	42.70
-5	3	-4	936.11	43.10
5	3	4	918.51	43.20
-6	-3	-4	1672.93	86.99
6	-3	4	1799.92	86.19
-6	-3	-4	1980.80	86.79
6	3	4	1859.41	87.19
-6	3	-4	1945.31	86.99
-6	3	-4	1733.43	86.59
6	3	4	1693.03	86.49
-6	3	-4	1691.13	86.49
-7	-3	-4	713.03	33.30
7	-3	4	678.03	32.80
-7	-3	-4	629.14	33.80
-7	3	-4	637.24	33.10
-7	3	-4	633.34	33.30
7	3	4	681.53	33.60
7	3	4	593.74	32.90
-7	3	-4	692.83	33.60
-8	-3	-4	417.16	20.50
8	-3	4	369.16	19.80
-8	-3	-4	380.36	20.10
-8	-3	-4	335.57	20.80
-8	3	-4	364.36	20.20
-8	3	-4	349.07	20.60
8	3	4	371.36	20.70
-8	3	-4	398.86	20.80
-9	-3	-4	67.69	8.30
-9	-3	-4	59.39	7.50
-9	-3	-4	78.49	9.20
9	-3	4	58.29	7.00
-9	3	-4	54.39	8.50
-9	3	-4	67.69	7.90
9	3	4	80.89	8.40
-9	3	-4	76.39	7.60
-10	-3	-4	519.45	27.30
10	-3	4	509.95	27.00
-10	-3	-4	495.45	28.70
10	3	4	525.95	28.00
-10	3	-4	482.65	28.10
-10	3	-4	515.35	27.30
-11	-3	-4	23.20	7.00
11	-3	4	28.00	5.90

-11	-3	-4	33.00	6.50
-11	3	-4	24.40	6.20
11	3	4	32.90	6.80
-11	3	-4	26.40	7.20
12	-3	4	297.97	18.20
-12	-3	-4	306.57	23.30
-12	-3	-4	281.17	20.50
-12	3	-4	235.68	19.40
-12	3	-4	295.37	17.80
12	3	4	303.87	19.20
-13	-3	-4	2.20	6.10
-13	3	-4	7.90	6.70
-14	-3	-4	265.97	19.20
-14	3	-4	274.67	18.80
-15	-3	-4	53.79	9.80
-15	3	-4	70.59	12.90
-16	-3	-4	160.98	16.50
-16	3	-4	119.49	15.60
-15	-4	4	-1.50	6.70
-15	4	4	2.60	7.20
-14	-4	4	5.90	6.30
-14	4	4	20.60	6.80
-13	-4	4	6.90	5.30
-13	4	4	16.10	5.90
13	4	-4	10.40	7.50
12	-4	-4	70.79	11.00
-12	-4	4	66.59	8.50
-12	4	4	43.30	12.40
12	4	-4	57.79	11.90
-11	-4	4	6.40	8.50
-11	-4	4	2.70	5.10
-11	-4	4	6.10	4.20
11	-4	-4	9.20	5.90
-11	4	4	11.40	4.60
11	4	-4	-0.40	6.20
-11	4	4	-1.80	6.80
-10	-4	4	49.99	7.90
-10	-4	4	40.20	6.40
-10	-4	4	37.40	8.20
10	-4	-4	62.49	9.00
-10	4	4	58.89	6.80
-10	4	4	66.79	11.00
10	4	-4	53.59	9.90
-9	-4	4	-4.10	5.00

-9	-4	4	4.80	3.80
9	-4	-4	11.30	4.30
9	-4	-4	3.60	4.20
-9	-4	4	7.50	3.20
9	4	-4	2.10	4.80
-9	4	4	8.80	3.80
-9	4	4	5.20	5.50
-8	-4	4	143.99	9.10
8	-4	-4	149.29	11.10
8	-4	-4	156.78	10.50
-8	-4	4	146.39	12.40
-8	-4	4	165.78	9.80
-8	4	4	150.88	9.70
-8	4	4	140.79	11.90
8	4	-4	148.89	11.90
-7	-4	4	9.50	2.50
7	-4	-4	2.30	3.30
7	-4	-4	6.00	3.30
-7	-4	4	0.10	4.80
-7	-4	4	6.50	2.70
-7	4	4	1.40	3.70
7	4	-4	-2.70	3.80
-7	4	4	9.60	2.80
6	-4	-4	9.00	3.20
6	-4	-4	7.50	3.10
-6	-4	4	-3.70	3.20
-6	-4	4	1.60	2.30
-6	-4	4	-1.70	2.00
-6	4	4	11.70	3.70
-6	4	4	3.40	2.40
6	4	-4	9.60	4.20
5	-4	-4	86.19	6.30
-5	-4	4	80.49	4.90
-5	-4	4	86.59	5.10
5	-4	-4	78.19	6.70
-5	-4	4	67.29	7.30
-5	4	4	82.59	7.20
-5	4	4	79.19	5.50
5	4	-4	76.09	7.40
-4	-4	4	767.32	40.90
4	-4	-4	821.72	40.70
4	-4	-4	845.62	40.40
-4	-4	4	876.91	39.60
-4	-4	4	795.62	39.50

4	4	-4	832.22	41.00
-4	4	4	837.02	40.10
-4	4	4	818.62	40.90
3	-4	-4	522.15	26.20
-3	-4	4	497.15	26.30
-3	-4	4	538.75	25.10
-3	-4	4	517.25	25.00
3	4	-4	540.05	26.50
-3	4	4	516.05	25.80
-3	4	4	533.45	26.40
2	-4	-4	64.39	4.80
-2	-4	4	66.19	4.90
2	-4	-4	69.59	4.50
2	4	-4	70.29	5.00
-2	4	4	66.19	4.90
-1	-4	4	79.39	5.00
1	-4	-4	88.49	4.80
1	-4	-4	75.69	5.00
-1	4	4	77.99	5.10
-1	4	4	81.39	5.00
1	4	-4	79.29	3.90
1	4	-4	74.49	3.90
0	-4	-4	24.20	2.60
0	-4	-4	18.40	2.10
0	-4	4	18.30	2.10
0	4	4	19.10	2.30
0	4	4	17.70	2.40
0	4	-4	17.00	1.50
0	4	-4	16.10	1.50
1	-4	4	85.99	4.90
-1	-4	-4	86.99	5.10
1	4	4	91.29	5.30
-1	4	-4	91.49	4.90
1	4	4	82.29	5.10
2	-4	4	564.34	27.20
-2	-4	-4	575.54	27.30
-2	-4	-4	540.85	27.60
-2	4	-4	567.04	26.80
-2	4	-4	576.64	27.20
2	4	4	517.05	27.70
-3	-4	-4	782.02	38.60
3	-4	4	781.02	37.50
3	-4	4	801.62	38.20
-3	4	-4	810.72	38.20

-3	4	-4	774.92	37.70
3	4	4	754.62	38.10
-4	-4	-4	346.57	20.10
4	-4	4	383.56	19.00
-4	-4	-4	439.76	19.80
4	-4	4	399.56	19.80
-4	4	-4	387.56	19.70
4	4	4	361.86	19.60
-4	4	-4	393.36	19.30
-5	-4	-4	80.09	5.90
5	-4	4	75.09	5.90
5	-4	4	81.49	5.10
-5	-4	-4	79.29	6.70
-5	4	-4	64.29	5.70
5	4	4	79.09	5.80
-5	4	-4	87.89	5.80
-6	-4	-4	35.70	5.70
-6	-4	-4	31.60	4.60
6	-4	4	18.10	2.90
-6	4	-4	24.20	4.10
-6	4	-4	15.20	3.40
-6	4	-4	29.70	5.30
6	4	4	33.10	4.50
7	-4	4	87.99	6.60
-7	-4	-4	102.59	7.50
-7	-4	-4	88.29	8.20
-7	4	-4	86.79	7.30
7	4	4	86.79	6.80
-7	4	-4	86.39	7.60
-7	4	-4	91.09	7.90
-8	-4	-4	2.90	3.70
8	-4	4	1.90	2.60
-8	-4	-4	3.10	3.80
-8	-4	-4	4.40	3.90
-8	4	-4	1.10	3.50
-8	4	-4	5.20	3.50
-8	4	-4	-3.10	3.40
-9	-4	-4	145.69	10.30
-9	-4	-4	128.49	9.90
-9	-4	-4	110.79	11.00
9	-4	4	114.39	9.00
-9	4	-4	125.59	10.70
-9	4	-4	127.29	9.70
-10	-4	-4	160.88	11.50

-10	-4	-4	139.19	13.40
10	-4	4	132.79	10.60
-10	4	-4	162.78	11.40
-10	4	-4	126.59	12.50
-11	-4	-4	27.40	7.80
-11	-4	-4	34.30	6.10
11	-4	4	25.90	5.50
-11	4	-4	31.60	7.40
-11	4	-4	33.50	6.40
-12	-4	-4	4.20	7.00
12	-4	4	10.60	5.30
-12	4	-4	28.70	6.90
-12	4	-4	17.30	6.80
-13	-4	-4	5.40	6.20
-13	4	-4	4.50	6.20
-14	-4	-4	15.80	7.60
-14	4	-4	15.10	7.10
-15	-4	-4	2.40	8.20
-15	4	-4	0.30	8.00
-15	-5	4	53.39	8.90
-15	5	4	85.89	11.80
-14	5	4	157.98	13.60
-13	-5	4	363.86	21.30
13	5	-4	359.16	24.80
-13	5	4	393.56	21.90
-12	-5	4	1.50	7.10
-12	-5	4	5.20	4.70
12	-5	-4	8.20	6.90
-12	-5	4	-9.50	8.90
-12	5	4	4.80	5.20
12	5	-4	-8.70	6.40
-12	5	4	11.00	8.90
-11	-5	4	564.24	27.70
-11	-5	4	442.76	30.40
11	-5	-4	505.15	28.70
-11	-5	4	519.55	26.70
-11	5	4	465.85	30.70
11	5	-4	511.15	29.50
-11	5	4	548.85	27.30
-10	-5	4	6.30	3.70
-10	-5	4	14.20	4.90
10	-5	-4	5.80	5.50
-10	-5	4	3.50	6.40
-10	5	4	9.10	4.40

-10	5	4	9.90	6.60
10	5	-4	3.40	5.90
-9	-5	4	455.15	22.40
9	-5	-4	441.96	23.70
-9	-5	4	404.66	21.70
-9	-5	4	385.56	25.00
9	-5	-4	405.26	23.20
-9	5	4	417.36	22.30
-9	5	4	409.66	24.90
9	5	-4	435.16	24.50
-8	-5	4	338.57	16.20
-8	-5	4	251.97	18.30
8	-5	-4	291.27	17.00
-8	-5	4	304.47	15.60
8	-5	-4	301.47	17.50
-8	5	4	292.47	16.10
8	5	-4	289.27	18.30
-8	5	4	277.67	18.30
7	-5	-4	776.82	40.00
-7	-5	4	882.31	39.30
7	-5	-4	803.22	40.40
-7	-5	4	741.13	41.10
-7	-5	4	782.42	38.90
7	5	-4	846.22	41.10
-7	5	4	792.32	41.10
-7	5	4	764.52	39.40
-6	-5	4	9.20	4.10
-6	-5	4	4.50	3.50
-6	-5	4	11.90	2.50
6	-5	-4	11.30	3.40
6	-5	-4	12.00	3.30
-6	5	4	13.80	2.90
6	5	-4	13.20	4.60
-6	5	4	10.80	3.80
-5	-5	4	1479.05	65.99
-5	-5	4	1344.47	65.89
5	-5	-4	1423.96	66.89
-5	-5	4	1211.48	68.39
5	-5	-4	1402.76	67.19
5	5	-4	1453.45	67.69
-5	5	4	1343.77	67.49
-5	5	4	1367.76	66.49
-4	-5	4	141.09	7.10
-4	-5	4	134.09	7.00

-4	-5	4	123.89	8.90
4	-5	-4	142.39	8.40
4	-5	-4	136.49	8.50
-4	5	4	144.29	8.00
-4	5	4	130.09	8.80
4	5	-4	129.89	9.00
3	-5	-4	1512.85	72.09
3	-5	-4	1536.75	71.89
-3	-5	4	1320.07	70.79
-3	-5	4	1443.26	70.79
-3	-5	4	1491.65	72.39
-3	5	4	1521.55	71.79
-3	5	4	1509.05	72.19
3	5	-4	1624.74	72.39
-2	-5	4	163.78	9.20
2	-5	-4	164.48	9.00
2	-5	-4	145.99	9.10
-2	5	4	159.98	9.00
-2	5	4	150.28	8.80
2	5	-4	158.28	9.00
1	-5	-4	5033.60	239.38
-1	-5	4	5158.88	239.38
1	-5	-4	5128.09	239.08
-1	5	4	4685.03	239.28
1	5	-4	5228.38	239.28
-1	5	4	5005.10	239.38
0	-5	-4	1895.51	89.09
0	-5	4	1880.21	88.69
0	-5	-4	1880.21	88.69
0	-5	4	1812.12	88.29
0	5	-4	1777.92	88.19
0	5	4	1897.61	88.99
0	5	-4	1872.91	88.69
1	-5	4	1133.09	53.59
-1	-5	-4	1143.79	53.79
-1	5	-4	1094.09	53.59
-1	5	-4	1116.39	53.59
1	5	4	1066.09	53.99
1	5	4	1168.28	54.09
-2	-5	-4	1504.35	71.19
2	-5	4	1535.35	71.09
-2	-5	-4	1481.25	71.59
-2	5	-4	1492.65	70.69
-2	5	-4	1466.25	70.99

2	5	4	1425.36	71.09
-3	-5	-4	1214.18	58.09
3	-5	4	1176.18	56.89
-3	-5	-4	1229.88	57.39
3	5	4	1173.78	57.49
-3	5	-4	1162.58	56.99
-3	5	-4	1197.58	57.39
4	-5	4	861.81	41.60
-4	-5	-4	820.52	42.90
-4	-5	-4	957.40	42.30
4	-5	4	882.71	42.40
-4	5	-4	865.81	41.90
4	5	4	847.42	42.30
-4	5	-4	826.02	42.20
5	-5	4	263.87	14.20
-5	-5	-4	264.57	13.80
5	-5	4	256.37	13.20
-5	-5	-4	249.08	14.70
-5	5	-4	258.97	13.70
5	5	4	238.58	13.80
-5	5	-4	263.97	14.00
6	-5	4	2306.57	109.19
-6	-5	-4	2498.65	108.99
6	-5	4	2241.78	108.39
-6	-5	-4	2167.08	109.69
6	5	4	2087.69	108.69
-6	5	-4	2280.67	108.99
-6	5	-4	2293.07	108.89
-7	-5	-4	14.50	3.70
7	-5	4	7.90	3.50
-7	-5	-4	17.60	4.60
7	-5	4	23.40	3.30
-7	5	-4	18.40	4.00
7	5	4	16.30	3.50
-7	5	-4	13.60	3.50
-8	-5	-4	323.57	16.60
8	-5	4	290.27	15.80
-8	-5	-4	253.07	17.50
-8	5	-4	280.87	16.10
8	5	4	287.97	16.20
-8	5	-4	274.77	16.80
-8	5	-4	301.77	16.80
-9	-5	-4	-4.70	4.40
9	-5	4	-1.90	3.00

-9	-5	-4	-0.60	4.00
-9	-5	-4	0.50	4.00
-9	5	-4	-2.30	3.70
9	5	4	-0.20	2.60
-9	5	-4	0.00	3.90
10	-5	4	1341.57	63.39
-10	-5	-4	1163.18	64.99
-10	-5	-4	1316.47	63.69
-10	5	-4	1341.47	63.79
-10	5	-4	1258.07	64.39
-11	-5	-4	4.60	5.20
-11	-5	-4	10.00	6.60
11	-5	4	3.70	4.30
-11	5	-4	1.50	6.20
-12	-5	-4	377.86	24.90
12	-5	4	384.86	21.90
-12	5	-4	380.96	22.30
-12	5	-4	363.16	23.90
-13	-5	-4	20.00	6.80
-13	5	-4	10.60	6.60
-14	-5	-4	264.47	18.80
-14	5	-4	235.08	18.10
-15	-5	-4	28.80	9.00
-15	5	-4	18.00	8.90
-15	-6	4	-2.70	7.00
-15	6	4	19.10	7.30
-14	-6	4	4.70	7.70
-14	6	4	11.10	6.10
-13	-6	4	6.60	5.60
13	6	-4	4.00	8.80
-13	6	4	11.00	5.60
-12	-6	4	-1.40	9.40
12	-6	-4	-2.20	6.10
-12	-6	4	-0.10	5.80
-12	-6	4	-5.60	4.50
-12	6	4	-4.10	8.70
-12	6	4	8.50	5.30
12	6	-4	-2.00	6.50
-11	-6	4	89.99	15.30
-11	-6	4	116.59	9.60
11	-6	-4	107.39	11.90
-11	-6	4	134.09	10.80
-11	6	4	110.59	15.10
11	6	-4	117.69	13.10

-11	6	4	143.89	10.60
-10	-6	4	119.69	9.80
10	-6	-4	113.19	11.00
-10	-6	4	103.99	8.40
-10	-6	4	94.69	13.10
-10	6	4	105.99	12.80
-10	6	4	123.19	9.50
10	6	-4	115.89	12.50
-9	-6	4	41.90	8.10
-9	-6	4	46.50	6.50
9	-6	-4	45.70	7.30
9	-6	-4	49.99	7.00
-9	-6	4	37.20	5.30
-9	6	4	36.60	7.20
9	6	-4	46.50	9.30
-9	6	4	48.70	6.60
8	-6	-4	26.60	5.30
8	-6	-4	33.90	5.60
-8	-6	4	24.00	6.30
-8	-6	4	27.40	4.00
-8	-6	4	20.50	3.40
-8	6	4	24.00	5.70
-8	6	4	31.30	5.50
8	6	-4	29.80	5.80
-7	-6	4	81.29	9.40
7	-6	-4	87.59	8.00
-7	-6	4	96.19	6.10
7	-6	-4	94.29	8.30
-7	-6	4	92.39	6.50
-7	6	4	86.99	9.00
-7	6	4	87.19	7.00
7	6	-4	88.09	9.50
6	-6	-4	258.37	14.30
-6	-6	4	245.78	15.30
-6	-6	4	207.98	14.20
6	-6	-4	240.28	14.30
-6	-6	4	241.98	12.70
6	6	-4	259.77	15.10
-6	6	4	263.07	15.10
-6	6	4	234.38	13.40
-5	-6	4	16.20	4.30
-5	-6	4	12.50	2.00
5	-6	-4	18.30	3.60
-5	-6	4	15.50	2.50

5	-6	-4	14.10	3.40
5	6	-4	14.80	3.90
-5	6	4	13.10	3.40
-5	6	4	13.10	2.90
-4	-6	4	593.24	28.80
4	-6	-4	583.54	30.20
-4	-6	4	566.74	28.80
4	-6	-4	611.94	30.10
-4	6	4	598.54	30.50
4	6	-4	668.73	30.80
-4	6	4	584.44	29.70
-3	-6	4	3.70	0.70
3	-6	-4	2.60	1.90
-3	-6	4	0.70	2.60
-3	-6	4	5.10	0.80
3	-6	-4	4.40	2.10
-3	6	4	3.00	2.10
3	6	-4	3.80	2.50
-3	6	4	3.20	2.30
2	-6	-4	136.39	8.40
-2	-6	4	133.99	6.80
-2	-6	4	154.88	8.70
2	-6	-4	134.19	8.00
2	6	-4	161.98	8.60
-2	6	4	139.09	8.40
-2	6	4	122.79	8.00
-1	-6	4	391.76	19.70
1	-6	-4	405.96	20.90
-1	-6	4	442.86	20.90
-1	6	4	391.46	20.70
1	6	-4	385.06	19.70
1	6	-4	446.26	20.90
-1	6	4	418.56	20.90
0	-6	4	292.87	14.30
0	-6	-4	275.17	15.20
0	-6	4	308.77	14.90
0	-6	-4	292.37	15.30
0	6	-4	289.57	14.20
0	6	4	291.27	15.20
0	6	4	284.37	15.00
1	-6	4	119.19	6.40
-1	6	-4	117.59	6.60
1	6	4	117.59	7.10
1	6	4	121.89	7.50

-1	6	-4	109.69	6.30
1	6	4	120.99	7.00
-2	-6	-4	429.26	21.50
-2	-6	-4	431.76	22.20
2	-6	4	427.46	21.50
2	6	4	454.35	22.40
2	6	4	403.96	21.50
-2	6	-4	451.85	21.30
-3	-6	-4	84.99	5.30
-3	-6	-4	76.39	6.30
3	-6	4	75.09	5.40
3	6	4	75.89	5.20
-3	6	-4	76.59	4.80
-3	6	-4	75.79	5.20
3	6	4	78.09	6.10
4	-6	4	6.20	1.50
-4	-6	-4	2.30	1.90
-4	-6	-4	2.20	2.50
4	-6	4	4.20	2.40
-4	6	-4	2.10	2.30
4	6	4	-1.40	2.40
4	6	4	5.20	2.10
5	-6	4	10.90	2.00
5	-6	4	13.30	3.20
-5	-6	-4	11.90	2.80
-5	-6	-4	11.70	3.50
5	6	4	15.00	2.90
-5	6	-4	10.60	2.80
-5	6	-4	13.50	2.50
6	-6	4	248.68	13.50
-6	-6	-4	210.08	14.00
-6	-6	-4	240.88	13.00
6	-6	4	225.88	12.20
6	6	4	207.28	12.70
-6	6	-4	227.68	13.00
6	6	4	230.88	13.70
-6	6	-4	220.48	12.80
-7	-6	-4	94.29	7.40
-7	-6	-4	89.69	8.90
7	-6	4	85.89	6.40
7	-6	4	81.19	7.30
7	6	4	76.69	7.00
7	6	4	92.19	8.30
-7	6	-4	86.09	7.50

-7	6	-4	83.39	7.30
8	-6	4	25.10	3.80
-8	-6	-4	23.60	4.40
8	-6	4	31.40	5.50
-8	-6	-4	18.90	5.80
8	6	4	20.20	4.90
-8	6	-4	17.50	4.30
8	6	4	16.70	4.20
-8	6	-4	18.40	4.50
-9	-6	-4	56.19	9.50
-9	-6	-4	46.50	7.50
-9	-6	-4	65.09	8.00
9	-6	4	55.59	6.50
-9	6	-4	55.49	8.40
-9	6	-4	51.59	6.80
9	6	4	54.79	8.40
-10	-6	-4	14.10	5.10
-10	-6	-4	15.90	6.30
10	-6	4	6.10	4.10
-10	6	-4	8.90	5.50
10	6	4	12.80	5.50
-10	6	-4	14.40	5.30
-11	-6	-4	38.50	9.50
11	-6	4	43.20	7.80
-11	-6	-4	55.39	8.70
-11	6	-4	44.90	7.80
11	6	4	44.00	7.40
-11	6	-4	27.70	6.20
-12	-6	-4	37.10	15.80
12	-6	4	18.10	5.40
-12	6	-4	20.50	6.70
12	6	4	15.40	6.40
-12	6	-4	25.70	7.60
-13	-6	-4	78.29	11.90
-13	6	-4	78.49	11.40
-14	-6	-4	8.50	7.70
-14	6	-4	6.40	7.40
-15	-6	-4	34.40	8.90
-15	6	-4	33.20	8.90
-15	-7	4	101.39	13.20
-15	7	4	122.79	13.40
-14	-7	4	23.20	6.90
-14	7	4	30.30	7.70
-13	-7	4	147.79	12.30

13	7	-4	136.19	16.40
-13	7	4	153.78	12.50
-12	-7	4	44.90	11.50
-12	-7	4	61.09	10.20
12	-7	-4	76.89	11.60
-12	-7	4	72.19	8.80
12	7	-4	102.59	14.00
-12	7	4	89.99	9.70
11	-7	-4	245.38	17.20
-11	-7	4	209.88	20.40
-11	-7	4	271.37	16.00
-11	-7	4	251.77	14.90
-11	7	4	251.07	19.40
-11	7	4	264.77	20.30
11	7	-4	245.38	18.60
-10	-7	4	24.20	4.30
-10	-7	4	28.10	5.20
-10	-7	4	19.40	7.80
10	-7	-4	21.50	6.20
-10	7	4	12.80	6.60
-10	7	4	27.90	5.30
10	7	-4	21.40	7.00
-9	-7	4	454.05	24.40
-9	-7	4	538.25	25.00
9	-7	-4	471.85	26.30
-9	-7	4	457.65	28.10
9	7	-4	491.25	27.50
-9	7	4	467.25	25.20
-9	7	4	465.65	27.70
-8	-7	4	87.69	6.50
-8	-7	4	98.69	7.10
8	-7	-4	87.79	8.90
-8	-7	4	61.59	10.30
8	-7	-4	89.99	9.20
8	7	-4	91.49	10.10
-8	7	4	91.69	7.20
-8	7	4	72.79	9.80
7	-7	-4	592.94	30.10
-7	-7	4	574.64	28.50
-7	-7	4	510.65	30.90
-7	-7	4	626.74	29.00
7	-7	-4	595.84	30.20
7	7	-4	574.84	30.80
-7	7	4	591.84	29.30

-7	7	4	537.55	30.70
-6	-7	4	48.60	4.20
-6	-7	4	53.09	7.60
6	-7	-4	44.20	6.40
6	-7	-4	49.00	6.30
-6	-7	4	38.70	4.10
-6	7	4	46.50	6.90
6	7	-4	53.39	7.40
-6	7	4	46.40	5.30
5	-7	-4	1844.22	88.29
-5	-7	4	1725.93	88.99
-5	-7	4	1807.92	86.89
5	-7	-4	1852.71	88.39
-5	-7	4	1779.82	86.89
-5	7	4	1818.62	87.79
5	7	-4	1917.21	88.89
-5	7	4	1859.61	88.79
4	-7	-4	126.19	7.90
-4	-7	4	104.09	5.90
4	-7	-4	110.59	7.80
-4	-7	4	119.69	8.90
-4	-7	4	101.59	5.80
-4	7	4	108.29	7.00
4	7	-4	120.69	8.50
-4	7	4	107.89	8.00
3	-7	-4	493.05	24.40
3	-7	-4	491.65	24.20
-3	-7	4	451.75	24.70
-3	-7	4	474.25	22.90
3	7	-4	483.25	24.60
-3	7	4	473.85	24.00
-3	7	4	476.75	24.50
3	7	-4	471.25	22.80
2	-7	-4	1533.35	71.39
2	-7	-4	1516.25	70.99
-2	-7	4	1439.56	69.99
-2	-7	4	1482.65	71.69
2	7	-4	1566.54	71.49
2	7	-4	1371.76	69.99
-2	7	4	1510.85	71.39
-2	7	4	1387.56	70.99
-1	-7	4	977.40	51.89
1	-7	-4	1078.69	51.39
1	-7	-4	1133.19	50.99

-1	-7	4	1038.40	50.19
1	7	-4	1007.90	50.09
-1	7	4	1092.09	51.39
-1	7	4	1019.70	51.09
1	7	-4	1077.49	51.29
0	-7	4	561.44	26.10
0	-7	-4	557.34	26.60
0	-7	-4	585.44	27.30
0	-7	4	468.65	27.00
0	7	4	553.94	27.10
0	7	4	524.25	26.80
0	7	4	537.65	27.30
0	7	-4	528.25	26.00
0	7	-4	536.55	26.80
1	-7	4	232.98	11.90
-1	-7	-4	242.58	12.20
-1	7	-4	230.08	11.80
1	7	4	240.28	12.70
1	7	4	221.38	12.90
1	7	4	238.18	12.40
-1	7	-4	226.78	12.20
2	-7	4	251.57	12.90
-2	-7	-4	226.28	13.70
-2	-7	-4	237.68	12.80
-2	7	-4	262.27	12.80
-2	7	-4	257.67	12.70
2	7	4	244.58	13.00
2	7	4	241.98	13.90
3	-7	4	18.20	3.30
-3	-7	-4	12.70	2.30
-3	-7	-4	10.80	3.10
-3	7	-4	12.30	2.60
3	7	4	11.10	3.40
-3	7	-4	7.90	2.10
3	7	4	10.50	2.30
4	-7	4	571.24	27.40
-4	-7	-4	535.45	28.80
4	-7	4	587.54	28.20
-4	7	-4	550.64	27.70
4	7	4	582.74	28.90
4	7	4	530.35	27.80
-4	7	-4	553.84	27.90
-5	-7	-4	196.88	10.60
5	-7	4	177.68	11.00

-5	-7	-4	173.98	11.90
5	-7	4	178.58	9.90
5	7	4	190.18	11.90
-5	7	-4	183.58	10.50
-5	7	-4	167.28	10.50
5	7	4	185.48	10.70
6	-7	4	1025.40	49.30
6	-7	4	972.80	48.30
-6	-7	-4	1061.59	48.90
-6	-7	-4	911.81	49.90
6	7	4	1040.80	49.90
-6	7	-4	984.30	48.90
-6	7	-4	992.90	49.00
6	7	4	968.90	48.80
7	-7	4	375.06	21.40
-7	-7	-4	424.86	21.00
7	-7	4	402.46	20.40
-7	-7	-4	365.76	22.40
-7	7	-4	393.56	21.20
7	7	4	376.26	20.80
-7	7	-4	362.16	20.90
7	7	4	448.86	22.40
8	-7	4	467.15	23.60
8	-7	4	423.56	24.40
-8	-7	-4	407.16	25.70
-8	-7	-4	485.15	24.20
8	7	4	507.15	25.60
-8	7	-4	469.85	24.30
8	7	4	451.35	24.00
-8	7	-4	427.66	24.50
9	-7	4	0.70	3.20
-9	-7	-4	5.00	5.50
-9	-7	-4	2.40	4.40
9	7	4	5.40	4.20
-9	7	-4	0.30	4.40
9	7	4	1.10	5.20
-9	7	-4	0.70	4.40
-10	-7	-4	678.93	41.00
-10	-7	-4	806.12	39.40
10	-7	4	770.02	38.60
-10	7	-4	715.23	39.90
10	7	4	806.32	40.60
-10	7	-4	780.82	39.20
-11	-7	-4	21.50	8.00

-11	-7	-4	18.70	5.90
11	-7	4	25.80	5.40
-11	7	-4	25.90	6.80
11	7	4	42.00	8.20
-11	7	-4	24.70	6.10
-12	-7	-4	310.47	21.00
12	-7	4	292.87	19.00
12	7	4	296.87	21.00
-12	7	-4	313.67	19.30
-12	7	-4	278.27	20.60
-13	-7	-4	27.30	7.60
-13	7	-4	28.80	7.20
-14	-7	-4	304.77	21.00
-14	7	-4	295.77	20.30
-15	-7	-4	87.89	14.50
-15	7	-4	84.29	13.40
-15	-8	4	80.49	12.80
-15	8	4	89.29	12.70
-14	-8	4	131.09	12.90
-14	8	4	131.49	13.40
-13	-8	4	112.79	11.10
13	8	-4	125.19	16.70
-13	8	4	129.29	12.00
-12	-8	4	282.87	18.00
-12	-8	4	277.07	16.70
-12	-8	4	245.38	23.70
12	-8	-4	285.27	19.30
12	8	-4	288.47	21.10
-12	8	4	292.67	17.80
-11	-8	4	0.80	8.30
11	-8	-4	10.50	5.80
-11	-8	4	1.20	4.10
-11	-8	4	0.90	5.10
-11	8	4	14.70	8.10
11	8	-4	8.00	7.40
-11	8	4	2.00	5.20
-10	-8	4	589.84	37.10
10	-8	-4	648.34	34.80
-10	-8	4	657.83	32.90
-10	-8	4	721.63	33.50
-10	8	4	634.44	33.60
-10	8	4	597.64	36.40
10	8	-4	708.63	36.50
-9	-8	4	170.78	15.60

9	-8	-4	171.88	13.20
-9	-8	4	185.68	11.00
-9	-8	4	201.08	11.70
-9	8	4	160.28	14.90
9	8	-4	205.68	15.00
-9	8	4	175.48	11.60
-8	-8	4	484.65	29.80
-8	-8	4	498.75	26.30
8	-8	-4	541.55	28.30
-8	-8	4	564.14	26.60
8	-8	-4	502.85	28.30
8	8	-4	555.54	29.30
-8	8	4	522.75	29.40
-8	8	4	533.95	27.30
7	-8	-4	23.50	4.60
7	-8	-4	22.60	4.90
-7	-8	4	22.80	6.00
-7	-8	4	23.90	3.20
-7	8	4	29.20	5.40
-7	8	4	28.80	5.80
7	8	-4	29.20	6.10
-6	-8	4	1843.42	95.19
6	-8	-4	1961.60	94.39
-6	-8	4	1868.71	92.79
6	-8	-4	1925.21	94.29
-6	-8	4	1984.40	92.79
-6	8	4	1948.31	94.89
6	8	-4	2084.99	95.09
-6	8	4	1945.51	93.69
-5	-8	4	2.90	1.50
-5	-8	4	2.50	3.60
5	-8	-4	5.50	3.10
5	-8	-4	5.10	3.30
-5	-8	4	4.60	1.70
-5	8	4	3.80	3.40
5	8	-4	9.40	4.20
-5	8	4	4.20	2.60
-4	-8	4	2987.40	152.78
4	-8	-4	3247.07	154.28
4	-8	-4	3394.06	154.28
-4	-8	4	3266.17	154.98
4	8	-4	2932.21	152.78
-4	8	4	3236.08	153.88
4	8	-4	3480.65	154.78

-4	8	4	3256.77	154.58
3	-8	-4	6.00	2.50
-3	-8	4	5.70	1.10
3	-8	-4	2.50	2.50
-3	-8	4	3.70	2.80
-3	8	4	3.20	2.60
3	8	-4	6.20	1.00
-3	8	4	3.60	2.50
3	8	-4	6.40	3.00
2	-8	-4	222.58	12.70
-2	-8	4	246.18	13.10
-2	-8	4	224.38	11.10
2	-8	-4	236.28	12.40
2	8	-4	238.38	12.90
-2	8	4	212.98	12.70
2	8	-4	211.98	11.00
-2	8	4	211.98	12.30
1	-8	-4	949.90	44.80
-1	-8	4	900.31	43.40
1	-8	-4	940.01	44.20
-1	-8	4	904.21	44.60
-1	8	4	847.42	44.30
1	8	-4	871.41	43.30
1	8	-4	919.21	44.60
-1	8	4	933.01	44.70
0	-8	4	3851.31	186.68
0	-8	-4	4074.79	187.18
0	-8	4	3900.31	187.38
0	8	4	4005.80	187.88
0	8	-4	3674.53	186.48
0	8	4	4083.79	187.68
0	8	-4	4044.30	187.48
0	8	4	3854.01	187.38
-1	-8	-4	515.65	25.00
1	-8	4	504.65	24.80
1	8	4	502.65	25.20
-1	8	-4	503.65	24.60
1	8	4	514.85	25.60
-1	8	-4	487.15	25.10
1	8	4	499.05	25.90
-2	-8	-4	465.85	22.90
-2	-8	-4	435.66	23.70
2	8	4	478.75	24.10
-2	8	-4	455.75	22.90

-2	8	-4	459.45	22.70
-2	8	-4	481.85	22.90
2	8	4	425.86	22.90
-3	-8	-4	1143.09	58.29
3	-8	4	1206.28	57.49
-3	-8	-4	1244.28	57.39
3	8	4	1236.78	58.49
-3	8	-4	1179.68	57.29
-3	8	-4	1171.88	57.29
3	8	4	1122.69	57.39
4	-8	4	290.97	16.00
4	-8	4	297.07	15.30
-4	-8	-4	290.77	17.00
-4	-8	-4	309.27	15.70
4	8	4	295.27	15.70
4	8	4	328.97	17.20
-4	8	-4	280.27	15.60
-4	8	-4	290.87	15.60
-5	-8	-4	1692.43	85.49
-5	-8	-4	1844.12	84.29
5	-8	4	1724.83	83.89
5	-8	4	1782.92	84.69
-5	8	-4	1717.83	84.29
5	8	4	1922.71	85.59
-5	8	-4	1681.83	84.29
5	8	4	1634.74	84.19
-6	-8	-4	44.40	5.30
6	-8	4	43.00	4.60
6	-8	4	40.30	5.60
-6	-8	-4	44.00	7.20
-6	8	-4	43.70	5.60
-6	8	-4	35.10	5.20
6	8	4	46.10	5.40
6	8	4	60.39	7.40
-7	-8	-4	1022.70	47.40
7	-8	4	943.71	46.80
7	-8	4	932.51	47.80
-7	-8	-4	933.51	48.90
7	8	4	1017.30	48.60
7	8	4	899.81	47.20
-7	8	-4	974.10	47.70
-7	8	-4	929.21	47.40
-8	-8	-4	12.40	3.90
-8	-8	-4	6.10	5.10

8	-8	4	6.00	3.00
8	-8	4	9.00	6.20
-8	8	-4	7.60	3.90
8	8	4	9.90	4.80
-8	8	-4	14.80	4.40
8	8	4	8.60	3.90
-9	-8	-4	586.14	34.30
-9	-8	-4	667.73	32.50
9	-8	4	644.34	31.80
-9	8	-4	605.14	32.30
-9	8	-4	604.84	33.00
9	8	4	685.83	34.10
9	8	4	552.84	32.00
-10	-8	-4	39.80	8.30
10	-8	4	47.80	7.20
-10	-8	-4	54.89	8.80
-10	8	-4	51.69	8.40
-10	8	-4	32.00	6.80
10	8	4	52.49	10.40
11	-8	4	288.87	17.40
-11	-8	-4	306.17	18.50
-11	-8	-4	264.17	21.30
-11	8	-4	274.07	19.50
-11	8	-4	285.17	18.30
11	8	4	321.97	20.70
-12	-8	-4	8.70	6.40
12	8	4	12.80	7.70
-12	8	-4	-0.90	6.80
-12	8	-4	-4.10	5.40
-13	-8	-4	270.77	18.70
-13	8	-4	249.18	18.20
-14	-8	-4	19.90	8.20
-14	8	-4	19.40	7.10
-15	-8	-4	125.89	16.60
-15	8	-4	146.99	15.30
-15	-9	4	30.60	7.80
-15	9	4	15.30	7.50
-14	-9	4	14.80	6.90
-14	9	4	24.00	7.60
-13	-9	4	13.30	5.80
-13	9	4	-3.60	5.90
13	9	-4	19.20	8.80
-12	-9	4	-2.20	5.00
12	-9	-4	11.20	6.30

-12	-9	4	12.30	13.40
-12	-9	4	0.70	5.90
12	9	-4	14.90	9.20
-12	9	4	6.60	5.60
11	-9	-4	59.59	10.50
-11	-9	4	69.29	8.90
-11	-9	4	72.09	7.90
-11	-9	4	56.59	11.10
-11	9	4	43.40	9.50
11	9	-4	69.09	12.90
-11	9	4	69.19	9.20
-10	-9	4	56.29	7.70
10	-9	-4	44.00	9.00
-10	-9	4	48.30	6.40
-10	-9	4	37.20	9.10
-10	9	4	51.99	8.10
-10	9	4	62.29	11.90
10	9	-4	42.40	8.20
9	-9	-4	117.09	10.90
-9	-9	4	112.29	8.30
-9	-9	4	91.49	13.50
-9	-9	4	132.39	9.00
-9	9	4	119.09	9.80
-9	9	4	125.19	13.10
9	9	-4	124.69	12.80
-8	-9	4	6.60	3.00
-8	-9	4	10.00	6.00
8	-9	-4	6.90	4.10
-8	-9	4	2.40	2.80
8	9	-4	10.10	5.50
-8	9	4	14.20	3.80
-8	9	4	11.40	5.70
7	-9	-4	25.70	5.00
-7	-9	4	22.70	4.20
7	-9	-4	26.40	5.60
-7	-9	4	25.30	6.30
-7	9	4	29.90	5.50
7	9	-4	42.50	8.40
-7	9	4	19.40	5.30
6	-9	-4	11.50	3.70
-6	-9	4	6.90	2.20
-6	-9	4	8.00	2.50
6	-9	-4	13.10	3.90
-6	-9	4	8.30	5.00

-6	9	4	10.30	4.20
6	9	-4	12.80	4.70
-5	-9	4	341.87	16.90
5	-9	-4	371.96	19.00
5	-9	-4	331.57	18.70
-5	-9	4	307.87	19.70
-5	9	4	344.27	19.30
-5	9	4	353.16	18.30
5	9	-4	336.07	19.40
4	-9	-4	34.70	5.00
4	-9	-4	32.20	5.20
-4	-9	4	32.60	2.60
-4	-9	4	37.60	6.50
-4	9	4	26.90	4.70
-4	9	4	35.00	5.60
4	9	-4	28.10	2.60
4	9	-4	31.00	6.30
-3	-9	4	10.90	1.50
3	-9	-4	11.20	3.00
-3	-9	4	10.40	3.60
3	-9	-4	7.80	2.90
3	9	-4	9.40	1.30
-3	9	4	8.70	3.10
-3	9	4	5.20	2.80
3	9	-4	8.60	3.20
2	-9	-4	57.39	5.70
-2	-9	4	65.69	6.10
2	-9	-4	60.19	5.40
-2	-9	4	58.89	3.70
2	9	-4	58.89	3.50
-2	9	4	43.60	5.20
2	9	-4	57.59	5.80
-2	9	4	59.09	5.80
-1	-9	4	46.10	3.40
-1	-9	4	46.00	5.20
1	-9	-4	47.40	5.50
1	-9	-4	61.39	4.60
-1	9	4	56.39	5.30
1	9	-4	42.30	3.20
1	9	-4	42.90	5.10
-1	9	4	45.50	4.90
0	-9	4	8.20	2.40
0	-9	4	11.30	1.90
0	-9	-4	9.30	2.40

0	9	4	10.20	2.90
0	9	4	6.70	2.40
0	9	4	4.00	3.40
0	9	-4	9.00	2.70
0	9	-4	9.80	1.70
-1	-9	-4	413.96	19.30
1	-9	4	392.86	19.00
1	9	4	375.16	19.50
-1	9	-4	341.17	19.20
-1	9	-4	359.86	19.50
1	9	4	373.66	20.40
1	9	4	389.86	19.90
-2	-9	-4	2.20	2.70
-2	-9	-4	8.80	2.30
2	-9	4	2.30	2.00
2	9	4	5.00	2.30
-2	9	-4	2.40	1.80
2	9	4	5.30	3.50
-2	9	-4	0.20	1.90
-2	9	-4	4.00	1.80
-3	-9	-4	35.10	3.90
-3	-9	-4	33.70	5.60
3	-9	4	30.90	4.30
-3	9	-4	34.60	3.90
-3	9	-4	25.20	3.90
3	9	4	29.60	3.90
3	9	4	33.30	5.10
4	-9	4	143.89	8.80
-4	-9	-4	107.69	9.80
-4	-9	-4	148.49	8.40
-4	9	-4	136.09	8.40
-4	9	-4	137.69	8.30
4	9	4	140.79	8.50
4	9	4	116.39	9.80
-5	-9	-4	61.59	7.90
5	-9	4	71.59	5.30
-5	-9	-4	73.99	6.00
5	-9	4	57.09	6.70
-5	9	-4	69.79	6.00
-5	9	-4	73.89	6.20
5	9	4	65.49	8.10
5	9	4	72.49	6.20
-6	-9	-4	110.99	10.40
6	-9	4	114.09	9.20

6	-9	4	124.59	7.80
-6	-9	-4	131.09	8.50
-6	9	-4	123.09	8.80
-6	9	-4	119.09	8.60
6	9	4	115.69	8.40
6	9	4	124.39	10.30
7	-9	4	52.29	5.50
-7	-9	-4	49.00	8.80
7	-9	4	53.49	7.50
-7	-9	-4	56.19	6.30
-7	9	-4	46.30	6.70
7	9	4	50.89	8.40
-7	9	-4	51.79	6.70
7	9	4	60.29	6.50
-8	-9	-4	144.29	9.50
8	-9	4	112.09	8.30
-8	-9	-4	99.69	11.70
-8	9	-4	118.29	9.50
8	9	4	109.89	9.20
-8	9	-4	111.79	10.00
8	9	4	108.19	11.00
-9	-9	-4	65.19	7.90
-9	-9	-4	45.10	8.60
9	-9	4	44.30	6.70
9	9	4	53.39	10.20
9	9	4	52.29	7.60
-9	9	-4	48.40	7.00
-9	9	-4	53.49	8.70
10	-9	4	11.20	4.30
-10	-9	-4	3.50	6.50
-10	9	-4	3.30	5.30
10	9	4	1.90	6.40
10	9	4	16.10	5.10
-11	-9	-4	15.70	6.40
11	-9	4	11.20	5.10
-11	-9	-4	11.40	7.80
11	9	4	8.90	7.00
-11	9	-4	7.30	6.70
-11	9	-4	19.50	6.20
-12	-9	-4	31.50	7.60
-12	9	-4	33.80	7.50
12	9	4	29.30	13.30
-13	-9	-4	4.20	7.20
-13	9	-4	11.10	7.00

-14	-9	-4	41.90	9.70
-14	9	-4	19.40	8.00
-15	-9	-4	35.50	10.10
-15	9	-4	29.30	8.50
-15	10	4	215.58	17.60
-14	-10	4	120.59	12.60
-14	10	4	143.99	13.50
-13	-10	4	120.79	12.00
13	10	-4	166.88	18.10
-13	10	4	138.09	13.30
-12	-10	4	676.53	36.30
12	10	-4	760.12	41.20
-12	10	4	707.83	37.40
-11	-10	4	30.70	10.90
-11	-10	4	38.10	5.70
11	-10	-4	54.39	10.30
-11	-10	4	43.70	6.30
-11	10	4	29.70	6.50
-11	10	4	49.90	11.00
11	10	-4	42.40	10.00
10	-10	-4	533.95	29.20
-10	-10	4	592.44	27.40
-10	-10	4	526.35	26.90
-10	-10	4	443.56	31.60
-10	10	4	496.25	30.90
10	10	-4	537.55	30.90
-10	10	4	518.75	31.70
-9	-10	4	795.82	47.80
-9	-10	4	925.41	45.70
-9	-10	4	903.41	43.80
9	-10	-4	914.61	45.90
-9	10	4	883.91	47.30
9	10	-4	912.11	47.00
-9	10	4	898.81	44.80
-8	-10	4	1183.58	58.79
-8	-10	4	1285.07	58.89
8	-10	-4	1210.38	60.69
-8	-10	4	1117.09	62.19
8	10	-4	1309.17	62.09
-8	10	4	1204.18	59.79
-8	10	4	1197.68	61.79
-7	-10	4	88.59	11.40
7	-10	-4	97.59	9.30
-7	-10	4	106.39	6.90

7	-10	-4	90.29	9.80
-7	10	4	107.99	10.50
7	10	-4	118.39	11.20
-7	10	4	102.19	8.40
-6	-10	4	2385.46	128.99
-6	-10	4	2695.03	128.09
6	-10	-4	2763.32	129.89
-6	-10	4	2560.24	130.89
6	-10	-4	2687.03	129.99
-6	10	4	2708.73	130.39
6	10	-4	2970.70	130.79
-6	10	4	2770.62	129.19
-5	-10	4	229.48	15.90
-5	-10	4	252.47	12.70
5	-10	-4	262.37	15.00
5	-10	-4	241.18	15.00
5	10	-4	235.78	12.70
-5	10	4	270.67	15.50
-5	10	4	262.77	14.30
5	10	-4	260.27	15.80
4	-10	-4	1872.71	90.09
-4	-10	4	1874.81	90.89
-4	-10	4	1848.02	88.39
4	-10	-4	1870.91	90.09
-4	10	4	1834.52	90.29
4	10	-4	1974.60	90.59
4	10	-4	1733.33	88.29
-4	10	4	1858.51	89.49
-3	-10	4	16.30	1.90
-3	-10	4	22.70	4.50
3	-10	-4	21.80	4.00
3	-10	-4	19.20	3.90
3	10	-4	18.10	4.00
3	10	-4	17.90	2.20
-3	10	4	14.00	3.60
-3	10	4	11.40	3.40
2	-10	-4	1724.73	79.59
-2	-10	4	1616.64	78.39
-2	-10	4	1623.44	80.19
2	-10	-4	1653.83	79.89
-2	10	4	1612.94	79.49
2	10	-4	1506.65	78.29
2	10	-4	1725.93	80.19
-2	10	4	1712.83	80.09

-1	-10	4	2530.35	123.89
1	-10	-4	2682.83	123.39
1	-10	-4	2623.14	124.19
-1	-10	4	2502.95	122.59
-1	10	4	2664.73	123.99
-1	10	4	2538.05	123.59
1	10	-4	2466.55	122.49
1	10	-4	2623.54	123.99
0	-10	4	1731.83	85.69
0	-10	4	1760.72	84.89
0	-10	-4	1858.01	85.39
0	10	-4	1716.93	84.69
0	10	4	1706.53	85.49
0	10	-4	1783.22	85.89
0	10	4	1820.12	86.39
0	10	4	1836.72	86.09
-1	-10	-4	1325.97	61.19
1	-10	4	1154.58	61.29
1	10	4	1342.27	61.99
-1	10	-4	1257.97	61.59
1	10	4	1302.67	61.39
1	10	4	1220.28	63.39
-1	10	-4	1251.47	60.69
2	-10	4	24.90	3.60
-2	-10	-4	19.90	3.90
-2	-10	-4	23.90	3.60
2	10	4	22.30	4.70
2	10	4	21.30	3.80
-2	10	-4	18.20	3.60
-2	10	-4	15.40	2.90
-2	10	-4	20.80	3.40
-3	-10	-4	381.66	21.30
3	-10	4	395.86	20.00
-3	-10	-4	393.46	19.70
-3	10	-4	371.96	19.60
3	10	4	421.86	21.60
-3	10	-4	381.76	19.90
3	10	4	351.66	19.80
4	-10	4	458.35	23.90
-4	-10	-4	488.45	23.50
-4	-10	-4	439.06	25.00
-4	10	-4	457.45	23.60
4	10	4	505.05	25.40
4	10	4	431.16	23.50

-4	10	-4	443.96	23.40
5	-10	4	1627.44	79.09
-5	-10	-4	1534.95	79.99
5	-10	4	1626.64	78.29
-5	-10	-4	1712.63	78.59
-5	10	-4	1608.64	78.69
5	10	4	1581.84	78.59
5	10	4	1705.13	80.09
-5	10	-4	1610.54	78.59
6	-10	4	156.78	11.50
-6	-10	-4	180.48	10.60
-6	-10	-4	166.08	12.80
6	-10	4	172.48	10.20
-6	10	-4	180.68	11.00
6	10	4	176.28	13.00
-6	10	-4	170.98	11.00
6	10	4	160.98	10.70
7	-10	4	514.25	26.10
-7	-10	-4	544.45	26.50
7	-10	4	512.15	27.30
-7	-10	-4	469.85	28.40
-7	10	-4	528.15	26.70
7	10	4	492.95	26.50
-7	10	-4	482.55	26.90
7	10	4	530.15	28.30
8	-10	4	25.60	4.10
-8	-10	-4	30.60	5.30
-8	-10	-4	26.10	6.70
-8	10	-4	15.00	4.60
8	10	4	23.10	4.90
8	10	4	22.30	6.40
-8	10	-4	22.80	5.10
-9	-10	-4	492.95	24.30
-9	-10	-4	391.16	26.30
9	-10	4	442.16	23.30
-9	10	-4	421.86	23.60
9	10	4	419.96	23.70
-9	10	-4	432.46	24.90
9	10	4	451.15	26.00
-10	-10	-4	-0.10	6.90
10	-10	4	1.10	3.80
-10	10	-4	2.90	5.70
10	10	4	-3.40	6.50
-10	10	-4	8.60	5.40

10	10	4	-1.10	4.70
-11	-10	-4	811.32	39.70
-11	-10	-4	636.24	42.90
-11	10	-4	754.62	39.00
11	10	4	801.42	41.50
-11	10	-4	710.03	40.40
-12	-10	-4	13.00	7.00
-12	10	-4	15.30	6.50
-13	-10	-4	166.28	15.50
-13	10	-4	181.68	15.00
-14	-10	-4	12.60	8.40
-14	10	-4	17.20	8.10
-15	-10	-4	163.18	17.50
-15	10	-4	136.59	14.60
-14	-11	4	4.70	6.50
-14	11	4	15.30	7.10
-13	-11	4	64.59	10.00
-13	11	4	83.89	11.40
13	11	-4	81.19	16.40
-12	-11	4	53.89	8.70
-12	11	4	85.69	10.50
12	11	-4	46.80	10.30
-11	-11	4	130.19	10.40
-11	-11	4	85.89	17.80
11	-11	-4	142.89	13.30
-11	-11	4	143.99	11.00
11	11	-4	156.58	16.60
-11	11	4	140.69	11.80
-10	-11	4	6.50	3.90
-10	-11	4	3.30	8.90
-10	-11	4	8.30	4.40
10	-11	-4	7.90	5.70
-10	11	4	10.00	7.20
-10	11	4	4.40	5.20
10	11	-4	4.90	7.10
9	-11	-4	116.49	10.90
-9	-11	4	104.79	8.40
-9	-11	4	94.69	7.90
-9	-11	4	111.99	15.00
9	11	-4	129.99	13.60
-9	11	4	116.29	9.90
-9	11	4	94.99	12.60
-8	-11	4	15.00	3.30
-8	-11	4	19.30	6.90

8	-11	-4	12.60	4.90
-8	-11	4	17.00	3.40
8	11	-4	24.90	6.80
-8	11	4	14.20	4.80
-8	11	4	7.30	5.50
-7	-11	4	19.10	3.20
7	-11	-4	15.70	4.50
-7	-11	4	17.50	4.10
-7	-11	4	21.50	6.50
-7	11	4	20.80	6.00
7	11	-4	22.30	6.00
-7	11	4	20.00	4.30
-6	-11	4	83.49	5.60
-6	-11	4	90.89	11.00
6	-11	-4	69.79	9.10
6	-11	-4	78.69	8.60
6	11	-4	95.99	10.00
-6	11	4	95.59	7.40
-6	11	4	86.39	9.50
5	-11	-4	695.03	35.80
-5	-11	4	688.53	37.00
-5	-11	4	685.23	33.80
5	-11	-4	687.33	35.90
5	11	-4	801.62	36.80
5	11	-4	653.33	33.80
-5	11	4	687.13	35.00
-5	11	4	702.73	36.30
-4	-11	4	126.49	10.80
-4	-11	4	134.49	7.00
4	-11	-4	144.99	9.80
4	-11	-4	113.79	9.40
4	11	-4	137.39	10.40
-4	11	4	118.59	8.60
4	11	-4	119.79	7.00
-4	11	4	135.29	10.00
3	-11	-4	601.84	31.10
3	-11	-4	624.24	31.40
-3	-11	4	606.84	31.90
-3	-11	4	596.14	29.50
-3	11	4	591.34	30.70
3	11	-4	672.33	31.90
-3	11	4	601.14	31.40
3	11	-4	581.04	29.40
-2	-11	4	125.79	9.30

-2	-11	4	116.09	6.80
2	-11	-4	130.59	8.60
2	-11	-4	126.69	9.00
2	11	-4	111.69	6.60
-2	11	4	124.09	9.00
-2	11	4	115.89	8.40
2	11	-4	141.99	9.60
-1	-11	4	519.15	26.50
-1	-11	4	497.65	24.90
1	-11	-4	514.15	25.80
-1	11	4	503.95	26.80
-1	11	4	508.65	26.50
-1	11	4	482.85	25.90
1	11	-4	538.35	26.60
1	11	-4	493.45	24.70
0	-11	4	57.09	4.70
0	-11	-4	72.89	5.50
0	11	-4	60.59	4.50
0	11	-4	71.79	6.30
0	11	4	66.99	6.40
0	11	4	60.99	7.00
0	11	4	60.69	5.80
1	-11	4	177.58	10.50
-1	-11	-4	207.18	10.90
-1	11	-4	192.78	11.50
1	11	4	190.08	11.60
1	11	4	188.58	12.60
-1	11	-4	191.38	11.10
-1	11	-4	184.88	10.20
1	11	4	192.48	11.10
-2	-11	-4	753.42	38.30
-2	-11	-4	758.52	36.90
2	-11	4	764.52	37.00
-2	11	-4	731.83	37.30
-2	11	-4	761.32	37.20
2	11	4	750.32	37.70
-2	11	-4	722.43	36.60
2	11	4	718.73	37.10
2	11	4	791.22	38.70
-3	-11	-4	3.20	3.80
-3	-11	-4	7.60	2.60
3	-11	4	6.10	3.00
-3	11	-4	7.90	2.50
-3	11	-4	5.30	2.60

-3	11	-4	8.30	2.80
3	11	4	4.70	2.80
3	11	4	2.10	4.10
-4	-11	-4	443.16	25.00
4	-11	4	465.95	23.80
-4	-11	-4	488.45	23.30
4	11	4	433.86	23.40
-4	11	-4	449.06	23.20
-4	11	-4	446.66	23.50
-5	-11	-4	28.20	5.80
5	-11	4	34.80	5.20
-5	-11	-4	39.20	5.10
-5	11	-4	31.50	5.30
5	11	4	42.70	5.40
5	11	4	29.10	6.90
-5	11	-4	35.80	5.30
-6	-11	-4	65.29	6.50
6	-11	4	54.99	5.80
-6	-11	-4	74.69	9.60
6	-11	4	57.89	7.60
6	11	4	65.29	9.30
-6	11	-4	61.29	6.70
-6	11	-4	57.69	6.70
6	11	4	52.29	6.40
-7	-11	-4	35.80	5.00
7	-11	4	43.20	7.30
-7	-11	-4	56.09	9.90
7	-11	4	39.60	5.50
-7	11	-4	46.10	6.60
7	11	4	50.09	9.20
7	11	4	36.20	6.30
-7	11	-4	30.40	5.10
-8	-11	-4	167.68	11.20
8	-11	4	153.28	10.50
-8	-11	-4	148.89	14.30
-8	11	-4	148.09	12.00
8	11	4	139.79	10.90
-8	11	-4	162.48	11.40
8	11	4	170.88	14.10
-9	-11	-4	22.40	7.30
9	-11	4	32.30	6.60
-9	11	-4	29.50	5.90
9	11	4	30.50	7.60
9	11	4	36.60	5.90

-9	11	-4	25.80	6.10
-10	-11	-4	42.50	9.50
-10	11	-4	59.09	10.60
-10	11	-4	39.90	7.00
10	11	4	33.60	6.40
10	11	4	46.30	9.70
11	-11	4	49.60	9.30
-11	-11	-4	62.99	11.10
11	11	4	42.80	9.60
-11	11	-4	69.59	9.10
-11	11	-4	36.10	8.70
-12	-11	-4	63.39	9.70
-12	11	-4	77.59	11.20
-13	-11	-4	6.00	8.00
-13	11	-4	7.20	6.80
-14	-11	-4	34.10	10.10
-14	11	-4	38.50	8.10
-14	12	4	62.79	9.80
-13	-12	4	43.10	7.10
-13	12	4	72.29	11.20
-12	-12	4	250.67	15.60
12	12	-4	233.18	21.80
-12	12	4	253.07	17.10
-11	-12	4	12.80	7.00
-11	-12	4	9.20	4.60
-11	-12	4	31.70	11.60
11	-12	-4	7.70	6.30
-11	12	4	13.10	5.60
11	12	-4	6.50	8.10
-10	-12	4	210.68	12.60
-10	-12	4	199.28	19.80
-10	-12	4	208.28	12.50
10	-12	-4	185.08	15.30
10	12	-4	199.78	17.50
-10	12	4	184.08	13.90
-10	12	4	206.98	18.30
-9	-12	4	102.59	7.70
9	-12	-4	103.29	10.80
-9	-12	4	104.79	7.90
-9	-12	4	89.99	14.80
9	12	-4	69.79	13.10
-9	12	4	89.19	14.20
-9	12	4	99.49	12.90
-8	-12	4	265.47	21.40

8	-12	-4	304.17	18.90
-8	-12	4	312.77	16.40
-8	-12	4	317.67	16.20
-8	12	4	283.17	20.20
8	12	-4	332.67	20.40
-8	12	4	316.47	17.80
-7	-12	4	52.89	5.20
7	-12	-4	50.89	8.00
-7	-12	4	46.20	5.00
-7	-12	4	59.09	11.20
7	12	-4	61.09	9.60
-7	12	4	54.69	7.10
-7	12	4	44.60	9.00
-6	-12	4	991.10	54.79
-6	-12	4	1055.79	51.49
6	-12	-4	1090.59	53.69
6	-12	-4	1092.79	55.69
-6	12	4	1112.89	52.89
-6	12	4	1025.60	54.09
6	12	-4	1125.19	54.59
5	-12	-4	77.89	8.10
-5	-12	4	76.79	4.90
5	-12	-4	67.79	8.50
-5	-12	4	54.69	9.50
-5	12	4	78.19	8.70
5	12	-4	76.09	9.30
-5	12	4	77.89	7.30
5	12	-4	68.69	5.00
-4	-12	4	750.12	41.40
-4	-12	4	805.82	38.60
4	-12	-4	846.72	40.80
4	-12	-4	811.52	40.70
-4	12	4	777.02	40.00
4	12	-4	777.12	38.60
-4	12	4	813.62	41.00
4	12	-4	831.42	41.20
3	-12	-4	102.09	8.20
-3	-12	4	93.29	9.10
-3	-12	4	101.39	5.90
3	-12	-4	104.79	8.50
3	12	-4	89.59	8.80
3	12	-4	95.39	5.70
-3	12	4	106.39	8.70
-3	12	4	87.79	7.40

2	-12	-4	272.67	15.70
-2	-12	4	264.67	13.50
2	-12	-4	266.27	14.90
-2	-12	4	253.67	15.70
-2	12	4	285.07	15.70
2	12	-4	256.27	15.70
-2	12	4	259.87	14.90
2	12	-4	256.07	13.30
-1	-12	4	596.24	32.00
-1	-12	4	622.94	30.50
1	-12	-4	633.44	31.30
-1	12	4	645.84	32.20
1	12	-4	624.54	32.10
-1	12	4	618.84	31.50
-1	12	4	617.34	32.50
0	-12	-4	847.62	40.10
0	-12	4	786.02	40.10
0	12	-4	791.22	39.30
0	12	-4	796.22	40.80
0	12	4	833.12	41.00
0	12	4	826.32	40.40
0	12	4	805.02	41.40
1	-12	4	1443.16	68.49
-1	-12	-4	1470.05	68.59
-1	12	-4	1374.36	69.19
1	12	4	1382.26	69.99
-1	12	-4	1460.35	68.99
1	12	4	1428.06	68.89
-1	12	-4	1372.36	68.09
2	-12	4	641.74	31.20
-2	-12	-4	649.64	31.10
-2	12	-4	633.44	31.50
-2	12	-4	602.74	31.60
2	12	4	634.34	32.00
2	12	4	617.24	31.30
-2	12	-4	598.84	30.70
2	12	4	600.94	32.60
-3	-12	-4	304.37	19.00
-3	-12	-4	358.76	17.30
3	-12	4	358.76	17.80
-3	12	-4	327.77	17.60
3	12	4	311.67	17.40
-3	12	-4	322.87	17.10
3	12	4	321.87	19.40

-3	12	-4	302.07	17.70
-4	-12	-4	20.70	5.40
4	-12	4	19.50	3.90
-4	-12	-4	14.90	3.20
4	12	4	17.60	3.50
-4	12	-4	18.60	3.40
4	12	4	15.80	5.90
-4	12	-4	17.40	3.50
-5	-12	-4	628.24	29.70
5	-12	4	601.24	30.50
-5	-12	-4	557.64	31.70
5	12	4	586.74	32.00
-5	12	-4	566.54	30.00
5	12	4	578.74	29.80
-5	12	-4	584.54	29.80
6	-12	4	485.75	24.60
6	-12	4	474.05	25.80
-6	-12	-4	500.95	24.80
-6	-12	-4	449.16	27.20
6	12	4	512.25	27.30
6	12	4	446.86	24.70
-6	12	-4	476.95	24.90
-6	12	-4	453.45	25.20
-7	-12	-4	206.48	13.10
7	-12	4	221.68	12.60
-7	-12	-4	197.48	16.10
7	12	4	192.38	13.00
7	12	4	203.88	15.80
-7	12	-4	204.38	13.70
-7	12	-4	226.88	13.30
-8	-12	-4	17.70	4.80
8	-12	4	25.00	4.40
-8	-12	-4	11.30	8.30
8	12	4	22.00	7.30
8	12	4	20.00	5.00
-8	12	-4	17.30	4.80
-8	12	-4	13.70	5.40
9	-12	4	217.68	13.50
9	12	4	231.68	17.50
-9	12	-4	183.18	16.20
-9	12	-4	194.28	15.20
9	12	4	199.88	13.70
10	-12	4	8.00	7.00
-10	-12	-4	3.30	7.90

10	12	4	2.20	5.10
-10	12	-4	8.20	5.80
-10	12	-4	7.30	6.30
10	12	4	6.00	8.20
11	12	4	360.36	25.40
11	12	4	326.57	21.10
-11	12	-4	385.36	21.80
-11	12	-4	356.46	29.50
-12	-12	-4	100.69	13.50
-12	12	-4	89.69	12.00
-13	-12	-4	320.77	21.80
-13	12	-4	311.97	20.80
-14	-12	-4	19.10	9.50
-14	12	-4	11.30	7.40
-14	13	4	86.09	13.00
-13	13	4	114.89	13.00
-12	-13	4	108.19	10.40
12	13	-4	109.89	17.70
-12	13	4	101.99	12.20
-11	-13	4	343.97	29.40
-11	-13	4	413.56	21.80
-11	13	4	410.66	22.90
11	13	-4	411.56	27.50
-10	-13	4	54.89	6.80
10	-13	-4	32.20	7.20
-10	-13	4	43.20	6.80
-10	-13	4	37.20	11.10
-10	13	4	32.60	6.40
10	13	-4	40.80	9.50
-9	-13	4	380.86	19.70
-9	-13	4	389.06	19.80
-9	-13	4	310.27	25.90
9	-13	-4	378.46	22.40
-9	13	4	358.96	24.30
-9	13	4	376.26	21.30
9	13	-4	401.06	24.70
-8	-13	4	14.80	7.80
-8	-13	4	13.60	3.30
8	-13	-4	4.90	5.30
-8	-13	4	9.20	3.30
-8	13	4	9.80	4.60
8	13	-4	10.30	6.60
-8	13	4	14.10	6.80
-7	-13	4	1339.47	73.79

7	-13	-4	1502.75	72.39
-7	-13	4	1427.36	69.89
-7	13	4	1433.66	73.09
-7	13	4	1470.75	71.39
7	13	-4	1574.24	73.49
-6	-13	4	12.10	2.50
-6	-13	4	11.10	6.10
6	-13	-4	9.90	4.40
-6	13	4	18.40	4.60
-6	13	4	12.10	5.00
6	13	-4	11.20	2.70
6	13	-4	13.10	5.50
-5	-13	4	883.91	42.40
-5	-13	4	847.62	45.80
5	-13	-4	835.22	44.80
5	-13	-4	897.21	44.70
5	13	-4	994.50	45.70
-5	13	4	919.91	45.30
5	13	-4	738.03	42.90
-5	13	4	928.01	44.00
-4	-13	4	51.19	9.00
4	-13	-4	57.09	7.10
-4	-13	4	60.99	4.40
4	-13	-4	64.79	7.60
4	13	-4	56.59	8.70
-4	13	4	61.79	7.80
-4	13	4	59.39	6.70
4	13	-4	55.79	4.40
3	-13	-4	644.54	32.70
-3	-13	4	616.04	33.20
-3	-13	4	621.14	30.60
3	-13	-4	593.54	32.10
3	13	-4	604.24	30.50
-3	13	4	604.54	31.90
3	13	-4	691.23	33.30
-3	13	4	651.33	32.90
-2	-13	4	292.57	17.60
-2	-13	4	301.07	15.20
2	-13	-4	297.87	16.70
-2	13	4	291.87	16.60
2	13	-4	299.27	17.50
-2	13	4	296.77	17.20
1	-13	-4	445.26	22.00
-1	-13	4	395.96	22.60

-1	13	4	395.46	23.10
1	13	-4	421.06	22.90
1	13	-4	416.66	20.80
-1	13	4	415.86	22.10
-1	13	4	428.06	22.80
0	-13	-4	585.04	28.60
0	-13	4	559.34	28.10
0	13	4	570.54	28.90
0	13	4	573.64	30.20
0	13	4	553.44	29.50
0	13	-4	545.35	27.80
0	13	-4	568.14	29.60
-1	-13	-4	627.44	30.60
1	-13	4	621.54	30.50
-1	13	-4	582.94	30.00
1	13	4	607.84	31.60
-1	13	-4	608.74	31.50
1	13	4	604.74	30.90
1	13	4	626.24	32.40
-1	13	-4	595.74	30.90
2	-13	4	123.89	8.50
-2	-13	-4	124.29	8.30
-2	13	-4	125.89	9.30
2	13	4	128.39	10.90
2	13	4	134.19	9.70
-2	13	-4	117.19	7.90
-2	13	-4	137.79	8.90
2	13	4	122.79	8.70
3	-13	4	878.11	43.70
-3	-13	-4	917.21	43.30
-3	-13	-4	871.11	45.10
-3	13	-4	892.61	43.80
-3	13	-4	867.31	43.90
3	13	4	900.01	45.30
-3	13	-4	849.22	43.10
3	13	4	856.41	43.40
-4	-13	-4	391.16	23.10
-4	-13	-4	432.16	21.00
4	-13	4	402.46	21.60
-4	13	-4	383.96	20.80
4	13	4	405.06	23.20
4	13	4	392.06	21.10
-4	13	-4	393.86	21.40
-5	-13	-4	393.16	19.70

5	-13	4	368.26	20.70
-5	-13	-4	344.47	22.10
-5	13	-4	365.86	19.80
5	13	4	364.46	19.80
-5	13	-4	348.57	20.10
5	13	4	392.06	22.40
-6	-13	-4	681.13	32.00
-6	-13	-4	619.84	34.60
6	-13	4	594.34	32.90
-6	13	-4	606.24	32.00
6	13	4	605.24	32.10
-6	13	-4	601.14	32.50
6	13	4	689.63	34.50
-7	-13	-4	152.78	14.80
-7	-13	-4	179.68	11.20
7	-13	4	165.88	10.80
-7	13	-4	144.29	11.80
-7	13	-4	157.58	11.40
7	13	4	168.98	14.10
7	13	4	150.88	11.20
-8	-13	-4	310.27	22.10
8	-13	4	332.67	18.40
-8	-13	-4	346.17	18.60
-8	13	-4	303.37	19.70
8	13	4	291.47	18.50
8	13	4	365.76	21.80
-8	13	-4	327.37	18.80
-9	-13	-4	6.70	7.20
-9	13	-4	5.00	5.70
9	13	4	3.50	4.70
-9	13	-4	6.60	5.40
9	13	4	9.90	7.30
10	-13	4	556.14	28.80
-10	-13	-4	485.45	33.70
-10	13	-4	555.14	29.60
10	13	4	511.15	29.00
10	13	4	598.94	32.60
-10	13	-4	526.95	31.10
11	13	4	4.90	8.20
11	13	4	2.30	6.90
-11	13	-4	-3.50	6.20
-12	-13	-4	415.86	24.80
-12	13	-4	365.46	23.50
-13	-13	-4	1.50	8.60

-13	13	-4	11.70	7.80
-14	-13	-4	128.49	17.20
-14	13	-4	121.59	14.70
-13	14	4	12.80	7.10
-12	-14	4	4.00	5.30
12	14	-4	8.20	9.90
-12	14	4	10.00	7.10
-11	-14	4	28.60	5.60
-11	14	4	17.90	6.40
11	14	-4	21.50	9.60
-10	-14	4	57.49	7.30
10	-14	-4	53.69	10.50
-10	-14	4	60.39	13.40
10	14	-4	57.29	10.90
-10	14	4	52.19	8.60
9	-14	-4	52.89	10.00
-9	-14	4	47.10	6.20
-9	-14	4	56.99	6.30
-9	-14	4	58.99	11.20
-9	14	4	48.60	8.70
9	14	-4	37.90	8.90
-9	14	4	29.00	8.60
-8	-14	4	8.60	3.50
-8	-14	4	11.40	8.40
8	-14	-4	7.70	5.40
-8	-14	4	7.90	3.10
8	14	-4	15.50	7.50
-8	14	4	12.30	6.00
-8	14	4	8.20	6.80
-7	-14	4	14.20	7.10
7	-14	-4	10.80	5.00
-7	14	4	12.30	4.70
7	14	-4	11.00	6.70
-7	14	4	11.20	5.60
6	-14	-4	69.99	8.70
-6	-14	4	53.79	11.00
6	14	-4	64.69	10.30
-6	14	4	61.09	9.70
-6	14	4	74.59	8.00
6	14	-4	63.09	5.50
-5	-14	4	35.00	7.00
5	-14	-4	31.70	5.60
-5	-14	4	47.60	4.10
-5	14	4	41.00	5.90

-5	14	4	36.10	6.30
5	14	-4	46.90	9.30
5	14	-4	41.20	4.40
-4	-14	4	227.18	16.80
4	-14	-4	260.67	15.90
-4	-14	4	278.97	13.30
4	-14	-4	239.38	15.90
-4	14	4	247.68	15.10
4	14	-4	257.57	16.80
4	14	-4	239.28	14.60
-4	14	4	269.77	16.50
-3	-14	4	30.20	6.10
3	-14	-4	25.70	5.60
-3	-14	4	25.70	3.80
3	-14	-4	30.40	5.50
-3	14	4	37.40	7.10
-3	14	4	28.30	5.00
3	14	-4	29.90	3.70
3	14	-4	36.80	6.20
-2	-14	4	10.10	4.30
2	-14	-4	2.10	3.80
2	14	-4	6.80	4.50
-2	14	4	10.90	4.80
2	14	-4	9.00	2.40
-2	14	4	11.00	4.40
-2	14	4	6.00	3.70
-1	-14	4	6.60	3.80
1	-14	-4	14.50	3.80
-1	-14	4	7.50	2.60
-1	14	4	4.30	3.90
1	14	-4	2.80	4.10
-1	14	4	8.50	4.70
-1	14	4	10.20	4.00
1	14	-4	10.80	2.40
0	-14	-4	13.60	3.40
0	-14	4	6.60	2.90
0	14	4	3.50	3.40
0	14	-4	4.90	2.40
0	14	-4	8.40	4.20
0	14	4	9.10	4.60
1	-14	4	44.30	5.80
-1	-14	-4	65.69	5.70
-1	14	-4	52.19	5.00
1	14	4	50.19	6.10

-1	14	-4	50.19	7.10
1	14	4	51.09	7.10
1	14	4	60.19	8.30
-1	14	-4	51.19	6.20
2	-14	4	107.09	8.10
-2	-14	-4	121.39	7.80
2	14	4	109.99	8.20
-2	14	-4	116.89	8.40
2	14	4	99.89	8.80
2	14	4	92.79	10.10
-2	14	-4	89.29	8.70
-2	14	-4	104.69	7.30
-3	-14	-4	32.20	5.00
3	-14	4	32.20	5.90
-3	-14	-4	35.80	6.60
3	14	4	32.00	5.50
-3	14	-4	33.70	4.80
3	14	4	25.10	6.10
-3	14	-4	28.00	4.50
-3	14	-4	29.90	4.80
4	-14	4	25.70	5.20
-4	-14	-4	24.90	4.00
-4	-14	-4	13.20	5.90
-4	14	-4	18.30	4.20
-4	14	-4	21.10	4.20
4	14	4	18.00	6.30
4	14	4	17.00	4.20
-4	14	-4	15.20	3.90
-5	-14	-4	38.50	7.70
5	-14	4	52.29	8.30
-5	-14	-4	59.69	6.40
-5	14	-4	59.49	7.20
5	14	4	53.39	6.90
-5	14	-4	49.80	6.60
5	14	4	48.70	9.50
-6	-14	-4	63.49	6.80
-6	-14	-4	61.39	10.50
-6	14	-4	52.19	7.50
6	14	4	43.20	7.10
-6	14	-4	42.20	7.10
6	14	4	53.89	10.40
-7	-14	-4	44.10	8.60
-7	-14	-4	82.69	8.10
-7	14	-4	68.69	8.10

-7	14	-4	53.09	8.50
7	14	4	68.09	11.50
7	14	4	58.79	8.00
8	-14	4	15.20	4.60
-8	-14	-4	-3.00	7.00
8	14	4	3.70	6.60
-8	14	-4	6.20	5.10
8	14	4	18.60	5.00
-8	14	-4	10.90	5.30
-9	-14	-4	94.39	15.00
9	-14	4	66.39	10.40
9	14	4	61.59	9.20
9	14	4	52.79	10.00
-9	14	-4	64.99	11.00
-9	14	-4	85.29	9.70
-10	14	-4	23.80	6.40
-10	14	-4	39.80	8.30
10	14	4	16.90	5.80
10	14	4	25.50	9.30
-11	14	-4	9.50	6.20
11	14	4	12.20	9.80
-12	-14	-4	13.40	8.40
-12	14	-4	8.20	7.10
-13	-14	-4	12.40	9.30
-13	14	-4	13.90	7.10
-13	15	4	206.58	16.70
12	15	-4	30.80	12.30
-12	15	4	31.00	8.10
-11	-15	4	281.77	16.70
-11	15	4	280.27	18.00
11	15	-4	276.97	23.90
-10	-15	4	92.79	8.40
-10	15	4	88.99	9.80
10	15	-4	56.99	11.20
-9	-15	4	62.99	6.40
-9	-15	4	56.69	18.90
-9	-15	4	59.09	6.80
9	-15	-4	55.69	10.70
9	15	-4	49.20	10.20
-9	15	4	52.39	7.80
-8	-15	4	92.99	15.10
-8	-15	4	89.69	7.60
-8	-15	4	94.89	7.10
8	-15	-4	100.09	11.20

-8	15	4	100.39	10.00
-8	15	4	112.79	12.90
8	15	-4	87.79	12.90
-7	-15	4	706.73	32.70
7	-15	-4	696.23	35.60
-7	-15	4	568.54	37.60
7	15	-4	734.23	37.00
-7	15	4	628.64	34.70
7	15	-4	648.64	32.80
-7	15	4	667.33	36.70
-6	-15	4	7.40	2.50
6	-15	-4	0.30	5.00
-6	-15	4	10.80	6.30
6	15	-4	4.20	5.80
6	15	-4	2.70	2.50
-6	15	4	-3.40	5.00
-6	15	4	2.30	4.00
5	-15	-4	492.75	26.50
-5	-15	4	424.76	27.50
-5	15	4	493.35	25.60
-5	15	4	485.75	26.90
5	15	-4	448.76	23.70
5	15	-4	517.35	27.50
4	-15	-4	9.30	4.70
-4	-15	4	2.10	5.30
-4	15	4	4.80	4.40
4	15	-4	6.80	2.50
-4	15	4	-4.60	3.90
4	15	-4	-0.30	4.80
-3	-15	4	1882.01	98.09
3	-15	-4	1942.11	97.29
-3	-15	4	2012.50	95.99
-3	15	4	2049.99	96.99
-3	15	4	2125.09	97.99
3	15	-4	1911.11	95.39
3	15	-4	2078.19	98.19
2	-15	-4	1.30	3.80
-2	-15	4	16.50	5.00
-2	-15	4	5.10	2.60
2	15	-4	7.20	2.30
-2	15	4	5.10	4.40
2	15	-4	5.30	4.50
-2	15	4	5.20	5.30
-2	15	4	0.20	4.00

1	-15	-4	1281.47	63.19
-1	-15	4	1311.47	62.49
-1	15	4	1286.77	64.59
1	15	-4	1264.77	62.19
-1	15	4	1301.37	64.89
-1	15	4	1261.27	63.39
1	15	-4	1334.77	64.49
0	-15	4	399.56	20.60
0	-15	-4	405.46	21.10
0	15	4	375.26	22.90
0	15	-4	385.86	22.30
0	15	-4	398.16	20.20
0	15	4	407.56	22.30
0	15	4	395.06	21.50
1	-15	4	1868.71	89.19
-1	-15	-4	1934.41	89.29
-1	15	-4	1818.22	89.89
1	15	4	1885.41	91.29
1	15	4	1911.21	90.49
-1	15	-4	1771.02	88.69
-1	15	-4	1759.22	90.19
1	15	4	1846.42	89.59
2	-15	4	111.39	8.60
-2	-15	-4	120.79	8.10
-2	15	-4	106.79	7.50
2	15	4	91.59	10.80
2	15	4	91.79	9.40
-2	15	-4	106.59	8.70
2	15	4	108.99	8.50
-2	15	-4	98.99	9.50
3	-15	4	409.46	21.30
-3	-15	-4	392.86	20.50
-3	-15	-4	386.56	23.00
-3	15	-4	388.86	20.20
3	15	4	389.96	20.80
-3	15	-4	377.66	21.50
3	15	4	381.46	23.10
3	15	4	371.96	21.80
-3	15	-4	381.06	21.20
-4	-15	-4	1133.49	59.29
-4	-15	-4	1205.88	56.99
4	-15	4	1178.28	57.89
-4	15	-4	1164.68	57.99
-4	15	-4	1147.39	57.69

-4	15	-4	1114.69	56.89
4	15	4	1219.48	59.59
4	15	4	1157.78	57.19
-5	-15	-4	429.46	26.20
-5	-15	-4	462.85	23.40
5	-15	4	430.76	24.60
5	15	4	434.66	23.70
5	15	4	486.95	26.70
-5	15	-4	432.26	24.20
-5	15	-4	437.26	23.50
-6	-15	-4	1343.57	64.99
-6	-15	-4	1295.87	67.89
-6	15	-4	1294.67	65.79
6	15	4	1406.56	67.69
-6	15	-4	1335.47	65.19
6	15	4	1297.47	65.19
-7	-15	-4	88.99	8.00
-7	-15	-4	74.49	12.50
7	15	4	72.69	12.30
7	15	4	71.39	8.60
-7	15	-4	70.19	8.30
-7	15	-4	64.39	9.30
-8	-15	-4	377.16	27.40
8	-15	4	454.55	26.00
-8	15	-4	431.26	25.10
-8	15	-4	420.56	27.80
8	15	4	461.35	27.20
8	15	4	443.76	23.90
9	-15	4	39.40	8.10
-9	-15	-4	54.49	11.50
9	15	4	34.20	6.60
9	15	4	44.00	9.90
-9	15	-4	42.90	8.20
-9	15	-4	43.90	7.40
10	15	4	592.84	33.50
10	15	4	666.63	37.30
-10	15	-4	649.43	33.90
11	15	4	-1.40	5.80
-11	15	-4	5.50	6.70
-12	-15	-4	394.86	24.40
-12	15	-4	324.17	22.10
-13	-15	-4	24.20	10.20
-13	15	-4	14.90	8.40
-13	16	4	9.50	7.40

12	16	-4	17.00	10.90
-12	16	4	16.30	7.50
-11	16	4	20.80	6.60
11	16	-4	14.90	10.50
-10	-16	4	10.00	4.70
10	16	-4	11.70	9.60
-10	16	4	16.40	6.50
-9	-16	4	2.10	3.90
9	-16	-4	2.30	5.90
-9	16	4	-5.00	5.60
9	16	-4	-7.00	8.50
-8	-16	4	11.50	3.30
8	-16	-4	6.00	5.70
-8	16	4	7.70	5.60
8	16	-4	3.80	7.40
-8	16	4	3.00	8.00
7	-16	-4	5.10	5.70
-7	-16	4	18.90	9.40
-7	-16	4	12.30	3.00
7	16	-4	11.40	3.30
-7	16	4	18.90	6.60
7	16	-4	18.50	7.10
-7	16	4	6.90	4.70
-6	-16	4	24.10	4.10
6	-16	-4	18.80	5.60
-6	-16	4	18.20	7.20
6	16	-4	24.10	7.50
6	16	-4	18.70	3.20
-6	16	4	12.40	6.50
-6	16	4	25.90	5.40
-5	-16	4	4.10	6.50
5	-16	-4	2.50	4.70
-5	-16	4	0.00	2.40
5	16	-4	5.60	2.50
-5	16	4	-4.00	4.70
-5	16	4	-3.20	4.40
-4	-16	4	252.37	13.20
4	-16	-4	241.68	16.30
-4	-16	4	242.58	17.70
-4	16	4	229.78	15.10
4	16	-4	239.28	13.10
-4	16	4	250.47	16.50
4	16	-4	271.57	17.50
-3	-16	4	48.90	9.80

-3	-16	4	57.39	5.50
3	16	-4	71.49	5.10
-3	16	4	51.29	7.70
3	16	-4	57.69	9.60
-3	16	4	66.99	8.80
2	-16	-4	195.78	13.90
-2	-16	4	237.88	15.60
-2	-16	4	220.88	12.40
-2	16	4	211.78	14.80
-2	16	4	236.78	15.90
-2	16	4	220.58	14.00
2	16	-4	213.68	12.00
2	16	-4	243.98	15.60
1	-16	-4	48.60	7.00
-1	-16	4	34.10	5.40
1	16	-4	43.30	4.80
-1	16	4	36.70	6.70
-1	16	4	63.39	10.70
1	16	-4	46.60	8.40
-1	16	4	43.40	7.60
0	-16	4	159.18	10.60
0	-16	-4	171.88	11.00
0	16	-4	159.88	10.00
0	16	4	172.98	12.70
0	16	-4	156.48	11.40
0	16	4	173.48	13.90
0	16	-4	180.08	12.90
0	16	4	174.88	11.60
1	-16	4	137.39	10.20
-1	-16	-4	163.08	10.30
-1	16	-4	136.79	10.50
1	16	4	150.68	13.30
1	16	4	167.18	10.80
-1	16	-4	149.79	11.80
-1	16	-4	146.89	9.40
1	16	4	156.28	11.80
-2	-16	-4	46.40	5.90
2	-16	4	34.70	6.50
-2	16	-4	36.40	7.40
2	16	4	43.80	6.50
-2	16	-4	37.50	5.40
2	16	4	52.49	8.10
-2	16	-4	34.80	6.40
2	16	4	47.30	8.00

-3	-16	-4	17.20	3.90
3	-16	4	5.10	4.40
-3	16	-4	15.30	4.90
-3	16	-4	9.00	3.50
3	16	4	17.60	6.80
-3	16	-4	9.40	4.30
3	16	4	9.90	4.00
3	16	4	16.80	4.90
-4	-16	-4	60.19	10.30
-4	-16	-4	61.09	6.70
4	-16	4	45.50	8.40
4	16	4	43.60	7.00
-4	16	-4	56.09	8.30
4	16	4	51.09	10.60
-4	16	-4	46.60	7.20
-4	16	-4	51.89	6.50
-5	-16	-4	53.99	8.80
-5	-16	-4	67.19	7.30
-5	16	-4	74.49	8.80
5	16	4	60.79	7.80
-5	16	-4	65.59	8.20
-5	16	-4	60.69	7.50
5	16	4	80.39	11.50
-6	-16	-4	54.39	7.10
-6	-16	-4	65.99	11.90
-6	16	-4	38.30	8.00
6	16	4	44.10	7.30
-6	16	-4	38.00	6.10
6	16	4	50.49	9.00
-7	-16	-4	165.08	16.90
-7	16	-4	168.68	12.00
-7	16	-4	140.09	12.60
7	16	4	146.79	11.60
7	16	4	182.78	15.90
-8	-16	-4	80.39	15.50
8	16	4	67.89	10.80
-8	16	-4	76.09	10.80
-8	16	-4	83.09	9.60
8	16	4	73.29	9.30
9	16	4	87.29	9.60
-9	16	-4	68.09	12.70
9	16	4	81.79	14.80
-9	16	-4	80.99	10.30
10	16	4	8.60	6.20

-10	16	-4	10.40	6.40
10	16	4	5.20	8.80
-11	16	-4	39.40	8.50
11	16	4	35.30	7.30
-12	16	-4	17.90	7.80
-13	-16	-4	105.09	17.50
-13	16	-4	85.59	13.20
-12	17	4	11.40	7.80
-11	17	4	317.87	20.60
11	17	-4	318.37	26.30
-10	-17	4	12.00	5.50
10	17	-4	13.90	10.20
-10	17	4	22.10	6.90
-9	-17	4	240.48	13.60
9	17	-4	190.78	19.90
-9	17	4	220.68	15.90
-8	-17	4	4.50	4.70
8	-17	-4	8.60	5.90
-8	17	4	1.90	5.90
8	17	-4	2.60	8.10
7	-17	-4	364.96	22.10
-7	-17	4	371.86	18.40
-7	17	4	364.86	20.70
7	17	-4	373.96	23.80
7	17	-4	337.57	18.60
-7	17	4	332.47	23.20
-6	-17	4	50.79	5.30
6	-17	-4	65.79	10.20
-6	17	4	52.59	9.60
6	17	-4	40.50	8.50
6	17	-4	56.89	5.60
-6	17	4	51.59	8.50
-5	-17	4	573.94	27.70
5	-17	-4	555.34	30.80
5	17	-4	570.34	31.80
-5	17	4	546.65	29.60
-5	17	4	543.75	31.10
5	17	-4	553.94	27.70
-4	-17	4	14.50	3.10
-4	-17	4	7.30	7.40
-4	17	4	12.40	6.30
-4	17	4	9.60	4.80
4	17	-4	10.00	2.90
4	17	-4	12.70	6.30

-3	-17	4	285.07	20.50
-3	-17	4	335.97	17.00
-3	17	4	333.57	20.10
-3	17	4	320.67	18.70
3	17	-4	310.37	20.40
3	17	-4	326.27	16.70
-3	17	4	340.07	21.10
-2	-17	4	191.68	11.00
-2	-17	4	168.88	13.80
2	17	-4	180.18	10.40
-2	17	4	179.48	12.40
-2	17	4	183.88	14.80
2	17	-4	188.28	14.30
-1	-17	4	53.69	6.40
1	-17	-4	64.69	8.00
-1	17	4	45.00	7.80
1	17	-4	64.89	9.20
-1	17	4	66.29	9.20
1	17	-4	65.99	5.60
-1	17	4	63.09	10.70
0	-17	-4	273.57	16.00
0	-17	4	281.47	15.70
0	17	-4	263.97	16.40
0	17	-4	267.17	17.60
0	17	4	284.07	17.40
0	17	-4	280.27	14.90
0	17	4	264.57	18.30
0	17	4	280.47	16.50
-1	-17	-4	416.06	22.00
1	-17	4	410.56	22.00
1	17	4	407.96	23.40
-1	17	-4	397.96	21.20
1	17	4	412.16	24.80
1	17	4	400.16	22.30
-1	17	-4	410.36	23.60
-1	17	-4	418.46	22.70
2	-17	4	53.99	6.60
-2	-17	-4	67.89	7.00
-2	17	-4	50.69	7.30
2	17	4	47.00	8.10
2	17	4	44.80	7.10
2	17	4	40.00	8.00
-2	17	-4	58.09	6.20
-2	17	-4	52.39	7.40

3	-17	4	54.59	8.20
-3	-17	-4	67.99	6.90
-3	17	-4	56.49	7.50
3	17	4	47.80	10.00
3	17	4	66.99	8.50
-3	17	-4	44.00	6.20
3	17	4	47.80	7.40
-3	17	-4	44.80	8.30
4	-17	4	104.19	11.10
-4	-17	-4	141.89	9.30
-4	-17	-4	114.69	12.80
4	17	4	121.09	9.60
4	17	4	118.39	13.40
-4	17	-4	121.59	10.20
-4	17	-4	117.09	11.00
-4	17	-4	113.29	9.10
-5	-17	-4	65.79	10.40
-5	-17	-4	67.69	7.40
5	17	4	61.99	8.00
5	17	4	52.59	8.90
-5	17	-4	61.39	8.30
-5	17	-4	54.79	7.70
-6	-17	-4	144.49	16.20
6	17	4	150.68	15.30
-6	17	-4	165.38	11.50
6	17	4	167.58	11.60
-6	17	-4	149.29	12.40
-7	-17	-4	82.49	14.30
-7	17	-4	74.89	10.10
7	17	4	70.39	13.00
7	17	4	69.29	8.80
-8	-17	-4	55.69	10.90
8	17	4	47.80	8.90
8	17	4	46.00	9.80
-8	17	-4	36.50	7.40
-8	17	-4	67.09	9.30
-9	17	-4	19.10	6.60
9	17	4	7.20	5.60
9	17	4	11.30	9.80
-10	17	-4	133.49	12.80
10	17	4	99.59	13.50
-11	17	-4	2.80	6.50
11	17	4	6.70	6.60
12	17	4	182.98	16.20

-12	17	-4	214.28	15.80
-12	18	4	215.68	17.60
-11	18	4	11.10	6.90
-10	18	4	472.55	27.80
10	18	-4	502.35	33.00
-9	-18	4	11.60	4.40
9	18	-4	-3.40	8.80
-9	18	4	4.90	6.40
-8	18	4	320.47	20.40
8	18	-4	341.17	24.10
7	-18	-4	18.70	6.90
-7	-18	4	12.70	3.30
-7	18	4	7.50	5.80
7	18	-4	18.30	7.90
7	18	-4	4.40	3.30
-6	-18	4	411.46	20.50
6	-18	-4	409.86	24.30
6	18	-4	445.96	25.80
6	18	-4	372.36	20.60
-6	18	4	397.96	22.90
-6	18	4	381.76	25.00
-5	-18	4	62.49	5.90
5	18	-4	63.79	11.90
5	18	-4	61.59	6.10
-5	18	4	67.19	11.30
-4	-18	4	172.58	10.20
4	18	-4	164.38	10.00
-4	18	4	153.48	12.10
-4	18	4	165.18	14.20
4	18	-4	197.88	15.50
-3	-18	4	45.70	6.10
-3	18	4	67.69	11.60
-3	18	4	45.80	8.40
3	18	-4	60.09	5.50
3	18	-4	62.69	11.00
-3	18	4	51.89	10.90
-2	-18	4	110.49	8.30
2	18	-4	106.59	7.30
-2	18	4	114.19	12.90
-2	18	4	102.69	12.50
2	18	-4	112.49	12.00
-2	18	4	94.29	11.00
-1	-18	4	250.97	14.40
-1	18	4	249.58	18.20

1	18	-4	254.47	17.40
-1	18	4	250.47	16.70
1	18	-4	243.78	13.80
-1	18	4	248.38	15.70
0	-18	4	325.37	18.20
0	18	-4	328.47	20.50
0	18	4	316.67	19.90
0	18	-4	324.17	17.50
0	18	4	343.57	19.10
0	18	4	326.87	21.60
0	18	-4	316.47	19.10
1	-18	4	303.67	17.20
-1	18	-4	282.27	17.60
1	18	4	292.97	18.70
-1	18	-4	290.67	16.20
-1	18	-4	296.17	18.80
1	18	4	306.87	20.30
1	18	4	293.17	17.50
2	-18	4	189.38	13.10
-2	-18	-4	200.18	12.80
-2	18	-4	190.08	13.30
-2	18	-4	187.98	14.40
2	18	4	197.48	16.00
2	18	4	191.78	13.00
2	18	4	191.38	14.00
-2	18	-4	198.78	12.00
-3	-18	-4	238.18	14.30
3	-18	4	203.98	14.40
3	18	4	216.18	17.10
3	18	4	185.48	14.80
-3	18	-4	212.28	13.90
-3	18	-4	207.78	12.70
3	18	4	194.88	13.50
-3	18	-4	197.58	15.00
4	18	4	54.39	8.80
4	18	4	68.59	9.20
4	18	4	54.49	7.60
-4	18	-4	43.10	7.00
-4	18	-4	50.89	8.20
-5	-18	-4	197.48	17.50
-5	18	-4	208.28	13.00
5	18	4	190.98	16.70
5	18	4	181.68	13.10
-5	18	-4	203.98	14.90

-5	18	-4	193.28	14.00
-6	-18	-4	85.39	14.70
6	18	4	79.99	9.70
-6	18	-4	110.09	11.70
-6	18	-4	102.79	10.70
-6	18	-4	92.99	9.70
6	18	4	132.69	14.10
-7	-18	-4	172.18	18.80
-7	18	-4	143.09	13.30
7	18	4	150.38	12.40
7	18	4	177.58	17.20
-7	18	-4	170.38	12.50
8	18	4	39.40	10.00
-8	18	-4	33.40	6.90
8	18	4	25.20	6.40
-9	18	-4	116.59	11.80
9	18	4	96.19	11.20
9	18	4	94.79	16.40
10	18	4	-9.40	6.10
-10	18	-4	-7.40	6.60
-11	18	-4	163.38	14.90
11	18	4	143.49	14.40
12	18	4	-6.50	7.20
-12	18	-4	3.20	8.20
11	19	-4	113.79	20.50
-11	19	4	82.09	12.90
10	19	-4	24.10	12.20
-10	19	4	40.70	8.30
-9	19	4	14.00	7.20
9	19	-4	10.50	19.70
8	19	-4	9.90	8.80
-8	19	4	5.80	6.40
8	19	-4	4.20	4.50
7	-19	-4	18.70	9.10
7	19	-4	8.10	8.30
-7	19	4	24.40	6.70
7	19	-4	15.80	3.80
-6	-19	4	22.30	3.60
6	-19	-4	19.60	6.90
-6	19	4	18.20	6.30
6	19	-4	28.00	8.70
6	19	-4	19.10	3.70
-5	-19	4	69.19	6.60
-5	19	4	76.29	10.00

5	19	-4	73.99	6.50
-5	19	4	66.89	11.80
5	19	-4	71.49	13.00
-4	-19	4	0.60	3.30
4	19	-4	-4.90	6.20
-4	19	4	0.10	7.30
4	19	-4	0.80	3.00
-4	19	4	2.80	10.40
-3	-19	4	39.90	6.20
-3	19	4	32.30	8.70
-3	19	4	35.40	6.90
-3	19	4	38.90	7.70
3	19	-4	37.00	8.20
3	19	-4	30.40	5.20
-2	-19	4	45.20	6.50
2	19	-4	38.70	5.60
-2	19	4	32.60	6.30
-2	19	4	39.70	8.20
2	19	-4	28.70	7.20
-2	19	4	50.39	8.00
-1	-19	4	8.90	4.40
-1	19	4	16.70	5.90
-1	19	4	4.00	5.10
1	19	-4	15.20	5.10
-1	19	4	4.40	7.00
1	19	-4	5.50	5.60
1	19	-4	9.70	3.40
0	-19	4	34.50	5.50
0	19	4	28.00	6.20
0	19	4	38.00	8.20
0	19	-4	36.50	5.80
0	19	-4	33.80	6.20
0	19	-4	27.10	6.70
0	19	4	23.70	6.30
1	-19	4	39.70	6.00
-1	19	-4	35.90	6.30
1	19	4	15.10	7.10
1	19	4	28.00	6.90
-1	19	-4	24.70	6.40
-1	19	-4	28.40	4.60
1	19	4	26.50	5.80
2	-19	4	70.29	9.40
-2	19	-4	73.39	7.60
2	19	4	74.49	8.70

2	19	4	59.89	11.90
-2	19	-4	75.79	12.30
-2	19	-4	68.49	8.90
2	19	4	87.29	10.60
3	-19	4	3.20	7.30
-3	19	-4	5.90	4.00
3	19	4	-7.10	6.20
-3	19	-4	-3.50	4.00
3	19	4	4.80	5.40
-3	19	-4	-3.60	5.30
3	19	4	1.30	4.60
-4	19	-4	4.30	4.50
-4	19	-4	5.90	5.50
-4	19	-4	3.40	4.50
4	19	4	8.80	5.50
4	19	4	7.40	4.90
4	19	4	-0.90	6.70
-5	-19	-4	6.50	7.70
-5	19	-4	4.60	5.20
5	19	4	6.60	5.20
-5	19	-4	4.80	6.20
-5	19	-4	7.70	5.00
5	19	4	-4.20	7.30
-6	-19	-4	94.69	15.30
6	19	4	82.79	9.60
6	19	4	96.59	14.70
-6	19	-4	100.49	12.30
-6	19	-4	120.29	14.70
-6	19	-4	77.99	10.70
-7	-19	-4	44.00	11.50
7	19	4	64.29	9.40
7	19	4	66.19	11.70
-7	19	-4	64.09	9.30
-7	19	-4	60.39	11.80
-8	19	-4	38.80	7.10
8	19	4	14.70	6.20
8	19	4	19.00	9.70
-9	19	-4	0.00	5.80
9	19	4	-5.10	10.40
9	19	4	1.60	6.70
10	19	4	28.70	7.60
-10	19	-4	39.50	7.60
-11	19	-4	9.30	7.70
11	19	4	7.30	7.30

-11	20	4	19.00	7.90
-10	20	4	242.78	17.80
10	20	-4	202.48	23.50
9	20	-4	123.49	19.40
-9	20	4	140.79	13.70
8	20	-4	322.77	24.70
-8	20	4	303.87	20.20
7	20	-4	15.00	4.00
-7	20	4	10.30	6.80
7	20	-4	-1.50	12.30
-6	20	4	856.61	43.10
6	20	-4	876.61	52.79
6	20	-4	765.82	40.50
-5	-20	4	51.29	6.50
-5	20	4	46.30	8.00
5	20	-4	43.70	6.00
5	20	-4	50.39	9.60
-4	-20	4	661.83	33.10
4	20	-4	681.23	37.40
-4	20	4	659.93	37.20
4	20	-4	627.84	32.90
-4	20	4	688.93	35.30
-4	20	4	662.73	37.90
-3	-20	4	18.50	4.40
3	20	-4	14.40	3.80
-3	20	4	10.90	7.40
-3	20	4	1.60	9.60
3	20	-4	18.40	7.50
-3	20	4	11.20	6.50
-2	-20	4	611.04	30.90
-2	20	4	618.14	32.50
2	20	-4	601.24	34.20
-2	20	4	620.34	33.70
2	20	-4	579.34	30.40
-2	20	4	601.34	34.90
-1	-20	4	174.98	11.90
-1	20	4	174.18	14.70
1	20	-4	159.38	13.20
1	20	-4	175.58	10.90
1	20	-4	188.88	16.30
-1	20	4	165.68	16.10
-1	20	4	150.58	13.00
0	-20	4	648.14	33.50
0	20	4	665.53	34.30

0	20	-4	662.93	32.90
0	20	4	647.64	35.60
0	20	4	658.03	37.20
0	20	-4	617.84	34.60
1	-20	4	108.29	10.60
1	20	4	111.79	14.70
-1	20	-4	135.39	9.50
-1	20	-4	123.99	13.30
1	20	4	132.39	11.20
-1	20	-4	128.49	11.20
1	20	4	129.69	12.80
2	-20	4	206.78	14.60
2	20	4	223.98	15.90
2	20	4	218.58	18.20
-2	20	-4	207.98	13.20
2	20	4	214.18	14.50
-2	20	-4	195.18	14.70
-2	20	-4	215.58	16.60
-3	20	-4	105.09	10.80
3	20	4	106.79	14.40
-3	20	-4	108.49	9.40
-3	20	-4	109.89	12.80
3	20	4	110.59	10.30
3	20	4	93.49	12.00
-4	20	-4	500.85	28.30
-4	20	-4	479.85	25.70
-4	20	-4	457.85	26.90
4	20	4	535.95	30.40
4	20	4	434.96	27.60
4	20	4	476.75	26.10
-5	-20	-4	748.83	44.90
-5	20	-4	776.72	42.70
-5	20	-4	858.31	43.60
5	20	4	837.42	41.40
5	20	4	888.51	45.40
5	20	4	752.72	42.70
-5	20	-4	799.62	41.10
-6	-20	-4	97.69	17.00
-6	20	-4	102.59	10.10
-6	20	-4	80.69	12.90
6	20	4	88.39	10.30
6	20	4	87.29	15.50
-7	20	-4	490.25	27.50
7	20	4	480.85	27.50

-7	20	-4	509.25	30.00
7	20	4	524.55	32.20
8	20	4	3.30	10.20
-8	20	-4	9.00	6.60
-9	20	-4	458.05	25.50
9	20	4	412.46	25.10
10	20	4	0.70	6.70
-10	20	-4	7.40	7.10
-11	20	-4	464.25	27.60
11	20	4	458.15	27.00
10	21	-4	-8.00	13.40
-10	21	4	19.40	8.30
-9	21	4	8.90	7.20
9	21	-4	15.30	11.20
8	21	-4	6.30	9.90
-8	21	4	-1.40	6.60
7	21	-4	3.90	9.40
-7	21	4	5.90	7.10
6	21	-4	18.30	9.60
6	21	-4	10.80	3.90
-6	21	4	7.30	6.80
-5	21	4	125.09	12.60
5	21	-4	104.99	8.70
-5	21	4	131.89	17.20
5	21	-4	147.39	16.20
4	21	-4	-1.70	7.40
-4	21	4	-5.20	6.20
4	21	-4	-4.40	3.30
-3	-21	4	167.88	14.30
3	21	-4	215.48	17.70
-3	21	4	200.08	14.80
-3	21	4	226.28	18.60
3	21	-4	186.28	11.70
-2	-21	4	-3.60	4.30
-2	21	4	10.80	6.30
-2	21	4	0.20	7.20
-2	21	4	10.10	7.10
2	21	-4	1.40	7.00
2	21	-4	8.90	3.90
-1	-21	4	104.09	9.80
1	21	-4	108.19	11.50
-1	21	4	119.19	15.00
-1	21	4	121.59	13.10
1	21	-4	115.69	9.10

1	21	-4	114.89	14.00
-1	21	4	126.99	12.60
0	-21	4	3.90	5.10
0	21	-4	9.50	4.30
0	21	-4	16.20	7.10
0	21	4	0.10	7.80
0	21	4	5.80	5.60
0	21	-4	8.10	5.10
0	21	4	11.00	6.60
1	-21	4	159.88	13.40
1	21	4	207.38	18.20
-1	21	-4	196.78	12.20
-1	21	-4	197.78	16.30
1	21	4	171.48	15.50
1	21	4	175.68	13.70
-1	21	-4	185.68	14.00
-2	21	-4	285.87	16.70
-2	21	-4	260.07	18.00
-2	21	-4	301.67	20.20
2	21	4	268.37	19.40
2	21	4	277.57	17.50
2	21	4	303.87	21.80
-3	21	-4	21.50	5.30
3	21	4	19.70	6.80
3	21	4	5.80	8.80
-3	21	-4	5.30	7.70
3	21	4	12.60	5.50
-3	21	-4	9.10	7.10
-4	21	-4	84.99	9.20
4	21	4	65.79	9.60
-4	21	-4	75.79	12.50
4	21	4	68.99	12.00
4	21	4	97.39	15.10
-5	21	-4	16.40	5.90
5	21	4	18.60	5.90
5	21	4	11.00	7.40
5	21	4	35.30	10.80
-5	21	-4	11.90	7.40
-6	21	-4	50.29	9.10
-6	21	-4	54.39	9.20
6	21	4	38.40	7.00
6	21	4	45.70	10.80
7	21	4	1.40	9.20
-7	21	-4	6.50	7.40

-7	21	-4	7.20	6.30
8	21	4	180.78	21.90
-8	21	-4	179.18	14.40
-8	21	-4	199.68	18.10
8	21	4	153.88	14.40
-9	21	-4	5.60	7.00
9	21	4	3.40	6.50
10	21	4	37.60	8.30
-10	21	-4	78.99	12.50
-9	22	4	23.80	7.90
9	22	-4	33.30	12.70
-8	22	4	250.37	18.90
8	22	-4	297.77	25.30
7	22	-4	-5.50	9.50
-7	22	4	0.50	7.20
6	22	-4	317.07	24.30
-6	22	4	307.17	20.20
-6	22	4	285.07	24.60
5	22	-4	-9.60	8.20
-5	22	4	6.20	7.10
-5	22	4	3.00	9.00
5	22	-4	3.90	3.70
4	22	-4	291.37	22.30
-4	22	4	316.07	19.60
4	22	-4	293.07	16.40
-4	22	4	290.27	23.10
3	22	-4	12.30	4.10
3	22	-4	11.50	7.90
-3	22	4	8.90	8.70
-3	22	4	1.10	6.30
-2	-22	4	134.89	11.10
-2	22	4	138.29	16.60
2	22	-4	138.19	9.80
-2	22	4	129.19	12.60
2	22	-4	114.09	15.30
-1	-22	4	136.39	11.30
1	22	-4	145.09	15.30
-1	22	4	125.29	16.90
1	22	-4	134.29	10.10
-1	22	4	128.39	12.60
0	-22	4	472.35	26.60
0	22	-4	488.85	25.70
0	22	-4	466.75	29.40
0	22	4	519.85	31.20

0	22	4	496.15	28.70
-1	22	-4	197.58	12.80
1	22	4	167.58	18.20
1	22	4	183.48	19.00
-1	22	-4	192.78	17.20
2	22	4	65.99	11.70
2	22	4	66.89	9.80
-2	22	-4	83.39	9.00
-2	22	-4	74.29	13.00
3	22	4	196.88	14.00
3	22	4	179.58	19.60
-3	22	-4	184.58	13.30
-3	22	-4	197.28	17.20
4	22	4	68.59	9.90
4	22	4	57.49	11.50
-4	22	-4	55.89	9.40
-5	22	-4	165.58	13.40
5	22	4	177.48	19.70
-5	22	-4	202.98	17.50
-6	22	-4	99.99	11.30
-6	22	-4	104.59	14.90
6	22	4	92.89	17.50
6	22	4	79.19	11.00
-7	22	-4	155.38	13.50
7	22	4	152.98	13.50
-7	22	-4	153.68	17.20
7	22	4	143.99	19.90
-8	22	-4	7.80	6.70
8	22	4	2.10	6.40
-8	22	-4	3.70	8.40
-9	22	-4	117.39	18.10
9	22	4	125.09	13.20
-9	22	-4	113.29	13.50
-8	23	4	-2.70	7.00
8	23	-4	16.60	11.60
-7	23	4	368.46	24.20
7	23	-4	419.06	29.30
-7	23	4	365.46	29.90
-6	23	4	-0.40	9.90
-6	23	4	-3.50	7.10
6	23	-4	6.80	10.50
-5	23	4	307.47	25.60
-5	23	4	293.87	20.30
5	23	-4	303.07	24.00

-4	23	4	29.30	8.20
-4	23	4	16.70	10.80
4	23	-4	24.10	4.70
4	23	-4	22.50	9.70
-3	23	4	230.58	21.70
-3	23	4	233.48	17.00
3	23	-4	207.78	13.50
3	23	-4	251.07	20.50
2	23	-4	75.99	8.10
-2	23	4	85.29	11.70
-2	23	4	79.09	12.60
2	23	-4	85.29	15.00
1	23	-4	236.98	14.60
1	23	-4	229.28	19.60
-1	23	4	248.28	17.00
0	23	-4	163.28	11.90
0	23	4	181.18	19.00
0	23	-4	162.18	17.00
0	23	4	168.48	14.10
1	23	4	223.78	16.50
1	23	4	254.97	21.60
-1	23	-4	243.68	19.90
-1	23	-4	233.98	15.10
2	23	4	47.40	10.70
2	23	4	57.19	8.30
-2	23	-4	64.69	10.50
-2	23	-4	56.19	7.20
-3	23	-4	48.10	10.00
3	23	4	23.60	10.60
-3	23	-4	47.10	7.20
3	23	4	41.70	7.40
4	23	4	310.07	25.40
-4	23	-4	312.27	19.00
-4	23	-4	317.97	23.20
4	23	4	309.47	19.40
-5	23	-4	188.78	17.90
5	23	4	176.68	21.10
5	23	4	145.99	13.70
-5	23	-4	169.68	13.30
-6	23	-4	226.68	17.10
-6	23	-4	253.17	20.90
6	23	4	247.48	17.10
6	23	4	255.37	24.20
-7	23	-4	6.30	8.30

-7	23	-4	3.70	6.50
7	23	4	5.90	6.80
-8	23	-4	170.38	19.50
-8	23	-4	167.98	14.90
8	23	4	164.28	14.70
-7	24	4	9.70	13.20
7	24	-4	17.60	11.80
-7	24	4	8.50	8.20
-6	24	4	14.50	13.20
-6	24	4	22.70	8.30
6	24	-4	29.20	12.30
-5	24	4	6.90	10.40
5	24	-4	-3.30	9.60
-5	24	4	0.50	7.80
4	24	-4	83.19	17.00
-4	24	4	73.79	12.30
-4	24	4	76.39	14.60
-3	24	4	-5.30	7.20
-3	24	4	-9.70	10.50
3	24	-4	4.30	4.40
3	24	-4	-3.20	8.80
-2	24	4	-8.20	9.00
2	24	-4	2.10	4.40
-2	24	4	0.30	6.70
2	24	-4	-10.60	9.10
-1	24	4	3.30	9.50
-1	24	4	-0.80	6.70
1	24	-4	-8.30	7.70
1	24	-4	-0.20	4.50
0	24	4	11.40	9.90
0	24	4	19.00	7.10
0	24	-4	15.40	8.50
0	24	-4	20.70	5.50
1	24	4	13.10	6.60
-1	24	-4	8.80	9.00
-1	24	-4	7.50	5.40
1	24	4	-5.80	12.10
-2	24	-4	17.40	6.00
-2	24	-4	6.60	8.30
2	24	4	35.30	7.40
3	24	4	-1.10	9.70
-3	24	-4	-6.40	8.60
3	24	4	0.70	6.00
-3	24	-4	-1.60	5.50

-4	24	-4	-0.70	5.80
-4	24	-4	-11.70	7.80
4	24	4	-3.60	10.00
4	24	4	3.70	6.10
-5	24	-4	23.10	7.10
5	24	4	20.30	7.00
-5	24	-4	11.40	8.80
5	24	4	36.20	11.80
-6	24	-4	98.49	16.10
6	24	4	35.80	12.90
-6	24	-4	74.29	11.50
6	24	4	62.09	11.20
-7	24	-4	-11.00	9.70
7	24	4	8.60	7.00
-7	24	-4	0.60	6.90
6	25	-4	45.10	13.30
-6	25	4	36.50	13.10
-6	25	4	31.40	9.10
5	25	-4	297.67	25.90
-5	25	4	287.27	20.70
-5	25	4	277.67	26.90
-4	25	4	3.80	7.40
-4	25	4	-1.40	10.60
4	25	-4	-9.90	9.80
-3	25	4	207.28	22.90
-3	25	4	178.38	16.20
3	25	-4	184.38	20.30
-2	25	4	26.40	8.40
-2	25	4	31.80	12.20
2	25	-4	38.50	6.10
2	25	-4	27.60	10.70
1	25	-4	269.77	22.80
-1	25	4	274.77	24.90
1	25	-4	276.27	16.80
-1	25	4	287.37	19.80
0	25	-4	199.78	13.80
0	25	4	173.08	21.90
0	25	4	205.28	16.00
0	25	-4	199.98	20.10
-1	25	-4	218.08	15.20
-1	25	-4	217.08	20.60
1	25	4	225.88	22.90
1	25	4	222.68	16.90
-2	25	-4	38.20	7.10

-2	25	-4	41.60	11.10
2	25	4	38.30	8.30
2	25	4	36.80	13.40
-3	25	-4	157.18	13.70
3	25	4	169.08	21.70
-3	25	-4	162.08	18.50
3	25	4	172.48	14.90
4	25	4	283.47	20.40
4	25	4	328.97	27.20
-4	25	-4	315.77	20.00
-4	25	-4	343.07	24.90
5	25	4	139.49	14.50
-5	25	-4	171.08	19.50
5	25	4	168.38	23.10
-5	25	-4	183.38	14.80
-6	25	-4	299.77	20.20
6	25	4	322.97	20.40
-6	25	-4	304.47	25.10
-5	26	4	-3.10	8.20
5	26	-4	-9.30	10.60
-5	26	4	-15.50	13.50
4	26	-4	89.09	14.80
-4	26	4	78.49	13.80
-4	26	4	56.79	14.10
3	26	-4	-1.50	10.50
-3	26	4	1.60	12.20
-3	26	4	-10.40	7.10
2	26	-4	46.10	11.40
-2	26	4	53.09	10.00
-2	26	4	61.69	15.10
-1	26	4	17.90	8.30
1	26	-4	10.90	9.80
-1	26	4	18.50	12.80
0	26	-4	130.79	18.30
0	26	4	88.09	15.10
0	26	4	93.29	13.60
0	26	-4	106.39	11.00
1	26	4	73.79	12.10
-1	26	-4	54.49	9.70
1	26	4	55.79	13.50
-1	26	-4	46.30	11.60
2	26	4	24.80	8.00
-2	26	-4	12.70	9.90
-2	26	-4	33.00	7.10

2	26	4	4.20	12.60
3	26	4	93.49	13.00
-3	26	-4	93.29	17.40
3	26	4	82.39	14.90
-3	26	-4	87.19	11.60
-4	26	-4	-0.30	6.80
4	26	4	7.30	12.30
4	26	4	0.00	7.10
-4	26	-4	13.70	9.90
-5	26	-4	5.80	10.60
-5	26	-4	16.20	7.50
5	26	4	2.20	7.30
-3	27	4	126.89	23.90
3	27	-4	103.09	20.30
-3	27	4	123.89	15.50
-2	27	4	40.30	9.70
-2	27	4	21.20	14.20
2	27	-4	34.80	11.40
1	27	-4	20.40	10.90
-1	27	4	43.00	9.10
-1	27	4	33.70	14.40
0	27	-4	44.80	11.90
0	27	4	72.69	13.00
0	27	4	59.29	15.50
-1	27	-4	236.38	23.50
-1	27	-4	240.08	17.20
1	27	4	327.57	28.40
1	27	4	252.47	19.20
2	27	4	-13.30	12.20
-2	27	-4	4.50	6.60
-2	27	-4	-6.40	10.30
2	27	4	-4.90	7.30
3	27	4	-2.40	12.60
3	27	4	-7.60	7.10
-3	27	-4	11.30	7.00
-3	27	-4	9.90	10.00
-16	0	5	17.90	8.20
-15	0	5	14.20	6.90
-14	0	5	5.90	6.00
-13	0	5	11.10	5.60
13	0	-5	-1.90	7.80
-12	0	5	4.10	9.50
12	0	-5	1.60	6.90
-12	0	5	1.30	4.70

11	0	-5	6.30	6.10
-11	0	5	2.80	7.60
11	0	-5	10.30	6.60
-11	0	5	11.80	4.30
-10	0	5	-1.30	3.50
10	0	-5	3.20	4.60
-10	0	5	-0.30	6.00
10	0	-5	-6.50	4.00
-9	0	5	5.90	3.20
9	0	-5	1.50	4.30
9	0	-5	10.20	3.80
-9	0	5	5.40	6.20
8	0	-5	-5.60	3.60
8	0	-5	-0.60	2.90
-8	0	5	-1.30	2.60
-7	0	5	-1.40	4.00
7	0	-5	3.60	2.60
-7	0	5	5.50	2.40
7	0	-5	7.50	4.30
6	0	-5	-0.30	2.10
-6	0	5	-0.50	3.30
-6	0	5	1.50	2.00
6	0	-5	-2.10	3.10
-5	0	5	-1.10	3.40
5	0	-5	6.60	3.50
-5	0	5	6.50	2.00
5	0	-5	7.00	2.20
-4	0	5	1.30	1.40
4	0	-5	-0.40	2.60
-4	0	5	0.40	2.40
4	0	-5	-1.00	1.30
-3	0	5	5.80	1.40
3	0	-5	4.00	1.20
3	0	-5	0.80	2.20
-3	0	5	0.70	1.90
-2	0	5	9.10	1.90
2	0	-5	3.10	1.00
-2	0	5	0.20	1.00
2	0	-5	-0.20	1.30
1	0	-5	0.70	1.10
-1	0	5	2.00	1.20
-1	0	5	1.70	0.90
1	0	-5	1.10	0.80
0	0	5	1.30	0.90

0	0	-5	2.10	0.90
0	0	5	1.60	0.90
0	0	-5	1.50	0.90
1	0	5	1.30	0.60
1	0	5	1.10	0.80
1	0	5	1.30	1.10
-1	0	-5	0.70	1.00
-2	0	-5	-0.20	1.10
2	0	5	1.60	1.40
2	0	5	0.80	0.60
-2	0	-5	1.80	0.90
2	0	5	0.40	0.80
3	0	5	2.20	1.10
3	0	5	2.30	1.60
-3	0	-5	2.60	1.20
-3	0	-5	1.30	1.70
-4	0	-5	3.80	1.70
-4	0	-5	4.80	2.10
4	0	5	2.60	2.00
4	0	5	1.30	1.50
-5	0	-5	0.10	2.00
5	0	5	0.40	2.00
5	0	5	0.70	1.80
-5	0	-5	-0.20	2.30
-6	0	-5	3.80	2.80
-6	0	-5	7.10	2.90
6	0	5	-1.40	2.20
-6	0	-5	-0.30	2.50
7	0	5	-2.70	2.40
-7	0	-5	-0.70	2.80
-7	0	-5	1.70	3.20
-7	0	-5	-3.00	2.80
8	0	5	3.10	3.10
-8	0	-5	4.40	3.40
-8	0	-5	2.30	3.80
-8	0	-5	9.90	4.00
-9	0	-5	1.60	4.40
-9	0	-5	0.10	3.60
-9	0	-5	1.60	4.10
9	0	5	-1.80	3.30
10	0	5	-1.70	4.30
-10	0	-5	10.10	4.90
-10	0	-5	1.40	5.10
11	0	5	-3.60	4.90

-11	0	-5	2.60	6.00
-11	0	-5	6.00	5.00
12	0	5	3.90	5.40
-12	0	-5	4.50	5.60
-12	0	-5	-3.10	5.80
-13	0	-5	3.60	6.30
-14	0	-5	11.80	7.40
-15	0	-5	8.50	8.40
-16	0	-5	12.70	9.40
-16	-1	5	142.09	14.70
-16	1	5	141.99	15.20
-15	-1	5	210.28	16.30
-15	1	5	222.48	16.30
-14	-1	5	39.30	7.10
-14	1	5	63.19	10.10
13	-1	-5	221.28	18.00
-13	-1	5	223.88	14.40
13	1	-5	219.68	17.70
-13	1	5	193.28	17.00
12	-1	-5	8.20	6.50
-12	-1	5	-2.00	8.90
-12	-1	5	8.50	4.90
12	1	-5	8.20	6.70
-12	1	5	7.30	5.00
-12	1	5	10.30	8.90
11	-1	-5	724.83	38.60
-11	-1	5	726.93	36.40
-11	-1	5	670.73	40.00
-11	-1	5	773.22	37.60
-11	1	5	746.33	36.60
-11	1	5	663.63	40.00
11	1	-5	707.83	38.60
-10	-1	5	59.49	10.90
-10	-1	5	40.30	6.10
10	-1	-5	50.69	7.80
10	-1	-5	46.20	8.40
-10	1	5	61.09	11.30
10	1	-5	41.60	7.20
-10	1	5	52.19	6.30
9	-1	-5	624.74	32.30
9	-1	-5	665.13	33.70
-9	-1	5	601.34	34.60
-9	-1	5	637.24	31.60
-9	1	5	560.34	35.90

9	1	-5	650.83	33.70
9	1	-5	650.43	32.10
-9	1	5	633.74	31.70
8	-1	-5	-0.50	3.90
-8	-1	5	3.70	2.60
-8	-1	5	2.70	5.20
8	-1	-5	4.10	3.40
-8	1	5	2.40	2.80
-8	1	5	-3.60	4.40
8	1	-5	-2.40	3.80
8	1	-5	-0.70	3.00
-7	-1	5	1246.78	59.49
-7	-1	5	1054.49	62.29
7	-1	-5	1275.37	61.09
7	-1	-5	1256.57	59.99
7	1	-5	1274.27	61.19
-7	1	5	1214.98	61.59
7	1	-5	1278.97	59.79
-7	1	5	1248.18	59.59
6	-1	-5	5.70	2.50
6	-1	-5	4.80	3.30
-6	-1	5	2.70	1.90
-6	-1	5	4.50	3.60
6	1	-5	5.20	3.50
-6	1	5	5.30	3.60
-6	1	5	4.60	2.20
6	1	-5	6.40	2.20
5	-1	-5	2112.69	99.09
-5	-1	5	1948.31	99.09
5	-1	-5	2016.60	100.89
-5	-1	5	2100.39	100.39
-5	1	5	2008.30	98.89
-5	1	5	2136.69	100.29
5	1	-5	2149.28	100.09
5	1	-5	2101.39	98.89
-4	-1	5	9.70	1.60
-4	-1	5	10.40	2.90
4	-1	-5	9.20	1.90
4	1	-5	9.00	1.70
4	1	-5	6.90	2.70
-4	1	5	9.20	2.70
-4	1	5	5.20	2.00
3	-1	-5	3056.69	143.09
3	-1	-5	3075.09	143.79

-3	-1	5	2906.71	142.89
-3	-1	5	3107.79	143.99
-3	1	5	3132.59	143.99
3	1	-5	2932.61	144.49
3	1	-5	2944.01	142.89
-3	1	5	2943.71	143.09
2	-1	-5	2022.20	92.99
-2	-1	5	2047.80	93.69
2	-1	-5	2043.30	93.59
-2	-1	5	1856.11	92.89
-2	1	5	1785.52	93.09
2	1	-5	1818.92	93.89
2	1	-5	1977.10	92.79
-2	1	5	2089.89	93.69
-1	-1	5	1192.38	55.29
-1	-1	5	1083.59	54.79
1	-1	-5	1137.69	55.19
1	-1	-5	1167.28	54.89
-1	1	5	1180.68	55.29
-1	1	5	1159.08	55.09
1	1	-5	1096.19	54.69
1	1	-5	1189.98	55.19
0	-1	5	1084.29	50.99
0	-1	-5	1071.99	50.99
0	-1	-5	1071.49	51.09
0	-1	5	1029.20	50.89
0	1	-5	1060.49	50.89
0	1	-5	1088.69	50.89
0	1	5	1062.79	51.09
0	1	5	1065.89	51.09
-1	-1	-5	3053.89	142.29
-1	-1	-5	3051.39	142.39
1	-1	5	2972.30	142.39
-1	1	-5	3099.19	142.39
1	1	5	3005.50	142.29
1	1	5	2847.22	142.39
1	1	5	2960.20	142.19
2	-1	5	952.00	46.70
-2	-1	-5	962.60	46.60
-2	-1	-5	1015.90	46.80
2	1	5	992.60	46.60
-2	1	-5	1005.50	46.70
2	1	5	967.90	46.40
-2	1	-5	970.40	46.20

2	1	5	900.71	46.90
-3	-1	-5	340.07	17.60
3	-1	5	374.56	17.10
3	-1	5	322.77	17.70
3	1	5	329.47	17.70
-3	1	-5	359.66	17.90
-3	1	-5	354.16	17.20
3	1	5	374.36	17.50
4	-1	5	1731.83	84.69
-4	-1	-5	1712.33	84.59
4	-1	5	1741.23	84.19
-4	-1	-5	1901.71	84.79
-4	1	-5	1944.81	84.89
4	1	5	1738.23	84.49
4	1	5	1704.03	84.69
-4	1	-5	1655.53	84.29
5	-1	5	282.97	14.30
-5	-1	-5	276.67	14.70
-5	-1	-5	302.27	14.90
-5	1	-5	281.77	14.70
5	1	5	253.47	14.60
-5	1	-5	253.47	14.60
-5	1	-5	258.27	14.40
5	1	5	281.47	14.60
-6	-1	-5	1443.66	64.69
6	-1	5	1328.57	64.19
-6	-1	-5	1260.87	64.49
-6	1	-5	1273.77	64.39
6	1	5	1288.57	64.39
6	1	5	1329.27	64.39
-6	1	-5	1221.08	64.29
-6	1	-5	1474.15	64.79
-7	-1	-5	38.90	5.60
7	-1	5	23.10	4.70
-7	-1	-5	36.40	5.70
-7	-1	-5	34.90	5.40
-7	1	-5	23.90	4.10
-7	1	-5	22.60	4.30
7	1	5	34.00	5.20
-7	1	-5	14.90	3.80
-8	-1	-5	979.20	53.59
8	-1	5	1070.89	53.09
-8	-1	-5	1081.99	53.39
-8	-1	-5	1205.48	53.79

-8	1	-5	1200.08	53.89
8	1	5	1067.19	53.39
-8	1	-5	1001.40	53.49
-8	1	-5	1037.80	53.29
-9	-1	-5	2.10	4.30
9	-1	5	2.70	3.30
-9	-1	-5	5.80	4.10
-9	-1	-5	-1.50	3.80
-9	1	-5	-2.10	4.00
9	1	5	-1.40	3.20
-9	1	-5	-7.40	3.90
-9	1	-5	6.30	4.10
-10	-1	-5	911.41	44.00
-10	-1	-5	824.12	44.70
10	-1	5	888.41	43.70
-10	1	-5	883.71	43.90
-10	1	-5	790.72	44.30
10	1	5	878.21	44.00
-11	-1	-5	30.50	7.70
11	-1	5	32.10	5.90
-11	-1	-5	52.59	8.70
11	1	5	36.30	6.70
-11	1	-5	39.40	7.50
-11	1	-5	41.20	6.90
12	-1	5	344.47	20.60
-12	-1	-5	354.26	20.90
-12	-1	-5	322.07	22.30
-12	1	-5	357.46	20.80
-12	1	-5	312.57	21.90
12	1	5	353.86	21.10
-13	-1	-5	-0.90	5.80
-13	1	-5	9.70	6.70
-14	-1	-5	233.98	18.30
-14	1	-5	266.37	18.30
-15	-1	-5	198.88	17.30
-15	1	-5	190.48	17.20
-16	-1	-5	103.79	15.60
-16	1	-5	151.58	16.60
-16	-2	5	69.49	13.30
-16	2	5	90.49	13.20
-15	-2	5	21.10	7.60
-15	2	5	20.40	7.50
-14	-2	5	23.40	6.70
-14	2	5	36.70	7.30

13	-2	-5	38.80	8.10
-13	-2	5	36.60	6.40
-13	2	5	46.60	8.60
13	2	-5	35.90	8.30
12	-2	-5	359.56	22.50
-12	-2	5	353.06	19.80
-12	-2	5	319.77	25.00
-12	2	5	381.56	20.10
12	2	-5	360.46	23.10
-12	2	5	307.07	24.80
-11	-2	5	9.60	7.80
11	-2	-5	7.20	5.90
-11	-2	5	12.50	4.30
-11	-2	5	5.80	5.50
11	2	-5	10.90	6.20
-11	2	5	9.60	7.30
-11	2	5	12.10	4.50
-10	-2	5	277.57	16.50
10	-2	-5	273.87	16.60
-10	-2	5	263.17	15.40
-10	-2	5	226.18	18.80
10	-2	-5	291.27	17.70
-10	2	5	288.57	15.70
10	2	-5	258.57	17.90
-10	2	5	278.37	19.50
9	-2	-5	10.70	5.10
-9	-2	5	14.30	3.50
9	-2	-5	8.30	4.20
-9	-2	5	7.80	4.20
-9	-2	5	5.30	5.60
-9	2	5	10.40	3.60
9	2	-5	11.30	5.30
-9	2	5	0.70	6.60
-8	-2	5	423.16	22.30
8	-2	-5	443.16	23.20
-8	-2	5	412.36	25.10
8	-2	-5	460.55	24.40
8	2	-5	456.05	24.60
-8	2	5	422.06	25.10
8	2	-5	444.46	22.80
-8	2	5	454.35	22.70
7	-2	-5	5.50	3.10
-7	-2	5	3.30	2.40
-7	-2	5	4.10	5.30

7	-2	-5	2.50	3.90
-7	2	5	8.60	2.70
-7	2	5	6.80	4.30
7	2	-5	3.30	2.60
7	2	-5	1.60	3.70
6	-2	-5	399.36	22.60
-6	-2	5	359.36	21.90
6	-2	-5	415.96	20.40
-6	-2	5	405.06	19.80
6	2	-5	416.36	21.60
-6	2	5	407.76	20.10
-6	2	5	423.26	22.00
6	2	-5	323.07	21.50
-5	-2	5	106.79	9.10
-5	-2	5	126.49	6.90
5	-2	-5	130.59	7.70
-5	2	5	119.89	8.90
5	2	-5	106.99	8.70
5	2	-5	134.59	7.10
-5	2	5	125.69	7.30
4	-2	-5	708.83	38.50
4	-2	-5	786.62	38.90
-4	-2	5	798.22	39.20
-4	-2	5	786.02	37.60
-4	2	5	797.82	39.10
-4	2	5	785.92	38.00
4	2	-5	806.52	39.00
4	2	-5	796.62	37.70
3	-2	-5	142.99	7.60
-3	-2	5	123.59	8.40
-3	-2	5	138.59	7.00
3	-2	-5	147.39	8.30
-3	2	5	128.59	8.00
-3	2	5	138.99	8.40
3	2	-5	125.59	8.30
3	2	-5	144.79	7.00
2	-2	-5	60.89	4.30
2	-2	-5	54.59	3.60
-2	-2	5	53.39	3.10
-2	-2	5	53.49	4.30
2	2	-5	55.39	4.50
2	2	-5	56.39	3.00
-2	2	5	51.59	3.80
-2	2	5	58.39	4.30

-1	-2	5	174.28	9.50
-1	-2	5	172.48	8.90
1	-2	-5	179.48	9.30
1	-2	-5	188.08	9.60
-1	2	5	181.58	9.50
1	2	-5	185.48	8.90
-1	2	5	189.08	9.70
0	-2	-5	1356.86	65.29
0	-2	-5	1337.87	65.19
0	-2	5	1363.26	64.99
0	-2	5	1348.67	64.99
0	2	5	1365.36	65.29
0	2	-5	1394.36	65.09
0	2	5	1382.06	65.29
0	2	-5	1373.86	64.89
1	-2	5	368.96	18.40
-1	-2	-5	386.96	18.50
-1	2	-5	377.36	18.40
1	2	5	379.16	18.60
1	2	5	368.16	18.40
1	2	5	363.26	18.60
-1	2	-5	384.86	18.00
2	-2	5	487.85	24.80
-2	-2	-5	509.65	24.90
-2	-2	-5	504.45	24.80
-2	2	-5	521.25	24.80
2	2	5	492.75	24.60
2	2	5	533.15	24.90
-2	2	-5	493.95	24.10
3	-2	5	42.70	3.70
-3	-2	-5	46.30	3.80
-3	-2	-5	51.29	3.80
3	-2	5	43.10	2.40
-3	2	-5	36.90	3.60
-3	2	-5	38.80	2.80
3	2	5	47.80	3.90
-3	2	-5	43.80	4.00
3	2	5	38.80	3.60
4	-2	5	110.99	5.90
-4	-2	-5	113.89	6.80
-4	-2	-5	95.19	6.80
4	2	5	103.89	6.80
-4	2	-5	105.39	6.70
-4	2	-5	106.69	6.20

-4	2	-5	115.09	6.90
4	2	5	104.69	6.70
5	-2	5	828.12	39.20
-5	-2	-5	887.91	39.90
-5	-2	-5	770.82	39.90
-5	2	-5	772.12	39.70
5	2	5	768.82	39.70
-5	2	-5	856.91	40.00
-5	2	-5	766.52	39.50
5	2	5	817.92	39.80
-6	-2	-5	94.79	7.50
-6	-2	-5	101.39	7.10
6	-2	5	94.39	6.50
-6	2	-5	102.59	7.60
6	2	5	85.79	6.90
6	2	5	100.59	7.20
-6	2	-5	94.19	7.10
-6	2	-5	85.39	6.90
-7	-2	-5	656.53	34.90
-7	-2	-5	759.02	34.90
7	-2	5	699.93	34.20
-7	-2	-5	671.73	34.60
7	2	5	642.24	34.40
-7	2	-5	666.43	34.60
7	2	5	700.63	34.70
-7	2	-5	721.13	34.90
-7	2	-5	659.23	34.60
-8	-2	-5	36.80	6.10
-8	-2	-5	34.80	5.40
8	-2	5	31.10	5.20
-8	-2	-5	36.80	6.50
-8	2	-5	26.50	4.60
-8	2	-5	45.10	6.70
-8	2	-5	32.00	5.90
8	2	5	22.90	4.30
-9	-2	-5	280.07	16.30
-9	-2	-5	286.17	16.40
-9	-2	-5	238.18	16.80
9	-2	5	278.87	15.80
-9	2	-5	254.67	16.60
-9	2	-5	278.37	16.30
9	2	5	277.67	16.50
-9	2	-5	306.07	18.30
10	-2	5	10.30	4.60

-10	-2	-5	30.70	5.60
-10	-2	-5	13.70	5.80
-10	2	-5	26.80	5.80
-10	2	-5	13.80	5.70
10	2	5	18.40	5.30
-11	-2	-5	361.66	20.80
-11	-2	-5	331.77	22.10
11	-2	5	351.56	20.30
-11	2	-5	374.56	20.80
11	2	5	345.17	21.10
-11	2	-5	336.77	21.60
12	-2	5	-4.70	4.90
-12	-2	-5	7.00	6.10
-12	-2	-5	19.90	6.90
-12	2	-5	-0.50	5.40
-12	2	-5	5.00	6.00
12	2	5	8.80	5.90
-13	-2	-5	171.58	14.10
-13	2	-5	163.78	13.40
-14	-2	-5	86.39	12.30
-14	2	-5	48.90	9.00
-15	-2	-5	8.90	8.00
-15	2	-5	39.60	9.10
-15	-3	5	15.70	7.20
-15	3	5	22.60	7.40
-14	-3	5	78.99	10.40
-14	3	5	69.79	11.00
-13	-3	5	125.49	10.80
13	-3	-5	125.49	13.80
-13	3	5	125.29	11.40
13	3	-5	91.49	14.10
12	-3	-5	21.10	6.90
-12	-3	5	13.80	5.00
-12	-3	5	11.20	10.10
12	3	-5	12.60	7.50
-12	3	5	13.60	8.60
11	-3	-5	394.06	23.00
-11	-3	5	366.26	20.20
-11	-3	5	390.96	21.50
-11	-3	5	347.97	24.80
11	3	-5	389.46	23.20
-11	3	5	368.66	20.70
-11	3	5	323.67	24.60
-10	-3	5	4.20	4.90

10	-3	-5	25.00	5.40
-10	-3	5	13.20	4.00
10	-3	-5	24.10	6.40
-10	-3	5	8.90	6.90
-10	3	5	18.50	10.00
10	3	-5	16.40	6.10
-9	-3	5	83.09	8.00
-9	-3	5	84.09	6.90
-9	-3	5	70.09	11.20
9	-3	-5	91.19	9.60
9	-3	-5	93.89	8.20
-9	3	5	70.89	10.50
9	3	-5	82.49	10.00
-9	3	5	84.39	7.30
-8	-3	5	0.50	4.70
8	-3	-5	11.30	4.30
-8	-3	5	2.20	3.20
-8	-3	5	5.50	2.60
8	-3	-5	8.50	3.70
-8	3	5	9.40	3.10
-8	3	5	4.60	4.80
8	3	-5	3.80	4.50
-7	-3	5	384.06	19.50
-7	-3	5	354.46	21.80
7	-3	-5	380.46	22.00
7	-3	-5	388.96	20.40
-7	3	5	398.56	20.00
7	3	-5	396.46	21.70
-7	3	5	391.26	22.00
6	-3	-5	144.19	8.90
-6	-3	5	111.29	11.30
-6	-3	5	137.49	7.80
6	3	-5	128.39	10.20
-6	3	5	136.69	8.20
-6	3	5	144.99	10.40
6	3	-5	138.69	8.00
5	-3	-5	284.77	14.90
-5	-3	5	250.67	15.80
-5	-3	5	278.57	13.90
5	-3	-5	271.67	15.60
-5	3	5	290.07	16.00
5	3	-5	294.47	16.00
-5	3	5	272.47	14.50
5	3	-5	279.47	14.10

4	-3	-5	7.70	2.20
-4	-3	5	4.30	2.70
-4	-3	5	9.40	1.80
4	-3	-5	7.70	2.50
4	3	-5	12.00	1.90
4	3	-5	5.90	3.20
-4	3	5	2.00	1.80
-4	3	5	6.10	2.60
3	-3	-5	639.04	31.00
3	-3	-5	650.83	30.50
-3	-3	5	622.54	31.20
-3	-3	5	585.84	29.80
3	3	-5	628.44	31.10
-3	3	5	603.74	30.50
3	3	-5	608.54	29.80
-3	3	5	648.24	31.20
2	-3	-5	5.60	1.40
-2	-3	5	5.50	1.70
2	-3	-5	8.80	1.90
2	3	-5	7.40	0.90
-2	3	5	6.80	1.90
2	3	-5	3.50	1.70
-2	3	5	1.40	1.80
-1	-3	5	138.69	8.10
1	-3	-5	159.18	8.40
1	-3	-5	152.78	8.00
-1	3	5	148.09	8.30
1	3	-5	151.38	8.30
-1	3	5	159.08	8.40
1	3	-5	151.08	7.40
0	-3	5	719.43	34.60
0	-3	5	711.43	34.70
0	-3	-5	732.93	34.90
0	-3	-5	725.93	35.20
0	3	5	740.43	35.10
0	3	5	721.63	35.10
0	3	-5	711.73	34.60
-1	-3	-5	12.90	2.10
-1	-3	-5	13.90	2.50
1	-3	5	15.90	1.90
-1	3	-5	13.10	1.90
1	3	5	14.10	2.30
1	3	5	17.80	2.10
1	3	5	10.50	2.40

-1	3	-5	14.10	1.50
-2	-3	-5	30.90	2.90
-2	-3	-5	30.40	3.10
-2	3	-5	27.60	2.70
2	3	5	26.70	2.90
2	3	5	29.50	2.70
-2	3	-5	26.60	1.60
2	3	5	29.70	3.20
-2	3	-5	28.40	2.80
3	-3	5	240.48	11.50
-3	-3	-5	227.68	12.70
-3	-3	-5	250.77	12.60
-3	3	-5	239.68	12.60
-3	3	-5	240.48	11.80
3	3	5	230.18	12.50
3	3	5	243.18	12.70
4	-3	5	32.20	2.50
-4	-3	-5	33.50	3.50
-4	-3	-5	31.50	4.10
-4	3	-5	39.20	4.10
-4	3	-5	27.60	3.60
4	3	5	36.80	4.00
-4	3	-5	34.40	3.20
4	3	5	37.10	3.90
5	-3	5	155.68	8.60
-5	-3	-5	170.88	9.50
-5	-3	-5	165.98	10.00
-5	3	-5	147.19	9.40
-5	3	-5	158.58	9.20
5	3	5	157.88	9.50
5	3	5	163.38	9.80
-5	3	-5	165.18	9.70
-6	-3	-5	1291.57	67.59
-6	-3	-5	1554.04	67.49
6	-3	5	1373.76	66.69
6	3	5	1412.36	67.59
-6	3	-5	1290.77	67.19
6	3	5	1301.47	67.19
-6	3	-5	1551.44	67.69
-6	3	-5	1321.17	67.09
-7	-3	-5	32.50	5.00
7	-3	5	24.70	4.20
-7	-3	-5	33.60	6.00
-7	3	-5	19.90	4.30

-7	3	-5	34.00	5.40
-7	3	-5	23.30	4.30
7	3	5	30.80	4.80
7	3	5	37.00	5.60
-8	-3	-5	41.60	7.10
-8	-3	-5	48.00	6.40
-8	-3	-5	52.29	6.80
8	-3	5	48.50	5.50
-8	3	-5	52.09	6.50
8	3	5	53.39	6.80
-8	3	-5	38.30	6.70
-8	3	-5	42.80	6.30
9	-3	5	10.70	3.60
-9	-3	-5	9.60	4.20
-9	-3	-5	9.90	4.60
-9	-3	-5	9.80	4.40
9	3	5	8.80	4.50
-9	3	-5	7.10	3.90
-9	3	-5	5.30	4.40
-10	-3	-5	36.10	6.00
10	-3	5	29.90	5.20
-10	-3	-5	37.60	6.90
-10	3	-5	26.90	6.20
-10	3	-5	30.40	6.10
10	3	5	48.80	8.10
-11	-3	-5	18.30	6.60
-11	-3	-5	23.10	5.70
11	-3	5	23.80	5.40
-11	3	-5	13.90	6.50
11	3	5	13.60	5.50
-11	3	-5	24.10	6.10
-12	-3	-5	279.27	20.50
-12	-3	-5	311.07	18.60
12	-3	5	274.57	17.80
12	3	5	285.07	19.10
-12	3	-5	273.07	19.60
-12	3	-5	300.87	18.60
-13	-3	-5	19.60	6.90
-13	3	-5	16.00	6.90
-14	-3	-5	44.70	9.30
-14	3	-5	38.10	8.30
-15	-3	-5	36.70	9.00
-15	3	-5	44.10	9.70
-15	-4	5	192.08	15.40

-15	4	5	190.38	15.40
-14	4	5	202.88	15.00
-13	-4	5	10.30	5.40
13	-4	-5	7.20	6.70
13	4	-5	0.50	7.60
-13	4	5	10.70	5.70
-12	-4	5	368.56	21.70
-12	-4	5	326.57	18.40
12	-4	-5	325.87	21.20
-12	-4	5	294.67	24.30
-12	4	5	273.87	23.30
-12	4	5	313.67	23.80
12	4	-5	337.57	22.20
-11	-4	5	75.39	8.10
-11	-4	5	63.39	9.50
-11	-4	5	67.59	11.10
11	-4	-5	100.29	11.80
-11	4	5	68.99	13.40
-11	4	5	104.19	9.30
11	4	-5	92.39	12.20
-10	-4	5	506.65	32.50
-10	-4	5	639.54	30.10
-10	-4	5	590.54	29.20
10	-4	-5	582.74	31.50
-10	4	5	461.15	38.00
-10	4	5	596.84	29.70
10	4	-5	601.44	32.10
-9	-4	5	144.19	10.00
-9	-4	5	126.09	13.20
9	-4	-5	126.19	10.80
9	-4	-5	160.18	12.10
-9	-4	5	140.39	9.10
-9	4	5	145.39	13.00
9	4	-5	145.09	12.20
-9	4	5	135.29	9.60
8	-4	-5	697.93	34.70
-8	-4	5	617.34	31.40
8	-4	-5	624.24	32.60
-8	-4	5	682.63	32.00
-8	-4	5	569.94	34.00
-8	4	5	619.94	34.30
8	4	-5	636.04	33.80
-8	4	5	638.24	32.00
-7	-4	5	52.49	8.40

-7	-4	5	73.19	5.80
7	-4	-5	66.09	6.70
-7	-4	5	71.09	5.20
7	4	-5	68.39	8.60
-7	4	5	72.79	6.00
-7	4	5	78.09	8.50
-6	-4	5	2031.10	104.19
6	-4	-5	2249.97	103.29
-6	-4	5	2295.97	102.59
6	-4	-5	1994.70	104.69
-6	4	5	2142.19	104.29
6	4	-5	2189.28	104.19
-6	4	5	2115.69	102.79
5	-4	-5	246.08	14.00
-5	-4	5	243.08	12.40
-5	-4	5	208.88	15.40
-5	-4	5	257.57	12.60
5	-4	-5	258.17	13.60
-5	4	5	243.98	13.10
5	4	-5	240.58	14.40
-5	4	5	258.67	14.60
4	-4	-5	2126.59	98.49
4	-4	-5	2093.69	98.89
-4	-4	5	1849.81	99.29
-4	-4	5	1955.50	97.49
-4	-4	5	2101.09	97.69
-4	4	5	2065.99	98.29
-4	4	5	2119.19	99.19
4	4	-5	2128.19	99.19
-3	-4	5	28.00	1.90
3	-4	-5	41.10	4.00
3	-4	-5	39.30	3.30
-3	-4	5	30.90	4.00
3	4	-5	38.20	4.20
-3	4	5	28.10	3.40
-2	-4	5	1982.20	96.79
2	-4	-5	2005.50	96.79
2	-4	-5	2061.29	96.39
-2	4	5	2024.10	96.79
2	4	-5	2135.69	96.89
-2	4	5	1909.91	96.59
-1	-4	5	316.47	16.10
1	-4	-5	335.57	16.00
1	-4	-5	316.77	16.30

1	4	-5	325.77	15.20
-1	4	5	313.97	16.20
1	4	-5	321.17	15.20
1	4	-5	325.07	16.10
-1	4	5	286.47	16.20
0	-4	-5	531.15	26.50
0	-4	5	564.84	26.40
0	4	-5	562.14	26.40
0	4	-5	528.15	26.10
0	4	-5	529.95	26.10
0	4	5	546.55	26.80
-1	-4	-5	1214.78	57.49
-1	4	-5	1213.08	57.09
-1	4	-5	1153.18	57.19
1	4	5	1223.08	57.59
1	4	5	1149.19	57.49
-1	4	-5	1230.08	57.29
-2	-4	-5	776.92	39.30
2	4	5	816.42	39.10
2	4	5	799.92	39.10
-2	4	-5	779.62	38.90
-2	4	-5	807.62	38.30
-2	4	-5	842.22	39.00
-3	-4	-5	1404.36	65.49
-3	-4	-5	1369.96	65.89
3	4	5	1467.45	67.79
-3	4	-5	1316.37	65.39
3	4	5	1284.97	65.49
-3	4	-5	1351.66	65.99
-3	4	-5	1337.57	64.79
-4	-4	-5	182.48	9.60
-4	-4	-5	152.88	10.00
4	-4	5	168.18	8.50
-4	4	-5	177.48	9.70
-4	4	-5	168.28	8.90
4	4	5	160.48	9.60
4	4	5	158.78	9.90
-4	4	-5	165.08	9.60
-5	-4	-5	1048.80	53.39
-5	-4	-5	1165.58	52.99
5	-4	5	1069.79	52.19
5	4	5	1025.10	52.89
-5	4	-5	1073.49	52.59
5	4	5	1138.39	53.29

-5	4	-5	1136.49	53.19
-5	4	-5	1040.40	52.89
6	-4	5	486.45	23.90
-6	-4	-5	468.35	25.30
-6	-4	-5	501.95	24.70
6	4	5	451.85	24.60
-6	4	-5	502.05	25.10
6	4	5	502.65	25.10
-6	4	-5	448.76	24.60
-6	4	-5	481.05	24.50
7	-4	5	541.55	26.50
-7	-4	-5	549.74	27.20
-7	-4	-5	508.25	27.90
7	4	5	500.05	27.00
-7	4	-5	506.75	27.10
7	4	5	528.35	27.60
-7	4	-5	495.85	27.10
-7	4	-5	593.14	27.80
8	-4	5	155.88	9.50
-8	-4	-5	145.59	11.40
-8	-4	-5	158.28	10.50
-8	4	-5	155.48	10.70
8	4	5	157.08	11.00
8	4	5	131.99	10.20
-8	4	-5	147.89	10.30
-8	4	-5	154.78	10.70
-9	-4	-5	447.76	22.90
-9	-4	-5	372.16	23.60
-9	-4	-5	423.26	22.80
9	-4	5	423.76	22.00
9	4	5	422.06	23.20
-9	4	-5	411.16	22.60
-9	4	-5	395.16	23.10
-10	-4	-5	125.59	10.60
10	-4	5	121.29	9.70
-10	-4	-5	132.99	12.50
-10	4	-5	123.09	10.80
-10	4	-5	116.29	11.60
10	4	5	134.59	11.60
-11	-4	-5	486.35	30.50
-11	-4	-5	569.34	29.30
11	-4	5	536.05	28.50
11	4	5	561.44	29.90
-11	4	-5	518.65	30.00

-12	-4	-5	10.80	7.10
12	-4	5	4.20	5.00
-12	-4	-5	2.10	5.80
-12	4	-5	-8.10	6.30
-12	4	-5	0.10	5.60
12	4	5	-3.50	5.60
-13	-4	-5	220.08	16.30
-13	4	-5	211.28	16.30
-14	-4	-5	13.80	8.10
-14	4	-5	20.60	7.40
-15	-4	-5	264.97	19.70
-15	4	-5	227.78	19.00
-15	-5	5	3.20	6.80
-15	5	5	11.10	7.10
-14	-5	5	21.60	7.30
-14	5	5	29.30	7.20
13	-5	-5	-9.20	8.60
-13	-5	5	-3.40	5.00
-13	5	5	7.90	5.70
13	5	-5	0.00	7.70
-12	-5	5	29.40	5.40
-12	-5	5	22.80	9.90
-12	-5	5	32.90	6.50
12	-5	-5	29.40	7.50
-12	5	5	29.40	5.80
-12	5	5	23.00	8.90
12	5	-5	32.80	8.90
-11	-5	5	-3.90	4.00
-11	-5	5	4.90	5.10
11	-5	-5	9.40	6.20
-11	-5	5	-4.20	8.00
11	5	-5	-3.60	6.60
-11	5	5	3.40	4.20
-11	5	5	6.70	8.10
-10	-5	5	11.40	3.80
-10	-5	5	13.60	4.90
-10	-5	5	8.30	7.10
10	-5	-5	13.20	5.50
10	5	-5	17.70	6.40
-10	5	5	13.50	6.90
-10	5	5	19.80	4.70
9	-5	-5	22.00	6.40
-9	-5	5	13.40	3.30
-9	-5	5	13.40	4.20

9	-5	-5	6.40	4.80
-9	-5	5	6.70	6.30
-9	5	5	16.40	6.40
9	5	-5	15.10	5.40
-9	5	5	18.10	4.10
-8	-5	5	65.29	5.60
-8	-5	5	70.79	10.00
-8	-5	5	66.39	6.20
8	-5	-5	64.69	7.50
8	5	-5	56.99	9.00
-8	5	5	54.69	6.30
-8	5	5	65.39	9.70
-7	-5	5	0.20	2.40
7	-5	-5	6.10	4.20
7	-5	-5	-0.80	3.30
-7	-5	5	1.80	2.10
-7	-5	5	-2.90	4.80
7	5	-5	0.50	4.00
-7	5	5	-2.30	3.70
-7	5	5	0.00	2.50
-6	-5	5	117.49	10.20
-6	-5	5	129.89	7.70
6	-5	-5	144.09	9.80
-6	-5	5	124.69	7.50
6	-5	-5	142.69	9.10
6	5	-5	138.59	10.60
-6	5	5	130.89	10.30
-6	5	5	142.99	8.50
5	-5	-5	94.89	7.30
-5	-5	5	86.39	5.10
-5	-5	5	82.09	7.70
5	-5	-5	87.59	6.70
-5	-5	5	77.29	4.90
5	5	-5	94.29	7.80
-5	5	5	86.49	7.70
-5	5	5	77.69	5.80
-4	-5	5	4.10	3.10
-4	-5	5	5.00	1.20
-4	-5	5	7.10	1.30
4	-5	-5	7.90	2.70
4	-5	-5	14.50	2.90
-4	5	5	12.10	2.60
4	5	-5	2.00	3.20
-4	5	5	5.60	2.70

3	-5	-5	92.59	6.60
3	-5	-5	93.59	6.00
-3	5	5	87.49	6.00
-3	5	5	96.79	6.80
3	5	-5	102.89	6.90
-2	-5	5	130.19	8.00
2	-5	-5	124.69	7.90
2	-5	-5	140.69	7.60
2	5	-5	139.29	8.00
-2	5	5	127.69	7.90
-2	5	5	119.69	7.70
-1	-5	5	319.97	17.60
1	-5	-5	360.86	18.00
1	5	-5	363.76	16.60
1	5	-5	334.87	17.50
-1	5	5	322.67	17.70
1	5	-5	349.87	16.60
-1	5	5	378.06	17.90
0	-5	-5	173.48	10.10
0	-5	-5	177.98	10.70
0	-5	5	176.38	9.60
0	5	5	197.78	10.10
0	5	5	177.68	9.90
0	5	-5	185.58	9.10
0	5	-5	176.48	9.60
0	5	-5	195.48	9.20
-1	5	-5	11.00	2.00
1	5	5	7.90	2.00
1	5	5	14.00	2.20
-1	5	-5	10.00	1.60
-1	5	-5	13.40	1.90
-2	-5	-5	97.99	5.80
-2	-5	-5	89.79	6.30
2	-5	5	93.79	5.70
-2	5	-5	88.09	5.70
2	5	5	89.29	5.90
-2	5	-5	96.09	5.10
2	5	5	96.49	6.00
-3	-5	-5	454.05	21.90
-3	-5	-5	422.86	22.40
3	-5	5	439.56	21.80
-3	5	-5	431.86	21.80
3	5	5	426.66	21.90
-3	5	-5	435.96	21.20

-4	-5	-5	125.49	8.40
-4	-5	-5	127.49	7.70
4	-5	5	120.39	7.80
4	-5	5	125.39	6.60
-4	5	-5	127.29	7.10
-4	5	-5	129.49	7.80
-4	5	-5	126.79	7.90
4	5	5	115.09	7.60
-5	-5	-5	284.87	16.50
-5	-5	-5	321.47	15.90
5	-5	5	294.57	14.80
-5	5	-5	281.87	15.80
-5	5	-5	305.77	15.40
5	5	5	271.47	15.70
-5	5	-5	305.57	16.10
6	-5	5	18.70	3.20
-6	-5	-5	16.80	3.40
-6	-5	-5	23.40	4.40
6	5	5	22.50	3.70
-6	5	-5	22.50	4.50
-6	5	-5	20.80	4.30
-6	5	-5	14.40	3.80
7	-5	5	0.10	2.10
-7	-5	-5	2.20	3.00
-7	-5	-5	5.60	3.80
-7	5	-5	-0.30	3.10
-7	5	-5	-3.20	3.20
-7	5	-5	7.00	3.50
7	5	5	4.70	3.20
-8	-5	-5	1.60	4.10
8	-5	5	9.50	2.90
-8	-5	-5	6.40	3.90
-8	5	-5	3.90	3.90
-8	5	-5	5.80	3.80
-8	5	-5	7.80	3.60
8	5	5	2.20	3.30
-9	-5	-5	11.00	4.50
9	-5	5	7.20	3.30
-9	-5	-5	7.20	5.10
-9	-5	-5	10.50	4.80
-9	5	-5	9.20	4.80
-9	5	-5	14.50	4.50
10	-5	5	6.20	3.90
-10	-5	-5	13.20	5.30

-10	-5	-5	-2.80	5.50
-10	5	-5	14.90	5.70
-10	5	-5	4.60	4.70
10	5	5	16.90	7.20
-11	-5	-5	49.60	8.80
11	-5	5	26.50	5.30
-11	-5	-5	4.80	6.40
11	5	5	16.60	6.50
-11	5	-5	23.50	7.20
-11	5	-5	25.30	6.20
-12	-5	-5	3.00	8.10
12	-5	5	1.80	4.90
-12	-5	-5	0.10	6.00
-12	5	-5	-0.70	5.50
-12	5	-5	4.50	6.50
12	5	5	8.10	6.50
-13	-5	-5	5.50	6.80
-13	5	-5	4.00	6.00
-14	-5	-5	10.30	7.40
-14	5	-5	-4.20	7.10
-15	-5	-5	28.20	9.20
-15	5	-5	12.70	8.30
-15	-6	5	60.29	11.40
-15	6	5	67.59	11.50
-14	-6	5	173.68	14.00
-14	6	5	195.68	14.90
-13	-6	5	19.80	5.80
-13	6	5	23.90	6.50
13	6	-5	12.60	8.20
-12	-6	5	370.96	21.60
-12	-6	5	366.16	20.40
12	-6	-5	378.76	23.70
-12	-6	5	316.77	26.70
12	6	-5	375.76	24.60
-12	6	5	381.86	21.20
-11	-6	5	14.20	5.50
-11	-6	5	-11.70	8.40
11	-6	-5	14.60	6.80
-11	-6	5	5.40	4.20
11	6	-5	3.40	7.30
-11	6	5	9.20	13.60
-11	6	5	11.70	4.70
-10	-6	5	411.26	20.20
10	-6	-5	306.57	24.40

-10	-6	5	358.96	19.00
-10	-6	5	313.27	23.50
-10	6	5	370.46	20.00
10	6	-5	371.56	22.50
-10	6	5	347.07	23.40
-9	-6	5	438.26	21.20
9	-6	-5	423.16	24.30
-9	-6	5	350.26	24.20
-9	-6	5	372.16	20.40
9	6	-5	411.86	23.80
-9	6	5	395.86	21.30
-9	6	5	380.26	24.00
-8	-6	5	648.04	37.30
8	-6	-5	699.83	36.00
-8	-6	5	679.13	34.40
8	-6	-5	682.13	37.70
-8	-6	5	778.52	34.80
-8	6	5	699.53	37.40
8	6	-5	726.73	37.00
-8	6	5	677.83	35.00
-7	-6	5	109.19	10.30
-7	-6	5	127.39	7.80
7	-6	-5	131.79	9.30
-7	-6	5	119.09	11.50
7	-6	-5	137.49	10.40
7	6	-5	133.29	10.90
-7	6	5	139.39	8.80
-7	6	5	138.89	10.90
6	-6	-5	608.54	30.30
-6	-6	5	632.44	29.00
6	-6	-5	594.44	30.50
-6	-6	5	554.14	31.30
-6	-6	5	576.74	28.80
-6	6	5	584.04	29.60
6	6	-5	590.04	31.00
-6	6	5	586.24	31.10
-5	-6	5	8.00	1.60
-5	-6	5	7.60	4.10
-5	-6	5	6.70	1.60
5	-6	-5	14.40	3.50
5	-6	-5	6.30	3.30
-5	6	5	11.20	2.90
-5	6	5	10.30	3.50
5	6	-5	10.80	4.00

4	-6	-5	2678.63	125.99
-4	-6	5	2536.75	126.29
-4	-6	5	2588.74	124.39
-4	-6	5	2497.35	124.39
4	-6	-5	2727.53	125.69
-4	6	5	2661.93	126.19
-4	6	5	2600.74	125.39
4	6	-5	2709.73	126.19
3	-6	-5	344.47	18.20
-3	-6	5	334.37	18.40
3	-6	-5	359.16	17.90
-3	6	5	347.57	18.40
3	6	-5	350.26	18.40
-3	6	5	334.77	17.80
2	-6	-5	164.88	8.90
-2	-6	5	161.68	9.40
2	-6	-5	149.99	9.30
-2	6	5	143.99	9.10
-2	6	5	153.98	9.30
2	6	-5	167.38	9.30
-1	-6	5	2258.27	109.59
1	-6	-5	2306.47	108.39
1	6	-5	2188.98	107.49
1	6	-5	2336.47	108.59
-1	6	5	2305.17	108.79
-1	6	5	2229.68	108.69
0	-6	-5	1992.10	93.59
0	-6	5	1934.71	92.99
0	-6	-5	2020.80	94.09
0	6	-5	1945.91	93.59
0	6	5	2021.70	93.99
0	6	5	1925.01	93.79
0	6	-5	1868.31	92.99
-1	-6	-5	1189.78	55.89
1	-6	5	1198.28	55.59
1	6	5	1136.29	56.49
1	6	5	1108.09	55.99
-1	6	-5	1152.78	55.69
1	6	5	1185.98	56.29
-1	6	-5	1151.38	55.49
2	-6	5	1285.07	59.49
-2	-6	-5	1282.17	59.69
-2	-6	-5	1263.97	60.29
-2	6	-5	1255.77	59.19

2	6	5	1192.28	59.69
2	6	5	1267.27	59.99
-2	6	-5	1171.98	59.19
-2	6	-5	1187.98	59.89
-3	-6	-5	1150.18	53.79
3	-6	5	1145.19	53.89
-3	-6	-5	1112.29	54.49
3	6	5	1075.29	53.89
-3	6	-5	1103.89	53.69
-3	6	-5	1086.49	53.29
4	-6	5	220.08	11.80
-4	-6	-5	229.58	11.70
-4	-6	-5	198.98	12.40
4	-6	5	211.58	10.70
-4	6	-5	215.78	11.00
4	6	5	185.38	11.50
-4	6	-5	201.68	11.60
-5	-6	-5	692.73	36.60
5	-6	5	726.73	35.00
5	-6	5	729.63	36.00
-5	-6	-5	759.12	35.80
-5	6	-5	733.03	35.40
-5	6	-5	707.03	35.80
5	6	5	695.83	35.80
-6	-6	-5	302.87	17.80
6	-6	5	313.17	15.80
6	-6	5	310.47	17.10
-6	-6	-5	343.17	16.90
6	6	5	272.97	16.40
-6	6	-5	321.47	16.70
-6	6	-5	293.17	16.80
-7	-6	-5	1526.45	67.09
-7	-6	-5	1278.37	67.79
7	-6	5	1384.76	66.19
7	6	5	1294.67	66.79
-7	6	-5	1345.57	66.89
-7	6	-5	1345.67	66.89
-7	6	-5	1422.76	67.39
8	-6	5	139.89	8.70
-8	-6	-5	151.68	9.90
-8	-6	-5	120.89	11.20
8	6	5	117.09	9.20
-8	6	-5	141.89	10.10
-8	6	-5	123.99	9.70

-8	6	-5	109.49	9.80
-9	-6	-5	733.63	41.60
-9	-6	-5	884.11	40.60
9	-6	5	817.52	39.60
9	6	5	733.63	40.00
-9	6	-5	821.82	40.40
-9	6	-5	761.92	40.70
10	-6	5	64.19	7.50
-10	-6	-5	54.29	10.90
-10	-6	-5	70.29	9.00
-10	6	-5	72.09	8.10
-10	6	-5	59.49	9.70
11	-6	5	632.54	31.70
-11	-6	-5	526.85	34.00
-11	-6	-5	655.93	32.60
-11	6	-5	623.94	32.30
-11	6	-5	568.24	33.20
12	-6	5	14.80	6.90
-12	-6	-5	12.40	6.60
-12	6	-5	12.90	6.80
-12	6	-5	9.30	7.70
-13	-6	-5	329.97	21.10
-13	6	-5	300.37	20.50
-14	-6	-5	25.50	8.30
-14	6	-5	30.20	8.30
-15	-6	-5	141.99	16.60
-15	6	-5	124.29	14.80
-15	-7	5	56.29	8.70
-15	7	5	61.99	9.50
-14	-7	5	111.99	11.70
-14	7	5	125.19	12.70
-13	-7	5	98.79	10.30
-13	7	5	93.69	10.90
13	7	-5	107.89	15.80
12	-7	-5	4.30	9.70
-12	-7	5	15.60	6.20
-12	-7	5	4.70	4.60
-12	-7	5	-3.30	9.70
12	7	-5	13.10	7.60
-12	7	5	15.50	5.40
-11	-7	5	465.75	31.70
-11	-7	5	512.05	26.80
-11	-7	5	562.64	27.70
11	7	-5	514.65	30.60

-10	-7	5	4.70	3.50
10	-7	-5	10.60	11.10
-10	-7	5	5.20	4.50
-10	-7	5	4.80	8.20
-10	7	5	4.00	7.00
10	7	-5	1.30	5.90
-10	7	5	10.20	4.70
9	-7	-5	269.17	19.20
-9	-7	5	265.77	15.20
-9	-7	5	297.97	16.00
-9	-7	5	263.97	19.80
-9	7	5	282.47	19.40
9	7	-5	318.67	19.30
-9	7	5	281.47	16.30
-8	-7	5	8.10	3.10
8	-7	-5	8.90	4.80
-8	-7	5	7.90	2.70
8	-7	-5	5.00	4.90
-8	-7	5	8.90	5.90
-8	7	5	10.10	3.80
-8	7	5	-2.10	4.90
8	7	-5	8.10	5.30
-7	-7	5	716.33	40.00
7	-7	-5	745.13	38.80
-7	-7	5	813.12	37.40
7	-7	-5	786.62	39.10
-7	-7	5	738.63	37.10
7	7	-5	817.22	39.90
-7	7	5	762.02	38.00
-7	7	5	727.03	39.70
-6	-7	5	181.38	10.10
6	-7	-5	200.58	12.60
-6	-7	5	201.58	10.30
6	-7	-5	201.18	12.20
-6	-7	5	190.88	13.40
-6	7	5	198.18	11.20
6	7	-5	189.18	13.00
-6	7	5	183.18	12.90
-5	-7	5	246.98	13.10
5	-7	-5	281.17	15.30
5	-7	-5	262.17	14.90
-5	-7	5	235.08	13.00
-5	-7	5	275.67	16.00
-5	7	5	265.87	14.40

5	7	-5	290.77	15.80
-5	7	5	263.07	15.60
-4	-7	5	268.97	13.80
-4	-7	5	268.27	16.20
-4	-7	5	276.67	13.80
4	-7	-5	303.57	16.00
4	-7	-5	307.17	15.70
-4	7	5	292.87	15.30
4	7	-5	289.97	16.10
-4	7	5	279.27	16.10
3	-7	-5	17.80	3.20
-3	-7	5	30.20	4.70
3	-7	-5	19.20	3.20
-3	7	5	20.20	4.20
3	7	-5	22.00	4.40
-3	7	5	11.20	2.80
2	-7	-5	57.79	4.40
2	-7	-5	47.10	5.70
-2	-7	5	54.99	5.10
-2	7	5	47.00	4.90
-2	7	5	37.30	4.70
2	7	-5	60.89	5.10
1	-7	-5	711.63	34.50
-1	-7	5	699.13	33.70
1	-7	-5	668.63	35.90
-1	-7	5	726.13	35.00
-1	7	5	716.63	35.10
-1	7	5	682.73	34.90
1	7	-5	761.02	35.00
1	7	-5	683.13	33.70
0	-7	5	933.21	45.70
0	-7	-5	968.40	45.70
0	-7	-5	968.00	46.30
0	-7	5	936.31	45.10
0	7	5	941.51	46.00
0	7	-5	946.61	45.80
0	7	-5	895.31	45.00
0	7	5	962.40	46.10
0	7	5	926.21	46.30
-1	-7	-5	849.81	41.20
1	-7	5	861.71	41.00
1	7	5	821.12	41.50
-1	7	-5	817.92	40.80
1	7	5	884.41	42.10

1	7	5	864.41	41.70
-1	7	-5	843.22	41.20
-2	-7	-5	47.10	4.00
2	-7	5	46.90	3.80
-2	-7	-5	41.10	4.90
-2	7	-5	42.30	3.90
2	7	5	44.10	4.20
-2	7	-5	45.50	3.40
2	7	5	39.10	5.10
-2	7	-5	41.00	3.50
-3	-7	-5	27.90	3.70
-3	-7	-5	26.70	4.90
3	-7	5	25.80	3.30
-3	7	-5	21.90	3.40
3	7	5	25.10	4.80
3	7	5	22.20	3.40
-3	7	-5	23.80	2.90
-4	-7	-5	96.19	6.30
-4	-7	-5	104.09	7.80
4	-7	5	86.59	6.50
4	7	5	74.69	6.20
-4	7	-5	89.39	5.70
4	7	5	79.09	7.20
-4	7	-5	83.19	6.20
-5	-7	-5	750.32	34.20
-5	-7	-5	680.33	35.20
5	-7	5	660.33	34.20
5	-7	5	692.83	33.30
-5	7	-5	715.23	33.80
5	7	5	627.64	34.00
-5	7	-5	656.03	34.00
6	-7	5	346.27	17.90
-6	-7	-5	393.76	18.90
6	-7	5	321.77	19.00
-6	-7	-5	353.56	20.10
6	7	5	377.06	19.80
-6	7	-5	319.87	18.60
-6	7	-5	368.46	18.70
6	7	5	331.37	18.70
7	-7	5	7.70	2.70
-7	-7	-5	14.00	3.80
7	-7	5	11.40	4.30
-7	-7	-5	13.10	4.40
7	7	5	10.40	3.80

-7	7	-5	7.90	3.80
-7	7	-5	6.00	3.50
7	7	5	17.80	3.90
-8	-7	-5	234.48	16.10
8	-7	5	241.48	13.30
8	-7	5	231.58	14.90
-8	-7	-5	256.87	14.40
8	7	5	213.28	14.10
-8	7	-5	245.88	14.80
-8	7	-5	240.88	14.50
-8	7	-5	229.38	15.10
-9	-7	-5	14.30	4.80
-9	-7	-5	18.30	6.10
9	-7	5	15.70	3.80
9	7	5	11.00	4.70
9	7	5	23.20	5.00
-9	7	-5	21.50	5.30
-9	7	-5	14.80	5.10
-10	-7	-5	351.36	23.40
-10	-7	-5	392.96	21.80
10	-7	5	373.76	20.40
-10	7	-5	373.76	22.10
-10	7	-5	349.47	20.80
-11	-7	-5	41.50	9.30
11	-7	5	33.90	7.50
-11	-7	-5	35.50	7.20
-11	7	-5	39.90	7.80
-11	7	-5	39.00	7.10
-12	-7	-5	416.76	24.00
-12	7	-5	399.46	23.50
-12	7	-5	375.86	25.30
-13	-7	-5	15.70	7.10
-13	7	-5	17.30	6.80
-14	-7	-5	58.59	10.30
-14	7	-5	38.00	8.30
-15	-7	-5	37.30	10.10
-15	7	-5	34.50	9.00
-15	-8	5	13.60	7.30
-15	8	5	30.30	7.90
-14	-8	5	81.09	10.80
-14	8	5	93.89	12.10
-13	-8	5	88.79	10.10
-13	8	5	90.89	11.00
-12	-8	5	295.57	17.80

-12	-8	5	276.87	16.60
-12	-8	5	231.68	23.40
-12	8	5	291.97	17.70
12	8	-5	291.07	21.60
11	-8	-5	40.50	8.70
-11	-8	5	32.30	6.00
-11	-8	5	38.10	10.50
-11	-8	5	36.50	6.90
-11	8	5	34.70	10.60
11	8	-5	58.39	12.00
-11	8	5	41.80	7.30
-10	-8	5	133.79	9.40
-10	-8	5	161.38	10.60
10	-8	-5	124.79	12.90
-10	-8	5	147.59	16.20
-10	8	5	140.99	11.00
-10	8	5	135.19	15.20
10	8	-5	132.39	13.90
-9	-8	5	61.69	6.30
-9	-8	5	73.99	7.30
9	-8	-5	67.19	9.30
-9	-8	5	78.09	12.80
9	8	-5	87.99	11.20
-9	8	5	87.09	8.00
-9	8	5	64.99	11.20
-8	-8	5	67.79	10.20
-8	-8	5	72.29	5.80
8	-8	-5	74.09	8.90
-8	-8	5	80.79	6.20
8	-8	-5	57.79	8.70
-8	8	5	80.49	10.30
8	8	-5	80.59	10.20
-8	8	5	77.59	7.40
7	-8	-5	107.89	9.50
-7	-8	5	107.89	11.30
-7	-8	5	112.69	7.10
7	-8	-5	101.99	9.70
-7	-8	5	126.09	7.30
-7	8	5	108.69	10.80
-7	8	5	107.09	8.00
7	8	-5	129.79	10.70
-6	-8	5	771.52	36.10
6	-8	-5	791.32	38.00
-6	-8	5	665.83	38.70

-6	-8	5	752.32	36.10
6	-8	-5	745.33	38.00
-6	8	5	742.13	37.20
6	8	-5	774.52	38.80
-6	8	5	723.23	38.50
-5	-8	5	219.18	11.60
-5	-8	5	207.38	14.50
5	-8	-5	254.27	14.10
5	-8	-5	235.28	13.70
-5	-8	5	222.78	11.60
5	8	-5	233.98	14.40
-5	8	5	245.68	14.40
-5	8	5	241.08	13.10
4	-8	-5	532.35	26.50
-4	-8	5	497.75	24.70
-4	-8	5	522.25	24.70
-4	-8	5	464.45	27.10
4	-8	-5	519.25	26.70
-4	8	5	548.45	27.10
4	8	-5	508.55	26.90
-4	8	5	531.55	26.20
-3	-8	5	57.99	6.30
3	-8	-5	64.39	5.50
3	-8	-5	58.79	6.00
3	8	-5	61.19	6.00
-3	8	5	51.69	5.40
-3	8	5	51.59	6.00
-2	-8	5	145.69	9.30
-2	-8	5	143.99	7.00
2	-8	-5	144.59	8.40
-2	8	5	126.49	8.50
2	8	-5	143.69	9.10
2	8	-5	137.09	7.00
-2	8	5	153.38	9.20
1	-8	-5	39.30	3.70
-1	-8	5	32.40	2.50
-1	-8	5	25.80	4.30
1	-8	-5	36.10	4.60
-1	8	5	23.10	4.20
1	8	-5	37.20	2.30
1	8	-5	27.00	4.30
-1	8	5	37.80	4.60
0	-8	-5	1218.68	56.09
0	-8	5	1140.29	54.69

0	-8	-5	1190.38	55.29
0	-8	5	1095.29	55.39
0	8	5	1126.89	55.59
0	8	-5	1082.19	54.59
0	8	5	1207.88	55.99
0	8	-5	1102.19	55.49
0	8	5	1138.79	56.09
-1	-8	-5	135.79	7.90
1	-8	5	136.99	7.60
-1	8	-5	136.59	7.50
1	8	5	141.09	8.60
-1	8	-5	126.19	8.00
1	8	5	142.59	8.30
1	8	5	133.69	9.10
2	-8	5	5.20	3.50
-2	-8	-5	4.20	2.00
-2	-8	-5	6.90	2.80
2	8	5	4.60	2.20
-2	8	-5	7.60	1.70
2	8	5	7.40	2.50
-2	8	-5	8.20	1.90
2	8	5	8.20	3.30
-2	8	-5	7.10	2.10
3	-8	5	603.24	28.30
-3	-8	-5	538.25	29.10
-3	-8	-5	597.74	28.30
3	8	5	578.14	29.40
-3	8	-5	560.94	27.90
3	8	5	529.85	28.20
-3	8	-5	553.54	28.10
-4	-8	-5	143.19	10.20
4	-8	5	136.79	9.00
-4	-8	-5	156.88	8.90
-4	8	-5	133.79	8.70
-4	8	-5	143.59	8.40
4	8	5	150.78	10.30
4	8	5	139.19	8.80
-5	-8	-5	605.54	33.50
5	-8	5	657.53	32.70
5	-8	5	652.23	31.70
-5	-8	-5	680.93	32.50
5	8	5	663.53	33.60
5	8	5	626.64	32.50
-5	8	-5	654.23	32.20

-5	8	-5	640.04	32.40
6	-8	5	388.46	19.60
-6	-8	-5	357.46	21.70
6	-8	5	382.96	20.90
-6	-8	-5	415.66	20.50
-6	8	-5	383.66	20.60
-6	8	-5	387.16	20.40
6	8	5	403.96	21.70
6	8	5	367.16	20.40
7	-8	5	419.56	22.70
-7	-8	-5	372.26	23.70
7	-8	5	413.76	21.40
-7	-8	-5	483.95	22.50
7	8	5	417.46	22.30
-7	8	-5	412.96	22.40
7	8	5	426.06	23.50
-7	8	-5	412.76	22.50
8	-8	5	0.70	2.70
-8	-8	-5	-1.20	5.30
-8	-8	-5	2.50	4.10
8	8	5	0.40	3.80
-8	8	-5	-0.50	4.00
8	8	5	6.40	4.60
-8	8	-5	4.80	4.00
-9	-8	-5	74.89	11.30
9	-8	5	84.79	8.30
-9	8	-5	94.39	8.60
9	8	5	110.49	10.80
9	8	5	96.39	8.80
-9	8	-5	78.39	9.60
10	-8	5	9.80	6.60
-10	-8	-5	6.00	6.90
-10	8	-5	6.70	5.90
10	8	5	9.70	6.20
10	8	5	10.30	5.10
-10	8	-5	11.10	5.20
11	-8	5	246.08	15.00
-11	-8	-5	217.38	19.20
-11	-8	-5	260.17	16.80
11	8	5	279.97	18.40
-11	8	-5	226.08	16.20
-11	8	-5	208.48	17.70
-12	-8	-5	32.30	7.70
-12	8	-5	17.10	9.10

-12	8	-5	36.60	7.40
-13	-8	-5	110.89	13.20
-13	8	-5	100.49	12.60
-14	-8	-5	36.80	9.40
-14	8	-5	40.80	8.30
-15	-8	-5	56.39	10.70
-15	8	-5	48.60	9.50
-15	9	5	230.18	17.60
-14	-9	5	161.58	13.70
-14	9	5	184.78	15.20
-13	-9	5	342.97	20.20
13	9	-5	371.06	25.80
-13	9	5	340.07	21.30
12	-9	-5	44.50	8.50
-12	-9	5	59.79	14.10
-12	-9	5	63.39	10.80
-12	-9	5	49.40	8.00
-12	9	5	64.29	9.60
12	9	-5	45.70	10.10
-11	-9	5	553.14	27.00
-11	-9	5	441.16	31.60
11	-9	-5	509.45	29.10
-11	-9	5	507.05	26.20
-11	9	5	446.16	31.80
-11	9	5	509.15	27.10
11	9	-5	551.04	31.00
-10	-9	5	47.10	9.80
10	-9	-5	52.89	9.70
-10	-9	5	53.99	7.60
-10	-9	5	49.00	6.30
10	9	-5	61.09	12.00
-10	9	5	55.29	8.10
-10	9	5	42.80	9.30
-9	-9	5	980.00	49.90
9	-9	-5	1029.70	52.19
-9	-9	5	925.91	53.89
-9	-9	5	1119.99	50.39
9	9	-5	1086.69	53.49
-9	9	5	1007.80	53.59
-9	9	5	1032.20	50.99
-8	-9	5	20.00	3.30
8	-9	-5	20.30	5.10
-8	-9	5	25.90	4.60
-8	-9	5	21.50	6.50

8	9	-5	20.80	6.70
-8	9	5	22.30	6.30
-8	9	5	34.60	6.20
-7	-9	5	1051.29	51.59
7	-9	-5	1094.79	53.49
-7	-9	5	989.40	54.49
7	-9	-5	1062.79	53.49
-7	-9	5	1106.19	51.49
-7	9	5	1025.40	54.19
-7	9	5	1073.09	52.49
7	9	-5	1126.69	54.29
-6	-9	5	121.99	7.00
6	-9	-5	133.69	9.90
-6	-9	5	123.09	7.10
-6	-9	5	100.69	10.80
6	-9	-5	134.99	10.10
-6	9	5	123.19	8.80
6	9	-5	129.09	10.60
-6	9	5	128.69	10.70
5	-9	-5	2125.99	103.29
5	-9	-5	2223.58	103.29
-5	-9	5	1982.90	101.29
-5	-9	5	2038.40	101.29
-5	-9	5	2063.49	103.99
-5	9	5	2171.28	103.69
-5	9	5	2111.09	102.59
5	9	-5	2359.96	103.89
4	-9	-5	288.47	16.10
-4	-9	5	270.87	16.90
-4	-9	5	286.07	14.00
4	-9	-5	285.47	16.20
-4	9	5	283.67	15.70
-4	9	5	297.37	16.60
4	9	-5	298.17	16.70
-3	-9	5	1874.41	92.09
-3	-9	5	1838.62	89.89
3	-9	-5	1991.70	91.49
3	-9	-5	1774.92	92.59
-3	9	5	1981.60	91.99
3	9	-5	1737.73	89.89
3	9	-5	2042.70	91.99
-3	9	5	1938.41	91.39
2	-9	-5	2164.98	111.19
-2	-9	5	2259.17	108.59

2	-9	-5	2424.76	109.89
-2	-9	5	2296.67	110.39
2	9	-5	2108.09	108.59
2	9	-5	2439.96	110.39
-2	9	5	2390.06	110.39
-2	9	5	2247.68	109.99
-1	-9	5	543.55	27.00
1	-9	-5	544.15	26.50
1	-9	-5	523.75	27.20
-1	-9	5	520.65	25.60
-1	9	5	547.15	27.20
1	9	-5	557.24	27.10
-1	9	5	489.75	26.80
1	9	-5	509.95	25.50
0	-9	-5	1878.51	88.69
0	-9	5	1828.62	88.19
0	-9	5	1820.42	88.89
0	9	5	1918.41	89.39
0	9	5	1865.41	89.69
0	9	-5	1705.83	87.99
0	9	5	1890.41	89.09
0	9	-5	1895.51	89.09
1	-9	5	312.57	15.70
-1	-9	-5	332.57	16.20
-1	9	-5	305.67	16.30
1	9	5	276.47	17.50
1	9	5	313.67	16.40
1	9	5	306.87	17.20
2	-9	5	1500.95	72.29
-2	-9	-5	1619.24	72.49
-2	-9	-5	1465.15	73.39
-2	9	-5	1455.95	72.09
2	9	5	1545.75	73.69
-2	9	-5	1492.95	72.29
2	9	5	1453.75	72.59
-2	9	-5	1435.36	72.39
2	9	5	1573.84	73.09
-3	-9	-5	26.40	4.70
3	-9	5	34.40	4.30
-3	-9	-5	30.40	4.10
3	9	5	29.60	4.20
3	9	5	25.70	4.90
-3	9	-5	23.10	3.80
-3	9	-5	26.80	3.60

-4	-9	-5	3243.88	159.28
-4	-9	-5	3514.65	158.08
4	-9	5	3396.46	158.28
-4	9	-5	3173.38	157.88
-4	9	-5	3293.27	157.98
4	9	5	3123.59	158.08
4	9	5	3440.76	159.28
-5	-9	-5	6.80	2.80
5	-9	5	3.50	3.20
-5	-9	-5	4.10	4.00
5	-9	5	5.20	2.10
5	9	5	11.20	4.30
-5	9	-5	0.10	2.70
-5	9	-5	3.80	2.50
5	9	5	2.40	2.60
-6	-9	-5	914.11	42.00
6	-9	5	812.02	42.20
-6	-9	-5	795.02	43.30
6	-9	5	847.72	41.20
-6	9	-5	845.72	41.90
6	9	5	837.02	42.00
6	9	5	877.21	43.20
-6	9	-5	815.62	41.90
7	-9	5	52.99	7.80
-7	-9	-5	60.59	8.90
-7	-9	-5	67.29	6.90
7	-9	5	63.69	5.60
7	9	5	53.49	6.70
-7	9	-5	57.19	7.00
7	9	5	54.59	8.40
-7	9	-5	55.39	7.20
8	-9	5	505.95	25.80
-8	-9	-5	458.25	28.10
-8	-9	-5	530.45	26.40
-8	9	-5	485.25	27.20
-8	9	-5	500.35	26.40
8	9	5	448.46	26.10
8	9	5	545.45	27.90
-9	-9	-5	56.79	11.50
9	9	5	73.49	8.20
-9	9	-5	69.99	8.40
-9	9	-5	64.69	12.20
9	9	5	53.29	10.20
10	-9	5	369.86	20.20

-10	-9	-5	329.37	23.70
10	9	5	420.26	23.60
10	9	5	335.07	21.00
-10	9	-5	387.46	25.70
-11	-9	-5	12.80	8.30
11	-9	5	1.00	4.30
-11	-9	-5	9.40	6.40
11	9	5	2.40	6.40
-11	9	-5	9.90	5.90
-12	-9	-5	199.28	15.80
-12	9	-5	194.18	15.30
-13	-9	-5	14.10	8.00
-13	9	-5	14.90	7.10
-14	-9	-5	184.58	16.70
-14	9	-5	132.49	14.90
-15	-9	-5	111.79	16.80
-15	9	-5	98.79	14.00
-14	-10	5	10.70	6.30
-14	10	5	20.10	7.40
-13	-10	5	11.10	5.70
-13	10	5	16.20	7.00
13	10	-5	12.00	10.20
-12	-10	5	8.60	5.00
-12	10	5	13.40	6.30
12	10	-5	11.20	8.60
-11	-10	5	16.30	4.40
11	-10	-5	28.70	7.00
-11	-10	5	23.80	11.10
11	10	-5	18.60	8.20
-10	-10	5	11.00	4.40
10	-10	-5	18.10	6.10
-10	-10	5	10.30	3.70
-10	-10	5	32.60	9.60
10	10	-5	26.20	7.70
-10	10	5	4.50	7.20
-10	10	5	15.90	5.50
-9	-10	5	17.30	3.60
-9	-10	5	16.20	7.50
-9	10	5	21.90	7.30
9	10	-5	16.70	6.60
-8	-10	5	-4.90	6.10
8	-10	-5	2.20	4.50
-8	-10	5	2.90	2.70
-8	-10	5	1.80	2.50

-8	10	5	3.40	5.30
8	10	-5	5.10	5.70
-8	10	5	-0.50	3.60
-7	-10	5	35.00	7.00
7	-10	-5	43.80	7.90
7	-10	-5	35.40	5.80
-7	-10	5	36.90	4.10
-7	-10	5	30.70	5.50
-7	10	5	37.50	6.40
7	10	-5	55.89	9.10
-7	10	5	40.60	7.20
-6	-10	5	125.99	11.50
-6	-10	5	108.79	6.40
6	-10	-5	99.89	9.20
6	-10	-5	113.19	9.60
-6	-10	5	104.29	6.60
-6	10	5	120.09	8.70
6	10	-5	134.19	10.70
-6	10	5	120.19	10.40
5	-10	-5	124.59	9.60
-5	-10	5	108.79	10.50
-5	-10	5	114.99	6.50
-5	-10	5	114.69	6.60
5	-10	-5	115.39	9.10
5	10	-5	139.39	10.60
-5	10	5	130.49	10.10
-5	10	5	131.69	8.50
-4	-10	5	13.80	1.90
4	-10	-5	14.80	4.00
-4	-10	5	7.80	4.10
4	-10	-5	11.90	3.90
4	10	-5	8.20	1.30
-4	10	5	8.10	3.80
4	10	-5	15.30	4.50
-4	10	5	12.60	3.50
-3	-10	5	61.59	6.90
3	-10	-5	45.40	5.40
-3	-10	5	50.79	3.20
3	10	-5	49.99	3.10
-3	10	5	44.40	5.70
3	10	-5	60.79	6.70
-3	10	5	51.09	6.20
-2	-10	5	3.10	1.20
2	-10	-5	2.00	2.50

-2	-10	5	7.10	3.10
2	-10	-5	2.90	3.00
2	10	-5	2.20	3.00
-2	10	5	2.60	2.80
2	10	-5	3.80	1.10
-2	10	5	1.20	2.80
-1	-10	5	28.30	2.80
1	-10	-5	34.10	5.60
-1	-10	5	31.20	4.90
1	-10	-5	31.70	4.40
-1	10	5	23.20	3.80
1	10	-5	28.20	4.80
-1	10	5	25.90	4.80
1	10	-5	21.30	2.40
0	-10	-5	4.90	2.40
0	-10	5	2.00	2.30
0	-10	5	0.20	1.50
0	10	-5	-2.10	1.40
0	10	5	2.40	3.70
0	10	-5	2.90	2.50
0	10	5	-1.10	2.40
0	10	5	-1.90	2.50
1	-10	5	84.39	5.90
-1	-10	-5	94.49	6.20
-1	10	-5	87.09	5.80
1	10	5	85.39	7.40
1	10	5	106.49	8.20
1	10	5	91.39	6.90
-1	10	-5	97.29	6.80
2	-10	5	185.58	10.20
-2	-10	-5	191.78	10.40
-2	10	-5	183.68	10.20
-2	10	-5	162.38	10.30
-2	10	-5	173.78	9.90
2	10	5	168.38	12.00
2	10	5	181.28	10.60
2	10	5	186.08	11.20
-3	-10	-5	-0.10	3.40
3	-10	5	6.10	2.70
-3	-10	-5	8.50	2.50
-3	10	-5	3.20	2.10
-3	10	-5	3.70	2.10
3	10	5	4.00	3.70
3	10	5	5.30	2.70

-3	10	-5	4.10	2.10
-4	-10	-5	143.89	8.80
-4	-10	-5	137.69	10.80
4	-10	5	139.79	9.20
-4	10	-5	132.69	8.60
4	10	5	151.28	11.10
-4	10	-5	139.29	8.70
4	10	5	123.19	8.80
-5	-10	-5	102.89	7.20
-5	-10	-5	85.09	9.30
5	-10	5	78.59	7.50
5	10	5	94.79	7.30
5	10	5	103.19	9.60
-5	10	-5	91.49	7.20
-5	10	-5	87.89	7.00
-6	-10	-5	8.20	5.00
-6	-10	-5	0.30	3.10
6	-10	5	4.00	2.40
6	-10	5	-4.40	3.60
6	10	5	2.50	3.30
6	10	5	2.60	4.30
-6	10	-5	-1.30	3.40
-6	10	-5	3.70	3.00
7	-10	5	68.39	8.30
-7	-10	-5	76.99	10.00
-7	-10	-5	75.69	7.50
7	-10	5	74.79	6.20
-7	10	-5	70.69	7.60
7	10	5	71.09	9.60
-7	10	-5	60.79	8.40
7	10	5	72.99	7.50
-8	-10	-5	18.30	5.10
-8	-10	-5	10.10	5.80
8	10	5	10.90	6.00
8	10	5	10.30	4.60
-8	10	-5	25.50	5.10
-9	-10	-5	9.20	6.70
9	-10	5	3.40	3.40
9	10	5	6.80	4.70
-9	10	-5	6.80	4.80
9	10	5	1.60	5.70
-10	-10	-5	12.60	7.20
10	-10	5	11.50	4.10
-10	10	-5	5.50	8.00

10	10	5	8.30	5.30
-10	10	-5	10.70	5.40
10	10	5	16.70	7.10
-11	-10	-5	8.90	7.20
11	-10	5	2.10	4.30
-11	10	-5	2.50	7.60
11	10	5	11.00	8.00
-11	10	-5	5.10	5.60
-12	-10	-5	21.40	7.60
-12	10	-5	13.00	6.50
-13	-10	-5	-4.90	7.60
-13	10	-5	5.20	6.80
-14	-10	-5	5.70	8.70
-14	10	-5	8.30	7.20
-14	11	5	95.39	12.60
-13	-11	5	258.17	16.60
13	11	-5	263.97	23.20
-13	11	5	268.37	18.20
-12	-11	5	4.00	4.90
12	11	-5	-0.30	8.40
-12	11	5	15.50	6.40
-11	-11	5	617.64	43.90
11	-11	-5	739.53	40.60
-11	-11	5	832.62	38.30
-11	-11	5	785.92	37.80
-11	11	5	748.03	39.10
11	11	-5	773.82	42.20
-10	-11	5	3.90	4.00
10	-11	-5	8.60	5.60
-10	-11	5	7.90	3.60
-10	-11	5	12.30	8.90
10	11	-5	3.90	6.90
-10	11	5	17.30	8.00
-10	11	5	8.90	5.10
-9	-11	5	958.20	42.90
-9	-11	5	760.82	46.90
-9	-11	5	886.21	42.70
9	-11	-5	896.81	45.40
-9	11	5	825.22	46.60
9	11	-5	903.81	46.50
-9	11	5	854.51	44.00
8	-11	-5	-2.70	5.10
-8	-11	5	-13.30	6.30
-8	-11	5	-3.60	2.50

-8	-11	5	2.50	2.50
-8	11	5	1.10	4.00
-8	11	5	-3.40	5.40
8	11	-5	6.00	6.50
-7	-11	5	478.05	28.30
-7	-11	5	468.05	25.60
7	-11	-5	495.65	26.70
-7	-11	5	483.15	24.00
-7	11	5	473.05	27.40
-7	11	5	491.15	25.70
7	11	-5	520.25	27.90
6	-11	-5	71.59	8.90
-6	-11	5	79.69	5.30
6	-11	-5	77.49	8.80
-6	-11	5	64.29	10.10
-6	-11	5	76.99	5.00
-6	11	5	87.49	9.50
6	11	-5	83.19	9.90
-6	11	5	80.79	7.50
5	-11	-5	1531.35	74.79
5	-11	-5	1551.84	74.49
-5	-11	5	1499.25	72.29
-5	-11	5	1406.96	75.19
-5	11	5	1520.55	74.89
-5	11	5	1528.55	73.79
5	11	-5	1585.24	74.99
-4	-11	5	147.59	11.60
-4	-11	5	149.29	7.60
4	-11	-5	145.49	10.30
4	11	-5	139.39	7.60
4	11	-5	148.19	11.00
-4	11	5	150.08	10.80
-4	11	5	147.59	9.60
3	-11	-5	510.55	28.60
-3	-11	5	530.15	25.60
3	-11	-5	555.44	27.50
-3	-11	5	495.45	28.00
3	11	-5	545.15	28.00
-3	11	5	546.35	27.90
3	11	-5	519.85	25.50
-3	11	5	538.35	27.30
-2	-11	5	1237.68	59.89
2	-11	-5	1250.17	61.69
-2	-11	5	1264.07	61.89

2	-11	-5	1293.77	61.19
-2	11	5	1340.87	61.89
2	11	-5	1266.97	61.69
-2	11	5	1221.88	61.29
2	11	-5	1162.88	59.69
-1	-11	5	311.77	17.60
1	-11	-5	333.87	17.10
1	-11	-5	346.77	18.20
-1	-11	5	315.87	16.00
-1	11	5	293.87	17.30
-1	11	5	314.87	18.20
-1	11	5	324.27	17.80
1	11	-5	315.67	15.80
1	11	-5	320.77	17.70
0	-11	-5	1002.50	46.10
0	-11	5	905.51	46.30
0	-11	5	957.60	45.40
0	11	-5	903.41	46.50
0	11	5	926.81	46.40
0	11	5	945.61	47.30
1	-11	5	1069.09	52.59
-1	-11	-5	1154.78	52.99
-1	11	-5	1092.99	53.39
1	11	5	1083.69	53.69
-1	11	-5	1030.80	52.39
1	11	5	1116.49	54.39
1	11	5	1073.79	53.29
-2	-11	-5	149.09	9.20
2	-11	5	150.38	9.10
-2	11	-5	138.09	9.40
-2	11	-5	170.78	8.90
2	11	5	154.88	9.60
2	11	5	145.09	10.10
-2	11	-5	160.08	9.30
-3	-11	-5	76.59	6.20
3	-11	5	73.79	6.30
-3	-11	-5	75.19	7.90
-3	11	-5	75.49	6.20
3	11	5	77.69	6.40
3	11	5	82.89	8.90
-3	11	-5	80.29	6.20
-3	11	-5	73.99	5.80
-4	-11	-5	774.02	37.30
-4	-11	-5	700.63	38.70

4	-11	5	786.92	37.60
4	11	5	779.22	39.10
4	11	5	717.23	37.30
-4	11	-5	735.23	37.20
-4	11	-5	755.62	37.20
-5	-11	-5	106.09	7.50
5	-11	5	82.69	8.10
-5	-11	-5	92.99	9.70
-5	11	-5	101.49	7.60
5	11	5	93.69	7.60
5	11	5	97.99	9.80
-5	11	-5	94.89	7.50
-6	-11	-5	329.37	21.30
-6	-11	-5	373.96	19.20
6	-11	5	373.96	18.60
6	-11	5	354.46	19.90
-6	11	-5	343.27	19.30
6	11	5	327.67	19.10
6	11	5	354.76	21.10
-6	11	-5	356.16	19.30
-7	-11	-5	24.60	4.90
-7	-11	-5	25.40	6.60
7	-11	5	25.70	5.10
7	11	5	32.30	6.90
7	11	5	14.40	4.40
-7	11	-5	26.50	6.10
-7	11	-5	25.20	5.10
-8	-11	-5	630.14	36.40
-8	-11	-5	679.13	34.20
8	-11	5	595.74	34.70
-8	11	-5	686.13	34.40
8	11	5	741.83	36.40
8	11	5	657.63	34.20
9	-11	5	22.00	4.40
-9	-11	-5	12.90	7.00
9	11	5	13.40	6.90
9	11	5	32.10	5.90
-9	11	-5	20.00	7.20
-9	11	-5	28.70	5.80
-10	-11	-5	590.64	37.90
10	-11	5	689.03	34.40
10	11	5	645.24	35.00
-10	11	-5	641.94	36.70
10	11	5	753.52	38.00

-10	11	-5	695.83	35.40
11	-11	5	24.60	8.60
-11	11	-5	27.10	6.90
11	11	5	25.00	8.40
-11	11	-5	17.70	8.50
-12	-11	-5	381.76	22.90
-12	11	-5	327.57	21.80
-13	-11	-5	1.70	8.30
-13	11	-5	2.70	7.00
-14	-11	-5	155.98	16.80
-14	11	-5	144.79	13.90
-14	12	5	21.50	7.70
13	12	-5	9.20	10.70
-13	12	5	12.50	6.60
-12	-12	5	393.16	22.30
-12	12	5	441.26	24.20
12	12	-5	398.76	28.20
-11	-12	5	20.60	4.60
11	-12	-5	20.90	7.20
-11	-12	5	4.70	10.70
-11	12	5	15.90	5.90
11	12	-5	13.50	8.40
-10	-12	5	245.08	13.30
-10	-12	5	215.08	12.90
-10	-12	5	196.28	21.30
10	-12	-5	214.98	16.60
-10	12	5	228.58	19.60
-10	12	5	227.28	15.10
10	12	-5	208.68	18.20
9	-12	-5	3.30	5.90
-9	-12	5	11.30	8.50
-9	-12	5	8.50	3.50
-9	-12	5	7.00	3.40
9	12	-5	-0.30	6.80
-9	12	5	2.70	6.70
-9	12	5	8.50	5.10
-8	-12	5	279.27	14.50
8	-12	-5	261.67	17.70
-8	-12	5	243.68	20.10
-8	-12	5	289.37	14.60
-8	12	5	255.67	18.80
8	12	-5	292.77	19.50
-8	12	5	279.67	16.10
-7	-12	5	16.50	2.60

-7	-12	5	12.60	4.30
-7	-12	5	17.10	6.50
7	-12	-5	16.90	5.60
-7	12	5	14.30	5.80
7	12	-5	19.60	6.90
-7	12	5	18.50	4.80
-6	-12	5	483.75	24.90
-6	-12	5	485.55	29.00
-6	-12	5	506.35	25.10
6	-12	-5	506.05	27.70
-6	12	5	497.95	26.50
-6	12	5	501.15	28.20
6	12	-5	602.84	29.00
5	-12	-5	162.68	11.80
-5	-12	5	140.49	12.70
5	-12	-5	141.49	12.60
-5	-12	5	159.28	8.30
-5	12	5	165.58	12.20
-5	12	5	171.78	10.50
5	12	-5	146.79	8.30
5	12	-5	174.28	12.60
4	-12	-5	40.50	6.60
-4	-12	5	26.10	2.80
-4	-12	5	42.00	7.60
4	12	-5	33.90	5.70
4	12	-5	26.30	2.80
-4	12	5	22.70	4.50
-4	12	5	28.70	5.30
-3	-12	5	317.57	19.30
-3	-12	5	327.27	16.40
3	-12	-5	350.06	19.00
3	-12	-5	346.47	18.50
-3	12	5	352.56	19.20
-3	12	5	307.77	18.20
3	12	-5	346.77	19.20
3	12	-5	317.87	16.30
2	-12	-5	443.96	22.60
-2	-12	5	429.86	21.10
2	-12	-5	443.46	23.50
-2	-12	5	414.46	23.40
-2	12	5	440.96	23.40
2	12	-5	408.36	20.90
-2	12	5	405.46	22.70
2	12	-5	454.35	23.40

-1	-12	5	223.48	13.70
1	-12	-5	233.98	13.00
-1	-12	5	221.08	11.90
-1	12	5	223.28	13.40
1	12	-5	235.78	13.90
1	12	-5	218.88	11.60
-1	12	5	225.78	14.60
-1	12	5	240.58	14.00
0	-12	5	118.59	7.90
0	12	-5	135.29	7.60
0	12	-5	151.28	9.70
0	12	5	136.69	10.40
0	12	5	132.69	9.10
0	12	5	119.09	9.60
-1	-12	-5	1327.67	61.99
1	-12	5	1240.78	61.59
-1	12	-5	1289.17	62.59
1	12	5	1288.47	62.89
-1	12	-5	1222.28	61.29
1	12	5	1338.17	63.59
1	12	5	1232.08	62.29
2	-12	5	154.28	9.50
-2	-12	-5	157.58	9.50
-2	12	-5	149.99	9.10
2	12	5	165.08	12.30
2	12	5	154.38	9.90
2	12	5	150.38	10.50
-2	12	-5	162.78	9.80
-2	12	-5	160.08	10.20
3	-12	5	155.78	9.80
-3	-12	-5	163.88	9.50
-3	-12	-5	144.19	11.20
3	12	5	153.88	12.20
-3	12	-5	152.08	9.80
3	12	5	145.79	9.70
-3	12	-5	140.29	9.50
-3	12	-5	150.38	9.10
4	-12	5	132.09	9.00
-4	-12	-5	125.79	11.00
-4	-12	-5	128.19	8.40
4	12	5	121.79	11.40
-4	12	-5	113.79	8.50
-4	12	-5	123.59	8.40
4	12	5	123.99	8.70

5	-12	5	324.47	19.20
-5	-12	-5	359.46	18.70
-5	-12	-5	349.57	20.90
-5	12	-5	333.47	18.70
-5	12	-5	342.47	18.70
5	12	5	367.86	19.00
-6	-12	-5	21.60	4.30
6	-12	5	11.70	4.80
-6	-12	-5	17.80	5.90
-6	12	-5	19.30	4.60
6	12	5	21.20	7.70
6	12	5	18.30	4.30
-6	12	-5	13.60	4.00
-7	-12	-5	530.05	31.00
-7	-12	-5	553.24	28.50
7	12	5	521.15	28.50
-7	12	-5	515.55	28.40
-7	12	-5	545.15	32.20
7	12	5	614.64	31.00
-8	-12	-5	6.40	6.30
8	-12	5	4.60	3.40
8	12	5	7.20	4.70
-8	12	-5	9.60	4.70
8	12	5	4.60	6.90
-8	12	-5	6.60	8.80
9	-12	5	114.99	9.40
-9	-12	-5	131.69	15.00
-9	12	-5	131.39	12.70
9	12	5	125.79	14.00
9	12	5	101.29	10.30
-10	-12	-5	24.70	8.90
10	-12	5	3.90	4.50
10	12	5	4.20	5.20
-10	12	-5	3.00	5.60
10	12	5	1.00	7.50
-10	12	-5	5.20	6.80
11	12	5	402.36	26.60
-11	12	-5	463.35	26.20
-11	12	-5	461.35	28.30
11	12	5	494.25	30.20
-12	-12	-5	9.50	7.30
-12	12	-5	3.80	6.80
-13	-12	-5	89.69	14.40
-13	12	-5	88.09	12.70

-14	-12	-5	36.20	10.70
-14	12	-5	29.50	8.90
-14	13	5	75.99	12.50
-13	13	5	83.39	12.00
-12	-13	5	53.19	8.40
-12	13	5	61.59	10.70
12	13	-5	40.60	11.00
-11	-13	5	140.79	10.30
-11	13	5	129.49	12.70
11	13	-5	154.28	17.50
-10	-13	5	29.80	4.60
-10	-13	5	23.00	4.30
10	-13	-5	25.20	7.30
-10	-13	5	34.30	11.10
10	13	-5	29.20	8.80
-10	13	5	24.80	6.40
-9	-13	5	144.19	9.00
-9	-13	5	114.99	17.00
-9	-13	5	143.39	8.80
9	-13	-5	120.39	12.70
-9	13	5	118.29	14.60
9	13	-5	120.29	14.70
-9	13	5	134.09	11.20
8	-13	-5	87.49	10.70
-8	-13	5	76.59	6.40
-8	-13	5	57.99	10.20
-8	-13	5	84.09	6.20
-8	13	5	83.59	11.90
8	13	-5	95.09	12.50
-8	13	5	97.29	9.10
-7	-13	5	187.48	17.60
-7	-13	5	258.37	13.20
-7	-13	5	242.38	12.50
7	-13	-5	250.87	16.30
7	13	-5	248.08	17.50
-7	13	5	234.28	17.20
-7	13	5	244.68	15.00
-6	-13	5	129.09	7.30
-6	-13	5	137.49	13.10
6	-13	-5	127.59	11.10
-6	13	5	126.39	11.60
-6	13	5	119.09	9.40
6	13	-5	136.49	12.50
-5	-13	5	482.25	23.30

-5	-13	5	437.36	27.00
5	13	-5	509.15	26.90
-5	13	5	476.85	25.20
-5	13	5	490.05	26.60
5	13	-5	464.55	23.30
4	-13	-5	6.40	4.20
-4	-13	5	4.50	1.70
4	-13	-5	2.80	4.60
-4	-13	5	1.40	4.30
4	13	-5	3.60	4.60
-4	13	5	8.00	4.30
-4	13	5	4.00	3.90
4	13	-5	3.60	1.60
-3	-13	5	112.09	6.30
-3	-13	5	98.19	9.90
3	-13	-5	113.59	8.50
3	-13	-5	118.49	9.90
-3	13	5	122.79	9.70
3	13	-5	107.49	6.20
-3	13	5	93.49	8.70
3	13	-5	97.69	9.70
2	-13	-5	145.99	9.60
-2	-13	5	128.29	10.40
-2	-13	5	139.39	7.60
2	-13	-5	139.79	10.70
-2	13	5	126.99	9.40
-2	13	5	128.79	10.40
2	13	-5	132.09	7.40
2	13	-5	130.99	10.40
-1	-13	5	452.55	22.10
1	-13	-5	473.65	23.10
-1	-13	5	403.16	23.60
1	13	-5	444.16	22.00
-1	13	5	467.25	23.70
1	13	-5	426.66	23.90
-1	13	5	429.56	24.40
0	-13	-5	311.77	16.50
0	-13	5	298.67	15.80
0	13	5	286.17	18.20
0	13	5	334.77	17.80
0	13	-5	300.97	17.40
0	13	5	300.87	17.00
0	13	-5	296.77	15.50
1	-13	5	110.09	7.40

-1	-13	-5	114.49	7.60
-1	13	-5	112.49	6.90
-1	13	-5	93.39	8.60
1	13	5	111.29	8.90
1	13	5	107.99	8.30
-1	13	-5	109.29	7.90
1	13	5	103.49	10.10
2	-13	5	138.89	8.80
-2	-13	-5	131.69	8.60
2	13	5	126.69	8.90
2	13	5	134.89	9.90
-2	13	-5	138.49	8.90
2	13	5	118.99	10.80
-2	13	-5	108.99	9.20
-2	13	-5	133.89	8.00
-3	-13	-5	5.90	2.90
3	-13	5	4.70	3.50
3	13	5	3.80	3.50
-3	13	-5	3.40	2.70
-3	13	-5	5.10	2.90
-3	13	-5	5.00	3.30
3	13	5	-0.80	3.20
3	13	5	1.60	4.60
4	-13	5	229.38	13.80
-4	-13	-5	240.38	13.20
-4	-13	-5	197.48	19.80
-4	13	-5	218.28	13.50
4	13	5	218.08	13.30
4	13	5	233.08	16.10
-4	13	-5	236.38	13.10
-4	13	-5	226.48	13.30
-5	-13	-5	96.89	11.80
-5	-13	-5	123.79	8.30
5	-13	5	89.99	9.10
-5	13	-5	95.99	8.10
5	13	5	102.99	11.40
-5	13	-5	96.19	8.40
5	13	5	95.29	8.20
-6	-13	-5	738.43	44.00
-6	-13	-5	846.92	40.60
6	-13	5	806.32	41.40
6	13	5	851.81	43.10
-6	13	-5	830.02	40.70
6	13	5	828.82	40.80

-6	13	-5	753.32	40.80
-7	-13	-5	71.99	13.80
-7	-13	-5	91.89	8.30
-7	13	-5	63.39	12.60
-7	13	-5	87.39	8.60
7	13	5	80.29	8.50
7	13	5	92.49	12.50
8	-13	5	99.49	8.30
8	13	5	78.59	9.30
8	13	5	80.89	12.80
-8	13	-5	97.09	9.30
-8	13	-5	72.09	12.00
9	-13	5	0.40	3.90
-9	-13	-5	9.50	8.10
9	13	5	0.90	4.80
-9	13	-5	3.90	6.20
-9	13	-5	8.50	5.20
9	13	5	-0.40	7.40
10	-13	5	273.87	16.10
10	13	5	253.97	21.30
10	13	5	241.78	16.80
-10	13	-5	258.87	19.40
-10	13	-5	272.87	17.30
11	13	5	17.70	6.30
-11	13	-5	23.20	7.10
-12	-13	-5	205.18	17.30
-12	13	-5	190.78	15.70
-13	-13	-5	44.20	10.10
-13	13	-5	44.90	8.90
-14	-13	-5	37.60	10.80
-14	13	-5	31.80	9.20
-13	14	5	36.60	8.30
12	14	-5	373.26	27.20
-12	14	5	318.17	20.70
-11	-14	5	108.39	9.10
11	14	-5	89.99	17.00
-11	14	5	110.99	11.70
-10	-14	5	425.46	22.40
10	-14	-5	422.76	25.80
-10	14	5	441.76	24.50
10	14	-5	425.66	28.30
-9	-14	5	27.50	5.00
9	-14	-5	22.60	6.70
-9	-14	5	14.70	14.80

-9	-14	5	21.90	4.00
-9	14	5	28.20	6.10
9	14	-5	18.70	8.20
-9	14	5	12.80	7.80
-8	-14	5	920.31	45.70
8	-14	-5	998.90	49.10
-8	-14	5	827.22	50.79
-8	-14	5	963.20	46.00
-8	14	5	957.10	47.70
-8	14	5	914.91	49.90
8	14	-5	1026.10	50.29
-7	-14	5	33.20	8.30
-7	-14	5	36.90	4.00
-7	-14	5	33.20	4.80
7	-14	-5	43.50	9.00
7	14	-5	38.00	7.80
-7	14	5	28.10	6.80
-7	14	5	31.20	5.80
-6	-14	5	661.93	39.30
-6	-14	5	725.03	35.20
6	-14	-5	697.33	38.80
-6	14	5	768.32	39.10
6	14	-5	819.12	39.30
6	14	-5	698.43	35.30
-6	14	5	733.03	37.20
-5	-14	5	1.40	1.90
-5	-14	5	8.40	5.50
5	14	-5	4.90	5.10
-5	14	5	1.20	5.10
-5	14	5	-0.20	4.10
5	14	-5	2.00	1.90
4	-14	-5	2068.19	98.09
-4	-14	5	1824.72	98.39
4	-14	-5	2080.99	98.89
-4	-14	5	1994.20	95.49
4	14	-5	2162.78	98.49
-4	14	5	2062.69	98.29
4	14	-5	1835.12	95.39
-4	14	5	2024.50	97.29
3	-14	-5	170.98	12.30
3	-14	-5	166.98	11.30
-3	-14	5	172.28	8.90
-3	-14	5	133.59	12.30
3	14	-5	157.88	12.30

-3	14	5	145.69	11.10
3	14	-5	161.28	8.70
-3	14	5	174.88	12.30
-2	-14	5	968.40	51.49
2	-14	-5	1065.99	50.79
-2	-14	5	1037.40	49.30
2	14	-5	1038.60	51.59
-2	14	5	1024.10	52.09
-2	14	5	1000.30	50.89
1	-14	-5	275.77	15.00
-1	-14	5	236.78	15.60
-1	-14	5	233.28	14.50
1	14	-5	264.47	13.50
-1	14	5	246.98	15.30
1	14	-5	289.37	16.20
-1	14	5	274.27	16.10
-1	14	5	254.27	16.70
0	-14	5	772.92	39.00
0	-14	-5	840.62	39.70
0	14	-5	802.42	40.70
0	14	5	766.12	41.30
0	14	-5	766.82	38.70
0	14	5	792.62	40.10
0	14	5	834.72	40.90
1	-14	5	788.22	38.60
-1	-14	-5	785.82	38.80
-1	14	-5	749.73	38.10
1	14	5	795.72	40.80
1	14	5	796.62	40.00
-1	14	-5	764.92	39.70
1	14	5	778.52	39.30
-1	14	-5	783.72	39.20
2	-14	5	688.83	34.90
-2	-14	-5	751.82	35.00
2	14	5	709.13	37.10
-2	14	-5	656.73	34.30
2	14	5	710.93	36.00
-2	14	-5	680.43	35.20
2	14	5	690.93	35.20
-2	14	-5	681.43	35.60
3	-14	5	478.35	25.50
-3	-14	-5	522.15	25.20
-3	-14	-5	469.45	27.10
3	14	5	487.85	26.30

3	14	5	486.95	25.50
-3	14	-5	471.55	24.80
3	14	5	521.25	27.80
-3	14	-5	490.85	25.60
-3	14	-5	461.05	25.70
4	-14	5	37.00	5.50
-4	-14	-5	44.10	5.80
-4	-14	-5	43.70	8.60
4	14	5	33.40	5.00
-4	14	-5	38.10	6.00
-4	14	-5	27.90	4.50
-4	14	-5	42.10	6.40
4	14	5	31.40	6.50
-5	-14	-5	1388.96	70.89
5	-14	5	1391.56	69.39
-5	-14	-5	1475.15	68.79
5	14	5	1523.55	71.19
-5	14	-5	1336.97	68.99
5	14	5	1370.76	68.89
-5	14	-5	1384.66	68.59
-6	-14	-5	81.09	11.90
-6	-14	-5	115.89	8.70
6	14	5	113.39	12.00
-6	14	-5	90.19	8.60
-6	14	-5	93.79	9.10
6	14	5	84.39	8.80
-7	-14	-5	751.92	41.80
7	14	5	815.82	41.60
-7	14	-5	777.92	39.10
7	14	5	726.83	39.10
-7	14	-5	769.32	43.30
-8	-14	-5	39.00	9.30
8	14	5	38.60	8.80
-8	14	-5	39.40	7.10
-8	14	-5	29.90	7.70
8	14	5	33.90	6.30
9	-14	5	568.14	30.10
-9	-14	-5	526.05	34.10
-9	14	-5	549.35	32.10
-9	14	-5	623.34	31.10
9	14	5	648.24	34.10
9	14	5	538.25	30.50
-10	14	-5	72.19	13.30
10	14	5	71.29	10.20

10	14	5	78.89	15.10
-10	14	-5	70.59	10.30
-11	14	-5	421.96	24.00
11	14	5	379.36	23.50
-12	-14	-5	-1.00	8.40
-12	14	-5	-7.70	6.70
-13	-14	-5	342.37	23.70
-13	14	-5	280.27	20.70
-13	15	5	9.80	7.90
-12	15	5	31.30	7.50
12	15	-5	19.80	10.80
11	15	-5	8.10	9.60
-11	15	5	10.50	6.80
-10	-15	5	5.70	3.90
10	15	-5	9.60	9.00
-10	15	5	3.10	6.30
-9	-15	5	4.60	3.80
-9	-15	5	8.00	3.50
9	-15	-5	0.10	6.10
9	15	-5	-2.80	8.00
-9	15	5	-0.40	5.30
-8	-15	5	4.90	3.00
-8	-15	5	6.80	2.80
8	-15	-5	13.80	6.00
-8	15	5	6.00	5.30
-8	15	5	17.00	7.90
8	15	-5	3.70	7.10
-7	-15	5	71.99	6.30
-7	-15	5	69.29	6.30
-7	-15	5	81.49	13.90
7	-15	-5	68.29	10.30
-7	15	5	70.59	11.70
6	-15	-5	23.60	8.00
-6	-15	5	14.10	7.60
-6	-15	5	18.30	2.60
-6	15	5	6.10	5.50
-6	15	5	15.70	4.90
6	15	-5	19.90	6.70
5	-15	-5	52.59	10.30
-5	-15	5	43.50	4.20
-5	-15	5	49.70	9.00
-5	15	5	42.50	7.70
-5	15	5	39.90	7.10
5	15	-5	61.89	9.70

5	15	-5	46.00	4.30
-4	-15	5	90.99	5.70
4	-15	-5	86.79	9.90
-4	-15	5	76.79	11.00
4	15	-5	73.59	5.70
-4	15	5	84.99	8.70
-4	15	5	94.39	10.20
4	15	-5	85.99	10.50
3	-15	-5	3.90	4.80
3	-15	-5	5.30	5.10
-3	-15	5	7.90	2.40
-3	-15	5	11.60	5.20
3	15	-5	2.70	4.80
-3	15	5	-2.90	4.20
-2	-15	5	-0.50	4.20
2	-15	-5	1.10	4.10
-2	15	5	-4.20	4.10
2	15	-5	2.20	2.20
2	15	-5	3.20	4.70
-2	15	5	-3.30	4.70
-2	15	5	2.00	4.70
-1	-15	5	55.69	5.50
1	-15	-5	63.09	7.20
-1	-15	5	75.39	8.10
1	15	-5	74.79	8.50
-1	15	5	62.99	9.50
-1	15	5	59.39	8.30
1	15	-5	58.89	5.00
-1	15	5	72.99	7.80
0	-15	5	5.40	3.00
0	-15	-5	8.70	3.80
0	15	5	3.80	4.30
0	15	5	5.20	5.70
0	15	5	0.40	3.80
0	15	-5	3.60	2.60
0	15	-5	4.20	4.40
1	-15	5	20.70	4.00
-1	15	-5	13.90	4.70
1	15	5	14.20	5.70
1	15	5	10.80	4.70
1	15	5	16.90	4.40
-1	15	-5	16.70	3.20
-1	15	-5	30.20	5.80
2	-15	5	33.20	6.20

-2	-15	-5	44.90	5.80
2	15	5	45.80	9.20
2	15	5	34.60	6.40
-2	15	-5	34.50	5.10
2	15	5	39.80	6.00
-2	15	-5	36.70	5.10
-2	15	-5	37.80	7.00
3	-15	5	20.80	4.70
-3	-15	-5	35.00	5.50
-3	15	-5	21.10	4.90
3	15	5	22.70	4.60
-3	15	-5	15.40	4.00
3	15	5	19.70	6.50
3	15	5	21.60	5.10
-3	15	-5	25.00	3.90
-4	-15	-5	27.20	6.60
-4	-15	-5	32.40	6.00
4	-15	5	28.50	5.50
-4	15	-5	22.50	4.40
-4	15	-5	34.00	6.70
4	15	5	27.50	4.80
-4	15	-5	33.80	6.20
4	15	5	29.30	6.90
5	-15	5	6.00	4.80
-5	-15	-5	5.80	4.00
-5	-15	-5	-5.00	5.60
5	15	5	0.40	5.80
5	15	5	7.30	4.40
-5	15	-5	4.70	4.00
-5	15	-5	6.90	4.20
-5	15	-5	6.80	4.00
-6	-15	-5	25.90	6.90
-6	-15	-5	14.40	4.50
6	15	5	10.70	6.60
-6	15	-5	12.20	4.50
6	15	5	15.80	4.80
-6	15	-5	14.20	4.70
-7	-15	-5	0.20	6.70
-7	15	-5	18.40	5.10
-7	15	-5	6.20	7.70
7	15	5	8.20	7.00
7	15	5	5.50	4.70
-8	-15	-5	141.59	15.80
-8	15	-5	124.19	13.20

8	15	5	119.79	11.20
8	15	5	141.89	15.30
-8	15	-5	123.09	12.40
-9	15	-5	11.20	7.20
9	15	5	7.80	8.30
9	15	5	9.30	5.40
-9	15	-5	20.40	6.20
-10	15	-5	-0.10	5.70
10	15	5	13.00	8.60
11	15	5	11.90	6.30
-11	15	-5	10.50	6.60
-12	15	-5	7.60	7.10
12	15	5	12.10	7.40
-13	-15	-5	30.40	10.70
-13	15	-5	38.20	9.00
-13	16	5	25.20	8.70
-12	16	5	189.18	15.50
12	16	-5	161.28	22.00
11	16	-5	20.60	10.60
-11	16	5	24.00	7.60
-10	-16	5	196.98	12.10
10	16	-5	171.28	20.00
-10	16	5	190.18	14.80
-9	-16	5	36.80	4.90
9	-16	-5	32.40	7.70
9	16	-5	33.70	10.00
-9	16	5	35.80	7.00
-8	-16	5	203.68	11.00
8	-16	-5	205.78	16.10
-8	-16	5	210.18	11.60
-8	16	5	196.48	14.30
8	16	-5	185.18	17.50
-8	16	5	181.68	17.40
7	-16	-5	6.10	6.50
-7	16	5	6.30	5.40
-7	16	5	15.20	6.80
7	16	-5	9.10	6.90
-6	-16	5	391.36	19.20
-6	16	5	367.56	23.70
-6	16	5	392.86	22.80
6	16	-5	380.66	23.80
-5	-16	5	42.70	4.30
-5	-16	5	33.80	7.70
5	-16	-5	49.20	7.70

-5	16	5	46.10	9.40
5	16	-5	44.10	7.70
-5	16	5	38.80	6.50
4	-16	-5	736.23	37.50
-4	-16	5	607.44	37.90
-4	-16	5	732.93	34.40
4	16	-5	745.23	38.10
-4	16	5	705.73	36.40
4	16	-5	705.03	34.80
-3	-16	5	18.00	6.00
-3	16	5	2.40	4.70
3	16	-5	7.50	2.50
-3	16	5	-3.50	4.10
3	16	-5	8.00	5.30
-2	-16	5	402.26	24.00
-2	-16	5	423.56	21.30
-2	16	5	433.06	24.80
-2	16	5	404.76	23.10
2	16	-5	409.46	21.00
2	16	-5	415.06	24.10
-2	16	5	454.95	24.30
-1	-16	5	375.66	19.40
1	-16	-5	377.06	20.50
-1	16	5	370.06	22.60
-1	16	5	364.56	20.90
1	16	-5	368.16	19.00
1	16	-5	371.46	21.70
-1	16	5	378.96	21.80
0	-16	5	265.17	15.10
0	-16	-5	295.67	16.00
0	16	-5	269.47	14.60
0	16	5	272.77	17.20
0	16	5	266.37	18.30
0	16	5	284.47	16.50
0	16	-5	263.67	17.10
-1	-16	-5	534.45	26.70
1	-16	5	496.35	26.40
-1	16	-5	505.05	25.80
1	16	5	502.45	29.00
-1	16	-5	530.45	28.00
-1	16	-5	508.75	27.20
1	16	5	497.35	27.90
1	16	5	533.15	27.30
-2	-16	-5	453.75	23.20

2	-16	5	450.65	23.30
-2	16	-5	440.26	23.70
-2	16	-5	429.86	22.60
-2	16	-5	429.56	24.40
2	16	5	422.66	24.50
2	16	5	450.85	23.70
2	16	5	432.16	25.80
3	-16	5	315.57	18.20
-3	-16	-5	339.57	17.80
-3	16	-5	318.27	18.30
-3	16	-5	323.37	17.30
3	16	5	323.77	20.80
3	16	5	298.97	19.00
-3	16	-5	313.97	18.90
3	16	5	318.97	18.10
-4	-16	-5	42.60	7.80
-4	-16	-5	60.99	6.70
4	-16	5	37.10	6.30
4	16	5	49.40	8.40
-4	16	-5	50.09	7.10
-4	16	-5	37.10	6.40
4	16	5	44.40	7.00
4	16	5	42.40	7.50
-4	16	-5	36.90	7.50
-5	-16	-5	821.92	39.80
-5	-16	-5	766.12	42.50
-5	16	-5	793.42	40.60
-5	16	-5	769.82	39.50
5	16	5	826.02	42.50
5	16	5	776.12	39.90
-5	16	-5	756.12	40.20
-6	-16	-5	175.28	15.90
6	16	5	182.48	15.50
-6	16	-5	161.78	11.80
6	16	5	162.08	12.10
-6	16	-5	162.58	12.50
-7	-16	-5	403.46	27.00
7	16	5	423.96	23.30
7	16	5	427.36	26.40
-7	16	-5	408.26	28.60
-7	16	-5	426.56	23.30
-8	-16	-5	22.10	9.40
8	16	5	7.10	5.30
8	16	5	7.10	7.90

-8	16	-5	6.30	5.50
-8	16	-5	10.10	6.60
9	16	5	376.56	25.50
9	16	5	336.87	21.50
-9	16	-5	414.46	22.10
-9	16	-5	338.27	23.30
-10	16	-5	52.79	8.40
10	16	5	65.79	10.30
-11	16	-5	390.56	22.90
11	16	5	341.97	22.50
12	16	5	8.10	7.30
-12	16	-5	21.00	8.00
-13	16	-5	238.78	18.50
12	17	-5	19.70	14.20
-12	17	5	16.10	8.00
11	17	-5	188.28	28.70
-11	17	5	193.38	15.80
-10	17	5	28.90	7.70
10	17	-5	27.40	11.70
-9	-17	5	58.99	6.70
9	17	-5	66.99	11.60
-9	17	5	57.29	10.30
-8	-17	5	1.10	3.40
8	-17	-5	-6.10	5.90
-8	17	5	-5.80	5.50
8	17	-5	1.60	7.70
7	-17	-5	119.29	12.90
-7	-17	5	122.39	9.60
-7	17	5	117.29	11.30
-7	17	5	97.49	16.50
7	17	-5	147.39	14.70
-6	17	5	122.89	11.20
-6	17	5	93.29	17.30
6	17	-5	142.99	14.10
6	17	-5	120.29	7.90
5	-17	-5	243.68	17.10
-5	17	5	232.78	15.50
5	17	-5	221.48	12.70
5	17	-5	255.67	17.70
-4	-17	5	19.00	4.00
4	17	-5	20.20	3.10
4	17	-5	13.50	7.00
-4	17	5	10.10	5.80
-4	17	5	12.80	5.50

-3	-17	5	59.39	5.50
-3	17	5	67.09	11.00
-3	17	5	57.79	9.80
3	17	-5	63.19	10.20
-3	17	5	53.49	8.60
3	17	-5	60.59	5.10
-2	-17	5	-1.20	3.00
2	17	-5	7.10	5.40
-2	17	5	2.20	4.90
-2	17	5	3.90	4.90
-2	17	5	4.40	6.20
2	17	-5	5.70	2.50
-1	-17	5	168.78	11.00
-1	17	5	199.58	15.30
-1	17	5	196.28	13.80
1	17	-5	189.68	14.00
1	17	-5	174.98	10.40
-1	17	5	184.98	12.80
0	-17	5	65.19	7.10
0	17	-5	77.29	9.70
0	17	-5	76.49	6.30
0	17	5	74.19	8.70
0	17	5	71.69	10.80
0	17	5	79.19	9.40
0	17	-5	80.59	8.10
1	-17	5	210.18	13.20
-1	-17	-5	230.08	13.50
1	17	5	210.98	14.70
-1	17	-5	203.98	12.30
-1	17	-5	205.28	13.70
1	17	5	237.98	16.90
-1	17	-5	231.38	15.20
1	17	5	209.58	13.90
2	-17	5	44.20	7.30
-2	-17	-5	41.40	6.20
2	17	5	45.70	6.20
-2	17	-5	48.80	5.60
-2	17	-5	30.40	6.10
2	17	5	43.20	8.30
2	17	5	29.40	7.00
-2	17	-5	42.10	6.90
-3	-17	-5	17.30	4.50
3	-17	5	16.50	5.00
-3	17	-5	8.50	4.90

3	17	5	15.80	4.90
-3	17	-5	10.40	3.80
-3	17	-5	10.90	4.30
3	17	5	14.00	6.90
3	17	5	7.10	5.30
4	-17	5	114.59	10.80
4	17	5	122.49	13.40
-4	17	-5	137.49	11.20
-4	17	-5	127.89	10.40
4	17	5	124.49	11.20
-4	17	-5	123.09	9.40
4	17	5	113.89	10.20
-5	-17	-5	493.05	29.00
-5	17	-5	450.75	26.10
5	17	5	468.55	25.70
-5	17	-5	487.45	26.70
-5	17	-5	461.95	25.30
5	17	5	511.25	29.00
-6	-17	-5	147.49	16.50
6	17	5	178.58	15.80
6	17	5	161.28	12.30
-6	17	-5	181.88	13.30
-6	17	-5	152.78	12.70
-6	17	-5	159.48	11.90
-7	-17	-5	7.10	8.10
7	17	5	-4.50	4.90
7	17	5	4.70	7.10
8	17	5	154.78	16.40
8	17	5	116.29	11.90
-8	17	-5	151.98	12.10
-8	17	-5	127.59	14.00
9	17	5	-1.50	9.20
-9	17	-5	8.00	6.20
-10	17	-5	129.99	12.80
10	17	5	112.49	12.40
-11	17	-5	13.50	7.40
11	17	5	10.20	7.10
12	17	5	234.88	19.10
-12	17	-5	282.87	19.10
-12	18	5	190.98	16.80
-11	18	5	18.00	7.80
11	18	-5	21.80	13.20
10	18	-5	36.10	15.30
-10	18	5	70.39	11.50

-9	18	5	11.90	6.70
9	18	-5	4.80	9.60
-8	-18	5	22.50	4.10
8	18	-5	20.70	9.30
-8	18	5	12.80	6.40
7	-18	-5	50.69	9.60
7	18	-5	61.19	5.90
7	18	-5	82.09	13.40
-7	18	5	63.89	10.00
-6	-18	5	243.48	13.40
6	-18	-5	268.37	20.70
-6	18	5	245.18	16.60
6	18	-5	266.97	19.30
6	18	-5	230.38	13.10
-6	18	5	221.78	19.50
-5	-18	5	12.70	3.00
5	-18	-5	10.40	6.50
-5	18	5	5.80	5.60
-5	18	5	11.70	6.30
5	18	-5	10.70	3.00
5	18	-5	12.20	6.70
-4	-18	5	16.70	3.30
4	18	-5	15.40	3.00
4	18	-5	8.00	6.20
-4	18	5	21.50	7.00
-4	18	5	9.90	5.80
-3	-18	5	80.89	6.40
-3	18	5	69.59	9.30
-3	18	5	77.59	10.60
3	18	-5	70.59	11.40
3	18	-5	73.79	6.00
-3	18	5	62.19	11.80
-2	-18	5	92.19	7.30
-2	18	5	94.09	10.80
-2	18	5	59.79	9.50
2	18	-5	78.39	11.20
2	18	-5	90.59	6.50
-2	18	5	89.89	12.30
-1	-18	5	127.29	9.00
-1	18	5	125.89	12.20
-1	18	5	119.49	11.90
-1	18	5	120.39	13.60
1	18	-5	127.79	12.30
1	18	-5	124.59	8.20

0	-18	5	192.68	11.90
0	18	-5	185.38	11.00
0	18	5	209.78	14.70
0	18	-5	178.88	12.80
0	18	5	186.68	15.90
0	18	5	178.58	13.10
0	18	-5	169.38	14.00
1	-18	5	203.48	12.90
1	18	5	204.98	13.70
-1	18	-5	204.78	13.50
-1	18	-5	202.48	11.90
1	18	5	209.58	16.50
-1	18	-5	193.38	14.70
1	18	5	186.48	14.40
2	-18	5	1.90	4.70
-2	18	-5	5.80	5.30
2	18	5	16.90	5.50
2	18	5	3.10	6.40
-2	18	-5	11.50	4.40
-2	18	-5	9.20	3.60
2	18	5	10.70	4.80
3	-18	5	11.80	5.30
3	18	5	9.80	6.60
3	18	5	21.40	5.50
-3	18	-5	14.20	4.70
3	18	5	19.80	5.30
-3	18	-5	23.20	9.10
-3	18	-5	16.10	4.40
4	18	5	12.80	5.00
-4	18	-5	8.80	4.30
-4	18	-5	7.60	5.50
4	18	5	16.80	7.70
-4	18	-5	12.80	4.90
4	18	5	7.80	5.50
-5	-18	-5	109.39	15.00
5	18	5	128.79	10.70
-5	18	-5	125.09	11.00
-5	18	-5	130.69	11.80
5	18	5	121.19	14.50
-5	18	-5	114.89	10.20
-6	-18	-5	69.09	11.10
6	18	5	65.79	10.60
6	18	5	83.89	9.30
-6	18	-5	72.39	9.00

-6	18	-5	62.79	9.70
-6	18	-5	70.29	10.50
-7	-18	-5	102.29	16.70
7	18	5	107.19	11.10
-7	18	-5	117.59	10.80
-7	18	-5	117.39	17.50
7	18	5	122.69	15.80
8	18	5	-5.50	7.90
-8	18	-5	-3.00	5.90
8	18	5	-3.40	5.40
9	18	5	33.70	7.30
-9	18	-5	42.10	7.70
9	18	5	35.90	11.70
10	18	5	-5.60	6.10
-10	18	-5	6.10	7.00
-11	18	-5	86.39	12.20
11	18	5	68.59	12.40
-12	18	-5	25.60	8.10
12	18	5	12.60	8.00
-11	19	5	267.77	19.90
11	19	-5	288.17	27.30
10	19	-5	19.20	12.50
-10	19	5	2.30	7.70
9	19	-5	362.66	27.10
-9	19	5	360.26	22.30
8	19	-5	-7.80	9.40
-8	19	5	-9.70	5.90
-7	19	5	250.57	16.90
7	19	-5	233.28	25.00
7	19	-5	225.88	12.80
6	-19	-5	74.89	12.60
6	19	-5	69.79	13.50
6	19	-5	48.20	5.60
-6	19	5	65.19	8.80
-5	-19	5	479.45	23.80
-5	19	5	495.65	26.60
5	19	-5	444.36	23.70
-5	19	5	450.65	28.30
5	19	-5	500.55	28.90
-4	-19	5	13.30	3.40
4	19	-5	-0.90	6.80
-4	19	5	6.10	5.70
-4	19	5	5.50	6.30
4	19	-5	9.80	3.20

-4	19	5	1.80	7.00
-3	-19	5	485.65	25.20
3	19	-5	493.35	29.10
-3	19	5	493.55	27.60
-3	19	5	527.75	29.00
3	19	-5	483.15	24.90
-3	19	5	502.05	30.00
-2	-19	5	709.13	35.90
2	19	-5	734.03	39.20
-2	19	5	709.13	39.00
-2	19	5	737.43	37.70
2	19	-5	686.93	35.50
-2	19	5	757.72	40.20
-1	-19	5	230.38	13.70
-1	19	5	234.78	16.80
1	19	-5	223.68	16.70
1	19	-5	225.28	12.90
-1	19	5	224.28	18.00
-1	19	5	227.38	15.30
0	-19	5	493.15	25.90
0	19	-5	454.25	26.80
0	19	5	494.65	29.30
0	19	-5	491.65	28.20
0	19	-5	504.15	25.20
0	19	5	527.15	27.30
0	19	5	482.15	28.00
1	-19	5	472.75	24.70
1	19	5	473.65	25.40
-1	19	-5	442.56	25.30
1	19	5	471.45	26.60
1	19	5	474.35	28.00
-1	19	-5	478.25	23.90
-1	19	-5	389.76	28.40
2	-19	5	458.65	25.20
2	19	5	448.56	26.40
-2	19	-5	424.06	27.20
2	19	5	484.25	25.50
2	19	5	484.45	28.30
-2	19	-5	491.25	24.30
-2	19	-5	449.16	25.70
3	-19	5	65.79	9.10
-3	19	-5	50.89	10.10
3	19	5	67.79	8.90
3	19	5	63.79	12.20

-3	19	-5	58.39	8.50
3	19	5	65.99	10.20
-3	19	-5	60.49	7.40
4	19	5	444.86	28.10
-4	19	-5	458.65	24.20
-4	19	-5	476.25	26.60
4	19	5	423.96	26.20
-4	19	-5	450.75	25.50
4	19	5	467.75	25.00
-5	-19	-5	8.10	8.00
-5	19	-5	0.30	5.70
-5	19	-5	-0.30	4.70
5	19	5	4.80	6.00
5	19	5	2.10	7.40
-5	19	-5	-5.10	4.50
5	19	5	3.70	5.00
-6	-19	-5	551.24	34.40
-6	19	-5	532.15	31.20
6	19	5	554.04	30.50
6	19	5	642.44	34.70
-6	19	-5	583.14	32.10
7	19	5	7.40	5.70
7	19	5	3.60	8.70
-7	19	-5	8.40	6.50
-7	19	-5	6.20	5.60
8	19	5	182.98	19.00
-8	19	-5	193.08	13.90
8	19	5	146.39	14.70
-9	19	-5	-10.80	5.90
9	19	5	1.10	6.10
-10	19	-5	268.27	18.00
10	19	5	220.28	17.60
-11	19	-5	-0.20	7.60
11	19	5	-4.90	7.10
10	20	-5	2.10	11.40
-10	20	5	6.90	7.80
9	20	-5	16.50	11.40
-9	20	5	14.10	7.10
8	20	-5	8.30	9.50
-8	20	5	3.80	6.60
-7	20	5	18.50	7.60
7	20	-5	38.30	9.70
6	20	-5	40.30	9.00
-6	20	5	30.10	7.60

6	20	-5	30.00	5.20
-5	20	5	3.50	6.00
5	20	-5	2.70	3.10
5	20	-5	4.20	7.80
-4	-20	5	7.90	3.60
4	20	-5	7.50	3.30
-4	20	5	6.60	8.10
-4	20	5	19.80	7.10
-4	20	5	6.00	6.00
4	20	-5	18.10	9.50
3	20	-5	-3.00	3.00
3	20	-5	4.10	11.30
-3	20	5	-1.40	7.20
-3	20	5	1.40	6.70
-3	20	5	-10.80	5.70
-2	-20	5	6.90	4.10
2	20	-5	11.20	8.10
2	20	-5	8.90	3.40
-2	20	5	10.90	7.40
-2	20	5	6.70	6.50
-1	-20	5	4.00	4.40
1	20	-5	5.40	4.80
1	20	-5	0.40	3.50
-1	20	5	-0.70	5.90
-1	20	5	7.30	7.40
1	20	-5	5.00	6.90
-1	20	5	2.80	5.60
0	-20	5	78.89	8.90
0	20	5	87.19	11.60
0	20	-5	106.09	12.30
0	20	-5	84.79	7.80
0	20	-5	94.09	9.90
0	20	5	100.89	14.30
0	20	5	84.99	10.60
1	-20	5	3.20	4.90
-1	20	-5	1.60	4.70
-1	20	-5	5.20	3.80
1	20	5	6.30	6.10
1	20	5	-3.10	6.90
1	20	5	2.40	5.20
-1	20	-5	3.90	6.00
2	-20	5	10.50	5.60
2	20	5	8.80	6.10
-2	20	-5	5.60	6.50

2	20	5	6.40	5.30
-2	20	-5	2.40	4.60
-2	20	-5	15.90	4.40
2	20	5	8.10	7.50
-3	20	-5	18.30	4.70
-3	20	-5	14.10	6.80
3	20	5	20.50	6.50
3	20	5	18.40	8.10
3	20	5	13.80	5.60
-3	20	-5	16.00	5.40
4	20	5	1.40	5.20
-4	20	-5	-3.60	5.70
4	20	5	-2.80	5.90
-4	20	-5	3.80	4.70
4	20	5	-2.90	8.20
-4	20	-5	3.30	5.00
-5	20	-5	6.50	5.40
-5	20	-5	8.80	5.20
5	20	5	15.50	6.70
-5	20	-5	6.20	5.90
5	20	5	9.50	8.30
5	20	5	7.00	5.50
6	20	5	14.30	5.80
-6	20	-5	4.20	5.30
-6	20	-5	1.20	6.20
6	20	5	8.00	8.80
7	20	5	13.20	7.60
-7	20	-5	14.60	6.20
7	20	5	13.00	9.20
-7	20	-5	6.40	7.50
-8	20	-5	26.20	8.40
8	20	5	30.70	7.10
-8	20	-5	33.10	7.30
8	20	5	30.10	11.30
-9	20	-5	13.30	6.70
9	20	5	9.90	6.80
10	20	5	7.30	7.10
-10	20	-5	15.70	7.30
-10	21	5	22.20	8.80
10	21	-5	8.10	12.60
-9	21	5	171.08	14.40
9	21	-5	151.58	21.00
-8	21	5	-3.10	6.50
8	21	-5	3.30	9.90

-7	21	5	359.96	22.50
7	21	-5	339.97	25.80
6	21	-5	29.20	9.60
-6	21	5	42.30	8.60
5	21	-5	631.44	32.90
5	21	-5	658.83	38.10
-5	21	5	703.03	36.10
-5	21	5	675.53	39.40
-4	21	5	-4.40	6.30
4	21	-5	3.20	3.30
-4	21	5	-8.90	8.90
4	21	-5	4.90	7.80
-3	-21	5	410.26	22.50
-3	21	5	432.06	28.00
-3	21	5	433.96	24.80
3	21	-5	427.96	26.80
3	21	-5	410.86	21.70
-2	-21	5	186.58	16.30
2	21	-5	226.48	17.60
2	21	-5	199.48	12.00
-2	21	5	207.78	16.90
-2	21	5	192.28	15.00
-2	21	5	202.68	18.60
-1	-21	5	193.28	12.70
1	21	-5	171.38	16.30
1	21	-5	185.18	11.60
-1	21	5	194.98	18.10
-1	21	5	178.98	16.00
1	21	-5	195.88	14.70
-1	21	5	179.68	14.60
0	-21	5	179.18	12.70
0	21	-5	183.08	11.70
0	21	-5	172.78	13.60
0	21	-5	186.78	16.00
0	21	5	183.28	17.90
0	21	5	175.88	15.90
1	-21	5	474.15	26.60
-1	21	-5	464.55	27.30
-1	21	-5	497.05	25.60
1	21	5	477.05	28.70
1	21	5	499.55	27.40
-1	21	-5	496.25	29.10
1	21	5	514.35	30.90
2	21	5	464.45	28.10

-2	21	-5	445.86	27.00
-2	21	-5	464.05	28.50
-2	21	-5	498.35	25.40
2	21	5	509.15	26.80
2	21	5	485.25	30.00
3	21	5	85.19	12.40
3	21	5	96.79	10.50
-3	21	-5	93.39	9.30
3	21	5	103.69	15.90
-3	21	-5	82.29	12.50
-4	21	-5	460.05	25.40
-4	21	-5	496.15	28.40
4	21	5	470.75	26.30
4	21	5	487.75	30.20
4	21	5	442.36	28.00
5	21	5	52.89	11.90
-5	21	-5	24.60	6.10
-5	21	-5	34.90	8.60
-6	21	-5	465.75	28.10
6	21	5	469.75	30.30
-6	21	-5	454.65	25.20
6	21	5	425.76	25.50
7	21	5	69.79	10.90
7	21	5	88.49	17.20
-7	21	-5	69.69	10.30
-7	21	-5	69.79	13.30
-8	21	-5	326.67	20.70
-8	21	-5	359.46	23.70
8	21	5	309.97	20.80
-9	21	-5	11.30	7.00
-9	21	-5	4.90	8.70
9	21	5	6.60	7.00
10	21	5	306.67	20.80
-10	21	-5	314.67	21.10
-9	22	5	4.10	7.60
9	22	-5	21.00	13.80
8	22	-5	56.49	13.50
-8	22	5	67.29	11.30
7	22	-5	-1.40	9.30
-7	22	5	-15.70	6.90
-6	22	5	452.85	26.60
-6	22	5	427.16	31.20
6	22	-5	457.15	30.00
-5	22	5	26.30	8.30

5	22	-5	20.80	9.00
-5	22	5	17.00	10.30
4	22	-5	245.28	14.50
-4	22	5	268.17	22.70
-4	22	5	264.37	18.50
4	22	-5	284.27	21.70
3	22	-5	26.50	4.30
-3	22	5	19.40	7.50
3	22	-5	29.20	9.50
-2	22	5	200.18	18.90
2	22	-5	205.58	17.80
-2	22	5	167.98	14.90
2	22	-5	178.38	11.50
-1	-22	5	83.99	9.70
-1	22	5	86.49	11.30
-1	22	5	98.99	15.80
1	22	-5	99.89	13.90
1	22	-5	91.79	8.30
0	-22	5	136.59	12.20
0	22	-5	172.58	16.10
0	22	5	157.48	13.60
0	22	5	161.78	18.30
0	22	-5	155.58	10.90
1	-22	5	502.55	28.80
-1	22	-5	546.75	31.60
1	22	5	536.45	29.60
-1	22	-5	525.75	27.80
1	22	5	555.84	33.30
2	22	5	19.00	6.60
-2	22	-5	22.30	5.40
2	22	5	8.30	9.00
-2	22	-5	20.30	8.40
-3	22	-5	88.49	9.60
3	22	5	111.99	16.90
3	22	5	108.89	11.10
-3	22	-5	83.09	13.50
-4	22	-5	64.49	12.10
4	22	5	68.89	12.80
-4	22	-5	72.59	12.90
4	22	5	60.79	10.00
5	22	5	324.17	24.80
-5	22	-5	264.37	18.50
-5	22	-5	336.07	22.40
5	22	5	316.37	19.20

-6	22	-5	7.80	6.40
-6	22	-5	10.10	8.30
6	22	5	6.50	9.90
6	22	5	16.60	6.60
-7	22	-5	210.68	18.60
-7	22	-5	207.48	15.10
7	22	5	163.18	15.10
7	22	5	218.28	27.30
8	22	5	11.40	7.10
-8	22	-5	3.30	8.20
-8	22	-5	0.80	6.90
-9	22	-5	41.70	10.20
-9	22	-5	42.90	8.70
9	22	5	36.00	8.40
-8	23	5	6.10	7.50
8	23	-5	2.00	11.50
7	23	-5	21.80	11.10
-7	23	5	35.60	12.20
-7	23	5	28.00	8.70
6	23	-5	9.90	10.90
-6	23	5	10.30	10.80
-6	23	5	4.60	7.60
-5	23	5	67.69	13.60
5	23	-5	74.39	12.10
-5	23	5	111.59	13.00
-4	23	5	14.20	7.70
4	23	-5	9.40	9.60
-4	23	5	26.20	11.60
-3	23	5	35.30	8.50
-3	23	5	21.60	11.00
3	23	-5	33.20	4.80
3	23	-5	19.40	8.90
-2	23	5	62.69	11.30
2	23	-5	76.49	7.50
-2	23	5	47.70	11.30
2	23	-5	59.19	10.40
1	23	-5	61.99	7.80
1	23	-5	66.59	11.70
-1	23	5	83.79	14.10
-1	23	5	73.59	11.50
0	23	5	146.89	13.90
0	23	-5	152.88	16.50
0	23	5	148.99	18.80
0	23	-5	144.19	10.90

-1	23	-5	85.09	9.40
1	23	5	104.49	16.80
-1	23	-5	94.59	14.30
1	23	5	114.79	12.20
-2	23	-5	10.80	7.60
-2	23	-5	18.40	5.50
2	23	5	14.10	6.40
2	23	5	10.10	9.90
-3	23	-5	0.40	7.40
3	23	5	-3.90	5.80
3	23	5	-3.70	9.50
-3	23	-5	-0.60	5.10
4	23	5	42.90	7.60
-4	23	-5	36.70	6.80
4	23	5	21.90	11.20
-4	23	-5	45.40	10.20
5	23	5	12.10	6.80
-5	23	-5	10.50	6.00
-5	23	-5	8.60	8.00
5	23	5	9.40	11.60
6	23	5	144.99	13.90
6	23	5	186.18	21.10
-6	23	-5	145.69	13.20
-6	23	-5	149.39	17.30
-7	23	-5	-5.00	6.70
-7	23	-5	-6.50	7.90
7	23	5	-1.30	6.80
-8	23	-5	71.79	11.90
-8	23	-5	60.79	12.10
8	23	5	73.49	12.00
-7	24	5	3.20	8.10
-7	24	5	0.80	12.50
7	24	-5	14.50	12.20
6	24	-5	179.28	21.40
-6	24	5	164.58	23.20
-6	24	5	133.79	15.20
-5	24	5	34.20	12.10
-5	24	5	29.30	9.00
5	24	-5	21.30	10.40
-4	24	5	323.97	26.70
-4	24	5	323.97	21.60
4	24	-5	296.57	24.40
-3	24	5	28.20	11.90
3	24	-5	29.30	10.90

-3	24	5	36.70	8.60
2	24	-5	151.38	17.80
-2	24	5	151.28	19.60
-2	24	5	151.38	14.70
2	24	-5	144.49	10.60
-1	24	5	224.88	22.50
1	24	-5	229.68	20.10
1	24	-5	216.28	14.00
-1	24	5	236.68	17.40
0	24	5	397.56	23.40
0	24	-5	358.86	20.90
0	24	-5	400.16	26.10
0	24	5	351.76	30.10
-1	24	-5	150.28	17.60
1	24	5	145.39	24.90
1	24	5	169.98	14.80
-1	24	-5	164.38	12.20
-2	24	-5	243.18	15.60
2	24	5	243.18	23.40
2	24	5	228.58	17.20
-2	24	-5	237.28	20.40
-3	24	-5	243.18	16.90
-3	24	-5	254.77	21.20
3	24	5	278.67	18.10
3	24	5	272.67	24.30
-4	24	-5	10.10	6.20
-4	24	-5	15.20	9.30
4	24	5	13.40	10.80
4	24	5	15.80	7.20
-5	24	-5	238.18	17.20
5	24	5	258.07	18.20
5	24	5	261.97	24.40
-5	24	-5	255.37	21.50
6	24	5	16.90	7.40
-6	24	-5	23.00	7.40
-6	24	-5	18.80	9.80
-7	24	-5	194.58	20.20
7	24	5	185.88	16.00
-7	24	-5	196.68	15.60
6	25	-5	9.10	11.60
-6	25	5	14.60	8.10
-6	25	5	-20.40	11.30
5	25	-5	11.00	11.50
-5	25	5	8.70	13.60

-5	25	5	1.00	7.90
-4	25	5	63.89	13.70
-4	25	5	77.09	13.30
4	25	-5	71.49	12.70
-3	25	5	1.40	7.80
3	25	-5	3.10	9.30
-3	25	5	-3.30	10.90
-2	25	5	2.90	7.90
-2	25	5	10.30	11.20
2	25	-5	11.90	10.40
-1	25	5	14.20	11.10
1	25	-5	20.20	5.30
-1	25	5	16.20	8.00
1	25	-5	18.80	9.40
0	25	-5	2.00	8.60
0	25	-5	14.80	5.40
0	25	5	3.90	7.70
0	25	5	1.30	11.20
1	25	5	8.80	7.30
-1	25	-5	0.90	8.80
-1	25	-5	8.70	5.50
1	25	5	14.00	11.10
-2	25	-5	-1.70	8.10
2	25	5	32.20	12.90
2	25	5	22.30	7.40
-3	25	-5	-8.10	8.50
3	25	5	4.80	7.30
3	25	5	-16.80	11.00
-3	25	-5	-1.90	5.90
4	25	5	19.90	7.40
4	25	5	22.70	18.40
-4	25	-5	19.80	7.00
-4	25	-5	28.90	9.70
5	25	5	14.90	7.80
-5	25	-5	22.50	7.50
-5	25	-5	28.60	10.90
6	25	5	-0.30	7.10
-6	25	-5	6.00	7.10
-6	25	-5	6.90	9.20
-5	26	5	-9.10	8.00
-5	26	5	6.00	13.10
5	26	-5	-7.60	11.00
-4	26	5	301.17	21.40
-4	26	5	297.47	28.60

4	26	-5	262.97	25.60
-3	26	5	-11.70	11.60
-3	26	5	-12.40	8.00
3	26	-5	0.40	11.30
2	26	-5	74.59	12.80
-2	26	5	55.59	15.00
-2	26	5	61.69	10.10
1	26	-5	43.90	11.10
-1	26	5	67.89	14.50
-1	26	5	61.69	10.30
0	26	-5	113.19	18.10
0	26	5	156.88	15.50
0	26	5	164.88	22.50
1	26	5	130.29	14.00
-1	26	-5	107.39	12.90
1	26	5	131.59	22.30
-1	26	-5	90.49	17.50
-2	26	-5	75.59	10.50
2	26	5	89.09	13.10
2	26	5	76.49	15.30
-2	26	-5	69.79	12.80
3	26	5	93.49	12.90
-3	26	-5	96.09	11.50
3	26	5	67.89	14.90
-3	26	-5	94.09	17.50
4	26	5	2.10	7.40
-4	26	-5	19.70	10.80
4	26	5	-2.00	12.90
-4	26	-5	9.70	6.80
5	26	5	338.97	22.90
-5	26	-5	343.57	22.10
-5	26	-5	406.16	30.40
-2	27	5	2.20	8.70
-2	27	5	1.80	14.70
2	27	-5	10.10	10.00
-1	27	5	92.29	17.70
-1	27	5	93.69	14.20
1	27	-5	93.59	18.90
0	27	5	38.70	15.70
0	27	5	27.60	9.00
0	27	-5	19.00	10.70
-1	27	-5	6.40	9.60
1	27	5	38.10	14.70
1	27	5	9.00	8.20

-2	27	-5	88.09	18.10
-2	27	-5	74.39	12.20
2	27	5	76.59	15.80
2	27	5	71.39	12.90
-15	0	6	52.49	8.60
-14	0	6	157.68	13.10
-13	0	6	34.50	6.30
13	0	-6	48.20	9.10
-12	0	6	945.71	55.29
12	0	-6	1052.59	53.49
-12	0	6	1062.29	50.89
-11	0	6	-0.60	7.90
-11	0	6	4.40	4.30
11	0	-6	3.00	5.60
11	0	-6	-7.10	5.20
-10	0	6	948.41	45.40
10	0	-6	901.81	47.40
10	0	-6	914.11	46.00
-10	0	6	899.71	48.90
-9	0	6	3.90	6.00
-9	0	6	-1.60	2.90
9	0	-6	-0.60	3.50
9	0	-6	4.50	4.60
-8	0	6	919.61	45.20
-8	0	6	866.61	47.70
8	0	-6	966.30	47.20
8	0	-6	948.61	45.80
7	0	-6	104.59	7.40
7	0	-6	112.09	9.30
-7	0	6	101.49	9.90
-7	0	6	106.99	7.00
6	0	-6	752.22	37.70
6	0	-6	745.93	36.50
-6	0	6	729.13	36.20
-5	0	6	1252.47	61.39
-5	0	6	1295.07	63.09
5	0	-6	1288.67	62.59
-4	0	6	3359.86	162.78
4	0	-6	3382.06	162.78
-4	0	6	3537.65	164.08
4	0	-6	3426.96	163.78
-3	0	6	244.08	13.10
3	0	-6	240.18	11.90
3	0	-6	251.77	13.00

-3	0	6	206.38	11.90
2	0	-6	434.66	21.50
2	0	-6	447.66	22.30
-2	0	6	468.85	22.50
-2	0	6	424.56	21.70
1	0	-6	1384.16	66.79
-1	0	6	1358.66	66.59
-1	0	6	1470.35	66.99
1	0	-6	1367.86	66.49
0	0	6	4032.60	191.28
-1	0	-6	4769.72	219.08
1	0	6	4469.85	219.08
2	0	6	2432.76	120.99
-2	0	-6	2506.65	120.49
-2	0	-6	2640.54	120.89
2	0	6	2620.84	120.39
2	0	6	2488.35	120.29
3	0	6	387.26	19.30
-3	0	-6	368.26	18.80
3	0	6	353.36	18.60
-3	0	-6	418.66	19.30
4	0	6	82.79	5.90
4	0	6	82.19	5.20
-4	0	-6	80.79	5.40
-4	0	-6	86.19	5.90
5	0	6	3800.92	178.88
-5	0	-6	3686.43	179.08
-5	0	-6	4047.09	179.38
5	0	6	3508.45	179.18
-6	0	-6	1127.39	57.29
-6	0	-6	1108.99	57.49
-6	0	-6	1246.98	57.69
6	0	6	1226.38	57.19
-7	0	-6	2466.45	124.79
-7	0	-6	2836.72	125.19
-7	0	-6	2470.75	124.79
7	0	6	2621.14	124.59
-8	0	-6	3.00	3.60
8	0	6	1.80	2.80
-8	0	-6	-1.30	3.90
-8	0	-6	2.60	3.60
9	0	6	1103.69	53.09
-9	0	-6	1008.00	53.49
-9	0	-6	1093.19	53.29

10	0	6	15.00	4.70
-10	0	-6	8.30	5.20
-10	0	-6	17.00	5.50
-11	0	-6	710.13	35.90
-11	0	-6	608.84	36.40
11	0	6	710.83	35.60
-12	0	-6	-1.80	6.40
-12	0	-6	7.00	5.60
12	0	6	-4.50	4.80
-13	0	-6	540.15	30.50
-14	0	-6	171.88	15.70
-15	0	-6	170.78	16.70
-15	-1	6	30.40	7.60
-15	1	6	29.40	7.50
-14	-1	6	34.30	6.70
-14	1	6	25.50	6.70
-13	-1	6	49.90	8.60
13	-1	-6	59.89	9.90
-13	1	6	36.70	11.40
13	1	-6	30.30	8.50
-12	-1	6	24.00	9.70
12	1	-6	29.10	7.70
-12	1	6	19.30	11.10
-11	-1	6	23.40	4.90
-11	-1	6	26.80	9.60
-11	-1	6	28.30	6.80
11	-1	-6	37.90	7.80
11	1	-6	31.60	6.90
-11	1	6	42.60	6.80
-11	1	6	19.90	9.30
-10	-1	6	72.39	12.80
-10	-1	6	99.09	8.00
10	-1	-6	93.79	10.80
10	-1	-6	87.79	9.30
-10	1	6	107.99	8.00
10	1	-6	89.19	10.80
-10	1	6	91.49	13.10
10	1	-6	97.59	9.00
-9	-1	6	11.20	6.30
9	-1	-6	14.10	5.10
-9	-1	6	19.70	3.70
9	-1	-6	17.00	4.30
9	1	-6	24.00	6.00
9	1	-6	25.80	4.30

-9	1	6	24.50	4.10
-9	1	6	26.60	7.20
-8	-1	6	9.20	2.70
8	-1	-6	5.20	4.10
-8	-1	6	3.60	5.70
8	-1	-6	5.20	3.20
8	1	-6	0.40	4.20
-8	1	6	1.10	5.10
-8	1	6	7.90	3.00
8	1	-6	6.20	3.30
7	-1	-6	149.59	10.80
7	-1	-6	156.28	9.40
-7	-1	6	144.89	11.70
-7	-1	6	147.69	8.70
7	1	-6	149.79	9.20
-7	1	6	140.59	11.90
-7	1	6	143.09	8.70
7	1	-6	143.09	11.00
-6	-1	6	32.50	6.80
6	-1	-6	44.60	4.70
-6	-1	6	41.30	3.90
6	-1	-6	36.20	6.20
6	1	-6	42.70	4.30
6	1	-6	35.90	6.30
-6	1	6	43.20	7.00
-6	1	6	44.40	4.30
-5	-1	6	60.49	4.40
5	-1	-6	61.49	6.30
5	-1	-6	56.69	4.80
5	1	-6	57.69	6.30
5	1	-6	57.69	4.40
-5	1	6	59.39	6.60
-5	1	6	52.89	4.50
4	-1	-6	16.90	2.90
4	-1	-6	16.50	3.20
-4	-1	6	9.30	3.20
-4	-1	6	15.00	2.40
-4	1	6	16.90	2.90
4	1	-6	15.50	2.40
4	1	-6	8.30	2.90
-4	1	6	18.50	3.80
-3	-1	6	75.69	4.90
-3	-1	6	99.89	6.40
3	-1	-6	85.69	5.10

3	-1	-6	86.59	6.10
-3	1	6	74.19	5.20
-3	1	6	83.69	6.20
3	1	-6	85.49	6.20
3	1	-6	85.79	4.80
-2	-1	6	7.40	1.30
-2	-1	6	7.30	2.20
2	-1	-6	6.50	1.80
2	-1	-6	6.50	1.30
2	1	-6	7.20	2.00
-2	1	6	5.40	1.90
-2	1	6	7.10	1.70
2	1	-6	6.00	1.10
-1	-1	6	1518.95	75.69
1	-1	-6	1589.54	75.79
1	-1	-6	1584.04	76.19
-1	-1	6	1630.34	76.19
1	1	-6	1548.74	75.69
-1	1	6	1632.24	76.29
1	1	-6	1627.74	76.19
-1	1	6	1601.54	75.99
0	-1	-6	558.84	26.70
0	-1	-6	556.84	26.80
0	-1	6	559.04	26.70
0	-1	6	530.85	26.60
0	1	-6	550.54	26.60
0	1	6	536.85	26.80
0	1	6	542.95	26.80
0	1	-6	567.64	26.70
1	-1	6	31.40	1.90
1	-1	6	32.60	1.90
-1	-1	-6	25.10	2.60
-1	-1	-6	27.90	2.50
1	-1	6	26.50	2.60
1	1	6	29.00	2.30
-1	1	-6	27.90	2.00
1	1	6	22.80	2.70
1	1	6	31.60	2.60
-1	1	-6	23.70	2.50
-2	-1	-6	7.10	1.40
-2	-1	-6	9.40	1.70
2	1	6	6.30	1.40
-2	1	-6	8.20	1.30
2	1	6	7.80	1.20

2	1	6	4.80	1.70
-2	1	-6	6.60	1.70
3	-1	6	74.09	5.10
-3	-1	-6	83.69	5.30
-3	-1	-6	73.29	4.90
3	-1	6	75.59	4.10
3	-1	6	79.09	4.00
-3	1	-6	69.59	4.40
3	1	6	72.09	4.70
-3	1	-6	86.19	5.30
3	1	6	72.89	5.20
-4	-1	-6	344.67	17.00
4	-1	6	341.57	16.10
-4	-1	-6	316.57	16.70
4	-1	6	302.57	16.80
-4	1	-6	343.77	17.00
4	1	6	334.07	16.50
-4	1	-6	318.37	16.30
4	1	6	295.77	16.80
-5	-1	-6	31.40	4.20
5	-1	6	22.40	3.20
-5	-1	-6	29.10	4.50
-5	1	-6	26.30	4.40
-5	1	-6	21.50	4.10
5	1	6	28.80	4.00
-5	1	-6	20.80	3.70
5	1	6	26.00	3.90
6	-1	6	194.48	10.80
-6	-1	-6	193.38	11.60
-6	-1	-6	213.58	11.80
-6	1	-6	189.28	11.30
6	1	6	202.28	11.30
-6	1	-6	206.58	11.80
-6	1	-6	177.08	11.30
6	1	6	176.18	11.20
-7	-1	-6	140.29	9.30
-7	-1	-6	158.08	9.50
-7	-1	-6	127.19	9.60
7	-1	6	125.49	8.70
-7	1	-6	125.89	9.30
-7	1	-6	149.39	9.80
-7	1	-6	133.79	9.60
7	1	6	132.99	9.20
-8	-1	-6	88.89	8.10

8	-1	6	90.69	7.30
-8	-1	-6	91.59	8.40
-8	-1	-6	96.19	8.40
-8	1	-6	87.49	8.10
8	1	6	91.89	7.70
-8	1	-6	87.69	7.60
-8	1	-6	99.69	8.60
-9	-1	-6	65.09	7.80
-9	-1	-6	64.19	8.50
9	-1	6	57.49	7.00
-9	1	-6	55.89	8.10
9	1	6	58.99	7.40
-9	1	-6	71.39	7.80
10	-1	6	78.19	8.50
-10	-1	-6	64.39	10.00
-10	-1	-6	99.49	9.60
-10	1	-6	92.69	9.30
10	1	6	84.19	9.10
-10	1	-6	64.89	9.70
11	-1	6	3.30	4.80
-11	-1	-6	16.30	5.60
-11	-1	-6	5.30	5.80
-11	1	-6	3.40	5.60
11	1	6	0.00	5.10
-11	1	-6	4.70	5.80
-12	-1	-6	10.40	6.40
12	-1	6	15.60	5.50
-12	-1	-6	14.50	6.90
12	1	6	9.00	5.60
-12	1	-6	10.00	6.50
-12	1	-6	-2.70	6.30
-13	-1	-6	9.90	6.50
-13	1	-6	9.70	6.90
-14	-1	-6	8.30	7.70
-14	1	-6	21.70	8.10
-15	-1	-6	11.80	8.50
-15	1	-6	16.50	8.30
-15	-2	6	110.69	12.90
-15	2	6	137.19	12.90
-14	-2	6	176.28	13.70
-14	2	6	181.08	14.20
-13	-2	6	1.40	5.00
13	-2	-6	1.40	6.30
-13	2	6	6.70	5.30

13	2	-6	-1.80	7.10
-12	-2	6	391.46	21.30
-12	-2	6	311.87	26.60
12	-2	-6	377.66	24.20
12	2	-6	402.96	24.50
-12	2	6	413.66	21.60
-12	2	6	392.16	27.80
11	-2	-6	24.20	6.90
-11	-2	6	14.70	5.90
-11	-2	6	20.20	4.80
-11	-2	6	16.10	8.60
-11	2	6	26.20	5.10
11	2	-6	12.30	7.00
-11	2	6	21.50	8.30
-10	-2	6	465.15	30.70
10	-2	-6	523.05	28.40
-10	-2	6	604.84	28.60
10	-2	-6	544.35	29.60
-10	-2	6	537.45	27.30
-10	2	6	565.54	27.60
10	2	-6	536.45	30.00
-10	2	6	478.25	30.70
-9	-2	6	6.60	3.20
9	-2	-6	14.30	4.40
-9	-2	6	12.20	6.50
9	-2	-6	15.10	5.00
-9	2	6	15.50	3.80
9	2	-6	10.20	3.60
9	2	-6	12.50	5.30
-9	2	6	18.20	6.50
-8	-2	6	662.43	32.30
-8	-2	6	601.84	35.00
8	-2	-6	643.94	34.10
8	-2	-6	674.73	33.20
-8	2	6	626.84	35.10
8	2	-6	670.73	32.80
-8	2	6	671.73	32.60
8	2	-6	667.23	34.60
7	-2	-6	311.67	17.80
-7	-2	6	290.37	18.90
-7	-2	6	313.77	15.90
7	-2	-6	309.87	16.80
7	2	-6	302.87	18.30
-7	2	6	306.57	16.20

7	2	-6	318.07	16.30
6	-2	-6	1384.86	66.99
-6	-2	6	1368.86	65.39
6	-2	-6	1397.66	66.09
-6	-2	6	1281.27	67.39
6	2	-6	1360.76	67.09
-6	2	6	1372.86	65.69
-6	2	6	1378.96	67.49
5	-2	-6	421.76	21.20
-5	-2	6	427.96	20.60
-5	-2	6	406.06	22.80
5	-2	-6	425.06	22.10
5	2	-6	409.46	22.20
5	2	-6	410.66	20.70
-5	2	6	410.06	21.00
-5	2	6	424.46	22.50
4	-2	-6	363.16	18.30
-4	-2	6	306.67	19.50
4	-2	-6	356.56	17.60
-4	-2	6	336.57	16.90
4	2	-6	358.66	18.50
-4	2	6	342.87	18.50
-4	2	6	335.97	17.40
4	2	-6	338.47	16.90
3	-2	-6	248.38	12.20
-3	-2	6	215.48	11.70
3	-2	-6	230.18	12.80
-3	-2	6	237.58	13.10
-3	2	6	250.77	13.20
3	2	-6	240.58	11.70
-3	2	6	210.38	12.30
3	2	-6	249.78	13.00
-2	-2	6	627.84	30.40
-2	-2	6	621.84	31.40
2	-2	-6	650.83	30.80
2	-2	-6	638.74	31.30
2	2	-6	633.54	31.30
-2	2	6	591.64	31.00
2	2	-6	637.24	30.30
-2	2	6	671.83	31.50
-1	-2	6	512.95	24.80
1	-2	-6	506.35	25.00
-1	-2	6	505.25	25.30
1	-2	-6	525.45	25.40

1	2	-6	508.95	25.30
-1	2	6	507.65	25.30
-1	2	6	537.15	25.50
1	2	-6	511.95	24.70
0	-2	-6	2233.48	105.49
0	-2	-6	2267.97	105.29
0	-2	6	2173.08	105.19
0	-2	6	2182.48	105.19
0	2	6	2166.28	105.49
0	2	6	2217.78	105.39
0	2	-6	2165.78	105.09
0	2	-6	2296.27	105.19
1	-2	6	2588.84	127.29
-1	-2	-6	2765.82	127.49
-1	-2	-6	2706.23	127.39
1	2	6	2644.84	127.49
1	2	6	2652.03	127.29
-1	2	-6	2715.23	127.29
-2	-2	-6	12.60	2.30
2	2	6	8.90	1.90
2	2	6	12.90	2.40
-2	2	-6	12.40	2.30
2	2	6	15.30	2.00
-3	-2	-6	1350.46	66.39
-3	-2	-6	1416.56	66.49
3	-2	6	1347.57	66.49
3	2	6	1401.56	66.39
-3	2	-6	1324.27	65.69
3	2	6	1336.97	66.49
-3	2	-6	1494.95	66.49
-4	-2	-6	250.47	13.20
4	-2	6	258.77	12.10
-4	-2	-6	249.78	13.10
4	2	6	265.97	13.00
-4	2	-6	236.28	13.00
-4	2	-6	226.28	13.10
-4	2	-6	245.28	12.40
4	2	6	208.38	12.90
-5	-2	-6	1644.74	81.89
-5	-2	-6	1824.42	81.89
5	-2	6	1739.53	81.09
-5	2	-6	1839.52	81.99
5	2	6	1693.53	81.69
5	2	6	1581.44	81.69

-5	2	-6	1603.04	81.29
-5	2	-6	1653.33	81.79
6	-2	6	55.09	4.80
-6	-2	-6	67.49	6.20
-6	-2	-6	69.29	6.20
-6	2	-6	44.70	5.50
6	2	6	65.89	5.90
6	2	6	70.29	5.90
-6	2	-6	51.09	5.50
-6	2	-6	49.99	5.80
-7	-2	-6	560.34	30.30
7	-2	6	599.44	29.50
-7	-2	-6	656.93	30.40
-7	2	-6	573.24	30.10
-7	2	-6	555.44	29.90
7	2	6	581.44	30.00
7	2	6	567.54	30.10
-7	2	-6	623.04	30.40
8	-2	6	16.00	3.30
-8	-2	-6	16.30	4.40
-8	-2	-6	11.60	4.30
-8	-2	-6	14.30	4.30
-8	2	-6	18.90	4.50
-8	2	-6	11.10	3.90
8	2	6	15.80	4.10
-8	2	-6	14.30	4.10
-9	-2	-6	275.37	17.50
9	-2	6	296.77	16.30
-9	-2	-6	285.27	16.70
9	2	6	302.67	17.00
-9	2	-6	283.17	16.70
-9	2	-6	270.57	17.20
-10	-2	-6	232.18	17.20
-10	-2	-6	288.97	16.70
10	-2	6	255.87	15.50
-10	2	-6	231.98	16.70
-10	2	-6	279.37	16.40
10	2	6	264.97	16.40
11	-2	6	581.74	30.50
-11	-2	-6	524.35	32.00
-11	-2	-6	625.94	31.40
-11	2	-6	528.45	31.60
11	2	6	613.14	31.30
-12	-2	-6	53.39	9.80

-12	-2	-6	29.30	7.60
12	-2	6	32.00	6.30
12	2	6	39.60	7.30
-12	2	-6	20.60	7.30
-12	2	-6	33.60	6.80
-13	-2	-6	185.38	14.90
-13	2	-6	175.08	14.20
-14	-2	-6	26.50	8.70
-14	2	-6	39.00	8.40
-15	-2	-6	158.18	16.50
-15	2	-6	140.09	15.70
-15	-3	6	194.08	15.70
-15	3	6	211.78	15.90
-14	-3	6	59.99	9.50
-14	3	6	83.99	10.50
-13	-3	6	183.78	13.50
13	-3	-6	200.28	17.10
13	3	-6	192.48	17.70
-13	3	6	218.38	14.20
-12	-3	6	5.20	9.90
-12	-3	6	16.20	4.90
12	-3	-6	16.60	6.70
-12	3	6	11.80	9.50
12	3	-6	12.60	7.30
-12	3	6	9.20	5.10
-11	-3	6	747.33	37.70
-11	-3	6	800.92	38.80
11	-3	-6	771.82	40.20
-11	-3	6	658.63	41.80
-11	3	6	726.13	42.00
-11	3	6	809.42	38.20
11	3	-6	730.03	40.60
-10	-3	6	22.30	7.80
-10	-3	6	31.70	5.80
10	-3	-6	49.90	8.10
10	-3	-6	45.80	8.90
-10	-3	6	36.60	6.10
-10	3	6	45.10	6.50
-10	3	6	36.60	8.10
10	3	-6	33.90	7.10
-9	-3	6	410.56	21.70
-9	-3	6	452.55	22.60
9	-3	-6	434.06	24.00
9	-3	-6	424.96	23.00

-9	-3	6	384.26	25.40
9	3	-6	427.36	24.50
-9	3	6	428.46	22.20
-9	3	6	411.26	25.50
-8	-3	6	2.50	2.40
8	-3	-6	4.60	3.50
-8	-3	6	1.00	4.80
8	-3	-6	0.20	3.80
8	3	-6	-2.90	4.10
-8	3	6	3.10	3.00
-8	3	6	3.30	5.30
7	-3	-6	839.42	41.90
7	-3	-6	900.71	42.90
-7	-3	6	830.72	41.00
-7	-3	6	798.02	43.50
-7	3	6	783.72	44.40
7	3	-6	861.01	41.30
-7	3	6	849.22	41.50
7	3	-6	882.71	43.20
-6	-3	6	300.27	19.30
6	-3	-6	344.27	18.90
6	-3	-6	360.36	18.00
-6	-3	6	337.97	17.00
6	3	-6	336.47	17.20
-6	3	6	345.07	17.50
6	3	-6	312.97	19.00
-6	3	6	357.36	19.50
5	-3	-6	675.13	33.40
-5	-3	6	655.63	34.50
-5	-3	6	682.43	32.60
5	-3	-6	671.73	34.10
5	3	-6	686.73	32.70
-5	3	6	663.93	33.20
-5	3	6	696.43	34.60
5	3	-6	659.73	34.30
4	-3	-6	9.60	2.80
4	-3	-6	15.00	2.60
-4	-3	6	10.00	1.50
-4	-3	6	11.60	3.40
4	3	-6	13.00	2.00
-4	3	6	16.70	3.50
4	3	-6	11.90	3.30
-4	3	6	13.20	2.50
3	-3	-6	1286.27	59.79

-3	-3	6	1156.08	60.69
-3	-3	6	1175.08	58.59
-3	3	6	1205.48	59.29
3	3	-6	1217.08	58.49
-3	3	6	1302.87	59.99
3	3	-6	1272.87	59.89
-2	-3	6	3011.40	145.29
2	-3	-6	3091.39	145.29
2	-3	-6	3178.08	144.89
2	3	-6	3052.29	144.29
-2	3	6	3117.59	145.39
2	3	-6	3110.49	145.39
-2	3	6	2766.52	145.09
1	-3	-6	249.28	12.80
1	-3	-6	245.48	13.20
-1	-3	6	256.77	12.30
-1	-3	6	259.47	13.00
-1	3	6	225.18	13.60
1	3	-6	245.58	12.20
1	3	-6	272.47	13.10
-1	3	6	261.27	13.20
0	-3	-6	2308.97	104.69
0	-3	6	2168.38	104.09
0	-3	6	2231.18	104.19
0	-3	-6	2242.18	104.39
0	3	-6	2244.78	104.29
0	3	6	1950.80	104.99
0	3	-6	2169.58	104.09
0	3	6	2219.28	104.59
-1	-3	-6	98.19	5.80
-1	-3	-6	92.79	6.00
1	-3	6	99.39	5.70
1	3	6	99.49	5.80
-1	3	-6	106.59	5.20
1	3	6	101.69	6.10
1	3	6	93.99	6.00
-1	3	-6	102.59	5.60
-2	-3	-6	2634.54	124.59
-2	-3	-6	2535.75	124.59
2	3	6	2568.74	124.69
2	3	6	2594.34	124.49
2	3	6	2722.33	124.79
-2	3	-6	2628.34	124.49
-3	-3	-6	127.49	7.40

-3	-3	-6	123.89	7.60
3	3	6	114.79	7.40
-3	3	-6	122.79	7.30
3	3	6	130.79	7.60
-3	3	-6	116.59	7.40
-3	3	-6	131.39	6.60
-4	-3	-6	1090.79	49.70
4	-3	6	975.80	48.60
-4	-3	-6	977.90	49.80
-4	3	-6	1137.29	49.90
-4	3	-6	931.91	49.60
4	3	6	1057.79	49.70
4	3	6	976.60	49.70
5	-3	6	23.00	2.50
-5	-3	-6	23.90	4.50
-5	-3	-6	19.60	3.20
5	3	6	25.50	4.20
-5	3	-6	25.80	3.80
-5	3	-6	23.20	3.60
-5	3	-6	21.50	3.50
6	-3	6	1477.55	70.79
-6	-3	-6	1587.34	71.79
-6	-3	-6	1395.16	71.89
-6	3	-6	1589.74	71.99
-6	3	-6	1445.16	71.69
6	3	6	1380.76	71.59
-6	3	-6	1439.96	71.29
6	3	6	1499.45	71.79
7	-3	6	29.00	4.10
-7	-3	-6	28.30	5.90
-7	-3	-6	29.00	5.10
-7	3	-6	29.90	5.50
7	3	6	28.90	5.50
-7	3	-6	26.80	4.20
7	3	6	32.30	5.40
-7	3	-6	28.80	5.30
-8	-3	-6	105.59	8.70
-8	-3	-6	97.09	8.10
8	-3	6	93.69	7.20
-8	-3	-6	93.79	9.10
-8	3	-6	88.99	8.40
8	3	6	89.79	8.30
-8	3	-6	95.59	8.80
8	3	6	111.29	8.60

-8	3	-6	98.99	8.30
9	-3	6	54.39	6.30
-9	-3	-6	44.00	8.40
-9	-3	-6	62.59	10.70
-9	-3	-6	57.49	7.00
9	3	6	41.50	7.60
-9	3	-6	41.60	7.90
-9	3	-6	55.99	7.40
10	-3	6	766.72	38.30
-10	-3	-6	800.72	39.30
-10	-3	-6	720.73	40.00
-10	3	-6	716.83	39.40
-10	3	-6	735.83	39.00
10	3	6	801.52	39.50
-11	-3	-6	17.20	5.70
11	-3	6	3.40	4.40
-11	-3	-6	14.30	6.20
11	3	6	5.70	5.20
-11	3	-6	12.50	8.20
-11	3	-6	1.20	5.60
-12	-3	-6	361.46	21.50
-12	-3	-6	346.47	23.40
-12	3	-6	387.96	21.90
12	3	6	365.16	22.10
-12	3	-6	325.07	22.50
-13	-3	-6	24.50	7.60
-13	3	-6	21.90	7.30
-14	-3	-6	158.58	15.30
-14	3	-6	143.29	13.60
-15	-3	-6	67.39	10.90
-15	3	-6	91.29	13.80
-15	-4	6	-3.70	6.70
-15	4	6	17.40	7.00
-14	-4	6	66.19	11.90
-14	4	6	92.59	11.30
-13	-4	6	7.10	5.30
13	-4	-6	15.40	7.70
-13	4	6	13.50	5.80
13	4	-6	6.10	7.90
12	-4	-6	72.39	11.90
-12	-4	6	55.99	8.00
-12	-4	6	60.59	11.60
-12	-4	6	69.79	8.40
-12	4	6	92.39	15.30

12	4	-6	71.89	12.90
-12	4	6	49.30	11.50
-11	-4	6	68.59	9.20
-11	-4	6	60.89	7.30
-11	-4	6	32.40	9.80
11	4	-6	67.29	11.60
-10	-4	6	-1.20	3.20
-10	-4	6	-2.90	6.90
-10	-4	6	0.80	4.50
10	-4	-6	2.40	5.10
-10	4	6	3.90	4.10
-10	4	6	-1.40	6.10
10	4	-6	1.90	5.60
-9	-4	6	40.20	8.30
9	-4	-6	60.89	8.50
-9	-4	6	51.09	5.50
-9	-4	6	49.90	6.80
9	-4	-6	55.19	7.80
9	4	-6	46.50	9.40
-9	4	6	64.09	6.60
-9	4	6	40.40	8.00
-8	-4	6	40.00	5.50
-8	-4	6	40.60	7.20
-8	-4	6	44.70	4.70
8	-4	-6	45.40	7.70
8	-4	-6	35.10	6.20
8	4	-6	35.30	6.20
-8	4	6	39.90	5.60
7	-4	-6	80.19	7.20
7	-4	-6	78.39	8.30
-7	-4	6	78.89	6.00
-7	-4	6	78.69	5.40
-7	-4	6	54.89	8.90
-7	4	6	82.99	6.50
7	4	-6	66.29	8.40
-7	4	6	78.89	9.10
6	-4	-6	201.18	10.90
6	-4	-6	189.48	11.70
-6	-4	6	152.68	12.20
-6	-4	6	159.58	9.70
6	4	-6	169.78	12.00
-6	4	6	178.48	12.20
-6	4	6	180.08	10.20
-5	-4	6	46.20	6.60

5	-4	-6	65.89	5.50
5	-4	-6	60.19	6.30
-5	-4	6	54.49	3.80
-5	4	6	62.89	4.90
-5	4	6	61.19	6.80
5	4	-6	43.50	6.50
5	4	-6	52.89	3.90
-4	-4	6	150.58	7.70
-4	-4	6	127.99	9.70
4	-4	-6	165.88	9.00
4	-4	-6	166.58	9.70
-4	4	6	149.79	8.80
-4	4	6	158.08	10.00
4	4	-6	143.09	9.80
4	4	-6	152.88	7.80
3	-4	-6	66.49	4.80
3	-4	-6	66.89	5.60
-3	-4	6	63.89	3.40
-3	-4	6	52.09	5.50
-3	4	6	66.79	5.70
-3	4	6	58.69	4.90
3	4	-6	62.89	5.60
3	4	-6	68.29	3.40
2	-4	-6	676.93	32.60
2	-4	-6	686.03	33.10
2	4	-6	655.53	31.70
-2	4	6	687.13	33.20
-2	4	6	615.94	32.80
2	4	-6	684.93	33.00
1	-4	-6	436.36	21.70
-1	-4	6	460.15	20.70
-1	-4	6	402.76	21.40
-1	4	6	430.16	21.60
1	4	-6	424.16	20.60
1	4	-6	426.86	21.40
-1	4	6	428.26	21.70
0	-4	-6	16.50	2.90
0	-4	6	19.20	2.20
0	-4	-6	16.70	2.90
0	4	-6	17.30	2.20
0	4	6	16.70	2.90
0	4	6	17.70	2.70
0	4	-6	20.20	1.90
0	4	-6	16.50	1.80

-1	-4	-6	91.99	5.60
-1	-4	-6	91.09	5.90
1	4	6	89.59	5.70
1	4	6	91.09	5.70
-1	4	-6	92.89	5.20
-1	4	-6	91.19	5.30
-1	4	-6	89.69	4.90
-2	-4	-6	40.50	4.00
-2	-4	-6	48.10	3.90
2	4	6	37.40	4.10
-2	4	-6	45.70	2.70
2	4	6	45.60	3.90
-2	4	-6	46.40	3.60
-2	4	-6	44.50	2.50
-3	-4	-6	115.59	7.80
-3	-4	-6	128.99	7.50
3	4	6	127.09	7.90
-3	4	-6	125.49	6.40
3	4	6	123.99	7.60
-3	4	-6	127.19	7.50
-3	4	-6	128.29	7.50
-4	-4	-6	331.37	17.80
4	-4	6	332.97	16.20
-4	-4	-6	359.46	17.50
-4	4	-6	362.66	17.70
4	4	6	302.47	17.30
-4	4	-6	329.37	17.50
4	4	6	333.57	17.70
-4	4	-6	332.87	16.80
-5	-4	-6	1.40	2.70
-5	-4	-6	2.20	2.60
-5	4	-6	3.30	2.50
-5	4	-6	3.60	2.80
5	4	6	8.40	2.60
5	4	6	2.10	2.60
6	-4	6	7.30	2.20
-6	-4	-6	9.00	3.30
6	4	6	13.10	3.50
6	4	6	9.90	3.40
-6	4	-6	12.90	3.70
-6	4	-6	19.30	3.80
7	-4	6	189.68	10.40
-7	-4	-6	201.98	11.70
-7	-4	-6	173.28	12.10

-7	4	-6	176.38	11.30
7	4	6	169.48	11.20
7	4	6	189.58	11.90
-7	4	-6	193.08	12.10
-7	4	-6	181.98	11.60
8	-4	6	200.98	11.20
-8	-4	-6	214.58	12.60
-8	-4	-6	174.18	13.10
-8	4	-6	192.28	12.30
-8	4	-6	169.28	12.30
-8	4	-6	192.08	13.00
8	4	6	221.28	13.00
8	4	6	168.98	11.80
9	-4	6	13.90	3.50
-9	-4	-6	19.00	5.30
-9	-4	-6	14.50	4.60
9	4	6	17.00	4.50
-9	4	-6	15.00	4.50
-9	4	-6	12.90	5.00
-10	-4	-6	19.10	5.40
10	-4	6	10.50	4.20
-10	-4	-6	7.80	5.40
-10	4	-6	16.80	5.70
10	4	6	13.90	5.50
-10	4	-6	19.90	5.60
-11	-4	-6	263.87	16.30
-11	-4	-6	204.38	17.20
-11	4	-6	215.88	16.90
-11	4	-6	242.28	15.90
11	4	6	243.08	16.50
-12	-4	-6	-1.70	5.90
12	-4	6	2.50	4.90
-12	-4	-6	16.70	7.20
-12	4	-6	12.30	6.20
12	4	6	2.00	5.50
-12	4	-6	1.30	6.70
-13	-4	-6	-2.20	6.70
-13	4	-6	2.90	6.40
-14	-4	-6	8.60	7.60
-14	4	-6	-2.00	6.90
-15	-4	-6	112.29	15.60
-15	4	-6	123.29	14.70
-15	-5	6	143.49	14.00
-15	5	6	179.88	14.60

-14	-5	6	41.00	7.20
-14	5	6	60.49	10.40
13	-5	-6	235.48	20.60
-13	-5	6	254.57	15.70
-13	5	6	265.77	16.70
13	5	-6	237.78	20.70
12	-5	-6	101.79	12.20
-12	-5	6	80.39	8.60
-12	-5	6	64.29	12.20
-12	-5	6	61.79	10.90
12	5	-6	68.49	13.20
-12	5	6	85.39	14.70
-12	5	6	87.29	9.30
-11	-5	6	1325.87	64.39
-11	-5	6	1145.59	68.19
-11	-5	6	1454.25	65.39
11	-5	-6	1362.86	66.89
11	5	-6	1355.26	67.49
-11	5	6	1359.66	65.09
-11	5	6	1232.38	68.59
10	-5	-6	72.89	9.80
-10	-5	6	61.39	6.60
-10	-5	6	64.19	10.40
-10	-5	6	62.89	8.20
-10	5	6	81.89	7.90
-10	5	6	69.79	10.50
10	5	-6	81.79	11.00
9	-5	-6	620.54	32.20
-9	-5	6	583.74	29.80
9	-5	-6	579.34	31.40
-9	-5	6	530.95	33.10
-9	-5	6	670.33	30.50
-9	5	6	595.14	30.50
9	5	-6	606.24	32.60
-9	5	6	583.24	33.30
-8	-5	6	31.10	5.00
-8	-5	6	24.90	4.10
-8	-5	6	25.90	6.70
8	-5	-6	49.40	7.70
8	-5	-6	38.20	5.60
-8	5	6	31.30	5.40
8	5	-6	22.90	5.80
7	-5	-6	622.74	31.70
-7	-5	6	625.84	31.60

-7	-5	6	613.54	30.40
7	-5	-6	628.24	32.40
-7	-5	6	582.04	33.10
7	5	-6	636.44	32.90
-7	5	6	626.54	31.10
-7	5	6	627.04	33.20
-6	-5	6	524.45	30.40
-6	-5	6	615.94	28.20
-6	-5	6	557.54	27.90
6	-5	-6	579.64	29.30
6	-5	-6	571.04	29.90
6	5	-6	598.34	30.40
-6	5	6	565.74	28.80
-6	5	6	582.44	30.60
5	-5	-6	3078.89	140.99
-5	-5	6	3019.60	139.89
-5	-5	6	2737.93	139.69
-5	-5	6	2830.32	141.79
5	-5	-6	2991.20	141.39
5	5	-6	3079.99	141.79
-5	5	6	2859.31	140.59
-5	5	6	2976.00	141.79
-4	-5	6	280.77	13.30
-4	-5	6	257.17	15.40
4	-5	-6	286.67	14.70
-4	-5	6	254.77	13.20
4	-5	-6	288.67	15.30
-4	5	6	262.87	14.50
4	5	-6	264.27	15.40
-4	5	6	283.47	15.40
3	-5	-6	32.20	3.60
-3	-5	6	19.70	4.20
3	-5	-6	27.90	4.40
-3	5	6	25.10	4.30
3	5	-6	25.30	4.40
-3	5	6	19.90	3.10
2	-5	-6	668.93	32.70
2	-5	-6	678.03	32.20
-2	-5	6	642.54	32.60
2	5	-6	685.33	32.70
-2	5	6	669.13	32.80
-2	5	6	601.14	32.50
-1	-5	6	891.41	43.60
1	-5	-6	928.11	43.30

1	-5	-6	904.71	43.80
-1	5	6	814.92	43.60
1	5	-6	934.11	43.50
-1	5	6	925.21	43.80
1	5	-6	880.41	42.50
1	5	-6	877.81	42.50
0	-5	-6	318.97	15.90
0	-5	-6	331.67	16.40
0	5	-6	315.87	15.40
0	5	-6	312.47	15.30
0	5	6	309.37	16.20
0	5	6	300.97	16.20
0	5	-6	303.37	15.70
-1	-5	-6	1691.93	80.79
-1	5	-6	1751.32	80.59
-1	5	-6	1652.13	80.39
1	5	6	1694.73	80.99
-1	5	-6	1637.24	80.49
1	5	6	1703.03	81.09
-2	-5	-6	587.34	30.60
-2	-5	-6	634.84	30.30
-2	5	-6	580.84	30.60
2	5	6	643.24	30.50
-2	5	-6	610.04	29.60
-2	5	-6	658.03	30.20
2	5	6	598.14	30.40
-3	-5	-6	181.38	11.00
-3	-5	-6	198.28	10.60
-3	5	-6	198.78	10.60
-3	5	-6	183.68	10.40
3	5	6	186.78	10.70
-3	5	-6	194.28	9.60
-4	-5	-6	822.32	38.60
-4	-5	-6	734.13	39.00
-4	5	-6	822.32	38.80
-4	5	-6	758.42	38.50
4	5	6	738.33	38.60
4	5	6	810.92	39.40
-4	5	-6	806.42	37.90
5	-5	6	195.88	10.00
-5	-5	-6	188.48	12.00
-5	-5	-6	204.08	11.40
-5	5	-6	181.18	11.40
5	5	6	210.28	12.00

-5	5	-6	207.18	10.90
5	5	6	186.28	11.40
-5	5	-6	198.98	11.70
6	-5	6	2663.33	130.49
-6	-5	-6	2666.03	132.19
-6	-5	-6	2975.70	131.69
-6	5	-6	2588.74	131.49
6	5	6	2582.94	131.49
-6	5	-6	2663.43	131.59
6	5	6	2834.52	131.99
-6	5	-6	2980.50	131.99
7	-5	6	9.70	2.40
-7	-5	-6	8.70	3.70
-7	-5	-6	6.70	3.60
-7	5	-6	6.60	4.20
7	5	6	14.40	4.20
-7	5	-6	4.00	3.70
7	5	6	9.20	3.60
-8	-5	-6	844.82	46.10
-8	-5	-6	927.21	45.30
8	-5	6	927.51	44.40
8	5	6	957.10	46.00
-8	5	-6	949.01	45.80
8	5	6	825.12	45.00
-8	5	-6	899.51	45.30
-9	-5	-6	98.49	10.70
9	-5	6	118.09	11.40
9	5	6	113.69	9.50
-9	5	-6	99.69	15.70
-9	5	-6	102.29	9.50
9	5	6	101.59	10.00
-10	-5	-6	483.25	29.50
10	-5	6	513.65	28.70
-10	-5	-6	557.44	28.60
-10	5	-6	471.15	31.20
10	5	6	542.35	28.90
-10	5	-6	548.25	28.30
-11	-5	-6	1.80	6.10
-11	-5	-6	11.40	5.80
11	-5	6	4.00	4.20
-11	5	-6	6.60	7.10
11	5	6	-5.80	5.30
-11	5	-6	1.80	5.50
-12	-5	-6	599.84	31.60

12	-5	6	559.84	30.00
12	5	6	610.04	32.30
-12	5	-6	559.94	31.10
-12	5	-6	495.05	35.40
-13	-5	-6	24.20	7.50
-13	5	-6	15.60	7.00
-14	-5	-6	137.99	14.60
-14	5	-6	118.49	14.00
-15	-5	-6	236.08	19.30
-15	5	-6	218.78	18.20
-15	-6	6	15.90	7.00
-15	6	6	13.50	7.20
-14	-6	6	86.09	10.70
-14	6	6	105.29	11.60
-13	-6	6	7.40	5.10
13	6	-6	-3.40	8.00
-13	6	6	9.60	5.90
-12	-6	6	12.90	11.10
12	-6	-6	27.80	7.20
-12	-6	6	22.70	6.60
-12	-6	6	33.20	5.40
-12	6	6	38.50	6.70
12	6	-6	36.80	8.80
11	-6	-6	17.30	6.70
-11	-6	6	4.80	9.60
-11	-6	6	15.90	4.20
-11	-6	6	8.90	5.40
-11	6	6	17.60	9.20
11	6	-6	8.20	6.70
-10	-6	6	44.60	5.90
-10	-6	6	41.10	9.40
10	-6	-6	60.99	9.50
-10	-6	6	35.20	5.60
-10	6	6	42.90	8.70
10	6	-6	25.60	7.30
-10	6	6	41.00	7.10
-9	-6	6	11.40	4.00
-9	-6	6	5.00	6.30
-9	-6	6	12.90	3.20
9	-6	-6	14.60	5.00
-9	6	6	21.40	4.40
-9	6	6	22.00	7.10
9	6	-6	13.70	6.00
-8	-6	6	15.60	5.90

8	-6	-6	20.10	5.10
8	-6	-6	20.40	5.20
-8	-6	6	20.80	3.10
-8	-6	6	26.10	3.70
-8	6	6	24.00	7.70
-8	6	6	19.30	4.10
8	6	-6	18.50	5.90
-7	-6	6	26.80	6.10
-7	-6	6	25.00	3.30
7	-6	-6	24.20	4.60
-7	-6	6	23.90	3.00
7	-6	-6	23.30	4.80
-7	6	6	27.20	5.80
7	6	-6	18.60	5.10
-7	6	6	26.70	4.00
-6	-6	6	15.90	4.80
6	-6	-6	29.50	4.50
6	-6	-6	23.90	4.60
-6	-6	6	21.20	3.10
-6	-6	6	23.20	2.80
-6	6	6	26.60	5.00
-6	6	6	20.80	3.40
6	6	-6	21.80	4.80
-5	-6	6	5.00	1.50
-5	-6	6	2.60	1.30
-5	-6	6	5.70	3.80
5	-6	-6	8.20	3.00
5	-6	-6	11.30	3.60
-5	6	6	10.70	4.00
5	6	-6	4.90	3.70
-5	6	6	8.40	2.80
-4	-6	6	424.46	23.40
-4	-6	6	425.56	21.00
4	-6	-6	449.66	22.60
4	-6	-6	455.05	23.10
-4	6	6	448.36	23.30
4	6	-6	461.25	23.30
-4	6	6	415.36	22.30
-3	-6	6	80.19	6.60
3	-6	-6	89.39	6.70
3	-6	-6	82.69	5.90
3	6	-6	88.49	6.60
-3	6	6	68.09	5.80
-3	6	6	79.19	6.60

2	-6	-6	420.96	20.90
2	-6	-6	415.46	21.40
-2	-6	6	421.36	21.40
2	6	-6	448.26	21.40
-2	6	6	373.66	21.20
-2	6	6	405.26	21.40
1	-6	-6	57.39	5.20
1	-6	-6	71.49	4.40
-1	-6	6	62.99	4.80
-1	6	6	63.79	5.00
1	6	-6	53.49	3.10
1	6	-6	55.59	3.20
1	6	-6	56.99	4.60
-1	6	6	59.19	4.90
0	-6	6	8.30	1.70
0	-6	-6	5.40	2.00
0	-6	-6	8.40	1.80
0	6	6	5.70	2.10
0	6	-6	9.90	1.90
0	6	-6	5.20	1.20
0	6	-6	5.70	1.20
0	6	6	9.20	2.50
-1	-6	-6	468.65	21.40
-1	6	-6	442.76	21.00
-1	6	-6	431.16	21.00
1	6	6	418.36	21.50
1	6	6	352.86	22.10
-1	6	-6	432.56	20.90
-2	-6	-6	293.47	16.40
-2	-6	-6	310.47	15.90
-2	6	-6	318.77	15.20
-2	6	-6	327.47	15.80
2	6	6	288.67	15.90
-2	6	-6	307.47	15.70
2	6	6	308.67	16.10
-2	6	-6	300.67	15.20
-3	-6	-6	100.89	7.40
-3	-6	-6	102.99	6.40
3	6	6	101.19	6.80
-3	6	-6	100.99	5.70
-3	6	-6	98.19	6.60
-3	6	-6	93.99	6.40
-4	-6	-6	56.39	4.80
-4	-6	-6	49.50	5.80

-4	6	-6	53.69	5.20
-4	6	-6	56.49	4.10
-4	6	-6	52.79	4.90
4	6	6	43.00	4.70
-5	-6	-6	582.64	28.80
5	-6	6	572.34	27.80
-5	-6	-6	586.74	29.80
-5	6	-6	526.65	28.80
-5	6	-6	569.94	29.10
5	6	6	551.94	28.90
-5	6	-6	616.64	28.40
-6	-6	-6	130.79	9.70
-6	-6	-6	132.19	8.50
6	-6	6	114.59	7.10
6	6	6	129.09	8.60
-6	6	-6	108.69	8.10
-6	6	-6	110.49	8.40
-6	6	-6	107.19	8.50
-7	-6	-6	69.99	8.00
-7	-6	-6	62.59	6.60
7	-6	6	53.69	6.90
-7	6	-6	50.49	6.70
-7	6	-6	64.59	6.80
-7	6	-6	61.09	7.30
7	6	6	50.79	6.50
-8	-6	-6	71.59	9.20
-8	-6	-6	68.59	7.90
8	6	6	61.29	6.90
-8	6	-6	75.59	7.90
-8	6	-6	77.49	7.30
8	6	6	77.39	9.90
-8	6	-6	65.29	8.00
9	-6	6	178.68	11.70
-9	-6	-6	193.68	14.60
9	6	6	217.28	14.20
9	6	6	179.48	12.40
-9	6	-6	175.78	14.00
-9	6	-6	185.78	12.90
-10	-6	-6	14.20	5.90
-10	-6	-6	7.20	5.40
10	-6	6	4.40	3.70
-10	6	-6	8.70	5.10
10	6	6	3.90	5.10
-10	6	-6	0.20	6.20

-11	-6	-6	75.39	10.10
11	-6	6	60.39	7.90
-11	-6	-6	65.69	11.90
-11	6	-6	55.69	10.70
11	6	6	64.79	10.90
-11	6	-6	61.69	9.70
-12	-6	-6	19.70	7.20
12	-6	6	20.40	5.70
12	6	6	42.00	11.30
-12	6	-6	5.80	8.40
-12	6	-6	24.00	7.00
-13	-6	-6	15.20	7.20
-13	6	-6	25.90	8.00
-14	-6	-6	4.70	7.80
-14	6	-6	0.30	6.90
-15	-6	-6	27.50	9.90
-15	6	-6	22.60	8.50
-15	-7	6	78.09	11.70
-15	7	6	110.79	13.00
-14	-7	6	13.20	6.10
-14	7	6	18.50	7.10
-13	-7	6	292.97	17.70
13	7	-6	288.17	23.20
-13	7	6	313.47	18.90
-12	-7	6	-2.70	4.20
-12	-7	6	3.30	5.80
12	-7	-6	2.20	6.70
-12	-7	6	-10.60	10.10
-12	7	6	11.40	5.50
12	7	-6	3.90	8.50
-11	-7	6	272.47	15.70
-11	-7	6	197.78	20.20
11	-7	-6	274.17	18.10
-11	-7	6	239.68	14.40
11	7	-6	240.48	19.00
-11	7	6	241.98	20.70
-10	-7	6	98.39	9.20
10	-7	-6	115.99	11.70
-10	-7	6	67.99	14.40
-10	-7	6	102.69	7.80
-10	7	6	115.49	9.40
10	7	-6	88.19	12.40
-10	7	6	107.69	13.70
-9	-7	6	528.85	33.90

9	-7	-6	639.64	32.90
-9	-7	6	694.63	30.90
-9	-7	6	580.64	30.10
9	7	-6	622.34	33.60
-9	7	6	545.75	35.90
-9	7	6	617.84	31.20
-8	-7	6	-1.40	2.20
8	-7	-6	5.20	4.60
-8	-7	6	3.90	2.80
8	-7	-6	2.80	4.40
-8	-7	6	7.30	5.90
-8	7	6	-3.20	5.70
8	7	-6	3.20	4.90
-8	7	6	3.60	3.50
-7	-7	6	207.18	10.70
7	-7	-6	192.98	13.30
7	-7	-6	199.78	12.60
-7	-7	6	170.58	14.00
-7	7	6	195.58	14.20
-7	7	6	207.58	11.80
7	7	-6	179.38	13.70
-6	-7	6	45.90	3.70
6	-7	-6	57.09	7.30
-6	-7	6	39.90	7.90
6	-7	-6	47.80	6.20
-6	-7	6	42.50	3.50
-6	7	6	52.09	5.20
6	7	-6	42.70	7.30
-6	7	6	51.79	7.60
-5	-7	6	1076.79	53.49
-5	-7	6	1030.20	55.89
5	-7	-6	1145.79	55.49
-5	-7	6	1106.09	53.49
5	-7	-6	1151.28	55.19
-5	7	6	1157.48	55.99
-5	7	6	1157.28	54.79
5	7	-6	1162.78	55.99
4	-7	-6	15.60	3.50
-4	-7	6	15.00	3.70
4	-7	-6	13.90	3.30
-4	7	6	17.40	3.60
-4	7	6	15.00	3.40
4	7	-6	21.30	4.20
3	-7	-6	225.28	12.50

3	-7	-6	224.88	11.90
-3	-7	6	199.58	12.40
-3	7	6	193.28	11.80
3	7	-6	216.98	12.40
-3	7	6	208.48	12.50
2	-7	-6	888.31	42.80
-2	-7	6	845.12	42.70
2	-7	-6	932.81	42.20
-2	7	6	796.42	42.40
-2	7	6	869.91	42.70
2	7	-6	846.82	42.50
1	-7	-6	2413.56	116.09
-1	-7	6	2435.16	115.79
1	-7	-6	2523.75	115.49
1	7	-6	2351.56	114.59
-1	7	6	2326.27	115.89
1	7	-6	2428.26	115.69
1	7	-6	2299.17	114.59
-1	7	6	2573.04	116.09
0	-7	-6	532.55	26.20
0	-7	6	506.25	25.50
0	-7	-6	529.25	25.60
0	7	6	484.95	25.90
0	7	-6	507.35	24.90
0	7	-6	506.95	25.60
0	7	6	532.85	26.10
-1	-7	-6	422.56	19.70
1	-7	6	398.16	19.20
1	7	6	369.46	19.80
-1	7	-6	392.86	19.40
1	7	6	371.86	20.30
-1	7	-6	389.26	19.20
1	7	6	359.56	20.60
-2	-7	-6	497.55	23.60
-2	-7	-6	458.15	24.20
2	-7	6	467.95	23.50
-2	7	-6	469.45	23.10
-2	7	-6	476.75	23.30
2	7	6	465.75	23.90
-2	7	-6	495.65	23.10
2	7	6	445.56	24.30
2	7	6	446.46	23.70
-3	-7	-6	1707.73	78.69
3	-7	6	1672.03	78.59

-3	-7	-6	1624.74	79.39
-3	7	-6	1605.34	78.49
3	7	6	1566.14	78.79
-4	-7	-6	1216.38	62.99
-4	-7	-6	1350.76	62.29
4	-7	6	1332.27	62.29
-4	7	-6	1296.57	62.19
4	7	6	1234.98	62.29
-4	7	-6	1268.97	61.59
-5	-7	-6	4.20	3.30
5	-7	6	3.40	1.90
-5	-7	-6	2.50	2.50
5	-7	6	2.10	3.00
-5	7	-6	4.80	2.20
-5	7	-6	-3.10	2.70
5	7	6	5.70	2.80
5	7	6	1.00	2.30
-6	-7	-6	1214.08	54.69
6	-7	6	1103.49	54.79
-6	-7	-6	1025.10	55.49
-6	7	-6	1147.29	54.99
-6	7	-6	1092.79	54.59
6	7	6	1066.59	54.59
-6	7	-6	1143.99	54.39
-7	-7	-6	140.49	11.20
-7	-7	-6	146.69	9.90
7	-7	6	131.09	8.90
7	7	6	135.59	9.70
-7	7	-6	126.29	9.80
-7	7	-6	129.19	10.40
-7	7	-6	150.18	9.90
-8	-7	-6	290.97	19.40
8	-7	6	310.07	16.60
-8	-7	-6	342.97	18.20
8	7	6	308.17	18.00
-8	7	-6	308.37	18.20
-8	7	-6	325.57	18.00
-9	-7	-6	5.80	5.40
9	-7	6	10.00	3.40
9	7	6	10.50	4.70
-9	7	-6	17.50	5.00
-9	7	-6	13.30	5.30
-10	-7	-6	782.42	43.00
10	-7	6	824.82	40.00

-10	7	-6	821.72	41.20
-10	7	-6	783.12	41.80
10	7	6	772.62	40.80
-11	-7	-6	34.30	8.40
11	-7	6	25.50	5.10
-11	-7	-6	31.10	8.60
-11	7	-6	30.40	6.70
-11	7	-6	18.00	7.50
-12	-7	-6	443.26	24.50
-12	7	-6	364.66	25.70
-12	7	-6	392.96	23.70
-13	-7	-6	66.79	12.00
-13	7	-6	68.69	10.50
-14	-7	-6	120.59	15.10
-14	7	-6	131.59	13.20
-15	-7	-6	139.69	16.60
-15	7	-6	107.29	13.90
-15	8	6	45.10	8.50
-14	-8	6	213.38	15.30
-14	8	6	240.08	16.80
-13	-8	6	50.39	8.80
-13	8	6	68.29	10.60
13	8	-6	43.90	10.30
-12	-8	6	408.76	22.80
12	-8	-6	415.56	26.10
-12	-8	6	350.26	29.40
-12	-8	6	487.45	25.30
12	8	-6	428.56	27.50
-12	8	6	453.85	24.00
-11	-8	6	69.79	12.70
-11	-8	6	46.10	8.20
-11	-8	6	42.90	6.70
11	-8	-6	42.70	8.20
-11	8	6	48.50	10.00
-11	8	6	50.19	8.40
11	8	-6	56.09	10.20
10	-8	-6	980.70	50.79
-10	-8	6	1095.99	49.00
-10	-8	6	958.10	48.30
-10	-8	6	851.11	52.39
-10	8	6	1021.70	49.40
-10	8	6	995.90	52.79
10	8	-6	998.70	51.89
9	-8	-6	8.00	5.10

-9	-8	6	0.60	3.50
-9	-8	6	5.80	6.80
-9	-8	6	0.50	2.80
-9	8	6	6.90	7.40
9	8	-6	17.60	6.70
-9	8	6	10.40	4.50
-8	-8	6	505.55	25.60
-8	-8	6	546.35	26.30
-8	-8	6	479.15	29.50
8	-8	-6	537.35	28.40
-8	8	6	473.25	30.50
-8	8	6	514.05	26.90
8	8	-6	561.84	29.20
-7	-8	6	179.58	14.20
-7	-8	6	166.28	9.90
7	-8	-6	187.08	13.10
-7	-8	6	191.58	10.10
7	-8	-6	184.78	12.70
-7	8	6	189.18	11.60
7	8	-6	198.58	13.80
-7	8	6	189.88	13.80
-6	-8	6	391.26	20.30
-6	-8	6	403.66	20.30
-6	-8	6	419.16	23.80
6	-8	-6	413.76	22.50
6	-8	-6	425.56	22.90
-6	8	6	421.36	23.40
6	8	-6	463.45	23.70
-6	8	6	397.56	21.70
5	-8	-6	607.04	29.80
-5	-8	6	537.45	30.70
5	-8	-6	598.94	30.20
-5	-8	6	555.54	27.80
-5	-8	6	545.05	27.80
5	8	-6	624.94	30.80
-5	8	6	596.24	29.50
-5	8	6	593.84	30.60
4	-8	-6	453.95	22.80
-4	-8	6	414.26	23.50
4	-8	-6	453.55	23.30
-4	8	6	420.46	22.60
4	8	-6	456.85	23.40
-4	8	6	423.46	23.30
3	-8	-6	80.59	6.00

-3	-8	6	75.29	7.00
3	-8	-6	84.89	7.10
-3	8	6	77.29	6.80
3	8	-6	72.39	6.70
-3	8	6	69.19	6.40
2	-8	-6	107.49	6.70
-2	-8	6	104.29	7.60
2	-8	-6	99.99	7.60
-2	8	6	97.39	7.60
-2	8	6	89.09	7.30
2	8	-6	108.59	7.40
1	-8	-6	811.92	38.30
-1	-8	6	782.92	38.60
1	-8	-6	775.72	38.90
-1	-8	6	763.52	37.30
1	8	-6	807.92	38.60
1	8	-6	755.42	37.20
-1	8	6	756.62	38.70
-1	8	6	792.72	38.90
0	-8	6	532.05	25.70
0	-8	6	511.65	24.90
0	-8	-6	513.85	25.60
0	-8	-6	521.75	26.30
0	8	6	496.35	25.90
0	8	-6	481.65	24.80
0	8	-6	534.75	25.80
0	8	6	509.05	26.50
1	-8	6	157.28	8.30
-1	-8	-6	155.48	8.80
1	8	6	147.99	9.20
1	8	6	145.29	9.30
-1	8	-6	157.48	8.70
1	8	6	139.09	9.70
-1	8	-6	146.99	8.20
2	-8	6	8.00	2.30
-2	-8	-6	10.10	2.50
2	8	6	8.20	2.60
-2	8	-6	6.80	2.20
2	8	6	13.50	3.60
-2	8	-6	7.10	1.80
2	8	6	11.80	2.90
-2	8	-6	7.90	2.00
-3	-8	-6	138.19	9.50
3	-8	6	132.89	8.30

-3	-8	-6	134.29	8.30
3	8	6	134.19	8.50
-3	8	-6	123.39	8.10
3	8	6	149.39	9.90
4	-8	6	10.80	3.30
-4	-8	-6	8.10	3.30
-4	-8	-6	10.50	3.00
4	8	6	12.80	3.90
4	8	6	11.80	3.20
-4	8	-6	12.00	2.40
-4	8	-6	7.40	3.00
-5	-8	-6	448.26	25.30
5	-8	6	483.95	24.50
-5	-8	-6	479.25	24.20
-5	8	-6	449.56	24.20
5	8	6	452.95	24.30
-5	8	-6	493.75	23.90
5	8	6	490.15	25.40
-6	-8	-6	201.88	11.70
6	-8	6	181.08	11.00
6	-8	6	205.48	12.30
-6	-8	-6	193.58	13.40
-6	8	-6	206.98	11.60
6	8	6	186.78	11.90
-6	8	-6	184.28	11.90
6	8	6	187.08	12.80
-7	-8	-6	667.53	32.20
7	-8	6	629.24	32.60
-7	-8	-6	606.74	33.60
7	-8	6	635.84	31.10
-7	8	-6	660.23	32.20
7	8	6	605.84	32.20
-7	8	-6	606.14	32.60
-7	8	-6	621.34	32.30
8	-8	6	-0.30	2.40
-8	-8	-6	-0.10	3.70
-8	-8	-6	1.70	4.80
8	8	6	-1.00	3.80
-8	8	-6	0.80	4.10
-8	8	-6	-1.40	4.20
8	8	6	3.60	3.70
-9	-8	-6	329.77	22.20
9	-8	6	370.16	18.90
-9	8	-6	365.16	20.90

9	8	6	329.37	20.00
-9	8	-6	357.06	20.30
-10	-8	-6	11.30	7.10
10	-8	6	4.10	3.60
10	8	6	1.70	4.40
10	8	6	13.40	5.30
-10	8	-6	2.40	5.90
11	-8	6	359.26	20.20
-11	-8	-6	366.36	24.50
-11	8	-6	373.96	21.80
-11	8	-6	348.67	23.00
-12	-8	-6	70.29	11.60
-12	8	-6	45.30	9.30
-12	8	-6	64.19	10.70
-13	-8	-6	387.26	24.00
-13	8	-6	355.96	23.10
-14	-8	-6	31.70	9.00
-14	8	-6	13.10	7.80
-15	-8	-6	145.39	17.40
-15	8	-6	145.29	15.90
-15	9	6	38.60	8.70
-14	-9	6	8.30	6.30
-14	9	6	22.10	7.00
-13	-9	6	12.90	5.30
-13	9	6	28.60	7.20
13	9	-6	17.10	9.40
12	-9	-6	17.90	7.30
-12	-9	6	24.00	5.10
-12	-9	6	19.90	13.30
-11	-9	6	7.50	9.70
-11	-9	6	35.70	5.00
-11	-9	6	43.70	6.00
11	-9	-6	30.90	7.60
-11	9	6	27.30	6.10
-11	9	6	22.90	10.00
11	9	-6	22.70	8.20
10	-9	-6	24.50	6.70
-10	-9	6	18.50	4.80
-10	-9	6	17.40	9.00
-10	9	6	24.10	8.50
10	9	-6	13.60	7.80
-9	-9	6	17.30	3.40
-9	-9	6	20.20	4.00
-9	-9	6	17.30	7.70

9	-9	-6	19.30	5.80
-9	9	6	17.60	4.90
9	9	-6	15.80	6.40
-9	9	6	25.20	9.90
8	-9	-6	9.30	5.20
-8	-9	6	6.00	2.50
-8	-9	6	-0.40	5.80
-8	9	6	-0.60	3.80
8	9	-6	6.40	5.50
-8	9	6	-0.50	6.40
-7	-9	6	53.89	4.30
-7	-9	6	43.80	7.60
7	-9	-6	51.79	7.90
-7	-9	6	52.29	4.30
7	-9	-6	56.49	8.10
-7	9	6	62.99	6.70
7	9	-6	51.79	9.00
-7	9	6	39.50	6.80
-6	-9	6	217.58	11.20
-6	-9	6	210.78	11.20
-6	-9	6	203.18	15.30
6	-9	-6	220.58	13.80
6	-9	-6	225.28	14.20
-6	9	6	244.98	15.20
-6	9	6	213.78	13.10
6	9	-6	242.98	15.00
5	-9	-6	131.09	9.60
-5	-9	6	93.09	10.20
-5	-9	6	112.29	6.00
-5	-9	6	113.99	5.90
5	-9	-6	132.69	9.10
5	9	-6	107.99	9.60
-5	9	6	119.09	9.90
-5	9	6	118.59	8.50
-4	-9	6	264.27	16.80
4	-9	-6	287.57	16.00
4	-9	-6	290.87	16.50
-4	9	6	284.77	16.60
-4	9	6	294.17	15.90
4	9	-6	300.77	16.80
-3	-9	6	299.47	17.90
3	-9	-6	340.37	17.20
3	-9	-6	336.17	17.80
-3	9	6	317.57	17.30

-3	9	6	316.47	17.70
3	9	-6	298.67	17.50
2	-9	-6	181.28	10.20
-2	-9	6	173.08	11.00
2	-9	-6	185.78	11.20
-2	-9	6	180.68	8.60
2	9	-6	166.38	10.70
2	9	-6	170.78	8.50
-2	9	6	188.68	11.10
-2	9	6	157.18	10.70
1	-9	-6	6.20	2.80
1	-9	-6	7.00	2.30
-1	-9	6	9.10	1.40
-1	-9	6	7.90	2.60
1	9	-6	12.20	2.90
-1	9	6	8.00	3.00
-1	9	6	9.40	3.20
1	9	-6	8.70	1.60
0	-9	-6	1074.99	50.69
0	-9	6	1006.70	50.79
0	-9	6	1085.09	50.09
0	9	6	1050.39	51.69
0	9	6	1049.70	51.19
0	9	-6	1003.90	50.89
0	9	6	1058.09	51.59
0	9	-6	1014.10	49.90
-1	-9	-6	25.40	3.60
1	-9	6	25.60	3.20
-1	9	-6	20.70	3.80
1	9	6	22.90	4.40
1	9	6	23.10	4.00
1	9	6	26.00	4.50
-2	-9	-6	13.70	2.80
2	-9	6	17.40	3.20
2	9	6	11.80	3.00
2	9	6	14.60	4.00
-2	9	-6	17.50	3.20
2	9	6	15.00	3.00
-2	9	-6	12.90	2.30
-3	-9	-6	20.30	4.50
-3	-9	-6	24.10	4.10
3	-9	6	20.90	3.30
3	9	6	19.80	3.40
3	9	6	17.90	4.50

3	9	6	22.50	3.70
-3	9	-6	17.70	3.30
-3	9	-6	19.90	3.20
-3	9	-6	23.40	3.30
-4	-9	-6	174.88	12.50
-4	-9	-6	211.28	11.10
4	-9	6	204.08	11.50
4	9	6	190.98	11.20
4	9	6	172.78	12.40
-4	9	-6	193.28	11.20
-4	9	-6	185.98	10.80
-5	-9	-6	3.10	3.80
-5	-9	-6	4.10	3.00
5	-9	6	3.90	3.20
5	9	6	-1.50	3.70
5	9	6	4.00	3.00
-5	9	-6	3.20	3.20
-5	9	-6	0.60	2.50
-6	-9	-6	189.08	10.90
-6	-9	-6	150.78	12.40
6	-9	6	167.68	9.60
6	-9	6	166.68	11.30
-6	9	-6	168.18	10.90
6	9	6	169.18	11.00
-6	9	-6	171.48	10.70
6	9	6	169.98	12.30
-7	-9	-6	75.09	7.40
-7	-9	-6	69.29	9.40
7	-9	6	60.59	5.50
7	-9	6	71.99	8.00
7	9	6	70.59	8.90
-7	9	-6	59.29	7.30
-7	9	-6	59.99	7.30
7	9	6	57.79	7.30
8	-9	6	24.30	4.80
-8	-9	-6	24.90	6.10
-8	-9	-6	29.40	5.20
8	9	6	24.10	5.90
8	9	6	26.90	5.20
-8	9	-6	27.30	5.30
-8	9	-6	29.40	5.20
-9	-9	-6	-3.10	6.10
9	-9	6	2.30	3.10
-9	9	-6	7.30	4.50

9	9	6	-7.90	5.30
9	9	6	2.50	4.40
-9	9	-6	1.10	5.00
10	-9	6	77.69	7.90
-10	-9	-6	80.69	12.90
10	9	6	91.19	12.20
-10	9	-6	67.19	8.70
-10	9	-6	73.89	11.10
10	9	6	80.19	9.50
11	-9	6	24.00	5.20
-11	-9	-6	32.20	8.70
-11	-9	-6	33.00	14.40
11	9	6	25.50	7.30
-11	9	-6	24.10	7.20
-11	9	-6	24.70	6.50
-12	-9	-6	12.10	7.20
-12	9	-6	11.80	7.10
-13	-9	-6	11.90	8.10
-13	9	-6	9.90	6.70
-14	-9	-6	2.00	9.10
-14	9	-6	15.90	7.90
-15	-9	-6	17.90	10.00
-15	9	-6	16.90	9.10
-14	10	6	317.87	20.70
-13	-10	6	37.30	6.30
-13	10	6	51.29	8.40
13	10	-6	41.40	10.40
-12	-10	6	644.14	32.80
12	10	-6	647.24	38.00
-12	10	6	637.44	34.20
-11	-10	6	14.80	4.30
-11	-10	6	18.20	5.10
11	-10	-6	16.70	7.30
-11	-10	6	3.60	11.10
-11	10	6	23.90	5.90
11	10	-6	27.80	8.50
-10	-10	6	1170.88	52.39
-10	-10	6	1061.89	51.99
10	-10	-6	1058.99	54.89
-10	-10	6	917.41	56.79
10	10	-6	1140.99	56.09
-10	10	6	1083.39	53.29
-10	10	6	1022.90	56.39
-9	-10	6	59.09	5.60

9	-10	-6	56.69	9.90
-9	-10	6	31.60	8.90
-9	-10	6	63.79	6.20
-9	10	6	62.29	8.00
-9	10	6	42.80	9.80
9	10	-6	65.89	11.30
8	-10	-6	406.76	23.20
-8	-10	6	419.76	20.40
-8	-10	6	398.46	20.20
-8	-10	6	354.96	24.50
8	10	-6	410.56	24.10
-8	10	6	411.06	21.90
-7	-10	6	177.68	9.50
7	-10	-6	179.38	13.20
-7	-10	6	171.78	9.50
-7	-10	6	163.48	14.60
-7	10	6	169.88	13.90
7	10	-6	198.38	14.20
-7	10	6	181.08	11.60
6	-10	-6	1471.95	70.89
-6	-10	6	1414.96	68.29
-6	-10	6	1372.46	68.19
6	-10	-6	1426.26	70.59
-6	-10	6	1309.47	71.39
6	10	-6	1533.45	71.39
-6	10	6	1447.26	71.39
-6	10	6	1494.25	69.99
5	-10	-6	1200.98	59.29
-5	-10	6	1161.88	57.09
-5	-10	6	1184.78	57.09
-5	-10	6	1115.89	60.19
5	-10	-6	1263.17	59.69
5	10	-6	1232.28	59.99
-5	10	6	1222.08	59.99
-5	10	6	1230.48	58.89
-4	-10	6	1268.17	68.09
4	-10	-6	1409.86	67.39
4	-10	-6	1392.36	67.79
-4	10	6	1381.66	67.19
-4	10	6	1389.26	67.99
4	10	-6	1431.36	67.99
-3	-10	6	227.78	14.70
3	-10	-6	262.67	14.70
3	-10	-6	246.68	13.80

-3	10	6	242.08	14.60
3	10	-6	250.57	14.50
-3	10	6	232.48	14.10
-2	-10	6	80.29	4.20
2	-10	-6	74.29	6.20
2	-10	-6	79.39	7.20
-2	-10	6	77.29	7.20
2	10	-6	73.49	4.10
-2	10	6	83.79	7.30
-2	10	6	60.09	6.30
2	10	-6	79.89	6.90
-1	-10	6	753.62	37.80
1	-10	-6	764.22	37.30
1	-10	-6	787.02	38.30
-1	-10	6	751.12	36.30
-1	10	6	723.53	37.80
1	10	-6	780.22	37.80
1	10	-6	731.03	36.20
0	-10	6	284.57	15.70
0	-10	6	287.87	14.70
0	-10	-6	311.47	15.60
0	10	6	272.67	15.90
0	10	-6	277.17	14.50
0	10	6	291.57	17.10
0	10	6	304.07	16.40
0	10	-6	302.07	15.90
-1	-10	-6	2913.11	134.19
1	-10	6	2802.42	133.79
-1	10	-6	2799.82	134.39
-1	10	-6	2661.13	133.59
1	10	6	2863.51	135.39
1	10	6	2895.81	134.89
1	10	6	2725.43	134.49
-2	-10	-6	138.29	8.30
2	-10	6	123.09	7.80
2	10	6	126.09	8.90
2	10	6	134.89	9.90
-2	10	-6	123.99	8.10
-2	10	-6	135.09	7.90
-2	10	-6	119.79	7.60
2	10	6	123.09	8.50
-3	-10	-6	788.12	41.80
3	-10	6	844.52	40.60
-3	-10	-6	866.21	40.70

-3	10	-6	784.42	40.40
3	10	6	815.12	41.20
3	10	6	845.02	42.10
-3	10	-6	831.32	40.40
3	10	6	804.52	40.90
-3	10	-6	845.42	40.40
-4	-10	-6	4.70	3.70
-4	-10	-6	13.40	3.40
4	-10	6	9.60	3.40
4	10	6	6.20	3.10
-4	10	-6	6.70	3.30
-4	10	-6	11.30	3.20
4	10	6	7.90	4.00
-5	-10	-6	851.81	41.90
-5	-10	-6	798.72	43.30
5	-10	6	837.32	42.20
5	10	6	824.52	42.00
-5	10	-6	872.01	41.80
-5	10	-6	847.22	41.90
5	10	6	883.81	43.40
-6	-10	-6	44.60	8.50
-6	-10	-6	52.49	6.10
6	-10	6	43.30	6.90
-6	10	-6	49.60	6.30
6	10	6	50.39	6.50
-6	10	-6	37.60	6.30
6	10	6	57.69	8.40
7	-10	6	1051.49	50.69
7	-10	6	1022.40	52.09
-7	-10	-6	981.20	53.29
-7	-10	-6	1055.19	51.59
-7	10	-6	1052.89	51.69
7	10	6	987.20	51.59
-7	10	-6	1034.10	51.69
7	10	6	1140.19	53.19
8	-10	6	47.20	5.40
-8	-10	-6	33.30	7.00
-8	10	-6	32.20	5.60
-8	10	-6	36.20	5.90
8	10	6	32.00	6.70
8	10	6	43.00	7.20
-9	-10	-6	298.67	21.20
9	-10	6	332.27	17.40
9	10	6	284.27	18.40

-9	10	-6	317.37	19.20
9	10	6	323.97	20.40
-9	10	-6	318.77	18.80
-10	-10	-6	64.69	10.60
10	-10	6	64.79	7.50
-10	10	-6	63.99	10.40
10	10	6	48.20	8.50
-10	10	-6	52.39	8.50
10	10	6	56.89	9.00
11	-10	6	498.95	25.90
11	10	6	457.55	27.20
-11	10	-6	456.05	28.10
11	10	6	517.65	29.50
-11	10	-6	488.85	27.10
-12	-10	-6	66.29	12.10
-12	10	-6	40.80	8.00
-13	-10	-6	325.87	22.20
-13	10	-6	304.57	20.90
-14	-10	-6	124.09	16.20
-14	10	-6	94.59	12.30
-14	11	6	-0.70	6.80
-13	11	6	178.88	14.70
13	11	-6	178.18	19.70
-12	-11	6	8.00	4.70
12	11	-6	27.40	10.00
-12	11	6	21.90	6.70
-11	-11	6	11.50	12.70
-11	-11	6	10.40	4.10
11	-11	-6	27.30	7.90
-11	11	6	16.40	5.70
11	11	-6	21.30	8.80
-10	-11	6	6.10	3.40
-10	-11	6	4.90	3.80
10	-11	-6	12.20	6.90
-10	-11	6	3.40	9.40
10	11	-6	15.50	7.30
-10	11	6	14.10	5.20
-10	11	6	11.60	7.80
9	-11	-6	63.09	10.80
-9	-11	6	75.39	6.00
-9	-11	6	65.09	13.70
-9	-11	6	77.69	6.40
-9	11	6	52.99	9.90
9	11	-6	92.09	12.00

-9	11	6	71.39	11.30
-8	-11	6	113.69	7.30
8	-11	-6	127.29	11.50
-8	-11	6	108.69	13.40
-8	-11	6	108.79	7.40
8	11	-6	131.69	13.00
-8	11	6	122.89	9.80
-7	-11	6	221.68	11.60
7	-11	-6	223.68	15.20
-7	-11	6	219.88	11.50
-7	-11	6	205.28	16.40
7	11	-6	248.58	16.60
-7	11	6	225.38	13.70
-7	11	6	213.28	16.30
-6	-11	6	126.29	7.20
6	-11	-6	145.49	11.30
6	-11	-6	130.69	11.00
-6	-11	6	133.59	12.70
-6	-11	6	128.69	7.10
-6	11	6	121.29	11.60
6	11	-6	161.68	12.50
-6	11	6	151.98	10.10
-5	-11	6	15.50	1.90
-5	-11	6	15.80	1.90
-5	-11	6	10.90	5.20
5	-11	-6	11.40	4.50
5	-11	-6	13.00	4.50
5	11	-6	12.00	5.00
-5	11	6	9.20	4.10
-5	11	6	19.30	5.10
-4	-11	6	22.60	1.80
-4	-11	6	17.00	4.80
4	-11	-6	20.10	4.50
4	-11	-6	22.80	4.60
-4	11	6	16.40	4.10
-4	11	6	20.90	4.60
4	11	-6	17.90	5.00
-3	-11	6	119.39	9.70
3	-11	-6	119.59	8.50
-3	-11	6	114.09	6.10
3	-11	-6	128.29	9.50
-3	11	6	109.89	8.50
3	11	-6	113.19	6.00
3	11	-6	137.29	9.70

-3	11	6	121.49	9.70
-2	-11	6	8.90	3.70
-2	-11	6	3.20	1.30
2	-11	-6	5.60	3.10
2	-11	-6	10.00	3.40
-2	11	6	-0.50	3.10
2	11	-6	2.80	1.10
2	11	-6	2.90	3.40
-1	-11	6	26.10	5.20
-1	-11	6	24.60	2.80
1	-11	-6	31.90	5.70
1	-11	-6	31.00	4.30
-1	11	6	21.60	4.00
1	11	-6	34.10	5.40
1	11	-6	27.20	2.60
-1	11	6	29.50	5.30
0	-11	6	54.19	5.50
0	-11	6	46.70	4.10
0	-11	-6	61.09	5.30
0	11	6	47.30	6.10
0	11	6	44.40	5.80
0	11	-6	42.60	3.90
0	11	-6	60.59	6.00
0	11	6	67.19	7.80
-1	-11	-6	74.19	6.00
1	-11	6	68.89	5.20
-1	11	-6	75.89	6.30
1	11	6	68.89	6.50
1	11	6	70.59	6.80
-1	11	-6	62.09	5.00
2	-11	6	248.08	13.60
-2	-11	-6	262.57	13.90
2	11	6	260.37	15.90
2	11	6	249.28	14.10
-2	11	-6	257.47	13.80
2	11	6	250.07	14.70
-2	11	-6	238.08	13.30
-2	11	-6	251.77	14.00
3	-11	6	178.48	10.30
-3	-11	-6	170.68	10.30
-3	-11	-6	165.48	11.80
-3	11	-6	162.98	10.10
3	11	6	168.98	11.10
-3	11	-6	164.98	10.00

-3	11	-6	164.78	9.80
3	11	6	167.98	10.60
3	11	6	175.08	12.40
4	-11	6	188.28	11.80
-4	-11	-6	202.68	13.60
-4	-11	-6	205.18	11.70
4	11	6	184.78	11.70
-4	11	-6	182.78	11.40
4	11	6	216.38	13.80
5	-11	6	36.80	6.20
-5	-11	-6	42.70	6.80
-5	-11	-6	49.60	5.90
5	11	6	45.00	8.40
5	11	6	44.60	5.90
-5	11	-6	40.70	5.90
-5	11	-6	45.20	5.80
6	-11	6	202.18	13.40
-6	-11	-6	221.18	12.90
-6	-11	-6	215.28	15.20
6	11	6	226.28	15.10
-6	11	-6	189.18	12.80
-6	11	-6	207.18	12.80
6	11	6	194.98	12.90
-7	-11	-6	14.10	6.00
7	-11	6	31.60	5.00
-7	-11	-6	25.90	4.90
-7	11	-6	21.80	4.70
7	11	6	25.10	4.80
-7	11	-6	23.40	5.20
7	11	6	27.90	6.10
8	-11	6	55.39	6.10
-8	-11	-6	38.30	7.80
-8	11	-6	65.69	8.20
-8	11	-6	66.39	8.50
8	11	6	59.29	8.00
8	11	6	66.69	10.40
-9	-11	-6	15.50	7.40
9	-11	6	26.30	4.30
9	11	6	15.70	5.40
-9	11	-6	22.30	6.00
9	11	6	30.30	7.50
-9	11	-6	16.60	6.00
-10	-11	-6	72.79	13.70
10	-11	6	75.19	8.10

-10	11	-6	75.39	10.90
10	11	6	62.59	9.80
-10	11	-6	68.39	9.10
10	11	6	81.99	12.80
-11	11	-6	42.40	8.30
-11	11	-6	60.09	10.30
11	11	6	59.19	13.30
11	11	6	52.69	9.80
-12	-11	-6	32.20	8.90
-12	11	-6	25.40	7.50
-13	-11	-6	-8.80	8.00
-13	11	-6	1.80	6.60
-14	-11	-6	5.10	9.40
-14	11	-6	10.20	8.30
-14	12	6	133.89	14.10
-13	12	6	9.60	6.90
13	12	-6	6.30	10.00
-12	-12	6	411.06	21.90
12	12	-6	399.26	28.70
-12	12	6	410.16	23.90
-11	-12	6	18.90	4.60
11	-12	-6	30.90	8.30
-11	12	6	33.60	6.90
11	12	-6	27.60	9.20
10	-12	-6	421.66	25.50
-10	-12	6	421.16	21.30
-10	-12	6	420.16	21.20
-10	12	6	400.36	23.20
10	12	-6	380.66	26.10
-10	12	6	414.56	27.60
-9	-12	6	29.40	4.80
9	-12	-6	37.20	7.40
-9	-12	6	44.80	5.20
-9	-12	6	30.90	10.60
9	12	-6	24.40	7.80
-9	12	6	34.90	8.60
-9	12	6	47.20	6.70
-8	-12	6	372.86	19.10
-8	-12	6	386.46	20.10
-8	-12	6	333.57	24.30
8	-12	-6	381.86	22.70
8	12	-6	401.96	24.20
-8	12	6	400.76	21.40
-7	-12	6	76.79	12.00

-7	-12	6	105.89	6.10
-7	-12	6	105.19	6.30
7	-12	-6	112.29	10.90
-7	12	6	97.89	8.60
7	12	-6	108.09	11.60
-6	-12	6	61.79	10.40
-6	-12	6	63.79	4.00
6	-12	-6	57.39	8.60
-6	-12	6	60.49	3.90
6	12	-6	63.39	9.40
-6	12	6	49.90	7.20
-6	12	6	43.40	8.20
5	-12	-6	397.26	21.90
-5	-12	6	392.96	18.70
5	-12	-6	365.96	21.30
-5	-12	6	390.76	18.60
-5	-12	6	385.96	22.90
-5	12	6	404.46	21.10
-5	12	6	334.27	23.20
5	12	-6	389.46	22.30
-4	-12	6	464.35	27.60
-4	-12	6	516.25	24.20
4	-12	-6	515.65	26.60
4	-12	-6	528.45	27.20
4	12	-6	485.05	24.10
-4	12	6	530.85	26.60
4	12	-6	531.15	27.40
-4	12	6	448.66	28.80
3	-12	-6	126.79	9.00
3	-12	-6	126.79	9.80
-3	-12	6	108.69	9.90
-3	-12	6	117.99	6.30
3	12	-6	118.69	6.20
3	12	-6	122.89	9.80
-3	12	6	111.39	9.10
-2	-12	6	537.85	26.00
2	-12	-6	582.04	28.50
-2	-12	6	525.45	28.30
2	-12	-6	546.05	27.60
2	12	-6	542.75	28.30
2	12	-6	511.05	25.90
-2	12	6	536.55	28.40
-2	12	6	512.85	27.80
-1	-12	6	539.65	28.90

1	-12	-6	581.74	28.40
-1	-12	6	564.14	27.30
1	12	-6	577.14	29.20
-1	12	6	523.55	28.90
-1	12	6	545.55	29.80
1	12	-6	567.64	27.10
-1	12	6	567.14	29.30
0	-12	-6	595.34	29.50
0	-12	6	580.84	28.80
0	12	-6	574.14	30.10
0	12	6	605.04	30.60
0	12	-6	572.64	28.60
0	12	6	570.14	30.00
0	12	6	605.94	31.20
1	-12	6	525.05	25.80
-1	-12	-6	557.94	26.40
1	12	6	487.05	27.90
-1	12	-6	504.45	26.70
-1	12	-6	497.05	25.50
1	12	6	537.05	27.20
1	12	6	504.45	26.70
-2	-12	-6	454.75	22.20
2	-12	6	443.56	22.00
-2	12	-6	410.76	22.40
2	12	6	412.96	22.40
2	12	6	423.76	23.00
-2	12	-6	436.86	22.20
2	12	6	432.76	24.20
-2	12	-6	410.26	21.60
3	-12	6	529.45	26.10
-3	-12	-6	521.75	26.00
-3	-12	-6	491.95	27.50
3	12	6	526.65	27.00
3	12	6	508.25	26.40
-3	12	-6	465.25	26.00
-3	12	-6	517.75	26.00
-3	12	-6	509.55	25.70
4	-12	6	7.70	3.60
-4	-12	-6	5.20	4.90
-4	-12	-6	11.20	3.40
-4	12	-6	9.30	3.60
-4	12	-6	13.50	3.40
4	12	6	8.30	5.00
-4	12	-6	5.20	2.80

4	12	6	6.30	3.50
-5	-12	-6	1171.68	63.19
5	-12	6	1256.57	61.99
-5	-12	-6	1321.27	61.69
5	12	6	1199.48	61.79
5	12	6	1319.47	63.59
-5	12	-6	1321.87	61.69
-5	12	-6	1232.08	61.59
-6	-12	-6	9.90	5.50
-6	-12	-6	10.60	4.00
6	-12	6	4.50	4.90
6	12	6	15.30	4.50
6	12	6	23.00	6.10
-6	12	-6	8.20	3.80
-6	12	-6	6.20	4.30
-7	-12	-6	746.03	43.30
-7	-12	-6	889.11	41.70
7	12	6	783.22	41.40
7	12	6	858.01	43.40
-7	12	-6	815.82	41.40
-7	12	-6	819.12	41.60
8	-12	6	10.70	3.50
-8	-12	-6	-0.40	6.20
-8	12	-6	11.70	4.60
8	12	6	6.10	6.30
-8	12	-6	8.00	4.80
8	12	6	7.30	5.00
9	-12	6	222.88	13.40
-9	-12	-6	194.38	17.90
9	12	6	214.18	14.90
9	12	6	229.48	17.60
-9	12	-6	235.18	15.70
-9	12	-6	211.98	17.10
-10	-12	-6	52.19	15.40
10	-12	6	96.69	9.00
-10	12	-6	91.59	12.00
10	12	6	98.49	14.00
-10	12	-6	118.59	10.90
10	12	6	101.89	10.60
-11	12	-6	257.87	17.00
11	12	6	241.38	16.50
-11	12	-6	204.78	18.10
-12	-12	-6	28.70	8.70
-12	12	-6	36.90	8.40

-13	-12	-6	43.00	10.50
-13	12	-6	28.60	8.40
-14	-12	-6	34.00	11.20
-14	12	-6	15.30	8.90
-13	13	6	229.28	17.20
13	13	-6	221.68	28.90
-12	13	6	21.80	6.90
12	13	-6	9.40	10.00
-11	-13	6	565.54	29.60
11	13	-6	592.84	35.30
-11	13	6	601.84	31.80
10	-13	-6	35.50	8.00
-10	-13	6	52.49	5.80
10	13	-6	26.00	8.90
-10	13	6	40.30	7.10
-9	-13	6	339.97	17.70
-9	-13	6	335.47	17.70
9	-13	-6	357.16	22.10
-9	-13	6	268.37	26.40
-9	13	6	353.46	23.70
-9	13	6	361.76	20.20
9	13	-6	365.16	23.30
-8	-13	6	17.10	8.50
-8	-13	6	22.10	3.80
8	-13	-6	22.00	6.50
-8	13	6	24.50	8.80
8	13	-6	28.40	7.90
-7	-13	6	482.25	24.00
-7	-13	6	436.46	28.90
7	-13	-6	501.15	27.60
-7	-13	6	472.55	23.80
7	13	-6	551.54	28.60
-7	13	6	492.85	26.00
-6	-13	6	15.80	2.70
-6	-13	6	14.20	2.50
6	-13	-6	19.60	5.50
-6	-13	6	13.50	6.20
6	13	-6	17.40	6.00
-6	13	6	14.90	4.70
-5	-13	6	428.56	21.70
5	-13	-6	456.65	25.00
-5	-13	6	430.76	26.00
-5	13	6	454.25	24.10
5	13	-6	477.35	25.50

-4	-13	6	439.56	27.00
4	-13	-6	516.75	26.60
-4	-13	6	494.15	23.40
4	-13	-6	472.65	26.00
-4	13	6	510.65	25.80
4	13	-6	493.95	26.70
-4	13	6	484.45	29.10
4	13	-6	463.05	23.30
-3	-13	6	610.44	34.10
-3	-13	6	639.24	31.20
3	-13	-6	653.13	33.30
3	-13	-6	669.43	33.90
-3	13	6	642.54	33.40
3	13	-6	621.44	31.10
3	13	-6	676.43	34.10
-3	13	6	674.33	34.20
2	-13	-6	388.76	21.50
-2	-13	6	373.16	18.90
2	-13	-6	421.26	20.70
-2	-13	6	372.46	21.50
-2	13	6	380.76	21.50
2	13	-6	363.36	18.70
-2	13	6	353.36	20.80
2	13	-6	416.16	21.60
-1	-13	6	948.11	45.90
1	-13	-6	976.60	47.00
-1	-13	6	920.71	47.60
1	13	-6	885.51	45.80
-1	13	6	993.70	48.10
-1	13	6	939.61	47.60
-1	13	6	965.50	48.60
1	13	-6	988.50	47.90
0	-13	-6	1311.57	60.79
0	-13	6	1193.28	60.49
0	13	-6	1235.58	61.49
0	13	6	1265.17	62.39
0	13	6	1236.58	61.39
0	13	-6	1214.98	59.79
0	13	6	1274.87	61.89
1	-13	6	236.78	13.50
-1	-13	-6	263.37	14.10
1	13	6	255.17	16.30
1	13	6	243.78	15.20
-1	13	-6	239.28	13.20

-1	13	-6	263.27	14.90
1	13	6	262.27	14.90
2	-13	6	414.36	21.70
-2	-13	-6	421.46	21.80
2	13	6	428.96	22.50
-2	13	-6	404.56	21.30
-2	13	-6	420.46	22.00
-2	13	-6	414.26	22.40
2	13	6	439.06	24.20
2	13	6	422.06	23.00
3	-13	6	15.50	3.80
-3	-13	-6	13.20	3.50
-3	13	-6	9.50	3.30
3	13	6	12.10	4.30
-3	13	-6	15.90	3.50
-3	13	-6	15.00	3.20
3	13	6	11.40	4.00
3	13	6	5.70	5.40
4	-13	6	380.56	21.00
-4	-13	-6	394.46	20.70
-4	-13	-6	391.06	22.90
-4	13	-6	386.86	20.90
4	13	6	434.56	23.40
4	13	6	372.96	21.00
-4	13	-6	383.96	20.90
-4	13	-6	379.16	20.50
-5	-13	-6	11.20	5.30
5	-13	6	4.20	4.40
-5	-13	-6	10.80	3.70
-5	13	-6	12.10	3.90
5	13	6	7.10	6.50
5	13	6	11.70	4.20
-5	13	-6	5.90	3.90
6	-13	6	907.51	48.70
-6	-13	-6	992.00	48.30
-6	-13	-6	966.10	50.59
-6	13	-6	1003.20	48.30
-6	13	-6	997.80	48.50
6	13	6	949.51	48.40
-7	-13	-6	12.00	6.30
-7	13	-6	4.20	6.30
7	13	6	5.90	4.70
7	13	6	7.30	6.40
-7	13	-6	6.60	4.60

-8	-13	-6	284.47	21.80
8	13	6	324.47	21.30
8	13	6	314.17	18.80
-8	13	-6	323.17	18.80
-8	13	-6	334.07	19.40
9	-13	6	-0.60	3.80
-9	-13	-6	12.40	7.90
-9	13	-6	1.70	5.40
9	13	6	-3.30	5.10
-9	13	-6	-8.90	4.60
9	13	6	-7.70	6.70
10	-13	6	387.96	21.90
-10	13	-6	406.26	23.40
10	13	6	431.06	26.80
-10	13	-6	388.36	24.50
-11	13	-6	20.60	7.20
11	13	6	13.00	6.40
-12	13	-6	337.07	20.60
12	13	6	284.67	20.20
-13	-13	-6	31.10	10.20
-13	13	-6	25.00	7.70
-13	14	6	19.00	7.60
-12	14	6	14.90	7.40
12	14	-6	30.00	10.90
-11	14	6	28.70	7.40
11	14	-6	30.80	10.60
10	-14	-6	8.20	6.80
-10	-14	6	13.70	3.70
-10	14	6	7.80	6.10
10	14	-6	2.20	8.60
-9	-14	6	9.60	3.10
-9	-14	6	5.30	4.40
9	-14	-6	9.30	7.20
-9	14	6	2.00	5.20
9	14	-6	4.80	7.50
-9	14	6	4.10	8.10
-8	-14	6	40.60	4.10
8	-14	-6	60.39	11.00
-8	14	6	37.30	6.50
-8	14	6	34.50	8.40
8	14	-6	22.00	7.60
-7	-14	6	28.20	3.90
-7	-14	6	35.90	3.50
7	-14	-6	33.30	7.10

-7	-14	6	22.20	8.20
-7	14	6	21.80	5.70
-7	14	6	23.50	6.90
7	14	-6	30.00	7.60
-6	-14	6	52.69	4.00
-6	-14	6	48.40	3.80
6	-14	-6	49.50	9.30
-6	-14	6	43.40	8.40
6	14	-6	51.89	8.50
-6	14	6	49.10	7.10
-6	14	6	47.90	8.40
-5	-14	6	5.20	1.60
5	-14	-6	0.80	4.70
-5	-14	6	5.30	6.00
-5	14	6	12.70	5.30
-5	14	6	-0.80	4.20
5	14	-6	1.10	1.50
5	14	-6	2.10	5.20
-4	-14	6	388.36	18.30
4	-14	-6	401.16	21.60
-4	-14	6	304.67	21.90
-4	14	6	387.36	22.00
4	14	-6	356.96	18.20
-4	14	6	402.46	21.10
4	14	-6	366.56	22.00
3	-14	-6	99.69	9.60
-3	-14	6	97.79	5.80
3	-14	-6	93.09	8.90
-3	-14	6	90.19	10.00
-3	14	6	115.29	10.10
-3	14	6	84.29	8.50
3	14	-6	112.39	10.20
3	14	-6	96.49	5.60
2	-14	-6	2.00	4.20
-2	-14	6	2.40	3.90
2	-14	-6	6.90	4.10
-2	-14	6	0.50	1.90
2	14	-6	3.90	1.80
2	14	-6	5.20	4.00
-2	14	6	-6.40	3.60
-2	14	6	-2.40	3.90
-1	-14	6	168.08	12.00
-1	-14	6	176.48	9.80
1	-14	-6	195.28	11.50

-1	14	6	172.08	13.40
1	14	-6	165.98	12.20
1	14	-6	179.48	9.60
-1	14	6	164.78	11.70
-1	14	6	185.58	12.60
0	-14	-6	36.90	5.30
0	-14	6	21.60	3.30
0	14	-6	17.10	4.50
0	14	6	25.40	5.00
0	14	6	28.30	5.30
0	14	-6	18.90	3.60
0	14	6	18.60	5.40
1	-14	6	150.68	9.10
-1	-14	-6	148.79	9.40
-1	14	-6	144.49	9.70
1	14	6	132.99	12.20
-1	14	-6	129.99	10.40
-1	14	-6	131.59	8.40
1	14	6	145.29	11.00
1	14	6	150.88	10.40
-2	-14	-6	7.00	3.20
2	-14	6	6.80	3.30
-2	14	-6	11.40	3.40
2	14	6	5.10	4.50
2	14	6	-0.10	5.10
-2	14	-6	1.10	3.60
-2	14	-6	5.50	2.70
2	14	6	5.80	3.40
-3	-14	-6	41.00	5.70
3	-14	6	35.50	6.10
-3	14	-6	32.90	5.60
-3	14	-6	30.70	6.00
3	14	6	38.30	5.50
3	14	6	24.70	6.10
3	14	6	32.80	5.50
-3	14	-6	31.70	5.10
4	-14	6	25.20	5.00
-4	-14	-6	29.50	7.00
-4	-14	-6	50.19	6.20
-4	14	-6	30.50	5.50
4	14	6	31.40	6.80
4	14	6	28.70	5.50
-4	14	-6	27.90	4.80
4	14	6	27.40	4.80

-4	14	-6	30.80	5.90
-5	-14	-6	141.09	9.70
5	-14	6	127.49	10.80
-5	-14	-6	117.69	14.40
5	14	6	113.49	12.50
-5	14	-6	114.59	11.20
5	14	6	134.29	10.00
-5	14	-6	132.39	9.60
-5	14	-6	117.29	9.60
-6	-14	-6	70.09	11.50
-6	-14	-6	26.50	8.60
6	14	6	32.10	7.80
-6	14	-6	33.90	5.80
-6	14	-6	41.10	7.20
6	14	6	40.30	7.20
-7	-14	-6	2.30	7.30
-7	14	-6	5.70	4.60
7	14	6	6.60	4.60
-7	14	-6	1.60	4.50
7	14	6	11.90	7.10
-8	-14	-6	42.30	8.90
-8	14	-6	53.29	9.20
8	14	6	50.29	8.60
8	14	6	56.79	10.40
-8	14	-6	50.19	8.50
-9	-14	-6	38.90	10.30
-9	14	-6	48.40	9.90
9	14	6	29.00	6.50
9	14	6	38.50	9.70
-9	14	-6	35.90	6.70
-10	14	-6	39.70	8.20
10	14	6	44.30	7.80
10	14	6	35.40	11.30
-11	14	-6	106.89	12.30
11	14	6	83.69	11.60
12	14	6	6.10	7.00
-12	14	-6	24.20	7.60
-13	-14	-6	26.60	10.40
-13	14	-6	8.10	7.70
-13	15	6	31.50	8.10
-12	15	6	42.60	8.60
12	15	-6	27.10	11.70
11	15	-6	630.14	39.20
-11	15	6	675.93	35.40

-10	-15	6	20.70	4.10
-10	15	6	12.40	6.80
10	15	-6	14.40	9.10
-9	-15	6	195.98	11.20
9	-15	-6	192.08	16.40
9	15	-6	193.08	18.50
-9	15	6	194.78	13.80
-8	-15	6	7.20	2.30
8	-15	-6	2.70	6.30
-8	15	6	-2.10	6.90
8	15	-6	2.70	7.40
-8	15	6	-0.50	4.90
-7	-15	6	878.01	42.10
7	-15	-6	860.01	45.70
-7	-15	6	856.01	41.90
-7	15	6	878.11	44.50
-7	15	6	843.72	46.50
7	15	-6	925.21	46.70
6	-15	-6	3.00	5.20
-6	-15	6	9.10	2.00
-6	15	6	12.00	5.70
6	15	-6	3.90	5.80
-6	15	6	4.10	4.70
-5	-15	6	1147.99	64.99
5	-15	-6	1289.17	64.19
-5	-15	6	1275.67	61.09
5	15	-6	1385.26	64.89
-5	15	6	1362.36	63.59
-5	15	6	1321.37	64.89
5	15	-6	1188.38	61.09
-4	-15	6	69.99	4.70
-4	-15	6	63.69	10.40
4	-15	-6	77.09	9.30
4	15	-6	67.59	4.60
4	15	-6	75.29	10.20
-4	15	6	74.89	9.40
-4	15	6	58.39	8.20
-3	-15	6	297.17	20.00
-3	-15	6	322.37	16.40
3	-15	-6	335.67	19.70
3	15	-6	312.77	16.20
-3	15	6	322.57	19.00
3	15	-6	345.97	20.00
-3	15	6	351.46	20.00

2	-15	-6	129.99	9.40
-2	-15	6	117.19	7.30
-2	-15	6	118.09	10.80
-2	15	6	118.99	10.90
2	15	-6	130.19	11.00
-2	15	6	104.49	11.40
-2	15	6	128.09	10.30
-1	-15	6	239.88	15.50
-1	-15	6	233.08	13.10
1	-15	-6	261.87	14.70
-1	15	6	257.27	15.80
1	15	-6	241.98	12.80
1	15	-6	244.38	15.70
-1	15	6	250.47	16.90
-1	15	6	231.78	15.10
0	-15	-6	912.91	44.50
0	-15	6	896.31	43.70
0	15	6	870.51	44.90
0	15	6	904.51	45.70
0	15	-6	870.01	43.30
0	15	6	916.41	46.60
0	15	-6	884.71	45.40
1	-15	6	701.93	35.50
-1	-15	-6	737.63	36.00
1	15	6	723.43	38.30
-1	15	-6	688.03	36.20
-1	15	-6	694.33	35.00
1	15	6	722.03	37.30
1	15	6	725.63	36.60
-1	15	-6	724.53	37.00
2	-15	6	1449.65	69.79
-2	-15	-6	1498.55	69.99
-2	15	-6	1401.86	69.29
2	15	6	1419.56	70.99
2	15	6	1443.66	72.09
2	15	6	1440.36	70.39
-2	15	-6	1405.06	70.69
-2	15	-6	1429.26	70.19
-3	-15	-6	28.20	4.50
3	-15	6	27.40	4.90
-3	15	-6	32.40	5.40
-3	15	-6	26.80	4.10
3	15	6	21.70	4.60
3	15	6	20.40	5.10

-3	15	-6	28.60	4.60
3	15	6	27.30	7.30
-4	-15	-6	642.54	35.30
-4	-15	-6	656.63	32.90
4	-15	6	629.84	33.40
4	15	6	621.74	33.90
-4	15	-6	636.14	32.60
-4	15	-6	639.14	33.50
4	15	6	673.23	33.40
-4	15	-6	639.04	33.20
4	15	6	685.93	35.80
-5	-15	-6	178.78	13.00
-5	-15	-6	190.88	15.10
5	-15	6	161.58	12.90
-5	15	-6	171.28	11.80
5	15	6	191.38	15.10
5	15	6	187.38	12.40
-5	15	-6	170.68	12.20
-6	-15	-6	1045.60	56.29
6	15	6	1153.68	56.49
6	15	6	1064.49	53.99
-6	15	-6	1054.79	54.19
-6	15	-6	1104.59	53.79
-7	-15	-6	14.00	8.00
-7	15	-6	14.70	5.30
-7	15	-6	14.90	5.50
7	15	6	19.80	7.30
7	15	6	16.40	5.20
-8	-15	-6	516.25	32.30
8	15	6	621.14	32.20
-8	15	-6	505.15	28.70
-8	15	-6	504.65	29.10
8	15	6	515.85	28.70
-9	15	-6	99.89	12.10
9	15	6	108.79	11.40
9	15	6	103.29	15.50
-9	15	-6	110.49	11.40
-10	15	-6	349.27	20.90
10	15	6	320.87	20.50
-11	15	-6	28.60	7.50
11	15	6	26.60	7.60
-12	15	-6	402.36	23.90
12	15	6	352.56	23.70
-13	-15	-6	67.99	12.10

-13	15	-6	48.80	9.30
12	16	-6	0.90	11.50
-12	16	6	7.90	7.10
-11	16	6	11.80	6.70
11	16	-6	9.90	15.10
-10	16	6	11.90	6.90
10	16	-6	16.00	11.10
-9	-16	6	0.40	3.10
9	-16	-6	2.50	7.10
-9	16	6	-5.90	5.50
9	16	-6	-8.60	8.60
-8	-16	6	68.19	5.90
8	-16	-6	93.59	12.80
-8	16	6	83.19	14.70
8	16	-6	49.00	9.80
-8	16	6	64.89	8.90
-7	-16	6	4.60	2.10
-7	-16	6	1.90	2.50
7	-16	-6	0.90	6.60
-7	16	6	-2.10	5.40
-7	16	6	6.00	6.70
7	16	-6	-7.60	7.00
6	-16	-6	92.39	11.40
-6	-16	6	85.89	5.60
6	16	-6	121.09	12.80
-6	16	6	87.39	11.90
-6	16	6	82.79	12.60
-5	-16	6	24.10	3.30
5	-16	-6	25.10	6.00
-5	16	6	33.80	7.00
5	16	-6	23.70	7.10
-5	16	6	12.70	5.70
5	16	-6	18.80	3.10
4	-16	-6	223.18	15.70
-4	-16	6	223.28	11.60
4	16	-6	205.48	11.50
-4	16	6	220.18	16.00
4	16	-6	256.27	16.50
-4	16	6	228.28	14.80
-3	-16	6	79.39	5.30
3	16	-6	54.89	6.50
-3	16	6	73.79	9.50
3	16	-6	74.29	10.10
-3	16	6	56.89	8.80

-2	-16	6	211.18	17.30
-2	16	6	179.38	13.90
2	16	-6	199.68	14.10
-2	16	6	211.58	15.40
2	16	-6	173.58	10.10
-2	16	6	169.38	13.00
-1	-16	6	117.29	8.30
1	16	-6	142.89	11.50
-1	16	6	123.19	10.80
-1	16	6	144.69	11.30
1	16	-6	132.39	7.90
-1	16	6	141.99	12.80
0	-16	-6	61.19	7.80
0	-16	6	52.29	5.90
0	16	-6	46.90	5.10
0	16	-6	56.99	8.40
0	16	6	50.69	8.40
0	16	6	51.39	7.90
0	16	6	55.29	9.90
1	-16	6	240.38	14.30
-1	-16	-6	260.67	14.70
1	16	6	244.78	16.20
-1	16	-6	239.68	15.00
1	16	6	263.87	17.60
-1	16	-6	239.98	13.60
-1	16	-6	280.87	16.30
1	16	6	251.27	15.40
2	-16	6	389.56	20.60
-2	-16	-6	384.96	20.40
2	16	6	401.36	21.40
-2	16	-6	381.26	21.80
-2	16	-6	369.76	19.80
-2	16	-6	370.86	21.00
2	16	6	395.06	23.40
2	16	6	360.96	21.90
-3	-16	-6	147.29	10.10
3	-16	6	144.99	10.80
3	16	6	146.99	10.90
3	16	6	146.89	13.20
-3	16	-6	136.89	10.50
3	16	6	148.49	11.70
-3	16	-6	143.59	9.50
-3	16	-6	148.69	11.30
4	-16	6	75.59	9.00

4	16	6	69.69	11.30
-4	16	-6	79.79	8.40
-4	16	-6	79.29	8.90
4	16	6	79.39	8.40
4	16	6	78.39	9.70
-4	16	-6	77.79	7.40
-5	-16	-6	570.14	32.60
5	16	6	620.04	33.00
-5	16	-6	530.55	29.40
-5	16	-6	579.94	34.40
5	16	6	560.74	30.00
-6	-16	-6	29.90	8.80
6	16	6	13.20	7.50
-6	16	-6	24.10	5.60
-6	16	-6	20.00	5.70
6	16	6	32.90	6.00
-6	16	-6	22.60	5.20
-7	-16	-6	22.50	8.20
-7	16	-6	30.60	5.80
7	16	6	18.70	5.70
7	16	6	34.90	9.10
-7	16	-6	24.70	6.00
-8	-16	-6	5.00	8.30
8	16	6	1.10	7.90
-8	16	-6	11.50	5.70
-8	16	-6	1.30	5.30
8	16	6	7.20	5.20
9	16	6	68.69	11.90
9	16	6	77.19	15.60
-9	16	-6	91.59	12.40
-9	16	-6	109.59	11.20
-10	16	-6	9.10	6.30
10	16	6	-1.10	6.10
-11	16	-6	29.60	7.80
11	16	6	28.50	7.70
12	16	6	45.00	9.30
-12	16	-6	69.29	11.60
-12	17	6	8.30	7.30
12	17	-6	4.40	10.90
-11	17	6	373.26	22.70
11	17	-6	341.17	28.00
-10	17	6	5.70	6.50
10	17	-6	-8.30	12.90
9	17	-6	63.99	11.30

-9	17	6	53.09	8.40
8	-17	-6	9.60	7.10
8	17	-6	0.50	7.70
-8	17	6	-1.10	5.80
-7	-17	6	170.38	9.50
-7	-17	6	189.58	10.20
7	-17	-6	178.68	15.40
7	17	-6	186.28	16.90
-7	17	6	179.68	13.80
-7	17	6	157.98	16.30
6	-17	-6	100.99	11.60
-6	-17	6	95.79	5.90
-6	17	6	85.59	12.50
-6	17	6	81.29	10.50
6	17	-6	94.49	13.10
-5	-17	6	116.69	6.90
5	-17	-6	115.69	12.10
5	17	-6	99.29	12.80
5	17	-6	114.59	6.80
-5	17	6	122.49	12.80
4	-17	-6	62.09	9.90
-4	-17	6	65.59	5.10
4	17	-6	55.99	5.20
-4	17	6	64.49	9.20
-4	17	6	57.69	10.50
4	17	-6	60.09	10.90
-3	-17	6	367.26	20.60
3	17	-6	396.36	23.80
3	17	-6	392.46	19.90
-3	17	6	395.16	22.60
-3	17	6	415.56	24.70
-3	17	6	423.86	23.90
-2	-17	6	293.87	15.60
-2	17	6	299.07	20.30
2	17	-6	283.17	19.00
2	17	-6	294.27	15.30
-2	17	6	316.97	19.10
-2	17	6	291.27	18.20
-1	-17	6	89.09	6.90
1	17	-6	69.49	9.90
-1	17	6	75.09	11.40
-1	17	6	77.49	9.30
-1	17	6	77.79	9.80
1	17	-6	80.99	6.10

0	-17	6	200.18	12.10
0	17	6	202.78	16.00
0	17	6	205.18	13.80
0	17	6	207.48	14.60
0	17	-6	202.88	14.50
0	17	-6	201.28	11.40
1	-17	6	360.26	19.20
-1	17	-6	345.47	19.90
1	17	6	354.86	21.20
-1	17	-6	349.87	18.40
1	17	6	365.86	22.70
-1	17	-6	337.77	20.70
1	17	6	352.46	20.30
2	-17	6	37.50	5.40
2	17	6	36.10	7.60
2	17	6	37.20	5.90
-2	17	-6	44.10	5.70
-2	17	-6	47.30	7.00
2	17	6	32.30	6.50
-2	17	-6	44.10	6.80
3	-17	6	-7.10	4.10
-3	17	-6	-0.10	4.80
-3	17	-6	-0.50	3.40
-3	17	-6	0.30	3.80
3	17	6	-10.50	6.10
3	17	6	1.00	4.50
3	17	6	3.30	5.20
4	-17	6	181.88	12.70
4	17	6	160.38	13.10
-4	17	-6	171.18	12.40
-4	17	-6	180.08	11.40
4	17	6	185.18	12.50
4	17	6	165.78	15.30
-5	-17	-6	-7.00	6.40
5	17	6	-0.10	4.40
5	17	6	1.70	6.90
-5	17	-6	-4.00	4.30
-5	17	-6	4.30	4.70
5	17	6	1.80	4.90
-6	-17	-6	300.37	23.60
6	17	6	352.06	23.40
6	17	6	364.16	20.50
-6	17	-6	360.56	20.00
-6	17	-6	343.47	21.00

-6	17	-6	373.16	21.10
-7	-17	-6	0.80	8.10
7	17	6	2.60	5.00
7	17	6	0.10	7.30
-7	17	-6	-4.40	5.30
-8	17	-6	83.29	11.40
-8	17	-6	114.99	10.70
8	17	6	84.99	14.40
8	17	6	75.89	12.40
-9	17	-6	15.30	6.50
9	17	6	4.80	9.20
9	17	6	0.10	5.80
-10	17	-6	330.37	20.10
10	17	6	292.37	20.10
11	17	6	19.70	7.90
-11	17	-6	35.30	8.40
-12	17	-6	111.89	13.70
12	17	6	74.09	10.80
11	18	-6	-11.80	10.40
-11	18	6	-4.50	7.20
-10	18	6	322.77	21.20
10	18	-6	373.06	27.20
9	18	-6	1.20	9.00
-9	18	6	2.60	6.80
-8	18	6	221.38	16.20
-7	-18	6	76.79	8.60
7	-18	-6	83.39	12.60
7	18	-6	101.09	14.60
-7	18	6	82.59	10.90
-6	-18	6	186.08	10.40
6	-18	-6	214.08	16.00
-6	18	6	202.98	16.90
6	18	-6	180.28	10.40
6	18	-6	216.58	17.40
-6	18	6	202.08	14.70
5	-18	-6	505.95	28.30
-5	-18	6	479.75	24.00
5	18	-6	534.75	29.00
-5	18	6	504.05	28.90
5	18	-6	428.26	24.30
-5	18	6	485.85	27.20
-4	-18	6	149.59	9.30
-4	18	6	158.68	14.40
4	18	-6	160.58	9.90

4	18	-6	157.38	14.50
-3	-18	6	150.58	9.30
3	18	-6	156.78	9.00
-3	18	6	154.18	12.70
-3	18	6	141.19	13.50
3	18	-6	168.58	14.30
-3	18	6	169.48	15.40
-2	-18	6	30.60	4.00
-2	18	6	34.20	7.00
2	18	-6	26.20	6.80
2	18	-6	30.60	4.50
-2	18	6	30.00	7.70
-2	18	6	23.40	6.10
-1	-18	6	265.17	14.80
1	18	-6	259.97	14.20
-1	18	6	276.87	18.10
-1	18	6	254.57	17.00
-1	18	6	275.37	19.40
1	18	-6	268.27	17.80
0	-18	6	264.47	15.20
0	18	6	287.27	19.60
0	18	-6	250.87	16.20
0	18	6	255.77	16.80
0	18	-6	261.47	17.60
0	18	6	283.87	18.00
0	18	-6	266.27	14.50
1	-18	6	110.49	9.50
1	18	6	122.39	11.00
-1	18	-6	118.89	11.60
-1	18	-6	126.69	10.10
1	18	6	121.69	13.80
-1	18	-6	121.89	8.40
1	18	6	120.19	11.90
2	-18	6	-2.70	4.30
2	18	6	2.90	4.70
-2	18	-6	10.50	3.70
2	18	6	14.20	6.60
-2	18	-6	5.90	4.40
-2	18	-6	5.20	5.70
2	18	6	10.00	5.40
3	-18	6	70.89	9.10
3	18	6	73.69	8.90
-3	18	-6	71.09	9.80
-3	18	-6	68.09	7.20

-3	18	-6	51.19	8.20
3	18	6	79.29	9.90
3	18	6	62.49	11.80
4	18	6	8.90	5.80
4	18	6	17.00	8.10
4	18	6	3.40	4.80
-4	18	-6	-1.50	4.30
-4	18	-6	4.10	4.10
-4	18	-6	0.00	4.70
-5	-18	-6	334.87	22.50
-5	18	-6	322.37	19.50
-5	18	-6	277.37	21.20
5	18	6	309.67	18.50
5	18	6	280.17	19.80
-5	18	-6	299.47	17.80
5	18	6	333.17	22.20
-6	-18	-6	88.49	14.70
-6	18	-6	84.49	10.80
6	18	6	69.89	9.70
6	18	6	78.49	14.00
-6	18	-6	64.69	10.70
-6	18	-6	72.89	9.20
-7	18	-6	161.98	14.00
-7	18	-6	148.59	13.50
7	18	6	148.69	12.60
-7	18	-6	170.58	12.60
7	18	6	155.38	17.50
-8	18	-6	3.30	6.00
8	18	6	-2.00	8.40
8	18	6	5.00	5.70
-9	18	-6	320.17	19.10
9	18	6	256.87	18.90
-10	18	-6	10.90	7.10
10	18	6	8.40	7.10
11	18	6	213.98	17.40
-11	18	-6	217.18	17.20
-11	19	6	4.80	8.00
11	19	-6	18.10	12.90
-10	19	6	18.20	7.60
10	19	-6	15.00	12.20
9	19	-6	16.20	10.20
-9	19	6	26.90	8.00
8	19	-6	0.50	8.30
-8	19	6	2.50	6.80

7	-19	-6	22.50	8.20
-7	19	6	22.10	6.80
7	19	-6	13.30	9.20
6	-19	-6	30.00	7.40
6	19	-6	20.60	8.30
6	19	-6	27.70	4.20
-6	19	6	23.00	6.80
-5	-19	6	6.10	3.00
-5	19	6	10.00	6.50
-5	19	6	9.50	7.80
-4	-19	6	65.69	7.30
-4	19	6	82.99	12.40
4	19	-6	69.69	5.90
-4	19	6	79.69	13.50
-4	19	6	65.29	10.40
-3	-19	6	-0.60	3.10
-3	19	6	-0.40	6.00
-3	19	6	0.00	6.20
3	19	-6	4.00	2.70
3	19	-6	-2.80	6.60
-3	19	6	6.60	7.20
-2	-19	6	3.40	3.50
-2	19	6	-2.80	5.80
2	19	-6	-0.80	6.80
-2	19	6	-8.10	6.20
2	19	-6	0.50	2.80
-2	19	6	-4.30	5.30
-1	-19	6	-5.70	3.60
1	19	-6	0.70	8.60
1	19	-6	2.00	3.00
-1	19	6	-0.90	7.10
-1	19	6	6.40	5.50
-1	19	6	4.70	5.70
0	-19	6	42.30	5.60
0	19	6	50.49	10.40
0	19	-6	49.90	8.00
0	19	-6	51.79	6.00
0	19	6	49.00	8.80
0	19	6	31.50	8.20
1	-19	6	21.60	5.00
1	19	6	9.90	7.10
-1	19	-6	15.60	6.20
1	19	6	20.60	6.10
-1	19	-6	13.90	3.80

-1	19	-6	8.10	4.60
1	19	6	3.00	5.90
2	-19	6	7.80	5.00
2	19	6	10.70	7.20
-2	19	-6	7.30	4.60
2	19	6	14.30	6.10
-2	19	-6	2.30	5.20
-2	19	-6	11.50	3.90
2	19	6	2.90	5.40
3	-19	6	195.58	13.30
3	19	6	171.48	14.30
3	19	6	187.18	13.40
3	19	6	189.48	17.00
-3	19	-6	179.98	14.70
-3	19	-6	175.48	11.70
-3	19	-6	170.08	13.10
-4	19	-6	27.10	5.60
4	19	6	53.59	9.60
-4	19	-6	25.20	5.30
-4	19	-6	19.70	6.10
4	19	6	21.30	5.70
4	19	6	20.50	8.00
-5	19	-6	2.00	5.50
-5	19	-6	11.10	6.10
-5	19	-6	14.60	5.10
5	19	6	-4.30	6.00
5	19	6	10.70	7.70
5	19	6	4.00	5.40
-6	-19	-6	177.28	18.30
-6	19	-6	159.68	14.20
-6	19	-6	146.29	14.20
6	19	6	149.99	17.40
6	19	6	172.48	12.90
-7	19	-6	24.20	6.30
-7	19	-6	18.10	6.90
7	19	6	18.70	6.60
7	19	6	18.50	9.90
8	19	6	-7.30	6.00
8	19	6	-3.00	8.70
-8	19	-6	7.10	6.80
-8	19	-6	6.90	6.00
9	19	6	23.40	7.50
-9	19	-6	2.90	6.60
-10	19	-6	68.19	12.00

10	19	6	50.59	8.80
-11	19	-6	37.70	8.60
11	19	6	31.40	8.70
10	20	-6	412.26	31.50
-10	20	6	392.66	25.10
9	20	-6	1.90	11.50
-9	20	6	-6.00	6.90
8	20	-6	226.58	21.40
-8	20	6	202.68	16.70
-7	20	6	6.30	6.70
7	20	-6	7.80	8.80
6	20	-6	916.71	49.90
-6	20	6	920.91	47.70
5	20	-6	17.20	3.40
-5	20	6	12.00	6.80
5	20	-6	5.20	7.50
-4	-20	6	699.93	35.60
4	20	-6	641.84	34.00
-4	20	6	720.83	39.90
-4	20	6	710.63	40.20
-4	20	6	694.23	37.20
4	20	-6	726.33	39.00
-3	-20	6	122.29	8.50
-3	20	6	109.89	12.10
-3	20	6	113.49	15.50
3	20	-6	114.89	13.80
3	20	-6	106.79	7.70
-3	20	6	114.09	13.80
-2	-20	6	219.28	13.00
2	20	-6	204.58	17.20
-2	20	6	223.58	17.50
-2	20	6	226.48	19.00
-2	20	6	223.28	16.10
2	20	-6	212.88	12.30
-1	-20	6	385.46	21.60
1	20	-6	386.46	21.00
1	20	-6	440.36	25.20
-1	20	6	406.56	25.00
-1	20	6	421.96	26.80
0	-20	6	651.93	33.00
0	20	6	686.83	37.60
0	20	6	644.94	35.60
0	20	-6	646.24	35.70
0	20	-6	589.14	34.20

0	20	6	639.64	34.80
0	20	-6	664.63	32.40
1	-20	6	825.32	41.70
1	20	6	871.41	43.00
-1	20	-6	827.62	41.00
1	20	6	793.02	43.70
-1	20	-6	837.22	43.90
1	20	6	856.41	45.60
-1	20	-6	774.02	42.60
2	-20	6	43.40	6.60
2	20	6	26.30	6.50
2	20	6	18.70	8.30
-2	20	-6	36.80	6.50
-2	20	-6	37.80	7.80
2	20	6	41.80	8.40
-2	20	-6	23.80	5.50
-3	20	-6	408.66	23.90
3	20	6	403.66	25.00
-3	20	-6	399.46	25.10
3	20	6	446.96	27.80
-3	20	-6	420.26	22.50
3	20	6	434.86	24.10
4	20	6	0.20	6.30
-4	20	-6	16.50	5.90
4	20	6	15.50	5.90
-4	20	-6	15.00	6.70
4	20	6	19.00	9.60
-4	20	-6	20.50	5.10
5	20	6	478.85	30.10
-5	20	-6	497.45	27.80
-5	20	-6	426.16	28.10
-5	20	-6	469.75	25.40
6	20	6	42.10	10.40
6	20	6	40.30	7.30
-6	20	-6	23.40	7.30
-6	20	-6	34.60	6.70
-7	20	-6	561.54	31.20
7	20	6	629.34	36.20
-7	20	-6	585.54	33.20
7	20	6	558.24	31.50
8	20	6	5.10	6.60
-8	20	-6	-6.60	7.00
-8	20	-6	4.40	6.20
-9	20	-6	207.48	17.90

-9	20	-6	211.88	16.10
9	20	6	192.88	16.50
10	20	6	97.99	13.20
-10	20	-6	86.39	13.10
10	21	-6	-0.40	12.50
-10	21	6	15.40	8.60
9	21	-6	117.19	20.60
-9	21	6	120.19	14.30
8	21	-6	-9.30	9.70
-8	21	6	1.40	7.60
-7	21	6	181.28	16.00
7	21	-6	180.58	20.00
6	21	-6	7.90	8.70
-6	21	6	11.10	7.50
-5	21	6	48.20	11.40
-5	21	6	71.99	11.30
5	21	-6	59.79	11.20
4	21	-6	1.90	3.10
-4	21	6	1.50	6.70
-4	21	6	9.20	8.80
4	21	-6	5.90	7.80
3	21	-6	23.10	3.80
-3	21	6	46.70	10.70
-3	21	6	13.20	6.80
3	21	-6	27.70	8.10
-2	-21	6	31.50	5.10
-2	21	6	36.60	8.10
-2	21	6	27.70	7.30
-2	21	6	31.60	9.80
2	21	-6	36.60	8.50
2	21	-6	40.90	5.60
-1	-21	6	28.90	5.80
1	21	-6	32.80	7.80
1	21	-6	38.20	6.10
-1	21	6	37.10	9.50
-1	21	6	38.70	8.50
-1	21	6	39.20	7.90
0	-21	6	35.70	6.00
0	21	6	47.50	10.30
0	21	-6	48.10	9.80
0	21	-6	50.69	6.90
0	21	6	40.70	8.40
0	21	6	57.39	10.50
0	21	-6	63.99	11.80

1	-21	6	54.69	8.60
-1	21	-6	43.40	7.40
-1	21	-6	51.49	9.10
1	21	6	40.10	8.50
1	21	6	51.19	11.10
1	21	6	47.00	7.90
-1	21	-6	60.19	7.30
2	-21	6	49.30	9.40
2	21	6	50.99	10.40
-2	21	-6	57.69	7.80
-2	21	-6	72.29	12.00
2	21	6	58.59	10.00
2	21	6	67.69	12.30
-2	21	-6	62.19	10.90
3	21	6	71.39	10.70
-3	21	-6	72.89	8.30
-3	21	-6	66.79	12.00
3	21	6	64.69	11.20
3	21	6	83.99	12.80
-4	21	-6	265.47	18.60
-4	21	-6	240.28	15.30
4	21	6	210.28	19.00
4	21	6	268.67	21.30
4	21	6	242.48	16.40
-5	21	-6	-1.50	6.80
5	21	6	3.00	5.90
5	21	6	11.60	10.50
-5	21	-6	0.50	6.10
-6	21	-6	161.58	15.80
6	21	6	151.38	13.70
6	21	6	209.68	19.70
-6	21	-6	147.59	12.70
7	21	6	6.20	11.10
-7	21	-6	7.60	5.90
7	21	6	-1.80	6.10
-7	21	-6	-0.90	7.00
8	21	6	35.20	7.80
-8	21	-6	40.50	8.90
-8	21	-6	35.90	7.60
9	21	6	10.10	7.40
-9	21	-6	10.90	7.30
-9	21	-6	-8.50	8.00
-10	21	-6	88.69	13.10
10	21	6	72.69	13.10

-9	22	6	29.30	8.90
9	22	-6	53.49	13.90
-8	22	6	82.49	12.70
8	22	-6	71.09	13.60
-7	22	6	24.10	8.60
7	22	-6	29.70	10.80
-6	22	6	134.19	14.50
6	22	-6	128.89	18.10
-6	22	6	152.28	20.10
-5	22	6	53.19	12.60
-5	22	6	54.89	9.70
5	22	-6	86.09	15.70
-4	22	6	242.18	22.50
4	22	-6	256.97	20.40
-4	22	6	230.08	18.10
-3	22	6	-11.40	8.40
-3	22	6	-3.50	6.90
3	22	-6	3.20	7.70
3	22	-6	6.30	3.40
-2	22	6	119.29	13.30
2	22	-6	107.39	15.10
-2	22	6	128.09	17.40
2	22	-6	136.59	9.00
1	22	-6	244.98	14.00
-1	22	6	232.78	21.00
1	22	-6	233.98	19.20
-1	22	6	245.68	17.60
0	-22	6	153.18	14.20
0	22	6	127.19	17.80
0	22	6	142.59	13.50
0	22	-6	149.99	10.50
0	22	-6	153.48	15.50
1	-22	6	138.39	11.50
-1	22	-6	113.39	14.50
-1	22	-6	129.19	10.00
1	22	6	135.49	13.00
1	22	6	108.49	16.50
-2	22	-6	55.99	7.90
-2	22	-6	42.40	9.30
2	22	6	55.79	8.30
2	22	6	48.90	10.70
3	22	6	137.49	13.20
3	22	6	139.89	18.70
-3	22	-6	152.78	11.40

-3	22	-6	143.59	15.00
-4	22	-6	10.80	8.00
4	22	6	4.70	6.20
4	22	6	-5.80	9.40
-4	22	-6	5.20	5.30
5	22	6	538.95	32.90
5	22	6	515.95	28.20
-5	22	-6	476.05	30.10
-5	22	-6	482.65	27.20
-6	22	-6	6.10	7.50
-6	22	-6	2.10	5.90
6	22	6	5.40	10.00
6	22	6	-1.30	6.40
-7	22	-6	193.58	18.00
-7	22	-6	199.08	15.10
7	22	6	177.18	15.80
-8	22	-6	-2.90	8.20
8	22	6	7.80	7.20
-8	22	-6	-5.20	6.40
9	22	6	114.49	14.30
-9	22	-6	149.49	14.10
-9	22	-6	110.59	19.30
-8	23	6	5.60	8.00
8	23	-6	5.40	11.60
-7	23	6	239.08	18.90
-7	23	6	266.17	26.90
7	23	-6	253.67	23.60
-6	23	6	34.30	8.90
6	23	-6	47.70	12.30
-6	23	6	25.70	12.50
5	23	-6	314.47	23.70
-5	23	6	250.67	19.90
-5	23	6	280.37	25.00
-4	23	6	90.89	12.70
4	23	-6	81.99	16.10
-4	23	6	102.89	18.30
3	23	-6	359.56	25.20
-3	23	6	338.17	22.70
-3	23	6	362.96	27.10
-2	23	6	150.48	14.50
2	23	-6	138.59	16.80
2	23	-6	148.19	10.00
1	23	-6	241.68	19.90
1	23	-6	235.78	13.80

-1	23	6	215.08	17.30
-1	23	6	233.18	22.40
0	23	6	219.98	21.60
0	23	-6	224.48	18.40
0	23	-6	198.88	13.00
0	23	6	196.88	16.20
1	23	6	168.18	20.50
-1	23	-6	188.88	12.50
-1	23	-6	181.28	17.30
1	23	6	187.78	15.50
2	23	6	232.48	17.00
-2	23	-6	232.28	19.50
-2	23	-6	233.08	14.90
2	23	6	227.18	22.50
-3	23	-6	3.00	7.30
-3	23	-6	10.60	5.30
3	23	6	-6.70	9.90
3	23	6	19.70	7.10
-4	23	-6	208.88	14.60
4	23	6	236.58	22.90
-4	23	-6	209.58	18.30
4	23	6	202.58	16.10
5	23	6	75.99	14.20
-5	23	-6	86.19	14.60
-5	23	-6	63.19	9.90
5	23	6	68.39	11.10
-6	23	-6	270.77	21.30
6	23	6	265.57	18.70
-6	23	-6	282.87	18.30
7	23	6	6.40	7.10
-7	23	-6	7.40	8.60
-7	23	-6	0.10	6.40
-8	23	-6	163.78	18.80
8	23	6	146.79	15.20
-8	23	-6	164.78	14.90
-7	24	6	10.80	8.80
7	24	-6	-0.70	11.10
-7	24	6	-5.70	12.40
-6	24	6	11.00	12.40
-6	24	6	11.90	7.90
6	24	-6	5.40	11.70
-5	24	6	-2.40	7.60
-5	24	6	21.70	13.30
5	24	-6	-2.60	10.50

-4	24	6	13.30	11.30
4	24	-6	19.50	9.70
-4	24	6	15.20	8.40
3	24	-6	-4.50	8.40
-3	24	6	-6.30	10.10
-3	24	6	-7.40	7.00
-2	24	6	0.10	10.20
2	24	-6	-13.10	8.20
-2	24	6	-3.90	7.50
1	24	-6	88.49	15.80
-1	24	6	108.09	13.20
-1	24	6	102.69	19.60
1	24	-6	94.79	8.80
0	24	6	14.50	16.40
0	24	6	-4.50	7.30
0	24	-6	7.60	4.70
0	24	-6	-13.00	7.90
-1	24	-6	11.30	5.30
1	24	6	10.30	6.90
-1	24	-6	18.10	8.80
1	24	6	18.80	11.50
2	24	6	4.40	6.70
-2	24	-6	-13.00	7.30
2	24	6	-1.00	11.10
-2	24	-6	8.20	5.20
3	24	6	11.50	7.10
3	24	6	6.50	10.80
-3	24	-6	15.20	8.60
-4	24	-6	12.40	8.50
-4	24	-6	12.90	6.30
4	24	6	-1.20	10.60
4	24	6	12.60	7.30
5	24	6	31.70	8.20
-5	24	-6	49.50	8.00
5	24	6	41.00	14.10
-5	24	-6	39.10	9.50
-6	24	-6	3.10	6.50
-6	24	-6	4.50	8.90
6	24	6	2.50	7.20
-7	24	-6	19.40	7.30
-7	24	-6	4.30	9.50
7	24	6	8.50	7.50
6	25	-6	7.50	11.80
-6	25	6	-7.00	14.00

-6	25	6	22.30	9.30
-5	25	6	395.26	25.10
-5	25	6	399.26	32.20
5	25	-6	357.76	29.10
4	25	-6	97.79	18.10
-4	25	6	68.19	15.10
-4	25	6	79.89	11.80
-3	25	6	176.88	16.40
3	25	-6	157.48	19.00
-3	25	6	143.29	22.30
2	25	-6	111.39	17.10
-2	25	6	82.69	15.20
-2	25	6	112.09	14.20
1	25	-6	102.39	16.80
-1	25	6	81.59	13.50
-1	25	6	76.99	14.40
0	25	-6	380.06	26.70
0	25	6	398.66	24.50
0	25	6	355.66	29.80
0	25	-6	380.06	21.50
1	25	6	242.38	18.70
-1	25	-6	243.98	15.80
1	25	6	235.08	24.80
-1	25	-6	268.97	21.70
-2	25	-6	309.07	17.90
-2	25	-6	274.57	22.70
2	25	6	309.47	20.40
2	25	6	214.58	32.80
3	25	6	-6.50	21.70
-3	25	-6	-2.40	5.70
3	25	6	-4.90	7.00
-3	25	-6	-1.30	8.70
4	25	6	187.18	23.40
4	25	6	211.08	17.00
-4	25	-6	201.28	15.30
-4	25	-6	218.18	20.20
5	25	6	42.00	9.00
-5	25	-6	28.60	10.00
-5	25	-6	42.00	8.10
-6	25	-6	415.16	25.20
6	25	6	410.46	25.90
-6	25	-6	463.35	31.20
-4	26	6	33.20	9.60
4	26	-6	33.60	11.60

-4	26	6	57.49	16.30
-3	26	6	-5.10	12.50
-3	26	6	-8.50	7.80
3	26	-6	11.50	10.60
-2	26	6	56.79	10.60
-2	26	6	61.49	14.70
2	26	-6	54.29	12.60
-1	26	6	62.79	14.80
-1	26	6	46.90	9.60
1	26	-6	58.39	12.50
0	26	6	3.90	12.30
0	26	-6	9.60	9.70
0	26	6	27.60	8.50
-1	26	-6	37.50	10.30
1	26	6	34.60	9.20
1	26	6	38.40	14.40
-2	26	-6	34.10	6.80
2	26	6	17.00	12.90
2	26	6	35.00	8.80
-2	26	-6	22.20	9.90
-3	26	-6	33.80	7.00
3	26	6	29.40	8.70
3	26	6	25.30	13.10
-3	26	-6	17.90	9.90
-4	26	-6	16.40	9.90
-4	26	-6	14.10	6.90
4	26	6	8.80	7.70
1	27	-6	-8.60	11.00
-1	27	6	8.30	14.20
-1	27	6	-14.80	8.20
0	27	6	233.68	18.90
0	27	6	216.28	27.50
0	27	-6	215.08	22.40
1	27	6	63.49	10.80
1	27	6	63.99	15.60
-1	27	-6	51.49	12.40
-15	0	7	5.70	6.90
-14	0	7	9.60	5.90
13	0	-7	11.00	7.60
-13	0	7	13.70	5.30
-12	0	7	0.20	9.40
-12	0	7	-2.10	4.20
12	0	-7	-4.70	6.80
11	0	-7	8.20	6.20

-11	0	7	17.40	9.60
10	0	-7	-4.00	5.30
-10	0	7	0.10	7.00
10	0	-7	-2.30	4.40
-10	0	7	1.40	3.40
9	0	-7	-1.10	4.30
-9	0	7	3.00	5.90
-9	0	7	7.10	3.10
9	0	-7	6.50	3.70
-8	0	7	-1.00	4.80
8	0	-7	-2.10	3.80
-8	0	7	0.40	2.50
8	0	-7	1.30	3.00
7	0	-7	7.80	4.30
-7	0	7	3.30	4.80
-7	0	7	9.20	2.60
7	0	-7	12.90	3.20
6	0	-7	-1.30	3.20
6	0	-7	2.10	2.20
-6	0	7	4.30	2.10
-6	0	7	-4.80	3.60
5	0	-7	1.30	2.20
5	0	-7	-2.00	2.80
-5	0	7	-0.30	1.90
-5	0	7	-1.00	3.30
-4	0	7	0.10	2.60
4	0	-7	0.90	2.40
4	0	-7	0.80	1.60
3	0	-7	0.10	1.30
-3	0	7	-1.50	1.50
3	0	-7	-0.30	2.10
-2	0	7	-0.50	1.80
2	0	-7	1.10	1.50
2	0	-7	1.30	1.10
-2	0	7	1.80	1.40
-1	0	7	1.60	1.20
1	0	-7	-0.10	1.30
-1	0	7	0.00	1.30
1	0	-7	1.50	1.00
0	0	7	1.60	1.30
0	0	7	1.10	1.10
0	0	-7	2.30	1.20
0	0	-7	0.10	1.10
1	0	7	3.00	1.00

-1	0	-7	1.40	0.90
-1	0	-7	-0.30	1.20
1	0	7	0.10	0.90
2	0	7	1.90	1.70
2	0	7	0.10	0.90
2	0	7	2.10	0.80
-3	0	-7	3.10	1.80
3	0	7	1.20	1.10
-3	0	-7	2.10	1.30
3	0	7	0.60	1.90
3	0	7	3.30	0.90
-4	0	-7	2.20	1.70
4	0	7	3.10	1.50
-4	0	-7	0.90	2.10
4	0	7	4.80	2.30
-5	0	-7	1.90	2.10
-5	0	-7	6.00	2.70
5	0	7	0.10	2.60
5	0	7	3.90	1.80
-6	0	-7	14.60	3.40
6	0	7	8.20	3.00
6	0	7	2.30	2.30
-6	0	-7	5.60	2.90
-7	0	-7	-4.00	3.20
-7	0	-7	2.30	3.00
7	0	7	0.00	2.50
-7	0	-7	5.00	3.70
-8	0	-7	-3.40	3.90
-8	0	-7	1.80	3.30
-8	0	-7	2.10	3.60
8	0	7	1.70	2.90
-9	0	-7	5.10	4.30
-9	0	-7	3.50	4.20
9	0	7	0.20	3.30
10	0	7	3.30	4.30
-10	0	-7	10.30	5.50
-11	0	-7	3.20	7.20
-11	0	-7	1.30	5.70
11	0	7	6.40	4.70
-12	0	-7	4.10	6.60
12	0	7	-1.00	4.90
-12	0	-7	13.30	6.20
-13	0	-7	15.00	7.60
-14	0	-7	13.50	7.90

-15	0	-7	19.70	8.20
-15	-1	7	177.98	14.80
-15	1	7	194.38	14.90
-14	-1	7	87.29	10.80
-14	1	7	92.49	10.50
-13	-1	7	359.76	19.80
13	-1	-7	322.67	23.30
13	1	-7	334.47	23.20
-12	-1	7	12.50	4.90
12	-1	-7	11.70	6.60
-12	-1	7	18.20	10.00
-12	1	7	-5.20	10.00
12	1	-7	16.10	7.10
-12	1	7	10.50	4.90
11	-1	-7	1037.10	51.39
-11	-1	7	905.11	52.79
-11	-1	7	1023.90	48.80
-11	1	7	912.61	52.79
11	1	-7	954.40	49.50
11	1	-7	1028.40	51.59
-11	1	7	1024.40	48.90
10	-1	-7	90.79	10.80
-10	-1	7	79.39	13.70
10	-1	-7	89.69	9.10
-10	-1	7	90.19	7.70
10	1	-7	98.89	11.40
-10	1	7	96.59	12.90
-10	1	7	91.39	7.90
10	1	-7	93.19	9.00
-9	-1	7	245.98	14.00
-9	-1	7	241.18	18.30
9	-1	-7	272.77	15.20
9	-1	-7	260.77	16.70
-9	1	7	255.87	14.30
9	1	-7	266.17	14.90
-9	1	7	241.08	17.90
9	1	-7	253.27	16.60
8	-1	-7	20.30	3.90
8	-1	-7	14.20	5.00
-8	-1	7	9.10	5.40
-8	-1	7	11.90	3.00
-8	1	7	9.70	5.40
8	1	-7	11.60	3.50
8	1	-7	14.70	5.10

-8	1	7	10.50	3.00
7	-1	-7	1239.08	58.79
-7	-1	7	1172.08	60.69
7	-1	-7	1212.08	59.99
-7	-1	7	1216.88	58.19
7	1	-7	1214.68	60.09
-7	1	7	1209.08	58.39
7	1	-7	1196.28	58.49
-7	1	7	1187.88	60.69
-6	-1	7	677.33	33.50
6	-1	-7	709.33	34.00
6	-1	-7	708.83	35.20
-6	-1	7	661.93	35.70
-6	1	7	655.13	35.70
6	1	-7	708.63	35.40
6	1	-7	680.93	33.70
-6	1	7	686.73	33.70
-5	-1	7	940.01	47.70
5	-1	-7	965.00	47.30
-5	-1	7	947.11	45.90
5	-1	-7	967.30	46.30
5	1	-7	951.20	46.00
5	1	-7	935.61	47.30
-5	1	7	953.20	47.70
-5	1	7	954.90	46.10
-4	-1	7	275.67	15.50
-4	-1	7	258.27	13.80
4	-1	-7	256.97	14.50
4	-1	-7	292.67	15.20
4	1	-7	281.67	13.80
4	1	-7	270.77	15.20
-4	1	7	270.67	14.10
3	-1	-7	1110.89	51.99
3	-1	-7	1114.29	52.79
-3	-1	7	1020.70	51.89
-3	-1	7	1020.40	53.79
3	1	-7	1101.79	52.79
3	1	-7	1109.59	51.79
-3	1	7	1093.09	53.29
-2	-1	7	488.15	28.10
-2	-1	7	536.55	26.70
2	-1	-7	561.04	26.70
2	-1	-7	570.84	27.40
-2	1	7	546.45	27.00

2	1	-7	580.74	27.40
-2	1	7	566.24	27.60
2	1	-7	546.25	26.50
1	-1	-7	914.71	43.50
-1	-1	7	835.92	43.00
-1	-1	7	942.71	43.50
1	-1	-7	910.61	43.10
-1	1	7	866.71	43.20
-1	1	7	929.01	43.60
1	1	-7	894.91	43.40
1	1	-7	874.31	42.90
0	-1	-7	2201.78	102.29
0	-1	-7	2120.39	102.39
0	-1	7	2122.69	102.29
0	-1	7	2228.08	102.39
0	1	7	2036.80	102.39
0	1	-7	2148.29	102.29
0	1	-7	2155.88	102.19
0	1	7	2176.98	102.39
1	-1	7	565.54	26.80
1	-1	7	554.64	27.30
1	-1	7	587.94	26.80
-1	-1	-7	556.54	27.20
-1	-1	-7	566.34	27.20
1	1	7	527.75	27.30
1	1	7	563.14	27.20
-1	1	-7	560.74	27.20
1	1	7	539.35	27.00
-1	1	-7	549.05	26.80
2	-1	7	637.34	31.40
-2	-1	-7	639.04	31.40
2	1	7	656.53	31.10
-2	1	-7	646.84	31.40
2	1	7	621.94	31.40
-3	-1	-7	603.34	30.40
3	-1	7	589.24	30.70
3	-1	7	649.83	29.70
-3	-1	-7	636.54	30.70
3	1	7	601.54	30.70
3	1	7	613.54	30.30
-3	1	-7	677.33	30.80
-3	1	-7	602.84	30.10
3	1	7	626.94	30.10
-4	-1	-7	2192.08	107.99

4	-1	7	2110.19	108.19
-4	-1	-7	2485.65	108.29
4	-1	7	2292.37	107.29
-4	1	-7	2153.48	107.69
-4	1	-7	2444.86	108.29
4	1	7	2122.79	108.19
4	1	7	2272.87	107.79
-5	-1	-7	7.40	2.70
5	-1	7	4.20	1.60
-5	-1	-7	8.20	2.50
5	-1	7	8.30	2.90
5	1	7	6.00	2.80
5	1	7	6.00	2.10
-5	1	-7	0.60	2.80
-5	1	-7	5.20	2.10
-6	-1	-7	2508.75	109.79
-6	-1	-7	2205.98	109.49
6	-1	7	2299.97	108.89
6	1	7	2098.29	109.49
6	1	7	2251.57	109.19
-6	1	-7	2174.08	109.19
-6	1	-7	2504.25	109.89
-6	1	-7	2189.58	109.59
-7	-1	-7	11.90	4.00
7	-1	7	6.60	2.60
-7	-1	-7	3.90	3.20
-7	-1	-7	6.50	3.30
-7	1	-7	13.30	3.40
7	1	7	8.40	2.90
-7	1	-7	5.80	3.40
-7	1	-7	11.30	4.00
7	1	7	8.70	3.50
-8	-1	-7	406.76	23.90
-8	-1	-7	490.65	24.40
-8	-1	-7	428.06	23.90
8	-1	7	450.25	23.10
-8	1	-7	474.35	24.20
8	1	7	455.65	23.50
-8	1	-7	434.06	23.90
-8	1	-7	428.46	23.80
-9	-1	-7	89.29	8.30
-9	-1	-7	70.59	9.00
9	-1	7	82.29	7.60
-9	1	-7	96.59	8.90

9	1	7	87.59	8.10
-9	1	-7	72.39	9.00
-10	-1	-7	1306.97	62.89
-10	-1	-7	1218.18	63.29
10	-1	7	1316.17	62.19
-10	1	-7	1255.57	62.69
10	1	7	1303.47	62.59
-10	1	-7	1163.68	62.99
11	-1	7	127.09	10.70
-11	-1	-7	125.79	12.70
-11	1	-7	127.99	13.00
-11	1	-7	105.49	12.60
11	1	7	130.89	11.30
-12	-1	-7	497.45	30.50
-12	-1	-7	532.15	29.50
12	1	7	549.25	29.00
-12	1	-7	475.45	30.30
-12	1	-7	558.44	29.60
-13	-1	-7	268.27	19.10
-13	1	-7	279.17	19.10
-14	-1	-7	129.09	14.70
-14	1	-7	130.19	14.30
-15	-1	-7	328.07	22.70
-15	1	-7	320.07	22.20
-15	-2	7	5.60	6.70
-15	2	7	5.90	6.80
-14	-2	7	54.79	10.00
-14	2	7	82.89	10.70
-13	-2	7	6.30	5.10
13	-2	-7	10.50	7.50
-13	2	7	16.30	5.40
13	2	-7	4.90	7.80
-12	-2	7	153.98	11.30
12	-2	-7	162.98	15.10
-12	2	7	166.18	11.80
-12	2	7	134.59	19.00
12	2	-7	151.18	15.00
-11	-2	7	6.80	9.00
-11	-2	7	8.40	5.70
11	-2	-7	11.90	6.00
-11	-2	7	12.00	4.10
-11	2	7	21.80	8.90
11	2	-7	5.90	6.50
-11	2	7	16.90	4.50

-10	-2	7	473.95	30.40
-10	-2	7	531.15	26.30
10	-2	-7	542.05	28.80
10	-2	-7	504.35	27.60
10	2	-7	535.45	29.10
-10	2	7	506.85	26.40
-10	2	7	477.25	30.40
9	-2	-7	9.90	5.10
-9	-2	7	4.40	2.90
-9	-2	7	7.90	6.30
9	-2	-7	7.40	4.10
-9	2	7	2.50	5.80
-9	2	7	7.80	3.40
9	2	-7	9.70	3.70
9	2	-7	2.20	4.80
-8	-2	7	173.08	14.90
8	-2	-7	197.58	12.10
8	-2	-7	202.48	13.60
-8	-2	7	192.88	10.90
8	2	-7	205.58	13.90
-8	2	7	195.28	11.40
-8	2	7	200.88	15.10
8	2	-7	186.28	11.50
7	-2	-7	144.19	11.00
-7	-2	7	148.99	8.50
-7	-2	7	134.99	12.40
7	-2	-7	160.78	9.70
-7	2	7	140.09	12.10
7	2	-7	140.99	11.30
-7	2	7	158.98	9.10
7	2	-7	133.29	8.90
6	-2	-7	581.34	28.20
-6	-2	7	571.04	27.50
-6	-2	7	555.74	30.00
6	-2	-7	554.94	29.20
-6	2	7	556.74	29.90
-6	2	7	542.95	27.80
6	2	-7	557.44	29.40
-5	-2	7	512.15	28.20
5	-2	-7	565.04	27.70
5	-2	-7	555.14	26.70
-5	-2	7	524.35	26.10
5	2	-7	506.65	26.20
-5	2	7	547.35	26.60

5	2	-7	543.55	27.90
-5	2	7	519.25	28.20
-4	-2	7	923.51	47.70
4	-2	-7	1040.00	49.00
4	-2	-7	1051.19	48.20
-4	-2	7	996.90	49.30
4	2	-7	989.60	47.70
-4	2	7	948.11	48.10
4	2	-7	1009.40	49.10
-3	-2	7	734.13	36.40
-3	-2	7	703.23	35.10
3	-2	-7	762.42	36.20
3	-2	-7	745.53	35.50
-3	2	7	639.64	35.60
3	2	-7	756.72	36.30
3	2	-7	731.13	35.00
-3	2	7	757.02	36.50
-2	-2	7	45.80	2.90
2	-2	-7	46.00	3.30
-2	-2	7	38.60	5.80
2	-2	-7	41.10	4.00
2	2	-7	39.60	4.10
-2	2	7	35.10	4.10
-2	2	7	38.70	3.90
2	2	-7	42.80	2.70
1	-2	-7	684.13	32.60
-1	-2	7	636.04	32.30
-1	-2	7	696.73	33.10
1	-2	-7	687.53	33.00
1	2	-7	667.73	32.20
-1	2	7	699.13	33.10
-1	2	7	593.94	33.20
1	2	-7	713.33	32.90
0	-2	-7	204.78	10.50
0	-2	7	203.98	10.30
0	-2	-7	196.58	10.60
0	-2	7	209.38	10.30
0	2	-7	208.38	10.30
0	2	-7	203.58	10.20
0	2	7	198.18	10.60
0	2	7	182.38	10.60
1	-2	7	468.15	22.80
1	-2	7	467.55	22.00
-1	-2	-7	491.45	23.90

-1	-2	-7	470.35	22.80
1	2	7	433.66	22.70
1	2	7	450.85	23.00
-1	2	-7	469.75	22.20
1	2	7	447.06	22.80
-1	2	-7	459.15	22.60
-2	-2	-7	22.20	3.00
2	-2	7	24.20	3.10
2	2	7	28.30	3.10
-2	2	-7	27.80	3.00
2	2	7	22.70	2.40
2	2	7	21.90	3.20
3	-2	7	33.70	3.90
-3	-2	-7	42.20	4.00
-3	-2	-7	46.30	3.90
-3	2	-7	37.50	3.90
3	2	7	40.60	3.30
-3	2	-7	43.40	2.80
3	2	7	48.20	3.90
3	2	7	41.40	4.00
-4	-2	-7	583.74	27.40
4	-2	7	504.15	27.30
4	-2	7	555.74	26.10
-4	-2	-7	532.75	27.30
4	2	7	523.35	27.40
-4	2	-7	528.05	26.60
4	2	7	553.94	27.10
-4	2	-7	586.24	27.50
-5	-2	-7	392.66	20.80
5	-2	7	414.66	19.70
-5	-2	-7	430.66	21.00
-5	2	-7	393.16	20.30
-5	2	-7	430.46	21.00
5	2	7	348.67	20.60
-5	2	-7	374.96	20.70
5	2	7	423.36	20.60
6	-2	7	252.87	13.20
-6	-2	-7	260.37	14.30
-6	-2	-7	247.58	14.40
6	2	7	234.48	14.30
6	2	7	259.37	14.10
-6	2	-7	282.07	14.70
-6	2	-7	235.88	14.20
-7	-2	-7	388.66	21.60

-7	-2	-7	433.16	21.70
7	-2	7	411.16	20.40
7	2	7	414.96	21.20
7	2	7	363.56	21.30
-7	2	-7	372.26	21.20
-7	2	-7	410.16	21.70
-8	-2	-7	5.00	4.10
-8	-2	-7	12.10	4.60
8	-2	7	12.00	3.10
-8	-2	-7	16.00	4.50
-8	2	-7	5.70	4.30
-8	2	-7	11.70	4.20
8	2	7	8.50	3.50
-8	2	-7	9.30	4.10
-9	-2	-7	469.65	27.40
-9	-2	-7	501.85	26.90
9	-2	7	513.95	26.10
-9	2	-7	469.85	27.10
9	2	7	537.25	26.90
-9	2	-7	521.85	27.00
10	-2	7	70.69	11.80
-10	-2	-7	112.99	10.20
-10	-2	-7	99.49	11.00
-10	2	-7	75.89	10.50
10	2	7	100.09	9.80
-10	2	-7	85.89	9.10
-11	-2	-7	81.09	11.40
11	-2	7	83.39	10.90
11	2	7	79.49	10.00
-11	2	-7	76.09	9.80
-11	2	-7	81.99	11.30
-12	-2	-7	16.80	7.60
-12	-2	-7	22.10	7.10
-12	2	-7	8.70	6.10
12	2	7	14.10	5.70
-12	2	-7	0.20	7.30
-13	-2	-7	218.68	17.20
-13	2	-7	232.18	17.00
-14	-2	-7	39.00	9.40
-14	2	-7	43.70	8.60
-15	-2	-7	136.69	15.90
-15	2	-7	116.79	14.60
-15	-3	7	102.79	12.20
-15	3	7	102.79	13.00

-14	3	7	26.50	6.70
13	-3	-7	37.90	8.30
-13	-3	7	42.00	6.50
-13	3	7	57.19	8.80
13	3	-7	35.10	9.50
-12	-3	7	25.70	10.20
-12	-3	7	24.70	5.10
12	-3	-7	21.70	6.70
-12	-3	7	17.10	6.80
12	3	-7	22.20	7.70
-12	3	7	20.80	9.80
-12	3	7	44.10	7.70
-11	-3	7	442.96	24.00
11	-3	-7	434.96	25.40
-11	-3	7	351.26	27.30
-11	-3	7	435.76	22.70
-11	3	7	403.16	28.10
11	3	-7	435.66	26.30
-11	3	7	469.25	23.30
-10	-3	7	47.00	8.00
-10	-3	7	48.70	6.10
10	-3	-7	57.09	8.70
-10	-3	7	24.50	8.70
10	-3	-7	54.39	9.40
10	3	-7	53.09	10.50
-10	3	7	50.79	6.80
-9	-3	7	317.77	22.60
9	-3	-7	371.96	20.10
9	-3	-7	361.16	21.20
-9	-3	7	354.76	18.60
-9	-3	7	393.66	19.70
-9	3	7	346.57	22.70
9	3	-7	355.26	21.50
-9	3	7	352.76	19.20
-8	-3	7	37.40	4.30
-8	-3	7	17.80	6.10
8	-3	-7	33.60	6.10
8	-3	-7	21.80	5.10
-8	3	7	29.10	5.00
-8	3	7	23.90	6.30
8	3	-7	20.90	5.60
8	3	-7	30.40	5.00
-7	-3	7	474.65	23.80
7	-3	-7	501.95	25.00

7	-3	-7	494.25	26.00
-7	-3	7	446.66	26.90
-7	3	7	470.85	26.70
7	3	-7	475.05	26.20
-7	3	7	496.45	24.50
7	3	-7	485.35	24.20
-6	-3	7	237.28	16.00
-6	-3	7	268.67	13.20
6	-3	-7	272.37	14.30
6	-3	-7	262.47	15.20
6	3	-7	235.28	15.30
-6	3	7	252.57	15.90
-6	3	7	257.07	13.90
6	3	-7	253.87	13.40
-5	-3	7	460.95	22.80
5	-3	-7	512.35	23.80
5	-3	-7	489.95	24.60
-5	-3	7	443.56	25.00
5	3	-7	462.65	22.90
-5	3	7	443.96	26.00
5	3	-7	445.46	24.60
-5	3	7	467.95	23.50
-4	-3	7	346.67	20.00
4	-3	-7	384.36	19.80
-4	-3	7	365.96	18.20
4	-3	-7	399.06	19.00
4	3	-7	376.46	20.00
4	3	-7	381.46	18.20
-4	3	7	346.17	21.20
-4	3	7	369.16	19.00
3	-3	-7	203.28	11.50
3	-3	-7	213.48	10.80
-3	-3	7	205.78	11.80
-3	-3	7	200.18	10.10
-3	3	7	181.08	11.00
-3	3	7	201.18	11.80
3	3	-7	202.88	11.70
3	3	-7	202.28	10.00
2	-3	-7	1555.24	72.19
-2	-3	7	1487.05	72.09
-2	-3	7	1486.85	70.99
2	3	-7	1524.75	72.09
2	3	-7	1498.05	70.99
-2	3	7	1509.25	72.19

-2	3	7	1402.76	71.89
1	-3	-7	415.96	22.20
-1	-3	7	458.35	21.50
1	-3	-7	453.35	22.30
1	3	-7	443.76	22.10
-1	3	7	461.35	22.40
1	3	-7	441.66	21.40
-1	3	7	428.96	22.20
0	-3	-7	1345.57	64.49
0	-3	7	1347.97	63.99
0	-3	7	1335.67	63.99
0	3	-7	1317.07	63.89
0	3	-7	1354.06	64.09
0	3	7	1347.47	64.49
0	3	7	1336.57	64.49
-1	-3	-7	6.60	1.80
-1	-3	-7	4.10	1.50
1	-3	7	9.50	1.70
1	3	7	3.80	1.70
-1	3	-7	4.40	1.40
-1	3	-7	5.70	1.00
1	3	7	7.40	2.00
-2	-3	-7	1345.17	61.99
2	-3	7	1248.28	61.89
2	3	7	1285.47	61.79
2	3	7	1315.67	62.09
2	3	7	1212.18	61.99
-2	3	-7	1315.37	61.79
-3	-3	-7	67.79	5.30
-3	-3	-7	69.29	5.30
3	-3	7	73.59	5.30
-3	3	-7	65.29	5.10
3	3	7	78.69	5.40
3	3	7	69.99	5.20
3	3	7	64.79	4.70
-3	3	-7	75.19	3.80
-3	3	-7	76.19	5.30
-4	-3	-7	292.47	16.60
-4	-3	-7	360.46	16.70
-4	3	-7	337.57	16.70
-4	3	-7	304.77	16.60
-4	3	-7	299.27	15.60
4	3	7	310.97	16.70
4	3	7	301.87	16.50

-5	-3	-7	195.58	11.40
-5	-3	-7	191.08	11.60
-5	3	-7	179.98	10.60
-5	3	-7	220.68	11.70
5	3	7	202.78	11.30
5	3	7	188.18	11.50
-5	3	-7	196.28	11.50
6	-3	7	435.56	24.60
-6	-3	-7	474.05	25.40
-6	-3	-7	540.05	25.40
6	3	7	508.55	25.20
-6	3	-7	465.55	24.60
-6	3	-7	542.85	25.60
6	3	7	466.35	25.20
-6	3	-7	474.85	25.20
-7	-3	-7	146.79	9.50
7	-3	7	123.49	10.10
-7	-3	-7	134.09	10.00
-7	3	-7	121.29	9.30
7	3	7	155.08	9.80
7	3	7	137.49	9.50
-7	3	-7	128.99	9.30
-7	3	-7	123.99	9.90
-8	-3	-7	167.88	12.60
-8	-3	-7	204.78	12.60
-8	3	-7	193.58	12.70
8	3	7	183.38	12.20
8	3	7	200.38	12.30
-8	3	-7	187.78	12.20
-8	3	-7	165.98	12.20
-9	-3	-7	445.66	23.20
9	-3	7	405.26	25.00
-9	-3	-7	405.16	23.80
-9	3	-7	381.66	24.50
-9	3	-7	431.76	23.10
9	3	7	429.86	23.20
-10	-3	-7	581.24	29.50
-10	-3	-7	517.55	30.10
10	-3	7	548.05	28.10
-10	3	-7	446.66	35.00
-10	3	-7	542.25	28.90
10	3	7	577.64	29.40
-11	-3	-7	18.90	6.90
11	-3	7	24.00	4.90

-11	3	-7	21.30	5.80
11	3	7	25.10	6.10
12	-3	7	69.49	9.40
-12	-3	-7	103.39	11.60
-12	-3	-7	96.39	13.00
-12	3	-7	109.89	11.70
-12	3	-7	46.90	15.80
12	3	7	103.59	11.40
-13	-3	-7	21.00	7.70
-13	3	-7	36.60	8.30
-14	-3	-7	41.20	9.20
-14	3	-7	66.49	12.10
-15	-3	-7	181.08	17.80
-15	3	-7	179.58	16.50
-15	-4	7	52.09	8.50
-15	4	7	91.39	11.90
-14	-4	7	298.57	18.50
-14	4	7	308.67	19.10
13	-4	-7	7.30	6.90
-13	-4	7	12.30	5.40
-13	4	7	9.80	5.60
13	4	-7	11.20	9.10
-12	-4	7	665.83	33.50
12	-4	-7	680.23	36.40
-12	-4	7	656.83	34.60
-12	-4	7	582.44	38.60
12	4	-7	698.73	37.20
-12	4	7	638.24	39.20
11	-4	-7	281.67	17.90
-11	-4	7	244.28	15.90
-11	-4	7	226.18	14.30
-11	-4	7	212.88	20.80
-11	4	7	256.37	15.10
-11	4	7	232.68	20.60
11	4	-7	249.58	18.80
-10	-4	7	815.32	39.90
-10	-4	7	740.83	43.80
10	-4	-7	830.42	42.50
-10	-4	7	911.11	41.00
-10	4	7	735.43	44.00
10	4	-7	807.32	42.80
-10	4	7	804.12	40.60
-9	-4	7	3.90	6.20
9	-4	-7	5.20	4.70

-9	-4	7	1.40	2.80
-9	-4	7	-2.50	3.80
9	-4	-7	5.00	4.80
-9	4	7	10.70	7.10
-9	4	7	10.10	3.80
9	4	-7	7.30	5.70
8	-4	-7	978.10	47.90
-8	-4	7	1049.49	46.30
-8	-4	7	901.21	45.60
8	-4	-7	921.21	46.90
-8	-4	7	875.01	48.70
8	4	-7	921.81	48.10
-8	4	7	942.01	46.30
-8	4	7	928.71	48.80
7	-4	-7	706.03	35.10
7	-4	-7	654.03	34.10
-7	-4	7	654.23	32.80
-7	-4	7	623.64	35.70
-7	4	7	686.73	33.60
-7	4	7	696.03	35.90
7	4	-7	698.33	35.50
-6	-4	7	1103.89	57.19
6	-4	-7	1181.88	55.79
-6	-4	7	1117.59	54.69
6	-4	-7	1155.18	56.49
-6	4	7	1168.98	57.29
6	4	-7	1117.89	54.89
6	4	-7	1132.79	56.79
-6	4	7	1149.78	55.49
-5	-4	7	741.63	39.10
5	-4	-7	829.02	38.10
5	-4	-7	771.72	38.60
-5	-4	7	738.93	36.90
5	4	-7	744.73	37.00
5	4	-7	759.02	39.00
-5	4	7	789.12	37.80
4	-4	-7	679.43	32.40
-4	-4	7	644.44	32.70
-4	-4	7	603.74	30.70
4	-4	-7	698.13	31.80
-4	4	7	616.54	31.70
4	4	-7	640.54	32.50
-4	4	7	595.14	33.70
4	4	-7	641.54	30.70

3	-4	-7	504.95	23.60
-3	-4	7	477.15	22.70
3	-4	-7	468.85	24.20
-3	-4	7	475.65	24.40
3	4	-7	477.45	24.30
3	4	-7	467.35	22.60
-3	4	7	477.05	24.40
-3	4	7	431.36	23.80
2	-4	-7	381.56	18.80
-2	-4	7	382.06	18.00
2	-4	-7	393.66	19.40
-2	-4	7	366.56	19.20
-2	4	7	379.76	19.40
-2	4	7	330.87	19.10
2	4	-7	372.86	17.90
2	4	-7	381.26	19.30
-1	-4	7	2123.89	100.59
1	-4	-7	2201.48	101.19
1	-4	-7	2140.19	101.59
-1	-4	7	2057.19	101.19
-1	4	7	2134.59	101.59
1	4	-7	2078.99	101.29
1	4	-7	2085.69	100.49
-1	4	7	2133.89	101.59
0	-4	7	1230.18	56.39
0	-4	-7	1257.27	56.89
0	-4	-7	1210.98	57.19
0	-4	7	1110.89	56.79
0	4	-7	1196.88	56.59
0	4	7	1055.69	57.39
0	4	-7	1191.88	56.29
0	4	7	1188.18	57.09
-1	-4	-7	4549.65	213.68
-1	-4	-7	4504.35	213.98
1	-4	7	4579.04	213.48
1	4	7	4353.56	213.78
1	4	7	4381.06	213.88
1	4	7	4482.85	214.08
-1	4	-7	4645.84	213.38
-2	-4	-7	13.80	2.30
2	4	7	8.70	1.90
2	4	7	13.40	2.50
-2	4	-7	17.50	3.10
2	4	7	18.10	3.30

-2	4	-7	14.30	2.70
-3	-4	-7	594.74	28.60
-3	-4	-7	550.24	28.80
3	4	7	573.94	28.40
3	4	7	558.74	28.70
3	4	7	585.94	28.90
-3	4	-7	570.44	28.50
-3	4	-7	587.34	27.50
-3	4	-7	580.94	28.50
-4	-4	-7	638.54	33.20
-4	-4	-7	712.23	33.10
4	4	7	713.23	33.30
4	4	7	590.84	32.90
-4	4	-7	657.03	32.00
-4	4	-7	633.74	32.90
-4	4	-7	701.13	33.10
-5	-4	-7	1987.00	100.09
5	-4	7	2003.40	98.39
-5	-4	-7	2203.58	99.89
-5	4	-7	2016.70	99.79
-5	4	-7	2194.18	99.99
5	4	7	2019.70	99.89
5	4	7	2189.98	99.99
-5	4	-7	1989.00	98.99
-6	-4	-7	260.47	13.80
-6	-4	-7	232.88	14.20
6	-4	7	234.88	12.20
-6	4	-7	259.67	14.20
-6	4	-7	220.48	13.80
6	4	7	256.67	14.00
-6	4	-7	221.08	13.10
6	4	7	231.48	13.80
-7	-4	-7	1057.29	48.80
7	-4	7	977.50	47.40
-7	-4	-7	954.10	49.10
-7	4	-7	951.40	48.20
-7	4	-7	936.21	48.60
-7	4	-7	1009.60	48.90
7	4	7	935.21	48.60
7	4	7	1016.00	48.80
8	-4	7	5.50	2.50
-8	-4	-7	1.80	4.00
-8	-4	-7	4.20	4.20
-8	4	-7	5.50	3.80

-8	4	-7	4.10	3.90
8	4	7	2.70	3.50
-8	4	-7	-3.30	4.20
8	4	7	2.40	3.80
9	-4	7	487.85	24.90
-9	-4	-7	466.65	27.00
-9	-4	-7	491.85	26.40
9	4	7	442.26	25.90
-9	4	-7	478.65	26.30
-9	4	-7	475.95	26.20
9	4	7	543.75	26.60
10	-4	7	159.08	10.60
-10	-4	-7	179.18	12.30
-10	-4	-7	151.08	13.60
10	4	7	150.78	12.70
-10	4	-7	140.99	13.20
-10	4	-7	146.99	11.90
-11	-4	-7	720.23	42.10
11	-4	7	795.42	39.80
-11	4	-7	815.12	41.10
11	4	7	832.52	41.50
-11	4	-7	772.32	41.70
12	-4	7	172.38	12.90
-12	-4	-7	214.38	15.20
-12	4	-7	139.79	16.20
12	4	7	224.78	22.20
-12	4	-7	204.88	15.20
-13	-4	-7	259.47	18.60
-13	4	-7	253.07	18.20
-14	-4	-7	124.49	14.90
-14	4	-7	101.69	13.60
-15	-4	-7	121.39	15.70
-15	4	-7	96.29	13.00
-15	-5	7	7.80	6.90
-15	5	7	26.50	7.60
-14	-5	7	3.20	5.70
-14	5	7	6.20	6.40
-13	-5	7	0.00	4.90
13	-5	-7	7.10	7.70
13	5	-7	-12.60	7.50
-13	5	7	1.20	5.80
-12	-5	7	16.00	11.20
12	-5	-7	16.20	6.40
-12	-5	7	9.80	6.10

-12	-5	7	8.70	4.40
12	5	-7	3.30	7.50
-12	5	7	14.70	5.40
-11	-5	7	58.29	9.10
-11	-5	7	66.09	7.20
11	-5	-7	73.39	11.30
-11	-5	7	40.90	10.30
11	5	-7	68.89	10.70
-11	5	7	59.39	10.90
-11	5	7	77.19	8.70
10	-5	-7	6.00	5.40
-10	-5	7	11.30	3.40
-10	-5	7	10.70	4.70
-10	-5	7	1.50	7.50
10	5	-7	0.90	5.80
-10	5	7	8.20	6.80
-10	5	7	11.50	4.40
9	-5	-7	12.60	4.70
-9	-5	7	2.80	2.80
-9	-5	7	2.50	3.80
9	-5	-7	3.90	5.00
-9	-5	7	4.40	6.40
-9	5	7	12.10	4.00
-9	5	7	5.50	5.80
9	5	-7	11.60	5.60
8	-5	-7	67.59	8.30
-8	-5	7	50.39	8.10
8	-5	-7	69.59	7.60
-8	-5	7	66.69	6.10
-8	-5	7	60.59	5.20
-8	5	7	80.99	6.70
-8	5	7	66.29	10.20
8	5	-7	58.89	9.20
7	-5	-7	258.77	14.90
-7	-5	7	252.27	12.70
7	-5	-7	228.58	13.90
-7	-5	7	224.18	12.20
-7	-5	7	193.78	15.40
-7	5	7	243.68	15.90
-7	5	7	233.48	13.30
7	5	-7	232.38	15.50
-6	-5	7	2.90	1.70
6	-5	-7	7.20	3.30
6	-5	-7	3.30	3.60

-6	-5	7	2.00	2.00
-6	-5	7	1.50	4.30
-6	5	7	8.00	3.00
-6	5	7	6.80	4.00
6	5	-7	1.60	4.40
5	-5	-7	9.70	3.10
5	-5	-7	12.10	3.70
-5	-5	7	8.50	3.90
5	5	-7	2.00	3.50
-5	5	7	10.30	3.10
-5	5	7	8.20	6.40
-4	-5	7	54.79	2.90
-4	-5	7	39.40	5.80
-4	-5	7	43.70	2.80
4	-5	-7	51.09	5.40
4	-5	-7	47.80	4.90
-4	5	7	43.00	7.10
4	5	-7	36.80	5.70
-4	5	7	40.20	4.80
-3	-5	7	25.20	4.40
3	-5	-7	23.30	4.40
3	-5	-7	28.70	3.60
-3	5	7	23.20	3.80
3	5	-7	29.50	4.50
-3	5	7	28.40	4.20
2	-5	-7	133.19	7.00
-2	-5	7	105.19	7.40
2	-5	-7	115.19	7.50
-2	5	7	106.29	7.60
2	5	-7	111.89	7.40
-2	5	7	102.39	7.30
1	-5	-7	9.30	2.00
-1	-5	7	9.10	2.10
1	-5	-7	7.00	2.20
1	5	-7	6.40	1.20
1	5	-7	13.20	2.30
-1	5	7	9.60	2.60
-1	5	7	7.10	2.60
1	5	-7	8.30	1.10
0	-5	7	216.68	11.00
0	-5	-7	215.08	11.80
0	-5	-7	215.88	11.20
0	5	7	206.38	11.50
0	5	-7	210.58	10.60

0	5	7	180.48	13.40
0	5	-7	224.88	11.10
0	5	-7	209.58	10.70
-1	-5	-7	128.99	7.80
-1	-5	-7	140.49	8.40
-1	5	-7	144.79	7.50
1	5	7	132.89	8.20
-1	5	-7	135.09	7.60
-1	5	-7	135.99	7.30
1	5	7	143.09	8.10
-2	-5	-7	25.40	3.30
-2	5	-7	21.90	2.00
-2	5	-7	20.60	3.00
2	5	7	21.50	3.60
-2	5	-7	20.80	3.30
2	5	7	18.00	3.80
2	5	7	22.10	3.20
-3	-5	-7	1.10	2.00
-3	-5	-7	2.30	2.00
3	5	7	2.90	2.20
3	5	7	0.90	2.30
-3	5	-7	0.80	1.90
-3	5	-7	3.30	0.90
-3	5	-7	0.00	2.00
3	5	7	0.20	1.70
-4	-5	-7	9.40	3.00
-4	-5	-7	8.40	2.60
4	5	7	7.80	2.80
-4	5	-7	14.10	2.30
-4	5	-7	10.90	3.00
-4	5	-7	12.80	2.70
4	5	7	8.30	2.70
-5	-5	-7	2.60	2.60
-5	-5	-7	1.60	2.80
-5	5	-7	0.30	2.00
-5	5	-7	7.40	3.10
-5	5	-7	-0.90	2.90
5	5	7	6.10	3.10
5	5	7	3.50	2.90
-6	-5	-7	30.00	5.10
-6	-5	-7	21.20	4.40
6	-5	7	22.90	2.70
-6	5	-7	26.90	4.40
6	5	7	29.20	5.20

-6	5	-7	24.10	4.30
6	5	7	26.30	4.30
-6	5	-7	19.20	3.20
-7	-5	-7	9.10	3.80
7	-5	7	3.40	2.10
-7	-5	-7	8.90	4.10
-7	5	-7	3.20	3.30
7	5	7	6.60	3.30
7	5	7	6.80	3.60
-7	5	-7	12.60	4.10
-7	5	-7	11.50	4.30
-8	-5	-7	10.50	4.60
8	-5	7	15.50	2.90
-8	-5	-7	3.20	4.20
-8	5	-7	12.30	4.40
8	5	7	8.00	4.00
-8	5	-7	5.70	4.80
-8	5	-7	11.80	4.20
8	5	7	11.20	4.40
9	-5	7	35.50	5.40
-9	-5	-7	28.20	6.30
9	5	7	48.50	7.50
-9	5	-7	31.00	5.80
9	5	7	41.60	6.60
-9	5	-7	37.80	6.80
10	-5	7	0.90	3.50
-10	-5	-7	0.20	5.30
-10	-5	-7	2.20	5.40
-10	5	-7	6.00	5.10
-10	5	-7	1.50	5.40
10	5	7	9.30	5.40
-11	-5	-7	17.30	6.90
-11	-5	-7	22.00	8.00
-11	5	-7	23.40	6.30
-11	5	-7	4.80	6.50
11	5	7	7.50	5.50
12	-5	7	24.30	5.40
-12	-5	-7	30.30	7.50
-12	5	-7	28.70	6.80
-12	5	-7	31.30	8.00
-13	-5	-7	2.50	7.10
-13	5	-7	1.30	7.00
-14	-5	-7	16.30	8.40
-14	5	-7	17.90	7.80

-15	-5	-7	8.80	8.80
-15	5	-7	10.40	8.30
-15	-6	7	13.70	7.00
-15	6	7	15.10	7.40
-14	-6	7	171.28	13.10
-14	6	7	177.98	14.40
-13	-6	7	-5.10	4.80
-13	6	7	6.70	5.60
13	6	-7	-3.10	8.40
12	-6	-7	310.87	21.20
-12	-6	7	236.48	24.70
-12	-6	7	316.87	18.80
-12	-6	7	308.67	17.30
-12	6	7	330.57	18.50
12	6	-7	306.17	22.50
-11	-6	7	52.09	8.70
11	-6	-7	58.99	10.60
-11	-6	7	30.60	10.40
-11	-6	7	32.90	5.10
-11	6	7	61.09	8.10
-11	6	7	49.40	10.70
11	6	-7	43.70	9.30
-10	-6	7	482.25	23.50
10	-6	-7	456.85	25.50
-10	-6	7	373.86	27.20
-10	-6	7	417.66	22.40
-10	6	7	401.96	27.10
-10	6	7	447.06	23.50
10	6	-7	460.65	26.60
-9	-6	7	42.50	5.10
-9	-6	7	43.50	8.60
-9	-6	7	43.90	6.50
-9	6	7	60.09	6.60
9	6	-7	60.49	10.20
-9	6	7	66.59	11.40
-8	-6	7	200.78	16.30
8	-6	-7	241.58	14.60
-8	-6	7	243.68	12.80
8	-6	-7	227.78	15.10
-8	-6	7	220.68	12.20
-8	6	7	237.18	13.50
8	6	-7	234.58	16.10
-8	6	7	229.18	16.60
-7	-6	7	107.59	6.90

7	-6	-7	125.09	9.60
7	-6	-7	123.89	9.00
-7	-6	7	85.69	10.60
7	6	-7	103.59	10.50
-7	6	7	93.89	10.60
-7	6	7	104.89	7.90
6	-6	-7	371.56	20.50
-6	-6	7	411.66	19.10
-6	-6	7	352.76	21.90
6	-6	-7	396.06	21.20
-6	-6	7	377.66	18.80
-6	6	7	376.36	20.00
6	6	-7	401.16	21.80
-6	6	7	383.16	21.80
-5	-6	7	364.26	21.50
5	-6	-7	383.66	20.80
-5	-6	7	372.16	18.70
5	-6	-7	426.76	20.50
-5	-6	7	404.26	18.80
-5	6	7	322.47	24.90
5	6	-7	392.86	21.50
-5	6	7	400.66	20.20
4	-6	-7	443.56	22.20
-4	-6	7	407.76	23.00
4	-6	-7	452.05	22.90
-4	6	7	435.76	22.40
4	6	-7	423.96	23.00
3	-6	-7	197.78	11.30
-3	-6	7	175.48	11.20
3	-6	-7	213.08	10.70
-3	6	7	148.19	12.00
-3	6	7	178.58	10.90
3	6	-7	196.78	11.40
2	-6	-7	3927.71	180.78
2	-6	-7	3796.62	181.38
-2	-6	7	3791.02	181.28
-2	6	7	3489.85	181.18
-2	6	7	3825.82	182.88
2	6	-7	3966.50	181.28
1	-6	-7	1215.88	55.49
1	-6	-7	1187.18	56.09
-1	-6	7	1138.29	55.69
1	6	-7	1104.19	54.59
1	6	-7	1164.98	55.59

-1	6	7	1137.99	55.99
1	6	-7	1097.99	54.59
0	-6	7	672.73	32.00
0	-6	-7	662.43	32.80
0	-6	-7	675.23	32.20
0	6	7	598.84	32.50
0	6	7	654.63	32.60
0	6	-7	687.73	32.10
0	6	-7	640.34	31.60
0	6	-7	650.83	31.60
-1	-6	-7	985.00	47.30
1	6	7	987.00	47.70
-1	6	-7	1003.00	47.00
-1	6	-7	964.10	46.90
-1	6	-7	963.50	46.90
1	6	7	949.40	47.60
-2	-6	-7	338.57	17.20
2	6	7	315.87	17.30
-2	6	-7	335.97	16.40
2	6	7	347.47	17.50
-2	6	-7	317.77	17.40
-2	6	-7	340.27	17.00
-3	-6	-7	1251.97	59.69
-3	-6	-7	1175.78	60.09
-3	6	-7	1334.77	59.69
3	6	7	1230.78	59.79
3	6	7	1171.18	59.79
-3	6	-7	1252.77	59.59
-3	6	-7	1204.98	58.69
-4	-6	-7	16.50	3.10
-4	-6	-7	9.70	3.10
-4	6	-7	11.30	3.10
-4	6	-7	11.30	2.40
4	6	7	20.70	3.50
4	6	7	20.10	3.90
-4	6	-7	15.30	3.20
-5	-6	-7	2634.24	131.89
-5	-6	-7	2849.22	131.39
-5	6	-7	2719.63	130.59
-5	6	-7	2874.31	131.59
5	6	7	2568.04	131.39
-5	6	-7	2681.73	131.29
5	6	7	2861.81	131.89
-6	-6	-7	34.10	6.30

6	-6	7	33.60	3.20
-6	-6	-7	40.80	5.60
6	6	7	36.30	6.10
6	6	7	36.40	5.20
-6	6	-7	35.00	5.10
-6	6	-7	37.00	4.70
-6	6	-7	33.80	5.50
7	-6	7	1859.71	88.99
-7	-6	-7	1947.11	90.29
-7	-6	-7	1753.02	90.99
-7	6	-7	1783.12	89.79
-7	6	-7	1961.00	90.69
7	6	7	1975.30	90.89
7	6	7	1760.72	90.19
-7	6	-7	1857.41	90.29
-8	-6	-7	5.40	4.50
8	-6	7	2.80	2.40
-8	-6	-7	-0.70	3.90
-8	6	-7	1.60	3.60
8	6	7	4.50	4.70
8	6	7	7.50	4.30
-8	6	-7	2.90	4.20
9	-6	7	403.86	19.60
-9	-6	-7	327.47	22.20
-9	6	-7	346.67	21.00
9	6	7	400.46	22.00
-9	6	-7	407.76	21.40
9	6	7	348.77	21.00
10	-6	7	66.29	7.40
-10	-6	-7	87.49	10.00
-10	-6	-7	77.49	11.60
10	6	7	92.99	9.60
-10	6	-7	83.49	9.70
10	6	7	77.89	10.60
-10	6	-7	66.79	10.10
-11	-6	-7	698.33	36.60
-11	-6	-7	641.64	37.50
11	-6	7	684.03	34.40
-11	6	-7	685.53	35.90
11	6	7	693.83	36.80
-11	6	-7	644.84	36.30
-12	-6	-7	296.67	18.90
-12	6	-7	219.98	18.60
-12	6	-7	288.57	18.40

-13	-6	-7	194.88	16.80
-13	6	-7	199.58	16.10
-14	-6	-7	120.49	15.20
-14	6	-7	95.99	12.40
-15	-6	-7	109.59	16.40
-15	6	-7	119.09	15.50
-15	7	7	138.29	14.30
-14	-7	7	-4.90	5.50
-14	7	7	3.00	6.40
-13	-7	7	89.09	9.40
13	7	-7	80.79	15.40
12	-7	-7	-6.20	6.50
-12	-7	7	-4.40	6.00
-12	-7	7	-4.70	4.10
-12	-7	7	-4.30	10.70
-12	7	7	2.00	5.20
12	7	-7	-5.40	7.20
-11	-7	7	136.59	10.90
11	-7	-7	159.98	13.60
-11	-7	7	94.99	16.70
-11	-7	7	118.89	9.10
-11	7	7	113.09	12.00
11	7	-7	130.79	14.70
-11	7	7	124.29	17.40
-10	-7	7	-1.30	4.40
-10	-7	7	7.20	8.70
10	-7	-7	16.10	5.90
-10	-7	7	8.90	3.40
-10	7	7	3.30	7.20
10	7	-7	6.90	6.70
-9	-7	7	95.89	7.30
-9	-7	7	111.19	8.40
9	-7	-7	114.49	10.90
-9	-7	7	92.49	13.40
-9	7	7	123.29	13.30
9	7	-7	123.49	12.60
-9	7	7	118.79	9.10
8	-7	-7	58.69	7.40
-8	-7	7	56.69	4.70
-8	-7	7	45.60	8.00
8	-7	-7	48.70	8.00
-8	7	7	56.29	6.60
-8	7	7	48.30	10.20
8	7	-7	51.79	9.70

-7	-7	7	120.59	7.00
7	-7	-7	111.79	9.30
-7	-7	7	105.59	11.30
7	-7	-7	109.19	9.50
-7	-7	7	110.49	6.60
-7	7	7	119.09	10.80
-7	7	7	111.79	8.40
7	7	-7	124.09	10.80
-6	-7	7	8.40	1.80
-6	-7	7	9.80	4.80
6	-7	-7	9.30	4.10
6	-7	-7	10.20	3.60
-6	-7	7	8.30	1.60
-6	7	7	14.00	4.80
-6	7	7	12.90	3.60
6	7	-7	4.10	4.30
-5	-7	7	453.05	25.50
-5	-7	7	451.95	22.50
5	-7	-7	477.95	25.00
5	-7	-7	494.75	24.50
-5	-7	7	453.75	22.50
5	7	-7	496.65	25.50
-5	7	7	467.85	26.70
-5	7	7	475.75	24.30
4	-7	-7	448.46	22.70
4	-7	-7	438.76	23.10
-4	-7	7	418.66	23.50
-4	7	7	434.46	22.70
4	7	-7	445.66	23.50
3	-7	-7	571.04	27.70
-3	-7	7	544.65	28.40
3	-7	-7	539.35	28.20
3	7	-7	594.64	28.40
-3	7	7	504.55	27.90
2	-7	-7	38.90	5.00
-2	-7	7	39.30	4.90
2	-7	-7	40.90	3.90
2	7	-7	38.20	4.80
-2	7	7	31.40	4.80
1	-7	-7	274.47	14.90
-1	-7	7	285.97	14.60
1	-7	-7	269.57	14.10
-1	7	7	272.57	14.80
-1	7	7	243.18	14.70

1	7	-7	285.87	14.40
1	7	-7	261.67	13.10
1	7	-7	258.77	13.00
0	-7	-7	288.17	14.50
0	-7	-7	274.77	15.10
0	-7	7	273.87	14.30
0	7	7	282.37	15.00
0	7	7	268.67	14.80
0	7	-7	270.17	13.60
0	7	-7	261.97	13.60
0	7	-7	276.07	14.30
-1	-7	-7	697.63	34.60
-1	7	-7	738.73	34.30
1	7	7	672.23	35.30
-1	7	-7	686.03	34.10
1	7	7	720.53	35.00
-1	7	-7	718.63	34.20
1	7	7	690.13	34.90
-2	-7	-7	252.17	12.80
-2	7	-7	227.18	12.40
2	7	7	236.68	13.10
-2	7	-7	235.38	12.10
-2	7	-7	218.58	12.90
2	7	7	232.08	13.00
-3	-7	-7	3.80	2.40
-3	-7	-7	3.00	2.70
-3	7	-7	5.10	1.50
-3	7	-7	2.60	2.30
3	7	7	1.90	2.00
-3	7	-7	5.30	1.60
3	7	7	6.40	2.70
-3	7	-7	3.70	2.30
3	7	7	5.10	2.30
-4	-7	-7	871.11	40.10
-4	-7	-7	779.82	40.70
-4	7	-7	795.62	39.90
-4	7	-7	818.12	39.20
-4	7	-7	843.22	40.10
4	7	7	753.72	40.10
-5	-7	-7	321.47	18.00
-5	-7	-7	323.67	17.00
-5	7	-7	298.77	16.70
-5	7	-7	304.47	17.10
-5	7	-7	336.47	16.20

5	7	7	287.67	16.90
-6	-7	-7	951.50	50.09
6	-7	7	990.00	47.80
-6	-7	-7	1058.09	49.30
-6	7	-7	951.50	49.10
6	7	7	950.90	49.20
-6	7	-7	1051.29	48.70
-6	7	-7	1014.60	49.50
-7	-7	-7	113.49	8.80
-7	-7	-7	98.69	9.90
7	-7	7	107.19	6.60
7	7	7	92.49	8.60
-7	7	-7	112.99	8.20
-7	7	-7	93.89	9.30
7	7	7	122.89	10.90
-7	7	-7	103.19	8.40
8	-7	7	665.83	32.10
-8	-7	-7	701.33	33.90
-8	-7	-7	625.64	34.80
-8	7	-7	628.04	33.50
8	7	7	594.74	33.40
-8	7	-7	637.74	33.20
8	7	7	693.23	34.50
9	-7	7	-1.20	2.80
-9	-7	-7	3.10	5.70
9	7	7	12.40	5.40
-9	7	-7	2.90	4.30
-9	7	-7	3.80	4.40
9	7	7	7.80	4.60
-10	-7	-7	243.08	18.10
10	-7	7	242.88	14.40
-10	7	-7	252.87	16.80
10	7	7	287.77	17.90
10	7	7	238.48	16.20
11	-7	7	46.00	7.50
-11	-7	-7	65.79	12.50
-11	-7	-7	74.69	10.30
-11	7	-7	51.09	8.50
-11	7	-7	76.59	10.40
11	7	7	76.09	11.40
-12	-7	-7	263.97	18.20
-12	7	-7	214.58	18.30
-12	7	-7	270.47	17.70
-13	-7	-7	48.00	9.60

-13	7	-7	40.20	8.40
-14	-7	-7	69.99	11.30
-14	7	-7	86.09	13.20
-15	-7	-7	178.78	19.00
-15	7	-7	178.88	17.30
-15	8	7	12.10	7.40
-14	-8	7	48.90	7.30
-14	8	7	59.69	11.00
-13	-8	7	8.80	4.90
13	8	-7	4.00	9.40
12	-8	-7	61.39	9.90
-12	-8	7	42.50	7.40
-12	-8	7	25.20	11.50
-12	8	7	58.79	9.10
12	8	-7	44.00	11.00
11	-8	-7	12.10	6.90
-11	-8	7	19.30	5.10
-11	-8	7	8.10	3.80
-11	-8	7	13.10	10.00
-11	8	7	13.10	8.90
-11	8	7	12.60	5.00
11	8	-7	15.20	7.80
-10	-8	7	507.95	26.20
10	-8	-7	506.35	29.20
-10	-8	7	587.24	27.10
10	8	-7	506.05	30.30
-10	8	7	479.45	31.40
-10	8	7	525.05	27.40
-9	-8	7	19.20	3.80
-9	-8	7	13.50	7.30
9	-8	-7	12.40	5.30
-9	-8	7	13.90	3.00
9	8	-7	9.60	6.50
-9	8	7	11.60	4.60
-9	8	7	17.40	7.50
-8	-8	7	171.38	15.50
-8	-8	7	178.58	10.30
8	-8	-7	193.18	13.90
-8	-8	7	207.48	10.80
-8	8	7	192.18	12.20
-8	8	7	180.88	15.10
8	8	-7	204.88	14.80
-7	-8	7	53.09	10.00
-7	-8	7	63.59	4.40

-7	-8	7	63.79	4.70
7	-8	-7	61.39	7.40
7	-8	-7	65.59	8.40
-7	8	7	49.20	9.40
-7	8	7	59.29	6.80
7	8	-7	75.69	9.50
-6	-8	7	314.77	15.60
6	-8	-7	329.97	18.10
-6	-8	7	307.87	15.60
6	-8	-7	322.27	18.50
-6	-8	7	281.47	19.20
-6	8	7	344.87	17.60
6	8	-7	323.47	19.10
-6	8	7	329.57	19.30
5	-8	-7	187.68	11.30
-5	-8	7	175.08	12.80
5	-8	-7	187.18	11.90
5	8	-7	177.48	12.50
-5	8	7	187.08	11.10
-5	8	7	182.38	12.60
4	-8	-7	249.18	13.40
4	-8	-7	235.78	13.80
-4	-8	7	213.68	14.20
4	8	-7	243.58	14.20
-4	8	7	235.28	15.20
-4	8	7	209.18	13.20
3	-8	-7	301.27	15.30
3	-8	-7	297.67	16.10
-3	-8	7	259.37	16.00
-3	8	7	283.17	17.10
-3	8	7	269.57	15.70
3	8	-7	289.67	16.00
2	-8	-7	42.00	5.20
-2	-8	7	48.70	5.50
2	-8	-7	39.30	4.50
-2	8	7	35.70	5.00
-2	8	7	45.20	5.50
2	8	-7	47.20	5.10
1	-8	-7	513.85	25.40
-1	-8	7	496.45	25.80
1	-8	-7	539.25	26.40
-1	8	7	517.55	26.20
1	8	-7	527.25	25.80
1	8	-7	503.95	24.40

1	8	-7	495.55	24.40
-1	8	7	476.45	26.00
0	-8	-7	329.27	16.90
0	-8	7	324.47	16.80
0	-8	-7	325.37	17.70
0	8	7	334.77	17.90
0	8	-7	332.77	16.90
0	8	-7	315.47	16.00
0	8	7	319.87	17.30
0	8	-7	320.37	16.10
0	8	7	319.67	17.40
-1	-8	-7	787.62	37.90
1	8	7	781.52	38.30
1	8	7	716.43	38.70
-1	8	-7	812.42	37.50
-1	8	-7	737.33	37.40
1	8	7	815.02	38.50
-1	8	-7	747.33	37.70
-2	-8	-7	103.09	6.80
-2	8	-7	100.09	6.30
2	8	7	96.09	7.10
2	8	7	94.79	7.90
-2	8	-7	99.29	6.20
-2	8	-7	99.09	6.40
2	8	7	94.69	6.90
-2	8	-7	98.19	6.50
-3	-8	-7	148.69	8.80
3	-8	7	142.29	8.50
-3	-8	-7	130.09	9.70
-3	8	-7	143.19	7.80
-3	8	-7	132.99	7.80
-3	8	-7	139.69	8.50
3	8	7	145.09	9.10
3	8	7	130.99	8.80
-3	8	-7	146.19	8.60
-4	-8	-7	7.60	3.40
4	-8	7	8.50	3.00
-4	-8	-7	3.20	2.70
-4	8	-7	1.60	2.70
-4	8	-7	7.00	2.90
4	8	7	8.70	3.10
4	8	7	3.20	2.80
-4	8	-7	5.20	1.90
-5	-8	-7	145.09	11.10

-5	-8	-7	175.98	10.10
5	8	7	156.38	10.30
-5	8	-7	153.68	10.30
-5	8	-7	162.58	9.20
-5	8	-7	153.18	10.00
-6	-8	-7	107.59	9.90
-6	-8	-7	128.49	8.60
-6	8	-7	105.49	7.60
-6	8	-7	106.19	8.50
6	8	7	106.79	8.60
-6	8	-7	96.39	8.60
-7	-8	-7	283.47	15.80
-7	-8	-7	258.07	17.00
7	-8	7	276.87	13.90
-7	8	-7	260.67	16.30
-7	8	-7	269.07	15.10
-7	8	-7	248.68	15.60
7	8	7	260.57	15.70
-8	-8	-7	48.20	7.60
8	-8	7	44.10	5.00
8	8	7	46.10	7.30
-8	8	-7	51.49	6.70
-8	8	-7	45.20	7.00
9	-8	7	334.07	16.90
-9	-8	-7	306.97	20.40
-9	8	-7	307.07	18.60
9	8	7	298.37	18.80
-9	8	-7	312.17	18.60
10	-8	7	141.19	9.70
-10	-8	-7	141.79	14.20
-10	8	-7	121.39	12.30
10	8	7	133.69	11.60
-10	8	-7	142.69	12.00
-11	-8	-7	115.49	12.50
-11	-8	-7	105.19	14.80
11	-8	7	94.79	8.80
11	8	7	103.99	12.60
-11	8	-7	82.89	12.30
-11	8	-7	88.89	11.30
-12	-8	-7	94.99	12.90
-12	8	-7	54.49	9.70
-12	8	-7	88.79	10.70
-13	-8	-7	183.28	16.70
-13	8	-7	177.28	15.60

-14	-8	-7	3.00	8.70
-14	8	-7	10.30	7.90
-15	-8	-7	31.40	11.20
-15	8	-7	22.00	9.20
-14	9	7	6.90	7.00
-13	-9	7	548.95	29.60
13	9	-7	588.94	35.50
-13	9	7	578.04	31.10
-12	-9	7	1.10	4.20
12	-9	-7	8.10	7.20
-12	9	7	3.30	5.40
12	9	-7	15.70	8.70
-11	-9	7	404.56	21.70
11	-9	-7	432.46	25.60
-11	-9	7	334.77	28.60
-11	-9	7	455.65	22.70
-11	9	7	388.56	31.70
-11	9	7	436.36	23.50
11	9	-7	446.16	26.90
-10	-9	7	202.68	19.30
-10	-9	7	226.48	13.40
10	-9	-7	219.68	16.30
-10	-9	7	214.08	12.40
-10	9	7	229.48	19.40
10	9	-7	242.98	18.60
-9	-9	7	677.43	30.50
-9	-9	7	499.25	34.50
9	-9	-7	604.94	32.90
-9	-9	7	601.84	29.90
9	9	-7	631.84	34.10
-9	9	7	641.44	31.70
-9	9	7	585.94	34.60
-8	-9	7	1.10	2.50
-8	-9	7	-0.30	5.80
8	-9	-7	8.60	4.90
-8	9	7	-3.50	4.00
8	9	-7	1.80	5.40
-8	9	7	-5.90	5.70
-7	-9	7	762.92	36.40
-7	-9	7	671.53	40.00
-7	-9	7	727.93	36.30
7	-9	-7	759.42	39.10
7	-9	-7	732.83	38.90
-7	9	7	773.12	38.10

7	9	-7	851.51	40.20
-7	9	7	770.42	40.20
-6	-9	7	500.85	25.10
6	-9	-7	518.85	27.50
-6	-9	7	461.55	28.60
6	-9	-7	552.44	28.00
-6	-9	7	487.85	25.10
6	9	-7	572.64	28.90
-6	9	7	544.55	27.00
-6	9	7	555.04	28.90
5	-9	-7	479.45	24.90
5	-9	-7	482.95	25.30
-5	-9	7	426.46	25.80
5	9	-7	499.05	25.90
-5	9	7	462.25	25.70
-5	9	7	470.75	24.60
4	-9	-7	66.49	6.80
-4	-9	7	43.90	7.10
4	-9	-7	72.19	6.30
-4	9	7	49.50	5.90
4	9	-7	53.39	7.10
-4	9	7	59.99	7.00
-3	-9	7	313.77	18.60
3	-9	-7	339.47	17.90
3	-9	-7	347.97	18.60
-3	9	7	337.27	18.60
-3	9	7	321.27	18.20
3	9	-7	345.17	18.50
-2	-9	7	929.01	47.20
2	-9	-7	999.00	46.70
2	-9	-7	963.30	47.40
-2	9	7	945.91	47.30
-2	9	7	929.21	47.20
2	9	-7	941.61	47.00
1	-9	-7	239.78	13.90
-1	-9	7	244.98	13.40
1	-9	-7	237.28	12.80
1	9	-7	225.38	11.50
1	9	-7	226.58	11.70
-1	9	7	219.38	13.50
-1	9	7	238.88	13.70
1	9	-7	257.97	13.40
0	-9	7	3268.77	158.48
0	-9	-7	3385.76	159.38

0	-9	7	3234.98	157.78
0	-9	-7	3489.15	158.58
0	9	-7	3076.39	157.68
0	9	7	3443.46	159.58
0	9	-7	3333.57	158.58
0	9	7	3376.06	159.18
0	9	7	3310.27	159.08
1	-9	7	135.99	8.10
-1	-9	-7	139.19	8.60
1	9	7	162.88	9.70
-1	9	-7	121.09	8.50
1	9	7	144.69	9.40
-1	9	-7	157.88	8.80
1	9	7	154.08	10.20
2	-9	7	1091.69	52.09
-2	-9	-7	1144.09	52.39
-2	9	-7	1061.19	52.09
2	9	7	1060.79	53.49
2	9	7	1059.59	52.89
2	9	7	1045.50	52.59
-2	9	-7	1060.19	51.89
-3	-9	-7	141.69	9.90
-3	-9	-7	142.69	8.80
3	-9	7	141.49	8.60
3	9	7	128.19	9.10
3	9	7	133.09	9.00
-3	9	-7	135.29	8.10
-3	9	-7	139.69	8.50
-3	9	-7	142.79	8.10
3	9	7	140.49	10.20
-4	-9	-7	1232.88	58.29
-4	-9	-7	1140.79	59.19
4	-9	7	1220.08	58.29
4	9	7	1143.29	58.39
4	9	7	1237.28	59.49
-4	9	-7	1163.48	57.59
-4	9	-7	1227.78	58.19
-5	-9	-7	7.90	3.30
-5	-9	-7	6.10	3.80
5	-9	7	4.70	3.20
-5	9	-7	1.10	3.10
5	9	7	0.40	3.80
5	9	7	3.70	3.20
-5	9	-7	3.40	2.30

-6	-9	-7	1288.17	60.29
-6	-9	-7	1183.68	61.49
6	-9	7	1257.57	60.59
-6	9	-7	1190.68	60.09
-6	9	-7	1246.28	59.79
-6	9	-7	1230.08	60.59
6	9	7	1162.68	60.29
6	9	7	1263.67	61.29
-7	-9	-7	77.79	9.40
7	-9	7	71.99	5.40
-7	-9	-7	81.99	7.90
-7	9	-7	69.69	7.30
-7	9	-7	52.09	7.50
7	9	7	67.69	7.80
-7	9	-7	65.89	7.90
7	9	7	55.79	7.80
-8	-9	-7	240.28	17.20
8	-9	7	263.57	13.60
-8	9	-7	263.87	15.20
8	9	7	224.78	14.80
-8	9	-7	235.38	15.20
9	-9	7	38.40	6.90
-9	-9	-7	44.70	8.10
9	9	7	50.99	8.30
-9	9	-7	41.20	8.50
-9	9	-7	53.89	8.40
10	-9	7	771.32	36.60
-10	-9	-7	666.73	40.10
10	9	7	676.53	37.90
-10	9	-7	763.22	38.20
-10	9	-7	755.72	38.60
11	-9	7	116.79	9.60
-11	9	-7	92.89	11.50
-11	9	-7	80.39	12.40
11	9	7	109.49	11.50
-12	-9	-7	473.85	27.10
-12	9	-7	431.26	26.10
-12	9	-7	416.46	34.00
-13	-9	-7	34.70	8.90
-13	9	-7	31.60	8.10
-14	-9	-7	99.29	15.20
-14	9	-7	91.99	12.20
-14	10	7	5.40	7.30
-13	-10	7	40.40	9.40

13	10	-7	40.20	11.50
-13	10	7	79.99	11.30
-12	-10	7	-3.90	4.20
-12	10	7	6.40	6.00
12	10	-7	6.40	8.70
11	-10	-7	15.40	7.00
-11	-10	7	35.90	6.00
-11	10	7	22.50	6.30
11	10	-7	17.40	8.80
-10	-10	7	6.90	3.80
-10	-10	7	-3.50	9.30
-10	-10	7	2.80	3.10
10	-10	-7	8.50	5.80
10	10	-7	9.10	7.20
-10	10	7	6.80	5.40
-10	10	7	6.50	8.00
-9	-10	7	76.29	6.00
9	-10	-7	81.09	10.20
-9	-10	7	77.29	7.20
9	10	-7	90.19	12.30
-9	10	7	88.79	8.80
-9	10	7	66.59	12.50
-8	-10	7	30.40	4.00
8	-10	-7	41.80	8.20
-8	-10	7	36.50	7.90
-8	-10	7	26.40	4.20
-8	10	7	27.40	5.40
-8	10	7	34.90	7.30
8	10	-7	54.99	10.40
-7	-10	7	63.49	4.20
-7	-10	7	59.09	4.20
-7	-10	7	61.99	10.90
7	-10	-7	75.19	8.70
-7	10	7	55.99	9.70
-7	10	7	61.89	6.80
7	10	-7	74.49	10.30
-6	-10	7	17.90	5.70
6	-10	-7	25.20	5.10
6	-10	-7	13.90	4.70
-6	10	7	19.00	5.50
-6	10	7	22.50	4.80
6	10	-7	12.90	5.40
-5	-10	7	7.00	4.50
5	-10	-7	7.10	3.90

5	-10	-7	5.60	3.80
5	10	-7	9.60	5.00
-5	10	7	1.90	3.40
-5	10	7	13.80	4.60
4	-10	-7	29.60	5.80
4	-10	-7	14.90	4.00
-4	-10	7	24.80	5.30
4	10	-7	23.00	4.60
-4	10	7	14.50	4.30
-4	10	7	11.10	4.30
-3	-10	7	16.80	4.50
3	-10	-7	22.30	4.30
3	-10	-7	22.70	3.90
3	10	-7	16.60	4.00
-3	10	7	15.60	4.30
-3	10	7	25.60	4.70
2	-10	-7	5.90	2.60
2	-10	-7	6.60	3.40
-2	-10	7	5.00	3.10
-2	10	7	9.70	3.70
-2	10	7	2.20	3.50
2	10	-7	3.70	3.20
1	-10	-7	71.79	7.10
-1	-10	7	69.69	4.30
-1	-10	7	79.59	6.60
1	-10	-7	67.59	5.60
-1	10	7	58.29	6.40
-1	10	7	77.79	7.00
1	10	-7	61.09	4.00
1	10	-7	90.39	6.80
0	-10	7	116.49	6.70
0	-10	-7	122.59	7.60
0	-10	7	106.29	7.70
0	10	7	122.49	9.60
0	10	-7	128.39	8.10
0	10	7	113.49	8.40
0	10	7	105.69	8.30
0	10	-7	112.29	6.40
1	-10	7	2.60	2.00
-1	-10	-7	6.80	2.70
-1	10	-7	3.00	2.30
-1	10	-7	3.90	2.00
1	10	7	1.70	3.60
1	10	7	5.40	2.90

1	10	7	6.10	2.90
2	-10	7	73.39	5.60
-2	-10	-7	76.59	5.80
2	10	7	67.39	8.10
-2	10	-7	72.89	6.10
-2	10	-7	71.69	5.40
2	10	7	67.39	6.50
-2	10	-7	75.19	5.90
2	10	7	71.39	6.70
3	-10	7	121.49	7.80
-3	-10	-7	108.99	9.20
-3	-10	-7	112.59	7.90
-3	10	-7	116.49	7.40
-3	10	-7	111.99	7.50
3	10	7	108.69	8.00
-3	10	-7	119.89	7.50
3	10	7	108.79	8.40
3	10	7	131.09	9.90
-4	-10	-7	222.88	15.10
4	-10	7	247.48	13.80
-4	-10	-7	259.97	13.90
-4	10	-7	245.68	13.60
4	10	7	247.68	15.40
4	10	7	242.78	13.90
-4	10	-7	229.88	13.10
5	-10	7	69.69	7.00
-5	-10	-7	54.29	8.20
-5	-10	-7	71.59	6.60
-5	10	-7	66.89	6.60
5	10	7	83.39	7.20
-5	10	-7	69.19	5.90
5	10	7	62.79	8.50
6	-10	7	11.80	4.10
-6	-10	-7	11.40	3.90
-6	-10	-7	10.20	4.80
6	10	7	7.30	4.00
-6	10	-7	4.60	4.10
6	10	7	5.00	4.50
-6	10	-7	2.90	2.90
-7	-10	-7	2.20	3.80
7	-10	7	2.60	5.30
-7	-10	-7	8.80	5.00
-7	10	-7	6.40	4.30
7	10	7	7.10	4.10

-7	10	-7	4.20	3.60
7	10	7	15.20	5.10
-8	-10	-7	12.10	5.90
8	-10	7	12.10	3.10
-8	10	-7	10.10	5.00
8	10	7	11.70	5.40
8	10	7	5.60	4.50
-8	10	-7	12.90	4.50
9	-10	7	5.50	3.10
-9	-10	-7	10.90	6.80
-9	10	-7	-0.20	4.80
9	10	7	7.80	5.20
-9	10	-7	3.40	4.90
9	10	7	1.70	5.60
-10	-10	-7	18.60	7.90
10	-10	7	2.70	3.90
-10	10	-7	8.70	6.10
-10	10	-7	5.50	5.30
10	10	7	12.10	5.70
10	10	7	6.50	6.30
11	-10	7	21.80	4.70
-11	10	-7	19.30	6.20
11	10	7	15.60	6.40
-11	10	-7	19.80	6.90
-12	-10	-7	9.10	7.70
-12	10	-7	9.70	7.10
-13	-10	-7	0.90	8.40
-13	10	-7	2.90	7.20
-14	-10	-7	3.50	9.30
-14	10	-7	-1.20	7.90
-14	11	7	31.30	7.90
-13	11	7	398.66	24.00
13	11	-7	414.66	29.30
-12	-11	7	18.70	4.50
-12	11	7	21.10	6.90
12	11	-7	23.30	9.60
-11	-11	7	848.12	42.70
11	-11	-7	867.81	46.30
-11	11	7	871.91	44.50
11	11	-7	896.01	48.00
10	-11	-7	60.29	10.70
-10	-11	7	40.90	6.00
-10	-11	7	5.40	15.50
-10	-11	7	35.20	5.30

10	11	-7	25.80	8.30
-10	11	7	26.50	9.20
-10	11	7	53.69	8.80
-9	-11	7	228.68	12.10
9	-11	-7	230.78	16.10
-9	11	7	195.38	18.00
9	11	-7	235.38	17.60
8	-11	-7	267.67	16.60
-8	-11	7	235.58	12.60
-8	-11	7	245.08	12.70
-8	-11	7	221.78	18.60
8	11	-7	238.38	17.70
-8	11	7	257.17	15.00
-8	11	7	252.17	18.40
-7	-11	7	608.14	37.20
-7	-11	7	678.23	32.80
7	-11	-7	702.93	36.10
-7	-11	7	663.43	32.80
-7	11	7	694.63	37.20
-7	11	7	709.53	35.10
7	11	-7	729.83	37.00
-6	-11	7	902.51	52.09
6	-11	-7	1046.50	51.49
6	11	-7	1091.09	52.19
-6	11	7	1029.10	50.59
-6	11	7	1015.00	52.09
5	-11	-7	745.13	38.40
5	-11	-7	761.72	38.10
-5	-11	7	668.63	38.90
-5	11	7	760.62	37.70
5	11	-7	781.42	38.90
-5	11	7	741.53	38.80
-4	-11	7	243.78	16.70
4	-11	-7	276.97	16.20
4	-11	-7	273.37	15.40
-4	11	7	263.87	16.40
-4	11	7	271.47	15.80
4	11	-7	275.77	16.50
-3	-11	7	437.56	25.50
3	-11	-7	503.75	24.80
3	-11	-7	482.75	25.50
3	11	-7	484.25	25.40
-3	11	7	454.35	25.10
-3	11	7	475.95	25.60

2	-11	-7	765.82	36.50
-2	-11	7	736.33	34.90
2	-11	-7	746.13	37.40
-2	-11	7	693.33	37.10
-2	11	7	715.03	37.10
2	11	-7	690.63	34.80
-2	11	7	736.13	37.30
2	11	-7	760.22	37.10
1	-11	-7	426.86	23.20
-1	-11	7	436.76	20.90
1	-11	-7	440.56	22.10
-1	-11	7	411.56	22.60
-1	11	7	440.56	23.10
-1	11	7	407.36	22.70
1	11	-7	436.46	22.80
1	11	-7	422.66	20.80
0	-11	7	1896.91	89.39
0	-11	7	1760.92	90.29
0	-11	-7	1981.20	90.19
0	11	7	1826.82	92.79
0	11	7	1935.91	91.09
0	11	-7	1896.71	90.59
0	11	-7	1815.52	89.19
0	11	7	1869.21	90.79
1	-11	7	332.27	17.00
-1	-11	-7	335.67	17.40
1	11	7	342.37	18.40
1	11	7	340.27	19.20
1	11	7	313.87	18.00
-1	11	-7	307.87	16.50
-1	11	-7	319.07	17.60
2	-11	7	497.65	24.80
-2	-11	-7	519.95	25.20
2	11	7	493.95	25.60
-2	11	-7	508.05	24.90
-2	11	-7	477.55	24.60
2	11	7	465.05	26.60
-2	11	-7	473.65	25.10
2	11	7	499.45	25.90
-3	-11	-7	7.20	3.10
3	-11	7	3.10	3.00
-3	11	-7	3.40	2.50
3	11	7	3.60	3.40
-3	11	-7	1.80	2.40

-3	11	-7	0.40	2.60
3	11	7	3.20	3.50
3	11	7	0.30	4.10
-4	-11	-7	1018.50	46.60
-4	-11	-7	929.81	47.90
4	-11	7	950.80	46.50
-4	11	-7	919.21	46.20
-4	11	-7	894.71	46.10
4	11	7	934.01	46.80
4	11	7	909.41	47.00
-4	11	-7	927.81	46.00
4	11	7	986.10	48.20
-5	-11	-7	9.40	3.40
-5	-11	-7	-2.20	4.30
5	-11	7	4.50	3.80
-5	11	-7	2.40	3.50
-5	11	-7	7.10	2.80
5	11	7	6.00	3.80
5	11	7	3.00	4.40
-6	-11	-7	1881.61	99.39
-6	-11	-7	2096.89	98.09
6	-11	7	1987.00	98.39
-6	11	-7	2033.60	97.69
6	11	7	2004.10	98.19
-6	11	-7	2040.80	97.99
6	11	7	2139.39	99.59
-7	-11	-7	5.30	5.80
-7	11	-7	4.80	4.30
-7	11	-7	7.00	3.90
7	11	7	2.00	5.00
7	11	7	10.70	4.40
-8	-11	-7	336.37	22.50
8	-11	7	379.46	18.90
-8	11	-7	363.96	20.50
-8	11	-7	345.17	20.40
8	11	7	336.77	20.40
8	11	7	373.16	22.20
-9	-11	-7	19.20	7.40
9	-11	7	35.70	5.80
9	11	7	18.30	6.90
-9	11	-7	26.70	6.30
9	11	7	34.20	6.60
10	-11	7	575.34	28.70
-10	-11	-7	504.85	33.20

-10	11	-7	544.95	30.90
-10	11	-7	581.14	30.30
10	11	7	576.34	32.30
-11	11	-7	165.48	15.30
11	11	7	169.48	14.10
-11	11	-7	184.58	13.90
-12	-11	-7	357.26	23.50
-12	11	-7	337.47	21.50
-13	-11	-7	99.59	14.80
-13	11	-7	67.19	12.60
-14	-11	-7	54.39	11.30
-14	11	-7	36.30	9.40
-14	12	7	98.79	13.60
-13	12	7	14.90	7.50
13	12	-7	4.00	10.50
-12	12	7	189.38	14.40
12	12	-7	199.78	20.50
-11	-12	7	7.00	3.60
11	-12	-7	13.60	6.60
-11	12	7	20.80	6.40
11	12	-7	21.00	9.80
10	-12	-7	256.67	18.00
-10	-12	7	233.78	13.20
-10	12	7	236.98	15.60
10	12	-7	259.07	19.80
9	-12	-7	25.10	6.60
-9	-12	7	23.70	3.80
-9	-12	7	23.50	4.30
-9	12	7	22.70	8.50
-9	12	7	37.60	6.40
9	12	-7	15.30	7.30
-8	-12	7	151.88	8.60
-8	-12	7	144.09	15.80
8	-12	-7	167.98	13.30
-8	-12	7	155.08	8.60
-8	12	7	169.48	12.10
-8	12	7	163.48	14.90
8	12	-7	182.78	15.00
-7	-12	7	-2.30	6.20
7	-12	-7	11.40	5.10
-7	-12	7	8.20	1.60
-7	-12	7	10.00	1.70
-7	12	7	14.60	5.00
-7	12	7	6.00	6.00

7	12	-7	17.30	6.40
-6	-12	7	318.87	21.30
6	-12	-7	340.17	20.20
6	12	-7	370.96	21.10
-6	12	7	330.47	20.80
-6	12	7	328.97	18.80
-5	-12	7	371.86	24.10
5	-12	-7	424.66	23.50
-5	12	7	418.56	24.00
5	12	-7	444.56	24.10
-5	12	7	425.56	22.60
4	-12	-7	946.71	47.80
-4	-12	7	909.81	48.80
4	-12	-7	946.51	48.30
-4	12	7	930.81	47.80
-4	12	7	967.80	48.70
4	12	-7	1037.60	48.70
3	-12	-7	253.77	13.90
3	-12	-7	229.68	14.70
-3	-12	7	212.58	14.60
3	12	-7	240.68	14.60
-3	12	7	237.18	14.80
-3	12	7	213.08	14.20
-2	-12	7	124.09	9.30
2	-12	-7	124.59	8.40
-2	-12	7	110.69	5.90
2	-12	-7	108.59	9.50
2	12	-7	120.79	9.20
-2	12	7	87.89	8.70
-2	12	7	111.69	9.40
2	12	-7	98.89	5.70
-1	-12	7	356.66	18.30
1	-12	-7	383.26	19.70
1	-12	-7	378.26	20.80
-1	-12	7	369.06	20.20
-1	12	7	374.26	20.70
1	12	-7	348.67	18.10
-1	12	7	371.26	21.20
1	12	-7	393.56	20.40
-1	12	7	361.96	20.50
0	-12	7	242.28	12.80
0	-12	-7	245.68	13.50
0	12	-7	229.08	13.20
0	12	7	243.98	14.90

0	12	7	236.88	14.50
0	12	7	250.57	16.00
0	12	-7	255.47	14.40
-1	-12	-7	306.07	16.40
1	-12	7	303.67	15.80
-1	12	-7	309.57	16.90
1	12	7	308.67	18.50
1	12	7	296.77	17.50
1	12	7	306.27	17.20
-1	12	-7	290.77	15.50
-2	-12	-7	160.98	10.00
2	-12	7	164.08	9.60
2	12	7	168.88	10.70
-2	12	-7	157.88	9.70
-2	12	-7	141.69	9.10
2	12	7	165.18	11.10
-2	12	-7	147.09	10.00
2	12	7	154.88	12.60
3	-12	7	947.91	46.70
-3	-12	-7	985.70	46.80
3	12	7	931.71	47.20
-3	12	-7	936.61	46.80
3	12	7	1037.20	48.90
-3	12	-7	933.41	46.80
3	12	7	929.21	47.50
-3	12	-7	895.51	46.30
4	-12	7	28.40	4.50
-4	-12	-7	37.90	5.50
-4	-12	-7	31.30	6.30
-4	12	-7	25.30	5.00
4	12	7	28.60	6.00
4	12	7	29.80	4.80
4	12	7	31.00	6.10
-4	12	-7	24.50	3.90
-4	12	-7	28.90	5.20
-5	-12	-7	312.47	17.10
5	-12	7	305.97	17.70
-5	-12	-7	312.17	19.20
5	12	7	345.77	19.60
-5	12	-7	312.87	17.10
-5	12	-7	293.47	16.70
5	12	7	275.27	17.30
6	-12	7	0.30	4.60
-6	-12	-7	8.60	5.30

-6	-12	-7	10.70	4.00
6	12	7	7.00	4.20
-6	12	-7	0.50	4.00
6	12	7	6.40	5.40
-6	12	-7	5.30	3.30
-7	-12	-7	795.32	43.50
7	12	7	891.01	43.60
-7	12	-7	791.22	41.40
7	12	7	782.22	41.60
-7	12	-7	837.22	41.40
-8	-12	-7	50.19	8.70
-8	12	-7	40.00	8.30
8	12	7	59.09	10.70
-8	12	-7	42.60	8.10
8	12	7	40.60	6.90
-9	-12	-7	244.38	19.90
9	-12	7	277.77	15.20
-9	12	-7	267.37	16.90
9	12	7	278.27	19.40
-9	12	-7	269.17	17.40
9	12	7	239.98	16.70
10	-12	7	101.19	8.80
-10	12	-7	110.39	11.20
10	12	7	81.89	11.20
10	12	7	117.39	14.10
-10	12	-7	87.19	11.70
-11	12	-7	158.28	15.30
11	12	7	152.78	13.80
-11	12	-7	179.98	14.40
-12	12	-7	6.40	7.10
12	12	7	3.10	6.90
-13	-12	-7	122.09	16.40
-13	12	-7	86.69	13.10
-14	-12	-7	83.59	13.30
-14	12	-7	86.39	13.70
13	13	-7	27.20	11.60
-12	13	7	21.90	7.00
12	13	-7	20.10	11.20
-11	13	7	389.06	22.20
11	13	-7	339.37	26.20
10	-13	-7	20.60	7.20
-10	-13	7	31.30	5.00
10	13	-7	28.40	9.00
-10	13	7	30.60	6.70

-9	-13	7	237.58	12.60
9	-13	-7	246.68	17.50
9	13	-7	238.98	19.00
-9	13	7	254.77	16.10
-9	13	7	234.08	20.10
8	-13	-7	38.20	7.20
-8	-13	7	24.80	3.30
-8	-13	7	25.00	3.40
8	13	-7	24.20	7.60
-8	13	7	23.10	7.60
-8	13	7	26.20	6.20
-7	-13	7	135.89	15.20
-7	-13	7	159.48	8.10
7	-13	-7	156.18	12.80
-7	-13	7	163.68	8.10
7	13	-7	159.38	14.60
-7	13	7	162.98	11.90
-7	13	7	167.28	14.60
-6	-13	7	98.49	13.00
6	-13	-7	111.29	10.60
-6	13	7	103.19	9.70
6	13	-7	108.69	11.50
-6	13	7	101.49	11.50
-5	-13	7	405.26	26.60
5	-13	-7	471.15	25.80
5	13	-7	455.65	26.10
-5	13	7	466.85	26.30
-5	13	7	512.85	25.30
-4	-13	7	23.60	5.90
4	-13	-7	38.60	7.60
-4	13	7	19.80	5.20
4	13	-7	19.80	5.50
-4	13	7	30.00	5.80
3	-13	-7	51.09	7.60
-3	-13	7	50.89	3.10
3	-13	-7	55.99	6.80
-3	-13	7	43.10	7.90
3	13	-7	47.90	2.90
-3	13	7	52.39	8.10
3	13	-7	38.50	7.60
-3	13	7	38.90	7.30
-2	-13	7	312.57	19.10
2	-13	-7	339.57	18.20
-2	-13	7	335.47	16.40

2	-13	-7	354.96	19.60
2	13	-7	311.77	16.20
2	13	-7	344.27	19.20
-2	13	7	316.17	18.70
-2	13	7	347.57	19.50
-1	-13	7	4.90	3.30
-1	-13	7	5.80	2.00
1	-13	-7	2.70	3.20
-1	13	7	1.00	4.80
1	13	-7	2.60	1.70
-1	13	7	4.60	4.00
-1	13	7	2.30	3.90
1	13	-7	2.80	3.70
0	-13	-7	240.18	13.20
0	13	7	221.38	15.20
0	13	7	233.48	14.20
0	13	7	242.88	14.60
0	13	-7	223.28	11.90
0	13	-7	204.78	13.70
-1	-13	-7	151.68	9.30
1	-13	7	146.49	8.90
1	13	7	151.28	12.20
1	13	7	155.78	11.10
1	13	7	146.29	10.60
-1	13	-7	130.69	10.00
-1	13	-7	136.89	8.30
-2	-13	-7	336.17	17.70
2	-13	7	347.57	17.60
2	13	7	321.07	18.40
-2	13	-7	322.77	18.20
2	13	7	322.07	18.80
-2	13	-7	318.27	17.10
2	13	7	351.16	20.60
-2	13	-7	320.37	17.70
-3	-13	-7	101.49	7.60
3	-13	7	94.19	7.60
3	13	7	90.49	8.50
-3	13	-7	87.89	6.90
3	13	7	90.49	8.20
-3	13	-7	80.59	10.40
-3	13	-7	75.69	7.40
3	13	7	87.59	10.80
-4	-13	-7	118.29	11.60
-4	-13	-7	138.49	9.40

4	-13	7	144.59	9.90
4	13	7	117.09	13.80
-4	13	-7	138.59	9.10
4	13	7	123.29	9.70
-4	13	-7	123.89	9.10
-4	13	-7	131.59	9.20
4	13	7	122.49	10.00
-5	-13	-7	92.09	7.70
5	-13	7	75.89	8.40
-5	-13	-7	81.69	10.30
-5	13	-7	76.79	7.80
-5	13	-7	70.69	7.30
-5	13	-7	83.59	7.30
5	13	7	82.19	8.30
5	13	7	85.49	11.20
6	-13	7	164.38	13.10
-6	-13	-7	150.38	13.90
6	13	7	154.08	13.80
-6	13	-7	163.38	11.30
-6	13	-7	147.99	10.70
6	13	7	154.68	11.50
-7	-13	-7	44.00	8.40
-7	13	-7	43.90	7.70
7	13	7	34.00	7.60
7	13	7	43.70	7.80
-7	13	-7	48.50	7.70
-8	-13	-7	32.30	8.00
8	13	7	43.60	6.90
8	13	7	38.90	8.00
-8	13	-7	44.00	8.10
-8	13	-7	43.30	6.80
-9	-13	-7	73.29	11.80
-9	13	-7	95.59	9.40
9	13	7	68.89	12.90
-9	13	-7	77.49	10.70
9	13	7	90.69	10.20
10	-13	7	153.08	10.70
-10	13	-7	137.69	13.70
10	13	7	139.69	15.80
-10	13	-7	165.18	13.10
10	13	7	131.29	12.80
-11	13	-7	94.19	12.10
11	13	7	88.39	11.80
12	13	7	107.99	13.80

-12	13	-7	128.29	13.70
-13	-13	-7	5.90	9.70
-13	13	-7	8.10	7.60
-13	14	7	5.40	7.40
-12	14	7	582.54	31.60
12	14	-7	537.75	38.30
11	14	-7	42.00	11.00
-11	14	7	68.39	10.90
10	-14	-7	683.83	37.90
-10	-14	7	696.63	33.90
-10	14	7	695.23	36.50
10	14	-7	673.63	39.70
-9	-14	7	15.10	3.20
9	-14	-7	13.20	6.30
-9	14	7	4.00	5.30
-9	14	7	-0.60	9.90
9	14	-7	15.80	8.90
8	-14	-7	546.65	30.80
-8	-14	7	531.65	26.50
-8	-14	7	536.25	26.60
8	14	-7	605.64	32.50
-8	14	7	543.95	29.50
-8	14	7	542.05	32.50
7	-14	-7	184.38	14.50
7	14	-7	196.88	15.90
-7	14	7	187.18	16.20
6	-14	-7	1085.69	55.89
-6	-14	7	1003.70	57.99
-6	14	7	1071.59	56.49
6	14	-7	1194.98	56.79
-6	14	7	1140.89	54.99
-5	-14	7	550.54	35.20
5	-14	-7	656.03	34.70
-5	14	7	666.43	35.30
5	14	-7	696.43	35.20
-5	14	7	671.23	33.80
4	-14	-7	404.76	23.20
-4	-14	7	351.26	23.80
-4	14	7	413.46	23.60
4	14	-7	430.16	23.70
-4	14	7	435.16	22.90
-3	-14	7	154.58	12.70
3	-14	-7	156.98	11.90
-3	-14	7	152.48	8.00

3	14	-7	146.99	7.90
3	14	-7	161.98	12.20
-3	14	7	140.69	11.10
-3	14	7	169.78	12.40
2	-14	-7	695.43	33.60
2	-14	-7	679.93	34.70
-2	-14	7	587.44	34.20
-2	-14	7	655.63	31.70
2	14	-7	688.63	34.50
2	14	-7	616.74	31.50
-2	14	7	682.93	34.70
-2	14	7	640.74	34.00
-1	-14	7	1265.37	70.69
-1	-14	7	1449.95	69.09
1	-14	-7	1543.75	70.39
-1	14	7	1472.05	71.39
1	14	-7	1448.16	71.09
-1	14	7	1447.66	71.09
-1	14	7	1467.65	72.09
0	-14	-7	327.87	17.10
0	-14	7	306.57	15.90
0	14	7	292.37	17.80
0	14	-7	310.67	17.80
0	14	-7	298.07	15.50
0	14	7	295.47	18.20
0	14	7	303.07	19.10
-1	-14	-7	1244.08	59.19
1	-14	7	1211.18	58.59
-1	14	-7	1205.68	59.79
1	14	7	1237.68	59.89
1	14	7	1187.48	60.19
1	14	7	1211.28	61.19
-1	14	-7	1162.58	58.19
2	-14	7	222.58	13.10
-2	-14	-7	235.28	13.30
2	14	7	236.48	14.30
-2	14	-7	219.18	12.50
2	14	7	233.18	16.00
-2	14	-7	221.28	13.30
-2	14	-7	219.18	13.80
2	14	7	235.58	14.60
3	-14	7	533.75	27.50
-3	-14	-7	549.45	27.50
3	14	7	538.25	29.80

3	14	7	521.95	28.40
-3	14	-7	503.35	26.80
3	14	7	552.24	28.10
-3	14	-7	523.05	27.80
-4	-14	-7	8.70	6.10
-4	-14	-7	10.10	3.70
4	-14	7	9.80	4.60
-4	14	-7	20.30	4.30
4	14	7	13.40	4.70
4	14	7	14.90	4.40
4	14	7	15.80	6.80
-4	14	-7	14.80	4.00
-4	14	-7	8.90	3.80
-5	-14	-7	1037.60	55.99
5	-14	7	1078.59	54.49
5	14	7	1065.89	54.39
5	14	7	1178.28	56.59
-5	14	-7	1115.29	54.19
-5	14	-7	1054.49	53.99
-5	14	-7	1131.69	53.99
-6	-14	-7	66.09	10.60
-6	14	-7	26.10	5.30
6	14	7	30.20	11.70
6	14	7	30.80	5.70
-6	14	-7	38.90	5.50
-7	-14	-7	687.93	40.10
7	14	7	722.43	38.00
7	14	7	847.62	46.80
-7	14	-7	712.03	37.80
-7	14	-7	728.23	37.60
-8	-14	-7	24.10	8.60
-8	14	-7	19.00	5.80
8	14	7	15.20	8.00
8	14	7	16.30	5.90
-8	14	-7	22.00	9.20
-9	14	-7	360.26	21.00
9	14	7	360.86	24.30
-9	14	-7	330.87	21.40
-10	14	-7	111.29	13.30
10	14	7	115.79	12.00
-10	14	-7	112.09	12.10
-11	14	-7	634.84	32.80
11	14	7	542.55	32.40
-12	14	-7	162.68	14.70

12	14	7	131.09	14.50
-13	-14	-7	179.48	18.80
-13	14	-7	157.18	15.70
-13	15	7	9.40	7.90
12	15	-7	27.20	12.20
-12	15	7	18.70	7.20
11	15	-7	34.80	14.50
10	15	-7	17.70	9.30
-10	15	7	14.90	6.40
-9	-15	7	66.39	5.20
9	-15	-7	81.79	12.40
-9	15	7	74.99	10.20
9	15	-7	77.19	14.30
-8	-15	7	4.10	1.90
8	-15	-7	3.20	6.10
-8	-15	7	3.40	1.60
-8	15	7	4.30	5.10
8	15	-7	6.00	7.50
-8	15	7	-0.20	7.40
7	-15	-7	6.00	5.60
7	15	-7	21.20	7.40
-7	15	7	10.50	6.90
-7	15	7	6.70	5.50
6	-15	-7	22.80	6.10
6	15	-7	15.60	6.80
-6	15	7	18.50	5.90
-6	15	7	16.50	6.40
5	-15	-7	44.10	8.80
5	15	-7	52.29	10.00
-5	15	7	45.10	6.80
-5	15	7	35.30	7.30
4	-15	-7	102.09	10.60
-4	-15	7	95.99	5.40
-4	15	7	103.79	10.90
-4	15	7	93.59	9.30
4	15	-7	99.99	11.00
-3	-15	7	2.30	1.80
-3	-15	7	0.40	5.00
3	-15	-7	7.50	4.80
-3	15	7	8.60	5.30
3	15	-7	5.80	4.70
3	15	-7	3.60	1.60
-3	15	7	-3.50	4.50
-2	-15	7	14.00	2.50

-2	-15	7	15.40	5.20
2	15	-7	19.00	5.20
-2	15	7	19.70	6.40
-2	15	7	19.30	5.30
-2	15	7	13.90	4.90
2	15	-7	14.60	2.20
-1	-15	7	8.90	3.00
-1	-15	7	5.30	3.90
-1	15	7	-0.80	5.10
-1	15	7	2.80	5.10
1	15	-7	6.80	2.30
-1	15	7	6.50	5.00
1	15	-7	13.80	4.80
0	-15	-7	14.80	4.00
0	-15	7	4.40	2.80
0	15	7	8.70	5.60
0	15	-7	1.60	2.40
0	15	7	6.50	4.60
0	15	-7	9.80	4.30
0	15	7	7.70	4.70
1	-15	7	-0.40	3.00
-1	-15	-7	5.50	3.50
-1	15	-7	2.20	3.30
1	15	7	0.90	4.20
-1	15	-7	3.80	2.60
-1	15	-7	-0.20	4.00
1	15	7	-5.90	5.00
1	15	7	0.40	4.50
-2	-15	-7	39.80	5.90
2	-15	7	37.80	6.00
2	15	7	33.20	6.10
2	15	7	26.60	5.30
-2	15	-7	30.70	4.90
-2	15	-7	34.30	5.60
2	15	7	35.50	7.00
-2	15	-7	38.90	6.10
3	-15	7	1.10	3.90
-3	-15	-7	7.20	3.80
3	15	7	5.30	4.10
3	15	7	5.40	4.30
3	15	7	1.50	6.10
-3	15	-7	7.50	4.00
-3	15	-7	1.80	3.80
-3	15	-7	6.20	3.30

4	-15	7	22.20	4.80
-4	15	-7	6.80	4.00
-4	15	-7	7.20	3.80
4	15	7	3.40	4.30
4	15	7	4.90	5.80
-4	15	-7	7.50	3.70
4	15	7	6.80	4.70
-5	-15	-7	4.80	6.00
5	-15	7	-6.10	4.50
5	15	7	3.60	4.60
5	15	7	3.00	6.10
-5	15	-7	2.00	4.00
-5	15	-7	7.50	3.90
-5	15	-7	3.90	3.80
5	15	7	8.00	4.60
-6	-15	-7	3.30	6.20
-6	15	-7	12.60	4.60
-6	15	-7	12.20	4.30
6	15	7	14.10	5.00
6	15	7	11.10	7.30
-6	15	-7	8.50	4.60
-7	-15	-7	6.80	7.30
-7	15	-7	7.70	5.00
7	15	7	10.00	7.60
-7	15	-7	10.80	5.10
7	15	7	15.60	5.40
-8	-15	-7	3.70	7.60
8	15	7	15.00	5.80
8	15	7	4.50	8.50
-8	15	-7	18.70	6.00
-8	15	-7	9.10	5.30
-9	15	-7	10.80	6.10
9	15	7	-6.40	8.50
-9	15	-7	-0.10	5.70
9	15	7	0.50	5.70
-10	15	-7	14.90	7.00
10	15	7	19.90	6.50
-11	15	-7	21.80	7.60
11	15	7	13.40	6.90
-12	15	-7	27.40	7.90
12	15	7	8.20	7.60
-13	-15	-7	15.70	11.10
-13	15	-7	10.00	8.50
12	16	-7	303.37	26.30

-12	16	7	284.47	20.00
-11	16	7	35.40	8.10
11	16	-7	23.90	11.60
-10	16	7	250.67	17.00
9	-16	-7	31.90	7.50
-9	16	7	30.70	7.60
9	16	-7	31.20	10.70
8	-16	-7	295.47	20.60
-8	16	7	330.97	19.40
8	16	-7	294.67	22.20
7	-16	-7	134.69	13.20
-7	16	7	113.99	14.50
-7	16	7	128.29	11.70
7	16	-7	128.69	14.20
6	-16	-7	486.15	28.20
-6	16	7	465.75	28.90
-6	16	7	506.65	27.30
6	16	-7	513.55	29.20
5	-16	-7	135.69	12.40
-5	16	7	113.09	10.90
-5	16	7	116.79	12.50
5	16	-7	122.39	12.90
4	-16	-7	591.84	30.80
-4	-16	7	530.55	26.50
4	16	-7	498.25	26.40
-4	16	7	572.84	29.80
4	16	-7	562.64	30.70
-4	16	7	559.14	30.70
3	-16	-7	76.59	9.70
-3	-16	7	80.89	5.30
3	16	-7	74.99	4.90
-3	16	7	79.79	9.50
3	16	-7	77.89	10.30
-3	16	7	86.09	10.50
-2	-16	7	382.86	19.60
-2	16	7	397.96	23.20
2	16	-7	382.86	22.90
-2	16	7	386.46	22.20
-2	16	7	411.56	24.20
-1	-16	7	536.75	26.60
-1	16	7	537.85	30.20
-1	16	7	560.34	29.60
1	16	-7	538.25	29.20
-1	16	7	519.45	28.60

1	16	-7	516.65	26.30
0	-16	7	345.97	18.10
0	16	7	341.97	21.90
0	16	-7	344.07	17.60
0	16	-7	335.37	20.20
0	16	7	335.47	20.10
0	16	7	358.26	20.80
1	-16	7	1136.49	54.59
-1	16	-7	1068.99	55.39
1	16	7	1117.89	57.49
-1	16	-7	1093.59	56.09
-1	16	-7	1109.49	54.09
1	16	7	1165.78	55.99
1	16	7	1137.29	56.59
2	-16	7	149.69	10.40
-2	16	-7	153.48	9.50
-2	16	-7	154.08	11.60
-2	16	-7	149.19	10.70
2	16	7	152.38	11.30
2	16	7	156.78	13.60
2	16	7	144.39	12.00
3	-16	7	247.48	14.20
-3	16	-7	220.98	14.00
3	16	7	218.18	16.90
3	16	7	234.18	14.70
-3	16	-7	219.88	14.60
3	16	7	221.28	15.10
-3	16	-7	226.68	13.10
4	-16	7	8.60	4.80
4	16	7	-2.40	4.20
4	16	7	-5.40	6.40
-4	16	-7	0.70	4.00
-4	16	-7	7.00	3.80
4	16	7	3.60	5.10
-4	16	-7	2.70	4.40
-5	-16	-7	1493.75	77.59
-5	16	-7	1500.55	75.49
-5	16	-7	1587.34	75.09
5	16	7	1538.85	75.79
5	16	7	1689.43	78.29
5	16	7	1482.35	76.29
-5	16	-7	1497.15	75.99
-6	-16	-7	10.80	7.20
6	16	7	5.10	6.90

-6	16	-7	2.90	4.80
-6	16	-7	8.20	4.70
-6	16	-7	10.00	4.80
6	16	7	14.10	5.20
-7	-16	-7	291.37	22.10
7	16	7	304.67	18.70
-7	16	-7	276.47	18.60
-7	16	-7	311.57	18.30
7	16	7	342.37	22.20
-8	16	-7	6.80	5.70
8	16	7	-6.10	7.00
-8	16	-7	-1.80	5.10
-9	16	-7	299.27	20.90
9	16	7	353.86	26.10
-9	16	-7	345.67	20.30
9	16	7	314.17	20.30
10	16	7	29.70	7.40
-10	16	-7	42.70	8.00
-11	16	-7	339.07	21.20
11	16	7	298.47	21.10
12	16	7	45.00	9.40
-12	16	-7	48.50	9.60
-12	17	7	16.20	8.30
12	17	-7	-4.40	11.80
-11	17	7	144.09	14.60
11	17	-7	166.08	20.70
-10	17	7	5.00	6.70
10	17	-7	0.10	9.80
-9	17	7	98.89	11.90
9	17	-7	92.59	16.30
8	-17	-7	123.69	14.10
-8	17	7	117.19	12.20
7	-17	-7	129.79	13.80
-7	17	7	139.09	12.00
-7	17	7	134.89	16.90
7	17	-7	161.28	15.90
6	-17	-7	13.90	6.20
-6	17	7	20.70	7.30
6	17	-7	14.40	7.10
-6	17	7	8.90	5.90
-5	-17	7	167.78	9.00
5	-17	-7	180.38	14.90
5	17	-7	199.18	15.80
-5	17	7	171.88	13.60

-5	17	7	181.68	15.60
-4	-17	7	45.50	4.10
4	-17	-7	51.19	9.90
-4	17	7	49.10	8.30
4	17	-7	68.99	10.90
4	17	-7	46.10	3.80
-4	17	7	57.79	10.50
-3	-17	7	188.78	10.60
3	17	-7	183.48	10.40
3	17	-7	208.78	15.20
-3	17	7	195.98	14.40
-3	17	7	207.98	15.50
-3	17	7	230.88	17.10
2	17	-7	116.19	11.40
2	17	-7	102.69	6.70
-2	17	7	113.99	12.40
-2	17	7	91.19	9.70
-2	17	7	104.99	11.40
-1	-17	7	88.09	6.70
-1	17	7	91.29	11.90
-1	17	7	81.59	10.60
1	17	-7	82.79	6.10
1	17	-7	89.79	10.20
-1	17	7	86.39	9.90
0	-17	7	108.09	8.40
0	17	-7	111.19	7.50
0	17	7	119.69	11.40
0	17	-7	119.79	11.00
0	17	7	114.69	12.70
0	17	7	118.09	10.90
1	-17	7	421.46	22.50
1	17	7	468.95	26.60
-1	17	-7	395.66	23.30
1	17	7	435.96	24.80
-1	17	-7	427.06	24.30
1	17	7	429.76	23.90
-1	17	-7	428.66	21.90
2	-17	7	195.38	12.50
-2	17	-7	211.98	14.10
-2	17	-7	181.88	12.70
2	17	7	181.88	13.40
-2	17	-7	192.18	11.40
2	17	7	177.68	13.90
2	17	7	210.68	16.10

3	-17	7	10.80	4.90
-3	17	-7	9.20	4.30
3	17	7	17.80	7.30
-3	17	-7	17.70	4.00
3	17	7	19.30	5.50
-3	17	-7	18.40	7.30
3	17	7	19.40	5.70
4	-17	7	209.78	14.50
-4	17	-7	236.58	15.10
-4	17	-7	202.78	14.60
4	17	7	224.28	15.40
4	17	7	225.18	14.80
4	17	7	262.67	17.90
-4	17	-7	208.28	13.50
-5	-17	-7	168.28	15.70
5	17	7	142.29	13.00
-5	17	-7	167.28	11.70
-5	17	-7	156.48	12.30
5	17	7	164.68	12.50
-5	17	-7	168.98	12.70
5	17	7	179.88	16.10
-6	-17	-7	136.69	15.90
-6	17	-7	148.89	11.50
6	17	7	163.08	15.90
-6	17	-7	144.49	12.00
-6	17	-7	164.78	12.60
6	17	7	154.58	12.40
-7	-17	-7	-1.60	8.80
-7	17	-7	-3.40	5.10
7	17	7	-7.60	7.00
-7	17	-7	-1.40	6.00
7	17	7	-4.20	6.00
-7	17	-7	-1.60	5.10
8	17	7	452.55	28.40
-8	17	-7	400.26	24.90
-8	17	-7	459.15	24.40
8	17	7	399.66	24.50
9	17	7	-0.50	6.00
-9	17	-7	5.80	6.30
10	17	7	158.18	15.10
-10	17	-7	192.38	15.10
11	17	7	-2.20	7.30
-11	17	-7	16.70	7.20
-12	17	-7	142.89	15.30

12	17	7	144.79	15.70
-11	18	7	11.60	7.30
11	18	-7	15.20	11.60
-10	18	7	219.98	16.70
10	18	-7	189.78	22.00
-9	18	7	14.40	6.90
9	18	-7	15.00	10.20
8	18	-7	42.00	10.40
-8	18	7	44.40	8.00
7	-18	-7	105.29	13.50
7	18	-7	82.29	14.60
-7	18	7	109.69	12.10
6	-18	-7	28.80	7.40
-6	18	7	27.30	7.00
-6	18	7	27.50	8.40
5	-18	-7	103.19	12.40
-5	-18	7	93.29	5.50
5	18	-7	81.89	5.10
-5	18	7	85.29	13.70
-5	18	7	78.29	10.90
5	18	-7	97.39	16.10
-4	-18	7	125.29	7.10
4	18	-7	112.89	6.80
4	18	-7	91.09	12.80
-4	18	7	124.99	11.90
-4	18	7	136.69	13.90
-3	-18	7	173.98	9.60
-3	18	7	169.68	15.60
3	18	-7	157.38	14.40
-3	18	7	169.58	14.60
-3	18	7	162.78	17.10
-2	-18	7	28.60	4.90
2	18	-7	23.20	6.50
-2	18	7	32.20	7.20
2	18	-7	25.70	4.10
-2	18	7	23.30	6.30
-2	18	7	34.50	8.20
-1	-18	7	52.49	6.00
-1	18	7	49.80	10.40
-1	18	7	58.49	11.50
-1	18	7	48.90	9.10
1	18	-7	46.20	5.20
1	18	-7	53.29	9.80
0	-18	7	53.09	6.80

0	18	7	62.29	12.10
0	18	-7	57.89	5.70
0	18	-7	68.19	10.00
0	18	7	58.19	8.50
0	18	7	58.39	9.80
1	-18	7	163.68	11.20
1	18	7	172.78	13.00
-1	18	-7	150.08	10.00
1	18	7	169.88	13.60
-1	18	-7	172.48	11.90
1	18	7	154.68	15.00
2	-18	7	42.90	7.30
2	18	7	42.20	7.00
-2	18	-7	51.69	7.60
-2	18	-7	41.30	6.20
-2	18	-7	39.50	5.90
2	18	7	39.80	6.90
2	18	7	35.70	8.20
3	-18	7	222.78	14.40
-3	18	-7	214.48	13.10
3	18	7	221.48	15.80
3	18	7	251.17	15.20
3	18	7	211.18	17.90
-3	18	-7	212.58	14.40
-3	18	-7	206.08	15.30
-4	18	-7	11.80	5.60
4	18	7	12.70	8.30
4	18	7	12.00	5.90
-4	18	-7	10.50	4.30
-4	18	-7	4.00	5.40
4	18	7	15.40	6.00
5	18	7	196.58	13.90
-5	18	-7	188.08	12.80
-5	18	-7	175.88	14.00
-5	18	-7	187.68	13.80
5	18	7	184.38	17.10
-6	-18	-7	12.20	8.10
-6	18	-7	10.30	5.10
-6	18	-7	10.30	5.30
6	18	7	13.60	5.90
-6	18	-7	9.00	5.70
6	18	7	10.20	8.00
-7	18	-7	173.88	14.40
7	18	7	171.68	13.90

-7	18	-7	162.48	14.20
-7	18	-7	189.88	13.30
7	18	7	163.98	17.80
8	18	7	9.30	6.40
-8	18	-7	6.80	6.30
-8	18	-7	2.20	5.70
8	18	7	3.60	9.20
9	18	7	93.19	12.40
-9	18	-7	91.89	11.50
-10	18	-7	125.59	13.00
10	18	7	105.19	13.20
11	18	7	50.79	9.50
-11	18	-7	49.30	9.10
11	19	-7	281.07	26.80
-11	19	7	286.67	20.40
10	19	-7	14.60	11.30
-10	19	7	9.50	7.40
-9	19	7	299.37	20.30
9	19	-7	301.97	25.00
8	19	-7	6.40	8.90
-8	19	7	-3.20	6.30
7	-19	-7	421.66	28.30
7	19	-7	436.96	28.30
-7	19	7	448.86	25.80
6	-19	-7	171.58	16.80
-6	19	7	213.68	15.80
6	19	-7	209.28	18.20
5	-19	-7	423.86	25.10
5	19	-7	361.36	19.50
-5	19	7	406.76	23.90
-5	19	7	401.96	26.00
5	19	-7	429.76	25.80
-4	-19	7	173.38	9.60
4	19	-7	170.38	15.60
-4	19	7	177.68	16.30
-4	19	7	187.18	17.70
4	19	-7	159.18	9.30
-4	19	7	173.58	14.00
-3	19	7	508.55	29.80
3	19	-7	445.56	23.40
-3	19	7	459.35	28.20
-3	19	7	474.75	26.90
3	19	-7	478.85	27.90
-2	-19	7	240.48	13.00

2	19	-7	224.58	12.50
2	19	-7	242.18	17.30
-2	19	7	247.38	17.70
-2	19	7	192.28	18.20
-2	19	7	232.58	19.30
-1	-19	7	312.07	17.00
-1	19	7	323.57	20.70
-1	19	7	332.07	22.40
1	19	-7	313.37	20.40
-1	19	7	285.57	19.70
1	19	-7	309.27	16.40
0	-19	7	961.40	47.00
0	19	-7	968.40	49.40
0	19	-7	879.51	48.10
0	19	7	929.31	49.50
0	19	-7	936.41	46.40
0	19	7	1016.30	51.29
0	19	7	975.40	48.90
1	-19	7	90.79	8.70
-1	19	-7	91.09	7.50
1	19	7	90.39	10.60
-1	19	-7	104.99	11.40
-1	19	-7	78.49	9.20
1	19	7	88.59	11.70
1	19	7	109.49	14.10
2	-19	7	355.86	19.90
-2	19	-7	350.86	18.80
2	19	7	329.87	21.70
-2	19	-7	350.96	21.70
-2	19	-7	341.17	20.40
2	19	7	352.86	21.00
2	19	7	367.26	23.80
3	-19	7	-5.10	5.00
3	19	7	3.00	6.20
-3	19	-7	-2.40	5.20
3	19	7	3.50	7.20
-3	19	-7	7.70	4.10
3	19	7	6.50	5.70
-3	19	-7	3.60	4.80
-4	19	-7	704.63	41.40
4	19	7	775.72	40.10
4	19	7	724.33	40.80
-4	19	-7	811.92	40.90
4	19	7	827.62	43.20

-4	19	-7	774.32	38.80
-5	19	-7	37.40	7.10
-5	19	-7	37.30	6.70
5	19	7	26.40	8.90
-5	19	-7	39.30	6.30
-6	19	-7	881.01	45.10
6	19	7	856.11	44.30
-6	19	-7	761.22	44.00
6	19	7	944.91	47.90
-7	19	-7	24.20	7.00
7	19	7	18.80	10.70
7	19	7	23.10	7.00
-7	19	-7	23.70	6.20
-8	19	-7	159.68	14.70
-8	19	-7	167.88	13.30
8	19	7	142.49	14.10
9	19	7	23.30	7.70
-9	19	-7	12.30	6.80
10	19	7	357.16	23.50
-10	19	-7	383.96	23.50
-11	19	-7	63.79	10.10
11	19	7	36.90	9.20
10	20	-7	-1.40	12.60
-10	20	7	1.40	7.70
-9	20	7	-8.70	7.60
9	20	-7	6.50	11.90
-8	20	7	8.90	7.60
8	20	-7	33.20	10.90
7	20	-7	9.10	9.00
-7	20	7	8.30	7.30
6	-20	-7	9.70	7.30
-6	20	7	6.30	6.60
6	20	-7	9.90	8.10
-5	20	7	5.90	7.20
5	20	-7	13.10	8.50
-4	20	7	36.40	7.90
4	20	-7	29.50	8.40
-4	20	7	18.10	9.40
-3	-20	7	27.10	4.10
3	20	-7	36.30	8.10
-3	20	7	27.80	8.50
3	20	-7	29.00	3.60
-3	20	7	34.20	7.80
-3	20	7	23.00	9.20

-2	-20	7	18.30	4.00
-2	20	7	13.90	7.80
2	20	-7	15.80	3.40
-2	20	7	4.40	7.00
-2	20	7	11.70	9.20
2	20	-7	22.30	7.10
-1	-20	7	8.80	4.10
-1	20	7	5.70	8.20
-1	20	7	11.70	6.90
-1	20	7	11.00	6.50
1	20	-7	2.90	3.20
1	20	-7	7.70	7.00
0	-20	7	5.20	4.30
0	20	-7	4.00	3.50
0	20	-7	4.50	6.20
0	20	7	-14.50	6.00
0	20	7	4.60	7.80
0	20	-7	1.40	4.70
0	20	7	2.70	6.70
1	-20	7	5.90	4.70
1	20	7	0.80	8.50
-1	20	-7	7.80	5.00
-1	20	-7	7.30	3.90
-1	20	-7	12.10	6.50
1	20	7	12.80	6.70
2	-20	7	33.80	6.10
-2	20	-7	26.40	4.70
2	20	7	28.40	9.00
-2	20	-7	10.10	6.30
2	20	7	23.40	7.20
-2	20	-7	12.10	5.50
-3	20	-7	108.09	12.10
-3	20	-7	82.89	10.40
3	20	7	83.89	12.70
3	20	7	120.99	15.80
-3	20	-7	107.89	9.10
-4	20	-7	19.00	5.20
4	20	7	15.00	9.90
4	20	7	21.80	6.70
-4	20	-7	14.60	6.90
-4	20	-7	11.10	10.30
5	20	7	7.60	8.50
-5	20	-7	9.20	6.50
-5	20	-7	7.00	4.90

-5	20	-7	9.50	5.70
5	20	7	8.20	6.20
-6	20	-7	16.70	5.80
6	20	7	23.00	6.70
6	20	7	23.50	9.60
-6	20	-7	16.90	6.90
7	20	7	17.70	11.50
-7	20	-7	10.40	6.10
7	20	7	15.90	6.40
-7	20	-7	13.60	7.00
8	20	7	9.30	6.90
-8	20	-7	7.10	7.20
-8	20	-7	1.10	6.50
-9	20	-7	12.80	6.90
9	20	7	-3.60	7.20
-9	20	-7	-4.10	7.30
10	20	7	25.10	8.20
-10	20	-7	22.60	7.80
-9	21	7	343.17	22.90
9	21	-7	322.37	27.80
-8	21	7	5.40	7.90
8	21	-7	17.50	10.50
-7	21	7	358.76	22.70
7	21	-7	346.07	26.20
6	21	-7	235.28	20.60
-6	21	7	224.98	17.90
-5	21	7	319.97	20.90
-5	21	7	275.27	28.00
5	21	-7	338.57	23.30
-4	21	7	133.59	13.60
4	21	-7	120.89	15.60
-4	21	7	135.59	18.00
3	21	-7	150.08	9.10
-3	21	7	144.19	14.10
-3	21	7	161.58	17.80
3	21	-7	145.09	15.80
2	21	-7	363.86	23.60
-2	21	7	354.56	22.40
-2	21	7	344.57	25.80
2	21	-7	345.37	18.30
-2	21	7	363.36	25.60
-1	-21	7	98.89	9.00
-1	21	7	86.89	15.80
1	21	-7	78.49	7.20

1	21	-7	82.39	12.90
-1	21	7	85.19	13.90
-1	21	7	81.19	12.00
0	-21	7	801.82	41.40
0	21	7	891.21	43.70
0	21	-7	798.52	40.80
0	21	-7	835.92	44.30
0	21	7	786.52	44.30
0	21	7	847.62	46.60
1	-21	7	164.88	12.30
-1	21	-7	168.18	11.10
1	21	7	173.68	18.20
1	21	7	144.59	15.20
1	21	7	172.18	14.30
-1	21	-7	169.38	15.20
2	-21	7	313.87	19.60
-2	21	-7	333.67	22.10
2	21	7	314.97	22.40
-2	21	-7	354.06	18.70
2	21	7	340.27	20.90
2	21	7	336.47	24.60
3	21	7	22.20	6.70
-3	21	-7	18.60	7.10
3	21	7	27.50	10.00
3	21	7	29.40	8.00
-3	21	-7	26.20	5.20
-4	21	-7	445.56	25.00
4	21	7	482.85	30.80
4	21	7	454.55	26.40
-4	21	-7	484.85	27.90
5	21	7	13.10	10.30
5	21	7	15.70	6.80
-5	21	-7	11.70	7.40
-6	21	-7	584.14	32.50
6	21	7	665.43	37.90
-6	21	-7	631.44	34.90
6	21	7	586.64	33.40
7	21	7	28.80	7.40
-7	21	-7	16.70	6.80
-7	21	-7	18.80	7.90
8	21	7	307.77	20.30
-8	21	-7	281.67	21.30
-8	21	-7	294.57	19.20
-9	21	-7	32.70	9.30

9	21	7	35.70	8.80
-9	21	-7	26.80	7.90
-9	22	7	4.60	8.20
9	22	-7	16.60	13.00
-8	22	7	117.79	14.50
8	22	-7	137.69	20.40
7	22	-7	27.80	10.40
-7	22	7	5.40	7.70
-6	22	7	79.89	14.40
6	22	-7	115.49	17.00
-6	22	7	95.29	13.50
5	22	-7	23.30	9.40
-5	22	7	12.20	10.50
-5	22	7	23.70	8.30
4	22	-7	147.69	16.90
-4	22	7	133.39	21.00
-4	22	7	135.59	14.70
3	22	-7	102.59	15.50
-3	22	7	120.79	14.00
-3	22	7	144.69	18.60
2	22	-7	38.10	5.70
-2	22	7	34.00	8.20
-2	22	7	44.20	11.60
2	22	-7	34.20	8.90
1	22	-7	145.69	10.00
1	22	-7	167.88	16.10
-1	22	7	167.78	19.20
-1	22	7	139.99	14.20
0	22	-7	101.39	8.70
0	22	-7	119.29	14.20
0	22	7	135.39	17.30
0	22	7	82.69	12.60
1	22	7	300.77	20.00
1	22	7	304.47	23.80
-1	22	-7	324.17	21.40
-1	22	-7	277.47	16.80
-2	22	-7	68.79	12.30
2	22	7	60.79	12.20
2	22	7	59.19	9.00
-2	22	-7	61.79	7.90
-3	22	-7	305.07	21.60
3	22	7	362.16	25.80
-3	22	-7	294.87	18.10
3	22	7	310.87	20.20

-4	22	-7	3.70	7.20
-4	22	-7	15.00	5.50
4	22	7	-17.70	8.90
4	22	7	17.80	6.70
-5	22	-7	109.19	14.00
5	22	7	86.79	11.90
-5	22	-7	88.09	10.50
5	22	7	111.29	18.10
-6	22	-7	-3.50	5.80
-6	22	-7	0.50	7.40
6	22	7	-3.40	6.50
-7	22	-7	269.67	18.10
7	22	7	267.97	19.10
-7	22	-7	286.27	21.00
8	22	7	1.10	7.30
-8	22	-7	6.30	6.80
-8	22	-7	-7.40	8.10
9	22	7	122.69	14.80
-9	22	-7	156.48	14.70
-8	23	7	16.40	8.40
8	23	-7	7.70	11.60
7	23	-7	-0.90	10.30
-7	23	7	3.10	12.70
-7	23	7	3.30	8.10
-6	23	7	113.79	22.30
-6	23	7	122.49	14.80
6	23	-7	132.09	18.80
-5	23	7	280.07	25.60
-5	23	7	239.88	19.20
5	23	-7	238.78	21.90
-4	23	7	13.50	10.80
4	23	-7	3.80	9.00
-4	23	7	14.00	7.90
3	23	-7	58.19	10.80
-3	23	7	77.99	12.40
-3	23	7	77.09	13.60
2	23	-7	83.49	12.30
-2	23	7	69.09	12.70
-2	23	7	61.49	14.50
-1	23	7	2.70	7.60
1	23	-7	2.40	3.90
-1	23	7	-7.00	9.60
1	23	-7	-0.60	7.60
0	23	7	66.39	12.20

0	23	-7	71.49	13.80
0	23	-7	63.79	7.70
0	23	7	59.49	12.80
-1	23	-7	47.30	7.50
1	23	7	39.70	8.40
-1	23	-7	31.10	9.70
1	23	7	39.60	12.90
-2	23	-7	91.99	9.30
-2	23	-7	74.49	14.30
2	23	7	107.09	12.60
2	23	7	96.19	15.20
-3	23	-7	-9.60	7.00
-3	23	-7	8.10	5.30
3	23	7	-0.90	6.60
3	23	7	13.90	11.60
4	23	7	68.69	13.80
4	23	7	66.59	11.50
-4	23	-7	66.79	9.60
-4	23	-7	64.49	13.30
5	23	7	1.50	10.80
-5	23	-7	5.40	6.00
5	23	7	6.90	6.80
-5	23	-7	12.80	8.10
6	23	7	77.99	12.50
-6	23	-7	71.39	10.60
-6	23	-7	51.89	10.30
7	23	7	8.40	7.40
-7	23	-7	8.10	6.80
-7	23	-7	0.60	8.10
8	23	7	44.00	9.30
-8	23	-7	52.99	9.20
-7	24	7	57.79	10.30
-7	24	7	46.80	15.30
7	24	-7	70.19	13.90
-6	24	7	346.37	30.00
6	24	-7	327.47	26.30
-6	24	7	297.57	22.10
-5	24	7	270.67	27.90
5	24	-7	303.77	24.70
-5	24	7	321.57	21.60
-4	24	7	265.57	26.20
4	24	-7	267.37	22.60
-4	24	7	244.78	19.80
-3	24	7	52.49	13.90

-3	24	7	51.99	10.60
3	24	-7	57.89	11.30
2	24	-7	100.29	16.40
-2	24	7	89.59	13.50
-2	24	7	102.89	20.40
1	24	-7	327.17	23.60
-1	24	7	314.07	21.40
-1	24	7	235.28	35.80
0	24	7	169.68	21.40
0	24	7	129.79	14.90
0	24	-7	142.09	17.30
0	24	-7	145.79	10.70
1	24	7	332.47	22.00
-1	24	-7	328.27	18.70
1	24	7	313.87	26.80
-1	24	-7	336.17	23.90
2	24	7	9.60	11.40
-2	24	-7	0.30	5.00
-2	24	-7	8.20	7.90
2	24	7	2.70	7.00
3	24	7	253.17	24.80
3	24	7	248.78	18.10
-3	24	-7	226.78	19.90
-3	24	-7	204.18	16.70
-4	24	-7	7.10	8.30
-4	24	-7	20.30	6.50
4	24	7	14.90	12.30
4	24	7	17.00	7.40
-5	24	-7	513.05	31.30
5	24	7	499.25	29.20
-5	24	-7	487.45	27.70
6	24	7	-6.10	7.20
-6	24	-7	0.00	6.80
-6	24	-7	-2.10	8.30
7	24	7	355.96	23.60
-7	24	-7	370.26	22.60
-5	25	7	3.60	13.40
5	25	-7	-3.90	10.00
-5	25	7	1.60	8.50
-4	25	7	12.60	13.10
4	25	-7	6.30	9.90
-4	25	7	-4.10	8.40
3	25	-7	18.80	10.40
-3	25	7	2.80	8.00

-3	25	7	12.00	14.10
-2	25	7	-8.70	8.00
-2	25	7	6.50	12.90
2	25	-7	-0.40	9.80
-1	25	7	-25.50	12.20
-1	25	7	3.30	8.00
0	25	-7	12.20	8.80
0	25	7	28.10	12.20
0	25	7	25.50	8.40
-1	25	-7	1.10	5.20
1	25	7	-4.10	15.40
1	25	7	-6.80	7.40
-1	25	-7	-1.80	8.40
2	25	7	32.80	14.60
2	25	7	15.70	8.10
-2	25	-7	9.10	8.80
-2	25	-7	21.60	6.00
3	25	7	9.70	7.80
3	25	7	-7.70	12.40
-3	25	-7	-0.80	5.40
-4	25	-7	4.10	6.20
4	25	7	17.60	8.20
-4	25	-7	5.90	8.80
5	25	7	-2.50	7.70
-5	25	-7	-7.20	6.20
-5	25	-7	-1.40	8.90
4	26	-7	153.08	20.50
-4	26	7	150.08	17.00
-4	26	7	119.59	25.00
3	26	-7	28.50	11.00
-3	26	7	59.39	16.90
-3	26	7	53.59	10.80
2	26	-7	58.39	12.10
-2	26	7	76.99	15.80
-2	26	7	83.69	14.60
1	26	-7	328.27	26.10
-1	26	7	333.87	30.90
-1	26	7	395.36	24.50
0	26	7	48.90	15.10
0	26	-7	44.10	10.90
0	26	7	30.50	9.30
1	26	7	270.37	28.00
-1	26	-7	261.47	22.80
1	26	7	268.37	20.10

2	26	7	21.90	12.20
2	26	7	30.50	8.90
-2	26	-7	17.10	10.00
-3	26	-7	107.09	11.50
3	26	7	122.09	14.40
-3	26	-7	100.99	17.50
4	26	7	3.40	7.80
-4	26	-7	2.10	6.30
-15	0	8	16.60	7.40
-14	0	8	363.86	21.10
13	0	-8	-7.60	7.40
-13	0	8	4.30	5.10
-12	0	8	237.08	22.20
12	0	-8	210.08	17.80
-12	0	8	222.28	14.00
11	0	-8	6.30	5.50
-11	0	8	12.50	4.40
11	0	-8	15.10	6.70
-11	0	8	17.50	9.40
10	0	-8	955.70	48.10
-10	0	8	861.71	49.60
-10	0	8	947.11	45.60
10	0	-8	921.31	46.30
9	0	-8	1.50	3.80
-9	0	8	8.90	6.70
-9	0	8	7.30	3.20
9	0	-8	5.90	5.00
-8	0	8	735.53	42.00
-8	0	8	811.52	39.10
8	0	-8	806.82	39.60
8	0	-8	824.72	41.30
-7	0	8	732.73	36.10
7	0	-8	767.62	38.10
7	0	-8	753.52	36.50
-7	0	8	682.63	38.50
6	0	-8	738.03	34.90
-6	0	8	688.53	36.80
-6	0	8	692.13	34.60
6	0	-8	708.13	36.30
-5	0	8	186.68	12.60
5	0	-8	197.08	10.70
-5	0	8	194.68	10.70
5	0	-8	204.08	12.30
4	0	-8	1180.38	56.29

4	0	-8	1189.38	57.39
-4	0	8	1099.69	56.39
-4	0	8	1216.48	57.89
3	0	-8	218.28	12.30
3	0	-8	232.38	11.10
-3	0	8	198.18	11.50
-3	0	8	212.18	12.50
-2	0	8	1538.95	75.99
2	0	-8	1599.54	76.49
2	0	-8	1590.64	75.79
-2	0	8	1628.64	76.79
1	0	-8	2784.62	132.99
-1	0	8	2674.93	132.79
-1	0	8	2913.91	133.19
1	0	-8	2791.22	132.59
0	0	8	48.20	3.50
0	0	-8	40.00	3.30
0	0	-8	35.60	3.20
1	0	8	2416.16	112.09
1	0	8	2337.67	112.09
1	0	8	2426.76	112.49
-1	0	-8	2273.27	112.09
-1	0	-8	2333.37	112.39
2	0	8	490.55	23.80
-2	0	-8	496.25	24.50
2	0	8	507.75	24.00
2	0	8	477.05	24.60
-3	0	-8	851.31	41.10
3	0	8	870.81	40.90
3	0	8	838.22	40.80
3	0	8	846.02	41.80
-3	0	-8	859.31	41.60
4	0	8	187.98	11.20
4	0	8	210.98	10.10
-4	0	-8	184.58	11.00
4	0	8	186.58	10.20
-4	0	-8	219.18	11.40
5	0	8	4504.95	207.78
-5	0	-8	3940.51	208.58
5	0	8	4167.38	208.48
-5	0	-8	4886.11	208.68
6	0	8	98.09	6.20
6	0	8	68.79	6.60
-6	0	-8	75.59	7.20

-6	0	-8	77.29	7.10
-7	0	-8	1427.56	70.79
-7	0	-8	1386.16	70.59
-7	0	-8	1526.15	70.89
7	0	8	1448.66	69.99
8	0	8	9.20	3.10
-8	0	-8	5.00	3.80
-8	0	-8	3.90	4.00
-8	0	-8	4.90	3.80
-9	0	-8	1539.15	75.59
-9	0	-8	1479.85	75.59
9	0	8	1588.44	74.89
-10	0	-8	726.53	34.50
-10	0	-8	652.63	34.60
10	0	8	547.85	36.70
11	0	8	644.04	32.40
-11	0	-8	563.84	33.20
-11	0	-8	600.34	32.50
-12	0	-8	494.45	31.60
-12	0	-8	613.34	31.20
12	0	8	546.85	30.10
-13	0	-8	278.27	19.50
-14	0	-8	402.86	25.40
-15	0	-8	90.69	14.80
-15	-1	8	18.80	7.30
-15	1	8	14.40	7.20
-14	-1	8	23.70	6.40
-14	1	8	13.00	6.30
13	-1	-8	37.20	9.30
-13	-1	8	36.30	6.10
13	1	-8	34.60	9.40
12	-1	-8	12.80	6.90
-12	-1	8	8.80	4.60
-12	-1	8	9.60	9.60
-12	1	8	11.90	4.70
-12	1	8	-9.90	10.80
12	1	-8	3.00	7.30
-11	-1	8	65.59	7.20
11	-1	-8	80.39	11.60
-11	-1	8	63.09	11.60
-11	1	8	68.99	12.10
11	1	-8	49.70	9.30
-11	1	8	68.09	7.80
11	1	-8	60.49	11.40

10	-1	-8	75.69	9.10
-10	-1	8	74.49	7.20
10	-1	-8	72.49	10.90
10	1	-8	70.39	10.60
10	1	-8	70.89	8.40
-10	1	8	89.29	7.60
-10	1	8	74.39	13.80
9	-1	-8	1.60	3.80
-9	-1	8	0.60	6.70
9	-1	-8	-0.70	4.50
-9	-1	8	1.80	2.80
9	1	-8	4.10	3.70
-9	1	8	5.20	3.30
-9	1	8	7.10	6.80
9	1	-8	-0.10	4.70
-8	-1	8	22.20	6.80
8	-1	-8	27.10	5.80
8	-1	-8	45.70	5.90
-8	-1	8	41.40	4.60
8	1	-8	33.70	5.50
-8	1	8	28.10	7.30
-8	1	8	44.50	5.10
8	1	-8	31.50	6.50
7	-1	-8	166.48	10.10
-7	-1	8	143.09	12.70
-7	-1	8	166.18	9.30
7	-1	-8	163.88	11.60
7	1	-8	165.78	9.70
7	1	-8	161.18	12.00
-7	1	8	170.48	9.60
-7	1	8	139.59	12.70
6	-1	-8	47.20	4.90
6	-1	-8	38.00	6.60
-6	-1	8	42.30	4.10
-6	-1	8	39.20	6.50
6	1	-8	34.30	6.50
6	1	-8	42.90	4.40
-6	1	8	40.60	6.30
-6	1	8	40.20	4.50
5	-1	-8	59.49	6.50
5	-1	-8	60.39	4.70
-5	-1	8	53.99	4.70
-5	-1	8	67.99	7.40
-5	1	8	50.79	4.90

5	1	-8	51.69	6.60
5	1	-8	55.79	4.50
-5	1	8	65.69	7.10
4	-1	-8	247.28	12.70
4	-1	-8	244.18	13.80
-4	-1	8	231.58	12.50
-4	-1	8	234.78	14.20
4	1	-8	243.18	12.30
4	1	-8	236.58	13.80
-4	1	8	235.58	12.80
-4	1	8	249.18	14.30
-3	-1	8	23.60	3.70
-3	-1	8	16.60	2.70
3	-1	-8	21.80	2.70
3	-1	-8	14.40	2.90
3	1	-8	21.80	4.10
3	1	-8	22.70	2.40
-3	1	8	17.10	2.90
-3	1	8	23.30	4.40
2	-1	-8	41.40	3.20
-2	-1	8	31.20	4.20
-2	-1	8	39.10	3.10
2	-1	-8	40.60	4.10
2	1	-8	33.90	4.00
-2	1	8	38.40	3.80
2	1	-8	36.80	2.80
-2	1	8	40.30	4.30
1	-1	-8	1.90	1.30
-1	-1	8	4.20	1.80
1	-1	-8	5.70	2.00
-1	-1	8	4.70	1.40
-1	1	8	6.70	2.20
-1	1	8	6.50	2.00
1	1	-8	1.70	1.70
1	1	-8	5.20	1.30
0	-1	8	204.08	10.60
0	-1	8	199.08	10.60
0	-1	-8	199.08	10.70
0	-1	-8	206.38	10.60
0	1	8	198.58	10.80
0	1	-8	212.48	10.60
0	1	8	198.88	10.70
0	1	-8	203.48	10.40
-1	-1	-8	289.47	14.10

-1	-1	-8	281.87	14.30
1	-1	8	282.47	14.30
1	-1	8	236.98	14.30
-1	1	-8	274.77	13.70
1	1	8	290.27	14.00
1	1	8	273.87	14.40
-1	1	-8	283.27	14.10
1	1	8	277.77	14.10
-2	-1	-8	75.49	5.00
2	-1	8	71.39	5.20
2	1	8	74.09	4.30
2	1	8	72.29	5.10
2	1	8	77.09	4.80
-2	1	-8	68.09	4.20
-3	-1	-8	3.50	2.20
-3	-1	-8	1.90	1.60
3	-1	8	1.60	2.10
-3	1	-8	4.40	1.30
-3	1	-8	5.70	2.30
3	1	8	2.40	1.50
3	1	8	6.50	2.40
3	1	8	1.80	1.20
4	-1	8	507.55	24.60
4	-1	8	478.95	25.80
4	-1	8	494.45	24.90
-4	-1	-8	506.35	25.50
-4	-1	-8	543.15	25.90
4	1	8	477.85	25.90
-4	1	-8	545.35	26.00
-4	1	-8	535.05	25.20
4	1	8	519.65	25.30
4	1	8	510.25	25.00
5	-1	8	544.05	27.90
5	-1	8	546.25	29.00
-5	-1	-8	561.24	28.70
-5	-1	-8	586.74	28.90
-5	1	-8	630.84	29.10
5	1	8	535.95	28.90
5	1	8	574.34	28.40
-5	1	-8	593.54	28.40
-6	-1	-8	617.64	30.40
-6	-1	-8	589.54	30.10
6	-1	8	563.64	29.40
6	1	8	603.34	29.70

-6	1	-8	588.84	29.80
-6	1	-8	636.44	30.50
-6	1	-8	601.24	30.40
6	1	8	559.94	30.30
7	-1	8	33.20	6.00
-7	-1	-8	46.90	6.30
-7	-1	-8	45.40	6.40
-7	1	-8	42.60	6.00
-7	1	-8	29.80	6.00
7	1	8	39.40	5.70
7	1	8	35.00	5.30
-7	1	-8	39.10	6.50
-8	-1	-8	64.09	7.40
-8	-1	-8	51.09	7.30
-8	-1	-8	49.70	7.50
-8	1	-8	47.10	7.00
-8	1	-8	59.89	7.60
8	1	8	55.99	6.20
-8	1	-8	47.10	7.20
9	-1	8	5.30	4.40
-9	-1	-8	15.00	5.10
-9	-1	-8	8.20	5.10
-9	1	-8	8.10	4.90
9	1	8	8.90	3.90
-9	1	-8	2.60	5.90
10	-1	8	18.50	4.70
-10	-1	-8	16.00	6.10
-10	1	-8	44.90	8.10
10	1	8	29.40	5.40
-10	1	-8	28.00	9.20
11	-1	8	31.70	5.60
-11	-1	-8	37.90	7.90
-11	-1	-8	39.60	7.30
-11	1	-8	52.79	9.50
11	1	8	31.70	6.30
-11	1	-8	28.20	12.00
12	-1	8	148.59	12.10
-12	-1	-8	146.09	15.50
-12	-1	-8	163.68	13.40
-12	1	-8	187.48	14.00
-12	1	-8	121.69	20.00
-13	-1	-8	67.59	12.00
-13	1	-8	63.49	11.60
-14	-1	-8	26.80	8.10

-14	1	-8	28.80	8.70
-15	-1	-8	12.30	8.90
-15	1	-8	30.50	9.30
-15	-2	8	8.10	6.80
-15	2	8	20.50	7.50
-14	-2	8	181.68	14.90
-14	2	8	189.78	14.30
-13	-2	8	10.40	4.90
13	-2	-8	11.90	8.30
-13	2	8	3.80	5.30
13	2	-8	6.90	8.60
12	-2	-8	470.75	28.10
-12	-2	8	454.85	24.70
-12	2	8	497.25	25.30
-12	2	8	461.15	31.90
12	2	-8	455.15	28.50
-11	-2	8	73.99	14.90
-11	-2	8	68.79	7.40
-11	-2	8	64.79	9.50
11	-2	-8	65.79	11.30
-11	2	8	79.09	7.90
-11	2	8	61.69	11.40
11	2	-8	58.59	11.90
-10	-2	8	886.91	51.29
10	-2	-8	1026.90	50.09
-10	-2	8	975.60	47.30
10	-2	-8	982.70	48.50
10	2	-8	968.80	50.09
-10	2	8	911.91	51.29
10	2	-8	950.00	47.90
-10	2	8	993.80	47.70
9	-2	-8	10.90	4.20
9	-2	-8	6.80	4.90
-9	-2	8	9.40	3.10
-9	-2	8	10.30	6.70
-9	2	8	3.30	3.30
9	2	-8	11.80	3.80
9	2	-8	3.30	5.00
-9	2	8	6.80	6.50
8	-2	-8	707.33	35.60
-8	-2	8	662.23	37.90
8	-2	-8	740.23	37.00
-8	-2	8	688.43	34.60
-8	2	8	693.23	38.00

8	2	-8	706.03	37.10
-8	2	8	706.03	35.00
8	2	-8	723.63	35.10
-7	-2	8	335.97	17.70
-7	-2	8	308.47	20.80
7	-2	-8	357.86	19.90
7	-2	-8	350.06	18.60
7	2	-8	348.87	20.20
-7	2	8	360.66	21.20
7	2	-8	362.86	18.10
-7	2	8	355.26	18.30
-6	-2	8	788.52	41.60
6	-2	-8	845.32	39.80
-6	-2	8	789.82	39.10
6	-2	-8	814.42	40.90
-6	2	8	819.02	41.60
6	2	-8	789.82	41.00
-6	2	8	791.52	39.50
5	-2	-8	1117.09	54.09
-5	-2	8	1055.29	52.99
-5	-2	8	1046.70	54.59
5	-2	-8	1168.18	53.19
5	2	-8	1072.49	54.19
-5	2	8	1101.09	53.09
5	2	-8	1104.19	52.59
-5	2	8	1084.19	54.49
-4	-2	8	1384.36	69.29
-4	-2	8	1356.06	67.69
4	-2	-8	1426.56	68.99
4	-2	-8	1471.05	68.09
4	2	-8	1451.25	69.09
4	2	-8	1406.76	67.59
-4	2	8	1357.16	68.19
-4	2	8	1473.85	69.39
3	-2	-8	1444.46	67.29
-3	-2	8	1353.46	66.99
-3	-2	8	1387.46	68.29
3	-2	-8	1445.26	68.09
-3	2	8	1455.15	68.39
3	2	-8	1409.86	68.09
-3	2	8	1284.47	67.59
3	2	-8	1446.76	66.89
-2	-2	8	604.44	30.30
-2	-2	8	650.03	31.40

2	-2	-8	632.54	31.20
2	2	-8	666.93	31.30
-2	2	8	655.33	31.40
2	2	-8	604.34	30.10
-2	2	8	580.74	30.90
-1	-2	8	1356.36	67.09
-1	-2	8	1359.66	67.59
1	-2	-8	1433.86	67.79
-1	2	8	1452.95	67.79
1	2	-8	1399.76	66.99
-1	2	8	1384.46	67.69
1	2	-8	1458.85	67.69
0	-2	-8	263.07	13.70
0	-2	8	259.87	13.30
0	-2	8	266.47	13.30
0	-2	-8	234.28	13.80
0	2	8	274.57	13.80
0	2	8	267.47	13.60
0	2	-8	256.07	13.20
0	2	-8	277.17	13.40
-1	-2	-8	4542.25	212.58
-1	-2	-8	4573.84	212.58
1	2	8	4366.76	212.38
1	2	8	4358.06	212.68
-1	2	-8	4530.05	212.38
1	2	8	4483.85	212.58
-2	-2	-8	254.97	12.90
2	-2	8	235.58	12.80
2	2	8	232.68	13.00
-2	2	-8	229.98	12.70
-2	2	-8	235.38	11.90
2	2	8	243.88	12.40
2	2	8	271.47	12.90
3	-2	8	96.59	6.70
-3	-2	-8	107.59	6.70
-3	-2	-8	99.29	6.40
-3	2	-8	98.59	5.30
3	2	8	104.59	5.90
-3	2	-8	102.99	6.60
3	2	8	98.59	6.80
3	2	8	99.49	6.40
-4	-2	-8	6.60	2.60
4	-2	8	1.80	2.40
-4	-2	-8	6.50	2.30

4	2	8	3.60	1.60
-4	2	-8	4.80	2.60
4	2	8	5.60	2.50
-4	2	-8	5.10	1.60
4	2	8	7.90	2.20
-5	-2	-8	1779.62	79.59
5	-2	8	1623.84	78.19
-5	-2	-8	1591.14	79.39
5	2	8	1661.83	79.19
5	2	8	1521.15	79.49
-5	2	-8	1762.72	79.69
-5	2	-8	1618.04	78.79
-5	2	-8	1602.34	79.59
-6	-2	-8	128.49	9.20
-6	-2	-8	136.89	9.00
6	-2	8	131.29	7.50
-6	2	-8	137.99	9.50
6	2	8	137.39	9.30
6	2	8	130.49	8.70
-6	2	-8	126.29	8.50
-7	-2	-8	1597.14	71.59
7	-2	8	1456.25	70.29
-7	-2	-8	1432.06	71.49
-7	2	-8	1437.66	71.49
-7	2	-8	1560.24	71.69
-7	2	-8	1389.56	70.89
7	2	8	1371.16	71.39
7	2	8	1456.25	71.09
8	-2	8	94.39	7.10
-8	-2	-8	97.69	9.10
-8	-2	-8	99.79	8.80
-8	2	-8	116.49	9.10
-8	2	-8	93.09	8.70
8	2	8	94.79	8.30
8	2	8	95.59	8.90
-8	2	-8	110.19	9.30
-9	-2	-8	295.77	18.30
9	-2	8	287.67	16.40
-9	-2	-8	325.27	18.20
-9	2	-8	272.37	17.80
-9	2	-8	280.97	17.80
9	2	8	322.97	17.50
10	-2	8	194.88	12.40
-10	-2	-8	222.98	14.40

-10	-2	-8	180.68	14.60
-10	2	-8	215.68	13.70
-10	2	-8	172.68	14.70
10	2	8	179.68	13.40
11	-2	8	388.86	22.00
-11	-2	-8	373.46	24.10
-11	-2	-8	434.46	23.60
-11	2	-8	374.86	24.20
11	2	8	422.86	23.10
-11	2	-8	398.16	23.40
12	-2	8	142.99	12.10
-12	-2	-8	192.68	14.90
-12	-2	-8	154.98	15.60
-12	2	-8	147.59	15.90
-12	2	-8	192.38	14.70
-13	-2	-8	85.89	12.60
-13	2	-8	65.19	10.10
-14	-2	-8	198.38	17.50
-14	2	-8	207.98	17.30
-15	-2	-8	22.80	9.40
-15	2	-8	27.70	9.30
-15	-3	8	199.78	15.30
-15	3	8	201.78	15.70
-14	-3	8	-7.10	6.20
-14	3	8	4.80	6.30
-13	-3	8	396.56	22.10
13	-3	-8	385.96	25.60
-13	3	8	416.96	22.40
13	3	-8	378.76	26.20
-12	-3	8	25.60	10.60
12	-3	-8	19.00	7.60
-12	-3	8	13.00	4.60
-12	-3	8	14.40	6.10
12	3	-8	16.40	8.20
-12	3	8	8.90	11.00
-12	3	8	24.30	5.60
-11	-3	8	838.32	39.20
11	-3	-8	770.32	40.70
-11	-3	8	752.22	37.80
-11	-3	8	666.23	42.50
-11	3	8	761.72	38.40
11	3	-8	778.12	41.30
-11	3	8	714.63	42.80
10	-3	-8	127.59	12.60

-10	-3	8	87.69	13.00
-10	-3	8	115.79	10.20
10	-3	-8	137.79	12.20
-10	-3	8	110.19	8.40
10	3	-8	108.59	12.50
-10	3	8	127.89	9.50
-10	3	8	124.19	15.20
9	-3	-8	497.35	27.70
9	-3	-8	515.95	26.60
-9	-3	8	470.85	29.20
-9	-3	8	471.55	25.10
9	3	-8	521.55	28.30
9	3	-8	515.55	25.80
-9	3	8	492.65	29.40
-9	3	8	506.75	25.70
8	-3	-8	102.49	9.70
8	-3	-8	114.59	8.60
-8	-3	8	79.69	11.40
-8	-3	8	95.99	6.70
8	3	-8	101.19	7.50
8	3	-8	93.29	10.30
-8	3	8	108.99	7.80
-8	3	8	103.49	11.60
-7	-3	8	379.06	23.00
7	-3	-8	390.26	22.00
7	-3	-8	403.46	21.00
-7	-3	8	394.36	19.80
7	3	-8	399.86	22.60
-7	3	8	388.86	22.90
7	3	-8	396.86	20.20
-7	3	8	400.96	20.50
-6	-3	8	1000.60	49.60
6	-3	-8	1058.79	50.39
-6	-3	8	948.01	51.89
6	-3	-8	1022.20	51.29
-6	3	8	1074.19	52.19
6	3	-8	1036.20	49.70
6	3	-8	1034.70	51.69
-6	3	8	1058.09	50.19
5	-3	-8	691.83	32.40
5	-3	-8	661.13	33.30
-5	-3	8	627.54	33.80
-5	-3	8	603.24	31.50
-5	3	8	623.74	32.30

5	3	-8	664.13	33.50
-5	3	8	698.43	34.00
5	3	-8	640.94	31.60
4	-3	-8	513.35	23.70
-4	-3	8	451.05	24.90
-4	-3	8	463.15	23.00
4	-3	-8	481.05	24.60
-4	3	8	467.55	24.90
4	3	-8	474.45	23.00
4	3	-8	459.15	24.60
-4	3	8	463.75	23.90
-3	-3	8	69.19	6.20
-3	-3	8	70.69	4.00
3	-3	-8	69.39	4.80
3	-3	-8	68.89	5.90
3	3	-8	71.29	3.90
-3	3	8	62.09	6.10
3	3	-8	59.79	5.80
-3	3	8	57.49	5.30
2	-3	-8	847.52	40.40
-2	-3	8	799.22	39.20
2	-3	-8	827.22	39.70
-2	-3	8	812.42	40.30
-2	3	8	847.12	40.50
2	3	-8	824.32	40.30
-2	3	8	789.42	40.20
2	3	-8	810.72	39.10
-1	-3	8	338.57	16.80
-1	-3	8	332.97	16.10
1	-3	-8	316.27	16.50
1	-3	-8	312.07	16.90
-1	3	8	327.67	17.00
1	3	-8	332.17	16.00
-1	3	8	329.17	17.00
1	3	-8	329.57	16.80
0	-3	-8	2114.89	97.39
0	-3	8	1983.90	97.19
0	-3	8	1957.20	97.09
0	-3	-8	2035.40	97.69
0	3	8	2093.69	97.69
0	3	-8	2083.19	97.29
0	3	-8	2014.60	96.99
0	3	8	2037.20	97.59
-1	-3	-8	80.69	5.40

1	-3	8	70.59	5.60
-1	-3	-8	84.99	5.40
1	3	8	83.79	5.60
1	3	8	75.19	5.40
1	3	8	80.89	5.20
-1	3	-8	82.29	5.00
-1	3	-8	78.29	4.50
2	-3	8	1369.76	65.09
-2	-3	-8	1423.26	65.19
2	3	8	1342.07	64.89
2	3	8	1381.76	65.29
-2	3	-8	1271.27	65.39
2	3	8	1320.77	65.29
-3	-3	-8	41.40	4.00
3	-3	8	30.70	4.20
-3	-3	-8	42.50	4.20
3	3	8	36.50	4.30
3	3	8	40.90	3.60
-3	3	-8	35.90	4.10
3	3	8	40.00	4.20
-4	-3	-8	2310.07	113.39
-4	-3	-8	2486.75	113.49
4	3	8	2236.08	113.49
4	3	8	2457.15	113.39
-4	3	-8	2303.17	113.39
-4	3	-8	2266.17	112.29
-4	3	-8	2524.65	113.49
4	3	8	2347.36	112.99
-5	-3	-8	218.98	12.70
-5	-3	-8	239.18	12.60
5	3	8	223.28	12.40
-5	3	-8	231.38	12.70
-5	3	-8	202.68	11.50
5	3	8	210.68	12.50
-5	3	-8	216.28	12.80
-6	-3	-8	1380.36	63.79
-6	-3	-8	1295.17	63.89
6	-3	8	1296.67	62.29
-6	3	-8	1292.27	62.89
-6	3	-8	1265.67	63.69
6	3	8	1147.89	63.49
6	3	8	1365.76	63.59
-6	3	-8	1387.66	63.99
-7	-3	-8	35.70	6.10

-7	-3	-8	32.20	6.30
7	-3	8	18.70	2.80
7	3	8	32.20	5.40
-7	3	-8	32.10	5.70
7	3	8	31.00	5.60
-7	3	-8	22.40	4.70
-8	-3	-8	705.33	37.30
-8	-3	-8	732.43	37.00
8	-3	8	728.13	35.50
8	3	8	683.83	36.80
8	3	8	740.63	36.80
-8	3	-8	701.13	36.80
-8	3	-8	684.23	36.30
-8	3	-8	798.72	37.60
-9	-3	-8	293.17	18.80
9	-3	8	317.87	16.70
-9	-3	-8	320.97	18.80
-9	3	-8	328.47	18.40
9	3	8	310.07	18.10
-9	3	-8	279.87	17.90
-10	-3	-8	493.15	29.60
-10	-3	-8	530.55	29.00
10	-3	8	542.75	27.50
-10	3	-8	563.44	29.00
-10	3	-8	516.65	28.90
10	3	8	546.65	28.90
11	-3	8	70.69	8.50
-11	-3	-8	94.09	11.90
-11	3	-8	57.19	11.20
-11	3	-8	83.59	9.70
11	3	8	98.29	10.60
12	-3	8	343.47	20.20
-12	-3	-8	370.56	22.50
-12	3	-8	356.26	22.90
-12	3	-8	358.36	21.70
-13	-3	-8	119.19	13.80
-13	3	-8	122.89	13.60
-14	-3	-8	17.90	8.80
-14	3	-8	44.70	9.30
-15	-3	-8	131.89	16.30
-15	3	-8	171.68	16.60
-15	-4	8	18.30	6.80
-15	4	8	30.20	7.80
-14	-4	8	0.80	5.50

-14	4	8	12.20	6.50
13	-4	-8	14.00	7.70
-13	-4	8	18.10	5.30
13	4	-8	18.40	8.50
-12	-4	8	30.50	11.80
12	-4	-8	28.30	7.70
-12	-4	8	31.20	5.30
-12	-4	8	31.70	7.20
12	4	-8	42.60	9.70
-12	4	8	16.80	13.40
-11	-4	8	83.39	9.80
11	-4	-8	98.09	12.20
-11	-4	8	90.29	7.80
-11	4	8	96.69	9.00
11	4	-8	65.99	12.80
10	-4	-8	147.49	12.60
-10	-4	8	138.69	10.90
-10	-4	8	146.29	9.20
-10	-4	8	108.99	15.60
10	4	-8	137.99	13.60
-10	4	8	125.99	15.80
-10	4	8	147.59	10.30
9	-4	-8	56.79	8.40
9	-4	-8	55.69	8.70
-9	-4	8	57.99	7.10
-9	-4	8	48.10	5.40
-9	4	8	70.29	6.80
9	4	-8	59.79	10.30
-9	4	8	55.99	9.40
8	-4	-8	10.90	5.10
8	-4	-8	6.90	4.00
-8	-4	8	5.50	2.40
-8	-4	8	-0.60	5.60
-8	4	8	14.20	5.70
-8	4	8	9.40	3.50
8	4	-8	5.40	5.10
-7	-4	8	7.80	2.20
7	-4	-8	6.50	3.40
7	-4	-8	13.00	4.70
-7	-4	8	3.70	5.20
-7	4	8	5.80	4.80
7	4	-8	3.80	4.50
-7	4	8	7.90	3.10
-6	-4	8	70.29	4.70

6	-4	-8	76.99	6.50
-6	-4	8	54.19	7.80
6	-4	-8	66.89	7.50
6	4	-8	53.89	7.90
-6	4	8	64.59	8.30
-6	4	8	75.89	6.10
6	4	-8	61.19	4.90
-5	-4	8	311.37	19.40
5	-4	-8	368.86	18.00
-5	-4	8	331.87	16.70
5	-4	-8	353.56	18.80
5	4	-8	321.87	19.00
5	4	-8	335.77	16.80
-5	4	8	340.47	19.30
-5	4	8	336.37	17.80
-4	-4	8	4.60	1.30
-4	-4	8	-1.70	2.90
4	-4	-8	3.00	2.20
4	-4	-8	6.50	3.00
4	4	-8	-1.00	2.70
-4	4	8	0.40	2.60
4	4	-8	4.00	1.20
-4	4	8	1.30	3.10
-3	-4	8	277.17	16.20
3	-4	-8	300.67	16.10
3	-4	-8	320.97	15.40
-3	-4	8	294.47	14.40
3	4	-8	295.97	14.40
3	4	-8	294.37	16.20
-3	4	8	310.97	16.50
-3	4	8	275.07	15.80
2	-4	-8	8.10	1.80
-2	-4	8	4.50	2.30
2	-4	-8	5.50	2.30
-2	4	8	4.40	2.50
2	4	-8	8.70	1.20
2	4	-8	2.30	2.30
-2	4	8	2.40	2.30
1	-4	-8	44.80	4.60
-1	-4	8	49.20	3.10
1	-4	-8	44.90	3.80
-1	-4	8	41.50	3.90
1	4	-8	43.80	2.80
-1	4	8	46.60	4.50

-1	4	8	42.00	4.30
1	4	-8	36.50	4.00
0	-4	8	327.07	16.10
0	-4	-8	325.17	16.80
0	-4	-8	327.67	16.40
0	-4	8	315.17	16.10
0	4	8	326.87	16.70
0	4	8	306.87	16.70
0	4	-8	324.57	16.30
0	4	-8	324.27	15.90
-1	-4	-8	648.04	31.40
-1	-4	-8	665.23	31.80
1	-4	8	650.73	31.30
1	4	8	603.54	31.60
1	4	8	630.74	31.80
1	4	8	653.43	31.50
-1	4	-8	652.23	31.10
-1	4	-8	613.54	30.80
-2	-4	-8	205.48	10.70
2	-4	8	187.68	10.50
-2	4	-8	181.08	10.80
2	4	8	200.28	10.50
2	4	8	190.28	10.90
-2	4	-8	188.88	10.50
2	4	8	183.08	10.70
-3	-4	-8	92.59	6.40
-3	-4	-8	94.69	6.70
3	4	8	89.59	6.70
3	4	8	97.29	6.90
-3	4	-8	104.59	6.50
3	4	8	98.89	6.20
-3	4	-8	94.89	6.60
-4	-4	-8	34.80	4.70
-4	-4	-8	30.90	4.10
4	4	8	30.00	4.70
-4	4	-8	29.10	4.50
4	4	8	30.50	4.60
4	4	8	32.30	3.70
-4	4	-8	30.10	4.50
-5	-4	-8	404.26	20.10
-5	-4	-8	380.96	20.30
-5	4	-8	354.36	18.90
-5	4	-8	390.96	20.10
5	4	8	399.76	20.10

-5	4	-8	357.76	19.90
5	4	8	370.26	20.10
-6	-4	-8	41.80	5.70
6	-4	8	35.40	2.90
-6	-4	-8	42.30	6.00
6	4	8	46.70	5.90
-6	4	-8	29.80	4.40
-6	4	-8	37.80	5.40
-6	4	-8	36.40	5.30
6	4	8	42.30	5.60
-7	-4	-8	362.56	18.30
-7	-4	-8	310.07	18.50
7	-4	8	330.27	16.30
-7	4	-8	346.67	18.40
7	4	8	318.37	18.20
-7	4	-8	312.97	17.90
7	4	8	320.97	18.10
-7	4	-8	287.37	17.20
-8	-4	-8	3.10	3.90
-8	-4	-8	6.20	5.00
8	-4	8	-0.40	2.40
-8	4	-8	5.80	4.20
8	4	8	4.90	3.90
8	4	8	10.90	4.20
-8	4	-8	0.70	3.60
-9	-4	-8	45.80	8.60
9	-4	8	33.60	5.30
-9	4	-8	38.80	6.40
-9	4	-8	39.50	7.20
9	4	8	30.90	5.80
9	4	8	43.50	7.70
-10	-4	-8	124.69	11.60
10	-4	8	106.69	8.80
-10	-4	-8	119.59	12.20
-10	4	-8	130.69	11.10
10	4	8	119.49	11.40
-10	4	-8	95.99	10.70
-11	-4	-8	62.09	9.30
-11	-4	-8	59.19	11.20
11	-4	8	50.19	7.70
-11	4	-8	56.59	8.80
11	4	8	58.59	10.10
-12	-4	-8	6.40	7.10
12	-4	8	-0.50	4.40

-12	4	-8	15.10	6.80
-12	4	-8	-0.90	6.40
-13	-4	-8	11.00	7.80
-13	4	-8	10.50	7.10
-14	-4	-8	55.19	10.80
-14	4	-8	73.89	12.50
-15	-4	-8	31.50	10.00
-15	4	-8	29.40	9.50
-15	-5	8	274.37	19.70
-15	5	8	325.07	20.30
-14	-5	8	4.00	5.70
-14	5	8	16.60	6.80
13	-5	-8	223.68	20.40
-13	-5	8	223.28	14.50
-13	5	8	233.58	15.50
13	5	-8	233.08	20.10
-12	-5	8	-0.70	5.80
12	-5	-8	14.50	7.90
-12	-5	8	-3.40	10.60
-12	5	8	8.70	5.40
12	5	-8	15.50	7.90
-11	-5	8	510.85	26.00
11	-5	-8	520.25	29.10
-11	-5	8	411.66	31.60
-11	-5	8	581.64	27.40
-11	5	8	464.65	31.80
11	5	-8	503.45	30.00
-11	5	8	522.45	27.00
-10	-5	8	1.60	4.40
-10	-5	8	6.60	7.80
10	-5	-8	7.10	5.00
-10	-5	8	1.30	3.10
-10	5	8	5.80	7.50
-10	5	8	9.50	4.30
10	5	-8	1.80	6.50
-9	-5	8	991.20	48.80
-9	-5	8	889.71	52.49
9	-5	-8	1045.70	51.49
-9	-5	8	1098.79	49.60
9	-5	-8	1031.60	50.89
-9	5	8	1015.70	49.80
9	5	-8	1003.80	51.89
-9	5	8	970.30	52.69
-8	-5	8	104.69	6.50

8	-5	-8	102.19	9.60
-8	-5	8	81.79	11.70
8	-5	-8	108.59	9.10
-8	-5	8	117.29	7.50
8	5	-8	89.49	10.70
-8	5	8	89.99	11.70
-8	5	8	95.89	7.90
7	-5	-8	1446.66	70.69
-7	-5	8	1539.85	68.79
-7	-5	8	1284.87	71.09
-7	-5	8	1384.66	68.39
7	-5	-8	1474.65	69.89
-7	5	8	1433.36	71.49
-7	5	8	1461.45	69.39
7	5	-8	1405.86	70.89
-6	-5	8	847.92	38.50
-6	-5	8	787.12	38.20
-6	-5	8	709.43	40.90
6	-5	-8	822.92	39.70
6	-5	-8	819.12	40.50
-6	5	8	781.82	39.30
6	5	-8	795.02	40.80
-6	5	8	784.12	41.10
5	-5	-8	2210.78	100.69
5	-5	-8	2110.99	101.29
-5	-5	8	2005.50	99.39
-5	-5	8	1975.30	101.59
5	5	-8	2138.99	101.59
-5	5	8	2087.69	101.69
-5	5	8	2116.49	100.59
4	-5	-8	653.13	30.60
-4	-5	8	575.84	31.50
-4	-5	8	615.84	29.30
4	-5	-8	628.04	31.30
4	5	-8	608.34	29.30
-4	5	8	589.04	30.80
4	5	-8	609.44	31.50
-4	5	8	625.74	31.70
3	-5	-8	1582.94	77.49
3	-5	-8	1660.73	76.89
-3	-5	8	1565.24	77.59
-3	5	8	1475.55	77.19
3	5	-8	1653.33	77.59
-3	5	8	1653.23	77.79

2	-5	-8	3338.97	157.48
-2	-5	8	3333.87	155.88
-2	-5	8	3190.18	157.18
2	-5	-8	3395.86	156.78
2	5	-8	3331.27	157.28
-2	5	8	3418.16	157.58
-2	5	8	3130.69	157.28
2	5	-8	3207.68	155.78
1	-5	-8	89.39	6.00
-1	-5	8	103.49	5.20
-1	-5	8	99.09	6.30
1	-5	-8	92.29	6.80
1	5	-8	92.39	5.00
-1	5	8	88.29	6.70
-1	5	8	87.89	6.50
1	5	-8	102.49	6.40
0	-5	-8	3158.78	147.99
0	-5	-8	3164.58	147.49
0	-5	8	3128.49	147.19
0	5	8	3048.40	147.89
0	5	-8	3028.80	146.99
0	5	-8	3154.18	147.29
0	5	-8	2997.50	146.89
0	5	8	3087.39	147.79
-1	-5	-8	436.56	22.30
-1	-5	-8	439.66	21.90
-1	5	-8	440.06	21.50
-1	5	-8	459.85	21.30
-1	5	-8	427.06	21.60
1	5	8	424.86	22.10
1	5	8	421.66	22.20
-2	-5	-8	1453.35	68.59
-2	5	-8	1413.56	68.29
-2	5	-8	1408.56	68.79
2	5	8	1376.66	68.69
-2	5	-8	1418.86	67.49
2	5	8	1421.56	68.99
2	5	8	1455.95	68.59
-3	-5	-8	4.10	2.40
-3	-5	-8	3.90	2.40
-3	5	-8	1.30	2.30
3	5	8	7.30	2.80
3	5	8	2.10	2.40
3	5	8	4.60	2.00

-3	5	-8	-0.20	2.10
-4	-5	-8	1524.75	74.59
-4	-5	-8	1589.64	74.29
-4	5	-8	1467.65	74.19
-4	5	-8	1530.05	73.09
4	5	8	1468.65	74.39
4	5	8	1605.44	74.69
-4	5	-8	1598.24	74.29
4	5	8	1507.85	74.09
-5	-5	-8	10.20	3.50
-5	-5	-8	14.40	3.40
5	5	8	7.40	3.30
-5	5	-8	5.20	2.00
-5	5	-8	5.80	3.20
5	5	8	8.80	3.20
-5	5	-8	2.10	3.10
6	-5	8	1128.49	52.99
-6	-5	-8	1067.39	55.19
-6	-5	-8	1155.28	54.79
-6	5	-8	1106.09	54.79
-6	5	-8	1199.98	55.09
6	5	8	1169.68	55.09
-6	5	-8	1067.09	53.89
6	5	8	1015.30	54.69
7	-5	8	17.10	3.00
-7	-5	-8	28.80	5.20
-7	-5	-8	24.20	4.60
7	5	8	25.30	4.60
-7	5	-8	17.30	4.90
7	5	8	28.70	4.80
-7	5	-8	25.30	4.00
-7	5	-8	12.90	4.30
-8	-5	-8	736.03	39.70
8	-5	8	792.32	37.30
8	5	8	810.62	39.40
8	5	8	739.93	38.90
-8	5	-8	775.42	39.00
-8	5	-8	726.33	38.40
9	-5	8	135.79	8.60
-9	-5	-8	120.69	12.00
-9	5	-8	123.59	10.40
9	5	8	147.49	11.40
-9	5	-8	127.79	10.40
9	5	8	131.29	10.80

10	-5	8	782.22	36.80
-10	-5	-8	694.23	39.50
-10	-5	-8	770.12	38.80
10	5	8	793.82	39.00
-10	5	-8	718.73	38.30
-10	5	-8	651.73	39.40
-11	-5	-8	439.76	27.80
11	-5	8	484.05	24.60
-11	-5	-8	507.95	27.20
11	5	8	485.75	27.20
-11	5	-8	424.26	26.60
-11	5	-8	460.85	26.50
12	-5	8	412.26	25.00
-12	-5	-8	450.25	25.20
-12	5	-8	359.86	24.80
-12	5	-8	423.66	24.60
-13	-5	-8	136.69	15.00
-13	5	-8	136.69	13.90
-14	-5	-8	145.39	15.80
-14	5	-8	102.79	13.90
-15	-5	-8	411.56	27.20
-15	5	-8	406.26	26.60
-15	6	8	-3.40	7.20
-14	-6	8	-1.10	5.50
-14	6	8	1.40	6.40
-13	-6	8	17.40	5.20
-13	6	8	19.50	6.30
13	6	-8	18.80	8.90
-12	-6	8	44.80	12.40
-12	-6	8	84.69	12.10
12	-6	-8	77.79	12.10
-12	-6	8	69.89	7.80
12	6	-8	50.49	9.80
-12	6	8	96.39	9.30
-11	-6	8	88.59	16.10
-11	-6	8	93.79	10.10
11	-6	-8	106.99	12.70
-11	-6	8	112.39	8.40
-11	6	8	121.69	10.10
-11	6	8	124.39	17.10
11	6	-8	104.49	14.40
-10	-6	8	2.50	4.60
-10	-6	8	6.20	8.10
10	-6	-8	20.00	5.70

-10	-6	8	3.90	3.40
-10	6	8	12.40	8.00
10	6	-8	11.40	7.30
-10	6	8	15.80	4.90
-9	-6	8	2.60	2.70
-9	-6	8	0.60	6.80
9	-6	-8	5.00	4.80
-9	-6	8	1.90	3.50
-9	6	8	10.20	6.50
-9	6	8	17.20	4.40
9	6	-8	4.20	5.80
-8	-6	8	20.20	2.80
8	-6	-8	22.60	5.20
-8	-6	8	26.40	4.90
-8	-6	8	15.70	6.40
8	-6	-8	26.20	5.50
-8	6	8	27.90	4.60
8	6	-8	16.30	5.80
-8	6	8	14.70	6.10
-7	-6	8	2.70	2.20
7	-6	-8	9.20	4.30
-7	-6	8	2.50	5.30
-7	-6	8	2.50	1.90
7	-6	-8	6.20	3.60
-7	6	8	11.00	3.50
-7	6	8	-4.80	4.60
7	6	-8	8.40	4.90
-6	-6	8	219.28	11.50
6	-6	-8	248.28	14.50
6	-6	-8	230.58	13.50
-6	-6	8	230.48	11.70
-6	-6	8	206.28	15.30
-6	6	8	235.68	15.30
-6	6	8	222.48	12.90
6	6	-8	229.98	15.00
5	-6	-8	9.80	3.30
-5	-6	8	-3.60	3.70
-5	-6	8	7.50	1.30
-5	-6	8	4.90	1.30
5	-6	-8	10.30	3.90
-5	6	8	12.40	4.20
5	6	-8	1.80	3.90
-5	6	8	9.00	3.20
4	-6	-8	3.90	2.90

-4	-6	8	2.20	3.30
4	-6	-8	0.10	2.30
4	6	-8	6.70	3.30
-4	6	8	-1.40	2.90
-4	6	8	1.60	3.10
-3	-6	8	67.39	6.70
3	-6	-8	78.99	6.80
3	-6	-8	85.19	5.90
-3	6	8	74.39	6.80
-3	6	8	78.89	6.60
3	6	-8	66.39	6.60
2	-6	-8	106.89	7.60
2	-6	-8	110.49	6.70
-2	-6	8	94.19	7.20
2	6	-8	104.09	7.40
-2	6	8	106.79	7.60
-2	6	8	83.69	7.30
1	-6	-8	19.40	3.50
-1	-6	8	24.30	3.70
1	-6	-8	24.90	3.20
1	6	-8	19.90	3.70
1	6	-8	18.70	2.00
1	6	-8	20.70	1.80
-1	6	8	23.00	4.00
-1	6	8	23.50	3.80
0	-6	8	880.61	42.70
0	-6	-8	884.81	43.60
0	-6	-8	923.31	43.00
0	6	8	902.91	43.40
0	6	-8	874.01	42.40
0	6	-8	859.21	42.30
0	6	8	830.32	43.40
0	6	-8	904.21	42.80
-1	-6	-8	1077.79	51.09
-1	-6	-8	1079.79	50.59
1	6	8	989.20	50.79
-1	6	-8	1029.30	50.19
-1	6	-8	1063.29	49.99
-1	6	-8	1039.20	50.09
1	6	8	1007.30	50.69
-2	-6	-8	96.59	6.70
2	6	8	96.69	6.70
2	6	8	89.49	6.90
-2	6	-8	100.69	6.30

-2	6	-8	101.19	5.50
-2	6	-8	100.69	6.40
-3	-6	-8	574.54	29.90
-3	-6	-8	608.64	29.60
-3	6	-8	594.34	29.40
-3	6	-8	584.44	28.40
3	6	8	621.54	30.20
3	6	8	592.94	29.50
-3	6	-8	615.14	29.40
3	6	8	538.05	29.60
-3	6	-8	579.14	28.30
-4	-6	-8	-1.40	2.90
-4	-6	-8	3.50	2.70
4	6	8	2.30	2.60
-4	6	-8	0.20	2.60
-4	6	-8	3.30	1.30
4	6	8	-0.60	2.20
4	6	8	0.90	2.90
-4	6	-8	-1.20	2.30
-5	-6	-8	627.24	33.60
-5	-6	-8	671.83	33.10
-5	6	-8	654.53	33.20
-5	6	-8	677.63	32.00
5	6	8	623.24	33.20
-5	6	-8	617.94	33.00
5	6	8	722.43	33.80
-6	-6	-8	14.50	4.00
-6	-6	-8	13.20	4.00
-6	6	-8	14.10	4.10
6	6	8	16.40	3.80
6	6	8	10.20	3.80
-6	6	-8	13.80	4.00
7	-6	8	416.16	20.00
-7	-6	-8	397.46	22.80
-7	-6	-8	412.76	21.90
7	6	8	460.85	22.70
-7	6	-8	382.66	22.00
-7	6	-8	399.76	21.20
7	6	8	362.26	21.80
-7	6	-8	405.16	22.50
8	-6	8	134.89	8.10
-8	-6	-8	135.69	11.70
-8	6	-8	144.89	10.80
-8	6	-8	121.59	10.00

8	6	8	139.39	10.30
8	6	8	153.48	11.20
9	-6	8	85.39	6.70
-9	-6	-8	86.39	10.80
9	6	8	83.69	9.40
9	6	8	101.19	10.30
-9	6	-8	92.09	9.30
-9	6	-8	97.09	9.60
-10	-6	-8	25.00	6.60
10	-6	8	29.00	4.30
10	6	8	50.19	9.80
-10	6	-8	18.00	5.90
10	6	8	27.30	6.00
-11	-6	-8	64.49	10.90
11	-6	8	45.00	7.10
-11	-6	-8	44.90	8.50
-11	6	-8	58.89	10.80
-11	6	-8	80.49	10.30
11	6	8	57.29	11.10
-12	-6	-8	112.69	13.10
-12	6	-8	106.39	12.00
-12	6	-8	82.59	13.00
-13	-6	-8	47.90	9.80
-13	6	-8	35.10	8.10
-14	-6	-8	38.70	9.50
-14	6	-8	51.09	9.60
-15	-6	-8	2.60	8.90
-15	6	-8	24.60	9.00
-15	7	8	101.49	12.90
-14	-7	8	1.90	5.70
-14	7	8	13.00	6.70
-13	-7	8	177.48	12.20
-13	7	8	191.68	14.10
13	7	-8	141.49	18.60
-12	-7	8	1.90	4.20
12	-7	-8	11.00	7.30
12	7	-8	8.10	8.80
-12	7	8	5.60	5.50
-11	-7	8	402.16	21.60
-11	-7	8	452.05	22.90
11	-7	-8	431.96	25.50
-11	7	8	418.86	23.30
11	7	-8	405.36	26.50
-11	7	8	380.46	28.60

-10	-7	8	159.18	10.90
-10	-7	8	139.09	9.30
-10	-7	8	113.29	16.50
-10	7	8	163.18	16.70
10	7	-8	147.39	14.90
-10	7	8	170.18	11.30
-9	-7	8	244.18	13.80
-9	-7	8	242.08	19.90
9	-7	-8	269.87	17.40
-9	-7	8	308.47	14.80
-9	7	8	244.28	19.40
-9	7	8	249.28	15.30
9	7	-8	265.87	18.40
-8	-7	8	46.30	5.10
8	-7	-8	40.20	8.30
-8	-7	8	50.59	8.60
-8	-7	8	49.80	4.40
8	-7	-8	58.39	9.00
-8	7	8	55.69	6.70
8	7	-8	43.60	7.60
-8	7	8	37.30	7.50
7	-7	-8	162.88	12.20
-7	-7	8	172.58	9.10
-7	-7	8	147.99	13.40
7	-7	-8	163.98	11.60
-7	-7	8	159.48	8.70
-7	7	8	175.38	10.90
-7	7	8	179.68	13.60
7	7	-8	159.18	13.20
-6	-7	8	910.61	41.10
6	-7	-8	867.11	43.60
6	-7	-8	877.41	42.90
-6	-7	8	828.92	41.00
-6	-7	8	779.32	44.10
-6	7	8	864.71	44.20
-6	7	8	883.81	42.70
6	7	-8	848.32	44.00
-5	-7	8	853.11	47.90
5	-7	-8	975.50	47.00
5	-7	-8	952.70	47.60
5	7	-8	977.60	48.00
-5	7	8	985.20	47.00
-5	7	8	955.70	48.10
4	-7	-8	108.49	7.40

4	-7	-8	99.09	8.00
-4	-7	8	88.19	8.40
-4	7	8	92.69	7.70
4	7	-8	97.99	8.30
-4	7	8	103.09	8.30
-3	-7	8	861.51	45.90
3	-7	-8	942.91	46.10
3	-7	-8	982.00	45.30
3	7	-8	950.20	46.00
-3	7	8	938.41	46.20
-3	7	8	871.51	45.80
2	-7	-8	103.39	6.20
-2	-7	8	78.69	6.60
2	-7	-8	78.09	7.00
-2	7	8	66.59	6.50
2	7	-8	92.09	6.70
-2	7	8	80.19	6.90
1	-7	-8	7.80	2.80
-1	-7	8	12.00	2.60
1	-7	-8	7.50	2.30
-1	7	8	10.20	3.00
1	7	-8	8.90	1.20
-1	7	8	9.00	3.30
1	7	-8	9.50	1.30
1	7	-8	11.40	2.60
0	-7	8	970.30	48.40
0	-7	-8	1032.70	48.70
0	-7	-8	1009.30	49.30
0	7	-8	992.50	47.90
0	7	8	1016.20	49.20
0	7	-8	990.00	48.50
0	7	8	1023.70	49.10
0	7	-8	968.70	47.90
-1	-7	-8	798.62	37.50
-1	7	-8	748.23	37.00
1	7	8	771.72	37.90
-1	7	-8	753.72	36.90
-1	7	-8	736.33	37.10
1	7	8	764.52	37.80
-2	-7	-8	1201.88	55.39
-2	7	-8	1125.89	54.59
-2	7	-8	1144.09	55.09
-2	7	-8	1123.29	54.99
2	7	8	1129.99	55.59

2	7	8	1109.99	55.59
-3	-7	-8	35.30	5.40
-3	-7	-8	40.10	4.80
-3	7	-8	39.30	3.20
3	7	8	38.80	4.70
3	7	8	42.20	5.00
-3	7	-8	36.00	4.40
-3	7	-8	37.50	4.70
-3	7	-8	42.80	3.10
-4	-7	-8	829.32	39.80
-4	-7	-8	779.72	40.40
-4	7	-8	841.82	39.80
4	7	8	884.91	44.00
-4	7	-8	774.22	39.60
4	7	8	768.32	39.70
4	7	8	760.42	39.90
-4	7	-8	794.12	38.70
-5	-7	-8	12.30	3.80
-5	-7	-8	12.40	3.30
-5	7	-8	21.00	3.40
-5	7	-8	14.60	3.60
-5	7	-8	13.80	3.70
5	7	8	15.00	3.90
5	7	8	24.10	4.40
-6	-7	-8	934.21	50.39
-6	-7	-8	1074.59	49.70
-6	7	-8	991.80	49.60
6	7	8	916.61	49.60
-6	7	-8	1060.09	49.99
-6	7	-8	1003.10	48.80
6	7	8	1036.30	50.29
-7	-7	-8	151.68	12.10
7	-7	8	152.78	8.40
-7	-7	-8	158.18	10.70
-7	7	-8	154.18	10.20
-7	7	-8	164.18	11.20
-7	7	-8	141.69	11.10
7	7	8	173.18	11.80
7	7	8	151.98	10.70
8	-7	8	305.27	14.90
-8	-7	-8	272.57	18.10
8	7	8	298.97	17.80
-8	7	-8	269.37	16.40
-8	7	-8	280.37	16.70

8	7	8	290.57	17.20
9	-7	8	81.39	6.40
-9	-7	-8	77.89	10.70
9	7	8	84.59	10.30
-9	7	-8	88.29	9.20
9	7	8	67.79	9.10
-9	7	-8	63.49	8.80
10	-7	8	411.06	21.60
-10	-7	-8	396.56	25.20
-10	7	-8	397.96	23.80
10	7	8	466.95	25.00
-10	7	-8	418.16	23.80
10	7	8	397.36	23.60
11	-7	8	225.68	13.90
-11	-7	-8	215.58	18.20
-11	-7	-8	265.17	17.60
11	7	8	247.08	17.50
-11	7	-8	251.77	16.70
-11	7	-8	209.08	16.60
-12	-7	-8	336.67	20.90
-12	7	-8	249.08	19.80
-12	7	-8	310.27	20.00
-13	-7	-8	105.69	14.10
-13	7	-8	108.09	13.40
-14	-7	-8	105.29	15.10
-14	7	-8	53.79	10.10
-15	-7	-8	152.68	17.70
-15	7	-8	109.69	15.30
-14	8	8	96.09	12.10
-13	-8	8	-0.40	4.70
-13	8	8	13.70	6.70
13	8	-8	7.10	9.40
12	-8	-8	213.08	17.60
-12	-8	8	222.28	13.50
-12	8	8	235.28	15.50
12	8	-8	230.68	19.60
-11	-8	8	32.10	5.80
-11	-8	8	14.30	10.60
-11	-8	8	38.40	5.70
11	-8	-8	25.60	7.10
-11	8	8	25.90	9.50
11	8	-8	23.30	8.30
-11	8	8	48.50	8.40
-10	-8	8	381.56	28.70

-10	-8	8	420.66	22.10
10	-8	-8	448.56	25.80
-10	-8	8	479.25	23.10
-10	8	8	457.75	24.00
-10	8	8	419.96	28.40
10	8	-8	436.46	26.80
-9	-8	8	6.30	3.40
-9	-8	8	5.10	2.50
9	-8	-8	1.90	5.20
-9	-8	8	6.10	8.00
-9	8	8	11.10	7.10
-9	8	8	17.60	5.00
9	8	-8	1.60	6.10
-8	-8	8	294.27	21.90
-8	-8	8	355.26	17.50
8	-8	-8	329.67	20.40
-8	8	8	343.97	19.00
8	8	-8	391.76	21.90
-8	8	8	334.17	21.90
-7	-8	8	264.97	13.00
-7	-8	8	237.28	12.80
-7	-8	8	224.18	17.40
7	-8	-8	275.77	15.90
7	-8	-8	254.67	16.20
-7	8	8	277.57	15.10
7	8	-8	253.17	17.10
-7	8	8	264.27	17.60
6	-8	-8	709.13	35.90
-6	-8	8	621.24	36.40
6	-8	-8	710.13	35.30
-6	-8	8	655.53	32.90
-6	-8	8	659.13	33.00
6	8	-8	758.82	36.60
-6	8	8	716.23	35.00
-6	8	8	711.83	36.70
5	-8	-8	1166.28	58.49
5	-8	-8	1228.78	57.99
-5	-8	8	1086.69	58.89
-5	8	8	1192.58	59.09
5	8	-8	1184.58	58.79
-5	8	8	1221.28	57.99
4	-8	-8	308.67	17.50
4	-8	-8	318.47	16.70
-4	-8	8	278.47	17.60

-4	8	8	306.17	17.10
4	8	-8	333.47	17.90
-4	8	8	289.37	17.60
3	-8	-8	591.94	28.40
3	-8	-8	551.44	29.10
-3	-8	8	527.75	29.10
-3	8	8	523.45	28.80
-3	8	8	577.64	29.30
3	8	-8	597.14	29.10
2	-8	-8	622.04	30.40
2	-8	-8	613.14	31.20
-2	-8	8	608.64	31.00
-2	8	8	619.84	31.20
-2	8	8	545.55	30.90
2	8	-8	645.54	30.90
1	-8	-8	388.66	19.40
1	-8	-8	389.36	20.40
-1	-8	8	370.36	19.70
1	8	-8	391.16	19.60
-1	8	8	350.76	20.10
-1	8	8	385.06	20.10
1	8	-8	370.56	18.20
1	8	-8	370.66	18.20
0	-8	-8	20.50	3.80
0	-8	-8	23.10	3.90
0	-8	8	28.50	3.70
0	8	8	15.40	3.20
0	8	8	26.50	4.40
0	8	8	26.60	5.50
0	8	-8	19.30	2.60
0	8	-8	16.00	2.50
-1	-8	-8	1442.06	69.29
-1	8	-8	1467.15	68.89
1	8	8	1409.96	70.19
1	8	8	1427.16	69.79
1	8	8	1410.46	69.79
-1	8	-8	1456.75	69.09
-2	-8	-8	126.69	8.20
-2	8	-8	131.89	7.40
2	8	8	126.59	8.50
2	8	8	125.29	8.60
-2	8	-8	122.99	7.70
-2	8	-8	127.69	7.70
-3	-8	-8	1042.70	53.09

-3	-8	-8	1131.99	52.49
-3	8	-8	1045.50	51.49
-3	8	-8	1050.69	51.59
-3	8	-8	1144.19	52.29
3	8	8	1023.20	52.59
-3	8	-8	1070.59	52.19
3	8	8	1075.19	52.69
-4	-8	-8	17.00	4.30
-4	-8	-8	12.60	3.10
-4	8	-8	8.80	3.10
4	8	8	15.50	3.80
-4	8	-8	9.50	3.20
-4	8	-8	12.00	2.10
4	8	8	15.00	3.50
-5	-8	-8	522.95	28.00
-5	-8	-8	552.04	27.10
5	8	8	489.55	27.10
-5	8	-8	508.95	26.90
-5	8	-8	546.65	26.10
-5	8	-8	528.05	27.20
-6	-8	-8	14.50	4.90
-6	-8	-8	5.10	3.70
-6	8	-8	7.30	4.00
-6	8	-8	5.70	4.20
-6	8	-8	6.40	2.80
6	8	8	5.50	4.00
-7	-8	-8	1027.50	54.89
-7	-8	-8	1067.79	53.79
-7	8	-8	1143.29	53.29
7	8	8	1166.08	55.39
7	8	8	1043.00	53.99
-7	8	-8	1080.39	54.19
-7	8	-8	1075.99	53.89
8	-8	8	20.10	3.80
-8	-8	-8	17.90	5.50
8	8	8	11.90	4.60
-8	8	-8	22.00	5.00
-8	8	-8	16.90	4.30
8	8	8	26.80	5.90
-9	-8	-8	360.96	23.40
9	-8	8	384.36	19.80
9	8	8	426.16	23.20
9	8	8	359.06	21.80
-9	8	-8	376.76	21.50

-9	8	-8	392.86	21.90
10	-8	8	208.48	11.70
-10	-8	-8	172.38	15.80
10	8	8	198.68	15.60
10	8	8	188.08	15.60
-10	8	-8	179.88	14.10
-10	8	-8	191.78	14.20
11	-8	8	372.66	20.20
-11	8	-8	404.66	23.00
11	8	8	398.96	24.60
11	8	8	360.06	22.30
-11	8	-8	348.87	22.80
-12	-8	-8	119.49	13.90
-12	8	-8	97.39	12.50
-12	8	-8	84.69	13.00
-13	-8	-8	157.98	16.80
-13	8	-8	184.18	15.50
-14	-8	-8	127.39	16.30
-14	8	-8	131.29	13.60
-14	9	8	37.00	7.90
-13	-9	8	19.00	7.80
13	9	-8	11.00	9.40
-13	9	8	15.20	6.50
-12	-9	8	10.80	4.20
12	-9	-8	11.50	7.60
12	9	-8	9.80	8.60
-12	9	8	13.40	6.30
-11	-9	8	143.69	9.90
11	-9	-8	179.28	15.10
11	9	-8	127.69	16.50
-11	9	8	169.88	12.30
-10	-9	8	9.60	8.60
-10	-9	8	12.80	3.10
10	-9	-8	14.90	6.10
-10	-9	8	4.20	3.90
-10	9	8	21.40	6.30
10	9	-8	12.60	8.10
-10	9	8	10.10	8.30
9	-9	-8	4.90	5.20
-9	-9	8	15.10	8.10
-9	-9	8	9.20	2.70
-9	9	8	-1.70	6.90
9	9	-8	1.80	6.20
-9	9	8	7.30	4.40

-8	-9	8	21.60	7.00
8	-9	-8	12.30	5.20
-8	-9	8	20.20	2.80
-8	-9	8	18.90	3.20
-8	9	8	17.80	6.40
8	9	-8	22.00	6.20
-8	9	8	19.40	4.90
7	-9	-8	270.57	16.60
-7	-9	8	251.77	13.00
-7	-9	8	259.47	12.90
-7	-9	8	227.78	17.90
7	9	-8	276.17	17.60
-7	9	8	260.47	18.10
-7	9	8	294.47	15.70
6	-9	-8	38.40	7.20
-6	-9	8	25.40	6.00
6	-9	-8	45.90	7.40
-6	9	8	40.20	5.80
6	9	-8	42.30	8.50
-6	9	8	42.20	8.70
-5	-9	8	13.40	4.90
5	-9	-8	25.20	5.20
5	-9	-8	24.70	4.30
-5	9	8	21.20	4.50
-5	9	8	15.20	4.80
5	9	-8	11.20	4.80
4	-9	-8	18.10	4.30
4	-9	-8	14.10	3.50
-4	-9	8	18.90	4.60
4	9	-8	17.40	4.70
-4	9	8	18.90	4.30
-4	9	8	28.60	5.40
3	-9	-8	14.70	3.90
3	-9	-8	26.60	4.60
-3	-9	8	16.60	4.40
-3	9	8	7.40	4.00
-3	9	8	17.80	4.00
3	9	-8	20.50	4.50
-2	-9	8	191.28	11.80
2	-9	-8	199.38	11.10
2	-9	-8	201.38	12.20
-2	9	8	168.28	11.60
-2	9	8	198.08	12.20
2	9	-8	175.48	11.50

1	-9	-8	249.48	14.60
-1	-9	8	239.08	14.10
1	-9	-8	263.07	13.80
1	9	-8	255.27	14.00
-1	9	8	245.08	14.60
1	9	-8	248.48	12.30
1	9	-8	247.48	12.40
-1	9	8	264.07	14.70
0	-9	8	168.28	10.20
0	-9	-8	185.28	11.50
0	-9	-8	193.88	10.40
0	9	-8	162.78	10.30
0	9	8	186.08	11.10
0	9	-8	176.18	9.30
0	9	-8	189.08	9.40
0	9	8	168.48	11.70
0	9	8	171.48	11.00
-1	-9	-8	117.59	7.80
-1	9	-8	115.29	7.50
-1	9	-8	116.39	7.00
1	9	8	123.99	8.50
-1	9	-8	123.39	7.10
1	9	8	110.89	9.20
1	9	8	113.59	8.40
-2	-9	-8	83.79	6.20
-2	9	-8	80.49	5.70
2	9	8	70.19	7.50
2	9	8	89.29	7.10
-2	9	-8	67.79	5.70
-2	9	-8	90.29	5.90
2	9	8	78.89	6.90
-3	-9	-8	33.50	4.80
-3	9	-8	26.00	3.20
-3	9	-8	24.70	3.60
-3	9	-8	16.90	3.50
3	9	8	24.40	4.20
3	9	8	20.70	3.70
-3	9	-8	26.60	4.10
3	9	8	24.60	4.70
-4	-9	-8	93.99	6.90
-4	-9	-8	73.09	7.90
-4	9	-8	81.49	5.50
4	9	8	69.59	7.80
-4	9	-8	76.69	6.70

-4	9	-8	71.69	5.40
4	9	8	78.69	7.20
-4	9	-8	81.69	6.80
4	9	8	76.29	6.90
-5	-9	-8	0.20	3.70
-5	-9	-8	3.90	3.40
5	9	8	-1.20	2.50
-5	9	-8	6.40	2.50
-5	9	-8	3.70	3.40
5	9	8	6.70	3.70
-5	9	-8	4.70	3.40
-6	-9	-8	214.28	12.90
-6	-9	-8	200.38	14.10
6	9	8	223.38	13.20
-6	9	-8	190.08	12.90
-6	9	-8	189.18	12.80
-6	9	-8	204.38	11.70
-7	-9	-8	4.90	5.00
-7	9	-8	3.20	3.30
-7	9	-8	1.20	4.20
7	9	8	4.20	4.30
-7	9	-8	-0.90	4.80
-8	-9	-8	66.39	10.30
8	-9	8	84.79	5.80
-8	9	-8	67.09	7.80
-8	9	-8	84.79	8.80
8	9	8	64.29	8.30
9	-9	8	16.60	3.30
-9	-9	-8	19.70	6.30
-9	9	-8	9.90	5.10
9	9	8	14.50	5.10
-9	9	-8	19.90	5.80
-10	-9	-8	37.70	8.10
10	-9	8	41.50	6.10
-10	9	-8	48.40	9.00
-10	9	-8	30.70	6.40
10	9	8	34.20	7.70
11	-9	8	7.90	4.10
11	9	8	11.30	6.30
-11	9	-8	18.50	6.80
-11	9	-8	13.90	6.50
-12	-9	-8	117.29	13.80
-12	9	-8	92.49	12.20
-13	-9	-8	26.00	9.30

-13	9	-8	24.80	8.30
-14	-9	-8	11.50	9.90
-14	9	-8	10.80	8.40
-14	10	8	344.67	21.90
-13	10	8	31.00	7.80
13	10	-8	22.50	10.20
-12	-10	8	270.67	15.60
-12	10	8	281.67	18.10
12	10	-8	298.77	23.00
-11	-10	8	59.39	6.60
11	-10	-8	76.09	11.90
11	10	-8	104.69	15.50
10	-10	-8	790.42	42.50
-10	-10	8	759.62	38.90
-10	-10	8	866.51	39.40
10	10	-8	826.12	43.80
-10	10	8	791.82	40.80
-10	10	8	755.12	45.00
-9	-10	8	51.79	9.60
-9	-10	8	65.99	7.60
9	-10	-8	58.39	9.90
-9	-10	8	54.39	5.30
-9	10	8	54.89	8.20
-9	10	8	71.69	10.80
9	10	-8	58.49	11.60
8	-10	-8	867.81	44.20
-8	-10	8	846.52	40.90
-8	-10	8	752.82	45.40
-8	-10	8	835.82	40.80
-8	10	8	868.51	45.70
-8	10	8	878.51	42.90
8	10	-8	904.21	45.10
-7	-10	8	342.47	22.70
7	-10	-8	349.17	21.10
7	10	-8	409.46	22.40
-7	10	8	370.16	20.10
-7	10	8	331.17	22.10
6	-10	-8	365.56	21.30
6	-10	-8	354.86	21.10
-6	-10	8	335.57	22.30
-6	10	8	378.66	20.50
6	10	-8	422.36	22.50
-6	10	8	368.06	22.20
5	-10	-8	654.23	33.60

5	-10	-8	649.54	33.00
-5	-10	8	572.84	34.00
5	10	-8	666.53	34.10
-5	10	8	655.53	33.00
-5	10	8	657.73	34.20
4	-10	-8	701.63	35.50
-4	-10	8	628.84	35.60
4	-10	-8	720.43	34.80
-4	10	8	691.63	35.70
-4	10	8	676.03	35.00
4	10	-8	690.73	35.50
3	-10	-8	1421.26	69.49
3	-10	-8	1462.95	68.69
-3	-10	8	1285.77	69.29
-3	10	8	1391.86	69.29
-3	10	8	1429.06	69.59
3	10	-8	1481.05	69.39
2	-10	-8	299.07	16.90
-2	-10	8	278.47	16.50
2	-10	-8	297.67	15.70
-2	10	8	288.47	16.80
2	10	-8	309.47	16.50
-2	10	8	267.27	16.60
1	-10	-8	2704.73	127.19
1	-10	-8	2746.23	126.19
-1	-10	8	2581.44	126.49
1	10	-8	2522.55	124.89
-1	10	8	2777.82	127.09
1	10	-8	2713.83	126.49
1	10	-8	2547.55	124.99
-1	10	8	2485.85	126.79
0	-10	8	359.46	18.80
0	-10	-8	363.76	18.80
0	-10	-8	370.76	20.00
0	10	8	343.57	19.60
0	10	-8	365.56	19.00
0	10	-8	346.27	17.70
0	10	-8	350.86	17.80
0	10	8	353.26	20.30
0	10	8	370.36	19.70
-1	-10	-8	1865.81	86.19
-1	10	-8	1774.62	86.09
1	10	8	1881.71	87.59
1	10	8	1871.41	86.99

-1	10	-8	1751.12	85.49
1	10	8	1821.52	86.79
-1	10	-8	1560.44	85.99
-2	-10	-8	151.68	9.40
2	10	8	158.58	11.20
-2	10	-8	145.89	9.00
2	10	8	139.09	9.70
-2	10	-8	143.69	8.90
2	10	8	148.89	10.00
-2	10	-8	147.49	8.70
-2	10	-8	152.38	8.80
-3	-10	-8	1316.57	61.79
3	-10	8	1305.87	61.59
-3	10	-8	1245.68	61.09
-3	10	-8	1271.77	61.59
3	10	8	1308.57	63.09
-3	10	-8	1275.37	61.29
-3	10	-8	1259.57	61.39
3	10	8	1230.28	62.19
3	10	8	1195.58	61.99
4	-10	8	113.99	8.20
-4	-10	-8	117.09	8.20
-4	-10	-8	107.69	9.50
-4	10	-8	107.39	7.20
4	10	8	108.79	8.50
-4	10	-8	120.59	8.10
-4	10	-8	108.89	7.10
4	10	8	112.39	8.70
4	10	8	116.49	9.90
-4	10	-8	105.49	8.10
-5	-10	-8	1581.44	73.09
-5	-10	-8	1407.86	73.99
5	10	8	1475.95	73.19
5	10	8	1564.74	74.29
-5	10	-8	1453.15	72.19
-5	10	-8	1522.15	73.09
-5	10	-8	1485.15	72.79
-6	-10	-8	223.88	15.40
-6	-10	-8	248.28	13.90
-6	10	-8	213.58	13.90
-6	10	-8	232.38	12.90
6	10	8	230.88	15.30
6	10	8	231.28	14.10
-6	10	-8	194.58	14.10

-7	-10	-8	1046.00	55.19
7	10	8	1031.40	53.89
-7	10	-8	1091.89	53.79
-7	10	-8	1149.89	53.29
-8	-10	-8	2.90	6.20
-8	10	-8	14.60	5.40
8	10	8	-4.40	3.70
8	10	8	4.40	4.60
-8	10	-8	7.10	4.10
9	-10	8	667.33	30.30
-9	-10	-8	586.14	34.20
-9	10	-8	629.64	32.10
-9	10	-8	548.75	32.70
9	10	8	578.84	32.20
10	-10	8	347.37	18.00
-10	-10	-8	327.67	23.00
-10	10	-8	316.57	20.40
-10	10	-8	354.86	20.20
10	10	8	321.37	20.20
11	-10	8	427.36	22.20
-11	10	-8	378.06	24.80
11	10	8	413.36	24.80
-11	10	-8	468.55	25.20
-12	-10	-8	224.88	18.30
-12	10	-8	216.28	17.10
-13	-10	-8	157.18	16.80
-13	10	-8	142.89	14.80
-14	-10	-8	181.68	19.00
-14	10	-8	150.98	16.40
-14	11	8	11.60	7.50
-13	11	8	11.40	6.90
13	11	-8	11.80	10.40
-12	11	8	14.10	6.80
12	11	-8	3.70	9.10
-11	-11	8	38.90	5.80
11	-11	-8	74.29	12.30
-11	11	8	71.49	9.90
11	11	-8	42.00	11.00
10	-11	-8	55.79	10.90
-10	-11	8	36.80	4.90
-10	11	8	20.70	9.80
-10	11	8	63.59	9.10
10	11	-8	68.29	13.20
-9	-11	8	135.69	8.50

-9	-11	8	145.29	8.30
9	-11	-8	147.29	13.20
-9	11	8	143.59	11.50
-9	11	8	143.59	16.10
9	11	-8	166.18	15.30
-8	-11	8	23.80	2.70
8	-11	-8	27.70	6.30
-8	-11	8	24.60	7.90
-8	-11	8	27.40	3.00
-8	11	8	24.80	7.40
8	11	-8	23.50	7.10
-8	11	8	26.20	5.70
7	-11	-8	144.49	12.90
-7	-11	8	167.18	15.30
-7	11	8	174.98	12.20
7	11	-8	204.28	14.70
-7	11	8	171.98	14.90
6	-11	-8	48.10	8.10
-6	-11	8	34.20	6.90
-6	11	8	53.99	8.20
6	11	-8	61.79	9.70
-6	11	8	43.30	6.60
5	-11	-8	184.18	12.50
5	-11	-8	189.48	13.40
-5	-11	8	178.58	14.40
5	11	-8	225.88	14.30
-5	11	8	202.48	14.20
-5	11	8	193.78	13.00
4	-11	-8	95.29	8.20
-4	-11	8	83.79	9.50
4	-11	-8	112.99	9.40
-4	11	8	98.29	9.70
4	11	-8	110.49	9.60
-4	11	8	82.29	8.70
-3	-11	8	20.90	4.60
3	-11	-8	15.00	3.60
3	-11	-8	17.70	4.30
3	11	-8	17.00	4.30
-3	11	8	19.00	4.70
-3	11	8	16.50	4.60
2	-11	-8	289.17	17.20
2	-11	-8	305.17	16.30
-2	-11	8	287.97	17.00
2	11	-8	323.97	17.00

-2	11	8	266.77	17.00
-2	11	8	294.67	17.40
1	-11	-8	286.77	16.40
-1	-11	8	272.37	15.60
1	-11	-8	286.37	15.10
1	11	-8	264.77	13.60
-1	11	8	268.57	16.10
1	11	-8	312.17	15.80
1	11	-8	258.67	13.40
-1	11	8	247.68	15.80
0	-11	8	706.63	37.00
0	-11	-8	737.23	36.90
0	11	-8	712.53	35.90
0	11	8	757.82	37.90
0	11	8	784.32	38.70
0	11	-8	778.12	37.30
0	11	8	735.63	37.70
1	-11	8	103.19	6.70
-1	-11	-8	109.39	7.60
-1	11	-8	93.39	6.40
1	11	8	111.29	8.60
-1	11	-8	104.19	7.50
1	11	8	96.09	8.30
1	11	8	101.39	9.50
2	-11	8	18.90	3.30
-2	-11	-8	31.60	4.50
-2	11	-8	28.60	4.40
2	11	8	19.40	4.00
2	11	8	37.10	7.10
-2	11	-8	26.30	4.60
2	11	8	21.30	4.10
-2	11	-8	22.00	4.10
3	-11	8	6.00	3.10
-3	-11	-8	4.00	2.90
3	11	8	10.60	4.80
3	11	8	0.20	3.30
-3	11	-8	4.10	2.80
3	11	8	6.40	3.80
-3	11	-8	3.10	2.50
-3	11	-8	-1.20	2.70
4	-11	8	47.90	6.20
-4	-11	-8	52.49	5.80
-4	-11	-8	73.19	8.20
-4	11	-8	54.59	5.30

-4	11	-8	45.50	5.10
4	11	8	76.59	8.80
4	11	8	48.70	6.60
4	11	8	49.50	6.70
-4	11	-8	51.19	6.00
-5	-11	-8	25.90	4.70
5	-11	8	17.20	4.40
-5	-11	-8	7.40	5.00
5	11	8	13.80	4.30
5	11	8	3.20	4.70
-5	11	-8	14.40	3.10
-5	11	-8	10.40	3.80
-6	-11	-8	362.36	22.30
6	-11	8	367.56	21.00
-6	-11	-8	411.46	22.10
6	11	8	433.26	22.70
-6	11	-8	363.26	20.60
-6	11	-8	343.77	21.00
6	11	8	355.16	20.90
-6	11	-8	371.26	19.90
-7	-11	-8	100.29	11.60
-7	11	-8	94.89	8.40
-7	11	-8	98.69	9.30
7	11	8	91.39	9.40
7	11	8	114.99	11.30
-8	-11	-8	170.88	14.90
-8	11	-8	184.38	12.10
8	11	8	183.38	14.50
8	11	8	156.38	12.50
-8	11	-8	164.18	12.00
-9	-11	-8	58.99	12.00
-9	11	-8	44.20	8.60
9	11	8	61.69	10.80
-9	11	-8	61.79	9.20
9	11	8	47.40	7.50
10	-11	8	60.99	6.70
-10	11	-8	47.90	7.70
10	11	8	41.00	7.70
-10	11	-8	68.19	10.00
11	-11	8	24.50	5.80
-11	11	-8	27.10	7.30
11	11	8	16.60	6.60
-11	11	-8	33.50	7.50
-12	11	-8	99.09	12.90

12	11	8	68.49	12.20
-13	-11	-8	53.89	11.40
-13	11	-8	55.49	9.80
-14	-11	-8	-7.80	10.10
-14	11	-8	11.80	8.60
-13	12	8	18.80	7.70
12	12	-8	272.67	23.80
-12	12	8	305.37	19.20
-11	12	8	67.29	10.30
11	12	-8	52.99	11.70
10	-12	-8	351.86	23.30
-10	-12	8	372.86	18.80
-10	12	8	387.56	21.70
10	12	-8	357.36	24.80
-9	-12	8	26.40	3.40
9	-12	-8	33.10	7.60
-9	-12	8	30.30	3.70
-9	12	8	19.70	5.90
-9	12	8	25.10	9.10
9	12	-8	28.50	7.90
8	-12	-8	258.87	16.80
-8	12	8	225.58	17.90
-8	12	8	210.18	19.00
8	12	-8	200.48	17.40
7	-12	-8	284.17	17.60
-7	-12	8	229.38	19.10
-7	12	8	275.07	16.00
-7	12	8	267.97	19.10
7	12	-8	274.47	18.90
6	-12	-8	521.85	28.90
-6	-12	8	460.85	30.00
6	12	-8	561.34	29.80
-6	12	8	519.45	29.80
-6	12	8	572.34	28.30
5	-12	-8	686.13	34.50
-5	-12	8	553.74	34.80
-5	12	8	671.83	35.00
5	12	-8	663.03	34.80
-5	12	8	679.73	33.90
4	-12	-8	357.66	19.80
-4	-12	8	323.67	20.90
4	-12	-8	366.36	20.70
-4	12	8	359.76	21.00
-4	12	8	374.76	20.30

4	12	-8	356.46	20.80
-3	-12	8	159.38	12.10
3	-12	-8	161.08	10.90
3	-12	-8	169.38	12.10
-3	12	8	175.78	12.10
-3	12	8	149.39	11.30
3	12	-8	177.28	11.90
2	-12	-8	318.27	18.10
-2	-12	8	295.57	17.70
2	-12	-8	324.87	16.90
-2	12	8	314.97	18.00
-2	12	8	302.27	17.90
2	12	-8	310.57	17.70
2	12	-8	282.87	14.70
2	12	-8	299.17	14.80
1	-12	-8	897.31	43.60
1	-12	-8	872.41	42.20
-1	-12	8	796.02	42.60
-1	-12	8	867.61	41.00
-1	12	8	852.21	43.30
1	12	-8	832.22	40.80
1	12	-8	838.22	42.80
-1	12	8	855.31	43.20
-1	12	8	858.01	44.00
0	-12	8	21.20	2.80
0	-12	-8	19.30	3.90
0	-12	8	14.80	3.40
0	12	8	17.30	4.20
0	12	8	15.90	5.30
0	12	8	10.50	4.20
0	12	-8	14.50	3.70
-1	-12	-8	1139.29	53.69
1	-12	8	1144.59	53.09
1	12	8	1009.00	56.79
1	12	8	1112.49	54.49
1	12	8	1086.09	54.59
-1	12	-8	1114.29	53.99
-1	12	-8	1063.69	52.79
2	-12	8	86.59	6.60
-2	-12	-8	86.09	6.70
-2	12	-8	77.49	7.10
2	12	8	83.29	8.10
2	12	8	75.79	9.60
-2	12	-8	87.79	6.80

-2	12	-8	75.89	6.10
2	12	8	78.99	7.40
3	-12	8	659.83	32.40
-3	-12	-8	676.73	32.60
3	12	8	643.54	33.30
3	12	8	637.44	33.00
-3	12	-8	599.74	32.30
-3	12	-8	629.34	32.10
-3	12	-8	648.64	32.10
3	12	8	651.43	34.50
4	-12	8	14.30	4.10
-4	-12	-8	19.50	5.50
-4	-12	-8	17.90	4.30
4	12	8	15.20	4.40
-4	12	-8	6.30	3.30
4	12	8	5.40	5.00
-4	12	-8	8.70	2.90
4	12	8	13.90	4.40
-4	12	-8	10.90	3.20
-5	-12	-8	816.62	45.30
-5	-12	-8	936.11	44.00
5	-12	8	902.61	44.10
-5	12	-8	854.11	43.30
-5	12	-8	917.61	43.80
-5	12	-8	877.41	43.40
5	12	8	843.52	44.30
5	12	8	903.01	45.70
5	12	8	849.02	44.20
6	-12	8	7.40	4.60
-6	-12	-8	5.80	5.40
6	12	8	11.10	4.50
6	12	8	11.20	5.40
-6	12	-8	5.50	3.30
-6	12	-8	2.90	4.50
-7	-12	-8	415.16	26.30
7	12	8	427.06	24.80
-7	12	-8	458.35	24.40
7	12	8	481.15	26.50
-7	12	-8	453.05	23.90
-8	-12	-8	43.40	8.70
-8	12	-8	40.40	6.80
8	12	8	51.29	8.30
8	12	8	37.30	6.90
-8	12	-8	40.60	7.90

-9	-12	-8	152.08	16.60
-9	12	-8	206.48	14.00
9	12	8	195.98	16.10
-9	12	-8	182.08	13.80
9	12	8	182.08	19.10
-10	12	-8	147.09	12.70
-10	12	-8	124.29	12.80
10	12	8	121.99	12.30
10	12	8	121.89	15.60
-11	12	-8	269.27	17.70
-11	12	-8	221.88	18.00
11	12	8	244.48	17.70
12	12	8	150.38	14.80
-12	12	-8	165.38	14.90
-13	-12	-8	170.28	17.50
-13	12	-8	118.19	14.60
-13	13	8	166.68	15.10
13	13	-8	207.08	22.50
-12	13	8	18.70	7.60
12	13	-8	9.90	15.80
-11	13	8	457.75	26.60
11	13	-8	478.35	31.10
10	-13	-8	29.00	8.10
-10	13	8	20.90	6.80
10	13	-8	-0.40	8.00
-9	-13	8	237.08	12.40
9	-13	-8	258.87	17.90
-9	-13	8	246.88	12.40
9	13	-8	272.67	20.10
-9	13	8	255.27	16.40
-9	13	8	238.88	20.80
8	-13	-8	244.58	16.70
-8	13	8	201.08	18.10
8	13	-8	237.58	18.30
-8	13	8	226.58	15.20
7	-13	-8	323.57	20.90
-7	13	8	331.47	19.80
-7	13	8	345.27	22.50
7	13	-8	358.26	22.30
-6	-13	8	417.26	28.80
6	-13	-8	472.55	26.60
-6	13	8	488.85	25.90
6	13	-8	503.25	27.50
-6	13	8	478.25	27.70

-5	-13	8	562.34	35.30
5	-13	-8	655.03	34.90
-5	13	8	664.33	34.20
-5	13	8	663.03	35.50
5	13	-8	731.63	35.50
4	-13	-8	295.57	18.20
-4	-13	8	266.17	18.70
-4	13	8	303.37	17.90
4	13	-8	331.87	18.80
-4	13	8	300.37	18.70
-3	-13	8	366.66	22.50
3	-13	-8	386.96	21.80
3	-13	-8	415.96	22.50
3	13	-8	412.26	22.40
-3	13	8	380.26	21.80
-3	13	8	396.36	22.70
-2	-13	8	441.26	26.10
2	-13	-8	511.15	25.40
2	-13	-8	518.45	26.80
2	13	-8	515.75	26.30
-2	13	8	479.15	26.20
2	13	-8	457.45	23.30
-2	13	8	478.25	26.50
-1	-13	8	80.89	7.70
-1	-13	8	82.69	5.20
1	-13	-8	78.99	6.80
-1	13	8	62.09	8.40
1	13	-8	81.29	8.10
1	13	-8	77.99	4.80
-1	13	8	81.29	8.60
-1	13	8	78.29	9.50
0	-13	-8	883.61	43.80
0	-13	8	876.51	42.90
0	13	8	895.41	44.90
0	13	-8	854.11	42.60
0	13	-8	900.31	44.40
0	13	8	853.01	44.60
0	13	8	925.11	45.90
1	-13	8	107.29	7.60
-1	-13	-8	122.19	8.60
1	13	8	121.09	9.80
1	13	8	111.89	11.70
-1	13	-8	132.49	9.10
-1	13	-8	111.09	7.20

1	13	8	107.79	9.70
2	-13	8	1489.55	71.09
-2	-13	-8	1500.25	71.49
-2	13	-8	1447.86	71.49
2	13	8	1460.85	72.39
-2	13	-8	1399.96	70.69
-2	13	-8	1496.95	71.69
2	13	8	1465.65	72.09
2	13	8	1494.95	76.19
-3	-13	-8	3.90	3.50
3	-13	8	11.50	3.80
3	13	8	6.70	4.20
-3	13	-8	4.40	3.20
3	13	8	8.40	3.90
-3	13	-8	8.20	3.20
3	13	8	3.50	5.40
-3	13	-8	6.80	3.10
4	-13	8	1442.46	68.29
-4	-13	-8	1404.76	68.19
4	13	8	1503.95	70.39
-4	13	-8	1349.07	67.79
-4	13	-8	1401.06	68.09
4	13	8	1369.06	68.59
4	13	8	1349.47	68.89
-4	13	-8	1358.96	67.79
5	-13	8	6.60	4.60
-5	-13	-8	10.70	5.50
5	13	8	3.50	4.20
-5	13	-8	11.70	3.50
5	13	8	8.10	4.10
5	13	8	8.90	6.40
-5	13	-8	7.00	4.30
-5	13	-8	10.20	3.70
-6	-13	-8	1446.16	74.59
-6	13	-8	1444.66	72.29
6	13	8	1434.66	73.09
6	13	8	1598.34	74.89
-6	13	-8	1506.25	72.69
-7	-13	-8	36.50	7.80
7	13	8	41.10	6.70
7	13	8	45.80	8.00
-7	13	-8	50.49	6.80
-7	13	-8	35.30	7.20
-8	-13	-8	151.28	15.00

-8	13	-8	148.89	12.00
8	13	8	165.78	15.00
-8	13	-8	160.68	12.00
8	13	8	125.99	12.20
-9	13	-8	137.19	12.20
-9	13	-8	133.99	11.80
9	13	8	112.79	12.00
9	13	8	134.99	15.00
-10	13	-8	385.86	23.00
10	13	8	338.37	22.40
-10	13	-8	394.46	22.70
11	13	8	195.98	15.70
-11	13	-8	192.68	15.00
-12	13	-8	229.08	17.60
12	13	8	219.58	17.70
-13	-13	-8	106.79	16.70
-13	13	-8	96.19	13.50
-13	14	8	4.10	8.00
13	14	-8	-9.70	11.90
-12	14	8	63.59	11.40
12	14	-8	57.89	14.00
-11	14	8	25.20	7.00
10	-14	-8	21.30	7.30
-10	14	8	25.20	7.00
10	14	-8	8.80	9.40
9	-14	-8	9.30	6.80
-9	14	8	9.30	5.50
9	14	-8	8.90	8.60
8	-14	-8	5.20	5.90
8	14	-8	9.60	7.50
-8	14	8	4.30	7.70
-8	14	8	7.40	5.60
7	-14	-8	16.10	5.80
-7	14	8	21.10	7.90
7	14	-8	21.60	7.70
-7	14	8	11.70	5.50
6	-14	-8	-1.70	4.70
-6	14	8	-3.60	5.00
-6	14	8	1.20	5.80
6	14	-8	4.30	5.70
5	-14	-8	15.00	5.10
-5	14	8	13.40	5.40
-5	14	8	14.80	5.80
5	14	-8	13.00	6.10

4	-14	-8	11.30	4.70
-4	-14	8	8.70	6.80
-4	14	8	13.10	5.60
4	14	-8	15.10	5.70
-4	14	8	11.90	5.40
-3	-14	8	34.50	6.60
3	-14	-8	40.20	7.70
-3	14	8	39.40	6.80
-3	14	8	28.60	5.70
3	14	-8	23.90	5.60
2	-14	-8	11.30	5.00
-2	-14	8	9.60	1.90
-2	-14	8	1.80	4.00
2	14	-8	8.30	1.50
2	14	-8	14.10	4.70
-2	14	8	4.00	4.90
-2	14	8	-1.00	4.30
1	-14	-8	37.00	6.00
-1	-14	8	28.80	5.00
-1	-14	8	28.40	3.70
-1	14	8	24.90	6.20
1	14	-8	26.00	5.20
1	14	-8	27.60	3.20
-1	14	8	35.60	6.30
-1	14	8	19.30	5.20
0	-14	8	509.45	25.20
0	-14	-8	505.55	25.90
0	14	-8	491.85	26.60
0	14	8	537.65	28.50
0	14	8	486.65	26.80
0	14	-8	487.35	24.60
0	14	8	488.75	27.20
1	-14	8	166.78	9.90
-1	-14	-8	178.18	10.80
1	14	8	177.78	12.10
-1	14	-8	163.08	9.40
1	14	8	156.08	11.90
-1	14	-8	140.59	11.00
1	14	8	158.38	13.30
-2	-14	-8	-0.90	3.00
2	-14	8	-1.60	3.10
-2	14	-8	-1.50	2.60
2	14	8	4.60	4.20
2	14	8	-0.40	5.80

-2	14	-8	-2.70	3.40
2	14	8	-0.70	4.00
3	-14	8	11.30	4.00
-3	-14	-8	8.00	4.20
-3	14	-8	15.60	3.50
3	14	8	18.30	4.90
3	14	8	10.90	4.80
3	14	8	20.80	6.40
-3	14	-8	20.50	3.90
-3	14	-8	16.50	4.20
4	-14	8	11.00	4.50
-4	14	-8	9.40	3.40
-4	14	-8	12.40	4.00
4	14	8	8.10	4.80
4	14	8	7.40	4.10
-4	14	-8	10.60	3.80
-5	-14	-8	49.20	9.90
5	-14	8	71.49	8.50
-5	14	-8	45.40	6.70
5	14	8	50.39	7.90
5	14	8	56.19	8.20
-5	14	-8	48.10	6.70
5	14	8	43.30	8.20
-5	14	-8	48.70	6.90
-6	-14	-8	23.60	7.00
-6	14	-8	27.40	5.40
6	14	8	21.80	5.40
6	14	8	16.20	7.20
-6	14	-8	14.50	4.40
-7	-14	-8	55.39	9.50
7	14	8	33.80	6.30
7	14	8	40.90	8.10
-7	14	-8	44.60	8.10
-7	14	-8	35.10	5.70
-8	-14	-8	22.50	8.00
-8	14	-8	20.30	5.60
8	14	8	16.50	6.20
8	14	8	5.10	7.00
9	14	8	8.00	6.10
9	14	8	11.00	7.70
-9	14	-8	11.70	6.00
-9	14	-8	21.70	6.60
-10	14	-8	12.20	6.70
10	14	8	9.10	6.40

-10	14	-8	23.50	7.10
-11	14	-8	30.20	7.80
11	14	8	35.90	7.70
12	14	8	6.00	7.70
-12	14	-8	19.90	7.90
-13	-14	-8	28.20	11.60
-13	14	-8	8.40	8.60
-12	15	8	5.90	7.30
12	15	-8	17.70	11.30
11	15	-8	370.16	27.60
-11	15	8	357.96	22.60
-10	15	8	1.10	6.00
10	15	-8	-3.00	13.80
9	-15	-8	584.74	33.10
9	15	-8	548.45	34.30
-9	15	8	603.14	31.60
8	-15	-8	69.19	11.00
8	15	-8	65.39	13.00
-8	15	8	55.99	9.50
-8	15	8	54.19	10.30
7	-15	-8	343.47	21.10
-7	15	8	295.77	22.30
-7	15	8	308.17	19.60
7	15	-8	342.57	22.00
6	-15	-8	225.58	16.30
-6	15	8	235.78	17.90
6	15	-8	270.57	17.70
-6	15	8	179.38	17.80
5	-15	-8	330.17	20.70
-5	15	8	332.97	21.70
-5	15	8	331.87	20.10
5	15	-8	353.56	21.50
4	-15	-8	267.77	17.50
-4	15	8	274.17	18.10
-4	15	8	260.37	16.60
4	15	-8	254.37	17.40
3	-15	-8	445.76	25.10
3	15	-8	442.66	25.00
-3	15	8	458.15	25.60
-3	15	8	415.16	24.40
-2	-15	8	1496.25	71.59
2	15	-8	1533.95	74.19
-2	15	8	1584.94	74.39
-2	15	8	1484.95	74.49

-2	15	8	1529.75	75.29
2	15	-8	1375.06	71.29
-1	-15	8	43.50	4.40
-1	15	8	30.80	6.10
-1	15	8	39.20	7.70
1	15	-8	42.80	6.50
-1	15	8	43.60	7.10
0	-15	8	1582.04	77.19
0	15	8	1585.14	79.29
0	15	8	1612.64	80.19
0	15	8	1635.44	79.09
0	15	-8	1535.25	76.79
0	15	-8	1691.63	79.09
1	-15	8	409.06	21.00
1	15	8	387.36	22.90
1	15	8	411.16	22.90
1	15	8	428.66	24.50
-1	15	-8	372.86	20.40
-1	15	-8	422.76	22.50
2	-15	8	571.54	28.50
-2	15	-8	541.45	27.90
2	15	8	537.45	30.10
2	15	8	579.04	29.80
2	15	8	547.05	31.40
-2	15	-8	574.24	29.50
3	-15	8	32.80	5.20
3	15	8	43.60	6.50
3	15	8	39.10	8.10
3	15	8	41.10	6.70
-3	15	-8	42.30	5.60
-3	15	-8	35.50	6.50
-3	15	-8	28.10	4.40
4	-15	8	1100.19	52.99
-4	15	-8	1058.89	52.39
-4	15	-8	1009.90	52.59
4	15	8	1038.10	53.79
4	15	8	1130.69	55.59
4	15	8	1069.49	53.49
-4	15	-8	1062.09	53.09
5	-15	8	87.79	9.60
5	15	8	102.49	9.90
-5	15	-8	85.79	8.60
-5	15	-8	90.19	8.90
5	15	8	92.59	10.00

5	15	8	106.89	13.20
-5	15	-8	92.59	12.70
-6	15	-8	1006.50	50.39
6	15	8	1121.29	53.69
-6	15	-8	1017.30	50.29
6	15	8	967.10	50.69
-6	15	-8	933.41	50.09
-7	15	-8	17.60	5.30
7	15	8	19.70	5.80
-7	15	-8	9.50	4.70
8	15	8	428.96	28.00
8	15	8	481.05	31.00
-8	15	-8	512.05	27.30
-8	15	-8	521.75	27.00
9	15	8	170.98	25.50
9	15	8	128.69	13.10
-9	15	-8	137.99	13.40
-9	15	-8	160.18	13.10
-10	15	-8	640.64	33.90
-10	15	-8	640.34	40.30
10	15	8	580.14	33.80
11	15	8	168.98	16.00
-11	15	-8	200.28	16.00
12	15	8	202.88	18.20
-12	15	-8	270.17	18.60
12	16	-8	55.19	13.50
-12	16	8	60.69	9.90
-11	16	8	9.00	7.40
11	16	-8	16.10	10.60
10	16	-8	25.00	11.20
-10	16	8	32.80	7.70
9	-16	-8	9.70	7.10
9	16	-8	2.20	8.70
-9	16	8	8.20	6.60
8	-16	-8	4.50	7.10
-8	16	8	10.50	6.40
7	-16	-8	13.80	6.40
-7	16	8	-5.80	7.40
7	16	-8	8.00	7.80
-7	16	8	-3.00	5.50
6	-16	-8	58.59	10.10
-6	16	8	51.59	9.20
6	16	-8	75.59	12.00
-6	16	8	50.59	9.60

5	-16	-8	27.50	6.80
-5	16	8	19.20	7.70
-5	16	8	22.20	9.50
5	16	-8	35.50	7.70
4	-16	-8	19.40	6.30
-4	16	8	12.20	6.30
4	16	-8	11.30	6.10
-4	16	8	12.50	5.80
3	-16	-8	30.90	6.70
-3	-16	8	37.20	3.20
-3	16	8	31.90	6.80
-3	16	8	26.10	6.20
3	16	-8	43.30	7.50
3	16	-8	33.60	2.70
-2	-16	8	76.59	5.50
2	16	-8	83.79	10.00
-2	16	8	78.69	10.20
2	16	-8	80.59	5.00
-2	16	8	75.69	9.90
-2	16	8	82.89	11.70
-1	16	8	173.88	14.30
-1	16	8	151.88	12.80
1	16	-8	160.98	12.20
-1	16	8	127.99	12.10
1	16	-8	137.49	8.40
0	-16	8	342.97	18.00
0	16	8	337.47	20.60
0	16	-8	338.47	17.50
0	16	8	361.46	22.20
0	16	-8	348.37	20.20
0	16	8	326.07	20.30
1	-16	8	280.27	15.60
-1	16	-8	268.87	16.40
-1	16	-8	264.47	14.90
1	16	8	311.17	19.70
1	16	8	268.77	17.50
-1	16	-8	314.97	17.50
1	16	8	268.97	17.90
2	-16	8	49.20	6.80
2	16	8	39.30	6.70
-2	16	-8	54.69	5.60
-2	16	-8	39.80	6.20
2	16	8	52.19	8.30
2	16	8	51.89	8.50

-2	16	-8	37.40	7.90
3	-16	8	49.40	7.20
3	16	8	46.90	8.40
-3	16	-8	48.70	7.70
-3	16	-8	46.40	7.40
-3	16	-8	47.20	6.10
3	16	8	44.10	8.40
3	16	8	51.59	8.40
4	-16	8	-3.20	4.50
4	16	8	-12.90	6.00
-4	16	-8	-4.40	3.50
4	16	8	-6.20	4.80
4	16	8	-3.20	4.60
-4	16	-8	-0.20	3.70
-5	16	-8	177.98	12.60
-5	16	-8	178.98	12.00
5	16	8	171.28	13.10
5	16	8	210.08	16.40
-5	16	-8	147.19	12.00
5	16	8	175.78	17.20
-6	-16	-8	14.20	7.20
-6	16	-8	15.40	5.50
-6	16	-8	11.40	4.70
-6	16	-8	16.10	5.00
6	16	8	14.70	5.50
6	16	8	8.20	7.70
-7	-16	-8	269.47	20.40
-7	16	-8	270.77	16.70
-7	16	-8	245.48	16.40
-7	16	-8	224.48	16.30
7	16	8	291.97	21.10
8	16	8	119.49	12.30
-8	16	-8	122.69	12.30
-8	16	-8	139.69	12.00
8	16	8	153.98	17.30
-9	16	-8	119.79	11.70
9	16	8	89.79	12.20
-9	16	-8	110.99	12.60
-10	16	-8	17.60	7.40
10	16	8	7.10	6.70
-11	16	-8	73.29	12.20
11	16	8	50.49	8.90
12	16	8	44.30	9.30
-12	16	-8	30.40	8.30

12	17	-8	14.40	13.60
-11	17	8	225.18	17.40
11	17	-8	236.28	23.60
10	17	-8	60.39	12.50
-10	17	8	101.99	12.40
9	17	-8	170.48	19.50
-9	17	8	189.68	14.50
8	-17	-8	71.09	12.80
8	17	-8	100.09	15.60
-8	17	8	94.19	11.40
7	-17	-8	108.79	13.30
7	17	-8	124.09	15.10
-7	17	8	145.99	12.80
6	-17	-8	372.16	23.60
-6	17	8	389.16	25.20
-6	17	8	384.26	22.80
5	-17	-8	502.75	28.60
-5	17	8	475.45	29.30
-5	17	8	512.65	27.80
5	17	-8	473.95	29.00
4	-17	-8	184.48	15.30
-4	17	8	184.08	15.30
-4	17	8	215.88	16.00
4	17	-8	193.98	15.70
-3	-17	8	187.38	9.70
-3	17	8	198.18	16.50
3	17	-8	174.88	9.40
3	17	-8	175.28	14.60
-3	17	8	186.28	15.00
-3	17	8	176.48	14.00
-2	-17	8	64.99	5.20
-2	17	8	57.49	9.50
-2	17	8	66.99	10.60
2	17	-8	51.69	4.90
-2	17	8	55.79	9.60
2	17	-8	56.49	9.80
-1	-17	8	27.50	4.90
1	17	-8	27.30	6.30
-1	17	8	29.40	8.10
-1	17	8	28.20	6.50
1	17	-8	26.50	4.00
-1	17	8	38.00	7.00
0	-17	8	966.30	45.60
0	17	-8	899.41	47.60

0	17	8	927.31	48.10
0	17	8	943.41	47.70
0	17	-8	919.31	45.10
0	17	8	921.11	49.30
1	-17	8	3.90	3.70
-1	17	-8	4.10	3.90
-1	17	-8	2.50	4.80
1	17	8	-4.20	6.10
1	17	8	2.60	5.50
1	17	8	3.50	5.10
-1	17	-8	2.90	2.90
2	-17	8	532.85	27.80
-2	17	-8	530.05	27.10
2	17	8	577.74	29.40
-2	17	-8	494.35	32.80
2	17	8	559.34	31.30
2	17	8	520.35	29.60
-2	17	-8	533.65	28.50
3	-17	8	6.30	4.50
-3	17	-8	-1.60	4.40
3	17	8	9.20	5.50
-3	17	-8	5.00	3.50
3	17	8	-6.60	5.80
-3	17	-8	5.90	4.60
3	17	8	3.50	5.10
4	-17	8	309.07	18.20
-4	17	-8	312.27	18.80
-4	17	-8	310.87	17.30
4	17	8	294.77	19.20
4	17	8	303.67	21.70
4	17	8	339.87	19.00
-4	17	-8	285.47	17.90
5	17	8	2.10	5.40
-5	17	-8	-0.30	4.40
-5	17	-8	6.30	4.60
5	17	8	-2.30	6.90
-5	17	-8	2.00	4.80
-6	-17	-8	398.36	26.40
-6	17	-8	414.86	23.50
6	17	8	441.76	26.80
6	17	8	400.66	26.50
-6	17	-8	437.46	22.70
-6	17	-8	361.66	22.80
7	17	8	2.10	8.10

-7	17	-8	3.40	5.00
-7	17	-8	5.40	5.50
-7	17	-8	6.00	5.40
7	17	8	3.10	5.70
8	17	8	105.09	16.20
-8	17	-8	90.19	11.50
8	17	8	104.99	11.80
-8	17	-8	95.19	11.80
-8	17	-8	121.49	11.50
-9	17	-8	67.09	10.40
9	17	8	30.10	7.80
-10	17	-8	139.29	13.70
10	17	8	116.19	14.00
11	17	8	17.40	8.20
-11	17	-8	69.69	12.20
12	17	8	268.07	20.20
11	18	-8	55.79	13.50
-11	18	8	36.70	9.50
10	18	-8	186.28	20.80
-10	18	8	165.18	15.40
9	18	-8	18.30	10.50
-9	18	8	2.00	7.00
8	18	-8	357.66	25.70
-8	18	8	356.96	22.50
7	-18	-8	164.18	15.60
7	18	-8	146.69	16.60
-7	18	8	167.18	14.30
6	-18	-8	357.86	23.20
6	18	-8	372.56	24.10
-6	18	8	347.77	22.10
-6	18	8	358.46	30.20
5	-18	-8	346.07	22.00
-5	18	8	330.67	20.80
5	18	-8	345.67	22.50
-5	18	8	298.37	22.50
4	-18	-8	384.06	24.00
4	18	-8	375.56	24.20
-4	18	8	382.76	23.10
-4	18	8	398.26	24.80
-3	-18	8	180.98	9.40
3	18	-8	138.09	16.70
-3	18	8	182.48	14.30
3	18	-8	165.78	9.00
-3	18	8	182.98	15.40

-3	18	8	174.68	16.70
-2	-18	8	28.90	4.90
-2	18	8	42.40	7.90
2	18	-8	32.30	4.90
-2	18	8	30.90	12.90
-2	18	8	27.60	8.10
2	18	-8	29.40	9.90
-1	-18	8	180.28	10.60
1	18	-8	154.48	16.10
1	18	-8	165.88	9.80
-1	18	8	158.08	13.70
-1	18	8	181.28	16.40
-1	18	8	203.68	15.20
0	-18	8	6.60	3.70
0	18	8	7.90	6.90
0	18	-8	9.40	5.50
0	18	8	15.70	6.80
0	18	-8	8.00	3.00
0	18	8	-5.00	5.40
1	-18	8	633.94	32.20
-1	18	-8	629.04	31.50
1	18	8	601.14	34.40
-1	18	-8	660.63	34.20
1	18	8	650.53	34.00
-1	18	-8	588.14	33.00
1	18	8	674.53	36.20
2	-18	8	73.89	8.20
2	18	8	65.59	12.70
2	18	8	80.19	9.90
-2	18	-8	74.69	10.00
-2	18	-8	68.09	8.60
2	18	8	63.49	10.50
-2	18	-8	76.79	6.90
3	-18	8	371.76	21.40
-3	18	-8	386.06	20.50
3	18	8	397.26	22.70
-3	18	-8	390.46	22.10
-3	18	-8	395.36	22.70
3	18	8	349.67	22.90
3	18	8	420.06	25.60
-4	18	-8	0.30	4.00
4	18	8	0.00	5.20
4	18	8	8.30	9.70
4	18	8	-1.90	7.10

-4	18	-8	5.90	5.30
-4	18	-8	-4.40	4.10
-5	18	-8	528.85	30.70
5	18	8	636.94	34.40
-5	18	-8	557.94	30.20
-5	18	-8	602.04	31.50
6	18	8	17.50	8.30
-6	18	-8	10.30	5.20
6	18	8	14.40	6.00
-6	18	-8	10.30	5.80
-6	18	-8	9.10	5.30
-7	18	-8	554.94	29.10
7	18	8	545.55	32.40
-7	18	-8	521.15	28.10
7	18	8	496.15	28.70
-7	18	-8	461.95	28.30
-8	18	-8	0.60	6.00
-8	18	-8	22.20	6.50
8	18	8	1.90	6.40
9	18	8	294.47	20.50
-9	18	-8	332.77	20.20
10	18	8	71.69	12.40
-10	18	-8	88.79	12.40
11	18	8	155.68	16.40
-11	18	-8	205.48	16.70
-10	19	8	30.70	8.60
10	19	-8	3.70	11.40
-9	19	8	9.70	7.90
9	19	-8	6.30	10.90
-8	19	8	27.70	8.20
8	19	-8	24.70	10.90
7	-19	-8	19.50	8.30
7	19	-8	3.30	8.60
-7	19	8	9.40	6.80
6	-19	-8	34.30	8.10
-6	19	8	40.80	8.10
6	19	-8	42.40	9.20
5	-19	-8	37.20	8.70
-5	19	8	22.90	7.40
-5	19	8	15.60	11.80
5	19	-8	25.30	8.40
-4	-19	8	29.80	3.10
-4	19	8	20.00	7.40
-4	19	8	35.30	10.50

-4	19	8	22.30	7.90
4	19	-8	14.20	7.00
-3	-19	8	13.60	2.90
3	19	-8	10.70	6.60
3	19	-8	12.90	2.40
-3	19	8	14.80	8.40
-3	19	8	8.10	7.00
-3	19	8	17.30	8.20
-2	-19	8	53.39	6.70
-2	19	8	64.29	11.60
2	19	-8	53.49	5.10
-2	19	8	54.19	9.70
-2	19	8	49.90	8.70
2	19	-8	46.90	8.20
-1	-19	8	-3.60	3.30
-1	19	8	-4.60	6.90
-1	19	8	-3.30	6.10
1	19	-8	0.70	2.80
-1	19	8	2.70	6.30
1	19	-8	1.50	5.60
0	-19	8	112.59	9.40
0	19	-8	101.79	12.30
0	19	8	137.69	13.10
0	19	-8	129.69	8.40
0	19	8	150.48	16.00
0	19	-8	125.49	10.70
1	-19	8	9.10	4.50
1	19	8	-5.00	5.80
-1	19	-8	-2.20	4.30
-1	19	-8	4.60	3.40
1	19	8	0.50	8.10
-1	19	-8	-1.60	5.60
2	-19	8	19.70	5.30
-2	19	-8	21.00	4.20
2	19	8	14.00	6.60
-2	19	-8	9.50	4.70
-2	19	-8	8.60	5.90
2	19	8	11.00	7.60
3	-19	8	2.20	5.30
3	19	8	-7.80	5.50
-3	19	-8	2.70	5.60
-3	19	-8	3.00	3.90
-3	19	-8	1.20	5.20
3	19	8	3.90	7.60

-4	19	-8	6.90	4.40
4	19	8	14.60	8.00
4	19	8	4.40	5.70
-4	19	-8	4.40	4.90
-4	19	-8	14.80	5.80
-5	19	-8	53.59	8.00
-5	19	-8	50.39	10.40
5	19	8	50.99	7.90
-5	19	-8	45.80	9.20
5	19	8	41.40	10.00
6	19	8	105.89	11.60
-6	19	-8	85.89	10.80
-6	19	-8	86.29	13.80
-6	19	-8	97.89	11.70
6	19	8	110.69	15.90
7	19	8	23.60	6.90
-7	19	-8	24.20	6.30
7	19	8	0.50	9.70
-7	19	-8	15.80	6.90
-8	19	-8	17.20	6.80
-8	19	-8	-0.40	6.40
8	19	8	6.80	7.10
-9	19	-8	28.00	7.70
-9	19	-8	19.10	7.60
9	19	8	28.00	7.90
10	19	8	37.50	8.90
-10	19	-8	75.49	12.30
-10	20	8	410.86	26.10
10	20	-8	412.06	31.90
-9	20	8	-3.80	6.90
9	20	-8	5.30	12.20
8	20	-8	436.86	30.10
-8	20	8	417.46	25.50
7	20	-8	234.48	20.80
-7	20	8	216.68	17.60
6	-20	-8	138.19	15.00
6	20	-8	125.89	16.60
-6	20	8	125.09	14.00
-5	20	8	325.77	22.30
5	20	-8	369.96	24.00
-4	20	8	601.14	34.90
4	20	-8	541.55	32.70
-4	20	8	546.05	31.70
-3	20	8	402.46	26.60

-3	20	8	428.46	25.10
3	20	-8	418.86	25.90
-3	20	8	402.56	28.10
-2	-20	8	229.88	13.40
2	20	-8	250.47	18.00
-2	20	8	248.78	20.20
-2	20	8	238.28	18.50
2	20	-8	224.28	12.40
-2	20	8	208.18	17.10
-1	-20	8	1008.40	49.30
1	20	-8	957.60	48.80
-1	20	8	980.50	52.69
-1	20	8	1026.00	54.29
1	20	-8	1007.30	52.39
-1	20	8	1066.19	52.19
0	-20	8	193.58	12.20
0	20	-8	181.38	13.60
0	20	-8	185.18	11.10
0	20	8	183.18	18.40
0	20	8	158.58	16.00
0	20	-8	198.18	15.80
0	20	8	200.48	15.30
1	-20	8	870.41	44.00
-1	20	-8	920.31	46.50
-1	20	-8	816.02	45.00
1	20	8	912.31	48.70
1	20	8	885.91	45.90
1	20	8	892.21	46.90
-1	20	-8	874.81	43.40
2	-20	8	149.88	11.30
-2	20	-8	139.69	9.70
2	20	8	122.39	14.10
-2	20	-8	130.39	11.90
2	20	8	125.89	12.80
-2	20	-8	130.89	13.30
2	20	8	125.89	16.80
3	-20	8	1054.09	51.19
-3	20	-8	994.30	50.89
-3	20	-8	871.81	49.90
3	20	8	1012.00	50.39
3	20	8	1006.80	53.29
-3	20	-8	949.01	48.40
4	20	8	79.59	10.70
-4	20	-8	72.59	10.00

4	20	8	73.09	11.90
-4	20	-8	67.89	8.70
-4	20	-8	70.09	11.40
-5	20	-8	478.35	26.30
-5	20	-8	430.76	27.70
-5	20	-8	507.05	28.50
5	20	8	487.85	27.70
5	20	8	560.04	32.20
-6	20	-8	173.58	15.30
6	20	8	197.88	14.70
-6	20	-8	151.58	13.10
6	20	8	177.58	19.10
7	20	8	600.34	33.40
-7	20	-8	610.64	34.10
-7	20	-8	611.74	32.50
8	20	8	30.00	7.90
-8	20	-8	24.10	7.30
-8	20	-8	33.70	8.30
9	20	8	176.08	16.50
-9	20	-8	208.18	16.00
-9	20	-8	208.68	17.90
-10	20	-8	396.46	29.20
10	20	8	341.17	23.70
-10	20	-8	384.46	23.80
-9	21	8	21.20	9.50
9	21	-8	22.60	12.70
8	21	-8	17.60	10.70
-8	21	8	5.30	7.80
7	21	-8	125.39	18.20
-7	21	8	104.39	13.80
6	21	-8	30.60	9.50
-6	21	8	14.10	7.30
5	21	-8	171.48	17.50
-5	21	8	127.49	14.90
-4	21	8	129.99	18.60
-4	21	8	120.49	13.00
4	21	-8	111.99	15.30
-3	21	8	-4.70	6.60
-3	21	8	-3.00	10.10
3	21	-8	-7.60	7.20
-2	21	8	124.99	13.80
2	21	-8	118.79	8.00
2	21	-8	134.69	14.50
-2	21	8	119.19	16.90

1	21	-8	182.78	10.70
-1	21	8	181.58	15.80
-1	21	8	152.38	16.90
1	21	-8	205.08	16.50
-1	21	8	174.08	19.00
0	21	-8	105.39	8.40
0	21	8	118.89	16.80
0	21	-8	137.19	14.20
0	21	8	115.79	12.80
0	21	8	109.09	14.10
1	-21	8	0.70	4.90
1	21	8	-14.60	7.50
-1	21	-8	0.10	3.80
1	21	8	3.70	6.80
-1	21	-8	-3.20	6.10
1	21	8	0.10	8.60
2	-21	8	82.09	10.20
-2	21	-8	106.79	13.20
-2	21	-8	100.09	8.70
2	21	8	101.59	17.00
2	21	8	98.29	12.10
3	21	8	-2.40	6.00
-3	21	-8	-4.80	6.30
-3	21	-8	1.40	4.40
3	21	8	0.70	9.00
-4	21	-8	43.00	8.20
-4	21	-8	64.39	11.80
4	21	8	63.29	12.50
4	21	8	43.40	8.20
-5	21	-8	14.70	7.10
5	21	8	4.70	9.60
-5	21	-8	12.20	10.10
5	21	8	8.30	6.70
-6	21	-8	181.08	14.00
6	21	8	187.48	15.80
-6	21	-8	215.88	17.00
7	21	8	47.80	8.80
-7	21	-8	28.10	8.30
-7	21	-8	28.90	6.90
8	21	8	85.09	13.20
-8	21	-8	88.79	12.10
-8	21	-8	79.99	13.80
9	21	8	29.30	8.80
-9	21	-8	41.30	8.80

-9	21	-8	24.30	8.80
8	22	-8	131.29	20.60
-8	22	8	110.59	15.20
7	22	-8	128.29	19.70
-7	22	8	105.99	14.50
-6	22	8	155.48	22.50
-6	22	8	138.79	15.30
6	22	-8	149.49	18.80
-5	22	8	243.28	18.60
-5	22	8	209.58	23.40
5	22	-8	233.88	20.90
4	22	-8	142.99	17.30
-4	22	8	171.28	15.80
-3	22	8	22.50	11.00
-3	22	8	36.50	9.30
3	22	-8	38.90	9.80
-2	22	8	1.20	9.80
-2	22	8	-7.00	6.90
2	22	-8	-3.70	6.90
1	22	-8	410.86	26.40
-1	22	8	441.06	29.80
1	22	-8	405.16	21.60
-1	22	8	426.06	25.80
0	22	-8	44.70	6.50
0	22	8	43.00	8.20
0	22	8	59.09	13.20
0	22	-8	51.79	9.70
-1	22	-8	375.36	24.70
1	22	8	403.86	28.50
-1	22	-8	370.86	20.80
1	22	8	411.56	24.20
-2	22	-8	1.10	6.70
2	22	8	-6.80	9.60
2	22	8	16.90	7.30
-2	22	-8	13.70	4.60
-3	22	-8	163.08	16.20
-3	22	-8	165.48	12.00
3	22	8	159.68	19.70
3	22	8	176.08	14.90
4	22	8	14.00	10.10
4	22	8	15.40	7.30
-4	22	-8	15.40	7.50
-4	22	-8	20.90	5.70
-5	22	-8	309.57	21.80

5	22	8	333.97	27.80
-5	22	-8	311.27	19.00
5	22	8	313.67	20.90
-6	22	-8	6.90	6.30
6	22	8	4.90	7.20
-6	22	-8	14.60	7.90
-7	22	-8	263.17	17.80
7	22	8	261.97	18.80
-7	22	-8	244.68	20.00
-8	22	-8	11.00	7.00
8	22	8	20.70	8.40
-8	22	-8	10.50	8.50
7	23	-8	199.58	22.40
-7	23	8	208.38	18.30
-6	23	8	180.98	23.90
-6	23	8	169.28	16.70
6	23	-8	167.28	20.00
-5	23	8	346.17	28.90
-5	23	8	290.77	22.50
5	23	-8	349.87	25.30
4	23	-8	191.48	19.60
-4	23	8	191.58	17.20
-4	23	8	190.68	22.20
-3	23	8	116.49	24.10
3	23	-8	186.28	17.80
-3	23	8	143.39	15.70
2	23	-8	300.77	22.30
-2	23	8	286.57	20.70
-2	23	8	274.97	25.00
-1	23	8	60.99	13.80
1	23	-8	46.50	9.30
-1	23	8	29.40	8.80
0	23	8	475.55	31.80
0	23	-8	471.15	28.60
0	23	-8	419.86	23.50
0	23	8	451.15	27.50
-1	23	-8	50.29	9.70
-1	23	-8	55.79	7.60
1	23	8	49.10	9.60
1	23	8	43.70	12.60
2	23	8	298.07	25.90
2	23	8	313.47	21.10
-2	23	-8	329.27	22.30
-2	23	-8	295.27	17.70

3	23	8	-5.90	6.70
-3	23	-8	-12.60	7.00
3	23	8	-3.50	10.00
-3	23	-8	-1.40	4.90
-4	23	-8	360.16	23.60
4	23	8	409.76	29.20
4	23	8	386.16	24.50
-4	23	-8	422.66	26.00
-5	23	-8	0.10	5.80
5	23	8	-3.90	6.90
-5	23	-8	11.80	7.80
-6	23	-8	201.38	18.10
-6	23	-8	164.58	14.50
6	23	8	179.18	16.00
7	23	8	24.30	8.90
-7	23	-8	24.90	9.10
-7	23	-8	25.10	7.50
-6	24	8	27.10	15.40
-6	24	8	7.00	8.60
6	24	-8	0.60	10.20
5	24	-8	67.29	12.80
-5	24	8	103.49	14.20
-5	24	8	126.09	23.50
-4	24	8	1.70	12.50
4	24	-8	2.80	9.00
-4	24	8	4.20	8.80
3	24	-8	4.60	8.80
-3	24	8	2.60	8.30
-3	24	8	1.40	11.40
-2	24	8	26.50	9.70
2	24	-8	35.00	10.00
-2	24	8	16.10	11.80
-1	24	8	81.19	13.30
-1	24	8	60.09	15.60
1	24	-8	50.59	11.00
0	24	-8	3.30	8.10
0	24	8	-0.40	7.40
0	24	8	10.00	12.00
-1	24	-8	15.50	4.80
1	24	8	8.10	11.10
1	24	8	4.10	7.90
-1	24	-8	11.30	8.60
-2	24	-8	-9.20	7.80
2	24	8	-8.00	11.30

-2	24	-8	-0.90	4.90
2	24	8	-1.30	7.30
-3	24	-8	5.10	5.40
3	24	8	5.30	7.60
-3	24	-8	10.30	7.80
3	24	8	1.70	11.10
4	24	8	29.80	8.70
-4	24	-8	28.00	6.50
-4	24	-8	17.20	9.10
5	24	8	11.60	8.00
-5	24	-8	10.00	8.90
-5	24	-8	17.10	6.50
6	24	8	9.90	8.10
-6	24	-8	-1.60	8.50
-6	24	-8	3.60	6.70
-5	25	8	218.88	27.00
-5	25	8	178.38	18.80
5	25	-8	196.38	21.80
-4	25	8	66.89	16.30
4	25	-8	50.69	11.70
-4	25	8	54.69	10.30
-3	25	8	114.09	18.10
-3	25	8	137.79	16.50
3	25	-8	176.78	19.00
2	25	-8	255.47	22.90
-2	25	8	301.57	21.40
-2	25	8	277.97	27.50
-1	25	8	7.70	9.00
-1	25	8	10.20	12.60
1	25	-8	6.70	8.80
0	25	8	364.16	30.30
0	25	-8	330.27	25.20
0	25	8	356.86	23.70
1	25	8	97.79	14.40
1	25	8	116.39	22.70
-1	25	-8	98.59	15.80
-2	25	-8	303.97	23.10
2	25	8	317.57	21.80
2	25	8	288.37	27.90
3	25	8	14.80	8.20
-3	25	-8	3.10	5.70
-3	25	-8	10.80	9.20
-4	25	-8	461.75	30.80
-4	25	-8	485.35	26.90

4	25	8	505.55	29.20
5	25	8	5.10	8.00
-5	25	-8	18.50	12.50
-5	25	-8	14.50	6.90
-3	26	8	-2.80	13.50
3	26	-8	16.80	11.30
-3	26	8	9.30	9.30
-2	26	8	41.80	15.10
-2	26	8	49.60	10.60
2	26	-8	32.80	11.50
-1	26	8	21.20	9.50
-1	26	8	-0.30	14.20
1	26	-8	18.10	10.30
0	26	-8	48.10	11.80
0	26	8	60.19	10.90
0	26	8	48.00	14.40
1	26	8	48.80	10.60
-1	26	-8	56.89	11.40
1	26	8	58.49	21.50
-2	26	-8	-13.00	8.30
2	26	8	8.30	8.60
3	26	8	45.90	9.90
-3	26	-8	64.99	13.50
-15	0	9	15.10	7.10
-14	0	9	-2.20	5.80
13	0	-9	-3.60	8.20
-13	0	9	9.70	5.20
-12	0	9	3.50	4.50
12	0	-9	-10.30	6.40
-12	0	9	-9.40	10.10
-11	0	9	22.30	9.90
-10	0	9	1.90	3.60
10	0	-9	-8.20	4.30
10	0	-9	-4.40	5.30
-10	0	9	-15.50	7.20
-9	0	9	4.90	3.20
-9	0	9	5.40	6.70
9	0	-9	5.20	3.90
9	0	-9	6.80	5.00
-8	0	9	1.30	2.80
-8	0	9	-1.50	5.70
8	0	-9	3.10	3.40
8	0	-9	1.40	4.90
-7	0	9	-2.80	4.70

-7	0	9	3.50	2.60
7	0	-9	5.80	2.90
7	0	-9	-1.00	4.00
6	0	-9	8.20	2.70
6	0	-9	2.30	3.60
-6	0	9	-6.70	4.00
-6	0	9	0.20	2.30
5	0	-9	0.50	3.20
-5	0	9	3.30	2.40
-5	0	9	-7.00	3.70
-4	0	9	-1.70	3.20
-4	0	9	7.30	2.50
4	0	-9	-0.20	2.90
3	0	-9	2.30	2.40
3	0	-9	7.20	1.70
-3	0	9	1.40	2.90
-3	0	9	-0.30	2.10
2	0	-9	-1.90	1.70
-2	0	9	-0.80	2.20
-2	0	9	1.00	2.20
2	0	-9	1.40	1.30
-1	0	9	1.60	1.90
1	0	-9	4.60	1.50
1	0	-9	-0.70	1.60
-1	0	9	4.40	1.80
0	0	9	1.00	1.80
0	0	9	0.40	1.50
0	0	-9	-2.20	1.40
0	0	-9	-0.70	1.30
-1	0	-9	2.90	1.40
-1	0	-9	2.90	1.70
1	0	9	1.60	1.90
-2	0	-9	0.30	1.90
2	0	9	1.40	1.10
2	0	9	-0.80	2.10
2	0	9	3.60	1.40
-3	0	-9	-2.60	1.80
3	0	9	1.10	2.10
3	0	9	1.80	1.20
3	0	9	1.20	0.90
-4	0	-9	-1.90	2.40
4	0	9	-0.70	2.50
4	0	9	0.00	1.40
-4	0	-9	0.30	1.90

4	0	9	-0.10	1.10
5	0	9	5.20	2.90
5	0	9	4.30	1.40
-5	0	-9	4.00	2.70
-5	0	-9	2.30	2.40
-6	0	-9	13.00	3.50
-6	0	-9	15.70	3.80
-7	0	-9	-1.10	3.60
-7	0	-9	1.40	3.50
7	0	9	1.20	2.30
-7	0	-9	0.30	3.30
7	0	9	0.80	3.30
-8	0	-9	-1.10	4.00
8	0	9	5.30	3.00
-8	0	-9	7.00	4.40
9	0	9	9.40	3.70
-9	0	-9	22.50	5.70
-9	0	-9	11.80	5.40
-10	0	-9	6.40	5.40
-10	0	-9	0.50	5.90
10	0	9	2.60	3.90
-11	0	-9	2.10	6.90
-11	0	-9	12.80	6.10
11	0	9	1.60	4.40
-12	0	-9	9.40	6.40
-12	0	-9	2.10	7.80
-13	0	-9	9.60	7.50
-14	0	-9	-3.20	8.10
-15	0	-9	23.30	8.80
-15	-1	9	292.77	19.60
-15	1	9	327.67	20.10
-14	-1	9	7.70	5.80
-14	1	9	4.90	5.90
-13	-1	9	238.28	14.90
13	-1	-9	226.08	19.70
13	1	-9	226.48	19.80
-12	-1	9	25.10	11.50
-12	-1	9	26.60	5.20
12	-1	-9	16.20	7.30
12	1	-9	18.30	7.80
-12	1	9	8.20	10.90
-12	1	9	23.90	5.20
-11	-1	9	628.74	39.80
11	-1	-9	698.13	37.80

-11	-1	9	717.43	34.80
-11	1	9	700.33	34.90
11	1	-9	721.43	38.20
-11	1	9	666.53	40.40
11	1	-9	675.33	35.80
-10	-1	9	193.58	18.90
-10	-1	9	226.08	12.60
10	-1	-9	210.88	14.20
10	-1	-9	221.48	15.80
-10	1	9	208.88	12.70
10	1	-9	199.18	13.60
-10	1	9	221.48	18.90
10	1	-9	202.08	16.30
9	-1	-9	604.84	30.70
-9	-1	9	600.04	29.80
9	-1	-9	605.54	32.30
-9	-1	9	559.54	33.90
9	1	-9	615.24	32.70
-9	1	9	558.24	33.90
9	1	-9	604.94	30.50
-9	1	9	598.14	30.00
-8	-1	9	125.39	13.20
8	-1	-9	160.38	10.20
-8	-1	9	152.98	9.20
8	-1	-9	146.79	12.00
8	1	-9	164.58	9.90
-8	1	9	136.49	13.80
8	1	-9	153.78	12.40
-8	1	9	154.58	9.50
7	-1	-9	830.92	40.30
7	-1	-9	836.22	41.80
-7	-1	9	783.32	42.60
-7	-1	9	793.02	39.70
-7	1	9	819.42	39.90
-7	1	9	788.92	42.60
7	1	-9	827.42	40.00
7	1	-9	822.32	41.90
6	-1	-9	893.51	42.40
-6	-1	9	841.72	44.40
-6	-1	9	855.91	42.00
6	-1	-9	894.91	43.80
-6	1	9	821.42	44.30
-6	1	9	843.92	42.20
6	1	-9	887.61	43.80

6	1	-9	858.91	42.10
5	-1	-9	2009.30	95.59
-5	-1	9	1903.71	94.29
-5	-1	9	1954.20	96.09
5	-1	-9	2046.90	94.49
-5	1	9	2023.20	96.19
5	1	-9	1996.80	95.69
-5	1	9	1867.01	94.49
-4	-1	9	1772.32	86.79
4	-1	-9	1798.22	86.39
4	-1	-9	1841.02	85.29
4	1	-9	1811.52	85.09
-4	1	9	1818.72	86.89
4	1	-9	1787.32	86.39
-4	1	9	1652.73	85.59
3	-1	-9	548.15	29.50
3	-1	-9	603.94	30.20
-3	-1	9	636.94	30.60
-3	-1	9	610.54	29.30
-3	1	9	569.54	29.60
-3	1	9	611.84	30.50
3	1	-9	601.54	28.90
3	1	-9	596.94	30.20
2	-1	-9	1351.56	66.99
-2	-1	9	1348.97	66.39
-2	-1	9	1457.35	67.29
2	1	-9	1336.07	66.09
-2	1	9	1347.67	66.69
2	1	-9	1411.36	66.99
-2	1	9	1446.76	67.29
-1	-1	9	1102.59	50.69
1	-1	-9	1033.20	50.49
-1	-1	9	973.20	50.09
-1	1	9	1075.79	50.69
1	1	-9	1067.49	50.49
-1	1	9	1027.70	50.39
1	1	-9	1011.40	49.90
0	-1	-9	4081.89	192.88
0	-1	9	4143.19	192.88
0	-1	-9	4028.40	192.98
0	-1	9	3910.41	192.88
0	1	9	4141.79	192.98
0	1	-9	4111.29	192.78
0	1	-9	4124.59	192.68

0	1	9	3938.21	192.98
-1	-1	-9	387.86	19.20
1	-1	9	416.66	18.80
-1	-1	-9	385.26	19.10
1	-1	9	365.76	19.20
-1	1	-9	335.97	19.40
1	1	9	384.46	19.00
1	1	9	404.36	19.10
-1	1	-9	375.26	18.80
1	1	9	367.56	19.40
2	-1	9	6137.89	302.57
-2	-1	-9	6584.24	302.47
-2	1	-9	6630.84	302.47
2	1	9	6145.09	302.67
-3	-1	-9	70.09	5.40
3	-1	9	64.99	5.50
3	1	9	72.59	5.80
3	1	9	77.49	4.50
3	1	9	77.79	4.90
-3	1	-9	74.49	5.40
4	-1	9	1747.23	86.79
-4	-1	-9	1784.82	86.39
-4	-1	-9	1826.42	86.79
4	1	9	1758.32	85.89
4	1	9	1738.43	86.89
-4	1	-9	1793.02	85.99
4	1	9	1847.92	86.09
-4	1	-9	1915.71	86.89
5	-1	9	428.66	20.70
-5	-1	-9	453.35	22.20
5	-1	9	427.76	20.50
-5	-1	-9	399.26	22.90
5	-1	9	407.36	22.10
-5	1	-9	425.16	21.30
5	1	9	408.96	21.30
5	1	9	396.06	22.10
-5	1	-9	452.75	22.20
5	1	9	427.56	21.00
-6	-1	-9	4273.97	190.18
6	-1	9	3969.80	188.88
6	-1	9	3802.52	189.98
-6	1	-9	4319.07	190.28
6	1	9	3674.73	189.98
-6	1	-9	3827.72	189.38

6	1	9	3973.10	189.28
7	-1	9	199.68	10.80
-7	-1	-9	207.08	12.40
-7	1	-9	186.48	12.50
7	1	9	184.98	11.40
7	1	9	192.68	12.60
-7	1	-9	178.88	11.80
-7	1	-9	213.88	12.70
8	-1	9	364.36	18.80
-8	-1	-9	370.46	20.40
8	1	9	355.66	20.00
-8	1	-9	359.86	19.80
-8	1	-9	340.97	20.00
9	-1	9	1360.36	67.09
-9	-1	-9	1441.36	68.69
-9	1	-9	1392.56	68.39
-9	1	-9	1332.97	68.09
10	-1	9	403.46	21.50
-10	-1	-9	402.36	23.10
-10	1	-9	368.96	23.00
10	1	9	420.26	22.10
-10	1	-9	386.36	23.10
11	-1	9	398.96	22.60
-11	-1	-9	447.96	24.60
11	1	9	424.26	23.20
-11	1	-9	386.56	24.50
-11	1	-9	411.36	23.90
-12	-1	-9	352.96	21.50
-12	1	-9	292.07	21.60
-12	1	-9	328.77	21.20
-13	-1	-9	342.47	22.50
-13	1	-9	337.07	22.30
-14	-1	-9	29.30	9.20
-14	1	-9	19.20	8.30
-15	-1	-9	302.87	22.40
-15	1	-9	288.97	21.70
-15	-2	9	9.00	6.90
-15	2	9	20.30	7.30
-14	-2	9	134.49	17.70
-14	2	9	164.48	13.20
-13	-2	9	12.70	5.10
13	-2	-9	14.80	8.80
13	2	-9	16.50	8.60
-13	2	9	18.50	5.90

-12	-2	9	112.29	9.50
-12	-2	9	86.09	15.60
12	-2	-9	149.69	14.70
12	2	-9	111.59	14.50
-12	2	9	124.09	10.10
-12	2	9	96.39	18.70
11	-2	-9	159.38	14.70
-11	-2	9	167.88	10.80
-11	-2	9	112.39	18.00
11	2	-9	145.49	14.90
-11	2	9	173.78	11.20
-11	2	9	159.98	18.20
10	-2	-9	174.28	14.10
10	-2	-9	157.88	12.40
-10	-2	9	168.08	17.20
-10	-2	9	171.58	10.40
-10	2	9	134.29	16.30
10	2	-9	149.69	14.20
10	2	-9	171.18	11.60
-10	2	9	162.88	10.80
9	-2	-9	70.59	9.30
-9	-2	9	55.59	5.80
9	-2	-9	80.19	8.10
-9	-2	9	65.29	12.50
-9	2	9	47.20	9.30
-9	2	9	68.59	6.50
9	2	-9	55.59	9.90
9	2	-9	56.39	7.00
8	-2	-9	545.45	27.10
-8	-2	9	475.05	29.70
-8	-2	9	514.05	26.00
8	-2	-9	536.15	28.40
-8	2	9	529.95	26.40
-8	2	9	497.25	29.70
8	2	-9	532.45	26.50
8	2	-9	518.15	28.70
-7	-2	9	318.87	17.00
-7	-2	9	316.67	20.70
7	-2	-9	347.87	18.10
7	-2	-9	341.17	19.60
-7	2	9	343.57	17.60
7	2	-9	328.07	17.40
-7	2	9	332.97	20.70
7	2	-9	329.47	19.70

-6	-2	9	269.57	14.60
-6	-2	9	263.57	17.70
6	-2	-9	309.37	17.00
6	-2	-9	292.67	15.40
-6	2	9	277.17	15.10
-6	2	9	298.97	17.80
6	2	-9	280.27	14.90
6	2	-9	264.87	16.90
5	-2	-9	513.35	26.70
5	-2	-9	536.35	25.60
-5	-2	9	489.25	27.40
5	2	-9	516.55	25.10
-5	2	9	485.05	25.60
5	2	-9	497.25	26.90
-5	2	9	523.75	27.50
-4	-2	9	343.17	19.00
4	-2	-9	341.87	17.30
4	-2	-9	335.47	18.30
-4	-2	9	331.67	17.10
4	2	-9	341.87	18.60
-4	2	9	310.37	17.70
-4	2	9	348.97	18.90
4	2	-9	354.36	16.90
-3	-2	9	311.37	17.70
3	-2	-9	323.47	17.40
3	-2	-9	343.47	16.50
-3	-2	9	319.17	16.20
-3	2	9	299.77	16.90
-3	2	9	332.37	17.80
3	2	-9	330.67	17.50
3	2	-9	333.17	16.00
-2	-2	9	1085.89	52.89
2	-2	-9	1088.99	52.99
2	-2	-9	1111.79	53.79
-2	-2	9	1115.49	53.89
2	2	-9	1120.09	53.69
2	2	-9	1066.69	52.69
-2	2	9	1077.59	53.49
-2	2	9	1158.98	53.99
-1	-2	9	1789.12	88.39
1	-2	-9	1841.12	88.49
-1	-2	9	1850.41	88.89
1	-2	-9	1852.81	88.99
1	2	-9	1815.72	88.19

-1	2	9	1852.11	88.89
-1	2	9	1914.71	89.09
1	2	-9	1915.81	88.89
0	-2	9	185.68	9.40
0	-2	9	162.18	9.40
0	-2	-9	166.28	9.60
0	-2	-9	166.58	9.40
0	2	9	173.08	9.60
0	2	-9	175.98	9.30
0	2	9	168.78	9.70
0	2	-9	180.58	9.10
1	-2	9	2267.27	108.99
1	-2	9	2339.47	109.79
-1	-2	-9	2415.96	109.79
-1	-2	-9	2300.07	109.79
1	2	9	2315.57	109.59
-1	2	-9	2287.87	109.19
1	2	9	2174.28	109.89
1	2	9	2390.36	109.89
-1	2	-9	2193.08	109.89
-2	-2	-9	-2.20	1.70
2	-2	9	0.60	2.00
2	2	9	4.20	1.90
-2	2	-9	1.90	1.00
-2	2	-9	-0.90	1.80
2	2	9	-2.00	2.00
2	2	9	2.00	1.30
3	-2	9	1501.15	75.49
-3	-2	-9	1618.04	75.49
-3	2	-9	1589.84	75.39
3	2	9	1578.94	74.99
3	2	9	1601.54	75.29
3	2	9	1511.45	75.69
-4	-2	-9	3.90	2.30
-4	-2	-9	3.90	2.50
4	-2	9	3.60	2.70
-4	2	-9	3.30	2.40
4	2	9	2.10	2.50
-4	2	-9	3.30	1.50
4	2	9	0.90	2.00
4	2	9	5.70	1.70
-5	-2	-9	886.11	42.90
5	-2	9	820.42	42.90
-5	-2	-9	855.21	42.70

5	2	9	887.71	42.40
-5	2	-9	875.41	42.00
5	2	9	794.72	42.80
-5	2	-9	941.11	43.10
-6	-2	-9	321.37	17.50
-6	-2	-9	359.06	17.60
6	-2	9	320.47	15.70
6	2	9	305.67	16.90
-6	2	-9	326.67	17.70
-6	2	-9	290.17	16.60
6	2	9	318.37	17.70
-6	2	-9	292.87	17.60
-7	-2	-9	139.39	10.40
-7	-2	-9	143.79	10.30
7	-2	9	144.19	8.20
-7	2	-9	142.39	9.40
7	2	9	151.48	9.60
7	2	9	132.59	9.90
-7	2	-9	146.89	10.40
-7	2	-9	141.49	10.30
-8	-2	-9	68.39	8.10
8	-2	9	55.39	5.40
8	2	9	62.99	7.00
-8	2	-9	51.89	6.90
-8	2	-9	60.39	7.70
8	2	9	56.79	7.00
-9	-2	-9	214.18	14.70
9	-2	9	201.98	12.30
-9	-2	-9	197.48	14.70
9	2	9	216.78	13.60
-9	2	-9	215.78	14.00
-9	2	-9	221.38	13.90
-10	-2	-9	110.79	11.70
-10	-2	-9	114.19	11.00
10	-2	9	96.89	8.50
-10	2	-9	91.49	10.60
-10	2	-9	100.69	11.20
10	2	9	105.99	12.10
-11	-2	-9	201.78	16.50
11	-2	9	206.78	13.40
-11	-2	-9	208.68	15.90
11	2	9	204.98	16.90
-11	2	-9	196.88	15.70
-11	2	-9	239.18	15.70

12	-2	9	179.98	17.00
-12	-2	-9	201.68	24.40
-12	2	-9	153.48	15.50
-12	2	-9	193.98	15.20
-13	-2	-9	28.30	8.10
-13	2	-9	32.70	8.10
-14	-2	-9	57.09	10.40
-14	2	-9	78.49	11.90
-15	-2	-9	66.29	11.50
-15	2	-9	59.99	10.90
-15	-3	9	185.58	15.00
-15	3	9	198.98	15.90
-14	-3	9	4.90	5.60
-14	3	9	9.40	6.40
-13	-3	9	283.57	16.80
13	-3	-9	277.17	21.30
-13	3	9	284.17	17.40
13	3	-9	276.37	21.40
-12	-3	9	47.40	12.70
-12	-3	9	57.79	10.30
12	-3	-9	78.69	13.10
-12	-3	9	73.99	7.90
-12	3	9	74.69	8.80
-12	3	9	47.10	13.10
12	3	-9	43.40	8.90
-11	-3	9	228.98	15.10
11	-3	-9	233.58	17.10
-11	-3	9	161.48	19.90
-11	-3	9	229.28	13.20
11	3	-9	226.98	17.70
-11	3	9	227.98	14.00
-11	3	9	215.38	20.00
10	-3	-9	57.59	10.20
-10	-3	9	58.99	6.30
10	3	-9	54.49	10.80
-10	3	9	64.89	7.50
-10	3	9	41.60	10.20
-9	-3	9	415.76	27.70
-9	-3	9	456.95	23.20
9	-3	-9	465.85	24.80
9	-3	-9	473.55	26.10
-9	3	9	489.55	24.10
-9	3	9	428.26	27.60
9	3	-9	454.75	26.40

9	3	-9	470.25	23.90
-8	-3	9	76.19	11.40
-8	-3	9	91.29	6.30
8	-3	-9	87.59	9.70
8	-3	-9	100.09	8.20
8	3	-9	81.19	10.30
-8	3	9	93.09	7.30
-8	3	9	71.19	11.30
8	3	-9	92.19	7.10
7	-3	-9	198.88	11.40
-7	-3	9	176.98	10.00
-7	-3	9	172.58	14.30
7	-3	-9	174.28	12.50
-7	3	9	186.38	10.90
-7	3	9	167.48	14.00
7	3	-9	183.18	13.10
7	3	-9	185.18	10.40
6	-3	-9	1539.45	71.39
-6	-3	9	1362.26	72.99
6	-3	-9	1532.35	72.49
-6	-3	9	1468.85	70.59
6	3	-9	1464.25	72.59
-6	3	9	1488.35	71.29
6	3	-9	1485.45	70.69
-6	3	9	1440.76	72.99
5	-3	-9	188.08	12.00
5	-3	-9	207.48	10.80
-5	-3	9	185.38	10.00
-5	-3	9	153.98	12.80
5	3	-9	168.18	12.30
-5	3	9	192.38	11.00
-5	3	9	180.38	12.70
5	3	-9	195.18	9.90
4	-3	-9	138.79	9.20
4	-3	-9	142.79	8.30
-4	-3	9	123.19	9.80
-4	-3	9	143.69	7.60
4	3	-9	140.19	7.40
-4	3	9	127.79	8.70
4	3	-9	139.89	9.60
-4	3	9	140.99	9.90
3	-3	-9	55.79	5.50
-3	-3	9	42.40	5.90
3	-3	-9	60.39	4.50

-3	-3	9	58.19	3.80
3	3	-9	58.79	5.80
3	3	-9	60.29	3.50
-3	3	9	49.00	5.40
-3	3	9	58.29	6.00
-2	-3	9	1777.02	88.39
-2	-3	9	1797.92	87.39
2	-3	-9	1868.61	87.79
2	-3	-9	1907.81	88.49
-2	3	9	1885.81	88.69
2	3	-9	1874.61	88.39
-2	3	9	1779.92	88.29
2	3	-9	1800.52	87.29
-1	-3	9	167.68	10.00
1	-3	-9	183.98	10.40
1	-3	-9	188.18	9.80
-1	-3	9	178.68	9.40
-1	3	9	184.28	10.40
1	3	-9	174.48	9.20
-1	3	9	182.48	10.40
1	3	-9	185.38	10.10
0	-3	-9	1353.26	64.59
0	-3	9	1288.47	63.99
0	-3	9	1331.07	64.09
0	-3	-9	1399.36	64.29
0	3	9	1295.97	64.59
0	3	-9	1313.97	63.89
0	3	-9	1349.96	64.09
0	3	9	1361.66	64.49
-1	-3	-9	74.39	5.30
-1	-3	-9	75.59	5.40
1	-3	9	80.39	5.30
1	3	9	67.89	5.40
1	3	9	75.39	5.70
-1	3	-9	66.59	4.30
1	3	9	78.39	5.10
-1	3	-9	72.49	4.90
2	-3	9	417.16	21.80
-2	-3	-9	456.35	21.90
-2	3	-9	428.46	20.70
2	3	9	439.96	21.60
2	3	9	409.96	21.90
2	3	9	421.06	22.00
-2	3	-9	453.75	21.70

-3	-3	-9	55.39	5.00
3	-3	9	54.59	4.80
-3	3	-9	49.50	4.60
3	3	9	46.30	4.00
3	3	9	47.70	4.90
3	3	9	47.80	5.10
-4	-3	-9	554.14	28.30
-4	-3	-9	570.34	28.30
4	-3	9	526.25	28.30
4	3	9	524.35	28.40
4	3	9	608.04	28.30
-4	3	-9	525.65	28.20
-4	3	-9	556.04	27.00
4	3	9	577.74	27.80
-4	3	-9	607.04	28.40
-5	-3	-9	400.86	22.00
-5	-3	-9	443.16	22.00
-5	3	-9	403.46	20.80
5	3	9	408.36	21.70
5	3	9	396.76	22.00
-5	3	-9	475.35	22.30
-5	3	-9	425.66	22.10
-6	-3	-9	545.65	27.10
-6	-3	-9	517.95	27.10
6	-3	9	522.45	25.00
-6	3	-9	503.25	26.00
-6	3	-9	501.95	27.10
6	3	9	487.75	27.00
-6	3	-9	562.84	27.20
6	3	9	528.45	26.70
-7	-3	-9	37.30	6.40
7	-3	9	26.30	3.40
-7	-3	-9	41.00	6.70
-7	3	-9	28.30	5.40
7	3	9	34.80	6.50
-7	3	-9	35.00	5.30
7	3	9	29.20	5.80
-7	3	-9	43.50	6.70
-8	-3	-9	33.40	5.60
8	-3	9	24.60	4.10
-8	3	-9	26.70	5.20
-8	3	-9	25.00	4.50
8	3	9	36.00	6.30
8	3	9	21.00	4.70

-9	-3	-9	-1.80	4.80
9	-3	9	1.70	2.80
-9	3	-9	1.60	4.80
9	3	9	8.80	4.60
9	3	9	0.70	4.20
-9	3	-9	0.90	4.10
10	-3	9	252.37	14.60
-10	-3	-9	283.37	17.60
-10	-3	-9	266.87	17.60
10	3	9	268.27	16.60
-10	3	-9	225.98	16.50
-10	3	-9	244.08	19.70
-11	-3	-9	151.38	14.40
11	-3	9	129.79	10.50
-11	-3	-9	162.58	13.50
11	3	9	163.98	12.80
-11	3	-9	144.09	13.60
-11	3	-9	168.48	13.50
12	-3	9	118.39	13.60
-12	-3	-9	142.89	12.80
-12	3	-9	142.99	13.50
-12	3	-9	92.59	14.00
-13	-3	-9	23.50	7.80
-13	3	-9	17.10	7.80
-14	-3	-9	15.20	8.60
-14	3	-9	20.00	8.20
-15	-3	-9	70.19	12.20
-15	3	-9	77.99	13.00
-15	-4	9	-6.50	7.30
-15	4	9	4.80	7.10
-14	-4	9	272.57	16.80
-14	4	9	254.27	17.80
-13	-4	9	3.10	4.90
13	-4	-9	15.80	7.80
13	4	-9	2.00	8.00
-13	4	9	13.30	5.80
-12	-4	9	636.04	32.20
-12	-4	9	677.93	33.70
12	-4	-9	636.94	35.80
-12	4	9	598.34	35.20
12	4	-9	614.34	36.10
11	-4	-9	143.49	13.60
-11	-4	9	85.29	17.10
-11	-4	9	111.79	10.90

-11	-4	9	128.89	9.20
-11	4	9	132.49	10.40
11	4	-9	122.29	14.20
-11	4	9	108.89	16.90
-10	-4	9	1086.49	53.59
10	-4	-9	1097.89	56.39
-10	-4	9	974.20	57.79
-10	-4	9	1218.18	54.69
10	4	-9	1142.09	57.09
-10	4	9	1141.89	54.49
-10	4	9	1047.10	58.39
9	-4	-9	357.36	20.00
-9	-4	9	342.47	18.00
-9	-4	9	375.06	19.10
9	-4	-9	354.16	21.10
-9	-4	9	294.97	22.90
9	4	-9	344.67	21.90
-9	4	9	353.26	23.40
-9	4	9	353.46	19.00
-8	-4	9	413.76	21.40
-8	-4	9	392.16	25.40
8	-4	-9	441.06	23.00
8	-4	-9	455.15	24.40
8	4	-9	428.96	21.90
8	4	-9	443.16	24.80
-8	4	9	421.56	22.30
-8	4	9	417.76	25.60
-7	-4	9	249.88	18.00
-7	-4	9	259.07	14.10
7	-4	-9	273.97	15.60
7	-4	-9	288.37	16.90
7	4	-9	272.17	17.40
-7	4	9	285.97	18.10
-7	4	9	288.37	15.20
6	-4	-9	425.56	22.50
-6	-4	9	404.76	20.20
-6	-4	9	373.26	23.10
6	-4	-9	438.06	21.40
6	4	-9	394.46	22.70
6	4	-9	409.76	20.30
-6	4	9	416.76	23.50
-6	4	9	387.46	21.20
-5	-4	9	2200.78	105.89
-5	-4	9	2105.79	107.99

5	-4	-9	2286.07	106.79
5	-4	-9	2252.27	107.59
5	4	-9	2226.28	107.79
-5	4	9	2230.28	106.89
5	4	-9	2150.78	105.79
-5	4	9	2348.47	108.29
4	-4	-9	608.04	29.90
-4	-4	9	566.34	28.00
-4	-4	9	562.04	30.10
4	-4	-9	609.74	28.90
4	4	-9	563.34	29.90
-4	4	9	570.84	29.30
4	4	-9	573.04	27.90
-4	4	9	588.14	30.20
3	-4	-9	1537.45	70.89
3	-4	-9	1524.35	71.79
-3	-4	9	1419.66	71.69
-3	-4	9	1403.56	70.19
3	4	-9	1499.15	70.09
-3	4	9	1550.04	71.99
3	4	-9	1503.45	71.79
-3	4	9	1363.46	71.39
-2	-4	9	213.58	10.20
-2	-4	9	205.08	11.60
2	-4	-9	197.98	11.70
2	-4	-9	205.28	10.90
-2	4	9	205.98	11.90
2	4	-9	201.18	11.60
2	4	-9	202.58	10.00
-2	4	9	180.68	11.50
1	-4	-9	1833.62	84.19
-1	-4	9	1792.92	83.69
1	-4	-9	1781.62	84.69
-1	-4	9	1747.13	84.19
1	4	-9	1656.63	83.49
-1	4	9	1768.72	84.59
-1	4	9	1666.93	84.59
1	4	-9	1801.42	84.39
0	-4	-9	557.04	28.60
0	-4	-9	543.25	28.10
0	-4	9	591.24	27.90
0	-4	9	590.04	27.90
0	4	9	536.65	28.60
0	4	-9	576.54	27.70

0	4	9	569.74	28.50
0	4	-9	596.94	28.10
-1	-4	-9	3113.19	147.59
1	-4	9	3106.19	147.09
-1	-4	-9	3182.78	147.29
-1	4	-9	3091.39	146.69
1	4	9	3017.70	147.59
1	4	9	3158.18	147.69
1	4	9	2975.10	147.29
-1	4	-9	3074.19	146.89
-2	-4	-9	86.49	6.00
2	-4	9	76.09	5.80
2	4	9	87.29	6.40
2	4	9	72.99	6.00
-2	4	-9	88.19	4.30
-2	4	-9	83.29	5.70
2	4	9	84.29	5.70
-3	-4	-9	967.10	46.80
-3	4	-9	969.90	46.70
3	4	9	929.71	47.00
-3	4	-9	918.31	46.80
3	4	9	949.31	46.60
3	4	9	1011.30	47.10
-4	-4	-9	197.68	12.00
-4	-4	-9	212.28	12.00
-4	4	-9	224.28	12.00
-4	4	-9	204.98	11.90
-4	4	-9	205.38	10.30
4	4	9	203.78	12.20
4	4	9	216.58	12.20
4	4	9	205.78	11.40
-5	-4	-9	1692.13	83.79
-5	-4	-9	1832.42	83.79
5	4	9	1818.42	83.69
-5	4	-9	1696.23	83.69
-5	4	-9	1657.13	82.39
-5	4	-9	1813.02	83.79
5	4	9	1598.54	83.69
-6	-4	-9	113.29	9.00
-6	-4	-9	131.09	8.80
-6	4	-9	125.89	9.10
-6	4	-9	134.29	9.20
6	4	9	101.29	8.40
6	4	9	118.09	8.90

-6	4	-9	105.99	7.30
-7	-4	-9	2415.86	110.49
-7	-4	-9	2222.68	110.69
7	-4	9	2294.27	108.49
7	4	9	2176.68	110.39
-7	4	-9	2393.36	110.69
-7	4	-9	2144.19	109.39
-7	4	-9	2248.97	110.39
7	4	9	2384.66	110.39
8	-4	9	328.87	16.00
-8	-4	-9	300.47	18.70
-8	4	-9	320.77	18.30
8	4	9	316.97	18.10
-8	4	-9	287.27	17.20
8	4	9	316.77	18.20
9	-4	9	227.58	11.90
-9	-4	-9	203.08	15.10
9	4	9	212.38	14.20
-9	4	-9	199.18	13.50
9	4	9	202.08	14.20
-9	4	-9	218.08	14.40
-10	-4	-9	162.68	13.70
-10	-4	-9	158.48	13.90
10	-4	9	157.18	10.10
10	4	9	159.58	12.90
-10	4	-9	144.09	12.30
-11	-4	-9	413.06	23.40
11	-4	9	357.96	20.30
-11	-4	-9	374.56	23.80
11	4	9	401.76	22.80
-11	4	-9	340.37	22.40
-11	4	-9	392.46	22.80
-12	-4	-9	294.67	18.80
-12	4	-9	206.38	18.00
-12	4	-9	274.27	17.60
-13	-4	-9	116.09	13.20
-13	4	-9	136.69	14.20
-14	-4	-9	284.97	20.30
-14	4	-9	265.07	20.10
-15	-4	-9	39.40	10.60
-15	4	-9	38.90	9.60
-15	5	9	-13.10	6.90
-14	-5	9	9.70	5.50
-14	5	9	4.40	6.60

-13	-5	9	-4.20	4.50
13	-5	-9	-5.90	7.90
-13	5	9	7.50	5.70
13	5	-9	-14.90	8.20
-12	-5	9	-9.80	17.60
12	-5	-9	17.90	7.20
-12	-5	9	15.10	4.40
12	5	-9	13.10	8.10
-12	5	9	23.00	5.70
-11	-5	9	-9.40	9.40
11	-5	-9	2.40	6.10
-11	-5	9	8.00	3.80
-11	-5	9	8.50	5.30
11	5	-9	-11.00	6.60
-11	5	9	-2.30	8.90
-11	5	9	9.40	4.90
-10	-5	9	102.89	9.30
10	-5	-9	106.89	11.90
-10	-5	9	91.69	7.50
-10	5	9	114.09	9.20
10	5	-9	104.39	13.20
-10	5	9	92.49	15.10
-9	-5	9	5.20	2.70
9	-5	-9	4.10	4.90
-9	-5	9	4.30	3.70
-9	-5	9	-2.80	7.50
-9	5	9	10.50	4.20
-9	5	9	-1.10	6.70
9	5	-9	7.60	6.00
-8	-5	9	0.10	5.80
-8	-5	9	7.30	3.10
-8	-5	9	6.00	2.30
8	-5	-9	5.00	4.60
8	-5	-9	14.30	4.70
8	5	-9	0.00	5.00
-8	5	9	3.60	5.90
-8	5	9	5.70	3.50
-7	-5	9	30.90	7.10
7	-5	-9	39.10	6.10
-7	-5	9	38.80	4.40
7	-5	-9	37.20	5.80
-7	5	9	29.90	5.70
-7	5	9	44.00	7.20
7	5	-9	34.90	6.20

6	-5	-9	33.90	5.30
-6	-5	9	28.60	3.10
6	-5	-9	36.80	6.60
-6	-5	9	21.50	6.20
-6	5	9	18.90	3.90
6	5	-9	24.10	5.90
-6	5	9	32.50	6.10
-5	-5	9	5.40	1.50
5	-5	-9	6.20	3.50
5	-5	-9	9.00	3.10
-5	-5	9	-3.30	3.90
-5	5	9	2.20	3.30
5	5	-9	-3.60	3.90
5	5	-9	6.60	1.60
-5	5	9	8.10	4.00
4	-5	-9	243.08	12.60
-4	-5	9	217.38	13.90
4	-5	-9	228.68	13.40
-4	-5	9	213.48	11.00
4	5	-9	217.58	11.00
4	5	-9	237.28	13.80
-4	5	9	212.48	13.10
-4	5	9	237.68	13.90
-3	-5	9	2.90	0.90
3	-5	-9	4.10	2.30
-3	-5	9	0.80	2.70
3	-5	-9	3.80	2.90
3	5	-9	3.60	0.90
3	5	-9	-0.40	2.60
-3	5	9	2.30	2.80
-3	5	9	-0.80	2.90
2	-5	-9	6.50	2.20
-2	-5	9	8.30	2.60
2	-5	-9	6.60	2.70
-2	-5	9	8.70	1.30
-2	5	9	1.00	2.90
-2	5	9	4.30	2.70
2	5	-9	7.10	1.30
2	5	-9	11.60	2.70
-1	-5	9	423.66	22.30
1	-5	-9	468.15	22.30
-1	-5	9	468.05	21.60
1	-5	-9	421.16	22.80
1	5	-9	435.26	22.40

1	5	-9	440.76	21.40
-1	5	9	427.46	22.90
-1	5	9	457.35	22.90
0	-5	-9	28.50	4.40
0	-5	9	33.70	3.20
0	-5	-9	35.80	3.80
0	-5	9	23.60	3.20
0	5	-9	31.10	2.70
0	5	-9	23.20	3.40
0	5	9	25.10	4.00
0	5	9	27.00	4.00
-1	-5	-9	360.26	18.20
-1	-5	-9	374.86	17.90
-1	5	-9	321.67	18.00
1	5	9	345.07	17.90
-1	5	-9	318.47	17.00
1	5	9	353.76	18.30
-1	5	-9	341.77	17.30
1	5	9	336.57	18.10
-2	-5	-9	87.59	6.40
2	5	9	91.79	6.90
2	5	9	92.09	6.80
-2	5	-9	93.09	6.20
-2	5	-9	101.39	6.00
2	5	9	94.59	6.30
-2	5	-9	80.09	4.80
-3	-5	-9	40.00	4.60
3	5	9	26.60	4.00
3	5	9	33.20	4.70
-3	5	-9	34.80	4.50
3	5	9	28.10	5.10
-3	5	-9	39.80	4.30
-4	-5	-9	3.40	2.80
-4	-5	-9	4.50	2.70
-4	5	-9	7.10	2.80
4	5	9	9.30	3.20
4	5	9	8.60	3.20
-4	5	-9	7.20	2.70
4	5	9	6.20	2.30
-5	-5	-9	236.68	13.20
-5	-5	-9	229.18	13.60
5	5	9	228.48	13.30
-5	5	-9	220.58	13.30
-5	5	-9	227.18	13.30

-5	5	-9	210.08	11.60
5	5	9	242.48	13.60
-6	-5	-9	12.70	4.00
-6	-5	-9	13.10	3.80
-6	5	-9	17.60	4.00
-6	5	-9	10.20	2.50
-6	5	-9	16.30	4.20
6	5	9	11.20	3.70
6	5	9	10.50	3.50
-7	-5	-9	39.60	7.00
-7	-5	-9	16.70	4.20
7	5	9	15.80	4.50
7	5	9	18.20	4.30
-7	5	-9	8.00	4.70
-7	5	-9	8.80	4.10
8	-5	9	43.40	4.10
-8	-5	-9	40.20	8.10
8	5	9	53.49	7.80
-8	5	-9	39.90	6.20
-8	5	-9	43.80	7.40
8	5	9	50.89	7.70
9	-5	9	3.80	2.60
-9	-5	-9	4.40	4.80
-9	5	-9	6.30	4.60
9	5	9	8.60	4.70
9	5	9	7.60	4.70
-9	5	-9	7.00	4.10
10	-5	9	-0.30	3.30
-10	-5	-9	7.00	5.60
-10	5	-9	-1.70	4.60
10	5	9	5.80	5.20
-10	5	-9	10.50	6.30
10	5	9	12.20	9.20
-11	-5	-9	9.20	6.80
-11	-5	-9	10.80	6.90
11	-5	9	5.00	3.90
-11	5	-9	10.70	5.90
11	5	9	0.10	5.60
-11	5	-9	-0.10	5.80
-12	-5	-9	15.90	7.50
-12	5	-9	37.50	8.00
-12	5	-9	18.30	7.10
-13	-5	-9	18.20	8.10
-13	5	-9	7.20	7.00

-14	-5	-9	10.90	8.90
-14	5	-9	14.60	8.00
-15	-5	-9	8.60	9.80
-15	5	-9	-0.80	8.20
-14	-6	9	157.78	14.90
-14	6	9	126.89	13.10
-13	-6	9	0.60	4.50
-13	6	9	8.80	6.00
13	6	-9	6.30	8.70
12	-6	-9	267.67	20.20
-12	-6	9	265.67	15.70
-12	6	9	280.77	17.10
12	6	-9	286.47	21.70
-11	-6	9	51.19	6.50
-11	-6	9	49.00	11.60
11	-6	-9	65.09	11.70
-11	-6	9	53.69	10.20
-11	6	9	57.59	11.50
11	6	-9	65.39	12.70
-10	-6	9	886.41	43.40
10	-6	-9	905.61	46.80
-10	-6	9	1005.20	44.40
-10	-6	9	745.23	48.20
10	6	-9	900.71	47.40
-10	6	9	851.91	48.60
-10	6	9	915.41	44.70
-9	-6	9	82.79	11.70
9	-6	-9	109.09	10.90
-9	-6	9	110.19	8.10
-9	-6	9	101.09	6.90
-9	6	9	109.79	13.50
-9	6	9	107.39	8.90
9	6	-9	101.59	11.80
-8	-6	9	641.54	32.60
8	-6	-9	647.84	33.40
-8	-6	9	572.24	35.30
-8	-6	9	632.04	31.30
8	-6	-9	644.94	34.20
-8	6	9	679.23	32.80
8	6	-9	665.23	35.00
-8	6	9	656.63	35.80
-7	-6	9	228.08	12.10
-7	-6	9	211.68	16.40
7	-6	-9	234.08	14.30

7	-6	-9	246.48	15.50
-7	-6	9	254.57	12.50
-7	6	9	243.28	13.90
7	6	-9	228.28	15.90
-7	6	9	243.18	16.90
-6	-6	9	1232.58	54.89
-6	-6	9	1006.70	57.59
6	-6	-9	1161.68	56.39
-6	-6	9	1121.49	54.79
6	-6	-9	1151.38	57.09
-6	6	9	1152.08	56.19
6	6	-9	1155.28	57.59
-6	6	9	1186.88	57.99
-5	-6	9	1262.27	69.69
5	-6	-9	1435.86	69.59
-5	-6	9	1462.75	67.19
5	-6	-9	1421.16	68.69
-5	-6	9	1385.86	67.19
5	6	-9	1441.26	69.89
-5	6	9	1452.45	70.09
-5	6	9	1459.15	68.99
4	-6	-9	774.72	37.30
-4	-6	9	718.93	38.30
4	-6	-9	751.82	38.20
4	6	-9	783.12	38.40
-4	6	9	727.23	37.80
-4	6	9	767.32	38.60
3	-6	-9	396.66	20.50
3	-6	-9	408.46	19.60
-3	-6	9	346.57	20.30
-3	6	9	354.16	20.20
-3	6	9	389.96	20.60
3	6	-9	381.06	20.40
2	-6	-9	5.90	2.30
-2	-6	9	3.80	2.50
2	-6	-9	7.20	2.90
-2	6	9	10.30	3.00
2	6	-9	7.90	1.20
2	6	-9	7.20	1.00
2	6	-9	2.90	2.40
-1	-6	9	2183.88	105.49
1	-6	-9	2283.57	106.09
1	-6	-9	2323.17	105.39
1	6	-9	2185.08	105.49

1	6	-9	2096.19	104.49
-1	6	9	2138.79	105.99
1	6	-9	2148.09	104.59
-1	6	9	2263.47	105.99
0	-6	-9	183.58	11.00
0	-6	-9	179.78	10.20
0	-6	9	192.18	10.00
0	6	-9	173.68	9.60
0	6	-9	190.68	10.10
0	6	-9	175.58	9.40
0	6	9	169.58	10.70
0	6	9	187.88	10.80
-1	-6	-9	4381.36	198.18
-1	-6	-9	4252.47	198.68
1	6	9	4085.69	198.48
-1	6	-9	3895.71	198.18
-1	6	-9	4160.38	197.58
1	6	9	4208.48	198.38
-1	6	-9	4154.78	197.68
-2	-6	-9	1.20	2.40
-2	6	-9	3.00	2.10
2	6	9	3.30	2.80
-2	6	-9	0.90	1.20
2	6	9	3.90	2.80
-2	6	-9	1.00	2.20
2	6	9	0.40	2.10
-3	-6	-9	1200.88	56.09
3	6	9	1129.29	56.29
-3	6	-9	1116.69	55.89
3	6	9	1141.89	55.99
3	6	9	1194.18	56.69
-3	6	-9	1176.38	55.89
-3	6	-9	1092.79	54.59
-3	6	-9	1154.98	54.69
-4	-6	-9	37.30	4.60
-4	-6	-9	27.00	5.30
4	6	9	28.30	4.60
-4	6	-9	31.80	2.60
-4	6	-9	36.40	5.10
4	6	9	35.90	4.60
-4	6	-9	36.60	5.00
4	6	9	40.70	5.30
-5	-6	-9	1844.52	90.69
-5	-6	-9	1974.10	90.29

-5	6	-9	1814.52	90.19
5	6	9	1767.32	90.29
-5	6	-9	1928.31	90.29
5	6	9	1954.00	90.69
-5	6	-9	1786.32	88.89
-6	-6	-9	146.89	9.80
-6	-6	-9	126.99	10.30
6	6	9	144.69	10.10
-6	6	-9	148.29	10.00
6	6	9	138.29	10.00
-6	6	-9	127.09	9.40
-6	6	-9	125.09	8.10
-7	-6	-9	923.41	45.60
-7	-6	-9	897.41	46.30
-7	6	-9	926.01	46.00
7	6	9	896.01	45.80
7	6	9	988.00	46.10
-7	6	-9	866.81	45.60
-7	6	-9	873.91	44.60
8	-6	9	193.58	9.90
-8	-6	-9	180.98	13.60
-8	6	-9	184.58	11.70
8	6	9	192.48	13.30
8	6	9	183.78	12.90
-8	6	-9	175.38	12.70
9	-6	9	514.15	25.00
-9	-6	-9	494.75	28.40
9	6	9	538.65	28.00
9	6	9	484.35	27.30
-9	6	-9	469.45	26.40
-9	6	-9	509.25	27.40
10	-6	9	511.65	25.10
-10	-6	-9	465.35	28.60
10	6	9	513.15	28.10
-10	6	-9	534.25	27.70
-10	6	-9	449.95	26.80
11	-6	9	361.76	20.10
-11	-6	-9	446.96	24.00
-11	-6	-9	377.46	24.40
11	6	9	405.16	23.60
-11	6	-9	334.17	22.40
-11	6	-9	378.06	22.50
-12	-6	-9	240.98	17.50
-12	6	-9	236.78	16.80

-12	6	-9	174.58	16.20
-13	-6	-9	303.27	20.90
-13	6	-9	251.67	19.40
-14	-6	-9	242.78	20.20
-14	6	-9	225.18	18.30
-14	7	9	2.80	6.80
-13	-7	9	67.09	8.50
13	7	-9	78.79	16.10
-13	7	9	98.99	10.90
12	-7	-9	21.00	7.80
-12	-7	9	23.00	4.50
-12	7	9	21.70	6.00
12	7	-9	8.10	8.50
11	-7	-9	205.28	16.00
-11	-7	9	181.38	11.10
-11	-7	9	178.68	12.60
-11	7	9	189.68	13.20
11	7	-9	194.18	17.70
-11	7	9	166.98	20.40
10	-7	-9	9.70	5.90
-10	-7	9	0.50	2.80
-10	-7	9	0.70	8.60
-10	-7	9	-3.20	4.20
10	7	-9	5.10	6.60
-10	7	9	-4.60	8.70
-10	7	9	9.30	4.80
-9	-7	9	154.08	16.70
-9	-7	9	209.98	11.10
-9	-7	9	168.28	10.00
9	-7	-9	190.48	14.20
9	7	-9	185.58	15.10
-9	7	9	185.78	12.40
-9	7	9	178.18	16.30
8	-7	-9	110.29	10.50
-8	-7	9	113.09	7.20
-8	-7	9	91.39	12.90
8	7	-9	102.19	11.60
-8	7	9	100.99	12.80
-8	7	9	108.29	8.90
-7	-7	9	247.18	18.70
7	-7	-9	288.37	17.00
-7	-7	9	320.07	14.80
7	-7	-9	290.87	17.70
-7	-7	9	273.67	14.40

-7	7	9	287.27	19.10
7	7	-9	322.17	18.90
-7	7	9	290.57	16.50
-6	-7	9	272.37	13.90
6	-7	-9	321.17	17.50
-6	-7	9	279.37	13.90
6	-7	-9	291.37	16.30
-6	-7	9	248.58	17.80
-6	7	9	290.17	16.10
-6	7	9	285.67	18.00
6	7	-9	295.47	18.10
-5	-7	9	363.56	22.40
5	-7	-9	392.86	21.10
5	-7	-9	409.86	22.10
5	7	-9	424.16	22.60
-5	7	9	407.06	22.60
-5	7	9	399.06	21.30
4	-7	-9	9.30	3.10
4	-7	-9	12.30	3.60
-4	-7	9	8.40	4.00
4	7	-9	11.60	3.90
-4	7	9	8.50	3.50
-4	7	9	7.40	3.70
-3	-7	9	447.06	25.30
3	-7	-9	489.85	24.50
3	-7	-9	495.15	25.50
-3	7	9	453.65	25.20
3	7	-9	532.75	25.50
-3	7	9	481.15	25.50
2	-7	-9	839.72	41.00
-2	-7	9	775.62	40.60
2	-7	-9	854.21	40.10
-2	7	9	839.42	41.00
2	7	-9	851.41	40.70
-2	7	9	740.33	40.80
-1	-7	9	9.70	2.60
1	-7	-9	8.30	2.30
1	-7	-9	5.20	3.00
-1	7	9	2.40	3.00
1	7	-9	3.70	1.10
1	7	-9	4.20	1.20
1	7	-9	8.60	2.60
-1	7	9	4.30	2.60
0	-7	-9	1634.34	78.49

0	-7	-9	1642.74	79.29
0	-7	9	1632.34	78.29
0	7	-9	1583.74	77.79
0	7	-9	1733.73	78.39
0	7	9	1602.04	79.19
0	7	9	1650.53	78.99
0	7	-9	1573.94	77.79
-1	-7	-9	47.20	5.90
-1	-7	-9	50.09	5.00
1	7	9	52.49	5.40
-1	7	-9	61.99	4.30
-1	7	-9	49.70	4.60
1	7	9	55.29	5.70
-1	7	-9	51.19	4.20
-2	-7	-9	859.51	42.40
2	7	9	876.81	42.70
-2	7	-9	886.51	42.10
2	7	9	816.92	42.70
-2	7	-9	846.12	42.10
-2	7	-9	893.61	41.60
-3	-7	-9	135.69	8.20
-3	7	-9	120.79	7.80
3	7	9	120.39	8.10
3	7	9	115.09	8.20
-3	7	-9	121.49	6.40
-3	7	-9	123.29	6.60
3	7	9	114.29	9.00
-3	7	-9	116.49	7.90
-4	-7	-9	1181.28	57.29
-4	-7	-9	1142.19	57.89
4	7	9	1162.28	57.39
-4	7	-9	1160.78	57.29
-4	7	-9	1192.18	55.99
-4	7	-9	1271.97	57.39
-4	7	-9	1111.59	55.99
4	7	9	1223.68	58.19
4	7	9	1119.59	57.59
-5	-7	-9	2.50	3.50
-5	-7	-9	10.90	3.60
-5	7	-9	5.70	3.50
-5	7	-9	6.60	1.90
5	7	9	8.80	3.70
-5	7	-9	4.40	3.20
5	7	9	2.70	2.60

5	7	9	1.70	3.30
-6	-7	-9	618.04	31.10
-6	-7	-9	573.34	31.70
-6	7	-9	609.24	31.20
-6	7	-9	594.64	31.10
6	7	9	562.94	31.20
-6	7	-9	606.54	29.90
6	7	9	661.23	31.90
-7	-7	-9	241.08	16.20
7	7	9	246.48	15.60
-7	7	-9	258.37	15.80
-7	7	-9	244.08	14.20
7	7	9	289.97	16.30
-7	7	-9	249.97	15.50
-8	-7	-9	74.59	9.70
8	-7	9	67.59	4.70
8	7	9	83.09	9.20
-8	7	-9	70.49	7.30
8	7	9	63.29	8.40
-8	7	-9	60.19	7.60
-9	-7	-9	11.50	5.40
9	-7	9	6.70	2.60
9	7	9	10.80	5.00
-9	7	-9	8.70	4.90
-9	7	-9	7.50	4.30
9	7	9	11.20	5.80
-10	-7	-9	245.48	18.00
10	-7	9	235.68	13.30
10	7	9	269.67	17.70
-10	7	-9	255.87	16.50
-10	7	-9	242.68	15.80
10	7	9	205.98	17.20
-11	-7	-9	386.16	21.80
11	-7	9	314.47	17.50
-11	7	-9	338.67	20.70
-11	7	-9	280.07	20.00
11	7	9	320.37	20.50
-12	-7	-9	292.87	19.60
-12	7	-9	259.87	18.30
-12	7	-9	232.98	18.30
-13	-7	-9	120.79	15.40
-13	7	-9	104.69	12.50
-14	-7	-9	34.60	9.40
-14	7	-9	19.70	8.40

-14	8	9	131.99	13.30
-13	8	9	27.80	6.90
13	8	-9	13.20	10.50
12	-8	-9	62.89	13.40
-12	-8	9	73.09	7.60
-12	8	9	91.19	10.30
12	8	-9	96.79	15.40
11	-8	-9	70.39	12.30
-11	-8	9	49.80	6.10
11	8	-9	35.60	9.10
-11	8	9	39.40	11.70
-10	-8	9	253.47	13.20
-10	-8	9	182.58	22.10
10	-8	-9	229.08	16.40
-10	-8	9	218.38	12.10
-10	8	9	200.18	19.80
10	8	-9	206.68	17.50
-10	8	9	206.78	17.80
9	-8	-9	10.10	5.50
-9	-8	9	9.30	8.30
-9	-8	9	3.30	2.50
-9	8	9	6.40	4.60
-9	8	9	-5.60	6.70
9	8	-9	0.70	5.90
-8	-8	9	119.79	7.20
-8	-8	9	132.29	13.70
-8	-8	9	137.09	7.70
8	-8	-9	121.79	11.60
8	8	-9	142.59	12.80
-8	8	9	134.19	9.90
-8	8	9	113.59	13.50
7	-8	-9	114.29	10.20
-7	-8	9	107.29	5.80
-7	-8	9	97.29	5.60
7	-8	-9	111.49	9.50
-7	-8	9	79.59	11.60
-7	8	9	107.39	11.80
7	8	-9	96.19	10.80
-7	8	9	99.29	8.60
6	-8	-9	103.99	9.50
-6	-8	9	85.49	10.50
6	-8	-9	112.19	8.80
6	8	-9	88.69	10.20
-6	8	9	118.19	8.30

-6	8	9	98.09	10.80
5	-8	-9	460.55	23.80
-5	-8	9	376.56	23.90
5	-8	-9	439.26	22.70
5	8	-9	444.86	24.10
-5	8	9	424.36	23.00
-5	8	9	438.56	24.20
4	-8	-9	209.68	11.60
-4	-8	9	169.48	12.50
4	-8	-9	205.58	12.60
4	8	-9	187.98	12.60
-4	8	9	185.08	12.20
-4	8	9	186.78	12.80
-3	-8	9	489.05	28.20
3	-8	-9	559.24	27.60
3	-8	-9	552.24	28.50
3	8	-9	568.64	28.40
-3	8	9	537.75	28.40
-3	8	9	558.24	28.70
2	-8	-9	530.65	25.70
2	-8	-9	516.45	26.60
-2	-8	9	504.05	26.30
2	8	-9	531.05	26.30
-2	8	9	502.95	26.50
-2	8	9	458.35	26.50
1	-8	-9	1017.10	48.00
-1	-8	9	924.21	47.20
1	-8	-9	991.60	47.00
1	8	-9	939.91	45.90
-1	8	9	984.10	47.80
1	8	-9	963.30	45.90
-1	8	9	896.41	47.70
1	8	-9	982.20	47.20
0	-8	-9	30.80	5.40
0	-8	9	34.80	4.00
0	-8	-9	43.80	4.60
0	8	-9	24.20	2.90
0	8	9	27.30	4.90
0	8	9	30.60	5.70
0	8	-9	32.00	4.10
0	8	9	33.50	4.90
0	8	-9	28.30	3.10
-1	-8	-9	802.12	38.50
1	8	9	695.73	39.20

-1	8	-9	803.62	38.00
1	8	9	743.43	39.00
-1	8	-9	786.82	38.00
1	8	9	816.22	39.00
-1	8	-9	807.82	37.90
-2	-8	-9	2.60	2.70
2	8	9	10.80	3.20
-2	8	-9	7.20	2.40
-2	8	-9	7.00	1.90
2	8	9	4.70	2.60
2	8	9	0.90	2.10
-2	8	-9	5.50	2.30
-3	-8	-9	112.79	8.00
-3	8	-9	119.89	6.50
3	8	9	107.69	8.40
-3	8	-9	122.49	7.80
-3	8	-9	112.39	7.70
-3	8	-9	114.99	6.70
3	8	9	112.49	8.10
-4	-8	-9	82.19	6.80
-4	-8	-9	74.29	7.70
-4	8	-9	88.79	7.00
-4	8	-9	79.69	7.00
4	8	9	83.89	7.20
-4	8	-9	90.29	5.30
-4	8	-9	87.59	5.20
4	8	9	84.19	7.00
-5	-8	-9	872.81	47.10
-5	-8	-9	979.40	46.50
-5	8	-9	946.01	46.40
5	8	9	961.20	47.30
-5	8	-9	971.90	45.20
5	8	9	878.01	46.30
5	8	9	899.81	46.70
-5	8	-9	972.40	46.50
-6	-8	-9	367.86	21.40
-6	-8	-9	397.66	20.60
-6	8	-9	367.76	20.50
6	8	9	352.56	20.50
-6	8	-9	381.76	20.80
6	8	9	382.76	21.30
-6	8	-9	366.26	19.20
-7	-8	-9	250.67	16.90
7	8	9	263.57	16.10

-7	8	-9	253.37	15.90
-7	8	-9	256.07	14.60
7	8	9	277.47	16.70
-7	8	-9	280.87	17.60
-8	-8	-9	1.10	4.90
-8	8	-9	2.10	3.60
8	8	9	7.60	4.90
-8	8	-9	9.40	4.80
8	8	9	2.00	4.40
-9	-8	-9	97.99	12.20
9	-8	9	119.69	7.30
9	8	9	107.89	10.30
-9	8	-9	125.19	11.00
-9	8	-9	117.89	10.20
9	8	9	124.19	12.10
10	-8	9	12.60	3.20
-10	-8	-9	6.00	6.80
-10	8	-9	18.60	5.90
-10	8	-9	10.80	5.60
10	8	9	12.90	6.70
10	8	9	18.20	6.40
11	-8	9	166.28	11.00
11	8	9	195.58	15.00
-11	8	-9	211.88	15.00
-11	8	-9	159.48	14.20
-12	-8	-9	121.89	14.60
-12	8	-9	121.89	13.20
-12	8	-9	90.79	12.80
-13	-8	-9	39.40	9.60
-13	8	-9	15.30	8.20
-14	-8	-9	55.49	11.40
-14	8	-9	42.40	9.90
-14	9	9	17.00	7.40
-13	9	9	294.97	18.70
13	9	-9	264.67	24.00
12	-9	-9	11.50	8.50
-12	-9	9	18.40	4.20
12	9	-9	30.20	10.50
-12	9	9	20.60	6.80
-11	-9	9	972.60	48.50
11	-9	-9	983.40	52.39
-11	9	9	1031.90	50.49
11	9	-9	1014.30	53.59
10	-9	-9	250.27	17.80

-10	-9	9	237.28	13.70
-10	-9	9	227.68	12.90
-10	9	9	264.47	21.60
-10	9	9	247.38	15.40
10	9	-9	242.38	19.30
9	-9	-9	396.56	24.10
-9	-9	9	436.46	20.50
-9	-9	9	349.87	26.50
-9	9	9	388.46	26.40
-9	9	9	406.26	22.40
9	9	-9	435.86	25.50
-8	-9	9	67.59	4.70
-8	-9	9	76.99	12.70
-8	-9	9	73.79	5.10
8	-9	-9	73.59	9.80
-8	9	9	89.49	8.60
-8	9	9	112.69	12.80
8	9	-9	102.29	12.00
-7	-9	9	73.79	11.30
7	-9	-9	75.69	9.30
-7	9	9	78.69	11.40
7	9	-9	94.19	10.40
-7	9	9	68.39	8.00
-6	-9	9	1224.08	71.19
6	-9	-9	1438.66	70.79
6	-9	-9	1482.05	70.19
6	9	-9	1495.85	71.39
-6	9	9	1510.45	70.09
-6	9	9	1427.16	71.49
5	-9	-9	824.82	41.20
-5	-9	9	712.53	41.30
5	-9	-9	811.42	40.30
-5	9	9	833.02	40.60
5	9	-9	847.62	41.40
-5	9	9	781.72	41.50
4	-9	-9	760.62	39.70
4	-9	-9	790.02	39.00
-4	-9	9	721.43	39.90
-4	9	9	803.22	40.20
-4	9	9	777.72	39.50
4	9	-9	826.42	40.00
3	-9	-9	233.68	13.20
3	-9	-9	229.58	14.30
-3	-9	9	222.08	14.10

3	9	-9	267.17	14.40
-3	9	9	207.78	14.10
-3	9	9	230.58	14.50
2	-9	-9	784.22	39.60
2	-9	-9	795.52	38.60
-2	-9	9	728.33	39.10
-2	9	9	789.42	39.50
-2	9	9	744.33	39.50
2	9	-9	835.52	39.20
1	-9	-9	107.79	8.20
-1	-9	9	101.09	7.30
1	-9	-9	97.89	6.60
-1	9	9	104.79	8.10
1	9	-9	85.39	5.00
1	9	-9	83.39	5.10
1	9	-9	107.69	7.30
-1	9	9	79.29	8.00
0	-9	9	1690.63	84.19
0	-9	-9	1852.61	85.39
0	-9	-9	1767.32	84.39
0	9	-9	1718.53	83.49
0	9	-9	1682.63	83.49
0	9	9	1790.42	85.59
0	9	9	1681.33	85.09
0	9	-9	1806.12	84.29
0	9	9	1775.52	85.09
-1	-9	-9	518.55	26.00
-1	9	-9	542.85	25.80
1	9	9	513.65	27.30
-1	9	-9	496.85	25.40
-1	9	-9	491.85	25.30
1	9	9	514.15	26.60
1	9	9	512.85	26.70
-2	-9	-9	1160.48	55.99
2	9	9	1142.09	56.39
2	9	9	1176.28	57.19
-2	9	-9	1157.18	55.39
-2	9	-9	1157.58	55.59
-2	9	-9	1120.29	55.49
2	9	9	1113.59	56.39
-3	-9	-9	173.48	10.50
3	9	9	172.88	11.00
-3	9	-9	173.48	9.60
-3	9	-9	171.98	9.40

-3	9	-9	168.08	10.30
3	9	9	183.58	11.30
-3	9	-9	171.68	10.40
-4	-9	-9	1164.28	55.09
-4	-9	-9	1113.99	55.99
4	9	9	1082.89	55.29
-4	9	-9	1129.19	54.89
-4	9	-9	1144.39	54.09
-4	9	-9	1163.58	55.09
-4	9	-9	1105.39	53.99
4	9	9	1074.89	55.39
-5	-9	-9	106.99	9.70
-5	-9	-9	117.89	8.80
5	9	9	108.79	8.30
-5	9	-9	104.69	8.50
5	9	9	111.59	9.00
-5	9	-9	101.59	6.90
-6	-9	-9	1842.92	92.09
6	9	9	1787.22	91.29
-6	9	-9	1860.11	90.99
-6	9	-9	1892.21	91.29
-6	9	-9	1996.40	90.19
-7	-9	-9	124.19	11.70
7	9	9	132.99	10.70
-7	9	-9	139.19	10.50
-7	9	-9	129.79	9.20
7	9	9	134.09	11.50
-8	-9	-9	632.74	35.60
-8	9	-9	666.43	33.40
8	9	9	591.04	34.00
8	9	9	708.53	35.40
-8	9	-9	672.53	34.30
-9	-9	-9	97.59	12.10
-9	9	-9	90.69	11.40
9	9	9	105.69	11.80
-9	9	-9	75.39	9.30
9	9	9	96.39	9.60
-10	-9	-9	302.77	21.80
10	-9	9	325.87	16.90
10	9	9	367.76	21.90
-10	9	-9	329.77	19.70
10	9	9	294.57	20.00
-10	9	-9	323.97	20.00
11	-9	9	161.68	10.30

11	9	9	158.18	13.80
-11	9	-9	185.78	14.50
-11	9	-9	136.99	13.80
-12	-9	-9	436.36	25.80
-12	9	-9	325.27	26.00
-12	9	-9	414.46	24.50
-13	-9	-9	207.18	18.80
-13	9	-9	184.88	16.50
-14	-9	-9	48.60	11.10
-14	9	-9	67.49	12.40
-14	10	9	5.20	7.50
13	10	-9	2.40	10.60
-13	10	9	4.00	7.00
12	10	-9	32.70	11.20
-12	10	9	17.60	6.50
11	-10	-9	87.89	13.50
-11	-10	9	83.09	6.90
11	10	-9	109.69	16.10
-10	-10	9	13.10	3.00
10	-10	-9	10.10	6.30
-10	10	9	5.40	9.30
-10	10	9	16.50	5.80
10	10	-9	17.80	7.90
-9	-10	9	17.50	2.70
9	-10	-9	11.40	6.20
-9	-10	9	11.40	2.20
9	10	-9	10.10	6.70
-9	10	9	12.00	8.00
8	-10	-9	92.79	10.40
-8	-10	9	84.69	13.40
8	10	-9	116.19	12.60
-8	10	9	84.59	9.30
-8	10	9	97.89	12.90
-7	-10	9	-2.10	6.50
7	-10	-9	11.00	4.90
7	10	-9	5.70	5.40
-7	10	9	4.20	5.70
-7	10	9	8.80	4.70
6	-10	-9	49.90	8.40
-6	-10	9	49.30	9.90
6	10	-9	57.59	9.60
-6	10	9	62.49	10.00
-6	10	9	65.49	7.90
5	-10	-9	113.29	10.20

5	-10	-9	124.79	9.00
-5	-10	9	100.19	10.80
-5	10	9	116.89	9.70
5	10	-9	124.19	11.00
-5	10	9	121.69	11.10
4	-10	-9	78.19	8.00
-4	-10	9	83.99	8.70
4	-10	-9	63.69	7.00
4	10	-9	85.59	8.60
-4	10	9	52.59	7.30
-4	10	9	71.89	8.50
-3	-10	9	18.90	4.10
3	-10	-9	18.90	3.70
3	-10	-9	15.40	4.60
3	10	-9	21.10	4.40
-3	10	9	7.30	4.10
-3	10	9	10.40	4.20
2	-10	-9	40.20	6.60
2	-10	-9	52.99	5.30
-2	-10	9	27.10	5.70
-2	10	9	46.20	6.40
2	10	-9	35.70	6.00
-2	10	9	40.10	5.90
1	-10	-9	53.49	7.00
1	-10	-9	60.89	5.10
-1	-10	9	61.19	6.00
-1	10	9	52.49	6.70
1	10	-9	43.80	3.30
1	10	-9	48.30	3.20
1	10	-9	62.69	6.10
-1	10	9	46.50	6.80
0	-10	9	2.50	2.30
0	-10	-9	2.10	2.70
0	-10	-9	-1.60	3.30
0	10	9	0.00	4.10
0	10	-9	1.00	2.50
0	10	9	-0.60	3.00
0	10	-9	0.90	1.60
0	10	-9	-0.10	1.60
0	10	9	-2.50	3.20
-1	-10	-9	59.99	5.60
-1	10	-9	54.89	4.70
1	10	9	51.49	7.20
1	10	9	55.09	6.40

-1	10	-9	50.19	5.20
1	10	9	46.50	6.60
-2	-10	-9	143.89	9.30
2	10	9	135.89	10.00
-2	10	-9	138.19	8.60
-2	10	-9	134.49	8.60
2	10	9	149.49	11.10
-2	10	-9	140.29	8.60
2	10	9	141.89	10.00
-3	-10	-9	4.50	3.40
3	10	9	2.30	4.20
-3	10	-9	10.30	3.00
-3	10	-9	9.00	3.10
3	10	9	8.60	3.30
-3	10	-9	9.60	2.30
-3	10	-9	7.30	2.40
3	10	9	5.90	3.50
-4	-10	-9	27.90	5.20
-4	-10	-9	40.60	5.80
4	10	9	26.50	4.60
-4	10	-9	28.90	4.20
-4	10	-9	30.40	3.90
4	10	9	27.20	5.70
-4	10	-9	38.20	5.40
-4	10	-9	35.20	5.40
4	10	9	27.30	5.40
-5	-10	-9	60.39	6.90
-5	-10	-9	52.79	8.30
5	10	9	60.49	7.30
-5	10	-9	54.89	6.70
-5	10	-9	46.90	5.00
-5	10	-9	53.99	6.70
5	10	9	50.39	6.60
-6	-10	-9	23.50	5.50
-6	10	-9	28.60	4.90
-6	10	-9	19.50	5.60
6	10	9	20.40	4.90
-6	10	-9	17.00	4.90
-7	-10	-9	31.30	6.70
7	10	9	28.80	5.80
-7	10	-9	30.20	5.70
-7	10	-9	36.50	5.90
-8	-10	-9	0.50	5.50
-8	10	-9	9.90	5.10

-8	10	-9	5.40	4.00
8	10	9	4.50	4.90
-9	-10	-9	6.50	6.30
9	10	9	6.20	6.70
-9	10	-9	7.40	9.20
-9	10	-9	1.30	4.60
9	10	9	6.10	7.90
10	-10	9	12.60	3.30
-10	10	-9	5.50	5.40
10	10	9	3.70	5.70
-10	10	-9	11.60	6.30
10	10	9	13.60	7.00
11	-10	9	6.20	4.50
-11	10	-9	8.10	6.40
11	10	9	9.40	6.80
-11	10	-9	14.10	7.10
-12	10	-9	30.10	8.00
12	10	9	34.70	8.70
-13	-10	-9	27.80	9.80
-13	10	-9	20.80	8.50
-14	-10	-9	11.80	10.60
-14	10	-9	7.50	8.90
-13	11	9	93.79	11.90
13	11	-9	81.09	16.80
-12	11	9	35.80	8.00
12	11	-9	46.10	11.70
11	-11	-9	640.34	36.10
11	11	-9	609.64	37.40
-11	11	9	662.63	34.20
10	-11	-9	103.89	12.80
-10	11	9	63.79	12.80
-10	11	9	76.89	10.10
10	11	-9	95.39	14.30
-9	-11	9	317.57	16.20
-9	-11	9	318.37	16.10
9	-11	-9	331.47	21.00
-9	11	9	350.96	19.70
-9	11	9	343.97	23.80
9	11	-9	328.17	22.30
8	-11	-9	178.58	13.80
-8	11	9	152.68	11.70
8	11	-9	180.18	15.20
-8	11	9	158.58	15.80
7	-11	-9	862.31	45.00

-7	-11	9	767.42	46.40
7	11	-9	954.20	46.00
-7	11	9	873.71	46.20
-7	11	9	883.81	43.90
6	-11	-9	440.56	24.60
-6	-11	9	374.46	25.50
-6	11	9	429.66	25.60
6	11	-9	445.46	25.30
-6	11	9	485.25	24.20
-5	-11	9	971.00	54.49
5	-11	-9	1084.79	54.19
5	11	-9	1148.69	54.59
-5	11	9	1032.70	54.49
-5	11	9	1126.39	53.69
4	-11	-9	880.31	44.30
4	-11	-9	881.61	45.20
-4	-11	9	794.62	45.30
-4	11	9	894.21	45.00
-4	11	9	929.11	45.70
4	11	-9	944.81	45.40
3	-11	-9	296.67	16.10
3	-11	-9	285.97	17.20
-3	-11	9	256.97	17.00
-3	11	9	282.07	17.20
3	11	-9	308.47	17.10
-3	11	9	281.37	17.10
-2	-11	9	1225.58	64.19
2	-11	-9	1367.66	63.69
2	-11	-9	1323.47	64.79
-2	11	9	1227.68	64.49
2	11	-9	1382.16	64.29
-2	11	9	1307.37	64.69
1	-11	-9	154.18	9.30
1	-11	-9	154.88	10.90
-1	-11	9	138.39	9.70
1	11	-9	135.09	7.20
1	11	-9	151.88	9.80
-1	11	9	142.99	10.30
1	11	-9	136.79	7.40
-1	11	9	117.79	10.10
0	-11	9	1636.44	84.79
0	-11	-9	1814.62	84.99
0	11	-9	1770.12	85.09
0	11	-9	1738.13	83.99

0	11	9	1817.32	86.59
0	11	9	1814.92	85.99
0	11	9	1782.72	85.79
0	11	-9	1704.83	83.79
-1	-11	-9	33.60	4.40
1	11	9	29.90	5.50
1	11	9	20.20	4.20
-1	11	-9	17.80	2.90
1	11	9	27.50	5.10
-1	11	-9	21.90	3.10
-1	11	-9	24.00	4.90
-2	-11	-9	3747.62	170.58
-2	11	-9	3433.56	169.98
-2	11	-9	3372.56	169.88
-2	11	-9	3555.84	170.28
2	11	9	3733.23	172.18
2	11	9	3518.65	171.18
2	11	9	3614.64	171.18
-2	11	-9	3583.94	170.08
-3	-11	-9	23.10	4.00
3	11	9	21.70	5.70
-3	11	-9	22.00	3.50
-3	11	-9	23.90	4.10
-3	11	-9	29.50	4.80
-3	11	-9	29.90	4.10
3	11	9	28.50	5.00
3	11	9	17.60	4.10
-4	-11	-9	729.23	35.00
-4	11	-9	692.33	34.80
4	11	9	686.73	35.40
-4	11	-9	660.43	33.90
-4	11	-9	682.03	34.10
4	11	9	647.34	35.20
-4	11	-9	684.93	34.60
4	11	9	733.13	36.60
-5	-11	-9	49.30	7.50
-5	-11	-9	71.89	7.60
-5	11	-9	47.60	5.20
5	11	9	52.99	8.80
-5	11	-9	55.09	6.60
-5	11	-9	53.79	5.40
-5	11	-9	39.00	6.70
5	11	9	49.99	7.00
5	11	9	50.29	7.20

-6	-11	-9	1227.48	64.79
6	11	9	1198.88	63.59
-6	11	-9	1277.87	63.59
-6	11	-9	1337.67	62.59
6	11	9	1390.06	65.09
-6	11	-9	1294.97	63.39
-7	-11	-9	162.18	14.50
-7	11	-9	202.18	13.10
7	11	9	176.18	13.10
-7	11	-9	200.98	11.90
-8	-11	-9	252.47	18.50
-8	11	-9	252.17	16.20
8	11	9	250.17	16.60
-8	11	-9	267.37	15.60
-9	-11	-9	377.26	25.60
-9	11	-9	455.25	23.80
-9	11	-9	402.66	22.90
9	11	9	390.76	25.20
-10	11	-9	169.58	13.60
10	11	9	134.39	13.30
-10	11	-9	151.58	13.00
-11	11	-9	353.76	23.80
-11	11	-9	450.85	24.60
11	11	9	392.96	24.10
-12	11	-9	307.27	20.20
12	11	9	267.57	20.10
-13	-11	-9	249.48	21.50
-13	11	-9	262.37	19.40
-13	12	9	-0.20	7.10
13	12	-9	11.50	10.70
-12	12	9	90.49	11.90
12	12	-9	91.69	17.70
11	-12	-9	86.79	14.70
11	12	-9	94.89	17.00
-11	12	9	83.59	11.00
10	-12	-9	241.18	18.60
10	12	-9	272.57	20.50
-10	12	9	237.18	16.60
9	-12	-9	8.90	6.80
9	12	-9	14.90	8.00
-9	12	9	-1.50	5.00
-9	12	9	-9.50	8.10
8	-12	-9	588.44	31.50
-8	12	9	518.05	30.10

-8	12	9	550.54	33.00
8	12	-9	583.84	32.20
7	-12	-9	87.99	10.60
7	12	-9	98.79	12.20
-7	12	9	98.29	10.00
-7	12	9	96.99	12.90
6	-12	-9	309.17	19.00
-6	-12	9	285.67	24.30
6	12	-9	340.37	20.10
-6	12	9	280.67	18.00
-6	12	9	286.17	19.90
5	-12	-9	359.86	20.80
-5	-12	9	285.27	21.10
-5	12	9	352.56	19.90
5	12	-9	381.16	21.20
-5	12	9	362.36	21.50
4	-12	-9	397.56	22.40
-4	-12	9	351.36	22.70
4	12	-9	419.56	22.60
-4	12	9	384.36	22.10
-4	12	9	393.46	22.90
-3	-12	9	64.39	8.70
3	-12	-9	84.79	7.60
3	-12	-9	95.59	9.30
3	12	-9	90.69	8.90
-3	12	9	78.89	9.00
-3	12	9	68.69	8.70
2	-12	-9	221.58	14.70
-2	-12	9	224.58	14.30
2	-12	-9	229.48	13.30
-2	12	9	198.08	14.60
2	12	-9	267.67	14.60
-2	12	9	224.08	14.90
1	-12	-9	1170.28	55.09
-1	-12	9	1090.39	55.49
1	-12	-9	1160.78	56.39
-1	12	9	1194.48	57.19
-1	12	9	1127.79	56.09
1	12	-9	1211.88	55.69
-1	12	9	1030.80	55.89
1	12	-9	1078.89	53.69
1	12	-9	1052.49	53.49
0	-12	9	12.20	3.20
0	-12	-9	3.00	3.40

0	12	-9	7.80	2.30
0	12	9	11.20	4.10
0	12	9	6.70	4.50
0	12	-9	19.20	4.00
-1	-12	-9	1329.17	62.49
-1	12	-9	1356.06	62.69
1	12	9	1297.67	63.39
-1	12	-9	1202.08	61.49
-1	12	-9	1212.78	61.69
1	12	9	1318.87	64.29
1	12	9	1251.07	63.29
-2	-12	-9	33.60	4.70
-2	12	-9	25.60	4.80
2	12	9	21.60	5.70
2	12	9	33.50	5.50
-2	12	-9	26.30	5.20
2	12	9	24.60	4.80
-2	12	-9	25.20	4.40
-2	12	-9	29.10	4.70
-3	-12	-9	629.84	31.80
-3	12	-9	613.54	31.20
3	12	9	677.53	33.90
3	12	9	633.54	32.50
-3	12	-9	595.44	31.10
3	12	9	566.04	32.30
-3	12	-9	649.54	31.50
-3	12	-9	633.44	31.50
-4	-12	-9	62.09	6.80
-4	12	-9	47.40	6.50
4	12	9	40.00	6.60
-4	12	-9	44.20	6.30
-4	12	-9	48.20	5.70
4	12	9	43.30	6.50
4	12	9	43.00	7.10
-4	12	-9	40.00	5.00
-5	-12	-9	842.72	44.90
-5	12	-9	874.91	42.70
5	12	9	867.01	43.90
5	12	9	814.62	43.80
-5	12	-9	847.82	43.40
-5	12	-9	880.01	43.20
5	12	9	973.40	45.60
-5	12	-9	829.42	42.50
-6	-12	-9	104.49	11.30

-6	12	-9	80.89	8.90
6	12	9	103.49	11.30
-6	12	-9	99.79	7.80
6	12	9	96.89	9.50
-6	12	-9	70.09	9.40
-7	-12	-9	164.28	14.50
7	12	9	177.78	14.50
7	12	9	156.78	12.80
-7	12	-9	187.88	11.70
-7	12	-9	165.38	12.40
-8	-12	-9	110.49	13.30
8	12	9	114.59	12.70
-8	12	-9	109.89	9.90
8	12	9	96.39	10.70
-8	12	-9	101.89	10.70
-9	-12	-9	73.99	14.20
9	12	9	73.89	10.00
-9	12	-9	90.79	10.10
-9	12	-9	82.59	9.90
9	12	9	67.99	11.70
-10	12	-9	177.78	14.20
-10	12	-9	153.38	13.60
10	12	9	157.18	14.00
11	12	9	122.49	13.30
-11	12	-9	116.79	13.30
-11	12	-9	115.59	13.30
-12	12	-9	118.69	13.90
12	12	9	109.59	13.80
-13	-12	-9	30.40	11.00
-13	13	9	110.89	13.60
13	13	-9	120.59	20.30
-12	13	9	34.60	8.30
12	13	-9	40.60	11.60
11	13	-9	158.08	25.20
-11	13	9	185.08	14.90
10	-13	-9	22.00	8.00
-10	13	9	26.70	7.00
10	13	-9	22.80	8.90
9	-13	-9	121.89	13.10
-9	13	9	116.19	11.40
9	13	-9	116.09	14.70
-9	13	9	79.99	15.60
8	-13	-9	37.70	7.00
-8	13	9	14.40	8.40

8	13	-9	40.60	8.20
-8	13	9	36.50	6.50
7	-13	-9	72.69	10.70
7	13	-9	69.49	12.00
-7	13	9	79.19	13.00
6	-13	-9	602.54	32.20
-6	13	9	577.64	32.80
6	13	-9	573.84	32.50
-6	13	9	581.54	31.00
5	-13	-9	102.59	10.30
5	13	-9	83.99	10.70
-5	13	9	93.69	10.80
-5	13	9	91.49	9.20
4	-13	-9	255.17	16.50
-4	-13	9	230.08	17.50
-4	13	9	254.37	16.30
4	13	-9	251.47	16.50
-4	13	9	272.97	17.10
3	-13	-9	171.98	12.20
-3	-13	9	147.49	12.30
-3	13	9	158.98	11.80
3	13	-9	153.88	12.10
-3	13	9	173.38	12.80
-2	-13	9	361.56	22.30
2	-13	-9	438.36	23.30
-2	13	9	383.26	22.70
2	13	-9	399.56	22.30
-2	13	9	432.56	23.20
-1	-13	9	15.30	4.10
1	-13	-9	13.10	5.40
1	-13	-9	16.40	4.00
-1	13	9	10.80	4.60
-1	13	9	13.00	5.20
1	13	-9	12.50	4.30
1	13	-9	11.40	2.10
-1	13	9	0.20	4.70
1	13	-9	13.10	1.80
0	-13	-9	444.16	22.20
0	13	9	431.16	23.40
0	13	9	412.86	24.40
0	13	-9	405.36	22.50
0	13	-9	416.96	20.80
0	13	-9	415.06	21.00
0	13	9	401.46	23.20

-1	-13	-9	112.29	7.90
1	-13	9	108.39	7.10
1	13	9	95.49	9.40
1	13	9	94.79	13.00
1	13	9	97.59	9.40
-1	13	-9	101.39	8.40
-1	13	-9	94.09	6.70
2	-13	9	114.49	7.90
-2	-13	-9	114.09	8.60
2	13	9	109.39	9.40
-2	13	-9	104.49	8.50
2	13	9	101.79	9.50
2	13	9	112.99	11.50
-2	13	-9	97.59	7.40
-2	13	-9	113.89	8.00
-3	-13	-9	4.10	6.00
3	-13	9	0.80	3.40
3	13	9	-2.40	4.00
-3	13	-9	1.50	3.00
-3	13	-9	-3.60	3.10
3	13	9	-2.50	4.90
-3	13	-9	3.00	2.90
4	-13	9	96.59	8.20
4	13	9	90.59	9.00
4	13	9	84.29	10.70
-4	13	-9	87.69	7.10
4	13	9	93.29	8.80
-4	13	-9	86.59	7.70
-4	13	-9	87.39	7.70
5	-13	9	222.78	13.20
-5	-13	-9	179.58	14.70
5	13	9	177.28	13.30
-5	13	-9	195.38	11.70
5	13	9	163.78	15.20
-5	13	-9	209.58	12.70
5	13	9	214.78	13.50
-5	13	-9	189.18	13.00
-5	13	-9	198.38	12.00
-6	-13	-9	316.47	20.30
-6	13	-9	320.97	18.00
-6	13	-9	294.57	17.20
-6	13	-9	293.47	17.20
6	13	9	332.17	20.70
6	13	9	307.77	18.60

-7	-13	-9	57.99	11.10
-7	13	-9	48.00	7.50
-7	13	-9	52.39	8.50
7	13	9	44.10	8.60
7	13	9	44.80	8.30
-8	-13	-9	13.60	7.20
-8	13	-9	13.60	5.10
8	13	9	1.60	6.80
-8	13	-9	15.30	7.20
-9	13	-9	15.40	6.40
9	13	9	13.30	7.20
-9	13	-9	6.60	5.30
9	13	9	11.40	6.40
-10	13	-9	264.37	18.90
-10	13	-9	314.07	19.40
10	13	9	296.77	19.50
11	13	9	51.29	8.60
-11	13	-9	47.50	9.30
12	13	9	51.19	9.80
-12	13	-9	66.09	12.40
-13	-13	-9	65.89	12.80
-13	13	-9	102.09	14.30
-13	14	9	4.40	7.70
13	14	-9	6.40	11.80
-12	14	9	274.97	19.30
12	14	-9	292.87	25.50
11	14	-9	59.69	12.10
-11	14	9	86.39	11.60
10	-14	-9	426.96	26.60
10	14	-9	443.56	30.00
-10	14	9	418.16	24.80
9	-14	-9	90.19	13.30
9	14	-9	92.59	14.80
-9	14	9	108.69	10.50
8	-14	-9	259.87	18.20
-8	14	9	249.08	16.50
-8	14	9	217.08	20.10
8	14	-9	241.28	19.10
7	-14	-9	278.17	19.10
-7	14	9	291.47	20.90
7	14	-9	297.47	20.40
-7	14	9	282.47	17.70
6	-14	-9	208.08	15.50
-6	14	9	198.68	16.70

-6	14	9	195.68	17.30
6	14	-9	236.18	16.70
5	-14	-9	864.61	45.30
-5	14	9	887.41	44.70
-5	14	9	840.52	45.80
5	14	-9	886.11	45.40
4	-14	-9	348.87	20.80
-4	14	9	332.97	20.40
4	14	-9	357.66	20.80
-4	14	9	326.97	21.20
3	-14	-9	580.34	31.50
-3	14	9	582.04	31.70
-3	14	9	591.74	31.30
3	14	-9	565.64	31.00
2	-14	-9	198.88	13.90
2	14	-9	172.28	9.10
-2	14	9	195.88	13.80
2	14	-9	195.48	13.20
-2	14	9	177.58	13.50
2	14	-9	179.58	9.20
-1	-14	9	587.14	32.00
-1	14	9	517.25	28.80
1	14	-9	492.25	25.80
1	14	-9	546.15	28.20
-1	14	9	542.25	29.80
1	14	-9	514.35	25.60
-1	14	9	518.15	28.70
0	14	-9	457.55	23.10
0	14	9	465.75	25.80
0	14	-9	492.95	25.30
0	14	9	481.45	27.10
0	14	9	447.46	25.80
1	-14	9	1793.62	85.89
1	14	9	1758.32	87.69
1	14	9	1760.52	87.49
-1	14	-9	1840.12	87.09
1	14	9	1853.71	88.89
-1	14	-9	1721.63	85.49
2	-14	9	1.10	3.30
2	14	9	3.00	4.60
-2	14	-9	3.50	3.60
2	14	9	7.00	6.00
-2	14	-9	8.60	3.20
-2	14	-9	4.00	2.90

2	14	9	8.60	4.40
3	-14	9	945.61	44.90
3	14	9	843.32	45.80
-3	14	-9	889.81	44.30
-3	14	-9	890.51	44.60
3	14	9	905.71	45.80
-3	14	-9	937.61	45.20
4	-14	9	23.10	5.20
-4	14	-9	33.70	6.10
4	14	9	38.90	7.30
-4	14	-9	22.80	4.60
-4	14	-9	26.90	5.50
4	14	9	20.20	5.30
4	14	9	31.80	7.20
-5	-14	-9	587.64	34.20
5	-14	9	597.94	32.40
-5	14	-9	638.34	31.90
-5	14	-9	591.94	31.50
5	14	9	633.14	32.70
5	14	9	676.53	35.00
5	14	9	606.14	33.70
-5	14	-9	643.84	32.00
-6	-14	-9	68.49	10.90
-6	14	-9	49.40	8.10
-6	14	-9	51.29	7.20
-6	14	-9	57.19	7.30
6	14	9	61.39	8.40
6	14	9	64.09	11.60
-7	-14	-9	919.51	52.99
-7	14	-9	1035.80	50.89
7	14	9	986.50	51.39
7	14	9	1114.69	53.59
-7	14	-9	1004.20	50.49
-8	-14	-9	147.99	18.20
8	14	9	162.08	15.70
8	14	9	153.48	13.20
-8	14	-9	156.78	12.30
-8	14	-9	154.18	12.70
-9	14	-9	318.57	19.30
9	14	9	290.27	19.70
-9	14	-9	316.37	19.50
-10	14	-9	212.28	16.90
10	14	9	240.38	17.20
-10	14	-9	236.58	16.90

-11	14	-9	227.78	17.20
11	14	9	201.08	17.30
12	14	9	128.89	15.30
-12	14	-9	168.58	15.20
13	14	9	90.69	14.70
12	15	-9	1.70	11.30
-12	15	9	0.90	7.70
11	15	-9	25.60	11.80
-11	15	9	4.20	7.40
10	15	-9	-3.20	8.30
-10	15	9	14.90	7.00
9	-15	-9	13.10	7.10
-9	15	9	6.20	6.80
9	15	-9	-0.70	8.20
8	-15	-9	11.10	6.70
-8	15	9	15.80	6.50
-8	15	9	16.50	9.60
7	-15	-9	103.09	12.30
7	15	-9	127.99	14.20
-7	15	9	88.39	11.10
-7	15	9	70.39	14.60
6	-15	-9	13.40	6.00
-6	15	9	17.60	7.20
-6	15	9	12.60	6.00
6	15	-9	17.80	7.50
5	-15	-9	7.00	5.50
-5	15	9	0.10	6.60
-5	15	9	0.20	6.10
5	15	-9	5.60	5.90
4	-15	-9	36.90	7.10
4	15	-9	59.59	9.90
-4	15	9	46.50	7.30
-4	15	9	51.19	10.50
3	-15	-9	12.70	5.40
3	15	-9	22.00	6.20
-3	15	9	6.20	5.80
-3	15	9	11.80	6.20
2	-15	-9	75.69	9.80
-2	15	9	74.19	11.20
2	15	-9	81.89	9.40
2	15	-9	72.99	4.50
-2	15	9	75.79	10.10
2	15	-9	71.79	4.30
-2	15	9	72.89	9.10

-1	15	9	0.30	5.30
1	15	-9	0.00	1.80
-1	15	9	-6.70	5.10
1	15	-9	4.00	4.50
-1	15	9	-5.30	4.80
0	-15	9	22.40	3.60
0	15	-9	39.10	7.50
0	15	9	24.10	6.20
0	15	9	41.80	8.10
0	15	9	18.20	6.90
0	15	-9	31.30	3.90
1	-15	9	3.20	3.40
-1	15	-9	3.80	4.00
-1	15	-9	2.40	2.60
1	15	9	-5.80	4.50
1	15	9	-4.80	6.00
1	15	9	1.80	4.80
2	-15	9	14.70	4.10
2	15	9	17.80	5.20
2	15	9	18.50	5.30
-2	15	-9	23.20	4.70
-2	15	-9	30.50	4.90
-2	15	-9	19.90	4.30
2	15	9	26.90	7.10
3	-15	9	12.20	4.20
-3	15	-9	9.60	3.50
-3	15	-9	2.60	4.00
3	15	9	-3.70	6.30
3	15	9	-0.20	5.00
3	15	9	7.30	4.80
-3	15	-9	10.30	3.70
4	-15	9	10.80	4.60
-4	15	-9	11.60	3.80
-4	15	-9	23.20	4.40
-4	15	-9	12.00	4.30
4	15	9	13.60	6.80
4	15	9	15.70	4.90
4	15	9	10.10	5.10
5	-15	9	-4.50	4.80
-5	15	-9	-0.70	4.30
-5	15	-9	-0.90	4.00
5	15	9	0.10	4.80
-5	15	-9	-2.80	3.70
-6	-15	-9	55.49	11.20

-6	15	-9	26.20	5.10
6	15	9	29.70	8.10
6	15	9	22.00	5.80
-6	15	-9	23.40	5.60
-6	15	-9	20.30	4.60
-7	-15	-9	30.30	8.30
-7	15	-9	20.40	5.40
-7	15	-9	24.00	5.30
7	15	9	26.10	8.60
7	15	9	18.50	11.10
-7	15	-9	32.30	6.20
8	15	9	-4.70	7.50
-8	15	-9	8.10	5.30
8	15	9	-3.20	5.50
-8	15	-9	19.10	6.10
9	15	9	15.00	7.10
-9	15	-9	18.50	6.40
-9	15	-9	22.80	6.40
-10	15	-9	9.30	6.50
-10	15	-9	15.20	7.30
10	15	9	10.50	7.00
11	15	9	3.40	7.50
-11	15	-9	18.10	7.60
-12	15	-9	16.40	7.90
12	15	9	16.20	8.40
-12	16	9	110.19	13.90
12	16	-9	105.89	22.10
11	16	-9	67.49	13.40
-11	16	9	109.59	12.80
10	16	-9	548.95	34.60
-10	16	9	537.85	30.60
9	-16	-9	29.90	8.10
-9	16	9	18.10	6.70
9	16	-9	29.10	9.80
8	-16	-9	357.96	24.20
-8	16	9	405.26	23.20
8	16	-9	377.76	25.60
7	-16	-9	117.69	13.40
-7	16	9	84.09	14.80
-7	16	9	104.29	10.90
7	16	-9	102.99	14.30
6	-16	-9	427.56	25.40
6	16	-9	354.26	30.90
-6	16	9	429.66	26.70

-6	16	9	428.26	24.00
5	-16	-9	664.73	36.00
-5	16	9	689.53	35.50
5	16	-9	655.43	36.20
-5	16	9	621.64	36.40
4	-16	-9	366.16	22.80
-4	16	9	370.66	23.50
4	16	-9	378.46	22.70
3	-16	-9	437.86	26.30
-3	16	9	486.65	26.40
-3	16	9	460.35	27.00
3	16	-9	454.25	26.20
-2	16	9	52.59	10.90
-2	16	9	47.10	7.40
-2	16	9	44.50	7.20
2	16	-9	40.00	3.20
2	16	-9	44.30	8.70
-1	-16	9	840.12	40.20
-1	16	9	815.22	43.00
-1	16	9	811.62	43.20
-1	16	9	833.72	44.30
1	16	-9	829.22	42.70
0	-16	9	131.99	8.40
0	16	9	119.79	13.30
0	16	9	124.19	12.00
0	16	-9	122.29	7.60
0	16	9	133.19	11.80
0	16	-9	121.29	11.00
1	-16	9	1312.37	63.09
-1	16	-9	1302.07	62.49
-1	16	-9	1342.97	64.49
1	16	9	1323.27	66.29
1	16	9	1321.17	64.79
-1	16	-9	1173.28	64.29
1	16	9	1299.77	65.09
2	-16	9	22.00	4.50
-2	16	-9	19.60	5.20
-2	16	-9	26.20	3.80
-2	16	-9	15.70	4.40
2	16	9	19.50	5.70
2	16	9	9.80	5.60
2	16	9	22.40	8.00
3	-16	9	339.27	18.70
-3	16	-9	322.77	18.50

3	16	9	352.56	20.10
3	16	9	314.47	22.00
3	16	9	309.77	20.20
-3	16	-9	348.97	18.00
4	-16	9	143.99	11.40
4	16	9	134.39	12.30
4	16	9	128.39	14.80
4	16	9	158.78	12.10
-4	16	-9	137.99	10.60
-4	16	-9	145.59	10.20
-4	16	-9	145.99	11.30
-5	16	-9	609.94	33.70
5	16	9	709.93	37.70
5	16	9	662.23	35.00
-5	16	-9	660.83	34.30
-5	16	-9	656.13	33.80
-6	-16	-9	4.50	7.00
-6	16	-9	3.10	4.30
-6	16	-9	6.10	4.60
-6	16	-9	-0.90	5.00
6	16	9	3.50	8.40
7	16	9	535.05	37.10
7	16	9	592.74	31.40
-7	16	-9	592.14	30.60
-7	16	-9	561.84	34.40
-7	16	-9	560.94	30.40
-8	16	-9	100.59	10.90
-8	16	-9	108.59	10.90
8	16	9	79.49	11.50
-8	16	-9	108.79	10.80
-9	16	-9	165.18	14.50
9	16	9	158.48	14.70
-9	16	-9	177.08	14.20
10	16	9	124.59	13.90
-10	16	-9	144.69	13.50
-11	16	-9	287.07	19.40
11	16	9	241.48	19.70
12	16	9	156.78	16.70
-12	16	-9	194.68	17.10
11	17	-9	100.49	19.50
-11	17	9	61.99	10.30
-10	17	9	8.70	7.20
10	17	-9	-2.10	10.00
-9	17	9	121.39	13.70

9	17	-9	152.98	18.60
8	-17	-9	41.10	8.60
-8	17	9	47.70	8.50
8	17	-9	41.90	10.00
7	-17	-9	317.87	22.80
-7	17	9	361.16	21.80
7	17	-9	352.66	23.90
6	-17	-9	51.89	9.20
6	17	-9	55.19	9.40
-6	17	9	57.69	11.00
-6	17	9	56.89	10.60
5	-17	-9	44.20	8.10
-5	17	9	29.60	8.70
-5	17	9	41.80	7.70
5	17	-9	74.69	11.80
4	-17	-9	105.49	12.90
-4	17	9	130.99	14.20
-4	17	9	128.59	11.70
4	17	-9	130.29	15.20
3	-17	-9	265.87	18.90
-3	17	9	303.67	20.90
-3	17	9	292.37	19.40
-3	17	9	261.27	22.10
-2	-17	9	355.06	17.60
-2	17	9	396.86	23.80
2	17	-9	314.67	17.20
-2	17	9	356.16	21.90
-2	17	9	361.76	22.30
2	17	-9	337.07	25.90
1	17	-9	95.19	17.10
-1	17	9	94.09	11.40
1	17	-9	105.19	6.70
-1	17	9	91.79	11.30
-1	17	9	127.89	13.30
0	-17	9	688.83	34.30
0	17	9	690.73	36.70
0	17	-9	674.33	33.80
0	17	9	731.43	38.60
0	17	9	672.33	37.00
1	-17	9	23.10	4.30
1	17	9	11.70	6.90
1	17	9	14.40	6.10
-1	17	-9	18.30	7.40
-1	17	-9	17.50	4.50

-1	17	-9	16.10	3.40
1	17	9	20.70	6.80
2	-17	9	137.09	10.20
2	17	9	134.29	11.90
2	17	9	141.89	14.70
-2	17	-9	141.79	11.50
-2	17	-9	137.59	11.00
2	17	9	129.49	12.50
-2	17	-9	126.99	8.90
3	-17	9	20.30	5.00
3	17	9	4.80	7.30
-3	17	-9	8.60	5.00
-3	17	-9	16.70	4.50
3	17	9	9.40	6.00
3	17	9	7.30	5.50
-3	17	-9	13.70	4.00
4	-17	9	619.54	32.90
4	17	9	712.73	36.80
-4	17	-9	669.43	33.50
-4	17	-9	566.04	32.60
4	17	9	626.34	33.80
-4	17	-9	612.04	32.10
-5	17	-9	72.39	8.60
-5	17	-9	72.69	9.40
-5	17	-9	67.09	8.50
5	17	9	66.69	13.00
-6	17	-9	374.36	21.40
-6	17	-9	398.16	22.20
6	17	9	363.76	22.60
-6	17	-9	331.67	21.40
6	17	9	456.65	26.50
-7	17	-9	47.40	9.20
7	17	9	57.29	11.30
-7	17	-9	63.79	9.70
7	17	9	60.69	10.70
-7	17	-9	58.69	9.20
-8	17	-9	70.89	10.40
-8	17	-9	56.69	10.60
-8	17	-9	75.79	10.30
8	17	9	64.69	11.20
9	17	9	69.79	11.80
-9	17	-9	55.09	8.70
-10	17	-9	88.59	12.40
10	17	9	83.69	12.70

-11	17	-9	185.08	16.40
11	17	9	180.58	17.10
-11	18	9	30.30	8.50
11	18	-9	36.10	12.30
10	18	-9	66.69	13.10
9	18	-9	19.20	11.10
-9	18	9	3.10	7.20
8	18	-9	97.09	17.40
-8	18	9	144.89	13.90
7	-18	-9	119.79	14.60
-7	18	9	109.19	12.70
7	18	-9	123.19	16.10
6	-18	-9	53.49	9.50
-6	18	9	33.30	8.00
6	18	-9	37.20	9.40
5	-18	-9	218.48	17.20
-5	18	9	165.28	18.20
-5	18	9	222.08	16.40
5	18	-9	227.28	17.70
4	-18	-9	73.09	12.00
4	18	-9	40.40	8.10
-4	18	9	59.59	12.10
-4	18	9	46.90	8.50
-3	18	9	13.60	8.50
-3	18	9	29.00	8.00
-3	18	9	4.20	6.90
3	18	-9	16.20	6.60
-2	-18	9	14.60	2.80
-2	18	9	1.70	6.50
2	18	-9	7.80	2.20
-2	18	9	8.00	8.60
-2	18	9	7.00	6.40
2	18	-9	7.30	5.90
-1	-18	9	339.97	17.10
1	18	-9	329.47	16.50
-1	18	9	351.16	21.10
-1	18	9	253.87	23.20
-1	18	9	311.87	22.30
1	18	-9	323.97	20.40
0	-18	9	70.19	6.80
0	18	9	42.30	7.90
0	18	-9	65.09	9.90
0	18	-9	64.99	5.60
0	18	9	59.39	10.40

1	-18	9	318.97	17.40
1	18	9	325.37	22.50
-1	18	-9	302.37	19.20
1	18	9	282.87	19.60
-1	18	-9	305.47	18.40
1	18	9	326.07	21.80
-1	18	-9	309.47	16.40
2	-18	9	5.00	4.40
2	18	9	3.40	6.60
-2	18	-9	-3.10	5.00
-2	18	-9	3.60	3.50
2	18	9	-1.00	5.80
2	18	9	3.30	7.70
-2	18	-9	1.90	4.90
3	-18	9	22.10	5.30
-3	18	-9	17.70	4.20
-3	18	-9	21.40	5.00
3	18	9	15.50	10.80
3	18	9	16.90	7.70
-3	18	-9	13.40	5.50
-4	18	-9	25.70	5.00
4	18	9	26.30	6.70
4	18	9	12.70	7.90
-4	18	-9	28.60	5.70
-4	18	-9	27.50	6.40
5	18	9	170.28	17.00
-5	18	-9	172.38	11.90
-5	18	-9	155.88	13.20
5	18	9	146.39	13.50
-5	18	-9	151.18	12.10
6	18	9	71.79	12.10
6	18	9	73.59	10.80
-6	18	-9	63.99	10.10
-6	18	-9	58.69	9.30
-6	18	-9	67.19	9.30
-7	18	-9	86.19	11.30
7	18	9	75.89	16.00
7	18	9	82.09	11.60
-7	18	-9	74.59	10.60
-7	18	-9	111.59	10.80
8	18	9	4.00	7.00
-8	18	-9	17.50	6.60
-8	18	-9	9.40	6.20
-9	18	-9	24.30	7.30

9	18	9	9.40	7.40
-9	18	-9	4.20	6.50
-10	18	-9	48.30	8.70
10	18	9	32.90	8.90
-11	18	-9	201.18	16.90
11	18	9	140.19	16.30
-10	19	9	109.39	13.70
10	19	-9	75.99	14.20
-9	19	9	290.67	20.50
9	19	-9	284.57	25.30
8	19	-9	180.08	19.70
-8	19	9	143.59	15.00
7	-19	-9	187.58	18.70
-7	19	9	153.28	15.20
7	19	-9	167.98	18.10
6	-19	-9	225.38	19.20
6	19	-9	251.97	20.00
-6	19	9	226.88	17.70
5	-19	-9	588.14	34.30
5	19	-9	620.34	34.70
-5	19	9	579.34	33.30
-4	19	9	433.86	27.70
-4	19	9	402.36	24.40
4	19	-9	400.96	25.10
-4	19	9	344.07	25.70
3	19	-9	105.19	13.00
-3	19	9	87.59	12.50
-3	19	9	111.09	15.60
-3	19	9	95.19	14.10
-2	-19	9	377.46	18.70
2	19	-9	394.16	23.20
-2	19	9	375.86	23.10
-2	19	9	336.37	23.40
-2	19	9	335.37	25.10
-1	-19	9	34.10	5.80
1	19	-9	44.30	4.70
1	19	-9	32.30	7.40
-1	19	9	42.20	8.20
-1	19	9	19.50	8.80
-1	19	9	38.80	8.40
0	-19	9	1051.99	50.69
0	19	9	1045.30	53.29
0	19	-9	996.00	50.09
0	19	-9	1061.49	53.29

0	19	9	1105.59	55.69
0	19	9	954.10	53.39
1	-19	9	97.09	8.70
1	19	9	88.89	11.30
1	19	9	81.59	12.50
-1	19	-9	89.69	9.70
-1	19	-9	98.49	11.40
1	19	9	101.99	14.60
-1	19	-9	90.79	7.50
2	-19	9	867.81	43.70
-2	19	-9	934.01	45.60
2	19	9	945.31	48.30
-2	19	-9	881.51	43.00
-2	19	-9	811.22	44.50
2	19	9	792.52	45.70
2	19	9	879.11	45.50
3	-19	9	39.50	6.50
-3	19	-9	26.90	6.70
-3	19	-9	25.20	5.00
3	19	9	37.30	9.90
3	19	9	33.40	7.80
-3	19	-9	32.70	6.00
-4	19	-9	631.34	36.50
4	19	9	687.43	37.50
-4	19	-9	692.53	35.70
4	19	9	770.92	41.00
-4	19	-9	745.93	37.60
-5	19	-9	26.40	6.00
-5	19	-9	45.40	7.60
-5	19	-9	23.50	5.60
5	19	9	26.60	7.20
5	19	9	39.50	10.60
-6	19	-9	711.83	36.50
-6	19	-9	583.74	35.30
-6	19	-9	653.23	36.50
6	19	9	745.83	40.10
6	19	9	683.93	36.30
-7	19	-9	84.39	11.80
7	19	9	79.09	12.00
-7	19	-9	78.09	10.50
-8	19	-9	232.28	17.60
8	19	9	230.88	17.90
-8	19	-9	242.98	17.00
-9	19	-9	168.58	14.80

9	19	9	155.68	15.10
-9	19	-9	133.99	14.90
-10	19	-9	122.19	14.30
-10	19	-9	150.28	15.40
10	19	9	118.09	14.90
-10	20	9	-9.50	8.60
10	20	-9	3.90	12.10
9	20	-9	-0.80	10.90
-9	20	9	-4.60	7.80
8	20	-9	100.89	17.90
-8	20	9	64.39	10.70
7	20	-9	5.30	8.70
-7	20	9	-7.20	7.10
6	-20	-9	6.30	8.10
-6	20	9	19.40	8.10
6	20	-9	17.70	8.80
-5	20	9	7.10	7.20
5	20	-9	13.70	7.90
-4	20	9	31.80	10.40
-4	20	9	25.20	8.40
4	20	-9	45.90	9.70
-3	20	9	4.60	8.80
3	20	-9	18.30	7.30
-3	20	9	-2.70	6.50
-3	20	9	7.80	8.40
2	20	-9	29.20	8.10
-2	20	9	12.30	7.90
2	20	-9	21.60	3.70
-2	20	9	21.80	9.40
-2	20	9	22.50	8.40
-1	20	9	59.39	9.80
1	20	-9	55.79	5.70
-1	20	9	75.29	11.80
1	20	-9	87.89	11.90
-1	20	9	42.30	9.10
0	-20	9	22.60	4.70
0	20	9	6.80	8.60
0	20	-9	15.40	7.20
0	20	9	27.00	8.70
0	20	9	18.30	7.20
0	20	-9	21.50	3.70
0	20	-9	14.40	5.20
1	-20	9	13.50	5.10
-1	20	-9	24.10	5.70

-1	20	-9	35.40	7.40
1	20	9	27.60	9.20
1	20	9	14.60	7.10
1	20	9	23.60	7.90
-1	20	-9	37.00	5.90
2	-20	9	1.50	5.00
-2	20	-9	-7.60	5.40
-2	20	-9	-1.70	5.90
2	20	9	-6.40	8.30
-2	20	-9	12.50	4.00
2	20	9	-0.40	6.60
3	-20	9	57.39	9.80
-3	20	-9	68.99	9.40
-3	20	-9	75.99	11.30
3	20	9	67.19	11.20
3	20	9	80.99	16.00
-3	20	-9	66.19	7.90
-4	20	-9	20.50	6.00
-4	20	-9	23.90	6.80
4	20	9	15.90	9.50
4	20	9	19.10	7.00
-4	20	-9	19.50	5.30
5	20	9	13.40	6.80
-5	20	-9	6.60	6.30
-5	20	-9	13.70	5.30
-5	20	-9	19.20	6.70
5	20	9	4.80	9.10
6	20	9	2.20	6.70
-6	20	-9	4.00	6.40
-6	20	-9	3.30	5.50
7	20	9	13.00	7.30
-7	20	-9	2.40	6.90
-7	20	-9	10.70	6.10
-8	20	-9	7.80	6.80
8	20	9	6.50	7.30
-8	20	-9	11.50	7.00
9	20	9	1.40	7.80
-9	20	-9	10.10	7.20
-9	20	-9	16.00	7.80
-9	21	9	113.79	15.20
9	21	-9	119.39	20.90
-8	21	9	36.00	8.80
8	21	-9	36.10	11.90
-7	21	9	125.99	14.60

7	21	-9	91.49	17.80
-6	21	9	243.68	19.20
6	21	-9	265.87	22.10
-5	21	9	306.97	26.50
-5	21	9	313.47	21.40
5	21	-9	271.07	22.40
-4	21	9	459.55	27.80
4	21	-9	452.65	28.90
-4	21	9	461.55	31.40
3	21	-9	86.19	13.80
-3	21	9	83.49	13.30
-3	21	9	88.79	13.70
-2	21	9	360.36	23.50
2	21	-9	360.26	23.70
-2	21	9	360.56	26.70
1	21	-9	18.80	8.00
-1	21	9	28.90	9.50
-1	21	9	9.00	8.00
-1	21	9	33.40	11.50
1	21	-9	16.70	3.40
0	21	9	765.02	42.50
0	21	9	639.24	39.30
0	21	-9	700.83	35.40
0	21	-9	751.12	39.50
0	21	9	729.03	39.10
-1	21	-9	21.40	7.50
1	21	9	19.90	7.80
-1	21	-9	29.80	4.70
1	21	9	34.50	11.10
2	21	9	357.16	26.70
-2	21	-9	363.66	20.00
2	21	9	365.66	23.10
-2	21	-9	398.76	23.80
3	21	9	50.29	8.50
-3	21	-9	53.79	9.10
-3	21	-9	57.39	7.80
3	21	9	61.19	12.30
-4	21	-9	440.66	24.30
-4	21	-9	454.75	27.10
4	21	9	465.65	30.90
4	21	9	448.76	26.70
5	21	9	40.00	14.30
-5	21	-9	65.49	12.60
-5	21	-9	51.89	8.70

5	21	9	44.60	8.40
-6	21	-9	512.85	29.10
6	21	9	484.85	28.40
-6	21	-9	463.15	26.80
-7	21	-9	114.89	14.10
7	21	9	130.89	13.90
-7	21	-9	114.69	12.10
8	21	9	191.48	16.90
-8	21	-9	185.78	17.70
-8	21	-9	203.88	15.60
-9	21	-9	70.59	13.70
9	21	9	72.49	13.20
-9	21	-9	66.69	12.70
8	22	-9	217.18	23.80
-8	22	9	205.58	18.70
7	22	-9	217.58	21.40
-7	22	9	172.08	16.90
-6	22	9	9.40	13.00
-6	22	9	20.70	9.00
6	22	-9	34.30	10.20
5	22	-9	272.57	21.70
-5	22	9	238.98	19.10
-5	22	9	231.48	25.00
-4	22	9	76.09	11.80
-4	22	9	83.29	16.10
4	22	-9	90.29	15.40
-3	22	9	164.38	16.40
-3	22	9	151.98	21.30
3	22	-9	187.38	17.50
-2	22	9	51.19	12.70
2	22	-9	54.29	9.60
-2	22	9	49.70	9.90
-1	22	9	297.87	21.80
-1	22	9	306.17	25.70
1	22	-9	337.37	22.50
0	22	-9	11.00	7.60
0	22	9	3.10	8.00
0	22	9	11.10	10.30
0	22	-9	8.20	3.70
1	22	9	312.57	23.60
-1	22	-9	233.78	14.70
-1	22	-9	258.67	19.50
1	22	9	242.98	18.80
-2	22	-9	-6.30	6.80

2	22	9	-19.70	9.20
-2	22	-9	-1.50	4.20
2	22	9	-6.60	7.10
-3	22	-9	258.77	19.40
3	22	9	255.17	18.80
-3	22	-9	244.68	15.60
3	22	9	284.67	24.10
4	22	9	10.30	11.50
4	22	9	17.70	7.70
-4	22	-9	13.40	7.70
-4	22	-9	11.60	5.60
-5	22	-9	345.27	22.60
5	22	9	350.06	22.00
-5	22	-9	300.47	19.60
-6	22	-9	37.20	8.60
6	22	9	27.80	8.50
-6	22	-9	34.70	7.40
-7	22	-9	222.18	16.40
7	22	9	217.48	17.70
-7	22	-9	212.58	18.80
-8	22	-9	45.00	8.30
-8	22	-9	32.30	10.00
8	22	9	42.20	9.30
7	23	-9	56.29	14.40
-7	23	9	76.09	12.00
6	23	-9	129.09	18.70
-6	23	9	100.09	15.00
-6	23	9	129.29	23.80
-5	23	9	24.50	13.00
-5	23	9	33.00	9.60
5	23	-9	49.40	10.90
-4	23	9	39.30	9.70
-4	23	9	41.20	14.10
4	23	-9	30.60	10.00
-3	23	9	3.50	23.70
-3	23	9	35.00	10.00
3	23	-9	25.40	10.00
-2	23	9	45.70	9.60
-2	23	9	21.00	12.60
2	23	-9	42.70	10.20
-1	23	9	7.40	11.10
1	23	-9	10.10	7.90
-1	23	9	12.80	8.70
0	23	9	267.87	19.70

0	23	9	250.87	24.80
0	23	-9	239.98	19.80
1	23	9	-1.40	11.10
-1	23	-9	7.30	4.20
1	23	9	-0.20	7.70
-1	23	-9	-6.00	7.40
2	23	9	48.10	8.80
2	23	9	42.60	11.60
-2	23	-9	45.20	7.60
-2	23	-9	37.30	9.20
-3	23	-9	3.00	5.00
3	23	9	-1.80	7.40
-3	23	-9	-5.80	7.00
3	23	9	2.50	10.10
4	23	9	89.89	13.60
-4	23	-9	114.79	14.50
5	23	9	-2.80	7.60
-5	23	-9	3.20	6.00
-5	23	-9	-3.60	6.90
-6	23	-9	73.29	11.20
6	23	9	68.09	10.40
-6	23	-9	39.20	9.80
7	23	9	6.00	8.10
-7	23	-9	-6.70	6.80
-7	23	-9	-1.50	10.30
-6	24	9	183.48	18.70
6	24	-9	206.98	21.90
-6	24	9	199.18	27.60
-5	24	9	181.78	18.10
5	24	-9	212.08	21.10
-5	24	9	225.78	27.50
4	24	-9	145.19	18.80
-4	24	9	190.38	24.80
-4	24	9	150.08	16.90
-3	24	9	268.67	27.70
3	24	-9	270.77	22.10
-3	24	9	261.97	20.70
-2	24	9	36.60	19.00
-2	24	9	6.60	8.70
2	24	-9	15.40	9.80
-1	24	9	382.06	25.90
-1	24	9	404.76	31.40
1	24	-9	405.56	26.90
0	24	9	8.00	11.70

0	24	9	18.40	9.10
0	24	-9	23.10	9.30
1	24	9	705.23	44.10
-1	24	-9	687.73	40.50
1	24	9	757.12	40.00
-2	24	-9	-2.10	4.80
2	24	9	-4.90	7.80
2	24	9	-3.80	12.30
-2	24	-9	-1.70	7.60
-3	24	-9	462.05	28.80
3	24	9	474.05	27.90
-3	24	-9	429.56	24.60
4	24	9	30.70	8.90
-4	24	-9	30.70	9.30
-4	24	-9	36.90	6.70
-5	24	-9	255.17	20.70
-5	24	-9	234.08	16.50
5	24	9	230.98	19.20
6	24	9	12.60	8.40
-6	24	-9	-7.20	6.50
4	25	-9	1.70	9.50
-4	25	9	-6.30	9.00
-4	25	9	-12.40	13.40
3	25	-9	-7.10	8.60
-3	25	9	-8.00	14.30
-3	25	9	-3.90	8.90
-2	25	9	6.20	9.50
-2	25	9	5.60	14.30
2	25	-9	11.70	9.00
1	25	-9	6.20	8.40
-1	25	9	-3.70	14.50
-1	25	9	-2.60	8.60
0	25	9	51.29	10.60
0	25	-9	36.30	9.50
1	25	9	5.20	8.80
-1	25	-9	-12.70	8.20
2	25	9	36.80	10.00
-2	25	-9	36.10	9.90
-3	25	-9	-10.10	8.20
3	25	9	7.70	8.40
4	25	9	92.99	14.20
-4	25	-9	71.89	10.60
-4	25	-9	75.39	15.80
2	26	-9	39.90	11.60

-2	26	9	20.40	10.60
-2	26	9	48.60	15.50
-1	26	9	225.98	20.20
1	26	-9	216.18	21.30
-1	26	9	220.28	35.00
0	26	9	68.19	11.60
0	26	-9	48.40	11.60
-1	26	-9	344.37	26.70
1	26	9	404.06	25.70
2	26	9	-14.00	8.60
-15	0	10	3.20	6.90
-14	0	10	407.16	23.10
-13	0	10	28.50	6.10
13	0	-10	34.30	9.90
-12	0	10	343.77	18.00
12	0	-10	302.37	21.80
-12	0	10	263.67	25.70
-11	0	10	610.54	39.20
-11	0	10	681.33	33.40
11	0	-10	680.93	36.50
11	0	-10	639.44	34.40
10	0	-10	1175.68	57.19
-10	0	10	986.90	58.39
-10	0	10	1147.69	54.39
10	0	-10	1119.89	55.09
-9	0	10	15.70	3.80
9	0	-10	16.90	4.50
9	0	-10	9.20	5.70
-9	0	10	14.10	7.50
-8	0	10	1078.49	51.69
-8	0	10	998.00	54.89
8	0	-10	1060.59	53.89
8	0	-10	1101.19	52.09
-7	0	10	492.25	29.20
7	0	-10	539.65	26.60
-7	0	10	521.65	26.30
7	0	-10	538.05	28.40
-6	0	10	1576.04	77.29
6	0	-10	1565.04	74.99
-6	0	10	1507.25	74.99
6	0	-10	1582.44	76.59
-5	0	10	2605.84	127.69
5	0	-10	2722.83	127.49
-5	0	10	2658.53	129.49

5	0	-10	2705.13	128.89
-4	0	10	136.89	9.10
4	0	-10	157.98	10.20
4	0	-10	166.98	8.70
-4	0	10	157.98	11.00
-3	0	10	1140.29	56.59
3	0	-10	1186.18	57.19
-3	0	10	1182.58	57.59
-2	0	10	891.31	44.00
2	0	-10	889.11	44.40
-2	0	10	933.31	44.80
-1	0	10	3440.66	170.68
1	0	-10	3551.14	170.88
-1	0	10	3773.12	171.18
0	0	10	6.20	2.00
0	0	-10	2.90	1.70
0	0	-10	5.70	1.90
1	0	10	7303.37	337.77
-1	0	-10	7338.87	337.97
1	0	10	6740.73	338.17
2	0	10	70.09	4.80
-2	0	-10	82.69	4.90
2	0	10	77.29	4.60
2	0	10	78.09	5.90
-2	0	-10	83.29	5.70
-3	0	-10	3214.28	147.89
3	0	10	3044.10	146.79
3	0	10	3032.00	147.99
4	0	10	439.86	23.50
4	0	10	464.95	22.00
-4	0	-10	443.96	22.60
-4	0	-10	482.65	23.50
4	0	10	446.26	22.20
5	0	10	2944.41	139.19
5	0	10	2851.51	138.99
-5	0	-10	2968.10	139.69
5	0	10	2777.72	140.39
-5	0	-10	3099.39	140.39
-6	0	-10	2301.97	100.89
-6	0	-10	2014.00	100.09
6	0	10	2057.49	99.29
6	0	10	2018.90	99.49
6	0	10	2032.30	100.79
7	0	10	542.65	30.30

7	0	10	600.14	29.00
-7	0	-10	595.94	30.40
-7	0	-10	590.24	29.80
8	0	10	1158.58	56.19
-8	0	-10	1158.88	57.09
-8	0	-10	1146.39	57.59
9	0	10	566.74	28.50
-9	0	-10	540.25	29.90
-9	0	-10	556.74	29.60
10	0	10	328.97	18.30
-10	0	-10	343.47	21.50
-10	0	-10	310.17	19.90
11	0	10	316.77	18.40
-11	0	-10	321.27	19.80
-11	0	-10	308.87	20.50
-12	0	-10	790.92	46.10
-12	0	-10	962.50	46.60
-13	0	-10	83.19	12.10
-14	0	-10	171.08	16.90
-15	0	-10	10.90	9.50
-15	-1	10	73.19	11.70
-15	1	10	86.79	12.40
-14	-1	10	32.80	6.70
-13	-1	10	39.40	6.20
13	-1	-10	25.60	9.70
13	1	-10	37.30	9.20
12	-1	-10	30.40	8.40
-12	-1	10	43.90	12.10
-12	-1	10	54.09	7.50
-12	1	10	57.79	8.00
12	1	-10	25.30	8.50
-12	1	10	45.90	12.30
-11	-1	10	118.39	17.80
11	-1	-10	114.19	13.60
-11	-1	10	128.99	9.40
-11	1	10	113.89	17.90
-11	1	10	142.79	10.00
11	1	-10	116.19	14.00
11	1	-10	101.79	11.30
-10	-1	10	80.59	7.40
10	-1	-10	82.59	9.00
-10	-1	10	54.69	11.60
10	-1	-10	87.29	11.60
10	1	-10	79.99	11.60

10	1	-10	84.49	8.80
-10	1	10	72.89	7.60
-10	1	10	53.29	10.80
9	-1	-10	218.88	13.80
-9	-1	10	186.58	17.70
-9	-1	10	207.18	12.40
9	-1	-10	229.58	15.80
9	1	-10	220.98	13.40
9	1	-10	223.48	16.10
-9	1	10	233.38	12.90
-9	1	10	213.58	18.30
8	-1	-10	261.07	14.80
-8	-1	10	227.08	18.00
8	-1	-10	266.27	16.90
-8	-1	10	252.67	13.90
8	1	-10	267.17	17.20
8	1	-10	263.07	14.50
-8	1	10	233.48	18.40
-8	1	10	259.47	14.20
7	-1	-10	8.80	4.60
-7	-1	10	2.80	2.60
-7	-1	10	3.70	5.90
7	-1	-10	10.60	3.40
-7	1	10	2.20	5.50
7	1	-10	4.50	4.30
7	1	-10	7.20	3.00
-7	1	10	5.60	3.00
-6	-1	10	1.40	5.20
6	-1	-10	10.50	4.40
-6	-1	10	8.80	2.70
6	-1	-10	14.40	3.10
6	1	-10	6.20	4.30
-6	1	10	13.00	5.20
6	1	-10	13.10	2.80
-6	1	10	11.30	3.00
-5	-1	10	35.30	4.30
5	-1	-10	24.90	4.50
-5	-1	10	36.40	7.30
-5	1	10	24.10	4.50
-5	1	10	29.10	5.80
5	1	-10	22.30	4.60
-4	-1	10	534.45	28.80
4	-1	-10	554.94	28.40
4	-1	-10	577.34	27.20

-4	-1	10	538.55	27.30
4	1	-10	564.74	28.50
-4	1	10	517.15	27.60
-4	1	10	552.54	28.90
4	1	-10	557.14	26.90
3	-1	-10	167.78	9.00
-3	-1	10	147.69	9.60
-3	-1	10	165.18	10.80
3	-1	-10	172.28	10.30
3	1	-10	169.38	8.70
-3	1	10	176.08	10.90
3	1	-10	157.58	10.20
-3	1	10	154.88	9.60
-2	-1	10	231.78	14.10
2	-1	-10	272.77	14.40
-2	-1	10	266.87	14.80
2	-1	-10	272.87	13.60
-2	1	10	283.97	14.90
2	1	-10	260.97	13.30
2	1	-10	265.37	14.40
-2	1	10	266.07	14.10
1	-1	-10	528.25	25.80
1	-1	-10	513.75	25.20
-1	-1	10	505.95	25.30
-1	-1	10	508.45	25.80
1	1	-10	511.65	25.10
1	1	-10	509.75	25.60
-1	1	10	520.05	25.90
-1	1	10	501.85	25.60
0	-1	-10	529.35	25.80
0	-1	-10	513.95	26.00
0	-1	10	556.24	25.90
0	-1	10	487.35	25.80
0	1	10	533.85	26.00
0	1	-10	522.25	25.80
0	1	10	509.95	26.10
-1	-1	-10	27.20	3.40
1	-1	10	35.40	3.10
-1	-1	-10	30.70	3.80
1	-1	10	32.80	3.80
-1	1	-10	28.60	3.00
1	1	10	40.20	3.70
1	1	10	32.60	3.30
-1	1	-10	31.70	3.60

1	1	10	27.80	4.10
2	-1	10	200.78	11.60
2	-1	10	207.28	10.30
-2	-1	-10	205.68	11.30
2	-1	10	211.78	10.50
-2	-1	-10	211.08	11.10
2	1	10	192.08	11.50
-2	1	-10	209.08	11.40
2	1	10	214.68	10.80
-2	1	-10	208.78	10.60
2	1	10	213.08	11.10
-3	-1	-10	3.70	2.40
3	-1	10	0.60	2.30
3	1	10	2.70	2.60
-3	1	-10	-0.50	2.30
3	1	10	-0.50	1.70
3	1	10	-0.60	1.20
4	-1	10	116.89	8.50
-4	-1	-10	125.69	7.90
-4	-1	-10	124.99	8.40
-4	1	-10	133.29	7.20
4	1	10	128.69	7.00
-4	1	-10	139.19	8.50
4	1	10	124.69	7.30
4	1	10	124.49	8.60
-5	-1	-10	7.20	3.00
5	-1	10	9.20	1.80
5	-1	10	10.10	1.50
-5	-1	-10	11.30	3.50
5	-1	10	8.50	3.40
-5	1	-10	5.10	3.10
5	1	10	10.90	3.30
5	1	10	6.20	1.90
-5	1	-10	5.90	2.30
-6	-1	-10	201.48	12.20
-6	-1	-10	185.58	11.80
6	-1	10	192.28	10.00
6	-1	10	200.48	12.20
6	-1	10	193.08	9.80
6	1	10	174.08	11.80
6	1	10	181.78	10.90
-6	1	-10	184.78	11.20
-6	1	-10	216.18	12.10
-7	-1	-10	5.50	3.60

7	-1	10	5.50	2.20
-7	-1	-10	11.70	4.00
7	1	10	9.50	3.80
7	1	10	5.20	2.90
-7	1	-10	4.30	4.20
-7	1	-10	1.50	3.10
-7	1	-10	0.60	3.70
-8	-1	-10	162.38	12.00
8	-1	10	160.78	9.70
-8	1	-10	165.18	12.00
8	1	10	181.08	10.60
8	1	10	137.49	11.20
-8	1	-10	161.88	11.20
9	-1	10	119.79	8.40
-9	-1	-10	119.49	11.30
-9	-1	-10	122.89	11.00
9	1	10	124.39	9.30
-9	1	-10	115.49	10.80
-9	1	-10	101.79	10.30
-10	-1	-10	102.29	12.10
10	-1	10	101.09	8.60
-10	-1	-10	111.99	13.20
-10	1	-10	98.89	11.10
-10	1	-10	115.79	11.40
10	1	10	111.29	9.50
-11	-1	-10	107.59	11.40
-11	-1	-10	76.29	12.20
11	-1	10	66.99	8.30
11	1	10	76.39	9.30
-11	1	-10	63.79	11.60
-11	1	-10	74.39	11.10
-12	-1	-10	144.89	14.00
-12	-1	-10	115.69	14.70
-12	1	-10	111.89	14.40
-12	1	-10	146.19	13.10
-13	-1	-10	46.10	9.20
-13	1	-10	24.40	8.80
-14	-1	-10	11.70	8.90
-14	1	-10	18.30	8.90
-15	-1	-10	97.29	15.80
-15	1	-10	112.19	16.00
-15	-2	10	21.90	7.00
-15	2	10	21.80	7.70
-14	-2	10	312.27	23.70

-14	2	10	295.77	19.00
-13	-2	10	9.10	5.10
13	-2	-10	4.40	8.40
-13	2	10	14.40	5.60
13	2	-10	-1.40	7.90
-12	-2	10	130.29	21.40
12	-2	-10	197.38	16.70
-12	-2	10	162.88	11.50
-12	2	10	163.88	21.30
12	2	-10	176.58	16.60
-12	2	10	170.88	12.30
-11	-2	10	235.68	13.30
11	-2	-10	232.28	17.90
-11	-2	10	174.28	20.40
11	2	-10	232.18	18.20
-11	2	10	203.78	20.50
-11	2	10	234.28	14.00
11	2	-10	217.08	14.70
10	-2	-10	666.03	35.10
-10	-2	10	643.14	32.00
10	-2	-10	632.04	33.40
-10	-2	10	586.64	36.80
-10	2	10	589.44	36.70
-10	2	10	652.93	32.50
10	2	-10	658.43	35.20
10	2	-10	656.23	32.80
-9	-2	10	193.88	17.10
-9	-2	10	198.98	11.50
9	-2	-10	210.18	13.20
9	-2	-10	216.28	15.20
-9	2	10	201.98	12.20
9	2	-10	197.08	12.40
9	2	-10	185.88	15.30
-9	2	10	170.98	17.10
-8	-2	10	119.59	8.20
8	-2	-10	140.39	9.50
-8	-2	10	88.29	12.90
8	-2	-10	140.79	11.70
8	2	-10	119.49	11.90
8	2	-10	138.09	8.90
-8	2	10	141.49	9.00
-8	2	10	120.29	13.40
7	-2	-10	554.04	28.30
7	-2	-10	579.04	29.90

-7	-2	10	512.05	30.90
-7	-2	10	570.54	27.70
-7	2	10	558.14	28.10
-7	2	10	535.45	30.80
7	2	-10	577.44	27.90
7	2	-10	560.14	30.10
6	-2	-10	437.36	23.50
-6	-2	10	401.56	24.60
6	-2	-10	456.25	22.10
-6	-2	10	427.16	21.50
-6	2	10	430.36	22.10
-6	2	10	421.86	24.30
6	2	-10	402.06	23.50
6	2	-10	428.56	21.50
-5	-2	10	1535.65	72.99
5	-2	-10	1599.34	73.19
5	-2	-10	1510.65	74.29
-5	-2	10	1433.46	74.79
5	2	-10	1546.35	72.79
-5	2	10	1509.25	74.89
5	2	-10	1525.55	74.49
-5	2	10	1487.95	73.49
-4	-2	10	257.27	13.70
4	-2	-10	260.27	13.70
4	-2	-10	258.67	15.00
-4	-2	10	260.07	15.60
4	2	-10	273.07	13.30
-4	2	10	262.27	15.60
4	2	-10	269.87	15.20
-4	2	10	245.68	14.40
3	-2	-10	1758.22	83.99
-3	-2	10	1690.33	82.89
-3	-2	10	1744.03	84.29
3	-2	-10	1749.72	83.09
-3	2	10	1774.72	84.39
3	2	-10	1741.93	82.69
3	2	-10	1782.82	84.09
-3	2	10	1646.54	83.59
2	-2	-10	107.89	7.60
-2	-2	10	124.09	7.90
-2	-2	10	110.49	6.50
2	-2	-10	105.09	6.70
-2	2	10	123.99	8.10
2	2	-10	108.19	6.20

-2	2	10	112.99	7.50
2	2	-10	115.39	7.60
1	-2	-10	2301.47	107.19
1	-2	-10	2293.67	106.59
-1	-2	10	1913.51	106.79
-1	-2	10	2259.97	106.99
-1	2	10	2373.56	107.29
1	2	-10	2297.47	106.89
-1	2	10	2229.18	106.99
1	2	-10	2205.68	106.29
0	-2	10	409.86	20.30
0	-2	10	381.66	20.10
0	-2	-10	416.56	20.30
0	-2	-10	414.66	20.60
0	2	10	398.36	20.50
0	2	-10	395.36	20.10
0	2	10	376.96	20.60
0	2	-10	429.56	20.00
1	-2	10	3222.08	155.78
-1	-2	-10	3292.87	155.88
1	-2	10	3079.29	155.08
-1	-2	-10	3439.16	155.88
1	2	10	3324.87	155.78
1	2	10	3377.06	155.98
1	2	10	3152.48	156.08
-1	2	-10	3236.18	155.38
-1	2	-10	3323.87	155.68
-2	-2	-10	67.99	5.20
-2	-2	-10	61.29	4.80
2	-2	10	61.69	5.30
2	2	10	61.49	5.10
2	2	10	52.19	4.90
-2	2	-10	68.09	4.90
-2	2	-10	64.69	3.80
2	2	10	62.89	4.40
3	-2	10	1158.38	59.69
-3	-2	-10	1290.37	59.79
3	2	10	1173.18	58.99
-3	2	-10	1290.87	59.69
3	2	10	1300.37	59.49
3	2	10	1166.28	59.89
-4	-2	-10	860.21	44.60
4	-2	10	894.21	45.10
-4	-2	-10	983.30	45.10

-4	2	-10	898.91	43.80
4	2	10	881.61	45.20
4	2	10	904.71	44.50
-4	2	-10	970.40	45.00
4	2	10	919.61	44.20
5	-2	10	515.95	27.80
-5	-2	-10	517.35	28.30
-5	-2	-10	576.24	27.90
-5	2	-10	550.64	26.60
5	2	10	559.84	27.20
5	2	10	502.05	27.80
5	2	10	540.45	26.80
-5	2	-10	569.24	27.90
-6	-2	-10	672.63	32.90
-6	2	-10	619.04	32.90
-6	2	-10	702.83	33.10
6	2	10	633.34	33.00
6	2	10	611.44	32.10
-6	2	-10	629.44	31.90
-7	-2	-10	686.83	32.50
7	-2	10	635.84	30.40
-7	2	-10	589.44	32.30
-7	2	-10	673.43	32.60
7	2	10	602.14	32.40
-7	2	-10	609.64	31.40
7	2	10	584.44	32.60
-8	-2	-10	146.19	13.50
8	-2	10	157.28	8.80
-8	2	-10	151.38	11.00
8	2	10	136.39	13.30
8	2	10	144.19	11.20
-8	2	-10	154.48	10.60
9	-2	10	50.99	5.50
-9	-2	-10	49.20	7.60
-9	-2	-10	47.20	8.10
-9	2	-10	50.19	7.90
9	2	10	48.10	7.40
-9	2	-10	55.09	8.60
-10	-2	-10	160.68	14.30
-10	-2	-10	130.19	13.20
10	-2	10	153.48	9.90
-10	2	-10	148.49	12.10
10	2	10	159.08	11.50
-10	2	-10	126.89	12.20

-11	-2	-10	76.39	12.80
-11	-2	-10	90.19	10.70
11	-2	10	66.09	7.90
11	2	10	95.59	9.70
-11	2	-10	69.99	11.20
-11	2	-10	73.89	11.10
-12	-2	-10	241.08	17.10
-12	2	-10	248.88	17.50
-12	2	-10	202.08	17.30
-13	-2	-10	50.49	9.70
-13	2	-10	48.80	9.60
-14	-2	-10	115.49	15.60
-14	2	-10	112.09	14.80
-15	-2	-10	27.30	9.50
-15	2	-10	19.50	9.40
-15	3	10	304.87	20.40
-14	-3	10	12.50	5.80
-14	3	10	4.60	6.30
-13	-3	10	301.87	17.80
13	-3	-10	303.67	22.60
13	3	-10	301.67	23.30
-13	3	10	316.97	18.70
12	-3	-10	104.69	14.10
-12	-3	10	72.79	8.20
-12	-3	10	78.59	12.00
-12	3	10	81.99	9.40
12	3	-10	69.99	14.00
-11	-3	10	565.84	28.50
-11	-3	10	530.45	27.10
11	-3	-10	562.74	30.80
-11	-3	10	433.86	33.40
-11	3	10	552.44	27.90
-11	3	10	478.35	33.00
11	3	-10	548.75	31.30
10	-3	-10	151.68	13.60
-10	-3	10	128.49	8.90
-10	3	10	143.39	10.10
10	3	-10	121.79	10.10
10	3	-10	127.09	13.70
-10	3	10	119.29	16.50
9	-3	-10	497.35	26.80
9	-3	-10	444.96	25.00
-9	-3	10	421.16	27.90
-9	-3	10	465.65	23.40

-9	3	10	464.05	24.30
9	3	-10	483.35	26.90
9	3	-10	480.25	24.10
-9	3	10	440.46	28.30
8	-3	-10	405.36	23.00
8	-3	-10	404.96	21.40
-8	-3	10	368.56	24.30
-8	-3	10	378.26	20.00
8	3	-10	403.66	20.50
-8	3	10	372.76	24.40
8	3	-10	413.66	23.50
-8	3	10	403.46	21.00
7	-3	-10	391.16	21.50
-7	-3	10	360.86	22.40
-7	-3	10	362.36	18.70
7	-3	-10	372.06	19.80
7	3	-10	374.76	19.00
-7	3	10	352.96	22.40
-7	3	10	368.86	19.60
7	3	-10	367.66	21.70
6	-3	-10	1342.97	63.19
-6	-3	10	1288.47	62.49
6	-3	-10	1296.07	64.29
-6	-3	10	1178.28	64.89
6	3	-10	1295.87	62.49
6	3	-10	1346.77	64.69
-6	3	10	1318.97	65.19
-6	3	10	1330.97	63.29
5	-3	-10	377.06	20.60
-5	-3	10	375.36	18.80
5	-3	-10	395.36	19.50
-5	-3	10	335.37	21.20
5	3	-10	390.46	18.70
-5	3	10	382.16	21.50
5	3	-10	370.36	20.90
-5	3	10	364.56	19.80
4	-3	-10	1731.93	81.69
4	-3	-10	1742.03	82.79
-4	-3	10	1636.24	83.09
-4	-3	10	1653.63	81.29
4	3	-10	1674.73	81.09
-4	3	10	1652.43	82.29
4	3	-10	1735.93	82.89
-4	3	10	1793.22	83.29

3	-3	-10	306.77	16.80
3	-3	-10	307.27	15.80
-3	-3	10	295.07	17.10
-3	-3	10	294.87	15.30
-3	3	10	302.57	16.60
3	3	-10	326.67	17.00
-3	3	10	310.97	17.20
3	3	-10	316.67	15.20
2	-3	-10	574.94	28.50
-2	-3	10	552.14	28.40
-2	-3	10	540.85	27.20
2	-3	-10	560.64	27.60
2	3	-10	550.34	27.00
-2	3	10	590.74	28.70
2	3	-10	567.34	28.30
-2	3	10	546.85	28.30
1	-3	-10	23.00	4.00
-1	-3	10	25.50	2.90
-1	-3	10	17.70	3.70
1	-3	-10	26.80	3.30
1	3	-10	19.10	3.50
1	3	-10	29.50	2.60
-1	3	10	24.10	4.10
-1	3	10	30.70	4.20
0	-3	-10	2315.97	106.79
0	-3	-10	2256.17	107.09
0	-3	10	2177.68	106.99
0	-3	10	2165.88	106.39
0	3	-10	2208.78	106.39
0	3	10	2295.17	106.99
0	3	10	2212.98	107.09
0	3	-10	2235.68	106.59
-1	-3	-10	3.60	2.20
1	-3	10	12.70	2.50
1	-3	10	7.90	1.20
1	3	10	9.70	2.60
-1	3	-10	10.40	2.10
-1	3	-10	13.60	1.80
1	3	10	9.30	2.10
1	3	10	11.90	2.60
-2	-3	-10	935.41	43.60
2	-3	10	845.12	43.50
-2	3	-10	920.41	43.40
2	3	10	910.31	43.70

-2	3	-10	869.51	42.50
2	3	10	885.71	43.30
2	3	10	872.51	43.80
-3	-3	-10	173.18	10.10
3	-3	10	157.48	9.90
3	3	10	169.08	9.40
3	3	10	177.08	10.10
3	3	10	169.18	10.40
-3	3	-10	169.48	9.90
4	-3	10	778.82	40.20
-4	-3	-10	834.72	40.20
-4	-3	-10	789.22	40.00
4	3	10	815.52	39.60
-4	3	-10	847.42	40.20
4	3	10	748.13	40.30
4	3	10	866.41	40.10
-5	-3	-10	380.06	21.00
-5	-3	-10	449.95	21.30
5	3	10	390.26	20.20
5	3	10	390.96	21.30
5	3	10	399.06	20.70
-5	3	-10	390.56	19.70
-5	3	-10	407.76	21.10
-5	3	-10	383.86	21.20
-6	-3	-10	1321.37	63.99
-6	-3	-10	1305.87	63.99
-6	3	-10	1262.97	64.09
6	3	10	1192.28	63.99
6	3	10	1360.16	63.59
-6	3	-10	1306.27	62.89
-6	3	-10	1391.56	64.19
7	-3	10	133.99	7.00
-7	-3	-10	144.59	10.00
-7	-3	-10	143.59	10.40
7	3	10	139.39	9.70
-7	3	-10	141.49	10.60
-7	3	-10	136.19	10.10
-7	3	-10	116.29	8.80
7	3	10	134.79	10.30
8	-3	10	132.29	7.30
-8	-3	-10	120.79	10.80
8	3	10	122.19	10.00
-8	3	-10	120.59	10.50
8	3	10	128.79	9.80

-8	3	-10	120.79	9.10
9	-3	10	151.68	9.00
-9	-3	-10	151.08	12.70
9	3	10	124.19	12.40
-9	3	-10	148.99	11.90
-9	3	-10	154.88	11.00
9	3	10	151.08	11.80
-10	-3	-10	230.68	16.90
10	-3	10	228.18	13.20
-10	-3	-10	244.38	16.90
10	3	10	252.07	16.10
-10	3	-10	218.78	15.70
-10	3	-10	229.18	15.50
-11	-3	-10	294.47	21.40
11	-3	10	306.57	17.30
-11	-3	-10	361.06	21.00
11	3	10	333.07	19.50
-11	3	-10	284.97	19.70
-11	3	-10	314.57	19.70
-12	-3	-10	134.19	14.00
-12	3	-10	105.69	13.30
-12	3	-10	118.69	12.20
-13	-3	-10	207.08	17.50
-13	3	-10	183.38	16.20
-14	-3	-10	36.20	10.00
-14	3	-10	38.60	9.30
-15	-3	-10	253.17	21.70
-15	3	-10	268.37	21.10
-14	-4	10	16.60	5.60
-14	4	10	7.40	6.50
13	-4	-10	43.00	9.70
-13	-4	10	21.30	5.20
-13	4	10	16.80	6.10
13	4	-10	32.70	9.70
-12	-4	10	5.30	4.10
12	-4	-10	8.00	7.00
12	4	-10	1.60	8.40
-12	4	10	14.30	5.40
-11	-4	10	99.79	10.40
-11	-4	10	106.09	8.30
11	-4	-10	100.49	13.10
11	4	-10	104.79	14.30
-11	4	10	115.49	9.90
-10	-4	10	70.49	6.50

-10	-4	10	76.19	8.50
10	-4	-10	69.09	10.60
-10	-4	10	45.40	11.40
-10	4	10	78.89	7.90
10	4	-10	64.69	12.10
-10	4	10	76.49	12.30
-9	-4	10	44.00	4.90
9	-4	-10	43.00	7.70
-9	-4	10	26.90	8.30
9	-4	-10	49.50	9.20
-9	4	10	36.80	6.80
9	4	-10	56.09	10.40
-9	4	10	20.40	7.90
8	-4	-10	15.20	5.20
8	-4	-10	24.30	4.70
-8	-4	10	19.60	3.00
-8	-4	10	5.80	6.30
-8	4	10	13.40	6.50
8	4	-10	11.70	5.70
8	4	-10	24.70	4.60
-8	4	10	19.40	4.20
-7	-4	10	22.20	6.40
7	-4	-10	35.20	5.90
7	-4	-10	41.10	7.50
-7	-4	10	34.80	4.10
-7	4	10	18.00	6.00
7	4	-10	27.30	5.90
-7	4	10	40.10	5.70
6	-4	-10	59.89	5.90
-6	-4	10	44.00	4.10
-6	-4	10	29.40	6.90
6	-4	-10	37.00	7.20
-6	4	10	48.30	5.80
6	4	-10	34.70	6.10
6	4	-10	44.90	4.00
-6	4	10	41.10	6.90
5	-4	-10	39.50	6.30
-5	-4	10	30.10	3.30
-5	-4	10	29.60	5.70
5	-4	-10	35.10	4.80
-5	4	10	29.20	5.70
5	4	-10	35.30	3.20
5	4	-10	31.50	5.50
-5	4	10	39.30	5.00

4	-4	-10	4.30	3.10
4	-4	-10	2.20	2.40
-4	-4	10	-3.40	3.40
-4	-4	10	3.70	1.50
4	4	-10	3.40	1.40
-4	4	10	-3.20	3.00
-4	4	10	1.20	3.40
4	4	-10	-1.70	3.20
3	-4	-10	146.99	8.30
3	-4	-10	146.19	9.40
-3	-4	10	140.19	7.40
-3	-4	10	135.09	9.50
-3	4	10	146.09	9.80
3	4	-10	146.39	7.20
-3	4	10	117.89	9.00
3	4	-10	139.19	9.40
-2	-4	10	11.80	3.00
2	-4	-10	14.70	3.30
2	-4	-10	19.20	3.30
-2	-4	10	14.00	2.30
2	4	-10	12.40	3.00
2	4	-10	15.00	1.90
-2	4	10	15.90	3.50
-2	4	10	13.80	3.60
-1	-4	10	12.70	2.50
1	-4	-10	9.50	2.80
1	-4	-10	21.80	3.30
-1	-4	10	16.40	2.60
1	4	-10	10.30	2.40
-1	4	10	17.60	3.40
1	4	-10	10.90	2.10
-1	4	10	8.60	2.70
0	-4	-10	121.79	8.00
0	-4	-10	124.79	7.50
0	-4	10	122.59	7.20
0	-4	10	109.19	6.90
0	4	10	129.09	7.80
0	4	-10	119.69	7.20
0	4	-10	115.69	6.80
0	4	10	136.19	8.20
1	-4	10	119.89	7.30
-1	-4	-10	110.89	7.40
-1	-4	-10	119.59	7.70
1	4	10	112.19	7.80

1	4	10	120.49	8.00
1	4	10	122.09	7.40
-1	4	-10	118.99	6.50
2	-4	10	85.49	6.10
-2	-4	-10	90.49	6.30
2	4	10	87.89	6.60
2	4	10	78.39	6.30
-2	4	-10	88.99	4.50
-2	4	-10	75.29	5.70
2	4	10	78.49	5.80
-3	-4	-10	4.90	2.60
3	-4	10	13.10	3.00
3	4	10	7.80	2.10
-3	4	-10	17.90	3.10
3	4	10	8.90	2.90
3	4	10	9.50	3.10
-4	-4	-10	6.40	2.80
-4	-4	-10	5.80	3.00
4	4	10	8.60	3.30
4	4	10	10.50	2.40
-4	4	-10	5.40	2.80
-4	4	-10	5.60	3.20
4	4	10	4.90	2.90
-5	-4	-10	157.28	9.50
-5	-4	-10	121.09	9.30
5	4	10	142.09	9.40
5	4	10	141.19	8.40
-5	4	-10	130.59	9.40
-5	4	-10	158.88	9.60
-5	4	-10	120.09	7.40
5	4	10	141.59	9.20
-6	-4	-10	3.70	3.50
-6	-4	-10	11.10	3.70
-6	4	-10	8.50	3.90
6	4	10	12.30	4.00
6	4	10	8.80	3.30
-6	4	-10	3.10	3.80
-7	-4	-10	10.30	4.00
-7	-4	-10	8.60	7.20
-7	4	-10	-4.20	2.90
7	4	10	3.70	4.10
-7	4	-10	5.90	4.00
7	4	10	5.20	3.60
-7	4	-10	4.30	4.30

-8	-4	-10	41.10	7.90
8	-4	10	30.60	3.20
8	4	10	39.00	5.80
-8	4	-10	37.10	6.80
-8	4	-10	27.80	5.70
8	4	10	37.00	6.70
-9	-4	-10	133.69	11.90
9	-4	10	131.89	7.80
9	4	10	116.99	11.00
-9	4	-10	110.19	9.90
9	4	10	151.18	11.10
-9	4	-10	133.89	10.70
10	-4	10	110.29	7.90
-10	-4	-10	130.79	12.10
-10	-4	-10	119.19	12.50
10	4	10	109.19	11.20
-10	4	-10	112.89	10.70
-10	4	-10	103.19	10.60
10	4	10	111.89	10.60
-11	-4	-10	6.00	6.90
-11	-4	-10	10.00	7.40
-11	4	-10	-0.20	5.70
-11	4	-10	10.50	6.40
-12	-4	-10	16.00	7.80
-12	4	-10	8.60	6.70
-12	4	-10	22.30	7.90
-13	-4	-10	57.59	10.00
-13	4	-10	36.20	8.80
-14	-4	-10	11.50	9.00
-14	4	-10	17.50	7.90
-14	5	10	14.10	6.80
-13	-5	10	292.37	17.70
13	-5	-10	309.97	27.80
13	5	-10	302.97	23.80
-13	5	10	341.17	19.40
12	-5	-10	202.58	17.50
-12	-5	10	189.98	12.50
-12	5	10	209.38	14.00
12	5	-10	205.38	18.30
-11	-5	10	527.85	28.10
11	-5	-10	556.54	32.00
-11	-5	10	623.64	29.70
-11	5	10	485.05	34.60
-11	5	10	584.04	29.40

11	5	-10	561.44	32.80
10	-5	-10	237.48	17.00
-10	-5	10	253.87	14.30
-10	-5	10	209.78	12.80
-10	5	10	224.58	20.30
-10	5	10	224.88	14.30
10	5	-10	236.38	18.10
-9	-5	10	233.28	19.60
-9	-5	10	298.07	15.30
9	-5	-10	269.67	17.70
-9	-5	10	266.27	14.20
-9	5	10	253.87	20.30
9	5	-10	268.97	18.70
-9	5	10	264.87	15.70
8	-5	-10	122.09	9.80
-8	-5	10	124.39	7.90
-8	-5	10	84.89	12.60
-8	-5	10	111.69	7.00
8	-5	-10	109.79	10.60
8	5	-10	118.59	11.80
-8	5	10	102.59	13.10
-8	5	10	106.99	8.80
-7	-5	10	720.73	42.70
7	-5	-10	811.82	42.00
7	-5	-10	852.31	41.00
7	5	-10	829.42	42.50
-7	5	10	852.71	40.80
-7	5	10	822.52	43.10
-6	-5	10	1062.19	50.59
6	-5	-10	1075.69	52.99
-6	-5	10	926.61	53.29
6	-5	-10	1087.69	51.89
6	5	-10	1079.09	53.39
-6	5	10	1084.49	53.79
-6	5	10	1092.49	52.09
6	5	-10	1033.20	50.69
-5	-5	10	387.56	19.30
5	-5	-10	388.06	21.60
-5	-5	10	375.56	22.30
5	-5	-10	398.06	20.60
5	5	-10	413.46	22.10
-5	5	10	405.26	22.40
-5	5	10	406.26	21.20
5	5	-10	389.06	19.30

-4	-5	10	1160.68	61.69
4	-5	-10	1306.57	60.69
4	-5	-10	1265.17	61.69
-4	-5	10	1221.88	59.59
-4	5	10	1338.07	62.19
-4	5	10	1250.07	61.39
4	5	-10	1263.37	61.79
4	5	-10	1216.38	59.59
3	-5	-10	702.43	35.60
3	-5	-10	718.03	34.60
-3	-5	10	672.43	35.50
-3	-5	10	720.23	33.70
-3	5	10	733.23	35.90
3	5	-10	719.33	33.60
3	5	-10	695.53	35.50
-3	5	10	659.13	35.40
-2	-5	10	1770.12	79.19
2	-5	-10	1687.03	79.89
-2	-5	10	1597.04	80.29
2	-5	-10	1689.53	80.69
-2	5	10	1572.94	80.59
2	5	-10	1682.43	80.39
-2	5	10	1707.33	80.79
2	5	-10	1595.14	78.89
-1	-5	10	607.84	28.40
-1	-5	10	575.04	29.00
1	-5	-10	551.84	29.60
1	-5	-10	586.44	29.00
1	5	-10	599.44	29.20
-1	5	10	586.54	29.70
-1	5	10	587.74	29.60
1	5	-10	576.74	28.20
0	-5	10	4111.39	197.48
0	-5	-10	4319.37	197.98
0	-5	10	4289.97	197.58
0	-5	-10	4196.78	198.48
0	5	-10	4024.10	197.68
0	5	10	4093.59	198.38
0	5	10	4132.19	198.18
0	5	-10	4111.49	197.28
-1	-5	-10	0.70	2.20
1	-5	10	0.10	2.10
-1	-5	-10	3.40	2.40
-1	5	-10	4.40	1.60

1	5	10	1.40	2.40
1	5	10	3.60	2.80
1	5	10	3.30	2.20
-2	-5	-10	3326.67	148.39
2	5	10	2977.20	148.59
-2	5	-10	3008.30	147.09
-2	5	-10	3187.88	147.99
-2	5	-10	2984.30	148.09
2	5	10	3182.38	148.79
2	5	10	3073.69	148.19
-3	-5	-10	294.27	15.70
3	5	10	286.97	16.10
-3	5	-10	309.37	15.70
-3	5	-10	290.17	15.70
3	5	10	284.37	15.50
3	5	10	287.47	16.20
-4	-5	-10	1592.44	79.59
-4	-5	-10	1696.33	79.49
4	5	10	1624.14	79.19
4	5	10	1565.84	79.69
-4	5	-10	1622.74	79.49
-4	5	-10	1674.23	79.39
4	5	10	1719.03	79.79
-5	-5	-10	385.26	19.60
-5	-5	-10	362.66	19.70
5	5	10	337.27	19.70
-5	5	-10	373.76	19.50
-5	5	-10	343.37	17.70
5	5	10	360.96	18.90
5	5	10	375.86	19.70
-5	5	-10	354.46	19.50
-6	-5	-10	2033.60	103.09
-6	-5	-10	2235.18	102.89
6	5	10	2213.88	102.89
-6	5	-10	2054.79	101.49
6	5	10	2018.60	102.99
-6	5	-10	2259.37	103.09
-6	5	-10	2083.09	102.89
-7	-5	-10	777.82	40.90
7	5	10	839.92	40.60
-7	5	-10	748.43	39.20
7	5	10	785.72	40.70
-7	5	-10	785.52	40.50
-7	5	-10	861.11	40.90

-8	-5	-10	79.19	9.40
8	5	10	56.69	8.60
8	5	10	92.89	8.90
-8	5	-10	78.19	7.00
-8	5	-10	60.89	8.50
9	-5	10	170.38	9.10
-9	-5	-10	172.58	13.50
-9	5	-10	177.28	12.80
9	5	10	164.98	12.70
9	5	10	161.28	12.60
-9	5	-10	145.79	11.20
10	-5	10	247.98	13.50
-10	-5	-10	265.07	18.00
-10	5	-10	234.38	15.80
10	5	10	237.18	16.80
10	5	10	278.27	17.10
-10	5	-10	248.48	16.40
-11	-5	-10	476.95	30.30
11	-5	10	521.95	26.40
-11	-5	-10	577.04	30.40
-11	5	-10	464.75	28.60
11	5	10	558.14	30.60
-11	5	-10	550.64	29.70
-12	-5	-10	557.84	29.40
-12	5	-10	418.26	27.60
-12	5	-10	492.45	28.30
-13	-5	-10	242.08	19.10
-13	5	-10	225.08	18.20
-14	-5	-10	22.40	9.10
-14	5	-10	11.20	8.00
-14	6	10	43.80	8.30
-13	-6	10	15.80	4.80
13	6	-10	2.10	9.00
12	-6	-10	16.00	8.00
-12	-6	10	15.50	4.40
12	6	-10	20.30	9.00
-12	6	10	29.50	6.40
11	-6	-10	18.10	7.20
-11	-6	10	14.40	3.70
-11	-6	10	15.50	5.10
11	6	-10	5.90	7.70
-11	6	10	13.30	10.80
-11	6	10	34.70	9.40
-10	-6	10	223.58	20.70

10	-6	-10	246.58	17.70
-10	-6	10	237.78	13.40
-10	-6	10	259.47	14.70
-10	6	10	245.68	21.30
10	6	-10	262.67	19.00
-10	6	10	242.88	15.30
9	-6	-10	14.80	5.60
-9	-6	10	19.60	3.00
-9	-6	10	20.30	3.90
-9	-6	10	16.60	8.30
-9	6	10	12.10	8.20
-9	6	10	16.20	4.70
9	6	-10	12.50	6.40
8	-6	-10	68.19	8.90
8	-6	-10	41.80	8.00
-8	-6	10	65.49	5.50
-8	-6	10	53.89	4.80
-8	-6	10	49.90	8.60
8	6	-10	65.99	9.80
-8	6	10	55.29	11.40
-8	6	10	57.59	7.20
7	-6	-10	114.39	9.00
-7	-6	10	108.89	6.70
7	-6	-10	112.39	10.40
-7	-6	10	104.59	6.40
-7	6	10	117.69	8.70
7	6	-10	101.39	11.00
-7	6	10	90.29	11.80
-6	-6	10	91.19	10.60
-6	-6	10	112.19	6.10
6	-6	-10	100.99	8.50
6	-6	-10	115.69	9.70
-6	-6	10	117.29	6.20
6	6	-10	102.49	10.60
-6	6	10	114.09	11.20
-6	6	10	107.29	8.80
5	-6	-10	82.49	7.80
-5	-6	10	66.99	3.80
-5	-6	10	70.89	3.90
-5	-6	10	56.49	8.50
5	-6	-10	72.49	6.50
5	6	-10	65.69	8.30
-5	6	10	74.79	8.50
-5	6	10	68.69	7.30

4	-6	-10	40.10	6.00
-4	-6	10	33.20	6.40
4	-6	-10	40.30	4.90
4	6	-10	39.00	6.50
-4	6	10	28.00	5.00
-4	6	10	44.90	6.80
3	-6	-10	222.38	13.60
-3	-6	10	226.28	13.60
3	-6	-10	232.48	12.60
3	6	-10	241.88	13.70
-3	6	10	228.18	13.80
-3	6	10	214.08	13.60
2	-6	-10	179.58	10.80
2	-6	-10	173.78	9.70
-2	-6	10	160.68	10.30
2	6	-10	165.58	10.40
2	6	-10	170.98	8.50
2	6	-10	168.28	8.30
-2	6	10	174.78	10.80
-2	6	10	149.49	10.80
-1	-6	10	125.99	8.00
1	-6	-10	122.79	7.80
1	-6	-10	122.79	8.80
-1	6	10	131.69	8.70
1	6	-10	125.59	6.90
1	6	-10	140.39	8.30
1	6	-10	117.69	6.60
-1	6	10	126.09	8.90
0	-6	-10	10.60	3.10
0	-6	-10	8.50	2.50
0	-6	10	9.90	2.20
0	6	-10	11.80	2.20
0	6	10	10.60	3.40
0	6	10	7.00	2.70
0	6	-10	13.60	2.50
0	6	-10	13.60	1.90
-1	-6	-10	226.38	12.10
-1	-6	-10	218.08	12.60
-1	6	-10	207.38	11.60
1	6	10	228.68	12.90
-1	6	-10	184.08	11.70
1	6	10	221.08	12.50
1	6	10	209.08	12.20
-1	6	-10	225.58	11.30

-2	-6	-10	34.70	4.60
2	6	10	40.40	4.60
-2	6	-10	39.10	4.10
2	6	10	31.60	5.40
2	6	10	40.40	5.10
-2	6	-10	33.30	2.80
-2	6	-10	35.00	4.40
-3	-6	-10	259.97	14.50
-3	6	-10	259.67	14.40
3	6	10	251.87	14.90
3	6	10	261.77	14.40
-3	6	-10	262.47	14.30
3	6	10	287.47	15.40
-4	-6	-10	124.99	8.30
-4	-6	-10	107.39	8.50
-4	6	-10	121.09	8.20
4	6	10	109.59	7.80
4	6	10	117.99	9.00
-4	6	-10	112.69	8.20
4	6	10	110.99	8.60
-5	-6	-10	170.38	11.20
-5	-6	-10	181.18	11.70
-5	6	-10	174.78	11.30
-5	6	-10	195.88	11.40
-5	6	-10	183.18	9.20
5	6	10	158.98	11.40
5	6	10	168.48	10.60
5	6	10	191.68	11.90
-6	-6	-10	13.60	4.20
-6	-6	-10	12.50	4.10
-6	6	-10	9.40	4.20
6	6	10	10.30	4.00
6	6	10	13.40	4.30
-6	6	-10	9.60	4.00
-7	-6	-10	268.57	17.10
7	6	10	318.97	17.00
-7	6	-10	264.67	16.50
-7	6	-10	280.37	17.00
7	6	10	254.47	16.30
-7	6	-10	271.97	14.80
-8	-6	-10	96.69	10.20
-8	6	-10	89.09	9.20
8	6	10	92.59	9.40
-8	6	-10	81.99	7.40

8	6	10	82.29	8.70
-9	-6	-10	55.39	9.60
9	-6	10	42.40	3.90
-9	6	-10	38.80	7.10
-9	6	-10	62.19	8.90
9	6	10	37.10	7.00
9	6	10	57.89	9.60
-10	-6	-10	177.78	14.40
10	-6	10	146.89	8.80
-10	6	-10	153.08	12.30
10	6	10	136.39	12.90
-10	6	-10	130.19	11.70
10	6	10	162.88	13.40
-11	-6	-10	26.40	7.70
-11	-6	-10	30.20	11.50
11	-6	10	20.90	3.90
-11	6	-10	28.20	6.40
-11	6	-10	30.60	7.50
-12	-6	-10	39.60	8.90
-12	6	-10	28.10	7.70
-12	6	-10	18.50	6.90
-13	-6	-10	33.80	9.60
-13	6	-10	37.50	8.40
-14	-6	-10	25.00	10.40
-14	6	-10	39.60	9.30
-14	7	10	11.60	7.00
13	7	-10	129.99	18.70
-13	7	10	172.08	13.90
-12	-7	10	29.30	4.80
12	-7	-10	51.39	9.90
12	7	-10	27.60	9.50
-12	7	10	38.90	6.90
11	-7	-10	492.85	29.10
-11	-7	10	482.55	24.50
11	7	-10	468.65	30.00
-11	7	10	529.05	26.40
-11	7	10	426.56	31.80
10	-7	-10	99.09	11.80
-10	-7	10	90.49	6.90
-10	-7	10	105.69	8.50
-10	7	10	94.39	9.30
10	7	-10	89.39	13.10
-10	7	10	86.79	15.50
9	-7	-10	284.87	18.20

-9	-7	10	229.68	20.30
-9	-7	10	273.07	14.10
-9	7	10	284.37	16.20
9	7	-10	283.47	19.30
-9	7	10	254.17	20.70
-8	-7	10	303.57	14.50
8	-7	-10	268.17	17.70
-8	-7	10	272.87	14.10
-8	7	10	280.97	16.20
8	7	-10	268.27	18.50
-8	7	10	257.47	19.30
7	-7	-10	193.88	13.80
-7	-7	10	190.98	9.80
-7	-7	10	209.18	10.00
7	-7	-10	195.88	12.60
-7	-7	10	159.08	15.00
-7	7	10	162.18	14.70
7	7	-10	181.28	14.50
-7	7	10	209.68	12.40
6	-7	-10	953.90	47.50
6	-7	-10	959.30	46.60
-6	-7	10	842.92	48.00
-6	7	10	981.60	46.80
-6	7	10	904.31	48.10
6	7	-10	973.40	48.00
5	-7	-10	423.76	22.70
5	-7	-10	415.76	21.50
-5	-7	10	354.76	22.80
-5	7	10	417.96	22.10
5	7	-10	424.26	23.00
-5	7	10	410.46	23.30
4	-7	-10	342.97	18.00
4	-7	-10	351.66	19.20
-4	-7	10	298.67	19.00
-4	7	10	357.16	19.60
-4	7	10	322.87	18.80
4	7	-10	351.76	19.30
-3	-7	10	56.39	6.80
3	-7	-10	69.09	5.70
3	-7	-10	73.59	7.10
-3	7	10	64.39	7.20
3	7	-10	71.59	7.10
-3	7	10	62.59	7.00
2	-7	-10	649.64	31.80

-2	-7	10	569.34	31.10
2	-7	-10	654.13	30.80
2	7	-10	636.44	31.30
-2	7	10	557.54	31.50
-2	7	10	632.54	31.70
-1	-7	10	11.20	2.80
1	-7	-10	10.90	2.70
1	-7	-10	8.70	3.20
-1	7	10	11.00	3.50
1	7	-10	11.00	1.50
1	7	-10	13.60	2.20
1	7	-10	14.70	3.10
-1	7	10	9.40	3.60
0	-7	10	1701.33	83.99
0	-7	-10	1860.81	84.49
0	-7	-10	1768.52	85.09
0	7	10	1661.83	84.99
0	7	-10	1770.52	84.19
0	7	10	1785.82	84.89
0	7	-10	1722.43	83.59
0	7	-10	1756.32	83.69
-1	-7	-10	334.77	17.10
-1	-7	-10	341.87	17.90
1	7	10	314.67	17.70
1	7	10	311.67	17.30
-1	7	-10	319.47	16.60
-1	7	-10	285.97	16.70
-1	7	-10	342.17	16.40
-2	-7	-10	1198.38	57.79
-2	7	-10	1176.48	57.39
2	7	10	1176.18	58.19
-2	7	-10	1190.38	56.69
-2	7	-10	1186.48	57.29
2	7	10	1161.38	58.49
2	7	10	1208.38	57.99
-3	-7	-10	49.70	5.40
-3	7	-10	41.40	3.00
3	7	10	47.10	5.90
-3	7	-10	48.70	3.00
-3	7	-10	51.29	5.10
3	7	10	41.20	5.20
3	7	10	37.50	6.20
-3	7	-10	58.59	5.50
-4	-7	-10	900.41	43.20

-4	-7	-10	835.42	43.60
-4	7	-10	898.81	43.10
-4	7	-10	862.31	43.00
4	7	10	860.31	43.10
4	7	10	826.82	43.40
4	7	10	892.51	43.90
-4	7	-10	875.51	41.50
-5	-7	-10	151.88	11.50
-5	-7	-10	169.78	11.00
-5	7	-10	160.68	8.90
5	7	10	168.48	10.60
5	7	10	164.28	11.70
-5	7	-10	180.18	11.10
-5	7	-10	181.88	11.10
5	7	10	177.98	11.50
-6	-7	-10	1651.33	79.49
-6	-7	-10	1552.24	80.09
-6	7	-10	1560.04	78.09
6	7	10	1586.34	79.19
6	7	10	1763.52	80.19
6	7	10	1588.44	79.69
-6	7	-10	1749.53	79.79
-6	7	-10	1607.44	79.49
-7	-7	-10	135.59	11.60
-7	7	-10	152.08	11.20
7	7	10	159.18	11.50
7	7	10	150.98	11.30
-7	7	-10	128.79	9.10
-8	-7	-10	123.29	11.80
8	7	10	146.49	11.60
-8	7	-10	124.39	10.80
-8	7	-10	126.59	9.20
8	7	10	138.59	11.30
-9	-7	-10	376.46	23.10
9	7	10	373.16	22.00
9	7	10	389.16	22.70
-9	7	-10	344.27	20.50
-9	7	-10	401.66	22.00
-10	-7	-10	222.18	17.50
10	-7	10	234.98	12.40
-10	7	-10	210.38	15.10
10	7	10	241.78	16.50
10	7	10	242.58	17.40
-10	7	-10	254.07	16.40

11	-7	10	190.28	13.10
-11	7	-10	156.68	13.80
11	7	10	208.98	15.40
-11	7	-10	195.18	15.30
-12	-7	-10	160.38	15.30
-12	7	-10	113.59	13.70
-12	7	-10	156.48	14.40
-13	-7	-10	177.28	17.60
-13	7	-10	158.28	15.90
-14	-7	-10	103.69	16.40
-14	7	-10	94.59	14.30
-14	8	10	244.68	18.00
-13	8	10	28.20	7.20
13	8	-10	15.50	10.00
-12	-8	10	215.38	12.90
12	-8	-10	229.38	18.70
12	8	-10	214.88	20.40
-12	8	10	239.48	15.60
11	-8	-10	161.48	15.40
-11	-8	10	143.69	9.70
-11	8	10	183.18	12.70
11	8	-10	165.48	17.10
-10	-8	10	145.49	9.10
-10	-8	10	164.28	10.20
10	-8	-10	169.68	14.80
-10	8	10	170.18	16.50
10	8	-10	156.48	15.70
-10	8	10	135.99	18.10
-9	-8	10	157.38	9.40
9	-8	-10	166.58	13.90
-9	8	10	161.48	11.70
9	8	-10	154.68	14.90
-9	8	10	126.59	16.10
8	-8	-10	515.55	27.80
-8	-8	10	457.65	29.30
-8	-8	10	520.05	24.10
-8	-8	10	476.15	23.90
8	8	-10	498.45	28.50
-8	8	10	467.75	29.10
-8	8	10	500.75	26.20
7	-8	-10	459.85	25.40
-7	-8	10	398.36	26.20
-7	8	10	449.46	26.60
7	8	-10	471.75	26.00

-7	8	10	463.95	24.20
-6	-8	10	293.27	20.60
6	-8	-10	358.76	20.20
6	-8	-10	338.37	19.00
-6	8	10	326.27	20.80
-6	8	10	351.46	19.20
6	8	-10	373.76	20.80
5	-8	-10	696.83	34.90
5	-8	-10	707.73	36.00
-5	-8	10	615.44	36.20
-5	8	10	686.43	36.50
5	8	-10	727.93	36.30
-5	8	10	727.53	35.50
-4	-8	10	501.85	28.70
4	-8	-10	529.15	28.60
4	-8	-10	544.95	27.60
-4	8	10	550.64	29.00
-4	8	10	553.44	28.60
4	8	-10	566.94	28.80
-3	-8	10	559.24	31.60
3	-8	-10	622.64	31.90
3	-8	-10	655.23	30.90
-3	8	10	580.84	31.80
3	8	-10	652.83	31.80
-3	8	10	615.44	32.00
2	-8	-10	59.09	5.20
2	-8	-10	56.29	6.90
-2	-8	10	75.29	6.50
-2	8	10	43.90	6.90
2	8	-10	71.69	6.60
-2	8	10	60.89	6.70
1	-8	-10	2487.45	116.29
1	-8	-10	2449.25	117.19
-1	-8	10	2413.96	116.49
1	8	-10	2552.74	116.59
1	8	-10	2321.67	115.19
-1	8	10	2510.05	117.09
-1	8	10	2393.76	117.19
1	8	-10	2297.57	115.19
0	-8	10	364.16	18.80
0	-8	-10	379.86	20.20
0	-8	-10	363.26	19.10
0	8	10	360.86	19.80
0	8	-10	404.96	19.10

0	8	-10	348.67	18.20
0	8	10	353.06	20.20
0	8	-10	364.26	18.30
0	8	10	373.66	20.50
-1	-8	-10	2410.56	110.99
-1	8	-10	1969.10	111.19
-1	8	-10	2475.25	110.49
-1	8	-10	2342.37	110.39
1	8	10	2384.46	111.49
1	8	10	2290.87	111.59
-2	-8	-10	13.60	3.10
2	8	10	12.00	3.10
-2	8	-10	9.40	2.70
-2	8	-10	16.70	2.90
2	8	10	12.10	3.90
-2	8	-10	9.20	2.80
-3	-8	-10	638.14	33.00
-3	8	-10	681.63	32.80
-3	8	-10	633.94	32.80
-3	8	-10	676.13	31.80
3	8	10	750.22	36.90
-3	8	-10	626.94	31.70
3	8	10	629.24	33.20
3	8	10	641.74	33.60
-4	-8	-10	12.10	3.50
4	8	10	4.80	4.00
4	8	10	9.90	3.00
-4	8	-10	10.10	3.50
4	8	10	6.90	3.80
-4	8	-10	7.10	3.50
-4	8	-10	12.50	2.30
-4	8	-10	9.60	1.80
-5	-8	-10	906.51	45.90
-5	-8	-10	899.91	46.70
5	8	10	868.01	46.30
-5	8	-10	939.31	46.00
5	8	10	999.30	47.10
-5	8	-10	984.20	44.50
-5	8	-10	915.01	46.00
5	8	10	880.31	45.90
-6	-8	-10	123.79	10.10
-6	8	-10	104.19	9.00
-6	8	-10	93.59	6.90
-6	8	-10	96.69	9.30

6	8	10	100.99	9.40
6	8	10	101.89	8.20
6	8	10	111.59	9.90
-7	-8	-10	624.94	35.50
7	8	10	668.43	35.10
7	8	10	741.53	35.60
-7	8	-10	657.93	34.70
-7	8	-10	665.03	33.30
-8	-8	-10	100.39	11.30
-8	8	-10	97.39	8.50
-8	8	-10	113.59	10.10
8	8	10	118.79	11.00
8	8	10	102.19	10.10
-9	-8	-10	533.55	31.50
9	8	10	646.24	31.40
-9	8	-10	502.25	33.90
-9	8	-10	543.45	29.10
-10	-8	-10	234.28	17.90
-10	8	-10	207.48	14.90
10	8	10	236.08	17.40
10	8	10	234.58	16.50
-10	8	-10	240.58	16.30
11	8	10	282.17	18.90
-11	8	-10	278.67	18.70
-11	8	-10	260.67	17.70
-12	-8	-10	323.57	21.70
-12	8	-10	315.97	20.60
-12	8	-10	266.77	19.70
-13	-8	-10	190.38	18.10
-13	8	-10	161.08	16.10
-14	-8	-10	315.07	23.30
-14	8	-10	278.47	21.70
-14	9	10	32.20	8.20
-13	9	10	0.90	6.70
12	-9	-10	25.50	9.40
12	9	-10	26.50	10.00
-12	9	10	16.60	6.30
-11	-9	10	26.00	4.00
11	-9	-10	33.10	8.40
11	9	-10	26.00	10.00
-11	9	10	35.80	6.60
-10	-9	10	8.00	2.90
10	-9	-10	10.10	6.40
10	9	-10	9.60	7.20

-10	9	10	10.20	5.60
-10	9	10	15.90	9.20
9	-9	-10	68.59	10.70
-9	-9	10	73.69	5.00
-9	-9	10	78.69	5.50
9	9	-10	74.99	12.20
-9	9	10	82.99	9.10
-9	9	10	60.49	10.80
8	-9	-10	56.69	9.50
8	9	-10	25.30	7.00
-8	9	10	30.00	8.50
-8	9	10	51.39	7.50
7	-9	-10	4.90	5.00
-7	-9	10	6.80	6.40
7	9	-10	2.60	5.50
-7	9	10	2.90	6.20
-7	9	10	3.80	4.40
6	-9	-10	56.99	8.50
-6	-9	10	35.40	7.30
6	9	-10	52.29	9.10
-6	9	10	44.90	8.90
-6	9	10	47.90	7.40
5	-9	-10	3.30	4.10
-5	-9	10	2.40	4.40
5	-9	-10	7.70	3.80
-5	9	10	4.20	4.80
5	9	-10	3.80	5.10
-5	9	10	3.30	4.40
-4	-9	10	165.58	12.80
4	-9	-10	195.18	11.30
4	-9	-10	196.28	12.70
-4	9	10	191.48	13.10
-4	9	10	179.58	12.50
4	9	-10	181.68	12.70
3	-9	-10	293.47	15.10
3	-9	-10	268.97	16.20
-3	-9	10	254.67	15.90
-3	9	10	272.07	16.20
-3	9	10	247.68	16.10
3	9	-10	276.27	16.00
2	-9	-10	65.99	5.70
-2	-9	10	54.19	6.30
2	-9	-10	65.19	7.30
-2	9	10	52.99	6.80

-2	9	10	51.69	6.80
2	9	-10	51.29	6.50
-1	-9	10	60.99	6.00
1	-9	-10	66.59	7.20
1	-9	-10	64.29	5.50
-1	9	10	54.49	7.40
1	9	-10	61.89	3.80
-1	9	10	70.89	6.90
1	9	-10	66.39	6.20
1	9	-10	62.39	3.80
0	-9	-10	9.10	2.90
0	-9	-10	11.90	4.10
0	-9	10	5.70	2.30
0	9	10	6.70	4.00
0	9	-10	6.30	1.80
0	9	-10	8.20	1.90
0	9	10	6.30	3.90
0	9	10	9.70	3.60
0	9	-10	9.40	2.70
-1	-9	-10	27.80	4.60
1	9	10	18.40	3.90
1	9	10	19.80	4.60
-1	9	-10	23.00	4.00
-1	9	-10	25.00	3.30
1	9	10	30.50	4.90
-2	-9	-10	237.98	13.10
-2	9	-10	207.98	12.00
2	9	10	230.58	13.80
2	9	10	225.68	13.50
-2	9	-10	219.28	12.40
-2	9	-10	236.98	12.40
-3	-9	-10	44.90	5.60
-3	9	-10	31.60	5.00
3	9	10	31.10	5.60
3	9	10	32.80	6.10
-3	9	-10	36.30	3.80
-3	9	-10	32.40	5.00
-3	9	-10	35.50	3.40
-4	-9	-10	55.39	6.60
4	9	10	54.89	6.20
-4	9	-10	58.89	6.30
-4	9	-10	59.29	4.30
-4	9	-10	57.49	6.40
-4	9	-10	56.19	4.50

4	9	10	60.19	7.10
-5	-9	-10	21.70	5.20
-5	-9	-10	25.00	4.70
-5	9	-10	25.20	3.60
5	9	10	14.80	3.80
-5	9	-10	26.90	4.60
5	9	10	26.20	5.10
-5	9	-10	22.80	3.70
-5	9	-10	28.80	5.00
5	9	10	19.90	5.10
-6	-9	-10	158.68	12.60
6	9	10	167.48	11.10
6	9	10	183.88	12.90
-6	9	-10	156.38	11.60
6	9	10	161.38	11.90
-6	9	-10	163.28	9.60
-6	9	-10	149.59	11.80
-7	-9	-10	4.10	5.00
-7	9	-10	7.30	3.20
7	9	10	16.20	5.40
-7	9	-10	9.90	4.90
7	9	10	10.90	5.00
-8	-9	-10	18.20	6.00
8	9	10	20.50	6.40
-8	9	-10	14.20	4.00
-8	9	-10	10.90	5.30
8	9	10	27.10	6.20
-9	-9	-10	11.10	6.10
-9	9	-10	13.80	4.90
9	9	10	24.80	6.70
9	9	10	19.40	7.00
-9	9	-10	25.90	9.40
-10	-9	-10	46.20	11.30
10	9	10	34.60	7.40
10	9	10	31.80	8.00
-10	9	-10	55.49	9.90
-10	9	-10	27.00	6.10
-11	9	-10	89.69	11.80
-11	9	-10	129.39	12.90
11	9	10	132.29	12.80
-12	-9	-10	29.50	9.40
12	9	10	29.90	7.70
-12	9	-10	25.80	7.80
-12	9	-10	17.60	7.50

-13	-9	-10	33.70	10.20
-13	9	-10	34.60	9.10
-14	-9	-10	25.60	10.40
-14	9	-10	16.10	9.00
-13	10	10	16.10	7.30
13	10	-10	3.90	10.10
-12	10	10	460.15	25.30
12	10	-10	396.16	34.00
11	-10	-10	304.67	22.10
11	10	-10	326.87	23.90
-11	10	10	323.27	19.60
-10	-10	10	536.85	30.00
10	-10	-10	639.24	34.30
-10	10	10	579.14	37.10
-10	10	10	634.84	32.20
10	10	-10	643.34	35.40
9	-10	-10	82.79	11.50
-9	-10	10	74.09	4.40
9	10	-10	64.89	12.80
-9	10	10	65.69	9.20
-9	10	10	76.79	14.50
8	-10	-10	581.34	32.10
-8	10	10	586.64	30.80
8	10	-10	625.64	33.20
-8	10	10	535.75	33.40
7	-10	-10	757.92	38.90
-7	-10	10	658.13	46.00
-7	10	10	701.93	39.70
7	10	-10	747.53	39.30
-7	10	10	775.52	37.90
6	-10	-10	534.35	28.90
-6	-10	10	430.16	29.10
-6	10	10	529.05	29.80
6	10	-10	563.34	29.40
-6	10	10	559.54	28.10
5	-10	-10	2226.78	98.49
-5	-10	10	1685.43	97.89
5	-10	-10	1978.50	97.79
-5	10	10	1977.00	98.29
5	10	-10	2048.40	97.99
-5	10	10	2098.49	97.49
4	-10	-10	300.67	17.60
4	-10	-10	293.67	16.50
-4	-10	10	249.28	17.60

4	10	-10	321.47	18.00
-4	10	10	285.87	18.00
-4	10	10	306.27	17.70
-3	-10	10	582.64	33.00
3	-10	-10	645.24	33.40
3	-10	-10	653.13	32.30
3	10	-10	677.93	33.20
-3	10	10	649.43	33.60
-3	10	10	616.14	33.40
2	-10	-10	313.57	18.40
-2	-10	10	318.17	17.90
2	-10	-10	341.27	17.30
2	10	-10	340.57	18.00
-2	10	10	288.27	18.30
-2	10	10	304.47	18.30
1	-10	-10	1951.20	91.69
1	-10	-10	1974.70	90.59
-1	-10	10	1775.42	90.69
1	10	-10	1954.90	90.89
1	10	-10	1858.81	89.19
-1	10	10	1921.31	91.49
1	10	-10	1831.82	89.19
-1	10	10	1760.52	91.49
0	-10	10	62.29	5.50
0	-10	-10	77.29	7.80
0	-10	-10	53.59	5.50
0	10	10	50.89	7.40
0	10	-10	56.49	4.30
0	10	10	63.19	8.20
0	10	-10	66.79	5.90
0	10	10	52.89	7.00
0	10	-10	57.79	4.30
-1	-10	-10	2586.24	118.99
1	10	10	2540.65	120.29
-1	10	-10	2481.85	118.59
-1	10	-10	2368.36	118.09
-1	10	-10	2415.96	118.29
1	10	10	2442.46	119.49
1	10	10	2511.75	119.79
-2	-10	-10	107.29	7.70
-2	10	-10	91.49	6.90
-2	10	-10	92.09	7.00
2	10	10	94.29	9.20
2	10	10	107.09	8.70

2	10	10	98.39	8.10
-2	10	-10	88.79	6.60
-3	-10	-10	785.42	39.00
-3	10	-10	772.72	37.90
-3	10	-10	812.52	38.20
3	10	10	762.22	39.40
-3	10	-10	815.32	38.80
3	10	10	741.13	39.50
3	10	10	741.83	40.20
-3	10	-10	806.42	38.70
-4	-10	-10	161.38	10.60
-4	10	-10	154.28	10.20
4	10	10	156.68	10.70
4	10	10	159.48	11.30
-4	10	-10	150.98	9.00
-4	10	-10	160.18	10.40
-4	10	-10	151.28	8.80
-5	-10	-10	2598.24	129.09
-5	10	-10	2697.63	128.19
5	10	10	2615.24	128.49
5	10	10	2589.24	128.29
-5	10	-10	2646.54	126.89
-5	10	-10	2763.72	126.99
-5	10	-10	2724.43	127.99
-6	-10	-10	244.38	16.50
6	10	10	259.07	15.70
-6	10	-10	224.58	15.40
-6	10	-10	253.67	15.20
-6	10	-10	252.67	13.60
-7	-10	-10	747.53	41.20
7	10	10	736.13	40.20
-7	10	-10	800.42	38.80
-7	10	-10	776.62	40.00
7	10	10	850.01	41.50
-8	-10	-10	406.06	25.30
-8	10	-10	417.66	22.60
8	10	10	405.66	24.20
-8	10	-10	430.86	24.00
8	10	10	466.25	25.40
-9	-10	-10	178.48	15.70
-9	10	-10	164.08	13.60
9	10	10	205.88	15.50
-9	10	-10	160.78	12.30
9	10	10	176.98	13.90

-10	10	-10	321.37	19.20
10	10	10	270.47	18.50
-10	10	-10	280.07	18.00
11	10	10	284.97	19.40
-11	10	-10	284.17	19.10
-11	10	-10	263.17	18.40
12	10	10	600.04	34.60
-12	10	-10	632.64	34.60
-13	-10	-10	65.09	11.70
-13	10	-10	49.80	9.70
-13	11	10	42.50	8.50
13	11	-10	55.19	12.40
-12	11	10	11.20	7.20
12	11	-10	16.00	10.20
11	-11	-10	143.19	14.90
-11	11	10	132.29	12.80
11	11	-10	125.49	18.20
10	-11	-10	102.49	13.10
10	11	-10	92.29	15.00
-10	11	10	105.09	10.90
9	-11	-10	43.20	8.10
-9	11	10	92.29	15.60
-9	11	10	55.49	8.70
9	11	-10	86.19	12.90
8	-11	-10	84.59	11.00
-8	11	10	65.19	13.70
8	11	-10	107.39	12.70
7	-11	-10	22.10	6.10
-7	11	10	16.80	5.80
-7	11	10	25.50	8.30
7	11	-10	33.90	7.60
6	-11	-10	232.48	15.70
-6	11	10	229.28	17.00
-6	11	10	228.38	15.10
6	11	-10	237.28	16.40
5	-11	-10	110.49	10.70
-5	-11	10	99.19	11.30
5	11	-10	122.89	11.40
-5	11	10	109.89	10.20
-5	11	10	111.49	11.50
4	-11	-10	190.48	13.60
-4	-11	10	166.98	13.90
-4	11	10	196.68	13.60
4	11	-10	218.58	14.00

-4	11	10	189.88	14.10
-3	-11	10	109.59	10.00
3	-11	-10	101.69	8.40
3	-11	-10	120.59	10.10
-3	11	10	105.19	10.00
3	11	-10	131.39	9.90
-3	11	10	86.19	9.50
-2	-11	10	379.56	22.00
2	-11	-10	420.86	22.90
2	-11	-10	416.26	21.40
-2	11	10	371.46	22.50
-2	11	10	398.26	22.70
2	11	-10	441.36	22.20
-1	-11	10	-0.80	2.90
1	-11	-10	0.40	3.10
1	-11	-10	-2.20	3.60
-1	11	10	0.10	3.50
1	11	-10	0.40	1.40
1	11	-10	2.50	1.20
-1	11	10	-3.90	4.10
1	11	-10	-1.60	2.90
0	-11	-10	111.79	8.20
0	-11	10	116.69	7.90
0	11	-10	98.59	6.50
0	11	-10	133.39	8.40
0	11	-10	99.69	6.40
0	11	10	122.09	10.60
0	11	10	104.99	9.20
0	11	10	103.39	9.30
-1	-11	-10	35.80	5.40
-1	11	-10	24.00	4.10
1	11	10	28.90	5.10
1	11	10	37.90	6.90
1	11	10	29.70	5.90
-1	11	-10	29.80	5.00
-1	11	-10	29.20	4.10
-2	-11	-10	149.19	10.20
-2	11	-10	147.29	9.40
2	11	10	178.88	12.50
2	11	10	147.29	10.70
-2	11	-10	139.79	9.20
-2	11	-10	167.28	9.70
2	11	10	146.79	10.80
-3	-11	-10	182.78	10.90

3	11	10	156.48	12.60
3	11	10	149.29	11.20
-3	11	-10	161.08	9.90
-3	11	-10	168.58	10.40
3	11	10	165.78	11.70
-3	11	-10	165.08	9.50
-3	11	-10	162.88	10.20
-4	11	-10	705.93	32.90
-4	11	-10	634.44	33.50
-4	11	-10	623.64	32.50
4	11	10	739.13	35.60
-4	11	-10	664.33	33.50
4	11	10	636.74	34.20
4	11	10	638.24	34.10
-5	-11	-10	64.59	9.00
-5	11	-10	54.39	5.40
-5	11	-10	56.39	7.50
-5	11	-10	57.89	5.50
5	11	10	52.79	7.40
5	11	10	40.40	7.80
5	11	10	66.89	7.90
-5	11	-10	55.89	7.30
-6	-11	-10	53.49	9.40
-6	11	-10	41.40	7.60
-6	11	-10	60.69	5.90
6	11	10	43.00	7.50
-6	11	-10	61.19	7.90
-7	-11	-10	17.90	6.40
-7	11	-10	19.60	5.50
7	11	10	13.90	5.70
-7	11	-10	17.70	4.00
-8	-11	-10	106.09	12.40
8	11	10	68.39	11.80
-8	11	-10	92.39	8.80
-8	11	-10	99.99	10.30
-9	-11	-10	37.10	8.10
-9	11	-10	22.10	6.10
9	11	10	20.20	6.90
9	11	10	8.40	6.00
-9	11	-10	13.00	5.10
-10	11	-10	30.90	6.30
-10	11	-10	36.30	7.40
10	11	10	35.90	7.90
11	11	10	53.49	9.00

-11	11	-10	60.59	11.30
-11	11	-10	79.69	11.60
-12	11	-10	68.09	12.50
12	11	10	31.00	8.30
-13	-11	-10	12.90	10.40
-13	11	-10	12.10	7.70
13	12	-10	6.80	12.10
-13	12	10	6.10	7.40
12	12	-10	205.38	21.60
-12	12	10	209.08	16.00
-11	12	10	196.18	15.10
11	12	-10	201.58	20.30
10	-12	-10	567.14	32.60
-10	12	10	603.54	30.70
10	12	-10	499.85	34.30
9	-12	-10	126.09	14.10
-9	12	10	119.29	17.60
9	12	-10	136.29	15.20
-9	12	10	146.89	12.20
8	-12	-10	285.17	19.10
-8	12	10	254.97	21.30
-8	12	10	275.97	17.50
8	12	-10	278.77	19.90
7	-12	-10	509.35	28.50
7	12	-10	526.25	29.30
-7	12	10	477.55	29.90
-7	12	10	498.75	27.80
6	-12	-10	224.28	16.20
6	12	-10	246.58	17.40
-6	12	10	238.48	15.70
-6	12	10	247.28	17.50
5	-12	-10	616.44	33.00
-5	12	10	587.74	33.60
5	12	-10	624.14	33.30
-5	12	10	627.54	32.80
4	-12	-10	53.19	8.50
-4	-12	10	30.40	7.10
4	12	-10	42.40	8.60
-4	12	10	51.69	7.50
-4	12	10	35.50	6.40
3	-12	-10	666.83	33.50
-3	-12	10	557.64	32.70
3	12	-10	622.44	32.80
-3	12	10	633.84	33.40

-3	12	10	643.74	33.30
2	-12	-10	17.60	4.90
-2	-12	10	15.50	4.40
2	-12	-10	16.00	4.10
-2	12	10	12.20	5.40
-2	12	10	13.50	4.80
2	12	-10	17.40	4.50
1	-12	-10	1267.87	60.39
-1	-12	10	1108.29	60.49
1	-12	-10	1257.97	61.69
-1	12	10	1307.87	62.59
1	12	-10	1209.48	58.89
-1	12	10	1238.48	61.69
1	12	-10	1281.77	60.79
1	12	-10	1187.28	58.69
-1	12	10	1256.67	61.49
0	-12	-10	28.30	4.50
0	-12	10	19.20	3.70
0	12	10	32.50	5.20
0	12	10	12.90	5.30
0	12	10	15.90	5.70
0	12	-10	20.90	2.90
0	12	-10	21.90	4.10
-1	-12	-10	2160.28	101.59
1	12	10	2126.79	102.59
-1	12	-10	2144.19	100.79
-1	12	-10	2042.90	100.59
1	12	10	2115.79	103.29
-1	12	-10	2116.89	101.49
1	12	10	2041.10	102.29
-2	-12	-10	17.70	4.10
2	12	10	34.20	6.70
-2	12	-10	15.80	3.20
2	12	10	13.30	4.70
-2	12	-10	15.70	3.40
-2	12	-10	17.40	3.60
-2	12	-10	12.50	3.00
2	12	10	11.00	4.10
-3	12	-10	517.85	25.70
3	12	10	491.05	26.90
-3	12	-10	491.85	25.30
3	12	10	511.45	27.10
-3	12	-10	498.95	25.80
3	12	10	492.55	28.00

-3	12	-10	507.85	25.70
4	12	10	239.18	15.10
-4	12	-10	244.78	14.20
4	12	10	241.08	16.60
-4	12	-10	240.48	13.50
4	12	10	221.68	15.00
-4	12	-10	226.38	13.00
-4	12	-10	251.47	13.90
-5	-12	-10	338.47	21.20
5	12	10	315.37	20.10
-5	12	-10	347.97	19.40
-5	12	-10	353.16	19.60
-5	12	-10	345.67	18.30
-5	12	-10	351.16	18.50
5	12	10	369.46	21.70
5	12	10	366.66	20.30
-6	-12	-10	683.73	38.30
-6	12	-10	730.73	35.70
-6	12	-10	709.23	35.60
-6	12	-10	719.03	36.70
6	12	10	749.13	38.70
6	12	10	697.53	37.20
-7	-12	-10	286.67	20.20
7	12	10	284.57	18.70
-7	12	-10	318.37	18.60
-7	12	-10	331.87	17.30
-8	-12	-10	91.49	12.90
-8	12	-10	101.49	9.40
-8	12	-10	117.69	11.10
8	12	10	106.49	11.40
-9	12	-10	112.09	11.60
9	12	10	104.69	11.90
-9	12	-10	103.29	10.50
-10	12	-10	315.67	19.30
10	12	10	291.07	19.30
-10	12	-10	262.87	18.20
11	12	10	79.99	12.70
-11	12	-10	109.89	12.70
-11	12	-10	88.59	12.20
-12	12	-10	114.69	14.00
12	12	10	111.49	14.20
-13	-12	-10	51.49	12.20
-13	12	-10	56.59	10.60
-13	13	10	218.68	17.80

13	13	-10	238.58	23.70
-12	13	10	32.00	8.10
12	13	-10	29.80	11.40
11	13	-10	504.45	32.50
-11	13	10	490.55	28.30
10	-13	-10	189.78	17.40
10	13	-10	201.38	19.50
-10	13	10	199.58	15.10
9	-13	-10	389.16	24.40
9	13	-10	360.06	28.20
-9	13	10	392.56	22.80
-9	13	10	398.96	32.50
8	-13	-10	171.88	14.70
-8	13	10	158.78	12.70
-8	13	10	153.58	17.20
8	13	-10	185.48	16.40
7	-13	-10	440.46	26.10
7	13	-10	471.15	26.80
-7	13	10	429.26	24.80
-7	13	10	425.86	27.70
6	-13	-10	657.53	36.40
-6	13	10	681.93	35.90
-6	13	10	654.93	37.50
6	13	-10	725.63	37.30
5	-13	-10	287.97	18.10
-5	13	10	257.17	17.50
5	13	-10	297.17	18.60
-5	13	10	258.77	18.70
4	-13	-10	724.63	38.30
4	13	-10	745.43	38.30
-4	13	10	736.73	38.10
-4	13	10	695.73	38.50
3	-13	-10	97.09	10.40
3	13	-10	121.29	10.30
-3	13	10	99.89	10.50
-3	13	10	92.19	9.80
2	-13	-10	1333.87	66.99
-2	13	10	1292.57	66.79
-2	13	10	1327.67	66.89
2	13	-10	1409.36	66.39
-1	-13	10	44.90	6.80
1	-13	-10	52.49	8.60
-1	13	10	38.40	8.00
1	13	-10	51.29	7.10

1	13	-10	43.10	3.60
-1	13	10	51.69	7.80
1	13	-10	44.00	3.30
-1	13	10	43.90	6.90
0	13	10	1768.52	85.19
0	13	-10	1662.53	81.89
0	13	-10	1770.62	83.39
0	13	10	1685.13	84.19
0	13	-10	1682.33	81.99
0	13	10	1710.13	84.19
1	13	10	3.60	4.30
1	13	10	-1.90	4.60
-1	13	-10	-2.10	2.60
-1	13	-10	0.80	2.40
-1	13	-10	-1.30	3.10
1	13	10	0.40	5.70
-2	13	-10	988.90	46.50
2	13	10	890.11	47.30
-2	13	-10	895.61	45.90
2	13	10	957.80	47.50
2	13	10	1010.50	48.90
-2	13	-10	888.51	45.60
-2	13	-10	920.61	45.90
-3	13	-10	0.50	3.30
3	13	10	-2.20	4.40
-3	13	-10	1.30	3.10
3	13	10	6.10	4.00
3	13	10	-4.40	4.90
-3	13	-10	-2.40	3.10
-3	13	-10	2.60	2.70
4	13	10	312.47	19.30
-4	13	-10	311.37	17.40
4	13	10	296.97	19.00
-4	13	-10	342.77	18.00
4	13	10	349.87	21.10
-4	13	-10	335.07	18.30
-4	13	-10	357.56	18.20
5	13	10	120.69	12.80
5	13	10	138.39	11.40
-5	13	-10	150.28	9.40
-5	13	-10	132.29	8.90
-5	13	-10	136.49	10.10
-5	13	-10	103.29	10.30
-6	-13	-10	517.65	30.10

-6	13	-10	524.45	27.10
-6	13	-10	521.75	28.00
6	13	10	498.35	28.70
6	13	10	582.34	30.60
-7	-13	-10	127.19	13.80
7	13	10	140.49	14.00
7	13	10	138.59	13.60
-7	13	-10	145.99	10.40
-7	13	-10	133.79	11.60
-8	-13	-10	284.57	20.40
8	13	10	254.87	17.70
-8	13	-10	252.37	22.40
-8	13	-10	304.47	17.00
8	13	10	290.87	20.20
9	13	10	84.49	11.70
-9	13	-10	91.29	10.40
-9	13	-10	117.69	11.60
-10	13	-10	247.68	17.20
10	13	10	240.68	18.00
-10	13	-10	266.97	18.10
11	13	10	234.98	17.50
-11	13	-10	183.78	18.40
-11	13	-10	233.98	17.10
12	13	10	261.57	19.90
-12	13	-10	267.77	19.50
-13	13	-10	122.89	15.00
13	13	10	101.69	15.40
-12	14	10	2.80	7.40
12	14	-10	0.40	10.90
11	14	-10	9.90	9.90
-11	14	10	24.70	7.80
10	-14	-10	37.10	8.70
10	14	-10	21.30	9.70
-10	14	10	32.20	7.40
9	-14	-10	13.50	7.50
9	14	-10	16.70	8.30
-9	14	10	22.90	6.50
8	-14	-10	-8.50	6.10
-8	14	10	-1.20	8.30
-8	14	10	-5.10	5.90
7	-14	-10	24.00	6.60
-7	14	10	24.00	8.20
7	14	-10	15.40	7.30
-7	14	10	22.10	6.30

6	-14	-10	10.10	6.10
6	14	-10	-0.40	6.60
-6	14	10	7.00	6.20
-6	14	10	4.00	7.20
5	-14	-10	87.49	10.40
-5	14	10	84.59	10.50
5	14	-10	61.29	10.90
-5	14	10	95.39	11.90
4	-14	-10	18.80	5.90
-4	14	10	28.20	7.20
-4	14	10	23.00	6.40
4	14	-10	12.10	5.60
3	-14	-10	11.10	5.70
-3	14	10	0.60	5.70
-3	14	10	8.40	6.00
3	14	-10	2.80	5.10
2	-14	-10	9.00	5.30
-2	14	10	-2.40	4.90
-2	14	10	16.80	5.40
2	14	-10	2.00	4.60
1	14	-10	3.40	1.50
-1	14	10	0.90	5.60
-1	14	10	-4.90	4.80
1	14	-10	8.90	4.30
1	14	-10	5.50	1.90
-1	14	10	7.30	4.70
0	14	10	9.60	6.30
0	14	10	12.00	5.00
0	14	10	10.00	5.70
0	14	-10	12.80	4.40
0	14	-10	4.10	4.00
0	14	-10	7.70	2.50
1	14	10	24.80	6.90
1	14	10	22.70	5.40
-1	14	-10	23.50	4.80
-1	14	-10	30.20	4.30
-1	14	-10	23.70	3.70
1	14	10	25.70	5.90
-2	14	-10	7.80	3.80
-2	14	-10	6.90	3.30
-2	14	-10	5.20	2.90
2	14	10	8.50	4.80
2	14	10	6.60	5.10
-2	14	-10	4.70	3.30

-3	14	-10	97.89	8.50
3	14	10	100.09	10.40
-3	14	-10	101.49	8.70
3	14	10	107.69	12.40
-3	14	-10	122.59	8.20
-3	14	-10	107.89	8.10
3	14	10	121.39	10.60
-4	14	-10	95.19	8.80
4	14	10	105.99	10.40
-4	14	-10	127.49	8.90
-4	14	-10	110.59	8.80
4	14	10	91.49	10.30
4	14	10	100.99	12.50
-4	14	-10	103.89	8.00
5	14	10	173.18	13.00
-5	14	-10	154.58	14.50
-5	14	-10	187.38	11.90
-5	14	-10	168.08	10.70
-5	14	-10	138.99	12.00
5	14	10	166.18	14.90
-6	-14	-10	21.80	6.60
-6	14	-10	12.80	5.20
6	14	10	18.70	5.70
6	14	10	11.20	6.80
-6	14	-10	10.20	4.00
-6	14	-10	17.70	4.30
-7	-14	-10	185.48	16.10
7	14	10	179.78	16.30
7	14	10	187.18	14.30
-7	14	-10	168.88	12.20
-7	14	-10	188.18	12.50
-7	14	-10	188.68	13.40
8	14	10	21.30	6.60
-8	14	-10	28.30	6.30
-8	14	-10	23.80	5.50
8	14	10	9.90	7.80
-9	14	-10	12.50	6.20
9	14	10	13.00	6.70
-9	14	-10	7.60	5.60
-10	14	-10	30.80	7.50
-10	14	-10	68.39	11.30
10	14	10	46.80	8.70
-11	14	-10	14.20	7.10
11	14	10	3.00	7.20

12	14	10	55.29	10.20
-12	14	-10	51.39	9.60
12	15	-10	53.99	13.30
-12	15	10	45.50	8.80
11	15	-10	356.26	28.00
-11	15	10	364.36	22.80
10	15	-10	190.78	20.20
-10	15	10	201.98	16.10
9	-15	-10	192.58	17.00
9	15	-10	164.08	18.40
-9	15	10	185.68	15.10
8	-15	-10	53.79	9.50
-8	15	10	41.60	8.10
8	15	-10	37.50	9.10
7	-15	-10	348.77	23.00
-7	15	10	371.76	25.40
-7	15	10	381.26	22.30
7	15	-10	358.06	24.60
6	-15	-10	897.61	47.80
-6	15	10	854.21	48.60
-6	15	10	981.80	47.30
5	-15	-10	453.05	26.70
-5	15	10	420.56	27.20
5	15	-10	499.55	27.20
-5	15	10	459.35	26.50
4	-15	-10	798.02	42.80
-4	15	10	774.12	43.10
4	15	-10	810.32	42.70
-4	15	10	870.71	43.00
3	-15	-10	581.24	31.50
-3	15	10	558.64	31.50
3	15	-10	571.94	30.80
-3	15	10	548.05	31.10
2	-15	-10	539.55	30.20
-2	15	10	533.95	30.00
-2	15	10	520.65	29.90
2	15	-10	551.14	29.30
-2	15	10	553.94	31.20
1	15	-10	163.58	8.90
1	15	-10	187.28	12.70
1	15	-10	171.98	9.20
-1	15	10	152.38	13.20
-1	15	10	196.28	15.20
-1	15	10	161.58	13.60

0	15	-10	1835.72	87.79
0	15	-10	1766.02	85.69
0	15	10	1824.62	88.59
0	15	10	1727.53	88.39
0	15	-10	1772.72	86.09
0	15	10	1850.91	89.69
-1	15	-10	17.90	3.70
-1	15	-10	23.30	4.20
1	15	10	20.10	5.50
1	15	10	14.10	6.30
1	15	10	15.90	6.10
-1	15	-10	24.00	4.90
-2	15	-10	828.12	41.40
2	15	10	773.22	42.20
2	15	10	863.41	44.10
-2	15	-10	793.82	40.10
-2	15	-10	819.52	40.50
-2	15	-10	794.12	40.80
2	15	10	852.01	42.50
-3	15	-10	20.20	4.60
3	15	10	21.60	6.00
-3	15	-10	23.90	4.30
3	15	10	20.00	7.50
3	15	10	20.50	5.50
-3	15	-10	25.20	4.90
-3	15	-10	26.90	4.40
-4	15	-10	1201.68	58.59
-4	15	-10	1130.69	58.89
4	15	10	1202.98	59.99
-4	15	-10	1144.79	58.49
4	15	10	1309.47	62.69
-5	15	-10	227.88	14.40
-5	15	-10	220.38	14.40
-5	15	-10	228.48	14.00
5	15	10	235.98	18.50
5	15	10	254.57	16.10
-6	-15	-10	1508.35	78.99
6	15	10	1707.93	79.69
-6	15	-10	1585.94	76.69
6	15	10	1499.25	81.19
-6	15	-10	1608.94	76.59
-6	15	-10	1480.25	76.19
7	15	10	171.68	14.10
-7	15	-10	170.08	13.20

-7	15	-10	176.28	12.40
7	15	10	180.18	16.80
-7	15	-10	166.78	12.40
-8	15	-10	142.09	12.00
-8	15	-10	149.69	11.60
8	15	10	124.29	13.10
-8	15	-10	151.58	12.60
-9	15	-10	135.39	12.50
-9	15	-10	119.29	12.20
9	15	10	105.09	12.80
10	15	10	250.57	18.90
-10	15	-10	253.87	18.30
-10	15	-10	287.67	18.70
-11	15	-10	419.16	24.60
11	15	10	361.26	24.90
12	15	10	202.68	18.60
-12	15	-10	236.98	18.50
-11	16	10	12.00	7.90
11	16	-10	24.70	11.50
-10	16	10	96.99	11.40
10	16	-10	57.19	12.00
9	-16	-10	25.90	9.60
9	16	-10	24.40	9.40
-9	16	10	24.70	7.80
8	-16	-10	85.59	13.60
-8	16	10	95.49	11.20
8	16	-10	112.79	15.60
7	-16	-10	86.89	12.80
-7	16	10	64.29	11.30
-7	16	10	81.49	11.50
7	16	-10	90.89	14.10
6	-16	-10	4.80	6.30
6	16	-10	-2.30	6.70
-6	16	10	3.60	6.00
-6	16	10	15.30	7.90
5	-16	-10	113.89	12.90
-5	16	10	123.09	12.70
5	16	-10	146.79	13.70
-5	16	10	112.29	14.50
4	-16	-10	184.88	15.20
-4	16	10	198.88	15.30
-4	16	10	195.68	16.20
3	-16	-10	294.57	20.00
3	16	-10	300.57	24.70

-3	16	10	307.27	20.20
-2	16	10	13.30	6.80
2	16	-10	20.70	6.00
-2	16	10	22.20	6.70
-2	16	10	22.50	7.40
1	16	-10	29.00	3.30
1	16	-10	21.80	3.70
1	16	-10	24.00	5.70
-1	16	10	20.60	6.80
-1	16	10	15.60	6.10
-1	16	10	22.90	7.20
0	16	-10	1.20	4.60
0	16	-10	1.40	2.70
0	16	10	0.20	5.80
0	16	10	-0.40	6.10
0	16	10	1.00	5.40
-1	16	-10	129.49	8.30
-1	16	-10	132.39	14.00
1	16	10	119.19	11.80
1	16	10	165.78	15.10
1	16	10	148.99	12.50
-1	16	-10	130.49	9.00
2	16	10	75.19	12.20
2	16	10	70.99	10.30
-2	16	-10	83.59	7.90
2	16	10	85.99	10.50
-2	16	-10	93.39	6.90
3	-16	10	675.93	34.90
3	16	10	674.63	38.40
-3	16	-10	667.73	34.30
3	16	10	677.33	36.40
-3	16	-10	651.53	34.90
3	16	10	705.13	38.50
-3	16	-10	759.12	35.80
4	-16	10	24.40	5.60
4	16	10	13.90	5.90
4	16	10	20.40	8.80
-4	16	-10	20.90	4.70
-4	16	-10	25.00	5.70
-4	16	-10	21.70	4.60
-4	16	-10	24.10	5.20
-5	16	-10	43.40	7.20
-5	16	-10	59.99	8.30
-5	16	-10	57.79	8.00

5	16	10	40.00	11.80
-6	16	-10	66.59	8.30
-6	16	-10	67.89	8.90
-6	16	-10	70.99	9.10
6	16	10	77.89	10.50
6	16	10	49.40	10.30
-7	16	-10	180.68	13.50
-7	16	-10	194.18	13.70
7	16	10	214.48	18.90
7	16	10	179.78	15.00
8	16	10	-2.60	6.10
-8	16	-10	6.10	5.10
-8	16	-10	-2.10	5.30
-8	16	-10	6.20	5.90
9	16	10	13.80	7.40
-9	16	-10	39.30	7.80
-9	16	-10	35.60	7.30
-10	16	-10	38.70	8.40
10	16	10	21.10	8.30
11	16	10	14.10	8.10
-11	16	-10	25.90	7.90
-11	17	10	345.07	21.80
11	17	-10	307.17	27.40
-10	17	10	157.28	15.70
10	17	-10	173.68	21.00
9	17	-10	245.88	22.80
-9	17	10	309.87	19.80
8	-17	-10	101.69	14.30
-8	17	10	120.89	13.40
8	17	-10	118.19	16.80
7	-17	-10	42.20	9.10
7	17	-10	53.49	10.70
-7	17	10	41.40	8.20
6	-17	-10	364.96	24.40
-6	17	10	401.46	23.60
-6	17	10	372.46	25.70
6	17	-10	400.76	25.20
5	-17	-10	108.79	12.80
5	17	-10	76.19	12.80
-5	17	10	83.29	13.90
-5	17	10	91.49	12.00
4	-17	-10	266.77	19.30
4	17	-10	284.37	19.30
-4	17	10	290.17	19.40

-4	17	10	285.57	20.50
3	-17	-10	-4.50	5.80
-3	17	10	-6.50	5.80
-3	17	10	1.60	7.10
3	17	-10	4.20	5.60
-3	17	10	-0.60	6.00
-2	17	10	221.58	17.20
2	17	-10	268.47	17.40
-2	17	10	272.57	19.90
-2	17	10	262.37	18.50
2	17	-10	251.77	12.60
-1	17	10	5.00	6.70
1	17	-10	2.80	2.60
-1	17	10	-4.30	5.90
1	17	-10	5.30	5.20
0	17	10	515.75	28.90
0	17	-10	507.05	25.50
0	17	10	485.25	30.20
0	17	-10	507.95	28.00
0	17	-10	503.65	25.10
0	17	10	518.45	29.80
1	-17	10	326.37	17.00
1	17	10	309.77	21.60
1	17	10	297.07	19.30
-1	17	-10	302.37	16.10
1	17	10	288.77	19.80
-1	17	-10	290.17	18.10
-1	17	-10	322.77	18.90
2	-17	10	561.44	28.20
2	17	10	580.14	32.20
-2	17	-10	538.35	29.30
-2	17	-10	528.45	28.50
2	17	10	508.75	30.30
2	17	10	550.64	32.20
-2	17	-10	547.35	27.40
3	-17	10	3.40	4.60
3	17	10	-13.20	7.60
-3	17	-10	6.10	4.00
-3	17	-10	3.00	3.70
-3	17	-10	-0.10	4.70
4	-17	10	372.96	21.20
-4	17	-10	391.26	21.70
-4	17	-10	339.97	20.60
4	17	10	392.86	24.90

4	17	10	358.56	22.20
-4	17	-10	390.36	20.40
5	17	10	42.20	10.60
5	17	10	40.70	7.80
-5	17	-10	42.70	6.10
-5	17	-10	41.50	6.60
-5	17	-10	36.90	5.70
-6	17	-10	399.36	22.30
-6	17	-10	350.16	21.40
6	17	10	392.46	26.70
-6	17	-10	378.16	21.70
6	17	10	397.56	23.20
7	17	10	80.69	12.00
-7	17	-10	88.99	10.30
-7	17	-10	98.99	11.00
-7	17	-10	105.39	10.80
-8	17	-10	155.18	12.90
8	17	10	107.29	13.40
-8	17	-10	136.89	12.80
-8	17	-10	138.49	12.50
-9	17	-10	146.39	13.10
9	17	10	117.59	14.50
-9	17	-10	127.09	13.60
10	17	10	74.49	12.90
-10	17	-10	75.99	12.50
-11	17	-10	208.78	17.90
11	17	10	223.58	18.60
10	18	-10	342.97	27.10
-10	18	10	301.87	21.70
9	18	-10	43.50	11.60
-9	18	10	69.39	12.70
-8	18	10	288.17	20.30
8	18	-10	313.67	23.60
7	-18	-10	104.29	14.70
7	18	-10	114.59	16.10
-7	18	10	98.59	13.10
6	-18	-10	167.18	16.20
-6	18	10	173.48	15.30
6	18	-10	183.08	17.50
5	-18	-10	424.76	26.70
-5	18	10	374.16	27.20
-5	18	10	462.85	26.00
5	18	-10	445.16	26.80
4	-18	-10	166.88	15.80

4	18	-10	194.28	15.50
-4	18	10	140.19	16.20
-4	18	10	168.68	15.10
-3	18	10	366.66	25.00
-3	18	10	378.76	23.60
3	18	-10	418.36	24.20
-3	18	10	397.36	26.60
2	18	-10	78.39	5.00
-2	18	10	88.89	12.40
2	18	-10	92.79	11.50
2	18	-10	74.39	4.70
-2	18	10	77.69	12.50
-2	18	10	79.09	11.90
-1	18	10	1424.96	69.49
-1	18	10	1256.17	67.59
-1	18	10	1320.97	67.39
1	18	-10	1270.67	63.99
1	18	-10	1416.16	67.29
0	18	-10	7.20	5.40
0	18	10	1.40	6.80
0	18	10	5.00	6.60
0	18	-10	4.00	2.80
0	18	10	-1.00	7.90
1	-18	10	1055.59	50.59
-1	18	-10	945.21	51.49
1	18	10	982.90	53.09
-1	18	-10	1008.50	49.90
1	18	10	1080.29	54.99
-1	18	-10	1095.29	52.49
1	18	10	1036.60	52.99
2	-18	10	18.60	4.90
2	18	10	4.80	6.20
2	18	10	14.20	7.80
-2	18	-10	10.20	4.40
-2	18	-10	11.10	5.50
-2	18	-10	14.80	3.90
3	-18	10	505.95	27.10
-3	18	-10	558.44	28.30
3	18	10	499.85	28.70
3	18	10	536.05	31.50
-3	18	-10	459.95	27.00
-3	18	-10	496.05	26.10
4	18	10	53.29	11.10
-4	18	-10	45.00	6.30

-4	18	-10	34.10	5.50
4	18	10	41.60	7.90
-4	18	-10	43.10	7.00
-5	18	-10	254.77	16.00
5	18	10	242.58	17.60
-5	18	-10	224.28	16.00
5	18	10	289.17	22.00
-5	18	-10	303.37	17.50
-6	18	-10	155.58	12.70
-6	18	-10	148.49	12.60
-6	18	-10	179.18	13.80
6	18	10	183.58	14.80
6	18	10	208.78	18.20
7	18	10	325.47	21.60
-7	18	-10	325.37	20.10
-7	18	-10	309.37	20.20
-7	18	-10	385.46	21.20
-8	18	-10	254.47	19.90
8	18	10	228.28	18.10
-8	18	-10	248.38	17.20
-8	18	-10	244.78	16.90
9	18	10	115.49	14.10
-9	18	-10	116.89	12.70
-9	18	-10	120.79	13.20
-10	18	-10	158.48	14.90
-10	18	-10	150.58	14.60
10	18	10	130.09	15.70
-10	19	10	25.00	9.40
10	19	-10	19.60	12.10
9	19	-10	20.80	11.10
-9	19	10	-3.40	7.40
8	19	-10	17.40	9.90
-8	19	10	22.10	8.40
7	-19	-10	35.80	12.30
7	19	-10	63.79	11.80
-7	19	10	24.30	8.40
6	-19	-10	33.80	9.10
6	19	-10	39.80	10.00
-6	19	10	67.19	12.20
5	-19	-10	120.59	14.70
-5	19	10	91.99	13.00
5	19	-10	91.79	14.60
-4	19	10	17.40	7.50
4	19	-10	8.80	7.70

-4	19	10	23.30	10.60
-4	19	10	16.70	8.30
-3	19	10	13.70	7.30
-3	19	10	14.60	8.90
-3	19	10	6.00	8.00
3	19	-10	14.40	6.70
-2	19	10	31.70	8.40
2	19	-10	29.30	3.20
-2	19	10	17.00	8.20
2	19	-10	41.10	8.10
-2	19	10	24.00	10.20
1	19	-10	1.00	2.60
-1	19	10	-4.10	6.90
-1	19	10	-11.50	6.70
1	19	-10	-0.20	5.70
0	-19	10	11.10	4.10
0	19	10	21.10	8.50
0	19	-10	10.00	3.10
0	19	-10	22.00	6.60
0	19	10	14.20	7.90
0	19	10	5.70	6.50
1	-19	10	13.10	4.50
1	19	10	2.50	6.70
-1	19	-10	-3.60	5.00
-1	19	-10	5.70	3.40
1	19	10	-15.30	8.90
-1	19	-10	1.50	5.80
2	-19	10	68.49	8.30
2	19	10	48.80	8.60
-2	19	-10	55.39	6.70
-2	19	-10	51.19	9.80
2	19	10	35.60	9.50
-2	19	-10	43.50	8.20
3	-19	10	17.00	5.80
-3	19	-10	3.70	5.60
-3	19	-10	8.90	4.10
-3	19	-10	4.80	4.70
3	19	10	3.40	7.90
3	19	10	5.80	6.90
4	19	10	77.99	14.80
-4	19	-10	52.99	10.40
4	19	10	60.99	11.10
-4	19	-10	65.99	7.90
-4	19	-10	57.39	8.90

5	19	10	32.80	7.90
-5	19	-10	19.10	6.40
-5	19	-10	45.90	8.00
-5	19	-10	22.30	5.70
5	19	10	20.90	9.30
-6	19	-10	82.69	11.30
-6	19	-10	59.09	9.20
6	19	10	79.09	11.60
-6	19	-10	53.69	9.80
-7	19	-10	3.90	6.20
-7	19	-10	3.00	5.90
7	19	10	5.20	6.80
-8	19	-10	0.10	6.10
-8	19	-10	0.60	6.60
8	19	10	12.40	7.30
9	19	10	0.00	7.60
-9	19	-10	9.00	7.10
-9	19	-10	6.90	7.30
-10	19	-10	-6.20	7.10
-10	19	-10	8.40	8.10
10	19	10	6.50	8.30
9	20	-10	63.89	13.40
-9	20	10	87.79	14.10
-8	20	10	450.15	26.90
8	20	-10	401.06	30.10
-7	20	10	417.76	26.00
7	20	-10	401.66	28.50
6	-20	-10	156.68	18.10
6	20	-10	165.38	18.00
-6	20	10	162.08	15.60
5	-20	-10	1052.09	56.89
5	20	-10	1069.79	56.59
-5	20	10	1088.19	55.79
-4	20	10	129.69	14.10
4	20	-10	148.29	15.70
-4	20	10	152.38	19.30
-3	20	10	276.77	19.60
-3	20	10	251.47	22.50
-3	20	10	233.38	22.10
3	20	-10	281.27	19.80
-2	20	10	85.49	11.40
2	20	-10	67.79	12.30
-2	20	10	82.59	13.30
-2	20	10	54.89	10.80

-1	20	10	870.21	44.30
-1	20	10	777.22	44.50
1	20	-10	847.02	43.80
1	20	-10	773.22	39.70
-1	20	10	843.22	46.70
0	20	10	1.20	7.60
0	20	-10	6.30	5.20
0	20	-10	6.80	3.20
0	20	10	-0.70	10.10
0	20	-10	10.40	6.80
-1	20	-10	912.91	45.40
-1	20	-10	849.32	47.30
1	20	10	985.00	51.59
1	20	10	935.61	48.50
-1	20	-10	963.40	48.50
2	-20	10	29.40	6.50
2	20	10	25.20	10.40
2	20	10	28.10	8.20
-2	20	-10	24.40	7.20
-2	20	-10	35.80	6.30
-2	20	-10	33.00	6.00
3	-20	10	452.75	24.30
3	20	10	464.55	26.50
3	20	10	450.75	29.50
-3	20	-10	370.76	24.40
-3	20	-10	466.45	26.00
-3	20	-10	437.06	23.30
4	20	10	33.80	11.20
-4	20	-10	42.30	7.70
-4	20	-10	36.60	6.00
-4	20	-10	24.90	6.00
4	20	10	49.80	8.80
5	20	10	665.73	36.00
-5	20	-10	622.24	37.90
-5	20	-10	643.64	33.90
-5	20	-10	678.23	35.80
-6	20	-10	157.38	13.30
-6	20	-10	164.78	14.90
6	20	10	188.68	15.50
7	20	10	503.85	29.80
-7	20	-10	495.45	28.40
-7	20	-10	537.75	29.80
8	20	10	112.49	14.10
-8	20	-10	116.09	13.10

-8	20	-10	99.69	13.70
-9	20	-10	33.50	8.40
-9	20	-10	19.80	8.00
9	20	10	28.60	9.20
8	21	-10	78.19	15.20
-8	21	10	95.69	14.80
7	21	-10	20.70	10.20
-7	21	10	28.90	9.50
6	21	-10	15.00	9.00
-6	21	10	-0.40	8.50
-5	21	10	63.79	13.60
-5	21	10	68.49	11.10
5	21	-10	76.99	14.80
-4	21	10	39.50	9.80
4	21	-10	32.20	8.90
-4	21	10	44.70	11.90
-3	21	10	20.20	11.00
-3	21	10	18.00	8.70
3	21	-10	34.90	8.10
2	21	-10	114.79	14.00
-2	21	10	106.69	18.70
-2	21	10	85.19	14.20
-1	21	10	0.00	7.90
-1	21	10	4.50	9.80
1	21	-10	16.30	7.40
0	21	-10	83.79	12.60
0	21	10	68.99	13.00
0	21	-10	64.89	6.60
0	21	10	100.09	17.10
1	21	10	-15.30	8.80
1	21	10	-14.90	6.90
-1	21	-10	1.40	3.50
-1	21	-10	-4.50	5.90
-2	21	-10	147.29	10.50
2	21	10	135.89	14.80
-2	21	-10	164.98	14.90
2	21	10	176.88	20.10
3	21	10	13.10	10.50
-3	21	-10	19.90	5.00
-3	21	-10	25.90	7.40
3	21	10	13.60	7.80
-4	21	-10	165.18	14.50
4	21	10	122.59	14.50
-4	21	-10	153.78	11.50

4	21	10	136.39	20.20
5	21	10	79.39	12.40
-5	21	-10	40.60	8.50
-5	21	-10	35.20	11.40
6	21	10	74.19	12.70
-6	21	-10	67.39	12.40
-6	21	-10	52.29	10.00
-7	21	-10	10.80	6.70
-7	21	-10	11.30	7.40
7	21	10	14.50	8.30
-8	21	-10	46.70	9.60
8	21	10	63.59	10.30
-8	21	-10	72.19	11.60
8	22	-10	100.19	15.40
-7	22	10	83.49	14.60
7	22	-10	80.29	14.00
-6	22	10	1.70	8.40
-6	22	10	39.50	14.40
6	22	-10	11.00	10.50
-5	22	10	313.57	28.30
-5	22	10	281.37	21.00
5	22	-10	307.27	23.50
-4	22	10	141.69	21.90
-4	22	10	112.89	15.40
4	22	-10	124.69	16.60
-3	22	10	183.38	22.60
3	22	-10	201.28	18.30
-3	22	10	177.18	17.70
-2	22	10	-6.20	8.20
-2	22	10	-1.40	11.80
2	22	-10	-14.60	7.30
-1	22	10	331.87	22.30
1	22	-10	303.17	22.20
-1	22	10	310.07	26.00
0	22	10	-2.70	10.20
0	22	10	-10.40	7.30
0	22	-10	1.20	7.00
-1	22	-10	393.76	25.40
1	22	10	431.86	30.20
-1	22	-10	381.56	21.30
1	22	10	432.16	25.90
2	22	10	-9.50	10.30
-2	22	-10	9.20	4.30
2	22	10	11.60	7.90

-2	22	-10	-0.20	6.80
3	22	10	248.38	18.30
-3	22	-10	219.68	14.40
3	22	10	244.58	23.60
-3	22	-10	216.68	18.10
4	22	10	46.30	8.70
-4	22	-10	38.50	6.50
-4	22	-10	43.00	8.60
-5	22	-10	119.09	11.50
5	22	10	114.89	14.50
-5	22	-10	129.49	14.40
-6	22	-10	73.99	11.30
-6	22	-10	100.39	13.90
6	22	10	88.39	13.60
-7	22	-10	189.58	14.90
7	22	10	180.58	16.80
-7	22	-10	160.58	17.00
8	22	10	45.70	9.60
6	23	-10	405.16	29.30
-6	23	10	415.86	27.10
-6	23	10	409.56	34.20
5	23	-10	84.99	16.90
-5	23	10	49.20	14.40
-5	23	10	68.99	11.70
-4	23	10	399.06	31.60
4	23	-10	366.46	26.10
-4	23	10	348.27	24.90
3	23	-10	12.70	9.10
-3	23	10	3.90	9.20
-3	23	10	19.80	12.90
-2	23	10	479.25	30.00
-2	23	10	480.45	34.60
2	23	-10	487.95	30.10
-1	23	10	-13.00	8.00
1	23	-10	13.00	8.50
-1	23	10	-4.00	11.00
0	23	10	594.54	34.60
0	23	-10	613.54	35.10
0	23	10	596.94	39.00
1	23	10	2.60	8.20
-1	23	-10	11.20	7.70
1	23	10	20.10	11.10
2	23	10	279.87	27.80
2	23	10	261.07	19.70

-2	23	-10	241.48	15.00
-2	23	-10	244.78	19.70
-3	23	-10	10.50	5.20
3	23	10	3.00	8.10
-3	23	-10	11.00	7.60
4	23	10	235.78	18.50
-4	23	-10	196.48	19.90
-4	23	-10	238.98	18.60
-5	23	-10	98.29	14.50
5	23	10	95.89	14.40
-5	23	-10	83.59	11.00
6	23	10	222.68	18.20
-6	23	-10	191.68	15.40
-6	23	-10	213.98	18.60
-5	24	10	-5.80	13.30
5	24	-10	4.30	10.00
-5	24	10	11.30	9.80
4	24	-10	8.80	9.80
-4	24	10	12.40	9.00
-4	24	10	20.50	14.40
3	24	-10	18.10	9.50
-3	24	10	-11.20	13.00
-3	24	10	-7.50	8.50
-2	24	10	-9.00	8.70
2	24	-10	1.60	8.60
1	24	-10	10.90	8.40
-1	24	10	6.00	8.50
-1	24	10	21.90	14.00
0	24	10	-24.10	11.30
0	24	-10	9.30	8.50
0	24	10	10.60	8.90
-1	24	-10	51.19	10.00
1	24	10	49.90	10.50
2	24	10	9.70	8.70
-2	24	-10	-1.30	8.50
-3	24	-10	5.10	8.00
3	24	10	17.00	8.50
-4	24	-10	1.00	7.90
4	24	10	6.30	8.60
-4	24	-10	-3.80	5.50
-5	24	-10	14.30	9.10
-5	24	-10	24.80	6.80
5	24	10	28.60	9.40
-4	25	10	162.58	26.40

-3	25	10	77.99	17.10
3	25	-10	67.99	12.20
-3	25	10	46.70	11.00
-2	25	10	330.37	23.70
-2	25	10	312.47	30.40
2	25	-10	332.27	25.30
-1	25	10	10.90	9.70
1	25	-10	6.70	9.20
0	25	10	664.43	37.80
0	25	-10	640.94	38.40
1	25	10	10.30	9.60
-1	25	-10	-7.50	8.30
2	25	10	298.67	22.20
-2	25	-10	284.97	22.60
-3	25	-10	29.60	10.70
3	25	10	22.10	9.50
-14	0	11	-0.60	5.90
-13	0	11	19.70	5.70
13	0	-11	13.20	8.40
-12	0	11	0.50	4.70
12	0	-11	-7.50	7.90
11	0	-11	12.40	5.90
11	0	-11	11.10	7.00
-11	0	11	15.70	11.10
-10	0	11	-7.30	8.20
-10	0	11	0.70	3.80
10	0	-11	-0.30	5.90
10	0	-11	-5.60	4.40
9	0	-11	17.70	6.30
-9	0	11	13.20	3.60
-9	0	11	6.60	7.40
9	0	-11	11.70	4.50
8	0	-11	4.00	5.10
-8	0	11	-2.80	6.00
8	0	-11	10.70	3.70
-8	0	11	3.40	3.20
-7	0	11	0.40	5.20
7	0	-11	3.40	4.50
-7	0	11	-3.40	2.70
7	0	-11	0.00	2.90
-6	0	11	7.60	5.80
6	0	-11	4.30	4.20
-6	0	11	13.80	3.20
-5	0	11	-3.00	2.70

5	0	-11	1.00	3.40
5	0	-11	3.60	2.30
-5	0	11	5.90	4.50
4	0	-11	-1.10	3.00
-4	0	11	-2.80	2.30
-4	0	11	-1.30	3.50
4	0	-11	1.30	1.90
3	0	-11	0.40	2.40
3	0	-11	0.60	1.60
-3	0	11	0.50	2.20
-3	0	11	0.20	2.90
2	0	-11	-3.20	2.10
-2	0	11	2.80	2.10
-2	0	11	-4.00	2.40
2	0	-11	4.10	1.60
1	0	-11	2.90	1.70
-1	0	11	-2.00	2.10
-1	0	11	1.40	2.00
1	0	-11	0.60	1.80
0	0	-11	1.80	1.80
0	0	-11	-0.40	1.70
0	0	11	-2.70	1.80
0	0	11	2.40	2.10
1	0	11	1.60	1.70
-1	0	-11	-1.90	1.80
1	0	11	1.00	2.20
-1	0	-11	3.00	1.60
2	0	11	1.00	2.40
2	0	11	0.30	1.60
2	0	11	0.60	1.40
-2	0	-11	0.90	2.30
-2	0	-11	1.90	1.50
3	0	11	3.80	2.70
-3	0	-11	2.40	2.40
3	0	11	-0.10	1.30
3	0	11	-0.40	1.10
4	0	11	2.60	1.40
4	0	11	2.50	1.20
-4	0	-11	0.90	2.90
4	0	11	7.60	3.30
-5	0	-11	1.80	2.30
5	0	11	5.30	3.40
5	0	11	2.90	1.60
5	0	11	1.50	1.30

-5	0	-11	7.70	3.10
-6	0	-11	4.70	3.00
6	0	11	6.20	3.90
6	0	11	4.50	1.60
-6	0	-11	-0.40	3.50
6	0	11	4.90	2.00
7	0	11	1.60	2.30
7	0	11	5.40	2.00
-7	0	-11	6.00	3.40
7	0	11	1.70	4.20
8	0	11	-0.50	2.60
-8	0	-11	3.30	4.00
-8	0	-11	2.40	4.60
-9	0	-11	-2.70	4.70
-9	0	-11	9.40	4.80
9	0	11	3.00	3.20
10	0	11	3.60	3.80
-10	0	-11	-4.50	5.00
-10	0	-11	-1.70	5.10
-11	0	-11	19.60	7.00
-11	0	-11	6.70	6.40
11	0	11	12.10	4.30
-12	0	-11	-1.00	7.20
-12	0	-11	0.00	6.70
-13	0	-11	16.90	8.00
-14	0	-11	-12.10	7.90
-14	-1	11	18.60	6.50
-14	1	11	29.90	6.90
-13	-1	11	393.76	21.30
13	-1	-11	369.26	25.80
13	1	-11	376.06	25.90
-13	1	11	367.86	23.00
-12	-1	11	193.78	12.40
12	-1	-11	181.08	17.50
-12	1	11	172.58	33.90
12	1	-11	172.18	17.50
-12	1	11	184.78	12.70
-11	-1	11	542.25	27.60
-11	-1	11	463.35	34.10
11	-1	-11	556.54	31.40
11	1	-11	579.74	31.60
-11	1	11	582.04	27.90
-11	1	11	448.66	34.00
11	1	-11	527.25	28.80

10	-1	-11	273.77	18.30
10	-1	-11	244.68	15.90
-10	-1	11	220.68	21.40
-10	-1	11	244.48	14.40
-10	1	11	240.38	21.20
-10	1	11	247.78	14.60
10	1	-11	264.57	15.50
10	1	-11	261.57	18.10
-9	-1	11	1026.50	49.60
-9	-1	11	895.71	53.39
9	-1	-11	1043.80	52.19
9	-1	-11	1031.90	50.29
9	1	-11	1022.80	50.09
-9	1	11	1037.30	49.80
-9	1	11	1002.30	53.89
9	1	-11	1032.40	52.19
-8	-1	11	667.33	33.10
8	-1	-11	682.53	35.50
8	-1	-11	686.33	33.70
-8	-1	11	606.24	36.60
-8	1	11	675.13	33.30
8	1	-11	690.63	33.50
8	1	-11	699.43	35.80
-8	1	11	603.54	36.60
-7	-1	11	123.49	8.30
7	-1	-11	128.29	11.20
-7	-1	11	113.69	12.50
7	-1	-11	135.09	8.90
7	1	-11	123.69	11.00
7	1	-11	122.49	8.40
-7	1	11	140.59	13.00
-7	1	11	133.39	8.80
-6	-1	11	2200.48	111.09
6	-1	-11	2307.57	108.89
-6	-1	11	2273.47	108.79
6	-1	-11	2283.27	110.39
-6	1	11	2281.37	109.09
-6	1	11	2227.58	111.09
6	1	-11	2331.47	108.69
6	1	-11	2268.27	110.39
-5	-1	11	560.64	27.90
5	-1	-11	575.44	27.90
5	-1	-11	547.85	29.30
-5	-1	11	564.34	30.20

5	1	-11	574.24	27.60
5	1	-11	561.14	29.40
-5	1	11	535.95	28.20
-5	1	11	548.55	30.10
4	-1	-11	1760.32	83.49
-4	-1	11	1740.93	82.39
-4	-1	11	1669.13	83.89
4	-1	-11	1781.82	82.19
-4	1	11	1743.83	83.99
4	1	-11	1740.13	83.49
4	1	-11	1635.04	82.29
-4	1	11	1658.83	82.79
-3	-1	11	204.18	12.40
3	-1	-11	201.48	11.80
-3	-1	11	199.78	10.90
3	-1	-11	201.08	10.50
3	1	-11	204.88	11.90
3	1	-11	161.08	11.20
-3	1	11	205.38	12.40
-3	1	11	201.98	11.40
-2	-1	11	3042.30	150.98
-2	-1	11	3324.37	151.98
2	-1	-11	3095.29	150.88
2	-1	-11	3188.98	151.68
-2	1	11	3298.77	152.08
2	1	-11	3157.08	151.58
-2	1	11	3104.89	151.38
1	-1	-11	196.68	10.20
-1	-1	11	189.58	11.00
-1	-1	11	175.58	10.30
1	-1	-11	188.58	10.70
1	1	-11	197.78	10.70
1	1	-11	191.78	10.10
-1	1	11	178.68	10.70
-1	1	11	187.88	11.00
0	-1	-11	3235.28	154.48
0	-1	11	3382.36	154.38
0	-1	-11	3302.27	154.28
0	-1	11	3121.29	154.28
0	1	-11	3221.58	154.28
0	1	-11	3235.58	154.08
0	1	11	3135.09	154.48
0	1	11	3284.97	154.48
-1	-1	-11	6.80	2.10

-1	-1	-11	10.40	2.40
1	-1	11	8.50	2.60
1	1	11	8.90	2.60
1	1	11	16.50	3.20
1	1	11	8.60	2.00
-1	1	-11	10.40	1.90
-1	1	-11	5.70	2.10
2	-1	11	1587.84	73.99
2	-1	11	1501.95	75.09
-2	-1	-11	1559.94	74.69
2	-1	11	1501.05	73.89
-2	-1	-11	1620.14	74.99
-2	1	-11	1594.44	74.89
-2	1	-11	1526.25	74.29
2	1	11	1633.84	74.69
2	1	11	1478.65	75.19
2	1	11	1549.24	74.39
3	-1	11	272.57	15.30
-3	-1	-11	285.17	15.20
3	1	11	285.37	14.20
-3	1	-11	308.47	15.20
3	1	11	274.37	15.40
3	1	11	266.67	14.70
-4	-1	-11	1340.27	62.19
4	-1	11	1222.88	62.19
-4	1	-11	1347.37	62.19
4	1	11	1302.87	61.19
4	1	11	1157.88	62.19
4	1	11	1278.07	60.99
5	-1	11	787.52	39.30
-5	-1	-11	827.62	39.20
-5	-1	-11	745.73	38.50
5	1	11	759.62	39.30
-5	1	-11	773.32	38.10
-5	1	-11	845.22	39.20
5	1	11	782.42	37.80
5	1	11	733.63	38.00
-6	-1	-11	1703.03	81.79
6	-1	11	1673.43	80.29
6	-1	11	1706.93	80.39
-6	-1	-11	1802.62	82.49
6	-1	11	1615.34	82.39
6	1	11	1690.13	80.99
6	1	11	1678.73	81.19

6	1	11	1574.04	82.29
-6	1	-11	1684.13	81.39
-6	1	-11	1797.62	82.49
-7	-1	-11	575.74	29.70
7	-1	11	581.54	30.20
7	-1	11	586.04	28.20
7	-1	11	591.74	28.10
-7	1	-11	581.44	30.30
-7	1	-11	591.94	29.40
7	1	11	558.34	28.60
7	1	11	569.04	29.00
7	1	11	562.24	30.20
-8	-1	-11	231.18	14.00
8	-1	11	207.48	11.70
-8	1	-11	218.78	13.30
-8	1	-11	199.18	14.00
8	1	11	199.48	14.10
8	1	11	224.98	12.70
-9	-1	-11	336.77	19.80
9	-1	11	338.57	17.40
-9	-1	-11	319.47	19.50
-9	1	-11	311.27	18.90
9	1	11	331.07	18.60
-9	1	-11	333.67	19.60
-10	-1	-11	155.38	12.90
-10	-1	-11	153.48	12.90
10	-1	11	121.99	9.40
10	1	11	150.48	10.60
-10	1	-11	121.99	11.90
-10	1	-11	135.49	12.60
-11	-1	-11	656.13	36.90
-11	-1	-11	731.93	37.20
11	-1	11	645.24	34.20
-11	1	-11	623.74	36.30
11	1	11	671.83	34.90
-11	1	-11	762.62	37.20
-12	-1	-11	63.09	11.10
-12	1	-11	64.29	9.60
-12	1	-11	99.19	12.70
-13	-1	-11	323.77	22.50
-13	1	-11	324.57	21.50
-14	-1	-11	26.90	9.10
-14	1	-11	37.40	10.00
-14	2	11	110.49	12.20

13	-2	-11	24.90	9.60
-13	-2	11	22.20	5.50
-13	2	11	23.60	6.10
13	2	-11	28.70	10.00
-12	-2	11	186.68	12.00
12	-2	-11	188.38	17.40
-12	2	11	173.78	12.70
12	2	-11	165.38	17.20
-11	-2	11	218.48	32.60
-11	-2	11	249.68	14.30
11	-2	-11	243.48	18.40
-11	2	11	262.57	15.10
11	2	-11	254.77	18.90
-11	2	11	231.18	22.50
11	2	-11	219.58	15.40
10	-2	-11	267.47	18.00
-10	-2	11	238.68	14.10
-10	-2	11	261.17	21.30
10	-2	-11	247.28	16.20
10	2	-11	238.18	15.10
-10	2	11	256.97	14.90
10	2	-11	241.78	18.20
-10	2	11	244.28	20.50
9	-2	-11	149.69	12.60
-9	-2	11	133.19	15.30
-9	-2	11	141.19	9.20
9	-2	-11	139.79	10.70
9	2	-11	143.79	13.50
-9	2	11	145.59	9.80
-9	2	11	127.79	15.10
9	2	-11	138.09	10.10
-8	-2	11	127.19	14.40
-8	-2	11	155.18	9.50
8	-2	-11	154.98	12.50
8	-2	-11	158.68	10.80
-8	2	11	158.68	10.30
8	2	-11	166.88	10.00
-8	2	11	156.98	14.90
8	2	-11	155.48	13.10
-7	-2	11	206.88	12.00
7	-2	-11	233.78	14.70
-7	-2	11	185.38	15.80
7	-2	-11	227.08	12.80
7	2	-11	223.28	12.20

-7	2	11	213.88	12.60
-7	2	11	193.28	15.90
7	2	-11	227.58	14.90
6	-2	-11	609.14	29.70
6	-2	-11	591.04	31.10
-6	-2	11	613.74	29.30
-6	-2	11	541.85	32.10
6	2	-11	597.14	31.40
6	2	-11	612.94	29.20
-6	2	11	568.54	32.00
-6	2	11	566.94	29.80
5	-2	-11	1783.42	86.49
-5	-2	11	1697.13	87.09
5	-2	-11	1845.22	85.29
-5	-2	11	1805.82	85.09
-5	2	11	1802.22	85.79
-5	2	11	1720.33	87.09
5	2	-11	1763.92	86.59
4	-2	-11	22.10	4.00
4	-2	-11	31.90	3.90
-4	-2	11	15.60	4.40
-4	-2	11	24.80	3.80
-4	2	11	20.40	4.60
4	2	-11	27.70	3.10
4	2	-11	26.80	4.80
-4	2	11	22.00	3.90
-3	-2	11	621.84	31.70
3	-2	-11	608.34	30.20
3	-2	-11	607.14	31.30
3	2	-11	610.34	29.80
-3	2	11	588.94	30.90
-3	2	11	658.23	31.90
3	2	-11	615.54	31.30
-2	-2	11	49.70	4.70
2	-2	-11	48.50	4.10
2	-2	-11	47.10	5.10
-2	-2	11	31.00	5.20
-2	2	11	36.40	4.90
2	2	-11	44.90	4.90
-2	2	11	46.30	5.60
2	2	-11	46.80	3.40
-1	-2	11	672.83	33.70
1	-2	-11	700.13	33.70
-1	-2	11	703.23	34.20

1	-2	-11	678.03	34.20
-1	2	11	725.93	34.50
1	2	-11	701.83	33.40
1	2	-11	699.93	34.00
-1	2	11	625.44	34.10
0	-2	-11	39.20	4.00
0	-2	-11	35.70	4.10
0	-2	11	24.10	3.70
0	-2	11	39.40	3.90
0	2	11	35.00	4.10
0	2	11	39.20	4.30
0	2	-11	35.00	3.60
0	2	-11	35.10	3.40
-1	-2	-11	1888.01	84.29
-1	-2	-11	1781.42	84.29
1	-2	11	1693.13	83.49
1	-2	11	1696.23	84.19
-1	2	-11	1799.42	83.99
1	2	11	1755.62	84.09
1	2	11	1792.32	84.29
-1	2	-11	1717.33	83.69
1	2	11	1656.83	84.39
2	-2	11	1.80	2.30
-2	-2	-11	2.70	1.90
-2	-2	-11	4.60	2.60
-2	2	-11	5.30	1.40
2	2	11	3.70	1.70
-2	2	-11	3.40	2.20
2	2	11	3.10	2.20
2	2	11	5.20	2.80
-3	-2	-11	66.39	5.80
3	-2	11	64.09	5.90
3	2	11	73.99	5.50
3	2	11	66.69	4.70
3	2	11	65.19	6.20
-3	2	-11	66.69	5.70
-4	-2	-11	212.28	11.70
4	-2	11	180.98	11.60
4	2	11	203.48	11.00
4	2	11	188.38	11.70
4	2	11	196.88	10.50
-4	2	-11	191.98	11.60
-5	-2	-11	273.77	15.10
5	-2	11	261.17	15.40

-5	-2	-11	274.57	15.40
5	2	11	262.87	14.10
5	2	11	258.77	15.70
-5	2	-11	289.17	14.00
-5	2	-11	287.07	15.50
-6	-2	-11	92.39	7.90
6	-2	11	80.69	8.00
-6	-2	-11	82.39	7.50
-6	2	-11	87.19	7.60
6	2	11	85.99	5.80
-6	2	-11	82.39	8.20
-6	2	-11	76.19	6.30
6	2	11	102.49	8.40
6	2	11	83.79	6.70
-7	-2	-11	586.04	29.60
7	2	11	582.34	28.80
7	2	11	512.65	29.60
-7	2	-11	576.04	28.60
-7	2	-11	568.54	29.90
8	-2	11	538.35	25.80
-8	-2	-11	513.55	28.20
8	2	11	520.25	28.40
8	2	11	530.05	27.30
-8	2	-11	515.25	27.30
-8	2	-11	545.25	28.60
-9	-2	-11	41.20	7.20
9	-2	11	37.20	4.70
-9	-2	-11	35.60	6.60
-9	2	-11	39.20	6.70
9	2	11	44.70	7.10
9	2	11	43.40	7.10
-9	2	-11	55.39	7.50
10	-2	11	93.59	7.40
-10	-2	-11	96.29	11.50
-10	-2	-11	105.89	11.70
10	2	11	93.59	9.40
-10	2	-11	88.19	10.30
-10	2	-11	101.09	11.30
-11	-2	-11	58.59	11.40
11	-2	11	42.00	6.60
-11	-2	-11	67.39	11.10
11	2	11	57.09	9.00
-11	2	-11	57.29	9.80
-11	2	-11	54.79	10.60

-12	-2	-11	222.58	17.30
-12	2	-11	233.08	16.90
-12	2	-11	177.48	16.50
-13	-2	-11	36.10	9.60
-13	2	-11	45.50	8.80
-14	-2	-11	143.39	16.30
-14	2	-11	124.59	15.60
-14	-3	11	28.80	6.30
-14	3	11	23.80	7.10
-13	-3	11	51.59	8.20
13	-3	-11	60.39	11.40
13	3	-11	48.90	10.80
-13	3	11	54.59	9.70
-12	-3	11	76.69	8.00
12	-3	-11	67.39	13.90
12	3	-11	67.39	13.70
-11	-3	11	285.07	17.70
11	-3	-11	307.47	20.20
-11	-3	11	288.87	16.00
-11	3	11	293.67	17.00
-11	3	11	257.87	23.80
11	3	-11	271.17	20.50
10	-3	-11	285.87	18.70
-10	-3	11	268.47	14.60
-10	-3	11	215.88	21.30
-10	3	11	208.68	21.00
10	3	-11	286.37	19.20
-10	3	11	270.87	15.50
10	3	-11	274.27	15.60
-9	-3	11	55.29	10.00
9	-3	-11	71.49	10.60
-9	-3	11	81.49	6.50
9	-3	-11	75.49	9.00
9	3	-11	80.69	7.40
-9	3	11	78.79	11.70
-9	3	11	80.39	7.80
9	3	-11	54.49	10.60
-8	-3	11	91.99	12.80
8	-3	-11	107.59	8.80
8	-3	-11	106.89	10.50
-8	-3	11	94.59	7.10
8	3	-11	109.79	7.60
-8	3	11	102.29	8.30
8	3	-11	110.39	11.00

-8	3	11	93.29	12.40
7	-3	-11	100.49	9.50
-7	-3	11	92.59	6.50
7	-3	-11	103.59	7.90
-7	-3	11	68.49	11.10
7	3	-11	96.99	6.70
-7	3	11	96.69	7.70
7	3	-11	70.89	9.90
-7	3	11	72.89	11.10
-6	-3	11	759.22	43.10
6	-3	-11	847.02	41.20
6	-3	-11	873.21	42.70
-6	-3	11	877.41	40.60
6	3	-11	821.22	42.70
-6	3	11	835.92	41.40
-6	3	11	835.52	43.40
6	3	-11	799.82	40.90
5	-3	-11	184.98	12.30
-5	-3	11	153.08	13.10
5	-3	-11	202.78	11.00
-5	3	11	191.28	11.80
5	3	-11	183.88	12.70
5	3	-11	187.88	10.00
-5	3	11	191.68	13.40
4	-3	-11	599.14	31.00
4	-3	-11	609.94	29.80
-4	-3	11	565.84	31.40
-4	-3	11	588.34	29.40
4	3	-11	607.44	31.10
-4	3	11	581.84	30.60
4	3	-11	635.94	29.20
-4	3	11	621.04	31.70
3	-3	-11	17.10	3.60
-3	-3	11	11.60	3.60
3	-3	-11	16.20	2.70
-3	-3	11	14.70	2.30
-3	3	11	16.00	3.70
3	3	-11	13.70	3.30
-3	3	11	18.40	4.20
3	3	-11	15.50	2.30
2	-3	-11	113.89	7.80
-2	-3	11	111.69	6.50
-2	-3	11	101.59	7.80
2	-3	-11	109.49	6.80

-2	3	11	110.39	7.80
2	3	-11	109.59	7.60
2	3	-11	108.29	6.10
-2	3	11	112.99	8.10
1	-3	-11	31.40	4.50
1	-3	-11	37.00	3.90
-1	-3	11	30.10	4.10
-1	3	11	35.30	4.60
1	3	-11	33.20	3.10
-1	3	11	43.20	4.70
1	3	-11	29.60	4.10
0	-3	-11	242.08	13.30
0	-3	11	238.58	12.80
0	-3	11	231.98	12.50
0	-3	-11	255.67	12.90
0	3	11	230.78	13.10
0	3	-11	239.78	12.60
0	3	11	240.38	13.40
-1	-3	-11	51.39	4.90
-1	-3	-11	60.59	5.00
1	-3	11	56.09	4.90
1	-3	11	54.59	3.40
1	3	11	62.79	4.80
1	3	11	55.09	5.30
1	3	11	57.29	5.50
-1	3	-11	59.19	4.50
-1	3	-11	50.59	3.90
-2	-3	-11	331.37	17.20
-2	-3	-11	329.87	17.20
2	-3	11	312.27	17.10
-2	3	-11	316.07	16.00
-2	3	-11	343.77	17.00
2	3	11	315.27	17.50
2	3	11	325.47	16.80
2	3	11	340.97	17.40
-3	-3	-11	137.59	8.80
3	-3	11	151.38	9.10
3	3	11	135.39	9.30
3	3	11	136.59	8.10
-3	3	-11	151.58	8.80
3	3	11	133.79	8.90
-4	-3	-11	125.69	8.10
4	-3	11	103.69	8.10
4	3	11	116.59	7.10

4	3	11	109.99	8.30
4	3	11	119.79	7.90
-4	3	-11	121.09	8.20
-5	-3	-11	-2.50	2.80
5	-3	11	4.30	3.40
-5	-3	-11	1.60	3.10
5	3	11	3.10	2.10
5	3	11	0.80	2.70
-5	3	-11	5.10	1.70
-5	3	-11	4.90	3.50
-5	3	-11	2.70	3.20
5	3	11	1.20	3.20
-6	-3	-11	589.54	29.20
-6	3	-11	525.85	29.10
-6	3	-11	562.64	27.70
6	3	11	547.25	27.90
6	3	11	526.65	29.10
6	3	11	576.94	28.50
-6	3	-11	587.34	29.20
-7	-3	-11	257.37	17.70
7	3	11	265.67	16.30
-7	3	-11	280.67	16.50
-7	3	-11	269.57	14.70
-8	-3	-11	47.90	8.70
-8	3	-11	39.90	7.30
8	3	11	46.00	7.30
-8	3	-11	39.80	6.20
8	3	11	39.30	6.90
-9	-3	-11	19.30	6.20
9	-3	11	16.80	2.60
9	3	11	18.80	5.00
-9	3	-11	20.20	6.00
-9	3	-11	15.40	4.70
9	3	11	11.60	5.60
-10	-3	-11	9.60	6.30
-10	-3	-11	7.80	6.40
-10	3	-11	9.50	6.30
-10	3	-11	-2.90	4.80
10	3	11	9.70	4.80
11	-3	11	172.38	10.90
-11	-3	-11	178.58	15.50
-11	-3	-11	182.98	16.60
-11	3	-11	207.58	15.30
11	3	11	181.78	13.40

-11	3	-11	156.18	14.00
-12	-3	-11	187.88	16.60
-12	3	-11	147.39	15.20
-12	3	-11	206.18	16.00
-13	-3	-11	123.29	15.00
-13	3	-11	115.29	14.40
-14	-3	-11	17.40	9.30
-14	3	-11	19.90	8.70
-14	4	11	385.96	22.80
-13	-4	11	138.09	10.60
13	-4	-11	133.59	16.40
-13	4	11	125.99	12.10
13	4	-11	112.29	17.10
12	-4	-11	230.88	18.30
-12	-4	11	210.88	13.10
12	4	-11	222.08	18.90
-12	4	11	193.68	14.20
11	-4	-11	308.97	21.00
-11	-4	11	312.77	18.10
-11	-4	11	298.77	16.50
-11	4	11	310.67	17.70
11	4	-11	299.47	21.70
-11	4	11	260.37	24.60
-10	-4	11	468.85	24.70
10	-4	-11	499.05	28.40
-10	-4	11	542.65	25.90
10	4	-11	471.45	28.90
-10	4	11	476.55	25.80
-10	4	11	457.45	31.00
9	-4	-11	500.35	27.30
-9	-4	11	465.25	23.70
9	-4	-11	474.05	27.20
9	4	-11	463.95	27.40
-9	4	11	472.25	24.90
9	4	-11	480.95	24.30
-9	4	11	441.26	29.00
8	-4	-11	390.76	21.30
-8	-4	11	381.26	19.60
-8	-4	11	356.56	24.10
8	-4	-11	387.86	22.60
8	4	-11	387.56	23.10
-8	4	11	378.56	20.80
-8	4	11	398.56	24.70
8	4	-11	401.16	20.00

-7	-4	11	1362.36	74.89
7	-4	-11	1534.65	74.29
7	-4	-11	1532.95	72.99
-7	-4	11	1517.15	71.89
7	4	-11	1499.65	74.49
-7	4	11	1538.15	72.89
-7	4	11	1496.35	75.29
-6	-4	11	45.80	9.00
6	-4	-11	86.69	6.80
-6	-4	11	78.69	6.40
6	-4	-11	55.89	7.80
6	4	-11	66.89	8.80
-6	4	11	56.29	9.40
6	4	-11	83.59	5.30
-6	4	11	70.59	7.00
-5	-4	11	1347.87	64.09
5	-4	-11	1357.36	64.89
-5	-4	11	1194.28	66.29
5	-4	-11	1374.06	66.09
-5	4	11	1358.36	65.49
-5	4	11	1398.76	66.79
5	4	-11	1308.57	63.99
5	4	-11	1365.26	66.19
-4	-4	11	166.28	8.90
4	-4	-11	166.78	9.80
-4	-4	11	161.78	11.60
4	-4	-11	173.78	11.20
4	4	-11	177.28	11.50
-4	4	11	165.28	11.90
-4	4	11	155.48	10.80
4	4	-11	176.78	8.80
3	-4	-11	945.01	47.60
3	-4	-11	967.40	46.60
-3	-4	11	912.61	47.60
-3	-4	11	960.10	46.00
3	4	-11	970.00	45.80
-3	4	11	931.01	47.50
-3	4	11	997.80	48.00
3	4	-11	985.00	47.60
2	-4	-11	6.20	2.40
-2	-4	11	10.40	1.90
2	-4	-11	6.20	2.90
-2	-4	11	13.10	3.10
2	4	-11	11.90	3.00

-2	4	11	9.50	3.50
2	4	-11	14.60	2.00
-2	4	11	5.60	2.90
-1	-4	11	1082.29	54.79
-1	-4	11	1139.39	54.29
1	-4	-11	1151.98	54.69
1	-4	-11	1133.69	55.29
1	4	-11	1133.89	54.09
1	4	-11	1146.29	54.89
-1	4	11	1138.09	55.29
-1	4	11	1110.19	55.39
0	-4	-11	369.66	18.20
0	-4	11	312.27	17.60
0	-4	-11	359.96	18.70
0	-4	11	375.96	18.00
0	4	-11	347.97	17.60
0	4	11	357.36	18.50
0	4	-11	344.97	17.90
0	4	11	350.16	18.80
-1	-4	-11	1883.91	86.29
1	-4	11	1781.02	86.09
-1	-4	-11	1811.42	86.59
1	4	11	1740.53	86.59
-1	4	-11	1764.02	85.49
1	4	11	1798.22	86.59
1	4	11	1754.72	86.19
-1	4	-11	1815.42	85.89
-2	-4	-11	23.20	4.30
2	-4	11	23.70	4.20
-2	4	-11	25.00	2.10
2	4	11	22.70	4.20
2	4	11	25.50	3.70
-2	4	-11	22.70	3.80
2	4	11	29.10	4.70
-3	-4	-11	1734.93	84.19
3	-4	11	1728.93	84.19
3	4	11	1718.43	83.89
-3	4	-11	1801.52	84.09
3	4	11	1860.81	84.49
3	4	11	1629.34	84.49
-4	-4	-11	200.18	11.50
4	4	11	187.98	11.90
4	4	11	178.58	11.30
4	4	11	193.38	10.60

-4	4	-11	189.08	11.30
-4	4	-11	180.98	11.40
-5	-4	-11	986.10	48.80
-5	-4	-11	968.30	48.80
5	4	11	969.00	48.10
-5	4	-11	1023.00	48.90
5	4	11	944.31	49.10
5	4	11	1060.99	48.80
-5	4	-11	949.90	48.90
-5	4	-11	992.30	47.10
-6	-4	-11	301.27	17.40
-6	-4	-11	316.07	17.20
6	4	11	290.37	16.20
6	4	11	302.27	17.40
-6	4	-11	287.87	15.50
-6	4	-11	288.07	17.40
6	4	11	307.17	16.90
-6	4	-11	341.57	17.70
-7	-4	-11	1060.29	53.39
-7	4	-11	1069.79	51.89
-7	4	-11	1059.99	53.39
7	4	11	1024.40	53.39
7	4	11	1126.79	52.99
-8	-4	-11	197.68	13.70
8	4	11	181.48	12.90
-8	4	-11	182.98	11.50
-8	4	-11	200.28	13.40
8	4	11	176.78	13.10
9	-4	11	143.29	7.60
-9	-4	-11	142.69	12.70
9	4	11	118.19	11.70
-9	4	-11	137.69	12.20
-9	4	-11	142.69	10.60
9	4	11	184.08	15.10
-10	-4	-11	1031.10	55.49
10	-4	11	919.91	46.50
-10	4	-11	1028.50	49.80
10	4	11	946.61	49.40
10	4	11	976.80	49.00
-10	4	-11	906.51	48.50
11	-4	11	193.08	11.60
-11	-4	-11	216.38	16.70
-11	4	-11	203.58	16.20
-11	4	-11	214.08	15.00

-12	-4	-11	415.46	24.10
-12	4	-11	352.36	23.00
-12	4	-11	314.57	22.20
-13	-4	-11	7.70	8.50
-13	4	-11	17.70	7.50
-14	-4	-11	249.38	21.00
-14	4	-11	270.47	20.30
-14	5	11	26.30	7.50
-13	-5	11	3.80	4.60
-13	5	11	4.90	6.30
13	5	-11	3.40	9.00
12	-5	-11	4.80	7.40
-12	-5	11	7.30	4.10
-12	5	11	18.30	5.70
12	5	-11	10.10	9.50
11	-5	-11	10.90	7.40
-11	-5	11	10.30	3.80
-11	-5	11	26.90	8.80
-11	5	11	13.00	6.10
-11	5	11	-0.90	11.10
11	5	-11	8.90	8.40
-10	-5	11	87.99	8.70
-10	-5	11	80.99	6.90
10	-5	-11	101.09	12.50
10	5	-11	92.39	13.30
-10	5	11	96.39	9.30
-10	5	11	82.79	15.90
-9	-5	11	30.40	4.60
9	-5	-11	14.10	6.00
-9	-5	11	6.10	7.80
-9	-5	11	25.80	4.50
-9	5	11	10.80	7.80
-9	5	11	29.30	5.30
9	5	-11	25.80	7.60
8	-5	-11	4.50	4.90
-8	-5	11	-8.20	6.60
-8	-5	11	7.90	2.50
8	-5	-11	1.10	4.10
8	5	-11	-0.60	5.20
-8	5	11	1.50	6.70
-8	5	11	7.10	4.00
7	-5	-11	10.00	4.90
-7	-5	11	4.90	6.40
7	-5	-11	12.60	4.10

-7	5	11	8.00	5.90
7	5	-11	-1.10	5.10
7	5	-11	6.80	2.40
-7	5	11	4.00	3.80
6	-5	-11	135.29	10.50
-6	-5	11	95.39	11.30
6	-5	-11	121.39	8.70
-6	-5	11	122.69	6.90
-6	5	11	120.59	9.30
-6	5	11	109.99	11.40
6	5	-11	117.29	6.90
6	5	-11	118.09	10.90
5	-5	-11	14.80	4.20
5	-5	-11	13.60	3.30
-5	-5	11	12.00	1.80
5	5	-11	14.30	2.50
5	5	-11	5.30	4.20
-5	5	11	9.30	4.20
-5	5	11	10.90	4.80
-4	-5	11	3.80	1.40
4	-5	-11	7.60	3.30
4	-5	-11	7.20	2.90
-4	-5	11	3.90	3.50
4	5	-11	1.10	3.30
4	5	-11	3.80	1.30
-4	5	11	1.60	3.70
-4	5	11	-3.00	3.30
3	-5	-11	-1.10	2.30
-3	-5	11	1.40	1.30
3	-5	-11	-1.60	2.80
-3	-5	11	-3.70	3.10
-3	5	11	-3.90	3.30
3	5	-11	2.40	1.10
-3	5	11	5.80	3.50
3	5	-11	-2.40	3.00
2	-5	-11	30.40	5.20
2	-5	-11	31.20	4.10
-2	-5	11	32.10	3.00
-2	-5	11	23.30	4.60
-2	5	11	25.90	5.20
-2	5	11	20.00	4.20
2	5	-11	27.30	2.30
2	5	-11	28.50	4.10
1	-5	-11	85.89	7.00

-1	-5	11	66.49	6.00
-1	-5	11	96.99	5.50
1	-5	-11	91.09	6.10
1	5	-11	85.09	6.30
-1	5	11	82.99	6.80
-1	5	11	80.19	7.10
1	5	-11	84.59	5.00
0	-5	-11	14.20	2.80
0	-5	11	6.40	2.50
0	-5	-11	6.10	2.70
0	-5	11	10.40	2.10
0	5	11	9.10	2.70
0	5	11	16.20	3.60
0	5	-11	9.20	2.30
0	5	-11	8.30	1.90
1	-5	11	28.00	3.90
-1	-5	-11	30.30	4.00
-1	-5	-11	30.90	4.70
1	5	11	21.70	4.00
-1	5	-11	23.50	3.40
1	5	11	29.40	4.90
1	5	11	23.30	4.90
-1	5	-11	26.00	3.00
-2	-5	-11	157.58	9.70
2	-5	11	164.48	9.60
-2	5	-11	168.68	8.10
2	5	11	147.09	9.90
2	5	11	154.58	9.40
2	5	11	174.08	10.40
-2	5	-11	148.29	9.50
-2	5	-11	153.88	9.10
-3	-5	-11	14.50	3.40
-3	5	-11	12.10	3.00
3	5	11	8.40	3.30
3	5	11	10.60	2.70
-3	5	-11	11.00	3.20
3	5	11	8.30	3.20
-4	-5	-11	3.80	3.10
4	5	11	1.60	2.90
4	5	11	2.70	3.40
-4	5	-11	5.10	2.90
4	5	11	0.10	2.10
-4	5	-11	0.30	3.00
-5	-5	-11	9.70	3.80

-5	-5	-11	16.50	3.90
5	5	11	6.50	3.50
5	5	11	7.40	4.00
-5	5	-11	9.70	3.70
5	5	11	5.40	2.60
-5	5	-11	6.50	3.90
-6	-5	-11	13.20	4.00
-6	-5	-11	14.30	4.40
-6	5	-11	10.10	4.40
-6	5	-11	7.60	2.40
6	5	11	13.50	4.00
6	5	11	12.20	3.10
-6	5	-11	13.10	4.60
6	5	11	22.40	4.30
-7	-5	-11	21.80	4.80
-7	5	-11	17.50	5.10
7	5	11	19.00	4.50
-7	5	-11	13.40	3.20
7	5	11	19.20	4.80
-8	-5	-11	81.39	10.00
-8	5	-11	87.39	9.40
8	5	11	99.79	9.50
8	5	11	81.69	9.40
-8	5	-11	83.89	7.30
-9	-5	-11	25.30	6.50
9	5	11	34.90	6.20
-9	5	-11	20.10	6.10
-9	5	-11	32.70	5.10
9	5	11	27.60	10.00
10	-5	11	17.70	4.40
-10	-5	-11	33.60	7.50
10	5	11	51.99	9.60
-10	5	-11	29.90	5.80
-10	5	-11	33.20	7.30
-11	-5	-11	15.20	8.90
-11	-5	-11	14.10	7.50
-11	5	-11	17.50	6.00
-11	5	-11	11.10	6.90
-12	-5	-11	31.40	8.80
-12	5	-11	32.70	7.70
-12	5	-11	11.60	6.80
-13	-5	-11	7.40	8.50
-13	5	-11	6.10	8.00
-14	-5	-11	4.40	8.50

-14	5	-11	5.40	7.70
-14	6	11	327.87	20.80
13	6	-11	60.09	11.90
-13	6	11	65.69	10.50
-12	-6	11	266.47	15.90
12	-6	-11	289.27	21.20
-12	6	11	317.47	18.00
12	6	-11	273.47	21.90
11	-6	-11	200.28	16.30
-11	-6	11	168.58	10.80
-11	6	11	142.59	20.50
11	6	-11	177.78	17.20
-11	6	11	186.78	13.00
-10	-6	11	519.55	24.80
-10	-6	11	461.25	23.80
10	-6	-11	461.55	27.80
10	6	-11	481.75	28.80
-10	6	11	467.35	25.50
-10	6	11	440.36	30.40
-9	-6	11	227.38	12.30
9	-6	-11	193.78	15.50
-9	-6	11	208.08	11.40
-9	6	11	194.88	17.90
-9	6	11	202.28	13.50
9	6	-11	224.28	16.90
-8	-6	11	702.93	43.80
8	-6	-11	847.42	42.90
-8	-6	11	905.71	40.10
8	6	-11	860.71	43.60
-8	6	11	764.42	44.20
-8	6	11	829.62	41.40
-7	-6	11	385.36	25.40
-7	-6	11	475.05	21.40
-7	-6	11	437.56	21.20
7	-6	-11	451.95	24.60
7	-6	-11	425.86	23.20
-7	6	11	419.76	23.10
-7	6	11	398.66	25.50
7	6	-11	432.26	25.00
6	-6	-11	69.79	8.70
6	-6	-11	84.29	7.30
-6	-6	11	74.59	10.10
-6	-6	11	79.49	4.70
-6	6	11	73.09	10.20

-6	6	11	85.39	8.00
6	6	-11	89.09	9.90
5	-6	-11	1263.17	59.59
-5	-6	11	1088.79	60.79
5	-6	-11	1264.97	60.79
-5	-6	11	1194.88	57.99
-5	6	11	1245.28	60.29
5	6	-11	1222.28	60.89
-5	6	11	1272.67	61.39
-4	-6	11	201.78	14.20
4	-6	-11	234.08	12.90
4	-6	-11	240.38	14.20
4	6	-11	239.18	14.40
-4	6	11	243.18	14.30
-4	6	11	220.68	14.50
3	-6	-11	1436.76	69.99
-3	-6	11	1345.87	70.79
3	-6	-11	1474.15	71.09
-3	-6	11	1577.04	68.89
-3	6	11	1399.76	71.09
-3	6	11	1416.86	71.09
3	6	-11	1466.25	68.79
3	6	-11	1475.65	70.89
2	-6	-11	137.19	7.90
-2	-6	11	138.59	6.90
-2	-6	11	114.59	8.60
2	-6	-11	124.79	9.00
-2	6	11	126.39	9.40
-2	6	11	120.69	9.30
2	6	-11	130.79	8.90
2	6	-11	123.99	6.50
1	-6	-11	1776.52	83.49
-1	-6	11	1668.23	83.49
1	-6	-11	1799.82	84.29
1	6	-11	1742.03	83.69
-1	6	11	1706.53	84.29
1	6	-11	1679.13	82.59
-1	6	11	1775.02	84.19
0	-6	-11	17.20	3.00
0	-6	11	22.60	3.40
0	-6	-11	16.70	3.50
0	6	11	16.00	3.80
0	6	-11	16.40	3.10
0	6	-11	15.90	2.70

0	6	11	9.80	2.90
0	6	-11	20.40	2.90
-1	-6	-11	1878.21	90.59
-1	-6	-11	1929.11	91.19
1	6	11	1913.21	91.29
-1	6	-11	1901.81	89.99
1	6	11	1810.22	91.09
-1	6	-11	1864.21	89.89
-1	6	-11	1893.41	90.19
1	6	11	1868.51	90.79
-2	-6	-11	70.99	6.20
-2	6	-11	79.09	4.40
2	6	11	73.89	6.80
-2	6	-11	68.49	6.00
-2	6	-11	68.99	5.50
2	6	11	65.39	6.00
2	6	11	83.59	7.20
-3	-6	-11	1458.15	67.89
3	6	11	1348.67	68.19
3	6	11	1416.26	68.39
3	6	11	1352.46	67.69
-3	6	-11	1356.16	67.69
-3	6	-11	1444.26	67.59
-4	-6	-11	331.87	17.70
-4	6	-11	330.67	17.80
4	6	11	331.37	18.30
4	6	11	312.07	17.30
-4	6	-11	342.77	17.70
4	6	11	302.37	18.10
-5	-6	-11	1229.38	58.79
-5	-6	-11	1161.48	58.99
5	6	11	1117.79	58.99
-5	6	-11	1268.97	58.79
5	6	11	1126.89	58.29
-5	6	-11	1182.98	58.79
5	6	11	1278.47	59.19
-6	-6	-11	751.82	39.50
-6	6	-11	751.92	37.20
6	6	11	727.93	38.50
6	6	11	779.12	39.50
-6	6	-11	822.72	39.40
6	6	11	822.02	39.50
-6	6	-11	755.12	39.20
-7	-6	-11	900.01	46.10

7	6	11	982.30	45.90
7	6	11	880.31	45.90
-7	6	-11	864.01	43.90
-7	6	-11	898.11	45.70
-8	-6	-11	197.48	14.80
8	6	11	210.88	14.40
8	6	11	224.28	14.50
-8	6	-11	205.78	12.30
-8	6	-11	218.68	14.40
-9	-6	-11	569.54	31.50
-9	6	-11	572.74	29.10
9	6	11	643.54	31.20
-9	6	-11	458.05	32.40
-10	-6	-11	455.85	27.90
-10	6	-11	515.75	27.20
10	6	11	491.65	27.20
10	6	11	494.75	27.20
-10	6	-11	418.56	25.30
-11	-6	-11	312.67	21.00
-11	6	-11	290.97	19.60
11	6	11	338.17	20.60
-11	6	-11	291.77	19.00
-12	-6	-11	312.87	21.10
-12	6	-11	231.88	18.70
-12	6	-11	322.17	20.30
-13	-6	-11	33.60	9.60
-13	6	-11	46.20	9.00
-14	-6	-11	259.97	21.30
-14	6	-11	239.08	20.20
-14	7	11	2.70	7.10
13	7	-11	90.29	20.00
-13	7	11	115.59	12.00
12	-7	-11	30.40	8.70
-12	-7	11	26.00	4.40
12	7	-11	20.10	9.70
-12	7	11	52.69	9.40
11	-7	-11	203.48	17.20
-11	-7	11	172.88	11.20
-11	7	11	217.08	13.90
-11	7	11	181.68	24.90
11	7	-11	196.58	18.20
10	-7	-11	141.29	14.00
-10	-7	11	128.29	9.30
-10	-7	11	121.59	8.10

10	7	-11	129.29	14.50
-10	7	11	135.79	12.80
-10	7	11	124.49	17.60
-9	-7	11	276.57	14.30
-9	-7	11	253.37	13.50
9	-7	-11	266.47	18.20
-9	7	11	258.07	15.80
-9	7	11	217.38	19.80
9	7	-11	259.47	18.70
-8	-7	11	15.00	8.30
-8	-7	11	15.10	2.50
8	-7	-11	16.10	5.80
-8	-7	11	16.00	2.90
8	7	-11	22.00	6.70
-8	7	11	19.80	4.90
-8	7	11	21.20	7.50
7	-7	-11	124.39	11.40
-7	-7	11	130.69	6.80
7	-7	-11	125.59	11.20
-7	-7	11	121.29	6.60
-7	-7	11	108.09	12.80
7	7	-11	127.89	11.90
-7	7	11	130.69	10.10
-7	7	11	104.79	12.50
-6	-7	11	287.67	20.30
6	-7	-11	341.97	18.60
6	-7	-11	332.87	19.70
-6	7	11	362.66	19.10
6	7	-11	334.87	20.20
-6	7	11	338.17	20.80
5	-7	-11	131.09	10.30
5	-7	-11	135.29	9.20
-5	-7	11	113.69	11.00
-5	7	11	116.39	10.10
5	7	-11	135.39	11.10
-5	7	11	139.39	11.30
-4	-7	11	926.31	51.69
4	-7	-11	1022.20	51.69
4	-7	-11	1052.79	50.69
-4	7	11	1061.99	51.69
4	7	-11	1088.49	51.89
-4	7	11	1054.19	52.19
-3	-7	11	14.80	3.70
3	-7	-11	18.80	4.00

3	-7	-11	23.80	3.60
3	7	-11	21.80	4.50
-3	7	11	17.90	4.30
-3	7	11	10.40	4.30
2	-7	-11	722.03	37.10
-2	-7	11	684.63	36.50
2	-7	-11	757.02	36.10
-2	7	11	729.83	37.10
2	7	-11	715.93	34.70
2	7	-11	770.72	36.70
2	7	-11	730.53	34.80
-2	7	11	715.43	37.20
1	-7	-11	91.49	6.80
1	-7	-11	104.19	8.20
-1	-7	11	95.39	7.00
-1	7	11	87.39	8.20
1	7	-11	104.29	5.70
-1	7	11	90.99	7.80
1	7	-11	99.99	7.30
1	7	-11	97.69	5.40
0	-7	-11	983.00	46.40
0	-7	-11	953.40	47.30
0	-7	11	934.31	45.90
0	7	-11	938.21	45.80
0	7	-11	964.90	46.20
0	7	-11	914.81	45.50
0	7	11	940.71	47.30
0	7	11	946.11	46.90
-1	-7	-11	47.60	5.10
-1	-7	-11	51.09	6.30
1	7	11	52.59	6.50
1	7	11	59.69	5.90
-1	7	-11	66.09	4.60
-1	7	-11	53.89	5.00
-1	7	-11	44.80	4.40
1	7	11	57.79	8.80
-2	-7	-11	798.72	38.40
-2	7	-11	775.42	37.80
2	7	11	797.42	39.20
-2	7	-11	769.32	38.00
2	7	11	742.13	38.80
-2	7	-11	771.32	37.20
2	7	11	741.13	38.40
-3	-7	-11	103.29	7.60

-3	7	-11	95.99	7.00
3	7	11	91.19	8.10
3	7	11	94.39	7.30
3	7	11	89.99	8.30
-3	7	-11	95.29	7.20
-4	-7	-11	278.57	15.80
4	7	11	305.07	16.90
4	7	11	259.27	16.30
4	7	11	276.67	15.70
-4	7	-11	282.17	15.90
-4	7	-11	303.67	15.90
-5	-7	-11	207.98	13.70
-5	-7	-11	215.78	13.10
-5	7	-11	208.18	13.10
5	7	11	246.58	14.10
5	7	11	218.38	13.70
5	7	11	195.18	12.50
-5	7	-11	204.68	13.10
-6	-7	-11	1072.79	53.99
-6	7	-11	1067.69	53.49
6	7	11	1208.18	54.09
6	7	11	1000.20	53.49
-6	7	-11	1110.99	51.49
6	7	11	987.70	52.99
-6	7	-11	1075.99	53.49
-7	-7	-11	267.57	17.10
-7	7	-11	248.78	14.20
7	7	11	288.77	16.90
-7	7	-11	272.97	16.50
7	7	11	264.97	16.40
-8	-7	-11	314.87	19.50
8	7	11	280.57	18.30
8	7	11	358.46	19.40
-8	7	-11	296.87	16.60
-8	7	-11	297.37	18.70
-9	-7	-11	380.06	23.30
9	7	11	366.26	22.70
-9	7	-11	362.96	20.60
9	7	11	417.26	23.00
-10	-7	-11	81.49	12.50
-10	7	-11	119.79	11.70
10	7	11	95.89	12.20
-10	7	-11	96.29	9.70
10	7	11	98.39	11.60

11	7	11	138.29	13.50
-11	7	-11	112.29	11.90
-11	7	-11	136.79	13.10
-12	-7	-11	95.79	14.00
-12	7	-11	94.19	12.80
-12	7	-11	78.89	11.60
-13	-7	-11	199.78	18.50
-13	7	-11	165.28	16.50
-14	-7	-11	32.80	10.90
-14	7	-11	25.50	9.50
-14	8	11	66.09	12.20
-13	8	11	24.60	7.30
13	8	-11	7.90	9.90
12	-8	-11	110.99	15.60
-12	8	11	131.49	11.90
12	8	-11	102.39	17.20
-11	-8	11	97.59	7.60
11	-8	-11	121.89	14.70
11	8	-11	110.59	15.40
-11	8	11	123.09	11.10
-10	-8	11	25.50	4.60
10	-8	-11	35.90	8.00
10	8	-11	34.70	8.90
-10	8	11	31.20	10.80
-10	8	11	39.70	6.30
-9	-8	11	123.29	7.50
9	-8	-11	110.69	12.20
-9	-8	11	105.59	6.90
-9	8	11	130.59	10.50
-9	8	11	107.29	15.70
9	8	-11	121.69	13.40
-8	-8	11	212.48	10.40
8	-8	-11	211.98	15.50
-8	-8	11	188.08	10.20
8	8	-11	207.78	16.10
-8	8	11	191.28	17.20
-8	8	11	211.38	13.50
-7	-8	11	97.29	12.80
7	-8	-11	110.99	11.20
-7	8	11	120.39	13.00
7	8	-11	114.59	11.80
-7	8	11	126.39	10.00
6	-8	-11	234.88	15.20
6	-8	-11	238.58	13.80

-6	-8	11	183.18	15.90
-6	8	11	249.68	14.70
6	8	-11	222.38	15.90
-6	8	11	227.18	16.30
-5	-8	11	547.35	33.70
5	-8	-11	658.33	32.60
5	-8	-11	666.23	33.70
-5	8	11	661.23	34.30
-5	8	11	653.73	33.40
5	8	-11	650.33	33.90
4	-8	-11	263.57	14.50
4	-8	-11	263.27	16.00
-4	-8	11	230.18	16.00
4	8	-11	273.47	16.20
-4	8	11	266.47	16.30
-4	8	11	263.97	16.00
3	-8	-11	163.28	11.10
3	-8	-11	162.78	9.60
-3	-8	11	125.59	10.40
-3	8	11	140.89	10.80
3	8	-11	162.98	10.70
-3	8	11	152.68	11.10
2	-8	-11	12.30	4.10
2	-8	-11	10.00	3.00
-2	-8	11	7.30	3.20
2	8	-11	7.00	3.30
-2	8	11	-2.00	3.80
-2	8	11	5.70	3.90
1	-8	-11	326.47	17.10
1	-8	-11	344.17	18.40
-1	-8	11	290.97	17.00
1	8	-11	321.37	15.70
-1	8	11	306.57	18.30
1	8	-11	323.37	17.30
1	8	-11	327.17	15.80
-1	8	11	322.27	18.00
0	-8	11	18.90	3.70
0	-8	-11	20.30	3.40
0	-8	-11	20.40	4.20
0	8	-11	19.60	3.20
0	8	11	18.10	3.90
0	8	-11	20.00	2.70
0	8	11	14.30	4.20
0	8	-11	16.30	2.30

0	8	11	21.40	4.70
-1	-8	-11	1833.82	88.69
-1	-8	-11	1909.31	89.69
1	8	11	1779.82	89.09
-1	8	-11	1869.91	88.19
1	8	11	1778.52	89.49
-1	8	-11	1892.51	88.09
-1	8	-11	1823.32	88.19
-2	-8	-11	89.59	7.00
-2	8	-11	78.49	5.30
-2	8	-11	80.99	6.40
2	8	11	84.99	7.70
-2	8	-11	88.69	6.30
2	8	11	74.29	7.00
-3	-8	-11	615.74	30.60
3	8	11	596.74	30.70
-3	8	-11	606.74	29.10
-3	8	-11	614.14	30.30
-3	8	-11	603.24	29.00
-3	8	-11	617.84	30.40
3	8	11	570.34	31.10
3	8	11	589.44	31.50
-4	-8	-11	47.40	6.30
4	8	11	35.80	6.10
4	8	11	36.40	7.20
-4	8	-11	49.00	6.10
-4	8	-11	48.70	6.20
4	8	11	48.50	5.90
-5	-8	-11	335.67	20.40
-5	-8	-11	413.16	22.10
-5	8	-11	368.46	19.80
5	8	11	359.16	20.20
-5	8	-11	371.26	19.80
-5	8	-11	358.16	17.60
5	8	11	335.57	19.50
-5	8	-11	336.37	17.50
5	8	11	375.76	20.90
-6	-8	-11	5.10	4.60
-6	8	-11	15.30	4.70
-6	8	-11	10.20	2.40
-6	8	-11	3.40	4.70
6	8	11	12.20	4.80
6	8	11	10.50	4.90
-7	-8	-11	372.16	22.50

-7	8	-11	355.06	19.60
-7	8	-11	412.76	21.80
7	8	11	412.86	22.40
7	8	11	376.56	21.80
-8	-8	-11	172.98	14.10
-8	8	-11	158.98	10.70
-8	8	-11	188.48	13.20
8	8	11	193.68	14.10
8	8	11	162.88	13.10
-9	-8	-11	19.30	6.90
9	8	11	12.80	6.30
-9	8	-11	10.00	5.60
9	8	11	9.30	6.40
-9	8	-11	3.10	4.20
-10	-8	-11	105.59	14.70
10	8	11	135.09	16.50
-10	8	-11	129.49	12.30
10	8	11	135.79	12.60
-10	8	-11	113.39	10.50
-11	8	-11	72.99	10.60
-11	8	-11	112.99	12.40
-12	-8	-11	136.39	15.70
-12	8	-11	80.49	12.00
-12	8	-11	113.79	13.40
-13	-8	-11	112.99	16.40
-13	8	-11	112.59	14.20
-14	-8	-11	118.19	17.60
-14	8	-11	122.49	15.60
-13	9	11	251.87	17.30
13	9	-11	220.08	22.40
12	-9	-11	132.49	21.80
-12	9	11	120.69	12.20
12	9	-11	91.89	19.20
11	-9	-11	293.97	21.10
-11	9	11	291.77	18.40
11	9	-11	284.47	22.60
-10	-9	11	238.78	15.00
10	-9	-11	264.17	19.40
-10	9	11	257.27	23.00
10	9	-11	262.07	20.10
-10	9	11	279.17	16.70
-9	-9	11	474.15	23.60
9	-9	-11	518.05	28.60
-9	-9	11	474.05	23.50

9	9	-11	519.65	29.20
-9	9	11	450.25	30.30
8	-9	-11	550.44	31.10
8	9	-11	569.24	32.00
-8	9	11	589.74	29.90
-8	9	11	535.85	32.90
7	-9	-11	331.57	20.30
-7	9	11	335.07	19.20
7	9	-11	329.27	21.00
-7	9	11	322.67	21.80
6	-9	-11	1326.67	65.29
-6	-9	11	1114.59	65.59
6	9	-11	1400.76	65.79
-6	9	11	1376.96	64.69
-6	9	11	1309.57	66.09
5	-9	-11	339.47	20.00
5	-9	-11	347.27	18.70
-5	-9	11	309.87	20.40
-5	9	11	316.57	20.40
-5	9	11	329.87	19.60
5	9	-11	367.46	20.40
-4	-9	11	740.93	42.70
4	-9	-11	856.61	41.80
4	-9	-11	875.91	43.00
4	9	-11	880.61	43.00
-4	9	11	838.82	42.90
-4	9	11	840.32	43.20
-3	-9	11	100.79	8.60
3	-9	-11	91.49	7.50
3	-9	-11	99.09	9.20
-3	9	11	85.89	9.30
-3	9	11	94.59	9.00
3	9	-11	115.59	8.90
2	-9	-11	1107.49	54.39
2	-9	-11	1129.39	53.19
-2	-9	11	985.50	53.59
-2	9	11	1037.50	54.39
-2	9	11	1081.19	54.19
2	9	-11	1178.78	53.89
-1	-9	11	57.99	6.00
1	-9	-11	65.69	5.60
1	-9	-11	68.49	7.40
-1	9	11	59.89	7.90
1	9	-11	60.99	3.90

1	9	-11	68.19	6.40
1	9	-11	61.49	3.80
-1	9	11	54.69	6.90
0	-9	-11	1209.98	58.39
0	-9	11	1176.28	57.99
0	-9	-11	1221.98	59.49
0	9	11	1128.19	59.29
0	9	-11	1181.88	57.49
0	9	-11	1287.67	58.39
0	9	11	1161.38	59.09
0	9	11	1241.38	59.89
0	9	-11	1175.28	57.39
-1	-9	-11	15.90	3.70
-1	9	-11	17.10	2.80
1	9	11	16.40	3.70
1	9	11	21.50	4.90
-1	9	-11	13.90	2.90
1	9	11	8.80	3.90
-1	9	-11	15.30	2.90
-2	-9	-11	1138.69	55.09
-2	9	-11	1158.78	54.19
2	9	11	1096.89	55.69
-2	9	-11	1123.79	54.59
-2	9	-11	1146.79	54.49
2	9	11	1071.39	55.39
-3	-9	-11	41.50	6.10
-3	9	-11	41.30	3.80
3	9	11	38.70	5.80
3	9	11	57.19	7.10
-3	9	-11	52.89	5.60
-3	9	-11	48.00	5.50
-3	9	-11	43.20	3.60
-4	-9	-11	2293.27	106.29
-4	9	-11	2220.68	104.69
-4	9	-11	2195.18	105.99
4	9	11	2322.97	107.39
-4	9	-11	2203.38	105.99
-4	9	-11	2160.18	104.59
4	9	11	2114.89	106.29
4	9	11	2100.09	106.59
-5	-9	-11	210.98	14.20
5	9	11	205.08	13.60
-5	9	-11	219.18	11.20
5	9	11	206.58	13.10

-5	9	-11	207.18	13.40
-5	9	-11	207.78	13.50
-5	9	-11	218.18	11.10
5	9	11	234.68	14.80
-6	-9	-11	1136.69	61.39
6	9	11	1170.78	61.09
-6	9	-11	1192.18	60.69
-6	9	-11	1222.28	60.69
-6	9	-11	1252.67	58.99
6	9	11	1393.56	61.99
-7	-9	-11	249.97	16.70
7	9	11	239.88	15.50
-7	9	-11	224.58	13.30
-7	9	-11	245.78	15.50
7	9	11	263.07	16.60
-8	-9	-11	375.76	23.40
-8	9	-11	382.66	20.50
8	9	11	449.56	23.70
8	9	11	334.57	23.10
-8	9	-11	389.16	22.00
-9	-9	-11	650.13	37.40
9	9	11	662.63	36.50
9	9	11	776.32	37.50
-9	9	-11	631.84	34.60
-9	9	-11	700.03	36.30
-10	9	-11	270.07	17.40
-10	9	-11	316.97	19.10
10	9	11	294.57	19.20
-11	9	-11	995.70	47.50
-11	9	-11	816.62	45.80
11	9	11	901.41	47.30
12	9	11	104.99	13.40
-12	9	-11	119.89	13.80
-12	9	-11	93.39	12.30
-13	-9	-11	489.55	30.90
-13	9	-11	478.65	29.10
13	10	-11	-0.80	10.20
-13	10	11	20.00	7.50
12	10	-11	12.80	9.60
-12	10	11	12.70	7.00
11	-10	-11	9.70	8.00
-11	10	11	21.70	6.80
10	-10	-11	14.80	7.20
-10	10	11	10.60	9.90

10	10	-11	17.60	8.60
9	-10	-11	69.39	11.60
-9	10	11	87.39	10.00
-9	10	11	79.59	15.90
9	10	-11	97.29	13.30
8	-10	-11	26.20	6.70
-8	10	11	21.00	7.90
8	10	-11	20.40	7.10
-8	10	11	21.00	5.80
7	-10	-11	16.30	5.70
7	10	-11	11.80	6.00
-7	10	11	8.90	5.00
-7	10	11	14.20	6.90
6	-10	-11	16.10	5.60
6	10	-11	4.50	5.20
-6	10	11	11.50	6.10
-6	10	11	13.40	5.30
-5	-10	11	7.90	5.70
5	-10	-11	1.90	4.40
5	10	-11	12.40	5.10
-5	10	11	-0.70	4.80
-5	10	11	9.60	5.60
4	-10	-11	99.19	9.50
-4	-10	11	72.19	9.50
4	-10	-11	89.09	7.70
-4	10	11	102.19	10.30
-4	10	11	91.29	9.70
4	10	-11	103.19	9.80
-3	-10	11	6.00	3.90
3	-10	-11	2.90	4.00
3	-10	-11	7.00	3.50
-3	10	11	-0.70	4.30
3	10	-11	5.40	4.00
-3	10	11	0.10	4.30
2	-10	-11	22.80	4.80
2	-10	-11	17.60	4.00
-2	-10	11	17.20	3.90
2	10	-11	17.30	4.50
-2	10	11	23.10	4.90
-2	10	11	9.40	4.90
1	-10	-11	128.89	10.20
-1	-10	11	129.59	8.90
1	-10	-11	140.49	8.50
-1	10	11	109.69	9.80

1	10	-11	122.39	6.60
1	10	-11	124.79	6.60
-1	10	11	118.19	10.00
0	-10	-11	6.20	3.40
0	-10	11	5.70	2.60
0	-10	-11	7.70	4.40
0	10	11	7.50	4.60
0	10	11	7.50	4.20
0	10	-11	9.90	2.20
0	10	-11	5.30	2.90
0	10	-11	6.20	2.00
0	10	11	6.50	3.60
-1	-10	-11	12.90	3.60
1	10	11	7.80	4.50
1	10	11	6.80	4.40
1	10	11	13.90	4.10
-1	10	-11	9.30	2.80
-1	10	-11	8.70	2.60
-1	10	-11	4.90	2.30
-2	-10	-11	187.08	11.40
-2	10	-11	174.38	10.50
2	10	11	176.18	12.30
-2	10	-11	175.28	10.60
2	10	11	177.08	11.80
2	10	11	178.18	12.90
-2	10	-11	186.88	10.30
-3	-10	-11	252.77	14.60
-3	10	-11	242.18	13.90
-3	10	-11	237.28	13.90
-3	10	-11	280.87	13.20
3	10	11	246.48	14.90
3	10	11	218.78	14.90
-3	10	-11	243.38	12.80
4	10	11	58.39	7.80
4	10	11	48.50	7.40
-4	10	-11	57.79	6.80
-4	10	-11	66.39	6.90
-4	10	-11	56.99	4.90
-4	10	-11	55.49	4.50
5	10	11	11.10	4.40
5	10	11	11.60	5.20
-5	10	-11	6.40	4.00
5	10	11	13.60	4.80
-5	10	-11	17.70	3.70

-5	10	-11	8.80	4.10
-6	-10	-11	17.60	5.70
-6	10	-11	20.80	5.60
6	10	11	13.40	5.10
-6	10	-11	17.70	3.10
6	10	11	11.90	5.70
-6	10	-11	24.40	5.10
-6	10	-11	15.60	2.90
-7	-10	-11	8.40	5.20
7	10	11	13.90	5.40
-7	10	-11	3.00	3.10
7	10	11	-0.20	5.00
-7	10	-11	13.80	5.00
-8	-10	-11	21.50	6.20
8	10	11	20.00	7.30
-8	10	-11	33.00	6.60
8	10	11	14.10	6.40
-8	10	-11	14.80	4.10
-9	-10	-11	22.10	6.60
9	10	11	12.90	6.40
-9	10	-11	22.30	6.10
-9	10	-11	13.40	4.70
9	10	11	9.70	6.00
-10	10	-11	25.40	7.20
10	10	11	30.40	7.60
-10	10	-11	18.60	5.60
-11	10	-11	71.29	12.00
11	10	11	67.89	12.40
12	10	11	13.50	8.20
-12	10	-11	3.50	7.60
-13	-10	-11	31.50	10.40
-13	10	-11	32.80	8.40
13	11	-11	281.87	25.50
-13	11	11	296.47	20.10
-12	11	11	194.08	15.50
12	11	-11	189.28	20.60
11	-11	-11	430.26	27.60
-11	11	11	449.06	25.00
11	11	-11	406.46	28.70
10	-11	-11	298.77	21.00
-10	11	11	285.27	18.60
9	-11	-11	521.35	30.00
-9	11	11	535.45	28.30
-9	11	11	487.65	32.50

9	11	-11	507.95	30.90
8	-11	-11	252.57	17.30
8	11	-11	238.18	18.20
-8	11	11	220.68	15.00
-8	11	11	210.08	19.20
7	-11	-11	81.79	11.10
-7	11	11	86.39	9.30
7	11	-11	108.19	12.40
-7	11	11	83.49	13.10
6	-11	-11	1020.20	52.79
-6	11	11	1082.89	52.39
6	11	-11	1046.80	53.19
-6	11	11	984.10	53.49
5	-11	-11	87.59	9.70
-5	11	11	63.69	10.20
5	11	-11	78.79	10.00
-5	11	11	74.59	9.50
4	-11	-11	1734.53	86.19
4	11	-11	1760.22	86.09
-4	11	11	1698.83	86.49
-4	11	11	1814.12	86.19
3	-11	-11	443.26	24.30
-3	-11	11	383.56	23.60
-3	11	11	427.96	24.10
-3	11	11	426.86	24.30
3	11	-11	464.85	24.00
-2	-11	11	1429.96	76.79
2	-11	-11	1607.34	76.39
2	-11	-11	1623.54	77.79
-2	11	11	1490.25	77.59
-2	11	11	1607.54	77.59
2	11	-11	1680.53	77.09
1	-11	-11	149.49	11.10
-1	-11	11	108.29	9.20
1	-11	-11	123.79	8.60
-1	11	11	131.89	10.20
1	11	-11	123.89	6.70
-1	11	11	110.49	10.90
1	11	-11	121.89	9.30
1	11	-11	131.59	6.60
0	-11	-11	2610.54	122.19
0	-11	-11	2698.43	123.59
0	-11	11	2354.36	121.79
0	11	11	2645.64	123.99

0	11	-11	2503.05	121.09
0	11	11	2555.14	123.09
0	11	11	2576.44	123.49
0	11	-11	2614.54	122.19
0	11	-11	2351.06	120.99
-1	-11	-11	20.40	4.10
-1	11	-11	13.60	3.00
-1	11	-11	18.00	3.30
1	11	11	19.40	4.60
1	11	11	36.20	6.40
-1	11	-11	24.90	3.80
1	11	11	23.10	5.50
-2	-11	-11	1444.46	70.19
2	11	11	1407.76	70.59
2	11	11	1495.35	71.69
2	11	11	1453.95	70.99
-2	11	-11	1440.36	69.39
-2	11	-11	1401.46	69.29
-2	11	-11	1410.46	69.29
3	11	11	368.36	21.20
3	11	11	386.06	22.00
3	11	11	357.86	20.70
-3	11	-11	376.76	19.80
-3	11	-11	379.66	19.80
-3	11	-11	376.36	19.20
-3	11	-11	365.76	18.90
-4	11	-11	1047.80	49.60
-4	11	-11	1065.79	48.70
-4	11	-11	954.60	48.40
-4	11	-11	1010.20	49.50
4	11	11	941.41	50.09
4	11	11	986.80	50.49
-5	11	-11	1317.07	64.69
-5	11	-11	1302.37	63.39
-5	11	-11	1330.97	63.59
-5	11	-11	1348.86	64.89
5	11	11	1289.07	65.49
-6	-11	-11	774.12	42.90
-6	11	-11	827.02	40.20
-6	11	-11	824.82	41.70
-6	11	-11	868.01	40.30
6	11	11	803.02	42.30
-7	-11	-11	597.24	35.90
-7	11	-11	651.03	33.20

7	11	11	713.63	36.50
-7	11	-11	701.63	34.90
7	11	11	663.53	35.40
-8	-11	-11	115.19	13.00
8	11	11	132.69	13.00
-8	11	-11	109.19	11.40
8	11	11	115.99	11.90
-8	11	-11	114.89	9.30
-9	11	-11	461.95	26.20
-9	11	-11	502.95	27.90
9	11	11	488.75	28.10
9	11	11	544.25	31.30
-10	11	-11	36.10	8.30
10	11	11	37.60	8.10
-10	11	-11	33.70	6.50
11	11	11	502.85	28.80
-11	11	-11	438.16	27.20
-11	11	-11	541.25	29.00
12	11	11	13.40	8.10
-12	11	-11	19.40	8.00
-13	-11	-11	178.68	19.60
-13	11	-11	162.08	16.90
13	12	-11	4.80	11.10
-13	12	11	16.30	7.60
12	12	-11	278.87	24.50
-12	12	11	265.07	18.80
-11	12	11	160.88	14.10
11	12	-11	161.88	19.30
10	-12	-11	270.07	21.10
10	12	-11	293.97	22.70
-10	12	11	281.77	18.50
9	-12	-11	75.69	12.40
-9	12	11	81.89	16.90
-9	12	11	63.19	10.50
8	-12	-11	107.09	12.90
-8	12	11	106.19	11.00
-8	12	11	104.39	15.80
7	-12	-11	369.86	22.30
7	12	-11	376.06	23.00
-7	12	11	330.67	25.10
-7	12	11	354.76	24.10
6	-12	-11	140.69	12.70
6	12	-11	139.89	13.20
-6	12	11	134.79	14.40

-6	12	11	127.89	12.00
5	-12	-11	801.12	42.00
-5	12	11	838.32	41.80
5	12	-11	834.32	42.30
-5	12	11	735.43	42.30
4	-12	-11	8.30	4.70
-4	12	11	6.50	5.50
4	12	-11	5.10	5.30
-4	12	11	-10.90	5.00
3	-12	-11	785.82	42.50
-3	12	11	805.82	42.70
3	12	-11	867.71	42.30
-3	12	11	824.82	42.70
2	-12	-11	4.60	4.30
-2	-12	11	9.60	6.80
-2	12	11	1.70	4.70
-2	12	11	-2.40	5.00
2	12	-11	5.70	4.20
1	-12	-11	727.93	36.70
-1	-12	11	640.64	35.10
-1	12	11	695.53	36.60
1	12	-11	679.23	33.20
-1	12	11	685.23	36.20
1	12	-11	764.52	35.60
1	12	-11	667.23	33.30
0	12	-11	51.59	6.40
0	12	11	46.10	7.60
0	12	11	42.20	7.50
0	12	-11	43.80	4.30
0	12	11	46.20	9.20
1	12	11	836.22	44.40
-1	12	-11	930.01	43.40
1	12	11	864.41	44.70
-1	12	-11	842.42	42.40
1	12	11	924.81	45.70
-1	12	-11	845.92	42.50
-2	12	-11	528.05	27.10
-2	12	-11	519.55	27.20
2	12	11	547.55	30.00
2	12	11	543.25	29.10
2	12	11	540.05	28.80
-2	12	-11	556.94	27.50
-3	12	-11	199.78	11.30
3	12	11	173.08	12.80

3	12	11	178.48	13.20
-3	12	-11	188.18	10.70
-3	12	-11	181.48	11.50
3	12	11	204.48	14.70
-3	12	-11	184.58	11.40
4	12	11	18.50	5.80
-4	12	-11	7.40	4.10
-4	12	-11	18.30	3.40
-4	12	-11	16.30	4.30
-4	12	-11	15.90	2.90
4	12	11	7.90	4.70
4	12	11	15.40	5.50
-5	12	-11	321.57	18.10
-5	12	-11	341.27	17.00
5	12	11	307.87	19.00
-5	12	-11	314.17	16.60
-5	12	-11	292.17	18.20
-6	-12	-11	278.47	18.40
-6	12	-11	265.77	16.70
-6	12	-11	279.97	15.00
6	12	11	255.37	17.30
-6	12	-11	270.77	14.90
-7	-12	-11	488.05	30.20
-7	12	-11	565.64	27.30
-7	12	-11	510.15	28.70
-7	12	-11	551.54	27.10
-8	-12	-11	423.66	26.30
-8	12	-11	436.76	23.10
8	12	11	432.66	25.00
9	12	11	74.19	11.10
-9	12	-11	104.99	11.10
-9	12	-11	64.49	8.90
-10	12	-11	188.38	14.20
-10	12	-11	205.48	16.00
10	12	11	196.08	16.20
11	12	11	52.69	9.50
-11	12	-11	71.59	12.40
-11	12	-11	55.69	11.10
-12	12	-11	155.38	15.40
12	12	11	135.69	15.40
-13	-12	-11	30.00	11.60
13	12	11	23.50	9.50
-13	12	-11	29.20	9.60
12	13	-11	149.88	20.70

-12	13	11	125.99	14.00
11	13	-11	100.39	18.20
-11	13	11	124.49	13.30
10	-13	-11	284.37	21.20
10	13	-11	263.87	22.50
-10	13	11	277.97	18.60
9	-13	-11	23.60	7.80
-9	13	11	27.50	7.30
9	13	-11	32.90	9.80
8	-13	-11	158.58	14.10
8	13	-11	117.39	17.50
-8	13	11	150.28	12.80
-8	13	11	116.59	16.70
7	-13	-11	4.30	6.10
-7	13	11	2.40	5.60
7	13	-11	10.20	6.90
-7	13	11	13.00	7.50
6	-13	-11	263.17	17.80
-6	13	11	245.98	19.10
6	13	-11	250.57	18.20
5	-13	-11	72.89	10.30
-5	13	11	57.59	9.90
-5	13	11	62.99	11.10
5	13	-11	56.19	10.40
4	-13	-11	306.27	18.90
4	13	-11	277.17	18.40
-4	13	11	275.47	19.00
-4	13	11	297.87	18.30
3	-13	-11	3.80	4.70
3	13	-11	4.20	4.40
-3	13	11	-2.10	5.20
-3	13	11	3.00	5.00
2	-13	-11	4.20	4.90
2	13	-11	8.80	4.70
-2	13	11	9.60	6.00
-2	13	11	3.20	4.80
1	-13	-11	3.10	4.70
-1	13	11	5.50	4.80
-1	13	11	-3.40	5.30
1	13	-11	0.90	1.60
-1	13	11	-1.90	5.60
1	13	-11	4.90	3.90
1	13	-11	4.20	1.40
0	13	11	456.45	25.70

0	13	11	465.75	25.40
0	13	-11	446.46	22.60
0	13	-11	448.86	24.20
-1	13	-11	2.60	2.60
1	13	11	2.90	4.40
1	13	11	8.90	5.80
-1	13	-11	0.80	2.70
1	13	11	1.10	4.70
-1	13	-11	2.20	3.50
2	13	11	330.47	19.30
-2	13	-11	314.77	17.00
2	13	11	306.27	20.30
2	13	11	322.07	18.90
-2	13	-11	314.07	17.10
-2	13	-11	313.37	16.80
-2	13	-11	326.27	17.60
3	13	11	58.89	8.70
3	13	11	39.50	7.40
-3	13	-11	51.29	6.10
3	13	11	40.60	7.80
-3	13	-11	42.50	6.40
-3	13	-11	56.09	6.20
-3	13	-11	53.39	5.50
-4	13	-11	44.10	6.70
-4	13	-11	37.00	5.60
4	13	11	49.90	8.10
4	13	11	48.20	8.50
-4	13	-11	39.00	5.10
-4	13	-11	41.10	5.90
-5	13	-11	14.80	3.40
5	13	11	9.80	6.80
-5	13	-11	23.70	5.30
-5	13	-11	20.10	3.90
-5	13	-11	17.40	5.20
5	13	11	20.00	5.50
-6	-13	-11	592.84	34.50
6	13	11	590.14	34.60
-6	13	-11	603.94	31.70
-6	13	-11	648.84	31.50
-6	13	-11	635.54	32.60
6	13	11	665.93	35.00
-7	-13	-11	86.99	12.20
-7	13	-11	74.29	9.90
-7	13	-11	68.79	7.70

-7	13	-11	71.29	7.50
7	13	11	76.59	10.40
8	13	11	-2.40	7.10
-8	13	-11	-0.80	4.20
-8	13	-11	9.00	5.70
8	13	11	9.50	6.20
-9	13	-11	135.09	12.60
9	13	11	121.79	13.20
-9	13	-11	117.39	11.00
-10	13	-11	8.80	5.80
10	13	11	9.20	7.10
-10	13	-11	12.20	7.00
11	13	11	86.39	13.20
-11	13	-11	93.89	13.00
-11	13	-11	67.09	11.30
-12	13	-11	48.70	9.90
12	13	11	73.29	13.50
-12	14	11	64.89	10.10
12	14	-11	47.90	12.90
-11	14	11	326.47	20.90
11	14	-11	284.77	24.90
10	-14	-11	231.68	21.90
-10	14	11	268.37	18.10
10	14	-11	242.38	21.80
9	-14	-11	237.38	19.50
9	14	-11	217.98	20.00
-9	14	11	259.77	16.80
8	-14	-11	288.77	20.70
-8	14	11	243.88	22.50
8	14	-11	316.27	21.70
-8	14	11	309.17	19.30
7	-14	-11	870.41	45.70
7	14	-11	851.41	46.10
-7	14	11	879.41	44.60
-7	14	11	827.12	46.80
6	-14	-11	65.29	11.20
-6	14	11	61.09	8.80
-6	14	11	43.30	8.80
5	-14	-11	657.73	35.60
5	14	-11	685.83	35.90
-5	14	11	612.74	36.10
4	-14	-11	77.89	10.50
-4	14	11	60.19	10.60
-4	14	11	87.89	11.50

4	14	-11	91.89	10.80
3	-14	-11	533.25	29.70
3	14	-11	552.14	29.20
-3	14	11	555.54	30.00
-3	14	11	494.85	29.80
2	-14	-11	3.30	5.10
-2	14	11	6.10	6.00
-2	14	11	3.60	5.30
2	14	-11	7.30	4.50
-1	14	11	831.02	43.50
1	14	-11	884.31	42.40
-1	14	11	810.62	43.10
1	14	-11	789.52	39.90
1	14	-11	782.22	39.70
-1	14	11	904.91	44.60
0	14	11	29.10	5.90
0	14	11	29.70	7.10
0	14	-11	21.30	4.10
0	14	11	27.80	8.30
0	14	-11	36.20	6.70
0	14	-11	31.20	3.90
1	14	11	1042.20	54.49
1	14	11	1082.69	54.79
-1	14	-11	1075.59	52.39
-1	14	-11	1050.49	52.09
-1	14	-11	1144.09	53.59
-2	14	-11	75.39	7.00
-2	14	-11	73.99	7.10
2	14	11	82.59	9.90
2	14	11	83.19	9.50
-2	14	-11	94.39	8.00
2	14	11	103.29	12.20
-2	14	-11	82.09	6.70
3	14	11	944.31	48.40
-3	14	-11	922.71	45.30
3	14	11	974.10	48.70
3	14	11	898.71	47.10
-3	14	-11	889.41	45.60
-3	14	-11	866.71	45.20
-3	14	-11	951.70	45.80
4	14	11	31.80	7.70
-4	14	-11	29.90	6.20
-4	14	-11	40.00	5.70
-4	14	-11	35.50	6.40

-4	14	-11	32.10	5.30
4	14	11	33.80	6.90
-5	14	-11	784.92	40.80
5	14	11	916.51	44.60
-5	14	-11	847.32	41.40
-5	14	-11	758.92	41.80
5	14	11	806.12	42.60
-5	14	-11	839.02	41.60
-6	14	-11	303.17	18.60
-6	14	-11	319.27	17.60
6	14	11	302.67	19.90
-6	14	-11	318.17	17.70
6	14	11	338.17	21.90
-7	-14	-11	730.23	46.80
7	14	11	921.91	45.90
-7	14	-11	824.42	43.10
-7	14	-11	863.71	42.20
-7	14	-11	874.71	42.20
7	14	11	820.82	43.90
8	14	11	136.09	13.90
-8	14	-11	157.08	13.10
-8	14	-11	160.98	11.90
-8	14	-11	165.68	11.50
9	14	11	164.38	15.60
-9	14	-11	216.78	15.30
-9	14	-11	188.28	14.00
-10	14	-11	624.94	32.80
10	14	11	578.04	33.00
-10	14	-11	551.24	31.60
11	14	11	80.69	13.80
-11	14	-11	116.79	13.80
-12	14	-11	463.15	27.80
12	14	11	433.36	28.00
12	15	-11	22.70	12.50
-12	15	11	4.40	8.10
11	15	-11	-11.80	11.20
-11	15	11	2.60	7.10
-10	15	11	43.20	8.90
10	15	-11	43.40	11.50
9	-15	-11	13.20	8.50
-9	15	11	-0.60	7.00
9	15	-11	7.20	8.40
8	-15	-11	19.00	8.50
8	15	-11	28.20	9.00

-8	15	11	18.00	7.50
7	-15	-11	6.40	6.90
-7	15	11	0.10	6.30
7	15	-11	9.10	7.50
-7	15	11	2.60	8.90
6	-15	-11	2.50	6.80
-6	15	11	18.80	8.50
6	15	-11	18.20	7.40
-6	15	11	16.00	7.40
5	-15	-11	-2.50	5.80
5	15	-11	2.80	6.10
-5	15	11	2.10	6.40
-5	15	11	9.70	7.30
4	-15	-11	8.30	5.70
-4	15	11	11.60	8.20
-4	15	11	9.00	6.60
3	-15	-11	47.70	9.40
-3	15	11	41.10	7.80
3	15	-11	29.20	7.70
-3	15	11	54.09	8.20
2	-15	-11	2.80	5.30
-2	15	11	1.40	5.90
2	15	-11	7.50	4.80
-2	15	11	-0.90	5.40
-2	15	11	-1.50	5.90
1	15	-11	3.50	1.60
-1	15	11	1.20	6.50
-1	15	11	-0.10	5.20
-1	15	11	-6.00	5.50
1	15	-11	4.00	1.90
1	15	-11	3.90	4.50
0	15	-11	75.19	8.40
0	15	-11	57.29	4.90
0	15	11	44.70	9.10
0	15	11	58.39	8.60
0	15	11	61.19	11.00
-1	15	-11	10.50	4.40
-1	15	-11	14.60	3.40
-1	15	-11	11.00	2.90
1	15	11	5.40	5.20
1	15	11	7.40	6.80
1	15	11	6.40	5.90
2	15	11	7.80	7.00
-2	15	-11	8.00	4.90

-2	15	-11	6.40	3.60
-2	15	-11	13.30	3.30
-2	15	-11	10.60	3.60
2	15	11	12.90	5.10
2	15	11	5.40	5.20
3	15	11	7.50	5.80
-3	15	-11	7.30	3.60
-3	15	-11	-1.40	7.20
-3	15	-11	7.30	4.20
3	15	11	-4.30	6.60
-3	15	-11	9.00	3.80
-4	15	-11	22.70	4.60
-4	15	-11	9.40	4.10
-4	15	-11	13.90	3.90
4	15	11	17.60	6.30
-4	15	-11	16.80	5.00
4	15	11	16.70	7.20
5	15	11	3.10	5.70
5	15	11	-5.30	6.70
-5	15	-11	5.40	4.40
-5	15	-11	6.00	3.60
-5	15	-11	4.00	3.90
6	15	11	12.70	6.40
-6	15	-11	9.80	4.00
-6	15	-11	13.10	4.50
6	15	11	11.10	7.50
-6	15	-11	11.50	5.10
7	15	11	39.10	7.90
7	15	11	32.60	8.80
-7	15	-11	33.00	5.70
-7	15	-11	38.90	7.90
-7	15	-11	37.90	7.60
-8	15	-11	21.30	5.90
8	15	11	19.70	7.60
-8	15	-11	25.50	6.40
-8	15	-11	24.70	5.60
-9	15	-11	4.10	5.70
-9	15	-11	-2.50	4.80
-9	15	-11	4.50	6.20
9	15	11	2.30	7.00
-10	15	-11	12.80	7.00
-10	15	-11	9.10	6.50
10	15	11	3.00	7.00
-11	15	-11	-2.90	7.80

11	15	11	-4.20	8.40
12	15	11	35.10	9.70
11	16	-11	146.09	21.20
-11	16	11	132.19	15.10
-10	16	11	464.65	27.30
10	16	-11	434.66	30.70
9	-16	-11	132.49	21.00
-9	16	11	128.99	14.00
9	16	-11	113.09	17.60
8	-16	-11	100.89	15.20
8	16	-11	122.29	16.10
-8	16	11	108.79	12.90
7	-16	-11	533.15	31.10
-7	16	11	524.75	30.00
7	16	-11	546.95	31.80
-7	16	11	473.55	40.00
6	-16	-11	40.00	8.40
-6	16	11	44.60	8.50
6	16	-11	37.50	8.60
-6	16	11	35.20	9.90
5	-16	-11	586.84	32.80
5	16	-11	574.34	32.80
-5	16	11	539.75	33.50
-5	16	11	614.74	32.50
4	-16	-11	159.18	13.80
4	16	-11	143.49	13.40
-4	16	11	123.69	14.00
-4	16	11	135.89	13.70
3	-16	-11	589.14	33.70
-3	16	11	564.34	33.70
-3	16	11	642.24	34.00
3	16	-11	637.24	33.30
2	16	-11	39.80	7.00
-2	16	11	38.40	8.60
-2	16	11	45.90	8.20
1	16	-11	835.82	41.80
-1	16	11	836.82	44.10
-1	16	11	795.72	42.60
1	16	-11	758.62	38.70
1	16	-11	787.82	38.90
-1	16	11	847.22	44.70
0	16	-11	38.90	4.20
0	16	11	46.70	8.40
0	16	-11	34.90	4.70

0	16	11	22.20	7.90
0	16	-11	38.50	7.80
0	16	11	31.60	7.10
1	16	11	834.42	43.40
-1	16	-11	815.32	39.90
1	16	11	767.82	42.70
-1	16	-11	859.31	42.00
1	16	11	854.01	44.60
-1	16	-11	788.32	40.30
-2	16	-11	11.90	3.60
-2	16	-11	7.90	4.00
-2	16	-11	19.90	5.10
2	16	11	2.00	6.90
-2	16	-11	18.70	4.20
2	16	11	23.00	7.60
-3	16	-11	576.84	31.40
-3	16	-11	621.34	32.10
3	16	11	656.13	36.10
-3	16	-11	625.74	31.10
-3	16	-11	602.74	31.50
-4	16	-11	204.98	13.00
-4	16	-11	188.78	12.10
-4	16	-11	186.48	12.20
-4	16	-11	175.08	13.10
4	16	11	180.68	14.50
-5	16	-11	482.95	25.70
-5	16	-11	502.95	26.00
-5	16	-11	440.26	25.20
5	16	11	493.95	29.90
5	16	11	493.25	27.60
-6	16	-11	290.97	18.10
-6	16	-11	275.47	17.20
-6	16	-11	316.67	17.90
6	16	11	299.57	19.50
6	16	11	304.17	22.50
-7	16	-11	347.87	19.70
7	16	11	346.87	21.70
-7	16	-11	306.77	20.20
-7	16	-11	352.96	19.90
-8	16	-11	335.97	19.50
-8	16	-11	341.17	20.40
8	16	11	319.07	21.40
-8	16	-11	310.37	19.60
-9	16	-11	137.19	12.40

-9	16	-11	150.38	13.90
9	16	11	138.29	14.60
-9	16	-11	158.68	13.40
10	16	11	225.58	18.60
-10	16	-11	267.67	18.50
11	16	11	38.90	10.20
-11	16	-11	80.59	13.60
11	17	-11	193.28	23.10
-11	17	11	172.58	16.90
10	17	-11	149.19	21.00
-10	17	11	166.68	15.40
-9	17	11	31.90	8.50
9	17	-11	65.29	12.10
8	-17	-11	39.00	9.80
-8	17	11	34.60	8.40
8	17	-11	42.70	10.20
7	-17	-11	101.89	14.60
7	17	-11	129.89	16.10
-7	17	11	113.89	13.70
6	-17	-11	519.75	32.30
-6	17	11	499.65	33.40
-6	17	11	596.44	31.70
6	17	-11	593.84	32.90
5	-17	-11	78.39	12.10
-5	17	11	59.19	9.70
-5	17	11	73.79	14.00
5	17	-11	85.99	12.80
4	-17	-11	493.45	29.50
-4	17	11	510.75	29.10
4	17	-11	557.54	29.40
-4	17	11	432.46	29.50
3	-17	-11	20.30	7.80
-3	17	11	4.20	6.50
-3	17	11	22.30	8.30
3	17	-11	25.90	6.90
-3	17	11	27.10	8.80
-2	17	11	259.07	21.20
-2	17	11	312.97	20.60
-2	17	11	318.17	22.60
2	17	-11	329.47	19.70
1	17	-11	5.60	2.30
-1	17	11	-1.00	7.00
1	17	-11	0.40	5.30
-1	17	11	1.10	6.00

-1	17	11	-4.00	6.90
1	17	-11	2.90	1.90
0	17	11	594.74	35.00
0	17	11	700.63	37.30
0	17	-11	687.03	34.40
0	17	-11	611.54	31.80
0	17	11	635.64	35.10
0	17	-11	641.34	31.40
1	17	11	-1.00	6.40
1	17	11	-10.50	9.80
1	17	11	2.60	8.20
-1	17	-11	5.50	4.00
-1	17	-11	2.10	4.70
-1	17	-11	6.50	3.70
-1	17	-11	7.40	3.10
-2	17	-11	396.66	22.10
-2	17	-11	402.86	21.50
-2	17	-11	373.06	22.50
2	17	11	454.05	27.20
2	17	11	433.66	25.10
-2	17	-11	464.05	23.80
-3	17	-11	43.20	7.40
-3	17	-11	42.30	6.40
3	17	11	35.00	7.90
-3	17	-11	51.19	6.70
-3	17	-11	41.30	7.10
3	17	11	35.70	8.70
-4	17	-11	199.48	12.70
-4	17	-11	186.48	12.80
-4	17	-11	219.28	14.10
4	17	11	197.28	15.30
-4	17	-11	179.78	13.50
5	17	11	101.29	12.00
-5	17	-11	121.79	10.00
-5	17	-11	120.79	10.90
-5	17	-11	108.79	9.80
5	17	11	108.99	16.40
-6	17	-11	315.57	19.80
6	17	11	338.57	21.40
-6	17	-11	373.66	20.20
-6	17	-11	320.17	19.40
7	17	11	35.70	8.00
-7	17	-11	36.40	7.40
-7	17	-11	43.30	8.90

-7	17	-11	45.50	7.10
8	17	11	72.79	12.40
-8	17	-11	69.99	10.10
-8	17	-11	56.09	10.30
-8	17	-11	62.49	10.70
-9	17	-11	116.59	13.20
9	17	11	114.99	14.20
-9	17	-11	127.29	14.80
-9	17	-11	133.09	12.30
-10	17	-11	27.10	7.10
10	17	11	25.20	8.80
-10	17	-11	26.30	8.60
-11	17	-11	71.99	11.00
11	17	11	56.39	10.40
-10	18	11	58.99	10.80
10	18	-11	58.69	13.90
-9	18	11	41.60	9.20
9	18	-11	32.20	10.90
8	18	-11	12.80	9.80
-8	18	11	24.60	8.70
7	-18	-11	19.90	8.50
7	18	-11	15.00	8.60
-7	18	11	13.30	7.40
6	-18	-11	54.19	9.80
6	18	-11	51.49	10.90
-6	18	11	70.49	11.20
5	-18	-11	194.48	17.90
5	18	-11	190.98	17.40
-5	18	11	217.08	19.80
-5	18	11	232.98	17.30
4	-18	-11	13.20	7.30
-4	18	11	14.00	9.00
4	18	-11	18.70	7.30
-4	18	11	22.10	8.00
-3	18	11	38.70	10.00
3	18	-11	47.40	8.40
-3	18	11	45.50	11.30
-3	18	11	51.99	9.80
-2	18	11	1.60	8.40
-2	18	11	-3.90	6.60
-2	18	11	6.60	8.00
2	18	-11	10.10	6.10
1	18	-11	404.06	23.70
1	18	-11	393.76	20.00

-1	18	11	407.56	27.10
-1	18	11	380.86	25.00
-1	18	11	418.06	24.60
0	18	11	23.30	8.00
0	18	11	31.80	9.00
0	18	-11	31.20	6.70
0	18	11	28.70	10.60
0	18	-11	27.90	4.40
0	18	-11	24.80	3.90
-1	18	-11	418.86	21.40
1	18	11	445.46	27.50
-1	18	-11	399.06	22.00
1	18	11	428.76	25.60
-1	18	-11	412.06	23.20
-1	18	-11	423.86	24.20
-2	18	-11	39.30	6.90
2	18	11	45.60	8.70
-2	18	-11	43.40	7.70
-2	18	-11	44.50	5.80
-2	18	-11	44.20	6.00
2	18	11	34.00	9.30
-3	18	-11	198.88	11.40
3	18	11	162.78	14.80
-3	18	-11	159.18	12.10
-3	18	-11	173.28	13.30
-3	18	-11	173.88	12.30
3	18	11	157.68	17.40
-4	18	-11	46.80	9.30
4	18	11	50.59	8.80
-4	18	-11	39.30	7.70
4	18	11	31.10	9.50
-4	18	-11	52.79	7.60
-5	18	-11	87.39	10.40
-5	18	-11	73.89	9.10
-5	18	-11	80.99	9.40
5	18	11	91.49	15.20
5	18	11	94.89	12.30
-6	18	-11	137.49	11.80
-6	18	-11	129.59	13.90
6	18	11	152.68	14.20
-6	18	-11	150.88	12.70
-7	18	-11	172.08	13.80
-7	18	-11	155.28	13.10
-7	18	-11	168.08	13.60

7	18	11	148.99	15.20
8	18	11	120.69	14.00
-8	18	-11	117.79	12.40
-8	18	-11	120.69	12.90
-8	18	-11	103.69	12.00
9	18	11	-4.30	7.40
-9	18	-11	-0.30	5.90
-9	18	-11	12.00	7.20
-10	18	-11	26.60	7.70
10	18	11	29.50	9.40
-10	18	-11	41.30	9.20
-9	19	11	335.27	23.40
9	19	-11	350.46	27.10
8	19	-11	269.47	23.60
-8	19	11	272.87	20.30
-7	19	11	176.98	16.20
7	19	-11	161.98	19.00
6	-19	-11	845.32	47.60
-6	19	11	870.51	46.40
6	19	-11	889.01	47.90
5	-19	-11	48.70	10.40
5	19	-11	57.19	10.30
-5	19	11	61.59	10.30
4	-19	-11	572.84	34.30
4	19	-11	604.04	33.70
-4	19	11	626.44	37.00
-4	19	11	518.75	34.70
-4	19	11	610.44	33.80
-3	19	11	-3.00	7.60
-3	19	11	7.10	8.00
-3	19	11	12.70	9.00
3	19	-11	9.80	7.10
-2	19	11	994.20	58.49
-2	19	11	1176.08	58.59
-2	19	11	1163.78	60.69
2	19	-11	1174.88	57.99
-1	19	11	103.49	13.80
-1	19	11	104.49	16.80
1	19	-11	111.19	12.40
0	19	-11	609.64	32.30
0	19	11	642.54	36.70
0	19	-11	693.63	35.90
0	19	11	702.53	39.10
-1	19	-11	17.60	3.60

1	19	11	12.00	8.80
-1	19	-11	14.20	6.00
1	19	11	-0.40	6.90
-1	19	-11	10.10	4.90
2	19	11	536.55	31.90
2	19	11	522.55	29.30
-2	19	-11	457.35	26.80
-2	19	-11	529.95	28.20
-2	19	-11	453.55	29.10
-2	19	-11	501.75	25.50
3	19	11	78.29	12.20
3	19	11	79.49	15.20
-3	19	-11	79.79	7.90
-3	19	-11	74.89	10.60
-3	19	-11	66.09	9.50
-3	19	-11	74.79	9.00
-4	19	-11	375.26	21.30
4	19	11	377.36	26.00
-4	19	-11	338.27	19.20
4	19	11	357.16	22.30
-4	19	-11	292.77	19.70
-5	19	-11	411.86	22.70
5	19	11	394.66	23.80
-5	19	-11	334.07	21.20
-5	19	-11	356.46	21.00
-6	19	-11	249.88	17.10
-6	19	-11	314.37	18.40
6	19	11	270.67	19.40
-6	19	-11	243.28	17.10
7	19	11	201.18	16.60
-7	19	-11	186.88	15.10
-7	19	-11	203.68	15.50
-8	19	-11	179.38	15.30
-8	19	-11	209.28	15.30
8	19	11	183.58	17.10
9	19	11	158.78	16.50
-9	19	-11	168.18	15.00
-9	19	-11	168.58	15.10
-9	20	11	7.90	8.40
9	20	-11	4.70	11.10
8	20	-11	14.50	10.80
-8	20	11	11.40	8.00
7	20	-11	2.70	9.40
-7	20	11	-14.60	8.10

6	-20	-11	14.10	10.30
-6	20	11	13.20	9.10
6	20	-11	34.10	10.90
5	-20	-11	11.40	8.70
5	20	-11	26.30	8.80
-5	20	11	14.70	8.70
-4	20	11	-13.00	7.70
4	20	-11	16.90	7.50
-4	20	11	-2.30	9.50
3	20	-11	108.79	13.70
-3	20	11	89.79	14.30
-3	20	11	91.79	17.40
-2	20	11	22.00	9.20
-2	20	11	23.30	11.30
2	20	-11	40.30	8.20
-1	20	11	76.29	12.60
-1	20	11	57.29	10.30
1	20	-11	84.99	12.00
0	20	11	-0.50	9.30
0	20	-11	7.80	3.00
0	20	11	-10.60	7.60
0	20	-11	4.70	6.20
1	20	11	34.40	11.00
-1	20	-11	17.30	3.80
1	20	11	20.30	8.50
-1	20	-11	17.20	5.10
-1	20	-11	25.00	7.00
2	20	11	-2.00	6.50
2	20	11	-10.50	8.40
-2	20	-11	-4.50	4.70
-2	20	-11	-0.30	6.00
-2	20	-11	2.20	3.80
3	20	11	15.90	7.80
-3	20	-11	20.10	4.70
3	20	11	8.50	10.00
-3	20	-11	11.20	6.20
-3	20	-11	13.20	5.30
-4	20	-11	4.40	6.10
4	20	11	-2.40	10.80
-4	20	-11	2.30	4.80
4	20	11	3.50	7.30
-4	20	-11	4.80	4.90
5	20	11	4.20	7.40
-5	20	-11	17.90	5.40

-5	20	-11	9.80	6.20
-6	20	-11	4.20	5.80
6	20	11	5.70	7.10
-6	20	-11	5.50	6.40
7	20	11	19.70	8.10
-7	20	-11	5.00	6.60
-7	20	-11	14.60	6.90
-8	20	-11	5.60	7.20
-8	20	-11	0.80	6.90
8	20	11	8.30	8.30
9	20	11	-8.90	8.10
-9	20	-11	0.20	8.40
-9	20	-11	-6.50	7.30
-8	21	11	165.28	17.40
8	21	-11	167.38	21.80
-7	21	11	75.19	14.00
7	21	-11	110.49	15.50
-6	21	11	310.77	23.20
6	21	-11	342.87	25.00
5	21	-11	97.99	16.10
-5	21	11	69.39	11.30
-5	21	11	119.39	21.30
4	21	-11	317.67	23.50
-4	21	11	335.77	23.30
-4	21	11	325.07	27.40
-3	21	11	91.59	15.40
-3	21	11	138.79	19.90
3	21	-11	132.79	15.30
2	21	-11	373.86	24.70
-2	21	11	383.76	29.10
-2	21	11	395.16	25.70
1	21	-11	28.10	7.70
-1	21	11	8.90	10.10
-1	21	11	13.70	8.30
0	21	11	634.94	36.40
0	21	11	648.54	39.50
0	21	-11	647.44	35.90
1	21	11	8.30	7.50
1	21	11	6.60	10.10
-1	21	-11	10.30	6.90
-1	21	-11	14.50	3.90
2	21	11	575.14	35.60
-2	21	-11	514.35	28.30
-2	21	-11	582.84	31.80

2	21	11	569.14	32.50
-3	21	-11	55.79	11.30
3	21	11	62.19	9.90
3	21	11	70.09	17.20
-3	21	-11	54.49	7.90
4	21	11	469.15	28.10
-4	21	-11	479.75	27.60
-4	21	-11	450.95	24.90
-5	21	-11	265.77	18.80
-5	21	-11	232.08	22.50
5	21	11	279.87	19.80
6	21	11	358.06	23.40
-6	21	-11	350.46	22.50
-6	21	-11	329.87	20.60
-7	21	-11	144.29	15.00
7	21	11	156.78	15.90
-7	21	-11	153.18	14.20
-8	21	-11	67.09	13.00
8	21	11	55.89	10.60
-8	21	-11	51.79	8.80
7	22	-11	67.19	12.70
-7	22	11	58.09	11.60
-6	22	11	129.59	24.80
6	22	-11	119.49	18.00
5	22	-11	287.77	22.70
-5	22	11	254.87	20.80
-5	22	11	240.68	31.10
-4	22	11	0.00	9.10
4	22	-11	9.00	8.20
-4	22	11	-1.10	11.80
3	22	-11	138.49	16.30
-3	22	11	128.69	16.20
-3	22	11	128.49	21.30
2	22	-11	-2.70	6.80
-2	22	11	-3.70	9.10
-2	22	11	-16.10	11.10
-1	22	11	112.69	15.20
-1	22	11	102.79	20.10
1	22	-11	138.89	15.00
0	22	11	7.70	9.00
0	22	-11	6.20	7.20
0	22	11	6.40	11.70
1	22	11	321.97	23.00
-1	22	-11	338.97	22.50

1	22	11	335.77	27.50
-2	22	-11	69.79	8.00
2	22	11	85.09	13.60
-2	22	-11	74.49	13.00
3	22	11	190.58	17.40
-3	22	-11	189.98	12.90
-3	22	-11	210.38	16.90
-4	22	-11	36.60	8.30
-4	22	-11	34.90	6.30
4	22	11	51.49	9.80
-5	22	-11	155.08	14.70
5	22	11	129.69	15.40
-5	22	-11	107.39	11.60
6	22	11	23.10	8.50
-6	22	-11	2.50	6.30
-6	22	-11	11.20	7.70
7	22	11	116.99	15.70
-7	22	-11	138.69	15.60
-7	22	-11	129.29	12.80
-6	23	11	80.39	12.90
6	23	-11	120.19	18.70
-6	23	11	88.19	17.00
5	23	-11	-7.30	9.20
-5	23	11	-8.70	12.80
-5	23	11	-1.00	9.10
-4	23	11	160.48	17.20
-4	23	11	136.49	25.60
4	23	-11	130.69	17.70
-3	23	11	9.80	12.50
-3	23	11	-16.90	8.80
3	23	-11	2.20	8.20
-2	23	11	16.30	12.20
2	23	-11	9.00	7.80
-2	23	11	6.10	9.60
-1	23	11	-0.60	12.60
1	23	-11	2.80	7.30
-1	23	11	-8.30	8.60
0	23	11	106.89	16.80
0	23	-11	155.48	16.20
0	23	11	170.48	17.00
-1	23	-11	45.90	9.40
1	23	11	47.40	10.20
2	23	11	150.48	16.00
-2	23	-11	112.89	14.80

3	23	11	23.10	9.50
-3	23	-11	19.20	7.80
-3	23	-11	20.50	6.60
4	23	11	8.20	8.40
-4	23	-11	13.00	7.90
-4	23	-11	19.30	7.00
5	23	11	52.99	10.60
-5	23	-11	29.80	9.10
-5	23	-11	42.70	7.30
-6	23	-11	210.18	26.10
6	23	11	201.18	18.20
-6	23	-11	164.48	14.50
-5	24	11	423.46	38.00
-4	24	11	34.00	10.70
-4	24	11	57.89	17.00
4	24	-11	80.29	13.20
3	24	-11	304.67	24.00
-3	24	11	309.47	23.30
-3	24	11	292.67	30.20
-2	24	11	-4.60	9.60
2	24	-11	8.70	8.00
-2	24	11	-21.20	18.50
-1	24	11	528.15	32.60
1	24	-11	527.05	32.30
0	24	11	-11.20	8.50
0	24	-11	8.70	8.40
1	24	11	453.55	28.90
-1	24	-11	446.46	28.70
-2	24	-11	3.00	8.00
2	24	11	-1.30	8.70
-3	24	-11	224.48	19.10
3	24	11	207.38	19.40
4	24	11	89.09	15.00
-4	24	-11	73.09	14.40
2	25	-11	30.60	10.20
-2	25	11	35.00	11.50
1	25	-11	19.60	9.70
-1	25	11	23.30	10.40
0	25	11	21.80	9.80
0	25	-11	36.00	10.10
1	25	11	12.40	9.80
-1	25	-11	3.30	8.80
2	25	11	17.10	9.90
-14	0	12	429.16	24.40

-13	0	12	191.58	13.30
13	0	-12	162.98	18.40
-12	0	12	121.39	10.20
12	0	-12	93.89	14.60
11	0	-12	441.86	26.30
-11	0	12	436.06	23.20
11	0	-12	422.76	26.80
10	0	-12	601.24	31.50
-10	0	12	540.95	35.90
10	0	-12	643.64	33.90
-10	0	12	613.64	30.70
9	0	-12	612.94	31.00
-9	0	12	549.85	35.10
-9	0	12	607.24	30.40
9	0	-12	621.84	33.30
8	0	-12	52.59	6.60
8	0	-12	50.59	9.00
-8	0	12	45.10	6.00
-8	0	12	38.50	8.00
-7	0	12	731.23	41.90
7	0	-12	817.02	39.00
7	0	-12	807.12	41.00
-7	0	12	784.52	39.00
-6	0	12	309.87	16.50
6	0	-12	292.57	18.00
-6	0	12	283.27	19.10
6	0	-12	321.37	16.20
5	0	-12	2603.84	123.99
-5	0	12	2593.24	122.89
-5	0	12	2468.55	124.49
5	0	-12	2589.84	122.39
-4	0	12	49.50	7.30
4	0	-12	29.40	3.80
4	0	-12	37.40	6.00
-4	0	12	31.80	5.00
-3	0	12	2429.86	119.49
3	0	-12	2423.46	118.89
-3	0	12	2559.94	120.69
3	0	-12	2576.54	120.19
-2	0	12	3.80	2.40
2	0	-12	6.80	2.00
-2	0	12	5.60	3.10
2	0	-12	8.60	2.90
1	0	-12	1707.13	82.19

1	0	-12	1702.73	81.59
-1	0	12	1780.92	82.39
-1	0	12	1627.34	81.89
0	0	-12	22.40	3.50
0	0	12	30.50	4.10
0	0	-12	23.70	3.60
0	0	12	21.20	3.90
-1	0	-12	2352.56	117.79
1	0	12	2539.45	117.79
1	0	12	2390.16	118.19
1	0	12	2511.95	117.69
-1	0	-12	2541.55	117.89
-2	0	-12	127.49	7.50
2	0	12	122.59	8.40
-2	0	-12	129.89	8.10
2	0	12	127.49	7.30
2	0	12	126.89	7.10
3	0	12	1640.24	77.09
3	0	12	1554.74	78.39
3	0	12	1631.44	77.09
-3	0	-12	1647.24	78.19
4	0	12	686.23	32.30
4	0	12	650.43	32.40
4	0	12	660.13	34.20
-4	0	-12	706.13	34.00
5	0	12	1053.79	49.80
-5	0	-12	1102.79	51.59
5	0	12	990.90	51.59
5	0	12	1042.00	49.70
-5	0	-12	1019.60	50.49
-6	0	-12	455.05	23.30
6	0	12	449.76	22.00
-6	0	-12	469.05	24.20
6	0	12	453.95	24.50
6	0	12	441.56	22.20
7	0	12	1734.43	85.59
7	0	12	1716.43	83.49
-7	0	-12	1772.72	84.69
7	0	12	1777.42	83.69
8	0	12	1503.55	72.19
-8	0	-12	1512.45	73.29
-8	0	-12	1533.05	74.39
8	0	12	1457.15	74.09
9	0	12	169.18	10.20

-9	0	-12	171.88	12.30
-9	0	-12	132.19	18.50
-10	0	-12	699.23	36.50
-10	0	-12	708.83	37.00
10	0	12	668.63	34.50
-11	0	-12	19.40	7.60
-11	0	-12	31.50	7.20
11	0	12	34.40	6.90
-12	0	-12	621.34	35.00
-13	0	-12	-1.00	8.00
-14	0	-12	255.47	20.80
-14	-1	12	22.10	6.50
-14	1	12	26.20	6.80
13	-1	-12	103.09	15.80
-13	-1	12	93.89	10.30
13	1	-12	118.39	16.90
-13	1	12	99.09	10.70
-12	-1	12	70.29	8.70
12	-1	-12	76.89	13.80
12	1	-12	74.19	13.80
-12	1	12	69.79	8.80
11	-1	-12	24.50	7.80
-11	-1	12	40.80	7.10
11	1	-12	30.70	8.10
11	1	-12	35.60	6.70
-11	1	12	43.70	7.30
10	-1	-12	95.29	12.10
-10	-1	12	62.49	7.40
10	-1	-12	62.59	9.60
-10	1	12	78.69	7.90
10	1	-12	68.49	8.80
10	1	-12	76.79	12.00
-10	1	12	66.49	12.10
9	-1	-12	23.00	6.40
9	-1	-12	15.90	4.80
-9	-1	12	25.20	9.00
-9	-1	12	20.80	4.10
9	1	-12	16.50	6.20
9	1	-12	27.80	4.90
-9	1	12	15.90	8.50
-9	1	12	21.40	4.40
-8	-1	12	24.10	7.50
-8	-1	12	29.80	4.40
8	-1	-12	30.00	6.20

8	-1	-12	32.30	4.90
8	1	-12	30.60	4.60
8	1	-12	40.30	7.10
-8	1	12	23.80	7.50
-8	1	12	37.20	6.10
7	-1	-12	73.49	6.90
7	-1	-12	68.79	9.50
-7	-1	12	65.69	6.40
7	1	-12	64.89	6.40
-7	1	12	80.89	7.10
7	1	-12	74.89	9.40
-6	-1	12	202.38	15.30
6	-1	-12	226.08	14.30
-6	-1	12	201.18	12.20
6	-1	-12	217.58	12.10
-6	1	12	220.48	12.60
6	1	-12	192.98	14.20
-6	1	12	221.38	15.40
6	1	-12	219.18	11.90
-5	-1	12	134.19	11.70
5	-1	-12	155.08	8.80
5	-1	-12	137.09	10.60
-5	-1	12	140.79	9.00
-5	1	12	142.39	9.50
5	1	-12	139.59	8.40
5	1	-12	140.69	10.80
-5	1	12	139.99	11.80
4	-1	-12	8.10	3.50
4	-1	-12	7.60	2.40
-4	-1	12	4.50	2.50
-4	-1	12	0.40	3.50
-4	1	12	4.70	3.00
-4	1	12	0.20	3.30
4	1	-12	6.90	2.10
4	1	-12	8.80	3.40
-3	-1	12	11.60	3.90
3	-1	-12	18.60	3.20
3	-1	-12	20.20	3.90
-3	-1	12	20.50	3.00
3	1	-12	17.70	3.80
3	1	-12	17.90	2.90
-3	1	12	16.20	4.30
-3	1	12	15.30	3.30
2	-1	-12	49.10	4.10

-2	-1	12	42.00	4.30
2	-1	-12	38.50	5.00
-2	-1	12	42.00	5.60
2	1	-12	44.60	5.10
2	1	-12	43.60	3.70
-2	1	12	46.70	5.80
-2	1	12	45.40	5.00
1	-1	-12	4.50	2.10
-1	-1	12	-1.40	2.30
1	-1	-12	2.50	2.50
-1	-1	12	2.50	2.10
-1	1	12	4.10	2.40
1	1	-12	2.40	1.80
1	1	-12	0.70	2.10
-1	1	12	2.40	2.50
0	-1	-12	2.90	2.10
0	-1	-12	8.40	2.40
0	-1	12	4.80	2.20
0	-1	12	6.80	2.50
0	1	-12	8.20	2.30
0	1	-12	6.00	2.10
0	1	12	8.30	2.70
0	1	12	7.00	2.40
1	-1	12	72.09	5.10
1	-1	12	76.99	5.90
-1	-1	-12	73.09	5.50
-1	-1	-12	70.89	5.60
1	1	12	78.59	5.70
-1	1	-12	75.89	5.60
1	1	12	78.89	6.20
1	1	12	72.79	5.40
-1	1	-12	69.79	5.10
-2	-1	-12	93.59	6.20
-2	-1	-12	91.29	6.70
2	-1	12	88.99	5.20
2	-1	12	97.49	5.30
2	-1	12	87.29	6.90
2	1	12	92.79	5.90
-2	1	-12	99.59	6.60
-2	1	-12	95.99	5.80
2	1	12	89.79	7.10
2	1	12	97.69	6.30
-3	-1	-12	18.30	3.30
3	-1	12	11.60	3.40

-3	1	-12	21.40	3.60
3	1	12	14.30	3.40
3	1	12	16.00	2.20
3	1	12	15.50	2.60
-4	-1	-12	227.08	12.90
4	-1	12	207.88	13.00
4	1	12	214.88	11.30
-4	1	-12	230.28	12.70
4	1	12	214.78	13.20
4	1	12	223.88	11.60
-5	-1	-12	162.48	9.50
-5	-1	-12	157.08	10.40
5	-1	12	148.09	10.60
5	1	12	149.99	8.80
5	1	12	153.48	8.40
-5	1	-12	167.68	10.40
5	1	12	152.38	10.70
6	-1	12	308.37	18.30
-6	-1	-12	314.97	18.20
-6	-1	-12	324.67	17.40
6	1	12	304.37	18.10
-6	1	-12	325.17	18.20
6	1	12	332.77	16.60
6	1	12	317.37	16.20
-6	1	-12	332.27	16.90
7	-1	12	122.29	6.40
-7	-1	-12	130.79	9.60
7	-1	12	107.29	9.60
7	1	12	108.89	7.30
-7	1	-12	114.29	9.80
7	1	12	128.39	8.20
-7	1	-12	131.59	8.80
7	1	12	134.59	10.40
8	-1	12	38.00	3.90
-8	-1	-12	28.70	5.40
8	1	12	27.80	5.40
-8	1	-12	36.60	6.40
8	1	12	20.90	5.40
-8	1	-12	45.50	7.50
-9	-1	-12	12.50	5.10
9	-1	12	21.20	4.20
-9	1	-12	10.80	8.20
-9	1	-12	19.30	5.00
9	1	12	18.70	4.10

10	-1	12	74.79	6.80
-10	-1	-12	78.09	10.60
-10	-1	-12	83.99	11.50
-10	1	-12	101.49	11.40
-10	1	-12	95.59	10.30
10	1	12	85.49	8.40
-11	-1	-12	84.19	11.70
11	-1	12	85.99	7.70
-11	-1	-12	92.69	12.50
-11	1	-12	86.49	11.50
11	1	12	86.79	8.80
-11	1	-12	98.69	12.20
-12	-1	-12	12.60	7.20
-12	1	-12	20.40	7.10
-12	1	-12	8.90	7.70
-13	-1	-12	40.60	9.90
-13	1	-12	26.30	8.60
-14	-1	-12	23.90	9.70
-14	1	-12	5.40	8.80
-14	-2	12	271.57	23.00
-14	2	12	276.37	18.20
13	-2	-12	74.69	15.40
-13	-2	12	67.59	9.10
-13	2	12	60.89	9.90
13	2	-12	82.39	15.50
12	-2	-12	152.78	16.40
-12	-2	12	145.99	11.10
12	2	-12	148.19	16.90
-12	2	12	154.68	11.70
11	-2	-12	516.75	30.30
-11	-2	12	519.95	26.50
11	2	-12	515.55	30.60
11	2	-12	498.35	27.90
-11	2	12	511.65	27.10
-10	-2	12	224.08	13.60
10	-2	-12	242.68	17.80
10	2	-12	243.98	18.30
-10	2	12	229.88	14.40
10	2	-12	257.07	14.90
-10	2	12	218.48	21.20
-9	-2	12	384.26	27.10
9	-2	-12	459.65	25.60
-9	-2	12	425.06	22.20
9	-2	-12	412.06	23.30

9	2	-12	459.95	25.90
9	2	-12	449.95	22.80
-9	2	12	376.96	27.30
-9	2	12	451.55	22.90
-8	-2	12	318.37	16.70
-8	-2	12	270.47	21.40
8	-2	-12	329.27	19.70
8	-2	-12	315.67	17.70
8	2	-12	318.47	19.90
-8	2	12	319.57	17.40
-8	2	12	278.77	21.10
8	2	-12	330.87	17.00
7	-2	-12	1551.14	72.59
7	-2	-12	1469.95	70.79
-7	-2	12	1362.66	73.29
-7	-2	12	1466.25	70.39
7	2	-12	1482.65	72.59
-7	2	12	1376.96	73.29
7	2	-12	1486.05	70.39
-7	2	12	1463.15	70.89
6	-2	-12	23.50	5.20
-6	-2	12	23.10	6.20
6	-2	-12	42.20	5.30
-6	-2	12	29.30	4.70
6	2	-12	32.60	4.40
-6	2	12	35.70	7.00
-6	2	12	35.50	5.90
6	2	-12	37.90	7.60
-5	-2	12	1034.80	49.00
5	-2	-12	1023.00	50.59
-5	-2	12	966.30	51.19
5	-2	-12	995.70	49.10
5	2	-12	1040.60	50.69
-5	2	12	1007.20	49.70
5	2	-12	1030.40	48.70
-5	2	12	961.10	51.09
-4	-2	12	8.00	4.10
4	-2	-12	8.60	3.50
-4	-2	12	5.70	2.60
4	-2	-12	8.70	2.60
-4	2	12	5.90	3.40
4	2	-12	6.40	3.20
-4	2	12	4.10	3.80
4	2	-12	6.10	3.60

3	-2	-12	540.95	26.80
3	-2	-12	549.95	28.00
-3	-2	12	532.65	28.30
-3	-2	12	535.15	26.80
-3	2	12	521.45	27.70
-3	2	12	542.25	28.50
3	2	-12	547.35	26.50
3	2	-12	569.74	28.10
-2	-2	12	370.36	20.70
2	-2	-12	390.06	19.70
-2	-2	12	389.56	19.80
2	-2	-12	394.06	20.70
-2	2	12	412.06	21.10
2	2	-12	390.66	20.50
-2	2	12	388.66	20.50
-1	-2	12	1112.49	55.29
1	-2	-12	1150.18	55.49
-1	-2	12	1108.59	54.89
1	-2	-12	1158.78	54.89
1	2	-12	1126.99	54.59
-1	2	12	1107.89	55.39
1	2	-12	1153.48	55.19
-1	2	12	1166.38	55.59
0	-2	12	98.49	6.60
0	-2	-12	102.99	6.60
0	-2	12	89.49	6.40
0	-2	-12	100.89	6.90
0	2	12	95.49	7.10
0	2	-12	101.49	6.20
0	2	12	97.69	6.80
0	2	-12	95.39	6.30
1	-2	12	1195.28	58.69
-1	-2	-12	1213.28	58.79
1	-2	12	1140.99	57.89
-1	-2	-12	1283.77	58.79
1	2	12	1237.48	58.59
1	2	12	1138.79	58.99
1	2	12	1252.97	58.89
-1	2	-12	1209.68	58.49
-1	2	-12	1224.48	58.19
-2	-2	-12	263.77	14.20
2	-2	12	269.47	14.50
-2	-2	-12	262.67	14.40
-2	2	-12	276.27	14.30

2	2	12	263.87	14.80
2	2	12	261.27	13.80
-2	2	-12	266.57	13.40
2	2	12	257.97	14.30
-3	-2	-12	827.32	40.80
3	-2	12	836.32	41.00
3	2	12	837.12	40.50
3	2	12	816.62	40.00
3	2	12	782.42	41.10
-3	2	-12	846.22	40.70
4	-2	12	221.08	13.10
-4	-2	-12	225.58	13.00
4	2	12	240.48	13.50
-4	2	-12	229.98	13.00
4	2	12	202.88	12.20
4	2	12	224.88	11.80
-5	-2	-12	479.25	24.70
-5	-2	-12	467.65	24.20
5	-2	12	459.75	24.80
5	2	12	428.26	24.70
5	2	12	468.35	23.30
-5	2	-12	498.95	24.70
5	2	12	486.35	23.80
-6	-2	-12	185.78	12.40
6	-2	12	194.38	12.70
-6	-2	-12	184.88	11.80
6	2	12	185.08	12.70
6	2	12	186.78	10.60
-6	2	-12	204.28	10.80
6	2	12	172.78	11.20
-6	2	-12	228.88	12.90
-7	-2	-12	457.05	23.50
7	2	12	453.35	22.70
-7	2	-12	408.26	24.10
7	2	12	423.56	23.90
-7	2	-12	436.96	22.50
-8	-2	-12	541.75	29.20
-8	2	-12	564.04	28.40
8	2	12	539.05	29.40
-8	2	-12	566.64	29.90
8	2	12	545.65	28.30
9	-2	12	67.79	4.80
-9	-2	-12	69.89	9.50
-9	2	-12	40.80	11.50

-9	2	-12	62.29	8.20
9	2	12	64.19	7.60
10	-2	12	460.35	24.50
-10	-2	-12	506.75	28.40
-10	-2	-12	483.25	27.90
-10	2	-12	542.95	28.20
10	2	12	493.75	26.10
-10	2	-12	479.35	26.80
-11	-2	-12	107.19	13.00
11	-2	12	70.49	7.10
-11	-2	-12	104.29	12.40
11	2	12	84.09	9.60
-11	2	-12	82.99	11.20
-11	2	-12	102.89	12.50
-12	-2	-12	362.96	23.00
-12	2	-12	362.36	22.00
-12	2	-12	291.97	21.40
-13	-2	-12	35.20	9.50
-13	2	-12	13.10	8.60
-14	-2	-12	98.79	15.60
-14	2	-12	90.39	15.00
-14	3	12	62.69	11.50
-13	-3	12	91.89	9.50
13	-3	-12	115.69	16.00
-13	3	12	87.49	10.80
13	3	-12	82.99	16.20
12	-3	-12	251.57	20.00
-12	-3	12	242.38	14.70
12	3	-12	239.78	20.60
11	-3	-12	304.87	20.80
-11	-3	12	291.37	16.00
11	3	-12	272.47	20.80
-11	3	12	287.97	16.80
-11	3	12	226.18	31.40
10	-3	-12	483.35	27.90
-10	-3	12	468.75	24.30
10	3	-12	486.05	28.60
-10	3	12	487.25	25.20
-10	3	12	412.06	30.50
10	3	-12	496.35	25.20
9	-3	-12	225.08	15.90
-9	-3	12	213.98	20.90
-9	-3	12	209.58	12.30
9	-3	-12	191.88	14.00

-9	3	12	223.38	13.50
9	3	-12	241.98	13.10
-9	3	12	200.18	18.50
9	3	-12	210.28	16.30
8	-3	-12	399.66	21.60
8	-3	-12	419.46	23.60
-8	-3	12	355.36	25.10
-8	-3	12	409.66	20.40
-8	3	12	410.96	21.40
-8	3	12	359.76	25.10
8	3	-12	402.56	23.60
8	3	-12	406.66	20.70
7	-3	-12	193.88	12.20
7	-3	-12	192.38	14.20
-7	-3	12	184.08	15.50
-7	-3	12	200.18	11.20
-7	3	12	204.48	12.40
7	3	-12	211.08	11.20
7	3	-12	182.98	14.40
-7	3	12	187.28	15.50
-6	-3	12	1165.48	63.19
6	-3	-12	1262.87	61.09
6	-3	-12	1313.37	62.69
-6	-3	12	1289.77	60.59
-6	3	12	1231.48	63.39
6	3	-12	1261.17	62.69
-6	3	12	1258.67	61.59
5	-3	-12	183.58	12.10
-5	-3	12	161.48	12.80
-5	-3	12	164.98	9.60
5	-3	-12	164.68	10.10
5	3	-12	169.28	9.20
5	3	-12	162.88	11.90
-5	3	12	171.88	13.00
-5	3	12	166.18	11.00
-4	-3	12	706.43	39.60
4	-3	-12	807.12	39.40
4	-3	-12	796.62	38.00
4	3	-12	781.02	37.40
-4	3	12	777.52	39.00
4	3	-12	804.22	39.40
-4	3	12	750.52	39.80
3	-3	-12	13.50	3.50
3	-3	-12	13.20	2.60

-3	-3	12	13.70	3.70
3	3	-12	16.60	3.60
-3	3	12	8.40	3.50
3	3	-12	15.90	2.50
-3	3	12	18.40	4.30
-2	-3	12	1431.56	69.39
-2	-3	12	1352.46	69.99
2	-3	-12	1494.05	70.19
2	-3	-12	1489.35	69.29
2	3	-12	1468.25	69.89
2	3	-12	1421.86	68.69
-2	3	12	1448.26	70.29
-2	3	12	1407.26	69.99
1	-3	-12	174.78	10.60
-1	-3	12	155.68	10.10
-1	-3	12	183.08	9.80
1	-3	-12	186.28	10.00
1	3	-12	169.28	9.70
-1	3	12	175.18	10.70
-1	3	12	171.38	10.70
1	3	-12	179.08	10.20
0	-3	12	1102.59	56.09
0	-3	-12	1157.78	56.89
0	-3	-12	1212.48	56.49
0	-3	12	1166.28	56.39
0	3	-12	1189.88	56.09
0	3	12	1122.59	56.89
0	3	12	1169.48	56.79
0	3	-12	1198.78	56.29
1	-3	12	73.39	5.80
1	-3	12	65.09	4.20
-1	-3	-12	73.79	5.90
-1	-3	-12	83.99	6.00
1	3	12	68.49	5.50
-1	3	-12	73.69	4.80
1	3	12	77.39	6.40
1	3	12	61.99	6.10
-1	3	-12	77.29	5.40
-2	-3	-12	1210.18	57.49
2	-3	12	1161.48	57.39
-2	-3	-12	1183.88	57.49
-2	3	-12	1194.88	57.19
-2	3	-12	1140.89	56.39
2	3	12	1164.38	57.09

2	3	12	1127.59	57.79
2	3	12	1258.67	57.59
3	-3	12	188.28	11.40
-3	-3	-12	195.68	11.40
3	3	12	191.38	11.80
-3	3	-12	186.58	11.10
3	3	12	202.58	11.30
3	3	12	183.18	10.50
4	-3	12	396.36	22.50
-4	-3	-12	452.35	22.60
4	3	12	390.26	22.70
4	3	12	433.46	21.60
4	3	12	463.45	22.20
-4	3	-12	430.66	22.40
-5	-3	-12	677.13	33.30
-5	-3	-12	654.43	33.00
5	-3	12	647.34	33.40
5	3	12	630.44	33.60
-5	3	-12	663.83	33.20
5	3	12	643.04	32.20
-6	-3	-12	1192.58	58.99
-6	-3	-12	1236.68	62.09
-6	3	-12	1174.18	59.29
6	3	12	1117.99	57.89
6	3	12	1224.88	58.49
-6	3	-12	1210.88	57.59
6	3	12	1132.89	59.29
-6	3	-12	1302.47	59.39
-7	-3	-12	680.43	34.20
7	3	12	669.33	33.40
-7	3	-12	650.83	34.40
-7	3	-12	659.53	32.70
7	3	12	630.64	34.20
-8	-3	-12	20.10	5.40
8	3	12	22.40	5.40
-8	3	-12	19.30	5.50
8	3	12	32.90	6.50
-8	3	-12	26.70	4.30
-9	-3	-12	362.86	21.20
9	3	12	334.97	21.60
-9	3	-12	347.77	19.50
9	3	12	352.16	19.90
-10	-3	-12	57.89	10.80
-10	-3	-12	46.40	7.90

-10	3	-12	52.19	8.90
-10	3	-12	55.19	10.40
10	3	12	75.49	8.80
-11	-3	-12	640.24	34.30
-11	-3	-12	573.84	33.60
-11	3	-12	575.54	32.40
-11	3	-12	627.34	33.80
-12	-3	-12	73.59	13.10
-12	3	-12	78.29	12.70
-12	3	-12	52.09	8.60
-13	-3	-12	237.68	19.10
-13	3	-12	194.78	18.30
-14	-3	-12	13.60	9.90
-14	3	-12	-2.50	8.60
-14	4	12	56.69	8.80
-13	-4	12	13.20	4.90
13	-4	-12	5.40	8.80
-13	4	12	7.70	6.10
13	4	-12	4.40	9.10
-12	-4	12	24.40	4.90
12	-4	-12	27.00	8.30
12	4	-12	15.00	8.50
-12	4	12	25.70	6.10
11	-4	-12	117.89	14.60
-11	-4	12	130.59	9.10
-11	4	12	117.99	10.80
-11	4	12	88.49	16.80
11	4	-12	125.29	14.70
-10	-4	12	30.50	5.50
10	-4	-12	55.59	11.20
10	4	-12	34.20	8.60
-10	4	12	20.80	10.30
-10	4	12	27.40	5.60
-9	-4	12	4.10	3.00
9	-4	-12	10.80	6.00
9	4	-12	3.30	5.80
9	4	-12	9.90	3.80
-9	4	12	6.40	8.30
-9	4	12	10.40	4.50
8	-4	-12	58.59	8.10
8	-4	-12	56.99	9.70
-8	-4	12	59.59	5.50
-8	-4	12	53.09	9.70
-8	4	12	50.49	7.40

8	4	-12	55.99	10.40
-8	4	12	62.29	12.10
8	4	-12	57.59	5.80
7	-4	-12	327.67	18.20
-7	-4	12	278.07	20.90
7	-4	-12	358.86	20.20
-7	-4	12	342.57	17.00
-7	4	12	317.47	21.20
-7	4	12	336.37	18.50
7	4	-12	325.67	20.30
6	-4	-12	10.10	4.90
-6	-4	12	-7.60	4.60
6	-4	-12	8.40	3.50
6	4	-12	-0.30	4.60
-6	4	12	3.90	5.30
6	4	-12	6.70	2.30
-6	4	12	6.40	4.20
5	-4	-12	406.36	20.60
-5	-4	12	405.96	19.70
-5	-4	12	358.96	22.30
5	-4	-12	380.76	21.80
-5	4	12	385.96	21.30
5	4	-12	396.26	22.10
5	4	-12	419.16	19.60
-5	4	12	390.56	22.70
4	-4	-12	30.20	5.20
-4	-4	12	27.20	5.40
-4	-4	12	30.20	3.30
4	-4	-12	34.50	4.60
4	4	-12	23.60	4.60
-4	4	12	24.00	4.90
4	4	-12	32.30	3.00
-4	4	12	28.40	5.20
-3	-4	12	135.49	10.20
3	-4	-12	150.78	9.00
3	-4	-12	160.58	10.40
-3	-4	12	156.78	8.30
3	4	-12	153.28	10.30
-3	4	12	150.08	10.70
3	4	-12	162.08	8.00
-3	4	12	142.29	10.30
-2	-4	12	10.90	2.20
-2	-4	12	7.40	3.00
2	-4	-12	10.20	3.40

2	-4	-12	10.80	2.70
2	4	-12	9.80	3.10
-2	4	12	10.10	3.70
2	4	-12	14.70	2.20
-2	4	12	17.40	4.10
-1	-4	12	204.48	11.00
1	-4	-12	203.38	12.00
1	-4	-12	205.78	11.20
-1	-4	12	180.68	11.20
1	4	-12	204.58	10.50
1	4	-12	201.48	11.40
-1	4	12	204.68	12.10
-1	4	12	198.58	12.00
0	-4	12	31.60	4.00
0	-4	12	25.40	3.50
0	-4	-12	29.90	4.10
0	-4	-12	31.50	4.80
0	4	12	26.60	5.00
0	4	12	30.20	4.40
0	4	-12	29.80	3.70
0	4	-12	27.50	3.30
-1	-4	-12	58.99	5.60
1	-4	12	57.99	5.20
-1	-4	-12	50.89	5.20
-1	4	-12	54.09	4.60
1	4	12	57.29	5.10
1	4	12	51.09	5.50
1	4	12	45.30	5.70
-1	4	-12	55.59	4.00
-2	-4	-12	122.59	7.90
2	-4	12	115.69	7.80
-2	4	-12	110.09	6.10
2	4	12	110.29	7.40
2	4	12	117.49	8.30
-2	4	-12	118.29	7.50
2	4	12	109.89	8.40
-3	-4	-12	33.40	4.80
3	-4	12	28.20	4.90
3	4	12	21.00	4.20
-3	4	-12	17.00	3.30
3	4	12	31.80	5.00
3	4	12	28.70	3.80
4	-4	12	1.90	3.10
-4	-4	-12	0.70	2.80

4	4	12	4.40	3.30
4	4	12	1.60	2.20
4	4	12	2.40	3.00
-4	4	-12	0.90	2.90
-4	4	-12	5.00	3.40
-5	-4	-12	85.99	7.50
-5	-4	-12	83.79	7.50
-5	4	-12	79.89	7.40
5	4	12	87.49	8.20
5	4	12	83.09	7.30
-5	4	-12	73.69	7.90
5	4	12	74.89	6.10
-6	4	-12	15.80	2.50
6	4	12	17.50	4.10
6	4	12	25.80	5.20
-6	4	-12	20.30	5.00
6	4	12	21.10	4.40
-6	4	-12	22.40	5.00
-7	-4	-12	39.80	7.20
-7	4	-12	27.60	3.80
-7	4	-12	29.20	5.60
7	4	12	33.30	5.80
7	4	12	40.30	7.20
-8	-4	-12	135.09	12.50
-8	4	-12	136.29	9.60
-8	4	-12	161.28	12.00
8	4	12	144.49	11.80
8	4	12	149.29	11.10
-9	-4	-12	123.99	12.90
9	4	12	129.29	11.50
9	4	12	136.49	10.90
-9	4	-12	97.59	9.50
-10	-4	-12	9.10	6.70
-10	4	-12	4.40	4.80
10	4	12	-0.10	4.90
10	4	12	6.60	5.80
-10	4	-12	-0.10	5.90
-11	-4	-12	25.90	8.30
-11	-4	-12	22.20	7.60
-11	4	-12	15.70	7.10
-12	-4	-12	218.88	17.70
-12	4	-12	146.09	15.10
-12	4	-12	209.78	15.90
-13	-4	-12	32.70	9.70

-13	4	-12	17.50	8.20
-14	-4	-12	-2.50	9.30
-14	4	-12	17.80	9.20
-14	5	12	48.10	8.50
-13	5	12	83.59	10.70
12	-5	-12	210.88	18.30
-12	-5	12	215.38	13.00
12	5	-12	213.88	19.50
-12	5	12	211.78	14.80
-11	-5	12	516.35	26.00
11	-5	-12	540.65	30.60
-11	5	12	435.26	34.20
11	5	-12	537.05	31.30
10	-5	-12	459.55	27.30
-10	-5	12	495.15	24.30
-10	-5	12	455.45	23.30
-10	5	12	404.06	30.30
10	5	-12	450.35	28.00
-10	5	12	465.45	24.80
-9	-5	12	171.98	10.00
9	-5	-12	174.28	14.40
-9	-5	12	188.28	10.90
-9	5	12	147.39	11.90
-9	5	12	143.19	17.10
9	5	-12	180.08	14.90
8	-5	-12	374.46	21.00
-8	-5	12	376.66	18.80
-8	-5	12	345.27	26.50
8	-5	-12	389.56	22.40
-8	5	12	351.56	24.30
-8	5	12	371.56	20.70
8	5	-12	370.16	22.70
7	-5	-12	20.30	4.70
-7	-5	12	7.20	6.60
7	-5	-12	15.10	5.80
-7	5	12	5.30	6.40
7	5	-12	8.40	5.30
7	5	-12	15.20	2.70
-7	5	12	7.10	4.20
6	-5	-12	864.11	42.40
-6	-5	12	866.61	41.10
-6	-5	12	758.32	44.20
6	-5	-12	878.81	43.80
-6	5	12	818.22	44.50

-6	5	12	899.21	43.00
6	5	-12	867.11	41.10
6	5	-12	860.11	44.00
5	-5	-12	176.78	12.00
5	-5	-12	174.28	10.30
-5	-5	12	135.69	12.20
-5	-5	12	166.98	8.70
-5	5	12	144.09	12.50
-5	5	12	175.68	11.50
5	5	-12	160.38	12.20
5	5	-12	174.18	8.70
4	-5	-12	2515.75	121.29
-4	-5	12	2320.17	122.39
-4	-5	12	2462.95	120.39
4	-5	-12	2637.74	122.49
4	5	-12	2550.14	120.29
-4	5	12	2575.74	122.29
4	5	-12	2653.13	122.59
-4	5	12	2561.64	122.89
-3	-5	12	131.69	7.00
3	-5	-12	120.49	8.00
-3	-5	12	111.79	9.10
3	-5	-12	131.99	9.40
3	5	-12	128.99	6.60
3	5	-12	130.49	9.40
-3	5	12	108.09	9.40
-3	5	12	135.79	9.90
2	-5	-12	715.53	36.90
-2	-5	12	683.03	36.40
-2	-5	12	774.82	35.40
2	-5	-12	744.63	35.90
-2	5	12	715.43	37.00
-2	5	12	743.73	37.10
2	5	-12	708.43	35.00
2	5	-12	745.03	36.60
-1	-5	12	170.68	9.10
-1	-5	12	147.09	9.50
1	-5	-12	170.88	10.50
1	-5	-12	165.08	9.50
-1	5	12	162.68	10.70
-1	5	12	156.58	10.30
1	5	-12	160.48	8.60
1	5	-12	158.58	9.80
0	-5	12	1999.40	91.19

0	-5	-12	1943.51	91.49
0	-5	-12	1893.71	92.09
0	-5	12	1872.01	90.99
0	5	12	1818.12	91.79
0	5	-12	1986.70	91.29
0	5	12	1812.22	92.09
-1	-5	-12	29.80	4.30
1	-5	12	35.40	4.40
-1	-5	-12	36.90	5.20
1	5	12	30.90	5.50
1	5	12	37.40	5.60
1	5	12	31.90	4.60
-1	5	-12	41.60	3.60
-1	5	-12	35.30	3.90
-2	-5	-12	2220.88	105.29
2	-5	12	2236.08	105.19
2	5	12	2126.39	105.09
2	5	12	2255.27	105.79
2	5	12	2110.69	105.69
-2	5	-12	2243.28	104.89
-2	5	-12	2143.59	103.99
-3	-5	-12	169.48	10.30
3	5	12	161.88	9.60
3	5	12	151.58	10.70
3	5	12	158.68	10.60
-3	5	-12	165.98	9.80
-3	5	-12	157.78	10.20
-4	-5	-12	768.92	36.90
4	5	12	642.34	37.00
4	5	12	782.72	37.20
4	5	12	710.63	36.30
-4	5	-12	718.43	36.90
-4	5	-12	773.52	36.80
-5	-5	-12	559.44	30.10
-5	-5	-12	593.54	30.10
-5	5	-12	619.44	30.10
5	5	12	599.74	30.10
-5	5	-12	562.74	30.20
5	5	12	568.44	29.40
5	5	12	576.34	30.50
-6	-5	-12	2211.38	109.29
6	5	12	2442.86	109.19
-6	5	-12	2258.07	107.29
6	5	12	2099.39	109.49

6	5	12	2299.67	109.59
-6	5	-12	2145.19	109.29
-6	5	-12	2353.86	109.39
-7	-5	-12	587.94	31.70
-7	5	-12	584.04	31.40
7	5	12	613.54	31.90
7	5	12	635.54	31.40
-7	5	-12	581.84	29.50
-8	-5	-12	509.05	28.20
-8	5	-12	507.15	27.90
8	5	12	480.95	27.70
-8	5	-12	534.85	25.90
-9	-5	-12	665.33	40.50
-9	5	-12	692.03	38.80
-9	5	-12	674.03	35.30
9	5	12	711.83	37.20
9	5	12	752.32	36.70
-10	5	-12	18.30	5.40
10	5	12	22.20	6.20
-10	5	-12	27.40	7.20
10	5	12	18.20	6.40
-11	-5	-12	699.43	36.80
-11	5	-12	599.44	34.40
-11	5	-12	672.93	35.70
-12	-5	-12	162.58	16.80
-12	5	-12	141.39	14.10
-12	5	-12	177.98	15.70
-13	-5	-12	451.75	28.50
-13	5	-12	445.46	27.80
-14	-5	-12	11.70	9.20
-14	5	-12	19.70	9.00
-14	6	12	38.10	8.30
-13	6	12	19.90	7.10
13	6	-12	4.40	9.30
12	-6	-12	43.10	9.40
-12	-6	12	45.50	7.70
-12	6	12	30.20	6.90
12	6	-12	49.70	10.50
-11	-6	12	21.70	4.10
11	-6	-12	37.80	8.30
-11	6	12	16.60	11.30
-11	6	12	39.40	6.80
11	6	-12	24.80	8.70
-10	-6	12	65.69	7.20

10	-6	-12	59.29	11.50
-10	-6	12	55.19	5.70
-10	6	12	54.39	8.30
-10	6	12	48.00	12.30
10	6	-12	68.79	12.60
-9	-6	12	13.00	4.60
9	-6	-12	17.40	6.20
-9	-6	12	15.40	3.00
-9	6	12	22.70	9.10
-9	6	12	11.50	5.30
9	6	-12	10.00	6.50
-8	-6	12	98.29	7.00
8	-6	-12	105.69	11.00
-8	6	12	94.39	13.90
-8	6	12	98.29	9.30
8	6	-12	96.39	11.50
-7	-6	12	234.88	12.00
7	-6	-12	234.28	14.40
-7	-6	12	241.98	12.30
7	-6	-12	230.68	15.90
-7	6	12	252.37	14.80
-7	6	12	215.08	17.20
7	6	-12	223.38	16.40
6	-6	-12	17.70	4.20
6	-6	-12	11.80	4.80
-6	-6	12	9.50	6.30
-6	-6	12	16.50	2.40
-6	6	12	2.20	4.30
6	6	-12	14.40	5.20
-6	6	12	15.30	5.50
-5	-6	12	432.36	20.60
5	-6	-12	436.36	23.80
5	-6	-12	436.26	22.30
-5	-6	12	379.06	24.00
5	6	-12	423.76	20.50
5	6	-12	445.56	24.00
-5	6	12	432.26	23.40
-5	6	12	423.06	24.30
-4	-6	12	8.10	1.40
4	-6	-12	9.70	2.90
-4	-6	12	3.10	4.40
4	-6	-12	9.40	4.30
-4	6	12	3.10	3.90
4	6	-12	5.90	4.10

-4	6	12	4.20	4.10
3	-6	-12	143.59	8.90
3	-6	-12	140.99	10.30
-3	-6	12	139.49	10.00
-3	-6	12	145.09	7.50
-3	6	12	129.29	10.50
3	6	-12	139.69	10.10
-3	6	12	141.19	10.60
3	6	-12	147.99	7.20
-2	-6	12	7.60	1.80
2	-6	-12	3.20	3.40
-2	-6	12	5.00	2.80
2	-6	-12	2.80	2.60
-2	6	12	3.70	3.80
2	6	-12	8.20	1.40
2	6	-12	6.50	3.10
-2	6	12	6.80	3.30
-1	-6	12	282.17	15.90
1	-6	-12	300.77	16.90
1	-6	-12	295.97	15.80
-1	-6	12	317.27	15.20
1	6	-12	299.47	16.10
1	6	-12	295.07	14.80
-1	6	12	306.37	17.20
-1	6	12	284.07	16.60
0	-6	12	188.58	11.10
0	-6	-12	215.28	11.80
0	-6	-12	215.88	12.70
0	-6	12	223.58	11.50
0	6	-12	205.98	10.90
0	6	12	207.78	12.90
0	6	12	210.58	12.30
0	6	-12	200.58	11.50
-1	-6	-12	157.58	10.40
-1	-6	-12	157.38	9.70
1	6	12	171.88	10.90
1	6	12	148.99	10.50
-1	6	-12	160.58	8.70
-1	6	-12	153.28	8.90
1	6	12	146.29	9.80
-1	6	-12	148.29	9.30
-2	-6	-12	87.49	6.60
2	6	12	73.19	7.30
2	6	12	60.59	6.00

-2	6	-12	67.89	5.70
2	6	12	75.89	7.20
-2	6	-12	70.79	4.30
-2	6	-12	68.89	6.20
-3	-6	-12	17.60	3.70
3	6	12	16.10	3.90
3	6	12	14.80	3.20
3	6	12	15.30	3.90
-3	6	-12	19.40	3.50
-3	6	-12	18.90	3.70
-4	-6	-12	1.00	3.20
4	6	12	1.20	2.70
-4	6	-12	4.70	3.20
4	6	12	-2.30	3.40
-4	6	-12	3.50	3.40
4	6	12	5.30	3.80
-5	6	-12	139.29	10.40
-5	6	-12	158.58	10.20
5	6	12	128.79	9.20
5	6	12	149.59	10.70
5	6	12	138.09	10.30
-6	-6	-12	271.17	16.30
6	6	12	299.57	16.30
-6	6	-12	257.07	16.00
-6	6	-12	251.07	16.10
6	6	12	246.98	16.30
-7	-6	-12	31.00	5.70
-7	6	-12	30.60	4.40
-7	6	-12	45.00	7.50
7	6	12	38.20	7.60
7	6	12	38.50	7.20
-8	-6	-12	32.30	6.30
8	6	12	26.80	6.10
-8	6	-12	28.40	6.40
-8	6	-12	17.30	3.70
8	6	12	26.10	5.70
-9	-6	-12	13.90	6.50
9	6	12	19.10	6.80
9	6	12	14.60	5.60
-9	6	-12	14.50	4.30
-9	6	-12	20.20	6.00
-10	-6	-12	72.69	12.40
10	6	12	87.69	11.00
-10	6	-12	71.69	8.80

-10	6	-12	85.29	11.20
10	6	12	89.19	11.00
11	6	12	72.29	11.80
-11	6	-12	73.89	11.60
-12	-6	-12	26.00	8.80
-12	6	-12	15.90	6.80
-12	6	-12	25.20	8.40
-13	-6	-12	37.20	10.20
-13	6	-12	34.70	9.20
-14	-6	-12	33.10	11.10
-14	6	-12	23.90	9.30
-14	7	12	18.50	7.90
-13	7	12	127.69	12.80
13	7	-12	99.59	18.30
12	-7	-12	141.49	16.00
12	7	-12	112.99	18.90
-12	7	12	123.79	12.30
11	-7	-12	185.98	17.00
-11	-7	12	171.28	10.80
11	7	-12	177.18	18.10
-11	7	12	210.78	13.80
-10	-7	12	251.57	12.80
10	-7	-12	248.78	18.10
-10	7	12	217.48	20.00
-10	7	12	199.28	21.80
10	7	-12	234.98	18.70
-9	-7	12	367.76	18.30
9	-7	-12	353.16	22.20
9	7	-12	351.56	22.80
-9	7	12	356.56	20.30
-9	7	12	324.77	24.50
8	-7	-12	546.35	29.80
-8	-7	12	563.04	26.00
-8	-7	12	512.65	25.70
8	7	-12	531.05	30.10
-8	7	12	527.15	28.20
-8	7	12	487.05	31.40
7	-7	-12	193.88	14.50
-7	-7	12	186.38	9.60
-7	-7	12	206.68	9.80
-7	7	12	205.98	13.30
7	7	-12	185.68	14.80
-7	7	12	173.18	15.70
-6	-7	12	618.64	38.60

6	-7	-12	768.82	38.50
6	-7	-12	743.63	37.00
-6	7	12	777.22	37.80
-6	7	12	731.73	39.30
6	7	-12	752.02	38.60
5	-7	-12	72.89	6.70
-5	-7	12	42.30	7.00
5	-7	-12	59.49	8.20
-5	7	12	63.29	9.50
-5	7	12	60.29	8.10
5	7	-12	60.09	8.80
-4	-7	12	1497.95	81.79
4	-7	-12	1704.33	80.89
4	-7	-12	1740.83	82.09
-4	7	12	1686.13	82.39
4	7	-12	1693.53	81.99
-4	7	12	1716.43	82.09
3	-7	-12	21.40	4.40
3	-7	-12	23.30	3.70
-3	-7	12	14.50	4.10
-3	7	12	13.50	4.80
3	7	-12	24.10	4.50
-3	7	12	16.60	4.90
-2	-7	12	925.21	50.59
2	-7	-12	1116.59	51.59
2	-7	-12	1036.50	50.29
-2	7	12	1052.09	51.39
-2	7	12	961.90	51.39
2	7	-12	1025.80	49.00
2	7	-12	1061.69	50.99
2	7	-12	1053.69	49.20
1	-7	-12	21.50	3.50
-1	-7	12	23.00	3.60
1	-7	-12	18.60	4.20
-1	7	12	20.70	3.90
-1	7	12	17.00	4.70
1	7	-12	22.10	3.70
1	7	-12	26.40	2.60
1	7	-12	26.40	3.20
0	-7	-12	1296.47	59.79
0	-7	-12	1238.08	60.59
0	-7	12	1196.98	59.09
0	7	12	1178.98	60.59
0	7	-12	1244.58	59.49

0	7	-12	1232.58	58.89
0	7	12	1167.88	60.09
0	7	-12	1260.67	59.39
-1	-7	-12	45.90	6.40
-1	-7	-12	55.49	5.60
-1	7	-12	42.60	4.90
1	7	12	46.80	6.60
1	7	12	41.80	6.50
-1	7	-12	38.20	4.30
-1	7	-12	47.80	4.40
1	7	12	48.10	5.50
-2	-7	-12	515.35	25.50
-2	7	-12	486.65	25.10
-2	7	-12	509.95	24.90
2	7	12	491.65	26.40
-2	7	-12	478.85	24.10
2	7	12	498.35	26.30
2	7	12	483.15	25.50
-3	-7	-12	9.90	3.40
3	7	12	4.90	2.70
-3	7	-12	9.90	3.10
-3	7	-12	7.90	3.30
3	7	12	4.90	3.60
3	7	12	10.60	4.00
-4	-7	-12	542.35	28.10
4	7	12	532.35	28.60
-4	7	-12	564.74	27.90
4	7	12	561.14	28.90
-4	7	-12	540.85	28.00
4	7	12	513.95	27.80
5	7	12	133.29	10.60
-5	7	-12	144.69	10.40
5	7	12	127.49	9.30
5	7	12	122.19	10.80
-5	7	-12	141.39	9.90
-6	-7	-12	1228.48	61.69
-6	7	-12	1263.37	61.29
6	7	12	1175.48	61.49
6	7	12	1338.77	61.79
-6	7	-12	1183.48	61.19
-7	-7	-12	299.37	18.60
7	7	12	327.87	18.50
-7	7	-12	314.37	18.10
7	7	12	285.87	18.30

-7	7	-12	283.97	15.30
-8	-7	-12	352.16	21.00
8	7	12	385.56	20.90
-8	7	-12	326.27	17.80
-8	7	-12	336.57	20.20
8	7	12	280.77	24.80
-9	-7	-12	188.08	15.20
-9	7	-12	193.28	14.50
9	7	12	202.78	14.70
-9	7	-12	182.18	11.80
9	7	12	195.08	15.10
-10	-7	-12	72.19	12.10
-10	7	-12	90.79	11.30
-10	7	-12	73.59	8.80
10	7	12	92.29	11.30
-11	7	-12	543.55	32.40
11	7	12	650.03	34.60
-11	7	-12	670.83	34.50
-12	-7	-12	160.38	16.00
-12	7	-12	103.29	12.70
-12	7	-12	125.59	13.30
-13	-7	-12	419.56	27.20
-13	7	-12	384.86	25.50
13	8	-12	43.10	12.20
-13	8	12	32.20	7.80
12	-8	-12	159.28	17.20
12	8	-12	170.18	18.90
-12	8	12	152.98	13.90
11	-8	-12	195.18	17.90
-11	8	12	201.58	14.40
10	-8	-12	168.18	15.10
-10	8	12	123.19	20.10
10	8	-12	155.18	16.60
-10	8	12	166.68	12.80
-9	-8	12	375.26	19.20
9	-8	-12	401.26	24.10
-9	-8	12	403.26	19.50
-9	8	12	352.76	26.40
-9	8	12	412.66	22.30
9	8	-12	400.26	24.90
8	-8	-12	359.76	21.70
8	8	-12	368.56	22.50
-8	8	12	317.17	23.40
-8	8	12	347.57	20.20

7	-8	-12	139.99	12.30
7	8	-12	166.68	13.50
-7	8	12	147.19	11.50
-7	8	12	129.59	14.30
6	-8	-12	9.30	5.10
-6	8	12	13.00	5.20
6	8	-12	10.20	5.60
-6	8	12	9.40	6.30
5	-8	-12	1366.16	66.09
5	-8	-12	1415.06	67.39
-5	-8	12	1135.09	67.19
-5	8	12	1325.07	67.79
5	8	-12	1448.36	67.59
-5	8	12	1432.86	67.19
-4	-8	12	104.29	10.20
4	-8	-12	112.09	8.40
4	-8	-12	111.09	10.10
-4	8	12	114.89	10.30
-4	8	12	109.99	10.50
4	8	-12	127.69	10.30
3	-8	-12	397.86	20.30
3	-8	-12	387.96	21.70
-3	-8	12	344.07	21.10
3	8	-12	427.56	21.60
-3	8	12	383.46	21.80
-3	8	12	379.16	22.00
2	-8	-12	109.49	7.60
-2	-8	12	102.79	8.50
2	-8	-12	115.59	9.60
-2	8	12	119.59	9.40
2	8	-12	125.49	8.90
2	8	-12	113.89	5.70
2	8	-12	116.49	5.80
-2	8	12	102.19	9.70
1	-8	-12	815.82	38.90
1	-8	-12	803.12	40.10
-1	-8	12	776.82	39.00
-1	8	12	749.82	39.60
1	8	-12	760.02	37.60
1	8	-12	767.12	37.80
-1	8	12	768.52	40.20
1	8	-12	834.12	39.20
0	-8	-12	61.29	7.10
0	-8	-12	53.89	5.90

0	-8	12	56.99	5.00
0	8	12	66.19	7.60
0	8	-12	52.49	5.50
0	8	-12	64.19	4.40
0	8	12	54.79	6.50
-1	-8	-12	2223.58	105.59
-1	-8	-12	2251.87	104.79
-1	8	-12	2151.18	103.99
-1	8	-12	2181.68	104.19
1	8	12	2087.39	104.99
-1	8	-12	2203.98	104.09
1	8	12	2139.29	105.59
-2	-8	-12	20.10	4.10
-2	8	-12	22.70	3.30
-2	8	-12	20.30	3.60
2	8	12	24.80	5.00
2	8	12	21.60	3.80
-2	8	-12	22.40	3.40
2	8	12	29.50	5.40
-3	-8	-12	634.84	32.80
3	8	12	640.34	32.90
-3	8	-12	668.93	32.50
-3	8	-12	649.14	32.60
3	8	12	686.23	34.00
-3	8	-12	627.54	31.00
3	8	12	625.44	33.50
-4	-8	-12	839.82	39.70
4	8	12	682.23	36.40
-4	8	-12	715.23	36.20
4	8	12	713.83	37.40
4	8	12	703.93	37.10
-4	8	-12	689.33	36.30
-5	8	-12	1217.68	60.99
-5	8	-12	1180.88	60.99
5	8	12	1392.56	62.29
5	8	12	1173.08	61.59
-6	-8	-12	583.14	32.80
6	8	12	657.53	33.10
-6	8	-12	632.14	32.40
-6	8	-12	622.54	32.40
6	8	12	603.24	32.80
-7	-8	-12	396.26	23.20
-7	8	-12	398.56	19.90
7	8	12	435.26	23.30

-7	8	-12	385.46	22.60
7	8	12	378.66	22.90
-8	-8	-12	602.64	34.30
8	8	12	690.63	34.40
8	8	12	590.94	35.40
-8	8	-12	670.13	33.80
-8	8	-12	610.44	31.40
-9	-8	-12	37.70	7.80
9	8	12	28.90	7.00
9	8	12	18.30	6.40
-9	8	-12	27.10	6.80
-9	8	-12	25.70	4.50
10	8	12	339.67	21.30
-10	8	-12	312.87	18.90
-10	8	-12	361.86	21.30
-11	8	-12	17.10	7.60
-11	8	-12	23.10	5.80
11	8	12	17.00	7.10
-12	-8	-12	459.55	27.10
-12	8	-12	334.87	23.10
12	8	12	404.66	25.40
-12	8	-12	423.96	25.20
-13	-8	-12	23.50	9.40
-13	8	-12	2.00	7.80
-13	9	12	15.90	7.20
13	9	-12	25.60	12.10
12	9	-12	14.00	9.70
-12	9	12	34.80	7.30
11	-9	-12	19.80	9.00
11	9	-12	11.20	9.50
-11	9	12	16.50	6.20
10	-9	-12	96.79	13.20
-10	9	12	85.49	10.30
-10	9	12	59.99	12.40
10	9	-12	75.29	15.50
9	-9	-12	35.80	7.40
-9	9	12	26.60	6.20
-9	9	12	26.90	9.80
9	9	-12	21.80	8.10
8	-9	-12	13.70	5.80
-8	9	12	0.30	7.50
8	9	-12	16.50	6.70
-8	9	12	18.50	5.80
7	-9	-12	12.60	5.50

-7	9	12	8.70	7.30
-7	9	12	0.50	4.80
7	9	-12	8.10	6.10
6	-9	-12	63.29	9.60
-6	9	12	63.99	8.70
-6	9	12	58.99	8.70
6	9	-12	65.89	10.10
5	-9	-12	9.70	5.10
5	9	-12	9.60	5.10
-5	9	12	8.90	5.40
-5	9	12	13.80	5.90
-4	-9	12	61.79	8.50
4	-9	-12	69.49	6.70
4	-9	-12	64.89	8.70
4	9	-12	59.49	8.30
-4	9	12	52.59	8.80
-4	9	12	55.39	8.90
-3	-9	12	-2.60	3.70
3	-9	-12	1.40	3.40
3	-9	-12	5.70	4.60
3	9	-12	-3.40	3.60
-3	9	12	-8.10	4.50
-3	9	12	6.20	4.40
2	-9	-12	24.10	4.20
-2	-9	12	22.80	4.30
2	-9	-12	26.00	5.10
-2	9	12	19.20	5.60
2	9	-12	18.80	4.30
-2	9	12	18.80	4.90
1	-9	-12	23.30	4.80
1	-9	-12	28.80	5.20
-1	-9	12	27.20	4.00
-1	9	12	24.60	4.60
1	9	-12	25.70	2.80
1	9	-12	26.10	5.30
1	9	-12	23.70	2.60
-1	9	12	18.00	5.30
0	-9	12	159.88	9.40
0	-9	-12	162.98	11.60
0	-9	-12	170.18	10.00
0	9	-12	145.29	9.90
0	9	12	157.88	11.00
0	9	-12	162.78	8.70
0	9	12	162.78	11.80

0	9	12	147.39	11.80
-1	-9	-12	31.30	5.10
1	9	12	40.80	6.10
1	9	12	30.70	5.80
-1	9	-12	30.30	4.70
-1	9	-12	22.90	4.00
-1	9	-12	31.60	4.50
-2	-9	-12	218.88	13.70
-2	9	-12	233.98	12.40
2	9	12	227.48	14.10
-2	9	-12	235.98	12.90
-2	9	-12	247.18	13.20
2	9	12	240.58	14.90
-3	-9	-12	127.19	9.00
3	9	12	113.29	9.30
3	9	12	123.49	10.10
-3	9	-12	113.09	8.80
-3	9	-12	136.09	7.00
-3	9	-12	114.99	6.70
-3	9	-12	122.19	8.50
3	9	12	129.89	10.80
-4	9	-12	126.09	9.10
-4	9	-12	122.69	9.10
4	9	12	109.59	9.10
4	9	12	126.59	10.30
4	9	12	122.79	10.90
-5	9	-12	401.46	22.00
-5	9	-12	405.86	21.80
5	9	12	404.76	22.70
5	9	12	388.46	23.00
-6	-9	-12	181.18	13.50
-6	9	-12	161.68	9.40
6	9	12	183.58	13.90
-6	9	-12	204.18	13.00
6	9	12	192.38	13.30
-6	9	-12	177.58	9.50
-6	9	-12	196.68	14.30
-7	-9	-12	51.79	9.50
-7	9	-12	84.69	9.00
7	9	12	57.29	10.00
7	9	12	76.09	9.40
-7	9	-12	51.49	5.20
-8	-9	-12	62.39	10.40
-8	9	-12	61.49	9.60

8	9	12	54.39	10.60
8	9	12	34.10	7.20
-9	-9	-12	68.69	11.40
-9	9	-12	63.09	7.40
-9	9	-12	81.49	10.30
9	9	12	78.79	10.50
9	9	12	62.99	11.40
-10	9	-12	70.49	9.20
-10	9	-12	121.79	12.20
10	9	12	112.89	12.50
-11	9	-12	60.79	9.70
11	9	12	90.89	12.70
-11	9	-12	102.29	12.60
-12	9	-12	8.00	7.90
-12	9	-12	4.30	6.20
12	9	12	16.10	7.90
-13	-9	-12	53.29	11.50
-13	9	-12	41.20	9.60
-13	10	12	79.39	12.70
13	10	-12	52.89	13.30
12	10	-12	167.78	20.10
-12	10	12	204.08	15.60
11	-10	-12	253.87	20.60
11	10	-12	253.37	21.80
-11	10	12	267.67	17.60
10	-10	-12	347.47	24.20
10	10	-12	353.26	24.90
-10	10	12	396.46	22.10
9	-10	-12	464.85	27.70
-9	10	12	425.06	30.30
9	10	-12	481.65	30.90
-9	10	12	470.65	25.90
8	-10	-12	328.07	20.60
-8	10	12	315.47	22.60
8	10	-12	301.77	21.30
7	-10	-12	714.73	38.30
-7	10	12	754.32	37.60
7	10	-12	730.63	38.80
-7	10	12	664.93	39.30
6	-10	-12	117.29	11.40
-6	10	12	103.29	12.80
6	10	-12	137.69	12.60
-6	10	12	119.89	11.30
5	-10	-12	1454.85	70.19

-5	10	12	1415.96	69.89
5	10	-12	1457.85	70.19
-5	10	12	1295.47	70.29
4	-10	-12	385.16	22.20
4	10	-12	401.66	22.10
-4	10	12	370.16	22.30
-4	10	12	358.96	22.10
3	-10	-12	1977.20	92.29
-3	-10	12	1669.73	91.49
-3	10	12	1841.22	92.09
3	10	-12	1960.20	91.69
-3	10	12	1947.11	92.39
2	-10	-12	42.90	5.30
-2	-10	12	31.10	6.30
2	-10	-12	39.70	6.20
-2	10	12	30.20	5.30
-2	10	12	32.70	6.40
2	10	-12	38.10	6.50
1	-10	-12	1156.48	58.09
-1	-10	12	1133.69	58.19
1	-10	-12	1213.28	59.49
-1	10	12	1167.58	59.09
1	10	-12	1188.78	56.69
-1	10	12	1190.38	59.69
1	10	-12	1283.87	58.59
1	10	-12	1175.18	56.69
0	-10	12	123.29	8.70
0	-10	-12	144.59	11.10
0	-10	-12	152.28	9.70
0	10	12	140.29	11.80
0	10	-12	137.79	7.90
0	10	12	129.79	11.40
0	10	-12	148.49	9.50
0	10	12	145.09	10.70
-1	-10	-12	4239.68	197.98
-1	10	-12	4044.10	197.08
-1	10	-12	3881.11	197.08
1	10	12	4185.38	199.08
1	10	12	4363.96	199.48
1	10	12	4110.29	198.48
-1	10	-12	4204.08	197.48
-2	-10	-12	252.67	16.10
2	10	12	207.18	13.60
2	10	12	219.18	14.50

-2	10	-12	218.78	12.40
-2	10	-12	205.68	11.90
-2	10	-12	224.38	12.50
-3	10	-12	1374.96	66.79
-3	10	-12	1439.16	65.99
3	10	12	1383.46	68.09
-3	10	-12	1309.57	65.79
-3	10	-12	1425.96	66.79
3	10	12	1314.47	67.59
4	10	12	652.13	35.30
4	10	12	711.13	35.70
-4	10	-12	647.94	32.30
-4	10	-12	670.73	33.90
4	10	12	654.33	34.80
-4	10	-12	681.33	32.40
-4	10	-12	662.83	33.90
-5	10	-12	523.95	29.00
-5	10	-12	578.94	27.00
5	10	12	601.44	30.70
-5	10	-12	527.85	29.00
-5	10	-12	578.44	27.00
5	10	12	505.05	29.60
-6	-10	-12	712.83	39.00
6	10	12	797.32	39.40
-6	10	-12	754.72	35.90
6	10	12	707.53	38.50
-6	10	-12	764.72	38.10
-6	10	-12	711.23	35.90
-7	-10	-12	707.43	39.20
7	10	12	817.42	39.70
-7	10	-12	731.83	38.20
7	10	12	708.93	43.80
-7	10	-12	719.43	36.00
-8	-10	-12	1521.35	78.59
8	10	12	1567.44	77.89
8	10	12	1802.02	78.89
-8	10	-12	1474.75	77.89
-8	10	-12	1481.95	75.49
-9	10	-12	17.90	4.60
-9	10	-12	16.90	6.90
9	10	12	27.00	7.20
-10	10	-12	806.02	44.70
-10	10	-12	967.00	47.00
10	10	12	929.31	47.30

11	10	12	2.60	7.10
-11	10	-12	3.50	5.50
-11	10	-12	15.10	7.90
-12	10	-12	459.95	26.00
-12	10	-12	328.17	26.60
12	10	12	430.36	26.00
-13	-10	-12	-11.30	9.60
-13	10	-12	1.60	8.10
-13	11	12	113.49	14.10
13	11	-12	113.89	20.40
-12	11	12	112.49	13.50
12	11	-12	137.59	19.10
11	-11	-12	40.20	9.20
11	11	-12	51.49	11.90
-11	11	12	38.70	7.60
10	-11	-12	82.09	13.70
-10	11	12	70.09	11.00
10	11	-12	75.79	15.10
9	-11	-12	14.00	6.80
9	11	-12	13.40	7.80
-9	11	12	4.60	6.10
-9	11	12	16.20	9.90
8	-11	-12	135.29	13.90
-8	11	12	124.29	16.80
8	11	-12	142.29	17.00
-8	11	12	150.28	12.60
7	-11	-12	80.39	10.80
-7	11	12	64.09	12.70
-7	11	12	65.79	12.50
6	-11	-12	290.07	19.40
-6	11	12	285.97	20.30
6	11	-12	338.77	20.10
-6	11	12	291.77	18.70
5	-11	-12	53.99	8.90
-5	11	12	33.50	7.10
-5	11	12	31.50	7.10
5	11	-12	34.60	6.70
4	-11	-12	100.79	10.40
-4	11	12	74.69	9.80
-4	11	12	92.69	11.00
4	11	-12	115.39	10.60
3	-11	-12	13.30	5.30
-3	11	12	2.90	4.40
-3	11	12	-2.00	5.50

3	11	-12	11.90	5.00
2	-11	-12	37.30	7.70
-2	11	12	13.00	5.60
-2	11	12	27.00	5.40
1	-11	-12	-2.60	4.00
-1	-11	12	3.50	3.20
1	11	-12	-1.00	3.40
-1	11	12	-5.80	4.50
1	11	-12	0.10	1.40
-1	11	12	0.10	3.90
1	11	-12	1.10	1.30
0	-11	-12	471.35	26.10
0	-11	12	437.66	23.80
0	11	12	517.85	26.90
0	11	-12	528.05	24.50
0	11	-12	428.76	23.00
0	11	12	441.26	25.30
0	11	12	448.86	26.00
1	11	12	16.70	5.80
-1	11	-12	6.90	2.90
1	11	12	8.50	5.00
-1	11	-12	7.00	2.70
-1	11	-12	9.20	3.30
1	11	12	9.50	4.00
-2	11	-12	156.68	9.80
-2	11	-12	186.58	9.70
2	11	12	147.19	11.60
2	11	12	166.38	12.90
-2	11	-12	142.29	9.70
2	11	12	137.29	11.30
3	11	12	56.99	7.90
-3	11	-12	69.29	5.70
-3	11	-12	78.89	7.00
-3	11	-12	75.89	7.00
3	11	12	78.79	8.40
-3	11	-12	72.79	5.40
-4	11	-12	576.74	30.60
4	11	12	603.84	32.00
-4	11	-12	611.44	30.60
-4	11	-12	557.54	29.20
-4	11	-12	636.24	29.40
5	11	12	4.10	4.70
-5	11	-12	6.30	2.60
-5	11	-12	7.30	4.50

5	11	12	1.30	6.80
-5	11	-12	-1.10	4.40
-5	11	-12	9.20	2.40
6	11	12	13.20	6.10
-6	11	-12	25.10	4.30
6	11	12	11.30	5.90
-6	11	-12	13.80	5.20
-6	11	-12	24.00	4.40
-7	-11	-12	326.67	21.00
-7	11	-12	322.67	19.60
7	11	12	363.16	21.50
-7	11	-12	322.07	17.30
7	11	12	312.57	20.30
-8	-11	-12	136.09	13.50
8	11	12	147.29	13.80
-8	11	-12	106.19	8.90
8	11	12	132.59	12.70
-9	11	-12	36.40	7.10
-9	11	-12	38.70	7.20
9	11	12	30.80	7.20
-10	11	-12	17.80	7.40
-10	11	-12	1.30	4.90
10	11	12	1.50	7.10
-11	11	-12	282.57	19.40
-11	11	-12	253.87	16.90
11	11	12	250.27	19.50
12	11	12	0.40	8.00
-12	11	-12	9.00	7.90
-13	-11	-12	36.10	11.30
-13	11	-12	35.00	10.20
-12	12	12	114.09	13.50
12	12	-12	68.89	13.60
-11	12	12	246.08	18.10
11	12	-12	252.87	22.50
10	-12	-12	235.98	19.30
-10	12	12	238.18	17.10
10	12	-12	238.68	20.80
9	-12	-12	212.58	17.50
-9	12	12	196.08	15.30
9	12	-12	185.48	18.00
-9	12	12	231.68	22.40
8	-12	-12	82.89	11.90
-8	12	12	80.19	11.00
-8	12	12	76.09	15.40

8	12	-12	91.49	13.30
7	-12	-12	631.94	33.90
-7	12	12	559.44	34.80
7	12	-12	594.74	39.60
-7	12	12	645.84	33.20
6	-12	-12	27.70	6.40
-6	12	12	20.70	7.30
6	12	-12	22.00	6.70
-6	12	12	26.30	7.50
5	-12	-12	1097.49	54.69
-5	12	12	995.10	54.89
-5	12	12	1123.89	54.49
5	12	-12	1053.29	54.49
4	-12	-12	13.50	5.20
-4	12	12	-1.40	6.00
4	12	-12	0.40	5.10
-4	12	12	4.70	5.80
3	-12	-12	669.73	35.50
-3	12	12	676.23	35.80
3	12	-12	686.63	35.00
-3	12	12	619.74	35.30
2	-12	-12	33.10	6.00
-2	12	12	18.10	5.90
-2	12	12	37.70	6.30
2	12	-12	37.40	7.20
1	-12	-12	595.84	30.60
1	12	-12	539.95	26.80
-1	12	12	602.24	31.50
-1	12	12	565.24	30.20
1	12	-12	576.34	29.20
1	12	-12	535.15	26.80
-1	12	12	497.95	30.10
0	12	-12	350.06	18.00
0	12	12	348.77	21.00
0	12	12	355.46	22.00
0	12	12	352.36	20.70
0	12	-12	378.16	19.70
-1	12	-12	348.47	18.10
1	12	12	352.96	20.40
1	12	12	363.16	21.80
-1	12	-12	351.26	19.10
1	12	12	368.56	21.20
-1	12	-12	344.67	18.20
2	12	12	62.09	8.20

2	12	12	44.50	7.50
-2	12	-12	56.59	6.00
2	12	12	81.99	9.10
-2	12	-12	60.99	6.20
-2	12	-12	57.79	6.10
3	12	12	39.90	7.10
3	12	12	39.10	8.30
-3	12	-12	34.30	5.20
-3	12	-12	44.00	4.60
3	12	12	37.40	6.60
-3	12	-12	37.50	6.10
-3	12	-12	39.50	5.90
-4	12	-12	452.25	23.20
-4	12	-12	403.66	21.70
-4	12	-12	415.36	23.10
-4	12	-12	441.76	22.10
4	12	12	454.75	24.70
-5	12	-12	291.57	16.90
-5	12	-12	289.77	15.10
5	12	12	269.97	17.80
-5	12	-12	260.77	16.80
-5	12	-12	291.57	14.90
-6	12	-12	332.57	18.00
-6	12	-12	352.66	19.90
-6	12	-12	342.67	17.80
-7	-12	-12	294.57	19.90
7	12	12	296.27	19.00
-7	12	-12	315.97	18.30
-7	12	-12	282.97	15.90
-7	12	-12	282.47	15.90
7	12	12	296.47	20.10
8	12	12	60.49	9.80
-8	12	-12	88.99	10.40
8	12	12	71.09	10.60
-8	12	-12	61.29	7.50
-9	12	-12	7.30	6.60
9	12	12	-2.80	6.70
-9	12	-12	5.20	4.40
10	12	12	188.98	15.60
-10	12	-12	156.38	12.90
-10	12	-12	203.28	15.60
-11	12	-12	92.99	13.00
-11	12	-12	52.99	10.10
11	12	12	54.49	9.60

12	12	12	106.99	14.80
-12	12	-12	122.19	14.90
12	13	-12	197.68	22.50
-12	13	12	161.08	15.60
-11	13	12	200.58	16.20
11	13	-12	184.48	20.60
10	-13	-12	168.78	17.10
10	13	-12	200.48	19.30
-10	13	12	174.58	14.90
9	-13	-12	170.58	16.80
9	13	-12	171.98	17.50
-9	13	12	173.38	14.50
8	-13	-12	181.68	16.20
-8	13	12	192.48	15.00
-8	13	12	164.28	19.20
8	13	-12	201.48	17.30
7	-13	-12	34.30	8.20
-7	13	12	22.60	8.70
-7	13	12	26.40	7.40
7	13	-12	29.20	7.70
6	-13	-12	933.21	47.70
-6	13	12	839.62	48.20
6	13	-12	883.61	52.59
-6	13	12	970.30	47.30
5	-13	-12	127.79	12.10
-5	13	12	86.09	16.30
5	13	-12	83.09	11.80
-5	13	12	99.39	12.30
4	-13	-12	589.44	32.30
4	13	-12	612.94	32.20
-4	13	12	591.94	32.10
-4	13	12	546.95	32.50
3	-13	-12	-0.40	4.80
3	13	-12	4.30	5.30
-3	13	12	-2.10	4.90
-3	13	12	-3.30	5.90
2	-13	-12	916.51	47.30
-2	13	12	891.91	47.30
-2	13	12	880.61	47.00
2	13	-12	963.90	46.40
1	-13	-12	-0.90	4.90
1	13	-12	0.70	1.30
-1	13	12	-2.40	6.00
-1	13	12	-2.40	4.40

1	13	-12	0.00	1.50
1	13	-12	-0.10	3.70
-1	13	12	-8.50	5.30
0	13	-12	925.01	44.80
0	13	12	842.52	45.70
0	13	12	963.70	47.30
0	13	-12	867.21	43.20
0	13	12	872.41	46.30
-1	13	-12	6.50	2.90
-1	13	-12	9.50	3.70
1	13	12	11.00	6.00
1	13	12	9.10	5.50
-1	13	-12	11.40	2.90
1	13	12	6.90	4.80
2	13	12	522.55	28.50
-2	13	-12	470.95	24.90
2	13	12	499.75	27.50
-2	13	-12	485.15	25.00
-2	13	-12	520.25	25.40
-2	13	-12	472.35	24.80
2	13	12	463.55	26.80
3	13	12	410.36	24.30
-3	13	-12	381.76	21.10
-3	13	-12	414.06	20.90
-3	13	-12	391.26	20.40
3	13	12	386.46	22.90
-3	13	-12	391.56	20.90
-4	13	-12	641.04	32.50
4	13	12	686.33	35.60
-4	13	-12	609.24	32.60
4	13	12	633.34	34.10
-4	13	-12	585.14	31.50
-4	13	-12	676.13	32.00
-5	13	-12	572.84	30.90
-5	13	-12	575.34	30.40
-5	13	-12	624.14	30.80
-5	13	-12	609.74	29.40
5	13	12	602.44	33.10
5	13	12	537.05	33.60
6	13	12	517.35	30.80
-6	13	-12	567.54	29.90
-6	13	-12	581.54	28.60
-6	13	-12	581.54	28.40
-7	-13	-12	364.36	25.00

7	13	12	383.26	23.60
-7	13	-12	411.36	21.10
-7	13	-12	424.86	21.20
-7	13	-12	395.06	22.90
-8	13	-12	239.38	13.90
-8	13	-12	232.38	16.30
8	13	12	223.38	17.00
-8	13	-12	233.68	14.10
-9	13	-12	242.48	16.20
-9	13	-12	195.48	13.80
9	13	12	208.88	16.80
10	13	12	1.70	7.10
-10	13	-12	-0.90	5.40
-10	13	-12	-5.10	6.80
-11	13	-12	299.57	19.80
11	13	12	329.17	22.50
-11	13	-12	353.46	22.20
12	13	12	24.50	9.00
-12	13	-12	25.40	9.30
-12	14	12	2.90	8.10
12	14	-12	12.80	11.40
11	14	-12	33.80	11.30
-11	14	12	38.90	8.80
10	-14	-12	19.50	12.40
-10	14	12	11.60	7.20
10	14	-12	4.60	9.50
9	-14	-12	15.10	8.20
9	14	-12	17.20	8.40
-9	14	12	6.60	6.70
8	-14	-12	62.59	10.40
-8	14	12	41.60	12.10
-8	14	12	63.49	9.30
8	14	-12	48.40	9.40
7	-14	-12	30.70	7.70
-7	14	12	24.40	7.50
-7	14	12	44.00	10.90
7	14	-12	27.20	7.80
6	-14	-12	-0.70	6.50
-6	14	12	-13.90	6.20
-6	14	12	8.50	8.20
6	14	-12	2.00	6.60
5	-14	-12	103.69	12.10
5	14	-12	92.69	12.00
-5	14	12	93.79	11.80

-5	14	12	82.99	13.90
4	-14	-12	35.80	7.60
4	14	-12	19.90	9.70
-4	14	12	27.00	6.90
3	-14	-12	14.50	5.70
3	14	-12	11.20	5.90
-3	14	12	0.60	6.20
-3	14	12	3.10	5.60
2	-14	-12	15.10	6.00
-2	14	12	3.20	6.40
-2	14	12	16.80	6.00
2	14	-12	10.00	4.90
-1	14	12	76.99	11.60
-1	14	12	56.99	8.50
1	14	-12	59.69	4.20
1	14	-12	65.79	3.90
-1	14	12	73.19	9.90
1	14	-12	71.49	8.40
0	14	12	130.99	12.80
0	14	12	142.39	12.40
0	14	12	157.28	15.10
0	14	-12	132.39	8.20
0	14	-12	145.79	8.20
0	14	-12	140.49	10.60
-1	14	-12	253.97	13.40
-1	14	-12	242.68	14.90
1	14	12	268.07	18.90
-1	14	-12	251.17	13.70
1	14	12	241.28	17.00
1	14	12	240.88	16.60
-2	14	-12	30.30	5.50
2	14	12	28.80	6.70
-2	14	-12	38.10	5.40
2	14	12	41.90	8.50
-2	14	-12	42.40	6.50
-2	14	-12	37.10	5.80
3	14	12	8.50	5.60
-3	14	-12	13.30	3.30
-3	14	-12	8.60	4.20
-3	14	-12	11.60	3.70
3	14	12	13.10	6.30
-3	14	-12	10.30	4.30
-4	14	-12	1.90	4.10
-4	14	-12	2.40	4.10

-4	14	-12	0.30	2.90
-4	14	-12	4.80	4.00
4	14	12	5.90	6.70
4	14	12	-0.60	7.00
-5	14	-12	7.20	3.30
5	14	12	8.00	6.60
5	14	12	-1.90	5.60
-5	14	-12	8.00	3.60
-5	14	-12	14.00	4.80
6	14	12	0.30	6.70
-6	14	-12	4.10	3.70
-6	14	-12	13.70	5.40
-6	14	-12	9.00	3.70
6	14	12	9.30	5.90
-7	14	-12	12.80	4.30
7	14	12	19.10	7.00
7	14	12	10.20	6.00
-7	14	-12	16.10	4.40
-7	14	-12	21.00	6.20
-8	14	-12	7.70	4.60
-8	14	-12	15.70	6.30
8	14	12	12.00	7.00
-8	14	-12	15.10	4.60
9	14	12	-2.50	6.80
-9	14	-12	8.30	4.70
-9	14	-12	6.20	5.10
-9	14	-12	4.90	6.60
10	14	12	16.70	8.30
-10	14	-12	11.60	7.50
-10	14	-12	7.30	5.70
-11	14	-12	36.20	9.10
11	14	12	37.00	8.90
12	14	12	24.40	8.90
-12	14	-12	15.70	9.30
11	15	-12	218.18	23.40
-11	15	12	230.28	18.00
-10	15	12	338.57	21.60
10	15	-12	290.47	25.40
9	-15	-12	190.78	18.50
9	15	-12	206.88	19.90
-9	15	12	212.98	16.70
8	-15	-12	69.69	10.70
8	15	-12	72.39	14.30
-8	15	12	51.69	9.40

7	-15	-12	19.90	8.00
7	15	-12	29.50	8.30
-7	15	12	29.30	10.10
-7	15	12	33.90	8.00
6	-15	-12	471.65	28.90
-6	15	12	530.55	28.60
-6	15	12	433.76	29.90
6	15	-12	520.25	29.40
5	-15	-12	392.36	23.90
5	15	-12	378.76	23.90
-5	15	12	357.86	24.40
-5	15	12	390.56	23.30
4	-15	-12	377.26	22.90
-4	15	12	313.77	22.60
-4	15	12	362.26	22.70
4	15	-12	387.56	22.60
3	-15	-12	0.10	5.60
-3	15	12	0.50	5.80
-3	15	12	-9.40	6.40
3	15	-12	4.20	4.90
2	-15	-12	531.05	29.50
2	15	-12	528.65	28.30
-2	15	12	523.95	30.70
-2	15	12	468.75	29.20
1	15	-12	13.10	1.80
-1	15	12	17.70	6.40
1	15	-12	7.70	2.00
-1	15	12	1.60	6.10
1	15	-12	15.10	5.20
-1	15	12	7.80	6.70
0	15	12	976.80	49.90
0	15	12	858.91	48.90
0	15	-12	969.80	46.00
0	15	-12	948.31	48.70
0	15	12	1032.00	51.19
-1	15	-12	1.30	3.00
-1	15	-12	2.80	2.70
1	15	12	-11.30	5.50
1	15	12	-0.10	7.20
-1	15	-12	4.50	4.10
-2	15	-12	1329.77	65.09
2	15	12	1426.16	69.09
2	15	12	1299.67	69.09
-2	15	-12	1254.07	65.19

-2	15	-12	1320.97	64.79
-2	15	-12	1411.16	65.89
-3	15	-12	200.48	12.90
3	15	12	212.28	17.30
-3	15	-12	193.18	12.20
-3	15	-12	200.88	12.40
-3	15	-12	214.28	13.10
-4	15	-12	322.17	17.40
-4	15	-12	283.67	16.60
-4	15	-12	316.77	17.30
-4	15	-12	301.07	17.90
4	15	12	287.77	19.40
4	15	12	304.97	21.70
5	15	12	165.48	14.10
5	15	12	163.78	16.40
-5	15	-12	190.68	11.90
-5	15	-12	167.28	11.10
-5	15	-12	175.98	12.40
6	15	12	623.14	34.30
-6	15	-12	575.64	30.90
-6	15	-12	603.44	30.10
-6	15	-12	563.64	29.70
6	15	12	543.25	32.00
7	15	12	360.86	22.90
-7	15	-12	381.36	26.70
-7	15	-12	374.16	20.50
-7	15	-12	366.96	20.50
8	15	12	106.59	13.70
-8	15	-12	127.59	12.60
-8	15	-12	139.09	11.10
-8	15	-12	139.69	10.80
9	15	12	442.96	28.20
-9	15	-12	516.75	27.80
-9	15	-12	495.65	26.00
-9	15	-12	456.25	26.20
10	15	12	7.50	8.10
-10	15	-12	27.40	6.40
-10	15	-12	23.90	7.70
-10	15	-12	25.40	6.20
-11	15	-12	348.27	22.40
11	15	12	286.17	22.60
-11	16	12	47.00	9.60
11	16	-12	41.50	13.00
10	16	-12	0.50	10.00

-10	16	12	10.70	8.20
-9	16	12	22.70	8.40
9	16	-12	17.50	9.60
8	-16	-12	70.89	11.50
8	16	-12	101.99	15.40
-8	16	12	80.59	11.00
7	-16	-12	77.79	13.60
7	16	-12	93.39	14.30
-7	16	12	65.69	12.30
6	-16	-12	14.80	7.50
-6	16	12	-0.50	8.60
6	16	-12	12.70	7.60
-6	16	12	1.70	7.40
5	-16	-12	97.99	12.70
-5	16	12	62.19	10.90
-5	16	12	78.39	11.40
5	16	-12	97.99	13.00
4	-16	-12	13.50	7.10
4	16	-12	3.50	6.20
-4	16	12	1.50	6.60
-4	16	12	4.60	7.50
3	-16	-12	22.60	7.80
-3	16	12	15.50	7.70
3	16	-12	32.20	6.80
-3	16	12	15.60	7.20
2	-16	-12	5.80	6.00
-2	16	12	-1.50	6.30
-2	16	12	-10.00	6.30
-2	16	12	-1.60	7.30
2	16	-12	-0.90	4.90
-1	16	12	242.38	18.00
-1	16	12	250.57	19.50
1	16	-12	234.28	12.40
1	16	-12	300.27	16.80
-1	16	12	260.27	18.20
1	16	-12	239.08	12.60
0	16	-12	89.09	6.00
0	16	12	81.09	11.90
0	16	-12	98.29	9.70
0	16	12	72.69	13.40
-1	16	-12	43.90	7.90
-1	16	-12	31.40	4.80
-1	16	-12	33.60	5.50
1	16	12	23.70	7.30

1	16	12	27.20	8.30
-2	16	-12	1.70	3.30
2	16	12	-2.40	5.90
-2	16	-12	4.50	4.30
2	16	12	-3.00	8.30
-2	16	-12	-3.00	3.70
-2	16	-12	5.20	3.70
-3	16	-12	70.89	7.50
-3	16	-12	93.99	9.00
-3	16	-12	78.19	7.50
3	16	12	71.39	10.90
-3	16	-12	66.99	8.20
-4	16	-12	27.10	5.20
-4	16	-12	32.20	5.40
-4	16	-12	26.90	6.20
-4	16	-12	37.00	6.30
4	16	12	26.20	7.20
4	16	12	18.10	8.40
5	16	12	126.69	15.70
5	16	12	96.99	12.40
-5	16	-12	115.19	10.00
-5	16	-12	134.49	10.10
-5	16	-12	104.59	9.20
-6	16	-12	35.40	7.20
6	16	12	33.70	7.60
-6	16	-12	40.60	7.80
-6	16	-12	30.60	6.40
6	16	12	29.50	11.80
-7	16	-12	16.50	4.90
-7	16	-12	17.80	6.30
-7	16	-12	25.80	5.50
7	16	12	11.00	6.60
-8	16	-12	6.80	5.50
-8	16	-12	0.20	4.80
-8	16	-12	8.50	6.60
8	16	12	-3.70	7.00
-9	16	-12	32.90	6.80
9	16	12	24.70	8.80
-9	16	-12	38.20	7.70
-9	16	-12	30.30	6.20
-10	16	-12	49.80	7.50
-10	16	-12	74.29	12.30
10	16	12	49.70	9.80
-11	16	-12	110.59	14.80

-11	16	-12	99.99	12.50
11	16	12	119.69	15.40
10	17	-12	167.48	20.70
-10	17	12	128.49	15.40
9	17	-12	117.29	18.20
-9	17	12	107.59	14.50
8	-17	-12	57.69	10.90
-8	17	12	56.09	9.80
8	17	-12	68.49	12.50
7	-17	-12	35.90	9.10
7	17	-12	26.20	9.00
-7	17	12	46.20	9.40
6	-17	-12	828.82	46.80
6	17	-12	857.21	46.90
-6	17	12	907.71	46.20
5	-17	-12	12.50	7.40
-5	17	12	4.80	8.10
5	17	-12	1.10	7.00
-5	17	12	9.00	7.40
4	-17	-12	558.34	31.50
-4	17	12	578.14	31.40
4	17	-12	542.05	30.70
-4	17	12	455.45	31.30
3	-17	-12	-0.10	6.20
-3	17	12	7.80	7.30
-3	17	12	4.50	8.80
3	17	-12	10.70	6.50
-2	17	12	557.84	32.70
-2	17	12	485.05	31.10
2	17	-12	541.35	29.60
-2	17	12	536.75	31.00
1	17	-12	55.19	4.30
1	17	-12	56.99	9.60
-1	17	12	49.20	9.70
1	17	-12	58.99	3.90
-1	17	12	58.19	9.80
0	17	12	223.88	17.80
0	17	-12	231.98	12.40
0	17	-12	235.68	12.90
0	17	-12	252.87	16.00
0	17	12	232.48	19.80
-1	17	-12	43.80	8.60
-1	17	-12	33.10	6.20
1	17	12	46.20	8.80

-1	17	-12	48.60	5.30
1	17	12	53.29	10.50
-2	17	-12	127.69	10.30
2	17	12	165.28	16.60
2	17	12	143.09	13.00
-2	17	-12	143.19	10.30
-2	17	-12	140.89	9.30
-2	17	-12	141.99	11.70
-3	17	-12	56.49	7.30
3	17	12	45.80	10.40
-3	17	-12	54.99	8.60
-3	17	-12	41.80	7.90
3	17	12	62.19	9.00
-3	17	-12	60.59	7.00
4	17	12	120.99	16.90
-4	17	-12	95.99	10.40
-4	17	-12	98.49	8.90
4	17	12	98.09	12.50
-4	17	-12	100.59	10.00
-4	17	-12	101.29	9.10
-5	17	-12	126.09	10.50
5	17	12	129.29	16.90
-5	17	-12	142.59	11.40
5	17	12	140.39	13.90
-5	17	-12	155.38	11.70
6	17	12	550.24	29.70
-6	17	-12	533.35	27.60
-6	17	-12	487.25	27.50
-6	17	-12	470.15	26.90
7	17	12	198.88	16.70
-7	17	-12	193.18	13.90
-7	17	-12	198.68	15.00
-7	17	-12	203.88	14.20
-8	17	-12	113.59	13.10
-8	17	-12	128.59	12.00
8	17	12	145.69	14.90
-8	17	-12	131.99	11.90
-9	17	-12	51.19	9.10
-9	17	-12	52.79	9.90
9	17	12	52.29	9.80
-9	17	-12	42.80	7.40
-10	17	-12	14.90	8.30
10	17	12	21.20	8.50
-10	17	-12	20.40	7.00

-10	18	12	79.19	13.30
10	18	-12	125.19	20.30
9	18	-12	327.57	26.50
-9	18	12	340.17	22.90
8	18	-12	107.09	17.20
-8	18	12	78.09	12.50
7	-18	-12	390.26	26.70
7	18	-12	396.46	27.20
-7	18	12	406.26	25.40
6	-18	-12	8.40	8.50
6	18	-12	15.20	9.00
-6	18	12	-3.10	8.10
5	-18	-12	775.82	44.00
-5	18	12	796.32	43.30
5	18	-12	822.32	43.80
4	-18	-12	7.90	7.50
-4	18	12	2.60	7.80
4	18	-12	10.00	7.30
-3	18	12	454.35	28.50
3	18	-12	465.35	27.60
-3	18	12	489.45	30.90
-2	18	12	-12.60	8.60
-2	18	12	-8.80	7.10
2	18	-12	2.20	6.10
1	18	-12	490.15	26.10
1	18	-12	388.26	21.60
-1	18	12	431.26	26.80
-1	18	12	500.45	29.70
0	18	-12	3.70	2.50
0	18	-12	2.20	5.40
0	18	12	-7.40	6.50
0	18	12	-9.60	7.90
-1	18	-12	472.05	28.00
-1	18	-12	568.04	29.20
-1	18	-12	494.15	29.70
-1	18	-12	522.75	26.40
1	18	12	571.14	32.90
1	18	12	557.14	30.90
-2	18	-12	189.98	13.40
-2	18	-12	159.58	11.50
-2	18	-12	158.18	11.80
-2	18	-12	165.28	10.40
2	18	12	145.19	14.20
2	18	12	166.28	18.00

3	18	12	207.08	19.30
-3	18	-12	197.38	12.20
-3	18	-12	176.78	13.00
3	18	12	195.28	16.30
-3	18	-12	226.98	14.50
-3	18	-12	172.58	12.70
-4	18	-12	177.58	12.40
4	18	12	218.28	19.00
-4	18	-12	165.28	12.60
4	18	12	190.68	15.90
-4	18	-12	199.98	13.90
-5	18	-12	286.27	18.40
-5	18	-12	362.96	19.60
-5	18	-12	302.47	18.60
5	18	12	323.47	21.50
6	18	12	307.87	20.20
-6	18	-12	255.77	17.30
-6	18	-12	306.87	18.40
-7	18	-12	175.68	14.80
-7	18	-12	221.88	14.50
-7	18	-12	188.18	14.10
7	18	12	165.18	16.30
8	18	12	171.08	16.20
-8	18	-12	160.48	14.30
-8	18	-12	172.68	13.70
-8	18	-12	147.99	13.60
-9	18	-12	15.60	6.70
9	18	12	12.00	8.30
-9	18	-12	15.50	7.90
-10	18	-12	187.48	15.30
10	18	12	147.99	17.20
-10	18	-12	172.28	16.30
9	19	-12	16.20	12.60
-9	19	12	15.70	8.90
-8	19	12	4.20	8.10
8	19	-12	12.50	9.70
-7	19	12	-11.80	8.00
7	19	-12	-5.00	8.70
6	-19	-12	17.10	8.90
-6	19	12	10.00	8.70
6	19	-12	27.10	9.90
5	-19	-12	1.00	8.10
5	19	-12	1.90	7.70
-5	19	12	-11.60	7.60

4	-19	-12	-8.80	8.40
4	19	-12	7.10	7.00
-4	19	12	8.00	10.20
-4	19	12	9.10	8.50
3	19	-12	8.50	7.50
-3	19	12	-2.10	8.60
-3	19	12	0.30	9.30
-2	19	12	30.30	11.60
2	19	-12	27.40	7.60
-2	19	12	15.20	8.70
1	19	-12	1.90	6.00
-1	19	12	5.30	10.00
-1	19	12	-11.10	8.00
0	19	12	38.80	10.70
0	19	12	44.40	9.80
0	19	-12	37.60	7.30
0	19	-12	37.00	4.70
-1	19	-12	-3.80	4.60
-1	19	-12	1.80	5.50
-1	19	-12	1.60	3.10
1	19	12	-2.70	7.70
1	19	12	-10.70	8.30
2	19	12	41.50	8.90
2	19	12	39.90	10.30
-2	19	-12	49.60	10.00
-2	19	-12	62.59	8.40
3	19	12	4.40	10.50
-3	19	-12	-4.10	5.40
-3	19	-12	0.50	4.10
-3	19	-12	0.00	4.40
3	19	12	5.50	7.40
-4	19	-12	3.30	5.50
-4	19	-12	6.90	4.70
-4	19	-12	10.60	4.70
4	19	12	-11.60	7.10
-5	19	-12	2.70	5.20
5	19	12	-10.40	6.60
-5	19	-12	2.30	5.50
-5	19	-12	3.60	5.00
6	19	12	10.20	7.70
-6	19	-12	3.60	5.20
-6	19	-12	2.50	5.70
-6	19	-12	9.60	5.80
-7	19	-12	40.60	7.40

-7	19	-12	55.89	10.30
-7	19	-12	48.30	8.40
7	19	12	49.10	9.70
-8	19	-12	6.70	6.10
8	19	12	0.70	7.60
-8	19	-12	6.60	6.90
-9	19	-12	7.80	7.40
9	19	12	18.60	9.00
-9	19	-12	-0.60	6.60
-8	20	12	103.49	15.20
8	20	-12	79.39	13.60
-7	20	12	90.89	13.20
7	20	-12	106.49	17.90
6	-20	-12	70.49	17.80
-6	20	12	74.29	11.60
6	20	-12	65.19	12.60
5	-20	-12	701.43	40.70
5	20	-12	717.23	40.50
-5	20	12	705.03	39.40
4	20	-12	24.70	8.80
-4	20	12	14.90	10.80
-4	20	12	18.90	9.70
-3	20	12	505.85	30.60
-3	20	12	489.35	33.20
3	20	-12	495.95	29.80
-2	20	12	11.60	9.20
-2	20	12	20.70	11.50
2	20	-12	22.30	7.70
1	20	-12	556.94	31.80
-1	20	12	556.74	35.90
-1	20	12	575.74	33.20
0	20	12	10.20	8.00
0	20	12	13.90	9.70
-1	20	-12	826.82	42.30
1	20	12	817.02	46.40
1	20	12	804.22	43.30
-1	20	-12	752.02	39.10
2	20	12	58.29	11.70
2	20	12	51.39	10.20
-2	20	-12	49.30	8.10
-2	20	-12	64.49	7.00
-2	20	-12	53.69	9.70
3	20	12	552.84	31.00
-3	20	-12	516.55	26.90

-3	20	-12	481.55	28.20
-3	20	-12	532.75	29.30
4	20	12	116.19	14.10
-4	20	-12	110.19	10.00
-4	20	-12	113.89	12.10
-4	20	-12	91.69	11.10
-5	20	-12	202.98	15.00
-5	20	-12	169.08	13.20
5	20	12	169.28	16.40
-6	20	-12	158.98	13.90
6	20	12	208.08	17.10
-6	20	-12	187.58	15.10
-7	20	-12	311.47	19.50
-7	20	-12	277.97	19.00
7	20	12	269.57	21.10
-8	20	-12	302.07	20.80
8	20	12	318.47	22.60
-8	20	-12	327.97	20.50
7	21	-12	20.10	10.50
-7	21	12	5.60	9.30
6	21	-12	135.59	18.00
-6	21	12	122.19	14.80
-5	21	12	50.39	14.60
-5	21	12	31.70	9.80
5	21	-12	43.20	10.30
-4	21	12	7.10	9.40
-4	21	12	11.80	12.00
4	21	-12	8.90	8.60
-3	21	12	6.60	9.30
-3	21	12	9.90	11.40
3	21	-12	16.60	7.70
2	21	-12	75.39	13.10
-2	21	12	95.99	19.50
-2	21	12	57.49	11.90
1	21	-12	1.30	6.50
-1	21	12	-2.50	8.60
0	21	12	146.69	16.60
0	21	-12	156.48	15.10
0	21	12	161.98	21.00
-1	21	-12	20.20	7.10
1	21	12	14.60	9.00
1	21	12	-2.10	10.10
2	21	12	82.59	14.20
-2	21	-12	104.59	12.60

-2	21	-12	79.29	7.90
-3	21	-12	17.50	7.40
3	21	12	15.80	8.10
-3	21	-12	16.40	5.00
4	21	12	72.89	11.00
-4	21	-12	67.99	11.60
-4	21	-12	60.09	8.90
-5	21	-12	34.30	7.60
5	21	12	27.90	8.90
-5	21	-12	36.00	7.90
-6	21	-12	89.39	12.50
6	21	12	101.79	14.40
-6	21	-12	71.99	11.00
-7	21	-12	37.50	7.90
7	21	12	34.60	9.20
-7	21	-12	32.30	8.00
-6	22	12	1.10	9.40
-6	22	12	6.50	14.00
6	22	-12	18.20	10.10
-5	22	12	306.27	23.40
5	22	-12	321.27	24.10
-4	22	12	34.70	14.00
4	22	-12	-3.70	8.40
-4	22	12	15.80	10.10
-3	22	12	237.38	20.40
3	22	-12	230.98	19.60
-3	22	12	215.98	25.40
-2	22	12	-11.40	8.50
2	22	-12	-0.10	7.80
-2	22	12	-19.00	11.10
-1	22	12	178.88	18.50
-1	22	12	178.48	22.70
1	22	-12	205.08	17.30
0	22	-12	78.69	13.30
0	22	12	89.19	14.70
-1	22	-12	223.58	18.10
1	22	12	242.08	19.50
2	22	12	12.90	8.40
-2	22	-12	0.40	6.90
3	22	12	11.50	8.60
-3	22	-12	23.00	5.30
-3	22	-12	21.60	7.40
4	22	12	71.19	11.20
-4	22	-12	61.19	12.40

-4	22	-12	71.19	9.00
-5	22	-12	159.88	14.80
-5	22	-12	120.69	11.90
5	22	12	119.19	15.70
6	22	12	101.99	15.30
-6	22	-12	93.09	11.80
-6	22	-12	109.59	13.70
5	23	-12	4.80	9.70
-5	23	12	12.00	10.40
-4	23	12	301.47	23.40
4	23	-12	298.67	23.60
3	23	-12	-0.70	8.00
-3	23	12	-8.30	9.40
-2	23	12	307.67	23.30
2	23	-12	304.17	22.40
1	23	-12	55.89	9.90
-1	23	12	47.10	11.40
0	23	12	321.17	23.90
0	23	-12	334.27	22.90
-1	23	-12	3.50	7.20
1	23	12	-1.80	9.10
-2	23	-12	192.18	17.40
2	23	12	214.88	19.00
3	23	12	43.60	10.90
-3	23	-12	36.40	9.00
-4	23	-12	163.68	16.30
4	23	12	150.58	17.20
-5	23	-12	67.39	10.70
5	23	12	87.59	15.60
-5	23	-12	87.09	14.30
4	24	-12	1.20	10.30
3	24	-12	-4.50	8.70
-3	24	12	-0.80	10.30
-2	24	12	-14.10	9.20
2	24	-12	-0.60	9.00
1	24	-12	18.00	8.90
-1	24	12	13.10	10.40
0	24	-12	7.20	8.10
0	24	12	22.00	10.30
-1	24	-12	22.70	8.90
1	24	12	33.30	10.50
2	24	12	-2.40	8.70
-2	24	-12	-11.00	7.50
-3	24	-12	-3.10	9.10

3	24	12	-3.60	8.70
4	24	12	3.40	9.40
-13	0	13	8.90	5.80
13	0	-13	5.60	9.90
-12	0	13	13.70	5.30
12	0	-13	11.30	8.40
-11	0	13	3.10	4.40
11	0	-13	7.70	7.60
-10	0	13	11.50	4.30
10	0	-13	18.20	7.40
10	0	-13	3.40	5.20
-9	0	13	-4.40	3.70
9	0	-13	2.60	4.20
9	0	-13	12.00	5.90
-8	0	13	-1.40	6.80
-8	0	13	1.30	3.60
8	0	-13	-2.50	3.60
8	0	-13	1.70	5.10
7	0	-13	-1.90	3.20
7	0	-13	-1.60	4.80
-7	0	13	2.30	6.20
-7	0	13	-4.70	3.20
6	0	-13	3.60	4.40
6	0	-13	11.70	3.00
-6	0	13	4.60	3.40
-6	0	13	-9.50	5.50
5	0	-13	-0.30	2.30
-5	0	13	0.80	3.00
5	0	-13	-1.30	3.90
-5	0	13	-3.60	4.20
-4	0	13	-3.30	2.70
4	0	-13	2.70	2.20
-4	0	13	-3.80	4.20
4	0	-13	2.30	3.30
-3	0	13	1.50	3.20
3	0	-13	1.20	2.00
3	0	-13	0.50	2.80
-3	0	13	1.80	2.70
2	0	-13	1.00	1.90
-2	0	13	-4.90	2.70
2	0	-13	-2.00	2.50
-2	0	13	-1.10	2.40
1	0	-13	-1.20	1.90
-1	0	13	0.80	2.40

1	0	-13	0.10	2.30
-1	0	13	-1.90	2.50
0	0	-13	3.20	2.20
0	0	-13	3.50	2.30
0	0	13	-1.30	2.30
0	0	13	6.10	2.70
-1	0	-13	2.00	2.00
1	0	13	6.30	2.50
-1	0	-13	-2.00	2.20
1	0	13	-1.60	1.80
1	0	13	-0.20	2.50
-2	0	-13	-0.60	2.50
2	0	13	-1.10	1.60
2	0	13	-0.30	1.80
-2	0	-13	0.90	1.70
2	0	13	-1.60	2.70
3	0	13	3.70	3.30
3	0	13	-0.70	1.50
3	0	13	0.80	1.40
-3	0	-13	-2.70	2.70
-4	0	-13	0.10	3.10
4	0	13	3.30	3.50
5	0	13	4.40	3.50
-5	0	-13	2.00	3.60
6	0	13	-0.70	3.50
-6	0	-13	0.80	2.80
7	0	13	9.40	4.80
-7	0	-13	7.00	3.40
7	0	13	6.40	1.60
7	0	13	4.40	1.90
8	0	13	-3.60	4.30
8	0	13	-0.30	2.20
-8	0	-13	-0.10	3.80
-8	0	-13	5.90	5.00
-9	0	-13	-3.10	5.10
9	0	13	3.30	2.70
-9	0	-13	0.40	4.40
-10	0	-13	4.50	5.20
10	0	13	1.60	3.20
-10	0	-13	1.30	5.80
11	0	13	6.70	3.70
-11	0	-13	13.80	6.20
-11	0	-13	16.70	7.40
-12	0	-13	10.10	7.90

-13	0	-13	5.80	7.90
-14	0	-13	-10.90	8.70
-14	-1	13	37.50	10.70
-14	1	13	38.00	7.90
-13	-1	13	105.39	10.90
13	-1	-13	122.99	17.10
13	1	-13	121.99	17.30
-13	1	13	122.99	11.30
-12	-1	13	370.36	19.90
12	-1	-13	359.26	24.60
12	1	-13	327.17	24.60
-12	1	13	346.27	20.10
11	-1	-13	197.38	17.90
-11	-1	13	206.48	13.10
-11	1	13	205.88	13.50
11	1	-13	228.28	18.40
10	-1	-13	698.53	37.40
-10	-1	13	737.83	36.60
10	-1	-13	732.93	39.70
10	1	-13	709.63	37.20
10	1	-13	728.23	39.90
-10	1	13	762.52	36.90
-9	-1	13	78.79	7.70
9	-1	-13	93.59	11.50
9	-1	-13	81.09	8.70
9	1	-13	103.69	8.60
-9	1	13	83.99	8.20
-9	1	13	66.89	13.30
9	1	-13	79.49	11.30
8	-1	-13	227.88	14.20
-8	-1	13	249.38	13.70
8	-1	-13	261.17	16.80
-8	-1	13	199.78	18.40
8	1	-13	242.18	13.90
8	1	-13	248.48	16.90
-8	1	13	238.78	14.10
-8	1	13	225.88	18.80
-7	-1	13	119.39	8.60
-7	-1	13	109.79	13.40
7	-1	-13	129.79	11.40
7	-1	-13	123.89	8.90
-7	1	13	114.29	13.50
7	1	-13	121.79	8.40
-7	1	13	123.79	9.20

7	1	-13	131.29	11.70
-6	-1	13	2190.48	103.89
6	-1	-13	2199.38	103.79
6	-1	-13	2257.47	105.49
-6	-1	13	1989.50	105.99
6	1	-13	2121.29	105.39
-6	1	13	2261.97	104.29
6	1	-13	2207.58	103.59
-6	1	13	2066.59	106.19
-5	-1	13	15.00	3.50
-5	-1	13	17.80	5.60
5	-1	-13	24.60	4.20
5	-1	-13	15.60	4.30
-5	1	13	17.00	3.90
5	1	-13	13.90	4.30
5	1	-13	23.20	3.00
-5	1	13	8.60	4.80
4	-1	-13	1684.73	81.49
-4	-1	13	1689.23	83.49
4	-1	-13	1738.03	82.99
-4	-1	13	1674.23	81.79
-4	1	13	1680.23	83.49
4	1	-13	1740.33	82.99
-4	1	13	1708.63	82.29
4	1	-13	1690.13	81.29
-3	-1	13	10.70	2.80
3	-1	-13	16.50	3.70
3	-1	-13	17.10	2.70
-3	-1	13	6.10	3.50
-3	1	13	12.30	3.40
3	1	-13	16.90	2.90
3	1	-13	11.10	3.40
-3	1	13	14.30	4.10
-2	-1	13	1208.88	59.09
2	-1	-13	1212.68	58.79
2	-1	-13	1211.38	57.89
-2	-1	13	1156.68	58.09
2	1	-13	1249.57	58.79
-2	1	13	1221.98	59.19
2	1	-13	1166.08	57.69
-2	1	13	1182.98	58.49
-1	-1	13	553.94	29.60
1	-1	-13	607.74	29.70
1	-1	-13	613.14	29.00

-1	-1	13	559.84	29.20
-1	1	13	590.04	29.80
1	1	-13	601.64	29.50
-1	1	13	556.64	29.50
1	1	-13	600.24	28.80
0	-1	13	1556.74	77.59
0	-1	-13	1607.14	77.79
0	-1	13	1634.14	77.59
0	-1	-13	1636.34	77.49
0	1	-13	1629.74	77.49
0	1	13	1669.23	77.89
0	1	13	1529.65	77.79
0	1	-13	1621.14	77.29
1	-1	13	393.46	19.50
-1	-1	-13	386.56	20.00
1	-1	13	367.66	19.40
1	-1	13	383.16	20.20
-1	-1	-13	393.06	20.00
1	1	13	373.26	20.40
1	1	13	385.76	19.80
-1	1	-13	415.96	19.90
-1	1	-13	387.96	19.60
1	1	13	388.06	20.00
2	-1	13	484.65	22.00
2	-1	13	432.36	23.40
2	-1	13	437.66	21.90
-2	-1	-13	451.85	22.90
-2	-1	-13	454.05	23.20
2	1	13	438.66	23.60
2	1	13	439.26	23.50
2	1	13	456.25	22.60
-2	1	-13	455.85	22.50
-2	1	-13	461.45	23.10
3	-1	13	499.65	27.40
-3	-1	-13	542.85	27.20
3	1	13	546.55	26.20
3	1	13	513.95	27.60
-3	1	-13	527.95	27.10
3	1	13	566.24	26.50
4	-1	13	369.86	20.50
-4	-1	-13	377.16	20.20
-4	1	-13	397.76	20.30
4	1	13	382.86	19.00
4	1	13	351.66	20.60

4	1	13	390.26	18.80
5	-1	13	374.36	21.50
-5	-1	-13	438.46	21.60
-5	1	-13	413.06	21.40
5	1	13	376.26	19.80
5	1	13	407.86	19.60
5	1	13	371.36	21.60
-6	-1	-13	601.34	29.80
6	-1	13	552.64	30.70
6	1	13	600.44	28.90
-6	1	-13	611.34	33.80
6	1	13	574.34	28.60
-6	1	-13	597.64	29.20
6	1	13	576.84	30.90
-7	-1	-13	1012.40	48.00
7	-1	13	945.21	48.70
7	1	13	932.81	48.90
7	1	13	941.21	46.90
-7	1	-13	996.40	47.50
-8	-1	-13	110.79	9.50
-8	1	-13	115.09	10.90
8	1	13	107.49	7.60
8	1	13	92.49	9.80
-8	1	-13	110.79	8.80
-9	-1	-13	213.48	15.60
9	-1	13	196.38	10.70
-9	-1	-13	218.28	14.40
9	1	13	201.68	12.10
-9	1	-13	198.28	14.60
-9	1	-13	212.88	13.60
10	-1	13	20.40	3.40
-10	-1	-13	25.30	7.80
-10	-1	-13	22.40	6.30
-10	1	-13	20.00	7.20
-10	1	-13	27.40	6.00
10	1	13	25.70	4.50
-11	-1	-13	558.74	33.50
-11	-1	-13	668.23	34.80
-11	1	-13	677.83	34.80
-11	1	-13	619.24	33.30
11	1	13	570.54	31.40
-12	-1	-13	2.20	7.40
-12	1	-13	9.30	8.10
-13	-1	-13	486.55	29.80

-13	1	-13	477.45	29.40
-14	-1	-13	23.70	9.90
-14	1	-13	15.90	9.70
-14	2	13	117.39	12.70
13	-2	-13	95.69	15.70
-13	-2	13	101.89	10.20
-13	2	13	118.29	11.40
12	-2	-13	21.50	8.50
-12	-2	13	14.00	4.90
-12	2	13	13.30	5.70
12	2	-13	13.20	8.30
11	-2	-13	157.48	15.80
-11	-2	13	132.09	10.20
-11	2	13	139.59	11.10
11	2	-13	130.49	15.80
10	-2	-13	77.09	11.50
-10	-2	13	55.49	7.00
10	2	-13	49.50	9.10
10	2	-13	55.19	8.10
-10	2	13	41.80	7.70
9	-2	-13	212.48	14.10
-9	-2	13	214.78	12.70
9	-2	-13	225.98	16.20
9	2	-13	216.68	16.70
-9	2	13	201.68	19.40
-9	2	13	223.08	13.60
9	2	-13	215.58	13.30
8	-2	-13	11.80	5.80
8	-2	-13	-0.80	4.00
-8	-2	13	-7.50	10.60
-8	-2	13	6.60	3.40
-8	2	13	-1.00	4.00
-8	2	13	-4.10	7.00
8	2	-13	3.80	5.10
8	2	-13	12.20	3.70
-7	-2	13	274.37	15.30
7	-2	-13	283.07	15.90
7	-2	-13	298.47	18.10
-7	-2	13	265.87	19.70
7	2	-13	274.97	15.20
7	2	-13	297.37	18.40
-7	2	13	275.47	19.70
-7	2	13	292.77	16.30
6	-2	-13	29.20	5.20

-6	-2	13	13.60	6.00
6	-2	-13	20.50	5.50
-6	-2	13	25.80	3.80
-6	2	13	24.50	6.90
6	2	-13	19.90	5.30
6	2	-13	28.40	4.40
-6	2	13	13.10	4.40
-5	-2	13	391.66	19.70
5	-2	-13	389.26	19.70
-5	-2	13	360.76	22.20
5	-2	-13	386.06	21.50
-5	2	13	391.26	20.70
5	2	-13	380.16	19.20
5	2	-13	369.16	21.50
-5	2	13	378.66	22.30
4	-2	-13	77.29	7.50
-4	-2	13	63.79	5.40
4	-2	-13	63.69	5.40
-4	-2	13	45.10	8.00
4	2	-13	68.09	7.50
-4	2	13	54.99	8.30
4	2	-13	69.29	4.70
-4	2	13	59.59	6.80
-3	-2	13	786.72	40.90
3	-2	-13	803.52	39.40
-3	-2	13	764.82	39.40
3	-2	-13	826.02	40.70
-3	2	13	828.92	41.20
-3	2	13	827.12	40.40
3	2	-13	839.02	40.60
3	2	-13	794.22	39.00
-2	-2	13	2.00	3.00
2	-2	-13	13.30	2.60
2	-2	-13	7.50	3.30
-2	-2	13	10.30	2.40
-2	2	13	7.20	3.20
2	2	-13	8.40	2.80
-2	2	13	11.70	4.00
1	-2	-13	378.86	19.70
-1	-2	13	371.56	19.00
-1	-2	13	357.46	19.40
1	-2	-13	362.76	18.90
1	2	-13	383.96	19.30
-1	2	13	387.26	19.90

1	2	-13	369.66	18.60
-1	2	13	343.57	19.50
0	-2	13	437.16	24.00
0	-2	-13	472.65	24.50
0	-2	13	451.25	24.20
0	-2	-13	517.25	24.20
0	2	13	477.05	24.70
0	2	-13	488.55	23.90
0	2	-13	509.35	24.10
0	2	13	472.95	24.50
1	-2	13	806.42	37.40
1	-2	13	751.22	38.20
1	-2	13	730.13	37.30
-1	-2	-13	770.62	38.30
-1	-2	-13	804.62	38.20
-1	2	-13	778.02	37.90
1	2	13	720.83	38.50
1	2	13	778.82	38.10
1	2	13	811.92	38.40
-1	2	-13	774.62	37.70
2	-2	13	164.38	10.40
-2	-2	-13	172.38	10.20
-2	-2	-13	167.68	10.00
-2	2	-13	171.48	10.00
-2	2	-13	169.18	9.10
2	2	13	168.38	10.80
2	2	13	178.18	10.20
2	2	13	162.68	9.50
3	-2	13	155.38	10.50
-3	-2	-13	155.68	10.20
3	2	13	177.98	10.00
3	2	13	175.78	9.30
3	2	13	154.28	10.80
-3	2	-13	174.38	10.20
4	-2	13	443.36	23.30
-4	-2	-13	452.65	23.10
4	2	13	430.56	21.80
-4	2	-13	436.66	22.90
4	2	13	437.26	22.20
4	2	13	433.76	23.50
5	-2	13	178.28	11.70
-5	-2	-13	166.88	11.20
5	2	13	170.08	11.80
-5	2	-13	174.98	11.30

5	2	13	175.78	9.70
5	2	13	189.58	10.30
-6	-2	-13	160.88	10.50
6	-2	13	153.98	11.40
-6	2	-13	168.88	9.00
6	2	13	149.99	9.60
6	2	13	156.08	11.50
6	2	13	150.18	8.90
-6	2	-13	152.58	11.60
-7	-2	-13	75.49	8.00
7	2	13	57.09	8.70
7	2	13	65.99	6.50
-7	2	-13	67.29	9.00
-7	2	-13	68.39	6.20
-8	-2	-13	75.79	8.80
8	2	13	87.79	9.70
8	2	13	69.39	7.30
-8	2	-13	74.99	9.70
-8	2	-13	75.39	7.40
-9	-2	-13	39.40	6.50
-9	2	-13	26.30	6.50
9	2	13	31.80	6.60
-9	2	-13	36.10	7.20
9	2	13	43.00	6.60
-10	-2	-13	255.67	17.70
-10	-2	-13	245.28	17.50
-10	2	-13	259.77	16.40
-10	2	-13	264.47	18.00
10	2	13	255.67	15.30
-11	-2	-13	13.30	7.20
-11	-2	-13	6.40	6.70
-11	2	-13	14.80	7.40
-11	2	-13	16.70	6.20
-12	-2	-13	97.69	13.00
-12	2	-13	109.99	13.70
-12	2	-13	100.49	12.70
-13	-2	-13	30.40	9.50
-13	2	-13	20.20	9.00
-14	-2	-13	108.49	16.50
-14	2	-13	105.19	16.00
-14	3	13	95.19	12.60
13	-3	-13	16.30	8.70
-13	-3	13	3.10	5.70
13	3	-13	-2.00	9.60

-13	3	13	3.90	6.10
-12	-3	13	248.78	14.50
12	-3	-13	255.87	19.90
-12	3	13	219.58	15.40
12	3	-13	226.48	19.90
-11	-3	13	63.89	7.60
11	-3	-13	72.59	13.30
11	3	-13	65.49	13.70
-11	3	13	73.99	9.00
10	-3	-13	170.28	15.10
-10	-3	13	159.38	10.30
10	3	-13	140.49	15.20
10	3	-13	165.28	11.30
-10	3	13	144.59	11.50
9	-3	-13	22.10	7.30
9	-3	-13	18.50	5.80
-9	-3	13	20.00	3.80
-9	3	13	13.20	4.80
9	3	-13	24.20	4.30
9	3	-13	24.00	7.10
-9	3	13	12.30	9.10
-8	-3	13	133.19	8.50
8	-3	-13	139.09	12.00
8	-3	-13	117.99	10.00
8	3	-13	132.39	8.90
8	3	-13	140.79	12.70
-8	3	13	116.19	14.90
-8	3	13	128.49	10.00
-7	-3	13	63.99	11.50
-7	-3	13	71.39	6.00
7	-3	-13	69.59	9.20
7	-3	-13	66.99	7.20
-7	3	13	52.69	7.60
7	3	-13	71.19	9.60
7	3	-13	59.59	5.70
-7	3	13	62.29	11.40
-6	-3	13	10.20	6.00
6	-3	-13	11.70	4.60
6	-3	-13	16.00	3.70
-6	-3	13	17.70	3.00
6	3	-13	15.20	5.50
-6	3	13	5.70	5.40
-6	3	13	16.50	4.60
6	3	-13	17.20	3.00

-5	-3	13	141.59	8.70
5	-3	-13	151.68	11.10
-5	-3	13	145.19	12.30
5	-3	-13	157.58	9.30
-5	3	13	144.79	10.30
5	3	-13	130.99	9.90
5	3	-13	143.79	11.20
-5	3	13	142.69	12.30
4	-3	-13	283.47	15.10
-4	-3	13	258.27	17.10
4	-3	-13	295.07	16.80
-4	-3	13	289.07	14.80
-4	3	13	271.17	17.30
-4	3	13	276.67	16.30
4	3	-13	285.87	16.70
4	3	-13	305.07	14.50
-3	-3	13	4.00	2.30
3	-3	-13	4.00	2.30
-3	-3	13	3.10	3.90
3	-3	-13	5.10	3.10
-3	3	13	3.30	3.40
3	3	-13	1.50	3.30
-3	3	13	4.50	3.50
3	3	-13	4.60	1.80
2	-3	-13	478.45	23.50
2	-3	-13	470.85	24.50
-2	-3	13	425.56	24.20
-2	-3	13	470.75	23.30
-2	3	13	461.35	24.70
-2	3	13	460.65	24.40
2	3	-13	491.25	24.20
1	-3	-13	76.89	5.70
-1	-3	13	78.19	5.60
1	-3	-13	76.79	6.60
-1	-3	13	60.49	6.00
1	3	-13	73.69	6.10
-1	3	13	69.59	6.70
-1	3	13	68.79	6.70
1	3	-13	86.19	5.10
0	-3	-13	496.75	25.50
0	-3	-13	507.05	25.00
0	-3	13	496.35	24.90
0	-3	13	463.95	24.50
0	3	13	481.05	25.60

0	3	-13	513.15	24.60
0	3	-13	514.55	24.80
0	3	13	480.95	25.30
1	-3	13	4.00	2.60
1	-3	13	3.50	1.40
-1	-3	-13	1.10	2.60
-1	-3	-13	3.70	2.50
1	-3	13	2.40	1.30
1	3	13	4.90	2.70
1	3	13	3.00	2.20
-1	3	-13	2.90	2.40
-1	3	-13	2.40	1.70
1	3	13	0.30	2.80
-2	-3	-13	592.74	29.70
2	-3	13	548.35	29.70
-2	-3	-13	595.44	29.80
2	3	13	578.74	29.30
2	3	13	581.04	30.20
-2	3	-13	613.54	29.50
2	3	13	624.34	29.90
-2	3	-13	579.94	28.60
3	-3	13	13.10	3.60
-3	-3	-13	12.00	3.40
3	3	13	11.70	3.30
3	3	13	11.20	3.80
3	3	13	9.80	2.50
-3	3	-13	12.40	3.20
4	-3	13	347.87	19.50
-4	-3	-13	360.16	19.30
-4	3	-13	361.36	19.20
4	3	13	343.97	19.80
4	3	13	381.76	19.30
4	3	13	342.07	18.20
-5	-3	-13	860.01	43.90
5	-3	13	894.81	44.20
5	3	13	872.11	44.40
5	3	13	891.81	43.30
-5	3	-13	897.51	43.90
5	3	13	854.01	42.80
-6	-3	-13	95.19	8.00
6	3	13	87.19	7.30
-6	3	-13	86.69	8.10
-6	3	-13	71.39	8.80
6	3	13	78.49	8.70

6	3	13	86.49	8.80
-7	-3	-13	287.07	16.60
7	3	13	286.37	17.40
-7	3	-13	280.97	14.80
7	3	13	262.57	15.80
-7	3	-13	275.37	17.20
-8	-3	-13	179.78	13.00
8	3	13	182.28	11.80
-8	3	-13	182.18	13.70
8	3	13	165.48	13.20
-8	3	-13	175.48	11.10
-9	-3	-13	217.28	15.60
9	3	13	224.08	15.60
-9	3	-13	219.38	15.00
9	3	13	202.88	13.80
-9	3	-13	220.68	13.60
-10	-3	-13	20.30	7.70
-10	-3	-13	22.90	6.90
-10	3	-13	20.80	5.40
10	3	13	26.20	7.10
-10	3	-13	25.40	7.30
-11	-3	-13	320.27	21.30
-11	-3	-13	290.47	20.50
-11	3	-13	319.67	20.90
-11	3	-13	292.37	19.10
-12	-3	-13	14.10	8.10
-12	3	-13	-2.90	6.10
-12	3	-13	8.50	7.90
-13	-3	-13	103.49	15.30
-13	3	-13	110.89	15.40
-14	-3	-13	4.10	9.80
-14	3	-13	1.70	9.20
-14	4	13	166.48	14.80
13	-4	-13	81.29	13.50
-13	4	13	78.39	11.30
13	4	-13	94.79	17.20
12	-4	-13	90.59	14.70
-12	-4	13	98.29	8.50
-12	4	13	70.09	10.20
-11	-4	13	498.95	24.90
11	-4	-13	482.15	29.30
11	4	-13	457.55	29.40
-11	4	13	476.15	26.10
10	-4	-13	114.79	13.20

-10	-4	13	98.79	7.90
-10	4	13	97.99	9.50
10	4	-13	113.99	13.80
9	-4	-13	177.28	14.10
-9	-4	13	153.98	9.70
-9	4	13	147.59	11.60
-9	4	13	136.59	17.10
9	4	-13	161.88	10.40
9	4	-13	170.68	14.60
8	-4	-13	309.37	20.00
8	-4	-13	303.47	18.20
-8	-4	13	314.87	16.70
8	4	-13	336.17	16.90
-8	4	13	347.27	18.40
-8	4	13	311.77	22.70
8	4	-13	303.17	20.20
7	-4	-13	737.73	37.90
7	-4	-13	769.22	39.60
-7	-4	13	689.13	43.00
-7	-4	13	784.32	37.00
-7	4	13	738.53	40.80
7	4	-13	769.92	39.90
7	4	-13	740.83	38.50
-7	4	13	801.52	38.50
-6	-4	13	65.29	10.00
6	-4	-13	62.79	6.60
-6	-4	13	62.19	5.40
6	-4	-13	60.59	8.40
6	4	-13	67.29	5.10
6	4	-13	72.09	9.20
-6	4	13	61.49	7.70
-6	4	13	68.09	10.40
5	-4	-13	1159.08	56.49
-5	-4	13	1133.79	54.59
-5	-4	13	992.90	56.69
5	-4	-13	1124.29	54.99
-5	4	13	1112.79	57.09
5	4	-13	1162.18	56.59
-5	4	13	1173.58	56.09
5	4	-13	1164.28	54.19
4	-4	-13	6.20	2.80
4	-4	-13	1.40	3.40
-4	-4	13	1.10	2.00
-4	-4	13	2.10	4.00

4	4	-13	4.10	1.70
-4	4	13	7.50	4.10
-4	4	13	-0.60	3.50
4	4	-13	3.20	3.70
3	-4	-13	1034.00	51.09
3	-4	-13	1108.19	52.39
-3	-4	13	971.10	52.19
-3	-4	13	1056.29	50.79
-3	4	13	1052.29	52.19
3	4	-13	1042.60	50.29
3	4	-13	1091.49	52.19
-3	4	13	1058.99	52.69
2	-4	-13	3.80	3.30
-2	-4	13	-0.60	2.80
-2	-4	13	5.10	2.40
2	-4	-13	0.50	2.40
2	4	-13	3.70	1.60
-2	4	13	-0.20	3.30
-2	4	13	0.60	3.10
2	4	-13	0.50	2.70
-1	-4	13	1832.02	94.89
1	-4	-13	2007.00	95.59
-1	-4	13	2023.20	94.59
1	-4	-13	1998.40	94.89
1	4	-13	2040.80	95.09
-1	4	13	1899.81	95.59
-1	4	13	2034.20	95.69
0	-4	-13	243.48	13.90
0	-4	13	244.88	13.00
0	-4	-13	251.27	13.30
0	-4	13	215.68	12.50
0	4	13	230.68	13.50
0	4	-13	255.67	12.70
0	4	13	231.98	14.00
0	4	-13	242.38	12.90
-1	-4	-13	2449.05	119.39
1	-4	13	2485.65	118.89
-1	-4	-13	2489.25	118.99
-1	4	-13	2511.35	118.59
-1	4	-13	2496.45	118.29
1	4	13	2593.64	119.59
1	4	13	2451.85	119.09
1	4	13	2387.76	119.49
2	-4	13	456.15	23.20

-2	-4	-13	451.05	23.20
-2	4	-13	458.85	22.80
2	4	13	444.06	23.50
-2	4	-13	440.36	21.80
2	4	13	446.16	22.80
2	4	13	434.56	23.70
3	-4	13	599.44	31.90
-3	-4	-13	641.14	31.90
3	4	13	675.13	32.20
3	4	13	584.74	32.30
3	4	13	618.54	31.30
-3	4	-13	662.73	31.70
4	-4	13	363.36	19.90
-4	-4	-13	354.16	19.70
4	4	13	357.86	20.40
-4	4	-13	372.46	19.60
4	4	13	385.96	19.80
4	4	13	361.36	18.90
-5	-4	-13	617.44	31.00
5	4	13	547.15	30.10
5	4	13	553.94	28.60
-5	4	-13	550.64	29.80
-5	4	-13	597.24	29.60
-6	-4	-13	776.82	40.00
6	4	13	805.02	39.50
-6	4	-13	763.52	40.30
6	4	13	774.12	40.50
-6	4	-13	821.32	40.40
-7	-4	-13	522.35	27.50
-7	4	-13	497.15	27.50
7	4	13	543.15	26.80
-7	4	-13	524.05	25.30
7	4	13	451.35	27.40
-8	-4	-13	1003.40	50.09
-8	4	-13	1015.30	50.29
8	4	13	1004.30	49.30
-8	4	-13	986.60	48.20
8	4	13	918.11	51.19
-9	-4	-13	68.59	10.30
-9	4	-13	78.59	7.90
-9	4	-13	75.19	10.40
9	4	13	78.79	9.30
9	4	13	65.49	9.90
-10	-4	-13	523.35	30.20

10	4	13	558.74	30.30
10	4	13	516.95	28.70
-10	4	-13	540.05	30.10
-10	4	-13	530.15	28.20
-11	-4	-13	75.19	13.00
-11	4	-13	80.99	12.00
-11	4	-13	58.79	8.00
-12	-4	-13	562.84	32.10
-12	4	-13	498.75	29.90
-12	4	-13	558.44	31.10
-13	-4	-13	-2.60	8.10
-13	4	-13	-1.60	7.70
-14	-4	-13	155.08	17.90
-14	4	-13	142.59	17.00
13	5	-13	-10.30	9.00
-13	5	13	1.50	6.40
12	-5	-13	9.80	8.20
-12	5	13	7.90	6.10
12	5	-13	-3.20	8.10
-11	-5	13	9.60	3.80
11	-5	-13	6.70	7.20
-11	5	13	4.30	5.50
11	5	-13	7.50	7.90
10	-5	-13	-1.10	6.10
-10	-5	13	-2.20	3.20
-10	5	13	5.50	5.10
10	5	-13	0.30	7.30
9	-5	-13	3.60	6.30
-9	-5	13	2.70	2.90
-9	5	13	-8.80	4.70
-9	5	13	0.90	8.20
9	5	-13	-0.60	5.90
-8	-5	13	2.10	2.70
8	-5	-13	1.90	5.40
-8	5	13	5.50	4.70
-8	5	13	9.30	8.10
8	5	-13	8.40	5.90
7	-5	-13	12.80	5.30
7	-5	-13	4.60	4.00
-7	5	13	8.00	6.60
-7	5	13	11.20	4.70
7	5	-13	22.00	3.80
7	5	-13	2.30	5.80
-6	-5	13	3.70	5.90

6	-5	-13	3.10	3.40
-6	-5	13	0.60	2.10
6	-5	-13	-2.00	4.10
-6	5	13	0.50	4.10
6	5	-13	-4.70	4.40
-6	5	13	-3.50	5.70
6	5	-13	5.90	2.10
-5	-5	13	4.30	1.80
5	-5	-13	3.10	4.10
-5	-5	13	-5.60	4.80
5	-5	-13	7.30	3.20
-5	5	13	2.50	4.70
5	5	-13	2.60	1.70
-5	5	13	-1.70	4.20
5	5	-13	-1.00	4.10
4	-5	-13	5.90	4.00
4	-5	-13	3.70	2.80
-4	-5	13	-4.20	3.60
-4	-5	13	2.80	1.80
-4	5	13	-4.20	3.90
4	5	-13	-1.30	3.40
4	5	-13	1.80	1.50
-4	5	13	-3.60	4.10
3	-5	-13	10.60	3.80
-3	-5	13	8.00	3.60
-3	-5	13	14.10	2.30
3	-5	-13	10.80	2.90
-3	5	13	11.80	4.50
-3	5	13	1.70	4.20
3	5	-13	11.10	3.90
3	5	-13	12.60	1.80
2	-5	-13	64.89	6.80
-2	-5	13	59.19	4.60
2	-5	-13	56.69	5.20
-2	-5	13	42.80	5.90
-2	5	13	52.79	6.80
2	5	-13	59.29	3.80
-2	5	13	46.30	6.60
2	5	-13	51.69	6.10
1	-5	-13	83.39	6.10
1	-5	-13	81.29	7.30
-1	-5	13	65.39	6.20
-1	-5	13	92.59	6.00
-1	5	13	78.89	7.10

1	5	-13	75.49	6.50
-1	5	13	84.59	7.70
1	5	-13	79.29	5.10
0	-5	-13	54.29	6.50
0	-5	13	65.99	5.20
0	-5	-13	63.29	5.60
0	-5	13	59.79	4.80
0	5	13	50.69	5.80
0	5	-13	61.29	5.20
0	5	-13	58.09	4.60
0	5	13	64.09	7.00
-1	-5	-13	15.60	3.50
1	-5	13	22.80	3.60
-1	-5	-13	18.50	4.00
-1	5	-13	20.00	2.60
1	5	13	18.60	4.00
1	5	13	21.60	4.40
1	5	13	19.70	4.30
-1	5	-13	20.30	3.10
-2	-5	-13	0.70	3.20
2	-5	13	4.90	2.80
2	5	13	2.70	3.60
2	5	13	4.40	2.50
-2	5	-13	1.90	2.50
-2	5	-13	5.40	1.40
2	5	13	9.00	3.40
3	-5	13	143.89	9.40
-3	-5	-13	139.39	9.20
-3	5	-13	143.29	9.10
-3	5	-13	127.09	9.50
3	5	13	130.99	10.10
3	5	13	141.49	8.80
3	5	13	138.19	9.90
-4	-5	-13	25.10	4.60
4	5	13	29.00	5.30
4	5	13	30.90	4.70
-4	5	-13	36.90	5.40
-4	5	-13	27.20	5.10
4	5	13	32.40	4.90
-5	5	-13	-0.90	3.90
5	5	13	-0.40	2.70
5	5	13	4.90	4.10
-5	5	-13	6.10	3.70
5	5	13	0.50	3.70

-6	-5	-13	23.40	5.80
6	5	13	25.10	5.60
6	5	13	30.00	6.90
-6	5	-13	25.10	5.80
-6	5	-13	23.50	5.30
-7	-5	-13	4.10	5.60
-7	5	-13	4.60	2.30
7	5	13	7.20	4.70
7	5	13	7.30	4.30
-7	5	-13	2.20	4.80
-8	-5	-13	19.80	6.40
-8	5	-13	17.70	6.00
8	5	13	22.40	5.30
-9	-5	-13	6.90	6.50
-9	5	-13	1.60	3.70
9	5	13	4.10	6.00
-9	5	-13	2.00	5.70
9	5	13	-2.60	4.80
-10	-5	-13	9.70	6.80
-10	5	-13	9.80	6.50
10	5	13	3.30	5.80
-10	5	-13	4.80	4.60
10	5	13	15.30	6.80
-11	-5	-13	2.50	7.90
-11	5	-13	2.40	5.50
-11	5	-13	13.70	6.90
11	5	13	3.70	6.60
-12	-5	-13	81.29	12.90
-12	5	-13	77.89	13.10
-13	-5	-13	10.70	9.10
-13	5	-13	17.90	8.70
13	6	-13	87.29	17.40
-13	6	13	74.79	11.70
12	-6	-13	83.49	14.60
-12	6	13	72.29	10.90
12	6	-13	66.49	12.10
11	-6	-13	409.36	26.00
-11	-6	13	402.16	20.90
-11	6	13	408.16	23.00
-10	-6	13	123.79	8.20
10	-6	-13	144.29	14.30
-10	6	13	111.89	12.10
10	6	-13	112.49	14.40
9	-6	-13	242.48	17.50

-9	-6	13	245.48	13.10
-9	6	13	241.68	15.60
9	6	-13	264.17	18.40
-9	6	13	207.68	20.50
8	-6	-13	95.39	11.00
-8	-6	13	103.99	7.50
-8	-6	13	99.19	6.90
-8	6	13	97.79	9.90
-8	6	13	76.19	14.60
8	6	-13	100.79	12.50
7	-6	-13	788.82	40.20
-7	-6	13	748.03	36.70
7	6	-13	741.63	40.30
-7	6	13	804.22	39.20
-7	6	13	726.23	41.30
6	-6	-13	81.99	9.30
-6	-6	13	88.49	4.90
6	-6	-13	73.09	7.70
6	6	-13	79.09	9.90
-6	6	13	81.49	10.80
-6	6	13	76.99	8.70
5	-6	-13	1802.72	85.89
5	-6	-13	1730.43	84.39
-5	-6	13	1821.62	83.09
-5	-6	13	1521.55	85.79
-5	6	13	1843.02	85.59
5	6	-13	1751.32	82.99
-5	6	13	1726.03	86.39
5	6	-13	1779.42	85.89
-4	-6	13	64.89	4.00
4	-6	-13	65.39	6.00
-4	-6	13	59.49	8.30
4	-6	-13	62.19	8.00
-4	6	13	60.99	8.00
4	6	-13	60.39	3.80
-4	6	13	65.19	8.80
4	6	-13	63.89	7.90
3	-6	-13	631.94	31.60
3	-6	-13	637.04	33.00
-3	-6	13	679.23	30.70
-3	-6	13	573.64	32.50
-3	6	13	625.14	33.10
-3	6	13	640.14	33.30
3	6	-13	639.44	30.40

3	6	-13	635.74	32.70
2	-6	-13	61.59	7.00
-2	-6	13	69.79	4.50
2	-6	-13	53.79	5.40
-2	-6	13	43.30	6.00
2	6	-13	50.79	6.30
-2	6	13	44.60	7.30
2	6	-13	57.19	3.60
-2	6	13	49.40	6.90
-1	-6	13	693.33	36.60
1	-6	-13	772.42	37.70
1	-6	-13	750.32	36.60
1	6	-13	737.43	35.70
-1	6	13	737.83	37.80
-1	6	13	704.53	37.40
1	6	-13	780.12	36.90
0	-6	-13	518.95	26.30
0	-6	-13	546.45	27.30
0	-6	13	477.15	25.50
0	-6	13	557.14	26.00
0	6	13	490.75	27.30
0	6	-13	515.25	26.00
0	6	13	512.05	26.80
0	6	-13	518.75	25.50
-1	-6	-13	1680.43	78.69
-1	-6	-13	1664.03	79.29
1	6	13	1599.94	79.39
-1	6	-13	1611.64	77.99
-1	6	-13	1632.74	77.79
1	6	13	1581.54	78.69
-1	6	-13	1582.84	78.29
1	6	13	1643.54	79.49
-2	-6	-13	279.97	14.80
2	6	13	264.77	15.60
-2	6	-13	245.38	14.50
-2	6	-13	271.07	13.00
2	6	13	246.38	15.30
2	6	13	261.07	14.60
-2	6	-13	253.37	14.10
-3	-6	-13	377.06	20.60
3	6	13	408.06	21.50
3	6	13	374.06	21.20
3	6	13	374.66	20.40
-3	6	-13	402.46	20.30

-3	6	-13	385.76	20.70
-4	-6	-13	416.86	21.80
-4	6	-13	407.66	21.30
4	6	13	410.36	22.10
4	6	13	379.96	20.90
4	6	13	400.96	22.20
-4	6	-13	385.26	21.50
-5	6	-13	1128.79	55.69
5	6	13	1225.98	56.29
5	6	13	1066.69	56.29
-5	6	-13	1086.49	55.89
-6	-6	-13	378.16	22.20
6	6	13	386.66	22.60
6	6	13	404.36	22.20
-6	6	-13	402.46	22.20
-6	6	-13	418.06	23.60
-7	-6	-13	480.95	27.00
7	6	13	448.66	26.70
-7	6	-13	508.85	27.10
7	6	13	527.55	26.90
-8	-6	-13	793.02	43.30
-8	6	-13	815.72	40.50
8	6	13	820.22	43.40
8	6	13	908.11	43.70
-8	6	-13	821.82	43.00
-9	6	-13	8.70	6.10
-9	6	-13	8.10	3.80
9	6	13	10.10	5.70
9	6	13	9.00	6.20
-10	-6	-13	378.66	25.30
-10	6	-13	366.76	20.80
-10	6	-13	407.16	23.30
10	6	13	358.06	23.20
11	6	13	2.40	6.60
-11	6	-13	8.60	5.40
-11	6	-13	7.10	7.50
-12	-6	-13	887.21	45.40
-12	6	-13	883.41	44.50
-12	6	-13	715.73	42.40
-13	-6	-13	10.70	9.50
-13	6	-13	8.80	8.00
13	7	-13	36.50	12.30
-13	7	13	41.90	8.50
12	-7	-13	72.89	14.20

-12	7	13	67.49	11.00
12	7	-13	46.10	11.00
11	-7	-13	35.50	9.10
11	7	-13	53.79	10.10
-11	7	13	70.59	10.10
-10	-7	13	134.89	8.60
10	-7	-13	144.39	15.00
-10	7	13	156.18	12.40
10	7	-13	121.39	18.80
9	-7	-13	43.90	7.90
-9	-7	13	41.20	4.60
-9	-7	13	42.00	5.10
-9	7	13	41.50	7.00
9	7	-13	40.40	8.90
-9	7	13	45.70	11.20
-8	-7	13	234.78	12.60
8	-7	-13	277.67	18.00
-8	-7	13	254.97	12.90
-8	7	13	268.87	16.10
-8	7	13	229.28	20.40
8	7	-13	252.17	18.30
7	-7	-13	5.90	5.20
7	7	-13	6.00	5.50
-7	7	13	2.10	6.50
-7	7	13	-5.30	4.70
6	-7	-13	324.37	20.20
6	7	-13	353.56	20.60
-6	7	13	315.07	21.10
-6	7	13	340.57	19.60
5	-7	-13	73.09	8.90
5	-7	-13	80.99	7.20
-5	7	13	54.99	8.50
-5	7	13	67.19	9.70
5	7	-13	61.89	9.10
4	-7	-13	336.57	18.50
4	-7	-13	372.16	20.20
-4	-7	13	305.97	19.80
4	7	-13	362.96	20.00
-4	7	13	349.17	20.50
-4	7	13	348.77	20.30
-3	-7	13	259.87	15.70
3	-7	-13	269.17	16.10
3	-7	-13	247.78	14.50
-3	7	13	246.78	16.10

3	7	-13	269.17	12.70
-3	7	13	257.87	16.50
3	7	-13	277.77	15.80
2	-7	-13	848.62	40.70
-2	-7	13	764.82	41.00
2	-7	-13	850.51	41.90
-2	7	13	807.72	42.10
2	7	-13	798.12	39.30
2	7	-13	844.52	39.60
2	7	-13	867.11	41.40
-2	7	13	804.52	41.80
1	-7	-13	259.17	13.50
-1	-7	13	224.48	13.30
1	-7	-13	244.58	14.70
1	7	-13	231.78	13.60
-1	7	13	232.58	14.90
1	7	-13	234.68	12.10
-1	7	13	231.98	14.30
1	7	-13	241.48	12.50
0	-7	-13	359.26	20.60
0	-7	13	379.56	18.90
0	-7	-13	378.86	19.70
0	7	13	347.47	20.10
0	7	13	368.56	20.90
0	7	-13	385.26	19.40
0	7	-13	376.16	19.10
-1	-7	-13	142.79	9.00
-1	-7	-13	132.19	9.90
1	7	13	138.29	10.40
1	7	13	108.79	9.00
-1	7	-13	134.39	8.60
1	7	13	139.19	10.40
-1	7	-13	134.39	8.00
-1	7	-13	128.29	7.90
-2	-7	-13	1249.07	60.49
-2	7	-13	1239.08	59.89
2	7	13	1304.87	61.49
2	7	13	1201.98	61.19
2	7	13	1166.68	60.39
-2	7	-13	1228.98	60.19
-2	7	-13	1258.17	59.09
-3	-7	-13	473.85	25.00
-3	7	-13	504.65	24.60
3	7	13	472.95	25.80

-3	7	-13	476.15	24.90
3	7	13	486.45	25.90
3	7	13	455.85	24.80
-4	7	-13	225.18	13.40
-4	7	-13	215.28	13.10
4	7	13	214.68	14.20
4	7	13	210.38	12.90
4	7	13	230.38	14.50
-5	7	-13	1082.39	54.79
-5	7	-13	1095.09	54.99
5	7	13	1187.98	55.59
5	7	13	1065.19	55.39
-6	-7	-13	285.07	17.50
6	7	13	301.97	17.70
6	7	13	264.57	17.20
-6	7	-13	293.37	17.30
-7	-7	-13	24.30	5.90
7	7	13	23.50	5.70
7	7	13	16.30	5.60
-7	7	-13	15.90	6.00
-8	-7	-13	9.50	5.50
-8	7	-13	10.60	5.40
8	7	13	11.90	5.80
8	7	13	5.20	5.50
-8	7	-13	5.80	2.80
-9	-7	-13	182.88	15.30
9	7	13	181.88	14.70
-9	7	-13	173.78	11.00
-9	7	-13	176.48	14.00
-10	7	-13	3.00	4.40
10	7	13	13.70	7.30
-10	7	-13	20.20	7.00
-11	7	-13	179.08	13.50
11	7	13	171.28	15.90
-11	7	-13	208.98	16.30
-12	-7	-13	7.20	9.00
-12	7	-13	0.40	6.10
-12	7	-13	11.10	7.30
-13	-7	-13	25.30	9.90
-13	7	-13	20.50	8.90
13	8	-13	55.79	12.80
-13	8	13	65.29	11.80
12	-8	-13	23.10	8.70
-12	8	13	25.50	7.40

12	8	-13	12.60	9.60
11	-8	-13	123.09	15.10
11	8	-13	91.09	15.40
-11	8	13	107.79	11.90
10	-8	-13	115.59	14.10
10	8	-13	76.49	14.90
-10	8	13	116.89	11.20
9	-8	-13	164.98	14.80
-9	8	13	136.29	17.60
9	8	-13	165.88	15.60
-9	8	13	122.59	17.40
8	-8	-13	152.28	13.90
-8	8	13	155.88	12.50
8	8	-13	177.58	14.90
7	-8	-13	167.08	13.40
7	8	-13	177.18	14.30
-7	8	13	150.78	12.60
-7	8	13	152.18	15.80
6	-8	-13	61.39	9.70
-6	8	13	79.59	11.00
-6	8	13	64.39	9.00
6	8	-13	70.99	10.10
5	-8	-13	251.47	16.10
5	8	-13	247.98	16.20
-5	8	13	258.77	15.90
-5	8	13	210.38	16.40
4	-8	-13	28.00	4.50
4	-8	-13	29.10	5.90
4	8	-13	17.10	5.30
-4	8	13	19.60	5.60
-4	8	13	14.20	5.80
3	-8	-13	497.85	25.50
-3	-8	13	388.26	24.50
3	-8	-13	481.95	23.90
3	8	-13	468.55	24.90
-3	8	13	480.95	25.60
-3	8	13	445.86	25.50
-2	-8	13	31.60	6.40
2	-8	-13	59.09	5.90
2	-8	-13	44.20	7.20
-2	8	13	36.30	7.00
2	8	-13	51.19	3.00
-2	8	13	50.99	7.20
2	8	-13	50.39	3.30

2	8	-13	41.90	6.50
1	-8	-13	350.16	18.10
1	-8	-13	337.67	19.40
-1	-8	13	314.87	17.90
1	8	-13	339.57	18.30
1	8	-13	326.57	16.50
-1	8	13	335.57	19.50
-1	8	13	323.07	18.90
1	8	-13	348.07	16.80
0	-8	-13	356.26	19.70
0	-8	-13	360.56	18.50
0	-8	13	326.87	17.70
0	8	-13	364.76	17.90
0	8	-13	336.37	18.30
0	8	13	332.47	19.70
0	8	-13	338.17	17.70
0	8	13	346.27	19.30
-1	-8	-13	613.34	30.50
-1	-8	-13	577.94	29.40
1	8	13	561.54	29.80
1	8	13	543.75	30.30
1	8	13	580.44	30.70
-1	8	-13	593.94	28.90
-1	8	-13	579.34	28.50
-1	8	-13	556.54	28.60
-2	-8	-13	76.89	6.90
-2	8	-13	79.39	6.90
2	8	13	69.49	7.20
2	8	13	74.39	8.00
-2	8	-13	67.69	6.20
-2	8	-13	83.99	5.30
2	8	13	103.89	9.20
3	8	13	123.99	10.70
-3	8	-13	136.59	9.60
-3	8	-13	144.99	9.30
3	8	13	155.68	11.30
3	8	13	127.09	9.70
-4	8	-13	440.56	23.30
-4	8	-13	455.55	23.60
4	8	13	421.76	24.30
4	8	13	445.36	24.70
5	8	13	13.00	5.10
-5	8	-13	18.90	4.90
-5	8	-13	6.90	4.60

5	8	13	14.30	5.00
-6	8	-13	77.99	9.00
6	8	13	77.79	9.90
6	8	13	77.19	8.80
-7	-8	-13	341.97	21.20
7	8	13	314.17	26.00
7	8	13	395.66	21.60
-7	8	-13	350.76	20.90
-8	-8	-13	223.98	16.80
8	8	13	263.77	17.00
-8	8	-13	238.88	12.80
8	8	13	241.88	16.70
-8	8	-13	231.58	16.60
-9	-8	-13	81.09	11.80
-9	8	-13	69.39	7.10
-9	8	-13	89.59	11.10
9	8	13	87.59	11.50
9	8	13	82.59	11.20
-10	8	-13	315.87	18.50
-10	8	-13	347.57	21.50
10	8	13	347.27	21.90
-11	8	-13	49.10	9.30
11	8	13	72.19	12.70
-11	8	-13	43.90	9.10
-12	8	-13	161.18	13.30
12	8	13	152.28	15.80
-12	8	-13	161.58	16.00
-13	-8	-13	9.50	9.90
-13	8	-13	17.90	8.50
13	9	-13	260.67	25.30
-13	9	13	296.77	19.70
-12	9	13	259.07	18.00
12	9	-13	255.17	22.70
11	-9	-13	167.68	17.60
11	9	-13	164.28	18.60
-11	9	13	187.68	14.40
10	-9	-13	481.05	29.30
-10	9	13	500.95	27.30
10	9	-13	478.35	30.40
9	-9	-13	288.57	19.60
-9	9	13	242.38	23.20
9	9	-13	267.87	22.90
-9	9	13	296.87	18.00
8	-9	-13	293.17	19.50

-8	9	13	276.37	22.10
-8	9	13	290.87	19.60
8	9	-13	270.27	19.80
7	-9	-13	315.07	20.10
-7	9	13	297.57	19.10
7	9	-13	335.87	20.60
-7	9	13	301.27	21.70
6	-9	-13	994.60	50.89
-6	9	13	1049.40	50.69
-6	9	13	924.41	51.69
6	9	-13	1007.60	51.19
5	-9	-13	5.40	5.00
5	9	-13	10.40	5.60
-5	9	13	5.60	5.60
-5	9	13	6.90	5.20
4	-9	-13	2169.88	105.79
-4	9	13	2062.99	105.89
-4	9	13	2204.58	105.89
4	9	-13	2230.48	105.69
3	-9	-13	4.30	4.20
3	-9	-13	10.10	4.60
3	9	-13	-0.10	3.80
-3	9	13	-6.90	5.00
-3	9	13	-1.80	4.20
2	-9	-13	1473.15	69.49
-2	-9	13	1259.47	68.29
2	-9	-13	1407.46	67.99
-2	9	13	1372.26	69.59
-2	9	13	1370.46	69.19
2	9	-13	1511.05	68.79
1	-9	-13	219.18	12.70
-1	-9	13	196.68	12.50
1	-9	-13	224.98	14.30
-1	9	13	181.28	14.00
-1	9	13	210.58	13.70
1	9	-13	213.78	12.80
1	9	-13	218.48	10.80
1	9	-13	218.08	10.90
0	-9	13	3028.40	148.79
0	-9	-13	3076.29	149.39
0	-9	-13	3284.87	150.78
0	9	-13	3214.18	149.39
0	9	13	3141.69	150.78
0	9	13	3035.40	150.08

0	9	-13	3096.99	148.59
0	9	-13	3107.09	148.49
-1	-9	-13	651.83	32.90
-1	9	-13	649.24	31.70
-1	9	-13	622.14	31.90
1	9	13	656.13	34.10
1	9	13	617.94	33.20
-1	9	-13	670.13	32.00
-2	9	-13	811.22	38.90
-2	9	-13	795.42	39.60
2	9	13	773.52	40.40
2	9	13	849.52	41.70
-2	9	-13	789.62	39.40
2	9	13	780.82	41.10
3	9	13	282.67	17.40
-3	9	-13	293.07	14.60
-3	9	-13	290.97	14.50
-3	9	-13	307.57	16.20
3	9	13	310.97	18.20
3	9	13	279.87	16.80
-3	9	-13	290.47	16.40
-4	9	-13	60.49	6.90
4	9	13	66.99	8.60
-4	9	-13	65.69	7.30
4	9	13	67.19	9.20
5	9	13	1451.65	76.49
-5	9	-13	1528.55	75.89
-5	9	-13	1544.45	75.89
5	9	13	1686.03	77.19
6	9	13	675.73	38.40
-6	9	-13	725.53	38.10
6	9	13	822.82	39.50
-7	-9	-13	961.70	50.89
-7	9	-13	1012.80	50.29
7	9	13	1088.79	51.19
7	9	13	903.81	51.09
-8	-9	-13	9.30	5.50
8	9	13	10.90	6.20
8	9	13	13.30	6.50
-8	9	-13	13.50	2.80
-9	9	-13	473.55	26.40
-9	9	-13	445.26	23.30
9	9	13	451.45	26.70
-10	9	-13	13.60	7.30

-10	9	-13	25.10	4.90
10	9	13	14.40	7.20
-11	9	-13	616.84	31.40
-11	9	-13	499.35	28.30
11	9	13	500.05	31.00
-12	9	-13	25.10	9.00
12	9	13	24.10	9.10
-12	9	-13	22.50	6.40
-13	-9	-13	285.67	22.90
-13	9	-13	235.48	20.30
-13	10	13	34.00	8.50
13	10	-13	33.40	13.00
-12	10	13	15.40	7.50
12	10	-13	5.70	9.90
11	-10	-13	25.60	9.00
-11	10	13	29.60	7.60
11	10	-13	14.40	10.20
10	-10	-13	11.80	7.90
-10	10	13	15.60	6.60
10	10	-13	6.80	8.10
9	-10	-13	17.30	7.10
-9	10	13	24.00	6.90
-9	10	13	17.50	10.70
9	10	-13	23.60	8.10
8	-10	-13	15.30	6.60
-8	10	13	11.00	8.50
8	10	-13	20.70	10.00
-8	10	13	-0.50	5.70
7	-10	-13	15.60	6.30
7	10	-13	15.80	6.60
-7	10	13	27.70	8.90
-7	10	13	19.00	6.40
6	-10	-13	28.90	6.70
-6	10	13	21.60	7.20
6	10	-13	19.90	6.30
-6	10	13	22.20	6.00
5	-10	-13	23.40	6.10
5	10	-13	50.69	9.40
-5	10	13	32.00	6.80
-5	10	13	34.20	7.10
4	-10	-13	7.20	4.60
4	10	-13	2.80	4.80
-4	10	13	2.90	5.60
-4	10	13	2.70	5.20

3	-10	-13	8.40	4.80
-3	10	13	3.60	5.90
3	10	-13	5.60	4.30
-3	10	13	6.40	4.90
2	-10	-13	48.60	8.20
2	10	-13	52.49	7.20
-2	10	13	41.00	7.20
-2	10	13	47.10	8.00
1	-10	-13	131.89	11.10
-1	-10	13	128.39	9.30
1	10	-13	133.79	7.10
1	10	-13	139.59	7.00
-1	10	13	118.79	10.40
-1	10	13	112.59	11.50
0	-10	13	58.99	5.70
0	-10	-13	53.89	8.50
0	10	13	60.59	9.00
0	10	13	59.09	7.80
0	10	-13	57.69	6.50
0	10	-13	61.59	5.60
0	10	13	53.89	9.10
0	10	-13	56.49	4.60
1	10	13	9.00	5.10
-1	10	-13	12.60	3.20
1	10	13	2.70	3.80
1	10	13	-1.30	3.30
-1	10	-13	8.80	2.60
-1	10	-13	6.50	3.00
2	10	13	12.30	4.90
2	10	13	13.60	4.10
-2	10	-13	21.60	4.20
-2	10	-13	17.70	3.60
-2	10	-13	19.90	3.90
3	10	13	48.20	8.10
-3	10	-13	46.80	6.30
-3	10	-13	37.40	5.90
-3	10	-13	36.40	3.60
3	10	13	33.10	6.30
-3	10	-13	47.30	4.10
4	10	13	8.90	4.80
4	10	13	12.80	5.20
-4	10	-13	9.70	4.00
-4	10	-13	9.00	4.20
-5	10	-13	6.60	4.70

-5	10	-13	22.00	5.00
5	10	13	8.20	5.10
5	10	13	11.40	5.70
6	10	13	91.59	11.20
-6	10	-13	61.19	9.20
6	10	13	62.59	10.30
-7	-10	-13	11.50	5.70
-7	10	-13	19.80	6.00
7	10	13	16.30	5.90
7	10	13	-1.20	6.10
-8	-10	-13	18.90	6.70
-8	10	-13	24.90	4.50
8	10	13	20.60	7.20
-8	10	-13	8.60	6.90
8	10	13	9.80	6.30
-9	10	-13	36.50	6.10
-9	10	-13	61.99	10.30
9	10	13	26.80	7.60
-10	10	-13	11.70	4.30
10	10	13	13.60	7.40
-10	10	-13	4.20	6.90
-11	10	-13	6.70	5.10
-11	10	-13	3.70	7.80
11	10	13	17.90	8.00
-12	10	-13	7.90	7.90
12	10	13	8.70	8.30
-13	-10	-13	-1.40	11.20
-13	10	-13	4.70	8.50
12	11	-13	283.37	25.20
-12	11	13	310.77	20.40
11	-11	-13	187.28	18.50
-11	11	13	192.48	15.60
11	11	-13	171.98	19.90
10	-11	-13	519.25	31.50
10	11	-13	497.55	32.10
-10	11	13	547.35	29.40
9	-11	-13	149.69	15.60
-9	11	13	145.39	13.50
9	11	-13	168.08	16.90
8	-11	-13	587.44	32.70
8	11	-13	598.84	33.20
-8	11	13	490.55	34.20
-8	11	13	607.74	31.50
7	-11	-13	121.09	12.70

7	11	-13	113.49	15.70
-7	11	13	93.69	15.20
-7	11	13	114.59	11.60
6	-11	-13	2023.10	99.79
6	11	-13	1922.71	100.19
-6	11	13	2216.18	99.79
-6	11	13	1943.21	100.59
5	-11	-13	6.80	5.30
-5	11	13	10.30	6.80
-5	11	13	2.30	6.30
5	11	-13	4.80	6.00
4	-11	-13	1959.00	97.09
-4	11	13	2048.70	97.39
4	11	-13	2020.90	96.79
-4	11	13	1871.21	97.19
3	-11	-13	50.39	8.80
-3	11	13	41.30	8.00
3	11	-13	37.40	6.30
-3	11	13	55.09	8.70
2	-11	-13	926.61	46.70
-2	11	13	883.21	46.50
-2	11	13	853.81	46.80
2	11	-13	955.70	45.80
1	-11	-13	758.12	37.70
1	11	-13	730.93	34.10
1	11	-13	733.73	36.30
-1	11	13	647.54	37.60
-1	11	13	686.83	37.00
1	11	-13	699.33	34.10
0	-11	-13	1610.64	76.49
0	-11	13	1595.64	75.09
0	11	13	1666.93	77.19
0	11	13	1430.16	76.19
0	11	-13	1463.45	73.59
0	11	-13	1639.74	74.99
0	11	13	1463.15	75.79
0	11	-13	1455.85	74.29
-1	11	-13	1073.79	51.49
1	11	13	1072.79	54.39
-1	11	-13	1095.69	51.99
1	11	13	1012.80	53.09
-1	11	-13	1053.09	51.39
1	11	13	1056.09	53.89
-2	11	-13	368.16	19.90

2	11	13	368.16	22.20
-2	11	-13	376.66	20.00
2	11	13	383.76	22.30
-2	11	-13	411.16	19.90
-3	11	-13	500.05	25.10
-3	11	-13	483.95	24.00
-3	11	-13	495.75	25.00
-3	11	-13	456.65	23.80
3	11	13	472.45	26.70
-4	11	-13	93.69	9.00
-4	11	-13	111.39	6.50
4	11	13	118.69	11.00
-4	11	-13	114.29	9.20
4	11	13	116.09	11.90
-4	11	-13	109.29	6.70
5	11	13	1050.59	51.59
-5	11	-13	985.20	47.60
5	11	13	982.20	50.69
-5	11	-13	974.00	49.70
-5	11	-13	980.30	49.70
-6	11	-13	451.35	25.60
6	11	13	523.75	27.70
6	11	13	432.76	26.90
-7	-11	-13	317.07	21.60
-7	11	-13	360.26	20.60
-7	11	-13	321.77	17.30
7	11	13	368.06	22.40
7	11	13	365.56	21.60
8	11	13	9.90	6.50
-8	11	-13	8.50	3.00
-8	11	-13	8.20	6.20
-8	11	-13	5.50	3.00
-9	11	-13	175.18	10.90
9	11	13	162.18	15.00
-9	11	-13	186.38	14.80
-10	11	-13	26.60	7.50
10	11	13	27.10	7.80
-10	11	-13	23.20	4.90
-11	11	-13	273.77	19.60
-11	11	-13	334.47	21.30
11	11	13	297.17	21.00
12	11	13	13.90	8.50
-12	11	-13	12.60	8.30
-12	12	13	42.80	8.70

12	12	-13	41.60	12.40
-11	12	13	177.28	15.10
11	12	-13	126.49	19.30
10	-12	-13	88.49	15.50
10	12	-13	110.69	16.20
-10	12	13	94.99	12.20
9	-12	-13	340.77	23.90
-9	12	13	350.96	22.10
9	12	-13	377.06	25.10
8	-12	-13	59.59	9.70
8	12	-13	46.40	9.00
-8	12	13	56.69	8.60
-8	12	13	26.60	10.20
7	-12	-13	481.35	28.50
-7	12	13	486.95	27.70
-7	12	13	435.76	29.90
7	12	-13	531.55	29.20
6	-12	-13	51.49	8.20
-6	12	13	28.30	7.40
6	12	-13	55.69	9.10
-6	12	13	37.90	9.60
5	-12	-13	646.44	34.60
-5	12	13	586.04	35.20
-5	12	13	658.33	34.50
4	-12	-13	70.99	10.30
-4	12	13	72.89	9.60
4	12	-13	55.19	9.70
-4	12	13	78.79	11.30
3	-12	-13	649.04	34.80
-3	12	13	654.23	35.20
3	12	-13	679.53	34.10
-3	12	13	586.84	34.50
2	-12	-13	7.70	4.90
-2	12	13	4.40	5.20
2	12	-13	10.80	4.40
-2	12	13	-8.90	5.60
1	-12	-13	767.02	38.80
1	12	-13	696.03	34.80
-1	12	13	692.13	38.00
-1	12	13	692.93	38.70
1	12	-13	780.22	37.30
1	12	-13	673.53	34.80
-1	12	13	805.22	39.80
0	12	-13	131.49	8.10

0	12	-13	156.38	10.50
0	12	13	137.49	12.70
0	12	13	163.88	13.80
0	12	13	138.99	11.70
1	12	13	344.47	21.20
-1	12	-13	363.36	19.00
-1	12	-13	365.66	19.00
1	12	13	364.16	22.20
1	12	13	379.26	22.90
-1	12	-13	407.66	20.00
-2	12	-13	296.67	16.30
-2	12	-13	289.67	16.30
2	12	13	295.37	18.80
2	12	13	292.87	19.60
-2	12	-13	325.57	16.40
-3	12	-13	573.84	29.40
-3	12	-13	598.44	30.40
-3	12	-13	592.84	30.40
3	12	13	569.54	32.00
-3	12	-13	643.84	29.80
-4	12	-13	304.37	16.20
-4	12	-13	309.57	18.00
-4	12	-13	345.47	16.50
-4	12	-13	316.27	18.00
4	12	13	310.97	19.80
-5	12	-13	69.19	5.30
5	12	13	83.19	12.90
-5	12	-13	52.89	8.40
-5	12	-13	66.39	5.10
-5	12	-13	46.60	8.20
-6	12	-13	237.48	15.70
-6	12	-13	245.78	12.90
6	12	13	257.57	18.20
6	12	13	238.28	17.00
-7	12	-13	42.40	5.10
-7	12	-13	38.20	7.10
7	12	13	38.20	7.90
7	12	13	32.50	7.80
-7	12	-13	44.20	5.10
-8	12	-13	183.78	14.50
8	12	13	170.48	14.50
-8	12	-13	166.68	10.90
-8	12	-13	208.08	11.10
9	12	13	38.10	8.70

-9	12	-13	34.60	4.90
-9	12	-13	29.90	7.30
-10	12	-13	140.39	13.70
10	12	13	131.69	14.60
-10	12	-13	119.89	10.10
-11	12	-13	26.80	8.50
11	12	13	17.60	8.40
12	12	13	179.38	17.90
-12	12	-13	204.88	17.90
12	13	-13	57.99	13.40
-12	13	13	94.59	13.30
11	13	-13	14.30	10.20
-11	13	13	42.50	9.00
10	-13	-13	88.89	15.60
-10	13	13	84.59	12.40
10	13	-13	94.59	16.70
9	-13	-13	10.90	7.90
-9	13	13	8.60	6.80
9	13	-13	0.10	9.20
8	-13	-13	99.59	13.50
8	13	-13	74.39	13.80
-8	13	13	74.99	10.10
7	-13	-13	2.70	6.50
-7	13	13	-3.30	6.70
7	13	-13	2.30	6.60
-7	13	13	-16.80	8.30
6	-13	-13	43.70	8.20
-6	13	13	38.20	8.10
6	13	-13	35.30	7.70
-6	13	13	16.20	8.80
5	-13	-13	32.50	7.00
5	13	-13	33.20	7.30
-5	13	13	26.50	8.10
-5	13	13	24.90	7.60
4	-13	-13	134.39	12.90
4	13	-13	127.69	13.50
-4	13	13	145.79	18.60
-4	13	13	130.59	13.80
3	-13	-13	6.90	5.70
3	13	-13	15.00	8.60
-3	13	13	20.00	7.20
-3	13	13	16.30	7.30
2	-13	-13	200.68	14.90
-2	13	13	174.68	15.10

-2	13	13	183.58	14.60
2	13	-13	184.98	13.50
1	-13	-13	117.59	11.60
1	13	-13	126.29	10.00
-1	13	13	102.69	11.80
-1	13	13	111.39	11.10
1	13	-13	108.09	6.00
1	13	-13	108.59	5.90
-1	13	13	128.49	14.00
0	13	-13	31.30	5.10
0	13	-13	32.10	3.80
0	13	-13	31.10	5.50
0	13	13	35.30	6.90
0	13	13	33.00	7.40
0	13	13	21.40	6.40
1	13	13	186.98	15.10
-1	13	-13	162.78	10.00
-1	13	-13	184.48	11.30
-1	13	-13	165.38	9.90
1	13	13	156.58	13.50
-2	13	-13	43.70	6.00
2	13	13	33.50	7.40
-2	13	-13	39.00	5.50
2	13	13	33.40	6.90
-2	13	-13	36.60	5.70
-3	13	-13	30.10	4.60
3	13	13	22.00	7.40
-3	13	-13	30.00	5.00
-3	13	-13	24.00	4.50
3	13	13	20.20	6.20
-3	13	-13	23.70	4.00
-4	13	-13	40.00	5.40
-4	13	-13	42.90	6.90
4	13	13	23.60	6.70
-4	13	-13	39.20	4.80
-4	13	-13	34.50	5.50
-5	13	-13	13.50	3.80
5	13	13	10.20	8.30
-5	13	-13	12.50	4.90
-5	13	-13	9.50	2.90
5	13	13	10.00	6.00
-5	13	-13	14.50	8.70
6	13	13	64.79	10.50
-6	13	-13	49.50	5.50

-6	13	-13	43.90	5.70
-6	13	-13	62.79	9.30
-7	13	-13	26.20	4.00
7	13	13	19.30	7.10
-7	13	-13	23.70	4.00
7	13	13	33.40	12.10
-7	13	-13	26.30	6.40
-8	13	-13	143.99	9.90
8	13	13	160.68	14.50
-8	13	-13	143.09	9.90
-8	13	-13	159.38	13.70
-9	13	-13	27.20	4.70
-9	13	-13	15.50	4.70
9	13	13	15.10	7.70
-9	13	-13	25.30	7.80
-10	13	-13	4.00	7.40
10	13	13	4.60	8.00
-11	13	-13	147.89	13.00
11	13	13	198.48	17.40
-11	13	-13	214.58	17.20
-12	13	-13	20.10	9.60
12	13	13	20.10	9.80
-11	14	13	383.96	24.00
11	14	-13	358.16	28.30
-10	14	13	50.29	9.20
10	14	-13	61.49	13.00
9	-14	-13	99.89	14.70
9	14	-13	89.89	16.00
-9	14	13	95.49	12.60
8	-14	-13	51.39	9.80
-8	14	13	51.39	9.40
8	14	-13	70.49	13.80
7	-14	-13	169.68	15.70
7	14	-13	181.88	16.40
-7	14	13	169.38	15.00
-7	14	13	143.99	28.00
6	-14	-13	39.40	8.40
6	14	-13	37.70	8.00
-6	14	13	51.49	9.20
-6	14	13	51.29	9.80
5	-14	-13	1106.29	56.49
-5	14	13	1000.60	56.69
-5	14	13	1148.79	56.39
5	14	-13	1111.19	56.19

4	-14	-13	-5.40	5.80
-4	14	13	-1.70	6.70
-4	14	13	3.10	6.50
4	14	-13	2.10	5.50
3	-14	-13	457.95	26.30
3	14	-13	492.05	25.80
-3	14	13	410.36	28.50
-3	14	13	408.76	26.20
2	-14	-13	-0.60	5.90
-2	14	13	-11.40	9.20
2	14	-13	2.70	4.80
-2	14	13	-3.30	5.30
1	-14	-13	1675.13	79.49
-1	14	13	1736.23	80.69
1	14	-13	1476.25	75.29
1	14	-13	1459.15	75.19
-1	14	13	1506.35	79.19
1	14	-13	1722.53	78.69
-1	14	13	1542.75	79.59
0	14	13	33.20	8.40
0	14	-13	52.69	5.20
0	14	-13	46.30	9.50
0	14	13	45.60	8.60
0	14	-13	41.00	4.30
1	14	13	729.93	38.30
-1	14	-13	661.33	33.70
-1	14	-13	651.13	33.50
-1	14	-13	705.73	37.60
1	14	13	675.83	37.10
2	14	13	381.46	23.50
2	14	13	351.76	25.60
-2	14	-13	345.97	19.10
-2	14	-13	362.86	18.80
-2	14	-13	348.77	20.40
-2	14	-13	352.66	19.00
-3	14	-13	605.54	30.80
-3	14	-13	564.44	30.10
-3	14	-13	641.34	30.60
3	14	13	561.04	34.10
-3	14	-13	597.14	30.50
3	14	13	635.74	34.30
-4	14	-13	120.09	8.60
4	14	13	119.89	13.80
-4	14	-13	110.19	9.90

-4	14	-13	106.79	8.00
-4	14	-13	128.99	9.70
4	14	13	129.39	12.30
5	14	13	457.05	27.50
-5	14	-13	429.26	22.80
5	14	13	442.56	26.20
-5	14	-13	438.86	24.30
-5	14	-13	467.55	23.10
-6	14	-13	580.24	28.90
-6	14	-13	569.64	30.40
-6	14	-13	578.84	28.70
6	14	13	547.75	31.80
-7	14	-13	283.97	15.60
-7	14	-13	288.47	15.60
7	14	13	252.67	18.40
-8	14	-13	316.97	16.90
8	14	13	305.07	20.60
-8	14	-13	303.17	19.40
-8	14	-13	279.67	16.80
-9	14	-13	35.90	5.70
9	14	13	34.00	8.30
-9	14	-13	31.90	7.90
-9	14	-13	50.19	7.50
-10	14	-13	318.57	21.00
-10	14	-13	300.87	17.70
10	14	13	289.77	21.30
11	14	13	104.89	15.00
-11	14	-13	89.89	14.50
-11	14	-13	84.39	10.50
11	15	-13	12.60	11.10
-11	15	13	3.30	8.50
10	15	-13	-2.80	9.50
-10	15	13	-3.10	7.50
9	-15	-13	2.80	8.00
9	15	-13	1.70	8.60
-9	15	13	-7.70	7.20
8	-15	-13	-13.60	7.10
-8	15	13	-12.80	7.10
8	15	-13	3.80	8.00
7	-15	-13	15.20	7.80
-7	15	13	9.40	7.40
7	15	-13	22.10	8.20
6	-15	-13	-1.70	6.90
6	15	-13	13.80	7.00

-6	15	13	9.20	7.30
5	-15	-13	11.20	6.70
-5	15	13	5.60	7.10
5	15	-13	4.30	6.40
-5	15	13	22.50	9.70
4	-15	-13	33.70	8.10
4	15	-13	16.70	6.70
-4	15	13	9.00	7.40
-4	15	13	18.30	7.90
3	-15	-13	4.80	6.30
-3	15	13	-5.70	6.80
3	15	-13	-1.10	5.70
-3	15	13	10.00	7.90
2	-15	-13	10.40	6.10
-2	15	13	22.10	8.70
-2	15	13	5.00	6.60
1	15	-13	157.98	11.80
1	15	-13	138.39	7.20
-1	15	13	123.69	13.60
1	15	-13	134.39	7.10
-1	15	13	144.99	15.30
0	15	-13	5.00	2.60
0	15	13	3.50	8.40
0	15	13	4.40	6.90
0	15	-13	5.30	3.00
0	15	-13	8.50	4.60
1	15	13	-8.80	5.60
1	15	13	-1.60	7.70
-1	15	-13	-0.40	2.80
-1	15	-13	0.60	4.00
-1	15	-13	1.00	3.00
-2	15	-13	39.20	6.50
2	15	13	57.89	9.90
-2	15	-13	57.19	7.30
-2	15	-13	48.20	6.00
2	15	13	33.60	7.40
-2	15	-13	47.00	6.30
-3	15	-13	8.00	3.80
-3	15	-13	5.80	3.30
-3	15	-13	-0.70	3.80
3	15	13	-4.60	5.80
3	15	13	-5.30	7.30
-3	15	-13	0.40	4.10
-4	15	-13	5.00	5.10

-4	15	-13	3.70	3.30
4	15	13	0.40	6.30
-4	15	-13	6.60	3.90
-4	15	-13	10.90	4.40
4	15	13	9.00	7.90
5	15	13	13.50	8.30
-5	15	-13	7.00	5.00
5	15	13	7.30	6.70
-5	15	-13	6.80	4.00
-5	15	-13	12.20	3.60
6	15	13	21.50	7.30
-6	15	-13	9.70	3.90
-6	15	-13	12.90	4.30
-6	15	-13	10.00	5.40
-7	15	-13	10.80	6.30
-7	15	-13	17.90	4.60
-7	15	-13	17.90	4.50
7	15	13	15.10	7.10
8	15	13	2.60	7.10
-8	15	-13	3.30	4.40
-8	15	-13	5.60	6.70
-8	15	-13	7.10	4.30
-9	15	-13	12.00	4.90
-9	15	-13	25.50	7.90
9	15	13	16.50	7.80
10	15	13	-12.30	7.90
-10	15	-13	7.00	5.60
-10	15	-13	4.00	7.50
-10	15	-13	13.90	5.30
-11	15	-13	10.80	8.70
-11	15	-13	0.50	5.70
11	15	13	-3.90	9.00
10	16	-13	48.90	13.40
-10	16	13	63.09	10.00
9	16	-13	137.19	18.40
-9	16	13	105.79	14.30
8	-16	-13	72.99	14.90
-8	16	13	85.49	13.20
8	16	-13	79.29	15.80
7	-16	-13	416.26	27.50
7	16	-13	436.86	28.10
-7	16	13	447.96	26.50
6	-16	-13	49.20	9.50
-6	16	13	35.30	9.00

6	16	-13	65.49	10.60
5	-16	-13	762.22	42.00
5	16	-13	744.63	41.70
-5	16	13	799.52	42.00
4	-16	-13	32.80	7.70
4	16	-13	36.80	7.70
-4	16	13	31.50	9.30
3	-16	-13	288.57	20.90
-3	16	13	325.57	21.60
3	16	-13	313.77	19.90
2	-16	-13	14.30	7.40
-2	16	13	1.90	7.70
2	16	-13	14.40	5.80
-2	16	13	-2.60	7.30
-1	16	13	715.23	40.00
1	16	-13	652.73	33.60
-1	16	13	718.23	38.90
1	16	-13	738.33	37.10
0	16	13	73.09	10.40
0	16	-13	62.09	8.70
0	16	13	56.59	9.80
0	16	-13	59.59	5.00
-1	16	-13	839.02	42.50
1	16	13	854.41	46.10
-1	16	-13	847.22	42.90
-1	16	-13	909.51	44.40
1	16	13	934.21	48.30
-2	16	-13	93.79	9.20
-2	16	-13	95.99	7.60
-2	16	-13	87.79	8.30
-2	16	-13	93.29	8.20
2	16	13	98.69	12.60
-3	16	-13	328.17	18.20
3	16	13	349.87	23.60
3	16	13	336.67	21.90
-3	16	-13	337.67	19.00
-3	16	-13	311.57	18.20
-3	16	-13	335.87	18.70
-4	16	-13	84.99	10.10
-4	16	-13	107.09	8.60
4	16	13	118.79	15.10
-4	16	-13	117.29	9.40
4	16	13	94.29	12.40
-4	16	-13	119.49	9.60

5	16	13	311.57	21.00
-5	16	-13	332.67	19.00
-5	16	-13	303.57	17.80
5	16	13	316.27	23.90
-5	16	-13	350.06	18.50
-6	16	-13	276.07	16.10
-6	16	-13	260.77	15.60
6	16	13	295.27	19.40
-6	16	-13	249.48	17.20
-7	16	-13	224.68	16.90
-7	16	-13	252.27	15.20
-7	16	-13	266.87	15.40
7	16	13	250.37	18.80
-8	16	-13	339.77	21.30
8	16	13	309.67	22.10
-8	16	-13	326.47	19.20
-8	16	-13	349.57	19.20
-9	16	-13	37.90	8.30
-9	16	-13	39.40	6.20
9	16	13	39.60	9.50
10	16	13	257.97	20.70
-10	16	-13	309.67	20.40
-10	16	-13	293.77	17.30
-10	16	-13	230.38	17.10
-10	17	13	176.68	17.40
10	17	-13	169.68	21.90
9	17	-13	35.90	11.40
-9	17	13	41.80	9.70
8	-17	-13	181.38	19.10
-8	17	13	212.08	17.60
8	17	-13	203.58	19.80
7	-17	-13	4.90	8.20
-7	17	13	15.60	8.10
7	17	-13	10.70	8.20
6	-17	-13	222.28	19.80
-6	17	13	207.08	18.00
6	17	-13	271.67	19.90
5	-17	-13	19.00	8.40
-5	17	13	18.20	9.00
5	17	-13	18.30	8.20
4	-17	-13	184.88	17.40
4	17	-13	237.08	17.50
-4	17	13	216.78	18.10
3	-17	-13	-4.20	6.60

3	17	-13	2.30	5.90
-3	17	13	-6.10	7.60
-3	17	13	-6.50	7.80
2	17	-13	370.86	22.00
-2	17	13	320.07	23.40
-2	17	13	369.66	25.50
-1	17	13	95.79	14.90
1	17	-13	107.09	11.20
-1	17	13	83.19	13.80
0	17	-13	275.07	15.40
0	17	13	314.87	21.50
0	17	13	299.87	23.30
0	17	-13	338.37	19.00
1	17	13	0.80	6.70
-1	17	-13	11.60	4.00
-1	17	-13	7.30	3.10
-1	17	-13	10.80	5.30
1	17	13	10.70	8.90
-2	17	-13	246.28	14.50
-2	17	-13	257.27	15.20
2	17	13	314.27	22.90
2	17	13	264.57	19.50
-2	17	-13	225.58	15.30
-2	17	-13	303.07	16.80
-3	17	-13	25.70	4.80
-3	17	-13	35.40	6.00
3	17	13	27.70	7.60
-3	17	-13	35.00	6.80
-3	17	-13	38.40	6.10
3	17	13	22.40	11.30
4	17	13	211.08	16.70
4	17	13	224.18	19.30
-4	17	-13	178.08	15.40
-4	17	-13	189.68	12.60
-4	17	-13	192.08	13.00
-5	17	-13	144.59	10.80
5	17	13	117.49	14.00
-5	17	-13	120.49	11.10
-5	17	-13	118.69	9.90
-6	17	-13	37.00	7.70
-6	17	-13	28.40	7.00
-6	17	-13	53.89	8.40
6	17	13	38.90	8.50
-7	17	-13	81.49	9.70

7	17	13	71.79	13.10
-7	17	-13	74.59	11.10
-7	17	-13	85.79	9.60
-8	17	-13	58.69	9.30
-8	17	-13	49.30	8.80
-8	17	-13	31.40	7.90
8	17	13	29.40	9.00
-9	17	-13	123.49	11.20
9	17	13	99.49	15.10
-9	17	-13	113.49	13.60
-9	17	-13	92.49	11.80
10	17	13	16.80	9.00
-10	17	-13	23.30	6.80
-10	17	-13	42.20	9.20
9	18	-13	60.59	12.50
-8	18	13	7.50	9.00
8	18	-13	7.80	9.10
7	-18	-13	152.18	17.50
-7	18	13	142.89	15.70
7	18	-13	146.49	17.80
6	-18	-13	14.30	8.60
6	18	-13	15.50	9.10
-6	18	13	7.50	7.70
5	-18	-13	359.86	24.80
-5	18	13	378.76	24.40
5	18	-13	331.57	23.90
4	-18	-13	43.60	9.60
-4	18	13	14.40	8.40
4	18	-13	26.50	7.80
3	18	-13	239.68	17.40
-3	18	13	222.98	21.40
-3	18	13	217.88	18.10
2	18	-13	103.49	12.10
-2	18	13	86.19	12.30
-2	18	13	88.49	16.40
-1	18	13	86.89	16.10
-1	18	13	86.29	12.90
1	18	-13	94.79	11.50
0	18	-13	78.09	10.40
0	18	13	68.69	12.00
0	18	13	68.29	10.60
0	18	-13	68.79	5.50
1	18	13	49.10	10.90
1	18	13	43.20	9.00

-1	18	-13	46.30	5.50
-1	18	-13	66.89	8.30
-1	18	-13	51.49	9.70
2	18	13	1.70	7.50
-2	18	-13	9.60	5.60
-2	18	-13	18.70	4.80
2	18	13	15.80	9.60
-2	18	-13	21.00	4.00
3	18	13	151.68	17.40
3	18	13	123.29	14.20
-3	18	-13	118.39	9.30
-3	18	-13	127.99	11.50
-3	18	-13	106.69	10.00
-4	18	-13	93.79	10.20
-4	18	-13	90.99	9.10
-4	18	-13	90.79	9.00
4	18	13	73.49	13.10
-5	18	-13	50.29	9.00
5	18	13	42.40	8.80
-5	18	-13	47.40	8.00
-5	18	-13	50.19	8.70
-6	18	-13	55.39	9.20
-6	18	-13	57.19	8.70
-6	18	-13	55.79	10.10
6	18	13	66.79	10.40
-7	18	-13	144.39	12.10
-7	18	-13	115.39	12.80
7	18	13	143.89	15.30
-7	18	-13	146.79	12.00
8	18	13	49.00	10.40
-8	18	-13	41.60	7.00
-8	18	-13	42.80	8.60
-9	18	-13	45.80	7.60
9	18	13	37.90	9.70
-9	18	-13	37.70	8.80
-9	19	13	30.80	9.80
9	19	-13	24.20	11.20
-8	19	13	175.58	17.60
8	19	-13	153.18	19.80
-7	19	13	69.59	11.20
7	19	-13	69.59	12.10
6	-19	-13	692.83	40.70
-6	19	13	695.03	39.60
6	19	-13	735.23	40.90

5	-19	-13	-4.50	8.00
5	19	-13	-2.70	8.10
-5	19	13	-2.20	8.30
4	-19	-13	575.14	36.80
-4	19	13	637.74	36.70
-4	19	13	663.63	39.60
4	19	-13	659.33	36.40
-3	19	13	31.40	10.30
-3	19	13	43.80	11.80
3	19	-13	46.20	8.90
-2	19	13	543.65	34.80
-2	19	13	514.65	31.60
2	19	-13	548.65	30.70
-1	19	13	44.60	12.20
1	19	-13	52.29	8.10
-1	19	13	44.60	10.80
0	19	13	656.13	38.20
0	19	13	656.03	40.40
0	19	-13	706.03	36.40
1	19	13	141.59	18.70
-1	19	-13	165.08	9.80
-1	19	-13	155.18	12.30
-1	19	-13	185.68	13.90
1	19	13	145.59	16.30
-2	19	-13	467.15	24.90
-2	19	-13	374.86	23.50
2	19	13	465.75	29.70
2	19	13	423.86	27.10
-2	19	-13	422.86	22.10
-3	19	-13	145.99	12.50
-3	19	-13	115.99	10.90
-3	19	-13	139.69	10.00
3	19	13	140.99	15.20
-4	19	-13	185.38	13.60
-4	19	-13	158.18	12.20
4	19	13	153.88	16.10
-4	19	-13	159.18	11.70
5	19	13	370.46	23.80
-5	19	-13	317.97	20.10
-5	19	-13	374.46	21.10
-5	19	-13	349.96	20.40
-6	19	-13	551.04	28.30
-6	19	-13	456.45	27.50
-6	19	-13	511.65	28.10

6	19	13	534.55	30.70
7	19	13	318.67	22.70
-7	19	-13	322.07	19.90
-7	19	-13	320.37	19.70
-7	19	-13	323.97	20.50
8	19	13	28.00	9.20
-8	19	-13	55.69	10.50
-8	19	-13	22.10	7.80
8	20	-13	-8.70	9.40
-8	20	13	-2.80	8.80
7	20	-13	6.50	9.70
-7	20	13	-7.80	8.80
-6	20	13	-0.90	8.40
6	20	-13	-3.90	9.50
5	-20	-13	13.10	10.30
-5	20	13	5.10	9.20
5	20	-13	19.10	9.00
-4	20	13	1.20	10.60
4	20	-13	-4.30	7.50
-3	20	13	1.40	9.20
-3	20	13	9.50	11.20
3	20	-13	19.10	8.20
-2	20	13	-1.50	9.20
-2	20	13	17.00	11.40
2	20	-13	5.00	6.90
-1	20	13	12.30	10.80
-1	20	13	12.10	8.70
1	20	-13	13.40	6.90
0	20	-13	15.60	6.80
0	20	13	2.10	9.80
0	20	13	20.80	9.20
1	20	13	15.10	9.00
1	20	13	-2.80	12.50
-1	20	-13	20.50	6.70
-2	20	-13	30.40	7.00
2	20	13	20.70	8.90
-2	20	-13	16.10	4.20
3	20	13	35.00	9.40
-3	20	-13	52.09	10.30
-3	20	-13	46.60	7.30
-4	20	-13	19.40	9.50
-4	20	-13	27.50	7.00
-4	20	-13	22.00	5.60
4	20	13	9.40	8.70

-5	20	-13	37.40	7.00
-5	20	-13	41.20	7.30
5	20	13	41.00	9.90
-6	20	-13	22.50	6.40
6	20	13	23.10	8.50
-6	20	-13	18.00	6.80
-7	20	-13	2.70	6.50
-7	20	-13	11.20	7.20
7	20	13	5.10	8.60
-8	20	-13	-0.50	6.60
-8	20	-13	4.40	7.40
8	20	13	0.60	8.60
7	21	-13	21.40	10.70
-7	21	13	19.30	10.30
6	21	-13	454.25	30.80
-6	21	13	467.35	29.70
-5	21	13	21.40	10.00
-5	21	13	10.50	12.60
5	21	-13	13.50	9.90
-4	21	13	375.56	26.50
-4	21	13	413.06	31.20
4	21	-13	361.06	25.70
-3	21	13	-9.20	9.30
3	21	-13	-1.50	7.90
-3	21	13	-1.80	11.40
-2	21	13	308.47	23.30
-2	21	13	320.37	27.50
2	21	-13	299.07	21.70
-1	21	13	103.09	16.30
-1	21	13	93.49	18.20
1	21	-13	116.79	13.70
0	21	-13	514.85	30.10
0	21	13	526.85	32.00
1	21	13	97.79	15.20
-1	21	-13	123.39	13.40
2	21	13	362.26	24.60
-2	21	-13	356.36	22.80
-3	21	-13	74.99	11.70
-3	21	-13	58.89	8.00
3	21	13	82.39	14.10
4	21	13	10.90	8.60
-4	21	-13	26.70	7.20
-4	21	-13	20.80	5.60
-5	21	-13	224.08	15.60

-5	21	-13	240.68	17.10
5	21	13	217.48	19.40
-6	21	-13	224.58	16.10
6	21	13	217.18	19.00
-6	21	-13	236.78	17.10
7	21	13	329.07	23.20
-7	21	-13	287.47	20.10
-7	21	-13	313.77	20.50
6	22	-13	-2.70	9.80
-6	22	13	-23.30	9.50
5	22	-13	90.39	17.20
-5	22	13	85.99	13.30
-4	22	13	-23.80	9.10
4	22	-13	-14.10	8.30
3	22	-13	162.58	17.40
-3	22	13	148.49	18.20
-2	22	13	2.10	8.80
2	22	-13	1.20	8.10
1	22	-13	205.48	17.70
-1	22	13	192.28	19.50
0	22	-13	21.80	7.90
0	22	13	28.00	10.20
-1	22	-13	117.49	13.80
1	22	13	108.09	16.30
2	22	13	15.70	9.70
-2	22	-13	17.60	6.90
-3	22	-13	128.49	14.00
3	22	13	108.59	16.10
4	22	13	33.20	9.80
-4	22	-13	36.40	8.20
-5	22	-13	32.70	6.90
-5	22	-13	44.10	8.40
5	22	13	27.90	10.40
-6	22	-13	178.08	14.60
6	22	13	193.48	18.30
-4	23	13	150.48	18.70
4	23	-13	124.39	18.10
3	23	-13	13.40	9.70
-3	23	13	-14.50	9.60
-2	23	13	57.59	11.50
2	23	-13	60.99	10.70
1	23	-13	57.79	10.40
-1	23	13	49.10	10.80
0	23	-13	21.20	8.20

0	23	13	10.70	10.30
-1	23	-13	13.60	7.90
1	23	13	-2.80	8.80
-2	23	-13	35.60	8.90
2	23	13	47.80	11.20
3	23	13	-4.20	9.20
-3	23	-13	1.80	7.60
-4	23	-13	70.49	13.20
4	23	13	83.19	12.70
-2	24	13	2.50	10.00
2	24	-13	1.40	9.10
-1	24	13	337.27	25.40
1	24	-13	329.57	24.20
0	24	13	75.29	13.10
0	24	-13	112.59	15.90
-1	24	-13	384.96	27.70
1	24	13	376.26	27.40
2	24	13	208.38	20.80
-13	0	14	364.46	22.30
13	0	-14	338.67	25.60
12	0	-14	5.50	9.30
-12	0	14	13.10	5.60
-11	0	14	612.54	31.80
11	0	-14	615.14	35.30
-10	0	14	15.10	4.90
10	0	-14	23.50	7.60
9	0	-14	373.36	22.10
9	0	-14	430.56	25.00
-9	0	14	424.26	22.00
8	0	-14	8.40	4.20
-8	0	14	7.20	4.10
8	0	-14	6.80	5.50
7	0	-14	1328.47	65.99
-7	0	14	1379.26	66.29
7	0	-14	1365.66	68.09
-7	0	14	1367.86	73.69
-6	0	14	44.90	6.00
6	0	-14	51.89	5.40
-6	0	14	30.50	7.20
6	0	-14	39.50	8.20
5	0	-14	442.96	22.30
5	0	-14	448.86	24.30
-5	0	14	425.86	25.30
-5	0	14	440.86	23.00

4	0	-14	492.15	25.10
-4	0	14	494.15	25.90
-4	0	14	507.85	27.70
4	0	-14	531.55	27.00
3	0	-14	962.40	47.70
3	0	-14	930.51	46.30
-3	0	14	978.50	48.40
-3	0	14	962.70	47.10
-2	0	14	39.90	5.10
2	0	-14	46.30	4.30
2	0	-14	43.80	5.60
-2	0	14	37.10	6.20
1	0	-14	1008.60	48.70
-1	0	14	957.80	49.10
1	0	-14	1012.50	49.30
-1	0	14	1034.60	49.60
0	0	14	944.51	49.90
0	0	-14	1065.59	49.90
0	0	14	1014.50	49.90
0	0	-14	1049.40	49.60
1	0	14	1810.42	86.09
1	0	14	1883.71	86.09
1	0	14	1735.73	86.59
-1	0	-14	1735.73	86.19
-1	0	-14	1795.52	86.19
-2	0	-14	597.84	29.40
-2	0	-14	593.64	29.80
2	0	14	576.94	29.00
2	0	14	596.34	30.30
2	0	14	597.04	29.10
3	0	14	417.46	20.50
-3	0	-14	434.76	22.10
3	0	14	428.46	20.60
3	0	14	402.96	22.50
4	0	14	888.31	45.90
-4	0	-14	946.31	45.60
5	0	14	453.35	25.40
-5	0	-14	489.85	25.10
6	0	14	964.50	48.70
7	0	14	45.80	7.80
-7	0	-14	55.69	6.40
-8	0	-14	402.56	21.80
-9	0	-14	9.30	4.80
9	0	14	5.50	3.30

-9	0	-14	8.50	6.70
-10	0	-14	913.11	47.40
-10	0	-14	950.90	48.90
-11	0	-14	7.70	6.10
-11	0	-14	-2.10	6.60
-12	0	-14	637.64	36.30
-13	0	-14	5.60	8.20
-13	-1	14	14.00	5.70
13	-1	-14	22.00	12.70
-13	1	14	5.10	6.10
13	1	-14	4.50	9.60
-12	-1	14	18.00	5.40
12	-1	-14	21.50	9.40
-12	1	14	11.30	5.90
-11	-1	14	15.20	4.90
11	-1	-14	13.00	7.20
11	1	-14	8.10	7.70
-11	1	14	6.40	5.10
-10	-1	14	152.58	10.90
10	-1	-14	170.58	15.70
10	-1	-14	142.09	12.60
10	1	-14	128.69	11.50
10	1	-14	175.88	15.50
-10	1	14	155.88	11.40
9	-1	-14	8.50	6.10
9	-1	-14	4.20	4.70
-9	-1	14	3.70	3.90
-9	1	14	6.20	4.20
9	1	-14	1.40	4.30
9	1	-14	3.20	6.00
-8	-1	14	-0.20	3.50
8	-1	-14	10.70	5.90
8	-1	-14	5.00	4.20
8	1	-14	5.10	6.10
-8	1	14	3.30	4.10
8	1	-14	3.30	3.70
-7	-1	14	19.60	4.00
7	-1	-14	8.30	5.30
7	-1	-14	15.60	3.90
-7	1	14	9.10	7.60
-7	1	14	15.10	4.30
7	1	-14	11.10	5.70
7	1	-14	32.60	5.40
-6	-1	14	306.67	16.20

-6	-1	14	269.07	19.30
6	-1	-14	314.87	18.50
6	-1	-14	302.87	16.00
-6	1	14	290.47	19.80
6	1	-14	294.07	15.70
-6	1	14	295.67	16.80
6	1	-14	284.37	18.20
5	-1	-14	48.90	7.60
5	-1	-14	47.30	5.00
-5	-1	14	36.90	6.80
-5	-1	14	45.10	5.70
-5	1	14	36.70	6.70
5	1	-14	41.40	4.70
-5	1	14	37.30	6.30
5	1	-14	46.40	7.70
4	-1	-14	71.69	5.40
-4	-1	14	62.59	5.90
4	-1	-14	72.49	7.50
-4	-1	14	51.09	8.50
-4	1	14	62.29	6.50
4	1	-14	66.39	5.00
4	1	-14	69.29	7.60
-4	1	14	71.09	8.80
-3	-1	14	2.10	2.80
3	-1	-14	1.60	3.10
-3	-1	14	1.80	3.50
3	-1	-14	6.10	2.30
3	1	-14	4.10	3.30
-3	1	14	1.90	3.10
3	1	-14	5.20	2.10
-3	1	14	0.30	3.80
-2	-1	14	5.60	2.70
2	-1	-14	3.80	2.30
-2	-1	14	5.70	3.40
2	-1	-14	4.30	3.10
-2	1	14	7.60	3.60
2	1	-14	6.70	2.20
-2	1	14	0.10	2.80
2	1	-14	5.00	3.00
1	-1	-14	186.88	11.20
1	-1	-14	196.98	10.50
-1	-1	14	165.68	11.20
-1	-1	14	182.18	10.80
-1	1	14	177.08	11.10

-1	1	14	173.18	11.40
1	1	-14	199.08	11.10
1	1	-14	187.18	10.30
0	-1	-14	91.49	6.90
0	-1	14	87.29	6.90
0	-1	14	91.19	6.70
0	-1	-14	85.49	6.50
0	1	-14	91.89	6.40
0	1	14	95.29	7.00
0	1	14	87.19	7.20
0	1	-14	91.19	6.60
1	-1	14	36.30	5.10
1	-1	14	29.50	4.00
-1	-1	-14	34.00	4.70
-1	-1	-14	34.20	4.50
1	-1	14	27.50	3.90
1	1	14	41.90	4.90
1	1	14	32.00	5.30
-1	1	-14	33.70	4.10
-1	1	-14	37.00	4.50
1	1	14	35.90	4.50
-2	-1	-14	253.87	13.90
2	-1	14	240.48	14.20
2	-1	14	232.08	12.40
-2	-1	-14	243.98	13.50
2	-1	14	266.87	12.50
-2	1	-14	249.68	13.10
2	1	14	221.08	14.20
2	1	14	265.07	13.50
2	1	14	249.78	13.20
-2	1	-14	246.08	13.80
-3	-1	-14	100.69	7.70
3	-1	14	88.99	8.00
3	1	14	101.79	6.10
3	1	14	96.79	8.10
3	1	14	98.59	6.50
-3	1	-14	89.99	7.40
-4	-1	-14	64.19	6.70
4	-1	14	68.49	7.30
-4	1	-14	69.69	6.90
4	1	14	62.99	7.50
4	1	14	69.59	4.90
4	1	14	66.79	4.60
-5	-1	-14	23.80	5.10

5	-1	14	29.90	5.40
5	1	14	22.50	4.80
5	1	14	22.50	2.80
-5	1	-14	25.90	4.70
5	1	14	18.50	2.80
6	-1	14	127.89	9.90
6	1	14	121.99	7.30
6	1	14	122.69	6.90
6	1	14	125.39	10.50
7	-1	14	42.90	6.90
-7	-1	-14	46.90	6.60
7	1	14	48.10	4.60
7	1	14	45.90	7.40
-7	1	-14	48.30	5.70
-8	-1	-14	62.89	8.00
-8	1	-14	56.59	7.10
8	1	14	55.19	5.30
-8	1	-14	47.50	9.20
-9	-1	-14	168.18	12.60
-9	-1	-14	170.68	14.10
9	1	14	151.68	13.50
-9	1	-14	175.98	12.00
-9	1	-14	156.38	14.10
9	1	14	155.58	9.80
-10	-1	-14	9.30	5.60
-10	-1	-14	9.60	7.00
-10	1	-14	11.10	5.40
-10	1	-14	12.30	6.60
-11	-1	-14	76.89	12.90
-11	-1	-14	73.39	11.80
-11	1	-14	93.29	12.80
-11	1	-14	74.49	10.70
-12	-1	-14	12.60	8.30
-12	1	-14	13.00	7.90
-13	-1	-14	154.78	15.30
-13	1	-14	135.39	16.30
13	2	-14	104.39	17.50
-13	2	14	120.29	12.00
12	-2	-14	60.49	11.40
-12	-2	14	69.19	8.70
12	2	-14	74.49	12.40
-12	2	14	79.49	14.70
-11	-2	14	260.47	15.50
11	-2	-14	263.07	19.90

-11	2	14	255.97	16.20
11	2	-14	259.17	20.30
-10	-2	14	183.68	12.00
10	-2	-14	190.68	16.60
10	2	-14	183.68	16.60
-10	2	14	184.38	12.80
-9	-2	14	205.08	12.50
9	-2	-14	176.88	13.70
9	-2	-14	229.78	16.40
9	2	-14	210.98	13.00
9	2	-14	213.78	16.60
-9	2	14	206.98	13.40
8	-2	-14	176.88	13.90
8	-2	-14	172.28	11.60
-8	-2	14	176.48	10.70
-8	2	14	167.58	11.70
8	2	-14	166.28	10.80
8	2	-14	157.78	13.90
-7	-2	14	424.36	21.30
7	-2	-14	409.46	21.70
7	-2	-14	414.36	23.80
-7	2	14	407.26	22.30
-7	2	14	364.76	25.40
7	2	-14	434.66	24.10
7	2	-14	408.96	21.20
6	-2	-14	21.10	3.80
6	-2	-14	16.80	5.60
-6	-2	14	8.80	6.40
-6	-2	14	22.60	3.70
6	2	-14	12.90	5.10
6	2	-14	23.90	3.40
-6	2	14	8.80	6.40
-6	2	14	17.90	4.70
5	-2	-14	1196.08	57.79
-5	-2	14	1206.48	57.79
-5	-2	14	1094.69	60.09
5	-2	-14	1232.78	59.59
5	2	-14	1221.98	57.49
5	2	-14	1230.78	59.59
-5	2	14	1199.88	58.69
-5	2	14	1147.49	60.19
-4	-2	14	1.60	2.50
4	-2	-14	6.80	2.60
-4	-2	14	3.40	4.70

4	-2	-14	5.00	3.80
4	2	-14	7.30	2.20
-4	2	14	2.60	3.70
-4	2	14	0.40	4.10
4	2	-14	1.60	3.70
-3	-2	14	915.11	47.90
3	-2	-14	999.60	47.80
3	-2	-14	900.91	46.30
-3	-2	14	918.81	46.40
-3	2	14	977.80	48.30
3	2	-14	930.71	46.00
3	2	-14	1004.50	47.70
-3	2	14	990.90	47.40
2	-2	-14	51.09	4.80
2	-2	-14	57.29	6.30
-2	-2	14	34.80	5.00
-2	-2	14	41.80	4.90
-2	2	14	50.49	6.70
2	2	-14	48.10	5.70
-2	2	14	44.20	5.80
2	2	-14	54.99	4.20
1	-2	-14	1350.86	64.69
-1	-2	14	1282.67	64.69
-1	-2	14	1290.47	65.19
1	-2	-14	1347.57	65.39
-1	2	14	1393.76	65.59
-1	2	14	1301.97	65.29
1	2	-14	1346.37	64.39
1	2	-14	1400.86	65.09
0	-2	14	211.78	12.20
0	-2	-14	214.38	12.40
0	-2	-14	216.08	12.10
0	-2	14	191.08	11.80
0	2	-14	225.58	12.00
0	2	14	209.08	12.70
0	2	-14	226.18	11.80
0	2	14	216.48	12.50
-1	-2	-14	399.76	20.90
1	-2	14	424.26	19.90
-1	-2	-14	407.96	20.80
1	-2	14	375.36	19.80
1	-2	14	389.56	20.90
1	2	14	396.76	20.70
1	2	14	426.36	21.10

1	2	14	367.36	21.20
-1	2	-14	410.66	20.50
-1	2	-14	395.46	20.20
2	-2	14	341.57	19.20
-2	-2	-14	365.06	19.00
-2	-2	-14	378.16	19.10
-2	2	-14	349.07	18.10
-2	2	-14	371.26	18.90
2	2	14	348.87	19.60
2	2	14	361.86	18.50
-3	-2	-14	275.17	14.80
3	-2	14	242.08	14.90
3	2	14	293.87	14.40
3	2	14	242.18	15.20
3	2	14	252.27	13.70
-3	2	-14	255.07	14.60
4	-2	14	448.56	24.90
-4	-2	-14	453.65	24.60
4	2	14	475.95	23.50
-4	2	-14	492.95	24.70
4	2	14	464.15	25.10
4	2	14	492.35	23.90
5	-2	14	59.49	7.40
5	2	14	47.70	4.40
5	2	14	62.89	5.40
-5	2	-14	48.00	6.90
5	2	14	47.30	7.30
6	-2	14	387.76	21.50
6	2	14	370.46	19.50
6	2	14	378.16	21.40
-7	-2	-14	457.05	23.90
-7	2	-14	470.55	22.70
7	2	14	455.95	22.90
-7	2	-14	419.86	25.00
7	2	14	419.66	24.60
-8	-2	-14	359.56	20.30
8	2	14	353.76	18.80
-8	2	-14	379.36	19.10
8	2	14	311.47	23.60
-8	2	-14	355.66	22.80
-9	-2	-14	23.20	5.90
-9	2	-14	31.40	5.20
9	2	14	19.10	4.10
9	2	14	13.20	6.20

-9	2	-14	11.00	6.30
-10	-2	-14	299.97	19.60
-10	-2	-14	265.57	18.00
-10	2	-14	288.17	19.10
10	2	14	250.37	15.40
-10	2	-14	263.07	16.80
-11	-2	-14	26.40	8.90
-11	-2	-14	31.00	7.50
-11	2	-14	28.80	7.90
-11	2	-14	20.90	6.30
-12	-2	-14	292.27	21.10
-12	2	-14	244.88	21.30
-12	2	-14	297.77	20.80
-13	-2	-14	23.20	8.70
-13	2	-14	7.50	8.90
13	-3	-14	39.60	12.30
-13	3	14	66.99	11.00
13	3	-14	60.69	11.60
12	-3	-14	209.58	19.60
-12	-3	14	210.78	13.70
-12	3	14	228.58	15.40
12	3	-14	219.68	19.90
-11	-3	14	141.49	10.20
11	-3	-14	128.39	15.10
-11	3	14	129.29	11.40
10	-3	-14	392.26	24.50
-10	-3	14	379.86	20.40
10	3	-14	374.36	24.80
-10	3	14	388.46	21.70
-9	-3	14	134.49	9.10
9	-3	-14	131.09	13.00
9	3	-14	128.99	9.60
-9	3	14	131.09	10.80
9	3	-14	132.49	13.80
8	-3	-14	169.18	11.70
8	-3	-14	158.08	13.80
-8	-3	14	160.48	10.00
-8	3	14	167.68	11.70
8	3	-14	161.38	10.20
8	3	-14	155.18	14.00
7	-3	-14	15.90	5.60
-7	-3	14	16.30	3.50
7	-3	-14	20.90	4.40
7	3	-14	20.00	6.20

7	3	-14	20.60	3.40
-7	3	14	30.10	7.60
-7	3	14	10.00	4.60
6	-3	-14	617.94	32.20
-6	-3	14	640.54	31.70
6	-3	-14	664.73	34.20
-6	3	14	590.84	35.10
6	3	-14	656.53	31.60
-6	3	14	678.53	33.30
6	3	-14	637.34	34.20
5	-3	-14	7.80	3.00
-5	-3	14	-2.40	4.90
5	-3	-14	2.30	4.00
-5	-3	14	0.60	2.30
-5	3	14	-0.60	4.90
5	3	-14	4.20	2.20
-5	3	14	0.70	3.90
5	3	-14	-0.10	3.90
-4	-3	14	1519.35	77.69
4	-3	-14	1651.93	77.39
-4	-3	14	1555.34	75.59
4	-3	-14	1545.85	75.89
4	3	-14	1538.75	75.59
-4	3	14	1588.44	77.99
4	3	-14	1621.14	77.29
-4	3	14	1590.84	76.99
3	-3	-14	9.30	3.90
-3	-3	14	5.60	3.90
3	-3	-14	7.80	2.70
-3	-3	14	4.50	2.50
-3	3	14	3.60	3.80
-3	3	14	6.60	3.90
3	3	-14	8.30	3.60
3	3	-14	11.30	2.10
-2	-3	14	1377.46	71.49
2	-3	-14	1461.65	70.69
2	-3	-14	1523.25	71.79
-2	-3	14	1488.15	70.59
-2	3	14	1432.56	71.59
2	3	-14	1448.36	70.19
2	3	-14	1517.45	71.49
-2	3	14	1488.45	71.99
1	-3	-14	301.77	16.30
1	-3	-14	299.87	15.40

-1	-3	14	254.07	15.60
-1	-3	14	288.57	15.30
-1	3	14	284.07	16.30
-1	3	14	283.87	16.30
1	3	-14	296.97	15.70
1	3	-14	293.27	14.80
0	-3	-14	1100.19	53.49
0	-3	14	1057.29	53.09
0	-3	-14	1128.29	54.09
0	-3	14	1096.59	53.49
0	3	14	1069.09	54.19
0	3	14	1104.49	53.89
0	3	-14	1081.09	53.09
0	3	-14	1147.19	53.39
1	-3	14	372.86	18.00
-1	-3	-14	378.96	19.30
1	-3	14	366.26	19.30
-1	-3	-14	380.96	19.70
1	-3	14	331.87	17.90
-1	3	-14	380.96	18.60
-1	3	-14	377.06	18.90
1	3	14	366.86	19.70
1	3	14	342.37	19.90
1	3	14	368.86	19.30
-2	-3	-14	413.26	21.80
2	-3	14	399.46	21.90
-2	-3	-14	400.36	21.70
2	3	14	422.76	21.40
2	3	14	388.56	22.30
-2	3	-14	437.26	21.60
2	3	14	464.05	22.20
-2	3	-14	410.66	20.70
-3	-3	-14	359.96	19.50
3	-3	14	362.46	19.70
3	3	14	366.06	20.60
3	3	14	360.06	18.70
-3	3	-14	371.96	19.30
3	3	14	355.86	20.10
4	-3	14	58.19	6.70
-4	-3	-14	38.90	6.10
4	3	14	44.50	4.80
4	3	14	61.09	6.10
-4	3	-14	47.40	6.20
4	3	14	46.80	7.00

5	-3	14	848.02	42.20
5	3	14	850.41	41.10
5	3	14	819.22	42.40
5	3	14	780.52	40.60
-5	3	-14	867.01	42.00
6	3	14	230.58	12.70
6	3	14	200.08	14.10
-6	3	-14	203.78	14.30
-7	-3	-14	528.15	27.70
7	3	14	525.95	28.70
-7	3	-14	520.85	25.90
-7	3	-14	527.15	28.60
7	3	14	510.05	26.80
-8	-3	-14	194.18	13.40
8	3	14	198.48	12.10
-8	3	-14	160.68	14.10
-8	3	-14	188.58	11.20
8	3	14	176.28	14.00
-9	-3	-14	268.67	17.40
9	3	14	272.17	18.10
-9	3	-14	277.87	15.70
9	3	14	258.17	15.80
-9	3	-14	269.97	18.20
-10	-3	-14	10.00	6.00
-10	-3	-14	2.30	6.40
10	3	14	10.10	4.60
-10	3	-14	9.00	4.80
10	3	14	4.90	6.20
-10	3	-14	8.40	6.90
-11	-3	-14	376.06	23.50
-11	3	-14	325.87	20.70
-11	3	-14	350.26	22.90
-12	-3	-14	6.80	8.80
-12	3	-14	29.30	8.80
-12	3	-14	19.20	6.80
-13	-3	-14	270.77	21.60
-13	3	-14	288.17	21.50
13	-4	-14	6.60	10.90
13	4	-14	18.80	10.00
-13	4	14	12.70	6.90
12	-4	-14	7.80	8.50
-12	4	14	9.50	6.00
12	4	-14	-1.10	8.50
-11	-4	14	36.20	6.50

11	-4	-14	22.70	8.40
11	4	-14	19.50	8.50
10	-4	-14	15.30	7.20
-10	-4	14	7.10	3.80
10	4	-14	0.80	7.70
-10	4	14	6.00	5.20
9	-4	-14	107.29	12.60
-9	-4	14	89.29	7.30
9	4	-14	96.49	8.00
9	4	-14	95.59	12.70
-9	4	14	87.69	9.60
-8	-4	14	11.30	3.20
8	-4	-14	6.00	8.20
8	-4	-14	10.40	5.70
8	4	-14	12.40	3.40
8	4	-14	20.60	6.90
-8	4	14	5.70	4.90
7	-4	-14	59.19	9.80
7	-4	-14	59.19	7.80
-7	-4	14	73.99	5.80
-7	4	14	53.59	8.00
7	4	-14	78.39	5.90
-7	4	14	80.99	12.00
7	4	-14	69.59	10.20
6	-4	-14	9.80	5.20
6	-4	-14	7.30	3.50
-6	-4	14	7.80	2.80
6	4	-14	10.10	2.70
-6	4	14	8.40	6.80
6	4	-14	2.30	5.10
-6	4	14	7.40	4.70
-5	-4	14	163.78	14.30
-5	-4	14	176.38	10.90
5	-4	-14	192.68	11.40
5	-4	-14	207.58	13.40
-5	4	14	179.98	13.00
5	4	-14	205.88	10.30
5	4	-14	201.88	13.40
-5	4	14	184.58	14.60
4	-4	-14	16.20	3.40
-4	-4	14	10.20	4.40
-4	-4	14	12.40	3.30
4	-4	-14	11.10	4.60
-4	4	14	11.30	4.80

-4	4	14	10.10	5.20
4	4	-14	11.90	2.20
4	4	-14	9.30	4.20
3	-4	-14	10.10	3.80
3	-4	-14	13.70	3.00
-3	-4	14	9.20	4.10
-3	-4	14	8.70	2.60
-3	4	14	7.30	4.20
3	4	-14	14.30	2.10
-3	4	14	5.80	4.00
3	4	-14	11.40	3.70
2	-4	-14	81.09	7.70
2	-4	-14	87.79	6.40
-2	-4	14	65.19	7.00
-2	-4	14	90.09	6.10
2	4	-14	78.29	7.10
-2	4	14	84.19	8.00
-2	4	14	70.09	7.60
-1	-4	14	123.89	7.70
1	-4	-14	121.59	8.00
1	-4	-14	117.99	9.00
-1	-4	14	103.49	8.00
-1	4	14	118.89	9.20
1	4	-14	121.19	8.10
1	4	-14	122.69	7.10
-1	4	14	123.39	9.10
0	-4	14	150.38	9.50
0	-4	-14	173.28	10.30
0	-4	-14	167.28	11.00
0	-4	14	183.48	10.20
0	4	-14	173.98	9.90
0	4	14	168.98	11.30
0	4	14	162.58	10.60
0	4	-14	178.68	9.60
-1	-4	-14	132.49	8.80
1	-4	14	117.29	8.20
-1	-4	-14	119.19	8.30
-1	4	-14	125.49	7.80
1	4	14	121.19	9.30
1	4	14	126.79	9.20
-1	4	-14	121.69	7.30
1	4	14	110.79	8.20
-2	-4	-14	117.59	8.10
2	-4	14	112.99	8.10

-2	-4	-14	113.29	8.30
2	4	14	109.19	8.90
-2	4	-14	113.19	7.70
-2	4	-14	112.69	6.40
2	4	14	104.99	8.60
2	4	14	110.59	7.60
-3	-4	-14	-0.10	3.50
3	-4	14	2.70	3.20
3	4	14	2.60	3.10
3	4	14	0.30	2.40
-3	4	-14	4.00	3.10
3	4	14	2.40	3.90
4	-4	14	4.00	3.70
-4	-4	-14	2.40	3.60
4	4	14	1.80	3.90
4	4	14	2.80	4.10
-4	4	-14	2.50	3.60
4	4	14	-2.30	2.20
5	4	14	14.00	4.80
5	4	14	7.50	3.80
-5	4	-14	8.00	4.30
-5	4	-14	2.00	4.70
6	4	14	17.00	5.40
-6	4	-14	14.50	5.40
6	4	14	11.80	4.20
-7	-4	-14	89.99	9.20
7	4	14	88.09	8.60
7	4	14	92.79	10.00
-7	4	-14	74.19	9.10
-8	-4	-14	70.29	9.50
8	4	14	44.70	7.90
-8	4	-14	53.69	5.80
8	4	14	73.79	9.80
-8	4	-14	61.39	10.90
-9	-4	-14	54.29	10.20
-9	4	-14	44.90	6.80
-9	4	-14	42.90	7.90
9	4	14	36.40	6.20
9	4	14	42.30	7.80
-10	-4	-14	-2.90	5.90
10	4	14	0.20	5.70
10	4	14	1.20	5.90
-10	4	-14	-0.30	4.60
-10	4	-14	3.00	6.80

-11	-4	-14	24.80	8.30
-11	4	-14	19.20	8.00
-12	-4	-14	100.09	15.00
-12	4	-14	79.89	11.80
-12	4	-14	100.69	13.90
-13	-4	-14	1.70	9.20
-13	4	-14	28.90	9.20
13	5	-14	111.99	18.80
-13	5	14	119.99	12.60
12	-5	-14	373.46	26.10
-12	5	14	405.96	22.70
12	5	-14	381.16	26.90
11	-5	-14	24.20	8.50
-11	-5	14	27.60	4.50
-11	5	14	27.80	6.40
11	5	-14	27.50	9.00
-10	-5	14	361.06	19.50
10	-5	-14	384.46	24.30
-10	5	14	382.86	21.70
-9	-5	14	128.99	8.30
9	-5	-14	137.29	13.70
9	5	-14	126.69	14.20
-9	5	14	113.39	11.00
8	-5	-14	597.34	33.10
-8	-5	14	584.14	29.60
8	5	-14	607.54	33.40
-8	5	14	617.74	31.70
7	-5	-14	86.99	8.20
7	-5	-14	77.49	10.10
7	5	-14	64.99	10.50
7	5	-14	75.89	5.70
-7	5	14	71.69	9.00
-7	5	14	67.59	12.50
6	-5	-14	1317.67	63.49
6	-5	-14	1214.28	61.69
-6	-5	14	1238.98	60.59
-6	5	14	1354.16	62.89
6	5	-14	1265.87	60.59
-6	5	14	1185.38	64.19
6	5	-14	1280.17	63.59
5	-5	-14	7.00	3.20
-5	-5	14	5.90	2.10
5	-5	-14	-1.90	4.10
-5	5	14	0.20	4.30

-5	5	14	4.50	5.30
5	5	-14	3.90	4.80
5	5	-14	6.50	1.90
-4	-5	14	644.44	38.60
4	-5	-14	787.12	39.00
-4	-5	14	782.52	36.50
4	-5	-14	756.52	37.40
4	5	-14	776.82	38.80
-4	5	14	735.33	39.30
-4	5	14	761.12	38.90
4	5	-14	780.82	36.30
3	-5	-14	157.78	9.50
3	-5	-14	155.98	11.20
-3	-5	14	166.68	8.80
-3	-5	14	140.49	10.90
-3	5	14	152.68	11.50
-3	5	14	155.38	11.60
3	5	-14	150.18	8.10
3	5	-14	158.28	10.80
2	-5	-14	1023.70	50.29
-2	-5	14	1056.69	50.29
2	-5	-14	1075.49	51.59
-2	-5	14	898.61	50.69
2	5	-14	1015.70	49.50
-2	5	14	1053.29	51.69
-2	5	14	1054.59	51.69
2	5	-14	1081.79	51.09
1	-5	-14	837.02	40.90
-1	-5	14	863.51	40.60
-1	-5	14	732.03	40.80
1	-5	-14	853.51	41.90
-1	5	14	840.42	41.80
1	5	-14	817.92	40.20
-1	5	14	831.02	42.10
1	5	-14	869.21	41.20
0	-5	-14	1684.03	81.39
0	-5	14	1751.32	81.19
0	-5	14	1628.04	80.69
0	-5	-14	1726.33	82.19
0	5	-14	1706.73	81.09
0	5	14	1626.24	82.19
0	5	14	1684.03	81.89
0	5	-14	1679.73	80.79
1	-5	14	208.08	12.10

-1	-5	-14	204.58	12.80
-1	-5	-14	205.18	12.20
-1	5	-14	218.18	11.60
1	5	14	198.38	13.10
1	5	14	215.48	12.30
1	5	14	199.08	13.00
-1	5	-14	215.58	11.20
-2	-5	-14	921.21	46.30
2	-5	14	953.10	46.30
2	5	14	1005.70	47.00
-2	5	-14	922.51	44.90
2	5	14	893.81	47.00
2	5	14	903.01	46.10
-2	5	-14	957.40	45.90
3	-5	14	209.48	12.20
-3	-5	-14	200.78	12.30
3	5	14	192.58	13.00
3	5	14	201.98	12.70
3	5	14	182.78	11.50
-3	5	-14	207.98	11.80
4	5	14	641.54	35.10
-4	5	-14	640.14	34.80
4	5	14	654.23	34.20
4	5	14	770.42	35.20
-4	5	-14	700.53	34.50
-5	5	-14	1206.98	59.79
-5	5	-14	1219.58	59.49
5	5	14	1189.38	59.99
6	5	14	249.97	14.30
-6	5	-14	217.38	14.70
6	5	14	204.18	14.90
-7	-5	-14	391.26	22.20
7	5	14	400.86	21.50
7	5	14	352.26	22.10
-7	5	-14	387.66	22.30
-8	-5	-14	218.28	15.30
8	5	14	206.88	15.50
8	5	14	232.08	14.80
-8	5	-14	224.98	11.80
-8	5	-14	177.58	19.80
-9	-5	-14	306.67	20.00
-9	5	-14	341.07	20.30
9	5	14	308.67	20.20
-9	5	-14	318.27	17.00

9	5	14	296.07	18.90
-10	-5	-14	5.70	6.40
-10	5	-14	9.10	6.80
10	5	14	12.90	6.80
-10	5	-14	6.80	4.40
-11	-5	-14	929.81	47.80
11	5	14	901.91	47.20
-11	5	-14	880.41	46.70
-11	5	-14	837.32	44.70
-12	-5	-14	16.70	9.10
-12	5	-14	14.30	6.40
-12	5	-14	8.20	8.20
-13	-5	-14	255.87	22.20
-13	5	-14	285.27	21.10
-13	6	14	7.30	7.20
13	6	-14	7.70	11.10
12	-6	-14	27.80	9.30
12	6	-14	16.70	10.20
11	-6	-14	25.60	8.70
11	6	-14	37.20	9.50
-11	6	14	51.79	9.60
-10	-6	14	8.30	3.20
10	-6	-14	11.10	7.50
-10	6	14	14.00	6.90
10	6	-14	3.70	7.20
-9	-6	14	88.79	6.60
9	-6	-14	90.49	11.90
9	6	-14	90.89	13.90
-9	6	14	87.99	10.20
-8	-6	14	5.60	2.60
-8	-6	14	3.10	2.80
8	-6	-14	2.30	5.60
-8	6	14	-4.20	5.00
8	6	-14	-1.10	5.70
-7	-6	14	98.99	5.90
7	-6	-14	92.79	10.90
-7	6	14	95.99	10.00
7	6	-14	96.99	11.30
-7	6	14	96.19	13.60
-6	-6	14	67.49	4.50
6	-6	-14	69.29	9.30
6	-6	-14	57.69	7.40
-6	6	14	57.59	8.80
6	6	-14	80.89	4.60

6	6	-14	71.19	10.00
-6	6	14	68.49	9.70
-5	-6	14	161.98	8.20
5	-6	-14	160.18	12.10
5	-6	-14	155.28	10.20
5	6	-14	149.29	8.00
5	6	-14	155.18	12.40
-5	6	14	140.69	13.10
-5	6	14	154.18	12.10
4	-6	-14	102.09	7.60
-4	-6	14	101.69	5.70
4	-6	-14	96.69	9.50
-4	6	14	80.29	10.20
4	6	-14	86.39	9.20
4	6	-14	99.49	5.40
-4	6	14	81.49	9.70
-3	-6	14	215.98	10.70
3	-6	-14	214.68	11.80
-3	-6	14	192.48	13.00
3	-6	-14	200.18	13.50
-3	6	14	190.28	13.70
3	6	-14	193.48	10.10
-3	6	14	189.68	13.60
3	6	-14	212.08	13.10
-2	-6	14	7.00	3.50
2	-6	-14	6.90	4.00
-2	-6	14	17.30	2.80
2	-6	-14	18.60	3.60
2	6	-14	12.30	3.70
-2	6	14	10.90	4.60
2	6	-14	16.60	2.40
-2	6	14	5.60	4.60
-1	-6	14	7.20	3.00
-1	-6	14	9.30	2.80
1	-6	-14	18.40	3.30
1	-6	-14	4.90	3.60
1	6	-14	10.90	3.30
-1	6	14	9.10	4.60
-1	6	14	9.00	3.50
1	6	-14	11.00	2.30
0	-6	14	130.49	8.10
0	-6	-14	151.38	9.10
0	-6	-14	144.89	10.20
0	-6	14	143.59	8.60

0	6	-14	136.29	7.90
0	6	14	131.99	10.50
0	6	14	115.29	9.30
0	6	-14	132.39	8.50
-1	-6	-14	149.88	10.10
-1	-6	-14	143.69	10.70
1	-6	14	158.78	9.70
-1	6	-14	153.38	9.10
-1	6	-14	164.98	8.80
1	6	14	144.39	10.00
1	6	14	160.68	11.20
1	6	14	151.08	11.20
-2	-6	-14	150.68	10.30
-2	6	-14	162.48	8.10
2	6	14	152.08	11.10
2	6	14	157.08	11.10
-2	6	-14	138.89	10.00
-2	6	-14	157.38	9.40
2	6	14	135.19	9.70
-3	-6	-14	-5.90	4.00
-3	6	-14	-1.60	3.60
3	6	14	1.40	3.80
-3	6	-14	2.20	3.00
3	6	14	-0.80	4.00
3	6	14	2.40	2.80
-4	6	-14	24.10	4.60
4	6	14	16.80	5.00
4	6	14	22.40	5.40
-4	6	-14	18.00	4.70
5	6	14	17.90	5.00
-5	6	-14	6.60	4.10
5	6	14	26.20	5.50
-5	6	-14	3.50	4.70
-6	6	-14	2.50	4.50
6	6	14	5.40	4.90
-7	-6	-14	93.89	15.90
-7	6	-14	111.29	10.90
7	6	14	125.59	10.80
-8	-6	-14	15.10	6.30
-8	6	-14	9.30	2.50
8	6	14	18.50	5.70
8	6	14	14.70	5.80
-9	-6	-14	6.90	6.60
-9	6	-14	12.90	3.60

-9	6	-14	9.70	6.60
9	6	14	11.00	5.80
9	6	14	12.50	6.30
-10	6	-14	21.10	4.80
10	6	14	15.00	7.00
-10	6	-14	23.50	7.50
-11	6	-14	121.19	13.50
11	6	14	105.39	13.60
-11	6	-14	64.19	9.80
-12	-6	-14	95.09	15.40
-12	6	-14	69.99	10.50
-12	6	-14	64.49	10.50
-13	-6	-14	20.20	10.30
-13	6	-14	36.30	9.30
13	7	-14	56.29	12.60
-13	7	14	61.39	9.70
12	-7	-14	247.18	20.80
-12	7	14	248.68	17.40
12	7	-14	249.48	22.20
11	-7	-14	33.90	9.10
-11	7	14	43.90	7.50
11	7	-14	35.50	10.10
10	-7	-14	440.76	27.40
10	7	-14	434.86	27.90
-10	7	14	438.26	25.00
-9	-7	14	38.40	3.90
9	-7	-14	39.10	8.20
9	7	-14	22.50	9.00
-9	7	14	33.30	8.00
8	-7	-14	214.18	16.00
-8	7	14	195.48	14.20
8	7	-14	194.08	16.20
7	-7	-14	29.20	6.80
-7	7	14	19.00	5.80
-7	7	14	22.40	8.60
7	7	-14	17.50	6.60
6	-7	-14	463.35	25.90
-6	7	14	469.75	25.40
6	7	-14	472.85	26.20
-6	7	14	406.16	26.80
5	-7	-14	126.59	10.90
5	-7	-14	108.69	10.60
-5	7	14	88.79	11.70
5	7	-14	114.59	11.10

-5	7	14	112.29	10.90
4	-7	-14	1496.05	72.09
4	-7	-14	1402.46	70.49
-4	7	14	1481.65	72.19
-4	7	14	1403.36	72.39
4	7	-14	1489.05	71.89
-3	-7	14	41.20	7.60
3	-7	-14	40.90	5.70
3	-7	-14	43.40	7.50
3	7	-14	45.20	3.20
3	7	-14	38.10	2.70
3	7	-14	35.90	5.70
-3	7	14	26.80	6.30
-3	7	14	36.10	7.60
2	-7	-14	470.25	24.10
-2	-7	14	425.86	24.40
2	-7	-14	491.75	25.60
-2	7	14	446.06	25.30
-2	7	14	458.45	25.90
2	7	-14	495.65	23.00
2	7	-14	470.25	22.60
2	7	-14	474.05	24.70
1	-7	-14	738.23	35.30
-1	-7	14	603.14	33.60
1	-7	-14	715.93	33.90
-1	7	14	657.93	34.70
1	7	-14	678.03	34.00
-1	7	14	643.54	35.10
1	7	-14	688.43	32.70
0	-7	14	1059.49	52.99
0	-7	-14	1087.29	53.79
0	-7	-14	1084.89	54.79
0	7	-14	1111.79	53.29
0	7	14	1065.59	54.39
0	7	-14	1109.09	53.49
0	7	14	1164.48	55.29
0	7	-14	1125.39	52.89
0	7	14	1077.09	54.99
-1	-7	-14	145.99	10.20
-1	-7	-14	167.28	11.30
1	7	14	162.28	11.70
-1	7	-14	156.18	9.10
-1	7	-14	162.68	9.90
1	7	14	144.29	10.60

1	7	14	158.28	11.70
-1	7	-14	160.18	9.30
-2	-7	-14	326.57	18.60
-2	7	-14	349.57	16.70
2	7	14	325.97	19.50
-2	7	-14	330.27	17.70
2	7	14	312.67	18.30
2	7	14	365.66	19.70
-2	7	-14	337.87	18.20
3	7	14	589.44	30.70
3	7	14	603.34	30.70
-3	7	-14	579.84	29.20
3	7	14	530.35	30.20
-3	7	-14	570.94	29.60
4	7	14	22.80	5.50
-4	7	-14	18.20	4.60
4	7	14	27.30	5.50
-4	7	-14	25.20	4.60
5	7	14	471.45	26.40
5	7	14	488.15	26.30
-5	7	-14	465.35	25.80
-5	7	-14	485.25	25.50
-6	7	-14	58.19	8.60
6	7	14	51.19	8.90
6	7	14	60.99	9.00
-7	-7	-14	245.08	16.80
7	7	14	237.78	17.20
-7	7	-14	278.17	16.80
-8	-7	-14	51.69	10.20
8	7	14	48.40	9.00
8	7	14	60.49	10.00
-9	7	-14	167.28	13.30
-9	7	-14	161.68	9.70
9	7	14	134.59	13.70
9	7	14	160.48	18.60
10	7	14	7.50	6.40
-10	7	-14	-1.20	3.80
-10	7	-14	-0.30	6.70
-11	7	-14	274.27	17.70
11	7	14	316.47	21.30
-11	7	-14	330.37	21.20
-12	-7	-14	23.90	9.60
-12	7	-14	22.20	8.30
-12	7	-14	28.00	7.00

-13	-7	-14	234.68	21.60
-13	7	-14	235.38	19.90
13	8	-14	124.09	20.70
-13	8	14	173.88	15.40
12	-8	-14	109.69	16.90
12	8	-14	94.69	17.30
-12	8	14	108.19	13.30
11	-8	-14	307.77	22.80
-11	8	14	309.17	19.70
11	8	-14	317.07	23.70
10	-8	-14	76.59	13.50
-10	8	14	73.99	10.50
10	8	-14	53.29	10.10
9	-8	-14	440.16	26.90
-9	8	14	441.56	24.90
9	8	-14	434.16	27.30
8	-8	-14	77.09	11.10
-8	8	14	61.19	9.90
7	-8	-14	655.33	35.60
-7	8	14	596.94	37.20
7	8	-14	681.93	36.10
-7	8	14	690.13	34.90
6	-8	-14	32.10	6.50
-6	8	14	25.70	7.40
6	8	-14	29.60	6.50
-6	8	14	23.80	6.50
5	-8	-14	1704.13	83.59
-5	8	14	1797.32	83.59
-5	8	14	1568.74	83.99
5	8	-14	1695.93	83.49
4	-8	-14	104.89	10.20
-4	8	14	91.39	10.80
-4	8	14	89.19	10.40
4	8	-14	106.69	10.10
3	-8	-14	1080.69	51.29
3	-8	-14	1032.40	51.99
3	8	-14	1049.70	51.59
-3	8	14	941.51	51.89
-3	8	14	1037.30	52.39
2	-8	-14	465.15	24.20
-2	-8	14	408.36	24.00
2	-8	-14	432.36	22.40
-2	8	14	418.76	24.40
2	8	-14	423.06	23.00

-2	8	14	416.16	23.80
2	8	-14	428.86	20.70
2	8	-14	435.86	21.00
-1	-8	14	948.81	48.20
1	-8	-14	980.20	49.60
1	-8	-14	1003.70	48.40
1	8	-14	976.80	47.10
1	8	-14	997.40	48.50
1	8	-14	1028.50	47.40
-1	8	14	969.20	49.90
-1	8	14	939.31	49.20
0	-8	-14	769.72	38.40
0	-8	-14	799.62	39.60
0	-8	14	727.33	37.40
0	8	14	811.02	41.60
0	8	14	733.63	39.00
0	8	-14	786.92	37.70
0	8	14	756.92	39.80
0	8	-14	761.32	37.30
0	8	-14	769.22	38.10
-1	-8	-14	291.27	17.20
1	8	14	310.17	18.00
1	8	14	274.57	17.40
-1	8	-14	300.67	15.10
-1	8	-14	281.77	15.50
-1	8	-14	275.57	15.00
1	8	14	263.57	16.40
2	8	14	992.70	53.19
2	8	14	1136.39	54.49
-2	8	-14	1081.69	52.79
-2	8	-14	1079.99	51.69
2	8	14	1080.69	54.19
-2	8	-14	1087.79	52.49
-3	8	-14	226.68	13.60
3	8	14	241.18	15.20
-3	8	-14	231.38	13.10
3	8	14	199.68	14.40
-4	8	-14	207.98	13.00
4	8	14	219.48	14.50
-4	8	-14	214.18	13.30
4	8	14	199.08	14.60
5	8	14	49.30	8.40
-5	8	-14	33.40	5.60
5	8	14	58.29	9.00

-5	8	-14	34.90	6.00
-6	8	-14	393.56	22.00
6	8	14	353.56	22.50
6	8	14	406.96	22.80
-7	-8	-14	106.09	11.30
-7	8	-14	110.69	11.20
7	8	14	111.49	11.40
7	8	14	123.09	11.90
-8	-8	-14	184.58	14.80
8	8	14	171.78	17.10
8	8	14	187.08	14.60
-8	8	-14	171.58	15.50
-9	8	-14	8.90	6.60
9	8	14	9.30	6.60
-9	8	-14	5.40	2.90
10	8	14	326.97	22.50
-10	8	-14	375.66	22.60
-10	8	-14	343.87	18.70
-11	8	-14	23.20	8.70
-11	8	-14	23.50	5.80
11	8	14	18.60	7.40
-12	8	-14	149.69	16.10
12	8	14	157.08	16.40
-13	-8	-14	1.60	10.40
-13	8	-14	21.40	8.70
12	9	-14	29.70	10.80
-12	9	14	27.40	8.10
11	-9	-14	10.10	8.30
11	9	-14	0.90	8.50
-11	9	14	33.20	7.90
10	-9	-14	64.79	13.40
10	9	-14	45.90	10.20
-10	9	14	62.99	10.90
9	-9	-14	11.70	7.00
9	9	-14	7.30	7.60
-9	9	14	2.30	6.10
8	-9	-14	6.10	6.10
-8	9	14	5.40	6.00
8	9	-14	12.00	6.90
7	-9	-14	16.80	6.00
-7	9	14	6.00	9.00
-7	9	14	2.00	5.90
6	-9	-14	47.00	9.50
-6	9	14	32.20	7.30

6	9	-14	31.10	6.70
-6	9	14	35.90	8.20
5	-9	-14	137.79	12.00
-5	9	14	131.29	12.30
5	9	-14	129.49	12.20
-5	9	14	118.99	12.80
4	-9	-14	81.49	9.70
4	9	-14	72.19	9.20
-4	9	14	67.99	10.40
-4	9	14	73.69	10.30
3	-9	-14	4.00	4.80
-3	9	14	-0.10	4.60
3	9	-14	-1.10	4.00
2	-9	-14	18.80	5.40
2	9	-14	20.10	4.50
-2	9	14	8.10	5.00
-2	9	14	16.80	6.20
2	9	-14	21.70	1.80
-1	-9	14	-1.60	3.70
1	-9	-14	1.60	4.30
1	9	-14	0.50	1.60
1	9	-14	-0.50	3.40
-1	9	14	-3.00	4.10
1	9	-14	2.60	2.10
-1	9	14	-6.40	4.70
0	-9	-14	147.29	11.10
0	-9	14	126.69	8.10
0	9	14	130.29	11.00
0	9	-14	123.59	9.10
0	9	14	113.29	10.20
0	9	-14	146.49	8.40
1	9	14	24.70	6.20
-1	9	-14	19.70	3.70
1	9	14	29.60	5.80
-1	9	-14	21.60	3.30
-1	9	-14	31.80	4.70
1	9	14	22.30	4.80
2	9	14	114.59	11.10
2	9	14	125.89	10.90
-2	9	-14	118.99	7.30
-2	9	-14	136.19	8.90
-2	9	-14	119.29	8.20
3	9	14	2.20	4.50
-3	9	-14	8.80	3.90

3	9	14	4.10	4.90
-3	9	-14	-2.60	3.30
-4	9	-14	11.30	4.30
-4	9	-14	16.30	4.70
4	9	14	21.20	5.70
4	9	14	10.90	5.40
5	9	14	96.79	11.00
-5	9	-14	114.79	10.10
-5	9	-14	106.89	9.40
5	9	14	107.39	10.70
6	9	14	241.78	16.90
-6	9	-14	233.58	15.60
6	9	14	221.98	20.50
-7	9	-14	92.39	10.60
7	9	14	106.29	10.60
7	9	14	91.69	11.60
-8	-9	-14	90.19	11.90
8	9	14	104.49	12.20
-8	9	-14	98.19	11.50
8	9	14	102.09	12.00
-9	9	-14	80.59	6.60
9	9	14	86.79	12.20
-9	9	-14	109.79	11.80
10	9	14	4.60	7.40
-10	9	-14	6.50	6.40
-10	9	-14	12.50	7.50
-11	9	-14	71.99	12.90
11	9	14	59.19	10.30
12	9	14	15.00	8.10
-12	9	-14	5.20	5.70
-12	9	-14	10.70	8.70
-12	10	14	21.90	8.40
12	10	-14	39.50	11.70
11	-10	-14	398.06	27.20
11	10	-14	392.66	28.10
-11	10	14	422.56	24.70
10	-10	-14	42.20	9.40
-10	10	14	71.19	11.00
10	10	-14	49.60	11.50
9	-10	-14	612.04	35.00
-9	10	14	606.14	33.60
9	10	-14	638.24	35.90
8	-10	-14	94.99	12.60
8	10	-14	89.29	13.10

-8	10	14	62.79	10.90
7	-10	-14	986.10	50.09
7	10	-14	967.80	50.19
-7	10	14	916.61	50.79
6	-10	-14	81.19	10.80
-6	10	14	66.19	10.60
-6	10	14	75.29	13.00
5	-10	-14	1288.57	65.49
-5	10	14	1231.88	65.89
-5	10	14	1363.86	65.59
5	10	-14	1313.07	65.39
4	-10	-14	27.40	6.10
4	10	-14	27.20	6.40
-4	10	14	23.00	7.30
-4	10	14	13.30	6.20
3	-10	-14	1325.17	65.69
-3	10	14	1241.68	65.49
3	10	-14	1363.06	65.09
-3	10	14	1302.07	65.99
2	-10	-14	54.79	8.60
-2	10	14	41.30	7.70
2	10	-14	72.89	7.80
-2	10	14	63.79	8.90
1	-10	-14	1626.14	77.49
-1	10	14	1466.95	77.49
1	10	-14	1716.73	76.39
1	10	-14	1492.15	74.39
1	10	-14	1557.34	74.49
-1	10	14	1516.75	76.99
0	-10	14	1533.55	76.89
0	-10	-14	1717.23	79.29
0	10	-14	1640.04	76.69
0	10	-14	1632.24	77.59
0	10	14	1507.35	78.49
0	10	14	1565.24	79.29
-1	10	-14	614.84	30.90
1	10	14	584.94	33.40
-1	10	-14	650.03	31.20
-1	10	-14	628.04	30.70
-2	10	-14	347.47	18.90
2	10	14	383.66	22.10
-2	10	-14	381.46	19.40
-2	10	-14	381.36	19.80
2	10	14	366.86	22.30

-3	10	-14	441.46	21.90
3	10	14	449.46	25.40
-3	10	-14	451.05	23.70
-3	10	-14	423.96	21.80
-3	10	-14	438.66	23.40
3	10	14	495.05	26.10
-4	10	-14	1742.13	83.59
-4	10	-14	1684.03	83.39
4	10	14	1811.92	85.29
4	10	14	1632.94	84.59
-5	10	-14	-4.60	4.60
-5	10	-14	15.20	5.20
5	10	14	8.70	6.00
5	10	14	4.60	5.80
-6	10	-14	928.41	47.50
6	10	14	915.31	48.40
6	10	14	970.30	48.90
7	10	14	86.09	11.10
-7	10	-14	68.39	10.20
7	10	14	100.89	12.10
8	10	14	296.07	20.00
-8	10	-14	313.67	19.60
-9	10	-14	-1.80	6.00
9	10	14	0.90	6.50
-10	10	-14	478.05	27.40
10	10	14	444.66	27.90
-11	10	-14	-3.70	7.50
-11	10	-14	2.00	4.50
11	10	14	-5.40	7.20
-12	10	-14	310.47	21.50
12	10	14	290.07	21.60
-12	10	-14	271.67	17.60
12	11	-14	28.40	11.30
-12	11	14	33.70	9.00
11	-11	-14	27.30	9.60
11	11	-14	18.90	9.90
-11	11	14	18.60	7.90
10	-11	-14	135.29	16.00
-10	11	14	135.29	13.70
10	11	-14	128.39	17.60
9	-11	-14	5.80	7.50
9	11	-14	8.50	8.10
-9	11	14	-2.10	6.60
8	-11	-14	66.19	12.40

-8	11	14	63.49	11.00
7	-11	-14	2.30	5.90
-7	11	14	2.80	6.50
7	11	-14	10.70	7.20
6	-11	-14	136.39	13.50
6	11	-14	174.08	14.30
-6	11	14	111.99	14.90
5	-11	-14	3.50	5.30
5	11	-14	4.80	6.70
-5	11	14	-4.00	6.00
-5	11	14	10.80	6.90
4	-11	-14	528.75	28.90
-4	11	14	461.85	28.90
-4	11	14	533.55	29.20
4	11	-14	506.25	29.50
3	-11	-14	77.79	9.90
-3	11	14	74.19	10.30
-3	11	14	68.89	9.60
3	11	-14	71.89	9.40
2	-11	-14	48.00	8.60
-2	11	14	21.80	5.70
-2	11	14	27.70	7.40
1	-11	-14	124.69	11.50
-1	11	14	96.99	12.10
-1	11	14	114.89	10.80
1	11	-14	144.99	9.90
1	11	-14	114.29	6.50
1	11	-14	114.79	6.40
0	-11	-14	559.94	30.20
0	11	14	574.34	31.00
0	11	-14	549.45	27.10
0	11	14	511.05	30.10
0	11	-14	603.24	28.50
0	11	-14	492.15	27.10
-1	11	-14	25.20	3.70
-1	11	-14	25.60	5.20
1	11	14	17.70	6.10
-1	11	-14	17.80	3.30
-2	11	-14	12.60	3.70
2	11	14	6.00	5.50
-2	11	-14	11.40	3.00
-2	11	-14	13.40	3.50
-3	11	-14	276.57	16.40
-3	11	-14	310.07	15.10

3	11	14	320.87	19.50
-3	11	-14	273.77	14.80
3	11	14	306.37	18.70
-3	11	-14	269.37	16.20
4	11	14	5.00	5.50
-4	11	-14	-2.20	3.80
4	11	14	2.30	5.90
-4	11	-14	2.60	4.00
5	11	14	38.10	7.50
-5	11	-14	38.60	6.20
5	11	14	40.80	7.70
-5	11	-14	36.60	6.20
-6	11	-14	81.89	9.90
6	11	14	59.59	11.80
6	11	14	94.49	11.00
7	11	14	206.08	15.40
-7	11	-14	201.28	15.00
7	11	14	212.18	16.50
-8	11	-14	-1.70	6.20
8	11	14	-2.10	6.60
-9	11	-14	36.40	6.10
9	11	14	35.10	8.40
-9	11	-14	42.10	8.30
-9	11	-14	42.20	4.90
-10	11	-14	2.80	6.90
10	11	14	7.30	7.80
-10	11	-14	1.70	3.60
-11	11	-14	7.70	8.30
11	11	14	18.50	8.70
-11	11	-14	16.10	4.80
12	11	14	10.20	9.30
-12	11	-14	5.00	8.20
-12	11	-14	3.00	5.50
-12	12	14	41.10	9.40
12	12	-14	49.99	13.60
11	12	-14	352.16	27.80
-11	12	14	425.86	24.70
10	-12	-14	73.69	15.00
-10	12	14	65.09	12.30
10	12	-14	93.19	17.10
9	-12	-14	351.26	23.70
9	12	-14	326.27	23.80
-9	12	14	338.67	21.90
8	-12	-14	7.00	7.00

8	12	-14	-0.80	7.50
-8	12	14	-3.90	6.40
7	-12	-14	391.56	24.80
7	12	-14	412.16	25.40
-7	12	14	401.36	24.20
6	-12	-14	9.40	6.20
6	12	-14	4.70	6.00
-6	12	14	-5.40	6.60
5	-12	-14	596.74	32.80
-5	12	14	580.94	33.40
5	12	-14	594.24	32.70
4	-12	-14	64.49	10.40
-4	12	14	57.29	10.80
4	12	-14	52.39	9.50
-4	12	14	40.60	9.90
3	-12	-14	484.65	27.00
-3	12	14	448.56	27.50
3	12	-14	498.65	32.40
-3	12	14	452.45	28.20
2	-12	-14	42.40	8.80
-2	12	14	48.40	8.80
-2	12	14	47.20	9.70
2	12	-14	53.39	9.60
1	-12	-14	24.70	6.60
1	12	-14	29.70	2.60
-1	12	14	26.90	7.00
1	12	-14	22.80	2.60
-1	12	14	29.50	7.60
1	12	-14	28.10	5.10
0	12	-14	479.15	24.60
0	12	-14	502.45	26.30
0	12	14	485.95	28.50
0	12	-14	517.45	24.80
0	12	14	505.85	29.00
1	12	14	377.56	23.30
1	12	14	360.56	22.50
-1	12	-14	375.26	20.00
-1	12	-14	375.96	19.10
-1	12	-14	368.56	19.20
-2	12	-14	305.77	16.70
-2	12	-14	312.27	16.70
2	12	14	293.37	19.60
-2	12	-14	314.57	16.70
3	12	14	69.69	10.40

-3	12	-14	73.89	7.70
-3	12	-14	83.09	5.80
-3	12	-14	82.89	7.80
-3	12	-14	79.39	6.30
-4	12	-14	76.89	8.00
-4	12	-14	79.79	8.30
-4	12	-14	73.49	5.20
-4	12	-14	82.19	5.60
4	12	14	63.09	11.30
4	12	14	77.79	12.10
5	12	14	15.20	6.30
5	12	14	3.80	6.70
-5	12	-14	11.60	5.00
6	12	14	70.59	10.30
6	12	14	69.09	10.10
-6	12	-14	79.09	9.60
-7	12	-14	111.09	11.40
7	12	14	107.89	12.70
8	12	14	89.89	11.50
-8	12	-14	89.49	11.40
9	12	14	-2.80	7.30
-9	12	-14	2.10	3.10
-9	12	-14	0.00	6.50
-9	12	-14	2.80	2.90
-10	12	-14	195.78	11.60
10	12	14	192.98	16.80
-10	12	-14	191.58	16.10
-10	12	-14	172.08	11.40
-11	12	-14	34.40	5.60
-11	12	-14	48.60	10.00
11	12	14	33.10	9.80
12	12	14	168.78	18.20
11	13	-14	40.30	11.40
-11	13	14	31.10	9.10
10	-13	-14	323.27	24.00
10	13	-14	344.77	25.40
-10	13	14	313.57	21.60
9	-13	-14	59.69	10.10
9	13	-14	31.60	9.70
-9	13	14	36.70	8.70
8	-13	-14	217.58	18.40
8	13	-14	238.18	19.40
-8	13	14	238.28	17.60
7	-13	-14	-7.10	6.60

7	13	-14	2.30	7.00
-7	13	14	-15.40	6.80
6	-13	-14	589.44	34.80
6	13	-14	651.03	35.30
-6	13	14	641.64	34.80
5	-13	-14	2.20	5.90
-5	13	14	-8.20	6.90
5	13	-14	3.30	5.70
4	-13	-14	414.46	25.60
-4	13	14	408.96	26.10
4	13	-14	470.75	25.40
3	-13	-14	0.10	5.40
-3	13	14	-15.80	11.00
3	13	-14	5.90	5.10
2	-13	-14	578.94	32.60
-2	13	14	545.35	36.00
2	13	-14	622.84	31.40
1	-13	-14	424.06	24.40
-1	13	14	390.96	25.20
1	13	-14	394.86	19.60
1	13	-14	421.06	23.10
1	13	-14	399.96	19.60
-1	13	14	415.16	25.60
0	13	14	408.66	24.40
0	13	-14	401.56	19.60
0	13	-14	363.66	19.00
0	13	14	347.07	22.90
-1	13	-14	381.86	20.30
1	13	14	378.36	24.30
1	13	14	433.16	25.10
-1	13	-14	395.96	20.30
2	13	14	501.35	28.30
-2	13	-14	457.95	24.10
-2	13	-14	456.25	24.10
3	13	14	218.98	16.60
-3	13	-14	240.28	13.80
-3	13	-14	217.08	12.50
-3	13	-14	243.98	13.90
4	13	14	143.39	13.40
-4	13	-14	131.79	10.80
-4	13	-14	139.09	8.50
-4	13	-14	156.78	9.60
-4	13	-14	149.69	10.80
-5	13	-14	328.37	18.60

5	13	14	335.97	28.50
5	13	14	279.77	20.00
-5	13	-14	312.77	16.00
-5	13	-14	316.87	16.00
6	13	14	165.28	15.10
-6	13	-14	198.28	10.10
-6	13	-14	188.98	14.00
6	13	14	199.78	16.90
7	13	14	302.17	20.60
-8	13	-14	29.20	4.60
-8	13	-14	31.20	7.40
8	13	14	23.60	7.80
-9	13	-14	233.38	13.30
9	13	14	222.48	18.60
-9	13	-14	253.17	18.00
-9	13	-14	259.97	13.60
-10	13	-14	7.20	4.00
-10	13	-14	6.40	4.10
-10	13	-14	5.40	7.90
10	13	14	15.00	8.30
-11	13	-14	355.46	20.70
-11	13	-14	421.46	25.40
11	13	14	379.26	25.60
-11	14	14	22.20	9.00
11	14	-14	29.90	12.00
-10	14	14	-1.70	7.50
10	14	-14	5.20	9.60
9	-14	-14	-9.20	8.00
-9	14	14	-12.40	6.90
9	14	-14	0.30	9.00
8	-14	-14	7.40	7.70
8	14	-14	-1.90	7.60
-8	14	14	-5.40	7.30
7	-14	-14	1.10	6.90
-7	14	14	-1.00	7.50
7	14	-14	8.60	7.30
6	-14	-14	15.10	7.10
6	14	-14	20.00	7.90
-6	14	14	-1.20	7.50
5	-14	-14	12.30	6.80
-5	14	14	17.00	7.30
5	14	-14	15.30	6.80
4	-14	-14	2.20	6.20
-4	14	14	-9.00	6.80

4	14	-14	-3.90	5.10
3	-14	-14	42.30	7.60
3	14	-14	35.60	6.70
-3	14	14	46.80	9.40
2	-14	-14	3.40	5.80
2	14	-14	2.00	4.90
-2	14	14	-14.10	6.50
1	-14	-14	8.40	6.50
-1	14	14	3.80	7.10
1	14	-14	14.20	5.00
-1	14	14	12.50	12.60
0	14	14	-12.80	6.10
0	14	14	-4.50	6.40
0	14	-14	2.50	2.40
0	14	-14	2.50	3.70
0	14	-14	8.00	4.40
-1	14	-14	41.80	5.20
-1	14	-14	47.90	7.10
-1	14	-14	41.20	5.30
1	14	14	32.20	7.90
1	14	14	44.70	8.50
-2	14	-14	6.10	3.80
2	14	14	5.40	6.20
-2	14	-14	8.00	3.70
-2	14	-14	10.40	3.30
-2	14	-14	10.80	4.00
2	14	14	-2.40	6.40
-3	14	-14	28.60	5.70
-3	14	-14	27.00	4.90
3	14	14	19.10	7.20
3	14	14	15.10	6.80
-3	14	-14	18.60	3.50
-3	14	-14	18.40	4.40
-4	14	-14	8.20	3.00
-4	14	-14	14.10	4.80
-4	14	-14	14.30	3.50
4	14	14	2.90	6.80
4	14	14	9.40	6.70
-4	14	-14	8.70	5.30
5	14	14	6.50	7.10
-5	14	-14	0.80	2.70
-5	14	-14	5.20	5.10
5	14	14	0.30	6.40
-5	14	-14	1.10	2.90

-6	14	-14	10.80	3.10
-6	14	-14	15.90	3.10
-6	14	-14	15.80	5.70
6	14	14	9.90	6.80
7	14	14	105.59	13.20
-7	14	-14	94.79	7.00
-7	14	-14	82.09	8.40
-7	14	-14	92.49	10.90
-8	14	-14	39.30	5.80
-8	14	-14	26.90	4.40
8	14	14	38.60	8.40
-8	14	-14	51.39	8.50
-9	14	-14	5.80	3.90
9	14	14	5.80	7.70
-9	14	-14	1.90	3.70
-9	14	-14	10.50	7.20
10	14	14	20.20	8.70
-10	14	-14	9.20	4.50
-10	14	-14	8.80	4.20
-10	14	-14	7.10	8.10
-11	14	-14	18.10	9.10
-11	14	-14	12.30	5.20
-11	14	-14	15.10	5.30
11	14	14	19.60	9.60
10	15	-14	190.18	21.40
-10	15	14	190.48	16.80
9	-15	-14	180.08	18.60
9	15	-14	169.88	19.20
-9	15	14	161.48	15.80
8	-15	-14	161.98	17.30
8	15	-14	166.08	18.00
-8	15	14	173.98	16.20
7	-15	-14	10.30	7.70
-7	15	14	-3.20	6.70
7	15	-14	8.30	7.70
6	-15	-14	679.33	39.30
-6	15	14	727.73	39.20
6	15	-14	723.73	39.40
5	-15	-14	5.40	6.90
-5	15	14	4.80	7.80
5	15	-14	-1.80	6.30
4	-15	-14	465.95	28.30
4	15	-14	464.55	27.40
-4	15	14	480.05	28.60

3	-15	-14	12.40	7.10
3	15	-14	13.40	6.00
-3	15	14	1.70	7.70
2	-15	-14	1027.70	54.59
2	15	-14	1051.89	53.39
-2	15	14	1063.09	55.09
-2	15	14	1059.29	55.99
-1	15	14	198.58	17.00
1	15	-14	189.58	14.00
-1	15	14	204.48	18.80
0	15	-14	593.14	27.50
0	15	-14	503.55	27.10
0	15	14	525.35	31.60
0	15	-14	570.44	29.30
-1	15	-14	114.79	8.20
1	15	14	116.19	15.10
-1	15	-14	125.19	10.10
1	15	14	120.19	12.80
-1	15	-14	119.99	7.90
-2	15	-14	409.66	22.10
-2	15	-14	412.86	21.80
2	15	14	416.46	25.70
2	15	14	478.65	27.40
-2	15	-14	379.66	22.00
-2	15	-14	439.16	22.70
-3	15	-14	73.19	7.70
-3	15	-14	86.29	7.90
-3	15	-14	69.59	8.30
3	15	14	69.49	13.40
3	15	14	74.49	10.90
-3	15	-14	75.19	6.90
4	15	14	219.58	17.00
-4	15	-14	200.78	14.30
4	15	14	237.28	18.90
-4	15	-14	232.78	13.00
-4	15	-14	211.18	13.80
-4	15	-14	203.58	12.50
-5	15	-14	715.43	36.30
5	15	14	750.02	40.10
-5	15	-14	757.02	36.50
-5	15	-14	719.23	37.80
-6	15	-14	310.07	16.20
-6	15	-14	311.07	16.20
6	15	14	285.27	19.90

-6	15	-14	272.77	18.10
-7	15	-14	523.05	26.40
7	15	14	529.45	30.60
-7	15	-14	516.35	28.00
-7	15	-14	512.95	28.70
-8	15	-14	173.98	11.60
-8	15	-14	172.98	15.30
8	15	14	203.08	16.70
-8	15	-14	207.68	11.80
-9	15	-14	249.38	15.70
-9	15	-14	289.47	15.90
9	15	14	295.77	20.90
-9	15	-14	290.67	19.60
10	15	14	80.59	14.20
-10	15	-14	70.19	8.60
-10	15	-14	65.49	8.20
-10	15	-14	75.19	13.20
10	16	-14	1.30	10.20
-10	16	14	-7.20	7.80
-9	16	14	18.30	8.20
9	16	-14	14.20	9.80
8	-16	-14	7.70	8.90
8	16	-14	30.10	10.00
-8	16	14	7.80	8.10
7	-16	-14	26.50	9.10
7	16	-14	35.10	9.70
-7	16	14	15.30	8.50
6	-16	-14	31.10	8.80
6	16	-14	31.10	8.60
-6	16	14	8.60	8.10
5	-16	-14	104.09	14.60
-5	16	14	110.89	14.80
5	16	-14	128.69	14.30
4	-16	-14	44.60	8.60
4	16	-14	38.10	8.10
-4	16	14	36.90	9.50
3	-16	-14	44.70	8.70
3	16	-14	57.29	10.90
-3	16	14	37.90	9.80
2	-16	-14	-3.70	7.00
-2	16	14	-13.40	7.40
-2	16	14	-15.20	7.30
2	16	-14	-4.10	5.30
-1	16	14	77.09	11.70

-1	16	14	90.69	15.20
1	16	-14	99.89	10.60
0	16	-14	-0.50	4.80
0	16	-14	5.40	3.60
0	16	14	-2.50	8.10
0	16	14	-9.20	7.30
1	16	14	61.59	10.10
1	16	14	66.99	15.00
-1	16	-14	69.19	5.80
-1	16	-14	48.00	6.20
-2	16	-14	37.10	5.70
-2	16	-14	38.50	6.40
2	16	14	24.40	8.00
-2	16	-14	32.60	4.50
2	16	14	37.10	9.90
-2	16	-14	35.60	5.20
-3	16	-14	2.90	3.60
3	16	14	4.40	7.70
3	16	14	-7.50	6.70
-3	16	-14	-3.10	4.50
-3	16	-14	7.00	4.10
-3	16	-14	5.90	4.20
-4	16	-14	45.10	6.60
-4	16	-14	60.09	7.40
-4	16	-14	53.39	8.10
-4	16	-14	51.39	7.10
4	16	14	60.69	11.70
4	16	14	53.19	9.40
-5	16	-14	20.00	5.80
5	16	14	5.50	7.40
-5	16	-14	15.40	4.20
-5	16	-14	22.90	4.80
-6	16	-14	79.49	8.00
-6	16	-14	61.59	10.20
-6	16	-14	92.99	8.00
6	16	14	77.69	13.00
7	16	14	94.39	13.60
-7	16	-14	99.29	8.60
-7	16	-14	94.09	11.40
-8	16	-14	60.79	7.90
-8	16	-14	51.89	8.60
8	16	14	39.60	9.40
-8	16	-14	68.29	7.80
9	16	14	21.80	9.20

-9	16	-14	17.10	5.00
-9	16	-14	22.10	5.00
-9	16	-14	13.30	7.80
10	16	14	30.10	10.00
-10	16	-14	34.30	8.80
-10	16	-14	24.40	5.80
-10	16	-14	42.10	8.50
-9	17	14	31.00	9.20
9	17	-14	39.40	11.40
8	-17	-14	71.99	13.10
8	17	-14	92.39	16.60
-8	17	14	79.39	14.20
7	-17	-14	4.60	8.40
7	17	-14	17.70	8.80
-7	17	14	3.10	8.10
6	-17	-14	318.17	23.50
-6	17	14	326.87	23.00
6	17	-14	308.47	22.80
5	-17	-14	-3.20	8.20
-5	17	14	-17.40	7.00
5	17	-14	8.20	7.40
4	-17	-14	277.37	21.70
4	17	-14	311.77	21.10
-4	17	14	309.07	22.30
3	-17	-14	2.30	7.40
-3	17	14	15.10	9.70
3	17	-14	-5.90	6.10
-3	17	14	-14.30	8.00
-2	17	14	351.56	23.80
-2	17	14	383.16	26.30
2	17	-14	354.26	22.30
-1	17	14	248.48	21.60
1	17	-14	262.57	17.10
-1	17	14	229.58	19.70
0	17	14	563.04	34.10
0	17	-14	541.25	27.60
0	17	-14	584.34	30.20
0	17	14	520.65	32.50
1	17	14	278.57	22.40
1	17	14	244.18	19.60
-1	17	-14	239.38	13.40
-1	17	-14	252.87	16.20
-2	17	-14	299.47	17.60
-2	17	-14	293.27	15.70

-2	17	-14	259.67	16.50
2	17	14	287.37	20.10
2	17	14	301.37	24.00
3	17	14	236.98	23.20
-3	17	-14	252.37	14.30
3	17	14	249.68	19.10
-3	17	-14	216.88	14.20
-3	17	-14	244.88	15.20
-4	17	-14	57.19	8.30
4	17	14	56.49	10.00
-4	17	-14	59.09	7.30
-4	17	-14	68.19	8.30
-5	17	-14	221.48	14.40
-5	17	-14	242.68	15.70
-5	17	-14	250.57	14.90
5	17	14	251.97	18.80
-6	17	-14	84.19	8.80
-6	17	-14	87.79	8.70
6	17	14	99.59	13.60
-6	17	-14	62.09	13.10
-7	17	-14	160.08	14.00
-7	17	-14	172.58	11.80
7	17	14	156.68	16.30
-8	17	-14	3.90	4.80
8	17	14	-7.90	8.10
-8	17	-14	13.90	7.20
-8	17	-14	8.50	4.80
-9	17	-14	139.59	12.10
-9	17	-14	205.58	16.00
-9	17	-14	175.88	12.40
9	17	14	147.79	17.10
-9	18	14	184.38	17.50
9	18	-14	189.48	21.60
-8	18	14	34.50	9.60
8	18	-14	37.20	11.00
7	-18	-14	333.07	25.50
-7	18	14	338.47	24.50
7	18	-14	390.56	26.10
6	-18	-14	21.80	9.30
-6	18	14	-0.30	8.80
6	18	-14	20.30	8.70
5	-18	-14	413.16	28.20
-5	18	14	430.16	27.80
5	18	-14	444.16	27.80

4	-18	-14	40.50	10.20
-4	18	14	21.10	9.90
4	18	-14	23.30	8.40
-3	18	14	319.87	23.10
-3	18	14	293.37	25.60
3	18	-14	321.67	21.30
-2	18	14	3.00	9.60
-2	18	14	6.60	9.20
2	18	-14	10.30	6.60
-1	18	14	371.46	25.40
1	18	-14	403.06	23.70
-1	18	14	401.06	27.90
0	18	14	203.48	18.90
0	18	-14	243.48	15.90
0	18	14	168.88	20.60
1	18	14	480.35	30.60
-1	18	-14	500.95	25.80
-1	18	-14	404.76	24.50
-1	18	-14	429.16	22.80
1	18	14	473.75	28.70
-2	18	-14	162.38	10.40
2	18	14	186.58	24.30
-2	18	-14	159.78	11.80
-2	18	-14	157.28	12.80
2	18	14	178.68	16.70
3	18	14	188.98	16.60
-3	18	-14	169.68	11.50
-3	18	-14	154.68	11.80
-3	18	-14	189.08	13.10
-4	18	-14	193.58	13.80
-4	18	-14	233.18	14.10
4	18	14	206.28	18.20
-4	18	-14	244.88	14.80
-5	18	-14	132.89	12.10
5	18	14	146.69	15.60
-5	18	-14	169.98	11.90
-5	18	-14	140.09	11.30
-6	18	-14	166.38	12.10
-6	18	-14	161.98	11.90
-6	18	-14	149.88	13.60
6	18	14	167.58	16.60
-7	18	-14	33.90	8.10
-7	18	-14	43.20	6.20
7	18	14	53.59	10.40

-8	18	-14	259.67	16.00
8	18	14	249.78	20.10
-8	18	-14	236.28	15.90
-8	18	-14	254.87	18.10
9	18	14	7.60	8.90
-9	18	-14	8.80	7.60
-9	18	-14	-5.70	5.60
-9	18	-14	2.00	5.50
8	19	-14	-3.70	10.00
-8	19	14	-6.30	9.00
7	19	-14	5.90	9.60
-7	19	14	-8.90	8.90
6	-19	-14	32.90	10.10
6	19	-14	20.00	10.00
-6	19	14	47.80	10.90
5	-19	-14	-0.90	8.90
-5	19	14	-1.90	9.50
5	19	-14	-4.50	8.40
4	-19	-14	57.69	11.00
-4	19	14	83.39	14.60
-4	19	14	75.39	14.60
4	19	-14	65.29	13.60
-3	19	14	10.60	11.90
3	19	-14	-2.10	6.90
-3	19	14	-15.90	7.90
-2	19	14	17.60	10.10
-2	19	14	12.80	8.90
2	19	-14	16.30	7.00
-1	19	14	21.30	9.80
1	19	-14	29.20	7.30
-1	19	14	14.80	10.50
0	19	-14	31.70	7.10
0	19	14	24.50	10.10
0	19	14	22.20	9.00
-1	19	-14	12.10	6.00
1	19	14	0.90	8.30
-1	19	-14	-4.90	4.40
2	19	14	18.40	8.80
-2	19	-14	22.10	5.30
-2	19	-14	25.20	4.30
-2	19	-14	15.50	5.90
-3	19	-14	27.40	5.60
-3	19	-14	40.20	6.90
-3	19	-14	37.70	6.70

3	19	14	28.40	9.00
-4	19	-14	21.00	5.60
-4	19	-14	21.50	5.90
4	19	14	25.50	9.40
-4	19	-14	23.30	5.30
5	19	14	48.70	10.60
-5	19	-14	42.90	7.00
-5	19	-14	42.10	8.50
-5	19	-14	54.09	9.30
-6	19	-14	3.10	5.40
-6	19	-14	11.40	6.30
6	19	14	20.00	8.20
-6	19	-14	6.90	5.30
7	19	14	49.80	10.10
-7	19	-14	37.60	8.20
-7	19	-14	56.59	9.50
-7	19	-14	41.30	6.60
-8	19	-14	12.70	7.60
8	19	14	28.80	9.90
-8	19	-14	15.80	6.10
7	20	-14	347.07	26.70
-7	20	14	356.96	25.50
-6	20	14	14.50	10.20
6	20	-14	30.90	10.40
5	-20	-14	413.96	29.40
5	20	-14	422.06	28.00
-5	20	14	431.46	28.30
-4	20	14	-4.70	9.10
4	20	-14	15.80	9.10
-3	20	14	457.15	30.20
3	20	-14	472.45	28.70
2	20	-14	9.60	7.30
-2	20	14	19.90	10.20
-1	20	14	756.82	44.00
1	20	-14	814.42	42.30
0	20	14	247.78	21.40
0	20	-14	263.77	18.30
-1	20	-14	406.26	23.70
1	20	14	368.56	26.20
-2	20	-14	181.28	14.60
2	20	14	169.88	16.80
3	20	14	241.38	19.40
-3	20	-14	228.58	14.20
-3	20	-14	242.88	16.70

4	20	14	405.36	26.40
-4	20	-14	384.56	21.80
-4	20	-14	395.86	23.10
-5	20	-14	121.19	11.60
-5	20	-14	90.09	12.90
5	20	14	86.79	15.30
6	20	14	720.43	39.40
-6	20	-14	657.63	36.30
-6	20	-14	694.93	36.30
7	20	14	36.40	10.10
-7	20	-14	70.49	10.50
-7	20	-14	55.69	8.70
6	21	-14	137.59	18.90
-6	21	14	128.79	17.70
-5	21	14	-0.60	10.00
5	21	-14	21.40	10.10
4	21	-14	186.18	17.80
-4	21	14	142.89	18.40
3	21	-14	14.00	8.60
-3	21	14	-8.30	9.90
2	21	-14	52.99	9.40
-2	21	14	44.90	11.50
-1	21	14	12.80	10.30
1	21	-14	3.20	6.80
0	21	14	175.38	18.50
0	21	-14	168.78	15.40
-1	21	-14	4.70	6.60
1	21	14	0.60	9.30
-2	21	-14	122.39	13.10
2	21	14	109.59	16.20
3	21	14	59.69	11.40
-3	21	-14	76.19	11.20
-4	21	-14	2.80	5.90
4	21	14	-6.20	8.70
-5	21	-14	25.60	6.50
5	21	14	31.10	10.10
-5	21	-14	29.90	7.30
-6	21	-14	46.30	8.10
-6	21	-14	64.29	11.20
6	21	14	55.09	11.30
5	22	-14	300.57	24.50
-5	22	14	291.37	24.30
-4	22	14	6.50	10.70
4	22	-14	19.10	9.80

-3	22	14	77.39	13.10
3	22	-14	73.39	11.50
-2	22	14	56.39	11.40
2	22	-14	63.79	10.90
-1	22	14	60.79	11.70
1	22	-14	55.79	9.60
0	22	14	140.89	17.90
0	22	-14	117.79	14.30
1	22	14	201.08	19.20
-1	22	-14	180.48	16.40
2	22	14	52.29	11.50
-2	22	-14	39.90	8.50
3	22	14	52.09	11.50
-3	22	-14	44.00	8.30
4	22	14	161.68	18.10
-4	22	-14	157.08	14.70
5	22	14	-5.30	9.80
-5	22	-14	11.10	7.60
-3	23	14	-1.50	10.00
3	23	-14	-5.90	8.60
-2	23	14	125.69	18.40
2	23	-14	114.79	16.40
1	23	-14	53.89	10.00
-1	23	14	62.69	12.90
0	23	-14	249.28	19.40
0	23	14	233.38	21.30
-1	23	-14	87.79	14.10
1	23	14	100.39	14.50
2	23	14	144.49	18.50
-2	23	-14	137.29	15.50
-3	23	-14	101.39	20.60
3	23	14	81.69	13.70
13	0	-15	9.80	10.20
-12	0	15	11.50	5.60
12	0	-15	-3.50	8.60
11	0	-15	8.70	10.50
-11	0	15	4.60	5.10
-10	0	15	13.90	4.80
10	0	-15	15.10	7.40
-9	0	15	-8.20	4.00
9	0	-15	-0.60	6.00
9	0	-15	-8.40	4.50
8	0	-15	-0.40	3.80
-8	0	15	-2.00	3.90

8	0	-15	-2.40	5.30
7	0	-15	4.50	3.60
7	0	-15	5.50	5.60
-7	0	15	-5.80	3.60
6	0	-15	4.20	4.90
6	0	-15	2.10	3.10
-6	0	15	0.80	3.60
5	0	-15	3.50	2.70
-5	0	15	2.20	3.30
5	0	-15	2.10	4.30
4	0	-15	0.70	2.30
4	0	-15	-1.50	3.50
-4	0	15	-4.10	4.20
-4	0	15	2.00	3.10
3	0	-15	0.90	3.20
-3	0	15	-1.20	2.90
-3	0	15	2.80	3.90
3	0	-15	2.30	2.30
2	0	-15	1.90	2.20
-2	0	15	4.20	2.80
2	0	-15	0.30	3.00
-2	0	15	7.00	3.80
1	0	-15	1.80	2.40
1	0	-15	0.40	2.70
-1	0	15	5.50	3.10
-1	0	15	6.30	3.20
0	0	15	1.50	2.80
0	0	-15	-0.30	2.40
0	0	15	2.80	3.10
0	0	-15	6.70	2.70
1	0	15	-0.50	2.50
1	0	15	1.50	3.20
1	0	15	0.50	2.40
-1	0	-15	0.90	2.80
-1	0	-15	0.70	2.40
2	0	15	-1.90	3.20
-2	0	-15	3.40	2.30
2	0	15	5.20	2.20
2	0	15	1.20	2.30
-2	0	-15	-2.30	2.80
-3	0	-15	-0.60	2.90
3	0	15	1.40	1.60
3	0	15	-0.70	1.50
3	0	15	0.70	3.60

-4	0	-15	-0.80	3.60
4	0	15	0.10	3.80
5	0	15	2.40	4.40
6	0	15	3.10	4.80
7	0	15	7.30	5.10
-8	0	-15	-2.20	3.80
8	0	15	-0.60	4.90
-9	0	-15	10.30	6.50
-9	0	-15	11.20	4.80
-10	0	-15	-4.20	6.90
-10	0	-15	-0.40	5.20
-11	0	-15	3.20	8.20
-11	0	-15	14.80	6.30
-12	0	-15	0.90	7.60
-13	0	-15	13.70	10.10
13	-1	-15	24.50	10.30
13	1	-15	59.29	11.50
-13	1	15	47.30	7.80
12	-1	-15	294.97	23.20
-12	-1	15	318.67	18.40
12	1	-15	310.07	23.60
-12	1	15	303.77	18.70
-11	-1	15	14.80	5.20
-11	1	15	16.50	5.60
11	1	-15	10.80	8.10
-10	-1	15	857.21	42.70
10	-1	-15	846.82	45.80
-10	1	15	870.31	43.10
10	1	-15	837.52	47.50
9	-1	-15	30.00	7.10
-9	-1	15	36.60	6.80
9	-1	-15	35.80	6.20
9	1	-15	35.20	5.50
9	1	-15	34.10	7.70
-9	1	15	25.60	5.40
8	-1	-15	454.95	25.70
8	-1	-15	426.56	23.00
-8	-1	15	419.76	22.70
8	1	-15	442.96	25.60
-8	1	15	419.26	23.10
8	1	-15	422.66	22.60
-7	-1	15	28.20	4.40
7	-1	-15	21.30	6.00
7	-1	-15	29.30	4.60

7	1	-15	20.70	6.00
7	1	-15	27.30	4.20
-7	1	15	26.50	5.20
-6	-1	15	965.90	48.60
6	-1	-15	966.50	48.40
6	-1	-15	998.30	50.49
6	1	-15	981.80	48.20
6	1	-15	1001.10	50.49
-6	1	15	1027.60	49.20
-5	-1	15	53.39	6.10
5	-1	-15	60.79	5.70
5	-1	-15	54.39	8.00
5	1	-15	64.39	8.10
-5	1	15	51.79	6.90
-5	1	15	35.80	8.30
5	1	-15	59.39	5.30
-4	-1	15	1077.09	52.49
4	-1	-15	1040.30	52.09
-4	-1	15	1046.40	54.39
4	-1	-15	1117.99	53.89
-4	1	15	1049.00	54.49
-4	1	15	1072.89	52.99
4	1	-15	1074.09	51.89
4	1	-15	1125.39	53.79
3	-1	-15	140.09	9.70
-3	-1	15	103.89	10.00
-3	-1	15	129.39	8.40
3	-1	-15	131.89	7.90
-3	1	15	110.39	10.50
3	1	-15	125.69	7.60
-3	1	15	134.19	9.00
3	1	-15	126.29	9.50
2	-1	-15	953.20	46.20
-2	-1	15	937.31	46.60
-2	-1	15	912.81	47.50
2	-1	-15	982.00	47.40
2	1	-15	1000.60	47.30
-2	1	15	938.31	47.70
-2	1	15	938.71	47.00
2	1	-15	941.71	46.10
1	-1	-15	733.03	35.70
1	-1	-15	731.93	35.00
-1	-1	15	705.03	35.30
-1	-1	15	669.33	35.60

1	1	-15	725.73	34.80
-1	1	15	700.33	35.90
-1	1	15	659.93	35.50
1	1	-15	736.33	35.50
0	-1	-15	1048.20	51.59
0	-1	15	1053.39	51.69
0	-1	-15	1059.19	51.99
0	-1	15	1031.00	51.89
0	1	-15	1097.89	51.49
0	1	15	1013.90	52.09
0	1	15	1084.49	51.99
0	1	-15	1073.79	51.69
-1	-1	-15	888.31	42.60
1	-1	15	787.22	41.80
1	-1	15	833.82	42.70
1	-1	15	920.71	42.00
-1	-1	-15	868.71	42.40
-1	1	-15	854.31	42.20
1	1	15	857.31	42.30
-1	1	-15	870.81	42.30
1	1	15	896.61	42.60
1	1	15	821.32	42.90
-2	-1	-15	1151.68	56.29
-2	-1	-15	1206.08	56.59
2	-1	15	1083.59	56.79
2	-1	15	1228.78	55.39
2	-1	15	1088.59	55.19
2	1	15	1083.99	56.99
-2	1	-15	1141.69	55.99
2	1	15	1162.68	55.99
2	1	15	1257.97	56.29
-2	1	-15	1157.68	56.39
3	-1	15	721.83	38.20
-3	-1	-15	748.13	37.80
3	1	15	752.42	36.80
3	1	15	800.82	37.00
-3	1	-15	783.12	37.80
3	1	15	724.03	38.30
4	-1	15	57.59	7.40
-4	-1	-15	42.80	6.70
-4	1	-15	63.39	6.90
4	1	15	46.80	7.30
4	1	15	67.29	4.60
4	1	15	56.09	4.30

5	-1	15	1377.06	66.19
5	1	15	1304.87	63.79
5	1	15	1358.06	63.79
5	1	15	1313.97	66.19
6	-1	15	56.79	8.40
6	1	15	53.79	7.70
7	-1	15	541.35	29.50
7	1	15	544.15	29.70
-8	-1	-15	79.29	8.20
8	1	15	67.89	9.70
-8	1	-15	71.29	7.30
8	1	15	62.89	5.40
-8	1	-15	60.69	10.00
-9	-1	-15	637.24	35.60
-9	-1	-15	638.44	33.80
9	1	15	657.73	35.40
-9	1	-15	657.03	33.30
-9	1	-15	654.33	35.50
-10	-1	-15	37.00	6.90
-10	-1	-15	38.50	8.70
-10	1	-15	37.90	6.30
-10	1	-15	30.90	8.40
-11	-1	-15	389.76	25.60
-11	-1	-15	410.86	24.20
-11	1	-15	403.86	23.50
-11	1	-15	405.96	25.60
-12	-1	-15	5.10	8.60
-12	1	-15	17.00	8.50
-13	-1	-15	362.56	25.20
-13	1	-15	347.37	25.20
13	-2	-15	154.38	18.80
13	2	-15	122.49	19.20
-13	2	15	134.49	13.30
12	-2	-15	42.40	9.80
-12	2	15	39.90	8.50
12	2	-15	33.70	9.80
-11	-2	15	339.37	18.90
11	-2	-15	334.67	26.40
-11	2	15	346.37	19.90
11	2	-15	320.07	23.40
10	-2	-15	14.80	7.20
-10	-2	15	18.50	4.80
-10	2	15	15.80	5.40
9	-2	-15	276.27	19.20

-9	-2	15	287.67	15.70
9	-2	-15	261.17	17.00
-9	2	15	280.07	16.60
9	2	-15	261.47	15.90
9	2	-15	275.27	19.60
8	-2	-15	11.20	6.20
8	-2	-15	24.50	5.20
-8	-2	15	24.20	4.40
8	2	-15	19.30	6.50
-8	2	15	12.70	5.00
8	2	-15	26.00	4.50
-7	-2	15	721.43	34.90
7	-2	-15	703.93	37.30
7	-2	-15	681.43	35.20
-7	2	15	688.23	35.80
7	2	-15	692.13	34.70
7	2	-15	712.83	37.50
-6	-2	15	272.97	15.20
6	-2	-15	269.57	15.40
6	-2	-15	289.77	17.90
6	2	-15	275.67	17.80
6	2	-15	275.87	14.90
-6	2	15	294.47	16.70
5	-2	-15	394.06	20.70
5	-2	-15	412.26	22.70
-5	-2	15	380.56	20.60
-5	2	15	421.36	25.10
5	2	-15	399.16	20.20
5	2	-15	401.06	22.70
-5	2	15	397.96	21.80
-4	-2	15	133.99	8.70
-4	-2	15	113.39	12.30
4	-2	-15	143.09	8.70
4	-2	-15	137.29	10.60
4	2	-15	143.19	10.60
4	2	-15	142.49	8.10
-4	2	15	137.29	11.90
-4	2	15	133.79	10.10
-3	-2	15	89.39	6.60
3	-2	-15	85.39	6.10
3	-2	-15	80.89	7.90
-3	-2	15	79.59	8.70
3	2	-15	82.79	5.60
3	2	-15	87.79	7.80

-3	2	15	81.49	7.50
-3	2	15	82.99	9.00
-2	-2	15	70.59	8.10
2	-2	-15	94.79	6.60
2	-2	-15	97.19	8.00
-2	-2	15	81.89	6.60
-2	2	15	89.69	8.60
-2	2	15	87.59	7.70
2	2	-15	89.79	5.90
2	2	-15	91.69	7.50
-1	-2	15	520.85	27.90
1	-2	-15	576.74	28.70
1	-2	-15	573.24	27.80
-1	-2	15	519.35	28.30
-1	2	15	545.55	28.60
-1	2	15	552.94	28.80
1	2	-15	566.24	27.50
1	2	-15	572.44	28.20
0	-2	-15	221.68	12.60
0	-2	15	213.68	12.30
0	-2	15	183.58	11.80
0	-2	-15	219.88	12.10
0	2	-15	222.58	12.00
0	2	15	214.28	12.90
0	2	-15	214.08	11.70
0	2	15	202.78	12.50
-1	-2	-15	393.46	20.80
-1	-2	-15	391.06	20.90
1	-2	15	422.46	19.90
1	-2	15	382.76	19.80
1	-2	15	386.36	20.90
1	2	15	433.56	21.30
-1	2	-15	393.96	20.30
1	2	15	399.96	20.80
1	2	15	368.36	21.40
-1	2	-15	397.66	20.50
-2	-2	-15	231.98	13.00
2	-2	15	222.58	13.40
-2	-2	-15	219.98	13.10
-2	2	-15	219.18	12.90
2	2	15	222.98	12.60
2	2	15	242.38	13.20
-2	2	-15	224.98	12.10
2	2	15	224.78	13.80

-3	-2	-15	123.59	8.90
3	-2	15	110.19	9.10
-3	2	-15	125.09	8.60
3	2	15	123.99	8.30
3	2	15	115.89	9.60
3	2	15	114.09	7.60
-4	-2	-15	39.00	7.00
4	-2	15	42.40	6.80
4	2	15	42.70	4.30
-4	2	-15	41.50	6.10
4	2	15	46.40	5.20
4	2	15	36.90	7.00
5	-2	15	419.06	22.90
5	2	15	394.66	22.90
5	2	15	421.66	21.00
6	-2	15	483.35	26.50
6	2	15	474.95	24.30
6	2	15	498.65	26.50
7	2	15	31.50	5.10
7	2	15	24.80	5.80
-7	2	-15	26.90	6.60
-8	-2	-15	314.17	18.50
-8	2	-15	339.27	17.20
-8	2	-15	317.57	19.60
8	2	15	316.47	20.00
8	2	15	299.17	16.70
-9	-2	-15	10.80	5.10
-9	2	-15	1.80	6.40
9	2	15	10.00	3.50
-9	2	-15	7.50	4.10
9	2	15	2.80	6.20
-10	-2	-15	211.88	16.90
-10	-2	-15	200.18	15.20
-10	2	-15	210.78	15.80
-10	2	-15	197.28	13.80
-11	-2	-15	-4.30	7.70
-11	2	-15	4.50	5.60
-11	2	-15	8.30	8.00
-12	-2	-15	258.47	20.50
-12	2	-15	232.38	19.70
-13	-2	-15	44.60	11.00
-13	2	-15	42.80	10.60
13	-3	-15	24.80	10.00
13	3	-15	40.60	11.10

-13	3	15	35.10	7.90
12	-3	-15	141.39	19.00
12	3	-15	165.78	18.80
-12	3	15	177.18	13.80
-11	-3	15	12.60	4.70
11	-3	-15	14.60	8.10
11	3	-15	11.90	8.30
-11	3	15	12.40	6.10
10	-3	-15	241.08	18.60
-10	-3	15	236.98	14.10
10	3	-15	245.28	19.20
-10	3	15	225.78	15.40
-9	-3	15	3.80	3.60
9	-3	-15	8.50	6.80
9	3	-15	9.20	7.40
-9	3	15	8.70	5.20
8	-3	-15	87.09	9.00
-8	-3	15	91.99	7.20
8	-3	-15	70.89	11.10
-8	3	15	76.09	8.80
8	3	-15	92.99	11.30
8	3	-15	85.89	7.50
7	-3	-15	6.30	5.70
7	-3	-15	10.50	4.10
-7	-3	15	7.70	3.20
7	3	-15	4.90	5.30
-7	3	15	1.90	4.50
7	3	-15	3.50	3.00
-6	-3	15	383.26	19.90
6	-3	-15	369.76	20.40
6	-3	-15	385.56	22.50
-6	3	15	381.26	21.60
6	3	-15	395.06	19.70
6	3	-15	401.66	22.70
5	-3	-15	252.57	16.20
5	-3	-15	259.77	14.20
-5	-3	15	253.67	13.70
5	3	-15	265.17	13.40
-5	3	15	240.68	17.70
5	3	-15	244.78	16.30
-5	3	15	260.87	15.80
-4	-3	15	205.68	11.90
4	-3	-15	219.78	14.10
4	-3	-15	208.58	12.20

4	3	-15	225.98	14.00
-4	3	15	225.48	13.90
-4	3	15	210.48	15.00
4	3	-15	223.78	11.50
3	-3	-15	44.60	4.90
3	-3	-15	44.40	6.70
-3	-3	15	43.50	4.80
-3	-3	15	29.20	5.30
3	3	-15	45.30	3.90
-3	3	15	38.30	5.70
-3	3	15	36.60	7.70
3	3	-15	42.20	6.50
-2	-3	15	135.29	10.90
2	-3	-15	169.18	11.20
-2	-3	15	174.08	9.90
2	-3	-15	176.08	10.00
2	3	-15	168.68	9.10
-2	3	15	162.18	11.30
2	3	-15	167.78	10.70
-2	3	15	165.58	11.70
-1	-3	15	89.59	6.80
1	-3	-15	97.39	6.70
1	-3	-15	90.89	7.80
-1	-3	15	74.89	7.10
-1	3	15	75.29	7.80
1	3	-15	94.89	6.10
-1	3	15	87.99	7.90
1	3	-15	91.29	7.00
0	-3	15	380.36	19.80
0	-3	15	352.46	19.20
0	-3	-15	372.36	19.70
0	-3	-15	376.86	20.40
0	3	15	378.16	20.60
0	3	-15	402.86	19.60
0	3	15	373.26	20.20
0	3	-15	373.46	19.30
1	-3	15	118.89	8.20
-1	-3	-15	113.09	8.10
-1	-3	-15	121.49	8.40
1	-3	15	97.89	6.20
1	-3	15	111.39	6.40
1	3	15	107.89	8.00
-1	3	-15	120.39	7.60
1	3	15	114.89	9.10

1	3	15	109.69	8.70
-1	3	-15	111.09	7.20
-2	-3	-15	131.89	9.20
-2	-3	-15	131.89	9.20
2	-3	15	140.99	9.40
2	3	15	133.09	8.70
-2	3	-15	141.29	7.80
-2	3	-15	131.99	8.90
2	3	15	135.09	9.90
-3	-3	-15	142.09	10.10
3	-3	15	152.48	10.30
3	3	15	153.48	9.90
-3	3	-15	145.49	9.50
3	3	15	136.59	8.80
3	3	15	137.09	10.70
4	-3	15	86.49	8.50
-4	3	-15	96.39	8.10
4	3	15	92.59	9.00
4	3	15	93.59	7.50
4	3	15	98.39	7.80
5	-3	15	262.37	16.50
5	3	15	255.67	16.60
-5	3	-15	293.07	17.00
5	3	15	272.87	15.10
6	-3	15	186.68	13.10
-6	3	-15	170.38	13.30
6	3	15	184.18	13.30
6	3	15	194.88	11.40
-7	3	-15	299.97	18.70
7	3	15	276.57	21.40
7	3	15	304.07	16.40
-8	-3	-15	4.60	4.80
8	3	15	9.80	5.60
-8	3	-15	2.40	2.90
-8	3	-15	5.90	5.80
8	3	15	-2.10	3.60
-9	-3	-15	57.99	9.70
9	3	15	54.49	8.80
9	3	15	51.99	7.30
-9	3	-15	69.39	11.00
-9	3	-15	63.09	7.20
-10	-3	-15	6.20	6.00
-10	3	-15	15.20	7.20
-10	3	-15	1.80	4.50

10	3	15	2.70	4.50
10	3	15	21.30	7.30
-11	-3	-15	191.38	17.50
-11	3	-15	192.38	14.30
-11	3	-15	197.68	16.90
-12	-3	-15	4.20	9.00
-12	3	-15	0.40	6.10
-12	3	-15	0.00	7.50
-13	-3	-15	74.39	12.40
-13	3	-15	35.10	10.00
13	4	-15	113.19	19.10
-13	4	15	98.09	12.90
12	-4	-15	22.70	13.10
12	4	-15	26.20	10.00
-12	4	15	33.70	7.40
11	-4	-15	280.47	21.60
-11	-4	15	288.67	17.60
11	4	-15	278.47	21.80
-11	4	15	288.87	18.10
10	-4	-15	119.59	14.50
-10	-4	15	128.79	9.00
10	4	-15	108.49	14.90
-10	4	15	111.19	11.00
-9	-4	15	489.85	24.90
9	-4	-15	478.95	28.70
-9	4	15	502.05	26.70
8	-4	-15	9.20	6.20
-8	-4	15	16.10	3.50
-8	4	15	6.20	5.00
8	4	-15	10.40	6.20
8	4	-15	11.30	3.60
7	-4	-15	417.86	23.40
7	-4	-15	455.75	25.60
-7	-4	15	433.56	22.30
7	4	-15	443.16	22.30
-7	4	15	457.05	24.50
7	4	-15	448.46	25.90
-6	-4	15	6.70	2.70
6	-4	-15	7.60	3.70
6	-4	-15	11.00	4.90
6	4	-15	3.60	5.40
-6	4	15	6.70	5.00
-5	-4	15	1379.96	65.99
5	-4	-15	1330.27	66.59

5	-4	-15	1458.45	68.39
5	4	-15	1422.46	68.39
5	4	-15	1336.57	66.29
-5	4	15	1267.27	69.39
-5	4	15	1415.96	67.89
-4	-4	15	9.40	2.80
4	-4	-15	10.90	3.30
4	-4	-15	11.00	4.20
-4	4	15	5.70	4.90
-4	4	15	5.40	4.80
4	4	-15	9.00	3.30
4	4	-15	12.20	4.20
3	-4	-15	1479.15	72.29
3	-4	-15	1565.94	73.79
-3	-4	15	1374.16	74.39
-3	-4	15	1542.55	72.09
-3	4	15	1517.05	73.69
-3	4	15	1473.65	74.09
3	4	-15	1537.55	73.49
2	-4	-15	25.30	4.60
-2	-4	15	24.20	3.40
-2	-4	15	19.80	4.00
2	-4	-15	15.50	3.60
2	4	-15	33.10	3.30
-2	4	15	16.70	4.40
2	4	-15	22.40	3.90
-2	4	15	25.30	5.20
-1	-4	15	622.14	33.60
1	-4	-15	679.33	34.50
-1	-4	15	705.83	33.50
1	-4	-15	660.33	33.50
-1	4	15	656.43	34.60
1	4	-15	694.33	33.80
-1	4	15	684.23	34.60
1	4	-15	682.23	32.90
0	-4	-15	708.13	33.80
0	-4	15	689.83	33.70
0	-4	-15	691.23	34.50
0	-4	15	585.44	33.00
0	4	-15	712.23	33.50
0	4	15	678.03	34.80
0	4	-15	677.43	33.20
0	4	15	659.83	34.20
-1	-4	-15	1125.69	55.49

-1	-4	-15	1118.99	54.99
1	-4	15	1139.99	55.09
-1	4	-15	1133.69	54.29
-1	4	-15	1133.69	54.59
1	4	15	1058.79	55.09
1	4	15	1192.18	55.79
1	4	15	1090.19	55.79
2	-4	15	630.74	30.80
-2	-4	-15	626.94	30.80
-2	-4	-15	609.14	30.90
2	4	15	624.74	31.20
-2	4	-15	590.24	29.30
-2	4	-15	589.34	30.20
2	4	15	570.34	30.30
2	4	15	572.54	31.20
3	-4	15	480.05	25.00
-3	-4	-15	459.95	24.80
3	4	15	468.55	24.30
3	4	15	497.95	27.10
3	4	15	452.95	25.50
-3	4	-15	475.45	24.40
4	-4	15	1585.34	77.29
-4	4	-15	1582.54	76.89
4	4	15	1654.23	76.99
4	4	15	1500.45	77.59
-5	4	-15	107.29	9.40
5	4	15	122.89	9.00
5	4	15	104.99	9.60
-5	4	-15	92.99	9.90
6	4	15	728.53	38.10
6	4	15	760.42	37.30
-6	4	-15	690.33	37.70
-7	4	-15	280.07	18.80
7	4	15	324.77	17.50
-8	-4	-15	559.14	30.00
-8	4	-15	544.35	30.60
8	4	15	545.05	28.90
8	4	15	572.24	30.90
-9	-4	-15	4.30	5.50
-9	4	-15	5.50	3.50
9	4	15	-1.80	4.50
9	4	15	-5.90	5.50
-9	4	-15	6.90	6.60
-10	-4	-15	448.76	26.80

-10	4	-15	453.75	24.40
-10	4	-15	481.35	27.60
10	4	15	460.35	27.50
-11	-4	-15	15.20	8.60
-11	4	-15	0.40	8.10
-11	4	-15	2.30	5.30
-12	-4	-15	481.25	30.10
-12	4	-15	459.65	26.50
-12	4	-15	495.95	29.50
-13	-4	-15	-2.70	9.50
-13	4	-15	-0.70	9.10
13	5	-15	-4.90	10.70
-13	5	15	3.00	7.40
12	5	-15	5.30	9.00
-12	5	15	6.20	6.60
11	-5	-15	25.80	8.60
-11	5	15	15.80	6.50
11	5	-15	5.20	8.50
10	-5	-15	23.80	8.20
-10	-5	15	13.70	3.80
10	5	-15	7.10	7.50
9	-5	-15	8.20	6.60
-9	-5	15	7.40	3.20
9	5	-15	-2.90	6.50
-9	5	15	0.90	5.20
8	-5	-15	2.20	5.50
-8	-5	15	4.00	2.90
8	5	-15	0.90	6.70
-8	5	15	-1.80	5.00
7	-5	-15	40.60	7.30
-7	5	15	37.40	6.60
7	5	-15	30.00	4.40
7	5	-15	20.50	6.30
6	-5	-15	-2.50	3.40
-6	-5	15	0.80	2.80
6	-5	-15	-2.80	4.60
6	5	-15	2.30	2.20
6	5	-15	-5.80	4.70
-6	5	15	-8.90	4.60
5	-5	-15	79.69	7.50
5	-5	-15	100.09	10.10
-5	-5	15	91.59	6.10
-5	5	15	79.29	12.50
-5	5	15	98.09	9.70

5	5	-15	95.79	5.80
5	5	-15	85.09	9.80
4	-5	-15	8.10	3.20
4	-5	-15	9.50	4.40
-4	-5	15	9.70	2.70
-4	5	15	-2.50	4.60
4	5	-15	8.80	1.90
-4	5	15	-1.80	4.40
4	5	-15	5.10	4.30
3	-5	-15	6.80	4.20
3	-5	-15	2.50	2.90
-3	-5	15	7.40	3.50
-3	5	15	-2.20	4.00
3	5	-15	1.00	3.50
-3	5	15	1.90	4.20
3	5	-15	4.40	1.70
-2	-5	15	24.40	4.20
2	-5	-15	27.80	4.70
2	-5	-15	36.30	6.60
-2	-5	15	28.90	4.60
2	5	-15	30.40	5.60
-2	5	15	35.70	5.70
-2	5	15	37.50	6.10
-1	-5	15	190.78	12.40
1	-5	-15	222.68	13.70
1	-5	-15	220.88	12.50
-1	-5	15	236.98	12.20
-1	5	15	201.58	13.50
1	5	-15	220.58	11.50
1	5	-15	221.18	12.70
-1	5	15	220.28	14.10
0	-5	15	5.50	2.30
0	-5	-15	5.50	3.60
0	-5	15	5.70	3.00
0	-5	-15	2.60	3.20
0	5	-15	5.50	2.60
0	5	15	0.20	3.10
0	5	15	9.40	4.10
0	5	-15	7.50	2.50
-1	-5	-15	0.30	3.10
-1	-5	-15	3.30	3.30
1	-5	15	-0.10	2.90
1	5	15	-3.20	2.80
1	5	15	2.20	4.20

-1	5	-15	1.70	2.10
1	5	15	14.50	4.40
-1	5	-15	-0.30	2.50
-2	-5	-15	2.40	3.50
2	-5	15	3.80	3.40
-2	5	-15	4.50	3.00
-2	5	-15	5.10	1.70
2	5	15	7.80	4.00
2	5	15	1.30	4.20
2	5	15	0.80	2.80
3	-5	15	8.90	3.80
-3	5	-15	13.20	3.60
3	5	15	6.00	4.50
3	5	15	3.30	4.00
4	5	15	61.29	8.10
-4	5	-15	54.39	7.70
4	5	15	62.89	9.50
-4	5	-15	59.49	6.80
5	5	15	-5.40	4.50
5	5	15	4.90	4.10
-5	5	-15	-2.40	4.50
-5	5	-15	-0.90	4.10
6	5	15	117.69	9.60
-6	5	-15	103.79	10.40
6	5	15	77.79	9.50
-7	5	-15	28.00	6.40
7	5	15	45.10	8.00
7	5	15	35.90	6.80
-8	-5	-15	45.10	7.30
8	5	15	69.99	10.10
-8	5	-15	43.80	7.60
8	5	15	46.40	8.80
-9	-5	-15	18.10	6.30
-9	5	-15	15.30	3.40
9	5	15	2.60	5.80
-9	5	-15	21.30	7.10
9	5	15	11.40	5.30
-10	5	-15	4.70	4.20
-10	5	-15	2.20	7.20
10	5	15	17.90	7.70
-11	5	-15	46.80	8.60
-11	5	-15	41.10	9.10
11	5	15	68.19	12.30
-12	-5	-15	16.00	9.00

-12	5	-15	4.50	9.60
-12	5	-15	5.90	7.70
-13	-5	-15	8.10	9.10
-13	5	-15	16.60	9.40
13	6	-15	171.08	21.30
-13	6	15	202.38	16.60
12	6	-15	4.50	10.20
-12	6	15	18.70	7.10
11	-6	-15	490.25	30.80
-11	6	15	518.85	28.00
11	6	-15	492.65	31.30
10	-6	-15	76.29	13.40
-10	6	15	67.19	10.20
10	6	-15	66.99	13.80
9	-6	-15	661.13	35.60
-9	6	15	638.24	33.70
9	6	-15	603.54	35.60
8	-6	-15	8.50	6.90
-8	-6	15	12.10	2.60
8	6	-15	12.80	7.50
-8	6	15	3.10	5.70
7	-6	-15	377.16	22.50
-7	-6	15	362.26	18.30
7	6	-15	364.86	22.70
-7	6	15	379.76	21.70
6	-6	-15	5.80	5.00
-6	-6	15	6.00	2.00
-6	6	15	0.70	5.40
6	6	-15	6.20	1.90
6	6	-15	0.80	5.00
-5	-6	15	464.65	21.90
5	-6	-15	457.05	25.20
5	-6	-15	411.56	23.50
5	6	-15	432.66	25.20
5	6	-15	444.66	21.70
-5	6	15	465.95	30.70
-5	6	15	457.75	25.20
4	-6	-15	36.30	7.50
4	-6	-15	35.60	5.40
-4	-6	15	30.70	3.60
-4	6	15	20.30	6.00
-4	6	15	27.10	6.40
4	6	-15	21.30	5.00
4	6	-15	33.20	3.00

3	-6	-15	1079.89	54.89
3	-6	-15	1160.08	56.49
-3	-6	15	1232.28	54.19
3	6	-15	1129.59	55.99
-3	6	15	1121.09	56.59
-3	6	15	1040.40	56.49
3	6	-15	1108.19	53.69
2	-6	-15	116.29	8.50
-2	-6	15	132.09	8.50
2	-6	-15	129.89	10.10
2	6	-15	124.59	9.10
2	6	-15	123.49	6.70
-2	6	15	116.39	10.20
-2	6	15	92.79	10.30
-1	-6	15	392.06	19.00
-1	-6	15	322.07	19.20
1	-6	-15	370.06	19.50
1	-6	-15	374.06	20.70
1	6	-15	367.06	18.40
-1	6	15	387.26	21.20
-1	6	15	339.27	20.40
1	6	-15	390.56	19.70
0	-6	-15	847.02	41.70
0	-6	15	908.91	41.40
0	-6	15	783.02	40.70
0	-6	-15	875.21	42.70
0	6	-15	842.32	40.80
0	6	15	840.32	42.90
0	6	-15	837.42	41.20
0	6	15	778.22	42.10
1	-6	15	1703.03	79.69
-1	-6	-15	1687.83	80.69
-1	-6	-15	1624.54	79.89
-1	6	-15	1679.83	78.99
1	6	15	1613.44	80.89
1	6	15	1518.65	79.99
1	6	15	1748.23	80.99
-1	6	-15	1605.94	79.19
-2	6	-15	341.57	16.50
2	6	15	317.77	19.00
-2	6	-15	344.57	17.60
2	6	15	317.57	19.20
-2	6	-15	324.87	18.20
3	6	15	153.68	11.20

-3	6	-15	131.19	9.30
-3	6	-15	136.79	10.30
3	6	15	131.89	10.90
-4	6	-15	798.82	42.60
-4	6	-15	870.71	42.30
4	6	15	804.12	43.40
4	6	15	909.11	43.30
-5	6	-15	44.30	7.50
-5	6	-15	57.99	8.30
5	6	15	56.09	8.50
5	6	15	78.09	11.20
6	6	15	348.87	22.30
6	6	15	400.66	21.40
-6	6	-15	367.66	21.60
7	6	15	63.49	9.10
7	6	15	49.40	8.70
-7	6	-15	49.70	7.90
-8	-6	-15	737.83	38.70
-8	6	-15	719.83	38.80
8	6	15	693.43	38.80
8	6	15	775.52	38.30
-9	-6	-15	12.30	6.30
-9	6	-15	16.50	2.80
9	6	15	8.30	6.10
-9	6	-15	0.00	6.20
-10	6	-15	422.36	22.80
-10	6	-15	480.25	26.90
10	6	15	426.26	26.70
-11	6	-15	3.60	7.00
11	6	15	1.80	6.90
-12	-6	-15	546.75	32.20
-12	6	-15	438.46	28.20
-12	6	-15	590.24	31.40
-13	-6	-15	13.50	10.30
-13	6	-15	16.10	9.10
12	-7	-15	312.87	32.00
-12	7	15	293.37	19.70
12	7	-15	294.17	24.40
11	-7	-15	16.20	8.60
-11	7	15	21.10	7.00
11	7	-15	17.90	9.40
10	-7	-15	367.86	24.70
10	7	-15	372.06	25.10
-10	7	15	364.66	22.20

9	-7	-15	1.70	7.40
-9	7	15	3.00	6.20
9	7	-15	8.80	7.10
8	-7	-15	215.18	16.30
8	7	-15	194.58	16.60
-8	7	15	194.48	14.50
7	-7	-15	14.20	6.80
-7	7	15	8.90	5.80
6	-7	-15	415.16	24.10
-6	7	15	399.16	23.80
6	7	-15	417.46	24.30
5	-7	-15	239.18	15.60
-5	7	15	218.28	15.70
5	7	-15	215.18	15.50
4	-7	-15	42.80	8.30
4	7	-15	30.30	5.60
-4	7	15	39.90	7.10
-4	7	15	28.80	6.40
3	-7	-15	156.08	11.80
3	-7	-15	153.78	10.10
3	7	-15	152.98	11.20
3	7	-15	156.38	7.80
-3	7	15	132.79	12.10
-3	7	15	147.49	12.50
2	-7	-15	13.40	3.80
2	-7	-15	14.90	5.00
-2	7	15	9.30	4.80
-2	7	15	11.40	5.70
2	7	-15	14.00	2.00
2	7	-15	14.80	4.10
1	-7	-15	213.28	13.80
-1	-7	15	201.58	12.30
1	-7	-15	219.08	12.40
1	7	-15	220.98	11.80
-1	7	15	198.58	13.50
-1	7	15	208.98	14.50
1	7	-15	221.68	12.60
1	7	-15	169.58	12.00
0	-7	-15	142.19	10.80
0	-7	15	138.79	8.30
0	-7	-15	134.59	10.30
0	7	-15	135.99	9.00
0	7	-15	134.89	9.00
0	7	15	127.99	11.20

0	7	-15	140.89	8.20
0	7	15	161.78	11.70
0	7	15	130.29	10.40
-1	-7	-15	159.48	11.50
-1	7	-15	144.79	10.00
-1	7	-15	157.68	9.10
-1	7	-15	153.08	9.30
1	7	15	171.18	12.30
1	7	15	148.59	11.90
-2	7	-15	54.29	6.70
-2	7	-15	51.19	5.70
2	7	15	45.50	8.20
-2	7	-15	53.59	3.90
2	7	15	38.90	7.40
3	7	15	89.99	8.90
-3	7	-15	76.19	7.40
3	7	15	99.29	9.50
-3	7	-15	79.39	8.10
-4	7	-15	38.80	6.40
4	7	15	46.30	8.40
4	7	15	53.19	8.20
-4	7	-15	42.00	6.00
-5	7	-15	124.49	10.80
5	7	15	118.09	10.80
5	7	15	136.99	11.50
-5	7	-15	122.69	10.10
-6	7	-15	37.20	6.70
7	7	15	199.88	15.40
7	7	15	265.97	15.90
-7	7	-15	206.98	15.70
-8	-7	-15	54.79	8.60
8	7	15	34.80	7.40
-8	7	-15	32.30	7.30
8	7	15	65.09	10.10
-9	-7	-15	46.20	8.70
9	7	15	58.79	10.30
-9	7	-15	45.60	8.20
10	7	15	34.20	8.30
-10	7	-15	40.60	9.00
11	7	15	219.78	18.20
-11	7	-15	234.08	18.00
-11	7	-15	199.98	13.50
-12	-7	-15	22.20	10.20
12	7	15	23.40	8.70

-12	7	-15	26.30	6.10
-12	7	-15	37.80	9.30
12	8	-15	5.00	9.80
-12	8	15	14.90	7.90
11	-8	-15	190.48	19.00
11	8	-15	206.28	20.00
-11	8	15	221.58	16.20
10	-8	-15	22.00	8.70
10	8	-15	22.50	9.00
-10	8	15	15.50	6.80
9	-8	-15	105.79	13.90
9	8	-15	93.49	14.10
-9	8	15	81.89	11.20
8	-8	-15	20.00	6.90
8	8	-15	11.70	7.00
7	-8	-15	244.88	16.90
7	8	-15	219.88	17.20
-7	8	15	229.28	16.40
6	-8	-15	119.49	11.70
-6	8	15	81.89	10.90
5	-8	-15	355.76	21.50
5	8	-15	346.57	21.40
-5	8	15	367.26	21.80
4	-8	-15	83.59	9.50
-4	8	15	62.99	10.20
4	8	-15	78.29	9.50
3	-8	-15	20.80	5.50
-3	8	15	17.50	6.60
-3	8	15	25.70	6.80
3	8	-15	16.30	4.60
2	-8	-15	32.80	5.80
2	8	-15	48.60	3.80
-2	8	15	38.70	7.40
2	8	-15	34.70	6.50
2	8	-15	42.80	3.10
-2	8	15	38.60	6.80
1	-8	-15	269.67	16.60
-1	8	15	260.87	17.50
-1	8	15	268.37	17.30
1	8	-15	264.67	13.50
1	8	-15	263.57	15.30
0	-8	-15	232.88	14.60
0	-8	15	192.18	11.70
0	8	-15	234.88	12.30

0	8	15	200.38	14.90
0	8	15	219.28	15.20
0	8	-15	216.18	12.70
0	8	-15	233.28	11.70
-1	-8	-15	26.80	5.20
1	8	15	18.70	5.60
1	8	15	32.60	6.40
-1	8	-15	25.20	3.40
-1	8	-15	23.40	4.10
-1	8	-15	20.20	3.20
2	8	15	160.28	12.00
-2	8	-15	158.88	8.60
2	8	15	152.98	12.50
-2	8	-15	153.78	9.90
-2	8	-15	161.58	10.60
3	8	15	73.09	9.40
-3	8	-15	79.89	7.30
3	8	15	79.19	9.70
-3	8	-15	78.69	8.00
-4	8	-15	311.77	17.00
4	8	15	302.97	18.80
4	8	15	289.37	18.60
-4	8	-15	292.47	17.50
-5	8	-15	283.37	18.20
5	8	15	304.07	18.70
-5	8	-15	285.27	18.20
5	8	15	340.97	19.50
-6	8	-15	364.56	21.20
6	8	15	373.66	21.90
6	8	15	347.77	21.80
7	8	15	51.69	9.30
-7	8	-15	62.79	8.50
-8	-8	-15	157.58	14.50
8	8	15	179.48	14.60
-8	8	-15	149.88	13.70
8	8	15	158.48	17.20
-9	8	-15	6.10	6.90
9	8	15	3.60	6.60
10	8	15	111.39	13.00
-10	8	-15	121.59	13.70
-11	8	-15	5.80	4.20
11	8	15	-6.50	7.70
-11	8	-15	9.60	8.00
-12	8	-15	332.97	23.20

-12	8	-15	311.07	19.20
12	8	15	364.56	23.70
-12	9	15	387.86	23.80
12	9	-15	364.26	27.80
11	-9	-15	52.19	11.00
-11	9	15	78.19	12.30
11	9	-15	77.99	16.20
10	-9	-15	830.72	46.30
10	9	-15	800.62	46.50
-10	9	15	911.21	44.50
9	-9	-15	2.90	7.20
-9	9	15	-1.50	6.10
9	9	-15	-1.50	7.30
8	-9	-15	750.72	41.10
-8	9	15	776.12	40.00
8	9	-15	744.83	41.40
7	-9	-15	168.48	14.60
7	9	-15	160.48	14.90
6	-9	-15	933.01	49.50
-6	9	15	969.20	49.30
6	9	-15	958.30	49.60
5	-9	-15	178.88	14.00
-5	9	15	165.68	14.20
4	-9	-15	1437.96	71.09
-4	9	15	1440.86	71.39
4	9	-15	1388.96	70.59
3	-9	-15	74.89	9.50
-3	9	15	66.29	9.90
3	9	-15	82.29	8.80
2	-9	-15	371.36	21.00
-2	9	15	316.07	21.30
2	9	-15	336.27	16.80
2	9	-15	343.67	17.00
2	9	-15	373.36	19.70
1	-9	-15	921.81	46.10
1	9	-15	930.51	43.40
1	9	-15	863.91	43.00
-1	9	15	894.41	46.50
1	9	-15	870.71	44.70
0	-9	15	271.87	14.00
0	-9	-15	256.17	16.60
0	9	-15	255.37	14.60
0	9	-15	261.37	13.50
0	9	15	233.58	17.00

0	9	-15	258.17	13.90
1	9	15	339.57	21.10
-1	9	-15	347.57	18.10
-1	9	-15	350.06	18.30
1	9	15	360.76	21.40
-1	9	-15	351.06	18.50
-2	9	-15	353.56	19.60
-2	9	-15	351.96	19.20
2	9	15	379.66	22.10
2	9	15	364.06	21.80
-2	9	-15	378.36	19.40
3	9	15	709.53	38.10
-3	9	-15	725.13	36.20
3	9	15	739.63	38.30
-3	9	-15	710.43	36.50
-4	9	-15	6.20	4.60
-4	9	-15	1.80	3.90
4	9	15	9.10	5.60
4	9	15	12.50	5.60
-5	9	-15	549.85	34.40
5	9	15	618.24	32.60
-5	9	-15	594.34	31.10
6	9	15	151.38	12.60
6	9	15	173.48	13.90
-6	9	-15	129.19	12.50
-7	9	-15	501.65	28.40
7	9	15	499.25	29.20
7	9	15	538.65	29.30
-8	9	-15	31.80	7.30
8	9	15	49.50	8.70
-9	9	-15	184.28	15.10
9	9	15	165.68	15.50
-10	9	-15	29.50	8.10
10	9	15	19.20	8.50
-11	9	-15	319.87	21.80
11	9	15	290.07	21.80
-11	9	-15	312.67	17.00
12	9	15	0.10	8.60
-12	9	-15	4.90	4.80
-12	9	-15	9.50	9.10
-12	10	15	30.10	8.60
12	10	-15	20.60	11.00
11	-10	-15	14.60	9.20
-11	10	15	13.50	7.90

11	10	-15	4.00	9.50
10	-10	-15	37.20	9.50
-10	10	15	71.89	11.60
10	10	-15	14.70	9.10
9	-10	-15	6.70	7.30
9	10	-15	8.90	7.80
-9	10	15	11.90	7.30
8	-10	-15	28.50	8.20
-8	10	15	19.30	7.20
8	10	-15	21.10	7.60
7	-10	-15	19.40	7.60
7	10	-15	23.30	7.40
-7	10	15	13.30	7.00
6	-10	-15	2.80	5.60
6	10	-15	9.40	6.50
5	-10	-15	-4.00	5.00
5	10	-15	-0.40	4.90
-5	10	15	-11.90	5.50
4	-10	-15	67.29	9.70
4	10	-15	49.70	9.50
-4	10	15	53.79	9.00
3	-10	-15	33.20	6.30
3	10	-15	37.70	6.80
-3	10	15	21.50	7.40
2	-10	-15	5.10	5.00
2	10	-15	2.90	4.00
-2	10	15	-4.10	6.10
1	-10	-15	70.69	9.20
1	10	-15	69.99	4.80
-1	10	15	56.59	10.30
1	10	-15	62.59	4.30
1	10	-15	48.90	7.40
0	-10	-15	0.00	4.80
0	10	15	7.70	5.80
0	10	15	3.70	4.90
0	10	-15	3.40	2.80
0	10	-15	3.20	2.20
0	10	-15	5.40	3.30
1	10	15	124.49	11.40
-1	10	-15	132.29	8.30
-1	10	-15	126.89	8.70
-1	10	-15	139.69	8.90
1	10	15	125.69	14.40
-2	10	-15	1.30	2.40

2	10	15	-3.10	5.10
-2	10	-15	0.70	3.20
-2	10	-15	4.70	3.60
2	10	15	-2.60	4.80
-3	10	-15	23.10	4.70
3	10	15	15.70	6.00
-3	10	-15	18.90	4.20
3	10	15	20.80	6.00
-4	10	-15	17.70	4.80
4	10	15	27.80	6.90
-4	10	-15	11.20	4.30
4	10	15	1.90	8.50
-5	10	-15	44.00	6.50
5	10	15	35.00	7.10
5	10	15	33.20	7.80
6	10	15	32.10	7.40
6	10	15	25.90	7.30
-6	10	-15	28.70	6.60
7	10	15	-4.80	6.00
-7	10	-15	2.10	6.00
7	10	15	3.70	7.50
-8	10	-15	1.30	6.30
8	10	15	-1.80	6.20
-9	10	-15	41.70	8.40
9	10	15	39.10	8.40
-10	10	-15	-8.80	6.80
10	10	15	1.10	7.10
11	10	15	5.50	8.00
-11	10	-15	2.50	3.80
-11	10	-15	19.00	8.80
-12	10	-15	3.60	4.60
-12	10	-15	-10.30	8.90
12	10	15	8.80	9.10
11	-11	-15	46.00	13.20
11	11	-15	40.70	11.50
-11	11	15	46.00	8.90
10	-11	-15	746.03	43.50
10	11	-15	775.82	44.00
-10	11	15	804.92	41.60
9	-11	-15	1.20	7.70
9	11	-15	8.00	8.10
-9	11	15	-6.10	6.70
8	-11	-15	335.27	22.80
8	11	-15	331.87	22.80

-8	11	15	317.67	21.20
7	-11	-15	69.49	12.20
7	11	-15	64.69	9.80
-7	11	15	68.69	10.00
6	-11	-15	626.64	35.50
6	11	-15	659.23	35.70
-6	11	15	649.43	35.40
5	-11	-15	60.59	10.60
5	11	-15	37.30	7.10
-5	11	15	35.80	9.40
4	-11	-15	258.77	17.30
4	11	-15	243.88	16.80
-4	11	15	236.58	18.20
3	-11	-15	258.27	17.20
-3	11	15	232.38	18.00
3	11	-15	246.68	16.20
2	-11	-15	643.04	33.70
-2	11	15	587.54	34.20
1	-11	-15	763.82	38.90
1	11	-15	668.93	37.90
1	11	-15	724.93	35.10
-1	11	15	719.53	39.30
-1	11	15	811.42	40.00
1	11	-15	700.53	35.00
0	-11	-15	315.17	19.30
0	11	15	272.87	19.60
0	11	-15	300.47	15.70
0	11	15	313.07	20.00
0	11	-15	304.57	17.10
-1	11	-15	458.55	23.90
1	11	15	436.36	26.40
-1	11	-15	475.05	23.70
-1	11	-15	474.15	23.40
-2	11	-15	336.67	17.70
-2	11	-15	324.57	17.40
2	11	15	311.07	20.10
2	11	15	329.47	21.70
-2	11	-15	317.77	17.00
-3	11	-15	292.47	15.20
-3	11	-15	300.87	16.80
3	11	15	337.37	20.30
-3	11	-15	310.97	17.10
3	11	15	272.57	24.30
-3	11	-15	290.57	15.10

-4	11	-15	23.40	5.20
4	11	15	15.50	6.50
-4	11	-15	21.20	4.90
4	11	15	26.10	6.90
5	11	15	437.16	26.40
-5	11	-15	432.66	24.30
5	11	15	448.76	25.90
6	11	15	2.00	6.60
6	11	15	-1.70	5.90
-6	11	-15	7.40	5.70
7	11	15	408.26	25.00
-8	11	-15	23.10	7.40
8	11	15	31.60	8.40
-9	11	-15	126.59	13.70
9	11	15	131.69	14.80
-10	11	-15	13.70	7.60
10	11	15	15.80	8.40
11	11	15	275.37	21.70
-11	11	-15	318.97	21.80
11	12	-15	258.57	23.90
-11	12	15	286.07	20.20
10	-12	-15	7.90	9.60
10	12	-15	12.80	9.00
-10	12	15	14.10	8.20
9	-12	-15	195.28	18.40
-9	12	15	193.88	16.40
9	12	-15	201.48	19.20
8	-12	-15	16.90	8.60
8	12	-15	22.10	8.80
7	-12	-15	320.37	21.80
-7	12	15	305.87	20.90
7	12	-15	323.27	21.90
6	-12	-15	60.39	12.10
-6	12	15	76.99	10.50
6	12	-15	68.29	11.80
5	-12	-15	268.17	18.70
-5	12	15	243.48	18.90
5	12	-15	273.87	18.40
4	-12	-15	361.46	21.90
-4	12	15	336.87	22.70
4	12	-15	337.07	21.20
3	-12	-15	39.30	7.40
3	12	-15	46.40	8.70
2	-12	-15	0.60	5.00

2	12	-15	4.30	4.40
-2	12	15	2.90	7.10
1	-12	-15	199.58	14.90
1	12	-15	178.98	9.30
-1	12	15	150.18	15.10
-1	12	15	201.58	16.20
1	12	-15	203.58	12.80
1	12	-15	179.28	9.40
0	12	-15	313.27	18.50
0	12	-15	309.27	16.00
0	12	15	330.07	21.10
0	12	15	285.37	20.70
-1	12	-15	203.28	11.40
-1	12	-15	197.08	11.10
1	12	15	192.58	15.50
1	12	15	157.38	18.70
-2	12	-15	14.30	4.10
2	12	15	10.80	9.70
-2	12	-15	14.10	4.50
-2	12	-15	18.80	3.80
3	12	15	10.60	7.00
-3	12	-15	16.80	3.10
3	12	15	17.20	6.70
-3	12	-15	11.20	2.40
-3	12	-15	12.20	4.40
-3	12	-15	7.50	4.10
-4	12	-15	113.49	10.20
4	12	15	136.79	12.90
4	12	15	152.28	13.90
-4	12	-15	110.19	10.20
5	12	15	131.59	14.00
-5	12	-15	126.29	11.10
5	12	15	108.29	11.80
-6	12	-15	196.18	14.30
6	12	15	183.08	15.90
7	12	15	4.20	6.70
-7	12	-15	9.40	7.40
8	12	15	199.38	17.20
-8	12	-15	217.38	16.40
-9	12	-15	0.30	6.90
9	12	15	-1.30	8.00
10	12	15	124.79	15.30
-10	12	-15	127.39	14.80
11	12	15	3.20	8.70

-11	12	-15	7.70	9.10
11	13	-15	32.10	12.00
-11	13	15	24.80	9.00
10	-13	-15	91.59	15.90
10	13	-15	41.60	10.70
-10	13	15	54.49	10.10
9	-13	-15	13.60	8.70
9	13	-15	7.90	8.30
-9	13	15	-3.60	7.90
8	-13	-15	22.00	8.80
-8	13	15	14.80	8.20
8	13	-15	28.10	9.50
7	-13	-15	3.80	7.20
7	13	-15	3.90	7.20
-7	13	15	-3.70	7.30
6	-13	-15	161.88	15.20
6	13	-15	131.39	14.50
-6	13	15	132.59	14.60
5	-13	-15	34.10	8.30
-5	13	15	11.80	8.10
5	13	-15	19.80	7.10
4	-13	-15	70.69	11.30
4	13	-15	91.49	11.00
-4	13	15	84.69	11.30
3	-13	-15	61.29	10.70
-3	13	15	50.29	9.40
3	13	-15	66.89	9.80
2	-13	-15	156.48	13.70
2	13	-15	141.99	11.70
-2	13	15	121.99	14.40
1	-13	-15	14.10	6.30
1	13	-15	14.20	2.00
1	13	-15	11.40	1.90
1	13	-15	15.10	4.90
-1	13	15	-1.70	6.70
-1	13	15	19.30	8.00
0	13	15	260.57	18.40
0	13	-15	241.08	12.40
0	13	-15	240.38	14.40
0	13	15	200.08	16.60
0	13	-15	202.68	12.70
-1	13	-15	238.78	12.60
1	13	15	219.58	17.80
-1	13	-15	231.88	13.70

-1	13	-15	215.48	12.40
1	13	15	197.88	16.90
-2	13	-15	296.57	15.90
2	13	15	289.47	19.40
-2	13	-15	293.27	15.80
-2	13	-15	288.47	15.90
2	13	15	242.88	19.30
-3	13	-15	9.40	4.30
3	13	15	9.80	6.70
-3	13	-15	9.50	3.20
-3	13	-15	6.00	4.10
-3	13	-15	5.80	2.60
-4	13	-15	49.90	4.50
4	13	15	58.69	10.40
-4	13	-15	49.90	4.90
-4	13	-15	53.59	7.10
-4	13	-15	62.59	8.20
4	13	15	61.29	9.40
5	13	15	371.26	24.60
-5	13	-15	386.06	21.80
5	13	15	377.26	23.30
-6	13	-15	48.60	9.10
6	13	15	68.89	12.00
-7	13	-15	187.18	14.30
7	13	15	159.28	15.50
-8	13	-15	39.20	8.10
8	13	15	48.20	9.30
9	13	15	11.40	8.30
-9	13	-15	23.90	8.40
10	13	15	7.00	8.30
-10	13	-15	11.90	8.40
11	13	15	151.88	17.90
-11	13	-15	192.68	17.60
-10	14	15	16.70	8.20
10	14	-15	22.50	11.20
9	-14	-15	353.86	25.50
-9	14	15	357.06	23.20
9	14	-15	348.67	25.60
8	-14	-15	25.40	9.80
-8	14	15	16.50	8.90
8	14	-15	33.60	9.80
7	-14	-15	267.57	20.60
-7	14	15	266.17	19.60
7	14	-15	255.57	20.40

6	-14	-15	13.20	7.50
-6	14	15	0.80	8.10
6	14	-15	14.60	7.60
5	-14	-15	915.01	49.60
-5	14	15	926.01	49.60
5	14	-15	964.00	49.30
4	-14	-15	8.80	6.50
-4	14	15	-9.40	6.90
4	14	-15	7.50	6.00
3	-14	-15	383.96	24.50
-3	14	15	386.36	25.30
3	14	-15	419.16	23.70
2	-14	-15	89.49	12.40
2	14	-15	84.39	10.10
-2	14	15	69.39	12.80
-2	14	15	96.29	15.60
1	-14	-15	564.24	32.20
-1	14	15	554.04	32.70
1	14	-15	564.84	30.20
-1	14	15	573.34	33.90
0	14	-15	171.48	10.60
0	14	15	173.18	16.50
0	14	15	204.08	17.80
0	14	-15	214.78	11.50
0	14	-15	204.58	13.40
1	14	15	714.43	40.10
-1	14	-15	707.93	36.40
-1	14	-15	796.62	37.80
-1	14	-15	695.63	36.40
1	14	15	802.52	41.60
-2	14	-15	464.25	23.90
2	14	15	475.35	28.60
-2	14	-15	482.35	24.40
2	14	15	473.75	27.90
-2	14	-15	430.46	23.70
-2	14	-15	461.75	24.00
3	14	15	276.07	19.20
-3	14	-15	301.87	16.40
3	14	15	263.17	20.10
-3	14	-15	261.27	15.20
-3	14	-15	285.17	16.50
-3	14	-15	309.07	15.80
-4	14	-15	378.26	22.90
-4	14	-15	423.76	22.90

-4	14	-15	453.95	21.30
4	14	15	421.46	25.90
-4	14	-15	401.96	21.00
-5	14	-15	104.29	6.40
-5	14	-15	85.29	10.40
5	14	15	76.89	11.70
-5	14	-15	101.89	6.40
6	14	15	431.86	26.80
-6	14	-15	440.26	24.80
-7	14	-15	272.97	17.80
7	14	15	250.07	19.30
8	14	15	329.97	23.40
-8	14	-15	372.16	22.40
9	14	15	9.00	8.60
-9	14	-15	2.10	7.40
10	14	15	444.66	29.40
-10	14	-15	509.65	29.00
-10	15	15	4.10	8.20
10	15	-15	-2.90	9.90
9	-15	-15	14.00	9.40
9	15	-15	1.40	9.40
-9	15	15	3.70	8.00
8	-15	-15	-11.80	8.90
-8	15	15	-1.50	8.00
8	15	-15	-3.20	8.20
7	-15	-15	6.70	8.60
-7	15	15	-16.00	7.40
7	15	-15	4.80	8.40
6	-15	-15	-3.60	7.10
-6	15	15	9.00	8.20
6	15	-15	8.80	7.30
5	-15	-15	15.60	7.50
-5	15	15	9.10	8.90
5	15	-15	29.30	8.10
4	-15	-15	-0.10	6.50
4	15	-15	6.00	6.10
-4	15	15	-0.80	7.50
3	-15	-15	5.40	7.10
3	15	-15	-3.40	5.60
-3	15	15	-17.10	7.40
2	-15	-15	24.30	7.40
2	15	-15	28.60	6.40
-2	15	15	21.10	9.10
-2	15	15	30.90	9.30

-1	15	15	7.50	8.20
1	15	-15	27.00	6.00
-1	15	15	21.40	9.50
0	15	-15	29.50	5.90
0	15	15	22.10	8.40
0	15	-15	11.90	3.60
0	15	15	15.10	8.50
-1	15	-15	8.10	4.70
-1	15	-15	3.20	3.00
1	15	15	-9.20	7.10
-1	15	-15	1.20	3.10
1	15	15	1.70	8.40
2	15	15	47.10	9.10
-2	15	-15	58.99	7.40
2	15	15	31.50	8.10
-2	15	-15	36.40	6.20
-2	15	-15	38.60	5.20
-2	15	-15	40.00	6.20
-3	15	-15	-5.40	4.50
-3	15	-15	6.10	4.10
3	15	15	-3.00	7.20
-3	15	-15	-0.10	3.00
3	15	15	-15.80	6.70
-3	15	-15	-0.50	3.70
-4	15	-15	45.50	5.90
-4	15	-15	57.09	8.30
-4	15	-15	52.89	8.80
4	15	15	33.90	8.60
-4	15	-15	67.09	6.50
-5	15	-15	11.00	5.70
-5	15	-15	13.60	3.30
-5	15	-15	11.50	3.10
5	15	15	8.50	7.60
-6	15	-15	26.40	4.20
6	15	15	14.90	7.80
-6	15	-15	23.10	4.00
-6	15	-15	13.70	6.40
-7	15	-15	16.60	7.00
7	15	15	16.60	8.10
-8	15	-15	1.50	7.10
8	15	15	20.10	8.20
9	15	15	11.90	8.80
-9	15	-15	-13.80	7.30
-10	15	-15	6.40	8.10

10	15	15	-0.80	9.10
-9	16	15	386.26	25.00
9	16	-15	364.36	27.60
8	-16	-15	21.00	9.90
8	16	-15	26.10	10.20
-8	16	15	26.70	8.60
7	-16	-15	111.89	17.10
7	16	-15	155.78	17.10
-7	16	15	138.59	15.80
6	-16	-15	13.00	8.30
6	16	-15	30.60	8.80
-6	16	15	35.60	10.20
5	-16	-15	708.23	39.80
5	16	-15	691.83	39.10
-5	16	15	725.23	39.50
4	-16	-15	3.10	7.20
4	16	-15	-0.60	6.30
-4	16	15	-5.00	8.60
3	-16	-15	568.14	33.70
3	16	-15	575.84	32.40
-3	16	15	605.74	34.70
2	-16	-15	42.80	9.20
-2	16	15	31.50	9.20
2	16	-15	46.90	7.70
-2	16	15	32.20	10.80
1	16	-15	495.25	27.40
-1	16	15	463.45	30.00
-1	16	15	508.15	31.30
0	16	15	199.58	18.70
0	16	-15	239.28	15.70
0	16	15	246.88	21.70
1	16	15	559.84	32.90
1	16	15	561.84	34.40
-1	16	-15	578.94	29.60
-1	16	-15	524.25	27.50
-2	16	-15	219.08	13.70
2	16	15	190.38	17.70
-2	16	-15	209.38	12.30
2	16	15	201.28	18.80
-2	16	-15	201.58	12.70
-3	16	-15	227.28	13.80
3	16	15	238.58	17.80
-3	16	-15	237.78	14.00
-3	16	-15	206.48	13.20

-4	16	-15	529.25	30.10
-4	16	-15	609.94	31.20
4	16	15	647.94	34.90
-4	16	-15	630.04	30.50
-5	16	-15	11.30	3.60
-5	16	-15	11.40	3.80
5	16	15	1.40	7.60
-5	16	-15	8.20	5.70
6	16	15	353.06	22.90
-6	16	-15	360.46	17.90
-6	16	-15	313.57	18.80
-7	16	-15	146.69	9.00
7	16	15	159.78	16.00
-7	16	-15	137.39	13.30
-8	16	-15	216.98	17.00
8	16	15	194.28	18.40
-9	16	-15	2.60	7.60
9	16	15	-15.80	8.40
9	17	-15	52.59	13.00
-9	17	15	40.10	11.00
8	-17	-15	38.60	16.00
8	17	-15	52.09	11.20
-8	17	15	40.70	10.10
7	-17	-15	3.60	8.70
7	17	-15	11.20	8.80
-7	17	15	-3.90	9.20
6	-17	-15	321.37	23.80
6	17	-15	320.17	23.30
-6	17	15	324.67	23.30
5	-17	-15	11.20	8.80
-5	17	15	-10.70	9.20
5	17	-15	9.30	7.60
4	-17	-15	191.38	18.30
4	17	-15	225.58	17.40
-4	17	15	176.08	18.30
3	-17	-15	65.09	10.80
3	17	-15	53.49	9.00
-3	17	15	45.90	11.40
-3	17	15	50.89	10.50
-2	17	15	248.08	21.90
-2	17	15	205.08	19.60
2	17	-15	231.18	16.90
1	17	-15	30.60	6.70
-1	17	15	19.70	10.40

-1	17	15	28.90	9.80
0	17	15	245.58	21.00
0	17	-15	248.48	16.20
0	17	15	193.68	19.30
-1	17	-15	266.07	16.30
1	17	15	265.97	23.00
1	17	15	238.88	20.10
-1	17	-15	228.38	13.30
-2	17	-15	40.50	6.20
-2	17	-15	49.00	7.10
2	17	15	51.79	10.70
3	17	15	101.29	14.60
-3	17	-15	114.39	9.70
-3	17	-15	92.49	8.60
-3	17	-15	101.49	8.80
4	17	15	23.70	8.60
-4	17	-15	27.50	4.80
-4	17	-15	19.00	5.40
-4	17	-15	39.50	5.50
-5	17	-15	186.88	14.00
5	17	15	195.48	17.80
-5	17	-15	212.78	12.50
-5	17	-15	189.68	12.30
-6	17	-15	109.79	8.50
6	17	15	89.39	14.40
-6	17	-15	75.99	11.30
-6	17	-15	105.89	9.20
-7	17	-15	257.77	18.00
-7	17	-15	252.97	14.90
7	17	15	272.07	20.80
-7	17	-15	268.27	14.70
-8	17	-15	-3.60	3.80
-8	17	-15	0.60	3.60
-8	17	-15	-4.50	7.20
8	17	15	-9.10	8.30
-9	17	-15	19.70	8.60
-9	17	-15	34.90	6.00
-9	17	-15	29.20	5.70
9	17	15	32.30	10.10
-8	18	15	-10.90	9.00
8	18	-15	6.20	10.40
7	-18	-15	79.69	16.10
7	18	-15	62.79	11.60
-7	18	15	97.09	15.40

6	-18	-15	36.60	11.10
6	18	-15	29.20	9.10
-6	18	15	12.10	9.30
5	-18	-15	96.69	16.30
5	18	-15	100.19	14.60
-5	18	15	72.29	12.20
4	-18	-15	3.00	9.20
4	18	-15	10.80	7.60
-4	18	15	-10.40	8.70
3	18	-15	-3.60	6.70
-3	18	15	7.90	10.10
-3	18	15	11.80	10.10
-2	18	15	12.20	11.00
2	18	-15	1.60	6.70
-2	18	15	-5.40	9.10
-1	18	15	43.40	15.50
-1	18	15	20.00	9.40
1	18	-15	44.10	7.80
0	18	15	178.28	18.10
0	18	-15	160.78	13.90
1	18	15	110.19	15.70
-1	18	-15	103.59	10.00
-1	18	-15	115.89	11.50
-2	18	-15	70.69	7.10
2	18	15	59.19	11.10
-2	18	-15	82.49	9.90
-2	18	-15	71.79	8.30
-3	18	-15	11.30	4.40
-3	18	-15	5.40	4.20
-3	18	-15	2.80	5.10
3	18	15	-11.40	8.40
-4	18	-15	34.00	6.00
4	18	15	36.20	10.30
-4	18	-15	34.40	5.40
-4	18	-15	43.70	8.10
-5	18	-15	9.50	4.70
-5	18	-15	7.20	5.80
-5	18	-15	7.00	4.50
5	18	15	2.10	8.30
-6	18	-15	101.99	9.30
6	18	15	119.49	14.90
-6	18	-15	81.59	11.80
-6	18	-15	104.89	9.40
-7	18	-15	3.50	4.60

7	18	15	10.40	8.50
-7	18	-15	2.10	4.50
-7	18	-15	6.00	7.10
-8	18	-15	28.10	8.50
-8	18	-15	30.20	5.30
8	18	15	48.40	11.10
-8	18	-15	37.10	7.20
-7	19	15	-10.00	8.80
7	19	-15	12.50	9.90
-6	19	15	258.17	22.10
6	19	-15	323.57	23.30
5	19	-15	5.80	8.00
-5	19	15	-7.00	8.50
4	-19	-15	549.35	44.50
-4	19	15	613.94	36.60
4	19	-15	640.24	35.50
-3	19	15	64.39	11.60
3	19	-15	64.49	13.00
-2	19	15	283.07	22.60
2	19	-15	291.17	20.20
1	19	-15	321.17	20.80
-1	19	15	311.57	24.20
0	19	15	390.76	27.30
0	19	-15	406.36	24.00
-1	19	-15	216.68	15.60
1	19	15	207.58	19.90
-2	19	-15	312.37	20.00
2	19	15	367.86	25.20
-2	19	-15	385.16	21.20
-3	19	-15	246.38	16.90
-3	19	-15	289.57	16.40
3	19	15	301.97	22.50
-3	19	-15	320.77	18.10
-4	19	-15	86.29	9.80
4	19	15	78.39	12.10
-4	19	-15	65.09	8.90
-4	19	-15	67.09	9.00
-5	19	-15	403.06	20.40
5	19	15	377.86	24.90
-5	19	-15	304.27	20.40
-5	19	-15	336.07	20.60
-6	19	-15	24.60	5.70
6	19	15	31.00	10.00
-6	19	-15	32.00	5.80

-6	19	-15	11.10	6.80
-7	19	-15	368.56	20.90
7	19	15	396.16	26.20
-7	19	-15	370.56	23.00
-7	19	-15	375.36	21.10
-6	20	15	-15.70	9.50
6	20	-15	9.80	9.30
5	-20	-15	-15.80	9.90
-5	20	15	-1.40	9.50
5	20	-15	7.70	8.10
-4	20	15	11.60	10.00
4	20	-15	7.80	8.20
-3	20	15	-5.50	9.90
3	20	-15	-1.40	7.80
-2	20	15	40.70	12.10
2	20	-15	44.80	9.30
-1	20	15	19.50	10.50
1	20	-15	30.50	7.60
0	20	-15	14.60	6.80
0	20	15	4.50	9.10
1	20	15	1.30	9.30
-1	20	-15	0.20	5.90
2	20	15	-7.20	9.00
-2	20	-15	12.30	6.00
3	20	15	21.60	10.00
-3	20	-15	37.00	7.10
-4	20	-15	45.00	9.40
4	20	15	47.30	10.80
-5	20	-15	23.30	6.00
5	20	15	19.60	9.70
-5	20	-15	3.20	10.30
-6	20	-15	9.00	6.70
6	20	15	16.30	9.50
-6	20	-15	21.00	6.10
5	21	-15	9.30	9.40
-5	21	15	10.80	10.50
-4	21	15	415.56	27.90
4	21	-15	375.66	27.10
3	21	-15	8.40	8.50
-3	21	15	3.60	10.50
2	21	-15	81.49	13.80
-2	21	15	68.29	13.20
-1	21	15	136.39	18.60
1	21	-15	163.48	15.40

0	21	15	165.98	19.00
0	21	-15	144.79	14.60
-1	21	-15	105.99	12.90
1	21	15	111.79	15.80
2	21	15	65.69	12.50
-2	21	-15	55.29	10.90
3	21	15	240.38	20.90
-3	21	-15	232.28	16.80
-4	21	-15	5.10	6.20
4	21	15	10.00	9.70
5	21	15	300.27	23.80
-5	21	-15	320.07	19.60
-4	22	15	35.00	12.00
4	22	-15	36.50	11.20
3	22	-15	126.99	16.80
-3	22	15	113.29	18.90
-2	22	15	-12.00	10.10
2	22	-15	4.90	8.20
-1	22	15	136.49	19.10
1	22	-15	178.08	16.60
0	22	15	94.09	15.60
0	22	-15	74.99	13.40
-1	22	-15	51.79	9.10
1	22	15	46.90	11.90
-2	22	-15	168.08	15.00
2	22	15	132.89	17.90
3	22	15	13.80	11.30
-3	22	-15	31.90	7.40
4	22	15	67.29	13.10
-4	22	-15	95.09	13.80
-1	23	15	85.59	14.70
0	23	15	230.18	22.70
1	23	15	94.99	18.00
13	0	-16	248.18	22.70
12	0	-16	59.89	11.30
-12	0	16	75.29	10.00
11	0	-16	696.73	39.90
-11	0	16	704.83	36.10
-10	0	16	39.50	6.20
10	0	-16	35.50	8.50
9	0	-16	901.31	46.70
-9	0	16	962.90	46.60
8	0	-16	14.40	4.50
-8	0	16	10.00	4.60

8	0	-16	2.00	5.60
7	0	-16	203.18	15.50
-7	0	16	213.48	13.20
7	0	-16	211.78	12.60
6	0	-16	290.87	18.50
-6	0	16	301.07	16.80
6	0	-16	294.87	16.00
5	0	-16	1088.69	53.29
5	0	-16	1031.20	51.19
-5	0	16	1042.80	51.89
-4	0	16	906.01	45.50
4	0	-16	915.51	44.70
4	0	-16	937.71	46.60
-3	0	16	338.77	19.00
3	0	-16	349.27	18.20
3	0	-16	377.26	20.00
2	0	-16	219.58	12.80
2	0	-16	204.38	11.40
-2	0	16	201.68	17.90
-2	0	16	191.28	12.20
1	0	-16	739.93	36.80
-1	0	16	729.33	37.80
1	0	-16	767.12	37.60
-1	0	16	752.32	37.50
0	0	16	843.62	41.60
0	0	-16	834.42	41.40
0	0	-16	857.21	41.00
0	0	16	798.22	41.40
1	0	16	813.52	41.10
1	0	16	792.52	41.90
-1	0	-16	837.82	41.30
-1	0	-16	828.22	41.30
1	0	16	906.01	41.40
2	0	16	551.94	29.60
-2	0	-16	606.44	30.60
2	0	16	644.74	29.90
-2	0	-16	621.54	30.20
2	0	16	586.24	31.10
-3	0	-16	15.70	4.20
3	0	16	17.20	4.70
4	0	16	790.22	40.40
5	0	16	36.50	6.40
6	0	16	1306.67	65.19
8	0	16	924.31	48.00

-9	0	-16	50.99	8.10
-9	0	-16	62.19	11.40
-10	0	-16	219.28	18.30
-10	0	-16	251.57	15.90
-11	0	-16	7.30	6.00
-11	0	-16	15.80	8.00
-12	0	-16	485.25	30.20
-13	0	-16	68.29	11.70
13	-1	-16	-4.30	10.00
13	1	-16	15.40	11.10
12	-1	-16	93.59	16.90
12	1	-16	88.59	17.00
-12	1	16	86.99	10.80
-11	-1	16	16.60	5.50
11	-1	-16	14.00	8.20
-11	1	16	25.20	6.20
11	1	-16	14.90	9.00
10	-1	-16	28.80	7.80
-10	-1	16	30.50	5.70
10	1	-16	28.20	8.40
-10	1	16	20.20	5.80
9	-1	-16	21.80	5.50
9	-1	-16	20.50	11.30
-9	-1	16	4.20	4.60
9	1	-16	5.70	6.90
-9	1	16	14.20	5.30
-8	-1	16	82.49	8.10
8	-1	-16	78.39	11.50
8	-1	-16	75.19	8.30
-8	1	16	72.29	8.80
8	1	-16	83.49	11.50
8	1	-16	98.49	8.30
-7	-1	16	2.70	3.60
7	-1	-16	-0.20	5.00
7	-1	-16	-0.90	3.60
7	1	-16	3.20	3.50
-7	1	16	-0.30	4.10
7	1	-16	2.90	5.20
6	-1	-16	7.80	3.50
-6	-1	16	1.90	3.40
6	-1	-16	3.40	4.90
6	1	-16	0.50	4.70
-6	1	16	2.20	4.20
6	1	-16	6.10	3.20

5	-1	-16	27.70	4.90
-5	-1	16	23.70	4.10
5	-1	-16	21.50	5.20
-5	1	16	21.70	4.80
5	1	-16	21.60	3.40
5	1	-16	15.70	5.10
-4	-1	16	148.29	9.60
4	-1	-16	145.99	9.00
4	-1	-16	142.79	11.10
4	1	-16	145.79	11.10
4	1	-16	145.59	8.60
-4	1	16	145.09	10.20
3	-1	-16	84.49	6.40
3	-1	-16	82.89	8.20
-3	-1	16	83.59	7.10
3	1	-16	95.59	8.20
-3	1	16	80.59	7.70
3	1	-16	88.19	6.00
-2	-1	16	240.08	13.70
2	-1	-16	238.48	13.20
2	-1	-16	244.18	14.40
2	1	-16	252.57	14.30
2	1	-16	239.18	12.90
-2	1	16	229.78	14.10
1	-1	-16	133.49	8.40
-1	-1	16	130.39	8.90
-1	-1	16	120.59	9.30
1	-1	-16	124.19	9.20
-1	1	16	133.39	9.20
1	1	-16	127.39	8.10
1	1	-16	131.29	8.90
-1	1	16	115.89	9.60
0	-1	-16	150.48	9.40
0	-1	16	135.79	9.60
0	-1	-16	156.28	10.00
0	-1	16	154.48	10.10
0	1	-16	160.08	9.30
0	1	16	146.79	10.00
0	1	-16	151.98	9.70
0	1	16	140.39	10.20
-1	-1	-16	77.99	6.90
1	-1	16	73.49	6.20
-1	-1	-16	86.49	7.00
1	-1	16	82.99	6.50

1	-1	16	89.39	7.70
1	1	16	81.59	7.30
-1	1	-16	81.19	6.50
1	1	16	80.89	6.80
1	1	16	93.09	7.80
-1	1	-16	83.49	6.70
2	-1	16	28.40	4.90
2	-1	16	27.50	2.90
-2	-1	-16	25.70	4.30
2	-1	16	31.10	3.30
-2	-1	-16	26.00	4.80
-2	1	-16	23.70	4.10
2	1	16	26.70	4.40
2	1	16	33.80	5.30
2	1	16	19.30	4.50
-2	1	-16	36.90	4.70
-3	-1	-16	33.70	6.10
3	-1	16	39.30	6.60
3	1	16	36.40	5.60
-3	1	-16	27.30	4.50
3	1	16	36.40	4.10
3	1	16	44.20	4.90
4	-1	16	5.20	4.50
4	1	16	9.10	4.50
4	1	16	8.70	2.20
4	1	16	7.10	1.90
-4	1	-16	9.30	4.20
5	-1	16	441.96	24.40
5	1	16	442.16	24.60
6	-1	16	132.99	11.40
6	1	16	129.09	10.90
7	1	16	33.70	8.20
8	1	16	25.20	6.60
-8	1	-16	25.60	7.50
-9	-1	-16	78.69	9.50
-9	-1	-16	75.79	11.20
9	1	16	89.49	10.90
-9	1	-16	99.69	12.20
-9	1	-16	93.09	8.70
-10	-1	-16	10.10	5.50
-10	-1	-16	10.20	7.30
-10	1	-16	5.10	7.20
-10	1	-16	1.90	4.80
-11	-1	-16	42.00	9.80

-11	-1	-16	38.50	8.30
-11	1	-16	37.00	6.90
-11	1	-16	50.89	9.80
-12	-1	-16	11.40	9.10
-12	1	-16	7.70	8.50
-13	-1	-16	15.60	9.40
-13	1	-16	9.10	9.20
13	-2	-16	159.98	19.20
-13	2	16	147.79	13.80
13	2	-16	134.49	19.40
12	-2	-16	10.50	9.20
12	2	-16	24.50	9.60
-12	2	16	21.80	6.90
11	-2	-16	308.37	22.60
-11	-2	16	297.27	17.80
11	2	-16	313.77	22.90
-11	2	16	309.47	19.00
-10	-2	16	3.70	4.40
10	-2	-16	5.80	7.00
10	2	-16	4.30	7.30
-10	2	16	10.60	5.70
-9	-2	16	430.56	23.30
9	-2	-16	437.86	27.00
9	2	-16	442.76	26.90
-9	2	16	447.16	24.30
8	-2	-16	9.90	6.40
-8	-2	16	6.50	3.90
8	-2	-16	7.00	4.80
-8	2	16	0.20	4.60
8	2	-16	7.20	4.10
-7	-2	16	415.36	21.10
7	-2	-16	394.36	23.70
7	-2	-16	370.56	21.20
-7	2	16	406.56	22.20
7	2	-16	395.06	23.80
7	2	-16	411.16	20.80
-6	-2	16	174.98	10.60
6	-2	-16	152.38	10.70
6	-2	-16	163.68	13.30
-6	2	16	169.08	12.10
6	2	-16	180.58	10.20
6	2	-16	171.08	13.40
5	-2	-16	1208.38	59.69
5	-2	-16	1243.78	61.59

-5	-2	16	1243.78	59.79
5	2	-16	1203.28	59.29
5	2	-16	1230.08	61.59
-5	2	16	1259.17	60.89
-4	-2	16	46.60	5.40
4	-2	-16	41.60	5.20
4	-2	-16	47.60	7.60
-4	2	16	39.20	6.70
4	2	-16	41.30	7.40
4	2	-16	50.29	4.60
3	-2	-16	328.37	17.20
-3	-2	16	324.87	17.40
3	-2	-16	342.17	18.90
3	2	-16	313.07	16.70
3	2	-16	333.37	18.70
-3	2	16	328.97	18.50
-2	-2	16	69.89	6.40
2	-2	-16	74.29	6.10
2	-2	-16	75.59	7.60
2	2	-16	73.99	7.10
-2	2	16	67.49	7.50
2	2	-16	75.39	5.50
-1	-2	16	157.08	9.90
1	-2	-16	158.58	10.50
1	-2	-16	154.18	9.50
-1	-2	16	139.19	10.30
1	2	-16	160.88	10.00
-1	2	16	151.48	11.00
1	2	-16	155.48	9.10
-1	2	16	147.99	10.70
0	-2	-16	1383.46	66.49
0	-2	16	1191.38	65.59
0	-2	16	1382.26	66.19
0	-2	-16	1396.96	65.99
0	2	-16	1414.06	65.99
0	2	16	1311.97	66.39
0	2	16	1336.07	66.69
0	2	-16	1458.95	65.79
-1	-2	-16	218.08	12.50
1	-2	16	200.68	11.20
1	-2	16	230.78	11.60
-1	-2	-16	205.68	12.60
1	-2	16	198.28	12.70
1	2	16	210.38	12.50

1	2	16	196.08	13.20
-1	2	-16	203.48	12.20
-1	2	-16	211.78	12.00
1	2	16	246.58	13.20
-2	-2	-16	842.02	42.60
2	-2	16	873.11	43.00
-2	-2	-16	831.32	42.60
-2	2	-16	850.51	41.80
-2	2	-16	861.31	42.40
2	2	16	840.42	42.30
2	2	16	864.01	43.40
2	2	16	901.41	42.70
3	-2	16	143.89	10.80
-3	-2	-16	148.49	10.70
3	2	16	160.88	11.60
-3	2	-16	155.78	10.30
3	2	16	177.08	10.20
4	-2	16	863.41	43.60
-4	2	-16	859.01	43.00
4	2	16	816.72	43.60
4	2	16	892.81	42.00
5	-2	16	21.80	5.20
5	2	16	12.40	5.10
5	2	16	19.00	3.20
6	-2	16	769.22	39.10
6	2	16	757.02	39.30
6	2	16	732.73	36.60
7	-2	16	7.90	6.20
-7	2	-16	-1.10	6.30
7	2	16	0.90	5.40
7	2	16	5.50	2.60
8	2	16	106.99	11.80
-8	2	-16	108.69	12.10
-9	-2	-16	32.30	6.40
-9	2	-16	33.90	7.50
-9	2	-16	35.00	6.60
9	2	16	44.30	8.30
-10	-2	-16	314.57	19.30
-10	-2	-16	298.37	21.40
-10	2	-16	298.07	17.80
-10	2	-16	302.37	21.00
-11	-2	-16	65.89	11.40
-11	2	-16	70.59	10.30
-11	2	-16	102.99	13.70

-12	-2	-16	288.87	21.50
-12	2	-16	244.88	19.50
-13	-2	-16	11.40	9.40
-13	2	-16	18.90	9.60
13	-3	-16	65.59	14.10
-13	3	16	54.19	8.80
13	3	-16	52.49	12.80
12	-3	-16	305.67	24.20
12	3	-16	318.77	25.00
-12	3	16	329.77	20.20
11	-3	-16	27.80	9.40
11	3	-16	43.00	9.90
10	-3	-16	465.65	35.50
-10	-3	16	550.34	28.20
10	3	-16	549.25	32.60
-10	3	16	606.54	29.70
-9	-3	16	10.10	4.00
9	-3	-16	11.20	6.60
-9	3	16	1.10	5.40
9	3	-16	14.10	7.10
8	-3	-16	863.21	44.80
-8	-3	16	827.72	41.70
-8	3	16	890.21	43.20
8	3	-16	838.72	41.70
8	3	-16	812.02	45.80
7	-3	-16	36.80	7.10
-7	-3	16	41.20	5.50
7	-3	-16	32.40	5.30
7	3	-16	40.40	5.60
-7	3	16	41.50	6.50
7	3	-16	45.90	8.00
6	-3	-16	807.62	41.20
-6	-3	16	796.22	38.70
6	-3	-16	720.23	39.00
6	3	-16	785.42	38.40
-6	3	16	842.22	40.30
6	3	-16	766.92	41.10
5	-3	-16	196.88	11.90
-5	-3	16	189.18	11.40
5	-3	-16	208.58	14.40
5	3	-16	196.98	14.10
5	3	-16	197.58	11.00
-5	3	16	213.08	13.70
-4	-3	16	918.61	46.70

4	-3	-16	979.10	48.60
4	-3	-16	905.11	46.80
4	3	-16	954.20	46.30
4	3	-16	1005.00	48.50
-4	3	16	996.30	48.40
3	-3	-16	8.40	3.10
3	-3	-16	7.30	4.10
-3	-3	16	5.80	3.00
-3	3	16	7.20	4.50
3	3	-16	9.30	4.00
3	3	-16	11.30	2.30
-2	-3	16	201.78	11.70
2	-3	-16	213.68	13.10
2	-3	-16	201.78	11.80
2	3	-16	202.58	11.00
-2	3	16	188.98	13.10
2	3	-16	213.68	12.60
1	-3	-16	635.14	31.90
1	-3	-16	616.64	31.00
-1	-3	16	537.65	31.20
-1	-3	16	607.14	31.00
1	3	-16	638.44	31.30
-1	3	16	610.04	32.00
-1	3	16	685.83	36.40
1	3	-16	626.24	30.50
0	-3	-16	378.66	20.10
0	-3	16	344.57	19.50
0	-3	-16	388.96	20.80
0	-3	16	365.16	20.10
0	3	-16	382.66	19.60
0	3	16	381.46	21.10
0	3	-16	382.96	19.90
0	3	16	434.16	22.30
-1	-3	-16	1023.90	51.39
1	-3	16	1040.60	49.70
-1	-3	-16	1055.49	51.09
1	-3	16	934.51	49.60
1	-3	16	1041.80	51.19
1	3	16	970.40	51.69
-1	3	-16	1045.50	50.69
-1	3	-16	1031.40	50.39
1	3	16	1156.18	53.39
1	3	16	1091.79	51.69
-2	-3	-16	453.75	23.40

2	-3	16	425.56	23.50
-2	-3	-16	453.45	23.50
2	3	16	474.75	23.80
-2	3	-16	439.66	23.00
-2	3	-16	438.06	22.20
2	3	16	417.86	24.00
3	-3	16	718.43	37.50
3	3	16	698.13	38.00
3	3	16	797.42	37.30
-3	3	-16	737.23	37.00
4	-3	16	61.09	7.80
4	3	16	72.59	7.00
4	3	16	61.49	8.10
-4	3	-16	61.99	7.30
5	-3	16	820.62	43.40
5	3	16	845.92	43.80
5	3	16	886.51	42.10
6	-3	16	11.30	5.30
6	3	16	5.80	3.50
-6	3	-16	1.70	5.40
6	3	16	10.40	5.60
7	3	16	459.05	23.50
7	3	16	450.55	26.00
-7	3	-16	458.85	26.40
8	3	16	3.30	3.50
8	3	16	3.00	5.80
-8	3	-16	2.90	6.20
-9	-3	-16	115.69	11.20
9	3	16	113.39	12.80
9	3	16	101.19	8.60
-9	3	-16	113.99	11.80
-10	-3	-16	159.78	13.70
-10	3	-16	145.59	10.90
-10	3	-16	129.79	14.60
10	3	16	157.28	15.00
-11	-3	-16	424.26	27.10
-11	3	-16	423.36	26.50
-11	3	-16	409.16	23.20
-12	-3	-16	11.10	9.10
-12	3	-16	15.60	8.70
12	-4	-16	29.80	9.90
-12	4	16	18.90	7.20
12	4	-16	17.10	10.00
11	-4	-16	19.30	9.30

-11	4	16	19.30	6.80
11	4	-16	17.30	8.70
-10	-4	16	12.10	4.00
10	4	-16	2.30	7.60
-10	4	16	14.70	6.00
-9	-4	16	39.30	5.90
9	-4	-16	35.50	7.90
-9	4	16	25.30	6.40
9	4	-16	31.40	8.00
8	-4	-16	18.70	6.60
-8	-4	16	11.50	3.50
8	4	-16	10.10	6.60
-8	4	16	7.90	5.60
-7	-4	16	15.30	3.20
7	-4	-16	14.70	6.30
7	4	-16	9.10	3.10
-7	4	16	8.70	5.40
6	-4	-16	115.39	9.50
6	-4	-16	127.59	11.90
-6	-4	16	136.99	8.30
6	4	-16	136.59	8.10
-6	4	16	141.89	11.50
6	4	-16	125.89	12.00
5	-4	-16	20.20	4.10
5	-4	-16	22.70	5.80
-5	-4	16	24.50	4.20
5	4	-16	21.70	3.10
-5	4	16	21.70	5.80
5	4	-16	16.70	5.20
4	-4	-16	15.20	4.80
4	-4	-16	13.60	3.50
-4	-4	16	19.50	3.10
4	4	-16	20.40	3.40
4	4	-16	15.70	4.70
-4	4	16	9.20	4.90
3	-4	-16	38.70	7.20
-3	-4	16	39.00	4.90
3	-4	-16	51.29	5.50
3	4	-16	43.60	3.90
3	4	-16	40.70	6.70
-3	4	16	34.50	6.20
-2	-4	16	5.80	2.90
2	-4	-16	2.00	3.80
2	-4	-16	4.30	2.90

2	4	-16	6.50	2.10
-2	4	16	3.90	4.40
2	4	-16	5.20	3.40
1	-4	-16	5.50	3.20
1	-4	-16	10.20	4.20
-1	-4	16	5.60	2.90
-1	-4	16	0.40	3.60
1	4	-16	7.80	2.40
-1	4	16	7.60	4.30
1	4	-16	7.00	3.20
0	-4	16	30.20	4.20
0	-4	-16	23.20	4.30
0	-4	16	22.20	4.20
0	-4	-16	32.10	6.20
0	4	-16	23.30	3.70
0	4	-16	24.70	3.60
0	4	16	22.80	5.20
-1	-4	-16	190.88	12.00
-1	-4	-16	180.38	11.40
1	-4	16	192.78	11.60
1	4	16	183.48	12.60
1	4	16	177.78	12.50
-1	4	-16	187.78	10.50
-1	4	-16	174.68	10.80
-2	-4	-16	201.68	13.20
2	-4	16	195.38	12.30
-2	4	-16	187.58	11.50
-2	4	-16	192.58	10.40
2	4	16	192.28	13.20
3	-4	16	5.30	4.00
3	4	16	8.50	4.50
-3	4	-16	4.30	3.40
3	4	16	2.70	4.00
4	-4	16	51.19	7.50
-4	4	-16	48.30	7.00
4	4	16	47.10	7.40
4	4	16	51.99	7.30
5	-4	16	8.50	4.70
5	4	16	6.00	5.20
5	4	16	-3.80	3.80
-5	4	-16	2.60	4.90
6	4	16	49.10	8.50
6	4	16	54.49	7.30
-6	4	-16	58.19	9.30

7	4	16	15.70	4.60
-7	4	-16	16.10	6.40
7	4	16	7.10	5.80
8	4	16	8.60	6.50
-8	4	-16	2.60	6.00
8	4	16	10.80	4.10
-9	-4	-16	28.40	6.60
-9	4	-16	26.50	7.20
9	4	16	29.30	5.20
9	4	16	29.40	7.50
-10	-4	-16	45.70	10.50
-10	4	-16	31.00	8.10
10	4	16	37.50	8.50
-10	4	-16	31.00	4.90
-11	-4	-16	4.00	8.90
-11	4	-16	5.80	7.30
-12	-4	-16	0.40	9.10
-12	4	-16	18.80	8.70
-12	4	-16	8.10	5.80
12	-5	-16	373.26	26.90
-12	5	16	383.66	22.90
12	5	-16	341.07	27.10
11	-5	-16	4.80	9.00
-11	5	16	7.00	6.70
11	5	-16	1.60	8.30
-10	5	16	560.94	30.80
10	5	-16	569.44	33.50
-9	-5	16	-2.70	3.00
9	-5	-16	3.20	7.00
9	5	-16	-3.60	6.70
-9	5	16	-8.80	5.60
-8	-5	16	113.39	7.40
8	-5	-16	96.29	12.50
8	5	-16	90.99	12.90
-8	5	16	116.49	11.20
-7	-5	16	4.30	3.20
7	-5	-16	6.30	5.50
7	5	-16	4.20	5.50
7	5	-16	6.20	2.80
-7	5	16	-2.10	5.30
6	-5	-16	726.53	37.20
6	5	-16	599.24	38.20
6	5	-16	694.53	33.70
-6	5	16	749.33	36.70

5	-5	-16	310.87	19.40
5	-5	-16	307.97	17.30
5	5	-16	303.97	19.30
5	5	-16	325.57	16.00
-5	5	16	329.47	19.50
4	-5	-16	316.67	18.10
4	-5	-16	350.56	20.20
-4	-5	16	351.76	17.80
4	5	-16	332.97	19.80
-4	5	16	349.07	20.20
4	5	-16	334.47	16.90
-3	-5	16	61.49	5.60
3	-5	-16	54.59	8.00
3	-5	-16	59.99	6.10
3	5	-16	63.19	7.50
-3	5	16	58.49	8.60
2	-5	-16	500.45	26.30
-2	-5	16	504.45	24.50
2	-5	-16	446.86	24.70
-2	5	16	492.65	26.50
2	5	-16	464.35	23.70
2	5	-16	493.95	25.50
-1	-5	16	258.17	14.10
1	-5	-16	265.57	15.70
1	-5	-16	256.57	14.40
-1	5	16	244.58	15.90
1	5	-16	248.88	14.50
1	5	-16	255.77	13.40
0	-5	16	1641.64	76.99
0	-5	-16	1553.84	76.99
0	-5	-16	1596.84	77.89
0	-5	16	1488.85	76.19
0	5	16	1595.54	78.19
0	5	-16	1668.63	76.79
0	5	-16	1587.64	76.39
-1	-5	-16	669.53	33.50
-1	-5	-16	644.44	33.90
1	-5	16	659.43	33.20
-1	5	-16	663.33	32.40
-1	5	-16	669.33	32.70
1	5	16	608.34	34.20
1	5	16	681.53	34.40
2	-5	16	522.05	26.80
2	5	16	487.35	27.60

-2	5	-16	495.85	25.20
-2	5	-16	504.95	26.20
2	5	16	559.94	27.80
3	-5	16	349.57	19.20
3	5	16	322.47	20.00
3	5	16	342.87	23.60
-3	5	-16	353.06	18.60
4	5	16	193.08	14.10
-4	5	-16	190.08	12.60
4	5	16	238.68	13.70
-4	5	-16	177.38	13.50
-5	5	-16	1112.09	58.39
5	5	16	1118.69	58.79
5	5	16	1278.77	58.19
-6	5	-16	219.38	16.10
6	5	16	273.47	15.40
6	5	16	224.08	21.20
7	5	16	2046.30	93.49
-7	5	-16	1839.02	94.69
7	5	16	1879.81	94.79
8	5	16	5.00	6.10
-8	5	-16	-2.30	5.90
8	5	16	-1.10	4.80
-9	-5	-16	478.45	28.10
-9	5	-16	499.05	28.70
9	5	16	521.35	29.20
10	5	16	37.40	8.80
-10	5	-16	32.00	8.60
-11	5	-16	592.94	33.70
-11	5	-16	563.94	29.80
11	5	16	587.64	33.90
-12	-5	-16	20.00	10.00
-12	5	-16	14.90	6.00
-12	5	-16	20.20	9.10
12	-6	-16	15.40	9.50
-12	6	16	31.30	7.90
12	6	-16	26.10	10.90
11	-6	-16	53.29	10.50
-11	6	16	70.39	10.90
11	6	-16	49.99	10.90
10	6	-16	-3.30	8.40
-10	6	16	0.70	6.20
9	-6	-16	15.30	7.90
9	6	-16	15.80	7.80

8	-6	-16	8.40	6.10
-8	-6	16	9.00	2.50
8	6	-16	12.30	7.20
-8	6	16	-1.30	6.00
-7	-6	16	16.20	2.50
7	-6	-16	17.20	6.50
-7	6	16	15.30	6.30
7	6	-16	14.10	6.50
7	6	-16	17.60	2.60
-6	-6	16	61.69	4.50
6	-6	-16	59.09	10.10
-6	6	16	56.49	9.60
6	6	-16	52.39	9.60
6	6	-16	58.59	4.30
5	-6	-16	31.70	6.60
-5	-6	16	29.80	3.70
5	6	-16	25.40	6.20
5	6	-16	25.90	3.20
-5	6	16	31.80	6.70
-4	-6	16	25.60	3.10
4	-6	-16	21.90	5.40
-4	6	16	12.90	6.40
4	6	-16	11.80	4.80
4	6	-16	22.60	2.90
3	-6	-16	13.90	4.30
3	-6	-16	12.20	4.90
3	6	-16	13.60	4.20
3	6	-16	12.20	2.00
-3	6	16	12.90	5.50
2	-6	-16	4.60	3.50
-2	-6	16	4.90	2.90
2	-6	-16	0.90	3.90
2	6	-16	2.10	3.50
-2	6	16	0.50	4.90
1	-6	-16	299.47	16.50
-1	-6	16	302.67	15.90
1	-6	-16	305.97	17.80
1	6	-16	292.97	15.10
-1	6	16	260.57	17.80
1	6	-16	301.17	16.40
0	-6	-16	114.49	9.90
0	-6	16	121.29	8.20
0	-6	16	103.39	7.20
0	6	-16	113.99	7.40

0	6	16	135.79	10.90
0	6	16	110.59	10.30
0	6	-16	113.59	8.10
-1	-6	-16	64.69	8.10
1	-6	16	78.89	7.10
-1	6	-16	57.59	6.00
1	6	16	68.69	8.70
1	6	16	74.39	9.00
-1	6	-16	81.29	5.70
2	6	16	31.00	6.20
-2	6	-16	29.00	5.40
2	6	16	25.70	5.50
-2	6	-16	27.50	5.00
-2	6	-16	27.90	3.30
3	6	16	402.36	23.20
-3	6	-16	379.16	22.10
3	6	16	439.56	23.10
-3	6	-16	403.66	21.60
-4	6	-16	51.19	7.30
-4	6	-16	56.89	8.30
4	6	16	56.99	9.10
4	6	16	63.39	8.80
5	6	16	63.69	9.50
-5	6	-16	62.39	8.90
5	6	16	63.89	8.90
6	6	16	158.68	11.90
6	6	16	127.39	12.40
-6	6	-16	134.49	12.10
7	6	16	151.98	13.70
-7	6	-16	151.88	14.00
7	6	16	169.48	12.80
8	6	16	87.69	10.50
8	6	16	78.79	11.40
-8	6	-16	83.59	11.40
-9	-6	-16	16.40	6.60
9	6	16	20.40	7.40
-9	6	-16	21.60	7.20
10	6	16	221.68	18.20
-10	6	-16	230.38	17.90
11	6	16	20.50	8.50
-11	6	-16	8.60	8.30
-11	6	-16	-3.80	4.10
-12	-6	-16	66.79	12.10
-12	6	-16	93.19	14.50

-12	6	-16	96.29	9.90
12	-7	-16	183.08	19.40
12	7	-16	172.78	20.60
-12	7	16	192.28	16.40
11	-7	-16	8.40	8.70
11	7	-16	-2.70	9.70
-11	7	16	3.50	7.00
10	7	-16	283.67	21.70
-10	7	16	290.17	19.30
9	-7	-16	11.20	7.80
9	7	-16	-3.40	7.10
-9	7	16	-7.20	5.90
8	-7	-16	167.38	14.90
8	7	-16	162.08	15.40
-8	7	16	154.48	19.90
7	-7	-16	60.99	10.70
-7	7	16	47.80	7.90
7	7	-16	57.09	9.00
6	-7	-16	391.56	23.40
-6	7	16	407.06	23.50
6	7	-16	377.76	23.50
5	-7	-16	168.48	13.20
-5	7	16	154.48	13.40
4	-7	-16	268.47	17.10
4	7	-16	255.67	12.60
-4	7	16	254.67	17.40
4	7	-16	251.87	16.70
3	-7	-16	42.60	8.10
3	7	-16	41.40	6.10
3	7	-16	41.20	3.10
-3	7	16	29.00	7.10
2	-7	-16	152.48	11.90
2	7	-16	153.18	10.60
-2	7	16	131.99	12.20
2	7	-16	150.48	8.00
1	-7	-16	644.34	33.20
1	7	-16	610.84	30.50
1	7	-16	643.84	31.20
1	7	-16	604.74	31.80
-1	7	16	628.44	33.60
0	-7	16	306.77	16.80
0	-7	-16	351.76	19.60
0	7	-16	335.47	17.70
0	7	16	355.86	20.20

0	7	-16	322.87	17.70
0	7	16	321.37	19.90
0	7	-16	329.57	17.00
-1	-7	-16	268.77	17.00
1	7	16	298.07	17.90
-1	7	-16	262.17	15.00
-1	7	-16	277.67	15.80
1	7	16	276.67	17.70
-1	7	-16	297.27	14.80
-2	7	-16	524.05	26.80
-2	7	-16	506.45	27.30
2	7	16	542.55	28.90
2	7	16	528.05	29.00
-2	7	-16	530.75	25.70
-3	7	-16	250.37	14.50
-3	7	-16	246.78	15.30
3	7	16	256.57	16.40
3	7	16	246.48	16.40
-4	7	-16	106.49	10.40
4	7	16	128.19	11.00
-4	7	-16	121.59	9.90
4	7	16	133.79	11.60
5	7	16	790.82	40.20
-5	7	-16	727.13	39.00
-6	7	-16	9.90	6.00
6	7	16	18.40	6.00
6	7	16	11.90	5.80
-7	7	-16	462.65	28.70
7	7	16	512.15	27.40
7	7	16	491.25	28.20
-8	7	-16	3.80	6.10
8	7	16	11.40	6.80
-9	7	-16	178.08	15.00
9	7	16	164.38	14.70
-10	7	-16	22.00	8.10
10	7	16	22.10	8.50
-11	7	-16	274.37	20.30
11	7	16	252.27	20.40
-11	7	-16	282.77	14.90
-12	-7	-16	21.30	10.50
-12	7	-16	29.60	9.80
-12	7	-16	9.60	5.00
12	7	16	19.00	9.80
-12	8	16	-1.70	7.80

12	8	-16	1.90	9.70
11	-8	-16	329.77	24.80
11	8	-16	349.87	25.90
-11	8	16	351.16	22.30
-10	8	16	22.00	7.40
10	8	-16	15.90	9.10
9	-8	-16	395.46	26.00
9	8	-16	407.96	26.30
-9	8	16	415.06	24.20
8	-8	-16	49.00	8.60
-8	8	16	55.99	10.40
8	8	-16	46.10	8.80
7	-8	-16	428.16	25.80
-7	8	16	439.56	30.00
7	8	-16	435.16	26.30
6	-8	-16	232.78	16.70
6	8	-16	217.08	16.60
-6	8	16	220.18	16.50
5	-8	-16	399.96	23.60
-5	8	16	384.66	23.50
5	8	-16	391.56	23.30
4	-8	-16	115.59	10.60
4	8	-16	66.69	11.80
-4	8	16	87.19	11.70
3	-8	-16	92.99	10.20
-3	8	16	71.39	10.80
3	8	-16	69.59	8.90
2	-8	-16	61.59	8.90
2	8	-16	53.39	7.40
-2	8	16	49.00	8.10
2	8	-16	65.99	4.10
2	8	-16	64.49	4.90
1	-8	-16	560.14	30.20
1	8	-16	566.84	27.80
1	8	-16	580.04	28.80
-1	8	16	510.95	30.40
0	-8	-16	276.27	16.70
0	8	-16	265.07	14.60
0	8	-16	257.47	14.50
0	8	16	259.67	17.50
0	8	16	241.18	17.20
0	8	-16	261.87	13.80
-1	-8	-16	266.17	16.40
-1	8	-16	249.38	14.50

1	8	16	265.97	17.30
-1	8	-16	269.37	13.90
-1	8	-16	249.58	13.90
1	8	16	231.78	16.90
-2	8	-16	500.65	23.90
-2	8	-16	493.95	25.00
-2	8	-16	469.15	25.50
2	8	16	479.75	27.40
2	8	16	485.85	27.30
3	8	16	163.28	13.30
3	8	16	190.38	13.70
-3	8	-16	152.78	10.90
-3	8	-16	171.58	11.80
-4	8	-16	307.57	18.60
4	8	16	374.56	21.00
4	8	16	332.37	20.60
-4	8	-16	318.07	19.20
5	8	16	69.79	10.30
-5	8	-16	49.99	9.00
5	8	16	54.79	9.60
6	8	16	410.86	22.70
6	8	16	349.57	22.50
-6	8	-16	349.67	21.70
7	8	16	31.50	7.90
7	8	16	44.20	7.40
8	8	16	316.47	20.70
-8	8	-16	299.67	20.10
-9	8	-16	1.20	6.70
9	8	16	4.00	7.10
-10	8	-16	183.28	16.00
10	8	16	162.88	15.30
11	8	16	9.30	8.80
-11	8	-16	11.50	7.90
12	8	16	225.08	19.50
-12	8	-16	214.68	19.50
-12	8	-16	218.88	13.20
12	9	-16	1.20	10.20
-12	9	16	0.20	7.80
11	-9	-16	15.00	9.40
11	9	-16	29.50	10.30
-11	9	16	27.60	8.50
10	9	-16	24.20	10.30
-10	9	16	24.90	7.80
9	-9	-16	7.50	7.70

-9	9	16	-4.90	7.00
9	9	-16	6.00	7.70
8	-9	-16	71.89	12.30
-8	9	16	59.69	11.00
8	9	-16	47.10	9.50
7	-9	-16	6.50	6.20
-7	9	16	-3.90	6.50
7	9	-16	8.60	6.40
6	-9	-16	36.00	7.50
6	9	-16	17.80	6.80
-6	9	16	17.60	12.60
5	-9	-16	32.50	6.70
5	9	-16	19.30	6.20
-5	9	16	23.80	7.90
4	-9	-16	23.20	6.30
-4	9	16	21.20	7.00
4	9	-16	19.40	5.90
3	-9	-16	2.20	4.80
3	9	-16	-3.10	4.70
-3	9	16	-12.20	5.30
2	-9	-16	190.98	13.90
2	9	-16	145.49	15.60
2	9	-16	196.38	9.50
2	9	-16	184.88	9.20
-2	9	16	168.08	14.80
1	-9	-16	185.88	13.20
1	9	-16	155.88	11.20
1	9	-16	174.58	9.10
-1	9	16	153.08	13.80
0	-9	-16	8.40	4.90
0	9	16	-1.20	5.80
0	9	16	-1.00	5.40
0	9	-16	6.40	2.40
0	9	-16	4.20	3.40
0	9	-16	10.90	3.20
-1	9	-16	47.30	5.30
1	9	16	46.50	7.80
-1	9	-16	48.40	5.30
1	9	16	46.50	7.60
-1	9	-16	50.49	6.20
2	9	16	12.90	6.20
-2	9	-16	23.10	3.60
-2	9	-16	15.50	4.40
-2	9	-16	18.10	3.90

2	9	16	16.50	5.80
-3	9	-16	319.57	17.90
3	9	16	317.57	20.20
3	9	16	326.37	20.60
-3	9	-16	330.07	18.50
4	9	16	1.90	6.00
-4	9	-16	4.40	4.90
-4	9	-16	-1.40	4.30
4	9	16	4.90	6.80
5	9	16	27.50	6.80
-5	9	-16	39.50	6.50
5	9	16	24.90	6.60
-6	9	-16	76.49	10.20
6	9	16	74.09	11.10
6	9	16	67.69	11.60
7	9	16	68.39	11.40
8	9	16	18.10	7.20
-8	9	-16	11.10	6.70
9	9	16	13.30	8.00
-9	9	-16	9.80	7.20
-10	9	-16	31.20	8.40
10	9	16	51.09	10.10
-11	9	-16	63.79	10.50
11	9	16	55.59	9.90
12	9	16	7.00	9.40
11	-10	-16	700.93	42.70
-11	10	16	793.22	40.70
11	10	-16	746.03	43.50
-10	10	16	35.80	8.80
10	10	-16	37.50	10.50
9	-10	-16	431.76	28.60
-9	10	16	462.85	27.10
9	10	-16	471.95	29.30
8	-10	-16	27.80	8.20
-8	10	16	25.10	8.10
8	10	-16	32.70	8.10
7	-10	-16	432.36	26.90
7	10	-16	455.15	27.20
-7	10	16	448.66	26.50
6	-10	-16	181.18	15.20
6	10	-16	181.98	15.50
-6	10	16	190.78	15.80
5	-10	-16	772.12	42.50
5	10	-16	833.42	42.50

4	-10	-16	553.84	30.70
-4	10	16	563.54	31.40
4	10	-16	524.15	29.90
3	-10	-16	124.49	11.80
-3	10	16	96.19	13.00
3	10	-16	136.89	11.10
2	-10	-16	105.89	10.90
-2	10	16	93.29	12.40
2	10	-16	107.89	9.60
1	-10	-16	469.85	25.70
-1	10	16	410.66	26.10
1	10	-16	436.76	21.70
1	10	-16	454.15	22.00
0	-10	-16	438.16	24.80
0	10	-16	435.86	21.40
0	10	-16	395.46	23.20
0	10	-16	461.35	21.90
0	10	16	399.96	25.30
1	10	16	276.27	18.40
-1	10	-16	266.17	14.40
-1	10	-16	247.08	14.00
-1	10	-16	262.17	14.60
1	10	16	247.18	17.30
-2	10	-16	852.41	42.20
-2	10	-16	842.62	41.10
-2	10	-16	825.22	41.80
2	10	16	872.01	44.90
2	10	16	843.02	44.80
-3	10	-16	68.79	8.10
3	10	16	68.79	13.60
-3	10	-16	57.89	7.00
3	10	16	63.39	10.80
-4	10	-16	482.45	25.60
4	10	16	448.56	26.90
-4	10	-16	479.35	25.30
4	10	16	479.95	27.70
5	10	16	122.89	13.20
5	10	16	117.19	12.60
-5	10	-16	123.59	11.20
6	10	16	775.22	42.90
-6	10	-16	795.72	42.00
6	10	16	867.01	43.70
-7	10	-16	7.70	7.50
7	10	16	-0.50	6.70

-8	10	-16	321.87	21.30
8	10	16	344.17	22.40
9	10	16	44.60	9.30
-9	10	-16	46.20	9.30
-10	10	-16	247.88	18.90
10	10	16	218.98	18.20
11	10	16	29.00	9.60
-11	10	-16	14.10	8.80
11	11	-16	26.30	10.80
-11	11	16	35.60	9.20
10	-11	-16	44.70	14.70
-10	11	16	23.00	8.30
10	11	-16	29.10	10.30
9	-11	-16	16.00	9.30
-9	11	16	7.10	7.80
9	11	-16	19.70	9.10
8	-11	-16	20.30	8.30
-8	11	16	13.90	7.60
8	11	-16	19.40	8.90
7	-11	-16	27.50	8.00
7	11	-16	34.30	7.80
-7	11	16	19.90	8.10
6	-11	-16	11.80	6.60
-6	11	16	-2.60	7.80
6	11	-16	16.80	6.90
5	-11	-16	7.50	6.10
-5	11	16	-4.60	7.10
5	11	-16	2.40	5.70
4	-11	-16	61.59	10.70
4	11	-16	59.19	9.80
-4	11	16	48.10	9.80
3	-11	-16	-4.40	5.30
3	11	-16	4.00	4.90
2	-11	-16	183.38	14.50
-2	11	16	174.88	17.10
2	11	-16	178.28	12.80
1	-11	-16	72.59	10.00
1	11	-16	67.39	4.60
-1	11	16	47.10	9.50
1	11	-16	62.79	7.90
1	11	-16	63.49	4.30
-1	11	16	72.59	11.80
0	-11	-16	19.20	5.90
0	11	16	7.70	7.80

0	11	-16	14.20	2.80
0	11	-16	16.20	3.30
0	11	-16	16.50	4.20
-1	11	-16	74.19	8.60
-1	11	-16	70.19	6.30
-1	11	-16	78.79	6.90
-2	11	-16	269.17	15.80
-2	11	-16	294.67	16.40
-2	11	-16	290.47	15.90
2	11	16	283.67	19.70
2	11	16	303.97	20.00
3	11	16	97.69	12.00
-3	11	-16	100.19	8.60
-3	11	-16	119.79	9.60
3	11	16	105.49	12.60
-4	11	-16	20.60	5.50
4	11	16	11.30	6.70
-4	11	-16	21.70	5.30
4	11	16	20.00	7.20
5	11	16	271.37	19.00
-5	11	-16	251.77	16.70
5	11	16	260.57	18.50
-6	11	-16	121.49	11.70
6	11	16	109.59	13.10
7	11	16	13.50	7.30
-7	11	-16	14.80	6.70
-8	11	-16	12.80	7.20
8	11	16	4.00	7.30
9	11	16	94.19	13.70
-9	11	-16	118.09	13.40
10	11	16	-6.10	7.80
-10	11	-16	4.40	7.90
11	11	16	61.09	11.60
-11	11	-16	60.39	11.00
-11	12	16	273.27	20.80
11	12	-16	280.87	25.00
10	-12	-16	25.10	10.50
10	12	-16	14.70	10.20
-10	12	16	6.50	8.30
9	12	-16	233.28	20.50
-9	12	16	219.68	18.00
8	-12	-16	8.30	7.70
-8	12	16	-3.30	7.80
8	12	-16	18.20	8.70

7	-12	-16	248.48	19.70
7	12	-16	261.87	19.90
-7	12	16	266.67	19.50
6	-12	-16	123.29	13.80
6	12	-16	125.89	13.70
-6	12	16	92.99	14.00
5	-12	-16	319.07	21.30
5	12	-16	331.87	21.30
-5	12	16	323.07	22.10
4	-12	-16	125.39	13.10
-4	12	16	77.59	13.70
4	12	-16	114.39	11.90
3	-12	-16	188.08	15.10
3	12	-16	163.58	13.40
-3	12	16	170.88	16.10
2	-12	-16	129.79	12.60
-2	12	16	98.99	14.00
2	12	-16	124.29	10.90
1	-12	-16	256.47	17.50
1	12	-16	233.88	12.00
-1	12	16	209.28	18.00
1	12	-16	249.48	15.20
-1	12	16	274.97	19.40
1	12	-16	231.48	12.00
0	12	16	330.37	21.50
0	12	-16	301.27	17.70
0	12	16	265.77	20.80
0	12	-16	330.67	16.20
-1	12	-16	115.79	8.30
-1	12	-16	127.39	9.30
-1	12	-16	136.19	8.80
1	12	16	125.39	13.30
2	12	16	190.98	15.80
2	12	16	179.68	17.80
-2	12	-16	193.48	11.50
-2	12	-16	191.18	11.90
-2	12	-16	168.48	10.90
-3	12	-16	37.50	4.90
-3	12	-16	34.30	3.80
3	12	16	19.50	7.10
-3	12	-16	36.60	5.30
-3	12	-16	42.40	7.50
3	12	16	35.20	8.00
-4	12	-16	365.76	20.90

-4	12	-16	386.86	21.20
4	12	16	375.36	24.10
4	12	16	360.46	23.50
5	12	16	9.70	6.30
-5	12	-16	1.10	5.40
6	12	16	386.96	25.10
-6	12	-16	408.26	23.30
7	12	16	33.70	8.90
-7	12	-16	30.80	7.60
8	12	16	287.17	21.10
-8	12	-16	287.37	19.60
-9	12	-16	11.50	7.70
9	12	16	12.90	8.60
10	12	16	235.08	20.10
-10	12	-16	270.47	19.90
-11	12	-16	12.00	9.30
11	12	16	19.20	9.80
10	-13	-16	408.56	31.20
10	13	-16	480.35	31.40
-10	13	16	485.25	28.30
9	13	-16	5.10	9.20
-9	13	16	-6.00	8.30
8	-13	-16	246.98	21.00
8	13	-16	266.57	21.10
-8	13	16	273.87	20.00
7	-13	-16	-2.20	7.70
-7	13	16	-3.50	8.00
7	13	-16	2.30	7.50
6	-13	-16	313.27	22.20
6	13	-16	318.97	22.00
-6	13	16	343.27	22.50
5	-13	-16	124.49	13.70
5	13	-16	110.89	13.30
-5	13	16	106.69	14.60
4	-13	-16	429.86	26.60
-4	13	16	452.15	27.70
4	13	-16	439.36	25.90
3	-13	-16	71.89	10.30
3	13	-16	70.19	10.30
-3	13	16	74.49	11.30
2	-13	-16	288.27	20.40
2	13	-16	324.37	19.10
-2	13	16	311.17	21.90
1	-13	-16	159.98	14.60

-1	13	16	147.19	15.80
1	13	-16	186.48	12.80
-1	13	16	180.58	17.00
0	13	16	285.97	20.60
0	13	16	245.88	19.60
0	13	-16	268.47	14.30
0	13	-16	294.47	16.60
-1	13	-16	331.07	18.10
1	13	16	331.27	22.60
-1	13	-16	316.17	17.00
-1	13	-16	315.07	17.20
1	13	16	331.97	22.10
-2	13	-16	460.45	22.40
-2	13	-16	402.56	22.30
-2	13	-16	424.36	22.50
2	13	16	421.06	26.30
-3	13	-16	124.59	9.80
-3	13	-16	125.79	10.10
-3	13	-16	122.19	7.60
3	13	16	123.39	13.60
3	13	16	124.19	16.20
-3	13	-16	120.49	8.00
4	13	16	31.30	12.10
-4	13	-16	14.30	5.40
4	13	16	11.40	7.50
-4	13	-16	17.20	5.50
-5	13	-16	850.71	42.60
5	13	16	806.92	44.50
-6	13	-16	26.10	6.80
6	13	16	12.50	7.80
-7	13	-16	406.76	23.90
7	13	16	395.96	25.80
-8	13	-16	0.80	6.90
9	13	16	199.78	18.40
-9	13	-16	216.78	17.40
-10	13	-16	4.20	8.20
10	13	16	-0.90	8.50
10	14	-16	9.70	9.80
-10	14	16	15.60	8.50
9	-14	-16	44.50	12.00
-9	14	16	48.20	9.50
9	14	-16	47.80	11.10
8	14	-16	12.10	9.20
-8	14	16	-6.40	8.50

7	-14	-16	14.80	8.40
-7	14	16	9.30	8.10
7	14	-16	21.00	8.90
6	-14	-16	21.20	8.40
-6	14	16	13.70	8.90
6	14	-16	31.20	8.40
5	-14	-16	-3.90	6.80
-5	14	16	-5.10	8.30
5	14	-16	5.40	7.00
4	-14	-16	8.00	6.60
-4	14	16	-16.00	7.90
4	14	-16	-0.10	6.20
3	-14	-16	-1.90	6.10
3	14	-16	2.30	5.70
-3	14	16	-8.90	7.90
2	-14	-16	14.50	7.70
-2	14	16	13.10	8.50
2	14	-16	24.00	5.90
-2	14	16	10.80	16.90
1	-14	-16	2.20	11.80
-1	14	16	4.00	7.90
1	14	-16	2.40	4.70
-1	14	16	-9.20	7.30
0	14	16	44.70	9.40
0	14	-16	47.90	6.70
0	14	16	37.30	9.40
0	14	-16	38.00	7.80
-1	14	-16	5.50	3.20
-1	14	-16	4.90	4.30
1	14	16	9.40	7.50
-1	14	-16	5.80	4.20
1	14	16	0.20	7.20
2	14	16	-6.90	6.60
2	14	16	8.20	8.40
-2	14	-16	2.30	3.80
-2	14	-16	4.50	3.80
-2	14	-16	1.30	3.70
-2	14	-16	5.90	3.20
-3	14	-16	72.69	6.10
3	14	16	66.39	12.60
-3	14	-16	76.79	8.20
-3	14	-16	72.69	8.30
-3	14	-16	75.79	6.80
-4	14	-16	135.49	7.70

-4	14	-16	144.69	11.10
-4	14	-16	132.29	11.20
-4	14	-16	124.79	7.80
4	14	16	122.69	14.90
-5	14	-16	23.50	6.30
5	14	16	34.20	8.30
-6	14	-16	35.90	7.40
6	14	16	30.90	8.80
7	14	16	15.20	8.50
-7	14	-16	17.30	7.10
-8	14	-16	9.50	7.60
8	14	16	13.60	8.90
9	14	16	-5.10	8.60
-9	14	-16	-5.10	7.60
10	14	16	36.10	10.50
-10	14	-16	39.40	9.40
9	-15	-16	-7.50	9.40
9	15	-16	-14.70	8.40
-9	15	16	6.60	8.80
8	-15	-16	318.57	27.40
-8	15	16	343.87	23.30
8	15	-16	345.07	24.80
-7	15	16	-8.10	8.90
7	15	-16	22.70	9.70
6	-15	-16	590.14	42.20
-6	15	16	719.83	38.50
6	15	-16	682.43	38.00
5	-15	-16	133.69	16.30
5	15	-16	114.29	14.30
-5	15	16	98.39	15.00
4	-15	-16	798.92	46.70
-4	15	16	841.02	46.00
4	15	-16	863.41	44.60
3	-15	-16	27.90	12.40
-3	15	16	12.80	8.90
3	15	-16	27.40	6.80
-2	15	16	115.09	16.10
2	15	-16	166.68	13.30
-2	15	16	157.08	19.20
-1	15	16	239.68	30.10
1	15	-16	243.98	16.10
-1	15	16	226.38	19.70
0	15	16	647.74	39.70
0	15	-16	730.73	36.70

0	15	16	736.63	41.10
-1	15	-16	659.43	33.40
-1	15	-16	636.24	31.80
1	15	16	643.84	37.70
1	15	16	640.14	37.10
-2	15	-16	350.86	20.20
2	15	16	393.96	25.30
-2	15	-16	415.86	21.00
-2	15	-16	373.36	20.20
3	15	16	274.57	20.40
-3	15	-16	280.47	15.30
-3	15	-16	285.27	15.90
-3	15	-16	241.98	14.70
4	15	16	59.99	10.40
-4	15	-16	73.99	9.30
-4	15	-16	91.19	6.60
-4	15	-16	69.29	6.20
-5	15	-16	735.83	38.20
5	15	16	735.03	40.80
6	15	16	196.78	17.90
-7	15	-16	600.84	32.90
7	15	16	591.84	34.80
-8	15	-16	0.10	7.40
8	15	16	8.70	8.80
-9	15	-16	183.98	16.90
9	15	16	183.58	18.10
9	16	-16	162.28	19.80
-9	16	16	131.69	17.20
8	-16	-16	6.40	10.20
8	16	-16	2.80	8.90
7	-16	-16	76.49	12.70
-7	16	16	92.29	12.90
7	16	-16	98.59	15.90
6	-16	-16	7.50	12.00
-6	16	16	12.30	9.10
6	16	-16	27.30	9.50
5	16	-16	41.70	8.90
-5	16	16	48.50	11.40
4	16	-16	9.40	7.00
-4	16	16	2.00	9.00
3	-16	-16	29.00	10.30
-3	16	16	60.19	12.10
-3	16	16	22.70	9.30
3	16	-16	49.30	8.20

2	-16	-16	40.20	9.70
-2	16	16	33.10	10.20
2	16	-16	46.00	7.60
-2	16	16	17.90	10.30
-1	16	16	95.19	14.60
1	16	-16	89.89	10.80
-1	16	16	76.39	12.50
0	16	16	42.60	13.20
0	16	16	21.80	9.60
0	16	-16	41.40	6.80
1	16	16	5.10	8.30
-1	16	-16	8.20	3.20
-1	16	-16	11.50	5.20
-2	16	-16	63.89	8.40
2	16	16	46.60	10.10
-2	16	-16	66.39	6.80
-2	16	-16	48.00	6.80
-3	16	-16	145.99	10.00
3	16	16	113.39	15.30
-3	16	-16	121.59	9.80
-3	16	-16	106.39	8.80
-4	16	-16	102.89	8.40
-4	16	-16	122.39	10.70
4	16	16	128.39	15.70
-4	16	-16	145.49	9.20
-5	16	-16	42.80	5.40
-5	16	-16	47.10	5.60
5	16	16	45.30	10.20
-5	16	-16	39.20	7.00
-6	16	-16	46.20	8.00
6	16	16	35.60	9.80
-7	16	-16	11.30	7.40
7	16	16	1.80	8.10
-8	16	-16	50.49	9.30
8	16	16	43.10	9.70
9	16	16	-6.30	8.80
-9	16	-16	7.50	7.90
-8	17	16	115.99	16.60
8	17	-16	101.59	17.60
7	-17	-16	16.40	11.00
-7	17	16	9.50	9.20
7	17	-16	18.70	9.70
6	-17	-16	153.98	17.50
-6	17	16	143.99	17.10

6	17	-16	123.49	16.70
5	-17	-16	91.59	15.40
-5	17	16	83.79	15.30
5	17	-16	115.29	14.70
4	-17	-16	301.67	23.40
4	17	-16	317.87	21.80
-4	17	16	333.07	24.40
3	-17	-16	-3.20	7.80
-3	17	16	3.80	10.00
3	17	-16	2.50	6.50
-2	17	16	168.78	19.10
2	17	-16	197.68	15.50
1	17	-16	276.67	18.50
-1	17	16	272.87	22.10
0	17	-16	258.57	16.90
0	17	16	229.38	20.80
1	17	16	207.28	18.10
-1	17	-16	202.58	14.60
-2	17	-16	159.98	11.40
2	17	16	156.98	16.50
-2	17	-16	180.78	12.50
-2	17	-16	167.08	10.70
-3	17	-16	58.29	7.20
-3	17	-16	59.39	7.70
3	17	16	51.29	10.30
-3	17	-16	56.49	8.10
4	17	16	-1.40	8.00
-4	17	-16	14.00	5.30
-4	17	-16	18.70	4.60
-4	17	-16	17.90	4.20
-5	17	-16	587.54	29.40
5	17	16	571.54	34.30
-5	17	-16	600.94	31.20
-5	17	-16	573.44	29.30
-6	17	-16	-6.10	6.30
6	17	16	-2.30	8.20
7	17	16	303.17	22.90
-7	17	-16	317.57	20.60
8	17	16	1.40	9.10
-8	17	-16	7.50	7.70
7	-18	-16	227.48	21.60
-7	18	16	246.98	20.80
7	18	-16	219.28	21.10
6	-18	-16	123.19	18.40

-6	18	16	127.39	17.40
6	18	-16	143.19	16.90
5	-18	-16	439.56	30.70
-5	18	16	459.95	30.30
5	18	-16	478.55	29.10
4	-18	-16	107.39	16.70
-4	18	16	70.19	12.10
4	18	-16	65.49	13.40
-3	18	16	325.97	24.60
3	18	-16	326.87	22.00
2	18	-16	127.39	13.40
-2	18	16	101.39	17.20
1	18	-16	367.46	22.20
-1	18	16	334.17	24.80
0	18	16	338.17	25.50
0	18	-16	354.86	21.50
-1	18	-16	226.08	15.70
1	18	16	271.47	20.90
-1	18	-16	283.07	17.20
-2	18	-16	227.68	15.30
-2	18	-16	262.87	16.40
2	18	16	280.37	21.60
-3	18	-16	47.50	7.40
3	18	16	40.40	11.00
-3	18	-16	48.70	7.60
-3	18	-16	50.49	8.50
-4	18	-16	373.66	19.10
-4	18	-16	304.37	19.00
4	18	16	334.17	24.40
-4	18	-16	330.37	19.30
-5	18	-16	3.40	5.80
-5	18	-16	7.50	4.20
-5	18	-16	0.40	4.20
5	18	16	-3.80	8.60
-6	18	-16	424.66	22.20
-6	18	-16	450.25	22.10
-6	18	-16	413.26	24.70
6	18	16	413.56	28.00
7	18	16	10.80	9.20
-7	18	-16	-0.90	6.80
7	19	-16	-7.50	9.20
6	-19	-16	16.40	10.10
6	19	-16	10.00	9.20
-6	19	16	15.10	10.80

5	-19	-16	-1.60	9.70
-5	19	16	12.20	10.30
5	19	-16	10.90	8.50
4	-19	-16	21.20	10.20
4	19	-16	34.30	8.60
-4	19	16	19.60	10.90
3	19	-16	-3.10	7.10
-3	19	16	0.20	10.30
-2	19	16	-4.70	9.90
2	19	-16	0.50	6.60
1	19	-16	108.09	13.20
-1	19	16	86.59	13.50
0	19	-16	6.70	6.20
0	19	16	-6.80	9.60
-1	19	-16	14.60	6.30
1	19	16	3.40	9.20
2	19	16	30.80	10.60
-2	19	-16	32.40	6.50
-2	19	-16	34.80	6.40
3	19	16	-8.60	9.50
-3	19	-16	15.00	5.60
-3	19	-16	14.80	4.90
4	19	16	-3.50	9.20
-4	19	-16	11.90	5.20
-5	19	-16	63.39	11.90
-5	19	-16	60.49	8.50
-5	19	-16	71.89	8.50
5	19	16	84.39	15.40
-6	19	-16	3.50	6.70
-6	19	-16	7.40	4.70
6	19	16	17.30	9.90
-6	19	-16	1.10	4.30
5	-20	-16	469.05	33.30
5	20	-16	525.85	31.70
-5	20	16	495.35	32.30
-4	20	16	132.99	19.00
4	20	-16	146.19	17.20
3	20	-16	104.29	15.20
-3	20	16	122.69	17.80
2	20	-16	44.00	9.00
-2	20	16	60.59	12.80
-1	20	16	289.17	24.60
1	20	-16	300.07	20.50
0	20	16	509.55	32.60

0	20	-16	487.15	28.60
-1	20	-16	287.57	18.70
1	20	16	267.07	23.20
2	20	16	537.25	34.10
-2	20	-16	550.64	30.00
-3	20	-16	33.00	6.60
3	20	16	25.70	10.70
4	20	16	236.18	21.50
-4	20	-16	253.27	16.10
-5	20	-16	1.20	5.20
5	20	16	1.00	9.50
-5	20	-16	-1.90	7.10
-4	21	16	30.20	11.60
4	21	-16	51.49	10.40
-3	21	16	14.60	12.00
3	21	-16	34.20	9.60
2	21	-16	118.49	15.30
-2	21	16	79.69	14.20
-1	21	16	44.60	11.90
1	21	-16	48.40	9.20
0	21	16	16.20	10.60
0	21	-16	28.80	8.20
1	21	16	61.29	13.20
-1	21	-16	74.99	12.10
2	21	16	13.40	9.80
-2	21	-16	22.50	7.00
-3	21	-16	40.10	7.50
3	21	16	28.60	11.40
-4	21	-16	42.40	6.90
4	21	16	30.10	11.50
-2	22	16	30.90	12.60
1	22	-16	28.20	11.50
-1	22	16	15.20	11.90
0	22	-16	247.38	21.10
0	22	16	249.18	23.70
1	22	16	4.00	10.90
-1	22	-16	25.90	8.50
2	22	16	19.50	11.60
-2	22	-16	23.10	11.70
12	0	-17	10.20	10.40
11	0	-17	-6.40	7.90
-11	0	17	8.10	5.70
10	0	-17	18.90	7.90
-10	0	17	10.30	5.30

-9	0	17	-1.10	5.00
9	0	-17	-0.70	6.50
8	0	-17	-0.50	4.50
-8	0	17	8.90	4.80
8	0	-17	9.20	6.30
7	0	-17	0.80	3.70
-7	0	17	-0.20	4.20
7	0	-17	0.00	8.60
6	0	-17	7.20	3.40
6	0	-17	6.00	5.20
-6	0	17	-1.60	3.90
-5	0	17	2.60	3.70
5	0	-17	4.10	2.90
5	0	-17	1.20	4.50
4	0	-17	0.40	3.80
4	0	-17	1.40	2.80
-4	0	17	4.30	3.80
-3	0	17	-1.30	3.50
3	0	-17	1.80	2.60
3	0	-17	-2.60	3.30
2	0	-17	0.20	2.70
-2	0	17	6.70	3.60
2	0	-17	-2.00	3.10
1	0	-17	-1.50	3.00
1	0	-17	3.20	2.80
-1	0	17	-1.10	3.40
0	0	17	-0.30	3.50
0	0	-17	0.00	3.00
0	0	-17	-3.40	2.80
-1	0	-17	0.70	2.60
1	0	17	0.00	3.60
-1	0	-17	-4.70	2.90
1	0	17	-0.30	3.10
2	0	17	3.10	3.80
-2	0	-17	2.40	3.60
-2	0	-17	1.10	2.50
2	0	17	4.30	2.80
2	0	17	4.50	3.90
-3	0	-17	-1.70	3.80
3	0	17	-0.70	3.90
4	0	17	1.60	4.30
5	0	17	6.60	4.80
6	0	17	-0.40	5.00
7	0	17	13.00	6.30

8	0	17	-6.00	6.20
-9	0	-17	-2.50	6.90
-10	0	-17	-7.60	4.70
-10	0	-17	-11.70	6.80
-11	0	-17	12.20	8.70
-11	0	-17	13.40	6.30
-12	0	-17	0.20	8.60
12	-1	-17	319.97	24.80
12	1	-17	312.17	24.70
-12	1	17	315.57	25.80
11	-1	-17	51.09	10.60
-11	-1	17	68.09	9.40
-11	1	17	72.09	10.10
11	1	-17	54.99	11.00
-10	-1	17	540.55	28.30
10	-1	-17	549.95	32.20
-10	1	17	543.45	28.80
10	1	-17	505.85	31.80
9	-1	-17	-4.60	6.20
-9	-1	17	1.10	4.70
-9	1	17	0.40	5.20
9	1	-17	-6.10	6.10
8	-1	-17	632.44	34.20
-8	-1	17	686.33	34.20
8	-1	-17	675.63	36.90
-8	1	17	687.93	34.70
8	1	-17	674.13	36.90
8	1	-17	663.03	34.60
7	-1	-17	139.99	10.40
-7	-1	17	157.88	10.60
7	1	-17	147.19	13.80
7	1	-17	148.49	10.20
-7	1	17	163.08	11.50
6	-1	-17	522.85	28.10
-6	-1	17	511.85	25.90
6	-1	-17	477.15	25.60
-6	1	17	523.25	26.60
6	1	-17	496.25	25.40
6	1	-17	462.25	30.40
5	-1	-17	913.91	45.10
-5	-1	17	932.11	45.50
5	-1	-17	936.11	47.20
-5	1	17	910.81	46.10
5	1	-17	921.01	47.20

5	1	-17	910.41	44.90
-4	-1	17	608.24	30.80
4	-1	-17	584.04	30.20
4	-1	-17	613.04	32.20
-4	1	17	599.34	31.30
4	1	-17	598.74	30.00
4	1	-17	641.54	32.20
3	-1	-17	57.29	7.30
-3	-1	17	50.69	6.10
3	-1	-17	53.29	5.50
-3	1	17	45.90	6.80
3	1	-17	58.09	7.20
3	1	-17	56.09	5.10
2	-1	-17	292.07	16.40
2	-1	-17	272.77	14.90
-2	-1	17	279.77	15.60
2	1	-17	266.97	14.70
-2	1	17	261.97	15.90
2	1	-17	286.07	16.10
1	-1	-17	1222.18	60.19
-1	-1	17	1252.27	60.69
1	-1	-17	1245.58	61.09
-1	1	17	1201.28	60.99
1	1	-17	1281.77	60.89
1	1	-17	1237.98	60.09
0	-1	-17	236.98	13.40
0	-1	17	224.78	14.00
0	-1	-17	237.28	13.90
0	1	17	242.78	14.40
0	1	-17	232.68	13.60
0	1	-17	236.08	13.20
-1	-1	-17	1060.59	51.89
1	-1	17	1005.20	52.29
1	-1	17	1126.09	51.59
-1	-1	-17	1043.30	52.09
-1	1	-17	1036.80	51.79
1	1	17	1031.40	52.59
1	1	17	1114.69	52.19
-1	1	-17	1038.10	51.79
-2	-1	-17	195.08	11.70
-2	-1	-17	186.58	12.10
2	-1	17	194.18	12.80
-2	1	-17	188.98	11.90
2	1	17	222.98	11.90

-2	1	-17	195.78	11.20
2	1	17	173.28	12.80
3	-1	17	188.08	12.90
3	1	17	193.98	13.10
-3	1	-17	183.98	12.20
3	1	17	199.28	10.90
4	-1	17	36.10	6.10
4	1	17	43.90	6.60
5	-1	17	1391.86	68.99
5	1	17	1386.66	69.09
6	-1	17	12.10	5.60
6	1	17	3.50	6.10
7	-1	17	637.94	34.40
7	1	17	635.14	34.50
-8	1	-17	17.70	7.10
8	1	17	11.90	6.60
-9	1	-17	160.18	14.40
9	1	17	173.38	15.30
-10	-1	-17	74.69	10.20
-10	-1	-17	58.59	10.70
-10	1	-17	81.49	13.40
-11	-1	-17	432.46	27.50
-11	1	-17	401.26	26.70
-11	1	-17	430.76	23.90
-12	-1	-17	22.10	9.50
-12	1	-17	48.10	10.40
12	-2	-17	12.70	9.50
-12	2	17	16.10	7.00
12	2	-17	5.00	10.00
11	-2	-17	252.37	21.40
11	2	-17	252.87	21.20
-11	2	17	256.27	17.10
10	-2	-17	17.30	8.60
-10	-2	17	12.10	5.00
-10	2	17	15.70	6.10
10	2	-17	19.40	8.10
9	-2	-17	330.17	22.70
-9	-2	17	339.37	19.10
-9	2	17	353.06	20.30
9	2	-17	352.46	23.00
8	-2	-17	65.69	8.90
-8	-2	17	72.09	7.60
8	-2	-17	55.49	11.60
-8	2	17	72.59	9.30

8	2	-17	87.89	11.80
7	-2	-17	82.99	12.70
-7	-2	17	65.49	7.20
7	-2	-17	63.49	8.10
-7	2	17	77.59	9.00
7	2	-17	80.39	11.00
7	2	-17	77.49	7.20
6	-2	-17	133.99	9.70
6	-2	-17	137.49	12.50
-6	-2	17	147.29	9.50
6	2	-17	133.29	9.00
-6	2	17	150.08	11.30
5	-2	-17	411.96	23.30
-5	-2	17	397.56	21.10
5	-2	-17	395.66	21.10
5	2	-17	403.66	20.60
5	2	-17	398.06	23.30
-5	2	17	421.76	22.50
4	-2	-17	448.96	23.90
4	-2	-17	486.85	25.90
-4	-2	17	457.05	24.20
4	2	-17	481.95	25.80
-4	2	17	507.25	25.50
4	2	-17	445.56	23.50
3	-2	-17	147.99	10.90
-3	-2	17	140.69	9.20
3	-2	-17	133.59	8.80
3	2	-17	137.09	10.30
-3	2	17	135.09	10.30
3	2	-17	128.39	8.10
2	-2	-17	144.99	9.10
2	-2	-17	151.18	10.80
-2	-2	17	144.99	9.50
-2	2	17	135.49	10.70
2	2	-17	142.09	10.10
2	2	-17	146.99	8.60
1	-2	-17	62.79	7.40
1	-2	-17	68.89	6.30
-1	-2	17	66.69	6.70
-1	2	17	70.89	7.60
1	2	-17	72.09	5.80
1	2	-17	58.49	6.60
0	-2	17	401.16	21.10
0	-2	-17	384.16	20.70

0	-2	-17	391.86	21.30
0	2	-17	396.36	20.40
0	2	-17	403.46	20.70
0	2	17	374.96	21.50
1	-2	17	49.50	4.40
-1	-2	-17	43.70	6.20
1	-2	17	48.50	6.80
1	-2	17	54.29	5.20
-1	-2	-17	50.59	6.30
-1	2	-17	52.99	5.90
1	2	17	38.30	7.10
1	2	17	61.99	7.40
-1	2	-17	54.69	5.50
-2	-2	-17	288.37	16.50
-2	-2	-17	288.87	16.80
2	-2	17	268.47	16.80
2	2	17	338.37	17.00
2	2	17	291.57	17.50
-2	2	-17	287.47	15.60
-2	2	-17	300.17	16.40
3	-2	17	-0.30	4.00
3	2	17	5.20	3.50
-3	2	-17	1.20	3.80
3	2	17	2.80	4.50
4	-2	17	319.47	19.00
4	2	17	319.37	19.40
4	2	17	333.67	17.10
5	-2	17	22.70	5.90
5	2	17	28.80	6.30
5	2	17	21.70	4.30
6	-2	17	135.99	11.80
6	2	17	132.39	8.00
7	-2	17	41.10	7.60
7	2	17	27.20	6.90
-7	2	-17	33.00	7.60
8	2	17	295.27	19.80
-8	2	-17	304.07	20.00
-9	2	-17	12.90	7.80
9	2	17	29.50	8.20
-10	-2	-17	54.09	10.20
-10	-2	-17	59.39	10.50
-10	2	-17	76.49	12.50
-11	-2	-17	31.50	9.70
-11	2	-17	52.19	10.20

-12	-2	-17	126.29	15.90
-12	2	-17	151.18	17.00
12	-3	-17	50.49	12.00
12	3	-17	42.90	11.20
-12	3	17	38.00	8.30
11	-3	-17	40.80	9.90
-11	3	17	47.20	8.00
11	3	-17	52.09	10.60
10	-3	-17	292.17	21.10
-10	-3	17	259.77	17.10
10	3	-17	252.87	21.10
-10	3	17	280.37	17.80
-9	-3	17	14.90	4.30
9	-3	-17	18.20	7.40
-9	3	17	8.00	5.90
9	3	-17	16.50	7.50
-8	-3	17	276.47	15.60
8	-3	-17	279.57	20.70
8	3	-17	285.07	19.60
-8	3	17	276.57	17.60
7	-3	-17	-5.20	5.30
-7	-3	17	-0.30	3.20
7	-3	-17	10.30	6.50
-7	3	17	0.30	5.10
7	3	-17	6.10	3.40
7	3	-17	1.60	5.60
-6	-3	17	210.78	12.10
6	-3	-17	195.88	12.80
6	-3	-17	222.38	15.40
6	3	-17	199.28	16.10
6	3	-17	216.58	11.90
-6	3	17	222.38	14.50
5	-3	-17	61.69	6.90
-5	-3	17	65.69	6.30
5	-3	-17	73.09	9.60
5	3	-17	62.19	11.20
-5	3	17	61.19	8.90
5	3	-17	76.39	5.80
4	-3	-17	506.85	27.70
-4	-3	17	543.15	27.60
4	-3	-17	554.34	29.60
4	3	-17	545.45	27.10
-4	3	17	561.44	29.30
4	3	-17	585.74	29.60

3	-3	-17	73.39	8.40
-3	-3	17	62.29	6.20
3	-3	-17	73.59	6.30
-3	3	17	60.89	8.40
3	3	-17	72.29	5.40
3	3	-17	76.49	8.00
2	-3	-17	15.00	3.70
-2	-3	17	17.20	3.60
2	-3	-17	17.70	4.70
-2	3	17	14.20	4.70
2	3	-17	22.20	3.80
2	3	-17	15.30	3.80
-1	-3	17	440.66	22.60
1	-3	-17	430.46	22.50
1	-3	-17	432.96	23.60
-1	3	17	420.86	23.60
1	3	-17	421.96	21.90
1	3	-17	449.16	22.90
0	-3	17	128.69	9.30
0	-3	-17	130.39	9.80
0	-3	-17	126.49	9.20
0	3	-17	134.39	8.90
0	3	17	135.39	10.50
0	3	-17	130.69	8.60
1	-3	17	248.98	14.80
-1	-3	-17	252.87	14.60
-1	-3	-17	242.08	14.90
-1	3	-17	255.07	14.10
-1	3	-17	246.48	13.80
1	3	17	245.88	15.60
1	3	17	269.87	15.70
-2	-3	-17	173.68	11.40
2	-3	17	159.18	11.80
2	3	17	173.58	12.50
-2	3	-17	180.38	10.10
2	3	17	189.08	12.20
-2	3	-17	184.88	11.10
3	-3	17	285.07	16.40
3	3	17	256.07	16.80
-3	3	-17	251.07	15.70
3	3	17	304.17	16.10
4	-3	17	19.60	5.30
4	3	17	16.70	5.70
4	3	17	18.60	4.20

5	-3	17	104.89	10.20
5	3	17	98.49	7.90
5	3	17	101.39	10.60
6	-3	17	20.20	5.90
-6	3	-17	19.00	6.50
6	3	17	12.00	6.10
6	3	17	18.10	3.80
7	3	17	161.48	10.30
-7	3	-17	166.28	13.80
7	3	17	184.68	14.40
8	3	17	7.60	6.30
-8	3	-17	6.10	6.60
-9	3	-17	156.68	14.80
9	3	17	142.59	13.60
-10	-3	-17	42.60	8.30
-10	3	-17	70.69	12.70
10	3	17	40.10	9.20
-11	-3	-17	189.38	18.00
-11	3	-17	165.18	12.60
-11	3	-17	200.78	17.30
-12	-3	-17	-3.10	8.90
-12	3	-17	18.20	6.00
-12	3	-17	13.20	8.60
12	-4	-17	10.50	9.40
-12	4	17	-4.80	7.10
12	4	-17	-4.80	9.40
11	-4	-17	451.25	30.10
-11	4	17	504.85	27.30
11	4	-17	473.45	30.60
10	-4	-17	25.60	8.50
10	4	-17	13.80	8.10
9	-4	-17	350.96	23.00
-9	-4	17	334.17	18.30
9	4	-17	342.97	23.20
-9	4	17	347.07	20.70
-8	-4	17	15.10	3.70
8	-4	-17	9.50	7.30
-8	4	17	12.50	6.30
8	4	-17	7.00	6.50
-7	-4	17	1023.70	50.59
7	-4	-17	968.30	54.19
-7	4	17	1104.59	52.79
7	4	-17	1066.09	53.79
7	4	-17	1040.60	50.79

-6	-4	17	74.69	6.40
6	-4	-17	88.19	10.70
6	4	-17	76.59	10.60
-6	4	17	83.19	9.60
6	4	-17	80.69	6.20
5	-4	-17	1074.59	53.39
5	-4	-17	977.00	51.39
-5	-4	17	1046.70	50.99
5	4	-17	1043.10	50.59
5	4	-17	1023.20	57.09
-5	4	17	1135.69	53.19
4	-4	-17	212.48	13.40
-4	-4	17	228.28	13.00
4	-4	-17	251.87	15.70
4	4	-17	241.98	12.30
4	4	-17	239.48	15.50
-4	4	17	240.88	15.70
-3	-4	17	603.04	30.00
3	-4	-17	578.84	30.10
3	-4	-17	600.34	31.70
-3	4	17	616.54	31.80
3	4	-17	570.94	29.20
3	4	-17	626.64	31.40
2	-4	-17	358.66	19.50
2	-4	-17	380.56	21.00
-2	-4	17	368.66	19.40
2	4	-17	356.96	18.60
-2	4	17	360.06	21.10
2	4	-17	390.06	20.30
1	-4	-17	1065.49	53.69
1	-4	-17	1111.79	54.89
-1	-4	17	1121.69	53.79
1	4	-17	1134.29	54.09
1	4	-17	1087.69	53.09
-1	4	17	1067.69	54.99
0	-4	17	1774.92	86.29
0	-4	-17	1792.92	87.09
0	-4	-17	1812.82	86.29
0	4	-17	1843.82	86.09
0	4	17	1681.73	87.19
0	4	-17	1795.62	85.69
1	-4	17	243.38	15.10
-1	-4	-17	258.27	15.60
1	4	17	260.97	16.30

-1	4	-17	249.08	14.50
-1	4	-17	260.77	14.10
1	4	17	305.37	16.70
2	-4	17	1133.19	55.09
2	4	17	1051.89	55.69
2	4	17	1232.48	55.69
-2	4	-17	1065.19	54.39
-2	4	-17	1083.29	53.69
3	-4	17	278.87	16.90
3	4	17	302.67	17.20
3	4	17	272.87	17.80
-3	4	-17	289.57	16.20
4	-4	17	1069.09	54.89
4	4	17	1189.48	54.59
4	4	17	1034.30	55.29
5	-4	17	10.00	5.00
5	4	17	2.20	5.30
-5	4	-17	-0.40	5.20
5	4	17	-5.70	3.80
6	4	17	1366.46	69.99
6	4	17	1453.55	68.09
-6	4	-17	1371.96	69.59
-7	4	-17	14.80	6.80
7	4	17	22.80	4.70
7	4	17	15.40	6.30
-8	4	-17	339.77	21.60
8	4	17	356.96	22.00
8	4	17	326.17	18.70
9	4	17	76.69	12.10
-9	4	-17	70.09	12.30
-10	4	-17	587.74	32.50
10	4	17	525.35	32.10
-11	-4	-17	169.88	17.70
-11	4	-17	143.99	16.70
-12	-4	-17	324.67	24.30
-12	4	-17	313.27	18.60
-12	4	-17	310.77	23.30
12	-5	-17	2.30	9.40
-12	5	17	0.90	7.50
12	5	-17	1.50	10.90
11	-5	-17	6.10	8.70
-11	5	17	11.30	7.20
11	5	-17	26.30	10.00
10	-5	-17	7.80	7.70

-10	5	17	2.20	6.30
10	5	-17	4.30	8.30
9	-5	-17	27.40	7.60
9	5	-17	21.90	8.50
-9	5	17	14.10	8.30
8	-5	-17	3.70	9.90
-8	-5	17	6.30	3.20
-8	5	17	-5.20	5.60
8	5	-17	-4.90	5.90
7	-5	-17	6.60	6.60
-7	-5	17	11.60	3.30
-7	5	17	14.30	6.10
7	5	-17	10.80	6.60
-6	-5	17	7.20	3.30
6	-5	-17	11.90	5.80
6	5	-17	12.10	2.80
6	5	-17	-1.10	5.30
-6	5	17	6.50	5.70
-5	-5	17	42.90	5.20
5	-5	-17	41.60	7.20
5	5	-17	38.10	4.20
-5	5	17	36.20	6.70
5	5	-17	33.40	6.70
4	-5	-17	2.80	4.50
-4	-5	17	3.70	3.00
-4	5	17	-6.00	5.00
-3	-5	17	31.10	4.70
3	-5	-17	29.70	5.90
3	-5	-17	38.10	6.40
-3	5	17	23.50	6.10
3	5	-17	22.60	3.40
3	5	-17	27.70	5.20
2	-5	-17	12.80	4.30
-2	-5	17	9.90	3.40
2	-5	-17	11.20	4.50
-2	5	17	1.00	5.20
2	5	-17	7.70	3.70
2	5	-17	10.80	2.40
-1	-5	17	0.80	3.00
1	-5	-17	1.60	3.90
-1	5	17	1.40	4.70
1	5	-17	2.70	2.40
1	5	-17	-1.90	2.90
0	-5	-17	42.70	7.20

0	-5	17	50.09	6.10
0	5	-17	38.60	5.40
0	5	-17	40.70	5.10
0	5	17	56.49	8.60
0	5	17	43.50	8.00
-1	-5	-17	90.49	9.10
1	-5	17	98.29	8.40
-1	5	-17	111.89	7.20
-1	5	-17	103.29	7.70
1	5	17	102.39	10.10
1	5	17	121.99	10.60
2	-5	17	159.28	11.10
-2	5	-17	154.58	8.80
-2	5	-17	154.08	10.20
2	5	17	161.78	12.30
3	-5	17	65.19	8.00
-3	5	-17	74.79	7.30
3	5	17	50.49	8.40
3	5	17	65.89	8.90
-4	5	-17	71.49	8.20
4	5	17	68.89	9.00
4	5	17	73.99	9.60
-4	5	-17	48.10	9.00
-5	5	-17	7.20	5.30
5	5	17	11.10	4.90
6	5	17	17.20	6.20
-6	5	-17	12.20	5.90
6	5	17	6.20	4.90
7	5	17	39.30	7.90
-7	5	-17	41.80	7.70
7	5	17	54.19	8.20
8	5	17	5.60	6.80
8	5	17	10.10	5.20
-8	5	-17	16.10	7.00
9	5	17	40.10	8.50
-9	5	-17	52.29	9.40
10	5	17	28.10	8.40
-10	5	-17	24.20	8.70
-11	5	-17	14.30	8.40
11	5	17	10.20	8.50
-12	-5	-17	9.80	10.10
-12	5	-17	26.70	9.70
-12	5	-17	23.00	5.20
12	-6	-17	-5.60	10.20

12	6	-17	0.40	10.50
-12	6	17	-14.10	7.40
11	-6	-17	194.38	19.90
-11	6	17	187.28	15.80
11	6	-17	193.38	19.50
10	-6	-17	14.70	8.30
-10	6	17	-1.40	6.70
10	6	-17	5.90	8.30
9	-6	-17	300.27	21.80
9	6	-17	306.97	22.20
-9	6	17	340.37	20.20
8	-6	-17	18.60	10.90
8	6	-17	15.90	7.30
-8	6	17	7.80	6.70
7	-6	-17	555.84	32.30
-7	-6	17	568.64	27.60
-7	6	17	592.64	31.30
7	6	-17	550.34	32.00
-6	-6	17	205.78	10.40
6	-6	-17	182.58	14.80
-6	6	17	187.38	14.80
6	6	-17	182.48	15.00
6	6	-17	191.58	10.10
5	-6	-17	769.52	42.20
-5	-6	17	860.21	39.20
5	6	-17	790.22	42.10
5	6	-17	787.72	38.90
-5	6	17	830.62	42.40
4	-6	-17	347.87	20.70
-4	-6	17	368.56	18.20
4	6	-17	325.57	16.90
4	6	-17	331.47	20.30
-4	6	17	331.27	21.00
3	-6	-17	518.15	28.80
-3	-6	17	577.34	26.50
-3	6	17	526.85	29.20
3	6	-17	494.75	25.80
2	-6	-17	93.49	9.60
-2	-6	17	104.69	7.10
2	6	-17	96.29	8.50
2	6	-17	94.99	6.00
-2	6	17	82.99	10.40
1	-6	-17	838.72	42.80
-1	-6	17	863.81	41.20

1	6	-17	841.32	41.60
-1	6	17	839.22	43.20
1	6	-17	785.52	40.40
0	-6	17	574.04	28.50
0	-6	-17	546.95	29.80
0	6	-17	556.74	28.30
0	6	17	598.34	30.70
0	6	-17	532.55	27.80
0	6	17	546.55	30.30
1	-6	17	277.07	16.10
-1	-6	-17	274.87	17.20
-1	6	-17	295.47	15.00
1	6	17	307.97	18.10
-1	6	-17	279.37	15.40
1	6	17	260.87	17.70
2	-6	17	482.35	24.30
-2	6	-17	418.46	24.20
2	6	17	455.85	25.30
-2	6	-17	442.96	22.30
2	6	17	477.15	25.70
-2	6	-17	445.86	23.50
-3	6	-17	122.89	9.40
3	6	17	155.18	12.70
3	6	17	118.39	11.40
-3	6	-17	120.49	10.50
4	6	17	1290.27	58.69
-4	6	-17	1137.19	58.09
-4	6	-17	1094.39	57.49
4	6	17	1149.98	59.09
-5	6	-17	12.80	5.80
5	6	17	25.80	6.90
5	6	17	32.00	6.20
6	6	17	1272.07	59.89
-6	6	-17	1135.39	60.29
6	6	17	1192.18	61.09
7	6	17	22.10	7.40
-7	6	-17	29.60	7.50
7	6	17	45.30	8.30
8	6	17	378.96	24.40
-8	6	-17	399.76	24.10
-9	6	-17	2.80	7.30
9	6	17	3.50	6.90
10	6	17	334.87	23.00
-10	6	-17	329.07	22.60

-11	6	-17	114.99	15.20
11	6	17	117.69	15.20
-12	-6	-17	269.47	22.50
-12	6	-17	257.87	14.70
-12	6	-17	255.37	21.40
12	-7	-17	45.60	11.30
12	7	-17	46.10	11.60
-12	7	17	42.60	9.30
11	-7	-17	5.70	9.20
-11	7	17	2.10	7.50
11	7	-17	-6.10	9.20
10	-7	-17	198.68	18.20
-10	7	17	182.98	15.60
10	7	-17	183.28	18.90
9	-7	-17	18.20	7.60
-9	7	17	4.20	6.80
9	7	-17	21.10	8.10
8	-7	-17	8.20	11.30
8	7	-17	10.00	7.00
-8	7	17	-7.60	6.10
7	-7	-17	11.70	7.50
7	7	-17	18.90	7.10
-7	7	17	21.20	7.30
6	-7	-17	224.18	16.00
-6	7	17	200.68	15.70
6	7	-17	194.78	15.80
5	-7	-17	1.50	5.30
-5	7	17	-9.80	5.90
5	7	-17	2.60	5.30
4	-7	-17	441.16	25.10
-4	7	17	436.46	25.60
4	7	-17	438.46	24.70
4	7	-17	436.46	21.20
-3	-7	17	139.39	8.10
3	-7	-17	126.19	11.60
3	7	-17	130.59	7.10
3	7	-17	124.99	10.80
-3	7	17	131.09	12.90
-2	-7	17	145.29	8.50
2	-7	-17	129.89	11.30
2	7	-17	106.49	8.40
-2	7	17	120.99	12.20
1	-7	-17	50.49	8.30
1	7	-17	44.00	6.30

1	7	-17	48.60	4.40
-1	7	17	34.30	7.00
0	-7	-17	157.08	12.30
0	7	17	170.28	13.40
0	7	-17	161.58	10.30
0	7	-17	159.48	10.60
0	7	17	176.18	13.70
0	7	-17	161.28	9.60
-1	-7	-17	80.79	9.10
1	7	17	88.59	10.50
1	7	17	86.09	10.20
-1	7	-17	75.49	6.60
-1	7	-17	83.19	6.20
-1	7	-17	69.99	7.70
2	7	17	26.00	6.10
-2	7	-17	29.50	3.60
2	7	17	38.10	6.70
-2	7	-17	31.20	5.40
-2	7	-17	28.20	4.40
-3	7	-17	373.76	21.20
3	7	17	391.66	23.50
3	7	17	447.66	23.70
-3	7	-17	382.96	22.00
4	7	17	13.60	5.70
-4	7	-17	3.80	4.40
-4	7	-17	3.30	5.00
5	7	17	219.18	15.40
-5	7	-17	203.18	14.50
5	7	17	202.68	15.80
-6	7	-17	13.30	6.20
6	7	17	24.60	7.30
6	7	17	21.50	6.40
7	7	17	335.97	22.20
-7	7	-17	332.97	21.40
7	7	17	373.66	21.70
-8	7	-17	70.59	11.80
8	7	17	66.39	10.20
9	7	17	176.28	16.00
-9	7	-17	167.88	15.50
10	7	17	9.30	8.30
-10	7	-17	-1.00	7.60
11	7	17	99.09	14.10
-11	7	-17	131.59	15.50
11	-8	-17	171.08	19.30

11	8	-17	182.98	20.40
-11	8	17	186.28	16.40
10	-8	-17	10.90	8.20
-10	8	17	8.90	7.80
10	8	-17	10.10	8.70
9	-8	-17	240.28	19.40
-9	8	17	254.77	17.90
9	8	-17	212.58	19.30
8	-8	-17	-0.10	7.50
8	8	-17	6.60	7.20
-8	8	17	-6.60	6.40
7	-8	-17	34.90	11.40
7	8	-17	38.00	8.20
-7	8	17	50.39	8.80
6	-8	-17	33.50	7.90
6	8	-17	20.80	6.70
-6	8	17	20.60	8.50
5	-8	-17	297.47	19.50
5	8	-17	290.77	19.20
-5	8	17	302.17	20.10
-4	8	17	45.30	8.30
4	8	-17	46.40	9.00
3	-8	-17	12.10	5.90
3	8	-17	9.30	4.60
-3	8	17	3.00	6.20
3	8	-17	10.40	1.60
3	8	-17	10.60	2.00
2	-8	-17	91.89	10.30
2	8	-17	96.69	6.70
-2	8	17	72.19	11.10
2	8	-17	91.49	5.50
2	8	-17	93.59	8.80
1	-8	-17	56.59	9.00
-1	8	17	43.20	8.30
1	8	-17	45.40	4.30
1	8	-17	44.30	5.70
0	-8	-17	78.99	9.50
0	8	-17	82.29	7.40
0	8	-17	76.39	6.10
0	8	17	81.59	11.00
0	8	-17	83.39	7.20
0	8	17	81.39	9.80
-1	-8	-17	223.68	15.60
-1	8	-17	211.38	12.60

1	8	17	232.08	16.20
1	8	17	235.88	16.60
-1	8	-17	225.98	12.40
-1	8	-17	221.38	13.50
2	8	17	263.77	18.60
-2	8	-17	287.17	14.20
-2	8	-17	276.97	15.50
-2	8	-17	288.47	16.30
2	8	17	266.17	18.40
3	8	17	171.08	13.50
-3	8	-17	136.79	10.90
3	8	17	135.29	18.00
-3	8	-17	127.09	9.90
4	8	17	81.29	11.00
-4	8	-17	64.99	8.30
-4	8	-17	73.89	9.00
4	8	17	85.29	10.80
5	8	17	11.40	6.40
5	8	17	15.90	6.50
-5	8	-17	9.40	5.80
-6	8	-17	290.67	20.00
6	8	17	354.46	20.70
6	8	17	328.77	21.10
7	8	17	9.60	6.70
-7	8	-17	8.40	6.60
8	8	17	249.18	18.40
-8	8	-17	237.78	17.80
9	8	17	15.00	8.20
-9	8	-17	8.80	7.30
-10	8	-17	251.47	18.60
10	8	17	198.48	17.80
11	8	17	81.99	12.30
11	-9	-17	56.49	11.40
-11	9	17	51.59	9.40
11	9	-17	44.30	12.30
10	-9	-17	351.96	25.70
-10	9	17	377.36	23.60
10	9	-17	347.37	25.60
9	-9	-17	16.60	8.30
9	9	-17	1.20	8.80
-9	9	17	-2.30	7.00
8	-9	-17	271.47	20.10
8	9	-17	275.07	20.20
-8	9	17	236.28	18.90

7	9	-17	16.90	7.20
-7	9	17	17.20	7.50
6	-9	-17	475.95	29.20
-6	9	17	523.05	29.30
6	9	-17	508.65	29.10
5	-9	-17	254.07	17.90
5	9	-17	249.68	17.60
4	-9	-17	409.46	24.70
-4	9	17	433.76	25.60
4	9	-17	409.76	24.10
3	-9	-17	19.90	6.50
-3	9	17	14.40	6.80
3	9	-17	15.80	5.10
2	-9	-17	252.97	16.50
2	9	-17	221.08	11.60
-2	9	17	217.18	16.50
2	9	-17	237.48	14.80
2	9	-17	239.88	12.00
1	-9	-17	1400.66	69.09
-1	9	17	1323.47	69.59
1	9	-17	1413.36	66.39
1	9	-17	1369.16	67.49
0	-9	-17	385.06	23.10
0	9	-17	370.36	19.60
0	9	17	364.76	23.50
0	9	-17	380.76	21.60
0	9	17	423.86	24.20
0	9	-17	399.26	20.40
1	9	17	741.33	37.80
-1	9	-17	676.63	34.10
-1	9	-17	651.03	34.70
1	9	17	664.43	37.20
-1	9	-17	714.03	34.80
2	9	17	207.08	16.10
-2	9	-17	224.28	11.00
-2	9	-17	193.38	12.30
-2	9	-17	192.38	13.00
3	9	17	700.63	35.20
3	9	17	626.84	34.80
-3	9	-17	613.64	32.40
-3	9	-17	612.84	33.00
4	9	17	106.89	11.90
4	9	17	133.39	12.80
-4	9	-17	88.49	9.80

-4	9	-17	90.79	9.40
5	9	17	796.82	40.30
-5	9	-17	723.13	38.40
5	9	17	693.73	39.40
6	9	17	83.39	12.10
-6	9	-17	71.39	10.70
-7	9	-17	580.94	32.20
7	9	17	581.04	33.10
-8	9	-17	23.60	8.00
8	9	17	16.40	7.80
-9	9	-17	263.37	19.30
9	9	17	257.77	19.80
-10	9	-17	47.10	9.70
10	9	17	37.40	10.50
11	9	17	335.57	24.50
-11	9	-17	398.16	25.30
11	-10	-17	15.40	10.50
11	10	-17	0.70	11.40
-11	10	17	13.60	8.60
10	-10	-17	16.40	9.90
10	10	-17	15.20	8.90
-10	10	17	2.50	8.10
9	-10	-17	-6.30	8.20
9	10	-17	7.60	8.30
-9	10	17	-6.10	8.00
8	-10	-17	1.60	7.20
8	10	-17	5.10	7.60
-8	10	17	-17.00	6.70
7	-10	-17	12.70	11.40
7	10	-17	9.30	7.60
-7	10	17	-8.80	6.80
6	-10	-17	53.99	13.20
-6	10	17	42.20	9.30
6	10	-17	35.50	8.20
5	-10	-17	53.29	10.70
5	10	-17	26.70	7.60
-5	10	17	35.00	9.30
4	-10	-17	8.30	6.00
4	10	-17	2.30	5.00
-4	10	17	-7.90	7.10
3	-10	-17	15.00	6.30
3	10	-17	14.20	5.10
2	-10	-17	9.50	6.20
2	10	-17	9.40	4.60

-2	10	17	-8.90	11.60
1	-10	-17	11.10	5.50
1	10	-17	8.10	4.30
1	10	-17	17.90	3.10
-1	10	17	0.40	8.40
0	10	17	23.90	7.40
0	10	-17	34.10	5.40
0	10	-17	26.30	4.30
0	10	-17	26.20	4.50
-1	10	-17	97.29	7.50
1	10	17	74.99	12.80
-1	10	-17	108.79	8.50
-1	10	-17	103.29	7.60
1	10	17	125.09	13.10
-2	10	-17	-1.50	3.50
-2	10	-17	-1.70	4.00
2	10	17	-1.90	6.00
2	10	17	-3.00	6.20
-3	10	-17	-4.70	3.80
-3	10	-17	-0.90	4.60
3	10	17	-7.20	6.10
3	10	17	-2.30	6.00
4	10	17	-3.10	6.20
4	10	17	-6.30	6.30
-4	10	-17	2.30	4.60
-4	10	-17	9.00	5.10
5	10	17	46.80	9.10
5	10	17	42.70	9.10
-5	10	-17	50.29	7.70
6	10	17	-8.30	6.70
-6	10	-17	3.50	5.80
-7	10	-17	5.40	6.40
7	10	17	7.60	7.50
-8	10	-17	54.79	9.20
8	10	17	87.19	13.30
-9	10	-17	5.20	7.80
9	10	17	-2.30	8.10
10	10	17	15.20	8.70
-10	10	-17	20.10	8.60
-11	10	-17	6.70	8.80
11	10	17	13.00	9.40
10	-11	-17	362.86	27.70
10	11	-17	387.76	27.60
-10	11	17	417.16	25.20

9	-11	-17	11.60	9.50
-9	11	17	-4.30	7.90
9	11	-17	2.20	8.20
8	-11	-17	243.98	19.80
-8	11	17	246.38	18.90
8	11	-17	246.98	20.00
7	-11	-17	208.08	19.20
-7	11	17	193.98	17.00
7	11	-17	198.88	17.30
6	11	-17	457.75	28.00
-6	11	17	483.35	28.60
5	-11	-17	522.25	36.90
-5	11	17	549.95	32.10
5	11	-17	561.44	31.00
4	-11	-17	218.48	18.80
-4	11	17	200.08	18.10
4	11	-17	240.88	16.20
3	-11	-17	78.49	11.90
3	11	-17	82.39	9.80
-3	11	17	65.79	10.40
2	-11	-17	238.68	20.70
2	11	-17	278.27	16.50
-2	11	17	255.87	19.50
1	11	-17	235.68	14.90
1	11	-17	243.18	12.20
1	11	-17	248.68	12.50
-1	11	17	230.78	18.50
0	-11	-17	68.39	11.20
0	11	17	57.49	9.90
0	11	-17	58.09	5.80
0	11	-17	59.59	5.10
0	11	-17	51.79	7.10
-1	11	-17	816.42	39.40
-1	11	-17	776.92	39.00
1	11	17	841.22	43.20
-1	11	-17	794.82	39.30
1	11	17	737.53	42.50
2	11	17	195.08	16.20
2	11	17	211.48	17.00
-2	11	-17	208.78	11.10
-2	11	-17	185.38	12.00
-2	11	-17	194.78	12.70
3	11	17	210.98	17.20
-3	11	-17	205.68	13.30

3	11	17	209.48	17.30
-3	11	-17	229.58	14.10
-4	11	-17	63.99	9.30
-4	11	-17	63.29	8.60
4	11	17	78.29	12.30
4	11	17	59.99	11.30
5	11	17	976.80	52.59
-5	11	-17	1043.80	51.29
6	11	17	-7.40	6.80
-6	11	-17	-0.60	6.10
-7	11	-17	777.92	41.10
7	11	17	779.72	42.60
-8	11	-17	1.20	7.20
8	11	17	2.50	8.20
-9	11	-17	290.17	21.00
9	11	17	309.87	21.80
-10	11	-17	54.69	10.50
10	11	17	28.30	9.60
10	-12	-17	9.00	9.50
-10	12	17	11.80	8.70
10	12	-17	5.80	9.40
9	-12	-17	87.19	16.10
-9	12	17	46.70	9.80
9	12	-17	59.89	11.00
8	-12	-17	33.30	9.20
-8	12	17	23.30	9.20
8	12	-17	31.90	9.70
7	-12	-17	137.39	15.40
7	12	-17	159.98	15.50
-7	12	17	116.19	15.20
6	-12	-17	42.10	10.60
6	12	-17	59.29	12.10
-6	12	17	59.69	10.80
5	12	-17	200.58	15.90
-5	12	17	156.98	16.90
-4	12	17	84.79	13.70
4	12	-17	66.39	10.80
-3	12	17	480.65	30.00
3	12	-17	500.25	27.60
2	-12	-17	115.39	19.10
2	12	-17	128.59	11.00
-2	12	17	95.49	13.20
1	-12	-17	29.20	7.40
-1	12	17	34.60	9.00

-1	12	17	62.79	10.40
1	12	-17	37.90	3.40
1	12	-17	39.10	3.20
1	12	-17	55.59	8.00
0	12	-17	260.57	13.90
0	12	-17	276.67	14.30
0	12	17	232.18	19.40
0	12	-17	284.97	16.00
1	12	17	273.47	20.00
-1	12	-17	307.17	16.40
-1	12	-17	287.77	15.70
-1	12	-17	295.47	16.10
2	12	17	330.97	21.80
2	12	17	297.37	21.20
-2	12	-17	305.47	16.20
-2	12	-17	296.87	16.80
-2	12	-17	300.47	17.20
3	12	17	12.70	7.60
3	12	17	-4.90	6.80
-3	12	-17	4.80	4.70
-3	12	-17	1.30	4.40
-4	12	-17	392.46	21.60
-4	12	-17	352.26	22.40
4	12	17	377.56	24.20
-5	12	-17	15.30	6.30
5	12	17	16.20	7.30
6	12	17	328.57	22.00
-6	12	-17	330.87	20.70
-7	12	-17	8.80	7.00
7	12	17	9.10	7.80
8	12	17	220.88	18.30
-8	12	-17	244.18	17.80
9	12	17	36.50	9.70
-9	12	-17	73.39	13.10
10	12	17	127.59	17.00
-10	12	-17	157.08	16.30
-10	13	17	345.97	22.80
10	13	-17	276.07	24.60
9	-13	-17	14.80	9.30
-9	13	17	3.20	8.80
9	13	-17	14.00	9.40
8	-13	-17	20.60	9.30
-8	13	17	17.00	8.90
8	13	-17	38.80	9.70

7	-13	-17	52.19	9.90
7	13	-17	57.29	10.60
-7	13	17	51.69	10.80
6	-13	-17	90.09	14.30
-6	13	17	90.09	14.40
6	13	-17	96.39	13.70
5	-13	-17	86.09	13.40
-5	13	17	63.79	11.50
5	13	-17	59.69	11.80
4	-13	-17	122.99	14.00
4	13	-17	117.19	12.90
-4	13	17	137.09	15.80
3	-13	-17	127.19	13.90
3	13	-17	130.19	12.70
-3	13	17	140.19	16.00
2	-13	-17	2.70	6.90
-2	13	17	17.00	9.10
-2	13	17	-1.80	8.10
2	13	-17	5.40	5.00
1	-13	-17	199.98	16.50
-1	13	17	199.58	17.80
-1	13	17	198.18	18.10
1	13	-17	185.38	13.60
0	13	17	37.80	9.80
0	13	-17	28.70	4.70
0	13	17	29.00	9.10
0	13	-17	35.90	5.70
1	13	17	113.99	14.60
-1	13	-17	132.49	8.60
-1	13	-17	110.79	9.40
2	13	17	197.88	17.20
2	13	17	211.28	23.80
-2	13	-17	187.58	11.50
-2	13	-17	168.38	12.80
-2	13	-17	202.88	12.20
3	13	17	100.39	14.10
-3	13	-17	108.69	6.90
-3	13	-17	118.89	7.20
-3	13	-17	125.89	9.70
-3	13	-17	131.99	10.30
-4	13	-17	-0.30	5.20
4	13	17	-1.30	7.30
5	13	17	166.58	15.90
-5	13	-17	161.08	13.30

6	13	17	8.00	7.60
7	13	17	214.48	17.50
-7	13	-17	197.18	16.40
-8	13	-17	-2.50	7.50
8	13	17	6.60	7.80
-9	13	-17	90.79	14.70
9	13	17	103.69	15.00
-10	13	-17	98.89	15.40
10	13	17	95.69	16.00
9	-14	-17	265.97	23.50
-9	14	17	309.57	22.10
9	14	-17	286.97	23.60
8	-14	-17	12.80	9.30
-8	14	17	7.00	9.20
8	14	-17	4.20	9.50
7	-14	-17	435.76	29.40
7	14	-17	469.55	29.00
-7	14	17	474.55	29.00
6	-14	-17	105.99	15.30
6	14	-17	89.49	14.20
-6	14	17	86.19	14.60
5	-14	-17	316.17	23.00
5	14	-17	355.66	22.70
-5	14	17	328.37	23.80
4	-14	-17	164.18	15.80
4	14	-17	145.49	14.10
-4	14	17	114.59	16.60
3	-14	-17	498.85	30.30
3	14	-17	496.15	28.70
-3	14	17	536.35	31.80
2	-14	-17	103.09	13.60
-2	14	17	93.59	15.40
2	14	-17	126.59	11.50
-2	14	17	87.99	16.40
1	-14	-17	559.74	33.10
-1	14	17	549.15	33.50
-1	14	17	586.94	34.80
1	14	-17	588.54	30.80
0	14	17	995.10	54.19
0	14	17	976.80	55.09
0	14	-17	993.10	50.89
0	14	-17	1091.69	49.60
1	14	17	189.48	18.20
-1	14	-17	231.18	13.50

-1	14	-17	194.18	11.80
-2	14	-17	600.64	29.70
2	14	17	617.14	34.50
-2	14	-17	577.24	29.60
-2	14	-17	547.35	29.10
-3	14	-17	85.09	9.10
-3	14	-17	101.49	7.00
3	14	17	94.89	14.10
-3	14	-17	86.09	6.40
4	14	17	584.34	34.50
-4	14	-17	609.44	31.50
-5	14	-17	1.30	5.80
5	14	17	-6.00	7.30
6	14	17	889.71	46.80
-6	14	-17	829.92	47.40
-7	14	-17	-7.00	7.00
7	14	17	6.80	7.80
-8	14	-17	259.27	19.10
8	14	17	230.78	19.30
9	14	17	41.80	10.40
-9	14	-17	51.49	10.20
9	15	-17	4.60	9.70
-9	15	17	1.60	9.10
8	-15	-17	3.00	10.50
8	15	-17	20.70	9.10
-8	15	17	-0.30	8.60
7	-15	-17	13.50	9.80
-7	15	17	-10.80	9.30
7	15	-17	9.20	8.40
6	-15	-17	-1.30	7.80
6	15	-17	0.30	7.60
-6	15	17	-14.00	9.30
5	-15	-17	1.80	7.70
5	15	-17	-0.80	6.60
-5	15	17	-5.10	9.30
4	-15	-17	14.20	7.80
4	15	-17	10.80	7.20
-4	15	17	-4.60	9.30
3	-15	-17	7.50	7.50
3	15	-17	13.50	6.50
-3	15	17	-14.00	9.10
2	-15	-17	6.30	7.30
-2	15	17	13.00	10.10
2	15	-17	21.80	6.20

1	15	-17	20.10	5.80
-1	15	17	1.00	8.70
0	15	17	18.70	10.00
0	15	-17	32.30	6.10
1	15	17	-6.60	8.40
-1	15	-17	5.00	3.20
-1	15	-17	5.80	4.80
-2	15	-17	2.20	3.80
-2	15	-17	2.30	3.30
-2	15	-17	8.30	4.20
2	15	17	-1.90	8.20
-3	15	-17	8.00	3.60
3	15	17	-6.00	7.50
-3	15	-17	1.70	2.90
-3	15	-17	-3.80	4.30
4	15	17	-4.40	7.90
-4	15	-17	0.10	5.10
-5	15	-17	13.20	6.30
5	15	17	14.10	8.70
-6	15	-17	16.20	7.30
6	15	17	30.80	9.40
-7	15	-17	11.70	7.70
7	15	17	-3.70	8.30
-8	15	-17	6.40	8.00
8	15	17	-12.20	8.10
-9	15	-17	7.90	8.10
9	15	17	8.30	8.80
8	-16	-17	-5.80	9.50
-8	16	17	-9.90	9.60
8	16	-17	-1.50	8.90
7	-16	-17	298.97	24.40
7	16	-17	311.57	23.50
-7	16	17	318.17	23.30
6	-16	-17	292.77	24.30
-6	16	17	335.77	24.10
6	16	-17	356.96	23.90
5	-16	-17	419.66	27.80
5	16	-17	399.96	26.30
-5	16	17	418.96	27.80
4	-16	-17	240.28	20.20
-4	16	17	245.38	21.70
4	16	-17	247.58	18.90
3	-16	-17	194.28	18.80
3	16	-17	205.78	16.40

-3	16	17	218.88	20.60
2	-16	-17	114.29	16.20
-2	16	17	92.09	16.60
2	16	-17	138.89	12.90
-1	16	17	97.29	16.50
1	16	-17	110.69	11.60
0	16	17	895.21	48.40
0	16	-17	845.22	44.30
1	16	17	108.99	14.60
-1	16	-17	99.29	10.10
2	16	17	222.98	19.80
-2	16	-17	223.58	12.90
-2	16	-17	204.58	12.90
-2	16	-17	212.98	13.70
3	16	17	51.69	11.40
-3	16	-17	46.00	7.50
-3	16	-17	35.40	6.00
-3	16	-17	38.30	6.60
-4	16	-17	484.85	25.50
-4	16	-17	549.35	27.50
4	16	17	531.45	32.00
-4	16	-17	491.85	25.40
5	16	17	-9.70	8.30
-5	16	-17	-6.20	5.80
-6	16	-17	553.74	29.90
6	16	17	517.95	32.80
-7	16	-17	-0.70	7.30
7	16	17	-17.30	8.60
-8	16	-17	212.28	17.30
8	16	17	206.28	20.00
7	-17	-17	17.60	10.50
-7	17	17	24.60	10.80
7	17	-17	48.00	11.20
6	-17	-17	274.97	23.40
-6	17	17	261.67	22.60
6	17	-17	280.87	21.70
5	-17	-17	31.10	9.60
-5	17	17	19.10	11.30
5	17	-17	30.20	9.10
4	-17	-17	256.17	21.80
4	17	-17	255.97	19.40
-4	17	17	225.28	20.50
3	-17	-17	235.88	20.80
-3	17	17	246.88	21.80

3	17	-17	241.88	18.20
2	17	-17	56.29	10.90
-2	17	17	39.10	12.00
1	17	-17	177.78	14.00
-1	17	17	122.89	18.40
0	17	-17	53.69	9.70
0	17	17	38.20	11.30
-1	17	-17	318.97	19.00
1	17	17	304.27	24.30
-2	17	-17	94.49	9.00
2	17	17	78.39	13.60
-2	17	-17	127.49	10.30
-3	17	-17	169.38	11.80
-3	17	-17	185.58	11.70
3	17	17	173.98	18.50
-3	17	-17	151.98	11.00
-4	17	-17	15.90	3.90
-4	17	-17	5.50	3.60
4	17	17	-5.10	8.80
-4	17	-17	5.90	5.20
5	17	17	135.59	17.00
-5	17	-17	149.19	12.70
-6	17	-17	22.30	7.30
6	17	17	30.50	9.30
-7	17	-17	75.49	12.50
7	17	17	55.79	11.50
7	-18	-17	16.80	16.50
7	18	-17	5.30	9.20
-7	18	17	5.30	10.40
6	-18	-17	76.39	13.20
-6	18	17	52.19	12.00
6	18	-17	93.79	16.50
5	-18	-17	124.99	18.90
5	18	-17	104.89	15.70
-5	18	17	129.79	17.70
4	-18	-17	113.39	17.90
-4	18	17	106.79	18.20
4	18	-17	123.29	14.70
-3	18	17	-3.80	9.80
3	18	-17	7.60	7.30
-2	18	17	8.80	9.90
2	18	-17	5.40	6.70
1	18	-17	6.60	6.50
-1	18	17	-3.30	9.00

0	18	17	50.49	12.00
0	18	-17	67.49	10.50
-1	18	-17	87.79	9.30
-1	18	-17	86.09	10.70
1	18	17	87.29	13.60
-2	18	-17	63.09	8.30
-2	18	-17	53.49	9.40
2	18	17	67.69	12.40
-3	18	-17	4.90	4.90
-3	18	-17	3.60	4.30
3	18	17	1.70	8.80
-4	18	-17	167.98	11.40
-4	18	-17	178.38	11.80
4	18	17	175.38	18.90
5	18	17	9.70	9.40
-5	18	-17	12.00	6.40
-5	18	-17	17.00	4.00
-5	18	-17	14.70	3.90
6	18	17	286.87	23.40
-6	18	-17	295.87	19.40
7	18	17	77.29	13.30
6	-19	-17	347.57	27.20
6	19	-17	317.67	24.70
-6	19	17	319.57	25.80
5	-19	-17	183.78	22.00
5	19	-17	221.98	19.90
-5	19	17	225.78	22.00
4	-19	-17	400.66	30.00
4	19	-17	411.36	27.30
-4	19	17	462.05	28.90
3	19	-17	122.89	15.20
-3	19	17	133.29	18.90
2	19	-17	238.28	18.00
-2	19	17	228.28	21.90
1	19	-17	524.45	30.00
0	19	-17	160.08	13.90
0	19	17	101.09	18.70
1	19	17	516.25	34.60
-1	19	-17	557.14	29.90
2	19	17	9.00	10.50
-2	19	-17	21.20	6.20
-3	19	-17	297.27	17.70
3	19	17	270.47	23.10
4	19	17	1.20	9.80

-4	19	-17	-4.80	4.30
-4	19	-17	0.30	4.70
-5	19	-17	801.02	39.40
5	19	17	781.72	45.30
-5	19	-17	792.52	39.40
6	19	17	9.30	10.30
-4	20	17	-16.20	10.90
4	20	-17	4.90	8.20
3	20	-17	18.40	8.40
-3	20	17	5.60	10.60
2	20	-17	14.70	7.90
-2	20	17	33.30	12.30
-1	20	17	7.30	10.90
1	20	-17	8.40	7.60
0	20	-17	32.20	7.90
0	20	17	53.19	13.20
-1	20	-17	-7.40	6.30
1	20	17	-3.50	10.00
2	20	17	17.10	11.50
-2	20	-17	22.90	6.90
3	20	17	-2.60	10.10
-3	20	-17	0.80	5.40
-4	20	-17	6.80	5.50
4	20	17	-13.20	10.80
2	21	-17	56.99	12.60
-2	21	17	21.80	11.70
-1	21	17	190.38	22.30
1	21	-17	240.58	18.80
0	21	17	41.40	12.60
0	21	-17	40.60	8.60
-1	21	-17	188.08	16.00
1	21	17	219.58	21.80
-2	21	-17	86.19	11.90
2	21	17	84.49	15.10
3	21	17	40.80	12.10
12	0	-18	29.40	11.00
11	0	-18	183.28	18.90
-10	0	18	74.79	9.90
10	0	-18	72.69	11.80
9	0	-18	1021.40	53.89
-9	0	18	1022.20	51.09
8	0	-18	107.99	13.20
-8	0	18	115.79	10.20
7	0	-18	229.98	17.00

7	0	-18	236.78	14.10
-7	0	18	240.08	14.80
6	0	-18	857.71	44.10
-6	0	18	850.81	42.40
6	0	-18	820.02	41.50
5	0	-18	1276.87	64.39
-5	0	18	1346.47	65.29
5	0	-18	1375.26	66.79
4	0	-18	869.51	43.70
-4	0	18	918.91	44.70
3	0	-18	327.87	19.10
3	0	-18	304.77	16.50
-3	0	18	309.27	17.70
-2	0	18	403.86	22.30
2	0	-18	402.76	21.30
2	0	-18	441.16	22.90
1	0	-18	29.40	5.20
-1	0	18	18.60	4.50
1	0	-18	21.50	4.50
0	0	-18	891.41	44.20
0	0	18	870.21	44.90
0	0	-18	925.61	44.70
1	0	18	73.29	7.90
-1	0	-18	73.29	6.90
-1	0	-18	67.89	7.20
-2	0	-18	855.11	43.10
2	0	18	822.72	43.50
2	0	18	901.11	42.00
-2	0	-18	853.41	42.50
3	0	18	40.40	7.50
4	0	18	1277.57	63.69
5	0	18	64.79	9.10
6	0	18	420.16	24.40
7	0	18	126.09	12.70
8	0	18	406.76	24.90
-9	0	-18	79.89	12.90
-10	0	-18	357.46	24.10
-11	0	-18	204.98	18.90
-12	0	-18	204.08	19.20
12	-1	-18	12.70	10.10
12	1	-18	18.90	10.00
11	-1	-18	87.99	16.50
11	1	-18	42.80	10.50
-11	1	18	72.89	10.60

-10	-1	18	38.20	6.40
10	-1	-18	41.10	9.90
10	1	-18	38.20	9.30
-10	1	18	31.90	7.00
9	-1	-18	82.69	13.50
-9	-1	18	63.89	8.80
-9	1	18	61.19	9.80
-8	-1	18	3.00	4.70
8	-1	-18	7.10	6.50
-8	1	18	6.00	5.30
8	1	-18	9.00	6.50
7	-1	-18	15.20	6.70
7	-1	-18	21.50	4.80
-7	-1	18	10.90	4.60
7	1	-18	19.40	4.50
7	1	-18	12.90	6.10
-7	1	18	23.90	5.70
6	-1	-18	88.79	8.30
6	-1	-18	104.89	11.50
-6	-1	18	116.49	8.90
6	1	-18	92.29	11.40
-6	1	18	114.39	9.90
6	1	-18	112.99	8.10
5	-1	-18	87.29	7.50
-5	-1	18	97.79	8.20
5	-1	-18	97.89	10.70
5	1	-18	89.89	10.30
-5	1	18	104.89	9.10
5	1	-18	87.19	7.20
4	-1	-18	32.00	5.50
-4	-1	18	42.10	6.20
4	1	-18	39.00	5.00
4	1	-18	31.20	7.50
-4	1	18	44.80	7.20
3	-1	-18	22.40	5.30
-3	-1	18	12.10	4.10
3	-1	-18	24.80	3.80
3	1	-18	28.20	3.60
-3	1	18	17.20	4.80
3	1	-18	29.90	6.90
2	-1	-18	2.50	3.80
-2	-1	18	3.50	3.50
2	-1	-18	5.90	3.30
2	1	-18	6.80	3.00

-2	1	18	2.00	4.00
2	1	-18	6.50	3.90
1	-1	-18	10.40	3.90
-1	-1	18	1.40	3.60
1	-1	-18	6.20	3.40
1	1	-18	9.10	3.70
-1	1	18	5.80	4.20
1	1	-18	5.50	3.00
0	-1	18	7.60	4.10
0	-1	-18	5.90	3.50
0	-1	-18	6.00	3.50
0	1	-18	7.20	3.50
0	1	18	8.10	4.00
0	1	-18	4.80	3.30
-1	-1	-18	148.89	10.90
-1	-1	-18	171.58	11.10
1	-1	18	170.98	11.50
1	-1	18	182.38	10.60
1	1	18	195.88	11.90
-1	1	-18	161.18	10.60
1	1	18	166.08	12.00
-1	1	-18	149.69	10.70
-2	-1	-18	52.99	6.20
2	-1	18	47.80	7.30
2	1	18	54.49	6.60
2	1	18	49.60	7.70
-2	1	-18	40.00	6.60
-2	1	-18	52.19	5.60
3	-1	18	66.29	8.30
3	1	18	56.29	8.50
3	1	18	73.39	5.80
4	-1	18	0.20	4.50
4	1	18	2.10	4.70
5	-1	18	117.79	11.10
5	1	18	116.79	10.90
6	-1	18	23.90	7.60
6	1	18	14.60	6.30
7	-1	18	137.49	12.90
7	1	18	129.99	13.00
-8	1	-18	44.60	8.60
8	1	18	47.20	8.60
-9	1	-18	-6.00	7.10
9	1	18	-9.00	6.80
-10	-1	-18	10.20	8.40

-10	1	-18	13.20	8.40
-11	-1	-18	38.20	9.80
-11	1	-18	57.79	10.70
-12	-1	-18	38.50	10.80
-12	1	-18	42.90	9.90
12	-2	-18	4.50	10.30
12	2	-18	-3.00	10.50
11	-2	-18	329.97	24.80
11	2	-18	340.47	24.90
10	-2	-18	77.69	14.00
-10	2	18	40.90	7.80
10	2	-18	59.59	10.40
9	-2	-18	573.04	32.60
-9	-2	18	537.65	28.70
-9	2	18	558.34	29.90
9	2	-18	541.95	32.30
-8	-2	18	7.50	4.40
8	-2	-18	9.80	7.00
8	2	-18	11.10	6.70
-8	2	18	12.90	5.80
7	-2	-18	330.27	20.10
-7	-2	18	363.46	19.90
7	-2	-18	358.56	22.90
7	2	-18	388.36	23.10
-7	2	18	411.06	21.50
6	-2	-18	182.48	12.40
6	-2	-18	206.18	15.30
-6	-2	18	207.08	12.30
6	2	-18	200.98	11.70
-6	2	18	201.58	13.80
6	2	-18	197.68	15.00
5	-2	-18	307.87	18.10
5	-2	-18	329.87	21.60
-5	-2	18	332.17	18.20
5	2	-18	331.87	17.50
5	2	-18	354.66	20.60
-5	2	18	359.26	19.60
-4	-2	18	956.40	45.70
4	-2	-18	869.11	45.30
4	2	-18	928.61	44.90
4	2	-18	922.41	47.20
-4	2	18	946.81	46.70
-3	-2	18	98.29	7.70
3	-2	-18	95.89	7.20

3	-2	-18	98.89	9.80
-3	2	18	80.69	9.00
3	2	-18	79.49	6.40
2	-2	-18	372.66	20.50
2	-2	-18	352.66	19.00
-2	-2	18	346.77	19.40
2	2	-18	379.86	20.40
-2	2	18	345.47	20.30
2	2	-18	360.86	18.60
-1	-2	18	296.07	16.60
1	-2	-18	280.97	16.00
1	-2	-18	285.67	17.10
-1	2	18	301.47	17.30
1	2	-18	284.07	15.60
1	2	-18	302.17	16.70
0	-2	-18	1305.37	63.39
0	-2	-18	1276.57	62.69
0	-2	18	1245.68	63.09
0	2	-18	1324.87	62.89
0	2	18	1245.88	63.59
0	2	-18	1314.47	62.49
1	-2	18	142.49	11.20
1	-2	18	177.38	9.70
-1	-2	-18	187.68	13.20
-1	-2	-18	171.98	11.20
-1	2	-18	157.38	10.30
-1	2	-18	151.68	10.50
1	2	18	181.68	12.20
1	2	18	149.29	11.80
2	-2	18	403.16	23.00
-2	-2	-18	399.26	22.80
2	2	18	394.26	23.40
2	2	18	469.65	22.80
-2	2	-18	430.86	22.50
-2	2	-18	424.06	21.60
3	-2	18	-0.20	4.50
3	2	18	1.10	3.50
3	2	18	-1.70	4.70
4	-2	18	248.08	15.80
4	2	18	226.58	16.10
4	2	18	256.27	13.30
5	-2	18	66.89	8.80
5	2	18	37.30	6.10
5	2	18	38.20	7.80

6	2	18	769.32	40.70
7	-2	18	24.50	7.10
7	2	18	13.60	6.30
-7	2	-18	12.30	6.80
-8	2	-18	401.76	24.80
8	2	18	410.46	25.00
-9	2	-18	43.90	9.60
9	2	18	53.69	9.60
-10	-2	-18	349.67	23.60
-10	2	-18	324.27	23.00
-11	-2	-18	24.80	9.00
-11	2	-18	39.00	9.10
-12	-2	-18	183.48	19.50
-12	2	-18	186.78	19.10
12	-3	-18	294.97	24.20
12	3	-18	271.27	23.70
-12	3	18	286.07	19.40
11	-3	-18	28.00	9.90
-11	3	18	11.20	7.00
11	3	-18	19.20	9.50
10	-3	-18	691.33	38.60
-10	3	18	672.43	36.10
10	3	-18	661.53	38.90
-9	-3	18	-0.50	4.10
9	-3	-18	2.80	8.00
-9	3	18	-2.80	5.80
9	3	-18	7.00	7.40
-8	-3	18	426.26	23.20
8	-3	-18	452.15	27.20
8	3	-18	448.66	27.10
-8	3	18	465.25	25.40
-7	-3	18	147.39	10.00
7	-3	-18	148.79	14.00
7	3	-18	162.58	14.30
-7	3	18	141.99	12.70
6	-3	-18	685.33	36.00
-6	-3	18	636.84	33.20
6	3	-18	648.54	32.90
6	3	-18	685.83	36.00
-6	3	18	679.03	35.10
5	-3	-18	505.45	32.60
-5	-3	18	587.04	28.80
5	-3	-18	556.54	28.90
-5	3	18	601.04	30.70

5	3	-18	586.24	31.10
5	3	-18	571.04	28.20
4	-3	-18	443.46	24.80
-4	-3	18	513.35	24.90
4	3	-18	496.45	26.70
-4	3	18	495.55	26.50
4	3	-18	478.65	24.10
3	-3	-18	734.93	38.90
3	-3	-18	794.92	40.90
-3	-3	18	810.52	39.10
-3	3	18	813.62	40.60
3	3	-18	813.92	41.30
3	3	-18	757.62	38.30
2	-3	-18	77.59	8.30
-2	-3	18	64.39	6.80
2	-3	-18	53.39	6.60
2	3	-18	75.89	5.60
-2	3	18	58.99	8.60
2	3	-18	53.69	10.10
1	-3	-18	1503.85	72.19
1	-3	-18	1416.26	71.09
-1	-3	18	1448.36	71.29
1	3	-18	1446.16	70.59
-1	3	18	1452.75	72.29
1	3	-18	1526.75	71.59
0	-3	-18	172.68	12.60
0	-3	18	161.18	10.90
0	-3	-18	144.59	11.40
0	3	-18	156.28	9.90
0	3	18	159.78	12.00
0	3	-18	168.28	10.40
-1	-3	-18	864.61	43.90
1	-3	18	827.82	43.50
1	3	18	833.12	44.30
1	3	18	978.00	44.40
-1	3	-18	864.71	42.70
-1	3	-18	843.92	42.90
2	-3	18	101.99	9.00
2	3	18	100.99	9.80
-2	3	-18	98.39	8.30
-2	3	-18	101.69	7.00
3	-3	18	333.27	19.60
-3	3	-18	305.87	18.70
3	3	18	329.67	20.10

3	3	18	380.56	19.20
4	-3	18	9.30	5.00
4	3	18	13.50	5.90
4	3	18	14.20	4.10
5	-3	18	581.64	30.60
5	3	18	546.95	35.30
5	3	18	559.44	28.30
6	3	18	74.59	9.60
6	3	18	51.19	6.10
-7	3	-18	867.11	45.70
7	3	18	883.91	46.20
-8	3	-18	70.59	11.60
8	3	18	71.59	11.60
9	3	18	193.38	17.40
-9	3	-18	243.48	17.90
-10	3	-18	128.79	15.00
10	3	18	97.89	14.20
-11	-3	-18	303.97	22.30
-11	3	-18	312.97	22.60
11	-4	-18	8.50	10.20
-11	4	18	4.00	7.20
11	4	-18	11.50	9.40
10	-4	-18	46.10	9.70
-10	4	18	36.20	7.70
10	4	-18	34.20	9.40
9	-4	-18	-1.00	7.40
9	4	-18	-3.00	7.90
-9	4	18	3.20	6.40
8	-4	-18	-4.80	6.90
-8	-4	18	9.40	3.60
-8	4	18	1.30	6.00
8	4	-18	3.30	6.80
7	-4	-18	78.29	11.80
-7	-4	18	91.39	7.40
-7	4	18	97.29	11.30
7	4	-18	94.19	12.20
6	-4	-18	50.99	10.00
-6	-4	18	37.60	5.60
6	4	-18	41.50	5.60
6	4	-18	22.70	6.40
-6	4	18	26.40	6.90
5	-4	-18	2.60	6.40
-5	-4	18	8.80	3.40
5	4	-18	5.20	5.20

5	4	-18	12.50	2.90
-5	4	18	7.50	5.50
-4	-4	18	185.78	11.30
4	4	-18	191.18	10.50
-4	4	18	197.58	13.90
4	4	-18	185.08	13.60
3	-4	-18	66.09	9.60
-3	-4	18	73.49	6.50
3	4	-18	68.79	8.30
-3	4	18	67.09	9.40
3	4	-18	69.39	5.40
2	-4	-18	124.59	10.70
-2	-4	18	132.59	8.90
2	4	-18	117.19	7.70
2	4	-18	116.89	13.10
-2	4	18	127.29	11.00
-1	-4	18	0.00	3.30
1	-4	-18	4.10	4.20
1	4	-18	4.00	2.70
-1	4	18	-3.20	4.70
1	4	-18	-3.60	3.80
0	-4	-18	99.39	9.80
0	-4	18	111.89	8.80
0	4	-18	110.39	7.80
0	4	-18	119.49	8.20
0	4	18	105.69	10.50
0	4	18	102.49	11.00
-1	-4	-18	20.30	5.60
1	-4	18	36.60	6.30
1	4	18	30.60	6.20
-1	4	-18	17.70	4.00
1	4	18	31.50	6.20
-1	4	-18	24.30	4.80
2	-4	18	40.90	7.00
2	4	18	36.10	6.30
2	4	18	37.70	6.50
-2	4	-18	36.60	4.40
-2	4	-18	34.90	6.00
3	-4	18	30.10	5.80
-3	4	-18	23.60	4.80
3	4	18	29.30	6.40
3	4	18	35.10	5.80
4	-4	18	153.38	11.80
4	4	18	134.19	11.00

4	4	18	152.58	12.60
5	-4	18	346.37	20.50
-5	4	-18	318.37	20.40
5	4	18	346.87	21.10
5	4	18	349.57	19.00
-6	4	-18	172.58	15.80
6	4	18	179.58	11.70
6	4	18	184.78	14.80
7	4	18	23.80	7.20
7	4	18	19.20	4.40
-7	4	-18	12.50	6.60
-8	4	-18	97.09	12.60
8	4	18	111.59	13.20
-9	4	-18	9.60	7.40
9	4	18	12.40	7.40
-10	4	-18	112.39	14.40
10	4	18	111.89	14.70
-11	-4	-18	14.20	9.50
-11	4	-18	29.70	9.20
11	-5	-18	11.70	9.40
11	5	-18	10.40	10.00
-11	5	18	-5.80	7.40
10	-5	-18	695.63	39.30
10	5	-18	660.93	39.30
-10	5	18	691.43	36.80
9	-5	-18	11.20	7.90
9	5	-18	4.30	7.20
-9	5	18	-0.70	6.50
8	-5	-18	651.43	36.50
-8	5	18	651.53	35.00
8	5	-18	646.24	36.50
-7	-5	18	231.18	13.00
7	-5	-18	236.28	17.60
7	5	-18	228.38	17.60
-7	5	18	247.38	16.80
6	-5	-18	796.22	42.80
-6	-5	18	802.52	39.50
-6	5	18	844.02	42.40
6	5	-18	792.62	42.60
5	-5	-18	212.38	17.00
-5	-5	18	219.08	12.00
5	5	-18	190.78	15.00
-5	5	18	217.78	15.60
-4	-5	18	1451.25	66.79

-4	5	18	1389.36	68.99
4	5	-18	1354.56	68.59
4	5	-18	1335.47	66.19
-3	-5	18	594.34	28.80
3	-5	-18	529.75	32.60
3	5	-18	603.74	30.40
-3	5	18	568.24	31.10
3	5	-18	557.74	28.00
-2	-5	18	664.33	31.00
2	-5	-18	546.25	33.10
2	5	-18	635.24	31.90
2	5	-18	582.54	30.10
-2	5	18	644.44	33.10
-1	-5	18	756.42	36.50
1	-5	-18	744.03	38.60
1	5	-18	711.73	35.70
-1	5	18	697.43	38.10
0	-5	18	32.20	4.90
0	-5	-18	28.50	7.70
0	5	18	35.30	6.60
0	5	-18	29.80	5.70
0	5	18	44.50	6.90
0	5	-18	26.90	5.00
1	-5	18	1671.53	81.49
-1	5	-18	1684.03	80.69
-1	5	-18	1678.03	80.89
1	5	18	1597.64	82.69
1	5	18	1753.32	83.79
2	-5	18	356.16	19.70
-2	5	-18	331.07	17.80
2	5	18	327.77	20.80
2	5	18	411.06	21.20
-2	5	-18	334.77	18.90
3	-5	18	1380.36	67.69
3	5	18	1281.47	68.39
3	5	18	1508.85	68.29
-3	5	-18	1318.67	66.99
-4	5	-18	1.30	5.40
4	5	18	18.20	5.50
5	5	18	1289.37	59.79
-5	5	-18	1118.79	60.29
5	5	18	1205.18	61.19
-6	5	-18	9.20	6.40
6	5	18	8.00	4.60

6	5	18	6.50	6.60
7	5	18	908.51	48.20
7	5	18	914.11	46.10
-7	5	-18	949.60	48.10
-8	5	-18	5.90	7.00
8	5	18	9.40	7.60
-9	5	-18	409.16	25.70
9	5	18	420.56	25.70
10	5	18	227.88	18.80
-10	5	-18	211.28	18.00
-11	5	-18	408.56	26.30
11	5	18	381.06	26.40
11	-6	-18	15.70	9.50
11	6	-18	10.40	9.60
-11	6	18	14.00	8.00
10	-6	-18	17.60	9.20
-10	6	18	9.30	7.30
10	6	-18	12.70	8.70
9	-6	-18	1.30	7.50
-9	6	18	2.70	6.90
9	6	-18	11.00	8.00
8	-6	-18	10.90	7.80
-8	6	18	-6.30	6.60
8	6	-18	8.70	7.20
7	-6	-18	69.79	11.60
7	6	-18	60.49	9.40
-7	6	18	52.69	8.60
6	-6	-18	8.40	6.50
-6	-6	18	3.20	2.60
-6	6	18	7.40	6.40
6	6	-18	3.60	6.20
5	-6	-18	210.58	15.90
-5	-6	18	213.38	11.50
5	6	-18	193.18	15.10
5	6	-18	198.38	10.70
-5	6	18	210.68	15.70
-4	-6	18	14.00	4.60
4	6	-18	20.40	3.30
4	6	-18	9.10	5.30
-4	6	18	5.10	6.40
-3	-6	18	32.90	5.30
-3	6	18	43.40	7.70
3	6	-18	28.30	4.40
3	6	-18	36.70	7.40

-2	-6	18	-0.20	3.40
2	-6	-18	10.30	9.50
2	6	-18	6.20	2.40
2	6	-18	7.50	4.00
-2	6	18	2.80	5.80
-1	-6	18	395.86	20.30
-1	6	18	397.36	23.00
1	6	-18	385.26	20.80
1	6	-18	361.56	19.40
0	-6	18	68.59	7.20
0	6	18	60.79	9.80
0	6	-18	63.99	6.10
0	6	18	89.09	11.00
-1	-6	-18	25.80	5.60
1	-6	18	39.40	6.80
-1	6	-18	23.90	4.80
1	6	18	32.00	6.50
-1	6	-18	34.60	4.50
1	6	18	33.60	6.80
2	-6	18	103.69	9.50
-2	6	-18	103.49	8.40
2	6	18	111.59	11.30
-2	6	-18	113.79	9.70
-2	6	-18	111.39	6.80
-3	6	-18	127.89	10.80
3	6	18	115.69	11.90
3	6	18	144.79	12.40
-3	6	-18	129.49	9.90
4	6	18	181.88	13.40
-4	6	-18	134.99	12.20
4	6	18	150.18	13.50
5	6	18	199.28	15.80
5	6	18	216.98	14.90
-5	6	-18	195.08	15.00
6	6	18	362.86	23.10
6	6	18	410.26	22.80
-6	6	-18	335.37	22.20
7	6	18	7.50	7.00
-7	6	-18	0.20	6.20
7	6	18	14.40	6.90
8	6	18	128.99	14.40
-8	6	-18	148.09	14.30
-9	6	-18	16.10	8.10
9	6	18	28.00	8.30

10	6	18	57.99	10.70
-10	6	-18	90.69	13.70
-11	6	-18	9.00	8.90
11	6	18	26.30	10.00
11	-7	-18	6.00	10.70
-11	7	18	-0.80	7.70
11	7	-18	1.70	9.00
10	-7	-18	370.86	26.10
10	7	-18	354.46	25.80
-10	7	18	386.46	23.40
9	-7	-18	18.50	8.20
9	7	-18	12.00	8.10
-9	7	18	-0.90	7.00
8	-7	-18	214.58	18.30
8	7	-18	225.38	18.40
-8	7	18	225.98	17.30
7	-7	-18	55.99	9.00
7	7	-18	54.29	9.30
-7	7	18	49.30	8.80
6	-7	-18	441.36	26.70
6	7	-18	441.96	26.40
-6	7	18	466.35	27.10
5	-7	-18	333.17	21.50
-5	7	18	369.06	21.80
5	7	-18	314.37	20.80
4	-7	-18	423.66	26.50
-4	7	18	440.56	25.80
4	7	-18	432.66	21.00
4	7	-18	424.36	24.50
3	7	-18	348.37	17.20
3	7	-18	344.27	20.10
-3	7	18	349.77	21.90
-2	-7	18	211.18	11.40
2	7	-18	197.28	12.90
2	7	-18	180.28	10.20
-2	7	18	170.68	15.00
-1	7	18	894.01	45.80
1	7	-18	877.91	43.60
1	7	-18	853.01	42.40
0	-7	-18	240.08	16.70
0	7	-18	239.68	14.80
0	7	18	248.98	17.40
0	7	18	296.17	18.30
0	7	-18	256.47	14.50

0	7	-18	252.47	13.80
-1	-7	-18	725.73	38.10
1	7	18	677.03	38.50
1	7	18	793.32	39.20
-1	7	-18	713.03	36.80
-1	7	-18	723.63	36.10
2	7	18	48.00	7.70
-2	7	-18	44.70	6.30
-2	7	-18	48.40	4.30
-2	7	-18	52.79	8.10
2	7	18	45.90	8.00
-3	7	-18	336.87	20.40
3	7	18	339.77	22.70
3	7	18	391.46	22.20
-3	7	-18	357.16	19.60
4	7	18	45.50	8.10
-4	7	-18	52.69	9.10
4	7	18	65.89	10.40
-5	7	-18	552.94	31.00
5	7	18	554.94	32.00
5	7	18	607.34	31.60
-6	7	-18	28.10	7.20
6	7	18	22.70	7.70
6	7	18	19.30	6.30
-7	7	-18	617.84	34.10
7	7	18	627.54	34.90
-8	7	-18	31.50	8.00
8	7	18	21.20	8.30
9	7	18	138.59	15.60
-9	7	-18	138.79	14.80
10	7	18	60.39	10.70
-10	7	-18	57.29	10.50
11	7	18	193.98	18.20
-11	7	-18	211.98	18.80
11	-8	-18	142.09	19.30
-11	8	18	150.58	15.50
11	8	-18	122.59	19.00
10	-8	-18	4.20	8.90
-10	8	18	-4.10	7.60
10	8	-18	3.40	9.40
9	-8	-18	298.17	22.40
9	8	-18	309.37	22.70
-9	8	18	309.27	21.20
8	-8	-18	6.60	7.70

-8	8	18	2.40	7.40
8	8	-18	1.60	7.70
7	-8	-18	387.96	24.60
-7	8	18	403.16	24.30
7	8	-18	363.66	24.20
6	-8	-18	186.88	15.70
-6	8	18	173.48	15.70
6	8	-18	187.78	15.50
5	-8	-18	293.17	19.40
5	8	-18	271.17	18.70
4	-8	-18	466.55	26.70
-4	8	18	454.85	27.10
4	8	-18	443.06	25.70
3	-8	-18	230.28	17.50
-3	8	18	235.08	17.70
3	8	-18	225.98	11.90
3	8	-18	253.77	12.40
3	8	-18	246.68	15.50
2	-8	-18	310.87	20.80
2	8	-18	310.57	15.80
-2	8	18	308.37	20.80
2	8	-18	335.17	18.50
1	-8	-18	86.19	10.10
1	8	-18	64.99	5.40
1	8	-18	71.39	7.70
1	8	-18	77.79	7.00
-1	8	18	55.99	10.60
0	-8	-18	855.61	44.40
0	8	18	920.71	45.50
0	8	-18	873.31	42.40
0	8	-18	800.42	41.50
0	8	18	813.02	44.80
0	8	-18	857.61	42.30
-1	-8	-18	110.29	11.40
-1	8	-18	108.69	9.50
-1	8	-18	121.39	7.90
-1	8	-18	118.69	8.30
1	8	18	101.59	13.50
1	8	18	136.49	13.20
-2	8	-18	342.67	19.30
2	8	18	289.07	23.80
-2	8	-18	321.67	17.10
-2	8	-18	354.36	18.40
2	8	18	379.46	22.00

3	8	18	14.60	6.50
-3	8	-18	2.80	4.30
3	8	18	3.00	6.30
-3	8	-18	2.50	4.90
4	8	18	336.37	22.40
-4	8	-18	330.57	20.80
4	8	18	409.56	22.80
5	8	18	-1.10	6.70
5	8	18	-5.00	5.90
-5	8	-18	-7.40	5.50
-6	8	-18	558.64	31.40
6	8	18	575.14	32.40
7	8	18	3.70	7.40
-7	8	-18	-6.40	6.60
-8	8	-18	283.57	19.30
8	8	18	252.07	19.60
9	8	18	39.90	9.60
-9	8	-18	40.80	9.20
10	8	18	150.28	16.30
-10	8	-18	155.88	16.50
11	8	18	45.00	11.10
-11	8	-18	31.40	9.70
11	-9	-18	33.30	10.70
10	-9	-18	26.80	10.10
10	9	-18	30.40	10.80
-10	9	18	31.50	9.60
9	-9	-18	8.40	8.30
-9	9	18	10.70	8.30
9	9	-18	-1.60	7.70
8	-9	-18	12.00	8.30
8	9	-18	7.20	7.30
-8	9	18	-1.70	7.70
7	-9	-18	12.60	7.70
-7	9	18	7.90	7.90
7	9	-18	5.70	6.70
6	-9	-18	26.00	7.40
6	9	-18	32.10	8.10
-6	9	18	7.50	7.60
5	-9	-18	39.80	7.70
-5	9	18	31.20	8.40
5	9	-18	35.40	7.40
4	-9	-18	68.89	11.00
4	9	-18	48.70	9.50
-4	9	18	53.29	9.80

3	-9	-18	-2.60	5.90
3	9	-18	-1.20	4.50
2	-9	-18	6.40	5.80
2	9	-18	3.30	2.40
2	9	-18	-2.80	4.30
2	9	-18	5.80	1.80
1	-9	-18	15.90	5.70
-1	9	18	22.70	7.90
1	9	-18	11.50	4.30
1	9	-18	24.50	3.90
1	9	-18	18.50	2.70
0	-9	-18	37.00	6.90
0	9	18	37.70	7.60
0	9	18	29.70	13.10
0	9	-18	34.60	4.80
0	9	-18	32.30	4.90
0	9	-18	37.80	4.90
-1	9	-18	153.58	9.80
1	9	18	134.39	13.80
1	9	18	167.18	14.80
-1	9	-18	152.48	9.50
-1	9	-18	159.98	11.00
-2	9	-18	-3.90	3.50
2	9	18	2.40	6.40
-2	9	-18	1.80	2.90
-2	9	-18	5.90	4.60
2	9	18	-4.10	6.10
-3	9	-18	15.50	4.80
3	9	18	25.90	7.50
-3	9	-18	26.80	5.70
3	9	18	22.60	7.30
-4	9	-18	14.20	5.60
4	9	18	11.80	8.50
4	9	18	19.20	6.90
-5	9	-18	75.89	10.60
5	9	18	71.19	11.50
6	9	18	7.50	7.30
-6	9	-18	7.70	7.40
-7	9	-18	190.98	16.00
7	9	18	226.58	17.70
8	9	18	9.10	8.40
-8	9	-18	18.20	7.60
9	9	18	14.50	8.50
-9	9	-18	6.40	7.60

10	9	18	26.30	9.60
-10	9	-18	23.90	9.00
10	-10	-18	5.20	10.50
-10	10	18	-7.20	8.50
10	10	-18	2.80	9.40
9	-10	-18	547.95	34.40
9	10	-18	549.85	34.20
-9	10	18	606.24	33.20
8	-10	-18	25.80	9.00
8	10	-18	38.70	9.60
-8	10	18	35.30	9.70
7	-10	-18	357.36	24.20
7	10	-18	372.56	24.10
-7	10	18	370.56	24.00
6	-10	-18	265.97	19.40
-6	10	18	266.07	19.70
6	10	-18	255.67	19.00
5	-10	-18	703.93	39.50
-5	10	18	741.93	40.10
5	10	-18	728.63	39.10
4	-10	-18	406.96	25.00
-4	10	18	432.16	26.20
4	10	-18	388.96	23.90
3	-10	-18	473.85	27.30
3	10	-18	450.85	25.70
-3	10	18	454.35	28.00
2	-10	-18	815.12	41.70
2	10	-18	735.33	37.10
2	10	-18	785.22	40.00
-2	10	18	789.92	42.50
1	-10	-18	75.29	10.90
-1	10	18	115.19	15.40
1	10	-18	62.19	6.10
1	10	-18	80.09	7.00
1	10	-18	71.39	8.20
-1	10	18	69.09	10.60
0	-10	-18	1063.49	53.79
0	10	18	999.50	54.39
0	10	18	1110.59	54.89
0	10	-18	983.40	50.39
0	10	-18	1046.70	51.39
0	10	-18	1057.79	50.89
1	10	18	231.88	18.60
-1	10	-18	246.98	14.20

1	10	18	294.17	19.70
-1	10	-18	260.67	14.20
-1	10	-18	251.87	15.00
-2	10	-18	597.04	28.40
-2	10	-18	562.54	29.40
2	10	18	529.05	32.30
2	10	18	609.34	33.30
-2	10	-18	591.04	30.10
3	10	18	0.50	6.50
-3	10	-18	0.00	4.30
3	10	18	7.70	6.80
-3	10	-18	4.60	4.90
-4	10	-18	929.31	46.70
4	10	18	905.01	48.60
-5	10	-18	15.60	6.70
5	10	18	26.70	8.50
6	10	18	642.04	36.40
-7	10	-18	1.30	6.80
7	10	18	10.40	8.30
-8	10	-18	562.14	31.70
8	10	18	544.15	32.60
-9	10	-18	99.29	13.90
9	10	18	85.19	14.80
-10	10	-18	328.37	23.10
10	10	18	296.97	23.50
10	-11	-18	1.00	9.60
10	11	-18	15.50	10.30
-10	11	18	5.20	8.80
9	-11	-18	21.80	10.60
9	11	-18	-7.10	8.10
-9	11	18	-12.10	7.70
8	-11	-18	38.90	9.40
8	11	-18	44.70	10.30
-8	11	18	34.00	10.00
7	-11	-18	9.60	7.60
-7	11	18	2.90	8.50
7	11	-18	14.60	7.70
6	-11	-18	106.39	14.50
-6	11	18	80.89	14.30
6	11	-18	119.29	13.80
5	-11	-18	70.39	12.50
5	11	-18	59.19	9.30
-5	11	18	57.99	11.20
4	-11	-18	39.40	8.10

-4	11	18	41.60	10.30
4	11	-18	50.69	10.40
3	-11	-18	39.10	7.60
3	11	-18	37.50	6.60
-3	11	18	19.90	9.20
2	-11	-18	112.19	12.30
-2	11	18	78.69	13.40
2	11	-18	101.59	10.10
1	-11	-18	13.60	6.50
1	11	-18	23.60	5.10
-1	11	18	-1.40	7.60
1	11	-18	23.80	3.20
0	-11	-18	123.79	13.00
0	11	-18	133.29	8.60
0	11	-18	120.79	9.60
0	11	18	118.29	14.60
0	11	18	120.89	14.60
0	11	-18	110.39	8.00
-1	11	-18	329.17	17.70
-1	11	-18	335.47	18.40
-1	11	-18	331.97	18.00
1	11	18	384.26	23.40
1	11	18	305.07	22.60
2	11	18	15.60	7.60
-2	11	-18	29.20	5.50
-2	11	-18	31.00	4.40
2	11	18	21.20	7.80
-2	11	-18	30.00	4.90
-3	11	-18	35.60	6.20
3	11	18	31.30	8.10
-3	11	-18	33.30	5.40
4	11	18	-7.20	6.60
-4	11	-18	0.40	5.40
-5	11	-18	132.69	12.30
5	11	18	117.19	14.40
6	11	18	0.70	7.10
-7	11	-18	88.79	12.70
7	11	18	69.99	12.50
-8	11	-18	23.80	8.20
8	11	18	42.10	9.80
-9	11	-18	68.19	10.70
9	11	18	47.60	10.90
-10	11	-18	1.10	8.20
10	11	18	5.20	9.00

9	-12	-18	146.89	18.60
-9	12	18	145.79	16.50
9	12	-18	112.69	17.50
8	-12	-18	27.10	9.10
8	12	-18	8.50	8.70
-8	12	18	6.10	9.10
7	-12	-18	126.19	16.10
7	12	-18	156.18	16.30
-7	12	18	148.79	16.50
6	-12	-18	173.38	16.70
-6	12	18	131.49	16.50
6	12	-18	162.38	15.90
5	-12	-18	464.75	28.20
-5	12	18	468.35	28.80
5	12	-18	424.96	26.80
4	-12	-18	138.79	14.70
4	12	-18	136.99	13.30
-4	12	18	126.89	15.60
3	-12	-18	49.80	8.50
-3	12	18	44.70	10.20
3	12	-18	49.70	9.50
2	-12	-18	146.99	14.20
-2	12	18	125.79	15.90
2	12	-18	133.49	11.80
1	-12	-18	236.98	17.60
1	12	-18	230.38	14.70
-1	12	18	207.68	18.50
1	12	-18	221.48	11.60
-1	12	18	222.98	19.00
0	12	-18	489.55	23.40
0	12	18	444.96	28.30
0	12	-18	447.06	24.60
-1	12	-18	54.49	6.70
1	12	18	56.19	9.90
-1	12	-18	44.00	6.90
2	12	18	573.64	34.40
-2	12	-18	600.54	29.60
-2	12	-18	614.84	30.80
-3	12	-18	4.20	4.90
3	12	18	-2.50	7.30
-4	12	-18	388.26	22.30
4	12	18	389.56	24.80
5	12	18	52.09	9.70
-5	12	-18	55.09	7.90

6	12	18	662.93	36.50
-6	12	-18	600.44	35.70
-7	12	-18	-4.10	7.00
7	12	18	3.90	7.80
8	12	18	136.19	17.00
-8	12	-18	152.88	15.60
-9	12	-18	47.70	9.70
9	12	18	77.79	12.20
9	-13	-18	-3.20	9.40
9	13	-18	-1.90	8.60
-9	13	18	-3.30	9.50
8	-13	-18	276.27	23.10
8	13	-18	271.67	22.00
-8	13	18	286.27	21.80
7	-13	-18	24.60	9.00
7	13	-18	27.30	8.70
-7	13	18	21.30	9.70
6	-13	-18	595.94	35.50
6	13	-18	599.34	34.70
-6	13	18	621.24	35.80
5	-13	-18	258.07	20.30
5	13	-18	256.27	19.20
-5	13	18	275.37	21.40
4	-13	-18	614.54	36.70
4	13	-18	638.74	35.50
-4	13	18	698.63	38.00
3	-13	-18	205.18	17.00
3	13	-18	178.98	14.60
-3	13	18	179.58	18.30
2	-13	-18	98.69	13.80
-2	13	18	118.59	15.90
2	13	-18	139.19	11.80
1	-13	-18	458.25	26.90
1	13	-18	417.06	23.80
-1	13	18	407.36	27.90
0	13	18	58.99	11.30
0	13	-18	57.09	5.70
0	13	-18	75.39	8.40
1	13	18	752.72	42.60
-1	13	-18	767.12	37.80
-1	13	-18	769.12	38.30
2	13	18	59.29	10.30
-2	13	-18	67.29	6.30
-2	13	-18	62.49	7.90

3	13	18	262.27	20.20
-3	13	-18	283.87	16.80
4	13	18	-7.70	7.10
-4	13	-18	-0.90	5.50
5	13	18	721.73	41.00
-5	13	-18	743.93	38.50
-6	13	-18	3.30	6.70
6	13	18	-0.20	7.80
7	13	18	451.55	28.90
-7	13	-18	445.36	26.90
-8	13	-18	2.80	8.20
8	13	18	4.30	9.10
-9	13	-18	171.78	16.60
9	13	18	154.48	18.30
9	-14	-18	16.40	10.30
8	-14	-18	-6.00	10.10
-8	14	18	4.80	9.60
8	14	-18	7.80	9.30
7	-14	-18	5.50	9.80
-7	14	18	-2.70	8.50
7	14	-18	8.10	7.90
6	-14	-18	26.90	9.30
6	14	-18	44.60	9.20
-6	14	18	14.50	9.60
5	-14	-18	38.00	9.50
5	14	-18	30.80	7.80
-5	14	18	19.70	10.50
4	-14	-18	98.59	14.50
-4	14	18	81.39	15.20
4	14	-18	68.39	12.00
3	-14	-18	-3.30	6.80
-3	14	18	5.60	9.70
3	14	-18	21.60	6.60
2	-14	-18	7.30	7.40
-2	14	18	-9.90	8.50
2	14	-18	10.60	5.60
-1	14	18	23.50	9.80
1	14	-18	25.10	6.00
0	14	-18	134.29	10.90
0	14	18	110.19	16.50
1	14	18	-0.70	8.90
-1	14	-18	1.50	4.20
-1	14	-18	0.10	3.30
2	14	18	0.80	8.60

-2	14	-18	-5.20	3.30
-2	14	-18	-1.50	4.00
-2	14	-18	6.80	3.20
3	14	18	-9.60	8.20
-3	14	-18	2.70	4.80
-3	14	-18	0.20	2.90
-3	14	-18	1.80	2.30
-4	14	-18	7.20	5.70
4	14	18	-3.50	8.00
5	14	18	2.70	7.70
-5	14	-18	-0.60	6.00
6	14	18	56.49	10.80
-6	14	-18	67.99	11.80
7	14	18	20.60	9.80
-7	14	-18	21.00	8.00
-8	14	-18	7.90	8.30
8	14	18	17.00	10.30
8	-15	-18	237.28	23.10
8	15	-18	271.17	22.30
-8	15	18	266.47	21.60
7	-15	-18	45.80	11.10
-7	15	18	54.79	11.80
7	15	-18	43.90	9.40
6	-15	-18	174.68	18.70
6	15	-18	168.08	16.70
-6	15	18	122.39	18.00
5	-15	-18	259.17	21.50
5	15	-18	244.18	19.60
-5	15	18	269.37	22.20
4	-15	-18	475.25	30.40
-4	15	18	488.15	31.10
4	15	-18	511.55	29.10
3	-15	-18	221.18	19.80
-3	15	18	251.77	21.60
3	15	-18	214.28	16.70
2	-15	-18	79.09	14.60
2	15	-18	100.99	11.70
-2	15	18	92.59	13.80
1	15	-18	652.73	34.90
-1	15	18	662.93	38.50
0	15	18	50.19	12.00
0	15	-18	63.99	9.20
1	15	18	427.86	27.40
-1	15	-18	404.16	22.50

2	15	18	154.48	18.00
-2	15	-18	172.68	11.00
-2	15	-18	169.18	11.00
-2	15	-18	154.08	10.40
-3	15	-18	402.66	20.40
-3	15	-18	365.96	20.60
-3	15	-18	430.26	22.00
3	15	18	410.46	27.40
4	15	18	8.10	9.20
-4	15	-18	0.30	5.50
5	15	18	510.05	31.90
-5	15	-18	503.15	28.10
-6	15	-18	24.10	8.00
6	15	18	51.49	10.70
7	15	18	412.26	27.50
-7	15	-18	407.06	25.30
8	15	18	-1.90	9.80
-8	15	-18	3.50	8.00
7	-16	-18	22.00	10.80
-7	16	18	41.20	11.20
7	16	-18	49.90	10.60
6	-16	-18	41.80	11.20
-6	16	18	46.60	12.30
6	16	-18	52.59	10.60
5	-16	-18	36.30	9.70
-5	16	18	28.30	11.30
5	16	-18	28.60	8.20
4	-16	-18	40.00	10.10
-4	16	18	37.90	11.10
4	16	-18	47.10	9.20
3	-16	-18	6.40	8.30
-3	16	18	1.10	9.90
3	16	-18	11.50	6.80
-2	16	18	39.90	11.50
2	16	-18	41.20	7.60
-1	16	18	54.69	12.20
1	16	-18	81.29	10.60
0	16	18	3.60	10.00
1	16	18	4.70	9.80
-1	16	-18	21.60	5.40
-2	16	-18	123.99	9.70
-2	16	-18	101.49	8.70
2	16	18	112.39	14.60
-3	16	-18	-1.00	4.90

-3	16	-18	3.10	3.40
3	16	18	-9.80	7.90
-3	16	-18	1.30	3.50
4	16	18	-6.10	9.00
-4	16	-18	12.60	5.50
5	16	18	5.80	9.40
-5	16	-18	13.70	6.50
-6	16	-18	125.99	13.40
6	16	18	135.49	17.70
7	16	18	1.60	9.60
-7	16	-18	-0.20	7.40
7	-17	-18	6.30	11.50
6	-17	-18	257.97	22.70
6	17	-18	214.28	20.50
-6	17	18	237.88	21.50
5	-17	-18	195.38	20.40
-5	17	18	179.38	19.80
5	17	-18	169.08	17.80
4	-17	-18	245.28	22.30
-4	17	18	279.87	23.40
4	17	-18	244.48	19.50
3	-17	-18	54.09	11.00
-3	17	18	54.39	12.20
3	17	-18	48.80	8.80
-2	17	18	32.80	11.70
2	17	-18	38.50	7.70
1	17	-18	166.58	14.10
-1	17	18	166.68	19.50
0	17	-18	46.20	7.30
0	17	18	27.90	10.80
1	17	18	631.24	36.50
-1	17	-18	552.44	30.80
-2	17	-18	34.80	6.90
2	17	18	21.60	9.80
-2	17	-18	28.00	5.50
-3	17	-18	186.68	11.50
3	17	18	186.28	19.70
-3	17	-18	156.88	10.90
4	17	18	-10.60	9.90
-4	17	-18	1.80	2.70
5	17	18	463.35	29.90
-5	17	-18	438.66	25.60
6	17	18	-4.40	9.30
-6	17	-18	6.10	7.00

6	-18	-18	146.69	20.90
6	18	-18	159.78	18.20
-6	18	18	131.39	19.60
5	-18	-18	318.27	25.80
-5	18	18	279.47	25.30
5	18	-18	319.57	23.20
4	-18	-18	117.79	18.60
4	18	-18	107.59	15.50
-4	18	18	104.09	15.00
-3	18	18	113.29	19.60
3	18	-18	168.68	15.60
-2	18	18	235.68	21.60
2	18	-18	247.48	18.00
-1	18	18	63.89	12.80
1	18	-18	83.39	11.70
0	18	18	413.36	29.60
0	18	-18	432.26	24.60
1	18	18	-2.90	10.90
-1	18	-18	9.10	5.50
-2	18	-18	242.78	15.80
2	18	18	264.77	23.10
-2	18	-18	296.77	16.80
3	18	18	3.30	10.40
-3	18	-18	1.10	4.00
-3	18	-18	8.50	4.60
-4	18	-18	260.47	13.90
4	18	18	211.48	20.70
-4	18	-18	242.98	13.80
5	18	18	-3.90	9.80
6	18	18	257.87	23.00
4	-19	-18	-7.70	11.70
4	19	-18	10.10	8.40
-4	19	18	-10.70	11.20
3	19	-18	21.00	8.40
-3	19	18	3.50	11.90
2	19	-18	2.00	7.20
-2	19	18	-11.00	10.50
1	19	-18	32.00	7.60
-1	19	18	3.50	10.60
0	19	-18	-6.90	5.90
0	19	18	0.40	10.50
1	19	18	82.29	14.10
-1	19	-18	50.79	10.30
-2	19	-18	4.00	5.40

2	19	18	-1.70	10.40
3	19	18	-5.60	10.90
-3	19	-18	-0.30	4.40
-3	19	-18	3.60	4.80
-4	19	-18	-2.30	4.10
-4	19	-18	3.50	4.30
4	19	18	9.30	10.70
-3	20	18	216.28	22.50
-2	20	18	282.07	25.30
2	20	-18	280.17	21.80
1	20	-18	10.60	7.60
-1	20	18	3.10	12.20
0	20	-18	291.57	19.90
0	20	18	307.07	26.60
1	20	18	47.00	14.10
-1	20	-18	42.00	7.60
-2	20	-18	202.38	15.00
2	20	18	214.08	22.60
-3	20	-18	0.60	5.30
3	20	18	-18.00	10.30
11	0	-19	4.20	9.60
-10	0	19	9.00	6.70
10	0	-19	10.10	9.00
-9	0	19	5.10	5.50
9	0	-19	11.00	7.40
-8	0	19	-9.20	4.90
8	0	-19	2.50	6.70
-7	0	19	-6.00	4.50
7	0	-19	-4.60	6.10
-6	0	19	-3.00	4.30
6	0	-19	-1.10	5.00
6	0	-19	-0.60	3.80
-5	0	19	7.70	4.40
5	0	-19	2.80	5.10
5	0	-19	2.80	3.40
4	0	-19	3.90	3.20
-4	0	19	-0.90	4.00
4	0	-19	2.80	4.50
3	0	-19	-1.60	3.90
-3	0	19	-0.20	3.90
3	0	-19	1.90	3.00
2	0	-19	3.20	4.00
2	0	-19	4.60	3.20
-2	0	19	3.80	4.10

1	0	-19	1.00	3.20
1	0	-19	0.40	3.60
-1	0	19	-1.40	3.90
0	0	-19	4.50	3.40
0	0	19	3.80	4.40
0	0	-19	2.10	3.70
-1	0	-19	1.80	3.90
1	0	19	-1.70	3.90
1	0	19	-4.40	4.10
-1	0	-19	4.40	3.30
2	0	19	6.10	2.90
2	0	19	1.90	4.50
-2	0	-19	5.20	3.30
3	0	19	-2.60	4.80
4	0	19	-0.70	4.90
6	0	19	1.50	5.80
7	0	19	18.40	7.00
8	0	19	2.10	7.20
-9	0	-19	-14.90	7.70
-10	0	-19	5.50	8.50
-11	0	-19	7.90	9.40
11	-1	-19	48.60	11.10
11	1	-19	38.50	10.20
10	-1	-19	388.36	26.60
10	1	-19	389.96	26.70
-10	1	19	404.06	23.00
9	-1	-19	344.17	23.80
-9	-1	19	338.47	19.80
9	1	-19	361.76	23.90
-9	1	19	340.87	20.70
-8	-1	19	158.18	12.10
8	-1	-19	163.88	15.70
8	1	-19	181.58	15.90
-8	1	19	170.68	13.10
-7	-1	19	99.49	9.40
7	-1	-19	104.39	12.90
7	1	-19	116.09	12.70
-7	1	19	90.09	10.10
6	-1	-19	172.48	12.00
-6	-1	19	197.58	12.50
6	-1	-19	222.48	15.50
-6	1	19	202.88	13.30
6	1	-19	188.38	15.00
6	1	-19	176.28	11.70

5	-1	-19	700.13	37.10
-5	-1	19	751.02	37.70
5	-1	-19	781.72	39.60
-5	1	19	739.13	38.30
5	1	-19	789.92	39.60
5	1	-19	735.23	36.90
4	-1	-19	614.44	31.50
4	-1	-19	537.15	29.20
-4	-1	19	566.54	30.00
-4	1	19	597.54	30.60
4	1	-19	634.84	31.60
4	1	-19	550.44	29.00
3	-1	-19	357.36	20.10
-3	-1	19	329.07	18.90
-3	1	19	348.47	19.60
3	1	-19	359.46	20.00
3	1	-19	317.07	17.70
2	-1	-19	4.00	3.50
2	-1	-19	6.20	4.20
-2	-1	19	7.00	4.20
2	1	-19	8.60	3.20
-2	1	19	6.40	4.50
2	1	-19	7.60	4.10
-1	-1	19	874.71	44.10
1	-1	-19	862.51	43.40
1	-1	-19	900.11	44.40
-1	1	19	854.41	44.30
1	1	-19	862.71	43.20
1	1	-19	913.91	44.20
0	-1	-19	63.89	7.50
0	-1	19	60.69	7.80
0	-1	-19	62.59	6.80
0	1	19	68.79	8.40
0	1	-19	60.39	6.60
0	1	-19	60.09	7.00
-1	-1	-19	1181.08	58.89
1	-1	19	1159.88	59.29
1	-1	19	1242.58	58.19
-1	1	-19	1170.88	58.59
-1	1	-19	1164.98	58.49
1	1	19	1315.27	59.19
1	1	19	1129.59	59.39
2	-1	19	-0.80	4.20
-2	-1	-19	2.20	3.40

-2	1	-19	2.70	2.90
2	1	19	3.90	4.80
2	1	19	5.60	3.80
3	-1	19	563.54	29.60
3	1	19	534.95	29.70
3	1	19	553.34	27.10
4	-1	19	7.40	5.60
4	1	19	9.10	5.40
5	-1	19	483.55	33.20
5	1	19	531.15	28.90
6	-1	19	61.79	10.40
6	1	19	51.79	8.30
7	-1	19	813.82	43.50
7	1	19	830.02	43.60
-8	1	-19	76.49	12.80
8	1	19	90.49	12.20
9	1	19	54.09	10.00
-9	1	-19	57.09	10.10
-10	-1	-19	112.69	15.60
-10	1	-19	115.09	15.60
-11	-1	-19	402.16	26.50
-11	1	-19	371.16	26.20
11	-2	-19	93.79	17.00
11	2	-19	67.59	12.50
-11	2	19	85.09	12.80
10	-2	-19	16.50	8.70
-10	2	19	21.20	7.20
10	2	-19	23.40	9.40
9	-2	-19	163.88	17.00
9	2	-19	144.29	16.50
-9	2	19	176.88	13.90
-8	-2	19	79.09	8.70
8	-2	-19	97.79	13.30
-8	2	19	90.89	11.00
8	2	-19	93.39	13.30
-7	-2	19	162.38	11.60
7	-2	-19	188.88	15.50
7	2	-19	181.68	15.20
-7	2	19	170.48	13.50
-6	-2	19	424.46	23.10
6	-2	-19	449.26	25.90
6	-2	-19	420.26	23.30
6	2	-19	443.26	25.80
-6	2	19	460.75	24.60

5	-2	-19	45.00	6.60
-5	-2	19	57.49	6.90
5	-2	-19	53.09	9.50
5	2	-19	44.10	9.30
5	2	-19	57.09	5.90
-5	2	19	44.30	6.80
4	-2	-19	161.18	13.10
4	-2	-19	162.78	10.60
-4	-2	19	172.38	11.10
4	2	-19	163.28	12.80
-4	2	19	177.58	12.40
4	2	-19	163.88	9.90
3	-2	-19	19.60	5.50
3	-2	-19	5.70	3.60
-3	-2	19	13.80	4.10
3	2	-19	13.80	4.90
3	2	-19	11.20	3.20
-3	2	19	11.90	5.00
2	-2	-19	347.17	19.70
2	-2	-19	321.87	18.10
-2	-2	19	337.67	18.70
-2	2	19	344.77	19.60
2	2	-19	350.06	19.30
2	2	-19	326.07	17.60
-1	-2	19	13.50	4.30
1	-2	-19	10.20	3.90
1	-2	-19	10.00	4.50
1	2	-19	13.40	3.50
-1	2	19	8.10	5.00
1	2	-19	6.60	3.80
0	-2	-19	655.03	33.70
0	-2	19	641.24	33.40
0	2	19	710.83	34.50
0	2	-19	637.64	33.00
0	2	-19	628.14	32.60
0	2	19	634.64	34.00
1	-2	19	0.20	2.70
-1	-2	-19	3.30	3.70
1	-2	19	1.40	4.30
1	2	19	8.30	4.60
-1	2	-19	-5.90	3.50
-1	2	-19	-1.90	3.00
1	2	19	6.40	5.00
-2	-2	-19	203.38	12.90

2	-2	19	194.78	13.70
2	2	19	234.58	13.40
2	2	19	200.88	14.20
-2	2	-19	187.88	11.80
-2	2	-19	203.28	13.40
3	-2	19	3.90	4.70
3	2	19	18.60	4.10
3	2	19	8.90	5.50
4	-2	19	412.26	22.10
4	2	19	351.06	22.30
4	2	19	366.46	19.00
5	-2	19	16.00	6.10
5	2	19	11.80	6.20
6	-2	19	357.56	21.30
6	2	19	325.47	21.30
7	-2	19	50.09	8.80
7	2	19	31.10	8.00
-7	2	-19	30.60	8.20
8	2	19	70.49	12.50
-8	2	-19	72.49	12.10
-9	2	-19	28.80	9.10
9	2	19	22.50	8.80
-10	-2	-19	100.59	15.70
-10	2	-19	129.09	15.60
-11	-2	-19	122.79	15.40
-11	2	-19	100.19	15.70
11	-3	-19	21.50	10.10
-11	3	19	21.40	7.70
11	3	-19	19.40	9.80
10	-3	-19	104.39	16.40
-10	3	19	113.49	14.20
10	3	-19	95.79	15.80
9	-3	-19	14.40	7.80
-9	3	19	4.30	6.80
9	3	-19	5.50	8.20
8	-3	-19	157.78	15.70
-8	-3	19	159.18	11.00
8	3	-19	179.68	16.10
-8	3	19	179.88	14.10
7	-3	-19	87.59	12.30
-7	-3	19	76.49	7.70
7	3	-19	86.79	12.00
-7	3	19	72.89	10.70
6	-3	-19	52.59	10.60

-6	-3	19	51.09	6.70
-6	3	19	55.39	7.80
6	3	-19	53.19	10.30
-5	-3	19	199.28	12.20
5	-3	-19	216.58	15.30
-5	3	19	193.18	14.40
5	3	-19	202.88	14.90
5	3	-19	194.68	11.40
4	-3	-19	286.97	17.80
-4	-3	19	256.47	15.20
4	3	-19	266.07	14.40
-4	3	19	269.27	17.30
4	3	-19	282.07	17.30
-3	-3	19	138.79	9.70
3	-3	-19	137.09	11.60
-3	3	19	137.29	11.60
3	3	-19	142.59	11.00
3	3	-19	130.19	8.40
-2	-3	19	80.89	7.40
2	-3	-19	70.69	8.90
2	3	-19	78.49	8.20
-2	3	19	62.19	9.20
2	3	-19	71.89	6.20
1	-3	-19	247.78	15.40
-1	-3	19	235.78	14.30
1	3	-19	238.38	14.30
1	3	-19	230.08	13.30
-1	3	19	240.88	15.60
0	-3	19	7.20	4.10
0	-3	-19	13.60	4.70
0	3	19	10.10	5.50
0	3	19	9.20	5.10
0	3	-19	8.70	3.50
0	3	-19	9.80	3.50
-1	-3	-19	300.07	18.30
1	-3	19	321.37	18.30
1	3	19	285.57	19.00
-1	3	-19	312.57	17.40
-1	3	-19	332.27	17.20
1	3	19	347.37	19.60
2	-3	19	139.49	11.00
-2	3	-19	118.19	8.80
2	3	19	168.98	11.60
2	3	19	134.49	11.80

-2	3	-19	139.69	10.00
3	-3	19	154.88	12.00
3	3	19	152.58	11.30
3	3	19	153.98	12.80
4	-3	19	10.70	5.50
4	3	19	17.30	4.20
5	-3	19	612.84	30.60
5	3	19	557.24	30.80
5	3	19	504.15	28.10
6	-3	19	122.49	11.80
-6	3	-19	81.29	11.90
6	3	19	119.49	12.60
7	3	19	441.86	26.70
-7	3	-19	449.26	26.60
8	3	19	-7.70	6.40
-8	3	-19	-0.50	7.10
9	3	19	98.09	13.80
-9	3	-19	93.69	13.80
-10	3	-19	105.39	14.70
10	3	19	88.79	14.60
-11	-3	-19	242.58	20.60
-11	3	-19	227.28	20.30
11	-4	-19	454.75	30.20
-11	4	19	464.95	26.90
11	4	-19	435.96	30.10
10	-4	-19	57.49	10.90
10	4	-19	49.10	10.30
-10	4	19	55.59	8.90
9	-4	-19	650.23	35.30
-9	4	19	539.55	34.80
9	4	-19	614.74	35.10
8	-4	-19	80.69	12.90
-8	4	19	61.29	11.10
8	4	-19	74.59	13.00
7	-4	-19	356.36	22.70
-7	-4	19	329.27	18.30
-7	4	19	357.16	21.50
7	4	-19	351.06	22.50
6	-4	-19	288.07	19.40
-6	-4	19	281.97	15.70
6	4	-19	280.87	19.10
-6	4	19	301.07	18.90
5	-4	-19	802.42	43.00
-5	-4	19	808.02	40.40

-5	4	19	817.82	42.60
5	4	-19	846.92	42.90
4	-4	-19	1059.79	53.69
-4	-4	19	1067.39	51.59
-4	4	19	1032.90	53.49
4	4	-19	1082.29	53.39
4	4	-19	1030.30	50.89
-3	-4	19	171.68	10.90
3	-4	-19	167.58	13.10
3	4	-19	189.08	12.50
-3	4	19	167.58	13.50
3	4	-19	158.18	9.70
2	-4	-19	552.94	30.00
-2	-4	19	578.34	28.50
-2	4	19	547.95	30.10
2	4	-19	542.35	27.50
2	4	-19	552.24	29.10
1	-4	-19	577.14	30.90
-1	-4	19	584.74	29.60
-1	4	19	569.44	31.00
1	4	-19	563.24	28.70
1	4	-19	584.04	29.80
0	-4	19	1412.66	69.79
0	-4	-19	1422.46	70.59
0	4	19	1478.45	74.19
0	4	-19	1437.56	69.09
0	4	-19	1448.06	69.49
0	4	19	1368.36	70.89
1	-4	19	81.69	9.00
-1	-4	-19	103.39	9.50
1	4	19	94.09	10.50
-1	4	-19	97.69	8.10
-1	4	-19	97.19	7.50
1	4	19	133.79	11.70
2	-4	19	1199.68	59.39
-2	4	-19	1189.08	58.69
2	4	19	1156.68	60.29
-2	4	-19	1103.69	58.09
2	4	19	1345.27	60.19
3	-4	19	18.30	5.50
3	4	19	19.90	6.30
3	4	19	19.60	5.80
4	-4	19	733.63	38.90
4	4	19	704.03	39.50

4	4	19	798.32	38.10
5	-4	19	70.89	9.90
-5	4	-19	58.09	8.40
5	4	19	74.49	10.90
5	4	19	60.99	7.70
6	4	19	1140.69	59.09
-6	4	-19	1207.78	60.59
6	4	19	1239.08	61.09
-7	4	-19	83.49	12.30
7	4	19	96.49	12.70
-8	4	-19	752.02	39.50
8	4	19	683.83	39.10
9	4	19	189.48	16.90
-9	4	-19	173.88	16.80
-10	4	-19	176.98	16.50
10	4	19	149.39	16.80
-11	-4	-19	223.08	20.50
-11	4	-19	205.68	19.40
11	-5	-19	33.30	11.50
-11	5	19	23.60	8.50
11	5	-19	10.40	10.80
10	-5	-19	-0.50	8.80
-10	5	19	-7.50	7.10
10	5	-19	12.60	8.60
9	-5	-19	49.60	9.60
9	5	-19	75.99	14.20
-9	5	19	26.10	8.20
8	-5	-19	13.30	7.20
-8	5	19	11.80	9.90
8	5	-19	7.70	7.00
7	-5	-19	11.60	7.00
7	5	-19	8.50	6.60
-7	5	19	-3.10	6.60
6	-5	-19	32.20	7.10
-6	-5	19	36.50	5.50
6	5	-19	32.70	7.20
-6	5	19	19.20	7.00
5	-5	-19	17.80	6.20
-5	-5	19	16.70	3.60
-5	5	19	10.90	6.60
5	5	-19	13.50	5.80
4	-5	-19	9.00	5.50
-4	-5	19	17.00	3.80
4	5	-19	12.30	5.30

4	5	-19	10.40	2.70
-4	5	19	14.70	6.20
3	-5	-19	3.70	5.20
-3	-5	19	0.60	3.50
3	5	-19	4.60	2.60
-3	5	19	0.80	5.70
3	5	-19	8.00	4.70
-2	-5	19	84.79	7.40
2	-5	-19	76.49	9.50
2	5	-19	79.19	8.20
-2	5	19	64.79	10.30
2	5	-19	71.69	5.90
1	-5	-19	78.29	9.70
-1	-5	19	96.39	8.10
1	5	-19	90.89	8.10
1	5	-19	89.69	6.80
-1	5	19	96.59	10.90
0	-5	-19	34.60	6.20
0	-5	19	39.90	6.70
0	5	-19	32.40	5.50
0	5	19	52.89	8.00
0	5	-19	34.80	5.90
0	5	19	43.50	7.20
1	-5	19	23.10	4.90
-1	-5	-19	16.20	5.30
-1	5	-19	21.10	4.20
1	5	19	15.50	6.40
-1	5	-19	35.90	5.10
1	5	19	20.60	5.90
2	-5	19	8.00	4.80
2	5	19	6.80	5.50
-2	5	-19	-2.20	4.40
2	5	19	-0.10	5.50
-2	5	-19	-0.80	2.50
3	-5	19	22.80	5.70
3	5	19	27.30	6.40
4	5	19	18.20	6.00
4	5	19	13.90	6.60
-4	5	-19	5.20	5.80
5	5	19	0.80	6.30
5	5	19	-3.10	4.70
-5	5	-19	3.70	6.10
6	5	19	1.80	4.80
6	5	19	0.60	6.10

-6	5	-19	7.30	6.60
7	5	19	5.10	7.20
-7	5	-19	14.30	7.30
8	5	19	2.60	7.40
-8	5	-19	25.10	8.00
9	5	19	5.00	8.10
-9	5	-19	14.00	7.80
10	5	19	16.60	8.60
-10	5	-19	2.30	8.50
11	5	19	10.80	9.60
-11	5	-19	11.80	10.10
11	-6	-19	421.16	30.50
11	6	-19	460.35	30.90
-11	6	19	476.45	27.60
10	-6	-19	136.59	17.60
10	6	-19	119.39	17.20
-10	6	19	108.29	14.30
9	-6	-19	549.35	32.80
-9	6	19	547.45	31.00
9	6	-19	533.15	32.60
8	-6	-19	8.40	7.30
8	6	-19	12.40	7.90
-8	6	19	11.30	7.50
7	-6	-19	406.86	25.00
7	6	-19	434.46	24.90
-7	6	19	331.87	24.60
6	-6	-19	368.46	23.10
6	6	-19	346.97	22.70
-6	6	19	381.26	23.10
5	-6	-19	981.90	51.39
-5	6	19	1004.90	51.49
5	6	-19	996.00	51.09
-4	-6	19	659.63	31.10
4	-6	-19	614.04	34.10
4	6	-19	599.04	33.30
-4	6	19	621.44	34.20
-3	-6	19	207.18	11.50
3	-6	-19	196.38	14.50
-3	6	19	182.58	15.10
3	6	-19	173.98	10.20
3	6	-19	201.78	13.40
2	-6	-19	459.75	26.50
-2	-6	19	502.15	24.30
-2	6	19	480.25	27.10

2	6	-19	445.46	23.20
2	6	-19	455.65	25.10
1	-6	-19	77.59	10.20
-1	-6	19	97.79	8.00
1	6	-19	94.19	8.10
1	6	-19	82.49	6.60
-1	6	19	91.09	11.20
0	-6	19	1465.55	69.89
0	-6	-19	1414.56	71.19
0	6	19	1527.45	72.19
0	6	-19	1370.86	69.09
0	6	19	1399.06	71.69
0	6	-19	1422.36	69.59
1	-6	19	99.29	9.20
-1	-6	-19	101.69	10.20
1	6	19	85.49	17.80
1	6	19	97.19	11.10
-1	6	-19	87.59	7.70
-1	6	-19	97.49	7.20
2	-6	19	1400.56	65.89
-2	6	-19	1234.28	64.09
2	6	19	1536.15	67.39
-2	6	-19	1232.68	65.69
-2	6	-19	1283.37	64.99
3	6	19	5.20	5.90
3	6	19	0.60	5.80
-3	6	-19	-3.90	4.90
-3	6	-19	1.20	4.80
-4	6	-19	527.75	31.40
4	6	19	644.94	32.00
4	6	19	583.14	32.70
-5	6	-19	44.10	7.60
5	6	19	46.60	8.30
5	6	19	33.40	7.80
6	6	19	663.63	35.60
6	6	19	600.04	34.90
-6	6	-19	618.34	34.20
-7	6	-19	48.50	8.40
7	6	19	34.90	8.80
8	6	19	392.26	25.50
-8	6	-19	427.36	25.60
9	6	19	197.58	18.00
-9	6	-19	189.58	17.40
10	6	19	176.48	17.90

-10	6	-19	184.58	17.90
11	6	19	208.18	19.90
10	-7	-19	253.67	22.10
-10	7	19	250.47	19.00
10	7	-19	208.28	20.80
9	-7	-19	28.90	9.90
-9	7	19	9.90	8.10
9	7	-19	28.60	9.30
8	-7	-19	296.57	21.90
-8	7	19	320.27	21.10
8	7	-19	298.57	21.70
7	-7	-19	110.39	13.50
7	7	-19	76.89	12.70
-7	7	19	66.69	10.20
6	-7	-19	192.88	16.20
6	7	-19	207.48	15.90
-6	7	19	178.48	16.40
5	-7	-19	199.18	15.70
5	7	-19	176.68	14.90
4	-7	-19	110.89	12.50
-4	7	19	118.79	12.80
4	7	-19	124.89	11.50
-3	-7	19	42.30	5.90
3	-7	-19	37.90	7.10
3	7	-19	44.30	6.40
-3	7	19	41.90	8.20
3	7	-19	26.20	4.80
-2	-7	19	157.28	9.90
2	-7	-19	161.48	13.10
-2	7	19	142.59	14.00
2	7	-19	141.39	11.20
2	7	-19	147.09	8.50
1	-7	-19	200.88	14.80
1	7	-19	200.08	12.70
-1	7	19	192.58	15.80
1	7	-19	201.18	11.10
0	-7	-19	0.10	5.00
0	7	-19	6.30	3.50
0	7	19	5.20	6.30
0	7	-19	2.30	4.10
0	7	19	8.50	6.20
0	7	-19	5.80	3.10
-1	-7	-19	591.74	32.20
-1	7	-19	558.64	30.80

-1	7	-19	570.84	29.90
1	7	19	664.23	33.50
-1	7	-19	589.84	29.60
-2	7	-19	121.29	10.60
-2	7	-19	105.39	9.00
-2	7	-19	115.29	7.10
2	7	19	135.69	15.90
2	7	19	131.29	12.80
-3	7	-19	233.48	14.90
3	7	19	242.48	17.90
3	7	19	284.27	18.10
-3	7	-19	238.18	16.00
4	7	19	84.99	11.70
4	7	19	66.29	9.50
-4	7	-19	59.59	10.00
5	7	19	533.35	32.00
-5	7	-19	469.45	27.50
5	7	19	479.45	28.90
-6	7	-19	0.30	6.10
6	7	19	7.40	6.80
7	7	19	255.77	19.50
-7	7	-19	257.77	18.50
8	7	19	16.00	8.30
-8	7	-19	8.70	7.80
-9	7	-19	159.88	16.40
9	7	19	161.28	17.00
10	7	19	97.79	15.10
-10	7	-19	104.09	15.20
10	-8	-19	8.80	9.50
-10	8	19	31.20	9.00
10	8	-19	1.20	8.70
9	-8	-19	140.69	17.60
-9	8	19	155.78	15.50
9	8	-19	135.99	17.00
8	-8	-19	9.80	7.80
8	8	-19	3.70	7.60
-8	8	19	3.20	7.80
7	-8	-19	66.09	13.30
-7	8	19	69.79	10.70
7	8	-19	83.29	12.80
6	-8	-19	262.77	19.00
-6	8	19	235.28	19.10
6	8	-19	250.47	18.30
5	-8	-19	92.69	12.20

-5	8	19	81.79	13.20
5	8	-19	75.19	11.60
4	-8	-19	143.99	13.70
-4	8	19	141.39	14.50
4	8	-19	136.39	12.70
3	-8	-19	108.49	12.00
-3	8	19	112.49	15.70
3	8	-19	110.19	6.80
3	8	-19	88.79	10.30
2	-8	-19	145.19	13.50
2	8	-19	149.79	11.70
2	8	-19	178.08	10.20
1	-8	-19	160.58	13.30
-1	8	19	123.79	19.90
1	8	-19	156.68	10.90
1	8	-19	141.89	8.80
1	8	-19	158.48	10.30
0	-8	-19	472.55	27.30
0	8	19	506.45	29.00
0	8	-19	477.15	25.20
0	8	-19	481.05	24.90
0	8	-19	485.75	24.30
0	8	19	455.85	28.30
-1	8	-19	-3.80	2.80
-1	8	-19	-1.30	3.40
1	8	19	-5.30	6.20
-1	8	-19	-8.60	4.20
1	8	19	-1.80	6.60
2	8	19	60.59	12.10
-2	8	-19	77.09	7.70
-2	8	-19	71.89	9.20
2	8	19	68.29	11.70
-2	8	-19	78.69	5.30
3	8	19	4.30	6.50
-3	8	-19	-0.40	4.90
-3	8	-19	-2.90	4.20
3	8	19	-2.70	6.50
-4	8	-19	233.88	16.00
4	8	19	239.28	18.20
5	8	19	136.29	14.40
-5	8	-19	133.39	12.70
6	8	19	452.25	27.00
-6	8	-19	408.66	25.20
-7	8	-19	33.00	8.20

7	8	19	35.20	8.90
8	8	19	153.18	16.10
-8	8	-19	142.09	15.40
-9	8	-19	39.20	9.50
9	8	19	32.10	9.00
-10	8	-19	76.99	11.90
10	8	19	71.69	12.10
10	-9	-19	489.15	31.20
10	9	-19	430.46	30.40
-10	9	19	481.85	29.00
9	-9	-19	86.49	16.10
9	9	-19	115.59	15.80
-9	9	19	81.99	14.20
8	-9	-19	205.88	19.70
-8	9	19	255.07	19.40
8	9	-19	230.38	19.10
7	-9	-19	137.39	15.90
-7	9	19	123.79	15.50
7	9	-19	155.58	15.20
6	-9	-19	283.47	20.70
6	9	-19	276.77	20.20
-6	9	19	327.57	21.60
5	-9	-19	744.03	41.20
5	9	-19	723.03	40.50
-5	9	19	823.02	42.00
4	-9	-19	474.95	28.20
4	9	-19	465.45	27.00
-4	9	19	491.75	29.10
3	-9	-19	667.23	36.50
3	9	-19	644.04	35.00
-3	9	19	686.03	37.40
2	-9	-19	117.19	12.20
2	9	-19	111.09	7.70
-2	9	19	79.79	13.60
2	9	-19	100.29	9.80
1	-9	-19	494.35	28.30
-1	9	19	543.15	30.10
1	9	-19	487.95	26.10
1	9	-19	489.75	25.00
-1	9	19	449.16	29.00
0	-9	-19	144.79	13.70
0	9	-19	172.68	10.80
0	9	19	142.19	14.60
0	9	19	187.28	16.00

0	9	-19	168.18	10.90
0	9	-19	149.09	9.60
-1	9	-19	1201.08	58.99
-1	9	-19	1171.78	58.29
-1	9	-19	1234.38	58.19
1	9	19	1116.39	61.29
1	9	19	1269.17	62.09
2	9	19	5.20	6.70
-2	9	-19	9.10	4.20
2	9	19	3.30	6.80
-2	9	-19	12.00	5.30
-2	9	-19	6.40	2.20
-3	9	-19	509.25	27.00
3	9	19	496.35	29.00
-3	9	-19	478.95	26.10
4	9	19	19.70	7.20
-4	9	-19	7.90	6.10
5	9	19	850.61	45.50
-5	9	-19	835.82	43.80
6	9	19	79.99	13.10
-6	9	-19	65.29	11.30
-7	9	-19	824.22	43.50
7	9	19	810.62	44.80
-8	9	-19	93.79	13.70
8	9	19	93.29	13.60
-9	9	-19	203.98	18.00
9	9	19	207.58	18.70
10	9	19	131.19	17.20
-10	9	-19	153.28	16.70
10	-10	-19	20.00	11.10
10	10	-19	11.80	9.40
9	-10	-19	19.50	9.60
-9	10	19	-17.10	8.40
9	10	-19	-2.30	8.80
8	-10	-19	9.20	8.90
8	10	-19	1.80	8.40
-8	10	19	0.90	8.50
7	-10	-19	2.90	7.70
7	10	-19	-0.10	6.90
-7	10	19	-14.30	8.20
6	-10	-19	0.40	6.90
6	10	-19	4.00	6.70
-6	10	19	7.50	8.20
5	-10	-19	8.40	7.00

-5	10	19	-7.20	8.00
5	10	-19	9.70	5.90
4	-10	-19	0.50	6.50
4	10	-19	8.60	6.10
-4	10	19	-9.10	8.10
3	-10	-19	86.59	12.20
3	10	-19	83.19	10.10
-3	10	19	75.39	13.50
2	-10	-19	-0.30	5.80
2	10	-19	1.60	4.70
-2	10	19	-5.30	8.00
1	-10	-19	-2.50	5.70
-1	10	19	-1.50	7.10
1	10	-19	-1.60	3.20
1	10	-19	-1.50	4.10
-1	10	19	5.50	8.60
0	-10	-19	3.80	5.90
0	10	19	1.70	7.60
0	10	19	-5.00	8.30
0	10	-19	0.40	3.60
0	10	-19	8.20	3.90
-1	10	-19	29.20	5.20
-1	10	-19	30.10	4.30
1	10	19	26.00	8.50
-2	10	-19	6.50	4.00
2	10	19	-10.10	7.20
-2	10	-19	1.00	2.20
-2	10	-19	1.90	4.70
-3	10	-19	23.70	5.30
3	10	19	31.90	8.70
-3	10	-19	22.60	6.00
4	10	19	16.50	8.50
-4	10	-19	23.30	6.50
-5	10	-19	-3.50	6.20
5	10	19	4.70	7.90
6	10	19	6.60	8.70
-6	10	-19	11.40	7.10
7	10	19	23.80	8.30
-7	10	-19	12.60	7.70
8	10	19	14.20	9.10
-8	10	-19	3.10	7.80
9	10	19	-8.40	8.00
-9	10	-19	-6.90	7.80
9	-11	-19	52.29	12.40

9	11	-19	39.70	11.00
8	-11	-19	262.57	21.90
-8	11	19	295.07	21.20
8	11	-19	250.07	21.00
7	-11	-19	82.99	14.40
7	11	-19	66.99	10.90
-7	11	19	68.59	11.70
6	-11	-19	473.05	30.20
-6	11	19	503.45	30.50
6	11	-19	503.75	29.40
5	-11	-19	398.86	26.00
5	11	-19	398.56	24.90
-5	11	19	417.86	26.70
4	-11	-19	674.13	37.60
-4	11	19	699.03	38.70
4	11	-19	650.03	36.30
3	-11	-19	494.35	28.90
3	11	-19	474.65	27.10
-3	11	19	482.15	30.10
2	-11	-19	60.69	11.60
2	11	-19	56.99	8.90
-2	11	19	47.90	11.00
1	-11	-19	783.22	41.10
1	11	-19	757.52	38.50
-1	11	19	715.83	41.80
0	-11	-19	56.69	11.50
0	11	-19	44.80	5.80
0	11	-19	59.19	6.40
0	11	19	49.90	10.10
1	11	19	1627.14	82.69
-1	11	-19	1630.94	78.59
-1	11	-19	1631.14	78.99
-2	11	-19	2.60	2.50
-2	11	-19	1.50	4.70
2	11	19	-4.70	8.00
3	11	19	450.35	29.00
-3	11	-19	487.15	25.80
-4	11	-19	14.70	6.10
4	11	19	8.60	8.20
-5	11	-19	651.73	35.00
5	11	19	654.43	37.50
-6	11	-19	39.70	8.30
6	11	19	46.00	9.40
-7	11	-19	490.05	29.00

7	11	19	501.95	30.90
-8	11	-19	106.89	14.80
8	11	19	102.79	15.60
-9	11	-19	93.99	14.90
9	11	19	98.59	16.00
9	-12	-19	305.27	24.90
9	12	-19	290.77	23.90
-9	12	19	305.87	22.80
8	-12	-19	46.00	10.70
8	12	-19	47.90	10.20
-8	12	19	36.90	10.40
7	-12	-19	319.67	24.80
-7	12	19	370.46	25.20
7	12	-19	376.26	24.50
6	-12	-19	153.88	16.80
6	12	-19	137.69	15.30
-6	12	19	127.59	17.00
5	-12	-19	111.39	15.70
5	12	-19	151.58	14.40
-5	12	19	120.19	16.30
4	-12	-19	130.39	15.00
-4	12	19	99.59	16.40
4	12	-19	122.29	13.10
3	-12	-19	194.78	17.00
3	12	-19	184.08	14.60
-3	12	19	175.68	18.40
2	-12	-19	299.97	20.80
-2	12	19	283.67	21.70
2	12	-19	298.47	18.40
1	-12	-19	60.49	12.40
-1	12	19	74.09	12.30
1	12	-19	84.49	9.20
0	12	-19	624.64	31.50
0	12	19	566.94	35.10
0	12	-19	622.44	30.20
1	12	19	-4.90	8.20
-1	12	-19	5.10	4.20
-1	12	-19	5.00	3.60
-2	12	-19	218.98	11.80
2	12	19	177.88	18.70
-2	12	-19	235.28	14.00
-3	12	-19	23.30	5.90
3	12	19	6.20	8.80
4	12	19	428.96	28.00

-4	12	-19	444.06	24.80
-5	12	-19	-2.20	6.20
5	12	19	1.00	7.80
6	12	19	187.58	18.00
-6	12	-19	186.88	15.70
7	12	19	43.00	11.00
-7	12	-19	31.50	8.90
8	12	19	118.39	16.20
-8	12	-19	96.39	14.10
-9	12	-19	21.60	9.20
9	12	19	4.90	9.80
8	-13	-19	98.19	17.90
-8	13	19	88.19	16.20
8	13	-19	56.89	11.40
7	-13	-19	46.00	11.60
7	13	-19	45.00	9.20
-7	13	19	62.09	11.70
6	-13	-19	48.40	10.20
-6	13	19	60.79	12.00
6	13	-19	31.60	8.40
5	-13	-19	219.18	19.30
5	13	-19	220.98	17.70
-5	13	19	213.38	20.70
4	-13	-19	198.68	17.90
4	13	-19	177.48	15.70
-4	13	19	199.88	19.80
3	-13	-19	109.99	14.10
3	13	-19	94.79	11.60
-3	13	19	106.29	16.60
2	-13	-19	17.80	8.70
-2	13	19	8.10	9.90
2	13	-19	15.40	5.90
1	-13	-19	550.74	30.40
1	13	-19	469.45	27.00
-1	13	19	483.75	31.50
0	13	19	-14.50	8.70
0	13	-19	-6.30	3.90
1	13	19	96.99	14.80
-1	13	-19	136.39	9.20
-1	13	-19	122.59	9.40
-2	13	-19	30.90	4.70
-2	13	-19	30.70	5.50
-2	13	-19	35.70	5.30
2	13	19	14.70	9.70

3	13	19	94.29	13.40
-3	13	-19	91.79	10.10
-4	13	-19	-0.20	5.80
4	13	19	1.10	8.70
-5	13	-19	113.29	12.70
5	13	19	119.49	14.70
6	13	19	48.90	10.90
-6	13	-19	68.39	9.70
-7	13	-19	35.20	9.10
7	13	19	55.59	11.00
-8	13	-19	2.30	8.20
8	13	19	-9.50	9.20
8	-14	-19	87.59	18.40
8	14	-19	106.79	16.90
-8	14	19	108.89	16.60
7	-14	-19	325.17	25.10
7	14	-19	298.87	23.20
-7	14	19	322.67	24.10
6	-14	-19	250.07	22.30
6	14	-19	266.77	20.30
-6	14	19	250.77	21.90
5	-14	-19	415.46	27.20
5	14	-19	384.76	25.10
-5	14	19	391.06	28.00
4	-14	-19	643.04	38.10
4	14	-19	635.54	36.30
-4	14	19	717.03	39.80
3	-14	-19	149.49	16.50
-3	14	19	141.99	18.60
3	14	-19	161.18	13.80
2	-14	-19	392.96	26.30
2	14	-19	403.26	23.30
-2	14	19	398.46	28.00
-1	14	19	91.79	14.20
1	14	-19	98.89	10.40
0	14	19	730.83	42.40
0	14	-19	738.03	37.70
1	14	19	111.59	16.90
-1	14	-19	100.39	9.00
-2	14	-19	636.44	38.30
-2	14	-19	797.82	37.30
-2	14	-19	818.82	38.20
2	14	19	778.42	42.90
3	14	19	0.30	9.00

-3	14	-19	-2.30	5.10
4	14	19	473.95	30.80
-4	14	-19	486.25	26.60
-5	14	-19	6.60	6.60
5	14	19	7.60	9.20
-6	14	-19	828.82	43.20
6	14	19	809.12	45.80
-7	14	-19	43.00	9.30
7	14	19	42.00	11.80
-8	14	-19	314.27	22.40
7	-15	-19	14.10	10.40
-7	15	19	-0.40	9.50
7	15	-19	14.60	8.90
6	-15	-19	19.90	10.60
6	15	-19	22.60	8.30
-6	15	19	1.60	10.30
5	-15	-19	14.90	8.80
5	15	-19	1.60	6.90
-5	15	19	-0.20	9.80
4	-15	-19	-8.70	8.60
-4	15	19	-23.20	9.80
4	15	-19	9.20	6.90
3	-15	-19	4.30	8.60
-3	15	19	-8.80	9.80
3	15	-19	6.00	6.30
2	-15	-19	36.20	9.30
2	15	-19	49.10	10.00
-2	15	19	33.80	11.00
-1	15	19	20.90	10.90
1	15	-19	45.50	9.10
0	15	-19	-1.70	4.60
0	15	19	-15.10	9.70
1	15	19	19.70	10.20
-1	15	-19	33.80	5.50
2	15	19	-1.60	9.40
-2	15	-19	-3.60	3.60
-3	15	-19	-4.20	6.20
3	15	19	-18.90	8.70
-4	15	-19	8.90	5.80
4	15	19	-12.40	9.00
-5	15	-19	8.80	6.90
5	15	19	15.50	10.00
-6	15	-19	1.60	7.10
6	15	19	-1.70	8.60

7	15	19	3.60	10.10
-7	15	-19	5.60	8.00
6	-16	-19	121.79	18.80
-6	16	19	136.99	18.90
6	16	-19	124.59	16.50
5	-16	-19	386.46	28.20
5	16	-19	356.56	25.60
-5	16	19	460.55	28.90
4	-16	-19	448.16	30.00
4	16	-19	431.36	27.30
-4	16	19	482.85	31.40
3	-16	-19	78.89	12.40
3	16	-19	61.39	11.50
-3	16	19	49.40	12.70
2	16	-19	238.68	17.30
-2	16	19	244.48	21.40
-1	16	19	53.99	12.40
1	16	-19	86.49	10.70
0	16	19	611.54	38.80
0	16	-19	657.83	33.70
-1	16	-19	57.19	8.60
1	16	19	63.19	13.30
-2	16	-19	339.97	18.70
2	16	19	340.77	25.10
-3	16	-19	-0.30	2.80
-3	16	-19	0.40	3.00
3	16	19	-9.30	9.80
4	16	19	306.57	24.10
5	16	19	3.30	10.00
6	16	19	488.55	32.00
-6	16	-19	460.25	27.50
5	-17	-19	157.58	19.60
5	17	-19	138.59	16.70
-5	17	19	150.08	20.00
4	-17	-19	39.20	11.00
4	17	-19	34.00	8.90
-4	17	19	25.60	11.90
3	-17	-19	68.09	12.60
-3	17	19	47.30	12.80
3	17	-19	47.20	8.90
-2	17	19	71.89	13.50
2	17	-19	98.39	12.40
1	17	-19	231.68	16.60
-1	17	19	233.08	22.60

0	17	-19	12.90	5.80
0	17	19	3.10	10.40
-1	17	-19	158.48	12.20
1	17	19	137.59	19.90
-2	17	-19	55.09	7.70
-2	17	-19	37.60	5.60
2	17	19	24.40	11.70
-3	17	-19	91.59	7.40
-3	17	-19	80.89	7.20
3	17	19	59.49	12.60
4	17	19	-4.60	10.40
5	17	19	287.77	24.60
4	-18	-19	61.19	13.50
4	18	-19	76.89	14.50
-4	18	19	40.70	13.30
3	18	-19	110.69	14.10
-3	18	19	105.19	19.90
-2	18	19	72.49	14.00
2	18	-19	75.49	12.80
1	18	-19	0.20	6.20
-1	18	19	7.10	10.90
0	18	19	353.16	26.90
0	18	-19	329.27	20.70
-1	18	-19	4.60	5.20
1	18	19	-0.70	10.50
2	18	19	151.28	19.90
-2	18	-19	135.79	11.00
-2	18	-19	129.69	10.40
-3	18	-19	6.60	4.00
-3	18	-19	9.50	4.20
3	18	19	-4.50	10.70
4	18	19	104.59	19.40
-3	19	19	121.59	20.40
-2	19	19	40.20	13.70
2	19	-19	48.00	10.20
-1	19	19	139.09	21.30
1	19	-19	194.18	15.80
0	19	19	27.00	12.20
0	19	-19	36.30	7.30
-1	19	-19	613.54	33.20
1	19	19	632.54	39.40
-2	19	-19	77.29	10.00
2	19	19	78.19	14.80
-3	19	-19	253.67	15.10

3	19	19	260.77	24.50
-3	19	-19	238.48	14.70
10	0	-20	141.29	17.70
-9	0	20	194.28	13.90
9	0	-20	174.18	17.40
8	0	-20	33.30	9.20
-8	0	20	29.00	6.30
-7	0	20	486.85	26.00
7	0	-20	467.85	28.40
6	0	-20	462.45	26.20
-6	0	20	424.86	24.10
5	0	-20	228.98	15.60
-5	0	20	207.18	13.60
5	0	-20	190.48	12.30
-4	0	20	284.77	16.60
4	0	-20	285.47	17.80
4	0	-20	259.77	15.10
3	0	-20	69.59	8.90
3	0	-20	54.49	6.30
-3	0	20	53.29	7.90
2	0	-20	841.02	43.40
-2	0	20	894.81	44.60
2	0	-20	913.81	45.00
1	0	-20	219.18	13.70
1	0	-20	186.98	12.30
-1	0	20	211.88	13.70
0	0	20	1899.61	86.79
0	0	20	1675.53	86.69
0	0	-20	1754.52	86.49
1	0	20	3.80	4.90
-1	0	-20	0.50	3.40
1	0	20	11.30	4.30
2	0	20	748.33	39.80
-2	0	-20	807.02	38.60
2	0	20	739.83	38.70
3	0	20	35.50	6.80
4	0	20	395.56	28.70
5	0	20	103.69	11.90
6	0	20	926.41	48.40
7	0	20	230.08	17.50
8	0	20	333.67	22.60
-9	0	-20	220.58	18.70
-10	0	-20	351.86	24.70
10	-1	-20	139.09	17.40

10	1	-20	123.79	16.90
9	-1	-20	81.79	14.50
-9	1	20	58.99	10.50
9	1	-20	39.40	9.40
-8	-1	20	9.80	5.30
8	-1	-20	17.10	7.80
8	1	-20	20.30	8.00
-8	1	20	-8.50	5.60
-7	-1	20	26.90	5.40
7	-1	-20	33.20	8.30
-7	1	20	21.90	6.20
7	1	-20	27.40	8.00
6	-1	-20	1.90	6.30
-6	-1	20	12.10	4.90
-6	1	20	-1.00	5.20
6	1	-20	1.30	5.60
5	-1	-20	4.70	5.20
5	-1	-20	17.50	4.20
-5	-1	20	10.60	4.60
-5	1	20	17.90	5.50
5	1	-20	12.60	5.80
5	1	-20	10.60	4.60
-4	-1	20	34.30	5.40
4	-1	-20	32.40	6.20
4	-1	-20	26.00	4.50
-4	1	20	28.90	5.70
4	1	-20	29.10	6.10
4	1	-20	26.20	4.10
-3	-1	20	61.69	7.80
3	-1	-20	74.19	9.30
3	-1	-20	63.09	6.90
3	1	-20	81.49	9.00
3	1	-20	59.29	6.40
-3	1	20	72.89	8.80
2	-1	-20	10.70	3.80
2	-1	-20	10.60	5.00
-2	-1	20	3.50	4.10
-2	1	20	5.90	5.10
2	1	-20	4.80	4.20
2	1	-20	2.90	3.30
1	-1	-20	27.40	5.30
-1	-1	20	43.80	6.10
1	1	-20	37.20	7.00
1	1	-20	30.40	4.60

-1	1	20	36.70	6.10
0	-1	-20	49.10	7.40
0	-1	20	48.40	7.80
0	-1	20	44.20	8.20
0	1	20	46.70	8.30
0	1	-20	43.40	7.00
0	1	-20	57.49	6.70
0	1	20	57.69	9.40
1	-1	20	180.78	13.50
1	-1	20	200.88	11.90
-1	-1	-20	179.88	12.80
1	1	20	183.28	14.00
-1	1	-20	203.18	12.50
1	1	20	234.98	13.80
2	-1	20	98.99	10.20
-2	-1	-20	96.09	8.60
-2	1	-20	102.39	8.20
2	1	20	105.69	10.50
2	1	20	105.69	8.60
3	-1	20	65.99	9.40
3	1	20	57.89	9.50
4	-1	20	4.50	5.50
4	1	20	6.60	6.50
5	-1	20	19.60	6.30
5	1	20	4.10	5.90
6	-1	20	7.50	6.30
6	1	20	8.50	6.90
7	-1	20	81.49	12.20
7	1	20	66.79	12.30
8	1	20	53.89	9.70
-8	1	-20	87.29	13.10
9	1	20	145.99	16.00
-9	1	-20	170.68	16.30
-10	-1	-20	25.80	10.00
-10	1	-20	31.10	9.80
10	-2	-20	101.89	17.00
-10	2	20	119.29	14.90
10	2	-20	108.49	16.80
9	-2	-20	303.17	22.60
9	2	-20	327.87	22.90
-9	2	20	294.37	19.70
-8	-2	20	170.68	13.80
8	-2	-20	201.08	16.90
8	2	-20	187.88	16.50

-8	2	20	166.48	14.20
7	-2	-20	139.19	14.20
-7	-2	20	117.69	10.20
-7	2	20	133.59	12.20
7	2	-20	126.29	13.90
6	-2	-20	324.17	20.70
-6	-2	20	309.57	17.70
-6	2	20	308.17	19.20
6	2	-20	303.77	20.40
-5	-2	20	202.18	13.10
5	-2	-20	202.98	15.60
5	2	-20	219.38	15.60
-5	2	20	212.68	14.70
4	-2	-20	350.66	21.30
-4	-2	20	360.66	19.30
4	2	-20	327.77	18.20
-4	2	20	339.67	20.50
4	2	-20	371.36	21.10
-3	-2	20	191.48	12.40
3	-2	-20	203.78	14.00
-3	2	20	187.48	13.70
3	2	-20	200.58	13.60
3	2	-20	169.18	10.90
2	-2	-20	270.67	16.70
-2	-2	20	245.88	15.50
2	2	-20	247.78	14.30
2	2	-20	288.97	16.20
-2	2	20	255.97	16.60
1	-2	-20	168.38	12.40
-1	-2	20	181.38	11.90
1	2	-20	176.18	11.80
1	2	-20	162.68	10.60
-1	2	20	158.58	12.50
0	-2	20	455.85	23.40
0	-2	20	423.56	23.90
0	-2	-20	440.76	24.20
0	2	20	474.05	25.30
0	2	-20	450.15	23.50
0	2	-20	421.96	23.00
0	2	20	408.96	24.40
-1	-2	-20	4.80	4.00
1	-2	20	3.90	4.70
1	2	20	5.20	5.40
-1	2	-20	-0.30	3.20

-1	2	-20	3.60	4.10
1	2	20	2.70	5.20
2	-2	20	572.04	31.30
2	2	20	633.04	30.80
2	2	20	582.14	31.90
-2	2	-20	574.44	29.80
3	-2	20	14.60	5.60
3	2	20	9.40	4.10
3	2	20	15.20	6.20
4	-2	20	463.85	25.90
4	2	20	447.46	26.30
5	-2	20	55.29	8.80
5	2	20	71.49	10.90
6	-2	20	732.73	39.40
6	2	20	735.53	39.70
7	-2	20	22.20	7.40
7	2	20	2.30	7.20
-7	2	-20	3.00	7.00
8	2	20	263.77	19.60
-8	2	-20	239.38	18.90
9	2	20	124.59	15.60
-9	2	-20	127.99	15.40
-10	-2	-20	103.09	15.50
-10	2	-20	84.49	12.50
10	-3	-20	359.46	26.00
10	3	-20	346.77	25.30
-10	3	20	360.36	22.50
9	-3	-20	158.38	17.20
9	3	-20	156.58	16.90
8	-3	-20	75.69	13.40
-8	3	20	87.49	11.60
8	3	-20	67.09	13.10
-7	-3	20	224.98	13.70
7	-3	-20	238.28	18.00
7	3	-20	229.18	17.50
-7	3	20	211.88	16.20
6	-3	-20	255.47	17.80
-6	-3	20	228.08	14.00
6	3	-20	231.48	17.20
-6	3	20	228.98	16.50
5	-3	-20	583.04	32.70
-5	-3	20	578.14	30.20
-5	3	20	624.04	32.10
5	3	-20	588.44	32.50

4	-3	-20	354.06	21.20
-4	-3	20	332.57	18.80
4	3	-20	328.47	18.20
-4	3	20	347.37	20.80
4	3	-20	360.96	20.80
-3	-3	20	496.15	26.00
3	-3	-20	521.95	27.80
-3	3	20	492.35	27.50
3	3	-20	468.55	24.90
3	3	-20	509.65	27.20
-2	-3	20	153.18	10.90
2	-3	-20	157.88	12.40
2	3	-20	145.49	9.40
2	3	-20	158.98	11.50
-2	3	20	143.29	12.30
-1	-3	20	549.74	28.60
1	-3	-20	558.64	29.60
1	3	-20	526.75	27.60
1	3	-20	568.84	28.70
-1	3	20	528.05	29.70
0	-3	-20	5.20	4.20
0	-3	20	5.60	4.40
0	-3	20	5.40	3.80
0	3	20	1.30	5.80
0	3	-20	6.30	3.50
0	3	20	2.40	5.20
0	3	-20	6.50	3.70
-1	-3	-20	689.73	35.80
1	-3	20	683.73	35.60
1	3	20	638.74	36.30
-1	3	-20	684.13	34.60
1	3	20	770.12	36.60
-1	3	-20	670.03	34.70
2	-3	20	12.80	5.10
2	3	20	13.30	6.00
2	3	20	8.90	5.20
-2	3	-20	13.40	3.30
3	-3	20	889.61	45.30
3	3	20	776.62	51.19
3	3	20	956.40	44.40
4	-3	20	75.39	9.80
4	3	20	65.09	10.70
4	3	20	48.90	7.10
5	-3	20	668.33	36.10

5	3	20	657.33	36.10
6	-3	20	80.19	11.60
-6	3	-20	70.99	9.90
6	3	20	94.59	12.10
7	3	20	453.45	26.70
-7	3	-20	410.76	26.00
8	3	20	81.29	13.70
-9	3	-20	117.29	14.80
9	3	20	114.39	15.20
10	3	20	197.08	18.80
-10	3	-20	176.68	18.00
10	-4	-20	10.20	8.90
10	4	-20	21.70	10.20
-10	4	20	12.50	8.00
9	-4	-20	23.00	8.90
-9	4	20	22.10	8.20
9	4	-20	25.80	8.60
8	-4	-20	6.60	7.10
8	4	-20	14.40	7.90
7	-4	-20	6.20	7.40
7	4	-20	6.00	6.40
-7	4	20	0.00	6.60
-6	-4	20	3.60	3.70
6	-4	-20	-0.10	6.30
6	4	-20	5.10	5.90
-6	4	20	-1.90	6.20
-5	-4	20	54.19	6.70
5	-4	-20	51.69	10.60
5	4	-20	48.30	7.90
-5	4	20	52.59	9.90
-4	-4	20	5.70	3.90
4	-4	-20	4.90	5.50
4	4	-20	6.90	5.20
-4	4	20	2.30	6.00
3	-4	-20	15.00	5.40
-3	-4	20	1.40	3.90
3	4	-20	7.40	4.60
-3	4	20	12.00	6.10
3	4	-20	7.80	3.00
-2	-4	20	158.48	10.80
2	-4	-20	155.68	12.60
2	4	-20	162.78	11.50
2	4	-20	154.78	9.50
-2	4	20	134.49	13.10

-1	-4	20	35.40	5.40
1	-4	-20	40.00	6.70
-1	4	20	36.00	6.90
1	4	-20	31.20	5.20
1	4	-20	46.00	5.60
0	-4	20	246.48	14.60
0	-4	-20	226.98	15.40
0	-4	20	233.28	13.00
0	4	20	210.98	16.00
0	4	-20	226.88	13.40
0	4	-20	245.08	14.00
0	4	20	231.98	17.00
1	-4	20	95.19	9.60
-1	-4	-20	94.89	9.70
-1	4	-20	88.59	7.80
-1	4	-20	104.19	8.60
1	4	20	95.49	11.10
1	4	20	123.09	12.10
2	-4	20	230.38	15.10
2	4	20	202.78	19.40
-2	4	-20	228.08	12.70
2	4	20	244.58	16.10
3	-4	20	42.00	6.90
3	4	20	40.50	7.50
3	4	20	63.79	9.40
4	-4	20	86.49	10.20
4	4	20	60.59	9.40
4	4	20	96.69	9.20
5	4	20	41.00	8.70
6	4	20	71.79	11.60
-6	4	-20	49.20	8.40
7	4	20	1.90	7.20
-7	4	-20	0.30	6.70
-8	4	-20	145.89	15.50
8	4	20	154.38	15.80
9	4	20	70.79	11.70
-9	4	-20	93.39	14.30
10	4	20	12.60	9.90
-10	4	-20	30.60	9.60
10	-5	-20	503.85	32.70
10	5	-20	504.15	32.00
-10	5	20	512.15	29.80
9	-5	-20	36.10	9.90
9	5	-20	24.60	8.90

-9	5	20	10.90	7.80
8	-5	-20	522.95	31.90
8	5	-20	538.05	31.80
-8	5	20	546.75	30.70
7	-5	-20	222.58	17.40
-7	5	20	171.28	18.40
7	5	-20	194.68	16.60
6	-5	-20	473.65	28.60
6	5	-20	485.35	28.30
-6	5	20	503.55	28.30
-5	-5	20	893.91	44.30
5	-5	-20	908.31	47.40
5	5	-20	884.51	46.90
-5	5	20	926.91	47.10
4	-5	-20	538.95	30.70
-4	-5	20	552.64	27.90
4	5	-20	556.64	30.00
-4	5	20	545.95	30.70
3	-5	-20	565.04	31.00
-3	-5	20	566.14	28.70
-3	5	20	559.84	31.30
3	5	-20	556.04	30.10
-2	-5	20	289.97	15.60
2	-5	-20	263.87	17.60
2	5	-20	244.88	14.20
2	5	-20	293.37	16.40
-2	5	20	244.88	18.00
-1	-5	20	1586.44	74.09
1	-5	-20	1560.24	75.49
1	5	-20	1429.56	73.09
1	5	-20	1559.14	74.19
-1	5	20	1461.15	75.69
0	-5	-20	4.20	5.20
0	-5	20	15.20	4.70
0	5	-20	11.00	3.70
0	5	20	19.80	7.60
0	5	20	12.10	6.10
0	5	-20	8.80	3.60
1	-5	20	2331.57	110.69
-1	-5	-20	2303.87	111.39
1	5	20	2610.84	112.39
1	5	20	2061.09	112.89
-1	5	-20	2149.78	109.69
-1	5	-20	2258.77	109.69

2	-5	20	29.70	6.00
2	5	20	44.30	7.60
2	5	20	27.80	7.10
-2	5	-20	26.30	4.60
-2	5	-20	38.80	7.20
3	-5	20	394.56	23.20
3	5	20	380.86	24.40
3	5	20	445.06	23.90
4	5	20	7.10	5.60
-4	5	-20	-0.70	5.60
4	5	20	14.80	7.10
5	5	20	1245.08	66.89
5	5	20	1386.56	64.89
-6	5	-20	311.67	21.10
6	5	20	348.97	22.30
-7	5	-20	617.84	35.80
7	5	20	683.13	36.70
-8	5	-20	240.08	19.20
8	5	20	258.77	19.90
-9	5	-20	157.28	16.20
9	5	20	160.78	17.10
-10	5	-20	192.48	18.90
10	5	20	207.28	19.60
10	-6	-20	14.60	9.80
-10	6	20	21.30	8.90
10	6	-20	24.10	9.30
9	-6	-20	37.90	9.70
-9	6	20	39.30	9.10
9	6	-20	44.10	10.30
8	-6	-20	9.70	9.20
-8	6	20	17.40	8.20
8	6	-20	23.70	8.10
7	-6	-20	4.60	7.30
-7	6	20	-2.40	7.00
7	6	-20	0.20	6.90
6	-6	-20	11.70	7.60
-6	6	20	4.00	7.30
6	6	-20	11.40	6.50
5	-6	-20	61.09	9.50
5	6	-20	51.59	10.70
-4	-6	20	59.69	6.50
4	-6	-20	65.59	10.80
-4	6	20	56.79	9.60
4	6	-20	50.09	9.40

-3	-6	20	59.29	7.00
3	-6	-20	59.99	10.20
-3	6	20	60.59	9.20
3	6	-20	67.99	8.90
-2	-6	20	84.59	7.80
2	-6	-20	74.49	10.50
2	6	-20	70.09	6.70
-2	6	20	81.29	11.70
2	6	-20	91.19	8.80
1	-6	-20	2.70	5.10
-1	-6	20	13.90	4.60
1	6	-20	7.00	4.20
-1	6	20	5.50	6.50
1	6	-20	10.20	3.30
0	-6	20	136.29	10.50
0	-6	-20	134.39	12.20
0	6	-20	118.59	9.00
0	6	-20	130.89	9.50
0	6	20	149.88	14.50
-1	-6	-20	124.79	11.80
1	-6	20	151.28	11.00
-1	6	-20	115.59	8.80
-1	6	-20	131.59	9.60
1	6	20	112.99	12.60
1	6	20	152.98	14.10
2	6	20	333.97	22.20
-2	6	-20	338.97	17.90
2	6	20	417.56	22.70
-2	6	-20	333.17	20.40
-2	6	-20	349.37	19.50
3	6	20	31.70	7.20
-3	6	-20	24.40	6.30
3	6	20	28.90	7.80
4	6	20	87.99	12.70
-4	6	-20	89.19	11.40
4	6	20	126.79	11.90
5	6	20	21.60	7.70
-5	6	-20	21.60	8.40
-6	6	-20	111.29	12.60
6	6	20	108.59	13.90
-7	6	-20	9.10	7.40
7	6	20	10.20	8.10
8	6	20	197.78	17.70
-8	6	-20	178.08	16.70

9	6	20	33.40	9.90
-9	6	-20	35.10	9.40
10	6	20	54.59	11.10
-10	6	-20	67.89	11.50
10	-7	-20	283.07	23.30
-10	7	20	290.87	21.00
10	7	-20	234.48	22.20
9	-7	-20	148.79	17.60
-9	7	20	177.98	16.60
9	7	-20	123.59	16.90
8	-7	-20	261.37	20.80
-8	7	20	267.07	19.70
8	7	-20	262.07	20.50
7	-7	-20	158.18	15.90
7	7	-20	152.48	15.60
-7	7	20	166.88	15.70
6	-7	-20	76.89	13.00
6	7	-20	76.59	12.30
-6	7	20	74.09	13.30
5	-7	-20	508.25	29.60
5	7	-20	499.35	28.80
-5	7	20	520.05	30.10
4	-7	-20	542.85	31.30
-4	7	20	574.44	31.90
4	7	-20	543.35	30.40
-3	-7	20	625.34	29.40
3	-7	-20	583.54	32.60
-3	7	20	555.74	33.30
3	7	-20	567.44	31.20
2	-7	-20	5.90	5.40
-2	7	20	1.60	8.10
2	7	-20	5.40	4.40
1	-7	-20	465.35	26.20
-1	7	20	447.26	26.90
1	7	-20	467.35	24.10
1	7	-20	419.96	23.50
0	-7	-20	2.10	5.30
0	7	-20	2.60	3.30
0	7	20	4.60	6.80
0	7	20	-4.40	6.20
0	7	-20	-1.40	3.50
0	7	-20	-1.60	4.50
-1	-7	-20	1038.60	52.19
-1	7	-20	1070.49	49.80

-1	7	-20	992.00	49.90
1	7	20	984.80	52.89
-1	7	-20	1002.10	50.89
2	7	20	2.40	6.50
2	7	20	10.10	6.70
-2	7	-20	17.20	2.70
-2	7	-20	21.00	5.80
-2	7	-20	16.40	4.90
-3	7	-20	402.86	23.40
3	7	20	420.86	25.40
-4	7	-20	56.89	8.30
4	7	20	58.39	9.70
5	7	20	569.24	31.80
-5	7	-20	513.95	30.40
6	7	20	128.09	14.60
-6	7	-20	128.79	13.50
7	7	20	256.37	20.00
-7	7	-20	241.58	18.60
8	7	20	53.29	10.50
-8	7	-20	56.09	10.20
-9	7	-20	85.99	14.40
9	7	20	54.99	10.60
-10	7	-20	184.18	17.70
10	7	20	144.39	18.30
9	-8	-20	328.77	24.60
-9	8	20	341.77	22.90
9	8	-20	278.37	23.10
8	-8	-20	144.39	16.60
-8	8	20	152.88	16.30
8	8	-20	135.89	16.00
7	-8	-20	181.68	17.10
7	8	-20	170.38	16.10
-7	8	20	156.38	17.00
6	-8	-20	481.85	28.80
-6	8	20	504.05	28.80
6	8	-20	434.36	27.80
5	-8	-20	229.88	18.10
5	8	-20	250.87	17.20
-5	8	20	215.88	18.60
4	-8	-20	563.74	32.40
4	8	-20	552.64	31.40
-4	8	20	605.44	33.10
3	-8	-20	119.39	13.10
3	8	-20	116.39	11.10

-3	8	20	124.29	14.50
2	-8	-20	497.25	28.50
2	8	-20	509.35	25.10
2	8	-20	487.55	26.60
-2	8	20	469.35	29.40
1	-8	-20	5.80	5.80
1	8	-20	0.60	3.80
-1	8	20	-2.10	7.40
1	8	-20	4.90	3.90
0	-8	-20	1153.68	58.19
0	8	-20	1158.98	55.89
0	8	20	1122.29	58.99
0	8	-20	1120.49	56.09
-1	8	-20	11.70	5.20
-1	8	-20	12.80	3.50
-1	8	-20	5.70	3.90
1	8	20	16.10	7.70
-2	8	-20	509.75	26.80
2	8	20	530.95	30.40
-2	8	-20	503.15	25.20
-2	8	-20	524.25	27.80
3	8	20	6.10	6.70
-3	8	-20	13.10	6.00
4	8	20	506.35	30.50
-4	8	-20	521.95	28.60
5	8	20	128.89	14.60
-5	8	-20	92.09	12.50
6	8	20	397.86	25.60
-6	8	-20	377.56	23.90
7	8	20	27.70	9.30
-7	8	-20	16.30	7.90
8	8	20	219.18	19.00
-8	8	-20	219.38	18.40
-9	8	-20	49.80	10.10
9	8	20	73.99	12.50
9	-9	-20	17.60	10.10
9	9	-20	38.80	10.40
-9	9	20	22.30	10.00
8	-9	-20	2.40	8.40
8	9	-20	-0.20	7.70
-8	9	20	-6.00	8.80
7	-9	-20	12.50	7.80
7	9	-20	7.70	7.70
-7	9	20	0.80	8.70

6	-9	-20	-2.80	6.80
-6	9	20	-1.00	8.40
6	9	-20	4.30	6.90
5	-9	-20	7.40	6.90
-5	9	20	3.80	8.80
5	9	-20	10.50	6.70
4	-9	-20	74.89	12.20
4	9	-20	60.19	10.60
-4	9	20	110.39	14.50
3	-9	-20	33.00	8.00
-3	9	20	36.70	9.90
3	9	-20	30.40	6.10
2	-9	-20	8.80	6.60
2	9	-20	7.40	4.80
-2	9	20	-5.10	7.50
1	-9	-20	-5.90	5.70
1	9	-20	3.70	4.10
-1	9	20	-0.80	8.10
1	9	-20	10.00	4.00
0	-9	-20	19.10	6.60
0	9	20	21.70	8.10
0	9	-20	20.30	4.50
0	9	-20	26.40	5.10
-1	9	-20	14.00	3.50
-1	9	-20	9.80	5.10
1	9	20	4.80	7.40
-2	9	-20	57.99	9.20
2	9	20	49.20	9.80
-3	9	-20	96.39	10.70
3	9	20	78.89	13.50
4	9	20	3.30	7.70
-4	9	-20	4.40	6.00
5	9	20	24.80	8.40
-5	9	-20	30.80	7.80
-6	9	-20	-4.10	6.60
6	9	20	4.10	8.30
7	9	20	123.59	15.70
-7	9	-20	115.79	13.90
-8	9	-20	-6.70	8.00
8	9	20	-1.00	8.60
-9	9	-20	14.50	8.80
9	9	20	27.40	9.70
9	-10	-20	294.07	24.40
-9	10	20	345.27	23.20

9	10	-20	290.97	23.90
8	-10	-20	86.19	16.50
-8	10	20	67.19	11.90
8	10	-20	113.19	15.00
7	-10	-20	257.07	20.80
-7	10	20	261.57	21.00
7	10	-20	233.38	19.40
6	-10	-20	593.24	34.50
-6	10	20	631.64	35.00
6	10	-20	553.64	33.30
5	-10	-20	230.18	18.80
5	10	-20	241.18	17.60
-5	10	20	236.08	20.10
4	-10	-20	766.32	41.10
-4	10	20	767.52	41.80
4	10	-20	705.23	39.40
3	-10	-20	115.39	13.50
3	10	-20	112.29	11.30
-3	10	20	104.59	15.50
2	-10	-20	413.16	25.60
2	10	-20	427.36	23.30
-2	10	20	394.96	26.80
1	-10	-20	118.49	13.50
-1	10	20	84.19	15.00
1	10	-20	129.99	8.60
1	10	-20	121.19	10.20
0	-10	-20	1698.33	83.39
0	10	-20	1658.73	80.59
0	10	20	1576.54	83.99
0	10	-20	1713.13	80.39
-1	10	-20	28.90	5.60
1	10	20	0.20	8.50
-1	10	-20	27.30	4.10
-2	10	-20	736.73	37.50
2	10	20	720.53	40.90
-3	10	-20	4.10	5.60
3	10	20	-3.80	7.50
-4	10	-20	479.55	27.70
4	10	20	513.15	30.40
5	10	20	176.88	17.80
-5	10	-20	172.98	15.10
6	10	20	967.00	51.89
-6	10	-20	957.00	49.99
-7	10	-20	152.58	15.10

7	10	20	118.99	16.70
-8	10	-20	348.57	23.20
8	10	20	310.07	24.20
9	10	20	108.49	17.10
-9	10	-20	146.19	16.00
8	-11	-20	22.40	9.70
8	11	-20	31.30	9.40
-8	11	20	-0.40	9.40
7	-11	-20	46.20	10.40
-7	11	20	64.69	11.80
7	11	-20	61.59	10.60
6	-11	-20	3.30	7.90
-6	11	20	-13.20	8.90
6	11	-20	4.30	7.10
5	-11	-20	99.99	14.70
-5	11	20	90.99	13.10
5	11	-20	84.79	12.60
4	-11	-20	41.50	8.80
-4	11	20	31.10	10.40
4	11	-20	51.89	8.20
3	-11	-20	66.99	12.60
3	11	-20	65.89	10.10
-3	11	20	51.29	11.20
2	-11	-20	6.60	7.30
2	11	-20	2.40	4.90
-2	11	20	-6.90	8.30
1	-11	-20	81.29	13.10
1	11	-20	81.49	8.80
-1	11	20	72.19	12.40
0	11	20	-0.20	8.40
0	11	-20	-4.10	3.80
0	11	-20	6.80	3.90
-1	11	-20	81.39	7.30
-1	11	-20	76.29	8.60
1	11	20	88.39	13.00
-2	11	-20	21.30	5.70
2	11	20	12.20	8.90
-2	11	-20	23.10	3.60
-3	11	-20	118.59	11.30
3	11	20	90.39	14.60
4	11	20	60.89	11.70
-4	11	-20	59.59	10.90
-5	11	-20	129.69	13.10
5	11	20	104.09	15.70

6	11	20	-6.10	8.60
-6	11	-20	13.40	7.40
-7	11	-20	72.69	13.40
7	11	20	61.39	11.80
-8	11	-20	25.30	9.30
8	11	20	22.70	9.40
8	-12	-20	57.09	11.80
8	12	-20	57.49	11.10
-8	12	20	44.00	11.20
7	-12	-20	133.29	17.50
-7	12	20	137.59	17.60
7	12	-20	124.79	15.70
6	-12	-20	203.18	19.10
6	12	-20	165.98	16.80
-6	12	20	207.08	19.80
5	-12	-20	122.39	16.00
5	12	-20	123.39	13.80
-5	12	20	96.59	13.70
4	-12	-20	372.76	24.80
-4	12	20	368.96	26.10
4	12	-20	340.77	22.30
3	-12	-20	77.09	11.40
3	12	-20	75.79	10.60
-3	12	20	66.09	12.90
2	-12	-20	320.37	21.80
2	12	-20	294.07	18.50
-2	12	20	280.57	23.40
1	-12	-20	-0.30	7.30
1	12	-20	-3.60	4.70
-1	12	20	3.10	9.50
0	12	20	445.06	29.40
0	12	-20	459.65	24.20
-1	12	-20	11.20	4.60
1	12	20	7.10	8.90
-1	12	-20	13.30	3.80
-2	12	-20	535.15	28.00
-2	12	-20	531.25	26.00
2	12	20	517.85	32.70
-3	12	-20	11.90	5.90
3	12	20	7.00	9.20
-4	12	-20	226.28	16.00
4	12	20	197.78	19.50
5	12	20	143.49	17.20
-5	12	-20	146.99	16.20

6	12	20	444.36	29.00
-6	12	-20	432.46	26.30
-7	12	-20	53.09	9.80
7	12	20	40.80	11.00
-8	12	-20	248.38	20.10
8	12	20	257.07	22.00
7	-13	-20	189.68	20.70
-7	13	20	212.48	20.10
7	13	-20	170.38	18.20
6	-13	-20	139.39	17.90
-6	13	20	149.19	18.70
6	13	-20	170.08	16.10
5	-13	-20	306.37	22.50
-5	13	20	294.77	23.90
5	13	-20	278.17	20.60
4	-13	-20	124.09	16.00
4	13	-20	132.79	13.80
-4	13	20	126.69	18.40
3	-13	-20	462.45	29.00
-3	13	20	496.75	30.70
3	13	-20	431.16	26.40
2	-13	-20	32.40	8.70
2	13	-20	50.59	9.20
-2	13	20	41.30	11.20
1	-13	-20	618.04	34.50
-1	13	20	570.54	35.70
1	13	-20	562.04	30.80
0	13	-20	7.90	4.50
0	13	20	19.20	10.70
-1	13	-20	830.82	40.80
1	13	20	809.22	46.40
-2	13	-20	0.20	2.70
-2	13	-20	1.20	2.50
-2	13	-20	1.90	5.10
2	13	20	4.60	9.60
3	13	20	367.06	26.40
-3	13	-20	400.46	22.20
-4	13	-20	37.40	7.50
4	13	20	28.70	10.60
5	13	20	321.27	24.40
6	13	20	84.39	12.90
-6	13	-20	83.79	13.30
-7	13	-20	281.27	20.10
7	13	20	233.28	22.40

7	-14	-20	26.90	10.40
7	14	-20	31.30	10.10
-7	14	20	13.40	11.10
6	-14	-20	46.20	12.10
6	14	-20	49.30	10.20
-6	14	20	52.89	12.60
5	-14	-20	49.20	11.30
-5	14	20	42.40	12.40
5	14	-20	38.80	8.60
4	-14	-20	32.40	10.10
-4	14	20	31.40	11.10
4	14	-20	36.50	8.40
3	-14	-20	-1.20	8.40
-3	14	20	-12.20	10.30
3	14	-20	7.90	6.20
2	-14	-20	14.50	9.40
-2	14	20	10.70	10.90
2	14	-20	12.30	5.80
1	14	-20	36.60	6.50
-1	14	20	33.30	12.20
0	14	-20	6.60	4.50
0	14	20	3.40	10.00
-1	14	-20	-1.80	3.90
1	14	20	-1.50	9.50
-2	14	-20	19.10	3.40
-2	14	-20	25.90	5.00
2	14	20	6.00	10.40
3	14	20	7.70	9.70
4	14	20	-9.30	9.40
-4	14	-20	0.20	6.10
5	14	20	34.00	10.60
6	14	20	2.60	10.00
-6	14	-20	18.20	7.90
-7	14	-20	1.70	8.20
7	14	20	3.80	10.70
6	-15	-20	138.19	19.50
-6	15	20	137.99	19.10
6	15	-20	149.59	16.60
5	-15	-20	675.83	39.90
-5	15	20	731.33	41.10
5	15	-20	630.44	37.70
4	-15	-20	140.59	18.10
-4	15	20	162.08	20.30
4	15	-20	168.38	15.30

3	-15	-20	169.08	19.10
-3	15	20	184.38	20.90
3	15	-20	159.38	14.50
2	-15	-20	158.28	18.30
-2	15	20	191.78	21.10
2	15	-20	160.08	13.90
1	15	-20	640.74	35.30
-1	15	20	720.83	41.30
0	15	-20	1.80	4.70
0	15	20	3.30	11.20
-1	15	-20	741.73	37.50
1	15	20	745.13	44.00
-2	15	-20	10.30	3.50
2	15	20	-4.70	9.40
-2	15	-20	11.20	3.60
3	15	20	353.86	27.00
4	15	20	-16.70	8.50
5	15	20	798.42	46.00
-5	15	-20	791.92	46.20
6	15	20	115.19	16.80
-6	15	-20	112.59	13.90
5	-16	-20	63.69	12.80
5	16	-20	67.79	13.70
-5	16	20	67.99	13.70
4	-16	-20	26.70	11.90
4	16	-20	68.39	12.80
-4	16	20	33.50	11.60
3	-16	-20	8.50	9.90
-3	16	20	23.20	12.50
3	16	-20	41.70	8.00
-2	16	20	40.70	12.60
2	16	-20	32.90	7.30
-1	16	20	-7.20	10.40
1	16	-20	10.10	5.60
0	16	-20	11.40	5.20
0	16	20	10.70	10.90
1	16	20	-8.20	10.80
-1	16	-20	12.40	4.80
2	16	20	111.19	18.80
-2	16	-20	104.99	8.40
-2	16	-20	93.99	8.10
3	16	20	0.70	10.00
4	16	20	140.19	19.90
5	16	20	3.00	10.20

4	-17	-20	132.39	19.80
-4	17	20	127.79	20.50
4	17	-20	123.69	18.80
3	-17	-20	242.98	23.30
-3	17	20	306.07	25.50
3	17	-20	249.88	19.20
-2	17	20	15.10	11.90
2	17	-20	24.10	7.10
-1	17	20	486.95	32.30
1	17	-20	447.26	26.40
0	17	20	-5.70	10.90
0	17	-20	0.20	5.20
-1	17	-20	344.37	20.60
1	17	20	411.46	28.20
-2	17	-20	3.80	4.20
-2	17	-20	7.00	4.20
2	17	20	8.00	11.70
3	17	20	200.38	22.80
4	17	20	25.80	11.60
2	18	-20	318.17	25.30
-2	18	20	321.57	27.60
-1	18	20	81.69	15.70
1	18	-20	140.39	12.90
0	18	20	576.84	37.70
0	18	-20	559.84	30.80
1	18	20	14.80	12.50
-1	18	-20	24.70	6.00
-2	18	-20	290.77	16.90
2	18	20	329.57	25.30
-2	18	-20	289.37	17.20
10	0	-21	18.30	10.30
9	0	-21	2.80	7.60
-8	0	21	3.30	5.60
8	0	-21	-3.30	7.40
7	0	-21	-2.00	6.30
-7	0	21	0.90	5.20
6	0	-21	2.90	6.50
-6	0	21	0.40	5.20
-5	0	21	3.90	4.80
5	0	-21	-2.40	5.10
-4	0	21	-0.30	4.70
4	0	-21	1.00	3.80
4	0	-21	3.90	5.10
3	0	-21	0.70	5.00

-3	0	21	1.90	4.70
3	0	-21	-0.20	3.60
2	0	-21	-0.20	3.80
2	0	-21	3.80	4.50
-2	0	21	-3.30	4.60
1	0	-21	-5.40	4.10
-1	0	21	3.40	5.00
0	0	21	10.70	5.60
0	0	21	9.80	5.50
0	0	-21	6.60	4.10
1	0	21	-9.00	4.40
1	0	21	3.40	5.10
-1	0	-21	4.20	4.00
2	0	21	4.40	5.60
-2	0	-21	3.20	3.70
3	0	21	7.50	6.90
4	0	21	-2.40	5.80
5	0	21	-10.70	6.00
6	0	21	3.80	6.90
7	0	21	6.10	7.60
8	0	21	5.10	7.80
-9	0	-21	2.80	8.80
-10	0	-21	-8.30	8.90
10	-1	-21	345.37	25.10
10	1	-21	315.97	24.80
9	-1	-21	164.98	17.50
9	1	-21	139.99	17.00
8	-1	-21	199.98	17.80
8	1	-21	196.48	17.60
-8	1	21	188.58	14.70
-7	-1	21	313.97	19.40
7	-1	-21	360.56	22.70
-7	1	21	356.16	20.50
7	1	-21	326.17	22.50
-6	-1	21	39.00	6.20
6	-1	-21	38.00	8.10
-6	1	21	34.00	6.70
6	1	-21	39.40	8.00
-5	-1	21	672.73	35.10
5	-1	-21	686.63	37.00
-5	1	21	671.03	35.60
5	1	-21	698.73	36.90
4	-1	-21	234.68	16.70
-4	-1	21	242.18	15.30

-4	1	21	253.47	16.10
4	1	-21	248.18	16.70
3	-1	-21	700.53	35.80
-3	-1	21	665.63	34.60
-3	1	21	691.63	35.20
3	1	-21	688.53	35.60
3	1	-21	623.84	33.40
-2	-1	21	24.30	5.20
2	-1	-21	16.50	5.30
-2	1	21	12.00	5.60
2	1	-21	25.80	5.40
2	1	-21	17.10	4.20
-1	-1	21	752.22	39.70
1	-1	-21	823.72	40.20
-1	1	21	774.72	40.20
1	1	-21	794.82	39.80
1	1	-21	734.93	38.60
0	-1	21	4.70	4.80
0	-1	-21	-4.00	3.90
0	-1	21	-2.80	4.80
0	1	-21	0.10	3.80
0	1	21	2.60	5.70
0	1	21	-4.30	4.80
1	-1	21	932.11	47.90
-1	-1	-21	925.61	47.30
1	1	21	909.81	48.00
-1	1	-21	927.91	47.00
1	1	21	1010.20	47.50
2	-1	21	11.20	5.30
2	1	21	1.20	5.40
-2	1	-21	8.00	3.60
3	-1	21	958.80	47.40
3	1	21	873.21	49.00
4	-1	21	16.40	6.50
4	1	21	31.80	7.20
5	-1	21	570.64	31.90
5	1	21	575.74	32.10
6	-1	21	392.36	24.50
6	1	21	401.26	24.70
7	-1	21	699.13	37.20
7	1	21	630.74	37.00
8	1	21	112.49	15.20
-8	1	-21	131.49	15.40
-9	-1	-21	223.38	20.00

-9	1	-21	240.28	19.60
9	1	21	252.17	19.90
-10	-1	-21	299.37	23.60
-10	1	-21	314.17	23.60
10	-2	-21	161.88	19.20
10	2	-21	142.09	18.60
9	-2	-21	97.59	15.50
-9	2	21	105.89	12.80
9	2	-21	125.19	15.90
8	-2	-21	7.40	7.40
-8	2	21	15.00	6.80
8	2	-21	9.40	7.20
-7	-2	21	80.99	9.70
7	-2	-21	77.79	12.90
-7	2	21	91.39	11.40
7	2	-21	82.49	13.10
-6	-2	21	174.48	12.70
6	-2	-21	194.28	16.10
-6	2	21	199.68	14.70
6	2	-21	197.38	15.70
5	-2	-21	129.79	13.10
-5	-2	21	129.89	10.40
-5	2	21	113.49	12.10
5	2	-21	127.49	12.60
4	-2	-21	425.26	24.60
-4	-2	21	389.56	22.60
4	2	-21	435.96	24.20
-4	2	21	414.06	23.80
-3	-2	21	59.09	8.10
3	-2	-21	72.79	9.80
3	2	-21	63.69	9.10
-3	2	21	48.60	7.50
3	2	-21	61.19	7.00
2	-2	-21	380.66	22.00
-2	-2	21	366.46	20.90
-2	2	21	368.96	22.10
2	2	-21	357.56	19.70
2	2	-21	402.46	21.60
-1	-2	21	13.20	4.90
1	-2	-21	11.00	5.20
-1	2	21	4.20	5.40
1	2	-21	11.50	4.50
1	2	-21	12.80	4.10
0	-2	21	796.12	39.00

0	-2	-21	775.82	39.90
0	-2	21	734.53	39.60
0	2	-21	744.63	38.70
0	2	21	859.21	41.00
0	2	21	745.13	40.20
0	2	-21	756.12	39.10
1	-2	21	7.80	5.10
-1	-2	-21	-0.50	4.00
1	2	21	8.60	5.40
1	2	21	-1.60	5.50
-1	2	-21	8.10	3.80
2	-2	21	445.36	25.20
2	2	21	487.75	24.30
2	2	21	383.26	26.60
-2	2	-21	450.35	23.30
3	-2	21	43.70	7.50
3	2	21	49.50	8.40
4	-2	21	104.79	13.40
4	2	21	111.29	12.50
5	-2	21	42.70	7.80
5	2	21	30.80	8.00
6	-2	21	385.56	23.40
6	2	21	352.06	23.70
7	-2	21	155.78	14.90
-7	2	-21	144.29	15.50
7	2	21	134.39	15.30
-8	2	-21	147.59	16.00
8	2	21	162.48	16.40
-9	2	-21	92.09	15.20
9	2	21	114.59	14.80
-10	-2	-21	21.20	10.80
-10	2	-21	21.50	10.10
10	-3	-21	107.49	17.20
10	3	-21	105.29	16.80
9	-3	-21	28.00	9.40
9	3	-21	43.50	9.30
-9	3	21	28.50	8.10
8	-3	-21	8.40	7.90
-8	3	21	13.00	8.60
8	3	-21	23.10	8.80
7	-3	-21	62.39	12.70
-7	3	21	38.60	8.20
7	3	-21	40.80	8.90
-6	-3	21	307.37	16.80

6	-3	-21	281.27	20.30
6	3	-21	294.17	19.90
-6	3	21	287.17	19.10
-5	-3	21	306.17	18.00
5	-3	-21	331.07	21.00
5	3	-21	336.47	20.50
-5	3	21	313.87	20.20
4	-3	-21	112.29	12.10
-4	-3	21	122.09	9.70
4	3	-21	116.09	11.70
-4	3	21	102.39	11.70
-3	-3	21	332.07	18.40
3	-3	-21	336.77	20.30
3	3	-21	318.07	19.50
-3	3	21	300.57	20.00
-2	-3	21	37.40	5.70
2	-3	-21	25.40	5.90
-2	3	21	27.50	6.60
2	3	-21	41.30	5.60
2	3	-21	26.00	5.40
1	-3	-21	239.28	16.00
-1	-3	21	225.98	14.90
1	3	-21	258.07	15.00
1	3	-21	224.58	13.70
-1	3	21	251.47	16.70
0	-3	21	7.20	4.80
0	-3	-21	-1.10	4.50
0	3	21	12.50	6.50
0	3	-21	3.80	4.00
0	3	-21	3.20	4.00
0	3	21	3.90	5.70
1	-3	21	715.23	37.10
-1	-3	-21	715.03	37.20
1	3	21	771.52	38.30
1	3	21	672.33	37.80
-1	3	-21	702.93	36.00
2	-3	21	52.69	9.40
-2	3	-21	49.30	6.10
2	3	21	46.40	8.90
2	3	21	50.89	7.40
3	-3	21	301.77	18.20
3	3	21	283.07	16.80
3	3	21	270.07	18.90
4	3	21	8.80	6.70

5	-3	21	234.48	17.00
5	3	21	233.38	17.70
6	-3	21	29.30	8.10
-6	3	-21	28.90	7.90
6	3	21	18.10	7.50
-7	3	-21	276.67	20.00
7	3	21	263.37	20.00
8	3	21	109.89	14.40
-8	3	-21	91.19	14.10
9	3	21	23.90	9.20
-9	3	-21	40.70	10.10
10	3	21	134.19	17.40
10	-4	-21	150.98	19.20
10	4	-21	129.69	17.50
9	-4	-21	561.64	33.90
9	4	-21	520.55	33.20
-9	4	21	579.54	31.70
8	-4	-21	351.96	23.40
8	4	-21	322.17	23.00
-8	4	21	309.27	21.60
7	-4	-21	228.08	18.30
7	4	-21	214.38	17.90
6	-4	-21	692.33	38.90
6	4	-21	717.93	38.60
-6	4	21	713.03	38.10
5	-4	-21	201.88	16.00
-5	-4	21	192.88	12.30
5	4	-21	193.78	15.10
-5	4	21	185.18	15.60
4	-4	-21	705.23	38.20
-4	-4	21	705.53	35.80
-4	4	21	719.43	38.00
4	4	-21	712.63	37.60
3	-4	-21	266.27	17.70
-3	-4	21	272.87	15.40
3	4	-21	268.07	16.70
-3	4	21	210.98	17.50
-2	-4	21	785.72	39.20
2	-4	-21	784.42	40.80
-2	4	21	740.03	40.70
2	4	-21	787.82	39.80
1	-4	-21	19.00	5.80
-1	-4	21	28.00	5.50
-1	4	21	25.30	7.20

1	4	-21	30.50	5.20
1	4	-21	35.60	5.70
0	-4	21	1103.49	55.09
0	-4	-21	1104.49	55.89
0	4	21	1253.07	57.19
0	4	-21	1033.60	54.09
0	4	21	1051.79	56.39
0	4	-21	1103.99	54.59
1	-4	21	10.00	5.10
-1	-4	-21	0.10	4.70
-1	4	-21	-0.90	3.30
-1	4	-21	2.30	4.20
1	4	21	9.50	9.60
1	4	21	12.30	6.50
2	-4	21	1255.47	63.79
2	4	21	1253.17	64.79
-2	4	-21	1204.48	61.99
2	4	21	1418.66	64.39
3	-4	21	41.60	7.20
3	4	21	36.60	6.60
3	4	21	51.39	8.60
4	-4	21	576.24	32.00
4	4	21	586.04	31.90
4	4	21	561.24	32.30
-5	4	-21	204.48	16.30
5	4	21	237.48	17.80
-6	4	-21	719.83	40.30
6	4	21	785.52	41.30
7	4	21	102.89	14.40
-7	4	-21	76.99	13.00
8	4	21	330.57	22.40
-8	4	-21	281.07	21.40
9	4	21	380.26	25.90
-9	4	-21	387.76	25.90
9	-5	-21	15.80	8.90
-9	5	21	11.00	8.60
9	5	-21	9.30	8.50
8	-5	-21	15.50	8.70
-8	5	21	5.10	7.80
8	5	-21	11.00	7.80
7	-5	-21	-1.40	6.90
7	5	-21	10.50	7.00
-7	5	21	-4.60	7.60
6	-5	-21	3.90	6.80

-6	5	21	-4.90	7.10
6	5	-21	9.00	6.40
5	-5	-21	2.80	6.10
5	5	-21	7.90	6.20
-4	-5	21	6.60	4.10
4	-5	-21	4.80	5.90
-4	5	21	0.30	7.90
4	5	-21	7.00	5.60
3	-5	-21	23.80	6.40
-3	-5	21	30.80	5.10
3	5	-21	18.70	5.40
-3	5	21	13.70	7.10
-2	-5	21	4.60	4.40
2	-5	-21	-0.80	5.00
-2	5	21	-10.50	6.90
2	5	-21	0.00	4.20
-1	-5	21	11.70	4.90
1	-5	-21	7.70	5.50
1	5	-21	14.30	4.40
0	-5	-21	7.10	5.10
0	-5	21	7.00	4.90
0	5	-21	7.30	4.10
0	5	21	4.60	6.60
0	5	-21	7.60	3.90
-1	-5	-21	5.50	5.20
1	-5	21	0.10	4.90
-1	5	-21	11.80	3.50
1	5	21	0.70	7.40
1	5	21	4.10	6.40
-1	5	-21	9.90	4.40
2	-5	21	4.90	5.50
2	5	21	11.40	6.70
-2	5	-21	-0.20	2.80
2	5	21	7.10	6.80
3	-5	21	39.40	7.10
3	5	21	23.80	7.60
3	5	21	38.70	8.20
-4	5	-21	4.10	6.50
4	5	21	19.90	7.80
5	5	21	11.40	7.40
-5	5	-21	10.80	6.70
6	5	21	-2.60	7.80
-6	5	-21	-2.90	7.10
7	5	21	4.30	8.40

-7	5	-21	14.70	8.00
-8	5	-21	12.90	8.30
8	5	21	-12.40	7.60
9	5	21	15.10	9.00
-9	5	-21	22.10	9.00
9	-6	-21	494.95	31.40
9	6	-21	472.15	30.60
-9	6	21	503.35	29.80
8	-6	-21	419.86	27.00
8	6	-21	398.36	26.20
-8	6	21	394.56	25.60
7	-6	-21	146.79	15.60
7	6	-21	131.49	14.80
-7	6	21	122.49	15.10
6	-6	-21	542.95	32.00
6	6	-21	539.85	31.50
-6	6	21	572.74	31.80
5	-6	-21	105.49	13.10
5	6	-21	102.19	12.30
-5	6	21	107.19	13.50
-4	-6	21	945.81	44.40
4	-6	-21	842.12	46.10
-4	6	21	873.81	46.30
4	6	-21	834.72	45.10
-3	-6	21	57.49	7.10
3	-6	-21	57.89	10.90
-3	6	21	58.29	9.50
3	6	-21	64.49	9.00
2	-6	-21	873.61	46.00
-2	-6	21	932.41	43.90
-2	6	21	833.02	46.30
2	6	-21	871.01	44.60
1	-6	-21	1.70	5.60
-1	-6	21	1.80	4.70
-1	6	21	-5.90	6.60
1	6	-21	1.40	3.90
0	-6	21	1296.67	60.79
0	-6	-21	1229.08	62.09
0	6	-21	1223.48	60.19
0	6	21	1162.78	62.59
-1	-6	-21	6.30	5.50
1	-6	21	13.30	5.40
-1	6	-21	4.90	3.30
-1	6	-21	3.90	4.10

1	6	21	4.10	7.00
-2	6	-21	524.95	26.20
2	6	21	532.15	30.70
-2	6	-21	529.15	28.80
3	6	21	3.50	6.80
-3	6	-21	12.00	6.30
-4	6	-21	460.95	26.80
4	6	21	487.45	28.70
5	6	21	402.56	24.50
-5	6	-21	340.47	22.80
6	6	21	567.74	32.90
-6	6	-21	542.15	31.40
7	6	21	164.28	16.00
-7	6	-21	138.59	15.60
-8	6	-21	537.25	32.00
8	6	21	552.74	32.90
9	6	21	322.77	24.60
-9	6	-21	341.67	23.60
9	-7	-21	54.09	11.20
9	7	-21	41.90	10.80
-9	7	21	52.79	10.70
8	-7	-21	113.99	16.00
-8	7	21	100.59	15.70
8	7	-21	107.79	15.10
7	-7	-21	53.59	9.90
-7	7	21	35.70	9.60
7	7	-21	47.60	9.70
6	-7	-21	106.39	14.00
6	7	-21	97.39	12.90
-6	7	21	77.29	14.10
5	-7	-21	341.17	22.80
5	7	-21	336.07	21.80
-5	7	21	366.46	23.30
4	-7	-21	90.09	12.60
-4	7	21	88.29	11.30
4	7	-21	74.19	10.90
3	-7	-21	363.86	22.70
-3	7	21	349.37	23.30
3	7	-21	348.57	20.90
2	-7	-21	19.70	7.20
-2	7	21	12.40	7.50
2	7	-21	16.20	4.90
1	-7	-21	294.27	19.30
-1	7	21	277.17	20.10

1	7	-21	295.27	16.80
1	7	-21	267.47	16.70
0	-7	-21	52.59	8.20
0	7	-21	55.69	7.00
0	7	-21	43.70	6.90
0	7	21	39.10	8.80
-1	7	-21	552.84	30.30
-1	7	-21	583.74	28.80
1	7	21	576.14	32.80
-2	7	-21	-2.80	2.40
2	7	21	-0.40	7.30
-2	7	-21	1.90	5.60
3	7	21	38.70	8.80
-3	7	-21	37.20	7.40
-4	7	-21	40.10	7.80
4	7	21	34.00	9.00
-5	7	-21	154.08	14.80
5	7	21	185.48	16.80
6	7	21	46.10	10.10
-6	7	-21	50.59	9.10
7	7	21	366.06	24.90
-7	7	-21	356.86	23.60
-8	7	-21	109.89	15.10
8	7	21	122.99	16.30
9	7	21	69.79	12.70
-9	7	-21	75.59	11.50
9	-8	-21	54.79	11.40
-9	8	21	95.39	15.80
9	8	-21	83.79	16.10
8	-8	-21	73.39	12.70
-8	8	21	82.29	14.70
8	8	-21	68.49	14.00
7	-8	-21	51.49	10.30
7	8	-21	13.70	7.60
-7	8	21	30.60	10.00
6	-8	-21	105.59	14.50
-6	8	21	93.59	15.10
6	8	-21	117.59	13.30
5	-8	-21	93.29	13.60
5	8	-21	86.69	12.10
-5	8	21	98.49	14.20
4	-8	-21	282.27	19.60
4	8	-21	252.47	17.80
-4	8	21	263.27	20.80

3	-8	-21	34.50	7.90
-3	8	21	38.20	9.80
3	8	-21	32.50	6.60
2	-8	-21	27.90	7.10
-2	8	21	44.50	9.80
2	8	-21	43.50	7.90
1	-8	-21	4.30	6.10
-1	8	21	-10.90	7.00
1	8	-21	0.80	4.00
1	8	-21	10.30	4.60
0	-8	-21	546.25	30.20
0	8	21	499.25	31.10
0	8	-21	530.85	27.20
0	8	-21	521.25	27.70
-1	8	-21	11.60	5.60
-1	8	-21	6.20	3.50
1	8	21	11.80	8.00
-2	8	-21	322.07	16.00
-2	8	-21	328.47	19.60
2	8	21	307.37	22.10
3	8	21	74.69	11.20
-3	8	-21	56.39	10.10
-4	8	-21	120.49	12.40
4	8	21	101.69	14.20
5	8	21	15.10	9.10
-5	8	-21	21.10	7.40
-6	8	-21	212.48	17.70
6	8	21	225.88	19.00
7	8	21	70.69	12.10
-7	8	-21	63.59	10.20
-8	8	-21	53.89	10.30
8	8	21	66.79	11.60
9	8	21	105.59	16.40
8	-9	-21	263.77	22.30
-8	9	21	287.97	22.30
8	9	-21	266.37	21.20
7	-9	-21	390.96	25.80
7	9	-21	338.47	24.20
-7	9	21	388.46	25.40
6	-9	-21	50.49	10.20
-6	9	21	24.60	9.80
6	9	-21	39.60	8.50
5	-9	-21	596.44	33.40
-5	9	21	586.14	33.70

5	9	-21	519.85	31.60
4	-9	-21	292.07	20.90
-4	9	21	299.77	21.90
4	9	-21	283.57	19.00
3	-9	-21	415.36	26.10
-3	9	21	442.36	27.50
3	9	-21	412.46	24.10
2	-9	-21	15.40	7.50
-2	9	21	16.20	9.20
2	9	-21	8.60	5.00
1	-9	-21	636.64	36.10
1	9	-21	702.63	34.60
1	9	-21	655.23	33.70
-1	9	21	611.04	37.30
0	-9	-21	53.99	8.80
0	9	21	47.90	10.30
0	9	-21	59.09	8.20
0	9	-21	55.79	7.20
-1	9	-21	817.42	40.40
1	9	21	738.73	43.50
-1	9	-21	811.62	39.10
2	9	21	-7.80	8.10
-2	9	-21	-0.10	5.70
3	9	21	614.14	35.80
-3	9	-21	629.14	33.40
-4	9	-21	49.70	8.30
4	9	21	60.99	11.30
-5	9	-21	620.84	34.50
5	9	21	636.74	36.60
6	9	21	90.39	15.50
-6	9	-21	95.39	13.70
7	9	21	423.16	27.50
-7	9	-21	426.46	26.50
-8	9	-21	49.90	10.30
8	9	21	57.29	11.80
8	-10	-21	17.70	9.70
-8	10	21	-4.60	9.60
8	10	-21	-2.00	9.20
7	-10	-21	34.00	9.80
-7	10	21	24.40	10.50
7	10	-21	29.90	8.70
6	-10	-21	-1.90	7.70
-6	10	21	-7.10	9.00
6	10	-21	-5.00	6.70

5	-10	-21	27.00	8.10
5	10	-21	17.40	7.30
-5	10	21	20.70	10.50
4	-10	-21	2.80	8.20
4	10	-21	-4.00	5.50
-4	10	21	3.20	9.40
3	-10	-21	3.40	7.60
-3	10	21	7.90	9.30
3	10	-21	6.80	5.50
2	-10	-21	20.00	7.20
-2	10	21	16.20	10.20
2	10	-21	12.60	5.20
1	-10	-21	1.20	6.50
-1	10	21	-0.80	9.60
1	10	-21	0.70	4.50
0	-10	-21	10.30	6.90
0	10	-21	1.90	5.20
0	10	-21	3.30	3.80
0	10	21	-1.60	8.30
-1	10	-21	-0.90	3.30
1	10	21	3.50	9.00
-1	10	-21	-7.40	4.90
2	10	21	0.50	7.80
-2	10	-21	4.60	5.40
3	10	21	5.30	8.90
-3	10	-21	7.70	6.20
4	10	21	24.20	9.70
-4	10	-21	38.00	7.70
5	10	21	13.40	9.30
-5	10	-21	10.30	7.50
-6	10	-21	1.80	7.60
6	10	21	-9.90	8.40
-7	10	-21	10.70	8.40
7	10	21	8.20	9.90
8	10	21	5.80	9.40
-8	10	-21	23.20	8.90
8	-11	-21	164.38	19.90
7	-11	-21	327.97	24.80
7	11	-21	298.77	22.70
-7	11	21	323.77	24.50
6	-11	-21	23.90	9.20
6	11	-21	17.50	7.50
-6	11	21	17.70	10.20
5	-11	-21	456.35	29.50

5	11	-21	445.56	27.80
-5	11	21	518.05	30.80
4	-11	-21	186.78	17.70
4	11	-21	186.48	15.50
-4	11	21	223.08	20.00
3	-11	-21	562.44	34.40
-3	11	21	632.04	36.40
3	11	-21	595.64	32.30
2	-11	-21	25.60	8.40
2	11	-21	28.50	6.00
-2	11	21	36.60	11.60
1	-11	-21	815.82	42.20
1	11	-21	737.43	38.90
-1	11	21	738.23	43.40
0	11	-21	4.50	4.10
0	11	21	-3.70	8.50
-1	11	-21	783.62	39.30
-1	11	-21	740.73	35.80
1	11	21	623.14	40.90
2	11	21	-11.10	8.70
-2	11	-21	6.10	5.50
-3	11	-21	477.55	26.20
3	11	21	459.85	30.40
4	11	21	150.58	16.90
-4	11	-21	142.99	13.40
5	11	21	486.65	32.10
-5	11	-21	525.75	29.30
6	11	21	379.56	26.80
-6	11	-21	398.26	24.80
7	11	21	435.76	30.80
-7	11	-21	495.65	28.90
7	-12	-21	105.49	17.20
-7	12	21	89.69	13.90
7	12	-21	68.49	10.90
6	-12	-21	186.58	19.50
6	12	-21	175.48	16.70
-6	12	21	155.18	19.50
5	-12	-21	35.70	9.80
5	12	-21	36.90	8.60
-5	12	21	41.90	12.00
4	-12	-21	471.15	29.30
4	12	-21	413.96	26.40
-4	12	21	493.25	30.80
3	-12	-21	-6.10	7.60

3	12	-21	2.90	5.70
-3	12	21	-14.80	9.20
2	-12	-21	126.39	15.50
2	12	-21	127.29	11.50
-2	12	21	92.99	17.70
1	-12	-21	39.60	9.60
-1	12	21	7.60	10.10
1	12	-21	34.90	5.90
0	12	21	386.96	28.70
0	12	-21	438.86	22.80
1	12	21	-9.80	9.70
-1	12	-21	7.50	3.80
2	12	21	207.58	20.10
-3	12	-21	16.20	6.40
3	12	21	15.40	10.10
4	12	21	84.69	16.00
-4	12	-21	91.99	11.70
-5	12	-21	35.20	8.10
5	12	21	44.10	11.70
6	12	21	227.68	21.90
-6	12	-21	243.88	18.70
-7	12	-21	49.10	9.80
7	12	21	52.49	12.10
6	-13	-21	21.90	10.00
6	13	-21	16.80	8.10
-6	13	21	33.30	12.20
5	-13	-21	226.28	20.70
5	13	-21	211.38	18.40
-5	13	21	273.67	22.80
4	-13	-21	19.60	8.70
-4	13	21	42.70	11.60
4	13	-21	29.00	7.40
3	-13	-21	95.89	15.30
3	13	-21	82.19	11.10
-3	13	21	101.59	15.30
2	-13	-21	10.30	7.90
-2	13	21	-13.50	10.30
2	13	-21	2.80	5.40
1	-13	-21	78.39	12.30
1	13	-21	79.69	9.40
-1	13	21	63.49	13.80
0	13	21	-18.70	10.70
0	13	-21	0.40	4.40
-1	13	-21	465.15	23.70

1	13	21	438.66	30.70
2	13	21	37.40	11.00
3	13	21	95.79	17.40
4	13	21	7.30	10.00
-4	13	-21	8.60	6.60
5	13	21	106.99	18.30
-5	13	-21	138.79	14.00
-6	13	-21	42.70	9.30
6	13	21	42.30	12.00
6	-14	-21	280.27	24.70
6	14	-21	315.27	26.20
-6	14	21	298.47	25.10
5	-14	-21	154.78	18.80
-5	14	21	165.98	20.60
5	14	-21	143.59	15.80
4	-14	-21	495.85	31.90
4	14	-21	468.95	29.00
-4	14	21	546.55	33.60
3	-14	-21	88.29	15.60
3	14	-21	89.49	11.70
-3	14	21	69.19	13.80
2	-14	-21	542.15	33.20
2	14	-21	498.35	29.40
-2	14	21	586.54	35.40
1	14	-21	6.70	5.20
-1	14	21	-4.90	10.70
0	14	-21	783.02	38.70
0	14	21	726.53	44.60
-1	14	-21	-0.30	4.00
1	14	21	-14.90	9.50
2	14	21	470.95	32.20
3	14	21	12.70	11.20
4	14	21	499.95	32.90
5	14	21	178.28	21.20
6	14	21	265.97	23.50
5	-15	-21	9.30	10.10
-5	15	21	1.00	12.00
4	-15	-21	5.00	9.00
-4	15	21	-13.50	11.00
4	15	-21	4.10	7.10
3	-15	-21	0.20	9.60
3	15	-21	6.50	6.80
-3	15	21	7.90	10.80
2	-15	-21	6.60	9.40

-2	15	21	7.80	12.00
2	15	-21	16.30	6.40
-1	15	21	59.19	14.20
1	15	-21	54.09	9.30
0	15	21	2.20	11.90
0	15	-21	13.20	5.20
1	15	21	21.60	12.60
-1	15	-21	39.80	7.20
-2	15	-21	17.50	3.30
2	15	21	1.60	10.90
-2	15	-21	21.50	3.50
3	15	21	61.49	13.10
4	15	21	-16.50	10.90
5	15	21	15.10	11.40
4	-16	-21	326.97	26.20
3	-16	-21	68.19	13.20
3	16	-21	48.90	8.80
-3	16	21	51.99	14.00
-2	16	21	296.57	26.50
2	16	-21	266.97	18.60
1	16	-21	8.50	5.90
-1	16	21	11.10	11.50
0	16	-21	464.95	26.10
0	16	21	503.85	33.70
1	16	21	-12.00	10.80
-1	16	-21	13.40	4.60
2	16	21	643.54	39.90
-2	16	-21	581.44	30.60
-2	16	-21	642.84	30.70
3	16	21	-0.70	12.10
4	16	21	295.37	25.80
-2	17	21	16.70	13.60
1	17	-21	218.88	16.20
-1	17	21	194.08	23.70
0	17	-21	-1.60	5.50
0	17	21	-4.30	12.20
1	17	21	326.47	27.80
-1	17	-21	301.97	18.00
-2	17	-21	5.40	3.90
2	17	21	-17.80	11.20
-2	17	-21	6.30	3.60
9	0	-22	239.98	20.60
8	0	-22	248.38	20.00
7	0	-22	30.50	8.10

-7	0	22	51.29	9.40
-6	0	22	568.04	30.20
6	0	-22	560.74	32.20
-5	0	22	273.27	17.00
5	0	-22	256.57	18.60
-4	0	22	1024.30	53.09
4	0	-22	1095.49	54.29
3	0	-22	8.30	5.20
-3	0	22	4.10	5.40
-2	0	22	652.23	34.30
2	0	-22	653.23	34.60
-1	0	22	8.70	5.70
1	0	-22	6.10	4.70
0	0	22	1077.09	52.19
0	0	22	985.50	52.29
0	0	-22	1037.20	51.89
-1	0	-22	18.30	5.10
1	0	22	24.00	6.70
2	0	22	837.52	45.40
3	0	22	408.26	24.20
4	0	22	207.38	16.20
5	0	22	385.56	24.10
6	0	22	508.65	30.00
7	0	22	189.68	17.40
8	0	22	371.86	25.10
-9	0	-22	232.38	19.90
9	-1	-22	2.70	9.10
9	1	-22	14.50	9.50
8	-1	-22	77.59	14.70
8	1	-22	88.89	14.40
7	-1	-22	124.69	14.80
-7	1	22	136.49	12.70
7	1	-22	123.69	14.50
-6	-1	22	21.50	5.80
6	-1	-22	5.80	6.50
6	1	-22	3.10	6.60
-6	1	22	12.60	6.40
5	-1	-22	270.07	18.60
-5	-1	22	245.88	16.20
-5	1	22	264.17	17.20
5	1	-22	265.47	18.30
-4	-1	22	26.70	6.00
4	-1	-22	30.80	6.60
4	1	-22	22.80	6.60

-4	1	22	14.20	6.20
-3	-1	22	100.69	10.30
3	-1	-22	109.69	11.50
3	1	-22	106.79	11.00
-3	1	22	105.49	11.30
-2	-1	22	10.30	5.50
2	-1	-22	16.30	5.60
2	1	-22	8.10	5.00
-2	1	22	9.00	5.70
1	-1	-22	132.09	11.30
-1	-1	22	135.19	11.70
-1	1	22	121.79	11.80
1	1	-22	121.39	10.80
0	-1	-22	16.00	5.20
0	-1	22	11.80	5.80
0	-1	22	15.30	5.90
0	1	-22	15.10	4.90
0	1	22	16.90	6.40
0	1	22	23.70	6.50
1	-1	22	23.70	6.50
-1	-1	-22	36.40	5.80
1	1	22	32.70	7.10
1	1	22	35.10	6.50
-1	1	-22	33.40	5.50
2	-1	22	15.00	6.30
2	1	22	26.40	7.40
3	-1	22	3.10	5.70
3	1	22	4.80	6.20
4	-1	22	42.90	8.20
4	1	22	49.80	8.80
5	-1	22	125.69	13.50
5	1	22	107.59	13.40
6	-1	22	124.99	14.70
6	1	22	136.89	14.60
7	-1	22	45.70	9.90
7	1	22	29.40	9.60
-8	1	-22	-2.50	8.10
8	1	22	9.40	8.10
-9	-1	-22	13.30	9.80
9	1	22	0.60	9.40
-9	1	-22	7.70	9.50
9	-2	-22	190.78	19.10
9	2	-22	204.68	19.20
8	-2	-22	143.79	16.40

8	2	-22	155.88	16.40
-8	2	22	149.88	14.30
7	-2	-22	43.80	9.10
-7	2	22	50.69	8.30
7	2	-22	69.29	12.50
6	-2	-22	533.05	30.40
-6	-2	22	492.55	28.30
6	2	-22	531.15	30.10
-6	2	22	515.45	28.80
-5	-2	22	53.79	8.60
5	-2	-22	77.79	11.70
-5	2	22	60.09	10.60
5	2	-22	67.99	11.20
-4	-2	22	277.67	17.20
4	-2	-22	290.27	19.20
4	2	-22	294.37	18.80
-4	2	22	289.87	18.80
-3	-2	22	5.10	5.00
3	-2	-22	2.70	5.00
3	2	-22	3.50	5.10
-3	2	22	6.90	5.80
-2	-2	22	461.25	25.20
2	-2	-22	454.05	26.10
2	2	-22	472.55	25.60
-2	2	22	457.95	26.00
-1	-2	22	61.69	9.10
1	-2	-22	65.09	9.50
1	2	-22	65.49	8.20
1	2	-22	80.09	8.90
-1	2	22	47.50	7.80
0	-2	-22	707.13	36.20
0	-2	22	654.43	36.00
0	2	22	679.23	36.60
0	2	-22	709.73	35.50
-1	-2	-22	15.20	5.20
1	-2	22	19.60	6.10
1	2	22	16.40	6.10
-1	2	-22	13.00	4.50
2	-2	22	643.04	33.90
-2	2	-22	622.44	32.00
2	2	22	628.64	34.40
3	2	22	-4.30	5.90
4	-2	22	188.68	15.00
4	2	22	171.98	15.60

5	-2	22	138.69	14.00
5	2	22	135.89	14.60
6	-2	22	311.87	21.40
6	2	22	295.07	21.50
-7	2	-22	210.38	18.20
7	2	22	218.48	18.30
8	2	22	143.89	16.30
-8	2	-22	107.39	15.60
-9	2	-22	192.78	19.10
9	2	22	245.08	20.50
9	-3	-22	158.48	19.10
9	3	-22	198.78	18.90
8	-3	-22	253.37	20.10
-8	3	22	223.28	17.70
8	3	-22	234.38	19.40
7	-3	-22	233.98	18.90
7	3	-22	221.88	18.20
6	-3	-22	52.79	9.40
-6	3	22	41.30	8.40
6	3	-22	53.89	9.00
-5	-3	22	617.84	31.80
5	-3	-22	627.34	34.40
5	3	-22	617.94	34.00
-5	3	22	605.14	33.70
-4	-3	22	61.29	8.60
4	-3	-22	56.29	10.70
4	3	-22	55.29	10.10
-4	3	22	64.79	8.90
-3	-3	22	509.65	27.20
3	-3	-22	523.15	28.90
-3	3	22	475.55	28.50
3	3	-22	531.15	28.20
2	-3	-22	6.80	5.50
-2	-3	22	-0.90	4.90
-2	3	22	5.60	6.30
2	3	-22	4.40	4.50
-1	-3	22	604.34	31.80
1	-3	-22	615.84	32.80
-1	3	22	576.64	33.00
1	3	-22	620.24	31.80
0	-3	22	79.89	9.70
0	-3	-22	74.59	9.80
0	3	-22	76.89	8.50
1	-3	22	1141.79	56.09

-1	-3	-22	1113.79	56.09
-1	3	-22	1100.09	54.89
1	3	22	1104.99	56.99
2	-3	22	64.69	10.00
-2	3	-22	55.99	6.80
2	3	22	52.99	9.90
2	3	22	69.89	10.90
3	-3	22	336.97	21.10
3	3	22	326.07	21.60
4	-3	22	34.20	7.80
4	3	22	31.50	8.40
5	-3	22	272.27	19.20
5	3	22	264.17	19.70
-6	3	-22	387.66	25.00
6	3	22	415.86	26.00
-7	3	-22	193.48	17.40
7	3	22	204.98	18.40
8	3	22	172.58	17.40
-8	3	-22	132.49	16.20
9	3	22	20.70	10.40
-9	3	-22	34.30	10.40
9	-4	-22	25.20	9.40
9	4	-22	34.00	9.30
-9	4	22	27.40	9.20
8	-4	-22	38.20	10.30
8	4	-22	42.80	9.90
-8	4	22	46.10	9.20
7	-4	-22	3.00	7.20
7	4	-22	4.80	7.10
-7	4	22	-4.80	7.60
6	-4	-22	64.19	12.80
6	4	-22	70.19	11.90
-6	4	22	71.19	12.70
5	-4	-22	18.70	7.20
5	4	-22	9.20	6.40
4	-4	-22	3.80	6.40
-4	-4	22	12.90	4.60
4	4	-22	8.00	5.60
3	-4	-22	7.20	5.60
-3	-4	22	4.60	4.90
-3	4	22	-4.80	10.70
3	4	-22	7.00	5.10
2	-4	-22	123.19	12.60
-2	-4	22	144.49	11.20

2	4	-22	140.29	11.30
1	-4	-22	10.30	5.50
-1	-4	22	11.00	5.30
1	4	-22	20.20	4.90
-1	4	22	25.10	8.30
0	-4	22	49.00	7.10
0	-4	-22	47.10	9.60
0	4	-22	53.09	7.30
0	4	22	42.50	8.50
-1	-4	-22	46.90	9.00
1	-4	22	54.59	9.30
-1	4	-22	36.70	6.70
1	4	22	40.00	7.90
2	-4	22	31.80	7.20
-2	4	-22	44.50	5.90
2	4	22	35.30	8.50
3	-4	22	8.90	6.30
3	4	22	6.80	6.90
4	-4	22	80.59	11.60
4	4	22	71.39	12.50
-5	4	-22	107.29	13.60
5	4	22	125.49	14.70
6	4	22	140.09	16.00
-6	4	-22	130.39	14.50
7	4	22	44.90	10.50
-7	4	-22	41.00	9.60
-8	4	-22	23.40	9.10
8	4	22	3.70	8.90
9	4	22	19.00	10.10
-9	4	-22	11.70	9.00
9	-5	-22	391.56	27.00
9	5	-22	365.76	26.10
8	-5	-22	84.39	15.50
-8	5	22	93.49	14.60
8	5	-22	79.99	14.30
7	-5	-22	333.07	23.10
7	5	-22	328.77	22.50
-7	5	22	329.17	22.40
6	-5	-22	63.39	12.90
6	5	-22	56.69	9.00
-6	5	22	69.09	10.30
5	-5	-22	855.11	46.30
-5	5	22	900.71	45.90
5	5	-22	846.92	45.40

4	-5	-22	151.88	13.80
-4	5	22	123.89	13.70
4	5	-22	112.69	12.20
-3	-5	22	1041.50	50.99
3	-5	-22	1025.40	53.19
-3	5	22	1005.10	53.39
3	5	-22	1046.10	52.29
2	-5	-22	9.60	6.10
-2	-5	22	3.30	4.90
-2	5	22	0.40	6.60
2	5	-22	9.50	4.90
1	-5	-22	579.94	32.50
-1	-5	22	620.64	31.20
-1	5	22	570.84	33.10
1	5	-22	594.14	31.00
0	-5	22	15.30	5.50
0	-5	-22	9.50	5.80
0	5	-22	5.60	4.10
0	5	22	2.30	6.60
1	-5	22	1363.26	64.79
-1	-5	-22	1306.57	65.29
-1	5	-22	1283.37	63.39
1	5	22	1251.67	66.09
2	-5	22	92.39	11.40
2	5	22	90.29	12.90
-2	5	-22	81.89	6.80
3	-5	22	570.24	30.30
3	5	22	518.85	31.40
-4	5	-22	291.27	21.30
4	5	22	359.76	22.40
-5	5	-22	531.05	30.90
5	5	22	562.44	32.40
6	5	22	292.87	21.30
-6	5	-22	251.87	19.70
-7	5	-22	595.94	36.00
7	5	22	693.43	37.70
-8	5	-22	341.77	23.40
8	5	22	336.37	24.40
8	-6	-22	48.30	10.40
-8	6	22	29.10	10.00
8	6	-22	43.50	9.90
7	-6	-22	62.99	11.00
-7	6	22	50.29	10.10
7	6	-22	47.20	8.70

6	-6	-22	156.88	16.10
6	6	-22	138.19	14.70
-6	6	22	161.78	16.20
5	-6	-22	6.70	6.90
5	6	-22	2.90	6.30
-5	6	22	-0.20	7.90
4	-6	-22	57.29	11.70
4	6	-22	39.90	7.00
-4	6	22	56.89	9.70
3	-6	-22	46.50	8.60
-3	-6	22	53.49	7.60
3	6	-22	30.40	6.30
-3	6	22	27.70	8.50
2	-6	-22	54.49	10.90
-2	-6	22	53.99	8.20
-2	6	22	63.59	10.00
2	6	-22	64.09	8.80
-1	-6	22	13.70	5.40
1	-6	-22	22.70	6.40
-1	6	22	16.00	7.90
1	6	-22	14.20	4.80
0	-6	22	212.98	13.70
0	-6	-22	178.78	14.80
0	6	22	165.88	16.50
0	6	-22	179.58	12.10
1	-6	22	1.00	5.60
-1	-6	-22	6.60	5.70
-1	6	-22	1.00	3.50
1	6	22	6.60	7.50
2	6	22	45.60	9.00
-2	6	-22	49.70	7.90
-2	6	-22	58.99	5.40
3	6	22	-9.70	7.30
-3	6	-22	0.30	6.30
4	6	22	231.78	18.70
5	6	22	46.20	10.30
-5	6	-22	43.90	8.70
-6	6	-22	94.69	13.70
6	6	22	139.09	16.20
7	6	22	84.49	14.80
-7	6	-22	54.89	10.00
-8	6	-22	32.50	9.80
8	6	22	7.00	9.90
8	-7	-22	140.89	17.70

8	7	-22	131.79	16.40
-8	7	22	117.79	16.80
7	-7	-22	158.08	17.20
7	7	-22	157.78	15.80
-7	7	22	150.48	17.50
6	-7	-22	12.40	8.30
-6	7	22	-2.20	8.50
6	7	-22	-6.10	6.70
5	-7	-22	642.64	36.60
5	7	-22	615.44	35.30
-5	7	22	688.53	36.90
4	-7	-22	12.10	6.90
4	7	-22	11.80	5.80
-4	7	22	13.10	8.70
3	-7	-22	284.77	19.80
-3	7	22	275.47	20.60
3	7	-22	277.27	17.70
2	-7	-22	0.40	6.00
2	7	-22	4.80	4.70
-2	7	22	-2.40	8.30
1	-7	-22	423.56	25.40
1	7	-22	434.26	23.20
1	7	-22	421.26	23.00
-1	7	22	393.66	26.50
0	-7	-22	98.29	12.20
0	7	-22	85.69	10.30
0	7	22	101.79	14.10
0	7	-22	86.89	8.50
1	7	22	623.04	36.00
-1	7	-22	620.84	33.50
-1	7	-22	654.13	31.90
-2	7	-22	13.70	6.40
-2	7	-22	13.80	2.80
2	7	22	14.40	8.30
-3	7	-22	214.98	15.90
3	7	22	206.28	18.50
4	7	22	57.69	10.30
5	7	22	189.58	18.90
-5	7	-22	212.78	16.70
6	7	22	162.18	17.40
-6	7	-22	148.99	15.70
7	7	22	250.97	20.70
-7	7	-22	253.97	19.70
-8	7	-22	65.99	11.10

8	7	22	50.69	10.80
8	-8	-22	163.58	19.20
8	8	-22	169.48	18.00
-8	8	22	185.08	19.00
7	-8	-22	77.89	14.80
7	8	-22	50.29	9.40
-7	8	22	63.79	11.60
6	-8	-22	339.77	23.90
6	8	-22	336.67	22.60
-6	8	22	355.86	24.60
5	-8	-22	34.70	8.50
-5	8	22	39.80	10.40
5	8	-22	37.30	7.60
4	-8	-22	559.24	32.60
4	8	-22	538.95	30.80
-4	8	22	580.34	33.40
3	-8	-22	22.40	8.10
3	8	-22	27.50	6.10
-3	8	22	20.70	9.10
2	-8	-22	433.06	26.20
2	8	-22	418.46	23.70
-2	8	22	422.76	27.50
1	-8	-22	4.20	6.60
1	8	-22	5.10	4.40
-1	8	22	-3.40	8.50
0	-8	-22	845.52	44.80
0	8	-22	833.32	41.90
0	8	22	806.62	45.70
0	8	-22	869.81	42.60
-1	8	-22	29.30	6.50
1	8	22	21.10	8.90
-1	8	-22	31.30	5.70
2	8	22	298.87	23.40
-2	8	-22	360.46	20.60
-3	8	-22	16.90	6.90
3	8	22	14.70	8.30
4	8	22	496.55	31.00
-4	8	-22	490.85	28.50
5	8	22	129.69	16.30
-5	8	-22	112.39	13.90
-6	8	-22	279.57	21.00
6	8	22	310.17	22.90
-7	8	-22	246.58	19.70
7	8	22	236.88	21.20

8	8	22	155.08	18.90
-8	8	-22	149.59	16.80
7	-9	-22	33.80	11.00
7	9	-22	17.60	8.20
-7	9	22	18.80	10.40
6	-9	-22	-3.80	7.30
-6	9	22	-4.60	9.30
6	9	-22	-0.80	6.70
5	-9	-22	78.09	14.10
5	9	-22	74.69	12.10
-5	9	22	89.59	15.90
4	-9	-22	12.20	7.60
4	9	-22	-0.40	5.80
-4	9	22	-13.10	8.80
3	-9	-22	4.30	7.10
-3	9	22	-6.70	8.80
3	9	-22	10.70	5.60
2	-9	-22	-2.50	6.60
-2	9	22	-7.50	8.70
2	9	-22	-2.80	4.60
1	-9	-22	101.69	13.80
1	9	-22	113.09	9.70
-1	9	22	91.29	15.90
0	-9	-22	40.70	8.60
0	9	-22	39.10	7.20
0	9	22	47.50	11.00
-1	9	-22	9.70	6.00
1	9	22	3.40	9.30
-1	9	-22	17.90	3.70
2	9	22	-0.20	8.30
-2	9	-22	24.10	6.90
3	9	22	8.40	9.50
-3	9	-22	4.10	6.70
4	9	22	9.70	8.60
-4	9	-22	2.10	7.00
-5	9	-22	20.70	8.30
5	9	22	22.60	10.30
6	9	22	57.59	11.80
-6	9	-22	44.90	9.50
-7	9	-22	-7.00	8.20
7	9	22	30.60	10.60
7	-10	-22	55.59	11.30
-7	10	22	92.09	16.60
7	10	-22	91.89	14.30

6	-10	-22	376.46	25.60
-6	10	22	354.56	25.80
6	10	-22	342.07	23.60
5	-10	-22	202.08	18.20
-5	10	22	138.79	18.50
5	10	-22	170.08	15.30
4	-10	-22	793.22	44.10
4	10	-22	744.43	41.90
-4	10	22	871.61	45.30
3	-10	-22	-0.90	7.60
3	10	-22	-0.80	5.50
-3	10	22	-3.70	9.60
2	-10	-22	392.26	25.10
2	10	-22	369.96	21.90
-2	10	22	391.46	26.70
1	-10	-22	-11.80	6.60
1	10	-22	-1.90	4.20
-1	10	22	-14.50	8.70
0	10	-22	1012.20	50.09
1	10	22	-6.00	9.90
-1	10	-22	7.70	3.60
-2	10	-22	1280.47	61.19
2	10	22	1168.08	64.59
-3	10	-22	238.38	16.90
3	10	22	237.68	20.90
-4	10	-22	251.27	17.60
4	10	22	218.38	21.30
5	10	22	188.58	19.30
-5	10	-22	194.88	16.60
-6	10	-22	317.37	22.00
6	10	22	306.17	24.40
7	10	22	200.58	21.10
-7	10	-22	222.18	18.60
6	-11	-22	12.90	9.00
-6	11	22	-6.30	10.60
6	11	-22	22.00	8.30
5	-11	-22	28.70	9.20
5	11	-22	31.90	8.00
-5	11	22	21.50	10.70
4	-11	-22	6.20	7.80
4	11	-22	1.20	6.00
-4	11	22	-14.40	10.70
3	-11	-22	30.20	8.80
-3	11	22	42.60	11.80

3	11	-22	47.50	9.80
2	-11	-22	0.70	7.70
2	11	-22	11.70	5.30
-2	11	22	-4.10	9.40
1	-11	-22	132.49	15.40
-1	11	22	98.39	18.30
1	11	-22	129.79	10.60
0	11	-22	45.70	7.40
0	11	22	50.89	11.90
-1	11	-22	107.59	8.20
1	11	22	101.79	17.70
2	11	22	3.40	10.10
-3	11	-22	48.30	7.80
3	11	22	27.90	10.60
-4	11	-22	5.50	6.80
4	11	22	4.90	9.80
-5	11	-22	227.68	17.60
5	11	22	216.38	21.20
6	11	22	6.80	10.20
-6	11	-22	13.00	8.20
6	-12	-22	409.46	29.60
6	12	-22	436.06	29.40
-6	12	22	457.35	30.30
5	-12	-22	27.90	10.30
-5	12	22	23.80	11.80
5	12	-22	8.10	7.50
4	-12	-22	503.35	31.70
4	12	-22	480.65	28.80
-4	12	22	537.35	33.20
3	-12	-22	20.30	9.40
3	12	-22	13.40	6.20
-3	12	22	6.10	11.20
2	-12	-22	315.47	22.90
-2	12	22	305.47	25.20
2	12	-22	303.67	18.90
1	-12	-22	2.10	7.90
1	12	-22	5.90	5.00
-1	12	22	6.80	10.50
0	12	-22	528.65	27.50
0	12	22	519.75	34.20
1	12	22	120.09	16.10
-1	12	-22	122.29	8.80
2	12	22	268.17	23.50
3	12	22	34.10	11.00

-4	12	-22	115.89	16.70
4	12	22	96.59	17.60
5	12	22	282.87	24.50
-5	12	-22	292.27	20.20
-6	12	-22	176.38	17.30
6	12	22	210.98	21.30
5	-13	-22	445.06	30.40
-5	13	22	479.95	31.50
5	13	-22	451.15	28.70
4	-13	-22	22.20	9.30
4	13	-22	13.40	6.80
-4	13	22	13.40	12.00
3	-13	-22	697.43	40.40
3	13	-22	655.63	37.10
-3	13	22	745.73	42.40
2	-13	-22	38.70	10.20
2	13	-22	52.29	9.50
-2	13	22	23.70	12.70
-1	13	22	350.36	27.70
1	13	-22	343.67	20.10
0	13	-22	43.20	7.70
0	13	22	36.20	13.20
1	13	22	450.95	32.80
-1	13	-22	507.85	25.00
2	13	22	56.39	13.50
3	13	22	240.58	21.70
4	13	22	120.59	19.50
5	13	22	207.08	22.20
4	-14	-22	2.90	10.00
4	14	-22	15.60	9.00
-4	14	22	-0.30	11.20
3	-14	-22	0.60	8.80
3	14	-22	-4.20	5.90
-3	14	22	-7.40	11.10
2	-14	-22	-4.50	8.60
-2	14	22	18.50	12.30
2	14	-22	12.60	5.90
1	14	-22	20.80	5.90
-1	14	22	-9.60	10.90
0	14	22	19.90	11.40
1	14	22	-12.60	10.40
-1	14	-22	-0.70	3.90
2	14	22	14.20	10.80
3	14	22	34.40	12.90

4	14	22	-3.50	11.50
3	-15	-22	489.35	32.70
-3	15	22	523.95	35.40
-2	15	22	20.10	13.00
2	15	-22	1.30	5.70
1	15	-22	425.56	24.80
-1	15	22	480.95	33.10
0	15	22	-10.80	12.60
0	15	-22	13.10	5.20
-1	15	-22	820.62	41.00
1	15	22	835.82	49.10
2	15	22	9.60	13.20
3	15	22	373.86	29.50
0	16	-22	275.87	16.90
0	16	22	251.17	25.70
8	0	-23	5.60	7.50
7	0	-23	4.80	7.80
6	0	-23	-4.20	7.10
-6	0	23	-0.50	5.70
-5	0	23	2.20	5.90
5	0	-23	0.40	5.80
-4	0	23	1.10	5.90
4	0	-23	0.10	5.70
-3	0	23	4.50	5.90
3	0	-23	1.70	5.40
-2	0	23	-4.30	5.80
2	0	-23	5.10	5.20
-1	0	23	-6.80	5.50
1	0	-23	-1.90	4.70
0	0	-23	6.40	4.80
0	0	23	0.70	6.00
-1	0	-23	-1.90	4.20
2	0	23	5.40	6.40
3	0	23	-3.30	6.50
4	0	23	3.30	6.80
5	0	23	1.00	7.80
6	0	23	-4.20	7.40
7	0	23	-6.40	8.50
8	0	23	-1.00	8.70
8	-1	-23	126.89	16.30
8	1	-23	160.08	17.00
7	-1	-23	431.96	27.10
-7	1	23	419.36	29.50
7	1	-23	420.46	26.70

6	-1	-23	7.20	6.90
-6	1	23	6.60	6.30
6	1	-23	6.60	6.90
5	-1	-23	765.92	40.60
-5	-1	23	725.73	38.50
-5	1	23	752.12	39.30
5	1	-23	753.72	40.30
-4	-1	23	1.20	5.50
4	-1	-23	0.10	5.80
-4	1	23	4.40	6.10
4	1	-23	7.80	5.90
-3	-1	23	515.65	28.30
3	-1	-23	539.95	29.20
3	1	-23	525.55	28.90
-3	1	23	491.45	28.60
-2	-1	23	0.70	5.60
2	-1	-23	9.50	5.50
2	1	-23	2.10	4.90
-2	1	23	11.00	6.10
-1	-1	23	709.43	37.40
1	-1	-23	716.13	37.50
1	1	-23	719.43	37.10
-1	1	23	693.03	37.60
0	-1	23	134.59	12.80
0	-1	-23	177.38	12.80
0	1	-23	146.29	12.00
0	1	23	141.59	15.50
1	-1	23	683.53	37.20
-1	-1	-23	695.33	36.40
-1	1	-23	705.13	36.00
1	1	23	712.23	37.70
2	-1	23	107.09	11.70
2	1	23	97.89	12.80
3	-1	23	202.68	16.30
3	1	23	212.58	16.80
4	-1	23	91.29	12.70
4	1	23	95.19	12.90
5	-1	23	366.56	23.60
5	1	23	360.46	24.00
6	-1	23	332.37	23.00
6	1	23	346.07	23.40
7	-1	23	223.38	19.00
7	1	23	210.38	18.70
8	1	23	102.09	15.60

-8	1	-23	56.99	11.60
8	-2	-23	188.08	17.80
8	2	-23	134.09	16.80
7	-2	-23	1.10	7.20
7	2	-23	9.30	7.50
-7	2	23	5.90	7.50
6	-2	-23	188.28	16.90
-6	2	23	202.28	15.90
6	2	-23	196.88	16.70
5	-2	-23	20.10	7.50
-5	-2	23	17.70	7.30
5	2	-23	19.40	7.00
-5	2	23	19.40	7.30
4	-2	-23	183.78	15.30
-4	-2	23	180.18	13.60
4	2	-23	208.28	15.10
-4	2	23	174.78	15.10
-3	-2	23	13.90	5.70
3	-2	-23	20.00	6.60
3	2	-23	18.80	6.10
-3	2	23	1.80	6.80
-2	-2	23	216.88	15.10
2	-2	-23	232.58	16.00
2	2	-23	217.08	15.10
-2	2	23	200.28	16.10
1	-2	-23	31.10	6.60
-1	-2	23	35.10	6.80
1	2	-23	43.60	6.50
0	-2	-23	270.47	17.50
0	-2	23	255.87	17.70
0	2	23	267.47	18.30
0	2	-23	275.97	16.60
-1	-2	-23	30.90	6.20
1	-2	23	38.70	7.30
1	2	23	20.30	7.40
-1	2	-23	36.90	5.70
2	2	23	291.07	19.90
3	-2	23	109.49	12.20
3	2	23	92.89	13.20
4	-2	23	206.08	16.60
4	2	23	185.58	16.90
5	-2	23	292.37	20.00
5	2	23	259.77	20.40
6	-2	23	55.49	10.20

6	2	23	40.50	9.60
-7	2	-23	37.40	9.40
7	2	23	70.89	11.80
8	2	23	38.10	10.30
-8	2	-23	36.30	10.10
8	-3	-23	65.79	11.00
8	3	-23	74.19	14.50
-8	3	23	63.49	12.90
7	-3	-23	103.89	15.30
-7	3	23	129.09	14.40
7	3	-23	132.19	14.80
6	-3	-23	2.40	7.10
6	3	-23	6.60	6.70
-6	3	23	3.70	7.40
5	-3	-23	107.89	13.60
5	3	-23	120.89	12.90
-4	-3	23	20.40	5.70
4	-3	-23	12.10	6.60
4	3	-23	24.00	6.70
-3	-3	23	378.16	21.60
3	-3	-23	366.26	23.20
3	3	-23	391.36	22.50
-2	-3	23	13.50	5.70
2	-3	-23	23.70	6.60
2	3	-23	20.70	5.50
-2	3	23	20.50	8.10
1	-3	-23	306.67	19.40
-1	-3	23	292.47	18.60
1	3	-23	317.97	18.30
-1	3	23	276.77	19.60
0	-3	-23	39.60	7.00
0	-3	23	36.70	7.00
0	3	23	31.80	7.90
0	3	-23	43.70	6.20
1	-3	23	367.86	22.40
-1	-3	-23	388.06	22.30
-1	3	-23	375.26	20.70
1	3	23	365.96	23.50
2	-3	23	12.80	6.60
2	3	23	10.10	7.70
3	-3	23	204.78	15.60
3	3	23	166.98	16.10
4	-3	23	148.19	14.40
4	3	23	155.68	15.60

5	-3	23	40.40	9.40
5	3	23	59.89	10.20
-6	3	-23	165.48	16.80
6	3	23	185.18	17.60
7	3	23	69.19	11.80
-7	3	-23	63.59	10.40
-8	3	-23	58.19	10.80
8	3	23	64.59	11.60
8	-4	-23	186.48	19.20
-8	4	23	206.48	18.10
8	4	-23	221.78	18.90
7	-4	-23	112.19	15.50
-7	4	23	122.39	14.90
7	4	-23	108.39	14.60
6	-4	-23	790.12	43.30
6	4	-23	775.92	42.60
-6	4	23	809.22	42.50
5	-4	-23	35.40	8.00
5	4	-23	39.30	7.80
-5	4	23	21.30	8.30
4	-4	-23	735.83	39.60
-4	4	23	723.43	39.40
4	4	-23	725.13	38.70
3	-4	-23	4.50	5.90
-3	-4	23	-0.70	5.10
-3	4	23	-5.70	6.90
3	4	-23	3.10	4.80
2	-4	-23	698.23	37.50
-2	-4	23	714.73	35.80
2	4	-23	694.03	36.20
-2	4	23	657.03	37.60
1	-4	-23	11.40	5.90
-1	-4	23	12.00	5.90
-1	4	23	16.30	7.50
1	4	-23	19.60	5.10
0	-4	-23	1163.08	57.69
0	-4	23	1182.38	56.99
0	4	-23	1126.29	56.19
0	4	23	1060.79	57.99
-1	-4	-23	168.98	13.80
1	-4	23	175.58	14.20
1	4	23	170.68	15.70
-1	4	-23	172.38	11.60
2	-4	23	721.23	36.90

2	4	23	653.53	38.00
3	-4	23	71.19	11.70
3	4	23	69.99	12.70
4	-4	23	174.48	15.50
4	4	23	163.28	16.40
-5	4	-23	370.26	24.60
5	4	23	412.96	25.90
6	4	23	239.28	19.40
-6	4	-23	204.88	18.40
7	4	23	316.57	22.70
-7	4	-23	251.97	21.00
8	4	23	91.09	16.20
-8	4	-23	94.69	15.00
8	-5	-23	4.00	8.20
8	5	-23	9.30	7.80
7	-5	-23	0.10	8.00
-7	5	23	13.10	8.60
7	5	-23	-3.90	7.70
6	-5	-23	9.60	7.40
6	5	-23	14.80	6.80
-6	5	23	18.40	8.70
5	-5	-23	9.80	7.10
5	5	-23	1.10	6.20
-5	5	23	5.10	8.10
4	-5	-23	-2.50	6.60
-4	5	23	-3.40	7.80
4	5	-23	-2.00	5.60
3	-5	-23	2.40	6.80
-3	5	23	2.80	7.50
3	5	-23	2.00	5.20
2	-5	-23	27.20	7.50
-2	-5	23	34.80	6.30
-2	5	23	27.70	8.60
2	5	-23	28.10	6.00
-1	-5	23	0.30	5.40
1	-5	-23	5.40	6.10
-1	5	23	-0.10	7.20
1	5	-23	5.60	4.60
0	-5	23	-1.70	5.50
0	-5	-23	-0.40	5.70
0	5	23	1.10	7.50
0	5	-23	4.40	4.30
1	-5	23	16.80	6.60
-1	-5	-23	14.90	6.20

1	5	23	12.00	8.00
-1	5	-23	16.70	4.40
2	-5	23	3.80	6.60
2	5	23	-3.20	7.30
-3	5	-23	-4.10	10.30
3	5	23	-6.60	7.40
4	5	23	22.10	8.90
-4	5	-23	26.30	8.00
-5	5	-23	7.80	7.70
5	5	23	1.70	8.50
-6	5	-23	0.10	8.10
6	5	23	9.90	9.20
7	5	23	-2.70	8.80
-7	5	-23	-9.90	8.10
7	-6	-23	74.49	15.00
-7	6	23	95.19	15.40
7	6	-23	57.09	9.60
6	-6	-23	722.13	40.90
6	6	-23	679.33	39.60
-6	6	23	796.62	40.60
5	-6	-23	27.50	8.70
5	6	-23	18.40	6.40
-5	6	23	29.40	9.20
4	-6	-23	771.32	43.00
4	6	-23	776.52	41.70
-4	6	23	838.02	43.50
3	-6	-23	13.70	6.70
-3	6	23	-2.10	8.30
3	6	-23	4.30	5.40
2	-6	-23	455.15	27.50
2	6	-23	457.95	25.60
-2	6	23	476.35	28.60
1	-6	-23	21.70	6.90
-1	-6	23	14.40	5.70
-1	6	23	12.60	8.00
1	6	-23	14.80	4.90
0	-6	-23	692.93	38.80
0	-6	23	777.62	37.60
0	6	23	701.13	39.80
0	6	-23	710.03	36.60
1	-6	23	478.15	25.10
-1	6	-23	415.66	22.70
1	6	23	410.96	27.10
2	6	23	614.84	34.20

-2	6	-23	556.04	31.70
-3	6	-23	125.99	13.30
3	6	23	119.59	15.60
4	6	23	130.09	15.80
-4	6	-23	95.39	13.40
-5	6	-23	331.77	22.60
5	6	23	348.27	24.50
-6	6	-23	329.97	23.60
6	6	23	371.96	25.40
-7	6	-23	100.39	14.80
7	6	23	123.59	17.00
7	-7	-23	162.78	17.30
7	7	-23	138.39	15.80
-7	7	23	133.79	17.80
6	-7	-23	-10.60	7.40
6	7	-23	15.40	7.50
-6	7	23	-1.50	9.30
5	-7	-23	317.67	22.50
5	7	-23	310.77	20.90
-5	7	23	326.87	23.10
4	-7	-23	111.89	14.30
-4	7	23	100.49	15.80
4	7	-23	103.99	11.80
3	-7	-23	326.17	22.50
3	7	-23	334.77	20.40
-3	7	23	346.87	24.10
2	-7	-23	6.10	7.00
-2	7	23	0.90	7.90
2	7	-23	6.40	4.90
1	-7	-23	216.98	17.10
-1	7	23	232.18	19.00
1	7	-23	211.78	14.20
0	-7	-23	74.39	11.80
0	7	23	57.89	11.00
0	7	-23	55.79	7.90
0	7	-23	66.99	10.00
-1	7	-23	252.77	14.00
-1	7	-23	231.28	16.70
1	7	23	230.38	19.80
2	7	23	82.89	14.10
-2	7	-23	73.39	11.40
3	7	23	244.68	20.70
-3	7	-23	260.07	18.10
-4	7	-23	281.97	19.90

4	7	23	299.67	22.70
5	7	23	77.39	12.30
-5	7	-23	71.09	13.00
-6	7	-23	128.49	15.10
6	7	23	95.79	16.50
-7	7	-23	29.10	9.40
7	7	23	17.80	10.10
7	-8	-23	-9.80	8.80
-7	8	23	-5.80	9.70
7	8	-23	4.50	7.60
6	-8	-23	49.90	10.10
-6	8	23	54.99	11.20
6	8	-23	40.40	8.40
5	-8	-23	19.10	8.60
5	8	-23	13.70	7.10
-5	8	23	-2.10	9.40
4	-8	-23	163.98	16.40
4	8	-23	151.48	13.70
-4	8	23	171.58	18.70
3	-8	-23	19.50	7.70
-3	8	23	4.80	9.50
3	8	-23	18.30	5.80
2	-8	-23	233.18	18.30
2	8	-23	226.08	15.10
-2	8	23	217.88	20.20
1	-8	-23	28.80	7.60
1	8	-23	40.70	6.00
-1	8	23	39.10	10.50
0	-8	-23	175.38	16.00
0	8	23	176.28	18.20
0	8	-23	182.88	12.20
-1	8	-23	19.90	6.50
-1	8	-23	13.30	3.90
1	8	23	7.70	8.70
-2	8	-23	320.07	19.60
2	8	23	283.27	23.10
-3	8	-23	4.90	7.00
3	8	23	11.70	10.10
-4	8	-23	27.00	8.30
4	8	23	45.10	11.00
5	8	23	95.79	15.80
-5	8	-23	111.09	14.10
6	8	23	14.30	9.40
-6	8	-23	21.50	8.80

7	8	23	51.39	11.50
6	-9	-23	7.40	8.80
-6	9	23	3.00	10.20
6	9	-23	8.70	7.70
5	-9	-23	773.62	42.70
-5	9	23	833.62	43.60
5	9	-23	691.23	40.80
4	-9	-23	15.40	8.50
-4	9	23	6.00	9.80
4	9	-23	4.00	5.80
3	-9	-23	417.76	26.70
-3	9	23	404.76	28.20
3	9	-23	426.76	24.20
2	-9	-23	-1.00	6.70
-2	9	23	-18.30	9.40
2	9	-23	2.60	4.70
1	-9	-23	915.21	48.60
-1	9	23	897.51	49.90
1	9	-23	908.41	45.70
0	-9	-23	122.59	15.00
0	9	-23	139.39	10.30
0	9	23	137.59	17.70
1	9	23	929.81	50.79
-1	9	-23	916.61	45.20
-2	9	-23	90.59	11.60
2	9	23	83.09	12.80
-3	9	-23	190.08	15.50
3	9	23	191.88	19.40
4	9	23	205.98	20.20
-4	9	-23	189.58	16.40
-5	9	-23	202.88	17.50
5	9	23	205.08	20.10
-6	9	-23	542.55	31.90
6	9	23	544.55	34.70
6	-10	-23	13.20	8.90
6	10	-23	1.20	8.60
-6	10	23	2.80	10.90
5	-10	-23	19.70	9.30
5	10	-23	0.80	6.60
-5	10	23	2.40	10.60
4	-10	-23	-10.90	7.60
4	10	-23	13.30	6.30
-4	10	23	6.70	9.90
3	-10	-23	-0.20	7.40

3	10	-23	-1.30	5.30
-3	10	23	7.20	10.60
2	-10	-23	52.99	10.10
2	10	-23	75.59	9.20
-2	10	23	38.90	12.20
1	-10	-23	2.20	7.70
1	10	-23	0.90	4.60
-1	10	23	-4.20	9.90
0	10	-23	47.10	7.50
0	10	23	41.10	11.80
1	10	23	-9.50	9.80
-1	10	-23	-6.40	3.30
2	10	23	-8.40	10.10
-3	10	-23	-4.60	8.10
3	10	23	-1.90	10.00
-4	10	-23	10.60	7.80
4	10	23	3.00	9.30
-5	10	-23	3.80	8.10
5	10	23	2.20	10.20
6	10	23	4.00	10.70
5	-11	-23	567.44	35.50
5	11	-23	603.04	35.50
-5	11	23	611.14	37.20
4	-11	-23	4.70	9.10
4	11	-23	7.60	6.40
-4	11	23	-7.50	10.90
3	-11	-23	475.15	30.10
-3	11	23	538.75	32.70
3	11	-23	447.06	27.10
2	-11	-23	-5.80	8.00
2	11	-23	4.20	5.20
-2	11	23	-9.00	10.90
1	-11	-23	495.75	30.00
1	11	-23	476.45	25.80
-1	11	23	463.85	32.20
0	11	23	295.37	24.70
0	11	-23	308.97	17.50
-1	11	-23	446.16	22.60
1	11	23	429.16	30.70
2	11	23	103.49	18.50
3	11	23	178.78	20.40
4	11	23	113.99	18.60
5	11	23	185.78	21.50
-5	11	-23	218.38	18.10

4	-12	-23	115.49	17.10
4	12	-23	112.99	13.90
-4	12	23	126.09	19.80
3	-12	-23	4.90	9.30
3	12	-23	8.00	6.20
-3	12	23	-2.40	11.00
2	-12	-23	153.08	17.20
2	12	-23	125.89	12.10
-2	12	23	132.09	20.10
1	-12	-23	18.80	8.90
-1	12	23	14.80	12.30
1	12	-23	11.10	5.00
0	12	23	122.49	16.50
0	12	-23	127.19	10.00
1	12	23	49.70	13.90
-1	12	-23	59.59	6.60
2	12	23	204.28	22.00
3	12	23	34.90	12.50
4	12	23	112.99	16.90
3	-13	-23	204.38	20.20
-3	13	23	195.28	22.90
3	13	-23	201.88	18.60
2	-13	-23	15.50	9.20
2	13	-23	11.10	5.70
-2	13	23	-10.00	10.80
-1	13	23	89.89	15.80
1	13	-23	80.39	9.70
0	13	23	48.50	14.00
0	13	-23	53.09	8.10
1	13	23	138.89	20.80
-1	13	-23	134.39	9.30
2	13	23	-5.10	12.00
3	13	23	66.89	15.10
2	-14	-23	319.07	25.60
-2	14	23	338.07	28.60
-1	14	23	12.30	12.70
1	14	-23	17.70	5.60
0	14	23	697.53	42.70
0	14	-23	666.63	34.70
1	14	23	132.79	22.50
-1	14	-23	180.28	10.90
2	14	23	203.58	23.60
7	0	-24	1.20	7.50
6	0	-24	668.73	37.40

-5	0	24	46.80	8.00
5	0	-24	51.99	9.20
-4	0	24	691.13	37.50
4	0	-24	734.33	38.70
-3	0	24	1.40	6.30
3	0	-24	1.30	5.50
2	0	-24	335.67	20.70
-2	0	24	332.87	21.00
1	0	-24	243.68	16.40
-1	0	24	232.68	17.10
0	0	-24	749.13	37.10
0	0	24	650.83	39.30
1	0	24	329.27	21.30
-1	0	-24	325.27	19.50
2	0	24	544.95	30.90
3	0	24	126.29	14.50
4	0	24	311.87	21.70
5	0	24	452.25	28.00
6	0	24	68.19	11.10
7	0	24	223.78	20.20
7	-1	-24	38.60	9.50
7	1	-24	32.50	9.20
6	-1	-24	0.30	7.70
6	1	-24	3.00	7.20
5	-1	-24	123.99	13.90
5	1	-24	130.59	13.90
-5	1	24	146.19	13.50
-4	-1	24	8.10	5.90
4	-1	-24	3.10	6.40
4	1	-24	5.70	6.00
-4	1	24	5.70	6.70
-3	-1	24	67.69	10.70
3	-1	-24	65.09	11.20
3	1	-24	73.19	10.50
-3	1	24	62.49	9.10
2	-1	-24	69.29	10.30
-2	-1	24	55.19	10.50
-2	1	24	55.49	10.10
2	1	-24	61.39	10.00
-1	-1	24	33.40	7.40
1	-1	-24	33.80	6.90
1	1	-24	38.30	6.70
-1	1	24	31.40	9.10
0	-1	24	39.00	8.10

0	-1	-24	35.20	6.80
0	1	-24	52.49	8.70
0	1	24	37.30	8.20
-1	-1	-24	101.29	10.40
1	-1	24	86.79	12.20
-1	1	-24	97.59	9.60
1	1	24	79.89	12.30
2	-1	24	84.99	12.30
2	1	24	70.59	12.40
3	-1	24	8.50	7.30
3	1	24	11.80	7.70
4	-1	24	27.50	8.50
4	1	24	10.10	7.90
5	-1	24	1.70	8.20
5	1	24	9.90	8.70
6	-1	24	6.10	8.50
6	1	24	10.20	8.70
7	-1	24	-1.60	9.20
7	1	24	11.20	9.50
7	-2	-24	4.20	7.50
7	2	-24	1.10	7.60
6	-2	-24	291.27	20.70
-6	2	24	243.68	21.10
6	2	-24	283.67	20.10
5	-2	-24	3.90	6.30
5	2	-24	-4.60	6.10
4	-2	-24	619.94	34.30
4	2	-24	606.44	33.70
-3	-2	24	5.70	5.90
3	-2	-24	-3.30	5.80
-3	2	24	-2.60	7.80
3	2	-24	3.80	5.70
2	-2	-24	227.38	16.50
-2	-2	24	208.58	15.70
-2	2	24	205.88	16.90
2	2	-24	237.38	15.80
1	-2	-24	15.30	6.00
-1	-2	24	21.00	6.90
-1	2	24	11.80	7.20
1	2	-24	22.00	5.60
0	-2	-24	442.26	25.70
0	-2	24	446.16	25.90
0	2	-24	464.15	25.00
0	2	24	439.76	26.70

-1	-2	-24	71.69	10.20
1	-2	24	88.59	16.10
1	2	24	82.19	12.40
-1	2	-24	84.59	9.00
2	-2	24	368.66	22.90
2	2	24	359.66	23.50
3	-2	24	103.39	13.40
3	2	24	115.19	14.00
4	-2	24	68.39	10.10
4	2	24	76.59	11.00
5	-2	24	337.27	22.90
5	2	24	324.97	23.30
6	-2	24	112.69	15.70
6	2	24	98.09	15.70
-7	2	-24	147.89	17.20
7	2	24	208.18	18.90
7	-3	-24	261.97	21.40
7	3	-24	266.57	20.40
-7	3	24	280.97	24.50
6	-3	-24	10.90	7.30
6	3	-24	6.00	6.80
-6	3	24	10.80	8.10
5	-3	-24	545.05	31.80
-5	3	24	549.85	31.30
5	3	-24	554.74	31.30
4	-3	-24	9.90	6.40
4	3	-24	2.30	6.00
-4	3	24	-7.80	7.20
3	-3	-24	538.75	29.90
-3	-3	24	516.85	28.60
3	3	-24	500.35	28.80
-3	3	24	513.45	30.00
2	-3	-24	10.00	6.40
-2	-3	24	-1.90	5.70
-2	3	24	-3.90	7.00
2	3	-24	7.60	5.40
1	-3	-24	439.36	25.80
-1	-3	24	439.06	25.10
-1	3	24	422.36	26.50
1	3	-24	455.35	24.70
0	-3	24	90.29	12.00
0	-3	-24	107.49	11.70
0	3	24	94.19	13.80
0	3	-24	115.09	10.20

1	-3	24	558.44	30.00
-1	-3	-24	546.55	29.70
1	3	24	502.95	31.00
-1	3	-24	526.45	28.10
2	-3	24	167.88	15.20
2	3	24	179.98	16.50
3	-3	24	151.88	14.90
3	3	24	143.39	16.20
4	-3	24	245.68	18.70
4	3	24	234.08	19.70
5	-3	24	137.39	15.50
5	3	24	105.49	15.40
-6	3	-24	375.06	25.00
6	3	24	390.16	26.20
7	3	24	192.78	18.90
-7	3	-24	172.88	17.70
7	-4	-24	21.70	8.40
7	4	-24	7.80	7.60
-7	4	24	1.30	8.50
6	-4	-24	48.50	9.60
6	4	-24	70.89	12.60
-6	4	24	45.80	9.90
5	-4	-24	9.70	7.30
5	4	-24	5.90	6.40
-5	4	24	7.30	8.20
4	-4	-24	140.89	14.40
-4	4	24	135.29	15.00
4	4	-24	137.59	13.00
3	-4	-24	42.60	7.90
3	4	-24	35.70	7.00
-3	4	24	50.29	9.50
-2	-4	24	10.20	5.90
2	-4	-24	10.90	6.30
-2	4	24	0.10	7.80
2	4	-24	17.40	5.70
1	-4	-24	18.60	6.70
-1	-4	24	11.70	6.30
1	4	-24	17.50	5.60
-1	4	24	20.50	8.30
0	-4	24	41.80	7.60
0	-4	-24	30.60	7.00
0	4	24	29.30	9.00
0	4	-24	34.70	5.70
1	-4	24	159.68	14.20

-1	-4	-24	144.69	13.50
1	4	24	169.58	15.80
-1	4	-24	146.29	11.00
2	4	24	252.87	19.70
3	-4	24	34.90	8.30
3	4	24	45.00	9.80
4	-4	24	-2.30	7.40
4	4	24	7.20	8.90
5	4	24	58.89	11.30
-5	4	-24	39.10	9.30
6	4	24	34.10	10.50
-6	4	-24	40.00	9.70
6	-5	-24	38.60	8.90
6	5	-24	21.30	7.50
-6	5	24	40.70	10.30
5	-5	-24	316.47	22.10
5	5	-24	309.07	21.10
-5	5	24	315.37	22.70
4	-5	-24	0.80	6.80
-4	5	24	-18.20	7.70
4	5	-24	0.40	5.40
3	-5	-24	654.33	35.60
3	5	-24	612.34	33.90
-3	5	24	636.24	36.00
2	-5	-24	5.90	6.50
2	5	-24	14.30	5.50
-2	5	24	15.10	8.50
-1	-5	24	765.72	38.10
1	-5	-24	730.53	39.50
1	5	-24	721.43	37.70
-1	5	24	695.93	40.30
0	-5	-24	370.06	23.60
0	-5	24	401.46	22.90
0	5	-24	390.86	21.50
0	5	24	374.96	25.10
1	-5	24	382.76	22.90
-1	5	-24	376.76	20.60
1	5	24	378.16	25.10
2	-5	24	92.59	12.90
2	5	24	83.19	14.50
-3	5	-24	448.36	28.60
3	5	24	554.74	31.00
-4	5	-24	189.38	17.30
4	5	24	228.68	19.40

5	5	24	165.78	17.80
-5	5	-24	151.28	15.80
-6	5	-24	486.85	30.60
6	5	24	532.55	32.10
6	-6	-24	43.50	10.20
-6	6	24	40.00	10.70
6	6	-24	28.50	8.20
5	-6	-24	-1.40	7.30
-5	6	24	-17.50	8.90
5	6	-24	6.70	6.60
4	-6	-24	95.39	14.00
4	6	-24	107.69	12.10
-4	6	24	93.19	16.00
3	-6	-24	27.40	7.80
3	6	-24	39.30	6.80
-3	6	24	41.70	10.50
2	-6	-24	9.30	7.00
2	6	-24	10.60	5.40
-2	6	24	8.20	9.10
1	-6	-24	0.20	6.30
-1	6	24	-1.50	8.60
1	6	-24	8.40	5.00
0	-6	24	115.89	11.90
0	-6	-24	99.39	12.80
0	6	-24	97.09	9.60
0	6	24	91.99	14.90
1	6	24	21.60	9.50
-1	6	-24	10.70	6.80
-1	6	-24	19.60	4.40
2	6	24	20.80	9.80
-2	6	-24	12.00	7.10
3	6	24	45.40	11.00
-3	6	-24	27.50	8.30
-4	6	-24	2.50	7.60
4	6	24	-4.70	9.50
5	6	24	-12.00	8.90
-5	6	-24	7.80	8.40
-6	6	-24	44.40	10.40
6	6	24	60.09	12.30
6	-7	-24	4.60	8.10
-6	7	24	16.10	10.30
6	7	-24	6.30	8.20
5	-7	-24	506.05	30.80
5	7	-24	478.75	29.00

-5	7	24	520.95	31.50
4	-7	-24	8.70	7.50
4	7	-24	2.10	5.80
-4	7	24	-5.30	9.00
3	-7	-24	313.17	22.40
-3	7	24	334.37	24.20
3	7	-24	331.17	20.30
2	-7	-24	42.90	8.30
2	7	-24	49.10	8.90
-2	7	24	38.60	10.80
1	-7	-24	321.57	22.10
-1	7	24	337.87	24.20
1	7	-24	331.57	19.30
0	-7	-24	177.08	15.60
0	7	-24	171.78	12.10
0	7	24	160.68	17.90
-1	7	-24	306.57	25.60
-1	7	-24	298.67	16.60
1	7	24	289.17	22.80
-2	7	-24	93.89	12.30
2	7	24	87.69	16.00
3	7	24	126.79	17.10
-3	7	-24	136.39	17.20
4	7	24	186.68	18.80
-4	7	-24	167.88	16.20
-5	7	-24	90.99	14.20
5	7	24	94.99	16.40
6	7	24	245.18	22.30
5	-8	-24	9.60	8.60
5	8	-24	11.10	6.80
-5	8	24	9.70	10.50
4	-8	-24	391.76	25.80
4	8	-24	375.06	23.60
-4	8	24	414.66	27.80
3	-8	-24	-10.80	7.00
-3	8	24	-17.30	9.80
3	8	-24	1.10	5.70
2	-8	-24	207.48	17.60
2	8	-24	206.88	14.50
-2	8	24	197.48	20.30
1	-8	-24	155.88	15.70
1	8	-24	160.38	12.10
-1	8	24	151.18	18.70
0	-8	-24	434.16	26.50

0	8	-24	419.36	22.80
0	8	24	414.16	28.70
-1	8	-24	147.99	10.00
1	8	24	149.29	18.40
2	8	24	179.98	19.30
3	8	24	161.08	18.70
4	8	24	50.69	11.90
-4	8	-24	64.39	9.80
5	8	24	141.79	19.20
-5	8	-24	156.48	16.10
5	-9	-24	36.70	9.90
-5	9	24	36.30	11.60
4	-9	-24	0.40	7.70
-4	9	24	-1.70	10.50
4	9	-24	7.00	6.00
3	-9	-24	4.00	7.40
-3	9	24	6.40	10.60
3	9	-24	13.30	6.00
2	-9	-24	4.30	7.90
2	9	-24	3.20	5.20
-2	9	24	9.30	9.90
1	-9	-24	23.90	8.60
1	9	-24	28.50	5.40
-1	9	24	16.00	11.10
0	9	-24	-0.90	4.20
0	9	24	-6.70	10.00
-1	9	-24	70.19	7.00
1	9	24	73.79	13.50
2	9	24	8.70	10.60
3	9	24	-10.90	10.20
4	9	24	24.80	11.10
-4	9	-24	28.10	8.50
5	9	24	-4.70	10.80
4	-10	-24	454.05	30.20
-4	10	24	496.25	32.30
3	-10	-24	-4.20	7.70
3	10	-24	7.40	6.00
-3	10	24	-14.00	10.50
2	-10	-24	480.45	30.60
2	10	-24	484.35	27.20
-2	10	24	535.85	33.40
1	-10	-24	178.48	17.50
-1	10	24	147.49	20.30
1	10	-24	182.08	12.80

0	10	24	458.05	31.10
0	10	-24	443.66	23.90
1	10	24	87.59	14.90
-1	10	-24	118.19	8.20
2	10	24	197.28	21.30
3	10	24	179.18	20.80
4	10	24	142.39	20.50
3	-11	-24	65.99	11.70
-3	11	24	67.69	14.00
3	11	-24	49.30	10.00
2	-11	-24	13.90	8.90
-2	11	24	-1.80	11.30
2	11	-24	16.00	5.70
1	-11	-24	17.80	9.70
1	11	-24	14.80	5.30
-1	11	24	5.10	11.60
0	11	24	24.80	12.40
-1	11	-24	30.20	5.60
1	11	24	-3.10	11.10
2	11	24	28.20	11.90
3	11	24	4.90	11.00
2	-12	-24	124.99	17.20
-2	12	24	101.99	15.60
1	12	-24	51.29	8.70
-1	12	24	37.30	13.90
0	12	-24	322.07	18.80
0	12	24	366.36	27.90
-1	12	-24	137.79	8.80
1	12	24	109.89	20.80
2	12	24	213.08	23.50
6	0	-25	-9.90	7.10
5	0	-25	7.30	6.90
-4	0	25	-8.10	7.20
4	0	-25	-5.00	6.40
-3	0	25	-2.50	6.80
3	0	-25	1.50	5.90
2	0	-25	-4.00	5.20
-1	0	25	-3.20	6.80
1	0	-25	-3.00	5.10
0	0	-25	0.10	5.50
0	0	25	-3.90	6.90
-1	0	-25	0.10	5.00
1	0	25	1.80	7.40
2	0	25	-1.20	7.20

3	0	25	5.90	8.20
4	0	25	5.70	8.40
5	0	25	-6.20	8.50
6	-1	-25	16.70	7.90
6	1	-25	5.90	7.40
5	-1	-25	319.87	22.30
5	1	-25	337.07	22.00
4	-1	-25	15.40	7.20
4	1	-25	13.20	6.70
-4	1	25	11.60	8.80
-3	-1	25	508.85	28.40
3	-1	-25	495.75	29.20
-3	1	25	506.75	29.10
3	1	-25	516.05	28.90
2	-1	-25	70.39	10.80
-2	-1	25	72.99	11.80
-2	1	25	55.99	9.60
2	1	-25	79.89	10.40
1	-1	-25	221.28	16.10
-1	1	25	211.18	17.30
1	1	-25	230.38	15.90
0	-1	25	281.97	19.20
0	-1	-25	263.37	17.90
0	1	-25	265.77	17.30
0	1	25	265.57	19.40
1	-1	25	480.85	28.10
-1	-1	-25	490.65	26.90
1	1	25	465.65	28.50
-1	1	-25	477.55	26.30
2	-1	25	150.58	15.40
2	1	25	163.08	16.20
3	-1	25	242.78	19.00
3	1	25	248.08	19.10
4	-1	25	280.47	21.90
4	1	25	330.87	22.70
5	-1	25	30.30	9.60
5	1	25	35.70	10.30
6	-2	-25	347.27	23.80
5	-2	-25	24.10	7.90
-5	2	25	11.30	8.00
5	2	-25	25.60	7.30
4	-2	-25	161.48	15.20
-4	2	25	151.48	15.20
4	2	-25	149.69	14.50

3	-2	-25	33.60	7.60
3	2	-25	43.80	7.70
-3	2	25	38.90	9.00
-2	-2	25	109.99	12.50
2	-2	-25	120.69	12.70
2	2	-25	112.89	11.70
-2	2	25	115.89	14.00
-1	-2	25	59.69	9.00
1	-2	-25	76.49	11.00
-1	2	25	66.49	9.90
1	2	-25	84.89	10.20
0	-2	-25	328.27	20.40
0	2	-25	313.87	19.40
0	2	25	325.47	22.00
-1	-2	-25	248.28	17.60
1	-2	25	286.67	19.20
1	2	25	267.27	19.60
-1	2	-25	262.47	16.40
2	-2	25	81.79	13.10
2	2	25	66.09	10.80
3	-2	25	71.79	13.30
3	2	25	44.70	10.00
4	-2	25	69.39	10.70
4	2	25	84.29	14.50
5	-2	25	250.17	20.90
5	2	25	255.97	21.10
5	-3	-25	86.79	14.30
-5	3	25	100.59	14.70
5	3	-25	119.99	13.50
4	-3	-25	72.49	12.80
4	3	-25	62.19	11.50
-4	3	25	50.39	9.70
3	-3	-25	57.49	11.80
3	3	-25	62.59	10.50
-3	3	25	60.49	10.10
2	-3	-25	1.00	6.30
2	3	-25	3.10	5.30
-2	3	25	-13.50	7.70
1	-3	-25	54.69	10.70
-1	-3	25	57.39	8.50
1	3	-25	58.19	9.30
-1	3	25	62.99	10.00
0	-3	25	113.49	13.30
0	-3	-25	139.39	12.70

0	3	-25	117.69	11.10
0	3	25	103.59	15.00
-1	-3	-25	56.59	10.30
1	-3	25	47.90	9.10
-1	3	-25	50.49	8.10
1	3	25	52.99	9.90
2	-3	25	33.40	8.60
2	3	25	34.20	9.90
3	-3	25	15.50	8.40
3	3	25	20.60	9.70
4	-3	25	27.30	9.10
4	3	25	30.60	9.50
5	-3	25	37.50	10.10
-5	3	-25	36.20	9.50
5	3	25	35.80	10.20
5	-4	-25	9.60	7.60
5	4	-25	9.30	6.80
-5	4	25	-11.30	8.40
4	-4	-25	585.44	33.40
-4	4	25	590.74	33.40
4	4	-25	551.34	32.10
3	-4	-25	41.30	8.20
-3	4	25	61.69	10.60
3	4	-25	61.99	10.40
2	-4	-25	185.48	16.50
2	4	-25	223.78	15.00
-2	4	25	183.78	17.90
1	-4	-25	30.70	7.40
-1	-4	25	24.40	10.30
1	4	-25	33.80	6.10
-1	4	25	20.40	8.90
0	-4	25	348.87	21.50
0	-4	-25	338.57	21.80
0	4	25	350.96	23.60
0	4	-25	337.57	19.90
1	-4	25	205.88	24.90
1	4	25	197.58	17.90
-1	4	-25	192.18	12.90
2	-4	25	385.66	24.20
2	4	25	382.96	25.70
3	-4	25	257.37	19.30
3	4	25	223.68	20.10
4	4	25	142.59	17.20
-4	4	-25	104.89	15.00

-5	4	-25	411.16	27.50
5	4	25	473.35	29.10
5	-5	-25	-5.50	7.60
5	5	-25	11.80	8.70
-5	5	25	5.00	9.50
4	-5	-25	9.10	7.80
4	5	-25	5.70	5.90
-4	5	25	4.60	9.20
3	-5	-25	16.10	7.90
3	5	-25	10.30	6.10
-3	5	25	15.50	9.30
2	-5	-25	30.60	8.20
2	5	-25	27.70	6.40
-2	5	25	12.80	9.20
1	-5	-25	-4.90	6.30
1	5	-25	-1.10	4.90
-1	5	25	-1.20	8.60
0	-5	25	16.50	7.70
0	-5	-25	9.50	7.00
0	5	-25	39.10	7.90
0	5	25	17.00	9.20
1	-5	25	8.80	7.40
1	5	25	1.20	9.00
-1	5	-25	-0.70	4.00
2	-5	25	9.80	7.60
2	5	25	0.70	8.60
-3	5	-25	-9.00	7.50
3	5	25	-3.50	8.60
4	5	25	-7.00	8.80
-4	5	-25	0.40	7.80
-5	5	-25	27.10	9.40
5	5	25	50.29	11.10
5	-6	-25	36.50	8.90
4	-6	-25	287.77	22.00
-4	6	25	337.17	23.70
4	6	-25	311.37	20.50
3	-6	-25	39.90	9.10
-3	6	25	42.00	10.70
3	6	-25	52.89	10.20
2	-6	-25	106.09	13.70
-2	6	25	98.09	16.50
2	6	-25	92.49	11.00
1	-6	-25	31.60	8.00
1	6	-25	49.50	8.70

-1	6	25	41.70	11.00
0	-6	-25	481.15	28.40
0	6	25	462.85	30.10
0	6	-25	471.65	25.50
-1	6	-25	133.29	10.10
1	6	25	140.99	17.20
2	6	25	510.05	31.90
3	6	25	141.49	17.70
-3	6	-25	103.59	14.20
-4	6	-25	60.49	9.80
4	6	25	33.60	10.70
4	-7	-25	105.99	15.70
-4	7	25	124.19	18.40
3	-7	-25	54.89	9.60
-3	7	25	48.70	12.30
3	7	-25	71.29	10.70
2	-7	-25	26.70	8.60
-2	7	25	25.70	10.80
2	7	-25	17.10	5.90
1	-7	-25	101.69	14.50
1	7	-25	118.79	10.80
-1	7	25	88.19	13.40
0	-7	-25	75.79	13.60
0	7	-25	82.49	9.10
0	7	25	90.29	16.10
1	7	25	90.39	16.70
-1	7	-25	100.39	8.60
2	7	25	30.60	11.20
-3	7	-25	52.89	10.90
3	7	25	59.79	12.50
-4	7	-25	10.80	8.20
4	7	25	-1.00	10.30
3	-8	-25	21.20	8.40
3	8	-25	3.50	6.80
-3	8	25	-6.30	10.00
2	-8	-25	51.79	9.50
-2	8	25	48.90	12.30
2	8	-25	71.99	9.90
1	-8	-25	19.80	8.00
-1	8	25	6.80	10.40
1	8	-25	7.00	5.20
0	8	-25	136.29	10.70
0	8	25	104.19	18.40
-1	8	-25	135.99	9.30

1	8	25	94.89	18.30
2	8	25	25.60	11.20
3	8	25	75.39	13.50
3	-9	-25	557.14	33.70
2	-9	-25	29.70	8.90
-2	9	25	34.40	12.50
2	9	-25	48.90	9.80
1	-9	-25	139.69	16.60
-1	9	25	127.49	19.90
1	9	-25	153.28	11.90
0	9	25	102.79	18.40
0	9	-25	96.19	9.60
1	9	25	337.17	25.80
-1	9	-25	296.07	16.50
2	9	25	282.07	24.40
1	-10	-25	2.10	8.20
-1	10	25	-11.40	11.00
1	10	-25	2.80	5.10
0	10	25	-18.90	11.10
0	10	-25	7.60	4.60
1	10	25	-0.30	11.20
-1	10	-25	-4.20	3.10
3	0	-26	178.58	15.50
-2	0	26	91.99	13.30
2	0	-26	81.09	11.70
1	0	-26	59.59	11.00
-1	0	26	55.39	9.60
0	0	-26	443.16	25.70
0	0	26	442.46	27.20
1	0	26	544.85	31.40
-1	0	-26	525.65	29.30
2	0	26	76.99	11.60
3	0	26	192.28	18.50
4	0	26	15.80	9.20
4	-1	-26	6.90	11.50
3	-1	-26	91.59	12.70
-3	1	26	77.49	10.60
3	1	-26	94.09	12.40
-2	-1	26	14.30	10.10
2	-1	-26	42.00	7.80
2	1	-26	39.30	7.40
-2	1	26	29.30	8.90
-1	-1	26	18.10	8.10
1	-1	-26	37.60	7.40

-1	1	26	36.10	9.00
1	1	-26	37.30	7.30
0	-1	26	39.80	9.20
0	-1	-26	57.79	10.60
0	1	26	48.10	10.10
0	1	-26	58.19	10.20
1	-1	26	7.60	8.40
-1	-1	-26	4.60	5.70
-1	1	-26	0.00	5.50
1	1	26	-7.10	8.20
2	-1	26	-2.40	8.20
2	1	26	-13.60	8.20
3	-1	26	36.10	9.50
3	1	26	13.40	9.10
4	-1	26	39.80	10.10
4	1	26	22.40	10.20
-4	2	26	267.17	20.60
3	-2	-26	17.40	7.60
3	2	-26	14.90	6.80
-3	2	26	17.70	8.70
2	-2	-26	185.98	16.30
2	2	-26	195.08	15.30
-2	2	26	193.98	17.50
-1	-2	26	43.50	10.20
1	-2	-26	64.29	11.30
1	2	-26	57.59	10.30
-1	2	26	47.50	10.00
0	-2	-26	159.08	14.80
0	-2	26	192.48	16.00
0	2	26	177.88	17.40
0	2	-26	165.48	13.70
1	-2	26	310.07	21.50
1	2	26	318.67	22.50
-1	2	-26	301.57	18.70
2	-2	26	167.18	16.70
2	2	26	155.58	17.70
3	-2	26	139.99	16.70
3	2	26	141.99	16.60
3	-3	-26	222.78	18.00
-3	3	26	192.08	18.60
3	3	-26	211.38	18.70
2	-3	-26	87.79	13.00
-2	3	26	86.39	15.20
2	3	-26	98.49	11.70

1	-3	-26	223.08	16.90
-1	3	26	179.58	18.20
1	3	-26	210.58	15.30
0	-3	-26	309.37	20.40
0	-3	26	294.47	20.40
0	3	-26	305.57	18.80
0	3	26	291.27	22.40
1	-3	26	211.88	17.70
1	3	26	222.68	19.50
-1	3	-26	216.28	14.60
2	-3	26	184.38	16.90
2	3	26	155.28	17.80
3	-3	26	124.69	16.20
3	3	26	119.39	17.10
3	-4	-26	9.20	7.60
-3	4	26	12.00	9.40
2	-4	-26	24.90	7.90
2	4	-26	48.80	9.90
-2	4	26	31.80	10.30
1	-4	-26	33.30	8.00
-1	4	26	17.10	9.30
1	4	-26	33.80	6.50
0	-4	-26	17.70	7.00
0	4	-26	8.80	5.30
0	4	26	-3.20	9.00
1	-4	26	46.50	9.00
-1	4	-26	43.60	8.50
1	4	26	49.70	11.30
2	-4	26	2.90	8.30
2	4	26	6.90	9.20
3	-4	26	14.20	9.10
3	4	26	-9.20	9.80
3	-5	-26	387.76	29.90
-3	5	26	418.46	27.50
2	-5	-26	17.20	8.20
2	5	-26	11.60	6.90
-2	5	26	12.90	10.20
1	-5	-26	303.37	21.30
1	5	-26	298.17	18.90
-1	5	26	307.67	23.60
0	-5	-26	233.48	18.60
0	5	-26	255.77	16.00
0	5	26	243.78	21.30
1	5	26	547.35	32.80

```
  -1   5 -26  503.95   27.30
   2   5  26  319.07   24.80
   2  -6 -26   40.20   11.90
  -2   6  26   34.50   11.50
   1  -6 -26   50.49   10.70
   1   6 -26   83.09   10.20
  -1   6  26   57.69   11.80
   0  -6 -26    0.80    8.60
   0   6 -26   -1.60    4.80
   0   6  26    7.60   10.10
   1   6  26    9.70   10.60
  -1   6 -26    6.60    4.50
   2   6  26    7.20   10.20
   1   7 -26  122.09   14.30
   0   7  26  296.17   23.90
   0   7 -26  248.78   16.20
   0   0   0    0.00    0.00
```

```
_computing_structure_solution     'SHELXT 2014/5 (Sheldrick, 2014)'
;
_shelx_hkl_checksum               72090
_olex2_submission_special_instructions  'No  special  instructions  were
received'
```
