# Supplementary material for: Structure-property relationships of photofunctional diiridium(II) complexes with tetracationic charge and an unsupported Ir–Ir bond
Source: Commun Chem. 2022 Nov 23;5:159. doi: 10.1038/s42004-022-00775-4 (PMC9814866; doi:10.1038/s42004-022-00775-4)
Supplement: Supplementary file 1 — Wong_PR File [file 42004_2022_775_MOESM1_ESM.pdf]

nature portfolio

Peer Review File

Structure-property relationships of photofunctional diiridium (II) complexes with tetracationic charge and an unsupported Ir–Ir bond

Open Access This file is licensed under a Creative Commons Attribution 4.0 International License, which permits use, sharing, adaptation, distribution and reproduction in any medium or format, as long as you give appropriate credit to the original author(s) and the source, provide a link to the Creative Commons license, and indicate if changes were made. In the cases where the authors are anonymous, such as is the case for the reports of anonymous peer reviewers, author attribution should be to 'Anonymous Referee' followed by a clear attribution to the source work. The images or other third party material in this file are included in the article's Creative Commons license, unless indicated otherwise in a credit line to the material. If material is not included in the article's Creative Commons license and your intended use is not permitted by statutory regulation or exceeds the permitted use, you will need to obtain permission directly from the copyright holder. To view a copy of this license, visit http://creativecommons.org/licenses/by/4.0/.

Reviewers' comments:

Reviewer #1 (Remarks to the Author):

The manuscript by Wong, Chung and co-workers describes tetracationic diiridium complexes with an unsupported Ir(II)-Ir(II) bond. The complexes were synthesized and then characterized by various methods including X-ray crystallography, NMR, IR, HRMS and electrochemical techniques.These novel compounds were found to be luminescent in both solution and solid states. Detailed theoretical investigations were also performed to understand the metal-metal interaction, as well as origins of luminescent properties. It was discovered that London dispersion forces make a substantial amount of contribution of the calculated binding energy for the dinuclear compounds. Photoirridiations were carried out to cleveage the Ir-Ir bond, generating reactive Ir radicals, which afforded new iridium complexes in the presence of dihalogen or TEMPO.
I believe the strength of this manuscript lies in the synthesis and characterization of unprecedented tetracationic Ir(II)-Ir(II) species with unsupported metal-metal bond. Once the following concerns are appropriately addressed, I believe the manuscript will be suitable for publication in Communications Chemistry.
1. In coordination chemistry, shorter bonds do not necessarily mean strong bonding interactions, especially in the area of metal-metal (multiple)bond. Thus, it is not that unusal to find longer Ir-Ir bonds in complexes 1-3 having relatively stronger binding energy, in comparison to previously reported neutral or dicationic Ir2 compounds. In recent years, the critical role of dispersion force in stabliziing "structurally unusal" coordination species have been revealed by several research groups, and this seems to be also the case of diiridium complexes 1-3 in this manuscript, as determined by calculations. Hence, I suggest the authors soften the language when discussing this so called "counterintuitive" or "abnormal" relationship between Ir-Ir distance and calculated binding energy.
2. The chemdraw structure of [NNN] ligand for 1 and 1' in Scheme 1 is incorrect. The subscript‟3”for the t-butyl groups should be deleted.
3. The bond lengths and angles determined by crystallography should be reported with estimated standard deviation shown in parentheses following the values.
4. For thereotical investigation, the authors need to clarify what information can be obtained from the linear simulations between the calculated Ir-Ir bond distances and binding energy shown in Fig. S27? To me, all the R square values are all quite low.
5. For the trimerization of CH3CN with 1 and TEMPO, it would be helpful to guide the readers if a possible reaction mechanism is provided in the manuscript

Reviewer #2 (Remarks to the Author):

Comments: This manuscript describes the synthesis of a series of complexes featuring unsupported Ir(II)–Ir(II) single bond with long bond distances. Their photoreactivity involving the generation of Ir(II) radical monomer was also investigated. All these complexes were fully characterized through NMR, IR, HRMS, EPR, Normalized excitation spectra, UV-Vis, Luminescence, CV, Elemental analyses and Crystal techniques. The comparison between previous examples of diiridium(II) complexes and tetracationic diiridium(II) complexes in this study are clarity. All these results will be interesting not only to the organometallic community but also to the broader readership interested in photoelectric functional material. Furthermore, the manuscript is well presented, and the conclusions are reasonable. Thus, I will recommend it be published on Communications Chemistry after considering the following minor points.

1. Complexes 1–3 exhibit interesting luminescence behaviors, the reviewer wondered if these complexes have AIE phenomenon.
2. Is it possible to compare the quantum yields of complex 1-3 with other complexes with Ir-Ir bond as shown in Chart 1?
3. Why the luminescence intensity of 1 diminishes to around 50% in aerated CH3CN solution as these

complexes are stable toward air and moisture?
4. The authors try to confirm the formation of a radical monomer ([Ir(N^N^N)(CO)(PPh3)]2+.) by EPR and by the reactions with Br2, I2, and TEMPO. Is it possible to isolate the free radical monomer and characterized it by X-ray diffraction? How about the reactions of this radical monomer with other radical trapping reagents? Complexes 1–3 are EPR silent?
5. The structure of complex A in Chart 1 should be updated, the substituent groups (Cp2Fe) on the ligands were missing.
6. The authors point that the Ir-Ir bond length in this study is longer than others, is it possible to add the corresponding Ir-Ir bond lengths in Chart 1 for comparison?
7. The structures of I and J in chart 1 are unclear.
8. Scheme 1: "tBu3" on the ligands of 1 and 1' should be "tBu"
9. Page 25: where is the Figure 9b? Figure caption of Figure 8, e should be d?
10. "13C NMR" and "31P NMR" should be "13C{1H} NMR" and "31P{1H} NMR"
11. Please check the format of references carefully in both main text and SI.

Reviewer #3 (Remarks to the Author):

In this manuscript, the authors synthesized a series of tetracationic dinuclear iridium(II) complexes with the unsupported Ir(II)–Ir(II) single bond. More interestingly, the resulting complexes were reported to exhibit photoluminescence for the first time. The related properties were further studied with the help of theoretical calculation. In general, the work reported herein is interesting and the manuscript is well-written. It is can be recommended to be published after the following revisions.
1.As shown in the manuscript, the complexes show the PL in both solid-state and solutions, and the quantum yields are also measured. These data need to be provided in Table 1 for a better understanding of their photophysical properties.
2.The authors employed the TD-DFT data to further assign the absorption and PL spectra. It will be better to confirm this with the help of corresponding data of the used ligands.
3.Did the counter ion affect their PL properties as well as other characters?
4.In the synthesis part, the F NMR for complex 1' should be provided.

Responses to reviewers' comments

Reviewer #1 (Remarks to the Author):

1. In coordination chemistry, shorter bonds do not necessarily mean strong bonding interactions, especially in the area of metal-metal (multiple)bond. Thus, it is not that unusal to find longer Ir-Ir bonds in complexes 1-3 having relatively stronger binding energy, in comparison to previously reported neutral or dicationic Ir2 compounds. In recent years, the critical role of dispersion force in stabliziing "structurally unusal" coordination species have been revealed by several research groups, and this seems to be also the case of diiridium complexes 1-3 in this manuscript, as determined by calculations. Hence, I suggest the authors soften the language when discussing this so called "counterintuitive" or "abnormal" relationship between Ir-Ir distance and calculated binding energy.

Response: Thank you so much for the suggestions. We fully agree with you that dispersion force has been found in some "structurally unusual" coordination species recently. We have deleted "counterintuitive" or "abnormal" in our revised manuscript.

2. The chemdraw structure of [NNN] ligand for 1 and 1' in Scheme 1 is incorrect. The subscript "3" for the t-butyl groups should be deleted.

Response: Thanks for this comment. The error has been corrected.

3. The bond lengths and angles determined by crystallography should be reported with estimated standard deviation shown in parentheses following the values.

Response: Thanks for this comment. The estimated standard deviation for the bond lengths and angles has been added in parentheses.

4. For thereotical investigation, the authors need to clarify what information can be obtained from the linear simulations between the calculated Ir-Ir bond distances and binding energy shown in Fig. S27? To me, all the R square values are all quite low.

Response: Thank you for your comments. Generally, there is often a good correlation between the binding energies and bond distances. A good R^2 value (0.78) can be obtained for our current Ir-Ir systems containing similar ligand sets as well as same coordination mode.

While, a poor R^2 value (0.35) was found for the previous systems containing different ligand sets and coordination modes developed by different groups. Therefore, apart from the metal-metal bonding, our computational results show that the binding energies for the previous systems are also partly affected by the ligands/coordinate modes and their interactions. The main aim of this Figure shows longer Ir-Ir bond distances and higher binding energies for our system, compared to the previous systems.

5. For the trimerization of CH_3CN with 1 and TEMPO, it would be helpful to guide the readers if a possible reaction mechanism is provided in the manuscript.

Response: Thanks a lot for your helpful comments. A proposed mechanism has been added in our revised SI, Figure S45.

Reviewer #2 (Remarks to the Author):

1. Complexes 1–3 exhibit interesting luminescence behaviors, the reviewer wondered if these complexes have AIE phenomenon.

Response: Thanks for this suggestion. We tried to add poor solvent (diethyl ether) into the acetonitrile solution of **1** in order to study the possible AIE phenomenon with the diethyl ether content from 50%-90%. There is indeed an increase in the luminescence intensity upon increasing the diethyl ether content, however, the absorbance at the excitation wavelength of 450 nm was also found to increase (please see below). Based on the similar profile of plots of luminescence intensity at 620 nm and absorbance at 450 nm versus diethyl ether content, such effect is mainly due to the change of absorbance at the excitation wavelength of 450 nm. In view of this, no AIE phenomenon was observed in the present system of unsupported diiridium(II) complexes.

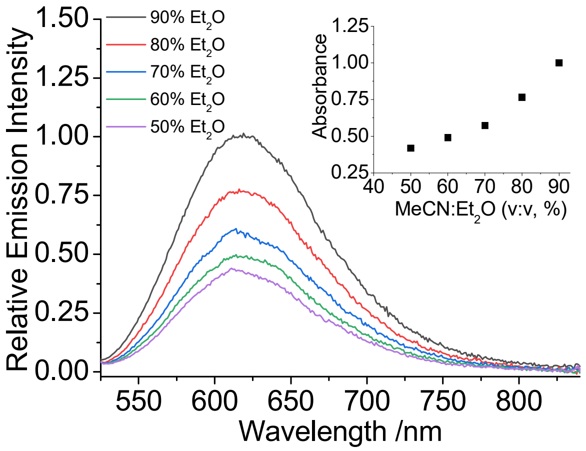

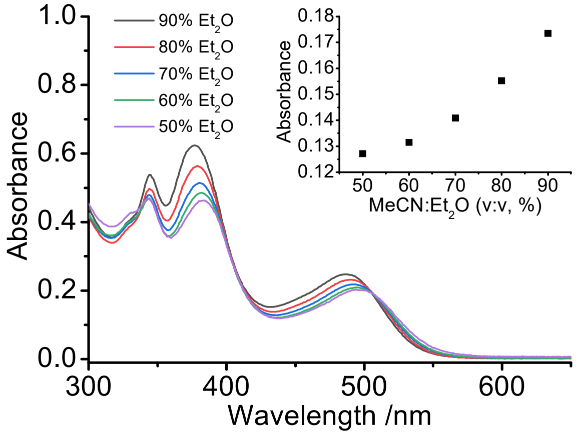

2. Is it possible to compare the quantum yields of complex 1-3 with other complexes with Ir-Ir bond as shown in Chart 1?

Response: Thanks for this comment. Since there is no report on the luminescence properties of other complexes in Chart 1, we cannot compare those with our complexes. This is also the novel of this study showing luminescence behaviours of the present complexes.

3. Why the luminescence intensity of 1 diminishes to around 50% in aerated CH_3CN solution as these complexes are stable toward air and moisture?

Response: Thanks for this comment. Because the luminescence (phosphorescence) of **1** is originated from its triplet state, it is common that the oxygen molecule in aerated solution would quench such longer-lived excited state and hence the diminishment of luminescence intensity.

4. The authors try to confirm the formation of a radical monomer ([Ir(N^N^N)(CO)(PPh3)]2+.) by EPR and by the reactions with Br2, I2, and TEMPO. Is it possible to isolate the free radical monomer and characterized it by X-ray diffraction? How about the reactions of this radical monomer with other radical trapping reagents? Complexes 1–3 are EPR silent?

Response: Thanks for this comment. We attempted to isolate the free radical monomer for characterization including X-ray diffraction, however, we failed to do so probably due to the highly reactive nature of this radical.

Apart from using TEMPO as the radical trapping reagent, we also tried to use butylated hydroxytoluene (BHT) and diphenylethylene (DPE) to trap the free radical monomer. However, no related trapped species could be isolated.
No EPR signal could be observed without the irradiation of light. All the related discussions have been added in the revised manuscript.

5. The structure of complex A in Chart 1 should be updated, the substituent groups (Cp_2Fe) on the ligands were missing.
Response: Thank you very much for your careful reading and important advice. We apologize for our mistake. We have performed additional calculations on complex A with the Cp2Fe group, as well as updated the structure of complex A in Chart 1. In addition, the corresponding data of complex A in Figure S26 of the revised SI, Tables S13, S42-47 and Cartesian coordinates have also been updated in our revised SI.

6. The authors point that the Ir-Ir bond length in this study is longer than others, is it possible to add the corresponding Ir-Ir bond lengths in Chart 1 for comparison?
Response: Thanks for this comment. The Ir-Ir bond lengths of the examples in Chart 1 have been added.

7. The structures of I and J in chart 1 are unclear.
Response: Thanks for this comment. The structures of I and J have been revised.

8. Scheme 1: "tBu3" on the ligands of 1 and 1' should be "tBu"
Response: Thanks for this comment. The error has been corrected.

9. Page 25: where is the Figure 9b? Figure caption of Figure 8, e should be d?
Response: Thanks for these comments. The text "Figure 9b" has been revised to "Figure 8b" in the main text. The Figure caption of Figure 8d has also been revised.

10. "13C NMR" and "31P NMR" should be "13C{1H} NMR" and "31P{1H} NMR"
Response: Thanks for this comment. The errors have been corrected.

11. Please check the format of references carefully in both main text and SI.

Response: Thanks for this comment. The format of all references in both main text and SI have been checked and revised if necessary.

Reviewer #3 (Remarks to the Author):

1. As shown in the manuscript, the complexes show the PL in both solid-state and solutions, and the quantum yields are also measured. These data need to be provided in Table 1 for a better understanding of their photophysical properties.

Response: Thanks for this comment. The emission quantum yield values in MeCN solution have been updated and added in the revised Table 1.

2.The authors employed the TD-DFT data to further assign the absorption and PL spectra. It will be better to confirm this with the help of corresponding data of the used ligands.

Response: Thank you for your suggestion. The following two sentences have been added or revised in the revised manuscript.

"This result further supports the assignment of such low-energy absorption band as MMLCT transition with the substantial lower-lying π* orbital energy level of bzimpy ligand in **3** (540 nm), relative **1** (496 nm) with terpyridine ligand."

"Qualitatively, our CPCM TD-B3LYP-D3//M06-L (including effect of spin-orbital coupling) calculations also supported the observed red-shift luminescence of **3** for the assignment of ^{3}MMLCT excited state (**1**: ~611 nm vs. **3**: ~644 nm; Tables S18–S20)."

3.Did the counter ion affect their PL properties as well as other characters?

Response: Thanks for this comment. The counter ion effect in **1'** with BF_4^- ion has been studied in more details. In principle, the photophysical and the electrochemical properties in solution state are essentially the same with those in **1** with OTf^- ion. Independent experiment has been conducted to study the photostability of **1** and **1'**.

However, the emission energies of **1'** in solid state at room temperature and 77K are found to be higher than those of **1**. This could be rationalized by the lower-lying HOMO in **1'** resulting from the shorter Ir-Ir bond, which was observed from the crystal structures. Since the

LUMOs of **1** and **1’** are suggested to be in similar energy level, larger HOMO-LUMO energy gap in **1’** could give rise to higher emission energy in solid state. This discussion has been added in the revised main text.

4.In the synthesis part, the F NMR for complex 1’ should be provided.

Response: Thanks for this comment. The ^{19}F NMR spectrum of complex **1’** has been added in Figure S12 in the revised SI.

REVIEWERS' COMMENTS:

Reviewer #1 (Remarks to the Author):

The authors have addressed my comments and I recommend publication.

Reviewer #2 (Remarks to the Author):

The authors have fully addressed my concerns and I find this paper ready for publication. The results presented will be of great interest to the community of Communications Chemistry.

Reviewer #3 (Remarks to the Author):

I am satisfied with the revision. It should be accepted to be published
